# Cross-cohort transcriptomic meta-analysis identifies conserved melanogenesis signatures and developmental background in black-boned chicken breast muscle

**DOI:** 10.5713/ab.260430

**Published:** 2026-06-24

**Authors:** Ming Qu, Yiming Diao, Linglu Ou, Ji Cao, Haiping Liang, Qing Wei, Jianzhen Huang, Yong Cui

**Affiliations:** 1 College of Animal Science and Technology, Jiangxi Agricultural University, Nanchang, China

**Keywords:** Breast Muscle, Chicken, Melanin, Meta-analysis, Muscle Development, Transcriptomics

## Abstract

**Objective:**

The purpose of this study was to identify conserved transcriptomic signatures associated with melanin deposition in black-boned chicken breast muscle and to distinguish robust pigmentation signals from normal breast muscle developmental and breed-background effects.

**Methods:**

RNA-seq datasets of public breast muscle were integrated from three pigmentation-related cohorts, comprising 53 samples alongside one ordinary breast muscle background cohort, which included 42 samples. Gene-level effects were estimated using Salmon, tximport, and DESeq2, followed by random-effects meta-analysis, leave-one-cohort-out analysis, background filtering, candidate layering, and exploratory signature score analysis.

**Results:**

In the pigmentation-related cohorts, 14,139 genes were eligible for meta-analysis, with 257 genes reached meta false discovery rate <0.05. Notably, *PMEL*, *TYRP1*, *DCT*, and *TYR* showed significant and directionally consistent upregulation in the breast muscle of black-boned chicken. Among the 227 significant and direction-consistent meta-analysis genes, 156 were identified by the background cohort at the prespecified threshold, including 145 background-development genes and 11 pigmentation-development background-overlap genes. *KIT* was the only melanogenesis-enriched gene not identified by the background filter. The 11 candidates related to muscle phenotype showed consistent association with pigmentation comparisons, whereas canonical myogenic regulators did not achieve meta-significant.

**Conclusion:**

The present study confirmed conserved melanogenesis anchors and identified 11 muscle-phenotype-related genes, rather than canonical myogenic regulators, as the primary reproducible muscle-context signals associated with melanin deposition in black-boned chicken breast muscle.

## INTRODUCTION

Black-boned chickens are poultry genetic resources characterized by extensive melanin deposition in skin, bone, connective tissue, and internal organs. In addition to these classic fibromelanotic tissues, visible pigmentation can also occur in breast muscle [1], a tissue that is central to carcass and meat-production traits. Breast muscle therefore provides a biologically relevant system for examining pigmentation-associated transcriptional signals within the broader context of muscle development and muscle phenotypic variation.

Previous transcriptomic studies have repeatedly implicated melanogenesis and melanosome-related genes in black-boned chicken breast muscle. Reported candidates include *PMEL*, *RAB29*, solute carrier genes, *PDZK1*, *EDNRB2*, *GPNMB*, *TRPM1*, *TYR*, *DCT*, and genes related to neural crest migration, melanocyte differentiation, melanosome formation, and melanin biosynthesis [2–5]. These studies established a clear pigmentation-related transcriptional component, but most were performed within individual breeds, stages, or analytical designs.

Together, these studies indicate that breast muscle pigmentation in black-boned chickens has a clear melanogenesis- and melanosome-related transcriptional component. However, two interpretive issues therefore remain. First, single-cohort candidate genes can be influenced by breed background, developmental stage, sample size, control structure, and analytical workflow. Second, breast muscle is a developmentally active tissue, so pigmentation comparisons may also capture ordinary muscle-development or breed-background expression changes. This distinction is important because hyperpigmentation has been associated with early skeletal muscle developmental phenotypes in Tengchong Snow chickens [6], and PRJNA729271 shows broad developmental and breed-associated expression variation in ordinary breast muscle [7].

Cross-study transcriptomic meta-analysis can address these issues by integrating effect sizes across independent datasets, evaluating heterogeneity, and testing whether candidate signals remain directionally consistent [8]. For black-boned chicken breast muscle, public RNA-seq datasets now include Yugan black-boned, Wuliangshan black-boned, Silkie, and ordinary breast muscle developmental cohorts. These datasets allow conserved pigmentation-associated signals to be evaluated separately from ordinary breast muscle developmental background.

The final goal of this study was therefore to distinguish conserved pigmentation-associated transcriptional signals from ordinary breast muscle developmental and breed-background variation in black-boned chickens. To achieve this goal, we integrated three pigmentation-related breast muscle RNA-seq cohorts and used PRJNA729271 as an independent background cohort, combining cohort-level DESeq2 effect sizes, random-effects meta-analysis, leave-one-cohort-out assessment, background filtering, sensitivity analysis, and targeted marker checks. This study provides a conservative and reusable public-transcriptome framework for prioritizing candidate genes associated with melanin deposition and breast muscle phenotypic traits, thereby offering a foundation for future functional validation and genetic improvement studies in black-boned chickens.

## MATERIALS AND METHODS

### Public RNA-seq datasets and study design

Public breast muscle RNA-seq datasets were collected to evaluate conserved pigmentation-related transcriptomic signatures in black-boned chickens. Three datasets were used as pigmentation-related cohorts: PRJNA827465, CRA004775, and the CRA014497 matched subset. PRJNA827465 included embryonic breast muscle samples from Yugan black-boned chickens [4]. CRA004775 included adult breast muscle samples from Wuliangshan black-boned chickens and control groups with ordinary or low pigmentation [3]. The CRA014497 matched subset included embryonic breast muscle samples from Silkie chickens and developmentally matched Wenchang control samples from PRJNA827465 [5]. PRJNA729271 was used as an ordinary breast muscle background dataset from the Arbor Acres and Lushi chicken breast muscle development study and was not included in the main pigmentation meta-analysis (Table 1) [7].

For each dataset, a sample sheet was curated to include sample identifiers, run accessions, developmental stage, breed, pigmentation status, replicate number, and comparison role (Supplement 1). The inventory of unique Salmon quantification directories was recorded separately to distinguish analysis records from reused control samples (Supplement 2). Samples were assigned as case, control, or background according to the dataset-specific comparison. The pigmentation-related cohorts were analysed separately at the cohort level, and their gene-level effect sizes were then combined by random-effects meta-analysis. PRJNA729271 was analysed independently to define expression changes associated with ordinary breast muscle development and breed background.

### Transcript-level quantification and gene annotation

Salmon quantification was performed against the chicken reference transcriptome derived from the GRCg7b chicken genome annotation [9]. Transcript-level abundance estimates were imported into R using the tximport package with Salmon input files and the transcript-to-gene mapping table [10]. Transcript-to-gene mapping was generated from the corresponding GTF annotation file and used for gene-level summarization. A gene identifier-to-symbol mapping table was also prepared from the same annotation and used to annotate downstream results.

### Cohort-level differential expression analysis

Cohort-level differential expression analysis was performed using DESeq2 [11]. For each pigmentation-related cohort, samples were first filtered to retain the relevant case and control groups. The comparison role was encoded as a two-level factor with control as the reference level. When a cohort included more than one developmental stage, the design formula included stage and condition (~stage+condition); otherwise, condition alone was used (~condition). Genes with counts of at least 10 in at least three samples were retained. DESeq2 was then used to estimate gene-level log2 fold changes, standard errors, test statistics, p-values, and adjusted p-values for the case-versus-control contrast.

The effect-size table generated for each cohort included cohort identifier, gene identifier, gene symbol, case group, control group, log2 fold change, standard error, statistic, p-value, adjusted p-value, and direction of change (Supplement 3). Variance-stabilizing transformation was also performed for each cohort and used in signature score analysis. The transformed expression matrices were also used to assess sample-level expression structure by principal component analysis (Supplement 4).

### Random-effects meta-analysis across pigmentation cohorts

Gene-level effect sizes from the three pigmentation-related cohorts were combined using a random-effects meta-analysis. Only genes with valid log2 fold change and standard error estimates in at least two cohorts were included. For each gene, the cohort-specific log2 fold changes were treated as effect sizes, and squared standard errors were used as within-cohort variances.

Between-cohort variance was estimated using the DerSimonian-Laird method [12]. Random-effects weights were calculated from within-cohort variance plus between-cohort variance, and a pooled meta log2 fold change and standard error were obtained for each gene. Two-sided p-values were calculated from the pooled z statistic and adjusted for multiple testing using the Benjamini-Hochberg method [13]. Heterogeneity was summarized using Q, tau^2^, and I^2^. Direction consistency was defined as the larger proportion of cohorts showing positive or negative effect estimates. Genes with meta FDR<0.05 were considered significant in the main meta-analysis (Supplement 5).

For genes detected in all three pigmentation-related cohorts, a leave-one-cohort-out analysis was also performed by repeating the random-effects meta-analysis after removing one cohort at a time (Supplement 6). This analysis was used to evaluate whether the main signal was dominated by a single cohort. The agreement between the full meta-analysis and the leave-one-cohort-out estimates was visualized as a robustness check (Supplement 7).

### Background analysis and candidate classification

PRJNA729271 was analysed separately to define ordinary breast muscle developmental and breed-background expression changes. Samples were modelled using DESeq2 with stage and breed in the design formula (~stage+breed) [11]. The same gene-level import and filtering strategy was used as described above. DESeq2 result tables were generated for all non-intercept coefficients, including stage-related contrasts and the breed-background contrast. For each gene, the strongest background effect was summarized as the contrast with the largest absolute log2 fold change (Supplement 8). A gene was flagged as a background-development gene if it had adjusted p-value<0.05 and absolute log2 fold change ≥1 in at least one PRJNA729271 background contrast. This cutoff was chosen as a conservative effect-size threshold to avoid treating very small but statistically significant developmental differences as biologically meaningful background signals. Sensitivity analyses were then performed using absolute log2 fold-change cutoffs of 0, 0.5, 1.0, 1.5, and 2.0 while retaining adjusted p-value<0.05 (Supplement 9). Background contrasts were classified as ordinary muscle-development contrasts when they corresponded to stage effects and as breed-growth background contrasts when they corresponded to the breed coefficient (Supplement 9).

The random-effects meta-analysis results were integrated with PRJNA729271 background flags and predefined biological gene sets. Gene sets included melanogenesis, melanoblast/neural crest, myogenesis, and oxidative/redox-related genes. Each meta-analysis gene was annotated for membership in these gene sets and for whether it was flagged by the PRJNA729271 background analysis (Supplement 10). Genes were classified into evidence classes based on background status and biological gene-set membership. Genes flagged by the background analysis and belonging to melanogenesis or melanoblast/neural crest gene sets were classified as pigmentation-development background overlap. Genes flagged by the background analysis but not belonging to these pigmentation-related gene sets were classified as background development. Genes not flagged by the background analysis but belonging to melanogenesis or melanoblast/neural crest gene sets were classified as melanogenesis-specific or enriched. Genes belonging to myogenesis gene sets were classified as myogenesis overlap when applicable, and all remaining significant meta-analysis genes were classified as other meta-signature genes.

A candidate priority score was calculated to rank conserved genes by combining meta-analysis significance, absolute effect size, direction consistency, heterogeneity, background status, and biological gene-set membership. The detailed scoring components are provided in Supplement 11.

### Signature score analysis

Signature score analysis was performed using variance-stabilized expression matrices from DESeq2. For each predefined gene set, genes were matched by gene symbol to the expression matrix. Gene expression values were z-transformed across samples for each gene, and the sample-level signature score was calculated as the mean z-score of genes in the set. Gene sets with fewer than two matched genes were not scored.

Melanogenesis and myogenesis signature scores were compared within each cohort. Pearson and Spearman correlations were calculated between the two scores, and a linear model was used to obtain a nominal p-value for the sample-level relationship (Supplement 12). Because composite mean z-score signatures can be insensitive to individual marker behavior, canonical myogenic regulators and markers were also inspected individually in the meta-analysis results (Supplement 13). Sample-level signature scores for all analyzed samples are provided in Supplement 14. All analyses and figures were generated in R [14].

## RESULTS

### Breast muscle RNA-seq cohorts captured pigmentation-related comparisons and ordinary muscle developmental background

This study included three breast muscle RNA-seq cohorts related to pigmentation deposition and one ordinary breast muscle background cohort (Figure 1A; Table 1). Across the three pigmentation-related cohorts, 62 cohort-specific sample records represented 53 unique samples: 24 embryonic breast muscle samples from PRJNA827465, 18 adult breast muscle samples from CRA004775, and a 20-sample CRA014497 matched subset comprising 11 Silkie samples and 9 Wenchang control samples reused from PRJNA827465 (Figure 1B; Supplement 1). PRJNA729271 was used as an ordinary breast muscle background cohort and included 42 samples across multiple developmental stages and breed backgrounds. In total, 95 unique Salmon quantification results were included in the current analysis (Supplement 2). Principal component analysis of variance-stabilized expression values showed that samples were structured primarily by developmental stage, breed, or comparison group within each cohort, supporting the use of cohort-specific modeling before effect-size integration (Supplement 4).

The sample structure showed that all three pigmentation-related cohorts compared black-boned chickens with corresponding controls, but differed in breed, developmental stage, and sample size. PRJNA827465 and the CRA014497 matched subset mainly represented embryonic breast muscle, whereas CRA004775 represented adult breast muscle (Figure 1C). PRJNA729271 was analysed separately from the pigmentation-related cohorts to describe the range of expression changes associated with ordinary breast muscle development and breed background.

### Cohort-level differential expression profiles varied among datasets

The three pigmentation-related cohorts differed in the number of detected differentially expressed genes. PRJNA827465, CRA004775, and the CRA014497 matched subset contained 14,362, 13,801, and 14,036 analyzable genes, respectively. At an adjusted p-value threshold of <0.05, 296, 158, and 3,699 genes were identified as differentially expressed in the three cohorts, respectively (Supplement 3). Cohort-level volcano plots further showed that the DEG burden and effect-size distribution differed among datasets, with the strongest single-cohort signal observed in the CRA014497 matched subset (Supplement 15). These differences indicate that single-cohort differential expression results were influenced by dataset-specific features, including sample source, developmental stage, breed background, and control structure. Therefore, subsequent analyses focused on genes with consistent direction and stable evidence across cohorts rather than on the differentially expressed gene list from any single dataset.

### Cross-cohort meta-analysis showed consistent upregulation of canonical melanogenesis genes in black-boned chicken breast muscle

Several canonical melanogenesis-related genes showed highly consistent upregulation in the cross-cohort integrated results. *PMEL*, *TYRP1*, *DCT*, and *TYR* were detected in all three pigmentation-related cohorts, with a direction consistency of 1. Their pooled effect sizes were high, with meta log2FC values of 8.92 for *PMEL*, 8.83 for *TYRP1*, 7.78 for *DCT*, and 6.70 for *TYR*. The corresponding meta FDR values were 9.40×10^−280^, 1.19×10^−184^, 7.58×10^−82^, and 2.65×10^−48^, respectively, and the I^2^ values were 0 for all four genes (Supplement 5).

Because these genes are established components of melanogenesis and melanosome formation, they were interpreted as positive anchors for the reliability of the analysis rather than as newly discovered candidates. In addition to *PMEL*, *TYRP1*, *DCT*, and *TYR*, genes such as *MLPH*, *OCA2*, *MLANA*, *GPNMB*, *TRPM1*, *EDN3*, *SLC24A5*, *SLC45A2*, and *KIT* also showed significant or relatively stable upregulation in the integrated results (Figure 2A).

### Random-effects meta-analysis identified 257 significant cross-cohort genes

The main meta-analysis included 14,139 genes, of which 257 reached meta FDR<0.05 (Supplement 5). The significant genes included canonical melanogenesis genes as well as genes related to pigment cell function, membrane transport, extracellular matrix, developmental processes, or incompletely annotated functions. Many of the top-ranked genes showed the same direction of effect across multiple cohorts, indicating a reproducible transcriptomic feature in black-boned chicken breast muscle.

Cohort-level effect sizes of representative genes further showed that *PMEL*, *TYRP1*, *DCT*, *TYR*, *MLPH*, *OCA2*, *MLANA*, and *GPNMB* generally retained the same direction across datasets (Figures 2B, 2C). However, the magnitude of effect and heterogeneity differed among genes. *PMEL*, *TYRP1*, *DCT*, *TYR*, *MLPH*, and *GPNMB* showed I^2^ values of 0, whereas *MLANA*, *SOX10*, *EDN3*, and RAB38 showed higher heterogeneity (I^2^ = 43.9, 45.9, 66.0, and 92.4, respectively). These differences likely reflect the mixture of embryonic and adult stages, breed composition, control structure, sequencing batches, and bulk-tissue cellular composition across the three public cohorts.

Leave-one-cohort-out analysis showed that pooled effect sizes from the full meta-analysis were highly concordant with estimates obtained after removing each pigmentation-related cohort. The major melanogenesis anchors retained the same direction and large effect sizes across these sensitivity analyses, indicating that the core meta-analysis signal was not driven by a single dataset (Supplements 6, 7).

### Background analysis showed that part of the conserved signature overlapped with ordinary breast muscle development

Using the independent ordinary breast muscle background cohort as a reference showed that some meta-significant genes also changed during ordinary breast muscle development or breed-background comparisons (Figure 3C). At the contrast level, 9,165 genes met the significance threshold in at least one ordinary muscle developmental contrast, whereas 212 genes met the threshold in the breed-background contrast (Figure 3A; Supplements 8, 9). These broad background expression changes provided the reference space used to flag conserved meta-analysis genes that were not specific to pigmentation-associated comparisons.

After applying the prespecified background filter to conserved meta-analysis genes with meta FDR<0.05 and direction consistency ≥0.67, 156 of 227 conserved genes were flagged by the PRJNA729271 background cohort. These comprised 145 background-development genes and 11 pigmentation-development background-overlap genes; 1 gene was classified as melanogenesis-enriched and not background-flagged, and 70 genes were classified as other conserved meta-signature genes (Figure 3B; Supplement 16). The only Class IV melanogenesis-enriched gene was *KIT* (meta log2FC = 0.691, meta FDR = 2.34×10^−12^), a melanoblast/neural crest-related gene that was not flagged by PRJNA729271.

Overall, the background analysis separated the conserved pigmentation-related signal into background-associated, background-overlapping, and relatively enriched classes. Sensitivity analysis supported this interpretation: when the background effect-size cutoff was varied from 0 to 2.0, 203, 194, 156, 125, and 103 of the 227 conserved genes were flagged, respectively (Supplement 9). *PMEL*, *TYRP1*, *DCT*, and *TYR* remained background-flagged at all tested cutoffs, indicating that the background annotation of canonical melanogenesis genes was not an artifact of a single threshold.

### Layered gene classification separated canonical melanogenesis anchors, background-overlapping genes, and other conserved signals

After integrating meta-analysis significance, effect size, direction consistency, heterogeneity, and background cohort annotation, conserved genes were grouped into five layers. Class I comprised four core conserved melanogenesis anchors: *PMEL*, *TYRP1*, *DCT*, and *TYR*. Class II comprised seven pigmentation-development background-overlap genes: *MLPH*, *OCA2*, *MLANA*, *TRPM1*, *EDN3*, *RAB38*, and *SOX10*. Class III comprised ordinary muscle-development or breed-background-associated conserved genes. Class IV contained one melanogenesis-enriched gene not flagged by the PRJNA729271 background cohort, KIT. Class V contained other conserved meta-signature genes (Figure 4A; Supplement 16).

### Sample-level coupling between melanogenesis and myogenesis signature scores was weak

To assess the relationship between melanogenesis-related and myogenesis-related expression features at the sample level, signature scores were compared across cohorts. The correlations were weak overall and differed among cohorts. The Pearson correlation coefficient was 0.294 in CRA004775, 0.120 in the CRA014497 matched subset, −0.067 in PRJNA 827465, and −0.067 in PRJNA729271; none of these relationships reached nominal significance (Figure 4B; Supplement 12).

Compared with the conserved signals observed in the gene-level meta-analysis, the sample-level signature scores did not show a stable strong correlation pattern. Individual marker inspection supported the same cautious interpretation: *PAX7*, *MYOD1*, *MYF5*, *MYOG*, *MYH7*, *MSTN*, *MEF2A*, and *MEF2C* did not reach meta FDR<0.05 (Supplement 13).

### A subset of conserved background-overlapping genes was related to muscle phenotype and metabolic context

Because the sample-level melanogenesis-myogenesis signature correlation was weak and canonical myogenic regulators were not meta-significant, we further examined whether more specific muscle-phenotype-related genes were embedded in the conserved meta-signature. A curated panel of 11 genes showed significant and directionally consistent meta-analysis evidence: *MYBPC2*, *CAPN1*, *CTSK*, *LRP4*, *SLN*, *SCN4A*, *TECRL*, *CD36*, *GPD1*, *THRSP*, and *PLTP* (Figure 5A–B; Supplement 17). These genes were related to structure/remodeling, calcium/excitability, and lipid-energy metabolism rather than to broad myogenic regulatory programs.

Background-overlap annotation further showed that ten of these genes met the background flag threshold in ordinary muscle-development contrasts, whereas *LRP4* showed conserved meta-analysis evidence without a background flag; none of the genes in this panel was flagged by the breed-growth background contrast under the same threshold (Figure 5C). As a specificity check, none of the eight selected canonical myogenic regulators or markers, including *PAX7*, *MYOD1*, *MYF5*, *MYOG*, *MYH7*, *MSTN*, *MEF2A*, and *MEF2C*, reached meta FDR<0.05, whereas all 11 genes in the curated muscle-phenotype panel were meta-significant (Figure 5D). Together, these 11 genes represent a focused muscle-phenotype-related candidate set that connects the conserved pigmentation-associated signature with skeletal-muscle structure/remodeling, calcium/excitability, and lipid-energy metabolism.

## DISCUSSION

This study integrated public breast muscle RNA-seq datasets from black-boned chickens and used an independent ordinary breast muscle developmental and breed-background cohort to refine the interpretation of pigmentation-associated expression signals. The main finding was that the conserved transcriptomic signature contained a strong melanogenesis-centered component, but this component was embedded within a broader breast muscle developmental background. By combining cohort-level effect sizes, random-effects meta-analysis, leave-one-cohort-out analysis, threshold sensitivity analysis, marker-level checks, and background filtering, the study separated expected melanogenesis anchors from muscle-context expression candidates.

The strongest and most consistent signals were canonical melanogenesis and melanosome-related genes, including *PMEL*, *TYRP1*, *DCT*, and *TYR*. These genes have established roles in melanogenic and melanosomal biology [15–18] and have already been implicated in previous black-boned chicken transcriptomic studies [2–5] and in recent studies of breast muscle pigmentation in Xuefeng, Xichuan, and other black-boned chicken populations [19–22]. Their repeated detection therefore supports the analytical reliability of the cross-cohort workflow rather than representing a new single-gene discovery. Their high pooled effect sizes, consistent direction across cohorts, and low heterogeneity indicate that the main cross-cohort signal captured a biologically expected pigmentation program. This result also supports the value of using these genes as positive anchors when evaluating more variable candidates.

Beyond these canonical anchors, the meta-analysis highlighted additional genes related to pigment cell function, melanosome transport, membrane trafficking, and developmental signaling. Genes such as *MLPH*, *OCA2*, *MLANA*, *TRPM1*, *EDN3*, *RAB38*, and *SOX10* formed a second layer of candidates with pigmentation relevance and evidence of overlap with the background cohort, whereas KIT was the only melanogenesis-enriched gene not flagged by PRJNA729271. The heterogeneity observed for MLANA, EDN3, RAB38, and SOX10 suggests that breed, stage, adult-versus-embryonic comparisons, control design, sequencing batch, and bulk-tissue composition influence the magnitude of these effects. The cross-cohort framework therefore extends earlier single-dataset observations by emphasizing direction consistency, between-cohort robustness, and the limits of breed- or stage-specific interpretation.

A central contribution of this analysis was the use of PRJNA729271 as a held-out ordinary breast muscle developmental and breed-background cohort. The background analysis showed that many meta-significant genes also changed during ordinary breast muscle development or breed-background contrasts. Sensitivity analysis across multiple log2 fold-change thresholds showed that the extent of background overlap changed gradually, but the major interpretation remained stable. This finding is important because breast muscle is a dynamic developmental tissue regulated by coordinated myogenic and postnatal growth programs [23], and pigmentation-associated comparisons can contain expression changes that are not specific to pigment biology. Together with the ordinary developmental variation observed in Arbor Acres and Lushi chickens [7], these findings indicate that muscle developmental background should be considered when interpreting transcriptomic signals in black-boned chicken breast muscle. This interpretation is also compatible with recent functional evidence that TNF-alpha signaling can affect myoblast proliferation, differentiation, migration, myotube fusion, and muscle regeneration, illustrating that muscle-related expression changes may reflect regulated tissue developmental context [24].

The muscle-phenotype panel further refines this interpretation. The absence of meta-significant changes in canonical myogenic regulators and markers (*PAX7*, *MYOD1*, *MYF5*, *MYOG*, *MYH7*, *MSTN*, *MEF2A*, and *MEF2C*) argues against presenting the dataset as evidence for a broad myogenesis program. Instead, the conserved genes pointed to more specific tissue-context features, including structure/remodeling, protein turnover, calcium/excitability, and lipid-energy metabolism. This distinction is the main biological message of the revision: the analysis does not simply rediscover melanogenesis genes, but identifies a reproducible muscle-tissue expression context in which pigmentation-related signals coexist with changes in genes relevant to breast muscle phenotype. The layered classification provides a practical way to organize candidate genes. Class I genes represent conserved melanogenesis anchors that define the expected pigmentation signal. Class II genes represent pigmentation-related candidates that also overlap with muscle developmental background and may be useful for exploring how pigment-related cellular programs coexist with muscle tissue development. Class III genes should not be treated as pigmentation-specific candidates, but they are useful for identifying the ordinary developmental background embedded in black-boned chicken comparisons. Class IV was represented only by KIT, which may be relatively enriched for pigmentation-related developmental biology in this analysis but still requires validation. This separation reduces the risk of overinterpreting broad developmental expression changes as direct pigmentation regulators.

The weak sample-level correlation between melanogenesis and myogenesis signature scores adds another perspective. Although some pigmentation-related genes overlapped with background developmental contrasts, the sample-level melanogenesis and myogenesis scores did not show a stable strong correlation across cohorts. This suggests that gene-level meta-analysis and background annotation were more informative than a simple sample-level signature-coupling analysis in this dataset. In practical terms, the pigmentation signal appears to be best interpreted as a conserved gene-level expression program with variable overlap with developmental background, rather than as a uniform sample-level coupling pattern.

The use of public bulk RNA-seq datasets defines the interpretive level of this study. Cohorts differed in breed, developmental stage, sequencing protocol, and control design, and bulk breast muscle transcriptomes represent mixed tissue cell populations [21,25]. No experimental validation was performed in the present study. Therefore, the 11 muscle-phenotype-related genes identified here should be interpreted as conserved cross-cohort expression candidates that link melanin deposition with breast muscle developmental and metabolic context, not as proof of direct causal regulation or physiological function. Functional validation will be required to determine whether these genes actively influence pigmentation-associated muscle phenotypes. Recent studies also emphasize that chicken breast muscle, meat-quality, and carcass phenotypes are shaped by multiple molecular and production-related layers, including protein side-chain modifications in growth-related breast myopathies, genetic architecture of carcass traits in native chickens, and dietary enzyme effects on growth and meat quality [26–28]. This wider context supports our cautious interpretation that pigmentation-associated transcriptomic signals in breast muscle should be separated from general muscle-phenotype and carcass-related background rather than treated as a single pigmentation-specific process.

## CONCLUSION

In summary, this study differs from previous single-cohort analyses by moving beyond the repeated identification of canonical melanogenesis genes and by extracting a defined set of muscle-phenotype-related candidates from cross-cohort evidence. Previous studies have established that melanogenesis and melanosome-related genes are repeatedly associated with breast muscle pigmentation in black-boned chickens [2–5,19–22]. The contribution of the present work is to show that, alongside these expected pigmentation anchors, 11 genes related to muscle structure/remodeling (*MYBPC2*, *CAPN1*, *CTSK*, and *LRP4*), calcium/excitability (*SLN*, *SCN4A*, and *TECRL*), and lipid-energy metabolism (*CD36*, *GPD1*, *THRSP*, and *PLTP*) are consistently embedded in the conserved expression signature, whereas canonical myogenic regulators are not. This gene-centered result provides a focused candidate set for interpreting pigmentation-associated muscle traits in black-boned chickens and for designing future functional validation studies.

## Figures and Tables

**Figure 1:**
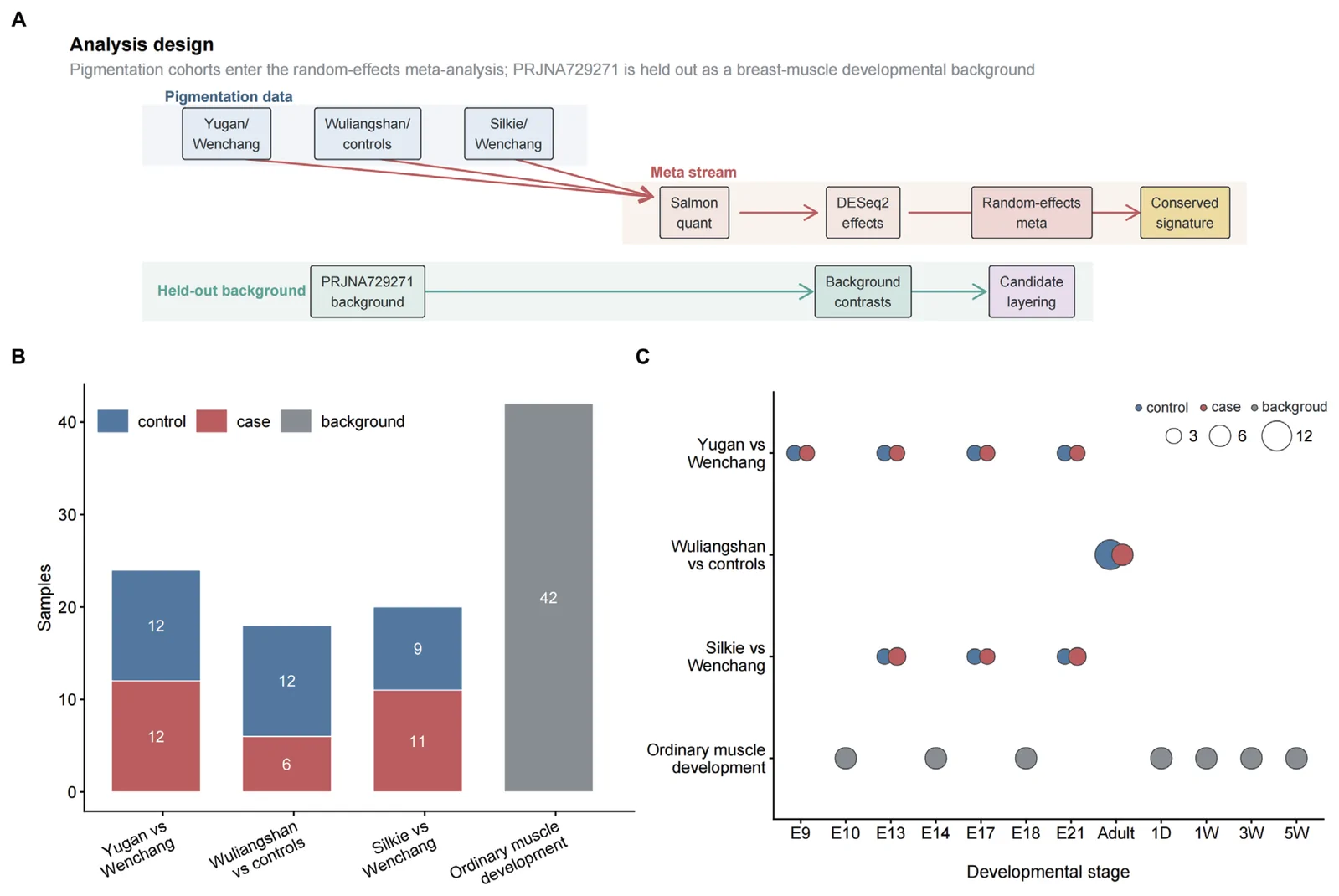
Study design and sample structure for cross-cohort transcriptomic meta-analysis. (A) Schematic overview of the study design. Three pigmentation-related breast muscle RNA-seq cohorts were included in the main meta-analysis to identify cross-cohort conserved expression signals. PRJNA729271 was used as an independent background cohort to describe expression changes associated with ordinary breast muscle development and breed background. (B) Number of cases, control, and background samples in ea ch cohort. PRJNA827465 contained 12 case and 12 control samples, CRA004775 contained 6 case and 12 control samples, the CRA014497 matched subset contained 11 case and 9 control samples, and PRJNA729271 contained 42 background samples. (C) Developmental stage coverage and sample numbers across cohorts. Dot size represents the number of samples at each stage, and colour represents the comparison role of the sample group in this study. Developmental stages are abbreviated as embryonic day (E), post-hatch day (D), and post-hatch week (W); Adult indicates adult breast muscle samples.

**Figure 2:**
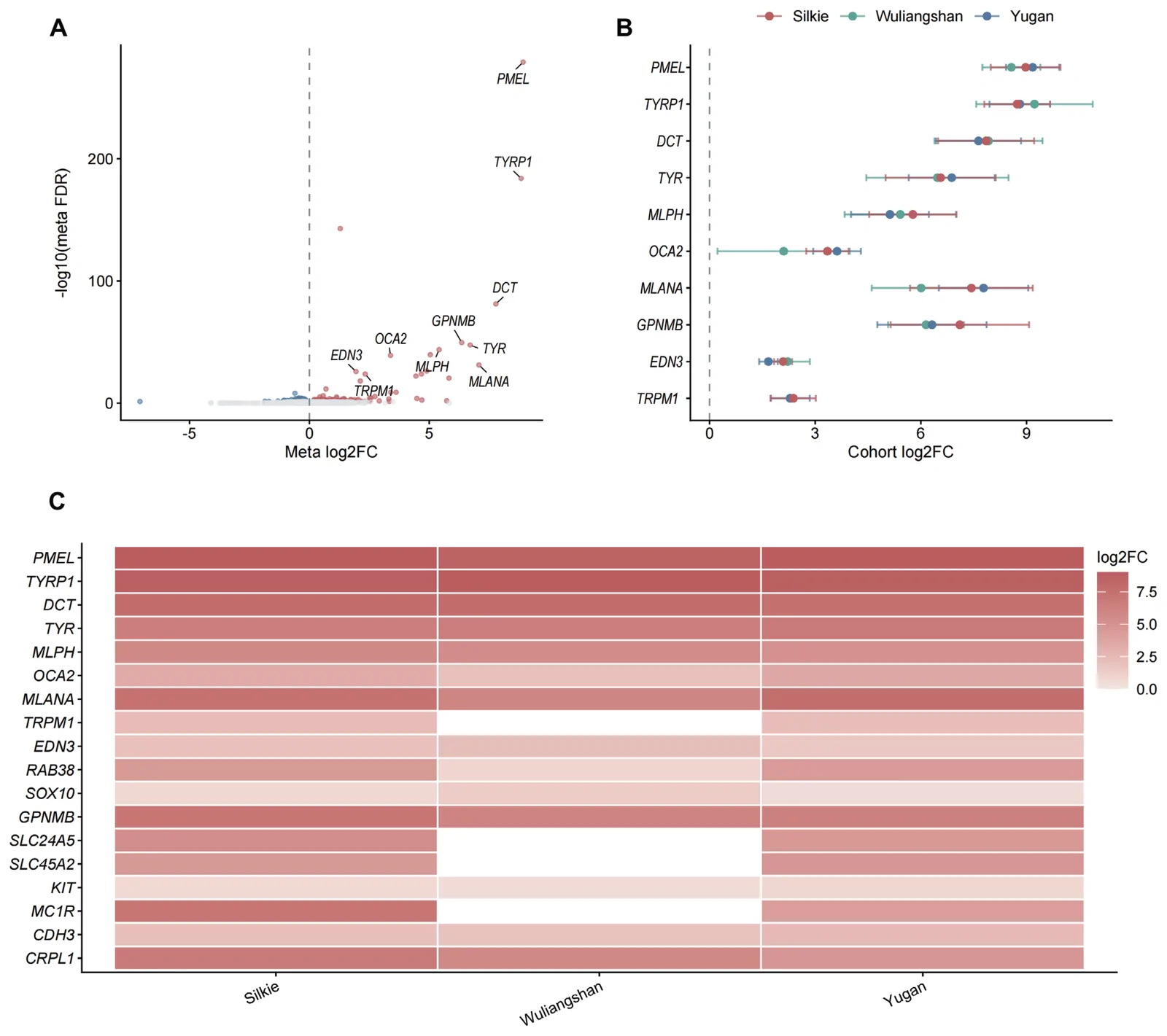
Conserved melanogenesis-related expression signatures across pigmentation cohorts. (A) Volcano plot of the random-effects meta-analysis. The x-axis shows meta log2 fold change, and the y-axis shows –log10 (meta FDR). Red points indicate significant genes upregulated in black-boned chicken breast muscle, blue points indicate significant downregulated genes, and grey points indicate genes that did not reach the significance threshold. (B) Cohort-level effect sizes of representative genes in the three pigmentation-related cohorts. Points represent cohort-specific log2 fold change, and horizontal lines represent 95% confidence intervals. The dashed vertical line indicates no expression difference. (C) Heatmap of log2 fold changes for representative pigmentation- and pigment-development-related genes across the three pigmentation-related cohorts. Colour intensity represents the effect size of each gene in each cohort. White cells indicate that the gene did not have a valid cohort-level effect-size estimate for that cohort after expression and model filtering; they should not be interpreted as zero expression or absence of biological expression.

**Figure 3:**
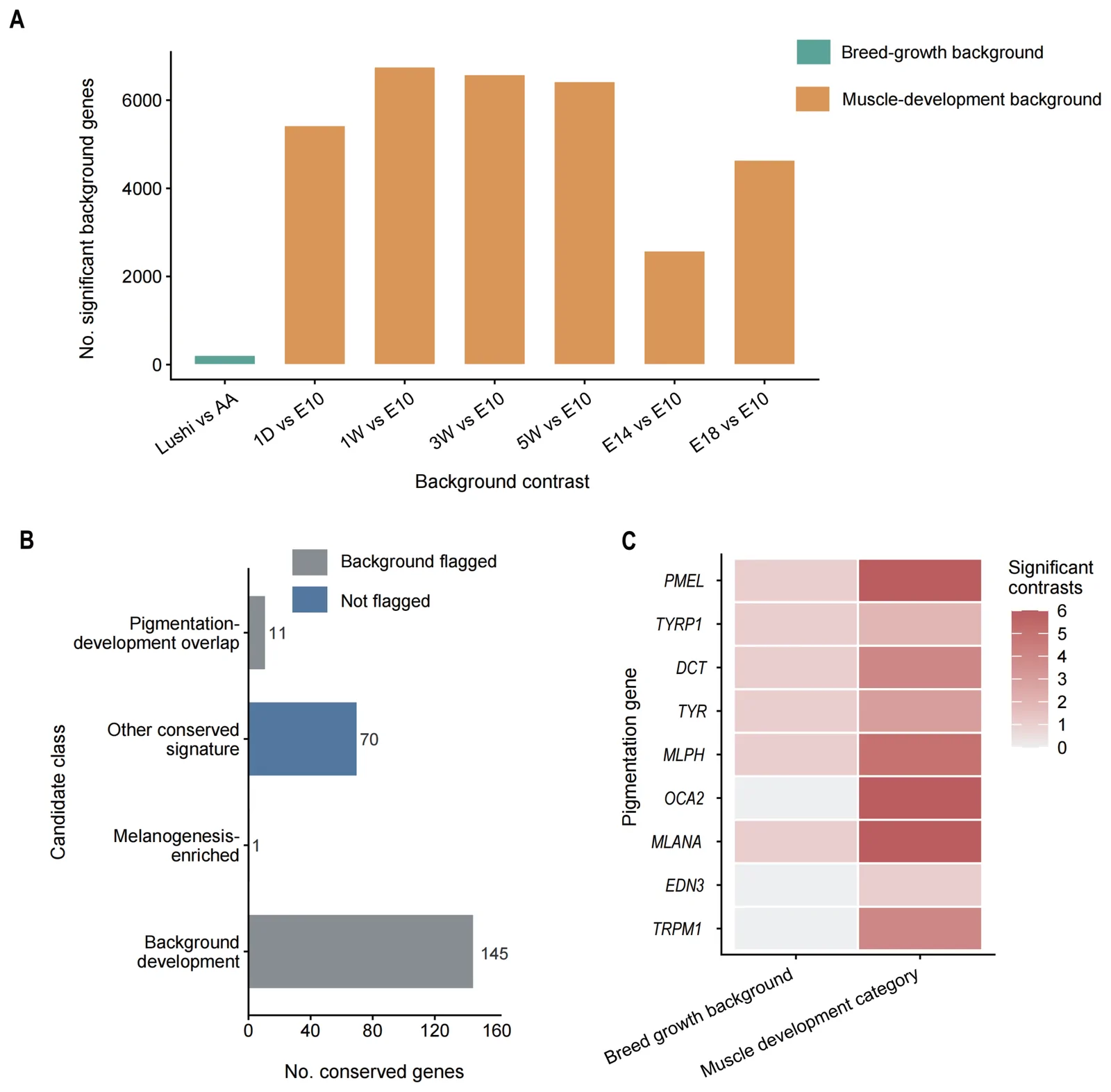
Background filtering separates pigmentation signatures from ordinary muscle-development background. (A) Number of significantly changed genes in each background contrast. Stage contrasts are shown relative to E10, and the breed contrast is shown as Lushi versus Arbor Acres. This panel shows the breadth of ordinary muscle-development and breed-background expression variation used to define the background filter. (B) Distribution of meta-significant and direction-consistent genes across evidence classes. Grey indicates genes flagged by the PRJNA729271 background cohort, and blue indicates genes not flagged by the background cohort. (C) Number of significant background contrasts for canonical pigmentation-related genes in PRJNA729271. Colour indicates the number of contrasts reaching the significance threshold for each gene within each background category. D, post-hatch day; E, embryonic day; W, post-hatch week.

**Figure 4:**
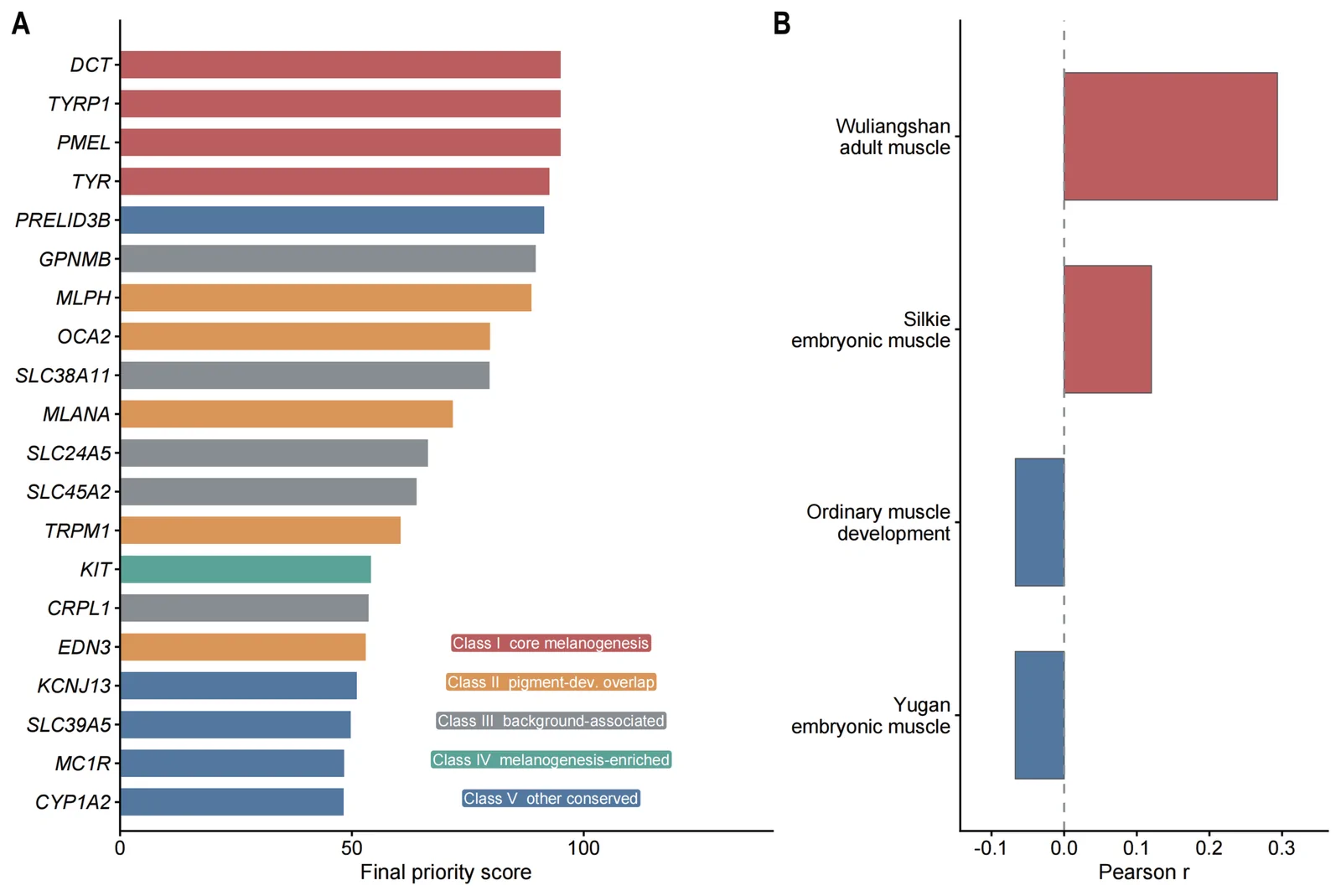
Layered conserved signatures and sample-level signature coupling. (A) Final candidate priority scores. Bar length represents the candidate priority score, and colour represents the gene layer, including core melanogenesis anchors, pigmentation-development background overlap genes, background-associated genes, melanogenesis-enriched genes, and other conserved genes. (B) Sample-level correlation between melanogenesis signature score and myogenesis signature score across cohorts. Bars represent Pearson correlation coefficients.

**Figure 5:**
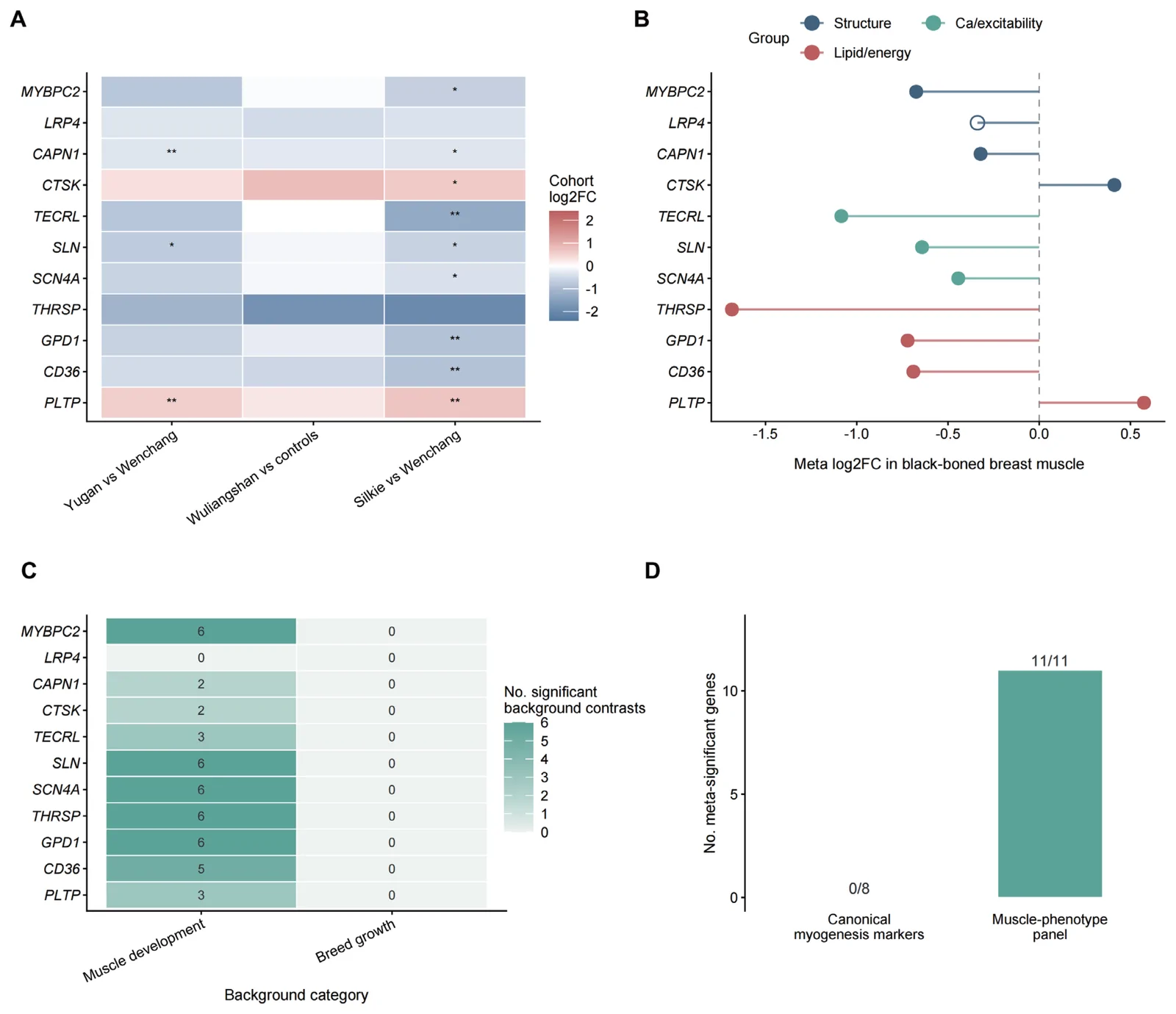
Muscle-phenotype-related background-overlapping genes and final interpretation framework. (A) Cohort-level log2 fold changes for curated muscle-phenotype-related genes across the three pigmentation-related cohorts. Asterisks indicate cohort-level adjusted p-value thresholds (* p<0.05; ** p<0.01). (B) Meta-analysis effect sizes for the same gene panel. The x-axis shows pooled meta log2 fold change in black-boned chicken breast muscle, colour indicates functional grouping, and the open circle indicates the gene not flagged by the background cohort. (C) Number of significant background contrasts for each muscle-phenotype-related gene. Values indicate how often each gene met the background flag threshold in ordinary muscle-development or breed-background contrasts. (D) Specificity check comparing the curated muscle-phenotype panel with canonical myogenesis markers. Bar labels indicate the number of meta-significant genes over the total number of genes tested in each panel. None of the selected canonical myogenic regulators or markers reached the meta-analysis significance threshold, whereas all genes in the curated muscle-phenotype panel were meta-significant.

**Table 1:** Public breast muscle RNA-seq cohorts used for cross-cohort transcriptomic meta-analysis and background filtering in black-boned chickens

Cohort/accession	Database	Tissue and stage	Breed or group comparison	Samples included	Role in this study
PRJNA827465	NCBI SRA	Breast muscle; embryonic stages E9, E13, E17, and E21	Yugan black-boned chicken vs. Wenchang chicken	24 Samples (12 case and 12 control)	Pigmentation cohort included in the main random-effects meta-analysis
CRA004775	NGDC GSA	Breast muscle; adult stage	Wuliangshan black-boned chicken vs. Chahua and Cobb controls	18 Samples (6 case and 12 control)	Independent adult pigmentation cohort included in the main random-effects meta-analysis
CRA014497 matched subset	NGDC GSA (CRA014497 cases) and NCBI SRA (PRJ NA827465 controls)	Breast muscle; matched embryonic stages E13, E17, and E21	Silkie chicken vs. Wenchang chicken	20 Samples (11 case and 9 matched controls)	Silkie embryonic pigmentation cohort included in the main random-effects meta-analysis
PRJNA729271	NCBI SRA	Breast muscle; E10, E14, E18, 1D, 1W, 3W, and 5W	Arbor Acres and Lushi chickens	42 Background samples	Held-out ordinary breast muscle developmental and breed-background cohort; not included in the main meta-analysis

RNA-seq, RNA sequencing; SRA, Sequence Read Archive; E, embryonic day; NGDC, National Genomics Data Center; GSA, Genome Sequence Archive; D, post-hatch day; W, post-hatch week.

## Data Availability

Upon reasonable request, the datasets of this study can be available from the corresponding author.

## SUPPLEMENTARY MATERIAL

**Supplement 1**. Curated sample metadata used in this study.

**Supplement 2**. Salmon quantification directory inventory used to distinguish unique quantification outputs from analysis records.

**Supplement 3**. Cohort-level DESeq2 effect-size results for the three pigmentation-related cohorts.

**Supplement 4**. Sample-level expression structure after variance-stabilizing transformation.

**Supplement 5**. Random-effects meta-analysis results across the three pigmentation-related cohorts.

**Supplement 6**. Leave-one-cohort-out meta-analysis results for genes detected in all three pigmentation-related cohorts.

**Supplement 7**. Leave-one-cohort-out robustness of meta-analysis effects.

**Supplement 8**. Gene-level summary of the strongest PRJNA729271 ordinary breast muscle background effect.

**Supplement 9**. Sensitivity analysis of PRJNA729271 background filtering using alternative absolute log2 fold-change cutoffs while retaining adjusted p-value <0.05.

**Supplement 10**. Long-format PRJNA729271 background contrast results across developmental and breed-background comparisons.

**Supplement 11**. Candidate annotation table integrating meta-analysis statistics, background flags, and predefined biological gene-set membership.

**Supplement 12**. Cohort-level correlation summary between melanogenesis and myogenesis signature scores.

**Supplement 13**. Meta-analysis results for canonical myogenic regulators and markers compared with the curated muscle-phenotype-related candidate panel.

**Supplement 14**. Sample-level biological signature scores for all analyzed samples.

**Supplement 15**. Cohort-level differential expression landscapes.

**Supplement 16**. Final layered conserved signature genes used for candidate prioritization.

**Supplement 17**. Curated muscle-phenotype-related conserved genes used in Figure 5.
