# Supplementary material for: Cross-cohort transcriptomic meta-analysis identifies conserved melanogenesis signatures and developmental background in black-boned chicken breast muscle
Source: Anim Biosci. 2026 Jun 24;39(10):260430. doi: 10.5713/ab.260430 (PMC13662945; doi:10.5713/ab.260430)
Supplement: Supplementary file 1 [file ab-260430-Supplementary-1.pdf]

Supplement 1. Curated sample metadata used in this study.

cohort_id	sample_id	run	biosample	source_accession	stage	breed	pigmentation	replicate	comparison_role
PRJNA827465	W1_1	SRR18778023	SAMN27610096		E9	Wenchang	normal	1	control
PRJNA827465	W1_2	SRR18778022	SAMN27610097		E9	Wenchang	normal	2	control
PRJNA827465	W1_3	SRR18778011	SAMN27610098		E9	Wenchang	normal	3	control
PRJNA827465	Y1_1	SRR18778019	SAMN27610108		E9	Yugan_black_boned	black_boned	1	case
PRJNA827465	Y1_2	SRR18778018	SAMN27610109		E9	Yugan_black_boned	black_boned	2	case
PRJNA827465	Y1_3	SRR18778017	SAMN27610110		E9	Yugan_black_boned	black_boned	3	case
PRJNA827465	W2_1	SRR18778006	SAMN27610099		E13	Wenchang	normal	1	control
PRJNA827465	W2_2	SRR18778005	SAMN27610100		E13	Wenchang	normal	2	control
PRJNA827465	W2_3	SRR18778004	SAMN27610101		E13	Wenchang	normal	3	control
PRJNA827465	Y2_1	SRR18778016	SAMN27610111		E13	Yugan_black_boned	black_boned	1	case
PRJNA827465	Y2_2	SRR18778015	SAMN27610112		E13	Yugan_black_boned	black_boned	2	case
PRJNA827465	Y2_3	SRR18778014	SAMN27610113		E13	Yugan_black_boned	black_boned	3	case
PRJNA827465	W3_1	SRR18778003	SAMN27610102		E17	Wenchang	normal	1	control
PRJNA827465	W3_2	SRR18778002	SAMN27610103		E17	Wenchang	normal	2	control
PRJNA827465	W3_3	SRR18778001	SAMN27610104		E17	Wenchang	normal	3	control
PRJNA827465	Y3_1	SRR18778013	SAMN27610114		E17	Yugan_black_boned	black_boned	1	case
PRJNA827465	Y3_2	SRR18778012	SAMN27610115		E17	Yugan_black_boned	black_boned	2	case
PRJNA827465	Y3_3	SRR18778010	SAMN27610116		E17	Yugan_black_boned	black_boned	3	case
PRJNA827465	W4_1	SRR18778000	SAMN27610105		E21	Wenchang	normal	1	control
PRJNA827465	W4_2	SRR18778021	SAMN27610106		E21	Wenchang	normal	2	control
PRJNA827465	W4_3	SRR18778020	SAMN27610107		E21	Wenchang	normal	3	control
PRJNA827465	Y4_1	SRR18778009	SAMN27610117		E21	Yugan_black_boned	black_boned	1	case
PRJNA827465	Y4_2	SRR18778008	SAMN27610118		E21	Yugan_black_boned	black_boned	2	case
PRJNA827465	Y4_3	SRR18778007	SAMN27610119		E21	Yugan_black_boned	black_boned	3	case
CRA004775	CH1	CRR314359	SAMC445057		adult	Chahua	normal_or_low_melanin	1	control
CRA004775	CH2	CRR314360	SAMC445058		adult	Chahua	normal_or_low_melanin	2	control
CRA004775	CH3	CRR314361	SAMC445059		adult	Chahua	normal_or_low_melanin	3	control
CRA004775	CH4	CRR314362	SAMC445060		adult	Chahua	normal_or_low_melanin	4	control
CRA004775	CH5	CRR314363	SAMC445061		adult	Chahua	normal_or_low_melanin	5	control
CRA004775	CH6	CRR314364	SAMC445062		adult	Chahua	normal_or_low_melanin	6	control
CRA004775	CB1	CRR314365	SAMC445063		adult	Cobb	normal_or_low_melanin	1	control
CRA004775	CB2	CRR314366	SAMC445064		adult	Cobb	normal_or_low_melanin	2	control
CRA004775	CB3	CRR314367	SAMC445065		adult	Cobb	normal_or_low_melanin	3	control
CRA004775	CB4	CRR314368	SAMC445066		adult	Cobb	normal_or_low_melanin	4	control
CRA004775	CB5	CRR314369	SAMC445067		adult	Cobb	normal_or_low_melanin	5	control
CRA004775	CB6	CRR314370	SAMC445068		adult	Cobb	normal_or_low_melanin	6	control
CRA004775	WL1	CRR314353	SAMC445051		adult	Wuliangshan_black_boned	black_boned	1	case
CRA004775	WL2	CRR314354	SAMC445052		adult	Wuliangshan_black_boned	black_boned	2	case
CRA004775	WL3	CRR314355	SAMC445053		adult	Wuliangshan_black_boned	black_boned	3	case
CRA004775	WL4	CRR314356	SAMC445054		adult	Wuliangshan_black_boned	black_boned	4	case
CRA004775	WL5	CRR314357	SAMC445055		adult	Wuliangshan_black_boned	black_boned	5	case
CRA004775	WL6	CRR314358	SAMC445056		adult	Wuliangshan_black_boned	black_boned	6	case
Yang2025_Silkie_Wenchang	E13-TH-BM-1	CRR1014974	SAMC3313436	CRA014497	E13	Silkie	black_boned	1	case
Yang2025_Silkie_Wenchang	E13-TH-BM-6	CRR1014975	SAMC3313437	CRA014497	E13	Silkie	black_boned	6	case
Yang2025_Silkie_Wenchang	E13-TH-BM-7	CRR1014976	SAMC3313438	CRA014497	E13	Silkie	black_boned	7	case
Yang2025_Silkie_Wenchang	E13-TH-BM-9	CRR1014977	SAMC3313439	CRA014497	E13	Silkie	black_boned	9	case
Yang2025_Silkie_Wenchang	W2_1	SRR18778006	SAMN27610099	PRJNA827465	E13	Wenchang	normal	1	control
Yang2025_Silkie_Wenchang	W2_2	SRR18778005	SAMN27610100	PRJNA827465	E13	Wenchang	normal	2	control
Yang2025_Silkie_Wenchang	W2_3	SRR18778004	SAMN27610101	PRJNA827465	E13	Wenchang	normal	3	control
Yang2025_Silkie_Wenchang	E17-TH-BM-5	CRR1014988	SAMC3313450	CRA014497	E17	Silkie	black_boned	5	case
Yang2025_Silkie_Wenchang	E17-TH-BM-6	CRR1014989	SAMC3313451	CRA014497	E17	Silkie	black_boned	6	case
Yang2025_Silkie_Wenchang	E17-TH-BM-8	CRR1014990	SAMC3313452	CRA014497	E17	Silkie	black_boned	8	case
Yang2025_Silkie_Wenchang	W3_1	SRR18778003	SAMN27610102	PRJNA827465	E17	Wenchang	normal	1	control
Yang2025_Silkie_Wenchang	W3_2	SRR18778002	SAMN27610103	PRJNA827465	E17	Wenchang	normal	2	control
Yang2025_Silkie_Wenchang	W3_3	SRR18778001	SAMN27610104	PRJNA827465	E17	Wenchang	normal	3	control
Yang2025_Silkie_Wenchang	E21-TH-BM-2	CRR1014998	SAMC3313460	CRA014497	E21	Silkie	black_boned	2	case
Yang2025_Silkie_Wenchang	E21-TH-BM-4	CRR1014999	SAMC3313461	CRA014497	E21	Silkie	black_boned	4	case
Yang2025_Silkie_Wenchang	E21-TH-BM-7	CRR1015000	SAMC3313462	CRA014497	E21	Silkie	black_boned	7	case
Yang2025_Silkie_Wenchang	E21-TH-BM-8	CRR1015001	SAMC3313463	CRA014497	E21	Silkie	black_boned	8	case
Yang2025_Silkie_Wenchang	W4_1	SRR18778000	SAMN27610105	PRJNA827465	E21	Wenchang	normal	1	control
Yang2025_Silkie_Wenchang	W4_2	SRR18778021	SAMN27610106	PRJNA827465	E21	Wenchang	normal	2	control
Yang2025_Silkie_Wenchang	W4_3	SRR18778020	SAMN27610107	PRJNA827465	E21	Wenchang	normal	3	control
PRJNA729271	AAE10P1	SRR14521328	SAMN19115447		E10	Arbor_Acres	background_not_melanogenesis	1	background
PRJNA729271	AAE10P2	SRR14521327	SAMN19115448		E10	Arbor_Acres	background_not_melanogenesis	2	background
PRJNA729271	AAE10P3	SRR14521316	SAMN19115449		E10	Arbor_Acres	background_not_melanogenesis	3	background
PRJNA729271	LSE10P1	SRR14521314	SAMN19115468		E10	Lushi	background_not_melanogenesis	1	background
PRJNA729271	LSE10P2	SRR14521313	SAMN19115469		E10	Lushi	background_not_melanogenesis	2	background
PRJNA729271	LSE10P3	SRR14521312	SAMN19115470		E10	Lushi	background_not_melanogenesis	3	background
PRJNA729271	AAE14P1	SRR14521305	SAMN19115450		E14	Arbor_Acres	background_not_melanogenesis	1	background
PRJNA729271	AAE14P2	SRR14521294	SAMN19115451		E14	Arbor_Acres	background_not_melanogenesis	2	background
PRJNA729271	AAE14P3	SRR14521291	SAMN19115452		E14	Arbor_Acres	background_not_melanogenesis	3	background
PRJNA729271	LSE14P1	SRR14521311	SAMN19115471		E14	Lushi	background_not_melanogenesis	1	background
PRJNA729271	LSE14P2	SRR14521310	SAMN19115472		E14	Lushi	background_not_melanogenesis	2	background
PRJNA729271	LSE14P3	SRR14521309	SAMN19115473		E14	Lushi	background_not_melanogenesis	3	background
PRJNA729271	AAE18P1	SRR14521290	SAMN19115453		E18	Arbor_Acres	background_not_melanogenesis	1	background
PRJNA729271	AAE18P2	SRR14521289	SAMN19115454		E18	Arbor_Acres	background_not_melanogenesis	2	background
PRJNA729271	AAE18P3	SRR14521288	SAMN19115455		E18	Arbor_Acres	background_not_melanogenesis	3	background
PRJNA729271	LSE18P1	SRR14521308	SAMN19115474		E18	Lushi	background_not_melanogenesis	1	background
PRJNA729271	LSE18P2	SRR14521307	SAMN19115475		E18	Lushi	background_not_melanogenesis	2	background
PRJNA729271	LSE18P3	SRR14521306	SAMN19115476		E18	Lushi	background_not_melanogenesis	3	background
PRJNA729271	AA1D_P1	SRR14521287	SAMN19115456		1D	Arbor_Acres	background_not_melanogenesis	1	background
PRJNA729271	AA1D_P2	SRR14521326	SAMN19115457		1D	Arbor_Acres	background_not_melanogenesis	2	background
PRJNA729271	AA1D_P3	SRR14521325	SAMN19115458		1D	Arbor_Acres	background_not_melanogenesis	3	background
PRJNA729271	LS1D_P1	SRR14521304	SAMN19115477		1D	Lushi	background_not_melanogenesis	1	background
PRJNA729271	LS1D_P2	SRR14521303	SAMN19115478		1D	Lushi	background_not_melanogenesis	2	background
PRJNA729271	LS1D_P3	SRR14521302	SAMN19115479		1D	Lushi	background_not_melanogenesis	3	background
PRJNA729271	AA1W_P1	SRR14521324	SAMN19115459		1W	Arbor_Acres	background_not_melanogenesis	1	background
PRJNA729271	AA1W_P2	SRR14521323	SAMN19115460		1W	Arbor_Acres	background_not_melanogenesis	2	background
PRJNA729271	AA1W_P3	SRR14521322	SAMN19115461		1W	Arbor_Acres	background_not_melanogenesis	3	background
PRJNA729271	LS1W_P1	SRR14521301	SAMN19115480		1W	Lushi	background_not_melanogenesis	1	background
PRJNA729271	LS1W_P2	SRR14521300	SAMN19115481		1W	Lushi	background_not_melanogenesis	2	background
PRJNA729271	LS1W_P3	SRR14521299	SAMN19115482		1W	Lushi	background_not_melanogenesis	3	background
PRJNA729271	AA3W_P1	SRR14521321	SAMN19115462		3W	Arbor_Acres	background_not_melanogenesis	1	background
PRJNA729271	AA3W_P2	SRR14521320	SAMN19115463		3W	Arbor_Acres	background_not_melanogenesis	2	background
PRJNA729271	AA3W_P3	SRR14521319	SAMN19115464		3W	Arbor_Acres	background_not_melanogenesis	3	background
PRJNA729271	LS3W_P1	SRR14521298	SAMN19115483		3W	Lushi	background_not_melanogenesis	1	background
PRJNA729271	LS3W_P2	SRR14521297	SAMN19115484		3W	Lushi	background_not_melanogenesis	2	background
PRJNA729271	LS3W_P3	SRR14521296	SAMN19115485		3W	Lushi	background_not_melanogenesis	3	background
PRJNA729271	AA5W_P1	SRR14521318	SAMN19115465		5W	Arbor_Acres	background_not_melanogenesis	1	background
PRJNA729271	AA5W_P2	SRR14521317	SAMN19115466		5W	Arbor_Acres	background_not_melanogenesis	2	background
PRJNA729271	AA5W_P3	SRR14521315	SAMN19115467		5W	Arbor_Acres	background_not_melanogenesis	3	background
PRJNA729271	LS5W_P1	SRR14521295	SAMN19115486		5W	Lushi	background_not_melanogenesis	1	background
PRJNA729271	LS5W_P2	SRR14521293	SAMN19115487		5W	Lushi	background_not_melanogenesis	2	background
PRJNA729271	LS5W_P3	SRR14521292	SAMN19115488		5W	Lushi	background_not_melanogenesis	3	background
