# Supplementary material for: Cross-cohort transcriptomic meta-analysis identifies conserved melanogenesis signatures and developmental background in black-boned chicken breast muscle
Source: Anim Biosci. 2026 Jun 24;39(10):260430. doi: 10.5713/ab.260430 (PMC13662945; doi:10.5713/ab.260430)
Supplement: Supplementary file 3 [file ab-260430-Supplementary-11.pdf]

Supplement1. Candidate annotation table integrating meta-analysis statistics, background flags, and predefined biological gene-set membership.

gene_id	symbol	k	meta_log2FC	meta_se	meta_z	meta_pvalue	tau2	Q	I2	direction_consistency	cohorts	meta_fdr	background_development_gen	melanogenesis	melanoblast_neural_crest	myogenesis	oxidative_redox	evidence_class	score_meta_fdr	score_effect	score_consistency	score_low_heterogeneity	score_background_penalty	score_biology_bonus	candidate_priority_score
ENSGALG00010007864	TYR	3	6.704858539	0.443324799	15.1240322	1.12442E-51	0	0.160698885	0	1	CRA004775;PRJNA827465;Yang2025_Silkie_Wenchang	2.64969E-48	TRUE	TRUE	FALSE	FALSE	FALSE	pigmentation_development_overlap	47.57680572	20	20	10	-10	5	92.57680572
ENSGALG00010016279	PRELID3B	3	1.286821488	0.049797398	25.84113883	3.0604E-147	0	1.664249548	0	1	CRA004775;PRJNA827465;Yang2025_Silkie_Wenchang	1.4423E-143	FALSE	FALSE	FALSE	FALSE	FALSE	other_meta_signature	50	6.434107438	20	10	5	0	91.43410744
ENSGALG00010020024	ENSGALG00010020024	2	4.690361764	1.087112901	4.314512097	1.59956E-05	0	0.088081952	0	1	PRJNA827465;Yang2025_Silkie_Wenchang	0.003185373	FALSE	FALSE	FALSE	FALSE	FALSE	other_meta_signature	2.496839729	20	20	10	5	0	57.49683973
ENSGALG00010029441	ENSGALG00010029441	2	-7.067735365	2.102087207	-3.362246504	0.000773111	0.213739327	1.024772863	2.417400379	1	PRJNA827465;Yang2025_Silkie_Wenchang	0.044616379	FALSE	FALSE	FALSE	FALSE	FALSE	other_meta_signature	1.350505682	20	20	9.758259962	5	0	56.10876564
ENSGALG00010002465	ENSGALG00010002465	2	3.315485858	0.687294216	4.823968804	1.40729E-06	0.292750606	1.447883474	30.9336685	1	CRA004775;Yang2025_Silkie_Wenchang	0.000462738	FALSE	FALSE	FALSE	FALSE	FALSE	other_meta_signature	3.334664807	16.57742929	20	6.90663315	5	0	51.81872725
ENSGALG00010022822	KCNJ13	2	4.483795155	0.879368507	5.098880754	3.41668E-07	1.214185762	4.652095426	78.50431024	1	PRJNA827465;Yang2025_Silkie_Wenchang	0.000146389	FALSE	FALSE	FALSE	FALSE	FALSE	other_meta_signature	3.834491325	20	20	2.149568976	5	0	50.9840603
ENSGALG00010013565	ENSGALG00010013565	2	-3.272102192	1.992857156	-1.641915068	0.100607607	0.956871226	1.13194181	11.65623614	1	PRJNA827465;Yang2025_Silkie_Wenchang	0.611088208	FALSE	FALSE	FALSE	FALSE	FALSE	other_meta_signature	0.213896097	16.36051096	20	8.834376386	5	0	50.40878344
ENSGALG00010024909	SLC39A5	2	2.527801801	0.633736666	3.988725816	6.64291E-05	0	0.543661011	0	1	CRA004775;PRJNA827465	0.009118852	FALSE	FALSE	FALSE	FALSE	FALSE	other_meta_signature	2.040059845	12.63900901	20	10	5	0	49.67906885
ENSGALG00010000571	ENSGALG00010000571	2	2.493056502	0.688053382	3.623347499	0.000290815	0	0.210419762	0	1	CRA004775;PRJNA827465	0.023991454	FALSE	FALSE	FALSE	FALSE	FALSE	other_meta_signature	1.619943433	12.46528251	20	10	5	0	49.08522594
ENSGALG00010009790	MC1R	2	5.730142408	1.478682318	3.875168004	0.000106551	3.761513276	7.139395547	85.99321198	1	PRJNA827465;Yang2025_Silkie_Wenchang	0.012333708	FALSE	FALSE	FALSE	FALSE	FALSE	other_meta_signature	1.908906333	20	20	1.400678802	5	0	48.30958514
ENSGALG00010021117	CYP1A2	2	2.031815692	0.441531225	4.601748593	4.18959E-06	0	0.497012559	0	1	CRA004775;PRJNA827465	0.001012003	FALSE	FALSE	FALSE	FALSE	FALSE	other_meta_signature	2.994818329	10.15907846	20	10	5	0	48.15389679
ENSGALG00010013965	SLC2A2	2	2.147457363	0.633429994	3.390204729	0.000698404	0	0.027965137	0	1	CRA004775;PRJNA827465	0.041665572	FALSE	FALSE	FALSE	FALSE	FALSE	other_meta_signature	1.380222652	10.73728681	20	10	5	0	47.11750947
ENSGALG00010022948	PDZK1IP1	2	1.876258093	0.423808395	4.427137633	9.54918E-06	0	0.212326572	0	1	CRA004775;PRJNA827465	0.002029388	FALSE	FALSE	FALSE	FALSE	FALSE	other_meta_signature	2.692634981	9.381290466	20	10	5	0	47.07392545
ENSGALG00010024342	HNF4A	2	2.077862829	0.567425071	3.661915792	0.000250336	0	0.921709634	0	1	CRA004775;PRJNA827465	0.021848783	FALSE	FALSE	FALSE	FALSE	FALSE	other_meta_signature	1.660572746	10.38931414	20	10	5	0	47.04988689
ENSGALG00010016241	TOPAZ1	2	1.974169494	0.509782669	3.872570829	0.000107693	0	0.393948323	0	1	PRJNA827465;Yang2025_Silkie_Wenchang	0.012333708	FALSE	FALSE	FALSE	FALSE	FALSE	other_meta_signature	1.908906333	9.87084747	20	10	5	0	46.7797538
ENSGALG00010000239	ENSGALG00010000239	3	-4.11305614	3.313322895	-1.241368943	0.214469487	28.38171597	16.61895222	87.96554696	1	CRA004775;PRJNA827465;Yang2025_Silkie_Wenchang	0.729547928	FALSE	FALSE	FALSE	FALSE	FALSE	other_meta_signature	0.136946172	20	20	1.203445304	5	0	46.34039148
ENSGALG00010029688	PIPOX	2	1.821276663	0.491975748	3.701964319	0.000213937	0	0.000923197	0	1	CRA004775;PRJNA827465	0.019515169	FALSE	FALSE	FALSE	FALSE	FALSE	other_meta_signature	1.709627691	9.106383317	20	10	5	0	45.81601101
ENSGALG00010007014	ENSGALG00010007014	3	1.429199532	0.287911255	4.964028011	6.9046E-07	0	0.718825319	0	1	CRA004775;PRJNA827465;Yang2025_Silkie_Wenchang	0.000263849	FALSE	FALSE	FALSE	FALSE	FALSE	other_meta_signature	3.578644641	7.145997662	20	10	5	0	45.7246423
ENSGALG00010025877	ENSGALG00010025877	2	-3.053353774	2.02376016	-1.508752783	0.131361971	4.316601025	1.925858223	48.07509775	1	PRJNA827465;Yang2025_Silkie_Wenchang	0.658603888	FALSE	FALSE	FALSE	FALSE	FALSE	other_meta_signature	0.18137571	15.26676887	20	5.192490225	5	0	45.6406348
ENSGALG00010000249	ENSGALG00010000249	3	-3.682614721	3.113941241	-1.182621776	0.236959078	24.62430973	14.86286489	86.54364408	1	CRA004775;PRJNA827465;Yang2025_Silkie_Wenchang	0.73427773	FALSE	FALSE	FALSE	FALSE	FALSE	other_meta_signature	0.134139618	18.41307361	20	1.345635592	5	0	44.89284882
ENSGALG00010022741	HNF1A	3	1.359969153	0.292412892	4.65085223	3.30566E-06	0	1.093573999	0	1	CRA004775;PRJNA827465;Yang2025_Silkie_Wenchang	0.000878972	FALSE	FALSE	FALSE	FALSE	FALSE	other_meta_signature	3.056024862	6.799845763	20	10	5	0	44.85587063
ENSGALG00010016414	KLB	3	1.449674242	0.343268478	4.223149909	2.40911E-05	0	0.83509063	0	1	CRA004775;PRJNA827465;Yang2025_Silkie_Wenchang	0.00415396	FALSE	FALSE	FALSE	FALSE	FALSE	other_meta_signature	2.381537692	7.248371209	20	10	5	0	44.6299089
ENSGALG00010007010	ENSGALG00010007010	2	1.679429017	0.664830873	2.526099621	0.011533677	0	0.388445323	0	1	PRJNA827465;Yang2025_Silkie_Wenchang	0.232637699	FALSE	FALSE	FALSE	FALSE	FALSE	other_meta_signature	0.633319906	8.397145084	20	10	5	0	44.03046499
ENSGALG00010027528	ENSGALG00010027528	2	1.574773711	0.503525811	3.127493522	0.001763037	0	0.962071173	0	1	CRA004775;PRJNA827465	0.076231134	FALSE	FALSE	FALSE	FALSE	FALSE	other_meta_signature	1.117867619	7.873868554	20	10	5	0	43.99173617
ENSGALG00010029676	FADS6	2	1.6583989	0.632751977	2.620930411	0.008769016	0	0.514919128	0	1	PRJNA827465;Yang2025_Silkie_Wenchang	0.201779961	FALSE	FALSE	FALSE	FALSE	FALSE	other_meta_signature	0.695121966	8.291994501	20	10	5	0	43.98711647
ENSGALG00010022774	A1CF	2	2.220858898	0.816756554	2.719119774	0.00654559	0.424546804	1.426456452	29.8962125	1	CRA004775;PRJNA827465	0.174949147	FALSE	FALSE	FALSE	FALSE	FALSE	other_meta_signature	0.75708817	11.10429449	20	7.01037875	5	0	43.87176141
ENSGALG00010011964	ENSGALG00010011964	3	-3.329625763	2.643848144	-1.25938616	0.207890886	16.90467882	11.88270589	83.16881678	1	CRA004775;PRJNA827465;Yang2025_Silkie_Wenchang	0.724136029	FALSE	FALSE	FALSE	FALSE	FALSE	other_meta_signature	0.140179844	16.64812882	20	1.683118322	5	0	43.47142698
ENSGALG00010022542	ENSGALG00010022542	2	1.489327137	0.496902509	2.99722201	0.002724522	0	0.944841291	0	1	PRJNA827465;Yang2025_Silkie_Wenchang	0.103000051	FALSE	FALSE	FALSE	FALSE	FALSE	other_meta_signature	0.98716256	7.446635683	20	10	5	0	43.43379824
ENSGALG00010007654	HTR1F	2	1.385719799	0.42625948	3.250883237	0.001150471	0	0.019458552	0	1	PRJNA827465;Yang2025_Silkie_Wenchang	0.059150942	FALSE	FALSE	FALSE	FALSE	FALSE	other_meta_signature	1.228038337	6.928598993	20	10	5	0	43.15663733
ENSGALG00010029474	ENSGALG00010029474	2	1.550666085	0.735198585	2.109179911	0.034929055	0	0.247685757	0	1	PRJNA827465;Yang2025_Silkie_Wenchang	0.398598214	FALSE	FALSE	FALSE	FALSE	FALSE	other_meta_signature	0.399464651	7.753330426	20	10	5	0	43.15279508
ENSGALG00010012961	ANKRD55	2	1.283843962	0.352338127	3.643783807	0.000268659	0	0.049341626	0	1	PRJNA827465;Yang2025_Silkie_Wenchang	0.023162019	FALSE	FALSE	FALSE	FALSE	FALSE	other_meta_signature	1.635223585	6.419219811	20	10	5	0	43.0544434
ENSGALG00010021083	SCD	3	0.92776779	0.201302885	4.60881517	4.0497E-06	0	1.150684558	0	1	CRA004775;PRJNA827465;Yang2025_Silkie_Wenchang	0.001004539	FALSE	FALSE	FALSE	FALSE	FALSE	other_meta_signature	2.998033164	4.638838952	20	10	5	0	42.63687212
ENSGALG00010023099	DNAJC22	2	1.698490752	0.745924822	2.277026721	0.02278463	0.165205761	1.155300562	13.44243805	1	CRA004775;PRJNA827465	0.327605279	FALSE	FALSE	FALSE	FALSE	FALSE	other_meta_signature	0.484649108	8.492453762	20	8.655756195	5	0	42.63285906
ENSGALG00010003767	ENSGALG00010003767	3	-1.491962154	1.053960892	-1.415576389	0.156899568	0	0.186700823	0	1	CRA004775;PRJNA827465;Yang2025_Silkie_Wenchang	0.685115194	FALSE	FALSE	FALSE	FALSE	FALSE	other_meta_signature	0.164236401	7.459810771	20	10	5	0	42.62404717
ENSGALG00010000234	ENSGALG00010000234	3	-3.054602178	2.508960965	-1.217476964	0.223422796	14.82476637	10.53975485	81.02422658	1	CRA004775;PRJNA827465;Yang2025_Silkie_Wenchang	0.732804055	FALSE	FALSE	FALSE	FALSE	FALSE	other_meta_signature	0.135012136	15.27301089	20	1.897577342	5	0	42.30560037
ENSGALG00010006123	MLH3	3	0.435845446	0.076881879	5.669026979	1.43611E-08	0	0.562081826	0	1	CRA004775;PRJNA827465;Yang2025_Silkie_Wenchang	8.46047E-06	FALSE	FALSE	FALSE	FALSE	FALSE	other_meta_signature	5.072605584	2.179227229	20	10	5	0	42.25183281
ENSGALG00010017466	SPIA4	2	1.397935675	0.794462944	1.759598337	0.078475934	0	0.022281183	0	1	CRA004775;PRJNA827465	0.555477525	FALSE	FALSE	FALSE	FALSE	FALSE	other_meta_signature	0.255333508	6.989678374	20	10	5	0	42.24501188
ENSGALG00010009993	ENSGALG00010009993	3	1.05424689	0.269615892	3.91018082	9.22271E-05	0	1.09773953	0	1	CRA004775;PRJNA827465;Yang2025_Silkie_Wenchang	0.01097502	FALSE	FALSE	FALSE	FALSE	FALSE	other_meta_signature	1.959594683	5.271234449	20	10	5	0	42.23082913
ENSGALG00010014789	F7	2	1.377845604	0.69568068	1.980571898	0.047639303	0	0.099587758	0	1	CRA004775;PRJNA827465	0.456349665	FALSE	FALSE	FALSE	FALSE	FALSE	other_meta_signature	0.340702264	6.889228018	20	10	5	0	42.22993028
ENSGALG00010000142	ENSGALG00010000142	3	-1.071050826	0.283278774	-3.780907438	0.000156258	0	0.659280577	0	1	CRA004775;PRJNA827465;Yang2025_Silkie_Wenchang	0.016126486	FALSE	FALSE	FALSE	FALSE	FALSE	other_meta_signature	1.79246026	5.355254128	20	10	5	0	42.14771439
ENSGALG00010029161	HNF1B	2	1.33182476	0.605253799	2.200440148	0.027775682	0	0.988340844	0	1	CRA004775;PRJNA827465	0.355403048	FALSE	FALSE	FALSE	FALSE	FALSE	other_meta_signature	0.449278852	6.659123798	20	10	5	0	42.10840265
ENSGALG00010022211	ENSGALG00010022211	3	1.009373226	0.230415847	4.380658887	1.18321E-05	0.010093534	2.127532467	5.994384067	1	CRA004775;PRJNA827465;Yang2025_Silkie_Wenchang	0.002424551	FALSE	FALSE	FALSE	FALSE	FALSE	other_meta_signature	2.615368647	5.046866131	20	9.400561593	5	0	42.06279637
ENSGALG00010028497	ADMP	2	1.23892015	0.500180303	2.476947097	0.013251154	0	0.111411152	0	1	CRA004775;Yang2025_Silkie_Wenchang	0.247174233	FALSE	FALSE	FALSE	FALSE	FALSE	other_meta_signature	0.606996805	6.19460075	20	10	5	0	41.80159755
ENSGALG00010021316	PLA2G12B	3	1.287896651	0.365962413	3.519204718	0.000432843	0.052059144	2.285454238	12.49004391	1	CRA004775;PRJNA827465;Yang2025_Silkie_Wenchang	0.030753573	FALSE	FALSE	FALSE	FALSE	FALSE	other_meta_signature	1.512104416	6.439483255	20	8.750995609	5	0	41.70258328
ENSGALG00010013069	ENSGALG00010013069	3	-1.279178149	1.303315923	-0.981479722	0.32635623	0	0.749057249	0	1	CRA004775;PRJNA827465;Yang2025_Silkie_Wenchang	0.774972145	FALSE	FALSE	FALSE	FALSE	FALSE	other_meta_signature	0.110713907	6.395890747	20	10	5	0	41.50660465
ENSGALG00010011436	UBAP2L2	3	-2.925899785	2.333097921	-1.254083576	0.209811629	13.72865425	13.35422545	85.02346686	1	CRA004775;PRJNA827465;Yang2025_Silkie_Wenchang	0.725312133	FALSE	FALSE	FALSE	FALSE	FALSE	other_meta_signature	0.139475057	14.62949892	20	1.497653314	5	0	41.26662729
ENSGALG00010018279	INSM1	2	1.11689547	0.539769896	2.069206671	0.038526697	0	0.085952755	0	1	CRA004775;PRJNA827465	0.415189763	FALSE	FALSE	FALSE	FALSE	FALSE	other_meta_signature	0.381753363	5.584477349	20	10	5	0	40.96623071
ENSGALG00010005478	ENSGALG00010005478	2	1.116981852	0.571751669	1.953613627	0.050746928	0	0.579794269	0	1	CRA004775;PRJNA827465	0.470424615	FALSE	FALSE	FALSE	FALSE	FALSE	other_meta_signature	0.327509962	5.584909258	20	10	5	0	40.91241922
ENSGALG00010002352	ENSGALG00010002352	3	-0.379516046	0.073840595	-5.139666656	2.75226E-07	0	0.336861992	0	1	CRA004775;PRJNA827465;Yang2025_Silkie_Wenchang	0.000129714	FALSE	FALSE	FALSE	FALSE	FALSE	other_meta_signature	3.887012643	1.897580229	20	10	5	0	40.78459287
ENSGALG00010026845	ENSGALG00010026845	2	-1.127859834	1.83473267	-0.614727068	0.538734981	0	0.225260199	0	1	PRJNA827465;Yang2025_Silkie_Wenchang	0.852136811	FALSE	FALSE	FALSE	FALSE	FALSE	other_meta_signature	0.069490673	5.639299171	20	10	5	0	40.70878984
ENSGALG00010017240	MELTF	3	0.627407471	0.146456455	4.283918177	1.8363E-05	0	1.576297331	0	1	CRA004775;PRJNA827465;Yang2025_Silkie_Wenchang	0.003606043	FALSE	FALSE	FALSE	FALSE	FALSE	other_meta_signature	2.442969062	3.137037353	20	10	5	0	40.58000641
ENSGALG00010002142	CLDN34	3	1.412284611	0.402745756	3.506640581	0.000453802	0.156374119	2.922031512	31.55446846	1	CRA004775;PRJNA827465;Yang2025_Silkie_Wenchang	0.031762888	FALSE	FALSE	FALSE	FALSE	FALSE	other_meta_signature	1.498080011	7.061423056	20	6.844553154	5	0	40.40405622
ENSGALG00010013520	CREG2	3	-0.450935734	0.097055257	-4.646175253	3.38146E-06	0	1.120133998	0	1	CRA004775;PRJNA827465;Yang2025_Silkie_Wenchang	0.000878972	FALSE	FALSE	FALSE	FALSE	FALSE	other_meta_signature	3.056024862	2.25467867	20	10	5	0	40.31070353
ENSGALG00010005520	ZIC2	2	-0.958834982	0.587719879	-1.631448953	0.102795623	0	0.273101211	0	1	CRA004775;PRJNA827465	0.613680652	FALSE	FALSE	FALSE	FALSE	FALSE	other_meta_signature	0.212057569	4.794174908	20	10	5	0	40.00623248
ENSGALG00010017967	ACSM3	2	0.946725064	0.554612939	1.707001403	0.087821792	0	0.208768377	0	1	PRJNA827465;Yang2025_Silkie_Wenchang	0.58378576	FALSE	FALSE	FALSE	FALSE	FALSE	other_meta_signature	0.233746502	4.733625322	20	10	5	0	39.96737182
ENSGALG00010022564	VTCN1	3	-0.720210797	0.220200746	-3.270700984	0.001072813	0	0.514148422	0	1	CRA004775;PRJNA827465;Yang2025_Silkie_Wenchang	0.056810862	FALSE	FALSE	FALSE	FALSE	FALSE	other_meta_signature	1.245568624	3.601053986	20	10	5	0	39.84662261
ENSGALG00010022625	ENSGALG00010022625	3	-0.355437669	0.077450938	-4.589197733	4.44953E-06	0	1.053768477	0	1	CRA004775;PRJNA827465;Yang2025_Silkie_Wenchang	0.001031342	FALSE	FALSE	FALSE	FALSE	FALSE	other_meta_signature	2.986597202	1.777188347	20	10	5	0	39.76378555
ENSGALG00010028478	PEA15	3	0.326434999	0.070159166	4.65277762	3.27493E-06	0	0.662938226	0	1	CRA004775;PRJNA827465;Yang2025_Silkie_Wenchang	0.000878972	FALSE	FALSE	FALSE	FALSE	FALSE	other_meta_signature	3.056024862	1.632174993	20	10	5	0	39.68819986
ENSGALG00010025782	ENSGALG00010025782	2	-0.925177594	1.953676326	-0.473557253	0.635815665	0	0.083974666	0	1	PRJNA827465;Yang2025_Silkie_Wenchang	0.88855018	FALSE	FALSE	FALSE	FALSE	FALSE	other_meta_signature	0.051318041	4.62588797	20	10	5	0	39.67720601
ENSGALG00010007857	ENSGALG00010007857	3	0.650050992	0.189210322	3.43560005	0.000591243	0	0.251119789	0	1	CRA004775;PRJNA827465;Yang2025_Silkie_Wenchang	0.037655794	FALSE	FALSE	FALSE	FALSE	FALSE	other_meta_signature	1.42416819	3.250254961	20	10	5	0	39.67442315
ENSGALG00010010880	ELP3	3	-0.229417252	0.046551685	-4.928226606	8.29793E-07	0	0.726052401	0	1	CRA004775;PRJNA827465;Yang2025_Silkie_Wenchang	0.000300832	FALSE	FALSE	FALSE	FALSE	FALSE	other_meta_signature	3.521675918	1.147086262	20	10	5	0	39.66876218
ENSGALG00010013319	ENSGALG00010013319	3	-2.692918959	2.315196534	-1.163149184	0.24476898	14.3389961	18.74609318	89.33111033	1	CRA004775;PRJNA827465;Yang2025_Silkie_Wenchang	0.738539945	FALSE	FALSE	FALSE	FALSE	FALSE	other_meta_signature	0.13162601	13.46459479	20	1.066888967	5	0	39.66310977

ENSGALG00010027218	IARS1	3	-0.104405386	0.043940931	-2.376039473	0.017499591	0	0.096868721	0	1	CRA004775;PRJNA827465;Yang2025_Silkie_Wenchang	0.287371327	FALSE	FALSE	FALSE	FALSE	FALSE	other_meta_signature
ENSGALG00010018240	SOUL	3	0.195198853	0.268423188	0.727205625	0.467100001	0	0.299739947	0	1	CRA004775;PRJNA827465;Yang2025_Silkie_Wenchang	0.82826404	FALSE	FALSE	FALSE	FALSE	FALSE	other_meta_signature
ENSGALG00010020429	ZNF148	3	0.117978647	0.052744067	2.236813615	0.025298524	0	1.390320908	0	1	CRA004775;PRJNA827465;Yang2025_Silkie_Wenchang	0.342973112	FALSE	FALSE	FALSE	FALSE	FALSE	other_meta_signature
ENSGALG00010025315	LRRC42	3	0.22406829	0.078780075	2.844225407	0.004451954	0.002087087	2.201576891	9.156023197	1	CRA004775;PRJNA827465;Yang2025_Silkie_Wenchang	0.14169643	FALSE	FALSE	FALSE	FALSE	FALSE	other_meta_signature
ENSGALG00010028081	UBQLN4	3	-0.108223886	0.046675635	-2.318657635	0.020413607	0	0.736027484	0	1	CRA004775;PRJNA827465;Yang2025_Silkie_Wenchang	0.308034145	FALSE	FALSE	FALSE	FALSE	FALSE	other_meta_signature
ENSGALG00010019934	TKFC	3	0.334150769	0.120985503	2.761907516	0.005746475	0.006617145	2.325710436	14.00477164	1	CRA004775;PRJNA827465;Yang2025_Silkie_Wenchang	0.165477424	FALSE	FALSE	FALSE	FALSE	FALSE	other_meta_signature
ENSGALG00010019068	SNRPA1	3	-0.142581106	0.07238044	-1.969884488	0.048851611	0	0.376699186	0	1	CRA004775;PRJNA827465;Yang2025_Silkie_Wenchang	0.462015333	FALSE	FALSE	FALSE	FALSE	FALSE	other_meta_signature
ENSGALG00010014974	LIG4	3	0.170017145	0.107724579	1.578257686	0.114506423	0	0.300389905	0	1	CRA004775;PRJNA827465;Yang2025_Silkie_Wenchang	0.635652261	FALSE	FALSE	FALSE	FALSE	FALSE	other_meta_signature
ENSGALG00010012159	WASHC1	3	-0.131890383	0.063529078	-2.076063229	0.037888104	0	0.19178264	0	1	CRA004775;PRJNA827465;Yang2025_Silkie_Wenchang	0.412394069	FALSE	FALSE	FALSE	FALSE	FALSE	other_meta_signature
ENSGALG00010028426	GPR21	3	0.157959443	0.090020115	1.754712754	0.079308453	0	1.795001038	0	1	CRA004775;PRJNA827465;Yang2025_Silkie_Wenchang	0.557371584	FALSE	FALSE	FALSE	FALSE	FALSE	other_meta_signature
ENSGALG00010017503	MED9	3	-0.122904984	0.05678822	-2.164269005	0.03044371	0	1.354643067	0	1	CRA004775;PRJNA827465;Yang2025_Silkie_Wenchang	0.373371282	FALSE	FALSE	FALSE	FALSE	FALSE	other_meta_signature
ENSGALG00010011639	ENSGALG00010011639	3	0.180788834	0.151721317	1.191584918	0.233424042	0	0.876208634	0	1	CRA004775;PRJNA827465;Yang2025_Silkie_Wenchang	0.734277773	FALSE	FALSE	FALSE	FALSE	FALSE	other_meta_signature
ENSGALG00010028871	PIP5K1A	3	-0.116048682	0.052404036	-2.214498955	0.026794475	0	0.488797904	0	1	CRA004775;PRJNA827465;Yang2025_Silkie_Wenchang	0.350025678	FALSE	FALSE	FALSE	FALSE	FALSE	other_meta_signature
ENSGALG00010016542	ENOX2	3	0.095999414	0.040084088	2.394950688	0.016622601	0	0.477117166	0	1	CRA004775;PRJNA827465;Yang2025_Silkie_Wenchang	0.278540968	FALSE	FALSE	FALSE	FALSE	FALSE	other_meta_signature
ENSGALG00010017649	ENSGALG00010017649	3	-0.147771575	0.079149452	-1.866994278	0.061902387	0	0.396545407	0	1	CRA004775;PRJNA827465;Yang2025_Silkie_Wenchang	0.507173571	FALSE	FALSE	FALSE	FALSE	FALSE	other_meta_signature
ENSGALG00010004717	ALKBH8	3	-0.164066071	0.100698284	-1.629283682	0.103252979	0	0.162249462	0	1	CRA004775;PRJNA827465;Yang2025_Silkie_Wenchang	0.613858001	FALSE	FALSE	FALSE	FALSE	FALSE	other_meta_signature
ENSGALG00010013085	LYSMD3	3	0.160340943	0.09642514	1.66285413	0.096341638	0	0.124200559	0	1	CRA004775;PRJNA827465;Yang2025_Silkie_Wenchang	0.603265909	FALSE	FALSE	FALSE	FALSE	FALSE	other_meta_signature
ENSGALG00010021025	CUEDC2	3	0.132145651	0.065369036	2.021532801	0.04322464	0	0.511643284	0	1	CRA004775;PRJNA827465;Yang2025_Silkie_Wenchang	0.437793079	FALSE	FALSE	FALSE	FALSE	FALSE	other_meta_signature
ENSGALG00010029178	NOTUM	3	0.141241515	0.075265023	1.876588997	0.060574447	0	0.562556564	0	1	CRA004775;PRJNA827465;Yang2025_Silkie_Wenchang	0.502232506	FALSE	FALSE	FALSE	FALSE	FALSE	other_meta_signature
ENSGALG00010020632	BLOC1S2	3	-0.130540265	0.065185407	-2.002599531	0.04522029	0	0.411764481	0	1	CRA004775;PRJNA827465;Yang2025_Silkie_Wenchang	0.446330405	FALSE	FALSE	FALSE	FALSE	FALSE	other_meta_signature
ENSGALG00010029701	MRPS7	3	-0.101301245	0.04407716	-2.298270698	0.021546387	0	0.084175192	0	1	CRA004775;PRJNA827465;Yang2025_Silkie_Wenchang	0.318999337	FALSE	FALSE	FALSE	FALSE	FALSE	other_meta_signature
ENSGALG00010009400	CNTFR	3	-0.149310362	0.085035798	-1.755853005	0.079113511	0	0.572538982	0	1	CRA004775;PRJNA827465;Yang2025_Silkie_Wenchang	0.557111902	FALSE	FALSE	FALSE	FALSE	FALSE	other_meta_signature
ENSGALG00010021215	SH3PXD2A	3	0.139692116	0.07456709	1.873374915	0.061016631	0	1.456093467	0	1	CRA004775;PRJNA827465;Yang2025_Silkie_Wenchang	0.503283102	FALSE	FALSE	FALSE	FALSE	FALSE	other_meta_signature
ENSGALG00010011381	RLIG1	3	0.138980007	0.074362444	1.868954249	0.06162918	0	0.731194985	0	1	CRA004775;PRJNA827465;Yang2025_Silkie_Wenchang	0.506318985	FALSE	FALSE	FALSE	FALSE	FALSE	other_meta_signature
ENSGALG00010016611	DNALI1	3	0.164054805	0.116553349	1.40755119	0.159264009	0	1.086676153	0	1	CRA004775;PRJNA827465;Yang2025_Silkie_Wenchang	0.68855483	FALSE	FALSE	FALSE	FALSE	FALSE	other_meta_signature
ENSGALG00010012992	HARS1	3	-0.08537605	0.035697872	-2.391628576	0.016773808	0	1.412752314	0	1	CRA004775;PRJNA827465;Yang2025_Silkie_Wenchang	0.279675549	FALSE	FALSE	FALSE	FALSE	FALSE	other_meta_signature
ENSGALG00010026030	IBA57	3	-0.13380334	0.070444447	-1.89941643	0.057509745	0	1.913233916	0	1	CRA004775;PRJNA827465;Yang2025_Silkie_Wenchang	0.492209614	FALSE	FALSE	FALSE	FALSE	FALSE	other_meta_signature
ENSGALG00010022765	KRT12	2	0.183077544	0.337654607	0.542203601	0.587678255	0	0.023071679	0	1	PRJNA827465;Yang2025_Silkie_Wenchang	0.869812793	FALSE	FALSE	FALSE	FALSE	FALSE	other_meta_signature
ENSGALG00010022149	NBEAL1	3	0.125089488	0.06245399	2.002906276	0.045187349	0	1.359737456	0	1	CRA004775;PRJNA827465;Yang2025_Silkie_Wenchang	0.446330405	FALSE	FALSE	FALSE	FALSE	FALSE	other_meta_signature
ENSGALG00010004326	ENSGALG00010004326	3	-0.810382458	0.460579453	-1.759484606	0.078495233	0.215226812	2.999319105	33.3181989	1	CRA004775;PRJNA827465;Yang2025_Silkie_Wenchang	0.555477525	FALSE	FALSE	FALSE	FALSE	FALSE	other_meta_signature
ENSGALG00010016598	AIFM1	3	-0.109538221	0.050917428	-2.151291341	0.031453211	0	0.078748882	0	1	CRA004775;PRJNA827465;Yang2025_Silkie_Wenchang	0.378160676	FALSE	FALSE	FALSE	FALSE	FALSE	other_meta_signature
ENSGALG00010005830	USP12	3	-0.168125496	0.150156173	-1.119670889	0.262854035	0	0.198638753	0	1	CRA004775;PRJNA827465;Yang2025_Silkie_Wenchang	0.746583607	FALSE	FALSE	FALSE	FALSE	FALSE	other_meta_signature
ENSGALG00010009253	SLC45A4	3	-0.15022161	0.091582694	-1.640283817	0.100946169	0	1.804069052	0	1	CRA004775;PRJNA827465;Yang2025_Silkie_Wenchang	0.611813162	FALSE	FALSE	FALSE	FALSE	FALSE	other_meta_signature
ENSGALG00010020614	FAIM	3	-0.131935664	0.069938555	-1.886451101	0.059234192	0	1.553037197	0	1	CRA004775;PRJNA827465;Yang2025_Silkie_Wenchang	0.499130355	FALSE	FALSE	FALSE	FALSE	FALSE	other_meta_signature
ENSGALG00010000186	THOC5	3	-0.089530272	0.038592605	-2.319881527	0.020347287	0	1.244668122	0	1	CRA004775;PRJNA827465;Yang2025_Silkie_Wenchang	0.307577531	FALSE	FALSE	FALSE	FALSE	FALSE	other_meta_signature
ENSGALG00010015052	ENSGALG00010015052	3	-0.13600191	0.074928354	-1.815092724	0.069509661	0	1.356216178	0	1	CRA004775;PRJNA827465;Yang2025_Silkie_Wenchang	0.527510598	FALSE	FALSE	FALSE	FALSE	FALSE	other_meta_signature
ENSGALG00010001702	PLEKHA1	3	0.105909045	0.048881841	2.166633712	0.030262795	0	1.712501702	0	1	CRA004775;PRJNA827465;Yang2025_Silkie_Wenchang	0.373371282	FALSE	FALSE	FALSE	FALSE	FALSE	other_meta_signature
ENSGALG00010025116	ANAPC7	3	-0.09718389	0.043498148	-2.23420756	0.025469419	0	0.930764426	0	1	CRA004775;PRJNA827465;Yang2025_Silkie_Wenchang	0.343290866	FALSE	FALSE	FALSE	FALSE	FALSE	other_meta_signature
ENSGALG00010016840	LRRC34	3	0.114230348	0.05573386	2.049568233	0.040406583	0	0.117960728	0	1	CRA004775;PRJNA827465;Yang2025_Silkie_Wenchang	0.422808261	FALSE	FALSE	FALSE	FALSE	FALSE	other_meta_signature
ENSGALG00010010925	FBXO16	2	-0.175265777	0.308880077	-0.56742338	0.570426569	0	0.000825448	0	1	PRJNA827465;Yang2025_Silkie_Wenchang	0.863134826	FALSE	FALSE	FALSE	FALSE	FALSE	other_meta_signature
ENSGALG00010004917	ENSGALG00010004917	3	-0.205412833	0.070457559	-2.915412284	0.003552189	0.001795288	2.222958176	10.02979626	1	CRA004775;PRJNA827465;Yang2025_Silkie_Wenchang	0.122272589	FALSE	FALSE	FALSE	FALSE	FALSE	other_meta_signature
ENSGALG00010018452	SPSB3	3	-0.121915448	0.062623886	-1.946788301	0.05156012	0	1.804036678	0	1	CRA004775;PRJNA827465;Yang2025_Silkie_Wenchang	0.472155789	FALSE	FALSE	FALSE	FALSE	FALSE	other_meta_signature
ENSGALG00010016603	ENSGALG00010016603	3	0.122692559	0.063689918	1.92640474	0.054053856	0	0.262115123	0	1	CRA004775;PRJNA827465;Yang2025_Silkie_Wenchang	0.481614646	FALSE	FALSE	FALSE	FALSE	FALSE	other_meta_signature
ENSGALG00010025365	SIKE1	3	-0.100295933	0.046385328	-2.162233967	0.030600147	0	1.755348437	0	1	CRA004775;PRJNA827465;Yang2025_Silkie_Wenchang	0.37426944	FALSE	FALSE	FALSE	FALSE	FALSE	other_meta_signature
ENSGALG00010009646	MVD	3	-0.148217983	0.099302235	-1.49259464	0.135543327	0	1.136100709	0	1	CRA004775;PRJNA827465;Yang2025_Silkie_Wenchang	0.664104722	FALSE	FALSE	FALSE	FALSE	FALSE	other_meta_signature
ENSGALG00010001469	ARHGEF26	3	0.133927652	0.076783081	1.744233885	0.081118315	0	0.054645015	0	1	CRA004775;PRJNA827465;Yang2025_Silkie_Wenchang	0.563418588	FALSE	FALSE	FALSE	FALSE	FALSE	other_meta_signature
ENSGALG00010020648	TET3	3	-0.126764919	0.068844613	-1.841319359	0.065574772	0	0.39361374	0	1	CRA004775;PRJNA827465;Yang2025_Silkie_Wenchang	0.519039076	FALSE	FALSE	FALSE	FALSE	FALSE	other_meta_signature
ENSGALG00010023665	C16orf87	3	-0.140983978	0.086401029	-1.631739564	0.102734362	0	0.001641374	0	1	CRA004775;PRJNA827465;Yang2025_Silkie_Wenchang	0.613680652	FALSE	FALSE	FALSE	FALSE	FALSE	other_meta_signature
ENSGALG00010028750	OTUD7B	3	0.109465201	0.053687324	2.038939429	0.041456073	0	1.860397891	0	1	CRA004775;PRJNA827465;Yang2025_Silkie_Wenchang	0.429726846	FALSE	FALSE	FALSE	FALSE	FALSE	other_meta_signature
ENSGALG00010008668	ENSGALG00010008668	3	0.164905179	0.213300652	0.77311146	0.439456419	0	0.077274875	0	1	CRA004775;PRJNA827465;Yang2025_Silkie_Wenchang	0.816978668	FALSE	FALSE	FALSE	FALSE	FALSE	other_meta_signature
ENSGALG00010029619	ENSGALG00010029619	3	-0.129917068	0.073057062	-1.778295829	0.075355281	0	1.332114379	0	1	CRA004775;PRJNA827465;Yang2025_Silkie_Wenchang	0.5461037	FALSE	FALSE	FALSE	FALSE	FALSE	other_meta_signature
ENSGALG00010016675	SKIL	3	0.125643514	0.068609231	1.831291679	0.067057019	0	1.638057388	0	1	CRA004775;PRJNA827465;Yang2025_Silkie_Wenchang	0.520153828	FALSE	FALSE	FALSE	FALSE	FALSE	other_meta_signature
ENSGALG00010009643	PSMC2	3	-0.108744903	0.053286343	-2.040764965	0.041274195	0	0.893742215	0	1	CRA004775;PRJNA827465;Yang2025_Silkie_Wenchang	0.428314172	FALSE	FALSE	FALSE	FALSE	FALSE	other_meta_signature
ENSGALG00010014935	PCYT1A	3	-0.130496243	0.073697675	-1.77069687	0.076611123	0	1.04206478	0	1	CRA004775;PRJNA827465;Yang2025_Silkie_Wenchang	0.550542847	FALSE	FALSE	FALSE	FALSE	FALSE	other_meta_signature
ENSGALG00010029604	ENSGALG00010029604	3	-0.120013847	0.06399693	-1.875306304	0.060750597	0	0.011794994	0	1	CRA004775;PRJNA827465;Yang2025_Silkie_Wenchang	0.502605433	FALSE	FALSE	FALSE	FALSE	FALSE	other_meta_signature
ENSGALG00010024423	SERGEF	3	-0.131548797	0.076609074	-1.717143801	0.085952901	0	0.590466811	0	1	CRA004775;PRJNA827465;Yang2025_Silkie_Wenchang	0.578157976	FALSE	FALSE	FALSE	FALSE	FALSE	other_meta_signature
ENSGALG00010019093	RPL32	3	-0.134114731	0.080320734	-1.669739855	0.094970845	0	0.215864836	0	1	CRA004775;PRJNA827465;Yang2025_Silkie_Wenchang	0.599193562	FALSE	FALSE	FALSE	FALSE	FALSE	other_meta_signature
ENSGALG00010023188	KIF1BP	3	-0.081032389	0.035490191	-2.283233392	0.022416624	0	0.880825996	0	1	CRA004775;PRJNA827465;Yang2025_Silkie_Wenchang	0.325844641	FALSE	FALSE	FALSE	FALSE	FALSE	other_meta_signature
ENSGALG00010020204	GSPT2	3	-0.087069928	0.039218518	-2.220122825	0.026410431	0	0.164797574	0	1	CRA004775;PRJNA827465;Yang2025_Silkie_Wenchang	0.349641467	FALSE	FALSE	FALSE	FALSE	FALSE	other_meta_signature
ENSGALG00010006346	NSUN3	3	-0.149486069	0.116957905	-1.278118567	0.201207623	0	0.489856233	0	1	CRA004775;PRJNA827465;Yang2025_Silkie_Wenchang	0.722423145	FALSE	FALSE	FALSE	FALSE	FALSE	other_meta_signature
ENSGALG00010028093	SFMBT1	3	-0.146817123	0.109228243	-1.344131516	0.178905862	0	0.963192184	0	1	CRA004775;PRJNA827465;Yang2025_Silkie_Wenchang	0.709949478	FALSE	FALSE	FALSE	FALSE	FALSE	other_meta_signature
ENSGALG00010025522	SUMO3	3	-0.101302457	0.049418521	-2.049888484	0.040375314	0	0.46019017	0	1	CRA004775;PRJNA827465;Yang2025_Silkie_Wenchang	0.422808261	FALSE	FALSE	FALSE	FALSE	FALSE	other_meta_signature
ENSGALG00010029759	ALDH3A2	3	-0.126443908	0.072617841	-1.74122373	0.081644369	0	1.539532038	0	1	CRA004775;PRJNA827465;Yang2025_Silkie_Wenchang	0.565426071	FALSE	FALSE	FALSE	FALSE	FALSE	other_meta_signature
ENSGALG00010020428	SEMA4C	3	0.110452401	0.056505415	1.954722406	0.050615844	0	0.164659702	0	1	CRA004775;PRJNA827465;Yang2025_Silkie_Wenchang	0.470424615	FALSE	FALSE	FALSE	FALSE	FALSE	other_meta_signature
ENSGALG00010012141	ENSGALG00010012141	3	0.141359655	0.096222113	1.469097392	0.141806375	0	1.1498263	0	1	CRA004775;PRJNA827465;Yang2025_Silkie_Wenchang	0.672367651	FALSE	FALSE	FALSE	FALSE	FALSE	other_meta_signature
ENSGALG00010012535	JMY	3	0.13209962	0.080172862	1.647684962	0.099417335	0	0.67967796	0	1	CRA004775;PRJNA827465;Yang2025_Silkie_Wenchang	0.608341948	FALSE	FALSE	FALSE	FALSE	FALSE	other_meta_signature
ENSGALG00010008024	MRS2	3	-0.12781777	0.074763009	-1.709639182	0.087332616	0	0.221384214	0	1	CRA004775;PRJNA827465;Yang2025_Silkie_Wenchang	0.582176262	FALSE	FALSE	FALSE	FALSE	FALSE	other_meta_signature
ENSGALG00010018423	IGFALS	3	-0.611613964	0.512381449	-1.19366922	0.232607385	0.18350885	2.607092283	23.2861831	1	CRA004775;PRJNA827465;Yang2025_Silkie_Wenchang	0.73395131	FALSE	FALSE	FALSE	FALSE	FALSE	other_meta_signature
ENSGALG00010007425	ARSA	3	-0.100109356	0.049270793	-2.031819436	0.042171939	0	0.385747243	0	1	CRA004775;PRJNA827465;Yang2025_Silkie_Wenchang	0.43333506	FALSE	FALSE	FALSE	FALSE	FALSE	other_meta_signature
ENSGALG00010022588	TCP11L1	3	-0.112031268	0.05934201	-1.88789136	0.059040537	0	0.091675898	0	1	CRA004775;PRJNA827465;Yang2025_Silkie_Wenchang	0.498524762	FALSE	FALSE	FALSE	FALSE	FALSE	other_meta_signature
ENSGALG00010018911	CNIH1	3	-0.081252364	0.036629105	-2.218245993	0.026538064	0	0.309768675	0	1	CRA004775;PRJNA827465;Yang2025_Silkie_Wenchang	0.349865188	FALSE	FALSE	FALSE	FALSE	FALSE	other_meta_signature
ENSGALG00010021835	NUDT8	3	-0.139562029	0.100234527	-1.392354842	0.163814972	0	0.016213535	0	1	CRA004775;PRJNA827465;Yang2025_Silkie_Wenchang	0.692450961	FALSE	FALSE	FALSE	FALSE	FALSE	other_meta_signature
ENSGALG00010008377	AKT2	3	-0.096498185	0.046991777	-2.053512153	0.040022932	0	1.226504245	0	1	CRA004775;PRJNA827465;Yang2025_Silkie_Wenchang	0.422462902	FALSE	FALSE	FALSE	FALSE	FALSE	other_meta_signature
ENSGALG00010018501	ATG14	3	0.107460675	0.055599943	1.932747942	0.053267256	0	1.290978192	0	1	CRA004775;PRJNA827465;Yang2025_Silkie_Wenchang	0.479323642	FALSE	FALSE	FALSE	FALSE	FALSE	other_meta_signature
ENSGALG00010023909	FOXRED1	3	-0.135981162	0.092648109	-1.46771654	0.142181236	0	0.368048376	0	1	CRA004775;PRJNA827465;Yang2025_Silkie_Wenchang	0.67251868	FALSE	FALSE	FALSE	FALSE	FALSE	other_meta_signature
ENSGALG00010012624	PDGFB	3	0.124988448	0.074771308	1.671609756	0.0946013	0	0.515438415	0	1	CRA004775;PRJNA827465;Yang2025_Silkie_Wenchang	0.598461398	FALSE	FALSE	FALSE	FALSE	FALSE	other_meta_signature
ENSGALG00010024288	CHID1	3	0.133594861	0.070021546	1.907910767	0.05640275	0.000248672	2.026516623	1.308482857	1	CRA004775;PRJNA827465;Yang2025_Silkie_Wenchang	0.488950637	FALSE	FALSE	FALSE	FALSE	FALSE	other_meta_signature
ENSGALG00010028405	S100A6	3	-0.143312315	0.126440113	-1.13344026	0.257029415	0	1.937333164	0	1	CRA004775;PRJNA827465;Yang2025_Silkie_Wenchang	0.744535853	FALSE	FALSE	FALSE	FALSE	FALSE	other_meta_signature
ENSGALG00010027088	AGBL4	3	0.149550109	0.173313567	0.862887492	0.388199328	0	0.62928014	0	1	CRA004775;PRJNA827465;Yang2025_Silkie_Wenchang	0.801233635	FALSE	FALSE	FALSE	FALSE	FALSE	other_meta_signature
ENSGALG00010017732	RHOJ	3	0.136425563	0.099475588	1.371447673	0.170235445	0	1.40494771	0	1	CRA004775;PRJNA827465;Yang2025_Silkie_Wenchang	0.6977362	FALSE	FALSE	FALSE	FALSE	FALSE	other_meta_signature
ENSGALG00010012772	TOMM22	3	-0.110002733	0.059536399	-1.847655131	0.064652249	0	0.783158629	0	1	CRA004775;PRJNA827465;Yang2025_Silkie_Wenchang	0.516391366	FALSE	FALSE	FALSE	FALSE	FALSE	other_meta_signature
ENSGALG00010027989	ENSGALG00010027989	3	-0.13286685	0.091276792	-1.455647667	0.145490044	0	0.836119326	0	1	CRA004775;PRJNA827465;Yang2025_Silkie_Wenchang	0.677188568	FALSE	FALSE	FALSE	FALSE	FALSE	other_meta_signature
ENSGALG00010004928	HDDC2	3	0.123951081	0.076034532	1.630194567	0.103060382	0	0.529328615	0	1	CRA004775;PRJNA827465;Yang2025_Silkie_Wenchang	0.613680652	FALSE	FALSE	FALSE	FALSE	FALSE	other_meta_signature
ENSGALG00010018014	LYRM1	3	0.086306439	0.040890025	2.110696681	0.034798391	0	0.067962132	0	1	CRA004775;PRJNA827465;Yang2025_Silkie_Wenchang	0.398151292	FALSE	FALSE	FALSE	FALSE	FALSE	other_meta_signature
ENSGALG00010028054	RPS15	3	-0.112916217	0.063239734	-1.785526425	0.074175969	0	1.92047167	0	1	CRA004775;PRJNA827465;Yang2025_Silkie_Wenchang	0.542101243	FALSE	FALSE	FALSE	FALSE	FALSE	other_meta_signature
ENSGALG00010010857	SEL1L3	3	0.107861747	0.057966093	1.8607731	0.062776226	0	0.407624954	0	1	CRA004775;PRJNA827465;Yang2025_Silkie_Wenchang	0.5118604	FALSE	FALSE	FALSE	FALSE	FALSE	other_meta_signature
ENSGALG00010020764	PRKAR2A	3	-0.10142692	0.052302304	-1.939243807	0.052471658	0	0.090119166	0	1	CRA004775;PRJNA827465;Yang2025_Silkie_Wenchang	0.475879903	FALSE	FALSE	FALSE	FALSE	FALSE	other_meta_signature
ENSGALG00010029186	ZNF302	3	0.234053649	0.083812954	2.7925713	0.005229094	0.003175122	2.259769062	11.49538093	1	CRA004775;PRJNA827465;Yang2025_Silkie_Wenchang	0.156640176	FALSE	FALSE	FALSE	FALSE	FALSE	other_meta_signature
ENSGALG00010021783	GIT2	3	-0.08074853	0.037567057	-2.149450478	0.031598708	0	0.67879463	0	1	CRA004775;PRJNA827465;Yang2025_Silkie_Wenchang	0.378943281	FALSE	FALSE	FALSE	FALSE	FALSE	other_meta_signature
ENSGALG00010009013	LIPG	3	0.137741552	0.111189879	1.238795771	0.21542114	0	0.168919683	0	1	CRA004775;PRJNA827465;Yang2025_Silkie_Wenchang	0.731012139	FALSE	FALSE	FALSE	FALSE	FALSE	other_meta_signature
ENSGALG00010020654	SAYSD1	3	-0.126175356	0.082521326	-1.529003014	0.126263697	0	1.698860898	0	1	CRA004775;PRJNA827465;Yang2025_Silkie_Wenchang	0.650008427	FALSE	FALSE	FALSE	FALSE	FALSE	other_meta_signature
ENSGALG00010019592	ALKBH3	3	-0.125888166	0.081724539	-1.540396156	0.123463818	0	0.009143556	0	1	CRA004775;PRJNA827465;Yang2025_Silkie_Wenchang	0.648071434	FALSE	FALSE	FALSE	FALSE	FALSE	other_meta_signature
ENSGALG00010013104	HLCS	3	-0.106361657	0.058299658	-1.8243959	0.068092241	0	1.167574443	0	1	CRA004775;PRJNA827465;Yang2025_Silkie_Wenchang	0.521211969	FALSE	FALSE	FALSE	FALSE	FALSE	other_meta_signature
ENSGALG00010012180	DDX59	3	-0.11536171	0.06717954	-1.717214954	0.085939904	0	0.162410785	0	1	CRA004775;PRJNA827465;Yang2025_Silkie_Wenchang	0.578157976	FALSE	FALSE	FALSE	FALSE	FALSE	other_meta_signature
ENSGALG00010021400	B4GALT2	3	-0.102356723	0.054322541	-1.884240338	0.059532474	0	0.820714308	0	1	CRA004775;PRJNA827465;Yang2025_Silkie_Wenchang	0.499130355	FALSE	FALSE	FALSE	FALSE	FALSE	other_meta_signature
ENSGALG00010008104	TCF25	3	-0.095497052	0.048474221	-1.970058528	0.048831663	0	0.021245699	0	1	CRA004775;PRJNA827465;Yang2025_Silkie_Wenchang	0.462015333	FALSE	FALSE	FALSE	FALSE	FALSE	other_meta_signature
ENSGALG00010024356	PARL	3	-0.105798553	0.057857423	-1.828608111	0.067458337	0	1.39870094	0	1	CRA004775;PRJNA827465;Yang2025_Silkie_Wenchang	0.520676908	FALSE	FALSE	FALSE	FALSE	FALSE	other_meta_signature
ENSGALG00010012113	WDR47	3	0.086685908	0.042017325	2.063099164	0.039103208	0	1.10677683	0	1	CRA004775;PRJNA827465;Yang2025_Silkie_Wenchang	0.418518087	FALSE	FALSE	FALSE	FALSE	FALSE	other_meta_signature
ENSGALG00010027330	ENSGALG00010027330	3	0.129790797	0.09323703	1.392052033	0.163906642	0	1.266662466	0	1	CRA004775;PRJNA827465;Yang2025_Silkie_Wenchang	0.692450961	FALSE	FALSE	FALSE	FALSE	FALSE	other_meta_signature
ENSGALG00010001835	MECP2	3	-0.121864574	0.077018136	-1.582284124	0.113584723	0	1.45470748	0	1	CRA004775;PRJNA827465;Yang2025_Silkie_Wenchang	0.634336062	FALSE	FALSE	FALSE	FALSE	FALSE	other_meta_signature
ENSGALG00010003113	WBP4	3	0.123510522	0.080099256	1.541968401	0.123081273	0	1.750334884	0	1	CRA004775;PRJNA827465;Yang2025_Silkie_Wenchang	0.648071434	FALSE	FALSE	FALSE	FALSE	FALSE	other_meta_signature
ENSGALG00010020132	SHC4	3	0.129555604	0.094179433	1.375625231	0.168937691	0	0.897446004	0	1	CRA004775;PRJNA827465;Yang2025_Silkie_Wenchang	0.696088052	FALSE	FALSE	FALSE	FALSE	FALSE	other_meta_signature
ENSGALG00010000870	DACH1	3	0.131452295	0.101898885	1.290026819	0.197041346	0	1.131960013	0	1	CRA004775;PRJNA827465;Yang2025_Silkie_Wenchang	0.720732808	FALSE	FALSE	FALSE	FALSE	FALSE	other_meta_signature
ENSGALG00010020166	HAPLN3	3	-0.133275894	0.116392049	-1.145060119	0.252184255	0	1.121210775	0	1	CRA004775;PRJNA827465;Yang2025_Silkie_Wenchang	0.743827862	FALSE	FALSE	FALSE	FALSE	FALSE	other_meta_signature
ENSGALG00010000262	HNRNPKL	3	-3.174337745	2.641798022	-1.2015823	0.229525402	17.20405033	13.74521603	85.44948296	0.666666667	CRA004775;PRJNA827465;Yang2025_Silkie_Wenchang	0.733660518	FALSE	FALSE	FALSE	FALSE	FALSE	other_meta_signature
ENSGALG00010019625	INTS1	3	-0.083732721	0.040718823	-2.056363983	0.039747445	0	0.856251595	0	1	CRA004775;PRJNA827465;Yang2025_Silkie_Wenchang	0.421862885	FALSE	FALSE	FALSE	FALSE	FALSE	other_meta_signature
ENSGALG00010006408	ENSGALG00010006408	2	0.147712744	0.306575796	0.481814763	0.629937541	0	0.105246938	0	1	PRJNA827465;Yang2025_Silkie_Wenchang	0.885800803	FALSE	FALSE	FALSE	FALSE	FALSE	other_meta_signature
ENSGALG00010016605	WDPCP	3	-0.124805093	0.087816291	-1.421206605	0.155256705	0	1.244461721	0	1	CRA004775;PRJNA827465;Yang2025_Silkie_Wenchang	0.682852683	FALSE	FALSE	FALSE	FALSE	FALSE	other_meta_signature
ENSGALG00010027668	RANBP3	3	-0.077877112	0.036909482	-2.10994866	0.034862778	0	1.661730165	0	1	CRA004775;PRJNA827465;Yang2025_Silkie_Wenchang	0.398484093	FALSE	FALSE	FALSE	FALSE	FALSE	other_meta_signature
ENSGALG00010000623	ENSGALG00010000623	3	-0.121362812	0.080479865	-1.507989756	0.131557149	0	0.009381239	0	1	CRA004775;PRJNA827465;Yang2025_Silkie_Wenchang	0.658603888	FALSE	FALSE	FALSE	FALSE	FALSE	other_meta_signature
ENSGALG00010012275	VIRMA	3	-0.080572661	0.038932571	-2.069543797	0.038495086	0	0.561658067	0	1	CRA004775;PRJNA827465;Yang2025_Silkie_Wenchang	0.415165537	FALSE	FALSE	FALSE	FALSE	FALSE	other_meta_signature
ENSGALG00010025904	RPL10A	3	-0.107095383	0.06220046	-1.721777987	0.085109744	0	1.243557923	0	1	CRA004775;PRJNA827465;Yang2025_Silkie_Wenchang	0.576049146	FALSE	FALSE	FALSE	FALSE	FALSE	other_meta_signature
ENSGALG00010012497	PLCXD3	2	0.143219978	0.269396806	0.531632056	0.594980858	0	0.071907803	0	1	PRJNA827465;Yang2025_Silkie_Wenchang	0.873293299	FALSE	FALSE	FALSE	FALSE	FALSE	other_meta_signature
ENSGALG00010022220	PPP1R14D	3	0.139349169	0.200643392	0.694511628	0.487361411	0	0.198460356	0	1	CRA004775;PRJNA827465;Yang2025_Silkie_Wenchang	0.835456916	FALSE	FALSE	FALSE	FALSE	FALSE	other_meta_signature
ENSGALG00010006557	COQ6	3	-0.118211389	0.077953985	-1.516425213	0.129411868	0	1.095874643	0	1	CRA004775;PRJNA827465;Yang2025_Silkie_Wenchang	0.656082898	FALSE	FALSE	FALSE	FALSE	FALSE	other_meta_signature
ENSGALG00010025353	SLC12A4	3	0.079194275	0.038636445	2.04972989	0.040390797	0	0.520370317	0	1	CRA004775;PRJNA827465;Yang2025_Silkie_Wenchang	0.422808261	FALSE	FALSE	FALSE	FALSE	FALSE	other_meta_signature
ENSGALG00010018317	ZMAT3	3	-0.124945122	0.097319221	-1.283868908	0.199187828	0	0.029056301	0	1	CRA004775;PRJNA827465;Yang2025_Silkie_Wenchang	0.721207861	FALSE	FALSE	FALSE	FALSE	FALSE	other_meta_signature
ENSGALG00010000174	ENSGALG00010000174	3	-0.13046546	0.130153722	-1.002395155	0.316152781	0	1.037937965	0	1	CRA004775;PRJNA827465;Yang2025_Silkie_Wenchang	0.769377654	FALSE	FALSE	FALSE	FALSE	FALSE	other_meta_signature
ENSGALG00010012751	AP3B1	3	0.122019155	0.091699272	1.330644753	0.18330593	0	0.163427231	0	1	CRA004775;PRJNA827465;Yang2025_Silkie_Wenchang	0.714968976	FALSE	FALSE	FALSE	FALSE	FALSE	other_meta_signature
ENSGALG00010003894	COPS4	3	-0.098085647	0.055108708	-1.779857481	0.075099288	0	0.887456925	0	1	CRA004775;PRJNA827465;Yang2025_Silkie_Wenchang	0.544527604	FALSE	FALSE	FALSE	FALSE	FALSE	other_meta_signature
ENSGALG00010026780	MYSM1	3	0.104217249	0.061177711	1.703516637	0.088471427	0	0.833437059	0	1	CRA004775;PRJNA827465;Yang2025_Silkie_Wenchang	0.585460093	FALSE	FALSE	FALSE	FALSE	FALSE	other_meta_signature
ENSGALG00010024850	CDK2AP1	3	-0.106821947	0.064348346	-1.660057383	0.096902909	0	0.3090308	0	1	CRA004775;PRJNA827465;Yang2025_Silkie_Wenchang	0.603786949	FALSE	FALSE	FALSE	FALSE	FALSE	other_meta_signature
ENSGALG00010012498	KCNT2	3	-0.130005977	0.143719134	-0.904583641	0.365686005	0	0.248149366	0	1	CRA004775;PRJNA827465;Yang2025_Silkie_Wenchang	0.788777182	FALSE	FALSE	FALSE	FALSE	FALSE	other_meta_signature
ENSGALG00010012988	RPL18A	3	-0.102250406	0.059225127	-1.726470012	0.08426289	0	1.923352958	0	1	CRA004775;PRJNA827465;Yang2025_Silkie_Wenchang	0.573817736	FALSE	FALSE	FALSE	FALSE	FALSE	other_meta_signature
ENSGALG00010015478	FBXO7	3	-0.080987356	0.040565511	-1.996458399	0.04588405	0	1.565281237	0	1	CRA004775;PRJNA827465;Yang2025_Silkie_Wenchang	0.449276024	FALSE	FALSE	FALSE	FALSE	FALSE	other_meta_signature

ENSGALG00010006478	ENSGALG00010006478	3	0.050773128	0.057920749	0.876596543	0.380705818	0	0.486359326	0	1	CRA004775;[illegible]	[illegible]	FALSE	other_meta_signature	0.098597745	0.25386564	20	10	5	0	35.35246338
ENSGALG00010012883	DCAF13	3	-0.044164608	0.038010955	-1.161891561	0.245279516	0	1.830760335	0	1	CRA004775;[illegible]	[illegible]	FALSE	other_meta_signature	0.131587425	0.220823039	20	10	5	0	35.35241046
ENSGALG00010017407	EPN2	3	0.042743434	0.034357418	1.244081684	0.213469501	0	1.032850046	0	1	CRA004775;[illegible]	[illegible]	FALSE	other_meta_signature	0.137631203	0.213717169	20	10	5	0	35.35134837
ENSGALG00010015085	PSMD2	3	-0.049956071	0.057204713	-0.873285942	0.382507241	0	1.595902876	0	1	CRA004775;[illegible]	[illegible]	FALSE	other_meta_signature	0.097806783	0.249780357	20	10	5	0	35.34758714
ENSGALG00010001300	MRPL49	3	0.138562549	0.116011352	1.194387845	0.232326289	0.002135062	2.100811144	4.798677118	1	CRA004775;[illegible]	[illegible]	FALSE	other_meta_signature	0.134372696	0.692812745	20	9.520132288	5	0	35.34731773
ENSGALG00010003737	SRP72	3	-0.043604491	0.038193494	-1.141673247	0.253589865	0	1.076443885	0	1	CRA004775;[illegible]	[illegible]	FALSE	other_meta_signature	0.128285937	0.218022453	20	10	5	0	35.34630839
ENSGALG00010019059	BRWD3	3	0.051597158	0.066294276	0.778304872	0.436389304	0	1.373925096	0	1	CRA004775;[illegible]	[illegible]	FALSE	other_meta_signature	0.087948017	0.257985789	20	10	5	0	35.34593381
ENSGALG00010015127	RPL11	3	-0.090731887	0.071140253	-1.27539449	0.202169648	0.001498221	2.051181506	2.495220733	1	CRA004775;[illegible]	[illegible]	FALSE	other_meta_signature	0.141208349	0.453659436	20	9.750477927	5	0	35.34534571
ENSGALG00010024457	CHD6	3	-0.046099018	0.04519381	-1.020029484	0.307714478	0	0.496996568	0	1	CRA004775;[illegible]	[illegible]	FALSE	other_meta_signature	0.114734975	0.230495092	20	10	5	0	35.34523007
ENSGALG00010018338	PDCD6IP	3	0.052091075	0.053674124	0.970506281	0.331794196	4.22591E-05	2.005121165	0.255404246	1	CRA004775;[illegible]	[illegible]	FALSE	other_meta_signature	0.109181365	0.260455374	20	9.974459575	5	0	35.34409631
ENSGALG00010006223	ATG10	3	0.05815293	0.121608208	0.478199059	0.632508532	0	0.105096674	0	1	CRA004775;[illegible]	[illegible]	FALSE	other_meta_signature	0.05206161	0.290764652	20	10	5	0	35.34282626
ENSGALG00010005356	DNAJC3	3	0.041602399	0.034534809	1.204651182	0.228337989	0	0.475706251	0	1	CRA004775;[illegible]	[illegible]	FALSE	other_meta_signature	0.134504852	0.208011994	20	10	5	0	35.34251685
ENSGALG00010014198	KDM3B	3	-0.040242893	0.031566401	-1.274864797	0.202357101	0	1.992700503	0	1	CRA004775;[illegible]	[illegible]	FALSE	other_meta_signature	0.141208349	0.201214465	20	10	5	0	35.34242281
ENSGALG00010009117	ERGIC1	3	-0.041566449	0.035278192	-1.178247709	0.238697868	0	1.063861343	0	1	CRA004775;[illegible]	[illegible]	FALSE	other_meta_signature	0.133639488	0.207832243	20	10	5	0	35.34147173
ENSGALG00010002834	MRPL53	3	-0.051953445	0.072076743	-0.720807343	0.471028057	0	1.172630655	0	1	CRA004775;[illegible]	[illegible]	FALSE	other_meta_signature	0.08146458	0.259767226	20	10	5	0	35.34123181
ENSGALG00010028111	TRUB2	3	-0.050336397	0.064034165	-0.786086571	0.431816771	0	1.282989842	0	1	CRA004775;[illegible]	[illegible]	FALSE	other_meta_signature	0.088290159	0.251681987	20	10	5	0	35.33997215
ENSGALG00010012366	INTS8	3	-0.046072742	0.047934582	-0.961158731	0.336472363	0	0.500378872	0	1	CRA004775;[illegible]	[illegible]	FALSE	other_meta_signature	0.10810411	0.230363711	20	10	5	0	35.33846782
ENSGALG00010020612	RPL27A	3	-0.050113229	0.066608017	-0.752360326	0.451834399	0	1.976122003	0	1	CRA004775;[illegible]	[illegible]	FALSE	other_meta_signature	0.08550811	0.250566145	20	10	5	0	35.33607426
ENSGALG00010024008	CCNE1	3	0.050540629	0.068932057	0.733194846	0.463439598	0	0.307007973	0	1	CRA004775;[illegible]	[illegible]	FALSE	other_meta_signature	0.082836053	0.252703144	20	10	5	0	35.3355392
ENSGALG00010007650	KLHL36	3	-0.048401285	0.05936421	-0.815327702	0.414884756	0	0.797674824	0	1	CRA004775;[illegible]	[illegible]	FALSE	other_meta_signature	0.091887095	0.242006426	20	10	5	0	35.33389352
ENSGALG00010009388	TMED7	3	-0.045202666	0.047212982	-0.95742027	0.338355171	0	0.207273627	0	1	CRA004775;[illegible]	[illegible]	FALSE	other_meta_signature	0.107283434	0.226013331	20	10	5	0	35.33329677
ENSGALG00010020890	RPS15A	3	-0.048675635	0.062100528	-0.783819983	0.433145747	0	1.126235539	0	1	CRA004775;[illegible]	[illegible]	FALSE	other_meta_signature	0.088290159	0.243378175	20	10	5	0	35.33166833
ENSGALG00010027392	RBBP5	3	-0.041714902	0.038734492	-1.076944598	0.281505017	0	1.250535114	0	1	CRA004775;[illegible]	[illegible]	FALSE	other_meta_signature	0.121532539	0.208574511	20	10	5	0	35.33010705
ENSGALG00010010986	NARS	3	-0.039071925	0.033010855	-1.183608383	0.23656812	0	0.812588458	0	1	CRA004775;[illegible]	[illegible]	FALSE	other_meta_signature	0.134139618	0.195359624	20	10	5	0	35.32949924
ENSGALG00010005384	MAPRE2	3	0.04719016	0.057105488	0.826368209	0.408595252	0	0.737081973	0	1	CRA004775;[illegible]	[illegible]	FALSE	other_meta_signature	0.093541793	0.235950798	20	10	5	0	35.32949259
ENSGALG00010005442	SASH1	3	-0.04678353	0.055199477	-0.847535758	0.396696565	0	0.63879853	0	1	CRA004775;[illegible]	[illegible]	FALSE	other_meta_signature	0.095325772	0.233917652	20	10	5	0	35.32924342
ENSGALG00010002985	ENSGALG00010002985	3	0.05296175	0.094555849	0.560110778	0.575403879	0	0.003820727	0	1	CRA004775;[illegible]	[illegible]	FALSE	other_meta_signature	0.063240538	0.26480875	20	10	5	0	35.32804929
ENSGALG00010023149	ADGRD1	3	-0.441806298	0.134505313	-3.28467544	0.001020999	0.017609061	2.918791494	31.47849019	1	CRA004775;[illegible]	[illegible]	FALSE	other_meta_signature	1.26543758	2.20903149	20	6.852150981	5	0	35.32662005
ENSGALG00010012867	A4GALT	3	-0.045983972	0.053306722	-0.862629904	0.388340983	0	0.157592521	0	1	CRA004775;[illegible]	[illegible]	FALSE	other_meta_signature	0.096240828	0.229919861	20	10	5	0	35.32616069
ENSGALG00010013352	ASXL1	3	-0.043444688	0.045844712	-0.947648851	0.343308248	0	1.831190472	0	1	CRA004775;[illegible]	[illegible]	FALSE	other_meta_signature	0.10618722	0.217223441	20	10	5	0	35.32341066
ENSGALG00010016904	EAF1	3	-0.040469611	0.038188239	-1.059740191	0.289262813	0	0.246126162	0	1	CRA004775;[illegible]	[illegible]	FALSE	other_meta_signature	0.119820073	0.202348057	20	10	5	0	35.32216813
ENSGALG00010000753	TRIO	3	0.046344837	0.057897401	0.800464903	0.42344149	0	1.488741703	0	1	CRA004775;[illegible]	[illegible]	FALSE	other_meta_signature	0.089508262	0.231724186	20	10	5	0	35.32123245
ENSGALG00010013880	ZMYM4	3	0.041624152	0.041673458	0.998816857	0.317883419	0	0.172935274	0	1	CRA004775;[illegible]	[illegible]	FALSE	other_meta_signature	0.112981985	0.20812076	20	10	5	0	35.32110274
ENSGALG00010028377	PDCL	3	-0.041206688	0.040715796	-1.012056555	0.311511013	0	1.661018474	0	1	CRA004775;[illegible]	[illegible]	FALSE	other_meta_signature	0.114734975	0.206033439	20	10	5	0	35.32076841
ENSGALG00010016576	MRPS26	3	-0.046945381	0.062829074	-0.747192002	0.454947671	0	1.199532357	0	1	CRA004775;[illegible]	[illegible]	FALSE	other_meta_signature	0.084727274	0.234726907	20	10	5	0	35.31945418
ENSGALG00010026977	ELK4	3	-0.049767618	0.079902493	-0.622854386	0.533380215	0	0.362548067	0	1	CRA004775;[illegible]	[illegible]	FALSE	other_meta_signature	0.070588658	0.24883809	20	10	5	0	35.31942675
ENSGALG00010006804	PSMA6	3	-0.045715045	0.056470814	-0.809534018	0.418208044	0	0.847237799	0	1	CRA004775;[illegible]	[illegible]	FALSE	other_meta_signature	0.09067311	0.228575223	20	10	5	0	35.31924833
ENSGALG00010008456	NHLRC1	3	-0.047985686	0.068373349	-0.701818564	0.48279232	0	0.557322735	0	1	CRA004775;[illegible]	[illegible]	FALSE	other_meta_signature	0.078945743	0.239928428	20	10	5	0	35.31887417
[illegible]																					

ENSGALG00010024940	RABL3	3	-0.147124152	0.087325281	-1.684783043	0.092030438	0.010699808	3.725057993	46.30956072	1	CRA004775;PRJNA827465;Yang2025_Silkie_Wenchang	0.591870389	FALSE	FALSE	FALSE	FALSE	FALSE	other_meta_signature	0.227773387	0.735620761	20	5.369043928	5	0	31.33243808
ENSGALG00010023991	TSPAN3	3	-0.20522932	0.086671123	-2.367908865	0.017888942	0.011846397	4.195681721	52.33194192	1	CRA004775;PRJNA827465;Yang2025_Silkie_Wenchang	0.290524057	FALSE	FALSE	FALSE	FALSE	FALSE	other_meta_signature	0.5368179	1.0261466	20	4.766805808	5	0	31.32977031
ENSGALG00010003861	NUDT15	3	-0.185803626	0.099875978	-1.860343505	0.062836942	0.014817764	3.917210019	48.94325322	1	CRA004775;PRJNA827465;Yang2025_Silkie_Wenchang	0.5118604	FALSE	FALSE	FALSE	FALSE	FALSE	other_meta_signature	0.290848468	0.929018131	20	5.105674678	5	0	31.32554128
ENSGALG00010019481	PYGL	3	-0.092467391	0.102050421	-0.906095151	0.364885492	0.013186086	3.481231954	42.54907382	1	CRA004775;PRJNA827465;Yang2025_Silkie_Wenchang	0.788777182	FALSE	FALSE	FALSE	FALSE	FALSE	other_meta_signature	0.103045662	0.462336957	20	5.745092618	5	0	31.31047524
ENSGALG00010011633	TADA2B	3	-0.312173466	0.147232123	-2.120280946	0.03398236	0.036374614	4.61185823	56.63353251	1	CRA004775;PRJNA827465;Yang2025_Silkie_Wenchang	0.392196231	FALSE	FALSE	FALSE	FALSE	FALSE	other_meta_signature	0.406496584	1.56086733	20	4.336646749	5	0	31.30401066
ENSGALG00010002424	CASK	3	0.108537928	0.07969653	1.361890267	0.173232518	0.008909355	3.571897494	44.00735174	1	CRA004775;PRJNA827465;Yang2025_Silkie_Wenchang	0.70060954	FALSE	FALSE	FALSE	FALSE	FALSE	other_meta_signature	0.154523954	0.542689642	20	5.599264826	5	0	31.29647842
ENSGALG00010026088	SNRPC	3	-0.207379819	0.131084087	-1.582036569	0.113641223	0.027083004	4.004299228	50.05368265	1	CRA004775;PRJNA827465;Yang2025_Silkie_Wenchang	0.634336062	FALSE	FALSE	FALSE	FALSE	FALSE	other_meta_signature	0.197680598	1.036899094	20	4.994631735	5	0	31.22921143
ENSGALG00010023670	EXTL2	3	-0.208343819	0.054440466	-3.82700286	0.000129713	0	1.292403231	0	0.666666667	CRA004775;PRJNA827465;Yang2025_Silkie_Wenchang	0.014107786	FALSE	FALSE	FALSE	FALSE	FALSE	other_meta_signature	1.850541127	1.041719093	13.33333333	10	5	0	31.22559355
ENSGALG00010002614	GPAT3	3	-0.162300278	0.136467705	-1.189294403	0.234323837	0.02629619	3.789266416	47.21933534	1	CRA004775;PRJNA827465;Yang2025_Silkie_Wenchang	0.734277773	FALSE	FALSE	FALSE	FALSE	FALSE	other_meta_signature	0.134139618	0.811501388	20	5.278066466	5	0	31.22370747
ENSGALG00010029528	TBL2	3	-0.18336663	0.137035995	-1.338090986	0.180866779	0.028774961	3.90014142	48.71980821	1	CRA004775;PRJNA827465;Yang2025_Silkie_Wenchang	0.712332977	FALSE	FALSE	FALSE	FALSE	FALSE	other_meta_signature	0.14731695	0.916833148	20	5.128019179	5	0	31.19216928
ENSGALG00010017495	NT5M	3	-0.218446993	0.101082607	-2.161074002	0.030689623	0.01578221	4.288111756	53.35942452	1	CRA004775;PRJNA827465;Yang2025_Silkie_Wenchang	0.374290312	FALSE	FALSE	FALSE	FALSE	FALSE	other_meta_signature	0.426791414	1.092234965	20	4.664057548	5	0	31.18308393
ENSGALG00010001668	UBE2E1	3	-0.249725844	0.098951801	-2.523711958	0.011612303	0.016361272	4.681284281	57.27668136	1	CRA004775;PRJNA827465;Yang2025_Silkie_Wenchang	0.232681791	FALSE	FALSE	FALSE	FALSE	FALSE	other_meta_signature	0.633237603	1.248629222	20	4.272331864	5	0	31.15419869
ENSGALG00010022627	CTCF	3	-0.057062034	0.05917096	-0.964358762	0.33486609	0.004632787	3.474777377	42.44235579	1	CRA004775;PRJNA827465;Yang2025_Silkie_Wenchang	0.778938719	FALSE	FALSE	FALSE	FALSE	FALSE	other_meta_signature	0.108496708	0.285310171	20	5.755764421	5	0	31.1495713
ENSGALG00010023208	BACE1	3	-0.107657453	0.058736491	-1.832888755	0.066819116	0.004610224	3.764576957	46.87318063	1	CRA004775;PRJNA827465;Yang2025_Silkie_Wenchang	0.520153828	FALSE	FALSE	FALSE	FALSE	FALSE	other_meta_signature	0.283868201	0.538287265	20	5.312681937	5	0	31.1348374
ENSGALG00010024898	NABP2	3	0.181831388	0.112623315	1.614509291	0.106417003	0.018953081	4.00035474	50.00443386	1	CRA004775;PRJNA827465;Yang2025_Silkie_Wenchang	0.620210226	FALSE	FALSE	FALSE	FALSE	FALSE	other_meta_signature	0.207461077	0.909156939	20	4.999556614	5	0	31.11617463
ENSGALG00010010368	ENSGALG00010010368	3	0.265563568	0.172642272	1.538230263	0.123992324	0.047528476	4.35126828	54.03638959	1	CRA004775;PRJNA827465;Yang2025_Silkie_Wenchang	0.648475836	FALSE	FALSE	FALSE	FALSE	FALSE	other_meta_signature	0.188106202	1.32781784	20	4.596361041	5	0	31.11228508
ENSGALG00010006071	TEX30	3	-0.278769832	0.227769695	-1.223910984	0.220985809	0.083946199	4.376673029	54.30318905	1	CRA004775;PRJNA827465;Yang2025_Silkie_Wenchang	0.732474544	FALSE	FALSE	FALSE	FALSE	FALSE	other_meta_signature	0.135207464	1.39384916	20	4.569681095	5	0	31.09873772
ENSGALG00010016303	ENSGALG00010016303	3	0.817397575	0.326640465	2.502438193	0.012334116	0.270643824	14.50016635	86.20705479	1	CRA004775;PRJNA827465;Yang2025_Silkie_Wenchang	0.238808216	FALSE	FALSE	FALSE	FALSE	FALSE	other_meta_signature	0.621950735	4.086987876	20	1.379294521	5	0	31.08823313
ENSGALG00010026163	CTNND1	3	-0.061983296	0.054586865	-1.135498372	0.256166564	0.003829856	3.545171041	43.5852325	1	CRA004775;PRJNA827465;Yang2025_Silkie_Wenchang	0.744535853	FALSE	FALSE	FALSE	FALSE	FALSE	other_meta_signature	0.128114384	0.30991648	20	5.64147675	5	0	31.07950761
ENSGALG00010021244	PPP3CB	3	0.083600776	0.067836575	1.232384974	0.217805311	0.006577974	3.636821534	45.00692483	1	CRA004775;PRJNA827465;Yang2025_Silkie_Wenchang	0.732474544	FALSE	FALSE	FALSE	FALSE	FALSE	other_meta_signature	0.135207464	0.418003879	20	5.499307517	5	0	31.05251886
ENSGALG00010024513	SPTY2D1	3	0.171375189	0.117530161	1.458137959	0.14480252	0.021107673	4.003969007	50.04956341	1	CRA004775;PRJNA827465;Yang2025_Silkie_Wenchang	0.676427194	FALSE	FALSE	FALSE	FALSE	FALSE	other_meta_signature	0.169778941	0.856875947	20	4.995043659	5	0	31.02169855
ENSGALG00010013285	ATP2B1	3	0.130430228	0.08885447	1.467908457	0.142129091	0.01116903	3.852038847	48.07944366	1	CRA004775;PRJNA827465;Yang2025_Silkie_Wenchang	0.67251868	FALSE	FALSE	FALSE	FALSE	FALSE	other_meta_signature	0.172295648	0.652151138	20	5.192055634	5	0	31.01650242
ENSGALG00010014186	MOCS3	3	-0.165270837	0.118358706	-1.396355557	0.162607451	0.021617184	3.995755481	49.94688715	1	CRA004775;PRJNA827465;Yang2025_Silkie_Wenchang	0.691876845	FALSE	FALSE	FALSE	FALSE	FALSE	other_meta_signature	0.159971204	0.826354184	20	5.005311285	5	0	30.99163667
ENSGALG00010013976	CDC40	3	-0.156243331	0.065241261	-2.394854557	0.016626959	0.006303954	4.298123992	53.4680711	1	CRA004775;PRJNA827465;Yang2025_Silkie_Wenchang	0.278540968	FALSE	FALSE	FALSE	FALSE	FALSE	other_meta_signature	0.55511092	0.781216654	20	4.65319289	5	0	30.98952046
ENSGALG00010008056	GTF2E2	3	-0.16568386	0.097666426	-1.696425945	0.089805244	0.014409629	4.06341075	50.78026508	1	CRA004775;PRJNA827465;Yang2025_Silkie_Wenchang	0.588940789	FALSE	FALSE	FALSE	FALSE	FALSE	other_meta_signature	0.229928366	0.828419298	20	4.921973492	5	0	30.98032116
ENSGALG00010007998	RPL13	3	-0.268471179	0.169451406	-1.584354982	0.11311296	0.049448274	4.50952664	55.64944705	1	CRA004775;PRJNA827465;Yang2025_Silkie_Wenchang	0.633889872	FALSE	FALSE	FALSE	FALSE	FALSE	other_meta_signature	0.197986187	1.342355894	20	4.435055295	5	0	30.97539738
ENSGALG00010024115	POLR2D	3	-0.175101312	0.136022622	-1.287295525	0.197991303	0.029849477	4.038534212	50.47708166	1	CRA004775;PRJNA827465;Yang2025_Silkie_Wenchang	0.720751552	FALSE	FALSE	FALSE	FALSE	FALSE	other_meta_signature	0.142214414	0.87550656	20	4.952291834	5	0	30.97001281
ENSGALG00010009695	ATAD2B	3	0.225192416	0.1501544	1.499739038	0.133682014	0.036499509	4.307229727	53.56644232	1	CRA004775;PRJNA827465;Yang2025_Silkie_Wenchang	0.662245476	FALSE	FALSE	FALSE	FALSE	FALSE	other_meta_signature	0.178981	1.125962079	20	4.643355768	5	0	30.94829885
ENSGALG00010018619	MAPK1IP1L	3	-0.154290748	0.065341229	-2.361307697	0.018210614	0.006997702	4.313829424	53.63748068	1	CRA004775;PRJNA827465;Yang2025_Silkie_Wenchang	0.291219217	FALSE	FALSE	FALSE	FALSE	FALSE	other_meta_signature	0.53577997	0.771453741	20	4.636251932	5	0	30.94348564
ENSGALG00010025470	ORAOV1	3	0.198262443	0.130071734	1.524254629	0.127445116	0.026269787	4.208830755	52.48086425	1	CRA004775;PRJNA827465;Yang2025_Silkie_Wenchang	0.652642701	FALSE	FALSE	FALSE	FALSE	FALSE	other_meta_signature	0.185324514	0.991312216	20	4.751913575	5	0	30.92855031
ENSGALG00010002464	NGLY1	3	0.114764356	0.096742571	1.186285986	0.235509379	0.013708276	3.834747249	47.84532408	1	CRA004775;PRJNA827465;Yang2025_Silkie_Wenchang	0.734277773	FALSE	FALSE	FALSE	FALSE	FALSE	other_meta_signature	0.134139618	0.57382178	20	5.215467592	5	0	30.92342899
ENSGALG00010020375	LRPPRC	3	-0.150625705	0.039520861	-3.811296192	0.00013824	0	1.724738888	0	0.666666667	CRA004775;PRJNA827465;Yang2025_Silkie_Wenchang	0.014586384	FALSE	FALSE	FALSE	FALSE	FALSE	other_meta_signature	1.836052359	0.753128526	13.33333333	10	5	0	30.92251422
ENSGALG00010014001	CDC16	3	-0.109923545	0.076717203	-1.432840877	0.151903317	0.008233301	3.843194176	47.95995445	1	CRA004775;PRJNA827465;Yang2025_Silkie_Wenchang	0.681329478	FALSE	FALSE	FALSE	FALSE	FALSE	other_meta_signature	0.166642821	0.549617724	20	5.204004555	5	0	30.9202651
ENSGALG00010023115	RPLP2	3	-0.174717628	0.158239388	-1.104134881	0.269534632	0.040622832	4.063897275	50.78615761	1	CRA004775;PRJNA827465;Yang2025_Silkie_Wenchang	0.749820979	FALSE	FALSE	FALSE	FALSE	FALSE	other_meta_signature	0.125042413	0.873588138	20	4.921384239	5	0	30.92001479
ENSGALG00010013114	PIGP	3	-0.30802801	0.174688255	-1.763301202	0.077849703	0.053780547	4.85423846	58.79889263	1	CRA004775;PRJNA827465;Yang2025_Silkie_Wenchang	0.554796848	FALSE	FALSE	FALSE	FALSE	FALSE	other_meta_signature	0.255866015	1.540140049	20	4.120110737	5	0	30.9161168
ENSGALG00010006598	ENSGALG00010006598	3	0.6616178	0.263473345	2.51113751	0.012034281	0.163925801	10.13540132	80.26718492	1	CRA004775;PRJNA827465;Yang2025_Silkie_Wenchang	0.235668556	FALSE	FALSE	FALSE	FALSE	FALSE	other_meta_signature	0.627698358	3.308089002	20	1.973281508	5	0	30.90906887
ENSGALG00010011759	CREB3	3	-0.131932075	0.115336236	-1.143890936	0.252668871	0.019256413	3.915248411	48.9176729	1	CRA004775;PRJNA827465;Yang2025_Silkie_Wenchang	0.744042702	FALSE	FALSE	FALSE	FALSE	FALSE	other_meta_signature	0.128402139	0.659660376	20	5.10823271	5	0	30.89629522
ENSGALG00010003639	ENSGALG00010003639	3	-0.589787023	0.483907089	-1.218802198	0.222919277	0.040613097	2.112932631	5.34482875	0.666666667	CRA004775;PRJNA827465;Yang2025_Silkie_Wenchang	0.732804055	FALSE	FALSE	FALSE	FALSE	FALSE	other_meta_signature	0.135012136	2.948935116	13.33333333	9.465517125	5	0	30.88279771
ENSGALG00010010771	ENSGALG00010010771	3	-0.216570233	0.127576814	-1.697567337	0.089589452	0.027514164	4.385518443	54.39535768	1	CRA004775;PRJNA827465;Yang2025_Silkie_Wenchang	0.588344292	FALSE	FALSE	FALSE	FALSE	FALSE	other_meta_signature	0.230368456	1.082851163	20	4.560464232	5	0	30.87368385
ENSGALG00010019302	USP34	3	0.11267312	0.105679476	1.066177884	0.286343238	0.017312789	3.853885355	48.10432029	1	CRA004775;PRJNA827465;Yang2025_Silkie_Wenchang	0.758891917	FALSE	FALSE	FALSE	FALSE	FALSE	other_meta_signature	0.119820073	0.563365602	20	5.189567971	5	0	30.87275365
ENSGALG00010021812	TEAD1	3	-0.192678557	0.053857955	-3.577531963	0.000346854	0	0.843570788	0	0.666666667	CRA004775;PRJNA827465;Yang2025_Silkie_Wenchang	0.026945968	FALSE	FALSE	FALSE	FALSE	FALSE	other_meta_signature	1.569506217	0.963392783	13.33333333	10	5	0	30.86623233
ENSGALG00010003245	ENSGALG00010003245	3	0.864916716	0.58128067	1.487950248	0.136764015	0.856504866	14.73803825	86.42967289	1	CRA004775;PRJNA827465;Yang2025_Silkie_Wenchang	0.664420216	FALSE	FALSE	FALSE	FALSE	FALSE	other_meta_signature	0.177557162	4.324583582	20	1.357032711	5	0	30.85917345
ENSGALG00010010958	LCORL	3	0.263147146	0.163477773	1.609681491	0.107467408	0.0441087	4.614986076	56.6629245	1	CRA004775;PRJNA827465;Yang2025_Silkie_Wenchang	0.623706433	FALSE	FALSE	FALSE	FALSE	FALSE	other_meta_signature	0.205019777	1.315735729	20	4.33370755	5	0	30.85446306
ENSGALG00010000486	ENSGALG00010000486	3	0.426424537	0.362319858	1.176928419	0.239224078	0.247916236	5.5734241	64.11541695	1	CRA004775;PRJNA827465;Yang2025_Silkie_Wenchang	0.735123848	FALSE	FALSE	FALSE	FALSE	FALSE	other_meta_signature	0.133639488	2.132122687	20	3.588458305	5	0	30.85422048
ENSGALG00010029482	DDX25	3	-0.380145865	0.182752649	-2.080111374	0.037515319	0.060734845	5.637529852	64.52346945	1	CRA004775;PRJNA827465;Yang2025_Silkie_Wenchang	0.410203552	FALSE	FALSE	FALSE	FALSE	FALSE	other_meta_signature	0.387000583	1.900729323	20	3.547653055	5	0	30.83538296
ENSGALG00010016400	ENSGALG00010016400	3	-0.166728564	0.11479644	-1.45238445	0.146394738	0.020203115	4.140801181	51.70016833	1	CRA004775;PRJNA827465;Yang2025_Silkie_Wenchang	0.677315181	FALSE	FALSE	FALSE	FALSE	FALSE	other_meta_signature	0.16920919	0.833642822	20	4.829983167	5	0	30.83283518
ENSGALG00010003680	TRAPPC5	3	-0.23378259	0.180857049	-1.292637422	0.19613649	0.056136292	4.430069042	54.85397674	1	CRA004775;PRJNA827465;Yang2025_Silkie_Wenchang	0.720732808	FALSE	FALSE	FALSE	FALSE	FALSE	other_meta_signature	0.142225708	1.16891295	20	4.514602326	5	0	30.82574098
ENSGALG00010025433	CHPF2	3	-0.073786322	0.089225202	-0.826967268	0.408255614	0.011268158	3.729110575	46.36790838	1	CRA004775;PRJNA827465;Yang2025_Silkie_Wenchang	0.8061908	FALSE	FALSE	FALSE	FALSE	FALSE	other_meta_signature	0.093562162	0.368931609	20	5.363209162	5	0	30.82570293
ENSGALG00010010125	AHCTF1	3	0.089873817	0.068974528	1.303000099	0.192574728	0.006585942	3.830585675	47.7886629	1	CRA004775;PRJNA827465;Yang2025_Silkie_Wenchang	0.720732808	FALSE	FALSE	FALSE	FALSE	FALSE	other_meta_signature	0.142225708	0.449369084	20	5.22113371	5	0	30.8127285
ENSGALG00010020596	SUMF1	3	-0.224384597	0.122731811	-1.828251334	0.06751184	0.025508378	4.55862191	56.12709192	1	CRA004775;PRJNA827465;Yang2025_Silkie_Wenchang	0.520676908	FALSE	FALSE	FALSE	FALSE	FALSE	other_meta_signature	0.283431683	1.121922987	20	4.387290808	5	0	30.79264548
ENSGALG00010003315	EPGN	2	0.402295318	0.432033244	0.931167504	0.351766924	0.239064456	2.722022056	63.2626048	1	PRJNA827465;Yang2025_Silkie_Wenchang	0.784982422	FALSE	FALSE	FALSE	FALSE	FALSE	other_meta_signature	0.105140068	2.011476589	20	3.67373952	5	0	30.79035618
ENSGALG00010014886	RABGAP1L	3	-0.169814869	0.092758272	-1.830724799	0.06714163	0.013314141	4.296548429	53.45100764	1	CRA004775;PRJNA827465;Yang2025_Silkie_Wenchang	0.520153828	FALSE	FALSE	FALSE	FALSE	FALSE	other_meta_signature	0.283868201	0.849074346	20	4.654899236	5	0	30.78784178
ENSGALG00010006177	NEK9	3	-0.193885105	0.123086634	-1.575192196	0.115212088	0.023937132	4.331575743	53.82742636	1	CRA004775;PRJNA827465;Yang2025_Silkie_Wenchang	0.637317572	FALSE	FALSE	FALSE	FALSE	FALSE	other_meta_signature	0.195644107	0.969425526	20	4.617257364	5	0	30.782327
ENSGALG00010024183	MTX2	3	-0.288752811	0.174434593	-1.655364377	0.097850608	0.054447044	4.856586614	58.81881332	1	CRA004775;PRJNA827465;Yang2025_Silkie_Wenchang	0.604390365	FALSE	FALSE	FALSE	FALSE	FALSE	other_meta_signature	0.218682468	1.443764057	20	4.118118668	5	0	30.78056519
ENSGALG00010011993	RELCH	3	-0.187273852	0.091030781	-2.057258539	0.039661363	0.012610453	4.503169899	55.58684116	1	CRA004775;PRJNA827465;Yang2025_Silkie_Wenchang	0.421633095	FALSE	FALSE	FALSE	FALSE	FALSE	other_meta_signature	0.375065308	0.93636926	20	4.441315884	5	0	30.75275045
ENSGALG00010000184	ENSGALG00010000184	3	0.475278685	1.590546286	0.298814747	0.765081399	0	0.395701113	0	0.666666667	CRA004775;PRJNA827465;Yang2025_Silkie_Wenchang	0.929758773	FALSE	FALSE	FALSE	FALSE	FALSE	other_meta_signature	0.031629715	2.376393427	13.33333333	10	5	0	30.74135647
ENSGALG00010010920	UBN2	3	0.141165353	0.130036494	1.085582579	0.277663711	0.0266852	4.072086108	50.88512504	1	CRA004775;PRJNA827465;Yang2025_Silkie_Wenchang	0.755279869	FALSE	FALSE	FALSE	FALSE	FALSE	other_meta_signature	0.121892091	0.705826764	20	4.911487496	5	0	30.73920635
ENSGALG00010000610	ENSGALG00010000610	3	-0.302100942	0.157694389	-1.915736784	0.055398608	0.045573282	5.115993231	60.90690684	1	CRA004775;PRJNA827465;Yang2025_Silkie_Wenchang	0.483625926	FALSE	FALSE	FALSE	FALSE	FALSE	other_meta_signature	0.315490426	1.510504709	20	3.909309316	5	0	30.73530445
ENSGALG00010025477	ENSGALG00010025477	3	0.104800564	0.086223196	1.215456732	0.224191942	0.011883656	3.943253179	49.28045678	1	CRA004775;PRJNA827465;Yang2025_Silkie_Wenchang	0.732804055	FALSE	FALSE	FALSE	FALSE	FALSE	other_meta_signature	0.135012136	0.524002819	20	5.071954322	5	0	30.73096928
ENSGALG00010004588	MINDY3	3	-0.149432822	0.105065865	-1.422277564	0.154945689	0.01659323	4.168809061	52.0246677	1	CRA004775;PRJNA827465;Yang2025_Silkie_Wenchang	0.682852683	FALSE	FALSE	FALSE	FALSE	FALSE	other_meta_signature	0.16567298	0.747164112	20	4.79753323	5	0	30.71037032
ENSGALG00010012216	BRD8	3	-0.08805295	0.066656276	-1.321000139	0.186501318	0.006660667	3.91859056	48.9612408	1	CRA004775;PRJNA827465;Yang2025_Silkie_Wenchang	0.716757878	FALSE	FALSE	FALSE	FALSE	FALSE	other_meta_signature	0.144627525	0.440264748	20	5.10387592	5	0	30.68876819
ENSGALG00010017535	LIMD1	3	-0.228403789	0.148039609	-1.542855935	0.122865734	0.037234964	4.59013326	56.42828026	1	CRA004775;PRJNA827465;Yang2025_Silkie_Wenchang	0.648071434	FALSE	FALSE	FALSE	FALSE	FALSE	other_meta_signature	0.188377121	1.142018946	20	4.357171974	5	0	30.68756804
ENSGALG00010016384	GNAS	3	0.077282111	0.082600689	0.935610972	0.349473523	0.010201685	3.861194753	48.20256093	1	CRA004775;PRJNA827465;Yang2025_Silkie_Wenchang	0.784982422	FALSE	FALSE	FALSE	FALSE	FALSE	other_meta_signature	0.105140068	0.386410557	20	5.179743907	5	0	30.67129453
ENSGALG00010007795	ZNF236	3	0.137879124	0.106022071	1.300475676	0.193437988	0.017730348	4.135022888	51.632674	1	CRA004775;PRJNA827465;Yang2025_Silkie_Wenchang	0.720732808	FALSE	FALSE	FALSE	FALSE	FALSE	other_meta_signature	0.142225708	0.689395622	20	4.8367326	5	0	30.66835393
ENSGALG00010012550	TAB1	3	-0.230826587	0.09855513	-2.342106261	0.019175255	0.016884142	5.017806982	60.14195032	1	CRA004775;PRJNA827465;Yang2025_Silkie_Wenchang	0.296953916	FALSE	FALSE	FALSE	FALSE	FALSE	other_meta_signature	0.527310943	1.154132934	20	3.985804968	5	0	30.66724885
ENSGALG00010007847	CMC2	3	-0.222430219	0.191189978	-1.163398942	0.244667679	0.060949936	4.526278294	55.81358746	1	CRA004775;PRJNA827465;Yang2025_Silkie_Wenchang	0.738539945	FALSE	FALSE	FALSE	FALSE	FALSE	other_meta_signature	0.13162601	1.112151093	20	4.418641254	5	0	30.66241836
ENSGALG00010020091	SUPT7L	3	-0.105978061	0.084316501	-1.256907717	0.208787054	0.010377473	4.018303883	50.22775633	1	CRA004775;PRJNA827465;Yang2025_Silkie_Wenchang	0.724136029	FALSE	FALSE	FALSE	FALSE	FALSE	other_meta_signature	0.140179844	0.529890307	20	4.977224367	5	0	30.64729452
ENSGALG00010013387	NDST1	3	-0.115598469	0.086897836	-1.330280181	0.183425975	0.01121722	4.062142222	50.76489472	1	CRA004775;PRJNA827465;Yang2025_Silkie_Wenchang	0.715067429	FALSE	FALSE	FALSE	FALSE	FALSE	other_meta_signature	0.145653003	0.577992343	20	4.923510528	5	0	30.64715587
ENSGALG00010021933	PRR14L	3	0.127585611	0.11157185	1.143528689	0.25281915	0.019628034	4.103523966	51.26140321	1	CRA004775;PRJNA827465;Yang2025_Silkie_Wenchang	0.744042702	FALSE	FALSE	FALSE	FALSE	FALSE	other_meta_signature	0.128402139	0.637928055	20	4.873859679	5	0	30.64018987
ENSGALG00010005695	KIF5B	3	0.153605542	0.096117929	1.59809459	0.110021927	0.014958283	4.282330805	53.29646188	1	CRA004775;PRJNA827465;Yang2025_Silkie_Wenchang	0.629288039	FALSE	FALSE	FALSE	FALSE	FALSE	other_meta_signature	0.201150523	0.768027711	20	4.670353812	5	0	30.63953205
ENSGALG00010024803	CLK1	3	0.253406473	0.149477143	1.695285769	0.090021223	0.039379056	4.837283766	58.65448262	1	CRA004775;PRJNA827465;Yang2025_Silkie_Wenchang	0.589283661	FALSE	FALSE	FALSE	FALSE	FALSE	other_meta_signature	0.2296756	1.267032366	20	4.134551738	5	0	30.6312597
ENSGALG00010000704	ENSGALG00010000704	3	-0.120338111	0.102506274	-1.173958493	0.240411652	0.016192262	4.088663284	51.08425759	1	CRA004775;PRJNA827465;Yang2025_Silkie_Wenchang	0.735123848	FALSE	FALSE	FALSE	FALSE	FALSE	other_meta_signature	0.133639488	0.601690553	20	4.891574241	5	0	30.62690428
ENSGALG00010011876	AMDHD1	3	0.418344699	0.263611898	1.586971991	0.112518988	0	1.809238427	0	0.666666667	CRA004775;PRJNA827465;Yang2025_Silkie_Wenchang	0.633574662	FALSE	FALSE	FALSE	FALSE	FALSE	other_meta_signature	0.198202199	2.091723497	13.33333333	10	5	0	30.62325903
ENSGALG00010013787	ZCCHC10	3	-0.148625351	0.107818674	-1.378475037	0.16805667	0.018223127	4.238267391	52.8109056	1	CRA004775;PRJNA827465;Yang2025_Silkie_Wenchang	0.695921937	FALSE	FALSE	FALSE	FALSE	FALSE	other_meta_signature	0.157439473	0.743126755	20	4.71890944	5	0	30.61947567
ENSGALG00010013347	ENSGALG00010013347	3	-0.431257007	0.394318061	-1.093678047	0.274096206	0	1.393078142	0	0.666666667	CRA004775;PRJNA827465;Yang2025_Silkie_Wenchang	0.754149077	FALSE	FALSE	FALSE	FALSE	FALSE	other_meta_signature	0.122542796	2.156285035	13.33333333	10	5	0	30.61216116
ENSGALG00010024212	RIMS4	3	-0.282046342	0.264519287	-1.066260026	0.286306114	0.118488593	4.899873646	59.18262093	1	CRA004775;PRJNA827465;Yang2025_Silkie_Wenchang	0.758891917	FALSE	FALSE	FALSE	FALSE	FALSE	other_meta_signature	0.119820073	1.410231708	20	4.081737907	5	0	30.61178969
ENSGALG00010029879	ENSGALG00010029879	3	0.49265686	0.364645246	1.351057954	0.176676869	0.266404592	6.708242968	70.18593379	1	CRA004775;PRJNA827465;Yang2025_Silkie_Wenchang	0.705546542	FALSE	FALSE	FALSE	FALSE	FALSE	other_meta_signature	0.151474333	2.4632843	20	2.981406621	5	0	30.59616525
ENSGALG00010000188	ENSGALG00010000188	3	-0.441800335	0.89706105	-0.492497512	0.62236768	0	0.829292857	0	0.666666667	CRA004775;PRJNA827465;Yang2025_Silkie_Wenchang	0.883816777	FALSE	FALSE	FALSE	FALSE	FALSE	other_meta_signature	0.053637759	2.209001677	13.33333333	10	5	0	30.59597277
ENSGALG00010014260	ADNP	3	0.107715666	0.091800936	1.173361308	0.240650946	0.01365848	4.0796724	50.97645585	1	CRA004775;PRJNA827465;Yang2025_Silkie_Wenchang	0.735123848	FALSE	FALSE	FALSE	FALSE	FALSE	other_meta_signature	0.133639488	0.538578332	20	4.902354415	5	0	30.57457223
ENSGALG00010004352	CHMP2A	3	-0.213027065	0.121827028	-1.748602656	0.080359727	0.025921138	4.701250951	57.45813144	1	CRA004775;PRJNA827465;Yang2025_Silkie_Wenchang	0.560812526	FALSE	FALSE	FALSE	FALSE	FALSE	other_meta_signature	0.251182295	1.065135323	20	4.254186856	5	0	30.57050447
ENSGALG00010014738	BRWD1	3	0.200901869	0.11925285	1.684671424	0.092051983	0.024275387	4.610607379	56.62176725	1	CRA004775;PRJNA827465;Yang2025_Silkie_Wenchang	0.591870389	FALSE	FALSE	FALSE	FALSE	FALSE	other_meta_signature	0.227773387	1.004509343	20	4.337823275	5	0	30.57010601
ENSGALG00010014994	ENSGALG00010014994	3	-0.257338653	0.116419896	-2.210435355	0.027074962	0.024860313	5.225879652	61.72893114	1	CRA004775;PRJNA827465;Yang2025_Silkie_Wenchang	0.350205925	FALSE	FALSE	FALSE	FALSE	FALSE	other_meta_signature	0.45567651	1.286693266	20	3.827106886	5	0	30.56947666
ENSGALG00010018298	ENSGALG00010018298	3	0.768063035	0.343929467	2.23319927	0.025535806	0.287875867	15.94509817	87.45696026	1	CRA004775;PRJNA827465;Yang2025_Silkie_Wenchang	0.343530702	FALSE	FALSE	FALSE	FALSE	FALSE	other_meta_signature	0.464034443	3.840315173	20	1.254303974	5	0	30.55865359
ENSGALG00010006623	ENSGALG00010006623	3	0.463364283	0.307344432	1.507638449	0.131647087	0.187832455	6.54486597	69.4416966	1	CRA004775;PRJNA827465;Yang2025_Silkie_Wenchang	0.658603888	FALSE	FALSE	FALSE	FALSE	FALSE	other_meta_signature	0.18137571	2.316821415	20	3.05583034	5	0	30.55402746
ENSGALG00010003676	POLR2B	3	-0.11842118	0.068832568	-1.720423695	0.085355453	0.007440182	4.237135857	52.79830368	1	CRA004775;PRJNA827465;Yang2025_Silkie_Wenchang	0.576484755	FALSE	FALSE	FALSE	FALSE	FALSE	other_meta_signature	0.239212173	0.592105901	20	4.720169632	5	0	30.55148771
ENSGALG00010014306	ENSGALG00010014306	3	0.387892797	0.213849597	1.813857979	0.069699594	0	1.511495902	0	0.666666667	CRA004775;PRJNA827465;Yang2025_Silkie_Wenchang	0.527560253	FALSE	FALSE	FALSE	FALSE	FALSE	other_meta_signature	0.277727932	1.939463987	13.33333333	10	5	0	30.55052525
ENSGALG00010009842	PHETA2	3	-0.253570701	0.11930333	-2.125428531	0.033550872	0.024754837	5.185770663	61.43292618	1	CRA004775;PRJNA827465;Yang2025_Silkie_Wenchang	0.389791105	FALSE	FALSE	FALSE	FALSE	FALSE	other_meta_signature	0.409168075	1.267853503	20	3.856707382	5	0	30.53372896
ENSGALG00010020212	JMJD7	3	-0.354972953	0.204958939	-1.731922283	0.083287401	0.081235815	5.694686237	64.87954004	1	CRA004775;PRJNA827465;Yang2025_Silkie_Wenchang	0.570542907	FALSE	FALSE	FALSE	FALSE	FALSE	other_meta_signature	0.24371169	1.774864766	20	3.512045996	5	0	30.53062245
ENSGALG00010019344	DNAJC24	3	0.190026183	0.058566695	3.244611703	0.001176109	0	1.916854003	0	0.666666667	CRA004775;PRJNA827465;Yang2025_Silkie_Wenchang	0.059727703	FALSE	FALSE	FALSE	FALSE	FALSE	other_meta_signature	1.223824189	0.950130914	13.33333333	10	5	0	30.50728844
ENSGALG00010001281	LRRN3	3	0.296111842	0.178062326	1.662967395	0.096318962	0.056742232	5.262366076	61.99428221	1	CRA004775;PRJNA827465;Yang2025_Silkie_Wenchang	0.603265909	FALSE	FALSE	FALSE	FALSE	FALSE	other_meta_signature	0.219491217	1.48055921	20	3.800571779	5	0	30.50062221
ENSGALG00010024694	GTF2H3	3	-0.174514107	0.1117482	-1.561672641	0.118365127	0.020836708	4.513473751	55.68823238	1	CRA004775;PRJNA827465;Yang2025_Silkie_Wenchang	0.641318343	FALSE	FALSE	FALSE	FALSE	FALSE	other_meta_signature	0.192926338	0.872570535	20	4.431176762	5	0	30.49667364
ENSGALG00010029294	PGS1	3	-0.168686929	0.091688223	-1.839788391	0.06579931	0.014257347	4.582467042	56.35538713	1	CRA004775;PRJNA827465;Yang2025_Silkie_Wenchang	0.519039076	FALSE	FALSE	FALSE	FALSE	FALSE	other_meta_signature	0.284799945	0.843434645	20	4.364461287	5	0	30.49269588
ENSGALG00010019008	HERPUD2	3	-0.133440171	0.069180326	-1.92887458	0.053746434	0.007835019	4.438402335	54.93874036	1	CRA004775;PRJNA827465;Yang2025_Silkie_Wenchang	0.480354509	FALSE	FALSE	FALSE	FALSE	FALSE	other_meta_signature	0.318438128	0.667200857	20	4.506125964	5	0	30.49176495
ENSGALG00010011746	SOD1	3	0.021347891	0.056206359	0.379812732	0.70408443	0	1.010840314	0	0.666666667	CRA004775;PRJNA827465;Yang2025_Silkie_Wenchang	0.908346522	FALSE	FALSE	FALSE	FALSE	TRUE	other_meta_signature	0.041748443	0.106739454	13.33333333	10	5	2	30.48182123
ENSGALG00010012135	CAMSAP2	3	0.191120792	0.148391406	1.287950544	0.197763179	0.037139144	4.566118531	56.19912215	1	CRA004775;PRJNA827465;Yang2025_Silkie_Wenchang	0.720732808	FALSE	FALSE	FALSE	FALSE	FALSE	other_meta_signature	0.142225708	0.95560396	20	4.380087785	5	0	30.47791745
ENSGALG00010003136	NUP98	3	-0.167938099	0.07598566	-2.210128839	0.027096222	0.009432717	4.782666426	58.18232296	1	CRA004775;PRJNA827465;Yang2025_Silkie_Wenchang	0.350205925	FALSE	FALSE	FALSE	FALSE	FALSE	other_meta_signature	0.45567651	0.839690493	20	4.181767704	5	0	30.47713471
ENSGALG00010017578	BSDC1	3	-0.162403251	0.115365179	-1.407731971	0.159210451	0.022573033	4.444019103	54.99569301	1	CRA004775;PRJNA827465;Yang2025_Silkie_Wenchang	0.68855483	FALSE	FALSE	FALSE	FALSE	FALSE	other_meta_signature	0.162061471	0.812016257	20	4.500430699	5	0	30.47450843
ENSGALG00010012704	SMIM15	3	-0.176641047	0.123444514	-1.430934774	0.15244891	0.024452872	4.519820376	55.75045392	1	CRA004775;PRJNA827465;Yang2025_Silkie_Wenchang	0.682072194	FALSE	FALSE	FALSE	FALSE	FALSE	other_meta_signature	0.166169655	0.883205237	20	4.424954608	5	0	30.4743295
ENSGALG00010022032	KBTBD4	3	-0.123323813	0.091388579	-1.349444474	0.177194244	0.013438297	4.273727537	53.20244488	1	CRA004775;PRJNA827465;Yang2025_Silkie_Wenchang	0.706465666	FALSE	FALSE	FALSE	FALSE	FALSE	other_meta_signature	0.15090894	0.616619066	20	4.679755512	5	0	30.44728352
ENSGALG00010018068	ADAM33	3	-0.159012486	0.109257382	-1.45539353	0.145560346	0.01785032	4.475737288	55.3146248	1	CRA004775;PRJNA827465;Yang2025_Silkie_Wenchang	0.677188568	FALSE	FALSE	FALSE	FALSE	FALSE	other_meta_signature	0.169290382	0.795062432	20	4.46853752	5	0	30.43289033
ENSGALG00010012964	CBR3	3	-0.221594384	0.13281327	-1.66846569	0.095223317	0.030972376	4.876865015	58.99004804	1	CRA004775;PRJNA827465;Yang2025_Silkie_Wenchang	0.599493285	FALSE	FALSE	FALSE	FALSE	FALSE	other_meta_signature	0.222215677	1.107971921	20	4.100995196	5	0	30.43118279
ENSGALG00010027374	NKTR	3	0.236510917	0.147362122	1.604964107	0.108501704	0.038033091	4.952031601	59.6125356	1	CRA004775;PRJNA827465;Yang2025_Silkie_Wenchang	0.625790073	FALSE	FALSE	FALSE	FALSE	FALSE	other_meta_signature	0.20357133	1.182554584	20	4.03874644	5	0	30.42487235
ENSGALG00010000283	ENSGALG00010000283	3	-0.386506533	0.278313497	-1.388745202	0.164910244	0	1.87244108	0	0.666666667	CRA004775;PRJNA827465;Yang2025_Silkie_Wenchang	0.693741724	FALSE	FALSE	FALSE	FALSE	FALSE	other_meta_signature	0.158802185	1.932532667	13.33333333	10	5	0	30.42466818
ENSGALG00010021029	PRDM2	3	0.232867372	0.140211094	1.660834145	0.096746761	0.035716695	4.950832942	59.60275729	1	CRA004775;PRJNA827465;Yang2025_Silkie_Wenchang	0.603786949	FALSE	FALSE	FALSE	FALSE	FALSE	other_meta_signature	0.219116278	1.164336862	20	4.039724271	5	0	30.42317741
ENSGALG00010005604	ENSGALG00010005604	3	0.390126641	0.316126082	1.234085585	0.217171014	0	1.856232046	0	0.666666667	CRA004775;PRJNA827465;Yang2025_Silkie_Wenchang	0.732474544	FALSE	FALSE	FALSE	FALSE	FALSE	other_meta_signature	0.135207464	1.950633204	13.33333333	10	5	0	30.419174
ENSGALG00010018007	DNAAF9	3	0.441140164	0.171345933	2.574558708	0.010036805	0.061247041	7.867985025	74.58053118	1	CRA004775;PRJNA827465;Yang2025_Silkie_Wenchang	0.218210914	FALSE	FALSE	FALSE	FALSE	FALSE	other_meta_signature	0.661123532	2.20570082	20	2.541946882	5	0	30.40877123
ENSGALG00010015072	MAP9	3	0.351857107	0.214562264	1.639883451	0.101029402	0.087008948	5.836875675	65.73509337	1	CRA004775;PRJNA827465;Yang2025_Silkie_Wenchang	0.611813162	FALSE	FALSE	FALSE	FALSE	FALSE	other_meta_signature	0.213381184	1.759285533	20	3.426490663	5	0	30.39915738
ENSGALG00010010457	AGGF1	3	0.24386007	0.149546585	1.630662918	0.102961465	0.040494707	5.062190294	60.49141016	1	CRA004775;PRJNA827465;Yang2025_Silkie_Wenchang	0.613680652	FALSE	FALSE	FALSE	FALSE	FALSE	other_meta_signature	0.212057569	1.219300351	20	3.950858984	5	0	30.3822169
ENSGALG00010018561	ACTR1A	3	-0.181677259	0.088665233	-2.049024785	0.040459691	0.014038803	4.88770426	59.08099399	1	CRA004775;PRJNA827465;Yang2025_Silkie_Wenchang	0.422808261	FALSE	FALSE	FALSE	FALSE	FALSE	other_meta_signature	0.373856536	0.908386297	20	4.091900601	5	0	30.37414343
ENSGALG00010002023	CLCN4	3	-0.374936377	0.270387831	-1.386661432	0.165545026	0.13835135	6.012356783	66.73517437	1	CRA004775;PRJNA827465;Yang2025_Silkie_Wenchang	0.693960232	FALSE	FALSE	FALSE	FALSE	FALSE	other_meta_signature	0.158665416	1.874681887	20	3.326482563	5	0	30.35982987
ENSGALG00010020590	INTS7	3	-0.152727256	0.1013009	-1.507659414	0.131641718	0.016228281	4.538293097	55.93056779	1	CRA004775;PRJNA827465;Yang2025_Silkie_Wenchang	0.658603888	FALSE	FALSE	FALSE	FALSE	FALSE	other_meta_signature	0.18137571	0.76363628	20	4.406943221	5	0	30.35195521
ENSGALG00010002925	INTS6	3	0.153195575	0.116955359	1.309863666	0.190241961	0.022703827	4.507319138	55.62772596	1	CRA004775;PRJNA827465;Yang2025_Silkie_Wenchang	0.719108624	FALSE	FALSE	FALSE	FALSE	FALSE	other_meta_signature	0.143205503	0.765977873	20	4.437227404	5	0	30.34641078
ENSGALG00010020303	EXD2	3	-0.099290334	0.079231586	-1.253166048	0.210145283	0.009781466	4.249214244	52.93247401	1	CRA004775;PRJNA827465;Yang2025_Silkie_Wenchang	0.725755779	FALSE	FALSE	FALSE	FALSE	FALSE	other_meta_signature	0.139209497	0.496451668	20	4.706752599	5	0	30.34241376
ENSGALG00010029432	ENSGALG00010029432	3	-0.393406353	1.362616299	-0.288713964	0.772800276	0	0.075171301	0	0.666666667	CRA004775;PRJNA827465;Yang2025_Silkie_Wenchang	0.931768237	FALSE	FALSE	FALSE	FALSE	FALSE	other_meta_signature	0.030692098	1.967031764	13.33333333	10	5	0	30.3310572
ENSGALG00010003495	ENSGALG00010003495	3	-0.330346201	0.167167683	-1.976136744	0.048139292	0	1.082319216	0	0.666666667	CRA004775;PRJNA827465;Yang2025_Silkie_Wenchang	0.458344409	FALSE	FALSE	FALSE	FALSE	FALSE	other_meta_signature	0.338808062	1.651731003	13.33333333	10	5	0	30.3238724
ENSGALG00010020313	FRA10AC1	3	0.159510548	0.128705905	1.239341336	0.215219117	0.02761745	4.57022408	56.2384696	1	CRA004775;PRJNA827465;Yang2025_Silkie_Wenchang	0.730959185	FALSE	FALSE	FALSE	FALSE	FALSE	other_meta_signature	0.136106872	0.79755274	20	4.37615304	5	0	30.30981265
ENSGALG00010003678	ZNFY1	3	0.343766867	0.21183255	1.622823629	0.104627113	0	1.359787306	0	0.666666667	CRA004775;PRJNA827465;Yang2025_Silkie_Wenchang	0.61638448	FALSE	FALSE	FALSE	FALSE	FALSE	other_meta_signature	0.210148305	1.718834335	13.33333333	10	5	0	30.26231597
ENSGALG00010021459	ARHGEF16	3	0.255756195	0.12037784	2.124611925	0.033619008	0.026615632	5.600937751	64.29169384	1	CRA004775;PRJNA827465;Yang2025_Silkie_Wenchang	0.389941882	FALSE	FALSE	FALSE	FALSE	FALSE	other_meta_signature	0.409000117	1.278780976	20	3.570830616	5	0	30.25861171
ENSGALG00010011637	CECR2	3	0.192235138	0.064734491	2.969593735	0.002981938	0	1.49893279	0	0.666666667	CRA004775;PRJNA827465;Yang2025_Silkie_Wenchang	0.110082571	FALSE	FALSE	FALSE	FALSE	FALSE	other_meta_signature	0.958281437	0.96117569	13.33333333	10	5	0	30.25279046
ENSGALG00010004542	ERMARD	3	-0.285333611	0.168847517	-1.689889299	0.091049135	0.054246041	5.560934741	64.0348234	1	CRA004775;PRJNA827465;Yang2025_Silkie_Wenchang	0.591870389	FALSE	FALSE	FALSE	FALSE	FALSE	other_meta_signature	0.227773387	1.426668056	20	3.59651766	5	0	30.2509591
ENSGALG00010029148	RALGDS	3	-0.18462627	0.111354296	-1.65800761	0.09731593	0.019588903	4.867827722	58.91391162	1	CRA004775;PRJNA827465;Yang2025_Silkie_Wenchang	0.604324105	FALSE	FALSE	FALSE	FALSE	FALSE	other_meta_signature	0.218730082	0.923131352	20	4.108608838	5	0	30.25047027
ENSGALG00010014077	SLITRK2	3	-0.200658178	0.068874748	-2.91337801	0.003575415	0	1.379901702	0	0.666666667	CRA004775;PRJNA827465;Yang2025_Silkie_Wenchang	0.122403858	FALSE	FALSE	FALSE	FALSE	FALSE	other_meta_signature	0.912204895	1.003290888	13.33333333	10	5	0	30.24882912
ENSGALG00010016016	ZDHHC2	3	-0.428110057	0.215886868	-1.983029637	0.047364119	0.092058692	7.250915218	72.41727506	1	CRA004775;PRJNA827465;Yang2025_Silkie_Wenchang	0.455593089	FALSE	FALSE	FALSE	FALSE	FALSE	other_meta_signature	0.341422872	2.140550286	20	2.758272494	5	0	30.24024565
ENSGALG00010020481	RPP30	3	-0.317371819	0.176902703	-1.794047314	0.072805617	0.06154028	5.920193917	66.21732281	1	CRA004775;PRJNA827465;Yang2025_Silkie_Wenchang	0.537304817	FALSE	FALSE	FALSE	FALSE	FALSE	other_meta_signature	0.269779266	1.586859095	20	3.378267719	5	0	30.23490608
ENSGALG00010001921	ENSGALG00010001921	3	0.365247301	0.560897141	0.651184102	0.514927652	0	1.512583581	0	0.666666667	CRA004775;PRJNA827465;Yang2025_Silkie_Wenchang	0.845309013	FALSE	FALSE	FALSE	FALSE	FALSE	other_meta_signature	0.0729845	1.826236505	13.33333333	10	5	0	30.23255434
ENSGALG00010022200	DNMBP	3	-0.166754864	0.077441213	-2.153309049	0.031294398	0.010320008	5.048510073	60.3843516	1	CRA004775;PRJNA827465;Yang2025_Silkie_Wenchang	0.376928949	FALSE	FALSE	FALSE	FALSE	FALSE	other_meta_signature	0.423740507	0.833774321	20	3.96156484	5	0	30.21907967
ENSGALG00010028010	ASB6	3	-0.127396264	0.120314702	-1.058858655	0.289664156	0.024732183	4.482498757	55.3820289	1	CRA004775;PRJNA827465;Yang2025_Silkie_Wenchang	0.758891917	FALSE	FALSE	FALSE	FALSE	FALSE	other_meta_signature	0.119820073	0.636981318	20	4.46179711	5	0	30.2185985
ENSGALG00010000434	TP53	3	-0.271228005	0.077391982	-3.504600854	0.000457292	0.002566407	2.216837143	9.781374496	0.666666667	CRA004775;PRJNA827465;Yang2025_Silkie_Wenchang	0.031762888	FALSE	FALSE	FALSE	FALSE	FALSE	other_meta_signature	1.498080011	1.356140024	13.33333333	9.02186255	5	0	30.20941592
ENSGALG00010019189	ICMT	3	-0.230322697	0.144725215	-1.591448295	0.111508723	0.038967828	5.188361847	61.45218743	1	CRA004775;PRJNA827465;Yang2025_Silkie_Wenchang	0.631406421	FALSE	FALSE	FALSE	FALSE	FALSE	other_meta_signature	0.199691006	1.151613487	20	3.854781257	5	0	30.20608575
ENSGALG00010028438	MED27	3	-0.298402165	0.126746587	-2.354321105	0.018556573	0.031999257	6.320868735	68.35877972	1	CRA004775;PRJNA827465;Yang2025_Silkie_Wenchang	0.293808949	FALSE	FALSE	FALSE	FALSE	FALSE	other_meta_signature	0.531934981	1.492010826	20	3.164122028	5	0	30.18806783
ENSGALG00010008196	PDCD6	3	-0.157324944	0.100048955	-1.572479632	0.115839359	0.017374225	4.758219689	57.96747248	1	CRA004775;PRJNA827465;Yang2025_Silkie_Wenchang	0.638973582	FALSE	FALSE	FALSE	FALSE	FALSE	other_meta_signature	0.194517097	0.786624719	20	4.203252752	5	0	30.18439457

ENSGALG00010020141	THOC7	3	-0.112552187	0.064547891	-1.743700454	0.081211336	0	0.521468926	0	0.666666667	CRA004775;PRJNA827465;Yang2025_Silkie_Wenchan	0.563418588	FALSE	FALSE	FALSE	FALSE	FALSE	other_meta_signature	0.249168829	0.562760937	13.33333333	10	5	0	29.1452631
ENSGALG00010008154	KCNQ3	3	-0.139068975	0.138054293	-1.007349873	0.313766671	0	0.222750513	0	0.666666667	CRA004775;PRJNA827465;Yang2025_Silkie_Wenchan	0.767829909	FALSE	FALSE	FALSE	FALSE	FALSE	other_meta_signature	0.114734975	0.695344873	13.33333333	10	5	0	29.14341318
ENSGALG00010028145	ENSGALG00010028145	3	-0.135031941	0.119353044	-1.131365711	0.257901197	0	1.042238875	0	0.666666667	CRA004775;PRJNA827465;Yang2025_Silkie_Wenchan	0.744535853	FALSE	FALSE	FALSE	FALSE	FALSE	other_meta_signature	0.128114384	0.675159705	13.33333333	10	5	0	29.13660742
ENSGALG00010011819	USP44	3	0.267008699	0.169947469	1.571124894	0.116153641	0.062730155	7.682167941	73.96568241	1	CRA004775;PRJNA827465;Yang2025_Silkie_Wenchan	0.638973582	FALSE	FALSE	FALSE	FALSE	FALSE	other_meta_signature	0.194517097	1.335043495	20	2.603431759	5	0	29.13299235
ENSGALG00010028535	TICAM1	3	-0.100808	0.045526691	-2.214261528	0.026810793	0.0002104	2.03266236	1.60687582	0.666666667	CRA004775;PRJNA827465;Yang2025_Silkie_Wenchan	0.350025678	FALSE	FALSE	FALSE	FALSE	FALSE	other_meta_signature	0.455900095	0.504040001	13.33333333	9.839312418	5	0	29.13258585
ENSGALG00010004177	CSPP1	3	0.437136589	0.224639993	1.945942853	0.051661605	0.124759968	12.34165503	83.79471801	1	CRA004775;PRJNA827465;Yang2025_Silkie_Wenchan	0.47224413	FALSE	FALSE	FALSE	FALSE	FALSE	other_meta_signature	0.325833432	2.185682943	20	1.620528199	5	0	29.13204457
ENSGALG00010014676	UBE2V1	3	-0.07403046	0.034206536	-2.164219702	0.030447492	0	1.510376173	0	0.666666667	CRA004775;PRJNA827465;Yang2025_Silkie_Wenchan	0.373371282	FALSE	FALSE	FALSE	FALSE	FALSE	other_meta_signature	0.427859089	0.370152299	13.33333333	10	5	0	29.13134472
ENSGALG00010022664	TRPC4AP	3	-0.081852007	0.039576951	-2.068173652	0.038623697	0	1.15386641	0	0.666666667	CRA004775;PRJNA827465;Yang2025_Silkie_Wenchan	0.415601561	FALSE	FALSE	FALSE	FALSE	FALSE	other_meta_signature	0.38132283	0.409260033	13.33333333	10	5	0	29.1239162
ENSGALG00010028686	TAGLN2	3	0.117774697	0.073662667	1.598838342	0.109856527	0	0.812821026	0	0.666666667	CRA004775;PRJNA827465;Yang2025_Silkie_Wenchan	0.629105483	FALSE	FALSE	FALSE	FALSE	FALSE	other_meta_signature	0.20127653	0.588873485	13.33333333	10	5	0	29.12348335
ENSGALG00010022209	ZFYVE19	3	-0.100557384	0.054391962	-1.84875451	0.064493269	0	0.667643088	0	0.666666667	CRA004775;PRJNA827465;Yang2025_Silkie_Wenchan	0.516231528	FALSE	FALSE	FALSE	FALSE	FALSE	other_meta_signature	0.287155475	0.502786921	13.33333333	10	5	0	29.12327573
ENSGALG00010019977	ZMIZ1	3	-0.113899526	0.06863831	-1.659416241	0.097031946	0	1.09561142	0	0.666666667	CRA004775;PRJNA827465;Yang2025_Silkie_Wenchan	0.603786949	FALSE	FALSE	FALSE	FALSE	FALSE	other_meta_signature	0.219116278	0.56949763	13.33333333	10	5	0	29.12194724
ENSGALG00010000150	B3GAT3	3	0.128323581	0.101259317	1.26727678	0.20505634	0	0.397009175	0	0.666666667	CRA004775;PRJNA827465;Yang2025_Silkie_Wenchan	0.72311575	FALSE	FALSE	FALSE	FALSE	FALSE	other_meta_signature	0.140792179	0.641617903	13.33333333	10	5	0	29.11574342
ENSGALG00010001542	MTO1	3	-0.087785168	0.044213572	-1.985480116	0.04709108	0	0.671717071	0	0.666666667	CRA004775;PRJNA827465;Yang2025_Silkie_Wenchan	0.455106482	FALSE	FALSE	FALSE	FALSE	FALSE	other_meta_signature	0.341886979	0.438925838	13.33333333	10	5	0	29.11414615
ENSGALG00010017544	SDE2	3	-0.075243149	0.035674143	-2.109178866	0.034929145	0	1.53928701	0	0.666666667	CRA004775;PRJNA827465;Yang2025_Silkie_Wenchan	0.398598214	FALSE	FALSE	FALSE	FALSE	FALSE	other_meta_signature	0.399464651	0.376215746	13.33333333	10	5	0	29.10901373
ENSGALG00010023518	METTL5	3	-0.091731849	0.047822819	-1.918160629	0.055090647	0	0.540637509	0	0.666666667	CRA004775;PRJNA827465;Yang2025_Silkie_Wenchan	0.482705868	FALSE	FALSE	FALSE	FALSE	FALSE	other_meta_signature	0.316317422	0.458659243	13.33333333	10	5	0	29.10831
ENSGALG00010009093	SLU7	3	0.190350381	0.134801442	1.412079715	0.157926491	0.036264391	6.687692049	70.09431676	1	CRA004775;PRJNA827465;Yang2025_Silkie_Wenchan	0.687476186	FALSE	FALSE	FALSE	FALSE	FALSE	other_meta_signature	0.162742341	0.951751907	20	2.990568324	5	0	29.10506257
ENSGALG00010007217	MSH3	3	0.127421565	0.106488077	1.196580581	0.231470073	0	0.738684364	0	0.666666667	CRA004775;PRJNA827465;Yang2025_Silkie_Wenchan	0.733660518	FALSE	FALSE	FALSE	FALSE	FALSE	other_meta_signature	0.134504852	0.637107823	13.33333333	10	5	0	29.10494601
ENSGALG00010021862	PDK1	3	-0.156497542	0.111427087	-1.40448383	0.160174823	0.022578772	6.331149932	68.41016211	1	CRA004775;PRJNA827465;Yang2025_Silkie_Wenchan	0.689218184	FALSE	FALSE	FALSE	FALSE	FALSE	other_meta_signature	0.161643273	0.782487709	20	3.158983789	5	0	29.10311477
ENSGALG00010024762	FAN1	3	0.108451622	0.064633341	1.67795167	0.093356534	0	1.014836264	0	0.666666667	CRA004775;PRJNA827465;Yang2025_Silkie_Wenchan	0.595384772	FALSE	FALSE	FALSE	FALSE	FALSE	other_meta_signature	0.225202278	0.542258109	13.33333333	10	5	0	29.10079372
ENSGALG00010005392	ENSGALG00010005392	3	-0.119404472	0.082580138	-1.445922411	0.148198965	0	1.339204663	0	0.666666667	CRA004775;PRJNA827465;Yang2025_Silkie_Wenchan	0.679217235	FALSE	FALSE	FALSE	FALSE	FALSE	other_meta_signature	0.167991303	0.597022362	13.33333333	10	5	0	29.098347
ENSGALG00010014066	BCL2L1	3	0.108306957	0.064688056	1.674296053	0.09407243	0	0.963795057	0	0.666666667	CRA004775;PRJNA827465;Yang2025_Silkie_Wenchan	0.598266301	FALSE	FALSE	FALSE	FALSE	FALSE	other_meta_signature	0.22310546	0.541534784	13.33333333	10	5	0	29.09797358
ENSGALG00010027348	ENSGALG00010027348	3	0.12345983	0.092239273	1.338473577	0.180742108	0	0.464137047	0	0.666666667	CRA004775;PRJNA827465;Yang2025_Silkie_Wenchan	0.712332977	FALSE	FALSE	FALSE	FALSE	FALSE	other_meta_signature	0.14731695	0.61729915	13.33333333	10	5	0	29.09794943
ENSGALG00010012074	KIAA0930	3	-0.164888836	0.11332137	-1.455055088	0.145654011	0.023403863	6.450132477	68.99288492	1	CRA004775;PRJNA827465;Yang2025_Silkie_Wenchan	0.677212123	FALSE	FALSE	FALSE	FALSE	FALSE	other_meta_signature	0.169275276	0.824444178	20	3.100711508	5	0	29.09443096
ENSGALG00010002581	ATP13A1	3	-0.15223275	0.116253748	-1.309486815	0.190369501	0.026768805	6.277190212	68.13861087	1	CRA004775;PRJNA827465;Yang2025_Silkie_Wenchan	0.719108624	FALSE	FALSE	FALSE	FALSE	FALSE	other_meta_signature	0.143205503	0.761163748	20	3.186138913	5	0	29.09050816

ENSGALG00010029668	CRCP	3	0.043990179	0.07778286	0.565551058	0.571699006	0	1.349322842	0	0.666666667	CRA004775;PRJNA827465;Yang2025_Silkie_Wenchang	0.863134826	FALSE	FALSE	FALSE	FALSE	FALSE	FALSE	FALSE	other_meta_signature	0.06392136	0.219950893	13.33333333	10	5	0	28.61720559
ENSGALG00010008129	TANGO6	3	0.040194442	0.054808755	0.733358055	0.463340075	0	0.947438405	0	0.666666667	CRA004775;PRJNA827465;Yang2025_Silkie_Wenchang	0.82634984	FALSE	FALSE	FALSE	FALSE	FALSE	FALSE	FALSE	other_meta_signature	0.082836053	0.20097221	13.33333333	10	5	0	28.6171416
ENSGALG00010013244	TRIM24	3	-0.040425213	0.056270471	-0.718408998	0.47250514	0	0.758231718	0	0.666666667	CRA004775;PRJNA827465;Yang2025_Silkie_Wenchang	0.829184957	FALSE	FALSE	FALSE	FALSE	FALSE	FALSE	FALSE	other_meta_signature	0.081348586	0.202126064	13.33333333	10	5	0	28.61680798
ENSGALG00010006061	RPL24	3	-0.068597452	0.059187132	-1.158992674	0.246459171	0.000502812	2.039021639	1.913743262	0.666666667	CRA004775;PRJNA827465;Yang2025_Silkie_Wenchang	0.740005569	FALSE	FALSE	FALSE	FALSE	FALSE	FALSE	FALSE	other_meta_signature	0.130765012	0.34298726	13.33333333	9.808625674	5	0	28.61571128
ENSGALG00010015388	PSMD1	3	-0.041622683	0.062434764	-0.666658771	0.50499012	0	0.999030082	0	0.666666667	CRA004775;PRJNA827465;Yang2025_Silkie_Wenchang	0.842995974	FALSE	FALSE	FALSE	FALSE	FALSE	FALSE	FALSE	other_meta_signature	0.0741745	0.208113416	13.33333333	10	5	0	28.61562125
ENSGALG00010021635	FOXJ2	3	-0.037490239	0.044834727	-0.836187519	0.403049419	0	0.598267082	0	0.666666667	CRA004775;PRJNA827465;Yang2025_Silkie_Wenchang	0.805038062	FALSE	FALSE	FALSE	FALSE	FALSE	FALSE	FALSE	other_meta_signature	0.094183586	0.187451196	13.33333333	10	5	0	28.61496812
ENSGALG00010029607	ZNF207	3	0.037655882	0.046192399	0.815196502	0.41495984	0	1.364104818	0	0.666666667	CRA004775;PRJNA827465;Yang2025_Silkie_Wenchang	0.809306269	FALSE	FALSE	FALSE	FALSE	FALSE	FALSE	FALSE	other_meta_signature	0.091887095	0.18827941	13.33333333	10	5	0	28.61349984
ENSGALG00010027985	PSMB4	3	-0.037555285	0.045814765	-0.819720123	0.412375676	0	0.779657121	0	0.666666667	CRA004775;PRJNA827465;Yang2025_Silkie_Wenchang	0.808459291	FALSE	FALSE	FALSE	FALSE	FALSE	FALSE	FALSE	other_meta_signature	0.092341844	0.187776423	13.33333333	10	5	0	28.6134516
ENSGALG00010005456	RPL7L1	3	0.038080053	0.048316451	0.78813845	0.430615727	0	0.666942316	0	0.666666667	CRA004775;PRJNA827465;Yang2025_Silkie_Wenchang	0.81572785	FALSE	FALSE	FALSE	FALSE	FALSE	FALSE	FALSE	other_meta_signature	0.08845471	0.190400263	13.33333333	10	5	0	28.61218831
ENSGALG00010020282	MAGI1	3	0.340701351	0.254192112	1.340330144	0.180138035	0.148928825	11.36060769	82.39530794	1	CRA004775;PRJNA827465;Yang2025_Silkie_Wenchang	0.711730573	FALSE	FALSE	FALSE	FALSE	FALSE	FALSE	FALSE	other_meta_signature	0.147684378	1.703506753	20	1.760469206	5	0	28.61166034
ENSGALG00010008035	TDP2	3	-0.043573037	0.081295297	-0.535984715	0.591969114	0	0.60613246	0	0.666666667	CRA004775;PRJNA827465;Yang2025_Silkie_Wenchang	0.871144272	FALSE	FALSE	FALSE	FALSE	FALSE	FALSE	FALSE	other_meta_signature	0.059909914	0.217865183	13.33333333	10	5	0	28.61110843
ENSGALG00010017500	ACAP2	3	0.039162276	0.054045528	0.724616399	0.468687396	0	0.077816124	0	0.666666667	CRA004775;PRJNA827465;Yang2025_Silkie_Wenchang	0.82826404	FALSE	FALSE	FALSE	FALSE	FALSE	FALSE	FALSE	other_meta_signature	0.081831194	0.19581138	13.33333333	10	5	0	28.61097591
ENSGALG00010003101	KLHL15	3	0.037778939	0.047776487	0.790743333	0.429093783	0	1.366095214	0	0.666666667	CRA004775;PRJNA827465;Yang2025_Silkie_Wenchang	0.815508786	FALSE	FALSE	FALSE	FALSE	FALSE	FALSE	FALSE	other_meta_signature	0.088571356	0.188894693	13.33333333	10	5	0	28.61079938
ENSGALG00010004147	SPRYD7	3	0.038915293	0.053251355	0.730785042	0.464910461	0	1.922788241	0	0.666666667	CRA004775;PRJNA827465;Yang2025_Silkie_Wenchang	0.826968056	FALSE	FALSE	FALSE	FALSE	FALSE	FALSE	FALSE	other_meta_signature	0.082511266	0.194576467	13.33333333	10	5	0	28.61042107
ENSGALG00010012190	CAST	3	-0.038994255	0.05388979	-0.723592631	0.469315866	0	0.279764661	0	0.666666667	CRA004775;PRJNA827465;Yang2025_Silkie_Wenchang	0.82826404	FALSE	FALSE	FALSE	FALSE	FALSE	FALSE	FALSE	other_meta_signature	0.081831194	0.194971273	13.33333333	10	5	0	28.6101358
ENSGALG00010024352	MAP6D1	3	0.05120441	0.263011809	0.194684832	0.845639687	0	0.191676814	0	0.666666667	CRA004775;PRJNA827465;Yang2025_Silkie_Wenchang	0.954077524	FALSE	FALSE	FALSE	FALSE	FALSE	FALSE	FALSE	other_meta_signature	0.020416335	0.256022049	13.33333333	10	5	0	28.60977172
ENSGALG00010001803	PPP4R1	3	-0.034353486	0.037273736	-0.92165395	0.356709108	0	1.140554782	0	0.666666667	CRA004775;PRJNA827465;Yang2025_Silkie_Wenchang	0.78657771	FALSE	FALSE	FALSE	FALSE	FALSE	FALSE	FALSE	other_meta_signature	0.104258365	0.171767428	13.33333333	10	5	0	28.60935913
ENSGALG00010003489	SGCE	3	0.037066843	0.046360226	0.799539724	0.423977522	0	1.53243432	0	0.666666667	CRA004775;PRJNA827465;Yang2025_Silkie_Wenchang	0.813751379	FALSE	FALSE	FALSE	FALSE	FALSE	FALSE	FALSE	other_meta_signature	0.089508262	0.185334213	13.33333333	10	5	0	28.60817581
ENSGALG00010027207	NOL8	3	0.19538819	0.154961564	1.260881634	0.207351496	0.051025817	8.029296263	75.09121678	1	CRA004775;PRJNA827465;Yang2025_Silkie_Wenchang	0.724136029	FALSE	FALSE	FALSE	FALSE	FALSE	FALSE	FALSE	other_meta_signature	0.140179844	0.976940951	20	2.490878322	5	0	28.60799912
ENSGALG00010020053	LFNG	3	0.040696765	0.0657168	0.619274914	0.535735268	0	1.636677871	0	0.666666667	CRA004775;PRJNA827465;Yang2025_Silkie_Wenchang	0.850714235	FALSE	FALSE	FALSE	FALSE	FALSE	FALSE	FALSE	other_meta_signature	0.0702163	0.203483827	13.33333333	10	5	0	28.60703346
ENSGALG00010028391	MED26	3	-0.037110104	0.047824311	-0.775967352	0.437768263	0	0.440369224	0	0.666666667	CRA004775;PRJNA827465;Yang2025_Silkie_Wenchang	0.816680119	FALSE	FALSE	FALSE	FALSE	FALSE	FALSE	FALSE	other_meta_signature	0.087948017	0.18555052	13.33333333	10	5	0	28.60683187
ENSGALG00010008231	ZCCHC6	3	0.358234488	0.232362372	1.54170611	0.123145027	0.133464553	12.29723111	83.7361762	1	CRA004775;PRJNA827465;Yang2025_Silkie_Wenchang	0.648071434	FALSE	FALSE	FALSE	FALSE	FALSE	FALSE	FALSE	other_meta_signature	0.188377121	1.791172442	20	1.62638238	5	0	28.60593194
ENSGALG00010006463	SCO2	3	0.041179693	0.070753336	0.582017691	0.560554765	0	1.408019996	0	0.666666667	CRA004775;PRJNA827465;Yang2025_Silkie_Wenchang	0.859930308	FALSE	FALSE	FALSE	FALSE	FALSE	FALSE	FALSE	other_meta_signature	0.065536744	0.205898465	13.33333333	10	5	0	28.60476854
ENSGALG00010016597	FRYL	3	-0.036094871	0.045234312	-0.797953364	0.424897554	0	0.157902491	0	0.666666667	CRA004775;PRJNA827465;Yang2025_Silkie_Wenchang	0.814337768	FALSE	FALSE	FALSE	FALSE	FALSE	FALSE	FALSE	other_meta_signature	0.089195423	0.180474357	13.33333333	10	5	0	28.60300311
ENSGALG00010010287	STAM2	3	0.03505443	0.041774632	0.839131991	0.401395246	0	0.919310558	0	0.666666667	CRA004775;PRJNA827465;Yang2025_Silkie_Wenchang	0.805038062	FALSE	FALSE	FALSE	FALSE	FALSE	FALSE	FALSE	other_meta_signature	0.094183586	0.17527215	13.33333333	10	5	0	28.60278907
ENSGALG00010029282	LUC7L3	3	0.539404644	0.372917661	1.446444348	0.148052611	0.37361167	27.12168805	92.62582773	1	CRA004775;PRJNA827465;Yang2025_Silkie_Wenchang	0.679004789	FALSE	FALSE	FALSE	FALSE	FALSE	FALSE	FALSE	other_meta_signature	0.168127163	2.697023219	20	0.737417227	5	0	28.60256761
ENSGALG00010022780	DYNC1I2	3	-0.032574684	0.035149869	-0.926737019	0.354063092	0	0.510145971	0	0.666666667	CRA004775;PRJNA827465;Yang2025_Silkie_Wenchang	0.786504015	FALSE	FALSE	FALSE	FALSE	FALSE	FALSE	FALSE	other_meta_signature	0.104299056	0.162873422	13.33333333	10	5	0	28.60050581
ENSGALG00010007374	CMIP	3	-0.042233316	0.082985656	-0.508923086	0.610806137	0	1.149002638	0	0.666666667	CRA004775;PRJNA827465;Yang2025_Silkie_Wenchang	0.879760007	FALSE	FALSE	FALSE	FALSE	FALSE	FALSE	FALSE	other_meta_signature	0.055635785	0.21116658	13.33333333	10	5	0	28.6001357
ENSGALG00010024804	WDR53	3	-0.039013321	0.061092198	-0.638597448	0.523084842	0	1.283361271	0	0.666666667	CRA004775;PRJNA827465;Yang2025_Silkie_Wenchang	0.848167051	FALSE	FALSE	FALSE	FALSE	FALSE	FALSE	FALSE	other_meta_signature	0.071518602	0.195066607	13.33333333	10	5	0	28.59991854
ENSGALG00010012081	CHIC1	3	-0.038666142	0.062009779	-0.623549108	0.532923742	0	1.561331853	0	0.666666667	CRA004775;PRJNA827465;Yang2025_Silkie_Wenchang	0.849985158	FALSE	FALSE	FALSE	FALSE	FALSE	FALSE	FALSE	other_meta_signature	0.070588658	0.193330712	13.33333333	10	5	0	28.5972527
ENSGALG00010027101	RNF220	3	-0.033550447	0.039150244	-0.856966497	0.391463401	0	1.141540361	0	0.666666667	CRA004775;PRJNA827465;Yang2025_Silkie_Wenchang	0.801760195	FALSE	FALSE	FALSE	FALSE	FALSE	FALSE	FALSE	other_meta_signature	0.095955509	0.167752237	13.33333333	10	5	0	28.59704108
ENSGALG00010001877	OAT	3	-0.256710504	0.132237853	-1.94127852	0.052224503	0.03999124	10.04864077	80.09681065	1	CRA004775;PRJNA827465;Yang2025_Silkie_Wenchang	0.475774649	FALSE	FALSE	FALSE	FALSE	FALSE	FALSE	FALSE	other_meta_signature	0.322598703	1.283552519	20	1.990318935	5	0	28.59647016
ENSGALG00010021065	CNNM2	3	0.038171812	0.060403059	0.631951643	0.527418476	0	1.668511692	0	0.666666667	CRA004775;PRJNA827465;Yang2025_Silkie_Wenchang	0.849493753	FALSE	FALSE	FALSE	FALSE	FALSE	FALSE	FALSE	other_meta_signature	0.07083981	0.190859062	13.33333333	10	5	0	28.59503221
ENSGALG00010010578	GLUD2	3	-0.040592203	0.077464099	-0.524013106	0.600269424	0	1.601389345	0	0.666666667	CRA004775;PRJNA827465;Yang2025_Silkie_Wenchang	0.87497004	FALSE	FALSE	FALSE	FALSE	FALSE	FALSE	FALSE	other_meta_signature	0.058006817	0.202961014	13.33333333	10	5	0	28.59430116
ENSGALG00010024784	ENSGALG00010024784	3	0.040762248	0.081931702	0.497514967	0.618825951	0	0.965640747	0	0.666666667	CRA004775;PRJNA827465;Yang2025_Silkie_Wenchang	0.882522345	FALSE	FALSE	FALSE	FALSE	FALSE	FALSE	FALSE	other_meta_signature	0.05427429	0.20381124	13.33333333	10	5	0	28.59141886
ENSGALG00010018199	BORCS7	3	-0.150739508	0.110256311	-1.367173519	0.171570924	0.02501727	7.456755381	73.17868298	1	CRA004775;PRJNA827465;Yang2025_Silkie_Wenchang	0.699471865	FALSE	FALSE	FALSE	FALSE	FALSE	FALSE	FALSE	other_meta_signature	0.15522975	0.753697541	20	2.682131702	5	0	28.59105899

ENSGALG00010012629	NUS1	3	-0.008268557	0.050275914	-0.164463589	0.869366208	0	1.04872638	0	0.666666667	CRA004775;PRJNA827465;Yang2025_Silkie_Wenchang	0.961839692	FALSE	FALSE	FALSE	FALSE	FALSE	other_meta_signature	0.016897305	0.041342786	13.33333333	10	5	0	28.39157342
ENSGALG00010016385	HTT	3	-0.008174761	0.050620273	-0.161491837	0.87170604	0	1.427775328	0	0.666666667	CRA004775;PRJNA827465;Yang2025_Silkie_Wenchang	0.962477257	FALSE	FALSE	FALSE	FALSE	FALSE	other_meta_signature	0.016609524	0.040873804	13.33333333	10	5	0	28.39081666
ENSGALG00010024752	MPP2	3	-0.009673453	0.108831808	-0.088884432	0.929173756	0	0.894386375	0	0.666666667	CRA004775;PRJNA827465;Yang2025_Silkie_Wenchang	0.979318494	FALSE	FALSE	FALSE	FALSE	FALSE	other_meta_signature	0.009076044	0.048367267	13.33333333	10	5	0	28.39077664
ENSGALG00010028609	MRPS21	3	-0.009140714	0.08529632	-0.107164223	0.914658698	0	0.182013185	0	0.666666667	CRA004775;PRJNA827465;Yang2025_Silkie_Wenchang	0.974809833	FALSE	FALSE	FALSE	FALSE	FALSE	other_meta_signature	0.011080099	0.045703569	13.33333333	10	5	0	28.390117
ENSGALG00010009386	TMEM60	3	-0.008522411	0.063851748	-0.133471851	0.893820225	0	1.853805275	0	0.666666667	CRA004775;PRJNA827465;Yang2025_Silkie_Wenchang	0.96808115	FALSE	FALSE	FALSE	FALSE	FALSE	other_meta_signature	0.014088236	0.042612055	13.33333333	10	5	0	28.39003362
ENSGALG00010029511	CUEDC1	3	-0.008002693	0.050415897	-0.158733525	0.873878824	0	1.189839044	0	0.666666667	CRA004775;PRJNA827465;Yang2025_Silkie_Wenchang	0.962477257	FALSE	FALSE	FALSE	FALSE	FALSE	other_meta_signature	0.016609524	0.040013466	13.33333333	10	5	0	28.38995632
ENSGALG00010005177	AASDHPPT	3	-0.00950798	0.107432406	-0.08850197	0.929477719	0	0.085491238	0	0.666666667	CRA004775;PRJNA827465;Yang2025_Silkie_Wenchang	0.979318494	FALSE	FALSE	FALSE	FALSE	FALSE	other_meta_signature	0.009076044	0.047539898	13.33333333	10	5	0	28.38994928
ENSGALG00010024086	GABARAPL1	3	0.007348092	0.038855231	0.189114623	0.850002981	0	1.830007057	0	0.666666667	CRA004775;PRJNA827465;Yang2025_Silkie_Wenchang	0.95575853	FALSE	FALSE	FALSE	FALSE	FALSE	other_meta_signature	0.019651817	0.036740461	13.33333333	10	5	0	28.38972561
ENSGALG00010019820	BBOX1	3	-0.009232601	0.103533632	-0.0891749	0.928942913	0	0.458393909	0	0.666666667	CRA004775;PRJNA827465;Yang2025_Silkie_Wenchang	0.979318494	FALSE	FALSE	FALSE	FALSE	FALSE	other_meta_signature	0.009076044	0.046163006	13.33333333	10	5	0	28.38857238
ENSGALG00010029521	ACTG1	3	0.007711607	0.048581385	0.158735838	0.873877002	0	1.641057196	0	0.666666667	CRA004775;PRJNA827465;Yang2025_Silkie_Wenchang	0.962477257	FALSE	FALSE	FALSE	FALSE	FALSE	other_meta_signature	0.016609524	0.038558034	13.33333333	10	5	0	28.38850089
ENSGALG00010020594	PQLC2	3	-0.008852429	0.085208482	-0.10389141	0.917255524	0	0.114072534	0	0.666666667	CRA004775;PRJNA827465;Yang2025_Silkie_Wenchang	0.975435564	FALSE	FALSE	FALSE	FALSE	FALSE	other_meta_signature	0.010801414	0.044262147	13.33333333	10	5	0	28.38839689
ENSGALG00010013117	ARSK	3	-0.009381529	0.126948488	-0.073900282	0.941089731	0	0.062714879	0	0.666666667	CRA004775;PRJNA827465;Yang2025_Silkie_Wenchang	0.98283974	FALSE	FALSE	FALSE	FALSE	FALSE	other_meta_signature	0.007517292	0.046907645	13.33333333	10	5	0	28.38775827
ENSGALG00010017384	ARID4A	3	0.170487392	0.171420671	0.994555626	0.319952438	0.065161053	8.255766227	75.77450784	1	CRA004775;PRJNA827465;Yang2025_Silkie_Wenchang	0.771329899	FALSE	FALSE	FALSE	FALSE	FALSE	other_meta_signature	0.112759834	0.852436962	20	2.422549216	5	0	28.38774601
ENSGALG00010019916	NOC3L	3	-0.007376885	0.04377027	-0.168536428	0.866161288	0	0.453954673	0	0.666666667	CRA004775;PRJNA827465;Yang2025_Silkie_Wenchang	0.96060415	FALSE	FALSE	FALSE	FALSE	FALSE	other_meta_signature	0.017455542	0.036884425	13.33333333	10	5	0	28.3876733
ENSGALG00010011775	VEZT	3	-0.0073264	0.045525623	-0.160929144	0.872149208	0	0.113821238	0	0.666666667	CRA004775;PRJNA827465;Yang2025_Silkie_Wenchang	0.962477257	FALSE	FALSE	FALSE	FALSE	FALSE	other_meta_signature	0.016609524	0.036631998	13.33333333	10	5	0	28.38657485
ENSGALG00010018061	NDUFAF5	3	-0.008342489	0.080493317	-0.103642006	0.917453451	0	1.827936958	0	0.666666667	CRA004775;PRJNA827465;Yang2025_Silkie_Wenchang	0.975475586	FALSE	FALSE	FALSE	FALSE	FALSE	other_meta_signature	0.010783596	0.041712444	13.33333333	10	5	0	28.38582937
ENSGALG00010022544	SPICE1	3	0.007369756	0.05199087	0.14175097	0.887276713	0	0.122221085	0	0.666666667	CRA004775;PRJNA827465;Yang2025_Silkie_Wenchang	0.966117785	FALSE	FALSE	FALSE	FALSE	FALSE	other_meta_signature	0.014969923	0.036848782	13.33333333	10	5	0	28.38515204
ENSGALG00010008120	CDC5L	3	-0.006414639	0.035626917	-0.180050343	0.857113046	0	0.072600302	0	0.666666667	CRA004775;PRJNA827465;Yang2025_Silkie_Wenchang	0.958153175	FALSE	FALSE	FALSE	FALSE	FALSE	other_meta_signature	0.018565057	0.032073194	13.33333333	10	5	0	28.38397158
ENSGALG00010027260	LEMD2	3	0.006491538	0.03882193	0.167213162	0.867202328	0	0.877453754	0	0.666666667	CRA004775;PRJNA827465;Yang2025_Silkie_Wenchang	0.961164993	FALSE	FALSE	FALSE	FALSE	FALSE	other_meta_signature	0.017202055	0.032457689	13.33333333	10	5	0	28.38299308
ENSGALG00010029727	ZSWIM7	3	-0.008236769	0.105068619	-0.078394192	0.937514494	0	1.518094853	0	0.666666667	CRA004775;PRJNA827465;Yang2025_Silkie_Wenchang	0.981988366	FALSE	FALSE	FALSE	FALSE	FALSE	other_meta_signature	0.007893657	0.041183847	13.33333333	10	5	0	28.38241084
ENSGALG00010012024	UBR5	3	0.006553958	0.042234485	0.155180245	0.876679222	0	1.039049817	0	0.666666667	CRA004775;PRJNA827465;Yang2025_Silkie_Wenchang	0.963326943	FALSE	FALSE	FALSE	FALSE	FALSE	other_meta_signature	0.016226293	0.032769789	13.33333333	10	5	0	28.38232941
ENSGALG00010021562	LRP6	3	0.007558547	0.087283054	0.086598101	0.930990975	0	0.97747726	0	0.666666667	CRA004775;PRJNA827465;Yang2025_Silkie_Wenchang	0.979504478	FALSE	FALSE	FALSE	FALSE	FALSE	other_meta_signature	0.008993574	0.037792734	13.33333333	10	5	0	28.38011964
ENSGALG00010019269	SOCS1	3	-0.007991907	0.128242739	-0.062318595	0.95030912	0	0.045924163	0	0.666666667	CRA004775;PRJNA827465;Yang2025_Silkie_Wenchang	0.984455786	FALSE	FALSE	FALSE	FALSE	FALSE	other_meta_signature	0.006803784	0.039959537	13.33333333	10	5	0	28.38009665
ENSGALG00010023227	RNPC3	3	0.423218383	0.279059353	1.516589137	0.12937045	0.206157507	18.50441787	89.19177024	1	CRA004775;PRJNA827465;Yang2025_Silkie_Wenchang	0.656082898	FALSE	FALSE	FALSE	FALSE	FALSE	other_meta_signature	0.183041283	2.116091914	20	1.080822976	5	0	28.37995617
ENSGALG00010023793	RBM45	3	0.006325875	0.045784942	0.138164978	0.890110033	0	1.865588125	0	0.666666667	CRA004775;PRJNA827465;Yang2025_Silkie_Wenchang	0.966800272	FALSE	FALSE	FALSE	FALSE	FALSE	other_meta_signature	0.014663236	0.031629377	13.33333333	10	5	0	28.37962595
ENSGALG00010017633	HLX	3	-0.007371971	0.085089974	-0.086637362	0.930959767	0	0.652924887	0	0.666666667	CRA004775;PRJNA827465;Yang2025_Silkie_Wenchang	0.979504478	FALSE	FALSE	FALSE	FALSE	FALSE	other_meta_signature	0.008993574	0.036859855	13.33333333	10	5	0	28.37918676
ENSGALG00010025473	PARS2	3	0.006866515	0.060717514	0.113089536	0.909959571	0	1.019867958	0	0.666666667	CRA004775;PRJNA827465;Yang2025_Silkie_Wenchang	0.974252466	FALSE	FALSE	FALSE	FALSE	FALSE	other_meta_signature	0.011328486	0.034332577	13.33333333	10	5	0	28.3789944
ENSGALG00010003900	RB1	3	0.006630897	0.053824437	0.123194908	0.901952758	0	0.011078116	0	0.666666667	CRA004775;PRJNA827465;Yang2025_Silkie_Wenchang	0.971635051	FALSE	FALSE	FALSE	FALSE	FALSE	other_meta_signature	0.012496827	0.033154483	13.33333333	10	5	0	28.37898464
ENSGALG00010012778	TBC1D15	3	0.006558074	0.052397604	0.125159815	0.90039703	0	0.942538758	0	0.666666667	CRA004775;PRJNA827465;Yang2025_Silkie_Wenchang	0.971378553	FALSE	FALSE	FALSE	FALSE	FALSE	other_meta_signature	0.012611489	0.032790372	13.33333333	10	5	0	28.37873519
ENSGALG00010010374	CMTM4	3	-0.007360184	0.089881547	-0.081887595	0.934736099	0	1.642037206	0	0.666666667	CRA004775;PRJNA827465;Yang2025_Silkie_Wenchang	0.981102536	FALSE	FALSE	FALSE	FALSE	FALSE	other_meta_signature	0.008285602	0.036800919	13.33333333	10	5	0	28.37841985
ENSGALG00010015701	UNC50	3	-0.006806249	0.065221008	-0.104356698	0.916886285	0	0.377832514	0	0.666666667	CRA004775;PRJNA827465;Yang2025_Silkie_Wenchang	0.975312608	FALSE	FALSE	FALSE	FALSE	FALSE	other_meta_signature	0.010856162	0.034031245	13.33333333	10	5	0	28.37822074
ENSGALG00010010651	NUAK1	3	0.006501728	0.053673867	0.12113396	0.903584931	0	1.006818242	0	0.666666667	CRA004775;PRJNA827465;Yang2025_Silkie_Wenchang	0.972222725	FALSE	FALSE	FALSE	FALSE	FALSE	other_meta_signature	0.01223423	0.03250864	13.33333333	10	5	0	28.37807621
ENSGALG00010022657	MRPS35	3	-0.006444667	0.055979909	-0.115124641	0.908346329	0	1.097803115	0	0.666666667	CRA004775;PRJNA827465;Yang2025_Silkie_Wenchang	0.973533913	FALSE	FALSE	FALSE	FALSE	FALSE	other_meta_signature	0.011648915	0.032223335	13.33333333	10	5	0	28.37720558
ENSGALG00010021290	ERCC3	3	0.005122813	0.031953712	0.160319819	0.872629147	0	1.969448832	0	0.666666667	CRA004775;PRJNA827465;Yang2025_Silkie_Wenchang	0.962477257	FALSE	FALSE	FALSE	FALSE	FALSE	other_meta_signature	0.016609524	0.025614067	13.33333333	10	5	0	28.37555692
ENSGALG00010024151	LEPROT	3	-0.156910142	0.125717882	-1.248113151	0.211989623	0.031008458	8.15562643	75.47705235	1	CRA004775;PRJNA827465;Yang2025_Silkie_Wenchang	0.727308175	FALSE	FALSE	FALSE	FALSE	FALSE	other_meta_signature	0.138281531	0.784550708	20	2.452294765	5	0	28.375127
ENSGALG00010003271	AKAP11	3	0.006441437	0.07344852	0.08770002	0.930115103	0	0.750400233	0	0.666666667	CRA004775;PRJNA827465;Yang2025_Silkie_Wenchang	0.979332751	FALSE	FALSE	FALSE	FALSE	FALSE	other_meta_signature	0.009069722	0.032207183	13.33333333	10	5	0	28.37461024
ENSGALG00010014762	STK40	3	-0.005370756	0.039850948	-0.134771093	0.892792862	0	1.57020622	0	0.666666667	CRA004775;PRJNA827465;Yang2025_Silkie_Wenchang	0.967888228	FALSE	FALSE	FALSE	FALSE	FALSE	other_meta_signature	0.014174792	0.026853779	13.33333333	10	5	0	28.3743619
ENSGALG00010007479	NFX1	3	-0.005929827	0.055625159	-0.106603327	0.915103679	0	0.052533011	0	0.666666667	CRA004775;PRJNA827465;Yang2025_Silkie_Wenchang	0.974809833	FALSE	FALSE	FALSE	FALSE	FALSE	other_meta_signature	0.011080099	0.029649135	13.33333333	10	5	0	28.37406257
ENSGALG00010008438	SYBU	3	-0.006797686	0.122355382	-0.055556905	0.955694796	0	0.539342855	0	0.666666667	CRA004775;PRJNA827465;Yang2025_Silkie_Wenchang	0.985476441	FALSE	FALSE	FALSE	FALSE	FALSE	other_meta_signature	0.006353754	0.033988432	13.33333333	10	5	0	28.37367552
ENSGALG00010016310	HDAC8	3	-0.005797308	0.057542731	-0.100747879	0.919750602	0	1.052218939	0	0.666666667	CRA004775;PRJNA827465;Yang2025_Silkie_Wenchang	0.975869927	FALSE	FALSE	FALSE	FALSE	FALSE	other_meta_signature	0.010608065	0.028986541	13.33333333	10	5	0	28.37292794
ENSGALG00010019305	SNRNP27	3	-0.005717348	0.055952157	-0.102182791	0.918611587	0	0.085598426	0	0.666666667	CRA004775;PRJNA827465;Yang2025_Silkie_Wenchang	0.975648054	FALSE	FALSE	FALSE	FALSE	FALSE	other_meta_signature	0.010706817	0.028586738	13.33333333	10	5	0	28.37262689
ENSGALG00010007324	ENSGALG00010007324	3	0.006614056	0.124662169	0.053055839	0.957687417	0	1.689489781	0	0.666666667	CRA004775;PRJNA827465;Yang2025_Silkie_Wenchang	0.985906358	FALSE	FALSE	FALSE	FALSE	FALSE	other_meta_signature	0.006164333	0.03307028	13.33333333	10	5	0	28.37256795
ENSGALG00010017843	NSD3	3	-0.004750301	0.034321487	-0.138406033	0.889919529	0	0.002859239	0	0.666666667	CRA004775;PRJNA827465;Yang2025_Silkie_Wenchang	0.966785003	FALSE	FALSE	FALSE	FALSE	FALSE	other_meta_signature	0.014670095	0.023751504	13.33333333	10	5	0	28.37175493
ENSGALG00010020025	LOXL2	3	-0.06224696	0.099604503	-0.624941219	0.532009638	0.0012603	2.071274356	3.441087172	0.666666667	CRA004775;PRJNA827465;Yang2025_Silkie_Wenchang	0.849856996	FALSE	FALSE	FALSE	FALSE	FALSE	other_meta_signature	0.070654146	0.311234799	13.33333333	9.655891283	5	0	28.37111356
ENSGALG00010013121	PCBD2	3	-0.00616568	0.097656293	-0.063136539	0.949657778	0	0.139289289	0	0.666666667	CRA004775;PRJNA827465;Yang2025_Silkie_Wenchang	0.984327492	FALSE	FALSE	FALSE	FALSE	FALSE	other_meta_signature	0.006860385	0.030828402	13.33333333	10	5	0	28.37102212
ENSGALG00010005548	EZR	3	0.006266682	0.113408735	0.055257491	0.955933328	0	1.037973331	0	0.666666667	CRA004775;PRJNA827465;Yang2025_Silkie_Wenchang	0.985486061	FALSE	FALSE	FALSE	FALSE	FALSE	other_meta_signature	0.006349514	0.031333411	13.33333333	10	5	0	28.37101626
ENSGALG00010026796	DCAKD	3	0.006089394	0.134238874	0.045362375	0.963818471	0	1.236077544	0	0.666666667	CRA004775;PRJNA827465;Yang2025_Silkie_Wenchang	0.986708374	FALSE	FALSE	FALSE	FALSE	FALSE	other_meta_signature	0.005811186	0.030446971	13.33333333	10	5	0	28.36959149
ENSGALG00010021767	CCDC58	3	0.005721124	0.084896194	0.067389642	0.946271515	0	1.344097472	0	0.666666667	CRA004775;PRJNA827465;Yang2025_Silkie_Wenchang	0.983919176	FALSE	FALSE	FALSE	FALSE	FALSE	other_meta_signature	0.007040575	0.02860562	13.33333333	10	5	0	28.36897953
ENSGALG00010008238	NTAQ1	3	-0.005768032	0.098181538	-0.058748644	0.953152314	0	1.900375922	0	0.666666667	CRA004775;PRJNA827465;Yang2025_Silkie_Wenchang	0.984742353	FALSE	FALSE	FALSE	FALSE	FALSE	other_meta_signature	0.006677383	0.028840161	13.33333333	10	5	0	28.36885088
ENSGALG00010017894	KAT6A	3	-0.004802142	0.043304711	-0.110891904	0.911702066	0	1.819133494	0	0.666666667	CRA004775;PRJNA827465;Yang2025_Silkie_Wenchang	0.974809833	FALSE	FALSE	FALSE	FALSE	FALSE	other_meta_signature	0.011080099	0.024010709	13.33333333	10	5	0	28.36842414
ENSGALG00010021016	FHIT	3	-0.005493783	0.110651005	-0.049649644	0.960401585	0	0.389696948	0	0.666666667	CRA004775;PRJNA827465;Yang2025_Silkie_Wenchang	0.986202903	FALSE	FALSE	FALSE	FALSE	FALSE	other_meta_signature	0.006033723	0.027468915	13.33333333	10	5	0	28.36683597
ENSGALG00010001426	ATE1	3	-0.104483056	0.105470746	-0.990635413	0.321863639	0.022976469	7.320317482	72.6787806	1	CRA004775;PRJNA827465;Yang2025_Silkie_Wenchang	0.772374405	FALSE	FALSE	FALSE	FALSE	FALSE	other_meta_signature	0.112172126	0.52241528	20	2.73212194	5	0	28.36670935
ENSGALG00010016672	DCTN4	3	-0.192682519	0.091494495	-2.105946589	0.035208992	0.017273586	9.974913832	79.94970149	1	CRA004775;PRJNA827465;Yang2025_Silkie_Wenchang	0.399762875	FALSE	FALSE	FALSE	FALSE	FALSE	other_meta_signature	0.39819754	0.963412595	20	2.005029851	5	0	28.36663999
ENSGALG00010014997	RAE1	3	0.004470379	0.048960191	0.091306411	0.927249124	0	0.720139363	0	0.666666667	CRA004775;PRJNA827465;Yang2025_Silkie_Wenchang	0.978973428	FALSE	FALSE	FALSE	FALSE	FALSE	other_meta_signature	0.009229096	0.022351897	13.33333333	10	5	0	28.36491433
ENSGALG00010002459	G3BP2	3	-0.004001304	0.03600265	-0.111139165	0.911505992	0	0.370807792	0	0.666666667	CRA004775;PRJNA827465;Yang2025_Silkie_Wenchang	0.974809833	FALSE	FALSE	FALSE	FALSE	FALSE	other_meta_signature	0.011080099	0.020006522	13.33333333	10	5	0	28.36441995
ENSGALG00010022230	NFS1	3	-0.004202066	0.04382044	-0.095892819	0.923605698	0	1.344118581	0	0.666666667	CRA004775;PRJNA827465;Yang2025_Silkie_Wenchang	0.977810802	FALSE	FALSE	FALSE	FALSE	FALSE	other_meta_signature	0.009745169	0.021010328	13.33333333	10	5	0	28.36408883
ENSGALG00010024945	APIP	3	-0.004666241	0.067144077	-0.069495941	0.944594864	0	0.647658054	0	0.666666667	CRA004775;PRJNA827465;Yang2025_Silkie_Wenchang	0.983656792	FALSE	FALSE	FALSE	FALSE	FALSE	other_meta_signature	0.007156405	0.023331204	13.33333333	10	5	0	28.36382094
ENSGALG00010019693	HAUS2	3	-0.004316904	0.05444577	-0.079288138	0.936803441	0	0.804704962	0	0.666666667	CRA004775;PRJNA827465;Yang2025_Silkie_Wenchang	0.981843465	FALSE	FALSE	FALSE	FALSE	FALSE	other_meta_signature	0.007957746	0.021584518	13.33333333	10	5	0	28.3628756
ENSGALG00010013791	AEBP2	3	-0.004038066	0.043975433	-0.091825503	0.926836682	0	0.599537951	0	0.666666667	CRA004775;PRJNA827465;Yang2025_Silkie_Wenchang	0.978973428	FALSE	FALSE	FALSE	FALSE	FALSE	other_meta_signature	0.009229096	0.020190331	13.33333333	10	5	0	28.36275276
ENSGALG00010023923	CSNK1G1	3	-0.0041586	0.057657536	-0.072125875	0.942501735	0	1.240126668	0	0.666666667	CRA004775;PRJNA827465;Yang2025_Silkie_Wenchang	0.983274891	FALSE	FALSE	FALSE	FALSE	FALSE	other_meta_signature	0.007325051	0.020793001	13.33333333	10	5	0	28.36145139
ENSGALG00010021204	NAGLU	3	-0.004141476	0.057043355	-0.072602249	0.942122637	0	1.622389372	0	0.666666667	CRA004775;PRJNA827465;Yang2025_Silkie_Wenchang	0.983274891	FALSE	FALSE	FALSE	FALSE	FALSE	other_meta_signature	0.007325051	0.020707379	13.33333333	10	5	0	28.36136576
ENSGALG00010006615	GRAMD1C	3	-0.00423804	0.069510068	-0.06097016	0.951382974	0	1.078611855	0	0.666666667	CRA004775;PRJNA827465;Yang2025_Silkie_Wenchang	0.984455786	FALSE	FALSE	FALSE	FALSE	FALSE	other_meta_signature	0.006803784	0.0211902	13.33333333	10	5	0	28.36132732
ENSGALG00010012945	CLINT1	3	-0.003345856	0.030893558	-0.108302704	0.913755577	0	0.886013543	0	0.666666667	CRA004775;PRJNA827465;Yang2025_Silkie_Wenchang	0.974809833	FALSE	FALSE	FALSE	FALSE	FALSE	other_meta_signature	0.011080099	0.016729279	13.33333333	10	5	0	28.36114271
ENSGALG00010020640	PTPN14	3	-0.004186828	0.070754534	-0.059173985	0.95281353	0	1.574899179	0	0.666666667	CRA004775;PRJNA827465;Yang2025_Silkie_Wenchang	0.984742353	FALSE	FALSE	FALSE	FALSE	FALSE	other_meta_signature	0.006677383	0.020934139	13.33333333	10	5	0	28.36094486
ENSGALG00010021870	ENSGALG00010021870	3	0.004492151	0.121363375	0.037014057	0.970473797	0	0.845787139	0	0.666666667	CRA004775;PRJNA827465;Yang2025_Silkie_Wenchang	0.98872525	FALSE	FALSE	FALSE	FALSE	FALSE	other_meta_signature	0.004924375	0.022460755	13.33333333	10	5	0	28.36071846
ENSGALG00010003121	ARMC1	3	0.003581953	0.040073272	0.089385092	0.928775871	0	1.571228302	0	0.666666667	CRA004775;PRJNA827465;Yang2025_Silkie_Wenchang	0.979318494	FALSE	FALSE	FALSE	FALSE	FALSE	other_meta_signature	0.009076044	0.017909765	13.33333333	10	5	0	28.36031914
ENSGALG00010003383	UBE2J1	3	0.003756239	0.052532098	0.071503688	0.94299689	0	1.249014277	0	0.666666667	CRA004775;PRJNA827465;Yang2025_Silkie_Wenchang	0.983323318	FALSE	FALSE	FALSE	FALSE	FALSE	other_meta_signature	0.007303662	0.018781194	13.33333333	10	5	0	28.35941819
ENSGALG00010027902	UBE2Q1	3	0.003308058	0.035473005	0.093255639	0.925700473	0	1.443717918	0	0.666666667	CRA004775;PRJNA827465;Yang2025_Silkie_Wenchang	0.978767867	FALSE	FALSE	FALSE	FALSE	FALSE	other_meta_signature	0.009320297	0.016540289	13.33333333	10	5	0	28.35919392
ENSGALG00010026492	SPECC1L	3	0.003820898	0.065694277	0.058161802	0.953619747	0	1.571937835	0	0.666666667	CRA004775;PRJNA827465;Yang2025_Silkie_Wenchang	0.984769864	FALSE	FALSE	FALSE	FALSE	FALSE	other_meta_signature	0.00666525	0.019104488	13.33333333	10	5	0	28.35910307
ENSGALG00010029364	ANAPC11	3	0.004009651	0.090828362	0.04414536	0.964788536	0	1.411830596	0	0.666666667	CRA004775;PRJNA827465;Yang2025_Silkie_Wenchang	0.986918722	FALSE	FALSE	FALSE	FALSE	FALSE	other_meta_signature	0.005718612	0.020048254	13.33333333	10	5	0	28.3591002
ENSGALG00010016918	RCOR1	3	-0.003462809	0.046091893	-0.075128382	0.940112566	0	1.312840273	0	0.666666667	CRA004775;PRJNA827465;Yang2025_Silkie_Wenchang	0.982794256	FALSE	FALSE	FALSE	FALSE	FALSE	other_meta_signature	0.00753739	0.017314047	13.33333333	10	5	0	28.35818477
ENSGALG00010019145	RAC1	3	0.003133629	0.036275405	0.086384392	0.931160854	0	1.235568511	0	0.666666667	CRA004775;PRJNA827465;Yang2025_Silkie_Wenchang	0.979504478	FALSE	FALSE	FALSE	FALSE	FALSE	other_meta_signature	0.008993574	0.015668144	13.33333333	10	5	0	28.35799505
ENSGALG00010013168	VPS26C	3	-0.003458	0.048992845	-0.070581732	0.94373065	0	0.982994644	0	0.666666667	CRA004775;PRJNA827465;Yang2025_Silkie_Wenchang	0.983337511	FALSE	FALSE	FALSE	FALSE	FALSE	other_meta_signature	0.007297393	0.017289999	13.33333333	10	5	0	28.35792073
ENSGALG00010003595	SNX13	3	-0.00325198	0.048882676	-0.066526216	0.946958886	0	1.461450539	0	0.666666667	CRA004775;PRJNA827465;Yang2025_Silkie_Wenchang	0.983959877	FALSE	FALSE	FALSE	FALSE	FALSE	other_meta_signature	0.00702261	0.016259898	13.33333333	10	5	0	28.35661584
ENSGALG00010026295	SMPD4	3	-0.003212498	0.054151599	-0.059324162	0.952693916	0	1.421443053	0	0.666666667	CRA004775;PRJNA827465;Yang2025_Silkie_Wenchang	0.984742353	FALSE	FALSE	FALSE	FALSE	FALSE	other_meta_signature	0.006677383	0.016062491	13.33333333	10	5	0	28.35607321
ENSGALG00010020176	ENSGALG00010020176	3	0.0038323	0.142625657	0.02686964	0.978563709	0	1.271629497	0	0.666666667	CRA004775;PRJNA827465;Yang2025_Silkie_Wenchang	0.992177288	FALSE	FALSE	FALSE	FALSE	FALSE	other_meta_signature	0.003410719	0.0191615	13.33333333	10	5	0	28.35590555

ENSGALG00010003729	RARS2	3	0.062455878	0.084862026	0.735969672	0.461749155	0.003959369	2.395999719	16.52753613	0.666666667	CRA004775;PRJNA827465;Yang2025_Silkie_Wenchan	0.825181557	FALSE	FALSE	FALSE	FALSE	FALSE	other_meta_signature	0.083450487	0.312279388	13.33333333	8.347246387	5	0
ENSGALG00010001398	WDR11	3	-0.065092908	0.048499456	-1.342136862	0.179551624	0.0013741	2.421541015	17.40796512	0.666666667	CRA004775;PRJNA827465;Yang2025_Silkie_Wenchan	0.710888159	FALSE	FALSE	FALSE	FALSE	FALSE	other_meta_signature	0.14819872	0.32546454	13.33333333	8.259203488	5	0
ENSGALG00010015782	ATG4A	3	-0.027270433	0.062504771	-0.436293618	0.662623712	0.002304457	2.342231588	14.61134713	0.666666667	CRA004775;PRJNA827465;Yang2025_Silkie_Wenchan	0.896410952	FALSE	FALSE	FALSE	FALSE	FALSE	other_meta_signature	0.047492846	0.136352163	13.33333333	8.538865287	5	0
ENSGALG00010018312	PRPF39	3	0.200004076	0.175977355	1.136533026	0.255733552	0.076570357	21.87606173	90.8575866	1	CRA004775;PRJNA827465;Yang2025_Silkie_Wenchan	0.744535853	FALSE	FALSE	FALSE	FALSE	FALSE	other_meta_signature	0.128114384	1.000020379	20	0.91424134	5	0
ENSGALG00010001906	SKAP2	3	-0.087531112	0.080281929	-1.090296573	0.275582524	0.004209635	2.45819371	18.63944683	0.666666667	CRA004775;PRJNA827465;Yang2025_Silkie_Wenchan	0.755279869	FALSE	FALSE	FALSE	FALSE	FALSE	other_meta_signature	0.121892091	0.437655558	13.33333333	8.136055317	5	0
ENSGALG00010011006	TMEM64	3	-0.21010461	0.209436723	-1.003188968	0.315769695	0.110752873	23.20575677	91.38144892	1	CRA004775;PRJNA827465;Yang2025_Silkie_Wenchan	0.769107272	FALSE	FALSE	FALSE	FALSE	FALSE	other_meta_signature	0.114013082	1.05052305	20	0.861855108	5	0
ENSGALG00010007905	MRPL32	3	0.074900911	0.089385214	0.837956383	0.402055201	0.005615148	2.433932948	17.82846765	0.666666667	CRA004775;PRJNA827465;Yang2025_Silkie_Wenchan	0.805038062	FALSE	FALSE	FALSE	FALSE	FALSE	other_meta_signature	0.094183586	0.374504553	13.33333333	8.217153235	5	0
ENSGALG00010026388	SNRPD3	3	-0.077859995	0.066147526	-1.177065869	0.239169216	0.00306765	2.451048262	18.40225951	0.666666667	CRA004775;PRJNA827465;Yang2025_Silkie_Wenchan	0.735123848	FALSE	FALSE	FALSE	FALSE	FALSE	other_meta_signature	0.133639488	0.389299975	13.33333333	8.159774049	5	0
ENSGALG00010024345	STARD3	3	-0.005430363	0.072284595	-0.07512476	0.940115448	0.003955665	2.314234838	13.57834705	0.666666667	CRA004775;PRJNA827465;Yang2025_Silkie_Wenchan	0.982794256	FALSE	FALSE	FALSE	FALSE	FALSE	other_meta_signature	0.00753739	0.027151814	13.33333333	8.642165295	5	0
ENSGALG00010017531	SACM1L	3	0.05108882	0.048512871	1.053098262	0.292295959	0.001434505	2.409524682	16.99607747	0.666666667	CRA004775;PRJNA827465;Yang2025_Silkie_Wenchan	0.759742234	FALSE	FALSE	FALSE	FALSE	FALSE	other_meta_signature	0.119333731	0.2554441	13.33333333	8.300392253	5	0
ENSGALG00010019684	PIGQ	3	-0.090103808	0.097077672	-0.928162021	0.353323532	0.005765923	2.463070352	18.8005329	0.666666667	CRA004775;PRJNA827465;Yang2025_Silkie_Wenchan	0.786273772	FALSE	FALSE	FALSE	FALSE	FALSE	other_meta_signature	0.104426211	0.450519041	13.33333333	8.11994671	5	0
ENSGALG00010020030	MRPL33	3	0.057532894	0.078747512	0.730599518	0.465023806	0.00435042	2.410408414	17.02650934	0.666666667	CRA004775;PRJNA827465;Yang2025_Silkie_Wenchan	0.826968056	FALSE	FALSE	FALSE	FALSE	FALSE	other_meta_signature	0.082511266	0.287664471	13.33333333	8.297349066	5	0
ENSGALG00010010780	PARG	3	-0.16739432	0.135570369	-1.234741198	0.216926838	0.047389526	19.59387459	89.79272838	1	CRA004775;PRJNA827465;Yang2025_Silkie_Wenchan	0.732178087	FALSE	FALSE	FALSE	FALSE	FALSE	other_meta_signature	0.135383273	0.836971599	20	1.020727162	5	0
ENSGALG00010028772	TTF1	3	0.011614113	0.053893652	0.215500578	0.829377062	0.001727498	2.33361839	14.29618448	0.666666667	CRA004775;PRJNA827465;Yang2025_Silkie_Wenchan	0.948887498	FALSE	FALSE	FALSE	FALSE	FALSE	other_meta_signature	0.022785275	0.058070566	13.33333333	8.570381552	5	0
ENSGALG00010012282	ENSGALG00010012282	3	-0.817130226	0.854577505	-0.95618036	0.338981121	1.214927192	4.490296701	55.45951341	0.666666667	CRA004775;PRJNA827465;Yang2025_Silkie_Wenchan	0.781358668	FALSE	FALSE	FALSE	FALSE	FALSE	other_meta_signature	0.107149566	4.085651132	13.33333333	4.454048659	5	0
ENSGALG00010019403	PACC1	3	0.038658639	0.081510104	0.474280325	0.63530002	0.003750301	2.380434583	15.98172811	0.666666667	CRA004775;PRJNA827465;Yang2025_Silkie_Wenchan	0.88839303	FALSE	FALSE	FALSE	FALSE	FALSE	other_meta_signature	0.051394858	0.193293193	13.33333333	8.401827189	5	0
ENSGALG00010027995	POLR2E	3	-0.016545168	0.075487587	-0.219177337	0.826511909	0.00466918	2.343362988	14.65257368	0.666666667	CRA004775;PRJNA827465;Yang2025_Silkie_Wenchan	0.947773875	FALSE	FALSE	FALSE	FALSE	FALSE	other_meta_signature	0.023295266	0.082725841	13.33333333	8.534742632	5	0
ENSGALG00010016673	LRPAP1	3	0.048189178	0.076629699	0.628857718	0.529442209	0.003885068	2.40221752	16.7435928	0.666666667	CRA004775;PRJNA827465;Yang2025_Silkie_Wenchan	0.849590825	FALSE	FALSE	FALSE	FALSE	FALSE	other_meta_signature	0.070790186	0.240945888	13.33333333	8.32564072	5	0
ENSGALG00010021807	CDC42	3	-0.302012485	0.228894408	-1.319440207	0.187021988	0.14695923	63.35889967	96.84337952	1	CRA004775;PRJNA827465;Yang2025_Silkie_Wenchan	0.716759278	FALSE	FALSE	FALSE	FALSE	FALSE	other_meta_signature	0.144626677	1.510062427	20	0.315662048	5	0
ENSGALG00010011210	NUP153	3	-0.033828784	0.053980308	-0.626687485	0.530864109	0.001828445	2.38333384	16.08393391	0.666666667	CRA004775;PRJNA827465;Yang2025_Silkie_Wenchan	0.849646601	FALSE	FALSE	FALSE	FALSE	FALSE	other_meta_signature	0.070761676	0.169143918	13.33333333	8.391606609	5	0
ENSGALG00010024410	MYG1	3	0.003400341	0.096100942	0.035383019	0.971774325	0.007223227	2.326176819	14.02201312	0.666666667	CRA004775;PRJNA827465;Yang2025_Silkie_Wenchan	0.989052489	FALSE	FALSE	FALSE	FALSE	FALSE	other_meta_signature	0.00478066	0.017001707	13.33333333	8.597798688	5	0
ENSGALG00010000369	ENSGALG00010000369	2	0.381694724	0.967647305	0.394456453	0.693244072	0	0.349471005	0	0.5	CRA004775;PRJNA827465	0.904091547	FALSE	FALSE	FALSE	FALSE	FALSE	other_meta_signature	0.043787591	1.908473618	10	10	5	0
ENSGALG00010006236	C3orf38	3	0.137495225	0.066362483	2.071881865	0.03827646	0.003541092	2.653292337	24.62195091	0.666666667	CRA004775;PRJNA827465;Yang2025_Silkie_Wenchan	0.414430377	FALSE	FALSE	FALSE	FALSE	FALSE	other_meta_signature	0.382548419	0.687476126	13.33333333	7.537804909	5	0
ENSGALG00010029656	MRPL27	3	-0.023685442	0.104507023	-0.22663972	0.820703904	0.008047355	2.363234083	15.37021178	0.666666667	CRA004775;PRJNA827465;Yang2025_Silkie_Wenchan	0.945561644	FALSE	FALSE	FALSE	FALSE	FALSE	other_meta_signature	0.024310153	0.118427212	13.33333333	8.462978822	5	0
ENSGALG00010005564	TM9SF2	3	-0.260285673	0.259635318	-1.002504881	0.31609981	0.185522973	38.49614586	94.80467471	1	CRA004775;PRJNA827465;Yang2025_Silkie_Wenchan	0.769377654	FALSE	FALSE	FALSE	FALSE	FALSE	other_meta_signature	0.113860431	1.301428366	20	0.519532529	5	0
ENSGALG00010005311	YME1L1	3	-0.148891243	0.130243189	-1.143178725	0.252964394	0.037059003	19.05757564	89.5054857	1	CRA004775;PRJNA827465;Yang2025_Silkie_Wenchan	0.744053165	FALSE	FALSE	FALSE	FALSE	FALSE	other_meta_signature	0.128396032	0.744456214	20	1.04945143	5	0
ENSGALG00010012667	SCAMP1	3	-0.231497771	0.213898934	-1.082276414	0.279129723	0.119188652	31.6301522	93.67691945	1	CRA004775;PRJNA827465;Yang2025_Silkie_Wenchan	0.755279869	FALSE	FALSE	FALSE	FALSE	FALSE	other_meta_signature	0.121892091	1.157488857	20	0.632308055	5	0
ENSGALG00010029673	TMEM248	3	-0.246758415	0.230515292	-1.070464404	0.284410323	0.138248313	35.92242282	94.4324468	1	CRA004775;PRJNA827465;Yang2025_Silkie_Wenchan	0.757823498	FALSE	FALSE	FALSE	FALSE	FALSE	other_meta_signature	0.120431932	1.233792074	20	0.55675532	5	0
ENSGALG00010027113	PIAS4	3	0.130532914	0.07205794	1.811499404	0.070063581	0.004351399	2.615899455	23.54446208	0.666666667	CRA004775;PRJNA827465;Yang2025_Silkie_Wenchan	0.528335453	FALSE	FALSE	FALSE	FALSE	FALSE	other_meta_signature	0.277090245	0.652664572	13.33333333	7.645553792	5	0
ENSGALG00010003915	MIOS	3	-0.025163077	0.06655088	-0.378102848	0.705354193	0.002914414	2.382437128	16.05234923	0.666666667	CRA004775;PRJNA827465;Yang2025_Silkie_Wenchan	0.909033172	FALSE	FALSE	FALSE	FALSE	FALSE	other_meta_signature	0.041420268	0.125815387	13.33333333	8.394765077	5	0
ENSGALG00010019373	DENND5A	3	-0.019559658	0.079254717	-0.246794878	0.805066974	0.005187718	2.37046412	15.62833697	0.666666667	CRA004775;PRJNA827465;Yang2025_Silkie_Wenchan	0.939864212	FALSE	FALSE	FALSE	FALSE	FALSE	other_meta_signature	0.026934887	0.097798291	13.33333333	8.437166303	5	0
ENSGALG00010017852	LETM2	3	0.097631725	0.073547684	1.327461578	0.184356042	0.00375195	2.523208075	20.73582755	0.666666667	CRA004775;PRJNA827465;Yang2025_Silkie_Wenchan	0.715592083	FALSE	FALSE	FALSE	FALSE	FALSE	other_meta_signature	0.145334473	0.488158623	13.33333333	7.926417245	5	0
ENSGALG00010005251	MTRF1L	3	-0.011612516	0.073805735	-0.157338937	0.874977735	0.002701071	2.357734194	15.17279576	0.666666667	CRA004775;PRJNA827465;Yang2025_Silkie_Wenchan	0.962693607	FALSE	FALSE	FALSE	FALSE	FALSE	other_meta_signature	0.016511912	0.058062579	13.33333333	8.482720424	5	0
ENSGALG00010005227	EXOSC9	3	0.03414567	0.066767262	0.511413369	0.609061635	0.002567668	2.405998071	16.87441383	0.666666667	CRA004775;PRJNA827465;Yang2025_Silkie_Wenchan	0.879624358	FALSE	FALSE	FALSE	FALSE	FALSE	other_meta_signature	0.055702753	0.170728352	13.33333333	8.312558617	5	0
ENSGALG00010011509	EXOC2	3	-0.191054347	0.167781452	-1.138709585	0.254824304	0.074766615	25.5984547	92.18702838	1	CRA004775;PRJNA827465;Yang2025_Silkie_Wenchan	0.744535853	FALSE	FALSE	FALSE	FALSE	FALSE	other_meta_signature	0.128114384	0.955271736	20	0.781297162	5	0
ENSGALG00010008012	CCDC90B	3	-0.043070698	0.068148856	-0.632009104	0.527380928	0.002697016	2.42567046	17.54856923	0.666666667	CRA004775;PRJNA827465;Yang2025_Silkie_Wenchan	0.849493753	FALSE	FALSE	FALSE	FALSE	FALSE	other_meta_signature	0.07083981	0.215353488	13.33333333	8.245143077	5	0

Gene	Score
RARS2	27.0763096
WDR11	27.06620008
ATG4A	27.05604363
PRPF39	27.0423761
SKAP2	27.0289363
TMEM64	27.02639124
MRPL32	27.01917471
SNRPD3	27.01604685
STARD3	27.01018783
SACM1L	27.00850342
PIGQ	27.0082253
MRPL33	27.00085814
PARG	26.99308203
TTF1	26.98457073
ENSGALG00010012282	26.98018269
PACC1	26.97984857
POLR2E	26.97409707
LRPAP1	26.97071013
CDC42	26.97035115
NUP153	26.96484554
MYG1	26.95291439
ENSGALG00010000369	26.95226121
C3orf38	26.94116279
MRPL27	26.93904952
TM9SF2	26.93482133
YME1L1	26.92230368
SCAMP1	26.911689
TMEM248	26.91097933
PIAS4	26.90864194
MIOS	26.89533407
DENND5A	26.89523281
LETM2	26.89324367
MTRF1L	26.89062825
EXOSC9	26.87232306
EXOC2	26.86468328
CCDC90B	26.86466971

ENSGALG00010021020	ZDHHC13	3	-0.092228189	0.106552474	-0.865565905	0.38672826	0.013575065	3.331865989	39.97357617	0.666666667	CRA004775;PRJNA827465;Yang2025_Silkie_Wenchan	0.800275179	FALSE	FALSE	FALSE	FALSE	FALSE	other_meta_signature	0.096760653	0.461140945	13.33333333	6.002642383	5	0	24.89387731
ENSGALG00010005799	UFSP2	3	-0.095227267	0.09799542	-0.971752229	0.331173827	0.01163467	3.348616181	40.27383575	0.666666667	CRA004775;PRJNA827465;Yang2025_Silkie_Wenchan	0.777514211	FALSE	FALSE	FALSE	FALSE	FALSE	other_meta_signature	0.109291664	0.476136337	13.33333333	5.972616425	5	0	24.89137776
ENSGALG00010026484	TNKS1BP1	3	0.024969974	0.089395725	0.279319549	0.779999605	0.00917154	3.123406228	35.9673429	0.666666667	CRA004775;PRJNA827465;Yang2025_Silkie_Wenchan	0.934350616	FALSE	FALSE	FALSE	FALSE	FALSE	other_meta_signature	0.029490124	0.124849868	13.33333333	6.40326571	5	0	24.89093904
ENSGALG00010015204	ENSGALG00010015204	3	0.229404778	0.13851187	1.656210242	0.09767925	0.027689057	3.853294923	48.09636844	0.666666667	CRA004775;PRJNA827465;Yang2025_Silkie_Wenchan	0.604390365	FALSE	FALSE	FALSE	FALSE	FALSE	other_meta_signature	0.218682468	1.147023892	13.33333333	5.190363156	5	0	24.88940285
ENSGALG00010022204	ENSGALG00010022204	3	0.311613565	0.361764642	0.861370982	0.389033753	0.199930493	4.084439642	51.03367475	0.666666667	CRA004775;PRJNA827465;Yang2025_Silkie_Wenchan	0.801233635	FALSE	FALSE	FALSE	FALSE	FALSE	other_meta_signature	0.096240828	1.558067826	13.33333333	4.896632525	5	0	24.88427451
ENSGALG00010022508	USF3	3	0.110053174	0.158778455	0.693124102	0.488231673	0.035347487	3.377784782	40.78959647	0.666666667	CRA004775;PRJNA827465;Yang2025_Silkie_Wenchan	0.835615889	FALSE	FALSE	FALSE	FALSE	FALSE	other_meta_signature	0.07799331	0.550265871	13.33333333	5.921040353	5	0	24.88263287
ENSGALG00010024201	IREB2	3	-0.077030211	0.065409327	-1.177664024	0.238930575	0.005344019	3.318582256	39.73330037	0.666666667	CRA004775;PRJNA827465;Yang2025_Silkie_Wenchan	0.735123848	FALSE	FALSE	FALSE	FALSE	FALSE	other_meta_signature	0.133639488	0.385151055	13.33333333	6.026669963	5	0	24.87879384
ENSGALG00010001586	ZBTB14	3	0.026179742	0.092244536	0.283808051	0.776557484	0.009759902	3.13443516	36.19265042	0.666666667	CRA004775;PRJNA827465;Yang2025_Silkie_Wenchan	0.933017188	FALSE	FALSE	FALSE	FALSE	FALSE	other_meta_signature	0.030110356	0.13089871	13.33333333	6.380734958	5	0	24.87507736
ENSGALG00010023770	SCRN2	3	-0.065862143	0.153160591	-0.430020168	0.667180971	0.028663	3.245574025	38.37761874	0.666666667	CRA004775;PRJNA827465;Yang2025_Silkie_Wenchan	0.897210552	FALSE	FALSE	FALSE	FALSE	FALSE	other_meta_signature	0.047105627	0.329310716	13.33333333	6.162238126	5	0	24.8719878
ENSGALG00010021251	RNF216	3	-0.027603251	0.075783265	-0.364239398	0.71567925	0.007041597	3.148395349	36.47557634	0.666666667	CRA004775;PRJNA827465;Yang2025_Silkie_Wenchan	0.912773397	FALSE	FALSE	FALSE	FALSE	FALSE	other_meta_signature	0.039637026	0.138016253	13.33333333	6.352442366	5	0	24.86342898
ENSGALG00010017429	TELO2	3	-0.081512097	0.095887732	-0.850078475	0.395281458	0.011459451	3.321462053	39.78555323	0.666666667	CRA004775;PRJNA827465;Yang2025_Silkie_Wenchan	0.802078722	FALSE	FALSE	FALSE	FALSE	FALSE	other_meta_signature	0.095783005	0.407560483	13.33333333	6.021444677	5	0	24.8581215
ENSGALG00010016814	HMGXB3	3	0.032252077	0.071210923	0.45290912	0.650614184	0.00577529	3.168529074	36.8792284	0.666666667	CRA004775;PRJNA827465;Yang2025_Silkie_Wenchan	0.89355503	FALSE	FALSE	FALSE	FALSE	FALSE	other_meta_signature	0.048878696	0.161260383	13.33333333	6.31207716	5	0	24.85554957
ENSGALG00010000153	NF2	3	0.081473061	0.078442058	1.038640027	0.298972183	0.007718856	3.337391649	40.07296085	0.666666667	CRA004775;PRJNA827465;Yang2025_Silkie_Wenchan	0.763944932	FALSE	FALSE	FALSE	FALSE	FALSE	other_meta_signature	0.116937946	0.407365304	13.33333333	5.992703915	5	0	24.8503405
ENSGALG00010002647	FAM168A	3	0.036004561	0.080268155	0.448553481	0.653753798	0.008126212	3.181702899	37.14057964	0.666666667	CRA004775;PRJNA827465;Yang2025_Silkie_Wenchan	0.89438074	FALSE	FALSE	FALSE	FALSE	FALSE	other_meta_signature	0.048477562	0.180022803	13.33333333	6.285942036	5	0	24.84777573
ENSGALG00010029198	PPM1D	3	-0.119289139	0.083613768	-1.426668631	0.153675432	0.008578158	3.47816692	42.49844686	0.666666667	CRA004775;PRJNA827465;Yang2025_Silkie_Wenchan	0.68233867	FALSE	FALSE	FALSE	FALSE	FALSE	other_meta_signature	0.166000015	0.596445696	13.33333333	5.750155314	5	0	24.84593436
ENSGALG00010004137	CROT	3	-0.04250633	0.079123576	-0.537214471	0.591119477	0.007589228	3.206021032	37.61737743	0.666666667	CRA004775;PRJNA827465;Yang2025_Silkie_Wenchan	0.871053488	FALSE	FALSE	FALSE	FALSE	FALSE	other_meta_signature	0.059955176	0.212531651	13.33333333	6.238262257	5	0	24.84408242
ENSGALG00010002673	SLC66A3	3	-0.167836597	0.142380007	-1.17879329	0.238480497	0.027087062	3.612186388	44.63187153	0.666666667	CRA004775;PRJNA827465;Yang2025_Silkie_Wenchan	0.735123848	FALSE	FALSE	FALSE	FALSE	FALSE	other_meta_signature	0.133639488	0.839182984	13.33333333	5.536812847	5	0	24.84296865
ENSGALG00010005182	NEPRO	3	0.040376026	0.115306524	0.350162543	0.726216716	0.015840719	3.192711044	37.35731256	0.666666667	CRA004775;PRJNA827465;Yang2025_Silkie_Wenchan	0.915610554	FALSE	FALSE	FALSE	FALSE	FALSE	other_meta_signature	0.03828921	0.201880128	13.33333333	6.264268744	5	0	24.83777142
ENSGALG00010016874	ADAMTSL3	3	0.06135866	0.111746556	0.549087705	0.582945262	0.014316983	3.259909998	38.64861295	0.666666667	CRA004775;PRJNA827465;Yang2025_Silkie_Wenchan	0.868305422	FALSE	FALSE	FALSE	FALSE	FALSE	other_meta_signature	0.061327487	0.3067933	13.33333333	6.135138705	5	0	24.83659283
ENSGALG00010002474	HPCAL1	3	0.012360866	0.100716246	0.122729609	0.902321217	0.010977763	3.112266274	35.73814629	0.666666667	CRA004775;PRJNA827465;Yang2025_Silkie_Wenchan	0.971661819	FALSE	FALSE	FALSE	FALSE	FALSE	other_meta_signature	0.012484862	0.061804328	13.33333333	6.426185371	5	0	24.83380789
ENSGALG00010010987	MARCHF7	3	-0.004184506	0.049543369	-0.084461471	0.932689535	0.002640088	3.091983484	35.31660146	0.666666667	CRA004775;PRJNA827465;Yang2025_Silkie_Wenchan	0.980031015	FALSE	FALSE	FALSE	FALSE	FALSE	other_meta_signature	0.00876018	0.020922529	13.33333333	6.468339854	5	0	24.8313559
ENSGALG00010013901	NFXL1	3	0.167445722	0.10451824	1.60207177	0.10913974	0.014622864	3.666567663	45.45307264	0.666666667	CRA004775;PRJNA827465;Yang2025_Silkie_Wenchan	0.627724048	FALSE	FALSE	FALSE	FALSE	FALSE	other_meta_signature	0.202231233	0.837228609	13.33333333	5.454692736	5	0	24.82748591
ENSGALG00010021127	SWAP70	3	-0.129105654	0.068804596	-1.876410331	0.060598957	0.006009632	3.609370861	44.58868105	0.666666667	CRA004775;PRJNA827465;Yang2025_Silkie_Wenchan	0.502232506	FALSE	FALSE	FALSE	FALSE	FALSE	other_meta_signature	0.299095182	0.645528271	13.33333333	5.541131895	5	0	24.81908868
ENSGALG00010000384	ENSGALG00010000384	3	0.105556153	0.146060824	0.722686275	0.469872649	0.026419122	3.4044736	41.25376681	0.666666667	CRA004775;PRJNA827465;Yang2025_Silkie_Wenchan	0.828279178	FALSE	FALSE	FALSE	FALSE	FALSE	other_meta_signature	0.081823256	0.527780763	13.33333333	5.874623319	5	0	24.81756067
ENSGALG00010010962	ANKDD1B	3	0.771684229	0.513635514	1.502396561	0.132994724	0.564837759	8.213583129	75.65009121	0.666666667	CRA004775;PRJNA827465;Yang2025_Silkie_Wenchan	0.661651092	FALSE	FALSE	FALSE	FALSE	FALSE	other_meta_signature	0.179370967	3.858421146	13.33333333	2.434990879	5	0	24.80611632
ENSGALG00010025531	ENSGALG00010025531	3	0.03287198	0.090802113	0.362017786	0.717338739	0.009623516	3.192966574	37.3623258	0.666666667	CRA004775;PRJNA827465;Yang2025_Silkie_Wenchan	0.913264832	FALSE	FALSE	FALSE	FALSE	FALSE	other_meta_signature	0.039403266	0.164359901	13.33333333	6.26376742	5	0	24.80086392
ENSGALG00010022266	COA3	3	0.044170755	0.167783517	0.263260395	0.792349883	0.037518192	3.216664209	37.82378669	0.666666667	CRA004775;PRJNA827465;Yang2025_Silkie_Wenchan	0.936723207	FALSE	FALSE	FALSE	FALSE	FALSE	other_meta_signature	0.02838872	0.220853775	13.33333333	6.217621331	5	0	24.80019716
ENSGALG00010016308	RPS6	3	-0.086478669	0.171717715	-0.503609478	0.614535831	0.036047359	3.350283568	40.30356059	0.666666667	CRA004775;PRJNA827465;Yang2025_Silkie_Wenchan	0.881194843	FALSE	FALSE	FALSE	FALSE	FALSE	other_meta_signature	0.054928053	0.432393343	13.33333333	5.969643941	5	0	24.79029867
ENSGALG00010029721	EXOC7	3	-0.016614029	0.059735945	-0.278124484	0.780916801	0.004252299	3.15321353	36.57264308	0.666666667	CRA004775;PRJNA827465;Yang2025_Silkie_Wenchan	0.934350616	FALSE	FALSE	FALSE	FALSE	FALSE	other_meta_signature	0.029490124	0.083070145	13.33333333	6.342735692	5	0	24.78862929
ENSGALG00010008292	REEP5	3	-0.086259556	0.093043888	-0.927084603	0.35388261	0.010798766	3.38012978	40.83067425	0.666666667	CRA004775;PRJNA827465;Yang2025_Silkie_Wenchan	0.78638225	FALSE	FALSE	FALSE	FALSE	FALSE	other_meta_signature	0.104366298	0.43129778	13.33333333	5.916932575	5	0	24.78592999
ENSGALG00010009286	ENSGALG00010009286	3	0.432318329	0.235975679	1.832046128	0.066944549	0.100623041	5.007380438	60.05895648	0.666666667	CRA004775;PRJNA827465;Yang2025_Silkie_Wenchan	0.520153828	FALSE	FALSE	FALSE	FALSE	FALSE	other_meta_signature	0.283868201	2.161591643	13.33333333	3.994104352	5	0	24.77289753
ENSGALG00010020736	LRRTM4	3	0.082644527	0.199008342	0.415281722	0.67793568	0.046966671	3.345321123	40.21500698	0.666666667	CRA004775;PRJNA827465;Yang2025_Silkie_Wenchan	0.899170887	FALSE	FALSE	FALSE	FALSE	FALSE	other_meta_signature	0.046157763	0.413222635	13.33333333	5.978499302	5	0	24.77121303
ENSGALG00010009186	PREB	3	0.034981572	0.127859134	0.273594625	0.784396165	0.021223075	3.211798819	37.72959912	0.666666667	CRA004775;PRJNA827465;Yang2025_Silkie_Wenchan	0.934935845	FALSE	FALSE	FALSE	FALSE	FALSE	other_meta_signature	0.029218189	0.174907859	13.33333333	6.227040088	5	0	24.76449947
ENSGALG00010003476	ENSGALG00010003476	3	-1.071281745	0.995021104	-1.076642235	0.281640128	2.668301356	20.99218995	90.47264719	0.666666667	CRA004775;PRJNA827465;Yang2025_Silkie_Wenchan	0.755905423	FALSE	FALSE	FALSE	FALSE	FALSE	other_meta_signature	0.121532539	5.356408726	13.33333333	0.952735281	5	0	24.76400988
ENSGALG00010029590	UTP6	3	0.050408748	0.077752914	0.648319727	0.516778178	0.007129702	3.282329647	39.06766793	0.666666667	CRA004775;PRJNA827465;Yang2025_Silkie_Wenchan	0.845666519	FALSE	FALSE	FALSE	FALSE	FALSE	other_meta_signature	0.072800863	0.252043739	13.33333333	6.093233207	5	0	24.75141114
ENSGALG00010011529	SCYL2	3	-0.013613531	0.079391234	-0.171473984	0.863851086	0.007184464	3.163671997	36.78232125	0.666666667	CRA004775;PRJNA827465;Yang2025_Silkie_Wenchan	0.960290736	FALSE	FALSE	FALSE	FALSE	FALSE	other_meta_signature	0.017597261	0.068067656	13.33333333	6.321767875	5	0	24.74076613

ENSGALG00010026298	SNF8	3	0.047223675	0.118140583	0.399724406	0.689359514	0.021633186	4.051259601	50.63263783	0.666666667	CRA004775;PRJNA827465;Yang2025_Silkie_Wenchang	0.903155502	FALSE	FALSE	FALSE	FALSE	FALSE	other_meta_signature	0.044237468	0.236118373	13.33333333	4.936736217	5	0	23.55042539
ENSGALG00010019212	RPL22	3	0.077956937	0.1291765	0.603491632	0.546181681	0.02707684	4.203604671	52.42178662	0.666666667	CRA004775;PRJNA827465;Yang2025_Silkie_Wenchang	0.855482603	FALSE	FALSE	FALSE	FALSE	FALSE	other_meta_signature	0.067788818	0.389784683	13.33333333	4.757821338	5	0	23.54872817
ENSGALG00010027323	ISG20L2	3	-0.057191303	0.106342987	-0.537800422	0.590714841	0.017693546	4.1077747	51.31183802	0.666666667	CRA004775;PRJNA827465;Yang2025_Silkie_Wenchang	0.871053488	FALSE	FALSE	FALSE	FALSE	FALSE	other_meta_signature	0.059955176	0.285956516	13.33333333	4.868816198	5	0	23.54806122
ENSGALG00010018609	ENSGALG00010018609	3	-0.047657231	0.115934238	-0.41107124	0.681020298	0.020743794	4.059446051	50.73219412	0.666666667	CRA004775;PRJNA827465;Yang2025_Silkie_Wenchang	0.899659371	FALSE	FALSE	FALSE	FALSE	FALSE	other_meta_signature	0.045921892	0.238286155	13.33333333	4.926780588	5	0	23.54432197
ENSGALG00010015175	MAPK9	3	-0.049563202	0.107271175	-0.462036537	0.644055119	0.017049833	4.074058465	50.90890282	0.666666667	CRA004775;PRJNA827465;Yang2025_Silkie_Wenchang	0.890953126	FALSE	FALSE	FALSE	FALSE	FALSE	other_meta_signature	0.050145144	0.247816012	13.33333333	4.909109718	5	0	23.54040421
ENSGALG00010015810	LIPT1	3	-0.027253442	0.111383417	-0.244681324	0.80670319	0.018776604	3.971627999	49.64281648	0.666666667	CRA004775;PRJNA827465;Yang2025_Silkie_Wenchang	0.940088204	FALSE	FALSE	FALSE	FALSE	FALSE	other_meta_signature	0.026831397	0.136267209	13.33333333	5.035718352	5	0	23.53215029
ENSGALG00010029191	GSG1L2	3	0.465409384	0.506002656	0.919776563	0.357689533	0.553533062	7.26089386	72.45518199	0.666666667	CRA004775;PRJNA827465;Yang2025_Silkie_Wenchang	0.787164224	FALSE	FALSE	FALSE	FALSE	FALSE	other_meta_signature	0.103934652	2.327046922	13.33333333	2.754481801	5	0	23.51879671
ENSGALG00010021689	MPRIP	3	0.032161142	0.110368887	0.291396816	0.770747849	0.018606831	4.011255455	50.14029841	0.666666667	CRA004775;PRJNA827465;Yang2025_Silkie_Wenchang	0.931419131	FALSE	FALSE	FALSE	FALSE	FALSE	other_meta_signature	0.030854846	0.160805712	13.33333333	4.985970159	5	0	23.51096405
ENSGALG00010001177	SEH1L	3	0.018940589	0.072799921	0.260173211	0.794730168	0.007673218	3.957929977	49.46853503	0.666666667	CRA004775;PRJNA827465;Yang2025_Silkie_Wenchang	0.936723207	FALSE	FALSE	FALSE	FALSE	FALSE	other_meta_signature	0.02838872	0.094702946	13.33333333	5.053146497	5	0	23.5095715
ENSGALG00010026006	ITGA2B	3	0.323351413	0.257754646	1.254493055	0.209662849	0.120702173	5.84883428	65.80515186	0.666666667	CRA004775;PRJNA827465;Yang2025_Silkie_Wenchang	0.724975059	FALSE	FALSE	FALSE	FALSE	FALSE	other_meta_signature	0.139676934	1.616757065	13.33333333	3.419484814	5	0	23.50925215
ENSGALG00010017038	ZNF598	3	0.111852419	0.103138338	1.084489253	0.278147929	0.017887234	4.454094131	55.09749141	0.666666667	CRA004775;PRJNA827465;Yang2025_Silkie_Wenchang	0.755279869	FALSE	FALSE	FALSE	FALSE	FALSE	other_meta_signature	0.121892091	0.559262093	13.33333333	4.490250859	5	0	23.50473838
ENSGALG00010022619	MADD	3	0.134825569	0.12459181	1.082138293	0.279191083	0.024892936	4.571665919	56.25227137	0.666666667	CRA004775;PRJNA827465;Yang2025_Silkie_Wenchang	0.755279869	FALSE	FALSE	FALSE	FALSE	FALSE	other_meta_signature	0.121892091	0.674127844	13.33333333	4.374772863	5	0	23.50412613
ENSGALG00010022027	EIF4ENIF1	3	-0.083687933	0.067642627	-1.237207022	0.216010235	0.007015618	4.334023528	53.85350386	0.666666667	CRA004775;PRJNA827465;Yang2025_Silkie_Wenchang	0.731012139	FALSE	FALSE	FALSE	FALSE	FALSE	other_meta_signature	0.136075411	0.418439666	13.33333333	4.614649614	5	0	23.50249802
ENSGALG00010024579	ZNF740	3	-0.015757606	0.104873765	-0.150253083	0.880564947	0.016740716	3.941966217	49.26389802	0.666666667	CRA004775;PRJNA827465;Yang2025_Silkie_Wenchang	0.964558251	FALSE	FALSE	FALSE	FALSE	FALSE	other_meta_signature	0.015671539	0.078788032	13.33333333	5.073610198	5	0	23.5014031
ENSGALG00010028823	GFI1B	3	0.144162831	0.229904079	0.627056429	0.530622246	0.088117948	4.575078721	56.2849052	0.666666667	CRA004775;PRJNA827465;Yang2025_Silkie_Wenchang	0.849646601	FALSE	FALSE	FALSE	FALSE	FALSE	other_meta_signature	0.070761676	0.720814156	13.33333333	4.37150948	5	0	23.49641865
ENSGALG00010005323	DNAJB14	3	0.071074724	0.106672185	0.666291064	0.505225076	0.018404914	4.225805316	52.67174301	0.666666667	CRA004775;PRJNA827465;Yang2025_Silkie_Wenchang	0.842995974	FALSE	FALSE	FALSE	FALSE	FALSE	other_meta_signature	0.0741745	0.355373619	13.33333333	4.732825699	5	0	23.49570715
ENSGALG00010000457	TPCN1	3	0.01699236	0.089909954	0.188993093	0.850098231	0.01186658	3.954323402	49.42244736	0.666666667	CRA004775;PRJNA827465;Yang2025_Silkie_Wenchang	0.95575853	FALSE	FALSE	FALSE	FALSE	FALSE	other_meta_signature	0.019651817	0.084961801	13.33333333	5.057755264	5	0	23.49570222
ENSGALG00010029219	TLCD1	3	-0.102731777	0.178988513	-0.573957377	0.565996645	0.051737432	4.366500991	54.19673546	0.666666667	CRA004775;PRJNA827465;Yang2025_Silkie_Wenchang	0.861485826	FALSE	FALSE	FALSE	FALSE	FALSE	other_meta_signature	0.064751864	0.513658886	13.33333333	4.580326454	5	0	23.49207054
ENSGALG00010027187	ENSGALG00010027187	3	-0.020038927	0.150193875	-0.133420403	0.89386091	0.034635807	3.968213067	49.59948052	0.666666667	CRA004775;PRJNA827465;Yang2025_Silkie_Wenchang	0.96808115	FALSE	FALSE	FALSE	FALSE	FALSE	other_meta_signature	0.014088236	0.100194637	13.33333333	5.040051948	5	0	23.48766815
ENSGALG00010029763	GEMIN4	3	-0.125827935	0.147090457	-0.855445945	0.392304318	0.037357575	4.519024261	55.74265849	0.666666667	CRA004775;PRJNA827465;Yang2025_Silkie_Wenchang	0.801809775	FALSE	FALSE	FALSE	FALSE	FALSE	other_meta_signature	0.095928653	0.629139673	13.33333333	4.425734151	5	0	23.48413581
ENSGALG00010023026	PHRF1	3	0.036990815	0.087752863	0.421534003	0.673365187	0.012069463	4.066202321	50.81405592	0.666666667	CRA004775;PRJNA827465;Yang2025_Silkie_Wenchang	0.899073436	FALSE	FALSE	FALSE	FALSE	FALSE	other_meta_signature	0.046204834	0.184954077	13.33333333	4.918594408	5	0	23.48308665
ENSGALG00010029307	SGSH	3	0.180406754	0.157671729	1.144192147	0.252543959	0.042517754	4.860212485	58.84953577	0.666666667	CRA004775;PRJNA827465;Yang2025_Silkie_Wenchang	0.744042702	FALSE	FALSE	FALSE	FALSE	FALSE	other_meta_signature	0.128402139	0.902033768	13.33333333	4.115046423	5	0	23.47881566
ENSGALG00010004746	LYN	3	-0.137972257	0.149505441	-0.922857762	0.356081335	0.034124202	4.60166171	56.5374396	0.666666667	CRA004775;PRJNA827465;Yang2025_Silkie_Wenchang	0.786517334	FALSE	FALSE	FALSE	FALSE	FALSE	other_meta_signature	0.104291701	0.689861285	13.33333333	4.34625604	5	0	23.47374236
ENSGALG00010029703	MIF4GD	3	-0.051074078	0.0820686	-0.622333975	0.533722286	0.010482249	4.154508715	51.85953052	0.666666667	CRA004775;PRJNA827465;Yang2025_Silkie_Wenchang	0.849985158	FALSE	FALSE	FALSE	FALSE	FALSE	other_meta_signature	0.070588658	0.25537039	13.33333333	4.814046948	5	0	23.47333933
ENSGALG00010017707	AK2	3	-0.020667063	0.08963469	-0.230569915	0.817648942	0.012358704	3.994410792	49.93003715	0.666666667	CRA004775;PRJNA827465;Yang2025_Silkie_Wenchang	0.944350465	FALSE	FALSE	FALSE	FALSE	FALSE	other_meta_signature	0.024866801	0.103335314	13.33333333	5.006996285	5	0	23.46853173
ENSGALG00010027530	PHF19	3	-0.091455534	0.155278479	-0.588977526	0.55587635	0.037871202	4.339698677	53.91385096	0.666666667	CRA004775;PRJNA827465;Yang2025_Silkie_Wenchang	0.858444105	FALSE	FALSE	FALSE	FALSE	FALSE	other_meta_signature	0.066287977	0.457277672	13.33333333	4.608614904	5	0	23.46551389
ENSGALG00010023903	TBKBP1	3	0.320293946	0.310474168	1.031628325	0.302246266	0.1894985	5.85733939	65.85480426	0.666666667	CRA004775;PRJNA827465;Yang2025_Silkie_Wenchang	0.765947595	FALSE	FALSE	FALSE	FALSE	FALSE	other_meta_signature	0.115800943	1.601469728	13.33333333	3.414519574	5	0	23.46512358
ENSGALG00010008274	NFAT5	3	0.198100809	0.196782425	1.006699706	0.314079103	0.068844476	4.967366592	59.73721764	0.666666667	CRA004775;PRJNA827465;Yang2025_Silkie_Wenchang	0.767829909	FALSE	FALSE	FALSE	FALSE	FALSE	other_meta_signature	0.114734975	0.990504045	13.33333333	4.026278236	5	0	23.46485059
ENSGALG00010002453	TOP2B	3	0.059273675	0.08170249	0.725481869	0.468156464	0.010609422	4.211520518	52.5112132	0.666666667	CRA004775;PRJNA827465;Yang2025_Silkie_Wenchang	0.82826404	FALSE	FALSE	FALSE	FALSE	FALSE	other_meta_signature	0.081831194	0.296368375	13.33333333	4.74887868	5	0	23.46041158
ENSGALG00010020609	AVPI1	3	0.183520604	0.2422763	0.757484758	0.448759494	0.10132738	4.852054791	58.78035005	0.666666667	CRA004775;PRJNA827465;Yang2025_Silkie_Wenchang	0.819423792	FALSE	FALSE	FALSE	FALSE	FALSE	other_meta_signature	0.086491431	0.917603021	13.33333333	4.121964995	5	0	23.45939278
ENSGALG00010018394	KLHL28	3	-0.014568816	0.084744057	-0.171915489	0.86350397	0.011014106	3.973432224	49.66568228	0.666666667	CRA004775;PRJNA827465;Yang2025_Silkie_Wenchang	0.960253797	FALSE	FALSE	FALSE	FALSE	FALSE	other_meta_signature	0.017613967	0.07284408	13.33333333	5.033431772	5	0	23.45722315
ENSGALG00010003204	PKD2	3	0.016952575	0.127052731	0.13342944	0.893853764	0.023743332	3.980734801	49.75801956	0.666666667	CRA004775;PRJNA827465;Yang2025_Silkie_Wenchang	0.96808115	FALSE	FALSE	FALSE	FALSE	FALSE	other_meta_signature	0.014088236	0.084762874	13.33333333	5.024198044	5	0	23.45638249
ENSGALG00010026005	DERL3	3	-0.01363093	0.12750382	-0.10690605	0.914863514	0.024709642	3.968510769	49.60326136	0.666666667	CRA004775;PRJNA827465;Yang2025_Silkie_Wenchang	0.974809833	FALSE	FALSE	FALSE	FALSE	FALSE	other_meta_signature	0.011080099	0.068154648	13.33333333	5.039673864	5	0	23.45224194
ENSGALG00010025491	ZMYND11	3	0.077173233	0.101689063	0.758913796	0.447904125	0.017016443	4.310357735	53.60013895	0.666666667	CRA004775;PRJNA827465;Yang2025_Silkie_Wenchang	0.81901853	FALSE	FALSE	FALSE	FALSE	FALSE	other_meta_signature	0.086706272	0.385866163	13.33333333	4.639986105	5	0	23.44589187
ENSGALG00010013306	ARSB	3	-0.102470604	0.186543738	-0.549311412	0.582791757	0.052500246	4.407804744	54.6259393	0.666666667	CRA004775;PRJNA827465;Yang2025_Silkie_Wenchang	0.868305422	FALSE	FALSE	FALSE	FALSE	FALSE	other_meta_signature	0.061327487	0.512353022	13.33333333	4.53740607	5	0	23.44441991
ENSGALG00010029398	RHBDF2	3	0.062465212	0.094328148	0.662211794	0.507835496	0.013657788	4.234755062	52.7717667	0.666666667	CRA004775;PRJNA827465;Yang2025_Silkie_Wenchang	0.844441501	FALSE	FALSE	FALSE	FALSE	FALSE	other_meta_signature	0.073430431	0.312326061	13.33333333	4.72282333	5	0	23.44191315

ENSGALG00010020595	ZFYVE27	3	-0.049796253	0.118902376	-0.418799475	0.675362688	0.025545809	5.131072548	61.02179454	0.666666667	CRA004775;PRJNA827465;Yang2025_Silkie_Wenchang	0.899170887	FALSE	FALSE	FALSE	FALSE	FALSE	other_meta_signature	0.046157763	0.248981263	13.33333333	3.897820546	5	0	22.52629291
ENSGALG00010010346	TFB2M	3	-0.08194044	0.148783454	-0.550736242	0.581814497	0.04122109	5.379341842	62.82073052	0.666666667	CRA004775;PRJNA827465;Yang2025_Silkie_Wenchang	0.867476027	FALSE	FALSE	FALSE	FALSE	FALSE	other_meta_signature	0.061742518	0.409702201	13.33333333	3.717926948	5	0	22.522705
ENSGALG00010024938	ENSGALG00010024938	3	0.20492224	0.228569796	0.896541202	0.369963785	0.105921258	6.532130304	69.38211721	0.666666667	CRA004775;PRJNA827465;Yang2025_Silkie_Wenchang	0.79136429	FALSE	FALSE	FALSE	FALSE	FALSE	other_meta_signature	0.101623551	1.0246112	13.33333333	3.061788279	5	0	22.52135636
ENSGALG00010016295	UBE2K	3	-0.086210681	0.095056928	-0.906937382	0.364439912	0.015301143	5.474826967	63.4691651	0.666666667	CRA004775;PRJNA827465;Yang2025_Silkie_Wenchang	0.788777182	FALSE	FALSE	FALSE	FALSE	FALSE	other_meta_signature	0.103045662	0.431053407	13.33333333	3.65308349	5	0	22.52051589
ENSGALG00010021301	SMURF1	3	0.005594285	0.111166608	0.050323429	0.959864654	0.020821131	4.81621641	58.47362681	0.666666667	CRA004775;PRJNA827465;Yang2025_Silkie_Wenchang	0.986087796	FALSE	FALSE	FALSE	FALSE	FALSE	other_meta_signature	0.006084416	0.027971424	13.33333333	4.152637319	5	0	22.52002649
ENSGALG00010014862	ENSGALG00010014862	3	0.258680818	0.509802973	0.507413317	0.611864844	0.532634859	7.04810051	71.62356017	0.666666667	CRA004775;PRJNA827465;Yang2025_Silkie_Wenchang	0.880077012	FALSE	FALSE	FALSE	FALSE	FALSE	other_meta_signature	0.055479323	1.293404089	13.33333333	2.837643983	5	0	22.51986073
ENSGALG00010022928	RNF111	3	0.162313735	0.159859043	1.015355353	0.309936467	0.05086258	6.135529177	67.40297467	0.666666667	CRA004775;PRJNA827465;Yang2025_Silkie_Wenchang	0.767829909	FALSE	FALSE	FALSE	FALSE	FALSE	other_meta_signature	0.114734975	0.811568675	13.33333333	3.259702533	5	0	22.51933952
ENSGALG00010007942	PGRMC2	3	-0.081116985	0.080227353	-1.011088879	0.311973892	0.01141974	5.461755611	63.38173762	0.666666667	CRA004775;PRJNA827465;Yang2025_Silkie_Wenchang	0.767829909	FALSE	FALSE	FALSE	FALSE	FALSE	other_meta_signature	0.114734975	0.405584925	13.33333333	3.661826238	5	0	22.51547947
ENSGALG00010027158	XRCC2	3	-0.042302763	0.131211774	-0.322400659	0.747149185	0.030209376	5.081844062	60.64420758	0.666666667	CRA004775;PRJNA827465;Yang2025_Silkie_Wenchang	0.923866711	FALSE	FALSE	FALSE	FALSE	FALSE	other_meta_signature	0.034390681	0.211513813	13.33333333	3.935579242	5	0	22.51481707
ENSGALG00010010604	HIVEP1	3	0.25013497	0.194634573	1.285151793	0.198739246	0.080433216	7.177672288	72.1358134	0.666666667	CRA004775;PRJNA827465;Yang2025_Silkie_Wenchang	0.720956764	FALSE	FALSE	FALSE	FALSE	FALSE	other_meta_signature	0.142090779	1.25067485	13.33333333	2.78641866	5	0	22.51251762
ENSGALG00010015660	KLHL3	3	0.129032577	0.146508073	0.880719909	0.378469438	0.041044758	5.8235419	65.65663931	0.666666667	CRA004775;PRJNA827465;Yang2025_Silkie_Wenchang	0.796289196	FALSE	FALSE	FALSE	FALSE	FALSE	other_meta_signature	0.098929177	0.645162885	13.33333333	3.434336069	5	0	22.51176146
ENSGALG00010001491	PHF3	3	0.223967461	0.192775069	1.161807188	0.245313794	0.077017613	6.836987381	70.74734984	0.666666667	CRA004775;PRJNA827465;Yang2025_Silkie_Wenchang	0.738605564	FALSE	FALSE	FALSE	FALSE	FALSE	other_meta_signature	0.131587425	1.119837306	13.33333333	2.925265016	5	0	22.51002308
ENSGALG00010024493	JAK1	3	-0.00243836	0.093333795	-0.026125155	0.979157513	0.01424861	4.806367505	58.38853359	0.666666667	CRA004775;PRJNA827465;Yang2025_Silkie_Wenchang	0.992352381	FALSE	FALSE	FALSE	FALSE	FALSE	other_meta_signature	0.003334084	0.012191799	13.33333333	4.161146641	5	0	22.51000586
ENSGALG00010008409	CWC15	3	-0.121808634	0.108344779	-1.124268612	0.26089909	0.02151527	5.815498608	65.60913974	0.666666667	CRA004775;PRJNA827465;Yang2025_Silkie_Wenchang	0.744969822	FALSE	FALSE	FALSE	FALSE	FALSE	other_meta_signature	0.12786132	0.60904317	13.33333333	3.439086026	5	0	22.50932385
ENSGALG00010004624	PHF10	3	-0.075906427	0.081384411	-0.932690003	0.350980048	0.011626621	5.421801555	63.11189224	0.666666667	CRA004775;PRJNA827465;Yang2025_Silkie_Wenchang	0.784982422	FALSE	FALSE	FALSE	FALSE	FALSE	other_meta_signature	0.105140068	0.379532134	13.33333333	3.688810776	5	0	22.50681631
ENSGALG00010010751	SLC30A5	3	0.060009663	0.140071594	0.428421365	0.668344373	0.036139514	5.22863284	61.74908315	0.666666667	CRA004775;PRJNA827465;Yang2025_Silkie_Wenchang	0.89740941	FALSE	FALSE	FALSE	FALSE	FALSE	other_meta_signature	0.047009381	0.300048317	13.33333333	3.825091685	5	0	22.50548272
ENSGALG00010001442	LDLRAD4	3	0.106879838	0.171135518	0.62453335	0.532277375	0.053302627	5.609361635	64.34531895	0.666666667	CRA004775;PRJNA827465;Yang2025_Silkie_Wenchang	0.849985158	FALSE	FALSE	FALSE	FALSE	FALSE	other_meta_signature	0.070588658	0.534399191	13.33333333	3.565468105	5	0	22.50378929
ENSGALG00010021903	MORC2	3	0.111084002	0.116775352	0.951262405	0.341471187	0.026439146	5.701271705	64.92010724	0.666666667	CRA004775;PRJNA827465;Yang2025_Silkie_Wenchang	0.782430115	FALSE	FALSE	FALSE	FALSE	FALSE	other_meta_signature	0.106554443	0.555420009	13.33333333	3.507989276	5	0	22.50329706
ENSGALG00010024532	TMEM86A	3	-0.02355189	0.12556054	-0.187573981	0.851210624	0.024202339	4.959908622	59.67667648	0.666666667	CRA004775;PRJNA827465;Yang2025_Silkie_Wenchang	0.956569815	FALSE	FALSE	FALSE	FALSE	FALSE	other_meta_signature	0.019283328	0.117759452	13.33333333	4.032332352	5	0	22.50270846
ENSGALG00010011450	PTDSS1	3	-0.141774773	0.11294261	-1.255281538	0.209376575	0.023320704	6.028684966	66.82526934	0.666666667	CRA004775;PRJNA827465;Yang2025_Silkie_Wenchang	0.724694098	FALSE	FALSE	FALSE	FALSE	FALSE	other_meta_signature	0.139845275	0.708873866	13.33333333	3.317473066	5	0	22.49952554
ENSGALG00010024701	EIF2B1	3	-0.185170354	0.164254326	-1.127339283	0.25959906	0.05512102	6.428144926	68.88682469	0.666666667	CRA004775;PRJNA827465;Yang2025_Silkie_Wenchang	0.744607475	FALSE	FALSE	FALSE	FALSE	FALSE	other_meta_signature	0.128072608	0.925851772	13.33333333	3.111317531	5	0	22.49857524
ENSGALG00010006041	QKI	3	0.101056269	0.136326815	0.741279467	0.458524009	0.035711783	5.598038953	64.27320323	0.666666667	CRA004775;PRJNA827465;Yang2025_Silkie_Wenchang	0.824249006	FALSE	FALSE	FALSE	FALSE	FALSE	other_meta_signature	0.083941568	0.505281344	13.33333333	3.572679677	5	0	22.49523592
ENSGALG00010017792	LSM1	3	0.243967494	0.236589498	1.031184798	0.302454168	0.118895451	7.081098043	71.75579284	0.666666667	CRA004775;PRJNA827465;Yang2025_Silkie_Wenchang	0.765947595	FALSE	FALSE	FALSE	FALSE	FALSE	other_meta_signature	0.115800943	1.219837471	13.33333333	2.824420716	5	0	22.49339246
ENSGALG00010009534	XPA	3	0.127436809	0.276818198	0.460362831	0.645255809	0.148352424	5.760310238	65.27964784	0.666666667	CRA004775;PRJNA827465;Yang2025_Silkie_Wenchang	0.891118567	FALSE	FALSE	FALSE	FALSE	FALSE	other_meta_signature	0.050064508	0.637184046	13.33333333	3.472035216	5	0	22.4926171
ENSGALG00010020934	ABCD3	3	-0.085185664	0.103460794	-0.823361779	0.410302291	0.017672785	5.494560694	63.60036568	0.666666667	CRA004775;PRJNA827465;Yang2025_Silkie_Wenchang	0.807076251	FALSE	FALSE	FALSE	FALSE	FALSE	other_meta_signature	0.093085432	0.425928319	13.33333333	3.639963432	5	0	22.49231052
ENSGALG00010012102	SON	3	0.105528028	0.121425446	0.869076717	0.384805171	0.0275466	5.663107034	64.6836977	0.666666667	CRA004775;PRJNA827465;Yang2025_Silkie_Wenchang	0.798852115	FALSE	FALSE	FALSE	FALSE	FALSE	other_meta_signature	0.097533611	0.527640139	13.33333333	3.53163023	5	0	22.49013731
ENSGALG00010012332	ENSGALG00010012332	2	-0.535390643	0.684084167	-0.782638553	0.433839395	0.495032534	2.117341935	52.77097273	0.5	PRJNA827465;Yang2025_Silkie_Wenchang	0.816237644	FALSE	FALSE	FALSE	FALSE	FALSE	other_meta_signature	0.08818338	2.676953213	10	4.722902727	5	0	22.48803932
ENSGALG00010000037	ND6	3	0.4386546	0.381836635	1.148801764	0.250637732	0.34799623	11.02218957	81.85478496	0.666666667	CRA004775;PRJNA827465;Yang2025_Silkie_Wenchang	0.742928069	FALSE	FALSE	FALSE	FALSE	FALSE	other_meta_signature	0.129053233	2.193273	13.33333333	1.814521504	5	0	22.47018107
ENSGALG00010009719	YIPF3	3	-0.167553352	0.125739658	-1.332541811	0.182682212	0.032020389	6.350692476	68.50737132	0.666666667	CRA004775;PRJNA827465;Yang2025_Silkie_Wenchang	0.714197397	FALSE	FALSE	FALSE	FALSE	FALSE	other_meta_signature	0.146181737	0.837766761	13.33333333	3.149262868	5	0	22.4665447
ENSGALG00010011593	WRNIP1	3	-0.158983157	0.163624071	-0.971636727	0.331231304	0.052800552	6.199042012	67.73695038	0.666666667	CRA004775;PRJNA827465;Yang2025_Silkie_Wenchang	0.777514211	FALSE	FALSE	FALSE	FALSE	FALSE	other_meta_signature	0.109291664	0.794915785	13.33333333	3.226304962	5	0	22.46384574
ENSGALG00010014916	DYNLT2B	3	-0.170158063	0.219807063	-0.774124638	0.438857089	0.097924491	6.268024616	68.09202065	0.666666667	CRA004775;PRJNA827465;Yang2025_Silkie_Wenchang	0.816680119	FALSE	FALSE	FALSE	FALSE	FALSE	other_meta_signature	0.087948017	0.850790315	13.33333333	3.190797935	5	0	22.4628696
ENSGALG00010009410	JMJD1C	3	0.152435403	0.155622533	0.979520121	0.327323052	0.048621367	6.142271488	67.4387561	0.666666667	CRA004775;PRJNA827465;Yang2025_Silkie_Wenchang	0.775602586	FALSE	FALSE	FALSE	FALSE	FALSE	other_meta_signature	0.110360752	0.762177014	13.33333333	3.25612439	5	0	22.46199549
ENSGALG00010012466	PDSS2	3	-0.020604693	0.090628956	-0.227352205	0.820149882	0.013399506	4.99998452	59.99987616	0.666666667	CRA004775;PRJNA827465;Yang2025_Silkie_Wenchang	0.94523143	FALSE	FALSE	FALSE	FALSE	FALSE	other_meta_signature	0.024461846	0.103023465	13.33333333	4.000012384	5	0	22.46083103
ENSGALG00010004868	NPAT	3	0.09036805	0.138539242	0.652292083	0.514212766	0.036903869	5.552013343	63.97703182	0.666666667	CRA004775;PRJNA827465;Yang2025_Silkie_Wenchang	0.845309013	FALSE	FALSE	FALSE	FALSE	FALSE	other_meta_signature	0.0729845	0.451840252	13.33333333	3.602296818	5	0	22.4604549
ENSGALG00010005491	GGACT	3	0.100956019	0.165147693	0.611307471	0.540996046	0.050133672	5.629436231	64.47246371	0.666666667	CRA004775;PRJNA827465;Yang2025_Silkie_Wenchang	0.85298597	FALSE	FALSE	FALSE	FALSE	FALSE	other_meta_signature	0.069058112	0.504780093	13.33333333	3.552753629	5	0	22.45992517
ENSGALG00010009144	CREBZF	3	0.416744885	0.322007773	1.294207533	0.195593746	0.247554487	10.53553486	81.01662586	0.666666667	CRA004775;PRJNA827465;Yang2025_Silkie_Wenchang	0.720732808	FALSE	FALSE	FALSE	FALSE	FALSE	other_meta_signature	0.142225708	2.083724427	13.33333333	1.898337414	5	0	22.45762088

ENSGALG00010009985	USP16	3	0.178596825	0.143885037	1.241246684	0.214514634	0.045627055	8.180121534	75.55048551	0.666666667	CRA004775;PRJNA827465;Yang2025_Silkie_Wenchang	0.729547928	FALSE	FALSE	FALSE	FALSE	FALSE	other_meta_signature	0.136946172	0.892984123	13.33333333	2.444951449	5	0	21.80821508
ENSGALG00010023978	TFDP2	3	0.18833483	0.195092269	0.965362857	0.334363099	0.083211469	8.257347069	75.77914573	0.666666667	CRA004775;PRJNA827465;Yang2025_Silkie_Wenchang	0.778938719	FALSE	FALSE	FALSE	FALSE	FALSE	other_meta_signature	0.108496708	0.941674149	13.33333333	2.422085427	5	0	21.80558962
ENSGALG00010019049	POLD2	3	0.074643051	0.15262002	0.489077713	0.624786679	0.047702001	6.569015227	69.55403617	0.666666667	CRA004775;PRJNA827465;Yang2025_Silkie_Wenchang	0.884543315	FALSE	FALSE	FALSE	FALSE	FALSE	other_meta_signature	0.053280895	0.373215253	13.33333333	3.044596383	5	0	21.80442586
ENSGALG00010021681	BTBD10	3	-0.057299522	0.137579732	-0.416482287	0.677057126	0.036907836	6.385580183	68.67943174	0.666666667	CRA004775;PRJNA827465;Yang2025_Silkie_Wenchang	0.899170887	FALSE	FALSE	FALSE	FALSE	FALSE	other_meta_signature	0.046157763	0.286497608	13.33333333	3.132056826	5	0	21.79804553
ENSGALG00010009115	COG5	3	-0.074254937	0.117928442	-0.629660969	0.528916424	0.026376708	6.617147148	69.77549455	0.666666667	CRA004775;PRJNA827465;Yang2025_Silkie_Wenchang	0.849590825	FALSE	FALSE	FALSE	FALSE	FALSE	other_meta_signature	0.070790186	0.371274686	13.33333333	3.022450545	5	0	21.79784875
ENSGALG00010022226	FBRSL1	3	0.352795664	0.282994497	1.246652029	0.212525115	0.197284617	12.80374366	84.37956857	0.666666667	CRA004775;PRJNA827465;Yang2025_Silkie_Wenchang	0.727707001	FALSE	FALSE	FALSE	FALSE	FALSE	other_meta_signature	0.138043447	1.763978322	13.33333333	1.562043143	5	0	21.79739825
ENSGALG00010027966	CLK2	3	0.046914373	0.091104496	0.514951237	0.606587096	0.015311044	6.305935855	68.28385118	0.666666667	CRA004775;PRJNA827465;Yang2025_Silkie_Wenchang	0.878668397	FALSE	FALSE	FALSE	FALSE	FALSE	other_meta_signature	0.056174993	0.234571865	13.33333333	3.171614882	5	0	21.79569507
ENSGALG00010020730	RRP12	3	-0.141332439	0.167568121	-0.843432735	0.39898647	0.059358012	7.524565849	73.42039341	0.666666667	CRA004775;PRJNA827465;Yang2025_Silkie_Wenchang	0.804589825	FALSE	FALSE	FALSE	FALSE	FALSE	other_meta_signature	0.094425464	0.706662193	13.33333333	2.657960659	5	0	21.79238165
ENSGALG00010024063	BRD7	3	0.121631632	0.148240058	0.820504481	0.411928576	0.045306461	7.251499854	72.41949886	0.666666667	CRA004775;PRJNA827465;Yang2025_Silkie_Wenchang	0.808116573	FALSE	FALSE	FALSE	FALSE	FALSE	other_meta_signature	0.092525987	0.608158158	13.33333333	2.758050114	5	0	21.79206759
ENSGALG00010017373	RP1-27O5.3	3	0.155391588	0.157902783	0.984096576	0.32506803	0.05012095	7.780846515	74.29585591	0.666666667	CRA004775;PRJNA827465;Yang2025_Silkie_Wenchang	0.774021031	FALSE	FALSE	FALSE	FALSE	FALSE	other_meta_signature	0.111247239	0.776957939	13.33333333	2.570414409	5	0	21.79195292
ENSGALG00010001903	NARS2	3	-0.092766316	0.150352159	-0.616993572	0.537238966	0.0475244	6.839836087	70.7595332	0.666666667	CRA004775;PRJNA827465;Yang2025_Silkie_Wenchang	0.851285637	FALSE	FALSE	FALSE	FALSE	FALSE	other_meta_signature	0.069924694	0.463831578	13.33333333	2.92404668	5	0	21.79113629
ENSGALG00010029182	HEATR6	3	-0.150764907	0.141792504	-1.063278398	0.287655718	0.042498369	7.7414752	74.1651307	0.666666667	CRA004775;PRJNA827465;Yang2025_Silkie_Wenchang	0.758891917	FALSE	FALSE	FALSE	FALSE	FALSE	other_meta_signature	0.119820073	0.753824535	13.33333333	2.58348693	5	0	21.79046487
ENSGALG00010014539	PAIP1	3	-0.087648145	0.09390753	-0.933345232	0.350641748	0.018253212	6.875360834	70.91061767	0.666666667	CRA004775;PRJNA827465;Yang2025_Silkie_Wenchang	0.784982422	FALSE	FALSE	FALSE	FALSE	FALSE	other_meta_signature	0.105140068	0.438240727	13.33333333	2.908938233	5	0	21.78565236
ENSGALG00010022279	EP400	3	0.077454368	0.1804605	0.429203996	0.667774775	0.066843786	6.632834806	69.84698009	0.666666667	CRA004775;PRJNA827465;Yang2025_Silkie_Wenchang	0.897394182	FALSE	FALSE	FALSE	FALSE	FALSE	other_meta_signature	0.047016751	0.387271838	13.33333333	3.015301991	5	0	21.78292391
ENSGALG00010010447	BTF3	3	0.135629209	0.236666855	0.573080707	0.566590049	0.121317804	7.390016438	72.93646074	0.666666667	CRA004775;PRJNA827465;Yang2025_Silkie_Wenchang	0.861677607	FALSE	FALSE	FALSE	FALSE	FALSE	other_meta_signature	0.064655193	0.678146044	13.33333333	2.706353926	5	0	21.7824885
ENSGALG00010019982	PLCE1	3	0.161965584	0.234416905	0.690929623	0.489609769	0.113992131	7.81070251	74.39410863	0.666666667	CRA004775;PRJNA827465;Yang2025_Silkie_Wenchang	0.835926821	FALSE	FALSE	FALSE	FALSE	FALSE	other_meta_signature	0.07783174	0.809827919	13.33333333	2.560589137	5	0	21.78158213
ENSGALG00010002340	RAB23	3	-0.020231244	0.126607746	-0.159794681	0.873042815	0.029771349	6.008324984	66.7128525	0.666666667	CRA004775;PRJNA827465;Yang2025_Silkie_Wenchang	0.962477257	FALSE	FALSE	FALSE	FALSE	FALSE	other_meta_signature	0.016609524	0.101156221	13.33333333	3.32871475	5	0	21.77981383
ENSGALG00010011860	CECR1	3	-0.185263056	0.189789182	-0.976151824	0.328989233	0.075894362	8.300213086	75.90423307	0.666666667	CRA004775;PRJNA827465;Yang2025_Silkie_Wenchang	0.776740299	FALSE	FALSE	FALSE	FALSE	FALSE	other_meta_signature	0.109724162	0.926315282	13.33333333	2.409576693	5	0	21.77894947
ENSGALG00010008191	ENSGALG00010008191	3	0.293691645	0.464879092	0.631759204	0.527544235	0.51278735	10.49801805	80.94878491	0.666666667	CRA004775;PRJNA827465;Yang2025_Silkie_Wenchang	0.849493753	FALSE	FALSE	FALSE	FALSE	FALSE	other_meta_signature	0.07083981	1.468458225	13.33333333	1.905121509	5	0	21.77775288
ENSGALG00010020037	MPP5	3	0.125081167	0.151401807	0.826153726	0.408716894	0.047662388	7.338888762	72.74791777	0.666666667	CRA004775;PRJNA827465;Yang2025_Silkie_Wenchang	0.806228613	FALSE	FALSE	FALSE	FALSE	FALSE	other_meta_signature	0.093541793	0.625405835	13.33333333	2.725208223	5	0	21.77748918
ENSGALG00010016778	MTIF2	3	0.115788129	0.107137819	1.080740025	0.279812773	0.021853289	7.292368765	72.57406935	0.666666667	CRA004775;PRJNA827465;Yang2025_Silkie_Wenchang	0.755279869	FALSE	FALSE	FALSE	FALSE	FALSE	other_meta_signature	0.121892091	0.578940647	13.33333333	2.742593065	5	0	21.77675914
ENSGALG00010014728	INTS13	3	0.02717614	0.095630481	0.284178636	0.776273487	0.017471311	6.10387143	67.2339101	0.666666667	CRA004775;PRJNA827465;Yang2025_Silkie_Wenchang	0.932913798	FALSE	FALSE	FALSE	FALSE	FALSE	other_meta_signature	0.030158483	0.135880698	13.33333333	3.27660899	5	0	21.7759815
ENSGALG00010008310	EXOSC5	3	0.041551705	0.157102127	0.264488495	0.791403529	0.047819546	6.24186293	67.95828389	0.666666667	CRA004775;PRJNA827465;Yang2025_Silkie_Wenchang	0.936723207	FALSE	FALSE	FALSE	FALSE	FALSE	other_meta_signature	0.02838872	0.207758525	13.33333333	3.204171611	5	0	21.77365219
ENSGALG00010024826	RBM33	3	0.34836372	0.325389131	1.070606504	0.284346398	0.261282899	12.69292333	84.24318852	0.666666667	CRA004775;PRJNA827465;Yang2025_Silkie_Wenchang	0.757823498	FALSE	FALSE	FALSE	FALSE	FALSE	other_meta_signature	0.120431932	1.741818601	13.33333333	1.575681148	5	0	21.77126501
ENSGALG00010006819	EMC8	3	-0.179897534	0.170158547	-1.057234779	0.290404451	0.064223625	8.274590119	75.82961849	0.666666667	CRA004775;PRJNA827465;Yang2025_Silkie_Wenchang	0.759391257	FALSE	FALSE	FALSE	FALSE	FALSE	other_meta_signature	0.119534407	0.899487668	13.33333333	2.417038151	5	0	21.76939356
ENSGALG00010003692	ZNFY2	3	-0.018843849	0.263858638	-0.071416457	0.943066312	0.138130658	6.002642287	66.68133958	0.666666667	CRA004775;PRJNA827465;Yang2025_Silkie_Wenchang	0.983323318	FALSE	FALSE	FALSE	FALSE	FALSE	other_meta_signature	0.007303662	0.094219245	13.33333333	3.331866042	5	0	21.76672228

ENSGALG00010018438	PPP2R2A	3	0.033817037	0.09622583	0.351434086	0.725262715	0.019086173	7.670297723	73.92539283	0.666666667	CRA004775;PRJNA827465;Yang2025_Silkie_Wenchang	0.915503178	FALSE	FALSE	FALSE	FALSE	FALSE	other_meta_signature	0.038340144	0.169085184	13.33333333	2.607460717	5	0	21.14821938
ENSGALG00010017870	RAB11A	3	-0.083471879	0.103440877	-0.806952548	0.419693822	0.023038319	8.668350422	76.92755942	0.666666667	CRA004775;PRJNA827465;Yang2025_Silkie_Wenchang	0.812327304	FALSE	FALSE	FALSE	FALSE	FALSE	other_meta_signature	0.090268949	0.417359395	13.33333333	2.307244058	5	0	21.14820573
ENSGALG00010021728	TMED5	3	0.055022462	0.115883915	0.474806726	0.634924738	0.029126353	8.04809848	75.14940945	0.666666667	CRA004775;PRJNA827465;Yang2025_Silkie_Wenchang	0.888385993	FALSE	FALSE	FALSE	FALSE	FALSE	other_meta_signature	0.051398297	0.275112311	13.33333333	2.485059055	5	0	21.144903
ENSGALG00010027920	SCAMP4	3	-0.229494299	0.162892879	-1.408866364	0.158874687	0.066331748	13.32587873	84.99160888	0.666666667	CRA004775;PRJNA827465;Yang2025_Silkie_Wenchang	0.68855483	FALSE	FALSE	FALSE	FALSE	FALSE	other_meta_signature	0.162061471	1.147471494	13.33333333	1.500839112	5	0	21.14370541
ENSGALG00010021543	PLEKHD1	3	0.27362394	0.492882242	0.555150737	0.578791561	0.605167876	14.50872692	86.21519303	0.666666667	CRA004775;PRJNA827465;Yang2025_Silkie_Wenchang	0.866556281	FALSE	FALSE	FALSE	FALSE	FALSE	other_meta_signature	0.062203225	1.368119699	13.33333333	1.378480697	5	0	21.14213695
ENSGALG00010022742	IFT46	3	-0.025915959	0.156880133	-0.165195927	0.868789771	0.053224834	7.512549208	73.37787821	0.666666667	CRA004775;PRJNA827465;Yang2025_Silkie_Wenchang	0.961701916	FALSE	FALSE	FALSE	FALSE	FALSE	other_meta_signature	0.016959519	0.129579795	13.33333333	2.662212179	5	0	21.14208483
ENSGALG00010007090	CEBPZ	3	0.110684566	0.134581459	0.82243547	0.410829099	0.039764476	9.251940099	78.38291236	0.666666667	CRA004775;PRJNA827465;Yang2025_Silkie_Wenchang	0.807419093	FALSE	FALSE	FALSE	FALSE	FALSE	other_meta_signature	0.092900985	0.553422829	13.33333333	2.161708764	5	0	21.14136591
ENSGALG00010020916	ARL6IP1	3	-0.340840321	0.353257907	-0.964848384	0.334620757	0.331889434	20.09822256	90.04887127	0.666666667	CRA004775;PRJNA827465;Yang2025_Silkie_Wenchang	0.778938719	FALSE	FALSE	FALSE	FALSE	FALSE	other_meta_signature	0.108496708	1.704201603	13.33333333	0.995112873	5	0	21.14114452
ENSGALG00010019024	CNPY3	3	0.247218412	0.20770077	1.190262373	0.233943285	0.107985857	13.91303092	85.62498703	0.666666667	CRA004775;PRJNA827465;Yang2025_Silkie_Wenchang	0.734277773	FALSE	FALSE	FALSE	FALSE	FALSE	other_meta_signature	0.134139618	1.23609206	13.33333333	1.437501297	5	0	21.14106631
ENSGALG00010018783	ENSGALG00010018783	3	0.025432756	0.182186157	0.139597631	0.888977912	0.062178609	7.503318934	73.34512877	0.666666667	CRA004775;PRJNA827465;Yang2025_Silkie_Wenchang	0.966441359	FALSE	FALSE	FALSE	FALSE	FALSE	other_meta_signature	0.014824493	0.127163779	13.33333333	2.665487123	5	0	21.14080873
ENSGALG00010023536	ENSGALG00010023536	3	0.034024742	0.289629926	0.117476609	0.906482379	0.183104923	7.618809387	73.74917919	0.666666667	CRA004775;PRJNA827465;Yang2025_Silkie_Wenchang	0.97313563	FALSE	FALSE	FALSE	FALSE	FALSE	other_meta_signature	0.011826626	0.170123708	13.33333333	2.625082081	5	0	21.14036575
ENSGALG00010012326	SGTB	3	-0.047728699	0.120597305	-0.395769198	0.692275302	0.031862332	7.923324813	74.7580713	0.666666667	CRA004775;PRJNA827465;Yang2025_Silkie_Wenchang	0.904075368	FALSE	FALSE	FALSE	FALSE	FALSE	other_meta_signature	0.043795363	0.238643494	13.33333333	2.52419287	5	0	21.13996506
ENSGALG00010027190	IPPK	3	0.173961322	0.137480105	1.265356342	0.205743618	0.044625326	11.1429647	82.0514553	0.666666667	CRA004775;PRJNA827465;Yang2025_Silkie_Wenchang	0.72361988	FALSE	FALSE	FALSE	FALSE	FALSE	other_meta_signature	0.14048951	0.869806612	13.33333333	1.79485447	5	0	21.13848393
ENSGALG00010024949	PAN2	3	-0.069985648	0.14085571	-0.496860571	0.619287378	0.042313605	8.33131623	75.99418934	0.666666667	CRA004775;PRJNA827465;Yang2025_Silkie_Wenchang	0.882522345	FALSE	FALSE	FALSE	FALSE	FALSE	other_meta_signature	0.05427429	0.349928241	13.33333333	2.400581066	5	0	21.13811693
ENSGALG00010007960	TSNAX	3	-0.039952951	0.107179558	-0.372766524	0.70932222	0.023247387	7.802506822	74.36721241	0.666666667	CRA004775;PRJNA827465;Yang2025_Silkie_Wenchang	0.910138998	FALSE	FALSE	FALSE	FALSE	FALSE	other_meta_signature	0.040892277	0.199764757	13.33333333	2.563278759	5	0	21.13726913
ENSGALG00010013431	TMEM175	3	-0.04190093	0.206824325	-0.202591888	0.839454031	0.094352991	7.779420464	74.29114406	0.666666667	CRA004775;PRJNA827465;Yang2025_Silkie_Wenchang	0.952830471	FALSE	FALSE	FALSE	FALSE	FALSE	other_meta_signature	0.020984363	0.209504652	13.33333333	2.570885594	5	0	21.13470794
ENSGALG00010024966	AGAP3	3	0.087265312	0.106284923	0.82105071	0.411617384	0.022652264	8.814119695	77.30913501	0.666666667	CRA004775;PRJNA827465;Yang2025_Silkie_Wenchang	0.807944877	FALSE	FALSE	FALSE	FALSE	FALSE	other_meta_signature	0.092618268	0.436326559	13.33333333	2.269086499	5	0	21.13136466
ENSGALG00010025723	SETD2	3	0.111117628	0.165136056	0.672885321	0.501020252	0.060104617	9.230423119	78.3325209	0.666666667	CRA004775;PRJNA827465;Yang2025_Silkie_Wenchang	0.841612597	FALSE	FALSE	FALSE	FALSE	FALSE	other_meta_signature	0.074887773	0.555588141	13.33333333	2.16674791	5	0	21.13055716
ENSGALG00010015756	PCM1	3	0.141697733	0.162416769	0.872432903	0.382972257	0.059255656	10.04831275	80.09616092	0.666666667	CRA004775;PRJNA827465;Yang2025_Silkie_Wenchang	0.798349794	FALSE	FALSE	FALSE	FALSE	FALSE	other_meta_signature	0.097806783	0.708488664	13.33333333	1.990383908	5	0	21.13001269
ENSGALG00010018459	SNX12	3	-0.120713423	0.171647005	-0.703265534	0.481890286	0.067824579	9.468414207	78.8771387	0.666666667	CRA004775;PRJNA827465;Yang2025_Silkie_Wenchang	0.833297144	FALSE	FALSE	FALSE	FALSE	FALSE	other_meta_signature	0.079200107	0.603567115	13.33333333	2.11228613	5	0	21.12838668
ENSGALG00010028485	USF1	3	0.046951104	0.179886768	0.261003654	0.794089689	0.070419187	7.899955101	74.68340042	0.666666667	CRA004775;PRJNA827465;Yang2025_Silkie_Wenchang	0.936723207	FALSE	FALSE	FALSE	FALSE	FALSE	other_meta_signature	0.02838872	0.234755519	13.33333333	2.531659958	5	0	21.12813753
ENSGALG00010013945	SCAF11	3	0.286121446	0.220285516	1.298866357	0.193989797	0.124857963	16.37702274	87.78776807	0.666666667	CRA004775;PRJNA827465;Yang2025_Silkie_Wenchang	0.720732808	FALSE	FALSE	FALSE	FALSE	FALSE	other_meta_signature	0.142225708	1.430607229	13.33333333	1.221223193	5	0	21.12738946
ENSGALG00010012843	ENSGALG00010012843	3	0.153282491	0.350031874	0.437910096	0.661451457	0.287624619	10.11650625	80.23032903	0.666666667	CRA004775;PRJNA827465;Yang2025_Silkie_Wenchang	0.896410952	FALSE	FALSE	FALSE	FALSE	FALSE	other_meta_signature	0.047492846	0.766412457	13.33333333	1.976967097	5	0	21.12420573
ENSGALG00010028855	SURF6	3	0.253268789	0.268076675	0.944762494	0.344780144	0.180433737	14.10671183	85.82235163	0.666666667	CRA004775;PRJNA827465;Yang2025_Silkie_Wenchang	0.783926329	FALSE	FALSE	FALSE	FALSE	FALSE	other_meta_signature	0.105724749	1.266343943	13.33333333	1.417764837	5	0	21.12316686
ENSGALG00010007783	NOL7	3	-0.082243802	0.179588718	-0.457956396	0.646983768	0.073048458	8.588011479	76.71172186	0.666666667	CRA004775;PRJNA827465;Yang2025_Silkie_Wenchang	0.892023744	FALSE	FALSE	FALSE	FALSE	FALSE	other_meta_signature	0.049623585	0.41121901	13.33333333	2.328827814	5	0	21.12300374
ENSGALG00010008295	DMAC2	3	0.126366144	0.322541644	0.391782416	0.695218989	0.241012104	9.469050113	78.87855723	0.666666667	CRA004775;PRJNA827465;Yang2025_Silkie_Wenchang	0.904561326	FALSE	FALSE	FALSE	FALSE	FALSE	other_meta_signature	0.043561984	0.631830722	13.33333333	2.112144277	5	0	21.12087032
ENSGALG00010015177	TC2N	3	0.036938359	0.22925452	0.1611238	0.871995896	0.1028954	7.743952345	74.17339479	0.666666667	CRA004775;PRJNA827465;Yang2025_Silkie_Wenchang	0.962477257	FALSE	FALSE	FALSE	FALSE	FALSE	other_meta_signature	0.016609524	0.184691797	13.33333333	2.582660521	5	0	21.11729518
ENSGALG00010027367	SEC22C	3	-0.160783617	0.177728823	-0.904656963	0.365647148	0.075560702	10.6623271	81.24236875	0.666666667	CRA004775;PRJNA827465;Yang2025_Silkie_Wenchang	0.788777182	FALSE	FALSE	FALSE	FALSE	FALSE	other_meta_signature	0.103045662	0.803918087	13.33333333	1.875763125	5	0	21.11606021
ENSGALG00010012758	NAB1	3	-0.07776335	0.100364114	-0.7748123	0.43845058	0.02026366	8.673667937	76.94170431	0.666666667	CRA004775;PRJNA827465;Yang2025_Silkie_Wenchang	0.816680119	FALSE	FALSE	FALSE	FALSE	FALSE	other_meta_signature	0.087948017	0.388816749	13.33333333	2.305829569	5	0	21.11592767
ENSGALG00010028174	GMPPB	3	0.110254787	0.223437753	0.493447438	0.621696471	0.11458473	9.188363284	78.23333778	0.666666667	CRA004775;PRJNA827465;Yang2025_Silkie_Wenchang	0.883700251	FALSE	FALSE	FALSE	FALSE	FALSE	other_meta_signature	0.053695022	0.551273933	13.33333333	2.176666222	5	0	21.11496851
ENSGALG00010012912	NKAPL	3	0.353888007	0.308703136	1.146369975	0.251642101	0.257453848	22.64370787	91.16752428	0.666666667	CRA004775;PRJNA827465;Yang2025_Silkie_Wenchang	0.74365156	FALSE	FALSE	FALSE	FALSE	FALSE	other_meta_signature	0.128630507	1.769440034	13.33333333	0.883247572	5	0	21.11465145
ENSGALG00010020153	AXL	3	-0.158835443	0.186124293	-0.853383725	0.393446544	0.074708727	10.57631948	81.08982994	0.666666667	CRA004775;PRJNA827465;Yang2025_Silkie_Wenchang	0.801809775	FALSE	FALSE	FALSE	FALSE	FALSE	other_meta_signature	0.095928653	0.794177213	13.33333333	1.891017006	5	0	21.11445621
ENSGALG00010005208	ENSGALG00010005208	3	-0.21838556	0.258862511	-0.84363533	0.398873215	0.16445259	12.58421054	84.10706819	0.666666667	CRA004775;PRJNA827465;Yang2025_Silkie_Wenchang	0.804589825	FALSE	FALSE	FALSE	FALSE	FALSE	other_meta_signature	0.094425464	1.0919278	13.33333333	1.589293181	5	0	21.10897978
ENSGALG00010005755	ZMYM2	3	0.072806755	0.151962815	0.479109018	0.631861074	0.0518864	8.477816709	76.40902052	0.666666667	CRA004775;PRJNA827465;Yang2025_Silkie_Wenchang	0.886776624	FALSE	FALSE	FALSE	FALSE	FALSE	other_meta_signature	0.052185764	0.364033776	13.33333333	2.359097948	5	0	21.10865082

ENSGALG00010004787	RIOK3	3	-0.040966313	0.13113838	-0.312389959	0.75474418	0.036840596	10.22662781	80.44321122	0.666666667	CRA004775;PRJNA827465;Yang2025_Silkie_Wenchan	0.927441599	FALSE	FALSE	FALSE	FALSE	FALSE	other_meta_signature	0.032713428	0.204831566	13.33333333	1.955678878	5	0	20.52655721
ENSGALG00010001262	ENSGALG00010001262	3	0.352818807	0.453030321	0.778797335	0.436099108	0.576182583	58.68218165	96.59181042	0.666666667	CRA004775;PRJNA827465;Yang2025_Silkie_Wenchan	0.816680119	FALSE	FALSE	FALSE	FALSE	FALSE	other_meta_signature	0.087948017	1.764094033	13.33333333	0.340818958	5	0	20.52619434
ENSGALG00010005769	TFG	3	0.080850919	0.158820909	0.509069739	0.610703342	0.059327616	11.54833777	82.68149027	0.666666667	CRA004775;PRJNA827465;Yang2025_Silkie_Wenchan	0.879760007	FALSE	FALSE	FALSE	FALSE	FALSE	other_meta_signature	0.055635785	0.404254594	13.33333333	1.731850973	5	0	20.52507468
ENSGALG00010016802	TMEM222	3	-0.142553604	0.181263976	-0.786441999	0.431608586	0.083018746	14.398808	86.10996132	0.666666667	CRA004775;PRJNA827465;Yang2025_Silkie_Wenchan	0.815975616	FALSE	FALSE	FALSE	FALSE	FALSE	other_meta_signature	0.088322819	0.712768018	13.33333333	1.389003868	5	0	20.52342804
ENSGALG00010015031	MYH9	3	0.163380003	0.263101002	0.620978261	0.534613927	0.160949124	15.35877528	86.97812838	0.666666667	CRA004775;PRJNA827465;Yang2025_Silkie_Wenchan	0.850368089	FALSE	FALSE	FALSE	FALSE	FALSE	other_meta_signature	0.070393045	0.816900014	13.33333333	1.302187162	5	0	20.52281356
ENSGALG00010005353	ENSGALG00010005353	3	-0.328487348	0.355043162	-0.925203984	0.354859811	0.349314495	45.6198211	95.61594072	0.666666667	CRA004775;PRJNA827465;Yang2025_Silkie_Wenchan	0.786517334	FALSE	FALSE	FALSE	FALSE	FALSE	other_meta_signature	0.104291701	1.642436742	13.33333333	0.438405928	5	0	20.5184677
ENSGALG00010012896	STK38L	3	-0.069861957	0.143345828	-0.487366513	0.62599862	0.042153112	11.23945477	82.20554253	0.666666667	CRA004775;PRJNA827465;Yang2025_Silkie_Wenchan	0.884565171	FALSE	FALSE	FALSE	FALSE	FALSE	other_meta_signature	0.053270165	0.349309783	13.33333333	1.779445747	5	0	20.51535903
ENSGALG00010001264	FAM3C	3	0.04107318	0.149507994	0.274722299	0.783529598	0.049009269	10.27694442	80.53896256	0.666666667	CRA004775;PRJNA827465;Yang2025_Silkie_Wenchan	0.934560964	FALSE	FALSE	FALSE	FALSE	FALSE	other_meta_signature	0.029392363	0.205365899	13.33333333	1.946103744	5	0	20.51419534
ENSGALG00010008716	TATDN1	3	0.180699482	0.202574566	0.892014656	0.372385077	0.098260318	17.01961966	88.24885609	0.666666667	CRA004775;PRJNA827465;Yang2025_Silkie_Wenchan	0.793781487	FALSE	FALSE	FALSE	FALSE	FALSE	other_meta_signature	0.100299034	0.903497408	13.33333333	1.175114391	5	0	20.51224417
ENSGALG00010022474	VCL	3	0.172091222	0.179414146	0.959184244	0.337465937	0.078986703	16.58068519	87.93777231	0.666666667	CRA004775;PRJNA827465;Yang2025_Silkie_Wenchan	0.780666047	FALSE	FALSE	FALSE	FALSE	FALSE	other_meta_signature	0.107534709	0.860456111	13.33333333	1.206222769	5	0	20.50754692
ENSGALG00010006015	CDC123	3	-0.250267426	0.241637465	-1.035714499	0.300335356	0.152836195	24.81654599	91.94086074	0.666666667	CRA004775;PRJNA827465;Yang2025_Silkie_Wenchan	0.764435932	FALSE	FALSE	FALSE	FALSE	FALSE	other_meta_signature	0.116658907	1.251337128	13.33333333	0.805913926	5	0	20.50724329
ENSGALG00010010933	ANAPC4	3	-0.118831632	0.153048524	-0.776431084	0.437494497	0.054209339	13.41669387	85.09319793	0.666666667	CRA004775;PRJNA827465;Yang2025_Silkie_Wenchan	0.816680119	FALSE	FALSE	FALSE	FALSE	FALSE	other_meta_signature	0.087948017	0.594158158	13.33333333	1.490680207	5	0	20.50611972
ENSGALG00010029667	TMEM104	3	-0.074175555	0.165147572	-0.449147113	0.653325536	0.065411307	11.41506639	82.47929594	0.666666667	CRA004775;PRJNA827465;Yang2025_Silkie_Wenchan	0.894256035	FALSE	FALSE	FALSE	FALSE	FALSE	other_meta_signature	0.04853812	0.370877776	13.33333333	1.752070406	5	0	20.50481964
ENSGALG00010028049	MAN1B1	3	-0.19938998	0.214734195	-0.928543219	0.35312586	0.111491828	18.75079115	89.33378339	0.666666667	CRA004775;PRJNA827465;Yang2025_Silkie_Wenchan	0.786027477	FALSE	FALSE	FALSE	FALSE	FALSE	other_meta_signature	0.104562272	0.996949901	13.33333333	1.066621661	5	0	20.50146717
ENSGALG00010018673	NAGPA	3	-0.149078618	0.171454732	-0.869492586	0.384577761	0.07497292	15.13163219	86.7826552	0.666666667	CRA004775;PRJNA827465;Yang2025_Silkie_Wenchan	0.798852115	FALSE	FALSE	FALSE	FALSE	FALSE	other_meta_signature	0.097533611	0.74539309	13.33333333	1.32173448	5	0	20.49799451
ENSGALG00010015156	CNOT6	3	-0.138217685	0.168791031	-0.818868656	0.412861355	0.066456912	14.48039917	86.18822606	0.666666667	CRA004775;PRJNA827465;Yang2025_Silkie_Wenchan	0.808510622	FALSE	FALSE	FALSE	FALSE	FALSE	other_meta_signature	0.09231427	0.691088424	13.33333333	1.381177394	5	0	20.49791342
ENSGALG00010022933	UNC119B	3	-0.053480683	0.139362302	-0.383752869	0.701161623	0.045646531	10.82600929	81.52597189	0.666666667	CRA004775;PRJNA827465;Yang2025_Silkie_Wenchan	0.907254657	FALSE	FALSE	FALSE	FALSE	FALSE	other_meta_signature	0.042270794	0.267403415	13.33333333	1.847402811	5	0	20.49041035
ENSGALG00010022807	ADCY6	3	0.176420463	0.207192499	0.851480936	0.394502249	0.105737819	16.96650754	88.21207019	0.666666667	CRA004775;PRJNA827465;Yang2025_Silkie_Wenchan	0.801809775	FALSE	FALSE	FALSE	FALSE	FALSE	other_meta_signature	0.095928653	0.882102315	13.33333333	1.178792981	5	0	20.49015728
ENSGALG00010023102	RNF10	3	-0.128827375	0.140289483	-0.91829674	0.358463533	0.046686605	14.28756444	86.00181291	0.666666667	CRA004775;PRJNA827465;Yang2025_Silkie_Wenchan	0.787164224	FALSE	FALSE	FALSE	FALSE	FALSE	other_meta_signature	0.103934652	0.644136877	13.33333333	1.399818709	5	0	20.48122357
ENSGALG00010000059	MFAP1	3	0.071425575	0.181691028	0.393115586	0.694234113	0.07997727	11.45905417	82.54655253	0.666666667	CRA004775;PRJNA827465;Yang2025_Silkie_Wenchan	0.904226333	FALSE	FALSE	FALSE	FALSE	FALSE	other_meta_signature	0.04372285	0.357127875	13.33333333	1.745344747	5	0	20.47952881
ENSGALG00010015095	RBFOX2	3	0.04296978	0.171020975	0.251254441	0.801617396	0.062961442	10.50523854	80.96187923	0.666666667	CRA004775;PRJNA827465;Yang2025_Silkie_Wenchan	0.938576506	FALSE	FALSE	FALSE	FALSE	FALSE	other_meta_signature	0.027530321	0.214848898	13.33333333	1.903812077	5	0	20.47952463
ENSGALG00010000056	UNC45A	3	0.127582533	0.181502382	0.702924838	0.482102591	0.083400448	14.00722024	85.72164951	0.666666667	CRA004775;PRJNA827465;Yang2025_Silkie_Wenchan	0.833306667	FALSE	FALSE	FALSE	FALSE	FALSE	other_meta_signature	0.079195143	0.637912663	13.33333333	1.427835049	5	0	20.47827619
ENSGALG00010024931	UBE3C	3	-0.099987406	0.15555331	-0.642785459	0.52036332	0.056964765	12.71979559	84.27647688	0.666666667	CRA004775;PRJNA827465;Yang2025_Silkie_Wenchan	0.847038585	FALSE	FALSE	FALSE	FALSE	FALSE	other_meta_signature	0.072096806	0.49993703	13.33333333	1.572352312	5	0	20.47771948
ENSGALG00010024825	UBXN7	3	-0.048201027	0.183714018	-0.262369891	0.793036284	0.076005393	10.68099882	81.27515943	0.666666667	CRA004775;PRJNA827465;Yang2025_Silkie_Wenchan	0.936723207	FALSE	FALSE	FALSE	FALSE	FALSE	other_meta_signature	0.02838872	0.241005134	13.33333333	1.872484057	5	0	20.47521124
ENSGALG00010018899	ACSS1	3	-0.140764549	0.237374452	-0.593006317	0.553176925	0.128955961	14.61917942	86.31934158	0.666666667	CRA004775;PRJNA827465;Yang2025_Silkie_Wenchan	0.857002226	FALSE	FALSE	FALSE	FALSE	FALSE	other_meta_signature	0.06701805	0.703822747	13.33333333	1.368065842	5	0	20.47223997
ENSGALG00010028031	NISCH	3	-0.013321631	0.120888748	-0.110197443	0.91225279	0.033268365	9.738504932	79.46296671	0.666666667	CRA004775;PRJNA827465;Yang2025_Silkie_Wenchan	0.974809833	FALSE	FALSE	FALSE	FALSE	FALSE	other_meta_signature	0.011080099	0.066608155	13.33333333	2.053703329	5	0	20.46472492
ENSGALG00010028464	ENSGALG00010028464	3	0.082789362	0.197360105	0.419483776	0.674862609	0.089676112	11.98972099	83.31904469	0.666666667	CRA004775;PRJNA827465;Yang2025_Silkie_Wenchan	0.899170887	FALSE	FALSE	FALSE	FALSE	FALSE	other_meta_signature	0.046157763	0.413946809	13.33333333	1.668095531	5	0	20.46153344
ENSGALG00010021713	RAB27A	3	0.122787043	0.160631898	0.764400125	0.444628834	0.064923088	14.03281735	85.74769449	0.666666667	CRA004775;PRJNA827465;Yang2025_Silkie_Wenchan	0.818036056	FALSE	FALSE	FALSE	FALSE	FALSE	other_meta_signature	0.087227554	0.613935216	13.33333333	1.425230551	5	0	20.45972665
ENSGALG00010008158	CLPTM1L	3	-0.065358434	0.110864615	-0.58953376	0.555503272	0.026864442	11.55680538	82.69417945	0.666666667	CRA004775;PRJNA827465;Yang2025_Silkie_Wenchan	0.858444105	FALSE	FALSE	FALSE	FALSE	FALSE	other_meta_signature	0.066287977	0.326792168	13.33333333	1.730582055	5	0	20.45699553
ENSGALG00010003218	MAP3K7	3	-0.076950627	0.11039502	-0.697047993	0.485772759	0.028340694	12.0519965	83.40523912	0.666666667	CRA004775;PRJNA827465;Yang2025_Silkie_Wenchan	0.835259765	FALSE	FALSE	FALSE	FALSE	FALSE	other_meta_signature	0.078178439	0.384753135	13.33333333	1.659476088	5	0	20.45574099
ENSGALG00010014944	FGB	3	0.178328869	1.110698953	0.160555539	0.872443475	3.232098653	16.49289319	87.87356483	0.666666667	CRA004775;PRJNA827465;Yang2025_Silkie_Wenchan	0.962477257	FALSE	FALSE	FALSE	FALSE	FALSE	other_meta_signature	0.016609524	0.891644345	13.33333333	1.212643517	5	0	20.45423072
ENSGALG00010014908	ENSGALG00010014908	3	-0.109514383	0.173986447	-0.629442031	0.529059709	0.066612838	13.31851241	84.98330791	0.666666667	CRA004775;PRJNA827465;Yang2025_Silkie_Wenchan	0.849590825	FALSE	FALSE	FALSE	FALSE	FALSE	other_meta_signature	0.070790186	0.547571914	13.33333333	1.501669209	5	0	20.45336464
ENSGALG00010028114	PFKFB4	3	0.071398664	0.165332871	0.431847967	0.665851915	0.06187402	11.65806785	82.84449854	0.666666667	CRA004775;PRJNA827465;Yang2025_Silkie_Wenchan	0.896630081	FALSE	FALSE	FALSE	FALSE	FALSE	other_meta_signature	0.047386695	0.356993321	13.33333333	1.715550146	5	0	20.4532635
ENSGALG00010025586	TCTN1	3	0.006566017	0.139136929	0.047191045	0.962360965	0.040847297	9.636209684	79.24495143	0.666666667	CRA004775;PRJNA827465;Yang2025_Silkie_Wenchan	0.98647409	FALSE	FALSE	FALSE	FALSE	FALSE	other_meta_signature	0.005914317	0.032830085	13.33333333	2.075504857	5	0	20.44758259
ENSGALG00010002518	MAST1	3	0.121812743	0.471863701	0.258152393	0.796289298	0.56828004	13.56978753	85.26137572	0.666666667	CRA004775;PRJNA827465;Yang2025_Silkie_Wenchan	0.936723207	FALSE	FALSE	FALSE	FALSE	FALSE	other_meta_signature	0.02838872	0.609063717	13.33333333	1.473862428	5	0	20.4446482
ENSGALG00010000134	ENSGALG00010000134	3	0.321096232	0.36138299	0.888520602	0.374260779	0.365313216	49.37988433	95.94976775	0.666666667	CRA004775;PRJNA827465;Yang2025_Silkie_Wenchan	0.794366713	FALSE	FALSE	FALSE	FALSE	FALSE	other_meta_signature	0.099978963	1.605481159	13.33333333	0.405023225	5	0	20.44381668

ENSGALG00010017866	RALA	3	-0.088993452	0.207905508	-0.428047593	0.668616471	0.115547154	26.2100165	92.36932949	0.666666667	CRA004775;PRJNA827465;Yang2025_Silkie_Wenchang	0.89743386	FALSE	FALSE	FALSE	FALSE	FALSE	other_meta_signature	0.046997549	0.44496726	13.33333333	0.763067051	5	0	19.58836519
ENSGALG00010005154	CWF19L2	3	0.022610991	0.265415457	0.085190936	0.932109597	0.182367187	17.73829948	88.72496204	0.666666667	CRA004775;PRJNA827465;Yang2025_Silkie_Wenchang	0.979880297	FALSE	FALSE	FALSE	FALSE	FALSE	other_meta_signature	0.008826975	0.113054956	13.33333333	1.127503796	5	0	19.58271906
ENSGALG00010017801	SMNDC1	3	-0.143560693	0.243848706	-0.588728541	0.556043389	0.16486663	43.0405147	95.35321542	0.666666667	CRA004775;PRJNA827465;Yang2025_Silkie_Wenchang	0.858444105	FALSE	FALSE	FALSE	FALSE	FALSE	other_meta_signature	0.066287977	0.717803464	13.33333333	0.464678458	5	0	19.58210323
ENSGALG00010008938	ZBED4	3	0.082232066	0.212678973	0.386648782	0.699016239	0.116856125	25.22818096	92.07235748	0.666666667	CRA004775;PRJNA827465;Yang2025_Silkie_Wenchang	0.906557084	FALSE	FALSE	FALSE	FALSE	FALSE	other_meta_signature	0.042604844	0.41116033	13.33333333	0.792764252	5	0	19.57986276
ENSGALG00010020399	ATP13A2	3	0.167436879	0.377479514	0.443565473	0.657356773	0.407342871	56.65485065	96.46985214	0.666666667	CRA004775;PRJNA827465;Yang2025_Silkie_Wenchang	0.895497391	FALSE	FALSE	FALSE	FALSE	FALSE	other_meta_signature	0.047935675	0.837184396	13.33333333	0.353014786	5	0	19.57146819
ENSGALG00010009047	SUPT5H	3	0.015585502	0.201139015	0.077486219	0.938236754	0.100710141	17.36388507	88.48184037	0.666666667	CRA004775;PRJNA827465;Yang2025_Silkie_Wenchang	0.981988366	FALSE	FALSE	FALSE	FALSE	FALSE	other_meta_signature	0.007893657	0.077927509	13.33333333	1.151815963	5	0	19.57097046
ENSGALG00010021168	IVNS1ABP	3	0.030454934	0.175211066	0.173818552	0.862008064	0.080752854	18.81725308	89.37145613	0.666666667	CRA004775;PRJNA827465;Yang2025_Silkie_Wenchang	0.960057662	FALSE	FALSE	FALSE	FALSE	FALSE	other_meta_signature	0.017702682	0.152274669	13.33333333	1.062854387	5	0	19.56616507
ENSGALG00010016704	CSTF2	3	0.034959386	0.199774409	0.174994318	0.861084098	0.104426285	19.28532366	89.62941958	0.666666667	CRA004775;PRJNA827465;Yang2025_Silkie_Wenchang	0.959482076	FALSE	FALSE	FALSE	FALSE	FALSE	other_meta_signature	0.017963134	0.174796932	13.33333333	1.037058042	5	0	19.56315144
ENSGALG00010002597	ENSGALG00010002597	3	0.020731274	0.239780762	0.086459287	0.931101319	0.151797054	17.91677673	88.83727787	0.666666667	CRA004775;PRJNA827465;Yang2025_Silkie_Wenchang	0.979504478	FALSE	FALSE	FALSE	FALSE	FALSE	other_meta_signature	0.008993574	0.103656369	13.33333333	1.116272213	5	0	19.56225549
ENSGALG00010000581	ENSGALG00010000581	3	0.150540497	0.220588021	0.682450917	0.494953869	0.129286425	50.21085566	96.01679761	0.666666667	CRA004775;PRJNA827465;Yang2025_Silkie_Wenchang	0.839154133	FALSE	FALSE	FALSE	FALSE	FALSE	other_meta_signature	0.076158262	0.752702486	13.33333333	0.398320239	5	0	19.56051432
ENSGALG00010017636	ZBTB8OS	3	0.057292648	0.291427075	0.19659343	0.844145713	0.223420385	21.97198595	90.89750009	0.666666667	CRA004775;PRJNA827465;Yang2025_Silkie_Wenchang	0.953651649	FALSE	FALSE	FALSE	FALSE	FALSE	other_meta_signature	0.020610236	0.286463241	13.33333333	0.910249991	5	0	19.5506568
ENSGALG00010006343	SEC23A	3	-0.032393156	0.130687767	-0.247866782	0.804237482	0.040843379	19.63780409	89.81556191	0.666666667	CRA004775;PRJNA827465;Yang2025_Silkie_Wenchang	0.939864212	FALSE	FALSE	FALSE	FALSE	FALSE	other_meta_signature	0.026934887	0.161965782	13.33333333	1.018443809	5	0	19.54067781
ENSGALG00010029254	MMD	3	-0.084316402	0.229017565	-0.368165657	0.71274971	0.137483323	27.34761638	92.68674837	0.666666667	CRA004775;PRJNA827465;Yang2025_Silkie_Wenchang	0.911172535	FALSE	FALSE	FALSE	FALSE	FALSE	other_meta_signature	0.040399379	0.421582012	13.33333333	0.731325163	5	0	19.52663989
ENSGALG00010022494	FHIP2A	3	-0.080751081	0.230399249	-0.350483267	0.725976046	0.130350586	26.66135579	92.49850602	0.666666667	CRA004775;PRJNA827465;Yang2025_Silkie_Wenchang	0.915610554	FALSE	FALSE	FALSE	FALSE	FALSE	other_meta_signature	0.03828921	0.403755407	13.33333333	0.750149398	5	0	19.52552735
ENSGALG00010001381	MDFIC	3	-0.146504622	0.325866158	-0.44958526	0.653009518	0.281779177	49.3018358	95.94335593	0.666666667	CRA004775;PRJNA827465;Yang2025_Silkie_Wenchang	0.894256035	FALSE	FALSE	FALSE	FALSE	FALSE	other_meta_signature	0.04853812	0.732523108	13.33333333	0.405664407	5	0	19.52005897
ENSGALG00010007251	SPAST	3	-0.13185768	0.197086576	-0.669034306	0.503473594	0.10978815	44.14582781	95.46956055	0.666666667	CRA004775;PRJNA827465;Yang2025_Silkie_Wenchang	0.8426408	FALSE	FALSE	FALSE	FALSE	FALSE	other_meta_signature	0.074357517	0.659288401	13.33333333	0.453043945	5	0	19.5200232
ENSGALG00010017368	ERLIN2	3	-0.021500047	0.12529244	-0.171598916	0.863752859	0.039631392	18.86599371	89.39891516	0.666666667	CRA004775;PRJNA827465;Yang2025_Silkie_Wenchang	0.960290736	FALSE	FALSE	FALSE	FALSE	FALSE	other_meta_signature	0.017597261	0.107500235	13.33333333	1.060108484	5	0	19.51853931
ENSGALG00010014209	CCDC50	3	-0.093963691	0.19484938	-0.482237569	0.629637192	0.101333978	30.18171537	93.3734714	0.666666667	CRA004775;PRJNA827465;Yang2025_Silkie_Wenchang	0.885779003	FALSE	FALSE	FALSE	FALSE	FALSE	other_meta_signature	0.052674619	0.469818457	13.33333333	0.66265286	5	0	19.51847927
ENSGALG00010005490	DCBLD2	3	-0.094709606	0.240764779	-0.393369854	0.694046331	0.157326516	30.4448635	93.43074736	0.666666667	CRA004775;PRJNA827465;Yang2025_Silkie_Wenchang	0.904226333	FALSE	FALSE	FALSE	FALSE	FALSE	other_meta_signature	0.04372285	0.473548029	13.33333333	0.656925264	5	0	19.50752948
ENSGALG00010005279	CCDC181	2	0.017180742	0.417872544	0.041114791	0.967204383	0.196790114	2.266172308	55.87272881	0.5	PRJNA827465;Yang2025_Silkie_Wenchang	0.987512768	FALSE	FALSE	FALSE	FALSE	FALSE	other_meta_signature	0.00545728	0.085903712	10	4.412727119	5	0	19.50408811
ENSGALG00010002441	USO1	3	0.080350319	0.159743597	0.502995552	0.614967399	0.066937285	28.19166991	92.90570581	0.666666667	CRA004775;PRJNA827465;Yang2025_Silkie_Wenchang	0.881230791	FALSE	FALSE	FALSE	FALSE	FALSE	other_meta_signature	0.054910337	0.401751593	13.33333333	0.709429419	5	0	19.49942468
ENSGALG00010029383	GNA13	3	-0.046388527	0.223241307	-0.207795448	0.835388687	0.12133789	21.95203859	90.88922885	0.666666667	CRA004775;PRJNA827465;Yang2025_Silkie_Wenchang	0.951700963	FALSE	FALSE	FALSE	FALSE	FALSE	other_meta_signature	0.021499491	0.231942636	13.33333333	0.911077115	5	0	19.49785258
ENSGALG00010029725	TSEN54	3	-0.049092235	0.211533067	-0.232078302	0.816477197	0.118702083	22.39157568	91.0680694	0.666666667	CRA004775;PRJNA827465;Yang2025_Silkie_Wenchang	0.943795853	FALSE	FALSE	FALSE	FALSE	FALSE	other_meta_signature	0.025121935	0.245461174	13.33333333	0.89319306	5	0	19.4971095
ENSGALG00010019312	KBTBD2	3	-0.032948093	0.136129953	-0.242034119	0.808753726	0.04501213	20.66057048	90.31972519	0.666666667	CRA004775;PRJNA827465;Yang2025_Silkie_Wenchang	0.941025884	FALSE	FALSE	FALSE	FALSE	FALSE	other_meta_signature	0.02639843	0.164740467	13.33333333	0.968027481	5	0	19.49249971
ENSGALG00010022868	VAC14	3	-0.010187691	0.154729081	-0.065842122	0.947503521	0.060283901	18.18836343	89.00395845	0.666666667	CRA004775;PRJNA827465;Yang2025_Silkie_Wenchang	0.984004959	FALSE	FALSE	FALSE	FALSE	FALSE	other_meta_signature	0.007002713	0.050938455	13.33333333	1.099604155	5	0	19.49087866
ENSGALG00010017034	ZMYM3	3	0.031740525	0.216786155	0.146413983	0.883594589	0.12359329	20.35778567	90.17574881	0.666666667	CRA004775;PRJNA827465;Yang2025_Silkie_Wenchang	0.965449961	FALSE	FALSE	FALSE	FALSE	FALSE	other_meta_signature	0.015270231	0.158702623	13.33333333	0.982425119	5	0	19.48973131
ENSGALG00010004116	SPRY1	3	0.027392531	0.238548683	0.114829942	0.908579916	0.146496827	20.19255687	90.09536032	0.666666667	CRA004775;PRJNA827465;Yang2025_Silkie_Wenchang	0.973650947	FALSE	FALSE	FALSE	FALSE	FALSE	other_meta_signature	0.011596709	0.136962657	13.33333333	0.990463968	5	0	19.47235667
ENSGALG00010007633	ENSGALG00010007633	3	0.061016024	0.294239869	0.207368309	0.83572223	0.236786765	24.61636139	91.8753224	0.666666667	CRA004775;PRJNA827465;Yang2025_Silkie_Wenchang	0.951850863	FALSE	FALSE	FALSE	FALSE	FALSE	other_meta_signature	0.021431092	0.305080121	13.33333333	0.81246776	5	0	19.47231231
ENSGALG00010025252	PPP1R9B	3	0.099806173	0.232915533	0.428508018	0.668281297	0.143453932	33.8606035	94.09343073	0.666666667	CRA004775;PRJNA827465;Yang2025_Silkie_Wenchang	0.89740941	FALSE	FALSE	FALSE	FALSE	FALSE	other_meta_signature	0.047009381	0.499030867	13.33333333	0.590656927	5	0	19.47003051
ENSGALG00010018520	ENSGALG00010018520	3	0.006782451	0.340479587	0.019920288	0.984106961	0.303040755	18.43956541	89.15375739	0.666666667	CRA004775;PRJNA827465;Yang2025_Silkie_Wenchang	0.994303868	FALSE	FALSE	FALSE	FALSE	FALSE	other_meta_signature	0.002480871	0.033912257	13.33333333	1.084624261	5	0	19.45435072
ENSGALG00010023084	ENSGALG00010023084	3	-0.054858795	0.513786656	-0.106773491	0.914968678	0.71286026	24.0779802	91.69365543	0.666666667	CRA004775;PRJNA827465;Yang2025_Silkie_Wenchang	0.974809833	FALSE	FALSE	FALSE	FALSE	FALSE	other_meta_signature	0.011080099	0.274293975	13.33333333	0.830634457	5	0	19.44934186
ENSGALG00010008405	LRATD2	3	-0.022092746	0.299523525	-0.073759635	0.941201646	0.232214944	20.08032387	90.04000128	0.666666667	CRA004775;PRJNA827465;Yang2025_Silkie_Wenchang	0.98283974	FALSE	FALSE	FALSE	FALSE	FALSE	other_meta_signature	0.007517292	0.110463729	13.33333333	0.995999872	5	0	19.44731423
ENSGALG00010015792	MARK3	3	-0.00700023	0.128200203	-0.054603894	0.956454036	0.043644808	18.72820614	89.32092062	0.666666667	CRA004775;PRJNA827465;Yang2025_Silkie_Wenchang	0.985487057	FALSE	FALSE	FALSE	FALSE	FALSE	other_meta_signature	0.006349075	0.035001152	13.33333333	1.067907938	5	0	19.4425915
ENSGALG00010012234	C1GALT1C1	3	-0.06398446	0.178888092	-0.3576787	0.720583775	0.08693776	26.84024683	92.5485037	0.666666667	CRA004775;PRJNA827465;Yang2025_Silkie_Wenchang	0.914714489	FALSE	FALSE	FALSE	FALSE	FALSE	other_meta_signature	0.038714442	0.3199223	13.33333333	0.74514963	5	0	19.43711971
ENSGALG00010004058	TMEM30A	3	-0.143324046	0.231704712	-0.618563365	0.536204043	0.145476835	65.96494744	96.96808672	0.666666667	CRA004775;PRJNA827465;Yang2025_Silkie_Wenchang	0.850714235	FALSE	FALSE	FALSE	FALSE	FALSE	other_meta_signature	0.0702163	0.716620232	13.33333333	0.303191328	5	0	19.42336119
ENSGALG00010024129	GID8	3	-0.151844015	0.320587217	-0.473643385	0.635754232	0.292925467	75.49971757	97.35098347	0.666666667	CRA004775;PRJNA827465;Yang2025_Silkie_Wenchang	0.88855018	FALSE	FALSE	FALSE	FALSE	FALSE	other_meta_signature	0.051318041	0.759220073	13.33333333	0.264901653	5	0	19.4087731
ENSGALG00010003063	GGH	3	-0.129249017	0.298908861	-0.43240276	0.665448712	0.243890089	52.51769219	96.19175954	0.666666667	CRA004775;PRJNA827465;Yang2025_Silkie_Wenchang	0.896630081	FALSE	FALSE	FALSE	FALSE	FALSE	other_meta_signature	0.047386695	0.646245083	13.33333333	0.380824046	5	0	19.40778916
ENSGALG00010015057	LAS1L	3	-0.038945995	0.235935653	-0.165070409	0.868888564	0.147163405	23.26745212	91.40430164	0.666666667	CRA004775;PRJNA827465;Yang2025_Silkie_Wenchang	0.96173598	FALSE	FALSE	FALSE	FALSE	FALSE	other_meta_signature	0.016944136	0.194729973	13.33333333	0.859569836	5	0	19.40457728
ENSGALG00010002432	RAVER1	3	0.115035064	0.351937562	0.326862139	0.743772158	0.352754372	43.49573283	95.40184779	0.666666667	CRA004775;PRJNA827465;Yang2025_Silkie_Wenchang	0.922330698	FALSE	FALSE	FALSE	FALSE	FALSE	other_meta_signature	0.035113337	0.575175322	13.33333333	0.459815221	5	0	19.40343721
ENSGALG00010006205	CGGBP1	3	-0.04542001	0.160403536	-0.2831609	0.777053499	0.066011138	24.74903608	91.91887719	0.666666667	CRA004775;PRJNA827465;Yang2025_Silkie_Wenchang	0.933271518	FALSE	FALSE	FALSE	FALSE	FALSE	other_meta_signature	0.029991988	0.227100049	13.33333333	0.808112281	5	0	19.39853765
ENSGALG00010017148	MOK	2	0.544477072	0.621114633	0.876612856	0.380696954	0.650729977	6.357973994	84.27171925	0.5	PRJNA827465;Yang2025_Silkie_Wenchang	0.796897116	FALSE	FALSE	FALSE	FALSE	FALSE	other_meta_signature	0.098597745	2.722385362	10	1.572828075	5	0	19.39381118
ENSGALG00010008257	DESI2	3	-0.116773121	0.192611573	-0.606262227	0.544340637	0.101137617	49.90448955	95.99234454	0.666666667	CRA004775;PRJNA827465;Yang2025_Silkie_Wenchang	0.854589415	FALSE	FALSE	FALSE	FALSE	FALSE	other_meta_signature	0.068242491	0.583865607	13.33333333	0.400765546	5	0	19.38620698
ENSGALG00010001295	ENSGALG00010001295	3	0.05447273	0.194060682	0.280699467	0.778940917	0.100620582	27.07740107	92.61376675	0.666666667	CRA004775;PRJNA827465;Yang2025_Silkie_Wenchang	0.934331354	FALSE	FALSE	FALSE	FALSE	FALSE	other_meta_signature	0.029499077	0.27236365	13.33333333	0.738623325	5	0	19.37381939
ENSGALG00010027814	ENSGALG00010027814	3	0.079570313	0.231668745	0.343465894	0.731247995	0.143606313	34.36694214	94.18045402	0.666666667	CRA004775;PRJNA827465;Yang2025_Silkie_Wenchang	0.916997157	FALSE	FALSE	FALSE	FALSE	FALSE	other_meta_signature	0.037632011	0.397851563	13.33333333	0.581954598	5	0	19.3507715
ENSGALG00010005988	NUP58	3	0.050117821	0.17611863	0.284568539	0.775974717	0.080439149	27.25403946	92.66163827	0.666666667	CRA004775;PRJNA827465;Yang2025_Silkie_Wenchang	0.932913798	FALSE	FALSE	FALSE	FALSE	FALSE	other_meta_signature	0.030158483	0.250589106	13.33333333	0.733836173	5	0	19.3479171
ENSGALG00010029735	SRP68	3	0.041057952	0.158657696	0.258783235	0.795802494	0.065475266	25.62103279	92.19391343	0.666666667	CRA004775;PRJNA827465;Yang2025_Silkie_Wenchang	0.936723207	FALSE	FALSE	FALSE	FALSE	FALSE	other_meta_signature	0.02838872	0.205289759	13.33333333	0.780608657	5	0	19.34762047
ENSGALG00010004494	WTAP	3	-0.10389914	0.167517901	-0.620227089	0.535108289	0.076576955	48.60539725	95.88523063	0.666666667	CRA004775;PRJNA827465;Yang2025_Silkie_Wenchang	0.850686418	FALSE	FALSE	FALSE	FALSE	FALSE	other_meta_signature	0.070230501	0.519495701	13.33333333	0.411476937	5	0	19.33453647
ENSGALG00010022821	ENSGALG00010022821	2	0.549013031	1.58709253	0.34592377	0.729400005	4.261843753	6.447190914	84.48936888	0.5	PRJNA827465;Yang2025_Silkie_Wenchang	0.916064799	FALSE	FALSE	FALSE	FALSE	FALSE	other_meta_signature	0.038073805	2.745065153	10	1.551063112	5	0	19.33420207
ENSGALG00010009798	YIPF4	3	-0.091393021	0.216535205	-0.422070033	0.6729739	0.12910856	42.0916247	95.24846091	0.666666667	CRA004775;PRJNA827465;Yang2025_Silkie_Wenchang	0.898977206	FALSE	FALSE	FALSE	FALSE	FALSE	other_meta_signature	0.04625132	0.456965105	13.33333333	0.475153909	5	0	19.31170367
ENSGALG00010015353	RELL1	3	-0.094885013	0.269101966	-0.352598735	0.724389286	0.201581311	45.97308853	95.64962881	0.666666667	CRA004775;PRJNA827465;Yang2025_Silkie_Wenchang	0.915490936	FALSE	FALSE	FALSE	FALSE	FALSE	other_meta_signature	0.038345951	0.474425063	13.33333333	0.435037119	5	0	19.28114147
ENSGALG00010029327	CRK	3	-0.088197981	0.191130379	-0.461454541	0.644472529	0.102113683	43.96025501	95.45043585	0.666666667	CRA004775;PRJNA827465;Yang2025_Silkie_Wenchang	0.890994141	FALSE	FALSE	FALSE	FALSE	FALSE	other_meta_signature	0.050125152	0.440989907	13.33333333	0.454956415	5	0	19.27940481
ENSGALG00010028523	SUGP1	3	-0.043297012	0.256962521	-0.168495437	0.866193533	0.181578797	28.50972995	92.98485112	0.666666667	CRA004775;PRJNA827465;Yang2025_Silkie_Wenchang	0.96060415	FALSE	FALSE	FALSE	FALSE	FALSE	other_meta_signature	0.017455542	0.216485061	13.33333333	0.701514888	5	0	19.26878882
ENSGALG00010018511	SOCS5	3	-0.038980711	0.214088363	-0.182077676	0.855521768	0.123149937	27.78490475	92.80184684	0.666666667	CRA004775;PRJNA827465;Yang2025_Silkie_Wenchang	0.957759014	FALSE	FALSE	FALSE	FALSE	FALSE	other_meta_signature	0.018743752	0.194903557	13.33333333	0.719815316	5	0	19.26679596
ENSGALG00010011817	KDSR	3	-0.094344233	0.26436761	-0.356867595	0.72119093	0.199938126	48.5455454	95.88015752	0.666666667	CRA004775;PRJNA827465;Yang2025_Silkie_Wenchang	0.914767969	FALSE	FALSE	FALSE	FALSE	FALSE	other_meta_signature	0.038689051	0.471721167	13.33333333	0.411984248	5	0	19.2557278
ENSGALG00010011545	USP12like	3	0.014182971	0.153121488	0.092625607	0.926201	0.062833753	23.8811812	91.62520487	0.666666667	CRA004775;PRJNA827465;Yang2025_Silkie_Wenchang	0.978827179	FALSE	FALSE	FALSE	FALSE	FALSE	other_meta_signature	0.00929398	0.070914854	13.33333333	0.837479513	5	0	19.25102168
ENSGALG00010013041	LRP12	3	-0.069082268	0.226317069	-0.305245503	0.760179178	0.140216257	37.6038403	94.68139428	0.666666667	CRA004775;PRJNA827465;Yang2025_Silkie_Wenchang	0.929129789	FALSE	FALSE	FALSE	FALSE	FALSE	other_meta_signature	0.031923615	0.345411338	13.33333333	0.531860572	5	0	19.24252886
ENSGALG00010014866	FAM26E	3	0.020450771	0.305069294	0.067036479	0.946552663	0.237383666	25.20888217	92.06628843	0.666666667	CRA004775;PRJNA827465;Yang2025_Silkie_Wenchang	0.983959877	FALSE	FALSE	FALSE	FALSE	FALSE	other_meta_signature	0.00702261	0.102253856	13.33333333	0.793371157	5	0	19.23598096
ENSGALG00010017190	STK26	3	0.056074631	0.186525706	0.300626828	0.763699072	0.095165835	34.49174598	94.20151128	0.666666667	CRA004775;PRJNA827465;Yang2025_Silkie_Wenchang	0.929610339	FALSE	FALSE	FALSE	FALSE	FALSE	other_meta_signature	0.031699055	0.280373157	13.33333333	0.579848872	5	0	19.22525442
ENSGALG00010024956	VPS37B	3	-0.026259643	0.225035578	-0.116691074	0.907104864	0.131366521	27.29977113	92.67393126	0.666666667	CRA004775;PRJNA827465;Yang2025_Silkie_Wenchang	0.97313563	FALSE	FALSE	FALSE	FALSE	FALSE	other_meta_signature	0.011826626	0.131298217	13.33333333	0.732606874	5	0	19.20906505
ENSGALG00010023706	CDK5RAP3	3	-0.035367135	0.256934896	-0.137650183	0.890516893	0.183607785	30.60500698	93.46512157	0.666666667	CRA004775;PRJNA827465;Yang2025_Silkie_Wenchang	0.966800272	FALSE	FALSE	FALSE	FALSE	FALSE	other_meta_signature	0.014663236	0.176835677	13.33333333	0.653487843	5	0	19.17832009
ENSGALG00010006016	MTMR6	3	-0.093017633	0.342034346	-0.27195407	0.785657335	0.332975916	58.93672009	96.60652986	0.666666667	CRA004775;PRJNA827465;Yang2025_Silkie_Wenchang	0.935287452	FALSE	FALSE	FALSE	FALSE	FALSE	other_meta_signature	0.029054892	0.465088163	13.33333333	0.339347014	5	0	19.1668234
ENSGALG00010015983	CNOT7	3	-0.073465695	0.208350411	-0.352606431	0.724383516	0.123542887	51.84222145	96.14214063	0.666666667	CRA004775;PRJNA827465;Yang2025_Silkie_Wenchang	0.915490936	FALSE	FALSE	FALSE	FALSE	FALSE	other_meta_signature	0.038345951	0.367328474	13.33333333	0.385785937	5	0	19.1247937
ENSGALG00010000711	SPRY2	3	0.044105908	0.309694845	0.142417314	0.886750387	0.250762634	36.0390022	94.45045679	0.666666667	CRA004775;PRJNA827465;Yang2025_Silkie_Wenchang	0.965929408	FALSE	FALSE	FALSE	FALSE	FALSE	other_meta_signature	0.015054611	0.22052954	13.33333333	0.554954321	5	0	19.12387181
ENSGALG00010010887	GABPA	3	-0.025771028	0.142647391	-0.180662458	0.856632528	0.051563775	31.70196594	93.69124299	0.666666667	CRA004775;PRJNA827465;Yang2025_Silkie_Wenchang	0.958093528	FALSE	FALSE	FALSE	FALSE	FALSE	other_meta_signature	0.018592094	0.128855142	13.33333333	0.630875701	5	0	19.11165627
ENSGALG00010016119	FETUB	2	-0.544328489	2.536875577	-0.21456649	0.830105322	11.11883259	7.341717319	86.37920862	0.5	PRJNA827465;Yang2025_Silkie_Wenchang	0.948887498	FALSE	FALSE	FALSE	FALSE	FALSE	other_meta_signature	0.022785275	2.721642444	10	1.362079138	5	0	19.10650686
ENSGALG00010014156	CD164	3	-0.07110652	0.280790731	-0.253236707	0.800085303	0.217427099	51.90586539	96.14687091	0.666666667	CRA004775;PRJNA827465;Yang2025_Silkie_Wenchang	0.938354512	FALSE	FALSE	FALSE	FALSE	FALSE	other_meta_signature	0.027633053	0.3555326	13.33333333	0.385312909	5	0	19.1018119
ENSGALG00010013799	SCRT2	2	-0.099317195	0.357265749	-0.27799249	0.781018124	0.170960155	2.817391846	64.50617967	0.5	PRJNA827465;Yang2025_Silkie_Wenchang	0.934350616	FALSE	FALSE	FALSE	FALSE	FALSE	other_meta_signature	0.029490124	0.496585975	10	3.549382033	5	0	19.07545813
ENSGALG00010008105	MMUT	3	0.02545129	0.247852505	0.102687241	0.918211201	0.164358643	33.64707709	94.05594728	0.666666667	CRA004775;PRJNA827465;Yang2025_Silkie_Wenchang	0.975648054	FALSE	FALSE	FALSE	FALSE	FALSE	other_meta_signature	0.010706817	0.127256449	13.33333333	0.594405272	5	0	19.06570187
ENSGALG00010016627	SH3RF2	2	0.283154908	1.4270332	0.198422089	0.842714837	3.119649931	3.893092386	74.31347883	0.5	CRA004775;PRJNA827465	0.953616496	FALSE	FALSE	FALSE	FALSE	FALSE	other_meta_signature	0.020626245	1.415774542	10	2.568652117	5	0	19.0050529
ENSGALG00010010417	OTOF	2	-0.638303618	1.813890009	-0.351897643	0.724915027	6.116859325	14.18100834	92.94831527	0.5	PRJNA827465;Yang2025_Silkie_Wenchang	0.915503178	FALSE	FALSE	FALSE	FALSE	FALSE	other_meta_signature	0.038340144	3.191518092	10	0.705168473	5	0	18.93502671
ENSGALG00010003762	EVI5L	3	0.016767655	0.28059523	0.059757447	0.952348818	0.215328753	41.07515302	95.13087633	0.666666667	CRA004775;PRJNA827465;Yang2025_Silkie_Wenchang	0.984742353	FALSE	FALSE	FALSE	FALSE	FALSE	other_meta_signature	0.006677383	0.083838273	13.33333333	0.486912367	5	0	18.91076136
ENSGALG00010016150	POLG	3	-0.029791999	0.315836954	-0.094327148	0.924849285	0.283998872	50.00850823	96.00068054	0.666666667	CRA004775;PRJNA827465;Yang2025_Silkie_Wenchang	0.978482792	FALSE	FALSE	FALSE	FALSE	FALSE	other_meta_signature	0.009446807	0.148959996	13.33333333	0.399931946	5	0	18.89167208
ENSGALG00010019047	GHRL	2	-0.084098192	0.547986504	-0.153467633	0.878029513	0.40329785	2.910772609	65.64486017	0.5	PRJNA827465;Yang2025_Silkie_Wenchang	0.963492987	FALSE	FALSE	FALSE	FALSE	FALSE	other_meta_signature	0.016151442	0.420490958	10	3.435513983	5	0	18.87215638
ENSGALG00010016344	SMIM14	3	-0.007289208	0.243025188	-0.029993631	0.976072133	0.166871627	43.01954298	95.35095015	0.666666667	CRA004775;PRJNA827465;Yang2025_Silkie_Wenchang	0.991072452	FALSE	FALSE	FALSE	FALSE	FALSE	other_meta_signature	0.003894596	0.036446038	13.33333333	0.464904985	5	0	18.83857895
ENSGALG00010015159	ENSGALG00010015159	2	-0.338704575	1.169282656	-0.28966869	0.772069712	2.199513867	4.929557341	79.7142029	0.5	CRA004775;Yang2025_Silkie_Wenchang	0.931425539	FALSE	FALSE	FALSE	FALSE	FALSE	other_meta_signature	0.030851858	1.693522874	10	2.02857971	5	0	18.75295444
ENSGALG00010005284	ENSGALG00010005284	2	0.293704953	0.393867625	0.745694579	0.455851935	0.247810594	4.796596221	79.15188284	0.5	PRJNA827465;Yang2025_Silkie_Wenchang	0.823230245	FALSE	FALSE	FALSE	FALSE	FALSE	other_meta_signature	0.084478682	1.468524764	10	2.084811716	5	0	18.63781516
ENSGALG00010026109	SPDEF	2	0.484006675	1.569754261	0.308332767	0.757829135	4.495982575	11.39997996	91.22805476	0.5	CRA004775;PRJNA827465	0.928544362	FALSE	FALSE	FALSE	FALSE	FALSE	other_meta_signature	0.032197343	2.420033375	10	0.877194524	5	0	18.32942524
ENSGALG00010006606	SULT	2	-0.447571503	2.470071455	-0.181197796	0.856212325	10.99391887	10.0964649	90.09554324	0.5	PRJNA827465;Yang2025_Silkie_Wenchang	0.957967139	FALSE	FALSE	FALSE	FALSE	FALSE	other_meta_signature	0.018649388	2.237857517	10	0.990445676	5	0	18.24695258
ENSGALG00010008442	WDR64	2	-0.463608206	0.932408877	-0.497215564	0.619037047	1.595433194	11.83102152	91.54764448	0.5	PRJNA827465;Yang2025_Silkie_Wenchang	0.882522345	FALSE	FALSE	FALSE	FALSE	FALSE	other_meta_signature	0.05427429	2.31804103	10	0.845235552	5	0	18.21755087
ENSGALG00010029463	ENSGALG00010029463	2	0.428455499	1.413996237	0.303010353	0.761881972	3.591442127	9.775787983	89.77064558	0.5	CRA004775;Yang2025_Silkie_Wenchang	0.929547529	FALSE	FALSE	FALSE	FALSE	FALSE	other_meta_signature	0.031728399	2.142277495	10	1.022935442	5	0	18.19694134
ENSGALG00010016570	ENSGALG00010016570	2	0.159906396	0.467646814	0.341938383	0.732397263	0.346101188	4.780000138	79.07949851	0.5	PRJNA827465;Yang2025_Silkie_Wenchang	0.9175369	FALSE	FALSE	FALSE	FALSE	FALSE	other_meta_signature	0.037376461	0.799531978	10	2.092050149	5	0	17.92895859
ENSGALG00010023399	SRRM4	2	-0.016050827	0.746292145	-0.021507431	0.982840876	0.808888273	3.630869338	72.45838649	0.5	PRJNA827465;Yang2025_Silkie_Wenchang	0.993948011	FALSE	FALSE	FALSE	FALSE	FALSE	other_meta_signature	0.002636331	0.080254135	10	2.754161351	5	0	17.83705182
ENSGALG00010016578	GRIK3	2	0.208743499	0.895446537	0.233116653	0.815670824	1.334550777	5.813625527	82.79902971	0.5	PRJNA827465;Yang2025_Silkie_Wenchang	0.943607411	FALSE	FALSE	FALSE	FALSE	FALSE	other_meta_signature	0.025208657	1.043717497	10	1.720097029	5	0	17.78902318
ENSGALG00010013588	ENSGALG00010013588	2	-0.174607288	0.728373229	-0.239722276	0.810545565	0.873547646	5.641816543	82.27521238	0.5	PRJNA827465;Yang2025_Silkie_Wenchang	0.942226732	FALSE	FALSE	FALSE	FALSE	FALSE	other_meta_signature	0.025844578	0.87303644	10	1.772478762	5	0	17.67134978
ENSGALG00010021853	ENSGALG00010021853	2	0.131911921	0.602918618	0.218788933	0.826814469	0.592035216	5.233389189	80.89192369	0.5	CRA004775;Yang2025_Silkie_Wenchang	0.947887439	FALSE	FALSE	FALSE	FALSE	FALSE	other_meta_signature	0.023243232	0.659559604	10	1.910807631	5	0	17.59361047
ENSGALG00010006923	ENSGALG00010006923	2	0.137360006	0.407195968	0.33733145	0.735867054	0.271030876	5.424982015	81.56675917	0.5	PRJNA827465;Yang2025_Silkie_Wenchang	0.918412887	FALSE	FALSE	FALSE	FALSE	FALSE	other_meta_signature	0.036962031	0.686800032	10	1.843324083	5	0	17.56708615
ENSGALG00010005585	ENSGALG00010005585	2	-0.005492254	1.163502469	-0.004720449	0.996233641	2.238359969	5.592065186	82.1175189	0.5	CRA004775;Yang2025_Silkie_Wenchang	0.998635055	FALSE	FALSE	FALSE	FALSE	FALSE	other_meta_signature	0.000593193	0.027461269	10	1.78824811	5	0	16.81630257
