# Supplementary material for: Cross-cohort transcriptomic meta-analysis identifies conserved melanogenesis signatures and developmental background in black-boned chicken breast muscle
Source: Anim Biosci. 2026 Jun 24;39(10):260430. doi: 10.5713/ab.260430 (PMC13662945; doi:10.5713/ab.260430)
Supplement: Supplementary file 4 [file ab-260430-Supplementary-12.pdf]

Supplement 12. Cohort-level correlation summary between melanogenesis and myogenesis signature scores.

cohort_id	n	pearson_r	spearman_r	lm_pvalue	cohort_label
CRA004775<br>adult breast muscle	18	0.293612801	0.267285862	0.236986271	Wuliangshan vs controls
PRJNA729271	42	-0.067064708	-8.10307E-05	0.673033667	PRJNA729271
PRJNA827465<br>embryonic breast muscle	24	-0.067213536	-0.171304348	0.755000474	Yugan vs Wenchang
Yang2025_Silkie_Wenchang<br>embryonic breast muscle	20	0.12010401	0.085714286	0.614002291	Silkie vs Wenchang
