# Supplementary material for: Cross-cohort transcriptomic meta-analysis identifies conserved melanogenesis signatures and developmental background in black-boned chicken breast muscle
Source: Anim Biosci. 2026 Jun 24;39(10):260430. doi: 10.5713/ab.260430 (PMC13662945; doi:10.5713/ab.260430)
Supplement: Supplementary file 5 [file ab-260430-Supplementary-13.pdf]

Supplement 13. Meta-analysis results for canonical myogenic regulators and markers compared with the curated muscle-phenotype-related candidate panel.

symbol	gene_category	gene_id	meta_log2FC	meta_fdr	I2	direction_consistency	meta_significant_and_direction_consistent
PAX7	Canonical myogenic regulator or marker	ENSGALG00010020440	0.068700836	0.8426408	8.010835503	0.666666667	No
MYOD1	Canonical myogenic regulator or marker	ENSGALG00010024369	-0.076113514	0.87497004	45.29392799	0.666666667	No
MYF5	Canonical myogenic regulator or marker	ENSGALG00010012264	-0.028209691	0.922962823	0	0.666666667	No
MYOG	Canonical myogenic regulator or marker	ENSGALG00010025215	0.092719063	0.799009727	0	0.666666667	No
MYH7	Canonical myogenic regulator or marker	ENSGALG00010029494	0.993549974	0.416257383	79.55467318	1	No
MSTN	Canonical myogenic regulator or marker	ENSGALG00010012852	-0.334827205	0.082569084	0	0.666666667	No
MEF2A	Canonical myogenic regulator or marker	ENSGALG00010018848	0.110712028	0.864305482	79.49663951	0.666666667	No
MEF2C	Canonical myogenic regulator or marker	ENSGALG00010007430	0.371120531	0.734277773	77.2082967	1	No
MYBPC2	Curated muscle-phenotype-related candidate	ENSGALG00010000224	-0.67421461	0.007297957	0	1	Yes
CAPN1	Curated muscle-phenotype-related candidate	ENSGALG00010001351	-0.321388065	9.62959E-05	0	1	Yes
CTSK	Curated muscle-phenotype-related candidate	ENSGALG00010028763	0.411918772	0.001834002	0	1	Yes
LRP4	Curated muscle-phenotype-related candidate	ENSGALG00010022929	-0.338364291	0.019568396	0	1	Yes
SLN	Curated muscle-phenotype-related candidate	ENSGALG00010004973	-0.642222	0.006748385	0	1	Yes
SCN4A	Curated muscle-phenotype-related candidate	ENSGALG00010023635	-0.443744128	0.003696156	0	1	Yes
TECRL	Curated muscle-phenotype-related candidate	ENSGALG00010003632	-1.084467047	0.011755221	0	1	Yes
CD36	Curated muscle-phenotype-related candidate	ENSGALG00010008392	-0.689932039	0.026043176	0	1	Yes
GPD1	Curated muscle-phenotype-related candidate	ENSGALG00010023167	-0.721409624	0.010177097	0	1	Yes
THRSP	Curated muscle-phenotype-related candidate	ENSGALG00010001976	-1.684290035	0.036365512	0	1	Yes
PLTP	Curated muscle-phenotype-related candidate	ENSGALG00010016006	0.574339269	8.12523E-07	0	1	Yes
