# Supplementary material for: Cross-cohort transcriptomic meta-analysis identifies conserved melanogenesis signatures and developmental background in black-boned chicken breast muscle
Source: Anim Biosci. 2026 Jun 24;39(10):260430. doi: 10.5713/ab.260430 (PMC13662945; doi:10.5713/ab.260430)
Supplement: Supplementary file 6 [file ab-260430-Supplementary-14.pdf]

Supplement 14. Sample-level biological signature scores for all analyzed samples.

sample_id	melanoblast_neural_crest	melanogenesis	myogenesis	oxidative_redox	cohort_id	run	biosample	stage	breed	pigmentation	replicate	comparison_role
CH1	-0.439675221	-0.593067351	0.081998061	0.170645659	CRA004775	CRR314359	SAMC445057	adult	Chahua	normal_or_low_melanin	1	control
CH2	-0.224541576	-0.919685604	-0.128948985	0.289774597	CRA004775	CRR314360	SAMC445058	adult	Chahua	normal_or_low_melanin	2	control
CH3	-0.991070908	-0.703770307	0.484637305	0.135569229	CRA004775	CRR314361	SAMC445059	adult	Chahua	normal_or_low_melanin	3	control
CH4	-0.187584193	-0.282801958	-2.498902876	-0.849105538	CRA004775	CRR314362	SAMC445060	adult	Chahua	normal_or_low_melanin	4	control
CH5	-0.405208887	-0.568588265	0.32757205	0.083623541	CRA004775	CRR314363	SAMC445061	adult	Chahua	normal_or_low_melanin	5	control
CH6	-0.544038168	-1.051358939	0.01844016	0.020566453	CRA004775	CRR314364	SAMC445062	adult	Chahua	normal_or_low_melanin	6	control
CB1	-0.813285205	-0.54855559	-0.683739886	0.853042932	CRA004775	CRR314365	SAMC445063	adult	Cobb	normal_or_low_melanin	1	control
CB2	0.110381033	-0.285921977	0.261233837	-0.387618838	CRA004775	CRR314366	SAMC445064	adult	Cobb	normal_or_low_melanin	2	control
CB3	-0.042962951	-0.579032457	0.363730458	-0.541565316	CRA004775	CRR314367	SAMC445065	adult	Cobb	normal_or_low_melanin	3	control
CB4	-0.761501893	-0.442636697	-0.337293904	0.379422707	CRA004775	CRR314368	SAMC445066	adult	Cobb	normal_or_low_melanin	4	control
CB5	0.144155306	-0.462606701	0.379092838	-0.261024347	CRA004775	CRR314369	SAMC445067	adult	Cobb	normal_or_low_melanin	5	control
CB6	0.108012386	-0.343297845	-0.394508392	-0.365343497	CRA004775	CRR314370	SAMC445068	adult	Cobb	normal_or_low_melanin	6	control
WL1	0.899350061	1.663810658	0.562405551	-0.050471278	CRA004775	CRR314353	SAMC445051	adult	Wuliangshan_black_boned	black_boned	1	case
WL2	0.294375029	0.904995979	0.08626972	-0.564358596	CRA004775	CRR314354	SAMC445052	adult	Wuliangshan_black_boned	black_boned	2	case
WL3	0.698805919	0.762860543	0.228986569	-0.056648742	CRA004775	CRR314355	SAMC445053	adult	Wuliangshan_black_boned	black_boned	3	case
WL4	0.72658566	1.114092352	0.30867922	-0.309632671	CRA004775	CRR314356	SAMC445054	adult	Wuliangshan_black_boned	black_boned	4	case
WL5	0.792545651	1.200262774	0.700645277	0.343761041	CRA004775	CRR314357	SAMC445055	adult	Wuliangshan_black_boned	black_boned	5	case
WL6	0.635657957	1.135301387	0.239702995	1.109362665	CRA004775	CRR314358	SAMC445056	adult	Wuliangshan_black_boned	black_boned	6	case
W1_1	-0.717210096	-0.827049355	-0.621159703	-0.086120314	PRJNA827465	SRR18778023	SAMN27610096	E9	Wenchang	normal	1	control
W1_2	-0.817629923	-0.724779208	-1.26174921	0.265211124	PRJNA827465	SRR18778022	SAMN27610097	E9	Wenchang	normal	2	control
W1_3	-0.540356984	-0.798438505	-0.651853814	-0.152309027	PRJNA827465	SRR18778011	SAMN27610098	E9	Wenchang	normal	3	control
Y1_1	0.634492398	0.413093904	-1.357547744	0.517894303	PRJNA827465	SRR18778019	SAMN27610108	E9	Yugan_black_boned	black_boned	1	case
Y1_2	0.514539134	0.645497226	-0.685343717	0.174779587	PRJNA827465	SRR18778018	SAMN27610109	E9	Yugan_black_boned	black_boned	2	case
Y1_3	0.524417448	0.400391142	-0.459784091	-0.166381445	PRJNA827465	SRR18778017	SAMN27610110	E9	Yugan_black_boned	black_boned	3	case
W2_1	-1.041400214	-0.938222502	0.675464372	-0.618973761	PRJNA827465	SRR18778006	SAMN27610099	E13	Wenchang	normal	1	control
W2_2	-0.629049	-0.748424593	0.533213593	-0.254307411	PRJNA827465	SRR18778005	SAMN27610100	E13	Wenchang	normal	2	control
W2_3	-1.389108888	-0.878691639	0.775226526	-0.501525708	PRJNA827465	SRR18778004	SAMN27610101	E13	Wenchang	normal	3	control
Y2_1	-0.183593914	0.193695127	0.61413463	-0.274528195	PRJNA827465	SRR18778016	SAMN27610111	E13	Yugan_black_boned	black_boned	1	case
Y2_2	-0.256977137	0.674198814	0.903432931	-0.254008221	PRJNA827465	SRR18778015	SAMN27610112	E13	Yugan_black_boned	black_boned	2	case
Y2_3	0.165421409	0.742277356	0.455020894	-0.56670156	PRJNA827465	SRR18778014	SAMN27610113	E13	Yugan_black_boned	black_boned	3	case
W3_1	0.044688774	-0.816222943	0.436146065	-0.030207862	PRJNA827465	SRR18778003	SAMN27610102	E17	Wenchang	normal	1	control
W3_2	0.165197185	-0.829498595	0.316205155	-0.193658151	PRJNA827465	SRR18778002	SAMN27610103	E17	Wenchang	normal	2	control
W3_3	0.121885641	-0.771646634	0.412599104	0.538793624	PRJNA827465	SRR18778001	SAMN27610104	E17	Wenchang	normal	3	control
Y3_1	0.337918175	0.318033991	0.360324645	-0.116908385	PRJNA827465	SRR18778013	SAMN27610114	E17	Yugan_black_boned	black_boned	1	case
Y3_2	0.813451676	1.168379981	-0.162008996	0.848270184	PRJNA827465	SRR18778012	SAMN27610115	E17	Yugan_black_boned	black_boned	2	case
Y3_3	0.992157072	1.521133521	0.337783276	-0.213205445	PRJNA827465	SRR18778010	SAMN27610116	E17	Yugan_black_boned	black_boned	3	case
W4_1	0.067020621	-0.795654898	-0.005135176	0.14776944	PRJNA827465	SRR18778000	SAMN27610105	E21	Wenchang	normal	1	control
W4_2	-0.320543053	-0.860853959	-0.239778991	0.318392422	PRJNA827465	SRR18778021	SAMN27610106	E21	Wenchang	normal	2	control
W4_3	-0.304364082	-0.716553359	0.095281192	0.23136087	PRJNA827465	SRR18778020	SAMN27610107	E21	Wenchang	normal	3	control
Y4_1	1.36045648	1.469579288	-0.177311379	0.21371052	PRJNA827465	SRR18778009	SAMN27610117	E21	Yugan_black_boned	black_boned	1	case
Y4_2	0.096171769	0.868525499	-0.110234584	-0.077459095	PRJNA827465	SRR18778008	SAMN27610118	E21	Yugan_black_boned	black_boned	2	case
Y4_3	0.362415507	1.29123034	-0.182924978	0.250112508	PRJNA827465	SRR18778007	SAMN27610119	E21	Yugan_black_boned	black_boned	3	case
E13-TH-BM-1	-0.106658127	-0.030753611	0.528310553	-0.642641686	Yang2025_Silkie_Wenchang	CRR1014974	SAMC3313436	E13	Silkie	black_boned	1	case
E13-TH-BM-6	-0.169120188	0.177876054	0.000763406	-0.683456348	Yang2025_Silkie_Wenchang	CRR1014975	SAMC3313437	E13	Silkie	black_boned	6	case
E13-TH-BM-7	-0.521282106	-0.035590386	0.168593952	-0.449910819	Yang2025_Silkie_Wenchang	CRR1014976	SAMC3313438	E13	Silkie	black_boned	7	case
E13-TH-BM-9	-0.093629829	0.215514533	0.193282373	-0.790666694	Yang2025_Silkie_Wenchang	CRR1014977	SAMC3313439	E13	Silkie	black_boned	9	case
W2_1	-0.987100795	-1.005163898	0.160575649	0.010104855	Yang2025_Silkie_Wenchang	SRR18778006	SAMN27610099	E13	Wenchang	normal	1	control
W2_2	-0.553018769	-0.775413236	0.020325207	0.432040242	Yang2025_Silkie_Wenchang	SRR18778005	SAMN27610100	E13	Wenchang	normal	2	control
W2_3	-1.335704753	-0.948862347	0.202446881	0.059552557	Yang2025_Silkie_Wenchang	SRR18778004	SAMN27610101	E13	Wenchang	normal	3	control
E17-TH-BM-5	1.030912958	1.020443562	-0.057282601	-0.365399129	Yang2025_Silkie_Wenchang	CRR1014988	SAMC3313450	E17	Silkie	black_boned	5	case
E17-TH-BM-6	0.978952345	1.117374222	0.321128311	-0.597275452	Yang2025_Silkie_Wenchang	CRR1014989	SAMC3313451	E17	Silkie	black_boned	6	case
E17-TH-BM-8	1.21825495	1.019187628	0.755254247	-0.586200856	Yang2025_Silkie_Wenchang	CRR1014990	SAMC3313452	E17	Silkie	black_boned	8	case
W3_1	-0.023798012	-0.886273675	-0.007003757	0.081097424	Yang2025_Silkie_Wenchang	SRR18778003	SAMN27610102	E17	Wenchang	normal	1	control
W3_2	0.10819456	-0.89385729	-0.062571846	-0.051130529	Yang2025_Silkie_Wenchang	SRR18778002	SAMN27610103	E17	Wenchang	normal	2	control
W3_3	0.00855476	-0.834614013	0.009416064	0.778135341	Yang2025_Silkie_Wenchang	SRR18778001	SAMN27610104	E17	Wenchang	normal	3	control
E21-TH-BM-2	0.382784218	0.973996711	0.0020552	0.728461615	Yang2025_Silkie_Wenchang	CRR1014998	SAMC3313460	E21	Silkie	black_boned	2	case
E21-TH-BM-4	0.975483759	1.123419308	0.058930802	-0.178349233	Yang2025_Silkie_Wenchang	CRR1014999	SAMC3313461	E21	Silkie	black_boned	4	case
E21-TH-BM-7	0.053550584	1.112095159	-0.54333448	0.941588211	Yang2025_Silkie_Wenchang	CRR1015000	SAMC3313462	E21	Silkie	black_boned	7	case
E21-TH-BM-8	-0.076869477	1.297364774	-0.502048022	0.630027486	Yang2025_Silkie_Wenchang	CRR1015001	SAMC3313463	E21	Silkie	black_boned	8	case
W4_1	0.006610607	-0.896860634	-0.355419068	0.200669465	Yang2025_Silkie_Wenchang	SRR18778000	SAMN27610105	E21	Wenchang	normal	1	control
W4_2	-0.401080776	-0.944962956	-0.574353936	0.268092192	Yang2025_Silkie_Wenchang	SRR18778021	SAMN27610106	E21	Wenchang	normal	2	control
W4_3	-0.495035907	-0.804919905	-0.319068933	0.215261357	Yang2025_Silkie_Wenchang	SRR18778020	SAMN27610107	E21	Wenchang	normal	3	control
AAE10P1	0.166787926	0.15396928	-0.422877421	-0.430067836	PRJNA729271	SRR14521328	SAMN19115447	E10	Arbor_Acres	background_not_melanogenesis	1	background
AAE10P2	0.154358536	0.855591894	-0.653078401	-0.256005354	PRJNA729271	SRR14521327	SAMN19115448	E10	Arbor_Acres	background_not_melanogenesis	2	background
AAE10P3	0.076518392	-0.096093917	-0.539336558	-0.102766826	PRJNA729271	SRR14521316	SAMN19115449	E10	Arbor_Acres	background_not_melanogenesis	3	background
LSE10P1	0.236202981	0.564902441	-0.565832976	-0.788533584	PRJNA729271	SRR14521314	SAMN19115468	E10	Lushi	background_not_melanogenesis	1	background
LSE10P2	0.319156495	0.982129601	-1.007139844	-0.182566234	PRJNA729271	SRR14521313	SAMN19115469	E10	Lushi	background_not_melanogenesis	2	background
LSE10P3	0.966647424	1.600643894	-1.099199778	-0.055882875	PRJNA729271	SRR14521312	SAMN19115470	E10	Lushi	background_not_melanogenesis	3	background
AAE14P1	-0.233343753	-0.197019104	0.881317531	-0.590305616	PRJNA729271	SRR14521305	SAMN19115450	E14	Arbor_Acres	background_not_melanogenesis	1	background
AAE14P2	-0.45606622	0.107012328	0.746014007	-0.296297017	PRJNA729271	SRR14521294	SAMN19115451	E14	Arbor_Acres	background_not_melanogenesis	2	background
AAE14P3	-0.867874679	-0.139092783	0.861230861	-0.082641278	PRJNA729271	SRR14521291	SAMN19115452	E14	Arbor_Acres	background_not_melanogenesis	3	background
LSE14P1	-0.249209003	0.284460935	0.707276008	-0.740101662	PRJNA729271	SRR14521311	SAMN19115471	E14	Lushi	background_not_melanogenesis	1	background
LSE14P2	-0.437032817	0.241652259	0.85292598	-0.228339367	PRJNA729271	SRR14521310	SAMN19115472	E14	Lushi	background_not_melanogenesis	2	background
LSE14P3	-0.153916633	0.213945124	0.758748667	-0.188024261	PRJNA729271	SRR14521309	SAMN19115473	E14	Lushi	background_not_melanogenesis	3	background
AAE18P1	0.752306512	-0.660024194	0.692974193	-0.17286066	PRJNA729271	SRR14521290	SAMN19115453	E18	Arbor_Acres	background_not_melanogenesis	1	background
AAE18P2	0.532740296	-0.643543404	0.654169628	0.262977453	PRJNA729271	SRR14521289	SAMN19115454	E18	Arbor_Acres	background_not_melanogenesis	2	background
AAE18P3	0.873688193	-0.569546202	0.781927554	-0.204737838	PRJNA729271	SRR14521288	SAMN19115455	E18	Arbor_Acres	background_not_melanogenesis	3	background
LSE18P1	0.509219699	0.98622429	0.501044513	-0.125940294	PRJNA729271	SRR14521308	SAMN19115474	E18	Lushi	background_not_melanogenesis	1	background
LSE18P2	0.617441586	0.519008756	0.667929466	-0.519346879	PRJNA729271	SRR14521307	SAMN19115475	E18	Lushi	background_not_melanogenesis	2	background
LSE18P3	0.525964361	1.015471781	0.599432606	-0.608294969	PRJNA729271	SRR14521306	SAMN19115476	E18	Lushi	background_not_melanogenesis	3	background
AA1D_P1	0.547778187	-0.510992499	0.030210513	-0.468872122	PRJNA729271	SRR14521287	SAMN19115456	1D	Arbor_Acres	background_not_melanogenesis	1	background
AA1D_P2	0.389970137	-0.707202909	0.140746069	-0.314215494	PRJNA729271	SRR14521326	SAMN19115457	1D	Arbor_Acres	background_not_melanogenesis	2	background
AA1D_P3	0.508827478	-0.373586426	0.071029913	-0.30868826	PRJNA729271	SRR14521325	SAMN19115458	1D	Arbor_Acres	background_not_melanogenesis	3	background
LS1D_P1	0.257222086	0.846104953	0.361095339	-0.354635124	PRJNA729271	SRR14521304	SAMN19115477	1D	Lushi	background_not_melanogenesis	1	background
LS1D_P2	0.076760771	0.996912778	0.169116932	-0.50913285	PRJNA729271	SRR14521303	SAMN19115478	1D	Lushi	background_not_melanogenesis	2	background
LS1D_P3	0.186549759	0.402771623	0.158152	-0.53410686	PRJNA729271	SRR14521302	SAMN19115479	1D	Lushi	background_not_melanogenesis	3	background
AA1W_P1	0.066506925	-0.527716925	-0.196266177	0.335879689	PRJNA729271	SRR14521324	SAMN19115459	1W	Arbor_Acres	background_not_melanogenesis	1	background
AA1W_P2	0.029538948	-0.504926172	-0.225657569	0.905107079	PRJNA729271	SRR14521323	SAMN19115460	1W	Arbor_Acres	background_not_melanogenesis	2	background
AA1W_P3	0.22269536	-0.380956224	-0.210579418	0.440412073	PRJNA729271	SRR14521322	SAMN19115461	1W	Arbor_Acres	background_not_melanogenesis	3	background
LS1W_P1	-0.218391217	0.081864615	-0.203486402	0.286433213	PRJNA729271	SRR14521301	SAMN19115480	1W	Lushi	background_not_melanogenesis	1	background
LS1W_P2	-0.363204578	-0.116330242	0.166778969	-0.055080564	PRJNA729271	SRR14521300	SAMN19115481	1W	Lushi	background_not_melanogenesis	2	background
LS1W_P3	-0.571936412	0.114745866	-0.230977268	0.384582419	PRJNA729271	SRR14521299	SAMN19115482	1W	Lushi	background_not_melanogenesis	3	background
AA3W_P1	-0.823246827	-0.078677545	-0.551469434	0.355112195	PRJNA729271	SRR14521321	SAMN19115462	3W	Arbor_Acres	background_not_melanogenesis	1	background
AA3W_P2	-0.111075119	-0.532056018	-0.546898668	0.594815631	PRJNA729271	SRR14521320	SAMN19115463	3W	Arbor_Acres	background_not_melanogenesis	2	background
AA3W_P3	-0.276493838	-0.392812474	-0.658635828	0.99039457	PRJNA729271	SRR14521319	SAMN19115464	3W	Arbor_Acres	background_not_melanogenesis	3	background
LS3W_P1	-0.80658645	-0.184346626	-0.446361923	0.467815966	PRJNA729271	SRR14521298	SAMN19115483	3W	Lushi	background_not_melanogenesis	1	background
LS3W_P2	-0.421781056	-0.286392852	-0.409731175	-0.072882693	PRJNA729271	SRR14521297	SAMN19115484	3W	Lushi	background_not_melanogenesis	2	background
LS3W_P3	-0.145777061	-0.299401741	-0.021871508	-0.292229539	PRJNA729271	SRR14521296	SAMN19115485	3W	Lushi	background_not_melanogenesis	3	background
AA5W_P1	-0.038409797	-0.392526506	-0.110210144	0.699304841	PRJNA729271	SRR14521318	SAMN19115465	5W	Arbor_Acres	background_not_melanogenesis	1	background
AA5W_P2	-0.206484077	-0.497230444	-0.078033082	0.395712097	PRJNA729271	SRR14521317	SAMN19115466	5W	Arbor_Acres	background_not_melanogenesis	2	background
AA5W_P3	-0.143834817	-0.580959589	-0.364062573	0.703166876	PRJNA729271	SRR14521315	SAMN19115467	5W	Arbor_Acres	background_not_melanogenesis	3	background
LS5W_P1	-0.546354598	-0.537782622	-0.563167298	0.795756079	PRJNA729271	SRR14521295	SAMN19115486	5W	Lushi	background_not_melanogenesis	1	background
LS5W_P2	-0.505974242	-0.452801004	-0.262534939	0.210198161	PRJNA729271	SRR14521293	SAMN19115487	5W	Lushi	background_not_melanogenesis	2	background
LS5W_P3	-0.439888858	-0.305393997	-0.434712364	0.654887714	PRJNA729271	SRR14521292	SAMN19115488	5W	Lushi	background_not_melanogenesis	3	background
