# Supplementary material for: Cross-cohort transcriptomic meta-analysis identifies conserved melanogenesis signatures and developmental background in black-boned chicken breast muscle
Source: Anim Biosci. 2026 Jun 24;39(10):260430. doi: 10.5713/ab.260430 (PMC13662945; doi:10.5713/ab.260430)
Supplement: Supplementary file 8 [file ab-260430-Supplementary-16.pdf]

Supplement 16. Final layered conserved signature genes used for candidate prioritization.

signature_layer	gene_id	symbol	meta_log2FC	meta_fdr	I2	direction_consistency	background_development_gene	evidence_class	candidate_priority_score	cohorts
Class I: core conserved melanogenesis anchors	ENSGALG00010024745	PMEL	8.91582778	9.3962E-280	0	1	TRUE	pigmentation_development_overlap	95	CRA004775;PRJNA827465;Yang2025_Silkie_Wenchang
Class I: core conserved melanogenesis anchors	ENSGALG00010014092	TYRP1	8.832436078	1.1884E-184	0	1	TRUE	pigmentation_development_overlap	95	CRA004775;PRJNA827465;Yang2025_Silkie_Wenchang
Class I: core conserved melanogenesis anchors	ENSGALG00010005140	DCT	7.779409913	7.5793E-82	0	1	TRUE	pigmentation_development_overlap	95	CRA004775;PRJNA827465;Yang2025_Silkie_Wenchang
Class I: core conserved melanogenesis anchors	ENSGALG00010007864	TYR	6.704858539	2.64969E-48	0	1	TRUE	pigmentation_development_overlap	92.57680572	CRA004775;PRJNA827465;Yang2025_Silkie_Wenchang
Class II: pigmentation -development background overlap	ENSGALG00010012325	MLPH	5.41105141	2.16012E-44	0	1	TRUE	pigmentation_development_overlap	88.66552202	CRA004775;PRJNA827465;Yang2025_Silkie_Wenchang
Class II: pigmentation -development background overlap	ENSGALG00010002896	OCA2	3.38211729	8.82411E-40	11.63318475	1	TRUE	pigmentation_development_overlap	79.80159695	CRA004775;PRJNA827465;Yang2025_Silkie_Wenchang
Class II: pigmentation -development background overlap	ENSGALG00010012923	MLANA	7.072751818	6.76399E-32	43.8899335	1	TRUE	pigmentation_development_overlap	71.78080375	CRA004775;PRJNA827465;Yang2025_Silkie_Wenchang
Class II: pigmentation -development background overlap	ENSGALG00010024748	TRPM1	2.328204354	1.32301E-24	0	1	TRUE	pigmentation_development_overlap	60.5194597	PRJNA827465;Yang2025_Silkie_Wenchang
Class II: pigmentation -development background overlap	ENSGALG00010016253	EDN3	1.944732901	1.34582E-26	66.02734752	1	TRUE	pigmentation_development_overlap	52.9919432	CRA004775;PRJNA827465;Yang2025_Silkie_Wenchang
Class II: pigmentation -development background overlap	ENSGALG00010007506	RAB38	3.332327553	0.037655794	92.44608534	1	TRUE	pigmentation_development_overlap	33.84119742	CRA004775;PRJNA827465;Yang2025_Silkie_Wenchang
Class II: pigmentation -development background overlap	ENSGALG00010010407	SOX10	0.663894197	0.000870057	45.90190075	1	TRUE	pigmentation_development_overlap	25.78973297	CRA004775;PRJNA827465;Yang2025_Silkie_Wenchang
Class III: ordinary muscle-development/background -associated conserved genes	ENSGALG00010001409	GPNMB	6.353508602	2.3918E-50	0	1	TRUE	background_development	89.62127425	CRA004775;PRJNA827465;Yang2025_Silkie_Wenchang
Class III: ordinary muscle-development/background -associated conserved genes	ENSGALG00010025640	SLC38A11	5.043745532	2.27894E-40	0	1	TRUE	background_development	79.6422678	PRJNA827465;Yang2025_Silkie_Wenchang
Class III: ordinary muscle-development/background -associated conserved genes	ENSGALG00010018219	SLC24A5	4.898060665	4.77201E-27	0	1	TRUE	background_development	66.32129856	PRJNA827465;Yang2025_Silkie_Wenchang
Class III: ordinary muscle-development/background -associated conserved genes	ENSGALG00010013190	SLC45A2	4.671732192	1.32301E-24	0	1	TRUE	background_development	63.87843793	PRJNA827465;Yang2025_Silkie_Wenchang
Class III: ordinary muscle-development/background -associated conserved genes	ENSGALG00010012311	ENSGALG00010012311	4.445685227	7.07845E-23	71.33614929	1	TRUE	background_development	55.01644678	CRA004775;PRJNA827465;Yang2025_Silkie_Wenchang
Class III: ordinary muscle-development/background -associated conserved genes	ENSGALG00010028874	CRPL1	5.822667805	3.28616E-21	69.70994824	1	TRUE	background_development	53.51231632	CRA004775;PRJNA827465;Yang2025_Silkie_Wenchang
Class III: ordinary muscle-development/background -associated conserved genes	ENSGALG00010008126	CDH3	2.121539826	8.99979E-19	0	1	TRUE	background_development	48.6534666	CRA004775;PRJNA827465;Yang2025_Silkie_Wenchang
Class III: ordinary muscle-development/background -associated conserved genes	ENSGALG00010022210	GPR143	3.394091803	1.35494E-09	0.857258125	1	TRUE	background_development	45.75280761	PRJNA827465;Yang2025_Silkie_Wenchang
Class III: ordinary muscle-development/background -associated conserved genes	ENSGALG00010011117	SLC6A15	3.617522337	1.30081E-09	18.47589505	1	TRUE	background_development	45.12580823	PRJNA827465;Yang2025_Silkie_Wenchang
Class III: ordinary muscle-development/background -associated conserved genes	ENSGALG00010007845	TFAP2B	2.732606089	4.10513E-06	0	1	TRUE	background_development	39.049703	PRJNA827465;Yang2025_Silkie_Wenchang
Class III: ordinary muscle-development/background -associated conserved genes	ENSGALG00010021707	DLX2	2.563205596	5.27527E-05	0	1	TRUE	background_development	37.09378356	PRJNA827465;Yang2025_Silkie_Wenchang
Class III: ordinary muscle-development/background -associated conserved genes	ENSGALG00010029213	APOH	2.516450083	9.39514E-05	5.061622352	1	TRUE	background_development	36.10318504	CRA004775;PRJNA827465;Yang2025_Silkie_Wenchang
Class III: ordinary muscle-development/background -associated conserved genes	ENSGALG00010011980	AvBD9	2.333742657	0.034941487	0	1	TRUE	background_development	33.12537191	CRA004775;PRJNA827465
Class III: ordinary muscle-development/background -associated conserved genes	ENSGALG00010013833	TMPRSS2	2.165028817	0.028931889	0	1	TRUE	background_development	32.36376729	CRA004775;PRJNA827465
Class III: ordinary muscle-development/background -associated conserved genes	ENSGALG00010007954	PSCA	1.724027682	0.001012003	0	1	TRUE	background_development	31.61495674	CRA004775;PRJNA827465;Yang2025_Silkie_Wenchang
Class III: ordinary muscle-development/background -associated conserved genes	ENSGALG00010026628	FYB2	1.964711858	0.023424158	0	1	TRUE	background_development	31.45389529	CRA004775;PRJNA827465
Class III: ordinary muscle-development/background -associated conserved genes	ENSGALG00010009259	MGAT4D	1.746174025	0.002029388	0	1	TRUE	background_development	31.42350511	CRA004775;Yang2025_Silkie_Wenchang
Class III: ordinary muscle-development/background -associated conserved genes	ENSGALG00010028466	MAMDC4	1.982211231	0.035345927	0	1	TRUE	background_development	31.36271678	CRA004775;PRJNA827465
Class III: ordinary muscle-development/background -associated conserved genes	ENSGALG00010001365	ENSGALG00010001365	-0.605539101	7.77434E-09	0	1	TRUE	background_development	31.12703206	CRA004775;PRJNA827465;Yang2025_Silkie_Wenchang
Class III: ordinary muscle-development/background -associated conserved genes	ENSGALG00010003339	ENSGALG00010003339	-1.850875603	0.030313479	0	1	TRUE	background_development	30.77274223	PRJNA827465;Yang2025_Silkie_Wenchang
Class III: ordinary muscle-development/background -associated conserved genes	ENSGALG00010015386	SLC24A4	1.135225986	1.00224E-05	0	1	TRUE	background_development	30.67515803	CRA004775;PRJNA827465;Yang2025_Silkie_Wenchang
Class III: ordinary muscle-development/background -associated conserved genes	ENSGALG00010022049	EPHX4	1.462157012	0.000461092	0	1	TRUE	background_development	30.64699765	CRA004775;PRJNA827465;Yang2025_Silkie_Wenchang
Class III: ordinary muscle-development/background -associated conserved genes	ENSGALG00010001976	THRSP	-1.684290035	0.036365512	0	1	TRUE	background_development	29.86076046	CRA004775;PRJNA827465;Yang2025_Silkie_Wenchang
Class III: ordinary muscle-development/background -associated conserved genes	ENSGALG00010011891	HAL	1.508276129	0.005143766	0	1	TRUE	background_development	29.83009946	PRJNA827465;Yang2025_Silkie_Wenchang
Class III: ordinary muscle-development/background -associated conserved genes	ENSGALG00010022482	PRTG	1.133459945	0.000142115	0	1	TRUE	background_development	29.51465865	CRA004775;PRJNA827465;Yang2025_Silkie_Wenchang
Class III: ordinary muscle-development/background -associated conserved genes	ENSGALG00010019935	ENSGALG00010019935	1.541443444	0.018119045	0	1	TRUE	background_development	29.44908191	CRA004775;PRJNA827465;Yang2025_Silkie_Wenchang
Class III: ordinary muscle-development/background -associated conserved genes	ENSGALG00010005345	NOX3	2.916781507	0.014371005	72.56775466	1	TRUE	background_development	29.16964493	PRJNA827465;Yang2025_Silkie_Wenchang
Class III: ordinary muscle-development/background -associated conserved genes	ENSGALG00010016006	PLTP	0.574339269	8.12523E-07	0	1	TRUE	background_development	28.96186043	CRA004775;PRJNA827465;Yang2025_Silkie_Wenchang
Class III: ordinary muscle-development/background -associated conserved genes	ENSGALG00010017452	ARMH4	-1.311385718	0.019568396	0	1	TRUE	background_development	28.26537337	CRA004775;PRJNA827465;Yang2025_Silkie_Wenchang
Class III: ordinary muscle-development/background -associated conserved genes	ENSGALG00010021784	ANGPTL7	1.316540471	0.023991454	0	1	TRUE	background_development	28.20264579	PRJNA827465;Yang2025_Silkie_Wenchang
Class III: ordinary muscle-development/background -associated conserved genes	ENSGALG00010006944	MAB21L2	1.29237895	0.004860124	6.25803606	1	TRUE	background_development	28.14944383	CRA004775;PRJNA827465;Yang2025_Silkie_Wenchang
Class III: ordinary muscle-development/background -associated conserved genes	ENSGALG00010014995	LRAT	1.000239239	0.001208589	0	1	TRUE	background_development	27.91891746	CRA004775;PRJNA827465;Yang2025_Silkie_Wenchang
Class III: ordinary muscle-development/background -associated conserved genes	ENSGALG00010014161	GFRA3	0.840720004	0.000295712	0	1	TRUE	background_development	27.73273153	CRA004775;PRJNA827465;Yang2025_Silkie_Wenchang
Class III: ordinary muscle-development/background -associated conserved genes	ENSGALG00010011044	MBL2	1.137833957	0.009648428	0	1	TRUE	background_development	27.70471321	PRJNA827465;Yang2025_Silkie_Wenchang
Class III: ordinary muscle-development/background -associated conserved genes	ENSGALG00010003512	ENSGALG00010003512	-1.054320794	0.004042433	0	1	TRUE	background_development	27.66496116	CRA004775;PRJNA827465;Yang2025_Silkie_Wenchang
Class III: ordinary muscle-development/background -associated conserved genes	ENSGALG00010009021	HNF4beta	1.113103944	0.01097502	0	1	TRUE	background_development	27.5251144	CRA004775;PRJNA827465;Yang2025_Silkie_Wenchang
Class III: ordinary muscle-development/background -associated conserved genes	ENSGALG00010000440	ENSGALG00010000440	1.062678213	0.007104388	0	1	TRUE	background_development	27.46186442	CRA004775;PRJNA827465;Yang2025_Silkie_Wenchang
Class III: ordinary muscle-development/background -associated conserved genes	ENSGALG00010003632	TECRL	-1.084467047	0.011755221	0	1	TRUE	background_development	27.35210443	PRJNA827465;Yang2025_Silkie_Wenchang
Class III: ordinary muscle-development/background -associated conserved genes	ENSGALG00010011020	SFTPA2	1.12092437	0.045877937	0	1	TRUE	background_development	26.94301797	PRJNA827465;Yang2025_Silkie_Wenchang
Class III: ordinary muscle-development/background -associated conserved genes	ENSGALG00010013928	CCNO	-0.920899596	0.006017467	0	1	TRUE	background_development	26.82508427	CRA004775;PRJNA827465;Yang2025_Silkie_Wenchang
Class III: ordinary muscle-development/background -associated conserved genes	ENSGALG00010012618	B3GALT2	-0.750117929	0.001346169	0	1	TRUE	background_development	26.62148997	CRA004775;PRJNA827465;Yang2025_Silkie_Wenchang
Class III: ordinary muscle-development/background -associated conserved genes	ENSGALG00010024860	NEU4	0.96613973	0.018839217	0	1	TRUE	background_development	26.5556358	PRJNA827465;Yang2025_Silkie_Wenchang
Class III: ordinary muscle-development/background -associated conserved genes	ENSGALG00010015320	TRPM6	0.982045579	0.023991454	0	1	TRUE	background_development	26.53017133	PRJNA827465;Yang2025_Silkie_Wenchang
Class III: ordinary muscle-development/background -associated conserved genes	ENSGALG00010008132	TDO2	1.022978635	0.042614956	0	1	TRUE	background_development	26.48533113	CRA004775;PRJNA827465;Yang2025_Silkie_Wenchang
Class III: ordinary muscle-development/background -associated conserved genes	ENSGALG00010016229	ENSGALG00010016229	0.866153627	0.009177882	0	1	TRUE	background_development	26.36802566	CRA004775;PRJNA827465;Yang2025_Silkie_Wenchang
Class III: ordinary muscle-development/background -associated conserved genes	ENSGALG00010007604	ENSGALG00010007604	-0.968890615	0.045984159	0	1	TRUE	background_development	26.18184483	CRA004775;PRJNA827465;Yang2025_Silkie_Wenchang
Class III: ordinary muscle-development/background -associated conserved genes	ENSGALG00010016199	RLBP1	0.948156819	0.038025573	0	1	TRUE	background_development	26.16070833	PRJNA827465;Yang2025_Silkie_Wenchang
Class III: ordinary muscle-development/background -associated conserved genes	ENSGALG00010022412	CHAC1	0.862111026	0.015730702	0	1	TRUE	background_development	26.11380703	CRA004775;PRJNA827465;Yang2025_Silkie_Wenchang
Class III: ordinary muscle-development/background -associated conserved genes	ENSGALG00010027947	GP9	-0.882279806	0.025673208	0	1	TRUE	background_development	26.0019189	CRA004775;PRJNA827465;Yang2025_Silkie_Wenchang
Class III: ordinary muscle-development/background -associated conserved genes	ENSGALG00010018229	AS3MT	-0.510545421	0.000387913	0	1	TRUE	background_development	25.96399251	CRA004775;PRJNA827465;Yang2025_Silkie_Wenchang
Class III: ordinary muscle-development/background -associated conserved genes	ENSGALG00010016958	TLR15	0.899282718	0.043606977	0	1	TRUE	background_development	25.85685761	CRA004775;PRJNA827465;Yang2025_Silkie_Wenchang
Class III: ordinary muscle-development/background -associated conserved genes	ENSGALG00010001420	FAM221A	0.905447121	0.049625521	0	1	TRUE	background_development	25.83153053	PRJNA827465;Yang2025_Silkie_Wenchang
Class III: ordinary muscle-development/background -associated conserved genes	ENSGALG00010002435	ATP7B	1.790864958	0.044066854	45.10062182	1	TRUE	background_development	25.80015056	PRJNA827465;Yang2025_Silkie_Wenchang
Class III: ordinary muscle-development/background -associated conserved genes	ENSGALG00010004929	ENSGALG00010004929	0.520621034	0.000729568	0	1	TRUE	background_development	25.74003914	CRA004775;PRJNA827465;Yang2025_Silkie_Wenchang
Class III: ordinary muscle-development/background -associated conserved genes	ENSGALG00010005145	AKAP7	-0.415642232	0.000231376	0	1	TRUE	background_development	25.71389305	CRA004775;PRJNA827465;Yang2025_Silkie_Wenchang
Class III: ordinary muscle-development/background -associated conserved genes	ENSGALG00010001351	CAPN1	-0.321388065	9.62959E-05	0	1	TRUE	background_development	25.62333245	CRA004775;PRJNA827465;Yang2025_Silkie_Wenchang
Class III: ordinary muscle-development/background -associated conserved genes	ENSGALG00010012026	SYT1	0.723460756	0.010276748	0	1	TRUE	background_development	25.6054481	PRJNA827465;Yang2025_Silkie_Wenchang
Class III: ordinary muscle-development/background -associated conserved genes	ENSGALG00010023167	GPD1	-0.721409624	0.010177097	0	1	TRUE	background_development	25.5994242	CRA004775;PRJNA827465;Yang2025_Silkie_Wenchang
Class III: ordinary muscle-development/background -associated conserved genes	ENSGALG00010018941	KLHL21	-0.340254554	0.000146389	0	1	TRUE	background_development	25.53576409	CRA004775;PRJNA827465;Yang2025_Silkie_Wenchang
Class III: ordinary muscle-development/background -associated conserved genes	ENSGALG00010000224	MYBPC2	-0.67421461	0.007297957	0	1	TRUE	background_development	25.50787174	CRA004775;PRJNA827465;Yang2025_Silkie_Wenchang
Class III: ordinary muscle-development/background -associated conserved genes	ENSGALG00010004973	SLN	-0.642222	0.006748385	0	1	TRUE	background_development	25.38191012	CRA004775;PRJNA827465;Yang2025_Silkie_Wenchang
Class III: ordinary muscle-development/background -associated conserved genes	ENSGALG00010024886	ABTB2	0.427751468	0.000624104	0	1	TRUE	background_development	25.34350007	CRA004775;PRJNA827465;Yang2025_Silkie_Wenchang
Class III: ordinary muscle-development/background -associated conserved genes	ENSGALG00010005519	PTBP3	0.578672898	0.004018016	0	1	TRUE	background_development	25.28935286	CRA004775;PRJNA827465;Yang2025_Silkie_Wenchang
Class III: ordinary muscle-development/background -associated conserved genes	ENSGALG00010022572	ENSGALG00010022572	0.771030642	0.040086841	0	1	TRUE	background_development	25.25215137	PRJNA827465;Yang2025_Silkie_Wenchang
Class III: ordinary muscle-development/background -associated conserved genes	ENSGALG00010024791	SHANK2	1.584352784	0.017651214	44.55574498	1	TRUE	background_development	25.21941483	PRJNA827465;Yang2025_Silkie_Wenchang
Class III: ordinary muscle-development/background -associated conserved genes	ENSGALG00010024665	MAP1LC3A	-0.360680639	0.000448315	0	1	TRUE	background_development	25.1518195	CRA004775;PRJNA827465;Yang2025_Silkie_Wenchang
Class III: ordinary muscle-development/background -associated conserved genes	ENSGALG00010008392	CD36	-0.689932039	0.026043176	0	1	TRUE	background_development	25.03396625	CRA004775;PRJNA827465;Yang2025_Silkie_Wenchang
Class III: ordinary muscle-development/background -associated conserved genes	ENSGALG00010020546	LRRC20	-0.390044479	0.000878972	0	1	TRUE	background_development	25.00624726	CRA004775;PRJNA827465;Yang2025_Silkie_Wenchang
Class III: ordinary muscle-development/background -associated conserved genes	ENSGALG00010002399	CSMD1	-0.663895219	0.021848783	0	1	TRUE	background_development	24.98004884	PRJNA827465;Yang2025_Silkie_Wenchang
Class III: ordinary muscle-development/background -associated conserved genes	ENSGALG00010000137	ENSGALG00010000137	-0.963238764	0.020510744	16.3933191	1	TRUE	background_development	24.86488051	CRA004775;PRJNA827465;Yang2025_Silkie_Wenchang
Class III: ordinary muscle-development/background -associated conserved genes	ENSGALG00010005190	ENSGALG00010005190	-0.598529168	0.014586384	0	1	TRUE	background_development	24.8286982	CRA004775;PRJNA827465;Yang2025_Silkie_Wenchang
Class III: ordinary muscle-development/background -associated conserved genes	ENSGALG00010003830	DMB2	-0.691791087	0.043606977	0	1	TRUE	background_development	24.81939946	CRA004775;PRJNA827465;Yang2025_Silkie_Wenchang
Class III: ordinary muscle-development/background -associated conserved genes	ENSGALG00010028763	CTSK	0.411918772	0.001834002	0	1	TRUE	background_development	24.79619406	CRA004775;PRJNA827465;Yang2025_Silkie_Wenchang
Class III: ordinary muscle-development/background -associated conserved genes	ENSGALG00010023635	SCN4A	-0.443744128	0.003696156	0	1	TRUE	background_development	24.65097035	CRA004775;PRJNA827465;Yang2025_Silkie_Wenchang
Class III: ordinary muscle-development/background -associated conserved genes	ENSGALG00010014865	TLR2	-0.648545092	0.041352297	0	1	TRUE	background_development	24.62622582	CRA004775;PRJNA827465;Yang2025_Silkie_Wenchang
Class III: ordinary muscle-development/background -associated conserved genes	ENSGALG00010020396	CHL1	0.60261296	0.02634328	0	1	TRUE	background_development	24.59239495	CRA004775;PRJNA827465;Yang2025_Silkie_Wenchang
Class III: ordinary muscle-development/background -associated conserved genes	ENSGALG00010004339	B4GALT6	0.474388627	0.006090275	0	1	TRUE	background_development	24.58730622	CRA004775;PRJNA827465;Yang2025_Silkie_Wenchang
Class III: ordinary muscle-development/background -associated conserved genes	ENSGALG00010008171	KIF21A	0.601635339	0.009956861	14.46522656	1	TRUE	background_development	24.58080529	CRA004775;PRJNA827465;Yang2025_Silkie_Wenchang
Class III: ordinary muscle-development/background -associated conserved genes	ENSGALG00010021533	SSX2IP	0.512281065	0.00983371	0	1	TRUE	background_development	24.56868794	PRJNA827465;Yang2025_Silkie_Wenchang
Class III: ordinary muscle-development/background -associated conserved genes	ENSGALG00010009214	PCDH10	0.710296327	0.010579298	10.33487592	1	TRUE	background_development	24.49553718	CRA004775;PRJNA827465;Yang2025_Silkie_Wenchang
Class III: ordinary muscle-development/background -associated conserved genes	ENSGALG00010021042	LRRC38	-0.29500299	0.001022418	0	1	TRUE	background_development	24.46538631	CRA004775;PRJNA827465;Yang2025_Silkie_Wenchang
Class III: ordinary muscle-development/background -associated conserved genes	ENSGALG00010025230	CHST8	0.558203985	0.025673208	0	1	TRUE	background_development	24.38153979	PRJNA827465;Yang2025_Silkie_Wenchang
Class III: ordinary muscle-development/background -associated conserved genes	ENSGALG00010015028	MCM9	-0.600910151	0.044066854	0	1	TRUE	background_development	24.36043871	CRA004775;PRJNA827465;Yang2025_Silkie_Wenchang
Class III: ordinary muscle-development/background -associated conserved genes	ENSGALG00010005068	PLCXD2	-0.463926877	0.009347809	0	1	TRUE	background_development	24.34892454	CRA004775;PRJNA827465;Yang2025_Silkie_Wenchang
Class III: ordinary muscle-development/background -associated conserved genes	ENSGALG00010007320	ENSGALG00010007320	-0.428058105	0.006271184	0	1	TRUE	background_development	24.342941	CRA004775;PRJNA827465;Yang2025_Silkie_Wenchang
Class III: ordinary muscle-development/background -associated conserved genes	ENSGALG00010001487	CDH19	0.446511778	0.007661662	0.54677995	1	TRUE	background_development	24.29355791	CRA004775;PRJNA827465;Yang2025_Silkie_Wenchang
Class III: ordinary muscle-development/background -associated conserved genes	ENSGALG00010012079	ENSGALG00010012079	-0.855622589	0.014107786	18.82319271	1	TRUE	background_development	24.2463348	CRA004775;PRJNA827465;Yang2025_Silkie_Wenchang
Class III: ordinary muscle-development/background -associated conserved genes	ENSGALG00010002377	ART1A	0.570242817	0.046403352	0	1	TRUE	background_development	24.18466473	CRA004775;PRJNA827465;Yang2025_Silkie_Wenchang
Class III: ordinary muscle-development/background -associated conserved genes	ENSGALG00010005350	ARHGAP20	-0.522296899	0.037273845	0	1	TRUE	background_development	24.0400803	CRA004775;PRJNA827465;Yang2025_Silkie_Wenchang
Class III: ordinary muscle-development/background -associated conserved genes	ENSGALG00010017848	CYP3A4	0.440708616	0.018633292	0	1	TRUE	background_development	23.93325349	CRA004775;PRJNA827465;Yang2025_Silkie_Wenchang
Class III: ordinary muscle-development/background -associated conserved genes	ENSGALG00010024277	ITGB3BP	-0.399739763	0.017680012	0	1	TRUE	background_development	23.75121625	CRA004775;PRJNA827465;Yang2025_Silkie_Wenchang
Class III: ordinary muscle-development/background -associated conserved genes	ENSGALG00010011365	PSMG4	-0.526377672	0.007035584	10.41459616	1	TRUE	background_development	23.74312858	CRA004775;PRJNA827465;Yang2025_Silkie_Wenchang
Class III: ordinary muscle-development/background -associated conserved genes	ENSGALG00010001267	BCHE	-0.282623486	0.004860124	0	1	TRUE	background_development	23.72647012	CRA004775;PRJNA827465;Yang2025_Silkie_Wenchang
Class III: ordinary muscle-development/background -associated conserved genes	ENSGALG00010008072	NRSN1	-0.454346232	0.035174871	0	1	TRUE	background_development	23.72549865	CRA004775;PRJNA827465;Yang2025_Silkie_Wenchang
Class III: ordinary muscle-development/background -associated conserved genes	ENSGALG00010011119	ENSGALG00010011119	-0.511668285	0.028615343	4.074197884	1	TRUE	background_development	23.69432269	CRA004775;PRJNA827465;Yang2025_Silkie_Wenchang
Class III: ordinary muscle-development/background -associated conserved genes	ENSGALG00010008139	ENSGALG00010008139	0.927746733	0.040086841	23.58332675	1	TRUE	background_development	23.67739915	CRA004775;PRJNA827465;Yang2025_Silkie_Wenchang
Class III: ordinary muscle-development/background -associated conserved genes	ENSGALG00010014238	PHLDA1	0.305564252	0.007190859	0	1	TRUE	background_development	23.67104048	CRA004775;PRJNA827465;Yang2025_Silkie_Wenchang
Class III: ordinary muscle-development/background -associated conserved genes	ENSGALG00010008284	BCKDHA	-0.269878484	0.006271184	0	1	TRUE	background_development	23.5520429	CRA004775;PRJNA827465;Yang2025_Silkie_Wenchang
Class III: ordinary muscle-development/background -associated conserved genes	ENSGALG00010017421	GPR157	-0.380508934	0.023424158	0	1	TRUE	background_development	23.53288067	CRA004775;PRJNA827465;Yang2025_Silkie_Wenchang
Class III: ordinary muscle-development/background -associated conserved genes	ENSGALG00010007970	SPRTN	0.229762692	0.004266288	0	1	TRUE	background_development	23.51876325	CRA004775;PRJNA827465;Yang2025_Silkie_Wenchang
Class III: ordinary muscle-development/background -associated conserved genes	ENSGALG00010025485	DENND2C	0.305817818	0.002045583	8.101443227	1	TRUE	background_development	23.4081277	CRA004775;PRJNA827465;Yang2025_Silkie_Wenchang
Class III: ordinary muscle-development/background -associated conserved genes	ENSGALG00010028380	RUSC1	-0.246122002	0.007130398	0	1	TRUE	background_development	23.37749627	CRA004775;PRJNA827465;Yang2025_Silkie_Wenchang
Class III: ordinary muscle-development/background -associated conserved genes	ENSGALG00010000614	KAT8	-0.364509786	0.033824796	0	1	TRUE	background_development	23.29331374	CRA004775;PRJNA827465;Yang2025_Silkie_Wenchang
Class III: ordinary muscle-development/background -associated conserved genes	ENSGALG00010023196	DGKZ	-0.176463182	0.004042433	0	1	TRUE	background_development	23.2756731	CRA004775;PRJNA827465;Yang2025_Silkie_Wenchang
Class III: ordinary muscle-development/background -associated conserved genes	ENSGALG00010024837	TNFRSF1A	-0.268375186	0.012370065	0	1	TRUE	background_development	23.24950396	CRA004775;PRJNA827465;Yang2025_Silkie_Wenchang
Class III: ordinary muscle-development/background -associated conserved genes	ENSGALG00010006531	FAM161B	0.296396833	0.018208234	0	1	TRUE	background_development	23.22171633	CRA004775;PRJNA827465;Yang2025_Silkie_Wenchang
Class III: ordinary muscle-development/background -associated conserved genes	ENSGALG00010004386	CFAP44	0.290726334	0.017301072	0	1	TRUE	background_development	23.21555865	CRA004775;PRJNA827465;Yang2025_Silkie_Wenchang
Class III: ordinary muscle-development/background -associated conserved genes	ENSGALG00010001780	ENSGALG00010001780	0.353744145	0.037799632	0	1	TRUE	background_development	23.19123316	CRA004775;PRJNA827465;Yang2025_Silkie_Wenchang
Class III: ordinary muscle-development/background -associated conserved genes	ENSGALG00010003589	SLC25A30	0.344562448	0.036365512	0	1	TRUE	background_development	23.16212253	CRA004775;PRJNA827465;Yang2025_Silkie_Wenchang
Class III: ordinary muscle-development/background -associated conserved genes	ENSGALG00010016547	SGCB	-0.298133472	0.021848783	0	1	TRUE	background_development	23.15124011	CRA004775;PRJNA827465;Yang2025_Silkie_Wenchang
Class III: ordinary muscle-development/background -associated conserved genes	ENSGALG00010020691	ENSGALG00010020691	0.334412806	0.035841831	0	1	TRUE	background_development	23.11767384	CRA004775;PRJNA827465;Yang2025_Silkie_Wenchang
Class III: ordinary muscle-development/background -associated conserved genes	ENSGALG00010010271	HUNK	0.280613097	0.019654451	0	1	TRUE	background_development	23.10960456	CRA004775;PRJNA827465;Yang2025_Silkie_Wenchang
Class III: ordinary muscle-development/background -associated conserved genes	ENSGALG00010015054	ENSGALG00010015054	-0.292238223	0.02338962	0	1	TRUE	background_development	23.09216796	CRA004775;PRJNA827465;Yang2025_Silkie_Wenchang
Class III: ordinary muscle-development/background -associated conserved genes	ENSGALG00010004835	HHLA2	0.973684219	0.002930645	43.26821393	1	TRUE	background_development	23.07463645	CRA004775;PRJNA827465;Yang2025_Silkie_Wenchang
Class III: ordinary muscle-development/background -associated conserved genes	ENSGALG00010018415	PELI2	0.222762512	0.011755221	0	1	TRUE	background_development	23.04358175	CRA004775;PRJNA827465;Yang2025_Silkie_Wenchang
Class III: ordinary muscle-development/background -associated conserved genes	ENSGALG00010010085	TDG	0.175712661	0.007548066	0	1	TRUE	background_development	23.00072763	CRA004775;PRJNA827465;Yang2025_Silkie_Wenchang
Class III: ordinary muscle-development/background -associated conserved genes	ENSGALG00010016856	NOD1	0.30552595	0.03431261	0	1	TRUE	background_development	22.992176	CRA004775;PRJNA827465;Yang2025_Silkie_Wenchang
Class III: ordinary muscle-development/background -associated conserved genes	ENSGALG00010000730	INTS5	-0.298044345	0.031762888	0	1	TRUE	background_development	22.98830174	CRA004775;PRJNA827465;Yang2025_Silkie_Wenchang
Class III: ordinary muscle-development/background -associated conserved genes	ENSGALG00010006603	MEST	-0.324156241	0.044492107	0	1	TRUE	background_development	22.97249823	CRA004775;PRJNA827465;Yang2025_Silkie_Wenchang
Class III: ordinary muscle-development/background -associated conserved genes	ENSGALG00010018832	EEPD1	-0.277544757	0.028615343	0	1	TRUE	background_development	22.93112483	CRA004775;PRJNA827465;Yang2025_Silkie_Wenchang
Class III: ordinary muscle-development/background -associated conserved genes	ENSGALG00010027914	BTBD2	-0.274277905	0.029565941	0	1	TRUE	background_development	22.90059782	CRA004775;PRJNA827465;Yang2025_Silkie_Wenchang
Class III: ordinary muscle-development/background -associated conserved genes	ENSGALG00010016373	SRL	-0.270360868	0.02906761	0	1	TRUE	background_development	22.88839501	CRA004775;PRJNA827465;Yang2025_Silkie_Wenchang
Class III: ordinary muscle-development/background -associated conserved genes	ENSGALG00010028042	DPP7	0.230853491	0.023424158	0	1	TRUE	background_development	22.78460346	CRA004775;PRJNA827465;Yang2025_Silkie_Wenchang
Class III: ordinary muscle-development/background -associated conserved genes	ENSGALG00010021774	FBXL20	-0.178568424	0.017046236	0	1	TRUE	background_development	22.66121363	CRA004775;PRJNA827465;Yang2025_Silkie_Wenchang
Class III: ordinary muscle-development/background -associated conserved genes	ENSGALG00010028422	RXRA	-0.264561293	0.049069007	0	1	TRUE	background_development	22.63199919	CRA004775;PRJNA827465;Yang2025_Silkie_Wenchang
Class III: ordinary muscle-development/background -associated conserved genes	ENSGALG00010023546	SLC11A2	-0.249124701	0.041175892	0	1	TRUE	background_development	22.63098049	CRA004775;PRJNA827465;Yang2025_Silkie_Wenchang
Class III: ordinary muscle-development/background -associated conserved genes	ENSGALG00010007695	RASGRP3	0.223579115	0.030753573	0	1	TRUE	background_development	22.62999999	CRA004775;PRJNA827465;Yang2025_Silkie_Wenchang
Class III: ordinary muscle-development/background -associated conserved genes	ENSGALG00010003803	LACTB2	-0.216520643	0.030247549	0	1	TRUE	background_development	22.60191303	CRA004775;PRJNA827465;Yang2025_Silkie_Wenchang
Class III: ordinary muscle-development/background -associated conserved genes	ENSGALG00010008752	GOLM1	-0.6379402	0.004042433	29.82813088	1	TRUE	background_development	22.6002451	CRA004775;PRJNA827465;Yang2025_Silkie_Wenchang
Class III: ordinary muscle-development/background -associated conserved genes	ENSGALG00010008298	ENSGALG00010008298	0.200023252	0.027360055	0	1	TRUE	background_development	22.5629993	CRA004775;PRJNA827465;Yang2025_Silkie_Wenchang
Class III: ordinary muscle-development/background -associated conserved genes	ENSGALG00010021666	CCDC47	-0.204210833	0.034941487	0	1	TRUE	background_development	22.47771278	CRA004775;PRJNA827465;Yang2025_Silkie_Wenchang
Class III: ordinary muscle-development/background -associated conserved genes	ENSGALG00010028134	INPP5E	-0.177878863	0.026629161	0	1	TRUE	background_development	22.46403683	CRA004775;PRJNA827465;Yang2025_Silkie_Wenchang
Class III: ordinary muscle-development/background -associated conserved genes	ENSGALG00010025891	NUP93	-0.219474176	0.04554129	0	1	TRUE	background_development	22.43896556	CRA004775;PRJNA827465;Yang2025_Silkie_Wenchang
Class III: ordinary muscle-development/background -associated conserved genes	ENSGALG00010028171	RNF123	-0.196836873	0.035345927	0	1	TRUE	background_development	22.43584499	CRA004775;PRJNA827465;Yang2025_Silkie_Wenchang
Class III: ordinary muscle-development/background -associated conserved genes	ENSGALG00010019588	CHMP7	-0.173254414	0.031762888	0	1	TRUE	background_development	22.36435208	CRA004775;PRJNA827465;Yang2025_Silkie_Wenchang
Class III: ordinary muscle-development/background -associated conserved genes	ENSGALG00010010828	SPDL1	0.195201184	0.048129738	0	1	TRUE	background_development	22.29359242	CRA004775;PRJNA827465;Yang2025_Silkie_Wenchang
Class III: ordinary muscle-development/background -associated conserved genes	ENSGALG00010010311	CTSV	0.18623485	0.04554129	0	1	TRUE	background_development	22.27276892	CRA004775;PRJNA827465;Yang2025_Silkie_Wenchang
Class III: ordinary muscle-development/background -associated conserved genes	ENSGALG00010024174	PLEKHB2	0.164139152	0.038061836	0	1	TRUE	background_development	22.24020603	CRA004775;PRJNA827465;Yang2025_Silkie_Wenchang
Class III: ordinary muscle-development/background -associated conserved genes	ENSGALG00010028888	WDR5B	-0.144146793	0.049288178	0	1	TRUE	background_development	22.0279912	CRA004775;PRJNA827465;Yang2025_Silkie_Wenchang
Class III: ordinary muscle-development/background -associated conserved genes	ENSGALG00010027712	STOM	-0.42260031	0.018633292	19.04233124	1	TRUE	background_development	21.93847883	CRA004775;PRJNA827465;Yang2025_Silkie_Wenchang
Class III: ordinary muscle-development/background -associated conserved genes	ENSGALG00010007214	MTHFSD	0.3092519	0.026870117	17.25324822	1	TRUE	background_development	21.39166512	CRA004775;PRJNA827465;Yang2025_Silkie_Wenchang
Class III: ordinary muscle-development/background -associated conserved genes	ENSGALG00010020941	ENSGALG00010020941	0.429347596	0.012660906	29.74023886	1	TRUE	background_development	21.07024933	CRA004775;PRJNA827465;Yang2025_Silkie_Wenchang
Class III: ordinary muscle-development/background -associated conserved genes	ENSGALG00010007767	VAT1L	0.814559553	0.009581207	53.70369694	1	TRUE	background_development	20.72100783	CRA004775;PRJNA827465;Yang2025_Silkie_Wenchang
Class III: ordinary muscle-development/background -associated conserved genes	ENSGALG00010009307	DIRAS2	0.933246304	0.023991454	55.75859105	1	TRUE	background_development	20.71031585	CRA004775;PRJNA827465;Yang2025_Silkie_Wenchang
Class III: ordinary muscle-development/background -associated conserved genes	ENSGALG00010021332	OIT3	0.805086348	0.029565941	77.45576229	1	TRUE	background_development	17.80906381	CRA004775;PRJNA827465;Yang2025_Silkie_Wenchang
Class IV: melanogenesis -enriched genes not flagged by PRJNA729271	ENSGALG00010013314	KIT	0.691366909	2.3408E-12	0	1	FALSE	melanogenesis_specific_or_enriched	54.08747062	CRA004775;PRJNA827465;Yang2025_Silkie_Wenchang
Class V: other conserved meta-signature genes	ENSGALG00010016279	PRELID3B	1.286821488	1.4423E-143	0	1	FALSE	other_meta_signature	91.43410744	CRA004775;PRJNA827465;Yang2025_Silkie_Wenchang
Class V: other conserved meta-signature genes	ENSGALG00010020024	ENSGALG00010020024	4.690361764	0.003185373	0	1	FALSE	other_meta_signature	57.49683973	PRJNA827465;Yang2025_Silkie_Wenchang
Class V: other conserved meta-signature genes	ENSGALG00010029441	ENSGALG00010029441	-7.067735365	0.044616379	2.417400379	1	FALSE	other_meta_signature	56.10876564	PRJNA827465;Yang2025_Silkie_Wenchang
Class V: other conserved meta-signature genes	ENSGALG00010002465	ENSGALG00010002465	3.315485858	0.000462738	30.9336685	1	FALSE	other_meta_signature	51.81872725	CRA004775;Yang2025_Silkie_Wenchang
Class V: other conserved meta-signature genes	ENSGALG00010022822	KCNJ13	4.483795155	0.000146389	78.50431024	1	FALSE	other_meta_signature	50.9840603	PRJNA827465;Yang2025_Silkie_Wenchang
Class V: other conserved meta-signature genes	ENSGALG00010024909	SLC39A5	2.527801801	0.009118852	0	1	FALSE	other_meta_signature	49.67906885	CRA004775;PRJNA827465
Class V: other conserved meta-signature genes	ENSGALG00010000571	ENSGALG00010000571	2.493056502	0.023991454	0	1	FALSE	other_meta_signature	49.08522594	CRA004775;PRJNA827465
Class V: other conserved meta-signature genes	ENSGALG00010009790	MC1R	5.730142408	0.012333708	85.99321198	1	FALSE	other_meta_signature	48.30958514	PRJNA827465;Yang2025_Silkie_Wenchang
Class V: other conserved meta-signature genes	ENSGALG00010021117	CYP1A2	2.031815692	0.001012003	0	1	FALSE	other_meta_signature	48.15389679	CRA004775;PRJNA827465
Class V: other conserved meta-signature genes	ENSGALG00010013965	SLC2A2	2.147457363	0.041665572	0	1	FALSE	other_meta_signature	47.11750947	CRA004775;PRJNA827465
Class V: other conserved meta-signature genes	ENSGALG00010022948	PDZK1IP1	1.876258093	0.002029388	0	1	FALSE	other_meta_signature	47.07392545	CRA004775;PRJNA827465
Class V: other conserved meta-signature genes	ENSGALG00010024342	HNF4A	2.077862829	0.021848783	0	1	FALSE	other_meta_signature	47.04988689	CRA004775;PRJNA827465
Class V: other conserved meta-signature genes	ENSGALG00010016241	TOPAZ1	1.974169494	0.012333708	0	1	FALSE	other_meta_signature	46.7797538	PRJNA827465;Yang2025_Silkie_Wenchang
Class V: other conserved meta-signature genes	ENSGALG00010029688	PIPOX	1.821276663	0.019515169	0	1	FALSE	other_meta_signature	45.81601101	CRA004775;PRJNA827465
Class V: other conserved meta-signature genes	ENSGALG00010007014	ENSGALG00010007014	1.429199532	0.000263849	0	1	FALSE	other_meta_signature	45.7246423	CRA004775;PRJNA827465;Yang2025_Silkie_Wenchang
Class V: other conserved meta-signature genes	ENSGALG00010022741	HNF1A	1.359969153	0.000878972	0	1	FALSE	other_meta_signature	44.85587063	CRA004775;PRJNA827465;Yang2025_Silkie_Wenchang
Class V: other conserved meta-signature genes	ENSGALG00010016414	KLB	1.449674242	0.00415396	0	1	FALSE	other_meta_signature	44.6299089	CRA004775;PRJNA827465;Yang2025_Silkie_Wenchang
Class V: other conserved meta-signature genes	ENSGALG00010012961	ANKRD55	1.283843962	0.023162019	0	1	FALSE	other_meta_signature	43.0544434	PRJNA827465;Yang2025_Silkie_Wenchang
Class V: other conserved meta-signature genes	ENSGALG00010021083	SCD	0.92776779	0.001004539	0	1	FALSE	other_meta_signature	42.63687212	CRA004775;PRJNA827465;Yang2025_Silkie_Wenchang
Class V: other conserved meta-signature genes	ENSGALG00010006123	MLH3	0.435845446	8.46047E-06	0	1	FALSE	other_meta_signature	42.25183281	CRA004775;PRJNA827465;Yang2025_Silkie_Wenchang
Class V: other conserved meta-signature genes	ENSGALG00010009993	ENSGALG00010009993	1.05424689	0.01097502	0	1	FALSE	other_meta_signature	42.23082913	CRA004775;PRJNA827465;Yang2025_Silkie_Wenchang
Class V: other conserved meta-signature genes	ENSGALG00010000142	ENSGALG00010000142	-1.071050826	0.016126486	0	1	FALSE	other_meta_signature	42.14771439	CRA004775;PRJNA827465;Yang2025_Silkie_Wenchang
Class V: other conserved meta-signature genes	ENSGALG00010022211	ENSGALG00010022211	1.009373226	0.002424551	5.994384067	1	FALSE	other_meta_signature	42.06279637	CRA004775;PRJNA827465;Yang2025_Silkie_Wenchang
Class V: other conserved meta-signature genes	ENSGALG00010021316	PLA2G12B	1.287896651	0.030753573	12.49004391	1	FALSE	other_meta_signature	41.70258328	CRA004775;PRJNA827465;Yang2025_Silkie_Wenchang
Class V: other conserved meta-signature genes	ENSGALG00010002352	ENSGALG00010002352	-0.379516046	0.000129714	0	1	FALSE	other_meta_signature	40.78459287	CRA004775;PRJNA827465;Yang2025_Silkie_Wenchang
Class V: other conserved meta-signature genes	ENSGALG00010017240	MELTF	0.627407471	0.003606043	0	1	FALSE	other_meta_signature	40.58000641	CRA004775;PRJNA827465;Yang2025_Silkie_Wenchang
Class V: other conserved meta-signature genes	ENSGALG00010002142	CLDN34	1.412284611	0.031762888	31.55446846	1	FALSE	other_meta_signature	40.40405622	CRA004775;PRJNA827465;Yang2025_Silkie_Wenchang
Class V: other conserved meta-signature genes	ENSGALG00010013520	CREG2	-0.450935734	0.000878972	0	1	FALSE	other_meta_signature	40.31070353	CRA004775;PRJNA827465;Yang2025_Silkie_Wenchang
Class V: other conserved meta-signature genes	ENSGALG00010022625	ENSGALG00010022625	-0.355437669	0.001031342	0	1	FALSE	other_meta_signature	39.76378555	CRA004775;PRJNA827465;Yang2025_Silkie_Wenchang
Class V: other conserved meta-signature genes	ENSGALG00010028478	PEA15	0.326434999	0.000878972	0	1	FALSE	other_meta_signature	39.68819986	CRA004775;PRJNA827465;Yang2025_Silkie_Wenchang
Class V: other conserved meta-signature genes	ENSGALG00010007857	ENSGALG00010007857	0.650050992	0.037655794	0	1	FALSE	other_meta_signature	39.67442315	CRA004775;PRJNA827465;Yang2025_Silkie_Wenchang
Class V: other conserved meta-signature genes	ENSGALG00010010880	ELP3	-0.229417252	0.000300832	0	1	FALSE	other_meta_signature	39.66876218	CRA004775;PRJNA827465;Yang2025_Silkie_Wenchang
Class V: other conserved meta-signature genes	ENSGALG00010005881	CORO2A	1.027727294	0.025974651	26.37257489	1	FALSE	other_meta_signature	39.08682926	CRA004775;PRJNA827465;Yang2025_Silkie_Wenchang
Class V: other conserved meta-signature genes	ENSGALG00010019999	ERLIN1	-0.180880719	0.000729568	0	1	FALSE	other_meta_signature	39.04133756	CRA004775;PRJNA827465;Yang2025_Silkie_Wenchang
Class V: other conserved meta-signature genes	ENSGALG00010012722	ENSGALG00010012722	0.393922743	0.017046236	0	1	FALSE	other_meta_signature	38.73798523	CRA004775;PRJNA827465;Yang2025_Silkie_Wenchang
Class V: other conserved meta-signature genes	ENSGALG00010002350	BEND6	0.540765299	0.000870057	21.41749833	1	FALSE	other_meta_signature	38.62252872	CRA004775;PRJNA827465;Yang2025_Silkie_Wenchang
Class V: other conserved meta-signature genes	ENSGALG00010023821	KANK4	0.235429532	0.003696156	0	1	FALSE	other_meta_signature	38.60939737	CRA004775;PRJNA827465;Yang2025_Silkie_Wenchang
Class V: other conserved meta-signature genes	ENSGALG00010021197	CHCHD1	0.302663454	0.008282513	0	1	FALSE	other_meta_signature	38.59515517	CRA004775;PRJNA827465;Yang2025_Silkie_Wenchang
Class V: other conserved meta-signature genes	ENSGALG00010002890	VXN	0.652817342	0.00394974	20.76088376	1	FALSE	other_meta_signature	38.59142981	CRA004775;PRJNA827465;Yang2025_Silkie_Wenchang
Class V: other conserved meta-signature genes	ENSGALG00010021146	AP5Z1	-0.221275486	0.00415396	0	1	FALSE	other_meta_signature	38.48791512	CRA004775;PRJNA827465;Yang2025_Silkie_Wenchang
Class V: other conserved meta-signature genes	ENSGALG00010021752	CSTB	-0.283352556	0.009177882	0	1	FALSE	other_meta_signature	38.45402031	CRA004775;PRJNA827465;Yang2025_Silkie_Wenchang
Class V: other conserved meta-signature genes	ENSGALG00010028127	MFSD12	0.336929077	0.018633292	0	1	FALSE	other_meta_signature	38.41435579	CRA004775;PRJNA827465;Yang2025_Silkie_Wenchang
Class V: other conserved meta-signature genes	ENSGALG00010022929	LRP4	-0.338364291	0.019568396	0	1	FALSE	other_meta_signature	38.40026623	CRA004775;PRJNA827465;Yang2025_Silkie_Wenchang
Class V: other conserved meta-signature genes	ENSGALG00010010352	MALT1	0.264265207	0.009581207	0	1	FALSE	other_meta_signature	38.3399058	CRA004775;PRJNA827465;Yang2025_Silkie_Wenchang
Class V: other conserved meta-signature genes	ENSGALG00010026909	ENSGALG00010026909	-0.265574837	0.01097502	0	1	FALSE	other_meta_signature	38.28746887	CRA004775;PRJNA827465;Yang2025_Silkie_Wenchang
Class V: other conserved meta-signature genes	ENSGALG00010014797	ZDHHC9	-0.202478413	0.006386264	0	1	FALSE	other_meta_signature	38.20714517	CRA004775;PRJNA827465;Yang2025_Silkie_Wenchang
Class V: other conserved meta-signature genes	ENSGALG00010007896	TIMMDC1	-0.241091737	0.010588107	0	1	FALSE	other_meta_signature	38.18064037	CRA004775;PRJNA827465;Yang2025_Silkie_Wenchang

Class V: other conserved meta-signature genes	ENSGALG00010002100	BTD	-0.288308411	0.018633292	0	1	FALSE	other_meta_signature	38.17125246	CRA004775;PRJNA827465;Yang2025_Silkie_Wenchang
Class V: other conserved meta-signature genes	ENSGALG00010020766	C2orf76	-0.322477585	0.030313479	0	1	FALSE	other_meta_signature	38.13075214	CRA004775;PRJNA827465;Yang2025_Silkie_Wenchang
Class V: other conserved meta-signature genes	ENSGALG00010013709	PSMF1	-0.230405749	0.010808649	0	1	FALSE	other_meta_signature	38.11825732	CRA004775;PRJNA827465;Yang2025_Silkie_Wenchang
Class V: other conserved meta-signature genes	ENSGALG00010002953	WDFY2	-0.287494879	0.025549313	0	1	FALSE	other_meta_signature	38.03009518	CRA004775;PRJNA827465;Yang2025_Silkie_Wenchang
Class V: other conserved meta-signature genes	ENSGALG00010015009	AGO4	-0.155771766	0.005869358	0	1	FALSE	other_meta_signature	38.0102682	CRA004775;PRJNA827465;Yang2025_Silkie_Wenchang
Class V: other conserved meta-signature genes	ENSGALG00010019458	ELP4	-0.216226033	0.012333708	0	1	FALSE	other_meta_signature	37.9900365	CRA004775;PRJNA827465;Yang2025_Silkie_Wenchang
Class V: other conserved meta-signature genes	ENSGALG00010020497	OSBPL11	-0.179597329	0.012370065	0	1	FALSE	other_meta_signature	37.80561467	CRA004775;PRJNA827465;Yang2025_Silkie_Wenchang
Class V: other conserved meta-signature genes	ENSGALG00010018259	WDR90	-0.23816415	0.029012643	0	1	FALSE	other_meta_signature	37.72823346	CRA004775;PRJNA827465;Yang2025_Silkie_Wenchang
Class V: other conserved meta-signature genes	ENSGALG00010008425	MAGI2	0.260917561	0.040510483	0	1	FALSE	other_meta_signature	37.69702039	CRA004775;PRJNA827465;Yang2025_Silkie_Wenchang
Class V: other conserved meta-signature genes	ENSGALG00010004899	GATAD1	-0.248322477	0.041175892	0	1	FALSE	other_meta_signature	37.62696938	CRA004775;PRJNA827465;Yang2025_Silkie_Wenchang
Class V: other conserved meta-signature genes	ENSGALG00010002689	COG6	-0.179644912	0.018795574	0	1	FALSE	other_meta_signature	37.62416896	CRA004775;PRJNA827465;Yang2025_Silkie_Wenchang
Class V: other conserved meta-signature genes	ENSGALG00010019896	DDB1	-0.172434474	0.018633292	0	1	FALSE	other_meta_signature	37.59188277	CRA004775;PRJNA827465;Yang2025_Silkie_Wenchang
Class V: other conserved meta-signature genes	ENSGALG00010005978	VPS16	-0.210346455	0.031762888	0	1	FALSE	other_meta_signature	37.54981229	CRA004775;PRJNA827465;Yang2025_Silkie_Wenchang
Class V: other conserved meta-signature genes	ENSGALG00010012419	HACE1	0.186299092	0.029818759	0	1	FALSE	other_meta_signature	37.45700589	CRA004775;PRJNA827465;Yang2025_Silkie_Wenchang
Class V: other conserved meta-signature genes	ENSGALG00010016924	LETM1	0.187253334	0.032504831	0	1	FALSE	other_meta_signature	37.42431876	CRA004775;PRJNA827465;Yang2025_Silkie_Wenchang
Class V: other conserved meta-signature genes	ENSGALG00010015469	TMEM33	-0.17956713	0.030313479	0	1	FALSE	other_meta_signature	37.41619986	CRA004775;PRJNA827465;Yang2025_Silkie_Wenchang
Class V: other conserved meta-signature genes	ENSGALG00010014887	MTMR12	0.324692103	0.010550347	13.88732943	1	FALSE	other_meta_signature	37.21146083	CRA004775;PRJNA827465;Yang2025_Silkie_Wenchang
Class V: other conserved meta-signature genes	ENSGALG00010024558	PFDN5	-0.151019036	0.041175892	0	1	FALSE	other_meta_signature	37.14045217	CRA004775;PRJNA827465;Yang2025_Silkie_Wenchang
Class V: other conserved meta-signature genes	ENSGALG00010024798	FBXO45	-0.151291482	0.017046236	4.309127865	1	FALSE	other_meta_signature	37.09391613	CRA004775;PRJNA827465;Yang2025_Silkie_Wenchang
Class V: other conserved meta-signature genes	ENSGALG00010024042	DCPS	-0.276897576	0.012333708	12.26435838	1	FALSE	other_meta_signature	37.06695838	CRA004775;PRJNA827465;Yang2025_Silkie_Wenchang
Class V: other conserved meta-signature genes	ENSGALG00010024502	VPS8	-0.119211011	0.045877937	0	1	FALSE	other_meta_signature	36.93445118	CRA004775;PRJNA827465;Yang2025_Silkie_Wenchang
Class V: other conserved meta-signature genes	ENSGALG00010016980	NPHP1	0.221772188	0.041352297	9.777589834	1	FALSE	other_meta_signature	36.51460231	CRA004775;PRJNA827465;Yang2025_Silkie_Wenchang
Class V: other conserved meta-signature genes	ENSGALG00010012066	STXBP3	0.366619128	0.039690063	38.93850694	1	FALSE	other_meta_signature	34.34056316	CRA004775;PRJNA827465;Yang2025_Silkie_Wenchang
