# Supplementary material for: Cross-cohort transcriptomic meta-analysis identifies conserved melanogenesis signatures and developmental background in black-boned chicken breast muscle
Source: Anim Biosci. 2026 Jun 24;39(10):260430. doi: 10.5713/ab.260430 (PMC13662945; doi:10.5713/ab.260430)
Supplement: Supplementary file 9 [file ab-260430-Supplementary-17.pdf]

Supplement 17. Curated muscle-phenotype-related conserved genes used in Figure 5.

symbol	functional_group	interpretation_note	gene_id	k	meta_log2FC	meta_se	meta_z	meta_pvalue	tau2	Q	I2	direction_consistency	cohorts	meta_fdr	background_development_gene	melanogenesis	melanoblast_neural_crest	myogenesis	oxidative_redox	evidence_class	score_meta_fdr	score_effect	score_consistency	score_low_heterogeneity	score_background_penalty	score_biology_bonus	candidate_priority_score	n_significant_background_contrasts	n_muscle_development_background_contrasts	swth_backgroun	meta_significan	trection_consis	terpretation_class
MYBPC2	Structure/remodeling	fast skeletal myosin-binding protein	ENSGALG00010000224	3	-0.67421461	0.166450562	-4.050539711	5.10996E-05	0	0.949756917	0	1	CRA004775;PRJNA827465;Yang2025_Silkie_Wenchan	0.007297957	TRUE	FALSE	FALSE	FALSE	FALSE	background_development	2.136798689	3.371073048	20	10	-10	0	25.50787174	6	6	0	TRUE	TRUE	nd background-overlapping
CTSK	Structure/remodeling	matrix and protease-related remodeling candidate	ENSGALG00010028763	3	0.411918772	0.092484359	4.453929017	8.4313E-06	0	1.836563676	0	1	CRA004775;PRJNA827465;Yang2025_Silkie_Wenchan	0.001834002	TRUE	FALSE	FALSE	FALSE	FALSE	background_development	2.736600198	2.059593861	20	10	-10	0	24.79619406	2	2	0	TRUE	TRUE	nd background-overlapping
LRP4	Structure/remodeling	neuromuscular junction and tissue interaction candidate	ENSGALG00010022929	3	-0.338364291	0.091479853	-3.698784803	0.000216634	0	0.10144386	0	1	CRA004775;PRJNA827465;Yang2025_Silkie_Wenchan	0.019568396	FALSE	FALSE	FALSE	FALSE	FALSE	other_meta_signature	1.708444777	1.691821453	20	10	5	0	38.40026623	0	0	0	TRUE	TRUE	without background flag
CAPN1	Structure/remodeling	calpain-related protein turnover/meat-quality axis	ENSGALG00010001351	3	-0.321388065	0.061785593	-5.201666765	1.97509E-07	0	0.033746742	0	1	CRA004775;PRJNA827465;Yang2025_Silkie_Wenchan	9.62959E-05	TRUE	FALSE	FALSE	FALSE	FALSE	background_development	4.016392122	1.606940325	20	10	-10	0	25.62333245	2	2	0	TRUE	TRUE	nd background-overlapping
TECRL	Calcium/excitability	calcium-associated membrane protein candidate	ENSGALG00010003632	2	-1.084467047	0.278838353	-3.889232004	0.000100562	0	0.820977549	0	1	PRJNA827465;Yang2025_Silkie_Wenchang	0.011755221	TRUE	FALSE	FALSE	FALSE	FALSE	background_development	1.929769193	5.422335234	20	10	-10	0	27.35210443	3	3	0	TRUE	TRUE	nd background-overlapping
SLN	Calcium/excitability	sarcoplasmic-reticulum calcium handling candidate	ENSGALG00010004973	3	-0.642222	0.157373335	-4.080881935	4.48651E-05	0	0.692066081	0	1	CRA004775;PRJNA827465;Yang2025_Silkie_Wenchan	0.006748385	TRUE	FALSE	FALSE	FALSE	FALSE	background_development	2.170800118	3.21111	20	10	-10	0	25.38190012	6	6	0	TRUE	TRUE	nd background-overlapping
SCN4A	Calcium/excitability	skeletal-muscle sodium channel candidate	ENSGALG00010023635	3	-0.443744128	0.103858045	-4.272602338	1.93205E-05	0	1.099224069	0	1	CRA004775;PRJNA827465;Yang2025_Silkie_Wenchan	0.003696156	TRUE	FALSE	FALSE	FALSE	FALSE	background_development	2.432249711	2.218720639	20	10	-10	0	24.65097035	6	6	0	TRUE	TRUE	nd background-overlapping
THRSP	Lipid/energy metabolism	lipogenic/metabolic candidate	ENSGALG00010001976	3	-1.684290035	0.488207472	-3.449947266	0.000560696	0	0.427651217	0	1	CRA004775;PRJNA827465;Yang2025_Silkie_Wenchan	0.036365512	TRUE	FALSE	FALSE	FALSE	FALSE	background_development	1.439310288	8.421450175	20	10	-10	0	29.86076046	6	6	0	TRUE	TRUE	nd background-overlapping
GPD1	Lipid/energy metabolism	glycerol-phosphate metabolism candidate	ENSGALG00010023167	3	-0.721409624	0.182880265	-3.944710083	7.98966E-05	0	0.896247978	0	1	CRA004775;PRJNA827465;Yang2025_Silkie_Wenchan	0.010177097	TRUE	FALSE	FALSE	FALSE	FALSE	background_development	1.992376082	3.607048121	20	10	-10	0	25.5994242	6	6	0	TRUE	TRUE	nd background-overlapping
CD36	Lipid/energy metabolism	fatty-acid uptake and metabolic candidate	ENSGALG00010008392	3	-0.689932039	0.19206253	-3.592226129	0.000327865	0	0.973592429	0	1	CRA004775;PRJNA827465;Yang2025_Silkie_Wenchan	0.026043176	TRUE	FALSE	FALSE	FALSE	FALSE	background_development	1.584306049	3.449660196	20	10	-10	0	25.03396625	5	5	0	TRUE	TRUE	nd background-overlapping
PLTP	Lipid/energy metabolism	lipid-transfer and metabolic candidate	ENSGALG00010016006	3	0.574339269	0.094590134	6.071872851	1.26427E-09	0	0.63497706	0	1	CRA004775;PRJNA827465;Yang2025_Silkie_Wenchan	8.12523E-07	TRUE	FALSE	FALSE	FALSE	FALSE	background_development	6.090164085	2.871696343	20	10	-10	0	28.96186043	3	3	0	TRUE	TRUE	nd background-overlapping
