# Supplementary material for: Cross-cohort transcriptomic meta-analysis identifies conserved melanogenesis signatures and developmental background in black-boned chicken breast muscle
Source: Anim Biosci. 2026 Jun 24;39(10):260430. doi: 10.5713/ab.260430 (PMC13662945; doi:10.5713/ab.260430)
Supplement: Supplementary file 10 [file ab-260430-Supplementary-2.pdf]

Supplement 2. Salmon quantification directory inventory used to distinguish unique quantification outputs from analysis records.

sample_id	quant_sf_exists	quant_sf_size_bytes	n_analysis_records	cohort_records	roles
AA1D_P1	TRUE	2291023	1	PRJNA729271	background
AA1D_P2	TRUE	2283357	1	PRJNA729271	background
AA1D_P3	TRUE	2290228	1	PRJNA729271	background
AA1W_P1	TRUE	2279983	1	PRJNA729271	background
AA1W_P2	TRUE	2281148	1	PRJNA729271	background
AA1W_P3	TRUE	2282498	1	PRJNA729271	background
AA3W_P1	TRUE	2285128	1	PRJNA729271	background
AA3W_P2	TRUE	2282672	1	PRJNA729271	background
AA3W_P3	TRUE	2282076	1	PRJNA729271	background
AA5W_P1	TRUE	2286033	1	PRJNA729271	background
AA5W_P2	TRUE	2285741	1	PRJNA729271	background
AA5W_P3	TRUE	2280830	1	PRJNA729271	background
AAE10P1	TRUE	2299749	1	PRJNA729271	background
AAE10P2	TRUE	2301443	1	PRJNA729271	background
AAE10P3	TRUE	2297242	1	PRJNA729271	background
AAE14P1	TRUE	2291359	1	PRJNA729271	background
AAE14P2	TRUE	2291383	1	PRJNA729271	background
AAE14P3	TRUE	2292107	1	PRJNA729271	background
AAE18P1	TRUE	2283341	1	PRJNA729271	background
AAE18P2	TRUE	2283016	1	PRJNA729271	background
AAE18P3	TRUE	2284895	1	PRJNA729271	background
CB1	TRUE	2288625	1	CRA004775	control
CB2	TRUE	2282447	1	CRA004775	control
CB3	TRUE	2282837	1	CRA004775	control
CB4	TRUE	2290159	1	CRA004775	control
CB5	TRUE	2282610	1	CRA004775	control
CB6	TRUE	2285543	1	CRA004775	control
CH1	TRUE	2274977	1	CRA004775	control
CH2	TRUE	2273515	1	CRA004775	control
CH3	TRUE	2271582	1	CRA004775	control
CH4	TRUE	2282495	1	CRA004775	control
CH5	TRUE	2271278	1	CRA004775	control
CH6	TRUE	2272844	1	CRA004775	control
E13-TH-BM-1	TRUE	2290605	1	Yang2025_Silkie_Wenchang	case
E13-TH-BM-6	TRUE	2293360	1	Yang2025_Silkie_Wenchang	case
E13-TH-BM-7	TRUE	2293944	1	Yang2025_Silkie_Wenchang	case
E13-TH-BM-9	TRUE	2292825	1	Yang2025_Silkie_Wenchang	case
E17-TH-BM-5	TRUE	2285404	1	Yang2025_Silkie_Wenchang	case
E17-TH-BM-6	TRUE	2285220	1	Yang2025_Silkie_Wenchang	case
E17-TH-BM-8	TRUE	2270841	1	Yang2025_Silkie_Wenchang	case
E21-TH-BM-2	TRUE	2279378	1	Yang2025_Silkie_Wenchang	case
E21-TH-BM-4	TRUE	2278630	1	Yang2025_Silkie_Wenchang	case
E21-TH-BM-7	TRUE	2283285	1	Yang2025_Silkie_Wenchang	case
E21-TH-BM-8	TRUE	2281099	1	Yang2025_Silkie_Wenchang	case
LS1D_P1	TRUE	2289992	1	PRJNA729271	background
LS1D_P2	TRUE	2290982	1	PRJNA729271	background
LS1D_P3	TRUE	2285183	1	PRJNA729271	background
LS1W_P1	TRUE	2283944	1	PRJNA729271	background
LS1W_P2	TRUE	2279779	1	PRJNA729271	background
LS1W_P3	TRUE	2282378	1	PRJNA729271	background
LS3W_P1	TRUE	2284471	1	PRJNA729271	background
LS3W_P2	TRUE	2285661	1	PRJNA729271	background
LS3W_P3	TRUE	2284145	1	PRJNA729271	background
LS5W_P1	TRUE	2281670	1	PRJNA729271	background
LS5W_P2	TRUE	2284579	1	PRJNA729271	background
LS5W_P3	TRUE	2281062	1	PRJNA729271	background
LSE10P1	TRUE	2300065	1	PRJNA729271	background
LSE10P2	TRUE	2298271	1	PRJNA729271	background
LSE10P3	TRUE	2299179	1	PRJNA729271	background
LSE14P1	TRUE	2295043	1	PRJNA729271	background
LSE14P2	TRUE	2294174	1	PRJNA729271	background
LSE14P3	TRUE	2291466	1	PRJNA729271	background
LSE18P1	TRUE	2282430	1	PRJNA729271	background
LSE18P2	TRUE	2284795	1	PRJNA729271	background
LSE18P3	TRUE	2283965	1	PRJNA729271	background
W1_1	TRUE	2289848	1	PRJNA827465	control
W1_2	TRUE	2290888	1	PRJNA827465	control
W1_3	TRUE	2290676	1	PRJNA827465	control
W2_1	TRUE	2279498	2	PRJNA827465;Yang2025_Silkie_Wenchang	control
W2_2	TRUE	2288539	2	PRJNA827465;Yang2025_Silkie_Wenchang	control
W2_3	TRUE	2288688	2	PRJNA827465;Yang2025_Silkie_Wenchang	control
W3_1	TRUE	2284077	2	PRJNA827465;Yang2025_Silkie_Wenchang	control
W3_2	TRUE	2282457	2	PRJNA827465;Yang2025_Silkie_Wenchang	control
W3_3	TRUE	2281168	2	PRJNA827465;Yang2025_Silkie_Wenchang	control
W4_1	TRUE	2280887	2	PRJNA827465;Yang2025_Silkie_Wenchang	control
W4_2	TRUE	2280747	2	PRJNA827465;Yang2025_Silkie_Wenchang	control
W4_3	TRUE	2280598	2	PRJNA827465;Yang2025_Silkie_Wenchang	control
WL1	TRUE	2277790	1	CRA004775	case
WL2	TRUE	2284222	1	CRA004775	case
WL3	TRUE	2281180	1	CRA004775	case
WL4	TRUE	2281087	1	CRA004775	case
WL5	TRUE	2278588	1	CRA004775	case
WL6	TRUE	2278066	1	CRA004775	case
Y1_1	TRUE	2292392	1	PRJNA827465	case
Y1_2	TRUE	2293153	1	PRJNA827465	case
Y1_3	TRUE	2290762	1	PRJNA827465	case
Y2_1	TRUE	2288288	1	PRJNA827465	case
Y2_2	TRUE	2286540	1	PRJNA827465	case
Y2_3	TRUE	2288619	1	PRJNA827465	case
Y3_1	TRUE	2285507	1	PRJNA827465	case
Y3_2	TRUE	2286299	1	PRJNA827465	case
Y3_3	TRUE	2285394	1	PRJNA827465	case
Y4_1	TRUE	2281241	1	PRJNA827465	case
Y4_2	TRUE	2280686	1	PRJNA827465	case
Y4_3	TRUE	2282192	1	PRJNA827465	case
