# Supplementary material for: Cross-cohort transcriptomic meta-analysis identifies conserved melanogenesis signatures and developmental background in black-boned chicken breast muscle
Source: Anim Biosci. 2026 Jun 24;39(10):260430. doi: 10.5713/ab.260430 (PMC13662945; doi:10.5713/ab.260430)
Supplement: Supplementary file 11 [file ab-260430-Supplementary-3.pdf]

Supplement 3. Cohort-level DESeq2 effect-size results for the three pigmentation-related cohorts.

cohort_id	gene_id	symbol	case	control	log2FoldChange	lfcSE	stat	pvalue	padj	direction
CRA004775	ENSGALG00010000007	ND1	case	control	-0.068207971	0.29046292	-0.234825053	0.814344522	0.958686054	down_in_case
CRA004775	ENSGALG00010000011	ND2	case	control	0.118544942	0.297994809	0.397808747	0.690771169	0.927867527	up_in_case
CRA004775	ENSGALG00010000017	COX1	case	control	0.168142254	0.261495013	0.643003673	0.520221717	0.865611895	up_in_case
CRA004775	ENSGALG00010000020	COX2	case	control	0.100969064	0.278356795	0.362732527	0.716804699	0.934011578	up_in_case
CRA004775	ENSGALG00010000022	ATP8	case	control	0.013361922	0.279570101	0.04779453	0.961879996	0.991860454	up_in_case
CRA004775	ENSGALG00010000023	ATP6	case	control	0.28937489	0.239655329	1.207462783	0.22725397	0.694715356	up_in_case
CRA004775	ENSGALG00010000024	COX3	case	control	0.109409038	0.251955548	0.434239449	0.664114569	0.918295135	up_in_case
CRA004775	ENSGALG00010000026	ND3	case	control	0.319547745	0.299211381	1.067966545	0.285535603	0.74152976	up_in_case
CRA004775	ENSGALG00010000028	ND4L	case	control	0.379011426	0.331494792	1.143340514	0.25289724	0.717347518	up_in_case
CRA004775	ENSGALG00010000029	ND4	case	control	0.429325249	0.319319516	1.344500504	0.178786594	0.644859234	up_in_case
CRA004775	ENSGALG00010000033	ND5	case	control	0.433267608	0.322067834	1.345268177	0.178538646	0.644548419	up_in_case
CRA004775	ENSGALG00010000034	CYTB	case	control	0.35033705	0.310015512	1.130062969	0.258449692	0.720037471	up_in_case
CRA004775	ENSGALG00010000037	ND6	case	control	0.458112606	0.332427072	1.378084534	0.16817719	0.63138062	up_in_case
CRA004775	ENSGALG00010000039	PDIA3	case	control	-0.195241996	0.251003731	-0.777844993	0.4366604	0.827068479	down_in_case
CRA004775	ENSGALG00010000042	CKMT1B	case	control	1.508947209	0.697420531	2.163611684	0.030494164	0.333062153	up_in_case
CRA004775	ENSGALG00010000043	PPIP5K1	case	control	-0.116673631	0.246538715	-0.473246693	0.636037189	0.910080596	down_in_case
CRA004775	ENSGALG00010000044	MAP1A	case	control	0.63355349	0.423416823	1.49628795	0.134578634	0.592420453	up_in_case
CRA004775	ENSGALG00010000045	TP53BP1	case	control	-0.281741397	0.300074077	-0.938906153	0.34777893	0.785270866	down_in_case
CRA004775	ENSGALG00010000046	TUBGCP4	case	control	-0.341053365	0.177530061	-1.92110205	0.054718843	0.430390248	down_in_case
CRA004775	ENSGALG00010000047	IQGAP1	case	control	0.050011101	0.394763341	0.126686285	0.899188702	0.978790377	up_in_case
CRA004775	ENSGALG00010000048	MAN2A2	case	control	-0.430279195	0.468007144	-0.919385955	0.357893733	0.790416653	down_in_case
CRA004775	ENSGALG00010000049	FES	case	control	-0.633722611	0.407481787	-1.555217022	0.119894392	0.572133279	down_in_case
CRA004775	ENSGALG00010000050	CIB1	case	control	-0.208285151	0.259629782	-0.802239053	0.42241469	0.820311312	down_in_case
CRA004775	ENSGALG00010000051	GDPGP1	case	control	-0.335835808	0.226017432	-1.485884537	0.137309664	0.596242097	down_in_case
CRA004775	ENSGALG00010000052	TTLL13	case	control	-0.01572041	0.315392759	-0.049843917	0.96024677	0.991431439	down_in_case
CRA004775	ENSGALG00010000053	HDDC3	case	control	-0.084910343	0.179582476	-0.472820874	0.636340981	0.910080596	down_in_case
CRA004775	ENSGALG00010000054	VPS33B	case	control	-0.293142966	0.30624303	-0.957223309	0.338454555	0.779253705	down_in_case
CRA004775	ENSGALG00010000055	PRC1	case	control	0.211976047	0.265549344	0.798254832	0.424722623	0.821775826	up_in_case
CRA004775	ENSGALG00010000056	UNC45A	case	control	-0.237386362	0.162633848	-1.459636872	0.1443899	0.606446401	down_in_case
CRA004775	ENSGALG00010000058	FURIN	case	control	-0.239738182	0.292146613	-0.820609143	0.411868938	0.817006008	down_in_case
CRA004775	ENSGALG00010000059	MFAP1	case	control	-0.255391456	0.171946717	-1.485294173	0.137465915	0.596242097	down_in_case
CRA004775	ENSGALG00010000060	HYPK	case	control	0.204496021	0.300617435	0.680253362	0.49634405	0.857009923	up_in_case
CRA004775	ENSGALG00010000061	SERINC4	case	control	-0.156616224	0.368910913	-0.424536706	0.671174442	0.921535334	down_in_case
CRA004775	ENSGALG00010000062	ENSGALG00010000062	case	control	-0.275929737	0.873060197	-0.316048926	0.751965373	0.941495694	down_in_case
CRA004775	ENSGALG00010000063	SERF2	case	control	-0.030338545	0.270517501	-0.112150027	0.910704451	0.98075825	down_in_case
CRA004775	ENSGALG00010000069	ENSGALG00010000069	case	control	0.624120205	0.492467869	1.267331828	0.205036664	0.674045742	up_in_case
CRA004775	ENSGALG00010000075	ENSGALG00010000075	case	control	-0.587499719	0.653139079	-0.899501711	0.368385485	0.794665838	down_in_case
CRA004775	ENSGALG00010000076	ENSGALG00010000076	case	control	-0.059721818	0.356898654	-0.167335511	0.867106064	0.973120718	down_in_case
CRA004775	ENSGALG00010000078	ENSGALG00010000078	case	control	-0.603801444	0.641020614	-0.941937639	0.346224568	0.784552667	down_in_case
CRA004775	ENSGALG00010000081	ENSGALG00010000081	case	control	0.028618089	0.686213145	0.041704374	0.966734367	0.993094598	up_in_case
CRA004775	ENSGALG00010000082	ENSGALG00010000082	case	control	-0.348974542	0.69116042	-0.504911062	0.613621304	0.902282137	down_in_case
CRA004775	ENSGALG00010000084	ENSGALG00010000084	case	control	-1.151209299	0.484648121	-2.375350792	0.017532279	0.263194728	down_in_case
CRA004775	ENSGALG00010000085	ENSGALG00010000085	case	control	-0.023616183	0.207011186	-0.114081677	0.909173047	0.98075825	down_in_case
CRA004775	ENSGALG00010000089	ENSGALG00010000089	case	control	0.23572622	0.369387575	0.638154166	0.523373329	0.866638286	up_in_case
CRA004775	ENSGALG00010000092	INPPL1	case	control	-0.630938133	0.374155673	-1.686298453	0.091738329	0.521493907	down_in_case
CRA004775	ENSGALG00010000093	ENSGALG00010000093	case	control	0.269213603	0.626047196	0.430021259	0.667180177	0.919278017	up_in_case
CRA004775	ENSGALG00010000098	ENSGALG00010000098	case	control	-0.423260256	0.30098448	-1.406252757	0.159649083	0.624907945	down_in_case
CRA004775	ENSGALG00010000101	ENSGALG00010000101	case	control	2.405828505	2.080033554	1.156629662	0.247423698	0.712046495	up_in_case
CRA004775	ENSGALG00010000105	CACNA1F	case	control	-0.047539138	0.296403743	-0.160386429	0.872576679	0.97401729	down_in_case
CRA004775	ENSGALG00010000106	ENSGALG00010000106	case	control	-0.143788462	0.638990898	-0.225024273	0.821960391	0.960766569	down_in_case
CRA004775	ENSGALG00010000107	ENSGALG00010000107	case	control	-0.564370066	0.882715019	-0.639357045	0.522590687	0.866638286	down_in_case
CRA004775	ENSGALG00010000121	ENSGALG00010000121	case	control	-0.270486143	0.27473612	-0.984530694	0.324854647	0.770185561	down_in_case
CRA004775	ENSGALG00010000123	ENSGALG00010000123	case	control	-1.169319031	0.368223341	-3.175570094	0.001495424	0.080693373	down_in_case
CRA004775	ENSGALG00010000124	ENSGALG00010000124	case	control	0.012609653	0.309078912	0.040797521	0.967457316	0.993391992	up_in_case
CRA004775	ENSGALG00010000126	ENSGALG00010000126	case	control	-0.430451306	0.434724188	-0.99017105	0.32209052	0.767876089	down_in_case
CRA004775	ENSGALG00010000127	ENSGALG00010000127	case	control	-0.537870196	0.411136789	-1.308251194	0.190788123	0.656209975	down_in_case
CRA004775	ENSGALG00010000133	USP39	case	control	-0.415153053	0.209576883	-1.980910517	0.047601309	0.405303781	down_in_case
CRA004775	ENSGALG00010000134	ENSGALG00010000134	case	control	-0.15998924	0.256868136	-0.622845802	0.533385857	0.870471032	down_in_case
CRA004775	ENSGALG00010000137	ENSGALG00010000137	case	control	-0.5535921	0.681727621	-0.812042938	0.416766994	0.818943344	down_in_case
CRA004775	ENSGALG00010000138	ENSGALG00010000138	case	control	0.489021044	0.378913756	1.290586672	0.19684704	0.66285976	up_in_case
CRA004775	ENSGALG00010000140	SEPHS3	case	control	-0.366457091	0.248462616	-1.474898302	0.140239887	0.600007375	down_in_case
CRA004775	ENSGALG00010000141	ENSGALG00010000141	case	control	-0.192914904	0.183646798	-1.050467019	0.293503447	0.746438942	down_in_case
CRA004775	ENSGALG00010000142	ENSGALG00010000142	case	control	-0.774610272	0.981812908	-0.788959145	0.430135886	0.824153229	down_in_case
CRA004775	ENSGALG00010000143	SCARF2	case	control	-0.223325623	0.325517357	-0.686063641	0.492672977	0.855763086	down_in_case
CRA004775	ENSGALG00010000145	ENSGALG00010000145	case	control	-1.310695739	0.437621478	-2.995044356	0.002744049	0.10984643	down_in_case
CRA004775	ENSGALG00010000146	ACADM	case	control	-0.461517042	0.428684726	-1.076588491	0.281664148	0.738260394	down_in_case
CRA004775	ENSGALG00010000147	ENSGALG00010000147	case	control	-0.330314938	0.193306258	-1.708764846	0.087494517	0.511973235	down_in_case
CRA004775	ENSGALG00010000148	ENSGALG00010000148	case	control	-0.510073134	0.336304946	-1.516698281	0.129342878	0.584172159	down_in_case
CRA004775	ENSGALG00010000149	ENSGALG00010000149	case	control	-0.050860714	0.152941421	-0.332550292	0.739473771	0.938064864	down_in_case
CRA004775	ENSGALG00010000150	B3GAT3	case	control	-0.001062307	0.321759241	-0.003301558	0.997365742	0.999619266	down_in_case
CRA004775	ENSGALG00010000151	TBX5	case	control	0.153691656	0.529462235	0.290278788	0.771602966	0.945997894	up_in_case
CRA004775	ENSGALG00010000153	NF2	case	control	-0.121115804	0.316514914	-0.382654334	0.701976078	0.930068722	down_in_case
CRA004775	ENSGALG00010000154	ENSGALG00010000154	case	control	0.266016224	1.099760503	0.241885595	0.808868812	0.955623701	up_in_case
CRA004775	ENSGALG00010000155	MAZ	case	control	-0.694134171	0.401791442	-1.727598198	0.084060286	0.503010645	down_in_case
CRA004775	ENSGALG00010000156	ENSGALG00010000156	case	control	-0.361286055	0.954978806	-0.378318401	0.705194078	0.930927733	down_in_case
CRA004775	ENSGALG00010000157	ENSGALG00010000157	case	control	0.74136312	1.561140755	0.474885508	0.634868581	0.909837348	up_in_case
CRA004775	ENSGALG00010000161	CDC42EP2	case	control	-0.18248058	0.224299706	-0.81355693	0.41589882	0.818943344	down_in_case
CRA004775	ENSGALG00010000166	PFDN6	case	control	0.007975368	0.307458034	0.025939695	0.979305439	0.996764329	up_in_case
CRA004775	ENSGALG00010000171	DPF2	case	control	-0.191839716	0.148700022	-1.290112223	0.197011696	0.66285976	down_in_case
CRA004775	ENSGALG00010000172	ENSGALG00010000172	case	control	-0.562627938	0.943393073	-0.596387608	0.550916317	0.877827378	down_in_case
CRA004775	ENSGALG00010000173	NIPSNAP1	case	control	-0.35187178	0.248682517	-1.414943778	0.157084981	0.621633948	down_in_case
CRA004775	ENSGALG00010000174	ENSGALG00010000174	case	control	-0.354237746	0.255349822	-1.387264509	0.165361121	0.630194031	down_in_case
CRA004775	ENSGALG00010000176	ENSGALG00010000176	case	control	-0.657744432	0.577801358	-1.138357366	0.254971289	0.71861246	down_in_case
CRA004775	ENSGALG00010000177	ENSGALG00010000177	case	control	-0.424094914	0.247202268	-1.715578571	0.086239203	0.508087097	down_in_case
CRA004775	ENSGALG00010000180	ENSGALG00010000180	case	control	-1.025566978	0.823958802	-1.244682351	0.213248536	0.682777582	down_in_case
CRA004775	ENSGALG00010000184	ENSGALG00010000184	case	control	-0.689022913	2.443516086	-0.281980101			down_in_case
CRA004775	ENSGALG00010000186	THOC5	case	control	-0.244203944	0.206368097	-1.183341552	0.236673811	0.703637928	down_in_case
CRA004775	ENSGALG00010000188	ENSGALG00010000188	case	control	0.646853049	1.494638936	0.432782148	0.665173044	0.918815167	up_in_case
CRA004775	ENSGALG00010000189	ENSGALG00010000189	case	control	-0.108693767	0.854563461	-0.127192154	0.898788315	0.978790377	down_in_case
CRA004775	ENSGALG00010000191	NEFH	case	control	0.082799661	0.390168488	0.212215143	0.831939194	0.963642489	up_in_case
CRA004775	ENSGALG00010000197	ENSGALG00010000197	case	control	-0.970759934	1.044226679	-0.929644831	0.352555008	0.786426886	down_in_case
CRA004775	ENSGALG00010000200	GAL3ST1	case	control	0.926036285	0.521802696	1.774686663	0.075949639	0.484761048	up_in_case
CRA004775	ENSGALG00010000202	ENSGALG00010000202	case	control	0.046273172	0.362957747	0.127489142	0.898553267	0.978790377	up_in_case
CRA004775	ENSGALG00010000205	RNF215	case	control	-0.226972545	0.170397064	-1.332021457	0.182853138	0.647378665	down_in_case
CRA004775	ENSGALG00010000210	CCDC157	case	control	-0.132142352	0.365755639	-0.361285891	0.71788574	0.934011578	down_in_case
CRA004775	ENSGALG00010000215	SF3A1	case	control	-0.359430514	0.124577793	-2.885189277	0.003911784	0.128995064	down_in_case
CRA004775	ENSGALG00010000221	TBC1D10A	case	control	-0.022705109	0.199657009	-0.113720573	0.909459303	0.98075825	down_in_case
CRA004775	ENSGALG00010000223	CASTOR1	case	control	0.481513997	0.35919172	1.340548712	0.180067019	0.644990528	up_in_case
CRA004775	ENSGALG00010000224	MYBPC2	case	control	-0.038286325	0.742909113	-0.051535679	0.958898672	0.991431439	down_in_case
CRA004775	ENSGALG00010000225	LIF	case	control	0.032977712	0.437206998	0.075428142	0.939874069	0.987076872	up_in_case
CRA004775	ENSGALG00010000226	ENSGALG00010000226	case	control	-0.527261406	0.301510916	-1.748730735	0.080337575	0.494445942	down_in_case
CRA004775	ENSGALG00010000227	ENSGALG00010000227	case	control	-0.373997279	0.369468762	-1.012256833	0.311415269	0.763050512	down_in_case
CRA004775	ENSGALG00010000228	ENSGALG00010000228	case	control	-0.434754128	1.00123274	-0.434218849	0.664129527	0.918295135	down_in_case
CRA004775	ENSGALG00010000231	ENSGALG00010000231	case	control	-0.55026319	0.490242209	-1.12243128	0.261679111	0.722422978	down_in_case
CRA004775	ENSGALG00010000232	ENSGALG00010000232	case	control	0.122866844	0.330661008	0.371579477	0.710205971	0.932234469	up_in_case
CRA004775	ENSGALG00010000234	ENSGALG00010000234	case	control	-0.412451434	1.216708555	-0.338989507	0.734617635	0.936819725	down_in_case
CRA004775	ENSGALG00010000239	ENSGALG00010000239	case	control	-0.32568538	1.168279906	-0.278773416	0.780418717	0.948399168	down_in_case
CRA004775	ENSGALG00010000241	ENSGALG00010000241	case	control	-1.919916349	0.836124769	-2.296207959	0.021663994	0.288534247	down_in_case
CRA004775	ENSGALG00010000242	ENSGALG00010000242	case	control	-0.210913568	0.590479162	-0.357190535	0.720949171	0.934511903	down_in_case
CRA004775	ENSGALG00010000246	ENSGALG00010000246	case	control	0.756079456	1.426502956	0.530023056	0.596095946	0.895574418	up_in_case
CRA004775	ENSGALG00010000247	ENSGALG00010000247	case	control	0.03719215	0.704687428	0.052778222	0.957908614	0.991431439	up_in_case
CRA004775	ENSGALG00010000249	ENSGALG00010000249	case	control	-0.038764129	1.232337216	-0.03145578	0.974906057	0.995106174	down_in_case
CRA004775	ENSGALG00010000250	ENSGALG00010000250	case	control	0.791663847	1.032591274	0.766676871	0.443273659	0.831667526	up_in_case
CRA004775	ENSGALG00010000255	ENSGALG00010000255	case	control	-0.115720238	0.378785779	-0.305503122	0.759982992	0.942236668	down_in_case
CRA004775	ENSGALG00010000256	ENSGALG00010000256	case	control	-0.169383578	0.693396634	-0.244280935	0.807013248	0.955397873	down_in_case
CRA004775	ENSGALG00010000262	HNRNPKL	case	control	0.038102878	1.116785561	0.034118348	0.972782777	0.994947809	up_in_case
CRA004775	ENSGALG00010000263	MTMR3	case	control	-0.113838642	0.214612805	-0.530437323	0.595808754	0.895308292	down_in_case
CRA004775	ENSGALG00010000267	ENSGALG00010000267	case	control	-0.202153453	0.257608392	-0.784731631	0.432610933	0.825615327	down_in_case
CRA004775	ENSGALG00010000271	RTN4R	case	control	0.779748095	0.667442474	1.168262622	0.242700847	0.709690398	up_in_case
CRA004775	ENSGALG00010000272	ENSGALG00010000272	case	control	1.17129757	0.544283426	2.151999336	0.031397407	0.337301359	up_in_case
CRA004775	ENSGALG00010000277	ENSGALG00010000277	case	control	-0.393283268	0.484944596	-0.810985978	0.417373723	0.818943344	down_in_case
CRA004775	ENSGALG00010000278	ENSGALG00010000278	case	control	0.938959428	1.463463625	0.641600797	0.521132415	0.86621283	up_in_case
CRA004775	ENSGALG00010000279	ENSGALG00010000279	case	control	-0.417799668	0.268059733	-1.558606597	0.119089521	0.570892125	down_in_case
CRA004775	ENSGALG00010000280	ENSGALG00010000280	case	control	0.98346357	0.855293495	1.14985508	0.250203565	0.714993603	up_in_case

CRA004775	ENSGALG00010000281	OSBP2	case	control	0.26721784	0.385354209	0.693434335	0.488037021	0.853655233	up_in_case
CRA004775	ENSGALG00010000282	ENSGALG00010000282	case	control	0.81821412	1.403038309	0.583173043	0.559776816	0.880918893	up_in_case
CRA004775	ENSGALG00010000283	ENSGALG00010000283	case	control	0.002798267	0.4411517	0.006343094	0.994938977	0.999001196	up_in_case
CRA004775	ENSGALG00010000284	ENSGALG00010000284	case	control	-0.753172399	0.231500909	-3.253431711	0.001140201	0.071593186	down_in_case
CRA004775	ENSGALG00010000285	MYLPF	case	control	-0.389898696	0.807987048	-0.482555627	0.629411292	0.908699404	down_in_case
CRA004775	ENSGALG00010000286	TCN2	case	control	0.114262698	0.228337251	0.500411988	0.616785014	0.903561837	up_in_case
CRA004775	ENSGALG00010000287	IQCD	case	control	-0.055934562	0.472744468	-0.118318807	0.905815056	0.979739788	down_in_case
CRA004775	ENSGALG00010000288	VARS2	case	control	-0.406863561	0.230283189	-1.766796623	0.077262294	0.488700005	down_in_case
CRA004775	ENSGALG00010000289	QPRT	case	control	-1.394811219	0.631473273	-2.208820671	0.027187117	0.31561562	down_in_case
CRA004775	ENSGALG00010000292	ABHD16A	case	control	-0.524798478	0.190510277	-2.754699052	0.005874614	0.154441997	down_in_case
CRA004775	ENSGALG00010000293	ENSGALG00010000293	case	control	-0.298409977	0.543677077	-0.548873568	0.583092219	0.889803356	down_in_case
CRA004775	ENSGALG00010000294	DUSP21	case	control	0.463359548	0.535093437	0.865941377	0.386522311	0.803905475	up_in_case
CRA004775	ENSGALG00010000295	ENSGALG00010000295	case	control	1.550559184	0.722796539	2.145222205	0.031935085	0.340418104	up_in_case
CRA004775	ENSGALG00010000298	SLC35E4	case	control	-0.738326544	0.453823844	-1.626901171	0.103758089	0.545612805	down_in_case
CRA004775	ENSGALG00010000301	UQCR10	case	control	0.193016369	0.395131986	0.488485811	0.625205774	0.906591226	up_in_case
CRA004775	ENSGALG00010000302	ENSGALG00010000302	case	control	-0.176785419	0.367072751	-0.481608669	0.630083967	0.908943555	down_in_case
CRA004775	ENSGALG00010000303	ENSGALG00010000303	case	control	-0.436030987	0.169598883	-2.570954354	0.010141869	0.202058223	down_in_case
CRA004775	ENSGALG00010000306	ENSGALG00010000306	case	control	-0.551884555	0.259144855	-2.129637319	0.033201568	0.347668706	down_in_case
CRA004775	ENSGALG00010000307	ZMAT5	case	control	-0.009677097	0.290537031	-0.03330762	0.973429278	0.994947809	down_in_case
CRA004775	ENSGALG00010000308	ENSGALG00010000308	case	control	-0.640473978	0.282816019	-2.264631193	0.023535321	0.297457904	down_in_case
CRA004775	ENSGALG00010000309	ENSGALG00010000309	case	control	0.53265612	1.171726228	0.454590934	0.649403558	0.915047477	up_in_case
CRA004775	ENSGALG00010000312	CABP7	case	control	-0.812415292	0.500498581	-1.623211979	0.104544098	0.547421908	down_in_case
CRA004775	ENSGALG00010000313	ENSGALG00010000313	case	control	0.674776811	1.422727068	0.474284089	0.635297336	0.909837348	up_in_case
CRA004775	ENSGALG00010000318	ENSGALG00010000318	case	control	0.270420196	1.19341571	0.226593461	0.820739877	0.960766569	up_in_case
CRA004775	ENSGALG00010000323	AP1B1	case	control	-0.328766496	0.222292042	-1.478984552	0.139144444	0.599104916	down_in_case
CRA004775	ENSGALG00010000324	ENSGALG00010000324	case	control	-0.211408642	0.316732758	-0.66746693	0.504473926	0.861471523	down_in_case
CRA004775	ENSGALG00010000326	ENSGALG00010000326	case	control	0.251078935	1.233278009	0.203586647	0.838676529	0.965800667	up_in_case
CRA004775	ENSGALG00010000331	ENSGALG00010000331	case	control	-0.15040738	0.354793063	-0.423929878	0.671616954	0.921605102	down_in_case
CRA004775	ENSGALG00010000338	ENSGALG00010000338	case	control	-0.053307062	0.275136484	-0.193747703	0.846373438	0.967696113	down_in_case
CRA004775	ENSGALG00010000340	NAA10	case	control	-0.210794321	0.268945067	-0.783782067	0.433167998	0.825828172	down_in_case
CRA004775	ENSGALG00010000341	EWSR1	case	control	-0.333730148	0.175815629	-1.898182484	0.057672047	0.43984932	down_in_case
CRA004775	ENSGALG00010000346	ENSGALG00010000346	case	control	-0.157257782	0.210337002	-0.747646779	0.454673241	0.838254622	down_in_case
CRA004775	ENSGALG00010000349	CHIR-IG1-5	case	control	-0.250947595	0.572565651	-0.438286151	0.661178864	0.917907174	down_in_case
CRA004775	ENSGALG00010000350	ENSGALG00010000350	case	control	-0.199535833	0.179761042	-1.110005993	0.266996444	0.727315996	down_in_case
CRA004775	ENSGALG00010000353	ENSGALG00010000353	case	control	0.069729139	0.405550947	0.171936818	0.863487201	0.971910432	up_in_case
CRA004775	ENSGALG00010000356	ENSGALG00010000356	case	control	-0.209952026	0.483095675	-0.434597197	0.66385483	0.918295135	down_in_case
CRA004775	ENSGALG00010000362	ENSGALG00010000362	case	control	0.66985326	0.513221932	1.305192194	0.191827411	0.656959697	up_in_case
CRA004775	ENSGALG00010000366	EMID1	case	control	0.031114487	0.359437762	0.086564326	0.931017823	0.985044263	up_in_case
CRA004775	ENSGALG00010000367	HSD17B10	case	control	-0.49535205	0.300653736	-1.647583219	0.099438226	0.537098329	down_in_case
CRA004775	ENSGALG00010000369	ENSGALG00010000369	case	control	0.700588533	1.107850753	0.632385301	0.527135136	0.86830001	up_in_case
CRA004775	ENSGALG00010000373	ENSGALG00010000373	case	control	-0.6071497	0.51326523	-1.182916092	0.236842403	0.703871614	down_in_case
CRA004775	ENSGALG00010000376	ASCC2	case	control	-0.298315722	0.215366765	-1.385152077	0.166005971	0.630194031	down_in_case
CRA004775	ENSGALG00010000377	ENSGALG00010000377	case	control	-0.332180582	0.332173477	-1.000021389	0.317300157	0.766199014	down_in_case
CRA004775	ENSGALG00010000381	ENSGALG00010000381	case	control	-0.296201285	0.451675961	-0.655782708	0.511963944	0.863170205	down_in_case
CRA004775	ENSGALG00010000382	ENSGALG00010000382	case	control	-0.218506987	0.546189443	-0.400057141	0.689114431	0.927420456	down_in_case
CRA004775	ENSGALG00010000384	ENSGALG00010000384	case	control	-0.630644333	0.439411997	-1.435200536	0.151229961	0.615293187	down_in_case
CRA004775	ENSGALG00010000386	ENSGALG00010000386	case	control	-0.028793316	0.378417185	-0.076088818	0.939348437	0.986950867	down_in_case
CRA004775	ENSGALG00010000387	DTX1	case	control	0.174773319	0.444209448	0.393448001	0.693988623	0.928504482	up_in_case
CRA004775	ENSGALG00010000388	ENSGALG00010000388	case	control	-0.354270793	0.224446409	-1.578420412	0.114469059	0.564488547	down_in_case
CRA004775	ENSGALG00010000391	ENSGALG00010000391	case	control	0.4405415	0.447878721	0.983617839	0.325303451	0.770430538	up_in_case
CRA004775	ENSGALG00010000402	PFKFB1	case	control	-0.010158035	0.67783223	-0.014986061	0.988043301	0.998065748	down_in_case
CRA004775	ENSGALG00010000404	RASAL1	case	control	2.590096366	0.740859501	3.496069582	0.000472166	0.044470881	up_in_case
CRA004775	ENSGALG00010000405	ENSGALG00010000405	case	control	-0.227787725	0.584822512	-0.3894989	0.696907125	0.929092636	down_in_case
CRA004775	ENSGALG00010000406	NSMCE3	case	control	-0.44891516	0.285718877	-1.571177814	0.116141352	0.566086328	down_in_case
CRA004775	ENSGALG00010000407	WSB2	case	control	-0.385104235	0.365689895	-1.053089625	0.292299918	0.746262643	down_in_case
CRA004775	ENSGALG00010000408	ENSGALG00010000408	case	control	-0.244962787	0.763505773	-0.320839469	0.748332047	0.940917938	down_in_case
CRA004775	ENSGALG00010000412	RFC5	case	control	-0.325995934	0.314789133	-1.03560098	0.300388335	0.752927916	down_in_case
CRA004775	ENSGALG00010000422	ITIH6	case	control	-0.439443984	0.343892645	-1.277852235	0.201301533	0.669255489	down_in_case
CRA004775	ENSGALG00010000423	ENSGALG00010000423	case	control	-0.201119304	0.403729727	-0.49815332	0.618375981	0.904143308	down_in_case
CRA004775	ENSGALG00010000428	GNL3L	case	control	-0.262478515	0.189710348	-1.383575106	0.166488595	0.630194031	down_in_case
CRA004775	ENSGALG00010000432	ENSGALG00010000432	case	control	0.067996449	0.243396643	0.27936478	0.779964897	0.94835413	up_in_case
CRA004775	ENSGALG00010000434	TP53	case	control	-0.428044261	0.176960964	-2.418862622	0.015569119	0.249884603	down_in_case
CRA004775	ENSGALG00010000435	P2RX6	case	control	0.48393927	0.360373822	1.342881309	0.17931041	0.644990528	up_in_case
CRA004775	ENSGALG00010000436	ENSGALG00010000436	case	control	-0.991517445	0.539051411	-1.839374548	0.065860115	0.460652311	down_in_case
CRA004775	ENSGALG00010000440	ENSGALG00010000440	case	control	0.747330156	0.53841728	1.388012948	0.165133102	0.630194031	up_in_case
CRA004775	ENSGALG00010000441	SLC7A4	case	control	-0.73065459	0.554935192	-1.316648503	0.187956473	0.652111233	down_in_case
CRA004775	ENSGALG00010000442	DGCR6	case	control	-0.362733858	0.21019444	-1.725706248	0.084400273	0.503614276	down_in_case
CRA004775	ENSGALG00010000443	TBX3	case	control	0.298244459	0.345710755	0.862699392	0.388302767	0.804270424	up_in_case
CRA004775	ENSGALG00010000444	RBM19	case	control	-0.037866115	0.240655995	-0.157345405	0.874972638	0.974301166	down_in_case
CRA004775	ENSGALG00010000447	SDSL	case	control	0.436970118	0.436253644	1.001642335	0.316516367	0.765866014	up_in_case
CRA004775	ENSGALG00010000449	PLBD2	case	control	-0.341867691	0.194681827	-1.75603289	0.079082793	0.491399489	down_in_case
CRA004775	ENSGALG00010000450	ENSGALG00010000450	case	control	-0.41923396	0.315105799	-1.330454598	0.183368536	0.647806087	down_in_case
CRA004775	ENSGALG00010000452	SLC8B1	case	control	-0.639556762	0.281026145	-2.275790964	0.022858524	0.294061167	down_in_case
CRA004775	ENSGALG00010000453	ACHE	case	control	-0.227882275	0.505674989	-0.450649685	0.652242051	0.915892481	down_in_case
CRA004775	ENSGALG00010000454	ENSGALG00010000454	case	control	-1.020281448	1.091191448	-0.935015986	0.349780061	0.785466132	down_in_case
CRA004775	ENSGALG00010000457	TPCN1	case	control	-0.378836719	0.242251925	-1.563813038	0.117861482	0.569050463	down_in_case
CRA004775	ENSGALG00010000458	ENSGALG00010000458	case	control	-0.491139949	0.239475518	-2.050898367	0.040276845	0.376363211	down_in_case
CRA004775	ENSGALG00010000462	ENSGALG00010000462	case	control	-0.021538364	0.366760095	-0.058726028	0.953170328	0.990670511	down_in_case
CRA004775	ENSGALG00010000465	ENSGALG00010000465	case	control	0.340354121	0.406181713	0.837935609	0.402066868	0.811868062	up_in_case
CRA004775	ENSGALG00010000466	FBXO21	case	control	-0.388892012	0.163841854	-2.373581615	0.017616499	0.264131992	down_in_case
CRA004775	ENSGALG00010000467	RNASEK	case	control	0.341811289	0.327890582	1.042455343	0.29720062	0.749872609	up_in_case
CRA004775	ENSGALG00010000468	TESC	case	control	-0.146141506	0.367555086	-0.397604364	0.690921843	0.927867527	down_in_case
CRA004775	ENSGALG00010000469	ENSGALG00010000469	case	control	-0.090907378	0.60566125	-0.150096078	0.880688814	0.974996282	down_in_case
CRA004775	ENSGALG00010000470	FBXW8	case	control	-0.438471537	0.264144609	-1.659967769	0.096920937	0.53104893	down_in_case
CRA004775	ENSGALG00010000471	ENSGALG00010000471	case	control	-0.399369994	0.273392542	-1.460793301	0.144072175	0.605852133	down_in_case
CRA004775	ENSGALG00010000475	RNFT2	case	control	-0.717390839	0.253808765	-2.826501433	0.004705953	0.141450672	down_in_case
CRA004775	ENSGALG00010000476	ENSGALG00010000476	case	control	-0.103599151	0.692149183	-0.149677488	0.88101907	0.974996282	down_in_case
CRA004775	ENSGALG00010000479	C12orf49	case	control	0.096380595	0.297153434	0.324346227	0.745675926	0.939421865	up_in_case
CRA004775	ENSGALG00010000483	ENSGALG00010000483	case	control	0.33625159	0.430003258	0.781974517	0.43422955	0.826182359	up_in_case
CRA004775	ENSGALG00010000485	MED13L	case	control	0.422479471	0.331178306	1.275685827	0.202066601	0.670354119	up_in_case
CRA004775	ENSGALG00010000486	ENSGALG00010000486	case	control	0.179459726	0.563764792	0.318323755	0.750239367	0.941495694	up_in_case
CRA004775	ENSGALG00010000491	ENSGALG00010000491	case	control	0.156575445	0.258685268	0.605273914	0.544997011	0.876418419	up_in_case
CRA004775	ENSGALG00010000495	ENSGALG00010000495	case	control	-0.71309853	0.647382042	-1.101511137	0.27067426	0.728665115	down_in_case
CRA004775	ENSGALG00010000501	ENSGALG00010000501	case	control	-0.15577692	0.25329584	-0.614999916	0.538554776	0.873206783	down_in_case
CRA004775	ENSGALG00010000507	TRPT1	case	control	-0.247205827	0.413099692	-0.598416875	0.549561809	0.877623839	down_in_case
CRA004775	ENSGALG00010000511	FERMT3	case	control	-0.451713486	0.508811198	-0.887782123	0.374657961	0.7983762	down_in_case
CRA004775	ENSGALG00010000526	POLD1	case	control	-0.138237106	0.348446478	-0.396724072	0.691570947	0.927867527	down_in_case
CRA004775	ENSGALG00010000529	STIP1	case	control	-0.121510089	0.272608804	-0.445730611	0.65579185	0.917054032	down_in_case
CRA004775	ENSGALG00010000532	ENSGALG00010000532	case	control	-0.31405645	0.552508515	-0.568419202	0.569750355	0.885169713	down_in_case
CRA004775	ENSGALG00010000546	FLRT1	case	control	-0.377839687	1.182386639	-0.319556797	0.74930433	0.941366343	down_in_case
CRA004775	ENSGALG00010000555	OTUB1	case	control	-0.373485614	0.292154611	-1.27838343	0.201114264	0.669025802	down_in_case
CRA004775	ENSGALG00010000561	ENSGALG00010000561	case	control	-0.020477974	0.242722855	-0.084367722	0.93276407	0.985090297	down_in_case
CRA004775	ENSGALG00010000564	NAA40	case	control	-0.436934949	0.264338139	-1.652939491	0.098343179	0.534301482	down_in_case
CRA004775	ENSGALG00010000569	SIPA1	case	control	-0.507798093	0.392376891	-1.29415902	0.195610499	0.661073909	down_in_case
CRA004775	ENSGALG00010000571	ENSGALG00010000571	case	control	2.64174099	0.760578232	3.473332365	0.000514038	0.046088692	up_in_case
CRA004775	ENSGALG00010000574	ENSGALG00010000574	case	control	0.08156301	0.314416211	0.259410956	0.795318177	0.952400963	up_in_case
CRA004775	ENSGALG00010000575	ENSGALG00010000575	case	control	-0.065312221	0.427157352	-0.152899675	0.878477392	0.974896507	down_in_case
CRA004775	ENSGALG00010000576	NOS1	case	control	-0.196239556	0.771291896	-0.254429688	0.799163618	0.952835572	down_in_case
CRA004775	ENSGALG00010000577	ENSGALG00010000577	case	control	0.366523111	0.447514857	0.819018867	0.41277565	0.817112606	up_in_case
CRA004775	ENSGALG00010000578	ENSGALG00010000578	case	control	0.439793314	0.321642557	1.367335589	0.171520142	0.636057362	up_in_case
CRA004775	ENSGALG00010000579	ENSGALG00010000579	case	control	-4.338252978	1.075095288	-4.035226484	5.45497E-05	0.01449308	down_in_case
CRA004775	ENSGALG00010000581	ENSGALG00010000581	case	control	-0.24593959	0.234973146	-1.046671051	0.29525132	0.747584126	down_in_case
CRA004775	ENSGALG00010000583	CCS	case	control	-0.280946475	0.281738938	-0.997187242	0.318673632	0.767037518	down_in_case
CRA004775	ENSGALG00010000584	RBM14	case	control	-0.239552535	0.204446447	-1.171712877	0.241312351	0.708581282	down_in_case
CRA004775	ENSGALG00010000585	SPTBN2	case	control	-1.127146094	0.622257097	-1.81138327	0.070081544	0.469724266	down_in_case
CRA004775	ENSGALG00010000586	ENSGALG00010000586	case	control	-0.585782803	0.497061861	-1.178490746	0.238601019	0.705288611	down_in_case
CRA004775	ENSGALG00010000589	ENSGALG00010000589	case	control	0.168531639	0.383790619	0.439123916	0.660571751	0.917907174	up_in_case
CRA004775	ENSGALG00010000591	SBK2	case	control	0.372776671	0.827017959	0.450747977	0.6521712	0.915892481	up_in_case
CRA004775	ENSGALG00010000592	CCT5	case	control	-0.026152871	0.180618946	-0.144795834	0.884872071	0.976476675	down_in_case
CRA004775	ENSGALG00010000593	ATPSCKMT	case	control	0.695073368	0.304292889	2.284224816	0.022358322	0.292422177	up_in_case
CRA004775	ENSGALG00010000595	MYO10	case	control	0.245575024	0.366149024	0.670696924	0.502413623	0.860091011	up_in_case
CRA004775	ENSGALG00010000596	ENSGALG00010000596	case	control	-2.784390038	1.613641042	-1.725532486	0.084431555	0.503614276	down_in_case
CRA004775	ENSGALG00010000598	ENSGALG00010000598	case	control	-1.008979499	0.917570126	-1.099621129	0.271497232	0.728910871	down_in_case
CRA004775	ENSGALG00010000599	RETREG1	case	control	1.054272345	0.722407756	1.459386803	0.144458676	0.606459064	up_in_case
CRA004775	ENSGALG00010000601	ENSGALG00010000601	case	control	0.272625745	0.71396563	0.381847155	0.702574739	0.930068722	up_in_case
CRA004775	ENSGALG00010000603	ZNF622	case	control	-0.29778858	0.183869726	-1.619562864	0.105326212	0.54902985	down_in_case

CRA004775	ENSGALG00010000604	ENSGALG00010000604	case	control	-0.666902373	0.208748541	-3.194764226	0.001399451	0.078533081	down_in_case
CRA004775	ENSGALG00010000606	UBE2QL1	case	control	-0.073187371	0.313081368	-0.233764697	0.815167658	0.958870945	down_in_case
CRA004775	ENSGALG00010000607	ENSGALG00010000607	case	control	-1.619050902	0.518659023	-3.121609441	0.001798654	0.085287202	down_in_case
CRA004775	ENSGALG00010000610	ENSGALG00010000610	case	control	-0.299191246	0.263322047	-1.136217985	0.255865345	0.719176609	down_in_case
CRA004775	ENSGALG00010000612	ENSGALG00010000612	case	control	-0.81326395	0.369056639	-2.203629101	0.027550439	0.317026013	down_in_case
CRA004775	ENSGALG00010000613	ENSGALG00010000613	case	control	-0.416208015	0.31488644	-1.321771795	0.186244153	0.6491093	down_in_case
CRA004775	ENSGALG00010000614	KAT8	case	control	-0.481547574	0.370604192	-1.299358141	0.19382105	0.657919837	down_in_case
CRA004775	ENSGALG00010000616	PSENEN	case	control	0.254216395	0.63221783	0.402102539	0.687608564	0.927111919	up_in_case
CRA004775	ENSGALG00010000619	ENSGALG00010000619	case	control	-0.448492862	0.370767221	-1.209634607	0.226419133	0.69451026	down_in_case
CRA004775	ENSGALG00010000621	UPP1	case	control	-0.028829328	0.457759319	-0.062979228	0.949783045	0.990168609	down_in_case
CRA004775	ENSGALG00010000623	ENSGALG00010000623	case	control	-0.105598955	0.233022264	-0.453171095	0.650425545	0.915428088	down_in_case
CRA004775	ENSGALG00010000625	ENSGALG00010000625	case	control	-0.337318511	0.389565084	-0.865884869	0.386553302	0.803905475	down_in_case
CRA004775	ENSGALG00010000627	ENSGALG00010000627	case	control	-0.130233523	0.47846923	-0.272187875	0.785477564	0.95041797	down_in_case
CRA004775	ENSGALG00010000630	ENSGALG00010000630	case	control	-0.767850146	0.374274002	-2.051572219	0.040211254	0.376363211	down_in_case
CRA004775	ENSGALG00010000634	ENSGALG00010000634	case	control	-0.459092038	0.316674824	-1.449726985	0.147134668	0.610295079	down_in_case
CRA004775	ENSGALG00010000635	ENSGALG00010000635	case	control	-0.046878504	0.29133327	-0.160910233	0.872164102	0.97401729	down_in_case
CRA004775	ENSGALG00010000636	ENSGALG00010000636	case	control	0.043722139	0.308533335	0.14170961	0.887309384	0.977118922	up_in_case
CRA004775	ENSGALG00010000639	SCN1B	case	control	0.549336743	0.355803603	1.543932491	0.122604688	0.57525466	up_in_case
CRA004775	ENSGALG00010000641	ENSGALG00010000641	case	control	0.206852094	0.730587045	0.283131347			up_in_case
CRA004775	ENSGALG00010000643	VKORC1	case	control	-0.996658656	0.497979175	-2.001406295	0.045348623	0.395678244	down_in_case
CRA004775	ENSGALG00010000646	ENSGALG00010000646	case	control	-2.466215937	0.873215479	-2.82429251	0.004738512	0.141624457	down_in_case
CRA004775	ENSGALG00010000649	PAPD7	case	control	0.580872985	0.296060467	1.962007936	0.04976156	0.413341381	up_in_case
CRA004775	ENSGALG00010000650	BCKDK	case	control	-0.508237912	0.406459043	-1.250403749	0.211152096	0.681585086	down_in_case
CRA004775	ENSGALG00010000651	ENSGALG00010000651	case	control	0.432497435	0.484680872	0.892334441	0.372213699	0.796004755	up_in_case
CRA004775	ENSGALG00010000652	SRD5A1	case	control	-0.642129222	0.590216833	-1.087954776	0.276615073	0.731346638	down_in_case
CRA004775	ENSGALG00010000653	NSUN2	case	control	0.024443566	0.226997157	0.107682256	0.914247745	0.981176987	up_in_case
CRA004775	ENSGALG00010000654	ENSGALG00010000654	case	control	-0.467805082	0.230776907	-2.027087927	0.042653421	0.388066397	down_in_case
CRA004775	ENSGALG00010000656	ENSGALG00010000656	case	control	-0.208670983	0.327620801	-0.636928369	0.524171503	0.866784927	down_in_case
CRA004775	ENSGALG00010000659	ENSGALG00010000659	case	control	-1.285049373	0.603337248	-2.129902267	0.033179683	0.347668706	down_in_case
CRA004775	ENSGALG00010000660	ENSGALG00010000660	case	control	-0.114181351	0.360328498	-0.316881266	0.751333697	0.941495694	down_in_case
CRA004775	ENSGALG00010000674	GRB10	case	control	0.834608737	0.3917319	2.130561073	0.033125319	0.347668706	up_in_case
CRA004775	ENSGALG00010000684	COBL	case	control	-0.006901134	0.433648041	-0.015914137	0.987302892	0.997897984	down_in_case
CRA004775	ENSGALG00010000685	ENSGALG00010000685	case	control	-0.333424151	0.620185671	-0.537619888	0.590839497	0.893804699	down_in_case
CRA004775	ENSGALG00010000686	ENSGALG00010000686	case	control	-0.423720455	0.268660715	-1.577158217	0.114759125	0.564684045	down_in_case
CRA004775	ENSGALG00010000687	IKZF1	case	control	-0.49844293	0.500125161	-0.996636381	0.31894104	0.767037518	down_in_case
CRA004775	ENSGALG00010000688	ENSGALG00010000688	case	control	-0.142433375	0.221707322	-0.642438748	0.520588348	0.865611895	down_in_case
CRA004775	ENSGALG00010000695	FIGNL1	case	control	-0.602078791	0.505867504	-1.190190685	0.233971453	0.701251407	down_in_case
CRA004775	ENSGALG00010000697	ENSGALG00010000697	case	control	-0.572515346	0.368446709	-1.553862016	0.120217334	0.572133279	down_in_case
CRA004775	ENSGALG00010000702	DDC	case	control	1.46528221	0.597250567	2.453379353	0.0141521	0.238037235	up_in_case
CRA004775	ENSGALG00010000704	ENSGALG00010000704	case	control	-0.403247172	0.174603502	-2.309502197	0.02091573	0.284579324	down_in_case
CRA004775	ENSGALG00010000710	ENSGALG00010000710	case	control	-1.382071302	0.669039185	-2.065755388	0.038851582	0.370839453	down_in_case
CRA004775	ENSGALG00010000711	SPRY2	case	control	1.093648029	0.345780857	3.162835667	0.001562405	0.080693373	up_in_case
CRA004775	ENSGALG00010000714	FBXL7	case	control	0.22546622	0.349839459	0.644484817	0.5192611	0.865611895	up_in_case
CRA004775	ENSGALG00010000717	NDFIP2	case	control	0.093854807	0.161822161	0.579987354	0.561923146	0.881532201	up_in_case
CRA004775	ENSGALG00010000718	EEF1G	case	control	-0.153303948	0.261781876	-0.58561712	0.558132841	0.880754991	down_in_case
CRA004775	ENSGALG00010000720	RBM26	case	control	0.337148649	0.17333548	1.945064276	0.051767243	0.419476344	up_in_case
CRA004775	ENSGALG00010000721	ENSGALG00010000721	case	control	-0.670597969	0.295145123	-2.272095715	0.02308073	0.295240109	down_in_case
CRA004775	ENSGALG00010000722	ANKH	case	control	-0.112533602	0.238181528	-0.472469896	0.636591425	0.910239023	down_in_case
CRA004775	ENSGALG00010000724	OBI1	case	control	-0.500508413	0.365170131	-1.370617063	0.170494361	0.634497418	down_in_case
CRA004775	ENSGALG00010000727	ENSGALG00010000727	case	control	-0.212519094	0.269793426	-0.787710424	0.430866107	0.824612364	down_in_case
CRA004775	ENSGALG00010000729	EDNRB	case	control	0.948354075	0.405903675	2.336401798	0.01947031	0.27573248	up_in_case
CRA004775	ENSGALG00010000730	INTS5	case	control	-0.18928301	0.353092092	-0.53607264	0.591908348	0.893804699	down_in_case
CRA004775	ENSGALG00010000731	ENSGALG00010000731	case	control	-0.649679452	0.873695138	-0.743599711	0.457118678	0.839688534	down_in_case
CRA004775	ENSGALG00010000732	ENSGALG00010000732	case	control	-0.11624467	0.324275248	-0.358475309	0.719987642	0.934189603	down_in_case
CRA004775	ENSGALG00010000735	ENSGALG00010000735	case	control	-0.047244841	0.835846931	-0.056523317			down_in_case
CRA004775	ENSGALG00010000741	FAM105A	case	control	-0.294198772	0.545349961	-0.539467852	0.589564073	0.893412703	down_in_case
CRA004775	ENSGALG00010000753	TRIO	case	control	0.395834179	0.318609254	1.242381299	0.214095912	0.683546061	up_in_case
CRA004775	ENSGALG00010000768	ENSGALG00010000768	case	control	-0.05683686	0.533984077	-0.106439242	0.915233859	0.981469218	down_in_case
CRA004775	ENSGALG00010000777	MYCBP2	case	control	0.104240495	0.358580859	0.290702899	0.771278554	0.945853152	up_in_case
CRA004775	ENSGALG00010000783	DAP	case	control	-0.462356592	0.374396099	-1.234939661	0.216852961	0.684403275	down_in_case
CRA004775	ENSGALG00010000784	FBXL3	case	control	0.293396987	0.211783478	1.385362968	0.165941509	0.630194031	up_in_case
CRA004775	ENSGALG00010000785	CLN5	case	control	-0.07013822	0.275202246	-0.254860638	0.798830739	0.952835572	down_in_case
CRA004775	ENSGALG00010000786	ANKRD33B	case	control	-0.257743162	0.686452184	-0.375471399	0.707309913	0.931617022	down_in_case
CRA004775	ENSGALG00010000787	SNRNP48	case	control	-0.220514821	0.213160694	-1.034500394	0.300902293	0.753440172	down_in_case
CRA004775	ENSGALG00010000788	GATD3AL1	case	control	-0.277381189	0.30704319	-0.903394693	0.366316456	0.793608387	down_in_case
CRA004775	ENSGALG00010000789	ROPN1L	case	control	-0.113772544	0.908956126	-0.125168356	0.900390269	0.978988423	down_in_case
CRA004775	ENSGALG00010000790	MARCHF6	case	control	0.159630741	0.197986562	0.806270584	0.420086848	0.819388688	up_in_case
CRA004775	ENSGALG00010000791	ACOD1	case	control	-0.126436437	0.603093227	-0.209646587	0.833943516	0.964019688	down_in_case
CRA004775	ENSGALG00010000795	ENSGALG00010000795	case	control	-0.672039441	0.508308871	-1.322108426	0.186132048	0.6491093	down_in_case
CRA004775	ENSGALG00010000796	ENSGALG00010000796	case	control	-0.62680185	0.482859765	-1.298103293	0.194251843	0.65892248	down_in_case
CRA004775	ENSGALG00010000797	ENSGALG00010000797	case	control	0.430170458	0.668841994	0.643157072	0.520122185	0.865611895	up_in_case
CRA004775	ENSGALG00010000799	ENSGALG00010000799	case	control	0.339907209	0.564145458	0.602516964	0.546830074	0.877099134	up_in_case
CRA004775	ENSGALG00010000800	LMO7	case	control	0.257854164	0.135343839	1.90517844	0.05675688	0.435477592	up_in_case
CRA004775	ENSGALG00010000802	SEMA5A	case	control	0.265850304	0.549389963	0.483900912	0.628456193	0.908417222	up_in_case
CRA004775	ENSGALG00010000803	MTRR	case	control	-0.262228857	0.210292424	-1.246972442	0.212407603	0.682275341	down_in_case
CRA004775	ENSGALG00010000805	FASTKD3	case	control	0.060269799	0.197940309	0.304484718	0.760758633	0.942236668	up_in_case
CRA004775	ENSGALG00010000806	CFAP90	case	control	0.003612066	0.290553074	0.012431689	0.990081203	0.998065748	up_in_case
CRA004775	ENSGALG00010000808	ADCY2	case	control	0.099548996	0.474091617	0.209978394	0.833684536	0.964019688	up_in_case
CRA004775	ENSGALG00010000810	UCHL3	case	control	-0.208375892	0.186287687	-1.118570394	0.26332346	0.723981064	down_in_case
CRA004775	ENSGALG00010000811	ENSGALG00010000811	case	control	-0.603912446	1.357414888	-0.444898941	0.656392788	0.91710603	down_in_case
CRA004775	ENSGALG00010000812	ENSGALG00010000812	case	control	0.201735904	0.19214165	1.049933234	0.293748811	0.746438942	up_in_case
CRA004775	ENSGALG00010000813	ENSGALG00010000813	case	control	-0.774144573	0.461039521	-1.679128444	0.093127014	0.524831789	down_in_case
CRA004775	ENSGALG00010000815	ENSGALG00010000815	case	control	-0.745341362	0.243086855	-3.066152476	0.002168327	0.094431421	down_in_case
CRA004775	ENSGALG00010000816	TBC1D4	case	control	-0.091002877	0.30033066	-0.303008948	0.761883042	0.942236668	down_in_case
CRA004775	ENSGALG00010000817	ENSGALG00010000817	case	control	-0.083384743	0.337041817	-0.247401773	0.804597303	0.954576316	down_in_case
CRA004775	ENSGALG00010000818	KLF12	case	control	0.280103177	0.361433937	0.774977524	0.438352941	0.827879589	up_in_case
CRA004775	ENSGALG00010000820	KLF5	case	control	1.232774371	0.447896943	2.752361653	0.005916714	0.154441997	up_in_case
CRA004775	ENSGALG00010000821	ENSGALG00010000821	case	control	-0.491420484	0.578452315	-0.849543639	0.395578858	0.808998414	down_in_case
CRA004775	ENSGALG00010000822	BASP1	case	control	0.119982466	0.42158166	0.284600773	0.775950019	0.946517228	up_in_case
CRA004775	ENSGALG00010000823	PIBF1	case	control	0.159489884	0.267342555	0.596574997	0.550791169	0.877827378	up_in_case
CRA004775	ENSGALG00010000843	DIS3	case	control	0.131062298	0.185742497	0.705612878	0.480428914	0.848385513	up_in_case
CRA004775	ENSGALG00010000853	ENSGALG00010000853	case	control	-0.752589387	0.428163919	-1.757713236	0.078796315	0.491399489	down_in_case
CRA004775	ENSGALG00010000857	ENSGALG00010000857	case	control	-0.83459794	0.808821814	-1.031868732	0.302133615	0.754801023	down_in_case
CRA004775	ENSGALG00010000859	BORA	case	control	0.349910294	0.458945438	0.762422425	0.445807927	0.833292411	up_in_case
CRA004775	ENSGALG00010000864	MZT1	case	control	-0.255852707	0.283020703	-0.904007034	0.365991671	0.793608387	down_in_case
CRA004775	ENSGALG00010000870	DACH1	case	control	0.571838922	0.457968055	1.248643689	0.211795425	0.682220401	up_in_case
CRA004775	ENSGALG00010001066	SLC33A1	case	control	-0.099147722	0.218091184	-0.454615908	0.649385588	0.915047477	down_in_case
CRA004775	ENSGALG00010001070	C3orf33	case	control	-0.438650414	0.38895978	-1.127752628	0.259424405	0.720037471	down_in_case
CRA004775	ENSGALG00010001082	P2RY1	case	control	-0.209376283	0.418961435	-0.499750729	0.617250607	0.903905787	down_in_case
CRA004775	ENSGALG00010001104	MBNL1	case	control	0.347820444	0.287528626	1.209689794	0.226397948	0.69451026	up_in_case
CRA004775	ENSGALG00010001111	SUCNR1	case	control	0.246313083	0.631171254	0.390247625	0.696353448	0.929092636	up_in_case
CRA004775	ENSGALG00010001112	TSC22D2	case	control	-0.117971732	0.192659225	-0.612333681	0.54031701	0.874311509	down_in_case
CRA004775	ENSGALG00010001113	IGSF10	case	control	0.065847206	0.525300335	0.12535154	0.900245251	0.978988423	up_in_case
CRA004775	ENSGALG00010001114	MED12L	case	control	-0.061897287	0.216472218	-0.285936402	0.774926833	0.946319184	down_in_case
CRA004775	ENSGALG00010001115	PFN2	case	control	-0.158631625	0.220559626	-0.719223315	0.472003336	0.845425322	down_in_case
CRA004775	ENSGALG00010001116	RNF13	case	control	0.087767092	0.137804703	0.636894753	0.524193401	0.866784927	up_in_case
CRA004775	ENSGALG00010001117	P2RY13	case	control	0.185837383	0.534613176	0.34761093	0.728132397	0.935650625	up_in_case
CRA004775	ENSGALG00010001118	P2RY12	case	control	0.089193871	0.643155771	0.138681599	0.889701759	0.977179624	up_in_case
CRA004775	ENSGALG00010001119	COMMD2	case	control	-0.227360693	0.186278157	-1.220544031	0.222258708	0.689907546	down_in_case
CRA004775	ENSGALG00010001121	WWTR1	case	control	0.303081435	0.317675598	0.954059541	0.340053505	0.780516732	up_in_case
CRA004775	ENSGALG00010001122	P2RY14	case	control	-0.181954537	0.417939033	-0.435361436	0.663300097	0.918295135	down_in_case
CRA004775	ENSGALG00010001123	CP	case	control	0.411923775	0.41461612	0.993506415	0.320463226	0.767037518	up_in_case
CRA004775	ENSGALG00010001124	GPR171	case	control	0.356516204	0.625658387	0.569825661	0.568795949	0.884663439	up_in_case
CRA004775	ENSGALG00010001125	CLRN1	case	control	-0.373040438	0.413503264	-0.902146296	0.366979159	0.793822622	down_in_case
CRA004775	ENSGALG00010001127	SIAH2	case	control	0.175962689	0.141017897	1.247803946	0.212102863	0.682275341	up_in_case
CRA004775	ENSGALG00010001129	TM4SF4	case	control	2.380806245	0.924309882	2.575766299	0.010001822	0.201425687	up_in_case
CRA004775	ENSGALG00010001131	HPS3	case	control	-0.105098762	0.18800681	-0.559015717	0.576150994	0.887937128	down_in_case
CRA004775	ENSGALG00010001132	SELENOT	case	control	-0.13983432	0.189989342	-0.73601139	0.461723766	0.842010079	down_in_case
CRA004775	ENSGALG00010001133	EIF2A	case	control	-0.064794598	0.213266072	-0.303820471	0.76126467	0.942236668	down_in_case
CRA004775	ENSGALG00010001134	ENSGALG00010001134	case	control	0.297122613	1.129694792	0.263011404	0.792541789	0.951284723	up_in_case
CRA004775	ENSGALG00010001135	GYG1	case	control	-0.037339894	0.369619244	-0.101022591	0.919532527	0.982170916	down_in_case
CRA004775	ENSGALG00010001136	SERP1	case	control	-0.066348576	0.167569877	-0.395945723	0.69214507	0.927867527	down_in_case
CRA004775	ENSGALG00010001140	NMD3	case	control	0.127316728	0.25977915	0.490096023	0.624065952	0.906441769	up_in_case
CRA004775	ENSGALG00010001141	MTAP	case	control	-0.028863822	0.311835492	-0.092561055	0.926252284	0.98417264	down_in_case
CRA004775	ENSGALG00010001142	PPM1L	case	control	0.318601599	0.399811116	0.796880292	0.425520558	0.822283278	up_in_case
CRA004775	ENSGALG00010001143	CDKN2A	case	control	0.508068176	0.519413948	0.978156589	0.327996883	0.771912569	up_in_case

CRA004775	ENSGALG00010001144	CDKN2B	case	control	2.371262543	0.483665886	4.902687186	9.45345E-07	0.000590883	up_in_case
CRA004775	ENSGALG00010001145	TRIM36	case	control	-1.046924698	0.666829341	-1.570003948	0.116414193	0.566086328	down_in_case
CRA004775	ENSGALG00010001146	DIPK1C	case	control	-0.153975982	0.48343473	-0.31850418	0.750102524	0.941495694	down_in_case
CRA004775	ENSGALG00010001147	PGGT1B	case	control	-0.162506037	0.19624266	-0.828087214	0.40762111	0.814105656	down_in_case
CRA004775	ENSGALG00010001149	CIDEA	case	control	-0.599335715	0.708053967	-0.846454851	0.397299051	0.809012179	down_in_case
CRA004775	ENSGALG00010001151	CCDC112	case	control	0.816678933	0.529811419	1.541452115	0.123206789	0.575549148	up_in_case
CRA004775	ENSGALG00010001152	FEM1C	case	control	0.262924965	0.332769759	0.790110754	0.42946309	0.823991481	up_in_case
CRA004775	ENSGALG00010001153	TSHZ1	case	control	-0.073917893	0.298649974	-0.247506778	0.804516047	0.954576316	down_in_case
CRA004775	ENSGALG00010001154	PTGR3	case	control	-0.09916326	0.22531244	-0.44011445	0.659854217	0.917907174	down_in_case
CRA004775	ENSGALG00010001155	CYB5A	case	control	-0.148856449	0.261629029	-0.568959986	0.569383295	0.884809383	down_in_case
CRA004775	ENSGALG00010001156	ALDH7A1	case	control	-0.696803548	0.372748307	-1.869367438	0.061571712	0.446401278	down_in_case
CRA004775	ENSGALG00010001157	TIMM21	case	control	0.186277196	0.365844579	0.509170306	0.610632855	0.901413878	up_in_case
CRA004775	ENSGALG00010001158	FRMPD1	case	control	0.013886317	0.559415889	0.024822886	0.980196237	0.996943672	up_in_case
CRA004775	ENSGALG00010001159	RTTN	case	control	0.010517421	0.490081914	0.021460537	0.982878283	0.997398088	up_in_case
CRA004775	ENSGALG00010001160	TRMT10B	case	control	-0.585271979	0.356439142	-1.641996939	0.100590639	0.538583505	down_in_case
CRA004775	ENSGALG00010001161	B3GALNT1	case	control	0.712326757	0.291673342	2.442207272	0.014597763	0.241689368	up_in_case
CRA004775	ENSGALG00010001162	ENSGALG00010001162	case	control	0.131646596	0.379652236	0.346755752	0.728774822	0.935965497	up_in_case
CRA004775	ENSGALG00010001164	KPNA4	case	control	0.342467606	0.221326006	1.547344625	0.121780167	0.573689301	up_in_case
CRA004775	ENSGALG00010001165	ENSGALG00010001165	case	control	-0.257970123	0.480633492	-0.536729394	0.591454549	0.893804699	down_in_case
CRA004775	ENSGALG00010001166	TRIM59	case	control	0.240510518	0.136883631	1.757043669	0.078910366	0.491399489	up_in_case
CRA004775	ENSGALG00010001167	SMC4	case	control	-0.093089322	0.456115536	-0.20409154	0.838281965	0.965677031	down_in_case
CRA004775	ENSGALG00010001169	ENSGALG00010001169	case	control	0.207882966	0.132447026	1.569555565	0.116518543	0.566086328	up_in_case
CRA004775	ENSGALG00010001170	DCAF10	case	control	-0.152992699	0.246628405	-0.620336896	0.535036009	0.870981055	down_in_case
CRA004775	ENSGALG00010001171	POLR1E	case	control	-0.252128223	0.240207748	-1.049625689	0.293890242	0.746438942	down_in_case
CRA004775	ENSGALG00010001172	TMEM200C	case	control	-0.673686898	0.51021801	-1.320390274	0.186704749	0.649968862	down_in_case
CRA004775	ENSGALG00010001173	IFT80	case	control	-0.206140122	0.187051895	-1.102047757	0.27044091	0.728665115	down_in_case
CRA004775	ENSGALG00010001174	ENSGALG00010001174	case	control	-0.347227284	0.39972047	-0.868675262	0.385024776	0.803182776	down_in_case
CRA004775	ENSGALG00010001177	SEH1L	case	control	-0.275232253	0.171422584	-1.605577553	0.108366761	0.555197961	down_in_case
CRA004775	ENSGALG00010001178	ZBTB5	case	control	-0.206752824	0.289561432	-0.714020587	0.475214449	0.845802989	down_in_case
CRA004775	ENSGALG00010001179	GRHPR	case	control	-1.161660525	0.440578706	-2.636669698	0.008372431	0.183326911	down_in_case
CRA004775	ENSGALG00010001180	SPIRE1	case	control	-0.165804065	0.329395278	-0.503358962	0.614711919	0.902639483	down_in_case
CRA004775	ENSGALG00010001181	MFSD1	case	control	-0.185591754	0.151916125	-1.221672508	0.221831497	0.689550502	down_in_case
CRA004775	ENSGALG00010001182	ZCCHC7	case	control	0.01925266	0.215160922	0.089480281	0.928700224	0.98473447	up_in_case
CRA004775	ENSGALG00010001183	RARRES1	case	control	0.375110642	0.362802779	1.033924391	0.301171511	0.753545086	up_in_case
CRA004775	ENSGALG00010001185	PTPN2	case	control	0.05789283	0.289849641	0.199734005	0.841688618	0.966478468	up_in_case
CRA004775	ENSGALG00010001187	GFM1	case	control	0.127828766	0.275190222	0.464510568	0.64228199	0.913111066	up_in_case
CRA004775	ENSGALG00010001188	PSMG2	case	control	-0.279939475	0.299654819	-0.934206484	0.350197392	0.785466132	down_in_case
CRA004775	ENSGALG00010001190	LXN	case	control	-0.500251854	0.643411133	-0.777499531	0.436864111	0.827138935	down_in_case
CRA004775	ENSGALG00010001191	TOMM5	case	control	-0.417127524	0.37693434	-1.10663179	0.268453157	0.727528758	down_in_case
CRA004775	ENSGALG00010001192	ENSGALG00010001192	case	control	0.327964081	0.394555335	0.83122455	0.405846789	0.813842524	up_in_case
CRA004775	ENSGALG00010001193	PRELID3A	case	control	0.35180383	0.394383365	0.892035166	0.372374084	0.796100128	up_in_case
CRA004775	ENSGALG00010001194	POT1	case	control	0.011816105	0.28029232	0.042156363	0.966374049	0.993094598	up_in_case
CRA004775	ENSGALG00010001195	ZNF608	case	control	0.070131876	0.360099722	0.194756817	0.84558333	0.966993336	up_in_case
CRA004775	ENSGALG00010001196	LSMEM1	case	control	0.470856917	0.602742216	0.781191203	0.434690047	0.826524175	up_in_case
CRA004775	ENSGALG00010001197	CEP76	case	control	-0.318830341	0.254958029	-1.25052089	0.211109329	0.681585086	down_in_case
CRA004775	ENSGALG00010001198	IFRD1	case	control	0.368467681	0.409124121	0.900625657	0.367787388	0.794321403	up_in_case
CRA004775	ENSGALG00010001199	AFG3L2	case	control	0.224072124	0.231550328	0.967703765	0.333192337	0.773910112	up_in_case
CRA004775	ENSGALG00010001200	CTTNBP2	case	control	0.557428961	0.339453177	1.642137999	0.100561408	0.538583505	up_in_case
CRA004775	ENSGALG00010001201	TUBB6	case	control	-0.162639904	0.269929089	-0.602528261	0.546822557	0.877099134	down_in_case
CRA004775	ENSGALG00010001202	CFTR	case	control	1.000250946	0.65474876	1.527686658	0.126590354	0.580923609	up_in_case
CRA004775	ENSGALG00010001203	TMX3	case	control	0.345331528	0.145518739	2.373106926	0.017639157	0.264131992	up_in_case
CRA004775	ENSGALG00010001204	SNX2	case	control	0.304637927	0.162929359	1.869754653	0.061517896	0.446401278	up_in_case
CRA004775	ENSGALG00010001205	CEP192	case	control	-0.14455337	0.340547567	-0.424473359	0.67122063	0.921535334	down_in_case
CRA004775	ENSGALG00010001206	RSRC1	case	control	-0.037487845	0.226763598	-0.165316856	0.868694592	0.973370973	down_in_case
CRA004775	ENSGALG00010001209	CDH7	case	control	-0.909197292	0.779403633	-1.166529451	0.243400448	0.710464777	down_in_case
CRA004775	ENSGALG00010001212	PAX5	case	control	2.135381712	0.777859839	2.745201135			up_in_case
CRA004775	ENSGALG00010001213	PPIC	case	control	-0.221693039	0.413422657	-0.536238242	0.591793907	0.893804699	down_in_case
CRA004775	ENSGALG00010001214	TES	case	control	-0.133295995	0.372802426	-0.357551308	0.720679123	0.934511903	down_in_case
CRA004775	ENSGALG00010001218	ENSGALG00010001218	case	control	-0.399057805	0.257058108	-1.552403105	0.120565801	0.572850296	down_in_case
CRA004775	ENSGALG00010001219	TFEC	case	control	-0.997949991	0.879696877	-1.134424843	0.256616383	0.719236059	down_in_case
CRA004775	ENSGALG00010001220	PLXNB2	case	control	0.496128207	0.266839318	1.859276999	0.062987886	0.450462537	up_in_case
CRA004775	ENSGALG00010001221	GRAMD2B	case	control	-0.49761091	0.279427532	-1.780822764	0.074941409	0.482673054	down_in_case
CRA004775	ENSGALG00010001222	ZNF800	case	control	0.061819019	0.356136539	0.173582355	0.8621937	0.971725725	up_in_case
CRA004775	ENSGALG00010001224	DNAJB9	case	control	0.718739095	0.271424068	2.648030075	0.008096232	0.182211603	up_in_case
CRA004775	ENSGALG00010001225	SOCS6	case	control	0.365650008	0.233066339	1.568866654	0.116679013	0.566504827	up_in_case
CRA004775	ENSGALG00010001228	THAP5	case	control	0.344413454	0.241498527	1.426151365	0.153824655	0.619153022	up_in_case
CRA004775	ENSGALG00010001229	ZNF407	case	control	0.395841875	0.209130356	1.892799698	0.058384512	0.443020311	up_in_case
CRA004775	ENSGALG00010001230	PTX3	case	control	-1.572368742	0.65318829	-2.407221264	0.016074428	0.25344686	down_in_case
CRA004775	ENSGALG00010001232	TSPAN12	case	control	-0.029953735	0.259637524	-0.11536751	0.90815383	0.980606464	down_in_case
CRA004775	ENSGALG00010001233	CNDP2	case	control	-0.098988204	0.210167345	-0.470997073	0.637642825	0.910421454	down_in_case
CRA004775	ENSGALG00010001234	AASS	case	control	0.528610354	0.317432322	1.665269468	0.095859007	0.529205137	up_in_case
CRA004775	ENSGALG00010001237	MELK	case	control	0.7720973	0.657179031	1.174866001	0.240048329	0.706680492	up_in_case
CRA004775	ENSGALG00010001238	GOLIM4	case	control	0.046795052	0.3630363	0.1288991	0.897437489	0.978790377	up_in_case
CRA004775	ENSGALG00010001239	ENSGALG00010001239	case	control	-1.064719323	0.664200735	-1.603008348	0.108932811	0.556232857	down_in_case
CRA004775	ENSGALG00010001244	ENSGALG00010001244	case	control	-0.087769469	0.240619581	-0.364764449	0.715287245	0.933870859	down_in_case
CRA004775	ENSGALG00010001245	SERPINI1	case	control	0.305220846	0.732933486	0.416437305	0.677090036	0.922924485	up_in_case
CRA004775	ENSGALG00010001248	PTPRZ1	case	control	1.85109987	0.515151813	3.593309437	0.000326504	0.035918102	up_in_case
CRA004775	ENSGALG00010001249	PDCD10	case	control	0.049921451	0.29643802	0.168404348	0.866265188	0.972806255	up_in_case
CRA004775	ENSGALG00010001251	ZNF277	case	control	0.061521103	0.190982976	0.322128726	0.747355177	0.940417372	up_in_case
CRA004775	ENSGALG00010001252	SNCAIP	case	control	-0.07482113	0.362174102	-0.206588847	0.836330971	0.965207162	down_in_case
CRA004775	ENSGALG00010001254	NETO1	case	control	0.040965485	1.115983151	0.036707978	0.970717847	0.99458618	up_in_case
CRA004775	ENSGALG00010001258	ENSGALG00010001258	case	control	-0.454172685	0.329715697	-1.377467587	0.168367729	0.631389806	down_in_case
CRA004775	ENSGALG00010001259	ENSGALG00010001259	case	control	0.518468429	0.458451946	1.130911176	0.258092479	0.720037471	up_in_case
CRA004775	ENSGALG00010001262	ENSGALG00010001262	case	control	-0.124849582	0.321862261	-0.387897549	0.698091852	0.929178049	down_in_case
CRA004775	ENSGALG00010001264	FAM3C	case	control	0.688700254	0.25325619	2.719381716	0.006540408	0.16028494	up_in_case
CRA004775	ENSGALG00010001265	OPN1LW	case	control	-0.719365414	0.618046197	-1.163934698	0.244450477	0.711765017	down_in_case
CRA004775	ENSGALG00010001267	BCHE	case	control	-0.787296536	0.651609439	-1.208233782	0.226957351	0.694715356	down_in_case
CRA004775	ENSGALG00010001268	CBLN2	case	control	0.623345398	1.04017436	0.599270105	0.54899278	0.877623839	up_in_case
CRA004775	ENSGALG00010001270	DRAP1	case	control	-0.161793567	0.292061111	-0.553971621	0.579598268	0.888438428	down_in_case
CRA004775	ENSGALG00010001271	ENSGALG00010001271	case	control	-0.208125838	0.306972711	-0.677994592	0.497775122	0.857810713	down_in_case
CRA004775	ENSGALG00010001273	ENSGALG00010001273	case	control	2.336624105	0.971005506	2.40639635	0.016110775	0.25344686	up_in_case
CRA004775	ENSGALG00010001276	ENSGALG00010001276	case	control	1.189921793	0.479725804	2.480420655	0.013122747	0.230460905	up_in_case
CRA004775	ENSGALG00010001277	ENSGALG00010001277	case	control	-0.222284675	0.28888379	-0.769460534	0.441619965	0.82961625	down_in_case
CRA004775	ENSGALG00010001279	LPIN2	case	control	-0.764231707	0.577427399	-1.323511335	0.185665389	0.6491093	down_in_case
CRA004775	ENSGALG00010001281	LRRN3	case	control	0.277240011	0.340859255	0.813356268	0.416013825	0.818943344	up_in_case
CRA004775	ENSGALG00010001283	ENSGALG00010001283	case	control	-0.282628865	0.181204444	-1.559723691	0.118825191	0.570892125	down_in_case
CRA004775	ENSGALG00010001285	ENSGALG00010001285	case	control	-0.094490121	0.222207513	-0.425233691	0.670666326	0.921535334	down_in_case
CRA004775	ENSGALG00010001286	SRFBP1	case	control	-0.010524529	0.31336291	-0.033585752	0.973207484	0.994947809	down_in_case
CRA004775	ENSGALG00010001287	PLPP4	case	control	0.267680527	0.688777465	0.388631367	0.69754886	0.929092636	up_in_case
CRA004775	ENSGALG00010001288	TGIF1	case	control	0.327631579	0.331267902	0.989023013	0.322651882	0.767880594	up_in_case
CRA004775	ENSGALG00010001289	ENSGALG00010001289	case	control	-0.180336565	0.32588789	-0.55336995	0.580010112	0.888438428	down_in_case
CRA004775	ENSGALG00010001291	ENSGALG00010001291	case	control	0.105565344	0.37755659	0.279601382	0.779783347	0.948291556	up_in_case
CRA004775	ENSGALG00010001292	LOX	case	control	-0.073488764	0.426348331	-0.172367895	0.863148311	0.971910432	down_in_case
CRA004775	ENSGALG00010001295	ENSGALG00010001295	case	control	-0.259133813	0.175869159	-1.473446589	0.140630656	0.600007375	down_in_case
CRA004775	ENSGALG00010001297	MYL12A	case	control	-0.285541932	0.16623165	-1.717735052	0.085844953	0.507503846	down_in_case
CRA004775	ENSGALG00010001298	DOCK4	case	control	0.297610714	0.357494254	0.832490902	0.405131912	0.81327583	up_in_case
CRA004775	ENSGALG00010001300	MRPL49	case	control	0.030087713	0.225432924	0.133466365	0.893824563	0.977834811	up_in_case
CRA004775	ENSGALG00010001301	GMPS	case	control	-0.11608451	0.252779684	-0.459231961	0.646067601	0.914043203	down_in_case
CRA004775	ENSGALG00010001304	SYVN1	case	control	-0.301862836	0.21820368	-1.383399378	0.166542441	0.630194031	down_in_case
CRA004775	ENSGALG00010001305	WNT16	case	control	1.327408225	0.585663822	2.266502001	0.023420665	0.296827246	up_in_case
CRA004775	ENSGALG00010001306	CEP120	case	control	0.270180031	0.257249067	1.05026632	0.293595685	0.746438942	up_in_case
CRA004775	ENSGALG00010001307	CALU	case	control	-0.293677222	0.164259824	-1.787882236	0.073795009	0.479562937	down_in_case
CRA004775	ENSGALG00010001308	MGMT	case	control	-0.059445683	0.235701122	-0.252207892	0.800880378	0.953541667	down_in_case
CRA004775	ENSGALG00010001313	EMILIN2	case	control	0.600775586	0.349037126	1.721236912	0.085207842	0.506107944	up_in_case
CRA004775	ENSGALG00010001320	CPED1	case	control	0.936019098	0.448151745	2.088620894	0.036741863	0.361658808	up_in_case
CRA004775	ENSGALG00010001330	ENSGALG00010001330	case	control	-0.247949154	0.21200478	-1.169545114	0.242184076	0.709690398	down_in_case
CRA004775	ENSGALG00010001333	PLCH1	case	control	0.977193048	0.63871281	1.529941209	0.126031282	0.580487602	up_in_case
CRA004775	ENSGALG00010001334	EBF3	case	control	0.032955076	0.354634769	0.092926805	0.925961711	0.98417264	up_in_case
CRA004775	ENSGALG00010001343	ENSGALG00010001343	case	control	-0.223165793	0.572310164	-0.389938546	0.69658199	0.929092636	down_in_case
CRA004775	ENSGALG00010001347	MYOM1	case	control	0.533406465	0.673061731	0.792507493	0.428064824	0.822916175	up_in_case
CRA004775	ENSGALG00010001349	ING3	case	control	0.221689722	0.264127545	0.839328297	0.401285108	0.811868062	up_in_case
CRA004775	ENSGALG00010001351	CAPN1	case	control	-0.279259005	0.27532156	-1.014301257	0.310439024	0.762244121	down_in_case
CRA004775	ENSGALG00010001353	GLRX3	case	control	0.019864362	0.267780687	0.07418146	0.940865998	0.987377172	up_in_case
CRA004775	ENSGALG00010001363	HIBADH	case	control	0.165966116	0.359459189	0.461710595	0.644288872	0.913739303	up_in_case
CRA004775	ENSGALG00010001364	SMCHD1	case	control	0.314593434	0.297732254	1.056632023	0.29067956	0.74447127	up_in_case
CRA004775	ENSGALG00010001365	ENSGALG00010001365	case	control	-0.403203162	0.448765632	-0.898471568	0.368934196	0.794896739	down_in_case

CRA004775	ENSGALG00010001367	ENSGALG00010001367	case	control	-1.25152286	0.693520934	-1.804592767	0.071138431	0.47030027	down_in_case
CRA004775	ENSGALG00010001368	WIPF3	case	control	0.251506523	0.602876388	0.417177596	0.676548511	0.922924485	up_in_case
CRA004775	ENSGALG00010001369	PPP2R2D	case	control	0.511543525	0.213305003	2.398178752	0.016476822	0.254704773	up_in_case
CRA004775	ENSGALG00010001372	ENSGALG00010001372	case	control	-0.509712036	0.365505582	-1.394539676	0.163154697	0.629717526	down_in_case
CRA004775	ENSGALG00010001373	IMPA2	case	control	0.185085355	0.291478894	0.634987161	0.525436789	0.867484846	up_in_case
CRA004775	ENSGALG00010001378	POLA2	case	control	-0.661494482	0.28442821	-2.325699277	0.020034599	0.280454072	down_in_case
CRA004775	ENSGALG00010001380	SCRN1	case	control	0.394370624	0.323410411	1.219412272	0.222687754	0.690301918	up_in_case
CRA004775	ENSGALG00010001381	MDFIC	case	control	0.362217921	0.349053023	1.037716042	0.299402274	0.752512244	up_in_case
CRA004775	ENSGALG00010001382	BNIP3	case	control	-0.426514173	0.407280494	-1.047224652	0.294995978	0.747369418	down_in_case
CRA004775	ENSGALG00010001385	MME	case	control	-0.123630004	0.644449692	-0.191838099	0.847869026	0.968053841	down_in_case
CRA004775	ENSGALG00010001386	FKBP14	case	control	-0.291298555	0.358540282	-0.81245698	0.416529462	0.818943344	down_in_case
CRA004775	ENSGALG00010001388	MPPE1	case	control	-0.564976772	0.321389193	-1.757920877	0.078760973	0.491399489	down_in_case
CRA004775	ENSGALG00010001395	GNAL	case	control	-0.117687652	0.311563146	-0.377732904	0.705629022	0.931021366	down_in_case
CRA004775	ENSGALG00010001397	KLHL7	case	control	0.02693159	0.159401834	0.168954076	0.865832764	0.972629493	up_in_case
CRA004775	ENSGALG00010001398	WDR11	case	control	0.02006979	0.263841653	0.076067557	0.939365351	0.986950867	up_in_case
CRA004775	ENSGALG00010001400	SNX24	case	control	-0.035512741	0.471151456	-0.075374362	0.939916857	0.987076872	down_in_case
CRA004775	ENSGALG00010001402	NUP42	case	control	-0.370385395	0.215688659	-1.717222396	0.085938545	0.507567605	down_in_case
CRA004775	ENSGALG00010001406	EML3	case	control	-0.427822638	0.176985834	-2.41727051	0.015637391	0.250325687	down_in_case
CRA004775	ENSGALG00010001407	FOXP2	case	control	0.483771112	0.329806743	1.466832087	0.142421739	0.602473513	up_in_case
CRA004775	ENSGALG00010001409	GPNMB	case	control	6.145820226	0.549273374	11.18900081	4.61606E-29	2.11585E-25	up_in_case
CRA004775	ENSGALG00010001411	FGFR2	case	control	0.299514367	0.532596091	0.562366815	0.573866124	0.886897708	up_in_case
CRA004775	ENSGALG00010001412	ENSGALG00010001412	case	control	-0.932524421	0.568007804	-1.64174579	0.100642698	0.538583505	down_in_case
CRA004775	ENSGALG00010001414	CCDC126	case	control	-0.369400049	0.233166754	-1.584274095	0.113131358	0.562886277	down_in_case
CRA004775	ENSGALG00010001415	MC5R	case	control	0.571902136	0.548110878	1.043405921	0.296760333	0.749174102	up_in_case
CRA004775	ENSGALG00010001416	AP4M1	case	control	-0.308469704	0.249301254	-1.237337157	0.215961939	0.683774974	down_in_case
CRA004775	ENSGALG00010001418	RNMT	case	control	0.154667985	0.259387121	0.596282439	0.550986561	0.877837585	up_in_case
CRA004775	ENSGALG00010001422	AP1S1	case	control	-0.209960813	0.23986087	-0.875344166	0.38138667	0.801753951	down_in_case
CRA004775	ENSGALG00010001423	FAM210A	case	control	0.62734548	0.340133597	1.844409033	0.065123552	0.457949961	up_in_case
CRA004775	ENSGALG00010001426	ATE1	case	control	-0.035208919	0.176637691	-0.199328462	0.842005816	0.966478468	down_in_case
CRA004775	ENSGALG00010001427	ASB11	case	control	0.29655026	0.704461038	0.420960484	0.673783937	0.922518087	up_in_case
CRA004775	ENSGALG00010001429	ENSGALG00010001429	case	control	2.0385118	0.652259302	3.125308898	0.001776184	0.085287202	up_in_case
CRA004775	ENSGALG00010001432	NSMCE4A	case	control	0.106911534	0.238617098	0.448046408	0.654119705	0.916560183	up_in_case
CRA004775	ENSGALG00010001440	CDCA7L	case	control	0.156220492	0.317646375	0.491806311	0.622856275	0.906338268	up_in_case
CRA004775	ENSGALG00010001441	HMGCLL1	case	control	-0.140322525	0.446825724	-0.314043077	0.753488322	0.941900693	down_in_case
CRA004775	ENSGALG00010001442	LDLRAD4	case	control	-0.154892851	0.341636958	-0.453384354	0.650272001	0.915428088	down_in_case
CRA004775	ENSGALG00010001443	ANKRD26	case	control	0.443012969	0.322473178	1.37379788	0.169504439	0.633147466	up_in_case
CRA004775	ENSGALG00010001448	GPR37	case	control	0.027667499	0.736483839	0.037567014	0.970032908	0.99440305	up_in_case
CRA004775	ENSGALG00010001453	TAX1BP1	case	control	0.245481092	0.268521263	0.914196103	0.360613811	0.792014139	up_in_case
CRA004775	ENSGALG00010001454	VEGFD	case	control	-0.275752661	0.598477032	-0.4607573	0.64497274	0.914043203	down_in_case
CRA004775	ENSGALG00010001456	LSM8	case	control	-0.101505602	0.289258303	-0.350916812	0.725650759	0.935238044	down_in_case
CRA004775	ENSGALG00010001457	NDC80	case	control	0.74847337	0.707181873	1.058388795	0.289878226	0.74395768	up_in_case
CRA004775	ENSGALG00010001458	JAZF1	case	control	-0.425248803	0.393852005	-1.079717246	0.280268112	0.73661445	down_in_case
CRA004775	ENSGALG00010001460	MALSU1	case	control	-0.095365334	0.255972653	-0.372560635	0.709475475	0.931893902	down_in_case
CRA004775	ENSGALG00010001461	ASB9	case	control	0.058516683	0.408077091	0.143396147	0.885977326	0.976819452	up_in_case
CRA004775	ENSGALG00010001462	DHX36	case	control	-0.200615111	0.121123135	-1.656290608	0.097662982	0.532395151	down_in_case
CRA004775	ENSGALG00010001463	DSEL	case	control	0.338675063	0.35568317	0.952181862	0.341004758	0.780875343	up_in_case
CRA004775	ENSGALG00010001465	TACC2	case	control	0.403411153	0.325187101	1.240550905	0.214771698	0.683649824	up_in_case
CRA004775	ENSGALG00010001466	PRKX	case	control	0.010413658	0.463538754	0.02246556	0.982076584	0.997086172	up_in_case
CRA004775	ENSGALG00010001468	ANOS1	case	control	0.4827719	0.44222213	1.091695478	0.274966973	0.731001618	up_in_case
CRA004775	ENSGALG00010001469	ARHGEF26	case	control	0.208161412	0.384784715	0.540981499	0.588520335	0.893041616	up_in_case
CRA004775	ENSGALG00010001470	GPR85	case	control	1.103493267	0.717257578	1.538489519	0.123928969	0.576485043	up_in_case
CRA004775	ENSGALG00010001471	BMP5	case	control	-0.046442394	0.652967177	-0.071125159	0.943298145	0.987862092	down_in_case
CRA004775	ENSGALG00010001472	ENSGALG00010001472	case	control	0.273085931	0.472389432	0.57809492	0.563200036	0.881532201	up_in_case
CRA004775	ENSGALG00010001475	BMT2	case	control	0.344209388	0.249241951	1.381025091	0.167271246	0.631096819	up_in_case
CRA004775	ENSGALG00010001476	IGF2BP3	case	control	0.224496411	0.526129182	0.426694468	0.669601881	0.920861632	up_in_case
CRA004775	ENSGALG00010001477	NLGN4	case	control	0.61552898	0.564421597	1.090548242	0.275471715	0.731001618	up_in_case
CRA004775	ENSGALG00010001479	TMEM168	case	control	-0.20433323	0.268015019	-0.7623947	0.445824469	0.833292411	down_in_case
CRA004775	ENSGALG00010001482	TRA2A	case	control	-0.071787506	0.205167488	-0.349897086	0.726415934	0.935238044	down_in_case
CRA004775	ENSGALG00010001486	WNT2	case	control	1.064861031	0.739500572	1.439973235	0.149874972	0.61366707	up_in_case
CRA004775	ENSGALG00010001487	CDH19	case	control	1.196954028	0.570482413	2.098143605	0.035892462	0.359151015	up_in_case
CRA004775	ENSGALG00010001491	PHF3	case	control	0.414680224	0.280043091	1.480772911	0.138667097	0.598309149	up_in_case
CRA004775	ENSGALG00010001492	ENSGALG00010001492	case	control	-0.429684334	0.498027242	-0.862772751	0.388262424	0.804270424	down_in_case
CRA004775	ENSGALG00010001495	CCNL1	case	control	0.206944783	0.238722024	0.866886011	0.386004469	0.803610044	up_in_case
CRA004775	ENSGALG00010001496	PTP4A1	case	control	0.253909889	0.234293342	1.08372644	0.278486109	0.73417609	up_in_case
CRA004775	ENSGALG00010001499	TIPARP	case	control	0.851607939	0.531673746	1.60174909	0.109211105	0.556276325	up_in_case
CRA004775	ENSGALG00010001501	CAPZA2	case	control	0.403911464	0.332002738	1.216590761	0.22375996	0.691598833	up_in_case
CRA004775	ENSGALG00010001504	RBMS3	case	control	0.200822931	0.308728314	0.650484333	0.515379419	0.864897625	up_in_case
CRA004775	ENSGALG00010001505	SSR3	case	control	-0.615582173	0.262261288	-2.347209452	0.018914619	0.272278915	down_in_case
CRA004775	ENSGALG00010001506	DPYSL4	case	control	1.183015822	0.47526226	2.489185282	0.012803622	0.227866767	up_in_case
CRA004775	ENSGALG00010001507	METTL4	case	control	-0.422500566	0.384230934	-1.099600601	0.27150618	0.728910871	down_in_case
CRA004775	ENSGALG00010001508	CD109	case	control	0.003193165	0.698007934	0.004574683	0.996349944	0.999606847	up_in_case
CRA004775	ENSGALG00010001510	AP1S2	case	control	0.059048487	0.269764263	0.218889213	0.826736349	0.961878235	up_in_case
CRA004775	ENSGALG00010001512	PLCL2	case	control	0.272637046	0.25351615	1.075422794	0.282185481	0.738291085	up_in_case
CRA004775	ENSGALG00010001513	ZRSR2	case	control	0.281771503	0.185562852	1.51846935	0.128896121	0.583811118	up_in_case
CRA004775	ENSGALG00010001514	MET	case	control	1.182317389	0.38088397	3.104140581	0.001908326	0.08697473	up_in_case
CRA004775	ENSGALG00010001516	CLTRN	case	control	0.756852879	0.867862348	0.872088622	0.383160033	0.802000938	up_in_case
CRA004775	ENSGALG00010001517	CAV2	case	control	0.661176081	0.254650622	2.596404741	0.009420502	0.195977803	up_in_case
CRA004775	ENSGALG00010001518	SLC17A5	case	control	0.244147473	0.442501329	0.551744044	0.58112373	0.888998848	up_in_case
CRA004775	ENSGALG00010001520	KCNAB1	case	control	-0.207225921	0.415942491	-0.498208107	0.618337369	0.904143308	down_in_case
CRA004775	ENSGALG00010001521	TBC1D5	case	control	0.003418691	0.3205938	0.010663621	0.991491822	0.998065748	up_in_case
CRA004775	ENSGALG00010001524	ACE2	case	control	2.365939661	0.879555231	2.689927339	0.007146758	0.167991567	up_in_case
CRA004775	ENSGALG00010001525	EEF1A1	case	control	-0.350208791	0.186993871	-1.872835669	0.061091081	0.445419429	down_in_case
CRA004775	ENSGALG00010001526	PPP1R3A	case	control	0.681071815	0.679770912	1.001913737	0.316385257	0.765818284	up_in_case
CRA004775	ENSGALG00010001527	STK32C	case	control	0.211250822	0.371992135	0.567890561	0.570109281	0.88518436	up_in_case
CRA004775	ENSGALG00010001528	NR1D2	case	control	0.282203692	0.286786926	0.984018677	0.32510633	0.770251058	up_in_case
CRA004775	ENSGALG00010001531	ENSGALG00010001531	case	control	-0.711276481	1.348543446	-0.527440538	0.597887699	0.896490702	down_in_case
CRA004775	ENSGALG00010001532	ENSGALG00010001532	case	control	0.078644878	0.348396447	0.22573387	0.821408414	0.960766569	up_in_case
CRA004775	ENSGALG00010001533	ENSGALG00010001533	case	control	-0.294304986	0.340617888	-0.864032677	0.38756994	0.804084828	down_in_case
CRA004775	ENSGALG00010001534	SLITRK3	case	control	0.64805786	0.724102533	0.894980794	0.370797355	0.795641647	up_in_case
CRA004775	ENSGALG00010001535	RAP2B	case	control	-0.226198312	0.279646106	-0.808873456	0.418587939	0.818943344	down_in_case
CRA004775	ENSGALG00010001540	TRABD	case	control	-0.214073685	0.31288826	-0.684185738	0.493857887	0.856049389	down_in_case
CRA004775	ENSGALG00010001542	MTO1	case	control	0.018162466	0.329545871	0.05511362	0.956047946	0.990926004	up_in_case
CRA004775	ENSGALG00010001545	SELENOO	case	control	-0.25482784	0.302700024	-0.841849421	0.399872245	0.811607858	down_in_case
CRA004775	ENSGALG00010001546	CGAS	case	control	-0.491917083	0.533146097	-0.92266845	0.356180012	0.789127613	down_in_case
CRA004775	ENSGALG00010001547	PWWP2B	case	control	0.09851441	0.232874003	0.42303739	0.672267981	0.921952322	up_in_case
CRA004775	ENSGALG00010001548	AKAP17A	case	control	0.049776578	0.197281964	0.252311852	0.800800028	0.953541667	up_in_case
CRA004775	ENSGALG00010001551	FHIP1B	case	control	-0.354245698	0.395772016	-0.895075156	0.370746914	0.795641647	down_in_case
CRA004775	ENSGALG00010001552	TUBGCP6	case	control	0.02273629	0.225877471	0.100657626	0.91982225	0.982171593	up_in_case
CRA004775	ENSGALG00010001553	CNGA4	case	control	0.022809792	0.481480933	0.047374237	0.962214962	0.991860454	up_in_case
CRA004775	ENSGALG00010001554	ENSGALG00010001554	case	control	-0.447934171	0.30949427	-1.44731006	0.147810103	0.611105449	down_in_case
CRA004775	ENSGALG00010001556	EPB41L3	case	control	0.501656746	0.311917397	1.608299991	0.107769493	0.55406723	up_in_case
CRA004775	ENSGALG00010001558	CCKBR	case	control	-0.083628044	0.45479091	-0.183882401	0.854105711	0.970073177	down_in_case
CRA004775	ENSGALG00010001559	HDAC10	case	control	0.078435381	0.320812304	0.244489942	0.806851391	0.955397873	up_in_case
CRA004775	ENSGALG00010001560	ENSGALG00010001560	case	control	-1.334354224	0.687747628	-1.940180045	0.052357813	0.420544558	down_in_case
CRA004775	ENSGALG00010001567	INPP5A	case	control	0.157713168	0.228604306	0.689895875	0.49025967	0.855184934	up_in_case
CRA004775	ENSGALG00010001570	SMPD1	case	control	-0.130827509	0.366833794	-0.356639742	0.721361522	0.934511903	down_in_case
CRA004775	ENSGALG00010001585	ENSGALG00010001585	case	control	0.23378669	0.201966322	1.157552844	0.247046563	0.712046495	up_in_case
CRA004775	ENSGALG00010001586	ZBTB14	case	control	0.308660806	0.204808187	1.507072598	0.13179205	0.587352231	up_in_case
CRA004775	ENSGALG00010001588	ENSGALG00010001588	case	control	0.096805418	0.761571621	0.127112691	0.898851207	0.978790377	up_in_case
CRA004775	ENSGALG00010001595	AKAIN1	case	control	1.162153039	0.546767239	2.12549867	0.033545025	0.348565623	up_in_case
CRA004775	ENSGALG00010001599	TBLX1	case	control	0.070382616	0.167883155	0.419235724	0.675043866	0.92288678	up_in_case
CRA004775	ENSGALG00010001604	ENSGALG00010001604	case	control	-0.557978078	0.64458888	-0.865634043	0.386690881	0.803905475	down_in_case
CRA004775	ENSGALG00010001605	APBB1	case	control	-0.233472077	0.285778411	-0.81696891	0.413946196	0.817589409	down_in_case
CRA004775	ENSGALG00010001606	PIGA	case	control	0.114257174	0.314187784	0.36365887	0.716112761	0.933921336	up_in_case
CRA004775	ENSGALG00010001609	ENSGALG00010001609	case	control	-0.601896846	0.972956517	-0.618626665	0.536162331	0.871588629	down_in_case
CRA004775	ENSGALG00010001611	MAPK12	case	control	-0.443105613	0.379017083	-1.169091403	0.242366807	0.709690398	down_in_case
CRA004775	ENSGALG00010001612	HPX	case	control	0.218485971	0.641945338	0.340349805	0.733593114	0.936819725	up_in_case
CRA004775	ENSGALG00010001617	TRIM3	case	control	-0.112520055	0.224269878	-0.5017172	0.615866464	0.9028981	down_in_case
CRA004775	ENSGALG00010001620	MOSPD2	case	control	-0.018379161	0.323180671	-0.056869616	0.954649058	0.990670511	down_in_case
CRA004775	ENSGALG00010001627	ARFIP2	case	control	-0.030691329	0.176618867	-0.173771521	0.862045027	0.97171745	down_in_case
CRA004775	ENSGALG00010001628	MAPK11	case	control	-0.033031308	0.344701494	-0.095825834	0.9236589	0.983448202	down_in_case
CRA004775	ENSGALG00010001632	FANCB	case	control	-1.156255273	0.679652739	-1.701244188	0.088897143	0.515249396	down_in_case
CRA004775	ENSGALG00010001633	B3GAT2	case	control	0.595119645	0.589796179	1.009025943	0.312962193	0.764938342	up_in_case
CRA004775	ENSGALG00010001635	TIMM10B	case	control	-0.426232689	0.268197856	-1.589247186	0.112004597	0.56149297	down_in_case
CRA004775	ENSGALG00010001639	ENSGALG00010001639	case	control	0.258763626	0.487039511	0.531299043	0.595211568	0.895305942	up_in_case
CRA004775	ENSGALG00010001642	ENSGALG00010001642	case	control	0.639995162	0.362976642	1.763185526	0.077869204	0.49028362	up_in_case

CRA004775	ENSGALG00010001647	ARL8BL	case	control	-0.202217785	0.255586078	-0.791192489	0.428831674	0.823726199	down_in_case
CRA004775	ENSGALG00010001649	UBE2E2	case	control	-0.197525884	0.475328959	-0.415556174	0.677734802	0.923000026	down_in_case
CRA004775	ENSGALG00010001654	RPS11	case	control	-0.360330643	0.24773444	-1.454503635	0.145806726	0.609044185	down_in_case
CRA004775	ENSGALG00010001657	SMAP1	case	control	-0.018803614	0.202788133	-0.092725418	0.926121704	0.98417264	down_in_case
CRA004775	ENSGALG00010001661	TCTN3	case	control	-0.451976343	0.277048319	-1.631398971	0.102806162	0.543350227	down_in_case
CRA004775	ENSGALG00010001666	PIEZO2	case	control	-0.13982106	0.475530392	-0.294031806	0.768733599	0.945220496	down_in_case
CRA004775	ENSGALG00010001668	UBE2E1	case	control	-0.511857365	0.203149861	-2.5196048	0.011748666	0.217806153	down_in_case
CRA004775	ENSGALG00010001670	SHOX	case	control	0.544317547	0.496364474	1.096608591	0.27281253	0.731001618	up_in_case
CRA004775	ENSGALG00010001672	ENSGALG00010001672	case	control	0.72918168	0.720209785	1.012457335	0.311319436	0.763050512	up_in_case
CRA004775	ENSGALG00010001674	ENSGALG00010001674	case	control	-0.113427941	0.206864675	-0.548319527	0.583472522	0.889906855	down_in_case
CRA004775	ENSGALG00010001677	SDHAF4	case	control	-0.178222783	0.304765219	-0.584787146	0.558690848	0.880754991	down_in_case
CRA004775	ENSGALG00010001678	SYAP1	case	control	0.152336586	0.185735011	0.820182397	0.412112136	0.817006008	up_in_case
CRA004775	ENSGALG00010001681	NAPG	case	control	-0.00839719	0.176943901	-0.047456793	0.962149165	0.991860454	down_in_case
CRA004775	ENSGALG00010001686	WASL	case	control	0.113118586	0.20129975	0.561941015	0.574156208	0.886904293	up_in_case
CRA004775	ENSGALG00010001688	ENSGALG00010001688	case	control	0.035195079	0.20822288	0.169025991	0.865776198	0.972629493	up_in_case
CRA004775	ENSGALG00010001689	ILK	case	control	-0.184715024	0.121397509	-1.521571786	0.128116411	0.5821156	down_in_case
CRA004775	ENSGALG00010001691	CTPS2	case	control	0.051269877	0.245277423	0.209028113	0.834426292	0.964202472	up_in_case
CRA004775	ENSGALG00010001692	ALDH18A1	case	control	-0.791877802	0.461234078	-1.716867507	0.086003383	0.507567605	down_in_case
CRA004775	ENSGALG00010001693	NKIRAS1	case	control	-0.213091423	0.538813542	-0.395482679	0.692486702	0.927867527	down_in_case
CRA004775	ENSGALG00010001694	ENSGALG00010001694	case	control	0.180435114	0.242486695	0.744103151	0.456814073	0.839688534	up_in_case
CRA004775	ENSGALG00010001695	LMOD2	case	control	-1.199718131	0.95978531	-1.249985927	0.211304688	0.681597647	down_in_case
CRA004775	ENSGALG00010001696	FAM135A	case	control	0.273737443	0.265852183	1.029660319	0.303169489	0.755679718	up_in_case
CRA004775	ENSGALG00010001697	TAF10	case	control	-0.318064605	0.274122116	-1.160302605	0.245925624	0.712046495	down_in_case
CRA004775	ENSGALG00010001698	RPL15	case	control	-0.397764564	0.27438802	-1.449642608	0.147158208	0.610295079	down_in_case
CRA004775	ENSGALG00010001699	ASB15	case	control	0.344206318	0.668989527	0.514516751	0.606890752	0.900898526	up_in_case
CRA004775	ENSGALG00010001700	ENSGALG00010001700	case	control	0.735831064	0.442169064	1.664139632	0.096084529	0.529546782	up_in_case
CRA004775	ENSGALG00010001701	NDUFA5	case	control	-0.118509657	0.378131453	-0.313408621	0.753970236	0.941900693	down_in_case
CRA004775	ENSGALG00010001702	PLEKHA1	case	control	0.245340385	0.156234892	1.570330302	0.116338288	0.566086328	up_in_case
CRA004775	ENSGALG00010001704	ENSGALG00010001704	case	control	-0.322644555	0.426024604	-0.757337845	0.448847483	0.834536953	down_in_case
CRA004775	ENSGALG00010001705	HTRA1	case	control	0.531138707	0.253187717	2.097805979	0.035922288	0.359151015	up_in_case
CRA004775	ENSGALG00010001706	IQUB	case	control	0.588058661	0.450951376	1.304040064	0.192219923	0.656959697	up_in_case
CRA004775	ENSGALG00010001707	ENSGALG00010001707	case	control	2.675398946	1.140578382	2.345651109	0.018993878	0.272278915	up_in_case
CRA004775	ENSGALG00010001708	AADAC	case	control	-0.24325935	0.351609048	-0.691846104	0.48903398	0.854256384	down_in_case
CRA004775	ENSGALG00010001710	LCMT2	case	control	-0.163780882	0.277182042	-0.590878401	0.554601899	0.879521649	down_in_case
CRA004775	ENSGALG00010001711	CREB5	case	control	1.272197902	0.555136778	2.291683693	0.021923902	0.289880363	up_in_case
CRA004775	ENSGALG00010001712	ATP6AP1	case	control	0.27437975	0.337893564	0.812030116	0.416774351	0.818943344	up_in_case
CRA004775	ENSGALG00010001713	PNPLA4	case	control	-0.075417593	0.381334246	-0.197772935	0.843222723	0.966478468	down_in_case
CRA004775	ENSGALG00010001716	TRIL	case	control	0.67446477	0.515156799	1.309241714	0.190452486	0.656023968	up_in_case
CRA004775	ENSGALG00010001717	STARD10	case	control	-0.284297087	0.594806331	-0.477965806	0.632674543	0.909651573	down_in_case
CRA004775	ENSGALG00010001721	TM4SF1	case	control	-0.37582418	0.360648924	-1.042077641	0.297375684	0.749916187	down_in_case
CRA004775	ENSGALG00010001725	MTA2	case	control	-0.341011639	0.17678778	-1.928932188	0.053739281	0.426902863	down_in_case
CRA004775	ENSGALG00010001727	COL9A1	case	control	-4.085348903	1.428304928	-2.860277818	0.0042327	0.133620227	down_in_case
CRA004775	ENSGALG00010001728	STS	case	control	0.253217564	0.394055712	0.642593309	0.520488026	0.865611895	up_in_case
CRA004775	ENSGALG00010001729	ENSGALG00010001729	case	control	0.504077692	0.297708083	1.69319451	0.090418449	0.518924915	up_in_case
CRA004775	ENSGALG00010001731	ENSGALG00010001731	case	control	-0.276039706	0.375460913	-0.735202244	0.462216333	0.842230074	down_in_case
CRA004775	ENSGALG00010001737	ENSGALG00010001737	case	control	0.279936409	0.82253574	0.34033343	0.733605445	0.936819725	up_in_case
CRA004775	ENSGALG00010001738	PUDP	case	control	-0.127125909	0.210926655	-0.602701963	0.546706975	0.877099134	down_in_case
CRA004775	ENSGALG00010001741	SATB1	case	control	0.462325453	0.276584894	1.671549905	0.09461311	0.526933666	up_in_case
CRA004775	ENSGALG00010001744	ENSGALG00010001744	case	control	-1.038543357	0.914867167	-1.135184859	0.256297872	0.719236059	down_in_case
CRA004775	ENSGALG00010001745	CADPS2	case	control	0.2311928	0.39828027	0.580477662	0.561592547	0.881532201	up_in_case
CRA004775	ENSGALG00010001749	VAPA	case	control	0.240813519	0.245707688	0.980081335	0.327045972	0.770998457	up_in_case
CRA004775	ENSGALG00010001752	ARRB1	case	control	-0.335412179	0.240228256	-1.396222843	0.1626474	0.629132038	down_in_case
CRA004775	ENSGALG00010001753	PNPLA8	case	control	0.357105622	0.477433398	0.747969504	0.454478552	0.838254622	up_in_case
CRA004775	ENSGALG00010001754	APCDD1	case	control	-0.92770587	0.639508127	-1.450655326	0.146875861	0.610295079	down_in_case
CRA004775	ENSGALG00010001755	CUZD1	case	control	2.523402258	0.880205603	2.86683276	0.00414602	0.131838709	up_in_case
CRA004775	ENSGALG00010001756	ENSGALG00010001756	case	control	-0.063289764	0.445764586	-0.141980243	0.887095611	0.977118922	down_in_case
CRA004775	ENSGALG00010001758	COL19A1	case	control	-1.897312585	0.808240053	-2.347461722	0.018901815	0.272278915	down_in_case
CRA004775	ENSGALG00010001759	C10orf88	case	control	0.085890739	0.256867672	0.334377381	0.738094806	0.937863632	up_in_case
CRA004775	ENSGALG00010001760	RAB5A	case	control	-0.333661856	0.309111958	-1.079420734	0.280400212	0.736726042	down_in_case
CRA004775	ENSGALG00010001761	ELOB	case	control	0.062116246	0.307943308	0.201713253	0.840140901	0.966461921	up_in_case
CRA004775	ENSGALG00010001763	ENSGALG00010001763	case	control	0.372299894	0.353211864	1.054041306	0.291864006	0.746127895	up_in_case
CRA004775	ENSGALG00010001764	ENSGALG00010001764	case	control	0.716128556	0.478645721	1.496155767	0.134613068	0.592420453	up_in_case
CRA004775	ENSGALG00010001766	ENSGALG00010001766	case	control	-0.815903455	0.638559052	-1.277725925	0.201346081	0.669255489	down_in_case
CRA004775	ENSGALG00010001767	ENSGALG00010001767	case	control	-0.124018156	0.37286066	-0.332612608	0.739426725	0.938064864	down_in_case
CRA004775	ENSGALG00010001771	ENSGALG00010001771	case	control	-0.292634276	0.262640542	-1.114200705	0.265193093	0.726330188	down_in_case
CRA004775	ENSGALG00010001773	NRCAM	case	control	2.000741309	0.689529792	2.901602412	0.003712594	0.12600076	up_in_case
CRA004775	ENSGALG00010001776	LMBRD1	case	control	-0.141276	0.345952011	-0.408368779	0.683002951	0.924861997	down_in_case
CRA004775	ENSGALG00010001778	KAT2B	case	control	0.249398248	0.256060501	0.973981724	0.330065614	0.77237154	up_in_case
CRA004775	ENSGALG00010001780	ENSGALG00010001780	case	control	0.241309683	0.627176916	0.384755366	0.700418671	0.929842957	up_in_case
CRA004775	ENSGALG00010001783	SGO1	case	control	0.402013775	0.617458706	0.651077993	0.514996142	0.864897625	up_in_case
CRA004775	ENSGALG00010001785	CNTN1	case	control	-0.230799978	0.527391259	-0.437625717	0.661657626	0.918136475	down_in_case
CRA004775	ENSGALG00010001786	P2RY8	case	control	-0.738154462	0.663239613	-1.112952918	0.265728651	0.726742745	down_in_case
CRA004775	ENSGALG00010001788	HOXA1	case	control	-0.202539039	0.43338982	-0.467336863	0.640258886	0.911610834	down_in_case
CRA004775	ENSGALG00010001790	HOXA2	case	control	0.393271145	0.288045366	1.365309746	0.172155717	0.636545649	up_in_case
CRA004775	ENSGALG00010001793	IKZF5	case	control	0.39619342	0.212365113	1.865623853	0.06209401	0.447747631	up_in_case
CRA004775	ENSGALG00010001795	ENSGALG00010001795	case	control	-0.400929369	0.544919973	-0.735758257	0.461877829	0.842010079	down_in_case
CRA004775	ENSGALG00010001796	SLCO2B1	case	control	-0.239346645	0.353703341	-0.676687543	0.498604222	0.858325821	down_in_case
CRA004775	ENSGALG00010001798	PSTK	case	control	0.338306923	0.434031608	0.779452273	0.435713342	0.826905159	up_in_case
CRA004775	ENSGALG00010001800	ENSGALG00010001800	case	control	-0.528670575	0.341407981	-1.548500926	0.121501739	0.573556613	down_in_case
CRA004775	ENSGALG00010001801	HOXA3	case	control	0.151658092	0.284007682	0.533992923	0.59334643	0.894450257	up_in_case
CRA004775	ENSGALG00010001803	PPP4R1	case	control	0.089727939	0.211283799	0.424679695	0.671070188	0.921535334	up_in_case
CRA004775	ENSGALG00010001804	ENSGALG00010001804	case	control	0.996445403	0.558955354	1.782692293	0.074636407	0.481462352	up_in_case
CRA004775	ENSGALG00010001805	ENSGALG00010001805	case	control	-0.526837012	0.367021308	-1.435439852	0.151161797	0.615293187	down_in_case
CRA004775	ENSGALG00010001806	ENSGALG00010001806	case	control	0.768619746	0.451372333	1.702850818	0.08859599	0.514477812	up_in_case
CRA004775	ENSGALG00010001807	ASMTL	case	control	0.020461527	0.288861766	0.070835014	0.943529065	0.987862092	up_in_case
CRA004775	ENSGALG00010001808	HOXA4	case	control	0.377230436	0.27000146	1.397142209	0.162370811	0.628415711	up_in_case
CRA004775	ENSGALG00010001810	OPN2SW	case	control	-1.085227956	0.684548549	-1.585319196	0.11289383	0.562886277	down_in_case
CRA004775	ENSGALG00010001811	ACADSB	case	control	0.469475496	0.285136348	1.646494738	0.099661943	0.537098329	up_in_case
CRA004775	ENSGALG00010001813	HOXA5	case	control	0.377621913	0.341547733	1.105619733	0.268891145	0.727586876	up_in_case
CRA004775	ENSGALG00010001817	SLC25A6	case	control	-0.173512972	0.316363957	-0.548459987	0.583376097	0.889906855	down_in_case
CRA004775	ENSGALG00010001819	HOXA6	case	control	0.02070526	0.365897454	0.056587604	0.954873709	0.990670511	up_in_case
CRA004775	ENSGALG00010001820	PGM2L1	case	control	-0.009073385	0.372108743	-0.024383692	0.980546556	0.996980737	down_in_case
CRA004775	ENSGALG00010001822	KCNE3	case	control	0.340209925	0.398444911	0.853844322	0.393191253	0.80770435	up_in_case
CRA004775	ENSGALG00010001823	RALBP1	case	control	0.253892308	0.16360069	1.551902425	0.120685573	0.572850296	up_in_case
CRA004775	ENSGALG00010001825	LIPT2	case	control	-0.242724591	0.467389158	-0.519320115	0.603537529	0.898692785	down_in_case
CRA004775	ENSGALG00010001826	HOXA7	case	control	-0.006186462	0.302405054	-0.020457536	0.983678386	0.99751066	down_in_case
CRA004775	ENSGALG00010001827	PDZRN4	case	control	0.468910806	0.580056306	0.808388429	0.418867011	0.818943344	up_in_case
CRA004775	ENSGALG00010001829	POLD3	case	control	-0.184864133	0.158445933	-1.166733214	0.243318125	0.710409979	down_in_case
CRA004775	ENSGALG00010001831	CSF2RA	case	control	-0.938505488	0.600595134	-1.56262586	0.118140622	0.569418751	down_in_case
CRA004775	ENSGALG00010001832	BUB3	case	control	-0.372514268	0.18776034	-1.983988034	0.047257174	0.403874082	down_in_case
CRA004775	ENSGALG00010001834	ZCRB1	case	control	0.126578299	0.235635773	0.53717777	0.591144825	0.893804699	up_in_case
CRA004775	ENSGALG00010001835	MECP2	case	control	-0.381360433	0.232212267	-1.642292363	0.100529429	0.538583505	down_in_case
CRA004775	ENSGALG00010001836	TWSG1	case	control	0.1667838	0.243148688	0.685933374	0.492755123	0.855763086	up_in_case
CRA004775	ENSGALG00010001837	TAB3	case	control	0.605554885	0.286511929	2.113541615	0.034554437	0.353803473	up_in_case
CRA004775	ENSGALG00010001838	PPHLN1	case	control	0.0501509	0.27044321	0.18543967	0.852884196	0.969725992	up_in_case
CRA004775	ENSGALG00010001839	ENSGALG00010001839	case	control	-0.164902754	0.333374388	-0.494647339	0.62084908	0.9054296	down_in_case
CRA004775	ENSGALG00010001840	HOXA9	case	control	0.223953855	0.359131143	0.623599092	0.532890907	0.869976453	up_in_case
CRA004775	ENSGALG00010001843	PRICKLE1	case	control	0.220786777	0.291015285	0.758677598	0.448045441	0.83429564	up_in_case
CRA004775	ENSGALG00010001844	HOXA10	case	control	0.521313273	0.252312784	2.066138957	0.038815361	0.370839453	up_in_case
CRA004775	ENSGALG00010001845	ANKRD12	case	control	0.758423194	0.279031794	2.71805296	0.006566733	0.160389237	up_in_case
CRA004775	ENSGALG00010001848	HOXA11	case	control	-0.568556377	0.323899588	-1.755347641	0.079199862	0.491399489	down_in_case
CRA004775	ENSGALG00010001849	NDUFV2	case	control	0.028379307	0.321350338	0.088312671	0.929628168	0.98473447	up_in_case
CRA004775	ENSGALG00010001850	MYOM2	case	control	0.474687972	0.714618036	0.664254116	0.506527683	0.8622397	up_in_case
CRA004775	ENSGALG00010001852	CPXM2	case	control	0.514600793	0.437105249	1.177292642	0.239078723	0.705941919	up_in_case
CRA004775	ENSGALG00010001853	ENSGALG00010001853	case	control	-0.539921496	0.288306659	-1.872733354	0.061105215	0.445419429	down_in_case
CRA004775	ENSGALG00010001854	CHRDL2	case	control	0.724177044	0.490877738	1.475269682	0.140140054	0.600007375	up_in_case
CRA004775	ENSGALG00010001856	CHST15	case	control	0.155196454	0.305766947	0.50756452	0.611758778	0.901413878	up_in_case
CRA004775	ENSGALG00010001857	HNRNPA2B1	case	control	-0.047153243	0.220914774	-0.213445404	0.830979569	0.963284108	down_in_case
CRA004775	ENSGALG00010001858	ADAMTS20	case	control	0.584402595	0.631805434	0.924972409	0.35498026	0.788279103	up_in_case
CRA004775	ENSGALG00010001860	RNF169	case	control	-0.373365053	0.295862932	-1.261952791	0.206965774	0.675685272	down_in_case
CRA004775	ENSGALG00010001861	CBX3	case	control	0.016938958	0.13917036	0.121713836	0.903125655	0.979310476	up_in_case
CRA004775	ENSGALG00010001864	ENSGALG00010001864	case	control	-0.262792626	0.287412731	-0.914338851	0.360538822	0.791975934	down_in_case
CRA004775	ENSGALG00010001865	CYBB	case	control	-0.417607279	0.824537525	-0.506474558	0.612523545	0.901413878	down_in_case
CRA004775	ENSGALG00010001866	PUS7L	case	control	0.292379804	0.24554717	1.190727647	0.233760521	0.701077628	up_in_case
CRA004775	ENSGALG00010001868	ENSGALG00010001868	case	control	-0.244598492	0.241034386	-1.01478671	0.31020751	0.762244121	down_in_case
CRA004775	ENSGALG00010001870	PLP2	case	control	-0.280454086	0.295516639	-0.949029764	0.342605474	0.782196227	down_in_case

CRA004775	ENSGALG00010001871	ENSGALG00010001871	case	control	-0.374560682	0.278182992	-1.346454285	0.178156054	0.644368088	down_in_case
CRA004775	ENSGALG00010001873	ENSGALG00010001873	case	control	-0.572329023	0.29089271	-1.967491808	0.049126539	0.410291029	down_in_case
CRA004775	ENSGALG00010001877	OAT	case	control	-0.246647491	0.177679043	-1.388163098	0.165087386	0.630194031	down_in_case
CRA004775	ENSGALG00010001878	XK	case	control	-0.004916505	0.494752505	-0.009937302	0.992071311	0.998065748	down_in_case
CRA004775	ENSGALG00010001879	IRAK4	case	control	-0.246593558	0.281848432	-0.87491549	0.38161989	0.801753951	down_in_case
CRA004775	ENSGALG00010001880	MTCL1	case	control	0.523215941	0.467623829	1.118882121	0.263190432	0.723981064	up_in_case
CRA004775	ENSGALG00010001883	KBTBD11	case	control	0.28509347	0.319278863	0.892929356	0.371895005	0.795818272	up_in_case
CRA004775	ENSGALG00010001885	SMPX	case	control	-0.748954639	0.663663421	-1.128515774	0.259102159	0.720037471	down_in_case
CRA004775	ENSGALG00010001886	NEU3	case	control	-0.593389401	0.195208119	-3.039778287	0.002367524	0.098653993	down_in_case
CRA004775	ENSGALG00010001888	SNX10	case	control	-0.3064432	0.249669491	-1.227395461	0.219673991	0.687155836	down_in_case
CRA004775	ENSGALG00010001890	KLHL34	case	control	0.608378262	0.644808524	0.943502202	0.34542409	0.784552667	up_in_case
CRA004775	ENSGALG00010001892	RAB12	case	control	0.430930593	0.290118486	1.485360685	0.137448304	0.596242097	up_in_case
CRA004775	ENSGALG00010001895	P4HA3	case	control	-0.614643228	0.553035817	-1.111398592	0.266396818	0.727311193	down_in_case
CRA004775	ENSGALG00010001896	ARHGEF10	case	control	0.194857473	0.339615352	0.573759319	0.566130681	0.883033678	up_in_case
CRA004775	ENSGALG00010001897	TLR7	case	control	-0.892317309	0.76432054	-1.167464777	0.243022725	0.709904257	down_in_case
CRA004775	ENSGALG00010001898	LHPP	case	control	-0.591239074	0.293671446	-2.013267147	0.044086533	0.392535833	down_in_case
CRA004775	ENSGALG00010001899	TWF1	case	control	0.266336583	0.259724805	1.02545686	0.305147671	0.757826553	up_in_case
CRA004775	ENSGALG00010001901	PRPS2	case	control	0.918804211	0.48162132	1.907731599	0.056425915	0.434685021	up_in_case
CRA004775	ENSGALG00010001902	WDR45	case	control	-0.473312664	0.274472767	-1.724443081	0.084627886	0.504429155	down_in_case
CRA004775	ENSGALG00010001903	NARS2	case	control	-0.238372327	0.172983696	-1.378004594	0.16820187	0.63138062	down_in_case
CRA004775	ENSGALG00010001906	SKAP2	case	control	-0.238232372	0.347703318	-0.685159904	0.493243022	0.856001758	down_in_case
CRA004775	ENSGALG00010001910	FAM53B	case	control	-0.08456732	0.324094075	-0.260934483	0.794143032	0.951843306	down_in_case
CRA004775	ENSGALG00010001911	USP35	case	control	-1.513457173	0.520426253	-2.908110736	0.003636196	0.12600076	down_in_case
CRA004775	ENSGALG00010001912	TMEM117	case	control	0.648807661	0.418949889	1.548652186	0.121465354	0.573556613	up_in_case
CRA004775	ENSGALG00010001914	ENSGALG00010001914	case	control	-0.007704535	0.370856661	-0.020774968	0.983425166	0.997424359	down_in_case
CRA004775	ENSGALG00010001915	EEF1AKMT2	case	control	0.50774542	0.254596141	1.99431703	0.046117419	0.399541954	up_in_case
CRA004775	ENSGALG00010001918	CNKSR2	case	control	0.667869407	0.381986846	1.748409437	0.080393155	0.494445942	up_in_case
CRA004775	ENSGALG00010001921	ENSGALG00010001921	case	control	1.23828628	1.183557405	1.046240997	0.295449779	0.747649966	up_in_case
CRA004775	ENSGALG00010001923	RAPGEF5	case	control	0.819953365	0.324820284	2.524329312	0.011591928	0.216871572	up_in_case
CRA004775	ENSGALG00010001928	KCTD21	case	control	-0.228527333	0.430222893	-0.531183571	0.595291577	0.895305942	down_in_case
CRA004775	ENSGALG00010001929	POLR2I	case	control	-0.231405679	0.284740508	-0.812689702	0.416395987	0.818943344	down_in_case
CRA004775	ENSGALG00010001935	ABRAXAS2	case	control	0.20832882	0.270482882	0.770210737	0.441174896	0.829353567	up_in_case
CRA004775	ENSGALG00010001938	ALG8	case	control	-0.63012057	0.280404852	-2.24718141	0.024628436	0.302160964	down_in_case
CRA004775	ENSGALG00010001940	PTPRM	case	control	0.109558462	0.266347281	0.411336887	0.680825525	0.923896221	up_in_case
CRA004775	ENSGALG00010001941	IL6	case	control	-0.325535694	0.85689506	-0.379901472	0.704018555	0.930465147	down_in_case
CRA004775	ENSGALG00010001947	TINAG	case	control	-0.356257616	0.64780643	-0.54994455	0.582357407	0.889579727	down_in_case
CRA004775	ENSGALG00010001948	EMX2	case	control	0.419686831	0.635831898	0.660059415	0.509215702	0.862971569	up_in_case
CRA004775	ENSGALG00010001952	ENSGALG00010001952	case	control	-0.139652152	0.189199137	-0.738122564	0.460439973	0.841265006	down_in_case
CRA004775	ENSGALG00010001957	NDUFC2	case	control	-0.039679109	0.393198535	-0.100913674	0.919618988	0.982170916	down_in_case
CRA004775	ENSGALG00010001958	RAB11FIP2	case	control	0.123311242	0.362456492	0.340209776	0.733698556	0.936819725	up_in_case
CRA004775	ENSGALG00010001961	ENSGALG00010001961	case	control	-1.17473024	0.782543609	-1.501169042	0.133311845	0.590393295	down_in_case
CRA004775	ENSGALG00010001966	ARHGAP6	case	control	0.028856291	0.299876304	0.096227313	0.923340039	0.983337118	up_in_case
CRA004775	ENSGALG00010001967	LAMA1	case	control	0.822239946	0.568759588	1.445672236	0.148269155	0.611192154	up_in_case
CRA004775	ENSGALG00010001969	PLEKHA8	case	control	-0.737021832	0.675579597	-1.09094744	0.275296011	0.731001618	down_in_case
CRA004775	ENSGALG00010001971	MLIP	case	control	0.299881789	0.585122964	0.512510716	0.608293623	0.900943922	up_in_case
CRA004775	ENSGALG00010001972	FAM204A	case	control	-0.028409682	0.253590824	-0.112029615	0.910799925	0.98075825	down_in_case
CRA004775	ENSGALG00010001974	ENSGALG00010001974	case	control	-0.750171105	0.659424799	-1.137614336	0.255281558	0.718871765	down_in_case
CRA004775	ENSGALG00010001976	THRSP	case	control	-1.802018346	0.683258518	-2.637388776	0.008354702	0.18329931	down_in_case
CRA004775	ENSGALG00010001982	DMD	case	control	0.840899529	0.461822285	1.820829259	0.068632815	0.46645716	up_in_case
CRA004775	ENSGALG00010001987	KCTD14	case	control	0.077188717	0.58008848	0.133063696	0.894143006	0.977834811	up_in_case
CRA004775	ENSGALG00010001991	TOMM7	case	control	0.060316913	0.332895522	0.181188719	0.856219449	0.970720887	up_in_case
CRA004775	ENSGALG00010001995	LRRC1	case	control	-0.027544378	0.490580466	-0.056146504	0.955225097	0.990670511	down_in_case
CRA004775	ENSGALG00010001997	INTS4	case	control	-0.236333998	0.214385906	-1.102376562	0.270297997	0.728665115	down_in_case
CRA004775	ENSGALG00010002005	COLQ	case	control	-0.32964916	0.553354175	-0.595729056	0.551356244	0.878019654	down_in_case
CRA004775	ENSGALG00010002006	ENSGALG00010002006	case	control	1.271041279	0.782406416	1.62452819	0.104263129	0.546673752	up_in_case
CRA004775	ENSGALG00010002012	MID1	case	control	-0.360919905	0.543738755	-0.663774473	0.506834666	0.8622397	down_in_case
CRA004775	ENSGALG00010002017	ARHGAP28	case	control	-0.034354376	0.23012797	-0.149283792	0.881329704	0.975011509	down_in_case
CRA004775	ENSGALG00010002023	CLCN4	case	control	-1.205868631	0.495682027	-2.432746327	0.014984795	0.244783262	down_in_case
CRA004775	ENSGALG00010002024	KLHL31	case	control	1.071939058	0.641578816	1.670783124	0.094764523	0.526933666	up_in_case
CRA004775	ENSGALG00010002027	ENSGALG00010002027	case	control	0.03698195	0.614265881	0.060205118	0.951992269	0.990168609	up_in_case
CRA004775	ENSGALG00010002034	WWC3	case	control	0.147355337	0.292998847	0.502921218	0.615019662	0.902639483	up_in_case
CRA004775	ENSGALG00010002039	AAMDC	case	control	0.13633679	0.37950122	0.359252574	0.719406149	0.93407508	up_in_case
CRA004775	ENSGALG00010002041	GCLC	case	control	0.359535781	0.359936792	0.998885885	0.317849975	0.766440025	up_in_case
CRA004775	ENSGALG00010002046	FAM126A	case	control	-0.328823155	0.39200404	-0.838825935	0.401566995	0.811868062	down_in_case
CRA004775	ENSGALG00010002050	ENSGALG00010002050	case	control	1.176827823	0.524801249	2.242425728	0.024933871	0.303958913	up_in_case
CRA004775	ENSGALG00010002054	RSF1	case	control	0.186796239	0.283259606	0.659452442	0.509605276	0.862971569	up_in_case
CRA004775	ENSGALG00010002062	GSTA4	case	control	0.344494629	0.576596374	0.597462358	0.550198731	0.877623839	up_in_case
CRA004775	ENSGALG00010002065	CLNS1A	case	control	-0.022314191	0.215299376	-0.103642617	0.917452966	0.981688766	down_in_case
CRA004775	ENSGALG00010002072	NEURL4	case	control	-0.052299166	0.199384027	-0.262303689	0.793087318	0.951455592	down_in_case
CRA004775	ENSGALG00010002079	ARX	case	control	-0.043488632	0.633391034	-0.068660006	0.945260253	0.988711172	down_in_case
CRA004775	ENSGALG00010002084	HACL1	case	control	-0.146740177	0.220970954	-0.66406998	0.506645523	0.8622397	down_in_case
CRA004775	ENSGALG00010002087	POLA1	case	control	-0.211318664	0.337117017	-0.626840693	0.530763666	0.869211096	down_in_case
CRA004775	ENSGALG00010002088	OZFL1	case	control	-0.112071638	0.294215373	-0.380917003	0.703264836	0.930068722	down_in_case
CRA004775	ENSGALG00010002090	AQP11	case	control	0.239355531	0.33033046	0.724594189	0.468701025	0.8440023	up_in_case
CRA004775	ENSGALG00010002097	PCYT1B	case	control	-1.427830021	0.810472327	-1.761725816	0.078115634	0.490500984	down_in_case
CRA004775	ENSGALG00010002098	ENSGALG00010002098	case	control	0.005184051	0.397892797	0.013028764	0.989604845	0.998065748	up_in_case
CRA004775	ENSGALG00010002100	BTD	case	control	-0.378470194	0.319442864	-1.184782122	0.236103603	0.70334936	down_in_case
CRA004775	ENSGALG00010002102	PAK1	case	control	-0.102112184	0.319175119	-0.319925265	0.749024985	0.941312609	down_in_case
CRA004775	ENSGALG00010002113	GDPD4	case	control	1.414008924	0.707366185	1.998977269	0.045610813	0.397461531	up_in_case
CRA004775	ENSGALG00010002118	ENSGALG00010002118	case	control	-0.536934594	0.464274273	-1.15650301	0.247475469	0.712046495	down_in_case
CRA004775	ENSGALG00010002122	ENSGALG00010002122	case	control	-1.575208524	0.817334088	-1.927251717	0.053948268	0.427596115	down_in_case
CRA004775	ENSGALG00010002126	ENSGALG00010002126	case	control	-0.292488183	0.736316195	-0.397231766	0.691196559	0.927867527	down_in_case
CRA004775	ENSGALG00010002134	ANKRD28	case	control	0.304421064	0.200902355	1.51526877	0.129704354	0.584172159	up_in_case
CRA004775	ENSGALG00010002135	ELOVL5	case	control	0.158602636	0.332149487	0.477503783	0.633003428	0.909723668	up_in_case
CRA004775	ENSGALG00010002136	ACAP1	case	control	-0.81406718	0.504125868	-1.61480938	0.106351981	0.552224847	down_in_case
CRA004775	ENSGALG00010002137	PDK3	case	control	0.332632909	0.309254491	1.075596049	0.282107955	0.738291085	up_in_case
CRA004775	ENSGALG00010002138	MYO7A	case	control	-0.782976541	0.610199952	-1.283147497	0.199440406	0.666548904	down_in_case
CRA004775	ENSGALG00010002142	CLDN34	case	control	2.604368609	0.887766758	2.933618078	0.003350362	0.12351428	up_in_case
CRA004775	ENSGALG00010002143	SLC16A13	case	control	-0.473231864	0.874384607	-0.541217058	0.588357981	0.89299234	down_in_case
CRA004775	ENSGALG00010002145	ENSGALG00010002145	case	control	0.121394335	0.666892507	0.182029838	0.85555931	0.970635695	up_in_case
CRA004775	ENSGALG00010002146	CACUL1	case	control	-0.11133466	0.269000829	-0.413882221	0.678960365	0.92338129	down_in_case
CRA004775	ENSGALG00010002148	POLR2A	case	control	-0.33370015	0.289421017	-1.152992112	0.248913622	0.713978143	down_in_case
CRA004775	ENSGALG00010002150	EIF3A	case	control	-0.032273898	0.211450284	-0.152631141	0.878689166	0.974974156	down_in_case
CRA004775	ENSGALG00010002159	GALNT15	case	control	-0.032709765	0.473121609	-0.069136062	0.944881317	0.988516661	down_in_case
CRA004775	ENSGALG00010002160	CAPN5	case	control	0.326087097	0.39305271	0.829626891	0.406749768	0.813842524	up_in_case
CRA004775	ENSGALG00010002161	CDKL5	case	control	-0.63988311	0.46744937	-1.368882173	0.171036109	0.635309977	down_in_case
CRA004775	ENSGALG00010002174	ENSGALG00010002174	case	control	0.999084841	0.951786383	1.049694404	0.293858637	0.746438942	up_in_case
CRA004775	ENSGALG00010002177	FBXO9	case	control	-0.004059708	0.323115375	-0.012564267	0.989975429	0.998065748	down_in_case
CRA004775	ENSGALG00010002181	FIS1	case	control	-0.504586565	0.360171658	-1.400961328	0.161225638	0.627121149	down_in_case
CRA004775	ENSGALG00010002185	ENSGALG00010002185	case	control	-0.448937793	0.331488961	-1.354306919	0.17563848	0.641148058	down_in_case
CRA004775	ENSGALG00010002186	CILK1	case	control	-0.104028111	0.294967577	-0.352676427	0.724331034	0.935238044	down_in_case
CRA004775	ENSGALG00010002192	ENSGALG00010002192	case	control	0.090053401	0.402245199	0.223876882	0.822853105	0.960875066	up_in_case
CRA004775	ENSGALG00010002194	SHROOM2	case	control	0.209063527	0.341249084	0.612642016	0.54011307	0.87429015	up_in_case
CRA004775	ENSGALG00010002196	ENSGALG00010002196	case	control	-0.078473664	0.242231001	-0.323962099	0.745966729	0.939616056	down_in_case
CRA004775	ENSGALG00010002197	GSTA2	case	control	-0.000297437	0.444506131	-0.00066914	0.999466104	0.999914248	down_in_case
CRA004775	ENSGALG00010002202	SFXN4	case	control	-0.077208711	0.27337997	-0.282422705	0.777619408	0.947570394	down_in_case
CRA004775	ENSGALG00010002206	DPH3P1	case	control	-0.064027983	0.276518463	-0.231550479	0.816887174	0.959594698	down_in_case
CRA004775	ENSGALG00010002211	ACER3	case	control	-0.25293596	0.348442018	-0.725905449	0.467896736	0.843870188	down_in_case
CRA004775	ENSGALG00010002215	YLEC18	case	control	-0.291302358	0.685957619	-0.424665242	0.671080725	0.921535334	down_in_case
CRA004775	ENSGALG00010002225	PRDX3	case	control	0.033413356	0.359216158	0.093017409	0.925889731	0.98417264	up_in_case
CRA004775	ENSGALG00010002226	OXNAD1	case	control	-0.219910714	0.226195818	-0.972213878	0.330944158	0.77237154	down_in_case
CRA004775	ENSGALG00010002231	TMEM14A	case	control	-0.246270006	0.292918779	-0.840745023	0.400490792	0.811868062	down_in_case
CRA004775	ENSGALG00010002235	MXRA5	case	control	0.246794352	0.559783806	0.440874404	0.659303925	0.917907174	up_in_case
CRA004775	ENSGALG00010002238	ENSGALG00010002238	case	control	-1.048349402	0.691889642	-1.515197422	0.129722416	0.584172159	down_in_case
CRA004775	ENSGALG00010002239	AGPAT5	case	control	0.365395449	0.247261995	1.477766323	0.139470335	0.59914295	up_in_case
CRA004775	ENSGALG00010002243	ARSH	case	control	-0.363687407	0.407146628	-0.893259043	0.371718466	0.795818272	down_in_case
CRA004775	ENSGALG00010002247	RFTN1	case	control	-0.239770348	0.382245966	-0.627267178	0.530484114	0.869211096	down_in_case
CRA004775	ENSGALG00010002251	GRK5	case	control	0.203348474	0.455236399	0.446687644	0.655100605	0.916967384	up_in_case
CRA004775	ENSGALG00010002253	ENSGALG00010002253	case	control	0.231259537	0.527129936	0.438714481	0.660868433	0.917907174	up_in_case
CRA004775	ENSGALG00010002254	ENSGALG00010002254	case	control	0.077767066	0.51041706	0.152359849	0.878903123	0.974981284	up_in_case
CRA004775	ENSGALG00010002259	TASL	case	control	0.049478526	0.968030496	0.051112569	0.95923582	0.991431439	up_in_case
CRA004775	ENSGALG00010002261	NR0B1	case	control	1.778911706	0.742855071	2.394695513	0.016634172	0.255187198	up_in_case
CRA004775	ENSGALG00010002269	MCPH1	case	control	-0.568594847	0.571884852	-0.994247084	0.32010259	0.767037518	down_in_case
CRA004775	ENSGALG00010002270	IMMP2L	case	control	-0.025941374	0.371709694	-0.06978934	0.944361332	0.988348632	down_in_case
CRA004775	ENSGALG00010002271	ENSGALG00010002271	case	control	-0.265582046	0.678878193	-0.391207214	0.695644077	0.929092636	down_in_case
CRA004775	ENSGALG00010002274	ENSGALG00010002274	case	control	-0.62532726	0.651845995	-0.959317484	0.33739883	0.778184725	down_in_case

CRA004775	ENSGALG00010002277	RGS10	case	control	-0.461746956	0.35398737	-1.304416471	0.192091622	0.656959697	down_in_case
CRA004775	ENSGALG00010002280	ST7	case	control	-0.122503101	0.331731977	-0.369283365	0.711916524	0.932428243	down_in_case
CRA004775	ENSGALG00010002281	TGFBR2	case	control	-0.187942995	0.324593656	-0.579010068	0.56258238	0.881532201	down_in_case
CRA004775	ENSGALG00010002282	TSKU	case	control	-0.150701836	0.44072523	-0.34194057	0.732395618	0.936819725	down_in_case
CRA004775	ENSGALG00010002285	CAV1	case	control	0.466269147	0.331260691	1.407559542	0.159261534	0.624907945	up_in_case
CRA004775	ENSGALG00010002288	ANGPT2	case	control	-0.16197119	0.38688453	-0.418655121	0.675468199	0.922924485	down_in_case
CRA004775	ENSGALG00010002289	ENSGALG00010002289	case	control	0.754758954	0.278394812	2.711109983	0.006705838	0.161492079	up_in_case
CRA004775	ENSGALG00010002291	RNASEH1	case	control	-0.110203821	0.19123465	-0.57627538	0.564429061	0.882478718	down_in_case
CRA004775	ENSGALG00010002293	GYG2	case	control	0.021612245	0.489505142	0.04415121	0.964783872	0.992871055	up_in_case
CRA004775	ENSGALG00010002294	DAZL	case	control	1.906385916	1.24327936	1.533352823	0.125188946	0.578063532	up_in_case
CRA004775	ENSGALG00010002295	YAF2	case	control	0.134156779	0.199843318	0.671309803	0.502023191	0.860091011	up_in_case
CRA004775	ENSGALG00010002303	ENSGALG00010002303	case	control	-1.060456245	0.943203465	-1.124313347	0.260880118	0.721659172	down_in_case
CRA004775	ENSGALG00010002308	GXYLT1	case	control	-0.032808793	0.279341728	-0.117450384	0.906503159	0.980055425	down_in_case
CRA004775	ENSGALG00010002309	GADL1	case	control	-0.147995487	0.776275732	-0.190648092	0.848801309	0.968223172	down_in_case
CRA004775	ENSGALG00010002310	PRRG1	case	control	0.523503618	0.208978754	2.505056655	0.012243178	0.222693044	up_in_case
CRA004775	ENSGALG00010002312	ENSGALG00010002312	case	control	0.515980398	0.307533477	1.677802373	0.093385686	0.525355698	up_in_case
CRA004775	ENSGALG00010002315	ENSGALG00010002315	case	control	0.226261003	0.273982188	0.825823768	0.408904069	0.814384687	up_in_case
CRA004775	ENSGALG00010002328	ENSGALG00010002328	case	control	-0.643673357	0.725971775	-0.88663689	0.375274426	0.798503808	down_in_case
CRA004775	ENSGALG00010002329	STT3B	case	control	0.043464841	0.124509935	0.349087329	0.727023749	0.935238044	up_in_case
CRA004775	ENSGALG00010002330	KHDRBS2	case	control	-0.158004117	1.065602634	-0.14827677	0.882124347	0.975346523	down_in_case
CRA004775	ENSGALG00010002333	ENSGALG00010002333	case	control	-0.599402853	0.732500421	-0.81829694	0.413187653	0.817112606	down_in_case
CRA004775	ENSGALG00010002334	OSBPL10	case	control	0.01636201	0.319794792	0.051164092	0.959194764	0.991431439	up_in_case
CRA004775	ENSGALG00010002335	TMEM47	case	control	0.66484743	0.317715647	2.092586362	0.036386095	0.360628495	up_in_case
CRA004775	ENSGALG00010002336	PRIM2	case	control	-0.300780211	0.350164669	-0.858967902	0.390358225	0.806479902	down_in_case
CRA004775	ENSGALG00010002338	CD99	case	control	0.203976	0.255302779	0.798957226	0.424315215	0.821240473	up_in_case
CRA004775	ENSGALG00010002339	ZNFY4	case	control	-0.304157711	0.409526732	-0.742705391	0.457660065	0.839704957	down_in_case
CRA004775	ENSGALG00010002340	RAB23	case	control	-0.116684694	0.276471289	-0.422049952	0.672988558	0.92201511	down_in_case
CRA004775	ENSGALG00010002341	FAM49A	case	control	0.248451546	0.357952987	0.69408988	0.487625845	0.853267336	up_in_case
CRA004775	ENSGALG00010002342	BAG2	case	control	0.352262175	0.413079418	0.852771065	0.393786273	0.80832289	up_in_case
CRA004775	ENSGALG00010002343	GPD1L	case	control	-0.467360091	0.330483942	-1.414168833	0.157312337	0.622106365	down_in_case
CRA004775	ENSGALG00010002345	SCML2	case	control	0.148513812	0.292255304	0.508164642	0.611337885	0.901413878	up_in_case
CRA004775	ENSGALG00010002346	FBXO15	case	control	-1.516418621	0.677766808	-2.237375161	0.025261831	0.30560733	down_in_case
CRA004775	ENSGALG00010002347	ZNF451	case	control	0.241639926	0.257609261	0.938009469	0.348239544	0.785270866	up_in_case
CRA004775	ENSGALG00010002348	THAP12	case	control	0.033428753	0.222333104	0.150354367	0.880485042	0.974996282	up_in_case
CRA004775	ENSGALG00010002349	ZBED1	case	control	0.230531531	0.238431769	0.966865831	0.33361111	0.774259303	up_in_case
CRA004775	ENSGALG00010002350	BEND6	case	control	0.950385116	0.299108673	3.177390702	0.001486067	0.080693373	up_in_case
CRA004775	ENSGALG00010002351	ENSGALG00010002351	case	control	-2.0656009	0.900365438	-2.294180576	0.021780129	0.288534247	down_in_case
CRA004775	ENSGALG00010002352	ENSGALG00010002352	case	control	-0.342063321	0.220088266	-1.554209712	0.120134402	0.572133279	down_in_case
CRA004775	ENSGALG00010002353	CMTM8	case	control	0.384328042	0.385712353	0.996411028	0.319050477	0.767037518	up_in_case
CRA004775	ENSGALG00010002355	UFM1	case	control	0.015778198	0.228710541	0.068987628	0.944999467	0.988565057	up_in_case
CRA004775	ENSGALG00010002356	WNT11	case	control	-1.209592474	0.596328716	-2.028398837	0.042519559	0.387723112	down_in_case
CRA004775	ENSGALG00010002357	RAB31	case	control	-0.01020824	0.345474344	-0.029548476	0.976427157	0.99560196	down_in_case
CRA004775	ENSGALG00010002358	FREM2	case	control	0.663493529	0.564932211	1.174465742	0.240208526	0.70694164	up_in_case
CRA004775	ENSGALG00010002359	CMTM6	case	control	0.297728885	0.312605789	0.952410017	0.340889081	0.780875343	up_in_case
CRA004775	ENSGALG00010002360	DHRSX	case	control	-0.129899048	0.255401752	-0.508606723	0.611027915	0.901413878	down_in_case
CRA004775	ENSGALG00010002361	ENSGALG00010002361	case	control	0.157735751	0.221972051	0.710610866	0.477325408	0.846532686	up_in_case
CRA004775	ENSGALG00010002362	TRPC4	case	control	1.365984913	0.570716774	2.393454991	0.016690529	0.255580695	up_in_case
CRA004775	ENSGALG00010002364	VSNL1	case	control	-0.464165406	0.641357968	-0.723722833	0.469235912	0.8440023	down_in_case
CRA004775	ENSGALG00010002365	RAI2	case	control	0.322117206	0.303335947	1.061915704	0.288273955	0.742851819	up_in_case
CRA004775	ENSGALG00010002366	ENSGALG00010002366	case	control	0.785670691	0.282960968	2.776604474	0.005492998	0.150521488	up_in_case
CRA004775	ENSGALG00010002368	UVRAG	case	control	0.184803936	0.158398334	1.166703788	0.243330012	0.710409979	up_in_case
CRA004775	ENSGALG00010002369	GEMIN8	case	control	-0.368887338	0.283003535	-1.303472545	0.192413484	0.656959697	down_in_case
CRA004775	ENSGALG00010002370	ENSGALG00010002370	case	control	-0.140449265	0.246509192	-0.56975265	0.568845475	0.884663439	down_in_case
CRA004775	ENSGALG00010002371	BAG3	case	control	0.606105457	0.464460522	1.304966575	0.19190423	0.656959697	up_in_case
CRA004775	ENSGALG00010002372	SMC6	case	control	0.4369147	0.302436596	1.444648914	0.148556526	0.611617003	up_in_case
CRA004775	ENSGALG00010002373	POSTN	case	control	0.170311393	0.588856722	0.289223824	0.772410101	0.946296525	up_in_case
CRA004775	ENSGALG00010002374	DST	case	control	0.488957498	0.357552498	1.367512467	0.171464732	0.636057362	up_in_case
CRA004775	ENSGALG00010002375	NPY	case	control	-0.354769346	0.795197303	-0.446140027	0.655496101	0.917033995	down_in_case
CRA004775	ENSGALG00010002376	GEN1	case	control	0.020449828	0.513084903	0.039856617	0.968207439	0.993568693	up_in_case
CRA004775	ENSGALG00010002377	ART1A	case	control	0.048941455	0.541217545	0.090428434	0.927946762	0.98473447	up_in_case
CRA004775	ENSGALG00010002378	ENSGALG00010002378	case	control	-0.043229877	0.360119936	-0.120043	0.904449085	0.979540372	down_in_case
CRA004775	ENSGALG00010002379	NHS	case	control	-0.258285565	0.390992589	-0.660589414	0.50887566	0.862971569	down_in_case
CRA004775	ENSGALG00010002380	CMPK2	case	control	-0.195329768	0.471676499	-0.414118083	0.67878763	0.92338129	down_in_case
CRA004775	ENSGALG00010002381	TDRD3	case	control	0.497191135	0.279037228	1.781809326	0.074780331	0.481867072	up_in_case
CRA004775	ENSGALG00010002382	ART1D	case	control	0.451521134	0.481915459	0.936930172	0.348794478	0.785311667	up_in_case
CRA004775	ENSGALG00010002383	INPP5F	case	control	-0.101264698	0.275188678	-0.367982791	0.712886066	0.932981748	down_in_case
CRA004775	ENSGALG00010002385	PALS2	case	control	-0.649747362	0.484021678	-1.342393104	0.17946857	0.644990528	down_in_case
CRA004775	ENSGALG00010002386	MHCY2B2	case	control	-0.014447914	0.566141183	-0.025519985	0.979640208	0.996764329	down_in_case
CRA004775	ENSGALG00010002387	MCMBP	case	control	0.020493226	0.19730293	0.103866811	0.917275046	0.981688766	up_in_case
CRA004775	ENSGALG00010002388	ENSGALG00010002388	case	control	0.098391697	0.577551307	0.170360098	0.864726951	0.972350383	up_in_case
CRA004775	ENSGALG00010002389	COL21A1	case	control	0.356628908	0.449436614	0.793502125	0.427485332	0.822776983	up_in_case
CRA004775	ENSGALG00010002391	REPS2	case	control	-0.337768757	0.37223663	-0.907403328	0.36419355	0.793608387	down_in_case
CRA004775	ENSGALG00010002392	DFNA5	case	control	0.078703825	0.260981785	0.301568267	0.762981202	0.94239774	up_in_case
CRA004775	ENSGALG00010002393	GPM6B	case	control	0.526132515	0.345056741	1.524771009	0.127316223	0.581272645	up_in_case
CRA004775	ENSGALG00010002395	CLN8	case	control	0.134804572	0.272672801	0.494382173	0.621036301	0.90544587	up_in_case
CRA004775	ENSGALG00010002396	SEC23IP	case	control	-0.236655705	0.168269205	-1.406411258	0.159602039	0.624907945	down_in_case
CRA004775	ENSGALG00010002397	ENSGALG00010002397	case	control	-0.258784916	0.465092086	-0.556416512	0.577926153	0.888378369	down_in_case
CRA004775	ENSGALG00010002398	ENSGALG00010002398	case	control	0.200947624	0.537671433	0.373736844	0.708600111	0.931801673	up_in_case
CRA004775	ENSGALG00010002400	RBBP7	case	control	-0.028658584	0.403122472	-0.071091507	0.943324928	0.987862092	down_in_case
CRA004775	ENSGALG00010002401	ZRANB1	case	control	0.015469815	0.429466626	0.036020994	0.971265619	0.994908384	up_in_case
CRA004775	ENSGALG00010002402	TXLNG	case	control	0.533628157	0.220422246	2.420936027	0.015480601	0.249560894	up_in_case
CRA004775	ENSGALG00010002403	ZNFY3	case	control	-0.320138071	0.465152966	-0.688242566	0.491300043	0.855746361	down_in_case
CRA004775	ENSGALG00010002404	OSBPL3	case	control	-0.048088603	0.45840194	-0.104904885	0.916451282	0.981626545	down_in_case
CRA004775	ENSGALG00010002405	KCNQ5	case	control	0.116914446	0.440579737	0.265365009	0.790728289	0.951128823	up_in_case
CRA004775	ENSGALG00010002406	DIAPH3	case	control	0.661730139	0.703212094	0.941010748	0.346699351	0.784552667	up_in_case
CRA004775	ENSGALG00010002408	OFD1	case	control	0.039224455	0.210559332	0.186286948	0.852219742	0.969500139	up_in_case
CRA004775	ENSGALG00010002409	CYCS	case	control	0.387301843	0.415161235	0.932895007	0.350874181	0.785681625	up_in_case
CRA004775	ENSGALG00010002410	RNF121	case	control	-0.079628245	0.263874389	-0.301765719	0.762830666	0.94239774	down_in_case
CRA004775	ENSGALG00010002411	ENSGALG00010002411	case	control	-0.382693539	0.306464195	-1.248738176	0.211760853	0.682220401	down_in_case
CRA004775	ENSGALG00010002413	TSSC1	case	control	-0.285501639	0.302869304	-0.942656239	0.345856764	0.784552667	down_in_case
CRA004775	ENSGALG00010002414	ENSGALG00010002414	case	control	-0.040064013	0.481210493	-0.083256731	0.933647404	0.985093894	down_in_case
CRA004775	ENSGALG00010002416	SH3BGRL2	case	control	-0.131874026	0.493969051	-0.266968195	0.78949365	0.950475151	down_in_case
CRA004775	ENSGALG00010002418	TRAPPC2	case	control	-0.193185932	0.221058654	-0.873912551	0.382165879	0.801753951	down_in_case
CRA004775	ENSGALG00010002419	CTBP2	case	control	0.050113073	0.220415709	0.227357085	0.820146088	0.960565501	up_in_case
CRA004775	ENSGALG00010002421	ENSGALG00010002421	case	control	-0.343040933	0.500123172	-0.685912895	0.492768038	0.855763086	down_in_case
CRA004775	ENSGALG00010002422	TRAPPC12	case	control	0.003237934	0.178174446	0.01817283	0.985500978	0.997562105	up_in_case
CRA004775	ENSGALG00010002423	ENSGALG00010002423	case	control	-1.35439824	0.637584975	-2.124263108	0.033648149	0.348565623	down_in_case
CRA004775	ENSGALG00010002424	CASK	case	control	0.427835709	0.197432123	2.167001506	0.030234739	0.331325011	up_in_case
CRA004775	ENSGALG00010002425	FDX2	case	control	-0.056797165	0.360509285	-0.157547025	0.87481375	0.974301166	down_in_case
CRA004775	ENSGALG00010002427	CYB5R4	case	control	0.064923096	0.225585397	0.287798309	0.773501132	0.946296525	up_in_case
CRA004775	ENSGALG00010002428	IBSP	case	control	1.052449272	0.59955749	1.75537674			up_in_case
CRA004775	ENSGALG00010002429	ENSGALG00010002429	case	control	-0.654846527	0.618089779	-1.059468299	0.289386559	0.74366559	down_in_case
CRA004775	ENSGALG00010002430	NUMA1	case	control	-0.181674271	0.244800348	-0.742132405	0.458007118	0.839796467	down_in_case
CRA004775	ENSGALG00010002431	BCKDHB	case	control	0.35780372	0.322745984	1.108623307	0.267592719	0.727430878	up_in_case
CRA004775	ENSGALG00010002432	RAVER1	case	control	-0.562040308	0.170468211	-3.297038819	0.0009771	0.066028181	down_in_case
CRA004775	ENSGALG00010002433	GRHL1	case	control	-0.102521977	0.275052928	-0.37273545	0.709345349	0.931874404	down_in_case
CRA004775	ENSGALG00010002434	GPR34	case	control	-0.161096264	0.406710606	-0.396095556	0.692034537	0.927867527	down_in_case
CRA004775	ENSGALG00010002436	ENSGALG00010002436	case	control	-0.358874096	0.586194225	-0.612210221	0.54039868	0.874340775	down_in_case
CRA004775	ENSGALG00010002437	TTK	case	control	-0.225497066	0.462759954	-0.487287337	0.62605472	0.907183337	down_in_case
CRA004775	ENSGALG00010002438	RARB	case	control	0.3715166	0.254266204	1.461132443	0.143979098	0.605645941	up_in_case
CRA004775	ENSGALG00010002439	GPR82	case	control	0.355030334	0.453606262	0.782683935	0.433812739	0.825998196	up_in_case
CRA004775	ENSGALG00010002440	LRRC51	case	control	-0.500097783	0.508175993	-0.984103518	0.325064617	0.770251058	down_in_case
CRA004775	ENSGALG00010002441	USO1	case	control	-0.170075007	0.166325056	-1.022545917	0.306522592	0.7585475	down_in_case
CRA004775	ENSGALG00010002442	ENSGALG00010002442	case	control	-2.040299183	0.781324812	-2.611332893	0.009019005	0.191685228	down_in_case
CRA004775	ENSGALG00010002443	TYK2	case	control	-0.887145045	0.190971936	-4.645421004	3.39384E-06	0.001866746	down_in_case
CRA004775	ENSGALG00010002444	KLF11	case	control	0.283623578	0.357697215	0.792915255	0.427827199	0.822830776	up_in_case
CRA004775	ENSGALG00010002445	EDRF1	case	control	0.289079078	0.216692639	1.334051212	0.182187073	0.647378665	up_in_case
CRA004775	ENSGALG00010002446	RIMS1	case	control	1.265714285	0.954853191	1.325559046	0.184985799	0.6491093	up_in_case
CRA004775	ENSGALG00010002447	YLEC12	case	control	-0.52966913	0.824152194	-0.642683638	0.5204294	0.865611895	down_in_case
CRA004775	ENSGALG00010002448	RAB9A	case	control	0.054799003	0.355619773	0.154094364	0.877535332	0.974479909	up_in_case
CRA004775	ENSGALG00010002449	ALG11	case	control	-0.102508661	0.291118537	-0.352120005	0.724748266	0.935238044	down_in_case
CRA004775	ENSGALG00010002450	ENSGALG00010002450	case	control	-0.84656029	0.373727512	-2.265180549	0.023501602	0.297457904	down_in_case
CRA004775	ENSGALG00010002453	TOP2B	case	control	0.177883333	0.200940172	0.885255208	0.376019002	0.798523018	up_in_case
CRA004775	ENSGALG00010002455	NYX	case	control	0.028123209	0.441641631	0.063678799	0.949225986	0.990168609	up_in_case
CRA004775	ENSGALG00010002457	ENSGALG00010002457	case	control	-0.036097813	0.677599752	-0.053273061	0.957514344	0.991431439	down_in_case
CRA004775	ENSGALG00010002458	ENSGALG00010002458	case	control	0.41678619	0.382162832	1.090598444	0.275449615	0.731001618	up_in_case

CRA004775	ENSGALG00010002459	G3BP2	case	control	-0.009924633	0.153460795	-0.064672107	0.948435072	0.990124756	down_in_case
CRA004775	ENSGALG00010002460	LAMTOR1	case	control	-0.205995162	0.350890715	-0.587063587	0.557161002	0.880622207	down_in_case
CRA004775	ENSGALG00010002461	TOMT	case	control	-0.309842754	0.233002608	-1.32978234	0.183589996	0.64798309	down_in_case
CRA004775	ENSGALG00010002462	RRM2	case	control	-0.076128557	0.514788013	-0.147883312	0.882434858	0.975352603	down_in_case
CRA004775	ENSGALG00010002463	ASRGL1	case	control	-0.451302754	0.240964931	-1.872898068	0.061082462	0.445419429	down_in_case
CRA004775	ENSGALG00010002464	NGLY1	case	control	0.120745558	0.181767306	0.664286449	0.506506993	0.8622397	up_in_case
CRA004775	ENSGALG00010002465	ENSGALG00010002465	case	control	2.656190069	0.783768889	3.388996558	0.000701489	0.055464216	up_in_case
CRA004775	ENSGALG00010002466	UROS	case	control	-0.612826272	0.2448006	-2.503369162	0.012301716	0.223462207	down_in_case
CRA004775	ENSGALG00010002467	NEK3	case	control	0.579858027	0.456427974	1.270426136	0.203932878	0.674045742	up_in_case
CRA004775	ENSGALG00010002468	ENSGALG00010002468	case	control	0.161553963	0.648668951	0.249054565	0.803318574	0.954058041	up_in_case
CRA004775	ENSGALG00010002470	OXSM	case	control	-0.082739788	0.22146443	-0.373603057	0.70869966	0.931801673	down_in_case
CRA004775	ENSGALG00010002471	OGFRL1	case	control	0.153535165	0.334960867	0.458367471	0.646688458	0.914043203	up_in_case
CRA004775	ENSGALG00010002472	CDC37	case	control	-0.313416666	0.243768149	-1.285716233	0.198542114	0.665405984	down_in_case
CRA004775	ENSGALG00010002473	ENSGALG00010002473	case	control	-0.105415482	0.703238739	-0.149899993	0.880843517	0.974996282	down_in_case
CRA004775	ENSGALG00010002474	HPCAL1	case	control	-0.492097602	0.346818385	-1.418891337	0.155930698	0.619442611	down_in_case
CRA004775	ENSGALG00010002475	ENSGALG00010002475	case	control	0.205162299	0.399996542	0.51291018	0.608014152	0.900943922	up_in_case
CRA004775	ENSGALG00010002476	DDX3X	case	control	0.869014575	0.359346974	2.418316107	0.015592525	0.249898376	up_in_case
CRA004775	ENSGALG00010002478	PPME1	case	control	-0.435848813	0.15260498	-2.856058906	0.004289355	0.134052107	down_in_case
CRA004775	ENSGALG00010002481	CKAP2	case	control	0.083209162	0.560886216	0.148353016	0.882064178	0.975346523	up_in_case
CRA004775	ENSGALG00010002483	COQ2	case	control	-0.44068133	0.211127392	-2.087276908	0.036863111	0.362074741	down_in_case
CRA004775	ENSGALG00010002487	BCCIP	case	control	0.000458108	0.239612117	0.001911872	0.998474547	0.999914248	up_in_case
CRA004775	ENSGALG00010002492	RPL8	case	control	-0.4149997	0.282268964	-1.470227877	0.141500047	0.601287746	down_in_case
CRA004775	ENSGALG00010002500	OSR1	case	control	0.657632614	0.469967414	1.399315345	0.16171844	0.627882156	up_in_case
CRA004775	ENSGALG00010002501	TCEANC	case	control	0.105507794	0.298129797	0.353898857	0.723414684	0.935238044	up_in_case
CRA004775	ENSGALG00010002504	ENSGALG00010002504	case	control	0.43892846	0.241821349	1.815093916	0.069509478	0.468451304	up_in_case
CRA004775	ENSGALG00010002507	C2CD3	case	control	-0.267502988	0.394667966	-0.6777925	0.497903267	0.857810713	down_in_case
CRA004775	ENSGALG00010002511	VPS36	case	control	-0.006879978	0.160031467	-0.042991406	0.965708385	0.993094598	down_in_case
CRA004775	ENSGALG00010002512	DAXX	case	control	0.113959497	0.287142526	0.396874328	0.691460136	0.927867527	up_in_case
CRA004775	ENSGALG00010002518	MAST1	case	control	0.004726083	0.348449272	0.013563188	0.989178474	0.998065748	up_in_case
CRA004775	ENSGALG00010002520	TTC32	case	control	-0.336351303	0.360137293	-0.933952996	0.35032814	0.785466132	down_in_case
CRA004775	ENSGALG00010002522	HPSE	case	control	-0.540661574	0.495919433	-1.090220585	0.275615988	0.731001618	down_in_case
CRA004775	ENSGALG00010002523	DNAJB13	case	control	0.022830675	0.784308035	0.029109322	0.976777401	0.99560196	up_in_case
CRA004775	ENSGALG00010002526	ZBTB22	case	control	0.047459238	0.30374177	0.156248638	0.87583704	0.974412785	up_in_case
CRA004775	ENSGALG00010002527	LRRC3B	case	control	0.922156058	0.646755339	1.425819011	0.153920592	0.619239923	up_in_case
CRA004775	ENSGALG00010002528	MED14	case	control	-0.152957245	0.208576302	-0.73333952	0.463351377	0.842456502	down_in_case
CRA004775	ENSGALG00010002530	STAG3	case	control	-0.202638725	0.232949291	-0.869883413	0.384364121	0.803182776	down_in_case
CRA004775	ENSGALG00010002533	LPAR2	case	control	0.046372866	0.291086827	0.159309393	0.873425121	0.974079175	up_in_case
CRA004775	ENSGALG00010002536	RGL2	case	control	-0.298921881	0.235519414	-1.26920272	0.204368771	0.674045742	down_in_case
CRA004775	ENSGALG00010002539	SNAP91	case	control	-0.570476821	0.515557782	-1.106523539	0.268499981	0.727528758	down_in_case
CRA004775	ENSGALG00010002543	UCP3	case	control	-0.000823703	0.99319443	-0.000829347	0.999338277	0.999914248	down_in_case
CRA004775	ENSGALG00010002544	THSD1	case	control	0.36533033	0.315631374	1.157458858	0.247084939	0.712046495	up_in_case
CRA004775	ENSGALG00010002546	ATP6AP2	case	control	-0.22634282	0.40907971	-0.553297595	0.580059647	0.888438428	down_in_case
CRA004775	ENSGALG00010002547	PBX4	case	control	-0.182210922	0.218928054	-0.832286767	0.405247098	0.81327583	down_in_case
CRA004775	ENSGALG00010002551	WDR35	case	control	-0.18246559	0.410343325	-0.44466567	0.656561382	0.91710603	down_in_case
CRA004775	ENSGALG00010002553	ENSGALG00010002553	case	control	-0.134760938	0.825845743	-0.163179309	0.870377257	0.973918682	down_in_case
CRA004775	ENSGALG00010002554	EGFL6	case	control	1.099426873	0.58746286	1.871483201	0.061278136	0.4456032	up_in_case
CRA004775	ENSGALG00010002556	ENSGALG00010002556	case	control	0.352221396	0.421395471	0.835845233	0.403241976	0.812213331	up_in_case
CRA004775	ENSGALG00010002557	ENSGALG00010002557	case	control	-0.675381469	0.45146082	-1.495991321	0.134655917	0.592420453	down_in_case
CRA004775	ENSGALG00010002561	PAAF1	case	control	-0.525933917	0.208262172	-2.525345394	0.011558463	0.216540083	down_in_case
CRA004775	ENSGALG00010002565	MRPS18C	case	control	0.034251667	0.307871351	0.111253179	0.911415582	0.98075825	up_in_case
CRA004775	ENSGALG00010002566	TMSB4X	case	control	-0.379027558	0.29285758	-1.294238509	0.195583049	0.661073909	down_in_case
CRA004775	ENSGALG00010002567	COA4	case	control	-0.52961959	0.387648287	-1.366237407	0.17186446	0.636057362	down_in_case
CRA004775	ENSGALG00010002569	GTPBP6	case	control	-0.154621556	0.322969558	-0.478749629	0.632116755	0.909651573	down_in_case
CRA004775	ENSGALG00010002570	ADAM12	case	control	0.656485355	0.536925787	1.22267429	0.221452741	0.689550502	up_in_case
CRA004775	ENSGALG00010002571	BCOR	case	control	-0.031582393	0.266888	-0.118335755	0.905801628	0.979739788	down_in_case
CRA004775	ENSGALG00010002573	MVB12A	case	control	-0.311438764	0.279832218	-1.112948203	0.265730676	0.726742745	down_in_case
CRA004775	ENSGALG00010002575	BGV	case	control	-0.196192701	0.466819248	-0.420275516	0.674284194	0.922533825	down_in_case
CRA004775	ENSGALG00010002576	HELQ	case	control	-0.164480891	0.267267178	-0.615417473	0.538279055	0.872880385	down_in_case
CRA004775	ENSGALG00010002577	MRPL48	case	control	-0.095133142	0.305393556	-0.311509985	0.755412953	0.941900693	down_in_case
CRA004775	ENSGALG00010002579	MATN3	case	control	-2.176331595	0.705395974	-3.085262283	0.002033727	0.090212204	down_in_case
CRA004775	ENSGALG00010002581	ATP13A1	case	control	-0.494421046	0.188435951	-2.623814846	0.008695104	0.188777515	down_in_case
CRA004775	ENSGALG00010002584	ENSGALG00010002584	case	control	-0.974582651	0.474944018	-2.051994793	0.040170168	0.376363211	down_in_case
CRA004775	ENSGALG00010002586	GPR63	case	control	0.435803226	0.320970802	1.35776595	0.174537962	0.639597725	up_in_case
CRA004775	ENSGALG00010002587	DOCK1	case	control	0.350256525	0.248458192	1.409720169	0.158622327	0.624453369	up_in_case
CRA004775	ENSGALG00010002588	FAM175A	case	control	-0.331300465	0.39132995	-0.846601353	0.39721736	0.808998414	down_in_case
CRA004775	ENSGALG00010002589	SLC4A7	case	control	0.390410474	0.39781403	0.981389405	0.326400749	0.770998457	up_in_case
CRA004775	ENSGALG00010002590	ATP6V1C2	case	control	0.829831384	0.943598224	0.879432966	0.379166564	0.800171797	up_in_case
CRA004775	ENSGALG00010002591	ENSGALG00010002591	case	control	0.328688113	0.282413165	1.163855489	0.244482581	0.711765017	up_in_case
CRA004775	ENSGALG00010002592	RPS6KA3	case	control	0.265658136	0.343855393	0.772586795	0.439766962	0.828842585	up_in_case
CRA004775	ENSGALG00010002597	ENSGALG00010002597	case	control	-0.455983385	0.144543529	-3.154644066	0.00160694	0.080802173	down_in_case
CRA004775	ENSGALG00010002598	NMT2	case	control	0.324961242	0.186436442	1.743013536	0.08133125	0.496834143	up_in_case
CRA004775	ENSGALG00010002599	KIFC1	case	control	-0.812780692	0.513018069	-1.584312018	0.113122732	0.562886277	down_in_case
CRA004775	ENSGALG00010002601	PLCXD1	case	control	-0.502013359	0.276620983	-1.814805778	0.069553762	0.468451304	down_in_case
CRA004775	ENSGALG00010002603	INSYN2A	case	control	0.65681464	0.376162187	1.74609427	0.080794572	0.495370226	up_in_case
CRA004775	ENSGALG00010002604	BLEC3	case	control	0.408621163	0.50125721	0.815192588	0.41496208	0.818789434	up_in_case
CRA004775	ENSGALG00010002605	EOMES	case	control	-0.498318251	0.408000019	-1.221368205	0.22194664	0.689550502	down_in_case
CRA004775	ENSGALG00010002606	ENSGALG00010002606	case	control	-0.111703607	0.319475986	-0.349646334	0.726604134	0.935238044	down_in_case
CRA004775	ENSGALG00010002608	RPP38	case	control	-0.63022245	0.304874756	-2.067151966	0.038719837	0.370564421	down_in_case
CRA004775	ENSGALG00010002609	PDIA6	case	control	0.184633922	0.13992774	1.319494783	0.187003753	0.650186754	up_in_case
CRA004775	ENSGALG00010002611	NPS	case	control	-0.571026466	0.655333682	-0.871352231	0.38356186	0.802427983	down_in_case
CRA004775	ENSGALG00010002612	JPH1	case	control	0.716331948	0.537441793	1.332854939	0.182579413	0.647378665	up_in_case
CRA004775	ENSGALG00010002613	ENSGALG00010002613	case	control	0.420770151	0.291455406	1.443686213	0.148827261	0.612367147	up_in_case
CRA004775	ENSGALG00010002614	GPAT3	case	control	-0.026083336	0.275183215	-0.094785346	0.924485326	0.983849734	down_in_case
CRA004775	ENSGALG00010002616	ZNFB3	case	control	-0.516478985	0.22619996	-2.283285041	0.022413584	0.292422177	down_in_case
CRA004775	ENSGALG00010002617	CLRN3	case	control	2.603146167	0.73158726	3.558216919	0.000373381	0.038604217	up_in_case
CRA004775	ENSGALG00010002619	RAB6A	case	control	-0.061816638	0.212284464	-0.291197182	0.770900517	0.945853152	down_in_case
CRA004775	ENSGALG00010002621	SLC25A15	case	control	-0.231816735	0.36704184	-0.631581226	0.527660557	0.86830001	down_in_case
CRA004775	ENSGALG00010002622	AZI2	case	control	0.08677024	0.168967667	0.513531619	0.607579499	0.900943922	up_in_case
CRA004775	ENSGALG00010002623	MID1IP1	case	control	0.0591873	0.464356671	0.12746086	0.89857565	0.978790377	up_in_case
CRA004775	ENSGALG00010002625	FHL5	case	control	-0.141661295	0.302793262	-0.46784824	0.63989312	0.911453315	down_in_case
CRA004775	ENSGALG00010002626	CHD7	case	control	0.625864893	0.279224033	2.241443498	0.024997362	0.304193557	up_in_case
CRA004775	ENSGALG00010002627	MRPS31	case	control	0.052242791	0.320905116	0.162798251	0.870677286	0.973918682	up_in_case
CRA004775	ENSGALG00010002628	TRIM7.2	case	control	-0.31680661	0.172708041	-1.834347777	0.066602386	0.462498477	down_in_case
CRA004775	ENSGALG00010002629	ENSGALG00010002629	case	control	0.494533607	0.517228092	0.956122869	0.339010162	0.779422963	up_in_case
CRA004775	ENSGALG00010002632	HPGD	case	control	1.198580012	0.516887908	2.318839334	0.02040375	0.281976732	up_in_case
CRA004775	ENSGALG00010002633	ENSGALG00010002633	case	control	-0.306865732	0.220567974	-1.391252443	0.16414889	0.629717526	down_in_case
CRA004775	ENSGALG00010002634	RGN	case	control	-0.411379895	0.458351431	-0.897520695	0.369441136	0.794896739	down_in_case
CRA004775	ENSGALG00010002638	FABP9	case	control	2.053839527	0.855760809	2.400015875	0.016394361	0.254445661	up_in_case
CRA004775	ENSGALG00010002646	FOXO1	case	control	0.666052218	0.401568667	1.658625964	0.097191187	0.531393157	up_in_case
CRA004775	ENSGALG00010002647	FAM168A	case	control	0.234691795	0.19213489	1.221494931	0.221898684	0.689550502	up_in_case
CRA004775	ENSGALG00010002649	UFL1	case	control	0.193257722	0.18471585	1.046243309	0.295448712	0.747649966	up_in_case
CRA004775	ENSGALG00010002653	EIF1AX	case	control	0.102586803	0.196029689	0.523322786	0.600749647	0.89768367	up_in_case
CRA004775	ENSGALG00010002655	FABP4	case	control	-0.236891755	0.653538401	-0.362475648	0.716996618	0.934011578	down_in_case
CRA004775	ENSGALG00010002658	FBXL12	case	control	-0.262496732	0.194788365	-1.347599646	0.177787184	0.643694464	down_in_case
CRA004775	ENSGALG00010002661	ACBD7	case	control	1.097883487	0.343590907	3.195321716	0.00139675	0.078533081	up_in_case
CRA004775	ENSGALG00010002664	ZNFB1	case	control	-0.345994138	0.342975362	-1.008801728	0.313069732	0.765065201	down_in_case
CRA004775	ENSGALG00010002667	ENSGALG00010002667	case	control	2.429771908	0.695387731	3.494125363	0.000475618	0.044491279	up_in_case
CRA004775	ENSGALG00010002669	IL4I1	case	control	-1.165332987	0.453100941	-2.571905911	0.010114037	0.202058223	down_in_case
CRA004775	ENSGALG00010002673	SLC66A3	case	control	0.276098953	0.363218921	0.760144743	0.44716807	0.834136489	up_in_case
CRA004775	ENSGALG00010002678	CCNI	case	control	0.138950936	0.338042836	0.411045351	0.681039281	0.923896221	up_in_case
CRA004775	ENSGALG00010002681	PLEKHB1	case	control	-0.16111624	0.202619578	-0.7951662	0.42651683	0.822585264	down_in_case
CRA004775	ENSGALG00010002682	UBL5	case	control	0.28935268	0.359323586	0.805270487	0.420663609	0.819388688	up_in_case
CRA004775	ENSGALG00010002685	CA8	case	control	0.215160697	0.487044321	0.441768208	0.658656946	0.917648599	up_in_case
CRA004775	ENSGALG00010002689	COG6	case	control	-0.075745097	0.188736522	-0.401327187	0.68817925	0.927123823	down_in_case
CRA004775	ENSGALG00010002693	MSC	case	control	1.101375845	0.56394825	1.952973246	0.050822765	0.416238146	up_in_case
CRA004775	ENSGALG00010002700	LHFPL6	case	control	-0.092667149	0.356235544	-0.260128867	0.794764371	0.952235329	down_in_case
CRA004775	ENSGALG00010002703	PIN1	case	control	-0.19509558	0.255564245	-0.763391533	0.445229928	0.833012432	down_in_case
CRA004775	ENSGALG00010002704	KCNB2	case	control	0.161052128	0.856199619	0.188101144	0.850797364	0.968989143	up_in_case
CRA004775	ENSGALG00010002705	JADE3	case	control	0.135060416	0.347987673	0.388118391	0.697928423	0.929122855	up_in_case
CRA004775	ENSGALG00010002706	ROCK2	case	control	0.961826592	0.438075265	2.195573843	0.02812246	0.319106625	up_in_case
CRA004775	ENSGALG00010002707	RELT	case	control	-0.091932591	0.580498481	-0.158368357	0.874166547	0.974115968	down_in_case
CRA004775	ENSGALG00010002712	PTPRE	case	control	0.563138839	0.357447905	1.57544311	0.1151542	0.565776219	up_in_case
CRA004775	ENSGALG00010002713	OLFM2	case	control	-0.498866237	0.457476065	-1.090475053	0.275503937	0.731001618	down_in_case
CRA004775	ENSGALG00010002716	NHLRC3	case	control	-0.355793681	0.447048122	-0.795873338	0.42610566	0.822541484	down_in_case
CRA004775	ENSGALG00010002722	TRIM7.1	case	control	-0.140054263	0.562472006	-0.248997748	0.803362523	0.954058041	down_in_case
CRA004775	ENSGALG00010002725	TSPAN7	case	control	-0.491226935	0.470448914	-1.04416637	0.296408423	0.748897794	down_in_case

CRA004775	ENSGALG00010002728	RP2	case	control	-0.220813031	0.313374205	-0.704630527	0.481040196	0.848743338	down_in_case
CRA004775	ENSGALG00010002730	E2F6	case	control	0.260472252	0.203517513	1.279851785	0.200597267	0.668058373	up_in_case
CRA004775	ENSGALG00010002733	RDH8	case	control	-0.195173578	0.483037979	-0.404054311	0.686172782	0.926725143	down_in_case
CRA004775	ENSGALG00010002741	PDK4	case	control	0.910775525	0.767584468	1.186547621	0.235406107	0.702489015	up_in_case
CRA004775	ENSGALG00010002742	PROSER1	case	control	0.11638988	0.187739518	0.619954081	0.535288019	0.870981055	up_in_case
CRA004775	ENSGALG00010002750	ENSGALG00010002750	case	control	0.087762738	0.691654214	0.12688817	0.89902891	0.978790377	up_in_case
CRA004775	ENSGALG00010002752	NDUFA4	case	control	0.024506326	0.406374216	0.060304825	0.951912859	0.990168609	up_in_case
CRA004775	ENSGALG00010002753	SLC9A7	case	control	-0.278762243	0.414182416	-0.673042196	0.500920446	0.859946721	down_in_case
CRA004775	ENSGALG00010002760	RAB2A	case	control	-0.026071921	0.205646232	-0.126780448	0.899114171	0.978790377	down_in_case
CRA004775	ENSGALG00010002762	PPAN	case	control	-0.350635521	0.210103186	-1.668872935	0.095142564	0.527976505	down_in_case
CRA004775	ENSGALG00010002763	SEPTIN11	case	control	0.138329943	0.197085654	0.70187728	0.482755699	0.849874999	up_in_case
CRA004775	ENSGALG00010002765	MAP7D2	case	control	1.571526161	0.768066599	2.046080591	0.040748442	0.378091653	up_in_case
CRA004775	ENSGALG00010002766	NANOS1	case	control	-0.977400633	0.703822272	-1.388703756	0.164922852	0.630194031	down_in_case
CRA004775	ENSGALG00010002768	TRIM39.2	case	control	-0.137463852	0.684095706	-0.200942428	0.840743592	0.966461921	down_in_case
CRA004775	ENSGALG00010002774	TBX18	case	control	0.831292591	0.623439303	1.333397793	0.182401296	0.647378665	up_in_case
CRA004775	ENSGALG00010002779	FBXO8	case	control	0.210692362	0.28956983	0.727604674	0.466855618	0.8434807	up_in_case
CRA004775	ENSGALG00010002781	EIF3G	case	control	-0.185440464	0.26547986	-0.698510478	0.484858008	0.851277875	down_in_case
CRA004775	ENSGALG00010002782	GSTA3	case	control	-0.138917064	0.273414332	-0.508082598	0.611395418	0.901413878	down_in_case
CRA004775	ENSGALG00010002784	TRIM27.2	case	control	-0.96978637	0.736919262	-1.316000843	0.18817376	0.652111233	down_in_case
CRA004775	ENSGALG00010002787	CEP44	case	control	0.209300989	0.291154459	0.71886582	0.472223598	0.845425322	up_in_case
CRA004775	ENSGALG00010002788	ENSGALG00010002788	case	control	-1.394537785	0.528248496	-2.639927599	0.008292374	0.183215969	down_in_case
CRA004775	ENSGALG00010002790	BCLAF3	case	control	0.462213325	0.278001969	1.662626082	0.096387307	0.530168743	up_in_case
CRA004775	ENSGALG00010002792	ENSGALG00010002792	case	control	-0.375415379	0.422009192	-0.889590525	0.373685796	0.797250404	down_in_case
CRA004775	ENSGALG00010002796	PRDM8	case	control	-0.327072883	0.592950824	-0.551602038	0.58122104	0.888998848	down_in_case
CRA004775	ENSGALG00010002800	TUBGCP5	case	control	0.013720916	0.231692211	0.059220445	0.952776525	0.99052166	up_in_case
CRA004775	ENSGALG00010002805	FAM171A1	case	control	-0.181163055	0.297080833	-0.609810648	0.541987247	0.875128455	down_in_case
CRA004775	ENSGALG00010002807	P2RY6	case	control	-0.595999515	0.341400767	-1.745747438	0.080854848	0.49547015	down_in_case
CRA004775	ENSGALG00010002809	SH3KBP1	case	control	0.269880918	0.316562364	0.852536336	0.393916481	0.808366404	up_in_case
CRA004775	ENSGALG00010002810	TRIM27.1	case	control	-0.722945763	0.45116753	-1.602388723	0.109069677	0.556276325	down_in_case
CRA004775	ENSGALG00010002812	TRIM13	case	control	-0.281872868	0.264272611	-1.066598871	0.28615301	0.741582427	down_in_case
CRA004775	ENSGALG00010002814	TERF1	case	control	-0.104244046	0.388190319	-0.268538501	0.788284843	0.95041797	down_in_case
CRA004775	ENSGALG00010002817	SHFM1	case	control	0.028987549	0.29308229	0.098905838	0.921213034	0.982481112	up_in_case
CRA004775	ENSGALG00010002819	BMP3	case	control	-0.280006485	0.614095645	-0.455965593	0.64841472	0.91477899	down_in_case
CRA004775	ENSGALG00010002825	TRIM41	case	control	-0.589754056	0.119540554	-4.933506137	8.07665E-07	0.000528867	down_in_case
CRA004775	ENSGALG00010002827	SBSPON	case	control	0.650951713	0.523136452	1.2443249	0.21338001	0.682777582	up_in_case
CRA004775	ENSGALG00010002832	LPIN1	case	control	0.149302726	0.503264644	0.296668418	0.766719671	0.94422015	up_in_case
CRA004775	ENSGALG00010002834	MRPL53	case	control	-0.300519519	0.285317521	-1.053280985	0.292212231	0.746262643	down_in_case
CRA004775	ENSGALG00010002835	CYFIP1	case	control	-0.053668091	0.25059312	-0.214164265	0.830418962	0.963232966	down_in_case
CRA004775	ENSGALG00010002837	DNMT1	case	control	-0.410461652	0.151483878	-2.709606181	0.006736314	0.161712092	down_in_case
CRA004775	ENSGALG00010002838	ENSGALG00010002838	case	control	0.413835166	0.396456024	1.043836241	0.296561161	0.748968786	up_in_case
CRA004775	ENSGALG00010002840	SRPX	case	control	0.10342443	0.41785252	0.247514195	0.804510307	0.954576316	up_in_case
CRA004775	ENSGALG00010002848	TRIB2	case	control	0.54363017	0.2129094	2.553340388	0.010669517	0.207813776	up_in_case
CRA004775	ENSGALG00010002853	CEP162	case	control	-0.157946279	0.329557739	-0.479267393	0.631748416	0.909651567	down_in_case
CRA004775	ENSGALG00010002855	RACK1	case	control	-0.481373786	0.246415837	-1.953501814	0.050760162	0.416238146	down_in_case
CRA004775	ENSGALG00010002857	DCAF15	case	control	-0.439238118	0.220303704	-1.99378453	0.046175607	0.399541954	down_in_case
CRA004775	ENSGALG00010002860	NIPA2	case	control	-0.156827613	0.188955038	-0.829973174	0.406553951	0.813842524	down_in_case
CRA004775	ENSGALG00010002864	RNASEH2B	case	control	-0.441935729	0.306723034	-1.440829936	0.149632734	0.61366707	down_in_case
CRA004775	ENSGALG00010002866	ENSGALG00010002866	case	control	-0.80591105	0.650835834	-1.238270862	0.215615644	0.683654538	down_in_case
CRA004775	ENSGALG00010002868	PPP1R9A	case	control	0.08813655	0.298139056	0.29562229	0.767518549	0.944590059	up_in_case
CRA004775	ENSGALG00010002870	FABP5	case	control	0.703458307	0.486737791	1.445251056	0.14838738	0.611192154	up_in_case
CRA004775	ENSGALG00010002878	P2RY2	case	control	-0.415088104	0.539129146	-0.769923324	0.441345377	0.829353567	down_in_case
CRA004775	ENSGALG00010002880	RASGEF1B	case	control	-0.13085388	0.392236646	-0.333609521	0.738674233	0.938064864	down_in_case
CRA004775	ENSGALG00010002881	ENSGALG00010002881	case	control	-0.659162428	0.651327263	-1.012029537	0.311523931	0.763050512	down_in_case
CRA004775	ENSGALG00010002883	MAP3K15	case	control	0.851974965	0.55821776	1.526241238	0.126949797	0.581120725	up_in_case
CRA004775	ENSGALG00010002884	ADHFE1	case	control	-0.145378949	0.423723613	-0.343098531	0.731524336	0.936574571	down_in_case
CRA004775	ENSGALG00010002886	HERC2	case	control	0.27771954	0.29343513	0.94644271	0.34392283	0.783503692	up_in_case
CRA004775	ENSGALG00010002890	VXN	case	control	0.451365295	0.328628353	1.37348251	0.169602395	0.633147466	up_in_case
CRA004775	ENSGALG00010002894	FCHSD2	case	control	0.173675233	0.188270379	0.922477736	0.356279438	0.789127613	up_in_case
CRA004775	ENSGALG00010002896	OCA2	case	control	2.104247976	0.957798547	2.196963007	0.028023088	0.318730759	up_in_case
CRA004775	ENSGALG00010002904	PON1	case	control	-0.184977641	0.297447529	-0.621883267	0.534018629	0.870956093	down_in_case
CRA004775	ENSGALG00010002908	S1PR2	case	control	-0.325473259	0.366888772	-0.887116979	0.375015924	0.798503808	down_in_case
CRA004775	ENSGALG00010002911	MYBL1	case	control	0.895328271	0.721149943	1.241528588	0.214410544	0.683562661	up_in_case
CRA004775	ENSGALG00010002912	RPS3	case	control	-0.338047703	0.185059113	-1.826701193	0.067744709	0.463543284	down_in_case
CRA004775	ENSGALG00010002913	NXNL1	case	control	1.150770916	0.711032147	1.618451318	0.10556537	0.549651422	up_in_case
CRA004775	ENSGALG00010002914	SLC27A1	case	control	0.208399651	0.566736929	0.367718496	0.713083148	0.933067501	up_in_case
CRA004775	ENSGALG00010002916	SDCBP	case	control	0.100483955	0.237941157	0.422305903	0.67280175	0.921952322	up_in_case
CRA004775	ENSGALG00010002917	PGLS	case	control	-0.240052472	0.162476152	-1.477462803	0.139551622	0.59930492	down_in_case
CRA004775	ENSGALG00010002918	LRATD1	case	control	0.057337541	0.38107579	0.150462304	0.880399889	0.974996282	up_in_case
CRA004775	ENSGALG00010002919	ENSGALG00010002919	case	control	0.275964296	0.234887354	1.174879325	0.240042998	0.706680492	up_in_case
CRA004775	ENSGALG00010002920	VCPIP1	case	control	0.008066675	0.346943996	0.023250656	0.981450332	0.997069916	up_in_case
CRA004775	ENSGALG00010002922	TMEM106B	case	control	0.262697235	0.312634026	0.840270775	0.400756584	0.811868062	up_in_case
CRA004775	ENSGALG00010002923	SERPINH1	case	control	-0.190438663	0.410783498	-0.463598622	0.642935342	0.913515591	down_in_case
CRA004775	ENSGALG00010002925	INTS6	case	control	0.164617294	0.192497167	0.855167356	0.392458505	0.80746892	up_in_case
CRA004775	ENSGALG00010002927	GABRA5	case	control	-0.254374833	1.266569235	-0.200837685	0.840825495	0.966461921	down_in_case
CRA004775	ENSGALG00010002928	ASB4	case	control	0.130067238	0.647444816	0.200893164	0.840782113	0.966461921	up_in_case
CRA004775	ENSGALG00010002929	PDHA2	case	control	0.066292687	0.260671905	0.254314659	0.799252476	0.952835572	up_in_case
CRA004775	ENSGALG00010002930	SERAF	case	control	0.938399593	0.719712598	1.303853226	0.192283631	0.656959697	up_in_case
CRA004775	ENSGALG00010002937	GALNT7	case	control	-0.015251511	0.210688914	-0.072388771	0.942292521	0.987651835	down_in_case
CRA004775	ENSGALG00010002939	HMGB2	case	control	0.371498725	0.268836361	1.381876783	0.167009538	0.63074654	up_in_case
CRA004775	ENSGALG00010002940	BG1	case	control	0.258524165	0.563491947	0.458789458	0.646385366	0.914043203	up_in_case
CRA004775	ENSGALG00010002943	SAP30	case	control	0.326822714	0.271870415	1.202126806	0.229314402	0.695782399	up_in_case
CRA004775	ENSGALG00010002946	GDPD5	case	control	-0.293546904	0.378216807	-0.776133949	0.4376699	0.827521409	down_in_case
CRA004775	ENSGALG00010002949	ENSGALG00010002949	case	control	4.375405596	1.220916854	3.583704806			up_in_case
CRA004775	ENSGALG00010002952	NBAS	case	control	0.081350305	0.239950277	0.339029844	0.734587248	0.936819725	up_in_case
CRA004775	ENSGALG00010002953	WDFY2	case	control	-0.32198789	0.275750861	-1.167676824	0.242937149	0.709865859	down_in_case
CRA004775	ENSGALG00010002954	CHST7	case	control	0.109192729	0.410038445	0.26629876	0.790009128	0.950891435	up_in_case
CRA004775	ENSGALG00010002955	BLEC1	case	control	-0.236499215	0.390285518	-0.605964618	0.544538248	0.875916944	down_in_case
CRA004775	ENSGALG00010002956	SCRG1	case	control	0.773791171	0.491407377	1.57464297	0.115338878	0.565776219	up_in_case
CRA004775	ENSGALG00010002957	ADGRG2	case	control	0.96133595	0.527206374	1.823452823	0.068234836	0.464963941	up_in_case
CRA004775	ENSGALG00010002958	ENSGALG00010002958	case	control	-1.071966993	0.542022124	-1.977718151	0.047960511	0.406854015	down_in_case
CRA004775	ENSGALG00010002960	BLEC2	case	control	-0.464224504	0.676597804	-0.68611589	0.492640031	0.855763086	down_in_case
CRA004775	ENSGALG00010002965	PRSS35	case	control	0.705906463	0.327403187	2.156076942	0.031077658	0.336379059	up_in_case
CRA004775	ENSGALG00010002967	OLFM4	case	control	3.248387439	1.268092795	2.561632281	0.010418156	0.204657231	up_in_case
CRA004775	ENSGALG00010002972	ME1	case	control	0.198709551	0.39203154	0.506871337	0.612245099	0.901413878	up_in_case
CRA004775	ENSGALG00010002978	DYNLT3	case	control	0.132061303	0.250994495	0.526152189	0.59878247	0.897129848	up_in_case
CRA004775	ENSGALG00010002982	HAND2	case	control	0.333464588	0.406321954	0.820690551	0.411822554	0.817006008	up_in_case
CRA004775	ENSGALG00010002983	NSMAF	case	control	-0.103832507	0.167350787	-0.620448278	0.534962696	0.870981055	down_in_case
CRA004775	ENSGALG00010002985	ENSGALG00010002985	case	control	0.038090753	0.284575176	0.133851284	0.893520174	0.977834811	up_in_case
CRA004775	ENSGALG00010002987	PCDH8	case	control	0.847013224	0.40320741	2.100688637	0.035668307	0.358272386	up_in_case
CRA004775	ENSGALG00010002991	BLB1	case	control	-0.272234605	0.347655202	-0.783059203	0.433592347	0.825828172	down_in_case
CRA004775	ENSGALG00010002992	ENSGALG00010002992	case	control	-0.551334269	0.40865031	-1.349159062	0.177285881	0.64323434	down_in_case
CRA004775	ENSGALG00010002994	DUSP4	case	control	-0.762155973	0.495200191	-1.539086589	0.123783159	0.576485043	down_in_case
CRA004775	ENSGALG00010002995	RWDD2A	case	control	-0.375278098	0.230997672	-1.624596886	0.104248481	0.546673752	down_in_case
CRA004775	ENSGALG00010002998	LECT1	case	control	-0.835261056	0.645453757	-1.294068006	0.195641932	0.661073909	down_in_case
CRA004775	ENSGALG00010002999	CMTM7	case	control	-0.703938826	0.491659393	-1.431761165	0.152212185	0.61669704	down_in_case
CRA004775	ENSGALG00010003001	MAP6	case	control	0.984494675	0.602477218	1.634077846	0.102242506	0.542513917	up_in_case
CRA004775	ENSGALG00010003002	PHKA2	case	control	-0.662516449	0.507784194	-1.304720503	0.191988037	0.656959697	down_in_case
CRA004775	ENSGALG00010003006	CYP7B1	case	control	-0.408783896	0.41508967	-0.984808648	0.324718072	0.770185561	down_in_case
CRA004775	ENSGALG00010003009	TNKS	case	control	0.331691901	0.161131929	2.058511326	0.039541075	0.37421151	up_in_case
CRA004775	ENSGALG00010003017	PGM3	case	control	-0.11218456	0.363592044	-0.308545145	0.757667554	0.941900693	down_in_case
CRA004775	ENSGALG00010003018	MOGAT2	case	control	2.54931676	0.665991945	3.827849242	0.000129268	0.024531539	up_in_case
CRA004775	ENSGALG00010003019	TAPBP	case	control	-0.493315171	0.282956247	-1.743432688	0.081258063	0.496834143	down_in_case
CRA004775	ENSGALG00010003022	BLB2	case	control	-0.624675393	0.308910317	-2.022190128	0.043156714	0.389657237	down_in_case
CRA004775	ENSGALG00010003024	SUGT1	case	control	0.113989201	0.264681435	0.430665646	0.666711501	0.919168997	up_in_case
CRA004775	ENSGALG00010003025	MFHAS1	case	control	-0.079788176	0.357883828	-0.222944347	0.823578821	0.960898591	down_in_case
CRA004775	ENSGALG00010003027	SCIN	case	control	0.534223284	0.409282079	1.305269182	0.191801204	0.656959697	up_in_case
CRA004775	ENSGALG00010003030	PPEF1	case	control	-0.401888481	0.776044147	-0.517868066	0.604550324	0.899304577	down_in_case
CRA004775	ENSGALG00010003034	ERI1	case	control	-0.240362512	0.194106877	-1.238299826	0.215604908	0.683654538	down_in_case
CRA004775	ENSGALG00010003035	DGAT2	case	control	-0.249611367	0.360442072	-0.692514517	0.488614273	0.854054691	down_in_case
CRA004775	ENSGALG00010003042	TOX	case	control	-0.523147251	0.51364098	-1.018507619	0.308436782	0.760910331	down_in_case
CRA004775	ENSGALG00010003051	ART1E	case	control	-0.325561737	0.390141251	-0.834471452	0.404015368	0.813056538	down_in_case
CRA004775	ENSGALG00010003052	ENSGALG00010003052	case	control	0.30099766	0.188667652	1.595385625	0.110626029	0.559477205	up_in_case
CRA004775	ENSGALG00010003057	DDX1	case	control	0.064677312	0.347991599	0.185858831	0.852555467	0.969725992	up_in_case
CRA004775	ENSGALG00010003061	ENSGALG00010003061	case	control	2.13822087	0.823213804	2.597406483	0.009393069	0.195703177	up_in_case
CRA004775	ENSGALG00010003063	GGH	case	control	0.338699563	0.267888691	1.264329458	0.206111801	0.674837165	up_in_case
CRA004775	ENSGALG00010003064	VAV1	case	control	-0.690715922	0.448970563	-1.538443674	0.123940171	0.576485043	down_in_case

CRA004775	ENSGALG00010003068	BRD2	case	control	0.203379428	0.178490432	1.139441627	0.254519002	0.718419134	up_in_case
CRA004775	ENSGALG00010003072	ELF1	case	control	-0.330400066	0.389452944	-0.848369673	0.396232127	0.808998414	down_in_case
CRA004775	ENSGALG00010003074	TTPA	case	control	0.046330407	0.774229093	0.059840694	0.952282514	0.990232672	up_in_case
CRA004775	ENSGALG00010003077	EIF2S3	case	control	-0.036665638	0.182318167	-0.201107975	0.840614146	0.966461921	down_in_case
CRA004775	ENSGALG00010003081	DOP1A	case	control	-0.100803156	0.279812186	-0.360252917	0.718658006	0.934011578	down_in_case
CRA004775	ENSGALG00010003087	TWISTNB	case	control	0.445200135	0.267307167	1.665500182	0.095813007	0.529205137	up_in_case
CRA004775	ENSGALG00010003090	DMA	case	control	-0.638298053	0.267701367	-2.384366058	0.017108575	0.258811894	down_in_case
CRA004775	ENSGALG00010003092	ART1F	case	control	-0.539946725	0.674617109	-0.800375083	0.423493513	0.820552247	down_in_case
CRA004775	ENSGALG00010003097	TFPI2	case	control	-0.399862474	0.315492673	-1.267422378	0.205004302	0.674045742	down_in_case
CRA004775	ENSGALG00010003101	KLHL15	case	control	-0.092065455	0.322207396	-0.285733525	0.775082226	0.946319184	down_in_case
CRA004775	ENSGALG00010003103	YTHDF3	case	control	0.244003141	0.232752246	1.048338506	0.294482675	0.746438942	up_in_case
CRA004775	ENSGALG00010003107	GNG11	case	control	-0.13992076	0.300262413	-0.465994922	0.641219136	0.912302569	down_in_case
CRA004775	ENSGALG00010003108	PPP1R3B	case	control	-0.410877523	0.332670136	-1.235089894	0.21679705	0.684403275	down_in_case
CRA004775	ENSGALG00010003109	ART5	case	control	-0.776874178	0.435579001	-1.783543688	0.074497843	0.4814003	down_in_case
CRA004775	ENSGALG00010003110	BF1	case	control	-0.43216428	0.185983038	-2.323675773	0.020142878	0.280632942	down_in_case
CRA004775	ENSGALG00010003113	WBP4	case	control	0.105886924	0.196575806	0.538656948	0.590123585	0.893771223	up_in_case
CRA004775	ENSGALG00010003114	CHRNA10	case	control	-0.679989621	0.822888816	-0.826344468	0.408608715	0.814384687	down_in_case
CRA004775	ENSGALG00010003115	UBE3D	case	control	-0.37890837	0.344082134	-1.101214891	0.270803142	0.728665115	down_in_case
CRA004775	ENSGALG00010003117	ID2	case	control	-0.231469871	0.423383064	-0.546714998	0.584574548	0.890296225	down_in_case
CRA004775	ENSGALG00010003121	ARMC1	case	control	0.107188994	0.260788117	0.411019474	0.681058255	0.923896221	up_in_case
CRA004775	ENSGALG00010003125	APOO	case	control	-0.050733846	0.333288375	-0.152222068	0.879011789	0.974981284	down_in_case
CRA004775	ENSGALG00010003126	ENSGALG00010003126	case	control	2.407640911	0.893132804	2.695725542			up_in_case
CRA004775	ENSGALG00010003131	TAP1	case	control	-0.642609508	0.33423958	-1.922601469	0.054530119	0.429956233	down_in_case
CRA004775	ENSGALG00010003132	TPBG	case	control	0.486798068	0.527774262	0.922360378	0.35634063	0.789127613	up_in_case
CRA004775	ENSGALG00010003133	SAT1	case	control	-0.201291799	0.227782337	-0.883702405	0.376856882	0.798523018	down_in_case
CRA004775	ENSGALG00010003136	NUP98	case	control	-0.217867285	0.181742019	-1.19877223	0.230616524	0.697177844	down_in_case
CRA004775	ENSGALG00010003152	ENSGALG00010003152	case	control	-0.079487077	0.275477353	-0.288543055	0.772931078	0.946296525	down_in_case
CRA004775	ENSGALG00010003154	MTFR1	case	control	0.505738785	0.202787999	2.493928573	0.012633796	0.226502393	up_in_case
CRA004775	ENSGALG00010003159	TAP2	case	control	-0.838356275	0.331764727	-2.526960245	0.011505453	0.216528087	down_in_case
CRA004775	ENSGALG00010003160	KIDINS220	case	control	0.085261529	0.32716894	0.260603984	0.794397917	0.951962157	up_in_case
CRA004775	ENSGALG00010003164	C3	case	control	-0.876010634	0.565842868	-1.548151762	0.121585762	0.573689301	down_in_case
CRA004775	ENSGALG00010003165	BMP2K	case	control	-0.526834105	0.48916695	-1.077002656	0.281479079	0.738104274	down_in_case
CRA004775	ENSGALG00010003166	PGAP2	case	control	-0.120686993	0.337226701	-0.357880892	0.720432451	0.934406529	down_in_case
CRA004775	ENSGALG00010003169	MTRF1	case	control	-0.198656368	0.209833282	-0.946734314	0.343774181	0.783449416	down_in_case
CRA004775	ENSGALG00010003170	C4	case	control	-0.378168024	0.405535134	-0.932516058	0.351069892	0.785991873	down_in_case
CRA004775	ENSGALG00010003171	PAQR3	case	control	-0.473535051	0.242160277	-1.955461302	0.050528647	0.416238146	down_in_case
CRA004775	ENSGALG00010003176	PRAG1	case	control	0.127146293	0.429252355	0.296204067	0.767074243	0.944367689	up_in_case
CRA004775	ENSGALG00010003178	CENPA	case	control	0.342450547	0.412331762	0.830521873	0.406243786	0.813842524	up_in_case
CRA004775	ENSGALG00010003180	IBTK	case	control	-0.036503555	0.199743879	-0.182751809	0.854992763	0.970530418	down_in_case
CRA004775	ENSGALG00010003183	NAA16	case	control	0.340014796	0.242777861	1.400518126	0.161358219	0.627121149	up_in_case
CRA004775	ENSGALG00010003184	BET1	case	control	-0.112305566	0.233656687	-0.480643493	0.630769898	0.909338686	down_in_case
CRA004775	ENSGALG00010003186	CYP21A1	case	control	-0.159264356	0.585119654	-0.27219109	0.785475092	0.95041797	down_in_case
CRA004775	ENSGALG00010003187	RHOG	case	control	-0.121358904	0.317391389	-0.382363567	0.70219171	0.930068722	down_in_case
CRA004775	ENSGALG00010003189	ANTXR2	case	control	-0.230875019	0.227221328	-1.01607988	0.309591349	0.762002042	down_in_case
CRA004775	ENSGALG00010003191	CC2D1A	case	control	-0.245671203	0.242871386	-1.011527982	0.311763796	0.763232972	down_in_case
CRA004775	ENSGALG00010003192	RGCC	case	control	-1.003930677	0.577243028	-1.739181989	0.082002757	0.498505705	down_in_case
CRA004775	ENSGALG00010003193	ACOT9	case	control	-0.010115879	0.208126709	-0.048604423	0.961234545	0.991860454	down_in_case
CRA004775	ENSGALG00010003198	PRKG2	case	control	-0.608753235	0.517800381	-1.175652351	0.239733827	0.706254731	down_in_case
CRA004775	ENSGALG00010003201	MBOAT2	case	control	0.789123839	0.462936282	1.704605729	0.088267982	0.513532836	up_in_case
CRA004775	ENSGALG00010003202	FAM46A	case	control	-0.518462439	0.486120913	-1.066529798	0.286184216	0.741582427	down_in_case
CRA004775	ENSGALG00010003204	PKD2	case	control	-0.227432776	0.283598388	-0.801953696	0.422579743	0.820450913	down_in_case
CRA004775	ENSGALG00010003205	ENSGALG00010003205	case	control	0.0353393	0.487871412	0.072435684	0.942255187	0.987651835	up_in_case
CRA004775	ENSGALG00010003208	ENSGALG00010003208	case	control	-0.400617025	0.576917217	-0.694409897	0.487425189	0.853180215	down_in_case
CRA004775	ENSGALG00010003210	EYA1	case	control	0.235783085	0.451753606	0.521928506	0.60172011	0.898185892	up_in_case
CRA004775	ENSGALG00010003212	LTB4R	case	control	-0.460329922	0.452786273	-1.016660506	0.30931496	0.761710245	down_in_case
CRA004775	ENSGALG00010003214	PRDX4	case	control	-0.223414649	0.249861761	-0.894153025	0.371240022	0.795818272	down_in_case
CRA004775	ENSGALG00010003218	MAP3K7	case	control	0.249163235	0.156830569	1.58874151	0.112118763	0.561860464	up_in_case
CRA004775	ENSGALG00010003220	SPP1	case	control	0.125469187	1.207628204	0.103897199	0.91725093	0.981688766	up_in_case
CRA004775	ENSGALG00010003222	ITGA8	case	control	0.539685569	0.331539866	1.627815006	0.103564118	0.545480965	up_in_case
CRA004775	ENSGALG00010003227	PLAC8L1	case	control	2.322526633	0.834795619	2.782150003	0.005400008	0.149708676	up_in_case
CRA004775	ENSGALG00010003229	STIM1	case	control	-0.254214053	0.169938183	-1.495920738	0.134674312	0.592420453	down_in_case
CRA004775	ENSGALG00010003237	ENSGALG00010003237	case	control	0.923024328	0.568859745	1.622586825	0.104677759	0.547517634	up_in_case
CRA004775	ENSGALG00010003239	RRM1	case	control	-0.649502705	0.29489124	-2.202516102	0.027628873	0.317397354	down_in_case
CRA004775	ENSGALG00010003240	PLACL2	case	control	-0.627551648	0.694141596	-0.904068638	0.365959007	0.793608387	down_in_case
CRA004775	ENSGALG00010003242	ASAP2	case	control	0.773110191	0.407980232	1.894969735	0.058096411	0.441372239	up_in_case
CRA004775	ENSGALG00010003243	VPS50	case	control	0.003823348	0.319935678	0.011950366	0.990465214	0.998065748	up_in_case
CRA004775	ENSGALG00010003245	ENSGALG00010003245	case	control	0.037386353	0.404016925	0.092536602	0.926271712	0.98417264	up_in_case
CRA004775	ENSGALG00010003246	VWA8	case	control	-0.00106584	0.263433424	-0.004045954	0.996771805	0.999606847	down_in_case
CRA004775	ENSGALG00010003247	ENSGALG00010003247	case	control	-0.15460182	0.444397674	-0.347890704	0.727922267	0.935569594	down_in_case
CRA004775	ENSGALG00010003248	MRI1	case	control	-0.122230827	0.205085514	-0.595999319	0.551175681	0.877935456	down_in_case
CRA004775	ENSGALG00010003249	YJU2B	case	control	-0.494955086	0.316652792	-1.563084545	0.11803271	0.569418751	down_in_case
CRA004775	ENSGALG00010003250	CD1G	case	control	1.627757303	0.672028025	2.422156879	0.015428688	0.249560894	up_in_case
CRA004775	ENSGALG00010003251	ELOF1	case	control	-0.104917363	0.228059425	-0.46004397	0.64548466	0.914043203	down_in_case
CRA004775	ENSGALG00010003252	PHEX	case	control	-0.132029145	0.555508301	-0.237672677	0.812134977	0.957585764	down_in_case
CRA004775	ENSGALG00010003253	ITGB1BP1	case	control	-0.208167593	0.195149496	-1.066708332	0.286103563	0.741582427	down_in_case
CRA004775	ENSGALG00010003254	DGKH	case	control	0.11502252	0.610551221	0.18839127	0.850569943	0.968989143	up_in_case
CRA004775	ENSGALG00010003255	CALCR	case	control	0.286406958	0.416634108	0.687430417	0.491811536	0.855746361	up_in_case
CRA004775	ENSGALG00010003256	ENSGALG00010003256	case	control	0.842879854	0.256804689	3.282182494	0.001030069	0.06744991	up_in_case
CRA004775	ENSGALG00010003257	ENSGALG00010003257	case	control	1.007958366	0.344467374	2.926135945	0.003432009	0.125102734	up_in_case
CRA004775	ENSGALG00010003259	VEGFC	case	control	0.240461637	0.375241521	0.64081831	0.521640735	0.866314221	up_in_case
CRA004775	ENSGALG00010003260	ACP5	case	control	0.372623997	0.768768543	0.484702451	0.627887428	0.90828391	up_in_case
CRA004775	ENSGALG00010003261	PEX2	case	control	0.095658943	0.258367092	0.3702443	0.711200472	0.932234469	up_in_case
CRA004775	ENSGALG00010003262	ENSGALG00010003262	case	control	-3.453602179	1.39533375	-2.475108324	0.013319577	0.232183685	down_in_case
CRA004775	ENSGALG00010003263	ENSGALG00010003263	case	control	0.490723469	0.898864127	0.545937315	0.585109027	0.890814241	up_in_case
CRA004775	ENSGALG00010003264	ENSGALG00010003264	case	control	-0.424615443	0.685620708	-0.619315371	0.535708621	0.871366127	down_in_case
CRA004775	ENSGALG00010003268	SMS	case	control	0.354519583	0.205829729	1.722392508	0.08499844	0.505542192	up_in_case
CRA004775	ENSGALG00010003269	ENSGALG00010003269	case	control	-1.526154926	0.760296945	-2.007314296	0.044716208	0.393998162	down_in_case
CRA004775	ENSGALG00010003271	AKAP11	case	control	0.116264898	0.366320358	0.31738585	0.750950843	0.941495694	up_in_case
CRA004775	ENSGALG00010003272	CPSF3	case	control	-0.100203122	0.158599965	-0.631797882	0.527518958	0.86830001	down_in_case
CRA004775	ENSGALG00010003273	NFIX	case	control	-0.62206739	0.331766086	-1.875018022	0.060790244	0.445397937	down_in_case
CRA004775	ENSGALG00010003274	BACH2	case	control	1.220307611	0.392032724	3.112769764	0.001853405	0.086393824	up_in_case
CRA004775	ENSGALG00010003275	MBTPS2	case	control	-0.409826726	0.295895104	-1.385040577	0.16604006	0.630194031	down_in_case
CRA004775	ENSGALG00010003276	TNFSF11	case	control	1.037656464	0.433031514	2.396260851	0.016563298	0.255167461	up_in_case
CRA004775	ENSGALG00010003279	ENSGALG00010003279	case	control	-0.502047164	0.75720026	-0.663030892	0.507310769	0.862729457	down_in_case
CRA004775	ENSGALG00010003280	ENSGALG00010003280	case	control	0.150311599	0.301570837	0.498428828	0.618181822	0.904143308	up_in_case
CRA004775	ENSGALG00010003292	GADD45GIP1	case	control	0.110627009	0.311614105	0.355012843	0.72257997	0.935185555	up_in_case
CRA004775	ENSGALG00010003294	ENSGALG00010003294	case	control	-0.686104904	2.704154166	-0.253722555	0.799709908	0.952847322	down_in_case
CRA004775	ENSGALG00010003295	IAH1	case	control	-0.389540122	0.337349802	-1.154706833	0.2482105	0.712818804	down_in_case
CRA004775	ENSGALG00010003298	IL7	case	control	0.208214653	0.285305407	0.729795678	0.465515087	0.843383332	up_in_case
CRA004775	ENSGALG00010003299	RAD23A	case	control	-0.045776685	0.393600172	-0.116302503	0.907412802	0.980354087	down_in_case
CRA004775	ENSGALG00010003300	CASP8AP2	case	control	0.406061549	0.387101645	1.048979137	0.29418772	0.746438942	up_in_case
CRA004775	ENSGALG00010003301	ENSGALG00010003301	case	control	1.073143079	0.379177938	2.830183329	0.004652134	0.140906372	up_in_case
CRA004775	ENSGALG00010003303	MTHFD2L	case	control	0.080306846	0.357261172	0.224784701	0.822146769	0.960766569	up_in_case
CRA004775	ENSGALG00010003305	CALR	case	control	-0.177048334	0.181305345	-0.976520211	0.328806737	0.77220987	down_in_case
CRA004775	ENSGALG00010003307	COL1A2	case	control	-1.115758335	0.734756886	-1.518540835	0.128878113	0.583811118	down_in_case
CRA004775	ENSGALG00010003308	MDN1	case	control	0.040428122	0.310373743	0.130256256	0.896363688	0.978634146	up_in_case
CRA004775	ENSGALG00010003311	ENSGALG00010003311	case	control	-0.669281331	2.194059854	-0.305042422	0.760333843	0.942236668	down_in_case
CRA004775	ENSGALG00010003317	LYRM2	case	control	-0.103927892	0.353034438	-0.294384572	0.768464055	0.945059483	down_in_case
CRA004775	ENSGALG00010003320	OR51E2	case	control	1.150704237	0.92424586	1.245019628	0.213124535	0.682777582	up_in_case
CRA004775	ENSGALG00010003323	STMN2	case	control	1.442743809	0.506318732	2.84947745	0.004379111	0.135624223	up_in_case
CRA004775	ENSGALG00010003324	ENSGALG00010003324	case	control	-0.047285557	0.535088899	-0.088369535	0.929582974	0.98473447	down_in_case
CRA004775	ENSGALG00010003330	HEY1	case	control	0.516468742	0.219126025	2.35694844	0.018425805	0.269546005	up_in_case
CRA004775	ENSGALG00010003331	EPSTI1	case	control	-0.455860649	0.415817897	-1.096298771	0.272948047	0.731001618	down_in_case
CRA004775	ENSGALG00010003333	ENSGALG00010003333	case	control	0.494564515	0.330718358	1.495425043	0.134803549	0.592420453	up_in_case
CRA004775	ENSGALG00010003344	ZFAND1	case	control	-0.227824406	0.292867841	-0.77790858	0.43662291	0.827068479	down_in_case
CRA004775	ENSGALG00010003346	ENSGALG00010003346	case	control	4.104255319	2.865442081	1.432328836	0.152049734	0.616583868	up_in_case
CRA004775	ENSGALG00010003348	ADAM17	case	control	-0.2934585	0.290455635	-1.010338464	0.312333159	0.764325516	down_in_case
CRA004775	ENSGALG00010003351	ENSGALG00010003351	case	control	1.489025481	3.064856685	0.485838535			up_in_case
CRA004775	ENSGALG00010003352	FARSA	case	control	-0.413825204	0.225944044	-1.831538451	0.067020214	0.462498477	down_in_case
CRA004775	ENSGALG00010003356	ENSGALG00010003356	case	control	0.833311248	0.267077327	3.120112281	0.001807821	0.085427315	up_in_case
CRA004775	ENSGALG00010003358	DNAJC15	case	control	0.000214461	0.285437768	0.000751342	0.999400516	0.999914248	up_in_case
CRA004775	ENSGALG00010003363	HBE1	case	control	0.865819208	1.025860311	0.843993279	0.398673161	0.810039516	up_in_case
CRA004775	ENSGALG00010003364	ANKRD6	case	control	-0.245689889	0.34539953	-0.711320855	0.476885431	0.846367006	down_in_case
CRA004775	ENSGALG00010003366	CASD1	case	control	0.011828651	0.229716883	0.051492301	0.958933236	0.991431439	up_in_case
CRA004775	ENSGALG00010003368	ENSGALG00010003368	case	control	2.318577441	2.296832359	1.009467422			up_in_case
CRA004775	ENSGALG00010003371	ENSGALG00010003371	case	control	-0.221434178	0.697531502	-0.317454017	0.750899125	0.941495694	down_in_case

CRA004775	ENSGALG00010003372	ENOX1	case	control	0.176446489	0.315379337	0.559473842	0.575838379	0.887937128	up_in_case
CRA004775	ENSGALG00010003374	SYCE2	case	control	-0.858296726	0.627922747	-1.366882678	0.171662082	0.636057362	down_in_case
CRA004775	ENSGALG00010003375	YWHAQ	case	control	-0.275046869	0.321305561	-0.856028972	0.391981755	0.80746892	down_in_case
CRA004775	ENSGALG00010003377	ENSGALG00010003377	case	control	-0.230685246	3.061959289	-0.075339096	0.939944915	0.987076872	down_in_case
CRA004775	ENSGALG00010003379	ENSGALG00010003379	case	control	0.910936249	0.930948675	0.97850319	0.327825514	0.771733617	up_in_case
CRA004775	ENSGALG00010003382	ASNS	case	control	-1.163799408	0.483925488	-2.404914467	0.016176251	0.25344686	down_in_case
CRA004775	ENSGALG00010003383	UBE2J1	case	control	0.176226647	0.19260742	0.914952537	0.360216548	0.791975934	up_in_case
CRA004775	ENSGALG00010003388	PXDN	case	control	0.46735605	0.491052199	0.951744134	0.341226761	0.781123554	up_in_case
CRA004775	ENSGALG00010003395	ENSGALG00010003395	case	control	0.745641343	0.411784565	1.81075593	0.070178641	0.469724266	up_in_case
CRA004775	ENSGALG00010003397	SPATA4	case	control	0.130005424	0.659954449	0.196991511	0.843834181	0.966478468	up_in_case
CRA004775	ENSGALG00010003400	PKIA	case	control	0.654852695	0.411155654	1.592712367	0.111224732	0.560478986	up_in_case
CRA004775	ENSGALG00010003401	ENSGALG00010003401	case	control	0.305979762	0.562970021	0.543509869	0.586778798	0.891776663	up_in_case
CRA004775	ENSGALG00010003403	GCDH	case	control	-0.422858003	0.303264901	-1.394351939	0.163211354	0.629717526	down_in_case
CRA004775	ENSGALG00010003406	C1GALT1	case	control	-0.069499022	0.23099979	-0.300861842	0.763519848	0.94239774	down_in_case
CRA004775	ENSGALG00010003407	CHMP4C	case	control	0.169183072	0.360268258	0.469603047	0.638638648	0.91099914	up_in_case
CRA004775	ENSGALG00010003408	ENSGALG00010003408	case	control	0.630015303	0.350124295	1.799404704	0.071954686	0.473280705	up_in_case
CRA004775	ENSGALG00010003412	ASB5	case	control	0.13493237	0.483586604	0.27902421	0.780226246	0.94835413	up_in_case
CRA004775	ENSGALG00010003420	ENSGALG00010003420	case	control	-0.215158844	0.533623128	-0.403203746	0.686798344	0.92689803	down_in_case
CRA004775	ENSGALG00010003428	SNX16	case	control	-0.601134806	0.561947831	-1.069734187	0.284738974	0.74120185	down_in_case
CRA004775	ENSGALG00010003429	ENSGALG00010003429	case	control	-6.543636395	2.731234905	-2.395852654	0.016581755	0.255167461	down_in_case
CRA004775	ENSGALG00010003431	PM20D2	case	control	0.010880294	0.619653439	0.017558676	0.985990924	0.997562105	up_in_case
CRA004775	ENSGALG00010003432	LACC1	case	control	-0.1649814	0.372762309	-0.44259142	0.658061292	0.917354438	down_in_case
CRA004775	ENSGALG00010003437	PNRC1	case	control	0.308924373	0.318086197	0.971197042	0.331450167	0.772629743	up_in_case
CRA004775	ENSGALG00010003438	KCNS3	case	control	1.064305112	0.486276204	2.188684337	0.028619791	0.322057735	up_in_case
CRA004775	ENSGALG00010003439	GMIP	case	control	-0.77107988	0.542539384	-1.421242222	0.155246354	0.619284425	down_in_case
CRA004775	ENSGALG00010003442	SPCS3	case	control	-0.184285254	0.221147696	-0.83331302	0.404668215	0.81313407	down_in_case
CRA004775	ENSGALG00010003447	ZC2HC1A	case	control	0.666031279	0.327253564	2.035214745	0.04182927	0.383462862	up_in_case
CRA004775	ENSGALG00010003449	BORCS6	case	control	-0.067784463	0.337681941	-0.20073464	0.840906072	0.966461921	down_in_case
CRA004775	ENSGALG00010003454	BPNT2	case	control	0.067972144	0.16406991	0.414287686	0.678663431	0.92338129	up_in_case
CRA004775	ENSGALG00010003456	CDS1	case	control	0.812379758	0.547363321	1.484169155	0.137764051	0.596852448	up_in_case
CRA004775	ENSGALG00010003466	TSC22D1	case	control	0.17615823	0.301338046	0.584586752	0.558825618	0.880754991	up_in_case
CRA004775	ENSGALG00010003468	ILVBL	case	control	-0.652845306	0.311698355	-2.094477865	0.036217433	0.360628495	down_in_case
CRA004775	ENSGALG00010003470	METTL3	case	control	0.086747434	0.320459751	0.270696815	0.786624227	0.95041797	up_in_case
CRA004775	ENSGALG00010003471	ADI1	case	control	0.212265992	0.250710787	0.846656799	0.397186445	0.808998414	up_in_case
CRA004775	ENSGALG00010003472	FAM110B	case	control	-0.325794239	0.439701018	-0.740944928	0.458726833	0.840276099	down_in_case
CRA004775	ENSGALG00010003473	ENSGALG00010003473	case	control	0.087352481	0.327561652	0.26667493	0.789719458	0.950663772	up_in_case
CRA004775	ENSGALG00010003474	RPS7	case	control	-0.230249234	0.205728695	-1.119188717	0.263059638	0.723900957	down_in_case
CRA004775	ENSGALG00010003475	ENSGALG00010003475	case	control	1.215781542	0.867150196	1.402042631	0.160902517	0.626790513	up_in_case
CRA004775	ENSGALG00010003476	ENSGALG00010003476	case	control	-1.489701426	0.645867933	-2.306510897	0.021082102	0.285392234	down_in_case
CRA004775	ENSGALG00010003477	ENSGALG00010003477	case	control	1.058884513	0.856285756	1.236601807	0.216234949	0.683774974	up_in_case
CRA004775	ENSGALG00010003478	COLEC11	case	control	0.744576076	0.40649315	1.831706333	0.066995185	0.462498477	up_in_case
CRA004775	ENSGALG00010003479	SYDE1	case	control	-0.204294547	0.260173962	-0.785222877	0.432322905	0.825447412	down_in_case
CRA004775	ENSGALG00010003485	RNGTT	case	control	-0.257323627	0.320897006	-0.801888525	0.422617445	0.820450913	down_in_case
CRA004775	ENSGALG00010003489	SGCE	case	control	0.209979438	0.294260645	0.713583149	0.47548498	0.846065472	up_in_case
CRA004775	ENSGALG00010003495	ENSGALG00010003495	case	control	0.196269809	0.705395008	0.278240996	0.780827367	0.948679724	up_in_case
CRA004775	ENSGALG00010003503	NUFIP1	case	control	0.319551713	0.249281733	1.281889808	0.199881304	0.667128108	up_in_case
CRA004775	ENSGALG00010003506	DYNC1I1	case	control	0.536204037	0.376862614	1.422810375	0.154791133	0.619284425	up_in_case
CRA004775	ENSGALG00010003507	WDFY3	case	control	0.045559126	0.28782934	0.158285203	0.874232068	0.974115968	up_in_case
CRA004775	ENSGALG00010003509	GPALPP1	case	control	-0.360742837	0.260308482	-1.385828207	0.165799367	0.630194031	down_in_case
CRA004775	ENSGALG00010003511	UBXN2B	case	control	-0.083543751	0.372031246	-0.224561113	0.82232072	0.960868728	down_in_case
CRA004775	ENSGALG00010003512	ENSGALG00010003512	case	control	-1.278523395	0.506152172	-2.525966431	0.011538051	0.216528087	down_in_case
CRA004775	ENSGALG00010003517	GTF2F2	case	control	-0.412317885	0.23970129	-1.720132108	0.085408431	0.506269697	down_in_case
CRA004775	ENSGALG00010003520	ENSGALG00010003520	case	control	0.499903495	0.784902115	0.636899156	0.524190532	0.866784927	up_in_case
CRA004775	ENSGALG00010003522	ENSGALG00010003522	case	control	-1.158114847	0.494818396	-2.340484621	0.019258732	0.274147845	down_in_case
CRA004775	ENSGALG00010003525	NOL10	case	control	-0.094004068	0.210526665	-0.446518581	0.655222694	0.916967384	down_in_case
CRA004775	ENSGALG00010003528	GDAP1	case	control	-0.494350873	0.465620778	-1.061702778	0.288370638	0.742851819	down_in_case
CRA004775	ENSGALG00010003530	ENSGALG00010003530	case	control	1.003140145	1.145500131	0.875722418	0.381180955	0.801753951	up_in_case
CRA004775	ENSGALG00010003531	PPM1K	case	control	-0.266911146	0.417869904	-0.63874221	0.522990648	0.866638286	down_in_case
CRA004775	ENSGALG00010003539	ENSGALG00010003539	case	control	-0.697563676	0.392782897	-1.775952266	0.075740785	0.484198762	down_in_case
CRA004775	ENSGALG00010003541	CNR1	case	control	-0.136413656	0.532375631	-0.256235725	0.797768824	0.952835572	down_in_case
CRA004775	ENSGALG00010003543	SOX11	case	control	0.730201309	0.350960811	2.080577905	0.037472558	0.365326975	up_in_case
CRA004775	ENSGALG00010003552	PI15	case	control	1.778582214	0.48265429	3.685002396	0.0002287	0.030954849	up_in_case
CRA004775	ENSGALG00010003556	ENSGALG00010003556	case	control	0.382748392	1.051010256	0.364171891	0.715729656	0.933921336	up_in_case
CRA004775	ENSGALG00010003558	TPT1	case	control	-0.019828507	0.398192538	-0.049796281	0.960284731	0.991431439	down_in_case
CRA004775	ENSGALG00010003560	ADGRL3	case	control	0.026435827	0.398148092	0.066396971	0.947061782	0.98933351	up_in_case
CRA004775	ENSGALG00010003571	RPL7	case	control	-0.33763127	0.210515081	-1.603834119	0.108750622	0.555936379	down_in_case
CRA004775	ENSGALG00010003577	ENSGALG00010003577	case	control	0.81911167	0.93577032	0.875334098	0.381392146	0.801753951	up_in_case
CRA004775	ENSGALG00010003582	RDH10	case	control	0.34275033	0.27080193	1.265686435	0.205625367	0.674045742	up_in_case
CRA004775	ENSGALG00010003583	RSAD2	case	control	-0.239348481	0.552441396	-0.433255875	0.66482889	0.918655755	down_in_case
CRA004775	ENSGALG00010003585	PNPLA6	case	control	-0.667289802	0.186704019	-3.574051627	0.0003515	0.037180544	down_in_case
CRA004775	ENSGALG00010003589	SLC25A30	case	control	0.077895916	0.754356863	0.103261361	0.917755541	0.981688766	up_in_case
CRA004775	ENSGALG00010003595	SNX13	case	control	0.250868836	0.218488518	1.148201465	0.250885405	0.715684776	up_in_case
CRA004775	ENSGALG00010003596	CRISPLD1	case	control	0.007763988	0.361437914	0.021480835	0.982862091	0.997398088	up_in_case
CRA004775	ENSGALG00010003597	RNF144A	case	control	-0.614860095	0.679045467	-0.905477062	0.365212707	0.793608387	down_in_case
CRA004775	ENSGALG00010003604	ODC	case	control	-0.510239963	0.32192149	-1.584982608	0.112970286	0.562886277	down_in_case
CRA004775	ENSGALG00010003617	COG3	case	control	-0.059219999	0.185661558	-0.318967476	0.749751176	0.941495694	down_in_case
CRA004775	ENSGALG00010003619	HNF4G	case	control	2.540493737	0.875071956	2.903182669	0.003693911	0.12600076	up_in_case
CRA004775	ENSGALG00010003627	MCOLN1	case	control	-0.400534374	0.137031783	-2.92293048	0.003467539	0.125102734	down_in_case
CRA004775	ENSGALG00010003634	CYP4F11	case	control	0.989640599	0.424977747	2.328688046	0.019875598	0.279171957	up_in_case
CRA004775	ENSGALG00010003639	ENSGALG00010003639	case	control	0.954586428	1.276561758	0.747779276	0.454593304	0.838254622	up_in_case
CRA004775	ENSGALG00010003641	ENSGALG00010003641	case	control	0.734850392	0.875433029	0.839413602	0.401237254	0.811868062	up_in_case
CRA004775	ENSGALG00010003652	MYCN	case	control	0.46055456	0.458451964	1.004586296	0.315096099	0.765098415	up_in_case
CRA004775	ENSGALG00010003659	ENSGALG00010003659	case	control	1.045676264	0.880298219	1.187865932	0.234886234	0.702145501	up_in_case
CRA004775	ENSGALG00010003660	IGFBP7	case	control	0.331939633	0.323514184	1.026043522	0.30487107	0.757550069	up_in_case
CRA004775	ENSGALG00010003665	RDH14	case	control	0.443995197	0.322157958	1.378190997	0.168144326	0.63138062	up_in_case
CRA004775	ENSGALG00010003666	SLC25A13	case	control	-0.222138296	0.378474818	-0.586930188	0.557250594	0.880622207	down_in_case
CRA004775	ENSGALG00010003669	ENSGALG00010003669	case	control	0.711192364	0.861020358	0.825987861	0.408810978	0.814384687	up_in_case
CRA004775	ENSGALG00010003671	ENSGALG00010003671	case	control	0.399868302	0.843056787	0.474307672	0.635280522	0.909837348	up_in_case
CRA004775	ENSGALG00010003674	AKIRIN2	case	control	0.377720465	0.195614311	1.930944948	0.053489861	0.425885384	up_in_case
CRA004775	ENSGALG00010003676	POLR2B	case	control	-0.074766476	0.142612521	-0.524263058	0.600095587	0.897206546	down_in_case
CRA004775	ENSGALG00010003678	ZNFY1	case	control	-0.03995135	0.54357411	-0.073497521	0.941410216	0.987577169	down_in_case
CRA004775	ENSGALG00010003680	TRAPPC5	case	control	-0.656942637	0.293815879	-2.235899027	0.025358386	0.30561189	down_in_case
CRA004775	ENSGALG00010003681	NT5C1B	case	control	-0.120853009	0.190920988	-0.633000124	0.526733562	0.86830001	down_in_case
CRA004775	ENSGALG00010003683	ENSGALG00010003683	case	control	1.064400268	0.871382467	1.221507556	0.221893906	0.689550502	up_in_case
CRA004775	ENSGALG00010003691	ERICH1	case	control	-0.293134454	0.385140312	-0.761110809	0.446590885	0.83381823	down_in_case
CRA004775	ENSGALG00010003692	ZNFY2	case	control	-0.495576253	0.333535005	-1.485829809	0.137324143	0.596242097	down_in_case
CRA004775	ENSGALG00010003695	NOA1	case	control	-0.175971771	0.245580468	-0.716554426	0.473649073	0.845445211	down_in_case
CRA004775	ENSGALG00010003696	ENSGALG00010003696	case	control	0.975136456	0.922566502	1.056982292	0.290519669	0.744351774	up_in_case
CRA004775	ENSGALG00010003700	REST	case	control	0.270490304	0.33984776	0.7959161	0.426080803	0.822541484	up_in_case
CRA004775	ENSGALG00010003701	SARAF	case	control	-0.117269171	0.180694278	-0.648992164	0.516343441	0.864913871	down_in_case
CRA004775	ENSGALG00010003703	TDRP	case	control	-0.238670418	0.373559997	-0.638907858	0.522882876	0.866638286	down_in_case
CRA004775	ENSGALG00010003707	ENSGALG00010003707	case	control	0.576424369	1.076643083	0.535390398	0.592379928	0.893965803	up_in_case
CRA004775	ENSGALG00010003713	SPERT	case	control	2.205824167	0.771801917	2.858018513	0.004262955	0.133620227	up_in_case
CRA004775	ENSGALG00010003714	ORC3	case	control	-0.475201786	0.250484985	-1.89712683	0.057811201	0.440178198	down_in_case
CRA004775	ENSGALG00010003715	ENSGALG00010003715	case	control	1.046565812	0.871686777	1.200621416	0.22989809	0.696021275	up_in_case
CRA004775	ENSGALG00010003716	YLEC8	case	control	-0.400490099	0.786957357	-0.50890953	0.610815639	0.901413878	down_in_case
CRA004775	ENSGALG00010003717	LEPROTL1	case	control	-0.321864844	0.189445298	-1.698985657	0.089321886	0.515997712	down_in_case
CRA004775	ENSGALG00010003718	SIAH3	case	control	2.444989893	1.860885159	1.313885428	0.188884763	0.653422485	up_in_case
CRA004775	ENSGALG00010003719	RPGR	case	control	0.448395137	0.23859513	1.879313868	0.060201649	0.445397937	up_in_case
CRA004775	ENSGALG00010003720	ENSGALG00010003720	case	control	0.779020708	0.920687268	0.846129555	0.397480475	0.809261772	up_in_case
CRA004775	ENSGALG00010003722	MHCY11	case	control	-1.068161989	0.541030998	-1.974308298	0.048346698	0.407414924	down_in_case
CRA004775	ENSGALG00010003724	ENSGALG00010003724	case	control	-0.052731151	0.20555793	-0.256526959	0.797543966	0.952835572	down_in_case
CRA004775	ENSGALG00010003726	ENPEP	case	control	1.386926317	1.222580021	1.134425799	0.256615982	0.719236059	up_in_case
CRA004775	ENSGALG00010003727	FBXO25	case	control	-0.407396076	0.274311652	-1.485157753	0.13750204	0.596242097	down_in_case
CRA004775	ENSGALG00010003729	RARS2	case	control	-0.195005876	0.263572472	-0.739856762	0.459386913	0.840589413	down_in_case
CRA004775	ENSGALG00010003730	ENSGALG00010003730	case	control	1.611590742	0.891236237	1.80826438	0.070565363	0.469895261	up_in_case
CRA004775	ENSGALG00010003731	ENSGALG00010003731	case	control	-0.459991915	0.510124872	-0.901724147	0.367203422	0.794067504	down_in_case
CRA004775	ENSGALG00010003733	STAU2	case	control	-0.039040524	0.194086848	-0.201149768	0.840581468	0.966461921	down_in_case
CRA004775	ENSGALG00010003734	ZC3H13	case	control	0.162174595	0.199070231	0.814660207	0.415266837	0.818921904	up_in_case
CRA004775	ENSGALG00010003736	ENSGALG00010003736	case	control	1.402514028	0.811423209	1.728461809	0.083905462	0.503010645	up_in_case
CRA004775	ENSGALG00010003737	SRP72	case	control	-0.170711417	0.128981187	-1.323537345	0.185656746	0.6491093	down_in_case
CRA004775	ENSGALG00010003740	PITX2	case	control	0.646800422	0.494891495	1.306954005	0.191228333	0.656959697	up_in_case
CRA004775	ENSGALG00010003742	ZFHX4	case	control	-0.067923074	0.40407907	-0.168093523	0.866509705	0.972842501	down_in_case
CRA004775	ENSGALG00010003743	ARL9	case	control	-0.327319999	0.327087057	-1.00071217	0.316965982	0.765974554	down_in_case
CRA004775	ENSGALG00010003745	ENSGALG00010003745	case	control	0.63326054	1.043445871	0.606893523	0.543921577	0.875729696	up_in_case
CRA004775	ENSGALG00010003746	HOPX	case	control	-0.518855744	0.438167706	-1.184148756	0.236354183	0.703419274	down_in_case

CRA004775	ENSGALG00010003749	MHCY15	case	control	-1.477309417	0.598158803	-2.469761225	0.013520326	0.232945019	down_in_case
CRA004775	ENSGALG00010003753	SLCO5A1	case	control	0.431225282	0.468484688	0.920468252	0.357328116	0.79022498	up_in_case
CRA004775	ENSGALG00010003754	FAM110C	case	control	0.265824242	0.422661135	0.628929939	0.529394925	0.869211096	up_in_case
CRA004775	ENSGALG00010003755	SLC35A1	case	control	0.08593926	0.250822633	0.342629605	0.731877127	0.936755276	up_in_case
CRA004775	ENSGALG00010003756	MHCY7	case	control	-0.919959044	0.585361429	-1.57160858	0.116041355	0.566086328	down_in_case
CRA004775	ENSGALG00010003757	ENSGALG00010003757	case	control	0.76438767	0.905009828	0.844618087	0.398324107	0.809780425	up_in_case
CRA004775	ENSGALG00010003761	UGT8	case	control	1.251624534	0.65625232	1.907230643	0.056490727	0.434721852	up_in_case
CRA004775	ENSGALG00010003762	EVI5L	case	control	-0.49115898	0.237323821	-2.06957303	0.038492346	0.370251617	down_in_case
CRA004775	ENSGALG00010003764	HDAC9	case	control	0.009668713	0.342905164	0.028196464	0.977505457	0.995827348	up_in_case
CRA004775	ENSGALG00010003765	ENSGALG00010003765	case	control	-1.361418318	0.725940296	-1.87538607			down_in_case
CRA004775	ENSGALG00010003767	ENSGALG00010003767	case	control	-1.304712579	2.035043477	-0.641122705	0.521442964	0.866299649	down_in_case
CRA004775	ENSGALG00010003768	ARSJ	case	control	-0.237233556	0.365101044	-0.64977507	0.515837525	0.864897625	down_in_case
CRA004775	ENSGALG00010003771	SPARCL1	case	control	0.480098785	0.616206496	0.779119966	0.43590905	0.826905159	up_in_case
CRA004775	ENSGALG00010003772	ENSGALG00010003772	case	control	-0.466625778	0.556490765	-0.838514865	0.401741603	0.811868062	down_in_case
CRA004775	ENSGALG00010003775	NCOA2	case	control	0.248235529	0.384044141	0.646372389	0.518038217	0.865435399	up_in_case
CRA004775	ENSGALG00010003777	ENSGALG00010003777	case	control	1.046326601	0.778804384	1.343503738	0.179108916	0.644923737	up_in_case
CRA004775	ENSGALG00010003778	LCA5	case	control	0.02041512	0.300110417	0.068025363	0.945765444	0.988886825	up_in_case
CRA004775	ENSGALG00010003785	ENSGALG00010003785	case	control	0.759148895	0.860665463	0.88204875	0.377750445	0.799125872	up_in_case
CRA004775	ENSGALG00010003786	ENSGALG00010003786	case	control	0.308669069	0.719716529	0.428875892	0.668013545	0.919688403	up_in_case
CRA004775	ENSGALG00010003787	SMIM8	case	control	0.137166033	0.404368102	0.339210813	0.734450924	0.936819725	up_in_case
CRA004775	ENSGALG00010003788	NUDT9	case	control	-0.141946361	0.263155052	-0.539401997	0.589609504	0.893412703	down_in_case
CRA004775	ENSGALG00010003790	ELOVL6	case	control	-0.18083912	0.42761184	-0.422904848	0.672364686	0.921952322	down_in_case
CRA004775	ENSGALG00010003791	ITGB8	case	control	1.030048055	0.399089049	2.580998042	0.009851514	0.201149915	up_in_case
CRA004775	ENSGALG00010003793	TRAM1L1	case	control	0.023585833	0.145897198	0.161660631	0.871573109	0.973918682	up_in_case
CRA004775	ENSGALG00010003795	LCP1	case	control	-0.765911342	0.513246927	-1.492286268	0.135624115	0.593592099	down_in_case
CRA004775	ENSGALG00010003796	ENSGALG00010003796	case	control	-1.850643022	0.498695019	-3.710971541	0.000206465	0.029269119	down_in_case
CRA004775	ENSGALG00010003797	ENSGALG00010003797	case	control	-0.010916919	0.347421224	-0.031422716	0.974932425	0.995106174	down_in_case
CRA004775	ENSGALG00010003803	LACTB2	case	control	-0.287711349	0.221393115	-1.299549667	0.19375536	0.657919837	down_in_case
CRA004775	ENSGALG00010003804	PSME1	case	control	-0.628634462	0.284562744	-2.209124258	0.027165999	0.31561562	down_in_case
CRA004775	ENSGALG00010003806	EGF	case	control	0.254190435	0.636922543	0.399091597	0.689825714	0.927796693	up_in_case
CRA004775	ENSGALG00010003808	TWIST1	case	control	0.003751989	0.258528128	0.014512885	0.9884208	0.998065748	up_in_case
CRA004775	ENSGALG00010003810	HMGN2P46	case	control	0.31502268	0.305564382	1.030953537	0.302562609	0.755164773	up_in_case
CRA004775	ENSGALG00010003812	BG8	case	control	-0.13303711	0.50019533	-0.265970315	0.790262071	0.951054854	down_in_case
CRA004775	ENSGALG00010003814	TPP1	case	control	-0.401620027	0.205592154	-1.953479346	0.050762822	0.416238146	down_in_case
CRA004775	ENSGALG00010003815	RASAL3	case	control	-0.899952673	0.498200087	-1.806408101	0.070854617	0.469911807	down_in_case
CRA004775	ENSGALG00010003816	KLHL8	case	control	-0.234755188	0.268593255	-0.874017435	0.382108759	0.801753951	down_in_case
CRA004775	ENSGALG00010003817	BF2	case	control	-0.348554124	0.245719205	-1.418505826	0.156043139	0.619442611	down_in_case
CRA004775	ENSGALG00010003818	GAB2	case	control	-0.032356416	0.456744622	-0.070841372	0.943524004	0.987862092	down_in_case
CRA004775	ENSGALG00010003819	PHIP	case	control	0.718497236	0.318984056	2.252455016	0.024293532	0.30145607	up_in_case
CRA004775	ENSGALG00010003820	SH3YL1	case	control	-0.359456385	0.443474014	-0.81054667	0.417626053	0.818943344	down_in_case
CRA004775	ENSGALG00010003821	RUBCNL	case	control	-0.402578332	0.330806814	-1.216959009	0.223619812	0.691474027	down_in_case
CRA004775	ENSGALG00010003822	ENSGALG00010003822	case	control	-0.447816811	0.288033596	-1.55473812	0.120008452	0.572133279	down_in_case
CRA004775	ENSGALG00010003823	PTPN13	case	control	0.228094679	0.334064891	0.682785545	0.494742365	0.856382445	up_in_case
CRA004775	ENSGALG00010003824	SP4	case	control	0.354576018	0.333679437	1.062624719	0.287952172	0.742682262	up_in_case
CRA004775	ENSGALG00010003825	DMB1	case	control	0.957167306	0.37936759	2.5230603	0.011633845	0.216997958	up_in_case
CRA004775	ENSGALG00010003826	ENSGALG00010003826	case	control	0.780610672	0.311435309	2.506493802	0.01219352	0.222250112	up_in_case
CRA004775	ENSGALG00010003829	ENSGALG00010003829	case	control	-0.033823349	0.350237114	-0.096572715	0.923065725	0.983159417	down_in_case
CRA004775	ENSGALG00010003830	DMB2	case	control	-0.545128516	0.293024138	-1.860353622	0.062835512	0.450026626	down_in_case
CRA004775	ENSGALG00010003831	IRAK1BP1	case	control	-0.592962846	0.276956119	-2.140999262	0.032274098	0.342175109	down_in_case
CRA004775	ENSGALG00010003832	ART1B	case	control	0.793175209	0.557567894	1.422562557	0.154863005	0.619284425	up_in_case
CRA004775	ENSGALG00010003833	ACP1	case	control	-0.594468795	0.248533716	-2.391904022	0.016761225	0.255907381	down_in_case
CRA004775	ENSGALG00010003835	ENSGALG00010003835	case	control	-0.309270439	0.417213842	-0.741275596	0.458526355	0.8401327	down_in_case
CRA004775	ENSGALG00010003836	CD1F	case	control	0.005821616	0.316834092	0.018374336	0.985340226	0.997562105	up_in_case
CRA004775	ENSGALG00010003837	ALKAL2	case	control	-0.229297685	0.703713703	-0.325839449	0.74454583	0.939218065	down_in_case
CRA004775	ENSGALG00010003838	GAR1	case	control	-0.211146253	0.296196931	-0.712857666	0.475933837	0.846243735	down_in_case
CRA004775	ENSGALG00010003839	ENSGALG00010003839	case	control	-0.120637787	0.532875687	-0.226390112	0.820898018	0.960766569	down_in_case
CRA004775	ENSGALG00010003841	UBE2W	case	control	0.042824319	0.379266046	0.112913666	0.910099002	0.98075825	up_in_case
CRA004775	ENSGALG00010003842	ENSGALG00010003842	case	control	-0.036345843	0.1991936	-0.182464915	0.855217886	0.970581465	down_in_case
CRA004775	ENSGALG00010003843	LRCH1	case	control	0.309713355	0.325077382	0.952737323	0.340723178	0.780875343	up_in_case
CRA004775	ENSGALG00010003844	TMEM18	case	control	0.00173081	0.206268468	0.008391055	0.993304985	0.998606291	up_in_case
CRA004775	ENSGALG00010003848	ESD	case	control	-0.091462687	0.204350881	-0.447576673	0.654458741	0.91662886	down_in_case
CRA004775	ENSGALG00010003850	PLA2G12A	case	control	-0.275857724	0.158160543	-1.744162728	0.081130719	0.496764413	down_in_case
CRA004775	ENSGALG00010003851	PHF14	case	control	0.135401937	0.185187362	0.731161867	0.464680289	0.843206474	up_in_case
CRA004775	ENSGALG00010003852	TCEB1	case	control	0.084180609	0.25068752	0.335798961	0.737022474	0.937564762	up_in_case
CRA004775	ENSGALG00010003853	ENSGALG00010003853	case	control	-0.022299714	0.470229673	-0.047423027	0.962176077	0.991860454	down_in_case
CRA004775	ENSGALG00010003858	SUCLA2	case	control	0.301240877	0.308802286	0.975513754	0.329305482	0.77237154	up_in_case
CRA004775	ENSGALG00010003860	TMEM70	case	control	0.252475424	0.234990625	1.074406367	0.28264059	0.738895581	up_in_case
CRA004775	ENSGALG00010003861	NUDT15	case	control	-0.215730092	0.170029743	-1.268778555	0.204520056	0.674045742	down_in_case
CRA004775	ENSGALG00010003862	PCK2	case	control	0.040289781	0.188477558	0.213764342	0.830730834	0.963284108	up_in_case
CRA004775	ENSGALG00010003863	HTR1B	case	control	0.615961512	0.442161952	1.393067653	0.163599334	0.629717526	up_in_case
CRA004775	ENSGALG00010003865	AKAP8L	case	control	-0.136871302	0.222409977	-0.61540091	0.538289991	0.872880385	down_in_case
CRA004775	ENSGALG00010003868	HBBA	case	control	-0.186282322	0.280420035	-0.664297479	0.506499934	0.8622397	down_in_case
CRA004775	ENSGALG00010003869	LY96	case	control	-0.265637516	0.475595598	-0.558536532	0.576478066	0.887937128	down_in_case
CRA004775	ENSGALG00010003870	MED4	case	control	-0.212658931	0.211872295	-1.003712783	0.315517074	0.765461123	down_in_case
CRA004775	ENSGALG00010003871	ENSGALG00010003871	case	control	-0.447594768	0.274933607	-1.628010384	0.103522684	0.545480965	down_in_case
CRA004775	ENSGALG00010003874	YES1	case	control	0.688808485	0.216894684	3.175773936	0.001494374	0.080693373	up_in_case
CRA004775	ENSGALG00010003875	AKAP8	case	control	-0.130675294	0.213090365	-0.613238867	0.539718409	0.874165823	down_in_case
CRA004775	ENSGALG00010003878	ARHGAP24	case	control	0.03123076	0.222447939	0.140395819	0.888347261	0.977118922	up_in_case
CRA004775	ENSGALG00010003879	CASP6	case	control	0.166326752	0.28606429	0.581431368	0.560949761	0.881008373	up_in_case
CRA004775	ENSGALG00010003882	ENSGALG00010003882	case	control	-0.111048472	0.157175415	-0.706525712	0.479861269	0.84760081	down_in_case
CRA004775	ENSGALG00010003884	TYMS	case	control	0.159053712	0.488471298	0.325615267	0.74471546	0.939218065	up_in_case
CRA004775	ENSGALG00010003887	GIPC1	case	control	0.030637141	0.221891725	0.138072481	0.890183134	0.977283345	up_in_case
CRA004775	ENSGALG00010003889	ITM2B	case	control	-0.145489822	0.179744134	-0.809427374	0.418269362	0.818943344	down_in_case
CRA004775	ENSGALG00010003894	COPS4	case	control	-0.310170421	0.253981787	-1.221230959	0.221998586	0.689550502	down_in_case
CRA004775	ENSGALG00010003895	THSD7A	case	control	0.902793585	0.544007349	1.65952461	0.097010126	0.53104893	up_in_case
CRA004775	ENSGALG00010003896	MCUB	case	control	-0.068915283	0.417955482	-0.164886659	0.869033193	0.973370973	down_in_case
CRA004775	ENSGALG00010003898	NDST4	case	control	0.705033388	0.768211223	0.917759811	0.358744627	0.79097996	up_in_case
CRA004775	ENSGALG00010003899	LIN54	case	control	0.46822749	0.355756999	1.316144141	0.188125668	0.652111233	up_in_case
CRA004775	ENSGALG00010003900	RB1	case	control	-0.016970125	0.231840797	-0.073197322	0.941649098	0.987577169	down_in_case
CRA004775	ENSGALG00010003902	ENSGALG00010003902	case	control	-0.265807653	0.322158352	-0.825083848	0.409323989	0.814878167	down_in_case
CRA004775	ENSGALG00010003903	COL28A1	case	control	0.557074757	0.603865091	0.922515253	0.356259878	0.789127613	up_in_case
CRA004775	ENSGALG00010003909	DNAJB1	case	control	-0.789735653	0.450377113	-1.753498637	0.079516453	0.491871682	down_in_case
CRA004775	ENSGALG00010003910	THAP9	case	control	-0.54780629	0.260198984	-2.105336004	0.03526207	0.355594571	down_in_case
CRA004775	ENSGALG00010003913	PSMB5	case	control	-0.234873192	0.327704461	-0.716722595	0.473545281	0.845445211	down_in_case
CRA004775	ENSGALG00010003914	CAMK2D	case	control	0.048582692	0.282797972	0.171792928	0.863600326	0.971956791	up_in_case
CRA004775	ENSGALG00010003915	MIOS	case	control	-0.206559282	0.189725129	-1.088729164	0.276273337	0.731001618	down_in_case
CRA004775	ENSGALG00010003920	LPAR6	case	control	0.307880149	0.363450945	0.847102348	0.396938076	0.808998414	up_in_case
CRA004775	ENSGALG00010003921	COLEC12	case	control	0.233692624	0.417158882	0.560200523	0.57534267	0.887739796	up_in_case
CRA004775	ENSGALG00010003923	ENSGALG00010003923	case	control	-0.305106941	0.275809326	-1.106224164	0.268629507	0.727528758	down_in_case
CRA004775	ENSGALG00010003925	SEC31A	case	control	-0.286393247	0.217843325	-1.314675337	0.188619039	0.652667439	down_in_case
CRA004775	ENSGALG00010003928	ENSGALG00010003928	case	control	-0.717149486	0.289878095	-2.473969223	0.013362121	0.232291431	down_in_case
CRA004775	ENSGALG00010003929	RCBTB2	case	control	0.381771883	0.418174096	0.912949624	0.361269037	0.792567091	up_in_case
CRA004775	ENSGALG00010003933	TECR	case	control	0.144760668	0.474622593	0.305001636	0.760364906	0.942236668	up_in_case
CRA004775	ENSGALG00010003937	ENSGALG00010003937	case	control	0.090094548	0.264129116	0.341100405	0.733027996	0.936819725	up_in_case
CRA004775	ENSGALG00010003948	SCD5	case	control	0.485265589	0.255920354	1.896158632	0.057939071	0.440420216	up_in_case
CRA004775	ENSGALG00010003958	CYSLTR2	case	control	0.404815234	0.443821074	0.912113591	0.361708925	0.792757982	up_in_case
CRA004775	ENSGALG00010003960	ASPH	case	control	0.244841711	0.365728412	0.669463194	0.503200052	0.860996832	up_in_case
CRA004775	ENSGALG00010003962	UMAD1	case	control	-0.407001416	0.316140403	-1.287407152	0.197952413	0.664272829	down_in_case
CRA004775	ENSGALG00010003968	DSN1	case	control	-0.126702557	0.280010897	-0.452491523	0.650914927	0.915614449	down_in_case
CRA004775	ENSGALG00010003972	PENK	case	control	0.221085228	0.713864866	0.309701791	0.75678774	0.941900693	up_in_case
CRA004775	ENSGALG00010003977	MYO6	case	control	-0.480814838	0.56215534	-0.855306004	0.392381764	0.80746892	down_in_case
CRA004775	ENSGALG00010003978	ANK2	case	control	0.238218043	0.575051803	0.414254928	0.678687418	0.92338129	up_in_case
CRA004775	ENSGALG00010003980	CHCHD7	case	control	-0.249365907	0.291469801	-0.855546291	0.392248789	0.80746892	down_in_case
CRA004775	ENSGALG00010003981	TMEM150C	case	control	-0.317978864	0.221199327	-1.437521838	0.150569772	0.614342929	down_in_case
CRA004775	ENSGALG00010003982	GLCCI1	case	control	-0.335269179	0.384634648	-0.87165621	0.383395956	0.80220295	down_in_case
CRA004775	ENSGALG00010003985	PRMT5	case	control	-0.413625566	0.31007341	-1.333960131	0.182216923	0.647378665	down_in_case
CRA004775	ENSGALG00010003986	FNDC3A	case	control	0.298946426	0.228111421	1.310528095	0.190017246	0.655361714	up_in_case
CRA004775	ENSGALG00010003988	ENOPH1	case	control	-0.476009563	0.171443181	-2.776485829	0.005495003	0.150521488	down_in_case
CRA004775	ENSGALG00010003990	ICA1	case	control	0.212875324	0.446777158	0.476468684	0.633740513	0.909837348	up_in_case
CRA004775	ENSGALG00010003993	ENSGALG00010003993	case	control	-0.091307282	0.304581659	-0.299779318	0.764345492	0.94239774	down_in_case
CRA004775	ENSGALG00010003994	SDR16C5	case	control	0.20301692	0.180952456	1.121935146	0.261890016	0.72256212	up_in_case
CRA004775	ENSGALG00010003995	MLNR	case	control	-0.173679924	0.813141082	-0.213591378	0.830865723	0.963284108	down_in_case
CRA004775	ENSGALG00010004000	ENSGALG00010004000	case	control	-0.10833051	0.283270177	-0.382428221	0.702143761	0.930068722	down_in_case
CRA004775	ENSGALG00010004001	LARP7	case	control	0.220487456	0.21910063	1.006329631	0.314257031	0.765098415	up_in_case
CRA004775	ENSGALG00010004002	CDADC1	case	control	-0.482937544	0.263703219	-1.831367647	0.067045687	0.462498477	down_in_case
CRA004775	ENSGALG00010004003	HNRNPDL	case	control	0.054421981	0.263829973	0.206276719	0.836574763	0.965269623	up_in_case

CRA004775	ENSGALG00010004006	NKAIN3	case	control	-0.272705306	0.435111516	-0.626748077	0.530824384	0.869211096	down_in_case
CRA004775	ENSGALG00010004007	ENSGALG00010004007	case	control	-0.032956039	0.359396197	-0.091698353	0.926937706	0.984216865	down_in_case
CRA004775	ENSGALG00010004016	ENSGALG00010004016	case	control	-0.31635365	0.426844767	-0.741144497	0.458605832	0.840166374	down_in_case
CRA004775	ENSGALG00010004018	HNRNPD	case	control	-0.309332804	0.214467531	-1.442329298	0.149209499	0.61265447	down_in_case
CRA004775	ENSGALG00010004029	ARL4A	case	control	0.302185758	0.335526754	0.900630887	0.367784606	0.794321403	up_in_case
CRA004775	ENSGALG00010004034	NEDD8	case	control	0.650374932	0.30423438	2.137743051	0.032537603	0.343644073	up_in_case
CRA004775	ENSGALG00010004038	SENP6	case	control	0.508155214	0.236584355	2.14788173	0.031723152	0.339235521	up_in_case
CRA004775	ENSGALG00010004039	ALPK1	case	control	-0.258353024	0.565282613	-0.457033381	0.647647052	0.914210065	down_in_case
CRA004775	ENSGALG00010004040	LGALS1B	case	control	0.28424252	0.417509186	0.680805429	0.495994615	0.856514121	up_in_case
CRA004775	ENSGALG00010004042	PLAG1	case	control	0.321017142	0.514037121	0.624501867	0.532298045	0.869886675	up_in_case
CRA004775	ENSGALG00010004043	CAB39L	case	control	-0.273911191	0.424522994	-0.645221094	0.518783918	0.865435399	down_in_case
CRA004775	ENSGALG00010004044	RNF138	case	control	0.07154203	0.235626177	0.303625134	0.761413501	0.942236668	up_in_case
CRA004775	ENSGALG00010004046	ENSGALG00010004046	case	control	-0.00766947	0.22908034	-0.033479391	0.9732923	0.994947809	down_in_case
CRA004775	ENSGALG00010004047	PDE7A	case	control	0.366311211	0.310562853	1.179507489	0.238196156	0.704790868	up_in_case
CRA004775	ENSGALG00010004049	SETDB2	case	control	-0.355941928	0.271478522	-1.311123713	0.18981597	0.655361714	down_in_case
CRA004775	ENSGALG00010004055	FILIP1	case	control	0.604193193	0.445998024	1.354699258	0.175513394	0.641031789	up_in_case
CRA004775	ENSGALG00010004056	SHROOM3	case	control	0.622425246	0.412488446	1.508951952	0.131311062	0.58691881	up_in_case
CRA004775	ENSGALG00010004057	ENSGALG00010004057	case	control	-0.295177761	0.376503413	-0.78399757	0.433041536	0.825787569	down_in_case
CRA004775	ENSGALG00010004058	TMEM30A	case	control	0.088636363	0.224503211	0.394811114	0.692982292	0.927867527	up_in_case
CRA004775	ENSGALG00010004060	ETV1	case	control	0.080319096	0.241625132	0.33241201	0.739578171	0.938064864	up_in_case
CRA004775	ENSGALG00010004061	MEP1B	case	control	2.256396587	0.760367786	2.967506814	0.003002256	0.114753923	up_in_case
CRA004775	ENSGALG00010004063	ENSGALG00010004063	case	control	0.78760296	0.415258856	1.896655419	0.057873431	0.440234772	up_in_case
CRA004775	ENSGALG00010004065	TIFA	case	control	0.057439958	0.374143499	0.153523869	0.877985169	0.974664895	up_in_case
CRA004775	ENSGALG00010004070	ARL11	case	control	-0.54210524	0.358717675	-1.511230917	0.130729628	0.586275434	down_in_case
CRA004775	ENSGALG00010004072	SGK3	case	control	-0.393592144	0.369259797	-1.065894925	0.286471143	0.741851239	down_in_case
CRA004775	ENSGALG00010004076	COX7A2	case	control	-0.525220668	0.37908877	-1.385482	0.165905133	0.630194031	down_in_case
CRA004775	ENSGALG00010004079	AP1AR	case	control	0.480455697	0.205747549	2.335170942	0.019534492	0.276072771	up_in_case
CRA004775	ENSGALG00010004081	RPS20	case	control	-0.542530307	0.292554935	-1.854456181	0.063673924	0.454139068	down_in_case
CRA004775	ENSGALG00010004082	FAM241A	case	control	0.043310158	0.215371725	0.20109491	0.840624362	0.966461921	up_in_case
CRA004775	ENSGALG00010004083	PHF11	case	control	-0.208910993	0.184483152	-1.132412314	0.25746113	0.719236059	down_in_case
CRA004775	ENSGALG00010004085	RB1CC1	case	control	0.804075393	0.39055284	2.058813331	0.039512124	0.374195055	up_in_case
CRA004775	ENSGALG00010004086	UTP3	case	control	-0.011593395	0.242964844	-0.047716348	0.961942305	0.991860454	down_in_case
CRA004775	ENSGALG00010004087	NCOA7	case	control	0.267511081	0.425135554	0.629237142	0.529193816	0.86920021	up_in_case
CRA004775	ENSGALG00010004095	ANKRD50	case	control	-0.410521182	0.322672528	-1.27225328	0.203283143	0.673096301	down_in_case
CRA004775	ENSGALG00010004096	COL12A1	case	control	-0.527347619	0.574473845	-0.917966281	0.358636519	0.79097996	down_in_case
CRA004775	ENSGALG00010004097	ENSGALG00010004097	case	control	-0.540186998	0.34121378	-1.583133592	0.113391018	0.562886277	down_in_case
CRA004775	ENSGALG00010004101	ARID1B	case	control	0.028972901	0.278497211	0.104033003	0.917143158	0.981688766	up_in_case
CRA004775	ENSGALG00010004102	PCMTD1	case	control	0.639617782	0.312077579	2.049547375	0.04040862	0.376363211	up_in_case
CRA004775	ENSGALG00010004104	SCARB2	case	control	-0.019542566	0.22854791	-0.085507526	0.931857912	0.985044263	down_in_case
CRA004775	ENSGALG00010004108	PERPB	case	control	1.247608566	0.443251132	2.814676549	0.004882635	0.142429514	up_in_case
CRA004775	ENSGALG00010004109	AGMO	case	control	1.534607043	0.715975843	2.14337824	0.032082739	0.340736995	up_in_case
CRA004775	ENSGALG00010004113	ECHDC1	case	control	-0.544069342	0.423627781	-1.284309873	0.199033554	0.666028746	down_in_case
CRA004775	ENSGALG00010004116	SPRY1	case	control	0.670916492	0.250344621	2.679971671	0.007362839	0.171004127	up_in_case
CRA004775	ENSGALG00010004117	STBD1	case	control	-0.214297163	0.622623822	-0.344184009	0.730707907	0.936394787	down_in_case
CRA004775	ENSGALG00010004122	TRIM55	case	control	0.164533562	0.623537438	0.263871184	0.791879181	0.951284723	up_in_case
CRA004775	ENSGALG00010004123	NDUFAF4	case	control	0.200391664	0.228514827	0.876930685	0.380524288	0.80151688	up_in_case
CRA004775	ENSGALG00010004125	MANEA	case	control	0.179947332	0.273943135	0.656878412	0.5112591	0.863170205	up_in_case
CRA004775	ENSGALG00010004127	EPHA7	case	control	0.252185371	0.804991178	0.313277186	0.754070081	0.941900693	up_in_case
CRA004775	ENSGALG00010004131	NUDT6	case	control	0.027038152	0.25947249	0.10420431	0.917007214	0.981688766	up_in_case
CRA004775	ENSGALG00010004133	CRH	case	control	-1.495898247	0.812300676	-1.841557308	0.065539931	0.45911339	down_in_case
CRA004775	ENSGALG00010004135	NUP54	case	control	-0.183283571	0.178175661	-1.028667833	0.303635791	0.755983298	down_in_case
CRA004775	ENSGALG00010004137	CROT	case	control	-0.316192535	0.235705216	-1.341474492	0.179766446	0.644990528	down_in_case
CRA004775	ENSGALG00010004139	PPEF2	case	control	0.639548228	0.777295119	0.822786883	0.410629197	0.816405743	up_in_case
CRA004775	ENSGALG00010004141	KPNA3	case	control	0.424699836	0.31092631	1.365917976	0.17196471	0.636057362	up_in_case
CRA004775	ENSGALG00010004142	RBBP8	case	control	0.159242837	0.337985449	0.471152936	0.637531525	0.910421454	up_in_case
CRA004775	ENSGALG00010004143	TCF24	case	control	-0.952249871	0.689788909	-1.38049461	0.167434408	0.631137759	down_in_case
CRA004775	ENSGALG00010004146	NAAA	case	control	-0.151442988	0.181150459	-0.836006647	0.403151164	0.812213331	down_in_case
CRA004775	ENSGALG00010004147	SPRYD7	case	control	0.153655507	0.27966932	0.54941853	0.58271826	0.889712708	up_in_case
CRA004775	ENSGALG00010004148	ENSGALG00010004148	case	control	-0.191962084	0.315242158	-0.608935318	0.542567313	0.875137006	down_in_case
CRA004775	ENSGALG00010004149	SDAD1	case	control	-0.126199426	0.191540055	-0.658867026	0.509981162	0.86310781	down_in_case
CRA004775	ENSGALG00010004154	COPS5	case	control	0.121250483	0.335441108	0.361465784	0.717751279	0.934011578	up_in_case
CRA004775	ENSGALG00010004158	JCHAIN	case	control	1.586814653	0.615964569	2.576145988	0.009990845	0.201425687	up_in_case
CRA004775	ENSGALG00010004172	ST8SIA6	case	control	0.435561609	0.421065202	1.034427938	0.300936149	0.753440172	up_in_case
CRA004775	ENSGALG00010004177	CSPP1	case	control	0.306906575	0.200729295	1.528957575	0.126274962	0.580543965	up_in_case
CRA004775	ENSGALG00010004181	BBS12	case	control	-0.397136762	0.291658928	-1.361647885	0.173309036	0.63806494	down_in_case
CRA004775	ENSGALG00010004187	ARFGEF1	case	control	0.001311125	0.22171003	0.005913691	0.995281585	0.999001196	up_in_case
CRA004775	ENSGALG00010004188	BMI1	case	control	0.225061188	0.146092395	1.540540061	0.123428765	0.575735738	up_in_case
CRA004775	ENSGALG00010004191	CABLES1	case	control	-0.168041765	0.272820411	-0.615942791	0.537932278	0.872603995	down_in_case
CRA004775	ENSGALG00010004200	ABCB1	case	control	0.982992673	0.355308762	2.766587204	0.005664643	0.152268906	up_in_case
CRA004775	ENSGALG00010004210	SYNCRIP	case	control	0.066787702	0.145296493	0.459664928	0.645756747	0.914043203	up_in_case
CRA004775	ENSGALG00010004211	RNF146	case	control	-0.027259358	0.168307953	-0.161961202	0.871336406	0.973918682	down_in_case
CRA004775	ENSGALG00010004212	RSPO3	case	control	-0.868185102	0.680496503	-1.275811261	0.202022246	0.670354119	down_in_case
CRA004775	ENSGALG00010004214	REPS1	case	control	0.216751448	0.171870912	1.261129325	0.207262257	0.676011217	up_in_case
CRA004775	ENSGALG00010004216	SNX14	case	control	0.123207694	0.175174026	0.703344535	0.481841063	0.848909035	up_in_case
CRA004775	ENSGALG00010004217	CCDC28A	case	control	-0.241094829	0.274857969	-0.877161505	0.380398922	0.801419576	down_in_case
CRA004775	ENSGALG00010004219	FGF2	case	control	0.284296246	0.377754909	0.752594446	0.451693656	0.836202977	up_in_case
CRA004775	ENSGALG00010004222	SPAG6	case	control	0.069189487	0.45848677	0.150908362	0.880048004	0.974996282	up_in_case
CRA004775	ENSGALG00010004224	NT5E	case	control	0.229516799	0.322018076	0.712745079	0.476003516	0.846243735	up_in_case
CRA004775	ENSGALG00010004225	CETN4	case	control	-0.224429227	0.21032687	-1.067049716	0.285949386	0.741582427	down_in_case
CRA004775	ENSGALG00010004226	RUNDC3B	case	control	0.110695444	0.325720329	0.339848127	0.733970903	0.936819725	up_in_case
CRA004775	ENSGALG00010004230	TAF1B	case	control	-0.452486824	0.274573374	-1.647963226	0.099360218	0.537098329	down_in_case
CRA004775	ENSGALG00010004233	NHSL1	case	control	0.068170708	0.484135208	0.140809235	0.888020647	0.977118922	up_in_case
CRA004775	ENSGALG00010004236	DBF4	case	control	0.111650534	0.377037066	0.296126148	0.767133746	0.944367689	up_in_case
CRA004775	ENSGALG00010004237	RUFY3	case	control	0.272383259	0.219424218	1.241354582	0.214474789	0.683562661	up_in_case
CRA004775	ENSGALG00010004238	CCNC	case	control	-0.054055804	0.302186293	-0.178882383	0.858030056	0.971038554	down_in_case
CRA004775	ENSGALG00010004240	ENSGALG00010004240	case	control	-0.121465488	0.312334268	-0.388895809	0.697353222	0.929092636	down_in_case
CRA004775	ENSGALG00010004241	PREX2	case	control	0.357775817	0.416826002	0.858333731	0.390708209	0.806479902	up_in_case
CRA004775	ENSGALG00010004242	ADAM22	case	control	0.114166302	0.501333606	0.227725212	0.819859871	0.960465449	up_in_case
CRA004775	ENSGALG00010004243	BLTP1	case	control	0.183440249	0.276090012	0.664421896	0.506420323	0.8622397	up_in_case
CRA004775	ENSGALG00010004244	TMEM241	case	control	-0.224145383	0.174974828	-1.28101502	0.200188391	0.667171435	down_in_case
CRA004775	ENSGALG00010004245	ARFGEF3	case	control	-0.16271627	0.6406128	-0.254000967	0.799494811	0.95283872	down_in_case
CRA004775	ENSGALG00010004247	MEOX2	case	control	0.171313566	0.416772744	0.411047912	0.681037403	0.923896221	up_in_case
CRA004775	ENSGALG00010004248	GRSF1	case	control	-0.057868558	0.244693528	-0.236494027	0.813049342	0.958114791	down_in_case
CRA004775	ENSGALG00010004249	USP45	case	control	0.181073437	0.273307549	0.66252629	0.507633992	0.862748056	up_in_case
CRA004775	ENSGALG00010004250	MOB1B	case	control	0.262672869	0.289403747	0.90763465	0.364071281	0.793608387	up_in_case
CRA004775	ENSGALG00010004251	CCN2	case	control	0.037795027	0.401629589	0.094104189	0.925026393	0.984140717	up_in_case
CRA004775	ENSGALG00010004255	RPS12	case	control	-0.269390933	0.241615399	-1.114957631	0.264868578	0.725683965	down_in_case
CRA004775	ENSGALG00010004257	ENSGALG00010004257	case	control	1.393525161	0.879003979	1.585345681	0.112887816	0.562886277	up_in_case
CRA004775	ENSGALG00010004265	PIP4K2A	case	control	0.066045347	0.141355042	0.467230214	0.640335179	0.911610834	up_in_case
CRA004775	ENSGALG00010004281	VIM	case	control	0.155974709	0.269272066	0.579245785	0.562423341	0.881532201	up_in_case
CRA004775	ENSGALG00010004283	DCK	case	control	0.456150009	0.278575746	1.637436195	0.101539361	0.54118905	up_in_case
CRA004775	ENSGALG00010004285	SLC18B1	case	control	-0.347591831	0.191312908	-1.816875996	0.069236103	0.468075542	down_in_case
CRA004775	ENSGALG00010004297	SULF1	case	control	0.448408022	0.438528635	1.022528488	0.306530837	0.7585475	up_in_case
CRA004775	ENSGALG00010004300	VNN2	case	control	-0.276067982	0.734236918	-0.375993056	0.706922059	0.931617022	down_in_case
CRA004775	ENSGALG00010004302	CRPPA	case	control	0.483157887	0.301619818	1.601877127	0.109182783	0.556276325	up_in_case
CRA004775	ENSGALG00010004306	PNISR	case	control	0.037394222	0.188272693	0.198617345	0.842562085	0.966478468	up_in_case
CRA004775	ENSGALG00010004307	NPC2	case	control	0.365604468	0.335037604	1.091234129	0.275169874	0.731001618	up_in_case
CRA004775	ENSGALG00010004308	DSG2	case	control	1.774502206	0.622591729	2.85018596	0.004369367	0.135624223	up_in_case
CRA004775	ENSGALG00010004309	MRPS28	case	control	-0.210361667	0.291759826	-0.72100971	0.470903541	0.845198132	down_in_case
CRA004775	ENSGALG00010004312	PDLIM5	case	control	0.350677577	0.558852718	0.62749552	0.530334472	0.869211096	up_in_case
CRA004775	ENSGALG00010004319	TAAR1	case	control	0.183973714	0.683424611	0.269193868	0.787780499	0.95041797	up_in_case
CRA004775	ENSGALG00010004323	TRDMT1	case	control	-0.834662857	0.315405859	-2.646313739	0.008137431	0.182592329	down_in_case
CRA004775	ENSGALG00010004325	LTBP2	case	control	-0.103417636	0.404781627	-0.255489946	0.798344708	0.952835572	down_in_case
CRA004775	ENSGALG00010004326	ENSGALG00010004326	case	control	-1.151601975	0.795559605	-1.447537014	0.147746577	0.611026522	down_in_case
CRA004775	ENSGALG00010004328	ENSGALG00010004328	case	control	-0.415696793	0.524044	-0.793247881	0.427633416	0.822776983	down_in_case
CRA004775	ENSGALG00010004329	COQ3	case	control	-0.039414016	0.28243589	-0.139550309	0.889015304	0.977162915	down_in_case
CRA004775	ENSGALG00010004330	STX7	case	control	0.157520249	0.174598195	0.902187157	0.366957456	0.793822622	up_in_case
CRA004775	ENSGALG00010004332	ENSGALG00010004332	case	control	-1.298089984	0.678029215	-1.914504501	0.055555725	0.431364635	down_in_case
CRA004775	ENSGALG00010004333	DIO2	case	control	0.661317278	0.605112638	1.092882939	0.2744452	0.731001618	up_in_case
CRA004775	ENSGALG00010004334	ENSGALG00010004334	case	control	0.578579727	0.500035032	1.157078385	0.247240337	0.712046495	up_in_case
CRA004775	ENSGALG00010004337	BMPR1B	case	control	-0.380540873	0.566925223	-0.671236448	0.502069913	0.860091011	down_in_case
CRA004775	ENSGALG00010004338	ENSGALG00010004338	case	control	0.217203273	0.194404989	1.117272115	0.263877995	0.723981064	up_in_case
CRA004775	ENSGALG00010004339	B4GALT6	case	control	0.211231298	0.864126992	0.24444474	0.806886395	0.955397873	up_in_case
CRA004775	ENSGALG00010004340	SLC4A4	case	control	0.042815884	0.452460488	0.094629	0.924609514	0.983850919	up_in_case
CRA004775	ENSGALG00010004341	ENSGALG00010004341	case	control	-0.009884195	0.296499693	-0.033336274	0.973406427	0.994947809	down_in_case
CRA004775	ENSGALG00010004342	ZNF438	case	control	-0.04824552	0.253307233	-0.190462467	0.848946752	0.968223172	down_in_case

CRA004775	ENSGALG00010004345	FAXC	case	control	-0.04901193	0.531885552	-0.092147512	0.92658084	0.984182984	down_in_case
CRA004775	ENSGALG00010004346	CEP128	case	control	0.02169062	0.567640116	0.038211923	0.969518715	0.994270502	up_in_case
CRA004775	ENSGALG00010004350	MOXD1	case	control	0.133427611	0.359307737	0.371346333	0.710379592	0.932234469	up_in_case
CRA004775	ENSGALG00010004352	CHMP2A	case	control	-0.332134938	0.219119096	-1.515773587	0.129576613	0.584172159	down_in_case
CRA004775	ENSGALG00010004355	ANKMY2	case	control	0.394931283	0.197309256	2.001585181	0.045329364	0.395678244	up_in_case
CRA004775	ENSGALG00010004356	TRAPPC8	case	control	0.083741268	0.209750863	0.399241588	0.689715202	0.927796693	up_in_case
CRA004775	ENSGALG00010004358	GC	case	control	1.796128381	0.811635431	2.21297434	0.026899415	0.31493941	up_in_case
CRA004775	ENSGALG00010004362	FBXL4	case	control	0.49098336	0.271136014	1.810837866	0.070165953	0.469724266	up_in_case
CRA004775	ENSGALG00010004365	TPD52	case	control	-0.50769419	0.449641798	-1.129108087	0.25885224	0.720037471	down_in_case
CRA004775	ENSGALG00010004366	UNC5C	case	control	0.418292744	0.851785482	0.491077569	0.623371586	0.906441769	up_in_case
CRA004775	ENSGALG00010004367	TSPAN13	case	control	-0.156506303	0.264797854	-0.591040675	0.554493168	0.879521649	down_in_case
CRA004775	ENSGALG00010004369	UBE2M	case	control	-0.03177201	0.373441841	-0.085078869	0.932198689	0.985044263	down_in_case
CRA004775	ENSGALG00010004370	ATP6V1H	case	control	0.007131967	0.2274766	0.031352528	0.974988399	0.995106174	up_in_case
CRA004775	ENSGALG00010004372	SYNJ2	case	control	-0.322891904	0.375132633	-0.860740645	0.389380902	0.80541167	down_in_case
CRA004775	ENSGALG00010004373	MMS22L	case	control	-0.169671642	0.572396217	-0.296423416	0.766906745	0.944281398	down_in_case
CRA004775	ENSGALG00010004374	ILDR1	case	control	0.664584344	0.821880612	0.808614212	0.418737087	0.818943344	up_in_case
CRA004775	ENSGALG00010004375	ENSGALG00010004375	case	control	0.452310527	0.873009209	0.518105104	0.604384939	0.899155826	up_in_case
CRA004775	ENSGALG00010004377	AGR2	case	control	2.711426754	0.769724304	3.522594703	0.000427344	0.042582695	up_in_case
CRA004775	ENSGALG00010004378	TRIM28	case	control	-0.204671829	0.143278952	-1.428484967	0.153152319	0.617472961	down_in_case
CRA004775	ENSGALG00010004379	ZBTB10	case	control	0.3185059	0.191291145	1.665032122	0.095906347	0.529205137	up_in_case
CRA004775	ENSGALG00010004380	ISCA2	case	control	-0.285033743	0.278982452	-1.021690578	0.306927372	0.7585475	down_in_case
CRA004775	ENSGALG00010004381	ENSGALG00010004381	case	control	-0.29137704	0.358662241	-0.812399544	0.416562408	0.818943344	down_in_case
CRA004775	ENSGALG00010004383	ENSGALG00010004383	case	control	1.243524927	0.806316418	1.542229453	0.123017845	0.575380405	up_in_case
CRA004775	ENSGALG00010004384	ENSGALG00010004384	case	control	-0.557011385	0.691929673	-0.80501156	0.420813009	0.819399559	down_in_case
CRA004775	ENSGALG00010004385	ADAMTS3	case	control	0.521148132	0.393864425	1.323166295	0.185780082	0.6491093	up_in_case
CRA004775	ENSGALG00010004386	CFAP44	case	control	0.512006959	0.414804192	1.234334098	0.217078435	0.684537009	up_in_case
CRA004775	ENSGALG00010004389	ENSGALG00010004389	case	control	-0.371249749	0.210807212	-1.76108657	0.078223752	0.490500984	down_in_case
CRA004775	ENSGALG00010004393	ZBTB45	case	control	-0.159009663	0.352035505	-0.451686437	0.65149489	0.915892481	down_in_case
CRA004775	ENSGALG00010004394	CCNY	case	control	0.349845332	0.281033128	1.244854422	0.213185267	0.682777582	up_in_case
CRA004775	ENSGALG00010004395	BOC	case	control	0.038453402	0.380083541	0.101170923	0.919414779	0.982170916	up_in_case
CRA004775	ENSGALG00010004397	ENSGALG00010004397	case	control	-0.131392175	0.306382274	-0.428850447	0.668032064	0.919688403	down_in_case
CRA004775	ENSGALG00010004398	COX18	case	control	-0.385133472	0.231332262	-1.664849804	0.095942725	0.529205137	down_in_case
CRA004775	ENSGALG00010004404	RGS20	case	control	1.133129434	0.816381945	1.387989336	0.165140292	0.630194031	up_in_case
CRA004775	ENSGALG00010004406	ENSGALG00010004406	case	control	-0.141269491	0.314787666	-0.448777086	0.65359247	0.916470479	down_in_case
CRA004775	ENSGALG00010004408	SERAC1	case	control	0.224779426	0.213861607	1.051050864	0.29323523	0.746438942	up_in_case
CRA004775	ENSGALG00010004416	STXBP6	case	control	0.373117684	0.460184337	0.810800486	0.417480255	0.818943344	up_in_case
CRA004775	ENSGALG00010004420	ENSGALG00010004420	case	control	-0.552782957	0.510912615	-1.081952063	0.279273829	0.735529913	down_in_case
CRA004775	ENSGALG00010004421	RAP1GDS1	case	control	0.058049756	0.165532043	0.350685914	0.725823994	0.935238044	up_in_case
CRA004775	ENSGALG00010004424	MRPL18	case	control	-0.020789521	0.339695878	-0.061200391	0.951199618	0.990168609	down_in_case
CRA004775	ENSGALG00010004425	CREM	case	control	0.560276221	0.456404019	1.227588274	0.219601566	0.687085582	up_in_case
CRA004775	ENSGALG00010004429	ENSGALG00010004429	case	control	-0.040500274	0.372506755	-0.108723597	0.913421725	0.980803023	down_in_case
CRA004775	ENSGALG00010004430	TCEA1	case	control	0.135014319	0.236857849	0.570022568	0.568662392	0.884663439	up_in_case
CRA004775	ENSGALG00010004433	AHR1A	case	control	-0.483595856	0.419881221	-1.151744427	0.24942611	0.714467496	down_in_case
CRA004775	ENSGALG00010004434	TCP1	case	control	-0.12370574	0.200647667	-0.616532162	0.537543352	0.872603995	down_in_case
CRA004775	ENSGALG00010004436	NOVA1	case	control	0.214727228	0.371025743	0.578739433	0.562765004	0.881532201	up_in_case
CRA004775	ENSGALG00010004440	GTF2H5	case	control	-0.149327829	0.221908308	-0.672925814	0.500994489	0.859964451	down_in_case
CRA004775	ENSGALG00010004443	ANKRD17	case	control	0.046124442	0.270567606	0.170472891	0.864638252	0.972350383	up_in_case
CRA004775	ENSGALG00010004446	ACAT2	case	control	0.153915401	0.196424685	0.783584819	0.433283766	0.825828172	up_in_case
CRA004775	ENSGALG00010004451	CLDND1	case	control	-0.265772603	0.144534258	-1.838820822	0.065941545	0.460987382	down_in_case
CRA004775	ENSGALG00010004454	SRI	case	control	-0.299117659	0.266146561	-1.123883238	0.261062563	0.721774856	down_in_case
CRA004775	ENSGALG00010004457	AGA	case	control	0.013750583	0.336282506	0.040889974	0.96738361	0.993390488	up_in_case
CRA004775	ENSGALG00010004468	LYPLA1	case	control	0.174120474	0.218100671	0.7983491	0.424667932	0.821775826	up_in_case
CRA004775	ENSGALG00010004471	C1H3ORF52	case	control	0.517582541	0.456027414	1.134981199	0.256383196	0.719236059	up_in_case
CRA004775	ENSGALG00010004472	NEIL3	case	control	-0.454070066	0.755528083	-0.60099694	0.547842024	0.877369241	down_in_case
CRA004775	ENSGALG00010004473	JDP2	case	control	-0.145146045	0.344420158	-0.421421458	0.673447353	0.922442329	down_in_case
CRA004775	ENSGALG00010004477	TULP4	case	control	0.168138146	0.228118524	0.737064854	0.461082911	0.841788517	up_in_case
CRA004775	ENSGALG00010004479	ENSGALG00010004479	case	control	0.856821029	0.663885771	1.290615143	0.196837163	0.66285976	up_in_case
CRA004775	ENSGALG00010004481	ZNF704	case	control	0.247130631	0.260075586	0.950226183	0.341997337	0.781616177	up_in_case
CRA004775	ENSGALG00010004483	STEAP4	case	control	-0.935173563	0.908572264	-1.029278133	0.303348996	0.755679718	down_in_case
CRA004775	ENSGALG00010004484	CLDN23	case	control	2.057651879	0.71119519	2.893230872	0.003813009	0.127263805	up_in_case
CRA004775	ENSGALG00010004487	ABHD10	case	control	-0.057935573	0.216022617	-0.268192165	0.788551405	0.95041797	down_in_case
CRA004775	ENSGALG00010004489	LIG1	case	control	-0.610577784	0.290030795	-2.10521708	0.035272416	0.355594571	down_in_case
CRA004775	ENSGALG00010004494	WTAP	case	control	0.156489152	0.148596152	1.053117123	0.292287316	0.746262643	up_in_case
CRA004775	ENSGALG00010004495	MRPL15	case	control	-0.21208941	0.259706126	-0.816651549	0.414127587	0.817612125	down_in_case
CRA004775	ENSGALG00010004501	TAGLN3	case	control	-0.066582675	0.84805925	-0.07851182	0.937420928	0.986688955	down_in_case
CRA004775	ENSGALG00010004503	ENSGALG00010004503	case	control	0.446574211	0.403496348	1.106761469	0.268397071	0.727528758	up_in_case
CRA004775	ENSGALG00010004505	ENSGALG00010004505	case	control	0.024531513	0.377176021	0.065039958	0.948142185	0.989972846	up_in_case
CRA004775	ENSGALG00010004507	CUL2	case	control	0.263919792	0.222802948	1.184543535	0.236197974	0.703419274	up_in_case
CRA004775	ENSGALG00010004515	ENSGALG00010004515	case	control	0.515788713	0.449366625	1.147812686	0.251045899	0.715684776	up_in_case
CRA004775	ENSGALG00010004516	ENSGALG00010004516	case	control	-0.252262117	0.537849109	-0.469020237	0.639055171	0.91121917	down_in_case
CRA004775	ENSGALG00010004522	TSPAN5	case	control	0.007281962	0.183139668	0.039761795	0.968283035	0.993572123	up_in_case
CRA004775	ENSGALG00010004533	PAG1	case	control	0.512714419	0.38157087	1.343693816	0.179047418	0.644923737	up_in_case
CRA004775	ENSGALG00010004534	CUBN	case	control	0.348231197	0.48136079	0.723430749	0.469415285	0.8440023	up_in_case
CRA004775	ENSGALG00010004542	ERMARD	case	control	-0.249069868	0.233929255	-1.064723044	0.287001279	0.741909972	down_in_case
CRA004775	ENSGALG00010004553	BATF	case	control	-0.409971854	0.46310764	-0.885262557	0.376015039	0.798523018	down_in_case
CRA004775	ENSGALG00010004565	ENSGALG00010004565	case	control	1.560259473	0.969959725	1.60858171	0.107707836	0.55406723	up_in_case
CRA004775	ENSGALG00010004568	RSU1	case	control	0.150246063	0.225085046	0.667507975	0.504447717	0.861471523	up_in_case
CRA004775	ENSGALG00010004576	STEAP2	case	control	0.128689977	0.391305351	0.328873542	0.742251273	0.938877854	up_in_case
CRA004775	ENSGALG00010004577	ZPLD1	case	control	-1.041753297	0.58603811	-1.777620396	0.075466222	0.483342345	down_in_case
CRA004775	ENSGALG00010004583	XKR4	case	control	0.535991388	0.541866379	0.989157861	0.322585912	0.767880594	up_in_case
CRA004775	ENSGALG00010004584	EIF4E	case	control	0.162563789	0.243516197	0.667568694	0.504408947	0.861471523	up_in_case
CRA004775	ENSGALG00010004588	MINDY3	case	control	-0.134428535	0.257919068	-0.52120433	0.602224438	0.898274026	down_in_case
CRA004775	ENSGALG00010004589	CDX2	case	control	2.986165165	0.771465404	3.870770028	0.000108492	0.022744947	up_in_case
CRA004775	ENSGALG00010004591	RRS1	case	control	-0.218939654	0.438604877	-0.499172866	0.617657608	0.903976249	down_in_case
CRA004775	ENSGALG00010004598	ENSGALG00010004598	case	control	2.694160884	0.874871321	3.079493887	0.002073526	0.091096037	up_in_case
CRA004775	ENSGALG00010004602	DYNLT2	case	control	0.540154292	0.548414068	0.984938796	0.324654135	0.770185561	up_in_case
CRA004775	ENSGALG00010004610	TGS1	case	control	-0.046186596	0.227498947	-0.203018944	0.839120225	0.965989303	down_in_case
CRA004775	ENSGALG00010004622	ENSGALG00010004622	case	control	-0.322075776	0.45100869	-0.714123215	0.475150992	0.84579952	down_in_case
CRA004775	ENSGALG00010004623	MBIP	case	control	-0.103470452	0.215696551	-0.479703783	0.631438037	0.909395104	down_in_case
CRA004775	ENSGALG00010004624	PHF10	case	control	0.069952313	0.186596392	0.374885666	0.7077455	0.931742212	up_in_case
CRA004775	ENSGALG00010004630	ENSGALG00010004630	case	control	-0.138952846	0.366979525	-0.378639234	0.704955783	0.930925979	down_in_case
CRA004775	ENSGALG00010004639	HBS1L	case	control	0.15620794	0.308767487	0.505907993	0.612921241	0.901859934	up_in_case
CRA004775	ENSGALG00010004641	METAP1	case	control	-0.129264118	0.17628077	-0.733285416	0.463384368	0.842456502	down_in_case
CRA004775	ENSGALG00010004650	GTPBP10	case	control	0.121112014	0.291199679	0.4159071	0.677477984	0.922924485	up_in_case
CRA004775	ENSGALG00010004666	C6orf120	case	control	0.363435778	0.16881212	2.152900981	0.031326461	0.337065861	up_in_case
CRA004775	ENSGALG00010004673	RAB18	case	control	0.21094523	0.258969852	0.814555163	0.415326984	0.818921904	up_in_case
CRA004775	ENSGALG00010004689	BBX	case	control	0.277711347	0.35180832	0.789382546	0.429888454	0.82413667	up_in_case
CRA004775	ENSGALG00010004705	ENSGALG00010004705	case	control	0.108284672	0.39550932	0.273785385	0.784249555	0.950165405	up_in_case
CRA004775	ENSGALG00010004707	OTUD1	case	control	1.292371071	0.700166232	1.845806054	0.064920371	0.457570489	up_in_case
CRA004775	ENSGALG00010004716	ADH6	case	control	2.178386377	0.700993219	3.107571255	0.001886315	0.086751567	up_in_case
CRA004775	ENSGALG00010004717	ALKBH8	case	control	-0.069088553	0.285445622	-0.24203753	0.808751083	0.955588258	down_in_case
CRA004775	ENSGALG00010004721	LAMA2	case	control	0.997937673	0.514048113	1.941331263	0.05221811	0.420135271	up_in_case
CRA004775	ENSGALG00010004728	ENSGALG00010004728	case	control	-0.035046969	0.44722564	-0.078365294	0.93753748	0.986688955	down_in_case
CRA004775	ENSGALG00010004729	ENSGALG00010004729	case	control	0.876939442	1.229953999	0.712985561	0.475854692	0.846243735	up_in_case
CRA004775	ENSGALG00010004730	CLDN12	case	control	0.03873362	0.271199151	0.142823529	0.886429554	0.976862703	up_in_case
CRA004775	ENSGALG00010004735	ENSGALG00010004735	case	control	0.031286812	0.272005455	0.115022736	0.908427101	0.98075825	up_in_case
CRA004775	ENSGALG00010004736	CD47	case	control	-0.060122658	0.280434561	-0.214391042	0.830242127	0.963232966	down_in_case
CRA004775	ENSGALG00010004746	LYN	case	control	-0.410947534	0.546025801	-0.752615597	0.451680943	0.836202977	down_in_case
CRA004775	ENSGALG00010004751	PTER	case	control	0.161695991	0.440647159	0.36695117	0.713655441	0.933244709	up_in_case
CRA004775	ENSGALG00010004757	TRMT10A	case	control	-0.142516204	0.227771131	-0.625699154	0.531512289	0.869580664	down_in_case
CRA004775	ENSGALG00010004758	FLT3	case	control	-0.354276775	0.526335742	-0.673100355	0.500883448	0.859946721	down_in_case
CRA004775	ENSGALG00010004760	THBS2	case	control	-0.567978369	0.63337231	-0.896752763	0.369850859	0.795234047	down_in_case
CRA004775	ENSGALG00010004762	IFT57	case	control	0.263184226	0.281117732	0.936206421	0.349166918	0.785311667	up_in_case
CRA004775	ENSGALG00010004765	ENSGALG00010004765	case	control	-1.649476916	0.561651858	-2.936831582	0.003315841	0.122900629	down_in_case
CRA004775	ENSGALG00010004769	HTR2A	case	control	-0.927651447	0.701817453	-1.321784521	0.186239914	0.6491093	down_in_case
CRA004775	ENSGALG00010004777	CDK14	case	control	0.126999396	0.332866864	0.381532107	0.702808452	0.930068722	up_in_case
CRA004775	ENSGALG00010004778	ENSGALG00010004778	case	control	1.366734502	1.199679151	1.139250024	0.254598886	0.718419134	up_in_case
CRA004775	ENSGALG00010004780	ENSGALG00010004780	case	control	0.521719043	0.472430696	1.104329265	0.269450332	0.72765348	up_in_case
CRA004775	ENSGALG00010004781	ENSGALG00010004781	case	control	-0.059487304	0.348878136	-0.170510268	0.86460886	0.972350383	down_in_case
CRA004775	ENSGALG00010004787	RIOK3	case	control	0.230561109	0.246881185	0.933895018	0.350358049	0.785466132	up_in_case
CRA004775	ENSGALG00010004791	FZD1	case	control	-0.075314232	0.345022843	-0.218287668	0.827204982	0.961901981	down_in_case
CRA004775	ENSGALG00010004798	PTPRK	case	control	0.235456448	0.378772576	0.621630135	0.534185101	0.870981055	up_in_case
CRA004775	ENSGALG00010004800	ZC3H12C	case	control	-0.55440225	0.482649124	-1.148665194	0.250694063	0.715557647	down_in_case
CRA004775	ENSGALG00010004803	RMC1	case	control	-0.182785479	0.283434518	-0.644894914	0.518995288	0.865435399	down_in_case
CRA004775	ENSGALG00010004808	THEMIS	case	control	0.188598183	0.776391213	0.242916432	0.808070136	0.955397873	up_in_case
CRA004775	ENSGALG00010004824	ATM	case	control	0.022334872	0.272266285	0.082033191	0.93462032	0.985731248	up_in_case

CRA004775	ENSGALG00010004832	C6orf58	case	control	1.975747441	0.786771437	2.511208908	0.012031847	0.220599905	up_in_case
CRA004775	ENSGALG00010004835	HHLA2	case	control	0.840670228	0.39502374	2.128151156	0.033324553	0.348211192	up_in_case
CRA004775	ENSGALG00010004837	CENPC	case	control	0.152602698	0.402487596	0.379148822	0.704577353	0.930616	up_in_case
CRA004775	ENSGALG00010004841	AKAP9	case	control	0.481922092	0.361669879	1.332491645	0.182698685	0.647378665	up_in_case
CRA004775	ENSGALG00010004846	TTLL2	case	control	0.643385182	0.758157108	0.848617227	0.396094318	0.808998414	up_in_case
CRA004775	ENSGALG00010004852	ENSGALG00010004852	case	control	-1.488573495	0.541342566	-2.749780988	0.005963511	0.154441997	down_in_case
CRA004775	ENSGALG00010004855	ZEB1	case	control	0.285795385	0.224899602	1.270768745	0.203810931	0.674045742	up_in_case
CRA004775	ENSGALG00010004859	NPC1	case	control	0.315496186	0.380156335	0.829911688	0.406588716	0.813842524	up_in_case
CRA004775	ENSGALG00010004866	SLC25A21	case	control	-1.549671548	0.689403324	-2.247844613	0.0245861	0.302160964	down_in_case
CRA004775	ENSGALG00010004868	NPAT	case	control	0.268678234	0.19284931	1.393202986	0.163558417	0.629717526	up_in_case
CRA004775	ENSGALG00010004871	C11orf87	case	control	-0.112550192	0.419413386	-0.268351455	0.788428803	0.95041797	down_in_case
CRA004775	ENSGALG00010004872	KIAA0408	case	control	-0.223418567	0.529107068	-0.422255874	0.672838262	0.921952322	down_in_case
CRA004775	ENSGALG00010004880	STAP1	case	control	-0.008651805	0.515437811	-0.016785352	0.986607856	0.997562105	down_in_case
CRA004775	ENSGALG00010004881	MIPOL1	case	control	0.149487504	0.416424138	0.358978961	0.719610828	0.93407508	up_in_case
CRA004775	ENSGALG00010004883	KRIT1	case	control	0.502917012	0.263362926	1.909596844	0.056185142	0.433802294	up_in_case
CRA004775	ENSGALG00010004887	MYH15	case	control	-0.41356727	0.94865691	-0.435950306	0.662872785	0.918295135	down_in_case
CRA004775	ENSGALG00010004890	ACAT1	case	control	-0.007277342	0.302002092	-0.024096991	0.980775244	0.997016366	down_in_case
CRA004775	ENSGALG00010004891	ANKIB1	case	control	0.487727085	0.300706859	1.621935353	0.104817191	0.547815057	up_in_case
CRA004775	ENSGALG00010004892	HEY2	case	control	0.416495721	0.420853545	0.989645273	0.322347533	0.767880594	up_in_case
CRA004775	ENSGALG00010004896	UBA6	case	control	-0.066390965	0.259390165	-0.255950201	0.797989291	0.952835572	down_in_case
CRA004775	ENSGALG00010004899	GATAD1	case	control	-0.355944329	0.326931544	-1.088742691	0.27626737	0.731001618	down_in_case
CRA004775	ENSGALG00010004901	CUL5	case	control	0.326230268	0.327446294	0.996286335	0.319111042	0.767037518	up_in_case
CRA004775	ENSGALG00010004902	ANKRD29	case	control	-0.026268256	0.435467833	-0.060321919	0.951899245	0.990168609	down_in_case
CRA004775	ENSGALG00010004907	ENSGALG00010004907	case	control	0.22711513	0.398120293	0.570468609	0.568359907	0.884508497	up_in_case
CRA004775	ENSGALG00010004917	ENSGALG00010004917	case	control	-0.585415648	0.266392506	-2.197568002	0.027979906	0.318730759	down_in_case
CRA004775	ENSGALG00010004921	RRAGD	case	control	0.185475011	0.264309185	0.70173502	0.482844429	0.849922394	up_in_case
CRA004775	ENSGALG00010004924	ENSGALG00010004924	case	control	0.053278627	0.688865155	0.077342607	0.938350997	0.986707289	up_in_case
CRA004775	ENSGALG00010004928	HDDC2	case	control	0.066513417	0.261852798	0.254010718	0.799487277	0.95283872	up_in_case
CRA004775	ENSGALG00010004929	ENSGALG00010004929	case	control	0.035462857	0.618625889	0.057325207	0.954286141	0.990670511	up_in_case
CRA004775	ENSGALG00010004933	SPTLC2	case	control	-0.199456123	0.291924227	-0.683246213	0.494451275	0.856382445	down_in_case
CRA004775	ENSGALG00010004934	CIP2A	case	control	-0.533303778	0.668477832	-0.797788277	0.424993365	0.822068331	down_in_case
CRA004775	ENSGALG00010004935	SVIL	case	control	0.109476107	0.217186745	0.504064403	0.614216121	0.902363507	up_in_case
CRA004775	ENSGALG00010004939	DDX10	case	control	0.41515518	0.262194818	1.583384381	0.113333881	0.562886277	up_in_case
CRA004775	ENSGALG00010004944	ALKBH1	case	control	-0.037646601	0.196426942	-0.191657012	0.848010881	0.968119355	down_in_case
CRA004775	ENSGALG00010004946	SH2D1B	case	control	-0.672217074	0.42820829	-1.569836666	0.116453115	0.566086328	down_in_case
CRA004775	ENSGALG00010004949	RAB39A	case	control	0.307074457	0.450659228	0.681389479	0.495625078	0.856382445	up_in_case
CRA004775	ENSGALG00010004950	SLIRP	case	control	0.065754493	0.298509734	0.220275876	0.825656307	0.961878235	up_in_case
CRA004775	ENSGALG00010004951	AFDN	case	control	-0.067366978	0.353113026	-0.190780212	0.848697792	0.968223172	down_in_case
CRA004775	ENSGALG00010004952	PEX1	case	control	0.463740493	0.246361764	1.882355791	0.059787724	0.445397937	up_in_case
CRA004775	ENSGALG00010004957	ENSGALG00010004957	case	control	0.805603522	0.445619027	1.807830173	0.070632936	0.469895261	up_in_case
CRA004775	ENSGALG00010004958	TPD52L1	case	control	1.749246759	0.75772176	2.308560809			up_in_case
CRA004775	ENSGALG00010004962	ENSGALG00010004962	case	control	-0.498996908	0.188694302	-2.644472579	0.008181834	0.182940477	down_in_case
CRA004775	ENSGALG00010004963	ENSGALG00010004963	case	control	-0.14476919	0.531920926	-0.272162991	0.785496697	0.95041797	down_in_case
CRA004775	ENSGALG00010004965	JCAD	case	control	0.220016033	0.352591521	0.623996949	0.532629589	0.869959553	up_in_case
CRA004775	ENSGALG00010004966	LAMA3	case	control	-0.602462421	0.601194906	-1.002108327	0.316291277	0.765818284	down_in_case
CRA004775	ENSGALG00010004967	SLC35F2	case	control	0.252709914	0.529468981	0.477289365	0.633156083	0.909723668	up_in_case
CRA004775	ENSGALG00010004968	TRAT1	case	control	0.0617271	0.576710625	0.107033054	0.914762757	0.981346752	up_in_case
CRA004775	ENSGALG00010004969	YTHDC1	case	control	0.085581332	0.174000003	0.491846731	0.622827698	0.906338268	up_in_case
CRA004775	ENSGALG00010004970	RBM48	case	control	-0.076420737	0.277856482	-0.275036725	0.783288023	0.94957186	down_in_case
CRA004775	ENSGALG00010004971	ENSGALG00010004971	case	control	-0.852624336	1.574887138	-0.54138758	0.588240466	0.892912535	down_in_case
CRA004775	ENSGALG00010004973	SLN	case	control	-0.115668954	0.719729308	-0.160711746	0.872320438	0.97401729	down_in_case
CRA004775	ENSGALG00010004974	MTPAP	case	control	0.059431458	0.205864564	0.288692027	0.772817065	0.946296525	up_in_case
CRA004775	ENSGALG00010004975	SNW1	case	control	-0.000542935	0.137734344	-0.003941901	0.996854827	0.999617204	down_in_case
CRA004775	ENSGALG00010004976	ENSGALG00010004976	case	control	-0.566583191	0.478785739	-1.183375244	0.236660464	0.703637928	down_in_case
CRA004775	ENSGALG00010004977	RNF217	case	control	0.211008413	0.253238968	0.833238323	0.404710333	0.81313407	up_in_case
CRA004775	ENSGALG00010004980	FNDC1	case	control	-0.539335978	0.66174253	-0.815023902	0.415058629	0.818834793	down_in_case
CRA004775	ENSGALG00010004981	ENSGALG00010004981	case	control	1.072670604	0.53377033	2.009610772	0.0444724	0.393273296	up_in_case
CRA004775	ENSGALG00010004983	CLXN	case	control	-1.124220147	0.618406608	-1.81793036	0.069074777	0.46744452	down_in_case
CRA004775	ENSGALG00010004987	MAP3K8	case	control	-0.400149095	0.503154002	-0.795281551	0.426449743	0.822571246	down_in_case
CRA004775	ENSGALG00010004988	HJURP	case	control	0.705077131	0.665647089	1.059235656	0.28949247	0.743798759	up_in_case
CRA004775	ENSGALG00010004990	ENSGALG00010004990	case	control	1.623605174	0.831324506	1.953034179	0.050815545	0.416238146	up_in_case
CRA004775	ENSGALG00010004991	FAM133B	case	control	0.39984973	0.154200936	2.593043476	0.009513074	0.197306616	up_in_case
CRA004775	ENSGALG00010004993	SULT1B	case	control	-0.017383534	0.259193216	-0.06706786	0.946527681	0.989137998	down_in_case
CRA004775	ENSGALG00010004995	PIT54	case	control	1.703381816	0.758556538	2.245556832	0.024732408	0.302344474	up_in_case
CRA004775	ENSGALG00010004996	GPR158	case	control	0.071971236	0.544817012	0.132101668	0.894903876	0.977834811	up_in_case
CRA004775	ENSGALG00010004999	ADCK1	case	control	-0.571051438	0.26201292	-2.179478163	0.029296165	0.325405143	down_in_case
CRA004775	ENSGALG00010005003	EXPH5	case	control	1.059048116	0.48983151	2.162066127	0.03061308	0.333830655	up_in_case
CRA004775	ENSGALG00010005004	CDK6	case	control	-0.255904354	0.381383527	-0.67098953	0.502227199	0.860091011	down_in_case
CRA004775	ENSGALG00010005010	NKAIN2	case	control	0.628666069	0.798014345	0.78778793	0.430820762	0.824612364	up_in_case
CRA004775	ENSGALG00010005012	ENSGALG00010005012	case	control	-0.301858219	0.574345987	-0.525568604	0.599187975	0.897206546	down_in_case
CRA004775	ENSGALG00010005014	SULT1B1	case	control	-0.204732693	0.298099961	-0.686792082	0.49221376	0.855746361	down_in_case
CRA004775	ENSGALG00010005016	STX11	case	control	-0.741675405	0.410717039	-1.805806273	0.070948605	0.469911807	down_in_case
CRA004775	ENSGALG00010005017	ENSGALG00010005017	case	control	-0.05722071	0.211072042	-0.271095638	0.786317476	0.95041797	down_in_case
CRA004775	ENSGALG00010005023	POGLUT3	case	control	-0.107059275	0.336502151	-0.318153316	0.750368643	0.941495694	down_in_case
CRA004775	ENSGALG00010005025	THOC1	case	control	-0.153211758	0.218992571	-0.699620803	0.484164147	0.850779152	down_in_case
CRA004775	ENSGALG00010005029	FAT4	case	control	0.091858531	0.321978902	0.285293633	0.77541919	0.946457419	up_in_case
CRA004775	ENSGALG00010005033	USP14	case	control	-0.176362581	0.280910193	-0.627825494	0.530118263	0.869211096	down_in_case
CRA004775	ENSGALG00010005035	NECTIN3	case	control	0.023108688	0.241106719	0.09584423	0.923644289	0.983448202	up_in_case
CRA004775	ENSGALG00010005037	THNSL1	case	control	-0.552766228	0.279749636	-1.975931893	0.048162491	0.407414924	down_in_case
CRA004775	ENSGALG00010005042	SLC46A3	case	control	0.707592573	0.53448582	1.323875297	0.185544464	0.6491093	up_in_case
CRA004775	ENSGALG00010005045	UTRN	case	control	0.511194562	0.244376574	2.091831279	0.036453611	0.360628495	up_in_case
CRA004775	ENSGALG00010005048	ROCK1	case	control	0.431224331	0.323214671	1.334173136	0.182147122	0.647378665	up_in_case
CRA004775	ENSGALG00010005050	ENSGALG00010005050	case	control	2.896777501	0.762796557	3.797575481	0.000146118	0.02543383	up_in_case
CRA004775	ENSGALG00010005057	FLVCR2	case	control	0.311822055	0.419980977	0.742467093	0.457804382	0.839704957	up_in_case
CRA004775	ENSGALG00010005060	POMP	case	control	-0.175585107	0.261665192	-0.671029667	0.50220163	0.860091011	down_in_case
CRA004775	ENSGALG00010005068	PLCXD2	case	control	-0.44696689	0.372338572	-1.200431336	0.229971865	0.696073276	down_in_case
CRA004775	ENSGALG00010005070	ALB	case	control	1.898009085	0.743588047	2.5525008	0.010695266	0.208020662	up_in_case
CRA004775	ENSGALG00010005075	TRDN	case	control	0.498138658	0.66435463	0.749808364	0.453370131	0.83804959	up_in_case
CRA004775	ENSGALG00010005077	ERG28	case	control	-0.358072839	0.204646238	-1.7497162	0.080167297	0.493676892	down_in_case
CRA004775	ENSGALG00010005081	GREB1L	case	control	-0.35596849	0.321403813	-1.107542835	0.268059303	0.727528758	down_in_case
CRA004775	ENSGALG00010005082	ENSGALG00010005082	case	control	0.191370187	0.199905398	0.95730375	0.338413963	0.779253705	up_in_case
CRA004775	ENSGALG00010005086	ENSGALG00010005086	case	control	2.633631558	0.663151422	3.971387935	7.14551E-05	0.017238232	up_in_case
CRA004775	ENSGALG00010005087	ENPP1	case	control	0.33555925	0.226157425	1.48374191	0.137877403	0.596924319	up_in_case
CRA004775	ENSGALG00010005092	ESCO1	case	control	0.851512294	0.311505119	2.733541895	0.006265715	0.158635228	up_in_case
CRA004775	ENSGALG00010005098	ENSGALG00010005098	case	control	2.435802796	0.893628057	2.725745658	0.006415641	0.15932286	up_in_case
CRA004775	ENSGALG00010005104	ENPP3	case	control	0.445253944	0.352221737	1.264129658	0.206183493	0.674837165	up_in_case
CRA004775	ENSGALG00010005107	TTLL5	case	control	-0.277940918	0.250705881	-1.108633418	0.267588356	0.727430878	down_in_case
CRA004775	ENSGALG00010005109	ABHD3	case	control	0.916746725	0.631389324	1.451951578	0.14651507	0.609922856	up_in_case
CRA004775	ENSGALG00010005111	RASSF6	case	control	-0.048229985	0.470564537	-0.102493879	0.918364671	0.981710522	down_in_case
CRA004775	ENSGALG00010005114	PHLDB2	case	control	0.728592004	0.332656548	2.190222948	0.028508072	0.321586952	up_in_case
CRA004775	ENSGALG00010005118	PRTFDC1	case	control	-0.645982878	0.337366949	-1.914778197	0.055520796	0.431364203	down_in_case
CRA004775	ENSGALG00010005119	MIB1	case	control	-0.155934187	0.279240256	-0.55842302	0.576555557	0.887937128	down_in_case
CRA004775	ENSGALG00010005122	IL8L1	case	control	-0.265002305	0.420801562	-0.629755992	0.528854242	0.86916155	down_in_case
CRA004775	ENSGALG00010005123	MTIF3	case	control	0.218529131	0.382445951	0.571398731	0.567729389	0.884152085	up_in_case
CRA004775	ENSGALG00010005126	MED23	case	control	-0.064888754	0.215435837	-0.301197584	0.763263832	0.94239774	down_in_case
CRA004775	ENSGALG00010005127	GATA6	case	control	1.141102711	0.448047585	2.54683375	0.010870521	0.210535959	up_in_case
CRA004775	ENSGALG00010005128	ENSGALG00010005128	case	control	2.526765167	0.93537971	2.701325613	0.006906369	0.16300527	up_in_case
CRA004775	ENSGALG00010005131	IL8L2	case	control	-1.182059394	0.739295366	-1.598900045	0.109842814	0.557566828	down_in_case
CRA004775	ENSGALG00010005132	LNX2	case	control	0.329009632	0.175467418	1.875046862	0.060786277	0.445397937	up_in_case
CRA004775	ENSGALG00010005140	DCT	case	control	7.919079008	0.782302799	10.12277984	4.37801E-24	1.20404E-20	up_in_case
CRA004775	ENSGALG00010005142	ENSGALG00010005142	case	control	2.58629837	0.795709087	3.25030644	0.001152807	0.071729642	up_in_case
CRA004775	ENSGALG00010005143	GTF3A	case	control	0.261026432	0.230358497	1.133131335	0.257159104	0.719236059	up_in_case
CRA004775	ENSGALG00010005145	AKAP7	case	control	-0.160247304	0.392585081	-0.408184904	0.68313793	0.924953691	down_in_case
CRA004775	ENSGALG00010005146	TGFB3	case	control	-1.007232727	0.404343369	-2.491033129	0.012737224	0.227466975	down_in_case
CRA004775	ENSGALG00010005148	TTC39C	case	control	-0.123125118	0.400860262	-0.307152217	0.758727514	0.942236668	down_in_case
CRA004775	ENSGALG00010005150	ALCAM	case	control	-0.20365175	0.365293268	-0.557502064	0.57718445	0.888189723	down_in_case
CRA004775	ENSGALG00010005154	CWF19L2	case	control	-0.446689021	0.245373672	-1.820443965	0.068691422	0.46645716	down_in_case
CRA004775	ENSGALG00010005157	IMPA1	case	control	-0.016307994	0.263155677	-0.0619709	0.950586006	0.990168609	down_in_case
CRA004775	ENSGALG00010005158	IFT43	case	control	-0.40779409	0.355297029	-1.147755418	0.251069546	0.715684776	down_in_case
CRA004775	ENSGALG00010005159	ARHGAP21	case	control	0.373150657	0.256978123	1.452071691	0.146481672	0.609922856	up_in_case
CRA004775	ENSGALG00010005165	METTL21C	case	control	0.021112022	0.692989998	0.030465119	0.975696112	0.995255743	up_in_case
CRA004775	ENSGALG00010005166	CBLB	case	control	-0.11893318	0.295871594	-0.401975663	0.687701937	0.927111919	down_in_case
CRA004775	ENSGALG00010005167	GUCY1A2	case	control	0.197197576	0.485433501	0.406229846	0.684573727	0.925894887	up_in_case
CRA004775	ENSGALG00010005170	GPATCH2L	case	control	-0.137202112	0.29707203	-0.461847963	0.644190353	0.913739303	down_in_case
CRA004775	ENSGALG00010005172	OSBPL1A	case	control	-0.289399984	0.228195096	-1.268212983	0.204721902	0.674045742	down_in_case
CRA004775	ENSGALG00010005174	TPP2	case	control	0.018843588	0.422790724	0.04456954	0.964450422	0.992825105	up_in_case

CRA004775	ENSGALG00010005176	BAMBI	case	control	0.804359432	0.328398029	2.449343055	0.014311707	0.239125488	up_in_case
CRA004775	ENSGALG00010005177	AASDHPPT	case	control	-0.004467358	0.306976562	-0.014552765	0.988388983	0.998065748	down_in_case
CRA004775	ENSGALG00010005178	EPHA5	case	control	-0.494958291	0.50683877	-0.976559649	0.328787204	0.77220987	down_in_case
CRA004775	ENSGALG00010005180	WAC	case	control	0.449345215	0.233397374	1.925236806	0.054199739	0.428826592	up_in_case
CRA004775	ENSGALG00010005181	IMPACT	case	control	0.142367145	0.405481079	0.351106754	0.725508261	0.935238044	up_in_case
CRA004775	ENSGALG00010005182	NEPRO	case	control	-0.173878786	0.216624958	-0.802671991	0.422164346	0.820119439	down_in_case
CRA004775	ENSGALG00010005186	ESR1	case	control	-0.215512756	0.491699185	-0.438302041	0.661167347	0.917907174	down_in_case
CRA004775	ENSGALG00010005187	KBTBD3	case	control	-0.872257082	0.626738433	-1.391740215	0.16400108	0.629717526	down_in_case
CRA004775	ENSGALG00010005188	ENSGALG00010005188	case	control	-0.511675235	0.933427134	-0.548168375	0.583576295	0.889906855	down_in_case
CRA004775	ENSGALG00010005190	ENSGALG00010005190	case	control	-0.639360492	0.694910314	-0.920061884	0.357540421	0.790309937	down_in_case
CRA004775	ENSGALG00010005191	MSANTD4	case	control	-0.098521706	0.236756293	-0.416131307	0.677313922	0.922924485	down_in_case
CRA004775	ENSGALG00010005193	EPB41L2	case	control	0.382409062	0.260221762	1.469550662	0.141683491	0.601695394	up_in_case
CRA004775	ENSGALG00010005194	ENSGALG00010005194	case	control	2.36312107	0.928085149	2.546233039	0.010889247	0.210602012	up_in_case
CRA004775	ENSGALG00010005198	TRPC3	case	control	-1.02917837	0.748020603	-1.375869015	0.16886219	0.632337016	down_in_case
CRA004775	ENSGALG00010005203	ENSGALG00010005203	case	control	-0.036349466	0.200075513	-0.181678737	0.855834854	0.970703965	down_in_case
CRA004775	ENSGALG00010005204	ITGBL1	case	control	-0.741910351	0.65064321	-1.140272179	0.254172925	0.718335926	down_in_case
CRA004775	ENSGALG00010005208	ENSGALG00010005208	case	control	0.047314166	0.272501047	0.1736293	0.862156804	0.971725725	up_in_case
CRA004775	ENSGALG00010005211	ESRRB	case	control	-0.911202488	0.452376064	-2.014258847	0.043982364	0.392535833	down_in_case
CRA004775	ENSGALG00010005212	TAF13	case	control	-0.297383451	0.28083501	-1.058925849	0.289633551	0.743883257	down_in_case
CRA004775	ENSGALG00010005213	ENSGALG00010005213	case	control	-0.333541361	0.541081074	-0.61643509	0.5376074	0.872603995	down_in_case
CRA004775	ENSGALG00010005214	BBS7	case	control	-0.007753624	0.223217373	-0.034735755	0.97229045	0.994947809	down_in_case
CRA004775	ENSGALG00010005215	VASH1	case	control	-0.092730199	0.300096017	-0.309001764	0.757320185	0.941900693	down_in_case
CRA004775	ENSGALG00010005216	ENSGALG00010005216	case	control	0.044099102	0.528017828	0.083518207	0.933439501	0.985090297	up_in_case
CRA004775	ENSGALG00010005217	CCNA2	case	control	0.310478862	0.208060083	1.492255786	0.135632103	0.593592099	up_in_case
CRA004775	ENSGALG00010005218	GTPBP8	case	control	-0.332978033	0.280198964	-1.188362825	0.234690496	0.702145501	down_in_case
CRA004775	ENSGALG00010005219	NALCN	case	control	-0.502101092	0.570839468	-0.879583703	0.379084871	0.800168929	down_in_case
CRA004775	ENSGALG00010005220	ANGEL1	case	control	-0.114836366	0.165870199	-0.692326692	0.488732191	0.854054691	down_in_case
CRA004775	ENSGALG00010005221	ENSGALG00010005221	case	control	0.398060565	0.483894802	0.82261798	0.410725271	0.816405743	up_in_case
CRA004775	ENSGALG00010005226	MYCT1	case	control	0.272004599	0.321233184	0.846751246	0.397133788	0.808998414	up_in_case
CRA004775	ENSGALG00010005227	EXOSC9	case	control	-0.148728068	0.207121892	-0.718070245	0.472713976	0.845425322	down_in_case
CRA004775	ENSGALG00010005228	ZNF521	case	control	-0.051313613	0.297867045	-0.172270192	0.863225117	0.971910432	down_in_case
CRA004775	ENSGALG00010005229	CIPC	case	control	-0.014593777	0.293854588	-0.049663261	0.960390734	0.99146644	down_in_case
CRA004775	ENSGALG00010005231	ENSGALG00010005231	case	control	0.218003615	0.455511386	0.478590923	0.632229677	0.909651573	up_in_case
CRA004775	ENSGALG00010005232	GATA3	case	control	-0.968116008	0.585260226	-1.654163336	0.09809433	0.533793778	down_in_case
CRA004775	ENSGALG00010005236	TMTC4	case	control	-0.155051002	0.327256021	-0.473791138	0.635648855	0.909837348	down_in_case
CRA004775	ENSGALG00010005237	RAP2A	case	control	-0.29281054	0.317510878	-0.922206325	0.356420964	0.789127613	down_in_case
CRA004775	ENSGALG00010005238	TMEM63C	case	control	-1.076022268	0.371782635	-2.894224113	0.003800968	0.127263805	down_in_case
CRA004775	ENSGALG00010005239	ENSGALG00010005239	case	control	0.479379082	0.574667908	0.834184536	0.404177004	0.81313407	up_in_case
CRA004775	ENSGALG00010005240	VIP	case	control	0.292433086	0.804737535	0.363389395	0.716314023	0.934006649	up_in_case
CRA004775	ENSGALG00010005241	C4orf54	case	control	0.261199468	0.552389184	0.47285406	0.636317302	0.910080596	up_in_case
CRA004775	ENSGALG00010005242	CCDC80	case	control	-0.053765377	0.440840885	-0.121960959	0.902929937	0.979310476	down_in_case
CRA004775	ENSGALG00010005243	NGB	case	control	-0.220768796	0.843590087	-0.261701506	0.79355158	0.951611387	down_in_case
CRA004775	ENSGALG00010005244	FBXO5	case	control	0.496177799	0.354637669	1.399111944	0.161779416	0.627882156	up_in_case
CRA004775	ENSGALG00010005245	KIAA1217	case	control	0.954881399	0.455193343	2.09774904	0.03592732	0.359151015	up_in_case
CRA004775	ENSGALG00010005246	ENSGALG00010005246	case	control	0.087157202	0.562537222	0.154935884	0.876871864	0.974479909	up_in_case
CRA004775	ENSGALG00010005247	THAP7	case	control	0.111215411	0.338069005	0.328972515	0.742176463	0.938877854	up_in_case
CRA004775	ENSGALG00010005248	TMEM200A	case	control	0.730906063	0.383233948	1.907205944	0.056493924	0.434721852	up_in_case
CRA004775	ENSGALG00010005249	PDGFD	case	control	-0.015070156	0.436862818	-0.034496312	0.972481383	0.994947809	down_in_case
CRA004775	ENSGALG00010005250	SLC35A5	case	control	-0.255901658	0.322438646	-0.793644499	0.427402419	0.822776983	down_in_case
CRA004775	ENSGALG00010005251	MTRF1L	case	control	0.031389823	0.227664144	0.137877764	0.890337024	0.977315147	up_in_case
CRA004775	ENSGALG00010005252	KLHL14	case	control	0.143539319	0.743085168	0.193166713	0.846828407	0.967891337	up_in_case
CRA004775	ENSGALG00010005253	POMT2	case	control	-0.146997459	0.258959331	-0.567646889	0.570274762	0.88518436	down_in_case
CRA004775	ENSGALG00010005254	ENSGALG00010005254	case	control	-0.109586149	0.728743016	-0.150376946	0.880467229	0.974996282	down_in_case
CRA004775	ENSGALG00010005255	BZW2	case	control	0.223342288	0.293708895	0.760420579	0.447003226	0.834117509	up_in_case
CRA004775	ENSGALG00010005258	ITIH5	case	control	0.492347965	0.568103444	0.866651962	0.386132734	0.803610044	up_in_case
CRA004775	ENSGALG00010005259	SLC27A5	case	control	1.884725797	0.53452931	3.525954075	0.00042196	0.042582695	up_in_case
CRA004775	ENSGALG00010005260	ANXA5	case	control	-0.027928481	0.297346363	-0.093925753	0.925168137	0.984140717	down_in_case
CRA004775	ENSGALG00010005261	ATG3	case	control	0.01306621	0.251410942	0.051971525	0.958551382	0.991431439	up_in_case
CRA004775	ENSGALG00010005262	ENSGALG00010005262	case	control	-0.171963282	0.273181213	-0.629484292	0.529032049	0.869191855	down_in_case
CRA004775	ENSGALG00010005263	ENSGALG00010005263	case	control	0.079662419	0.586327037	0.135866869	0.891926527	0.977819591	up_in_case
CRA004775	ENSGALG00010005264	ITIH2	case	control	0.507664363	0.319318611	1.589836437	0.111871678	0.561235844	up_in_case
CRA004775	ENSGALG00010005265	DYNC2H1	case	control	0.230508828	0.358725228	0.642577689	0.520498164	0.865611895	up_in_case
CRA004775	ENSGALG00010005266	SLC19A2	case	control	-0.431697296	0.390898187	-1.104372725	0.269431487	0.72765348	down_in_case
CRA004775	ENSGALG00010005267	CYP51A1	case	control	-0.037855325	0.204228702	-0.185357517	0.852948628	0.969725992	down_in_case
CRA004775	ENSGALG00010005268	MTTP	case	control	2.322104333	0.897919796	2.586093261	0.009707066	0.199823154	up_in_case
CRA004775	ENSGALG00010005269	KIN	case	control	-0.017566199	0.207505224	-0.084654251	0.932536268	0.985044263	down_in_case
CRA004775	ENSGALG00010005270	GSTZ1	case	control	0.541842965	0.330643609	1.638752269	0.101264864	0.540680709	up_in_case
CRA004775	ENSGALG00010005271	ENSGALG00010005271	case	control	-1.531504509	0.786336296	-1.947645706	0.051457371	0.418692489	down_in_case
CRA004775	ENSGALG00010005272	MBNL2	case	control	0.627700411	0.367538564	1.707849115	0.087664343	0.512530774	up_in_case
CRA004775	ENSGALG00010005273	ENSGALG00010005273	case	control	-0.975879598	0.515923202	-1.891521052	0.058554823	0.443329681	down_in_case
CRA004775	ENSGALG00010005275	L3MBTL3	case	control	0.105619375	0.392424206	0.269145923	0.787817393	0.95041797	up_in_case
CRA004775	ENSGALG00010005277	ENSGALG00010005277	case	control	-0.801302211	0.425245222	-1.884329725	0.059520389	0.445059745	down_in_case
CRA004775	ENSGALG00010005278	DAPP1	case	control	-0.240348861	0.461800625	-0.520460233	0.602742838	0.898657353	down_in_case
CRA004775	ENSGALG00010005281	TMED8	case	control	-0.216746269	0.31380824	-0.690696552	0.489756257	0.854649529	down_in_case
CRA004775	ENSGALG00010005282	FLT1	case	control	0.57155521	0.368973045	1.549043265	0.12137132	0.573531622	up_in_case
CRA004775	ENSGALG00010005283	ATP5C1	case	control	0.025708733	0.378243019	0.067968824	0.945810452	0.988886825	up_in_case
CRA004775	ENSGALG00010005285	MPP7	case	control	0.48203278	0.314141608	1.534444238	0.124920401	0.577423041	up_in_case
CRA004775	ENSGALG00010005286	ENSGALG00010005286	case	control	-0.055840143	0.599148367	-0.093199191	0.925745317	0.98417264	down_in_case
CRA004775	ENSGALG00010005288	ENSGALG00010005288	case	control	0.297420551	0.209199008	1.421711096	0.155110138	0.619284425	up_in_case
CRA004775	ENSGALG00010005290	ENSGALG00010005290	case	control	0.710156472	0.613527758	1.157496891	0.247069409	0.712046495	up_in_case
CRA004775	ENSGALG00010005291	IFNGR1	case	control	-0.076600872	0.304386251	-0.251656808	0.801306345	0.953541667	down_in_case
CRA004775	ENSGALG00010005292	TAF3	case	control	0.598165426	0.158258606	3.779670764	0.000157036	0.026992502	up_in_case
CRA004775	ENSGALG00010005293	BLZF1	case	control	-0.214001037	0.222776246	-0.960609761	0.336748419	0.777081307	down_in_case
CRA004775	ENSGALG00010005295	ENSGALG00010005295	case	control	0.784345635	0.603403727	1.299868728	0.193645965	0.657919837	up_in_case
CRA004775	ENSGALG00010005296	PAN3	case	control	0.063015917	0.389444441	0.161809775	0.871455655	0.973918682	up_in_case
CRA004775	ENSGALG00010005297	IL22RA2	case	control	-1.766586002	0.866302012	-2.039226478	0.041427429	0.380794507	down_in_case
CRA004775	ENSGALG00010005301	ENSGALG00010005301	case	control	0.528035531	0.380354788	1.388271025	0.165054532	0.630194031	up_in_case
CRA004775	ENSGALG00010005302	PSPC1	case	control	-0.379131707	0.24564157	-1.543434634	0.122725356	0.57525466	down_in_case
CRA004775	ENSGALG00010005303	ENSGALG00010005303	case	control	-0.248270098	0.433228394	-0.573069775	0.566597451	0.883033678	down_in_case
CRA004775	ENSGALG00010005304	ENSGALG00010005304	case	control	0.120833603	0.320410884	0.377120781	0.706083848	0.93131216	up_in_case
CRA004775	ENSGALG00010005305	SCAF8	case	control	0.201030221	0.191421779	1.050195132	0.293628407	0.746438942	up_in_case
CRA004775	ENSGALG00010005307	TIAM2	case	control	0.361866176	0.25491211	1.419572322	0.155732228	0.619442611	up_in_case
CRA004775	ENSGALG00010005308	ENSGALG00010005308	case	control	-1.00100276	0.738806002	-1.354892567	0.175451788	0.641031789	down_in_case
CRA004775	ENSGALG00010005309	ASXL3	case	control	0.187570553	0.475849304	0.394180577	0.693447725	0.928219572	up_in_case
CRA004775	ENSGALG00010005310	LAMTOR3	case	control	-0.166635589	0.223364423	-0.746025652	0.45565192	0.839192403	down_in_case
CRA004775	ENSGALG00010005311	YME1L1	case	control	-0.163128341	0.294928155	-0.553112134	0.580186628	0.888533949	down_in_case
CRA004775	ENSGALG00010005312	ENSGALG00010005312	case	control	-0.350050497	0.632655028	-0.553303904	0.580055328	0.888438428	down_in_case
CRA004775	ENSGALG00010005313	MPHOSPH8	case	control	0.161140184	0.235329833	0.684741844	0.493506839	0.856001758	up_in_case
CRA004775	ENSGALG00010005314	IL20RA	case	control	-0.491409345	0.642646087	-0.764665583	0.444470705	0.832504887	down_in_case
CRA004775	ENSGALG00010005315	ENSGALG00010005315	case	control	0.232258154	0.463966857	0.500592124	0.616658207	0.903534044	up_in_case
CRA004775	ENSGALG00010005316	PARP4	case	control	0.238620174	0.218631236	1.091427642	0.275084755	0.731001618	up_in_case
CRA004775	ENSGALG00010005317	CHCHD3	case	control	0.251420592	0.237696918	1.057736021	0.290175808	0.744282131	up_in_case
CRA004775	ENSGALG00010005320	ENSGALG00010005320	case	control	-1.037130924	0.669351742	-1.549455777	0.121272195	0.573473028	down_in_case
CRA004775	ENSGALG00010005321	HS6ST3	case	control	0.33556011	0.510627858	0.657151984	0.511083196	0.863170205	up_in_case
CRA004775	ENSGALG00010005322	TFB1M	case	control	-0.099061555	0.279675625	-0.354201604	0.723187801	0.935187451	down_in_case
CRA004775	ENSGALG00010005323	DNAJB14	case	control	0.037711467	0.197869741	0.190587338	0.848848911	0.968223172	up_in_case
CRA004775	ENSGALG00010005326	MASTL	case	control	-1.029266095	0.638276316	-1.612571342	0.106837669	0.552884225	down_in_case
CRA004775	ENSGALG00010005329	VIPAS39	case	control	-0.3078774	0.155045179	-1.985727013	0.047063644	0.402720701	down_in_case
CRA004775	ENSGALG00010005330	ENSGALG00010005330	case	control	-0.224869452	0.624214363	-0.360243956	0.718664706	0.934011578	down_in_case
CRA004775	ENSGALG00010005332	ENSGALG00010005332	case	control	0.143566147	0.543997033	0.263909797	0.791849426	0.951284723	up_in_case
CRA004775	ENSGALG00010005333	PLXNA4	case	control	-0.820896705	0.676806417	-1.212897344	0.225169071	0.692994606	down_in_case
CRA004775	ENSGALG00010005334	NME7	case	control	0.258904976	0.270710313	0.956391256	0.338874601	0.779398613	up_in_case
CRA004775	ENSGALG00010005335	AHSA1	case	control	0.002388972	0.258891871	0.009227683	0.992637478	0.998439571	up_in_case
CRA004775	ENSGALG00010005336	PEX7	case	control	-0.205939374	0.240586133	-0.855990208	0.392003196	0.80746892	down_in_case
CRA004775	ENSGALG00010005337	DCTN1	case	control	-0.373443907	0.229487549	-1.627294853	0.103674491	0.545522581	down_in_case
CRA004775	ENSGALG00010005338	PCGF1	case	control	-0.421169605	0.266684347	-1.579281309	0.114271547	0.564447197	down_in_case
CRA004775	ENSGALG00010005340	ENSGALG00010005340	case	control	-1.510246769	0.712646075	-2.119210113	0.034072714	0.35069902	down_in_case
CRA004775	ENSGALG00010005341	UGGT2	case	control	0.615853319	0.218495067	2.818614295	0.004823144	0.142429514	up_in_case
CRA004775	ENSGALG00010005342	MAP3K5	case	control	0.30349445	0.238272319	1.273729367	0.202759345	0.671841868	up_in_case
CRA004775	ENSGALG00010005344	ENSGALG00010005344	case	control	-0.568071686	0.910217549	-0.624105398	0.532558369	0.869946559	down_in_case
CRA004775	ENSGALG00010005346	FBXO30	case	control	0.455986207	0.549068281	0.830472681	0.406271587	0.813842524	up_in_case
CRA004775	ENSGALG00010005347	DOK1	case	control	-0.195441298	0.366984495	-0.532560095	0.594338132	0.894956597	down_in_case
CRA004775	ENSGALG00010005349	ACBD5	case	control	0.365313924	0.244329304	1.495170325	0.134869996	0.592523104	up_in_case
CRA004775	ENSGALG00010005350	ARHGAP20	case	control	-0.107117062	0.573921693	-0.186640552	0.851942469	0.969430695	down_in_case
CRA004775	ENSGALG00010005351	DTNA	case	control	0.056873919	0.457076355	0.1244298	0.90097498	0.979157637	up_in_case
CRA004775	ENSGALG00010005352	ENSGALG00010005352	case	control	0.44486356	0.403075191	1.103673881	0.26973463	0.72797291	up_in_case

CRA004775	ENSGALG00010005353	ENSGALG00010005353	case	control	0.042543451	0.272297649	0.156238776	0.875844813	0.974412785	up_in_case
CRA004775	ENSGALG00010005354	M1AP	case	control	0.742840524	0.339408605	2.18863197	0.0286236	0.322057735	up_in_case
CRA004775	ENSGALG00010005355	FDX1	case	control	0.165348666	0.184155394	0.89787577	0.369251784	0.794896739	up_in_case
CRA004775	ENSGALG00010005356	DNAJC3	case	control	0.013187516	0.196934418	0.066963996	0.946610366	0.989137998	up_in_case
CRA004775	ENSGALG00010005357	TNIP3	case	control	-1.334260035	0.662991125	-2.012485515	0.044168782	0.392535833	down_in_case
CRA004775	ENSGALG00010005358	SHPRH	case	control	-0.20201297	0.247226354	-0.817117459	0.413861306	0.817589409	down_in_case
CRA004775	ENSGALG00010005359	MEIS2	case	control	0.670028723	0.317743697	2.108708148	0.034969781	0.354362166	up_in_case
CRA004775	ENSGALG00010005360	CAMK1D	case	control	-0.572434218	0.37259989	-1.536324174	0.124458893	0.576754431	down_in_case
CRA004775	ENSGALG00010005361	ENSGALG00010005361	case	control	-0.834836687	0.68151373	-1.224974128	0.220584969	0.68855283	down_in_case
CRA004775	ENSGALG00010005362	ATP1B1	case	control	0.422038125	0.293015747	1.440325747	0.14977526	0.61366707	up_in_case
CRA004775	ENSGALG00010005363	RDX	case	control	0.348608626	0.174878973	1.993427917	0.046214609	0.399541954	up_in_case
CRA004775	ENSGALG00010005364	MAP7	case	control	1.690149033	0.827454046	2.042589605	0.041093081	0.379122508	up_in_case
CRA004775	ENSGALG00010005365	ENSGALG00010005365	case	control	0.246757933	0.726075887	0.339851436	0.733968411	0.936819725	up_in_case
CRA004775	ENSGALG00010005366	AUP1	case	control	-0.580914461	0.275313544	-2.11001047	0.034857454	0.354006537	down_in_case
CRA004775	ENSGALG00010005367	H2AZ1	case	control	0.090479629	0.239387271	0.377963408	0.705457778	0.930974079	up_in_case
CRA004775	ENSGALG00010005368	CCDC3	case	control	0.196261723	0.418464055	0.469004974	0.639066081	0.91121917	up_in_case
CRA004775	ENSGALG00010005369	DPT	case	control	0.167497784	0.597001092	0.28056529	0.779043842	0.94792594	up_in_case
CRA004775	ENSGALG00010005372	CDIN1	case	control	0.114225657	0.246906677	0.462626847	0.643631862	0.913647817	up_in_case
CRA004775	ENSGALG00010005374	ENSGALG00010005374	case	control	1.393999575	0.665978749	2.093159243	0.036334942	0.360628495	up_in_case
CRA004775	ENSGALG00010005375	ENSGALG00010005375	case	control	0.502702314	0.957323198	0.525112433	0.599505034	0.897206546	up_in_case
CRA004775	ENSGALG00010005376	HTRA2	case	control	-0.257065678	0.319886657	-0.803614883	0.421619425	0.819812626	down_in_case
CRA004775	ENSGALG00010005377	EMCN	case	control	0.145749903	0.365194656	0.39910196	0.689818078	0.927796693	up_in_case
CRA004775	ENSGALG00010005379	DZIP1	case	control	-0.144578842	0.354894004	-0.407385982	0.683724521	0.925304767	down_in_case
CRA004775	ENSGALG00010005380	XCL1	case	control	-0.183957261	0.496840338	-0.370254278	0.711193037	0.932234469	down_in_case
CRA004775	ENSGALG00010005381	FGF9	case	control	-0.674166919	0.916656835	-0.735462708	0.462057743	0.842114782	down_in_case
CRA004775	ENSGALG00010005382	RAB32	case	control	-0.211365604	0.362357325	-0.583307109	0.559686578	0.880918893	down_in_case
CRA004775	ENSGALG00010005383	OPTN	case	control	0.009403002	0.388043514	0.024231825	0.980667693	0.996980737	up_in_case
CRA004775	ENSGALG00010005384	MAPRE2	case	control	0.055219805	0.26232452	0.210501881	0.833275983	0.964019688	up_in_case
CRA004775	ENSGALG00010005385	PDSS1	case	control	-0.18329377	0.249188382	-0.735563068	0.461996646	0.842114782	down_in_case
CRA004775	ENSGALG00010005386	CPOX	case	control	-0.317824219	0.384846844	-0.825845981	0.408891467	0.814384687	down_in_case
CRA004775	ENSGALG00010005387	CLDN10	case	control	1.282052338	0.624309313	2.05355312	0.040018963	0.375888502	up_in_case
CRA004775	ENSGALG00010005388	ENSGALG00010005388	case	control	-0.003171927	0.399274756	-0.007944221	0.993661496	0.998818657	down_in_case
CRA004775	ENSGALG00010005389	MCM10	case	control	0.546173332	0.467275014	1.168847713	0.242464993	0.709690398	up_in_case
CRA004775	ENSGALG00010005392	ENSGALG00010005392	case	control	-0.196675119	0.273671781	-0.718653266	0.472354585	0.845425322	down_in_case
CRA004775	ENSGALG00010005393	CHIR2A15	case	control	1.11840419	0.694366007	1.610683961	0.107248623	0.553800541	up_in_case
CRA004775	ENSGALG00010005394	PHYH	case	control	-0.140620012	0.390429849	-0.360167166	0.718722127	0.934011578	down_in_case
CRA004775	ENSGALG00010005396	MICU2	case	control	0.40490279	0.223422067	1.812277523	0.069943326	0.469724266	up_in_case
CRA004775	ENSGALG00010005398	SEPHS1	case	control	0.276417727	0.226583455	1.219937825	0.222488445	0.6901691	up_in_case
CRA004775	ENSGALG00010005399	NDNF	case	control	0.72302264	0.549821525	1.315013339	0.18850542	0.652602726	up_in_case
CRA004775	ENSGALG00010005401	BCLAF1	case	control	0.00849595	0.243728495	0.034858254	0.972192769	0.994947809	up_in_case
CRA004775	ENSGALG00010005402	ABCC4	case	control	0.183046977	0.341371285	0.53621082	0.591812856	0.893804699	up_in_case
CRA004775	ENSGALG00010005403	ENSGALG00010005403	case	control	-0.578544138	0.44700861	-1.294257259	0.195576574	0.661073909	down_in_case
CRA004775	ENSGALG00010005404	BEND7	case	control	0.359895048	0.247144344	1.456213976	0.145333477	0.608365494	up_in_case
CRA004775	ENSGALG00010005405	CEBPD	case	control	0.442288477	0.673653963	0.656551436	0.511469384	0.863170205	up_in_case
CRA004775	ENSGALG00010005407	DPH6	case	control	-0.264241776	0.280116623	-0.943327723	0.3455133	0.784552667	down_in_case
CRA004775	ENSGALG00010005408	APBB1IP	case	control	-0.953100374	0.645243303	-1.477117811	0.13964406	0.599514664	down_in_case
CRA004775	ENSGALG00010005409	PRKCQ	case	control	0.587502215	0.406526845	1.445174466	0.148408887	0.611192154	up_in_case
CRA004775	ENSGALG00010005410	GR42L6	case	control	0.179782525	0.681383799	0.263849134	0.791896172	0.951284723	up_in_case
CRA004775	ENSGALG00010005411	STXBP5	case	control	-0.255943312	0.375805286	-0.681052986	0.495837964	0.856382445	down_in_case
CRA004775	ENSGALG00010005412	MTFR2	case	control	-0.795198098	0.588623495	-1.350945222	0.176712981	0.64251195	down_in_case
CRA004775	ENSGALG00010005414	ENSGALG00010005414	case	control	-0.488964292	0.549033013	-0.890591788	0.373148206	0.796804495	down_in_case
CRA004775	ENSGALG00010005415	PRKDC	case	control	-0.091053496	0.326415601	-0.278949584	0.780283516	0.94835413	down_in_case
CRA004775	ENSGALG00010005416	PPP3CA	case	control	0.085008994	0.317145235	0.268044368	0.788665167	0.95041797	up_in_case
CRA004775	ENSGALG00010005417	LOXL3	case	control	0.009074021	0.20218163	0.044880541	0.964202527	0.99271855	up_in_case
CRA004775	ENSGALG00010005418	ENSGALG00010005418	case	control	0.023399814	0.727370638	0.032170413	0.974336151	0.994947809	up_in_case
CRA004775	ENSGALG00010005420	DHRS4	case	control	-0.285427907	0.256950722	-1.110827418	0.266642642	0.727315996	down_in_case
CRA004775	ENSGALG00010005421	ENSGALG00010005421	case	control	-0.497472186	0.515429825	-0.965159782	0.334464787	0.774742828	down_in_case
CRA004775	ENSGALG00010005423	ST3GAL6	case	control	-0.1848988	0.265337313	-0.696844322	0.485900225	0.851645437	down_in_case
CRA004775	ENSGALG00010005424	ENSGALG00010005424	case	control	-0.201141082	0.394402346	-0.509989568	0.61005877	0.901413878	down_in_case
CRA004775	ENSGALG00010005425	ELAPOR2	case	control	-0.269041064	0.64889466	-0.414614391	0.678424211	0.92338129	down_in_case
CRA004775	ENSGALG00010005426	ENSGALG00010005426	case	control	-0.412736564	0.756433713	-0.545634809	0.585316992	0.890933579	down_in_case
CRA004775	ENSGALG00010005427	ENSGALG00010005427	case	control	-0.328781779	0.380876783	-0.863223473	0.38801461	0.804270424	down_in_case
CRA004775	ENSGALG00010005428	ZNF770	case	control	0.08871942	0.26606141	0.33345467	0.738791101	0.938064864	up_in_case
CRA004775	ENSGALG00010005429	ZDHHC20	case	control	-0.080858663	0.430246641	-0.187935606	0.850927131	0.968989143	down_in_case
CRA004775	ENSGALG00010005430	MCM4	case	control	-0.397671566	0.389813664	-1.0201581	0.307653486	0.759250374	down_in_case
CRA004775	ENSGALG00010005434	PRDM5	case	control	-0.048984045	0.598676262	-0.08182059	0.934789383	0.985758344	down_in_case
CRA004775	ENSGALG00010005435	MAD2L1	case	control	-0.714225973	0.520319258	-1.372668725	0.169855356	0.633147466	down_in_case
CRA004775	ENSGALG00010005436	AQR	case	control	-0.105014565	0.229699966	-0.457181456	0.647540626	0.914210065	down_in_case
CRA004775	ENSGALG00010005437	PDE7B	case	control	0.999850347	0.51207322	1.95255348	0.050872528	0.416397698	up_in_case
CRA004775	ENSGALG00010005438	GPR180	case	control	0.188118371	0.2490067	0.755475137	0.449963945	0.835337561	up_in_case
CRA004775	ENSGALG00010005440	ENSGALG00010005440	case	control	0.247143592	0.539164093	0.458382884	0.646677386	0.914043203	up_in_case
CRA004775	ENSGALG00010005441	UBE2V2	case	control	-0.016928063	0.273786209	-0.061829495	0.950698615	0.990168609	down_in_case
CRA004775	ENSGALG00010005442	SASH1	case	control	-0.049436628	0.378513424	-0.130607331	0.896085943	0.978486286	down_in_case
CRA004775	ENSGALG00010005443	BANK1	case	control	0.136702139	0.445643739	0.30675207	0.759032093	0.942236668	up_in_case
CRA004775	ENSGALG00010005444	ACTC1	case	control	-1.237652927	0.770494172	-1.606310563	0.108205691	0.554993081	down_in_case
CRA004775	ENSGALG00010005445	SFT2D2	case	control	-0.09924311	0.272284708	-0.364482864	0.715497468	0.933870859	down_in_case
CRA004775	ENSGALG00010005446	ENSGALG00010005446	case	control	-0.155801486	0.506827831	-0.307405151	0.758535008	0.942236668	down_in_case
CRA004775	ENSGALG00010005448	ENSGALG00010005448	case	control	-0.094793248	0.210404779	-0.450528018	0.652329756	0.915892481	down_in_case
CRA004775	ENSGALG00010005449	TIPRL	case	control	0.226830295	0.211375953	1.073113059	0.283220392	0.739728197	up_in_case
CRA004775	ENSGALG00010005450	ENSGALG00010005450	case	control	-0.926972828	0.582197809	-1.592195666	0.111340747	0.56070984	down_in_case
CRA004775	ENSGALG00010005453	UST	case	control	0.082871439	0.235172311	0.352386038	0.724548771	0.935238044	up_in_case
CRA004775	ENSGALG00010005454	GPR161	case	control	0.029162524	0.267690384	0.108941244	0.913249093	0.980803023	up_in_case
CRA004775	ENSGALG00010005455	ENSGALG00010005455	case	control	-0.713198552	0.687122281	-1.037949971	0.299293347	0.75239174	down_in_case
CRA004775	ENSGALG00010005456	RPL7L1	case	control	0.036789174	0.172144318	0.21371123	0.830772253	0.963284108	up_in_case
CRA004775	ENSGALG00010005457	SEMA3D	case	control	0.046514245	0.421222657	0.11042674	0.912070947	0.98075825	up_in_case
CRA004775	ENSGALG00010005458	PRKD1	case	control	0.477529392	0.224612379	2.12601547	0.033501971	0.348565623	up_in_case
CRA004775	ENSGALG00010005459	ZC3H12D	case	control	-0.360341174	0.419659489	-0.858651319	0.390532916	0.806479902	down_in_case
CRA004775	ENSGALG00010005460	TGDS	case	control	-0.0325481	0.253289688	-0.12850148	0.897752127	0.978790377	down_in_case
CRA004775	ENSGALG00010005461	ENSGALG00010005461	case	control	0.408224742	1.242248606	0.328617589	0.742444752	0.938877854	up_in_case
CRA004775	ENSGALG00010005462	AHI1	case	control	0.257412283	0.396077866	0.649903226	0.515754734	0.864897625	up_in_case
CRA004775	ENSGALG00010005463	SKA3	case	control	0.316317288	0.557156029	0.567735556	0.570214544	0.88518436	up_in_case
CRA004775	ENSGALG00010005465	ENSGALG00010005465	case	control	-0.493721375	0.310214371	-1.591549008	0.111486075	0.56070984	down_in_case
CRA004775	ENSGALG00010005466	LATS2	case	control	0.424045578	0.286939248	1.477823548	0.139455014	0.59914295	up_in_case
CRA004775	ENSGALG00010005467	ENSGALG00010005467	case	control	-1.367786169	0.44367342	-3.082867054	0.002050167	0.090649034	down_in_case
CRA004775	ENSGALG00010005468	PPIL4	case	control	-0.020367927	0.238452493	-0.085417126	0.931929778	0.985044263	down_in_case
CRA004775	ENSGALG00010005470	SEMA3A	case	control	0.115370389	0.450386031	0.256158897	0.797828146	0.952835572	up_in_case
CRA004775	ENSGALG00010005472	PFKP	case	control	-0.216301519	0.354780269	-0.60967742	0.542075515	0.875128455	down_in_case
CRA004775	ENSGALG00010005473	ENSGALG00010005473	case	control	0.280232961	0.605592817	0.462741553	0.64354963	0.913638565	up_in_case
CRA004775	ENSGALG00010005474	COL8A1	case	control	-0.449909081	0.500813219	-0.898357042	0.368995231	0.794896739	down_in_case
CRA004775	ENSGALG00010005475	TMEM181	case	control	0.007972486	0.248758342	0.032049119	0.974432879	0.994947809	up_in_case
CRA004775	ENSGALG00010005476	NFKB1	case	control	0.03589998	0.223849358	0.16037562	0.872585194	0.97401729	up_in_case
CRA004775	ENSGALG00010005477	TMEM161B	case	control	0.441292163	0.261574956	1.687058158	0.09159217	0.521441551	up_in_case
CRA004775	ENSGALG00010005478	ENSGALG00010005478	case	control	0.823220766	0.689737805	1.193527104			up_in_case
CRA004775	ENSGALG00010005481	ENSGALG00010005481	case	control	0.20669813	1.159657862	0.178240615	0.858534015	0.971262956	up_in_case
CRA004775	ENSGALG00010005482	XPO4	case	control	-0.124480664	0.234214409	-0.531481663	0.595085045	0.895305942	down_in_case
CRA004775	ENSGALG00010005483	FFAR2L7	case	control	0.851104101	0.875297552	0.972359741	0.330871613	0.77237154	up_in_case
CRA004775	ENSGALG00010005484	GINM1	case	control	-0.131574801	0.223721828	-0.588117851	0.556453192	0.87981923	down_in_case
CRA004775	ENSGALG00010005485	PITRM1	case	control	-0.208658993	0.224431102	-0.929724052	0.352513978	0.786426886	down_in_case
CRA004775	ENSGALG00010005486	IRF5	case	control	-0.200028461	0.408941617	-0.489136964	0.624744734	0.90648604	down_in_case
CRA004775	ENSGALG00010005487	ENSGALG00010005487	case	control	-0.175897281	0.332037666	-0.529750987	0.596284592	0.895574418	down_in_case
CRA004775	ENSGALG00010005489	G2E3	case	control	0.049384503	0.379662142	0.130074867	0.896507195	0.978653683	up_in_case
CRA004775	ENSGALG00010005490	DCBLD2	case	control	0.406415933	0.199408036	2.038112107	0.041538723	0.381307727	up_in_case
CRA004775	ENSGALG00010005491	GGACT	case	control	-0.469573262	0.302722317	-1.551168298	0.120861357	0.573473028	down_in_case
CRA004775	ENSGALG00010005492	TNPO3	case	control	-0.35259301	0.276332764	-1.275972509	0.201965237	0.670354119	down_in_case
CRA004775	ENSGALG00010005493	KLF6	case	control	1.253480688	0.455641243	2.751025523	0.005940901	0.154441997	up_in_case
CRA004775	ENSGALG00010005494	PPDPFL	case	control	-2.74716077	0.740946986	-3.707634718	0.000209204	0.029354755	down_in_case
CRA004775	ENSGALG00010005495	TXN	case	control	-0.292300881	0.309651834	-0.943966252	0.345186894	0.784183873	down_in_case
CRA004775	ENSGALG00010005497	MKX	case	control	0.242345764	0.332405815	0.729065958	0.465961317	0.843416358	up_in_case
CRA004775	ENSGALG00010005498	SMO	case	control	-0.390522744	0.401783362	-0.971973408	0.331063778	0.772451228	down_in_case
CRA004775	ENSGALG00010005499	PDE5A	case	control	0.531287541	0.317461307	1.673550538	0.094218966	0.526882882	up_in_case
CRA004775	ENSGALG00010005500	KATNA1	case	control	0.032124825	0.194562885	0.165112811	0.86885519	0.973370973	up_in_case
CRA004775	ENSGALG00010005501	ENSGALG00010005501	case	control	0.197162668	0.82584618	0.238740183	0.811307056	0.957303241	up_in_case
CRA004775	ENSGALG00010005502	SCFD1	case	control	-0.089750517	0.175049337	-0.51271555	0.608150311	0.900493922	down_in_case
CRA004775	ENSGALG00010005503	DYNLT1	case	control	-0.45567226	0.248416354	-1.834308622	0.066608195	0.462498477	down_in_case
CRA004775	ENSGALG00010005504	LATS1	case	control	0.218812904	0.255043537	0.85794334	0.390923751	0.806479902	up_in_case
CRA004775	ENSGALG00010005505	FABP2	case	control	2.558850078	0.883488069	2.896304056	0.003775864	0.12725956	up_in_case

CRA004775	ENSGALG00010005507	USP53	case	control	1.07483305	0.469378599	2.289906382	0.022026744	0.290827737	up_in_case
CRA004775	ENSGALG00010005508	SS18	case	control	0.039479399	0.242897795	0.162535023	0.870884551	0.973918682	up_in_case
CRA004775	ENSGALG00010005509	COCH	case	control	0.28431983	0.559341679	0.508311539	0.611234879	0.901413878	up_in_case
CRA004775	ENSGALG00010005510	MYOZ2	case	control	-1.118052818	0.781580588	-1.430502287	0.15257291	0.617257216	down_in_case
CRA004775	ENSGALG00010005511	AHCYL2	case	control	0.052798721	0.314979685	0.167625798	0.866877674	0.973062856	up_in_case
CRA004775	ENSGALG00010005512	SMARCAD1	case	control	-0.130674172	0.28770299	-0.45419817	0.6496862	0.915164406	down_in_case
CRA004775	ENSGALG00010005513	TMEM30CP	case	control	0.492658698	0.365215795	1.348952331	0.177352278	0.643283771	up_in_case
CRA004775	ENSGALG00010005514	EEF1AKMT1	case	control	0.111533912	0.374624752	0.297721685	0.765915591	0.943567936	up_in_case
CRA004775	ENSGALG00010005515	PCCA	case	control	0.274823751	0.230936943	1.190038058	0.234031434	0.701278329	up_in_case
CRA004775	ENSGALG00010005516	LARP4B	case	control	0.459473893	0.220508313	2.083703271	0.037187167	0.363933342	up_in_case
CRA004775	ENSGALG00010005517	ENSGALG00010005517	case	control	1.798694926	0.917497083	1.96043667	0.049944773	0.414161886	up_in_case
CRA004775	ENSGALG00010005518	HPGDS	case	control	1.113909201	0.50045836	2.225777987	0.026029056	0.308822734	up_in_case
CRA004775	ENSGALG00010005519	PTBP3	case	control	0.505278706	0.554353304	0.911474148	0.362045601	0.792867581	up_in_case
CRA004775	ENSGALG00010005520	ZIC2	case	control	-0.652779324	0.829699519	-0.786765942	0.431418894	0.825210907	down_in_case
CRA004775	ENSGALG00010005521	SYTL3	case	control	-0.322368826	0.545236653	-0.5912457	0.554355807	0.879486798	down_in_case
CRA004775	ENSGALG00010005522	SYNPO2	case	control	0.109425343	0.570458058	0.191820137	0.847883096	0.968053841	up_in_case
CRA004775	ENSGALG00010005523	ENSGALG00010005523	case	control	0.171303792	0.554606272	0.308874603	0.757416917	0.941900693	up_in_case
CRA004775	ENSGALG00010005524	STRIP2	case	control	-0.500528584	0.411138027	-1.217422255	0.2234436	0.691242507	down_in_case
CRA004775	ENSGALG00010005526	STRN3	case	control	0.393688274	0.213270055	1.845961323	0.064897822	0.457570489	up_in_case
CRA004775	ENSGALG00010005527	NUP43	case	control	-0.137556081	0.22475724	-0.612020687	0.540524071	0.874440764	down_in_case
CRA004775	ENSGALG00010005529	ENSGALG00010005529	case	control	-0.088076958	0.498114259	-0.176820793	0.859649156	0.971325143	down_in_case
CRA004775	ENSGALG00010005530	IL17D	case	control	-0.412429152	0.52257094	-0.789230936	0.429977044	0.824153229	down_in_case
CRA004775	ENSGALG00010005531	SUSD1	case	control	0.71058148	0.354822292	2.002640465	0.045215893	0.395272567	up_in_case
CRA004775	ENSGALG00010005532	GTPBP4	case	control	0.009073224	0.205963448	0.044052594	0.964862481	0.992887645	up_in_case
CRA004775	ENSGALG00010005534	ENSGALG00010005534	case	control	0.07744853	0.212065456	0.365210493	0.714954287	0.93365018	up_in_case
CRA004775	ENSGALG00010005535	AP4S1	case	control	0.132331781	0.309640368	0.42737251	0.669108027	0.920550723	up_in_case
CRA004775	ENSGALG00010005536	TAF4B	case	control	0.053929919	0.519668626	0.103777515	0.91734591	0.981688766	up_in_case
CRA004775	ENSGALG00010005537	GRIN3A	case	control	-0.118178477	0.727015914	-0.162552806	0.870870548	0.973918682	down_in_case
CRA004775	ENSGALG00010005538	ENSGALG00010005538	case	control	-0.748151485	0.751536074	-0.995496438	0.319494876	0.767037518	down_in_case
CRA004775	ENSGALG00010005539	IFT88	case	control	-0.028544724	0.212466833	-0.134349084	0.893126542	0.977834811	down_in_case
CRA004775	ENSGALG00010005540	PCMT1	case	control	0.259301944	0.343017235	0.75594436	0.449682556	0.835055345	up_in_case
CRA004775	ENSGALG00010005542	KCTD1	case	control	0.082247145	0.204712148	0.401769735	0.687853497	0.927111919	up_in_case
CRA004775	ENSGALG00010005543	SEC24D	case	control	-0.296786866	0.454720778	-0.652679358	0.513963012	0.863954532	down_in_case
CRA004775	ENSGALG00010005544	AQP4	case	control	0.997866821	0.633436282	1.575323121	0.11518188	0.565776219	up_in_case
CRA004775	ENSGALG00010005545	RPL34	case	control	-0.197462611	0.270449464	-0.730127572	0.465312209	0.843383332	down_in_case
CRA004775	ENSGALG00010005546	NRF1	case	control	0.601159488	0.189591906	3.170807759	0.001520157	0.080693373	up_in_case
CRA004775	ENSGALG00010005547	LRP11	case	control	-0.942882747	0.499557534	-1.887435748	0.059101741	0.44414236	down_in_case
CRA004775	ENSGALG00010005548	EZR	case	control	0.21674989	0.415464353	0.521705144	0.601875642	0.898185892	up_in_case
CRA004775	ENSGALG00010005549	RNF20	case	control	-0.142762344	0.200298653	-0.712747396	0.476002082	0.846243735	down_in_case
CRA004775	ENSGALG00010005550	ENSGALG00010005550	case	control	-1.2318944	0.590689319	-2.085520018	0.037022123	0.363117842	down_in_case
CRA004775	ENSGALG00010005551	METTL14	case	control	-0.283589769	0.233056392	-1.216828972	0.223669295	0.691474027	down_in_case
CRA004775	ENSGALG00010005552	WDR37	case	control	0.131588408	0.241847571	0.544096464	0.586375093	0.891503646	up_in_case
CRA004775	ENSGALG00010005553	PPP1R14C	case	control	-0.394731983	0.352061391	-1.121202136	0.262201831	0.723230258	down_in_case
CRA004775	ENSGALG00010005554	CRYL1	case	control	0.508617626	0.397558002	1.279354518	0.200772242	0.668317382	up_in_case
CRA004775	ENSGALG00010005555	PRSS12	case	control	0.74345681	0.579945895	1.281941672	0.199863108	0.667128108	up_in_case
CRA004775	ENSGALG00010005557	CMSS1	case	control	0.236218128	0.380807659	0.620308238	0.535054872	0.870981055	up_in_case
CRA004775	ENSGALG00010005558	TAGAP	case	control	-0.611177556	0.458464179	-1.333097731	0.182499734	0.647378665	down_in_case
CRA004775	ENSGALG00010005559	FILIP1L	case	control	0.398634514	0.379099795	1.051529228	0.293015593	0.746438942	up_in_case
CRA004775	ENSGALG00010005560	RSPH3	case	control	-0.112652532	0.666017849	-0.169143413	0.865683838	0.972629493	down_in_case
CRA004775	ENSGALG00010005561	CLYBL	case	control	0.376803136	0.517076222	0.728718747	0.466173723	0.843480361	up_in_case
CRA004775	ENSGALG00010005562	HECTD1	case	control	0.448406793	0.348317116	1.287352162	0.19797157	0.664272829	up_in_case
CRA004775	ENSGALG00010005564	TM9SF2	case	control	-0.059269605	0.202493442	-0.292698884	0.769752323	0.945437613	down_in_case
CRA004775	ENSGALG00010005565	PGAP4	case	control	0.174223145	0.347476533	0.501395428	0.616092857	0.9028981	up_in_case
CRA004775	ENSGALG00010005567	NDST3	case	control	0.260372377	0.715800413	0.363749967	0.716044727	0.933921336	up_in_case
CRA004775	ENSGALG00010005568	PLEKHG1	case	control	-0.237123093	0.417678259	-0.567717107	0.570227074	0.88518436	down_in_case
CRA004775	ENSGALG00010005572	ENSGALG00010005572	case	control	-0.68839891	0.390942521	-1.76086988	0.078260429	0.490500984	down_in_case
CRA004775	ENSGALG00010005573	CHST9	case	control	1.110192189	0.495657941	2.239835373	0.025101612	0.304882883	up_in_case
CRA004775	ENSGALG00010005575	ENSGALG00010005575	case	control	-1.452348007	0.66564014	-2.181881648	0.029118269	0.324740723	down_in_case
CRA004775	ENSGALG00010005578	HEATR5A	case	control	0.284708119	0.275796647	1.032311751	0.301926098	0.754801023	up_in_case
CRA004775	ENSGALG00010005581	GR42L5	case	control	0.242406933	0.533533516	0.454342465	0.649582356	0.915164406	up_in_case
CRA004775	ENSGALG00010005583	ALDOB	case	control	1.80908539	0.605467267	2.98791609	0.002808867	0.110041951	up_in_case
CRA004775	ENSGALG00010005585	ENSGALG00010005585	case	control	-1.2686459	0.84316971	-1.504615127	0.132423055	0.588922842	down_in_case
CRA004775	ENSGALG00010005587	LEF1	case	control	0.381023429	0.645884014	0.589925468	0.555240619	0.87981923	up_in_case
CRA004775	ENSGALG00010005588	PLAGL1	case	control	0.455740956	0.412031002	1.106084139	0.268690103	0.727528758	up_in_case
CRA004775	ENSGALG00010005590	TBC1D23	case	control	0.108245822	0.164972449	0.656144841	0.511730935	0.863170205	up_in_case
CRA004775	ENSGALG00010005593	ENSGALG00010005593	case	control	-0.518183255	0.718927422	-0.720772695	0.471049378	0.845198132	down_in_case
CRA004775	ENSGALG00010005594	MRPL50	case	control	-0.17774029	0.36349115	-0.488981067	0.624855101	0.90648604	down_in_case
CRA004775	ENSGALG00010005596	PARD3	case	control	-0.024751474	0.187536559	-0.131982126	0.894998429	0.977834811	down_in_case
CRA004775	ENSGALG00010005597	ENSGALG00010005597	case	control	-0.407572292	0.815526942	-0.499765576	0.617240152	0.903905787	down_in_case
CRA004775	ENSGALG00010005601	TMEM215	case	control	-1.646006816	0.774633478	-2.124884689	0.033596236	0.348565623	down_in_case
CRA004775	ENSGALG00010005604	ENSGALG00010005604	case	control	-0.169279872	0.592672739	-0.285621154	0.7751683	0.946319184	down_in_case
CRA004775	ENSGALG00010005609	GJB6	case	control	0.022744541	0.659008687	0.034513264	0.972467866	0.994947809	up_in_case
CRA004775	ENSGALG00010005610	MTHFD1L	case	control	-0.314103035	0.317002981	-0.990851993	0.321757857	0.767750105	down_in_case
CRA004775	ENSGALG00010005617	DTD2	case	control	0.29084663	0.426507755	0.681925771	0.495285889	0.856382445	up_in_case
CRA004775	ENSGALG00010005618	PHACTR2	case	control	0.204895901	0.169687972	1.207486299	0.227244918	0.694715356	up_in_case
CRA004775	ENSGALG00010005619	CELF2	case	control	0.090847777	0.208183305	0.436383584	0.662558447	0.918173506	up_in_case
CRA004775	ENSGALG00010005621	CDH2	case	control	0.659214599	0.454806311	1.449440307	0.147214659	0.610295079	up_in_case
CRA004775	ENSGALG00010005629	NRP1	case	control	0.069992949	0.495029922	0.14139135	0.887560788	0.977118922	up_in_case
CRA004775	ENSGALG00010005630	NIT2	case	control	-0.117322394	0.337895393	-0.347215134	0.728429702	0.935784458	down_in_case
CRA004775	ENSGALG00010005632	ITGB1	case	control	0.245336218	0.198030229	1.238882666	0.215388954	0.683654538	up_in_case
CRA004775	ENSGALG00010005634	HADH	case	control	0.216249049	0.339103371	0.637708344	0.523663553	0.866638286	up_in_case
CRA004775	ENSGALG00010005636	ENSGALG00010005636	case	control	0.005117691	0.805514471	0.00635332	0.994930818	0.999001196	up_in_case
CRA004775	ENSGALG00010005637	TOPORS	case	control	0.159593558	0.189939173	0.840235088	0.400776589	0.811868062	up_in_case
CRA004775	ENSGALG00010005645	FUCA2	case	control	-0.507067859	0.511822403	-0.990710559	0.321826934	0.767750105	down_in_case
CRA004775	ENSGALG00010005649	TOMM70	case	control	0.018967029	0.162202182	0.116934489	0.906911967	0.980273095	up_in_case
CRA004775	ENSGALG00010005651	USP6NL	case	control	0.171690632	0.23413951	0.733283471	0.463385554	0.842456502	up_in_case
CRA004775	ENSGALG00010005656	CYP2U1	case	control	0.0508139	0.219429373	0.231572918	0.816869744	0.959594698	up_in_case
CRA004775	ENSGALG00010005660	UBAC2	case	control	0.048179942	0.227646211	0.211643946	0.832384822	0.963965276	up_in_case
CRA004775	ENSGALG00010005670	SGMS2	case	control	0.618855144	0.525538093	1.17756477	0.238970162	0.705835663	up_in_case
CRA004775	ENSGALG00010005675	GJA3	case	control	-0.424920254	0.589635234	-0.720649359	0.471125277	0.845198132	down_in_case
CRA004775	ENSGALG00010005679	TMEM45A	case	control	-0.187654545	0.546843608	-0.343159438	0.731478518	0.936574571	down_in_case
CRA004775	ENSGALG00010005680	ENSGALG00010005680	case	control	0.237157856	0.569782337	0.416225355	0.677245108	0.922924485	up_in_case
CRA004775	ENSGALG00010005681	EPC1	case	control	-0.035141095	0.216352	-0.162425561	0.870970743	0.973918682	down_in_case
CRA004775	ENSGALG00010005688	ENSGALG00010005688	case	control	0.006572169	0.563198257	0.01166937	0.990689401	0.998065748	up_in_case
CRA004775	ENSGALG00010005692	SEC24B	case	control	0.258998663	0.235714206	1.098782575	0.271862914	0.729299051	up_in_case
CRA004775	ENSGALG00010005694	ENSGALG00010005694	case	control	-0.150368531	0.273416394	-0.549961649	0.582345678	0.889579727	down_in_case
CRA004775	ENSGALG00010005695	KIF5B	case	control	0.505389313	0.228276815	2.213931858	0.026833467	0.314835326	up_in_case
CRA004775	ENSGALG00010005696	NUBPL	case	control	-0.470425057	0.295678536	-1.591001714	0.11160919	0.560737293	down_in_case
CRA004775	ENSGALG00010005699	PRPF18	case	control	-0.125581784	0.190353664	-0.659728745	0.509427917	0.862971569	down_in_case
CRA004775	ENSGALG00010005704	AKAP12	case	control	0.757890061	0.429732196	1.763633415	0.077793718	0.49028362	up_in_case
CRA004775	ENSGALG00010005705	ECHDC3	case	control	-0.356083913	0.337508847	-1.05503579	0.291408957	0.745518988	down_in_case
CRA004775	ENSGALG00010005712	ENSGALG00010005712	case	control	0.046211136	0.359562836	0.128520334	0.897737207	0.978790377	up_in_case
CRA004775	ENSGALG00010005714	ARHGAP5	case	control	0.790999761	0.324914624	2.434484942	0.014913	0.244420343	up_in_case
CRA004775	ENSGALG00010005716	ZBTB2	case	control	0.351948087	0.195834009	1.797175518	0.07230776	0.474381682	up_in_case
CRA004775	ENSGALG00010005720	COL25A1	case	control	-0.608379462	0.987140521	-0.616304821	0.537693358	0.872603995	down_in_case
CRA004775	ENSGALG00010005724	PROSER2	case	control	1.081089183	0.442147524	2.445087046	0.014481719	0.240504965	up_in_case
CRA004775	ENSGALG00010005740	ENSGALG00010005740	case	control	-0.347177618	0.533482015	-0.65077661	0.515190701	0.864897625	down_in_case
CRA004775	ENSGALG00010005742	GPR183	case	control	-0.347381345	0.470840042	-0.737790573	0.460641723	0.841318147	down_in_case
CRA004775	ENSGALG00010005743	ETNPPL	case	control	0.918866276	0.695258066	1.321619008	0.18629505	0.6491093	up_in_case
CRA004775	ENSGALG00010005748	AIG1	case	control	-0.401504135	0.451307387	-0.889646718	0.373655612	0.797250404	down_in_case
CRA004775	ENSGALG00010005752	RMND1	case	control	0.360148805	0.255926385	1.407235933	0.15935744	0.624907945	up_in_case
CRA004775	ENSGALG00010005755	ZMYM2	case	control	-0.10631497	0.176779416	-0.601399031	0.547574244	0.877277621	down_in_case
CRA004775	ENSGALG00010005756	TET2	case	control	1.072407049	0.626222051	1.712502852	0.08680404	0.509281038	up_in_case
CRA004775	ENSGALG00010005757	ARHGAP12	case	control	0.794515072	0.222090711	3.57743495	0.000346983	0.037067336	up_in_case
CRA004775	ENSGALG00010005758	HIVEP2	case	control	0.484746721	0.263879816	1.836998106	0.066210175	0.462160465	up_in_case
CRA004775	ENSGALG00010005760	GPR18	case	control	0.972930136	0.647577526	1.502414918	0.132989986	0.589727603	up_in_case
CRA004775	ENSGALG00010005763	AKAP6	case	control	0.6547552	0.504820355	1.297006337	0.194629006	0.659287597	up_in_case
CRA004775	ENSGALG00010005769	TFG	case	control	0.007044972	0.206059753	0.034188974	0.972726459	0.994947809	up_in_case
CRA004775	ENSGALG00010005772	SVEP1	case	control	0.078671357	0.35685288	0.220458799	0.825513857	0.961878235	up_in_case
CRA004775	ENSGALG00010005777	ENSGALG00010005777	case	control	0.009573221	1.090115259	0.008781843	0.992993193	0.998511839	up_in_case
CRA004775	ENSGALG00010005779	ARMT1	case	control	-0.178054274	0.230787458	-0.771507587	0.440406127	0.829353567	down_in_case
CRA004775	ENSGALG00010005784	RASL11A	case	control	-1.06686342	0.517513198	-2.061519252	0.039253529	0.373030597	down_in_case
CRA004775	ENSGALG00010005785	RPL21	case	control	-0.122215035	0.252155502	-0.484681213	0.627902495	0.90828391	down_in_case
CRA004775	ENSGALG00010005789	RNASET2	case	control	-0.064239931	0.468159325	-0.137218096	0.890858408	0.977513084	down_in_case
CRA004775	ENSGALG00010005790	MUSK	case	control	0.289491244	0.624320108	0.463690406	0.642869572	0.913515591	up_in_case
CRA004775	ENSGALG00010005792	ADGRG6	case	control	0.421597968	0.367324215	1.147754357	0.251069985	0.715684776	up_in_case

CRA004775	ENSGALG00010005799	UFSP2	case	control	-0.313650855	0.202435877	-1.549383732	0.121289502	0.573473028	down_in_case
CRA004775	ENSGALG00010005800	FRMD4A	case	control	0.635351149	0.308531526	2.059274648	0.039467936	0.374034172	up_in_case
CRA004775	ENSGALG00010005814	UPF2	case	control	0.087154978	0.191880488	0.454214905	0.649674156	0.915164406	up_in_case
CRA004775	ENSGALG00010005817	VTA1	case	control	-0.073967248	0.197286341	-0.374923311	0.707717502	0.931742212	down_in_case
CRA004775	ENSGALG00010005821	DOCK9	case	control	0.843188373	0.418852671	2.013090594	0.0441051	0.392535833	up_in_case
CRA004775	ENSGALG00010005826	LRP2BP	case	control	-0.947204629	0.469865355	-2.015906512	0.043809753	0.391981676	down_in_case
CRA004775	ENSGALG00010005827	ENSGALG00010005827	case	control	-0.328511176	0.433445624	-0.757906316	0.448507069	0.83429564	down_in_case
CRA004775	ENSGALG00010005828	LPAR1	case	control	-0.51436919	0.464411376	-1.107572331	0.268046558	0.727528758	down_in_case
CRA004775	ENSGALG00010005829	NPAS3	case	control	-0.636300186	0.433143225	-1.469029526	0.141824781	0.602109467	down_in_case
CRA004775	ENSGALG00010005830	USP12	case	control	-0.070858033	0.399879926	-0.177198274	0.859352651	0.971325143	down_in_case
CRA004775	ENSGALG00010005831	SEC61A2	case	control	-0.175009383	0.273711337	-0.639393987	0.52256666	0.866638286	down_in_case
CRA004775	ENSGALG00010005836	NEBL	case	control	0.019180282	0.504200393	0.03804099	0.969655001	0.994312149	up_in_case
CRA004775	ENSGALG00010005839	RPS6KA2	case	control	-0.650612936	0.355324099	-1.831040837	0.067094448	0.462498477	down_in_case
CRA004775	ENSGALG00010005841	ENSGALG00010005841	case	control	0.360229387	0.594504932	0.605931704	0.544560105	0.875916944	up_in_case
CRA004775	ENSGALG00010005847	ENSGALG00010005847	case	control	-1.532442752	0.690467248	-2.219428593	0.02645758	0.312290288	down_in_case
CRA004775	ENSGALG00010005848	WASF3	case	control	-0.074799984	0.389686304	-0.191949224	0.847781979	0.968053841	down_in_case
CRA004775	ENSGALG00010005849	SNX25	case	control	0.346905226	0.216828237	1.599907979	0.109618999	0.557047618	up_in_case
CRA004775	ENSGALG00010005851	SLC15A1	case	control	2.690314009	0.9350233	2.877269484	0.004011329	0.130093822	up_in_case
CRA004775	ENSGALG00010005853	COMMD3	case	control	-0.435237135	0.278122722	-1.564910383	0.117603924	0.568272695	down_in_case
CRA004775	ENSGALG00010005854	ENSGALG00010005854	case	control	0.522087373	0.737749104	0.707676052	0.479146452	0.847184661	up_in_case
CRA004775	ENSGALG00010005858	CFAP97	case	control	0.06453039	0.302539185	0.213295974	0.831096114	0.963284108	up_in_case
CRA004775	ENSGALG00010005859	ENSGALG00010005859	case	control	-0.713259026	0.762382105	-0.935566328	0.349496518	0.785360568	down_in_case
CRA004775	ENSGALG00010005860	ABI3BP	case	control	0.336048911	0.591685728	0.567951693	0.57006777	0.88518436	up_in_case
CRA004775	ENSGALG00010005861	MPC1L	case	control	0.342060811	0.310810747	1.100543703	0.271095299	0.728765754	up_in_case
CRA004775	ENSGALG00010005866	DNAJC1	case	control	0.109257967	0.208007536	0.525259655	0.5994027	0.897206546	up_in_case
CRA004775	ENSGALG00010005871	EGLN3	case	control	0.6674839	0.448399497	1.48859199	0.136594842	0.595534456	up_in_case
CRA004775	ENSGALG00010005872	STK24	case	control	0.3503421	0.224842424	1.558167246	0.119193607	0.570892125	up_in_case
CRA004775	ENSGALG00010005875	CDK8	case	control	-0.12262455	0.329894901	-0.371707928	0.710110321	0.932234469	down_in_case
CRA004775	ENSGALG00010005879	FAM107B	case	control	0.230599649	0.266636725	0.864845791	0.387123434	0.803905475	up_in_case
CRA004775	ENSGALG00010005880	SLC25A4	case	control	0.083103213	0.622762704	0.133442823	0.893843181	0.977834811	up_in_case
CRA004775	ENSGALG00010005881	CORO2A	case	control	1.768238979	0.558914863	3.163700047	0.001557773	0.080693373	up_in_case
CRA004775	ENSGALG00010005884	MYB	case	control	0.980237002	0.633384573	1.547617424	0.121714434	0.573689301	up_in_case
CRA004775	ENSGALG00010005887	CCNH	case	control	-0.226394374	0.280773534	-0.806323767	0.42005619	0.819388688	down_in_case
CRA004775	ENSGALG00010005888	IMPG2	case	control	-0.58514572	0.414231808	-1.412604511	0.157772042	0.62262379	down_in_case
CRA004775	ENSGALG00010005890	SNX32	case	control	0.105132203	0.124659786	0.843352986	0.399031058	0.810498681	up_in_case
CRA004775	ENSGALG00010005891	RNF6	case	control	0.232153305	0.220173622	1.054410164	0.291695171	0.745973646	up_in_case
CRA004775	ENSGALG00010005892	ENSGALG00010005892	case	control	-0.536322807	0.743528302	-0.721321308	0.47071185	0.845117985	down_in_case
CRA004775	ENSGALG00010005893	SFT2D1	case	control	-0.183926343	0.174701315	-1.052804571	0.29243057	0.746262643	down_in_case
CRA004775	ENSGALG00010005895	MLLT10	case	control	0.260906716	0.226807945	1.15034205	0.250003018	0.714890768	up_in_case
CRA004775	ENSGALG00010005899	ENSGALG00010005899	case	control	0.27109115	0.181484394	1.493743593	0.13524265	0.593023494	up_in_case
CRA004775	ENSGALG00010005900	SPTSSA	case	control	0.183562813	0.218410604	0.840448265	0.400657097	0.811868062	up_in_case
CRA004775	ENSGALG00010005902	EAPP	case	control	0.173218725	0.208124272	0.832285074	0.405248054	0.81327583	up_in_case
CRA004775	ENSGALG00010005903	PRR18	case	control	-0.620880045	0.516090397	-1.203045142	0.228958852	0.695782399	down_in_case
CRA004775	ENSGALG00010005905	CITED2	case	control	0.511092195	0.24291685	2.103979999	0.035380192	0.35615887	up_in_case
CRA004775	ENSGALG00010005909	BRMS1L	case	control	0.188646013	0.217075661	0.869033461	0.384828829	0.803182776	up_in_case
CRA004775	ENSGALG00010005910	ENSGALG00010005910	case	control	-0.541419493	0.578432998	-0.936010729	0.349267664	0.785360568	down_in_case
CRA004775	ENSGALG00010005911	TBXT	case	control	-0.519165042	0.887695293	-0.584846001	0.55865127	0.880754991	down_in_case
CRA004775	ENSGALG00010005913	NFKBIA	case	control	0.19646387	0.288281358	0.681500431	0.495554894	0.856382445	up_in_case
CRA004775	ENSGALG00010005915	PAPSS1	case	control	0.053561237	0.201987729	0.265170745	0.790877931	0.95117511	up_in_case
CRA004775	ENSGALG00010005926	NUDT5	case	control	-0.319384331	0.368613562	-0.866447585	0.386244758	0.80363923	down_in_case
CRA004775	ENSGALG00010005935	ATP8A2	case	control	1.456618554	0.893428294	1.63036985	0.103023353	0.544156998	up_in_case
CRA004775	ENSGALG00010005941	TXLNB	case	control	0.036984616	0.532465956	0.069459118	0.944624173	0.988387362	up_in_case
CRA004775	ENSGALG00010005949	SLITRK1	case	control	0.686523064	0.805969971	0.851797323	0.394326593	0.808587082	up_in_case
CRA004775	ENSGALG00010005956	TXNL4B	case	control	-0.107466324	0.245195137	-0.438288969	0.661176821	0.917907174	down_in_case
CRA004775	ENSGALG00010005963	UGCG	case	control	0.901573249	0.286478945	3.147083803	0.001649077	0.081569987	up_in_case
CRA004775	ENSGALG00010005964	HSPA14	case	control	-0.292905828	0.248217242	-1.180038201	0.237985022	0.70473329	down_in_case
CRA004775	ENSGALG00010005966	PLXDC2	case	control	0.367338442	0.345409782	1.063485927	0.287561644	0.742456237	up_in_case
CRA004775	ENSGALG00010005967	GJD2	case	control	-0.557053168	1.044217102	-0.533464896	0.593711805	0.894601252	down_in_case
CRA004775	ENSGALG00010005969	HECA	case	control	0.270312946	0.204388088	1.322547458	0.185985917	0.6491093	up_in_case
CRA004775	ENSGALG00010005970	SHISA2	case	control	-0.43613627	0.353576492	-1.233499058	0.217389628	0.684603098	down_in_case
CRA004775	ENSGALG00010005975	ABRACL	case	control	-0.459430495	0.40825571	-1.125349832	0.260440821	0.721313541	down_in_case
CRA004775	ENSGALG00010005978	VPS16	case	control	-0.492459432	0.346160562	-1.422632982	0.154842578	0.619284425	down_in_case
CRA004775	ENSGALG00010005979	SUV39H2	case	control	0.155200386	0.189395465	0.819451437	0.412528898	0.817038226	up_in_case
CRA004775	ENSGALG00010005981	AREL1	case	control	-0.376525345	0.272978612	-1.379321781	0.167795564	0.63138062	down_in_case
CRA004775	ENSGALG00010005984	IDH3B	case	control	-0.419074299	0.417200631	-1.004491048	0.315141984	0.765098415	down_in_case
CRA004775	ENSGALG00010005985	ENSGALG00010005985	case	control	2.361314605	0.619342576	3.812614692	0.000137504	0.025433454	up_in_case
CRA004775	ENSGALG00010005988	NUP58	case	control	0.147589477	0.194743707	0.757865194	0.448531689	0.83429564	up_in_case
CRA004775	ENSGALG00010005989	FCF1	case	control	-0.182288371	0.221680184	-0.822303411	0.410904236	0.816405743	down_in_case
CRA004775	ENSGALG00010005990	SLC2A12	case	control	-0.536759871	0.417968069	-1.284212624	0.199067569	0.666028746	down_in_case
CRA004775	ENSGALG00010005991	FARP1	case	control	-0.037713119	0.21518029	-0.175262887	0.860873072	0.971325143	down_in_case
CRA004775	ENSGALG00010005997	ENSGALG00010005997	case	control	-1.023173333	0.674468022	-1.517007923	0.129264684	0.584172159	down_in_case
CRA004775	ENSGALG00010005998	YLPM1	case	control	0.117735679	0.216352254	0.544185126	0.586314086	0.891503646	up_in_case
CRA004775	ENSGALG00010005999	PDE10A	case	control	1.45539784	0.721264618	2.017841723	0.043607748	0.391416542	up_in_case
CRA004775	ENSGALG00010006001	NOP56	case	control	-0.386048635	0.192461006	-2.005853773	0.044871852	0.39452227	down_in_case
CRA004775	ENSGALG00010006004	DCLRE1C	case	control	-0.281519826	0.403181146	-0.698246504	0.485023048	0.851302844	down_in_case
CRA004775	ENSGALG00010006009	MALRD1	case	control	2.140977704	0.677378909	3.160679607	0.001574015	0.080693373	up_in_case
CRA004775	ENSGALG00010006010	IPO5	case	control	0.017451571	0.187810515	0.092921162	0.925966194	0.98417264	up_in_case
CRA004775	ENSGALG00010006013	TRMT10C	case	control	0.091159774	0.252603011	0.360881579	0.718187975	0.934011578	up_in_case
CRA004775	ENSGALG00010006015	CDC123	case	control	-0.313993476	0.245697961	-1.277965331	0.201261651	0.669255489	down_in_case
CRA004775	ENSGALG00010006016	MTMR6	case	control	0.046793747	0.202699309	0.230853018	0.817428991	0.959812855	up_in_case
CRA004775	ENSGALG00010006021	ENSGALG00010006021	case	control	-0.008450402	0.235931596	-0.035817171	0.971428141	0.994908384	down_in_case
CRA004775	ENSGALG00010006022	AFG2A	case	control	-0.194212381	0.218132669	-0.890340647	0.373283002	0.796804495	down_in_case
CRA004775	ENSGALG00010006024	AMER2	case	control	-0.158891684	0.678547335	-0.234164481	0.814857289	0.958848415	down_in_case
CRA004775	ENSGALG00010006025	TNFAIP3	case	control	0.196392903	0.353951454	0.554858302	0.578991585	0.888378369	up_in_case
CRA004775	ENSGALG00010006028	ARL5B	case	control	0.508821273	0.292040451	1.742297243	0.081456446	0.49694214	up_in_case
CRA004775	ENSGALG00010006029	HSDL2	case	control	-0.151363857	0.325309018	-0.465292534	0.641721981	0.912545911	down_in_case
CRA004775	ENSGALG00010006031	ERCC5	case	control	0.541124268	0.261519807	2.069152137	0.038531813	0.370251617	up_in_case
CRA004775	ENSGALG00010006033	NSUN6	case	control	-0.285970791	0.33779661	-0.846576853	0.39723102	0.808998414	down_in_case
CRA004775	ENSGALG00010006035	ADH1C	case	control	1.610381656	0.688195981	2.340004445	0.01928351	0.274216703	up_in_case
CRA004775	ENSGALG00010006037	METTL21EP	case	control	0.228608352	1.101678739	0.20750909	0.835612295	0.965077632	up_in_case
CRA004775	ENSGALG00010006038	ENSGALG00010006038	case	control	-0.08899965	0.360063605	-0.247177578	0.804770797	0.954576316	down_in_case
CRA004775	ENSGALG00010006039	PCNP	case	control	-0.073392849	0.21681065	-0.338511274	0.734977933	0.936931635	down_in_case
CRA004775	ENSGALG00010006041	QKI	case	control	0.404320815	0.20941505	1.930715174	0.053518285	0.425885384	up_in_case
CRA004775	ENSGALG00010006043	ENSGALG00010006043	case	control	1.994474337	0.887924748	2.246220011	0.024689919	0.302325091	up_in_case
CRA004775	ENSGALG00010006046	ENSGALG00010006046	case	control	2.617170524	0.836065452	3.130341672	0.001746031	0.084784105	up_in_case
CRA004775	ENSGALG00010006047	ZBTB11	case	control	0.157044006	0.212198178	0.740081782	0.459250372	0.840589413	up_in_case
CRA004775	ENSGALG00010006049	AIMP1	case	control	0.106509893	0.235017999	0.453198876	0.650405542	0.915428088	up_in_case
CRA004775	ENSGALG00010006052	RALGAPA1	case	control	0.14225002	0.286846471	0.495909952	0.619957944	0.905089892	up_in_case
CRA004775	ENSGALG00010006053	TMEM243	case	control	-0.003358041	0.543111414	-0.00618297	0.995066735	0.999001196	down_in_case
CRA004775	ENSGALG00010006054	ENSGALG00010006054	case	control	2.484313407	0.949339575	2.616885962	0.008873598	0.190031214	up_in_case
CRA004775	ENSGALG00010006055	ENSGALG00010006055	case	control	0.59471982	0.386577093	1.538424887	0.123944761	0.576485043	up_in_case
CRA004775	ENSGALG00010006059	BIVM	case	control	-0.077157309	0.152556413	-0.505762478	0.613023402	0.901859934	down_in_case
CRA004775	ENSGALG00010006060	ENSGALG00010006060	case	control	-0.467175289	0.48490928	-0.963428231	0.335332664	0.775897605	down_in_case
CRA004775	ENSGALG00010006061	RPL24	case	control	-0.260141695	0.243735376	-1.067312016	0.285830964	0.741582427	down_in_case
CRA004775	ENSGALG00010006062	ENSGALG00010006062	case	control	0.279230986	0.711017294	0.392720385	0.694526013	0.928767671	up_in_case
CRA004775	ENSGALG00010006063	POGLUT2	case	control	0.15711399	0.18507573	0.848917302	0.39592731	0.808998414	up_in_case
CRA004775	ENSGALG00010006064	DMTF1	case	control	0.226311722	0.174472838	1.297117217	0.194590858	0.659287597	up_in_case
CRA004775	ENSGALG00010006065	WDR54	case	control	-0.307580751	0.245276084	-1.254018519	0.209835274	0.67982426	down_in_case
CRA004775	ENSGALG00010006066	ARHGAP18	case	control	0.105734191	0.238798713	0.44277538	0.657928213	0.917354438	up_in_case
CRA004775	ENSGALG00010006068	PACRG	case	control	0.206061288	0.631608882	0.326248244	0.744236543	0.939218065	up_in_case
CRA004775	ENSGALG00010006069	TTR	case	control	3.239533727	1.226335279	2.64163788	0.008250622	0.183215969	up_in_case
CRA004775	ENSGALG00010006070	TMEM242	case	control	0.100282001	0.255584193	0.392363861	0.694789385	0.928924129	up_in_case
CRA004775	ENSGALG00010006071	TEX30	case	control	-0.930465612	0.400160958	-2.325228372	0.020059752	0.280454072	down_in_case
CRA004775	ENSGALG00010006072	ENSGALG00010006072	case	control	-0.136931213	0.445091139	-0.307647582	0.75835051	0.942236668	down_in_case
CRA004775	ENSGALG00010006073	CEP97	case	control	-0.104935289	0.236037776	-0.444569894	0.656630608	0.91710603	down_in_case
CRA004775	ENSGALG00010006075	SOX21	case	control	0.016582152	0.534340011	0.031032959	0.975243255	0.995245303	up_in_case
CRA004775	ENSGALG00010006076	PODXL	case	control	0.353282191	0.405794258	0.870594357	0.383975678	0.802757395	up_in_case
CRA004775	ENSGALG00010006077	TBCK	case	control	-0.059121812	0.350125853	-0.168858743	0.865907751	0.972629493	down_in_case
CRA004775	ENSGALG00010006078	SPATA13	case	control	-0.62593798	0.67286697	-0.930255174	0.352238981	0.786426886	down_in_case
CRA004775	ENSGALG00010006079	ENSGALG00010006079	case	control	1.431203353	1.288082499	1.111111559	0.266520333	0.727311193	up_in_case
CRA004775	ENSGALG00010006080	ENSGALG00010006080	case	control	-0.112280662	0.283572318	-0.395950715	0.692141387	0.927867527	down_in_case
CRA004775	ENSGALG00010006081	ZDHHC14	case	control	0.065539938	0.24888833	0.263330699	0.7922957	0.951284723	up_in_case
CRA004775	ENSGALG00010006083	CACNB2	case	control	0.789144946	0.650425144	1.213275582	0.225024475	0.692859731	up_in_case
CRA004775	ENSGALG00010006084	TMEM68	case	control	-0.563292887	0.450232856	-1.251114572	0.21089268	0.681387509	down_in_case
CRA004775	ENSGALG00010006085	DLST	case	control	-0.215108423	0.22999439	-0.935276827	0.349645654	0.785360568	down_in_case
CRA004775	ENSGALG00010006086	ENSGALG00010006086	case	control	0.57297247	0.538254318	1.064501391	0.287101625	0.741909972	up_in_case

CRA004775	ENSGALG00010006087	ENSGALG00010006087	case	control	2.565836254	0.852376458	3.010214828	0.00261063	0.106489884	up_in_case
CRA004775	ENSGALG00010006091	RTKN	case	control	-0.105903165	0.323518319	-0.327348278	0.743404482	0.939218065	down_in_case
CRA004775	ENSGALG00010006096	EDIL3	case	control	0.218523593	0.524078663	0.416967163	0.676702426	0.922924485	up_in_case
CRA004775	ENSGALG00010006100	RPS6KL1	case	control	0.229669718	0.308064497	0.745524785	0.455954533	0.839434251	up_in_case
CRA004775	ENSGALG00010006101	ENSGALG00010006101	case	control	0.054121242	0.371373012	0.145732835	0.884132299	0.976208708	up_in_case
CRA004775	ENSGALG00010006103	NXPE3	case	control	-0.463528397	0.44695956	-1.037070103	0.299703187	0.752512244	down_in_case
CRA004775	ENSGALG00010006107	PGF	case	control	0.019813712	0.414935223	0.047751338	0.961914419	0.991860454	up_in_case
CRA004775	ENSGALG00010006108	MKLN1	case	control	-0.103850875	0.194482492	-0.533985725	0.59335141	0.894450257	down_in_case
CRA004775	ENSGALG00010006111	WBP1	case	control	0.013960052	0.332273434	0.042013748	0.966487739	0.993094598	up_in_case
CRA004775	ENSGALG00010006113	EIF2B2	case	control	-0.409370396	0.219714941	-1.863188708	0.062435721	0.448843826	down_in_case
CRA004775	ENSGALG00010006118	NPNT	case	control	1.245744745	0.38040445	3.274790148	0.001057405	0.067945677	up_in_case
CRA004775	ENSGALG00010006119	ENSGALG00010006119	case	control	2.056886292	0.844826083	2.434686066	0.014904715	0.244420343	up_in_case
CRA004775	ENSGALG00010006120	MOGS	case	control	-0.233457445	0.291895987	-0.799796693	0.423828599	0.82073892	down_in_case
CRA004775	ENSGALG00010006121	ENSGALG00010006121	case	control	0.007317738	0.604445396	0.012106533	0.99034062	0.998065748	up_in_case
CRA004775	ENSGALG00010006123	MLH3	case	control	0.366687761	0.240340232	1.525702788	0.1270839	0.581154209	up_in_case
CRA004775	ENSGALG00010006126	ENSGALG00010006126	case	control	-1.305509418	0.579734555	-2.251908923	0.024328028	0.30145607	down_in_case
CRA004775	ENSGALG00010006128	ACYP1	case	control	-0.360069221	0.308144948	-1.168506002	0.24260272	0.709690398	down_in_case
CRA004775	ENSGALG00010006131	ENSGALG00010006131	case	control	-0.336628817	0.311619617	-1.080255538	0.280028403	0.73661445	down_in_case
CRA004775	ENSGALG00010006132	MIPEP	case	control	-0.361769929	0.220259985	-1.642467785	0.100493097	0.538583505	down_in_case
CRA004775	ENSGALG00010006134	K123	case	control	2.018021333	0.577980682	3.491503083	0.000480311	0.044626726	up_in_case
CRA004775	ENSGALG00010006135	ZC2HC1C	case	control	0.107751366	0.343004491	0.314139811	0.753414854	0.941900693	up_in_case
CRA004775	ENSGALG00010006139	GAREM1	case	control	0.922963273	0.395681524	2.332591282	0.019669607	0.277696884	up_in_case
CRA004775	ENSGALG00010006146	CCDC142	case	control	-0.298217683	0.289332089	-1.03071071	0.302676501	0.755164773	down_in_case
CRA004775	ENSGALG00010006147	ENSGALG00010006147	case	control	-0.824780015	0.595816846	-1.384284484	0.166271363	0.630194031	down_in_case
CRA004775	ENSGALG00010006151	ENSGALG00010006151	case	control	0.405820562	0.307541939	1.319561696	0.186981399	0.650186754	up_in_case
CRA004775	ENSGALG00010006155	TNFRSF19	case	control	-0.901351155	0.660628457	-1.364384392	0.172446618	0.63702643	down_in_case
CRA004775	ENSGALG00010006156	NFKBIZ	case	control	-0.319117876	0.41491576	-0.769114859	0.441825128	0.829654585	down_in_case
CRA004775	ENSGALG00010006157	SNX9	case	control	0.130859636	0.134763179	0.971034056	0.33153132	0.772629743	up_in_case
CRA004775	ENSGALG00010006163	ENSGALG00010006163	case	control	1.193254026	0.541159208	2.204996252	0.027454358	0.316243014	up_in_case
CRA004775	ENSGALG00010006172	ENSGALG00010006172	case	control	-0.402545205	0.192392432	-2.092313096	0.036410517	0.360628495	down_in_case
CRA004775	ENSGALG00010006173	TBPL1	case	control	0.084845591	0.273686026	0.310010679	0.756552835	0.941900693	up_in_case
CRA004775	ENSGALG00010006175	IL2RA	case	control	0.044550922	0.480417713	0.092733721	0.926115107	0.98417264	up_in_case
CRA004775	ENSGALG00010006176	MMR1L3	case	control	-0.138164338	0.565763879	-0.244208482	0.807069358	0.955397873	down_in_case
CRA004775	ENSGALG00010006177	NEK9	case	control	-0.295053807	0.314662077	-0.937684672	0.348406483	0.785270866	down_in_case
CRA004775	ENSGALG00010006178	RPS23	case	control	-0.430346008	0.299923231	-1.434853867	0.151328744	0.615293187	down_in_case
CRA004775	ENSGALG00010006180	CD200L	case	control	-0.930448255	0.868696974	-1.071084951	0.284131234	0.740820744	down_in_case
CRA004775	ENSGALG00010006183	PRKN	case	control	0.132227982	0.338565115	0.390554064	0.696126886	0.929092636	up_in_case
CRA004775	ENSGALG00010006184	LBX2	case	control	-0.354961867	0.566114064	-0.627014748	0.530649568	0.869211096	down_in_case
CRA004775	ENSGALG00010006186	CHIR1AB3	case	control	-0.862285989	0.684387319	-1.259938584	0.207691519	0.676460246	down_in_case
CRA004775	ENSGALG00010006187	TCF21	case	control	2.074593585	0.719944947	2.881600314	0.003956613	0.129233684	up_in_case
CRA004775	ENSGALG00010006188	TMED10	case	control	-0.285918712	0.131661678	-2.171616797	0.029884579	0.329809667	down_in_case
CRA004775	ENSGALG00010006193	RBM17	case	control	-0.010330366	0.146230907	-0.070644204	0.943680928	0.987862092	down_in_case
CRA004775	ENSGALG00010006197	ENSGALG00010006197	case	control	-1.141618847	0.824230935	-1.385071584	0.16603058	0.630194031	down_in_case
CRA004775	ENSGALG00010006198	ENSGALG00010006198	case	control	-0.146942505	0.52289108	-0.281019338	0.778695567	0.94792594	down_in_case
CRA004775	ENSGALG00010006200	GSTCD	case	control	-0.342877562	0.251344907	-1.364171511	0.172513592	0.63702643	down_in_case
CRA004775	ENSGALG00010006202	SACS	case	control	0.279790401	0.375338244	0.745435366	0.456008571	0.839434251	up_in_case
CRA004775	ENSGALG00010006203	MMR1L4	case	control	-0.832133445	0.600529987	-1.385665102	0.165849189	0.630194031	down_in_case
CRA004775	ENSGALG00010006205	CGGBP1	case	control	0.414256644	0.183942809	2.252094797	0.024316282	0.30145607	up_in_case
CRA004775	ENSGALG00010006207	ENSGALG00010006207	case	control	0.53994637	0.44129821	1.223540813	0.221125499	0.689187837	up_in_case
CRA004775	ENSGALG00010006212	FOS	case	control	-0.001123354	0.655348238	-0.001714133	0.99863232	0.999914248	down_in_case
CRA004775	ENSGALG00010006219	CLEC14A	case	control	0.013198209	0.450709563	0.02928318	0.976638742	0.99560196	up_in_case
CRA004775	ENSGALG00010006220	SGCG	case	control	0.296883488	0.451975996	0.656856759	0.511273024	0.863170205	up_in_case
CRA004775	ENSGALG00010006223	ATG10	case	control	0.152835145	0.317141149	0.481915216	0.629866176	0.908943555	up_in_case
CRA004775	ENSGALG00010006227	AGPAT4	case	control	-0.524256347	0.489104002	-1.0718709	0.283778022	0.740508942	down_in_case
CRA004775	ENSGALG00010006228	ENSGALG00010006228	case	control	0.099618804	0.76984851	0.129400529	0.897040729	0.978790377	up_in_case
CRA004775	ENSGALG00010006229	INTS12	case	control	0.060784601	0.233787812	0.259999015	0.794864533	0.952272364	up_in_case
CRA004775	ENSGALG00010006232	ZNF654	case	control	0.403395987	0.231798312	1.740288714	0.081808335	0.497763899	up_in_case
CRA004775	ENSGALG00010006236	C3orf38	case	control	-0.060292384	0.181234238	-0.332676565	0.739378442	0.938064864	down_in_case
CRA004775	ENSGALG00010006237	ENSGALG00010006237	case	control	-0.397038516	0.575909077	-0.689411805	0.490564157	0.855184934	down_in_case
CRA004775	ENSGALG00010006239	EYA4	case	control	0.670441977	0.370237135	1.810844765	0.070164885	0.469724266	up_in_case
CRA004775	ENSGALG00010006241	MRC1	case	control	-0.516359954	0.513336955	-1.005888918	0.314469008	0.765098415	down_in_case
CRA004775	ENSGALG00010006242	ENSGALG00010006242	case	control	1.118844456	0.679689059	1.646112206	0.09974066	0.537224369	up_in_case
CRA004775	ENSGALG00010006244	EPHA3	case	control	-0.434211217	0.429803421	-1.010255376	0.312372954	0.764325516	down_in_case
CRA004775	ENSGALG00010006245	PFKFB3	case	control	0.419894175	0.686884449	0.611302491	0.540999342	0.874592283	up_in_case
CRA004775	ENSGALG00010006246	SUPT20H	case	control	0.045171615	0.167931109	0.268988964	0.787938175	0.95041797	up_in_case
CRA004775	ENSGALG00010006249	C9orf72	case	control	-0.265705	0.229457791	-1.157968959	0.246876704	0.712046495	down_in_case
CRA004775	ENSGALG00010006251	SEMA3E	case	control	2.226527098	0.90086464	2.471544559	0.013453078	0.232695952	up_in_case
CRA004775	ENSGALG00010006252	COX7C	case	control	-0.035194183	0.391740301	-0.089840599	0.928413885	0.98473447	down_in_case
CRA004775	ENSGALG00010006254	PROS1	case	control	-0.104927927	0.313034818	-0.335195708	0.737477459	0.937597314	down_in_case
CRA004775	ENSGALG00010006259	PPA2	case	control	0.106613619	0.252506795	0.422220792	0.672863867	0.921952322	up_in_case
CRA004775	ENSGALG00010006260	MAP3K4	case	control	1.865257333	0.40597351	4.59452966	4.33726E-06	0.002293912	up_in_case
CRA004775	ENSGALG00010006263	EXOSC8	case	control	-3.28252E-05	0.290885662	-0.000112846	0.999909962	0.999914248	down_in_case
CRA004775	ENSGALG00010006266	CENPW	case	control	0.31756041	0.717628876	0.442513423	0.65811772	0.917354438	up_in_case
CRA004775	ENSGALG00010006270	STAM	case	control	0.280727584	0.286366228	0.980309675	0.326933281	0.770998457	up_in_case
CRA004775	ENSGALG00010006273	ARL13B	case	control	0.469544637	0.198230845	2.368675959	0.017851886	0.265690984	up_in_case
CRA004775	ENSGALG00010006277	GNG10	case	control	-0.01569045	0.330796098	-0.047432391	0.962168614	0.991860454	down_in_case
CRA004775	ENSGALG00010006279	HACD1	case	control	0.495823831	0.501604605	0.988475436	0.322919859	0.768114682	up_in_case
CRA004775	ENSGALG00010006281	TRMT11	case	control	0.100662885	0.181866173	0.553499769	0.579921238	0.888438428	up_in_case
CRA004775	ENSGALG00010006283	DNAJC25	case	control	0.362356183	0.214659073	1.688054356	0.091400795	0.521441551	up_in_case
CRA004775	ENSGALG00010006285	FZD8	case	control	0.026165383	0.330086397	0.079268286	0.936819231	0.98646154	up_in_case
CRA004775	ENSGALG00010006287	ECPAS	case	control	0.341918023	0.285265835	1.198594368	0.230685709	0.697177844	up_in_case
CRA004775	ENSGALG00010006288	ARF5	case	control	-0.272711708	0.413649136	-0.659282673	0.509714267	0.862971569	down_in_case
CRA004775	ENSGALG00010006289	SKIDA1	case	control	-0.137323349	0.34200672	-0.401522372	0.68803557	0.927111919	down_in_case
CRA004775	ENSGALG00010006291	ENSGALG00010006291	case	control	-1.314300898	0.430728749	-3.051342407	0.002278206	0.096690159	down_in_case
CRA004775	ENSGALG00010006293	SLC22A3	case	control	-0.260638296	0.729122844	-0.357468289	0.720741262	0.934511903	down_in_case
CRA004775	ENSGALG00010006300	GCC1	case	control	-0.094287734	0.158420219	-0.595174875	0.551726583	0.878258849	down_in_case
CRA004775	ENSGALG00010006301	ENSGALG00010006301	case	control	0.167060929	0.223955529	0.745955814	0.455694107	0.839192403	up_in_case
CRA004775	ENSGALG00010006310	ALG5	case	control	-0.18427956	0.32920141	-0.559777552	0.575631177	0.887937128	down_in_case
CRA004775	ENSGALG00010006311	ABCD4	case	control	-0.398343708	0.345941639	-1.151476618	0.249536209	0.714467496	down_in_case
CRA004775	ENSGALG00010006323	ANAPC10	case	control	0.209721215	0.256265459	0.818374886	0.413143158	0.817112606	up_in_case
CRA004775	ENSGALG00010006327	DENND6B	case	control	0.024267532	0.203560403	0.119215388	0.905104716	0.979544699	up_in_case
CRA004775	ENSGALG00010006328	SYNDIG1L	case	control	0.366641309	0.557570092	0.657569899	0.510814542	0.863170205	up_in_case
CRA004775	ENSGALG00010006330	STX19	case	control	1.747075467	0.692264236	2.523711867	0.011612306	0.216957641	up_in_case
CRA004775	ENSGALG00010006331	SMAD9	case	control	-0.068941116	0.525993725	-0.131068323	0.895721259	0.978243431	down_in_case
CRA004775	ENSGALG00010006339	ENSGALG00010006339	case	control	0.224996281	1.07676563	0.208955668	0.834482847	0.964202472	up_in_case
CRA004775	ENSGALG00010006341	HHIP	case	control	1.004863933	0.388036413	2.589612464	0.009608403	0.198088683	up_in_case
CRA004775	ENSGALG00010006342	RFXAP	case	control	0.261695848	0.256411142	1.020610283	0.307439116	0.759130055	up_in_case
CRA004775	ENSGALG00010006343	SEC23A	case	control	0.16926492	0.199024802	0.850471489	0.395063005	0.808998414	up_in_case
CRA004775	ENSGALG00010006344	HINT3	case	control	0.145552873	0.244923333	0.594279326	0.552325304	0.878258849	up_in_case
CRA004775	ENSGALG00010006345	SMC2	case	control	0.303788645	0.728856712	0.416801602	0.676823531	0.922924485	up_in_case
CRA004775	ENSGALG00010006346	NSUN3	case	control	-0.309718655	0.298654234	-1.037047596	0.299713676	0.752512244	down_in_case
CRA004775	ENSGALG00010006347	PPP6R2	case	control	0.3871223	0.237240768	1.631769712	0.102728008	0.543312631	up_in_case
CRA004775	ENSGALG00010006351	IGF2R	case	control	-0.243332992	0.427523712	-0.569168412	0.569241855	0.884809383	down_in_case
CRA004775	ENSGALG00010006352	GEMIN2	case	control	-0.102225138	0.333889919	-0.306164193	0.759479634	0.942236668	down_in_case
CRA004775	ENSGALG00010006353	ABCE1	case	control	-0.016531092	0.187324025	-0.088248648	0.929679053	0.98473447	down_in_case
CRA004775	ENSGALG00010006355	PTGR1	case	control	0.528031103	0.240530922	2.195273267	0.028144001	0.319106625	up_in_case
CRA004775	ENSGALG00010006359	SGK1	case	control	0.245011289	0.322189068	0.760458109	0.4469808	0.834117509	up_in_case
CRA004775	ENSGALG00010006361	SERTM1	case	control	0.005739372	0.485848603	0.011813087	0.990574739	0.998065748	up_in_case
CRA004775	ENSGALG00010006363	TRAPPC6A	case	control	-0.281793658	0.282863221	-0.996218796	0.319143849	0.767037518	down_in_case
CRA004775	ENSGALG00010006372	SBF1	case	control	-0.418942416	0.19446071	-2.15438078	0.031210323	0.336670173	down_in_case
CRA004775	ENSGALG00010006378	CAAP1	case	control	-0.332479865	0.238469106	-1.394226154	0.163249322	0.629717526	down_in_case
CRA004775	ENSGALG00010006381	SPART	case	control	0.214240147	0.41199803	0.520002844	0.603061593	0.898692785	up_in_case
CRA004775	ENSGALG00010006382	PNN	case	control	0.168845198	0.182780422	0.923759756	0.355611413	0.788778426	up_in_case
CRA004775	ENSGALG00010006387	OTUD4	case	control	0.027110231	0.196727482	0.137806017	0.890393729	0.977315147	up_in_case
CRA004775	ENSGALG00010006404	SMAD1	case	control	0.139364324	0.262008233	0.531908186	0.594789587	0.895238208	up_in_case
CRA004775	ENSGALG00010006410	ADM2	case	control	-0.06306231	0.738753451	-0.085363134	0.931972701	0.985044263	down_in_case
CRA004775	ENSGALG00010006421	LMF2	case	control	-0.275200258	0.141575671	-1.943838632	0.051914913	0.419683701	down_in_case
CRA004775	ENSGALG00010006434	MMAA	case	control	-0.08703861	0.206085179	-0.422342892	0.672774755	0.921952322	down_in_case
CRA004775	ENSGALG00010006446	LSAMP	case	control	0.085449408	0.393211856	0.217311371	0.827965696	0.962251207	up_in_case
CRA004775	ENSGALG00010006454	SOD2	case	control	0.081208238	0.285614307	0.284328327	0.77615878	0.946608123	up_in_case
CRA004775	ENSGALG00010006457	NCAPH2	case	control	0.117247211	0.445921876	0.262932181	0.792602852	0.951284723	up_in_case
CRA004775	ENSGALG00010006463	SCO2	case	control	-0.300520668	0.296746422	-1.012718759	0.311194515	0.763050512	down_in_case
CRA004775	ENSGALG00010006465	PLAA	case	control	-0.214384607	0.250837018	-0.854676909	0.392730037	0.807553294	down_in_case
CRA004775	ENSGALG00010006472	DCLK1	case	control	-1.033781185	0.718627065	-1.438550306	0.150277975	0.613766096	down_in_case
CRA004775	ENSGALG00010006478	ENSGALG00010006478	case	control	0.081747639	0.274827298	0.297450944	0.766122255	0.943612838	up_in_case
CRA004775	ENSGALG00010006480	IFT74	case	control	-0.072524983	0.299110354	-0.242468982	0.808416789	0.955397873	down_in_case

CRA004775	ENSGALG00010006486	CEP43	case	control	0.064110019	0.147059429	0.435946335	0.662875666	0.918295135	up_in_case
CRA004775	ENSGALG00010006492	GAP43	case	control	1.514641852	0.66019034	2.294250249	0.021776128	0.288534247	up_in_case
CRA004775	ENSGALG00010006496	UBE2H	case	control	0.599634087	0.255313277	2.348620856	0.018843083	0.272278915	up_in_case
CRA004775	ENSGALG00010006506	ZNF827	case	control	-0.096330806	0.347518046	-0.277196557	0.781629183	0.948910196	down_in_case
CRA004775	ENSGALG00010006515	FBXO33	case	control	-0.164541118	0.225617301	-0.729292998	0.465822454	0.843390341	down_in_case
CRA004775	ENSGALG00010006516	NBEA	case	control	0.380131268	0.28665903	1.326074634	0.184814977	0.6491093	up_in_case
CRA004775	ENSGALG00010006517	ZBTB20	case	control	0.424781066	0.401824444	1.057130975	0.290451817	0.74431661	up_in_case
CRA004775	ENSGALG00010006521	ZNF410	case	control	-0.380523355	0.208197495	-1.827703813	0.067594016	0.463543284	down_in_case
CRA004775	ENSGALG00010006524	TAB2	case	control	0.245366025	0.215096905	1.140723182	0.253985136	0.718204856	up_in_case
CRA004775	ENSGALG00010006526	LSM6	case	control	-0.13532141	0.305987866	-0.442244367	0.658312384	0.917354438	down_in_case
CRA004775	ENSGALG00010006528	ZC3HC1	case	control	-0.459189471	0.25223685	-1.820469415	0.06868755	0.46645716	down_in_case
CRA004775	ENSGALG00010006531	FAM161B	case	control	0.26245831	0.400661855	0.655061885	0.512427911	0.863317349	up_in_case
CRA004775	ENSGALG00010006532	KLHDC10	case	control	-0.214734775	0.266433936	-0.805958798	0.420266606	0.819388688	down_in_case
CRA004775	ENSGALG00010006538	TEK	case	control	0.415144224	0.308768408	1.344516518	0.178781419	0.644859234	up_in_case
CRA004775	ENSGALG00010006542	RFC3	case	control	-0.653169781	0.427045354	-1.529509161	0.12613827	0.580497774	down_in_case
CRA004775	ENSGALG00010006544	MOB3B	case	control	0.07548496	0.50461467	0.149589309	0.881088644	0.974996282	up_in_case
CRA004775	ENSGALG00010006546	SMOC2	case	control	0.200758986	0.371796095	0.539970667	0.589217262	0.893412703	up_in_case
CRA004775	ENSGALG00010006548	TMEM209	case	control	-0.393000216	0.204613845	-1.920692202	0.054770523	0.430390248	down_in_case
CRA004775	ENSGALG00010006549	ZDHHC23	case	control	-0.420038494	0.336014902	-1.250059122	0.211277951	0.681597647	down_in_case
CRA004775	ENSGALG00010006552	CPA2	case	control	2.458805697	1.385707988	1.774403928	0.075996361	0.484761048	up_in_case
CRA004775	ENSGALG00010006557	COQ6	case	control	-0.311774789	0.213578754	-1.459764992	0.144354673	0.606446401	down_in_case
CRA004775	ENSGALG00010006558	USP10	case	control	-0.017954485	0.185148882	-0.096973227	0.922747656	0.983159417	down_in_case
CRA004775	ENSGALG00010006564	SLC10A7	case	control	-0.207369353	0.374047436	-0.554393193	0.579309784	0.888378369	down_in_case
CRA004775	ENSGALG00010006566	CPA5	case	control	2.255939198	1.281053537	1.76100306	0.078237885	0.490500984	up_in_case
CRA004775	ENSGALG00010006568	ENTPD5	case	control	-0.128152914	0.331810754	-0.386222907	0.69933159	0.929347461	down_in_case
CRA004775	ENSGALG00010006570	ENSGALG00010006570	case	control	1.592995837	0.487441101	3.268078616	0.001082803	0.068615769	up_in_case
CRA004775	ENSGALG00010006574	CPA1	case	control	2.441601478	1.466440296	1.66498526	0.095915697	0.529205137	up_in_case
CRA004775	ENSGALG00010006575	SSBP2	case	control	0.037549626	0.336497175	0.111589721	0.911148723	0.98075825	up_in_case
CRA004775	ENSGALG00010006576	CCDC191	case	control	-0.412343556	0.243025301	-1.696710399	0.089751426	0.516522282	down_in_case
CRA004775	ENSGALG00010006580	ALDH6A1	case	control	0.049944126	0.321158833	0.15551223	0.876417513	0.974479909	up_in_case
CRA004775	ENSGALG00010006581	CEP41	case	control	-0.398635526	0.250665764	-1.590307029	0.111765615	0.56111317	down_in_case
CRA004775	ENSGALG00010006585	TTC29	case	control	-0.124970144	0.455887608	-0.274124898	0.783988641	0.950086173	down_in_case
CRA004775	ENSGALG00010006592	STARD13	case	control	0.343096841	0.262919273	1.304951276	0.191909439	0.656959697	up_in_case
CRA004775	ENSGALG00010006598	ENSGALG00010006598	case	control	0.856744257	0.275388969	3.111033314	0.001864339	0.086609886	up_in_case
CRA004775	ENSGALG00010006602	EDNRA	case	control	-0.008215271	0.280572215	-0.029280416	0.976640946	0.99560196	down_in_case
CRA004775	ENSGALG00010006603	MEST	case	control	-0.556697162	0.360306379	-1.545066072	0.122330282	0.57525466	down_in_case
CRA004775	ENSGALG00010006608	KL	case	control	1.112796211	0.886713071	1.25496764	0.209490507	0.679571589	up_in_case
CRA004775	ENSGALG00010006614	TMEM184C	case	control	0.192271242	0.177097746	1.085678652	0.277621189	0.732797725	up_in_case
CRA004775	ENSGALG00010006615	GRAMD1C	case	control	-0.174431772	0.199412366	-0.874728958	0.3817214	0.801753951	down_in_case
CRA004775	ENSGALG00010006616	COPG2	case	control	-0.287840203	0.15861256	-1.814737768	0.069564218	0.468451304	down_in_case
CRA004775	ENSGALG00010006619	LIN52	case	control	-0.282861877	0.364205806	-0.776653947	0.437362965	0.827373521	down_in_case
CRA004775	ENSGALG00010006621	DACT2	case	control	0.798257249	0.655267569	1.218215714	0.22314201	0.690760052	up_in_case
CRA004775	ENSGALG00010006623	ENSGALG00010006623	case	control	0.569325614	0.516595146	1.102073099	0.270429894	0.728665115	up_in_case
CRA004775	ENSGALG00010006625	ENSGALG00010006625	case	control	-1.122806957	0.478814368	-2.344973401	0.019028437	0.272278915	down_in_case
CRA004775	ENSGALG00010006627	SF3B5	case	control	0.039109224	0.267908251	0.145979917	0.883937242	0.976208708	up_in_case
CRA004775	ENSGALG00010006633	FBXO18	case	control	-0.064483858	0.154619631	-0.417048329	0.676643058	0.922924485	down_in_case
CRA004775	ENSGALG00010006634	PRMT9	case	control	0.157572229	0.211935776	0.743490468	0.45718479	0.839688534	up_in_case
CRA004775	ENSGALG00010006635	ENSGALG00010006635	case	control	-0.477915844	0.721300041	-0.662575651	0.507602369	0.862748056	down_in_case
CRA004775	ENSGALG00010006651	ENSGALG00010006651	case	control	-0.269453341	0.274049933	-0.983227176	0.325495643	0.770440423	down_in_case
CRA004775	ENSGALG00010006653	CFL2	case	control	0.133800275	0.350134237	0.382139934	0.702357572	0.930068722	up_in_case
CRA004775	ENSGALG00010006657	PDS5B	case	control	0.427605973	0.242620898	1.76244494	0.077994151	0.490500984	up_in_case
CRA004775	ENSGALG00010006670	ANKRD16	case	control	-0.663981252	0.250916954	-2.646219167	0.008139706	0.182592329	down_in_case
CRA004775	ENSGALG00010006673	BAZ1A	case	control	-0.081324883	0.406864686	-0.199881891	0.841572954	0.966478468	down_in_case
CRA004775	ENSGALG00010006678	ENSGALG00010006678	case	control	-2.529299195	1.875509665	-1.348592994	0.177467732	0.643283771	down_in_case
CRA004775	ENSGALG00010006681	CRISPLD2	case	control	-0.154582776	0.383721162	-0.402851839	0.687057223	0.926974477	down_in_case
CRA004775	ENSGALG00010006682	ARHGAP10	case	control	0.339003711	0.267273632	1.268376936	0.204663374	0.674045742	up_in_case
CRA004775	ENSGALG00010006685	ENSGALG00010006685	case	control	-0.182599019	0.258966386	-0.705107031	0.480743631	0.848616843	down_in_case
CRA004775	ENSGALG00010006715	IRF8	case	control	-0.860486438	0.300494369	-2.863569261	0.004188972	0.132724786	down_in_case
CRA004775	ENSGALG00010006716	ENSGALG00010006716	case	control	0.402812387	0.331783606	1.214081649	0.224716547	0.692859731	up_in_case
CRA004775	ENSGALG00010006720	SRP54	case	control	0.080653973	0.230329171	0.350168296	0.726212399	0.935238044	up_in_case
CRA004775	ENSGALG00010006728	ENSGALG00010006728	case	control	-0.276945876	0.459984225	-0.602076898	0.547122951	0.877099134	down_in_case
CRA004775	ENSGALG00010006730	HAPLN1	case	control	-3.525327063	1.247399525	-2.826141098	0.004711251	0.141450672	down_in_case
CRA004775	ENSGALG00010006735	NR3C2	case	control	0.534490342	0.270533401	1.975690766	0.048189811	0.407414924	up_in_case
CRA004775	ENSGALG00010006739	VCAN	case	control	0.257153219	0.424363521	0.605973904	0.544532083	0.875916944	up_in_case
CRA004775	ENSGALG00010006740	BRCA2	case	control	0.743954526	0.505557559	1.471552571	0.141141741	0.600619496	up_in_case
CRA004775	ENSGALG00010006748	FAM177A1	case	control	0.256395338	0.180545746	1.420112873	0.155574823	0.619442611	up_in_case
CRA004775	ENSGALG00010006752	ZAR1L	case	control	-0.067625503	0.912456996	-0.074113633	0.940919967	0.987377172	down_in_case
CRA004775	ENSGALG00010006760	TASOR2	case	control	-0.001221151	0.275763296	-0.004428258	0.996466773	0.999606847	down_in_case
CRA004775	ENSGALG00010006761	PPP2R3C	case	control	-0.080924267	0.240609053	-0.336330931	0.736621328	0.937549044	down_in_case
CRA004775	ENSGALG00010006767	ENSGALG00010006767	case	control	-0.201502212	0.638180845	-0.315744689	0.752196304	0.941597758	down_in_case
CRA004775	ENSGALG00010006794	PERPA	case	control	1.257024699	0.908370316	1.383824061	0.166412334	0.630194031	up_in_case
CRA004775	ENSGALG00010006803	COX4I1	case	control	0.192899268	0.336458002	0.573323467	0.566425699	0.883033678	up_in_case
CRA004775	ENSGALG00010006804	PSMA6	case	control	-0.114228859	0.248844797	-0.459036557	0.646207914	0.914043203	down_in_case
CRA004775	ENSGALG00010006814	ENSGALG00010006814	case	control	1.305961648	1.150023956	1.135595168	0.256126032	0.719236059	up_in_case
CRA004775	ENSGALG00010006816	ENSGALG00010006816	case	control	-0.423665715	0.65632259	-0.645514449	0.518593858	0.865435399	down_in_case
CRA004775	ENSGALG00010006819	EMC8	case	control	-0.299881679	0.213597854	-1.403954546	0.160332385	0.625455498	down_in_case
CRA004775	ENSGALG00010006821	XRCC4	case	control	-0.177761382	0.306750333	-0.579498579	0.562252805	0.881532201	down_in_case
CRA004775	ENSGALG00010006824	ENSGALG00010006824	case	control	-0.761303765	0.62482833	-1.218420691	0.223064146	0.690760052	down_in_case
CRA004775	ENSGALG00010006828	GTF2A1	case	control	0.380491424	0.227222968	1.674528885	0.094026702	0.526235729	up_in_case
CRA004775	ENSGALG00010006833	LTV1	case	control	-0.12196505	0.230350974	-0.52947486	0.59647608	0.895574418	down_in_case
CRA004775	ENSGALG00010006835	FRY	case	control	0.617884952	0.305462244	2.022786656	0.043095148	0.389478281	up_in_case
CRA004775	ENSGALG00010006838	ASB13	case	control	-0.588412619	0.326217573	-1.80374286	0.071271627	0.470954418	down_in_case
CRA004775	ENSGALG00010006840	ENSGALG00010006840	case	control	-0.030547423	0.524097699	-0.058285742	0.953521024	0.990670511	down_in_case
CRA004775	ENSGALG00010006843	STON2	case	control	0.357474735	0.313249161	1.141183374	0.253793621	0.718204856	up_in_case
CRA004775	ENSGALG00010006847	SEL1L	case	control	-0.058474151	0.304777982	-0.191858187	0.847853291	0.968053841	down_in_case
CRA004775	ENSGALG00010006850	IRF2BPL	case	control	-0.035251962	0.320290351	-0.110062518	0.912359794	0.98075825	down_in_case
CRA004775	ENSGALG00010006851	TMEM167A	case	control	-0.140531659	0.214236901	-0.655963832	0.511847395	0.863170205	down_in_case
CRA004775	ENSGALG00010006853	NET1	case	control	-0.063515187	0.252823506	-0.251223427	0.801641373	0.953543664	down_in_case
CRA004775	ENSGALG00010006855	DCLK2	case	control	-0.245480148	0.404309002	-0.607159741	0.543744907	0.875729696	down_in_case
CRA004775	ENSGALG00010006877	GSE1	case	control	0.006423925	0.294086469	0.021843661	0.982572666	0.997368918	up_in_case
CRA004775	ENSGALG00010006883	LRBA	case	control	0.034087205	0.386040673	0.088299518	0.929638622	0.98473447	up_in_case
CRA004775	ENSGALG00010006885	RHOU	case	control	0.351483193	0.388301323	0.905181549	0.365369215	0.793608387	up_in_case
CRA004775	ENSGALG00010006901	TUBAL3	case	control	-0.071618601	0.682024243	-0.105008878	0.916368764	0.981626545	down_in_case
CRA004775	ENSGALG00010006903	B3GLCT	case	control	0.103263656	0.308649858	0.334565699	0.737952723	0.937854704	up_in_case
CRA004775	ENSGALG00010006912	ENSGALG00010006912	case	control	-0.456251433	0.258416685	-1.765564912	0.077468871	0.489106724	down_in_case
CRA004775	ENSGALG00010006913	HSPH1	case	control	0.696047202	0.336222039	2.070201005	0.038433525	0.370251617	up_in_case
CRA004775	ENSGALG00010006922	SMYD3	case	control	0.04164807	0.265788551	0.156696252	0.875484241	0.974412785	up_in_case
CRA004775	ENSGALG00010006926	ID4	case	control	-0.186130279	0.365710644	-0.508955049	0.610783733	0.901413878	down_in_case
CRA004775	ENSGALG00010006939	FBXO31	case	control	-0.286034418	0.398994983	-0.716887255	0.473443667	0.845445211	down_in_case
CRA004775	ENSGALG00010006942	ALOX5AP	case	control	-1.190049387	0.375654268	-3.167937887	0.001535243	0.080693373	down_in_case
CRA004775	ENSGALG00010006944	MAB21L2	case	control	1.617287471	0.385886275	4.19109871	2.77607E-05	0.01060381	up_in_case
CRA004775	ENSGALG00010006953	MAP1LC3B	case	control	-0.098365018	0.282514669	-0.348176675	0.727707504	0.935548472	down_in_case
CRA004775	ENSGALG00010006955	HELT	case	control	-0.1702393	0.631345505	-0.269645224	0.787433205	0.95041797	down_in_case
CRA004775	ENSGALG00010006957	PALM2AKAP2	case	control	0.495681128	0.32027162	1.547689824	0.121696993	0.573689301	up_in_case
CRA004775	ENSGALG00010006958	KIF26B	case	control	-0.579457982	0.66992469	-0.864959884	0.387060808	0.803905475	down_in_case
CRA004775	ENSGALG00010006961	ZCCHC14	case	control	0.338980725	0.388530799	0.872468092	0.382953068	0.801925538	up_in_case
CRA004775	ENSGALG00010006963	ENSGALG00010006963	case	control	-0.373334838	0.592617513	-0.629976046	0.528710256	0.86916155	down_in_case
CRA004775	ENSGALG00010006983	GTPBP2	case	control	-0.142387975	0.156295328	-0.911018755	0.362285492	0.792899538	down_in_case
CRA004775	ENSGALG00010006988	USPL1	case	control	0.087842518	0.172976675	0.507828693	0.611573486	0.901413878	up_in_case
CRA004775	ENSGALG00010006992	SLC35B4	case	control	-0.432588319	0.192363755	-2.248803675	0.024524989	0.302160964	down_in_case
CRA004775	ENSGALG00010006998	POLH	case	control	0.047584683	0.280831202	0.169442293	0.86544876	0.972629493	up_in_case
CRA004775	ENSGALG00010007001	SNX30	case	control	0.092890305	0.270114124	0.34389281	0.730926899	0.936441634	up_in_case
CRA004775	ENSGALG00010007002	EXOC4	case	control	-0.219140691	0.248157184	-0.88307212	0.377197307	0.798610687	down_in_case
CRA004775	ENSGALG00010007007	ACSL1	case	control	0.271245046	0.312294779	0.868554532	0.385090833	0.803182776	up_in_case
CRA004775	ENSGALG00010007011	HMGB1	case	control	-0.22057362	0.328402467	-0.671656405	0.501802461	0.860091011	down_in_case
CRA004775	ENSGALG00010007014	ENSGALG00010007014	case	control	1.977029751	0.899784308	2.197226305	0.028004288	0.318730759	up_in_case
CRA004775	ENSGALG00010007017	SRD5A2	case	control	-0.30359537	0.546892321	-0.555128236	0.57880695	0.888378369	down_in_case
CRA004775	ENSGALG00010007021	SULT6B1	case	control	0.77428312	0.545616757	1.419097031	0.155870729	0.619442611	up_in_case
CRA004775	ENSGALG00010007028	KATNAL1	case	control	-0.100606484	0.220079666	-0.457136663	0.647572819	0.914210065	down_in_case
CRA004775	ENSGALG00010007033	CEBPZOS	case	control	0.002517534	0.340088659	0.007402582	0.994093648	0.998960959	up_in_case
CRA004775	ENSGALG00010007038	CENPU	case	control	-0.518413083	0.59638897	-0.869253305	0.384708597	0.803182776	down_in_case
CRA004775	ENSGALG00010007041	NAE1	case	control	-0.209893052	0.226061218	-0.928478815	0.353159252	0.787065859	down_in_case
CRA004775	ENSGALG00010007051	E2F3	case	control	-0.222667463	0.309859069	-0.718608829	0.472381972	0.845425322	down_in_case
CRA004775	ENSGALG00010007066	INIP	case	control	-0.35575715	0.271570493	-1.309999276	0.19019608	0.655814017	down_in_case
CRA004775	ENSGALG00010007067	CASP3	case	control	-0.282827741	0.376183076	-0.751835366	0.452150071	0.836699721	down_in_case
CRA004775	ENSGALG00010007070	UBL3	case	control	0.379639787	0.190073794	1.997328396	0.045789521	0.398261668	up_in_case

CRA004775	ENSGALG00010007085	PRIMPOL	case	control	-0.027452612	0.435733173	-0.063003265	0.949763905	0.990168609	down_in_case
CRA004775	ENSGALG00010007090	CEBPZ	case	control	0.18675769	0.209010607	0.893532114	0.371572282	0.795818272	up_in_case
CRA004775	ENSGALG00010007092	DYNC1LI2	case	control	0.20105885	0.185087937	1.086288242	0.277351488	0.732448686	up_in_case
CRA004775	ENSGALG00010007097	ZCCHC9	case	control	-0.453758464	0.256286083	-1.770515433	0.076641316	0.487462874	down_in_case
CRA004775	ENSGALG00010007098	NDUFAF7	case	control	-0.039427662	0.20094448	-0.196211721	0.844444455	0.966751844	down_in_case
CRA004775	ENSGALG00010007102	CMTM3	case	control	-0.155506087	0.269620424	-0.576759302	0.564102066	0.88227565	down_in_case
CRA004775	ENSGALG00010007106	SLC7A1	case	control	-0.997328686	0.423726056	-2.353711014	0.018587055	0.270466236	down_in_case
CRA004775	ENSGALG00010007108	MBOAT1	case	control	-0.578563985	0.46581588	-1.242044357	0.214220197	0.683562661	down_in_case
CRA004775	ENSGALG00010007110	ARL6	case	control	0.003384342	0.275517632	0.012283576	0.99019937	0.998065748	up_in_case
CRA004775	ENSGALG00010007114	CKLF	case	control	-0.540676521	0.376138398	-1.437440376	0.150592903	0.614342929	down_in_case
CRA004775	ENSGALG00010007115	ENSGALG00010007115	case	control	-0.678573154	0.364630155	-1.860990226	0.062745557	0.449850967	down_in_case
CRA004775	ENSGALG00010007118	ENSGALG00010007118	case	control	0.414015592	0.277158486	1.493786455	0.135231443	0.593023494	up_in_case
CRA004775	ENSGALG00010007123	ENSGALG00010007123	case	control	-0.555914646	0.39539147	-1.405985429	0.159728452	0.624907945	down_in_case
CRA004775	ENSGALG00010007126	ENSGALG00010007126	case	control	-0.106500631	0.889594178	-0.119718219	0.904706367	0.979540372	down_in_case
CRA004775	ENSGALG00010007130	IRF2	case	control	0.093168553	0.209678321	0.444340421	0.656796482	0.91710603	up_in_case
CRA004775	ENSGALG00010007133	BLVRA	case	control	-0.425679824	0.531442117	-0.800990003	0.423137433	0.820552247	down_in_case
CRA004775	ENSGALG00010007135	TK2	case	control	-0.068938545	0.247964766	-0.278017505	0.780998921	0.94872051	down_in_case
CRA004775	ENSGALG00010007137	ENSGALG00010007137	case	control	-1.026222899	0.337538311	-3.040315319	0.002363306	0.098653993	down_in_case
CRA004775	ENSGALG00010007141	PRKD3	case	control	-0.116803252	0.34320594	-0.340329924	0.733608085	0.936819725	down_in_case
CRA004775	ENSGALG00010007143	CKMT2	case	control	-0.033100564	0.944916284	-0.035030155	0.972055695	0.994947809	down_in_case
CRA004775	ENSGALG00010007146	ENSGALG00010007146	case	control	0.197868645	0.201885673	0.980102459	0.327035545	0.770998457	up_in_case
CRA004775	ENSGALG00010007160	ENSGALG00010007160	case	control	-0.805136168	0.397328623	-2.026373438	0.042726531	0.388066397	down_in_case
CRA004775	ENSGALG00010007162	ENSGALG00010007162	case	control	-0.88224927	0.770730448	-1.144692379	0.25233661	0.717019836	down_in_case
CRA004775	ENSGALG00010007165	ENSGALG00010007165	case	control	-0.436515913	0.393517816	-1.109265946	0.267315471	0.727315996	down_in_case
CRA004775	ENSGALG00010007166	RASGRF2	case	control	0.569066628	0.470845204	1.208606615	0.226814014	0.694715356	up_in_case
CRA004775	ENSGALG00010007168	MINA	case	control	-0.126663624	0.227866687	-0.55586723	0.578301622	0.888378369	down_in_case
CRA004775	ENSGALG00010007169	QPCT	case	control	0.066211082	0.295770214	0.223859871	0.822866341	0.960875066	up_in_case
CRA004775	ENSGALG00010007175	FOXC2	case	control	-0.780381445	0.761530333	-1.024754249	0.305479159	0.758032511	down_in_case
CRA004775	ENSGALG00010007181	GJB2	case	control	0.163456138	0.750258284	0.217866488	0.827533139	0.962155085	up_in_case
CRA004775	ENSGALG00010007182	ENSGALG00010007182	case	control	-0.171033788	0.35424076	-0.482817922	0.629225024	0.908586927	down_in_case
CRA004775	ENSGALG00010007183	VOPP1	case	control	-0.135274695	0.369068755	-0.366529796	0.713969783	0.933244709	down_in_case
CRA004775	ENSGALG00010007184	VGLL3	case	control	0.104777644	0.409162639	0.256078228	0.797890433	0.952835572	up_in_case
CRA004775	ENSGALG00010007187	ENPP6	case	control	0.140090604	0.248480418	0.563789313	0.572897526	0.886154542	up_in_case
CRA004775	ENSGALG00010007188	MAB21L1	case	control	0.187819938	0.429698979	0.437096543	0.662041334	0.918136475	up_in_case
CRA004775	ENSGALG00010007190	ENSGALG00010007190	case	control	-0.063970641	0.575464165	-0.111163553	0.911486653	0.98075825	down_in_case
CRA004775	ENSGALG00010007201	ENSGALG00010007201	case	control	-0.145715307	0.693930233	-0.209985529	0.833678968	0.964019688	down_in_case
CRA004775	ENSGALG00010007203	SLC30A6	case	control	-0.453840666	0.215876217	-2.102318964	0.035525344	0.35709723	down_in_case
CRA004775	ENSGALG00010007204	LANCL2	case	control	-0.377618644	0.230545028	-1.637938788	0.101434464	0.540839592	down_in_case
CRA004775	ENSGALG00010007207	EPM2A	case	control	0.141869148	0.421076777	0.336919906	0.736177279	0.937385622	up_in_case
CRA004775	ENSGALG00010007214	MTHFSD	case	control	0.488967768	0.255324687	1.91508222	0.055482019	0.431364203	up_in_case
CRA004775	ENSGALG00010007217	MSH3	case	control	-0.058000921	0.32089653	-0.180746488	0.856566568	0.970913248	down_in_case
CRA004775	ENSGALG00010007223	FOXF1	case	control	1.885690495	0.52197828	3.612584217	0.000303161	0.035211539	up_in_case
CRA004775	ENSGALG00010007224	DHFR	case	control	-0.367526976	0.506357972	-0.72582441	0.46794642	0.843870188	down_in_case
CRA004775	ENSGALG00010007226	ENSGALG00010007226	case	control	1.802797057	0.577018099	3.124333637	0.001782083	0.085287202	up_in_case
CRA004775	ENSGALG00010007230	ENSGALG00010007230	case	control	-0.385372777	0.287765886	-1.33918854	0.180509302	0.645561356	down_in_case
CRA004775	ENSGALG00010007243	BEAN1	case	control	-0.412938359	0.441068024	-0.936223748	0.349157998	0.785311667	down_in_case
CRA004775	ENSGALG00010007251	SPAST	case	control	0.141567456	0.117720581	1.202571839	0.229142051	0.695782399	up_in_case
CRA004775	ENSGALG00010007252	NFATC1	case	control	0.289904599	0.422006266	0.686967522	0.492103195	0.855746361	up_in_case
CRA004775	ENSGALG00010007259	ANKRD34B	case	control	0.30703409	0.196027802	1.566278291	0.117283481	0.567740281	up_in_case
CRA004775	ENSGALG00010007265	CD200R1A	case	control	-0.718980225	0.474473304	-1.515322821	0.129690672	0.584172159	down_in_case
CRA004775	ENSGALG00010007270	CDH5	case	control	0.526005336	0.38697692	1.35926798	0.174061687	0.639244835	up_in_case
CRA004775	ENSGALG00010007275	DPY30	case	control	-0.176737072	0.22479862	-0.786201765	0.431749292	0.825268907	down_in_case
CRA004775	ENSGALG00010007277	FAM151B	case	control	-1.096814236	0.425374739	-2.578465844	0.009924011	0.201425687	down_in_case
CRA004775	ENSGALG00010007283	STOX2	case	control	0.920575212	0.335487821	2.743989956	0.006069739	0.155408639	up_in_case
CRA004775	ENSGALG00010007284	ENSGALG00010007284	case	control	0.065760922	0.245876945	0.267454606	0.78911916	0.95041797	up_in_case
CRA004775	ENSGALG00010007287	ENSGALG00010007287	case	control	-1.261364436	0.758856992	-1.662189911	0.096474702	0.530437278	down_in_case
CRA004775	ENSGALG00010007294	TRAPPC11	case	control	0.007934221	0.176693497	0.044903865	0.964183936	0.99271855	up_in_case
CRA004775	ENSGALG00010007300	CCDC125	case	control	-1.127287691	0.727091683	-1.550406527	0.121043973	0.573473028	down_in_case
CRA004775	ENSGALG00010007302	CDH11	case	control	0.049708334	0.364426636	0.136401483	0.891503901	0.977782257	up_in_case
CRA004775	ENSGALG00010007304	F5	case	control	0.86893669	0.32118774	2.705385607	0.006822514	0.161752401	up_in_case
CRA004775	ENSGALG00010007318	RMDN2	case	control	0.353240963	0.304948176	1.158363912	0.24671556	0.712046495	up_in_case
CRA004775	ENSGALG00010007319	ATP9B	case	control	0.281511391	0.234252761	1.20174204	0.229463488	0.695782399	up_in_case
CRA004775	ENSGALG00010007320	ENSGALG00010007320	case	control	-0.399963955	0.437300383	-0.914620636	0.360390822	0.791975934	down_in_case
CRA004775	ENSGALG00010007321	CD200	case	control	-0.319315254	0.455349742	-0.70125274	0.483145302	0.850234328	down_in_case
CRA004775	ENSGALG00010007322	AK6	case	control	-0.163295938	0.268278693	-0.608680234	0.542736411	0.875137006	down_in_case
CRA004775	ENSGALG00010007324	ENSGALG00010007324	case	control	0.369711605	0.400205268	0.923804943	0.355587881	0.788778426	up_in_case
CRA004775	ENSGALG00010007326	RWDD4	case	control	0.31271034	0.284062569	1.100850214	0.270961853	0.728665115	up_in_case
CRA004775	ENSGALG00010007328	NOVA1	case	control	-0.037510684	0.402614467	-0.09316775	0.925770295	0.98417264	down_in_case
CRA004775	ENSGALG00010007330	MMP13	case	control	-0.562029402	0.830371824	-0.676840647	0.498507066	0.858266015	down_in_case
CRA004775	ENSGALG00010007333	QTRT2	case	control	-0.556200466	0.259220284	-2.145667218	0.031899538	0.340302989	down_in_case
CRA004775	ENSGALG00010007337	PRORP	case	control	-0.399661966	0.21399304	-1.867640021	0.061812264	0.446686038	down_in_case
CRA004775	ENSGALG00010007339	ENSGALG00010007339	case	control	-0.186045383	0.428944789	-0.43372804	0.664485942	0.918557471	down_in_case
CRA004775	ENSGALG00010007344	RAD17	case	control	0.064824705	0.174258045	0.372004091	0.709889803	0.932234469	up_in_case
CRA004775	ENSGALG00010007353	ING2	case	control	-0.12677184	0.254280912	-0.498550357	0.618096185	0.904143308	down_in_case
CRA004775	ENSGALG00010007357	NOX4	case	control	-0.16294757	0.524058285	-0.310934059	0.755850752	0.941900693	down_in_case
CRA004775	ENSGALG00010007360	ROBO1	case	control	-0.596186178	0.58337338	-1.02196329	0.306798276	0.7585475	down_in_case
CRA004775	ENSGALG00010007365	MEMO1	case	control	0.221260889	0.340639725	0.649545172	0.515986059	0.864897625	up_in_case
CRA004775	ENSGALG00010007369	ENSGALG00010007369	case	control	-0.162828997	0.352500582	-0.461925471	0.644134767	0.913739303	down_in_case
CRA004775	ENSGALG00010007374	CMIP	case	control	-0.183975956	0.342676442	-0.536879498	0.591350853	0.893804699	down_in_case
CRA004775	ENSGALG00010007375	CHORDC1	case	control	0.210517477	0.239556053	0.878781707	0.379519647	0.800591196	up_in_case
CRA004775	ENSGALG00010007376	CDKN2AIP	case	control	0.137467615	0.175774948	0.782066022	0.434175774	0.826182359	up_in_case
CRA004775	ENSGALG00010007381	EGFR	case	control	0.233955442	0.293532973	0.797032918	0.425431913	0.822283278	up_in_case
CRA004775	ENSGALG00010007382	EIF2AK2	case	control	-0.388575481	0.287835035	-1.349993692	0.177018006	0.643089023	down_in_case
CRA004775	ENSGALG00010007387	ENSGALG00010007387	case	control	-1.541511159	0.938742537	-1.642102172	0.100568832	0.538583505	down_in_case
CRA004775	ENSGALG00010007388	CYP1B1	case	control	1.099144726	0.477124254	2.303686546	0.021240246	0.286336139	up_in_case
CRA004775	ENSGALG00010007391	NANP	case	control	-0.07302388	0.20155744	-0.362298112	0.717129269	0.934011578	down_in_case
CRA004775	ENSGALG00010007392	NAALAD2	case	control	0.214500126	0.448144152	0.478640912	0.632194108	0.909651573	up_in_case
CRA004775	ENSGALG00010007393	PLCG2	case	control	0.244904614	0.619311245	0.395446741	0.692513219	0.927867527	up_in_case
CRA004775	ENSGALG00010007399	WWC2	case	control	0.50096781	0.365145943	1.371965978	0.170074029	0.63378857	up_in_case
CRA004775	ENSGALG00010007401	SEC61G	case	control	-0.120569467	0.287501785	-0.419369455	0.674946144	0.92288678	down_in_case
CRA004775	ENSGALG00010007403	SDR42E1	case	control	-0.065759538	0.549687809	-0.119630701	0.904775699	0.979540372	down_in_case
CRA004775	ENSGALG00010007404	FOLH1	case	control	0.341234397	0.392409827	0.869586778	0.384526266	0.803182776	up_in_case
CRA004775	ENSGALG00010007408	HCLS1	case	control	-0.743579813	0.357516648	-2.079846677	0.037539598	0.365326975	down_in_case
CRA004775	ENSGALG00010007413	GPATCH11	case	control	0.467761477	0.293999161	1.591029971	0.111602831	0.560737293	up_in_case
CRA004775	ENSGALG00010007416	DCTD	case	control	-0.581122132	0.525368675	-1.106122539	0.268673485	0.727528758	down_in_case
CRA004775	ENSGALG00010007418	NME6	case	control	-0.10256554	0.214580755	-0.477981075	0.632663675	0.909651573	down_in_case
CRA004775	ENSGALG00010007419	RABL2A	case	control	-0.324324039	0.299144917	-1.084170316	0.27828929	0.733841198	down_in_case
CRA004775	ENSGALG00010007423	HEATR5B	case	control	0.006469308	0.286550144	0.022576531	0.981988065	0.997069916	up_in_case
CRA004775	ENSGALG00010007425	ARSA	case	control	-0.210776737	0.259387566	-0.812593836	0.416450967	0.818943344	down_in_case
CRA004775	ENSGALG00010007429	C21orf91	case	control	0.599714496	0.275087953	2.18008273	0.02925133	0.325169793	up_in_case
CRA004775	ENSGALG00010007430	MEF2C	case	control	0.175154202	0.405438652	0.432011603	0.66573298	0.918888163	up_in_case
CRA004775	ENSGALG00010007432	NAT	case	control	-0.259788891	0.418287448	-0.621077425	0.534548682	0.870981055	down_in_case
CRA004775	ENSGALG00010007433	RNF144B	case	control	0.785544073	0.344651954	2.279238707	0.022652879	0.292762917	up_in_case
CRA004775	ENSGALG00010007434	ENSGALG00010007434	case	control	0.547014828	0.419654836	1.303487488	0.192408385	0.656959697	up_in_case
CRA004775	ENSGALG00010007436	PNAT3	case	control	-0.548899003	0.441748815	-1.242559087	0.214030354	0.683502083	down_in_case
CRA004775	ENSGALG00010007437	TUSC1	case	control	-0.315979957	0.364424017	-0.867066775	0.385905424	0.803541109	down_in_case
CRA004775	ENSGALG00010007441	STRN	case	control	0.00610747	0.259906027	0.023498761	0.981252426	0.997069916	up_in_case
CRA004775	ENSGALG00010007455	TRPC6	case	control	0.236690743	0.494528357	0.478619153	0.63220959	0.909651573	up_in_case
CRA004775	ENSGALG00010007457	SHANK3	case	control	-0.046559771	0.296356821	-0.157107136	0.875160414	0.974356282	down_in_case
CRA004775	ENSGALG00010007465	PNAT10	case	control	0.064455278	0.343020902	0.187904811	0.850951271	0.968989143	up_in_case
CRA004775	ENSGALG00010007467	CHODL	case	control	0.065844691	0.659339268	0.099864659	0.920451774	0.982481112	up_in_case
CRA004775	ENSGALG00010007479	NFX1	case	control	-0.031483667	0.406933951	-0.077368002	0.938330795	0.986707289	down_in_case
CRA004775	ENSGALG00010007480	TENM3	case	control	0.270097257	0.245749075	1.099077411	0.271734302	0.729164673	up_in_case
CRA004775	ENSGALG00010007485	CTSC	case	control	0.054257181	0.477513684	0.113624348	0.909535585	0.98075825	up_in_case
CRA004775	ENSGALG00010007500	MPHOSPH6	case	control	0.10634651	0.231911542	0.458564973	0.646546595	0.914043203	up_in_case
CRA004775	ENSGALG00010007504	TMPRSS15	case	control	2.570679211	0.831409814	3.091951968	0.00198845	0.089065729	up_in_case
CRA004775	ENSGALG00010007506	RAB38	case	control	0.921909719	0.623447	1.478729899	0.139212518	0.599104916	up_in_case
CRA004775	ENSGALG00010007513	GALNT12	case	control	0.289943508	0.273182473	1.061354723	0.288528725	0.742851819	up_in_case
CRA004775	ENSGALG00010007514	VIT	case	control	0.147350991	0.874109951	0.168572605	0.86613283	0.972737059	up_in_case
CRA004775	ENSGALG00010007521	SLC12A7	case	control	0.073083342	0.398528761	0.183382856	0.854497627	0.97035175	up_in_case
CRA004775	ENSGALG00010007529	CDH13	case	control	0.346515383	0.313199258	1.106373575	0.268564858	0.727528758	up_in_case
CRA004775	ENSGALG00010007532	DCUN1D5	case	control	-0.444477405	0.250024102	-1.777738229	0.075446858	0.483342345	down_in_case
CRA004775	ENSGALG00010007536	GABBR2	case	control	-0.472774996	0.729196814	-0.648350332	0.516758387	0.864913871	down_in_case
CRA004775	ENSGALG00010007539	RPS3A	case	control	-0.383559739	0.223436065	-1.716642022	0.0860446	0.507593003	down_in_case
CRA004775	ENSGALG00010007542	MRPL36	case	control	-0.191245004	0.413783372	-0.462186295	0.64394773	0.913727726	down_in_case
CRA004775	ENSGALG00010007544	MLYCD	case	control	0.242776039	0.214949165	1.129457934	0.258704705	0.720037471	up_in_case

CRA004775	ENSGALG00010007546	MMP10	case	control	-0.457293677	0.813636534	-0.562036803	0.574090944	0.886903109	down_in_case
CRA004775	ENSGALG00010007547	ENSGALG00010007547	case	control	0.785766218	0.759359016	1.034775648	0.300773698	0.753358675	up_in_case
CRA004775	ENSGALG00010007548	HSBP1	case	control	-0.223051637	0.215405655	-1.035495734	0.300437457	0.752927916	down_in_case
CRA004775	ENSGALG00010007549	SH3D19	case	control	0.174946147	0.2458967	0.711461956	0.476798018	0.846367006	up_in_case
CRA004775	ENSGALG00010007551	FEZ2	case	control	0.480060908	0.34192771	1.40398363	0.160323724	0.625455498	up_in_case
CRA004775	ENSGALG00010007555	NDUFS6	case	control	0.29870179	0.427633894	0.698498867	0.484865266	0.851277875	up_in_case
CRA004775	ENSGALG00010007566	FHIP1A	case	control	0.097736622	0.359550179	0.271830269	0.78575253	0.95041797	up_in_case
CRA004775	ENSGALG00010007567	TRIP13	case	control	-0.632810109	0.485124227	-1.304428997	0.192087354	0.656959697	down_in_case
CRA004775	ENSGALG00010007569	GPR89A	case	control	-0.521125599	0.228515791	-2.280479596	0.02257926	0.292594999	down_in_case
CRA004775	ENSGALG00010007572	PDZK1	case	control	2.066278688	0.631769855	3.270619314	0.001073123	0.068613766	up_in_case
CRA004775	ENSGALG00010007574	ENSGALG00010007574	case	control	0.276045254	0.515048976	0.535959233	0.591986725	0.893804699	up_in_case
CRA004775	ENSGALG00010007575	MMP27	case	control	-0.309352322	0.989838466	-0.312528087	0.754639221	0.941900693	down_in_case
CRA004775	ENSGALG00010007577	WFDC1	case	control	-0.282329431	0.304756233	-0.926410686	0.354232593	0.78730441	down_in_case
CRA004775	ENSGALG00010007579	GATB	case	control	-0.32335841	0.241502627	-1.338943658	0.180589016	0.645678511	down_in_case
CRA004775	ENSGALG00010007580	BRD9	case	control	0.219387916	0.218618876	1.003517718	0.315611133	0.765461123	up_in_case
CRA004775	ENSGALG00010007581	LPCAT1	case	control	-0.080907801	0.370294773	-0.218495661	0.827042939	0.961897808	down_in_case
CRA004775	ENSGALG00010007584	ELP2	case	control	-0.096749426	0.182362517	-0.530533509	0.595742082	0.895305942	down_in_case
CRA004775	ENSGALG00010007585	KCNG4	case	control	-0.092672913	0.852148422	-0.10875208	0.913399133	0.980803023	down_in_case
CRA004775	ENSGALG00010007586	MMP7	case	control	4.412302553	1.229368597	3.589080252	0.000331847	0.036216061	up_in_case
CRA004775	ENSGALG00010007588	TTC27	case	control	-0.199996157	0.220327739	-0.90772119	0.364025545	0.793608387	down_in_case
CRA004775	ENSGALG00010007590	FBXW7	case	control	0.936309004	0.269373454	3.47587704	0.000509186	0.046064555	up_in_case
CRA004775	ENSGALG00010007593	SEC61B	case	control	-0.623760293	0.332891228	-1.873766088	0.060962672	0.445419429	down_in_case
CRA004775	ENSGALG00010007595	ATP2C2	case	control	-0.663646057	0.467037732	-1.420968825	0.155325823	0.619284425	down_in_case
CRA004775	ENSGALG00010007596	ENSGALG00010007596	case	control	2.133952509	1.064538539	2.004579854			up_in_case
CRA004775	ENSGALG00010007597	SMKR1	case	control	-0.964342619	0.741701965	-1.300175359	0.193540874	0.657919837	down_in_case
CRA004775	ENSGALG00010007601	TMEM154	case	control	-0.836654147	0.533608645	-1.567917153	0.116900468	0.567018814	down_in_case
CRA004775	ENSGALG00010007603	ALG2	case	control	-0.035247192	0.176801971	-0.199359723	0.841981364	0.966478468	down_in_case
CRA004775	ENSGALG00010007604	ENSGALG00010007604	case	control	-0.392987745	1.006419227	-0.390481158	0.696180786	0.929092636	down_in_case
CRA004775	ENSGALG00010007610	TLDC1	case	control	-0.204945626	0.253008735	-0.810033797	0.417920752	0.818943344	down_in_case
CRA004775	ENSGALG00010007614	BIRC6	case	control	0.335864184	0.271615029	1.236544917	0.21625608	0.683774974	up_in_case
CRA004775	ENSGALG00010007624	TPPP	case	control	0.408997067	0.422658206	0.967678045	0.333205186	0.773910112	up_in_case
CRA004775	ENSGALG00010007626	DLK2	case	control	-1.688614007	0.728573345	-2.317699405	0.020465663	0.281976732	down_in_case
CRA004775	ENSGALG00010007633	ENSGALG00010007633	case	control	-0.49756821	0.19318018	-2.575669044	0.010004636	0.201425687	down_in_case
CRA004775	ENSGALG00010007640	TMEM123	case	control	-0.10685838	0.459588362	-0.232508891	0.816142783	0.959129938	down_in_case
CRA004775	ENSGALG00010007642	ZNF318	case	control	0.709294596	0.22897265	3.097726276	0.001950115	0.087921396	up_in_case
CRA004775	ENSGALG00010007643	COTL1	case	control	-0.298193832	0.338759432	-0.880252486	0.378722546	0.799971387	down_in_case
CRA004775	ENSGALG00010007650	KLHL36	case	control	-0.209270859	0.231721451	-0.903113882	0.366465457	0.793608387	down_in_case
CRA004775	ENSGALG00010007651	CTNNAL1	case	control	0.798064477	0.309240323	2.580725786	0.009859286	0.201149915	up_in_case
CRA004775	ENSGALG00010007655	SLC9A3	case	control	2.760685743	0.830619408	3.323647046	0.000888486	0.062654188	up_in_case
CRA004775	ENSGALG00010007658	BIRC2	case	control	-0.027623765	0.147767523	-0.186940703	0.851707125	0.969430695	down_in_case
CRA004775	ENSGALG00010007660	FAM98A	case	control	0.033666685	0.278955975	0.120688164	0.903938034	0.979540372	up_in_case
CRA004775	ENSGALG00010007663	ARFIP1	case	control	0.296642626	0.18938578	1.566340545	0.117268914	0.567740281	up_in_case
CRA004775	ENSGALG00010007671	FAM206A	case	control	-0.173019743	0.251037115	-0.689219769	0.490684978	0.855184934	down_in_case
CRA004775	ENSGALG00010007673	FHDC1	case	control	0.507724979	0.627950506	0.80854299	0.418778069	0.818943344	up_in_case
CRA004775	ENSGALG00010007678	CDH8	case	control	0.680806886	0.740453343	0.919446029	0.357862323	0.790416653	up_in_case
CRA004775	ENSGALG00010007679	TMEM135	case	control	0.338370139	0.335873321	1.007433811	0.31372635	0.765098415	up_in_case
CRA004775	ENSGALG00010007680	CHMP2B	case	control	-0.092042133	0.19078894	-0.482429083	0.629501165	0.908699404	down_in_case
CRA004775	ENSGALG00010007695	RASGRP3	case	control	0.348330161	0.276441716	1.26004919	0.207651618	0.676460246	up_in_case
CRA004775	ENSGALG00010007698	ADAMTS18	case	control	-2.398772924	0.933874642	-2.568624115	0.010210314	0.202829374	down_in_case
CRA004775	ENSGALG00010007699	SLC39A6	case	control	0.332584792	0.404421875	0.822370926	0.410865821	0.816405743	up_in_case
CRA004775	ENSGALG00010007707	ENSGALG00010007707	case	control	-0.029023073	0.481356144	-0.060294386	0.951921173	0.990168609	down_in_case
CRA004775	ENSGALG00010007713	SCLT1	case	control	0.254221692	0.265639399	0.957018021	0.338558159	0.779253705	up_in_case
CRA004775	ENSGALG00010007719	LTBP1	case	control	-0.066242205	0.4148473	-0.159678524	0.873134319	0.97407675	down_in_case
CRA004775	ENSGALG00010007724	RBM11	case	control	-0.655406236	0.323215353	-2.027769507	0.042583778	0.388051378	down_in_case
CRA004775	ENSGALG00010007730	COG2	case	control	-0.349588104	0.17845597	-1.958959982	0.050117473	0.414161886	down_in_case
CRA004775	ENSGALG00010007731	ENSGALG00010007731	case	control	-0.280965843	0.407278647	-0.689861462	0.490281313	0.855184934	down_in_case
CRA004775	ENSGALG00010007741	ENSGALG00010007741	case	control	-0.323134279	0.424989451	-0.760334823	0.447054472	0.834117509	down_in_case
CRA004775	ENSGALG00010007748	CSGALNACT1	case	control	-0.218419165	0.528877405	-0.41298638	0.679616592	0.923739354	down_in_case
CRA004775	ENSGALG00010007751	HSPA13	case	control	0.490520627	0.177192238	2.768296355	0.005635019	0.15193558	up_in_case
CRA004775	ENSGALG00010007753	PRSS23	case	control	0.409459836	0.483609934	0.846673749	0.397176994	0.808998414	up_in_case
CRA004775	ENSGALG00010007758	DEK	case	control	0.130322644	0.226973087	0.57417664	0.565848275	0.883033678	up_in_case
CRA004775	ENSGALG00010007759	AGT	case	control	1.308630917	0.718532727	1.821254436	0.068568189	0.46645716	up_in_case
CRA004775	ENSGALG00010007761	ENSGALG00010007761	case	control	-0.199112914	0.279368502	-0.712724994	0.476015947	0.846243735	down_in_case
CRA004775	ENSGALG00010007762	SH2D4A	case	control	-0.582990209	0.634033897	-0.919493756	0.35783737	0.790416653	down_in_case
CRA004775	ENSGALG00010007763	FZD4	case	control	0.28606209	0.267225757	1.070488464	0.284399499	0.741035375	up_in_case
CRA004775	ENSGALG00010007765	NUDT7	case	control	0.053797551	0.264597477	0.203318457	0.83888613	0.965800667	up_in_case
CRA004775	ENSGALG00010007766	ENSGALG00010007766	case	control	-0.386200418	0.360919781	-1.07004503	0.28459904	0.741197235	down_in_case
CRA004775	ENSGALG00010007767	VAT1L	case	control	0.562625027	0.407895315	1.379336823	0.167790928	0.63138062	up_in_case
CRA004775	ENSGALG00010007772	CLEC3A	case	control	-3.724610552	1.282105086	-2.905074313	0.003671659	0.12600076	down_in_case
CRA004775	ENSGALG00010007777	KIF13A	case	control	0.391617413	0.239368512	1.63604398	0.101830383	0.541925225	up_in_case
CRA004775	ENSGALG00010007780	SIRT5	case	control	-0.181151532	0.490353509	-0.36943048	0.711806883	0.932407723	down_in_case
CRA004775	ENSGALG00010007781	MBP	case	control	0.786287271	0.289404809	2.716911557	0.006589421	0.160658037	up_in_case
CRA004775	ENSGALG00010007783	NOL7	case	control	-0.448058334	0.193753165	-2.312521368	0.020748969	0.283942565	down_in_case
CRA004775	ENSGALG00010007784	INTS10	case	control	-0.340705554	0.181735029	-1.874737944	0.060828784	0.445397937	down_in_case
CRA004775	ENSGALG00010007786	SAMSN1	case	control	-0.37969792	0.536058263	-0.708314648	0.478749883	0.847161194	down_in_case
CRA004775	ENSGALG00010007787	C1orf198	case	control	-0.128190063	0.295105161	-0.434387737	0.664006902	0.918295135	down_in_case
CRA004775	ENSGALG00010007788	PALM2AKAP2	case	control	0.894002329	0.520706017	1.716904166	0.085996684	0.507567605	up_in_case
CRA004775	ENSGALG00010007789	WWOX	case	control	-0.390322028	0.220854232	-1.767328723	0.077173192	0.488700005	down_in_case
CRA004775	ENSGALG00010007792	ENSGALG00010007792	case	control	-0.014963772	0.328348093	-0.04557289	0.963650677	0.992373284	down_in_case
CRA004775	ENSGALG00010007795	ZNF236	case	control	0.439276959	0.198898175	2.208551986	0.027205818	0.31561562	up_in_case
CRA004775	ENSGALG00010007796	MCUR1	case	control	0.175572225	0.197153921	0.890533775	0.373179341	0.796804495	up_in_case
CRA004775	ENSGALG00010007798	HGSNAT	case	control	-0.03948305	0.231089327	-0.170856226	0.864336817	0.972350383	down_in_case
CRA004775	ENSGALG00010007800	MAF	case	control	0.603896244	0.266806386	2.263424996	0.023609503	0.297845774	up_in_case
CRA004775	ENSGALG00010007801	TTC13	case	control	0.239114866	0.189203856	1.263794883	0.206303658	0.674837165	up_in_case
CRA004775	ENSGALG00010007802	ENSGALG00010007802	case	control	3.901763581	1.478636138	2.638758434	0.008321025	0.183215969	up_in_case
CRA004775	ENSGALG00010007803	ME3	case	control	-0.389640635	0.407462423	-0.956261517	0.338940127	0.779398613	down_in_case
CRA004775	ENSGALG00010007805	ENSGALG00010007805	case	control	-0.354919376	1.153908295	-0.307580228	0.758401767	0.942236668	down_in_case
CRA004775	ENSGALG00010007806	DYNLRB2	case	control	0.100286772	0.970645561	0.103319663	0.91770927	0.981688766	up_in_case
CRA004775	ENSGALG00010007809	ARV1	case	control	-0.005307599	0.370135704	-0.014339604	0.988559044	0.998065748	down_in_case
CRA004775	ENSGALG00010007811	PRCP	case	control	-0.391338465	0.472911113	-0.827509555	0.407948309	0.814297749	down_in_case
CRA004775	ENSGALG00010007812	POMK	case	control	0.127531777	0.31606277	0.403501423	0.686579388	0.92689803	up_in_case
CRA004775	ENSGALG00010007817	CDYL2	case	control	-0.952419834	0.654713269	-1.454712894	0.145748762	0.609044185	down_in_case
CRA004775	ENSGALG00010007818	ENSGALG00010007818	case	control	1.028740509	0.476301071	2.159853446	0.030784017	0.334633212	up_in_case
CRA004775	ENSGALG00010007819	NRIP1	case	control	1.425533018	0.407568108	3.497655949	0.000469366	0.044470881	up_in_case
CRA004775	ENSGALG00010007821	FAM89A	case	control	0.144284077	0.198016237	0.728647708	0.466217187	0.843480361	up_in_case
CRA004775	ENSGALG00010007826	ENSGALG00010007826	case	control	0.073746555	1.833849346	0.040214075	0.967922457	0.993568693	up_in_case
CRA004775	ENSGALG00010007830	SMIM19	case	control	-0.124384739	0.323709839	-0.384247632	0.700794917	0.930068722	down_in_case
CRA004775	ENSGALG00010007835	GUCY1A1	case	control	0.274525214	0.422905056	0.649141479	0.516246933	0.864913871	up_in_case
CRA004775	ENSGALG00010007846	ENSGALG00010007846	case	control	-0.482419021	0.430680191	-1.120132831	0.262657162	0.723367482	down_in_case
CRA004775	ENSGALG00010007847	CMC2	case	control	-0.019558867	0.41690887	-0.046914009	0.962581763	0.991860454	down_in_case
CRA004775	ENSGALG00010007849	CXADR	case	control	-0.302182965	0.672511584	-0.44933496	0.653190042	0.916442743	down_in_case
CRA004775	ENSGALG00010007851	AvBD7	case	control	0.889816946	0.600220089	1.482484447			up_in_case
CRA004775	ENSGALG00010007852	SLC20A2	case	control	0.977364616	0.389060732	2.512113244	0.012001057	0.220535154	up_in_case
CRA004775	ENSGALG00010007853	C1orf131	case	control	0.78603318	0.320385394	2.453398924	0.01415133	0.238037235	up_in_case
CRA004775	ENSGALG00010007854	FAM181B	case	control	-0.358497499	0.386768828	-0.926903806	0.353976481	0.78730441	down_in_case
CRA004775	ENSGALG00010007856	PDCD2	case	control	0.050815401	0.304448211	0.166909837	0.867440993	0.973120718	up_in_case
CRA004775	ENSGALG00010007857	ENSGALG00010007857	case	control	0.543602205	0.468238858	1.160950646	0.245661971	0.712046495	up_in_case
CRA004775	ENSGALG00010007859	CENPN	case	control	-0.412713689	0.581821917	-0.7093471	0.478109106	0.846790098	down_in_case
CRA004775	ENSGALG00010007860	DEFB4A	case	control	0.661728273	0.593382922	1.115179167			up_in_case
CRA004775	ENSGALG00010007861	INTU	case	control	0.369488244	0.28841833	1.281084472	0.200163997	0.667171435	up_in_case
CRA004775	ENSGALG00010007862	ATMIN	case	control	0.173055955	0.217863282	0.794332818	0.427001704	0.822776983	up_in_case
CRA004775	ENSGALG00010007863	AvBD1	case	control	1.159420588	0.641390766	1.807666478			up_in_case
CRA004775	ENSGALG00010007864	TYR	case	control	6.469947628	1.029982589	6.281608737	3.35087E-10	4.60779E-07	up_in_case
CRA004775	ENSGALG00010007866	TBP	case	control	-0.01066688	0.18704399	-0.057028726	0.954522312	0.990670511	down_in_case
CRA004775	ENSGALG00010007867	ZNF516	case	control	-0.758566913	0.561675557	-1.35054286	0.176841915	0.642787581	down_in_case
CRA004775	ENSGALG00010007868	INHBA	case	control	0.462773244	0.536628023	0.86237249	0.388482574	0.804467665	up_in_case
CRA004775	ENSGALG00010007869	GCSH	case	control	-0.114266997	0.275512947	-0.414742749	0.678330234	0.92338129	down_in_case
CRA004775	ENSGALG00010007871	HSPA4L	case	control	0.184306865	0.249635291	0.738304524	0.460329418	0.841265006	up_in_case
CRA004775	ENSGALG00010007872	KLHL2	case	control	0.14495439	0.156592953	0.925676328	0.354614214	0.787789315	up_in_case
CRA004775	ENSGALG00010007873	ENSGALG00010007873	case	control	3.844687308	1.216948844	3.159284244			up_in_case
CRA004775	ENSGALG00010007874	ENSGALG00010007874	case	control	-0.398623134	0.527014034	-0.756380491	0.449421101	0.835020884	down_in_case
CRA004775	ENSGALG00010007877	MSMO1	case	control	0.409571455	0.266451137	1.537135321	0.124260175	0.576485043	up_in_case
CRA004775	ENSGALG00010007878	PSMB1	case	control	-0.232212235	0.247288218	-0.93903477	0.347712893	0.785252092	down_in_case
CRA004775	ENSGALG00010007879	PLK4	case	control	0.221335436	0.432292497	0.512003881	0.608648293	0.901007337	up_in_case
CRA004775	ENSGALG00010007880	BCO1	case	control	0.153886258	0.306402821	0.502235123	0.615502139	0.902802125	up_in_case
CRA004775	ENSGALG00010007882	CPE	case	control	0.056019552	0.48724279	0.114972562	0.908466871	0.98075825	up_in_case

CRA004775	ENSGALG00010007883	DHTKD1	case	control	0.215415642	0.343937368	0.626322296	0.531103565	0.869441746	up_in_case
CRA004775	ENSGALG00010007884	GLI3	case	control	-0.254469594	0.464798486	-0.547483699	0.584046467	0.889984814	down_in_case
CRA004775	ENSGALG00010007885	USP38	case	control	0.282641268	0.310137795	0.911340933	0.362115765	0.792867581	up_in_case
CRA004775	ENSGALG00010007887	CDT1	case	control	-0.179429347	0.29976786	-0.59856099	0.549465676	0.877623839	down_in_case
CRA004775	ENSGALG00010007888	MIOX	case	control	-0.072360711	0.674873053	-0.107221219	0.914613482	0.981303879	down_in_case
CRA004775	ENSGALG00010007889	DCAF6	case	control	0.136250611	0.352390665	0.386646482	0.699017942	0.929347461	up_in_case
CRA004775	ENSGALG00010007890	ENSGALG00010007890	case	control	-0.230156187	0.281227844	-0.818397581	0.413130203	0.817112606	down_in_case
CRA004775	ENSGALG00010007891	GDI1	case	control	-0.032713212	0.143880727	-0.227363408	0.820141171	0.960565501	down_in_case
CRA004775	ENSGALG00010007892	ENSGALG00010007892	case	control	2.533471967	0.651158956	3.890712005	9.99505E-05	0.022373664	up_in_case
CRA004775	ENSGALG00010007893	MFSD8	case	control	-0.173603261	0.266690145	-0.650954919	0.515075589	0.864897625	down_in_case
CRA004775	ENSGALG00010007894	CD88	case	control	-0.341307834	0.388327798	-0.878916821	0.379446377	0.800591196	down_in_case
CRA004775	ENSGALG00010007895	GOLGB1	case	control	-0.190905527	0.209478482	-0.911337173	0.362117746	0.792867581	down_in_case
CRA004775	ENSGALG00010007896	TIMMDC1	case	control	-0.123124375	0.314162506	-0.391913017	0.695122485	0.928924129	down_in_case
CRA004775	ENSGALG00010007897	EFHC1	case	control	-0.478351873	0.475649323	-1.005681812	0.314568655	0.765098415	down_in_case
CRA004775	ENSGALG00010007898	GAB1	case	control	0.629193294	0.231410909	2.718943959	0.00654907	0.16028494	up_in_case
CRA004775	ENSGALG00010007899	APRT	case	control	-0.43136126	0.288115981	-1.497179224	0.13434663	0.592420453	down_in_case
CRA004775	ENSGALG00010007900	FHOD3	case	control	-0.487781543	0.573453919	-0.850602859	0.394990001	0.808998414	down_in_case
CRA004775	ENSGALG00010007901	FSTL5	case	control	1.223739561	0.835080215	1.465415584	0.142807568	0.602997185	up_in_case
CRA004775	ENSGALG00010007902	C7orf25	case	control	0.232096064	0.442686855	0.524289487	0.600077208	0.897206546	up_in_case
CRA004775	ENSGALG00010007903	PSMA2	case	control	-0.106448963	0.244669474	-0.435072516	0.663509792	0.918295135	down_in_case
CRA004775	ENSGALG00010007904	ENSGALG00010007904	case	control	-0.18168464	0.235878117	-0.77024797	0.441152814	0.829353567	down_in_case
CRA004775	ENSGALG00010007905	MRPL32	case	control	-0.169219986	0.283242178	-0.59743922	0.550214175	0.877623839	down_in_case
CRA004775	ENSGALG00010007906	NAF1	case	control	-0.13911633	0.246204784	-0.565043161	0.572044407	0.885631912	down_in_case
CRA004775	ENSGALG00010007909	GALNS	case	control	0.041310145	0.37868784	0.109087592	0.913133016	0.980803023	up_in_case
CRA004775	ENSGALG00010007910	ANKS6	case	control	0.39966504	0.251301697	1.59037939	0.111749313	0.56111317	up_in_case
CRA004775	ENSGALG00010007911	IFI6	case	control	0.163118992	0.512826935	0.318078052	0.750425732	0.941495694	up_in_case
CRA004775	ENSGALG00010007912	TRAPPC2L	case	control	-0.393131788	0.226726846	-1.733944591	0.082927915	0.499930626	down_in_case
CRA004775	ENSGALG00010007914	PAQR8	case	control	-0.108598318	0.507164441	-0.214128416	0.830446917	0.963232966	down_in_case
CRA004775	ENSGALG00010007915	HECW1	case	control	0.223823333	0.345131848	0.648515442	0.516651626	0.864913871	up_in_case
CRA004775	ENSGALG00010007916	MCM3	case	control	-0.920727884	0.677250209	-1.359509191	0.173985292	0.639185614	down_in_case
CRA004775	ENSGALG00010007917	POGLUT1	case	control	-0.099847375	0.192079587	-0.519822939	0.60318699	0.898692785	down_in_case
CRA004775	ENSGALG00010007920	FAT3	case	control	-0.279023966	0.760514585	-0.366888382	0.713702278	0.933244709	down_in_case
CRA004775	ENSGALG00010007921	FAM120B	case	control	-0.273781832	0.267888986	-1.021997342	0.306782159	0.7585475	down_in_case
CRA004775	ENSGALG00010007923	YAP1	case	control	0.132009857	0.253978173	0.519768509	0.60322493	0.898692785	up_in_case
CRA004775	ENSGALG00010007924	ABHD18	case	control	0.389509614	0.189564699	2.054758168	0.039902368	0.375888502	up_in_case
CRA004775	ENSGALG00010007925	STK17A	case	control	-0.094302063	0.593487941	-0.158894657	0.873751871	0.974115968	down_in_case
CRA004775	ENSGALG00010007926	EIF3E	case	control	-0.151314784	0.196505583	-0.770027911	0.441283336	0.829353567	down_in_case
CRA004775	ENSGALG00010007927	DLL1	case	control	0.332243734	0.390696227	0.850388896	0.395108907	0.808998414	up_in_case
CRA004775	ENSGALG00010007929	LRRC14B	case	control	-0.18781207	0.601475867	-0.312252046	0.754848981	0.941900693	down_in_case
CRA004775	ENSGALG00010007930	CBFA2T3	case	control	-0.097566659	0.307930122	-0.316846752	0.751359888	0.941495694	down_in_case
CRA004775	ENSGALG00010007931	GBE1	case	control	0.660914249	0.211251999	3.128558562	0.00175666	0.084784105	up_in_case
CRA004775	ENSGALG00010007932	NPY5R	case	control	0.16368714	0.418055701	0.391543852	0.695395281	0.929017829	up_in_case
CRA004775	ENSGALG00010007935	LARP1B	case	control	0.192181039	0.234337481	0.820103717	0.412156983	0.817006008	up_in_case
CRA004775	ENSGALG00010007936	ENSGALG00010007936	case	control	1.006723056	0.595249972	1.69126099	0.090786967	0.520388321	up_in_case
CRA004775	ENSGALG00010007937	ANGPTL5	case	control	-1.165674144	0.940255793	-1.239741518	0.215071016	0.683649824	down_in_case
CRA004775	ENSGALG00010007938	BCL9	case	control	-0.059530247	0.2499221	-0.238195211	0.811729691	0.957566199	down_in_case
CRA004775	ENSGALG00010007940	GLIPR2	case	control	0.109198011	0.427259718	0.255577595	0.79827702	0.952835572	up_in_case
CRA004775	ENSGALG00010007941	MRPL19	case	control	-0.065069733	0.176487963	-0.368692183	0.712357176	0.932916526	down_in_case
CRA004775	ENSGALG00010007942	PGRMC2	case	control	-0.259456736	0.182175567	-1.424212587	0.154384942	0.619284425	down_in_case
CRA004775	ENSGALG00010007943	BLOC1S4	case	control	-0.140488357	0.308888505	-0.454818989	0.649239468	0.915047477	down_in_case
CRA004775	ENSGALG00010007944	ACSF3	case	control	-0.454580844	0.380302042	-1.195315287	0.231963869	0.698947772	down_in_case
CRA004775	ENSGALG00010007945	ENSGALG00010007945	case	control	0.21951234	0.372588766	0.589154478	0.555757651	0.87981923	up_in_case
CRA004775	ENSGALG00010007946	ENSGALG00010007946	case	control	-0.422197357	0.137310128	-3.07477214	0.002106634	0.092255823	down_in_case
CRA004775	ENSGALG00010007948	ENSGALG00010007948	case	control	-1.703031146	0.675084766	-2.522692309	0.011646025	0.216997958	down_in_case
CRA004775	ENSGALG00010007950	ARC	case	control	-1.561993934	0.86690032	-1.801814923	0.071574529	0.471598108	down_in_case
CRA004775	ENSGALG00010007951	TGFBR1	case	control	-0.119888094	0.347580157	-0.344922146	0.730152901	0.936393482	down_in_case
CRA004775	ENSGALG00010007952	EMC2	case	control	0.058098651	0.271332414	0.214123519	0.830450736	0.963232966	up_in_case
CRA004775	ENSGALG00010007954	PSCA	case	control	2.285632023	0.620742053	3.682096313	0.000231324	0.030954849	up_in_case
CRA004775	ENSGALG00010007955	TMA16	case	control	0.044687749	0.328464468	0.136050481	0.891781374	0.977819591	up_in_case
CRA004775	ENSGALG00010007956	HS1BP3	case	control	-0.121187637	0.27563887	-0.439660914	0.660182716	0.917907174	down_in_case
CRA004775	ENSGALG00010007957	CDH15	case	control	-0.113417613	0.581144103	-0.195162633	0.845265631	0.966993336	down_in_case
CRA004775	ENSGALG00010007958	ENSGALG00010007958	case	control	-0.817522517	0.713716254	-1.145444723	0.252024981	0.716772599	down_in_case
CRA004775	ENSGALG00010007960	TSNAX	case	control	-0.197210863	0.210305521	-0.937735069	0.348380577	0.785270866	down_in_case
CRA004775	ENSGALG00010007962	ADCY8	case	control	-0.643357716	0.687005346	-0.936466827	0.349032884	0.785311667	down_in_case
CRA004775	ENSGALG00010007963	LDAH	case	control	-0.178838614	0.347752321	-0.51427008	0.607063177	0.900898526	down_in_case
CRA004775	ENSGALG00010007964	RECQL4	case	control	0.511776624	0.500277491	1.02298551	0.306314697	0.7585475	up_in_case
CRA004775	ENSGALG00010007965	EGLN1	case	control	0.458536211	0.366773157	1.250190212	0.211230072	0.681597647	up_in_case
CRA004775	ENSGALG00010007966	TMEM74	case	control	-0.638627466	0.591994226	-1.078773134	0.280688874	0.737061584	down_in_case
CRA004775	ENSGALG00010007968	SLC22A31	case	control	0.384593028	0.604776495	0.635925885	0.52482473	0.867302346	up_in_case
CRA004775	ENSGALG00010007970	SPRTN	case	control	0.02479237	0.307083513	0.080734944	0.935652746	0.986264424	up_in_case
CRA004775	ENSGALG00010007972	EXOC8	case	control	0.385783963	0.228337706	1.689532448	0.091117439	0.521441551	up_in_case
CRA004775	ENSGALG00010007973	CARMIL1	case	control	0.598617586	0.250283049	2.3917624	0.016767693	0.255907381	up_in_case
CRA004775	ENSGALG00010007975	ANKRD11	case	control	0.299123906	0.269984326	1.107930636	0.267891774	0.727528758	up_in_case
CRA004775	ENSGALG00010007976	ENSGALG00010007976	case	control	-0.055621885	0.478213055	-0.116311933	0.907405329	0.980354087	down_in_case
CRA004775	ENSGALG00010007977	APOB	case	control	2.324881752	1.27840203	1.818584214	0.068974888	0.466998367	up_in_case
CRA004775	ENSGALG00010007978	JADE1	case	control	0.318252532	0.288859252	1.101756406	0.270567587	0.728665115	up_in_case
CRA004775	ENSGALG00010007979	RAB30	case	control	0.126042474	0.222472122	0.566554015	0.571017225	0.885321726	up_in_case
CRA004775	ENSGALG00010007980	MARCHF1	case	control	-0.350666897	0.408754924	-0.857890332	0.390953023	0.806479902	down_in_case
CRA004775	ENSGALG00010007982	RECK	case	control	0.651567842	0.253473724	2.57055379	0.010153606	0.202058223	up_in_case
CRA004775	ENSGALG00010007983	RCHY1	case	control	-0.211561853	0.253945243	-0.83310028	0.404788175	0.81313407	down_in_case
CRA004775	ENSGALG00010007984	EIF3H	case	control	0.023380508	0.169067059	0.138291326	0.890010181	0.977283345	up_in_case
CRA004775	ENSGALG00010007986	MYC	case	control	0.030212409	0.400431513	0.075449629	0.939856973	0.987076872	up_in_case
CRA004775	ENSGALG00010007987	ENSGALG00010007987	case	control	-0.020135583	0.321162444	-0.062695946	0.950008626	0.990168609	down_in_case
CRA004775	ENSGALG00010007988	TARBP1	case	control	-0.515506706	0.219641704	-2.347034719	0.018923491	0.272278915	down_in_case
CRA004775	ENSGALG00010007989	SPG7	case	control	-0.064968956	0.226675352	-0.286616762	0.774405778	0.946319184	down_in_case
CRA004775	ENSGALG00010007991	PCF11	case	control	0.415476682	0.232159532	1.789617162	0.073515478	0.478490829	up_in_case
CRA004775	ENSGALG00010007992	LAPTM4A	case	control	-0.267042358	0.20446633	-1.306045641	0.191537037	0.656959697	down_in_case
CRA004775	ENSGALG00010007993	LRRC14	case	control	-0.307463971	0.313134032	-0.981892542	0.326152791	0.770998457	down_in_case
CRA004775	ENSGALG00010007994	FAM65B	case	control	-0.113012048	0.376517041	-0.300151217	0.764061814	0.94239774	down_in_case
CRA004775	ENSGALG00010007996	NUDCD1	case	control	0.084431473	0.256310951	0.329410321	0.741845568	0.938877854	up_in_case
CRA004775	ENSGALG00010007997	TMEM192	case	control	-0.214539803	0.314161846	-0.682895791	0.494672694	0.856382445	down_in_case
CRA004775	ENSGALG00010007998	RPL13	case	control	-0.511638202	0.243772044	-2.09883871	0.035831121	0.359120808	down_in_case
CRA004775	ENSGALG00010007999	SDC1	case	control	-0.057006613	0.457039218	-0.124730244	0.900737113	0.979145745	down_in_case
CRA004775	ENSGALG00010008000	C8orf82	case	control	-0.362814728	0.213554799	-1.698930344	0.089332309	0.515997712	down_in_case
CRA004775	ENSGALG00010008001	ALDH5A1	case	control	0.14657001	0.303540648	0.482867818	0.629189594	0.908586927	up_in_case
CRA004775	ENSGALG00010008002	GJA8	case	control	0.471417717	0.489018061	0.964008805	0.335041511	0.775615458	up_in_case
CRA004775	ENSGALG00010008004	ENY2	case	control	-0.256573742	0.283972568	-0.903515941	0.366252132	0.793608387	down_in_case
CRA004775	ENSGALG00010008006	CPNE7	case	control	0.021936101	1.138791827	0.019262609	0.984631612	0.997562105	up_in_case
CRA004775	ENSGALG00010008007	CBR4	case	control	-0.074670307	0.255800589	-0.291908268	0.770356762	0.945754599	down_in_case
CRA004775	ENSGALG00010008008	BTC	case	control	-0.412770827	0.443892365	-0.929889449	0.352428326	0.786426886	down_in_case
CRA004775	ENSGALG00010008010	CERK	case	control	0.364353241	0.237107081	1.536661154	0.124376309	0.576634733	up_in_case
CRA004775	ENSGALG00010008011	ADNP2	case	control	-0.098157827	0.306158383	-0.320611266	0.748504999	0.940917938	down_in_case
CRA004775	ENSGALG00010008012	CCDC90B	case	control	0.01403277	0.223088007	0.062902394	0.949844228	0.990168609	up_in_case
CRA004775	ENSGALG00010008013	ENSGALG00010008013	case	control	-0.243133817	0.354371129	-0.686099394	0.492650433	0.855763086	down_in_case
CRA004775	ENSGALG00010008015	ARHGAP39	case	control	-0.150482629	0.408595918	-0.368292052	0.712655479	0.932981748	down_in_case
CRA004775	ENSGALG00010008016	GSR	case	control	-0.573129678	0.271954534	-2.107446673	0.03507888	0.354683583	down_in_case
CRA004775	ENSGALG00010008017	GMNN	case	control	-0.279861158	0.404040018	-0.692657028	0.488524813	0.854017887	down_in_case
CRA004775	ENSGALG00010008018	ENSGALG00010008018	case	control	-0.691460174	0.695605902	-0.994040119	0.320203336	0.767037518	down_in_case
CRA004775	ENSGALG00010008019	PPP2CB	case	control	-0.251552983	0.158625403	-1.585830379	0.112777792	0.562886277	down_in_case
CRA004775	ENSGALG00010008020	COA6	case	control	-0.060201155	0.29118611	-0.206744597	0.836209326	0.965207162	down_in_case
CRA004775	ENSGALG00010008022	SLC35F3	case	control	-0.07688439	0.656628336	-0.117089662	0.906789002	0.980273095	down_in_case
CRA004775	ENSGALG00010008023	ATAD2	case	control	-0.38970723	0.468576408	-0.83168342	0.405587662	0.813707034	down_in_case
CRA004775	ENSGALG00010008024	MRS2	case	control	-0.037447722	0.224738516	-0.166627968	0.867662786	0.973120718	down_in_case
CRA004775	ENSGALG00010008025	TBC1D22A	case	control	0.251321714	0.123438423	2.036008795	0.041749472	0.382986652	up_in_case
CRA004775	ENSGALG00010008026	PURG	case	control	0.384241839	0.321980302	1.193370638	0.232724249	0.699801257	up_in_case
CRA004775	ENSGALG00010008027	C6orf62	case	control	0.279505709	0.257814898	1.084133273	0.278305712	0.733841198	up_in_case
CRA004775	ENSGALG00010008028	KCNK1	case	control	0.107904521	0.400125074	0.269676979	0.787408772	0.95041797	up_in_case
CRA004775	ENSGALG00010008029	LY6E	case	control	0.176619053	0.275458782	0.641181419	0.52140482	0.866299649	up_in_case
CRA004775	ENSGALG00010008030	GJA5	case	control	0.157129822	0.362003806	0.434055717	0.664247981	0.918366578	up_in_case
CRA004775	ENSGALG00010008031	MAP3K21	case	control	-0.723360031	0.645437153	-1.12072884	0.262403302	0.723230258	down_in_case
CRA004775	ENSGALG00010008032	PUM2	case	control	0.090820713	0.295976494	0.3068511	0.758956711	0.942236668	up_in_case
CRA004775	ENSGALG00010008033	ACOT13	case	control	0.248422931	0.308852097	0.804342704	0.421199081	0.819803052	up_in_case
CRA004775	ENSGALG00010008034	DPEP1	case	control	1.518348367	0.549124531	2.76503467	0.005691675	0.152268906	up_in_case
CRA004775	ENSGALG00010008035	TDP2	case	control	0.102877297	0.205680711	0.500179605	0.616948618	0.90367069	up_in_case
CRA004775	ENSGALG00010008036	SPOCK3	case	control	0.575442966	0.423003066	1.360375402	0.173711158	0.639185614	up_in_case
CRA004775	ENSGALG00010008037	ENSGALG00010008037	case	control	6.631917796	1.245645094	5.32408294	1.01464E-07	9.26853E-05	up_in_case

CRA004775	ENSGALG00010008038	WRN	case	control	-0.085533516	0.279533225	-0.305986939	0.759614591	0.942236668	down_in_case
CRA004775	ENSGALG00010008039	CHMP1A	case	control	-0.602951011	0.192730583	-3.128465654	0.001757215	0.084784105	down_in_case
CRA004775	ENSGALG00010008041	ENSGALG00010008041	case	control	0.38085819	0.62089599	0.613400949	0.539611259	0.874095231	up_in_case
CRA004775	ENSGALG00010008042	ENSGALG00010008042	case	control	1.631931598	0.788905626	2.068601801	0.038583469	0.370251617	up_in_case
CRA004775	ENSGALG00010008043	IL17REL	case	control	1.368475081	0.681533441	2.007935339	0.044650163	0.393998162	up_in_case
CRA004775	ENSGALG00010008046	RHOB	case	control	0.281669159	0.255138698	1.103984463	0.269599877	0.727898624	up_in_case
CRA004775	ENSGALG00010008047	ASAP1	case	control	0.110612717	0.518385808	0.213379138	0.831031251	0.963284108	up_in_case
CRA004775	ENSGALG00010008048	ENSGALG00010008048	case	control	3.101716575	1.257490993	2.466591485	0.013640588	0.234005339	up_in_case
CRA004775	ENSGALG00010008050	CDK10	case	control	-0.598902505	0.22894857	-2.615882269	0.008899724	0.190031214	down_in_case
CRA004775	ENSGALG00010008051	ENSGALG00010008051	case	control	-0.081712989	0.194170221	-0.420831725	0.673877963	0.922518087	down_in_case
CRA004775	ENSGALG00010008052	RBFA	case	control	0.311356089	0.335850539	0.92706741	0.353891536	0.78730441	up_in_case
CRA004775	ENSGALG00010008056	GTF2E2	case	control	-0.32067858	0.255858106	-1.2533454	0.210080033	0.67982426	down_in_case
CRA004775	ENSGALG00010008057	BAG1	case	control	-0.004967162	0.217472054	-0.02284046	0.981777534	0.997069916	down_in_case
CRA004775	ENSGALG00010008058	USP25	case	control	0.765324728	0.362674678	2.110223774	0.034839086	0.354006537	up_in_case
CRA004775	ENSGALG00010008059	ENSGALG00010008059	case	control	-0.086669784	0.203052982	-0.426833348	0.669500715	0.920814596	down_in_case
CRA004775	ENSGALG00010008060	ENSGALG00010008060	case	control	2.346027681	0.954867033	2.456915571	0.014013562	0.236876171	up_in_case
CRA004775	ENSGALG00010008062	VPS9D1	case	control	-0.365167848	0.307122008	-1.188999283	0.234439949	0.701890647	down_in_case
CRA004775	ENSGALG00010008063	KCNV1	case	control	1.102649459	0.831940922	1.325393943	0.185040525	0.6491093	up_in_case
CRA004775	ENSGALG00010008064	DDX60	case	control	1.301754595	0.638646096	2.038303536	0.041519586	0.381307727	up_in_case
CRA004775	ENSGALG00010008065	CHMP5	case	control	-0.034602436	0.235421058	-0.146981055	0.883146974	0.975590781	down_in_case
CRA004775	ENSGALG00010008066	PCNX2	case	control	-1.330280112	0.732945363	-1.814978549	0.069527206	0.468451304	down_in_case
CRA004775	ENSGALG00010008067	MPC2	case	control	-0.026271868	0.366399455	-0.071702804	0.942838425	0.987862092	down_in_case
CRA004775	ENSGALG00010008069	DLG2	case	control	-0.552012469	0.379273603	-1.455446582	0.145545668	0.60888302	down_in_case
CRA004775	ENSGALG00010008071	SMIM18	case	control	-0.486634785	0.350955008	-1.386601626	0.165563272	0.630194031	down_in_case
CRA004775	ENSGALG00010008072	NRSN1	case	control	-0.57231274	0.504150512	-1.13520214	0.256290633	0.719236059	down_in_case
CRA004775	ENSGALG00010008073	ZNF276	case	control	-0.204265959	0.134255255	-1.521474586	0.128140784	0.5821156	down_in_case
CRA004775	ENSGALG00010008074	RHAG	case	control	-0.661047084	0.59584951	-1.109419531	0.267249241	0.727315996	down_in_case
CRA004775	ENSGALG00010008075	SDHA	case	control	0.026545053	0.297291294	0.089289708	0.928851673	0.98473447	up_in_case
CRA004775	ENSGALG00010008076	ENSGALG00010008076	case	control	2.510842389	1.484799864	1.691030859	0.090830909	0.520423263	up_in_case
CRA004775	ENSGALG00010008078	GUCY1B1	case	control	0.348895298	0.29715201	1.174130701	0.240342678	0.70694164	up_in_case
CRA004775	ENSGALG00010008079	CCDC127	case	control	0.378521825	0.188811647	2.004758876	0.044988831	0.394778076	up_in_case
CRA004775	ENSGALG00010008081	TOP1MT	case	control	-0.264385026	0.271310444	-0.974474191	0.329821148	0.77237154	down_in_case
CRA004775	ENSGALG00010008083	FANCA	case	control	0.223218349	0.515689896	0.432853835	0.66512096	0.918815167	up_in_case
CRA004775	ENSGALG00010008084	CYP2AC1	case	control	1.859916469	0.768954398	2.418760429	0.015573493	0.249884603	up_in_case
CRA004775	ENSGALG00010008086	RHPN1	case	control	-0.070457575	0.523241678	-0.134655891	0.892883949	0.977834811	down_in_case
CRA004775	ENSGALG00010008087	CPNE8	case	control	0.735536207	0.389855351	1.886690039	0.059202028	0.44414236	up_in_case
CRA004775	ENSGALG00010008088	SLC35A4	case	control	-0.475900319	0.277570828	-1.714518496	0.086433543	0.508360843	down_in_case
CRA004775	ENSGALG00010008091	ENSGALG00010008091	case	control	1.80297841	0.577407381	3.122541327	0.001792969	0.085287202	up_in_case
CRA004775	ENSGALG00010008092	SPIRE2	case	control	0.760555868	0.333217405	2.282461406	0.022462114	0.292594999	up_in_case
CRA004775	ENSGALG00010008093	EFR3A	case	control	0.088535831	0.239059678	0.37035033	0.711121478	0.932234469	up_in_case
CRA004775	ENSGALG00010008094	PIM3	case	control	-0.324749474	0.28900407	-1.123684777	0.261146777	0.721774856	down_in_case
CRA004775	ENSGALG00010008096	CPSF1	case	control	-0.292174081	0.210337403	-1.389073351	0.164810446	0.630194031	down_in_case
CRA004775	ENSGALG00010008098	ENSGALG00010008098	case	control	0.112127549	0.288275323	0.38895993	0.697305788	0.929092636	up_in_case
CRA004775	ENSGALG00010008099	PANK3	case	control	0.462580358	0.31288329	1.478443793	0.139289031	0.599104916	up_in_case
CRA004775	ENSGALG00010008100	ENSGALG00010008100	case	control	-0.860723436	0.448620683	-1.918599537	0.055035034	0.430875458	down_in_case
CRA004775	ENSGALG00010008101	CENPQ	case	control	-0.078843147	0.309912794	-0.2544043	0.79918323	0.952835572	down_in_case
CRA004775	ENSGALG00010008103	RBPMS	case	control	0.094866947	0.263082042	0.360598339	0.718399731	0.934011578	up_in_case
CRA004775	ENSGALG00010008104	TCF25	case	control	-0.069825246	0.214844669	-0.325003394	0.745178503	0.939384561	down_in_case
CRA004775	ENSGALG00010008105	MMUT	case	control	0.132192569	0.23646181	0.559044052	0.576131656	0.887937128	up_in_case
CRA004775	ENSGALG00010008106	ENSGALG00010008106	case	control	-1.11569126	0.564131552	-1.977714694	0.047960902	0.406854015	down_in_case
CRA004775	ENSGALG00010008107	TUBB3	case	control	-0.561215031	0.243766531	-2.302264504	0.02132026	0.286863893	down_in_case
CRA004775	ENSGALG00010008108	ENSGALG00010008108	case	control	-0.02516249	0.41748311	-0.060271875	0.951939102	0.990168609	down_in_case
CRA004775	ENSGALG00010008110	DEF8	case	control	-0.210497547	0.380019948	-0.553911836	0.579639185	0.888438428	down_in_case
CRA004775	ENSGALG00010008111	SYTL2	case	control	-0.434737571	0.657499039	-0.661198793	0.508484835	0.862971569	down_in_case
CRA004775	ENSGALG00010008113	DBNDD1	case	control	-0.040112749	0.247805551	-0.161871876	0.87140675	0.973918682	down_in_case
CRA004775	ENSGALG00010008115	ENSGALG00010008115	case	control	-0.709170498	0.375381696	-1.889198397	0.058865249	0.44414236	down_in_case
CRA004775	ENSGALG00010008116	URAH	case	control	1.232574445	0.69746116	1.767230228	0.077189679	0.488700005	up_in_case
CRA004775	ENSGALG00010008117	ENSGALG00010008117	case	control	0.087309378	0.54603156	0.159898044	0.87296139	0.97407675	up_in_case
CRA004775	ENSGALG00010008118	ENSGALG00010008118	case	control	0.298324611	0.4339446	0.687471654	0.491785558	0.855746361	up_in_case
CRA004775	ENSGALG00010008119	CCDC83	case	control	-0.361115244	1.040650508	-0.347009146	0.728584448	0.935823872	down_in_case
CRA004775	ENSGALG00010008120	CDC5L	case	control	0.017133231	0.171007776	0.100189775	0.920193664	0.982421046	up_in_case
CRA004775	ENSGALG00010008121	MPZL1	case	control	-0.400620889	0.341286583	-1.173854785	0.240453196	0.70711546	down_in_case
CRA004775	ENSGALG00010008123	ENSGALG00010008123	case	control	-0.649325113	0.275206595	-2.359409709	0.018304035	0.269196555	down_in_case
CRA004775	ENSGALG00010008124	ENSGALG00010008124	case	control	-0.2305287	0.429964908	-0.536157012	0.591850041	0.893804699	down_in_case
CRA004775	ENSGALG00010008126	CDH3	case	control	1.898037267	0.356083215	5.330319399	9.80402E-08	9.26853E-05	up_in_case
CRA004775	ENSGALG00010008127	TBC1D2	case	control	-0.185809746	0.362235008	-0.512953586	0.607983788	0.900943922	down_in_case
CRA004775	ENSGALG00010008128	RARS	case	control	-0.034824646	0.195339798	-0.178277268	0.85850523	0.971262956	down_in_case
CRA004775	ENSGALG00010008129	TANGO6	case	control	-0.13307321	0.302197404	-0.440351929	0.659682236	0.917907174	down_in_case
CRA004775	ENSGALG00010008130	RCSD1	case	control	-0.182312939	0.343130202	-0.531322914	0.595195029	0.895305942	down_in_case
CRA004775	ENSGALG00010008132	TDO2	case	control	0.45506065	0.574530275	0.792056867	0.428327519	0.823075979	up_in_case
CRA004775	ENSGALG00010008133	CREG1	case	control	-0.086786453	0.355526321	-0.244106971	0.807147974	0.955397873	down_in_case
CRA004775	ENSGALG00010008134	SLC2A13	case	control	-0.039734181	0.366396353	-0.108445897	0.913641995	0.980803023	down_in_case
CRA004775	ENSGALG00010008135	WWC1	case	control	0.089868363	0.264539862	0.339715769	0.734070585	0.936819725	up_in_case
CRA004775	ENSGALG00010008136	ELP1	case	control	-0.141687859	0.260396669	-0.544123162	0.586356723	0.891503646	down_in_case
CRA004775	ENSGALG00010008139	ENSGALG00010008139	case	control	0.369829683	0.617697732	0.598722747	0.549357786	0.877623839	up_in_case
CRA004775	ENSGALG00010008141	APTX	case	control	-0.206913956	0.256770042	-0.805833713	0.420338736	0.819388688	down_in_case
CRA004775	ENSGALG00010008142	CD247	case	control	-0.618115497	0.409700121	-1.508702256	0.131374889	0.58691881	down_in_case
CRA004775	ENSGALG00010008143	DCTN6	case	control	-0.295472902	0.318266525	-0.928381967	0.353209469	0.787065859	down_in_case
CRA004775	ENSGALG00010008144	NTPCR	case	control	-0.248904899	0.24823513	-1.002698126	0.316006535	0.765806859	down_in_case
CRA004775	ENSGALG00010008145	ENSGALG00010008145	case	control	-0.22670379	0.381549122	-0.59416672	0.552400609	0.878258849	down_in_case
CRA004775	ENSGALG00010008146	SOX4	case	control	0.273637929	0.271575383	1.007594748	0.313649051	0.765098415	up_in_case
CRA004775	ENSGALG00010008147	CDKAL1	case	control	-0.626520279	0.239725201	-2.613493601	0.008962176	0.191062954	down_in_case
CRA004775	ENSGALG00010008148	ABCD2	case	control	-0.078966205	0.227218003	-0.347534985	0.72818944	0.935650625	down_in_case
CRA004775	ENSGALG00010008149	DNAJA1	case	control	0.094198358	0.410308588	0.229579299	0.818418694	0.959941088	up_in_case
CRA004775	ENSGALG00010008150	TERT	case	control	1.292722586	0.618189349	2.091143415	0.03651521	0.360668554	up_in_case
CRA004775	ENSGALG00010008151	SIPA1L2	case	control	0.214694384	0.339857151	0.631719482	0.527570195	0.86830001	up_in_case
CRA004775	ENSGALG00010008152	ENSGALG00010008152	case	control	-0.778859887	0.461336899	-1.688267055	0.091359976	0.521441551	down_in_case
CRA004775	ENSGALG00010008153	SMU1	case	control	-0.106594877	0.152429934	-0.699304095	0.484362008	0.850851044	down_in_case
CRA004775	ENSGALG00010008154	KCNQ3	case	control	0.104608204	0.55132598	0.189739298	0.849513425	0.968413719	up_in_case
CRA004775	ENSGALG00010008155	MAP10	case	control	0.144480642	0.48175655	0.299903846	0.7642505	0.94239774	up_in_case
CRA004775	ENSGALG00010008157	CTSO	case	control	0.029909975	0.283737232	0.105414346	0.916047033	0.981626545	up_in_case
CRA004775	ENSGALG00010008158	CLPTM1L	case	control	-0.033399751	0.203611629	-0.164036559	0.869702364	0.973723922	down_in_case
CRA004775	ENSGALG00010008159	SUPT3H	case	control	-0.541784824	0.255767871	-2.118267718	0.034152401	0.35073164	down_in_case
CRA004775	ENSGALG00010008160	TXNL4A	case	control	-0.228719774	0.308479067	-0.741443419	0.458424627	0.840058241	down_in_case
CRA004775	ENSGALG00010008161	TMEFF1	case	control	-0.035489351	0.440524998	-0.080561491	0.935790692	0.986264424	down_in_case
CRA004775	ENSGALG00010008162	HSBP1L1	case	control	-0.196676599	0.358834601	-0.548098202	0.583624475	0.889906855	down_in_case
CRA004775	ENSGALG00010008163	CHTF8	case	control	-0.293334622	0.368470032	-0.796088139	0.425980808	0.822541484	down_in_case
CRA004775	ENSGALG00010008164	TG	case	control	0.685606459	0.721350783	0.95044807	0.341884628	0.781616177	up_in_case
CRA004775	ENSGALG00010008165	MRPL1	case	control	0.084159903	0.223617149	0.376357107	0.70665143	0.931565891	up_in_case
CRA004775	ENSGALG00010008166	RUNX2	case	control	-0.881652981	0.469495169	-1.877874447	0.060398343	0.445397937	down_in_case
CRA004775	ENSGALG00010008167	UTP4	case	control	-0.455380028	0.348962785	-1.304952987	0.191908857	0.656959697	down_in_case
CRA004775	ENSGALG00010008168	CAVIN4	case	control	0.302489325	0.548621547	0.551362457	0.581385231	0.888998848	up_in_case
CRA004775	ENSGALG00010008169	HTR4	case	control	1.216106857	0.648132588	1.876324197	0.060610776	0.445397937	up_in_case
CRA004775	ENSGALG00010008170	PICALM	case	control	0.351391148	0.227431098	1.545044416	0.12233552	0.57525466	up_in_case
CRA004775	ENSGALG00010008171	KIF21A	case	control	0.613482342	0.258628946	2.372055996	0.01768941	0.26439899	up_in_case
CRA004775	ENSGALG00010008172	SNTB2	case	control	-0.32107776	0.261512006	-1.22777445	0.219531649	0.687023148	down_in_case
CRA004775	ENSGALG00010008174	SLC26A2	case	control	-0.851630566	0.478415507	-1.780106526	0.075058529	0.482980734	down_in_case
CRA004775	ENSGALG00010008175	ENSGALG00010008175	case	control	0.212040367	0.938101906	0.226031272	0.821177098	0.960766569	up_in_case
CRA004775	ENSGALG00010008176	SLA	case	control	-0.988654687	0.53933381	-1.833103485	0.066787183	0.462498477	down_in_case
CRA004775	ENSGALG00010008177	NREP	case	control	-0.127059829	0.478278121	-0.265660969	0.790500326	0.951054854	down_in_case
CRA004775	ENSGALG00010008178	PGR	case	control	0.091659652	0.423613383	0.216375722	0.828694889	0.962854535	up_in_case
CRA004775	ENSGALG00010008179	SEMA6A	case	control	0.302977252	0.355150687	0.853094933	0.393606661	0.808074827	up_in_case
CRA004775	ENSGALG00010008180	POU2F1	case	control	0.43274566	0.46133587	0.938027341	0.34823036	0.785270866	up_in_case
CRA004775	ENSGALG00010008181	DISC1	case	control	-0.178048736	0.402187316	-0.442701023	0.657982003	0.917354438	down_in_case
CRA004775	ENSGALG00010008182	CLIC5	case	control	0.57611641	0.585570442	0.983855006	0.325186809	0.770309012	up_in_case
CRA004775	ENSGALG00010008183	ALG10	case	control	-0.315407451	0.306285862	-1.029781295	0.303112683	0.755679718	down_in_case
CRA004775	ENSGALG00010008184	SLC66A2	case	control	-0.159847705	0.266847076	-0.599023632	0.549157125	0.877623839	down_in_case
CRA004775	ENSGALG00010008185	PDGFC	case	control	-0.217074799	0.290941086	-0.746112562	0.455599421	0.839192403	down_in_case
CRA004775	ENSGALG00010008186	NOP53	case	control	-0.3967312	0.278445037	-1.424809739	0.154212206	0.619284425	down_in_case
CRA004775	ENSGALG00010008187	ENSGALG00010008187	case	control	-0.573178371	0.476931496	-1.2018044	0.22943932	0.695782399	down_in_case
CRA004775	ENSGALG00010008188	VPS4A	case	control	-0.0214245	0.33906717	-0.063186594	0.949617919	0.990168609	down_in_case
CRA004775	ENSGALG00010008189	EXOC3	case	control	-0.058376885	0.167710195	-0.348081909	0.72777867	0.935548472	down_in_case
CRA004775	ENSGALG00010008190	TSNARE1	case	control	-0.213160904	0.290152164	-0.734652125	0.462551384	0.842456502	down_in_case
CRA004775	ENSGALG00010008191	ENSGALG00010008191	case	control	-0.230752564	0.514936286	-0.448118671	0.654067554	0.916560183	down_in_case
CRA004775	ENSGALG00010008192	HNRNPU	case	control	-0.022798514	0.187144998	-0.121822726	0.903039415	0.979310476	down_in_case
CRA004775	ENSGALG00010008193	CNOT6L	case	control	0.255750273	0.260735437	0.980880375	0.326651736	0.770998457	up_in_case

CRA004775	ENSGALG00010008194	PDF	case	control	-0.168366413	0.385839299	-0.436364086	0.662572591	0.918173506	down_in_case
CRA004775	ENSGALG00010008195	ENSGALG00010008195	case	control	1.313977595	0.61527804	2.135583442	0.032713382	0.344970569	up_in_case
CRA004775	ENSGALG00010008196	PDCD6	case	control	-0.195973271	0.190040222	-1.031219969	0.302437678	0.755164773	down_in_case
CRA004775	ENSGALG00010008197	STARD4	case	control	0.232669977	0.366409078	0.635000579	0.525428037	0.867484846	up_in_case
CRA004775	ENSGALG00010008198	GLRB	case	control	3.066462815	1.054476922	2.908041655	0.003636999	0.12600076	up_in_case
CRA004775	ENSGALG00010008199	TPRKB	case	control	0.085443347	0.294119746	0.290505306	0.771429692	0.94586979	up_in_case
CRA004775	ENSGALG00010008200	COG8	case	control	-0.191020936	0.237794663	-0.803302032	0.421800184	0.819812626	down_in_case
CRA004775	ENSGALG00010008201	ENPP4	case	control	-0.369227163	0.502892531	-0.734206894	0.462822653	0.842456502	down_in_case
CRA004775	ENSGALG00010008202	KCNG2	case	control	0.360953493	0.400344543	0.901607126	0.367265604	0.794067504	up_in_case
CRA004775	ENSGALG00010008203	CXCL13	case	control	2.404392118	0.777069577	3.09417868	0.001973586	0.088688821	up_in_case
CRA004775	ENSGALG00010008204	ENSGALG00010008204	case	control	-0.028544354	0.340362638	-0.083864534	0.933164138	0.985090297	down_in_case
CRA004775	ENSGALG00010008205	ENSGALG00010008205	case	control	0.334299476	0.248444853	1.34556813	0.178441836	0.644368088	up_in_case
CRA004775	ENSGALG00010008206	CXCL13L2	case	control	1.333858225	0.840308276	1.587343911	0.112434775	0.562828756	up_in_case
CRA004775	ENSGALG00010008207	CAMK4	case	control	-0.035549287	0.427513851	-0.083153532	0.933729461	0.985093894	down_in_case
CRA004775	ENSGALG00010008208	ILDR2	case	control	0.241372113	0.290669675	0.830400051	0.406312637	0.813842524	up_in_case
CRA004775	ENSGALG00010008209	PPARGC1B	case	control	0.485900146	0.328328016	1.47992289	0.138893826	0.598723826	up_in_case
CRA004775	ENSGALG00010008210	CTDP1	case	control	0.461153153	0.224460092	2.054499526	0.039927369	0.375888502	up_in_case
CRA004775	ENSGALG00010008211	ADGRB1	case	control	0.155494302	0.575315956	0.270276359	0.786947651	0.95041797	up_in_case
CRA004775	ENSGALG00010008212	GTSE1	case	control	-0.279905107	0.672954727	-0.415934529	0.677457912	0.922924485	down_in_case
CRA004775	ENSGALG00010008213	ISCA1	case	control	-0.042875147	0.236311963	-0.181434519	0.856026526	0.970703965	down_in_case
CRA004775	ENSGALG00010008214	CCNG2	case	control	-0.007739958	0.233917407	-0.033088424	0.973604074	0.994947809	down_in_case
CRA004775	ENSGALG00010008215	SELENOW	case	control	-0.043352273	0.511662602	-0.084728242	0.932477443	0.985044263	down_in_case
CRA004775	ENSGALG00010008216	GPA33	case	control	2.566422023	0.840995675	3.051647112	0.002275895	0.096690159	up_in_case
CRA004775	ENSGALG00010008217	ARHGEF37	case	control	-0.33493072	0.477731245	-0.701086066	0.483249305	0.850257583	down_in_case
CRA004775	ENSGALG00010008218	ARHGAP42	case	control	0.387421483	0.263799954	1.468618466	0.141936303	0.602211079	up_in_case
CRA004775	ENSGALG00010008219	NIP7	case	control	-0.55299334	0.293422129	-1.884634064	0.05947926	0.444994181	down_in_case
CRA004775	ENSGALG00010008221	ENSGALG00010008221	case	control	-0.315999973	0.336362342	-0.939462995	0.347493083	0.785013534	down_in_case
CRA004775	ENSGALG00010008222	STYXL2	case	control	0.635540891	0.601042425	1.057397722	0.290330111	0.744282131	up_in_case
CRA004775	ENSGALG00010008223	ENSGALG00010008223	case	control	-0.588569766	0.324410457	-1.814274952	0.069635406	0.468596196	down_in_case
CRA004775	ENSGALG00010008224	ENSGALG00010008224	case	control	-0.263760823	0.310578796	-0.849255734	0.395739007	0.808998414	down_in_case
CRA004775	ENSGALG00010008226	COX20	case	control	-0.099455597	0.289920181	-0.343044752	0.731564793	0.936574571	down_in_case
CRA004775	ENSGALG00010008227	DUSP1	case	control	0.758967131	0.456769699	1.661596934	0.096593619	0.530629094	up_in_case
CRA004775	ENSGALG00010008229	PIGY	case	control	0.368433943	0.278494631	1.322948099	0.185852638	0.6491093	up_in_case
CRA004775	ENSGALG00010008230	TRMU	case	control	0.005549844	0.175832604	0.031563227	0.974820369	0.995106174	up_in_case
CRA004775	ENSGALG00010008231	ZCCHC6	case	control	0.313193945	0.226840506	1.380679093	0.167377652	0.631096819	up_in_case
CRA004775	ENSGALG00010008232	PYURF	case	control	-0.300666733	0.353308966	-0.851002273	0.39476809	0.808974685	down_in_case
CRA004775	ENSGALG00010008233	CYRIB	case	control	0.099211292	0.14080654	0.704592928	0.481063601	0.848743338	up_in_case
CRA004775	ENSGALG00010008234	COMMD10	case	control	-0.422430298	0.258205862	-1.63602133	0.101835123	0.541925225	down_in_case
CRA004775	ENSGALG00010008236	ENSGALG00010008236	case	control	0.504888623	0.40725492	1.239736092	0.215073023	0.683649824	up_in_case
CRA004775	ENSGALG00010008238	NTAQ1	case	control	-0.403486862	0.335588013	-1.202327995	0.229236475	0.695782399	down_in_case
CRA004775	ENSGALG00010008240	RCAN2	case	control	0.574037806	0.495882273	1.157609048	0.247023615	0.712046495	up_in_case
CRA004775	ENSGALG00010008241	HERC3	case	control	-0.214192534	0.184982111	-1.157909448	0.246900991	0.712046495	down_in_case
CRA004775	ENSGALG00010008243	YTHDC2	case	control	-0.166105623	0.300500388	-0.552763422	0.580425418	0.888718398	down_in_case
CRA004775	ENSGALG00010008245	FBXO32	case	control	-0.127209355	0.713061729	-0.178398798	0.858409793	0.971262956	down_in_case
CRA004775	ENSGALG00010008246	TERF2	case	control	-0.327409989	0.134147357	-2.440674166	0.014659875	0.241689368	down_in_case
CRA004775	ENSGALG00010008247	GASK1B	case	control	0.370823411	0.446330606	0.830826759	0.406071504	0.813842524	up_in_case
CRA004775	ENSGALG00010008248	TPK1	case	control	0.177492981	0.288037275	0.616215316	0.537752421	0.872603995	up_in_case
CRA004775	ENSGALG00010008250	KLHL38	case	control	-0.689961735	1.004478624	-0.686885434	0.492154927	0.855746361	down_in_case
CRA004775	ENSGALG00010008252	ZFP92	case	control	-0.45131011	0.250852743	-1.799103744	0.072002271	0.473280705	down_in_case
CRA004775	ENSGALG00010008255	MSANTD3	case	control	-0.384566795	0.254669721	-1.510060922	0.13102788	0.586487428	down_in_case
CRA004775	ENSGALG00010008257	DESI2	case	control	0.362147937	0.172959143	2.09383517	0.036274666	0.360628495	up_in_case
CRA004775	ENSGALG00010008258	CCDC82	case	control	-0.089566961	0.296405787	-0.302176828	0.762517267	0.94239774	down_in_case
CRA004775	ENSGALG00010008259	MCC	case	control	-0.058697488	0.389429306	-0.150726942	0.880191119	0.974996282	down_in_case
CRA004775	ENSGALG00010008261	CRX	case	control	0.100894355	0.663021886	0.15217349	0.879050102	0.974981284	up_in_case
CRA004775	ENSGALG00010008262	CYB5B	case	control	-0.159822186	0.192748251	-0.829175801	0.407004936	0.813842524	down_in_case
CRA004775	ENSGALG00010008264	CNTNAP2	case	control	-0.442994275	0.602043939	-0.735817183	0.461841963	0.842010079	down_in_case
CRA004775	ENSGALG00010008265	ENSGALG00010008265	case	control	2.24439231	0.723271477	3.103111875	0.001914972	0.08697473	up_in_case
CRA004775	ENSGALG00010008266	ECSCR	case	control	0.03113402	0.421971174	0.073782339	0.94118358	0.987516633	up_in_case
CRA004775	ENSGALG00010008267	MAML2	case	control	-0.090550737	0.463153313	-0.195509208	0.84499433	0.966990128	down_in_case
CRA004775	ENSGALG00010008268	ENSGALG00010008268	case	control	-0.694571808	0.40475819	-1.716016686	0.086158989	0.508049851	down_in_case
CRA004775	ENSGALG00010008270	ENSGALG00010008270	case	control	-0.559299605	0.23825364	-2.347496582	0.018900047	0.272278915	down_in_case
CRA004775	ENSGALG00010008272	DCP2	case	control	-0.245624289	0.400728886	-0.612943809	0.539913495	0.874206929	down_in_case
CRA004775	ENSGALG00010008273	TMEM144	case	control	0.031970882	0.246094274	0.12991315	0.89663514	0.978697397	up_in_case
CRA004775	ENSGALG00010008274	NFAT5	case	control	-0.06842556	0.391665823	-0.174703934	0.861312277	0.97165529	down_in_case
CRA004775	ENSGALG00010008275	CELSR1	case	control	-0.281453021	0.423927179	-0.663918321	0.50674259	0.8622397	down_in_case
CRA004775	ENSGALG00010008276	DNAJC18	case	control	-0.057575104	0.230753037	-0.249509627	0.802966595	0.954005463	down_in_case
CRA004775	ENSGALG00010008277	MZB1	case	control	1.152615944	0.711337198	1.620351006	0.105156898	0.54835514	up_in_case
CRA004775	ENSGALG00010008278	CYP39A1	case	control	-0.013879281	0.243485101	-0.057002589	0.954543133	0.990670511	down_in_case
CRA004775	ENSGALG00010008279	MAPK15	case	control	-0.304013366	0.362744696	-0.83809183	0.401979131	0.811868062	down_in_case
CRA004775	ENSGALG00010008282	FAM83H	case	control	0.92117639	0.396194998	2.325058101	0.020068854	0.280454072	up_in_case
CRA004775	ENSGALG00010008283	NQO1	case	control	0.29857066	0.407943644	0.731891927	0.464234534	0.842643327	up_in_case
CRA004775	ENSGALG00010008284	BCKDHA	case	control	-0.607936604	0.371114979	-1.638135454	0.101393441	0.540830567	down_in_case
CRA004775	ENSGALG00010008285	ADSS2	case	control	-0.192605784	0.297258038	-0.647941382	0.517022864	0.864913871	down_in_case
CRA004775	ENSGALG00010008286	ANXA13	case	control	2.017456584	0.706597181	2.855172139	0.004301351	0.134122166	up_in_case
CRA004775	ENSGALG00010008289	MTMR2	case	control	-0.382545283	0.275927406	-1.386398288	0.165625319	0.630194031	down_in_case
CRA004775	ENSGALG00010008290	FAM13A	case	control	0.075037269	0.458141543	0.163786215	0.869899444	0.973806302	up_in_case
CRA004775	ENSGALG00010008291	PTGFRN	case	control	0.166604947	0.42352831	0.393373815	0.694043407	0.928504482	up_in_case
CRA004775	ENSGALG00010008292	REEP5	case	control	-0.232461539	0.211393649	-1.099661889	0.271479466	0.728910871	down_in_case
CRA004775	ENSGALG00010008293	PLA2G7	case	control	-0.002937545	0.163652549	-0.017949886	0.985678832	0.997562105	down_in_case
CRA004775	ENSGALG00010008294	NOB1	case	control	-0.314968721	0.268910165	-1.171278599	0.241486811	0.708942172	down_in_case
CRA004775	ENSGALG00010008295	DMAC2	case	control	-0.398356909	0.34562494	-1.152569919	0.249086956	0.714300782	down_in_case
CRA004775	ENSGALG00010008296	PALLD	case	control	0.694252482	0.321004875	2.162747472	0.030560608	0.333522949	up_in_case
CRA004775	ENSGALG00010008297	ENSGALG00010008297	case	control	-0.443562428	0.362517446	-1.223561606	0.221117651	0.689187837	down_in_case
CRA004775	ENSGALG00010008298	ENSGALG00010008298	case	control	0.200808954	0.208070969	0.965098376	0.33449554	0.774742828	up_in_case
CRA004775	ENSGALG00010008299	ANKRD66	case	control	0.25465243	0.538208409	0.473148368	0.636107331	0.910080596	up_in_case
CRA004775	ENSGALG00010008300	CUL1	case	control	-0.025690037	0.208888268	-0.122984585	0.902119305	0.979310476	down_in_case
CRA004775	ENSGALG00010008301	FAM91A1	case	control	0.015712176	0.327276183	0.048008921	0.961709133	0.991860454	up_in_case
CRA004775	ENSGALG00010008302	ENSGALG00010008302	case	control	0.010309613	0.312808965	0.032958175	0.973707941	0.994947809	up_in_case
CRA004775	ENSGALG00010008303	TEX10	case	control	0.055984364	0.186795773	0.299708947	0.764399173	0.94239774	up_in_case
CRA004775	ENSGALG00010008305	SLC23A1	case	control	0.943213929	0.637068675	1.480552986	0.138725731	0.598374382	up_in_case
CRA004775	ENSGALG00010008306	ENSGALG00010008306	case	control	-0.467578879	0.693158944	-0.674562282	0.499953903	0.85956977	down_in_case
CRA004775	ENSGALG00010008307	ENSGALG00010008307	case	control	2.402320615	0.923913359	2.600157895	0.009318088	0.195703177	up_in_case
CRA004775	ENSGALG00010008308	WWP2	case	control	-0.366841596	0.226009352	-1.623125738	0.104562529	0.547421908	down_in_case
CRA004775	ENSGALG00010008309	ENSGALG00010008309	case	control	0.404766007	0.4810693	0.841388147	0.400130523	0.811658978	up_in_case
CRA004775	ENSGALG00010008310	EXOSC5	case	control	-0.267228778	0.27756541	-0.962759652	0.335668153	0.776282001	down_in_case
CRA004775	ENSGALG00010008311	SRP19	case	control	-0.085161254	0.282405465	-0.301556678	0.762990037	0.94239774	down_in_case
CRA004775	ENSGALG00010008312	ZBTB18	case	control	0.78499129	0.337580687	2.325344193	0.020053563	0.280454072	up_in_case
CRA004775	ENSGALG00010008313	PSMD7	case	control	0.140690753	0.248796982	0.565484163	0.571744493	0.885500402	up_in_case
CRA004775	ENSGALG00010008314	INVS	case	control	0.142619386	0.218099587	0.653918643	0.513164222	0.863903061	up_in_case
CRA004775	ENSGALG00010008316	ADGRF5	case	control	0.695576919	0.378377545	1.838314478	0.066016079	0.461115298	up_in_case
CRA004775	ENSGALG00010008317	CEP57	case	control	-0.407969248	0.273561272	-1.49132677	0.135875724	0.594283423	down_in_case
CRA004775	ENSGALG00010008318	ADPRH	case	control	-0.303383532	0.228028948	-1.330460606	0.183366558	0.647806087	down_in_case
CRA004775	ENSGALG00010008319	ERP44	case	control	-0.085576154	0.185845359	-0.460469686	0.645179125	0.914043203	down_in_case
CRA004775	ENSGALG00010008320	GPRIN3	case	control	-0.449262126	0.484296467	-0.927659308	0.353584322	0.78730441	down_in_case
CRA004775	ENSGALG00010008321	ZFHX3	case	control	-0.2745326	0.409144665	-0.670991517	0.502225934	0.860091011	down_in_case
CRA004775	ENSGALG00010008323	AKT3	case	control	0.155751822	0.277866098	0.560528338	0.575119116	0.887494441	up_in_case
CRA004775	ENSGALG00010008324	SCRIB	case	control	-0.103295776	0.372781031	-0.277095044	0.781707127	0.948910196	down_in_case
CRA004775	ENSGALG00010008325	AADAT	case	control	-0.456801542	0.366797066	-1.245379487	0.21299229	0.682777582	down_in_case
CRA004775	ENSGALG00010008327	TGFB1	case	control	-0.182699536	0.216012601	-0.845781848	0.397674454	0.809315551	down_in_case
CRA004775	ENSGALG00010008330	EZH2	case	control	-0.154813533	0.441332426	-0.350786672	0.725748397	0.935238044	down_in_case
CRA004775	ENSGALG00010008331	CCDC97	case	control	-0.354735945	0.305406529	-1.161520501	0.245430291	0.712046495	down_in_case
CRA004775	ENSGALG00010008332	DHX38	case	control	-0.354754828	0.15057187	-2.356049828	0.01847044	0.269821857	down_in_case
CRA004775	ENSGALG00010008333	APC	case	control	0.288977013	0.328553653	0.87954284	0.379107016	0.800168929	up_in_case
CRA004775	ENSGALG00010008334	STX17	case	control	-0.258091909	0.258389576	-0.998847991	0.317868334	0.766440025	down_in_case
CRA004775	ENSGALG00010008335	HNRNPUL1	case	control	-0.376212639	0.14361207	-2.619644991	0.008802135	0.189418079	down_in_case
CRA004775	ENSGALG00010008336	PAIP2B	case	control	-0.025822879	0.232555621	-0.111039583	0.911584958	0.98075825	down_in_case
CRA004775	ENSGALG00010008339	FAM76B	case	control	-0.119224304	0.237369846	-0.502272322	0.615475976	0.902802125	down_in_case
CRA004775	ENSGALG00010008340	PDIA4	case	control	0.175659434	0.258934924	0.678392206	0.497523049	0.857810713	up_in_case
CRA004775	ENSGALG00010008341	DHODH	case	control	-0.40943178	0.222968399	-1.836277169	0.066316675	0.462498477	down_in_case
CRA004775	ENSGALG00010008342	PLD3	case	control	-0.307072184	0.193719878	-1.58513513	0.112935635	0.562886277	down_in_case
CRA004775	ENSGALG00010008343	TNFRSF21	case	control	0.339797121	0.244080554	1.39215155	0.163876511	0.629717526	up_in_case
CRA004775	ENSGALG00010008344	MFAP3L	case	control	0.511624098	0.699023504	0.731912583	0.464221926	0.842643327	up_in_case
CRA004775	ENSGALG00010008346	NR4A3	case	control	0.510368289	0.83951104	0.607935173	0.543230471	0.875456081	up_in_case
CRA004775	ENSGALG00010008347	PUF60	case	control	-0.234197759	0.131021769	-1.787472125	0.073861213	0.479688056	down_in_case
CRA004775	ENSGALG00010008348	ENSGALG00010008348	case	control	0.392020516	0.270078901	1.451503672	0.146639661	0.609934051	up_in_case
CRA004775	ENSGALG00010008351	IST1	case	control	-0.27604522	0.154335167	-1.788608687	0.073677858	0.479028001	down_in_case

CRA004775	ENSGALG00010008352	ENSGALG00010008352	case	control	-0.650060235	0.286374979	-2.269961698	0.023209907	0.296238434	down_in_case
CRA004775	ENSGALG00010008354	TMEM71	case	control	-0.085668261	0.878578481	-0.097507807	0.922323135	0.9830428	down_in_case
CRA004775	ENSGALG00010008355	MAFA	case	control	0.881862008	0.619043005	1.424556938	0.154285315	0.619284425	up_in_case
CRA004775	ENSGALG00010008356	ENSGALG00010008356	case	control	0.079967091	0.620879736	0.128796427	0.897518733	0.978790377	up_in_case
CRA004775	ENSGALG00010008358	HPF1	case	control	-0.398348268	0.282982299	-1.407679101	0.159226113	0.624907945	down_in_case
CRA004775	ENSGALG00010008360	ZC3H3	case	control	-0.368430791	0.176206155	-2.090907617	0.036536346	0.360668554	down_in_case
CRA004775	ENSGALG00010008361	MATR3	case	control	0.122983532	0.159488142	0.771113955	0.44063939	0.829353567	up_in_case
CRA004775	ENSGALG00010008362	RAMP3	case	control	0.342988976	0.634683594	0.540409393	0.588914731	0.893412703	up_in_case
CRA004775	ENSGALG00010008366	RPRD1A	case	control	-0.287587233	0.253977333	-1.132334249	0.257493936	0.719236059	down_in_case
CRA004775	ENSGALG00010008368	SNCA	case	control	1.69700509	0.565475072	3.001025463	0.002690721	0.108504691	up_in_case
CRA004775	ENSGALG00010008372	CLCN3	case	control	-0.068882068	0.263438155	-0.261473392	0.793727466	0.951739309	down_in_case
CRA004775	ENSGALG00010008373	MACIR	case	control	0.2362149	0.221059987	1.068555659	0.285269938	0.741413527	up_in_case
CRA004775	ENSGALG00010008374	ZNF821	case	control	-0.227523842	0.259930323	-0.875326277	0.381396401	0.801753951	down_in_case
CRA004775	ENSGALG00010008375	ENSGALG00010008375	case	control	0.096649053	0.263955077	0.366157205	0.714247774	0.93334033	up_in_case
CRA004775	ENSGALG00010008376	MMRN1	case	control	-0.065416702	0.582951435	-0.112216383	0.910651839	0.98075825	down_in_case
CRA004775	ENSGALG00010008377	AKT2	case	control	-0.317009887	0.2915242	-1.087422201	0.276850264	0.731546499	down_in_case
CRA004775	ENSGALG00010008380	SIL1	case	control	-0.112763998	0.218848701	-0.515260074	0.606371295	0.900898526	down_in_case
CRA004775	ENSGALG00010008381	C18orf21	case	control	0.2242036	0.341503283	0.656519604	0.511489858	0.863170205	up_in_case
CRA004775	ENSGALG00010008382	ENSGALG00010008382	case	control	-0.647980952	0.325077507	-1.993312172	0.046227274	0.399541954	down_in_case
CRA004775	ENSGALG00010008385	CTNNA1	case	control	-0.018819215	0.179302911	-0.104957665	0.916409401	0.981626545	down_in_case
CRA004775	ENSGALG00010008386	SESN3	case	control	-0.514311495	0.487359314	-1.055302485	0.291287005	0.745345665	down_in_case
CRA004775	ENSGALG00010008387	PHF20L1	case	control	0.184804909	0.255381782	0.723641708	0.469285729	0.8440023	up_in_case
CRA004775	ENSGALG00010008389	GALNT1	case	control	-0.104758108	0.320601261	-0.326755135	0.743853095	0.939218065	down_in_case
CRA004775	ENSGALG00010008391	ENSGALG00010008391	case	control	-0.712344337	0.361498807	-1.970530253	0.048777631	0.409738062	down_in_case
CRA004775	ENSGALG00010008392	CD36	case	control	-0.545271785	0.545273812	-0.999996284	0.317312306	0.766199014	down_in_case
CRA004775	ENSGALG00010008394	CD2AP	case	control	0.327709835	0.245451131	1.335132715	0.181832914	0.647378665	up_in_case
CRA004775	ENSGALG00010008395	ENDOD1	case	control	-0.180159977	0.194818435	-0.924758361	0.355091614	0.788279103	down_in_case
CRA004775	ENSGALG00010008397	ATXN1L	case	control	-0.07471724	0.27124005	-0.275465367	0.78295873	0.94936527	down_in_case
CRA004775	ENSGALG00010008399	INO80C	case	control	0.237986033	0.244645744	0.972778145	0.330663577	0.77237154	up_in_case
CRA004775	ENSGALG00010008400	MAP1LC3C	case	control	0.119419668	0.58372913	0.204580621	0.837899798	0.965490877	up_in_case
CRA004775	ENSGALG00010008401	PSMD8	case	control	-0.181086785	0.336993136	-0.537360457	0.591018652	0.893804699	down_in_case
CRA004775	ENSGALG00010008405	LRATD2	case	control	0.586773688	0.30216936	1.941870244	0.05215281	0.420135271	up_in_case
CRA004775	ENSGALG00010008407	GNAI1	case	control	0.390938843	0.20039823	1.950809861	0.051079668	0.417596029	up_in_case
CRA004775	ENSGALG00010008408	SMARCA4	case	control	-0.303810097	0.15395249	-1.973401643	0.048449821	0.407981313	down_in_case
CRA004775	ENSGALG00010008409	CWC15	case	control	0.075271577	0.241825342	0.311264221	0.755599764	0.941900693	up_in_case
CRA004775	ENSGALG00010008410	NAPRT	case	control	-0.465270942	0.135815687	-3.425752598	0.000613099	0.051333788	down_in_case
CRA004775	ENSGALG00010008412	NEK1	case	control	0.160279626	0.215323891	0.744365267	0.456655526	0.839688534	up_in_case
CRA004775	ENSGALG00010008415	ENSGALG00010008415	case	control	0.184964215	0.562729031	0.328691439	0.742388926	0.938877854	up_in_case
CRA004775	ENSGALG00010008416	MSX2	case	control	0.306339615	0.831876979	0.368251103	0.712686009	0.932981748	up_in_case
CRA004775	ENSGALG00010008418	TMEM245	case	control	0.177056021	0.259483855	0.682339257	0.495024455	0.856382445	up_in_case
CRA004775	ENSGALG00010008422	EXO1	case	control	-0.2813346	0.71226598	-0.394985312	0.692853728	0.927867527	down_in_case
CRA004775	ENSGALG00010008424	PPIP5K2	case	control	0.047157109	0.315327169	0.149549781	0.881119831	0.974996282	up_in_case
CRA004775	ENSGALG00010008425	MAGI2	case	control	0.284430841	0.374503974	0.759486843	0.447561382	0.834136489	up_in_case
CRA004775	ENSGALG00010008426	AP1G1	case	control	-0.168081087	0.154180636	-1.090156916	0.275644028	0.731001618	down_in_case
CRA004775	ENSGALG00010008428	ENSGALG00010008428	case	control	-0.028339609	0.826553591	-0.034286475	0.97264871	0.994947809	down_in_case
CRA004775	ENSGALG00010008429	IGFBP3	case	control	0.509303701	0.184616405	2.75871314	0.005802945	0.154046903	up_in_case
CRA004775	ENSGALG00010008430	ENSGALG00010008430	case	control	0.107077283	0.851938559	0.125686626	0.899979989	0.978929349	up_in_case
CRA004775	ENSGALG00010008431	SH3RF1	case	control	0.378253342	0.219688387	1.721772127	0.085110806	0.505772986	up_in_case
CRA004775	ENSGALG00010008433	EBAG9	case	control	0.15276737	0.201471304	0.758258702	0.448296126	0.83429564	up_in_case
CRA004775	ENSGALG00010008437	SHKBP1	case	control	-0.318140264	0.207479474	-1.533357773	0.125187727	0.578063532	down_in_case
CRA004775	ENSGALG00010008438	SYBU	case	control	-0.138937456	0.306560687	-0.453213545	0.65039498	0.915428088	down_in_case
CRA004775	ENSGALG00010008441	PHLPP2	case	control	-0.150529565	0.285402082	-0.527429806	0.59789515	0.896490702	down_in_case
CRA004775	ENSGALG00010008443	RSFR	case	control	-0.427724208	0.331829899	-1.288986343	0.197402835	0.663190847	down_in_case
CRA004775	ENSGALG00010008444	SLC7A11	case	control	0.837729627	0.734400048	1.14069931	0.253995074	0.718204856	up_in_case
CRA004775	ENSGALG00010008447	FRRS1L	case	control	-0.471095903	0.276489989	-1.703844342	0.088410171	0.51361566	down_in_case
CRA004775	ENSGALG00010008448	TNS3	case	control	0.026825497	0.36680673	0.073132511	0.941700671	0.987577169	up_in_case
CRA004775	ENSGALG00010008449	SRA1	case	control	-0.358543694	0.34170669	-1.049273265	0.294052367	0.746438942	down_in_case
CRA004775	ENSGALG00010008450	GIN1	case	control	-0.238233315	0.359723458	-0.662267943	0.507799518	0.862748056	down_in_case
CRA004775	ENSGALG00010008451	OPN3	case	control	0.398188291	0.521940001	0.762900506	0.445522734	0.833012432	up_in_case
CRA004775	ENSGALG00010008453	AMOTL1	case	control	-0.277045893	0.463308765	-0.597972484	0.549858293	0.877623839	down_in_case
CRA004775	ENSGALG00010008455	FKBP1B	case	control	-0.230997992	0.573990454	-0.402442219	0.687358604	0.927111919	down_in_case
CRA004775	ENSGALG00010008456	NHLRC1	case	control	-0.274067761	0.330585972	-0.829036268	0.407083885	0.813842524	down_in_case
CRA004775	ENSGALG00010008457	MARVELD3	case	control	-0.150908191	0.194958414	-0.774053234	0.438899311	0.828368259	down_in_case
CRA004775	ENSGALG00010008459	EPB41L4B	case	control	1.564099045	0.461540563	3.388865837	0.000701823	0.055464216	up_in_case
CRA004775	ENSGALG00010008464	SF3B6	case	control	-0.307624858	0.251057302	-1.225317311	0.220455689	0.688504695	down_in_case
CRA004775	ENSGALG00010008468	EPPK1	case	control	0.565000114	0.583669617	0.968013578	0.333037588	0.773910112	up_in_case
CRA004775	ENSGALG00010008469	NOCT	case	control	0.099443608	0.75141602	0.132341613	0.894714094	0.977834811	up_in_case
CRA004775	ENSGALG00010008474	ENSGALG00010008474	case	control	0.369628216	0.349557018	1.057418954	0.290320425	0.744282131	up_in_case
CRA004775	ENSGALG00010008476	RNF166	case	control	-0.216065861	0.164655428	-1.312230421	0.1894424	0.654755848	down_in_case
CRA004775	ENSGALG00010008486	CTU2	case	control	-0.680658942	0.273001298	-2.493244349	0.01265817	0.226643879	down_in_case
CRA004775	ENSGALG00010008488	CCSER1	case	control	-0.366132426	0.384968009	-0.95107234	0.341567655	0.781513614	down_in_case
CRA004775	ENSGALG00010008492	ANKRD49	case	control	-0.102365478	0.297006472	-0.344657397	0.73035195	0.936393482	down_in_case
CRA004775	ENSGALG00010008497	PAM	case	control	0.432074893	0.3172982	1.361731312	0.173282696	0.63806494	up_in_case
CRA004775	ENSGALG00010008498	ENSGALG00010008498	case	control	0.083650784	0.307854332	0.271721967	0.78583581	0.95041797	up_in_case
CRA004775	ENSGALG00010008500	ENSGALG00010008500	case	control	0.298058843	0.313011775	0.952228851	0.340980932	0.780875343	up_in_case
CRA004775	ENSGALG00010008501	ENSGALG00010008501	case	control	0.393427288	0.285420939	1.378410741	0.168076509	0.63138062	up_in_case
CRA004775	ENSGALG00010008504	RNF139	case	control	0.245601604	0.268285072	0.915450128	0.359955373	0.791975934	up_in_case
CRA004775	ENSGALG00010008505	PTPN3	case	control	0.127060603	0.579577872	0.219229562	0.826471228	0.961878235	up_in_case
CRA004775	ENSGALG00010008509	MGARP	case	control	0.562686942	0.630028443	0.893113553	0.371796366	0.795818272	up_in_case
CRA004775	ENSGALG00010008510	ATOH1	case	control	2.973298375	0.909434124	3.269393898	0.001077781	0.068613766	up_in_case
CRA004775	ENSGALG00010008514	PIEZO1	case	control	-0.342817227	0.275031227	-1.246466561	0.212593158	0.682280269	down_in_case
CRA004775	ENSGALG00010008515	TAFA5	case	control	-0.204935094	0.473900225	-0.432443546	0.665419075	0.918877053	down_in_case
CRA004775	ENSGALG00010008516	MRE11	case	control	0.007511452	0.246242831	0.030504246	0.975664907	0.995255743	up_in_case
CRA004775	ENSGALG00010008522	NUDT12	case	control	-0.398502731	0.304899487	-1.30699705	0.191213713	0.656959697	down_in_case
CRA004775	ENSGALG00010008527	CHUK	case	control	-0.243717057	0.125078312	-1.948515718	0.051353286	0.418339211	down_in_case
CRA004775	ENSGALG00010008534	RAB33B	case	control	0.372675259	0.246244764	1.51343425	0.130169391	0.584671406	up_in_case
CRA004775	ENSGALG00010008542	MOCOS	case	control	0.456136046	0.346806117	1.315247984	0.188426573	0.652494034	up_in_case
CRA004775	ENSGALG00010008561	EEF1D	case	control	-0.213894705	0.310863168	-0.688067056	0.491410555	0.855746361	down_in_case
CRA004775	ENSGALG00010008580	PANX1	case	control	-0.620684435	0.417708461	-1.485927371	0.137298333	0.596242097	down_in_case
CRA004775	ENSGALG00010008588	PYCR3	case	control	-0.834382841	0.296375062	-2.815293685	0.004873268	0.142429514	down_in_case
CRA004775	ENSGALG00010008592	KIAA0513	case	control	-0.233060109	0.240340448	-0.969708227	0.332191948	0.772935571	down_in_case
CRA004775	ENSGALG00010008596	ENSGALG00010008596	case	control	-0.217929458	0.472878502	-0.460857192	0.644901066	0.914041905	down_in_case
CRA004775	ENSGALG00010008618	EIF4EBP3	case	control	0.268637398	0.236250183	1.137088633	0.255501235	0.718871765	up_in_case
CRA004775	ENSGALG00010008623	TRPS1	case	control	0.75617533	0.436016999	1.734279472	0.082868508	0.499850744	up_in_case
CRA004775	ENSGALG00010008636	AGTPBP1	case	control	0.168359744	0.379290807	0.443880371	0.657129077	0.917108353	up_in_case
CRA004775	ENSGALG00010008641	HEPHL1	case	control	-2.561781202	1.039798309	-2.463728955	0.013750005	0.234005339	down_in_case
CRA004775	ENSGALG00010008653	ENSGALG00010008653	case	control	0.350187529	0.282269779	1.240612899	0.214748785	0.683649824	up_in_case
CRA004775	ENSGALG00010008656	CCDC6	case	control	0.32164942	0.190915454	1.684774142	0.092032156	0.52187835	up_in_case
CRA004775	ENSGALG00010008658	TSTA3	case	control	-0.314857467	0.307895784	-1.022610518	0.306492034	0.7585475	down_in_case
CRA004775	ENSGALG00010008666	SLC16A9	case	control	0.294127179	0.583553471	0.504027813	0.614241833	0.902363507	up_in_case
CRA004775	ENSGALG00010008668	ENSGALG00010008668	case	control	0.042390595	0.506609469	0.083675094	0.933314759	0.985090297	up_in_case
CRA004775	ENSGALG00010008674	RXFP1	case	control	-0.385082527	0.540575201	-0.712356997	0.476243738	0.846367006	down_in_case
CRA004775	ENSGALG00010008686	ENSGALG00010008686	case	control	-1.089730173	1.023416536	-1.064796331	0.286968106	0.741909972	down_in_case
CRA004775	ENSGALG00010008688	HBEGF	case	control	0.054157383	0.586330549	0.09236664	0.926406744	0.984182984	up_in_case
CRA004775	ENSGALG00010008690	YIF1B	case	control	-0.385302391	0.188418517	-2.044928479	0.04086191	0.378415958	down_in_case
CRA004775	ENSGALG00010008693	C4orf46	case	control	-0.017993816	0.226416116	-0.079472327	0.936656942	0.98646154	down_in_case
CRA004775	ENSGALG00010008696	PFN4	case	control	-0.025947616	0.513691703	-0.05051204	0.959714355	0.991431439	down_in_case
CRA004775	ENSGALG00010008699	NAA35	case	control	-0.180930383	0.298192335	-0.606757323	0.544011974	0.875729696	down_in_case
CRA004775	ENSGALG00010008700	ENSGALG00010008700	case	control	-0.21201154	0.236874417	-0.895037728	0.37076692	0.795641647	down_in_case
CRA004775	ENSGALG00010008708	ZC3H4	case	control	-0.020709535	0.196650981	-0.105311121	0.916128939	0.981626545	down_in_case
CRA004775	ENSGALG00010008716	TATDN1	case	control	0.062415212	0.293230501	0.21285375	0.831441038	0.963521465	up_in_case
CRA004775	ENSGALG00010008729	TMEM254	case	control	-0.313282131	0.220678291	-1.419632757	0.155714624	0.619442611	down_in_case
CRA004775	ENSGALG00010008731	NDUFB9	case	control	-4.23068E-05	0.308976028	-0.000136926	0.999890749	0.999914248	down_in_case
CRA004775	ENSGALG00010008739	VSTM5	case	control	-0.520784239	0.604492952	-0.861522433	0.388950371	0.804884358	down_in_case
CRA004775	ENSGALG00010008745	GRAMD4	case	control	0.843647839	0.399506284	2.111726083	0.03470995	0.354006537	up_in_case
CRA004775	ENSGALG00010008750	PFDN1	case	control	-0.365794493	0.223255231	-1.638458779	0.101326026	0.540680709	down_in_case
CRA004775	ENSGALG00010008752	GOLM1	case	control	-0.318608516	0.275910471	-1.15475326	0.248191482	0.712818804	down_in_case
CRA004775	ENSGALG00010008756	ITSN2	case	control	-0.117421448	0.248651024	-0.472233922	0.636759829	0.910290509	down_in_case
CRA004775	ENSGALG00010008762	SAE1	case	control	-0.245688639	0.242535675	-1.013	0.311060161	0.762865752	down_in_case
CRA004775	ENSGALG00010008764	ETFDH	case	control	0.359917693	0.256546282	1.40293475	0.160636297	0.626285718	up_in_case
CRA004775	ENSGALG00010008768	PRXL2C	case	control	-0.110290647	0.361958627	-0.304705121	0.760590749	0.942236668	down_in_case
CRA004775	ENSGALG00010008775	MED17	case	control	-0.178911554	0.154510084	-1.15792801	0.246893416	0.712046495	down_in_case
CRA004775	ENSGALG00010008780	KARS1	case	control	0.122040855	0.138091041	0.883770983	0.376819853	0.798523018	up_in_case
CRA004775	ENSGALG00010008782	CCDC9	case	control	-0.110016863	0.266658325	-0.412576143	0.679917184	0.923896221	down_in_case
CRA004775	ENSGALG00010008790	GPN1	case	control	-0.11226914	0.189308892	-0.593047366	0.553149453	0.878636726	down_in_case
CRA004775	ENSGALG00010008793	C11orf54	case	control	-0.294399343	0.209340665	-1.406317036	0.159630004	0.624907945	down_in_case

CRA004775	ENSGALG00010008799	ENSGALG00010008799	case	control	0.77143998	0.395511121	1.950488723	0.051117896	0.417660244	up_in_case
CRA004775	ENSGALG00010008807	BRD1	case	control	0.27871829	0.230966414	1.206748137	0.227529155	0.694718454	up_in_case
CRA004775	ENSGALG00010008811	TERF2IP	case	control	0.125027892	0.18539991	0.674368677	0.500076951	0.85956977	up_in_case
CRA004775	ENSGALG00010008814	ENSGALG00010008814	case	control	-2.05802586	0.638037853	-3.225554487	0.001257289	0.074844075	down_in_case
CRA004775	ENSGALG00010008821	ENSGALG00010008821	case	control	-0.372646069	0.269430257	-1.383089167	0.166637527	0.630220864	down_in_case
CRA004775	ENSGALG00010008824	GREM2	case	control	-2.927379509	0.561499436	-5.213503918	1.85307E-07	0.000149891	down_in_case
CRA004775	ENSGALG00010008825	PPID	case	control	-0.146457488	0.262473102	-0.557990465	0.576850895	0.88797455	down_in_case
CRA004775	ENSGALG00010008830	DHX34	case	control	-0.462028564	0.29930282	-1.543682629	0.122665236	0.57525466	down_in_case
CRA004775	ENSGALG00010008832	MTSS1	case	control	-0.15741131	0.375431313	-0.41928125	0.675010598	0.92288678	down_in_case
CRA004775	ENSGALG00010008837	ENSGALG00010008837	case	control	-0.11590622	0.305418145	-0.379500111	0.704316522	0.930465147	down_in_case
CRA004775	ENSGALG00010008839	ZNF512	case	control	-0.04357744	0.171192891	-0.254551689	0.799069377	0.952835572	down_in_case
CRA004775	ENSGALG00010008846	SLC35D2	case	control	-0.896534664	0.669430804	-1.339249194	0.180489563	0.645561356	down_in_case
CRA004775	ENSGALG00010008850	CYSTM1	case	control	-0.103304057	0.282510825	-0.365664066	0.714615763	0.93334033	down_in_case
CRA004775	ENSGALG00010008852	KHDRBS3	case	control	0.325953516	0.359477454	0.906742586	0.364542937	0.793608387	up_in_case
CRA004775	ENSGALG00010008859	ENSGALG00010008859	case	control	-1.496154999	0.59853153	-2.49970958	0.012429515	0.224435632	down_in_case
CRA004775	ENSGALG00010008860	SQLE	case	control	0.012202248	0.575097032	0.02121772	0.983071979	0.997398088	up_in_case
CRA004775	ENSGALG00010008862	ZNF367	case	control	-0.103829193	0.369182154	-0.281241095	0.778525487	0.94792594	down_in_case
CRA004775	ENSGALG00010008864	ADAT1	case	control	-0.201433651	0.246576541	-0.816921391	0.413973352	0.817589409	down_in_case
CRA004775	ENSGALG00010008866	WASHC5	case	control	0.119215192	0.178136634	0.669234564	0.503345861	0.8610984	up_in_case
CRA004775	ENSGALG00010008871	PRR7	case	control	0.046682639	0.31743638	0.147061401	0.883083556	0.975590781	up_in_case
CRA004775	ENSGALG00010008877	FNDC4	case	control	-0.485631781	0.294129302	-1.651082627	0.098721704	0.535162165	down_in_case
CRA004775	ENSGALG00010008879	HABP4	case	control	0.051792443	0.335535887	0.154357387	0.877327951	0.974479909	up_in_case
CRA004775	ENSGALG00010008888	FBXO46	case	control	-0.008993204	0.285062262	-0.031548208	0.974832347	0.995106174	down_in_case
CRA004775	ENSGALG00010008892	ENSGALG00010008892	case	control	-0.197506173	0.212470627	-0.929569306	0.352594126	0.786426886	down_in_case
CRA004775	ENSGALG00010008893	ENSGALG00010008893	case	control	-0.137055114	0.597470953	-0.229392096	0.818564179	0.959941088	down_in_case
CRA004775	ENSGALG00010008897	NSMCE2	case	control	0.661081412	0.303047982	2.181441391	0.029150785	0.324790814	up_in_case
CRA004775	ENSGALG00010008903	ENSGALG00010008903	case	control	0.226358255	0.515537074	0.4390727	0.66060886	0.917907174	up_in_case
CRA004775	ENSGALG00010008905	GABARAPL2	case	control	-0.242959957	0.249521235	-0.973704528	0.330203268	0.77237154	down_in_case
CRA004775	ENSGALG00010008906	DBN1	case	control	-0.019905525	0.483946019	-0.041131706	0.967190898	0.993324759	down_in_case
CRA004775	ENSGALG00010008910	TRIB1	case	control	0.179789793	0.453936163	0.39606845	0.692054532	0.927867527	up_in_case
CRA004775	ENSGALG00010008918	PLAC9	case	control	-0.02957396	0.393823191	-0.075094512	0.940139514	0.987076872	down_in_case
CRA004775	ENSGALG00010008921	OPA3	case	control	0.072461025	0.36944998	0.196132166	0.84450672	0.966751844	up_in_case
CRA004775	ENSGALG00010008931	FNIP2	case	control	0.256303821	0.270275978	0.948304112	0.342974658	0.782518139	up_in_case
CRA004775	ENSGALG00010008932	VASP	case	control	0.26100489	0.426688309	0.611699183	0.540736802	0.874592283	up_in_case
CRA004775	ENSGALG00010008936	IFT172	case	control	-0.01047909	0.223923663	-0.046797598	0.962674543	0.991860454	down_in_case
CRA004775	ENSGALG00010008938	ZBED4	case	control	0.746354558	0.235667567	3.166980369	0.001540307	0.080693373	up_in_case
CRA004775	ENSGALG00010008939	RANBP9	case	control	-0.004604109	0.262831879	-0.017517315	0.98602392	0.997562105	down_in_case
CRA004775	ENSGALG00010008943	ENSGALG00010008943	case	control	-0.55261646	0.53887232	-1.025505373	0.305124791	0.757826553	down_in_case
CRA004775	ENSGALG00010008946	COL22A1	case	control	-1.777617079	0.911801499	-1.949565866	0.051227885	0.418062106	down_in_case
CRA004775	ENSGALG00010008955	KRTCAP3	case	control	1.122221769	0.395304125	2.838882011	0.004527189	0.138149936	up_in_case
CRA004775	ENSGALG00010008956	LVRN	case	control	1.284383707	0.573015434	2.241446967	0.024997137	0.304193557	up_in_case
CRA004775	ENSGALG00010008960	LRRC2	case	control	0.428720794	0.589478092	0.727288766	0.467049078	0.843719374	up_in_case
CRA004775	ENSGALG00010008966	NRBP1	case	control	0.364900389	0.250566907	1.456299211	0.145309923	0.608365494	up_in_case
CRA004775	ENSGALG00010008969	PPP1R37	case	control	-0.349455429	0.295709876	-1.181750956	0.237304533	0.703879272	down_in_case
CRA004775	ENSGALG00010008971	ANXA11	case	control	0.024953967	0.169056223	0.147607506	0.882652531	0.975436386	up_in_case
CRA004775	ENSGALG00010008974	SMCO4	case	control	0.006129194	0.323707424	0.018934363	0.984893466	0.997562105	up_in_case
CRA004775	ENSGALG00010008980	ENSGALG00010008980	case	control	-0.103543354	0.408930837	-0.253205053	0.800109762	0.952993446	down_in_case
CRA004775	ENSGALG00010008983	ENSGALG00010008983	case	control	-0.595056562	0.596939549	-0.996845599	0.318839461	0.767037518	down_in_case
CRA004775	ENSGALG00010008988	PPM1G	case	control	-0.044052692	0.124188668	-0.354723929	0.722796423	0.935185555	down_in_case
CRA004775	ENSGALG00010008991	ENSGALG00010008991	case	control	-0.254361767	0.271455147	-0.937030551	0.348742843	0.785311667	down_in_case
CRA004775	ENSGALG00010008995	ECD	case	control	0.067413587	0.213159186	0.316259358	0.751805657	0.941495694	up_in_case
CRA004775	ENSGALG00010008996	CHRM3	case	control	1.119199143	0.5068314	2.208227712	0.027228404	0.31561562	up_in_case
CRA004775	ENSGALG00010008997	CHST6	case	control	0.491859851	0.228074498	2.156575399	0.031038763	0.336379059	up_in_case
CRA004775	ENSGALG00010008999	TRAPPC9	case	control	-0.302571006	0.372484034	-0.812305973	0.416616084	0.818943344	down_in_case
CRA004775	ENSGALG00010009000	FAM149B1	case	control	-0.081374917	0.221380245	-0.367579848	0.713186544	0.933114002	down_in_case
CRA004775	ENSGALG00010009002	ENSGALG00010009002	case	control	-0.276883491	0.34459586	-0.803502083	0.421684593	0.819812626	down_in_case
CRA004775	ENSGALG00010009003	ALG12	case	control	-0.480549859	0.445617306	-1.078391373	0.280859135	0.737061584	down_in_case
CRA004775	ENSGALG00010009007	TMEM65	case	control	0.143051514	0.175260182	0.8162237	0.414372204	0.817977631	up_in_case
CRA004775	ENSGALG00010009011	ACP6	case	control	-0.061187543	0.175182067	-0.349279718	0.726879323	0.935238044	down_in_case
CRA004775	ENSGALG00010009013	LIPG	case	control	0.316516518	0.622436346	0.508512268	0.611094138	0.901413878	up_in_case
CRA004775	ENSGALG00010009015	TMEM231	case	control	0.03657061	0.146626271	0.249413762	0.803040741	0.954005463	up_in_case
CRA004775	ENSGALG00010009016	DNAJC9	case	control	0.114076184	0.31830593	0.358385357	0.720054949	0.934189603	up_in_case
CRA004775	ENSGALG00010009019	ZNF513	case	control	-0.315870965	0.289019674	-1.092904718	0.274435637	0.731001618	down_in_case
CRA004775	ENSGALG00010009020	ARHGAP35	case	control	-0.402280212	0.368990271	-1.09021902	0.275616677	0.731001618	down_in_case
CRA004775	ENSGALG00010009021	HNF4beta	case	control	1.762079863	0.640449335	2.751318122	0.005935597	0.154441997	up_in_case
CRA004775	ENSGALG00010009024	PMAIP1	case	control	-0.166471757	0.516753929	-0.322148991	0.747339825	0.940417372	down_in_case
CRA004775	ENSGALG00010009025	UTP23	case	control	0.202725993	0.266801984	0.759836905	0.44735208	0.834136489	up_in_case
CRA004775	ENSGALG00010009026	RAPGEF2	case	control	-0.108205996	0.343481345	-0.315027285	0.752740937	0.94167946	down_in_case
CRA004775	ENSGALG00010009028	PDLIM7	case	control	-0.208274443	0.271687598	-0.766595325	0.443322156	0.831667526	down_in_case
CRA004775	ENSGALG00010009029	IGSF3	case	control	0.140873159	0.488982185	0.288094666	0.773274275	0.946296525	up_in_case
CRA004775	ENSGALG00010009030	ZDHHC7	case	control	-0.043248451	0.172320491	-0.250976833	0.801832021	0.953543664	down_in_case
CRA004775	ENSGALG00010009031	SPIN1	case	control	0.251578299	0.261108494	0.963501014	0.335296155	0.775897605	up_in_case
CRA004775	ENSGALG00010009034	SNX17	case	control	-0.247309845	0.345446226	-0.71591416	0.474044355	0.845580999	down_in_case
CRA004775	ENSGALG00010009035	ENSGALG00010009035	case	control	-0.23574519	0.302147626	-0.780231811	0.435254441	0.826905159	down_in_case
CRA004775	ENSGALG00010009037	ENSGALG00010009037	case	control	-0.917338103	0.422754934	-2.169905134	0.030014033	0.330442732	down_in_case
CRA004775	ENSGALG00010009038	RAD21	case	control	-0.114938831	0.257831851	-0.445789885	0.655749029	0.917054032	down_in_case
CRA004775	ENSGALG00010009039	SLC36A4	case	control	-0.336889271	0.280375003	-1.201566714	0.229531444	0.695782399	down_in_case
CRA004775	ENSGALG00010009040	CRELD2	case	control	-0.294147415	0.37864407	-0.776844108	0.43725075	0.827286039	down_in_case
CRA004775	ENSGALG00010009043	TFAM	case	control	-0.048156864	0.170064667	-0.283167958	0.777048089	0.947261429	down_in_case
CRA004775	ENSGALG00010009047	SUPT5H	case	control	-0.481600652	0.241712694	-1.992450802	0.046321618	0.39985472	down_in_case
CRA004775	ENSGALG00010009050	PIK3CG	case	control	0.144701183	0.207330092	0.697926583	0.485223109	0.851302844	up_in_case
CRA004775	ENSGALG00010009051	TROJANZ	case	control	-1.028056407	0.482794026	-2.129389244	0.03322207	0.347668706	down_in_case
CRA004775	ENSGALG00010009057	NXNL2	case	control	-0.207758742	0.614844735	-0.337904401	0.735435231	0.937109578	down_in_case
CRA004775	ENSGALG00010009059	MED29	case	control	-0.520528761	0.477529598	-1.090045021	0.275693313	0.731001618	down_in_case
CRA004775	ENSGALG00010009063	ENSGALG00010009063	case	control	-0.904109505	0.553034419	-1.63481598	0.10208763	0.542100708	down_in_case
CRA004775	ENSGALG00010009065	EIF2B4	case	control	-0.466475309	0.291622836	-1.599584298	0.109690834	0.557206745	down_in_case
CRA004775	ENSGALG00010009067	ENSGALG00010009067	case	control	10.29373482	1.204401131	8.546766155			up_in_case
CRA004775	ENSGALG00010009068	AGO2	case	control	0.0167164	0.433194555	0.038588666	0.969218339	0.994160926	up_in_case
CRA004775	ENSGALG00010009070	ENSGALG00010009070	case	control	0.517100208	0.317291138	1.629734163	0.103157694	0.544537983	up_in_case
CRA004775	ENSGALG00010009073	PRKAR2B	case	control	-0.893821431	0.593004704	-1.507275447	0.131740068	0.587352231	down_in_case
CRA004775	ENSGALG00010009076	TMEM214	case	control	-0.298789984	0.195626773	-1.527347101	0.126674723	0.580923609	down_in_case
CRA004775	ENSGALG00010009078	PTTG2	case	control	-0.076347948	0.347360012	-0.219794869	0.826030916	0.961878235	down_in_case
CRA004775	ENSGALG00010009079	KCTD15	case	control	0.322254804	0.255891009	1.25934399	0.20790611	0.676506608	up_in_case
CRA004775	ENSGALG00010009080	TLL1	case	control	0.285260978	0.478064835	0.596699352	0.550708125	0.877827378	up_in_case
CRA004775	ENSGALG00010009081	HBP1	case	control	0.092343799	0.282213349	0.327212726	0.743506996	0.939218065	up_in_case
CRA004775	ENSGALG00010009083	MED30	case	control	-0.108007438	0.242613808	-0.445182568	0.656187823	0.91710603	down_in_case
CRA004775	ENSGALG00010009085	PAF1	case	control	-0.180760159	0.194925665	-0.927328679	0.353755908	0.78730441	down_in_case
CRA004775	ENSGALG00010009086	UBE2D1	case	control	0.299413751	0.382336124	0.783116561	0.433558667	0.825828172	up_in_case
CRA004775	ENSGALG00010009088	ENSGALG00010009088	case	control	0.437890855	0.464833623	0.942037826	0.346173274	0.784552667	up_in_case
CRA004775	ENSGALG00010009089	AGBL5	case	control	-0.410884506	0.178753988	-2.298603295	0.021527476	0.288534247	down_in_case
CRA004775	ENSGALG00010009091	EXT1	case	control	0.189697196	0.167685069	1.131270643	0.257941197	0.720037471	up_in_case
CRA004775	ENSGALG00010009092	EED	case	control	-0.330633064	0.271837646	-1.216288727	0.223874954	0.69173716	down_in_case
CRA004775	ENSGALG00010009093	SLU7	case	control	0.206243774	0.22938085	0.899132486	0.368582096	0.794831796	up_in_case
CRA004775	ENSGALG00010009094	DARS	case	control	-0.312664003	0.255395523	-1.224234468	0.22086379	0.688684348	down_in_case
CRA004775	ENSGALG00010009096	ENSGALG00010009096	case	control	2.431869153	0.902375718	2.694962978	0.007039645	0.165757126	up_in_case
CRA004775	ENSGALG00010009097	C1QTNF2	case	control	0.177843395	0.621939849	0.28594951	0.774916793	0.946319184	up_in_case
CRA004775	ENSGALG00010009098	CAD	case	control	-0.783713264	0.326498122	-2.400360712	0.016378923	0.254445661	down_in_case
CRA004775	ENSGALG00010009099	CXCR4	case	control	1.08793025	0.454207066	2.395229693	0.016609956	0.255187198	up_in_case
CRA004775	ENSGALG00010009100	ENSGALG00010009100	case	control	0.002448027	0.241269073	0.010146461	0.991904434	0.998065748	up_in_case
CRA004775	ENSGALG00010009101	HIKESHI	case	control	0.208004388	0.281548917	0.738785963	0.460036977	0.840995542	up_in_case
CRA004775	ENSGALG00010009102	HTRA3	case	control	0.390739928	0.288746511	1.353228224	0.175982731	0.641383126	up_in_case
CRA004775	ENSGALG00010009103	AP3S1	case	control	-0.119993803	0.268938474	-0.446175667	0.655470358	0.917033995	down_in_case
CRA004775	ENSGALG00010009104	MTR	case	control	-0.507088178	0.407273486	-1.245080261	0.213102249	0.682777582	down_in_case
CRA004775	ENSGALG00010009106	THSD7B	case	control	0.595840824	0.510102086	1.168081528	0.242773881	0.709690398	up_in_case
CRA004775	ENSGALG00010009109	ENSGALG00010009109	case	control	0.179831346	0.233473438	0.770243276	0.441155598	0.829353567	up_in_case
CRA004775	ENSGALG00010009111	LRFN1	case	control	-0.390444991	0.438279799	-0.89085783	0.373005446	0.796804495	down_in_case
CRA004775	ENSGALG00010009112	ENSGALG00010009112	case	control	-0.399333574	1.018720669	-0.391995162	0.695061788	0.928924129	down_in_case
CRA004775	ENSGALG00010009113	RPLP1	case	control	-0.024362567	0.296730746	-0.082103278	0.934564586	0.985731248	down_in_case
CRA004775	ENSGALG00010009114	SETD7	case	control	0.457914624	0.329070179	1.391540935	0.164061455	0.629717526	up_in_case
CRA004775	ENSGALG00010009115	COG5	case	control	0.020866933	0.266198056	0.078388749	0.937518823	0.986688955	up_in_case
CRA004775	ENSGALG00010009116	S1PR3	case	control	-0.035685235	0.415015927	-0.085985217	0.931478169	0.985044263	down_in_case
CRA004775	ENSGALG00010009117	ERGIC1	case	control	-0.026460301	0.119747167	-0.220968075	0.825117291	0.961785867	down_in_case
CRA004775	ENSGALG00010009118	ACTN2	case	control	0.445079344	0.711455373	0.625589968	0.53158392	0.869594396	up_in_case
CRA004775	ENSGALG00010009119	CISD1	case	control	0.065320904	0.285604927	0.228710703	0.819093776	0.960136264	up_in_case
CRA004775	ENSGALG00010009120	ATG12	case	control	-0.257709596	0.198139195	-1.30064925	0.193378541	0.657919837	down_in_case
CRA004775	ENSGALG00010009121	SYCN	case	control	1.513875977	0.45010932	3.363351769	0.000770022	0.058500383	up_in_case

CRA004775	ENSGALG00010009123	ENSGALG00010009123	case	control	0.12462966	0.269588765	0.462295452	0.643869461	0.913727726	up_in_case
CRA004775	ENSGALG00010009125	CD72L1	case	control	-0.0363274	0.658582334	-0.055159998	0.956010997	0.990926004	down_in_case
CRA004775	ENSGALG00010009126	JPH3	case	control	0.237228314	0.429366635	0.552507565	0.580600653	0.888772078	up_in_case
CRA004775	ENSGALG00010009127	SCOC	case	control	0.260323314	0.215606391	1.207400732	0.227277854	0.694715356	up_in_case
CRA004775	ENSGALG00010009129	ENSGALG00010009129	case	control	1.52605459	0.487697454	3.129100995	0.00175342	0.084784105	up_in_case
CRA004775	ENSGALG00010009130	HEATR1	case	control	-0.030930417	0.325427662	-0.095045445	0.92427873	0.983849734	down_in_case
CRA004775	ENSGALG00010009131	CDKL2	case	control	-0.210250632	0.566753172	-0.370973896	0.710656975	0.932234469	down_in_case
CRA004775	ENSGALG00010009133	BOD1L1	case	control	0.45065582	0.282220798	1.596820022	0.110305832	0.558567026	up_in_case
CRA004775	ENSGALG00010009135	TENM4	case	control	-0.079630494	0.492429427	-0.161709454	0.87153466	0.973918682	down_in_case
CRA004775	ENSGALG00010009136	KHK	case	control	1.534664116	0.42114932	3.643990491	0.000268443	0.033398296	up_in_case
CRA004775	ENSGALG00010009137	IPMK	case	control	0.427059753	0.227206123	1.879613749	0.060160738	0.445397937	up_in_case
CRA004775	ENSGALG00010009138	ENSGALG00010009138	case	control	0.488655211	0.245881373	1.987361647	0.046882335	0.402672695	up_in_case
CRA004775	ENSGALG00010009139	CKS2	case	control	0.907932127	0.929401992	0.976899269	0.328619024	0.77220987	up_in_case
CRA004775	ENSGALG00010009141	MAML3	case	control	-0.266961724	0.396103308	-0.673969943	0.500330423	0.859740701	down_in_case
CRA004775	ENSGALG00010009142	LGALS8	case	control	0.09962581	0.463682233	0.214857941	0.829878078	0.963232966	up_in_case
CRA004775	ENSGALG00010009143	MCCC2	case	control	0.228538716	0.342353919	0.667551045	0.504420216	0.861471523	up_in_case
CRA004775	ENSGALG00010009144	CREBZF	case	control	-0.053460776	0.335257083	-0.159462034	0.873304868	0.974079175	down_in_case
CRA004775	ENSGALG00010009146	CXCL13L3	case	control	1.675866033	1.059046062	1.582429786	0.113551489	0.562886277	up_in_case
CRA004775	ENSGALG00010009147	HNMT	case	control	0.593333631	0.331161365	1.791675282	0.073184996	0.477576895	up_in_case
CRA004775	ENSGALG00010009149	FUT4	case	control	-0.30066956	0.527028054	-0.570500105	0.568338551	0.884508497	down_in_case
CRA004775	ENSGALG00010009150	ENSGALG00010009150	case	control	-1.065306709	0.690388314	-1.543054377	0.122817583	0.57525466	down_in_case
CRA004775	ENSGALG00010009151	CCNJL	case	control	-0.625498958	0.52754198	-1.185685656	0.235746461	0.702894534	down_in_case
CRA004775	ENSGALG00010009152	RPP25L	case	control	-0.271327161	0.342768005	-0.791576683	0.428607547	0.823499005	down_in_case
CRA004775	ENSGALG00010009153	SECISBP2	case	control	0.031737353	0.18059567	0.175737063	0.860500517	0.971325143	up_in_case
CRA004775	ENSGALG00010009155	PAK4	case	control	-0.130142654	0.168087789	-0.774254066	0.438780561	0.828368259	down_in_case
CRA004775	ENSGALG00010009157	ABHD1	case	control	-0.413686411	0.439297023	-0.94170092	0.346345784	0.784552667	down_in_case
CRA004775	ENSGALG00010009159	PTK2	case	control	0.141755284	0.217131141	0.652855611	0.513849368	0.863954532	up_in_case
CRA004775	ENSGALG00010009160	ENSGALG00010009160	case	control	0.52987571	0.472187359	1.122172588	0.261789065	0.72256212	up_in_case
CRA004775	ENSGALG00010009163	SAMD12	case	control	0.193169387	0.25714436	0.75120989	0.452526349	0.837058088	up_in_case
CRA004775	ENSGALG00010009164	TMEM128	case	control	0.122043055	0.157044616	0.777123454	0.437085938	0.827232725	up_in_case
CRA004775	ENSGALG00010009166	CLGN	case	control	-0.077899652	0.58659177	-0.132800452	0.894351197	0.977834811	down_in_case
CRA004775	ENSGALG00010009167	DCTN3	case	control	-0.567393565	0.365850174	-1.550890516	0.120927923	0.573473028	down_in_case
CRA004775	ENSGALG00010009168	MRPS12	case	control	-0.177752103	0.338984516	-0.524366439	0.600023694	0.897206546	down_in_case
CRA004775	ENSGALG00010009170	RPL26L1	case	control	-0.325406842	0.264984674	-1.228021369	0.219438946	0.687020074	down_in_case
CRA004775	ENSGALG00010009172	SPOPL	case	control	0.303694439	0.295620125	1.027131141	0.304273037	0.756847621	up_in_case
CRA004775	ENSGALG00010009173	LYAR	case	control	0.347198604	0.265071492	1.309830046	0.190253336	0.655846986	up_in_case
CRA004775	ENSGALG00010009178	ENSGALG00010009178	case	control	-0.071707321	0.491636528	-0.145854338	0.884036379	0.976208708	down_in_case
CRA004775	ENSGALG00010009180	NFKBIB	case	control	-0.336317322	0.354053328	-0.949905833	0.342160103	0.781616177	down_in_case
CRA004775	ENSGALG00010009186	PREB	case	control	-0.236990293	0.263004432	-0.901088592	0.367541218	0.794321403	down_in_case
CRA004775	ENSGALG00010009189	ENSGALG00010009189	case	control	-1.964885802	0.874403085	-2.247116732	0.024632568	0.302160964	down_in_case
CRA004775	ENSGALG00010009190	ZBTB49	case	control	-0.285077829	0.291749912	-0.977130815	0.328504394	0.77220987	down_in_case
CRA004775	ENSGALG00010009192	LSM14A	case	control	0.05579639	0.162137185	0.344130742	0.730747964	0.936394787	up_in_case
CRA004775	ENSGALG00010009193	EDARADD	case	control	-0.2369428	0.442961402	-0.5349062	0.592714721	0.894176646	down_in_case
CRA004775	ENSGALG00010009195	ENSGALG00010009195	case	control	-0.03256542	0.527403078	-0.06174674	0.950764518	0.990168609	down_in_case
CRA004775	ENSGALG00010009197	FH	case	control	0.163369369	0.32103548	0.508882598	0.610834518	0.901413878	up_in_case
CRA004775	ENSGALG00010009198	KCNN2	case	control	-0.007485546	0.432052907	-0.01732553	0.986176918	0.997562105	down_in_case
CRA004775	ENSGALG00010009199	SEMA4D	case	control	-0.030468528	0.415958324	-0.073248993	0.94160798	0.987577169	down_in_case
CRA004775	ENSGALG00010009200	ADGRA3	case	control	0.044860105	0.338004572	0.132720409	0.894414502	0.977834811	up_in_case
CRA004775	ENSGALG00010009202	FABP6	case	control	0.448824246	0.315677805	1.421779545	0.15509026	0.619284425	up_in_case
CRA004775	ENSGALG00010009205	SIRT2	case	control	-0.336549637	0.303578093	-1.108609762	0.267598565	0.727430878	down_in_case
CRA004775	ENSGALG00010009208	SLC5A6	case	control	-0.668887123	0.332730539	-2.010296752	0.04439979	0.393136844	down_in_case
CRA004775	ENSGALG00010009209	SLC6A19	case	control	2.53648855	0.829838746	3.056604145	0.002238597	0.096690159	up_in_case
CRA004775	ENSGALG00010009210	PCDH15	case	control	-0.181202791	0.830307867	-0.218235666	0.827245497	0.961901981	down_in_case
CRA004775	ENSGALG00010009212	NXPH2	case	control	-0.317649962	0.496495591	-0.639784053	0.522313002	0.866593398	down_in_case
CRA004775	ENSGALG00010009213	DUS4L	case	control	-0.067199385	0.280218668	-0.239810521	0.81047715	0.956724552	down_in_case
CRA004775	ENSGALG00010009214	PCDH10	case	control	1.406721347	0.626113957	2.246749704	0.024656028	0.302179178	up_in_case
CRA004775	ENSGALG00010009216	NSG1	case	control	0.691492691	0.626142192	1.104370061	0.269432642	0.72765348	up_in_case
CRA004775	ENSGALG00010009219	TNFRSF11B	case	control	-0.06700123	0.309919087	-0.216189427	0.828840095	0.962881849	down_in_case
CRA004775	ENSGALG00010009220	GADD45G	case	control	1.082559562	0.656068248	1.650071568	0.098928299	0.535944002	up_in_case
CRA004775	ENSGALG00010009223	ENSGALG00010009223	case	control	0.094558657	0.369112288	0.256178567	0.797812957	0.952835572	up_in_case
CRA004775	ENSGALG00010009224	ENSGALG00010009224	case	control	-0.34169869	0.639663363	-0.534185182	0.59321342	0.894450257	down_in_case
CRA004775	ENSGALG00010009225	HNRNPL	case	control	-0.002107999	0.234518385	-0.008988631	0.992828207	0.998444544	down_in_case
CRA004775	ENSGALG00010009227	ENSGALG00010009227	case	control	-0.445249746	0.288539717	-1.543114237	0.122803061	0.57525466	down_in_case
CRA004775	ENSGALG00010009228	ATP6V0E1	case	control	-0.168519041	0.23689682	-0.711360501	0.476860869	0.846367006	down_in_case
CRA004775	ENSGALG00010009232	ECH1	case	control	-0.110686632	0.336314997	-0.329115957	0.742068044	0.938877854	down_in_case
CRA004775	ENSGALG00010009234	PCDH18	case	control	-0.074228179	0.349096152	-0.212629611	0.831615874	0.963561668	down_in_case
CRA004775	ENSGALG00010009235	KLHDC4	case	control	0.360678819	0.302776508	1.191237792	0.233560247	0.700782667	up_in_case
CRA004775	ENSGALG00010009236	DTWD2	case	control	0.40866573	0.417485866	0.978873212	0.32764263	0.771474966	up_in_case
CRA004775	ENSGALG00010009237	DENND3	case	control	0.162334134	0.436625665	0.37179247	0.710047371	0.932234469	up_in_case
CRA004775	ENSGALG00010009239	STX18	case	control	0.056088267	0.178101489	0.314923066	0.752820068	0.941692782	up_in_case
CRA004775	ENSGALG00010009243	BCAP29	case	control	-0.1604042	0.149998688	-1.069370684	0.284902672	0.74120185	down_in_case
CRA004775	ENSGALG00010009246	RUSC2	case	control	0.284441316	0.475095048	0.598704021	0.549370275	0.877623839	up_in_case
CRA004775	ENSGALG00010009250	ERO1B	case	control	0.041578717	0.276445665	0.150404664	0.880445362	0.974996282	up_in_case
CRA004775	ENSGALG00010009252	SIRT1	case	control	0.242143529	0.247037951	0.980187568	0.32699354	0.770998457	up_in_case
CRA004775	ENSGALG00010009253	SLC45A4	case	control	-0.68334338	0.457326138	-1.494214574	0.135119545	0.592957144	down_in_case
CRA004775	ENSGALG00010009256	CREBRF	case	control	-0.173253995	0.322369992	-0.537438346	0.590964862	0.893804699	down_in_case
CRA004775	ENSGALG00010009257	ENSGALG00010009257	case	control	0.099253153	0.682316255	0.145465028	0.884343726	0.976236592	up_in_case
CRA004775	ENSGALG00010009258	DNAJC12	case	control	0.108995768	0.307428116	0.354540661	0.722933737	0.935185555	up_in_case
CRA004775	ENSGALG00010009259	MGAT4D	case	control	1.625318132	0.687296317	2.364799711	0.018039824	0.267312092	up_in_case
CRA004775	ENSGALG00010009263	COLEC10	case	control	0.149717057	0.413692653	0.361904076	0.717423714	0.934011578	up_in_case
CRA004775	ENSGALG00010009266	PCDH7	case	control	1.022139562	0.874783035	1.168449228	0.242625608	0.709690398	up_in_case
CRA004775	ENSGALG00010009268	PWWP2A	case	control	-0.107174292	0.25502806	-0.420245098	0.674306412	0.922533825	down_in_case
CRA004775	ENSGALG00010009273	MAL2	case	control	0.945544305	0.61621919	1.534428529	0.124924264	0.577423041	up_in_case
CRA004775	ENSGALG00010009275	CBLL1	case	control	-0.130905231	0.148131291	-0.883710861	0.376852316	0.798523018	down_in_case
CRA004775	ENSGALG00010009276	ENSGALG00010009276	case	control	-0.511906029	0.478623871	-1.069537187	0.284827681	0.74120185	down_in_case
CRA004775	ENSGALG00010009277	ACTN4	case	control	0.049360871	0.233863107	0.211067372	0.8328347	0.964019688	up_in_case
CRA004775	ENSGALG00010009279	ELMOD2	case	control	-0.236400409	0.26959824	-0.876861844	0.380561683	0.80151688	down_in_case
CRA004775	ENSGALG00010009280	HSD17B4	case	control	-0.143355201	0.187509302	-0.764523142	0.444555551	0.832504887	down_in_case
CRA004775	ENSGALG00010009284	CCN3	case	control	-0.779096201	0.753626907	-1.033795626	0.301231717	0.753545086	down_in_case
CRA004775	ENSGALG00010009286	ENSGALG00010009286	case	control	-0.12316883	0.316177065	-0.389556497	0.696864526	0.929092636	down_in_case
CRA004775	ENSGALG00010009289	GPR137B	case	control	-0.3788752	0.331990345	-1.14122355	0.253776907	0.718204856	down_in_case
CRA004775	ENSGALG00010009294	ACOX3	case	control	-0.11701306	0.271584859	-0.430852663	0.666575505	0.919168997	down_in_case
CRA004775	ENSGALG00010009297	CTNNA3	case	control	0.533960386	0.527002003	1.013203712	0.310962868	0.762763182	up_in_case
CRA004775	ENSGALG00010009299	MAP4K1	case	control	-0.482762123	0.437014919	-1.104681104	0.269297794	0.72765348	down_in_case
CRA004775	ENSGALG00010009303	TTC1	case	control	0.010813843	0.273666813	0.039514632	0.968480088	0.993583205	up_in_case
CRA004775	ENSGALG00010009306	TBC1D9	case	control	0.089908808	0.315419094	0.285045547	0.775609247	0.946517228	up_in_case
CRA004775	ENSGALG00010009307	DIRAS2	case	control	1.227549957	0.400386131	3.06591528	0.002170048	0.094431421	up_in_case
CRA004775	ENSGALG00010009309	TRIM54	case	control	-0.407876551	0.538359831	-0.757628128	0.448673636	0.834421311	down_in_case
CRA004775	ENSGALG00010009311	BNIP1	case	control	-0.091556161	0.18497092	-0.494975972	0.620617082	0.905283281	down_in_case
CRA004775	ENSGALG00010009313	UNC13B	case	control	0.796533668	0.496070392	1.605686774	0.108342749	0.555197961	up_in_case
CRA004775	ENSGALG00010009320	GPR20	case	control	1.474113668	0.493137938	2.9892522	0.002796612	0.109874886	up_in_case
CRA004775	ENSGALG00010009322	ENSGALG00010009322	case	control	-0.0906509	0.376725378	-0.240628598	0.809842983	0.956221094	down_in_case
CRA004775	ENSGALG00010009323	RNF150	case	control	0.253119506	0.314675274	0.804383207	0.421175697	0.819803052	up_in_case
CRA004775	ENSGALG00010009324	NKX2-5	case	control	2.69934489	0.722997499	3.733546652	0.000188802	0.028219773	up_in_case
CRA004775	ENSGALG00010009326	DLD	case	control	0.33887126	0.318580977	1.063689564	0.287469354	0.742456237	up_in_case
CRA004775	ENSGALG00010009327	NID1	case	control	0.456518392	0.431813659	1.057211559	0.290415046	0.74431661	up_in_case
CRA004775	ENSGALG00010009328	ENSGALG00010009328	case	control	-0.34511276	0.36989166	-0.933010384	0.350814607	0.785681625	down_in_case
CRA004775	ENSGALG00010009330	EIF3K	case	control	-0.364152295	0.242501199	-1.501651522	0.13318713	0.590030999	down_in_case
CRA004775	ENSGALG00010009332	ENSGALG00010009332	case	control	-0.430330105	0.253004934	-1.700876331	0.088966212	0.515249396	down_in_case
CRA004775	ENSGALG00010009333	ADRA1B	case	control	0.893990786	0.393873772	2.26973932	0.023223404	0.296238434	up_in_case
CRA004775	ENSGALG00010009334	SS18L2	case	control	-0.354868288	0.330192484	-1.074731573	0.282494924	0.738659097	down_in_case
CRA004775	ENSGALG00010009335	PTP4A3	case	control	-0.055181455	0.482317831	-0.114408906	0.908913655	0.98075825	down_in_case
CRA004775	ENSGALG00010009336	LAMB1	case	control	0.623325663	0.425374246	1.465358255	0.142823201	0.602997185	up_in_case
CRA004775	ENSGALG00010009337	ZNF330	case	control	-0.215402104	0.250963289	-0.858301247	0.390726141	0.806479902	down_in_case
CRA004775	ENSGALG00010009338	KYNU	case	control	-0.283368835	1.007911616	-0.281144527	0.77859955	0.94792594	down_in_case
CRA004775	ENSGALG00010009340	SYK	case	control	0.628751999	0.458334656	1.371818586	0.17011992	0.63378857	up_in_case
CRA004775	ENSGALG00010009344	ENPP2	case	control	0.368470313	0.330715474	1.11416109	0.265210085	0.726330188	up_in_case
CRA004775	ENSGALG00010009345	ZFAT	case	control	0.194738359	0.245435333	0.793440607	0.42752116	0.822776983	up_in_case
CRA004775	ENSGALG00010009346	STC2	case	control	1.118958777	0.643964235	1.737610127	0.082279534	0.499148629	up_in_case
CRA004775	ENSGALG00010009347	TESK1	case	control	-0.13790748	0.392921964	-0.35097931	0.72560387	0.935238044	down_in_case
CRA004775	ENSGALG00010009348	ERGIC2	case	control	-0.184373222	0.167070035	-1.103568467	0.269780376	0.72797291	down_in_case
CRA004775	ENSGALG00010009351	GTF3C2	case	control	-0.384810988	0.317466975	-1.212129195	0.22546293	0.693278343	down_in_case
CRA004775	ENSGALG00010009352	HSF1	case	control	-0.25290893	0.231863704	-1.090765501	0.27537608	0.731001618	down_in_case
CRA004775	ENSGALG00010009353	CD72	case	control	2.670315244	0.575841388	4.637240916			up_in_case
CRA004775	ENSGALG00010009355	GARRE1	case	control	0.00205812	0.207596416	0.009914045	0.992089866	0.998065748	up_in_case
CRA004775	ENSGALG00010009357	LYST	case	control	-0.102856423	0.335365783	-0.306699218	0.759072325	0.942236668	down_in_case

CRA004775	ENSGALG00010009359	REEP3	case	control	0.126051435	0.420386798	0.299846321	0.764294381	0.94239774	up_in_case
CRA004775	ENSGALG00010009360	TNFAIP8	case	control	-0.480829429	0.404678454	-1.1881765	0.23476388	0.702145501	down_in_case
CRA004775	ENSGALG00010009361	LRRK2	case	control	0.327675114	0.295995711	1.107026562	0.268282444	0.727528758	up_in_case
CRA004775	ENSGALG00010009362	IL15	case	control	0.386924307	0.387073264	0.999615173	0.317496777	0.766264094	up_in_case
CRA004775	ENSGALG00010009363	TAF2	case	control	0.041748366	0.169140021	0.24682725	0.80504192	0.954689985	up_in_case
CRA004775	ENSGALG00010009365	CA5B	case	control	-0.327088253	0.254098775	-1.287248444	0.198007707	0.664272829	down_in_case
CRA004775	ENSGALG00010009367	DMXL1	case	control	0.267037569	0.345804101	0.7722221	0.439982894	0.829135916	up_in_case
CRA004775	ENSGALG00010009368	DSCC1	case	control	-0.508418731	0.582759114	-0.87243377	0.382971785	0.801925538	down_in_case
CRA004775	ENSGALG00010009370	ENSGALG00010009370	case	control	-0.520596472	0.514217547	-1.012405109	0.311344396	0.763050512	down_in_case
CRA004775	ENSGALG00010009371	PHTF2	case	control	0.231618071	0.256256288	0.90385322	0.366073237	0.793608387	up_in_case
CRA004775	ENSGALG00010009372	RAB28	case	control	0.007530664	0.229924083	0.032752827	0.973871697	0.994947809	up_in_case
CRA004775	ENSGALG00010009373	ARHGAP15	case	control	-0.298150301	0.495006064	-0.602316461	0.546963505	0.877099134	down_in_case
CRA004775	ENSGALG00010009376	CDO1	case	control	-0.74357134	0.623357418	-1.192849107	0.232928474	0.699803243	down_in_case
CRA004775	ENSGALG00010009378	ENSGALG00010009378	case	control	-0.095815197	0.232696642	-0.41176012	0.680515255	0.923896221	down_in_case
CRA004775	ENSGALG00010009379	RYR1	case	control	0.024040927	0.565078459	0.042544406	0.966064713	0.993094598	up_in_case
CRA004775	ENSGALG00010009381	INPP4B	case	control	0.00085782	0.540267191	0.001587769	0.998733144	0.999914248	up_in_case
CRA004775	ENSGALG00010009383	ASXL2	case	control	-0.127936677	0.347708907	-0.367941904	0.712916554	0.932981748	down_in_case
CRA004775	ENSGALG00010009385	GPI	case	control	0.26747795	0.527743602	0.506833146	0.612271897	0.901413878	up_in_case
CRA004775	ENSGALG00010009386	TMEM60	case	control	-0.087154658	0.268923114	-0.324087642	0.745871683	0.939582403	down_in_case
CRA004775	ENSGALG00010009387	DEPTOR	case	control	0.189198451	0.437912637	0.432046109	0.665707901	0.918888163	up_in_case
CRA004775	ENSGALG00010009388	TMED7	case	control	-0.034325691	0.261317263	-0.131356384	0.89549339	0.978072248	down_in_case
CRA004775	ENSGALG00010009389	CPEB4	case	control	0.632189365	0.285416168	2.214973912	0.026761855	0.314790198	up_in_case
CRA004775	ENSGALG00010009391	RSBN1L	case	control	0.083630059	0.26608053	0.314303565	0.753290491	0.941900693	up_in_case
CRA004775	ENSGALG00010009392	MAPRE3	case	control	-0.207199055	0.343660324	-0.602918172	0.546563127	0.877099134	down_in_case
CRA004775	ENSGALG00010009393	MARVELD2	case	control	0.192540534	0.442842949	0.434782883	0.663720031	0.918295135	up_in_case
CRA004775	ENSGALG00010009394	BANP	case	control	-0.037132304	0.211709886	-0.175392394	0.860771317	0.971325143	down_in_case
CRA004775	ENSGALG00010009396	AUH	case	control	0.23701854	0.275181991	0.861315593	0.38906425	0.804998872	up_in_case
CRA004775	ENSGALG00010009398	WTIP	case	control	0.561239257	0.204576449	2.743420657	0.006080274	0.155408639	up_in_case
CRA004775	ENSGALG00010009400	CNTFR	case	control	-0.60709712	0.622479157	-0.975289073	0.329416889	0.77237154	down_in_case
CRA004775	ENSGALG00010009404	GNG4	case	control	-0.481397496	0.774725364	-0.621378257	0.534350774	0.870981055	down_in_case
CRA004775	ENSGALG00010009407	PTPN12	case	control	0.225534325	0.286808475	0.786358652	0.4316574	0.825262449	up_in_case
CRA004775	ENSGALG00010009408	ENSGALG00010009408	case	control	-0.253025938	0.213544931	-1.184883841	0.236063377	0.70334936	down_in_case
CRA004775	ENSGALG00010009410	JMJD1C	case	control	0.356968662	0.207303328	1.721963006	0.08507622	0.505772986	up_in_case
CRA004775	ENSGALG00010009411	SMARCA5	case	control	-0.05156233	0.346071762	-0.148993172	0.881559021	0.975011509	down_in_case
CRA004775	ENSGALG00010009415	HMP19	case	control	1.480915363	0.86895178	1.70425494	0.088333469	0.513532836	up_in_case
CRA004775	ENSGALG00010009416	COL14A1	case	control	0.966848327	0.567304674	1.704284083	0.088328027	0.513532836	up_in_case
CRA004775	ENSGALG00010009431	SMN	case	control	-0.087521156	0.246981674	-0.354362957	0.723066892	0.935185555	down_in_case
CRA004775	ENSGALG00010009432	BOP1	case	control	-0.784034162	0.325248884	-2.410566799	0.015927754	0.252039758	down_in_case
CRA004775	ENSGALG00010009435	GTDC1	case	control	0.062955397	0.116936306	0.538373402	0.590319285	0.893804699	up_in_case
CRA004775	ENSGALG00010009437	DPYSL5	case	control	-0.244529288	0.555454821	-0.440232541	0.659768693	0.917907174	down_in_case
CRA004775	ENSGALG00010009439	B3GALNT2	case	control	0.409249616	0.193913301	2.110477283	0.034817266	0.354006537	up_in_case
CRA004775	ENSGALG00010009440	DOK3	case	control	0.593776207	0.305450584	1.94393541	0.051903241	0.419683701	up_in_case
CRA004775	ENSGALG00010009441	ZNF518B	case	control	0.344713012	0.188607865	1.827670402	0.067599033	0.463543284	up_in_case
CRA004775	ENSGALG00010009444	NFIL3	case	control	0.486607728	0.453991224	1.071843911	0.283790147	0.740508942	up_in_case
CRA004775	ENSGALG00010009446	ENSGALG00010009446	case	control	0.129799103	0.324646069	0.399817265	0.689291114	0.927531275	up_in_case
CRA004775	ENSGALG00010009447	DDX41	case	control	-0.441699129	0.153007088	-2.886788681	0.003891955	0.128824566	down_in_case
CRA004775	ENSGALG00010009451	ROR2	case	control	0.440225335	0.327503544	1.344184949	0.178888588	0.644923737	up_in_case
CRA004775	ENSGALG00010009452	TBCE	case	control	-0.118937405	0.238420581	-0.498855444	0.617881225	0.904143308	down_in_case
CRA004775	ENSGALG00010009453	GSAP	case	control	-0.133906478	0.245810945	-0.544753929	0.585922769	0.89145601	down_in_case
CRA004775	ENSGALG00010009457	BDP1	case	control	0.388791568	0.301656023	1.288857302	0.1974477	0.663190847	up_in_case
CRA004775	ENSGALG00010009458	SCX	case	control	-1.656089574	1.079630855	-1.533940574			down_in_case
CRA004775	ENSGALG00010009461	ZEB2	case	control	-0.261069143	0.31594439	-0.82631359	0.408626226	0.814384687	down_in_case
CRA004775	ENSGALG00010009462	NRBF2	case	control	0.469166446	0.268701834	1.746048541	0.080802517	0.495370226	up_in_case
CRA004775	ENSGALG00010009463	MRPL13	case	control	-0.156247342	0.291032643	-0.536872223	0.591355879	0.893804699	down_in_case
CRA004775	ENSGALG00010009464	SPTLC1	case	control	0.076581845	0.224085649	0.341752564	0.732537111	0.936819725	up_in_case
CRA004775	ENSGALG00010009465	GGPS1	case	control	0.353756356	0.25709754	1.375961654	0.168833505	0.632337016	up_in_case
CRA004775	ENSGALG00010009466	FAM193B	case	control	-0.265104504	0.146389412	-1.810954092	0.070147958	0.469724266	down_in_case
CRA004775	ENSGALG00010009467	EGR2	case	control	0.214465965	0.424632429	0.505062614	0.613514859	0.902282137	up_in_case
CRA004775	ENSGALG00010009469	DNMT3A	case	control	0.570022603	0.252250593	2.259747321	0.023836938	0.298798294	up_in_case
CRA004775	ENSGALG00010009470	ZNF469	case	control	-0.64830791	0.49872153	-1.299939687	0.193621642	0.657919837	down_in_case
CRA004775	ENSGALG00010009472	ADO	case	control	0.211448188	0.358030976	0.590586298	0.554797648	0.879629016	up_in_case
CRA004775	ENSGALG00010009474	ACVR2A	case	control	0.215551773	0.259228315	0.831513228	0.405683759	0.813707034	up_in_case
CRA004775	ENSGALG00010009477	ZNF365	case	control	0.460787941	0.352865297	1.30584658	0.191604736	0.656959697	up_in_case
CRA004775	ENSGALG00010009479	MTBP	case	control	-0.839896762	0.744198009	-1.128593132	0.259069509	0.720037471	down_in_case
CRA004775	ENSGALG00010009482	ARID4B	case	control	0.444298626	0.282405488	1.573264844	0.115657506	0.566086328	up_in_case
CRA004775	ENSGALG00010009484	RTKN2	case	control	0.117261386	0.492526054	0.23808159	0.811817813	0.957566199	up_in_case
CRA004775	ENSGALG00010009486	B4GALT1	case	control	-0.301576292	0.276653751	-1.090085676	0.275675405	0.731001618	down_in_case
CRA004775	ENSGALG00010009489	MROH1	case	control	-0.627997184	0.239164857	-2.625792063	0.008644761	0.188268829	down_in_case
CRA004775	ENSGALG00010009490	SNTB1	case	control	-0.115200575	0.335246182	-0.343629789	0.731124719	0.936495391	down_in_case
CRA004775	ENSGALG00010009491	RBM34	case	control	0.181754954	0.247929601	0.733090981	0.463502941	0.842456502	up_in_case
CRA004775	ENSGALG00010009492	CDC42SE2	case	control	0.197870226	0.368223622	0.537364292	0.591016003	0.893804699	up_in_case
CRA004775	ENSGALG00010009493	ENSGALG00010009493	case	control	0.886809039	0.403005435	2.200489027	0.027772218	0.318297866	up_in_case
CRA004775	ENSGALG00010009494	UBA2	case	control	0.102010077	0.214476948	0.475622569	0.634343294	0.909837348	up_in_case
CRA004775	ENSGALG00010009497	HS3ST1	case	control	0.51532558	0.407691497	1.264008653	0.206226921	0.674837165	up_in_case
CRA004775	ENSGALG00010009498	ORC4	case	control	-0.357372947	0.295714291	-1.208507528	0.226852102	0.694715356	down_in_case
CRA004775	ENSGALG00010009499	MXD3	case	control	-0.152012869	0.435967342	-0.348679485	0.727329947	0.935420324	down_in_case
CRA004775	ENSGALG00010009500	CCDC146	case	control	-0.017439696	0.590715493	-0.029523004	0.976447472	0.99560196	down_in_case
CRA004775	ENSGALG00010009501	HGH1	case	control	-0.292838457	0.267295869	-1.095559234	0.27327171	0.731001618	down_in_case
CRA004775	ENSGALG00010009502	ARID5B	case	control	0.299682166	0.360662976	0.830920237	0.406018691	0.813842524	up_in_case
CRA004775	ENSGALG00010009503	IL11RA	case	control	-0.340206278	0.346548517	-0.981698842	0.326248236	0.770998457	down_in_case
CRA004775	ENSGALG00010009506	HAS2	case	control	0.219895105	0.40031675	0.549302785	0.582797677	0.889712708	up_in_case
CRA004775	ENSGALG00010009508	TOMM20	case	control	0.037108383	0.297543646	0.124715764	0.900748577	0.979145745	up_in_case
CRA004775	ENSGALG00010009511	ZFPM1	case	control	-0.388946105	0.528049345	-0.736571513	0.461382964	0.842000948	down_in_case
CRA004775	ENSGALG00010009514	PRELID1	case	control	-0.314674022	0.222285325	-1.415631114	0.156883537	0.621625652	down_in_case
CRA004775	ENSGALG00010009515	MBD5	case	control	0.586446383	0.188378373	3.1131301	0.001851144	0.086393824	up_in_case
CRA004775	ENSGALG00010009518	FGL2	case	control	0.222705051	0.359970146	0.618676445	0.53612953	0.871588629	up_in_case
CRA004775	ENSGALG00010009522	TAF5L	case	control	0.119708916	0.161958369	0.739133872	0.45982571	0.840995542	up_in_case
CRA004775	ENSGALG00010009524	FAM185A	case	control	0.212048769	0.34962967	0.606495349	0.54418587	0.875729696	up_in_case
CRA004775	ENSGALG00010009525	ENSGALG00010009525	case	control	0.487716985	0.480730108	1.014533888	0.310328068	0.762244121	up_in_case
CRA004775	ENSGALG00010009526	ENSGALG00010009526	case	control	-1.000689043	0.489815934	-2.042989976	0.04105343	0.379122508	down_in_case
CRA004775	ENSGALG00010009530	KIF3C	case	control	-0.214697091	0.357444388	-0.600644739	0.548076633	0.877369241	down_in_case
CRA004775	ENSGALG00010009534	XPA	case	control	-0.461435695	0.374556646	-1.231951695	0.217967129	0.685245996	down_in_case
CRA004775	ENSGALG00010009538	CRIM1	case	control	0.448588807	0.352970968	1.270894343	0.203766239	0.674045742	up_in_case
CRA004775	ENSGALG00010009540	RAB10	case	control	0.320759178	0.250141862	1.28230907	0.199734247	0.667123059	up_in_case
CRA004775	ENSGALG00010009541	PMPCB	case	control	-0.458396799	0.245375301	-1.868145642	0.061741773	0.446686038	down_in_case
CRA004775	ENSGALG00010009542	WDR1	case	control	0.405936357	0.300270376	1.351902785	0.176406417	0.641736676	up_in_case
CRA004775	ENSGALG00010009543	MAF1	case	control	-0.682211667	0.393381475	-1.734224181	0.082878314	0.499850744	down_in_case
CRA004775	ENSGALG00010009548	PIK3C3	case	control	-0.066225619	0.202552231	-0.326955763	0.743701344	0.939218065	down_in_case
CRA004775	ENSGALG00010009551	HADHA	case	control	0.004500924	0.254509599	0.017684692	0.985890393	0.997562105	up_in_case
CRA004775	ENSGALG00010009554	ABCB10	case	control	-0.138345951	0.382296007	-0.361881757	0.717440393	0.934011578	down_in_case
CRA004775	ENSGALG00010009555	EPC2	case	control	0.2674496	0.278251889	0.961178022	0.336462665	0.776900397	up_in_case
CRA004775	ENSGALG00010009557	CIDEC	case	control	0.286427397	0.514012831	0.557237835	0.577364942	0.888268665	up_in_case
CRA004775	ENSGALG00010009558	TMEM26	case	control	0.010040008	0.477140985	0.021042015	0.98321214	0.997398088	up_in_case
CRA004775	ENSGALG00010009560	RAB24	case	control	-0.277370549	0.300774202	-0.922188626	0.356430195	0.789127613	down_in_case
CRA004775	ENSGALG00010009562	HADHB	case	control	-0.061209041	0.405889096	-0.150802379	0.880131609	0.974996282	down_in_case
CRA004775	ENSGALG00010009565	TMEM8B	case	control	-0.313746199	0.433988457	-0.722936738	0.469718752	0.844062961	down_in_case
CRA004775	ENSGALG00010009567	NSD1	case	control	-0.124122872	0.238064697	-0.521382941	0.602100033	0.898185892	down_in_case
CRA004775	ENSGALG00010009569	ZC3H18	case	control	-0.051381549	0.186971662	-0.274809285	0.783462764	0.949699971	down_in_case
CRA004775	ENSGALG00010009570	SHARPIN	case	control	-0.199418085	0.409287051	-0.487232822	0.626093348	0.907183337	down_in_case
CRA004775	ENSGALG00010009571	DNAJC2	case	control	0.526450923	0.327013369	1.609875844	0.107424963	0.553800541	up_in_case
CRA004775	ENSGALG00010009574	TAF1C	case	control	-0.586312246	0.263371189	-2.226182177	0.026001981	0.308767914	down_in_case
CRA004775	ENSGALG00010009575	RHOBTB1	case	control	0.960359438	0.530675166	1.809693575	0.070343319	0.469724266	up_in_case
CRA004775	ENSGALG00010009578	KIF5C	case	control	-0.214754571	0.359199622	-0.597869702	0.549926877	0.877623839	down_in_case
CRA004775	ENSGALG00010009582	TIGD5	case	control	-0.038005537	0.314473019	-0.120854685	0.903806135	0.979540372	down_in_case
CRA004775	ENSGALG00010009583	LYPD6B	case	control	-0.357752881	0.900771164	-0.397162892	0.691247344	0.927867527	down_in_case
CRA004775	ENSGALG00010009593	NCBP1	case	control	0.083874387	0.186741826	0.44914623	0.653326173	0.916442743	up_in_case
CRA004775	ENSGALG00010009594	HINT2	case	control	-0.481577001	0.413774286	-1.163864013	0.244479125	0.711765017	down_in_case
CRA004775	ENSGALG00010009595	ENSGALG00010009595	case	control	-0.252448099	0.389682193	-0.64783073	0.517094437	0.864913871	down_in_case
CRA004775	ENSGALG00010009596	URB2	case	control	-0.161078557	0.255947783	-0.629341482	0.529125519	0.869191855	down_in_case
CRA004775	ENSGALG00010009597	CDK1	case	control	0.503102097	0.502353689	1.001489803	0.316590068	0.765909574	up_in_case
CRA004775	ENSGALG00010009600	LRRC17	case	control	0.733968558	0.54149884	1.355438837	0.175277783	0.640706322	up_in_case
CRA004775	ENSGALG00010009601	ZHX2	case	control	0.1851361	0.235343624	0.786662909	0.431479222	0.825211514	up_in_case
CRA004775	ENSGALG00010009602	TSTD2	case	control	-0.211777501	0.312471767	-0.677749234	0.497930704	0.857810713	down_in_case
CRA004775	ENSGALG00010009603	HINT1	case	control	-0.504230273	0.438612579	-1.149602856	0.250307481	0.715141943	down_in_case
CRA004775	ENSGALG00010009605	ENSGALG00010009605	case	control	-0.012688941	0.250834379	-0.05058693	0.959654678	0.991431439	down_in_case
CRA004775	ENSGALG00010009606	CHSY3	case	control	-0.010278782	0.410754742	-0.025024134	0.980035713	0.996865238	down_in_case

CRA004775	ENSGALG00010009608	AFAP1	case	control	0.380481769	0.320416876	1.18745858	0.235046785	0.702145501	up_in_case
CRA004775	ENSGALG00010009609	MMADHC	case	control	-0.112654833	0.20460566	-0.550594899	0.581911406	0.889293593	down_in_case
CRA004775	ENSGALG00010009612	DERL1	case	control	-0.372689382	0.195051792	-1.910720113	0.056040559	0.433172413	down_in_case
CRA004775	ENSGALG00010009615	ENSGALG00010009615	case	control	-0.920346506	0.593659804	-1.550292777	0.12107126	0.573473028	down_in_case
CRA004775	ENSGALG00010009616	ARMC10	case	control	-0.167134763	0.311891208	-0.535875199	0.592044806	0.893804699	down_in_case
CRA004775	ENSGALG00010009617	SLC25A46	case	control	0.026853611	0.184752274	0.145349284	0.884435105	0.976236592	up_in_case
CRA004775	ENSGALG00010009619	ENSGALG00010009619	case	control	0.774085681	0.385687476	2.007028303	0.044746649	0.393998162	up_in_case
CRA004775	ENSGALG00010009620	TMOD1	case	control	-1.034507145	0.466837189	-2.215991293	0.026692098	0.314252091	down_in_case
CRA004775	ENSGALG00010009623	XPO5	case	control	-0.17509035	0.170085094	-1.029427951	0.30327862	0.755679718	down_in_case
CRA004775	ENSGALG00010009627	IL17C	case	control	-0.35208542	0.707653776	-0.497539096	0.61880894	0.904276486	down_in_case
CRA004775	ENSGALG00010009634	TBC1D31	case	control	-0.309380824	0.324728996	-0.952735443	0.340724131	0.780875343	down_in_case
CRA004775	ENSGALG00010009635	TDRD7	case	control	-0.234800547	0.241170451	-0.973587542	0.330261375	0.77237154	down_in_case
CRA004775	ENSGALG00010009637	CYBA	case	control	0.158223967	0.357595518	0.442466303	0.658151809	0.917354438	up_in_case
CRA004775	ENSGALG00010009638	RND3	case	control	-0.093588926	0.275754882	-0.339391727	0.73431465	0.936819725	down_in_case
CRA004775	ENSGALG00010009640	NPR2	case	control	-0.370841183	0.437320505	-0.847984896	0.396446382	0.808998414	down_in_case
CRA004775	ENSGALG00010009643	PSMC2	case	control	-0.201015225	0.248338297	-0.809441102	0.418261468	0.818943344	down_in_case
CRA004775	ENSGALG00010009644	SLC27A6	case	control	-0.594420112	0.718202717	-0.82764949	0.407869032	0.814297749	down_in_case
CRA004775	ENSGALG00010009646	MVD	case	control	-0.45266351	0.302460746	-1.496602504	0.134496718	0.592420453	down_in_case
CRA004775	ENSGALG00010009647	GALNT2	case	control	-0.100041198	0.345832982	-0.289276048	0.77237014	0.946296525	down_in_case
CRA004775	ENSGALG00010009649	TENM2	case	control	0.839168153	0.704590429	1.191001351	0.233653054	0.700908192	up_in_case
CRA004775	ENSGALG00010009650	KLHL29	case	control	0.045543898	0.357781309	0.127295354	0.898706637	0.978790377	up_in_case
CRA004775	ENSGALG00010009652	CIAO2B	case	control	-0.25141291	0.24544937	-1.024296419	0.305695289	0.758032511	down_in_case
CRA004775	ENSGALG00010009653	ABLIM2	case	control	-0.199435008	0.353257746	-0.564559476	0.572373435	0.885642749	down_in_case
CRA004775	ENSGALG00010009654	NDUFA12	case	control	-0.041438685	0.403591738	-0.10267476	0.918221106	0.981710522	down_in_case
CRA004775	ENSGALG00010009657	MSMP	case	control	-2.483817925	0.978609357	-2.538109725	0.011145303	0.214048978	down_in_case
CRA004775	ENSGALG00010009658	NAPEPLD	case	control	0.273098626	0.312926924	0.872723326	0.382813901	0.801925538	up_in_case
CRA004775	ENSGALG00010009659	APOLD1	case	control	1.117769504	0.400193006	2.793076059	0.005220941	0.147117143	up_in_case
CRA004775	ENSGALG00010009663	FER	case	control	0.373039055	0.305378068	1.221564658	0.2218723	0.689550502	up_in_case
CRA004775	ENSGALG00010009665	DGKQ	case	control	-0.354939539	0.517722698	-0.685578477	0.492978957	0.855820432	down_in_case
CRA004775	ENSGALG00010009666	RRAD	case	control	0.210835323	0.773807175	0.272464937	0.785264548	0.95041797	up_in_case
CRA004775	ENSGALG00010009672	DDX47	case	control	-0.255002175	0.225090128	-1.132889202	0.257260785	0.719236059	down_in_case
CRA004775	ENSGALG00010009676	ACO1	case	control	-0.260892209	0.294034433	-0.88728455	0.374925721	0.798503808	down_in_case
CRA004775	ENSGALG00010009677	SLC26A5	case	control	0.423755481	0.395899345	1.070361662	0.284456549	0.741035375	up_in_case
CRA004775	ENSGALG00010009678	JARID2	case	control	0.105844893	0.392147005	0.269911261	0.787228524	0.95041797	up_in_case
CRA004775	ENSGALG00010009679	ANK3	case	control	0.440893454	0.444693443	0.991454812	0.321463547	0.767650058	up_in_case
CRA004775	ENSGALG00010009683	ADAMTS19	case	control	-0.112325647	0.501025347	-0.224191547	0.822608261	0.960875066	down_in_case
CRA004775	ENSGALG00010009686	MAT2B	case	control	0.112320444	0.209940505	0.535010833	0.592642367	0.89416559	up_in_case
CRA004775	ENSGALG00010009689	ENSGALG00010009689	case	control	1.158421982	0.603806745	1.918531039	0.05504371	0.430875458	up_in_case
CRA004775	ENSGALG00010009690	RBM43	case	control	-0.266220655	0.339978368	-0.783051747	0.433596725	0.825828172	down_in_case
CRA004775	ENSGALG00010009695	ATAD2B	case	control	0.482838459	0.381071992	1.267053126	0.205136295	0.674045742	up_in_case
CRA004775	ENSGALG00010009697	ENSGALG00010009697	case	control	-0.796451342	0.454354392	-1.752929774	0.079614062	0.492032795	down_in_case
CRA004775	ENSGALG00010009699	POLR1C	case	control	-0.229445194	0.173401848	-1.323199243	0.185769128	0.6491093	down_in_case
CRA004775	ENSGALG00010009700	SH3TC1	case	control	0.137971707	0.491882512	0.28049728	0.779096012	0.94792594	up_in_case
CRA004775	ENSGALG00010009702	ENSGALG00010009702	case	control	-0.733751437	0.25990956	-2.823102915	0.00475613	0.141624457	down_in_case
CRA004775	ENSGALG00010009704	RGP1	case	control	-0.410569519	0.290638513	-1.41264664	0.157759648	0.62262379	down_in_case
CRA004775	ENSGALG00010009705	DTNBP1	case	control	-0.030694694	0.182525955	-0.168166189	0.866452539	0.972842501	down_in_case
CRA004775	ENSGALG00010009708	SMIM45	case	control	-0.064766435	0.655640273	-0.098783491	0.921310177	0.982481112	down_in_case
CRA004775	ENSGALG00010009710	ENSGALG00010009710	case	control	-0.060558295	0.280714744	-0.215728942	0.82919904	0.96297907	down_in_case
CRA004775	ENSGALG00010009712	UBXN2A	case	control	0.469497561	0.433859189	1.082142716	0.279189118	0.735529913	up_in_case
CRA004775	ENSGALG00010009715	NUDCD2	case	control	-0.211816047	0.220902459	-0.958866858	0.337625823	0.778184725	down_in_case
CRA004775	ENSGALG00010009717	EDN1	case	control	0.388963149	0.427397541	0.910073437	0.362783782	0.793608387	up_in_case
CRA004775	ENSGALG00010009718	ENSGALG00010009718	case	control	-0.753485925	0.334368867	-2.253457186	0.024230337	0.30145607	down_in_case
CRA004775	ENSGALG00010009719	YIPF3	case	control	-0.360184427	0.178323894	-2.019832673	0.043400747	0.389812978	down_in_case
CRA004775	ENSGALG00010009720	RELN	case	control	0.338140025	0.476014817	0.710356091	0.477483345	0.846563638	up_in_case
CRA004775	ENSGALG00010009721	ZHX1	case	control	0.323185989	0.271010544	1.192521826	0.233056698	0.69988265	up_in_case
CRA004775	ENSGALG00010009722	GBA2	case	control	-0.129708696	0.211626938	-0.612912027	0.53993451	0.874206929	down_in_case
CRA004775	ENSGALG00010009724	ISOC1	case	control	-0.056976522	0.248292691	-0.229473215	0.818501136	0.959941088	down_in_case
CRA004775	ENSGALG00010009726	PRR16	case	control	0.632999483	0.419228513	1.509915149	0.131065077	0.586487428	up_in_case
CRA004775	ENSGALG00010009727	CDH20	case	control	0.451196339	0.457266731	0.986724615	0.32377766	0.769171227	up_in_case
CRA004775	ENSGALG00010009728	CCDC149	case	control	-0.453438392	0.286431203	-1.583062137	0.113407302	0.562886277	down_in_case
CRA004775	ENSGALG00010009730	C8orf76	case	control	-0.210409936	0.243330291	-0.864709176	0.387198432	0.803905475	down_in_case
CRA004775	ENSGALG00010009731	NMI	case	control	-0.335376689	0.261262813	-1.283675568	0.199255496	0.666495337	down_in_case
CRA004775	ENSGALG00010009732	HMMR	case	control	-0.050010711	0.648811131	-0.077080538	0.938559475	0.986707289	down_in_case
CRA004775	ENSGALG00010009736	WISP1	case	control	-0.384832411	0.421957354	-0.91201731	0.361759606	0.792757982	down_in_case
CRA004775	ENSGALG00010009738	ENSGALG00010009738	case	control	-0.491814138	0.457251025	-1.075588923	0.282111144	0.738291085	down_in_case
CRA004775	ENSGALG00010009740	LRRC73	case	control	-1.655367243	0.439988618	-3.7622956	0.000168361	0.027735809	down_in_case
CRA004775	ENSGALG00010009741	C2H6orf52	case	control	0.054573961	0.278353535	0.19605988	0.844563298	0.966751844	up_in_case
CRA004775	ENSGALG00010009743	CCNG1	case	control	0.201888101	0.367569314	0.549251782	0.582832672	0.889712708	up_in_case
CRA004775	ENSGALG00010009744	DAPK1	case	control	-0.325780166	0.415777399	-0.783544674	0.43330733	0.825828172	down_in_case
CRA004775	ENSGALG00010009749	NDRG1	case	control	-0.00114209	0.272224447	-0.004195397	0.996652567	0.999606847	down_in_case
CRA004775	ENSGALG00010009751	ENSGALG00010009751	case	control	-0.144139167	0.480968931	-0.299684985	0.764417452	0.94239774	down_in_case
CRA004775	ENSGALG00010009761	PDP2	case	control	0.611458288	0.237043331	2.57952116	0.00989374	0.201255643	up_in_case
CRA004775	ENSGALG00010009762	MAN2A1	case	control	0.691387326	0.281151359	2.459128525	0.013927475	0.236148836	up_in_case
CRA004775	ENSGALG00010009765	STX10	case	control	-0.333733432	0.169042851	-1.97425345	0.048352931	0.407414924	down_in_case
CRA004775	ENSGALG00010009769	TMCC3	case	control	0.835879928	0.452394983	1.847677273	0.064649044	0.457298868	up_in_case
CRA004775	ENSGALG00010009772	ENSGALG00010009772	case	control	0.165847632	1.036335593	0.160032747	0.872855278	0.97407675	up_in_case
CRA004775	ENSGALG00010009774	PIAS2	case	control	-0.326513409	0.215014317	-1.518565896	0.128871801	0.583811118	down_in_case
CRA004775	ENSGALG00010009775	KIAA1614	case	control	-0.070220167	0.171140364	-0.410307453	0.681580425	0.924278508	down_in_case
CRA004775	ENSGALG00010009777	ST3GAL1	case	control	-0.564718981	0.385713799	-1.464088094	0.14316988	0.603744472	down_in_case
CRA004775	ENSGALG00010009778	ENSGALG00010009778	case	control	-0.181616107	0.180684363	-1.005156746	0.314821379	0.765098415	down_in_case
CRA004775	ENSGALG00010009779	PPP1R3E	case	control	-0.752790747	0.330875257	-2.27514972	0.02289695	0.294061167	down_in_case
CRA004775	ENSGALG00010009780	MFSD2B	case	control	-0.275123435	0.519641131	-0.529448918	0.596494071	0.895574418	down_in_case
CRA004775	ENSGALG00010009784	ENSGALG00010009784	case	control	0.106754838	0.288114512	0.370529192	0.71098823	0.932234469	up_in_case
CRA004775	ENSGALG00010009786	DIPK2B	case	control	0.034868029	0.352849577	0.098818397	0.921282462	0.982481112	up_in_case
CRA004775	ENSGALG00010009788	PAK1IP1	case	control	0.21809449	0.267299555	0.815917895	0.414547097	0.818205415	up_in_case
CRA004775	ENSGALG00010009793	TNFAIP6	case	control	0.484358135	0.481620827	1.005683533	0.314567827	0.765098415	up_in_case
CRA004775	ENSGALG00010009795	NDUFA6	case	control	-0.122818625	0.425307442	-0.288776103	0.772752721	0.946296525	down_in_case
CRA004775	ENSGALG00010009796	ENSGALG00010009796	case	control	-0.442786647	0.569518168	-0.777475893	0.436878052	0.827138935	down_in_case
CRA004775	ENSGALG00010009798	YIPF4	case	control	0.285875062	0.173356998	1.649054066	0.099136559	0.536492255	up_in_case
CRA004775	ENSGALG00010009801	ENSGALG00010009801	case	control	1.792521763	0.682714964	2.625578546	0.008650185	0.188268829	up_in_case
CRA004775	ENSGALG00010009804	XPR1	case	control	-0.148961854	0.138046921	-1.079066837	0.280557934	0.73695361	down_in_case
CRA004775	ENSGALG00010009806	WDCP	case	control	-0.116679917	0.45247144	-0.257872447	0.79650535	0.952835572	down_in_case
CRA004775	ENSGALG00010009809	SMDT1	case	control	0.190345494	0.272090182	0.699567665	0.484197342	0.850779152	up_in_case
CRA004775	ENSGALG00010009811	ENSGALG00010009811	case	control	0.081614287	0.229624239	0.3554254	0.722270923	0.935123572	up_in_case
CRA004775	ENSGALG00010009815	ENSGALG00010009815	case	control	-1.119520863	0.883185572	-1.267594149	0.204942923	0.674045742	down_in_case
CRA004775	ENSGALG00010009822	ENSGALG00010009822	case	control	-0.118049178	0.373432466	-0.316119216	0.751912022	0.941495694	down_in_case
CRA004775	ENSGALG00010009830	RNASEL	case	control	-0.134965464	0.446646642	-0.302175034	0.762518635	0.94239774	down_in_case
CRA004775	ENSGALG00010009831	N6AMT1	case	control	-0.445422044	0.228585136	-1.948604585	0.051342664	0.418339211	down_in_case
CRA004775	ENSGALG00010009833	SOD3	case	control	-0.085035102	0.368395941	-0.230825297	0.817450528	0.959812855	down_in_case
CRA004775	ENSGALG00010009840	ENSGALG00010009840	case	control	-0.399582064	0.301326114	-1.326078442	0.184813716	0.6491093	down_in_case
CRA004775	ENSGALG00010009841	GABRA1	case	control	1.889470429	0.710777252	2.658315842	0.007853226	0.178495383	up_in_case
CRA004775	ENSGALG00010009842	PHETA2	case	control	-0.092645665	0.259827911	-0.356565486	0.72141712	0.934511903	down_in_case
CRA004775	ENSGALG00010009847	ENSGALG00010009847	case	control	-0.019055789	0.164115326	-0.116112183	0.907563633	0.980354087	down_in_case
CRA004775	ENSGALG00010009850	RIF1	case	control	-0.452634225	0.401045092	-1.12863674	0.259051106	0.720037471	down_in_case
CRA004775	ENSGALG00010009852	ENSGALG00010009852	case	control	0.984826916	0.744737794	1.322380741	0.186041399	0.6491093	up_in_case
CRA004775	ENSGALG00010009857	ORC5	case	control	-0.384383844	0.330438008	-1.163255541	0.244725838	0.711765017	down_in_case
CRA004775	ENSGALG00010009858	ST8SIA5	case	control	-0.21792309	0.474412911	-0.459353203	0.645980548	0.914043203	down_in_case
CRA004775	ENSGALG00010009866	NAGA	case	control	-0.067781827	0.343686262	-0.197220064	0.84365533	0.966478468	down_in_case
CRA004775	ENSGALG00010009868	LTN1	case	control	0.225857765	0.192007576	1.176296113	0.239476568	0.706254731	up_in_case
CRA004775	ENSGALG00010009876	SELENOI	case	control	0.025434888	0.202223327	0.125776229	0.899909059	0.978929349	up_in_case
CRA004775	ENSGALG00010009883	ENSGALG00010009883	case	control	0.029920034	0.343312717	0.087150963	0.930551517	0.985044263	up_in_case
CRA004775	ENSGALG00010009884	GFOD1	case	control	0.726563307	0.348283111	2.086128454	0.036966989	0.362835878	up_in_case
CRA004775	ENSGALG00010009910	ENSGALG00010009910	case	control	-0.089842282	0.357525003	-0.251289507	0.801590287	0.953543664	down_in_case
CRA004775	ENSGALG00010009914	RWDD2B	case	control	-0.116440439	0.290702432	-0.400548556	0.688752528	0.927420456	down_in_case
CRA004775	ENSGALG00010009943	DHX15	case	control	-0.108823994	0.179015751	-0.607901782	0.543252617	0.875456081	down_in_case
CRA004775	ENSGALG00010009948	WBP2NL	case	control	-0.285929324	0.203436633	-1.405495754	0.159873911	0.624907945	down_in_case
CRA004775	ENSGALG00010009972	PTK2B	case	control	-0.995813322	0.543722182	-1.831474519	0.067029748	0.462498477	down_in_case
CRA004775	ENSGALG00010009985	USP16	case	control	-0.081558385	0.186322626	-0.437726684	0.661584424	0.918136475	down_in_case
CRA004775	ENSGALG00010009989	TBC1D7	case	control	-0.82055719	0.22524906	-3.642888413	0.000269596	0.033398296	down_in_case
CRA004775	ENSGALG00010009991	LHFPL3	case	control	-0.331829616	0.989780118	-0.335255892	0.737432064	0.937597314	down_in_case
CRA004775	ENSGALG00010009993	ENSGALG00010009993	case	control	0.914652663	0.95767429	0.955076975	0.339538772	0.779891494	up_in_case
CRA004775	ENSGALG00010009995	SEPTIN3	case	control	2.129732298	0.731217107	2.912585437	0.003584501	0.125102734	up_in_case
CRA004775	ENSGALG00010010006	PPARGC1A	case	control	-0.20035472	0.588780002	-0.340287916	0.733639716	0.936819725	down_in_case
CRA004775	ENSGALG00010010007	CTIF	case	control	0.145639994	0.323079279	0.450787171	0.652142949	0.915892481	up_in_case
CRA004775	ENSGALG00010010009	ELF2	case	control	0.251983896	0.204888904	1.229856234	0.218750946	0.686309227	up_in_case
CRA004775	ENSGALG00010010011	EPB41L4A	case	control	-0.082884039	0.527848392	-0.157022433	0.875227168	0.974356282	down_in_case

CRA004775	ENSGALG00010010014	GLUL	case	control	0.289695533	0.700069269	0.413809812	0.679013397	0.92338129	up_in_case
CRA004775	ENSGALG00010010017	CCDC59	case	control	0.124462409	0.210105208	0.592381361	0.553595245	0.878871229	up_in_case
CRA004775	ENSGALG00010010022	KMT2E	case	control	-0.05268395	0.331991599	-0.158690611	0.873912636	0.974115968	down_in_case
CRA004775	ENSGALG00010010035	ATP10B	case	control	1.652622104	0.731776705	2.258369381	0.02392264	0.299054748	up_in_case
CRA004775	ENSGALG00010010037	NAA15	case	control	0.45856709	0.228977522	2.002672949	0.045212404	0.395272567	up_in_case
CRA004775	ENSGALG00010010042	METTL25	case	control	-0.306627686	0.272724621	-1.124312448	0.260880499	0.721659172	down_in_case
CRA004775	ENSGALG00010010049	ZBTB7C	case	control	-1.200387364	0.833933161	-1.439428745	0.150029085	0.61366707	down_in_case
CRA004775	ENSGALG00010010052	ZNF346	case	control	-0.33065108	0.213681398	-1.547402267	0.121766275	0.573689301	down_in_case
CRA004775	ENSGALG00010010059	ENSGALG00010010059	case	control	0.038063631	0.261948428	0.145309638	0.884466405	0.976236592	up_in_case
CRA004775	ENSGALG00010010061	CYP2D6	case	control	0.026856982	0.45114384	0.059530862	0.952529285	0.990414381	up_in_case
CRA004775	ENSGALG00010010062	RGS7BP	case	control	0.709295981	0.583828466	1.214904758	0.224402419	0.692494987	up_in_case
CRA004775	ENSGALG00010010064	ENSGALG00010010064	case	control	0.314661836	0.24202554	1.30011831	0.193560423	0.657919837	up_in_case
CRA004775	ENSGALG00010010071	GLT8D2	case	control	0.053488514	0.467399507	0.114438534	0.908890169	0.98075825	up_in_case
CRA004775	ENSGALG00010010077	CCT8	case	control	-0.0749693	0.207662277	-0.361015497	0.718087862	0.934011578	down_in_case
CRA004775	ENSGALG00010010080	CDC42EP3	case	control	0.373012829	0.297520126	1.253739821	0.209936589	0.67982426	up_in_case
CRA004775	ENSGALG00010010084	HK3	case	control	-0.015803178	0.381333385	-0.041441895	0.966943614	0.993235351	down_in_case
CRA004775	ENSGALG00010010085	TDG	case	control	0.104086856	0.336628147	0.309204256	0.757166157	0.941900693	up_in_case
CRA004775	ENSGALG00010010094	SLC2A9	case	control	-0.181594341	0.773111961	-0.234887507	0.814296047	0.958686054	down_in_case
CRA004775	ENSGALG00010010095	C12orf73	case	control	-0.538862164	0.332183415	-1.622182626	0.104764251	0.547761677	down_in_case
CRA004775	ENSGALG00010010100	IRF2BP2	case	control	0.249505408	0.32705741	0.762879543	0.445535237	0.833012432	up_in_case
CRA004775	ENSGALG00010010106	C7orf73	case	control	-0.697987442	0.326883926	-2.135276123	0.032738462	0.344970569	down_in_case
CRA004775	ENSGALG00010010107	MGST2	case	control	0.956059876	0.31712091	3.014811849	0.002571387	0.105549686	up_in_case
CRA004775	ENSGALG00010010111	CNOT4	case	control	-0.051979043	0.315015542	-0.165004693	0.868940288	0.973370973	down_in_case
CRA004775	ENSGALG00010010115	NEB	case	control	0.784103007	0.548427712	1.429729005	0.152794812	0.617472961	up_in_case
CRA004775	ENSGALG00010010119	SRPK2	case	control	-0.389456555	0.284308066	-1.369839978	0.17073686	0.635056142	down_in_case
CRA004775	ENSGALG00010010122	THBS4	case	control	-0.500242888	0.80000343	-0.625300929	0.531773571	0.869801162	down_in_case
CRA004775	ENSGALG00010010124	ENSGALG00010010124	case	control	-0.056997299	0.414187338	-0.137612364	0.890546783	0.977405126	down_in_case
CRA004775	ENSGALG00010010125	AHCTF1	case	control	0.404615772	0.186394475	2.17074981	0.02995009	0.330139205	up_in_case
CRA004775	ENSGALG00010010127	FAM13C	case	control	0.473399081	0.590430193	0.801786707	0.422676349	0.820450913	up_in_case
CRA004775	ENSGALG00010010136	EPHX2	case	control	0.191378125	0.223073931	0.857913446	0.390940259	0.806479902	up_in_case
CRA004775	ENSGALG00010010142	ENSGALG00010010142	case	control	1.718477315	0.548621905	3.132352719	0.001734114	0.084784105	up_in_case
CRA004775	ENSGALG00010010150	NTRK2	case	control	-0.070583253	0.640526266	-0.11019572	0.912254157	0.98075825	down_in_case
CRA004775	ENSGALG00010010153	ENSGALG00010010153	case	control	-0.206305618	0.486526306	-0.424037951	0.671538136	0.921605102	down_in_case
CRA004775	ENSGALG00010010156	ENSGALG00010010156	case	control	-0.097914517	0.222899932	-0.439275671	0.6604618	0.917907174	down_in_case
CRA004775	ENSGALG00010010161	SEPSECS	case	control	-0.098194209	0.207323137	-0.473628804	0.635764632	0.909908353	down_in_case
CRA004775	ENSGALG00010010167	PUS7	case	control	-0.074763033	0.208387901	-0.35876859	0.719768212	0.934081982	down_in_case
CRA004775	ENSGALG00010010168	SNCB	case	control	-0.890427767	0.726557109	-1.225544084	0.220370291	0.688394336	down_in_case
CRA004775	ENSGALG00010010171	ENSGALG00010010171	case	control	0.359298579	0.560196503	0.641379546	0.521276119	0.866299649	up_in_case
CRA004775	ENSGALG00010010178	LGI2	case	control	-1.118480357	0.521731458	-2.143785542	0.032050074	0.340736995	down_in_case
CRA004775	ENSGALG00010010179	CDHR2	case	control	2.158131439	0.862521709	2.502118401	0.012345263	0.223662335	up_in_case
CRA004775	ENSGALG00010010183	NKX3-2	case	control	-0.503938765	0.435687381	-1.156652194	0.247414489	0.712046495	down_in_case
CRA004775	ENSGALG00010010185	PHYHIPL	case	control	1.662178852	0.610090251	2.724480273	0.006440278	0.159568035	up_in_case
CRA004775	ENSGALG00010010186	TNPO1	case	control	0.380570201	0.167280045	2.27504841	0.022903026	0.294061167	up_in_case
CRA004775	ENSGALG00010010188	DSP	case	control	1.838074742	0.651201205	2.822591126	0.004763728	0.141624457	up_in_case
CRA004775	ENSGALG00010010190	RNF44	case	control	0.147168428	0.277947385	0.529483047	0.596470402	0.895574418	up_in_case
CRA004775	ENSGALG00010010191	RINT1	case	control	-0.368046889	0.229062921	-1.606750182	0.10810918	0.554704978	down_in_case
CRA004775	ENSGALG00010010195	ENSGALG00010010195	case	control	2.052910788	0.800442758	2.564719046	0.010325938	0.20410795	up_in_case
CRA004775	ENSGALG00010010196	EFCAB10	case	control	0.191956043	0.753616016	0.254713328	0.798944522	0.952835572	up_in_case
CRA004775	ENSGALG00010010197	SCCPDH	case	control	0.003164064	0.133195986	0.023754947	0.981048077	0.997069916	up_in_case
CRA004775	ENSGALG00010010201	RIOK1	case	control	-0.103804671	0.22881015	-0.453671618	0.650065198	0.915428088	down_in_case
CRA004775	ENSGALG00010010205	ARL5A	case	control	-0.011152144	0.182407822	-0.061138517	0.951248895	0.990168609	down_in_case
CRA004775	ENSGALG00010010206	SCARA5	case	control	-0.083876898	0.266149758	-0.315149255	0.752648332	0.94167946	down_in_case
CRA004775	ENSGALG00010010214	FAF2	case	control	-0.270602062	0.166493342	-1.625302601	0.104098098	0.546564696	down_in_case
CRA004775	ENSGALG00010010215	ENSGALG00010010215	case	control	0.03849592	0.194941659	0.197474058	0.843456581	0.966478468	up_in_case
CRA004775	ENSGALG00010010216	ATXN7L1	case	control	0.269701261	0.240947464	1.119336376	0.262996663	0.723872521	up_in_case
CRA004775	ENSGALG00010010218	PBK	case	control	-0.134457683	0.414357661	-0.324496675	0.745562039	0.939421865	down_in_case
CRA004775	ENSGALG00010010223	ENSGALG00010010223	case	control	-0.582179488	0.50890052	-1.143994681	0.252625843	0.717153621	down_in_case
CRA004775	ENSGALG00010010226	BICC1	case	control	0.333275273	0.270475285	1.232183832	0.217880421	0.685219057	up_in_case
CRA004775	ENSGALG00010010231	C1orf53	case	control	-0.524305838	0.356112431	-1.472304228	0.140938742	0.600572867	down_in_case
CRA004775	ENSGALG00010010234	ENSGALG00010010234	case	control	0.003093102	0.511461587	0.006047574	0.995174763	0.999001196	up_in_case
CRA004775	ENSGALG00010010243	ESCO2	case	control	-0.156441527	0.244418037	-0.640057211	0.522135406	0.866416245	down_in_case
CRA004775	ENSGALG00010010246	CLTB	case	control	-0.383241286	0.313634414	-1.221936335	0.221731704	0.689550502	down_in_case
CRA004775	ENSGALG00010010252	WDR7	case	control	0.03692909	0.23947904	0.154205936	0.877447362	0.974479909	up_in_case
CRA004775	ENSGALG00010010253	IL10RB	case	control	0.002530675	0.17675861	0.014317123	0.988576979	0.998065748	up_in_case
CRA004775	ENSGALG00010010254	WDR41	case	control	-0.18351358	0.271240748	-0.676570835	0.498678289	0.858345869	down_in_case
CRA004775	ENSGALG00010010255	CACNB4	case	control	-0.110340355	0.400729944	-0.275348416	0.783048571	0.94936527	down_in_case
CRA004775	ENSGALG00010010257	ENSGALG00010010257	case	control	-0.126129128	0.324058373	-0.389217311	0.697115399	0.929092636	down_in_case
CRA004775	ENSGALG00010010259	IGF1	case	control	-2.525849004	0.912114707	-2.769222977	0.005619017	0.151801761	down_in_case
CRA004775	ENSGALG00010010262	HSPA9	case	control	0.215104917	0.285454369	0.753552722	0.451117843	0.83602715	up_in_case
CRA004775	ENSGALG00010010263	RAB4A	case	control	0.135740611	0.252828611	0.536887855	0.591345081	0.893804699	up_in_case
CRA004775	ENSGALG00010010266	EFNA5	case	control	-0.181120219	0.475573119	-0.380846208	0.703317369	0.930068722	down_in_case
CRA004775	ENSGALG00010010267	CCSER2	case	control	1.019761674	0.340733343	2.9928438	0.002763912	0.10984643	up_in_case
CRA004775	ENSGALG00010010270	SSR1	case	control	-0.027563007	0.227513348	-0.121148966	0.903573045	0.979468548	down_in_case
CRA004775	ENSGALG00010010271	HUNK	case	control	0.422330602	0.300703303	1.404476101	0.160177123	0.625204549	up_in_case
CRA004775	ENSGALG00010010274	MIS18A	case	control	0.040191795	0.280037815	0.143522742	0.885877352	0.976802138	up_in_case
CRA004775	ENSGALG00010010275	PDE8B	case	control	0.253897815	0.583866649	0.434855829	0.663667078	0.918295135	up_in_case
CRA004775	ENSGALG00010010279	CCSAP	case	control	-0.035397961	0.422857407	-0.083711342	0.93328594	0.985090297	down_in_case
CRA004775	ENSGALG00010010280	HSP90B1	case	control	0.120452406	0.317486514	0.379393771	0.704395475	0.930465147	up_in_case
CRA004775	ENSGALG00010010284	CCDC25	case	control	0.034520731	0.313497651	0.110114798	0.912318332	0.98075825	up_in_case
CRA004775	ENSGALG00010010285	ENSGALG00010010285	case	control	-0.216472311	0.698808861	-0.309773278	0.756733374	0.941900693	down_in_case
CRA004775	ENSGALG00010010287	STAM2	case	control	0.077078693	0.206184472	0.373833648	0.708528084	0.931801673	up_in_case
CRA004775	ENSGALG00010010288	ENSGALG00010010288	case	control	-0.137732804	0.306649357	-0.449154062	0.653320523	0.916442743	down_in_case
CRA004775	ENSGALG00010010290	ENSGALG00010010290	case	control	-0.765228965	0.333155513	-2.296912209	0.021623778	0.288534247	down_in_case
CRA004775	ENSGALG00010010291	TCF20	case	control	0.082220963	0.27921309	0.294473884	0.768395817	0.945059483	up_in_case
CRA004775	ENSGALG00010010293	ENSGALG00010010293	case	control	-0.807273764	0.811518367	-0.994769554	0.319848357	0.767037518	down_in_case
CRA004775	ENSGALG00010010295	ENSGALG00010010295	case	control	-0.251093503	0.879692467	-0.285433277	0.775312216	0.946410855	down_in_case
CRA004775	ENSGALG00010010300	DENND1B	case	control	0.324439485	0.245260646	1.322835485	0.185890094	0.6491093	up_in_case
CRA004775	ENSGALG00010010302	WDR36	case	control	-0.052089761	0.222401863	-0.23421459	0.814818389	0.958848415	down_in_case
CRA004775	ENSGALG00010010303	ETF1	case	control	0.340649666	0.301661288	1.129245546	0.258794265	0.720037471	up_in_case
CRA004775	ENSGALG00010010305	URB1	case	control	0.240424605	0.297592827	0.807897849	0.41914939	0.818943344	up_in_case
CRA004775	ENSGALG00010010308	PACRGL	case	control	0.043373497	0.560540244	0.077378024	0.938322822	0.986707289	up_in_case
CRA004775	ENSGALG00010010310	NT5DC3	case	control	0.120432107	0.290479211	0.414598025	0.678436194	0.92338129	up_in_case
CRA004775	ENSGALG00010010311	CTSV	case	control	0.443662974	0.475143391	0.933745437	0.350435221	0.785466132	up_in_case
CRA004775	ENSGALG00010010317	ENSGALG00010010317	case	control	-0.292767835	0.359619473	-0.814104511	0.415585082	0.818943344	down_in_case
CRA004775	ENSGALG00010010320	SYPL1	case	control	0.184310023	0.613108763	0.300615543	0.763707678	0.94239774	up_in_case
CRA004775	ENSGALG00010010321	NUP133	case	control	-0.553911571	0.29348886	-1.88733423	0.059115385	0.44414236	down_in_case
CRA004775	ENSGALG00010010322	ENSGALG00010010322	case	control	0.093434488	0.291617136	0.320401227	0.748664196	0.940945193	up_in_case
CRA004775	ENSGALG00010010323	FBP2	case	control	-0.200220116	0.684378	-0.292557791	0.769860181	0.945462833	down_in_case
CRA004775	ENSGALG00010010327	FBP1	case	control	-0.684548177	0.540794354	-1.265819755	0.205577621	0.674045742	down_in_case
CRA004775	ENSGALG00010010330	ALPK2	case	control	0.678232932	0.43877776	1.545732245	0.122169246	0.574931316	up_in_case
CRA004775	ENSGALG00010010333	WAPL	case	control	0.273584528	0.198147365	1.380712422	0.1673674	0.631096819	up_in_case
CRA004775	ENSGALG00010010334	ZBTB41	case	control	0.443172853	0.283253062	1.564582742	0.117680778	0.568397745	up_in_case
CRA004775	ENSGALG00010010336	FMNL2	case	control	0.083500872	0.304035982	0.274641413	0.783591746	0.949772596	up_in_case
CRA004775	ENSGALG00010010337	SCARA3	case	control	-0.205665193	0.350485941	-0.586800121	0.557337955	0.880622207	down_in_case
CRA004775	ENSGALG00010010338	CNST	case	control	-0.089646476	0.318465435	-0.281495153	0.778330645	0.947907599	down_in_case
CRA004775	ENSGALG00010010345	ENSGALG00010010345	case	control	-0.332769322	0.475274473	-0.700162413	0.483825882	0.850349167	down_in_case
CRA004775	ENSGALG00010010346	TFB2M	case	control	0.264534352	0.259458877	1.019561771	0.307936344	0.759812069	up_in_case
CRA004775	ENSGALG00010010347	FBXW11	case	control	0.172934175	0.200699346	0.861657887	0.388875806	0.804884358	up_in_case
CRA004775	ENSGALG00010010349	RREB1	case	control	0.041700619	0.303705485	0.13730611	0.890788841	0.977513084	up_in_case
CRA004775	ENSGALG00010010351	NAMPTP1	case	control	0.926264991	0.365772401	2.532353423	0.011329973	0.216086625	up_in_case
CRA004775	ENSGALG00010010352	MALT1	case	control	0.231145893	0.296741941	0.778945815	0.436011634	0.82694408	up_in_case
CRA004775	ENSGALG00010010356	STAB2	case	control	-0.343669061	0.532493526	-0.645395755	0.518670754	0.865435399	down_in_case
CRA004775	ENSGALG00010010358	ASPM	case	control	0.166700698	0.579926187	0.287451578	0.773766573	0.946296525	up_in_case
CRA004775	ENSGALG00010010361	PIGM	case	control	-0.343209175	0.378829096	-0.905973638	0.364949806	0.793608387	down_in_case
CRA004775	ENSGALG00010010366	CLU	case	control	0.137170783	0.473742881	0.2895469	0.772162895	0.946296525	up_in_case
CRA004775	ENSGALG00010010368	ENSGALG00010010368	case	control	0.036213199	0.339079158	0.106798657	0.914948713	0.981393117	up_in_case
CRA004775	ENSGALG00010010371	SOCS1L	case	control	-0.204120071	0.28431012	-0.717948663	0.472788941	0.845425322	down_in_case
CRA004775	ENSGALG00010010374	CMTM4	case	control	-0.27881474	0.342988288	-0.812898719	0.416276129	0.818943344	down_in_case
CRA004775	ENSGALG00010010379	SLC35F6	case	control	-0.39537813	0.329185131	-1.201081374	0.229719638	0.695787387	down_in_case
CRA004775	ENSGALG00010010381	MAST4	case	control	0.891778899	0.386312106	2.308441504	0.020974593	0.284887644	up_in_case
CRA004775	ENSGALG00010010382	SEC11C	case	control	-0.587645237	0.365340058	-1.60848838	0.107728259	0.55406723	down_in_case
CRA004775	ENSGALG00010010383	SLIT2	case	control	-0.27351245	0.462214761	-0.591743217	0.554022552	0.879382329	down_in_case
CRA004775	ENSGALG00010010386	PRPF40A	case	control	0.121553048	0.227812711	0.533565699	0.593642045	0.894594166	up_in_case
CRA004775	ENSGALG00010010390	KCNK3	case	control	-0.534607291	0.861604222	-0.62047896	0.534942502	0.870981055	down_in_case
CRA004775	ENSGALG00010010393	FANCC	case	control	-0.803782272	0.52820265	-1.521730858	0.128076532	0.5821156	down_in_case
CRA004775	ENSGALG00010010394	MRPL42	case	control	-0.055204173	0.28048898	-0.196814054	0.843973053	0.966478468	down_in_case

CRA004775	ENSGALG00010010395	GRP	case	control	1.658038197	0.661566981	2.506228763	0.012202664	0.222250112	up_in_case
CRA004775	ENSGALG00010010397	PICK1	case	control	-0.195235187	0.208179821	-0.93781994	0.348336952	0.785270866	down_in_case
CRA004775	ENSGALG00010010399	FGF18	case	control	0.437948189	0.603491849	0.725690313	0.468028641	0.843870188	up_in_case
CRA004775	ENSGALG00010010404	COP1	case	control	0.212187925	0.162519882	1.305612107	0.191684501	0.656959697	up_in_case
CRA004775	ENSGALG00010010405	EVA1C	case	control	0.136776199	0.325336382	0.420414706	0.674182527	0.922533825	up_in_case
CRA004775	ENSGALG00010010406	PTCH1	case	control	-0.018819377	0.262660161	-0.071649147	0.942881127	0.987862092	down_in_case
CRA004775	ENSGALG00010010407	SOX10	case	control	1.381615481	0.487692933	2.832961863	0.004611889	0.139995768	up_in_case
CRA004775	ENSGALG00010010408	GAS8	case	control	-0.312530467	0.192412572	-1.624272592	0.104317644	0.546673752	down_in_case
CRA004775	ENSGALG00010010409	RAX	case	control	1.056815925	0.608461542	1.736865608	0.082410895	0.499148629	up_in_case
CRA004775	ENSGALG00010010410	CACNA2D1	case	control	0.900493713	0.410234763	2.195069246	0.028158631	0.319106625	up_in_case
CRA004775	ENSGALG00010010411	CD180	case	control	-1.823618526	0.671835068	-2.714384249	0.00663991	0.160913119	down_in_case
CRA004775	ENSGALG00010010416	SLC35B3	case	control	-0.12654819	0.262032535	-0.482948387	0.629132384	0.908586927	down_in_case
CRA004775	ENSGALG00010010420	NPM1	case	control	0.012825734	0.18687824	0.068631499	0.945282945	0.988711172	up_in_case
CRA004775	ENSGALG00010010421	POLR2F	case	control	-0.131381409	0.318711883	-0.412226266	0.680173587	0.923896221	down_in_case
CRA004775	ENSGALG00010010422	CDH1	case	control	2.618766589	0.709143983	3.69285597	0.00022175	0.030492791	up_in_case
CRA004775	ENSGALG00010010423	C21orf59	case	control	-0.12247835	0.272669913	-0.449181756	0.653300547	0.916442743	down_in_case
CRA004775	ENSGALG00010010425	BLOC1S5	case	control	0.098104714	0.217748539	0.450541321	0.652320166	0.915892481	up_in_case
CRA004775	ENSGALG00010010431	SOCS2	case	control	0.043414647	0.277754097	0.156306055	0.875791784	0.974412785	up_in_case
CRA004775	ENSGALG00010010434	PROM1	case	control	-0.781790894	0.685873627	-1.139846851	0.254350111	0.718335926	down_in_case
CRA004775	ENSGALG00010010435	GAS1	case	control	-0.009616903	0.373986038	-0.025714605	0.979484975	0.996764329	down_in_case
CRA004775	ENSGALG00010010438	LDB3	case	control	0.39090532	0.714701733	0.54694889	0.584413846	0.89025771	up_in_case
CRA004775	ENSGALG00010010439	HGF	case	control	0.81979288	0.715559168	1.145667496	0.251932758	0.716658534	up_in_case
CRA004775	ENSGALG00010010444	TXNDC5	case	control	-0.120561292	0.315669845	-0.381922106	0.702519142	0.930068722	down_in_case
CRA004775	ENSGALG00010010445	LMAN1L	case	control	-0.174890011	0.382938049	-0.456705755	0.647882553	0.914309626	down_in_case
CRA004775	ENSGALG00010010446	C22orf23	case	control	-2.147501085	0.875395512	-2.453178085	0.014160022	0.238037235	down_in_case
CRA004775	ENSGALG00010010447	BTF3	case	control	-0.174240827	0.258101226	-0.675087174	0.499620382	0.859322061	down_in_case
CRA004775	ENSGALG00010010448	GALNT13	case	control	-0.48373709	0.358397868	-1.34972089	0.177105529	0.643089023	down_in_case
CRA004775	ENSGALG00010010449	PAPPA2	case	control	-1.145247429	1.030702608	-1.111132756	0.26651121	0.727311193	down_in_case
CRA004775	ENSGALG00010010456	BMP6	case	control	0.340036288	0.269557037	1.261463218	0.207142004	0.675932139	up_in_case
CRA004775	ENSGALG00010010457	AGGF1	case	control	0.265712618	0.187627475	1.416171152	0.1567254	0.621254244	up_in_case
CRA004775	ENSGALG00010010460	SEMA3C	case	control	0.853883602	0.335732014	2.543348762	0.010979558	0.211456439	up_in_case
CRA004775	ENSGALG00010010461	SLC7A5	case	control	0.059371218	0.543895957	0.109159147	0.913076263	0.980803023	up_in_case
CRA004775	ENSGALG00010010463	ACAA2	case	control	-0.196999403	0.279885828	-0.7038563	0.481522268	0.848818238	down_in_case
CRA004775	ENSGALG00010010465	BMPR1A	case	control	0.501165027	0.224978324	2.227614719	0.025906218	0.30842979	up_in_case
CRA004775	ENSGALG00010010466	CRADD	case	control	0.072803879	0.207527634	0.350815349	0.725726881	0.935238044	up_in_case
CRA004775	ENSGALG00010010468	ENSGALG00010010468	case	control	1.776194976	1.151079469	1.54306894	0.12281405	0.57525466	up_in_case
CRA004775	ENSGALG00010010471	KCNJ3	case	control	-0.589269958	0.519383842	-1.134555814	0.256561476	0.719236059	down_in_case
CRA004775	ENSGALG00010010472	MMRN2	case	control	0.557116683	0.439671303	1.267120867	0.205112075	0.674045742	up_in_case
CRA004775	ENSGALG00010010473	TRIM35	case	control	-0.211695771	0.297325038	-0.712001157	0.476464059	0.846367006	down_in_case
CRA004775	ENSGALG00010010475	CCDC71L	case	control	0.195048974	0.2615708	0.745683287	0.455858758	0.839383205	up_in_case
CRA004775	ENSGALG00010010483	FGFBP1	case	control	2.332746951	0.74002624	3.152248969	0.001620181	0.080802173	up_in_case
CRA004775	ENSGALG00010010486	ENSGALG00010010486	case	control	0.482675016	1.054419206	0.45776387	0.647122096	0.914210065	up_in_case
CRA004775	ENSGALG00010010494	ENSGALG00010010494	case	control	0.776612136	0.534317832	1.453464752	0.146094759	0.609511236	up_in_case
CRA004775	ENSGALG00010010496	MRPL17	case	control	-0.518312289	0.411958926	-1.258164967	0.208332102	0.677150891	down_in_case
CRA004775	ENSGALG00010010499	NR4A2	case	control	0.838497571	0.593983089	1.411652261	0.158052376	0.623101556	up_in_case
CRA004775	ENSGALG00010010505	CD38	case	control	-0.232118944	0.20089218	-1.155440417	0.247910119	0.712818804	down_in_case
CRA004775	ENSGALG00010010512	PLXNC1	case	control	0.430191209	0.527577525	0.815408519	0.41483851	0.818663081	up_in_case
CRA004775	ENSGALG00010010515	RXRG	case	control	-1.017746847	0.373281078	-2.726489254	0.006401204	0.15932286	down_in_case
CRA004775	ENSGALG00010010521	SNCG	case	control	0.515220939	0.826337245	0.623499596	0.532956268	0.869976453	up_in_case
CRA004775	ENSGALG00010010526	ENSGALG00010010526	case	control	-0.359376265	0.649305885	-0.553477604	0.579936412	0.888438428	down_in_case
CRA004775	ENSGALG00010010528	GPD2	case	control	0.146998156	0.39074357	0.376201087	0.706767408	0.931617022	up_in_case
CRA004775	ENSGALG00010010534	SYNJ1	case	control	-0.198779692	0.322692711	-0.616003043	0.537892512	0.872603995	down_in_case
CRA004775	ENSGALG00010010535	LMX1A	case	control	0.341551151	0.977885079	0.349275347	0.726882604	0.935238044	up_in_case
CRA004775	ENSGALG00010010537	ENSGALG00010010537	case	control	-1.14743096	0.677087344	-1.694657226	0.090140464	0.517978072	down_in_case
CRA004775	ENSGALG00010010544	ENSGALG00010010544	case	control	-0.645495396	0.395557798	-1.631861131	0.102708744	0.543312631	down_in_case
CRA004775	ENSGALG00010010546	MICALL1	case	control	-0.300991058	0.247020717	-1.218485077	0.223039692	0.690760052	down_in_case
CRA004775	ENSGALG00010010547	BST1	case	control	0.094577398	0.419179225	0.225625204	0.821492937	0.960766569	up_in_case
CRA004775	ENSGALG00010010567	ERMN	case	control	-0.676770187	0.490139367	-1.380770926	0.167349406	0.631096819	down_in_case
CRA004775	ENSGALG00010010572	AQP3	case	control	0.083396394	0.501447966	0.166311162	0.867912083	0.97330444	up_in_case
CRA004775	ENSGALG00010010573	CEP83	case	control	0.07895665	0.567325562	0.13917344	0.889313096	0.977162915	up_in_case
CRA004775	ENSGALG00010010575	PAXBP1	case	control	0.247333707	0.213055675	1.16088767	0.245687584	0.712046495	up_in_case
CRA004775	ENSGALG00010010577	PBX1	case	control	0.286229968	0.182061848	1.572157874	0.115913942	0.566086328	up_in_case
CRA004775	ENSGALG00010010578	GLUD2	case	control	0.089962801	0.241044764	0.373220309	0.708984482	0.931872072	up_in_case
CRA004775	ENSGALG00010010585	FBXL5	case	control	-0.066831908	0.408425891	-0.163632887	0.870020154	0.973841851	down_in_case
CRA004775	ENSGALG00010010586	CYTIP	case	control	-0.576282164	0.414550726	-1.390136667	0.164487381	0.63006979	down_in_case
CRA004775	ENSGALG00010010588	SLC25A3	case	control	-0.025479864	0.293701524	-0.08675428	0.93086683	0.985044263	down_in_case
CRA004775	ENSGALG00010010593	EIF3L	case	control	-0.258740379	0.142258064	-1.818809931	0.068940433	0.466995021	down_in_case
CRA004775	ENSGALG00010010596	SHLD2	case	control	0.016706661	0.295559912	0.056525464	0.954923211	0.990670511	up_in_case
CRA004775	ENSGALG00010010599	F2RL1	case	control	0.795176711	0.297724517	2.670847258	0.007566007	0.173689745	up_in_case
CRA004775	ENSGALG00010010604	HIVEP1	case	control	0.426670753	0.256322695	1.664584376	0.095995705	0.529285058	up_in_case
CRA004775	ENSGALG00010010605	NUF2	case	control	0.440300921	0.475260059	0.926442085	0.354216281	0.78730441	up_in_case
CRA004775	ENSGALG00010010614	IKBIP	case	control	-0.042937341	0.209957045	-0.204505359	0.837958605	0.965490877	down_in_case
CRA004775	ENSGALG00010010618	APAF1	case	control	-0.213207876	0.362091036	-0.588823956	0.555979374	0.87981923	down_in_case
CRA004775	ENSGALG00010010619	LRRC52	case	control	-0.854249111	0.488122641	-1.750070657	0.080106122	0.493676892	down_in_case
CRA004775	ENSGALG00010010620	ANKRD54	case	control	-0.337890226	0.283221904	-1.193022931	0.232860392	0.699803243	down_in_case
CRA004775	ENSGALG00010010623	SMAD2	case	control	-0.21277939	0.175514135	-1.212320534	0.225389706	0.693278343	down_in_case
CRA004775	ENSGALG00010010626	ADTRP	case	control	0.762615438	0.744863884	1.023831944	0.30591466	0.758225033	up_in_case
CRA004775	ENSGALG00010010635	ALDH9A1	case	control	-0.319784885	0.181883326	-1.758186924	0.078715709	0.491399489	down_in_case
CRA004775	ENSGALG00010010638	ACVR1C	case	control	-1.268401136	0.467547561	-2.712881515	0.006670095	0.160913119	down_in_case
CRA004775	ENSGALG00010010642	CC2D2A	case	control	0.139283781	0.279657284	0.498051684	0.618447614	0.904143308	up_in_case
CRA004775	ENSGALG00010010645	FAM219A	case	control	-0.107653991	0.427352828	-0.251908925	0.801111461	0.953541667	down_in_case
CRA004775	ENSGALG00010010646	MCM3AP	case	control	-0.255016541	0.194536778	-1.310891149	0.189894542	0.655361714	down_in_case
CRA004775	ENSGALG00010010648	CENPM	case	control	0.100704096	0.403732991	0.249432418	0.803026312	0.954005463	up_in_case
CRA004775	ENSGALG00010010651	NUAK1	case	control	-0.342627677	0.355877145	-0.962769547	0.335663187	0.776282001	down_in_case
CRA004775	ENSGALG00010010655	C1QTNF7	case	control	0.36775054	0.340525774	1.079949207	0.280164799	0.73661445	up_in_case
CRA004775	ENSGALG00010010657	TMEM170B	case	control	-0.812904471	0.596247728	-1.363366991	0.172766879	0.637223104	down_in_case
CRA004775	ENSGALG00010010659	ADAMTS1	case	control	1.27444101	0.436107757	2.922307595	0.003474482	0.125102734	up_in_case
CRA004775	ENSGALG00010010661	CPEB2	case	control	0.136161698	0.393674508	0.345873799	0.72943756	0.936339759	up_in_case
CRA004775	ENSGALG00010010662	MGST3	case	control	-0.057669276	0.256331331	-0.224979428	0.821995278	0.960766569	down_in_case
CRA004775	ENSGALG00010010663	EFR3B	case	control	-0.313817627	0.447673248	-0.700997053	0.483304854	0.850257583	down_in_case
CRA004775	ENSGALG00010010666	C12orf75	case	control	-0.169950766	0.571368721	-0.297444994	0.766126797	0.943612838	down_in_case
CRA004775	ENSGALG00010010671	SIGMAR1	case	control	-0.319750534	0.221067394	-1.446393918	0.148066747	0.611192154	down_in_case
CRA004775	ENSGALG00010010672	TNFRSF13C	case	control	3.076091877	0.64059306	4.801943806			up_in_case
CRA004775	ENSGALG00010010678	KCNIP1	case	control	-0.526320093	0.720853036	-0.730135084	0.465307618	0.843383332	down_in_case
CRA004775	ENSGALG00010010680	ENSGALG00010010680	case	control	-0.913891169	0.791218781	-1.15504231	0.248073101	0.712818804	down_in_case
CRA004775	ENSGALG00010010682	SMIM13	case	control	0.007125949	0.305044293	0.023360376	0.981362812	0.997069916	up_in_case
CRA004775	ENSGALG00010010685	STIM2	case	control	0.119876253	0.244989845	0.489311112	0.624621456	0.90646861	up_in_case
CRA004775	ENSGALG00010010688	IQGAP2	case	control	-0.278147147	0.460079984	-0.604562591	0.545469669	0.876563448	down_in_case
CRA004775	ENSGALG00010010689	POFUT2	case	control	-0.376979215	0.213229384	-1.767951527	0.077069008	0.488700005	down_in_case
CRA004775	ENSGALG00010010690	MTPN	case	control	-0.193441403	0.385657967	-0.501587986	0.615957372	0.9028981	down_in_case
CRA004775	ENSGALG00010010693	KCNMB1	case	control	0.51430236	0.47692408	1.078373648	0.280867042	0.737061584	up_in_case
CRA004775	ENSGALG00010010697	NEDD9	case	control	0.415206484	0.303793833	1.366737701	0.171707536	0.636057362	up_in_case
CRA004775	ENSGALG00010010698	GPRIN2	case	control	-0.840613821	0.816611062	-1.029393134	0.303294974	0.755679718	down_in_case
CRA004775	ENSGALG00010010699	DNAJC27	case	control	-0.330730001	0.212695181	-1.55494826	0.119958392	0.572133279	down_in_case
CRA004775	ENSGALG00010010706	SREBF2	case	control	-0.210972676	0.241526349	-0.873497558	0.382391936	0.8018649	down_in_case
CRA004775	ENSGALG00010010710	UCK2	case	control	-0.390279892	0.222565771	-1.753548585	0.079507888	0.491871682	down_in_case
CRA004775	ENSGALG00010010711	ENSGALG00010010711	case	control	0.767915417	0.320498869	2.396000398	0.016575072	0.255167461	up_in_case
CRA004775	ENSGALG00010010712	ADAMTS5	case	control	-0.036172466	0.423684623	-0.085375923	0.931962534	0.985044263	down_in_case
CRA004775	ENSGALG00010010713	NSA2	case	control	0.114446961	0.249892384	0.457984988	0.646963226	0.914163628	up_in_case
CRA004775	ENSGALG00010010719	SH3BP4	case	control	-0.442692595	0.332336676	-1.33206061	0.182840273	0.647378665	down_in_case
CRA004775	ENSGALG00010010722	TBC1D19	case	control	0.494841411	0.383904976	1.288968474	0.197409047	0.663190847	up_in_case
CRA004775	ENSGALG00010010724	ADCY3	case	control	0.027064912	0.360160471	0.075146813	0.940097902	0.987076872	up_in_case
CRA004775	ENSGALG00010010733	GFM2	case	control	0.066250027	0.198604611	0.333577486	0.73869841	0.938064864	up_in_case
CRA004775	ENSGALG00010010734	AMN1	case	control	-0.456084989	0.368826862	-1.236582896	0.216241973	0.683774974	down_in_case
CRA004775	ENSGALG00010010740	CCDC134	case	control	-0.372210073	0.264817093	-1.405536436	0.159861823	0.624907945	down_in_case
CRA004775	ENSGALG00010010742	LCP2	case	control	-0.464213178	0.461875633	-1.005060983	0.314867486	0.765098415	down_in_case
CRA004775	ENSGALG00010010743	KLHL20	case	control	-0.219704534	0.186871997	-1.175695326	0.239716647	0.706254731	down_in_case
CRA004775	ENSGALG00010010745	ATVR1	case	control	0.202035846	0.133777292	1.510240211	0.130982142	0.586487428	up_in_case
CRA004775	ENSGALG00010010748	SF3B1	case	control	-0.239482616	0.191076045	-1.253336677	0.210083206	0.67982426	down_in_case
CRA004775	ENSGALG00010010749	NCOA4	case	control	-0.147767247	0.288241709	-0.512650468	0.608195844	0.900943922	down_in_case
CRA004775	ENSGALG00010010751	SLC30A5	case	control	-0.234748337	0.185574268	-1.264983231	0.205877339	0.674534022	down_in_case
CRA004775	ENSGALG00010010752	ENSGALG00010010752	case	control	-0.899348083	0.538960438	-1.668671796	0.095182442	0.527976505	down_in_case
CRA004775	ENSGALG00010010753	ENSGALG00010010753	case	control	0.611484994	0.366627799	1.667863143	0.095342898	0.528014574	up_in_case
CRA004775	ENSGALG00010010754	DNAI1	case	control	-0.742426092	0.931701192	-0.796849997	0.425538154	0.822283278	down_in_case
CRA004775	ENSGALG00010010756	CENPL	case	control	0.67752518	0.484257777	1.399100257	0.161782921	0.627882156	up_in_case
CRA004775	ENSGALG00010010759	ENSGALG00010010759	case	control	-0.382756595	0.720827608	-0.530996026	0.595421533	0.895305942	down_in_case

CRA004775	ENSGALG00010010761	MEI1	case	control	-1.290642851	0.664700862	-1.94168975	0.05217467	0.420135271	down_in_case
CRA004775	ENSGALG00010010762	MRPL39	case	control	0.016847475	0.273443624	0.061612243	0.950871627	0.990168609	up_in_case
CRA004775	ENSGALG00010010766	CENPO	case	control	-0.188740109	0.481250859	-0.392186539	0.69492039	0.928924129	down_in_case
CRA004775	ENSGALG00010010768	ENSGALG00010010768	case	control	-0.09575264	0.286538398	-0.334170361	0.738251008	0.937887067	down_in_case
CRA004775	ENSGALG00010010769	HEXB	case	control	-0.146561605	0.402019661	-0.364563277	0.715437432	0.933870859	down_in_case
CRA004775	ENSGALG00010010771	ENSGALG00010010771	case	control	-0.294599034	0.204160413	-1.442978246	0.149026599	0.612469669	down_in_case
CRA004775	ENSGALG00010010774	KPTN	case	control	-0.509442707	0.359901012	-1.415507849	0.156919649	0.621625652	down_in_case
CRA004775	ENSGALG00010010776	GCAT	case	control	-0.039111094	0.40433946	-0.096728362	0.922942116	0.983159417	down_in_case
CRA004775	ENSGALG00010010778	UPP2	case	control	-0.196180744	0.553504489	-0.354433881	0.723013747	0.935185555	down_in_case
CRA004775	ENSGALG00010010779	DOCK2	case	control	-0.097900289	0.542429152	-0.180484934	0.856771881	0.970913248	down_in_case
CRA004775	ENSGALG00010010780	PARG	case	control	-0.134881173	0.139012774	-0.97027898	0.331907452	0.772935571	down_in_case
CRA004775	ENSGALG00010010781	NAPA	case	control	-0.188887153	0.237731759	-0.794538994	0.426881717	0.822776983	down_in_case
CRA004775	ENSGALG00010010783	F2RL2	case	control	0.25530081	0.401629929	0.635661815	0.52499687	0.867302346	up_in_case
CRA004775	ENSGALG00010010784	CCDC148	case	control	0.584859518	0.534207305	1.094817523	0.273596588	0.731001618	up_in_case
CRA004775	ENSGALG00010010786	NABP1	case	control	-0.348152928	0.502415493	-0.692958184	0.488335794	0.853845967	down_in_case
CRA004775	ENSGALG00010010789	ENSGALG00010010789	case	control	-0.607986841	0.319610801	-1.902272512	0.057135537	0.436969285	down_in_case
CRA004775	ENSGALG00010010791	H-10	case	control	0.09739593	0.320727499	0.303671903	0.761377866	0.942236668	up_in_case
CRA004775	ENSGALG00010010792	XRCC6	case	control	-0.386642167	0.278166735	-1.38996551	0.164539351	0.63006979	down_in_case
CRA004775	ENSGALG00010010796	CAVIN2	case	control	0.276050741	0.284846863	0.969119821	0.332485409	0.773343515	up_in_case
CRA004775	ENSGALG00010010799	DARS2	case	control	-0.218762284	0.199908443	-1.094312376	0.273817999	0.731001618	down_in_case
CRA004775	ENSGALG00010010800	BICRA	case	control	-0.197940649	0.1971982	-1.003764988	0.315491904	0.765461123	down_in_case
CRA004775	ENSGALG00010010805	PTRHD1	case	control	-0.025088969	0.319426356	-0.078543828	0.937395469	0.986688955	down_in_case
CRA004775	ENSGALG00010010809	CENPH	case	control	0.468171463	0.312760908	1.49689891	0.134419564	0.592420453	up_in_case
CRA004775	ENSGALG00010010810	GALT	case	control	-0.451771585	0.380840336	-1.186249306	0.23552386	0.702687914	down_in_case
CRA004775	ENSGALG00010010813	EHD2	case	control	0.216393579	0.338631931	0.639022959	0.522807996	0.866638286	up_in_case
CRA004775	ENSGALG00010010816	AP2S1	case	control	-0.511205436	0.315852167	-1.618495894	0.105555771	0.549651422	down_in_case
CRA004775	ENSGALG00010010821	RGS8	case	control	0.552878291	0.563411839	0.981304001	0.32644285	0.770998457	up_in_case
CRA004775	ENSGALG00010010823	TMEFF2	case	control	0.586800107	0.328424356	1.786713126	0.073983867	0.479781244	up_in_case
CRA004775	ENSGALG00010010824	PKP4	case	control	0.024629318	0.220059078	0.111921393	0.910885734	0.98075825	up_in_case
CRA004775	ENSGALG00010010825	MAK	case	control	0.8981121	0.626100215	1.434454227	0.151442682	0.615574438	up_in_case
CRA004775	ENSGALG00010010826	NCOA1	case	control	0.394046155	0.295227844	1.334718805	0.181968396	0.647378665	up_in_case
CRA004775	ENSGALG00010010828	SPDL1	case	control	0.223606689	0.291571026	0.766902982	0.443139201	0.831664685	up_in_case
CRA004775	ENSGALG00010010829	LAMB4	case	control	-0.38211943	0.806179503	-0.473988025	0.635508447	0.909837348	down_in_case
CRA004775	ENSGALG00010010831	DESI1	case	control	-0.377752514	0.164794797	-2.292259953	0.021890647	0.28971924	down_in_case
CRA004775	ENSGALG00010010834	KGD4	case	control	0.085662207	0.373598973	0.229289192	0.818644153	0.959941088	up_in_case
CRA004775	ENSGALG00010010836	DIP2A	case	control	0.007497889	0.277788926	0.026991317	0.978466659	0.996363673	up_in_case
CRA004775	ENSGALG00010010837	RBPJ	case	control	-0.131371127	0.390543683	-0.33638011	0.736584246	0.937549044	down_in_case
CRA004775	ENSGALG00010010838	JAM2	case	control	0.506836665	0.357790511	1.416573804	0.156607572	0.62114529	up_in_case
CRA004775	ENSGALG00010010840	RGS16	case	control	-1.842858527	0.957360013	-1.924937852	0.054237134	0.428875691	down_in_case
CRA004775	ENSGALG00010010841	PMM1	case	control	-0.74372439	0.260194412	-2.858341135	0.004258623	0.133620227	down_in_case
CRA004775	ENSGALG00010010843	ZNF395	case	control	0.157072799	0.20871098	0.752585222	0.451699201	0.836202977	up_in_case
CRA004775	ENSGALG00010010844	CDC14B	case	control	0.257564418	0.213294531	1.207552845	0.227219306	0.694715356	up_in_case
CRA004775	ENSGALG00010010845	FKRP	case	control	-0.278003056	0.338044742	-0.822385387	0.410857593	0.816405743	down_in_case
CRA004775	ENSGALG00010010849	ACBD6	case	control	-0.402268959	0.213776942	-1.881722866	0.059873653	0.445397937	down_in_case
CRA004775	ENSGALG00010010850	ENSGALG00010010850	case	control	0.016751887	0.468333341	0.035769153	0.97146643	0.994908384	up_in_case
CRA004775	ENSGALG00010010851	PNOC	case	control	1.279653629	1.063743477	1.20297201	0.228987152	0.695782399	up_in_case
CRA004775	ENSGALG00010010853	SMIM20	case	control	0.079790762	0.389392382	0.204910949	0.837641701	0.965490877	up_in_case
CRA004775	ENSGALG00010010855	ATP5PF	case	control	0.038961166	0.318421852	0.122357075	0.902616233	0.979310476	up_in_case
CRA004775	ENSGALG00010010857	SEL1L3	case	control	0.175599465	0.122986219	1.427797904	0.153350036	0.617667355	up_in_case
CRA004775	ENSGALG00010010859	ENSGALG00010010859	case	control	0.480879863	0.399597381	1.203410949	0.228817333	0.695782399	up_in_case
CRA004775	ENSGALG00010010861	ENSGALG00010010861	case	control	0.131489515	0.431357492	0.304827243	0.760497732	0.942236668	up_in_case
CRA004775	ENSGALG00010010862	STRN4	case	control	-0.458666052	0.147533238	-3.108899788	0.001877854	0.086751567	down_in_case
CRA004775	ENSGALG00010010865	ENSGALG00010010865	case	control	0.08898332	0.274268176	0.324439098	0.745605624	0.939421865	up_in_case
CRA004775	ENSGALG00010010867	QSOX1	case	control	-0.147682674	0.36022863	-0.409969287	0.681828478	0.924278508	down_in_case
CRA004775	ENSGALG00010010868	SLC39A10	case	control	-0.207424601	0.36160015	-0.573629742	0.566218381	0.883033678	down_in_case
CRA004775	ENSGALG00010010869	ENSGALG00010010869	case	control	1.620204301	0.432570592	3.745525774	0.000180016	0.027735809	up_in_case
CRA004775	ENSGALG00010010870	NOL6	case	control	-0.24125855	0.243535548	-0.990650242	0.321856395	0.767750105	down_in_case
CRA004775	ENSGALG00010010874	ENSGALG00010010874	case	control	-0.346755153	0.447084883	-0.775591316	0.43799033	0.827682376	down_in_case
CRA004775	ENSGALG00010010876	ENSGALG00010010876	case	control	-0.252496796	0.34103119	-0.74039209	0.459062117	0.840589413	down_in_case
CRA004775	ENSGALG00010010880	ELP3	case	control	-0.387751546	0.217551197	-1.782346183	0.074692796	0.481528662	down_in_case
CRA004775	ENSGALG00010010882	CEP350	case	control	-0.091320587	0.212977788	-0.428779864	0.668083434	0.919688403	down_in_case
CRA004775	ENSGALG00010010885	SNRPD2	case	control	-0.27727821	0.261354804	-1.060926397	0.288723352	0.742851819	down_in_case
CRA004775	ENSGALG00010010887	GABPA	case	control	0.292506948	0.183754554	1.591835092	0.111421763	0.56070984	up_in_case
CRA004775	ENSGALG00010010888	GNPAT	case	control	-0.122108262	0.32066212	-0.380800395	0.703351366	0.930068722	down_in_case
CRA004775	ENSGALG00010010889	CCL19	case	control	-0.04657065	0.669256157	-0.069585687	0.944523429	0.988367831	down_in_case
CRA004775	ENSGALG00010010890	NOL12	case	control	0.062332082	0.213360551	0.292144363	0.770176249	0.945754599	up_in_case
CRA004775	ENSGALG00010010891	POLR3H	case	control	-0.41132698	0.244254382	-1.684010647	0.092179611	0.52187835	down_in_case
CRA004775	ENSGALG00010010893	CCL21	case	control	0.052417747	0.77579942	0.067566107	0.946131037	0.988996418	up_in_case
CRA004775	ENSGALG00010010894	SPAG1	case	control	0.120688708	0.306286879	0.394038128	0.69355289	0.928269981	up_in_case
CRA004775	ENSGALG00010010896	LY86	case	control	-0.626941657	0.468868794	-1.337136668	0.18117803	0.647292985	down_in_case
CRA004775	ENSGALG00010010897	CEP170	case	control	0.57052284	0.222935524	2.559138305	0.010493199	0.20539647	up_in_case
CRA004775	ENSGALG00010010900	SLC34A2	case	control	2.476125221	0.915035944	2.706041482	0.006809054	0.161712092	up_in_case
CRA004775	ENSGALG00010010904	TOR1AIP1	case	control	-0.318484295	0.327012147	-0.973921912	0.330095313	0.77237154	down_in_case
CRA004775	ENSGALG00010010905	ENC1	case	control	0.276234806	0.400637284	0.689488515	0.490515898	0.855184934	up_in_case
CRA004775	ENSGALG00010010906	ATP6V1C1	case	control	0.164107491	0.335938824	0.488504095	0.625192826	0.906591226	up_in_case
CRA004775	ENSGALG00010010908	STK17B	case	control	-0.208259599	0.360030173	-0.578450402	0.562960074	0.881532201	down_in_case
CRA004775	ENSGALG00010010909	SLIT3	case	control	-0.614706557	0.534820351	-1.149370169	0.250403375	0.715267307	down_in_case
CRA004775	ENSGALG00010010911	ENSGALG00010010911	case	control	0.559213576	0.495094025	1.129509845	0.258682819	0.720037471	up_in_case
CRA004775	ENSGALG00010010912	BAALC	case	control	-0.953934091	0.73656863	-1.295105509	0.195283835	0.660927396	down_in_case
CRA004775	ENSGALG00010010913	IFT56	case	control	-0.335772334	0.449271787	-0.747370175	0.454840144	0.838386795	down_in_case
CRA004775	ENSGALG00010010915	APP	case	control	0.291373943	0.267418941	1.089578553	0.275898835	0.731001618	up_in_case
CRA004775	ENSGALG00010010917	TANC1	case	control	0.183690322	0.215570785	0.852111397	0.394152267	0.808470737	up_in_case
CRA004775	ENSGALG00010010918	TOR1AIP2	case	control	-0.26588462	0.23936865	-1.110774614	0.266665376	0.727315996	down_in_case
CRA004775	ENSGALG00010010920	UBN2	case	control	0.034980013	0.228023099	0.153405566	0.878078456	0.974689768	up_in_case
CRA004775	ENSGALG00010010921	ENSGALG00010010921	case	control	-0.342796073	0.278363325	-1.23146996	0.218147145	0.68549849	down_in_case
CRA004775	ENSGALG00010010922	DYM	case	control	-0.199236643	0.266764197	-0.746864257	0.455145503	0.838631357	down_in_case
CRA004775	ENSGALG00010010923	F13A1	case	control	-1.412687609	0.635428812	-2.22320358	0.026202076	0.309806312	down_in_case
CRA004775	ENSGALG00010010924	ENSGALG00010010924	case	control	-0.078717317	0.249010264	-0.316120774	0.751910839	0.941495694	down_in_case
CRA004775	ENSGALG00010010928	HECW2	case	control	-0.118074377	0.327233663	-0.360825889	0.718229608	0.934011578	down_in_case
CRA004775	ENSGALG00010010929	FMC1	case	control	0.024309665	0.332727315	0.073061824	0.94175692	0.987577169	up_in_case
CRA004775	ENSGALG00010010931	CYYR1	case	control	0.539005348	0.340028429	1.585177303	0.112926056	0.562886277	up_in_case
CRA004775	ENSGALG00010010932	GHITM	case	control	-0.015534582	0.283057203	-0.054881424	0.956232931	0.99096835	down_in_case
CRA004775	ENSGALG00010010933	ANAPC4	case	control	0.042390292	0.235753869	0.179807409	0.857303767	0.970913248	up_in_case
CRA004775	ENSGALG00010010934	ENSGALG00010010934	case	control	-0.221292356	0.199996654	-1.106480296	0.268518688	0.727528758	down_in_case
CRA004775	ENSGALG00010010935	LGALS1A	case	control	0.498943672	0.513757323	0.971166053	0.331465595	0.772629743	up_in_case
CRA004775	ENSGALG00010010937	CDK7	case	control	-0.375399326	0.250587586	-1.49807631	0.134113425	0.592323374	down_in_case
CRA004775	ENSGALG00010010938	FZD6	case	control	-0.028867942	0.244028112	-0.118297608	0.905831853	0.979739788	down_in_case
CRA004775	ENSGALG00010010939	ZCCHC4	case	control	-0.291376284	0.289136659	-1.007745907	0.31357646	0.765098415	down_in_case
CRA004775	ENSGALG00010010941	RIPK4	case	control	2.286734169	0.694834092	3.291050619	0.00099814	0.066306359	up_in_case
CRA004775	ENSGALG00010010942	NRN1	case	control	0.289253366	0.58861631	0.491412421	0.623134781	0.906441769	up_in_case
CRA004775	ENSGALG00010010943	WDSUB1	case	control	-0.206993465	0.224346942	-0.922648926	0.35619019	0.789127613	down_in_case
CRA004775	ENSGALG00010010945	PI4K2B	case	control	-0.026655434	0.348967137	-0.076383795	0.939113762	0.986950867	down_in_case
CRA004775	ENSGALG00010010947	LUC7L2	case	control	0.033297006	0.193646791	0.171947108	0.863479111	0.971910432	up_in_case
CRA004775	ENSGALG00010010948	PRDM15	case	control	0.102506137	0.257787882	0.397637532	0.690897391	0.927867527	up_in_case
CRA004775	ENSGALG00010010949	FZD3	case	control	0.487926133	0.543156177	0.898316459	0.36901686	0.794896739	up_in_case
CRA004775	ENSGALG00010010951	ENSGALG00010010951	case	control	0.006976728	0.343160692	0.020330791	0.983779494	0.99751066	up_in_case
CRA004775	ENSGALG00010010952	ENSGALG00010010952	case	control	0.160558323	0.58494482	0.274484562	0.783712266	0.949834952	up_in_case
CRA004775	ENSGALG00010010953	EXTL3	case	control	-0.160893386	0.200449829	-0.802661628	0.422170337	0.820119439	down_in_case
CRA004775	ENSGALG00010010954	MTERF2	case	control	0.462681868	0.444283488	1.041411351	0.297684676	0.750344577	up_in_case
CRA004775	ENSGALG00010010955	GTF3C3	case	control	-0.414221628	0.189686271	-2.183719602	0.028982858	0.323755716	down_in_case
CRA004775	ENSGALG00010010957	ZMAT2	case	control	-0.075161522	0.243575791	-0.308575503	0.757644458	0.941900693	down_in_case
CRA004775	ENSGALG00010010958	LCORL	case	control	0.67682207	0.362578245	1.866692442	0.06194455	0.447373689	up_in_case
CRA004775	ENSGALG00010010959	TMEM263	case	control	0.054832809	0.165689927	0.330936286	0.740692612	0.938450627	up_in_case
CRA004775	ENSGALG00010010960	POLDIP3	case	control	-0.225707238	0.154667602	-1.459305214	0.144481121	0.606459064	down_in_case
CRA004775	ENSGALG00010010962	ANKDD1B	case	control	-0.50820041	0.788036974	-0.64489412	0.518995803	0.865435399	down_in_case
CRA004775	ENSGALG00010010963	TSPAN14	case	control	-0.129377911	0.371772212	-0.348003177	0.727837797	0.935548472	down_in_case
CRA004775	ENSGALG00010010964	INTS9	case	control	-0.275277015	0.191264768	-1.439245802	0.150080893	0.61366707	down_in_case
CRA004775	ENSGALG00010010965	BAZ2B	case	control	0.793637866	0.415714754	1.909092375	0.056250176	0.434060704	up_in_case
CRA004775	ENSGALG00010010966	ENSGALG00010010966	case	control	0.034136148	0.726662627	0.046976612	0.962531868	0.991860454	up_in_case
CRA004775	ENSGALG00010010967	PRXL2A	case	control	-0.609802063	0.375714878	-1.623044755	0.104579838	0.547421908	down_in_case
CRA004775	ENSGALG00010010968	RRP7A	case	control	-0.2341998	0.2495694	-0.938415525	0.34803091	0.785270866	down_in_case
CRA004775	ENSGALG00010010970	TPM2	case	control	-0.448127762	0.553092784	-0.810221675	0.417812782	0.818943344	down_in_case
CRA004775	ENSGALG00010010971	LCORL	case	control	0.855328427	0.336249848	2.543728811	0.01096762	0.211456439	up_in_case
CRA004775	ENSGALG00010010972	EXOSC3	case	control	0.090495246	0.257839546	0.350975046	0.725607069	0.935238044	up_in_case
CRA004775	ENSGALG00010010973	PGAP1	case	control	-0.426430582	0.339557889	-1.255840597	0.209173769	0.678704224	down_in_case
CRA004775	ENSGALG00010010974	NCAPG	case	control	0.86406918	0.573963415	1.505442956	0.132210232	0.588356927	up_in_case

CRA004775	ENSGALG00010010975	ATP8B1	case	control	2.417146601	0.596202646	4.054236621	5.02983E-05	0.013833034	up_in_case
CRA004775	ENSGALG00010010977	FARS2	case	control	-0.3429268	0.228229793	-1.502550547	0.132954984	0.589727603	down_in_case
CRA004775	ENSGALG00010010978	SERHL	case	control	-0.679931556	0.232975106	-2.918473003	0.003517503	0.125102734	down_in_case
CRA004775	ENSGALG00010010980	POLK	case	control	0.326463882	0.270076147	1.208784577	0.226745618	0.694715356	up_in_case
CRA004775	ENSGALG00010010981	RIC8B	case	control	0.232487512	0.145532659	1.597493739	0.110155692	0.558567026	up_in_case
CRA004775	ENSGALG00010010982	ZBTB21	case	control	0.647482364	0.230927098	2.803838835	0.005049814	0.143768101	up_in_case
CRA004775	ENSGALG00010010983	MED28	case	control	0.174366289	0.196340768	0.888079898	0.374497775	0.798266843	up_in_case
CRA004775	ENSGALG00010010984	MAT1A	case	control	-0.231464179	0.405275677	-0.571127733	0.567913061	0.884213371	down_in_case
CRA004775	ENSGALG00010010985	CERT1	case	control	0.300531039	0.235264047	1.277420169	0.201453948	0.669290466	up_in_case
CRA004775	ENSGALG00010010986	NARS	case	control	-0.158573606	0.215498141	-0.73584675	0.461823966	0.842010079	down_in_case
CRA004775	ENSGALG00010010987	MARCHF7	case	control	-0.282404417	0.168771178	-1.673297658	0.094268713	0.526933666	down_in_case
CRA004775	ENSGALG00010010988	ANKRD44	case	control	-0.241774441	0.424883866	-0.569036531	0.569331349	0.884809383	down_in_case
CRA004775	ENSGALG00010010989	TRIQK	case	control	-0.178566905	0.476827779	-0.374489309	0.708040309	0.931801673	down_in_case
CRA004775	ENSGALG00010010990	MED18	case	control	-0.454020685	0.25813447	-1.758853378	0.078602416	0.491399489	down_in_case
CRA004775	ENSGALG00010010991	C2CD2	case	control	-0.461004524	0.448060774	-1.028888379	0.30353213	0.755862064	down_in_case
CRA004775	ENSGALG00010010992	PCDHA6	case	control	-0.007651656	0.463444974	-0.016510387	0.986827215	0.997656315	down_in_case
CRA004775	ENSGALG00010010993	ENSGALG00010010993	case	control	-0.396321998	0.901095809	-0.439822263	0.660065842	0.917907174	down_in_case
CRA004775	ENSGALG00010010994	NAA20	case	control	-0.149120667	0.268733324	-0.554902032	0.578961672	0.888378369	down_in_case
CRA004775	ENSGALG00010010995	HEBP1	case	control	-0.359964901	0.256933091	-1.401006384	0.161212164	0.627121149	down_in_case
CRA004775	ENSGALG00010010997	LAP3	case	control	-0.714451985	0.250000223	-2.857805396	0.004265819	0.133620227	down_in_case
CRA004775	ENSGALG00010010998	SOAT1	case	control	0.004738991	0.265886303	0.017823375	0.985779757	0.997562105	up_in_case
CRA004775	ENSGALG00010011001	ANGPT1	case	control	0.122665176	0.44929702	0.273015778	0.784841087	0.950244571	up_in_case
CRA004775	ENSGALG00010011002	RPRM	case	control	-1.506953291	0.955095272	-1.577804157	0.114610608	0.564488547	down_in_case
CRA004775	ENSGALG00010011003	FECH	case	control	-0.255953654	0.314609086	-0.81356091	0.415896539	0.818943344	down_in_case
CRA004775	ENSGALG00010011006	TMEM64	case	control	-0.010260002	0.257315519	-0.039873233	0.968194191	0.993568693	down_in_case
CRA004775	ENSGALG00010011007	ENSGALG00010011007	case	control	-0.552682346	0.404532187	-1.366225887	0.171868075	0.636057362	down_in_case
CRA004775	ENSGALG00010011013	ENSGALG00010011013	case	control	-0.112129746	0.480744902	-0.233241676	0.815573746	0.958870945	down_in_case
CRA004775	ENSGALG00010011015	LYRM4	case	control	-0.448875038	0.322107986	-1.393554514	0.163452172	0.629717526	down_in_case
CRA004775	ENSGALG00010011018	QDPR	case	control	-0.69807939	0.212961639	-3.277958389	0.001045608	0.067571071	down_in_case
CRA004775	ENSGALG00010011019	NECAB1	case	control	-1.082662746	0.653182092	-1.657520558	0.097414276	0.531987176	down_in_case
CRA004775	ENSGALG00010011021	PPP1R3G	case	control	-0.096359851	0.327898854	-0.293870655	0.768856743	0.945220496	down_in_case
CRA004775	ENSGALG00010011022	C5orf22	case	control	-0.10988205	0.268047468	-0.409935041	0.681853599	0.924278508	down_in_case
CRA004775	ENSGALG00010011025	POLR3B	case	control	-0.202814069	0.234416236	-0.865187804	0.386935718	0.803905475	down_in_case
CRA004775	ENSGALG00010011027	FAM234B	case	control	-0.243032279	0.299219014	-0.812222046	0.416664232	0.818943344	down_in_case
CRA004775	ENSGALG00010011028	HMBOX1	case	control	-0.200547097	0.358801596	-0.558935911	0.57620546	0.887937128	down_in_case
CRA004775	ENSGALG00010011029	HMGCR	case	control	0.374343619	0.26342275	1.421075513	0.155294808	0.619284425	up_in_case
CRA004775	ENSGALG00010011033	DROSHA	case	control	-0.220142034	0.252231048	-0.872779288	0.382783391	0.801925538	down_in_case
CRA004775	ENSGALG00010011034	FAM180A	case	control	-0.779325817	0.72450437	-1.075667517	0.28207598	0.738291085	down_in_case
CRA004775	ENSGALG00010011040	CDH6	case	control	0.818329995	0.626748295	1.305675661	0.191662878	0.656959697	up_in_case
CRA004775	ENSGALG00010011045	ENSGALG00010011045	case	control	0.264517436	0.579753125	0.456258749	0.648203923	0.914575431	up_in_case
CRA004775	ENSGALG00010011049	ABL2	case	control	-0.100958192	0.324724672	-0.310903977	0.755873621	0.941900693	down_in_case
CRA004775	ENSGALG00010011055	TOR3A	case	control	-0.06217749	0.193790573	-0.320848887	0.74832491	0.940917938	down_in_case
CRA004775	ENSGALG00010011056	ENSGALG00010011056	case	control	1.447672432	1.148148117	1.260876024	0.207353518	0.6761485	up_in_case
CRA004775	ENSGALG00010011059	ENSGALG00010011059	case	control	0.289459115	0.442960461	0.653464903	0.513456609	0.863903061	up_in_case
CRA004775	ENSGALG00010011061	KIF13B	case	control	-0.519904999	0.358130567	-1.45171914	0.146579715	0.609922856	down_in_case
CRA004775	ENSGALG00010011062	TMEM55A	case	control	-0.087758	0.279768961	-0.31368026	0.753763895	0.941900693	down_in_case
CRA004775	ENSGALG00010011064	MYLIP	case	control	-0.263723336	0.262656815	-1.004060513	0.315349447	0.765461123	down_in_case
CRA004775	ENSGALG00010011067	TMTC2	case	control	0.289521678	0.220616028	1.312332932	0.189407825	0.654755848	up_in_case
CRA004775	ENSGALG00010011069	ENSGALG00010011069	case	control	-0.266303684	0.676460241	-0.393672338	0.693822967	0.928450722	down_in_case
CRA004775	ENSGALG00010011076	PKP2	case	control	0.594708709	0.66398036	0.895672139	0.370427895	0.795641647	up_in_case
CRA004775	ENSGALG00010011077	RASGEF1A	case	control	-0.335494821	0.45116359	-0.743621224	0.457105659	0.839688534	down_in_case
CRA004775	ENSGALG00010011080	EMP1	case	control	0.086936108	0.464386463	0.187206378	0.851498825	0.969284796	up_in_case
CRA004775	ENSGALG00010011085	UGT1A1	case	control	2.486405666	1.106259974	2.247578079	0.024603107	0.302160964	up_in_case
CRA004775	ENSGALG00010011086	OTUD6A	case	control	0.320786369	0.22695551	1.413432834	0.157528497	0.622106365	up_in_case
CRA004775	ENSGALG00010011091	YARS2	case	control	0.102754662	0.279158521	0.36808714	0.712808259	0.932981748	up_in_case
CRA004775	ENSGALG00010011094	CSGALNACT2	case	control	-0.293389978	0.277868802	-1.055857932	0.291033126	0.744957302	down_in_case
CRA004775	ENSGALG00010011095	TNN	case	control	-0.431282568	0.500559881	-0.861600348	0.38890748	0.804884358	down_in_case
CRA004775	ENSGALG00010011096	LDB2	case	control	0.479390896	0.316705583	1.513679963	0.130107029	0.584671406	up_in_case
CRA004775	ENSGALG00010011099	CCBE1	case	control	0.384320886	0.478245279	0.803606232	0.421624423	0.819812626	up_in_case
CRA004775	ENSGALG00010011101	ENSGALG00010011101	case	control	-0.563914838	0.697242512	-0.808778622	0.418642495	0.818943344	down_in_case
CRA004775	ENSGALG00010011104	ABCG1	case	control	-1.191867202	0.782788528	-1.522591555	0.127860924	0.5821156	down_in_case
CRA004775	ENSGALG00010011106	USP40	case	control	-0.295383342	0.247610751	-1.192934234	0.232895131	0.699803243	down_in_case
CRA004775	ENSGALG00010011107	ENSGALG00010011107	case	control	-0.522559992	0.350774872	-1.489730407	0.136295136	0.595518184	down_in_case
CRA004775	ENSGALG00010011109	ENSGALG00010011109	case	control	0.851717332	0.744565315	1.143912179	0.25266006	0.717153621	up_in_case
CRA004775	ENSGALG00010011111	MRPS14	case	control	-0.11672244	0.295584545	-0.394886817	0.69292642	0.927867527	down_in_case
CRA004775	ENSGALG00010011116	TAPT1	case	control	0.441085305	0.272118154	1.620933037	0.105032001	0.547911625	up_in_case
CRA004775	ENSGALG00010011118	GMPR2	case	control	-0.1671141	0.259927812	-0.642925043	0.520272739	0.865611895	down_in_case
CRA004775	ENSGALG00010011119	ENSGALG00010011119	case	control	-1.179062598	0.488421711	-2.414025771	0.015777346	0.25139547	down_in_case
CRA004775	ENSGALG00010011120	ENSGALG00010011120	case	control	0.863772617	1.30072672	0.66406925	0.50664599	0.8622397	up_in_case
CRA004775	ENSGALG00010011121	NUP35	case	control	-0.174905122	0.208987027	-0.836918561	0.402638346	0.811996248	down_in_case
CRA004775	ENSGALG00010011124	CACYBP	case	control	0.033840695	0.265877321	0.127279358	0.898719298	0.978790377	up_in_case
CRA004775	ENSGALG00010011126	ATXN1	case	control	0.259930653	0.35590973	0.730327469	0.465190042	0.843383332	up_in_case
CRA004775	ENSGALG00010011128	GCNT4	case	control	-0.062799508	0.355759753	-0.176522239	0.859883679	0.971325143	down_in_case
CRA004775	ENSGALG00010011129	ENSGALG00010011129	case	control	0.060444616	0.190418411	0.317430523	0.75091695	0.941495694	up_in_case
CRA004775	ENSGALG00010011130	DNM1L	case	control	0.123812552	0.15427007	0.802570144	0.422223231	0.820119439	up_in_case
CRA004775	ENSGALG00010011131	RET	case	control	-0.271005164	0.616152784	-0.439834357	0.660057082	0.917907174	down_in_case
CRA004775	ENSGALG00010011133	CDC37L1	case	control	0.260516802	0.295152627	0.882651137	0.37742479	0.798702414	up_in_case
CRA004775	ENSGALG00010011136	ENSGALG00010011136	case	control	-0.159490376	0.603546768	-0.264255206	0.791583274	0.951284723	down_in_case
CRA004775	ENSGALG00010011139	UBASH3A	case	control	-0.720702465	0.506418127	-1.42313718	0.154696392	0.619284425	down_in_case
CRA004775	ENSGALG00010011141	MSX1	case	control	0.10842967	0.722440827	0.15008796	0.880695219	0.974996282	up_in_case
CRA004775	ENSGALG00010011142	DUSP19	case	control	0.032862921	0.259311874	0.126731261	0.899153103	0.978790377	up_in_case
CRA004775	ENSGALG00010011143	AK3	case	control	0.307857696	0.352774723	0.872675041	0.382840226	0.801925538	up_in_case
CRA004775	ENSGALG00010011144	ATF7IP	case	control	-0.260187276	0.326551318	-0.796773009	0.425582873	0.822283278	down_in_case
CRA004775	ENSGALG00010011146	BMS1	case	control	0.016605499	0.206835993	0.080283411	0.936011851	0.986264424	up_in_case
CRA004775	ENSGALG00010011148	ENSGALG00010011148	case	control	-0.524951629	0.284449372	-1.845501097	0.064964679	0.457648208	down_in_case
CRA004775	ENSGALG00010011150	FGD4	case	control	-0.162665622	0.498587007	-0.326253232	0.74423277	0.939218065	down_in_case
CRA004775	ENSGALG00010011152	SLC18A3	case	control	-0.09827972	0.70036173	-0.140327085	0.888401566	0.977118922	down_in_case
CRA004775	ENSGALG00010011161	ENSGALG00010011161	case	control	0.165191608	0.147825825	1.117474621	0.263791445	0.723981064	up_in_case
CRA004775	ENSGALG00010011166	SOX7	case	control	0.188432421	0.386735516	0.487238471	0.626089346	0.907183337	up_in_case
CRA004775	ENSGALG00010011170	RBM24	case	control	0.584535825	0.357163791	1.63660438	0.10171316	0.541695453	up_in_case
CRA004775	ENSGALG00010011175	GPR52	case	control	-0.68762129	0.702303276	-0.979094522	0.327533278	0.771474966	down_in_case
CRA004775	ENSGALG00010011178	RSPO2	case	control	0.362852772	0.611658853	0.593227369	0.553028999	0.878546876	up_in_case
CRA004775	ENSGALG00010011181	ENSGALG00010011181	case	control	0.414300814	0.427165339	0.969883967	0.332104333	0.772935571	up_in_case
CRA004775	ENSGALG00010011184	FAM8A1	case	control	0.304832927	0.300851146	1.013235054	0.3109479	0.762763182	up_in_case
CRA004775	ENSGALG00010011190	ENSGALG00010011190	case	control	0.263630532	0.310076052	0.850212489	0.39520696	0.808998414	up_in_case
CRA004775	ENSGALG00010011192	RPL17	case	control	-0.387932474	0.328352727	-1.18145044	0.23742383	0.703927359	down_in_case
CRA004775	ENSGALG00010011193	FAM20B	case	control	-0.018785173	0.236721982	-0.079355425	0.936749923	0.98646154	down_in_case
CRA004775	ENSGALG00010011198	PINX1	case	control	-0.201361369	0.235449973	-0.855219334	0.392429735	0.80746892	down_in_case
CRA004775	ENSGALG00010011200	PCDHB4	case	control	0.820820611	0.728621151	1.126539643	0.259937171	0.720816008	up_in_case
CRA004775	ENSGALG00010011203	ENSGALG00010011203	case	control	-0.251003849	0.237358956	-1.05748632	0.290289695	0.744282131	down_in_case
CRA004775	ENSGALG00010011205	CYTL1	case	control	-6.007586635	1.322698995	-4.541915173	5.57455E-06	0.002839096	down_in_case
CRA004775	ENSGALG00010011210	NUP153	case	control	0.181573802	0.178775148	1.015654601	0.309793894	0.762070812	up_in_case
CRA004775	ENSGALG00010011211	SLC37A1	case	control	-0.038534269	0.263447218	-0.146269411	0.883708713	0.976132903	down_in_case
CRA004775	ENSGALG00010011212	NCKAP1	case	control	0.376489227	0.177062239	2.126310101	0.033477448	0.348565623	up_in_case
CRA004775	ENSGALG00010011213	XKR6	case	control	-0.154560194	0.269021701	-0.574526861	0.565611329	0.883033678	down_in_case
CRA004775	ENSGALG00010011218	ENSGALG00010011218	case	control	-0.145998028	1.057243682	-0.138093072	0.890166861	0.977283345	down_in_case
CRA004775	ENSGALG00010011222	RCL1	case	control	-0.106152722	0.282947858	-0.37516708	0.707536211	0.931742212	down_in_case
CRA004775	ENSGALG00010011231	PLBD1	case	control	-0.116666821	0.318374642	-0.366445081	0.714032986	0.933244709	down_in_case
CRA004775	ENSGALG00010011235	ENSGALG00010011235	case	control	-0.390960035	0.221213519	-1.767342417	0.0771709	0.488700005	down_in_case
CRA004775	ENSGALG00010011237	ENSGALG00010011237	case	control	-0.189427918	0.542119202	-0.349421156	0.726773153	0.935238044	down_in_case
CRA004775	ENSGALG00010011239	RALGPS2	case	control	-0.052103343	0.336955664	-0.15462967	0.877113278	0.974479909	down_in_case
CRA004775	ENSGALG00010011244	PDE9A	case	control	-0.072363966	0.375257232	-0.192838297	0.84708561	0.967891337	down_in_case
CRA004775	ENSGALG00010011246	DNAJB5	case	control	-0.626843206	0.640179135	-0.979168442	0.327496759	0.771474966	down_in_case
CRA004775	ENSGALG00010011247	ENSGALG00010011247	case	control	0.249426171	0.281631603	0.88564695	0.375807803	0.798523018	up_in_case
CRA004775	ENSGALG00010011250	ENSGALG00010011250	case	control	0.264727142	0.220256007	1.201906567	0.22939973	0.695782399	up_in_case
CRA004775	ENSGALG00010011251	FRZB	case	control	-0.157311818	0.514123198	-0.305980782	0.759619278	0.942236668	down_in_case
CRA004775	ENSGALG00010011253	KDM4C	case	control	0.149871071	0.305647253	0.49033999	0.623893334	0.906441769	up_in_case
CRA004775	ENSGALG00010011262	ENSGALG00010011262	case	control	-0.034058769	0.424003315	-0.080326657	0.935977458	0.986264424	down_in_case
CRA004775	ENSGALG00010011267	EVC2	case	control	0.084157376	0.295561276	0.284737492	0.775845265	0.946517228	up_in_case
CRA004775	ENSGALG00010011271	ANGPTL1	case	control	0.038264999	0.329319263	0.116194234	0.907498606	0.980354087	up_in_case
CRA004775	ENSGALG00010011273	RUNX1T1	case	control	0.155690448	0.356666085	0.436515986	0.662462403	0.918173506	up_in_case
CRA004775	ENSGALG00010011274	ENSGALG00010011274	case	control	-0.064179404	0.66168865	-0.096993357	0.92273167	0.983159417	down_in_case
CRA004775	ENSGALG00010011275	WDR4	case	control	-0.307080624	0.233391435	-1.315732191	0.188263946	0.65225939	down_in_case
CRA004775	ENSGALG00010011278	ENSGALG00010011278	case	control	1.106799139	0.609573216	1.815695162	0.069417147	0.468451304	up_in_case
CRA004775	ENSGALG00010011280	FAM199X	case	control	0.12570669	0.291729107	0.430902118	0.666539543	0.919168997	up_in_case
CRA004775	ENSGALG00010011281	ENSGALG00010011281	case	control	-0.63257561	1.198970732	-0.527598876	0.597777773	0.896490702	down_in_case

CRA004775	ENSGALG00010011282	CRY1	case	control	0.511784915	0.35106384	1.457811534	0.144892498	0.607785042	up_in_case
CRA004775	ENSGALG00010011283	LONRF3	case	control	0.097761964	0.324606133	0.301171032	0.763284078	0.94239774	up_in_case
CRA004775	ENSGALG00010011285	ENSGALG00010011285	case	control	0.190024059	0.414618362	0.458310767	0.64672919	0.914043203	up_in_case
CRA004775	ENSGALG00010011287	ENSGALG00010011287	case	control	0.108443079	0.24916185	0.435231473	0.66339442	0.918295135	up_in_case
CRA004775	ENSGALG00010011288	ENSGALG00010011288	case	control	-0.039107047	0.768034909	-0.050918319	0.959390608	0.991431439	down_in_case
CRA004775	ENSGALG00010011293	NDUFV3	case	control	0.483903924	0.333047266	1.452958703	0.14623522	0.609727264	up_in_case
CRA004775	ENSGALG00010011295	FAM167A	case	control	0.103255833	0.754864774	0.136787193	0.891199007	0.977652776	up_in_case
CRA004775	ENSGALG00010011297	OSR2	case	control	-0.312918091	0.497551301	-0.628916236	0.529403896	0.869211096	down_in_case
CRA004775	ENSGALG00010011300	EVC	case	control	0.712114778	0.243902847	2.919665709	0.00350407	0.125102734	up_in_case
CRA004775	ENSGALG00010011304	COMMD5	case	control	-0.037826937	0.241817678	-0.156427511	0.875696054	0.974412785	down_in_case
CRA004775	ENSGALG00010011306	ECI2	case	control	-0.093613341	0.295780078	-0.316496436	0.75162573	0.941495694	down_in_case
CRA004775	ENSGALG00010011307	ENSGALG00010011307	case	control	-1.492490393	0.934881509	-1.596448724	0.110388645	0.558567026	down_in_case
CRA004775	ENSGALG00010011308	SPIC	case	control	2.462695088	0.657169099	3.747429834	0.000178656	0.027735809	up_in_case
CRA004775	ENSGALG00010011311	DOCK8	case	control	-0.709719956	0.673263412	-1.054149005	0.291814703	0.746127895	down_in_case
CRA004775	ENSGALG00010011312	VPS13B	case	control	0.062211303	0.331790488	0.187501767	0.851267238	0.969261886	up_in_case
CRA004775	ENSGALG00010011313	RASSF9	case	control	0.426296842	0.637768775	0.668419117	0.503866096	0.861452528	up_in_case
CRA004775	ENSGALG00010011316	PARPBP	case	control	0.961187779	0.854850227	1.124393197	0.260846257	0.721659172	up_in_case
CRA004775	ENSGALG00010011317	BLK	case	control	-0.168016977	0.296754216	-0.566182274	0.571269878	0.885321726	down_in_case
CRA004775	ENSGALG00010011318	PRP4K	case	control	0.185003267	0.270207938	0.684669993	0.493552188	0.856001758	up_in_case
CRA004775	ENSGALG00010011321	MAP2	case	control	0.105599911	0.402938189	0.262074716	0.793263839	0.95151527	up_in_case
CRA004775	ENSGALG00010011322	GATA4	case	control	2.609081088	0.894718452	2.916091742	0.003544463	0.125102734	up_in_case
CRA004775	ENSGALG00010011323	GUCY2C	case	control	2.273421758	0.635589805	3.576869455	0.000347734	0.037067336	up_in_case
CRA004775	ENSGALG00010011326	COX6C	case	control	-0.112036208	0.308036472	-0.363710851	0.71607394	0.933921336	down_in_case
CRA004775	ENSGALG00010011329	PCDHGC5	case	control	0.048498847	0.355291988	0.136504195	0.891422709	0.977782257	up_in_case
CRA004775	ENSGALG00010011333	ENSGALG00010011333	case	control	0.028534379	0.263253437	0.108391287	0.913685312	0.980803023	up_in_case
CRA004775	ENSGALG00010011335	VCP	case	control	-0.158201923	0.309393385	-0.511329365	0.609120446	0.90115757	down_in_case
CRA004775	ENSGALG00010011339	ENSGALG00010011339	case	control	-0.050759823	0.551601116	-0.092022699	0.926680005	0.984182984	down_in_case
CRA004775	ENSGALG00010011340	PKNOX1	case	control	0.477566098	0.268770856	1.776852241	0.075592554	0.483926075	up_in_case
CRA004775	ENSGALG00010011342	RASAL2	case	control	-0.017717491	0.322615931	-0.054918215	0.95620362	0.99096835	down_in_case
CRA004775	ENSGALG00010011343	PXDC1	case	control	0.252785822	0.249100787	1.014793352	0.310204343	0.762244121	up_in_case
CRA004775	ENSGALG00010011345	CRMP1	case	control	-0.247953223	0.536733327	-0.461967257	0.644104801	0.913739303	down_in_case
CRA004775	ENSGALG00010011348	SLC22A23	case	control	-0.108495612	0.426094067	-0.25462831	0.799010191	0.952835572	down_in_case
CRA004775	ENSGALG00010011349	ENSGALG00010011349	case	control	0.46801685	0.245711613	1.904740458	0.056813817	0.435477592	up_in_case
CRA004775	ENSGALG00010011351	KANK1	case	control	0.648114373	0.237398902	2.730064746	0.006332189	0.158894029	up_in_case
CRA004775	ENSGALG00010011352	NTS	case	control	0.618248248	0.714586428	0.865183306	0.386938187	0.803905475	up_in_case
CRA004775	ENSGALG00010011353	ENSGALG00010011353	case	control	-0.480110747	0.830133227	-0.578353849	0.563025246	0.881532201	down_in_case
CRA004775	ENSGALG00010011354	ENSGALG00010011354	case	control	-0.033877766	1.396401027	-0.024260771	0.980644604	0.996980737	down_in_case
CRA004775	ENSGALG00010011357	UNC80	case	control	-0.193976548	0.520477537	-0.372689567	0.709379502	0.931874404	down_in_case
CRA004775	ENSGALG00010011359	RNF19A	case	control	0.36829343	0.245733367	1.498752226	0.133937923	0.592212338	up_in_case
CRA004775	ENSGALG00010011360	TCF4	case	control	0.211866273	0.324488984	0.652922854	0.513806015	0.863954532	up_in_case
CRA004775	ENSGALG00010011361	NUP37	case	control	-0.271304651	0.255405365	-1.062251183	0.288121669	0.742790532	down_in_case
CRA004775	ENSGALG00010011363	FDFT1	case	control	-0.355799681	0.250780819	-1.418767523	0.155966804	0.619442611	down_in_case
CRA004775	ENSGALG00010011364	OTP	case	control	-0.088497616	0.800092645	-0.110609211	0.911926243	0.98075825	down_in_case
CRA004775	ENSGALG00010011365	PSMG4	case	control	-0.214770732	0.305381179	-0.703287389	0.481876668	0.848909035	down_in_case
CRA004775	ENSGALG00010011367	IER3IP1	case	control	-0.363601454	0.270638403	-1.343495414	0.17911161	0.644923737	down_in_case
CRA004775	ENSGALG00010011371	ENSGALG00010011371	case	control	0.060872466	0.272440795	0.223433741	0.823197947	0.960898591	up_in_case
CRA004775	ENSGALG00010011372	VMA21	case	control	-0.148094788	0.145151761	-1.020275518	0.307597811	0.759249237	down_in_case
CRA004775	ENSGALG00010011374	ENSGALG00010011374	case	control	-0.358828019	0.206989211	-1.733559048	0.082996352	0.500123942	down_in_case
CRA004775	ENSGALG00010011375	C12orf50	case	control	-0.086635714	0.219253807	-0.395138926	0.692740362	0.927867527	down_in_case
CRA004775	ENSGALG00010011377	SEC16B	case	control	1.274867391	0.41766867	3.052341441	0.002270637	0.096690159	up_in_case
CRA004775	ENSGALG00010011378	TUBB2B	case	control	-0.091608776	0.226389708	-0.404650796	0.685734215	0.926686318	down_in_case
CRA004775	ENSGALG00010011381	RLIG1	case	control	0.277385976	0.249194071	1.113132329	0.265651601	0.726742745	up_in_case
CRA004775	ENSGALG00010011382	ENSGALG00010011382	case	control	0.226093091	0.350349002	0.645336764	0.518708973	0.865435399	up_in_case
CRA004775	ENSGALG00010011384	PUM3	case	control	0.060725173	0.227154028	0.267330382	0.789214797	0.95041797	up_in_case
CRA004775	ENSGALG00010011386	CTSB	case	control	0.022025324	0.240282141	0.091664425	0.926964663	0.984216865	up_in_case
CRA004775	ENSGALG00010011388	GPR50	case	control	0.501318356	0.333452074	1.503419518	0.132730897	0.589506365	up_in_case
CRA004775	ENSGALG00010011389	RC3H1	case	control	0.190622298	0.302403087	0.630358308	0.528460182	0.868929328	up_in_case
CRA004775	ENSGALG00010011394	SERPINC1	case	control	1.085049307	0.498385123	2.177130207	0.029470854	0.326554159	up_in_case
CRA004775	ENSGALG00010011398	ENSGALG00010011398	case	control	-0.102279639	0.314194259	-0.325529943	0.744780024	0.939218065	down_in_case
CRA004775	ENSGALG00010011399	JAKMIP1	case	control	0.03751447	0.806725836	0.046502131	0.962910036	0.991860454	up_in_case
CRA004775	ENSGALG00010011400	BPHL	case	control	0.072003336	0.468605327	0.15365454	0.877882131	0.974657764	up_in_case
CRA004775	ENSGALG00010011401	ANKRD46	case	control	-0.026835701	0.186913814	-0.143572595	0.885837982	0.976802138	down_in_case
CRA004775	ENSGALG00010011406	UBE2R2	case	control	-0.3470539	0.210192965	-1.651120434	0.098713986	0.535162165	down_in_case
CRA004775	ENSGALG00010011407	CEP290	case	control	0.659625532	0.340986446	1.934462613	0.053056274	0.423679925	up_in_case
CRA004775	ENSGALG00010011411	ENSGALG00010011411	case	control	0.492027419	0.580773586	0.847193176	0.396887457	0.808998414	up_in_case
CRA004775	ENSGALG00010011412	KANSL1L	case	control	0.308689034	0.207292591	1.489146488	0.136448798	0.595518184	up_in_case
CRA004775	ENSGALG00010011413	ZBTB37	case	control	0.058309691	0.46135625	0.126387562	0.899425148	0.978790377	up_in_case
CRA004775	ENSGALG00010011414	PABPC1	case	control	-0.250410203	0.221492863	-1.130556532	0.258241792	0.720037471	down_in_case
CRA004775	ENSGALG00010011416	ENSGALG00010011416	case	control	-0.657458962	0.777783731	-0.84529791	0.397944528	0.809455905	down_in_case
CRA004775	ENSGALG00010011417	WFS1	case	control	0.283998905	0.133842462	2.121889433	0.033847024	0.349587488	up_in_case
CRA004775	ENSGALG00010011418	RIPK1	case	control	-0.092346511	0.298092231	-0.309791742	0.756719332	0.941900693	down_in_case
CRA004775	ENSGALG00010011419	RNF180	case	control	0.10221063	0.414169357	0.246784627	0.805074909	0.954689985	up_in_case
CRA004775	ENSGALG00010011420	HMGB3	case	control	-0.109563022	0.286320465	-0.382658716	0.701972829	0.930068722	down_in_case
CRA004775	ENSGALG00010011421	ENSGALG00010011421	case	control	0.456647908	0.252502883	1.808485915	0.070530907	0.469895261	up_in_case
CRA004775	ENSGALG00010011422	KATNAL2	case	control	0.638240176	0.658118941	0.969794571	0.3321489	0.772935571	up_in_case
CRA004775	ENSGALG00010011423	GDF6	case	control	0.009915985	0.489529887	0.020256137	0.983839046	0.99751066	up_in_case
CRA004775	ENSGALG00010011424	ACADL	case	control	0.120762101	0.259381369	0.465577392	0.641518028	0.912444602	up_in_case
CRA004775	ENSGALG00010011425	WASHC3	case	control	0.067495218	0.191782213	0.3519368	0.72488566	0.935238044	up_in_case
CRA004775	ENSGALG00010011426	NQO2	case	control	-0.1940065	0.260675527	-0.744245165	0.456728169	0.839688534	down_in_case
CRA004775	ENSGALG00010011427	TMTC3	case	control	0.212960022	0.173727061	1.225831028	0.220262268	0.688374653	up_in_case
CRA004775	ENSGALG00010011428	UQCRB	case	control	0.181862863	0.435213977	0.417869997	0.67604217	0.922924485	up_in_case
CRA004775	ENSGALG00010011429	UBAP2	case	control	-0.343883945	0.295586089	-1.163396919	0.244668499	0.711765017	down_in_case
CRA004775	ENSGALG00010011430	ENSGALG00010011430	case	control	0.494369555	0.669150865	0.738801338	0.460027639	0.840995542	up_in_case
CRA004775	ENSGALG00010011431	DRAM1	case	control	0.287652458	0.39294463	0.732043234	0.46414218	0.842643327	up_in_case
CRA004775	ENSGALG00010011433	RNASE6	case	control	-0.713934834	0.381879787	-1.869527683	0.061549436	0.446401278	down_in_case
CRA004775	ENSGALG00010011434	NPL	case	control	-0.534272212	0.249311247	-2.142992818	0.032113675	0.340736995	down_in_case
CRA004775	ENSGALG00010011436	UBAP2L2	case	control	-0.435861706	1.085338756	-0.401590474	0.687985441	0.927111919	down_in_case
CRA004775	ENSGALG00010011437	DMRT1	case	control	0.143819026	0.534668507	0.268987277	0.787939474	0.95041797	up_in_case
CRA004775	ENSGALG00010011438	ENSGALG00010011438	case	control	1.242649792	0.321248795	3.868185072	0.000109648	0.022744947	up_in_case
CRA004775	ENSGALG00010011439	ENSGALG00010011439	case	control	-0.459097265	0.657923503	-0.697797331	0.485303948	0.851302844	down_in_case
CRA004775	ENSGALG00010011440	FUNDC1	case	control	-0.025609002	0.285419192	-0.089724175	0.928506405	0.98473447	down_in_case
CRA004775	ENSGALG00010011441	CD99L2	case	control	0.036391807	0.27168035	0.133950825	0.89344146	0.977834811	up_in_case
CRA004775	ENSGALG00010011442	ENSGALG00010011442	case	control	0.07117728	1.249662321	0.056957211	0.954579281	0.990670511	up_in_case
CRA004775	ENSGALG00010011443	ENSGALG00010011443	case	control	0.233990658	0.610615063	0.383204858	0.701567876	0.930068722	up_in_case
CRA004775	ENSGALG00010011445	MTERF3	case	control	0.084077276	0.26374298	0.318784886	0.74988964	0.941495694	up_in_case
CRA004775	ENSGALG00010011446	ENSGALG00010011446	case	control	0.100809254	0.36339243	0.27741154	0.781464122	0.948910196	up_in_case
CRA004775	ENSGALG00010011447	LAMC1	case	control	0.378060508	0.417417863	0.905712337	0.365088131	0.793608387	up_in_case
CRA004775	ENSGALG00010011449	DUSP22	case	control	-0.547975721	0.372861371	-1.469650018	0.141656566	0.601695394	down_in_case
CRA004775	ENSGALG00010011450	PTDSS1	case	control	0.23525009	0.257804393	0.912513894	0.361498259	0.792757982	up_in_case
CRA004775	ENSGALG00010011452	KITLG	case	control	0.726109145	0.390300779	1.86038354	0.062831282	0.450026626	up_in_case
CRA004775	ENSGALG00010011453	GNPTAB	case	control	0.385099653	0.212734175	1.810238781	0.070258766	0.469724266	up_in_case
CRA004775	ENSGALG00010011456	KIAA1328	case	control	-0.28727974	0.366225572	-0.784433862	0.432785575	0.825642959	down_in_case
CRA004775	ENSGALG00010011457	ENSGALG00010011457	case	control	-0.256799446	0.591037242	-0.43448945	0.663933055	0.918295135	down_in_case
CRA004775	ENSGALG00010011458	TPGS2	case	control	0.014787649	0.31083247	0.047574338	0.962055484	0.991860454	up_in_case
CRA004775	ENSGALG00010011459	ENSGALG00010011459	case	control	0.219679354	0.510809846	0.430060922	0.667151325	0.919278017	up_in_case
CRA004775	ENSGALG00010011460	LAMC2	case	control	1.936132761	0.643550736	3.008516117	0.002625269	0.106489884	up_in_case
CRA004775	ENSGALG00010011461	IRF4	case	control	-0.216374325	0.762785056	-0.283663561	0.776668222	0.946929223	down_in_case
CRA004775	ENSGALG00010011463	IMP3	case	control	-0.302068833	0.369406902	-0.817713017	0.413521076	0.817353502	down_in_case
CRA004775	ENSGALG00010011464	FANCG	case	control	-0.749965663	0.391275339	-1.916721013	0.055273384	0.430875458	down_in_case
CRA004775	ENSGALG00010011465	PPP2R2C	case	control	-0.284088868	0.636446312	-0.446367372	0.655331898	0.917011186	down_in_case
CRA004775	ENSGALG00010011466	MTMR1	case	control	0.011352091	0.133797388	0.084845388	0.932384309	0.985044263	up_in_case
CRA004775	ENSGALG00010011467	ENSGALG00010011467	case	control	0.219689366	1.01454389	0.216540032	0.828566824	0.962787088	up_in_case
CRA004775	ENSGALG00010011469	CPS1	case	control	-1.000582127	0.489137761	-2.045603933	0.040795354	0.378271689	down_in_case
CRA004775	ENSGALG00010011471	NMNAT2	case	control	-0.090230205	0.579014537	-0.155834092	0.876163798	0.974421894	down_in_case
CRA004775	ENSGALG00010011475	NDP	case	control	0.623654498	0.721614409	0.864248954	0.387451145	0.804080999	up_in_case
CRA004775	ENSGALG00010011477	SDC2	case	control	0.605495589	0.252876838	2.394428818	0.016646274	0.255187198	up_in_case
CRA004775	ENSGALG00010011478	HDAC3	case	control	-0.421556415	0.140953394	-2.990750371	0.002782929	0.109874886	down_in_case
CRA004775	ENSGALG00010011480	BLTP3B	case	control	0.164422992	0.194182408	0.846745049	0.397137243	0.808998414	up_in_case
CRA004775	ENSGALG00010011482	DMRT2	case	control	-0.164819104	0.570089304	-0.289111026	0.772496416	0.946296525	down_in_case
CRA004775	ENSGALG00010011484	CHPT1	case	control	-0.006169176	0.152897545	-0.040348428	0.967815345	0.993568693	down_in_case
CRA004775	ENSGALG00010011485	MTM1	case	control	0.074928337	0.171635607	0.436554734	0.662434297	0.918173506	up_in_case
CRA004775	ENSGALG00010011487	MAN2B2	case	control	-0.307354528	0.266320412	-1.154077995	0.248468194	0.713295645	down_in_case
CRA004775	ENSGALG00010011493	ENSGALG00010011493	case	control	-0.077122952	0.571240699	-0.135009555	0.89260432	0.977834811	down_in_case
CRA004775	ENSGALG00010011496	ACTR6	case	control	0.000527257	0.200488147	0.002629864	0.997901674	0.999646385	up_in_case
CRA004775	ENSGALG00010011497	CPQ	case	control	0.243429568	0.429838672	0.56632775	0.571171	0.885321726	up_in_case
CRA004775	ENSGALG00010011499	SMG7	case	control	0.097897269	0.256917185	0.381046015	0.703169105	0.930068722	up_in_case

CRA004775	ENSGALG00010011503	NCF2	case	control	1.531716017	0.85722655	1.786827552	0.073965365	0.479781244	up_in_case
CRA004775	ENSGALG00010011505	MYL1	case	control	-0.183709963	0.796477036	-0.230653183	0.817584248	0.959839579	down_in_case
CRA004775	ENSGALG00010011509	EXOC2	case	control	-0.0889932	0.155805982	-0.571179614	0.567877896	0.884213371	down_in_case
CRA004775	ENSGALG00010011510	ENSGALG00010011510	case	control	0.269380468	0.387045651	0.695991462	0.486434177	0.851987818	up_in_case
CRA004775	ENSGALG00010011515	SERINC5	case	control	-0.080233945	0.284893813	-0.281627545	0.778229117	0.947907599	down_in_case
CRA004775	ENSGALG00010011523	PIGO	case	control	-0.45820204	0.264451591	-1.732649968	0.083157902	0.500877929	down_in_case
CRA004775	ENSGALG00010011525	ENSGALG00010011525	case	control	0.037599656	0.373097359	0.100777063	0.919727435	0.982170916	up_in_case
CRA004775	ENSGALG00010011529	SCYL2	case	control	0.200214805	0.274771955	0.728658079	0.466210842	0.843480361	up_in_case
CRA004775	ENSGALG00010011530	ARPC5	case	control	-0.464365573	0.342429961	-1.356089201	0.175070788	0.640697583	down_in_case
CRA004775	ENSGALG00010011531	MTDH	case	control	0.140095739	0.234783127	0.596702756	0.550705852	0.877827378	up_in_case
CRA004775	ENSGALG00010011533	ENSGALG00010011533	case	control	0.599258678	0.353201445	1.696648432	0.089763147	0.516522282	up_in_case
CRA004775	ENSGALG00010011536	FCHSD1	case	control	-0.200156343	0.259940278	-0.770008962	0.441294577	0.829353567	down_in_case
CRA004775	ENSGALG00010011538	ENSGALG00010011538	case	control	-0.525101152	0.531951281	-0.987122638	0.323582521	0.769025795	down_in_case
CRA004775	ENSGALG00010011539	LANCL1	case	control	-0.310903177	0.23881228	-1.301872654	0.192959922	0.657594025	down_in_case
CRA004775	ENSGALG00010011544	STOML2	case	control	-0.388958807	0.283231387	-1.373289911	0.169662238	0.633147466	down_in_case
CRA004775	ENSGALG00010011545	USP12-like	case	control	0.356863015	0.127364408	2.801905344	0.005080178	0.143998699	up_in_case
CRA004775	ENSGALG00010011546	MAOA	case	control	0.007939855	0.321361728	0.024706908	0.980288745	0.996964022	up_in_case
CRA004775	ENSGALG00010011548	ENSGALG00010011548	case	control	0.847237852	0.694577086	1.219789523	0.222544674	0.690169557	up_in_case
CRA004775	ENSGALG00010011549	KIAA0232	case	control	0.349959537	0.281964817	1.241146113	0.214551777	0.683562661	up_in_case
CRA004775	ENSGALG00010011551	GMDS	case	control	-0.439930739	0.170202689	-2.584746111	0.009745073	0.200006709	down_in_case
CRA004775	ENSGALG00010011555	ENSGALG00010011555	case	control	-0.332177966	0.941568209	-0.352792249	0.724244195	0.935238044	down_in_case
CRA004775	ENSGALG00010011556	LAPTM4B	case	control	0.657606263	0.378718533	1.736398422	0.082493411	0.499281205	up_in_case
CRA004775	ENSGALG00010011558	MYBPC1	case	control	0.141493035	1.052993973	0.134372122	0.893108325	0.977834811	up_in_case
CRA004775	ENSGALG00010011559	MTX3	case	control	0.246517196	0.22524886	1.094421503	0.273770157	0.731001618	up_in_case
CRA004775	ENSGALG00010011564	ARAP3	case	control	-0.226670418	0.308845646	-0.73392784	0.462992718	0.842456502	down_in_case
CRA004775	ENSGALG00010011570	RGL1	case	control	0.084740711	0.244047569	0.347230303	0.728418307	0.935784458	up_in_case
CRA004775	ENSGALG00010011571	ATOSB	case	control	-0.530949165	0.235013614	-2.259227268	0.023869251	0.298869476	down_in_case
CRA004775	ENSGALG00010011572	PTCD2	case	control	0.102883801	0.345015622	0.298200413	0.765550207	0.94337135	up_in_case
CRA004775	ENSGALG00010011573	CMAS	case	control	0.224142652	0.207829878	1.078490994	0.280814699	0.737061584	up_in_case
CRA004775	ENSGALG00010011574	GAS2L3	case	control	0.079568814	0.495858514	0.160466769	0.872513397	0.97401729	up_in_case
CRA004775	ENSGALG00010011575	ENSGALG00010011575	case	control	0.550021383	0.846403075	0.649833867	0.51579954	0.864897625	up_in_case
CRA004775	ENSGALG00010011577	ENSGALG00010011577	case	control	0.403192427	0.40683528	0.991045878	0.321663179	0.767750105	up_in_case
CRA004775	ENSGALG00010011578	MYLK4	case	control	0.710065906	0.708384267	1.002373908	0.316163039	0.765818284	up_in_case
CRA004775	ENSGALG00010011586	ENSGALG00010011586	case	control	0.729127756	0.67039018	1.087616999	0.276764223	0.731499819	up_in_case
CRA004775	ENSGALG00010011588	COLGALT2	case	control	-0.066098034	0.28717918	-0.230163043	0.817965079	0.959889304	down_in_case
CRA004775	ENSGALG00010011589	RERG	case	control	-0.384013257	0.462185785	-0.830863409	0.406050797	0.813842524	down_in_case
CRA004775	ENSGALG00010011593	WRNIP1	case	control	-0.338694755	0.259841215	-1.303468179	0.192414973	0.656959697	down_in_case
CRA004775	ENSGALG00010011595	ENSGALG00010011595	case	control	-0.361492816	0.326570855	-1.106935326	0.268321891	0.727528758	down_in_case
CRA004775	ENSGALG00010011597	TBC1D14	case	control	-0.050216539	0.315378659	-0.159226181	0.873490678	0.974079175	down_in_case
CRA004775	ENSGALG00010011601	ANO4	case	control	-0.861533363	0.646848187	-1.331894223	0.18289495	0.647378665	down_in_case
CRA004775	ENSGALG00010011603	ENSGALG00010011603	case	control	0.021448235	0.365030937	0.058757306	0.953145414	0.990670511	up_in_case
CRA004775	ENSGALG00010011605	APPL2	case	control	-0.360971485	0.293853877	-1.228404704	0.219295083	0.687020074	down_in_case
CRA004775	ENSGALG00010011607	TSEN15	case	control	-0.042758707	0.303395666	-0.14093381	0.887922232	0.977118922	down_in_case
CRA004775	ENSGALG00010011617	MAMLD1	case	control	0.305892274	0.237033761	1.290500865	0.196876812	0.66285976	up_in_case
CRA004775	ENSGALG00010011619	PCDH1	case	control	0.012600118	0.380185761	0.033142003	0.973561347	0.994947809	up_in_case
CRA004775	ENSGALG00010011621	WASHC4	case	control	0.145473088	0.211983913	0.686245883	0.492558068	0.855763086	up_in_case
CRA004775	ENSGALG00010011622	BACH1	case	control	0.649241547	0.334513783	1.940851408	0.052276303	0.420135271	up_in_case
CRA004775	ENSGALG00010011625	ERBB4	case	control	0.42362227	0.544246663	0.778364478	0.436354174	0.82694408	up_in_case
CRA004775	ENSGALG00010011626	SERPINB1	case	control	-0.0578343	0.456585049	-0.126667091	0.899203894	0.978790377	down_in_case
CRA004775	ENSGALG00010011628	MATN2	case	control	1.129808874	0.317047604	3.563530712	0.0003659	0.038226803	up_in_case
CRA004775	ENSGALG00010011630	DELE1	case	control	0.315484303	0.247624165	1.274044895	0.202647506	0.671648534	up_in_case
CRA004775	ENSGALG00010011633	TADA2B	case	control	-0.538755058	0.263896286	-2.041540891	0.041197094	0.379270506	down_in_case
CRA004775	ENSGALG00010011634	AVD	case	control	-0.894776097	0.580141987	-1.542339836	0.122991033	0.575380405	down_in_case
CRA004775	ENSGALG00010011635	PCDH12	case	control	-0.210388393	0.414955342	-0.507014544	0.612144614	0.901413878	down_in_case
CRA004775	ENSGALG00010011636	GRIK1	case	control	0.406395021	0.486992533	0.834499491	0.403999574	0.813056538	up_in_case
CRA004775	ENSGALG00010011637	CECR2	case	control	-0.090978718	0.242732703	-0.374810301	0.707801552	0.931742212	down_in_case
CRA004775	ENSGALG00010011638	GRPEL1	case	control	-0.046890922	0.240557193	-0.194926295	0.845450648	0.966993336	down_in_case
CRA004775	ENSGALG00010011639	ENSGALG00010011639	case	control	0.044359878	0.292010891	0.151911724	0.879256561	0.974996282	up_in_case
CRA004775	ENSGALG00010011641	ENSGALG00010011641	case	control	1.169181153	0.914104521	1.279045368	0.20088108	0.668517845	up_in_case
CRA004775	ENSGALG00010011643	C1orf21	case	control	0.289682027	0.341728088	0.847697445	0.39660649	0.808998414	up_in_case
CRA004775	ENSGALG00010011647	FBXO38	case	control	0.260196608	0.191590769	1.358085307	0.174436617	0.639597725	up_in_case
CRA004775	ENSGALG00010011648	ENSGALG00010011648	case	control	-0.099609138	0.378744225	-0.262998433	0.792551787	0.951284723	down_in_case
CRA004775	ENSGALG00010011649	MRPS27	case	control	0.412797266	0.281039063	1.468825229	0.141880199	0.602158834	up_in_case
CRA004775	ENSGALG00010011650	RPL30	case	control	-0.185980265	0.249562652	-0.745224749	0.456135864	0.839556186	down_in_case
CRA004775	ENSGALG00010011651	SERPINB6	case	control	-0.294948011	0.352604758	-0.836483357	0.402883036	0.812204167	down_in_case
CRA004775	ENSGALG00010011653	NR2C1	case	control	-0.003745702	0.234383997	-0.015981047	0.987249512	0.997897984	down_in_case
CRA004775	ENSGALG00010011655	SORCS2	case	control	0.062844916	0.598288229	0.105041204	0.916343113	0.981626545	up_in_case
CRA004775	ENSGALG00010011657	ALDH1L2	case	control	-0.09348977	0.518746035	-0.180222621	0.856977801	0.970913248	down_in_case
CRA004775	ENSGALG00010011658	TENM1	case	control	0.407158238	0.696198551	0.584830631	0.558661606	0.880754991	up_in_case
CRA004775	ENSGALG00010011659	CD14	case	control	-0.65018393	0.457193802	-1.422118863	0.154991748	0.619284425	down_in_case
CRA004775	ENSGALG00010011660	ENSGALG00010011660	case	control	0.033761088	0.247965541	0.136152336	0.891700855	0.977819591	up_in_case
CRA004775	ENSGALG00010011662	RIDA	case	control	-0.082261169	0.249152597	-0.330163805	0.741276194	0.938778937	down_in_case
CRA004775	ENSGALG00010011663	UTP20	case	control	0.00966317	0.259804676	0.037193981	0.970330337	0.994414835	up_in_case
CRA004775	ENSGALG00010011664	SERPINB10B	case	control	-1.40612858	0.501411302	-2.804341616	0.005041945	0.143768101	down_in_case
CRA004775	ENSGALG00010011667	ENSGALG00010011667	case	control	0.1692225	0.581939053	0.290790761	0.771211352	0.945853152	up_in_case
CRA004775	ENSGALG00010011669	ENSGALG00010011669	case	control	0.376309508	1.458106378	0.25808097	0.796344418	0.952835572	up_in_case
CRA004775	ENSGALG00010011670	TMCO6	case	control	-0.462432745	0.26560766	-1.741036929	0.081677106	0.497331356	down_in_case
CRA004775	ENSGALG00010011671	SLC41A2	case	control	0.041140731	0.207939839	0.197849203	0.843163049	0.966478468	up_in_case
CRA004775	ENSGALG00010011673	POP1	case	control	-0.188191563	0.427445721	-0.440270083	0.659741506	0.917907174	down_in_case
CRA004775	ENSGALG00010011674	ENSGALG00010011674	case	control	-0.042525418	0.49379891	-0.086118899	0.9313719	0.985044263	down_in_case
CRA004775	ENSGALG00010011677	EDEM3	case	control	-0.060657654	0.348630859	-0.173988194	0.86187474	0.971668964	down_in_case
CRA004775	ENSGALG00010011681	MAP1B	case	control	0.27562231	0.47667595	0.578217361	0.563117379	0.881532201	up_in_case
CRA004775	ENSGALG00010011682	USP15	case	control	0.423364789	0.284014249	1.490646295	0.136054383	0.594875618	up_in_case
CRA004775	ENSGALG00010011685	NDUFA2	case	control	-0.074546326	0.352867518	-0.211258679	0.832685425	0.963988657	down_in_case
CRA004775	ENSGALG00010011688	PDE3A	case	control	0.275789955	0.432726337	0.637331105	0.523909194	0.866784927	up_in_case
CRA004775	ENSGALG00010011692	ARL1	case	control	-0.278998034	0.152215147	-1.832918997	0.066814618	0.462498477	down_in_case
CRA004775	ENSGALG00010011696	ZFC3H1	case	control	0.114563075	0.332498441	0.344552218	0.730431033	0.936393482	up_in_case
CRA004775	ENSGALG00010011699	TIAM1	case	control	-0.870387442	0.517787278	-1.680974945	0.092767781	0.524527039	down_in_case
CRA004775	ENSGALG00010011702	NUP205	case	control	0.091938387	0.257900011	0.356488494	0.721474767	0.934511903	up_in_case
CRA004775	ENSGALG00010011703	YWHAZ	case	control	-0.040428436	0.238867121	-0.169250737	0.865599423	0.972629493	down_in_case
CRA004775	ENSGALG00010011708	IK	case	control	-0.215646867	0.170638049	-1.263767774	0.206313391	0.674837165	down_in_case
CRA004775	ENSGALG00010011712	DCAF12L2	case	control	-0.347569692	0.164545016	-2.112307621	0.034660073	0.353831224	down_in_case
CRA004775	ENSGALG00010011720	IKZF2	case	control	-0.182938583	0.407949388	-0.448434508	0.653839642	0.916560183	down_in_case
CRA004775	ENSGALG00010011723	SPRY3	case	control	0.587695049	0.386149353	1.521937158	0.128024827	0.5821156	up_in_case
CRA004775	ENSGALG00010011724	ARHGEF28	case	control	0.289408754	0.364721305	0.793506577	0.427482739	0.822776983	up_in_case
CRA004775	ENSGALG00010011726	WDR55	case	control	-0.304217358	0.257691324	-1.180549481	0.237781743	0.70473329	down_in_case
CRA004775	ENSGALG00010011729	FAM129A	case	control	-0.092900619	0.363150819	-0.255818282	0.798091157	0.952835572	down_in_case
CRA004775	ENSGALG00010011734	ENSGALG00010011734	case	control	1.644339069	0.345767136	4.755625676	1.97833E-06	0.001133499	up_in_case
CRA004775	ENSGALG00010011738	FGD6	case	control	0.144438511	0.275575013	0.524135007	0.600184642	0.897206546	up_in_case
CRA004775	ENSGALG00010011745	AFAP1L1	case	control	-0.3266512	0.355864369	-0.917909261	0.358666373	0.79097996	down_in_case
CRA004775	ENSGALG00010011746	SOD1	case	control	0.156705391	0.276738146	0.56625873	0.571217911	0.885321726	up_in_case
CRA004775	ENSGALG00010011747	UTP15	case	control	-0.066145787	0.239907601	-0.275713594	0.782768054	0.94936527	down_in_case
CRA004775	ENSGALG00010011749	ENSGALG00010011749	case	control	-0.345892728	0.989461482	-0.349576749	0.726656363	0.935238044	down_in_case
CRA004775	ENSGALG00010011751	ZNF706	case	control	0.331872328	0.176218377	1.883301466	0.059659525	0.445373576	up_in_case
CRA004775	ENSGALG00010011752	RNF2	case	control	-0.14181362	0.262091248	-0.541084914	0.588449056	0.893032002	down_in_case
CRA004775	ENSGALG00010011754	IL17B	case	control	-0.33627967	0.67515127	-0.498080482	0.618427317	0.904143308	down_in_case
CRA004775	ENSGALG00010011758	SERPINB11	case	control	-1.646899855	0.832460493	-1.978351967	0.047889014	0.406815029	down_in_case
CRA004775	ENSGALG00010011759	CREB3	case	control	-0.524724973	0.253653818	-2.068665775	0.038577461	0.370251617	down_in_case
CRA004775	ENSGALG00010011764	PCYOX1L	case	control	-0.201308235	0.275196961	-0.731506026	0.464470125	0.842936346	down_in_case
CRA004775	ENSGALG00010011770	CHST11	case	control	0.165196057	0.399310713	0.413703043	0.679091598	0.92338461	up_in_case
CRA004775	ENSGALG00010011774	ANKRA2	case	control	-0.386937014	0.252110456	-1.534791613	0.124835024	0.577398725	down_in_case
CRA004775	ENSGALG00010011775	VEZT	case	control	-0.036114565	0.137602418	-0.26245589	0.792969988	0.951411771	down_in_case
CRA004775	ENSGALG00010011776	IL17RA	case	control	-1.220487741	0.493776022	-2.47174364	0.01344559	0.232695952	down_in_case
CRA004775	ENSGALG00010011784	GRPEL2	case	control	-0.187939826	0.266657851	-0.704797646	0.480936174	0.848743338	down_in_case
CRA004775	ENSGALG00010011789	TXNRD1	case	control	0.562478223	0.368589046	1.52603076	0.127002204	0.581154209	up_in_case
CRA004775	ENSGALG00010011790	AMY2A	case	control	2.443706509	1.058168227	2.3093743	0.02092282	0.284579324	up_in_case
CRA004775	ENSGALG00010011792	HDHD5	case	control	-0.067757212	0.269426614	-0.251486708	0.801437838	0.953543664	down_in_case
CRA004775	ENSGALG00010011793	TLN1	case	control	-0.428653849	0.255827504	-1.675558107	0.09382478	0.525859426	down_in_case
CRA004775	ENSGALG00010011796	METAP2	case	control	0.148377894	0.224774483	0.66011894	0.509177505	0.862971569	up_in_case
CRA004775	ENSGALG00010011797	SCAF4	case	control	0.117755524	0.186359085	0.631874339	0.527468993	0.86830001	up_in_case
CRA004775	ENSGALG00010011802	VPS4B	case	control	-0.079459354	0.163366925	-0.486385809	0.62669365	0.90758663	down_in_case
CRA004775	ENSGALG00010011808	SMARCA2	case	control	0.166610841	0.21248002	0.784124743	0.432966918	0.825787569	up_in_case
CRA004775	ENSGALG00010011810	FGFR4	case	control	0.057471993	0.347301989	0.165481323	0.868565149	0.973370973	up_in_case
CRA004775	ENSGALG00010011814	ENSGALG00010011814	case	control	-0.036246943	0.62572393	-0.057928011	0.953805971	0.990670511	down_in_case
CRA004775	ENSGALG00010011817	KDSR	case	control	0.304526622	0.118945189	2.56022647	0.010460397	0.205193897	up_in_case
CRA004775	ENSGALG00010011819	USP44	case	control	0.33748308	0.214901257	1.57040999	0.11631976	0.566086328	up_in_case

CRA004775	ENSGALG00010011823	ENSGALG00010011823	case	control	2.039775206	0.683192011	2.985654358	0.002829723	0.110544088	up_in_case
CRA004775	ENSGALG00010011837	VLDLR	case	control	0.312203812	0.320546091	0.973974792	0.330069056	0.77237154	up_in_case
CRA004775	ENSGALG00010011839	CBS	case	control	-0.685565654	0.71630769	-0.957082639	0.338525546	0.779253705	down_in_case
CRA004775	ENSGALG00010011841	KCNMB4	case	control	-0.266962603	0.528541664	-0.505092826	0.61349364	0.902282137	down_in_case
CRA004775	ENSGALG00010011843	BCL2	case	control	0.394251711	0.333617079	1.181749184	0.237305236	0.703879272	up_in_case
CRA004775	ENSGALG00010011846	UBAP1	case	control	0.22642274	0.300068385	0.754570462	0.450506752	0.835404673	up_in_case
CRA004775	ENSGALG00010011850	SPAG16	case	control	-1.101038472	0.740368132	-1.487150006	0.136975197	0.596242097	down_in_case
CRA004775	ENSGALG00010011856	NFYB	case	control	0.045451283	0.227078647	0.200156571	0.841358131	0.966461921	up_in_case
CRA004775	ENSGALG00010011857	ENSGALG00010011857	case	control	0.196309672	0.589498267	0.333011449	0.739125641	0.938064864	up_in_case
CRA004775	ENSGALG00010011859	NTN4	case	control	0.553681728	0.488818201	1.132694582	0.257342534	0.719236059	up_in_case
CRA004775	ENSGALG00010011860	CECR1	case	control	-0.613456538	0.320384542	-1.91475074	0.055524299	0.431364203	down_in_case
CRA004775	ENSGALG00010011864	ENSGALG00010011864	case	control	0.11773078	0.562132498	0.209435997	0.834107894	0.964019688	up_in_case
CRA004775	ENSGALG00010011866	KLF8	case	control	0.261624412	0.319145717	0.81976476	0.412350224	0.817035725	up_in_case
CRA004775	ENSGALG00010011867	ENSGALG00010011867	case	control	-0.437696681	0.364173563	-1.201890322	0.229406025	0.695782399	down_in_case
CRA004775	ENSGALG00010011868	SNRPF	case	control	-0.315907495	0.342083105	-0.923481722	0.355756221	0.788778426	down_in_case
CRA004775	ENSGALG00010011871	CCT2	case	control	0.033648831	0.2137303	0.157435942	0.874901289	0.974301166	up_in_case
CRA004775	ENSGALG00010011873	RFX3	case	control	0.729236195	0.450634706	1.618242414	0.105610366	0.549677569	up_in_case
CRA004775	ENSGALG00010011875	PSD2	case	control	-0.020361472	0.551109101	-0.036946354	0.97052778	0.994539645	down_in_case
CRA004775	ENSGALG00010011876	AMDHD1	case	control	-0.025131464	0.634486993	-0.039609108	0.968404766	0.993583205	down_in_case
CRA004775	ENSGALG00010011880	U2AF1	case	control	-0.181514536	0.212349672	-0.854790752	0.392666999	0.807553294	down_in_case
CRA004775	ENSGALG00010011881	RIOK2	case	control	-0.103846815	0.243609889	-0.42628325	0.669901462	0.921089391	down_in_case
CRA004775	ENSGALG00010011882	ENSGALG00010011882	case	control	0.073390484	0.578218395	0.126925197	0.898999603	0.978790377	up_in_case
CRA004775	ENSGALG00010011883	HCFC2	case	control	0.277106057	0.240470438	1.152349781	0.249177368	0.714300782	up_in_case
CRA004775	ENSGALG00010011885	NCALD	case	control	-0.42820815	0.468301263	-0.914386067	0.36051402	0.791975934	down_in_case
CRA004775	ENSGALG00010011886	NTNG1	case	control	0.453953538	0.482601036	0.940639376	0.346889696	0.784552667	up_in_case
CRA004775	ENSGALG00010011887	PGM5	case	control	0.067528939	0.343032248	0.196858864	0.843937985	0.966478468	up_in_case
CRA004775	ENSGALG00010011888	CAND1	case	control	-0.042243827	0.302287255	-0.139747297	0.888859656	0.977118922	down_in_case
CRA004775	ENSGALG00010011893	LIX1	case	control	-1.083166311	0.730836018	-1.482092131	0.138315782	0.5975433	down_in_case
CRA004775	ENSGALG00010011894	VWC2L	case	control	-0.089293687	0.712361857	-0.125348776	0.900247439	0.978988423	down_in_case
CRA004775	ENSGALG00010011896	SIK1	case	control	0.799189844	0.503328086	1.587810947	0.112329095	0.562709431	up_in_case
CRA004775	ENSGALG00010011898	ENSGALG00010011898	case	control	-0.163396639	0.258336417	-0.632495567	0.527063104	0.86830001	down_in_case
CRA004775	ENSGALG00010011899	PHLPP1	case	control	0.451407123	0.268777	1.679485683	0.093057427	0.524831789	up_in_case
CRA004775	ENSGALG00010011902	TP53I3	case	control	0.53643206	0.428957521	1.25054821	0.211099356	0.681585086	up_in_case
CRA004775	ENSGALG00010011903	LTA4H	case	control	-0.233792026	0.335784199	-0.696256782	0.486268033	0.851808837	down_in_case
CRA004775	ENSGALG00010011904	KIF24	case	control	-0.99414113	0.705683839	-1.408762784	0.158905323	0.624851901	down_in_case
CRA004775	ENSGALG00010011907	BIK	case	control	-0.329456292	0.54552098	-0.603929645	0.545890419	0.87682971	down_in_case
CRA004775	ENSGALG00010011908	ENSGALG00010011908	case	control	-0.65940783	0.300404147	-2.195068994	0.028158649	0.319106625	down_in_case
CRA004775	ENSGALG00010011909	C8orf48	case	control	-0.527363902	0.502654894	-1.049157002	0.294105865	0.746438942	down_in_case
CRA004775	ENSGALG00010011911	NUDT2	case	control	-0.177843951	0.29570773	-0.601417997	0.547561615	0.877277621	down_in_case
CRA004775	ENSGALG00010011912	TCP11X2	case	control	-0.159859725	0.281019824	-0.568855689	0.569454079	0.884809383	down_in_case
CRA004775	ENSGALG00010011913	CXXC5	case	control	-0.139916425	0.387877794	-0.360722957	0.718306561	0.934011578	down_in_case
CRA004775	ENSGALG00010011915	RRM2B	case	control	0.044451395	0.288320886	0.154173343	0.87747306	0.974479909	up_in_case
CRA004775	ENSGALG00010011916	MCAT	case	control	-0.248582957	0.228079365	-1.089896745	0.27575863	0.731001618	down_in_case
CRA004775	ENSGALG00010011917	HSF2BP	case	control	-0.284524744	0.408124106	-0.697152508	0.485707357	0.85158254	down_in_case
CRA004775	ENSGALG00010011921	MEGF10	case	control	0.746730761	0.367933937	2.029524013	0.042404946	0.386934578	up_in_case
CRA004775	ENSGALG00010011922	PIK3R1	case	control	0.249671636	0.368077049	0.678313513	0.497572933	0.857810713	up_in_case
CRA004775	ENSGALG00010011923	BARD1	case	control	-1.278119892	0.726954844	-1.758183335	0.07871632	0.491399489	down_in_case
CRA004775	ENSGALG00010011925	RRP1B	case	control	0.345398282	0.203214707	1.699671674	0.0891927	0.515997712	up_in_case
CRA004775	ENSGALG00010011926	DYRK2	case	control	0.954776312	0.315244744	3.028682728	0.002456225	0.101733579	up_in_case
CRA004775	ENSGALG00010011929	ELK3	case	control	-0.360515037	0.32440127	-1.111324369	0.266428754	0.727311193	down_in_case
CRA004775	ENSGALG00010011930	TSPO	case	control	-0.092128084	0.199813333	-0.461070755	0.644747843	0.913918935	down_in_case
CRA004775	ENSGALG00010011931	ENSGALG00010011931	case	control	0.35222152	1.088291828	0.323646205	0.746205902	0.939659099	up_in_case
CRA004775	ENSGALG00010011932	VAV3	case	control	0.076488359	0.548885138	0.139352214	0.889171832	0.977162915	up_in_case
CRA004775	ENSGALG00010011935	ST8SIA4	case	control	-0.502342004	0.44705575	-1.123667473	0.26115412	0.721774856	down_in_case
CRA004775	ENSGALG00010011936	CKAP4	case	control	0.005758283	0.403022702	0.014287738	0.988600422	0.998065748	up_in_case
CRA004775	ENSGALG00010011937	SLC1A1	case	control	1.760366253	0.496015877	3.549011906	0.00038668	0.039386897	up_in_case
CRA004775	ENSGALG00010011941	ENSGALG00010011941	case	control	-0.561099496	0.610936022	-0.918425949	0.35839591	0.790805867	down_in_case
CRA004775	ENSGALG00010011943	TTLL12	case	control	-0.464375438	0.247983991	-1.872602489	0.061123297	0.445419429	down_in_case
CRA004775	ENSGALG00010011947	THAP1	case	control	0.162960565	0.176043447	0.925683792	0.354610333	0.787789315	up_in_case
CRA004775	ENSGALG00010011950	PDXK	case	control	-0.337655104	0.41923483	-0.805408043	0.420584252	0.819388688	down_in_case
CRA004775	ENSGALG00010011952	UBE2D2	case	control	0.236010912	0.214940761	1.098027711	0.272192389	0.73004048	up_in_case
CRA004775	ENSGALG00010011953	CDK17	case	control	0.392104879	0.200938811	1.951364579	0.051013692	0.417304746	up_in_case
CRA004775	ENSGALG00010011954	ENSGALG00010011954	case	control	-0.182314543	0.376526821	-0.48420068	0.628243454	0.908314135	down_in_case
CRA004775	ENSGALG00010011957	ENSGALG00010011957	case	control	0.148305707	0.362492676	0.409127458	0.682446127	0.924564673	up_in_case
CRA004775	ENSGALG00010011960	CFAP54	case	control	0.587193545	0.962000804	0.61038779	0.541604954	0.87495415	up_in_case
CRA004775	ENSGALG00010011961	AGPAT3	case	control	-0.25173909	0.26265951	-0.958423663	0.337849168	0.778445695	down_in_case
CRA004775	ENSGALG00010011962	CPSF6	case	control	-0.101576179	0.178362321	-0.569493479	0.569021295	0.884809383	down_in_case
CRA004775	ENSGALG00010011963	ZCCHC2	case	control	0.376836209	0.250507979	1.504288248	0.132507164	0.589106372	up_in_case
CRA004775	ENSGALG00010011964	ENSGALG00010011964	case	control	-0.689362811	1.196377112	-0.576208625	0.564474176	0.882478718	down_in_case
CRA004775	ENSGALG00010011965	ATIC	case	control	-0.509922573	0.217349851	-2.346091199	0.018971465	0.272278915	down_in_case
CRA004775	ENSGALG00010011966	SLC25A24	case	control	0.130994458	0.242802684	0.539509924	0.589535051	0.893412703	up_in_case
CRA004775	ENSGALG00010011967	MTMR9	case	control	-0.03445484	0.262067372	-0.131473214	0.895400975	0.978048996	down_in_case
CRA004775	ENSGALG00010011968	ENSGALG00010011968	case	control	0.482002785	0.555181625	0.868189368	0.385290673	0.803415611	up_in_case
CRA004775	ENSGALG00010011969	TMEM173	case	control	0.113420072	0.234803581	0.483042344	0.62906567	0.908586927	up_in_case
CRA004775	ENSGALG00010011971	RNF170	case	control	0.09033948	0.208528971	0.433222683	0.664853001	0.918655755	up_in_case
CRA004775	ENSGALG00010011972	IPO11	case	control	0.002392035	0.195311431	0.012247285	0.990228325	0.998065748	up_in_case
CRA004775	ENSGALG00010011973	TRAPPC10	case	control	0.292305286	0.211481427	1.382179463	0.166916604	0.630568744	up_in_case
CRA004775	ENSGALG00010011976	TNFRSF11A	case	control	0.32184588	0.250207951	1.286313563	0.198333651	0.665114204	up_in_case
CRA004775	ENSGALG00010011977	GATD3A	case	control	0.215186117	0.330565762	0.650963111	0.5150703	0.864897625	up_in_case
CRA004775	ENSGALG00010011978	ENSGALG00010011978	case	control	-0.656794526	1.389127969	-0.47281067	0.636348261	0.910080596	down_in_case
CRA004775	ENSGALG00010011979	NEDD1	case	control	-0.083784681	0.164382861	-0.509692316	0.610267037	0.901413878	down_in_case
CRA004775	ENSGALG00010011980	AvBD9	case	control	2.439719435	0.730698721	3.338885595	0.000841152	0.06088574	up_in_case
CRA004775	ENSGALG00010011982	STK3	case	control	0.181523613	0.250599715	0.724356822	0.468846701	0.8440023	up_in_case
CRA004775	ENSGALG00010011983	LYZ	case	control	0.722574424	0.34918809	2.069298591	0.038518076	0.370251617	up_in_case
CRA004775	ENSGALG00010011984	FN1	case	control	-0.34684474	0.44067813	-0.787070464	0.431240618	0.825026746	down_in_case
CRA004775	ENSGALG00010011985	ENSGALG00010011985	case	control	-0.484178813	0.9744344	-0.496881896	0.619272339	0.904574831	down_in_case
CRA004775	ENSGALG00010011987	H2BC32	case	control	0.346956371	1.030904749	0.336555216	0.736452221	0.937549044	up_in_case
CRA004775	ENSGALG00010011988	HINTW	case	control	-0.360429322	1.136607293	-0.317109809	0.751160282	0.941495694	down_in_case
CRA004775	ENSGALG00010011989	H1-010	case	control	-0.160077381	0.402976572	-0.397237437	0.691192378	0.927867527	down_in_case
CRA004775	ENSGALG00010011991	CSNK1A1	case	control	0.02550597	0.180613647	0.1412184	0.887697411	0.977118922	up_in_case
CRA004775	ENSGALG00010011992	YEATS4	case	control	0.048832822	0.204631079	0.238638346	0.811386028	0.957303241	up_in_case
CRA004775	ENSGALG00010011993	RELCH	case	control	-0.068532346	0.322970318	-0.212193946	0.831955729	0.963642489	down_in_case
CRA004775	ENSGALG00010011994	TSPAN8	case	control	2.200953718	0.706292546	3.116206919	0.001831937	0.086270421	up_in_case
CRA004775	ENSGALG00010011996	LRRC70	case	control	0.10653406	0.516782343	0.2061488	0.83667468	0.965269623	up_in_case
CRA004775	ENSGALG00010011997	DENND5B	case	control	-0.086194819	0.303157191	-0.284323847	0.776162212	0.946608123	down_in_case
CRA004775	ENSGALG00010011998	MPPED1	case	control	0.434230139	0.306023825	1.418942262	0.15591585	0.619442611	up_in_case
CRA004775	ENSGALG00010012001	EEIG2	case	control	-0.569101561	0.355512666	-1.600791241	0.109423164	0.556651068	down_in_case
CRA004775	ENSGALG00010012004	LGR5	case	control	0.456029987	0.736182454	0.619452399	0.535618371	0.871322397	up_in_case
CRA004775	ENSGALG00010012005	ENSGALG00010012005	case	control	-3.584075894	1.574966868	-2.275651613	0.02286687	0.294061167	down_in_case
CRA004775	ENSGALG00010012007	MREG	case	control	-0.139018332	0.446395112	-0.311424406	0.755478003	0.941900693	down_in_case
CRA004775	ENSGALG00010012008	ZNF366	case	control	0.192248391	0.549983331	0.349553124	0.726674096	0.935238044	up_in_case
CRA004775	ENSGALG00010012010	HENMT1	case	control	-0.178044684	0.476708846	-0.373487267	0.70878582	0.931801673	down_in_case
CRA004775	ENSGALG00010012011	HOOK3	case	control	0.830768126	0.212280459	3.913540277	9.09527E-05	0.02119815	up_in_case
CRA004775	ENSGALG00010012013	FOXD1	case	control	0.079984843	0.597637079	0.133835141	0.893532938	0.977834811	up_in_case
CRA004775	ENSGALG00010012015	ENSGALG00010012015	case	control	-0.237200969	0.399035904	-0.594435156	0.552221101	0.878258849	down_in_case
CRA004775	ENSGALG00010012016	WDR91	case	control	-0.387075741	0.120518384	-3.21175682	0.00131926	0.076223292	down_in_case
CRA004775	ENSGALG00010012017	N4BP3	case	control	-0.10723766	0.215736461	-0.497077125	0.619134665	0.904574831	down_in_case
CRA004775	ENSGALG00010012019	ENSGALG00010012019	case	control	-0.068098121	0.568823446	-0.119717501	0.904706937	0.979540372	down_in_case
CRA004775	ENSGALG00010012021	ENSGALG00010012021	case	control	0.208505989	0.210969842	0.988321301	0.322995317	0.768161294	up_in_case
CRA004775	ENSGALG00010012024	UBR5	case	control	0.191698018	0.198502134	0.965722707	0.334182954	0.774540671	up_in_case
CRA004775	ENSGALG00010012028	TMEM140	case	control	-0.27245019	0.300908999	-0.905423869	0.365240875	0.793608387	down_in_case
CRA004775	ENSGALG00010012031	GRK6	case	control	-0.528531663	0.351501918	-1.503638063	0.132674585	0.589469537	down_in_case
CRA004775	ENSGALG00010012034	ARHGEF9	case	control	0.279685545	0.349680234	0.799832297	0.423807968	0.82073892	up_in_case
CRA004775	ENSGALG00010012040	B4GALT7	case	control	-0.468557596	0.20756705	-2.257379463	0.023984374	0.299554156	down_in_case
CRA004775	ENSGALG00010012041	ICOSLG	case	control	-0.348599969	0.35941103	-0.969920062	0.332086339	0.772935571	down_in_case
CRA004775	ENSGALG00010012043	NUP50	case	control	0.113363366	0.293807721	0.385842026	0.699613668	0.929506044	up_in_case
CRA004775	ENSGALG00010012045	ENSGALG00010012045	case	control	-0.47907316	0.502683213	-0.953031945	0.340573885	0.780875343	down_in_case
CRA004775	ENSGALG00010012047	LMAN2	case	control	-0.318819457	0.110597084	-2.882711244	0.003942687	0.129085442	down_in_case
CRA004775	ENSGALG00010012049	IFNAR1	case	control	-0.177816701	0.393422344	-0.451974079	0.651287656	0.915825397	down_in_case
CRA004775	ENSGALG00010012051	ARK2C	case	control	0.215179703	0.65697856	0.327529262	0.743267614	0.939218065	up_in_case
CRA004775	ENSGALG00010012052	KLF10	case	control	0.452051613	0.330169762	1.369149041	0.170952691	0.635171699	up_in_case
CRA004775	ENSGALG00010012053	ENSGALG00010012053	case	control	-2.022653023	1.00763828	-2.007320547	0.044715543	0.393998162	down_in_case
CRA004775	ENSGALG00010012054	FAM60A	case	control	0.083917947	0.368512918	0.227720502	0.819863533	0.960465449	up_in_case
CRA004775	ENSGALG00010012055	ENSGALG00010012055	case	control	0.653412947	0.420203338	1.554992281	0.119947908	0.572133279	up_in_case
CRA004775	ENSGALG00010012059	ARHGDIB	case	control	-0.480467126	0.414115857	-1.160223927	0.245957648	0.712046495	down_in_case
CRA004775	ENSGALG00010012060	ENSGALG00010012060	case	control	-0.054328695	0.289140511	-0.187897208	0.850957231	0.968989143	down_in_case

CRA004775	ENSGALG00010012061	AZIN1	case	control	0.290901806	0.299698593	0.970647887	0.331723651	0.772879011	up_in_case
CRA004775	ENSGALG00010012062	HMGA2	case	control	0.960165531	0.440946567	2.177509934	0.029442541	0.326503538	up_in_case
CRA004775	ENSGALG00010012063	PTPRD	case	control	0.386076915	0.612292768	0.630542994	0.528339382	0.868929328	up_in_case
CRA004775	ENSGALG00010012064	RGS14	case	control	-0.726111838	0.377733998	-1.922283518	0.054570093	0.43002484	down_in_case
CRA004775	ENSGALG00010012066	STXBP3	case	control	0.016342287	0.439429229	0.037189805	0.970333667	0.994414835	up_in_case
CRA004775	ENSGALG00010012067	ENSGALG00010012067	case	control	-0.18398645	0.192631514	-0.955121239	0.339516389	0.779891494	down_in_case
CRA004775	ENSGALG00010012070	DNAJC28	case	control	0.634888865	0.355911577	1.783838755	0.07444987	0.481316485	up_in_case
CRA004775	ENSGALG00010012071	ENSGALG00010012071	case	control	-0.082529102	0.26114118	-0.316032507	0.751977835	0.941495694	down_in_case
CRA004775	ENSGALG00010012072	CAPRIN2	case	control	-0.050546155	0.291938804	-0.173139556	0.862541734	0.971799376	down_in_case
CRA004775	ENSGALG00010012074	KIAA0930	case	control	-0.378080792	0.289863271	-1.304341838	0.192117056	0.656959697	down_in_case
CRA004775	ENSGALG00010012075	PIGN	case	control	-0.138517185	0.245254509	-0.564789555	0.572216912	0.885642749	down_in_case
CRA004775	ENSGALG00010012077	IFNGR2	case	control	0.036428837	0.189658534	0.192075921	0.847682736	0.968053841	up_in_case
CRA004775	ENSGALG00010012078	TMBIM4	case	control	-0.545704986	0.310564844	-1.757137023	0.078894457	0.491399489	down_in_case
CRA004775	ENSGALG00010012079	ENSGALG00010012079	case	control	-1.196671715	0.498970232	-2.398282781	0.016472143	0.254704773	down_in_case
CRA004775	ENSGALG00010012081	CHIC1	case	control	0.000616169	0.180577618	0.003412211	0.997277455	0.999619266	up_in_case
CRA004775	ENSGALG00010012082	HAUS1	case	control	-0.207254746	0.452561027	-0.457959774	0.646981341	0.914163628	down_in_case
CRA004775	ENSGALG00010012083	MGP	case	control	0.375237876	0.399049352	0.940329497	0.347048574	0.784653886	up_in_case
CRA004775	ENSGALG00010012084	TMEM50B	case	control	-0.129369758	0.168754599	-0.766614713	0.443310625	0.831667526	down_in_case
CRA004775	ENSGALG00010012085	IPO8	case	control	0.123282697	0.226600321	0.544053497	0.58640466	0.891503646	up_in_case
CRA004775	ENSGALG00010012086	CIBAR1	case	control	0.259853201	0.295899489	0.878180635	0.379845699	0.800802457	up_in_case
CRA004775	ENSGALG00010012087	TMEM171	case	control	-0.425826291	0.458145129	-0.929457206	0.352652193	0.786426886	down_in_case
CRA004775	ENSGALG00010012088	ATP5F1AZ	case	control	-0.146940903	0.359238518	-0.409034375	0.682514435	0.924564673	down_in_case
CRA004775	ENSGALG00010012090	HELB	case	control	-0.331621527	0.393025375	-0.843766201	0.398800066	0.810149167	down_in_case
CRA004775	ENSGALG00010012091	ENSGALG00010012091	case	control	-0.032398464	0.433233228	-0.074782963	0.940387398	0.987076872	down_in_case
CRA004775	ENSGALG00010012092	OC3	case	control	1.165209788	0.590118985	1.974533641	0.048321096	0.407414924	up_in_case
CRA004775	ENSGALG00010012093	ENSGALG00010012093	case	control	0.03993352	0.169307575	0.235863754	0.813538395	0.958280492	up_in_case
CRA004775	ENSGALG00010012094	ENSGALG00010012094	case	control	0.752115402	0.599706991	1.254138128	0.209791804	0.67982426	up_in_case
CRA004775	ENSGALG00010012095	RNF38	case	control	-0.37642602	0.254910957	-1.476696113	0.139757115	0.599625611	down_in_case
CRA004775	ENSGALG00010012096	ENSGALG00010012096	case	control	-0.109498542	0.476085707	-0.229997542	0.81809368	0.959889304	down_in_case
CRA004775	ENSGALG00010012097	GART	case	control	0.321648176	0.499302704	0.644194742	0.51944916	0.865611895	up_in_case
CRA004775	ENSGALG00010012100	UBE2F	case	control	0.317330472	0.315317583	1.006383686	0.314231038	0.765098415	up_in_case
CRA004775	ENSGALG00010012101	ENSGALG00010012101	case	control	0.149394145	0.228636991	0.653411964	0.513490728	0.863903061	up_in_case
CRA004775	ENSGALG00010012102	SON	case	control	0.445259975	0.240429439	1.851936168	0.064034989	0.455768706	up_in_case
CRA004775	ENSGALG00010012103	ENSGALG00010012103	case	control	-0.31934247	0.319783543	-0.998620713	0.317978463	0.76657115	down_in_case
CRA004775	ENSGALG00010012105	DONSON	case	control	-0.642852079	0.451935487	-1.422442133	0.154897939	0.619284425	down_in_case
CRA004775	ENSGALG00010012106	TMTC1	case	control	0.048134668	0.336989875	0.142837133	0.88641881	0.976862703	up_in_case
CRA004775	ENSGALG00010012107	CLCC1	case	control	0.143508798	0.249271583	0.575712625	0.56480944	0.882585553	up_in_case
CRA004775	ENSGALG00010012108	PSTPIP2	case	control	0.261909075	0.334979371	0.78186628	0.434293164	0.826182359	up_in_case
CRA004775	ENSGALG00010012110	ENSGALG00010012110	case	control	0.005416	0.273261162	0.019819866	0.98418707	0.997534931	up_in_case
CRA004775	ENSGALG00010012111	DRD1	case	control	-0.083374452	0.577791845	-0.144298423	0.885264823	0.976602443	down_in_case
CRA004775	ENSGALG00010012112	MBD2	case	control	-0.460858741	0.387388281	-1.189655867	0.234181678	0.701422839	down_in_case
CRA004775	ENSGALG00010012113	WDR47	case	control	0.322946969	0.230224207	1.402749841	0.160691449	0.626323163	up_in_case
CRA004775	ENSGALG00010012114	TAL2	case	control	-1.440809795	1.049301138	-1.373113726	0.169716995	0.633147466	down_in_case
CRA004775	ENSGALG00010012116	RBM12B	case	control	-0.158559425	0.235794539	-0.672447402	0.501298914	0.860057564	down_in_case
CRA004775	ENSGALG00010012118	ENSGALG00010012118	case	control	0.638170041	0.622925838	1.02447194			up_in_case
CRA004775	ENSGALG00010012119	EPG5	case	control	-0.321680214	0.285831577	-1.125418744	0.260411632	0.721313541	down_in_case
CRA004775	ENSGALG00010012120	ENSGALG00010012120	case	control	-0.246153854	0.615204521	-0.400117108	0.689070264	0.927420456	down_in_case
CRA004775	ENSGALG00010012121	SREK1	case	control	0.158557049	0.242660302	0.653411572	0.513490981	0.863903061	up_in_case
CRA004775	ENSGALG00010012122	GRIP1	case	control	0.626013689	0.563269985	1.111391883	0.266399705	0.727311193	up_in_case
CRA004775	ENSGALG00010012123	ENSGALG00010012123	case	control	0.324625335	0.38852297	0.835537046	0.403415397	0.812443633	up_in_case
CRA004775	ENSGALG00010012124	ERAP1	case	control	-0.211002092	0.289741622	-0.728242254	0.466465305	0.8434807	down_in_case
CRA004775	ENSGALG00010012125	RNF152	case	control	0.484580817	0.331269777	1.462798153	0.143522619	0.604835897	up_in_case
CRA004775	ENSGALG00010012127	ENSGALG00010012127	case	control	-0.205421648	0.152338392	-1.348456194	0.177511701	0.643283771	down_in_case
CRA004775	ENSGALG00010012128	ENSGALG00010012128	case	control	1.479956985	0.501855261	2.948971745	0.003188331	0.120778906	up_in_case
CRA004775	ENSGALG00010012129	CRYZL1	case	control	-0.325553119	0.270401821	-1.203960528	0.228604835	0.695782399	down_in_case
CRA004775	ENSGALG00010012130	TMEM67	case	control	0.25074897	0.402505933	0.622969624	0.533304483	0.870441537	up_in_case
CRA004775	ENSGALG00010012131	SLC14A1	case	control	-0.504736401	0.668853794	-0.754628897	0.45047168	0.835404673	down_in_case
CRA004775	ENSGALG00010012133	RAB3IP	case	control	0.198606948	0.589577981	0.3368629	0.736220254	0.937385622	up_in_case
CRA004775	ENSGALG00010012135	CAMSAP2	case	control	0.280489386	0.333714459	0.840507142	0.400624099	0.811868062	up_in_case
CRA004775	ENSGALG00010012136	ENSGALG00010012136	case	control	0.49262345	0.441312596	1.116268729	0.264307123	0.724455452	up_in_case
CRA004775	ENSGALG00010012137	ENSGALG00010012137	case	control	1.6300296	0.565411753	2.882907174	0.003940235	0.129085442	up_in_case
CRA004775	ENSGALG00010012138	DDX11	case	control	0.303048603	0.428375744	0.707436421	0.47929531	0.847254121	up_in_case
CRA004775	ENSGALG00010012141	ENSGALG00010012141	case	control	0.090745647	0.199363347	0.455177185	0.648981773	0.915047477	up_in_case
CRA004775	ENSGALG00010012144	H1-01	case	control	-0.189811973	0.355948148	-0.533256769	0.593855849	0.894720256	down_in_case
CRA004775	ENSGALG00010012145	ENSGALG00010012145	case	control	0.055294477	0.50671748	0.109122892	0.913105018	0.980803023	up_in_case
CRA004775	ENSGALG00010012147	ARFGAP3	case	control	-0.083258234	0.277114361	-0.300447201	0.763836065	0.94239774	down_in_case
CRA004775	ENSGALG00010012148	SETBP1	case	control	0.582794919	0.425853777	1.368532935	0.17114532	0.635543964	up_in_case
CRA004775	ENSGALG00010012149	SFXN1	case	control	-0.287971987	0.347607024	-0.828441218	0.407420672	0.814105656	down_in_case
CRA004775	ENSGALG00010012151	MYRFL	case	control	2.606427125	0.822728528	3.168028137	0.001534767	0.080693373	up_in_case
CRA004775	ENSGALG00010012154	CWC27	case	control	0.018116125	0.342922992	0.052828553	0.957868511	0.991431439	up_in_case
CRA004775	ENSGALG00010012157	H2BC32	case	control	-0.577509648	0.417599295	-1.382927735	0.166687025	0.630220864	down_in_case
CRA004775	ENSGALG00010012159	WASHC1	case	control	-0.156256807	0.251784583	-0.620597197	0.534864684	0.870981055	down_in_case
CRA004775	ENSGALG00010012163	PDP1	case	control	0.739982068	0.537100817	1.377734019	0.168285424	0.631389806	up_in_case
CRA004775	ENSGALG00010012169	H3C33	case	control	-0.328574407	0.906259111	-0.362561218	0.716932686	0.934011578	down_in_case
CRA004775	ENSGALG00010012171	CDH17	case	control	2.654439727	0.838708187	3.164914528	0.001551285	0.080693373	up_in_case
CRA004775	ENSGALG00010012172	CNOT2	case	control	-0.026824003	0.153082353	-0.175225967	0.860902081	0.971325143	down_in_case
CRA004775	ENSGALG00010012175	PACSIN2	case	control	0.382217	0.218286228	1.75098999	0.079947634	0.493409519	up_in_case
CRA004775	ENSGALG00010012179	ENSGALG00010012179	case	control	-0.448115953	0.26553024	-1.687626817	0.091482888	0.521441551	down_in_case
CRA004775	ENSGALG00010012180	DDX59	case	control	-0.04954631	0.266703133	-0.185773257	0.852622577	0.969725992	down_in_case
CRA004775	ENSGALG00010012186	KIF20A	case	control	0.579404491	0.567077392	1.021737948	0.306904945	0.7585475	up_in_case
CRA004775	ENSGALG00010012187	ENSGALG00010012187	case	control	0.769886658	1.159342271	0.664071929	0.506644275	0.8622397	up_in_case
CRA004775	ENSGALG00010012188	ADAMTS6	case	control	0.574935859	0.723474925	0.794686642	0.426795804	0.822776983	up_in_case
CRA004775	ENSGALG00010012190	CAST	case	control	0.020904115	0.197926033	0.105615794	0.915887193	0.981626545	up_in_case
CRA004775	ENSGALG00010012191	BEST3	case	control	0.246864401	0.615791777	0.400889408	0.688801549	0.927339918	up_in_case
CRA004775	ENSGALG00010012193	TTLL1	case	control	-0.171398132	0.483247742	-0.354679633	0.722829611	0.935185555	down_in_case
CRA004775	ENSGALG00010012194	LRRFIP1	case	control	-0.060790467	0.33854245	-0.179565272	0.85749387	0.970943344	down_in_case
CRA004775	ENSGALG00010012195	ITSN1	case	control	0.168120467	0.275921961	0.609304408	0.542322686	0.875135326	up_in_case
CRA004775	ENSGALG00010012196	CENPK	case	control	-0.337289189	0.576698957	-0.584861798	0.558640647	0.880754991	down_in_case
CRA004775	ENSGALG00010012198	TMEM5	case	control	0.139627915	0.258796016	0.539528842	0.589522001	0.893412703	up_in_case
CRA004775	ENSGALG00010012199	ENSGALG00010012199	case	control	0.301967785	0.340535902	0.886742875	0.37521735	0.798503808	up_in_case
CRA004775	ENSGALG00010012200	GEM	case	control	0.34743525	0.368840589	0.94196588	0.346210109	0.784552667	up_in_case
CRA004775	ENSGALG00010012207	GRIA3	case	control	1.564042016	0.668686019	2.338978191	0.019336562	0.274403578	up_in_case
CRA004775	ENSGALG00010012209	KIF14	case	control	0.824339493	0.71206271	1.157678224	0.246995374	0.712046495	up_in_case
CRA004775	ENSGALG00010012210	PPWD1	case	control	-0.195744834	0.251240371	-0.779113774	0.435912697	0.826905159	down_in_case
CRA004775	ENSGALG00010012211	ENSGALG00010012211	case	control	0.55959161	1.175933929	0.475869941	0.634167038	0.909837348	up_in_case
CRA004775	ENSGALG00010012212	RAD54B	case	control	-0.018361279	0.649900773	-0.028252434	0.977460817	0.995827348	down_in_case
CRA004775	ENSGALG00010012215	RAB17	case	control	0.57239862	0.496055535	1.153900279	0.248541055	0.713355885	up_in_case
CRA004775	ENSGALG00010012216	BRD8	case	control	-0.305694279	0.1286462	-2.376240249	0.017490071	0.262848048	down_in_case
CRA004775	ENSGALG00010012219	KDM5A	case	control	-0.063324326	0.305594292	-0.207216981	0.835840407	0.965107526	down_in_case
CRA004775	ENSGALG00010012226	SLCO4C1	case	control	-1.390397715	0.577907149	-2.40591887	0.016131847	0.25344686	down_in_case
CRA004775	ENSGALG00010012228	NHP2	case	control	-0.247923649	0.233324889	-1.062568377	0.287977734	0.742682262	down_in_case
CRA004775	ENSGALG00010012229	FSBP	case	control	0.018411305	0.446381918	0.041245634	0.967100074	0.993321864	up_in_case
CRA004775	ENSGALG00010012232	PCSK1	case	control	0.490648773	0.718558429	0.682823766	0.49471821	0.856382445	up_in_case
CRA004775	ENSGALG00010012234	C1GALT1C1	case	control	0.011993502	0.138548249	0.086565528	0.931016868	0.985044263	up_in_case
CRA004775	ENSGALG00010012236	PRLH	case	control	0.710472365	0.466198701	1.523968995	0.127516457	0.581777968	up_in_case
CRA004775	ENSGALG00010012240	TRIM14	case	control	0.210671113	0.509115758	0.413798059	0.679022006	0.92338129	up_in_case
CRA004775	ENSGALG00010012242	CCDC77	case	control	-0.343853576	0.615355224	-0.558788749	0.576305902	0.887937128	down_in_case
CRA004775	ENSGALG00010012243	ENSGALG00010012243	case	control	-1.162225054	1.13241992	-1.02631986	0.304740839	0.757500231	down_in_case
CRA004775	ENSGALG00010012244	ENSGALG00010012244	case	control	-0.020197683	0.388437092	-0.051997307	0.958530839	0.991431439	down_in_case
CRA004775	ENSGALG00010012245	ENSGALG00010012245	case	control	0.180224423	0.238071613	0.757017691	0.449039264	0.834536953	up_in_case
CRA004775	ENSGALG00010012247	TRIM23	case	control	0.323665574	0.295849821	1.094019843	0.273946276	0.731001618	up_in_case
CRA004775	ENSGALG00010012248	H2AC39	case	control	-1.072349954	0.384041082	-2.792279278	0.005233816	0.147178335	down_in_case
CRA004775	ENSGALG00010012250	NANS	case	control	-0.291368018	0.272229501	-1.070302875	0.284483001	0.741035375	down_in_case
CRA004775	ENSGALG00010012253	MYF6	case	control	-0.041092437	0.712600459	-0.057665466	0.954015102	0.990670511	down_in_case
CRA004775	ENSGALG00010012259	IL1RAPL2	case	control	0.891169465	0.458376785	1.944185425	0.051873095	0.419683701	up_in_case
CRA004775	ENSGALG00010012260	ENSGALG00010012260	case	control	-0.176287443	0.803399238	-0.219426949	0.82631748	0.961878235	down_in_case
CRA004775	ENSGALG00010012263	SHLD3	case	control	-0.0105373	0.312610917	-0.033707397	0.97311048	0.994947809	down_in_case
CRA004775	ENSGALG00010012264	MYF5	case	control	0.736901996	0.585804781	1.257931004	0.20841671	0.677206564	up_in_case
CRA004775	ENSGALG00010012266	H3C33	case	control	-0.632512874	0.836475483	-0.756164271	0.449550712	0.835036044	down_in_case
CRA004775	ENSGALG00010012268	ENSGALG00010012268	case	control	0.103490394	0.3886967	0.266249738	0.79004688	0.950891435	up_in_case
CRA004775	ENSGALG00010012271	ENSGALG00010012271	case	control	0.36773301	0.503835419	0.729867326	0.465471286	0.843383332	up_in_case
CRA004775	ENSGALG00010012274	B4GALNT3	case	control	-0.374181483	0.378503202	-0.988582079	0.322867658	0.768114682	down_in_case
CRA004775	ENSGALG00010012275	VIRMA	case	control	-0.108011782	0.272124858	-0.396919937	0.691426502	0.927867527	down_in_case
CRA004775	ENSGALG00010012277	H3C33	case	control	-0.319906654	0.271057047	-1.180218916	0.237913158	0.70473329	down_in_case
CRA004775	ENSGALG00010012282	ENSGALG00010012282	case	control	0.421356791	1.005688536	0.418973446	0.675235539	0.92288678	up_in_case
CRA004775	ENSGALG00010012283	CUPIN1A	case	control	0.774206547	0.603309397	1.283266183	0.199398836	0.666548904	up_in_case

CRA004775	ENSGALG00010012285	FBLN1	case	control	0.226433728	0.362946545	0.623876245	0.532708862	0.869976453	up_in_case
CRA004775	ENSGALG00010012286	ENSGALG00010012286	case	control	0.258084419	1.146965468	0.225014986	0.821967616	0.960766569	up_in_case
CRA004775	ENSGALG00010012288	MCTS1	case	control	0.267834236	0.296077874	0.9046074	0.365673414	0.793608387	up_in_case
CRA004775	ENSGALG00010012290	ENSGALG00010012290	case	control	-0.337565712	0.227233958	-1.485542547	0.137400162	0.596242097	down_in_case
CRA004775	ENSGALG00010012291	NR5A2	case	control	-0.077311482	0.625904547	-0.123519604	0.901695651	0.979157637	down_in_case
CRA004775	ENSGALG00010012293	SRGAP1	case	control	0.024465486	0.197769443	0.123707111	0.901547182	0.979157637	up_in_case
CRA004775	ENSGALG00010012295	CUL4B	case	control	0.202476924	0.277578828	0.729439365	0.465732945	0.843390341	up_in_case
CRA004775	ENSGALG00010012297	ABLIM3	case	control	-0.141748498	0.40586289	-0.349252176	0.726899998	0.935238044	down_in_case
CRA004775	ENSGALG00010012300	NINJ2	case	control	0.340318879	0.56803136	0.599119877	0.549092947	0.877623839	up_in_case
CRA004775	ENSGALG00010012303	CA9	case	control	0.597306617	0.548156277	1.089664831	0.275860814	0.731001618	up_in_case
CRA004775	ENSGALG00010012307	ENSGALG00010012307	case	control	-0.144030447	0.314296161	-0.458263462	0.646763171	0.914043203	down_in_case
CRA004775	ENSGALG00010012308	TRABD2A	case	control	-0.10059951	0.637616151	-0.157774406	0.874634567	0.974301166	down_in_case
CRA004775	ENSGALG00010012309	TRAPPC13	case	control	-0.122630962	0.224251354	-0.546846028	0.584484518	0.89025771	down_in_case
CRA004775	ENSGALG00010012311	ENSGALG00010012311	case	control	3.141007322	0.614570319	5.110899804	3.20628E-07	0.000238658	up_in_case
CRA004775	ENSGALG00010012312	SH3TC2	case	control	-0.372408522	0.483505517	-0.770226004	0.441165841	0.829353567	down_in_case
CRA004775	ENSGALG00010012314	ADRB2	case	control	-0.036165195	0.559985737	-0.06458235	0.948506538	0.990124756	down_in_case
CRA004775	ENSGALG00010012315	ATP6V1E1	case	control	-0.121854866	0.262976242	-0.463368344	0.643100365	0.913561273	down_in_case
CRA004775	ENSGALG00010012317	ENSGALG00010012317	case	control	-0.478391953	0.570180427	-0.839018546	0.401458903	0.811868062	down_in_case
CRA004775	ENSGALG00010012318	POLR1D	case	control	-0.612886662	0.293504756	-2.088166032	0.03678286	0.36180337	down_in_case
CRA004775	ENSGALG00010012320	DPY19L4	case	control	0.083730017	0.232992151	0.359368403	0.719319509	0.93407508	up_in_case
CRA004775	ENSGALG00010012322	ENSGALG00010012322	case	control	-1.050925322	0.684612388	-1.535066178	0.124767573	0.577326485	down_in_case
CRA004775	ENSGALG00010012323	SMAD7B	case	control	0.34967739	1.170967239	0.298622693	0.765227949	0.943143275	up_in_case
CRA004775	ENSGALG00010012324	ENSGALG00010012324	case	control	-0.617885799	0.287284144	-2.150782815	0.031493346	0.337804215	down_in_case
CRA004775	ENSGALG00010012325	MLPH	case	control	5.415812647	0.806495587	6.715241518	1.87755E-11	3.22727E-08	up_in_case
CRA004775	ENSGALG00010012326	SGTB	case	control	0.056982144	0.152303227	0.374136157	0.70830302	0.931801673	up_in_case
CRA004775	ENSGALG00010012327	UBTD2	case	control	-0.070818206	0.187256384	-0.378188471	0.705290589	0.930927733	down_in_case
CRA004775	ENSGALG00010012329	ATXN10	case	control	-0.111807484	0.292546401	-0.38218718	0.70232253	0.930068722	down_in_case
CRA004775	ENSGALG00010012331	ENSGALG00010012331	case	control	-0.619774668	0.550292647	-1.126263763	0.260053891	0.720822628	down_in_case
CRA004775	ENSGALG00010012334	ARHGEF39	case	control	-0.257917886	0.557300796	-0.462798345	0.643508918	0.913638565	down_in_case
CRA004775	ENSGALG00010012336	ENSGALG00010012336	case	control	0.224214557	1.006783643	0.222703814	0.823766033	0.960898591	up_in_case
CRA004775	ENSGALG00010012337	ACSS3	case	control	-0.092391313	0.296674332	-0.311423346	0.755478808	0.941900693	down_in_case
CRA004775	ENSGALG00010012338	H3C33	case	control	0.096799384	0.835097175	0.115913916	0.907720767	0.980372908	up_in_case
CRA004775	ENSGALG00010012339	WNK1	case	control	0.255447883	0.373033643	0.684785106	0.493479535	0.856001758	up_in_case
CRA004775	ENSGALG00010012340	ENHO	case	control	-0.18930965	0.266068278	-0.71150778	0.476769631	0.846367006	down_in_case
CRA004775	ENSGALG00010012341	GOPC	case	control	0.267801825	0.220774002	1.213013411	0.225124693	0.692994606	up_in_case
CRA004775	ENSGALG00010012343	BCL2L13	case	control	-0.24491356	0.322588123	-0.759214437	0.447724291	0.834136489	down_in_case
CRA004775	ENSGALG00010012345	H2BC32	case	control	-0.514758498	0.536899876	-0.958760695	0.337679314	0.778184725	down_in_case
CRA004775	ENSGALG00010012346	NLN	case	control	-0.264295283	0.2974504	-0.888535644	0.374252691	0.79800725	down_in_case
CRA004775	ENSGALG00010012347	ENSGALG00010012347	case	control	-0.175163972	0.765295498	-0.228884101	0.818958998	0.960060118	down_in_case
CRA004775	ENSGALG00010012350	H2AC32	case	control	-0.713690538	0.352496542	-2.02467387	0.042900864	0.38862304	down_in_case
CRA004775	ENSGALG00010012352	PTPRC	case	control	-0.187878839	0.419098597	-0.448292695	0.653941972	0.916560183	down_in_case
CRA004775	ENSGALG00010012353	PIGG	case	control	-0.434809561	0.227082037	-1.914768632	0.055522017	0.431364203	down_in_case
CRA004775	ENSGALG00010012354	DCBLD1	case	control	0.28316715	0.474743643	0.596463279	0.550865778	0.877827378	up_in_case
CRA004775	ENSGALG00010012358	STK10	case	control	-0.459209182	0.454828956	-1.009630491	0.312672356	0.7645017	down_in_case
CRA004775	ENSGALG00010012360	ROS1	case	control	2.227521767	0.81788018	2.723530686	0.006458822	0.159739667	up_in_case
CRA004775	ENSGALG00010012361	WIF1	case	control	-0.064475336	0.827911194	-0.077877116	0.937925803	0.986707289	down_in_case
CRA004775	ENSGALG00010012362	COL6A3	case	control	0.214094034	0.530842133	0.403310176	0.686720057	0.92689803	up_in_case
CRA004775	ENSGALG00010012366	INTS8	case	control	-0.094317031	0.229674693	-0.410654869	0.681325624	0.924046618	down_in_case
CRA004775	ENSGALG00010012367	RAD52	case	control	0.515962148	0.270690564	1.906095806	0.056637777	0.43534157	up_in_case
CRA004775	ENSGALG00010012371	ATP6V1G3	case	control	0.220721012	0.575735757	0.383372076	0.701443905	0.930068722	up_in_case
CRA004775	ENSGALG00010012372	LEMD3	case	control	0.050026178	0.171515167	0.291672036	0.770537392	0.945754599	up_in_case
CRA004775	ENSGALG00010012376	VGLL2	case	control	0.760868987	0.667087409	1.140583643	0.254043227	0.718204856	up_in_case
CRA004775	ENSGALG00010012378	RPS19BP1	case	control	-0.267709401	0.218595355	-1.22468019	0.220695741	0.688628803	down_in_case
CRA004775	ENSGALG00010012382	SH3PXD2B	case	control	-0.959741169	0.429175488	-2.23624414	0.025335783	0.30560733	down_in_case
CRA004775	ENSGALG00010012384	NRK	case	control	-0.4637376	0.485085735	-0.955991006	0.339076778	0.7794458	down_in_case
CRA004775	ENSGALG00010012387	ERBIN	case	control	0.220386061	0.205374044	1.073095983	0.283228053	0.739728197	up_in_case
CRA004775	ENSGALG00010012390	MMP16	case	control	-0.103171155	0.390712463	-0.264059033	0.791734431	0.951284723	down_in_case
CRA004775	ENSGALG00010012391	NEURL1B	case	control	-0.216041616	0.379779782	-0.568860234	0.569450994	0.884809383	down_in_case
CRA004775	ENSGALG00010012393	ENSGALG00010012393	case	control	0.511454467	1.146584565	0.446067811	0.655548263	0.917033995	up_in_case
CRA004775	ENSGALG00010012399	ERC1	case	control	0.365736823	0.348845339	1.048421124	0.294444625	0.746438942	up_in_case
CRA004775	ENSGALG00010012401	RIPK2	case	control	0.298273166	0.285302179	1.045464031	0.295808556	0.748282461	up_in_case
CRA004775	ENSGALG00010012404	VAMP7	case	control	-0.450334414	0.186421545	-2.415677941	0.015705946	0.250548106	down_in_case
CRA004775	ENSGALG00010012405	MSRB3	case	control	0.010955684	0.37592888	0.029142971	0.976750564	0.99560196	up_in_case
CRA004775	ENSGALG00010012406	ENSGALG00010012406	case	control	0.165922277	0.552115337	0.300521044	0.763779747	0.94239774	up_in_case
CRA004775	ENSGALG00010012407	ATF4	case	control	0.226113334	0.239307871	0.944863756	0.344728437	0.783972357	up_in_case
CRA004775	ENSGALG00010012412	PURA	case	control	0.294330145	0.261005771	1.127676769	0.259456452	0.720037471	up_in_case
CRA004775	ENSGALG00010012413	NEK7	case	control	-0.055062294	0.221303616	-0.248808832	0.803508659	0.954149185	down_in_case
CRA004775	ENSGALG00010012414	ENSGALG00010012414	case	control	0.458212555	0.630446359	0.726806568	0.467344459	0.843870188	up_in_case
CRA004775	ENSGALG00010012415	BID	case	control	-0.151157622	0.199379054	-0.758141939	0.448366015	0.83429564	down_in_case
CRA004775	ENSGALG00010012418	ENSGALG00010012418	case	control	0.116342886	0.925118743	0.125759949	0.899921946	0.978929349	up_in_case
CRA004775	ENSGALG00010012419	HACE1	case	control	0.460776964	0.244990086	1.880798405	0.059999347	0.445397937	up_in_case
CRA004775	ENSGALG00010012422	ENSGALG00010012422	case	control	0.193174074	1.328118217	0.145449458	0.884356018	0.976236592	up_in_case
CRA004775	ENSGALG00010012426	MIEF1	case	control	-0.520266959	0.234073273	-2.222667081	0.026238257	0.30996759	down_in_case
CRA004775	ENSGALG00010012427	C7	case	control	-1.08988113	0.577225098	-1.888138844	0.059007313	0.44414236	down_in_case
CRA004775	ENSGALG00010012428	CCNE2	case	control	0.351131886	0.638670504	0.549785663	0.582466394	0.889647382	up_in_case
CRA004775	ENSGALG00010012429	CXCR7	case	control	0.574940296	0.239226715	2.403328141	0.016246602	0.25344686	up_in_case
CRA004775	ENSGALG00010012430	F13B	case	control	0.297437833	0.816532765	0.364269317	0.71565691	0.933921336	up_in_case
CRA004775	ENSGALG00010012431	LAMP2	case	control	0.173544538	0.187637765	0.924891306	0.355022449	0.788279103	up_in_case
CRA004775	ENSGALG00010012433	ENSGALG00010012433	case	control	-0.203855915	1.22096078	-0.166963524	0.86739875	0.973120718	down_in_case
CRA004775	ENSGALG00010012436	ENSGALG00010012436	case	control	-0.390089487	0.468176311	-0.833210646	0.404725939	0.81313407	down_in_case
CRA004775	ENSGALG00010012437	ENSGALG00010012437	case	control	0.094034324	0.90801806	0.103559971	0.917518556	0.981688766	up_in_case
CRA004775	ENSGALG00010012439	TENT2	case	control	-0.082975085	0.198639338	-0.417717284	0.676153834	0.922924485	down_in_case
CRA004775	ENSGALG00010012441	FST	case	control	-0.093868475	0.386961037	-0.242578621	0.808331845	0.955397873	down_in_case
CRA004775	ENSGALG00010012442	CPLX1	case	control	1.302807973	0.955588978	1.363356006	0.172770339	0.637223104	up_in_case
CRA004775	ENSGALG00010012445	SOBP	case	control	0.05378632	0.447346546	0.120234125	0.904297686	0.979540372	up_in_case
CRA004775	ENSGALG00010012447	COPS8	case	control	0.001804668	0.295655783	0.00610395	0.995129783	0.999001196	up_in_case
CRA004775	ENSGALG00010012449	SUB1	case	control	0.253987322	0.169280479	1.500393449	0.133512515	0.590901384	up_in_case
CRA004775	ENSGALG00010012456	ENSGALG00010012456	case	control	-0.084612272	0.245096152	-0.34522073	0.729928436	0.936393482	down_in_case
CRA004775	ENSGALG00010012458	MRPS30	case	control	-0.048402261	0.251405879	-0.192526369	0.847329915	0.967904441	down_in_case
CRA004775	ENSGALG00010012464	ENSGALG00010012464	case	control	-0.039364328	0.689835376	-0.057063366	0.954494719	0.990670511	down_in_case
CRA004775	ENSGALG00010012466	PDSS2	case	control	0.003646088	0.265658679	0.01372471	0.989049609	0.998065748	up_in_case
CRA004775	ENSGALG00010012469	CETN2	case	control	-0.191875601	0.216944408	-0.884445943	0.376455531	0.798523018	down_in_case
CRA004775	ENSGALG00010012470	CHD1	case	control	0.674147649	0.282383002	2.387352087	0.01697023	0.257853739	up_in_case
CRA004775	ENSGALG00010012477	ENSGALG00010012477	case	control	0.355807407	0.345574801	1.029610393	0.303192934	0.755679718	up_in_case
CRA004775	ENSGALG00010012483	ENSGALG00010012483	case	control	-0.404260947	0.548624437	-0.736862814	0.46120578	0.841789313	down_in_case
CRA004775	ENSGALG00010012485	C6	case	control	1.057251971	0.72711936	1.454028084	0.14593852	0.609044185	up_in_case
CRA004775	ENSGALG00010012486	RADX	case	control	0.441965464	0.291685598	1.515211815	0.129718772	0.584172159	up_in_case
CRA004775	ENSGALG00010012498	KCNT2	case	control	-0.340096757	0.600776501	-0.566095306	0.571328994	0.885321726	down_in_case
CRA004775	ENSGALG00010012506	TP53INP1	case	control	-0.568829841	0.414371996	-1.37275165	0.169829566	0.633147466	down_in_case
CRA004775	ENSGALG00010012508	MDM1	case	control	-0.071485916	0.22825863	-0.313179469	0.754144316	0.941900693	down_in_case
CRA004775	ENSGALG00010012509	MICAL3	case	control	0.473541411	0.377848628	1.25325693	0.210112218	0.67982426	up_in_case
CRA004775	ENSGALG00010012511	OXCT1	case	control	0.393056031	0.212724782	1.847720922	0.064642726	0.457298868	up_in_case
CRA004775	ENSGALG00010012517	MGAT3	case	control	-0.407887665	0.414995597	-0.98287227	0.325670307	0.770657786	down_in_case
CRA004775	ENSGALG00010012519	ENSGALG00010012519	case	control	-0.224743069	1.019254128	-0.22049758	0.825483657	0.961878235	down_in_case
CRA004775	ENSGALG00010012521	HOMER1	case	control	0.708578512	0.317086793	2.234651609	0.02544023	0.305924309	up_in_case
CRA004775	ENSGALG00010012529	BEND3	case	control	0.049540562	0.251842204	0.196712711	0.844052363	0.966488804	up_in_case
CRA004775	ENSGALG00010012532	FAM174A	case	control	-0.072045039	0.34874517	-0.206583619	0.836335054	0.965207162	down_in_case
CRA004775	ENSGALG00010012535	JMY	case	control	0.120089921	0.40893772	0.293663105	0.769015349	0.945269515	up_in_case
CRA004775	ENSGALG00010012536	FBXO4	case	control	-0.460372079	0.410690716	-1.12097026	0.262300522	0.723230258	down_in_case
CRA004775	ENSGALG00010012538	RAP1B	case	control	-0.126384089	0.345544139	-0.365753819	0.714548783	0.93334033	down_in_case
CRA004775	ENSGALG00010012550	TAB1	case	control	-0.614517202	0.20923583	-2.936959704	0.003314472	0.122900629	down_in_case
CRA004775	ENSGALG00010012551	PCGF3	case	control	0.216629999	0.298254382	0.726326291	0.467638766	0.843870188	up_in_case
CRA004775	ENSGALG00010012553	ENSGALG00010012553	case	control	0.291932112	0.373336119	0.781955178	0.434240916	0.826182359	up_in_case
CRA004775	ENSGALG00010012555	ENSGALG00010012555	case	control	0.014042486	0.526983947	0.026646894	0.97874137	0.996495823	up_in_case
CRA004775	ENSGALG00010012561	OSGIN2	case	control	-0.346782188	0.437099228	-0.79337177	0.427561253	0.822776983	down_in_case
CRA004775	ENSGALG00010012565	SYNGR1	case	control	-0.163920395	0.344883692	-0.475291813	0.634578994	0.909837348	down_in_case
CRA004775	ENSGALG00010012569	C6orf203	case	control	0.270860009	0.391327272	0.692157252	0.488838581	0.854132062	up_in_case
CRA004775	ENSGALG00010012571	AGAP1	case	control	-0.027701382	0.256945044	-0.107810532	0.914145988	0.981144356	down_in_case
CRA004775	ENSGALG00010012574	PHAX	case	control	0.215855972	0.185250594	1.165210685	0.243933721	0.71125478	up_in_case
CRA004775	ENSGALG00010012578	NDUFS4	case	control	-0.195073433	0.362194094	-0.538588111	0.590171093	0.893771223	down_in_case
CRA004775	ENSGALG00010012582	CD24	case	control	-0.388803105	0.569648935	-0.682531084	0.494903194	0.856382445	down_in_case
CRA004775	ENSGALG00010012583	NDUFAF6	case	control	-0.129986843	0.317698052	-0.409152155	0.682428004	0.924564673	down_in_case
CRA004775	ENSGALG00010012586	CDC73	case	control	0.031578018	0.190831415	0.165475996	0.868569342	0.973370973	up_in_case
CRA004775	ENSGALG00010012587	MBLAC2	case	control	-0.306538277	0.370410531	-0.827563613	0.407917682	0.814297749	down_in_case
CRA004775	ENSGALG00010012588	PEX26	case	control	-0.184842418	0.176189179	-1.049113344	0.294125955	0.746438942	down_in_case
CRA004775	ENSGALG00010012593	TUBA8B	case	control	-3.707138	0.831908247	-4.456186142	8.34306E-06	0.004097336	down_in_case

CRA004775	ENSGALG00010012599	DMGDH	case	control	-0.230495306	0.388968988	-0.592580162	0.553462158	0.878871229	down_in_case
CRA004775	ENSGALG00010012600	NUP107	case	control	-0.120178162	0.250617424	-0.479528358	0.631562799	0.909479532	down_in_case
CRA004775	ENSGALG00010012601	TUBA8A	case	control	-0.252057695	0.508979004	-0.495222186	0.620443291	0.905283281	down_in_case
CRA004775	ENSGALG00010012604	ENSGALG00010012604	case	control	0.523359999	1.297794438	0.403268795	0.686750495	0.92689803	up_in_case
CRA004775	ENSGALG00010012606	ATP5PO	case	control	0.008924869	0.322649087	0.027661226	0.977932349	0.996114646	up_in_case
CRA004775	ENSGALG00010012607	LMNB1	case	control	0.22186816	0.458009276	0.484418486	0.628088901	0.90828391	up_in_case
CRA004775	ENSGALG00010012610	RPL3	case	control	-0.297814379	0.223237635	-1.334068868	0.182181287	0.647378665	down_in_case
CRA004775	ENSGALG00010012614	LHFPL2	case	control	0.245010618	0.370899718	0.660584535	0.508878789	0.862971569	up_in_case
CRA004775	ENSGALG00010012618	B3GALT2	case	control	-0.339389664	0.855035217	-0.396930626	0.691418619	0.927867527	down_in_case
CRA004775	ENSGALG00010012624	PDGFB	case	control	0.227991288	0.296303701	0.769451366	0.441625405	0.82961625	up_in_case
CRA004775	ENSGALG00010012629	NUS1	case	control	-0.206919296	0.206480379	-1.002125707	0.316282883	0.765818284	down_in_case
CRA004775	ENSGALG00010012631	GLRX2	case	control	-0.237141021	0.335115903	-0.707638817	0.479169581	0.847184661	down_in_case
CRA004775	ENSGALG00010012633	USP18	case	control	-0.183382163	0.443101305	-0.413860579	0.678976215	0.92338129	down_in_case
CRA004775	ENSGALG00010012640	PRRC1	case	control	-0.164654923	0.160358363	-1.026793489	0.304517716	0.757082464	down_in_case
CRA004775	ENSGALG00010012648	MRPS6	case	control	0.153952204	0.367007957	0.4194792	0.674865953	0.92288678	up_in_case
CRA004775	ENSGALG00010012650	DSE	case	control	-0.45738646	0.428946948	-1.066300768	0.286287703	0.741582427	down_in_case
CRA004775	ENSGALG00010012653	CBX7	case	control	-0.202033331	0.395250374	-0.511152789	0.609244074	0.90116325	down_in_case
CRA004775	ENSGALG00010012654	TENT5B	case	control	0.180307954	0.681919495	0.264412376	0.791462176	0.951284723	up_in_case
CRA004775	ENSGALG00010012662	SLC35E3	case	control	-0.159298877	0.226517288	-0.703252624	0.48189833	0.848909035	down_in_case
CRA004775	ENSGALG00010012667	SCAMP1	case	control	-0.046901672	0.238134324	-0.19695469	0.843862996	0.966478468	down_in_case
CRA004775	ENSGALG00010012669	ERCC8	case	control	-0.295662439	0.325187213	-0.909206843	0.363240953	0.793608387	down_in_case
CRA004775	ENSGALG00010012670	ENSGALG00010012670	case	control	0.464744167	0.739511296	0.628447691	0.529710703	0.869211096	up_in_case
CRA004775	ENSGALG00010012671	NBN	case	control	0.074086342	0.252874568	0.292976643	0.769540005	0.945407363	up_in_case
CRA004775	ENSGALG00010012672	RO60	case	control	0.264932326	0.284822292	0.930167102	0.352284572	0.786426886	up_in_case
CRA004775	ENSGALG00010012673	ENSGALG00010012673	case	control	0.1744378	0.431225195	0.404516717	0.685832787	0.926686318	up_in_case
CRA004775	ENSGALG00010012674	ENSGALG00010012674	case	control	-0.432349181	0.348599511	-1.24024609	0.214884386	0.683649824	down_in_case
CRA004775	ENSGALG00010012676	ENSGALG00010012676	case	control	0.20515929	0.336578473	0.609543706	0.542164112	0.875128455	up_in_case
CRA004775	ENSGALG00010012682	ENSGALG00010012682	case	control	0.306096156	0.332031334	0.921889366	0.356586287	0.789346109	up_in_case
CRA004775	ENSGALG00010012683	MDM2	case	control	0.188509703	0.227265682	0.829468407	0.406839407	0.813842524	up_in_case
CRA004775	ENSGALG00010012685	NPTXR	case	control	0.510693642	0.402980154	1.267292289	0.205050797	0.674045742	up_in_case
CRA004775	ENSGALG00010012688	NDUFAF2	case	control	-0.003380517	0.227970959	-0.014828718	0.988168829	0.998065748	down_in_case
CRA004775	ENSGALG00010012690	NT5DC1	case	control	0.201314656	0.586293742	0.34336825	0.731321442	0.936524599	up_in_case
CRA004775	ENSGALG00010012693	UCHL5	case	control	0.023796028	0.204171612	0.116549151	0.907217334	0.980354087	up_in_case
CRA004775	ENSGALG00010012696	DNAL4	case	control	-0.245259923	0.218509053	-1.122424538	0.261681976	0.722422978	down_in_case
CRA004775	ENSGALG00010012699	RNF128	case	control	-0.07500543	0.421790411	-0.177826304	0.858859389	0.971325143	down_in_case
CRA004775	ENSGALG00010012702	PTPRO	case	control	0.018061844	0.722171809	0.025010453	0.980046626	0.996865238	up_in_case
CRA004775	ENSGALG00010012704	SMIM15	case	control	-0.458333856	0.294412613	-1.556773849	0.119524188	0.571877909	down_in_case
CRA004775	ENSGALG00010012705	CPM	case	control	0.317209825	0.233315765	1.359573045	0.173965073	0.639185614	up_in_case
CRA004775	ENSGALG00010012706	TBC1D8B	case	control	0.176247268	0.198404331	0.888323695	0.374366658	0.798126498	up_in_case
CRA004775	ENSGALG00010012708	SPP2	case	control	1.827284219	0.691567458	2.642235687	0.008236072	0.183215969	up_in_case
CRA004775	ENSGALG00010012709	PPP1R8	case	control	-0.404229445	0.220946692	-1.829533816	0.067319678	0.463087992	down_in_case
CRA004775	ENSGALG00010012711	ELL2	case	control	0.146794486	0.366602293	0.400418899	0.688848007	0.927420456	up_in_case
CRA004775	ENSGALG00010012712	EEF1E1	case	control	0.117324811	0.334140424	0.351124265	0.725495125	0.935238044	up_in_case
CRA004775	ENSGALG00010012714	RGS2	case	control	-0.702345872	0.364076441	-1.929116503	0.053716401	0.426902863	down_in_case
CRA004775	ENSGALG00010012719	COL10A1	case	control	-6.719018714	2.392927205	-2.807865905	0.004987099	0.142684328	down_in_case
CRA004775	ENSGALG00010012720	ACO2	case	control	0.135510647	0.279109207	0.485511205	0.627313766	0.907733515	up_in_case
CRA004775	ENSGALG00010012722	ENSGALG00010012722	case	control	0.518282244	0.364644658	1.421335079	0.15521937	0.619284425	up_in_case
CRA004775	ENSGALG00010012723	ENSGALG00010012723	case	control	-0.87757469	0.776813528	-1.129710875	0.258598073	0.720037471	down_in_case
CRA004775	ENSGALG00010012725	ENSGALG00010012725	case	control	0.136540476	0.395071931	0.345609156	0.729636464	0.936339759	up_in_case
CRA004775	ENSGALG00010012726	IL7R	case	control	0.17975717	0.499169538	0.360112458	0.718763037	0.934011578	up_in_case
CRA004775	ENSGALG00010012730	CAP2	case	control	0.142401183	0.563301782	0.252797323	0.800424836	0.953286153	up_in_case
CRA004775	ENSGALG00010012732	MGST1	case	control	0.161615962	0.2271619	0.711457167	0.476800985	0.846367006	up_in_case
CRA004775	ENSGALG00010012734	TMEM19	case	control	-0.335885831	0.260253653	-1.290609478	0.196839128	0.66285976	down_in_case
CRA004775	ENSGALG00010012738	RAB21	case	control	-0.090432141	0.182343773	-0.495943127	0.619934538	0.905089892	down_in_case
CRA004775	ENSGALG00010012741	ENSGALG00010012741	case	control	-0.225072951	0.314318884	-0.716065635	0.473950822	0.84552384	down_in_case
CRA004775	ENSGALG00010012742	ENSGALG00010012742	case	control	0.099136387	0.242324245	0.409106348	0.682461618	0.924564673	up_in_case
CRA004775	ENSGALG00010012743	ENSGALG00010012743	case	control	-0.989560758	0.311994834	-3.171721615	0.001515382	0.080693373	down_in_case
CRA004775	ENSGALG00010012744	ARL4C	case	control	0.246874263	0.585916595	0.421347109	0.673501635	0.922442329	up_in_case
CRA004775	ENSGALG00010012745	MARCHF3	case	control	-0.11859648	0.486203782	-0.243923401	0.807290145	0.955397873	down_in_case
CRA004775	ENSGALG00010012747	TOB2	case	control	0.154998229	0.373914741	0.414528266	0.678487271	0.92338129	up_in_case
CRA004775	ENSGALG00010012749	RGS4	case	control	-0.146976727	0.228600826	-0.642940488	0.520262716	0.865611895	down_in_case
CRA004775	ENSGALG00010012751	AP3B1	case	control	0.175699381	0.222075771	0.791168619	0.428845601	0.823726199	up_in_case
CRA004775	ENSGALG00010012753	GTPBP1	case	control	-0.216283142	0.188298438	-1.148618886	0.250713166	0.715557647	down_in_case
CRA004775	ENSGALG00010012756	RPA2	case	control	-0.565111775	0.303512357	-1.861906981	0.062616203	0.449423965	down_in_case
CRA004775	ENSGALG00010012758	NAB1	case	control	0.027813653	0.225551895	0.123313763	0.901858643	0.979157637	up_in_case
CRA004775	ENSGALG00010012759	ATP1B4	case	control	0.267847154	0.727070821	0.36839211	0.71258088	0.932981748	up_in_case
CRA004775	ENSGALG00010012760	PLEKHF2	case	control	0.189200509	0.23494781	0.805287392	0.420653856	0.819388688	up_in_case
CRA004775	ENSGALG00010012761	SMPDL3A	case	control	-0.680740841	0.304407981	-2.236277906	0.025333573	0.30560733	down_in_case
CRA004775	ENSGALG00010012763	SMPDL3B	case	control	0.063722711	0.217475322	0.293011226	0.769513571	0.945407363	up_in_case
CRA004775	ENSGALG00010012765	PHF5A	case	control	-0.277420242	0.327480052	-0.847136308	0.39691915	0.808998414	down_in_case
CRA004775	ENSGALG00010012767	JOSD1	case	control	-0.468141493	0.201623878	-2.321855422	0.020240722	0.281415838	down_in_case
CRA004775	ENSGALG00010012768	EPS8	case	control	0.397714097	0.204137812	1.948262762	0.05138353	0.418339211	up_in_case
CRA004775	ENSGALG00010012769	NEMP2	case	control	0.057655995	0.236694189	0.243588554	0.807549494	0.955397873	up_in_case
CRA004775	ENSGALG00010012771	ITGA2	case	control	-0.129790624	0.684751822	-0.189544036	0.849666445	0.968413719	down_in_case
CRA004775	ENSGALG00010012772	TOMM22	case	control	-0.244429981	0.166241739	-1.470328588	0.141472782	0.601287746	down_in_case
CRA004775	ENSGALG00010012773	XKR8	case	control	-0.548261611	0.19284127	-2.843071982	0.004468098	0.137451483	down_in_case
CRA004775	ENSGALG00010012775	KCNE2	case	control	-0.050319497	0.476374527	-0.105630117	0.915875829	0.981626545	down_in_case
CRA004775	ENSGALG00010012778	TBC1D15	case	control	0.127359263	0.284500242	0.447659595	0.654398886	0.91662886	up_in_case
CRA004775	ENSGALG00010012780	FABP7	case	control	0.509220833	0.49126999	1.036539671	0.299950442	0.752667614	up_in_case
CRA004775	ENSGALG00010012781	SPIN1L	case	control	0.245418111	1.292262743	0.189913477	0.849376932	0.968413719	up_in_case
CRA004775	ENSGALG00010012786	TEF	case	control	-0.159472048	0.19591522	-0.813984987	0.415653552	0.818943344	down_in_case
CRA004775	ENSGALG00010012788	RGS1	case	control	1.432846215	0.627538448	2.283280364	0.022413859	0.292422177	up_in_case
CRA004775	ENSGALG00010012791	CFAP418	case	control	-0.453289668	0.29888247	-1.516615107	0.129363889	0.584172159	down_in_case
CRA004775	ENSGALG00010012792	CBY1	case	control	-0.331745067	0.442880468	-0.749062311	0.45381965	0.838210075	down_in_case
CRA004775	ENSGALG00010012795	TBCA	case	control	-0.362738863	0.305913321	-1.185757006	0.235718275	0.702894534	down_in_case
CRA004775	ENSGALG00010012796	MOCS2	case	control	-0.067593268	0.334848669	-0.201862138	0.840024502	0.966461921	down_in_case
CRA004775	ENSGALG00010012797	ZC3H7B	case	control	-0.108250945	0.150385438	-0.71982332	0.471633785	0.845425322	down_in_case
CRA004775	ENSGALG00010012798	MFSD6	case	control	-0.418302089	0.221542548	-1.888134328	0.059007919	0.44414236	down_in_case
CRA004775	ENSGALG00010012801	DMC1	case	control	0.37657472	0.431209692	0.873298368	0.38250047	0.801915531	up_in_case
CRA004775	ENSGALG00010012803	NUP155	case	control	-0.066661271	0.207978431	-0.320520115	0.748574085	0.940917938	down_in_case
CRA004775	ENSGALG00010012804	ENSGALG00010012804	case	control	-0.319481965	0.343765334	-0.929360623	0.352702228	0.786426886	down_in_case
CRA004775	ENSGALG00010012808	EYA3	case	control	-0.235529692	0.192873994	-1.221158369	0.222026063	0.689550502	down_in_case
CRA004775	ENSGALG00010012809	RHOBTB3	case	control	0.497544021	0.428589513	1.160887064	0.24568783	0.712046495	up_in_case
CRA004775	ENSGALG00010012812	DECR1	case	control	0.497494539	0.498513049	0.997956904	0.318300257	0.767037518	up_in_case
CRA004775	ENSGALG00010012818	RGS18	case	control	-0.849242054	0.417404521	-2.034577997	0.041893353	0.38379447	down_in_case
CRA004775	ENSGALG00010012819	RANGAP1	case	control	-0.301741306	0.189608828	-1.591388487	0.111522174	0.56070984	down_in_case
CRA004775	ENSGALG00010012821	ZBTB33	case	control	0.234102931	0.147893095	1.58291996	0.113439709	0.562886277	up_in_case
CRA004775	ENSGALG00010012824	HIBCH	case	control	0.116509048	0.275730027	0.422547554	0.672625398	0.921952322	up_in_case
CRA004775	ENSGALG00010012827	ENSGALG00010012827	case	control	-0.159425206	0.650161096	-0.245208774	0.806294783	0.955397873	down_in_case
CRA004775	ENSGALG00010012829	RGS5	case	control	0.0033526	0.315315539	0.010632523	0.991516634	0.998065748	up_in_case
CRA004775	ENSGALG00010012830	ARRDC3	case	control	0.230442271	0.393241101	0.586007591	0.557870414	0.880754991	up_in_case
CRA004775	ENSGALG00010012831	ENSGALG00010012831	case	control	-1.534925913	0.799160496	-1.920672908	0.054772957	0.430390248	down_in_case
CRA004775	ENSGALG00010012832	CHADL	case	control	-0.56044834	0.424609728	-1.319914037	0.18686372	0.650186754	down_in_case
CRA004775	ENSGALG00010012833	JAK2	case	control	0.157695135	0.28223297	0.558741011	0.576338486	0.887937128	up_in_case
CRA004775	ENSGALG00010012836	ENSGALG00010012836	case	control	1.625719516	0.447970778	3.629074921	0.000284439	0.034015459	up_in_case
CRA004775	ENSGALG00010012837	PKIB	case	control	-0.066022124	0.551170863	-0.119785221	0.904653289	0.979540372	down_in_case
CRA004775	ENSGALG00010012838	C2orf88	case	control	-0.163178981	0.300615047	-0.542817074	0.587255756	0.892195484	down_in_case
CRA004775	ENSGALG00010012839	TGFBI	case	control	-0.368129259	0.402602831	-0.914373251	0.360520753	0.791975934	down_in_case
CRA004775	ENSGALG00010012841	L3MBTL2	case	control	-0.264055285	0.178131844	-1.482358681	0.138244882	0.597424693	down_in_case
CRA004775	ENSGALG00010012842	DDX17	case	control	-0.179876265	0.215066038	-0.836376891	0.402942909	0.812204585	down_in_case
CRA004775	ENSGALG00010012843	ENSGALG00010012843	case	control	-0.551453449	0.400212325	-1.377902217	0.168233481	0.63138062	down_in_case
CRA004775	ENSGALG00010012845	HDHD2	case	control	-0.573773756	0.341095305	-1.682150843	0.092539593	0.523452056	down_in_case
CRA004775	ENSGALG00010012847	RLN3	case	control	0.782780906	0.828988632	0.94426012	0.34503674	0.783972357	up_in_case
CRA004775	ENSGALG00010012848	SERINC1	case	control	-0.223979421	0.285446539	-0.784663292	0.432651011	0.825615327	down_in_case
CRA004775	ENSGALG00010012850	CALB1	case	control	2.366972753	1.292956177	1.830667423	0.067150198	0.462616422	up_in_case
CRA004775	ENSGALG00010012851	ENSGALG00010012851	case	control	0.02040863	0.339813573	0.060058312	0.952109192	0.990176052	up_in_case
CRA004775	ENSGALG00010012852	MSTN	case	control	0.594578364	0.74357654	0.799619585	0.423931237	0.820822084	up_in_case
CRA004775	ENSGALG00010012854	LECT2	case	control	0.894309251	0.569482493	1.570389365			up_in_case
CRA004775	ENSGALG00010012857	NSDHL	case	control	0.096731943	0.570633824	0.169516666	0.865390265	0.972629493	up_in_case
CRA004775	ENSGALG00010012859	FBXL21	case	control	0.271434837	0.220580085	1.230550062	0.218491192	0.686265963	up_in_case
CRA004775	ENSGALG00010012861	WDR70	case	control	-0.298826958	0.183801771	-1.625811095	0.103989847	0.546356911	down_in_case
CRA004775	ENSGALG00010012865	EP300	case	control	0.150139375	0.305639485	0.491230295	0.623263574	0.906441769	up_in_case
CRA004775	ENSGALG00010012867	A4GALT	case	control	-0.032132266	0.194983039	-0.164795184	0.869105195	0.973372336	down_in_case
CRA004775	ENSGALG00010012868	CTHRC1	case	control	-0.398271264	0.835548958	-0.476658201	0.633605532	0.909837348	down_in_case
CRA004775	ENSGALG00010012869	ENSGALG00010012869	case	control	-0.674544734	0.391936691	-1.72105534	0.085240782	0.506107944	down_in_case
CRA004775	ENSGALG00010012870	KCNC2	case	control	-0.640479747	0.327318279	-1.956749097	0.050376974	0.415806588	down_in_case

CRA004775	ENSGALG00010012871	ENSGALG00010012871	case	control	0.587458508	0.521032799	1.127488536	0.259535985	0.720112858	up_in_case
CRA004775	ENSGALG00010012873	PTAFR	case	control	-0.666605029	0.404020242	-1.649929779	0.098957299	0.535944002	down_in_case
CRA004775	ENSGALG00010012875	HSF2	case	control	-0.102607736	0.230706152	-0.444755092	0.656496751	0.91710603	down_in_case
CRA004775	ENSGALG00010012876	KDELR3	case	control	-0.293107211	0.389999895	-0.751557152	0.452317419	0.836784182	down_in_case
CRA004775	ENSGALG00010012877	SLC25A32	case	control	0.122727855	0.167305311	0.733556242	0.463219239	0.842456502	up_in_case
CRA004775	ENSGALG00010012878	GDNF	case	control	0.582708701	0.538146434	1.082806954	0.278894118	0.735110795	up_in_case
CRA004775	ENSGALG00010012879	RCAN1	case	control	0.496454181	0.322949206	1.537251588	0.124231712	0.576485043	up_in_case
CRA004775	ENSGALG00010012880	CLK4	case	control	0.238841666	0.213104815	1.120770857	0.262385412	0.723230258	up_in_case
CRA004775	ENSGALG00010012883	DCAF13	case	control	-0.245189288	0.183299032	-1.337646384	0.181011737	0.647021677	down_in_case
CRA004775	ENSGALG00010012884	ENSGALG00010012884	case	control	-0.219669702	0.424832536	-0.51707363	0.605104761	0.899740096	down_in_case
CRA004775	ENSGALG00010012885	PMS1	case	control	0.884925473	0.280707477	3.152482733	0.001618884	0.080802173	up_in_case
CRA004775	ENSGALG00010012887	KCNJ4	case	control	-0.362292085	0.763338016	-0.47461554	0.635061028	0.909837348	down_in_case
CRA004775	ENSGALG00010012888	ENSGALG00010012888	case	control	0.268715787	1.454532633	0.184743732	0.853430043	0.970037736	up_in_case
CRA004775	ENSGALG00010012889	RIC1	case	control	0.29112155	0.25728775	1.131501789	0.257843951	0.719921253	up_in_case
CRA004775	ENSGALG00010012890	MFAP3	case	control	-0.333757983	0.24667683	-1.353017159	0.176050149	0.641458822	down_in_case
CRA004775	ENSGALG00010012892	ORMDL1	case	control	-0.795237259	0.252667268	-3.147369526	0.001647466	0.081569987	down_in_case
CRA004775	ENSGALG00010012893	ENSGALG00010012893	case	control	-0.115047249	0.380001917	-0.302754391	0.762077044	0.942385021	down_in_case
CRA004775	ENSGALG00010012894	CLIC6	case	control	-1.359489484	1.043942976	-1.302264123	0.192826112	0.657463888	down_in_case
CRA004775	ENSGALG00010012896	STK38L	case	control	-0.2640865	0.350611106	-0.753217725	0.45131909	0.836054284	down_in_case
CRA004775	ENSGALG00010012897	ENSGALG00010012897	case	control	-0.346135985	0.502167587	-0.689283805	0.490644687	0.855184934	down_in_case
CRA004775	ENSGALG00010012898	FAM114A2	case	control	0.014282855	0.251522705	0.056785549	0.954716026	0.990670511	up_in_case
CRA004775	ENSGALG00010012899	ENSGALG00010012899	case	control	0.307331546	0.260477279	1.179878519	0.238048535	0.70473329	up_in_case
CRA004775	ENSGALG00010012900	RBX1	case	control	-0.23951436	0.264097875	-0.906915133	0.364451678	0.793608387	down_in_case
CRA004775	ENSGALG00010012903	RGMB	case	control	0.081801194	0.276802853	0.295521498	0.767595533	0.944590059	up_in_case
CRA004775	ENSGALG00010012904	ERMP1	case	control	-0.450039113	0.369515828	-1.217915659	0.223256025	0.690817641	down_in_case
CRA004775	ENSGALG00010012905	NKAIN1	case	control	-0.300380057	0.433049424	-0.693639203	0.487908502	0.853591579	down_in_case
CRA004775	ENSGALG00010012908	STRAP	case	control	-0.101593058	0.194711108	-0.521763032	0.601835332	0.898185892	down_in_case
CRA004775	ENSGALG00010012910	XPNPEP3	case	control	-0.214674902	0.281357718	-0.762996314	0.445465594	0.833012432	down_in_case
CRA004775	ENSGALG00010012911	CSNK1E	case	control	0.145693642	0.294090165	0.495404674	0.620314496	0.905283281	up_in_case
CRA004775	ENSGALG00010012912	NKAPL	case	control	0.071931143	0.19076251	0.377071696	0.706120324	0.93131216	up_in_case
CRA004775	ENSGALG00010012913	EGFLAM	case	control	1.001846685	0.386219631	2.593981782	0.009487151	0.197066191	up_in_case
CRA004775	ENSGALG00010012915	ENSGALG00010012915	case	control	-0.252014401	0.453811889	-0.555327895	0.578670402	0.888378369	down_in_case
CRA004775	ENSGALG00010012917	RUNX1	case	control	-2.095924127	0.572409217	-3.661583471	0.000250661	0.032213474	down_in_case
CRA004775	ENSGALG00010012918	GLRX	case	control	0.303492108	0.354421346	0.856303128	0.391830131	0.80746892	up_in_case
CRA004775	ENSGALG00010012921	EBF1	case	control	0.053569303	0.340392246	0.157375215	0.874949146	0.974301166	up_in_case
CRA004775	ENSGALG00010012922	SNRNP40	case	control	-0.274965831	0.201085853	-1.367405145	0.17149835	0.636057362	down_in_case
CRA004775	ENSGALG00010012923	MLANA	case	control	6.007947288	0.716554994	8.384488753	5.09467E-17	1.16761E-13	up_in_case
CRA004775	ENSGALG00010012924	GLIPR1	case	control	-0.199992934	0.389535965	-0.513413271	0.607662264	0.900943922	down_in_case
CRA004775	ENSGALG00010012925	SETD4	case	control	-0.090530601	0.326132596	-0.277588326	0.781328395	0.948910196	down_in_case
CRA004775	ENSGALG00010012926	OSGEPL1	case	control	-0.130513686	0.464996874	-0.280676481	0.778958549	0.94792594	down_in_case
CRA004775	ENSGALG00010012927	ENSGALG00010012927	case	control	0.561921571	1.148204073	0.489391724	0.624564394	0.90646861	up_in_case
CRA004775	ENSGALG00010012930	IL6ST	case	control	0.096777856	0.277887681	0.348262492	0.72764306	0.935548472	up_in_case
CRA004775	ENSGALG00010012932	ATF6	case	control	-0.15980784	0.129078156	-1.238070368	0.21568997	0.683655659	down_in_case
CRA004775	ENSGALG00010012933	ZCCHC17	case	control	-0.296492045	0.200969585	-1.475308039	0.140129746	0.600007375	down_in_case
CRA004775	ENSGALG00010012934	NDUFA1	case	control	0.261548889	0.290529129	0.900250138	0.367987149	0.794533903	up_in_case
CRA004775	ENSGALG00010012938	UPF3B	case	control	0.545786329	0.22041642	2.476160033	0.013280404	0.232044256	up_in_case
CRA004775	ENSGALG00010012939	GJA1	case	control	0.44342959	0.33162908	1.337125168	0.181181783	0.647292985	up_in_case
CRA004775	ENSGALG00010012940	FBN2	case	control	0.973250165	0.657949104	1.479218011	0.139082057	0.599104916	up_in_case
CRA004775	ENSGALG00010012943	ASNSD1	case	control	-0.270905948	0.247406496	-1.094983166	0.273524012	0.731001618	down_in_case
CRA004775	ENSGALG00010012944	NCK1	case	control	0.074383273	0.149267721	0.498321222	0.618257652	0.904143308	up_in_case
CRA004775	ENSGALG00010012945	CLINT1	case	control	0.130263901	0.163891059	0.794820057	0.426718182	0.822776983	up_in_case
CRA004775	ENSGALG00010012948	ST13P5	case	control	-0.038746729	0.168487505	-0.229967967	0.818116662	0.959889304	down_in_case
CRA004775	ENSGALG00010012949	ENSGALG00010012949	case	control	-0.383512371	0.348556369	-1.100287946	0.271206683	0.728818271	down_in_case
CRA004775	ENSGALG00010012952	BRD10	case	control	0.383367699	0.392375454	0.977043021	0.328547854	0.77220987	up_in_case
CRA004775	ENSGALG00010012954	RIMS2	case	control	-0.967669156	0.743401221	-1.301678191	0.193026418	0.65765765	down_in_case
CRA004775	ENSGALG00010012955	RPL39L	case	control	-0.240904991	0.293200998	-0.821637691	0.411283131	0.816804498	down_in_case
CRA004775	ENSGALG00010012956	FABP3	case	control	-0.070506764	0.365344206	-0.192987224	0.846968974	0.967891337	down_in_case
CRA004775	ENSGALG00010012957	DERA	case	control	-0.250821055	0.335001228	-0.748716824	0.454027901	0.838254622	down_in_case
CRA004775	ENSGALG00010012958	OLFML2B	case	control	0.108184223	0.411653798	0.262803899	0.79270173	0.951284723	up_in_case
CRA004775	ENSGALG00010012959	TBC1D32	case	control	0.388537727	0.458563556	0.847293079	0.396831783	0.808998414	up_in_case
CRA004775	ENSGALG00010012962	SLC25A17	case	control	-0.069904736	0.264107099	-0.264683288	0.791253452	0.951284723	down_in_case
CRA004775	ENSGALG00010012963	UIMC1	case	control	-0.36577156	0.178100387	-2.053738157	0.040001041	0.375888502	down_in_case
CRA004775	ENSGALG00010012964	CBR3	case	control	-0.212597966	0.233879396	-0.909006821	0.363346525	0.793608387	down_in_case
CRA004775	ENSGALG00010012965	ENSGALG00010012965	case	control	0.386809897	0.284447372	1.359864548	0.173872791	0.639185614	up_in_case
CRA004775	ENSGALG00010012966	GLIPR1L	case	control	-0.774143136	0.51508564	-1.502940631	0.132854354	0.589506365	down_in_case
CRA004775	ENSGALG00010012967	ECRG4	case	control	-0.472884388	0.479284621	-0.986646278	0.323816075	0.769171227	down_in_case
CRA004775	ENSGALG00010012968	LSM11	case	control	-0.321653602	0.212893768	-1.510864338	0.130823018	0.586275434	down_in_case
CRA004775	ENSGALG00010012969	SLC12A2	case	control	0.584314424	0.242946491	2.405115717	0.016167346	0.25344686	up_in_case
CRA004775	ENSGALG00010012970	UNC5A	case	control	0.207603374	0.416940644	0.497920692	0.618539942	0.904143308	up_in_case
CRA004775	ENSGALG00010012971	ENSGALG00010012971	case	control	-0.74431672	0.764679868	-0.973370362	0.330369263	0.77237154	down_in_case
CRA004775	ENSGALG00010012973	GLDC	case	control	1.322557193	0.41116743	3.216590366	0.001297237	0.075586027	up_in_case
CRA004775	ENSGALG00010012974	SLC49A3	case	control	-0.2235403	0.440615323	-0.507336646	0.61191863	0.901413878	down_in_case
CRA004775	ENSGALG00010012975	KRR1	case	control	-0.080087032	0.206379591	-0.388056936	0.6979739	0.929122855	down_in_case
CRA004775	ENSGALG00010012976	SLC9A2	case	control	1.068087477	0.299790613	3.562778258	0.000366951	0.038226803	up_in_case
CRA004775	ENSGALG00010012977	NKRF	case	control	0.30399725	0.223490845	1.360222382	0.173759562	0.639185614	up_in_case
CRA004775	ENSGALG00010012978	HSD17B7	case	control	-0.003854058	0.222074832	-0.01735477	0.986153592	0.997562105	down_in_case
CRA004775	ENSGALG00010012979	HTR1A	case	control	-0.011719819	0.608208688	-0.019269403	0.984626192	0.997562105	down_in_case
CRA004775	ENSGALG00010012980	KICS2	case	control	-0.123018121	0.177911255	-0.691457781	0.489277904	0.854356883	down_in_case
CRA004775	ENSGALG00010012982	DDR2	case	control	-0.63244632	0.336804483	-1.877784746	0.060410618	0.445397937	down_in_case
CRA004775	ENSGALG00010012985	ARNTL2	case	control	0.438501062	0.23703934	1.849908382	0.064326756	0.456529144	up_in_case
CRA004775	ENSGALG00010012986	TMEM184B	case	control	-0.142108887	0.189775652	-0.748825706	0.453962264	0.838254622	down_in_case
CRA004775	ENSGALG00010012988	RPL18A	case	control	-0.366140666	0.255323925	-1.434024116	0.151565381	0.615709174	down_in_case
CRA004775	ENSGALG00010012991	MFSD9	case	control	-0.131580004	0.276746232	-0.475453641	0.634463669	0.909837348	down_in_case
CRA004775	ENSGALG00010012992	HARS1	case	control	-0.277736569	0.171265353	-1.621673993	0.10487317	0.547815057	down_in_case
CRA004775	ENSGALG00010012993	DOP1B	case	control	0.082224549	0.356301607	0.230772322	0.817491684	0.959812855	up_in_case
CRA004775	ENSGALG00010012995	SERINC2	case	control	-0.005249003	0.459285477	-0.011428629	0.990881472	0.998065748	down_in_case
CRA004775	ENSGALG00010012997	UBE2A	case	control	-0.105999199	0.16070104	-0.659604934	0.509507387	0.862971569	down_in_case
CRA004775	ENSGALG00010012998	MAFF	case	control	0.225847747	0.496394341	0.454976474	0.649126164	0.915047477	up_in_case
CRA004775	ENSGALG00010013001	ADGRV1	case	control	-0.215737804	0.749180465	-0.287965068	0.773373478	0.946296525	down_in_case
CRA004775	ENSGALG00010013004	UAP1	case	control	-0.549840038	0.251022932	-2.190397641	0.028495411	0.321586952	down_in_case
CRA004775	ENSGALG00010013005	SLC40A1	case	control	-0.020968961	0.399559629	-0.052480178	0.958146089	0.991431439	down_in_case
CRA004775	ENSGALG00010013008	STEEP1	case	control	-0.181350434	0.225978176	-0.802513046	0.422256245	0.820119439	down_in_case
CRA004775	ENSGALG00010013012	TMEM182	case	control	0.064102636	0.571035014	0.112256928	0.910619692	0.98075825	up_in_case
CRA004775	ENSGALG00010013013	DCSTAMP	case	control	-0.033066734	0.585834332	-0.056443831	0.95498824	0.990670511	down_in_case
CRA004775	ENSGALG00010013015	PLA2G6	case	control	-0.551551694	0.289708851	-1.903813748	0.056934445	0.435916236	down_in_case
CRA004775	ENSGALG00010013016	QRSL1	case	control	-0.404617695	0.280407517	-1.442963082	0.149030871	0.612469669	down_in_case
CRA004775	ENSGALG00010013017	SCMH1	case	control	-0.149017589	0.233552722	-0.638046895	0.523443154	0.866638286	down_in_case
CRA004775	ENSGALG00010013018	ID1	case	control	-0.10566545	0.40435223	-0.261320309	0.793845506	0.951797851	down_in_case
CRA004775	ENSGALG00010013019	DPYS	case	control	0.262349933	1.023831793	0.256243198	0.797763055	0.952835572	up_in_case
CRA004775	ENSGALG00010013021	SLC25A5	case	control	0.065901123	0.178140367	0.369939302	0.711427717	0.932320836	up_in_case
CRA004775	ENSGALG00010013027	UHMK1	case	control	-0.298945832	0.224454528	-1.331877039	0.182900597	0.647378665	down_in_case
CRA004775	ENSGALG00010013029	MYLK2	case	control	0.023881564	0.746281698	0.032000737	0.974471463	0.994947809	up_in_case
CRA004775	ENSGALG00010013032	BAIAP2L2	case	control	-0.257171626	0.540110805	-0.476146049	0.633970333	0.909837348	down_in_case
CRA004775	ENSGALG00010013033	MAP3K1	case	control	-0.621868309	0.526113873	-1.182003252	0.23720441	0.703879272	down_in_case
CRA004775	ENSGALG00010013036	ENSGALG00010013036	case	control	-0.901095457	0.650576492	-1.385072267	0.166030371	0.630194031	down_in_case
CRA004775	ENSGALG00010013037	MORC4	case	control	0.321277818	0.196455969	1.635368066	0.101971913	0.542023878	up_in_case
CRA004775	ENSGALG00010013040	SLC25A43	case	control	-0.174702304	0.413785881	-0.422204603	0.672875682	0.921952322	down_in_case
CRA004775	ENSGALG00010013041	LRP12	case	control	0.434799644	0.185393488	2.345280019	0.019012794	0.272278915	up_in_case
CRA004775	ENSGALG00010013042	SLC25A48	case	control	-0.351596788	0.619714412	-0.567352932	0.570474421	0.885244439	down_in_case
CRA004775	ENSGALG00010013044	ENSGALG00010013044	case	control	0.094071261	0.240882724	0.390527222	0.69614673	0.929092636	up_in_case
CRA004775	ENSGALG00010013045	FSD1L	case	control	0.36197396	0.410044854	0.882766742	0.377362313	0.798693115	up_in_case
CRA004775	ENSGALG00010013046	SLC12A5	case	control	-0.701383487	0.649536919	-1.079820817	0.280221979	0.73661445	down_in_case
CRA004775	ENSGALG00010013047	CTPS1	case	control	-0.37545587	0.283752258	-1.323181963	0.185774873	0.6491093	down_in_case
CRA004775	ENSGALG00010013048	WDR75	case	control	-0.357432152	0.246308807	-1.451154577	0.146736822	0.609969178	down_in_case
CRA004775	ENSGALG00010013051	XPOT	case	control	0.100435848	0.152924498	0.65676755	0.511330392	0.863170205	up_in_case
CRA004775	ENSGALG00010013053	MRTFA	case	control	0.234447583	0.300500402	0.780190582	0.435278705	0.826905159	up_in_case
CRA004775	ENSGALG00010013056	ENSGALG00010013056	case	control	0.009381175	0.315257422	0.029757191	0.9762607	0.995591879	up_in_case
CRA004775	ENSGALG00010013058	ENSGALG00010013058	case	control	0.000644598	0.543363157	0.001186311	0.999053461	0.999914248	up_in_case
CRA004775	ENSGALG00010013062	ENSGALG00010013062	case	control	-0.142782243	0.195563838	-0.730105548	0.46532567	0.843383332	down_in_case
CRA004775	ENSGALG00010013063	PARP12	case	control	-0.456739862	0.404906062	-1.128014382	0.259313845	0.720037471	down_in_case
CRA004775	ENSGALG00010013065	FKTN	case	control	0.464273447	0.257126372	1.805623608	0.070977152	0.469911807	up_in_case
CRA004775	ENSGALG00010013069	ENSGALG00010013069	case	control	-0.300625919	1.745292422	-0.172249598	0.863241307	0.971910432	down_in_case
CRA004775	ENSGALG00010013071	ENSGALG00010013071	case	control	-0.303514984	0.59360966	-0.511303983	0.609138216	0.90115757	down_in_case
CRA004775	ENSGALG00010013076	RTN4IP1	case	control	-0.456353006	0.286425351	-1.593270302	0.111099566	0.560403826	down_in_case
CRA004775	ENSGALG00010013078	COL5A2	case	control	-0.057510286	0.519873775	-0.110623556	0.911914867	0.98075825	down_in_case
CRA004775	ENSGALG00010013079	ENSGALG00010013079	case	control	-0.079945595	0.428654819	-0.186503432	0.852049988	0.969430695	down_in_case

CRA004775	ENSGALG00010013080	ARHGEF7	case	control	0.419415405	0.275419774	1.522822416	0.12780314	0.5821156	up_in_case
CRA004775	ENSGALG00010013081	NCOA5	case	control	-0.230422608	0.150893722	-1.527052321	0.126748002	0.580923609	down_in_case
CRA004775	ENSGALG00010013082	CHAF1B	case	control	-0.014229494	0.227159371	-0.062641019	0.950052365	0.990168609	down_in_case
CRA004775	ENSGALG00010013084	SGSM3	case	control	-0.11292025	0.246755354	-0.457620263	0.647225283	0.914210065	down_in_case
CRA004775	ENSGALG00010013085	LYSMD3	case	control	0.079820615	0.248874082	0.320726911	0.748417352	0.940917938	up_in_case
CRA004775	ENSGALG00010013086	CXCL14	case	control	-0.798224996	0.59394056	-1.34394761	0.178965329	0.644923737	down_in_case
CRA004775	ENSGALG00010013087	GPN2	case	control	-0.65597321	0.236476642	-2.773945054	0.005538103	0.151100111	down_in_case
CRA004775	ENSGALG00010013089	TBK1	case	control	0.070586731	0.226102105	0.312189623	0.754896418	0.941900693	up_in_case
CRA004775	ENSGALG00010013093	GPATCH3	case	control	0.130998139	0.19899174	0.658309431	0.510339319	0.863170205	up_in_case
CRA004775	ENSGALG00010013095	ADSL	case	control	-0.376795026	0.445652864	-0.845489968	0.397837331	0.809455905	down_in_case
CRA004775	ENSGALG00010013099	SIM2	case	control	1.026757935	0.614920443	1.669741098	0.094970599	0.527652809	up_in_case
CRA004775	ENSGALG00010013100	ZFPM2	case	control	-0.073448817	0.710824133	-0.103329099	0.917701782	0.981688766	down_in_case
CRA004775	ENSGALG00010013103	AIRIM	case	control	-0.197115868	0.234962899	-0.838923374	0.40151231	0.811868062	down_in_case
CRA004775	ENSGALG00010013104	HLCS	case	control	-0.075238062	0.307190331	-0.244923277	0.806515838	0.955397873	down_in_case
CRA004775	ENSGALG00010013106	SETD9	case	control	-0.682430812	0.335845059	-2.031981099	0.042155569	0.385939571	down_in_case
CRA004775	ENSGALG00010013110	ANKRD10	case	control	0.603648722	0.285445996	2.114756315	0.034450722	0.353267619	up_in_case
CRA004775	ENSGALG00010013111	RFESD	case	control	0.269356182	0.525290037	0.512776109	0.608107944	0.900943922	up_in_case
CRA004775	ENSGALG00010013112	CD40	case	control	-0.214552239	0.178674188	-1.200801535	0.229828196	0.6959629	down_in_case
CRA004775	ENSGALG00010013114	PIGP	case	control	-0.525906583	0.2743225	-1.91711064	0.055223877	0.430875458	down_in_case
CRA004775	ENSGALG00010013115	ENSGALG00010013115	case	control	-0.152479155	0.348739597	-0.437229257	0.661945094	0.918136475	down_in_case
CRA004775	ENSGALG00010013116	TARS	case	control	-0.317534085	0.288548126	-1.100454502	0.271134143	0.728765754	down_in_case
CRA004775	ENSGALG00010013117	ARSK	case	control	-0.084817922	0.406641496	-0.208581571	0.834774899	0.964344207	down_in_case
CRA004775	ENSGALG00010013119	RNF114	case	control	-0.150678733	0.168662889	-0.89337218	0.371657896	0.795818272	down_in_case
CRA004775	ENSGALG00010013121	PCBD2	case	control	-0.139076594	0.38834476	-0.358126614	0.720248563	0.934352641	down_in_case
CRA004775	ENSGALG00010013123	PPFIBP1	case	control	0.213714937	0.208663033	1.024210823	0.305735708	0.758032511	up_in_case
CRA004775	ENSGALG00010013129	RASSF3	case	control	0.272657764	0.180836021	1.507762454	0.131615335	0.587352231	up_in_case
CRA004775	ENSGALG00010013131	SPATA2	case	control	0.144492687	0.189635803	0.761948347	0.446090833	0.833494028	up_in_case
CRA004775	ENSGALG00010013132	SKIC3	case	control	0.133599776	0.208734911	0.64004519	0.52214322	0.866416245	up_in_case
CRA004775	ENSGALG00010013133	TXNDC15	case	control	-0.307399608	0.231083723	-1.330252099	0.183435224	0.647806087	down_in_case
CRA004775	ENSGALG00010013134	ENSGALG00010013134	case	control	0.354147912	1.028415031	0.344362832	0.730573437	0.936394787	up_in_case
CRA004775	ENSGALG00010013135	TTC3	case	control	0.308939344	0.216348341	1.42797186	0.153299958	0.617646565	up_in_case
CRA004775	ENSGALG00010013137	TMEM121	case	control	1.119721467	0.718026135	1.559443888	0.118891356	0.570892125	up_in_case
CRA004775	ENSGALG00010013138	ENSGALG00010013138	case	control	0.182939815	0.783825362	0.233393589	0.815455791	0.958870945	up_in_case
CRA004775	ENSGALG00010013139	TMEM185A	case	control	-0.042330111	0.172067736	-0.246008417	0.805675717	0.955171181	down_in_case
CRA004775	ENSGALG00010013143	ENSGALG00010013143	case	control	0.6639097	0.824499253	0.805227776	0.420688251	0.819388688	up_in_case
CRA004775	ENSGALG00010013144	TEDC1	case	control	-0.87320384	0.467552209	-1.86760713	0.061816852	0.446686038	down_in_case
CRA004775	ENSGALG00010013145	ENSGALG00010013145	case	control	-0.499376122	0.472090593	-1.057797231	0.290147895	0.744282131	down_in_case
CRA004775	ENSGALG00010013146	TNRC6B	case	control	-0.08597988	0.305819113	-0.281146196	0.778598271	0.94792594	down_in_case
CRA004775	ENSGALG00010013149	MCTP1	case	control	-0.116092602	0.645210217	-0.179929887	0.857207611	0.970913248	down_in_case
CRA004775	ENSGALG00010013150	GNS	case	control	0.274035557	0.287782486	0.952231531	0.340979573	0.780875343	up_in_case
CRA004775	ENSGALG00010013152	TBXAS1	case	control	-0.062278769	0.513299588	-0.121330253	0.903429459	0.979456447	down_in_case
CRA004775	ENSGALG00010013154	ENSGALG00010013154	case	control	0.183584005	1.012289293	0.181355277	0.856088721	0.970703965	up_in_case
CRA004775	ENSGALG00010013156	ABCC9	case	control	0.214446928	0.391855499	0.547260224	0.584199966	0.890120081	up_in_case
CRA004775	ENSGALG00010013158	CRIP1	case	control	-0.109896755	0.276902107	-0.396879447	0.691456361	0.927867527	down_in_case
CRA004775	ENSGALG00010013160	CRYBG1	case	control	1.952783293	0.788131343	2.477738399	0.013221805	0.231608964	up_in_case
CRA004775	ENSGALG00010013162	IDS	case	control	-0.327059822	0.258720349	-1.264144177	0.206178283	0.674837165	down_in_case
CRA004775	ENSGALG00010013164	FAM83F	case	control	1.813740437	0.781884508	2.319703765	0.020356908	0.281976732	up_in_case
CRA004775	ENSGALG00010013165	NUDC	case	control	-0.149517428	0.221694062	-0.67443136	0.500037111	0.85956977	down_in_case
CRA004775	ENSGALG00010013168	VPS26C	case	control	-0.177433717	0.205150078	-0.864897149	0.387095242	0.803905475	down_in_case
CRA004775	ENSGALG00010013170	COL3A1	case	control	0.124155115	0.603990089	0.205558199	0.837136033	0.965269623	up_in_case
CRA004775	ENSGALG00010013171	SLC9A8	case	control	-0.292748624	0.209048564	-1.400385721	0.161397844	0.627121149	down_in_case
CRA004775	ENSGALG00010013172	TNFSF13B	case	control	-0.541615977	0.560002956	-0.967166283	0.333460915	0.774166524	down_in_case
CRA004775	ENSGALG00010013173	KCNJ8	case	control	0.530859903	0.358105057	1.482413869	0.138230206	0.597424693	up_in_case
CRA004775	ENSGALG00010013174	ADAMTS12	case	control	0.241038665	0.487937945	0.493994507	0.621310059	0.90544587	up_in_case
CRA004775	ENSGALG00010013175	HIPK2	case	control	-0.035986875	0.407437361	-0.088324926	0.929618428	0.98473447	down_in_case
CRA004775	ENSGALG00010013177	OXR1	case	control	0.252875568	0.176220785	1.434992854	0.151289134	0.615293187	up_in_case
CRA004775	ENSGALG00010013179	TBC1D30	case	control	0.139738345	0.359408243	0.388801168	0.697423236	0.929092636	up_in_case
CRA004775	ENSGALG00010013180	C5orf24	case	control	0.14345475	0.283196373	0.506555746	0.612466566	0.901413878	up_in_case
CRA004775	ENSGALG00010013181	DYRK1A	case	control	0.312866629	0.206186786	1.517394177	0.129167193	0.584172159	up_in_case
CRA004775	ENSGALG00010013182	NR0B2	case	control	2.272372511	0.606953972	3.743895938	0.000181189	0.027735809	up_in_case
CRA004775	ENSGALG00010013183	CLTA	case	control	-0.519905103	0.374396245	-1.388649353	0.164939402	0.630194031	down_in_case
CRA004775	ENSGALG00010013184	LDHB	case	control	-0.296909329	0.293480139	-1.011684574	0.311688893	0.763232972	down_in_case
CRA004775	ENSGALG00010013185	ENSGALG00010013185	case	control	-0.356442067	0.470915099	-0.756913652	0.449101596	0.834540006	down_in_case
CRA004775	ENSGALG00010013186	DDX46	case	control	0.594786878	0.24139632	2.46394343	0.01374178	0.234005339	up_in_case
CRA004775	ENSGALG00010013187	KDF1	case	control	1.098818356	0.472590433	2.325096486	0.020066802	0.280454072	up_in_case
CRA004775	ENSGALG00010013188	ENSGALG00010013188	case	control	-0.005002934	0.772158442	-0.006479154	0.994830419	0.999001196	down_in_case
CRA004775	ENSGALG00010013191	ABRA	case	control	1.534355197	1.007111913	1.523520055	0.127628647	0.58187175	up_in_case
CRA004775	ENSGALG00010013192	B4GALT5	case	control	-0.057396887	0.307743523	-0.18650884	0.852045747	0.969430695	down_in_case
CRA004775	ENSGALG00010013194	GRAP2	case	control	-1.072335462	0.529772758	-2.024142324	0.04295551	0.388861893	down_in_case
CRA004775	ENSGALG00010013196	MRPS9	case	control	0.079675107	0.220023626	0.36212069	0.717261843	0.934011578	up_in_case
CRA004775	ENSGALG00010013198	CAMLG	case	control	-0.031057993	0.150692087	-0.20610235	0.836710963	0.965269623	down_in_case
CRA004775	ENSGALG00010013203	AMACR	case	control	-0.40488266	0.293681173	-1.378646974	0.168003626	0.63138062	down_in_case
CRA004775	ENSGALG00010013206	AFF2	case	control	-0.115924654	0.316085968	-0.366750397	0.713805211	0.933244709	down_in_case
CRA004775	ENSGALG00010013207	C1QTNF3	case	control	0.605634561	0.415884473	1.456256724	0.145321664	0.608365494	up_in_case
CRA004775	ENSGALG00010013208	ENSGALG00010013208	case	control	-1.150433286	0.829632081	-1.386678881	0.165539703	0.630194031	down_in_case
CRA004775	ENSGALG00010013209	KIAA1549	case	control	0.01355744	0.413869894	0.032757735	0.973867783	0.994947809	up_in_case
CRA004775	ENSGALG00010013212	ENSGALG00010013212	case	control	0.329828242	0.40376386	0.816884011	0.413994716	0.817589409	up_in_case
CRA004775	ENSGALG00010013213	SH2D1A	case	control	-0.820129513	0.532712055	-1.539536237	0.123673438	0.576340398	down_in_case
CRA004775	ENSGALG00010013214	RAI14	case	control	0.684422161	0.389642151	1.75654035	0.078996188	0.491399489	up_in_case
CRA004775	ENSGALG00010013215	SF3A3	case	control	-0.26007275	0.22185464	-1.172266446	0.241090099	0.708079869	down_in_case
CRA004775	ENSGALG00010013217	TGFBRAP1	case	control	0.377866588	0.299183271	1.262993705	0.206591442	0.675197427	up_in_case
CRA004775	ENSGALG00010013220	CSTF1	case	control	-0.037015885	0.144720594	-0.255774828	0.798124712	0.952835572	down_in_case
CRA004775	ENSGALG00010013221	C2orf49	case	control	0.283425971	0.210375499	1.347238496	0.177903433	0.643776346	up_in_case
CRA004775	ENSGALG00010013225	GNE	case	control	0.194493315	0.325973796	0.59665322	0.550738931	0.877827378	up_in_case
CRA004775	ENSGALG00010013226	DUSP6	case	control	0.232043208	0.320964474	0.722956048	0.469706888	0.844062961	up_in_case
CRA004775	ENSGALG00010013227	CITED4	case	control	-0.832965937	0.530273366	-1.570823637	0.11622362	0.566086328	down_in_case
CRA004775	ENSGALG00010013228	RTFDC1	case	control	0.056106948	0.210039631	0.267125534	0.789372509	0.950475151	up_in_case
CRA004775	ENSGALG00010013230	ENSGALG00010013230	case	control	-0.115289708	0.318887467	-0.361537281	0.717697841	0.934011578	down_in_case
CRA004775	ENSGALG00010013232	FHL2	case	control	0.617887788	0.877187173	0.704396744	0.481185733	0.848786191	up_in_case
CRA004775	ENSGALG00010013233	LRRCC1	case	control	0.006797324	0.393237227	0.017285557	0.986208808	0.997562105	up_in_case
CRA004775	ENSGALG00010013235	GCNT7	case	control	0.334179135	0.492793918	0.678131613	0.497688248	0.857810713	up_in_case
CRA004775	ENSGALG00010013236	MON2	case	control	0.163703828	0.24931093	0.656625156	0.511421969	0.863170205	up_in_case
CRA004775	ENSGALG00010013238	RAD1	case	control	-0.211649122	0.249048282	-0.849831688	0.395418669	0.808998414	down_in_case
CRA004775	ENSGALG00010013241	SEC24A	case	control	0.415804602	0.286530577	1.451170086	0.146732504	0.609969178	up_in_case
CRA004775	ENSGALG00010013242	SULT4A1	case	control	0.533338837	0.330420778	1.614120153	0.106501367	0.552224847	up_in_case
CRA004775	ENSGALG00010013244	TRIM24	case	control	0.133548914	0.312096757	0.427908689	0.6687176	0.920123584	up_in_case
CRA004775	ENSGALG00010013246	LNPEP	case	control	0.525716787	0.307487153	1.709719515	0.087317752	0.511374111	up_in_case
CRA004775	ENSGALG00010013247	ATG5	case	control	-0.133258086	0.181437789	-0.734456074	0.462670822	0.842456502	down_in_case
CRA004775	ENSGALG00010013248	ENSGALG00010013248	case	control	-0.438242858	1.17264404	-0.373721985	0.708611167	0.931801673	down_in_case
CRA004775	ENSGALG00010013249	AURKA	case	control	0.407930177	0.336500931	1.212270573	0.225408824	0.693278343	up_in_case
CRA004775	ENSGALG00010013250	E2F5	case	control	-0.301581999	0.185511853	-1.625675095	0.104018791	0.546356911	down_in_case
CRA004775	ENSGALG00010013251	MTA1	case	control	0.233172213	0.187161148	1.245836626	0.21282438	0.682777582	up_in_case
CRA004775	ENSGALG00010013252	PPM1H	case	control	-0.481403101	0.467826807	-1.029019913	0.303470317	0.755845016	down_in_case
CRA004775	ENSGALG00010013253	SHB	case	control	-0.366489159	0.262563297	-1.3958126	0.162770935	0.62925587	down_in_case
CRA004775	ENSGALG00010013254	SAR1B	case	control	0.423211808	0.350603424	1.207095478	0.227395376	0.694715356	up_in_case
CRA004775	ENSGALG00010013256	BRIX1	case	control	0.081015427	0.275029864	0.294569563	0.768322716	0.945059483	up_in_case
CRA004775	ENSGALG00010013257	STX12	case	control	0.04651264	0.169772716	0.27397005	0.784107638	0.950146645	up_in_case
CRA004775	ENSGALG00010013258	RBIS	case	control	-0.138482127	0.345563497	-0.400742926	0.688609403	0.927339918	down_in_case
CRA004775	ENSGALG00010013259	PTPRB	case	control	0.452911628	0.496001638	0.913125268	0.361176662	0.792567091	up_in_case
CRA004775	ENSGALG00010013260	FAM210B	case	control	-0.137070393	0.270518883	-0.506694361	0.612369287	0.901413878	down_in_case
CRA004775	ENSGALG00010013261	JADE2	case	control	0.365096842	0.26356859	1.38520619	0.165989429	0.630194031	up_in_case
CRA004775	ENSGALG00010013262	CA13	case	control	-0.390523008	0.372141609	-1.049393561	0.293997021	0.746438942	down_in_case
CRA004775	ENSGALG00010013263	ATP11A	case	control	0.227975318	0.497356731	0.458373845	0.646683879	0.914043203	up_in_case
CRA004775	ENSGALG00010013264	DNAJC21	case	control	0.380166903	0.336754584	1.128913816	0.258934192	0.720037471	up_in_case
CRA004775	ENSGALG00010013265	PACS2	case	control	-0.02358206	0.281962203	-0.083635536	0.933346213	0.985090297	down_in_case
CRA004775	ENSGALG00010013266	KIAA1755	case	control	-0.277193927	0.4149681	-0.667988521	0.50414092	0.861471523	down_in_case
CRA004775	ENSGALG00010013267	PNPLA3	case	control	0.146071618	0.337567234	0.432718593	0.665219221	0.918815167	up_in_case
CRA004775	ENSGALG00010013268	PTPRR	case	control	0.41896832	0.376606685	1.11248243	0.26593078	0.727145389	up_in_case
CRA004775	ENSGALG00010013269	INPP5B	case	control	-0.139744193	0.158198807	-0.883345429	0.377049666	0.798523018	down_in_case
CRA004775	ENSGALG00010013271	GULP1	case	control	0.28331343	0.313693644	0.903153237	0.366444573	0.793608387	up_in_case
CRA004775	ENSGALG00010013273	STAG2	case	control	0.646018599	0.318781886	2.026522294	0.042711291	0.388066397	up_in_case
CRA004775	ENSGALG00010013274	MTF1	case	control	0.036909922	0.316212053	0.116725221	0.907077803	0.980354087	up_in_case
CRA004775	ENSGALG00010013275	MAPRE1	case	control	-0.035362761	0.166663293	-0.212180859	0.831965939	0.963642489	down_in_case
CRA004775	ENSGALG00010013276	POC1B	case	control	-0.054249197	0.360926879	-0.150305229	0.880523808	0.974996282	down_in_case
CRA004775	ENSGALG00010013277	YRDC	case	control	-0.214655447	0.204756831	-1.048343273	0.294480479	0.746438942	down_in_case
CRA004775	ENSGALG00010013278	SAMM50	case	control	0.085539414	0.257856161	0.331733063	0.740090832	0.938317263	up_in_case

CRA004775	ENSGALG00010013280	FRS2	case	control	0.542085566	0.383934864	1.411920658	0.157973324	0.623088507	up_in_case
CRA004775	ENSGALG00010013281	BIRC8	case	control	-0.052044653	0.349281787	-0.149004772	0.881549868	0.975011509	down_in_case
CRA004775	ENSGALG00010013282	SEC22B	case	control	-0.728314349	0.229800847	-3.169328396	0.001527917	0.080693373	down_in_case
CRA004775	ENSGALG00010013283	PAWR	case	control	0.570832501	0.311758715	1.83100736	0.067099445	0.462498477	up_in_case
CRA004775	ENSGALG00010013284	MANEAL	case	control	-1.038446263	0.399797037	-2.597433613	0.009392327	0.195703177	down_in_case
CRA004775	ENSGALG00010013285	ATP2B1	case	control	0.411945563	0.228834921	1.800186615	0.071831177	0.472834135	up_in_case
CRA004775	ENSGALG00010013286	RNF14	case	control	0.11492483	0.276117383	0.416217294	0.677251006	0.922924485	up_in_case
CRA004775	ENSGALG00010013288	CA3A	case	control	2.499477355	1.024191124	2.44044036			up_in_case
CRA004775	ENSGALG00010013289	TM9SF4	case	control	-0.284818954	0.17963718	-1.585523405	0.112847464	0.562886277	down_in_case
CRA004775	ENSGALG00010013290	AKR1D1	case	control	0.179840786	0.308827402	0.58233429	0.560341533	0.880918893	up_in_case
CRA004775	ENSGALG00010013292	PRDM1	case	control	-0.668114583	0.688085929	-0.970975505	0.331560476	0.772629743	down_in_case
CRA004775	ENSGALG00010013293	TUBGCP3	case	control	-0.130845975	0.288642828	-0.453314485	0.650322304	0.915428088	down_in_case
CRA004775	ENSGALG00010013295	CA3B	case	control	-0.139857145	0.709237363	-0.197193707	0.843675955	0.966478468	down_in_case
CRA004775	ENSGALG00010013296	AGXT2	case	control	-0.102544477	0.665852376	-0.154004822	0.877605934	0.974479909	down_in_case
CRA004775	ENSGALG00010013297	FCHO2	case	control	0.118615082	0.443753976	0.267299198	0.789238804	0.95041797	up_in_case
CRA004775	ENSGALG00010013299	PLAGL2	case	control	-0.199533319	0.330686876	-0.6033905	0.54624894	0.877099134	down_in_case
CRA004775	ENSGALG00010013300	GNPDA1	case	control	-0.476482514	0.180171743	-2.644601782	0.008178711	0.182940477	down_in_case
CRA004775	ENSGALG00010013301	PARVB	case	control	-0.420900672	0.391634512	-1.074728245	0.282496414	0.738659097	down_in_case
CRA004775	ENSGALG00010013302	ENSGALG00010013302	case	control	-1.091868007	0.628461517	-1.737366535	0.082322494	0.499148629	down_in_case
CRA004775	ENSGALG00010013303	TFPI	case	control	0.173325293	0.553483279	0.31315362	0.754163953	0.941900693	up_in_case
CRA004775	ENSGALG00010013304	KCNJ15	case	control	0.877769776	0.544897736	1.610889011	0.107203916	0.553800541	up_in_case
CRA004775	ENSGALG00010013305	THOC2	case	control	0.426463138	0.21648871	1.969909368	0.048848759	0.41008503	up_in_case
CRA004775	ENSGALG00010013306	ARSB	case	control	-0.556612645	0.571514125	-0.973926314	0.330093127	0.77237154	down_in_case
CRA004775	ENSGALG00010013307	POFUT1	case	control	-0.235699486	0.180505212	-1.305776617	0.191628534	0.656959697	down_in_case
CRA004775	ENSGALG00010013308	NMU	case	control	0.024536011	1.383369457	0.017736412	0.985849133	0.997562105	up_in_case
CRA004775	ENSGALG00010013309	ENSGALG00010013309	case	control	0.502422659	0.713840112	0.703830803	0.481538148	0.848818238	up_in_case
CRA004775	ENSGALG00010013310	MYO16	case	control	-0.820237326	0.730584018	-1.122714576	0.261558735	0.7223728	down_in_case
CRA004775	ENSGALG00010013311	RSPO1	case	control	-0.097508011	0.733754781	-0.132889097	0.894281089	0.977834811	down_in_case
CRA004775	ENSGALG00010013312	ENSGALG00010013312	case	control	1.444993937	0.846376261	1.707271345	0.08777163	0.512706568	up_in_case
CRA004775	ENSGALG00010013313	GAK	case	control	-0.19085144	0.438179561	-0.435555322	0.663159392	0.918295135	down_in_case
CRA004775	ENSGALG00010013314	KIT	case	control	0.467791109	0.473209514	0.988549671	0.322883521	0.768114682	up_in_case
CRA004775	ENSGALG00010013315	UBXN11	case	control	-0.888111504	0.715630058	-1.241020404	0.214598211	0.683562661	down_in_case
CRA004775	ENSGALG00010013316	KDM7A	case	control	0.29821006	0.310272376	0.961123461	0.336490095	0.776900397	up_in_case
CRA004775	ENSGALG00010013317	PREP	case	control	-0.190470525	0.132949867	-1.432649233	0.151958105	0.61657595	down_in_case
CRA004775	ENSGALG00010013319	ENSGALG00010013319	case	control	-0.174788283	1.140860107	-0.153207464	0.878234672	0.974784484	down_in_case
CRA004775	ENSGALG00010013320	CALCRL	case	control	0.623616689	0.372861144	1.672517234	0.094422372	0.526933666	up_in_case
CRA004775	ENSGALG00010013322	IRS2	case	control	0.300540018	0.672230248	0.447078987	0.65481803	0.916851922	up_in_case
CRA004775	ENSGALG00010013323	NOTCH2	case	control	-0.498905124	0.361849402	-1.378764538	0.167967364	0.63138062	down_in_case
CRA004775	ENSGALG00010013324	PARVG	case	control	-0.484515251	0.365302199	-1.326340912	0.184726801	0.6491093	down_in_case
CRA004775	ENSGALG00010013325	CA2	case	control	0.05118471	0.392647397	0.13035795	0.896283234	0.978624007	up_in_case
CRA004775	ENSGALG00010013327	SLF1	case	control	-0.04477086	0.29781403	-0.150331601	0.880503002	0.974996282	down_in_case
CRA004775	ENSGALG00010013328	SLC37A3	case	control	-0.456010026	0.213268983	-2.138191966	0.032501166	0.343523087	down_in_case
CRA004775	ENSGALG00010013329	LIFR	case	control	0.538178339	0.365328876	1.473133866	0.140714943	0.600177167	up_in_case
CRA004775	ENSGALG00010013330	ERG	case	control	0.306202122	0.366150939	0.836272939	0.403001373	0.812204585	up_in_case
CRA004775	ENSGALG00010013331	KIF3B	case	control	-0.333736451	0.227416816	-1.46751	0.142237371	0.602473513	down_in_case
CRA004775	ENSGALG00010013332	LUM	case	control	0.445263419	0.552730625	0.80557038	0.42049061	0.819388688	up_in_case
CRA004775	ENSGALG00010013333	PPP1R12A	case	control	0.307005169	0.255078048	1.20357346	0.228754483	0.695782399	up_in_case
CRA004775	ENSGALG00010013334	SH3BGRL3	case	control	-0.235294395	0.2153009	-1.092863031	0.274453942	0.731001618	down_in_case
CRA004775	ENSGALG00010013335	POPDC3	case	control	0.57553088	0.539531697	1.066723017	0.28609693	0.741582427	up_in_case
CRA004775	ENSGALG00010013337	RAB19	case	control	-0.190122204	0.397396639	-0.478419255	0.632351831	0.909651573	down_in_case
CRA004775	ENSGALG00010013338	ENSGALG00010013338	case	control	2.178694007	0.883856787	2.464985324	0.013701886	0.234005339	up_in_case
CRA004775	ENSGALG00010013339	ATP6V0D2	case	control	1.075984179	0.95041964	1.132114841	0.257586156	0.719347529	up_in_case
CRA004775	ENSGALG00010013342	REG4A	case	control	2.533613331	0.666707975	3.800184529	0.000144588	0.02543383	up_in_case
CRA004775	ENSGALG00010013344	OSMR	case	control	0.720165553	0.262018352	2.748530964	0.005986298	0.154441997	up_in_case
CRA004775	ENSGALG00010013345	DCN	case	control	0.555621951	0.378979635	1.466099756	0.142621112	0.602997185	up_in_case
CRA004775	ENSGALG00010013346	HMGCS2	case	control	1.237877082	0.524474825	2.360222119	0.018263996	0.2691835	up_in_case
CRA004775	ENSGALG00010013347	ENSGALG00010013347	case	control	0.068151552	0.872643634	0.078097805	0.937750253	0.986707289	up_in_case
CRA004775	ENSGALG00010013348	MKRN4P	case	control	-0.305225487	0.171209101	-1.782764389	0.074624665	0.481462352	down_in_case
CRA004775	ENSGALG00010013349	ETS2	case	control	0.043899142	0.208617196	0.210429161	0.833332735	0.964019688	up_in_case
CRA004775	ENSGALG00010013351	EDAR	case	control	1.332175281	0.763900833	1.743911282	0.081174561	0.496765193	up_in_case
CRA004775	ENSGALG00010013352	ASXL1	case	control	-0.220335937	0.271316114	-0.812100445	0.416733998	0.818943344	down_in_case
CRA004775	ENSGALG00010013353	NDFIP1	case	control	-0.097108407	0.238095572	-0.40785474	0.683380323	0.925087467	down_in_case
CRA004775	ENSGALG00010013354	WWP1	case	control	0.6392855	0.347046861	1.842072562	0.065464537	0.45911339	up_in_case
CRA004775	ENSGALG00010013355	FAM171B	case	control	0.124099518	0.393552901	0.315331224	0.75251018	0.94167946	up_in_case
CRA004775	ENSGALG00010013356	AMER1	case	control	-0.236988736	0.228211502	-1.038460962	0.299055501	0.75239174	down_in_case
CRA004775	ENSGALG00010013357	RICTOR	case	control	0.752228563	0.421040462	1.786594473	0.074003056	0.479781244	up_in_case
CRA004775	ENSGALG00010013358	RPS16	case	control	-0.223446192	0.285808617	-0.781803554	0.434330032	0.826182359	down_in_case
CRA004775	ENSGALG00010013359	BTG1	case	control	-0.05318327	0.177108119	-0.300287027	0.763958228	0.94239774	down_in_case
CRA004775	ENSGALG00010013360	PRR5	case	control	-0.439721376	0.307844485	-1.428388025	0.153180204	0.617472961	down_in_case
CRA004775	ENSGALG00010013361	ENSGALG00010013361	case	control	0.260225563	0.425544482	0.611512013	0.540860668	0.874592283	up_in_case
CRA004775	ENSGALG00010013362	ENSGALG00010013362	case	control	-1.861188141	0.808041898	-2.303331232	0.021260214	0.286336139	down_in_case
CRA004775	ENSGALG00010013363	HS3ST5	case	control	0.325091689	0.368974804	0.881067447	0.378281315	0.799601151	up_in_case
CRA004775	ENSGALG00010013365	CEP85	case	control	0.142255785	0.504318081	0.282075521	0.777885603	0.947617375	up_in_case
CRA004775	ENSGALG00010013367	NOL4L	case	control	0.251704272	0.20279743	1.241161054	0.214546259	0.683562661	up_in_case
CRA004775	ENSGALG00010013369	MTMR8	case	control	-0.074888204	0.342650031	-0.218555952	0.826995969	0.961897808	down_in_case
CRA004775	ENSGALG00010013370	CNKSR1	case	control	-0.537445059	0.68350277	-0.786309994	0.431685899	0.825262449	down_in_case
CRA004775	ENSGALG00010013371	FAT1	case	control	0.442386897	0.364395685	1.214028913	0.224736683	0.692859731	up_in_case
CRA004775	ENSGALG00010013372	SPRY4	case	control	-0.266322795	0.38773644	-0.686865529	0.492167471	0.855746361	down_in_case
CRA004775	ENSGALG00010013373	ENSGALG00010013373	case	control	0.623560216	0.867020186	0.719199191	0.472018198	0.845425322	up_in_case
CRA004775	ENSGALG00010013374	RMDN1	case	control	-0.571871555	0.406788623	-1.405819931	0.159777602	0.624907945	down_in_case
CRA004775	ENSGALG00010013375	PSMG1	case	control	-0.119866652	0.205020174	-0.584657838	0.558777809	0.880754991	down_in_case
CRA004775	ENSGALG00010013376	ARHGAP8	case	control	-0.105548211	0.431490172	-0.244613245	0.806755907	0.955397873	down_in_case
CRA004775	ENSGALG00010013378	SH3RF3	case	control	0.236611552	0.318213481	0.743562314	0.45714131	0.839688534	up_in_case
CRA004775	ENSGALG00010013379	ZNF593	case	control	-0.464127297	0.230161666	-2.01652736	0.04374486	0.391981676	down_in_case
CRA004775	ENSGALG00010013380	PHGDH	case	control	-0.549891459	0.487223069	-1.128623609	0.259056647	0.720037471	down_in_case
CRA004775	ENSGALG00010013381	DENND2A	case	control	0.198653579	0.236463202	0.840103565	0.400850321	0.811868062	up_in_case
CRA004775	ENSGALG00010013382	ASB12	case	control	-0.566215441	0.788485217	-0.718105335	0.472692341	0.845425322	down_in_case
CRA004775	ENSGALG00010013383	SEPTIN10	case	control	0.505148357	0.285381272	1.770082365	0.076713421	0.487695909	up_in_case
CRA004775	ENSGALG00010013385	PGRMC1	case	control	-0.108847058	0.231526773	-0.470127305	0.638264067	0.91099914	down_in_case
CRA004775	ENSGALG00010013386	ITGAV	case	control	0.292687608	0.377852848	0.774607387	0.438571691	0.828178978	up_in_case
CRA004775	ENSGALG00010013387	NDST1	case	control	-0.426779325	0.180879837	-2.359463228	0.018301395	0.269196555	down_in_case
CRA004775	ENSGALG00010013388	SOWAHC	case	control	-0.371086787	0.452124402	-0.820762572	0.411781522	0.817006008	down_in_case
CRA004775	ENSGALG00010013389	NAP1L1	case	control	-0.057365722	0.157201032	-0.364919501	0.715171497	0.933884548	down_in_case
CRA004775	ENSGALG00010013390	SLC16A2	case	control	0.418866644	0.325124001	1.288328893	0.1976315	0.663646093	up_in_case
CRA004775	ENSGALG00010013391	HMGN1	case	control	0.477003341	0.277210805	1.720724205	0.085300882	0.506234134	up_in_case
CRA004775	ENSGALG00010013392	HDAC2	case	control	0.053174677	0.201452118	0.2639569	0.79181313	0.951284723	up_in_case
CRA004775	ENSGALG00010013393	ADCK2	case	control	-0.326768212	0.301374979	-1.084257933	0.278250451	0.733841198	down_in_case
CRA004775	ENSGALG00010013394	DNMT3B	case	control	1.732949354	0.624008709	2.777123667	0.005484231	0.150521488	up_in_case
CRA004775	ENSGALG00010013396	FYB1	case	control	-0.472938446	0.454284439	-1.041062395	0.297846591	0.750344577	down_in_case
CRA004775	ENSGALG00010013397	MATN1	case	control	-2.324700923	0.611251999	-3.803179258	0.000142851	0.02543383	down_in_case
CRA004775	ENSGALG00010013398	CHAMP1	case	control	0.410566377	0.164586094	2.49453867	0.012612098	0.226502393	up_in_case
CRA004775	ENSGALG00010013399	FGF1	case	control	0.11508054	0.484002669	0.2377684	0.812060729	0.957585764	up_in_case
CRA004775	ENSGALG00010013400	BBS10	case	control	-0.586380421	0.306163798	-1.915250675	0.055460543	0.431364203	down_in_case
CRA004775	ENSGALG00010013402	MARCKS	case	control	0.187990679	0.311829675	0.60286334	0.546599606	0.877099134	up_in_case
CRA004775	ENSGALG00010013403	CPNE3	case	control	0.081505349	0.241709674	0.337203505	0.735963495	0.937385622	up_in_case
CRA004775	ENSGALG00010013404	ENSGALG00010013404	case	control	0.649175356	0.550168343	1.179957669	0.238017051	0.70473329	up_in_case
CRA004775	ENSGALG00010013405	RPS14	case	control	-0.344478871	0.24838266	-1.386887758	0.165475991	0.630194031	down_in_case
CRA004775	ENSGALG00010013406	ENSGALG00010013406	case	control	-0.828031651	0.711968545	-1.163017183	0.244822531	0.711767728	down_in_case
CRA004775	ENSGALG00010013407	ZC3H15	case	control	0.626079211	0.273605796	2.288252733	0.022122807	0.290827737	up_in_case
CRA004775	ENSGALG00010013408	RLIM	case	control	0.175689503	0.144108111	1.219150692	0.222787003	0.690453927	up_in_case
CRA004775	ENSGALG00010013409	DAB2	case	control	-0.160134557	0.272595737	-0.587443366	0.556905976	0.880409028	down_in_case
CRA004775	ENSGALG00010013410	KIAA0825	case	control	0.315747836	0.401992852	0.785456343	0.432186057	0.825415344	up_in_case
CRA004775	ENSGALG00010013411	ENSGALG00010013411	case	control	2.883527808	0.855919348	3.368924673	0.00075462	0.057648813	up_in_case
CRA004775	ENSGALG00010013412	COMMD7	case	control	-0.280014649	0.239131525	-1.170965012	0.241612841	0.708952282	down_in_case
CRA004775	ENSGALG00010013413	WRB	case	control	-0.265341529	0.164802613	-1.610056563	0.107385509	0.553800541	down_in_case
CRA004775	ENSGALG00010013414	LAPTM5	case	control	-0.527398408	0.434597031	-1.21353431	0.224925605	0.692859731	down_in_case
CRA004775	ENSGALG00010013416	NDUFB2	case	control	-0.010977281	0.341098939	-0.032182101	0.97432683	0.994947809	down_in_case
CRA004775	ENSGALG00010013417	AKR1B1	case	control	-0.338514802	0.326397202	-1.037125318	0.299677457	0.752512244	down_in_case
CRA004775	ENSGALG00010013419	ENSGALG00010013419	case	control	0.510083969	0.535455855	0.952616289	0.340784521	0.780875343	up_in_case
CRA004775	ENSGALG00010013420	ENSGALG00010013420	case	control	0.193868706	1.148618241	0.168784283	0.865966321	0.972629493	up_in_case
CRA004775	ENSGALG00010013425	ENSGALG00010013425	case	control	-0.047309586	0.383810024	-0.123263028	0.901898817	0.979157637	down_in_case
CRA004775	ENSGALG00010013427	NEXMIF	case	control	0.393299502	0.781108654	0.50351446	0.614602616	0.902639483	up_in_case
CRA004775	ENSGALG00010013428	BPGM	case	control	0.495270976	0.455120518	1.088219398	0.276498263	0.731178388	up_in_case
CRA004775	ENSGALG00010013429	PDE4DIP	case	control	0.127998608	0.396737769	0.322627736	0.746977186	0.940371993	up_in_case
CRA004775	ENSGALG00010013431	TMEM175	case	control	-0.244241369	0.233909867	-1.044168731	0.296407331	0.748897794	down_in_case
CRA004775	ENSGALG00010013435	SDC3	case	control	0.494767864	0.305983523	1.616975512	0.105883567	0.55052342	up_in_case

CRA004775	ENSGALG00010013436	NPAS2	case	control	0.973615493	0.327270334	2.974957983	0.002930287	0.113497569	up_in_case
CRA004775	ENSGALG00010013437	ENSGALG00010013437	case	control	-0.257197708	0.28918153	-0.889398808	0.373788786	0.797265331	down_in_case
CRA004775	ENSGALG00010013438	RPL37	case	control	-0.241984476	0.280339629	-0.863183264	0.388036714	0.804270424	down_in_case
CRA004775	ENSGALG00010013440	EEA1	case	control	0.391408333	0.248847415	1.572884865	0.11574548	0.566086328	up_in_case
CRA004775	ENSGALG00010013442	ENSGALG00010013442	case	control	0.050452404	0.326626865	0.154464955	0.877243141	0.974479909	up_in_case
CRA004775	ENSGALG00010013444	OSBPL8	case	control	-0.239644239	0.427268045	-0.560875642	0.574882316	0.887328177	down_in_case
CRA004775	ENSGALG00010013446	ATP5I	case	control	0.0827318	0.363910411	0.22734112	0.820158501	0.960565501	up_in_case
CRA004775	ENSGALG00010013449	LCA5L	case	control	0.588650688	0.462985086	1.271424731	0.203577589	0.673741378	up_in_case
CRA004775	ENSGALG00010013450	CD74	case	control	-0.714126316	0.245082147	-2.913824307	0.003570308	0.125102734	down_in_case
CRA004775	ENSGALG00010013451	PRKAB2	case	control	-0.285691694	0.475743025	-0.600516831	0.548161847	0.877403511	down_in_case
CRA004775	ENSGALG00010013452	LSS	case	control	-0.220112861	0.366426713	-0.600700912	0.548039212	0.877369241	down_in_case
CRA004775	ENSGALG00010013453	PUM1	case	control	0.265775774	0.183285153	1.450067118	0.147039804	0.610295079	up_in_case
CRA004775	ENSGALG00010013457	SH3BGR	case	control	0.126021081	0.682806537	0.184563377	0.853571513	0.970050055	up_in_case
CRA004775	ENSGALG00010013458	LAMA4	case	control	0.495639709	0.419359002	1.181898343	0.23724604	0.703879272	up_in_case
CRA004775	ENSGALG00010013459	ATP10D	case	control	-0.100053919	0.460308819	-0.217362593	0.827925781	0.962251207	down_in_case
CRA004775	ENSGALG00010013461	S100B	case	control	2.085050403	0.740325398	2.816397234	0.004856558	0.142429514	up_in_case
CRA004775	ENSGALG00010013463	ENSGALG00010013463	case	control	-0.46383753	0.441864139	-1.04972884	0.2938428	0.746438942	down_in_case
CRA004775	ENSGALG00010013464	ABCB7	case	control	-0.469145529	0.308267272	-1.521879133	0.128039369	0.5821156	down_in_case
CRA004775	ENSGALG00010013465	CALD1	case	control	0.349453333	0.258187386	1.35348724	0.175900024	0.641310011	up_in_case
CRA004775	ENSGALG00010013466	BRAF	case	control	-0.103063106	0.376814888	-0.273511238	0.784460255	0.950165405	down_in_case
CRA004775	ENSGALG00010013468	DEPDC1B	case	control	0.324132449	0.728556221	0.444896961	0.656394219	0.91710603	up_in_case
CRA004775	ENSGALG00010013470	RPL31	case	control	-0.23518575	0.270327454	-0.870003198	0.384298656	0.803182776	down_in_case
CRA004775	ENSGALG00010013472	ENSGALG00010013472	case	control	-0.073276883	0.282329265	-0.259544059	0.795215492	0.952360933	down_in_case
CRA004775	ENSGALG00010013476	ZDHHC17	case	control	0.237312872	0.273431269	0.867906854	0.385445326	0.803446489	up_in_case
CRA004775	ENSGALG00010013477	RFC1	case	control	0.119885739	0.203432901	0.589313421	0.555651043	0.87981923	up_in_case
CRA004775	ENSGALG00010013480	TBC1D8	case	control	0.391865092	0.342914887	1.14274739	0.253143488	0.717579076	up_in_case
CRA004775	ENSGALG00010013482	ENSGALG00010013482	case	control	-0.58470027	0.439515453	-1.330329265	0.183409809	0.647806087	down_in_case
CRA004775	ENSGALG00010013483	ENSGALG00010013483	case	control	0.3683846	0.372043754	0.99016472	0.322093613	0.767876089	up_in_case
CRA004775	ENSGALG00010013484	TCOF1	case	control	-0.014164427	0.145312684	-0.0974755	0.922348789	0.9830428	down_in_case
CRA004775	ENSGALG00010013486	PLPP6	case	control	-0.821710794	0.437115071	-1.879850066	0.060128515	0.445397937	down_in_case
CRA004775	ENSGALG00010013487	ENSGALG00010013487	case	control	0.361145031	0.263941828	1.368275101	0.171225981	0.635671833	up_in_case
CRA004775	ENSGALG00010013488	UPRT	case	control	-0.013477678	0.197673465	-0.068181523	0.945641135	0.988886825	down_in_case
CRA004775	ENSGALG00010013491	CNOT11	case	control	0.081205265	0.249115232	0.325974707	0.744443491	0.939218065	up_in_case
CRA004775	ENSGALG00010013492	B3GALT5	case	control	0.630626755	0.877408886	0.718737598	0.472302613	0.845425322	up_in_case
CRA004775	ENSGALG00010013493	ENSGALG00010013493	case	control	1.213567557	0.349090386	3.476370606	0.000508249	0.046064555	up_in_case
CRA004775	ENSGALG00010013494	ASB1	case	control	0.198705454	0.376680123	0.527517759	0.597834088	0.896490702	up_in_case
CRA004775	ENSGALG00010013497	FAM172A	case	control	-0.144696844	0.279838035	-0.517073543	0.605104822	0.899740096	down_in_case
CRA004775	ENSGALG00010013498	KDR	case	control	0.184983543	0.429314703	0.430880987	0.666554909	0.919168997	up_in_case
CRA004775	ENSGALG00010013501	AKR1B10	case	control	-1.017647266	0.509765599	-1.996304316	0.045900809	0.398725217	down_in_case
CRA004775	ENSGALG00010013502	PAFAH2	case	control	-0.067982696	0.263640571	-0.257861285	0.796513965	0.952835572	down_in_case
CRA004775	ENSGALG00010013506	RNF149	case	control	0.27586692	0.25917126	1.064419411	0.287138744	0.741909972	up_in_case
CRA004775	ENSGALG00010013508	TUBE1	case	control	-0.854611449	0.4621556	-1.849185533	0.064431027	0.456696419	down_in_case
CRA004775	ENSGALG00010013509	ENSGALG00010013509	case	control	-0.035896885	0.486631122	-0.073766109	0.941196494	0.987516633	down_in_case
CRA004775	ENSGALG00010013510	MRPS33	case	control	-0.165097107	0.226263807	-0.729666439	0.4655941	0.843383332	down_in_case
CRA004775	ENSGALG00010013511	FAM78B	case	control	-0.286882731	0.237870884	-1.206043909	0.22780056	0.694718454	down_in_case
CRA004775	ENSGALG00010013512	ENSGALG00010013512	case	control	0.802511099	1.32319709	0.606494002	0.544186764	0.875729696	up_in_case
CRA004775	ENSGALG00010013514	LMBRD2	case	control	0.105242478	0.36349059	0.289532881	0.772173621	0.946296525	up_in_case
CRA004775	ENSGALG00010013515	RBP4L	case	control	0.09632918	0.605502165	0.159089736	0.873598175	0.974115968	up_in_case
CRA004775	ENSGALG00010013516	CSRP2	case	control	0.375066877	0.282096304	1.329570337	0.183659877	0.64798309	up_in_case
CRA004775	ENSGALG00010013517	STMN1	case	control	-0.098831365	0.453943609	-0.217717274	0.827649404	0.962208907	down_in_case
CRA004775	ENSGALG00010013520	CREG2	case	control	-0.202795193	0.347262402	-0.583982577	0.559232034	0.880918893	down_in_case
CRA004775	ENSGALG00010013523	TMEM178B	case	control	-0.250505859	0.528361112	-0.474118654	0.635415297	0.909837348	down_in_case
CRA004775	ENSGALG00010013524	ZDHHC15	case	control	-0.906478802	0.387468056	-2.339492991	0.019309934	0.274308782	down_in_case
CRA004775	ENSGALG00010013526	SIRPA	case	control	-1.286221367	0.587756204	-2.188358641	0.028643488	0.322057735	down_in_case
CRA004775	ENSGALG00010013527	ADARB1	case	control	-0.120314492	0.237544275	-0.506492913	0.612510663	0.901413878	down_in_case
CRA004775	ENSGALG00010013528	PRDX6	case	control	-0.293371135	0.350681372	-0.836574617	0.402831719	0.812204167	down_in_case
CRA004775	ENSGALG00010013531	ARSI	case	control	-0.920187102	0.70433453	-1.30646314	0.191395106	0.656959697	down_in_case
CRA004775	ENSGALG00010013532	WDR19	case	control	-0.334544429	0.282587214	-1.18386258	0.236467465	0.703419274	down_in_case
CRA004775	ENSGALG00010013533	SKP2	case	control	-0.215954583	0.178968152	-1.206664874	0.227561232	0.694718454	down_in_case
CRA004775	ENSGALG00010013538	E2F7	case	control	-0.078635652	0.667601839	-0.117788249	0.90623544	0.980044552	down_in_case
CRA004775	ENSGALG00010013539	NSFL1C	case	control	-0.363825758	0.26061488	-1.39602834	0.162705961	0.629181573	down_in_case
CRA004775	ENSGALG00010013541	GJA9	case	control	0.256621064	0.597647103	0.429385607	0.667642627	0.919546651	up_in_case
CRA004775	ENSGALG00010013544	ENSGALG00010013544	case	control	-0.514627487	0.40660806	-1.265659826	0.205634897	0.674045742	down_in_case
CRA004775	ENSGALG00010013546	IDUA	case	control	0.148678425	0.372959548	0.398644908	0.690154867	0.927867527	up_in_case
CRA004775	ENSGALG00010013549	PCP4	case	control	0.683321958	0.596407603	1.145729791	0.251906973	0.716658534	up_in_case
CRA004775	ENSGALG00010013550	KLHL5	case	control	0.05114126	0.189427346	0.26997823	0.787177002	0.95041797	up_in_case
CRA004775	ENSGALG00010013551	UBE2N	case	control	-0.036378483	0.206769087	-0.175937726	0.860342867	0.971325143	down_in_case
CRA004775	ENSGALG00010013552	FGF16	case	control	-0.159693647	0.697033926	-0.229104555	0.818787653	0.959941088	down_in_case
CRA004775	ENSGALG00010013553	TADA1	case	control	-0.117517336	0.223485369	-0.525839057	0.599000035	0.897206546	down_in_case
CRA004775	ENSGALG00010013554	ENSGALG00010013554	case	control	0.298096848	1.290095269	0.231065764	0.817263712	0.959812855	up_in_case
CRA004775	ENSGALG00010013556	RASL11B	case	control	0.78108153	0.343764682	2.272140133	0.023078048	0.295240109	up_in_case
CRA004775	ENSGALG00010013558	SNAPC3	case	control	-0.428345523	0.270164926	-1.585496422	0.112853589	0.562886277	down_in_case
CRA004775	ENSGALG00010013561	AGK	case	control	-0.387633193	0.249480584	-1.553760966	0.120241445	0.572133279	down_in_case
CRA004775	ENSGALG00010013562	RERGL	case	control	0.162852573	0.366660625	0.444150699	0.656933633	0.91710603	up_in_case
CRA004775	ENSGALG00010013563	FKBP1A	case	control	-0.099884455	0.269022179	-0.371287065	0.710423731	0.932234469	down_in_case
CRA004775	ENSGALG00010013566	ENSGALG00010013566	case	control	-1.426991218	0.928613862	-1.53668955	0.124369351	0.576634733	down_in_case
CRA004775	ENSGALG00010013569	ENSGALG00010013569	case	control	-0.555983117	0.936466516	-0.593703146	0.552710678	0.878345607	down_in_case
CRA004775	ENSGALG00010013572	DENND11	case	control	-0.233179804	0.329085443	-0.708569186	0.478591864	0.847099591	down_in_case
CRA004775	ENSGALG00010013573	ENSGALG00010013573	case	control	-0.322927866	0.597732915	-0.540254448	0.589021568	0.893412703	down_in_case
CRA004775	ENSGALG00010013574	NR2F1	case	control	0.382628621	0.362322067	1.05604559	0.290947387	0.74489248	up_in_case
CRA004775	ENSGALG00010013576	CAMK2A	case	control	-0.215295908	0.543384358	-0.396212928	0.691947955	0.927867527	down_in_case
CRA004775	ENSGALG00010013579	H2AC32	case	control	-1.040379056	0.312935722	-3.324577493	0.000885526	0.062654188	down_in_case
CRA004775	ENSGALG00010013580	RANBP3L	case	control	0.931282848	0.592858481	1.570834993	0.116220982	0.566086328	up_in_case
CRA004775	ENSGALG00010013582	MAP4K4	case	control	-0.216858607	0.391578463	-0.553806267	0.579711439	0.888438428	down_in_case
CRA004775	ENSGALG00010013583	FASLG	case	control	-0.266322527	0.443869389	-0.600002014	0.548504893	0.877623839	down_in_case
CRA004775	ENSGALG00010013587	SLC26A1	case	control	1.977008583	0.740946667	2.66821982	0.007625435	0.174466871	up_in_case
CRA004775	ENSGALG00010013592	H3C33	case	control	-0.45642739	0.341611585	-1.33610044	0.181516446	0.647378665	down_in_case
CRA004775	ENSGALG00010013595	WEE2	case	control	-1.27147959	1.261245307	-1.008114427	0.313399532	0.765098415	down_in_case
CRA004775	ENSGALG00010013596	SDCBP2	case	control	0.064542168	0.224744294	0.287180451	0.773974154	0.946296525	up_in_case
CRA004775	ENSGALG00010013598	PAQR7	case	control	-0.073391693	0.426307243	-0.172156805	0.863314255	0.971910432	down_in_case
CRA004775	ENSGALG00010013604	FYN	case	control	0.143128712	0.33648263	0.425367312	0.670568931	0.921535334	up_in_case
CRA004775	ENSGALG00010013609	PIGC	case	control	-0.319994642	0.489773823	-0.653351868	0.513529461	0.863903061	down_in_case
CRA004775	ENSGALG00010013612	TWIST2	case	control	-0.181670985	0.492989425	-0.368508888	0.712493819	0.932981748	down_in_case
CRA004775	ENSGALG00010013614	ATRX	case	control	0.466157507	0.338786036	1.375964345	0.168832672	0.632337016	up_in_case
CRA004775	ENSGALG00010013617	PIK3C2G	case	control	0.379425077	0.342556406	1.107628029	0.268022493	0.727528758	up_in_case
CRA004775	ENSGALG00010013619	IL1R2	case	control	0.404103435	0.434084177	0.930933344	0.351888045	0.786426886	up_in_case
CRA004775	ENSGALG00010013621	PDGFRA	case	control	0.195882395	0.310672011	0.630511885	0.528359729	0.868929328	up_in_case
CRA004775	ENSGALG00010013624	SST1	case	control	3.032056385	0.788928915	3.843256759	0.000121412	0.023828766	up_in_case
CRA004775	ENSGALG00010013625	SSBP1	case	control	-0.113970589	0.299247557	-0.380857206	0.703309208	0.930068722	down_in_case
CRA004775	ENSGALG00010013627	MYCBP	case	control	-0.039161979	0.264432319	-0.148098308	0.882265185	0.975346523	down_in_case
CRA004775	ENSGALG00010013635	BCL6	case	control	0.046445367	0.339270216	0.136897861	0.891111529	0.977634805	up_in_case
CRA004775	ENSGALG00010013638	COMMD8	case	control	0.065468962	0.222696678	0.293982661	0.768771153	0.945220496	up_in_case
CRA004775	ENSGALG00010013639	ENSGALG00010013639	case	control	-0.029190885	0.552125968	-0.052869974	0.957835508	0.991431439	down_in_case
CRA004775	ENSGALG00010013640	SNPH	case	control	0.811023451	0.860293356	0.942728949	0.345819562	0.784552667	up_in_case
CRA004775	ENSGALG00010013645	RRAGC	case	control	-0.229077895	0.227519707	-1.006848584	0.314007543	0.765098415	down_in_case
CRA004775	ENSGALG00010013646	CLDN1	case	control	1.01403164	0.465889645	2.176548998	0.029514234	0.326771523	up_in_case
CRA004775	ENSGALG00010013648	NAV3	case	control	-0.137862053	0.353214843	-0.390306512	0.696309909	0.929092636	down_in_case
CRA004775	ENSGALG00010013650	BACE2	case	control	-0.528831173	0.422847587	-1.250642525	0.211064929	0.681585086	down_in_case
CRA004775	ENSGALG00010013651	ENSGALG00010013651	case	control	-0.28469563	0.157656217	-1.805800219	0.070949551	0.469911807	down_in_case
CRA004775	ENSGALG00010013657	MAGT1	case	control	-0.513935198	0.24906413	-2.063465335	0.039068438	0.371740574	down_in_case
CRA004775	ENSGALG00010013660	CDX1	case	control	2.723854684	0.860404816	3.165782703	0.001546663	0.080693373	up_in_case
CRA004775	ENSGALG00010013663	OPA1	case	control	0.071898417	0.188601531	0.38121863	0.703041026	0.930068722	up_in_case
CRA004775	ENSGALG00010013664	REV3L	case	control	0.554967391	0.226418098	2.451073466	0.014243088	0.239125488	up_in_case
CRA004775	ENSGALG00010013666	COX7B	case	control	0.179186975	0.323049328	0.55467373	0.579117848	0.888378369	up_in_case
CRA004775	ENSGALG00010013667	SOWAHA	case	control	-0.285439185	0.58591972	-0.487164325	0.626141884	0.907183337	down_in_case
CRA004775	ENSGALG00010013669	NPR3	case	control	0.055040051	0.442614262	0.124352186	0.901036429	0.979157637	up_in_case
CRA004775	ENSGALG00010013672	ENSGALG00010013672	case	control	1.600493773	0.569256068	2.811553293	0.004930292	0.142429514	up_in_case
CRA004775	ENSGALG00010013675	CHRNA6	case	control	0.293146198	0.741556326	0.395312113	0.692612561	0.927867527	up_in_case
CRA004775	ENSGALG00010013678	ATP7A	case	control	0.139372566	0.206840636	0.673816173	0.500428192	0.859743636	up_in_case
CRA004775	ENSGALG00010013679	MFSD4B	case	control	0.016771627	0.306697138	0.054684656	0.956389694	0.9909743	up_in_case
CRA004775	ENSGALG00010013686	UQCRQ	case	control	0.313298194	0.361324437	0.867082772	0.385896659	0.803541109	up_in_case
CRA004775	ENSGALG00010013690	CREB3L2	case	control	-0.304878292	0.389428615	-0.782886208	0.433693937	0.82588635	down_in_case
CRA004775	ENSGALG00010013696	CETN3	case	control	-0.085860752	0.329785565	-0.260353276	0.794591281	0.952110901	down_in_case
CRA004775	ENSGALG00010013697	HDAC4	case	control	0.215231795	0.285785511	0.753123537	0.451375682	0.836054284	up_in_case
CRA004775	ENSGALG00010013704	LEAP2	case	control	1.853641605	0.694854135	2.667670107	0.007637921	0.174466871	up_in_case
CRA004775	ENSGALG00010013709	PSMF1	case	control	-0.484917606	0.289182966	-1.676853976	0.09357104	0.525355698	down_in_case

CRA004775	ENSGALG00010013715	IL1R1	case	control	0.172218079	0.33860871	0.508604988	0.611029132	0.901413878	up_in_case
CRA004775	ENSGALG00010013719	NCDN	case	control	-0.576388688	0.238565733	-2.416058167	0.015689555	0.250548106	down_in_case
CRA004775	ENSGALG00010013720	SLC16A10	case	control	0.649672522	0.446066577	1.456447436	0.145268971	0.608365494	up_in_case
CRA004775	ENSGALG00010013732	FNTA	case	control	-0.422487155	0.263676075	-1.602296133	0.10909014	0.556276325	down_in_case
CRA004775	ENSGALG00010013737	AFF4	case	control	0.352598874	0.315658992	1.117024647	0.263983787	0.723981064	up_in_case
CRA004775	ENSGALG00010013740	GNPDA2	case	control	-0.524948968	0.279659735	-1.877098852	0.060504547	0.445397937	down_in_case
CRA004775	ENSGALG00010013744	ENSGALG00010013744	case	control	-0.057384863	0.52923112	-0.108430629	0.913654106	0.980803023	down_in_case
CRA004775	ENSGALG00010013746	ENSGALG00010013746	case	control	0.455834289	0.263960787	1.726901535	0.084185349	0.503318579	up_in_case
CRA004775	ENSGALG00010013749	NELL2	case	control	0.985567669	0.539752757	1.825961343	0.067856085	0.463543284	up_in_case
CRA004775	ENSGALG00010013751	FUT10	case	control	-0.064955672	0.329148833	-0.197344379	0.843558053	0.966478468	down_in_case
CRA004775	ENSGALG00010013753	PLEKHA5	case	control	0.723880676	0.353246275	2.049223807	0.040440235	0.376363211	up_in_case
CRA004775	ENSGALG00010013755	ANGPT4	case	control	-0.266791473	0.279907872	-0.953140298	0.34051899	0.780875343	down_in_case
CRA004775	ENSGALG00010013757	DGKI	case	control	-0.70241361	1.064531233	-0.659833726	0.509360538	0.862971569	down_in_case
CRA004775	ENSGALG00010013760	RPF2	case	control	-0.330757866	0.205028787	-1.61322647	0.106695315	0.552875967	down_in_case
CRA004775	ENSGALG00010013765	PRLL	case	control	0.852699847	1.159212731	0.735585303			up_in_case
CRA004775	ENSGALG00010013766	DNM3	case	control	-0.055795896	0.264508748	-0.210941589	0.832932851	0.964019688	down_in_case
CRA004775	ENSGALG00010013767	PGK2	case	control	0.287658279	0.570439322	0.504274981	0.614068156	0.902363507	up_in_case
CRA004775	ENSGALG00010013768	FAM110A	case	control	-1.012019633	0.454554041	-2.226401137	0.025987324	0.308767914	down_in_case
CRA004775	ENSGALG00010013771	PELO	case	control	-0.120641824	0.244861053	-0.492695028	0.622228091	0.906040637	down_in_case
CRA004775	ENSGALG00010013772	KIAA0319L	case	control	-0.188613751	0.257334503	-0.732951657	0.463587915	0.842456502	down_in_case
CRA004775	ENSGALG00010013775	DBX2	case	control	-0.445685292	0.482468525	-0.923760347	0.355611105	0.788778426	down_in_case
CRA004775	ENSGALG00010013777	CPLANE1	case	control	0.23753074	0.257702717	0.921723847	0.356672638	0.78941018	up_in_case
CRA004775	ENSGALG00010013778	ENSGALG00010013778	case	control	0.745443062	1.414879157	0.526859879			up_in_case
CRA004775	ENSGALG00010013779	SLC52A3	case	control	-0.716686765	0.383344452	-1.869563422	0.061544469	0.446401278	down_in_case
CRA004775	ENSGALG00010013783	NDUFA10	case	control	0.188234144	0.256280842	0.734483867	0.462653889	0.842456502	up_in_case
CRA004775	ENSGALG00010013784	ANO6	case	control	0.498656646	0.305445262	1.632556495	0.102562307	0.542853845	up_in_case
CRA004775	ENSGALG00010013787	ZCCHC10	case	control	-0.017520919	0.224636718	-0.077996684	0.937830691	0.986707289	down_in_case
CRA004775	ENSGALG00010013791	AEBP2	case	control	0.125067537	0.195377243	0.640133598	0.522085747	0.866416245	up_in_case
CRA004775	ENSGALG00010013792	FGF10	case	control	0.014721446	0.577349198	0.02549834	0.979657473	0.996764329	up_in_case
CRA004775	ENSGALG00010013793	METTL13	case	control	-0.635195795	0.269424452	-2.357602626	0.01839337	0.269466431	down_in_case
CRA004775	ENSGALG00010013794	GTF3C6	case	control	-0.084399236	0.39993156	-0.211034197	0.832860587	0.964019688	down_in_case
CRA004775	ENSGALG00010013797	IL1RL1	case	control	0.258424197	0.438502183	0.589333889	0.555637316	0.87981923	up_in_case
CRA004775	ENSGALG00010013802	PYROXD1	case	control	-0.240825462	0.266839803	-0.902509518	0.366786268	0.793822622	down_in_case
CRA004775	ENSGALG00010013805	HSPA4	case	control	0.680344107	0.352325526	1.931009978	0.053481818	0.425885384	up_in_case
CRA004775	ENSGALG00010013808	PLAAT1	case	control	-0.600261212	0.323218334	-1.857138498	0.063291452	0.452349667	down_in_case
CRA004775	ENSGALG00010013809	MX1	case	control	-0.745332704	0.576661107	-1.292496919	0.196185112	0.662184948	down_in_case
CRA004775	ENSGALG00010013814	AMD1	case	control	0.44060072	0.335987387	1.311360893	0.189735865	0.655361714	up_in_case
CRA004775	ENSGALG00010013815	SRXN1	case	control	-0.413960113	0.361936384	-1.143737217	0.252732634	0.717153621	down_in_case
CRA004775	ENSGALG00010013820	MASP1	case	control	0.749381524	0.712819156	1.051292628	0.293124212	0.746438942	up_in_case
CRA004775	ENSGALG00010013823	GUF1	case	control	0.390546764	0.2225989	1.754486495	0.079347182	0.491399489	up_in_case
CRA004775	ENSGALG00010013828	PTN	case	control	0.185639972	0.755749811	0.245636809	0.805963393	0.955171181	up_in_case
CRA004775	ENSGALG00010013831	TCERG1	case	control	0.071661602	0.193165515	0.370985485	0.710648343	0.932234469	up_in_case
CRA004775	ENSGALG00010013833	TMPRSS2	case	control	1.943411511	0.793855104	2.448068295	0.014362443	0.23945185	up_in_case
CRA004775	ENSGALG00010013836	IL18R1	case	control	-0.324165961	0.511875006	-0.633291247	0.526543468	0.86830001	down_in_case
CRA004775	ENSGALG00010013837	IFI27L2	case	control	0.374537296	0.177418088	2.111043465	0.034768576	0.354006537	up_in_case
CRA004775	ENSGALG00010013842	CDK19	case	control	0.225381769	0.38316812	0.588205952	0.556394063	0.87981923	up_in_case
CRA004775	ENSGALG00010013846	YIPF7	case	control	0.160797847	0.735142279	0.218730239	0.826860192	0.961878235	up_in_case
CRA004775	ENSGALG00010013848	ITPA	case	control	-0.444764246	0.240378259	-1.850268189	0.064274905	0.456529144	down_in_case
CRA004775	ENSGALG00010013857	IL18RAP	case	control	-0.593382726	0.788027576	-0.752997413	0.451451468	0.836082039	down_in_case
CRA004775	ENSGALG00010013859	MYOC	case	control	-0.349943604	0.422605593	-0.828061934	0.407635426	0.814105656	down_in_case
CRA004775	ENSGALG00010013861	TCF15	case	control	0.227465813	0.5236034	0.434423866	0.66398067	0.918295135	up_in_case
CRA004775	ENSGALG00010013866	KCTD8	case	control	0.242940102	0.565099553	0.429906731	0.667263489	0.919300695	up_in_case
CRA004775	ENSGALG00010013867	RECQL	case	control	-0.216014479	0.279483149	-0.772906986	0.43957743	0.828842585	down_in_case
CRA004775	ENSGALG00010013871	ENSGALG00010013871	case	control	-0.202263205	0.259801385	-0.778530125	0.436256554	0.82694408	down_in_case
CRA004775	ENSGALG00010013872	GLS2	case	control	0.597604152	0.519254757	1.150888162	0.249778249	0.714767563	up_in_case
CRA004775	ENSGALG00010013878	ARID2	case	control	0.42743718	0.350481758	1.219570407	0.22262777	0.690271583	up_in_case
CRA004775	ENSGALG00010013880	ZMYM4	case	control	0.078298206	0.220424572	0.355215415	0.722428217	0.935185555	up_in_case
CRA004775	ENSGALG00010013889	ENSGALG00010013889	case	control	-0.395909606	0.27322914	-1.449002133	0.147336987	0.610443055	down_in_case
CRA004775	ENSGALG00010013890	SFPQ	case	control	-0.302185103	0.179810008	-1.680580004	0.092844523	0.524612711	down_in_case
CRA004775	ENSGALG00010013893	CSNK2A1	case	control	-0.085417555	0.165837336	-0.515068304	0.60650529	0.900898526	down_in_case
CRA004775	ENSGALG00010013897	AMOT	case	control	0.093991315	0.248788977	0.377795335	0.705582639	0.931021366	up_in_case
CRA004775	ENSGALG00010013898	EMB	case	control	-0.068379778	0.567219734	-0.120552538	0.904045464	0.979540372	down_in_case
CRA004775	ENSGALG00010013899	WNT5B	case	control	-0.195080076	0.391976683	-0.497682859	0.618707592	0.904224476	down_in_case
CRA004775	ENSGALG00010013901	NFXL1	case	control	-0.002072857	0.338745938	-0.006119208	0.995117609	0.999001196	down_in_case
CRA004775	ENSGALG00010013908	TBC1D20	case	control	-0.102083067	0.213720839	-0.477646765	0.63290164	0.909723668	down_in_case
CRA004775	ENSGALG00010013910	ADIPOR2	case	control	0.375740579	0.310413224	1.210452874	0.226105163	0.694166578	up_in_case
CRA004775	ENSGALG00010013911	TNIK	case	control	-0.231178291	0.223550645	-1.034120437	0.301079863	0.753545086	down_in_case
CRA004775	ENSGALG00010013914	NRG1	case	control	0.544258991	0.663237791	0.820609136	0.411868942	0.817006008	up_in_case
CRA004775	ENSGALG00010013915	ENSGALG00010013915	case	control	-0.767879043	0.448443498	-1.712320608	0.086837601	0.509281038	down_in_case
CRA004775	ENSGALG00010013921	PPP2R2B	case	control	0.294944294	0.242135344	1.218096826	0.22318718	0.690760052	up_in_case
CRA004775	ENSGALG00010013928	CCNO	case	control	-0.626757372	0.592181402	-1.058387463	0.289878833	0.74395768	down_in_case
CRA004775	ENSGALG00010013930	SLC22A16	case	control	-0.758183665	0.673281558	-1.126101934	0.260122375	0.720867147	down_in_case
CRA004775	ENSGALG00010013934	STAT1	case	control	-0.429312337	0.345923884	-1.241060121	0.21458354	0.683562661	down_in_case
CRA004775	ENSGALG00010013936	DLGAP3	case	control	-0.669354201	0.69876281	-0.957913317	0.338106471	0.778777567	down_in_case
CRA004775	ENSGALG00010013938	ENSGALG00010013938	case	control	0.164826728	0.590699726	0.279036404	0.780216887	0.94835413	up_in_case
CRA004775	ENSGALG00010013940	UPF3A	case	control	-1.404355165	0.684819815	-2.050692948	0.040296858	0.376363211	down_in_case
CRA004775	ENSGALG00010013942	CACNA2D4	case	control	2.202458336	0.989269519	2.226348123	0.025990872	0.308767914	up_in_case
CRA004775	ENSGALG00010013944	METTL24	case	control	1.363840619	0.500384845	2.725583387	0.006418796	0.15932286	up_in_case
CRA004775	ENSGALG00010013945	SCAF11	case	control	0.266355459	0.180674756	1.474226195	0.140420699	0.600007375	up_in_case
CRA004775	ENSGALG00010013947	ENSGALG00010013947	case	control	0.034820671	0.373480409	0.093232926	0.925718517	0.98417264	up_in_case
CRA004775	ENSGALG00010013949	PRRC2C	case	control	0.281635015	0.235670253	1.195038454	0.232072006	0.698952825	up_in_case
CRA004775	ENSGALG00010013953	DHX29	case	control	0.35304216	0.361398333	0.976878219	0.328629446	0.77220987	up_in_case
CRA004775	ENSGALG00010013954	LPL	case	control	1.067057051	0.46319943	2.303666591	0.021241367	0.286336139	up_in_case
CRA004775	ENSGALG00010013958	TUSC3	case	control	-0.362380943	0.487934408	-0.742683722	0.457673187	0.839704957	down_in_case
CRA004775	ENSGALG00010013959	STK32A	case	control	0.566748726	0.729561956	0.776834265	0.437256558	0.827286039	up_in_case
CRA004775	ENSGALG00010013964	SLC38A1	case	control	-0.44723597	0.458409785	-0.975624834	0.329250413	0.77237154	down_in_case
CRA004775	ENSGALG00010013965	SLC2A2	case	control	2.242160089	0.849670665	2.638857833	0.008318586	0.183215969	up_in_case
CRA004775	ENSGALG00010013967	MLLT3	case	control	0.737231983	0.373083198	1.976052491	0.048148832	0.407414924	up_in_case
CRA004775	ENSGALG00010013971	ENSGALG00010013971	case	control	-0.047848371	0.369795069	-0.129391587	0.897047804	0.978790377	down_in_case
CRA004775	ENSGALG00010013973	ENSGALG00010013973	case	control	0.453445271	0.443337876	1.022798403	0.306403173	0.7585475	up_in_case
CRA004775	ENSGALG00010013976	CDC40	case	control	-0.221377384	0.216350174	-1.023236451	0.306196062	0.7585475	down_in_case
CRA004775	ENSGALG00010013977	SLC38A2	case	control	0.208072902	0.367540417	0.566122506	0.571310505	0.885321726	up_in_case
CRA004775	ENSGALG00010013980	FMO4	case	control	-0.860013126	0.894338564	-0.961619191	0.336240928	0.776900397	down_in_case
CRA004775	ENSGALG00010013981	RLF	case	control	0.248535633	0.212759344	1.168153783	0.242744739	0.709690398	up_in_case
CRA004775	ENSGALG00010013989	ENSGALG00010013989	case	control	-0.588716986	0.255269941	-2.30625268	0.021096518	0.285392234	down_in_case
CRA004775	ENSGALG00010013995	REM1	case	control	0.149981072	0.36484349	0.411083317	0.681011442	0.923896221	up_in_case
CRA004775	ENSGALG00010014000	STAT4	case	control	-0.164854651	0.485469058	-0.339578082	0.734174286	0.936819725	down_in_case
CRA004775	ENSGALG00010014001	CDC16	case	control	-0.179546857	0.218419699	-0.82202685	0.411061616	0.816600445	down_in_case
CRA004775	ENSGALG00010014002	ZDHHC18	case	control	-0.01109216	0.254638554	-0.043560411	0.965254809	0.993058423	down_in_case
CRA004775	ENSGALG00010014010	EIF5A2	case	control	0.017236634	0.32402593	0.053195231	0.957576355	0.991431439	up_in_case
CRA004775	ENSGALG00010014014	PARP8	case	control	-0.333536771	0.472533068	-0.70584853	0.480282339	0.848235607	down_in_case
CRA004775	ENSGALG00010014018	DPYSL3	case	control	-0.227837042	0.393349508	-0.579222898	0.562438782	0.881532201	down_in_case
CRA004775	ENSGALG00010014019	IRAG2	case	control	0.344451498	0.379493472	0.907661194	0.364057252	0.793608387	up_in_case
CRA004775	ENSGALG00010014020	PDXP	case	control	-0.472477985	0.1759427	-2.685408293	0.007244125	0.169989702	down_in_case
CRA004775	ENSGALG00010014026	SH3BP1	case	control	-0.675557369	0.322058779	-2.097621346	0.035938608	0.359151015	down_in_case
CRA004775	ENSGALG00010014030	WASF1	case	control	0.056741278	0.377363886	0.150362237	0.880478833	0.974996282	up_in_case
CRA004775	ENSGALG00010014031	RPL22L1	case	control	0.094290688	0.341813626	0.275854092	0.782660136	0.94936527	up_in_case
CRA004775	ENSGALG00010014032	PSD3	case	control	0.066183358	0.330382596	0.200323379	0.841227679	0.966461921	up_in_case
CRA004775	ENSGALG00010014033	SLC38A4	case	control	-0.434949405	0.694694564	-0.626101639	0.531248277	0.869455011	down_in_case
CRA004775	ENSGALG00010014035	PSIP1	case	control	0.050580354	0.202130856	0.250235689	0.802405087	0.953744693	up_in_case
CRA004775	ENSGALG00010014041	SKIV2L2	case	control	0.143523506	0.246468849	0.582319049	0.560351797	0.880918893	up_in_case
CRA004775	ENSGALG00010014045	GOLT1B	case	control	-0.444637284	0.266533519	-1.668222767	0.095271513	0.528014574	down_in_case
CRA004775	ENSGALG00010014047	HM13	case	control	-0.406547132	0.131022473	-3.102880914	0.001916467	0.08697473	down_in_case
CRA004775	ENSGALG00010014054	ENSGALG00010014054	case	control	0.8427562	0.43728878	1.927230332	0.053950931	0.427596115	up_in_case
CRA004775	ENSGALG00010014056	FMO3	case	control	0.029487381	0.267586503	0.110197564	0.912252694	0.98075825	up_in_case
CRA004775	ENSGALG00010014062	GGA1	case	control	-0.380437704	0.223977241	-1.698555188	0.089403026	0.516112935	down_in_case
CRA004775	ENSGALG00010014066	BCL2L1	case	control	-0.044243036	0.168318035	-0.262853808	0.792663261	0.951284723	down_in_case
CRA004775	ENSGALG00010014076	PRRX1	case	control	-0.605919922	0.407714421	-1.486138068	0.137242605	0.596242097	down_in_case
CRA004775	ENSGALG00010014077	SLITRK2	case	control	0.029609608	0.209708619	0.141194045	0.88771665	0.977118922	up_in_case
CRA004775	ENSGALG00010014080	MYO1B	case	control	0.020399783	0.334772166	0.060936317	0.951409927	0.990168609	up_in_case
CRA004775	ENSGALG00010014082	HNRNPR	case	control	-0.067205592	0.186305612	-0.360727684	0.718303027	0.934011578	down_in_case
CRA004775	ENSGALG00010014083	ENSGALG00010014083	case	control	-0.380706847	0.286638497	-1.328177657	0.184119424	0.648687214	down_in_case
CRA004775	ENSGALG00010014089	FIG4	case	control	-0.279368492	0.340529467	-0.82039447	0.411991267	0.817006008	down_in_case
CRA004775	ENSGALG00010014091	CHIC2	case	control	-0.031352827	0.182716686	-0.171592578	0.863757843	0.972054513	down_in_case
CRA004775	ENSGALG00010014092	TYRP1	case	control	9.225334396	0.843873176	10.93213371	8.09229E-28	2.78193E-24	up_in_case
CRA004775	ENSGALG00010014093	PLD1	case	control	-0.350206016	0.351594527	-0.996050818	0.319225454	0.767037518	down_in_case

CRA004775	ENSGALG00010014096	JAKMIP2	case	control	0.391534292	0.44486251	0.88012427	0.378791993	0.799995192	up_in_case
CRA004775	ENSGALG00010014099	TPX2	case	control	-0.062804633	0.25031457	-0.250902826	0.80188924	0.953543664	down_in_case
CRA004775	ENSGALG00010014102	TRIM2	case	control	0.545210193	0.374295296	1.456631164	0.14521822	0.608365494	up_in_case
CRA004775	ENSGALG00010014104	ALDH1A1	case	control	2.195281407	0.532078785	4.125857804	3.69355E-05	0.013023094	up_in_case
CRA004775	ENSGALG00010014109	AMIGO2	case	control	0.2830284	0.33672357	0.840536349	0.40060773	0.811868062	up_in_case
CRA004775	ENSGALG00010014111	GORAB	case	control	-0.420760403	0.177500595	-2.3704732	0.017765332	0.26524547	down_in_case
CRA004775	ENSGALG00010014114	ANXA1	case	control	-0.591366257	0.554042427	-1.067366376	0.285806426	0.741582427	down_in_case
CRA004775	ENSGALG00010014120	SOX5	case	control	0.277537037	0.400453044	0.693057628	0.488273387	0.853845967	up_in_case
CRA004775	ENSGALG00010014122	ABCA1	case	control	-0.068306932	0.389872698	-0.175203169	0.860919995	0.971325143	down_in_case
CRA004775	ENSGALG00010014125	PLPP1	case	control	0.053444488	0.307300299	0.17391616	0.861931353	0.971668964	up_in_case
CRA004775	ENSGALG00010014128	COL4A1	case	control	0.782045633	0.406985906	1.921554586	0.054661828	0.430254604	up_in_case
CRA004775	ENSGALG00010014131	CDC23	case	control	-0.370310276	0.23132669	-1.600810853	0.109418819	0.556651068	down_in_case
CRA004775	ENSGALG00010014134	RPAP3	case	control	0.155055877	0.276109454	0.561573952	0.574406332	0.88709136	up_in_case
CRA004775	ENSGALG00010014135	ZBTB24	case	control	0.539928918	0.247575911	2.180862091	0.029193618	0.324790814	up_in_case
CRA004775	ENSGALG00010014143	NFATC2	case	control	-0.323533182	0.470158629	-0.68813622	0.491367003	0.855746361	down_in_case
CRA004775	ENSGALG00010014145	NTMT2	case	control	0.89640528	0.689865962	1.299390503	0.193809949	0.657919837	up_in_case
CRA004775	ENSGALG00010014147	ENSGALG00010014147	case	control	0.548736625	0.809490699	0.677878851	0.49784851	0.857810713	up_in_case
CRA004775	ENSGALG00010014148	KCNG1	case	control	-0.539228045	0.993542707	-0.542732628	0.587313906	0.892195484	down_in_case
CRA004775	ENSGALG00010014149	LGALS2	case	control	-0.081065831	0.317381546	-0.255420746	0.798398149	0.952835572	down_in_case
CRA004775	ENSGALG00010014151	ATP23	case	control	-0.129268447	0.226599227	-0.570471702	0.56835781	0.884508497	down_in_case
CRA004775	ENSGALG00010014153	FOCAD	case	control	0.188635028	0.217127511	0.868775344	0.384970022	0.803182776	up_in_case
CRA004775	ENSGALG00010014156	CD164	case	control	0.591782275	0.231445864	2.556892851	0.010561174	0.206054018	up_in_case
CRA004775	ENSGALG00010014159	CDC42EP1	case	control	-0.301271191	0.249533937	-1.20733554	0.227302949	0.694715356	down_in_case
CRA004775	ENSGALG00010014161	GFRA3	case	control	0.910234782	0.440652075	2.06565414	0.038861148	0.370839453	up_in_case
CRA004775	ENSGALG00010014162	COL18A1	case	control	0.567747371	0.627650319	0.904559997	0.365698536	0.793608387	up_in_case
CRA004775	ENSGALG00010014165	LUZP1	case	control	0.296762427	0.337795196	0.878527673	0.379657427	0.800637244	up_in_case
CRA004775	ENSGALG00010014166	FGF12	case	control	0.211430386	0.47443672	0.445645072	0.655853647	0.917054032	up_in_case
CRA004775	ENSGALG00010014167	SLC38A9	case	control	-0.346988594	0.249183084	-1.392504613	0.163769646	0.629717526	down_in_case
CRA004775	ENSGALG00010014170	CEMIP2	case	control	-0.298012826	0.381650937	-0.780851812	0.434889657	0.826789391	down_in_case
CRA004775	ENSGALG00010014171	DLC1	case	control	0.309059351	0.267103449	1.15707735	0.24724076	0.712046495	up_in_case
CRA004775	ENSGALG00010014173	MND1	case	control	-0.064625811	0.370474576	-0.17444061	0.861519201	0.97165529	down_in_case
CRA004775	ENSGALG00010014176	ABHD17B	case	control	0.283848916	0.207046749	1.370941186	0.170393291	0.634292946	up_in_case
CRA004775	ENSGALG00010014178	LRTM2	case	control	-0.637210422	1.046783823	-0.60873163	0.542702338	0.875137006	down_in_case
CRA004775	ENSGALG00010014179	CTBPL	case	control	-0.178304054	0.217565961	-0.819540209	0.412478271	0.817038226	down_in_case
CRA004775	ENSGALG00010014180	SLC19A1	case	control	-0.401212511	0.199066925	-2.015465455	0.043855902	0.391981676	down_in_case
CRA004775	ENSGALG00010014184	SLC44A1	case	control	0.850486596	0.377737745	2.251526643	0.024352201	0.30145607	up_in_case
CRA004775	ENSGALG00010014186	MOCS3	case	control	-0.0987309	0.242120768	-0.407775427	0.683438556	0.925087467	down_in_case
CRA004775	ENSGALG00010014190	HACD4	case	control	0.309096705	0.478275944	0.64627274	0.518102739	0.865435399	up_in_case
CRA004775	ENSGALG00010014192	KDM1A	case	control	-0.27258571	0.265732957	-1.025788119	0.304991468	0.757712316	down_in_case
CRA004775	ENSGALG00010014193	CARD10	case	control	-1.064040396	0.717573507	-1.482831218	0.13811926	0.597257214	down_in_case
CRA004775	ENSGALG00010014194	LRIG3	case	control	0.50015918	0.335954512	1.488770539	0.136547803	0.595518184	up_in_case
CRA004775	ENSGALG00010014197	SH3GL2	case	control	-0.479599494	0.672895186	-0.712740265	0.476006495	0.846243735	down_in_case
CRA004775	ENSGALG00010014198	KDM3B	case	control	-0.041603605	0.192857347	-0.215722168	0.82920432	0.96297907	down_in_case
CRA004775	ENSGALG00010014199	LNX1	case	control	0.370855275	0.381332183	0.972525507	0.330789182	0.77237154	up_in_case
CRA004775	ENSGALG00010014200	DCP1B	case	control	-0.105640886	0.339980233	-0.310726553	0.756008509	0.941900693	down_in_case
CRA004775	ENSGALG00010014203	TMEM38B	case	control	-0.712464881	0.380623512	-1.871836233	0.061229264	0.445483389	down_in_case
CRA004775	ENSGALG00010014204	KIFAP3	case	control	0.064616043	0.419440711	0.154052864	0.877568054	0.974479909	up_in_case
CRA004775	ENSGALG00010014205	DPM1	case	control	-0.106814393	0.215721561	-0.495149361	0.620494693	0.905283281	down_in_case
CRA004775	ENSGALG00010014209	CCDC50	case	control	0.350751287	0.183935571	1.906924723	0.056530336	0.434758754	up_in_case
CRA004775	ENSGALG00010014210	ENSGALG00010014210	case	control	0.736593718	0.718572639	1.025078993	0.305325917	0.757865363	up_in_case
CRA004775	ENSGALG00010014213	ETFRF1	case	control	0.217062081	0.284892309	0.761909236	0.446114177	0.833494028	up_in_case
CRA004775	ENSGALG00010014214	REEP2	case	control	0.15219267	0.232020148	0.655945923	0.511858919	0.863170205	up_in_case
CRA004775	ENSGALG00010014217	RASA3	case	control	-0.156144159	0.262504955	-0.594823662	0.551961349	0.878258849	down_in_case
CRA004775	ENSGALG00010014218	IL31RA	case	control	-0.399193782	0.504742768	-0.79088559	0.429010758	0.823883065	down_in_case
CRA004775	ENSGALG00010014220	C9orf85	case	control	-0.080033429	0.351250447	-0.227852888	0.819760609	0.960465449	down_in_case
CRA004775	ENSGALG00010014221	SCYL3	case	control	-0.007295683	0.26589225	-0.027438495	0.978109995	0.996148018	down_in_case
CRA004775	ENSGALG00010014222	TBX22	case	control	-0.127541717	0.681173844	-0.18723813	0.85147393	0.969284796	down_in_case
CRA004775	ENSGALG00010014225	ZMPSTE24	case	control	-0.228335882	0.21611985	-1.05652434	0.290728727	0.74447127	down_in_case
CRA004775	ENSGALG00010014226	SLC16A7	case	control	-0.751814071	0.52201932	-1.440203537	0.149809823	0.61366707	down_in_case
CRA004775	ENSGALG00010014236	WDR44	case	control	0.274334629	0.164177533	1.670963281	0.094728931	0.526933666	up_in_case
CRA004775	ENSGALG00010014238	PHLDA1	case	control	0.470527955	0.263676514	1.784489439	0.074344169	0.481085493	up_in_case
CRA004775	ENSGALG00010014240	PIGV	case	control	-0.339078082	0.212637618	-1.594628862	0.110795255	0.559715483	down_in_case
CRA004775	ENSGALG00010014241	ENSGALG00010014241	case	control	0.957303099	1.184702534	0.808053559	0.41905975	0.818943344	up_in_case
CRA004775	ENSGALG00010014243	ENSGALG00010014243	case	control	0.120842032	0.346895972	0.348352364	0.727575573	0.935548472	up_in_case
CRA004775	ENSGALG00010014244	SESN1	case	control	0.104159948	0.660467612	0.157706367	0.874688183	0.974301166	up_in_case
CRA004775	ENSGALG00010014246	KRAS	case	control	0.482247725	0.22071861	2.18489834	0.028896301	0.323755716	up_in_case
CRA004775	ENSGALG00010014249	ENSGALG00010014249	case	control	0.170088612	0.187172793	0.908725082	0.363495261	0.793608387	up_in_case
CRA004775	ENSGALG00010014250	ENSGALG00010014250	case	control	-0.120738727	0.588421917	-0.205190738	0.837423105	0.965409551	down_in_case
CRA004775	ENSGALG00010014257	NOP16	case	control	-0.508963203	0.255730175	-1.990235225	0.04656503	0.401451864	down_in_case
CRA004775	ENSGALG00010014258	ENSGALG00010014258	case	control	1.191222236	0.575940447	2.068308004	0.03861107	0.370251617	up_in_case
CRA004775	ENSGALG00010014260	ADNP	case	control	0.353080302	0.175473563	2.012156687	0.044203423	0.392535833	up_in_case
CRA004775	ENSGALG00010014263	ZNF131	case	control	0.496394231	0.246794476	2.011366864	0.044286721	0.392641326	up_in_case
CRA004775	ENSGALG00010014265	ZNF462	case	control	-0.057006901	0.260533995	-0.218807917	0.826799679	0.961878235	down_in_case
CRA004775	ENSGALG00010014267	SCFD2	case	control	-0.198578137	0.211072368	-0.940805939	0.346804317	0.784552667	down_in_case
CRA004775	ENSGALG00010014272	PARD6B	case	control	0.619217943	0.291620318	2.123370371	0.033722828	0.348925965	up_in_case
CRA004775	ENSGALG00010014274	FIRRM	case	control	-0.437396521	0.259734754	-1.684012304	0.092179291	0.52187835	down_in_case
CRA004775	ENSGALG00010014278	G3BP1	case	control	-0.167376303	0.300914506	-0.556225436	0.578056753	0.888378369	down_in_case
CRA004775	ENSGALG00010014284	TMEM131L	case	control	-0.408297591	0.415253277	-0.983249534	0.325484641	0.770440423	down_in_case
CRA004775	ENSGALG00010014288	ARMC2	case	control	-0.310203345	0.45158096	-0.686927422	0.492128465	0.855746361	down_in_case
CRA004775	ENSGALG00010014291	OSTN	case	control	-2.625361529	1.455987543	-1.803148345	0.07136492	0.471344389	down_in_case
CRA004775	ENSGALG00010014292	NIM1K	case	control	0.761450495	0.66574491	1.143757141	0.252724369	0.717153621	up_in_case
CRA004775	ENSGALG00010014294	TMPO	case	control	0.281324908	0.170108973	1.653792285	0.098169724	0.533793778	up_in_case
CRA004775	ENSGALG00010014295	CACNA1C	case	control	-0.269009924	0.402070987	-0.669060769	0.503456714	0.861180902	down_in_case
CRA004775	ENSGALG00010014298	METTL18	case	control	-0.161577899	0.374175137	-0.431824253	0.665869151	0.918888163	down_in_case
CRA004775	ENSGALG00010014300	ELFN2	case	control	-0.858201383	0.809389716	-1.060306754	0.289005068	0.743240824	down_in_case
CRA004775	ENSGALG00010014301	ARID1A	case	control	-0.053359425	0.25993696	-0.205278331	0.837354673	0.965409551	down_in_case
CRA004775	ENSGALG00010014302	FOXO3	case	control	0.340635865	0.547808462	0.621815632	0.534063106	0.870956093	up_in_case
CRA004775	ENSGALG00010014303	GABRA2	case	control	1.286072239	0.762478691	1.686699253	0.091661196	0.521441551	up_in_case
CRA004775	ENSGALG00010014305	ARL10	case	control	-0.476404868	0.390322409	-1.220541934	0.222259503	0.689907546	down_in_case
CRA004775	ENSGALG00010014306	ENSGALG00010014306	case	control	-0.056211625	0.501084945	-0.112179833	0.910680819	0.98075825	down_in_case
CRA004775	ENSGALG00010014308	ENSGALG00010014308	case	control	0.233485127	0.497088658	0.469705199	0.638565653	0.91099914	up_in_case
CRA004775	ENSGALG00010014310	COL6A1	case	control	0.061901054	0.505252672	0.122515046	0.902491131	0.979310476	up_in_case
CRA004775	ENSGALG00010014312	KIAA1191	case	control	-0.444379746	0.248891534	-1.785435359	0.074190728	0.48054484	down_in_case
CRA004775	ENSGALG00010014314	ENSGALG00010014314	case	control	0.397548667	0.420541537	0.945325568	0.344492689	0.783972357	up_in_case
CRA004775	ENSGALG00010014317	RAD23B	case	control	0.142581932	0.277702074	0.513434884	0.607647149	0.900943922	up_in_case
CRA004775	ENSGALG00010014318	ENSGALG00010014318	case	control	-0.731474186	0.514349079	-1.422135699	0.154986861	0.619284425	down_in_case
CRA004775	ENSGALG00010014319	ENSGALG00010014319	case	control	-0.195631829	0.272652891	-0.717512393	0.473057992	0.845445211	down_in_case
CRA004775	ENSGALG00010014323	KLF4	case	control	0.378037032	0.414403108	0.912244684	0.361639926	0.792757982	up_in_case
CRA004775	ENSGALG00010014324	COL4A2	case	control	0.742680776	0.393072229	1.889425713	0.058834808	0.44414236	up_in_case
CRA004775	ENSGALG00010014328	RASSF8	case	control	-0.198159002	0.349518714	-0.566948189	0.570749383	0.885321726	down_in_case
CRA004775	ENSGALG00010014329	IER5	case	control	-0.078121608	0.310471386	-0.251622569	0.801332812	0.953541667	down_in_case
CRA004775	ENSGALG00010014334	HMGCS1	case	control	0.093026173	0.231760338	0.401389531	0.688133356	0.927123823	up_in_case
CRA004775	ENSGALG00010014338	BHLHE41	case	control	0.319754218	0.284388287	1.124357901	0.260861224	0.721659172	up_in_case
CRA004775	ENSGALG00010014340	EFNB1	case	control	0.347663479	0.266173971	1.306151304	0.191501109	0.656959697	up_in_case
CRA004775	ENSGALG00010014344	LSG1	case	control	-0.373061334	0.183747149	-2.030297266	0.042326332	0.386779506	down_in_case
CRA004775	ENSGALG00010014346	AFG1L	case	control	-0.159661778	0.187983446	-0.849339561	0.395692374	0.808998414	down_in_case
CRA004775	ENSGALG00010014347	KIAA0040	case	control	-0.392979286	0.292932876	-1.341533568	0.179747279	0.644990528	down_in_case
CRA004775	ENSGALG00010014350	PTGER4	case	control	-0.417878464	0.315678032	-1.323748955	0.185586434	0.6491093	down_in_case
CRA004775	ENSGALG00010014355	RAB20	case	control	0.196615271	0.311190981	0.631815454	0.527507474	0.86830001	up_in_case
CRA004775	ENSGALG00010014359	TMEM271	case	control	0.201563539	0.35701702	0.564576835	0.572361626	0.885642749	up_in_case
CRA004775	ENSGALG00010014361	SSPN	case	control	0.308631647	0.437941918	0.704731915	0.480977086	0.848743338	up_in_case
CRA004775	ENSGALG00010014362	NAXD	case	control	-0.215485348	0.325059663	-0.662910143	0.507388105	0.862748056	down_in_case
CRA004775	ENSGALG00010014367	OPHN1	case	control	-0.380718738	0.235844993	-1.614275261	0.106467734	0.552224847	down_in_case
CRA004775	ENSGALG00010014371	TTC33	case	control	-0.248147131	0.25084752	-0.98923494	0.322548207	0.767880594	down_in_case
CRA004775	ENSGALG00010014372	CCDC171	case	control	0.304642165	0.225197996	1.352774759	0.176127599	0.641571022	up_in_case
CRA004775	ENSGALG00010014375	H2BC35	case	control	-0.485931967	0.417615791	-1.163586191	0.24459175	0.711765017	down_in_case
CRA004775	ENSGALG00010014379	CARS2	case	control	-0.339310965	0.265942541	-1.275880735	0.201997682	0.670354119	down_in_case
CRA004775	ENSGALG00010014380	SNX3	case	control	-0.078766006	0.196505097	-0.40083442	0.688542036	0.927339918	down_in_case
CRA004775	ENSGALG00010014388	MFNG	case	control	-1.063436785	0.420532621	-2.528785475	0.011445796	0.216528087	down_in_case
CRA004775	ENSGALG00010014389	COL6A2	case	control	0.024451048	0.513431739	0.047622782	0.962016875	0.991860454	up_in_case
CRA004775	ENSGALG00010014390	SPARC	case	control	0.048101438	0.506981635	0.094878067	0.924411678	0.983849734	up_in_case
CRA004775	ENSGALG00010014393	ATP8A1	case	control	0.12875288	0.42143463	0.305510915	0.759977058	0.942236668	up_in_case
CRA004775	ENSGALG00010014396	ING1	case	control	0.11333776	0.281325386	0.402870716	0.687043335	0.926974477	up_in_case
CRA004775	ENSGALG00010014397	PRKAA1	case	control	0.041744149	0.202957863	0.205678893	0.837041747	0.965269623	up_in_case
CRA004775	ENSGALG00010014399	GAS6	case	control	0.81646298	0.280226877	2.91357842	0.003573121	0.125102734	up_in_case
CRA004775	ENSGALG00010014400	RIPOR3	case	control	0.015799326	0.240631061	0.065657884	0.947650204	0.989681625	up_in_case

CRA004775	ENSGALG00010014403	H2BC37	case	control	0.706084154	0.413084698	1.709296322	0.087396074	0.511614907	up_in_case
CRA004775	ENSGALG00010014407	YIPF6	case	control	-0.294074583	0.224638901	-1.309099099	0.190500785	0.656023968	down_in_case
CRA004775	ENSGALG00010014408	ENSGALG00010014408	case	control	2.34598826	0.649632224	3.611255989	0.000304718	0.035211539	up_in_case
CRA004775	ENSGALG00010014411	ATP13A3	case	control	-0.175485111	0.400052241	-0.438655487	0.660911185	0.917907174	down_in_case
CRA004775	ENSGALG00010014414	TMEM255B	case	control	-1.376426517	0.780620522	-1.763246646	0.0778589	0.49028362	down_in_case
CRA004775	ENSGALG00010014416	H2BC32	case	control	-0.551484701	0.497024214	-1.109573107	0.267183025	0.727315996	down_in_case
CRA004775	ENSGALG00010014418	RAC2	case	control	-0.937813783	0.490760172	-1.910941101	0.05601215	0.433172413	down_in_case
CRA004775	ENSGALG00010014427	TMEM267	case	control	-0.308125324	0.228981892	-1.345631836	0.178421279	0.644368088	down_in_case
CRA004775	ENSGALG00010014431	ENSGALG00010014431	case	control	-0.593481412	0.426017723	-1.3930909	0.163592305	0.629717526	down_in_case
CRA004775	ENSGALG00010014439	ENSGALG00010014439	case	control	1.028548629	0.69407686	1.481894424	0.138368389	0.597582825	up_in_case
CRA004775	ENSGALG00010014449	ENSGALG00010014449	case	control	-1.391596113	0.506745617	-2.746143365	0.006030041	0.155279193	down_in_case
CRA004775	ENSGALG00010014453	NR2E1	case	control	0.42699542	0.402393116	1.061139973	0.288626294	0.742851819	up_in_case
CRA004775	ENSGALG00010014465	SIMC1	case	control	0.095638356	0.320928835	0.298004871	0.765699446	0.943470706	up_in_case
CRA004775	ENSGALG00010014469	TMCO3	case	control	0.027572327	0.233133443	0.118268433	0.905854968	0.979739788	up_in_case
CRA004775	ENSGALG00010014470	RPS6KA1	case	control	-0.708987717	0.375882253	-1.886196305	0.059268506	0.44414236	down_in_case
CRA004775	ENSGALG00010014474	PTPN1	case	control	-0.109884077	0.374647619	-0.293299814	0.769292996	0.945357282	down_in_case
CRA004775	ENSGALG00010014476	C5orf34	case	control	-0.774488658	0.458874289	-1.687801382	0.091449362	0.521441551	down_in_case
CRA004775	ENSGALG00010014477	ITPR2	case	control	-0.437129856	0.512904589	-0.852263491	0.394067864	0.808418201	down_in_case
CRA004775	ENSGALG00010014482	THOC3	case	control	-0.483448925	0.213518116	-2.264205656	0.023561469	0.29751493	down_in_case
CRA004775	ENSGALG00010014487	OSTM1	case	control	-0.581858333	0.354166339	-1.642895635	0.100404528	0.538583505	down_in_case
CRA004775	ENSGALG00010014488	ENSGALG00010014488	case	control	-0.655322139	0.722909957	-0.906505898	0.364668145	0.793608387	down_in_case
CRA004775	ENSGALG00010014492	CPLX2	case	control	0.164981903	0.414772632	0.39776468	0.690803655	0.927867527	up_in_case
CRA004775	ENSGALG00010014494	KLHL9	case	control	-0.12961779	0.229297958	-0.565281049	0.571882616	0.885500402	down_in_case
CRA004775	ENSGALG00010014504	SEC63	case	control	0.240364122	0.129705112	1.853158433	0.063859654	0.455062268	up_in_case
CRA004775	ENSGALG00010014505	CYTH4	case	control	-0.505076791	0.398116977	-1.268664286	0.204560825	0.674045742	down_in_case
CRA004775	ENSGALG00010014512	HMGN4	case	control	-0.164561928	0.239229957	-0.687881777	0.491527233	0.855746361	down_in_case
CRA004775	ENSGALG00010014515	ETNK1	case	control	-0.221887474	0.433327237	-0.512055219	0.608612364	0.901007337	down_in_case
CRA004775	ENSGALG00010014517	DCUN1D2	case	control	-0.15919534	0.279387851	-0.569800508	0.568813011	0.884663439	down_in_case
CRA004775	ENSGALG00010014537	GKAP1	case	control	0.215576752	0.348456927	0.618661117	0.53613963	0.871588629	up_in_case
CRA004775	ENSGALG00010014539	PAIP1	case	control	0.072073179	0.134387825	0.536307357	0.591746147	0.893804699	up_in_case
CRA004775	ENSGALG00010014547	DHDDS	case	control	-0.16611223	0.197417914	-0.841424302	0.400110276	0.811658978	down_in_case
CRA004775	ENSGALG00010014567	UTP11	case	control	-0.213732354	0.244611949	-0.873760889	0.382248484	0.801753951	down_in_case
CRA004775	ENSGALG00010014570	KIF27	case	control	-0.055607474	0.5401797	-0.102942546	0.91800857	0.981688766	down_in_case
CRA004775	ENSGALG00010014572	IL1RAP	case	control	-0.282405203	0.289078619	-0.976914878	0.328611296	0.77220987	down_in_case
CRA004775	ENSGALG00010014573	CEBPB	case	control	-0.088421988	0.503069413	-0.175764986	0.860478579	0.971325143	down_in_case
CRA004775	ENSGALG00010014576	HRH2	case	control	-0.962908702	0.427520955	-2.252307613	0.024302839	0.30145607	down_in_case
CRA004775	ENSGALG00010014578	ENSGALG00010014578	case	control	0.152442858	0.458483335	0.332493782	0.739516434	0.938064864	up_in_case
CRA004775	ENSGALG00010014579	NNT	case	control	0.521087437	0.262907655	1.982016983	0.04747734	0.404519261	up_in_case
CRA004775	ENSGALG00010014592	QNG1	case	control	-0.268056146	0.214060916	-1.252242358	0.210481566	0.680684888	down_in_case
CRA004775	ENSGALG00010014599	SSTR3	case	control	-0.679057911	0.675893212	-1.004682247	0.315049879	0.765098415	down_in_case
CRA004775	ENSGALG00010014602	FRK	case	control	2.657953191	0.778844963	3.412685858	0.00064326	0.052858455	up_in_case
CRA004775	ENSGALG00010014603	ENSGALG00010014603	case	control	0.222274921	0.367767239	0.604390214	0.54558424	0.876645114	up_in_case
CRA004775	ENSGALG00010014611	TMEM189	case	control	0.022541056	0.297639748	0.075732682	0.939631774	0.987076872	up_in_case
CRA004775	ENSGALG00010014614	GHR	case	control	0.322979001	0.344681987	0.937034753	0.348740682	0.785311667	up_in_case
CRA004775	ENSGALG00010014617	GRTP1	case	control	-0.122663662	0.342084005	-0.358577602	0.719911105	0.934179258	down_in_case
CRA004775	ENSGALG00010014626	C2CD5	case	control	0.102575939	0.181066319	0.566510324	0.571046917	0.885321726	up_in_case
CRA004775	ENSGALG00010014632	NWD2	case	control	0.033259322	0.496821695	0.066944182	0.94662614	0.989137998	up_in_case
CRA004775	ENSGALG00010014634	CCDC152	case	control	0.412724336	0.341149012	1.209806626	0.226353103	0.69451026	up_in_case
CRA004775	ENSGALG00010014641	DOCK11	case	control	-0.806022409	0.514577317	-1.56637765	0.117260232	0.567740281	down_in_case
CRA004775	ENSGALG00010014645	SLC36A1	case	control	-0.503369447	0.30104146	-1.672093428	0.0945059	0.526933666	down_in_case
CRA004775	ENSGALG00010014648	C1QTNF6	case	control	-1.540776145	0.753766031	-2.044103983	0.040943276	0.378415958	down_in_case
CRA004775	ENSGALG00010014653	FAM162B	case	control	0.800744036	0.460303143	1.739601495	0.081929017	0.498277712	up_in_case
CRA004775	ENSGALG00010014654	LAMP1	case	control	0.251392334	0.166801151	1.507137882	0.131775318	0.587352231	up_in_case
CRA004775	ENSGALG00010014655	FHL3	case	control	-0.040757548	0.286471069	-0.14227457	0.886863132	0.977118922	down_in_case
CRA004775	ENSGALG00010014658	SELENOP1	case	control	-0.29795884	0.355535717	-0.838055998	0.401999254	0.811868062	down_in_case
CRA004775	ENSGALG00010014668	GP5	case	control	-0.477556535	0.347784009	-1.373141154	0.169708469	0.633147466	down_in_case
CRA004775	ENSGALG00010014676	UBE2V1	case	control	0.048137162	0.195898169	0.245725429	0.805894786	0.955171181	up_in_case
CRA004775	ENSGALG00010014677	ENSGALG00010014677	case	control	-0.201215504	0.243894158	-0.825011576	0.409365019	0.814878167	down_in_case
CRA004775	ENSGALG00010014682	IL13RA1	case	control	-0.237545052	0.406668756	-0.584124177	0.55913677	0.880918893	down_in_case
CRA004775	ENSGALG00010014687	CUL4A	case	control	0.326778375	0.337521309	0.968171095	0.332958928	0.773910112	up_in_case
CRA004775	ENSGALG00010014690	CSF3R	case	control	-0.988695979	0.619970499	-1.594746815	0.110768865	0.559715483	down_in_case
CRA004775	ENSGALG00010014691	PABIR2	case	control	-0.063787891	0.129089058	-0.494138632	0.621208276	0.90544587	down_in_case
CRA004775	ENSGALG00010014696	SPINK5	case	control	1.423658541	0.501069163	2.841241581	0.004493826	0.137934366	up_in_case
CRA004775	ENSGALG00010014702	ENSGALG00010014702	case	control	-0.464997325	0.427194326	-1.088491341	0.276378257	0.731001618	down_in_case
CRA004775	ENSGALG00010014703	PCID2	case	control	-0.29581573	0.214578285	-1.378591172	0.16802084	0.63138062	down_in_case
CRA004775	ENSGALG00010014705	ARAP2	case	control	0.367467859	0.575706543	0.638290227	0.523284773	0.866638286	up_in_case
CRA004775	ENSGALG00010014707	KPNA5	case	control	0.517866947	0.291213591	1.778306241	0.075353572	0.483342345	up_in_case
CRA004775	ENSGALG00010014710	NPEPL1	case	control	-0.124507167	0.363604529	-0.342424686	0.732031314	0.936799657	down_in_case
CRA004775	ENSGALG00010014713	ST8SIA1	case	control	-0.639863307	0.646895787	-0.989128883	0.322600088	0.767880594	down_in_case
CRA004775	ENSGALG00010014716	TMEM35A	case	control	-0.332162801	0.303597205	-1.094090445	0.273915313	0.731001618	down_in_case
CRA004775	ENSGALG00010014717	ELOVL7	case	control	1.055853882	0.29204285	3.615407405	0.000299876	0.035211539	up_in_case
CRA004775	ENSGALG00010014722	KCTD17	case	control	0.071492403	0.752752243	0.09497468	0.924334939	0.983849734	up_in_case
CRA004775	ENSGALG00010014726	PWP2	case	control	-0.399573568	0.255127342	-1.566173049	0.11730811	0.567740281	down_in_case
CRA004775	ENSGALG00010014728	INTS13	case	control	0.4080469	0.182200861	2.239544295	0.025120523	0.304882883	up_in_case
CRA004775	ENSGALG00010014729	MRPS15	case	control	-0.37848954	0.318466329	-1.188475847	0.23464599	0.702145501	down_in_case
CRA004775	ENSGALG00010014734	ZUP1	case	control	-0.411187824	0.253123607	-1.624454666	0.104278808	0.546673752	down_in_case
CRA004775	ENSGALG00010014735	STX16	case	control	0.193460018	0.181349102	1.06678233	0.28607014	0.741582427	up_in_case
CRA004775	ENSGALG00010014736	KIF2A	case	control	0.153309096	0.227752426	0.673139244	0.500858709	0.859946721	up_in_case
CRA004775	ENSGALG00010014737	RMI1	case	control	0.334991052	0.268560199	1.247359265	0.212265796	0.682275341	up_in_case
CRA004775	ENSGALG00010014738	BRWD1	case	control	0.361703536	0.236212951	1.53126039	0.125705051	0.579473736	up_in_case
CRA004775	ENSGALG00010014739	TRMT2B	case	control	-0.552509929	0.292432137	-1.889361182	0.058843449	0.44414236	down_in_case
CRA004775	ENSGALG00010014744	IL2RB	case	control	-0.186472317	0.372662532	-0.500378494	0.616808593	0.903561837	down_in_case
CRA004775	ENSGALG00010014745	LSM10	case	control	-0.130825811	0.277565831	-0.471332551	0.637403274	0.910421454	down_in_case
CRA004775	ENSGALG00010014748	FGFR1OP2	case	control	-0.00475348	0.23938106	-0.019857375	0.984157148	0.997534931	down_in_case
CRA004775	ENSGALG00010014750	LONRF1	case	control	0.420270127	0.318970445	1.317583285	0.187643184	0.651750801	up_in_case
CRA004775	ENSGALG00010014754	DIMT1	case	control	0.027738033	0.245674187	0.112905771	0.910105261	0.98075825	up_in_case
CRA004775	ENSGALG00010014758	FXN	case	control	-0.373131238	0.246873089	-1.511429369	0.130679091	0.586275434	down_in_case
CRA004775	ENSGALG00010014762	STK40	case	control	-0.151679424	0.154049657	-0.984613834	0.324813792	0.770185561	down_in_case
CRA004775	ENSGALG00010014764	ENSGALG00010014764	case	control	2.74809005	0.937705667	2.930653132	0.003382502	0.124365749	up_in_case
CRA004775	ENSGALG00010014766	F10	case	control	0.299115406	0.339515815	0.881005812	0.378314674	0.799601151	up_in_case
CRA004775	ENSGALG00010014767	LRRC15	case	control	-0.646865644	0.585994901	-1.103875893	0.269646978	0.727898624	down_in_case
CRA004775	ENSGALG00010014769	CCDC69	case	control	-0.517537422	0.458907146	-1.127760651	0.259421016	0.720037471	down_in_case
CRA004775	ENSGALG00010014777	APCDD1L	case	control	0.727131108	0.387889863	1.874581363	0.060850339	0.445397937	up_in_case
CRA004775	ENSGALG00010014778	TMPRSS6	case	control	-0.297811713	0.745751211	-0.399344592	0.689639314	0.927796693	down_in_case
CRA004775	ENSGALG00010014780	EVA1B	case	control	0.005576033	0.301511672	0.018493588	0.985245092	0.997562105	up_in_case
CRA004775	ENSGALG00010014783	ENSGALG00010014783	case	control	-0.621730714	0.216901403	-2.866420902	0.004151419	0.131838709	down_in_case
CRA004775	ENSGALG00010014789	F7	case	control	1.709931854	1.261487004	1.355489076	0.175261787	0.640706322	up_in_case
CRA004775	ENSGALG00010014792	ENSGALG00010014792	case	control	-0.2518574	0.547198938	-0.460266611	0.645324864	0.914043203	down_in_case
CRA004775	ENSGALG00010014793	TLR2A	case	control	-1.214408487	0.641374157	-1.893447801	0.058298344	0.442661803	down_in_case
CRA004775	ENSGALG00010014795	THRAP3	case	control	-0.170900264	0.152966469	-1.11724004	0.263891705	0.723981064	down_in_case
CRA004775	ENSGALG00010014796	PRLR	case	control	1.12791178	0.904651756	1.246791124	0.212474096	0.682275341	up_in_case
CRA004775	ENSGALG00010014797	ZDHHC9	case	control	-0.412260404	0.254304818	-1.621126992	0.104990406	0.547902496	down_in_case
CRA004775	ENSGALG00010014801	MPST	case	control	-0.269294982	0.218564423	-1.232108038	0.217908729	0.685219057	down_in_case
CRA004775	ENSGALG00010014802	ENSGALG00010014802	case	control	-0.20751389	0.475011053	-0.436861181	0.662212026	0.918136475	down_in_case
CRA004775	ENSGALG00010014806	MAP7D1	case	control	-0.288148418	0.364626668	-0.790256017	0.429378268	0.823991481	down_in_case
CRA004775	ENSGALG00010014807	HES1	case	control	0.377199971	0.65159153	0.578890231	0.562663242	0.881532201	up_in_case
CRA004775	ENSGALG00010014810	ENTREP1	case	control	-0.154382312	0.416600049	-0.370576798	0.710952766	0.932234469	down_in_case
CRA004775	ENSGALG00010014812	RWDD1	case	control	0.185021026	0.206084918	0.89779023	0.369297394	0.794896739	up_in_case
CRA004775	ENSGALG00010014813	SASH3	case	control	-0.718514054	0.440005551	-1.632965884	0.102476172	0.54281581	down_in_case
CRA004775	ENSGALG00010014816	TST	case	control	0.128307518	0.362908153	0.353553695	0.723673382	0.935238044	up_in_case
CRA004775	ENSGALG00010014818	ENSGALG00010014818	case	control	0.160492842	1.389240972	0.115525561	0.908028562	0.980606464	up_in_case
CRA004775	ENSGALG00010014821	C3AR1L	case	control	-0.07655957	0.621103323	-0.123263822	0.901898188	0.979157637	down_in_case
CRA004775	ENSGALG00010014824	ANXA6	case	control	-0.174655008	0.231407748	-0.754750043	0.450398974	0.835404673	down_in_case
CRA004775	ENSGALG00010014829	ENSGALG00010014829	case	control	-0.59596008	0.571007458	-1.043699293	0.296624537	0.748968786	down_in_case
CRA004775	ENSGALG00010014831	VAPB	case	control	0.365060581	0.142348512	2.564554949	0.010330822	0.20410795	up_in_case
CRA004775	ENSGALG00010014837	APBA1	case	control	-0.764531088	0.554000017	-1.380019972	0.167580496	0.63138062	down_in_case
CRA004775	ENSGALG00010014839	TM7SF3	case	control	-0.629097662	0.334579148	-1.880265598	0.060071889	0.445397937	down_in_case
CRA004775	ENSGALG00010014840	NCF4	case	control	-0.519360282	0.459720076	-1.129731569	0.258589351	0.720037471	down_in_case
CRA004775	ENSGALG00010014847	PAICS	case	control	-0.094320587	0.233227569	-0.404414398	0.685908015	0.926696906	down_in_case
CRA004775	ENSGALG00010014856	MMGT1	case	control	0.008953919	0.189645954	0.047213869	0.962342774	0.991860454	up_in_case
CRA004775	ENSGALG00010014859	TRAPPC3	case	control	0.120123143	0.278376606	0.431513067	0.666095354	0.918888163	up_in_case
CRA004775	ENSGALG00010014861	MED21	case	control	-0.035491879	0.25864488	-0.137222429	0.890854983	0.977513084	down_in_case
CRA004775	ENSGALG00010014862	ENSGALG00010014862	case	control	-0.595358322	0.795960515	-0.747974693	0.454475422	0.838254622	down_in_case
CRA004775	ENSGALG00010014865	TLR2	case	control	-0.564526865	0.543711168	-1.038284476	0.299137634	0.75239174	down_in_case
CRA004775	ENSGALG00010014866	FAM26E	case	control	0.80855712	0.362896017	2.228068327	0.025875959	0.308336492	up_in_case
CRA004775	ENSGALG00010014867	TP63	case	control	0.390186726	0.556702984	0.700888512	0.483372594	0.850257583	up_in_case

CRA004775	ENSGALG00010014870	ENSGALG00010014870	case	control	2.448536564	0.885462366	2.765263276	0.005687687	0.152268906	up_in_case
CRA004775	ENSGALG00010014873	CALHM6	case	control	-0.409568947	0.515901309	-0.79389011	0.427259408	0.822776983	down_in_case
CRA004775	ENSGALG00010014876	PCNT	case	control	0.438214442	0.220675975	1.985782284	0.047057504	0.402720701	up_in_case
CRA004775	ENSGALG00010014877	ENSGALG00010014877	case	control	0.14104716	0.194453866	0.725350251	0.468237185	0.843870188	up_in_case
CRA004775	ENSGALG00010014878	TMCO1	case	control	-0.195074082	0.200482624	-0.973022389	0.330542175	0.77237154	down_in_case
CRA004775	ENSGALG00010014880	SFRP2	case	control	-0.280504144	0.544172541	-0.515469126	0.606225238	0.900898526	down_in_case
CRA004775	ENSGALG00010014882	XPNPEP2	case	control	1.614092744	0.731966196	2.205146568	0.027443811	0.316243014	up_in_case
CRA004775	ENSGALG00010014883	PPAT	case	control	-0.464139909	0.347577006	-1.335358499	0.181759041	0.647378665	down_in_case
CRA004775	ENSGALG00010014886	RABGAP1L	case	control	-0.169874569	0.21034408	-0.80760328	0.419318998	0.819158337	down_in_case
CRA004775	ENSGALG00010014887	MTMR12	case	control	0.300584409	0.311089673	0.966230754	0.333928727	0.774300343	up_in_case
CRA004775	ENSGALG00010014889	PVALB	case	control	-2.698745432	0.919593457	-2.934715783	0.003338533	0.123409064	down_in_case
CRA004775	ENSGALG00010014893	SLC9A6	case	control	-0.430204083	0.256561863	-1.676804486	0.09358072	0.525355698	down_in_case
CRA004775	ENSGALG00010014894	RAB22A	case	control	0.099543522	0.256954454	0.387397536	0.698461928	0.929347461	up_in_case
CRA004775	ENSGALG00010014897	COL8A2	case	control	-0.117917523	0.361332425	-0.32634083	0.7441665	0.939218065	down_in_case
CRA004775	ENSGALG00010014898	ENSGALG00010014898	case	control	0.289004798	0.556696542	0.519142435	0.603661419	0.898692785	up_in_case
CRA004775	ENSGALG00010014900	UBLCP1	case	control	-0.131665106	0.210255709	-0.626214179	0.531174468	0.869441746	down_in_case
CRA004775	ENSGALG00010014902	AASDH	case	control	-0.365318944	0.253761478	-1.439615447	0.149976227	0.61366707	down_in_case
CRA004775	ENSGALG00010014903	MCF2L	case	control	0.323106045	0.473042576	0.68303798	0.494582844	0.856382445	up_in_case
CRA004775	ENSGALG00010014906	IFT27	case	control	0.010077043	0.17271852	0.058343734	0.953474832	0.990670511	up_in_case
CRA004775	ENSGALG00010014907	ENSGALG00010014907	case	control	0.058880171	0.205475804	0.286555254	0.77445288	0.946319184	up_in_case
CRA004775	ENSGALG00010014908	ENSGALG00010014908	case	control	-0.105874742	0.3338639	-0.317119467	0.751152954	0.941495694	down_in_case
CRA004775	ENSGALG00010014909	FBXL14	case	control	-0.151126815	0.191848752	-0.787739373	0.43084917	0.824612364	down_in_case
CRA004775	ENSGALG00010014912	ENSGALG00010014912	case	control	-0.910845909	0.515794948	-1.765907002	0.077411452	0.48896871	down_in_case
CRA004775	ENSGALG00010014914	PTAR1	case	control	0.22852495	0.289043113	0.790625828	0.429162371	0.823883065	up_in_case
CRA004775	ENSGALG00010014916	DYNLT2B	case	control	-0.112174661	0.255861012	-0.438420298	0.661081635	0.917907174	down_in_case
CRA004775	ENSGALG00010014917	EIF3D	case	control	-0.485821016	0.236893448	-2.050799719	0.040286455	0.376363211	down_in_case
CRA004775	ENSGALG00010014918	PLRG1	case	control	-0.069437349	0.200953172	-0.34553995	0.729688482	0.936339759	down_in_case
CRA004775	ENSGALG00010014922	MAN1A1	case	control	-0.651358072	0.520296058	-1.25189892	0.210606699	0.680684888	down_in_case
CRA004775	ENSGALG00010014928	SUCO	case	control	-0.193917226	0.270992975	-0.715580271	0.47425056	0.845684862	down_in_case
CRA004775	ENSGALG00010014929	FHL1	case	control	-0.44169624	0.480678402	-0.918901782	0.358146948	0.790696227	down_in_case
CRA004775	ENSGALG00010014931	RNF145	case	control	0.100618481	0.138666475	0.725615052	0.468074791	0.843870188	up_in_case
CRA004775	ENSGALG00010014934	MAMDC2	case	control	-0.160385067	0.473062853	-0.339035428	0.734583041	0.936819725	down_in_case
CRA004775	ENSGALG00010014935	PCYT1A	case	control	-0.343584077	0.22298041	-1.540871129	0.123348154	0.575554958	down_in_case
CRA004775	ENSGALG00010014936	FOXRED2	case	control	-0.713942354	0.26258922	-2.718856292	0.006550806	0.16028494	down_in_case
CRA004775	ENSGALG00010014940	VAMP4	case	control	-0.237689878	0.203319255	-1.169047554	0.242384473	0.709690398	down_in_case
CRA004775	ENSGALG00010014942	PMEPA1	case	control	-0.219427504	0.421262869	-0.52088024	0.602450202	0.898318448	down_in_case
CRA004775	ENSGALG00010014943	APLN	case	control	0.458977996	0.523879866	0.876113067	0.38096857	0.801753951	up_in_case
CRA004775	ENSGALG00010014944	FGB	case	control	1.497673908	0.509326676	2.940497681	0.003276855	0.122856914	up_in_case
CRA004775	ENSGALG00010014945	TXN2	case	control	-0.269732821	0.30849466	-0.874351669	0.381926769	0.801753951	down_in_case
CRA004775	ENSGALG00010014946	PCK1	case	control	2.845353288	0.931779168	3.053677724	0.002260548	0.096690159	up_in_case
CRA004775	ENSGALG00010014948	RBM38	case	control	0.669945117	0.393080651	1.704345192	0.088316617	0.513532836	up_in_case
CRA004775	ENSGALG00010014949	AGO3	case	control	-0.19479541	0.334067399	-0.583102126	0.559824553	0.880918893	down_in_case
CRA004775	ENSGALG00010014953	ENSGALG00010014953	case	control	-0.355916896	0.659516537	-0.539663339	0.589429227	0.893412703	down_in_case
CRA004775	ENSGALG00010014956	FGG	case	control	1.498952251	0.817788588	1.832933686	0.066812433	0.462498477	up_in_case
CRA004775	ENSGALG00010014965	ABHD13	case	control	0.085414348	0.210063373	0.406612282	0.684292775	0.925605936	up_in_case
CRA004775	ENSGALG00010014967	HNRNPH1	case	control	-0.069380406	0.224906185	-0.308485984	0.757712564	0.941900693	down_in_case
CRA004775	ENSGALG00010014970	SLC51A	case	control	1.314974167	0.677357676	1.941329099	0.052218372	0.420135271	up_in_case
CRA004775	ENSGALG00010014971	SMC5	case	control	0.162972496	0.297402818	0.547985717	0.58370171	0.889906855	up_in_case
CRA004775	ENSGALG00010014972	FGA	case	control	1.717026118	0.821131835	2.091048044	0.036523757	0.360668554	up_in_case
CRA004775	ENSGALG00010014974	LIG4	case	control	0.306346857	0.334803866	0.915003941	0.360189561	0.791975934	up_in_case
CRA004775	ENSGALG00010014976	ITGB2	case	control	-0.312746759	0.518934869	-0.602670543	0.546727882	0.877099134	down_in_case
CRA004775	ENSGALG00010014980	ENSGALG00010014980	case	control	-0.605282489	0.566042795	-1.06932284	0.284924222	0.74120185	down_in_case
CRA004775	ENSGALG00010014982	ENSGALG00010014982	case	control	0.936454603	0.676589448	1.384081006	0.166333652	0.630194031	up_in_case
CRA004775	ENSGALG00010014984	LPP	case	control	0.371179586	0.368817834	1.006403573	0.314221475	0.765098415	up_in_case
CRA004775	ENSGALG00010014985	ENSGALG00010014985	case	control	0.195508997	0.206900299	0.944943037	0.344687958	0.783972357	up_in_case
CRA004775	ENSGALG00010014987	KLF9	case	control	0.595246747	0.50842896	1.170756966	0.24169648	0.708952282	up_in_case
CRA004775	ENSGALG00010014990	AGO1	case	control	0.090163444	0.20270416	0.444803128	0.656462034	0.91710603	up_in_case
CRA004775	ENSGALG00010014992	ARL15	case	control	0.122664159	0.180521696	0.679498151	0.496822279	0.857620281	up_in_case
CRA004775	ENSGALG00010014994	ENSGALG00010014994	case	control	-0.207271282	0.176771763	-1.172536149	0.240981868	0.708064458	down_in_case
CRA004775	ENSGALG00010014995	LRAT	case	control	0.770137644	0.538788948	1.429386491	0.152893179	0.617472961	up_in_case
CRA004775	ENSGALG00010014997	RAE1	case	control	-0.064037997	0.200714945	-0.319049472	0.749688998	0.941495694	down_in_case
CRA004775	ENSGALG00010015002	ADAM19	case	control	-0.61232418	0.573173629	-1.068304871	0.285383012	0.741413527	down_in_case
CRA004775	ENSGALG00010015006	HSPB3	case	control	-0.004876149	0.703162015	-0.006934603	0.994467032	0.999001196	down_in_case
CRA004775	ENSGALG00010015009	AGO4	case	control	-0.200699628	0.283391262	-0.708206831	0.478816825	0.847170633	down_in_case
CRA004775	ENSGALG00010015013	SNX18	case	control	0.09626487	0.320874326	0.300008016	0.764171041	0.94239774	up_in_case
CRA004775	ENSGALG00010015016	ARGLU1	case	control	0.110783571	0.169281423	0.654434305	0.512832039	0.863786547	up_in_case
CRA004775	ENSGALG00010015017	FAM184A	case	control	0.122747342	0.371962423	0.329999308	0.741400485	0.938778937	up_in_case
CRA004775	ENSGALG00010015018	MAP7D3	case	control	-0.06633816	0.173691757	-0.381930386	0.702513001	0.930068722	down_in_case
CRA004775	ENSGALG00010015024	CRACD	case	control	0.91402222	0.314874721	2.90281232	0.003698282	0.12600076	up_in_case
CRA004775	ENSGALG00010015025	TRAF3IP1	case	control	0.149968998	0.24940572	0.601305368	0.547636615	0.877277621	up_in_case
CRA004775	ENSGALG00010015027	CLSPN	case	control	0.957698135	0.419968516	2.280404597	0.022583703	0.292594999	up_in_case
CRA004775	ENSGALG00010015028	MCM9	case	control	-0.551742845	0.647913522	-0.851568654	0.394453544	0.808680497	down_in_case
CRA004775	ENSGALG00010015029	TFRC	case	control	-0.086168187	0.296344078	-0.29077074	0.771226665	0.945853152	down_in_case
CRA004775	ENSGALG00010015031	MYH9	case	control	-0.174096156	0.421560643	-0.412980099	0.679621195	0.923739354	down_in_case
CRA004775	ENSGALG00010015032	C1orf216	case	control	-0.339976299	0.285064861	-1.192627872	0.233015145	0.69988265	down_in_case
CRA004775	ENSGALG00010015033	BMP7	case	control	0.052218648	0.475115362	0.109907302	0.912482891	0.980787663	up_in_case
CRA004775	ENSGALG00010015035	SLC35F1	case	control	0.974935074	0.436416199	2.233957119	0.025485895	0.306075579	up_in_case
CRA004775	ENSGALG00010015037	CYFIP2	case	control	-0.379220662	0.588877292	-0.643972297	0.519593398	0.865611895	down_in_case
CRA004775	ENSGALG00010015038	PCSK5	case	control	0.325336282	0.347646734	0.935824361	0.349363627	0.785360568	up_in_case
CRA004775	ENSGALG00010015039	FAM114A1	case	control	-0.122935275	0.203786027	-0.603256644	0.546337971	0.877099134	down_in_case
CRA004775	ENSGALG00010015040	ZC4H2	case	control	-0.175639981	0.298347451	-0.588709506	0.55605616	0.87981923	down_in_case
CRA004775	ENSGALG00010015042	EFNB2	case	control	0.979198365	0.369527359	2.64986703	0.008052346	0.18152099	up_in_case
CRA004775	ENSGALG00010015043	COQ10B	case	control	0.07746749	0.424675109	0.182415895	0.855256353	0.970581465	up_in_case
CRA004775	ENSGALG00010015044	ZC3H12B	case	control	0.146928906	0.325414188	0.451513523	0.651619481	0.915892481	up_in_case
CRA004775	ENSGALG00010015046	PSMB2	case	control	-0.465566986	0.242805895	-1.91744515	0.055181403	0.430875458	down_in_case
CRA004775	ENSGALG00010015047	NPY2R	case	control	-0.155825029	0.498765362	-0.312421513	0.754720203	0.941900693	down_in_case
CRA004775	ENSGALG00010015049	DCK2	case	control	0.75324996	0.460505803	1.635701343	0.101902108	0.541943185	up_in_case
CRA004775	ENSGALG00010015050	HIVEP3	case	control	-0.142714946	0.363203622	-0.392933708	0.694368445	0.928765642	down_in_case
CRA004775	ENSGALG00010015052	ENSGALG00010015052	case	control	-0.17961775	0.333403112	-0.538740474	0.590065942	0.893771223	down_in_case
CRA004775	ENSGALG00010015054	ENSGALG00010015054	case	control	-0.462448837	0.380593504	-1.215072858	0.224338304	0.69245253	down_in_case
CRA004775	ENSGALG00010015055	ZNF335	case	control	-0.285618821	0.248648558	-1.148684809	0.250685972	0.715557647	down_in_case
CRA004775	ENSGALG00010015056	ENSGALG00010015056	case	control	-0.339590602	0.466665887	-0.727695363	0.46680009	0.8434807	down_in_case
CRA004775	ENSGALG00010015057	LAS1L	case	control	-0.489492337	0.218682887	-2.238365996	0.025197198	0.305505769	down_in_case
CRA004775	ENSGALG00010015059	ENSGALG00010015059	case	control	-0.460005716	0.27103454	-1.697221749	0.089654745	0.516522282	down_in_case
CRA004775	ENSGALG00010015061	MMP9	case	control	0.479398313	0.470341074	1.019256746			up_in_case
CRA004775	ENSGALG00010015062	ENSGALG00010015062	case	control	0.674093679	0.494680512	1.362684928	0.17298183	0.637372225	up_in_case
CRA004775	ENSGALG00010015063	EEF1AKMT4	case	control	-0.263321192	0.297943035	-0.883797107	0.376805748	0.798523018	down_in_case
CRA004775	ENSGALG00010015064	RFK	case	control	-0.166425082	0.268119457	-0.620712439	0.534788844	0.870981055	down_in_case
CRA004775	ENSGALG00010015066	RUFY1	case	control	0.158470488	0.240101798	0.660013752	0.509245004	0.862971569	up_in_case
CRA004775	ENSGALG00010015068	ESM1	case	control	1.135016719	0.567369571	2.000489235	0.045447461	0.396289181	up_in_case
CRA004775	ENSGALG00010015069	HSPD1	case	control	0.173090652	0.260448165	0.664587721	0.506314225	0.8622397	up_in_case
CRA004775	ENSGALG00010015070	EDN2	case	control	0.146355659	0.685339557	0.213552038	0.830896405	0.963284108	up_in_case
CRA004775	ENSGALG00010015071	SNAI1	case	control	-0.271831063	0.351316792	-0.773749133	0.439079159	0.828368259	down_in_case
CRA004775	ENSGALG00010015072	MAP9	case	control	0.16098345	0.403513278	0.398954531	0.689926708	0.927841776	up_in_case
CRA004775	ENSGALG00010015075	ENSGALG00010015075	case	control	-0.073917988	0.409301407	-0.18059549	0.856685096	0.970913248	down_in_case
CRA004775	ENSGALG00010015076	ITK	case	control	-0.637509129	0.549215577	-1.160763017	0.245738287	0.712046495	down_in_case
CRA004775	ENSGALG00010015080	ASF1A	case	control	-0.054634546	0.285465277	-0.19138771	0.848221848	0.968199438	down_in_case
CRA004775	ENSGALG00010015083	CEP135	case	control	-0.374575585	0.737166453	-0.508128909	0.611362943	0.901413878	down_in_case
CRA004775	ENSGALG00010015084	FOXO6	case	control	0.72425207	0.436706209	1.658442348	0.097228215	0.531393157	up_in_case
CRA004775	ENSGALG00010015085	PSMD2	case	control	-0.201461211	0.357274676	-0.563883265	0.57283358	0.886154542	down_in_case
CRA004775	ENSGALG00010015086	FOXN3	case	control	0.435902953	0.287578597	1.515769799	0.129577571	0.584172159	up_in_case
CRA004775	ENSGALG00010015088	ENSGALG00010015088	case	control	3.041936355	0.908656412	3.347730026	0.000814763	0.060328515	up_in_case
CRA004775	ENSGALG00010015090	GZMA	case	control	-0.030450045	0.655274022	-0.046469177	0.9629363	0.991860454	down_in_case
CRA004775	ENSGALG00010015091	MSN	case	control	-0.162243759	0.361876293	-0.448340392	0.653907554	0.916560183	down_in_case
CRA004775	ENSGALG00010015092	HSPE1	case	control	0.09161016	0.275692076	0.332291597	0.739669084	0.938064864	up_in_case
CRA004775	ENSGALG00010015094	CDK5RAP1	case	control	-0.801489854	0.278329781	-2.879641018	0.003981282	0.129424609	down_in_case
CRA004775	ENSGALG00010015095	RBFOX2	case	control	-0.077155506	0.318265693	-0.24242483	0.808450997	0.955397873	down_in_case
CRA004775	ENSGALG00010015097	MFSD2A	case	control	-0.129821771	0.439380417	-0.295465538	0.767638274	0.944590059	down_in_case
CRA004775	ENSGALG00010015098	HAVCR1	case	control	-0.367645741	0.546871624	-0.672270648	0.501411412	0.860091011	down_in_case
CRA004775	ENSGALG00010015100	MOB4	case	control	0.10065639	0.229499099	0.438591654	0.660957445	0.917907174	up_in_case
CRA004775	ENSGALG00010015101	CAP1	case	control	-0.482608259	0.406934821	-1.185959604	0.235638253	0.702876706	down_in_case
CRA004775	ENSGALG00010015103	ITPK1	case	control	0.542031911	0.300174264	1.805724124	0.070961443	0.469911807	up_in_case
CRA004775	ENSGALG00010015104	CNGA2	case	control	-0.815405794	0.737600013	-1.105485059	0.268949465	0.727586876	down_in_case
CRA004775	ENSGALG00010015105	ENSGALG00010015105	case	control	-0.671052437	0.529943348	-1.266272028	0.205415708	0.674045742	down_in_case
CRA004775	ENSGALG00010015107	MB	case	control	-2.849540234	0.852844223	-3.341220071	0.000834111	0.06088574	down_in_case
CRA004775	ENSGALG00010015108	ENSGALG00010015108	case	control	-0.191563236	0.285316003	-0.671407263	0.501961119	0.860091011	down_in_case

CRA004775	ENSGALG00010015109	PPT1	case	control	-0.091298811	0.248487858	-0.367417596	0.713307549	0.933183532	down_in_case
CRA004775	ENSGALG00010015110	ENSGALG00010015110	case	control	0.149260178	0.33109553	0.450806986	0.652128666	0.915892481	up_in_case
CRA004775	ENSGALG00010015111	ADAMTS2	case	control	-0.001715733	0.466152761	-0.003680623	0.997063294	0.999619266	down_in_case
CRA004775	ENSGALG00010015112	CBLN4	case	control	1.162843528	0.574234602	2.025032146	0.042864063	0.38854564	up_in_case
CRA004775	ENSGALG00010015115	CLDN2	case	control	1.638064511	0.288635709	5.675197005	1.38529E-08	1.58743E-05	up_in_case
CRA004775	ENSGALG00010015117	RASD2	case	control	0.712891005	0.421271203	1.692237685	0.090600664	0.51974756	up_in_case
CRA004775	ENSGALG00010015118	GPX8	case	control	-0.189897767	0.461661901	-0.411335149	0.6808268	0.923896221	down_in_case
CRA004775	ENSGALG00010015119	TLR1A	case	control	0.055465901	0.604822423	0.091706092	0.926931557	0.984216865	up_in_case
CRA004775	ENSGALG00010015122	ENSGALG00010015122	case	control	-0.0287485	0.270676507	-0.106209807	0.91541589	0.981478883	down_in_case
CRA004775	ENSGALG00010015123	CEP85L	case	control	1.677009237	0.510218786	3.286843375	0.001013172	0.066660879	up_in_case
CRA004775	ENSGALG00010015124	UXS1	case	control	0.219085086	0.234186155	0.935516813	0.349522023	0.785360568	up_in_case
CRA004775	ENSGALG00010015126	MCM5	case	control	-0.946020742	0.542406015	-1.744119194	0.081138308	0.496764413	down_in_case
CRA004775	ENSGALG00010015127	RPL11	case	control	-0.373354997	0.280178738	-1.332560061	0.182676219	0.647378665	down_in_case
CRA004775	ENSGALG00010015128	OCRL	case	control	-0.140198734	0.228349745	-0.613964925	0.539238505	0.873987916	down_in_case
CRA004775	ENSGALG00010015129	PRRG3	case	control	0.227132999	0.318219933	0.713761069	0.475374937	0.845979132	up_in_case
CRA004775	ENSGALG00010015131	DOK5	case	control	0.069544083	0.628570785	0.110638427	0.911903074	0.98075825	up_in_case
CRA004775	ENSGALG00010015132	GCNT1	case	control	1.370880725	0.532058126	2.576561956	0.009978832	0.201425687	up_in_case
CRA004775	ENSGALG00010015133	RFTN2	case	control	-0.27602608	0.395620407	-0.697704354	0.485362105	0.851302844	down_in_case
CRA004775	ENSGALG00010015134	PLN	case	control	-1.914227685	0.597717093	-3.202564735	0.001362097	0.077718645	down_in_case
CRA004775	ENSGALG00010015136	TCEB3	case	control	-0.170746069	0.220680649	-0.773724699	0.439093611	0.828368259	down_in_case
CRA004775	ENSGALG00010015137	AMN	case	control	0.59969231	0.702031946	0.854223677	0.392981067	0.807553294	up_in_case
CRA004775	ENSGALG00010015138	ENSGALG00010015138	case	control	0.749732626	0.704784363	1.063775909	0.287430227	0.742456237	up_in_case
CRA004775	ENSGALG00010015140	ASCC3	case	control	0.310736641	0.182098498	1.706420667	0.087929785	0.512984066	up_in_case
CRA004775	ENSGALG00010015141	PFDN4	case	control	0.197824709	0.290112013	0.681890785	0.495308012	0.856382445	up_in_case
CRA004775	ENSGALG00010015142	HNRNPAB	case	control	-0.171328746	0.211443747	-0.810280505	0.417778977	0.818943344	down_in_case
CRA004775	ENSGALG00010015143	EXOC1	case	control	-0.118013028	0.205782276	-0.573484898	0.566316422	0.883033678	down_in_case
CRA004775	ENSGALG00010015145	CLIC2	case	control	-0.312990682	0.212208032	-1.474923824	0.140233024	0.600007375	down_in_case
CRA004775	ENSGALG00010015146	ENSGALG00010015146	case	control	0.006109655	0.267545011	0.02283599	0.981781099	0.997069916	up_in_case
CRA004775	ENSGALG00010015148	ENSGALG00010015148	case	control	0.164809426	0.211387066	0.779657098	0.435592738	0.826905159	up_in_case
CRA004775	ENSGALG00010015149	ENSGALG00010015149	case	control	-0.176346551	0.387727962	-0.454820307	0.64923852	0.915047477	down_in_case
CRA004775	ENSGALG00010015152	BOLL	case	control	0.130705511	0.454637328	0.287494016	0.773734083	0.946296525	up_in_case
CRA004775	ENSGALG00010015153	ERCC6L	case	control	0.479634985	0.645629233	0.742895396	0.457545013	0.839704957	up_in_case
CRA004775	ENSGALG00010015154	CYP24A1	case	control	-0.537766456	0.570672706	-0.942337789	0.346019727	0.784552667	down_in_case
CRA004775	ENSGALG00010015155	HMOX1	case	control	-0.473035867	0.222647438	-2.124596045	0.033620334	0.348565623	down_in_case
CRA004775	ENSGALG00010015156	CNOT6	case	control	0.017141886	0.259287916	0.066111396	0.947289138	0.989454837	up_in_case
CRA004775	ENSGALG00010015157	CDC42BPB	case	control	0.418646029	0.236346923	1.771319989	0.076507505	0.487052593	up_in_case
CRA004775	ENSGALG00010015158	PIN4	case	control	-0.039896635	0.315587918	-0.126420032	0.899399446	0.978790377	down_in_case
CRA004775	ENSGALG00010015159	ENSGALG00010015159	case	control	-1.624647183	0.906287181	-1.792640586	0.073030412	0.47704647	down_in_case
CRA004775	ENSGALG00010015161	GFPT2	case	control	-0.033805812	0.350219717	-0.096527439	0.923101682	0.983159417	down_in_case
CRA004775	ENSGALG00010015163	PLK2	case	control	-0.172500651	0.444797611	-0.387818295	0.698150506	0.929178049	down_in_case
CRA004775	ENSGALG00010015164	ENSGALG00010015164	case	control	0.765357048	0.81562106	0.938373327	0.348052589	0.785270866	up_in_case
CRA004775	ENSGALG00010015165	KLF3	case	control	-0.376725598	0.266703616	-1.412525276	0.157795354	0.62262379	down_in_case
CRA004775	ENSGALG00010015166	YTHDF2	case	control	0.171074633	0.195710544	0.874120676	0.382052539	0.801753951	up_in_case
CRA004775	ENSGALG00010015169	ENSGALG00010015169	case	control	-1.400681135	0.780930502	-1.79360536	0.07287618	0.476411335	down_in_case
CRA004775	ENSGALG00010015171	NHSL2	case	control	-0.559730889	0.393978663	-1.420713713	0.155400004	0.619285223	down_in_case
CRA004775	ENSGALG00010015172	PPP4R3A	case	control	0.241274139	0.200386465	1.204044094	0.228572536	0.695782399	up_in_case
CRA004775	ENSGALG00010015173	ENS-1	case	control	2.257654039	0.850611443	2.654154323	0.007950746	0.179820234	up_in_case
CRA004775	ENSGALG00010015174	ST6GAL2	case	control	-0.071050861	0.526674399	-0.134904718	0.892687209	0.977834811	down_in_case
CRA004775	ENSGALG00010015175	MAPK9	case	control	0.533098533	0.317336448	1.67991586	0.092973687	0.524612711	up_in_case
CRA004775	ENSGALG00010015177	TC2N	case	control	0.887345323	0.520473402	1.70488121	0.088216581	0.513532836	up_in_case
CRA004775	ENSGALG00010015180	PITHD1	case	control	-0.188001793	0.324988978	-0.578486674	0.562935592	0.881532201	down_in_case
CRA004775	ENSGALG00010015181	VPS13A	case	control	0.753966084	0.356104101	2.11726313	0.034237522	0.351343409	up_in_case
CRA004775	ENSGALG00010015182	PHYKPL	case	control	-0.650376922	0.33497924	-1.941543962	0.052192333	0.420135271	down_in_case
CRA004775	ENSGALG00010015186	ENSGALG00010015186	case	control	0.616132391	0.574905633	1.071710477	0.283850093	0.740508942	up_in_case
CRA004775	ENSGALG00010015188	SULT1C3	case	control	2.217715737	1.00440012	2.208000271	0.027244254	0.31561562	up_in_case
CRA004775	ENSGALG00010015190	FBLN5	case	control	0.728499052	0.496239123	1.468040344	0.142093264	0.602343234	up_in_case
CRA004775	ENSGALG00010015191	TOM1	case	control	0.005826773	0.460589186	0.012650693	0.989906476	0.998065748	up_in_case
CRA004775	ENSGALG00010015192	ENSGALG00010015192	case	control	0.719786975	0.394437824	1.82484268	0.068024773	0.463992389	up_in_case
CRA004775	ENSGALG00010015193	LYPLA2	case	control	-0.410914353	0.174524826	-2.354475075	0.018548888	0.270196773	down_in_case
CRA004775	ENSGALG00010015194	TMLHE	case	control	-0.214823405	0.213627461	-1.005598268	0.314608857	0.765098415	down_in_case
CRA004775	ENSGALG00010015196	PDE4D	case	control	0.977014206	0.370876812	2.634336188	0.008430197	0.184298316	up_in_case
CRA004775	ENSGALG00010015198	NME5	case	control	0.204480846	0.352509466	0.58007193	0.561866112	0.881532201	up_in_case
CRA004775	ENSGALG00010015199	ENSGALG00010015199	case	control	-0.583182282	0.215497331	-2.706215797	0.006805481	0.161712092	down_in_case
CRA004775	ENSGALG00010015200	TBC1D1	case	control	0.076982905	0.194541817	0.395713921	0.692316084	0.927867527	up_in_case
CRA004775	ENSGALG00010015201	GCC2	case	control	0.506195102	0.314489757	1.609575799	0.107490495	0.553800541	up_in_case
CRA004775	ENSGALG00010015203	HMGXB4	case	control	0.109485676	0.153015291	0.715521143	0.474287081	0.845684862	up_in_case
CRA004775	ENSGALG00010015204	ENSGALG00010015204	case	control	-0.203494233	0.323751173	-0.628551338	0.529642827	0.869211096	down_in_case
CRA004775	ENSGALG00010015207	AKR1E2	case	control	-0.041764202	0.270664593	-0.154302422	0.877371288	0.974479909	down_in_case
CRA004775	ENSGALG00010015212	GNAQ	case	control	0.210451304	0.295850087	0.711344406	0.476870841	0.846367006	up_in_case
CRA004775	ENSGALG00010015213	RUBCN	case	control	-0.575256025	0.227284884	-2.530991131	0.011374072	0.21639381	down_in_case
CRA004775	ENSGALG00010015218	PLCL1	case	control	0.023835237	0.437971116	0.054421938	0.956599001	0.9909743	up_in_case
CRA004775	ENSGALG00010015227	FYTTD1	case	control	-0.219172758	0.203431687	-1.077377674	0.281311574	0.738078244	down_in_case
CRA004775	ENSGALG00010015234	TRIP11	case	control	0.835623846	0.289507712	2.886361264	0.003897245	0.128824566	up_in_case
CRA004775	ENSGALG00010015241	ENSGALG00010015241	case	control	-0.862904991	0.494072098	-1.746516338	0.080721271	0.495313785	down_in_case
CRA004775	ENSGALG00010015248	ENSGALG00010015248	case	control	-0.198630044	0.213154159	-0.931860983	0.351408374	0.786113067	down_in_case
CRA004775	ENSGALG00010015253	ENSGALG00010015253	case	control	-0.350367344	0.321678678	-1.089184232	0.276072651	0.731001618	down_in_case
CRA004775	ENSGALG00010015260	RASGEF1C	case	control	0.169956815	0.377244616	0.450521513	0.652334446	0.915892481	up_in_case
CRA004775	ENSGALG00010015261	IQCG	case	control	-0.053787622	0.442141279	-0.121652568	0.903174179	0.979310476	down_in_case
CRA004775	ENSGALG00010015285	ALG13	case	control	-0.303657719	0.813061674	-0.373474398	0.708795397	0.931801673	down_in_case
CRA004775	ENSGALG00010015291	ZFP64	case	control	0.03647793	0.142235613	0.256461299	0.79759466	0.952835572	up_in_case
CRA004775	ENSGALG00010015296	RPL35A	case	control	-0.22211633	0.241162741	-0.921022579	0.357038639	0.789838855	down_in_case
CRA004775	ENSGALG00010015298	ENSGALG00010015298	case	control	0.676779194	0.354326331	1.910044881	0.056127435	0.433600201	up_in_case
CRA004775	ENSGALG00010015299	GALE	case	control	-0.318629164	0.247130069	-1.289317667	0.197287672	0.663190847	down_in_case
CRA004775	ENSGALG00010015300	ATXN3	case	control	0.010832767	0.383185444	0.028270299	0.977446569	0.995827348	up_in_case
CRA004775	ENSGALG00010015306	BCAT1	case	control	0.06121373	0.317777343	0.192630884	0.847248056	0.967891337	up_in_case
CRA004775	ENSGALG00010015310	PGM2	case	control	-0.072640016	0.312081419	-0.232759823	0.815947913	0.959064856	down_in_case
CRA004775	ENSGALG00010015314	GMEB1	case	control	0.011820516	0.137875722	0.085733125	0.931678569	0.985044263	up_in_case
CRA004775	ENSGALG00010015318	SALL4	case	control	0.894447436	0.417019714	2.144856478	0.031964324	0.340465854	up_in_case
CRA004775	ENSGALG00010015327	LARGE1	case	control	0.402242615	0.396547972	1.01436054	0.310410746	0.762244121	up_in_case
CRA004775	ENSGALG00010015328	ENSGALG00010015328	case	control	-0.148651572	0.798147365	-0.186245772	0.852252031	0.969500139	down_in_case
CRA004775	ENSGALG00010015333	MED7	case	control	-0.223527579	0.228117791	-0.979877888	0.327146399	0.771098755	down_in_case
CRA004775	ENSGALG00010015339	ENSGALG00010015339	case	control	1.405006492	1.013748901	1.385951186	0.16576181	0.630194031	up_in_case
CRA004775	ENSGALG00010015348	CPSF2	case	control	0.138584385	0.20512901	0.675596226	0.499297038	0.858902178	up_in_case
CRA004775	ENSGALG00010015353	RELL1	case	control	0.335434264	0.198241921	1.692045059	0.090637383	0.51974756	up_in_case
CRA004775	ENSGALG00010015356	ENSGALG00010015356	case	control	-0.3383109	0.226690907	-1.492388488	0.135597331	0.593592099	down_in_case
CRA004775	ENSGALG00010015362	ATP9A	case	control	-0.945434972	0.548874269	-1.722498258	0.084979298	0.505542192	down_in_case
CRA004775	ENSGALG00010015364	CLOCK	case	control	0.908147546	0.29162999	3.11404032	0.001845443	0.086393824	up_in_case
CRA004775	ENSGALG00010015370	ENSGALG00010015370	case	control	0.828578776	0.355735747	2.329197396	0.019848611	0.279077963	up_in_case
CRA004775	ENSGALG00010015374	LIMS1	case	control	-0.556967691	0.38115112	-1.461277855	0.143939205	0.605645941	down_in_case
CRA004775	ENSGALG00010015384	TAF12	case	control	-0.389550085	0.246270515	-1.581797499	0.113695806	0.562992809	down_in_case
CRA004775	ENSGALG00010015386	SLC24A4	case	control	0.866297427	0.900824786	0.961671393	0.336214697	0.776900397	up_in_case
CRA004775	ENSGALG00010015388	PSMD1	case	control	-0.262990327	0.336157151	-0.782343397	0.434012789	0.826150314	down_in_case
CRA004775	ENSGALG00010015390	CCNB3	case	control	0.267864245	0.454353994	0.589549665	0.555492606	0.87981923	up_in_case
CRA004775	ENSGALG00010015391	SYN3	case	control	1.249709337	0.675018544	1.851370379	0.064116287	0.455932569	up_in_case
CRA004775	ENSGALG00010015392	TMEM165	case	control	-0.126006806	0.194948587	-0.646359166	0.518046779	0.865435399	down_in_case
CRA004775	ENSGALG00010015393	DCX	case	control	-0.90684797	0.780353581	-1.162098813	0.24519533	0.712046495	down_in_case
CRA004775	ENSGALG00010015397	SRD5A3	case	control	-0.214849748	0.236406287	-0.908815714	0.36344741	0.793608387	down_in_case
CRA004775	ENSGALG00010015407	TIMP3	case	control	0.534661769	0.40166509	1.331113364	0.183151712	0.647806087	up_in_case
CRA004775	ENSGALG00010015409	FAM13B	case	control	-0.281179785	0.258967141	-1.085773989	0.277578997	0.732797725	down_in_case
CRA004775	ENSGALG00010015410	HTR2B	case	control	0.565117979	0.456165886	1.238843141	0.215403594	0.683654538	up_in_case
CRA004775	ENSGALG00010015413	CARNMT1	case	control	0.278592665	0.220849032	1.261462016	0.207142437	0.675932139	up_in_case
CRA004775	ENSGALG00010015416	CAPN6	case	control	0.433912757	0.543705957	0.798065115	0.424832704	0.821873173	up_in_case
CRA004775	ENSGALG00010015422	ENSGALG00010015422	case	control	-0.616465981	0.395960956	-1.556885778	0.119497607	0.571877909	down_in_case
CRA004775	ENSGALG00010015430	HMGCL	case	control	-0.335312376	0.282375805	-1.187468505	0.235042872	0.702145501	down_in_case
CRA004775	ENSGALG00010015431	RIN3	case	control	-0.412895381	0.410731746	-1.005267755	0.314767937	0.765098415	down_in_case
CRA004775	ENSGALG00010015436	SLC30A9	case	control	0.082776462	0.17829225	0.464274033	0.642451426	0.913111066	up_in_case
CRA004775	ENSGALG00010015440	ENSGALG00010015440	case	control	-0.145344479	0.551993582	-0.263308277	0.792312981	0.951284723	down_in_case
CRA004775	ENSGALG00010015443	PAK3	case	control	0.471995624	0.247702969	1.905490381	0.056716357	0.435477592	up_in_case
CRA004775	ENSGALG00010015449	HDX	case	control	0.319438234	0.297040651	1.075402418	0.2821946	0.738291085	up_in_case
CRA004775	ENSGALG00010015466	NMRK1	case	control	0.324718162	0.35951489	0.903211999	0.366413391	0.793608387	up_in_case
CRA004775	ENSGALG00010015469	TMEM33	case	control	-0.226042195	0.230428239	-0.980965685	0.326609664	0.770998457	down_in_case
CRA004775	ENSGALG00010015472	LGMN	case	control	0.095818817	0.334773411	0.286219915	0.774709691	0.946319184	up_in_case
CRA004775	ENSGALG00010015477	ARMC9	case	control	-0.226321223	0.462861826	-0.488960659	0.62486955	0.90648604	down_in_case
CRA004775	ENSGALG00010015478	FBXO7	case	control	-0.209337646	0.115281754	-1.815878399	0.069389027	0.468451304	down_in_case
CRA004775	ENSGALG00010015481	GOLGA5	case	control	-0.143900757	0.209297049	-0.687543172	0.491740505	0.855746361	down_in_case
CRA004775	ENSGALG00010015483	SGCD	case	control	0.282286345	0.443204578	0.636921094	0.524176242	0.866784927	up_in_case

CRA004775	ENSGALG00010015485	BCAS1	case	control	1.052129714	0.37746346	2.787368387	0.005313803	0.148215235	up_in_case
CRA004775	ENSGALG00010015486	MTFR1L	case	control	-0.059686699	0.323921452	-0.184262877	0.853807235	0.970073177	down_in_case
CRA004775	ENSGALG00010015487	RPS6KA6	case	control	0.359915466	0.269525986	1.335364618	0.181757039	0.647378665	up_in_case
CRA004775	ENSGALG00010015490	RANBP2	case	control	0.311509889	0.210644133	1.478844367	0.139181915	0.599104916	up_in_case
CRA004775	ENSGALG00010015493	OSTF1	case	control	-0.528864362	0.365159619	-1.448310094	0.147530346	0.610831526	down_in_case
CRA004775	ENSGALG00010015494	CHRDL1	case	control	0.567100344	0.49494134	1.145793043	0.251880794	0.716658534	up_in_case
CRA004775	ENSGALG00010015498	MID2	case	control	-0.101308186	0.309269766	-0.327572227	0.743235124	0.939218065	down_in_case
CRA004775	ENSGALG00010015505	CHGA	case	control	2.26171119	0.562238006	4.02269353	5.75363E-05	0.014927967	up_in_case
CRA004775	ENSGALG00010015507	AMMECR1	case	control	0.075085655	0.341706791	0.219737088	0.826075918	0.961878235	up_in_case
CRA004775	ENSGALG00010015508	FUCA1	case	control	-0.248718706	0.210311806	-1.182618852	0.236960238	0.703871614	down_in_case
CRA004775	ENSGALG00010015516	SELENON	case	control	-0.197683701	0.227587801	-0.868604119	0.385063701	0.803182776	down_in_case
CRA004775	ENSGALG00010015520	UBE3A	case	control	0.393703937	0.214728391	1.833497356	0.066728641	0.462498477	up_in_case
CRA004775	ENSGALG00010015521	DKC1	case	control	-0.153401673	0.21025528	-0.729597242	0.465636408	0.843383332	down_in_case
CRA004775	ENSGALG00010015523	MRPL22	case	control	-0.122623423	0.231654935	-0.529336547	0.596572007	0.895574418	down_in_case
CRA004775	ENSGALG00010015532	NXT2	case	control	-0.122036797	0.311332986	-0.391981586	0.695071819	0.928924129	down_in_case
CRA004775	ENSGALG00010015533	PSAT1	case	control	-0.067269123	0.314360448	-0.213987234	0.830557012	0.963232966	down_in_case
CRA004775	ENSGALG00010015540	ACSL4	case	control	-0.049899393	0.561649598	-0.088843349	0.929205612	0.98473447	down_in_case
CRA004775	ENSGALG00010015542	CNGA3	case	control	1.092613919	0.705211415	1.549342362	0.121299442	0.573473028	up_in_case
CRA004775	ENSGALG00010015544	RTCB	case	control	-0.333439472	0.224864931	-1.482843367	0.138116031	0.597257214	down_in_case
CRA004775	ENSGALG00010015555	PNRC2	case	control	0.201840682	0.265105758	0.761359101	0.446442609	0.833749389	up_in_case
CRA004775	ENSGALG00010015557	IRS4	case	control	0.363908219	0.403598826	0.901658268	0.367238428	0.794067504	up_in_case
CRA004775	ENSGALG00010015558	MPP1	case	control	-0.323023057	0.379613013	-0.850927248	0.394809767	0.808974685	down_in_case
CRA004775	ENSGALG00010015562	IRF9	case	control	-0.094514988	0.349353104	-0.270542861	0.786742648	0.95041797	down_in_case
CRA004775	ENSGALG00010015563	B3GNT7	case	control	1.080212306	0.664753734	1.624981177	0.10416657	0.546673752	up_in_case
CRA004775	ENSGALG00010015564	PKD2L2	case	control	-0.633128063	0.76813296	-0.824242801	0.409801612	0.815275169	down_in_case
CRA004775	ENSGALG00010015565	ENSGALG00010015565	case	control	-1.264998978	2.174953353	-0.5816212	0.56082186	0.881008373	down_in_case
CRA004775	ENSGALG00010015567	ENSGALG00010015567	case	control	0.778726422	0.354047545	2.199496742	0.027842621	0.318522359	up_in_case
CRA004775	ENSGALG00010015570	LIMCH1	case	control	0.480828824	0.31759724	1.513957818	0.130036538	0.584671406	up_in_case
CRA004775	ENSGALG00010015571	DUSP15	case	control	-0.442592314	0.409856409	-1.079871643	0.280199343	0.73661445	down_in_case
CRA004775	ENSGALG00010015573	MAN1C1	case	control	-0.146614925	0.275295586	-0.532572743	0.594329374	0.894956597	down_in_case
CRA004775	ENSGALG00010015574	C14orf132	case	control	0.082898522	0.500361552	0.165677241	0.868410957	0.973370973	up_in_case
CRA004775	ENSGALG00010015580	GEMIN5	case	control	-0.021705073	0.243152291	-0.089265344	0.928871035	0.98473447	down_in_case
CRA004775	ENSGALG00010015583	PRDM4	case	control	-0.059509949	0.415458287	-0.143239287	0.886101203	0.976819452	down_in_case
CRA004775	ENSGALG00010015584	TTLL9	case	control	-0.183822061	0.418716351	-0.439013334	0.660651875	0.917907174	down_in_case
CRA004775	ENSGALG00010015585	BDKRB1	case	control	0.229768674	0.510640806	0.449961443	0.652738242	0.916272312	up_in_case
CRA004775	ENSGALG00010015588	BDKRB2	case	control	1.05756049	0.710160866	1.489184409	0.136438815	0.595518184	up_in_case
CRA004775	ENSGALG00010015589	FRMD3	case	control	0.857451263	0.433070728	1.979933549	0.047710996	0.405986328	up_in_case
CRA004775	ENSGALG00010015596	UCHL1	case	control	-1.192893766	0.707552751	-1.685943224	0.091806736	0.521667116	down_in_case
CRA004775	ENSGALG00010015605	LDLRAP1	case	control	-0.178883272	0.200152349	-0.893735562	0.371463394	0.795818272	down_in_case
CRA004775	ENSGALG00010015606	TMEM131	case	control	0.515285213	0.197524543	2.608714877	0.009088293	0.192562589	up_in_case
CRA004775	ENSGALG00010015610	ENSGALG00010015610	case	control	0.357579357	0.799273777	0.447380319	0.654600484	0.916640657	up_in_case
CRA004775	ENSGALG00010015613	PWP1	case	control	-0.098254505	0.232707097	-0.422223932	0.672861575	0.921952322	down_in_case
CRA004775	ENSGALG00010015614	ATG2B	case	control	0.227520559	0.310856228	0.731915715	0.464220014	0.842643327	up_in_case
CRA004775	ENSGALG00010015616	NADK2	case	control	0.146423395	0.187103504	0.782579649	0.433873996	0.82600046	up_in_case
CRA004775	ENSGALG00010015617	SRSF10	case	control	-0.045107285	0.148249467	-0.304266082	0.760925183	0.942236668	down_in_case
CRA004775	ENSGALG00010015618	NCL	case	control	-0.113985968	0.219148471	-0.520131249	0.6029721	0.898692785	down_in_case
CRA004775	ENSGALG00010015620	PDRG1	case	control	-0.137696951	0.345317973	-0.398754082	0.690074414	0.927867527	down_in_case
CRA004775	ENSGALG00010015621	IDNK	case	control	-0.043901426	0.383241602	-0.114552871	0.908799537	0.98075825	down_in_case
CRA004775	ENSGALG00010015624	ITGA1	case	control	0.83886516	0.337849732	2.482953456	0.013029811	0.229478386	up_in_case
CRA004775	ENSGALG00010015626	XKR7	case	control	0.140101232	0.595846603	0.235129699	0.814108069	0.958686054	up_in_case
CRA004775	ENSGALG00010015628	UBQLN1	case	control	0.104818208	0.215622186	0.486119772	0.626882249	0.90758663	up_in_case
CRA004775	ENSGALG00010015631	GSKIP	case	control	0.121515455	0.200948342	0.604709917	0.545371758	0.876563448	up_in_case
CRA004775	ENSGALG00010015632	COL4A5	case	control	0.986546244	0.525569205	1.877100551	0.060504314	0.445397937	up_in_case
CRA004775	ENSGALG00010015633	CCM2L	case	control	-0.259921358	0.386697639	-0.672156569	0.501484027	0.860091011	down_in_case
CRA004775	ENSGALG00010015635	ABTB3	case	control	-0.568025683	0.45535282	-1.247440794	0.212235917	0.682275341	down_in_case
CRA004775	ENSGALG00010015636	APBB2	case	control	0.16605641	0.324371671	0.51193253	0.608698231	0.901007337	up_in_case
CRA004775	ENSGALG00010015643	ENSGALG00010015643	case	control	0.247865217	0.337739949	0.733893687	0.463013535	0.842456502	up_in_case
CRA004775	ENSGALG00010015645	MACO1	case	control	0.212325451	0.228978464	0.927272578	0.353785028	0.78730441	up_in_case
CRA004775	ENSGALG00010015647	PDZD2	case	control	-0.809679162	0.436369659	-1.855489137	0.063526409	0.453557451	down_in_case
CRA004775	ENSGALG00010015653	HCK	case	control	0.193843591	0.353135631	0.548921077	0.583059613	0.889803356	up_in_case
CRA004775	ENSGALG00010015655	ENSGALG00010015655	case	control	0.335112135	0.442033218	0.758115276	0.448381975	0.83429564	up_in_case
CRA004775	ENSGALG00010015660	KLHL3	case	control	-0.149859251	0.240972744	-0.621892954	0.534012259	0.870956093	down_in_case
CRA004775	ENSGALG00010015668	ZNF217	case	control	-0.169922605	0.391327186	-0.434221315	0.664127736	0.918295135	down_in_case
CRA004775	ENSGALG00010015669	INPP4A	case	control	0.138943828	0.317993642	0.436939013	0.662155578	0.918136475	up_in_case
CRA004775	ENSGALG00010015670	ENSGALG00010015670	case	control	0.235311428	0.212836362	1.105597868	0.268900613	0.727586876	up_in_case
CRA004775	ENSGALG00010015674	RFKL	case	control	0.192910788	0.307614745	0.627118143	0.530581795	0.869211096	up_in_case
CRA004775	ENSGALG00010015676	TGM2	case	control	-0.132421337	0.345295998	-0.383500932	0.701348379	0.930068722	down_in_case
CRA004775	ENSGALG00010015677	GRHL3	case	control	0.038444929	0.469320804	0.081916097	0.934713434	0.985753848	up_in_case
CRA004775	ENSGALG00010015680	ENSGALG00010015680	case	control	-0.036486801	0.261863913	-0.139334973	0.889185455	0.977162915	down_in_case
CRA004775	ENSGALG00010015684	PDE6D	case	control	-0.117462623	0.234183246	-0.501584227	0.615960017	0.9028981	down_in_case
CRA004775	ENSGALG00010015686	CNOT8	case	control	-0.187223893	0.206307537	-0.907499047	0.364142953	0.793608387	down_in_case
CRA004775	ENSGALG00010015691	EXOC3L4	case	control	2.350679247	0.651524954	3.607965024	0.000308608	0.035363914	up_in_case
CRA004775	ENSGALG00010015693	COA5	case	control	-0.176158218	0.228576381	-0.770675503	0.440899295	0.829353567	down_in_case
CRA004775	ENSGALG00010015695	ASAH1	case	control	0.421820501	0.317732235	1.327597439	0.184311131	0.648746785	up_in_case
CRA004775	ENSGALG00010015697	FAXDC2	case	control	0.487789213	0.259370395	1.880666501	0.060017299	0.445397937	up_in_case
CRA004775	ENSGALG00010015698	TNFAIP2	case	control	-0.218958388	0.271353481	-0.806912029	0.419717168	0.819388688	down_in_case
CRA004775	ENSGALG00010015701	UNC50	case	control	-0.044204743	0.178815856	-0.247208185	0.804747111	0.954576316	down_in_case
CRA004775	ENSGALG00010015703	GK5	case	control	-0.045374193	0.424735716	-0.106829239	0.914924451	0.981393117	down_in_case
CRA004775	ENSGALG00010015706	ENSGALG00010015706	case	control	0.142238616	0.365853544	0.388785672	0.697434701	0.929092636	up_in_case
CRA004775	ENSGALG00010015709	HNRNPA0	case	control	-0.251078966	0.217932603	-1.152094558	0.249282219	0.714439307	down_in_case
CRA004775	ENSGALG00010015711	LBFABP	case	control	1.079367467	0.448609707	2.406027893	0.016127033	0.25344686	up_in_case
CRA004775	ENSGALG00010015716	MGAT4A	case	control	0.239305493	0.399162376	0.59951916	0.548826737	0.877623839	up_in_case
CRA004775	ENSGALG00010015719	XRN1	case	control	0.130950578	0.339024223	0.386257291	0.699306127	0.929347461	up_in_case
CRA004775	ENSGALG00010015729	RHCE	case	control	-0.584820527	0.659526538	-0.886727817	0.375225458	0.798503808	down_in_case
CRA004775	ENSGALG00010015731	PHF6	case	control	-0.023692061	0.210646282	-0.112473198	0.91044822	0.98075825	down_in_case
CRA004775	ENSGALG00010015733	COL4A6	case	control	0.364737021	0.554696638	0.657543234	0.510831681	0.863170205	up_in_case
CRA004775	ENSGALG00010015734	TSHZ2	case	control	0.226427637	0.363135154	0.623535437	0.532932722	0.869976453	up_in_case
CRA004775	ENSGALG00010015738	CRACDL	case	control	0.598553408	0.976859729	0.612732197	0.54005343	0.87429015	up_in_case
CRA004775	ENSGALG00010015742	HPRT1	case	control	-0.01364771	0.246983242	-0.055257635	0.955933213	0.990926004	down_in_case
CRA004775	ENSGALG00010015746	PAPOLB	case	control	0.203613982	0.178688481	1.13949137	0.254498266	0.718419134	up_in_case
CRA004775	ENSGALG00010015749	ATR	case	control	0.15491387	0.345921697	0.447829297	0.654276398	0.916560183	up_in_case
CRA004775	ENSGALG00010015751	MYOM3	case	control	-0.270832652	0.888993712	-0.304650807	0.760632119	0.942236668	down_in_case
CRA004775	ENSGALG00010015753	RPRD1B	case	control	0.001585893	0.1884906	0.008413642	0.993286964	0.998606291	up_in_case
CRA004775	ENSGALG00010015756	PCM1	case	control	0.228775143	0.243182699	0.940754191	0.346830841	0.784552667	up_in_case
CRA004775	ENSGALG00010015758	PCDH19	case	control	0.005083527	0.424774001	0.011967604	0.990451461	0.998065748	up_in_case
CRA004775	ENSGALG00010015759	EIF5	case	control	0.592900235	0.302556252	1.959636368	0.050038307	0.414161886	up_in_case
CRA004775	ENSGALG00010015764	TTI1	case	control	-0.250834765	0.191716345	-1.308364006	0.190749875	0.656209975	down_in_case
CRA004775	ENSGALG00010015765	ENSGALG00010015765	case	control	-0.062070356	1.219332648	-0.050905187	0.959401073	0.991431439	down_in_case
CRA004775	ENSGALG00010015766	TLE1	case	control	-0.146902274	0.267684882	-0.548788087	0.583150887	0.889803356	down_in_case
CRA004775	ENSGALG00010015769	TMEM50A	case	control	0.029371529	0.196798559	0.149246667	0.881358997	0.975011509	up_in_case
CRA004775	ENSGALG00010015774	PLS1	case	control	1.795986849	0.533074244	3.369112032	0.000754108	0.057648813	up_in_case
CRA004775	ENSGALG00010015776	SPOCK1	case	control	1.173091808	0.469196522	2.500214201	0.012411824	0.224435632	up_in_case
CRA004775	ENSGALG00010015778	PABIR3	case	control	-0.206397936	0.206597669	-0.999033228	0.317778595	0.766440025	down_in_case
CRA004775	ENSGALG00010015782	ATG4A	case	control	0.26638613	0.207406401	1.284367927	0.19901325	0.666028746	up_in_case
CRA004775	ENSGALG00010015785	FGL1	case	control	0.821341	0.353354907	2.324408077	0.020103633	0.280632942	up_in_case
CRA004775	ENSGALG00010015788	TRPC1	case	control	0.078263457	0.205849125	0.380198154	0.70379833	0.930301916	up_in_case
CRA004775	ENSGALG00010015792	MARK3	case	control	0.295338647	0.092025985	3.209296243	0.001330603	0.0764133	up_in_case
CRA004775	ENSGALG00010015793	RSRP1	case	control	0.228449528	0.34111702	0.669710141	0.503042585	0.860900883	up_in_case
CRA004775	ENSGALG00010015795	PSMD10	case	control	-0.518757366	0.161408127	-3.213948237	0.001309233	0.075963119	down_in_case
CRA004775	ENSGALG00010015796	LARP1	case	control	0.084763356	0.307528219	0.275627896	0.782833881	0.94936527	up_in_case
CRA004775	ENSGALG00010015797	PIP5K1B	case	control	0.156224144	0.254508719	0.613826296	0.539330119	0.873987916	up_in_case
CRA004775	ENSGALG00010015798	IL22RA1	case	control	0.928528198	0.50347669	1.844232747	0.065149228	0.457949961	up_in_case
CRA004775	ENSGALG00010015800	BVES	case	control	0.106620366	0.581785832	0.183263943	0.854590924	0.97035175	up_in_case
CRA004775	ENSGALG00010015801	VSTM2L	case	control	0.276593302	0.312491214	0.885123451	0.376090053	0.798523018	up_in_case
CRA004775	ENSGALG00010015803	SYF2	case	control	-0.144032088	0.233117156	-0.617852803	0.536672373	0.872114864	down_in_case
CRA004775	ENSGALG00010015806	CKB	case	control	-0.117303035	0.433848756	-0.270377715	0.786869683	0.95041797	down_in_case
CRA004775	ENSGALG00010015807	ENSGALG00010015807	case	control	-2.061301673	1.105065904	-1.865320126	0.062136546	0.447819518	down_in_case
CRA004775	ENSGALG00010015809	PCOLCE2	case	control	0.083596993	0.328543388	0.254447345	0.799149978	0.952835572	up_in_case
CRA004775	ENSGALG00010015810	LIPT1	case	control	-0.176714287	0.244815959	-0.72182503	0.470402058	0.844782383	down_in_case
CRA004775	ENSGALG00010015811	CTNNBL1	case	control	-0.204893286	0.161689088	-1.267205405	0.205081854	0.674045742	down_in_case
CRA004775	ENSGALG00010015813	ENSGALG00010015813	case	control	0.183982844	0.309625497	0.594210896	0.552371066	0.878258849	up_in_case
CRA004775	ENSGALG00010015814	PAQR9	case	control	0.520929875	0.450158127	1.157215306	0.247184406	0.712046495	up_in_case
CRA004775	ENSGALG00010015816	MITD1	case	control	-0.598034003	0.451498188	-1.324554602	0.185318922	0.6491093	down_in_case
CRA004775	ENSGALG00010015820	ENSGALG00010015820	case	control	-0.61453843	0.433462444	-1.417743193	0.156265755	0.6201473	down_in_case
CRA004775	ENSGALG00010015822	WFDC2	case	control	-0.147020131	0.754566543	-0.194840511	0.845517807	0.966993336	down_in_case
CRA004775	ENSGALG00010015825	RUNX3	case	control	-0.26711755	0.472521408	-0.565302534	0.571868005	0.885500402	down_in_case

CRA004775	ENSGALG00010015827	ENSGALG00010015827	case	control	-0.121246892	0.412677774	-0.293805238	0.768906732	0.945220496	down_in_case
CRA004775	ENSGALG00010015829	SMAD5	case	control	0.357696792	0.138418007	2.584178164	0.009761136	0.200037819	up_in_case
CRA004775	ENSGALG00010015831	LYG2	case	control	0.236612043	0.527270032	0.448749272	0.653612537	0.916470479	up_in_case
CRA004775	ENSGALG00010015832	RAB39B	case	control	-0.131993857	0.252823913	-0.522078213	0.601615875	0.898185892	down_in_case
CRA004775	ENSGALG00010015834	STARD8	case	control	-0.231070028	0.259172516	-0.891568411	0.372624308	0.796268311	down_in_case
CRA004775	ENSGALG00010015837	GDA	case	control	-0.153942323	0.587770057	-0.261909094	0.793391528	0.951585426	down_in_case
CRA004775	ENSGALG00010015838	IFNLR1	case	control	0.115285928	1.655438287	0.069640728	0.944479619	0.988367831	up_in_case
CRA004775	ENSGALG00010015839	U2SURP	case	control	0.371971917	0.189228778	1.96572593	0.049330278	0.410866536	up_in_case
CRA004775	ENSGALG00010015840	MTUS1	case	control	0.308242201	0.293473946	1.050322201	0.293570001	0.746438942	up_in_case
CRA004775	ENSGALG00010015843	ENSGALG00010015843	case	control	-0.193157725	0.426668308	-0.452711677	0.65075637	0.915614449	down_in_case
CRA004775	ENSGALG00010015845	SAP30L	case	control	0.162817075	0.375429958	0.433681626	0.664519651	0.918557471	up_in_case
CRA004775	ENSGALG00010015848	UBE2B	case	control	0.352538781	0.43046829	0.818965738	0.412805962	0.817112606	up_in_case
CRA004775	ENSGALG00010015852	ZFAND5	case	control	0.053438192	0.477207828	0.111980962	0.910838501	0.98075825	up_in_case
CRA004775	ENSGALG00010015855	NIPAL3	case	control	-0.363029338	0.257835422	-1.407988614	0.159134441	0.624907945	down_in_case
CRA004775	ENSGALG00010015856	TXNDC9	case	control	0.389271719	0.200562502	1.940899796	0.052270433	0.420135271	up_in_case
CRA004775	ENSGALG00010015858	CHST2	case	control	-0.575629683	0.230298039	-2.499498853	0.01243691	0.224435632	down_in_case
CRA004775	ENSGALG00010015861	PPP2CA	case	control	0.089564551	0.190802588	0.46940952	0.638776945	0.911090319	up_in_case
CRA004775	ENSGALG00010015862	PDGFRL	case	control	-0.201895441	0.475900018	-0.424239195	0.671391378	0.921605102	down_in_case
CRA004775	ENSGALG00010015863	ENSGALG00010015863	case	control	0.472019242	0.799095755	0.590691715	0.554727	0.879618425	up_in_case
CRA004775	ENSGALG00010015865	VRK1	case	control	-0.033903397	0.424208584	-0.079921525	0.93629967	0.986325359	down_in_case
CRA004775	ENSGALG00010015866	ARHGEF6	case	control	-0.125460188	0.253056555	-0.495779244	0.62005017	0.905128439	down_in_case
CRA004775	ENSGALG00010015867	PICALML	case	control	0.038621642	0.127284024	0.30342883	0.761563078	0.942236668	up_in_case
CRA004775	ENSGALG00010015869	SLC7A2	case	control	0.428811115	0.496865751	0.863032145	0.388119793	0.804270424	up_in_case
CRA004775	ENSGALG00010015870	EIF5B	case	control	0.445021738	0.24304333	1.831038678	0.06709477	0.462498477	up_in_case
CRA004775	ENSGALG00010015874	CLIC4	case	control	0.074447147	0.288374882	0.258160998	0.796282657	0.952835572	up_in_case
CRA004775	ENSGALG00010015875	DNTTIP1	case	control	-0.130721233	0.331581773	-0.394235281	0.69340734	0.928219572	down_in_case
CRA004775	ENSGALG00010015876	SKP1	case	control	-0.056670033	0.245822772	-0.23053207	0.817678346	0.959868101	down_in_case
CRA004775	ENSGALG00010015877	REV1	case	control	0.061551416	0.217020056	0.283620864	0.776700946	0.946929223	up_in_case
CRA004775	ENSGALG00010015879	TRMT61A	case	control	-0.832777805	0.223234976	-3.730498768	0.000191101	0.028256246	down_in_case
CRA004775	ENSGALG00010015881	GAB3	case	control	-0.259343257	0.407929371	-0.635755293	0.524935931	0.867302346	down_in_case
CRA004775	ENSGALG00010015886	AFF3	case	control	-0.908942633	0.558603787	-1.627168763	0.10370126	0.545522581	down_in_case
CRA004775	ENSGALG00010015887	SLC9A9	case	control	-0.421725623	0.42146146	-1.000626778	0.317007279	0.765974554	down_in_case
CRA004775	ENSGALG00010015889	SH3BP5	case	control	0.022095226	0.322884575	0.068430726	0.945442764	0.988803122	up_in_case
CRA004775	ENSGALG00010015892	UBE2C	case	control	-0.468402823	0.562611814	-0.832550636	0.405098209	0.81327583	down_in_case
CRA004775	ENSGALG00010015893	TCF7	case	control	-0.22637942	0.67306739	-0.336339902	0.736614564	0.937549044	down_in_case
CRA004775	ENSGALG00010015894	CEP78	case	control	0.172013919	0.470067608	0.365934423	0.714414009	0.93334033	up_in_case
CRA004775	ENSGALG00010015895	RBMXL1	case	control	-0.200061232	0.280044447	-0.714390999	0.474985436	0.84579952	down_in_case
CRA004775	ENSGALG00010015896	LONRF2	case	control	0.61985191	0.25469627	2.433690567	0.014945766	0.244665743	up_in_case
CRA004775	ENSGALG00010015898	ENSGALG00010015898	case	control	-0.331774208	0.66752854	-0.497018761	0.619175822	0.904574831	down_in_case
CRA004775	ENSGALG00010015899	SMARCA1	case	control	0.027804012	0.197735104	0.14061242	0.888176136	0.977118922	up_in_case
CRA004775	ENSGALG00010015900	ENSGALG00010015900	case	control	0.118531882	0.324194782	0.365619341	0.714649141	0.93334033	up_in_case
CRA004775	ENSGALG00010015902	ENSGALG00010015902	case	control	-0.54599537	0.494210811	-1.104782328	0.26925392	0.72765348	down_in_case
CRA004775	ENSGALG00010015904	MTMR7	case	control	1.139113094	0.521314424	2.185078798	0.02888307	0.323755716	up_in_case
CRA004775	ENSGALG00010015905	ANPEP	case	control	0.308262371	0.386731784	0.797096033	0.42539526	0.822283278	up_in_case
CRA004775	ENSGALG00010015906	BAG5	case	control	-0.131184823	0.314596412	-0.416994021	0.676682781	0.922924485	down_in_case
CRA004775	ENSGALG00010015908	CHRD	case	control	-0.285839504	0.378680518	-0.754830233	0.450350851	0.835404673	down_in_case
CRA004775	ENSGALG00010015909	TNNC2	case	control	-0.171680341	0.781205453	-0.219763367	0.826055451	0.961878235	down_in_case
CRA004775	ENSGALG00010015910	C5orf15	case	control	-0.039758074	0.163277695	-0.243499723	0.8076183	0.955397873	down_in_case
CRA004775	ENSGALG00010015911	TM9SF2L	case	control	-0.086694395	0.147158669	-0.589121905	0.5557795	0.87981923	down_in_case
CRA004775	ENSGALG00010015913	CHST10	case	control	-0.241381059	0.253381078	-0.952640428	0.340772286	0.780875343	down_in_case
CRA004775	ENSGALG00010015914	SRRM1	case	control	-0.275995538	0.18550237	-1.487827558	0.136796376	0.596224078	down_in_case
CRA004775	ENSGALG00010015916	COA8	case	control	0.296115896	0.324582746	0.912297093	0.361612344	0.792757982	up_in_case
CRA004775	ENSGALG00010015917	TMEM63A	case	control	-0.442446641	0.288165453	-1.535390992	0.124687815	0.577300385	down_in_case
CRA004775	ENSGALG00010015918	BICD1	case	control	0.480296706	0.492387683	0.975444192	0.329339972	0.77237154	up_in_case
CRA004775	ENSGALG00010015919	ENSGALG00010015919	case	control	-0.190919144	0.573016264	-0.333182767	0.738996326	0.938064864	down_in_case
CRA004775	ENSGALG00010015920	KCNE5	case	control	0.413333841	1.320942372	0.312908307	0.754350326	0.941900693	up_in_case
CRA004775	ENSGALG00010015921	SNX21	case	control	-0.080297172	0.473983968	-0.169409047	0.865474908	0.972629493	down_in_case
CRA004775	ENSGALG00010015923	RCAN3	case	control	-0.133355674	0.209062442	-0.637874851	0.52355515	0.866638286	down_in_case
CRA004775	ENSGALG00010015927	GLA	case	control	-0.407532862	0.312165182	-1.305503899	0.19172132	0.656959697	down_in_case
CRA004775	ENSGALG00010015928	CAPN7	case	control	-0.024096445	0.190255641	-0.126652987	0.899215057	0.978790377	down_in_case
CRA004775	ENSGALG00010015931	RBM47	case	control	-0.266346959	0.685476967	-0.388557124	0.697603789	0.929092636	down_in_case
CRA004775	ENSGALG00010015932	ACOT8	case	control	-0.502624239	0.29294587	-1.715758062	0.086206333	0.508087097	down_in_case
CRA004775	ENSGALG00010015935	EPHX1	case	control	0.451906385	0.254181939	1.77788551	0.075422661	0.483342345	up_in_case
CRA004775	ENSGALG00010015936	RPL36AL	case	control	-0.132912676	0.211416085	-0.628678166	0.529559775	0.869211096	down_in_case
CRA004775	ENSGALG00010015937	ENSGALG00010015937	case	control	0.021819632	0.350269116	0.062293908	0.950328779	0.990168609	up_in_case
CRA004775	ENSGALG00010015939	SUN2	case	control	-0.160805921	0.216566675	-0.742523848	0.457770008	0.839704957	down_in_case
CRA004775	ENSGALG00010015942	AP3S2	case	control	-0.341939135	0.243920248	-1.401848097	0.160960612	0.626839247	down_in_case
CRA004775	ENSGALG00010015946	TLE4	case	control	0.169124655	0.241803616	0.6994298	0.48428347	0.85082177	up_in_case
CRA004775	ENSGALG00010015954	CTSA	case	control	-0.27604923	0.248030786	-1.112963577	0.265724073	0.726742745	down_in_case
CRA004775	ENSGALG00010015957	ENSGALG00010015957	case	control	-1.273713618	0.598774033	-2.127202499	0.033403262	0.348503984	down_in_case
CRA004775	ENSGALG00010015959	TRNAU1AP	case	control	-0.001693491	0.366683472	-0.004618399	0.996315064	0.999606847	down_in_case
CRA004775	ENSGALG00010015960	CHRNA9	case	control	-3.170589668	1.083192084	-2.9270798	0.003421611	0.125102734	down_in_case
CRA004775	ENSGALG00010015963	DIPK2A	case	control	-0.151391689	0.445875301	-0.339538182	0.734204338	0.936819725	down_in_case
CRA004775	ENSGALG00010015964	KLC1	case	control	-0.08522739	0.268085454	-0.317911279	0.750552237	0.941495694	down_in_case
CRA004775	ENSGALG00010015967	ZSWIM1	case	control	-0.474715562	0.312417847	-1.519489257	0.12863939	0.58341697	down_in_case
CRA004775	ENSGALG00010015970	THPO	case	control	-0.021247633	0.556474005	-0.038182616	0.969542082	0.994270502	down_in_case
CRA004775	ENSGALG00010015971	ENSGALG00010015971	case	control	-0.50189305	0.417742368	-1.201441578	0.229579956	0.695782399	down_in_case
CRA004775	ENSGALG00010015972	FSTL4	case	control	-0.713523999	0.605959259	-1.177511506	0.238991408	0.705835663	down_in_case
CRA004775	ENSGALG00010015974	VPS37A	case	control	0.492207279	0.249198511	1.975161399	0.048249835	0.407414924	up_in_case
CRA004775	ENSGALG00010015975	BTK	case	control	-0.477525228	0.41831139	-1.141554447	0.253639268	0.718204856	down_in_case
CRA004775	ENSGALG00010015979	ENSGALG00010015979	case	control	0.075644024	0.56672544	0.13347561	0.893817252	0.977834811	up_in_case
CRA004775	ENSGALG00010015980	EXOSC7	case	control	-0.359990423	0.265150924	-1.357681194	0.174564867	0.639597725	down_in_case
CRA004775	ENSGALG00010015983	CNOT7	case	control	0.341786245	0.11704263	2.920185956	0.003498226	0.125102734	up_in_case
CRA004775	ENSGALG00010015984	PDCL3	case	control	-0.122102593	0.260803616	-0.468178298	0.63965709	0.911400337	down_in_case
CRA004775	ENSGALG00010015985	TIMM8A	case	control	-0.369452352	0.257229661	-1.436274301	0.150924304	0.614921512	down_in_case
CRA004775	ENSGALG00010015986	NEURL2	case	control	-0.378532354	0.545409233	-0.694033636	0.487661115	0.853267336	down_in_case
CRA004775	ENSGALG00010015987	POLR2H	case	control	-0.481223385	0.261784717	-1.838240946	0.066026909	0.461115298	down_in_case
CRA004775	ENSGALG00010015989	ENAH	case	control	1.237721918	0.300907989	4.113290318	3.90059E-05	0.013135644	up_in_case
CRA004775	ENSGALG00010015992	ATP10A	case	control	0.000745559	0.417870923	0.001784184	0.998576428	0.999914248	up_in_case
CRA004775	ENSGALG00010015994	ENSGALG00010015994	case	control	-0.41993612	0.227015888	-1.84980938	0.064341028	0.456529144	down_in_case
CRA004775	ENSGALG00010016004	XRCC3	case	control	0.193220822	0.436897658	0.442256484	0.658303617	0.917354438	up_in_case
CRA004775	ENSGALG00010016006	PLTP	case	control	0.251433115	0.646488666	0.38892115	0.697334476	0.929092636	up_in_case
CRA004775	ENSGALG00010016008	TAF7	case	control	-0.1826386	0.234417939	-0.779115291	0.435911803	0.826905159	down_in_case
CRA004775	ENSGALG00010016011	ADD1	case	control	0.092266276	0.160132443	0.576187276	0.564488605	0.882478718	up_in_case
CRA004775	ENSGALG00010016015	ZDHHC3	case	control	-0.115099807	0.181750134	-0.633285952	0.526546925	0.86830001	down_in_case
CRA004775	ENSGALG00010016016	ZDHHC2	case	control	-0.961364844	0.431200718	-2.229506592	0.025780217	0.308336492	down_in_case
CRA004775	ENSGALG00010016017	RAPGEF6	case	control	-0.005906037	0.398328357	-0.014827056	0.988170155	0.998065748	down_in_case
CRA004775	ENSGALG00010016021	ENSGALG00010016021	case	control	0.027889976	0.662842056	0.042076352	0.966437832	0.993094598	up_in_case
CRA004775	ENSGALG00010016022	EPHB3	case	control	-0.223517713	0.251713268	-0.887985426	0.374548591	0.798266843	down_in_case
CRA004775	ENSGALG00010016025	PCIF1	case	control	-0.114019412	0.120436159	-0.946720757	0.343781091	0.783449416	down_in_case
CRA004775	ENSGALG00010016027	COPS2	case	control	0.276766948	0.386079524	0.716865129	0.47345732	0.845445211	up_in_case
CRA004775	ENSGALG00010016030	TGM4	case	control	1.456551086	0.499918288	2.913578319	0.003573122	0.125102734	up_in_case
CRA004775	ENSGALG00010016039	ENSGALG00010016039	case	control	-0.316491047	0.580983705	-0.544750299	0.585925266	0.89145601	down_in_case
CRA004775	ENSGALG00010016040	RPSA2	case	control	-0.449815349	0.249624358	-1.801968978	0.071550286	0.471598108	down_in_case
CRA004775	ENSGALG00010016041	ENSGALG00010016041	case	control	0.184044292	0.281625992	0.653506058	0.513430085	0.863903061	up_in_case
CRA004775	ENSGALG00010016043	ADAMTSL1	case	control	0.275142096	0.437580573	0.628780418	0.529492822	0.869211096	up_in_case
CRA004775	ENSGALG00010016047	SRP9	case	control	-0.049919087	0.2715677	-0.183818206	0.854156073	0.970073177	down_in_case
CRA004775	ENSGALG00010016049	ZFYVE21	case	control	0.150368403	0.252864967	0.594658899	0.552071501	0.878258849	up_in_case
CRA004775	ENSGALG00010016053	DRP2	case	control	1.483803998	0.539769278	2.748959709	0.005978473	0.154441997	up_in_case
CRA004775	ENSGALG00010016057	SOHO1	case	control	0.297177971	0.785963808	0.378106432	0.705351531	0.930927733	up_in_case
CRA004775	ENSGALG00010016059	EPDR1	case	control	0.380990207	0.366499877	1.039537065	0.298555029	0.751772608	up_in_case
CRA004775	ENSGALG00010016060	ENSGALG00010016060	case	control	2.687674524	1.686708145	1.593443734	0.111060681	0.560403826	up_in_case
CRA004775	ENSGALG00010016066	C14orf2	case	control	0.222143046	0.317069946	0.700612117	0.483545114	0.850285021	up_in_case
CRA004775	ENSGALG00010016067	AHSG	case	control	1.757652723	0.775315978	2.267014706	0.023389327	0.296703543	up_in_case
CRA004775	ENSGALG00010016069	GALK2	case	control	0.07628618	0.238691911	0.319601026	0.749270797	0.941366343	up_in_case
CRA004775	ENSGALG00010016076	RAB3GAP2	case	control	0.054420983	0.153653684	0.354179488	0.723204374	0.935187451	up_in_case
CRA004775	ENSGALG00010016083	CDH22	case	control	-1.094084162	0.700250313	-1.562418669	0.118189392	0.569454215	down_in_case
CRA004775	ENSGALG00010016087	IARS2	case	control	-0.216792382	0.230113349	-0.94211128	0.34613567	0.784552667	down_in_case
CRA004775	ENSGALG00010016094	FGF7	case	control	0.497561442	0.510191578	0.975244327	0.329439079	0.77237154	up_in_case
CRA004775	ENSGALG00010016097	FNIP1	case	control	-0.003017176	0.267755864	-0.011268383	0.991009321	0.998065748	down_in_case
CRA004775	ENSGALG00010016100	SFRP4	case	control	0.121030886	0.590274211	0.205041798	0.837539469	0.965462757	up_in_case
CRA004775	ENSGALG00010016103	BPNT1	case	control	0.274495869	0.221622305	1.238575103	0.215502893	0.683654538	up_in_case
CRA004775	ENSGALG00010016107	UVSSA	case	control	-0.164466747	0.159575079	-1.030654333	0.302702947	0.755164773	down_in_case
CRA004775	ENSGALG00010016109	MLH1	case	control	-0.075923527	0.297013975	-0.255622744	0.798242155	0.952835572	down_in_case
CRA004775	ENSGALG00010016111	EPB41	case	control	-0.417922913	0.571033678	-0.731870868	0.464247389	0.842643327	down_in_case
CRA004775	ENSGALG00010016113	DTWD1	case	control	-0.101123184	0.278516133	-0.36307837	0.716546342	0.934011578	down_in_case

CRA004775	ENSGALG00010016114	HAUS6	case	control	-0.317694456	0.34051463	-0.932983278	0.350828602	0.785681625	down_in_case
CRA004775	ENSGALG00010016117	ENSGALG00010016117	case	control	-0.676769398	0.420350942	-1.610010422	0.107395581	0.553800541	down_in_case
CRA004775	ENSGALG00010016118	EPRS	case	control	0.143356826	0.181555543	0.789603136	0.429759576	0.824104894	up_in_case
CRA004775	ENSGALG00010016121	SLC35C2	case	control	-0.198230318	0.161190349	-1.229790236	0.218775665	0.686309227	down_in_case
CRA004775	ENSGALG00010016122	AFG2B	case	control	-0.040247133	0.239162531	-0.168283604	0.866360172	0.972833474	down_in_case
CRA004775	ENSGALG00010016127	ENSGALG00010016127	case	control	-2.281910739	0.718302555	-3.17681	0.001489046	0.080693373	down_in_case
CRA004775	ENSGALG00010016129	CENPI	case	control	0.269071341	0.572317302	0.470143643	0.638252395	0.91099914	up_in_case
CRA004775	ENSGALG00010016131	SMYD1	case	control	0.362131374	0.738839162	0.490135597	0.62403795	0.906441769	up_in_case
CRA004775	ENSGALG00010016137	STARD3NL	case	control	-0.206205797	0.296358302	-0.695798956	0.486554743	0.852090458	down_in_case
CRA004775	ENSGALG00010016139	LURAP1L	case	control	-0.134027096	0.497755239	-0.269263054	0.787727261	0.95041797	down_in_case
CRA004775	ENSGALG00010016141	RRP15	case	control	-0.027418942	0.242879403	-0.112891178	0.910116831	0.98075825	down_in_case
CRA004775	ENSGALG00010016147	FABP1	case	control	1.694789579	0.575610191	2.944335601	0.003236488	0.121931375	up_in_case
CRA004775	ENSGALG00010016150	POLG	case	control	-0.701154084	0.176226582	-3.978707841	6.92908E-05	0.017238232	down_in_case
CRA004775	ENSGALG00010016159	ASPG	case	control	-0.124611049	0.299439099	-0.416148224	0.677301544	0.922924485	down_in_case
CRA004775	ENSGALG00010016165	KK34	case	control	0.137728539	0.487998921	0.282231237	0.777766208	0.947617375	up_in_case
CRA004775	ENSGALG00010016168	PLIN2	case	control	-0.173834668	0.46885582	-0.370763592	0.710813621	0.932234469	down_in_case
CRA004775	ENSGALG00010016169	RHOH	case	control	0.040872835	0.239660443	0.170544769	0.864581729	0.972350383	up_in_case
CRA004775	ENSGALG00010016181	FANCI	case	control	-0.077483023	0.478346958	-0.161980801	0.871320973	0.973918682	down_in_case
CRA004775	ENSGALG00010016182	ELMO2	case	control	-0.058882629	0.13854813	-0.424997644	0.670838392	0.921535334	down_in_case
CRA004775	ENSGALG00010016190	THNSL2	case	control	-0.436793036	0.35127037	-1.24346678	0.213695875	0.683061827	down_in_case
CRA004775	ENSGALG00010016193	TCAIM	case	control	0.247460349	0.250204075	0.989034044	0.322646485	0.767880594	up_in_case
CRA004775	ENSGALG00010016197	SNRPN	case	control	-0.459488882	0.236200684	-1.945332561	0.051734966	0.419476344	down_in_case
CRA004775	ENSGALG00010016200	MICU3	case	control	0.331130953	0.267860903	1.236204872	0.21638242	0.684017163	up_in_case
CRA004775	ENSGALG00010016205	CD40LG	case	control	-0.122233215	0.425122893	-0.287524424	0.773710803	0.946296525	down_in_case
CRA004775	ENSGALG00010016206	KNG1	case	control	1.583007821	0.579782299	2.730348657	0.006326737	0.158894029	up_in_case
CRA004775	ENSGALG00010016209	ADAMTS7	case	control	-1.009174547	0.601217588	-1.678551273	0.09323953	0.524906446	down_in_case
CRA004775	ENSGALG00010016211	TACC3	case	control	0.289403016	0.45039992	0.642546775	0.520518229	0.865611895	up_in_case
CRA004775	ENSGALG00010016218	N4BP2	case	control	0.154086674	0.437698971	0.352038008	0.724809759	0.935238044	up_in_case
CRA004775	ENSGALG00010016221	TMEM200B	case	control	-0.136126585	0.449174591	-0.303059407	0.761844589	0.942236668	down_in_case
CRA004775	ENSGALG00010016224	MOSPD1	case	control	0.199456702	0.333824136	0.597490355	0.550180045	0.877623839	up_in_case
CRA004775	ENSGALG00010016225	P4HA2	case	control	0.278439781	0.260438278	1.069120033	0.285015586	0.741207705	up_in_case
CRA004775	ENSGALG00010016229	ENSGALG00010016229	case	control	0.820976558	0.556116479	1.476267273	0.139872156	0.599932008	up_in_case
CRA004775	ENSGALG00010016235	DDX27	case	control	0.070610222	0.172845932	0.408515382	0.68289534	0.924807349	up_in_case
CRA004775	ENSGALG00010016236	GPATCH2	case	control	0.128401494	0.298325924	0.430406758	0.66689978	0.919168997	up_in_case
CRA004775	ENSGALG00010016240	EIF4A2	case	control	0.128709768	0.1517958	0.847913898	0.396485924	0.808998414	up_in_case
CRA004775	ENSGALG00010016242	PDS5A	case	control	0.385305959	0.253485406	1.520032124	0.128502902	0.582990237	up_in_case
CRA004775	ENSGALG00010016245	FAM96A	case	control	-0.236718216	0.286364241	-0.826633293	0.408444941	0.814384687	down_in_case
CRA004775	ENSGALG00010016247	KIF26A	case	control	0.19444795	0.461640075	0.421211158	0.673600897	0.9224864	up_in_case
CRA004775	ENSGALG00010016251	MPDZ	case	control	0.598661621	0.252638885	2.369633721	0.017805715	0.265560073	up_in_case
CRA004775	ENSGALG00010016253	EDN3	case	control	2.217953407	0.321126623	6.906787689	4.95751E-12	9.73867E-09	up_in_case
CRA004775	ENSGALG00010016257	ENSGALG00010016257	case	control	-0.555106558	0.398320991	-1.393616132	0.163433554	0.629717526	down_in_case
CRA004775	ENSGALG00010016258	CTNNB1	case	control	-0.085367108	0.244220463	-0.349549367	0.726676916	0.935238044	down_in_case
CRA004775	ENSGALG00010016260	INTS6L	case	control	0.50594386	0.37113428	1.363236671	0.172807933	0.637223104	up_in_case
CRA004775	ENSGALG00010016262	SPG11	case	control	-0.062213976	0.1963998	-0.316772092	0.751416542	0.941495694	down_in_case
CRA004775	ENSGALG00010016266	PDLIM4	case	control	0.080752329	0.338279548	0.238714783	0.811326753	0.957303241	up_in_case
CRA004775	ENSGALG00010016270	RFC4	case	control	-0.354639051	0.243381499	-1.457132331	0.145079855	0.608229601	down_in_case
CRA004775	ENSGALG00010016274	ZNF831	case	control	-0.077412789	0.687077036	-0.112669737	0.910292395	0.98075825	down_in_case
CRA004775	ENSGALG00010016276	ACTR2	case	control	-0.268949433	0.297930081	-0.902726679	0.366670973	0.793780314	down_in_case
CRA004775	ENSGALG00010016279	PRELID3B	case	control	1.216571043	0.209676035	5.802146361	6.54714E-09	8.18452E-06	up_in_case
CRA004775	ENSGALG00010016281	SLC22A4	case	control	-1.1405821	0.692484875	-1.647085937	0.099540383	0.537098329	down_in_case
CRA004775	ENSGALG00010016286	ULK4	case	control	-0.57032675	0.564819048	-1.009751269	0.312614473	0.7645017	down_in_case
CRA004775	ENSGALG00010016287	BCL11B	case	control	-0.418976883	0.58432172	-0.717031163	0.473354868	0.845445211	down_in_case
CRA004775	ENSGALG00010016289	IRF1	case	control	-0.848073703	0.435822582	-1.945915007	0.05166495	0.419476344	down_in_case
CRA004775	ENSGALG00010016292	PHKA1	case	control	0.140531813	0.5451219	0.257798876	0.796562132	0.952835572	up_in_case
CRA004775	ENSGALG00010016295	UBE2K	case	control	0.242323377	0.316451256	0.765752616	0.443823513	0.832130395	up_in_case
CRA004775	ENSGALG00010016299	SRSF4	case	control	0.12047147	0.277268674	0.434493621	0.663930026	0.918295135	up_in_case
CRA004775	ENSGALG00010016303	ENSGALG00010016303	case	control	1.349727312	0.281443824	4.795725463	1.62087E-06	0.00096907	up_in_case
CRA004775	ENSGALG00010016304	EIF3J	case	control	0.373109189	0.327255933	1.140114362	0.254238659	0.718335926	up_in_case
CRA004775	ENSGALG00010016305	DENND4C	case	control	0.14583224	0.375187537	0.388691591	0.697504304	0.929092636	up_in_case
CRA004775	ENSGALG00010016307	RAD50	case	control	-0.153692775	0.323727067	-0.474760349	0.634957798	0.909837348	down_in_case
CRA004775	ENSGALG00010016308	RPS6	case	control	-0.522110886	0.286740389	-1.820848778	0.068629847	0.46645716	down_in_case
CRA004775	ENSGALG00010016310	HDAC8	case	control	-0.083100962	0.268456813	-0.309550577	0.756902745	0.941900693	down_in_case
CRA004775	ENSGALG00010016312	MECR	case	control	-0.574016785	0.356963993	-1.608052344	0.107823715	0.55406723	down_in_case
CRA004775	ENSGALG00010016314	ACER2	case	control	0.455266016	0.46841952	0.971919394	0.331090651	0.772451228	up_in_case
CRA004775	ENSGALG00010016315	RPS4Y1	case	control	-0.428724534	0.245006592	-1.749848974	0.080144377	0.493676892	down_in_case
CRA004775	ENSGALG00010016317	CTDSPL2	case	control	0.622956596	0.220296074	2.827815239	0.004686685	0.141330266	up_in_case
CRA004775	ENSGALG00010016319	RBM41	case	control	-0.368885974	0.248547868	-1.48416471	0.137765229	0.596852448	down_in_case
CRA004775	ENSGALG00010016322	EBF4	case	control	-0.031869542	0.321160633	-0.099232404	0.920953747	0.982481112	down_in_case
CRA004775	ENSGALG00010016324	TUBB1	case	control	0.168115166	0.317540568	0.529428939	0.596507928	0.895574418	up_in_case
CRA004775	ENSGALG00010016328	USH2A	case	control	0.479723341	0.737428306	0.650535567	0.515346335	0.864897625	up_in_case
CRA004775	ENSGALG00010016332	ENSGALG00010016332	case	control	-0.156932085	0.44135102	-0.355572046	0.722161081	0.935087834	down_in_case
CRA004775	ENSGALG00010016335	ABHD4	case	control	0.376181254	0.363015422	1.03626797	0.300077145	0.752710838	up_in_case
CRA004775	ENSGALG00010016338	PTPRU	case	control	-0.11430507	0.324719313	-0.352011925	0.724829319	0.935238044	down_in_case
CRA004775	ENSGALG00010016339	SETD3	case	control	-0.021382258	0.328812546	-0.065028717	0.948151136	0.989972846	down_in_case
CRA004775	ENSGALG00010016340	RGS12	case	control	0.822670009	0.276752437	2.972584516	0.002953039	0.113745767	up_in_case
CRA004775	ENSGALG00010016341	SLX4	case	control	0.045847414	0.327583263	0.139956521	0.888694343	0.977118922	up_in_case
CRA004775	ENSGALG00010016344	SMIM14	case	control	0.282782927	0.143708946	1.967747545	0.049097092	0.410291029	up_in_case
CRA004775	ENSGALG00010016345	CTSZ	case	control	0.25132812	0.264460989	0.950340998	0.341939013	0.781616177	up_in_case
CRA004775	ENSGALG00010016346	TRAP1	case	control	-0.189408729	0.278965481	-0.67896834	0.497157923	0.857772041	down_in_case
CRA004775	ENSGALG00010016347	ANO10	case	control	0.077298095	0.208076028	0.371489672	0.710272847	0.932234469	up_in_case
CRA004775	ENSGALG00010016350	CASC4	case	control	0.158264295	0.15332361	1.032223906	0.301967239	0.754801023	up_in_case
CRA004775	ENSGALG00010016353	C1orf232	case	control	-0.287201622	0.273134732	-1.051501653	0.293028251	0.746438942	down_in_case
CRA004775	ENSGALG00010016355	MCF2L2	case	control	0.033907999	0.705694641	0.04804911	0.961677104	0.991860454	up_in_case
CRA004775	ENSGALG00010016361	NELFCD	case	control	-0.003822316	0.182963963	-0.020891085	0.983332538	0.997403978	down_in_case
CRA004775	ENSGALG00010016362	NUP62	case	control	-0.11245276	0.163039786	-0.689725878	0.490366589	0.855184934	down_in_case
CRA004775	ENSGALG00010016366	KIF3A	case	control	-0.011054152	0.190375117	-0.058065107	0.953696768	0.990670511	down_in_case
CRA004775	ENSGALG00010016368	CCNK	case	control	-0.02306097	0.142442128	-0.16189712	0.871386871	0.973918682	down_in_case
CRA004775	ENSGALG00010016370	TMEM255A	case	control	0.419703145	0.476386638	0.88101368	0.378310416	0.799601151	up_in_case
CRA004775	ENSGALG00010016372	DNAAF6	case	control	-0.252156015	0.338784342	-0.744296544	0.456697091	0.839688534	down_in_case
CRA004775	ENSGALG00010016373	SRL	case	control	-0.32266857	0.726649021	-0.444050099	0.657006364	0.917108353	down_in_case
CRA004775	ENSGALG00010016374	FRMD5	case	control	0.424721409	0.655529594	0.647905774	0.517045896	0.864913871	up_in_case
CRA004775	ENSGALG00010016375	CCNI2	case	control	-0.490573774	0.459162995	-1.068408779	0.285336159	0.741413527	down_in_case
CRA004775	ENSGALG00010016377	FRMPD3	case	control	0.432725152	0.601345248	0.719595197	0.47177427	0.845425322	up_in_case
CRA004775	ENSGALG00010016378	WDR76	case	control	-0.352629151	0.253688788	-1.390006839	0.164526801	0.63006979	down_in_case
CRA004775	ENSGALG00010016379	TFAP4	case	control	0.083353984	0.192963292	0.431968088	0.665764607	0.918888163	up_in_case
CRA004775	ENSGALG00010016382	UGDH	case	control	0.045828669	0.224655813	0.203995028	0.838357384	0.965677031	up_in_case
CRA004775	ENSGALG00010016383	FAM110D	case	control	-0.617664142	0.393736486	-1.568724676	0.116712106	0.566504827	down_in_case
CRA004775	ENSGALG00010016384	GNAS	case	control	0.330474048	0.152714708	2.163996196	0.030464641	0.3330042	up_in_case
CRA004775	ENSGALG00010016385	HTT	case	control	0.350042945	0.311538286	1.12359527	0.261184763	0.721774856	up_in_case
CRA004775	ENSGALG00010016387	CCDC85C	case	control	0.237561729	0.30696736	0.773898988	0.438990528	0.828368259	up_in_case
CRA004775	ENSGALG00010016390	ENSGALG00010016390	case	control	0.190731201	0.503955727	0.378468169	0.705082836	0.930925979	up_in_case
CRA004775	ENSGALG00010016392	STMN3	case	control	0.518286706	0.668643312	0.775131818	0.438261772	0.827821102	up_in_case
CRA004775	ENSGALG00010016393	BLM	case	control	0.078611501	0.305593467	0.257242085	0.796991898	0.952835572	up_in_case
CRA004775	ENSGALG00010016394	PDIK1L	case	control	-0.205904898	0.230321566	-0.893988791	0.371327888	0.795818272	down_in_case
CRA004775	ENSGALG00010016395	ENSGALG00010016395	case	control	0.325105305	0.203111476	1.600624998	0.109460002	0.556651068	up_in_case
CRA004775	ENSGALG00010016397	LIAS	case	control	-0.229386989	0.244147244	-0.939543634	0.347451701	0.785013534	down_in_case
CRA004775	ENSGALG00010016398	SEPTIN6	case	control	-0.18964227	0.162450017	-1.167388431	0.243053541	0.709904257	down_in_case
CRA004775	ENSGALG00010016399	ACSM5	case	control	-0.419341909	0.684789289	-0.612366338	0.540295408	0.874311509	down_in_case
CRA004775	ENSGALG00010016400	ENSGALG00010016400	case	control	-0.05729463	0.336913669	-0.170057305	0.86496507	0.972458072	down_in_case
CRA004775	ENSGALG00010016402	SEPTIN8	case	control	-0.291137757	0.227294144	-1.28088543	0.200233911	0.667171435	down_in_case
CRA004775	ENSGALG00010016403	GRK4	case	control	-0.475813176	0.550145533	-0.864886011	0.387101356	0.803905475	down_in_case
CRA004775	ENSGALG00010016404	ESRRG	case	control	0.69787596	0.331896661	2.102690511	0.035492832	0.357031407	up_in_case
CRA004775	ENSGALG00010016407	RPL9	case	control	-0.320751482	0.22764593	-1.408992825	0.15883729	0.624764009	down_in_case
CRA004775	ENSGALG00010016409	GMEB2	case	control	-0.220295335	0.380424155	-0.579078201	0.562536408	0.881532201	down_in_case
CRA004775	ENSGALG00010016410	TRAK1	case	control	0.151535404	0.274412311	0.552217949	0.580799038	0.888901418	up_in_case
CRA004775	ENSGALG00010016411	CTSH	case	control	0.033022367	0.351309913	0.093997824	0.925110885	0.984140717	up_in_case
CRA004775	ENSGALG00010016412	TMSB15B	case	control	0.141630037	0.330227763	0.428885919	0.668006248	0.919688403	up_in_case
CRA004775	ENSGALG00010016414	KLB	case	control	1.04121427	0.796128167	1.307847546	0.190925024	0.65651663	up_in_case
CRA004775	ENSGALG00010016415	RASGRF1	case	control	0.277152643	0.411792018	0.673040347	0.500921623	0.859946721	up_in_case
CRA004775	ENSGALG00010016416	CYP46A1	case	control	0.355574853	0.252629943	1.407492905	0.15928128	0.624907945	up_in_case
CRA004775	ENSGALG00010016417	NOP14	case	control	0.210050959	0.241602507	0.8694072	0.384624446	0.803182776	up_in_case
CRA004775	ENSGALG00010016418	NFIB	case	control	0.294553351	0.302096149	0.975031798	0.329544487	0.77237154	up_in_case
CRA004775	ENSGALG00010016421	ENSGALG00010016421	case	control	0.796282516	0.560056982	1.421788392	0.15508769	0.619284425	up_in_case
CRA004775	ENSGALG00010016422	KCTD3	case	control	-0.338384353	0.333675674	-1.014111545	0.31052953	0.762244121	down_in_case
CRA004775	ENSGALG00010016423	TRMT44	case	control	-0.045968395	0.197009105	-0.23333132	0.81550414	0.958870945	down_in_case
CRA004775	ENSGALG00010016425	TRIM63	case	control	-0.148300974	0.762771683	-0.194423806	0.845844051	0.967171258	down_in_case

CRA004775	ENSGALG00010016426	HELZ2	case	control	-0.48229698	0.529530076	-0.910801865	0.36239978	0.793023452	down_in_case
CRA004775	ENSGALG00010016428	ADRA1D	case	control	0.969724174	0.532444716	1.821267344	0.068566228	0.46645716	up_in_case
CRA004775	ENSGALG00010016429	ENSGALG00010016429	case	control	-0.328947067	0.48632627	-0.67639173	0.498791966	0.858434084	down_in_case
CRA004775	ENSGALG00010016431	B3GNT5	case	control	-0.203101621	0.434064673	-0.467906361	0.639851554	0.911453315	down_in_case
CRA004775	ENSGALG00010016433	TMED3	case	control	0.091623105	0.214127321	0.427890773	0.668730645	0.920123584	up_in_case
CRA004775	ENSGALG00010016436	SLC30A2	case	control	-0.979406411	0.777929069	-1.258991919	0.208033251	0.676600102	down_in_case
CRA004775	ENSGALG00010016441	ENSGALG00010016441	case	control	0.08568554	0.761055623	0.112587749	0.910357398	0.98075825	up_in_case
CRA004775	ENSGALG00010016445	ZDHHC21	case	control	0.024400669	0.335738185	0.072677671	0.942062617	0.987651835	up_in_case
CRA004775	ENSGALG00010016447	ID3	case	control	0.233313845	0.292490146	0.797681045	0.425055606	0.822073086	up_in_case
CRA004775	ENSGALG00010016449	FMR1	case	control	0.29141158	0.210815124	1.382308697	0.166876937	0.630568744	up_in_case
CRA004775	ENSGALG00010016450	ENSGALG00010016450	case	control	-0.185365933	0.573953494	-0.322963332	0.746723012	0.9402175	down_in_case
CRA004775	ENSGALG00010016451	USP46	case	control	0.233425964	0.337365	0.691909251	0.488994321	0.854256384	up_in_case
CRA004775	ENSGALG00010016452	GLCE	case	control	-0.101139419	0.269210972	-0.375688323	0.70714862	0.931617022	down_in_case
CRA004775	ENSGALG00010016453	MYCL	case	control	-0.21083727	0.380182107	-0.554569155	0.579189392	0.888378369	down_in_case
CRA004775	ENSGALG00010016454	ANKS3	case	control	-0.237094684	0.168537583	-1.406776344	0.159493719	0.624907945	down_in_case
CRA004775	ENSGALG00010016455	RBM27	case	control	-0.039020156	0.229067602	-0.170343407	0.864740076	0.972350383	down_in_case
CRA004775	ENSGALG00010016456	LAMP3	case	control	1.150224186	0.745995224	1.541865348	0.123106318	0.575549148	up_in_case
CRA004775	ENSGALG00010016457	SMOX	case	control	0.011008607	0.205512549	0.053566591	0.957280475	0.991431439	up_in_case
CRA004775	ENSGALG00010016458	ZC3H12A	case	control	-0.410852104	0.26967828	-1.523489779	0.127636216	0.58187175	down_in_case
CRA004775	ENSGALG00010016459	PAQR5	case	control	0.12014437	0.274083293	0.438349849	0.661132696	0.917907174	up_in_case
CRA004775	ENSGALG00010016460	LBR	case	control	-0.368442761	0.429720187	-0.857401566	0.391222994	0.80679475	down_in_case
CRA004775	ENSGALG00010016462	RNF24	case	control	0.115607178	0.315367499	0.366579242	0.713932895	0.933244709	up_in_case
CRA004775	ENSGALG00010016463	FREM1	case	control	0.356359419	0.582901783	0.611354141	0.540965156	0.874592283	up_in_case
CRA004775	ENSGALG00010016464	PANK2	case	control	-0.473676973	0.176566101	-2.682717528	0.007302664	0.170964283	down_in_case
CRA004775	ENSGALG00010016465	STK35	case	control	-0.790628261	0.575255203	-1.374395672	0.169318879	0.633147466	down_in_case
CRA004775	ENSGALG00010016466	TMEM164	case	control	0.817224784	0.484393503	1.687109301	0.091582337	0.521441551	up_in_case
CRA004775	ENSGALG00010016468	TTC39B	case	control	0.093525966	0.315452728	0.296481716	0.766862228	0.944281398	up_in_case
CRA004775	ENSGALG00010016469	MAVS	case	control	-0.655309709	0.25833851	-2.536631916	0.011192457	0.21465477	down_in_case
CRA004775	ENSGALG00010016470	TNIP1	case	control	-0.620709869	0.325863077	-1.904818044	0.056803727	0.435477592	down_in_case
CRA004775	ENSGALG00010016471	POMGNT2	case	control	-0.149059644	0.321203799	-0.464065632	0.642600724	0.913228871	down_in_case
CRA004775	ENSGALG00010016472	TFDP1	case	control	0.359445338	0.153218547	2.345964924	0.018977893	0.272278915	up_in_case
CRA004775	ENSGALG00010016473	RAB9B	case	control	-0.158274001	0.17252513	-0.917396794	0.358934752	0.791216895	down_in_case
CRA004775	ENSGALG00010016474	MAPK8IP3	case	control	-0.293913138	0.238288628	-1.233433342	0.217414132	0.684603098	down_in_case
CRA004775	ENSGALG00010016475	MCCC1	case	control	0.137352661	0.245612144	0.559225853	0.576007591	0.887937128	up_in_case
CRA004775	ENSGALG00010016477	CNIH3	case	control	-0.127723654	0.646660887	-0.197512571	0.843426445	0.966478468	down_in_case
CRA004775	ENSGALG00010016479	ENSGALG00010016479	case	control	-0.834533506	0.831632622	-1.003488179	0.315625378	0.765461123	down_in_case
CRA004775	ENSGALG00010016480	GPX3	case	control	-0.255004379	0.330623986	-0.771282151	0.44053971	0.829353567	down_in_case
CRA004775	ENSGALG00010016482	EML1	case	control	-0.176313599	0.198641185	-0.887598407	0.374756811	0.798463109	down_in_case
CRA004775	ENSGALG00010016483	WDR26	case	control	-0.295694689	0.330822268	-0.893817368	0.371419615	0.795818272	down_in_case
CRA004775	ENSGALG00010016491	DNAJC8	case	control	-0.131645742	0.194339872	-0.677399552	0.498152483	0.857992359	down_in_case
CRA004775	ENSGALG00010016496	PLP1	case	control	1.768127905	0.544503276	3.247230979	0.001165338	0.07185901	up_in_case
CRA004775	ENSGALG00010016498	KIF23	case	control	0.36385812	0.447835877	0.812480953	0.416515712	0.818943344	up_in_case
CRA004775	ENSGALG00010016500	ENSGALG00010016500	case	control	0.190019052	0.377459184	0.503416159	0.614671713	0.902639483	up_in_case
CRA004775	ENSGALG00010016502	YIPF5	case	control	-0.361509987	0.332645017	-1.086774094	0.277136659	0.732162575	down_in_case
CRA004775	ENSGALG00010016504	SNRK	case	control	0.080895604	0.298735035	0.270793828	0.786549607	0.95041797	up_in_case
CRA004775	ENSGALG00010016507	SPATA18	case	control	-0.059239786	0.373482059	-0.158614811	0.873972359	0.974115968	down_in_case
CRA004775	ENSGALG00010016508	CREBBP	case	control	0.467791944	0.243157281	1.923824536	0.054376581	0.429731243	up_in_case
CRA004775	ENSGALG00010016510	THG1L	case	control	-0.195834815	0.261315607	-0.749418749	0.453604854	0.83804959	down_in_case
CRA004775	ENSGALG00010016514	GASK1A	case	control	-0.288059874	0.649237517	-0.443689507	0.657267082	0.917108353	down_in_case
CRA004775	ENSGALG00010016518	TMEM42	case	control	-0.483190555	0.203276832	-2.377007501	0.017453733	0.262588933	down_in_case
CRA004775	ENSGALG00010016519	MEX3B	case	control	0.418544251	0.332288328	1.259581562	0.20782035	0.676506608	up_in_case
CRA004775	ENSGALG00010016520	CHMP1B	case	control	-0.217841593	0.18351457	-1.18705339	0.235206562	0.702145501	down_in_case
CRA004775	ENSGALG00010016526	EIF1B	case	control	-0.033019435	0.324374082	-0.101794308	0.918919944	0.98189977	down_in_case
CRA004775	ENSGALG00010016527	MRPS34	case	control	-0.166657762	0.192655656	-0.865055121	0.387008535	0.803905475	down_in_case
CRA004775	ENSGALG00010016529	DCUN1D1	case	control	0.199278981	0.160731524	1.239825119	0.215040085	0.683649824	up_in_case
CRA004775	ENSGALG00010016533	CNIH4	case	control	-0.360785232	0.32729151	-1.102336056	0.2703156	0.728665115	down_in_case
CRA004775	ENSGALG00010016534	TRIT1	case	control	-0.400598278	0.21216977	-1.888102524	0.059012188	0.44414236	down_in_case
CRA004775	ENSGALG00010016535	BNC2	case	control	0.101720875	0.468730827	0.21701341	0.828197894	0.962439723	up_in_case
CRA004775	ENSGALG00010016541	UBE2J2	case	control	0.054308238	0.126474793	0.429399698	0.667632374	0.919546651	up_in_case
CRA004775	ENSGALG00010016542	ENOX2	case	control	0.190276587	0.179285958	1.061302225	0.288552575	0.742851819	up_in_case
CRA004775	ENSGALG00010016543	TCEA3	case	control	-0.135945493	0.531508309	-0.255773035	0.798126097	0.952835572	down_in_case
CRA004775	ENSGALG00010016544	ADCY9	case	control	-0.247343469	0.295555401	-0.836876839	0.4026618	0.811996248	down_in_case
CRA004775	ENSGALG00010016546	C1QTNF12	case	control	0.78438154	0.603483745	1.29975587	0.193684655	0.657919837	up_in_case
CRA004775	ENSGALG00010016547	SGCB	case	control	-0.094309404	0.352506587	-0.267539411	0.789053874	0.95041797	down_in_case
CRA004775	ENSGALG00010016548	EVL	case	control	0.173746469	0.238483117	0.728548295	0.466278016	0.843480361	up_in_case
CRA004775	ENSGALG00010016549	PTPRA	case	control	0.076840705	0.268575271	0.286104913	0.774797769	0.946319184	up_in_case
CRA004775	ENSGALG00010016550	FBLIM1	case	control	-0.498262572	0.37695034	-1.321825503	0.186226264	0.6491093	down_in_case
CRA004775	ENSGALG00010016552	ENSGALG00010016552	case	control	0.956541532	0.600082196	1.594017518	0.110932111	0.560201052	up_in_case
CRA004775	ENSGALG00010016553	SLC25A14	case	control	-0.465449362	0.275752011	-1.687927354	0.091425175	0.521441551	down_in_case
CRA004775	ENSGALG00010016556	ASAP3	case	control	-0.274667756	0.400342445	-0.686082026	0.492661385	0.855763086	down_in_case
CRA004775	ENSGALG00010016558	NR2F2	case	control	0.275972343	0.253080844	1.090451327	0.275514383	0.731001618	up_in_case
CRA004775	ENSGALG00010016559	ROGDI	case	control	-0.834259486	0.305534017	-2.730496242	0.006323905	0.158894029	down_in_case
CRA004775	ENSGALG00010016560	DCUN1D4	case	control	0.072559587	0.300391078	0.241550406	0.809128554	0.955623701	up_in_case
CRA004775	ENSGALG00010016561	DEGS2	case	control	1.981480518	0.793420097	2.497391391	0.012511078	0.225478163	up_in_case
CRA004775	ENSGALG00010016562	GLIS2	case	control	-0.006858246	0.358932594	-0.019107338	0.984755478	0.997562105	down_in_case
CRA004775	ENSGALG00010016564	SPEN	case	control	0.645206365	0.326365754	1.976942606	0.048048118	0.407342584	up_in_case
CRA004775	ENSGALG00010016565	NVL	case	control	-0.254006174	0.192345909	-1.32056967	0.186644891	0.649925019	down_in_case
CRA004775	ENSGALG00010016566	CNTLN	case	control	-0.255244684	0.847985173	-0.301001352	0.763413464	0.94239774	down_in_case
CRA004775	ENSGALG00010016567	E2F2	case	control	0.147676111	0.501223311	0.294631371	0.768275495	0.945059483	up_in_case
CRA004775	ENSGALG00010016568	PAM16	case	control	0.048063497	0.305620948	0.157265062	0.875035954	0.974301166	up_in_case
CRA004775	ENSGALG00010016569	YY1	case	control	0.497674957	0.20210071	2.462509696	0.013796844	0.234512243	up_in_case
CRA004775	ENSGALG00010016572	RAB33A	case	control	-0.040466165	0.19199965	-0.210761657	0.833073259	0.964019688	down_in_case
CRA004775	ENSGALG00010016573	DEGS1	case	control	-0.387089154	0.272511068	-1.420452962	0.155475853	0.61933617	down_in_case
CRA004775	ENSGALG00010016576	MRPS26	case	control	-0.164408269	0.315542316	-0.52103398	0.6023431	0.898318448	down_in_case
CRA004775	ENSGALG00010016577	ZBTB17	case	control	-0.16808906	0.290763471	-0.57809552	0.563199631	0.881532201	down_in_case
CRA004775	ENSGALG00010016580	MARS2	case	control	-0.224386071	0.201756683	-1.112161779	0.266068598	0.727311193	down_in_case
CRA004775	ENSGALG00010016581	H2AFYMACROH2A1	case	control	0.038048373	0.233051589	0.163261591	0.870312474	0.973918682	up_in_case
CRA004775	ENSGALG00010016583	VPS41	case	control	-0.138451993	0.181868787	-0.761274074	0.446493382	0.833749389	down_in_case
CRA004775	ENSGALG00010016584	EHBP1	case	control	0.192764279	0.269899714	0.714207051	0.475099158	0.84579952	up_in_case
CRA004775	ENSGALG00010016588	ATP11B	case	control	0.055847811	0.237458662	0.235189613	0.814061568	0.958686054	up_in_case
CRA004775	ENSGALG00010016589	FBXO28	case	control	-0.278893213	0.200863919	-1.388468446	0.164994447	0.630194031	down_in_case
CRA004775	ENSGALG00010016591	HOMER2	case	control	0.408026053	0.405860517	1.005335664	0.314735248	0.765098415	up_in_case
CRA004775	ENSGALG00010016592	TMEM129	case	control	-0.194152372	0.194879585	-0.996268401	0.319119753	0.767037518	down_in_case
CRA004775	ENSGALG00010016595	ENSGALG00010016595	case	control	-0.334602381	0.648307813	-0.516116534	0.605773021	0.900441554	down_in_case
CRA004775	ENSGALG00010016597	FRYL	case	control	0.007019422	0.374510191	0.018742941	0.985046172	0.997562105	up_in_case
CRA004775	ENSGALG00010016598	AIFM1	case	control	-0.068678895	0.239358728	-0.286928727	0.774166894	0.946319184	down_in_case
CRA004775	ENSGALG00010016599	ENSGALG00010016599	case	control	-0.844251523	0.796465385	-1.059997759	0.289145619	0.74346324	down_in_case
CRA004775	ENSGALG00010016600	GNL2	case	control	0.112127007	0.253498741	0.442317808	0.658259246	0.917354438	up_in_case
CRA004775	ENSGALG00010016602	HSPB7	case	control	-0.271459579	0.737867687	-0.367897366	0.712949764	0.932981748	down_in_case
CRA004775	ENSGALG00010016603	ENSGALG00010016603	case	control	0.16586299	0.312600956	0.530590157	0.595702818	0.895305942	up_in_case
CRA004775	ENSGALG00010016604	TP53BP2	case	control	0.030590759	0.17430146	0.175504891	0.860682928	0.971325143	up_in_case
CRA004775	ENSGALG00010016605	WDPCP	case	control	-0.397068937	0.318353816	-1.247256723	0.21230338	0.682275341	down_in_case
CRA004775	ENSGALG00010016606	VASN	case	control	-0.004348775	0.30586848	-0.014217793	0.988656225	0.998065748	down_in_case
CRA004775	ENSGALG00010016607	FAM103A1	case	control	-0.367420983	0.282179405	-1.302082915	0.192888043	0.657512017	down_in_case
CRA004775	ENSGALG00010016611	DNALI1	case	control	0.020132697	0.331068089	0.060811349	0.951509452	0.990168609	up_in_case
CRA004775	ENSGALG00010016612	GRIA1	case	control	-0.823645453	0.68945223	-1.194637448	0.232228711	0.699075527	down_in_case
CRA004775	ENSGALG00010016613	SNIP1	case	control	-0.089450083	0.166901916	-0.535944017	0.591997242	0.893804699	down_in_case
CRA004775	ENSGALG00010016614	DNAJC19	case	control	0.02342665	0.388391169	0.060317155	0.951903039	0.990168609	up_in_case
CRA004775	ENSGALG00010016615	BCORL1	case	control	-0.03456543	0.193308848	-0.178809351	0.858087403	0.971038554	down_in_case
CRA004775	ENSGALG00010016616	SLBP	case	control	0.058979353	0.571146828	0.103264783	0.917752825	0.981688766	up_in_case
CRA004775	ENSGALG00010016619	SLC25A47	case	control	0.09171256	0.498763237	0.183879951	0.854107634	0.970073177	up_in_case
CRA004775	ENSGALG00010016620	ENSGALG00010016620	case	control	-0.141383133	0.305271371	-0.463139181	0.643264607	0.913638565	down_in_case
CRA004775	ENSGALG00010016622	KIAA1143	case	control	-0.059850554	0.314077972	-0.190559542	0.84887069	0.968223172	down_in_case
CRA004775	ENSGALG00010016623	DNAJA3	case	control	0.100196425	0.262774577	0.381301821	0.702979302	0.930068722	up_in_case
CRA004775	ENSGALG00010016624	MDH1	case	control	0.207044205	0.288369335	0.717982739	0.47276793	0.845425322	up_in_case
CRA004775	ENSGALG00010016626	CAPN2	case	control	-0.101089407	0.347964897	-0.290516106	0.771421431	0.94586979	down_in_case
CRA004775	ENSGALG00010016627	SH3RF2	case	control	1.98205278	1.342768708	1.476093961	0.13991867	0.599944381	up_in_case
CRA004775	ENSGALG00010016629	LARS1	case	control	-0.073680494	0.154548298	-0.476747363	0.633542032	0.909837348	down_in_case
CRA004775	ENSGALG00010016630	ENSGALG00010016630	case	control	-0.727462934	0.408882742	-1.779148052	0.075215492	0.483342345	down_in_case
CRA004775	ENSGALG00010016631	FAM53A	case	control	0.700795417	0.203335706	3.446494616	0.00056791	0.049426131	up_in_case
CRA004775	ENSGALG00010016640	OCIAD1	case	control	0.196597656	0.218689046	0.898982643	0.368661906	0.794836919	up_in_case
CRA004775	ENSGALG00010016643	BTBD1	case	control	0.475215363	0.298576763	1.591601967	0.111474168	0.56070984	up_in_case
CRA004775	ENSGALG00010016644	NMRAL1	case	control	0.053750246	0.472884244	0.1136647	0.909503597	0.98075825	up_in_case
CRA004775	ENSGALG00010016647	EGR1	case	control	0.346169615	0.577172771	0.599767751	0.548661029	0.877623839	up_in_case
CRA004775	ENSGALG00010016648	ADRA2C	case	control	0.835802681	0.441614215	1.892608191	0.058409994	0.443020311	up_in_case
CRA004775	ENSGALG00010016651	ENSGALG00010016651	case	control	-0.684608209	0.372069085	-1.840002938	0.065767806	0.460330134	down_in_case

CRA004775	ENSGALG00010016652	FXR1	case	control	0.091509162	0.335215735	0.272985878	0.784864071	0.950244571	up_in_case
CRA004775	ENSGALG00010016653	EPHA2	case	control	-0.228236792	0.449973639	-0.507222585	0.61199865	0.901413878	down_in_case
CRA004775	ENSGALG00010016654	SMIM3	case	control	0.067350138	0.289780451	0.232417812	0.816213516	0.959131094	up_in_case
CRA004775	ENSGALG00010016655	TRANK1	case	control	-0.016301354	0.450444022	-0.036189523	0.97113124	0.994908384	down_in_case
CRA004775	ENSGALG00010016657	SLC25A29	case	control	-0.044906583	0.415520279	-0.108073144	0.913937671	0.980997339	down_in_case
CRA004775	ENSGALG00010016658	ZAR1	case	control	0.999951474	0.655085132	1.526445075	0.126899059	0.581085346	up_in_case
CRA004775	ENSGALG00010016661	UBE4B	case	control	-0.47744196	0.438476844	-1.088864707	0.276213552	0.731001618	down_in_case
CRA004775	ENSGALG00010016664	TM6SF1	case	control	-0.352088736	0.294060088	-1.197336022	0.23117561	0.69761014	down_in_case
CRA004775	ENSGALG00010016665	ENSGALG00010016665	case	control	0.384457831	0.532150696	0.722460448	0.470011434	0.84441171	up_in_case
CRA004775	ENSGALG00010016667	WARS1	case	control	-0.168807721	0.181997317	-0.927528625	0.353652137	0.78730441	down_in_case
CRA004775	ENSGALG00010016671	CASS4	case	control	-0.49255652	0.5278203	-0.9331898	0.35072198	0.785681625	down_in_case
CRA004775	ENSGALG00010016672	DCTN4	case	control	-0.49714615	0.201595121	-2.466062409	0.013660753	0.234005339	down_in_case
CRA004775	ENSGALG00010016673	LRPAP1	case	control	-0.141693759	0.233880106	-0.605839296	0.544621473	0.875916944	down_in_case
CRA004775	ENSGALG00010016674	SLC10A4	case	control	0.148690313	0.812207945	0.183069266	0.85474367	0.970407877	up_in_case
CRA004775	ENSGALG00010016675	SKIL	case	control	0.442291338	0.292216797	1.513572605	0.130134274	0.584671406	up_in_case
CRA004775	ENSGALG00010016676	NLRC3	case	control	-0.756410359	0.453939406	-1.666324511	0.095648797	0.529205137	down_in_case
CRA004775	ENSGALG00010016677	MEAF6	case	control	-0.29359148	0.229132028	-1.281320129	0.200081246	0.667171435	down_in_case
CRA004775	ENSGALG00010016678	NOX1	case	control	-0.464660136	0.480937483	-0.966154964	0.333966645	0.774300343	down_in_case
CRA004775	ENSGALG00010016681	SUSD4	case	control	-0.607781885	0.365858933	-1.661246535	0.096663943	0.530629094	down_in_case
CRA004775	ENSGALG00010016682	GPR141	case	control	-0.407566733	1.031553209	-0.395100058	0.692769046	0.927867527	down_in_case
CRA004775	ENSGALG00010016683	RBM22	case	control	-0.383462173	0.158271365	-2.4228146	0.015400784	0.249441914	down_in_case
CRA004775	ENSGALG00010016691	ENSGALG00010016691	case	control	-1.697299587	0.543621679	-3.12220732	0.001795005	0.085287202	down_in_case
CRA004775	ENSGALG00010016692	PRKCI	case	control	0.189934911	0.156197165	1.215994612	0.223986975	0.69173716	up_in_case
CRA004775	ENSGALG00010016696	DOK7	case	control	0.758143172	0.509710159	1.487400551	0.136909051	0.596242097	up_in_case
CRA004775	ENSGALG00010016699	SYNPO	case	control	-0.337855223	0.465260055	-0.72616426	0.467738079	0.843870188	down_in_case
CRA004775	ENSGALG00010016700	CLUAP1	case	control	-0.180880854	0.211754869	-0.854199268	0.392994589	0.807553294	down_in_case
CRA004775	ENSGALG00010016702	WDR25	case	control	-0.190177939	0.26356854	-0.721550223	0.470571052	0.844975521	down_in_case
CRA004775	ENSGALG00010016704	CSTF2	case	control	-0.391343666	0.181351795	-2.157925514	0.030933623	0.335993884	down_in_case
CRA004775	ENSGALG00010016706	RTN4	case	control	-0.228492904	0.2411172	-0.94764249	0.343311487	0.78289822	down_in_case
CRA004775	ENSGALG00010016707	KIF1B	case	control	0.558799821	0.3674318	1.520825965	0.128303517	0.58266237	up_in_case
CRA004775	ENSGALG00010016709	DNASE1	case	control	2.626396017	0.979778758	2.680601101	0.007349006	0.17099185	up_in_case
CRA004775	ENSGALG00010016712	ENSGALG00010016712	case	control	0.207602214	0.30018306	0.691585373	0.48919775	0.854325408	up_in_case
CRA004775	ENSGALG00010016720	INTS15	case	control	-0.103423892	0.199180671	-0.51924663	0.603588766	0.898692785	down_in_case
CRA004775	ENSGALG00010016722	BMP15	case	control	0.924786573	0.755255181	1.224469022	0.220775345	0.688641913	up_in_case
CRA004775	ENSGALG00010016724	PGD	case	control	-0.271150658	0.203852812	-1.330129593	0.183475577	0.647806087	down_in_case
CRA004775	ENSGALG00010016729	ELMO1	case	control	0.00228516	0.339855054	0.006723925	0.994635125	0.999001196	up_in_case
CRA004775	ENSGALG00010016731	C4orf48	case	control	1.136642042	0.497104498	2.28652536	0.022223542	0.291321191	up_in_case
CRA004775	ENSGALG00010016732	ARHGAP26	case	control	-0.151716851	0.26287311	-0.577148614	0.563839066	0.881964623	down_in_case
CRA004775	ENSGALG00010016733	PHC3	case	control	-0.359089072	0.400463131	-0.896684473	0.369887308	0.795234047	down_in_case
CRA004775	ENSGALG00010016735	ENSGALG00010016735	case	control	0.243443175	0.787232231	0.30923934	0.757139471	0.941900693	up_in_case
CRA004775	ENSGALG00010016736	CENPS	case	control	-0.106027555	0.402189819	-0.263625657	0.792068387	0.951284723	down_in_case
CRA004775	ENSGALG00010016738	NAA60	case	control	-0.097109981	0.188636558	-0.51479937	0.606693226	0.900898526	down_in_case
CRA004775	ENSGALG00010016741	NELFA	case	control	-0.093872242	0.322518961	-0.291059606	0.771005733	0.945853152	down_in_case
CRA004775	ENSGALG00010016742	TRMT12	case	control	-0.246954526	0.245502877	-1.005912963	0.31445744	0.765098415	down_in_case
CRA004775	ENSGALG00010016745	BEGAIN	case	control	1.24231046	0.750186277	1.656002647	0.097721283	0.532395151	up_in_case
CRA004775	ENSGALG00010016746	HDGFL3	case	control	-0.093157343	0.2871821	-0.324384227	0.74564716	0.939421865	down_in_case
CRA004775	ENSGALG00010016750	SYTL4	case	control	-0.138068137	0.233425623	-0.591486639	0.554194405	0.879382329	down_in_case
CRA004775	ENSGALG00010016751	DFFA	case	control	-0.285266508	0.180761718	-1.578135632	0.114534454	0.564488547	down_in_case
CRA004775	ENSGALG00010016752	ENSGALG00010016752	case	control	-0.583765592	0.405409568	-1.439940344	0.149884278	0.61366707	down_in_case
CRA004775	ENSGALG00010016754	ENSGALG00010016754	case	control	-0.368838603	0.521196231	-0.707677034	0.479145842	0.847184661	down_in_case
CRA004775	ENSGALG00010016756	NUDT16L1	case	control	-0.290828315	0.298649549	-0.973811332	0.330150226	0.77237154	down_in_case
CRA004775	ENSGALG00010016757	SRPX2	case	control	-0.633808592	0.46461473	-1.364159489	0.172517375	0.63702643	down_in_case
CRA004775	ENSGALG00010016758	NR3C1	case	control	0.345877824	0.286745459	1.20621901	0.227733056	0.694718454	up_in_case
CRA004775	ENSGALG00010016760	DLK1	case	control	0.621859158	0.642670561	0.967617308	0.33323553	0.773910112	up_in_case
CRA004775	ENSGALG00010016761	GPR160	case	control	-0.419653551	0.279614746	-1.500827681	0.133400137	0.590594103	down_in_case
CRA004775	ENSGALG00010016762	EML6	case	control	0.458474245	0.545953781	0.839767505	0.401038756	0.811868062	up_in_case
CRA004775	ENSGALG00010016763	UBALD1	case	control	0.208135325	0.254239545	0.818658345	0.412981369	0.817112606	up_in_case
CRA004775	ENSGALG00010016766	AOAH	case	control	-0.218118376	0.531817419	-0.410137705	0.681704935	0.924278508	down_in_case
CRA004775	ENSGALG00010016767	SLAIN2	case	control	0.607226069	0.257577135	2.357453305	0.018400769	0.269466431	up_in_case
CRA004775	ENSGALG00010016768	GALNT10	case	control	0.112215999	0.337911407	0.332087039	0.739823537	0.938064864	up_in_case
CRA004775	ENSGALG00010016772	TSPAN6	case	control	-0.280044528	0.234613875	-1.193640092	0.232618784	0.699790177	down_in_case
CRA004775	ENSGALG00010016773	RPS27A	case	control	-0.318242432	0.248717829	-1.279532045	0.200709762	0.668271173	down_in_case
CRA004775	ENSGALG00010016774	PEX14	case	control	-0.273212082	0.219510418	-1.244642895	0.213263046	0.682777582	down_in_case
CRA004775	ENSGALG00010016777	TNMD	case	control	-1.347148378	0.830859669	-1.621390986	0.104933813	0.547815057	down_in_case
CRA004775	ENSGALG00010016778	MTIF2	case	control	0.195966687	0.265213904	0.738900504	0.459967416	0.840995542	up_in_case
CRA004775	ENSGALG00010016779	CTNNA2	case	control	0.42305494	0.305760732	1.383614363	0.166476568	0.630194031	up_in_case
CRA004775	ENSGALG00010016780	PRORSD1P	case	control	-0.207869642	0.252130008	-0.824454195	0.409681532	0.81515421	down_in_case
CRA004775	ENSGALG00010016781	CDIP1	case	control	-0.402752257	0.406251074	-0.991387549	0.321496377	0.767650058	down_in_case
CRA004775	ENSGALG00010016783	CLBA1	case	control	0.053372285	0.478467718	0.11154835	0.911181527	0.98075825	up_in_case
CRA004775	ENSGALG00010016785	PDGFRB	case	control	-0.020474529	0.429804818	-0.047636807	0.962005697	0.991860454	down_in_case
CRA004775	ENSGALG00010016786	DISP1	case	control	0.271905093	0.17229383	1.578147595	0.114531706	0.564488547	up_in_case
CRA004775	ENSGALG00010016787	PPP2R5C	case	control	0.196679224	0.180337394	1.090618087	0.275440967	0.731001618	up_in_case
CRA004775	ENSGALG00010016788	ANLN	case	control	0.861676597	0.608352368	1.416410362	0.156655392	0.621155794	up_in_case
CRA004775	ENSGALG00010016789	DISP3	case	control	-0.038603528	0.314868414	-0.122602099	0.902422192	0.979310476	down_in_case
CRA004775	ENSGALG00010016792	HMOX2	case	control	0.105431242	0.1879496	0.560954863	0.574828308	0.887328177	up_in_case
CRA004775	ENSGALG00010016793	SLC9A1	case	control	-0.043289627	0.224677584	-0.192674437	0.847213944	0.967891337	down_in_case
CRA004775	ENSGALG00010016795	GUCA2B	case	control	2.498184623	1.078426384	2.316509184	0.020530484	0.282032647	up_in_case
CRA004775	ENSGALG00010016796	BROX	case	control	-0.202556177	0.241569766	-0.838499703	0.401750114	0.811868062	down_in_case
CRA004775	ENSGALG00010016797	SEC62	case	control	0.067415442	0.222265316	0.30331067	0.761653115	0.942236668	up_in_case
CRA004775	ENSGALG00010016799	WDTC1	case	control	-0.482265204	0.336103466	-1.434871261	0.151323786	0.615293187	down_in_case
CRA004775	ENSGALG00010016800	RPL14	case	control	-0.245726881	0.254240833	-0.966512256	0.333787917	0.774300343	down_in_case
CRA004775	ENSGALG00010016802	TMEM222	case	control	-0.554928618	0.167593956	-3.311149352	0.000929136	0.063882749	down_in_case
CRA004775	ENSGALG00010016804	CSF1R	case	control	-0.930070254	0.463401528	-2.007050469	0.044744289	0.393998162	down_in_case
CRA004775	ENSGALG00010016807	VSIG4	case	control	-0.2755551	0.424096211	-0.649746667	0.515855874	0.864897625	down_in_case
CRA004775	ENSGALG00010016809	SYTL1	case	control	-0.381336285	0.358132008	-1.064792524	0.286969829	0.741909972	down_in_case
CRA004775	ENSGALG00010016810	SH3GL3	case	control	0.042296851	0.404797065	0.104489027	0.916781276	0.981688766	up_in_case
CRA004775	ENSGALG00010016812	LRRC31	case	control	-0.036641448	0.376734955	-0.097260548	0.922519486	0.983072338	down_in_case
CRA004775	ENSGALG00010016813	ENSGALG00010016813	case	control	0.187665434	1.27479282	0.147212497	0.882964297	0.975577339	up_in_case
CRA004775	ENSGALG00010016814	HMGXB3	case	control	-0.203063465	0.193676381	-1.048467884	0.294423091	0.746438942	down_in_case
CRA004775	ENSGALG00010016816	MAP3K6	case	control	-0.389068998	0.225359975	-1.726433443	0.084269463	0.503602517	down_in_case
CRA004775	ENSGALG00010016817	GGCT	case	control	-0.880535974	0.50571505	-1.7411702	0.081653749	0.497331356	down_in_case
CRA004775	ENSGALG00010016820	EIF2AK3	case	control	0.067252887	0.259886358	0.258778058	0.795806489	0.952819767	up_in_case
CRA004775	ENSGALG00010016823	HSF3	case	control	0.36126863	0.172647477	2.092521919	0.036391853	0.360628495	up_in_case
CRA004775	ENSGALG00010016825	ENSGALG00010016825	case	control	-0.10136837	0.526193895	-0.192644519	0.847237376	0.967891337	down_in_case
CRA004775	ENSGALG00010016828	LACTBL1	case	control	-0.391638126	0.780673579	-0.50166694	0.615901823	0.9028981	down_in_case
CRA004775	ENSGALG00010016831	AIDA	case	control	0.020254164	0.184011575	0.110070053	0.912353818	0.98075825	up_in_case
CRA004775	ENSGALG00010016832	DYNC1H1	case	control	0.222074851	0.252013428	0.881202454	0.378208251	0.799601151	up_in_case
CRA004775	ENSGALG00010016834	ENSGALG00010016834	case	control	0.616275002	0.192839429	3.195793546	0.001394469	0.078533081	up_in_case
CRA004775	ENSGALG00010016835	CCDC88A	case	control	-0.162591626	0.498132564	-0.326402322	0.744119981	0.939218065	down_in_case
CRA004775	ENSGALG00010016836	HEPH	case	control	-0.398956786	0.443282217	-0.900006295	0.3681169	0.794533903	down_in_case
CRA004775	ENSGALG00010016840	LRRC34	case	control	0.154613947	0.278138381	0.555888569	0.578287033	0.888378369	up_in_case
CRA004775	ENSGALG00010016842	NPY7R	case	control	-0.654625606	0.416382531	-1.572173559	0.115910306	0.566086328	down_in_case
CRA004775	ENSGALG00010016845	MIA3	case	control	0.195517593	0.274756276	0.711603737	0.476710192	0.846367006	up_in_case
CRA004775	ENSGALG00010016847	EPHB2	case	control	0.166268667	0.272814714	0.609456377	0.542221979	0.875128455	up_in_case
CRA004775	ENSGALG00010016848	ENSGALG00010016848	case	control	-0.526480581	0.245539794	-2.144176196	0.032018772	0.340736995	down_in_case
CRA004775	ENSGALG00010016849	WASF2	case	control	0.194306865	0.161962736	1.199701055	0.230255463	0.696525332	up_in_case
CRA004775	ENSGALG00010016850	GPR83L	case	control	-0.594301908	0.848042862	-0.700792301	0.483432644	0.850257583	down_in_case
CRA004775	ENSGALG00010016851	TEC	case	control	0.552240259	0.369566096	1.494293618	0.135098892	0.592957144	up_in_case
CRA004775	ENSGALG00010016852	AHDC1	case	control	0.097733083	0.427847014	0.228429975	0.819311989	0.960310191	up_in_case
CRA004775	ENSGALG00010016854	TAF1A	case	control	-0.312214183	0.248160378	-1.258114549	0.208350332	0.677150891	down_in_case
CRA004775	ENSGALG00010016856	NOD1	case	control	0.192463354	0.345387356	0.557239143	0.577364049	0.888268665	up_in_case
CRA004775	ENSGALG00010016858	ENSGALG00010016858	case	control	-0.143110884	0.415309865	-0.344588212	0.730403969	0.936393482	down_in_case
CRA004775	ENSGALG00010016859	RPIA	case	control	0.318901724	0.314714133	1.013306016	0.310914015	0.762763182	up_in_case
CRA004775	ENSGALG00010016861	FGR	case	control	-0.382713866	0.229055311	-1.670836024	0.094754071	0.526933666	down_in_case
CRA004775	ENSGALG00010016862	TXK	case	control	-0.360853967	0.454555431	-0.793861303	0.42727618	0.822776983	down_in_case
CRA004775	ENSGALG00010016863	MYNN	case	control	-0.087775961	0.35530748	-0.247042254	0.804785523	0.954618192	down_in_case
CRA004775	ENSGALG00010016864	ENSGALG00010016864	case	control	-0.130011788	0.423223443	-0.307194202	0.758695559	0.942236668	down_in_case
CRA004775	ENSGALG00010016865	CFAP36	case	control	0.148648318	0.202837943	0.732842759	0.463654338	0.842456502	up_in_case
CRA004775	ENSGALG00010016866	ZNRF2	case	control	0.014693113	0.284374036	0.051668263	0.958793026	0.991431439	up_in_case
CRA004775	ENSGALG00010016867	FLT4	case	control	-0.108893993	0.351563351	-0.309742164	0.756757036	0.941900693	down_in_case
CRA004775	ENSGALG00010016868	NIPAL1	case	control	-0.080989336	0.613744121	-0.13195945	0.895016366	0.977834811	down_in_case
CRA004775	ENSGALG00010016869	HSP90AA1	case	control	0.727312618	0.250567459	2.902661905	0.003700058	0.12600076	up_in_case
CRA004775	ENSGALG00010016870	ENSGALG00010016870	case	control	-0.092152659	0.588874896	-0.156489366	0.875647301	0.974412785	down_in_case
CRA004775	ENSGALG00010016872	EDA2R	case	control	0.810515298	0.723442667	1.120358717	0.26256093	0.723367482	up_in_case
CRA004775	ENSGALG00010016873	ENTPD6	case	control	-0.115952582	0.265365738	-0.436953855	0.662144814	0.918136475	down_in_case
CRA004775	ENSGALG00010016874	ADAMTSL3	case	control	0.477413417	0.447750212	1.066249449	0.286310894	0.741582427	up_in_case

CRA004775	ENSGALG00010016875	SESN2	case	control	-0.179046577	0.341417387	-0.524421379	0.59998549	0.897206546	down_in_case
CRA004775	ENSGALG00010016876	MTURN	case	control	-0.828639155	0.423608317	-1.95614468	0.050448113	0.416143973	down_in_case
CRA004775	ENSGALG00010016877	UBE2D3	case	control	-0.050841993	0.207696021	-0.244790403	0.806618725	0.955397873	down_in_case
CRA004775	ENSGALG00010016878	SUCLG1	case	control	0.1771589	0.335589941	0.52790289	0.597566739	0.896490702	up_in_case
CRA004775	ENSGALG00010016879	RNF130	case	control	-0.354219818	0.237845268	-1.489286803	0.136411861	0.595518184	down_in_case
CRA004775	ENSGALG00010016880	AR	case	control	-0.452949594	0.434751514	-1.041858577	0.297477251	0.75002011	down_in_case
CRA004775	ENSGALG00010016881	C1QB	case	control	-0.708594357	0.390591992	-1.814154852	0.069653889	0.468596196	down_in_case
CRA004775	ENSGALG00010016882	ENSGALG00010016882	case	control	-0.099678012	0.238546953	-0.417854896	0.676053211	0.922924485	down_in_case
CRA004775	ENSGALG00010016883	TMEM251	case	control	-0.02007692	0.203844101	-0.098491543	0.921541987	0.982481112	down_in_case
CRA004775	ENSGALG00010016884	SBK1	case	control	0.796182609	0.349176147	2.280174682	0.02259733	0.292594999	up_in_case
CRA004775	ENSGALG00010016885	CDCA4	case	control	-0.141591008	0.26952884	-0.525327855	0.599355297	0.897206546	down_in_case
CRA004775	ENSGALG00010016887	TJP2	case	control	0.327610821	0.289269168	1.132546628	0.257404693	0.719236059	up_in_case
CRA004775	ENSGALG00010016888	ENSGALG00010016888	case	control	0.234156952	0.305903059	0.765461296	0.443996905	0.832130395	up_in_case
CRA004775	ENSGALG00010016889	FUNDC2	case	control	-0.218618597	0.357840516	-0.610938636	0.541240206	0.874673178	down_in_case
CRA004775	ENSGALG00010016890	UBR7	case	control	-0.152049095	0.155794223	-0.975961065	0.329083759	0.77237154	down_in_case
CRA004775	ENSGALG00010016891	ENSGALG00010016891	case	control	0.572648462	0.37234057	1.537969561	0.124056057	0.576485043	up_in_case
CRA004775	ENSGALG00010016892	MAL	case	control	1.374190847	0.513899634	2.674045193	0.007494235	0.172634743	up_in_case
CRA004775	ENSGALG00010016893	TBC1D9B	case	control	-0.109726844	0.166269026	-0.659935568	0.509295178	0.862971569	down_in_case
CRA004775	ENSGALG00010016894	BTBD7	case	control	0.375398248	0.319706455	1.174196647	0.240316269	0.70694164	up_in_case
CRA004775	ENSGALG00010016895	ENSGALG00010016895	case	control	-0.040107572	0.20762538	-0.19317278	0.846823656	0.967891337	down_in_case
CRA004775	ENSGALG00010016896	SPTBN1	case	control	0.410943921	0.263261541	1.560972105	0.118530333	0.570497237	up_in_case
CRA004775	ENSGALG00010016899	SAXO2	case	control	0.5600819	0.496166411	1.128818654	0.258974342	0.720037471	up_in_case
CRA004775	ENSGALG00010016900	CISD2	case	control	-0.195956625	0.273591704	-0.71623745	0.473844742	0.845497032	down_in_case
CRA004775	ENSGALG00010016901	MECOM	case	control	0.630410221	0.394771175	1.596900334	0.110287925	0.558567026	up_in_case
CRA004775	ENSGALG00010016902	MRNIP	case	control	-0.019679924	0.39326044	-0.050042979	0.960088139	0.991431439	down_in_case
CRA004775	ENSGALG00010016903	PPM1A	case	control	0.361314106	0.26013489	1.388949041	0.164848246	0.630194031	up_in_case
CRA004775	ENSGALG00010016904	EAF1	case	control	-0.003906518	0.179959776	-0.021707729	0.982681099	0.997398088	down_in_case
CRA004775	ENSGALG00010016905	C1QC	case	control	-0.825323394	0.381896465	-2.161118184	0.030686211	0.334098251	down_in_case
CRA004775	ENSGALG00010016907	SQSTM1	case	control	-0.345463171	0.327628725	-1.054434928	0.291683838	0.745973646	down_in_case
CRA004775	ENSGALG00010016908	CMC4	case	control	-0.032982877	0.258406836	-0.127639337	0.898434399	0.978790377	down_in_case
CRA004775	ENSGALG00010016909	MRPS5	case	control	-0.138176434	0.236172902	-0.585064726	0.558504196	0.880754991	down_in_case
CRA004775	ENSGALG00010016910	NSD2	case	control	-0.285821461	0.226260545	-1.263240402	0.206502797	0.675197427	down_in_case
CRA004775	ENSGALG00010016911	AXIN1	case	control	0.274831341	0.161585954	1.700836826	0.088973632	0.515249396	up_in_case
CRA004775	ENSGALG00010016912	CCDC88C	case	control	0.396409552	0.229457324	1.727595982	0.084060684	0.503010645	up_in_case
CRA004775	ENSGALG00010016913	BRCC3	case	control	-0.525517435	0.186748913	-2.814032091	0.004892434	0.142429514	down_in_case
CRA004775	ENSGALG00010016914	PDIA2	case	control	1.407361762	0.473203416	2.974115809	0.002938342	0.113497569	up_in_case
CRA004775	ENSGALG00010016915	PLOD2	case	control	0.128617737	0.343471498	0.374464076	0.708059079	0.931801673	up_in_case
CRA004775	ENSGALG00010016916	ACYP2	case	control	0.133291134	0.491868971	0.270989108	0.78639941	0.95041797	up_in_case
CRA004775	ENSGALG00010016917	METTL6	case	control	0.628619892	0.311831829	2.015893933	0.043811068	0.391981676	up_in_case
CRA004775	ENSGALG00010016918	RCOR1	case	control	-0.032764037	0.319751568	-0.102467166	0.918385874	0.981710522	down_in_case
CRA004775	ENSGALG00010016919	ARHGDIG	case	control	0.136828911	0.315073976	0.434275508	0.664088387	0.918295135	up_in_case
CRA004775	ENSGALG00010016920	C1QA	case	control	-0.692987041	0.353750411	-1.95897169	0.050116102	0.414161886	down_in_case
CRA004775	ENSGALG00010016921	EFL1	case	control	0.199700964	0.167889162	1.189480976	0.234250453	0.70147604	up_in_case
CRA004775	ENSGALG00010016922	ENSGALG00010016922	case	control	0.059006858	0.4567036	0.129201648	0.897198092	0.978790377	up_in_case
CRA004775	ENSGALG00010016923	ENSGALG00010016923	case	control	-0.090881102	0.438438701	-0.207283484	0.835788472	0.965107526	down_in_case
CRA004775	ENSGALG00010016924	LETM1	case	control	0.302698513	0.212109857	1.42708367	0.153555779	0.618314939	up_in_case
CRA004775	ENSGALG00010016925	PHACTR4	case	control	0.00886291	0.177398294	0.049960515	0.960153854	0.991431439	up_in_case
CRA004775	ENSGALG00010016926	ENSGALG00010016926	case	control	0.005451535	0.530964672	0.010267228	0.991808081	0.998065748	up_in_case
CRA004775	ENSGALG00010016927	MGAT4B	case	control	0.549558896	0.320905315	1.712526626	0.086799662	0.509281038	up_in_case
CRA004775	ENSGALG00010016928	RGS11	case	control	0.062235003	0.382327953	0.162779107	0.870692359	0.973918682	up_in_case
CRA004775	ENSGALG00010016930	C10H15ORF48	case	control	-0.506887372	0.433359916	-1.169668337	0.242134465	0.709690398	down_in_case
CRA004775	ENSGALG00010016931	SLC30A4	case	control	0.170432397	0.17089485	0.997293929	0.31862186	0.767037518	up_in_case
CRA004775	ENSGALG00010016932	OGT	case	control	-0.382562249	0.189298418	-2.020947946	0.043285154	0.389812978	down_in_case
CRA004775	ENSGALG00010016934	SLC25A38	case	control	-0.330818555	0.283717937	-1.166012127	0.243609542	0.710773354	down_in_case
CRA004775	ENSGALG00010016935	PRIMA1	case	control	0.71669322	0.571808581	1.253379617	0.210067586	0.67982426	up_in_case
CRA004775	ENSGALG00010016936	RCC1	case	control	-0.146473848	0.21467513	-0.682304691	0.495046308	0.856382445	down_in_case
CRA004775	ENSGALG00010016937	DHRS7	case	control	-0.118569754	0.306868762	-0.386385871	0.699210912	0.929347461	down_in_case
CRA004775	ENSGALG00010016938	PSME4	case	control	0.160622981	0.518979848	0.309497529	0.756943091	0.941900693	up_in_case
CRA004775	ENSGALG00010016939	FAM234A	case	control	-0.533335579	0.182294311	-2.925684165	0.003436997	0.125102734	down_in_case
CRA004775	ENSGALG00010016940	FGFR3	case	control	0.37293565	0.390857631	0.954147037	0.340009219	0.780516732	up_in_case
CRA004775	ENSGALG00010016941	SLC8A1	case	control	-0.34400502	0.497663059	-0.691240818	0.489414216	0.8544864	down_in_case
CRA004775	ENSGALG00010016942	ZBTB40	case	control	-0.311271667	0.291842496	-1.066574166	0.286164171	0.741582427	down_in_case
CRA004775	ENSGALG00010016943	PCNX4	case	control	-0.23821785	0.296718683	-0.802840751	0.422066785	0.820119439	down_in_case
CRA004775	ENSGALG00010016945	BDH2	case	control	-0.259172613	0.286544452	-0.904476117	0.365742993	0.793608387	down_in_case
CRA004775	ENSGALG00010016946	TAF1	case	control	0.164981802	0.181623901	0.908370547	0.363682482	0.793608387	up_in_case
CRA004775	ENSGALG00010016947	CANX	case	control	0.438761425	0.179799962	2.4402754	0.014676069	0.241689368	up_in_case
CRA004775	ENSGALG00010016948	CCR8	case	control	-0.37790965	0.457090308	-0.826772398	0.408366077	0.814384687	down_in_case
CRA004775	ENSGALG00010016949	LUC7L	case	control	0.160236443	0.191358229	0.837363742	0.40238814	0.811996248	up_in_case
CRA004775	ENSGALG00010016950	ENSGALG00010016950	case	control	-0.268897293	0.425740636	-0.631598843	0.527649043	0.86830001	down_in_case
CRA004775	ENSGALG00010016952	GINS1	case	control	0.068255094	0.505079325	0.135137374	0.892503261	0.977834811	up_in_case
CRA004775	ENSGALG00010016953	GJA4	case	control	0.069288818	0.322683053	0.214727169	0.829980039	0.963232966	up_in_case
CRA004775	ENSGALG00010016955	ENSGALG00010016955	case	control	-0.101864242	1.008709474	-0.100984719	0.919562591	0.982170916	down_in_case
CRA004775	ENSGALG00010016956	CENPE	case	control	-0.113069269	0.725364015	-0.155879347	0.876128125	0.974421894	down_in_case
CRA004775	ENSGALG00010016957	WNT4	case	control	-0.246541862	0.425892432	-0.578882938	0.562668164	0.881532201	down_in_case
CRA004775	ENSGALG00010016958	TLR15	case	control	0.827633847	0.448295773	1.846178117	0.064866347	0.457570489	up_in_case
CRA004775	ENSGALG00010016961	EIF4A3	case	control	-0.160224433	0.150177791	-1.066898315	0.286017756	0.741582427	down_in_case
CRA004775	ENSGALG00010016962	SQOR	case	control	0.034417741	0.191418872	0.179803282	0.857307007	0.970913248	up_in_case
CRA004775	ENSGALG00010016963	CD8A	case	control	-0.641518178	0.481641786	-1.331940451	0.182879757	0.647378665	down_in_case
CRA004775	ENSGALG00010016964	MAML1	case	control	-0.228953341	0.377503796	-0.606492818	0.54418755	0.875729696	down_in_case
CRA004775	ENSGALG00010016965	SLC25A33	case	control	-0.544324039	0.600816588	-0.90597372	0.364949762	0.793608387	down_in_case
CRA004775	ENSGALG00010016966	KDM3A	case	control	0.010295327	0.215167673	0.047847927	0.96183744	0.991860454	up_in_case
CRA004775	ENSGALG00010016967	ERLEC1	case	control	0.065127478	0.180767883	0.360282351	0.718635996	0.934011578	up_in_case
CRA004775	ENSGALG00010016968	C15orf61	case	control	-0.076603743	0.325188571	-0.235567143	0.813768572	0.958469519	down_in_case
CRA004775	ENSGALG00010016969	CHMP3	case	control	0.196588398	0.254401812	0.772747632	0.439671751	0.828842585	up_in_case
CRA004775	ENSGALG00010016970	SYNE3	case	control	-0.602328646	0.30343429	-1.985038162	0.047140226	0.403125153	down_in_case
CRA004775	ENSGALG00010016972	ASB3	case	control	-0.247304236	0.28313506	-0.873449708	0.382418007	0.8018649	down_in_case
CRA004775	ENSGALG00010016973	PLSCR1	case	control	-0.137693645	0.17669112	-0.779290125	0.43580883	0.826905159	down_in_case
CRA004775	ENSGALG00010016975	MALL	case	control	0.714450598	0.45407437	1.573421986	0.115621139	0.566086328	up_in_case
CRA004775	ENSGALG00010016976	ENSGALG00010016976	case	control	0.240306686	0.7921317	0.303367087	0.761610126	0.942236668	up_in_case
CRA004775	ENSGALG00010016977	CLCN7	case	control	-0.535454248	0.238528415	-2.244823735	0.02477945	0.302344474	down_in_case
CRA004775	ENSGALG00010016978	FAM181A	case	control	1.091830993	0.951579807	1.147387728	0.251221411	0.715759105	up_in_case
CRA004775	ENSGALG00010016980	NPHP1	case	control	0.300306641	0.198438719	1.513347006	0.130191538	0.584671406	up_in_case
CRA004775	ENSGALG00010016981	ENSGALG00010016981	case	control	2.22671647	1.071524953	2.078081769	0.037701829	0.365870038	up_in_case
CRA004775	ENSGALG00010016982	GLRX5	case	control	0.04431129	0.263880575	0.167921757	0.866644834	0.972914777	up_in_case
CRA004775	ENSGALG00010016983	ZFAND2A	case	control	-0.016145772	0.376482323	-0.04288587	0.965792513	0.993094598	down_in_case
CRA004775	ENSGALG00010016984	RNF103	case	control	0.320659555	0.276764411	1.158601115	0.246618814	0.712046495	up_in_case
CRA004775	ENSGALG00010016985	ENSGALG00010016985	case	control	0.142513365	0.273592638	0.520896199	0.602439083	0.898318448	up_in_case
CRA004775	ENSGALG00010016986	GFER	case	control	0.026020244	0.162783827	0.159845388	0.873002869	0.97407675	up_in_case
CRA004775	ENSGALG00010016987	ASB2	case	control	0.841536642	0.84847971	0.991817049	0.321286779	0.767549427	up_in_case
CRA004775	ENSGALG00010016988	ECI1	case	control	-0.332804202	0.347778864	-0.956942001	0.33859653	0.779253705	down_in_case
CRA004775	ENSGALG00010016989	RHOG2	case	control	0.173505217	0.214495476	0.808899188	0.418573136	0.818943344	up_in_case
CRA004775	ENSGALG00010016991	PDLIM3	case	control	0.436181557	0.73369974	0.594495995	0.55218042	0.878258849	up_in_case
CRA004775	ENSGALG00010016993	ENSGALG00010016993	case	control	0.220432664	0.303887117	0.725376798	0.468220903	0.843870188	up_in_case
CRA004775	ENSGALG00010016994	ATL2	case	control	0.219585436	0.26810184	0.819037408	0.412765071	0.817112606	up_in_case
CRA004775	ENSGALG00010016996	ZIC4	case	control	-0.028752256	1.267599199	-0.02268245	0.981903575	0.997069916	down_in_case
CRA004775	ENSGALG00010016998	DNASE1L2	case	control	1.094264806	0.860102919	1.272248684	0.203284775	0.673096301	up_in_case
CRA004775	ENSGALG00010016999	ITGB1BP2	case	control	-0.11182891	0.637862234	-0.175318281	0.860829548	0.971325143	down_in_case
CRA004775	ENSGALG00010017001	TPCN3	case	control	-0.254859595	0.233460287	-1.091661447	0.274981937	0.731001618	down_in_case
CRA004775	ENSGALG00010017003	CPZ	case	control	-0.775036791	0.962700271	-0.805065517	0.420781874	0.819399559	down_in_case
CRA004775	ENSGALG00010017004	CX3CR1	case	control	-0.266995511	0.368750212	-0.724055207	0.469031843	0.8440023	down_in_case
CRA004775	ENSGALG00010017006	DNAH3	case	control	0.055703856	0.400493833	0.139087924	0.889380671	0.977162915	up_in_case
CRA004775	ENSGALG00010017008	RTN1	case	control	0.150249797	0.519683229	0.28911804	0.772491049	0.946296525	up_in_case
CRA004775	ENSGALG00010017010	NONO	case	control	-0.150810145	0.140084089	-1.076568696	0.281672996	0.738260394	down_in_case
CRA004775	ENSGALG00010017012	SYNGR3	case	control	-0.701871146	0.447208878	-1.569448147	0.116543553	0.566086328	down_in_case
CRA004775	ENSGALG00010017017	ENSGALG00010017017	case	control	-0.151491415	0.383340792	-0.395187306	0.69270466	0.927867527	down_in_case
CRA004775	ENSGALG00010017018	HP1BP3	case	control	-0.159002986	0.185237347	-0.858374342	0.390685791	0.806479902	down_in_case
CRA004775	ENSGALG00010017022	CCR4	case	control	-0.242300227	0.462304391	-0.524114052	0.600199215	0.897206546	down_in_case
CRA004775	ENSGALG00010017024	BUB1	case	control	0.708076473	0.567207616	1.248355017	0.211901075	0.682275341	up_in_case
CRA004775	ENSGALG00010017033	LDAF1	case	control	-0.292593023	0.214406304	-1.364666138	0.172358007	0.63702643	down_in_case
CRA004775	ENSGALG00010017034	ZMYM3	case	control	-0.400416902	0.193709301	-2.067102098	0.038724534	0.370564421	down_in_case
CRA004775	ENSGALG00010017035	PIK3CD	case	control	-0.936998699	0.658708384	-1.422478782	0.154887307	0.619284425	down_in_case
CRA004775	ENSGALG00010017038	ZNF598	case	control	0.409447855	0.218145087	1.876951992	0.060524674	0.445397937	up_in_case
CRA004775	ENSGALG00010017039	MAP2K5	case	control	-0.11984444	0.179025717	-0.669425832	0.503223878	0.860996832	down_in_case
CRA004775	ENSGALG00010017041	JKAMP	case	control	-0.653620374	0.204709262	-3.192920368	0.001408418	0.078533081	down_in_case
CRA004775	ENSGALG00010017042	HNRNPLL	case	control	-0.123425051	0.215183604	-0.573580182	0.566251925	0.883033678	down_in_case
CRA004775	ENSGALG00010017045	GLB1	case	control	-0.018698117	0.190003915	-0.098409115	0.921607437	0.982481112	down_in_case

CRA004775	ENSGALG00010017049	TRAF3	case	control	1.002113735	0.389748364	2.571181375	0.010135223	0.202058223	up_in_case
CRA004775	ENSGALG00010017050	L3HYPDH	case	control	-1.130697425	0.34318608	-3.29470655	0.000985245	0.066028181	down_in_case
CRA004775	ENSGALG00010017054	GALM	case	control	-0.403266437	0.184640886	-2.184058172	0.028957974	0.323755716	down_in_case
CRA004775	ENSGALG00010017055	GPR135	case	control	-0.466009817	0.507235046	-0.918725589	0.358239122	0.790696227	down_in_case
CRA004775	ENSGALG00010017056	AGTR1	case	control	0.478277511	0.410505945	1.165092775	0.243981441	0.71125478	up_in_case
CRA004775	ENSGALG00010017057	NDUFAB1	case	control	-0.079571225	0.426410778	-0.186606975	0.851968798	0.969430695	down_in_case
CRA004775	ENSGALG00010017060	CLSTN1	case	control	0.280439076	0.201393201	1.392495254	0.163772478	0.629717526	up_in_case
CRA004775	ENSGALG00010017063	TMPPE	case	control	-0.193885281	0.429562457	-0.451355277	0.651733511	0.915892481	down_in_case
CRA004775	ENSGALG00010017065	VRK2	case	control	-0.15796863	0.284203643	-0.55582901	0.578327751	0.888378369	down_in_case
CRA004775	ENSGALG00010017070	BLOC1S6	case	control	0.0255659	0.246047337	0.103906427	0.917243607	0.981688766	up_in_case
CRA004775	ENSGALG00010017077	CTNNBIP1	case	control	0.141156786	0.228501244	0.617750626	0.536739735	0.872114864	up_in_case
CRA004775	ENSGALG00010017080	PALB2	case	control	-0.321764433	0.283689793	-1.134212231	0.256705535	0.719236059	down_in_case
CRA004775	ENSGALG00010017083	ENSGALG00010017083	case	control	1.082252977	0.393636944	2.749368403	0.005971023	0.154441997	up_in_case
CRA004775	ENSGALG00010017085	ENSGALG00010017085	case	control	-0.392634917	0.238916703	-1.64339668	0.100300887	0.538583505	down_in_case
CRA004775	ENSGALG00010017088	ENSGALG00010017088	case	control	-0.84004481	0.75820647	-1.107936747	0.267889134	0.727528758	down_in_case
CRA004775	ENSGALG00010017089	CRTAP	case	control	-0.104957302	0.219908686	-0.477276747	0.633165067	0.909723668	down_in_case
CRA004775	ENSGALG00010017092	ANKS4B	case	control	2.36940825	0.71869564	3.296817343	0.000977871	0.066028181	up_in_case
CRA004775	ENSGALG00010017094	SORBS2	case	control	0.753609336	0.246149729	3.061589139	0.002201654	0.095504567	up_in_case
CRA004775	ENSGALG00010017096	DCTN5	case	control	-0.419414885	0.140139368	-2.992841268	0.002763935	0.10984643	down_in_case
CRA004775	ENSGALG00010017098	DAAM1	case	control	0.38955008	0.261992681	1.486873903	0.137048118	0.596242097	up_in_case
CRA004775	ENSGALG00010017101	CSMD2	case	control	-1.057815862	0.38694002	-2.73379802	0.006260844	0.158635228	down_in_case
CRA004775	ENSGALG00010017104	FANCL	case	control	-0.023957624	0.455820469	-0.052559343	0.958083012	0.991431439	down_in_case
CRA004775	ENSGALG00010017105	PLK1	case	control	0.411780448	0.522531411	0.788049176	0.430667942	0.824577397	up_in_case
CRA004775	ENSGALG00010017106	CRYM	case	control	-0.146820072	0.3571223	-0.411119865	0.680984645	0.923896221	down_in_case
CRA004775	ENSGALG00010017109	ITGB1BP3	case	control	-0.125782957	0.239846403	-0.524431283	0.599978603	0.897206546	down_in_case
CRA004775	ENSGALG00010017110	BRF1	case	control	0.02099496	0.21209129	0.098990206	0.921146046	0.982481112	up_in_case
CRA004775	ENSGALG00010017111	GJB1	case	control	0.988070479	0.449717036	2.197093726	0.028013753	0.318730759	up_in_case
CRA004775	ENSGALG00010017112	LZIC	case	control	-0.058586052	0.163290836	-0.358783464	0.719757084	0.934081982	down_in_case
CRA004775	ENSGALG00010017115	LGALSL	case	control	0.008448409	0.379992423	0.022233098	0.982262016	0.997200811	up_in_case
CRA004775	ENSGALG00010017116	ENSGALG00010017116	case	control	-1.295914008	0.95448632	-1.35770831	0.174556259	0.639597725	down_in_case
CRA004775	ENSGALG00010017119	BTBD6	case	control	0.092697256	0.26209959	0.353671884	0.723584796	0.935238044	up_in_case
CRA004775	ENSGALG00010017120	ERN2	case	control	-0.164929561	0.247543668	-0.666264512	0.505242045	0.861769208	down_in_case
CRA004775	ENSGALG00010017122	NCBP2	case	control	-0.319205283	0.211769854	-1.507321641	0.131728233	0.587352231	down_in_case
CRA004775	ENSGALG00010017127	SOX8	case	control	0.61856398	0.378687084	1.633443563	0.102375741	0.542513917	up_in_case
CRA004775	ENSGALG00010017131	TLR3	case	control	0.422323307	0.471353842	0.895979346	0.370263794	0.795584784	up_in_case
CRA004775	ENSGALG00010017132	TMEM108	case	control	-1.450236805	0.74542407	-1.945519152	0.051712527	0.419476344	down_in_case
CRA004775	ENSGALG00010017133	WDR20	case	control	0.168122336	0.216556918	0.776342485	0.437546794	0.827521409	up_in_case
CRA004775	ENSGALG00010017135	ENSGALG00010017135	case	control	0.513415431	0.292460372	1.755504269	0.079173091	0.491399489	up_in_case
CRA004775	ENSGALG00010017136	ENSGALG00010017136	case	control	1.121060167	0.579281095	1.935261095	0.052958264	0.423142991	up_in_case
CRA004775	ENSGALG00010017139	COL9A2	case	control	-3.076199191	0.807307437	-3.810443273	0.000138718	0.025433454	down_in_case
CRA004775	ENSGALG00010017140	UQCRC2	case	control	-0.047008196	0.340099453	-0.138218971	0.890067362	0.977283345	down_in_case
CRA004775	ENSGALG00010017141	AFTPH	case	control	0.225910857	0.18014418	1.254055816	0.209821719	0.67982426	up_in_case
CRA004775	ENSGALG00010017142	RBP7	case	control	0.31621725	0.547607456	0.577452418	0.563633873	0.881844281	up_in_case
CRA004775	ENSGALG00010017143	FAM149A	case	control	0.131208593	0.396362172	0.331032078	0.740620256	0.938450627	up_in_case
CRA004775	ENSGALG00010017144	CPB1	case	control	2.627648841	1.209559017	2.172402342	0.029825329	0.329420155	up_in_case
CRA004775	ENSGALG00010017147	SRSF7L	case	control	-0.463003104	0.209316427	-2.211976913	0.02696826	0.31493941	down_in_case
CRA004775	ENSGALG00010017152	CYP4V2	case	control	0.199345623	0.300316487	0.663785146	0.506827834	0.8622397	up_in_case
CRA004775	ENSGALG00010017154	PIGZ	case	control	0.02727338	0.33570488	0.08124213	0.935249395	0.986167812	up_in_case
CRA004775	ENSGALG00010017162	BCL11A	case	control	0.43517559	0.348443545	1.248912759	0.211696984	0.682220401	up_in_case
CRA004775	ENSGALG00010017163	CDV3	case	control	-0.064884664	0.184448854	-0.351775914	0.725006323	0.935238044	down_in_case
CRA004775	ENSGALG00010017167	SMAP2	case	control	-0.580337325	0.267852132	-2.166633213	0.030262833	0.331325011	down_in_case
CRA004775	ENSGALG00010017174	DHX57	case	control	-0.349884677	0.258454698	-1.353756305	0.175814138	0.641310011	down_in_case
CRA004775	ENSGALG00010017183	NFYC	case	control	-0.049357648	0.155377795	-0.31766217	0.75074121	0.941495694	down_in_case
CRA004775	ENSGALG00010017184	CARD11	case	control	-0.658008337	0.466345415	-1.410989184	0.158247803	0.62369319	down_in_case
CRA004775	ENSGALG00010017185	NAA30	case	control	0.036284145	0.227654498	0.159382509	0.873367518	0.974079175	up_in_case
CRA004775	ENSGALG00010017187	ZNF839	case	control	0.30330823	0.311799399	0.972767206	0.330669015	0.77237154	up_in_case
CRA004775	ENSGALG00010017188	ENSGALG00010017188	case	control	-0.15039678	0.467020509	-0.322034638	0.747426454	0.940421005	down_in_case
CRA004775	ENSGALG00010017190	STK26	case	control	0.502437908	0.150562165	3.337079459	0.000846637	0.06088574	up_in_case
CRA004775	ENSGALG00010017198	TOPBP1	case	control	-0.374503854	0.311426619	-1.202542847	0.229153276	0.695782399	down_in_case
CRA004775	ENSGALG00010017200	KCNQ4	case	control	-0.66276054	0.599228267	-1.10602349	0.268716352	0.727528758	down_in_case
CRA004775	ENSGALG00010017201	AP5M1	case	control	-0.25285762	0.350790902	-0.720821489	0.471019352	0.845198132	down_in_case
CRA004775	ENSGALG00010017207	ENSGALG00010017207	case	control	0.012049922	0.684872667	0.017594398	0.985962426	0.997562105	up_in_case
CRA004775	ENSGALG00010017208	CINP	case	control	-0.428980515	0.289146147	-1.483611385	0.137912047	0.596924319	down_in_case
CRA004775	ENSGALG00010017209	ENSGALG00010017209	case	control	0.2169876	0.912960705	0.237674633	0.81213346	0.957585764	up_in_case
CRA004775	ENSGALG00010017211	UBFD1	case	control	0.354859964	0.337498489	1.051441637	0.293055801	0.746438942	up_in_case
CRA004775	ENSGALG00010017212	TINAGL1	case	control	0.389542931	0.398133098	0.978423881	0.327864722	0.771733617	up_in_case
CRA004775	ENSGALG00010017215	FRMD7	case	control	-0.054113915	0.752081144	-0.071952229	0.942639925	0.987825149	down_in_case
CRA004775	ENSGALG00010017219	EARS2	case	control	-0.75129974	0.346747556	-2.166705218	0.030257338	0.331325011	down_in_case
CRA004775	ENSGALG00010017221	SPPL2A	case	control	0.155188588	0.120811035	1.284556402	0.198947342	0.666028746	up_in_case
CRA004775	ENSGALG00010017223	NOC2L	case	control	-0.242714276	0.177698416	-1.365877541	0.171977403	0.636057362	down_in_case
CRA004775	ENSGALG00010017226	PEF1	case	control	-0.288333934	0.179697344	-1.604553117	0.108592186	0.555731727	down_in_case
CRA004775	ENSGALG00010017228	RAP2C	case	control	0.061566229	0.209851841	0.293379506	0.769232089	0.945357282	up_in_case
CRA004775	ENSGALG00010017240	MELTF	case	control	0.316832549	0.364598631	0.868989957	0.384852624	0.803182776	up_in_case
CRA004775	ENSGALG00010017246	GGA2	case	control	-0.44984846	0.240074232	-1.87378902	0.06095951	0.445419429	down_in_case
CRA004775	ENSGALG00010017248	TECPR2	case	control	0.055089378	0.245808507	0.224115019	0.822667806	0.960875066	up_in_case
CRA004775	ENSGALG00010017250	COL16A1	case	control	-0.061467385	0.387328131	-0.158695896	0.873908472	0.974115968	down_in_case
CRA004775	ENSGALG00010017252	EXOC5	case	control	-0.216457686	0.196508405	-1.101518717	0.270670963	0.728665115	down_in_case
CRA004775	ENSGALG00010017256	CARHSP1	case	control	0.358446586	0.268285179	1.336065551	0.181527848	0.647378665	up_in_case
CRA004775	ENSGALG00010017263	COG7	case	control	-0.437940327	0.180675236	-2.423908976	0.015354453	0.249441914	down_in_case
CRA004775	ENSGALG00010017264	PIAS1	case	control	0.03285621	0.266302168	0.12337943	0.901806645	0.979157637	up_in_case
CRA004775	ENSGALG00010017266	PMM2	case	control	-0.135542834	0.296472398	-0.457185341	0.647537834	0.914210065	down_in_case
CRA004775	ENSGALG00010017272	NRXN1	case	control	0.974744172	0.385540133	2.528256043	0.011463072	0.216528087	up_in_case
CRA004775	ENSGALG00010017278	CCR5	case	control	-1.155273132	0.600802344	-1.922883864	0.054494637	0.429956233	down_in_case
CRA004775	ENSGALG00010017280	SOS1	case	control	0.195276286	0.199873128	0.977001202	0.328568557	0.77220987	up_in_case
CRA004775	ENSGALG00010017283	INF2	case	control	0.661971585	0.478120799	1.384527898	0.166196872	0.630194031	up_in_case
CRA004775	ENSGALG00010017284	ENSGALG00010017284	case	control	0.739780776	0.359613465	2.057155388	0.039671281	0.375050449	up_in_case
CRA004775	ENSGALG00010017285	IL21R	case	control	-0.054619565	0.677978912	-0.080562336	0.935790021	0.986264424	down_in_case
CRA004775	ENSGALG00010017289	ADGRB2	case	control	0.000894931	0.315404203	0.00283741	0.997736077	0.999646385	up_in_case
CRA004775	ENSGALG00010017290	DACT1	case	control	-0.014109187	0.337147392	-0.041848722	0.966619295	0.993094598	down_in_case
CRA004775	ENSGALG00010017294	ENSGALG00010017294	case	control	0.355420671	0.633636788	0.560921773	0.574850866	0.887328177	up_in_case
CRA004775	ENSGALG00010017296	SCNN1G	case	control	-0.965505572	0.602781901	-1.60174944	0.109211028	0.556276325	down_in_case
CRA004775	ENSGALG00010017299	USP31	case	control	-0.283533332	0.448667118	-0.631945868	0.52742225	0.86830001	down_in_case
CRA004775	ENSGALG00010017300	TRPM7	case	control	0.240912345	0.261825588	0.920125289	0.357507291	0.790309937	up_in_case
CRA004775	ENSGALG00010017304	ANKRD9	case	control	-0.354841221	0.53226583	-0.666661658	0.504988275	0.861471523	down_in_case
CRA004775	ENSGALG00010017305	HS3ST2	case	control	0.629059201	0.469556966	1.339686654	0.180347238	0.645317427	up_in_case
CRA004775	ENSGALG00010017307	TMEM186	case	control	-0.240785556	0.377576691	-0.637712977	0.523660537	0.866638286	down_in_case
CRA004775	ENSGALG00010017310	SAMD11	case	control	-0.379992629	0.390566087	-0.972927866	0.330589154	0.77237154	down_in_case
CRA004775	ENSGALG00010017312	PTP4A2	case	control	0.033426505	0.378245495	0.088372512	0.929580608	0.98473447	up_in_case
CRA004775	ENSGALG00010017313	MAP4K3	case	control	0.521207299	0.224916524	2.31733663	0.020485401	0.281976732	up_in_case
CRA004775	ENSGALG00010017314	CALML4	case	control	-0.247404144	0.348333365	-0.710251066	0.477548459	0.846563638	down_in_case
CRA004775	ENSGALG00010017318	ADSS1	case	control	0.708511499	0.455982335	1.553813479	0.120228914	0.572133279	up_in_case
CRA004775	ENSGALG00010017320	ENSGALG00010017320	case	control	-1.149163025	0.73741836	-1.55835966	0.119148014	0.570892125	down_in_case
CRA004775	ENSGALG00010017322	KHDRBS1	case	control	-0.151130468	0.187247859	-0.80711453	0.419600502	0.819388688	down_in_case
CRA004775	ENSGALG00010017323	ENSGALG00010017323	case	control	-0.198967313	0.316723236	-0.628205606	0.529869258	0.869211096	down_in_case
CRA004775	ENSGALG00010017326	STON1	case	control	0.157743322	0.387295219	0.407294782	0.683791495	0.925304767	up_in_case
CRA004775	ENSGALG00010017327	CLN6	case	control	-0.315569769	0.478740442	-0.659166725	0.509788712	0.862971569	down_in_case
CRA004775	ENSGALG00010017332	FGFR1	case	control	0.147669854	0.229385121	0.643763875	0.519728563	0.865611895	up_in_case
CRA004775	ENSGALG00010017334	KDM8	case	control	-0.241427262	0.277165908	-0.871056848	0.383723114	0.802541516	down_in_case
CRA004775	ENSGALG00010017335	THUMPD2	case	control	-0.296019869	0.365111992	-0.810764576	0.417500881	0.818943344	down_in_case
CRA004775	ENSGALG00010017337	ABAT	case	control	0.495207984	0.498047819	0.994298067	0.320077777	0.767037518	up_in_case
CRA004775	ENSGALG00010017338	MBNL3	case	control	0.098446984	0.326909171	0.30114476	0.76330411	0.94239774	up_in_case
CRA004775	ENSGALG00010017340	SIVA1	case	control	-0.252161882	0.251021088	-1.004544614	0.315116178	0.765098415	down_in_case
CRA004775	ENSGALG00010017341	TA3	case	control	-0.278418166	0.302643338	-0.919954715	0.357596424	0.790309937	down_in_case
CRA004775	ENSGALG00010017343	DLG1	case	control	0.318192102	0.217118629	1.465521882	0.142778587	0.602997185	up_in_case
CRA004775	ENSGALG00010017344	RERE	case	control	0.300334036	0.309953853	0.968963713	0.332563294	0.773393854	up_in_case
CRA004775	ENSGALG00010017346	GM2A	case	control	-0.155323894	0.325459557	-0.47724484	0.633187784	0.909723668	down_in_case
CRA004775	ENSGALG00010017347	TMEM39B	case	control	-0.581372752	0.234900596	-2.474973508	0.013324606	0.232183685	down_in_case
CRA004775	ENSGALG00010017349	ENSGALG00010017349	case	control	-0.396382433	0.345488257	-1.147310872	0.251253162	0.715759105	down_in_case
CRA004775	ENSGALG00010017351	CCR2	case	control	-0.895342062	0.444960317	-2.012184072	0.044200537	0.392535833	down_in_case
CRA004775	ENSGALG00010017352	GPCPD1	case	control	0.29839101	0.473972393	0.629553567	0.528986711	0.869191855	up_in_case
CRA004775	ENSGALG00010017354	FEM1B	case	control	-0.318611599	0.128136619	-2.486499189	0.012900686	0.228267513	down_in_case
CRA004775	ENSGALG00010017356	NSMCE1	case	control	-0.403775575	0.235844091	-1.712044487	0.08688847	0.509293844	down_in_case
CRA004775	ENSGALG00010017359	EHD3	case	control	0.117247555	0.139950415	0.83777926	0.402154689	0.811926168	up_in_case
CRA004775	ENSGALG00010017361	METTL22	case	control	-0.024399341	0.301635159	-0.080890241	0.935529241	0.986264424	down_in_case
CRA004775	ENSGALG00010017362	ENO1	case	control	-0.032721334	0.261163899	-0.12529042	0.900293637	0.978988423	down_in_case
CRA004775	ENSGALG00010017363	TIMM9	case	control	-0.159688488	0.298010736	-0.535848106	0.592063532	0.893804699	down_in_case

CRA004775	ENSGALG00010017364	AKT1	case	control	0.195353042	0.143798998	1.358514628	0.174300444	0.639555285	up_in_case
CRA004775	ENSGALG00010017365	CD8BP	case	control	0.20992573	0.582607848	0.360320807	0.718607241	0.934011578	up_in_case
CRA004775	ENSGALG00010017367	TIMD4	case	control	-0.131498604	0.62062089	-0.211882336	0.832198831	0.963831056	down_in_case
CRA004775	ENSGALG00010017368	ERLIN2	case	control	0.263865536	0.143881581	1.833907675	0.0666677	0.462498477	up_in_case
CRA004775	ENSGALG00010017372	ENSGALG00010017372	case	control	-0.172779996	0.263662964	-0.655306278	0.51227058	0.863263816	down_in_case
CRA004775	ENSGALG00010017373	RP1-27O5.3	case	control	-0.354944683	0.311376021	-1.139922984	0.254318389	0.718335926	down_in_case
CRA004775	ENSGALG00010017375	BDH1A	case	control	0.050257932	0.316746746	0.158669133	0.873929558	0.974115968	up_in_case
CRA004775	ENSGALG00010017376	GALNT14	case	control	0.038051464	0.445374521	0.085437003	0.931913976	0.985044263	up_in_case
CRA004775	ENSGALG00010017377	USP8	case	control	0.393681419	0.178916298	2.200366449	0.027780906	0.318297866	up_in_case
CRA004775	ENSGALG00010017378	ENSGALG00010017378	case	control	-0.205597107	0.806015449	-0.25507837	0.79866257	0.952835572	down_in_case
CRA004775	ENSGALG00010017380	MARCKSL1	case	control	0.045369801	0.343662283	0.132018563	0.894969609	0.977834811	up_in_case
CRA004775	ENSGALG00010017381	CA6	case	control	-0.103540302	0.407200569	-0.25427347	0.799284294	0.952835572	down_in_case
CRA004775	ENSGALG00010017383	ENSGALG00010017383	case	control	-0.060350859	0.343340967	-0.175775293	0.860470481	0.971325143	down_in_case
CRA004775	ENSGALG00010017384	ARID4A	case	control	0.029856221	0.214381843	0.139266558	0.889239515	0.977162915	up_in_case
CRA004775	ENSGALG00010017387	HS6ST2	case	control	-0.339054619	0.2798954	-1.211361885	0.225756741	0.69359464	down_in_case
CRA004775	ENSGALG00010017388	PLPBP	case	control	-0.389012143	0.227907734	-1.706884344	0.087843551	0.512706568	down_in_case
CRA004775	ENSGALG00010017390	DDX24	case	control	-0.05298051	0.368024156	-0.143959327	0.885532587	0.97665693	down_in_case
CRA004775	ENSGALG00010017391	CRAMP1	case	control	-0.049449599	0.358271944	-0.138022526	0.890222615	0.977283345	down_in_case
CRA004775	ENSGALG00010017393	VDAC1	case	control	0.04197641	0.391668238	0.107173384	0.91465143	0.981303879	up_in_case
CRA004775	ENSGALG00010017395	BRF2	case	control	-0.264491403	0.218776453	-1.20895736	0.226679228	0.694715356	down_in_case
CRA004775	ENSGALG00010017396	CCR8L	case	control	0.306960343	0.391924311	0.783213325	0.433501851	0.825828172	up_in_case
CRA004775	ENSGALG00010017398	SLC22A5	case	control	-0.791372315	0.459957744	-1.720532647	0.085335665	0.506234134	down_in_case
CRA004775	ENSGALG00010017399	CPXM1	case	control	-0.666023407	0.575630508	-1.157032849	0.24725894	0.712046495	down_in_case
CRA004775	ENSGALG00010017400	GPC4	case	control	-0.111966453	0.256760795	-0.436073012	0.662783758	0.918295135	down_in_case
CRA004775	ENSGALG00010017402	SLC2A5	case	control	0.053794829	0.372959572	0.144237694	0.885312776	0.976602443	up_in_case
CRA004775	ENSGALG00010017403	ITGA11	case	control	-0.658548669	0.578305661	-1.138755356	0.254805207	0.718438877	down_in_case
CRA004775	ENSGALG00010017405	XCR1	case	control	-0.05750733	0.647007261	-0.088882048	0.929175651	0.98473447	down_in_case
CRA004775	ENSGALG00010017406	IFT140	case	control	0.119134544	0.288430823	0.413043733	0.679754573	0.923739354	up_in_case
CRA004775	ENSGALG00010017407	EPN2	case	control	0.225368037	0.184900357	1.218862096	0.222896538	0.690637741	up_in_case
CRA004775	ENSGALG00010017409	GPC3	case	control	-0.157646124	0.648297218	-0.243169521	0.807874077	0.955397873	down_in_case
CRA004775	ENSGALG00010017411	RAB11FIP1	case	control	0.079302796	0.327506507	0.242141133	0.808670807	0.955575515	up_in_case
CRA004775	ENSGALG00010017414	ENSGALG00010017414	case	control	-0.180820744	0.352607151	-0.512810768	0.608083697	0.900943922	down_in_case
CRA004775	ENSGALG00010017415	HDAC1	case	control	-0.420247947	0.277656381	-1.513554075	0.130138977	0.584671406	down_in_case
CRA004775	ENSGALG00010017417	PRLHRL	case	control	0.179932129	0.724283686	0.248427699	0.803803505	0.954334484	up_in_case
CRA004775	ENSGALG00010017418	CHGB	case	control	0.454599574	0.447801927	1.015180032	0.310020017	0.762244121	up_in_case
CRA004775	ENSGALG00010017419	B9D1	case	control	0.142323308	0.230375735	0.617787753	0.536715258	0.872114864	up_in_case
CRA004775	ENSGALG00010017421	GPR157	case	control	-0.557119673	0.570017775	-0.977372457	0.328384793	0.77220987	down_in_case
CRA004775	ENSGALG00010017422	ENSGALG00010017422	case	control	-0.251856921	0.340837502	-0.738935475	0.45994618	0.840995542	down_in_case
CRA004775	ENSGALG00010017423	TMEM204	case	control	0.320887708	0.302473973	1.06087709	0.288745762	0.742851819	up_in_case
CRA004775	ENSGALG00010017424	APOD	case	control	-0.683422051	0.490317619	-1.393835392	0.163367318	0.629717526	down_in_case
CRA004775	ENSGALG00010017425	H6PD	case	control	-0.322188574	0.452814623	-0.711524226	0.476759444	0.846367006	down_in_case
CRA004775	ENSGALG00010017428	PSMA3	case	control	-0.041409395	0.240232939	-0.172371845	0.863145205	0.971910432	down_in_case
CRA004775	ENSGALG00010017429	TELO2	case	control	-0.16733414	0.165637201	-1.010244915	0.312377965	0.764325516	down_in_case
CRA004775	ENSGALG00010017430	CACNA1H	case	control	0.11341942	0.371534895	0.305272591	0.760158549	0.942236668	up_in_case
CRA004775	ENSGALG00010017431	FYCO1	case	control	-0.347181149	0.510766408	-0.679725884	0.496678043	0.857478942	down_in_case
CRA004775	ENSGALG00010017433	RNPS1	case	control	-0.266588818	0.165866436	-1.60724993	0.107999553	0.55434933	down_in_case
CRA004775	ENSGALG00010017434	FGF13	case	control	0.197602642	0.495673058	0.398655199	0.690147283	0.927867527	up_in_case
CRA004775	ENSGALG00010017435	ACTR10	case	control	-0.599531217	0.205573769	-2.916379944	0.00354119	0.125102734	down_in_case
CRA004775	ENSGALG00010017437	DRG2	case	control	-0.1672071	0.201721722	-0.828899822	0.407161096	0.813842524	down_in_case
CRA004775	ENSGALG00010017439	PPP4R4	case	control	-0.461758158	0.715660175	-0.64521986	0.518784718	0.865435399	down_in_case
CRA004775	ENSGALG00010017441	SPSB1	case	control	0.133408869	0.482155059	0.276692874	0.782015944	0.949117497	up_in_case
CRA004775	ENSGALG00010017442	LCK	case	control	-0.796826154	0.414412956	-1.922782923	0.054507318	0.429956233	down_in_case
CRA004775	ENSGALG00010017445	F9	case	control	1.46392398	0.612011461	2.391987852	0.016757397	0.255907381	up_in_case
CRA004775	ENSGALG00010017447	CAPN13	case	control	1.989539518	0.734943223	2.707065601	0.006788084	0.161712092	up_in_case
CRA004775	ENSGALG00010017449	TRMT6	case	control	-0.234350558	0.247001817	-0.948780707	0.342732156	0.782225706	down_in_case
CRA004775	ENSGALG00010017450	ENSGALG00010017450	case	control	-0.109980296	0.262833463	-0.418440995	0.675624719	0.922924485	down_in_case
CRA004775	ENSGALG00010017451	GABPB1	case	control	0.047831629	0.239789976	0.199473011	0.841892753	0.966478468	up_in_case
CRA004775	ENSGALG00010017452	ARMH4	case	control	-1.795900391	1.143716896	-1.570231582	0.116361245	0.566086328	down_in_case
CRA004775	ENSGALG00010017453	PPP1R2	case	control	0.313790529	0.274101716	1.14479593	0.252293702	0.717019836	up_in_case
CRA004775	ENSGALG00010017454	ENSGALG00010017454	case	control	-0.354043259	0.23813836	-1.486712424	0.13709078	0.596242097	down_in_case
CRA004775	ENSGALG00010017455	CCR9	case	control	-0.311673839	0.366027702	-0.851503417	0.394489766	0.808680497	down_in_case
CRA004775	ENSGALG00010017457	ZMAT4	case	control	-0.682371839	0.684007887	-0.997608146	0.31846941	0.767037518	down_in_case
CRA004775	ENSGALG00010017458	ENSGALG00010017458	case	control	-0.050875734	0.313081731	-0.162499848	0.870912248	0.973918682	down_in_case
CRA004775	ENSGALG00010017459	FAM167B	case	control	-0.416630477	0.31469956	-1.32389914	0.185536544	0.6491093	down_in_case
CRA004775	ENSGALG00010017462	MCM8	case	control	-0.547151045	0.211899205	-2.582128819	0.009819293	0.200930196	down_in_case
CRA004775	ENSGALG00010017463	PPP1R36	case	control	-0.362451084	0.682326416	-0.531198962	0.595280912	0.895305942	down_in_case
CRA004775	ENSGALG00010017464	SH3BP2	case	control	-0.036622934	0.276556283	-0.132424886	0.894648231	0.977834811	down_in_case
CRA004775	ENSGALG00010017465	TMEM234	case	control	-0.619619694	0.305060514	-2.031136992	0.0422411	0.386465312	down_in_case
CRA004775	ENSGALG00010017466	SPIA4	case	control	1.345997444	0.867318415	1.551906914	0.120684498	0.572850296	up_in_case
CRA004775	ENSGALG00010017467	SFRP1	case	control	1.107960129	0.333672212	3.320504638	0.000898549	0.062720521	up_in_case
CRA004775	ENSGALG00010017468	AMDHD2	case	control	-0.656489753	0.243044751	-2.701106484	0.006910921	0.16300527	down_in_case
CRA004775	ENSGALG00010017470	HSPA2	case	control	0.963801423	0.423230561	2.27724912	0.022771353	0.294017728	up_in_case
CRA004775	ENSGALG00010017471	CRLS1	case	control	-0.066460055	0.305753612	-0.217364742	0.827924106	0.962251207	down_in_case
CRA004775	ENSGALG00010017472	LZTFL1	case	control	-0.015409634	0.271173679	-0.0568257	0.954684041	0.990670511	down_in_case
CRA004775	ENSGALG00010017473	EIF3I	case	control	-0.244574313	0.188620036	-1.296650758	0.194751379	0.659287597	down_in_case
CRA004775	ENSGALG00010017475	STUM	case	control	-0.591035181	0.475895026	-1.241944438	0.214257063	0.683562661	down_in_case
CRA004775	ENSGALG00010017476	ATP6V0C	case	control	-0.118808787	0.1860065	-0.638734598	0.522995601	0.866638286	down_in_case
CRA004775	ENSGALG00010017477	MCF2	case	control	-0.028386103	0.504068768	-0.056313949	0.955091707	0.990670511	down_in_case
CRA004775	ENSGALG00010017480	DCDC2B	case	control	-0.258042297	0.275552939	-0.936452711	0.349040149	0.785311667	down_in_case
CRA004775	ENSGALG00010017482	CORO2B	case	control	0.001871887	0.509132488	0.00367662	0.997066488	0.999619266	up_in_case
CRA004775	ENSGALG00010017484	IQCC	case	control	-0.481394206	0.232679296	-2.06891724	0.038553854	0.370251617	down_in_case
CRA004775	ENSGALG00010017485	LBH	case	control	-0.221233138	0.227701854	-0.971591287	0.331253919	0.772569986	down_in_case
CRA004775	ENSGALG00010017487	C1QTNF8	case	control	-0.86094083	0.855137034	-1.006786977	0.314037154	0.765098415	down_in_case
CRA004775	ENSGALG00010017489	TNFRSF1B	case	control	-0.634997008	0.503001571	-1.262415556	0.206799295	0.675462494	down_in_case
CRA004775	ENSGALG00010017490	SCNN1B	case	control	-0.950075873	0.777474358	-1.222002839	0.221706553	0.689550502	down_in_case
CRA004775	ENSGALG00010017491	CCDC28B	case	control	-0.1029607	0.365737726	-0.281515122	0.778315331	0.947907599	down_in_case
CRA004775	ENSGALG00010017493	YPEL5	case	control	0.094600434	0.362422033	0.261022856	0.794074882	0.951843306	up_in_case
CRA004775	ENSGALG00010017494	TBC1D24	case	control	-0.426878526	0.372524538	-1.145907136	0.251833578	0.716658534	down_in_case
CRA004775	ENSGALG00010017495	NT5M	case	control	-0.434405467	0.274704518	-1.581355376	0.113796805	0.563290086	down_in_case
CRA004775	ENSGALG00010017496	PARP1	case	control	-0.188171679	0.209029113	-0.900217569	0.368004478	0.794533903	down_in_case
CRA004775	ENSGALG00010017499	SNRPD1	case	control	-0.383400271	0.26234879	-1.461414288	0.143901782	0.605645941	down_in_case
CRA004775	ENSGALG00010017500	ACAP2	case	control	-0.005101034	0.202089046	-0.025241518	0.979862321	0.99682524	down_in_case
CRA004775	ENSGALG00010017502	NAT8B	case	control	-0.16301929	0.355263861	-0.458868204	0.646328813	0.914043203	down_in_case
CRA004775	ENSGALG00010017503	MED9	case	control	-0.323321784	0.419943707	-0.769916964	0.441349151	0.829353567	down_in_case
CRA004775	ENSGALG00010017505	TNFRSF8	case	control	-0.652883971	0.737480645	-0.885289635	0.376000439	0.798523018	down_in_case
CRA004775	ENSGALG00010017509	RASD1	case	control	1.00342181	0.433090481	2.316887241	0.020509875	0.282031291	up_in_case
CRA004775	ENSGALG00010017510	HDC	case	control	-0.138982968	0.60640944	-0.22918998	0.81872126	0.959941088	down_in_case
CRA004775	ENSGALG00010017513	TNIP2	case	control	-0.030498696	0.327190344	-0.093213926	0.925733612	0.98417264	down_in_case
CRA004775	ENSGALG00010017514	TXLNA	case	control	0.060585573	0.175865029	0.344500402	0.730469994	0.936393482	up_in_case
CRA004775	ENSGALG00010017515	ENSGALG00010017515	case	control	1.608196615	0.854232599	1.882621452	0.059751687	0.445397937	up_in_case
CRA004775	ENSGALG00010017516	AAK1	case	control	-0.06095021	0.268315734	-0.227158539	0.820300466	0.96064995	down_in_case
CRA004775	ENSGALG00010017517	LIN9	case	control	-0.059467608	0.361630856	-0.16444285	0.869382534	0.973496509	down_in_case
CRA004775	ENSGALG00010017518	PEMT	case	control	-0.220810004	0.432448684	-0.510603945	0.609628413	0.901246133	down_in_case
CRA004775	ENSGALG00010017519	NTN3	case	control	0.920261002	0.428456308	2.147852614	0.031725466	0.339235521	up_in_case
CRA004775	ENSGALG00010017520	ZBTB1	case	control	0.037093847	0.175408384	0.211471347	0.832519489	0.963988657	up_in_case
CRA004775	ENSGALG00010017521	MIIP	case	control	-0.33575771	0.238758795	-1.406263214	0.159645979	0.624907945	down_in_case
CRA004775	ENSGALG00010017522	XXYLT1	case	control	-0.295826727	0.21159316	-1.398092103	0.162085411	0.62801817	down_in_case
CRA004775	ENSGALG00010017524	ACBD3	case	control	-0.316721331	0.141590256	-2.236886488	0.02529376	0.30560733	down_in_case
CRA004775	ENSGALG00010017526	PLEKHA2	case	control	-0.285939082	0.378198447	-0.756055673	0.449615817	0.835044179	down_in_case
CRA004775	ENSGALG00010017528	RAI1	case	control	-0.095461489	0.354390256	-0.269368266	0.787646304	0.95041797	down_in_case
CRA004775	ENSGALG00010017529	MFN2	case	control	0.096824745	0.364632008	0.265540993	0.790592735	0.951054854	up_in_case
CRA004775	ENSGALG00010017530	TLR1B	case	control	-0.587618634	0.4792495	-1.226122581	0.220152549	0.688339633	down_in_case
CRA004775	ENSGALG00010017531	SACM1L	case	control	0.068205096	0.189878428	0.359204028	0.719442464	0.93407508	up_in_case
CRA004775	ENSGALG00010017532	MIXL1	case	control	0.30829689	0.736299824	0.418711074	0.675427302	0.922924485	up_in_case
CRA004775	ENSGALG00010017533	FAM193A	case	control	0.113511347	0.246843361	0.45985173	0.645622649	0.914043203	up_in_case
CRA004775	ENSGALG00010017534	ATP11C	case	control	-0.238990422	0.412902039	-0.578806591	0.562719684	0.881532201	down_in_case
CRA004775	ENSGALG00010017535	LIMD1	case	control	-0.217139854	0.285139798	-0.761520684	0.446346129	0.833749389	down_in_case
CRA004775	ENSGALG00010017536	GSC	case	control	0.46336218	0.553171293	0.837646828	0.402229084	0.811957155	up_in_case
CRA004775	ENSGALG00010017537	MANBA	case	control	0.06688098	0.460450364	0.145251226	0.884512522	0.976236592	up_in_case
CRA004775	ENSGALG00010017538	H3-3A	case	control	-0.031500829	0.178571293	-0.176404778	0.859975951	0.971325143	down_in_case
CRA004775	ENSGALG00010017539	KPNA6	case	control	-0.087704692	0.224789095	-0.390164356	0.696415017	0.929092636	down_in_case
CRA004775	ENSGALG00010017540	EIF4G1	case	control	-0.063610225	0.235326408	-0.270306361	0.786924572	0.95041797	down_in_case
CRA004775	ENSGALG00010017541	SREBF1	case	control	-0.319298117	0.351022423	-0.909623135	0.363021294	0.793608387	down_in_case
CRA004775	ENSGALG00010017542	PLOD1	case	control	0.198542103	0.22740696	0.873069596	0.382625145	0.801925538	up_in_case
CRA004775	ENSGALG00010017543	ZBTB25	case	control	-0.697130542	0.339360881	-2.054245441	0.039951942	0.375888502	down_in_case
CRA004775	ENSGALG00010017544	SDE2	case	control	0.033803992	0.189030718	0.178828036	0.858072731	0.971038554	up_in_case

CRA004775	ENSGALG00010017545	DICER1	case	control	0.433891215	0.375565869	1.155299913	0.247967632	0.712818804	up_in_case
CRA004775	ENSGALG00010017546	NFU1	case	control	-0.42403959	0.340589376	-1.245017078	0.213125473	0.682777582	down_in_case
CRA004775	ENSGALG00010017547	RNF4	case	control	0.034117405	0.165135223	0.206602836	0.836320046	0.965207162	up_in_case
CRA004775	ENSGALG00010017550	TM2D2	case	control	-0.416676046	0.267740609	-1.556267643	0.119644462	0.572133279	down_in_case
CRA004775	ENSGALG00010017551	NALF2	case	control	-0.651568071	0.395041475	-1.649366239	0.099072627	0.536357359	down_in_case
CRA004775	ENSGALG00010017552	LARS2	case	control	0.089479027	0.207138861	0.431976049	0.665758821	0.918888163	up_in_case
CRA004775	ENSGALG00010017553	EDA	case	control	0.360354674	0.339241062	1.062237785	0.28812775	0.742790532	up_in_case
CRA004775	ENSGALG00010017554	FAM131A	case	control	-0.198549542	0.568811951	-0.349060075	0.727044209	0.935238044	down_in_case
CRA004775	ENSGALG00010017555	CELA2A	case	control	2.445569227	1.347135417	1.81538485	0.069464788	0.468451304	up_in_case
CRA004775	ENSGALG00010017556	TOM1L2	case	control	-0.420389362	0.26247459	-1.601638325	0.109235611	0.556276325	down_in_case
CRA004775	ENSGALG00010017557	ENSGALG00010017557	case	control	-1.628513335	0.711351459	-2.289323112	0.022060586	0.290827737	down_in_case
CRA004775	ENSGALG00010017558	ENSGALG00010017558	case	control	0.0369972	0.175899623	0.210331322	0.83340909	0.964019688	up_in_case
CRA004775	ENSGALG00010017559	DUSP10	case	control	0.787683404	0.422081956	1.866185923	0.062015358	0.447495793	up_in_case
CRA004775	ENSGALG00010017562	SLC30A10	case	control	1.216044114	0.77855228	1.561929938	0.118304495	0.569809145	up_in_case
CRA004775	ENSGALG00010017563	ALK	case	control	0.454398297	0.501759316	0.905610085	0.365142269	0.793608387	up_in_case
CRA004775	ENSGALG00010017565	ENSGALG00010017565	case	control	0.007426914	0.386950604	0.019193442	0.984686789	0.997562105	up_in_case
CRA004775	ENSGALG00010017568	CDCP1	case	control	0.985443786	0.370781603	2.657747253	0.007866486	0.178501739	up_in_case
CRA004775	ENSGALG00010017569	CLMN	case	control	0.04598639	0.328465455	0.140003733	0.888657041	0.977118922	up_in_case
CRA004775	ENSGALG00010017571	ADAM9	case	control	-0.13711392	0.199490725	-0.687319775	0.49188124	0.855746361	down_in_case
CRA004775	ENSGALG00010017574	DYNC1LI1	case	control	0.148659718	0.16899887	0.879649179	0.379049388	0.800168929	up_in_case
CRA004775	ENSGALG00010017575	CLCN2	case	control	2.165084786	0.649018503	3.335936921	0.000850125	0.06088574	up_in_case
CRA004775	ENSGALG00010017576	ENSGALG00010017576	case	control	-0.926397721	0.397436946	-2.330930054	0.019757048	0.278359806	down_in_case
CRA004775	ENSGALG00010017577	CLPX	case	control	0.245301842	0.256802543	0.955215783	0.339468586	0.779891494	up_in_case
CRA004775	ENSGALG00010017578	BSDC1	case	control	-0.304109229	0.21259015	-1.430495392	0.152574887	0.617257216	down_in_case
CRA004775	ENSGALG00010017579	P3H2	case	control	-0.277313299	0.378706674	-0.732264091	0.464007393	0.842643327	down_in_case
CRA004775	ENSGALG00010017580	LYPLAL1	case	control	-0.050842242	0.359769986	-0.141318741	0.887618145	0.977118922	down_in_case
CRA004775	ENSGALG00010017582	CLCN6	case	control	-0.173969776	0.219374006	-0.793028212	0.427761386	0.822830776	down_in_case
CRA004775	ENSGALG00010017583	MTHFD1	case	control	-0.436861265	0.247364086	-1.766065847	0.077384802	0.48896871	down_in_case
CRA004775	ENSGALG00010017584	WNT11B	case	control	3.305988141	0.775047376	4.265530399	1.99428E-05	0.008271499	up_in_case
CRA004775	ENSGALG00010017585	ENSGALG00010017585	case	control	-0.195214452	0.198860408	-0.981665749	0.326264545	0.770998457	down_in_case
CRA004775	ENSGALG00010017586	ZFYVE28	case	control	0.30859331	0.320059525	0.964174741	0.334958326	0.77555345	up_in_case
CRA004775	ENSGALG00010017587	MB21D2	case	control	0.332249894	0.31198096	1.064968496	0.286890187	0.741909972	up_in_case
CRA004775	ENSGALG00010017588	NOX5	case	control	-1.067829233	0.841279104	-1.269292471	0.204336771	0.674045742	down_in_case
CRA004775	ENSGALG00010017589	ATPAF2	case	control	-0.299719429	0.31850811	-0.941010353	0.346699554	0.784552667	down_in_case
CRA004775	ENSGALG00010017590	ENSGALG00010017590	case	control	-0.514063505	0.491496739	-1.045914375	0.295600567	0.747893909	down_in_case
CRA004775	ENSGALG00010017591	ESR2	case	control	0.73791274	0.814541178	0.905924414	0.364975861	0.793608387	up_in_case
CRA004775	ENSGALG00010017592	GID4	case	control	-0.181787508	0.288845781	-0.629358364	0.52911447	0.869191855	down_in_case
CRA004775	ENSGALG00010017593	MTHFR	case	control	-0.282912899	0.136003882	-2.080182531	0.037508794	0.365326975	down_in_case
CRA004775	ENSGALG00010017594	CEP68	case	control	-0.298240994	0.363638982	-0.820156828	0.41212671	0.817006008	down_in_case
CRA004775	ENSGALG00010017595	CCNF	case	control	-0.226497776	0.371855063	-0.609102304	0.542456631	0.875137006	down_in_case
CRA004775	ENSGALG00010017596	TDP1	case	control	-0.480618237	0.326410499	-1.472434983	0.140903452	0.600572867	down_in_case
CRA004775	ENSGALG00010017597	CNOT10	case	control	-0.245775999	0.159989267	-1.536203046	0.124488589	0.576754431	down_in_case
CRA004775	ENSGALG00010017598	ENSGALG00010017598	case	control	-0.099204843	0.260204631	-0.381257021	0.703012542	0.930068722	down_in_case
CRA004775	ENSGALG00010017599	KCNK13	case	control	-1.071415485	0.70397887	-1.521942675	0.128023445	0.5821156	down_in_case
CRA004775	ENSGALG00010017600	MXD4	case	control	0.057302881	0.283404114	0.202194952	0.83976432	0.966461921	up_in_case
CRA004775	ENSGALG00010017601	ENSGALG00010017601	case	control	-0.157863585	0.236640144	-0.667103994	0.504705709	0.861471523	down_in_case
CRA004775	ENSGALG00010017603	CYP3A5	case	control	2.44546236	0.964558829	2.535316963	0.011234562	0.214862956	up_in_case
CRA004775	ENSGALG00010017604	GATM	case	control	0.077159693	0.752561615	0.102529403	0.918336476	0.981710522	up_in_case
CRA004775	ENSGALG00010017605	GFPT1	case	control	-0.242456828	0.120937256	-2.00481503	0.044982825	0.394778076	down_in_case
CRA004775	ENSGALG00010017606	ENSGALG00010017606	case	control	-1.23587393	0.580943321	-2.127357155	0.033390419	0.348503984	down_in_case
CRA004775	ENSGALG00010017608	RBBP4	case	control	-0.466412801	0.143816884	-3.243101843	0.001182359	0.072583143	down_in_case
CRA004775	ENSGALG00010017609	TRIM71	case	control	0.041633056	0.625367485	0.066573745	0.946921048	0.98933351	up_in_case
CRA004775	ENSGALG00010017610	PSMC1	case	control	-0.44524621	0.280050613	-1.58987765	0.111862387	0.561235844	down_in_case
CRA004775	ENSGALG00010017612	CLDN11	case	control	-0.393972405	0.623489839	-0.63188264	0.527463568	0.86830001	down_in_case
CRA004775	ENSGALG00010017613	HAUS3	case	control	-0.691398123	0.544663672	-1.269403777	0.20429709	0.674045742	down_in_case
CRA004775	ENSGALG00010017615	AGTRAP	case	control	-0.375187011	0.213526573	-1.757097518	0.078901189	0.491399489	down_in_case
CRA004775	ENSGALG00010017616	PDCD7	case	control	0.01514656	0.307938616	0.049186944	0.960770316	0.991783846	up_in_case
CRA004775	ENSGALG00010017619	POLN	case	control	0.39428449	0.794706516	0.496138489	0.619796706	0.905089892	up_in_case
CRA004775	ENSGALG00010017620	UBAP1L	case	control	0.29323825	0.420855009	0.696767874	0.485948074	0.851645437	up_in_case
CRA004775	ENSGALG00010017621	SYNE2	case	control	0.387975063	0.415042064	0.934784921	0.349899152	0.785466132	up_in_case
CRA004775	ENSGALG00010017622	RAB1B	case	control	-0.041689655	0.301799216	-0.138137055	0.890132101	0.977283345	down_in_case
CRA004775	ENSGALG00010017625	YBX1	case	control	0.381949299	0.184967167	2.064957279	0.038927043	0.371210654	up_in_case
CRA004775	ENSGALG00010017626	COPS7B	case	control	-0.016735196	0.217037809	-0.077107285	0.938538197	0.986707289	down_in_case
CRA004775	ENSGALG00010017628	NRDE2	case	control	-0.029765027	0.269683847	-0.110370078	0.912115883	0.98075825	down_in_case
CRA004775	ENSGALG00010017629	ENSGALG00010017629	case	control	0.294379295	0.161040085	1.827987705	0.067551397	0.463543284	up_in_case
CRA004775	ENSGALG00010017631	NAT8L	case	control	-0.312786078	0.699982538	-0.446848401	0.654984522	0.916967384	down_in_case
CRA004775	ENSGALG00010017632	AWAT1	case	control	-0.600485359	0.401299631	-1.496351636	0.134562046	0.592420453	down_in_case
CRA004775	ENSGALG00010017633	HLX	case	control	-0.233059058	0.335473639	-0.694716456	0.487233013	0.852952408	down_in_case
CRA004775	ENSGALG00010017634	PPIH	case	control	-0.367207712	0.255082537	-1.43956429	0.149990709	0.61366707	down_in_case
CRA004775	ENSGALG00010017636	ZBTB8OS	case	control	-0.569028466	0.272547465	-2.087814199	0.036814598	0.361856712	down_in_case
CRA004775	ENSGALG00010017638	GFRA2	case	control	0.358128435	0.590445427	0.606539434	0.544156605	0.875729696	up_in_case
CRA004775	ENSGALG00010017639	ENSGALG00010017639	case	control	0.284825487	0.309824429	0.919312553	0.357932114	0.790416653	up_in_case
CRA004775	ENSGALG00010017640	P2RY4	case	control	0.859862387	0.804786438	1.068435483	0.285324118	0.741413527	up_in_case
CRA004775	ENSGALG00010017641	GPRC5B	case	control	0.411833927	0.451078505	0.912998342	0.361243414	0.792567091	up_in_case
CRA004775	ENSGALG00010017642	MTARC1	case	control	0.033896285	0.421697968	0.08038048	0.935934651	0.986264424	up_in_case
CRA004775	ENSGALG00010017643	CALM1	case	control	0.322944119	0.324432475	0.995412433	0.319535714	0.767037518	up_in_case
CRA004775	ENSGALG00010017644	RBBP6	case	control	0.320537725	0.17217327	1.861715956	0.062643138	0.449423965	up_in_case
CRA004775	ENSGALG00010017645	SYNC	case	control	-0.576526369	0.411577702	-1.400771633	0.161282374	0.627121149	down_in_case
CRA004775	ENSGALG00010017646	MAEA	case	control	-0.167987896	0.204816581	-0.820186995	0.412109514	0.817006008	down_in_case
CRA004775	ENSGALG00010017647	ITGA9	case	control	0.323865076	0.362301967	0.893909239	0.371370454	0.795818272	up_in_case
CRA004775	ENSGALG00010017648	ENSGALG00010017648	case	control	-0.026545381	0.341770898	-0.077670104	0.938090476	0.986707289	down_in_case
CRA004775	ENSGALG00010017649	ENSGALG00010017649	case	control	-0.333813243	0.305942872	-1.091096651	0.275230356	0.731001618	down_in_case
CRA004775	ENSGALG00010017651	PPCS	case	control	-0.462710681	0.331788017	-1.394597326	0.163137302	0.629717526	down_in_case
CRA004775	ENSGALG00010017652	C1orf115	case	control	0.240417091	0.348686607	0.689493332	0.490512868	0.855184934	up_in_case
CRA004775	ENSGALG00010017653	ENSGALG00010017653	case	control	0.395909541	0.441232565	0.897280874	0.36956906	0.795047581	up_in_case
CRA004775	ENSGALG00010017654	KBTBD13	case	control	-0.754444944	0.476483	-1.583361722	0.113339042	0.562886277	down_in_case
CRA004775	ENSGALG00010017655	ENSGALG00010017655	case	control	0.116201673	0.325730075	0.35674223	0.721284788	0.934511903	up_in_case
CRA004775	ENSGALG00010017656	FSD2	case	control	0.123901403	0.376085285	0.329450282	0.741815367	0.938877854	up_in_case
CRA004775	ENSGALG00010017657	DIS3L2	case	control	-0.3819854	0.250360499	-1.525741489	0.127074257	0.581154209	down_in_case
CRA004775	ENSGALG00010017658	WHAMM	case	control	0.513468422	0.355267665	1.445300185	0.148373586	0.611192154	up_in_case
CRA004775	ENSGALG00010017659	TNRC6A	case	control	0.067046955	0.255147815	0.26277691	0.792722533	0.951284723	up_in_case
CRA004775	ENSGALG00010017660	CLIP4	case	control	0.190467358	0.455409504	0.418233165	0.67577665	0.922924485	up_in_case
CRA004775	ENSGALG00010017661	NHSL3	case	control	-0.117889641	0.372710876	-0.316303194	0.751772387	0.941495694	down_in_case
CRA004775	ENSGALG00010017662	GOLGA4	case	control	0.357941479	0.277621956	1.289312578	0.19728944	0.663190847	up_in_case
CRA004775	ENSGALG00010017663	ZMYND12	case	control	-0.663343685	0.370553039	-1.790145043	0.073430597	0.478325033	down_in_case
CRA004775	ENSGALG00010017664	ENSGALG00010017664	case	control	0.583754261	0.271082589	2.153418495	0.031285803	0.336891999	up_in_case
CRA004775	ENSGALG00010017665	CTBP1	case	control	0.176225338	0.144050941	1.223354299	0.221195907	0.689251057	up_in_case
CRA004775	ENSGALG00010017667	SLC2A1	case	control	-0.086978492	0.196620492	-0.442367382	0.658223379	0.917354438	down_in_case
CRA004775	ENSGALG00010017668	ENSGALG00010017668	case	control	0.246280426	0.422323752	0.583155516	0.559788614	0.880918893	up_in_case
CRA004775	ENSGALG00010017669	ENSGALG00010017669	case	control	0.556802089	0.272615822	2.04244231	0.041107676	0.379122508	up_in_case
CRA004775	ENSGALG00010017670	FAM179A	case	control	-0.095111871	0.634965277	-0.149790665	0.880929774	0.974996282	down_in_case
CRA004775	ENSGALG00010017671	ANTXR1	case	control	-0.199343331	0.519130085	-0.383994949	0.700982191	0.930068722	down_in_case
CRA004775	ENSGALG00010017672	SPRED2	case	control	0.462418871	0.257627802	1.794910595	0.072667946	0.476063325	up_in_case
CRA004775	ENSGALG00010017673	WDR89	case	control	0.071351767	0.166586929	0.428315517	0.668421423	0.919882205	up_in_case
CRA004775	ENSGALG00010017675	ENSGALG00010017675	case	control	0.058442067	0.596997595	0.097893304	0.922017017	0.98284155	up_in_case
CRA004775	ENSGALG00010017676	SPON2	case	control	0.822660947	0.360777406	2.280245195	0.02259315	0.292594999	up_in_case
CRA004775	ENSGALG00010017677	WDR43	case	control	0.199597289	0.229980605	0.867887482	0.385455932	0.803446489	up_in_case
CRA004775	ENSGALG00010017678	YARS	case	control	-0.570044376	0.225306991	-2.530078511	0.011403701	0.21639381	down_in_case
CRA004775	ENSGALG00010017679	RASL12	case	control	0.26528357	0.529239786	0.50125402	0.616192361	0.902947693	up_in_case
CRA004775	ENSGALG00010017681	ENSGALG00010017681	case	control	2.42167667	1.000449854	2.420587759	0.015495439	0.249560894	up_in_case
CRA004775	ENSGALG00010017683	MYO15A	case	control	-0.260646903	0.831943304	-0.313298877	0.754053603	0.941900693	down_in_case
CRA004775	ENSGALG00010017684	TRMT61B	case	control	-0.16671747	0.23185629	-0.719055195	0.472106912	0.845425322	down_in_case
CRA004775	ENSGALG00010017685	PHC2	case	control	-0.169541697	0.343611736	-0.49341067	0.621722444	0.905838666	down_in_case
CRA004775	ENSGALG00010017687	TTC7B	case	control	-0.003851646	0.224361813	-0.017167119	0.986303294	0.997562105	down_in_case
CRA004775	ENSGALG00010017688	SPDYA	case	control	-0.151482025	0.482261925	-0.314107371	0.753439492	0.941900693	down_in_case
CRA004775	ENSGALG00010017691	LZTS1	case	control	-0.500003443	0.513544574	-0.973632024	0.33023928	0.77237154	down_in_case
CRA004775	ENSGALG00010017692	EPCAM	case	control	2.605025051	1.087381903	2.395685494	0.016589318	0.255167461	up_in_case
CRA004775	ENSGALG00010017693	MTFMT	case	control	0.000594203	0.218379185	0.002720969	0.997828983	0.999646385	up_in_case
CRA004775	ENSGALG00010017694	ATP6V1B2	case	control	-0.479998689	0.291503294	-1.646632124	0.099633683	0.537098329	down_in_case
CRA004775	ENSGALG00010017695	FNDC5	case	control	-0.545420223	0.438825151	-1.242910122	0.213900955	0.683502083	down_in_case
CRA004775	ENSGALG00010017696	SLC18A1	case	control	-0.152828563	0.347466	-0.439837459	0.660054835	0.917907174	down_in_case
CRA004775	ENSGALG00010017699	RNF19B	case	control	-0.42893869	0.281570601	-1.523378824	0.127663957	0.58187175	down_in_case
CRA004775	ENSGALG00010017700	VPS35L	case	control	-0.272436226	0.17190162	-1.58483804	0.113003137	0.562886277	down_in_case
CRA004775	ENSGALG00010017701	PPP2R5E	case	control	0.021169938	0.173796637	0.121808674	0.903050544	0.979310476	up_in_case
CRA004775	ENSGALG00010017703	RPS6KA5	case	control	0.088326098	0.307231783	0.287490107	0.773737076	0.946296525	up_in_case
CRA004775	ENSGALG00010017704	ZNF362	case	control	0.004157767	0.222392821	0.0186956	0.985083938	0.997562105	up_in_case

CRA004775	ENSGALG00010017705	MAK16	case	control	0.184467008	0.242406179	0.760983108	0.446667157	0.833847417	up_in_case
CRA004775	ENSGALG00010017706	FGFRL1	case	control	0.296691143	0.433527465	0.684365276	0.493744537	0.856049389	up_in_case
CRA004775	ENSGALG00010017707	AK2	case	control	-0.281545666	0.167074992	-1.685145464	0.09196051	0.52187835	down_in_case
CRA004775	ENSGALG00010017708	MEIS1	case	control	0.11951913	0.208346725	0.573654951	0.566201319	0.883033678	up_in_case
CRA004775	ENSGALG00010017710	RAB41	case	control	-0.149173103	0.189079947	-0.788941953	0.430145934	0.824153229	down_in_case
CRA004775	ENSGALG00010017711	LRRFIP2	case	control	0.65793593	0.287572528	2.287895631	0.0221436	0.290827737	up_in_case
CRA004775	ENSGALG00010017712	MSH2	case	control	-0.227765049	0.230293942	-0.989018846	0.322653921	0.767880594	down_in_case
CRA004775	ENSGALG00010017713	ALKBH5	case	control	0.242662732	0.368075526	0.659274292	0.509719647	0.862971569	up_in_case
CRA004775	ENSGALG00010017714	ATAD3A	case	control	-0.189880018	0.157697667	-1.204076269	0.228560101	0.695782399	down_in_case
CRA004775	ENSGALG00010017715	CILP	case	control	-0.517940638	0.621512787	-0.833354758	0.404644682	0.81313407	down_in_case
CRA004775	ENSGALG00010017717	LLGL1	case	control	-0.162202521	0.244300366	-0.663947107	0.506724165	0.8622397	down_in_case
CRA004775	ENSGALG00010017718	ATOH8	case	control	-0.760460495	0.649791047	-1.170315441	0.24187405	0.709321831	down_in_case
CRA004775	ENSGALG00010017719	ENSGALG00010017719	case	control	2.645017381	1.329557879	1.989396192	0.04665749	0.401745235	up_in_case
CRA004775	ENSGALG00010017720	ENSGALG00010017720	case	control	-1.117008508	0.731077092	-1.527894282	0.126538788	0.580923609	down_in_case
CRA004775	ENSGALG00010017721	VWA1	case	control	0.435345604	0.318527317	1.366744956	0.171705261	0.636057362	up_in_case
CRA004775	ENSGALG00010017723	KCNK12	case	control	-0.373581859	0.777694388	-0.480371036	0.630963586	0.909357765	down_in_case
CRA004775	ENSGALG00010017724	FLII	case	control	0.114285767	0.283529309	0.403082728	0.686887366	0.9269272	up_in_case
CRA004775	ENSGALG00010017725	ENSGALG00010017725	case	control	-0.457434541	0.354867132	-1.289030457	0.197387498	0.663190847	down_in_case
CRA004775	ENSGALG00010017726	TTI2	case	control	-0.262394404	0.189992291	-1.381079217	0.167254605	0.631096819	down_in_case
CRA004775	ENSGALG00010017727	AZIN2	case	control	-0.220314154	0.166088523	-1.326486323	0.184678662	0.6491093	down_in_case
CRA004775	ENSGALG00010017729	MIEF2	case	control	-0.003886949	0.433969751	-0.008956727	0.992853661	0.998444544	down_in_case
CRA004775	ENSGALG00010017730	TARP	case	control	-0.410959569	0.419790854	-0.978962656	0.327598432	0.771474966	down_in_case
CRA004775	ENSGALG00010017732	RHOJ	case	control	0.573741416	0.505578283	1.134822116	0.256449858	0.719236059	up_in_case
CRA004775	ENSGALG00010017733	TRIM62	case	control	-0.162302496	0.259158289	-0.626267815	0.531139293	0.869441746	down_in_case
CRA004775	ENSGALG00010017734	TOP3A	case	control	-0.223448047	0.249462745	-0.895717104	0.370403873	0.795641647	down_in_case
CRA004775	ENSGALG00010017736	ENSGALG00010017736	case	control	2.574816147	1.188066703	2.167231974	0.03021717	0.331325011	up_in_case
CRA004775	ENSGALG00010017737	ENSGALG00010017737	case	control	-0.075237877	0.381721045	-0.197101728	0.843747931	0.966478468	down_in_case
CRA004775	ENSGALG00010017738	NDUFS5	case	control	-0.010129951	0.30184378	-0.033560243	0.973227826	0.994947809	down_in_case
CRA004775	ENSGALG00010017739	MRPL20	case	control	-0.445845801	0.286829566	-1.554392761	0.120090759	0.572133279	down_in_case
CRA004775	ENSGALG00010017741	SMCR8	case	control	-0.131420115	0.32212379	-0.407980159	0.683288241	0.925066123	down_in_case
CRA004775	ENSGALG00010017742	DGLUCY	case	control	-0.225752739	0.429502045	-0.525615051	0.599155697	0.897206546	down_in_case
CRA004775	ENSGALG00010017744	ETAA1	case	control	0.075890537	0.191367161	0.396570323	0.691684341	0.927867527	up_in_case
CRA004775	ENSGALG00010017745	SHMT1	case	control	-0.203408444	0.404441682	-0.502936402	0.615008987	0.902639483	down_in_case
CRA004775	ENSGALG00010017746	MSH6	case	control	-0.098952742	0.282515924	-0.350255448	0.726146997	0.935238044	down_in_case
CRA004775	ENSGALG00010017747	DUSP26	case	control	-0.480682392	0.608062032	-0.790515386	0.42922684	0.823883065	down_in_case
CRA004775	ENSGALG00010017749	TMEM88B	case	control	-1.091064192	0.831669241	-1.311896771	0.189554967	0.654917174	down_in_case
CRA004775	ENSGALG00010017751	ST3GAL5	case	control	0.502357837	0.325565938	1.543029469	0.122823626	0.57525466	up_in_case
CRA004775	ENSGALG00010017753	GNRHR	case	control	0.391457364	0.690407915	0.566994316	0.570718043	0.885321726	up_in_case
CRA004775	ENSGALG00010017754	PDZD11	case	control	-0.380268985	0.208369582	-1.824973595	0.068005014	0.463992389	down_in_case
CRA004775	ENSGALG00010017755	C1D	case	control	-0.07660587	0.342208811	-0.223857094	0.822868503	0.960875066	down_in_case
CRA004775	ENSGALG00010017756	EIF2B5	case	control	-0.242013093	0.17021512	-1.421807257	0.155082212	0.619284425	down_in_case
CRA004775	ENSGALG00010017757	ENSGALG00010017757	case	control	-0.069499184	0.313890756	-0.221412014	0.824771639	0.961627506	down_in_case
CRA004775	ENSGALG00010017759	ENSGALG00010017759	case	control	2.659702712	0.893857354	2.975533738	0.002924792	0.113497569	up_in_case
CRA004775	ENSGALG00010017760	ENSGALG00010017760	case	control	-0.660338565	0.653952891	-1.009764731	0.312608021	0.7645017	down_in_case
CRA004775	ENSGALG00010017761	PARP16	case	control	0.053402222	0.327507743	0.163056364	0.870474056	0.973918682	up_in_case
CRA004775	ENSGALG00010017762	FBXO11	case	control	0.175074204	0.159045026	1.1007839	0.270990721	0.728665115	up_in_case
CRA004775	ENSGALG00010017763	POLR1A	case	control	-0.192816868	0.2519782	-0.765212497	0.444145019	0.832130395	down_in_case
CRA004775	ENSGALG00010017764	IGDCC3	case	control	-0.71906294	0.43674446	-1.646415711	0.099678201	0.537098329	down_in_case
CRA004775	ENSGALG00010017765	SYT16	case	control	-0.36569905	0.552477112	-0.661926154	0.508018549	0.862971569	down_in_case
CRA004775	ENSGALG00010017766	DNAAF10	case	control	0.174440822	0.359164215	0.485685418	0.627190224	0.907733315	up_in_case
CRA004775	ENSGALG00010017767	KIF4B	case	control	0.445791001	0.630385482	0.707172062	0.479459558	0.847326614	up_in_case
CRA004775	ENSGALG00010017768	FOXN2	case	control	-0.047209917	0.318835054	-0.148070033	0.882287499	0.975346523	down_in_case
CRA004775	ENSGALG00010017770	PTCD3	case	control	-0.17459126	0.222311564	-0.78534493	0.43225136	0.82542542	down_in_case
CRA004775	ENSGALG00010017771	PNO1	case	control	0.120355738	0.312169102	0.385546605	0.699832485	0.929617127	up_in_case
CRA004775	ENSGALG00010017772	ENSGALG00010017772	case	control	0.335464798	0.494393228	0.678538417	0.497430374	0.857810713	up_in_case
CRA004775	ENSGALG00010017773	DVL3	case	control	-0.068725851	0.170371533	-0.403388115	0.686662729	0.92689803	down_in_case
CRA004775	ENSGALG00010017774	ASH2L	case	control	-0.145582609	0.231954064	-0.62763552	0.530242735	0.869211096	down_in_case
CRA004775	ENSGALG00010017775	PRPSAP2	case	control	-0.463888673	0.213352151	-2.174286368	0.029683636	0.328382681	down_in_case
CRA004775	ENSGALG00010017776	GPR68	case	control	0.45464583	0.335866125	1.353651934	0.17584745	0.641310011	up_in_case
CRA004775	ENSGALG00010017778	PPP3R1	case	control	0.216263092	0.2506186	0.862917167	0.388183011	0.804270424	up_in_case
CRA004775	ENSGALG00010017779	SNAPC1	case	control	-0.046802074	0.229780612	-0.203681564	0.83860235	0.965800667	down_in_case
CRA004775	ENSGALG00010017780	TAF9	case	control	-0.073257591	0.189208383	-0.387179412	0.698623391	0.929347461	down_in_case
CRA004775	ENSGALG00010017781	CYP26C1	case	control	-0.232414022	0.396418314	-0.586284774	0.557684161	0.880722248	down_in_case
CRA004775	ENSGALG00010017782	IGDCC4	case	control	-0.842662727	0.368189627	-2.288665039	0.022098822	0.290827737	down_in_case
CRA004775	ENSGALG00010017783	STAR	case	control	-0.1793474	0.421840717	-0.425154313	0.670724187	0.921535334	down_in_case
CRA004775	ENSGALG00010017785	SLC5A10	case	control	-1.015923307	1.203745973	-0.843968188	0.398687182	0.810039516	down_in_case
CRA004775	ENSGALG00010017786	KAZALD1	case	control	-0.519431197	0.458013368	-1.134096151	0.256754218	0.719236059	down_in_case
CRA004775	ENSGALG00010017787	CNRIP1	case	control	-0.481478804	0.31213844	-1.542516852	0.122948046	0.575380405	down_in_case
CRA004775	ENSGALG00010017789	IMMT	case	control	0.186110095	0.325704785	0.571407309	0.567723576	0.884152085	up_in_case
CRA004775	ENSGALG00010017790	R3HCC1L	case	control	-0.049072088	0.234205926	-0.20952539	0.834038117	0.964019688	down_in_case
CRA004775	ENSGALG00010017791	CCNL2	case	control	-0.181224989	0.199879593	-0.906670792	0.364580913	0.793608387	down_in_case
CRA004775	ENSGALG00010017792	LSM1	case	control	-0.251005973	0.284947882	-0.880883799	0.378380717	0.799617833	down_in_case
CRA004775	ENSGALG00010017793	FAM83G	case	control	1.681355402	0.711417427	2.363387988	0.0181087	0.268043846	up_in_case
CRA004775	ENSGALG00010017795	MACF1	case	control	-0.366473351	0.33608263	-1.090426337	0.275525386	0.731001618	down_in_case
CRA004775	ENSGALG00010017796	MXI1	case	control	-0.142419921	0.35520417	-0.400952278	0.688455259	0.927339918	down_in_case
CRA004775	ENSGALG00010017797	PPP1R21	case	control	-0.178305669	0.220655663	-0.808072027	0.41904912	0.818943344	down_in_case
CRA004775	ENSGALG00010017798	BAG4	case	control	-0.000112795	0.189876508	-0.000594047	0.999526019	0.999914248	down_in_case
CRA004775	ENSGALG00010017799	GRAPL	case	control	-0.261199164	0.376219488	-0.69427335	0.487510801	0.853221461	down_in_case
CRA004775	ENSGALG00010017801	SMNDC1	case	control	0.140226553	0.183408658	0.76455798	0.444534799	0.832504887	up_in_case
CRA004775	ENSGALG00010017803	ENSGALG00010017803	case	control	0.365886881	0.33262508	1.099997874	0.271333048	0.728872973	up_in_case
CRA004775	ENSGALG00010017804	DUSP5	case	control	-0.657910945	0.472738272	-1.391702309	0.164012563	0.629717526	down_in_case
CRA004775	ENSGALG00010017805	DPP8	case	control	0.096744402	0.288439649	0.335406046	0.737318808	0.937597314	up_in_case
CRA004775	ENSGALG00010017806	ENSGALG00010017806	case	control	2.363394977	0.774204706	3.052674519	0.002268118	0.096690159	up_in_case
CRA004775	ENSGALG00010017807	MRPL35	case	control	-0.078173404	0.285795509	-0.273529156	0.784446484	0.950165405	down_in_case
CRA004775	ENSGALG00010017808	SFRP5	case	control	0.956495288	0.574134216	1.665978548	0.095717687	0.529205137	up_in_case
CRA004775	ENSGALG00010017809	MOSMO	case	control	0.346626135	0.261127376	1.327421658	0.184369239	0.648746785	up_in_case
CRA004775	ENSGALG00010017810	HIF1A	case	control	-0.137211375	0.325188754	-0.42194379	0.673066046	0.922029409	down_in_case
CRA004775	ENSGALG00010017811	HACD3	case	control	-0.020004547	0.249353479	-0.080225657	0.936057785	0.986264424	down_in_case
CRA004775	ENSGALG00010017812	ENSGALG00010017812	case	control	2.569643565	0.998777582	2.572788588	0.010088281	0.202058223	up_in_case
CRA004775	ENSGALG00010017813	DDHD2	case	control	-0.172387597	0.19312387	-0.892627083	0.372056911	0.795917016	down_in_case
CRA004775	ENSGALG00010017814	ENSGALG00010017814	case	control	0.412962681	0.469016039	0.880487333	0.378595364	0.799935404	up_in_case
CRA004775	ENSGALG00010017815	VWA9	case	control	-0.283806759	0.176138804	-1.61126766	0.107121396	0.553769294	down_in_case
CRA004775	ENSGALG00010017816	PRKCH	case	control	0.005331722	0.411465891	0.012957871	0.989661404	0.998065748	up_in_case
CRA004775	ENSGALG00010017817	REEP1	case	control	-0.068907637	0.338591977	-0.203512314	0.838734622	0.965800667	down_in_case
CRA004775	ENSGALG00010017818	SLC38A6	case	control	-0.418649483	0.275863941	-1.517594077	0.129116761	0.584172159	down_in_case
CRA004775	ENSGALG00010017820	FLRT2	case	control	0.641134682	0.277327206	2.311834791	0.020786789	0.283942565	up_in_case
CRA004775	ENSGALG00010017821	ENSGALG00010017821	case	control	-0.162693142	0.449339475	-0.362071777	0.717298393	0.934011578	down_in_case
CRA004775	ENSGALG00010017823	PPIE	case	control	-0.167135853	0.224923044	-0.743080166	0.457433147	0.839704957	down_in_case
CRA004775	ENSGALG00010017824	YAE1	case	control	-0.07225332	0.215301565	-0.335591242	0.737179129	0.937564762	down_in_case
CRA004775	ENSGALG00010017825	PLPP5	case	control	-0.047777689	0.201074988	-0.237611297	0.812182588	0.957585764	down_in_case
CRA004775	ENSGALG00010017826	PABPC4	case	control	0.0147809	0.442286152	0.033419315	0.973340207	0.994947809	up_in_case
CRA004775	ENSGALG00010017828	ENSGALG00010017828	case	control	-0.703481674	0.239392025	-2.938617839	0.003296793	0.122856914	down_in_case
CRA004775	ENSGALG00010017829	TRNP1	case	control	-0.251155762	0.807571686	-0.311001199	0.75579971	0.941900693	down_in_case
CRA004775	ENSGALG00010017830	DENND4A	case	control	0.637446947	0.413189472	1.542747312	0.122892098	0.575379379	up_in_case
CRA004775	ENSGALG00010017831	PLEK	case	control	-0.172372672	0.55230709	-0.312095707	0.754967789	0.941900693	down_in_case
CRA004775	ENSGALG00010017833	ENSGALG00010017833	case	control	0.699815634	0.445559541	1.57064448	0.116265252	0.566086328	up_in_case
CRA004775	ENSGALG00010017838	B3GNT2	case	control	0.289440091	0.286660306	1.009697139	0.312640414	0.7645017	up_in_case
CRA004775	ENSGALG00010017841	GALC	case	control	0.948260402	0.462129732	2.051935497	0.040175932	0.376363211	up_in_case
CRA004775	ENSGALG00010017843	NSD3	case	control	-0.009880332	0.308635702	-0.032012927	0.974461742	0.994947809	down_in_case
CRA004775	ENSGALG00010017848	CYP3A4	case	control	0.62482387	0.3107598	2.010632877	0.044364249	0.393075248	up_in_case
CRA004775	ENSGALG00010017849	FBXO48	case	control	0.103245677	0.316834318	0.32586646	0.744525393	0.939218065	up_in_case
CRA004775	ENSGALG00010017850	TRMT5	case	control	-0.405454797	0.216251024	-1.874926596	0.060802823	0.445397937	down_in_case
CRA004775	ENSGALG00010017852	LETM2	case	control	-0.064372243	0.185514467	-0.346993113	0.728596493	0.935823872	down_in_case
CRA004775	ENSGALG00010017856	FASTKD5	case	control	-0.480595215	0.28383139	-1.693241944	0.090409424	0.518924915	down_in_case
CRA004775	ENSGALG00010017858	GPR65	case	control	0.217067144	0.460549548	0.471322022	0.637410792	0.910421454	up_in_case
CRA004775	ENSGALG00010017859	EXOC6	case	control	0.07206748	0.435576668	0.165453032	0.868587415	0.973370973	up_in_case
CRA004775	ENSGALG00010017860	CYSLTR1	case	control	-0.2394849	0.303212586	-0.789825063	0.42962994	0.824104894	down_in_case
CRA004775	ENSGALG00010017861	PRSS56	case	control	1.083003376	0.753472591	1.437349398	0.150618739	0.614342929	up_in_case
CRA004775	ENSGALG00010017865	UBOX5	case	control	-0.313854941	0.238481663	-1.316054816	0.188155645	0.652111233	down_in_case
CRA004775	ENSGALG00010017866	RALA	case	control	0.345162116	0.185189352	1.86383349	0.062345091	0.448843826	up_in_case
CRA004775	ENSGALG00010017867	APLF	case	control	0.171371945	0.199249687	0.860086397	0.389741419	0.805914925	up_in_case
CRA004775	ENSGALG00010017868	CHRND	case	control	-0.430008113	0.658101584	-0.653406895	0.513493995	0.863903061	down_in_case
CRA004775	ENSGALG00010017870	RAB11A	case	control	-0.014082967	0.167724554	-0.083964852	0.933084377	0.985090297	down_in_case
CRA004775	ENSGALG00010017873	HSPG2	case	control	0.11698181	0.3601563	0.324808452	0.745326048	0.93941141	up_in_case
CRA004775	ENSGALG00010017876	COMMD1	case	control	-0.163375539	0.273402193	-0.597564846	0.550130327	0.877623839	down_in_case
CRA004775	ENSGALG00010017878	CHRNG	case	control	-1.250409309	0.664664939	-1.881262627	0.059936202	0.445397937	down_in_case

CRA004775	ENSGALG00010017880	CDK13	case	control	0.308868156	0.319389349	0.967058411	0.333514834	0.774166524	up_in_case
CRA004775	ENSGALG00010017882	LZTS3	case	control	0.297768386	0.555614676	0.535926063	0.592009651	0.893804699	up_in_case
CRA004775	ENSGALG00010017886	DDRGK1	case	control	-0.293416025	0.163590572	-1.793599846	0.072877061	0.476411335	down_in_case
CRA004775	ENSGALG00010017887	PROKR1	case	control	0.007610567	0.718962374	0.010585487	0.991554161	0.998065748	up_in_case
CRA004775	ENSGALG00010017888	MPLKIP	case	control	-0.13517152	0.259785886	-0.520318951	0.602841289	0.898692785	down_in_case
CRA004775	ENSGALG00010017890	ANAPC1	case	control	-0.220408945	0.22486187	-0.980197066	0.326988853	0.770998457	down_in_case
CRA004775	ENSGALG00010017891	GOLGA7B	case	control	-0.961498958	0.829374684	-1.159305892	0.246331521	0.712046495	down_in_case
CRA004775	ENSGALG00010017894	KAT6A	case	control	-0.004784452	0.261006868	-0.018330752	0.985374995	0.997562105	down_in_case
CRA004775	ENSGALG00010017895	MNAT1	case	control	0.021117869	0.24596547	0.085857046	0.931580058	0.985044263	up_in_case
CRA004775	ENSGALG00010017898	NHERF2	case	control	0.247116935	0.246703229	1.00167694	0.316499647	0.765866014	up_in_case
CRA004775	ENSGALG00010017905	EIF4E2	case	control	-0.274303973	0.229409955	-1.19569342	0.23181622	0.698920857	down_in_case
CRA004775	ENSGALG00010017909	NTHL1	case	control	-0.309026199	0.311205052	-0.992998657	0.320710609	0.767037518	down_in_case
CRA004775	ENSGALG00010017915	BFSP1	case	control	1.663289974	0.559727816	2.971604997	0.002962476	0.113790507	up_in_case
CRA004775	ENSGALG00010017918	SIX4	case	control	0.424140155	0.432950673	0.979650065	0.327258883	0.771231688	up_in_case
CRA004775	ENSGALG00010017920	AP3M2	case	control	-0.488749059	0.239108055	-2.044051002	0.040948509	0.378415958	down_in_case
CRA004775	ENSGALG00010017921	SPATA7	case	control	0.617877664	0.34137297	1.809978289	0.070299154	0.469724266	up_in_case
CRA004775	ENSGALG00010017922	MERTK	case	control	-0.119440812	0.303655143	-0.393343614	0.694065709	0.928504482	down_in_case
CRA004775	ENSGALG00010017927	PCSK2	case	control	0.277360283	0.234025114	1.185173154	0.235948992	0.703274167	up_in_case
CRA004775	ENSGALG00010017930	TSC2	case	control	-0.106859212	0.209844951	-0.509229371	0.610591458	0.901413878	down_in_case
CRA004775	ENSGALG00010017933	SIX1	case	control	0.026040457	0.447038411	0.05825105	0.953548658	0.990670511	up_in_case
CRA004775	ENSGALG00010017937	PTPN21	case	control	0.38249879	0.453876186	0.842738178	0.39937489	0.810957489	up_in_case
CRA004775	ENSGALG00010017941	DIS3L	case	control	0.009060679	0.220664535	0.041060875	0.967247365	0.993324759	up_in_case
CRA004775	ENSGALG00010017946	PTGDR	case	control	0.321744388	0.283879678	1.133382955	0.257053469	0.719236059	up_in_case
CRA004775	ENSGALG00010017948	PLAT	case	control	0.518279309	0.230580508	2.247715183	0.024594357	0.302160964	up_in_case
CRA004775	ENSGALG00010017949	SFXN3	case	control	-0.147244155	0.326122536	-0.451499478	0.651629601	0.915892481	down_in_case
CRA004775	ENSGALG00010017950	FBLN7	case	control	-1.540823992	0.695132961	-2.216588882	0.026651198	0.314252091	down_in_case
CRA004775	ENSGALG00010017954	PTGER2	case	control	0.207067339	0.313586795	0.660319066	0.509049098	0.862971569	up_in_case
CRA004775	ENSGALG00010017955	IKBKB	case	control	-0.020034883	0.133174275	-0.15044109	0.880416625	0.974996282	down_in_case
CRA004775	ENSGALG00010017956	TIPIN	case	control	0.01499242	0.200674449	0.07471016	0.940445324	0.987076872	up_in_case
CRA004775	ENSGALG00010017957	THUMPD1	case	control	-0.029641808	0.239173562	-0.1239343	0.901367295	0.979157637	down_in_case
CRA004775	ENSGALG00010017958	CAPN10	case	control	-0.422547418	0.265430605	-1.591931785	0.111400033	0.56070984	down_in_case
CRA004775	ENSGALG00010017959	PDZD7	case	control	-0.748248948	0.625213255	-1.196789962	0.231388432	0.698074227	down_in_case
CRA004775	ENSGALG00010017962	TXNDC16	case	control	-0.16256396	0.272449152	-0.59667633	0.550723499	0.877827378	down_in_case
CRA004775	ENSGALG00010017963	LPAR4	case	control	-0.352871678	0.404765357	-0.871793181	0.383321215	0.802168624	down_in_case
CRA004775	ENSGALG00010017970	AMPH	case	control	-2.442885649	1.294211021	-1.887548173	0.059086633	0.44414236	down_in_case
CRA004775	ENSGALG00010017972	RNPEPL1	case	control	-0.117965255	0.208691014	-0.565262741	0.571895067	0.885500402	down_in_case
CRA004775	ENSGALG00010017973	PKD1	case	control	-0.08943888	0.310031847	-0.28848288	0.772977133	0.946296525	down_in_case
CRA004775	ENSGALG00010017974	MXRA8	case	control	-0.051472846	0.414021349	-0.124324134	0.901058639	0.979157637	down_in_case
CRA004775	ENSGALG00010017976	GPR137C	case	control	-0.017495317	0.258776878	-0.067607727	0.946097904	0.988996418	down_in_case
CRA004775	ENSGALG00010017979	ENSGALG00010017979	case	control	-0.367037609	0.334936623	-1.095841971	0.273147937	0.731001618	down_in_case
CRA004775	ENSGALG00010017983	SLC4A11	case	control	0.22347511	0.462378272	0.483316633	0.628870932	0.908586927	up_in_case
CRA004775	ENSGALG00010017985	ABCC5	case	control	-0.026661552	0.487501839	-0.054690157	0.956385311	0.9909743	down_in_case
CRA004775	ENSGALG00010017987	ERI2	case	control	-0.259308274	0.533967545	-0.485625533	0.62723269	0.907733515	down_in_case
CRA004775	ENSGALG00010017991	RAB26	case	control	-0.568493656	0.435334021	-1.305879231	0.19159363	0.656959697	down_in_case
CRA004775	ENSGALG00010017992	ENSGALG00010017992	case	control	-0.220695469	0.330963854	-0.666826504	0.504882961	0.861471523	down_in_case
CRA004775	ENSGALG00010017993	REXO5	case	control	-0.290044223	0.289117686	-1.003204708	0.315762102	0.765657673	down_in_case
CRA004775	ENSGALG00010017996	ZC3H14	case	control	0.071459457	0.210787002	0.339012637	0.73460021	0.936819725	up_in_case
CRA004775	ENSGALG00010017997	ENSGALG00010017997	case	control	1.164621969	0.91958034	1.266471148	0.205344453	0.674045742	up_in_case
CRA004775	ENSGALG00010017999	GPR174	case	control	-0.110827601	0.516431466	-0.214602728	0.830077067	0.963232966	down_in_case
CRA004775	ENSGALG00010018000	MAP2K1	case	control	-0.055240465	0.210187033	-0.262815762	0.792692586	0.951284723	down_in_case
CRA004775	ENSGALG00010018001	TRAF7	case	control	-0.206493004	0.132795342	-1.554971743	0.119952799	0.572133279	down_in_case
CRA004775	ENSGALG00010018002	ZC3H8	case	control	0.432347612	0.239146558	1.807877207	0.070625614	0.469895261	up_in_case
CRA004775	ENSGALG00010018004	CDC42BPA	case	control	0.382091804	0.305915677	1.249010213	0.211661338	0.682220401	up_in_case
CRA004775	ENSGALG00010018006	DCUN1D3	case	control	0.076773419	0.244716546	0.313723857	0.75373078	0.941900693	up_in_case
CRA004775	ENSGALG00010018007	DNAAF9	case	control	0.155169731	0.28764564	0.539447532	0.589578091	0.893412703	up_in_case
CRA004775	ENSGALG00010018008	ABCF3	case	control	-0.246233628	0.427289754	-0.576268506	0.564433707	0.882478718	down_in_case
CRA004775	ENSGALG00010018010	ENSGALG00010018010	case	control	-0.109130369	0.21652628	-0.50400519	0.61425773	0.902363507	down_in_case
CRA004775	ENSGALG00010018011	LIPA	case	control	1.259903389	0.542713485	2.321489005	0.020260467	0.281415838	up_in_case
CRA004775	ENSGALG00010018012	DVL1	case	control	0.229215733	0.257029032	0.891789271	0.372505893	0.796258129	up_in_case
CRA004775	ENSGALG00010018014	LYRM1	case	control	0.05234481	0.184447684	0.283792179	0.776569648	0.946929223	up_in_case
CRA004775	ENSGALG00010018016	IFIT5	case	control	0.117290443	0.455989308	0.257221916	0.797007467	0.952835572	up_in_case
CRA004775	ENSGALG00010018019	NAPB	case	control	-0.409997082	0.570150829	-0.719102843	0.472077556	0.845425322	down_in_case
CRA004775	ENSGALG00010018020	SNAPC5	case	control	-0.289271831	0.257439961	-1.123647742	0.261162494	0.721774856	down_in_case
CRA004775	ENSGALG00010018021	ATRN	case	control	-0.499347151	0.351912524	-1.418952487	0.155912869	0.619442611	down_in_case
CRA004775	ENSGALG00010018022	XPO6	case	control	-0.582156134	0.304455939	-1.912119492	0.055860869	0.432968819	down_in_case
CRA004775	ENSGALG00010018025	CPTP	case	control	-0.365772564	0.20370664	-1.79558489	0.072560561	0.475813195	down_in_case
CRA004775	ENSGALG00010018030	POLB	case	control	-0.339640187	0.32383317	-1.048812224	0.29426455	0.746438942	down_in_case
CRA004775	ENSGALG00010018032	ERO1A	case	control	-0.312032827	0.251122256	-1.242553452	0.214032432	0.683502083	down_in_case
CRA004775	ENSGALG00010018033	COQ8A	case	control	0.29577737	0.382779412	0.772709713	0.439694197	0.828842585	up_in_case
CRA004775	ENSGALG00010018035	ITM2A	case	control	-0.154313305	0.165524621	-0.932267984	0.351198049	0.786098731	down_in_case
CRA004775	ENSGALG00010018036	RPL4	case	control	-0.284268889	0.177501012	-1.60150573	0.109264952	0.556276325	down_in_case
CRA004775	ENSGALG00010018037	GZF1	case	control	-0.098870076	0.264856783	-0.373296372	0.708927876	0.931872072	down_in_case
CRA004775	ENSGALG00010018039	TTC14	case	control	0.196917828	0.293584002	0.670737596	0.502387708	0.860091011	up_in_case
CRA004775	ENSGALG00010018042	ZWILCH	case	control	-0.321200631	0.460341849	-0.697743713	0.485337486	0.851302844	down_in_case
CRA004775	ENSGALG00010018043	PSMC6	case	control	-0.377597789	0.27877459	-1.354491417	0.17557965	0.641103495	down_in_case
CRA004775	ENSGALG00010018044	INTS11	case	control	-0.216775368	0.188504869	-1.149972249	0.250155301	0.714993603	down_in_case
CRA004775	ENSGALG00010018045	PSEN2	case	control	-0.203808009	0.225417767	-0.904134626	0.36592402	0.793608387	down_in_case
CRA004775	ENSGALG00010018046	CAPN15	case	control	-0.388140603	0.224290311	-1.730527734	0.083536031	0.502115892	down_in_case
CRA004775	ENSGALG00010018048	GFRA4	case	control	0.982082454	0.46361124	2.118331846	0.034146973	0.35073164	up_in_case
CRA004775	ENSGALG00010018049	SUGCT	case	control	-0.472771779	0.605112555	-0.781295605	0.434628655	0.82652173	down_in_case
CRA004775	ENSGALG00010018051	GNPNAT1	case	control	0.006856803	0.20665146	0.033180519	0.973530634	0.994947809	up_in_case
CRA004775	ENSGALG00010018052	PPP1RL	case	control	-0.355945371	0.310296679	-1.147113052	0.2513349	0.715759105	down_in_case
CRA004775	ENSGALG00010018053	DIAPH2	case	control	0.274785492	0.255105379	1.077145032	0.281415478	0.738078244	up_in_case
CRA004775	ENSGALG00010018054	SMAD6	case	control	-0.065688944	0.230803624	-0.284609673	0.775943199	0.946517228	down_in_case
CRA004775	ENSGALG00010018057	SSTR4	case	control	-0.299345751	0.411775593	-0.726963317	0.467248428	0.843870188	down_in_case
CRA004775	ENSGALG00010018058	ITPKB	case	control	-0.018086603	0.260682866	-0.069381633	0.944685848	0.988387362	down_in_case
CRA004775	ENSGALG00010018060	CASKIN1	case	control	-0.954171709	0.543583522	-1.755335971	0.079201857	0.491399489	down_in_case
CRA004775	ENSGALG00010018061	NDUFAF5	case	control	-0.23075557	0.410497322	-0.562136603	0.574022951	0.886897708	down_in_case
CRA004775	ENSGALG00010018062	GOLGA7	case	control	-0.296538792	0.244803559	-1.211333664	0.225767552	0.69359464	down_in_case
CRA004775	ENSGALG00010018063	DECR2	case	control	-0.423618911	0.235102167	-1.801850306	0.07156896	0.471598108	down_in_case
CRA004775	ENSGALG00010018064	TTC8	case	control	-0.275595297	0.189784702	-1.452147064	0.146460718	0.609922856	down_in_case
CRA004775	ENSGALG00010018067	ENSGALG00010018067	case	control	-0.439299154	0.5684093	-0.772857085	0.439606965	0.828842585	down_in_case
CRA004775	ENSGALG00010018068	ADAM33	case	control	-0.63371396	0.40405216	-1.568396418	0.116788647	0.566676316	down_in_case
CRA004775	ENSGALG00010018069	ENSGALG00010018069	case	control	-0.056998436	0.341295684	-0.167006026	0.867365308	0.973120718	down_in_case
CRA004775	ENSGALG00010018071	GPAT4	case	control	-0.846893004	0.255275011	-3.317571119	0.000908038	0.062759842	down_in_case
CRA004775	ENSGALG00010018075	MLST8	case	control	-0.161224617	0.350396447	-0.460120583	0.645429671	0.914043203	down_in_case
CRA004775	ENSGALG00010018078	NKX6-3	case	control	0.775837735	0.679365276	1.142003812	0.253452435	0.717862912	up_in_case
CRA004775	ENSGALG00010018080	BRICD5	case	control	-0.130434855	0.355698603	-0.366700499	0.713842435	0.933244709	down_in_case
CRA004775	ENSGALG00010018081	DIO3	case	control	-0.397969436	0.477378687	-0.833655643	0.40447506	0.81313407	down_in_case
CRA004775	ENSGALG00010018082	RAB11FIP3	case	control	-0.032383331	0.287533646	-0.112624492	0.910328267	0.98075825	down_in_case
CRA004775	ENSGALG00010018085	PGP	case	control	0.320361791	0.339020082	0.944964052	0.344677229	0.783972357	up_in_case
CRA004775	ENSGALG00010018087	ENSGALG00010018087	case	control	-0.13553681	0.364433787	-0.371910658	0.709959369	0.932234469	down_in_case
CRA004775	ENSGALG00010018089	TASP1	case	control	-0.000568959	0.263830661	-0.002156532	0.998279338	0.999914248	down_in_case
CRA004775	ENSGALG00010018092	ENSGALG00010018092	case	control	-0.613375627	0.757754241	-0.80946512	0.418247658	0.818943344	down_in_case
CRA004775	ENSGALG00010018095	E4F1	case	control	-0.024875919	0.165937605	-0.14991128	0.880834612	0.974996282	down_in_case
CRA004775	ENSGALG00010018098	EGR4	case	control	0.363225391	0.752770232	0.482518271	0.629437822	0.908699404	up_in_case
CRA004775	ENSGALG00010018099	SMAD3	case	control	-0.293643498	0.312046706	-0.941024187	0.346692465	0.784552667	down_in_case
CRA004775	ENSGALG00010018102	ZDHHC4	case	control	-0.673881477	0.265781045	-2.535476056	0.01122946	0.214862956	down_in_case
CRA004775	ENSGALG00010018103	BTRC	case	control	-0.171029849	0.214071549	-0.798937782	0.42432649	0.821240473	down_in_case
CRA004775	ENSGALG00010018115	KLHL4	case	control	-0.899173792	0.804925669	-1.117089226	0.263956177	0.723981064	down_in_case
CRA004775	ENSGALG00010018121	FERMT2	case	control	0.313708533	0.238190676	1.317047914	0.187822564	0.652044454	up_in_case
CRA004775	ENSGALG00010018123	USP13	case	control	0.28936583	0.558178071	0.51841132	0.604171319	0.898935274	up_in_case
CRA004775	ENSGALG00010018128	SEPTIN12	case	control	-1.183550359	0.483097076	-2.449922422	0.0142887	0.239125488	down_in_case
CRA004775	ENSGALG00010018137	NDUFB5	case	control	0.250229519	0.364202827	0.687060892	0.492044357	0.855746361	up_in_case
CRA004775	ENSGALG00010018140	CHPF	case	control	-0.295060185	0.194963096	-1.513415568	0.130174133	0.584671406	down_in_case
CRA004775	ENSGALG00010018142	DDHD1	case	control	-0.188937376	0.369659948	-0.511111299	0.609273125	0.90116325	down_in_case
CRA004775	ENSGALG00010018143	MRPL47	case	control	0.058380888	0.30551965	0.191087178	0.848457293	0.968223172	up_in_case
CRA004775	ENSGALG00010018145	ENSGALG00010018145	case	control	-0.732246127	0.504410662	-1.451686457	0.146588807	0.609922856	down_in_case
CRA004775	ENSGALG00010018147	ISM1	case	control	0.950105931	0.451272981	2.105390687	0.035257314	0.355594571	up_in_case
CRA004775	ENSGALG00010018148	XRN2	case	control	-0.107550457	0.141268047	-0.761321897	0.446464825	0.833749389	down_in_case
CRA004775	ENSGALG00010018149	ENSGALG00010018149	case	control	0.231699804	0.429360272	0.539639597	0.589445604	0.893412703	up_in_case
CRA004775	ENSGALG00010018150	AAGAB	case	control	-0.237270833	0.153733899	-1.543386558	0.122737013	0.57525466	down_in_case
CRA004775	ENSGALG00010018157	FBXL2	case	control	-0.547919495	0.259155828	-2.114247241	0.034494156	0.353449432	down_in_case
CRA004775	ENSGALG00010018160	ENSGALG00010018160	case	control	-0.618652472	0.313312325	-1.974555175	0.04831865	0.407414924	down_in_case
CRA004775	ENSGALG00010018162	ENSGALG00010018162	case	control	0.297814647	0.593462841	0.501825264	0.61579044	0.9028981	up_in_case
CRA004775	ENSGALG00010018163	CCT7	case	control	-0.316231656	0.166737355	-1.896585539	0.057882661	0.440234772	down_in_case

CRA004775	ENSGALG00010018165	DACH2	case	control	0.175678496	0.368246173	0.477068083	0.633313641	0.909809432	up_in_case
CRA004775	ENSGALG00010018166	MSS51	case	control	-0.185530259	0.703559078	-0.263702458	0.792009201	0.951284723	down_in_case
CRA004775	ENSGALG00010018172	ACTL6A	case	control	-0.249429614	0.290491136	-0.858647934	0.390534784	0.806479902	down_in_case
CRA004775	ENSGALG00010018174	BMP4	case	control	0.214941291	0.3510088	0.612352998	0.540304232	0.874311509	up_in_case
CRA004775	ENSGALG00010018177	KIF20B	case	control	0.802785217	0.459162198	1.748369574	0.080400053	0.494445942	up_in_case
CRA004775	ENSGALG00010018179	PRADC1	case	control	-0.474097965	0.221724549	-2.138229465	0.032498124	0.343523087	down_in_case
CRA004775	ENSGALG00010018185	GNB4	case	control	0.029704889	0.273133141	0.108756076	0.913395963	0.980803023	up_in_case
CRA004775	ENSGALG00010018188	UBP1	case	control	-0.025189414	0.212067546	-0.118780144	0.90544954	0.979739788	down_in_case
CRA004775	ENSGALG00010018189	MIS18BP1	case	control	-0.110028435	0.372801146	-0.295139744	0.76788713	0.944811733	down_in_case
CRA004775	ENSGALG00010018192	IQCH	case	control	-0.396206916	0.473206933	-0.837280456	0.402434942	0.811996248	down_in_case
CRA004775	ENSGALG00010018193	ANK1	case	control	-0.648610315	0.435643177	-1.488856818	0.136525077	0.595518184	down_in_case
CRA004775	ENSGALG00010018198	BTBD3	case	control	0.421699488	0.258957245	1.628452171	0.103429043	0.545480965	up_in_case
CRA004775	ENSGALG00010018199	BORCS7	case	control	-0.552078154	0.186053856	-2.967302947	0.003004248	0.114753923	down_in_case
CRA004775	ENSGALG00010018206	KIZ	case	control	0.087839518	0.324640554	0.270574694	0.786718162	0.95041797	up_in_case
CRA004775	ENSGALG00010018209	GMPPA	case	control	-0.777100428	0.211196899	-3.679506816	0.000233685	0.030954849	down_in_case
CRA004775	ENSGALG00010018215	CFAP70	case	control	-0.461398941	0.438254457	-1.052810609	0.292427802	0.746262643	down_in_case
CRA004775	ENSGALG00010018216	FANCM	case	control	-0.075486267	0.240157694	-0.314319587	0.753278324	0.941900693	down_in_case
CRA004775	ENSGALG00010018226	JAG1	case	control	0.548447631	0.312551237	1.754744712	0.079302984	0.491399489	up_in_case
CRA004775	ENSGALG00010018229	AS3MT	case	control	-0.592330263	0.316914261	-1.86905525	0.061615128	0.446401278	down_in_case
CRA004775	ENSGALG00010018231	SMYD5	case	control	-0.530997758	0.131626511	-4.034124702	5.48062E-05	0.01449308	down_in_case
CRA004775	ENSGALG00010018233	RASSF2	case	control	-0.508240597	0.360900697	-1.408256069	0.159055259	0.624907945	down_in_case
CRA004775	ENSGALG00010018240	SOUL	case	control	0.287651679	0.755237899	0.380875588	0.703295568	0.930068722	up_in_case
CRA004775	ENSGALG00010018241	RALGAPA2	case	control	-0.144924526	0.359207625	-0.403456152	0.686612685	0.92689803	down_in_case
CRA004775	ENSGALG00010018244	CHM	case	control	0.311582317	0.181725921	1.714572779	0.086423583	0.508360843	up_in_case
CRA004775	ENSGALG00010018251	USP48	case	control	0.036466808	0.147249938	0.247652449	0.804403326	0.954576316	up_in_case
CRA004775	ENSGALG00010018255	MFN1	case	control	0.328705798	0.267462693	1.22897812	0.21908001	0.686718587	up_in_case
CRA004775	ENSGALG00010018258	OSCP1	case	control	-0.619320391	0.453392214	-1.365970504	0.171948221	0.636057362	down_in_case
CRA004775	ENSGALG00010018259	WDR90	case	control	-0.530885018	0.228913321	-2.319153008	0.020386741	0.281976732	down_in_case
CRA004775	ENSGALG00010018261	PRNP	case	control	0.109343735	0.293405052	0.372671617	0.709392863	0.931874404	up_in_case
CRA004775	ENSGALG00010018263	SLX4IP	case	control	0.357388361	0.553240999	0.645990377	0.518285587	0.865435399	up_in_case
CRA004775	ENSGALG00010018264	CFAP61	case	control	0.380972538	0.742156088	0.513332093	0.607719038	0.900943922	up_in_case
CRA004775	ENSGALG00010018265	PDCD4	case	control	-0.169789096	0.188924239	-0.898715256	0.368804349	0.794896739	down_in_case
CRA004775	ENSGALG00010018267	ADPRHL2	case	control	-0.105411003	0.251558838	-0.419031206	0.675193326	0.92288678	down_in_case
CRA004775	ENSGALG00010018274	ZNF711	case	control	0.018805262	0.176366116	0.106626277	0.915085471	0.981463249	up_in_case
CRA004775	ENSGALG00010018275	ZNF639	case	control	0.131222449	0.242145573	0.54191554	0.587876691	0.89265596	up_in_case
CRA004775	ENSGALG00010018276	ARHGAP17	case	control	0.357835738	0.231397169	1.546413633	0.122004704	0.574353537	up_in_case
CRA004775	ENSGALG00010018278	PUSL1	case	control	-0.167172519	0.28147851	-0.59390864	0.55257322	0.878345607	down_in_case
CRA004775	ENSGALG00010018279	INSM1	case	control	1.339292209	0.931014254	1.438530295	0.150283648	0.613766096	up_in_case
CRA004775	ENSGALG00010018283	POF1B	case	control	1.196658958	0.510063826	2.346096505	0.018971195	0.272278915	up_in_case
CRA004775	ENSGALG00010018284	ENSGALG00010018284	case	control	-0.295452036	0.529279807	-0.558215204	0.57669744	0.887937128	down_in_case
CRA004775	ENSGALG00010018285	BBIP1	case	control	0.266181831	0.267158157	0.996345513	0.319082297	0.767037518	up_in_case
CRA004775	ENSGALG00010018287	MKKS	case	control	-0.148666048	0.136334867	-1.090447738	0.275515963	0.731001618	down_in_case
CRA004775	ENSGALG00010018288	CRNKL1	case	control	0.260814948	0.146592129	1.779187942	0.075208955	0.483342345	up_in_case
CRA004775	ENSGALG00010018289	HEYL	case	control	0.011447036	0.337742795	0.033892762	0.972962665	0.994947809	up_in_case
CRA004775	ENSGALG00010018290	RHOT2	case	control	-0.436394304	0.226106905	-1.930035284	0.053602466	0.426308566	down_in_case
CRA004775	ENSGALG00010018294	FKBP3	case	control	0.084231807	0.332599618	0.253252866	0.800072817	0.952993446	up_in_case
CRA004775	ENSGALG00010018296	PIK3CA	case	control	0.474853207	0.275206494	1.72544332	0.08444761	0.503614276	up_in_case
CRA004775	ENSGALG00010018298	ENSGALG00010018298	case	control	1.723142648	0.448735024	3.84000035	0.000123034	0.023828766	up_in_case
CRA004775	ENSGALG00010018299	SHOC2	case	control	0.157701979	0.220043499	0.716685473	0.473568192	0.845445211	up_in_case
CRA004775	ENSGALG00010018301	ENSGALG00010018301	case	control	-0.332218684	0.46751826	-0.710600445	0.477331867	0.846532686	down_in_case
CRA004775	ENSGALG00010018302	RAB11FIP5	case	control	-0.4937154	0.314172419	-1.571479132	0.116071398	0.566086328	down_in_case
CRA004775	ENSGALG00010018306	DES	case	control	-0.463125851	0.634007654	-0.730473596	0.465100747	0.843383332	down_in_case
CRA004775	ENSGALG00010018308	ACAP3	case	control	-0.093498412	0.424527635	-0.22024105	0.825683428	0.961878235	down_in_case
CRA004775	ENSGALG00010018309	DPYSL2	case	control	0.12885111	0.275007849	0.468536119	0.639401248	0.911382154	up_in_case
CRA004775	ENSGALG00010018310	APOOL	case	control	0.07291339	0.156215754	0.466747995	0.640680187	0.911724439	up_in_case
CRA004775	ENSGALG00010018311	NKX2-3	case	control	0.878169324	0.525465206	1.671222594	0.094677719	0.526933666	up_in_case
CRA004775	ENSGALG00010018312	PRPF39	case	control	0.000957633	0.236255481	0.004053379	0.99676588	0.999606847	up_in_case
CRA004775	ENSGALG00010018313	MYEF2	case	control	-0.159418471	0.241214613	-0.660898893	0.508677156	0.862971569	down_in_case
CRA004775	ENSGALG00010018314	SLC24A3	case	control	-0.738088773	0.328747893	-2.245151341	0.024758419	0.302344474	down_in_case
CRA004775	ENSGALG00010018315	CLASP2	case	control	0.108691598	0.244542019	0.444470028	0.656702793	0.91710603	up_in_case
CRA004775	ENSGALG00010018317	ZMAT3	case	control	-0.132818384	0.288736502	-0.459998591	0.645517232	0.914043203	down_in_case
CRA004775	ENSGALG00010018319	STUB1	case	control	-0.031315263	0.274451034	-0.114101458	0.909157367	0.98075825	down_in_case
CRA004775	ENSGALG00010018322	NT5C1A	case	control	-0.26617614	0.708457748	-0.375712089	0.70713095	0.931617022	down_in_case
CRA004775	ENSGALG00010018323	GOT1	case	control	-0.136516659	0.487033378	-0.280302471	0.779245455	0.94795144	down_in_case
CRA004775	ENSGALG00010018325	ENSGALG00010018325	case	control	0.447151593	0.721218238	0.61999485	0.535261178	0.870981055	up_in_case
CRA004775	ENSGALG00010018326	LCMT1	case	control	-0.293027517	0.237345038	-1.234605614	0.216977318	0.684522675	down_in_case
CRA004775	ENSGALG00010018329	ENSGALG00010018329	case	control	-0.815181388	0.279820491	-2.913229787	0.003577113	0.125102734	down_in_case
CRA004775	ENSGALG00010018333	ENSGALG00010018333	case	control	0.548019617	0.247708647	2.212355617	0.026942103	0.31493941	up_in_case
CRA004775	ENSGALG00010018336	JMJD8	case	control	-0.224547448	0.218595556	-1.027227871	0.304313177	0.756847621	down_in_case
CRA004775	ENSGALG00010018337	TMEM151B	case	control	-0.236024729	0.80936445	-0.291617366	0.770579196	0.945754599	down_in_case
CRA004775	ENSGALG00010018338	PDCD6IP	case	control	0.121703649	0.165026649	0.737478765	0.460831252	0.841484611	up_in_case
CRA004775	ENSGALG00010018339	FAM76A	case	control	-0.188663006	0.269979341	-0.698805344	0.484673689	0.851181085	down_in_case
CRA004775	ENSGALG00010018340	DNPEP	case	control	-0.244241646	0.353682882	-0.690566771	0.489837835	0.854683425	down_in_case
CRA004775	ENSGALG00010018342	BNIP3L	case	control	0.163019545	0.23038413	0.70759885	0.479194407	0.847184661	up_in_case
CRA004775	ENSGALG00010018344	SFXN5	case	control	-0.08240378	0.304019286	-0.27104787	0.786354215	0.95041797	down_in_case
CRA004775	ENSGALG00010018345	WDR24	case	control	-0.148384579	0.31184773	-0.475823824	0.634199896	0.909837348	down_in_case
CRA004775	ENSGALG00010018348	TOGARAM1	case	control	0.071699323	0.248886129	0.288080832	0.773284865	0.946296525	up_in_case
CRA004775	ENSGALG00010018349	KCNMB2	case	control	0.110881059	0.396348026	0.279756808	0.779664091	0.948271795	up_in_case
CRA004775	ENSGALG00010018350	CNNM1	case	control	-1.015687312	0.50608489	-2.006950477	0.044754936	0.393998162	down_in_case
CRA004775	ENSGALG00010018351	RIN2	case	control	0.092033749	0.158879213	0.579268657	0.562407911	0.881532201	up_in_case
CRA004775	ENSGALG00010018354	AARS2	case	control	-0.498849192	0.141603353	-3.522862849	0.000426912	0.042582695	down_in_case
CRA004775	ENSGALG00010018355	PRKCB	case	control	-0.433643343	0.44630527	-0.971629447	0.331234927	0.772569986	down_in_case
CRA004775	ENSGALG00010018359	CACNG3	case	control	1.009940199	0.840110705	1.202151328	0.229304903	0.695782399	up_in_case
CRA004775	ENSGALG00010018361	ASIC4	case	control	0.35706472	0.595362271	0.599743614	0.548677117	0.877623839	up_in_case
CRA004775	ENSGALG00010018363	TCTE1	case	control	-0.70507266	0.652875515	-1.079949614	0.280164618	0.73661445	down_in_case
CRA004775	ENSGALG00010018365	HPSE2	case	control	-0.348317756	0.388049712	-0.897611169	0.369392882	0.794896739	down_in_case
CRA004775	ENSGALG00010018367	PAK5	case	control	-0.147835101	0.446465046	-0.331123573	0.740551146	0.938450627	down_in_case
CRA004775	ENSGALG00010018369	ENSGALG00010018369	case	control	3.196779765	0.821136751	3.893114955	9.89652E-05	0.022373664	up_in_case
CRA004775	ENSGALG00010018370	ZBTB8A	case	control	0.246624676	0.16034697	1.538068832	0.124031786	0.576485043	up_in_case
CRA004775	ENSGALG00010018371	ENSGALG00010018371	case	control	0.352368444	0.26135378	1.34824315	0.17758019	0.643283771	up_in_case
CRA004775	ENSGALG00010018372	NLGN3	case	control	-0.568521433	0.471461297	-1.205870847	0.227867293	0.694767882	down_in_case
CRA004775	ENSGALG00010018374	HPS1	case	control	-0.451334986	0.25954756	-1.738929798	0.082047112	0.498554944	down_in_case
CRA004775	ENSGALG00010018376	PYGB	case	control	0.264805066	0.252506176	1.048707284	0.294312861	0.746438942	up_in_case
CRA004775	ENSGALG00010018377	RAP1GAP	case	control	0.999057912	0.353146823	2.829015712	0.004669141	0.141110666	up_in_case
CRA004775	ENSGALG00010018378	ARPP21	case	control	0.133859721	0.431035783	0.310553615	0.756139995	0.941900693	up_in_case
CRA004775	ENSGALG00010018379	SRBD1	case	control	0.070041682	0.419857868	0.166822364	0.867509821	0.973120718	up_in_case
CRA004775	ENSGALG00010018380	SLC20A1	case	control	-0.650488602	0.173397369	-3.751432929	0.000175827	0.027735809	down_in_case
CRA004775	ENSGALG00010018381	HAPSTR1	case	control	0.214151413	0.184029007	1.163682925	0.244552532	0.711765017	up_in_case
CRA004775	ENSGALG00010018383	METRN	case	control	0.445345455	0.303307491	1.468296924	0.142023586	0.602343234	up_in_case
CRA004775	ENSGALG00010018384	LAMP5	case	control	-0.017097123	0.337205681	-0.050702358	0.959562697	0.991431439	down_in_case
CRA004775	ENSGALG00010018385	SPR	case	control	-0.152314116	0.253525799	-0.600783498	0.547984197	0.877369241	down_in_case
CRA004775	ENSGALG00010018387	NT5DC4	case	control	0.404645242	0.319397901	1.266900129	0.205191003	0.674045742	up_in_case
CRA004775	ENSGALG00010018388	SMC3	case	control	0.181508084	0.151818592	1.195559004	0.231868698	0.698920857	up_in_case
CRA004775	ENSGALG00010018391	STAC	case	control	0.136282034	0.817903233	0.166623665	0.867666172	0.973120718	up_in_case
CRA004775	ENSGALG00010018393	PRKCE	case	control	0.235333029	0.301647723	0.78015848	0.435297598	0.826905159	up_in_case
CRA004775	ENSGALG00010018394	KLHL28	case	control	-0.044281929	0.172412758	-0.25683673	0.797304815	0.952835572	down_in_case
CRA004775	ENSGALG00010018395	MED12	case	control	0.037146334	0.224778705	0.165257352	0.868741425	0.973370973	up_in_case
CRA004775	ENSGALG00010018396	TBL1XR1	case	control	0.15768895	0.240310274	0.656188965	0.511702548	0.863170205	up_in_case
CRA004775	ENSGALG00010018397	FAM173A	case	control	-0.574477823	0.504546728	-1.138601821	0.254869269	0.718472184	down_in_case
CRA004775	ENSGALG00010018398	LRFN5	case	control	-0.264437188	0.406765769	-0.650096956	0.515629595	0.864897625	down_in_case
CRA004775	ENSGALG00010018399	ABHD12	case	control	0.241551014	0.254293234	0.949891626	0.342167322	0.781616177	up_in_case
CRA004775	ENSGALG00010018401	ENSGALG00010018401	case	control	-0.253813956	0.152973627	-1.659200745	0.097075348	0.531191049	down_in_case
CRA004775	ENSGALG00010018404	IL2RG	case	control	-0.244233007	0.447448341	-0.545835094	0.585179297	0.890822597	down_in_case
CRA004775	ENSGALG00010018405	DCLK3	case	control	-0.442390608	0.464321724	-0.952767413	0.340707929	0.780875343	down_in_case
CRA004775	ENSGALG00010018406	TMEM260	case	control	-0.223722563	0.320739685	-0.697520678	0.485477003	0.851329141	down_in_case
CRA004775	ENSGALG00010018410	EXOC6B	case	control	-0.285118494	0.294321949	-0.968729973	0.332679934	0.773534286	down_in_case
CRA004775	ENSGALG00010018413	SLC15A2	case	control	-0.192730934	0.926175281	-0.208093369	0.835156065	0.964656115	down_in_case
CRA004775	ENSGALG00010018414	EPAS1	case	control	0.416942656	0.255977098	1.628827967	0.103349442	0.545340822	up_in_case
CRA004775	ENSGALG00010018415	PELI2	case	control	0.311445854	0.252664471	1.232646016	0.217707861	0.68505739	up_in_case
CRA004775	ENSGALG00010018416	DUT	case	control	-0.080381328	0.223592077	-0.359499896	0.719221156	0.93407508	down_in_case
CRA004775	ENSGALG00010018418	PLCB4	case	control	0.706553071	0.317073024	2.228360717	0.02585647	0.308336492	up_in_case
CRA004775	ENSGALG00010018419	USP7	case	control	0.360997744	0.212465128	1.699091745	0.089301899	0.515997712	up_in_case
CRA004775	ENSGALG00010018420	RHOQ	case	control	0.725679309	0.288909982	2.511783444	0.012012278	0.220535154	up_in_case
CRA004775	ENSGALG00010018422	ENSGALG00010018422	case	control	-0.5131011	0.515094019	-0.99613096	0.319186519	0.767037518	down_in_case
CRA004775	ENSGALG00010018423	IGFALS	case	control	-0.191782435	0.75290013	-0.254724933	0.798935558	0.952835572	down_in_case
CRA004775	ENSGALG00010018425	CYP26B1	case	control	0.275063984	0.345656823	0.795771892	0.426164632	0.822541484	up_in_case

CRA004775	ENSGALG00010018426	ENSGALG00010018426	case	control	1.928739516	0.575395387	3.352024641	0.000802229	0.060285388	up_in_case
CRA004775	ENSGALG00010018427	PIGF	case	control	0.141895739	0.220732793	0.642839413	0.520328306	0.865611895	up_in_case
CRA004775	ENSGALG00010018428	PPP2R5D	case	control	-0.200537829	0.146049472	-1.373081505	0.16972701	0.633147466	down_in_case
CRA004775	ENSGALG00010018430	LOXL4	case	control	-1.607300785	0.571497061	-2.812439286	0.00491673	0.142429514	down_in_case
CRA004775	ENSGALG00010018434	HSPBAP1	case	control	-0.104213755	0.304399642	-0.342358337	0.732081239	0.936799657	down_in_case
CRA004775	ENSGALG00010018436	FOXO4	case	control	-0.345107343	0.379938241	-0.908324843	0.363706621	0.793608387	down_in_case
CRA004775	ENSGALG00010018438	PPP2R2A	case	control	0.388962615	0.169475743	2.295093145	0.021727787	0.288534247	up_in_case
CRA004775	ENSGALG00010018440	MEA1	case	control	-0.252580649	0.429145701	-0.588566188	0.556152321	0.87981923	down_in_case
CRA004775	ENSGALG00010018441	CRIPT	case	control	0.267300716	0.235104689	1.136943368	0.255561961	0.718871765	up_in_case
CRA004775	ENSGALG00010018443	PLCB1	case	control	0.70650172	0.331223239	2.133007706	0.032924094	0.346660963	up_in_case
CRA004775	ENSGALG00010018444	NUBP2	case	control	-0.411630439	0.197010827	-2.089379787	0.036673549	0.361347294	down_in_case
CRA004775	ENSGALG00010018446	FBN1	case	control	0.577549394	0.483630514	1.194195522	0.232401494	0.699289485	up_in_case
CRA004775	ENSGALG00010018448	KLHDC3	case	control	-0.299434888	0.423658254	-0.706784028	0.479700701	0.847527617	down_in_case
CRA004775	ENSGALG00010018451	PIK3R4	case	control	0.034545329	0.274474871	0.125859713	0.899842973	0.978929349	up_in_case
CRA004775	ENSGALG00010018452	SPSB3	case	control	-0.131925811	0.28171889	-0.468288835	0.639578052	0.911382154	down_in_case
CRA004775	ENSGALG00010018453	TMX4	case	control	-0.512344736	0.418994878	-1.222794745	0.221407231	0.689550502	down_in_case
CRA004775	ENSGALG00010018455	RBM20	case	control	0.542641153	0.556950635	0.974307451	0.329903906	0.77237154	up_in_case
CRA004775	ENSGALG00010018458	SEMA5B	case	control	0.269517566	0.369396764	0.729615397	0.465625308	0.843383332	up_in_case
CRA004775	ENSGALG00010018459	SNX12	case	control	0.018927321	0.199822909	0.094720478	0.924536852	0.983849734	up_in_case
CRA004775	ENSGALG00010018460	IL1B	case	control	-1.235998059	0.688543157	-1.795091633	0.072639102	0.476063325	down_in_case
CRA004775	ENSGALG00010018462	KTN1	case	control	0.295797729	0.213579565	1.384953325	0.16606674	0.630194031	up_in_case
CRA004775	ENSGALG00010018465	NAALADL2	case	control	1.407306382	0.67923584	2.071896535	0.038275091	0.369867027	up_in_case
CRA004775	ENSGALG00010018466	DYSF	case	control	0.035501715	0.473942413	0.074907233	0.940288522	0.987076872	up_in_case
CRA004775	ENSGALG00010018467	TLX1	case	control	2.390054483	0.958248245	2.49419135	0.012624447	0.226502393	up_in_case
CRA004775	ENSGALG00010018469	KLHDC7A	case	control	-0.104200617	0.217787923	-0.478449932	0.632330002	0.909651573	down_in_case
CRA004775	ENSGALG00010018470	IQCB1	case	control	-0.049208192	0.322078351	-0.152783296	0.878569171	0.97491968	down_in_case
CRA004775	ENSGALG00010018471	HHEX	case	control	-0.416785622	0.404490863	-1.03039564	0.302824319	0.75533053	down_in_case
CRA004775	ENSGALG00010018472	LMF1	case	control	0.022807645	0.216696174	0.10525172	0.916176072	0.981626545	up_in_case
CRA004775	ENSGALG00010018476	ENSGALG00010018476	case	control	-0.032226517	0.240346165	-0.13408376	0.893336341	0.977834811	down_in_case
CRA004775	ENSGALG00010018480	EME2	case	control	-1.584912095	0.585583728	-2.706550778	0.006798618	0.161712092	down_in_case
CRA004775	ENSGALG00010018482	TWNK	case	control	-0.551216678	0.348661479	-1.580950894	0.113889266	0.563544907	down_in_case
CRA004775	ENSGALG00010018485	ATP2C1	case	control	-0.000749137	0.221337359	-0.003384592	0.997299491	0.999619266	down_in_case
CRA004775	ENSGALG00010018486	ENSGALG00010018486	case	control	0.262712309	0.169755717	1.547590348	0.121720957	0.573689301	up_in_case
CRA004775	ENSGALG00010018487	ENSGALG00010018487	case	control	-0.455664621	0.386822826	-1.177967251	0.238809662	0.705601991	down_in_case
CRA004775	ENSGALG00010018488	ENSGALG00010018488	case	control	-0.215057632	0.350486586	-0.613597327	0.539481451	0.873987916	down_in_case
CRA004775	ENSGALG00010018489	SLC7A3	case	control	-0.753635351	0.290051851	-2.598278022	0.00936926	0.195703177	down_in_case
CRA004775	ENSGALG00010018490	NME3	case	control	-0.772038598	0.305655707	-2.525843879	0.011542076	0.216528087	down_in_case
CRA004775	ENSGALG00010018491	TIA1	case	control	0.023539055	0.202429936	0.116282481	0.907428669	0.980354087	up_in_case
CRA004775	ENSGALG00010018492	ENSGALG00010018492	case	control	-0.750694339	0.552607773	-1.358457799	0.174318465	0.639555285	down_in_case
CRA004775	ENSGALG00010018494	HAO1	case	control	0.326024576	0.646916213	0.503967237	0.6142844	0.902363507	up_in_case
CRA004775	ENSGALG00010018495	ENSGALG00010018495	case	control	0.406875794	0.927754978	0.438559538	0.660980721	0.917907174	up_in_case
CRA004775	ENSGALG00010018497	ASTE1	case	control	-0.294522598	0.273620993	-1.076388895	0.281753367	0.738260394	down_in_case
CRA004775	ENSGALG00010018498	ENSGALG00010018498	case	control	0.158291824	0.582523285	0.271734758	0.785825974	0.95041797	up_in_case
CRA004775	ENSGALG00010018501	ATG14	case	control	0.155250894	0.171975058	0.902752387	0.366657326	0.793780314	up_in_case
CRA004775	ENSGALG00010018502	ARHGEF10L	case	control	-0.567392383	0.262682703	-2.159991415	0.030773334	0.334633212	down_in_case
CRA004775	ENSGALG00010018503	BMP2	case	control	-0.093364118	0.477725693	-0.195434576	0.84505275	0.966990128	down_in_case
CRA004775	ENSGALG00010018504	SLCO3A1	case	control	-0.433066989	0.361281528	-1.198696739	0.230645887	0.697177844	down_in_case
CRA004775	ENSGALG00010018505	CHTF18	case	control	-0.33928775	0.311244345	-1.090100933	0.275668686	0.731001618	down_in_case
CRA004775	ENSGALG00010018506	ENSGALG00010018506	case	control	-0.20503936	0.312993599	-0.655091226	0.512409021	0.863317349	down_in_case
CRA004775	ENSGALG00010018508	ENSGALG00010018508	case	control	-0.537727081	0.229385683	-2.344205075	0.019067684	0.272556884	down_in_case
CRA004775	ENSGALG00010018509	ENSGALG00010018509	case	control	-0.318206565	0.154505683	-2.059513664	0.039445057	0.374034172	down_in_case
CRA004775	ENSGALG00010018510	ENSGALG00010018510	case	control	-0.085995987	0.319590673	-0.269081653	0.787866848	0.95041797	down_in_case
CRA004775	ENSGALG00010018511	SOCS5	case	control	0.214929304	0.186879008	1.150098694	0.250103224	0.714993603	up_in_case
CRA004775	ENSGALG00010018512	FERMT1	case	control	-0.336627285	0.258326223	-1.303109228	0.192537474	0.656993806	down_in_case
CRA004775	ENSGALG00010018513	RPUSD1	case	control	-0.655817747	0.268213328	-2.445134814	0.014479801	0.240504965	down_in_case
CRA004775	ENSGALG00010018514	EAF2	case	control	-0.0552247	0.567785912	-0.097263244	0.922517345	0.983072338	down_in_case
CRA004775	ENSGALG00010018515	SEMA4G	case	control	-0.240964212	0.237014999	-1.016662292	0.30931411	0.761710245	down_in_case
CRA004775	ENSGALG00010018516	LRRN4	case	control	0.479875966	0.422730752	1.135181116	0.25629944	0.719236059	up_in_case
CRA004775	ENSGALG00010018517	ECT2	case	control	0.780074583	0.537362551	1.45167277	0.146592614	0.609922856	up_in_case
CRA004775	ENSGALG00010018518	ENSGALG00010018518	case	control	1.005549998	0.847147519	1.186983347	0.235234189	0.702145501	up_in_case
CRA004775	ENSGALG00010018519	OGDH	case	control	-0.148796408	0.409040412	-0.363769456	0.716030173	0.933921336	down_in_case
CRA004775	ENSGALG00010018520	ENSGALG00010018520	case	control	-0.945390787	0.297823005	-3.174337685	0.001501789	0.080693373	down_in_case
CRA004775	ENSGALG00010018521	ENSGALG00010018521	case	control	-0.131534591	0.256790742	-0.512224819	0.608493675	0.901007337	down_in_case
CRA004775	ENSGALG00010018522	NEK11	case	control	0.43916601	0.629159013	0.698020692	0.485164254	0.851302844	up_in_case
CRA004775	ENSGALG00010018523	TEX11	case	control	0.156872597	0.317226551	0.494512821	0.620944054	0.90544587	up_in_case
CRA004775	ENSGALG00010018524	SEC22A	case	control	-0.199101021	0.115845737	-1.718673703	0.085673803	0.507059728	down_in_case
CRA004775	ENSGALG00010018525	ST8SIA2	case	control	-0.123361187	0.692978138	-0.178015986	0.858710421	0.971325143	down_in_case
CRA004775	ENSGALG00010018526	ENSGALG00010018526	case	control	0.128910987	0.280163894	0.460127053	0.645425027	0.914043203	up_in_case
CRA004775	ENSGALG00010018527	CCT4	case	control	-0.135902536	0.195093965	-0.696600414	0.486052897	0.851645437	down_in_case
CRA004775	ENSGALG00010018529	UBR2	case	control	0.304475599	0.232664727	1.308645292	0.190654531	0.656098436	up_in_case
CRA004775	ENSGALG00010018530	FAM161A	case	control	-0.546248687	0.493122561	-1.107734122	0.267976658	0.727528758	down_in_case
CRA004775	ENSGALG00010018531	ADCY5	case	control	0.459259172	0.384814273	1.193456699	0.232690561	0.699801257	up_in_case
CRA004775	ENSGALG00010018532	MFSD13A	case	control	-0.686522742	0.275963133	-2.487733537	0.012856002	0.228267513	down_in_case
CRA004775	ENSGALG00010018533	PCYOX1	case	control	0.146776394	0.218668075	0.671229184	0.50207454	0.860091011	up_in_case
CRA004775	ENSGALG00010018534	NCEH1	case	control	0.006125239	0.364820301	0.016789744	0.986604352	0.997562105	up_in_case
CRA004775	ENSGALG00010018535	FAM174B	case	control	-0.173919334	0.276152023	-0.629795617	0.528828314	0.86916155	down_in_case
CRA004775	ENSGALG00010018536	ENSGALG00010018536	case	control	-0.517486819	0.346618215	-1.492959105	0.135447892	0.593544921	down_in_case
CRA004775	ENSGALG00010018539	CHD2	case	control	0.082286693	0.179156864	0.459299699	0.646018964	0.914043203	up_in_case
CRA004775	ENSGALG00010018540	TNFSF10	case	control	-0.001730614	0.39385241	-0.004394067	0.996494053	0.999606847	down_in_case
CRA004775	ENSGALG00010018542	ZMIZ2	case	control	-0.432786483	0.435719304	-0.993269013	0.320578874	0.767037518	down_in_case
CRA004775	ENSGALG00010018543	MRPL3	case	control	-0.057423151	0.25213423	-0.227748333	0.819841895	0.960465449	down_in_case
CRA004775	ENSGALG00010018544	HACD2	case	control	0.063986713	0.36298783	0.176277846	0.860075666	0.971325143	up_in_case
CRA004775	ENSGALG00010018546	SNRPGP15	case	control	-0.338428132	0.341101537	-0.992162436	0.321118293	0.767427349	down_in_case
CRA004775	ENSGALG00010018548	RGMA	case	control	0.182247684	0.327205992	0.556981498	0.57754007	0.888339319	up_in_case
CRA004775	ENSGALG00010018551	FBXO34	case	control	0.199535035	0.232321894	0.858873141	0.390410509	0.806479902	up_in_case
CRA004775	ENSGALG00010018552	DTD1	case	control	0.055374743	0.22270984	0.248640755	0.80363868	0.95422118	up_in_case
CRA004775	ENSGALG00010018554	PPIA	case	control	-0.076440828	0.171639878	-0.445355875	0.656062594	0.91710603	down_in_case
CRA004775	ENSGALG00010018555	UBN1	case	control	-0.17410488	0.215293086	-0.808687743	0.41869478	0.818943344	down_in_case
CRA004775	ENSGALG00010018556	MRPL2	case	control	-0.140926636	0.472484944	-0.298266936	0.765499438	0.94337135	down_in_case
CRA004775	ENSGALG00010018557	TBCC	case	control	-0.140945013	0.175213843	-0.804417111	0.421156122	0.819803052	down_in_case
CRA004775	ENSGALG00010018558	ENSGALG00010018558	case	control	0.190115649	0.343946651	0.552747493	0.580436327	0.888718398	up_in_case
CRA004775	ENSGALG00010018559	DLG3	case	control	-0.338178528	0.304724096	-1.109785975	0.267091264	0.727315996	down_in_case
CRA004775	ENSGALG00010018560	ENSGALG00010018560	case	control	-0.139783331	0.290347754	-0.481434176	0.630207952	0.908943555	down_in_case
CRA004775	ENSGALG00010018561	ACTR1A	case	control	-0.28046947	0.140678237	-1.993694797	0.046185418	0.399541954	down_in_case
CRA004775	ENSGALG00010018562	ENSGALG00010018562	case	control	1.25404519	1.227101168	1.021957458	0.306801036	0.7585475	up_in_case
CRA004775	ENSGALG00010018564	DLGAP5	case	control	0.386362545	0.410351495	0.941540484	0.346427953	0.784552667	up_in_case
CRA004775	ENSGALG00010018566	ENSGALG00010018566	case	control	-0.217148742	0.422214984	-0.514308468	0.607036342	0.900898526	down_in_case
CRA004775	ENSGALG00010018568	FAM136A	case	control	0.336954179	0.323326854	1.042147211	0.297343433	0.749916187	up_in_case
CRA004775	ENSGALG00010018573	SEC23B	case	control	-0.02434789	0.213452342	-0.114067103	0.9091846	0.98075825	down_in_case
CRA004775	ENSGALG00010018574	ENSGALG00010018574	case	control	0.147859194	0.360849408	0.409753185	0.681987011	0.924305479	up_in_case
CRA004775	ENSGALG00010018576	PTK7	case	control	-0.100867672	0.180651449	-0.558355176	0.576601874	0.887937128	down_in_case
CRA004775	ENSGALG00010018577	NARFL	case	control	-0.04216236	0.14727305	-0.286287003	0.774658312	0.946319184	down_in_case
CRA004775	ENSGALG00010018579	FNDC3B	case	control	0.33246568	0.356328421	0.933031608	0.350803649	0.785681625	up_in_case
CRA004775	ENSGALG00010018581	SUFU	case	control	-0.094759646	0.167474698	-0.5658147	0.571519752	0.885417769	down_in_case
CRA004775	ENSGALG00010018582	RCC2	case	control	-0.434309933	0.24209672	-1.79395216	0.072820805	0.476411335	down_in_case
CRA004775	ENSGALG00010018583	PPL	case	control	0.179425023	0.443015649	0.405008319	0.685471396	0.926686318	up_in_case
CRA004775	ENSGALG00010018585	GDPD2	case	control	2.147030201	0.641518966	3.346791469	0.000817527	0.060328515	up_in_case
CRA004775	ENSGALG00010018587	SRF	case	control	0.21866089	0.361953428	0.604113329	0.545768299	0.876735967	up_in_case
CRA004775	ENSGALG00010018588	POLR3F	case	control	0.160154527	0.220994388	0.724699522	0.468636389	0.8440023	up_in_case
CRA004775	ENSGALG00010018590	TGFA	case	control	-0.252581838	0.386529339	-0.653460973	0.513459142	0.863903061	down_in_case
CRA004775	ENSGALG00010018593	LGALS3	case	control	1.62655038	0.653488443	2.489027	0.012809324	0.227866767	up_in_case
CRA004775	ENSGALG00010018594	BICRAL	case	control	0.180484514	0.283244497	0.637203954	0.523992003	0.866784927	up_in_case
CRA004775	ENSGALG00010018598	ENSGALG00010018598	case	control	-0.378529759	0.211526365	-1.78951574	0.073531795	0.478490829	down_in_case
CRA004775	ENSGALG00010018602	MCTP2	case	control	-0.522729925	0.522296056	-1.000830695	0.316908667	0.765974554	down_in_case
CRA004775	ENSGALG00010018604	FAM43A	case	control	-0.250326619	0.363477358	-0.688699347	0.491012487	0.855538864	down_in_case
CRA004775	ENSGALG00010018606	DZANK1	case	control	-0.35870389	0.563577629	-0.636476453	0.524465924	0.86702704	down_in_case
CRA004775	ENSGALG00010018607	HAGHL	case	control	-0.286031835	0.165335589	-1.730007656	0.083628907	0.502394542	down_in_case
CRA004775	ENSGALG00010018608	TRIM8	case	control	-0.10772935	0.205926862	-0.523143747	0.600874224	0.897720715	down_in_case
CRA004775	ENSGALG00010018609	ENSGALG00010018609	case	control	-0.27391749	0.227719581	-1.202871922	0.229025887	0.695782399	down_in_case
CRA004775	ENSGALG00010018611	NUDCD3	case	control	-0.364688924	0.287398363	-1.26893181	0.204465386	0.674045742	down_in_case
CRA004775	ENSGALG00010018613	PADI3	case	control	0.969927052	0.402245632	2.411280511	0.015896617	0.252039758	up_in_case
CRA004775	ENSGALG00010018616	ENSGALG00010018616	case	control	4.391945108	1.105141445	3.974102255	7.06452E-05	0.017238232	up_in_case
CRA004775	ENSGALG00010018618	FAHD1	case	control	-0.1191744	0.25137391	-0.474092158	0.635434191	0.909837348	down_in_case
CRA004775	ENSGALG00010018619	MAPK1IP1L	case	control	-0.24266297	0.109818032	-2.209682386	0.027127213	0.31561562	down_in_case
CRA004775	ENSGALG00010018623	ARRDC4	case	control	0.24109327	0.297774577	0.809650281	0.418141201	0.818943344	up_in_case
CRA004775	ENSGALG00010018624	ENSGALG00010018624	case	control	-0.246379395	0.682862045	-0.360804056	0.718245931	0.934011578	down_in_case

CRA004775	ENSGALG00010018627	MYLK	case	control	0.703004168	0.253645476	2.77160144	0.005578129	0.15147564	up_in_case
CRA004775	ENSGALG00010018628	XDH	case	control	-0.466028024	0.770785377	-0.604614512	0.545435162	0.876563448	down_in_case
CRA004775	ENSGALG00010018631	KAT14	case	control	-0.19795177	0.158638447	-1.247817116	0.212098039	0.682275341	down_in_case
CRA004775	ENSGALG00010018633	ENSGALG00010018633	case	control	-0.255894092	0.500871151	-0.510898045	0.60942245	0.901246133	down_in_case
CRA004775	ENSGALG00010018638	SEC14L5	case	control	0.498863854	0.345045506	1.445791484	0.148235695	0.611192154	up_in_case
CRA004775	ENSGALG00010018644	ESF1	case	control	0.113275162	0.293730984	0.385642539	0.699761424	0.929612534	up_in_case
CRA004775	ENSGALG00010018649	HAGH	case	control	0.341672992	0.204824042	1.668129328	0.095290056	0.528014574	up_in_case
CRA004775	ENSGALG00010018654	ENSGALG00010018654	case	control	0.817236997	0.442506838	1.846834728	0.064771097	0.457570489	up_in_case
CRA004775	ENSGALG00010018655	BIRC5	case	control	-0.162142479	0.420955526	-0.385177221	0.700106119	0.929819314	down_in_case
CRA004775	ENSGALG00010018665	IL13RA2	case	control	0.495924367	0.43753965	1.133438689	0.257030075	0.719236059	up_in_case
CRA004775	ENSGALG00010018668	OVOL2	case	control	1.083547993	0.725202607	1.494131408	0.135141276	0.592957144	up_in_case
CRA004775	ENSGALG00010018670	MRAS	case	control	-0.815812356	0.429674067	-1.898677206	0.05760693	0.439596503	down_in_case
CRA004775	ENSGALG00010018672	PKDCC	case	control	1.004769963	0.361237183	2.781468822	0.005411353	0.149721353	up_in_case
CRA004775	ENSGALG00010018673	NAGPA	case	control	-0.538740571	0.158806736	-3.392428964	0.000692759	0.055384465	down_in_case
CRA004775	ENSGALG00010018685	ARL3	case	control	-0.280228052	0.199489127	-1.404728452	0.160102042	0.625090553	down_in_case
CRA004775	ENSGALG00010018693	ENSGALG00010018693	case	control	0.392192395	0.286382064	1.369472618	0.170851588	0.635139279	up_in_case
CRA004775	ENSGALG00010018694	MGME1	case	control	-0.00374868	0.279092771	-0.013431664	0.989283405	0.998065748	down_in_case
CRA004775	ENSGALG00010018698	MRPS16	case	control	-0.242434352	0.280305048	-0.864894706	0.387096584	0.803905475	down_in_case
CRA004775	ENSGALG00010018701	SFXN2	case	control	-0.545299549	0.385434927	-1.414764234	0.157137634	0.621633948	down_in_case
CRA004775	ENSGALG00010018704	PGPEP1L	case	control	1.25427825	0.781420539	1.605125778	0.108466127	0.555500081	up_in_case
CRA004775	ENSGALG00010018707	SLC45A1	case	control	-0.24246573	0.18861192	-1.28552708	0.19860816	0.66546316	down_in_case
CRA004775	ENSGALG00010018709	SNX5	case	control	-0.616540357	0.419299215	-1.470406658	0.141451649	0.601287746	down_in_case
CRA004775	ENSGALG00010018713	ENSGALG00010018713	case	control	-0.20915668	0.268143984	-0.780016307	0.435381277	0.826905159	down_in_case
CRA004775	ENSGALG00010018715	DNAJC13	case	control	0.433927567	0.205403405	2.112562678	0.034638216	0.353831224	up_in_case
CRA004775	ENSGALG00010018719	ERRFI1	case	control	-0.229179104	0.198490525	-1.154609793	0.248250255	0.712818804	down_in_case
CRA004775	ENSGALG00010018721	LRCH2	case	control	0.184873597	0.31400184	0.588765967	0.556018279	0.87981923	up_in_case
CRA004775	ENSGALG00010018724	RRBP1	case	control	-0.250623286	0.339579639	-0.738039791	0.46049027	0.841265006	down_in_case
CRA004775	ENSGALG00010018726	EBF2	case	control	0.261524949	0.508375054	0.514433088	0.606949231	0.900898526	up_in_case
CRA004775	ENSGALG00010018727	PARK7	case	control	0.106787476	0.446811909	0.238998724	0.811106572	0.957219917	up_in_case
CRA004775	ENSGALG00010018730	IGF1R	case	control	0.239379544	0.417621231	0.573197736	0.566510817	0.883033678	up_in_case
CRA004775	ENSGALG00010018732	ENSGALG00010018732	case	control	-0.111515505	0.527809623	-0.211279787	0.832668954	0.963988657	down_in_case
CRA004775	ENSGALG00010018736	EEF2KMT	case	control	-0.264579398	0.198920158	-1.330078368	0.183492452	0.647806087	down_in_case
CRA004775	ENSGALG00010018747	FLRT3	case	control	0.676548107	0.330605067	2.046393643	0.040717657	0.378061108	up_in_case
CRA004775	ENSGALG00010018750	SOCS4	case	control	-0.100140743	0.213250674	-0.469591683	0.638646768	0.91099914	down_in_case
CRA004775	ENSGALG00010018752	PLS3	case	control	0.441770611	0.283419563	1.558716011	0.119063611	0.570892125	up_in_case
CRA004775	ENSGALG00010018753	SYNM	case	control	0.577004109	0.468546136	1.23147768	0.21814426	0.68549849	up_in_case
CRA004775	ENSGALG00010018756	EML4	case	control	-0.067651771	0.318025859	-0.212724121	0.831542152	0.963557439	down_in_case
CRA004775	ENSGALG00010018758	ENSGALG00010018758	case	control	-0.64289771	0.278284612	-2.31021653	0.020876169	0.284507636	down_in_case
CRA004775	ENSGALG00010018764	TMEM177	case	control	-0.405222575	0.364553981	-1.111557125	0.266328616	0.727311193	down_in_case
CRA004775	ENSGALG00010018767	NDUFB6	case	control	0.171224194	0.404229975	0.423581141	0.671871312	0.921862144	up_in_case
CRA004775	ENSGALG00010018774	MSRB1	case	control	-0.328784644	0.323149973	-1.017436706	0.308945728	0.761414086	down_in_case
CRA004775	ENSGALG00010018780	WDHD1	case	control	-0.182681751	0.371414607	-0.491853976	0.622822576	0.906338268	down_in_case
CRA004775	ENSGALG00010018783	ENSGALG00010018783	case	control	-0.320453834	0.50238764	-0.6378617	0.523563712	0.866638286	down_in_case
CRA004775	ENSGALG00010018786	DSTN	case	control	-0.279618773	0.34523622	-0.809934639	0.417977742	0.818943344	down_in_case
CRA004775	ENSGALG00010018796	PER3	case	control	-0.107271221	0.366445269	-0.292734632	0.769724996	0.945437613	down_in_case
CRA004775	ENSGALG00010018799	ENSGALG00010018799	case	control	-0.353632163	0.376144499	-0.940149765	0.347140745	0.784733255	down_in_case
CRA004775	ENSGALG00010018806	GCH1	case	control	0.017563113	0.396195105	0.044329454	0.964641794	0.992871055	up_in_case
CRA004775	ENSGALG00010018807	RD3	case	control	0.144035264	0.652406194	0.220775439	0.825267289	0.961797651	up_in_case
CRA004775	ENSGALG00010018808	MATCAP2	case	control	0.026828092	0.216906538	0.123685032	0.901564663	0.979157637	up_in_case
CRA004775	ENSGALG00010018811	VAMP3	case	control	-0.23195539	0.327100264	-0.70912627	0.478246122	0.846814631	down_in_case
CRA004775	ENSGALG00010018815	ENSGALG00010018815	case	control	0.27516545	0.249369807	1.103443326	0.26983469	0.727976619	up_in_case
CRA004775	ENSGALG00010018819	LRRC28	case	control	0.043582029	0.246476339	0.176820335	0.859649515	0.971325143	up_in_case
CRA004775	ENSGALG00010018822	TRAF5	case	control	0.118795208	0.50653533	0.23452502	0.814577412	0.958758519	up_in_case
CRA004775	ENSGALG00010018823	RAB40C	case	control	0.088989005	0.124253424	0.716189559	0.473874309	0.845497032	up_in_case
CRA004775	ENSGALG00010018829	PTPN4	case	control	0.513143762	0.366811537	1.39893027	0.161833894	0.627882156	up_in_case
CRA004775	ENSGALG00010018831	WBP1L	case	control	0.095017575	0.505277187	0.188050395	0.850837146	0.968989143	up_in_case
CRA004775	ENSGALG00010018832	EEPD1	case	control	-0.095609856	0.38187447	-0.250369857	0.802301338	0.953744693	down_in_case
CRA004775	ENSGALG00010018834	SAMD4A	case	control	0.184390889	0.412141521	0.447397022	0.654588426	0.916640657	up_in_case
CRA004775	ENSGALG00010018835	CAMK2B	case	control	0.027559316	0.237764203	0.115910283	0.907723646	0.980372908	up_in_case
CRA004775	ENSGALG00010018837	RCOR3	case	control	0.179697636	0.203337619	0.88374024	0.376836453	0.798523018	up_in_case
CRA004775	ENSGALG00010018838	WFIKKN1	case	control	-0.135519884	0.966127633	-0.140271202	0.888445717	0.977118922	down_in_case
CRA004775	ENSGALG00010018839	RPL3L	case	control	-0.376798361	0.718029959	-0.524766907	0.599745239	0.897206546	down_in_case
CRA004775	ENSGALG00010018843	MCRIP2	case	control	0.326642646	0.389589561	0.838427614	0.401790586	0.811868062	up_in_case
CRA004775	ENSGALG00010018847	CGRRF1	case	control	-0.270618052	0.246870105	-1.096196126	0.272992955	0.731001618	down_in_case
CRA004775	ENSGALG00010018848	MEF2A	case	control	0.376941382	0.214324451	1.758741854	0.078621365	0.491399489	up_in_case
CRA004775	ENSGALG00010018852	NDUFB10	case	control	0.294985326	0.368813835	0.799821747	0.423814081	0.82073892	up_in_case
CRA004775	ENSGALG00010018854	LYSMD4	case	control	-0.101024341	0.49403701	-0.204487394	0.837972642	0.965490877	down_in_case
CRA004775	ENSGALG00010018857	ENSGALG00010018857	case	control	0.261182381	0.285189343	0.91582097	0.359760803	0.791966047	up_in_case
CRA004775	ENSGALG00010018858	EPB41L5	case	control	-0.143570529	0.301851883	-0.475632379	0.634336304	0.909837348	down_in_case
CRA004775	ENSGALG00010018860	AGTR2	case	control	0.359661239	0.476210121	0.755257444	0.450094527	0.835337561	up_in_case
CRA004775	ENSGALG00010018864	ABCA3	case	control	-0.117042297	0.306575999	-0.381772538	0.70263009	0.930068722	down_in_case
CRA004775	ENSGALG00010018865	ENSGALG00010018865	case	control	-0.487380499	0.778812664	-0.625799401	0.531446525	0.86957653	down_in_case
CRA004775	ENSGALG00010018867	RPS2	case	control	-0.337731114	0.319099761	-1.058387236	0.289878936	0.74395768	down_in_case
CRA004775	ENSGALG00010018872	KCTD9	case	control	-0.054320484	0.157885007	-0.344050933	0.730807982	0.936394787	down_in_case
CRA004775	ENSGALG00010018874	ENSGALG00010018874	case	control	-0.173987409	0.283344123	-0.614049822	0.539182405	0.873987916	down_in_case
CRA004775	ENSGALG00010018876	GMFB	case	control	-0.26611756	0.435231182	-0.611439553	0.540908624	0.874592283	down_in_case
CRA004775	ENSGALG00010018877	CST7	case	control	0.039431864	0.443007348	0.089009504	0.929074357	0.98473447	up_in_case
CRA004775	ENSGALG00010018880	TMEM17	case	control	-0.281200135	0.526906386	-0.533681396	0.593561983	0.894594166	down_in_case
CRA004775	ENSGALG00010018881	ENSGALG00010018881	case	control	2.809779919	1.149719533	2.44388291			up_in_case
CRA004775	ENSGALG00010018882	HHAT	case	control	-0.680600481	0.31436326	-2.165012796	0.030386705	0.332416535	down_in_case
CRA004775	ENSGALG00010018883	TBL3	case	control	-0.327399	0.234290248	-1.397407717	0.162291	0.628283654	down_in_case
CRA004775	ENSGALG00010018885	EFEMP1	case	control	0.460255744	0.28703531	1.603481273	0.10882844	0.555936379	up_in_case
CRA004775	ENSGALG00010018887	ADAMTS17	case	control	-0.478587184	0.600861152	-0.796502125	0.425740241	0.822471769	down_in_case
CRA004775	ENSGALG00010018892	APMAP	case	control	-0.260654753	0.275379669	-0.946528673	0.343879005	0.783503692	down_in_case
CRA004775	ENSGALG00010018894	SERTAD4	case	control	0.429568143	0.236147836	1.819064489	0.068901592	0.466961947	up_in_case
CRA004775	ENSGALG00010018895	SEPTIN7	case	control	0.137513004	0.129376931	1.062886583	0.287833388	0.74258854	up_in_case
CRA004775	ENSGALG00010018897	CCDC85A	case	control	0.550220035	0.361968957	1.520075202	0.128492076	0.582990237	up_in_case
CRA004775	ENSGALG00010018899	ACSS1	case	control	-0.049132289	0.398055812	-0.123430654	0.901766084	0.979157637	down_in_case
CRA004775	ENSGALG00010018901	TCF7L2	case	control	0.325114972	0.257435325	1.26289961	0.20662526	0.675197427	up_in_case
CRA004775	ENSGALG00010018902	NOXO1	case	control	0.682131299	0.776256915	0.878744248	0.379539961	0.800591196	up_in_case
CRA004775	ENSGALG00010018903	CAMTA1	case	control	0.217991288	0.334377867	0.651930972	0.514445704	0.864386959	up_in_case
CRA004775	ENSGALG00010018904	UGP2	case	control	0.002747577	0.473355317	0.005804471	0.995368728	0.999001196	up_in_case
CRA004775	ENSGALG00010018908	ENSGALG00010018908	case	control	-0.43835101	0.455547201	-0.962251572	0.335923249	0.776741314	down_in_case
CRA004775	ENSGALG00010018909	TTBK1	case	control	0.325013743	0.355778406	0.913528585	0.360964607	0.792531424	up_in_case
CRA004775	ENSGALG00010018911	CNIH1	case	control	-0.198243216	0.240750273	-0.823439217	0.410258269	0.815947564	down_in_case
CRA004775	ENSGALG00010018913	ENSGALG00010018913	case	control	-0.125017034	0.273614603	-0.456909219	0.647736298	0.914210065	down_in_case
CRA004775	ENSGALG00010018915	DOCK5	case	control	-0.324330649	0.640052081	-0.506725404	0.612347503	0.901413878	down_in_case
CRA004775	ENSGALG00010018916	CERS3	case	control	0.083891741	0.480895856	0.174448875	0.861512706	0.97165529	up_in_case
CRA004775	ENSGALG00010018917	DNAJC11	case	control	-0.079925464	0.215944224	-0.370120868	0.711292434	0.932234469	down_in_case
CRA004775	ENSGALG00010018919	RALB	case	control	0.024631795	0.247634147	0.099468491	0.920766305	0.982481112	up_in_case
CRA004775	ENSGALG00010018920	ACAD11	case	control	-0.094795058	0.232932194	-0.406964175	0.684034301	0.925529437	down_in_case
CRA004775	ENSGALG00010018922	EEF2K	case	control	0.35005599	0.306412024	1.142435555	0.253273019	0.717798287	up_in_case
CRA004775	ENSGALG00010018923	VPS54	case	control	-0.09771524	0.239920415	-0.40728189	0.683800963	0.925304767	down_in_case
CRA004775	ENSGALG00010018925	LINS1	case	control	-0.305663314	0.405260676	-0.75423877	0.450705861	0.835552293	down_in_case
CRA004775	ENSGALG00010018932	CDKN3	case	control	1.492585439	0.460635817	3.240272216			up_in_case
CRA004775	ENSGALG00010018933	PHF13	case	control	-0.015908775	0.153411436	-0.103700059	0.91740738	0.981688766	down_in_case
CRA004775	ENSGALG00010018936	SYT14	case	control	0.620093652	0.654440835	0.947516748	0.343375526	0.782914419	up_in_case
CRA004775	ENSGALG00010018937	INHBB	case	control	0.769701867	0.743283789	1.035542384	0.300415684	0.752927916	up_in_case
CRA004775	ENSGALG00010018941	KLHL21	case	control	-0.099182811	0.410576802	-0.241569446	0.809113799	0.955623701	down_in_case
CRA004775	ENSGALG00010018943	ACKR4	case	control	-0.198932474	0.450306852	-0.441770924	0.658654981	0.917648599	down_in_case
CRA004775	ENSGALG00010018944	NEFL	case	control	0.434491357	0.54770908	0.793288577	0.42760971	0.822776983	up_in_case
CRA004775	ENSGALG00010018945	HABP2	case	control	1.653454507	0.44228563	3.738431445	0.000185172	0.027981316	up_in_case
CRA004775	ENSGALG00010018947	ENSGALG00010018947	case	control	0.669838051	0.399876813	1.675111008	0.093912454	0.5260245	up_in_case
CRA004775	ENSGALG00010018948	POLR3E	case	control	0.292805826	0.21944646	1.334292776	0.182107924	0.647378665	up_in_case
CRA004775	ENSGALG00010018950	TTL	case	control	0.259172973	0.258406808	1.002964959	0.315877768	0.765677454	up_in_case
CRA004775	ENSGALG00010018951	YKT6	case	control	-0.376833827	0.183927554	-2.048816604	0.040480052	0.376363211	down_in_case
CRA004775	ENSGALG00010018955	ZBTB48	case	control	-0.201335872	0.292695606	-0.687867763	0.491536059	0.855746361	down_in_case
CRA004775	ENSGALG00010018958	PELI3	case	control	0.15656946	0.237902927	0.658123304	0.510458903	0.863170205	up_in_case
CRA004775	ENSGALG00010018959	UBA5	case	control	-0.065097821	0.288505305	-0.22563821	0.82148282	0.960766569	down_in_case
CRA004775	ENSGALG00010018960	RBFOX1	case	control	0.40686178	0.451630133	0.900873856	0.367655393	0.794321403	up_in_case
CRA004775	ENSGALG00010018961	ASB7	case	control	0.079096974	0.294970433	0.268152213	0.788582157	0.95041797	up_in_case
CRA004775	ENSGALG00010018968	GLI2	case	control	-0.127988724	0.390993663	-0.327342195	0.743409082	0.939218065	down_in_case
CRA004775	ENSGALG00010018969	DIEXF	case	control	-0.105930805	0.195240287	-0.542566325	0.58742843	0.892218236	down_in_case
CRA004775	ENSGALG00010018971	POLR1B	case	control	0.102072002	0.091362011	1.117225862	0.263897766	0.723981064	up_in_case
CRA004775	ENSGALG00010018974	TAS1R1	case	control	-0.317376749	0.270535302	-1.173143567	0.240738238	0.707499788	down_in_case
CRA004775	ENSGALG00010018977	NPHP3	case	control	-0.200443678	0.195540839	-1.025073224	0.305328639	0.757865363	down_in_case

CRA004775	ENSGALG00010018983	MDGA2	case	control	1.327586419	0.587024474	2.261552079	0.023725092	0.2983663	up_in_case
CRA004775	ENSGALG00010018985	NOL9	case	control	-0.219377529	0.227203735	-0.965554238	0.334267283	0.774605563	down_in_case
CRA004775	ENSGALG00010018986	CTDSP1	case	control	-0.191082553	0.282249743	-0.676998148	0.49840713	0.858201408	down_in_case
CRA004775	ENSGALG00010018987	MRPS10	case	control	-0.154780565	0.344876743	-0.448799661	0.653576184	0.916470479	down_in_case
CRA004775	ENSGALG00010018990	GCK	case	control	-0.36968176	0.39744038	-0.930156519	0.352290051	0.786426886	down_in_case
CRA004775	ENSGALG00010018991	RYBP	case	control	0.030750009	0.282993639	0.108659718	0.913472393	0.980803023	up_in_case
CRA004775	ENSGALG00010018993	ENSGALG00010018993	case	control	0.146725135	0.501325152	0.292674594	0.769770891	0.945437613	up_in_case
CRA004775	ENSGALG00010018998	RPS29	case	control	0.044916855	0.268041579	0.167574207	0.866918264	0.973062856	up_in_case
CRA004775	ENSGALG00010018999	THUMPD3	case	control	-0.366610145	0.196671281	-1.864075643	0.062311082	0.448842166	down_in_case
CRA004775	ENSGALG00010019000	TRERF1	case	control	-0.371228284	0.255118588	-1.455120486	0.145635909	0.609044185	down_in_case
CRA004775	ENSGALG00010019001	ALDH1A3	case	control	-0.219968651	0.342394675	-0.6424418	0.520586367	0.865611895	down_in_case
CRA004775	ENSGALG00010019004	ECE2	case	control	-0.364024807	0.361383051	-1.007310127	0.313785765	0.765098415	down_in_case
CRA004775	ENSGALG00010019005	ENSGALG00010019005	case	control	0.013142401	0.425733067	0.03087005	0.975373176	0.995245303	up_in_case
CRA004775	ENSGALG00010019006	ENSGALG00010019006	case	control	-0.090902169	0.111199446	-0.817469625	0.413660101	0.817510786	down_in_case
CRA004775	ENSGALG00010019008	HERPUD2	case	control	-0.151483161	0.116819533	-1.296728019	0.194724785	0.659287597	down_in_case
CRA004775	ENSGALG00010019009	NRAP	case	control	1.504961445	0.668059241	2.252736512	0.024275767	0.30145607	up_in_case
CRA004775	ENSGALG00010019011	VIL1	case	control	2.182130965	0.647161071	3.371851403	0.000746647	0.057648813	up_in_case
CRA004775	ENSGALG00010019012	ADAM28	case	control	0.916333922	0.987893101	0.927563844	0.35363386	0.78730441	up_in_case
CRA004775	ENSGALG00010019013	LRRK1	case	control	-0.402018338	0.282368239	-1.423737809	0.154522384	0.619284425	down_in_case
CRA004775	ENSGALG00010019016	PLEKHG5	case	control	0.078538982	0.347279865	0.226154724	0.821081084	0.960766569	up_in_case
CRA004775	ENSGALG00010019017	IRAK2	case	control	0.183234801	0.144344021	1.269431181	0.204287321	0.674045742	up_in_case
CRA004775	ENSGALG00010019018	SH3BGRL	case	control	-0.398109829	0.420041745	-0.947786341	0.343238235	0.782861001	down_in_case
CRA004775	ENSGALG00010019019	TBX20	case	control	-0.764134928	0.890359415	-0.858231985	0.390764378	0.806479902	down_in_case
CRA004775	ENSGALG00010019020	KCNK2	case	control	-2.598873082	0.748473983	-3.472229015	0.000516156	0.046088692	down_in_case
CRA004775	ENSGALG00010019021	LRR1	case	control	-0.466487554	0.238691494	-1.954353489	0.050659427	0.416238146	down_in_case
CRA004775	ENSGALG00010019023	AP2M1	case	control	-0.250514352	0.136711484	-1.832430924	0.066887244	0.462498477	down_in_case
CRA004775	ENSGALG00010019024	CNPY3	case	control	-0.062879425	0.204701533	-0.307176129	0.758709314	0.942236668	down_in_case
CRA004775	ENSGALG00010019025	DNAAF2	case	control	-0.191034795	0.323953052	-0.589699012	0.555392457	0.87981923	down_in_case
CRA004775	ENSGALG00010019026	CHSY1	case	control	-0.394846412	0.488676528	-0.807991359	0.419095556	0.818943344	down_in_case
CRA004775	ENSGALG00010019027	GNLY	case	control	-0.496677802	0.391110012	-1.269918402	0.204113697	0.674045742	down_in_case
CRA004775	ENSGALG00010019028	TNFRSF25	case	control	-0.165243265	0.356951559	-0.462929101	0.643415187	0.913638565	down_in_case
CRA004775	ENSGALG00010019029	CENPF	case	control	0.012465028	0.797534598	0.015629451	0.98753001	0.998054179	up_in_case
CRA004775	ENSGALG00010019030	CDR2	case	control	0.193071081	0.396695911	0.486697935	0.626472409	0.907566592	up_in_case
CRA004775	ENSGALG00010019032	GNMT	case	control	-0.056207302	0.437516995	-0.128468843	0.897777954	0.978790377	down_in_case
CRA004775	ENSGALG00010019034	CASP7	case	control	0.330227198	0.314824529	1.048924614	0.294212816	0.746438942	up_in_case
CRA004775	ENSGALG00010019035	ENSGALG00010019035	case	control	-0.204371217	0.472360865	-0.43265908	0.665262462	0.918815167	down_in_case
CRA004775	ENSGALG00010019036	REL	case	control	0.005962797	0.294989025	0.020213622	0.983872961	0.99751066	up_in_case
CRA004775	ENSGALG00010019038	PLEKHS1	case	control	0.016509504	0.786567963	0.020989291	0.983254198	0.997398088	up_in_case
CRA004775	ENSGALG00010019040	HMGN5	case	control	0.166642352	0.180025418	0.925660127	0.354622636	0.787789315	up_in_case
CRA004775	ENSGALG00010019041	KATNIP	case	control	0.307471845	0.322862855	0.952329573	0.340929864	0.780875343	up_in_case
CRA004775	ENSGALG00010019042	PEX6	case	control	-0.232802623	0.2471038	-0.94212482	0.346128739	0.784552667	down_in_case
CRA004775	ENSGALG00010019043	METTL9	case	control	0.090351376	0.209378079	0.431522614	0.666088413	0.918888163	up_in_case
CRA004775	ENSGALG00010019045	LBX1	case	control	0.262794615	0.604121809	0.435002695	0.663560472	0.918295135	up_in_case
CRA004775	ENSGALG00010019046	SELENOS	case	control	-0.160201583	0.18273731	-0.876676926	0.380662143	0.801605686	down_in_case
CRA004775	ENSGALG00010019049	POLD2	case	control	-0.148787334	0.213034158	-0.69842008	0.484914522	0.851277875	down_in_case
CRA004775	ENSGALG00010019050	TNS1	case	control	-0.165130317	0.43585803	-0.378862624	0.704789881	0.930807304	down_in_case
CRA004775	ENSGALG00010019051	IGSF6	case	control	-0.858668552	0.485472962	-1.768725797	0.076939645	0.488700005	down_in_case
CRA004775	ENSGALG00010019059	BRWD3	case	control	0.160758414	0.333965093	0.481362924	0.630258583	0.908943555	up_in_case
CRA004775	ENSGALG00010019062	PUS10	case	control	-0.283500836	0.255266158	-1.110608781	0.266736782	0.727315996	down_in_case
CRA004775	ENSGALG00010019064	ENSGALG00010019064	case	control	0.070279696	0.328400299	0.214006187	0.830542233	0.963232966	up_in_case
CRA004775	ENSGALG00010019065	CAND2	case	control	0.131042481	0.419531086	0.312354639	0.75477102	0.941900693	up_in_case
CRA004775	ENSGALG00010019067	GTF3C1	case	control	-0.237103385	0.204364307	-1.160199587	0.245967555	0.712046495	down_in_case
CRA004775	ENSGALG00010019068	SNRPA1	case	control	-0.29287486	0.464579057	-0.630409088	0.528426966	0.868929328	down_in_case
CRA004775	ENSGALG00010019072	AEBP1	case	control	-0.002393378	0.427858658	-0.005593853	0.995536775	0.999096934	down_in_case
CRA004775	ENSGALG00010019076	ESPN	case	control	0.906776668	0.385653718	2.351271684	0.018709368	0.271671082	up_in_case
CRA004775	ENSGALG00010019079	CAPG	case	control	-0.472706733	0.373380312	-1.266019438	0.205506123	0.674045742	down_in_case
CRA004775	ENSGALG00010019081	PCSK6	case	control	0.19789082	0.310564038	0.637198117	0.523995804	0.866784927	up_in_case
CRA004775	ENSGALG00010019082	POLR3K	case	control	0.189669998	0.384664442	0.493079103	0.621956696	0.905987979	up_in_case
CRA004775	ENSGALG00010019083	OTOA	case	control	0.287486799	0.480044883	0.598874834	0.549256355	0.877623839	up_in_case
CRA004775	ENSGALG00010019087	PEX13	case	control	0.048496822	0.196012928	0.247416444	0.804585949	0.954576316	up_in_case
CRA004775	ENSGALG00010019093	RPL32	case	control	-0.158064576	0.276134657	-0.572418463	0.567038511	0.88345191	down_in_case
CRA004775	ENSGALG00010019094	SNRNP25	case	control	-0.35727231	0.27287525	-1.309288074	0.190436788	0.656023968	down_in_case
CRA004775	ENSGALG00010019101	POLE2	case	control	-0.582841259	0.264915887	-2.200099308	0.02779985	0.318297866	down_in_case
CRA004775	ENSGALG00010019104	POLL	case	control	-0.254843222	0.222627404	-1.144707334	0.252330413	0.717019836	down_in_case
CRA004775	ENSGALG00010019108	KDELR2	case	control	-0.16024612	0.2267554	-0.706691525	0.479758197	0.847527617	down_in_case
CRA004775	ENSGALG00010019110	MBD4	case	control	-0.314657644	0.202411676	-1.554542956	0.120054959	0.572133279	down_in_case
CRA004775	ENSGALG00010019113	KLHDC1	case	control	0.026571206	0.219059868	0.121296548	0.903456154	0.979456447	up_in_case
CRA004775	ENSGALG00010019114	TENT5D	case	control	-0.283634798	0.510583463	-0.555511133	0.578545097	0.888378369	down_in_case
CRA004775	ENSGALG00010019115	DBNL	case	control	-0.288570724	0.199276782	-1.448090041	0.14759187	0.610831526	down_in_case
CRA004775	ENSGALG00010019120	DAGLB	case	control	-0.010126183	0.253711949	-0.039912123	0.968163186	0.993568693	down_in_case
CRA004775	ENSGALG00010019123	PLEKHO2	case	control	-0.107287112	0.219756163	-0.4882098	0.625401244	0.906779049	down_in_case
CRA004775	ENSGALG00010019124	BMPER	case	control	0.119692789	0.426079513	0.280916555	0.778774403	0.94792594	up_in_case
CRA004775	ENSGALG00010019125	ENSGALG00010019125	case	control	-0.436580707	0.469050492	-0.930775501	0.351969705	0.786426886	down_in_case
CRA004775	ENSGALG00010019127	TMBIM1	case	control	-0.257268919	0.202070866	-1.273161855	0.202960613	0.672346758	down_in_case
CRA004775	ENSGALG00010019130	RPS6KC1	case	control	-0.121066701	0.135999378	-0.890200406	0.373358287	0.796841503	down_in_case
CRA004775	ENSGALG00010019132	ENSGALG00010019132	case	control	-0.103410048	0.240261779	-0.430405738	0.666900522	0.919168997	down_in_case
CRA004775	ENSGALG00010019135	KIAA1841	case	control	0.083907282	0.313647146	0.267521266	0.789067843	0.95041797	up_in_case
CRA004775	ENSGALG00010019139	DPCD	case	control	-0.219126401	0.342059343	-0.640609315	0.521776546	0.86633051	down_in_case
CRA004775	ENSGALG00010019141	ACOT7	case	control	-0.097702356	0.261140032	-0.374137797	0.708301799	0.931801673	down_in_case
CRA004775	ENSGALG00010019144	IFT122	case	control	-0.144549904	0.197979202	-0.730126711	0.465312735	0.843383332	down_in_case
CRA004775	ENSGALG00010019145	RAC1	case	control	0.012034508	0.107990113	0.111440833	0.911266782	0.98075825	up_in_case
CRA004775	ENSGALG00010019146	KLHDC2	case	control	0.074029738	0.332712593	0.222503564	0.823921899	0.960901588	up_in_case
CRA004775	ENSGALG00010019149	COX5B	case	control	0.251314003	0.383110252	0.655983498	0.511834742	0.863170205	up_in_case
CRA004775	ENSGALG00010019157	ENSGALG00010019157	case	control	-0.005709542	0.812478191	-0.007027317	0.994393058	0.999001196	down_in_case
CRA004775	ENSGALG00010019158	ENSGALG00010019158	case	control	-0.061211125	0.293537634	-0.208529053	0.8348159	0.964344207	down_in_case
CRA004775	ENSGALG00010019166	PNPT1	case	control	-0.040740387	0.220432188	-0.1848205	0.853369828	0.970037736	down_in_case
CRA004775	ENSGALG00010019169	GPR153	case	control	-0.038663784	0.455579761	-0.084867213	0.932366958	0.985044263	down_in_case
CRA004775	ENSGALG00010019172	NEMF	case	control	-0.142036356	0.17576996	-0.808080951	0.419043983	0.818943344	down_in_case
CRA004775	ENSGALG00010019174	ENSGALG00010019174	case	control	-0.562266719	0.561540414	-1.001293415	0.316684975	0.765914152	down_in_case
CRA004775	ENSGALG00010019183	ANGEL2	case	control	0.212027655	0.220567603	0.961281947	0.336410422	0.776900397	up_in_case
CRA004775	ENSGALG00010019184	PNKD	case	control	-0.446011423	0.259527538	-1.718551437	0.085696081	0.507059728	down_in_case
CRA004775	ENSGALG00010019185	ANKDD1A	case	control	-0.817801568	0.432446898	-1.891102865	0.058610614	0.443329681	down_in_case
CRA004775	ENSGALG00010019189	ICMT	case	control	-0.28169288	0.203462526	-1.384495147	0.166206893	0.630194031	down_in_case
CRA004775	ENSGALG00010019193	ENSGALG00010019193	case	control	-0.148236162	0.52492406	-0.282395442	0.77764031	0.947570394	down_in_case
CRA004775	ENSGALG00010019194	ENSGALG00010019194	case	control	-0.181737101	0.315477655	-0.576069646	0.564568107	0.882502677	down_in_case
CRA004775	ENSGALG00010019204	ARF6	case	control	-0.07757124	0.148756645	-0.521464031	0.602043556	0.898185892	down_in_case
CRA004775	ENSGALG00010019206	RNF207	case	control	-0.179868997	0.467427459	-0.384806228	0.700380985	0.929842957	down_in_case
CRA004775	ENSGALG00010019208	PLXND1	case	control	-0.345242571	0.464798776	-0.742778572	0.45761575	0.839704957	down_in_case
CRA004775	ENSGALG00010019209	VCPKMT	case	control	-0.44095844	0.165611937	-2.662600583	0.007753939	0.176823247	down_in_case
CRA004775	ENSGALG00010019212	RPL22	case	control	-0.240205525	0.262040001	-0.916675026	0.359312957	0.791589131	down_in_case
CRA004775	ENSGALG00010019213	RHBDF1	case	control	-0.096159445	0.275205978	-0.349408998	0.726782279	0.935238044	down_in_case
CRA004775	ENSGALG00010019215	ENSGALG00010019215	case	control	-0.320898254	0.175920626	-1.824108185	0.068135718	0.464518723	down_in_case
CRA004775	ENSGALG00010019218	RMND5A	case	control	-0.116961617	0.325837139	-0.358957293	0.719627038	0.93407508	down_in_case
CRA004775	ENSGALG00010019221	VASH2	case	control	-0.064012222	0.497911166	-0.128561531	0.897704607	0.978790377	down_in_case
CRA004775	ENSGALG00010019223	AAMP	case	control	-0.41873848	0.217202116	-1.927874769	0.053870705	0.427596115	down_in_case
CRA004775	ENSGALG00010019227	CLCN5	case	control	-0.132331459	0.247279455	-0.53514943	0.592546532	0.8941191	down_in_case
CRA004775	ENSGALG00010019230	SPG21	case	control	-0.312287551	0.172429572	-1.811102049	0.070125056	0.469724266	down_in_case
CRA004775	ENSGALG00010019231	ENSGALG00010019231	case	control	-0.336753887	0.516001258	-0.652622221	0.513999856	0.863954532	down_in_case
CRA004775	ENSGALG00010019232	SOS2	case	control	0.213094363	0.228970223	0.930664082	0.352027355	0.786426886	up_in_case
CRA004775	ENSGALG00010019233	ARPC2	case	control	-0.222463761	0.189242998	-1.175545537	0.23977653	0.706254731	down_in_case
CRA004775	ENSGALG00010019236	MPG	case	control	-0.482064315	0.396077168	-1.217096955	0.223567329	0.691469713	down_in_case
CRA004775	ENSGALG00010019237	SORCS3	case	control	-0.916468042	0.625495108	-1.465188186	0.142869582	0.603007864	down_in_case
CRA004775	ENSGALG00010019239	CYTH3	case	control	0.645460708	0.274419033	2.352098909	0.01866781	0.271354185	up_in_case
CRA004775	ENSGALG00010019240	ENSGALG00010019240	case	control	-0.322407735	0.349096156	-0.923549942	0.355720686	0.788778426	down_in_case
CRA004775	ENSGALG00010019243	SERTAD2	case	control	0.319712484	0.332313527	0.962080859	0.336008988	0.776808944	up_in_case
CRA004775	ENSGALG00010019246	IL17RC	case	control	-0.16019576	0.390639994	-0.410085404	0.6817433	0.924278508	down_in_case
CRA004775	ENSGALG00010019248	RMI2	case	control	-0.245822071	0.360854872	-0.681221429	0.495731391	0.856382445	down_in_case
CRA004775	ENSGALG00010019250	CXCR1	case	control	-0.605896285	0.307363306	-1.971270718	0.048692919	0.409276483	down_in_case
CRA004775	ENSGALG00010019252	TATDN3	case	control	-0.285763272	0.251524183	-1.136126432	0.255903654	0.719176609	down_in_case
CRA004775	ENSGALG00010019253	HBAD	case	control	-0.578158463	0.327204614	-1.766963049	0.077234417	0.488700005	down_in_case
CRA004775	ENSGALG00010019255	ENSGALG00010019255	case	control	0.086038025	0.43040141	0.19990182	0.841557367	0.966478468	up_in_case
CRA004775	ENSGALG00010019256	BBS9	case	control	0.245562195	0.351390978	0.698828969	0.484658922	0.851181085	up_in_case
CRA004775	ENSGALG00010019257	SLC1A4	case	control	-0.694763692	0.384286429	-1.807931895	0.070617101	0.469895261	down_in_case
CRA004775	ENSGALG00010019259	L2HGDH	case	control	-0.255786186	0.259594381	-0.985330208	0.324461899	0.770087471	down_in_case
CRA004775	ENSGALG00010019260	TRAIL-LIKE	case	control	0.105292656	0.420730412	0.250261575	0.80238507	0.953744693	up_in_case
CRA004775	ENSGALG00010019263	SNRPB2	case	control	-0.138994879	0.194548462	-0.714448615	0.47494982	0.84579952	down_in_case

CRA004775	ENSGALG00010019264	HK2	case	control	-0.259770925	0.316844183	-0.819869636	0.412290428	0.817035725	down_in_case
CRA004775	ENSGALG00010019265	RUFY4	case	control	-0.731039259	0.28939553	-2.526090359	0.011533981	0.216528087	down_in_case
CRA004775	ENSGALG00010019266	ATP5S	case	control	-0.190212131	0.306417622	-0.620761072	0.534756839	0.870981055	down_in_case
CRA004775	ENSGALG00010019267	STARD5	case	control	0.216470908	0.278558766	0.77711038	0.43709365	0.827232725	up_in_case
CRA004775	ENSGALG00010019268	NSL1	case	control	-0.14888493	0.291132023	-0.511400045	0.609070964	0.90115757	down_in_case
CRA004775	ENSGALG00010019269	SOCS1	case	control	-0.053156721	0.311890239	-0.170434063	0.864668786	0.972350383	down_in_case
CRA004775	ENSGALG00010019270	ENSGALG00010019270	case	control	-0.243673616	0.43833403	-0.555908507	0.578273403	0.888378369	down_in_case
CRA004775	ENSGALG00010019271	CDKL1	case	control	-0.642591501	0.417002034	-1.540979298	0.123321824	0.575554958	down_in_case
CRA004775	ENSGALG00010019275	CTAGE1	case	control	3.069434517	0.956629909	3.20859142			up_in_case
CRA004775	ENSGALG00010019277	IGFBP5	case	control	0.820829088	0.285977682	2.870255753	0.004101399	0.131210386	up_in_case
CRA004775	ENSGALG00010019278	JPT2	case	control	-0.349113476	0.246368529	-1.417037626	0.156471927	0.620786344	down_in_case
CRA004775	ENSGALG00010019280	TLNRD1	case	control	0.174547072	0.185413551	0.941393288	0.346503352	0.784552667	up_in_case
CRA004775	ENSGALG00010019281	ENSGALG00010019281	case	control	2.650500025	0.834281751	3.176984301	0.001488151	0.080693373	up_in_case
CRA004775	ENSGALG00010019282	IGFBP2	case	control	-0.319546147	0.268146662	-1.191684224	0.233385086	0.700409935	down_in_case
CRA004775	ENSGALG00010019283	NT5C3A	case	control	0.355294847	0.317996672	1.117291088	0.263869885	0.723981064	up_in_case
CRA004775	ENSGALG00010019284	CHD5	case	control	0.194981901	0.195065014	0.999573922	0.317516749	0.766264094	up_in_case
CRA004775	ENSGALG00010019285	BATF3	case	control	-0.312381858	0.361277698	-0.86465857	0.387226215	0.803905475	down_in_case
CRA004775	ENSGALG00010019287	AHSA2	case	control	0.419670168	0.144607334	2.902136125	0.003706275	0.12600076	up_in_case
CRA004775	ENSGALG00010019288	RPL37A	case	control	-0.154164818	0.269032757	-0.573033635	0.56662192	0.883033678	down_in_case
CRA004775	ENSGALG00010019289	FKBP9	case	control	-0.291547078	0.234844475	-1.241447462	0.214440495	0.683562661	down_in_case
CRA004775	ENSGALG00010019291	ADD2	case	control	-0.162332745	0.367732759	-0.441442164	0.658892923	0.917791389	down_in_case
CRA004775	ENSGALG00010019292	ENSGALG00010019292	case	control	0.300172978	0.330276865	0.908852572	0.363427952	0.793608387	up_in_case
CRA004775	ENSGALG00010019294	SMARCAL1	case	control	0.056405742	0.204276896	0.276123942	0.782452872	0.94936527	up_in_case
CRA004775	ENSGALG00010019295	MAP4K5	case	control	0.151358936	0.333732398	0.45353384	0.650164382	0.915428088	up_in_case
CRA004775	ENSGALG00010019296	ENSGALG00010019296	case	control	0.116344491	0.253478373	0.458991784	0.646240066	0.914043203	up_in_case
CRA004775	ENSGALG00010019297	SLC25A26	case	control	-0.547690848	0.29349397	-1.866105961	0.062026542	0.447495793	down_in_case
CRA004775	ENSGALG00010019298	PGAP6	case	control	-0.425178069	0.250272558	-1.698860122	0.089345542	0.515997712	down_in_case
CRA004775	ENSGALG00010019300	MESDC2	case	control	0.106698149	0.142649108	0.747976281	0.454474464	0.838254622	up_in_case
CRA004775	ENSGALG00010019302	USP34	case	control	0.220531381	0.24081428	0.915773686	0.359785608	0.791966047	up_in_case
CRA004775	ENSGALG00010019305	SNRNP27	case	control	-0.021153439	0.247232607	-0.085560879	0.931815498	0.985044263	down_in_case
CRA004775	ENSGALG00010019307	ATF3	case	control	1.638088703	0.699343041	2.342325021	0.019164018	0.273365569	up_in_case
CRA004775	ENSGALG00010019310	CRELD1	case	control	-0.549671969	0.43143073	-1.274067726	0.202639415	0.671648534	down_in_case
CRA004775	ENSGALG00010019312	KBTBD2	case	control	0.218846211	0.195910152	1.11707438	0.263962524	0.723981064	up_in_case
CRA004775	ENSGALG00010019313	MRPL28	case	control	-0.329585768	0.257097416	-1.281948973	0.199860547	0.667128108	down_in_case
CRA004775	ENSGALG00010019314	GKN2	case	control	1.850266994	0.849347516	2.178456945	0.029372034	0.325984539	up_in_case
CRA004775	ENSGALG00010019315	ENSGALG00010019315	case	control	-0.186188135	0.682317916	-0.272875928	0.784948591	0.950244571	down_in_case
CRA004775	ENSGALG00010019316	CLEC16A	case	control	-0.009051224	0.303282037	-0.029844246	0.976191271	0.995591879	down_in_case
CRA004775	ENSGALG00010019318	NOG2	case	control	-0.426144858	0.60104421	-0.709007509	0.478319817	0.846836076	down_in_case
CRA004775	ENSGALG00010019319	ACTR3	case	control	-0.04509707	0.409064494	-0.110244401	0.91221555	0.98075825	down_in_case
CRA004775	ENSGALG00010019322	EGR3	case	control	0.568042365	0.423876822	1.340111879	0.180208975	0.644990528	up_in_case
CRA004775	ENSGALG00010019323	LRIG1	case	control	0.432222518	0.3373435	1.281253434	0.200104663	0.667171435	up_in_case
CRA004775	ENSGALG00010019325	SLITRK4	case	control	2.298491953	0.89103447	2.579576919	0.009892143	0.201255643	up_in_case
CRA004775	ENSGALG00010019331	CEMIP	case	control	-0.761981959	0.845844472	-0.900853508	0.367666213	0.794321403	down_in_case
CRA004775	ENSGALG00010019334	ATL1	case	control	-0.008698423	0.259324249	-0.033542652	0.973241854	0.994947809	down_in_case
CRA004775	ENSGALG00010019335	HIPK3	case	control	0.637439207	0.406129198	1.569547843	0.116520341	0.566086328	up_in_case
CRA004775	ENSGALG00010019336	DEXI	case	control	0.021389338	0.322276962	0.06636943	0.947083708	0.98933351	up_in_case
CRA004775	ENSGALG00010019337	XPO1	case	control	0.365924769	0.253901496	1.441207616	0.149526037	0.61366707	up_in_case
CRA004775	ENSGALG00010019344	DNAJC24	case	control	-0.234605887	0.327462543	-0.716435793	0.4737223	0.845445211	down_in_case
CRA004775	ENSGALG00010019345	ENSGALG00010019345	case	control	-0.049863839	0.338803596	-0.14717624	0.882992914	0.975577339	down_in_case
CRA004775	ENSGALG00010019346	POU3F4	case	control	0.849298714	0.568116575	1.494937398	0.134930781	0.59260082	up_in_case
CRA004775	ENSGALG00010019350	SAV1	case	control	0.216752801	0.172922475	1.25346807	0.210035412	0.67982426	up_in_case
CRA004775	ENSGALG00010019355	ENTPD4	case	control	-0.192380102	0.16637073	-1.156333823	0.247544639	0.712046495	down_in_case
CRA004775	ENSGALG00010019356	AVL9	case	control	-0.137550573	0.31344803	-0.438830554	0.660784319	0.917907174	down_in_case
CRA004775	ENSGALG00010019362	ENSGALG00010019362	case	control	0.745861045	0.619651307	1.203678643	0.22871381	0.695782399	up_in_case
CRA004775	ENSGALG00010019365	ENSGALG00010019365	case	control	0.338915486	0.636894025	0.532137959	0.594630449	0.895102715	up_in_case
CRA004775	ENSGALG00010019371	TMEM87A	case	control	0.050527345	0.16633266	0.303772844	0.761300957	0.942236668	up_in_case
CRA004775	ENSGALG00010019373	DENND5A	case	control	-0.253281736	0.305346929	-0.829488402	0.406828097	0.813842524	down_in_case
CRA004775	ENSGALG00010019376	ENSGALG00010019376	case	control	-0.452215059	0.486636724	-0.929266199	0.352751148	0.786426886	down_in_case
CRA004775	ENSGALG00010019388	GPR75	case	control	0.002615125	0.306476672	0.008532869	0.993191838	0.998606291	up_in_case
CRA004775	ENSGALG00010019390	TVP23A	case	control	0.1114024	0.442325265	0.251856289	0.801152147	0.953541667	up_in_case
CRA004775	ENSGALG00010019391	GANC	case	control	0.624017326	0.290212823	2.150205907	0.031538931	0.338029495	up_in_case
CRA004775	ENSGALG00010019392	NIN	case	control	0.118073692	0.437959949	0.269599293	0.787468545	0.95041797	up_in_case
CRA004775	ENSGALG00010019398	SLC35F5	case	control	0.269229204	0.2637543	1.02075759	0.307369302	0.759093979	up_in_case
CRA004775	ENSGALG00010019399	CD93	case	control	-0.261399145	0.393392357	-0.664474385	0.506386738	0.8622397	down_in_case
CRA004775	ENSGALG00010019403	PACC1	case	control	-0.425844326	0.318576931	-1.336707978	0.181317977	0.647378665	down_in_case
CRA004775	ENSGALG00010019411	LSM5	case	control	-0.166134278	0.303358095	-0.547650717	0.583931758	0.889908634	down_in_case
CRA004775	ENSGALG00010019418	ZNF703	case	control	-0.257818247	0.350292897	-0.736007634	0.461726052	0.842010079	down_in_case
CRA004775	ENSGALG00010019422	IMMP1L	case	control	-0.097081034	0.389707294	-0.249112693	0.803273611	0.954058041	down_in_case
CRA004775	ENSGALG00010019423	LYPD1	case	control	-0.26766783	0.357220217	-0.749307619	0.453671817	0.83804959	down_in_case
CRA004775	ENSGALG00010019426	GRM7	case	control	1.117899897	0.505498678	2.211479368	0.027002659	0.31493941	up_in_case
CRA004775	ENSGALG00010019430	ENSGALG00010019430	case	control	-0.689701874	0.281543478	-2.449717111	0.014296849	0.239125488	down_in_case
CRA004775	ENSGALG00010019441	LMCD1	case	control	0.603022964	0.351902483	1.713608152	0.086600715	0.509126309	up_in_case
CRA004775	ENSGALG00010019443	NCKAP5	case	control	1.05910993	0.430533739	2.459992875	0.013893977	0.235871708	up_in_case
CRA004775	ENSGALG00010019451	CAV3	case	control	-0.440807644	0.75288469	-0.585491576	0.558217229	0.880754991	down_in_case
CRA004775	ENSGALG00010019458	ELP4	case	control	-0.002557035	0.229409793	-0.011146144	0.991106848	0.998065748	down_in_case
CRA004775	ENSGALG00010019461	MGAT5	case	control	-0.512937143	0.508594515	-1.008538487	0.313196021	0.765098415	down_in_case
CRA004775	ENSGALG00010019470	TGFB2	case	control	-0.061363508	0.363289831	-0.168910613	0.865866951	0.972629493	down_in_case
CRA004775	ENSGALG00010019471	NUBP1	case	control	-0.456082083	0.221845161	-2.055857707	0.039796234	0.375850282	down_in_case
CRA004775	ENSGALG00010019472	CAPN3	case	control	0.61788203	0.4406466	1.402216721	0.16085054	0.626765592	up_in_case
CRA004775	ENSGALG00010019473	OXTR	case	control	-0.718555695	0.533742262	-1.346259696	0.178218779	0.644368088	down_in_case
CRA004775	ENSGALG00010019474	TMEM163	case	control	-1.145243467	1.221345123	-0.937690293	0.348403594	0.785270866	down_in_case
CRA004775	ENSGALG00010019477	NCAPH	case	control	0.187550682	0.441013185	0.42527228	0.670638199	0.921535334	up_in_case
CRA004775	ENSGALG00010019481	PYGL	case	control	-0.542156821	0.27578295	-1.965882306	0.049312207	0.410866536	down_in_case
CRA004775	ENSGALG00010019483	SCUBE2	case	control	0.087956218	0.609208771	0.144377794	0.885202151	0.976602443	up_in_case
CRA004775	ENSGALG00010019495	EMP2	case	control	0.728852792	0.193209311	3.772348185	0.000161718	0.027119381	up_in_case
CRA004775	ENSGALG00010019498	ENSGALG00010019498	case	control	-1.16151667	0.654614704	-1.774351634	0.076005005	0.484761048	down_in_case
CRA004775	ENSGALG00010019509	ZNF106	case	control	0.765593821	0.528600075	1.448342249	0.147521357	0.610831526	up_in_case
CRA004775	ENSGALG00010019515	ENSGALG00010019515	case	control	0.004537146	0.155212361	0.02923186	0.976679672	0.99560196	up_in_case
CRA004775	ENSGALG00010019520	CD6	case	control	-0.733417396	0.61983576	-1.18324473	0.23671217	0.703637928	down_in_case
CRA004775	ENSGALG00010019524	HHIPL2	case	control	0.396177899	0.557546156	0.710574172	0.477348153	0.846532686	up_in_case
CRA004775	ENSGALG00010019529	SNAP23	case	control	0.161604586	0.18274058	0.884338804	0.376513347	0.798523018	up_in_case
CRA004775	ENSGALG00010019534	PPP2R5A	case	control	0.162846455	0.28130653	0.578893261	0.562661198	0.881532201	up_in_case
CRA004775	ENSGALG00010019540	UNC5D	case	control	-0.010302822	0.700172694	-0.014714687	0.988259802	0.998065748	down_in_case
CRA004775	ENSGALG00010019546	HSD17B12	case	control	-0.137172648	0.186989064	-0.733586475	0.463200806	0.842456502	down_in_case
CRA004775	ENSGALG00010019550	ACMSD	case	control	-0.889933995	0.641967926	-1.386259281	0.165667746	0.630194031	down_in_case
CRA004775	ENSGALG00010019557	TRIM9	case	control	0.217019087	0.373196344	0.581514503	0.560893747	0.881008373	up_in_case
CRA004775	ENSGALG00010019572	NFKBIE	case	control	-0.33211454	0.29009311	-1.144854974	0.252269238	0.717019836	down_in_case
CRA004775	ENSGALG00010019577	MXD1	case	control	-0.188495756	0.191721909	-0.983172749	0.325522424	0.770440423	down_in_case
CRA004775	ENSGALG00010019580	CD5	case	control	-0.996698587	0.565237651	-1.763326603	0.077845421	0.49028362	down_in_case
CRA004775	ENSGALG00010019581	SLC35B2	case	control	-0.660641185	0.169897401	-3.888471413	0.000100878	0.022373664	down_in_case
CRA004775	ENSGALG00010019584	RAD18	case	control	-0.280382345	0.250829395	-1.117820919	0.263643485	0.723981064	down_in_case
CRA004775	ENSGALG00010019585	PDE1C	case	control	-0.323883626	0.412703661	-0.78478496	0.43257966	0.825615327	down_in_case
CRA004775	ENSGALG00010019586	GLYR1	case	control	-0.218266244	0.195527891	-1.116292121	0.264297113	0.724455452	down_in_case
CRA004775	ENSGALG00010019587	CCNT2	case	control	-0.215746406	0.306522026	-0.703852866	0.481524407	0.848818238	down_in_case
CRA004775	ENSGALG00010019588	CHMP7	case	control	-0.25775269	0.301004787	-0.85630761	0.391827653	0.80746892	down_in_case
CRA004775	ENSGALG00010019591	ENSGALG00010019591	case	control	-0.177500485	0.378680117	-0.468734631	0.63925933	0.911337928	down_in_case
CRA004775	ENSGALG00010019592	ALKBH3	case	control	-0.13251887	0.270783149	-0.489391124	0.624564819	0.90646861	down_in_case
CRA004775	ENSGALG00010019594	TMX1	case	control	-0.089592333	0.47381982	-0.189085237	0.850026012	0.968570409	down_in_case
CRA004775	ENSGALG00010019595	GRIN2A	case	control	0.448161914	1.102080891	0.406650653	0.684264589	0.925605936	up_in_case
CRA004775	ENSGALG00010019598	KCNAB2	case	control	-0.670483299	0.408595507	-1.640946334	0.100808556	0.539174815	down_in_case
CRA004775	ENSGALG00010019602	SLC29A1	case	control	-0.318509027	0.263115374	-1.210529899	0.226075625	0.694166578	down_in_case
CRA004775	ENSGALG00010019610	CORO7	case	control	-0.28888042	0.255770765	-1.12945051	0.258707836	0.720037471	down_in_case
CRA004775	ENSGALG00010019613	FRMD6	case	control	-0.235685574	0.357922545	-0.658482059	0.510228422	0.863170205	down_in_case
CRA004775	ENSGALG00010019614	MICALL2	case	control	-0.156746263	0.35500587	-0.441531469	0.658828285	0.917791389	down_in_case
CRA004775	ENSGALG00010019617	ANO3	case	control	-0.116251163	0.372609133	-0.311992253	0.755046411	0.941900693	down_in_case
CRA004775	ENSGALG00010019618	NPHP4	case	control	-0.500257583	0.22588523	-2.2146538	0.026783836	0.314790198	down_in_case
CRA004775	ENSGALG00010019621	ENSGALG00010019621	case	control	-0.208483761	0.406404738	-0.512995401	0.607954538	0.900943922	down_in_case
CRA004775	ENSGALG00010019622	C2orf42	case	control	-0.013183583	0.207243603	-0.063613944	0.949277629	0.990168609	down_in_case
CRA004775	ENSGALG00010019624	SRGAP3	case	control	-0.528309005	0.328609764	-1.607709397	0.10789884	0.55424615	down_in_case
CRA004775	ENSGALG00010019625	INTS1	case	control	-0.282678516	0.229127402	-1.233717637	0.21730814	0.68458287	down_in_case
CRA004775	ENSGALG00010019630	AJAP1	case	control	0.262126889	0.553049754	0.473966198	0.635524011	0.909837348	up_in_case
CRA004775	ENSGALG00010019634	GNG2	case	control	-0.371872908	0.450078063	-0.82624091	0.408667446	0.814384687	down_in_case
CRA004775	ENSGALG00010019635	LRRC57	case	control	-0.021968932	0.216306372	-0.10156396	0.919102787	0.982018836	down_in_case
CRA004775	ENSGALG00010019640	MGRN1	case	control	-0.119350813	0.198843952	-0.600223503	0.548357292	0.877614191	down_in_case
CRA004775	ENSGALG00010019641	CAPN11	case	control	-0.403305304	0.250594483	-1.609394186	0.107530176	0.553800541	down_in_case
CRA004775	ENSGALG00010019643	RAB3GAP1	case	control	-0.16828099	0.144650329	-1.163364034	0.244681835	0.711765017	down_in_case

CRA004775	ENSGALG00010019645	ENSGALG00010019645	case	control	-0.198077978	0.301771705	-0.656383534	0.511577382	0.863170205	down_in_case
CRA004775	ENSGALG00010019646	VPS37C	case	control	-0.029019563	0.369447446	-0.078548554	0.937391709	0.986688955	down_in_case
CRA004775	ENSGALG00010019648	PRDM16	case	control	0.564891212	0.388398891	1.454409952	0.145832683	0.609044185	up_in_case
CRA004775	ENSGALG00010019649	TMEM63B	case	control	0.152386199	0.271475685	0.561325408	0.574575724	0.887223154	up_in_case
CRA004775	ENSGALG00010019650	KBTBD8	case	control	-0.16210344	0.329429592	-0.492073099	0.622667668	0.906338268	down_in_case
CRA004775	ENSGALG00010019652	TNFRSF10B	case	control	-0.36435937	0.51550132	-0.706805891	0.479687113	0.847527617	down_in_case
CRA004775	ENSGALG00010019655	RTRAF	case	control	0.071041953	0.338161851	0.210082696	0.833603131	0.964019688	up_in_case
CRA004775	ENSGALG00010019657	PPP1R17	case	control	0.21112631	0.392775651	0.537523926	0.590905763	0.893804699	up_in_case
CRA004775	ENSGALG00010019659	MRPL14	case	control	0.026284136	0.31211521	0.084212928	0.932887139	0.985090297	up_in_case
CRA004775	ENSGALG00010019667	ITPRID1	case	control	-2.077491012	1.108289395	-1.874502293	0.060861226	0.445397937	down_in_case
CRA004775	ENSGALG00010019670	ENSGALG00010019670	case	control	-0.062301148	0.205613185	-0.303001716	0.761888554	0.942236668	down_in_case
CRA004775	ENSGALG00010019673	NID2	case	control	0.265837267	0.402472315	0.660510692	0.50892616	0.862971569	up_in_case
CRA004775	ENSGALG00010019675	MAFK	case	control	0.054040574	0.141378961	0.382239149	0.702283986	0.930068722	up_in_case
CRA004775	ENSGALG00010019679	RHOBTB2	case	control	-0.042081052	0.270984374	-0.155289591	0.876593021	0.974479909	down_in_case
CRA004775	ENSGALG00010019680	CCDC34	case	control	0.158900164	0.339020984	0.468703034	0.639281918	0.911337928	up_in_case
CRA004775	ENSGALG00010019682	TMEM158	case	control	1.223396465	0.515585163	2.372831013	0.017652338	0.264131992	up_in_case
CRA004775	ENSGALG00010019683	TMEM184A	case	control	-0.07452653	0.331262858	-0.224977017	0.821997154	0.960766569	down_in_case
CRA004775	ENSGALG00010019684	PIGQ	case	control	-0.227588351	0.226994636	-1.002615548	0.316046391	0.765806859	down_in_case
CRA004775	ENSGALG00010019690	ENSGALG00010019690	case	control	-0.19925988	0.339682085	-0.586606974	0.557467697	0.88071442	down_in_case
CRA004775	ENSGALG00010019692	MMEL1	case	control	1.105891997	0.520642823	2.124089582	0.033662653	0.348565623	up_in_case
CRA004775	ENSGALG00010019693	HAUS2	case	control	-0.141144223	0.222557189	-0.634193054	0.52595484	0.867878941	down_in_case
CRA004775	ENSGALG00010019697	EIF4EBP1	case	control	-0.249529279	0.358197503	-0.696624841	0.486037606	0.851645437	down_in_case
CRA004775	ENSGALG00010019700	HES5B	case	control	-0.152738649	0.806455794	-0.189394943	0.849783288	0.968413719	down_in_case
CRA004775	ENSGALG00010019706	NPRL3	case	control	-0.207896396	0.218510574	-0.951424878	0.341388736	0.781234235	down_in_case
CRA004775	ENSGALG00010019713	NUDT14	case	control	-0.254308619	0.215088823	-1.182342326	0.237069897	0.703871614	down_in_case
CRA004775	ENSGALG00010019716	VEGFA	case	control	0.293731887	0.238912091	1.229455929	0.218900912	0.686455288	up_in_case
CRA004775	ENSGALG00010019717	ZRANB3	case	control	0.095453316	0.306465503	0.311465123	0.755447053	0.941900693	up_in_case
CRA004775	ENSGALG00010019726	PEBP4	case	control	-0.431057999	0.672778906	-0.640712715	0.521709352	0.866323547	down_in_case
CRA004775	ENSGALG00010019731	PANK4	case	control	-0.171042198	0.268107664	-0.637960865	0.523499155	0.866638286	down_in_case
CRA004775	ENSGALG00010019732	HBA1	case	control	-0.280556881	0.282776504	-0.992150611	0.321124061	0.767427349	down_in_case
CRA004775	ENSGALG00010019735	ENSGALG00010019735	case	control	0.151438077	0.396955163	0.3814992	0.702832865	0.930068722	up_in_case
CRA004775	ENSGALG00010019738	ADRA2A	case	control	-0.028475287	0.621064316	-0.045849176	0.963430463	0.992220813	down_in_case
CRA004775	ENSGALG00010019743	KCNU1	case	control	-0.473207958	0.773845872	-0.611501561	0.540867586	0.874592283	down_in_case
CRA004775	ENSGALG00010019752	GPAM	case	control	-0.195427067	0.296153094	-0.659885279	0.509327452	0.862971569	down_in_case
CRA004775	ENSGALG00010019761	MRPS18A	case	control	-0.369341226	0.266955084	-1.383533216	0.16650143	0.630194031	down_in_case
CRA004775	ENSGALG00010019767	SUCLG2	case	control	-0.011465872	0.264931251	-0.043278668	0.965479396	0.993094598	down_in_case
CRA004775	ENSGALG00010019771	VWCE	case	control	0.561994928	0.645200642	0.87103901	0.383732854	0.802541516	up_in_case
CRA004775	ENSGALG00010019772	ENSGALG00010019772	case	control	-0.424934561	0.264207123	-1.608338778	0.107761002	0.55406723	down_in_case
CRA004775	ENSGALG00010019778	ABHD17C	case	control	0.44471444	0.250107103	1.778096	0.075388089	0.483342345	up_in_case
CRA004775	ENSGALG00010019785	ACSL5	case	control	0.317523042	0.541646443	0.586218273	0.557728844	0.880722248	up_in_case
CRA004775	ENSGALG00010019786	R3HDM1	case	control	0.485189301	0.22022767	2.203125978	0.027585871	0.317168318	up_in_case
CRA004775	ENSGALG00010019789	ARNT2	case	control	0.137047334	0.381752661	0.358995098	0.719598756	0.93407508	up_in_case
CRA004775	ENSGALG00010019794	RSPH9	case	control	-0.266904867	0.483951095	-0.551512062	0.5812827	0.888998848	down_in_case
CRA004775	ENSGALG00010019799	DOK2	case	control	-1.190219049	0.619331485	-1.921780303	0.054633408	0.430254604	down_in_case
CRA004775	ENSGALG00010019800	EXT2	case	control	0.113233428	0.235933344	0.479938217	0.631271326	0.909357765	up_in_case
CRA004775	ENSGALG00010019801	FIBIN	case	control	-0.083990288	0.360532939	-0.232961482	0.815791316	0.959044745	down_in_case
CRA004775	ENSGALG00010019803	FAH	case	control	-0.458091542	0.669520892	-0.684207987	0.493843839	0.856049389	down_in_case
CRA004775	ENSGALG00010019804	ELFN1	case	control	0.363229391	0.498808627	0.728193883	0.46649491	0.8434807	up_in_case
CRA004775	ENSGALG00010019807	ZDHHC6	case	control	-0.023303147	0.291720358	-0.079881799	0.936331266	0.986325359	down_in_case
CRA004775	ENSGALG00010019810	ZFAND6	case	control	0.014262911	0.284403396	0.050150282	0.960002631	0.991431439	up_in_case
CRA004775	ENSGALG00010019811	UBXN4	case	control	-0.138977683	0.328818928	-0.422657186	0.672545397	0.921952322	down_in_case
CRA004775	ENSGALG00010019813	ENSGALG00010019813	case	control	-0.37692391	0.339430964	-1.110458238	0.266801615	0.727315996	down_in_case
CRA004775	ENSGALG00010019816	ENSGALG00010019816	case	control	0.321516513	0.624599282	0.514756457	0.606723217	0.900898526	up_in_case
CRA004775	ENSGALG00010019820	BBOX1	case	control	0.103557209	0.417086226	0.24828729	0.803912132	0.954381053	up_in_case
CRA004775	ENSGALG00010019823	LCT	case	control	0.742798036	0.357941457	2.075194202	0.03796854	0.367679852	up_in_case
CRA004775	ENSGALG00010019825	BCL2A1	case	control	-0.775276251	0.409692207	-1.892338293	0.058445922	0.443048442	down_in_case
CRA004775	ENSGALG00010019830	JAG2	case	control	0.278290269	0.289695933	0.960628842	0.336738822	0.777081307	up_in_case
CRA004775	ENSGALG00010019832	MAD2L1BP	case	control	-0.608072347	0.459810721	-1.322440557	0.186021491	0.6491093	down_in_case
CRA004775	ENSGALG00010019833	MTHFS	case	control	-0.1302596	0.346599035	-0.375822165	0.707049109	0.931617022	down_in_case
CRA004775	ENSGALG00010019834	PLCH2	case	control	1.273073906	0.43839571	2.903937871	0.003685013	0.12600076	up_in_case
CRA004775	ENSGALG00010019838	ALX4	case	control	1.444716014	0.452539248	3.192465671	0.001410637	0.078533081	up_in_case
CRA004775	ENSGALG00010019843	MCM6	case	control	-0.085144303	0.274019594	-0.310723413	0.756010897	0.941900693	down_in_case
CRA004775	ENSGALG00010019846	ENSGALG00010019846	case	control	-0.269263491	0.18131936	-1.485023389	0.137537629	0.596242097	down_in_case
CRA004775	ENSGALG00010019848	MAD1L1	case	control	-0.348703449	0.172016931	-2.027146084	0.042647475	0.388066397	down_in_case
CRA004775	ENSGALG00010019854	CCDC14	case	control	-0.257436026	0.220157914	-1.16932442	0.242272948	0.709690398	down_in_case
CRA004775	ENSGALG00010019855	TTC7A	case	control	-0.086772719	0.405515458	-0.213981284	0.830561652	0.963232966	down_in_case
CRA004775	ENSGALG00010019859	BIN3	case	control	-0.447857914	0.159470411	-2.808407605	0.004978717	0.142684328	down_in_case
CRA004775	ENSGALG00010019865	ENSGALG00010019865	case	control	0.083514278	0.297664081	0.28056552	0.779043665	0.94792594	up_in_case
CRA004775	ENSGALG00010019867	PEX11A	case	control	-0.65797467	0.553696908	-1.188330044	0.234703406	0.702145501	down_in_case
CRA004775	ENSGALG00010019872	VTI1A	case	control	-0.170048272	0.214320321	-0.793430466	0.427527066	0.822776983	down_in_case
CRA004775	ENSGALG00010019873	LGR4	case	control	0.21020439	0.29919357	0.702569877	0.48232384	0.849435835	up_in_case
CRA004775	ENSGALG00010019875	PLEKHH1	case	control	-1.812829908	0.855611387	-2.118753835	0.034111276	0.35073164	down_in_case
CRA004775	ENSGALG00010019876	MRM2	case	control	0.290065948	0.325728198	0.890515314	0.373189249	0.796804495	up_in_case
CRA004775	ENSGALG00010019878	MCFD2	case	control	0.120231228	0.20295356	0.592407582	0.553577691	0.878871229	up_in_case
CRA004775	ENSGALG00010019891	PIGH	case	control	-0.29409325	0.288924594	-1.017889291	0.308730572	0.761225406	down_in_case
CRA004775	ENSGALG00010019896	DDB1	case	control	-0.226742184	0.216547108	-1.047080176	0.295062602	0.747369418	down_in_case
CRA004775	ENSGALG00010019898	NUDT1	case	control	-0.141386867	0.371521343	-0.380561896	0.70352836	0.93012388	down_in_case
CRA004775	ENSGALG00010019899	ENSGALG00010019899	case	control	-0.317669598	0.599405483	-0.529974461	0.596129638	0.895574418	down_in_case
CRA004775	ENSGALG00010019905	ENSGALG00010019905	case	control	0.372657509	0.222282991	1.676500333	0.093640231	0.525355698	up_in_case
CRA004775	ENSGALG00010019906	ENSGALG00010019906	case	control	0.513073946	0.351980565	1.457676921	0.144929616	0.607785042	up_in_case
CRA004775	ENSGALG00010019910	ENSGALG00010019910	case	control	-0.303048109	0.188996997	-1.603454623	0.10883432	0.555936379	down_in_case
CRA004775	ENSGALG00010019912	ARG2	case	control	0.48845561	0.65040056	0.751007364	0.452648224	0.837170912	up_in_case
CRA004775	ENSGALG00010019916	NOC3L	case	control	0.100700604	0.28619458	0.351860626	0.724942789	0.935238044	up_in_case
CRA004775	ENSGALG00010019919	LIN7C	case	control	0.329000789	0.181501365	1.812662891	0.069883831	0.469724266	up_in_case
CRA004775	ENSGALG00010019921	CALM2	case	control	0.287595206	0.347756793	0.827000973	0.40823651	0.814384687	up_in_case
CRA004775	ENSGALG00010019925	RDH11	case	control	0.000626754	0.40993826	0.001528898	0.998780116	0.999914248	up_in_case
CRA004775	ENSGALG00010019926	KIF7	case	control	-0.402987489	0.146526609	-2.750268312	0.005954648	0.154441997	down_in_case
CRA004775	ENSGALG00010019931	ZFYVE26	case	control	0.013138379	0.370071751	0.035502249	0.971679253	0.994947809	up_in_case
CRA004775	ENSGALG00010019932	KALRN	case	control	-0.101144033	0.210694909	-0.480049727	0.631192035	0.909357765	down_in_case
CRA004775	ENSGALG00010019934	TKFC	case	control	0.089345625	0.254556275	0.35098575	0.725599039	0.935238044	up_in_case
CRA004775	ENSGALG00010019935	ENSGALG00010019935	case	control	1.762952172	1.134640965	1.553753325	0.120243268	0.572133279	up_in_case
CRA004775	ENSGALG00010019947	ENSGALG00010019947	case	control	-0.58403659	0.50432492	-1.158056179	0.246841111	0.712046495	down_in_case
CRA004775	ENSGALG00010019948	TICRR	case	control	-0.008233093	0.301885866	-0.027272203	0.978242627	0.996209314	down_in_case
CRA004775	ENSGALG00010019954	CYB561A3	case	control	0.176390485	0.299189407	0.589561265	0.555484827	0.87981923	up_in_case
CRA004775	ENSGALG00010019956	SLC29A3	case	control	-0.166503192	0.410927678	-0.405188554	0.685338918	0.926656387	down_in_case
CRA004775	ENSGALG00010019961	HTATSF1	case	control	0.104507459	0.213308797	0.489935065	0.624179849	0.906441769	up_in_case
CRA004775	ENSGALG00010019962	SNX8	case	control	-0.416744485	0.253115992	-1.646456556	0.099669797	0.537098329	down_in_case
CRA004775	ENSGALG00010019967	CEP170B	case	control	0.304939821	0.264862831	1.151312248	0.2496038	0.714467496	up_in_case
CRA004775	ENSGALG00010019972	PPP1CB	case	control	-0.141841346	0.20892361	-0.678914872	0.497191802	0.857772041	down_in_case
CRA004775	ENSGALG00010019975	ENSGALG00010019975	case	control	-0.06977101	0.320743138	-0.217529238	0.827795924	0.962251207	down_in_case
CRA004775	ENSGALG00010019977	ZMIZ1	case	control	-0.001053642	0.310413774	-0.003394314	0.997291734	0.999619266	down_in_case
CRA004775	ENSGALG00010019982	PLCE1	case	control	0.180584796	0.421245275	0.428692751	0.668146836	0.919688403	up_in_case
CRA004775	ENSGALG00010019985	UMPS	case	control	-0.283358279	0.280129616	-1.011525603	0.311764934	0.763232972	down_in_case
CRA004775	ENSGALG00010019989	ENSGALG00010019989	case	control	0.28387864	0.507718478	0.559126075	0.57607568	0.887937128	up_in_case
CRA004775	ENSGALG00010019990	MARCHF8	case	control	0.133561706	0.297101017	0.44954981	0.653035084	0.916442743	up_in_case
CRA004775	ENSGALG00010019991	EIF3B	case	control	-0.072366311	0.163607483	-0.442316631	0.658260098	0.917354438	down_in_case
CRA004775	ENSGALG00010019992	CHAC2	case	control	-0.104371603	0.299291565	-0.348728849	0.727292884	0.935420324	down_in_case
CRA004775	ENSGALG00010019994	PLD4	case	control	-0.202483926	0.308970321	-0.655350731	0.512241965	0.863263816	down_in_case
CRA004775	ENSGALG00010019996	ITGB5	case	control	0.138923158	0.28313445	0.490661442	0.623665922	0.906441769	up_in_case
CRA004775	ENSGALG00010019997	FSHR	case	control	1.62340356	0.992525726	1.635628697	0.101917321	0.541943185	up_in_case
CRA004775	ENSGALG00010019998	PLEK2	case	control	1.9061647	0.645246823	2.954163635	0.003135177	0.119423328	up_in_case
CRA004775	ENSGALG00010019999	ERLIN1	case	control	-0.333168005	0.12618289	-2.640358017	0.008281849	0.183215969	down_in_case
CRA004775	ENSGALG00010020003	ENSGALG00010020003	case	control	-0.035940093	0.534401003	-0.067253041	0.94638026	0.989106556	down_in_case
CRA004775	ENSGALG00010020005	EOGT	case	control	0.420124545	0.306582451	1.370347662	0.170578402	0.63463842	up_in_case
CRA004775	ENSGALG00010020006	PLB1	case	control	1.09613946	0.646024835	1.696745078	0.089744866	0.516522282	up_in_case
CRA004775	ENSGALG00010020008	EIF2S1	case	control	-0.031289959	0.199472604	-0.156863443	0.875352472	0.974412785	down_in_case
CRA004775	ENSGALG00010020009	MACROD2	case	control	0.338084864	0.238957714	1.414831344	0.157117952	0.621633948	up_in_case
CRA004775	ENSGALG00010020010	SORBS3	case	control	0.246916884	0.342753823	0.720391334	0.471284084	0.845200296	up_in_case
CRA004775	ENSGALG00010020011	TLE3	case	control	-0.259114758	0.238248341	-1.08758263	0.276779402	0.731499819	down_in_case
CRA004775	ENSGALG00010020013	ADAM8	case	control	0.05514884	0.599549221	0.091983841	0.926710878	0.984182984	up_in_case
CRA004775	ENSGALG00010020015	ATP6V1D	case	control	-0.145690604	0.245174622	-0.594231992	0.552356958	0.878258849	down_in_case
CRA004775	ENSGALG00010020017	ENSGALG00010020017	case	control	0.555011891	0.342882206	1.618666356	0.105519069	0.549651422	up_in_case
CRA004775	ENSGALG00010020018	ENSGALG00010020018	case	control	-1.926036666	0.905675288	-2.126630473	0.033450798	0.348565623	down_in_case
CRA004775	ENSGALG00010020019	PEX10	case	control	-0.020759714	0.293584209	-0.070711276	0.943627547	0.987862092	down_in_case
CRA004775	ENSGALG00010020023	FOSL2	case	control	-0.206144163	0.460287098	-0.447859964	0.654254264	0.916560183	down_in_case
CRA004775	ENSGALG00010020025	LOXL2	case	control	0.585408652	0.467580682	1.25199495	0.210571704	0.680684888	up_in_case

CRA004775	ENSGALG00010020027	RER1	case	control	-0.029234647	0.190302594	-0.153621903	0.877907866	0.974657764	down_in_case
CRA004775	ENSGALG00010020030	MRPL33	case	control	-0.261264995	0.332182377	-0.786510703	0.431568351	0.825262449	down_in_case
CRA004775	ENSGALG00010020032	CHST12	case	control	-0.285517498	0.275853134	-1.035034455	0.30065282	0.753217716	down_in_case
CRA004775	ENSGALG00010020036	TMF1	case	control	0.353009105	0.251117844	1.405750777	0.159798143	0.624907945	up_in_case
CRA004775	ENSGALG00010020037	MPP5	case	control	0.618075642	0.239061735	2.585422723	0.009725967	0.199912967	up_in_case
CRA004775	ENSGALG00010020038	SKI	case	control	0.14205136	0.426257091	0.333252779	0.738943481	0.938064864	up_in_case
CRA004775	ENSGALG00010020041	CDH23	case	control	0.197645047	0.406483652	0.486231231	0.62680323	0.90758663	up_in_case
CRA004775	ENSGALG00010020046	BDNF	case	control	0.076424853	0.684628465	0.111629675	0.911117042	0.98075825	up_in_case
CRA004775	ENSGALG00010020048	ENSGALG00010020048	case	control	0.185075973	0.440360798	0.42028258	0.674279034	0.922533825	up_in_case
CRA004775	ENSGALG00010020053	LFNG	case	control	-0.002722297	0.243902684	-0.011161408	0.99109467	0.998065748	down_in_case
CRA004775	ENSGALG00010020055	R3HCC1	case	control	-0.235930928	0.154363502	-1.528411344	0.12641044	0.580747947	down_in_case
CRA004775	ENSGALG00010020056	UACA	case	control	0.192110586	0.409002597	0.469705051	0.638565759	0.91099914	up_in_case
CRA004775	ENSGALG00010020057	RBKS	case	control	-0.620231905	0.251100063	-2.470058741	0.013509087	0.232945019	down_in_case
CRA004775	ENSGALG00010020059	PRKCZ	case	control	0.264555574	0.203952478	1.297143217	0.194581914	0.659287597	up_in_case
CRA004775	ENSGALG00010020061	UBA3	case	control	0.029500114	0.196353226	0.150240027	0.880575248	0.974996282	up_in_case
CRA004775	ENSGALG00010020063	ENSGALG00010020063	case	control	-0.672202507	0.39379203	-1.706998763	0.087822282	0.512706568	down_in_case
CRA004775	ENSGALG00010020064	TMEM138	case	control	-0.326888278	0.188214518	-1.736785668	0.08242501	0.499148629	down_in_case
CRA004775	ENSGALG00010020065	TTYH3	case	control	-0.340681753	0.54496243	-0.625147229	0.531874433	0.86986267	down_in_case
CRA004775	ENSGALG00010020066	ENSGALG00010020066	case	control	-0.093023799	0.477749813	-0.194712371	0.845618127	0.966993336	down_in_case
CRA004775	ENSGALG00010020069	TMEM216	case	control	0.023325473	0.275000654	0.084819701	0.932404731	0.985044263	up_in_case
CRA004775	ENSGALG00010020070	ARL6IP5	case	control	-0.255403808	0.2076024	-1.230254603	0.218601778	0.686309227	down_in_case
CRA004775	ENSGALG00010020071	ENSGALG00010020071	case	control	-0.125289402	0.293425209	-0.426989224	0.669387176	0.920750531	down_in_case
CRA004775	ENSGALG00010020074	ENSGALG00010020074	case	control	-0.637219084	0.240361831	-2.651082666	0.00802342	0.181165927	down_in_case
CRA004775	ENSGALG00010020075	CPSF7	case	control	-0.384320057	0.118893339	-3.232477621	0.001227217	0.073691998	down_in_case
CRA004775	ENSGALG00010020080	SLC4A1AP	case	control	-0.132579229	0.188027707	-0.705104748	0.480745052	0.848616843	down_in_case
CRA004775	ENSGALG00010020084	ENSGALG00010020084	case	control	0.075813097	0.32411745	0.233906248	0.815057763	0.958870945	up_in_case
CRA004775	ENSGALG00010020087	ENSGALG00010020087	case	control	1.99307342	0.518358183	3.844973391	0.000120566	0.023828766	up_in_case
CRA004775	ENSGALG00010020088	LMOD3	case	control	-0.074992923	0.612584386	-0.12242056	0.902565957	0.979310476	down_in_case
CRA004775	ENSGALG00010020089	CASP14	case	control	-0.421088888	0.770365381	-0.546609309	0.584647173	0.890308225	down_in_case
CRA004775	ENSGALG00010020090	MORF4L2	case	control	0.097835453	0.19858818	0.492654968	0.622256402	0.906040637	up_in_case
CRA004775	ENSGALG00010020091	SUPT7L	case	control	-0.137747779	0.29442235	-0.467857753	0.639886317	0.911453315	down_in_case
CRA004775	ENSGALG00010020092	SDHAF2	case	control	-0.048621114	0.274935556	-0.176845492	0.859629755	0.971325143	down_in_case
CRA004775	ENSGALG00010020094	CLEC3B	case	control	-0.150928395	0.260066983	-0.580344315	0.561682449	0.881532201	down_in_case
CRA004775	ENSGALG00010020096	GPHN	case	control	0.173724045	0.366255469	0.474324782	0.635268322	0.909837348	up_in_case
CRA004775	ENSGALG00010020097	RETSAT	case	control	-0.518823638	0.413943993	-1.253366751	0.210072266	0.67982426	down_in_case
CRA004775	ENSGALG00010020098	GABRD	case	control	1.335856977	0.489300529	2.730135973	0.006330821	0.158894029	up_in_case
CRA004775	ENSGALG00010020100	MOCS1	case	control	0.209177569	0.343201849	0.609488467	0.542200715	0.875128455	up_in_case
CRA004775	ENSGALG00010020101	KIF15	case	control	-0.064174919	0.695678374	-0.092247972	0.926501024	0.984182984	down_in_case
CRA004775	ENSGALG00010020102	TMEM127	case	control	-0.581146648	0.272061314	-2.136087048	0.032672319	0.344802044	down_in_case
CRA004775	ENSGALG00010020103	PSAP	case	control	-0.357964733	0.31566237	-1.13401142	0.256789758	0.719236059	down_in_case
CRA004775	ENSGALG00010020104	BRAT1	case	control	-0.250560614	0.204054521	-1.227910132	0.219480706	0.687020074	down_in_case
CRA004775	ENSGALG00010020106	ENSGALG00010020106	case	control	-0.159067767	0.416849646	-0.381595063	0.702761747	0.930068722	down_in_case
CRA004775	ENSGALG00010020108	RPAP1	case	control	-0.271058819	0.250380613	-1.082587092	0.278991739	0.735227176	down_in_case
CRA004775	ENSGALG00010020109	WDFY4	case	control	0.253886335	0.5580159	0.454980467	0.649123291	0.915047477	up_in_case
CRA004775	ENSGALG00010020111	ADRA2B	case	control	-0.035441735	0.275031943	-0.128864068	0.897465209	0.978790377	down_in_case
CRA004775	ENSGALG00010020113	CIAO1	case	control	-0.402309349	0.265766774	-1.513768421	0.130084585	0.584671406	down_in_case
CRA004775	ENSGALG00010020116	GNA12	case	control	0.183135484	0.374135226	0.489490085	0.624494772	0.90646861	up_in_case
CRA004775	ENSGALG00010020117	AKAP13	case	control	-0.542118649	0.461187456	-1.175484376	0.239800985	0.706254731	down_in_case
CRA004775	ENSGALG00010020118	GNB1	case	control	0.07305691	0.232906767	0.313674483	0.753768283	0.941900693	up_in_case
CRA004775	ENSGALG00010020120	DAAM2	case	control	-0.011672061	0.383410347	-0.030442738	0.975713961	0.995255743	down_in_case
CRA004775	ENSGALG00010020121	ENSGALG00010020121	case	control	-0.167688508	0.639473538	-0.262229002	0.793144895	0.951455592	down_in_case
CRA004775	ENSGALG00010020123	ENSGALG00010020123	case	control	-0.007195213	0.411959774	-0.017465815	0.986065004	0.997562105	down_in_case
CRA004775	ENSGALG00010020125	NAV2	case	control	-0.00551333	0.388726363	-0.014183063	0.988683932	0.998065748	down_in_case
CRA004775	ENSGALG00010020126	NADK	case	control	-0.050486265	0.41424596	-0.121875094	0.90299794	0.979310476	down_in_case
CRA004775	ENSGALG00010020127	FRMD4B	case	control	0.135242737	0.359228465	0.376481126	0.706559245	0.931533669	up_in_case
CRA004775	ENSGALG00010020129	MGAT2	case	control	-0.178344883	0.16326228	-1.092382654	0.274664944	0.731001618	down_in_case
CRA004775	ENSGALG00010020130	PDXDC1	case	control	-0.094251063	0.184308217	-0.511377435	0.609086793	0.90115757	down_in_case
CRA004775	ENSGALG00010020131	SNRNP200	case	control	-0.309629275	0.166488944	-1.85975878	0.062919662	0.450394725	down_in_case
CRA004775	ENSGALG00010020132	SHC4	case	control	0.474616846	0.376966736	1.259041717	0.208015264	0.676600102	up_in_case
CRA004775	ENSGALG00010020136	KCNK17	case	control	0.362557024	0.430510557	0.842155942	0.399700671	0.811379381	up_in_case
CRA004775	ENSGALG00010020137	NTAN1	case	control	-0.126749577	0.234778408	-0.539868969	0.589287399	0.893412703	down_in_case
CRA004775	ENSGALG00010020138	ENSGALG00010020138	case	control	-0.169903356	0.189502333	-0.896576596	0.369944891	0.795234047	down_in_case
CRA004775	ENSGALG00010020140	SECISBP2L	case	control	0.645475439	0.369302554	1.74782284	0.080494709	0.494585677	up_in_case
CRA004775	ENSGALG00010020141	THOC7	case	control	0.004144374	0.231471092	0.017904501	0.985715038	0.997562105	up_in_case
CRA004775	ENSGALG00010020142	SLC35E2B	case	control	-0.222211752	0.236525435	-0.939483535	0.347482542	0.785013534	down_in_case
CRA004775	ENSGALG00010020143	KCNK5	case	control	0.191857283	0.519381942	0.369395367	0.71183305	0.932407723	up_in_case
CRA004775	ENSGALG00010020147	ABHD2	case	control	0.251357587	0.464250325	0.541426842	0.58821341	0.892912535	up_in_case
CRA004775	ENSGALG00010020149	FAHD2A	case	control	-0.428249981	0.409162236	-1.046650799	0.295260664	0.747584126	down_in_case
CRA004775	ENSGALG00010020152	ATXN7	case	control	0.433051957	0.312484354	1.385835649	0.165797094	0.630194031	up_in_case
CRA004775	ENSGALG00010020153	AXL	case	control	-0.106080382	0.349246871	-0.303740395	0.76132568	0.942236668	down_in_case
CRA004775	ENSGALG00010020155	MFGE8	case	control	0.001660831	0.280212628	0.005927038	0.995270935	0.999001196	up_in_case
CRA004775	ENSGALG00010020160	SYT7	case	control	-0.394415038	0.668504117	-0.589996423	0.555193048	0.87981923	down_in_case
CRA004775	ENSGALG00010020163	CDC2L1	case	control	0.014393856	0.198973537	0.072340556	0.942330891	0.987651835	up_in_case
CRA004775	ENSGALG00010020164	C10orf71	case	control	0.672579402	0.561404027	1.198030954	0.230904967	0.697294488	up_in_case
CRA004775	ENSGALG00010020166	HAPLN3	case	control	-0.41742017	0.48651926	-0.857972551	0.39090762	0.806479902	down_in_case
CRA004775	ENSGALG00010020168	COX7A2L	case	control	-0.237705304	0.268204962	-0.886282276	0.375465437	0.798503808	down_in_case
CRA004775	ENSGALG00010020170	SORCS1	case	control	0.36552661	0.623345698	0.586394694	0.557610309	0.880722248	up_in_case
CRA004775	ENSGALG00010020171	RRN3	case	control	0.168080312	0.144777184	1.160958569	0.245658748	0.712046495	up_in_case
CRA004775	ENSGALG00010020175	ACAN	case	control	-5.342995091	1.046498397	-5.10559319	3.29758E-07	0.000238658	down_in_case
CRA004775	ENSGALG00010020176	ENSGALG00010020176	case	control	0.413209396	0.435785984	0.948193404	0.343031005	0.782518139	up_in_case
CRA004775	ENSGALG00010020179	PSMD6	case	control	-0.159242423	0.313262804	-0.508334922	0.611218483	0.901413878	down_in_case
CRA004775	ENSGALG00010020180	TCF7L1	case	control	0.038537183	0.303538232	0.126959897	0.898972139	0.978790377	up_in_case
CRA004775	ENSGALG00010020181	LEUTX	case	control	0.654428014	0.740634068	0.883605066	0.376909444	0.798523018	up_in_case
CRA004775	ENSGALG00010020183	XPNPEP1	case	control	-0.183828586	0.200645506	-0.916185914	0.359569392	0.791966047	down_in_case
CRA004775	ENSGALG00010020184	RSL1D1	case	control	-0.124966581	0.263642567	-0.474000016	0.635499896	0.909837348	down_in_case
CRA004775	ENSGALG00010020186	MGA	case	control	0.283806913	0.37999263	0.746874782	0.455139149	0.838631357	up_in_case
CRA004775	ENSGALG00010020187	MMP23B	case	control	0.170496771	0.394123633	0.432597177	0.665307441	0.918815167	up_in_case
CRA004775	ENSGALG00010020193	VSTM4	case	control	0.450405745	0.622166063	0.723931715	0.469107658	0.8440023	up_in_case
CRA004775	ENSGALG00010020194	PRICKLE2	case	control	-0.078917489	0.334364729	-0.236022171	0.813415466	0.958280492	down_in_case
CRA004775	ENSGALG00010020199	DET1	case	control	-0.63960112	0.172213222	-3.714007034	0.000204003	0.029221322	down_in_case
CRA004775	ENSGALG00010020202	MAPKBP1	case	control	0.047811014	0.317744429	0.150470031	0.880393793	0.974996282	up_in_case
CRA004775	ENSGALG00010020204	GSPT2	case	control	-0.018433168	0.179632048	-0.102616254	0.918267542	0.981710522	down_in_case
CRA004775	ENSGALG00010020206	MRPS11	case	control	-0.158272182	0.241295685	-0.655926283	0.511871556	0.863170205	down_in_case
CRA004775	ENSGALG00010020211	ADD3	case	control	-0.146347913	0.298701391	-0.48994721	0.624171255	0.906441769	down_in_case
CRA004775	ENSGALG00010020212	JMJD7	case	control	-0.868336578	0.288503636	-3.009794228	0.002614247	0.106489884	down_in_case
CRA004775	ENSGALG00010020215	PRMT3	case	control	-0.216540282	0.209348291	-1.034354193	0.30097061	0.753440172	down_in_case
CRA004775	ENSGALG00010020224	MRPL46	case	control	-0.129123862	0.274073221	-0.471129073	0.637548565	0.910421454	down_in_case
CRA004775	ENSGALG00010020226	TCIM	case	control	0.464345561	0.285174989	1.628282911	0.103464911	0.545480965	up_in_case
CRA004775	ENSGALG00010020228	TM9SF3	case	control	0.041679891	0.208150238	0.200239461	0.841293307	0.966461921	up_in_case
CRA004775	ENSGALG00010020230	ENSGALG00010020230	case	control	-0.369064431	0.380787961	-0.969212445	0.332439203	0.773343515	down_in_case
CRA004775	ENSGALG00010020236	SLC35G1	case	control	0.29014517	0.454044672	0.639023399	0.52280771	0.866638286	up_in_case
CRA004775	ENSGALG00010020237	MIB2	case	control	0.085558827	0.225341658	0.379684908	0.704179325	0.930465147	up_in_case
CRA004775	ENSGALG00010020244	SLC25A37	case	control	-0.107069709	0.316977404	-0.337783413	0.735526411	0.937109578	down_in_case
CRA004775	ENSGALG00010020246	ADAMTS9	case	control	0.356957414	0.349612015	1.021010146	0.307249632	0.758947455	up_in_case
CRA004775	ENSGALG00010020248	NOLC1	case	control	0.104485196	0.205364299	0.508779749	0.610906616	0.901413878	up_in_case
CRA004775	ENSGALG00010020250	ENSGALG00010020250	case	control	0.983122231	0.480922113	2.04424418	0.040929431	0.378415958	up_in_case
CRA004775	ENSGALG00010020252	DMTN	case	control	0.016258259	0.311860154	0.052133172	0.958422582	0.991431439	up_in_case
CRA004775	ENSGALG00010020254	ENSGALG00010020254	case	control	-0.055352028	0.344904476	-0.160485097	0.872498961	0.97401729	down_in_case
CRA004775	ENSGALG00010020257	ZNF710	case	control	-0.071402042	0.19704041	-0.362372579	0.717073628	0.934011578	down_in_case
CRA004775	ENSGALG00010020259	ENSGALG00010020259	case	control	-0.342141267	0.441348733	-0.775217512	0.438211142	0.827821102	down_in_case
CRA004775	ENSGALG00010020261	SSU72	case	control	-0.17868848	0.218316531	-0.818483505	0.413081157	0.817112606	down_in_case
CRA004775	ENSGALG00010020265	ENSGALG00010020265	case	control	1.213260789	0.447194252	2.713050949	0.006666686	0.160913119	up_in_case
CRA004775	ENSGALG00010020268	CPLANE2	case	control	-0.16835183	0.30355392	-0.554602722	0.579166427	0.888378369	down_in_case
CRA004775	ENSGALG00010020269	ENSGALG00010020269	case	control	-0.549222792	0.780287366	-0.703872466	0.4815122	0.848818238	down_in_case
CRA004775	ENSGALG00010020272	ENSGALG00010020272	case	control	-0.208807984	0.227794894	-0.916649098	0.359326548	0.791589131	down_in_case
CRA004775	ENSGALG00010020277	IDH2	case	control	-0.259677791	0.375437503	-0.691667158	0.489146376	0.854325408	down_in_case
CRA004775	ENSGALG00010020282	MAGI1	case	control	0.391345012	0.363900078	1.075418873	0.282187236	0.738291085	up_in_case
CRA004775	ENSGALG00010020283	FBXO42	case	control	-0.088423551	0.200441379	-0.441144198	0.659108608	0.917907174	down_in_case
CRA004775	ENSGALG00010020284	HEG1	case	control	-0.140569802	0.455666105	-0.308492997	0.757707228	0.941900693	down_in_case
CRA004775	ENSGALG00010020285	ADAMTS14	case	control	0.188616912	0.558093884	0.337966276	0.735388602	0.937109578	up_in_case
CRA004775	ENSGALG00010020287	GALNT16	case	control	-0.151140125	0.394250531	-0.383360612	0.701452404	0.930068722	down_in_case
CRA004775	ENSGALG00010020291	SNX29	case	control	-0.273657322	0.458465363	-0.596898575	0.550575099	0.877827378	down_in_case
CRA004775	ENSGALG00010020292	NPM2	case	control	0.185946967	0.333515685	0.557535898	0.57716134	0.888189723	up_in_case
CRA004775	ENSGALG00010020293	SEMA4B	case	control	-0.089966347	0.216980788	-0.414628171	0.678414123	0.92338129	down_in_case
CRA004775	ENSGALG00010020295	LGI1	case	control	0.334105098	0.405966809	0.822986241	0.410515818	0.816341723	up_in_case
CRA004775	ENSGALG00010020298	ENSGALG00010020298	case	control	-0.283098341	0.303297427	-0.933401724	0.350612591	0.785681625	down_in_case

CRA004775	ENSGALG00010020300	EHD4	case	control	0.017915785	0.415434626	0.043125402	0.96560157	0.993094598	up_in_case
CRA004775	ENSGALG00010020303	EXD2	case	control	-0.338814109	0.198820506	-1.704120542	0.08835857	0.513532836	down_in_case
CRA004775	ENSGALG00010020304	ZFP36L2	case	control	0.524336611	0.299483999	1.750800086	0.079980352	0.493409519	up_in_case
CRA004775	ENSGALG00010020305	XPO7	case	control	0.033871447	0.170269277	0.198928708	0.842318512	0.966478468	up_in_case
CRA004775	ENSGALG00010020307	SHQ1	case	control	-0.514306719	0.388102441	-1.325182903	0.185110495	0.6491093	down_in_case
CRA004775	ENSGALG00010020309	GXYLT2	case	control	0.251943732	0.253734379	0.992942827	0.320737817	0.767037518	up_in_case
CRA004775	ENSGALG00010020311	SGPL1	case	control	0.334896459	0.331816927	1.009280814	0.312839979	0.764775564	up_in_case
CRA004775	ENSGALG00010020313	FRA10AC1	case	control	0.122077414	0.235409242	0.518575279	0.604056952	0.898935274	up_in_case
CRA004775	ENSGALG00010020314	THADA	case	control	-0.205071486	0.24936306	-0.822381172	0.410859992	0.816405743	down_in_case
CRA004775	ENSGALG00010020315	ANO5	case	control	0.211756259	0.248091371	0.85354141	0.393359134	0.807807863	up_in_case
CRA004775	ENSGALG00010020316	PDPK1	case	control	0.290387186	0.214555361	1.353437098	0.175916032	0.641310011	up_in_case
CRA004775	ENSGALG00010020319	NTRK3	case	control	0.883965774	0.469239177	1.883827733	0.059588281	0.445083352	up_in_case
CRA004775	ENSGALG00010020321	SZRD1	case	control	0.013893963	0.260507617	0.053334189	0.95746564	0.991431439	up_in_case
CRA004775	ENSGALG00010020323	ARHGAP25	case	control	-0.143665141	0.402557767	-0.356880808	0.721181038	0.934511903	down_in_case
CRA004775	ENSGALG00010020324	PLEKHH2	case	control	0.499274756	0.341065343	1.463868338	0.143229926	0.603787465	up_in_case
CRA004775	ENSGALG00010020329	PPP4R2	case	control	0.266598431	0.192828433	1.382568054	0.16679735	0.63046464	up_in_case
CRA004775	ENSGALG00010020336	OSBP	case	control	-0.171199051	0.290513342	-0.58929841	0.555661111	0.87981923	down_in_case
CRA004775	ENSGALG00010020338	NECAP2	case	control	-0.328580773	0.305253901	-1.076417934	0.281740385	0.738260394	down_in_case
CRA004775	ENSGALG00010020340	DCAF5	case	control	-0.121092461	0.181918072	-0.665642832	0.505639422	0.8622397	down_in_case
CRA004775	ENSGALG00010020341	DYNC2LI1	case	control	-0.164085381	0.410684733	-0.399540981	0.689494634	0.927714355	down_in_case
CRA004775	ENSGALG00010020342	CPPED1	case	control	0.221034712	0.326305294	0.677386229	0.498160934	0.857992359	up_in_case
CRA004775	ENSGALG00010020343	PATL1	case	control	-0.349541249	0.298159899	-1.172328171	0.241065326	0.708079869	down_in_case
CRA004775	ENSGALG00010020345	AGBL1	case	control	-0.485722814	0.782848797	-0.620455464	0.534957967	0.870981055	down_in_case
CRA004775	ENSGALG00010020346	PCBD1	case	control	0.050263628	0.361864517	0.138901788	0.889527758	0.977162915	up_in_case
CRA004775	ENSGALG00010020350	PDZRN3	case	control	0.038984366	0.378027143	0.103125838	0.917863099	0.981688766	up_in_case
CRA004775	ENSGALG00010020351	CROCC	case	control	-0.226437756	0.194706562	-1.162969311	0.244841954	0.711767728	down_in_case
CRA004775	ENSGALG00010020352	ABCG5	case	control	2.268028298	0.781819337	2.900962139	0.003720188	0.12600076	up_in_case
CRA004775	ENSGALG00010020356	STX3	case	control	0.054315838	0.311862937	0.174165738	0.861735211	0.97165529	up_in_case
CRA004775	ENSGALG00010020357	RBP4	case	control	0.961750002	0.49115996	1.958119718	0.050215967	0.41472658	up_in_case
CRA004775	ENSGALG00010020362	CDS2	case	control	-0.732277708	0.356656759	-2.053172101	0.040055889	0.37597852	down_in_case
CRA004775	ENSGALG00010020364	MRPL16	case	control	-0.140050461	0.333188458	-0.420334072	0.674241423	0.922533825	down_in_case
CRA004775	ENSGALG00010020365	UNC5B	case	control	-0.469923498	0.484251396	-0.970412273	0.331841034	0.772935571	down_in_case
CRA004775	ENSGALG00010020370	CEP55	case	control	-0.365348007	0.726381999	-0.502969522	0.614985701	0.902639483	down_in_case
CRA004775	ENSGALG00010020373	MS4A4A	case	control	-0.477282146	0.430161807	-1.109540965	0.267196882	0.727315996	down_in_case
CRA004775	ENSGALG00010020374	PCNA	case	control	-0.37751122	0.297445605	-1.269177334	0.204377823	0.674045742	down_in_case
CRA004775	ENSGALG00010020375	LRPPRC	case	control	0.041866159	0.202199602	0.207053619	0.835967984	0.965107526	up_in_case
CRA004775	ENSGALG00010020377	TMEM230	case	control	-0.597755272	0.298221823	-2.004398155	0.045027427	0.394778076	down_in_case
CRA004775	ENSGALG00010020379	COL13A1	case	control	-1.327173041	0.686910181	-1.932091091	0.053348264	0.42576435	down_in_case
CRA004775	ENSGALG00010020382	MFAP2	case	control	-0.178432713	0.410656568	-0.434505928	0.663921091	0.918295135	down_in_case
CRA004775	ENSGALG00010020383	BAAT	case	control	-0.076855266	0.234587842	-0.327618284	0.743200296	0.939218065	down_in_case
CRA004775	ENSGALG00010020385	SLC12A8	case	control	1.239721003	0.646196129	1.918490296	0.055048871	0.430875458	up_in_case
CRA004775	ENSGALG00010020386	PPM1B	case	control	0.320562209	0.135892092	2.358946754	0.018326885	0.269244656	up_in_case
CRA004775	ENSGALG00010020388	MYOF	case	control	0.539792162	0.366786774	1.471678373	0.141107751	0.600619496	up_in_case
CRA004775	ENSGALG00010020389	ACTN1	case	control	-0.239404379	0.556602721	-0.430117156	0.667110421	0.919278017	down_in_case
CRA004775	ENSGALG00010020390	KLHL25	case	control	0.243717441	0.430727539	0.565827393	0.571511123	0.885417769	up_in_case
CRA004775	ENSGALG00010020392	SLC23A2	case	control	0.303300484	0.386073182	0.785603606	0.432099751	0.825415344	up_in_case
CRA004775	ENSGALG00010020393	SHISA9	case	control	1.385190285	0.765950151	1.808460098	0.070534922	0.469895261	up_in_case
CRA004775	ENSGALG00010020396	CHL1	case	control	1.202922887	0.477862156	2.517301009	0.011825775	0.21809505	up_in_case
CRA004775	ENSGALG00010020399	ATP13A2	case	control	-0.466209993	0.206177452	-2.261207462	0.023746413	0.2983663	down_in_case
CRA004775	ENSGALG00010020402	SLC3A1	case	control	1.000098192	0.393184238	2.543586679	0.010972083	0.211456439	up_in_case
CRA004775	ENSGALG00010020405	UBE2I	case	control	-0.192596255	0.2275012	-0.84657248	0.397233459	0.808998414	down_in_case
CRA004775	ENSGALG00010020413	CYP26A1	case	control	0.875428554	0.465807773	1.8793773	0.060192994	0.445397937	up_in_case
CRA004775	ENSGALG00010020414	ENSGALG00010020414	case	control	1.509939769	0.779150062	1.937931911	0.05263153	0.422249808	up_in_case
CRA004775	ENSGALG00010020415	TSR3	case	control	-0.333543106	0.284894797	-1.170758853	0.241695721	0.708952282	down_in_case
CRA004775	ENSGALG00010020417	ENSGALG00010020417	case	control	-2.154006595	0.68692213	-3.135736208	0.001714233	0.084187217	down_in_case
CRA004775	ENSGALG00010020422	ERCC4	case	control	0.229333268	0.185311937	1.237552589	0.215882003	0.683774974	up_in_case
CRA004775	ENSGALG00010020428	SEMA4C	case	control	0.055631063	0.235214835	0.236511711	0.813035621	0.958114791	up_in_case
CRA004775	ENSGALG00010020429	ZNF148	case	control	0.093446332	0.279634377	0.334173263	0.738248818	0.937887067	up_in_case
CRA004775	ENSGALG00010020433	GNPTG	case	control	-0.368613597	0.199113491	-1.851273835	0.064130168	0.455932569	down_in_case
CRA004775	ENSGALG00010020436	CNTN4	case	control	0.195666362	0.365044364	0.536007076	0.59195366	0.893804699	up_in_case
CRA004775	ENSGALG00010020437	SNX4	case	control	0.092527817	0.191332797	0.48359622	0.628672458	0.908552282	up_in_case
CRA004775	ENSGALG00010020438	HTR7	case	control	-0.004237962	0.327014991	-0.012959533	0.989660078	0.998065748	down_in_case
CRA004775	ENSGALG00010020440	PAX7	case	control	0.151560651	0.549098601	0.276017187	0.782534866	0.94936527	up_in_case
CRA004775	ENSGALG00010020443	PREPL	case	control	0.177534236	0.349391746	0.508123725	0.611366578	0.901413878	up_in_case
CRA004775	ENSGALG00010020449	PRPF19	case	control	-0.40928537	0.186996472	-2.188733117	0.028616243	0.322057735	down_in_case
CRA004775	ENSGALG00010020450	ANKRD39	case	control	-0.066247798	0.445790975	-0.148607311	0.881863503	0.975269828	down_in_case
CRA004775	ENSGALG00010020453	ENSGALG00010020453	case	control	0.305903941	0.217931292	1.403671487	0.160416697	0.625606919	up_in_case
CRA004775	ENSGALG00010020457	IL5RA	case	control	-2.065856279	1.296743421	-1.593111055	0.11113528	0.560403826	down_in_case
CRA004775	ENSGALG00010020460	MAPK8	case	control	0.079240241	0.22343674	0.354642847	0.722857173	0.935185555	up_in_case
CRA004775	ENSGALG00010020462	CNNM4	case	control	-0.331068638	0.19943293	-1.660050009	0.096904392	0.53104893	down_in_case
CRA004775	ENSGALG00010020467	ALDH4A1	case	control	-0.328794842	0.293704239	-1.119475983	0.262937131	0.723853552	down_in_case
CRA004775	ENSGALG00010020473	TMEM109	case	control	-0.28079803	0.545666681	-0.514596254	0.606835184	0.900898526	down_in_case
CRA004775	ENSGALG00010020476	TMEM132A	case	control	-0.016944882	0.212926417	-0.07958093	0.936570563	0.98646154	down_in_case
CRA004775	ENSGALG00010020481	RPP30	case	control	-0.189677454	0.205135902	-0.924642891	0.355151695	0.788279103	down_in_case
CRA004775	ENSGALG00010020484	TRNT1	case	control	0.110221533	0.288497631	0.382053509	0.702421675	0.930068722	up_in_case
CRA004775	ENSGALG00010020485	LMAN2L	case	control	-0.665742588	0.229082048	-2.906131639	0.003659275	0.12600076	down_in_case
CRA004775	ENSGALG00010020486	MRTFB	case	control	0.233974008	0.357510569	0.6544534	0.512819741	0.863786547	up_in_case
CRA004775	ENSGALG00010020489	IFFO2	case	control	-0.052720516	0.312303808	-0.168811633	0.865944808	0.972629493	down_in_case
CRA004775	ENSGALG00010020493	DEPDC7	case	control	0.75151184	0.361314568	2.079937837	0.037531235	0.365326975	up_in_case
CRA004775	ENSGALG00010020497	OSBPL11	case	control	-0.180248225	0.265513281	-0.678867076	0.497222089	0.857772041	down_in_case
CRA004775	ENSGALG00010020498	FRMPD2	case	control	2.736706203	0.792605433	3.452797683	0.000554805	0.048593139	up_in_case
CRA004775	ENSGALG00010020507	ANKRD1	case	control	1.144896298	0.968386782	1.182271711	0.237097907	0.703871614	up_in_case
CRA004775	ENSGALG00010020509	CRBN	case	control	0.312622839	0.223526999	1.398590953	0.161935681	0.627882156	up_in_case
CRA004775	ENSGALG00010020514	CAMKMT	case	control	0.058705097	0.303648522	0.193332398	0.846698655	0.967891337	up_in_case
CRA004775	ENSGALG00010020517	UBR4	case	control	0.093157904	0.251203607	0.370846205	0.710752085	0.932234469	up_in_case
CRA004775	ENSGALG00010020520	ARHGAP22	case	control	-0.119138794	0.384178918	-0.310112785	0.75647519	0.941900693	down_in_case
CRA004775	ENSGALG00010020524	ENSGALG00010020524	case	control	1.127733646	0.390313608	2.889301381	0.003860988	0.128409904	up_in_case
CRA004775	ENSGALG00010020525	ENSGALG00010020525	case	control	0.200062747	0.645415332	0.309975201	0.756579815	0.941900693	up_in_case
CRA004775	ENSGALG00010020530	SRSF5A	case	control	-0.150271781	0.159874025	-0.939938686	0.347249012	0.784848975	down_in_case
CRA004775	ENSGALG00010020533	ENSGALG00010020533	case	control	-0.099750417	0.267820031	-0.372453161	0.709555479	0.931909971	down_in_case
CRA004775	ENSGALG00010020535	LMLN	case	control	-0.81276202	0.42396048	-1.917070245	0.055229008	0.430875458	down_in_case
CRA004775	ENSGALG00010020537	CCDC167	case	control	-0.48135881	0.306555802	-1.570215949	0.11636488	0.566086328	down_in_case
CRA004775	ENSGALG00010020540	PCGF5	case	control	0.460154247	0.304541862	1.510972069	0.130795567	0.586275434	up_in_case
CRA004775	ENSGALG00010020544	BMERB1	case	control	0.871919349	0.440483682	1.97945891	0.047764362	0.406189076	up_in_case
CRA004775	ENSGALG00010020545	STYX	case	control	0.449847508	0.318172201	1.413849189	0.157406187	0.622106365	up_in_case
CRA004775	ENSGALG00010020546	LRRC20	case	control	-0.128041947	0.526412361	-0.243235068	0.807823302	0.955397873	down_in_case
CRA004775	ENSGALG00010020548	CMTR1	case	control	-0.272278163	0.30539372	-0.89156438	0.37262647	0.796268311	down_in_case
CRA004775	ENSGALG00010020550	HECTD2	case	control	0.420194899	0.43997329	0.955046383	0.339554241	0.779891494	up_in_case
CRA004775	ENSGALG00010020554	ZFP36L1	case	control	0.173813633	0.278460806	0.624194246	0.532500025	0.869946559	up_in_case
CRA004775	ENSGALG00010020556	EMC1	case	control	-0.273733691	0.203836573	-1.342907641	0.179301882	0.644990528	down_in_case
CRA004775	ENSGALG00010020557	ENSGALG00010020557	case	control	-0.14362817	0.43833024	-0.327671141	0.743160326	0.939218065	down_in_case
CRA004775	ENSGALG00010020558	EIF4EBP2	case	control	-0.308811336	0.364462466	-0.84730628	0.396824427	0.808998414	down_in_case
CRA004775	ENSGALG00010020562	LRRN1	case	control	0.216325596	0.524388512	0.412529244	0.679951551	0.923896221	up_in_case
CRA004775	ENSGALG00010020568	PLA2G4F	case	control	-0.03220611	0.657353987	-0.048993557	0.96092443	0.991824137	down_in_case
CRA004775	ENSGALG00010020569	DTX3L	case	control	-0.265572348	0.344764535	-0.770300658	0.441121566	0.829353567	down_in_case
CRA004775	ENSGALG00010020571	MRTO4	case	control	-0.356432123	0.185835041	-1.918002763	0.055110661	0.430875458	down_in_case
CRA004775	ENSGALG00010020572	PPP1R3C	case	control	-0.48222401	0.495655278	-0.972901998	0.330602012	0.77237154	down_in_case
CRA004775	ENSGALG00010020574	DTL	case	control	0.189127757	0.539645422	0.350466713	0.725988467	0.935238044	up_in_case
CRA004775	ENSGALG00010020576	AKR7L	case	control	-0.073502645	0.217582964	-0.337814337	0.735503105	0.937109578	down_in_case
CRA004775	ENSGALG00010020578	TNKS2	case	control	0.231684877	0.234850664	0.986519999	0.323878006	0.769171227	up_in_case
CRA004775	ENSGALG00010020580	ENSGALG00010020580	case	control	-0.35361835	0.170313688	-2.076276749	0.037868362	0.366968184	down_in_case
CRA004775	ENSGALG00010020582	KANSL3	case	control	-0.10461938	0.238797318	-0.438109527	0.661306889	0.91799223	down_in_case
CRA004775	ENSGALG00010020584	PARP9	case	control	-0.500067446	0.404657671	-1.235778985	0.216540729	0.684157514	down_in_case
CRA004775	ENSGALG00010020586	C11orf16	case	control	0.018044286	0.216927282	0.083181264	0.93370741	0.985093894	up_in_case
CRA004775	ENSGALG00010020588	PALD1	case	control	-0.270268516	0.299517006	-0.902347816	0.366872133	0.793822622	down_in_case
CRA004775	ENSGALG00010020590	INTS7	case	control	-0.527906907	0.268368091	-1.967100127	0.049171669	0.410291029	down_in_case
CRA004775	ENSGALG00010020594	PQLC2	case	control	0.064090699	0.28990212	0.221077027	0.825032457	0.961768508	up_in_case
CRA004775	ENSGALG00010020595	ZFYVE27	case	control	-0.384539932	0.180518371	-2.130198327	0.033155243	0.347668706	down_in_case
CRA004775	ENSGALG00010020596	SUMF1	case	control	-0.494814879	0.19573573	-2.527974224	0.011472277	0.216528087	down_in_case
CRA004775	ENSGALG00010020597	ARID5A	case	control	-0.119616621	0.479272905	-0.249579352	0.802912668	0.954005463	down_in_case
CRA004775	ENSGALG00010020599	PARN	case	control	-0.091547467	0.345933433	-0.264638969	0.791287597	0.951284723	down_in_case
CRA004775	ENSGALG00010020601	AKIP1	case	control	-0.206058539	0.300973821	-0.684639409	0.493571492	0.856001758	down_in_case
CRA004775	ENSGALG00010020602	DENND2B	case	control	0.133776768	0.255653774	0.5232732	0.600784148	0.89768367	up_in_case
CRA004775	ENSGALG00010020603	VPS39	case	control	0.225504566	0.256141278	0.880391355	0.378647338	0.799935404	up_in_case
CRA004775	ENSGALG00010020605	STAMBP	case	control	-0.243136503	0.123773386	-1.964368194	0.049487409	0.411853997	down_in_case
CRA004775	ENSGALG00010020607	ENSGALG00010020607	case	control	-0.39724692	0.519718767	-0.764349771	0.444658833	0.832584915	down_in_case

CRA004775	ENSGALG00010020608	CAPZB	case	control	-0.087331157	0.305364222	-0.285990143	0.774885671	0.946319184	down_in_case
CRA004775	ENSGALG00010020609	AVPI1	case	control	-0.418603461	0.472378595	-0.886160943	0.375530807	0.798503808	down_in_case
CRA004775	ENSGALG00010020611	LPGAT1	case	control	-0.092222936	0.259378845	-0.355553039	0.722175318	0.935087834	down_in_case
CRA004775	ENSGALG00010020612	RPL27A	case	control	-0.360328457	0.252281805	-1.428277623	0.153211966	0.617472961	down_in_case
CRA004775	ENSGALG00010020613	ACTG2	case	control	1.179069058	0.47513824	2.481528444	0.013082027	0.230039581	up_in_case
CRA004775	ENSGALG00010020614	FAIM	case	control	-0.115345199	0.339952199	-0.339298287	0.734385033	0.936819725	down_in_case
CRA004775	ENSGALG00010020616	PI4K2A	case	control	-0.430776685	0.27280604	-1.579058457	0.114322649	0.564470646	down_in_case
CRA004775	ENSGALG00010020617	DGUOK	case	control	-0.026278812	0.349180609	-0.07525851	0.940009032	0.987076872	down_in_case
CRA004775	ENSGALG00010020619	QARS	case	control	-0.511290498	0.253130651	-2.019867985	0.043397083	0.389812978	down_in_case
CRA004775	ENSGALG00010020620	MARF1	case	control	0.117496114	0.189244406	0.620869681	0.534685371	0.870981055	up_in_case
CRA004775	ENSGALG00010020622	ABCG2	case	control	0.327692962	0.316035637	1.036886111	0.299788938	0.752512244	up_in_case
CRA004775	ENSGALG00010020623	NEK2	case	control	1.071925965	0.570756386	1.878079671	0.060370267	0.445397937	up_in_case
CRA004775	ENSGALG00010020624	MINOS1	case	control	0.248303995	0.247382987	1.003723003	0.315512146	0.765461123	up_in_case
CRA004775	ENSGALG00010020625	SLC30A1	case	control	-0.221316533	0.289038151	-0.76570007	0.443854785	0.832130395	down_in_case
CRA004775	ENSGALG00010020626	MORN4	case	control	-0.104580763	0.295120535	-0.354366269	0.72306441	0.935185555	down_in_case
CRA004775	ENSGALG00010020629	TRIM66	case	control	-0.245635206	0.510300954	-0.481353609	0.630265202	0.908943555	down_in_case
CRA004775	ENSGALG00010020630	QRICH1	case	control	0.204029052	0.210404978	0.969696888	0.332197602	0.772935571	up_in_case
CRA004775	ENSGALG00010020632	BLOC1S2	case	control	-0.07447986	0.259494398	-0.287019145	0.774097661	0.946319184	down_in_case
CRA004775	ENSGALG00010020633	HOGA1	case	control	-0.057253061	0.284640022	-0.201141991	0.840587549	0.966461921	down_in_case
CRA004775	ENSGALG00010020635	NDE1	case	control	0.51380423	0.328136938	1.565822593	0.117390156	0.567791779	up_in_case
CRA004775	ENSGALG00010020636	NBL1	case	control	-0.185192687	0.317836701	-0.582666152	0.560118063	0.880918893	down_in_case
CRA004775	ENSGALG00010020638	IMPDH2	case	control	-0.304699974	0.311672763	-0.977627852	0.328258417	0.77220987	down_in_case
CRA004775	ENSGALG00010020640	PTPN14	case	control	0.518890226	0.42354687	1.225106977	0.220534917	0.68855283	up_in_case
CRA004775	ENSGALG00010020642	ANKRD2	case	control	-1.669099416	0.700729049	-2.381946943	0.017221379	0.259661377	down_in_case
CRA004775	ENSGALG00010020646	CEP70	case	control	0.008818522	0.330311895	0.026697562	0.978700957	0.996495823	up_in_case
CRA004775	ENSGALG00010020648	TET3	case	control	-0.024705681	0.411764981	-0.05999947	0.952156057	0.990176052	down_in_case
CRA004775	ENSGALG00010020649	MYH11	case	control	1.064245663	0.422491314	2.518976433	0.011769653	0.217806153	up_in_case
CRA004775	ENSGALG00010020651	LMO1	case	control	-0.136877685	0.340407675	-0.402099292	0.687610954	0.927111919	down_in_case
CRA004775	ENSGALG00010020653	NDUFAF3	case	control	-0.334211165	0.353872669	-0.944439045	0.344945337	0.783972357	down_in_case
CRA004775	ENSGALG00010020654	SAYSD1	case	control	-0.538908441	0.374355316	-1.43956401	0.149990788	0.61366707	down_in_case
CRA004775	ENSGALG00010020655	RIC3	case	control	-0.179776403	0.445070739	-0.403927707	0.686265881	0.926725143	down_in_case
CRA004775	ENSGALG00010020657	DALRD3	case	control	-0.492158443	0.161896977	-3.039948312	0.002366188	0.098653993	down_in_case
CRA004775	ENSGALG00010020659	RAD51B	case	control	0.057970309	0.305577642	0.1897073	0.8495385	0.968413719	up_in_case
CRA004775	ENSGALG00010020662	BFAR	case	control	-0.09906271	0.302214288	-0.327789633	0.743070726	0.939218065	down_in_case
CRA004775	ENSGALG00010020664	ESYT3	case	control	0.444124809	0.502946889	0.883045146	0.37721188	0.798610687	up_in_case
CRA004775	ENSGALG00010020666	TMCO4	case	control	-0.587183956	0.299281965	-1.961975745	0.049765308	0.413341381	down_in_case
CRA004775	ENSGALG00010020667	UBTD1	case	control	-0.170735176	0.318555889	-0.535966158	0.59198194	0.893804699	down_in_case
CRA004775	ENSGALG00010020670	ENSGALG00010020670	case	control	0.065782591	0.216058179	0.304467025	0.760772111	0.942236668	up_in_case
CRA004775	ENSGALG00010020672	RNF186	case	control	1.699786847	0.815921283	2.083273083	0.037226339	0.363933342	up_in_case
CRA004775	ENSGALG00010020673	MMS19	case	control	-0.382423443	0.165428475	-2.311714738	0.020793409	0.283942565	down_in_case
CRA004775	ENSGALG00010020675	SMYD2	case	control	-0.483435884	0.245814103	-1.966672691	0.049220957	0.410453233	down_in_case
CRA004775	ENSGALG00010020676	CYP2C23a	case	control	2.538089214	0.934782371	2.715165896	0.006624258	0.160913119	up_in_case
CRA004775	ENSGALG00010020677	CYR61	case	control	0.333288326	0.45917511	0.725841446	0.467935976	0.843870188	up_in_case
CRA004775	ENSGALG00010020679	OTUD3	case	control	0.077507567	0.2657582	0.291646945	0.770556578	0.945754599	up_in_case
CRA004775	ENSGALG00010020681	BCL10	case	control	0.209235128	0.165297839	1.265806791	0.205582263	0.674045742	up_in_case
CRA004775	ENSGALG00010020682	ZDHHC16	case	control	-0.455781751	0.249343409	-1.827927808	0.067560387	0.463543284	down_in_case
CRA004775	ENSGALG00010020684	P4HTM	case	control	-0.350788301	0.371325238	-0.94469286	0.344815703	0.783972357	down_in_case
CRA004775	ENSGALG00010020685	RPL6	case	control	-0.160713138	0.211471497	-0.759975415	0.447269281	0.834136489	down_in_case
CRA004775	ENSGALG00010020686	C1orf52	case	control	0.088214283	0.234240996	0.376596261	0.706473667	0.931529615	up_in_case
CRA004775	ENSGALG00010020691	ENSGALG00010020691	case	control	0.435286068	0.344850936	1.262244126	0.206860955	0.675503441	up_in_case
CRA004775	ENSGALG00010020692	TUB	case	control	-0.037324572	0.280316949	-0.133151321	0.894073708	0.977834811	down_in_case
CRA004775	ENSGALG00010020693	PTGFR	case	control	-0.053246831	0.378528592	-0.140667924	0.888132286	0.977118922	down_in_case
CRA004775	ENSGALG00010020695	ENSGALG00010020695	case	control	-0.429984052	0.76976712	-0.558589788	0.576441711	0.887937128	down_in_case
CRA004775	ENSGALG00010020696	MOB1A	case	control	-0.72378835	0.297813362	-2.430342098	0.015084577	0.246059339	down_in_case
CRA004775	ENSGALG00010020699	PLA2G10	case	control	0.365257201	0.417306163	0.87527392	0.381424881	0.801753951	up_in_case
CRA004775	ENSGALG00010020700	MTHFD2	case	control	0.019194619	0.477133797	0.040229007	0.967910552	0.993568693	up_in_case
CRA004775	ENSGALG00010020701	PTPN11	case	control	0.226963018	0.205045972	1.106888448	0.268342161	0.727528758	up_in_case
CRA004775	ENSGALG00010020704	PROX1	case	control	0.068646545	0.566846193	0.121102595	0.903609773	0.979468548	up_in_case
CRA004775	ENSGALG00010020705	EXOSC1	case	control	0.009372287	0.341540628	0.027441206	0.978107833	0.996148018	up_in_case
CRA004775	ENSGALG00010020708	FAM214A	case	control	0.222024997	0.342500597	0.648247037	0.516825184	0.864913871	up_in_case
CRA004775	ENSGALG00010020711	PLA2G2E	case	control	1.641849469	0.454260629	3.614333634	0.000301121	0.035211539	up_in_case
CRA004775	ENSGALG00010020712	ZC3H7A	case	control	0.023200702	0.234288258	0.099026312	0.921117379	0.982481112	up_in_case
CRA004775	ENSGALG00010020714	PGAM1	case	control	-0.075167951	0.624332126	-0.120397377	0.904168369	0.979540372	down_in_case
CRA004775	ENSGALG00010020716	RRAS	case	control	0.161609272	0.387916173	0.416608751	0.676964608	0.922924485	up_in_case
CRA004775	ENSGALG00010020719	RPH3A	case	control	-0.06411959	0.51389664	-0.124771375	0.90070455	0.979145745	down_in_case
CRA004775	ENSGALG00010020720	ENSGALG00010020720	case	control	0.82085861	0.867580153	0.946147289	0.344073467	0.783503692	up_in_case
CRA004775	ENSGALG00010020721	PTDSS2	case	control	0.011318101	0.242668586	0.046640159	0.962800025	0.991860454	up_in_case
CRA004775	ENSGALG00010020723	RPS24	case	control	-0.04145965	0.239229821	-0.173304689	0.862411938	0.9717602	down_in_case
CRA004775	ENSGALG00010020724	TACR1	case	control	0.631211387	0.633187032	0.99687984	0.318822838	0.767037518	up_in_case
CRA004775	ENSGALG00010020725	FOPNL	case	control	-0.288275584	0.401275483	-0.718398198	0.472511797	0.845425322	down_in_case
CRA004775	ENSGALG00010020727	ANO9	case	control	1.801638895	0.529393029	3.403216132	0.000665976	0.054134577	up_in_case
CRA004775	ENSGALG00010020728	TMEM37	case	control	0.364534624	0.399891236	0.91158443	0.361987522	0.792867581	up_in_case
CRA004775	ENSGALG00010020730	RRP12	case	control	-0.328576599	0.244793381	-1.34226096	0.179511397	0.644990528	down_in_case
CRA004775	ENSGALG00010020732	ARIH2	case	control	0.329137337	0.30476143	1.07998357	0.280149497	0.73661445	up_in_case
CRA004775	ENSGALG00010020733	TMEM229B	case	control	-0.064980285	0.345074326	-0.188308084	0.850635148	0.968989143	down_in_case
CRA004775	ENSGALG00010020734	SIGIRR	case	control	2.602661999	0.633746898	4.106784597	4.01205E-05	0.013135644	up_in_case
CRA004775	ENSGALG00010020736	LRRTM4	case	control	0.215920817	0.643290419	0.335650603	0.73713436	0.937564762	up_in_case
CRA004775	ENSGALG00010020737	POLR3A	case	control	0.209261401	0.215063714	0.97302049	0.330543119	0.77237154	up_in_case
CRA004775	ENSGALG00010020739	PKP3	case	control	1.950239775	0.859942672	2.267871847	0.023337019	0.296313338	up_in_case
CRA004775	ENSGALG00010020742	THAP10	case	control	0.204028207	0.460279896	0.443269864	0.657570551	0.917343273	up_in_case
CRA004775	ENSGALG00010020743	PURB	case	control	0.003377187	0.29369669	0.011498895	0.990825412	0.998065748	up_in_case
CRA004775	ENSGALG00010020744	CHST3	case	control	-0.26988488	0.312810005	-0.862775728	0.388260786	0.804270424	down_in_case
CRA004775	ENSGALG00010020745	ABCC1	case	control	-0.08787533	0.20359475	-0.431618842	0.666018462	0.918888163	down_in_case
CRA004775	ENSGALG00010020747	DBI	case	control	0.090807166	0.266083144	0.341273651	0.732897582	0.936819725	up_in_case
CRA004775	ENSGALG00010020750	AIFM2	case	control	-0.261242075	0.462933183	-0.564319182	0.572536929	0.885796053	down_in_case
CRA004775	ENSGALG00010020753	SYDE2	case	control	0.35106972	0.196421228	1.787330852	0.07388403	0.479688056	up_in_case
CRA004775	ENSGALG00010020754	SLC25A20	case	control	-0.130481929	0.153152321	-0.851974871	0.394228039	0.808505558	down_in_case
CRA004775	ENSGALG00010020756	MACROH2A2	case	control	-0.18507717	0.325899983	-0.567895611	0.570105853	0.88518436	down_in_case
CRA004775	ENSGALG00010020759	ARHGAP19	case	control	0.291488873	0.457938377	0.63652423	0.524434793	0.86702704	up_in_case
CRA004775	ENSGALG00010020760	TXNDC11	case	control	-0.029075298	0.229575487	-0.126648093	0.899218931	0.978790377	down_in_case
CRA004775	ENSGALG00010020762	TTBK2	case	control	-0.09798139	0.440403235	-0.222481086	0.823939396	0.960901588	down_in_case
CRA004775	ENSGALG00010020764	PRKAR2A	case	control	-0.005590038	0.327853958	-0.017050391	0.986396415	0.997562105	down_in_case
CRA004775	ENSGALG00010020765	GLT1D1	case	control	-0.086282469	0.505191661	-0.170791554	0.86438767	0.972350383	down_in_case
CRA004775	ENSGALG00010020766	C2orf76	case	control	-0.422157618	0.256943631	-1.642997014	0.100383551	0.538583505	down_in_case
CRA004775	ENSGALG00010020767	UBXN10	case	control	0.33213295	0.277398179	1.197314818	0.231183871	0.69761014	up_in_case
CRA004775	ENSGALG00010020770	B4GALNT4	case	control	-0.430223497	0.515882618	-0.833956181	0.404305677	0.81313407	down_in_case
CRA004775	ENSGALG00010020771	LINGO1	case	control	0.193391725	0.580274208	0.333276445	0.738925618	0.938064864	up_in_case
CRA004775	ENSGALG00010020773	ZCCHC24	case	control	0.67146132	0.348625967	1.926022111	0.054101613	0.428296649	up_in_case
CRA004775	ENSGALG00010020775	UBR1	case	control	0.240592301	0.231790582	1.037972722	0.299282755	0.75239174	up_in_case
CRA004775	ENSGALG00010020776	MCOLN3	case	control	1.062748276	0.684802868	1.551903951	0.120685207	0.572850296	up_in_case
CRA004775	ENSGALG00010020778	SLIT1	case	control	0.549610847	0.356643034	1.541067103	0.123300455	0.575554958	up_in_case
CRA004775	ENSGALG00010020779	SLC15A4	case	control	0.002093741	0.122219787	0.017130947	0.98633215	0.997562105	up_in_case
CRA004775	ENSGALG00010020781	SNN	case	control	0.357276591	0.320099414	1.116142596	0.264361102	0.724455452	up_in_case
CRA004775	ENSGALG00010020782	PPIF	case	control	0.013773187	0.256765309	0.053641151	0.95722107	0.991431439	up_in_case
CRA004775	ENSGALG00010020786	DDX19	case	control	0.219849188	0.154558925	1.422429589	0.154901579	0.619284425	up_in_case
CRA004775	ENSGALG00010020790	PRPH2L	case	control	0.226870229	0.477093532	0.475525686	0.63441233	0.909837348	up_in_case
CRA004775	ENSGALG00010020794	STEAP3	case	control	-0.242237679	0.38790612	-0.624475013	0.532315675	0.869886675	down_in_case
CRA004775	ENSGALG00010020797	DGCR8	case	control	0.186008932	0.300078668	0.61986723	0.535345202	0.870981055	up_in_case
CRA004775	ENSGALG00010020798	C1QL2	case	control	-0.331658363	0.634241525	-0.522921238	0.601029064	0.89775697	down_in_case
CRA004775	ENSGALG00010020803	ABCC6	case	control	-0.081674838	0.320580551	-0.254771657	0.798899467	0.952835572	down_in_case
CRA004775	ENSGALG00010020806	HTD2	case	control	-0.397802286	0.337454171	-1.178833515	0.238464476	0.705036553	down_in_case
CRA004775	ENSGALG00010020809	ENSGALG00010020809	case	control	-0.400594861	0.629575497	-0.636293602	0.524585074	0.867119769	down_in_case
CRA004775	ENSGALG00010020810	IFITM5	case	control	-0.09656481	0.187630277	-0.514654731	0.606794312	0.900898526	down_in_case
CRA004775	ENSGALG00010020811	MCOLN2	case	control	-0.238198111	0.900649671	-0.264473656	0.791414962	0.951284723	down_in_case
CRA004775	ENSGALG00010020813	NPY4R	case	control	-0.595265888	0.64005489	-0.930023186	0.35235908	0.786426886	down_in_case
CRA004775	ENSGALG00010020816	TRMT2A	case	control	-0.320338321	0.213861302	-1.49787885	0.13416473	0.592323374	down_in_case
CRA004775	ENSGALG00010020818	TMEM62	case	control	-0.303187687	0.212162894	-1.429032577	0.15299487	0.617472961	down_in_case
CRA004775	ENSGALG00010020820	VWA5B1	case	control	-0.03047707	0.650246721	-0.046870009	0.962616831	0.991860454	down_in_case
CRA004775	ENSGALG00010020824	ENSGALG00010020824	case	control	2.12646192	0.733546157	2.898879503	0.003744988	0.126529062	up_in_case
CRA004775	ENSGALG00010020827	ENSGALG00010020827	case	control	-0.336352063	0.341463281	-0.985031429	0.324608633	0.770185561	down_in_case
CRA004775	ENSGALG00010020828	CAMK2N1	case	control	0.30759245	0.383691474	0.801666106	0.422746127	0.820450913	up_in_case
CRA004775	ENSGALG00010020831	MUL1	case	control	-0.132858292	0.254737856	-0.521549072	0.60198433	0.898185892	down_in_case
CRA004775	ENSGALG00010020833	RANBP1	case	control	0.144493024	0.156225089	0.924902817	0.355016461	0.788279103	up_in_case
CRA004775	ENSGALG00010020835	PTGR2	case	control	0.174864362	0.259574425	0.673657899	0.500528834	0.859809119	up_in_case
CRA004775	ENSGALG00010020839	PGGHG	case	control	-0.496062915	0.30255511	-1.639578705	0.101092794	0.54048484	down_in_case
CRA004775	ENSGALG00010020843	HMG20A	case	control	-0.658829427	0.373989502	-1.761625457	0.0781326	0.490500984	down_in_case

CRA004775	ENSGALG00010020845	RAB35	case	control	0.021987319	0.133755414	0.164384518	0.86942845	0.973496509	up_in_case
CRA004775	ENSGALG00010020847	MIDEAS	case	control	0.161056662	0.457988431	0.351660985	0.725092524	0.935238044	up_in_case
CRA004775	ENSGALG00010020854	ENSGALG00010020854	case	control	0.444692516	0.727853191	0.610964576	0.541223032	0.874673178	up_in_case
CRA004775	ENSGALG00010020855	DNAL1	case	control	0.006725158	0.237262864	0.028344757	0.977387184	0.995827348	up_in_case
CRA004775	ENSGALG00010020861	CDA	case	control	0.245572678	0.296239149	0.828967675	0.407122699	0.813842524	up_in_case
CRA004775	ENSGALG00010020866	LCOR	case	control	-0.043915471	0.250008156	-0.175656153	0.860564084	0.971325143	down_in_case
CRA004775	ENSGALG00010020870	RIOX1	case	control	-0.125726781	0.322383224	-0.389991697	0.696542687	0.929092636	down_in_case
CRA004775	ENSGALG00010020873	PINK1	case	control	-0.259381682	0.360128313	-0.720247957	0.471372342	0.845200296	down_in_case
CRA004775	ENSGALG00010020875	ENSGALG00010020875	case	control	-0.350133889	0.449088026	-0.779655364	0.435593759	0.826905159	down_in_case
CRA004775	ENSGALG00010020878	XYLT1	case	control	-0.745255412	0.483953867	-1.539930689	0.123577249	0.576232877	down_in_case
CRA004775	ENSGALG00010020880	PXK	case	control	0.071696634	0.2109975	0.339798499	0.734008278	0.936819725	up_in_case
CRA004775	ENSGALG00010020886	GBF1	case	control	-0.00556153	0.181234514	-0.030686926	0.975519218	0.995255743	down_in_case
CRA004775	ENSGALG00010020887	ARPP19	case	control	0.145019662	0.165959492	0.873825657	0.382213206	0.801753951	up_in_case
CRA004775	ENSGALG00010020889	PPRC1	case	control	0.060736004	0.423098938	0.143550359	0.885855542	0.976802138	up_in_case
CRA004775	ENSGALG00010020890	RPS15A	case	control	-0.341055848	0.283513934	-1.20295974	0.2289919	0.695782399	down_in_case
CRA004775	ENSGALG00010020894	PSMD13	case	control	-0.654512457	0.33258506	-1.967955076	0.049073207	0.410291029	down_in_case
CRA004775	ENSGALG00010020896	MARCO	case	control	0.192842085	0.625562288	0.308269998	0.757876893	0.94201981	up_in_case
CRA004775	ENSGALG00010020898	ENSGALG00010020898	case	control	-0.527765547	0.288545961	-1.829051927	0.06739183	0.463352525	down_in_case
CRA004775	ENSGALG00010020900	PDHB	case	control	0.155024966	0.226569195	0.684227907	0.493831262	0.856049389	up_in_case
CRA004775	ENSGALG00010020903	AGMAT	case	control	0.353367206	0.543682546	0.649951352	0.515723646	0.864897625	up_in_case
CRA004775	ENSGALG00010020905	KCTD6	case	control	-0.081606415	0.194110579	-0.420411991	0.67418451	0.922533825	down_in_case
CRA004775	ENSGALG00010020906	SIRT3	case	control	-0.527209129	0.341987036	-1.541605594	0.123169465	0.575549148	down_in_case
CRA004775	ENSGALG00010020912	PDE8A	case	control	0.311489454	0.439011607	0.709524416	0.477999106	0.846704329	up_in_case
CRA004775	ENSGALG00010020915	EN1	case	control	-0.358777747	0.718027073	-0.499671614	0.617306323	0.903905787	down_in_case
CRA004775	ENSGALG00010020916	ARL6IP1	case	control	-0.279991471	0.264976228	-1.056666378	0.290663875	0.74447127	down_in_case
CRA004775	ENSGALG00010020918	ENSGALG00010020918	case	control	0.472380209	0.566413288	0.833985041	0.404289413	0.81313407	up_in_case
CRA004775	ENSGALG00010020920	ACOX2	case	control	-0.295847699	0.531158946	-0.556985251	0.577537506	0.888339319	down_in_case
CRA004775	ENSGALG00010020921	LDB1	case	control	-0.406370731	0.239139707	-1.699302624	0.089262178	0.515997712	down_in_case
CRA004775	ENSGALG00010020922	INSIG2	case	control	-0.016806973	0.173826434	-0.096688244	0.922973976	0.983159417	down_in_case
CRA004775	ENSGALG00010020924	DNAJC16	case	control	-0.201775363	0.244350677	-0.825761423	0.408939442	0.814384687	down_in_case
CRA004775	ENSGALG00010020925	RIC8A	case	control	-0.674698788	0.293897768	-2.295692111	0.021693492	0.288534247	down_in_case
CRA004775	ENSGALG00010020927	ENSGALG00010020927	case	control	-0.166871689	0.426873933	-0.390915621	0.695859607	0.929092636	down_in_case
CRA004775	ENSGALG00010020928	AACS	case	control	-0.093867316	0.326144762	-0.287808748	0.773493141	0.946296525	down_in_case
CRA004775	ENSGALG00010020932	CASP9	case	control	-0.442998741	0.239059566	-1.853089374	0.06386955	0.455062268	down_in_case
CRA004775	ENSGALG00010020934	ABCD3	case	control	0.140388408	0.373976486	0.375393678	0.707367706	0.931617022	up_in_case
CRA004775	ENSGALG00010020935	BRI3BP	case	control	-0.078376979	0.303188581	-0.258509008	0.796014098	0.952835572	down_in_case
CRA004775	ENSGALG00010020936	BET1L	case	control	-0.401576144	0.25481715	-1.575938451	0.115039989	0.565575575	down_in_case
CRA004775	ENSGALG00010020938	SMG1	case	control	0.282654235	0.286754681	0.985700508	0.3242801	0.7698853	up_in_case
CRA004775	ENSGALG00010020939	CTRC	case	control	2.540513536	1.545134739	1.64420194	0.100134496	0.538583505	up_in_case
CRA004775	ENSGALG00010020940	ENSGALG00010020940	case	control	0.270721105	0.43117169	0.627873099	0.530087075	0.869211096	up_in_case
CRA004775	ENSGALG00010020941	ENSGALG00010020941	case	control	0.509668071	0.350103274	1.455764939	0.145457611	0.60869982	up_in_case
CRA004775	ENSGALG00010020943	FAM107A	case	control	-0.376942433	0.806688287	-0.467271484	0.640305656	0.911610834	down_in_case
CRA004775	ENSGALG00010020944	MYO5A	case	control	-0.082718096	0.469161716	-0.176310413	0.860050082	0.971325143	down_in_case
CRA004775	ENSGALG00010020945	NUMB	case	control	0.577570569	0.165616202	3.487403772	0.000487734	0.045012321	up_in_case
CRA004775	ENSGALG00010020946	CTBS	case	control	-0.079411459	0.403886732	-0.196618144	0.844126371	0.966493067	down_in_case
CRA004775	ENSGALG00010020949	HPS6	case	control	-0.596923287	0.257565512	-2.317559063	0.020473297	0.281976732	down_in_case
CRA004775	ENSGALG00010020950	EFHD2	case	control	-0.870751295	0.439224394	-1.982474804	0.047426125	0.404519261	down_in_case
CRA004775	ENSGALG00010020952	GNG5	case	control	-0.378757675	0.274708234	-1.378763459	0.167967696	0.63138062	down_in_case
CRA004775	ENSGALG00010020954	PLIN1	case	control	-2.396505531	0.684912469	-3.498995331	0.000467015	0.044470881	down_in_case
CRA004775	ENSGALG00010020957	RPF1	case	control	0.038892481	0.262476967	0.148174833	0.882204793	0.975346523	up_in_case
CRA004775	ENSGALG00010020959	ENSGALG00010020959	case	control	0.799105706	0.703691206	1.135591433	0.256127596	0.719236059	up_in_case
CRA004775	ENSGALG00010020960	CLEC19A	case	control	-0.597766928	0.719897642	-0.83034989	0.406340989	0.813842524	down_in_case
CRA004775	ENSGALG00010020963	CCDC93	case	control	0.230155281	0.219808818	1.047070283	0.295067164	0.747369418	up_in_case
CRA004775	ENSGALG00010020965	DHX37	case	control	-0.250194918	0.240770438	-1.03914301	0.29873823	0.752069968	down_in_case
CRA004775	ENSGALG00010020966	PAPLN	case	control	0.482708777	0.574137044	0.840755325	0.40048502	0.811868062	up_in_case
CRA004775	ENSGALG00010020967	VPS51	case	control	-0.517613029	0.226105066	-2.289258869	0.022064316	0.290827737	down_in_case
CRA004775	ENSGALG00010020969	RBP3	case	control	0.213671109	0.929393608	0.229903786	0.818166534	0.959889304	up_in_case
CRA004775	ENSGALG00010020970	ARMH3	case	control	-0.272327941	0.218913834	-1.24399603	0.213501024	0.682915232	down_in_case
CRA004775	ENSGALG00010020971	TMEM51	case	control	-0.155280307	0.395972396	-0.392149323	0.694947885	0.928924129	down_in_case
CRA004775	ENSGALG00010020973	GNB5	case	control	0.120605726	0.313360724	0.384878247	0.700327623	0.929842957	up_in_case
CRA004775	ENSGALG00010020975	HCCS	case	control	0.136764438	0.239667255	0.570642987	0.568241673	0.884508497	up_in_case
CRA004775	ENSGALG00010020976	GDF10	case	control	-0.149485739	0.513666827	-0.291016922	0.771038378	0.945853152	down_in_case
CRA004775	ENSGALG00010020978	BCL2L10	case	control	0.354824481	0.329920964	1.075483283	0.282158413	0.738291085	up_in_case
CRA004775	ENSGALG00010020979	ENSGALG00010020979	case	control	-0.19914612	0.271712442	-0.732929705	0.463601304	0.842456502	down_in_case
CRA004775	ENSGALG00010020981	CIT	case	control	-0.85392527	0.75693166	-1.128140511	0.259260583	0.720037471	down_in_case
CRA004775	ENSGALG00010020982	TLCD4	case	control	-0.324084105	0.436781936	-0.741981474	0.45809856	0.839796467	down_in_case
CRA004775	ENSGALG00010020984	PSEN1	case	control	0.055331214	0.25059275	0.220801337	0.825247122	0.961797651	up_in_case
CRA004775	ENSGALG00010020987	NADSYN1	case	control	-0.516532275	0.169774881	-3.042454059	0.002346576	0.098653993	down_in_case
CRA004775	ENSGALG00010020989	MAPK4	case	control	0.360621073	0.412305689	0.874644912	0.381767143	0.801753951	up_in_case
CRA004775	ENSGALG00010020990	UBB	case	control	-0.312730951	0.20910259	-1.495586212	0.134761518	0.592420453	down_in_case
CRA004775	ENSGALG00010020991	ALG14	case	control	-0.09991464	0.314483763	-0.317710011	0.750704917	0.941495694	down_in_case
CRA004775	ENSGALG00010020992	KCNH7	case	control	0.466170073	0.577843073	0.806741648	0.419815344	0.819388688	up_in_case
CRA004775	ENSGALG00010020994	DHCR7	case	control	-0.015877385	0.402199832	-0.039476359	0.968510601	0.993583205	down_in_case
CRA004775	ENSGALG00010020998	SYT17	case	control	0.478494756	0.34587869	1.383417857	0.166536778	0.630194031	up_in_case
CRA004775	ENSGALG00010020999	IFIH1	case	control	-0.34265285	0.396337716	-0.864547672	0.387287104	0.803905475	down_in_case
CRA004775	ENSGALG00010021000	NFKB2	case	control	-0.432158817	0.217356577	-1.98824817	0.04678425	0.402081388	down_in_case
CRA004775	ENSGALG00010021002	SLC44A3	case	control	0.004067252	0.350924329	0.01159011	0.990752637	0.998065748	up_in_case
CRA004775	ENSGALG00010021003	KAZN	case	control	-0.303694993	0.325138278	-0.934048724	0.35027876	0.785466132	down_in_case
CRA004775	ENSGALG00010021004	PSD	case	control	0.083665596	0.361524303	0.231424541	0.816985002	0.959627638	up_in_case
CRA004775	ENSGALG00010021010	LEO1	case	control	-0.151635751	0.171474416	-0.884305395	0.376531377	0.798523018	down_in_case
CRA004775	ENSGALG00010021011	SCARB1	case	control	0.163052155	0.279574801	0.583214776	0.559748725	0.880918893	up_in_case
CRA004775	ENSGALG00010021012	KCNIP2	case	control	-0.195058549	0.915285081	-0.213112344	0.831239337	0.96336891	down_in_case
CRA004775	ENSGALG00010021013	FAP	case	control	-0.113135712	0.549508616	-0.205885238	0.836880557	0.965269623	down_in_case
CRA004775	ENSGALG00010021014	FBXL15	case	control	-0.085322704	0.280610338	-0.304061158	0.761081298	0.942236668	down_in_case
CRA004775	ENSGALG00010021015	RBM25	case	control	0.154050793	0.23405337	0.658186607	0.510418229	0.863170205	up_in_case
CRA004775	ENSGALG00010021016	FHIT	case	control	0.164355392	0.373549361	0.439983062	0.659949375	0.917907174	up_in_case
CRA004775	ENSGALG00010021017	COQ7	case	control	0.253361026	0.272531354	0.929658267	0.352548049	0.786426886	up_in_case
CRA004775	ENSGALG00010021018	F3	case	control	1.072015988	0.466978644	2.29564243	0.021696335	0.288534247	up_in_case
CRA004775	ENSGALG00010021019	VSIG10	case	control	-0.397444223	0.27653191	-1.437245426	0.15064827	0.614342929	down_in_case
CRA004775	ENSGALG00010021020	ZDHHC13	case	control	-0.608436816	0.441426654	-1.378341814	0.168097779	0.63138062	down_in_case
CRA004775	ENSGALG00010021021	GCG	case	control	1.331653267	0.659073498	2.020492814	0.043332295	0.389812978	up_in_case
CRA004775	ENSGALG00010021022	ZFYVE1	case	control	0.175417115	0.244466269	0.717551405	0.47303393	0.845445211	up_in_case
CRA004775	ENSGALG00010021023	MGEA5	case	control	0.378517058	0.220427336	1.717196535	0.085943269	0.507567605	up_in_case
CRA004775	ENSGALG00010021024	ENSGALG00010021024	case	control	-0.413420133	0.278595671	-1.48394313	0.137824008	0.596919036	down_in_case
CRA004775	ENSGALG00010021025	CUEDC2	case	control	0.055048412	0.305662834	0.180095209	0.857077824	0.970913248	up_in_case
CRA004775	ENSGALG00010021026	ADGRA2	case	control	-0.115949455	0.373570216	-0.310381957	0.756270513	0.941900693	down_in_case
CRA004775	ENSGALG00010021027	PLA2G4A	case	control	-0.28251767	0.494063701	-0.571824382	0.567440957	0.88397877	down_in_case
CRA004775	ENSGALG00010021028	ENSGALG00010021028	case	control	-0.791235062	0.638991832	-1.238255361	0.21562139	0.683654538	down_in_case
CRA004775	ENSGALG00010021029	PRDM2	case	control	0.305672288	0.199794109	1.529936442	0.126032462	0.580487602	up_in_case
CRA004775	ENSGALG00010021030	CSRP3	case	control	-0.916165814	0.699677689	-1.309411217	0.190395094	0.656023968	down_in_case
CRA004775	ENSGALG00010021031	DDX54	case	control	-0.367774936	0.18468559	-1.991356965	0.046441657	0.400639413	down_in_case
CRA004775	ENSGALG00010021032	TMC7	case	control	-0.036404238	0.43545257	-0.083600925	0.933373731	0.985090297	down_in_case
CRA004775	ENSGALG00010021033	RNF122	case	control	-0.262501781	0.360713527	-0.727729239	0.466779348	0.8434807	down_in_case
CRA004775	ENSGALG00010021034	NPM3	case	control	-0.474390765	0.413192161	-1.148111724	0.250922445	0.715684776	down_in_case
CRA004775	ENSGALG00010021036	E2F8	case	control	-0.090287298	0.476515469	-0.189474013	0.849721321	0.968413719	down_in_case
CRA004775	ENSGALG00010021038	PTGS2	case	control	0.736647329	0.529310937	1.39171001	0.16401023	0.629717526	up_in_case
CRA004775	ENSGALG00010021041	GLTP	case	control	-0.517549896	0.361637285	-1.431129803	0.152393017	0.617173463	down_in_case
CRA004775	ENSGALG00010021042	LRRC38	case	control	-0.156052983	0.318257806	-0.490335131	0.623896771	0.906441769	down_in_case
CRA004775	ENSGALG00010021043	PTPRG	case	control	0.304416597	0.296104781	1.028070522	0.303916656	0.756271793	up_in_case
CRA004775	ENSGALG00010021044	EIF4G2	case	control	0.257486661	0.292095827	0.881514344	0.378039494	0.799511085	up_in_case
CRA004775	ENSGALG00010021045	DPP4	case	control	0.750988499	0.525661301	1.428654721	0.153103498	0.617472961	up_in_case
CRA004775	ENSGALG00010021046	ENSGALG00010021046	case	control	-0.510871476	0.53277115	-0.958894783	0.337611753	0.778184725	down_in_case
CRA004775	ENSGALG00010021047	TMC5	case	control	1.210093632	0.680615923	1.777939057	0.075413865	0.483342345	up_in_case
CRA004775	ENSGALG00010021048	API5	case	control	-0.012946255	0.16436646	-0.078764577	0.93721988	0.986688955	down_in_case
CRA004775	ENSGALG00010021050	CTR9	case	control	-0.040972878	0.235004931	-0.174349014	0.861591182	0.97165529	down_in_case
CRA004775	ENSGALG00010021051	GDE1	case	control	-0.164629957	0.318509887	-0.516875501	0.60524307	0.899848358	down_in_case
CRA004775	ENSGALG00010021052	CEP15	case	control	-0.083978411	0.304792575	-0.275526434	0.78291182	0.94936527	down_in_case
CRA004775	ENSGALG00010021055	IRAG1	case	control	0.613931971	0.381276582	1.610201099	0.107353962	0.553800541	up_in_case
CRA004775	ENSGALG00010021057	ZDHHC8	case	control	0.11310684	0.347905314	0.325108112	0.74509925	0.939383862	up_in_case
CRA004775	ENSGALG00010021058	CCP110	case	control	0.350452406	0.319315822	1.097510307	0.272418377	0.73036169	up_in_case
CRA004775	ENSGALG00010021062	MYO9A	case	control	0.084073694	0.256166618	0.32819926	0.742761007	0.939191413	up_in_case
CRA004775	ENSGALG00010021064	LYVE1	case	control	0.407819401	0.488243122	0.835279357	0.403560438	0.812550381	up_in_case
CRA004775	ENSGALG00010021065	CNNM2	case	control	-0.18777688	0.188345264	-0.996982224	0.318773138	0.767037518	down_in_case
CRA004775	ENSGALG00010021069	PGAM5	case	control	-0.294324432	0.205349161	-1.433287724	0.151775629	0.616199194	down_in_case
CRA004775	ENSGALG00010021070	RNF141	case	control	0.078631271	0.173716214	0.452642094	0.650806482	0.915614449	up_in_case
CRA004775	ENSGALG00010021071	HIF1AN	case	control	-0.139535238	0.317096285	-0.440040597	0.659907705	0.917907174	down_in_case

CRA004775	ENSGALG00010021072	ENSGALG00010021072	case	control	0.551124138	0.134800937	4.088429594	4.34303E-05	0.013292084	up_in_case
CRA004775	ENSGALG00010021073	NDUFB8	case	control	-0.209003734	0.347140262	-0.602072872	0.547125631	0.877099134	down_in_case
CRA004775	ENSGALG00010021074	TTC17	case	control	0.033860621	0.223808586	0.151292772	0.879744771	0.974996282	up_in_case
CRA004775	ENSGALG00010021075	DHRS3	case	control	-0.207741061	0.277993983	-0.747286176	0.454890835	0.838386795	down_in_case
CRA004775	ENSGALG00010021077	SEC31B	case	control	-0.217768566	0.211052758	-1.031820517	0.302156206	0.754801023	down_in_case
CRA004775	ENSGALG00010021078	ENSGALG00010021078	case	control	-0.581706269	0.515136021	-1.129228487	0.25880146	0.720037471	down_in_case
CRA004775	ENSGALG00010021080	TTLL7	case	control	0.392832985	0.40918282	0.960042716	0.337033717	0.777478719	up_in_case
CRA004775	ENSGALG00010021081	KCNH6	case	control	0.101291349	0.519069333	0.195140306	0.845283109	0.966993336	up_in_case
CRA004775	ENSGALG00010021082	SENP8	case	control	-0.018164832	0.341533608	-0.053186075	0.95758365	0.991431439	down_in_case
CRA004775	ENSGALG00010021083	SCD	case	control	0.750065822	0.559215188	1.341282994	0.179828589	0.644990528	up_in_case
CRA004775	ENSGALG00010021085	AMPD3	case	control	0.063469974	0.517910647	0.122550046	0.902463413	0.979310476	up_in_case
CRA004775	ENSGALG00010021086	PSMD14	case	control	-0.260049863	0.259815019	-1.000903889	0.316873276	0.765974554	down_in_case
CRA004775	ENSGALG00010021087	THBS1	case	control	0.449715317	0.462370491	0.972629796	0.330737328	0.77237154	up_in_case
CRA004775	ENSGALG00010021088	ANKLE2	case	control	0.261299511	0.196540084	1.329497299	0.183683956	0.64798309	up_in_case
CRA004775	ENSGALG00010021090	ADM	case	control	0.578931756	0.468130527	1.23668875	0.216202657	0.683774974	up_in_case
CRA004775	ENSGALG00010021091	TANK	case	control	-0.070585526	0.174304575	-0.404955096	0.685510519	0.926686318	down_in_case
CRA004775	ENSGALG00010021092	VPS13D	case	control	0.390335103	0.182432906	2.139609086	0.032386372	0.343100923	up_in_case
CRA004775	ENSGALG00010021093	GRAMD2	case	control	0.774989349	0.636180009	1.218191925	0.223151048	0.690760052	up_in_case
CRA004775	ENSGALG00010021094	ENSGALG00010021094	case	control	0.733203188	0.390092975	1.87956009	0.060168057	0.445397937	up_in_case
CRA004775	ENSGALG00010021095	NT5C2	case	control	0.120721554	0.163075836	0.740278617	0.459130953	0.840589413	up_in_case
CRA004775	ENSGALG00010021096	ZFAND4	case	control	0.130929859	0.215913966	0.606398284	0.544250307	0.875729696	up_in_case
CRA004775	ENSGALG00010021097	KATNBL1	case	control	0.285374988	0.196946919	1.448994427	0.147339139	0.610443055	up_in_case
CRA004775	ENSGALG00010021098	SDK1	case	control	0.605044024	0.534097063	1.132835333	0.257283411	0.719236059	up_in_case
CRA004775	ENSGALG00010021099	HMCN1	case	control	-0.577809674	0.550831904	-1.048976413	0.294188975	0.746438942	down_in_case
CRA004775	ENSGALG00010021100	EMC7	case	control	-0.337842987	0.202157185	-1.671189607	0.094684232	0.526933666	down_in_case
CRA004775	ENSGALG00010021102	GOLGA3	case	control	0.273354246	0.28693138	0.952681599	0.340751419	0.780875343	up_in_case
CRA004775	ENSGALG00010021103	RBMS1	case	control	0.299344711	0.138849006	2.155901007	0.031091396	0.336379059	up_in_case
CRA004775	ENSGALG00010021104	INA	case	control	0.534778263	0.494974904	1.080414903	0.279957463	0.73661445	up_in_case
CRA004775	ENSGALG00010021105	ENSGALG00010021105	case	control	0.987172226	0.644165311	1.532482748	0.125403352	0.578470811	up_in_case
CRA004775	ENSGALG00010021106	ITGB6	case	control	0.4757835	0.874691638	0.543944265	0.586479827	0.891519357	up_in_case
CRA004775	ENSGALG00010021108	PCGF6	case	control	-0.347691946	0.199476447	-1.743022557	0.081329675	0.496834143	down_in_case
CRA004775	ENSGALG00010021110	PKLR	case	control	0.022057533	0.591717271	0.037277148	0.970264026	0.994414835	up_in_case
CRA004775	ENSGALG00010021111	AVEN	case	control	0.323172335	0.353953997	0.91303485	0.361224213	0.792567091	up_in_case
CRA004775	ENSGALG00010021112	TAF5	case	control	-0.076714035	0.20405166	-0.375953986	0.706951104	0.931617022	down_in_case
CRA004775	ENSGALG00010021113	SYP	case	control	2.235618044	0.511580758	4.370019801	1.24235E-05	0.005510836	up_in_case
CRA004775	ENSGALG00010021114	SBF2	case	control	0.303694513	0.305240644	0.994934714	0.319768018	0.767037518	up_in_case
CRA004775	ENSGALG00010021115	ALPL	case	control	-0.044158645	0.415222425	-0.106349373	0.91530516	0.981469218	down_in_case
CRA004775	ENSGALG00010021116	FAM21C	case	control	-0.037248922	0.205892797	-0.180914156	0.856434958	0.970885169	down_in_case
CRA004775	ENSGALG00010021117	CYP1A2	case	control	2.191879678	0.496486435	4.414782606	1.01112E-05	0.004794431	up_in_case
CRA004775	ENSGALG00010021118	USMG5	case	control	0.24054378	0.279619811	0.860252996	0.389649597	0.805846234	up_in_case
CRA004775	ENSGALG00010021120	PDCD11	case	control	-0.306499831	0.276080884	-1.110181287	0.266920915	0.727315996	down_in_case
CRA004775	ENSGALG00010021121	PLA2R1	case	control	0.024909556	0.366233608	0.068015483	0.945773309	0.988886825	up_in_case
CRA004775	ENSGALG00010021122	CYP1A1	case	control	2.51543858	0.716031723	3.513026729	0.000443033	0.043515325	up_in_case
CRA004775	ENSGALG00010021123	RYR3	case	control	0.895533332	0.635572046	1.409019383	0.158829437	0.624764009	up_in_case
CRA004775	ENSGALG00010021124	CALHM2	case	control	-0.268558895	0.350982241	-0.765163769	0.444174031	0.832130395	down_in_case
CRA004775	ENSGALG00010021125	LY75	case	control	0.131470167	0.401409104	0.327521636	0.743273381	0.939218065	up_in_case
CRA004775	ENSGALG00010021126	ECE1	case	control	0.089675675	0.252991965	0.354460566	0.722993752	0.935185555	up_in_case
CRA004775	ENSGALG00010021127	SWAP70	case	control	0.38596204	0.289869838	1.331501212	0.183024146	0.647649264	up_in_case
CRA004775	ENSGALG00010021129	TXNRD3	case	control	-0.243490255	0.222676424	-1.093471192	0.274186971	0.731001618	down_in_case
CRA004775	ENSGALG00010021130	ENSGALG00010021130	case	control	0.272120434	0.560183517	0.485770155	0.627130137	0.907733515	up_in_case
CRA004775	ENSGALG00010021131	EDC3	case	control	-0.344161975	0.168556034	-2.041825302	0.041168863	0.379270506	down_in_case
CRA004775	ENSGALG00010021133	ENSGALG00010021133	case	control	-0.124028957	0.344979979	-0.359525086	0.719202314	0.93407508	down_in_case
CRA004775	ENSGALG00010021134	CHFR	case	control	0.002564426	0.314116795	0.008163927	0.993486201	0.998715458	up_in_case
CRA004775	ENSGALG00010021135	CLK3	case	control	-0.109734452	0.253302036	-0.433215831	0.664857979	0.918655755	down_in_case
CRA004775	ENSGALG00010021136	WEE1	case	control	0.528153075	0.337422839	1.565255858	0.117522929	0.568234105	up_in_case
CRA004775	ENSGALG00010021137	PLAU	case	control	0.629810891	0.4740552	1.328560241	0.183993096	0.648574486	up_in_case
CRA004775	ENSGALG00010021138	DDX18	case	control	0.039248154	0.213426663	0.183895272	0.854095614	0.970073177	up_in_case
CRA004775	ENSGALG00010021139	ARHGAP29	case	control	0.352568001	0.172882228	2.03935364	0.041414746	0.380794507	up_in_case
CRA004775	ENSGALG00010021140	SLC11A1	case	control	-0.96139181	0.579318691	-1.659521478	0.097010756	0.53104893	down_in_case
CRA004775	ENSGALG00010021141	SGSM1	case	control	0.237279971	0.418680501	0.566732794	0.570895737	0.885321726	up_in_case
CRA004775	ENSGALG00010021142	WNT6	case	control	-0.140491581	0.711650779	-0.197416465	0.843501646	0.966478468	down_in_case
CRA004775	ENSGALG00010021144	FOXK1	case	control	0.187197248	0.202468447	0.924574919	0.355187064	0.788279103	up_in_case
CRA004775	ENSGALG00010021145	EIF4G3	case	control	0.296076763	0.339971002	0.870888286	0.383815154	0.802591572	up_in_case
CRA004775	ENSGALG00010021146	AP5Z1	case	control	-0.384309228	0.206259752	-1.863229371	0.062430002	0.448843826	down_in_case
CRA004775	ENSGALG00010021147	ARID3B	case	control	-0.182705335	0.250047403	-0.730682796	0.464972927	0.843383332	down_in_case
CRA004775	ENSGALG00010021148	GPATCH8	case	control	-0.000508636	0.145632984	-0.00349259	0.997213322	0.999619266	down_in_case
CRA004775	ENSGALG00010021150	ENSGALG00010021150	case	control	-1.154871326	0.667762878	-1.729463204	0.083726227	0.502759538	down_in_case
CRA004775	ENSGALG00010021152	STK11IP	case	control	-0.774915436	0.337347594	-2.297083036	0.021614033	0.288534247	down_in_case
CRA004775	ENSGALG00010021153	PTPRF	case	control	0.795395695	0.336650791	2.362672883	0.018143677	0.268272793	up_in_case
CRA004775	ENSGALG00010021154	CHST13	case	control	0.575420103	0.387163089	1.486247322	0.137213715	0.596242097	up_in_case
CRA004775	ENSGALG00010021155	RADIL	case	control	-0.355711826	0.27863207	-1.276636338	0.201730667	0.670047922	down_in_case
CRA004775	ENSGALG00010021156	DDOST	case	control	-0.384014207	0.209319925	-1.834580282	0.066567902	0.462498477	down_in_case
CRA004775	ENSGALG00010021157	ENSGALG00010021157	case	control	-0.802425957	0.509856014	-1.573828562	0.115527089	0.566086328	down_in_case
CRA004775	ENSGALG00010021158	ACTG1L	case	control	-0.360855126	0.242910639	-1.485546815	0.137399032	0.596242097	down_in_case
CRA004775	ENSGALG00010021159	MMD2	case	control	-0.504698394	0.600845331	-0.839980554	0.40091929	0.811868062	down_in_case
CRA004775	ENSGALG00010021160	ZNF143	case	control	0.043549858	0.255770881	0.170269021	0.864798573	0.972350383	up_in_case
CRA004775	ENSGALG00010021161	ENSGALG00010021161	case	control	-0.203268283	0.338377375	-0.600714758	0.548029988	0.877369241	down_in_case
CRA004775	ENSGALG00010021162	WIPI2	case	control	-0.567823312	0.348067209	-1.63136112	0.102814144	0.543350227	down_in_case
CRA004775	ENSGALG00010021163	INHA	case	control	-0.937783991	0.804015953	-1.166374855	0.24346292	0.710496311	down_in_case
CRA004775	ENSGALG00010021164	SLC41A3	case	control	-0.182628168	0.584421932	-0.312493693	0.754665356	0.941900693	down_in_case
CRA004775	ENSGALG00010021165	PTRF	case	control	0.062944783	0.399332779	0.157624884	0.874752394	0.974301166	up_in_case
CRA004775	ENSGALG00010021166	NEURL1	case	control	-0.169905948	0.605753642	-0.280486879	0.779103991	0.94792594	down_in_case
CRA004775	ENSGALG00010021167	KIAA1671	case	control	-0.011691198	0.378693081	-0.030872488	0.975371231	0.995245303	down_in_case
CRA004775	ENSGALG00010021168	IVNS1ABP	case	control	-0.183459055	0.149188458	-1.229713458	0.218804425	0.686309227	down_in_case
CRA004775	ENSGALG00010021169	CAMK2G	case	control	0.459081631	0.276447118	1.660649006	0.096783961	0.530864077	up_in_case
CRA004775	ENSGALG00010021170	UBL7	case	control	-0.434271619	0.270760508	-1.603895717	0.108737041	0.555936379	down_in_case
CRA004775	ENSGALG00010021171	ENSGALG00010021171	case	control	0.355576142	0.266682408	1.333331828	0.182422933	0.647378665	up_in_case
CRA004775	ENSGALG00010021172	SLC29A4	case	control	-0.855565427	0.356783843	-2.397993752	0.016485146	0.254704773	down_in_case
CRA004775	ENSGALG00010021173	KLF15	case	control	0.492958372	0.52333987	0.941946907	0.346219823	0.784552667	up_in_case
CRA004775	ENSGALG00010021174	CRYBB3	case	control	0.513809076	0.71011614	0.723556397	0.469338119	0.8440023	up_in_case
CRA004775	ENSGALG00010021175	IPO7	case	control	0.15529032	0.166643288	0.931872635	0.351402351	0.786113067	up_in_case
CRA004775	ENSGALG00010021176	ENSGALG00010021176	case	control	0.056854716	0.290887292	0.195452732	0.845038538	0.966990128	up_in_case
CRA004775	ENSGALG00010021177	OBSL1	case	control	-0.530462656	0.621072209	-0.854107861	0.393045229	0.807553294	down_in_case
CRA004775	ENSGALG00010021178	NDST2	case	control	-0.174040276	0.235052425	-0.740431739	0.459038066	0.840589413	down_in_case
CRA004775	ENSGALG00010021180	TMEM198	case	control	-0.296282178	0.319767453	-0.926555142	0.354157555	0.78730441	down_in_case
CRA004775	ENSGALG00010021181	DAGLA	case	control	-0.614807032	0.327417187	-1.877748197	0.06041562	0.445397937	down_in_case
CRA004775	ENSGALG00010021182	ZSWIM8	case	control	-0.108631498	0.233430172	-0.465370423	0.641666211	0.912545911	down_in_case
CRA004775	ENSGALG00010021183	CFAP100	case	control	-0.522038086	0.808273019	-0.645868504	0.518364518	0.865435399	down_in_case
CRA004775	ENSGALG00010021184	DRAXIN	case	control	-0.141144673	0.387268312	-0.364462231	0.715512872	0.933870859	down_in_case
CRA004775	ENSGALG00010021185	ENSGALG00010021185	case	control	-0.575497786	0.538311976	-1.069078548	0.285034277	0.741207705	down_in_case
CRA004775	ENSGALG00010021186	SWT1	case	control	0.178529307	0.172194302	1.036789864	0.2998338	0.752512244	up_in_case
CRA004775	ENSGALG00010021187	SEMA7A	case	control	0.385429389	0.428919104	0.898606255	0.368862426	0.794896739	up_in_case
CRA004775	ENSGALG00010021188	TNRC18	case	control	-0.0146883	0.300057656	-0.048951592	0.960957874	0.991824137	down_in_case
CRA004775	ENSGALG00010021189	ENSGALG00010021189	case	control	-0.128646218	0.513476889	-0.250539451	0.8021702	0.953744693	down_in_case
CRA004775	ENSGALG00010021190	ENSGALG00010021190	case	control	0.002909933	0.225732726	0.012891057	0.98971471	0.998065748	up_in_case
CRA004775	ENSGALG00010021191	MYRF	case	control	-0.181702819	0.451775464	-0.402197184	0.687538915	0.927111919	down_in_case
CRA004775	ENSGALG00010021192	ENSGALG00010021192	case	control	-0.113786299	0.366989268	-0.310053478	0.756520289	0.941900693	down_in_case
CRA004775	ENSGALG00010021193	USP37	case	control	-0.018710306	0.216784037	-0.086308503	0.931221179	0.985044263	down_in_case
CRA004775	ENSGALG00010021194	CYP11A1	case	control	-0.397134881	0.382744681	-1.037597387	0.299457535	0.752512244	down_in_case
CRA004775	ENSGALG00010021195	GCLM	case	control	-0.020428381	0.265597346	-0.076914855	0.93869128	0.986770414	down_in_case
CRA004775	ENSGALG00010021196	ATP6V0A1	case	control	-0.427394515	0.256942882	-1.663383361	0.096235722	0.529546782	down_in_case
CRA004775	ENSGALG00010021197	CHCHD1	case	control	0.11827008	0.310715752	0.380637541	0.703472221	0.93012388	up_in_case
CRA004775	ENSGALG00010021199	TMEM258	case	control	-0.282972755	0.274543954	-1.030701101	0.302681008	0.755164773	down_in_case
CRA004775	ENSGALG00010021200	FTO	case	control	-0.035023246	0.49513492	-0.070734752	0.943608862	0.987862092	down_in_case
CRA004775	ENSGALG00010021201	TRMT1L	case	control	0.273274577	0.211789046	1.29031497	0.196941321	0.66285976	up_in_case
CRA004775	ENSGALG00010021203	FUT11	case	control	0.268627983	0.228895685	1.173582558	0.240562271	0.707284967	up_in_case
CRA004775	ENSGALG00010021204	NAGLU	case	control	-0.199788399	0.179371721	-1.113823282	0.265355007	0.726437726	down_in_case
CRA004775	ENSGALG00010021206	HSD17B1	case	control	-0.07987035	0.392687916	-0.20339396	0.838827121	0.965800667	down_in_case
CRA004775	ENSGALG00010021207	MAD2L2	case	control	-0.375718941	0.288261387	-1.303396703	0.192439361	0.656959697	down_in_case
CRA004775	ENSGALG00010021210	ENSGALG00010021210	case	control	-0.039241711	0.221782164	-0.176938085	0.859557023	0.971325143	down_in_case
CRA004775	ENSGALG00010021211	FEN1	case	control	-0.518771434	0.307549132	-1.686792058	0.091643343	0.521441551	down_in_case
CRA004775	ENSGALG00010021212	MYO18B	case	control	0.074177036	0.327877597	0.226233927	0.821019485	0.960766569	up_in_case
CRA004775	ENSGALG00010021213	FBXO44	case	control	-0.393692764	0.199538877	-1.973012829	0.048494101	0.408104271	down_in_case
CRA004775	ENSGALG00010021214	FBXL18	case	control	-0.36974244	0.280884485	-1.316350527	0.188056419	0.652111233	down_in_case
CRA004775	ENSGALG00010021215	SH3PXD2A	case	control	0.198072586	0.300524287	0.659090112	0.509837905	0.862971569	up_in_case
CRA004775	ENSGALG00010021217	FBXO2	case	control	-0.256878903	0.328053151	-0.783040497	0.433603331	0.825828172	down_in_case

CRA004775	ENSGALG00010021218	DPF3	case	control	0.746509032	0.32201715	2.318227563	0.020436957	0.281976732	up_in_case
CRA004775	ENSGALG00010021220	SYNPO2L	case	control	0.49607151	0.672258722	0.737917552	0.460564553	0.841288943	up_in_case
CRA004775	ENSGALG00010021221	ENSGALG00010021221	case	control	1.362003897	0.657332093	2.072017951	0.038263767	0.369867027	up_in_case
CRA004775	ENSGALG00010021222	ENSGALG00010021222	case	control	0.241668994	0.430975693	0.560748547	0.574968967	0.887362319	up_in_case
CRA004775	ENSGALG00010021223	DFFB	case	control	-0.110227225	0.282765224	-0.389818886	0.696670477	0.929092636	down_in_case
CRA004775	ENSGALG00010021224	MYOZ1	case	control	-0.352648085	0.77162157	-0.457022067	0.647655185	0.914210065	down_in_case
CRA004775	ENSGALG00010021227	CHCHD4	case	control	-0.216225893	0.275424361	-0.785064519	0.432415741	0.825510045	down_in_case
CRA004775	ENSGALG00010021228	ESRP2	case	control	2.231262833	0.731886883	3.048644382	0.002298764	0.097262482	up_in_case
CRA004775	ENSGALG00010021229	GLO1	case	control	0.372725396	0.41741414	0.892939074	0.3718898	0.795818272	up_in_case
CRA004775	ENSGALG00010021230	C1orf174	case	control	-0.072328824	0.194712156	-0.371465375	0.710290941	0.932234469	down_in_case
CRA004775	ENSGALG00010021231	STN1	case	control	-1.329005652	0.410034786	-3.241202205	0.001190267	0.072743843	down_in_case
CRA004775	ENSGALG00010021232	ACTB	case	control	-0.426337501	0.291611212	-1.462006549	0.143739415	0.605378468	down_in_case
CRA004775	ENSGALG00010021233	COASY	case	control	-0.109045748	0.167233371	-0.652057347	0.514364178	0.864355593	down_in_case
CRA004775	ENSGALG00010021234	ISLR2	case	control	-1.554308887	0.844001799	-1.841594281	0.065534518	0.45911339	down_in_case
CRA004775	ENSGALG00010021235	XRCC5	case	control	-0.097265345	0.386978047	-0.251345899	0.801546691	0.953543664	down_in_case
CRA004775	ENSGALG00010021236	BTBD9	case	control	0.232598698	0.176499125	1.317846185	0.187555144	0.651609596	up_in_case
CRA004775	ENSGALG00010021237	FSCN1	case	control	-0.77295385	0.470834458	-1.641667972	0.100658833	0.538583505	down_in_case
CRA004775	ENSGALG00010021238	NDUFS8	case	control	-0.000695647	0.393064663	-0.001769802	0.998587903	0.999914248	down_in_case
CRA004775	ENSGALG00010021239	USP54	case	control	0.474999189	0.240520958	1.974876503	0.048282164	0.407414924	up_in_case
CRA004775	ENSGALG00010021240	SLK	case	control	0.459410981	0.310772367	1.478287741	0.139330778	0.599104916	up_in_case
CRA004775	ENSGALG00010021241	TMEM43	case	control	-0.384965085	0.275262134	-1.398539928	0.161950991	0.627882156	down_in_case
CRA004775	ENSGALG00010021242	BCAR3	case	control	0.892964025	0.605830654	1.473949888	0.140495084	0.600007375	up_in_case
CRA004775	ENSGALG00010021243	DYL1	case	control	-0.293093073	0.238307369	-1.22989513	0.218736378	0.686309227	down_in_case
CRA004775	ENSGALG00010021244	PPP3CB	case	control	0.289153608	0.148288276	1.949942473	0.051182976	0.417943648	up_in_case
CRA004775	ENSGALG00010021245	ZFAND3	case	control	0.32143272	0.190876771	1.683980289	0.092185478	0.52187835	up_in_case
CRA004775	ENSGALG00010021246	PECR	case	control	-0.307187174	0.299665342	-1.025100774	0.305315641	0.757865363	down_in_case
CRA004775	ENSGALG00010021247	GNAO1	case	control	-1.660807673	0.608635085	-2.728741268	0.006357656	0.158952969	down_in_case
CRA004775	ENSGALG00010021248	ANXA7	case	control	0.088121117	0.268082694	0.328708713	0.742375868	0.938877854	up_in_case
CRA004775	ENSGALG00010021249	ENSGALG00010021249	case	control	0.280541046	0.639017017	0.439019681	0.660647276	0.917907174	up_in_case
CRA004775	ENSGALG00010021250	ISLR	case	control	0.019007951	0.303822055	0.062562776	0.950114672	0.990168609	up_in_case
CRA004775	ENSGALG00010021251	RNF216	case	control	-0.24907382	0.157715708	-1.579258169	0.114276852	0.564447197	down_in_case
CRA004775	ENSGALG00010021252	XPC	case	control	0.020549111	0.236791355	0.086781506	0.930845188	0.985044263	up_in_case
CRA004775	ENSGALG00010021253	TMEM169	case	control	-0.301181655	0.268440633	-1.121967457	0.261876277	0.72256212	down_in_case
CRA004775	ENSGALG00010021254	FNBP1L	case	control	0.55143733	0.203243955	2.713179486	0.0066641	0.160913119	up_in_case
CRA004775	ENSGALG00010021255	EIF3F	case	control	-0.208084145	0.209632252	-0.992615129	0.320897549	0.767152677	down_in_case
CRA004775	ENSGALG00010021256	ENSGALG00010021256	case	control	-0.198175797	0.331697536	-0.59745936	0.550200732	0.877623839	down_in_case
CRA004775	ENSGALG00010021257	RGS6	case	control	-1.046205892	0.7838819	-1.334647339	0.181991796	0.647378665	down_in_case
CRA004775	ENSGALG00010021258	LSM3	case	control	-0.419644863	0.304910135	-1.376290306	0.168731773	0.632337016	down_in_case
CRA004775	ENSGALG00010021259	CEP104	case	control	3.5289E-05	0.264433278	0.000133451	0.999893521	0.999914248	up_in_case
CRA004775	ENSGALG00010021260	KDM4A	case	control	-0.175863115	0.203520253	-0.864106211	0.387529547	0.804084828	down_in_case
CRA004775	ENSGALG00010021261	JUP	case	control	0.019844676	0.344153363	0.057662304	0.954017621	0.990670511	up_in_case
CRA004775	ENSGALG00010021262	MDGA1	case	control	0.493336158	0.649549915	0.759504614	0.447550755	0.834136489	up_in_case
CRA004775	ENSGALG00010021263	STOML1	case	control	-0.244191969	0.257080729	-0.949864931	0.342180888	0.781616177	down_in_case
CRA004775	ENSGALG00010021264	CBFB	case	control	-0.566935907	0.317942741	-1.78313839	0.074563778	0.481462352	down_in_case
CRA004775	ENSGALG00010021265	SIPA1L1	case	control	0.294936223	0.246239303	1.197762579	0.231009459	0.697389917	up_in_case
CRA004775	ENSGALG00010021266	COQ5	case	control	-0.405363094	0.233406217	-1.736727919	0.082435207	0.499148629	down_in_case
CRA004775	ENSGALG00010021267	LRRC47	case	control	-0.309135719	0.219880594	-1.405925433	0.159746268	0.624907945	down_in_case
CRA004775	ENSGALG00010021269	NUDT13	case	control	0.254169268	0.313857256	0.809824414	0.418041099	0.818943344	up_in_case
CRA004775	ENSGALG00010021271	ENSGALG00010021271	case	control	0.322203981	0.252049547	1.278335884	0.201131021	0.669025802	up_in_case
CRA004775	ENSGALG00010021272	ENSGALG00010021272	case	control	-0.444176934	0.35685611	-1.244694771	0.213243969	0.682777582	down_in_case
CRA004775	ENSGALG00010021275	MAP3K2	case	control	0.074566978	0.211315694	0.352870045	0.724185869	0.935238044	up_in_case
CRA004775	ENSGALG00010021277	ENSGALG00010021277	case	control	0.562312341	0.320254488	1.755829698	0.079117492	0.491399489	up_in_case
CRA004775	ENSGALG00010021284	ENSGALG00010021284	case	control	-2.181445713	0.988574574	-2.206657716	0.027337981	0.316243014	down_in_case
CRA004775	ENSGALG00010021287	COL17A1	case	control	1.212166337	0.764559496	1.585444094	0.11286547	0.562886277	up_in_case
CRA004775	ENSGALG00010021290	ERCC3	case	control	-0.008155013	0.145828071	-0.055922109	0.955403858	0.99078118	down_in_case
CRA004775	ENSGALG00010021293	PCNX1	case	control	0.005776855	0.290329442	0.019897586	0.984125071	0.997534931	up_in_case
CRA004775	ENSGALG00010021296	GATC	case	control	-0.029541557	0.299969125	-0.098481993	0.92154957	0.982481112	down_in_case
CRA004775	ENSGALG00010021298	RPGRIP1L	case	control	-0.410444044	0.36555216	-1.122805685	0.26152003	0.7223728	down_in_case
CRA004775	ENSGALG00010021301	SMURF1	case	control	-0.065400635	0.269226686	-0.242920328	0.808067118	0.955397873	down_in_case
CRA004775	ENSGALG00010021302	P4HA1	case	control	0.459117992	0.213136607	2.154102004	0.031232173	0.336670173	up_in_case
CRA004775	ENSGALG00010021307	MAP3K9	case	control	-0.363519711	0.387048233	-0.939210364	0.347622749	0.785177467	down_in_case
CRA004775	ENSGALG00010021309	ST3GAL3	case	control	-0.208220176	0.402352657	-0.51750665	0.604802528	0.899485135	down_in_case
CRA004775	ENSGALG00010021311	TRIAP1	case	control	-0.048614991	0.270284124	-0.179866247	0.857257574	0.970913248	down_in_case
CRA004775	ENSGALG00010021313	KPNA7	case	control	-1.263174138	1.104968174	-1.143176941	0.252965134	0.717347518	down_in_case
CRA004775	ENSGALG00010021314	ZP3	case	control	3.331773726	0.655375568	5.083762488	3.70031E-07	0.000254415	up_in_case
CRA004775	ENSGALG00010021315	COX6A1	case	control	0.171901864	0.196425202	0.875151774	0.381491329	0.801753951	up_in_case
CRA004775	ENSGALG00010021316	PLA2G12B	case	control	2.071095961	0.651769798	3.177649485	0.001484741	0.080693373	up_in_case
CRA004775	ENSGALG00010021318	TP73	case	control	-0.281964706	0.339674737	-0.830102079	0.406481072	0.813842524	down_in_case
CRA004775	ENSGALG00010021320	SLC6A6	case	control	-0.29389275	0.576541945	-0.509750856	0.610226019	0.901413878	down_in_case
CRA004775	ENSGALG00010021325	ARPC1A	case	control	-0.001804178	0.181383603	-0.009946752	0.992063771	0.998065748	down_in_case
CRA004775	ENSGALG00010021327	IGSF1	case	control	-0.647609745	0.590858325	-1.096049116	0.273057281	0.731001618	down_in_case
CRA004775	ENSGALG00010021329	PSMC3IP	case	control	-0.327447036	0.348158626	-0.940511055	0.346955482	0.784572411	down_in_case
CRA004775	ENSGALG00010021330	COMMD4	case	control	-0.22031784	0.247922341	-0.888656663	0.374187629	0.797992258	down_in_case
CRA004775	ENSGALG00010021332	OIT3	case	control	0.270767003	0.301154845	0.899095625	0.368601728	0.794831796	up_in_case
CRA004775	ENSGALG00010021335	NFATC3	case	control	0.396232649	0.237432118	1.668824977	0.095152071	0.527976505	up_in_case
CRA004775	ENSGALG00010021338	PDIA5	case	control	0.414838747	0.142041233	2.920551579	0.003494124	0.125102734	up_in_case
CRA004775	ENSGALG00010021341	L3MBTL1	case	control	-0.907152202	0.384112375	-2.361684398	0.018192122	0.268700183	down_in_case
CRA004775	ENSGALG00010021342	SFR1	case	control	-1.034984442	0.795262497	-1.301437507	0.193108742	0.657775158	down_in_case
CRA004775	ENSGALG00010021345	NEIL1	case	control	0.016244585	0.260645264	0.062324496	0.950304422	0.990168609	up_in_case
CRA004775	ENSGALG00010021346	RPS21	case	control	-0.104222743	0.281876923	-0.369745569	0.711572075	0.932407723	down_in_case
CRA004775	ENSGALG00010021348	SLC4A3	case	control	-0.427075068	0.367549079	-1.161953851	0.245254211	0.712046495	down_in_case
CRA004775	ENSGALG00010021350	CABLES2	case	control	0.223049122	0.357082338	0.624643391	0.532205135	0.869886675	up_in_case
CRA004775	ENSGALG00010021352	CFAP43	case	control	1.041995382	0.59643491	1.747039557	0.080630477	0.495198609	up_in_case
CRA004775	ENSGALG00010021353	IPO13	case	control	-0.394236019	0.319765782	-1.232889951	0.217616825	0.684927664	down_in_case
CRA004775	ENSGALG00010021354	ENSGALG00010021354	case	control	-0.082457219	0.253332951	-0.325489514	0.744810617	0.939218065	down_in_case
CRA004775	ENSGALG00010021358	MMP15	case	control	-0.446418607	0.371917462	-1.200316342	0.230016505	0.696073276	down_in_case
CRA004775	ENSGALG00010021360	PTPRN	case	control	-0.581195035	0.511274999	-1.136756221	0.255640209	0.718871765	down_in_case
CRA004775	ENSGALG00010021361	WRAP73	case	control	-0.043041506	0.244141578	-0.176297322	0.860060366	0.971325143	down_in_case
CRA004775	ENSGALG00010021362	ARPC1B	case	control	-0.438309135	0.286534892	-1.529688525	0.126093845	0.580487602	down_in_case
CRA004775	ENSGALG00010021363	ENSGALG00010021363	case	control	-0.453455647	0.249721539	-1.815845153	0.069394129	0.468451304	down_in_case
CRA004775	ENSGALG00010021367	GRIP2	case	control	-0.985446612	0.59974116	-1.643119863	0.100358136	0.538583505	down_in_case
CRA004775	ENSGALG00010021372	CD86	case	control	-1.109574656	0.57308004	-1.93616001	0.052848107	0.423142991	down_in_case
CRA004775	ENSGALG00010021373	CEP250	case	control	-0.000183639	0.297909305	-0.000616427	0.999508163	0.999914248	down_in_case
CRA004775	ENSGALG00010021375	TUBG1	case	control	-0.41488099	0.154838336	-2.679446198	0.007374405	0.171004127	down_in_case
CRA004775	ENSGALG00010021378	KIF11	case	control	0.785854751	0.572925018	1.371653754	0.170171251	0.63378857	up_in_case
CRA004775	ENSGALG00010021381	DNAJB2	case	control	0.009727248	0.423656486	0.022960224	0.981682001	0.997069916	up_in_case
CRA004775	ENSGALG00010021382	TPRG1L	case	control	-0.149023759	0.298862688	-0.498636214	0.618035688	0.904143308	down_in_case
CRA004775	ENSGALG00010021383	MSI1	case	control	0.00647112	0.30227045	0.021408377	0.982919891	0.997398088	up_in_case
CRA004775	ENSGALG00010021384	ARTN	case	control	0.396151612	0.537567916	0.736933139	0.46116301	0.841789313	up_in_case
CRA004775	ENSGALG00010021387	SLC2A14	case	control	-0.423537109	0.31598153	-1.34038565	0.180119999	0.644990528	down_in_case
CRA004775	ENSGALG00010021388	DPH2	case	control	-0.672924868	0.248919057	-2.703388305	0.006863651	0.16244762	down_in_case
CRA004775	ENSGALG00010021390	GSTO2	case	control	0.106081501	0.687753332	0.154243529	0.877417722	0.974479909	up_in_case
CRA004775	ENSGALG00010021391	ENSGALG00010021391	case	control	-0.062774512	0.28142635	-0.223058403	0.823490052	0.960898591	down_in_case
CRA004775	ENSGALG00010021393	CCDC174	case	control	0.077617017	0.196171432	0.395659123	0.692356515	0.927867527	up_in_case
CRA004775	ENSGALG00010021394	MAN2C1	case	control	-0.475834877	0.185300818	-2.567904888	0.010231522	0.202829374	down_in_case
CRA004775	ENSGALG00010021396	MED6	case	control	-0.210189802	0.194450464	-1.080942662	0.27972262	0.736447587	down_in_case
CRA004775	ENSGALG00010021397	ENSGALG00010021397	case	control	2.502826983	1.287334814	1.944192728	0.051872214	0.419683701	up_in_case
CRA004775	ENSGALG00010021400	B4GALT2	case	control	-0.270033514	0.261706338	-1.031818778	0.302157021	0.754801023	down_in_case
CRA004775	ENSGALG00010021401	PDAP1	case	control	0.162038265	0.191804556	0.84480926	0.398217344	0.809683083	up_in_case
CRA004775	ENSGALG00010021402	ENSGALG00010021402	case	control	0.125485488	0.235543986	0.532747578	0.594208327	0.894956597	up_in_case
CRA004775	ENSGALG00010021404	UQCC1	case	control	-0.175934389	0.229928458	-0.765170132	0.444170242	0.832130395	down_in_case
CRA004775	ENSGALG00010021405	IDE	case	control	0.444027705	0.335513691	1.323426486	0.185693589	0.6491093	up_in_case
CRA004775	ENSGALG00010021407	SIN3A	case	control	-0.301192095	0.202167909	-1.489811595	0.136273782	0.595518184	down_in_case
CRA004775	ENSGALG00010021408	TTC9	case	control	0.043032309	0.411853623	0.104484474	0.916784889	0.981688766	up_in_case
CRA004775	ENSGALG00010021409	MEGF6	case	control	-0.280291592	0.390972781	-0.716908199	0.473430743	0.845445211	down_in_case
CRA004775	ENSGALG00010021411	MCU	case	control	0.101373257	0.201623882	0.502783977	0.61511616	0.902639483	up_in_case
CRA004775	ENSGALG00010021412	ZNF319	case	control	-0.443766353	0.348316549	-1.274031779	0.202652154	0.671648534	down_in_case
CRA004775	ENSGALG00010021413	SYNJ2BP	case	control	-0.399936007	0.203289322	-1.967324218	0.049145845	0.410291029	down_in_case
CRA004775	ENSGALG00010021414	BUD31	case	control	0.009492452	0.288595288	0.032891916	0.97376078	0.994947809	up_in_case
CRA004775	ENSGALG00010021415	SIRT4	case	control	-0.493439356	0.33470437	-1.474254299	0.140413135	0.600007375	down_in_case
CRA004775	ENSGALG00010021416	PLEKHH3	case	control	-0.185567097	0.393991415	-0.470992741	0.637645918	0.910421454	down_in_case
CRA004775	ENSGALG00010021417	STK16	case	control	-0.697571323	0.322767072	-2.16122208	0.030678189	0.334098251	down_in_case
CRA004775	ENSGALG00010021419	MANSC1	case	control	-0.663890603	0.320544627	-2.07113315	0.038346355	0.370251617	down_in_case
CRA004775	ENSGALG00010021420	PLXNB3	case	control	0.102311084	0.328773438	0.311190235	0.755656006	0.941900693	up_in_case
CRA004775	ENSGALG00010021421	GLB1L	case	control	-0.368773022	0.242772649	-1.519005634	0.128761078	0.583776321	down_in_case
CRA004775	ENSGALG00010021424	PTCD1	case	control	0.075911278	0.236556264	0.320901577	0.748284979	0.940917938	up_in_case

CRA004775	ENSGALG00010021425	PTPN9	case	control	0.040859057	0.308160477	0.132590192	0.89451749	0.977834811	up_in_case
CRA004775	ENSGALG00010021426	SLC38A7	case	control	-0.235659144	0.371979944	-0.633526478	0.526389895	0.86830001	down_in_case
CRA004775	ENSGALG00010021427	RPLP0	case	control	-0.518052908	0.371639569	-1.393965959	0.163327885	0.629717526	down_in_case
CRA004775	ENSGALG00010021429	MED8	case	control	-0.042029567	0.185233533	-0.226900425	0.820501172	0.960766569	down_in_case
CRA004775	ENSGALG00010021431	CPSF4	case	control	-0.205832788	0.151792885	-1.356010775	0.17509574	0.640697583	down_in_case
CRA004775	ENSGALG00010021432	SYT9	case	control	0.850246326	0.640529029	1.327412634	0.184372223	0.648746785	up_in_case
CRA004775	ENSGALG00010021433	ENSGALG00010021433	case	control	-0.305480245	0.788973814	-0.387186799	0.698617922	0.929347461	down_in_case
CRA004775	ENSGALG00010021434	ANKZF1	case	control	-0.468679832	0.256132501	-1.829833503	0.067274839	0.46301117	down_in_case
CRA004775	ENSGALG00010021435	GCN1	case	control	0.356993656	0.476384627	0.749381142	0.453627514	0.83804959	up_in_case
CRA004775	ENSGALG00010021436	ENSGALG00010021436	case	control	0.042789263	0.799417136	0.053525577	0.957313152	0.991431439	up_in_case
CRA004775	ENSGALG00010021438	GOT2	case	control	-0.141252687	0.424773095	-0.332536802	0.739483955	0.938064864	down_in_case
CRA004775	ENSGALG00010021439	ITPRIP	case	control	-0.128717465	0.419712314	-0.306680221	0.759086786	0.942236668	down_in_case
CRA004775	ENSGALG00010021440	CCR10	case	control	0.104979169	0.879448315	0.119369345	0.904982747	0.979544699	up_in_case
CRA004775	ENSGALG00010021441	ENSGALG00010021441	case	control	-0.923608422	0.512520385	-1.802091095	0.071531075	0.471598108	down_in_case
CRA004775	ENSGALG00010021443	SNUPN	case	control	-0.410847381	0.224056423	-1.833678211	0.066701775	0.462498477	down_in_case
CRA004775	ENSGALG00010021444	ATG9A	case	control	-0.246013126	0.414244227	-0.593884259	0.552589528	0.878345607	down_in_case
CRA004775	ENSGALG00010021446	HOXB8	case	control	0.27867428	0.75149243	0.370827794	0.710765798	0.932234469	up_in_case
CRA004775	ENSGALG00010021447	OCSTAMP	case	control	0.614809609	0.600244264	1.024265697	0.305709795	0.758032511	up_in_case
CRA004775	ENSGALG00010021448	SZT2	case	control	-0.468785579	0.21495413	-2.180863326	0.029193527	0.324790814	down_in_case
CRA004775	ENSGALG00010021450	OLFML1	case	control	1.339176068	0.580270701	2.307847122	0.021007641	0.284887644	up_in_case
CRA004775	ENSGALG00010021452	SLC25A1	case	control	-0.304848234	0.176407509	-1.728091032	0.083971905	0.503010645	down_in_case
CRA004775	ENSGALG00010021454	ATP5J2	case	control	0.066044939	0.346335568	0.19069638	0.848763475	0.968223172	up_in_case
CRA004775	ENSGALG00010021457	USB1	case	control	0.181437102	0.413107062	0.439201163	0.660515783	0.917907174	up_in_case
CRA004775	ENSGALG00010021458	USP30	case	control	0.268348113	0.186041481	1.442410106	0.149186714	0.61265447	up_in_case
CRA004775	ENSGALG00010021459	ARHGEF16	case	control	0.37391652	0.222946605	1.677157269	0.093511732	0.525355698	up_in_case
CRA004775	ENSGALG00010021461	SNX33	case	control	-0.260418845	0.242209752	-1.075179025	0.282294584	0.73841218	down_in_case
CRA004775	ENSGALG00010021462	SETD5	case	control	-0.217402496	0.25298306	-0.859355944	0.390144168	0.806425511	down_in_case
CRA004775	ENSGALG00010021464	ENSGALG00010021464	case	control	0.123396652	0.717692686	0.171935222	0.863488456	0.971910432	up_in_case
CRA004775	ENSGALG00010021465	ABCB6	case	control	-0.18852612	0.345195535	-0.546142986	0.584967654	0.89069762	down_in_case
CRA004775	ENSGALG00010021466	HES4	case	control	-0.222628463	0.381392454	-0.583725401	0.559405075	0.880918893	down_in_case
CRA004775	ENSGALG00010021467	LHFPL4	case	control	-0.541837024	0.367735201	-1.473443452	0.140631501	0.600007375	down_in_case
CRA004775	ENSGALG00010021469	CSPG4	case	control	0.58576012	0.371119319	1.578360627	0.114482785	0.564488547	up_in_case
CRA004775	ENSGALG00010021470	ENSGALG00010021470	case	control	0.088812267	0.553664564	0.160408076	0.872559629	0.97401729	up_in_case
CRA004775	ENSGALG00010021473	SLC8A3	case	control	0.092951214	0.382945875	0.242726768	0.80821707	0.955397873	up_in_case
CRA004775	ENSGALG00010021474	PPFIBP2	case	control	0.545581792	0.427893354	1.27504152	0.202294547	0.670948459	up_in_case
CRA004775	ENSGALG00010021476	ALKBH2	case	control	-0.034740052	0.250269369	-0.138810642	0.889599784	0.977162915	down_in_case
CRA004775	ENSGALG00010021477	ZFAND2B	case	control	-0.140344414	0.320948102	-0.437280709	0.661907784	0.918136475	down_in_case
CRA004775	ENSGALG00010021478	SLC13A3	case	control	-0.240156972	0.40312998	-0.595730865	0.551355035	0.878019654	down_in_case
CRA004775	ENSGALG00010021479	SKAP1	case	control	-0.670917323	0.488711681	-1.372828497	0.169805669	0.633147466	down_in_case
CRA004775	ENSGALG00010021480	ENSGALG00010021480	case	control	-0.31809354	0.208090923	-1.528627658	0.126356775	0.580725942	down_in_case
CRA004775	ENSGALG00010021481	MICU1	case	control	0.210539229	0.284561504	0.739872492	0.459377367	0.840589413	up_in_case
CRA004775	ENSGALG00010021483	UNG	case	control	-0.31263839	0.395418721	-0.790651462	0.429147408	0.823883065	down_in_case
CRA004775	ENSGALG00010021484	MARCHF5	case	control	0.078525819	0.238653559	0.329036864	0.742127824	0.938877854	up_in_case
CRA004775	ENSGALG00010021485	SMOC1	case	control	0.327591039	0.432733617	0.757027016	0.449033677	0.834536953	up_in_case
CRA004775	ENSGALG00010021487	KLHL17	case	control	-0.547906406	0.271131839	-2.020811748	0.043299256	0.389812978	down_in_case
CRA004775	ENSGALG00010021488	HYI	case	control	-0.414970888	0.321272804	-1.291646484	0.196479598	0.662853522	down_in_case
CRA004775	ENSGALG00010021489	CNPPD1	case	control	-0.262998927	0.198058185	-1.327887189	0.184215378	0.648706783	down_in_case
CRA004775	ENSGALG00010021490	ACACB	case	control	-0.408868888	0.687436127	-0.594773641	0.551994789	0.878258849	down_in_case
CRA004775	ENSGALG00010021492	PARP6	case	control	0.250705458	0.323992484	0.773800227	0.439048938	0.828368259	up_in_case
CRA004775	ENSGALG00010021493	UNC93B1	case	control	-0.413149875	0.288513031	-1.431997277	0.152144601	0.616604893	down_in_case
CRA004775	ENSGALG00010021494	SLC2A10	case	control	-0.725507192	0.434028212	-1.671566898	0.094609757	0.526933666	down_in_case
CRA004775	ENSGALG00010021496	MTMR14	case	control	-0.334916684	0.327457404	-1.02277939	0.306412165	0.7585475	down_in_case
CRA004775	ENSGALG00010021498	B3GALT6	case	control	0.059403717	0.29543104	0.201074732	0.840640139	0.966461921	up_in_case
CRA004775	ENSGALG00010021501	SLC23A3	case	control	-0.186631597	0.491823737	-0.379468462	0.70434002	0.930465147	down_in_case
CRA004775	ENSGALG00010021502	DNAJB12	case	control	-0.030646678	0.255270316	-0.120055784	0.904438958	0.979540372	down_in_case
CRA004775	ENSGALG00010021503	KCNS1	case	control	-0.222526285	0.561628043	-0.396216477	0.691945337	0.927867527	down_in_case
CRA004775	ENSGALG00010021504	ODR4	case	control	-0.128367148	0.330178259	-0.388781345	0.697437902	0.929092636	down_in_case
CRA004775	ENSGALG00010021505	IHH	case	control	0.683214944	0.477477649	1.430883613	0.152463574	0.617173463	up_in_case
CRA004775	ENSGALG00010021506	MAX	case	control	-0.276192897	0.209288744	-1.319673918	0.186943912	0.650186754	down_in_case
CRA004775	ENSGALG00010021509	DDIT4	case	control	0.096627521	0.507275452	0.190483338	0.848930398	0.968223172	up_in_case
CRA004775	ENSGALG00010021510	NTSR1	case	control	-0.694906417	0.610597215	-1.138076624	0.255088488	0.718752938	down_in_case
CRA004775	ENSGALG00010021512	CPNE9	case	control	-1.402983879	0.579975296	-2.419040758	0.015561496	0.249884603	down_in_case
CRA004775	ENSGALG00010021513	SDF4	case	control	0.11941318	0.132357302	0.90220319	0.366948941	0.793822622	up_in_case
CRA004775	ENSGALG00010021514	TPR	case	control	0.210858424	0.232316707	0.907633491	0.364071894	0.793608387	up_in_case
CRA004775	ENSGALG00010021516	SRSF5	case	control	-0.512297853	0.371918015	-1.377448341	0.168373676	0.631389806	down_in_case
CRA004775	ENSGALG00010021518	CPEB3	case	control	0.551420188	0.313135263	1.760964838	0.078244355	0.490500984	up_in_case
CRA004775	ENSGALG00010021519	FNTB	case	control	-0.303369087	0.367455349	-0.825594423	0.4090342	0.814455442	down_in_case
CRA004775	ENSGALG00010021521	ENSGALG00010021521	case	control	0.641660193	0.384715598	1.667881929	0.095339168	0.528014574	up_in_case
CRA004775	ENSGALG00010021523	CELF6	case	control	-0.070553775	0.352208488	-0.200318213	0.84123172	0.966461921	down_in_case
CRA004775	ENSGALG00010021525	ENSGALG00010021525	case	control	0.210390721	0.408555949	0.514961834	0.606579691	0.900898526	up_in_case
CRA004775	ENSGALG00010021526	KCNJ12	case	control	0.572393687	0.568551067	1.00675862	0.314050785	0.765098415	up_in_case
CRA004775	ENSGALG00010021528	BRPF1	case	control	0.041763264	0.218613843	0.191036684	0.848496853	0.968223172	up_in_case
CRA004775	ENSGALG00010021529	BTAF1	case	control	0.162490014	0.277815625	0.584884359	0.558625476	0.880754991	up_in_case
CRA004775	ENSGALG00010021530	MLF2	case	control	-0.335328506	0.13237714	-2.53313002	0.011304901	0.215907911	down_in_case
CRA004775	ENSGALG00010021531	RAB15	case	control	-0.161996832	0.278183145	-0.582338776	0.560338512	0.880918893	down_in_case
CRA004775	ENSGALG00010021532	DPP10	case	control	0.423074297	0.258747901	1.635083009	0.102031647	0.542100708	up_in_case
CRA004775	ENSGALG00010021534	KIFC3	case	control	-0.107554366	0.325083763	-0.33085124	0.740756855	0.938450627	down_in_case
CRA004775	ENSGALG00010021535	SUSD6	case	control	-0.178460247	0.256537387	-0.695650054	0.486648012	0.85214527	down_in_case
CRA004775	ENSGALG00010021537	GPX2	case	control	1.267310076	0.676057496	1.87455961	0.060853334	0.445397937	up_in_case
CRA004775	ENSGALG00010021538	TNFRSF18	case	control	0.239332057	0.538800182	0.444194462	0.656901996	0.91710603	up_in_case
CRA004775	ENSGALG00010021539	HEXA	case	control	-0.254462181	0.539185986	-0.471937675	0.636971275	0.910342177	down_in_case
CRA004775	ENSGALG00010021540	STK4	case	control	-0.463254685	0.391267005	-1.183986073	0.236418576	0.703419274	down_in_case
CRA004775	ENSGALG00010021541	CYP27A1	case	control	-0.058851323	0.566600837	-0.103867343	0.917274624	0.981688766	down_in_case
CRA004775	ENSGALG00010021543	PLEKHD1	case	control	-0.629035626	0.534786786	-1.17623629	0.239500467	0.706254731	down_in_case
CRA004775	ENSGALG00010021545	CYB5R2	case	control	0.376370359	0.380423192	0.989346517	0.322493632	0.767880594	up_in_case
CRA004775	ENSGALG00010021546	OGG1	case	control	-0.277015104	0.338603906	-0.818109596	0.41329461	0.817140789	down_in_case
CRA004775	ENSGALG00010021549	ANAPC16	case	control	-0.397668767	0.348452303	-1.141243046	0.253768796	0.718204856	down_in_case
CRA004775	ENSGALG00010021550	MAP2K3	case	control	-0.004123586	0.314922836	-0.013093957	0.989552832	0.998065748	down_in_case
CRA004775	ENSGALG00010021552	ENSGALG00010021552	case	control	0.381257079	0.422167765	0.903093771	0.36647613	0.793608387	up_in_case
CRA004775	ENSGALG00010021553	SLC39A9	case	control	-0.127419843	0.338363853	-0.376576403	0.706488426	0.931529615	down_in_case
CRA004775	ENSGALG00010021554	SNX11	case	control	-0.460591877	0.230912114	-1.994663115	0.046079635	0.399541954	down_in_case
CRA004775	ENSGALG00010021556	CAMK1	case	control	-0.397748299	0.276320798	-1.439443944	0.150024781	0.61366707	down_in_case
CRA004775	ENSGALG00010021558	CHURC1	case	control	-0.057695327	0.436282961	-0.132242907	0.894792164	0.977834811	down_in_case
CRA004775	ENSGALG00010021561	TGIF2	case	control	0.060881251	0.19844671	0.306788915	0.759004046	0.942236668	up_in_case
CRA004775	ENSGALG00010021562	LRP6	case	control	-0.073363658	0.36962703	-0.198480229	0.842669352	0.966478468	down_in_case
CRA004775	ENSGALG00010021564	PRKAG3	case	control	-0.076755087	0.694552426	-0.110510142	0.912004807	0.98075825	down_in_case
CRA004775	ENSGALG00010021565	CBX1	case	control	-0.120368007	0.152938981	-0.787032883	0.431262617	0.825026746	down_in_case
CRA004775	ENSGALG00010021566	ENSGALG00010021566	case	control	-0.10566605	0.330634442	-0.31958573	0.749282393	0.941366343	down_in_case
CRA004775	ENSGALG00010021568	TADA3	case	control	-0.339485028	0.337955581	-1.004525584	0.315125346	0.765098415	down_in_case
CRA004775	ENSGALG00010021570	KATNB1	case	control	-0.63822433	0.241889163	-2.638499067	0.008327393	0.183215969	down_in_case
CRA004775	ENSGALG00010021572	MYL9	case	control	0.125961777	0.336557866	0.374264843	0.708207286	0.931801673	up_in_case
CRA004775	ENSGALG00010021573	NATD1	case	control	-0.113518691	0.203535848	-0.557733156	0.577026613	0.888145619	down_in_case
CRA004775	ENSGALG00010021574	TTLL4	case	control	-0.053586801	0.30278397	-0.17698031	0.859523856	0.971325143	down_in_case
CRA004775	ENSGALG00010021575	ADGRL2	case	control	0.212389595	0.311755107	0.681270619	0.495700271	0.856382445	up_in_case
CRA004775	ENSGALG00010021576	ERH	case	control	-0.505554493	0.291857924	-1.732193819	0.083239059	0.501147243	down_in_case
CRA004775	ENSGALG00010021577	ARIH1	case	control	7.10141E-05	0.191954039	0.000369954	0.99970482	0.999914248	up_in_case
CRA004775	ENSGALG00010021578	ENSGALG00010021578	case	control	0.343686882	0.481791928	0.713351268	0.47562842	0.846211205	up_in_case
CRA004775	ENSGALG00010021579	C20H20ORF24	case	control	-0.002279159	0.232408834	-0.009806682	0.992175525	0.998065748	down_in_case
CRA004775	ENSGALG00010021580	KCTD10	case	control	-0.353359948	0.237734239	-1.486365405	0.137182495	0.596242097	down_in_case
CRA004775	ENSGALG00010021582	STK36	case	control	-0.103894896	0.275375012	-0.377285126	0.705961725	0.931281627	down_in_case
CRA004775	ENSGALG00010021583	NFE2L1	case	control	-0.103296611	0.33831405	-0.305327582	0.760116671	0.942236668	down_in_case
CRA004775	ENSGALG00010021584	OVCH2	case	control	0.742697831	0.544850791	1.363121507	0.17284422	0.637223104	up_in_case
CRA004775	ENSGALG00010021586	BBS4	case	control	-0.429527912	0.258215255	-1.663449017	0.096222588	0.529546782	down_in_case
CRA004775	ENSGALG00010021588	RNF25	case	control	-0.456002806	0.259181227	-1.759397513	0.078510014	0.491393356	down_in_case
CRA004775	ENSGALG00010021593	DCAF7	case	control	-0.197695584	0.194188433	-1.018060554	0.30864918	0.761161204	down_in_case
CRA004775	ENSGALG00010021594	ADPGK	case	control	-0.330834054	0.257553242	-1.284526849	0.198957675	0.666028746	down_in_case
CRA004775	ENSGALG00010021595	UBE3B	case	control	-0.355573851	0.233206974	-1.524713626	0.127330542	0.581272645	down_in_case
CRA004775	ENSGALG00010021597	GGT7	case	control	-0.094776347	0.341931743	-0.277179141	0.781642556	0.948910196	down_in_case
CRA004775	ENSGALG00010021599	ARPC4	case	control	-0.529665109	0.207551289	-2.551972144	0.010711508	0.208042302	down_in_case
CRA004775	ENSGALG00010021600	FMN1	case	control	0.158860743	0.344380541	0.461294192	0.644587551	0.913880134	up_in_case
CRA004775	ENSGALG00010021601	LPAR5	case	control	0.017328884	0.498382838	0.034770226	0.972262963	0.994947809	up_in_case
CRA004775	ENSGALG00010021602	PLEKHG4	case	control	-0.165325898	0.315633824	-0.523790182	0.600424483	0.897438811	down_in_case
CRA004775	ENSGALG00010021604	SPTB	case	control	-0.278737342	0.230042696	-1.211676556	0.225636217	0.69359464	down_in_case
CRA004775	ENSGALG00010021605	MAP3K3	case	control	-0.143812499	0.302421661	-0.475536371	0.634404716	0.909837348	down_in_case
CRA004775	ENSGALG00010021606	RNF8	case	control	0.190045244	0.202715927	0.937495374	0.348503802	0.785311667	up_in_case
CRA004775	ENSGALG00010021607	SLC9A5	case	control	-0.317840662	0.279504691	-1.137156805	0.25547274	0.718871765	down_in_case

CRA004775	ENSGALG00010021609	ENSGALG00010021609	case	control	0.382014666	0.513741564	0.743593069	0.457122698	0.839688534	up_in_case
CRA004775	ENSGALG00010021610	TMEM11	case	control	-0.362883649	0.294446857	-1.232424937	0.21779039	0.685160297	down_in_case
CRA004775	ENSGALG00010021611	GREM1	case	control	-1.090112687	0.570112485	-1.91210106	0.055863233	0.432968819	down_in_case
CRA004775	ENSGALG00010021612	BCS1L	case	control	-0.287025668	0.344788403	-0.83246903	0.405144252	0.81327583	down_in_case
CRA004775	ENSGALG00010021613	ADGRL4	case	control	0.497159365	0.401350295	1.238716831	0.215450383	0.683654538	up_in_case
CRA004775	ENSGALG00010021615	FLVCR1	case	control	-0.072890505	0.328140808	-0.222131791	0.82421129	0.961055665	down_in_case
CRA004775	ENSGALG00010021616	ENSGALG00010021616	case	control	0.724428786	0.573678741	1.262777814	0.20666904	0.675197427	up_in_case
CRA004775	ENSGALG00010021617	SORBS1	case	control	0.298296689	0.290683852	1.026189405	0.304802314	0.757516108	up_in_case
CRA004775	ENSGALG00010021618	NEO1	case	control	0.197603541	0.247033036	0.799907352	0.423764478	0.82073892	up_in_case
CRA004775	ENSGALG00010021619	FHOD1	case	control	-0.159859631	0.424114383	-0.376925749	0.706228786	0.93136588	down_in_case
CRA004775	ENSGALG00010021621	ZNF518A	case	control	0.109037336	0.237430933	0.459238123	0.646063177	0.914043203	up_in_case
CRA004775	ENSGALG00010021622	PTHLH	case	control	0.420132568	0.590939193	0.71095736	0.477110659	0.846532686	up_in_case
CRA004775	ENSGALG00010021623	FAR1	case	control	0.66300275	0.269875557	2.456698032	0.01402205	0.236876171	up_in_case
CRA004775	ENSGALG00010021624	HSD11B2	case	control	1.009655326	0.47986902	2.104022731	0.035376464	0.35615887	up_in_case
CRA004775	ENSGALG00010021626	ZNF142	case	control	-0.202185993	0.371276299	-0.544570157	0.586049184	0.89145601	down_in_case
CRA004775	ENSGALG00010021627	NCOA6	case	control	0.241245798	0.255486609	0.944260049	0.345036776	0.783972357	up_in_case
CRA004775	ENSGALG00010021628	REC114	case	control	0.357905836	0.444025514	0.806047906	0.420215227	0.819388688	up_in_case
CRA004775	ENSGALG00010021629	DHRS7B	case	control	-0.445339735	0.213881501	-2.082179775	0.037326052	0.364280015	down_in_case
CRA004775	ENSGALG00010021632	LIMD2	case	control	-0.917577572	0.574727173	-1.596544614	0.110367253	0.558567026	down_in_case
CRA004775	ENSGALG00010021633	CCNJ	case	control	-0.117135838	0.388753621	-0.301311246	0.763177166	0.94239774	down_in_case
CRA004775	ENSGALG00010021634	PLCD4	case	control	-0.132602439	0.458289598	-0.289342023	0.772319657	0.946296525	down_in_case
CRA004775	ENSGALG00010021635	FOXJ2	case	control	0.098646585	0.196717086	0.501464246	0.616044435	0.9028981	up_in_case
CRA004775	ENSGALG00010021636	C1orf159	case	control	-0.139326681	0.295519106	-0.471464209	0.637309273	0.910421454	down_in_case
CRA004775	ENSGALG00010021637	ATP6V0D1	case	control	-0.249589346	0.136284385	-1.831386237	0.067042914	0.462498477	down_in_case
CRA004775	ENSGALG00010021638	SRC	case	control	-0.241820645	0.271603444	-0.890344544	0.37328091	0.796804495	down_in_case
CRA004775	ENSGALG00010021639	SCG5	case	control	0.393987167	0.476566858	0.826719611	0.408396003	0.814384687	up_in_case
CRA004775	ENSGALG00010021640	PLEKHG3	case	control	0.103979092	0.237886874	0.437094702	0.66204267	0.918136475	up_in_case
CRA004775	ENSGALG00010021641	ENSGALG00010021641	case	control	-0.769793016	0.710045064	-1.084146703	0.278299758	0.733841198	down_in_case
CRA004775	ENSGALG00010021642	NPTN	case	control	0.207956125	0.242000941	0.859319491	0.390164274	0.806425511	up_in_case
CRA004775	ENSGALG00010021643	ENSGALG00010021643	case	control	-0.596700385	0.204699723	-2.91500338	0.003556847	0.125102734	down_in_case
CRA004775	ENSGALG00010021644	TARDBP	case	control	0.029022562	0.228861212	0.126812936	0.899088457	0.978790377	up_in_case
CRA004775	ENSGALG00010021645	ASCC1	case	control	-0.364448908	0.304592916	-1.196511438	0.231497038	0.698248687	down_in_case
CRA004775	ENSGALG00010021646	RPUSD3	case	control	-0.40393666	0.356666641	-1.132532771	0.257410515	0.719236059	down_in_case
CRA004775	ENSGALG00010021647	ARHGAP11B	case	control	0.071486238	0.410155887	0.17429041	0.861637235	0.97165529	up_in_case
CRA004775	ENSGALG00010021648	BRE	case	control	-0.056544126	0.19689917	-0.287173002	0.773979857	0.946296525	down_in_case
CRA004775	ENSGALG00010021649	PXN	case	control	-0.143417545	0.228528432	-0.627569814	0.530285788	0.869211096	down_in_case
CRA004775	ENSGALG00010021650	CD276	case	control	0.153262379	0.218174706	0.702475469	0.482382695	0.849435835	up_in_case
CRA004775	ENSGALG00010021651	RNF113B	case	control	0.01114267	0.180852291	0.061611994	0.950871825	0.990168609	up_in_case
CRA004775	ENSGALG00010021652	BORCS5	case	control	-0.417659694	0.183451885	-2.276671585	0.022805845	0.294061167	down_in_case
CRA004775	ENSGALG00010021655	USP22	case	control	-0.143747776	0.255898476	-0.561737524	0.574294864	0.887018834	down_in_case
CRA004775	ENSGALG00010021656	SETD6	case	control	-0.602821307	0.290262046	-2.076817534	0.037818403	0.366742499	down_in_case
CRA004775	ENSGALG00010021657	JAGN1	case	control	-0.43487302	0.312802811	-1.390246524	0.16445403	0.63006979	down_in_case
CRA004775	ENSGALG00010021658	STRADA	case	control	-0.269449129	0.177767528	-1.515738742	0.129585427	0.584172159	down_in_case
CRA004775	ENSGALG00010021659	SPOCK2	case	control	0.128026989	0.271536706	0.471490544	0.637290471	0.910421454	up_in_case
CRA004775	ENSGALG00010021660	RNF223	case	control	1.403403317	0.494397319	2.838614334	0.004530988	0.138149936	up_in_case
CRA004775	ENSGALG00010021661	DUSP16	case	control	0.481834827	0.328712262	1.465825534	0.142695823	0.602997185	up_in_case
CRA004775	ENSGALG00010021662	RASGRP1	case	control	-0.147892482	0.46089966	-0.320877829	0.748302976	0.940917938	down_in_case
CRA004775	ENSGALG00010021663	CNOT9	case	control	-0.060788929	0.199719836	-0.304371015	0.760845248	0.942236668	down_in_case
CRA004775	ENSGALG00010021664	TYSND1	case	control	-0.734440817	0.447046255	-1.64287433	0.100408937	0.538583505	down_in_case
CRA004775	ENSGALG00010021665	CNOT1	case	control	-0.04482178	0.195519031	-0.2292451	0.818678421	0.959941088	down_in_case
CRA004775	ENSGALG00010021666	CCDC47	case	control	-0.165798834	0.204624345	-0.81025957	0.417791007	0.818943344	down_in_case
CRA004775	ENSGALG00010021667	TBX15	case	control	0.316915212	0.552533815	0.573567089	0.566260788	0.883033678	up_in_case
CRA004775	ENSGALG00010021668	PYGO1	case	control	-0.199517691	0.304995097	-0.654166879	0.513004297	0.863903061	down_in_case
CRA004775	ENSGALG00010021669	ENSGALG00010021669	case	control	-0.34689471	0.186337854	-1.861643798	0.062653315	0.449423965	down_in_case
CRA004775	ENSGALG00010021670	BLCAP	case	control	-0.228967123	0.347325637	-0.659228971	0.509748746	0.862971569	down_in_case
CRA004775	ENSGALG00010021671	TNFRSF13B	case	control	0.209035885	0.59416605	0.351813916	0.724977821	0.935238044	up_in_case
CRA004775	ENSGALG00010021674	ING4	case	control	-0.250169923	0.282677372	-0.885001587	0.376155775	0.798523018	down_in_case
CRA004775	ENSGALG00010021675	DDX42	case	control	-0.405352293	0.192213893	-2.10886054	0.034956621	0.354362166	down_in_case
CRA004775	ENSGALG00010021676	TP53INP2	case	control	0.107880862	0.484061093	0.222866212	0.823639634	0.960898591	up_in_case
CRA004775	ENSGALG00010021677	ENSGALG00010021677	case	control	0.145989935	0.188638735	0.773912815	0.438982351	0.828368259	up_in_case
CRA004775	ENSGALG00010021678	DNAAF4	case	control	0.181240578	0.470866605	0.38490854	0.700305178	0.929842957	up_in_case
CRA004775	ENSGALG00010021680	PIGT	case	control	-0.179480404	0.213753458	-0.839660821	0.401098587	0.811868062	down_in_case
CRA004775	ENSGALG00010021681	BTBD10	case	control	0.227757795	0.252834241	0.900818629	0.367684761	0.794321403	up_in_case
CRA004775	ENSGALG00010021682	MYL4	case	control	1.994178324	0.846646471	2.355384911	0.018503528	0.269821857	up_in_case
CRA004775	ENSGALG00010021684	BIN1	case	control	0.295240366	0.391523639	0.754080562	0.450800848	0.835552293	up_in_case
CRA004775	ENSGALG00010021685	DBNDD2	case	control	0.008109477	0.479836649	0.016900496	0.986515997	0.997562105	up_in_case
CRA004775	ENSGALG00010021686	CCPG1	case	control	-0.115191632	0.513530589	-0.224313087	0.822513693	0.960868728	down_in_case
CRA004775	ENSGALG00010021687	ZNF384	case	control	-0.365900783	0.149835307	-2.442019778	0.014605346	0.241689368	down_in_case
CRA004775	ENSGALG00010021688	ITPR1	case	control	0.15374971	0.205459014	0.74832302	0.454265343	0.838254622	up_in_case
CRA004775	ENSGALG00010021689	MPRIP	case	control	0.07418365	0.291150946	0.254794466	0.79888185	0.952835572	up_in_case
CRA004775	ENSGALG00010021691	PIGB	case	control	-0.543714727	0.308435353	-1.762815842	0.077931555	0.490451628	down_in_case
CRA004775	ENSGALG00010021693	PPA1	case	control	-0.121646488	0.231742548	-0.524920819	0.599638236	0.897206546	down_in_case
CRA004775	ENSGALG00010021694	ENSGALG00010021694	case	control	0.335044968	1.158403032	0.289230051			up_in_case
CRA004775	ENSGALG00010021695	FNBP4	case	control	-0.241410815	0.162740406	-1.48341043	0.137965398	0.596967334	down_in_case
CRA004775	ENSGALG00010021697	PIANP	case	control	-0.197999956	0.329191961	-0.601472636	0.547525232	0.877277621	down_in_case
CRA004775	ENSGALG00010021698	GYPC	case	control	0.351207451	0.307016737	1.143935844	0.252650245	0.717153621	up_in_case
CRA004775	ENSGALG00010021699	SYS1	case	control	-0.213430409	0.228450946	-0.934250493	0.350174695	0.785466132	down_in_case
CRA004775	ENSGALG00010021700	FLCN	case	control	-0.365188872	0.239090924	-1.527405835	0.126660126	0.580923609	down_in_case
CRA004775	ENSGALG00010021701	DR1	case	control	0.042787145	0.227114204	0.188394842	0.850567143	0.968989143	up_in_case
CRA004775	ENSGALG00010021702	PLD6	case	control	-1.00289571	0.399457987	-2.510641277	0.012051209	0.220660685	down_in_case
CRA004775	ENSGALG00010021703	CD82	case	control	-0.00343237	0.350496994	-0.009792867	0.992186548	0.998065748	down_in_case
CRA004775	ENSGALG00010021704	COPS7A	case	control	-0.078357157	0.274904127	-0.285034486	0.775617721	0.946517228	down_in_case
CRA004775	ENSGALG00010021705	CCDC18	case	control	0.109692835	0.501136001	0.218888355	0.826737018	0.961878235	up_in_case
CRA004775	ENSGALG00010021706	ENSGALG00010021706	case	control	-0.12774327	0.316096222	-0.404127797	0.686118745	0.926725143	down_in_case
CRA004775	ENSGALG00010021710	ENSGALG00010021710	case	control	0.35395478	0.501952774	0.705155541	0.480713445	0.848616843	up_in_case
CRA004775	ENSGALG00010021713	RAB27A	case	control	0.182603137	0.156355419	1.167872136	0.242858345	0.70978642	up_in_case
CRA004775	ENSGALG00010021714	TSPAN18	case	control	-0.169090367	0.383905332	-0.44044808	0.659612608	0.917907174	down_in_case
CRA004775	ENSGALG00010021715	MMAB	case	control	-0.242797202	0.286681684	-0.846922616	0.397038256	0.808998414	down_in_case
CRA004775	ENSGALG00010021716	TFCP2L1	case	control	-0.65220894	0.781021273	-0.835071927	0.403677212	0.812613869	down_in_case
CRA004775	ENSGALG00010021717	SDC4	case	control	0.267216522	0.320785005	0.833008143	0.404840137	0.81313407	up_in_case
CRA004775	ENSGALG00010021719	BHLHE40	case	control	-0.507153518	0.409357145	-1.238902323	0.215381673	0.683654538	down_in_case
CRA004775	ENSGALG00010021720	CACNB1	case	control	-0.489265802	0.49273571	-0.992957871	0.320730485	0.767037518	down_in_case
CRA004775	ENSGALG00010021721	TP53I11	case	control	0.261330267	0.255057256	1.024594519	0.305554552	0.758032511	up_in_case
CRA004775	ENSGALG00010021722	AARS1	case	control	-0.099419408	0.258125909	-0.385158576	0.700119931	0.929819314	down_in_case
CRA004775	ENSGALG00010021723	COPS3	case	control	0.19618573	0.265112728	0.740008718	0.459294704	0.840589413	up_in_case
CRA004775	ENSGALG00010021725	SH2D6	case	control	0.182628354	0.499313113	0.365759178	0.714544784	0.93334033	up_in_case
CRA004775	ENSGALG00010021726	ARNTL	case	control	0.435104218	0.43382142	1.002956971	0.315881623	0.765677454	up_in_case
CRA004775	ENSGALG00010021727	MVK	case	control	-0.545874031	0.189494248	-2.880689191	0.003968067	0.129300694	down_in_case
CRA004775	ENSGALG00010021728	TMED5	case	control	0.166673175	0.161370631	1.032859411	0.301669696	0.754503454	up_in_case
CRA004775	ENSGALG00010021729	RBPJL	case	control	1.767205492	0.649708874	2.719995929	0.006528272	0.16028494	up_in_case
CRA004775	ENSGALG00010021730	ARL8B	case	control	-0.254657054	0.349232912	-0.729189733	0.46588561	0.843390341	down_in_case
CRA004775	ENSGALG00010021731	SRM	case	control	-0.022895994	0.229848157	-0.099613565	0.920651124	0.982481112	down_in_case
CRA004775	ENSGALG00010021732	CASR	case	control	-1.385070661	0.787112308	-1.759686193	0.078461028	0.491310381	down_in_case
CRA004775	ENSGALG00010021733	ENSGALG00010021733	case	control	-0.081954681	0.415669839	-0.197162923	0.843700044	0.966478468	down_in_case
CRA004775	ENSGALG00010021734	PARD3B	case	control	-0.020362707	0.335807058	-0.060638114	0.951647419	0.990168609	down_in_case
CRA004775	ENSGALG00010021735	PIGBOS1	case	control	-0.008316082	0.394401839	-0.021085301	0.98317761	0.997398088	down_in_case
CRA004775	ENSGALG00010021736	EDEM1	case	control	-0.214417766	0.312640644	-0.685828188	0.492821459	0.855763086	down_in_case
CRA004775	ENSGALG00010021738	PRDM11	case	control	-0.370617069	0.255047929	-1.453127145	0.146188456	0.609717153	down_in_case
CRA004775	ENSGALG00010021739	EXOSC10	case	control	-0.16494381	0.262961463	-0.627254687	0.530492301	0.869211096	down_in_case
CRA004775	ENSGALG00010021740	PDLIM1	case	control	0.148485312	0.218782109	0.678690376	0.497334065	0.857810713	up_in_case
CRA004775	ENSGALG00010021741	RPL19	case	control	-0.192740813	0.220494255	-0.874130771	0.382047042	0.801753951	down_in_case
CRA004775	ENSGALG00010021742	MATN4	case	control	-1.088551731	0.602695643	-1.806138379	0.070896727	0.469911807	down_in_case
CRA004775	ENSGALG00010021744	LITAF	case	control	-0.087273001	0.346778809	-0.251667629	0.80129798	0.953541667	down_in_case
CRA004775	ENSGALG00010021745	RSL24D1	case	control	0.08697447	0.324002861	0.268437351	0.788362692	0.95041797	up_in_case
CRA004775	ENSGALG00010021746	MTOR	case	control	0.010485746	0.243008319	0.043149741	0.965582168	0.993094598	up_in_case
CRA004775	ENSGALG00010021747	AKTIP	case	control	-0.204871133	0.299246335	-0.684623699	0.493581408	0.856001758	down_in_case
CRA004775	ENSGALG00010021748	CDCA7	case	control	-0.659850904	0.554584981	-1.189810266	0.234120974	0.701393793	down_in_case
CRA004775	ENSGALG00010021749	CLASP1	case	control	0.200368028	0.268012487	0.747607065	0.454697202	0.838254622	up_in_case
CRA004775	ENSGALG00010021750	HELLS	case	control	0.270642482	0.477066503	0.567305565	0.570506597	0.885244439	up_in_case
CRA004775	ENSGALG00010021751	MTCH2	case	control	-0.337274575	0.217111594	-1.553461831	0.120312841	0.572266302	down_in_case
CRA004775	ENSGALG00010021752	CSTB	case	control	-0.398694518	0.207989595	-1.91689646	0.055251087	0.430875458	down_in_case
CRA004775	ENSGALG00010021753	MTF2	case	control	-0.157140918	0.31246693	-0.502904156	0.615031659	0.902639483	down_in_case
CRA004775	ENSGALG00010021754	SLC17A9	case	control	1.558343516	0.476130957	3.27293047			up_in_case
CRA004775	ENSGALG00010021755	ENSGALG00010021755	case	control	0.237651559	0.42818295	0.555023405	0.578878652	0.888378369	up_in_case
CRA004775	ENSGALG00010021756	USP42	case	control	0.281184301	0.300562731	0.935526171	0.349517202	0.785360568	up_in_case
CRA004775	ENSGALG00010021758	RASSF10	case	control	-0.351915819	0.560965926	-0.627339029	0.530437025	0.869211096	down_in_case

CRA004775	ENSGALG00010021759	NIFK	case	control	-0.230164164	0.16201308	-1.420651742	0.155418028	0.619285223	down_in_case
CRA004775	ENSGALG00010021760	CSTA	case	control	1.472545253	1.012551476	1.454291745	0.145865438	0.609044185	up_in_case
CRA004775	ENSGALG00010021761	MAFB	case	control	-0.269462573	0.46129237	-0.584147041	0.559121388	0.880918893	down_in_case
CRA004775	ENSGALG00010021762	LAMB2	case	control	-0.563307664	0.382119734	-1.47416533	0.140437082	0.600007375	down_in_case
CRA004775	ENSGALG00010021763	ENSGALG00010021763	case	control	2.54145749	0.905026739	2.808157352	0.004982587	0.142684328	up_in_case
CRA004775	ENSGALG00010021764	FAM222A	case	control	0.412012086	0.651248739	0.632649341	0.526962658	0.86830001	up_in_case
CRA004775	ENSGALG00010021765	EIF2AK1	case	control	-0.166433465	0.192517814	-0.864509423	0.387308106	0.803905475	down_in_case
CRA004775	ENSGALG00010021766	AGRN	case	control	0.245676315	0.422348057	0.581691594	0.560774435	0.881008373	up_in_case
CRA004775	ENSGALG00010021767	CCDC58	case	control	0.052351315	0.297762391	0.175815741	0.860438703	0.971325143	up_in_case
CRA004775	ENSGALG00010021768	DIPK1A	case	control	-0.089924783	0.232723029	-0.386402599	0.699198526	0.929347461	down_in_case
CRA004775	ENSGALG00010021769	ENSGALG00010021769	case	control	-0.251006912	0.257858377	-0.973429351	0.330339957	0.77237154	down_in_case
CRA004775	ENSGALG00010021770	RALGAPB	case	control	0.071940841	0.267858371	0.268577907	0.788254516	0.95041797	up_in_case
CRA004775	ENSGALG00010021771	ANKRD61	case	control	-0.171478717	0.40551821	-0.422863173	0.672395093	0.921952322	down_in_case
CRA004775	ENSGALG00010021772	TSN	case	control	-0.172318411	0.201515728	-0.855111475	0.392489438	0.80746892	down_in_case
CRA004775	ENSGALG00010021774	FBXL20	case	control	-0.275588071	0.203005214	-1.357541838	0.174609109	0.639597725	down_in_case
CRA004775	ENSGALG00010021775	CCDC36	case	control	-0.526798666	0.44586815	-1.181512215	0.237399304	0.703927359	down_in_case
CRA004775	ENSGALG00010021776	PMS2	case	control	0.585338779	0.241418485	2.424581442	0.015326045	0.249406448	up_in_case
CRA004775	ENSGALG00010021778	RBL2	case	control	0.240728657	0.215410665	1.117533603	0.26376624	0.723981064	up_in_case
CRA004775	ENSGALG00010021779	UNC13C	case	control	0.131772405	0.583862367	0.22569087	0.82144186	0.960766569	up_in_case
CRA004775	ENSGALG00010021781	RAPGEF4	case	control	-0.296320252	0.438415669	-0.675888825	0.499111233	0.858876056	down_in_case
CRA004775	ENSGALG00010021782	ADAM11	case	control	-0.282336991	0.634590882	-0.44491183	0.656383473	0.91710603	down_in_case
CRA004775	ENSGALG00010021783	GIT2	case	control	-0.144414166	0.217440257	-0.664155607	0.506590723	0.8622397	down_in_case
CRA004775	ENSGALG00010021785	CCDC71	case	control	-0.203634391	0.196960466	-1.033884593	0.301190119	0.753545086	down_in_case
CRA004775	ENSGALG00010021786	FAM162A	case	control	0.133088969	0.403289491	0.330008522	0.741393523	0.938778937	up_in_case
CRA004775	ENSGALG00010021788	RPL5	case	control	-0.038003786	0.228101248	-0.166609285	0.867677487	0.973120718	down_in_case
CRA004775	ENSGALG00010021789	CHD9	case	control	0.409976482	0.294940287	1.390032152	0.164519114	0.63006979	up_in_case
CRA004775	ENSGALG00010021790	PHF21A	case	control	0.458601029	0.173699167	2.640202806	0.008285643	0.183215969	up_in_case
CRA004775	ENSGALG00010021791	PLEKHM2	case	control	-0.510692863	0.432840537	-1.179863759	0.238054406	0.70473329	down_in_case
CRA004775	ENSGALG00010021792	KLHDC8B	case	control	-1.194421617	0.507039653	-2.355676937	0.01848899	0.269821857	down_in_case
CRA004775	ENSGALG00010021793	ENSGALG00010021793	case	control	0.102817416	0.41138331	0.249930937	0.802640758	0.953859914	up_in_case
CRA004775	ENSGALG00010021794	ENSGALG00010021794	case	control	0.149726947	0.414656426	0.361086764	0.718034588	0.934011578	up_in_case
CRA004775	ENSGALG00010021795	NDRG3	case	control	-0.374737949	0.195244747	-1.919324101	0.05494333	0.430875458	down_in_case
CRA004775	ENSGALG00010021797	SLC25A34	case	control	0.185678481	0.428256767	0.43356812	0.664602089	0.918579086	up_in_case
CRA004775	ENSGALG00010021798	RANBP10	case	control	-0.332036315	0.304224138	-1.091420021	0.275088107	0.731001618	down_in_case
CRA004775	ENSGALG00010021799	ENSGALG00010021799	case	control	-0.391205839	0.276497958	-1.414859773	0.157109615	0.621633948	down_in_case
CRA004775	ENSGALG00010021800	TMEM82	case	control	0.854086179	0.417771639	2.044385252	0.040915503	0.378415958	up_in_case
CRA004775	ENSGALG00010021801	VAMP1	case	control	-0.414325321	0.556819065	-0.744093273	0.456820049	0.839688534	down_in_case
CRA004775	ENSGALG00010021803	TLL2	case	control	0.800910788	0.52081459	1.537804055	0.124096532	0.576485043	up_in_case
CRA004775	ENSGALG00010021805	FUT8	case	control	0.255344585	0.403758779	0.632418657	0.527113346	0.86830001	up_in_case
CRA004775	ENSGALG00010021807	CDC42	case	control	-0.089635297	0.166031424	-0.539869468	0.589287055	0.893412703	down_in_case
CRA004775	ENSGALG00010021810	ANKRD13A	case	control	-0.379286027	0.3277771	-1.157146204	0.247212632	0.712046495	down_in_case
CRA004775	ENSGALG00010021812	TEAD1	case	control	0.128042185	0.422225586	0.303255392	0.761695239	0.942236668	up_in_case
CRA004775	ENSGALG00010021817	EVI5	case	control	0.292425163	0.176862543	1.653403587	0.098248753	0.533999449	up_in_case
CRA004775	ENSGALG00010021821	PPP1R19L	case	control	0.214998237	0.280344322	0.766907764	0.443136357	0.831664685	up_in_case
CRA004775	ENSGALG00010021823	ENSGALG00010021823	case	control	-0.574756864	0.203547072	-2.823704896	0.004747207	0.141624457	down_in_case
CRA004775	ENSGALG00010021826	FBXO40	case	control	0.588970983	0.639112814	0.921544632	0.35676615	0.789490076	up_in_case
CRA004775	ENSGALG00010021827	CCZ1	case	control	0.040757249	0.227996017	0.178762986	0.85812381	0.971038554	up_in_case
CRA004775	ENSGALG00010021829	ERCC6	case	control	-0.027626288	0.214308362	-0.128909052	0.897429614	0.978790377	down_in_case
CRA004775	ENSGALG00010021831	ENSGALG00010021831	case	control	-0.274828898	0.452448531	-0.607425771	0.54356839	0.875657091	down_in_case
CRA004775	ENSGALG00010021835	NUDT8	case	control	-0.094396559	0.370433196	-0.254827484	0.798856347	0.952835572	down_in_case
CRA004775	ENSGALG00010021837	ENSGALG00010021837	case	control	-1.210969006	0.554746861	-2.182921783	0.029041571	0.324148245	down_in_case
CRA004775	ENSGALG00010021839	C1orf50	case	control	-0.554965825	0.212920471	-2.606446542	0.00914871	0.193544486	down_in_case
CRA004775	ENSGALG00010021840	ENSGALG00010021840	case	control	0.163206626	0.487705275	0.334641912	0.737895225	0.937854704	up_in_case
CRA004775	ENSGALG00010021841	CENPT	case	control	-0.740398844	0.467472374	-1.583834435	0.113231401	0.562886277	down_in_case
CRA004775	ENSGALG00010021843	ENSGALG00010021843	case	control	-1.026651145	0.482342884	-2.128467483	0.033298343	0.348201914	down_in_case
CRA004775	ENSGALG00010021847	P3H1	case	control	-0.310660966	0.236575603	-1.313157243	0.189129968	0.654106185	down_in_case
CRA004775	ENSGALG00010021848	LARGE2	case	control	-0.13105033	0.36454043	-0.359494637	0.719225089	0.93407508	down_in_case
CRA004775	ENSGALG00010021851	ENSGALG00010021851	case	control	0.256907108	0.320879766	0.800633555	0.423343819	0.820552247	up_in_case
CRA004775	ENSGALG00010021853	ENSGALG00010021853	case	control	-0.423711908	0.283184539	-1.496239556	0.13459124	0.592420453	down_in_case
CRA004775	ENSGALG00010021854	TAPBPL	case	control	-0.131990324	0.398188875	-0.331476676	0.740284456	0.938450627	down_in_case
CRA004775	ENSGALG00010021860	ENSGALG00010021860	case	control	-0.70607442	0.332787286	-2.121698905	0.033863031	0.349587488	down_in_case
CRA004775	ENSGALG00010021862	PDK1	case	control	-0.053020435	0.296274241	-0.178957289	0.857971239	0.971038554	down_in_case
CRA004775	ENSGALG00010021863	CLDN19	case	control	1.091694057	0.558442064	1.95489224	0.050595791	0.416238146	up_in_case
CRA004775	ENSGALG00010021864	GFI1	case	control	-1.547314808	0.676287629	-2.287953736	0.022140215	0.290827737	down_in_case
CRA004775	ENSGALG00010021866	SCNN1A	case	control	-0.046800401	0.563531132	-0.083048475	0.933812995	0.985106444	down_in_case
CRA004775	ENSGALG00010021867	CXCL12	case	control	0.366014326	0.36843723	0.993423837	0.32050345	0.767037518	up_in_case
CRA004775	ENSGALG00010021869	GJC3	case	control	-0.015281705	0.342743319	-0.044586441	0.964436951	0.992825105	down_in_case
CRA004775	ENSGALG00010021870	ENSGALG00010021870	case	control	-0.046728898	0.269396852	-0.173457477	0.862291849	0.971756697	down_in_case
CRA004775	ENSGALG00010021871	ENSGALG00010021871	case	control	-0.612958529	0.803060074	-0.76327855	0.445297292	0.833012432	down_in_case
CRA004775	ENSGALG00010021874	TMCC1	case	control	-0.264898305	0.263053213	-1.007014141	0.313927979	0.765098415	down_in_case
CRA004775	ENSGALG00010021877	PLEKHN1	case	control	-1.750215911	0.635277659	-2.755040864	0.005868481	0.154441997	down_in_case
CRA004775	ENSGALG00010021879	CREBL2	case	control	0.061731377	0.254618533	0.242446517	0.808434194	0.955397873	up_in_case
CRA004775	ENSGALG00010021881	PHF20	case	control	0.310530316	0.191960666	1.617676804	0.105732267	0.550103825	up_in_case
CRA004775	ENSGALG00010021883	ENSGALG00010021883	case	control	0.029822072	0.671373643	0.044419486	0.964570029	0.992871055	up_in_case
CRA004775	ENSGALG00010021887	NUTF2	case	control	-0.089991083	0.229834059	-0.391548075	0.695392161	0.929017829	down_in_case
CRA004775	ENSGALG00010021892	NDUFV1	case	control	-0.115658005	0.341129054	-0.339044721	0.734576041	0.936819725	down_in_case
CRA004775	ENSGALG00010021893	FAM43B	case	control	-0.039605669	0.441029187	-0.089802829	0.9284439	0.98473447	down_in_case
CRA004775	ENSGALG00010021897	ENSGALG00010021897	case	control	-0.274898611	0.373618785	-0.735772991	0.461868861	0.842010079	down_in_case
CRA004775	ENSGALG00010021902	LMTK2	case	control	0.806693059	0.314165518	2.567732651	0.010236607	0.202829374	up_in_case
CRA004775	ENSGALG00010021903	MORC2	case	control	0.171802002	0.172501644	0.995944138	0.319277288	0.767037518	up_in_case
CRA004775	ENSGALG00010021905	RBSN	case	control	0.269543354	0.129392227	2.083149504	0.037237598	0.363933342	up_in_case
CRA004775	ENSGALG00010021908	EDC4	case	control	-0.104953101	0.158767046	-0.661050916	0.508579662	0.862971569	down_in_case
CRA004775	ENSGALG00010021913	ENSGALG00010021913	case	control	0.590967834	0.348132809	1.697535592	0.089595448	0.516522282	up_in_case
CRA004775	ENSGALG00010021916	MRPS25	case	control	0.17349721	0.268843074	0.645347516	0.518702007	0.865435399	up_in_case
CRA004775	ENSGALG00010021919	PARVA	case	control	0.260668531	0.300777424	0.866649256	0.386134216	0.803610044	up_in_case
CRA004775	ENSGALG00010021922	MRPL51	case	control	-0.105531052	0.218120051	-0.483820957	0.628512941	0.908417222	down_in_case
CRA004775	ENSGALG00010021924	ENSGALG00010021924	case	control	0.490158423	0.757189903	0.647338826	0.517412678	0.865038509	up_in_case
CRA004775	ENSGALG00010021928	ENSGALG00010021928	case	control	-3.598259885	2.051037167	-1.754361131	0.079368647	0.491399489	down_in_case
CRA004775	ENSGALG00010021931	ITGA6	case	control	1.095112972	0.392834718	2.787719418	0.005308049	0.148215235	up_in_case
CRA004775	ENSGALG00010021932	RPAP2	case	control	0.004156286	0.312651491	0.01329367	0.989393498	0.998065748	up_in_case
CRA004775	ENSGALG00010021933	PRR14L	case	control	0.175803741	0.226518024	0.776113698	0.437681856	0.827521409	up_in_case
CRA004775	ENSGALG00010021934	EFTUD2	case	control	-0.344148369	0.142614546	-2.413136527	0.015815894	0.251718007	down_in_case
CRA004775	ENSGALG00010021935	CETP	case	control	-0.325283925	0.658158048	-0.494233757	0.621141102	0.90544587	down_in_case
CRA004775	ENSGALG00010021938	CABP2	case	control	-0.179817306	0.25172967	-0.714327024	0.475024985	0.84579952	down_in_case
CRA004775	ENSGALG00010021943	OCM3	case	control	0.056976229	1.011644807	0.056320389	0.955086577	0.990670511	up_in_case
CRA004775	ENSGALG00010021945	C1QTNF4	case	control	0.272911549	0.564053448	0.483839874	0.628499515	0.908417222	up_in_case
CRA004775	ENSGALG00010021947	CDK2AP2	case	control	0.131069105	0.246265079	0.532227735	0.594568276	0.895102715	up_in_case
CRA004775	ENSGALG00010021949	ENSGALG00010021949	case	control	0.939542387	0.371373147	2.52991471	0.011409026	0.21639381	up_in_case
CRA004775	ENSGALG00010021951	PSKH1	case	control	-0.648308115	0.295414201	-2.19457329	0.028194221	0.319106625	down_in_case
CRA004775	ENSGALG00010021953	PITPNM1	case	control	-0.367309517	0.326533415	-1.12487574	0.260641693	0.721579208	down_in_case
CRA004775	ENSGALG00010021958	BHLHA15	case	control	0.515393333	0.834646441	0.617498988	0.536905648	0.872281377	up_in_case
CRA004775	ENSGALG00010021960	CDKN1B	case	control	0.057163937	0.233792396	0.244507255	0.806837983	0.955397873	up_in_case
CRA004775	ENSGALG00010021965	PEX16	case	control	-0.357939239	0.248785399	-1.438746967	0.150222227	0.613766096	down_in_case
CRA004775	ENSGALG00010021966	ADGRA1	case	control	0.082408672	0.402381062	0.20480256	0.837726387	0.965490877	up_in_case
CRA004775	ENSGALG00010021969	CCDC103	case	control	-0.014796248	0.575680448	-0.025702189	0.979494878	0.996764329	down_in_case
CRA004775	ENSGALG00010021970	ERGIC3	case	control	-0.31646261	0.238805356	-1.325190588	0.185107946	0.6491093	down_in_case
CRA004775	ENSGALG00010021971	ICOS	case	control	-0.23880298	0.530451709	-0.450187974	0.652574907	0.916136554	down_in_case
CRA004775	ENSGALG00010021972	PISD	case	control	-0.406542382	0.480674159	-0.845775405	0.397678049	0.809315551	down_in_case
CRA004775	ENSGALG00010021974	GFAP	case	control	-0.373372892	0.55570244	-0.671893563	0.501651459	0.860091011	down_in_case
CRA004775	ENSGALG00010021978	NR2C2	case	control	0.300734867	0.19701689	1.526442056	0.126899811	0.581085346	up_in_case
CRA004775	ENSGALG00010021980	GLMN	case	control	0.026418147	0.298716977	0.08843872	0.929527988	0.98473447	up_in_case
CRA004775	ENSGALG00010021983	CTLA4	case	control	-0.684583775	0.707590093	-0.967486377	0.333300948	0.773931331	down_in_case
CRA004775	ENSGALG00010021988	FAM180B	case	control	0.062065123	0.629124578	0.098653153	0.921413666	0.982481112	up_in_case
CRA004775	ENSGALG00010021989	NCAPD2	case	control	-0.270103666	0.401113569	-0.673384514	0.500702698	0.859946721	down_in_case
CRA004775	ENSGALG00010021995	CD28	case	control	-0.931744219	0.809818892	-1.150558759	0.249913808	0.714890768	down_in_case
CRA004775	ENSGALG00010021999	ANKRD22	case	control	0.456604907	0.76220431	0.599058417	0.54913393	0.877623839	up_in_case
CRA004775	ENSGALG00010022002	KIF18B	case	control	-0.286137692	0.528469805	-0.541445678	0.58820043	0.892912535	down_in_case
CRA004775	ENSGALG00010022005	NLRC5	case	control	-0.223603947	0.252494143	-0.885580729	0.3758435	0.798523018	down_in_case
CRA004775	ENSGALG00010022006	SFI1	case	control	0.007569064	0.29498564	0.025659092	0.979529253	0.996764329	up_in_case
CRA004775	ENSGALG00010022017	CNP1	case	control	-0.413979657	0.865754843	-0.478171922	0.632527845	0.909651573	down_in_case
CRA004775	ENSGALG00010022019	BTBD8	case	control	0.353078056	0.369012391	0.956818971	0.338658635	0.779266214	up_in_case
CRA004775	ENSGALG00010022021	TECPR1	case	control	0.165099868	0.229762742	0.718566757	0.472407902	0.845425322	up_in_case
CRA004775	ENSGALG00010022022	STAMBPL1	case	control	-0.227869762	0.478367104	-0.476349147	0.633825657	0.909837348	down_in_case
CRA004775	ENSGALG00010022024	MICAL2	case	control	0.406557814	0.758680754	0.535874689	0.592045158	0.893804699	up_in_case
CRA004775	ENSGALG00010022027	EIF4ENIF1	case	control	0.002271438	0.196880125	0.011537162	0.990794881	0.998065748	up_in_case
CRA004775	ENSGALG00010022028	FGD5	case	control	0.445128228	0.332079156	1.340428087	0.180106209	0.644990528	up_in_case

CRA004775	ENSGALG00010022029	ENSGALG00010022029	case	control	-0.458098272	1.11338672	-0.411445784	0.680745688	0.923896221	down_in_case
CRA004775	ENSGALG00010022030	ZNF341	case	control	-0.264789939	0.202823785	-1.3055172	0.191716794	0.656959697	down_in_case
CRA004775	ENSGALG00010022032	KBTBD4	case	control	-0.441993649	0.196373889	-2.250776067	0.024399724	0.30145607	down_in_case
CRA004775	ENSGALG00010022033	RAPH1	case	control	-0.211948073	0.343938468	-0.616238348	0.537737222	0.872603995	down_in_case
CRA004775	ENSGALG00010022034	ENSGALG00010022034	case	control	0.060577787	0.305187544	0.198493641	0.842658859	0.966478468	up_in_case
CRA004775	ENSGALG00010022037	DRG1	case	control	-0.220125062	0.238930821	-0.921292034	0.356897979	0.789654724	down_in_case
CRA004775	ENSGALG00010022038	GAPDH	case	control	-0.348736593	0.621346549	-0.561259404	0.574620712	0.887223154	down_in_case
CRA004775	ENSGALG00010022041	MAPK8IP1	case	control	-0.124561941	0.461951228	-0.269643057	0.787434872	0.95041797	down_in_case
CRA004775	ENSGALG00010022042	AIP	case	control	-0.210514055	0.123431058	-1.705519333	0.08809761	0.513317894	down_in_case
CRA004775	ENSGALG00010022044	PXMP4	case	control	-0.116384702	0.197232334	-0.590089363	0.55513074	0.87981923	down_in_case
CRA004775	ENSGALG00010022046	CRY2	case	control	0.051971745	0.237320906	0.21899354	0.82665508	0.961878235	up_in_case
CRA004775	ENSGALG00010022049	EPHX4	case	control	1.732188731	1.49389043	1.159515247	0.246246225	0.712046495	up_in_case
CRA004775	ENSGALG00010022050	ENSGALG00010022050	case	control	-0.312873644	0.312249777	-1.001997975	0.316344571	0.765818284	down_in_case
CRA004775	ENSGALG00010022051	BAIAP2L1	case	control	0.285314109	0.241970226	1.179128993	0.238346815	0.704840228	up_in_case
CRA004775	ENSGALG00010022052	IFFO1	case	control	-0.330809492	0.300438723	-1.101088065	0.270858331	0.728665115	down_in_case
CRA004775	ENSGALG00010022054	ENSGALG00010022054	case	control	-0.280391125	0.454159695	-0.61738443	0.536981189	0.872301043	down_in_case
CRA004775	ENSGALG00010022055	E2F1	case	control	-0.039968931	0.296968204	-0.134589934	0.892936101	0.977834811	down_in_case
CRA004775	ENSGALG00010022058	NLRP3	case	control	-0.111665449	0.243435347	-0.458706797	0.646444732	0.914043203	down_in_case
CRA004775	ENSGALG00010022059	NPTX2	case	control	0.622259055	0.470023068	1.323890458	0.185539428	0.6491093	up_in_case
CRA004775	ENSGALG00010022062	MITF	case	control	0.626441142	0.34187459	1.832371172	0.06689614	0.462498477	up_in_case
CRA004775	ENSGALG00010022065	PABPC1L	case	control	-0.75497701	0.310047853	-2.435033826	0.014890398	0.244420343	down_in_case
CRA004775	ENSGALG00010022066	DAD1	case	control	-0.584162653	0.206043566	-2.835141446	0.00458054	0.139351783	down_in_case
CRA004775	ENSGALG00010022067	TMEM130	case	control	-0.138092836	0.442060494	-0.312384477	0.754748346	0.941900693	down_in_case
CRA004775	ENSGALG00010022069	NOP2	case	control	-0.065940469	0.202413538	-0.325771041	0.74459759	0.939218065	down_in_case
CRA004775	ENSGALG00010022071	ACTA2	case	control	0.309306541	0.407266365	0.75946989	0.447571519	0.834136489	up_in_case
CRA004775	ENSGALG00010022072	SMTN	case	control	0.022048634	0.350840034	0.062845262	0.949889723	0.990168609	up_in_case
CRA004775	ENSGALG00010022073	PTPMT1	case	control	-0.38051353	0.425105503	-0.895103749	0.37073163	0.795641647	down_in_case
CRA004775	ENSGALG00010022076	YWHAB	case	control	0.002201482	0.221425705	0.009942305	0.992067319	0.998065748	up_in_case
CRA004775	ENSGALG00010022079	ABI2	case	control	-0.005378515	0.311136316	-0.017286684	0.986207909	0.997562105	down_in_case
CRA004775	ENSGALG00010022081	FAS	case	control	0.143947358	0.287064486	0.501446069	0.616057224	0.9028981	up_in_case
CRA004775	ENSGALG00010022082	TGFBR3	case	control	0.438844314	0.325603316	1.347788221	0.177726508	0.643644247	up_in_case
CRA004775	ENSGALG00010022084	SELENOM	case	control	-0.205890739	0.271360662	-0.758734658	0.4480113	0.83429564	down_in_case
CRA004775	ENSGALG00010022085	MLLT6	case	control	-0.248902734	0.2032062	-1.224877657	0.22062132	0.68855283	down_in_case
CRA004775	ENSGALG00010022087	SLC35C1	case	control	-0.306039738	0.265741319	-1.151645287	0.249466864	0.714467496	down_in_case
CRA004775	ENSGALG00010022088	DKK3	case	control	-0.388733825	0.3426977	-1.134334503	0.256654262	0.719236059	down_in_case
CRA004775	ENSGALG00010022090	TRRAP	case	control	0.043933654	0.306767382	0.14321488	0.886120478	0.976819452	up_in_case
CRA004775	ENSGALG00010022092	CTRL	case	control	2.666875147	1.329994934	2.005176921	0.044944136	0.394778076	up_in_case
CRA004775	ENSGALG00010022093	CHD4	case	control	-0.117193846	0.200844953	-0.58350406	0.559554025	0.880918893	down_in_case
CRA004775	ENSGALG00010022097	INPP5J	case	control	0.420408147	0.376123867	1.117738552	0.263678672	0.723981064	up_in_case
CRA004775	ENSGALG00010022098	CDC7	case	control	0.472061896	0.580356038	0.813400509	0.415988468	0.818943344	up_in_case
CRA004775	ENSGALG00010022100	ENTPD1	case	control	-0.701246347	0.364664977	-1.922987923	0.054481567	0.429956233	down_in_case
CRA004775	ENSGALG00010022101	ENSGALG00010022101	case	control	-0.506909154	0.462607643	-1.095764763	0.273181733	0.731001618	down_in_case
CRA004775	ENSGALG00010022104	EXOC3L1	case	control	-0.326284492	0.294333154	-1.10855501	0.267622196	0.727430878	down_in_case
CRA004775	ENSGALG00010022105	DLG5	case	control	0.549063277	0.293559585	1.870363993	0.061433289	0.446401278	up_in_case
CRA004775	ENSGALG00010022108	USP47	case	control	0.493543677	0.21466982	2.299082741	0.021500241	0.288534247	up_in_case
CRA004775	ENSGALG00010022111	PCGF2	case	control	0.28004281	0.398306223	0.703084195	0.482003281	0.848985157	up_in_case
CRA004775	ENSGALG00010022115	E2F4	case	control	-0.13878372	0.17177043	-0.807960482	0.419113332	0.818943344	down_in_case
CRA004775	ENSGALG00010022116	CYP20A1	case	control	-0.186180921	0.155765207	-1.195266417	0.231982956	0.698947772	down_in_case
CRA004775	ENSGALG00010022117	FAM20C	case	control	0.051915495	0.297319985	0.174611522	0.861384895	0.97165529	up_in_case
CRA004775	ENSGALG00010022122	PSMB3	case	control	-0.465073272	0.24368702	-1.908486021	0.056328428	0.434420761	down_in_case
CRA004775	ENSGALG00010022124	ENSGALG00010022124	case	control	-0.325034886	0.223452153	-1.4546062	0.145778314	0.609044185	down_in_case
CRA004775	ENSGALG00010022125	CBFA2T2	case	control	0.137509304	0.26827228	0.512573658	0.608249583	0.900943922	up_in_case
CRA004775	ENSGALG00010022128	PLA2G3	case	control	-0.766914642	0.777466838	-0.986427465	0.323923393	0.769171227	down_in_case
CRA004775	ENSGALG00010022129	ELMO3	case	control	-0.192674337	0.386015226	-0.499136623	0.617683139	0.903976249	down_in_case
CRA004775	ENSGALG00010022134	CH25H	case	control	-0.169442396	0.328289116	-0.516137722	0.605758223	0.900441554	down_in_case
CRA004775	ENSGALG00010022135	CHST1	case	control	-0.045110089	0.36593187	-0.123274557	0.901889688	0.979157637	down_in_case
CRA004775	ENSGALG00010022137	PDDC1	case	control	-0.304500108	0.258175993	-1.179428438	0.238227617	0.704790868	down_in_case
CRA004775	ENSGALG00010022138	SPAG17	case	control	-0.9853846	0.686148615	-1.43610958	0.150971162	0.614930229	down_in_case
CRA004775	ENSGALG00010022142	TMEM208	case	control	-0.267621653	0.331935629	-0.806245639	0.420101228	0.819388688	down_in_case
CRA004775	ENSGALG00010022143	MATCAP1	case	control	-0.465658287	0.248227464	-1.87593379	0.060664372	0.445397937	down_in_case
CRA004775	ENSGALG00010022144	SNTA1	case	control	-0.480550289	0.435931489	-1.102352782	0.270308331	0.728665115	down_in_case
CRA004775	ENSGALG00010022145	P2RX2	case	control	-0.763489069	1.048988543	-0.727833563	0.466715476	0.8434807	down_in_case
CRA004775	ENSGALG00010022148	TALDO1	case	control	-0.599086773	0.35549092	-1.685237907	0.09194268	0.52187835	down_in_case
CRA004775	ENSGALG00010022149	NBEAL1	case	control	0.182322508	0.355788437	0.512446411	0.608338616	0.900943922	up_in_case
CRA004775	ENSGALG00010022150	CPNE2	case	control	-0.522437355	0.317576003	-1.645078188	0.099953688	0.538160989	down_in_case
CRA004775	ENSGALG00010022151	PIP4K2B	case	control	-0.236437586	0.249071236	-0.949276961	0.342479768	0.782039071	down_in_case
CRA004775	ENSGALG00010022152	ZNF644	case	control	0.786474901	0.242859239	3.238398109	0.001202029	0.072815451	up_in_case
CRA004775	ENSGALG00010022154	CPN1	case	control	-1.040550367	0.592885168	-1.755062234	0.079248663	0.491399489	down_in_case
CRA004775	ENSGALG00010022157	EPS8L2	case	control	0.099515947	0.386473241	0.257497638	0.796794638	0.952835572	up_in_case
CRA004775	ENSGALG00010022158	CWC25	case	control	-0.126725192	0.188959088	-0.670648834	0.502444265	0.860091011	down_in_case
CRA004775	ENSGALG00010022159	KIAA2013	case	control	-0.050651694	0.236064516	-0.214567165	0.830104796	0.963232966	down_in_case
CRA004775	ENSGALG00010022161	CELF1	case	control	0.500685066	0.139144822	3.598301817	0.000320302	0.035519918	up_in_case
CRA004775	ENSGALG00010022162	POLE	case	control	-0.507960098	0.57865787	-0.877824574	0.380038926	0.800906556	down_in_case
CRA004775	ENSGALG00010022164	PDGFA	case	control	0.577870872	0.266710246	2.166661687	0.03026066	0.331325011	up_in_case
CRA004775	ENSGALG00010022165	FAM192A	case	control	0.20619729	0.218239865	0.944819544	0.344751012	0.783972357	up_in_case
CRA004775	ENSGALG00010022166	SDHB	case	control	-0.012153583	0.375688337	-0.032350175	0.974192796	0.994947809	down_in_case
CRA004775	ENSGALG00010022167	WDR3	case	control	-0.151123389	0.182804921	-0.82669213	0.408411583	0.814384687	down_in_case
CRA004775	ENSGALG00010022169	TOX2	case	control	-0.439313272	0.491258752	-0.894260449	0.371182556	0.795818272	down_in_case
CRA004775	ENSGALG00010022170	RSPRY1	case	control	-0.015476238	0.213390963	-0.072525275	0.942183892	0.987651835	down_in_case
CRA004775	ENSGALG00010022171	FOXP1	case	control	-0.071716739	0.304040263	-0.235879085	0.813526498	0.958280492	down_in_case
CRA004775	ENSGALG00010022172	GIPC2	case	control	1.00274507	0.438542845	2.286538434	0.022222778	0.291321191	up_in_case
CRA004775	ENSGALG00010022173	RPL23	case	control	-0.139596983	0.271059312	-0.515005303	0.606549314	0.900898526	down_in_case
CRA004775	ENSGALG00010022174	KCNMA1	case	control	0.194932521	0.365028482	0.534020031	0.593327676	0.894450257	up_in_case
CRA004775	ENSGALG00010022176	ARL2BPL	case	control	-0.335547648	0.355302022	-0.944401176	0.344964681	0.783972357	down_in_case
CRA004775	ENSGALG00010022178	ENSGALG00010022178	case	control	-0.751504342	0.582267137	-1.290652166	0.196824318	0.66285976	down_in_case
CRA004775	ENSGALG00010022180	PLLP	case	control	1.204627614	0.596014553	2.02113792	0.04326549	0.389812978	up_in_case
CRA004775	ENSGALG00010022181	ENSGALG00010022181	case	control	-0.407839532	0.688222435	-0.592598426	0.553449933	0.878871229	down_in_case
CRA004775	ENSGALG00010022182	CNTNAP1	case	control	-0.086999028	0.481985307	-0.180501411	0.856758947	0.970913248	down_in_case
CRA004775	ENSGALG00010022184	PRKAR1B	case	control	0.301266379	0.198742349	1.515864035	0.129553736	0.584172159	up_in_case
CRA004775	ENSGALG00010022186	DNAJB4	case	control	1.134919447	0.446436364	2.542175187	0.011016494	0.211871065	up_in_case
CRA004775	ENSGALG00010022190	ACVR1B	case	control	-0.191693111	0.288777821	-0.663808287	0.506813021	0.8622397	down_in_case
CRA004775	ENSGALG00010022193	GDAP2	case	control	-0.421553956	0.152670802	-2.761195658	0.005759016	0.153176468	down_in_case
CRA004775	ENSGALG00010022194	ENSGALG00010022194	case	control	-0.565601858	0.203881529	-2.774169204	0.005534289	0.151100111	down_in_case
CRA004775	ENSGALG00010022196	PLXNA1	case	control	-0.002988759	0.267033431	-0.011192454	0.991069901	0.998065748	down_in_case
CRA004775	ENSGALG00010022199	RBM39	case	control	0.060142601	0.160924112	0.373732687	0.708603204	0.931801673	up_in_case
CRA004775	ENSGALG00010022200	DNMBP	case	control	-0.367257262	0.159917463	-2.296542567	0.021644878	0.288534247	down_in_case
CRA004775	ENSGALG00010022202	CX3CL1	case	control	-0.01259845	0.452850917	-0.027820303	0.977805472	0.996059193	down_in_case
CRA004775	ENSGALG00010022203	DNAAF5	case	control	-0.567485354	0.287208588	-1.975864854	0.048170086	0.407414924	down_in_case
CRA004775	ENSGALG00010022204	ENSGALG00010022204	case	control	-0.178739779	0.548090195	-0.326113804	0.744338253	0.939218065	down_in_case
CRA004775	ENSGALG00010022205	GALNT18	case	control	0.604664545	0.286524503	2.110341481	0.034828953	0.354006537	up_in_case
CRA004775	ENSGALG00010022207	ENSGALG00010022207	case	control	-0.355453214	0.444832521	-0.799072004	0.424248662	0.821240473	down_in_case
CRA004775	ENSGALG00010022208	CCL17	case	control	-0.533309471	0.604011225	-0.88294629	0.377265291	0.798610687	down_in_case
CRA004775	ENSGALG00010022209	ZFYVE19	case	control	0.040881818	0.181717095	0.224975083	0.821998659	0.960766569	up_in_case
CRA004775	ENSGALG00010022210	EZH1	case	control	-0.259907921	0.114552458	-2.268898673	0.023274488	0.296313338	down_in_case
CRA004775	ENSGALG00010022211	ENSGALG00010022211	case	control	1.638969391	0.494036161	3.317508962	0.00090824	0.062759842	up_in_case
CRA004775	ENSGALG00010022213	IDH1	case	control	-0.34926925	0.194213523	-1.798377606	0.072117189	0.473809587	down_in_case
CRA004775	ENSGALG00010022214	ENSGALG00010022214	case	control	0.181900742	0.541955228	0.33563795	0.737143902	0.937564762	up_in_case
CRA004775	ENSGALG00010022215	ENSGALG00010022215	case	control	0.247260004	0.256630286	0.963487233	0.335303068	0.775897605	up_in_case
CRA004775	ENSGALG00010022216	ROMO1	case	control	-0.069446501	0.280091753	-0.247941971	0.804179305	0.954576316	down_in_case
CRA004775	ENSGALG00010022218	ENSGALG00010022218	case	control	-0.114757684	0.524642562	-0.218734987	0.826856493	0.961878235	down_in_case
CRA004775	ENSGALG00010022219	EIF4E3	case	control	-0.342680105	0.248877892	-1.376900547	0.168542998	0.631852444	down_in_case
CRA004775	ENSGALG00010022220	PPP1R14D	case	control	0.3110711	0.439379629	0.707977976	0.478958935	0.847184661	up_in_case
CRA004775	ENSGALG00010022221	CUTC	case	control	-0.556245837	0.246474286	-2.256810824	0.024019898	0.299725607	down_in_case
CRA004775	ENSGALG00010022222	ENSGALG00010022222	case	control	-0.007674967	0.237607597	-0.032301017	0.974231998	0.994947809	down_in_case
CRA004775	ENSGALG00010022223	DOK4	case	control	-0.352165517	0.347145881	-1.01445973	0.310363436	0.762244121	down_in_case
CRA004775	ENSGALG00010022224	NHEJ1	case	control	-0.491179395	0.298022194	-1.64813026	0.099325944	0.537098329	down_in_case
CRA004775	ENSGALG00010022226	FBRSL1	case	control	0.429643551	0.245812099	1.747853556	0.080489389	0.494585677	up_in_case
CRA004775	ENSGALG00010022227	TMEM80	case	control	-0.329185856	0.261313249	-1.259736569	0.207764409	0.676506608	down_in_case
CRA004775	ENSGALG00010022228	FUBP1	case	control	0.066373572	0.268036588	0.247628777	0.804421643	0.954576316	up_in_case
CRA004775	ENSGALG00010022229	RAPSN	case	control	-0.002352929	0.559046091	-0.004208829	0.99664185	0.999606847	down_in_case
CRA004775	ENSGALG00010022230	NFS1	case	control	-0.114245016	0.228847359	-0.499219289	0.617624907	0.903976249	down_in_case
CRA004775	ENSGALG00010022231	ZNF503	case	control	-0.122356743	0.415703715	-0.294336419	0.768500847	0.945059483	down_in_case
CRA004775	ENSGALG00010022232	GPR27	case	control	0.609891816	0.276176003	2.208344711	0.027220253	0.31561562	up_in_case
CRA004775	ENSGALG00010022233	FAM46C	case	control	0.25459034	0.432339778	0.588866333	0.555950944	0.87981923	up_in_case
CRA004775	ENSGALG00010022234	COX15	case	control	-0.282751672	0.188092003	-1.503262591	0.132771343	0.589506365	down_in_case
CRA004775	ENSGALG00010022235	RAMP2	case	control	-0.086857634	0.2236376	-0.388385648	0.697730663	0.929092636	down_in_case

CRA004775	ENSGALG00010022236	SHF	case	control	-0.250480676	0.339235616	-0.738367862	0.460290938	0.841265006	down_in_case
CRA004775	ENSGALG00010022237	SUN1	case	control	0.126628245	0.178169168	0.710719176	0.477258274	0.846532686	up_in_case
CRA004775	ENSGALG00010022238	COMTD1	case	control	0.088495458	0.290357007	0.304781548	0.760532536	0.942236668	up_in_case
CRA004775	ENSGALG00010022239	DEAF1	case	control	-0.2808069	0.197910574	-1.41885749	0.155940568	0.619442611	down_in_case
CRA004775	ENSGALG00010022240	ENSGALG00010022240	case	control	0.262289707	0.20722568	1.265720091	0.205613313	0.674045742	up_in_case
CRA004775	ENSGALG00010022241	POLR2C	case	control	-0.416108888	0.247021523	-1.684504582	0.092084195	0.52187835	down_in_case
CRA004775	ENSGALG00010022242	USP19	case	control	-0.481406451	0.193742755	-2.484771367	0.012963466	0.228832637	down_in_case
CRA004775	ENSGALG00010022243	ENSGALG00010022243	case	control	1.530628558	0.54382508	2.814560442	0.004884399	0.142429514	up_in_case
CRA004775	ENSGALG00010022244	ENTPD7	case	control	-0.222156309	0.232720355	-0.954606267	0.339776847	0.780142164	down_in_case
CRA004775	ENSGALG00010022245	PIKFYVE	case	control	0.197102584	0.38079826	0.517603689	0.604734807	0.899481702	up_in_case
CRA004775	ENSGALG00010022246	RHOV	case	control	1.519428234	0.737687794	2.059717196	0.039425585	0.374034172	up_in_case
CRA004775	ENSGALG00010022247	GALNT9	case	control	-0.674048216	0.602707571	-1.118366931	0.263410312	0.723981064	down_in_case
CRA004775	ENSGALG00010022248	FIGNL2	case	control	-0.847308532	0.499649154	-1.695806999	0.089922437	0.517157435	down_in_case
CRA004775	ENSGALG00010022249	DRD4	case	control	-0.011898376	0.833907865	-0.014268214	0.988615998	0.998065748	down_in_case
CRA004775	ENSGALG00010022250	SLC25A28	case	control	-0.53790404	0.33304731	-1.615097988	0.106289477	0.552224847	down_in_case
CRA004775	ENSGALG00010022251	WNK4	case	control	-0.403702958	0.30493494	-1.323898659	0.185536704	0.6491093	down_in_case
CRA004775	ENSGALG00010022252	COQ9	case	control	-0.096892603	0.545324926	-0.177678661	0.858975344	0.971325143	down_in_case
CRA004775	ENSGALG00010022253	ALPK3	case	control	0.320707679	0.576213023	0.556578324	0.577815567	0.888378369	up_in_case
CRA004775	ENSGALG00010022255	NOC4L	case	control	-0.320839664	0.242800299	-1.321413793	0.186363429	0.6491093	down_in_case
CRA004775	ENSGALG00010022256	PSMC3	case	control	-0.162105135	0.237375758	-0.682905182	0.494666759	0.856382445	down_in_case
CRA004775	ENSGALG00010022258	ENSGALG00010022258	case	control	-0.973135804	0.567474604	-1.714853487	0.086372092	0.508360843	down_in_case
CRA004775	ENSGALG00010022259	GET4	case	control	0.131068193	0.186645728	0.702229804	0.482535861	0.849596752	up_in_case
CRA004775	ENSGALG00010022264	SCN8A	case	control	-1.017280827	0.583766651	-1.742615521	0.081400797	0.496834143	down_in_case
CRA004775	ENSGALG00010022266	COA3	case	control	-0.296477769	0.327482807	-0.905323158	0.365294211	0.793608387	down_in_case
CRA004775	ENSGALG00010022270	ENSGALG00010022270	case	control	-1.942241578	0.868481872	-2.236363983	0.025327938	0.30560733	down_in_case
CRA004775	ENSGALG00010022271	SLC18A2	case	control	-0.562282062	0.490193591	-1.147061227	0.251356317	0.715759105	down_in_case
CRA004775	ENSGALG00010022274	CCDC91	case	control	0.077137871	0.290937592	0.265135458	0.790905113	0.95117511	up_in_case
CRA004775	ENSGALG00010022275	VDAC2	case	control	0.248163183	0.279759225	0.887059874	0.375046666	0.798503808	up_in_case
CRA004775	ENSGALG00010022276	BECN1	case	control	0.2709699	0.223318529	1.213378488	0.224985147	0.692859731	up_in_case
CRA004775	ENSGALG00010022279	EP400	case	control	0.138180829	0.264924073	0.521586534	0.601958241	0.898185892	up_in_case
CRA004775	ENSGALG00010022280	PDZD8	case	control	0.251008037	0.258170456	0.972257014	0.330922703	0.77237154	up_in_case
CRA004775	ENSGALG00010022282	TMT1A	case	control	-0.863893388	0.597496294	-1.445855642	0.148217696	0.611192154	down_in_case
CRA004775	ENSGALG00010022290	NEXN	case	control	0.937897574	0.602983658	1.555427849	0.119844206	0.572133279	up_in_case
CRA004775	ENSGALG00010022293	DNAJC10	case	control	0.120783666	0.17903338	0.674643277	0.499902431	0.85956977	up_in_case
CRA004775	ENSGALG00010022296	CIAPIN1	case	control	-0.14175412	0.259906835	-0.545403587	0.585475974	0.891076936	down_in_case
CRA004775	ENSGALG00010022297	VPS18	case	control	-0.218294668	0.255462861	-0.854506469	0.392824426	0.807553294	down_in_case
CRA004775	ENSGALG00010022299	MRGBP	case	control	0.187676191	0.188298584	0.996694649	0.318912748	0.767037518	up_in_case
CRA004775	ENSGALG00010022302	ENSGALG00010022302	case	control	-0.682736489	0.349315582	-1.954497663	0.050642391	0.416238146	down_in_case
CRA004775	ENSGALG00010022303	DUSP13A	case	control	0.186830382	0.547901586	0.340992591	0.733109159	0.936819725	up_in_case
CRA004775	ENSGALG00010022304	ADAP1	case	control	-0.053437174	0.417278444	-0.128061189	0.89810055	0.978790377	down_in_case
CRA004775	ENSGALG00010022307	ENSGALG00010022307	case	control	-0.31774375	0.602445167	-0.527423519	0.597899515	0.896490702	down_in_case
CRA004775	ENSGALG00010022308	OGFR	case	control	-0.403593543	0.283855664	-1.421826633	0.155076586	0.619284425	down_in_case
CRA004775	ENSGALG00010022309	WSCD2	case	control	1.132167028	0.412503863	2.744621638	0.00605807	0.155408639	up_in_case
CRA004775	ENSGALG00010022310	DCLRE1A	case	control	-0.272233593	0.2187664	-1.244403131	0.213351231	0.682777582	down_in_case
CRA004775	ENSGALG00010022311	DLL4	case	control	0.625213532	0.463194326	1.349786681	0.177084418	0.643089023	up_in_case
CRA004775	ENSGALG00010022312	ZNF592	case	control	-0.069949175	0.194406688	-0.35980848	0.718990361	0.93407508	down_in_case
CRA004775	ENSGALG00010022313	ENSGALG00010022313	case	control	0.217915179	0.334427957	0.651605749	0.514655538	0.864633879	up_in_case
CRA004775	ENSGALG00010022314	DUSP13B	case	control	-0.2255314	0.984124128	-0.229169668	0.818737046	0.959941088	down_in_case
CRA004775	ENSGALG00010022316	INO80	case	control	0.112280424	0.203647186	0.55134778	0.581395291	0.888998848	up_in_case
CRA004775	ENSGALG00010022317	PSME3	case	control	-0.444917116	0.16963661	-2.622765905	0.008721918	0.18887416	down_in_case
CRA004775	ENSGALG00010022320	COL9A3	case	control	-2.641178585	0.699801384	-3.774183142	0.000160533	0.027119381	down_in_case
CRA004775	ENSGALG00010022322	ENSGALG00010022322	case	control	-0.495347861	0.263549697	-1.879523547	0.060173041	0.445397937	down_in_case
CRA004775	ENSGALG00010022323	DUSP29	case	control	0.142817601	0.827632018	0.172561716	0.862995947	0.971910432	up_in_case
CRA004775	ENSGALG00010022325	MIGA1	case	control	0.000727474	0.275711649	0.002638531	0.99789476	0.999646385	up_in_case
CRA004775	ENSGALG00010022326	COX19	case	control	-0.476627587	0.29477283	-1.616931881	0.105892986	0.55052342	down_in_case
CRA004775	ENSGALG00010022328	ENSGALG00010022328	case	control	-0.796606658	0.362991965	-2.194557275	0.028195371	0.319106625	down_in_case
CRA004775	ENSGALG00010022332	KAT6B	case	control	0.090386454	0.194429646	0.46488	0.642017394	0.912871594	up_in_case
CRA004775	ENSGALG00010022333	ENSGALG00010022333	case	control	0.228647271	0.305620207	0.748141861	0.454374594	0.838254622	up_in_case
CRA004775	ENSGALG00010022334	PDE1A	case	control	0.231697287	0.396451888	0.584427252	0.558932896	0.880802917	up_in_case
CRA004775	ENSGALG00010022339	ENSGALG00010022339	case	control	2.528082382	0.818836681	3.087407342	0.002019107	0.089985187	up_in_case
CRA004775	ENSGALG00010022340	GFOD2	case	control	-0.283587202	0.217930678	-1.301272517	0.193165191	0.657804492	down_in_case
CRA004775	ENSGALG00010022343	GATA5	case	control	1.658097114	0.428865211	3.866242991	0.000110525	0.022744947	up_in_case
CRA004775	ENSGALG00010022345	USP33	case	control	0.131697757	0.274203786	0.480291534	0.631020108	0.909357765	up_in_case
CRA004775	ENSGALG00010022347	SEC11A	case	control	-0.507299111	0.308099567	-1.64654276	0.099652064	0.537098329	down_in_case
CRA004775	ENSGALG00010022348	G6PC1	case	control	1.096394591	0.366028488	2.99538049	0.002741027	0.10984643	up_in_case
CRA004775	ENSGALG00010022353	SLC39A13	case	control	-0.185552349	0.202187126	-0.917725836	0.358762418	0.79097996	down_in_case
CRA004775	ENSGALG00010022354	FAR2	case	control	-0.025414643	0.200832953	-0.126546182	0.899299595	0.978790377	down_in_case
CRA004775	ENSGALG00010022356	TCFL5	case	control	-0.316838076	0.575313176	-0.550722788	0.58182372	0.889258417	down_in_case
CRA004775	ENSGALG00010022357	ENSGALG00010022357	case	control	0.068721521	0.733016861	0.093751623	0.925306461	0.98417264	up_in_case
CRA004775	ENSGALG00010022359	ZZZ3	case	control	0.272828397	0.21020414	1.29792114	0.194314435	0.65892248	up_in_case
CRA004775	ENSGALG00010022363	KIF18A	case	control	-0.24800901	0.555354619	-0.446577739	0.655179972	0.916967384	down_in_case
CRA004775	ENSGALG00010022365	PLCG1	case	control	-0.490848253	0.331574788	-1.480354573	0.138778646	0.598414915	down_in_case
CRA004775	ENSGALG00010022366	AARSD1	case	control	-0.237629665	0.218226609	-1.08891242	0.276192509	0.731001618	down_in_case
CRA004775	ENSGALG00010022373	AK5	case	control	3.072383221	0.924302401	3.324002207	0.000887355	0.062654188	up_in_case
CRA004775	ENSGALG00010022375	NMB	case	control	0.169469036	0.59329475	0.285640546	0.775153445	0.946319184	up_in_case
CRA004775	ENSGALG00010022377	ZHX3	case	control	-0.007278912	0.305182719	-0.023850997	0.980971462	0.997068414	down_in_case
CRA004775	ENSGALG00010022379	METTL15	case	control	-0.140533724	0.352993615	-0.398119735	0.690541928	0.927867527	down_in_case
CRA004775	ENSGALG00010022385	ENSGALG00010022385	case	control	-0.462694435	0.628585445	-0.736088369	0.461676921	0.842010079	down_in_case
CRA004775	ENSGALG00010022386	SPI1	case	control	-0.656274454	0.581186892	-1.129196929	0.258814769	0.720037471	down_in_case
CRA004775	ENSGALG00010022388	CYP2W1	case	control	1.502028235	0.579692087	2.591079415	0.009567541	0.197747835	up_in_case
CRA004775	ENSGALG00010022391	ENSGALG00010022391	case	control	0.043669157	0.33107548	0.131900909	0.895062669	0.977834811	up_in_case
CRA004775	ENSGALG00010022392	EMILIN3	case	control	-0.376630074	0.843510645	-0.446503048	0.655233911	0.916967384	down_in_case
CRA004775	ENSGALG00010022395	PTGES3L	case	control	-0.233308431	0.403925049	-0.577603275	0.563531995	0.881785214	down_in_case
CRA004775	ENSGALG00010022396	PPP1R1C	case	control	1.622562309	0.727001238	2.231856322	0.02562446	0.307471166	up_in_case
CRA004775	ENSGALG00010022397	ATF1	case	control	0.04062058	0.170587571	0.23812157	0.811786805	0.957566199	up_in_case
CRA004775	ENSGALG00010022400	NHLRC2	case	control	0.215391245	0.231361423	0.93097303	0.351867515	0.786426886	up_in_case
CRA004775	ENSGALG00010022401	LCAT	case	control	-0.273394831	0.597644436	-0.457453988	0.647344767	0.914210065	down_in_case
CRA004775	ENSGALG00010022406	ITPRID2	case	control	0.09313155	0.413443599	0.225258173	0.821778437	0.960766569	up_in_case
CRA004775	ENSGALG00010022407	RUNDC1	case	control	-0.225035138	0.259450533	-0.867352766	0.385748754	0.803541109	down_in_case
CRA004775	ENSGALG00010022408	RAD51AP1	case	control	-0.124932301	0.309094002	-0.404188694	0.686073967	0.926725143	down_in_case
CRA004775	ENSGALG00010022409	CARNS1	case	control	-0.047731579	0.625603625	-0.076296839	0.939182941	0.986950867	down_in_case
CRA004775	ENSGALG00010022411	ADRB1	case	control	-0.482640015	0.416866045	-1.157782026	0.246953	0.712046495	down_in_case
CRA004775	ENSGALG00010022412	CHAC1	case	control	0.465055047	0.948478492	0.490316913	0.623909661	0.906441769	up_in_case
CRA004775	ENSGALG00010022415	DIP2B	case	control	-0.331933075	0.412745717	-0.804207196	0.421277324	0.819812626	down_in_case
CRA004775	ENSGALG00010022416	CCDC186	case	control	0.325639344	0.217521186	1.497046566	0.134381142	0.592420453	up_in_case
CRA004775	ENSGALG00010022421	CMKLR1	case	control	0.494468957	0.390719031	1.26553589	0.205679291	0.674045742	up_in_case
CRA004775	ENSGALG00010022422	RPS6KB2	case	control	-0.456297143	0.402708058	-1.133071799	0.257184103	0.719236059	down_in_case
CRA004775	ENSGALG00010022426	EXD1	case	control	-0.053316256	0.369964464	-0.14411183	0.885412163	0.976602443	down_in_case
CRA004775	ENSGALG00010022427	RPL27	case	control	-0.245412604	0.239755581	-1.023594958	0.306026628	0.75836586	down_in_case
CRA004775	ENSGALG00010022429	ADK	case	control	-0.180235538	0.249670282	-0.721894239	0.470359504	0.844782383	down_in_case
CRA004775	ENSGALG00010022431	LARP4	case	control	0.098233648	0.263499411	0.37280405	0.709294288	0.931874404	up_in_case
CRA004775	ENSGALG00010022433	LNPK	case	control	-0.436322174	0.221251694	-1.972062518	0.04860247	0.408766096	down_in_case
CRA004775	ENSGALG00010022435	PIGK	case	control	0.220669058	0.333888234	0.660906961	0.508671982	0.862971569	up_in_case
CRA004775	ENSGALG00010022436	AFAP1L2	case	control	-0.053719235	0.289946015	-0.18527323	0.853014734	0.969725992	down_in_case
CRA004775	ENSGALG00010022438	RPN2	case	control	-0.245117574	0.276585414	-0.886227408	0.375494997	0.798503808	down_in_case
CRA004775	ENSGALG00010022439	IFI35	case	control	-0.233536895	0.222165837	-1.051182746	0.293174666	0.746438942	down_in_case
CRA004775	ENSGALG00010022440	SORD	case	control	-0.802374337	0.35669191	-2.249488464	0.024481436	0.302160964	down_in_case
CRA004775	ENSGALG00010022441	FICD	case	control	-0.232438073	0.176695497	-1.315472534	0.188351142	0.652397118	down_in_case
CRA004775	ENSGALG00010022448	FERRY3	case	control	-0.270234083	0.272125465	-0.993049596	0.320685785	0.767037518	down_in_case
CRA004775	ENSGALG00010022449	ENSGALG00010022449	case	control	-0.433540881	0.231323779	-1.874173434	0.060906524	0.445419429	down_in_case
CRA004775	ENSGALG00010022451	CHP1	case	control	0.157767029	0.152290731	1.035959496	0.300221039	0.752927916	up_in_case
CRA004775	ENSGALG00010022452	AP3M1	case	control	-0.181942889	0.186449595	-0.975828822	0.329149299	0.77237154	down_in_case
CRA004775	ENSGALG00010022453	ENSGALG00010022453	case	control	-0.696961645	0.281887188	-2.472484291	0.013417761	0.232695952	down_in_case
CRA004775	ENSGALG00010022454	ABLIM1	case	control	-0.071226892	0.445610736	-0.15984106	0.873006279	0.97407675	down_in_case
CRA004775	ENSGALG00010022455	C7orf50	case	control	-0.355698504	0.280481747	-1.268169884	0.204737289	0.674045742	down_in_case
CRA004775	ENSGALG00010022456	ATP5G3	case	control	-0.365062972	0.385843358	-0.946142947	0.344075681	0.783503692	down_in_case
CRA004775	ENSGALG00010022460	VAT1	case	control	-0.00888919	0.380259358	-0.023376649	0.981349831	0.997069916	down_in_case
CRA004775	ENSGALG00010022462	ITGA5	case	control	-0.074993049	0.347819122	-0.215609333	0.82929228	0.96297907	down_in_case
CRA004775	ENSGALG00010022463	DUSP12	case	control	-0.260416562	0.237847005	-1.094891072	0.273564361	0.731001618	down_in_case
CRA004775	ENSGALG00010022466	NEDD4	case	control	-0.267771023	0.263580583	-1.015898135	0.309677897	0.762058118	down_in_case
CRA004775	ENSGALG00010022467	SART3	case	control	0.004023037	0.181987625	0.022106101	0.98236332	0.997230032	up_in_case
CRA004775	ENSGALG00010022468	MYBPC3	case	control	-2.830957669	0.879394883	-3.219210986	0.001285439	0.075498328	down_in_case
CRA004775	ENSGALG00010022470	ENSGALG00010022470	case	control	-0.251707431	0.485525013	-0.518423199	0.604163032	0.898935274	down_in_case
CRA004775	ENSGALG00010022471	ST6GALNAC5	case	control	0.334721621	0.630246757	0.531096141	0.595352158	0.895305942	up_in_case
CRA004775	ENSGALG00010022472	QSER1	case	control	0.424967845	0.2961015	1.43521004	0.151227254	0.615293187	up_in_case
CRA004775	ENSGALG00010022473	ZNF385A	case	control	-1.322972955	0.389682315	-3.395003835	0.000686276	0.055187057	down_in_case

CRA004775	ENSGALG00010022474	VCL	case	control	0.886799284	0.22837826	3.883028458	0.000103163	0.022517478	up_in_case
CRA004775	ENSGALG00010022476	OIP5	case	control	-0.274373173	0.226472677	-1.211506732	0.225701256	0.69359464	down_in_case
CRA004775	ENSGALG00010022478	ATP6V1A	case	control	-0.425637763	0.344930555	-1.233981033	0.217209972	0.68458287	down_in_case
CRA004775	ENSGALG00010022479	ENSGALG00010022479	case	control	2.020253042	0.538903733	3.74881991	0.000177669	0.027735809	up_in_case
CRA004775	ENSGALG00010022480	PIF1	case	control	0.458207826	0.718031866	0.638144138	0.523379857	0.866638286	up_in_case
CRA004775	ENSGALG00010022481	CORO1B	case	control	-0.049040254	0.17739196	-0.27645139	0.78220139	0.949258787	down_in_case
CRA004775	ENSGALG00010022482	PRTG	case	control	0.701845572	0.539556389	1.300782617	0.193332874	0.657919837	up_in_case
CRA004775	ENSGALG00010022483	TOP2A	case	control	0.566742595	0.569764967	0.994695405	0.31988443	0.767037518	up_in_case
CRA004775	ENSGALG00010022485	ATF2	case	control	0.062152202	0.244648046	0.254047408	0.799458933	0.95283872	up_in_case
CRA004775	ENSGALG00010022489	NUSAP1	case	control	0.495806016	0.566279875	0.87554942	0.381275033	0.801753951	up_in_case
CRA004775	ENSGALG00010022493	ENSGALG00010022493	case	control	-0.436478003	0.248781299	-1.754464682	0.079350916	0.491399489	down_in_case
CRA004775	ENSGALG00010022494	FHIP2A	case	control	0.174781644	0.333872796	0.523497708	0.600627946	0.897645352	up_in_case
CRA004775	ENSGALG00010022496	TUBGCP2	case	control	-0.40607758	0.173089701	-2.346052814	0.018973419	0.272278915	down_in_case
CRA004775	ENSGALG00010022497	CORO1C	case	control	-0.576049451	0.424997723	-1.355417737	0.175284502	0.640706322	down_in_case
CRA004775	ENSGALG00010022498	NAA50	case	control	0.292839881	0.294449289	0.994534176	0.319962876	0.767037518	up_in_case
CRA004775	ENSGALG00010022500	ENSGALG00010022500	case	control	1.512313935	0.414708368	3.646692593	0.000265637	0.033398296	up_in_case
CRA004775	ENSGALG00010022502	ENSGALG00010022502	case	control	2.335901849	0.710659332	3.286950224	0.001012787	0.066660879	up_in_case
CRA004775	ENSGALG00010022504	RAD9A	case	control	-0.260840732	0.387051606	-0.673917193	0.500363961	0.859740701	down_in_case
CRA004775	ENSGALG00010022505	ZNF511	case	control	-0.312414519	0.279396334	-1.118176875	0.263491459	0.723981064	down_in_case
CRA004775	ENSGALG00010022506	EBNA1BP2	case	control	0.046155859	0.190281953	0.242565614	0.808341922	0.955397873	up_in_case
CRA004775	ENSGALG00010022507	ENSGALG00010022507	case	control	-0.611651951	0.376032946	-1.626591388	0.10382391	0.545750223	down_in_case
CRA004775	ENSGALG00010022508	USF3	case	control	-0.182107127	0.353186141	-0.515612324	0.606125201	0.900867665	down_in_case
CRA004775	ENSGALG00010022509	ENSGALG00010022509	case	control	2.465027025	0.601549448	4.097796172	4.17102E-05	0.013292084	up_in_case
CRA004775	ENSGALG00010022511	IGFBP4	case	control	-0.958892415	0.492931182	-1.945286584	0.051740496	0.419476344	down_in_case
CRA004775	ENSGALG00010022512	PRAP1	case	control	2.287921929	0.761223322	3.005585697	0.002650699	0.107205178	up_in_case
CRA004775	ENSGALG00010022513	NDUFAF1	case	control	0.144796779	0.281842719	0.513750291	0.607426586	0.900943922	up_in_case
CRA004775	ENSGALG00010022515	CHRNA1	case	control	-0.538197714	0.426175881	-1.262853526	0.206641824	0.675197427	down_in_case
CRA004775	ENSGALG00010022516	TRUB1	case	control	-0.745296501	0.320970321	-2.322010642	0.020232363	0.281415838	down_in_case
CRA004775	ENSGALG00010022517	HNRNPA1	case	control	-0.230172904	0.194432692	-1.183817911	0.23648515	0.703419274	down_in_case
CRA004775	ENSGALG00010022518	ECHS1	case	control	-0.259755917	0.347164737	-0.748220914	0.454326918	0.838254622	down_in_case
CRA004775	ENSGALG00010022520	TMOD2	case	control	1.00651652	0.443363991	2.270181027	0.023196602	0.296238434	up_in_case
CRA004775	ENSGALG00010022521	RTF1	case	control	0.304447416	0.176651683	1.723433434	0.084810173	0.505209623	up_in_case
CRA004775	ENSGALG00010022522	TNS4	case	control	1.243363083	0.485035826	2.563445867	0.010363886	0.204467428	up_in_case
CRA004775	ENSGALG00010022524	SOX18	case	control	0.046961568	0.332035647	0.14143532	0.887526053	0.977118922	up_in_case
CRA004775	ENSGALG00010022525	ISCU	case	control	-0.216992044	0.347357464	-0.624693771	0.532172062	0.869886675	down_in_case
CRA004775	ENSGALG00010022526	PPP1CA	case	control	-0.039238891	0.405137681	-0.096853225	0.922842956	0.983159417	down_in_case
CRA004775	ENSGALG00010022528	SIDT1	case	control	0.072449767	0.839663317	0.086284307	0.931240414	0.985044263	up_in_case
CRA004775	ENSGALG00010022529	CCR7	case	control	-0.348743706	0.745746295	-0.467643901	0.640039265	0.911484465	down_in_case
CRA004775	ENSGALG00010022531	ENSGALG00010022531	case	control	0.258870442	0.440061208	0.588260082	0.556357735	0.87981923	up_in_case
CRA004775	ENSGALG00010022532	CBX5	case	control	-0.38664977	0.196850059	-1.964184169	0.049508738	0.411853997	down_in_case
CRA004775	ENSGALG00010022533	TMEM119	case	control	-0.638290345	0.36008743	-1.772598239	0.076295304	0.486386983	down_in_case
CRA004775	ENSGALG00010022536	SMUG1	case	control	-0.224681194	0.305942863	-0.734389394	0.462711449	0.842456502	down_in_case
CRA004775	ENSGALG00010022538	WIPF1	case	control	-0.553885052	0.435589435	-1.271575954	0.203523825	0.673725594	down_in_case
CRA004775	ENSGALG00010022539	LYSMD2	case	control	0.117017536	0.217939062	0.53692778	0.591317501	0.893804699	up_in_case
CRA004775	ENSGALG00010022540	GPR146	case	control	0.442623077	0.283854433	1.55933121	0.118918008	0.570892125	up_in_case
CRA004775	ENSGALG00010022541	ITPKA	case	control	0.068564045	0.273736574	0.25047455	0.802220383	0.953744693	up_in_case
CRA004775	ENSGALG00010022544	SPICE1	case	control	0.025907711	0.173661863	0.149184805	0.88140781	0.975011509	up_in_case
CRA004775	ENSGALG00010022545	CSTF3	case	control	-0.019761607	0.180972515	-0.109196733	0.913046451	0.980803023	down_in_case
CRA004775	ENSGALG00010022546	MTG1	case	control	-0.594851126	0.446367499	-1.332648832	0.182647072	0.647378665	down_in_case
CRA004775	ENSGALG00010022547	SMARCE1	case	control	-0.007564309	0.136322522	-0.055488328	0.955749428	0.990926004	down_in_case
CRA004775	ENSGALG00010022548	ENSGALG00010022548	case	control	0.138610942	0.371798115	0.372812386	0.709288084	0.931874404	up_in_case
CRA004775	ENSGALG00010022550	TRIM45	case	control	-0.732521263	0.282019423	-2.597414231	0.009392857	0.195703177	down_in_case
CRA004775	ENSGALG00010022552	GPR155	case	control	0.129037943	0.192246324	0.671211496	0.502085806	0.860091011	up_in_case
CRA004775	ENSGALG00010022553	PRPF6	case	control	-0.137299	0.207328173	-0.66223031	0.507823632	0.862748056	down_in_case
CRA004775	ENSGALG00010022554	SCG3	case	control	0.769161203	0.719012584	1.06974651	0.284733425	0.74120185	up_in_case
CRA004775	ENSGALG00010022557	WNT1	case	control	-0.307013722	0.353114127	-0.869446162	0.384603143	0.803182776	down_in_case
CRA004775	ENSGALG00010022559	SSH1	case	control	-0.188514413	0.268755407	-0.701434865	0.48303167	0.850143158	down_in_case
CRA004775	ENSGALG00010022564	VTCN1	case	control	-1.015457677	0.542245087	-1.872691335	0.061111021	0.445419429	down_in_case
CRA004775	ENSGALG00010022565	ARF3	case	control	-0.45043111	0.354844618	-1.269375629	0.204307124	0.674045742	down_in_case
CRA004775	ENSGALG00010022567	PAOX	case	control	-0.173944494	0.255235524	-0.681505818	0.495551487	0.856382445	down_in_case
CRA004775	ENSGALG00010022568	SCRN3	case	control	0.455692059	0.416670853	1.093649955	0.274108531	0.731001618	up_in_case
CRA004775	ENSGALG00010022569	KIAA1549L	case	control	-0.285733699	1.037485477	-0.275409831	0.783001392	0.94936527	down_in_case
CRA004775	ENSGALG00010022571	RIPOR1	case	control	-0.012036357	0.197569786	-0.060922055	0.951421284	0.990168609	down_in_case
CRA004775	ENSGALG00010022573	AP4E1	case	control	0.224758793	0.204046593	1.10150721	0.270675968	0.728665115	up_in_case
CRA004775	ENSGALG00010022574	ITPRIPL2	case	control	-0.170425982	0.327693433	-0.520077503	0.603009558	0.898692785	down_in_case
CRA004775	ENSGALG00010022575	TMEM41B	case	control	0.408549244	0.243843571	1.675456287	0.093844741	0.525859426	up_in_case
CRA004775	ENSGALG00010022578	ZNF512B	case	control	0.065068378	0.140675667	0.462541811	0.643692826	0.913647817	up_in_case
CRA004775	ENSGALG00010022579	TNFAIP8L3	case	control	0.005166762	0.372015522	0.013888565	0.988918885	0.998065748	up_in_case
CRA004775	ENSGALG00010022581	TMEM125	case	control	1.118539184	0.698957252	1.600296987	0.109532715	0.556814922	up_in_case
CRA004775	ENSGALG00010022582	ENSGALG00010022582	case	control	-0.168146828	0.430527756	-0.390559785	0.696122656	0.929092636	down_in_case
CRA004775	ENSGALG00010022583	FKBP11	case	control	-0.264226612	0.315695741	-0.836966032	0.402611662	0.811996248	down_in_case
CRA004775	ENSGALG00010022585	CIR1	case	control	0.070916666	0.209469844	0.338553104	0.734946416	0.936931635	up_in_case
CRA004775	ENSGALG00010022588	TCP11L1	case	control	-0.059240742	0.186584023	-0.31750169	0.750862958	0.941495694	down_in_case
CRA004775	ENSGALG00010022590	ENSGALG00010022590	case	control	-0.873342555	0.382932126	-2.28067194	0.022567867	0.292594999	down_in_case
CRA004775	ENSGALG00010022592	CCDC65	case	control	0.134336689	0.622413544	0.215831886	0.829118792	0.96297907	up_in_case
CRA004775	ENSGALG00010022593	MANBAL	case	control	-0.461152298	0.270860492	-1.702545451	0.088653166	0.514592522	down_in_case
CRA004775	ENSGALG00010022597	SLCO4A1	case	control	-0.383451771	0.561880939	-0.682443102	0.494958809	0.856382445	down_in_case
CRA004775	ENSGALG00010022599	RNLS	case	control	-0.473689426	0.344986327	-1.373067248	0.169731442	0.633147466	down_in_case
CRA004775	ENSGALG00010022603	ATRNL1	case	control	0.275285661	0.385206445	0.714644483	0.474828751	0.84579952	up_in_case
CRA004775	ENSGALG00010022608	P3H4	case	control	-0.503428385	0.399568952	-1.259928689	0.207695088	0.676460246	down_in_case
CRA004775	ENSGALG00010022614	DAO	case	control	0.564739644	0.418561199	1.349240314	0.17725979	0.64323434	up_in_case
CRA004775	ENSGALG00010022616	GLDN	case	control	1.92490635	0.451866367	4.259901798	2.04517E-05	0.008271499	up_in_case
CRA004775	ENSGALG00010022619	MADD	case	control	-0.199467091	0.305548347	-0.652816789	0.513874399	0.863954532	down_in_case
CRA004775	ENSGALG00010022622	MPPED2	case	control	0.181308413	0.232416991	0.780099651	0.435332222	0.826905159	up_in_case
CRA004775	ENSGALG00010022623	TOP1	case	control	0.067244752	0.242288267	0.277540274	0.781365286	0.948910196	up_in_case
CRA004775	ENSGALG00010022625	ENSGALG00010022625	case	control	-0.502988485	0.229283967	-2.193735969	0.028254396	0.319511682	down_in_case
CRA004775	ENSGALG00010022627	CTCF	case	control	-0.006924359	0.130778523	-0.052947217	0.957773963	0.991431439	down_in_case
CRA004775	ENSGALG00010022630	ENSGALG00010022630	case	control	0.563984875	0.943780393	0.597580623	0.550119797	0.877623839	up_in_case
CRA004775	ENSGALG00010022637	DMXL2	case	control	-0.541509482	0.4541392	-1.192386569	0.233109704	0.699888984	down_in_case
CRA004775	ENSGALG00010022642	SEC13	case	control	-0.044372281	0.180515569	-0.245808612	0.80583039	0.955171181	down_in_case
CRA004775	ENSGALG00010022643	SP3	case	control	0.236620045	0.197237469	1.199670866	0.230267192	0.696525332	up_in_case
CRA004775	ENSGALG00010022644	ARMC8	case	control	-0.132967975	0.20237075	-0.657051351	0.511147898	0.863170205	down_in_case
CRA004775	ENSGALG00010022645	ENSGALG00010022645	case	control	1.774818966	0.667441964	2.659136018	0.007834133	0.178356226	up_in_case
CRA004775	ENSGALG00010022647	SVIP	case	control	-0.065876436	0.341487211	-0.192910404	0.847029137	0.967891337	down_in_case
CRA004775	ENSGALG00010022649	PATZ1	case	control	-0.116408801	0.353849667	-0.328978127	0.742172221	0.938877854	down_in_case
CRA004775	ENSGALG00010022650	NR1H3	case	control	-0.177210394	0.265566944	-0.667290858	0.504586365	0.861471523	down_in_case
CRA004775	ENSGALG00010022652	CARMIL2	case	control	0.144640992	0.518434477	0.278995705	0.780248121	0.94835413	up_in_case
CRA004775	ENSGALG00010022655	NRIP3	case	control	-0.110518605	0.600477315	-0.184051258	0.853973244	0.970073177	down_in_case
CRA004775	ENSGALG00010022656	FKBP10	case	control	-0.532622184	0.429845927	-1.239100223	0.215308384	0.683654538	down_in_case
CRA004775	ENSGALG00010022657	MRPS35	case	control	-0.15025735	0.318254331	-0.472129788	0.636834151	0.910302122	down_in_case
CRA004775	ENSGALG00010022659	TIE1	case	control	0.214548814	0.313199422	0.68502302	0.493329395	0.856001758	up_in_case
CRA004775	ENSGALG00010022660	METAP1D	case	control	-0.096609607	0.315684932	-0.306031733	0.759580485	0.942236668	down_in_case
CRA004775	ENSGALG00010022661	ENSGALG00010022661	case	control	-0.321446876	0.753727479	-0.426476259	0.669760844	0.920988136	down_in_case
CRA004775	ENSGALG00010022662	GFRA1	case	control	1.551669792	0.477706688	3.248164259	0.001161522	0.07185901	up_in_case
CRA004775	ENSGALG00010022664	TRPC4AP	case	control	0.105054196	0.188854647	0.556270112	0.578026216	0.888378369	up_in_case
CRA004775	ENSGALG00010022666	ENSGALG00010022666	case	control	-0.280939195	0.146932054	-1.912034765	0.055871735	0.432968819	down_in_case
CRA004775	ENSGALG00010022667	PTEN	case	control	0.295658965	0.180430901	1.63862711	0.101290943	0.540680709	up_in_case
CRA004775	ENSGALG00010022671	DBR1	case	control	-0.197893834	0.192548729	-1.02775975	0.304062854	0.756498698	down_in_case
CRA004775	ENSGALG00010022673	KLHL42	case	control	-0.018425622	0.296484127	-0.062147078	0.950445707	0.990168609	down_in_case
CRA004775	ENSGALG00010022674	ENSGALG00010022674	case	control	1.170604698	0.35239905	3.3218157	0.000894337	0.062720521	up_in_case
CRA004775	ENSGALG00010022681	PPP2R3A	case	control	0.337921892	0.361259081	0.935400409	0.349581986	0.785360568	up_in_case
CRA004775	ENSGALG00010022682	EIF1	case	control	-0.209532161	0.349945541	-0.598756482	0.549335286	0.877623839	down_in_case
CRA004775	ENSGALG00010022684	ENSGALG00010022684	case	control	-0.248213275	0.676487263	-0.366914928	0.713682476	0.933244709	down_in_case
CRA004775	ENSGALG00010022685	ATAD1	case	control	-0.27710413	0.16079752	-1.723310973	0.084832305	0.505209623	down_in_case
CRA004775	ENSGALG00010022689	MPL	case	control	-0.399179558	0.471396267	-0.846802544	0.39710519	0.808998414	down_in_case
CRA004775	ENSGALG00010022690	AICDA	case	control	2.924157783	0.970669456	3.012516533			up_in_case
CRA004775	ENSGALG00010022691	ASIC2	case	control	0.482417244	0.862188237	0.55952659	0.57580239	0.887937128	up_in_case
CRA004775	ENSGALG00010022695	PIK3IP1	case	control	0.08473334	0.416743948	0.203322305	0.838883123	0.965800667	up_in_case
CRA004775	ENSGALG00010022698	SEMA6D	case	control	0.716363999	0.336267792	2.130337834	0.033143732	0.347668706	up_in_case
CRA004775	ENSGALG00010022702	CDC20	case	control	0.43885532	0.436648966	1.005052925	0.314871366	0.765098415	up_in_case
CRA004775	ENSGALG00010022708	SPRED1	case	control	0.087808904	0.234260249	0.374834845	0.707783298	0.931742212	up_in_case
CRA004775	ENSGALG00010022709	RNF5	case	control	-0.146081207	0.15638594	-0.934107038	0.350248682	0.785466132	down_in_case
CRA004775	ENSGALG00010022711	LRRC49	case	control	-0.086343425	0.33406616	-0.258462052	0.796050332	0.952835572	down_in_case
CRA004775	ENSGALG00010022712	DDX23	case	control	-0.282898324	0.230806688	-1.225693785	0.22031393	0.688374653	down_in_case
CRA004775	ENSGALG00010022713	PNLIPRP1	case	control	2.349327007	1.399570406	1.678605804	0.093228895	0.524906446	up_in_case
CRA004775	ENSGALG00010022717	ELOVL1	case	control	-0.191607853	0.451974687	-0.423934919	0.671613277	0.921605102	down_in_case

CRA004775	ENSGALG00010022718	C12orf43	case	control	-0.56833128	0.267763365	-2.122513208	0.033794665	0.349406342	down_in_case
CRA004775	ENSGALG00010022719	MSL2	case	control	0.074750216	0.249020425	0.300177046	0.764042113	0.94239774	up_in_case
CRA004775	ENSGALG00010022724	LUZP2	case	control	-0.057511992	0.652238846	-0.088176275	0.929736574	0.98473447	down_in_case
CRA004775	ENSGALG00010022727	ACP2	case	control	-0.14914169	0.182190505	-0.818602981	0.413012965	0.817112606	down_in_case
CRA004775	ENSGALG00010022729	ENSGALG00010022729	case	control	-0.521635228	0.276218811	-1.88848553	0.058960799	0.44414236	down_in_case
CRA004775	ENSGALG00010022731	LRRC8B	case	control	0.241165052	0.38524214	0.626009014	0.531309029	0.869455011	up_in_case
CRA004775	ENSGALG00010022733	PCCB	case	control	0.112162989	0.244744097	0.4582868	0.646746406	0.914043203	up_in_case
CRA004775	ENSGALG00010022734	ENSGALG00010022734	case	control	0.130154701	0.372883281	0.349049442	0.727052192	0.935238044	up_in_case
CRA004775	ENSGALG00010022737	CYLD	case	control	0.057362119	0.204979453	0.279843262	0.779597759	0.948271795	up_in_case
CRA004775	ENSGALG00010022739	RND1	case	control	0.193314205	0.416307827	0.464354001	0.64239414	0.913111066	up_in_case
CRA004775	ENSGALG00010022741	HNF1A	case	control	1.577357959	0.601247058	2.623477216	0.008703727	0.188777515	up_in_case
CRA004775	ENSGALG00010022742	IFT46	case	control	-0.446383164	0.193639883	-2.305223272	0.021154072	0.285746215	down_in_case
CRA004775	ENSGALG00010022743	ZNF326	case	control	-0.05486971	0.300714063	-0.18246473	0.855218031	0.970581465	down_in_case
CRA004775	ENSGALG00010022745	PAPSS2	case	control	0.984676841	0.348895616	2.822267735	0.004768535	0.141624457	up_in_case
CRA004775	ENSGALG00010022747	NT5C3B	case	control	-0.4823491	0.184368917	-2.616217027	0.008891003	0.190031214	down_in_case
CRA004775	ENSGALG00010022748	ENSGALG00010022748	case	control	-0.39378714	0.585327578	-0.672763687	0.501097643	0.86003416	down_in_case
CRA004775	ENSGALG00010022749	HSPA12A	case	control	0.34032263	0.474398252	0.717377495	0.473141202	0.845445211	up_in_case
CRA004775	ENSGALG00010022751	SLC25A12	case	control	0.050228508	0.529158901	0.094921407	0.924377253	0.983849734	up_in_case
CRA004775	ENSGALG00010022754	SNX20	case	control	-0.220562557	0.536782158	-0.410897706	0.681147545	0.923896221	down_in_case
CRA004775	ENSGALG00010022755	MINPP1	case	control	-0.778941218	0.321424184	-2.423405754	0.015375742	0.249441914	down_in_case
CRA004775	ENSGALG00010022759	THSD4	case	control	0.06107572	0.395516473	0.154420167	0.877278454	0.974479909	up_in_case
CRA004775	ENSGALG00010022764	MFAP5	case	control	0.120301544	0.549420934	0.218960612	0.82668073	0.961878235	up_in_case
CRA004775	ENSGALG00010022767	CACNB3	case	control	-0.51556198	0.640049733	-0.805502999	0.420529476	0.819388688	down_in_case
CRA004775	ENSGALG00010022769	FXYD6	case	control	-0.311428506	0.352223609	-0.884178395	0.376599919	0.798523018	down_in_case
CRA004775	ENSGALG00010022770	HAT1	case	control	-0.198287467	0.236800472	-0.837360946	0.402389711	0.811996248	down_in_case
CRA004775	ENSGALG00010022771	SGMS1	case	control	-0.057659713	0.482547693	-0.119490186	0.904887015	0.979540372	down_in_case
CRA004775	ENSGALG00010022774	A1CF	case	control	2.856266989	0.803920337	3.552922919	0.000380976	0.039095538	up_in_case
CRA004775	ENSGALG00010022776	CDH4	case	control	0.247710712	0.44897387	0.551726343	0.581135859	0.888998848	up_in_case
CRA004775	ENSGALG00010022778	GDF11	case	control	0.263156154	0.457602179	0.57507627	0.565239716	0.882950982	up_in_case
CRA004775	ENSGALG00010022780	DYNC1I2	case	control	-0.14044417	0.211301992	-0.66466089	0.506267414	0.8622397	down_in_case
CRA004775	ENSGALG00010022782	TMPRSS13	case	control	-0.679785322	0.322530198	-2.107664104	0.035060054	0.354683583	down_in_case
CRA004775	ENSGALG00010022783	STAG1	case	control	0.139312379	0.154839423	0.899721638	0.368268405	0.794665838	up_in_case
CRA004775	ENSGALG00010022786	KRT40	case	control	2.291134872	0.823399738	2.782530485	0.00539368	0.149708676	up_in_case
CRA004775	ENSGALG00010022787	ENO4	case	control	0.763292644	0.92063531	0.829093383	0.407051568	0.813842524	up_in_case
CRA004775	ENSGALG00010022788	CYBRD1	case	control	0.177326081	0.29806101	0.594932162	0.551888818	0.878258849	up_in_case
CRA004775	ENSGALG00010022789	ENSGALG00010022789	case	control	-0.883794414	0.499842777	-1.768144815	0.077036697	0.488700005	down_in_case
CRA004775	ENSGALG00010022792	DDB2	case	control	-0.425396159	0.212248246	-2.004238751	0.045044491	0.394778076	down_in_case
CRA004775	ENSGALG00010022795	TAF4	case	control	0.036778042	0.240418034	0.152975387	0.878417685	0.974896507	up_in_case
CRA004775	ENSGALG00010022796	RIMKLB	case	control	-0.397810175	0.322444243	-1.233733224	0.217302329	0.68458287	down_in_case
CRA004775	ENSGALG00010022797	EFHD1	case	control	-0.128334661	0.271283678	-0.473064438	0.636167207	0.910080596	down_in_case
CRA004775	ENSGALG00010022799	DCAF17	case	control	-0.134210368	0.286554059	-0.468359684	0.639527394	0.911382154	down_in_case
CRA004775	ENSGALG00010022800	KLHL10	case	control	-0.940106083	0.541864099	-1.734948089	0.08275	0.499833536	down_in_case
CRA004775	ENSGALG00010022801	LSM14B	case	control	-0.153847776	0.214967864	-0.715678023	0.474190184	0.845684862	down_in_case
CRA004775	ENSGALG00010022803	ENSGALG00010022803	case	control	-0.694643828	0.501283022	-1.385731807	0.165828812	0.630194031	down_in_case
CRA004775	ENSGALG00010022804	SNX19	case	control	-0.561742739	0.207481935	-2.707429626	0.006780644	0.161712092	down_in_case
CRA004775	ENSGALG00010022805	ENSGALG00010022805	case	control	-0.612686593	0.473479358	-1.294009089	0.195662281	0.661073909	down_in_case
CRA004775	ENSGALG00010022806	SPPL3	case	control	-0.041999373	0.19182957	-0.218941076	0.826695948	0.961878235	down_in_case
CRA004775	ENSGALG00010022807	ADCY6	case	control	-0.169504439	0.260972038	-0.649511878	0.516007571	0.864897625	down_in_case
CRA004775	ENSGALG00010022808	PSMA7	case	control	-0.136382068	0.259029894	-0.526510923	0.598533265	0.897049692	down_in_case
CRA004775	ENSGALG00010022809	MYO1E	case	control	0.554458961	0.281666374	1.968495401	0.049011065	0.410291029	up_in_case
CRA004775	ENSGALG00010022811	GIGYF2	case	control	-0.116877587	0.203015974	-0.575706358	0.564813677	0.882585553	down_in_case
CRA004775	ENSGALG00010022813	USP24	case	control	0.368836777	0.410900931	0.89762945	0.369383133	0.794896739	up_in_case
CRA004775	ENSGALG00010022815	LRRC8D	case	control	-0.797574282	0.575343748	-1.386256973	0.16566845	0.630194031	down_in_case
CRA004775	ENSGALG00010022816	METTL8	case	control	0.190654141	0.290499757	0.656297077	0.511632998	0.863170205	up_in_case
CRA004775	ENSGALG00010022819	PACSIN3	case	control	-0.287732344	0.353787338	-0.813291809	0.416050772	0.818943344	down_in_case
CRA004775	ENSGALG00010022820	SHTN1	case	control	0.271774915	0.822456035	0.330443091	0.741065186	0.93868712	up_in_case
CRA004775	ENSGALG00010022823	IGHMBP2	case	control	-0.223734046	0.279291543	-0.801077054	0.423087039	0.820552247	down_in_case
CRA004775	ENSGALG00010022824	PUS1	case	control	0.090529569	0.174237022	0.519577116	0.603358351	0.898692785	up_in_case
CRA004775	ENSGALG00010022825	SS18L1	case	control	-0.509081365	0.523274283	-0.972876714	0.33061458	0.77237154	down_in_case
CRA004775	ENSGALG00010022826	SNORC	case	control	-2.173756896	0.904879385	-2.402261485	0.016294057	0.253461053	down_in_case
CRA004775	ENSGALG00010022827	CMTR2	case	control	-0.499982435	0.273820449	-1.825949947	0.067857802	0.463543284	down_in_case
CRA004775	ENSGALG00010022828	PRKG1	case	control	0.729540047	0.343438491	2.124223305	0.033651475	0.348565623	up_in_case
CRA004775	ENSGALG00010022830	MTG2	case	control	-0.175802512	0.288780652	-0.608775244	0.542673425	0.875137006	down_in_case
CRA004775	ENSGALG00010022831	LRRC8C	case	control	-0.043341608	0.27380964	-0.158291024	0.874227481	0.974115968	down_in_case
CRA004775	ENSGALG00010022834	CHRNA7	case	control	1.454974009	0.789437615	1.843051282	0.065321523	0.458517748	up_in_case
CRA004775	ENSGALG00010022837	ITGB3	case	control	0.478309637	0.299523736	1.596900608	0.110287864	0.558567026	up_in_case
CRA004775	ENSGALG00010022838	ARFGAP2	case	control	-0.453327276	0.202552475	-2.238073252	0.025216279	0.305505769	down_in_case
CRA004775	ENSGALG00010022839	SYT12	case	control	-0.221971466	0.296109175	-0.749627112	0.453479317	0.83804959	down_in_case
CRA004775	ENSGALG00010022840	MARVELD1	case	control	-0.577845161	0.306590158	-1.884747911	0.059463881	0.444994181	down_in_case
CRA004775	ENSGALG00010022842	PHC1	case	control	-0.219192134	0.186817005	-1.173298617	0.240676077	0.707468304	down_in_case
CRA004775	ENSGALG00010022845	HYDIN	case	control	-1.854294474	0.508489342	-3.646673237	0.000265657	0.033398296	down_in_case
CRA004775	ENSGALG00010022850	ULK1	case	control	0.590052098	0.401323484	1.470265562	0.141489844	0.601287746	up_in_case
CRA004775	ENSGALG00010022858	HERC4	case	control	0.878781944	0.34132445	2.574623482	0.010034926	0.201740156	up_in_case
CRA004775	ENSGALG00010022860	MOB3C	case	control	-0.573796246	0.317019748	-1.809970038	0.070300434	0.469724266	down_in_case
CRA004775	ENSGALG00010022862	ACADS	case	control	-0.089186933	0.269625668	-0.330780573	0.740810236	0.938450627	down_in_case
CRA004775	ENSGALG00010022863	FAM81A	case	control	-0.041930783	0.841736243	-0.049814634	0.960270105	0.991431439	down_in_case
CRA004775	ENSGALG00010022864	CSTPP1	case	control	-0.458608711	0.329261479	-1.392840463	0.16366804	0.629717526	down_in_case
CRA004775	ENSGALG00010022866	KLHL11	case	control	-0.398968838	0.36207258	-1.101902933	0.270503873	0.728665115	down_in_case
CRA004775	ENSGALG00010022867	ATPAF1	case	control	-0.041676921	0.318208531	-0.130973612	0.895796182	0.978247562	down_in_case
CRA004775	ENSGALG00010022868	VAC14	case	control	-0.561666318	0.177783388	-3.15927335	0.001581631	0.080693373	down_in_case
CRA004775	ENSGALG00010022872	KRT13	case	control	0.33865226	0.996051549	0.339994713	0.73386051	0.936819725	up_in_case
CRA004775	ENSGALG00010022873	NGEF	case	control	-0.464852501	0.543372719	-0.855494736	0.392277317	0.80746892	down_in_case
CRA004775	ENSGALG00010022874	EFCAB14	case	control	-0.091862898	0.156678683	-0.586313951	0.557664558	0.880722248	down_in_case
CRA004775	ENSGALG00010022875	SPON1	case	control	-0.542053691	0.494847463	-1.095395515	0.273343398	0.731001618	down_in_case
CRA004775	ENSGALG00010022877	KYAT3	case	control	0.071941086	0.268072903	0.268363886	0.788419235	0.95041797	up_in_case
CRA004775	ENSGALG00010022879	ENSGALG00010022879	case	control	-0.593801196	0.493292782	-1.20375002	0.228686213	0.695782399	down_in_case
CRA004775	ENSGALG00010022881	CCNB1	case	control	-0.438680379	0.5684355	-0.771732903	0.440272639	0.829353567	down_in_case
CRA004775	ENSGALG00010022885	M6PR	case	control	-0.266859855	0.147876132	-1.804617502	0.071134557	0.47030027	down_in_case
CRA004775	ENSGALG00010022886	PLA2G15	case	control	0.114104417	0.578123958	0.197370159	0.84353788	0.966478468	up_in_case
CRA004775	ENSGALG00010022888	OSBPL2	case	control	0.112209017	0.138373323	0.810915101	0.417414427	0.818943344	up_in_case
CRA004775	ENSGALG00010022890	MYPN	case	control	0.508400238	0.634232549	0.801599096	0.422784901	0.820450913	up_in_case
CRA004775	ENSGALG00010022891	NTM	case	control	0.647866221	0.475302318	1.363061354	0.172863175	0.637223104	up_in_case
CRA004775	ENSGALG00010022900	MMP17	case	control	-0.090560748	0.511453412	-0.177065489	0.859456949	0.971325143	down_in_case
CRA004775	ENSGALG00010022911	SMPD3	case	control	-0.948366174	0.468871582	-2.022656548	0.04310857	0.389478281	down_in_case
CRA004775	ENSGALG00010022917	CYP4B7	case	control	0.787183556	0.419915756	1.874622576	0.060844665	0.445397937	up_in_case
CRA004775	ENSGALG00010022919	ACLY	case	control	-0.436877175	0.325889669	-1.340567734	0.180060839	0.644990528	down_in_case
CRA004775	ENSGALG00010022921	ENSGALG00010022921	case	control	-0.142420976	0.243125169	-0.58579281	0.558014756	0.880754991	down_in_case
CRA004775	ENSGALG00010022923	CD63	case	control	-0.09449722	0.207418289	-0.455587696	0.648686493	0.91497466	down_in_case
CRA004775	ENSGALG00010022928	RNF111	case	control	0.101873808	0.190701126	0.534206643	0.593198574	0.894450257	up_in_case
CRA004775	ENSGALG00010022929	LRP4	case	control	-0.450711653	0.430360641	-1.047288273	0.294966643	0.747369418	down_in_case
CRA004775	ENSGALG00010022930	GTF2B	case	control	0.150832749	0.234463231	0.643310887	0.520022393	0.865611895	up_in_case
CRA004775	ENSGALG00010022931	RDH5	case	control	-0.455504082	0.400271577	-1.137987576	0.25512567	0.718752938	down_in_case
CRA004775	ENSGALG00010022932	OPCML	case	control	0.444668104	0.547020034	0.812891809	0.416280091	0.818943344	up_in_case
CRA004775	ENSGALG00010022933	UNC119B	case	control	0.047652188	0.171163665	0.278401309	0.780704315	0.948614035	up_in_case
CRA004775	ENSGALG00010022937	BLOC1S1	case	control	0.455555838	0.338456732	1.345979545	0.178309114	0.644368088	up_in_case
CRA004775	ENSGALG00010022942	NEU2	case	control	-1.160771594	0.572680505	-2.026909565	0.042671662	0.388066397	down_in_case
CRA004775	ENSGALG00010022946	KRT17	case	control	0.990948842	0.578552193	1.71280803	0.086747862	0.509281038	up_in_case
CRA004775	ENSGALG00010022948	PDZK1IP1	case	control	2.047959225	0.564324643	3.62904447	0.000284472	0.034015459	up_in_case
CRA004775	ENSGALG00010022950	ITGA7	case	control	-0.468355058	0.596058625	-0.785753345	0.432012004	0.825415344	down_in_case
CRA004775	ENSGALG00010022954	ADRM1	case	control	-0.580691339	0.319221638	-1.819085142	0.068898442	0.466961947	down_in_case
CRA004775	ENSGALG00010022956	PBLD	case	control	-0.102021402	0.278989193	-0.365682271	0.714602177	0.93334033	down_in_case
CRA004775	ENSGALG00010022957	SLTM	case	control	0.440404567	0.28012739	1.572158176	0.115913872	0.566086328	up_in_case
CRA004775	ENSGALG00010022958	SFSWAP	case	control	0.152385367	0.149101752	1.022022649	0.306770181	0.7585475	up_in_case
CRA004775	ENSGALG00010022959	ADAMTS8	case	control	0.590949612	0.611890631	0.965776531	0.334156014	0.774540671	up_in_case
CRA004775	ENSGALG00010022967	ADAMTS15	case	control	0.367460809	0.408273774	0.900035301	0.368101465	0.794533903	up_in_case
CRA004775	ENSGALG00010022969	TTN	case	control	0.965237274	0.610067106	1.582182131	0.113607998	0.562963454	up_in_case
CRA004775	ENSGALG00010022971	CKAP5	case	control	0.029305785	0.2638422	0.111073152	0.911558339	0.98075825	up_in_case
CRA004775	ENSGALG00010022972	ENSGALG00010022972	case	control	1.479773286	0.700496882	2.112462346	0.034646812	0.353831224	up_in_case
CRA004775	ENSGALG00010022974	INPP5D	case	control	-0.592765877	0.442085202	-1.340840801	0.179972146	0.644990528	down_in_case
CRA004775	ENSGALG00010022976	MINDY2	case	control	0.128461421	0.289531515	0.443687178	0.657268767	0.917108353	up_in_case
CRA004775	ENSGALG00010022978	ARL14EP	case	control	0.054594671	0.166719615	0.327463992	0.743316973	0.939218065	up_in_case
CRA004775	ENSGALG00010022979	LAMA5	case	control	0.1171122	0.370878458	0.315769757	0.752177275	0.941597758	up_in_case
CRA004775	ENSGALG00010022981	HNRNPH3	case	control	0.014712271	0.270014958	0.054486873	0.956547267	0.9909743	up_in_case
CRA004775	ENSGALG00010022983	PKN2	case	control	0.048200197	0.265794742	0.181343682	0.856097821	0.970703965	up_in_case
CRA004775	ENSGALG00010022986	ALG9	case	control	-0.29812765	0.133421076	-2.234486931	0.025451052	0.305924309	down_in_case
CRA004775	ENSGALG00010022987	F2	case	control	1.253049672	0.453280889	2.764399961	0.005702759	0.152269204	up_in_case

CRA004775	ENSGALG00010022990	SLC25A22	case	control	-0.373209461	0.323871338	-1.152338652	0.24918194	0.714300782	down_in_case
CRA004775	ENSGALG00010022991	TAL1	case	control	-0.403592239	0.504153734	-0.800534068	0.423401433	0.820552247	down_in_case
CRA004775	ENSGALG00010022992	SLC7A6	case	control	-0.618823657	0.392298264	-1.577431547	0.114696261	0.564684045	down_in_case
CRA004775	ENSGALG00010022997	ARHGAP1	case	control	-0.357571769	0.227095224	-1.574545525	0.115361385	0.565776219	down_in_case
CRA004775	ENSGALG00010023002	ATG16L1	case	control	-0.255582133	0.324610473	-0.787350236	0.431076869	0.824900923	down_in_case
CRA004775	ENSGALG00010023003	ENSGALG00010023003	case	control	-0.226511515	1.401187672	-0.1616568	0.871576126	0.973918682	down_in_case
CRA004775	ENSGALG00010023005	IRF7	case	control	-0.606333248	0.294762886	-2.057020329	0.03968427	0.375050449	down_in_case
CRA004775	ENSGALG00010023007	LMO4	case	control	0.234859118	0.283273026	0.829091003	0.407052914	0.813842524	up_in_case
CRA004775	ENSGALG00010023008	CDHR5	case	control	2.41834458	0.87904909	2.751091614	0.005939703	0.154441997	up_in_case
CRA004775	ENSGALG00010023012	ENSGALG00010023012	case	control	-1.691429625	0.70881529	-2.386277001	0.017019926	0.258038592	down_in_case
CRA004775	ENSGALG00010023013	ENSGALG00010023013	case	control	-0.153833206	0.688049316	-0.22357875	0.8230851	0.960898591	down_in_case
CRA004775	ENSGALG00010023015	METTL2B	case	control	-0.122273356	0.185657312	-0.658597043	0.510154562	0.863170205	down_in_case
CRA004775	ENSGALG00010023017	BUB1B	case	control	0.538813634	0.511819431	1.052741653	0.292459413	0.746262643	up_in_case
CRA004775	ENSGALG00010023019	CABP1	case	control	-0.73764286	0.547992508	-1.346082017	0.178276067	0.644368088	down_in_case
CRA004775	ENSGALG00010023024	PRMT7	case	control	-0.322047438	0.176280963	-1.826898558	0.067715023	0.463543284	down_in_case
CRA004775	ENSGALG00010023026	PHRF1	case	control	0.094126029	0.190967634	0.49288996	0.622090342	0.906040637	up_in_case
CRA004775	ENSGALG00010023028	TMEM61	case	control	0.277742025	0.397960326	0.697913854	0.48523107	0.851302844	up_in_case
CRA004775	ENSGALG00010023030	HS2ST1	case	control	0.124630786	0.17240958	0.722876222	0.469755933	0.844062961	up_in_case
CRA004775	ENSGALG00010023041	CD151	case	control	0.003296803	0.285928817	0.011530152	0.990800474	0.998065748	up_in_case
CRA004775	ENSGALG00010023042	ENSGALG00010023042	case	control	1.131182662	0.735678859	1.537603873	0.1241455	0.576485043	up_in_case
CRA004775	ENSGALG00010023043	CNP	case	control	0.102257401	0.219562152	0.46573328	0.641406428	0.912380241	up_in_case
CRA004775	ENSGALG00010023046	SLC7A6OS	case	control	-0.254960903	0.14364266	-1.774966458	0.075903426	0.484761048	down_in_case
CRA004775	ENSGALG00010023048	ARFGAP1	case	control	0.08128808	0.232414523	0.349754736	0.726522772	0.935238044	up_in_case
CRA004775	ENSGALG00010023050	RUFY2	case	control	0.410554624	0.221196042	1.856066775	0.063444041	0.453204678	up_in_case
CRA004775	ENSGALG00010023052	RAD54L	case	control	0.070523481	0.434370012	0.162358079	0.871023881	0.973918682	up_in_case
CRA004775	ENSGALG00010023053	MAN1A2	case	control	-0.01495649	0.306068708	-0.048866446	0.961025729	0.991824137	down_in_case
CRA004775	ENSGALG00010023054	AQP9	case	control	-0.245619118	0.524366528	-0.46841113	0.639490611	0.911382154	down_in_case
CRA004775	ENSGALG00010023056	SAG	case	control	-0.04445518	0.635564856	-0.069945938	0.944236689	0.988293402	down_in_case
CRA004775	ENSGALG00010023057	PAFAH1B2	case	control	-0.199479022	0.181170818	-1.10105493	0.270872751	0.728665115	down_in_case
CRA004775	ENSGALG00010023058	PPARA	case	control	0.332430834	0.434101491	0.765790583	0.443800918	0.832130395	up_in_case
CRA004775	ENSGALG00010023059	SELENOF	case	control	-0.280680961	0.182095737	-1.541392273	0.123221343	0.575549148	down_in_case
CRA004775	ENSGALG00010023060	ALDH1A2	case	control	-0.307908894	0.354677755	-0.868137034	0.385319319	0.803415611	down_in_case
CRA004775	ENSGALG00010023062	STX2	case	control	0.579313282	0.203137437	2.851829242	0.004346844	0.135234068	up_in_case
CRA004775	ENSGALG00010023063	ENSGALG00010023063	case	control	-0.078864281	0.327150939	-0.241063901	0.809505591	0.955986893	down_in_case
CRA004775	ENSGALG00010023064	ATG13	case	control	-0.241690503	0.188037593	-1.285330763	0.198676724	0.665530728	down_in_case
CRA004775	ENSGALG00010023065	DNA2	case	control	0.41542769	0.479823719	0.865792318	0.386604062	0.803905475	up_in_case
CRA004775	ENSGALG00010023066	TLR21	case	control	-0.416296082	0.26666104	-1.561143247	0.118489956	0.570497237	down_in_case
CRA004775	ENSGALG00010023068	CDPF1	case	control	-0.19749796	0.193385938	-1.02126329	0.307129714	0.758911177	down_in_case
CRA004775	ENSGALG00010023069	ENSGALG00010023069	case	control	-0.33001237	0.599091657	-0.550854558	0.58173338	0.889219177	down_in_case
CRA004775	ENSGALG00010023070	SIDT2	case	control	-0.398068565	0.270318217	-1.472592448	0.140860962	0.600572867	down_in_case
CRA004775	ENSGALG00010023071	POLR2M	case	control	-0.047105421	0.237599836	-0.198255275	0.842845343	0.966478468	down_in_case
CRA004775	ENSGALG00010023073	POMGNT1	case	control	-0.359246503	0.257563445	-1.394788391	0.163079661	0.629717526	down_in_case
CRA004775	ENSGALG00010023074	CCNT1	case	control	-0.004561951	0.173295624	-0.026324675	0.978998374	0.996609908	down_in_case
CRA004775	ENSGALG00010023075	PKDREJ	case	control	-0.270265714	0.576041008	-0.469177906	0.638942476	0.91121917	down_in_case
CRA004775	ENSGALG00010023076	DNAJC7	case	control	0.161804098	0.242717426	0.666635687	0.505004868	0.861471523	up_in_case
CRA004775	ENSGALG00010023077	KANSL2	case	control	-0.076760694	0.204402225	-0.375537468	0.707260786	0.931617022	down_in_case
CRA004775	ENSGALG00010023078	TAGLN	case	control	0.580279015	0.446950234	1.298307887	0.194181557	0.65892248	up_in_case
CRA004775	ENSGALG00010023079	SLC25A16	case	control	0.125505542	0.257561532	0.487283721	0.626057282	0.907183337	up_in_case
CRA004775	ENSGALG00010023080	MCRS1	case	control	-0.31748246	0.355484637	-0.893097554	0.371804933	0.795818272	down_in_case
CRA004775	ENSGALG00010023081	PRLHR2	case	control	-0.65661432	0.874832852	-0.750559742	0.452917657	0.837556576	down_in_case
CRA004775	ENSGALG00010023083	HARBI1	case	control	-0.457725371	0.311808555	-1.467969253	0.142112575	0.602343234	down_in_case
CRA004775	ENSGALG00010023084	ENSGALG00010023084	case	control	-0.9769857	0.397523289	-2.45768167	0.013983706	0.236810279	down_in_case
CRA004775	ENSGALG00010023086	PCSK7	case	control	-0.431968468	0.140262832	-3.079707308	0.002072041	0.091096037	down_in_case
CRA004775	ENSGALG00010023087	SH3GLB1	case	control	0.331898186	0.271699013	1.221565668	0.221871918	0.689550502	up_in_case
CRA004775	ENSGALG00010023090	LRRC41	case	control	-0.344849566	0.164631389	-2.094676896	0.036199724	0.360628495	down_in_case
CRA004775	ENSGALG00010023091	ENSGALG00010023091	case	control	-0.408787878	0.156124407	-2.618347042	0.008835689	0.18984307	down_in_case
CRA004775	ENSGALG00010023093	POP5	case	control	-0.520774563	0.328120951	-1.587142062	0.112480473	0.562852616	down_in_case
CRA004775	ENSGALG00010023094	CCDC32	case	control	-0.234747823	0.189441456	-1.239157614	0.215287134	0.683654538	down_in_case
CRA004775	ENSGALG00010023096	SPATS2	case	control	-0.087586753	0.166651406	-0.525568642	0.599187949	0.897206546	down_in_case
CRA004775	ENSGALG00010023097	LURAP1	case	control	0.271216306	0.348228308	0.778846232	0.4360703	0.82694408	up_in_case
CRA004775	ENSGALG00010023099	DNAJC22	case	control	2.241369443	0.828200068	2.706314005	0.006803468	0.161712092	up_in_case
CRA004775	ENSGALG00010023101	CLCA2	case	control	2.441577006	0.756877454	3.225855114	0.001255969	0.074844075	up_in_case
CRA004775	ENSGALG00010023102	RNF10	case	control	0.095735472	0.197595319	0.484502733	0.628029125	0.90828391	up_in_case
CRA004775	ENSGALG00010023103	DGKD	case	control	0.219620396	0.372536326	0.589527466	0.555507493	0.87981923	up_in_case
CRA004775	ENSGALG00010023104	ENSGALG00010023104	case	control	-0.196067405	0.324177548	-0.604814883	0.545302004	0.876563448	down_in_case
CRA004775	ENSGALG00010023107	ZC3HAV1	case	control	-0.127171315	0.380973511	-0.333806188	0.738525814	0.938064864	down_in_case
CRA004775	ENSGALG00010023108	TET1	case	control	0.187837353	0.35404476	0.530546908	0.595732795	0.895305942	up_in_case
CRA004775	ENSGALG00010023110	LIMA1	case	control	0.040108662	0.249660775	0.160652638	0.872366994	0.97401729	up_in_case
CRA004775	ENSGALG00010023112	PCMT1L	case	control	-0.068096704	0.221435498	-0.307523881	0.758444648	0.942236668	down_in_case
CRA004775	ENSGALG00010023113	MYZAP	case	control	0.234751851	0.450059633	0.521601658	0.601947709	0.898185892	up_in_case
CRA004775	ENSGALG00010023114	UQCRHL	case	control	-0.033506319	0.388571781	-0.086229418	0.931284046	0.985044263	down_in_case
CRA004775	ENSGALG00010023115	RPLP2	case	control	-0.273402493	0.247082794	-1.10652178	0.268500742	0.727528758	down_in_case
CRA004775	ENSGALG00010023116	PROC	case	control	0.195868074	0.574811811	0.340751651	0.733290551	0.936819725	up_in_case
CRA004775	ENSGALG00010023117	RPUSD2	case	control	0.019037004	0.223242493	0.085275002	0.932042764	0.985044263	up_in_case
CRA004775	ENSGALG00010023118	PIDD1	case	control	0.128401745	0.334095072	0.384326964	0.700736125	0.930068722	up_in_case
CRA004775	ENSGALG00010023119	NDRG4	case	control	0.647947772	0.331765717	1.953028111	0.050816264	0.416238146	up_in_case
CRA004775	ENSGALG00010023120	CHRM4	case	control	-0.43645519	0.456738512	-0.955590952	0.339278934	0.779780148	down_in_case
CRA004775	ENSGALG00010023122	ENSGALG00010023122	case	control	0.062545559	0.294561296	0.212334614	0.831845993	0.963642489	up_in_case
CRA004775	ENSGALG00010023123	RNF214	case	control	0.143264321	0.203644648	0.703501527	0.481743255	0.848909035	up_in_case
CRA004775	ENSGALG00010023124	NSUN4	case	control	-0.028426548	0.272352117	-0.104374248	0.916872358	0.981688766	down_in_case
CRA004775	ENSGALG00010023125	COL20A1	case	control	0.923396055	0.696247244	1.326247339	0.184757784	0.6491093	up_in_case
CRA004775	ENSGALG00010023126	CGNL1	case	control	0.248471649	0.503726228	0.493267246	0.621823769	0.905890311	up_in_case
CRA004775	ENSGALG00010023127	CERS5	case	control	-0.271022681	0.301951019	-0.897571672	0.369413947	0.794896739	down_in_case
CRA004775	ENSGALG00010023128	IWS1	case	control	0.2613656	0.21538462	1.213483119	0.224945165	0.692859731	up_in_case
CRA004775	ENSGALG00010023131	SOX6	case	control	0.134234183	0.388241988	0.345748753	0.729531541	0.936339759	up_in_case
CRA004775	ENSGALG00010023134	KNL1	case	control	0.106770773	0.790717917	0.135030168	0.892588022	0.977834811	up_in_case
CRA004775	ENSGALG00010023135	GINS3	case	control	-0.34502603	0.235227065	-1.466778619	0.142436288	0.602473513	down_in_case
CRA004775	ENSGALG00010023136	MDK	case	control	-0.34741055	0.627397853	-0.553732449	0.579761964	0.888438428	down_in_case
CRA004775	ENSGALG00010023137	ETV6	case	control	-0.352786141	0.163782398	-2.153993011	0.03124072	0.336670173	down_in_case
CRA004775	ENSGALG00010023140	CCAR1	case	control	0.267678183	0.18979333	1.410366652	0.158431447	0.624136085	up_in_case
CRA004775	ENSGALG00010023142	TLK2	case	control	0.3511507	0.260398686	1.348511799	0.177493828	0.643283771	up_in_case
CRA004775	ENSGALG00010023144	C11orf58	case	control	-0.13815125	0.186190553	-0.741988507	0.458094299	0.839796467	down_in_case
CRA004775	ENSGALG00010023147	RAD51	case	control	-0.438318309	0.338514059	-1.294830443	0.195378728	0.661073909	down_in_case
CRA004775	ENSGALG00010023149	ADGRD1	case	control	-0.444275007	0.522764508	-0.849856867	0.395404669	0.808998414	down_in_case
CRA004775	ENSGALG00010023151	ENSGALG00010023151	case	control	0.53220698	0.802645292	0.663066221	0.507288144	0.862729457	up_in_case
CRA004775	ENSGALG00010023158	ENSGALG00010023158	case	control	-0.074076682	0.479007955	-0.154646037	0.877100374	0.974479909	down_in_case
CRA004775	ENSGALG00010023160	ENSGALG00010023160	case	control	-0.087434263	0.243046922	-0.359742315	0.719039845	0.93407508	down_in_case
CRA004775	ENSGALG00010023162	CHRNA4	case	control	-1.972824492	0.681844212	-2.893365462	0.003811375	0.127263805	down_in_case
CRA004775	ENSGALG00010023163	CRTAC1	case	control	-0.305782746	0.36039496	-0.848465657	0.396178691	0.808998414	down_in_case
CRA004775	ENSGALG00010023164	NKIRAS2	case	control	-0.275223409	0.321424582	-0.856261235	0.391853299	0.80746892	down_in_case
CRA004775	ENSGALG00010023165	CSNK2A2	case	control	-0.29512612	0.155003642	-1.903994741	0.056910869	0.435916236	down_in_case
CRA004775	ENSGALG00010023166	KDM2A	case	control	-0.353470916	0.198132009	-1.784017224	0.074420866	0.481316485	down_in_case
CRA004775	ENSGALG00010023167	GPD1	case	control	-0.230396253	0.753697734	-0.305687867	0.759842312	0.942236668	down_in_case
CRA004775	ENSGALG00010023169	RAN	case	control	-0.313957846	0.208457241	-1.506101898	0.132041017	0.588174937	down_in_case
CRA004775	ENSGALG00010023170	TCF12	case	control	0.115067882	0.198292911	0.580292465	0.561717408	0.881532201	up_in_case
CRA004775	ENSGALG00010023171	ENSGALG00010023171	case	control	-0.339448752	0.216669157	-1.566668539	0.117192188	0.567740281	down_in_case
CRA004775	ENSGALG00010023172	DHX58	case	control	-0.547749537	0.310956613	-1.761498273	0.078154105	0.490500984	down_in_case
CRA004775	ENSGALG00010023175	GRK2	case	control	-0.174917748	0.206904147	-0.845404749	0.397884894	0.809455905	down_in_case
CRA004775	ENSGALG00010023177	LZTS2	case	control	-0.215310362	0.421711376	-0.510563323	0.609656863	0.901246133	down_in_case
CRA004775	ENSGALG00010023178	CFAP20	case	control	-0.197259927	0.144729032	-1.362960313	0.172895019	0.637223104	down_in_case
CRA004775	ENSGALG00010023179	KAT2A	case	control	-0.626317186	0.150671771	-4.156831637	3.22692E-05	0.01199279	down_in_case
CRA004775	ENSGALG00010023180	EEF1A2	case	control	-0.273532376	0.712894549	-0.383692618	0.701206285	0.930068722	down_in_case
CRA004775	ENSGALG00010023181	PLEKHA7	case	control	0.140007368	0.360625608	0.388234682	0.69784237	0.929122855	up_in_case
CRA004775	ENSGALG00010023182	EPHB6	case	control	-0.242399575	0.416687857	-0.581729395	0.560748968	0.881008373	down_in_case
CRA004775	ENSGALG00010023184	SMARCD1	case	control	-0.358724541	0.118502909	-3.027137016	0.00246882	0.10194819	down_in_case
CRA004775	ENSGALG00010023185	FBXW4	case	control	-0.489042107	0.440808466	-1.109420858	0.267248668	0.727315996	down_in_case
CRA004775	ENSGALG00010023186	MIER1	case	control	0.14175746	0.248788482	0.569791086	0.568819402	0.884663439	up_in_case
CRA004775	ENSGALG00010023187	ENSGALG00010023187	case	control	1.323041544	0.726944575	1.820003324	0.068758499	0.466664825	up_in_case
CRA004775	ENSGALG00010023188	KIF1BP	case	control	-0.192368944	0.175444498	-1.096466095	0.272874853	0.731001618	down_in_case
CRA004775	ENSGALG00010023189	RMDN3	case	control	-0.188190421	0.238566612	-0.78883805	0.430206667	0.824153229	down_in_case
CRA004775	ENSGALG00010023190	SF3B3	case	control	-0.343688263	0.174657796	-1.967780833	0.04909326	0.410291029	down_in_case
CRA004775	ENSGALG00010023193	PPDPF	case	control	0.426688298	0.502281279	0.849500699	0.395602741	0.808998414	up_in_case
CRA004775	ENSGALG00010023194	MRC2	case	control	-0.388731474	0.4327964	-0.898185552	0.369086635	0.794896739	down_in_case
CRA004775	ENSGALG00010023195	ENSGALG00010023195	case	control	0.905033922	0.463248033	1.953670297	0.050740221	0.416238146	up_in_case
CRA004775	ENSGALG00010023196	DGKZ	case	control	-0.344365055	0.172453337	-1.996859324	0.045840467	0.398452757	down_in_case
CRA004775	ENSGALG00010023199	RPS13	case	control	-0.091856579	0.228702304	-0.401642562	0.687947102	0.927111919	down_in_case

CRA004775	ENSGALG00010023200	VPS26A	case	control	-0.103791191	0.228296918	-0.454632469	0.649373672	0.915047477	down_in_case
CRA004775	ENSGALG00010023201	ZNF280D	case	control	0.266184383	0.212479704	1.252752041	0.21029596	0.680258704	up_in_case
CRA004775	ENSGALG00010023202	PID1	case	control	-0.0106156	0.274955406	-0.038608441	0.969202572	0.994160926	down_in_case
CRA004775	ENSGALG00010023203	COG4	case	control	-0.414899385	0.152804838	-2.715224132	0.006623093	0.160913119	down_in_case
CRA004775	ENSGALG00010023204	SRGN	case	control	-0.94025374	0.557497766	-1.686560552	0.091687883	0.521441551	down_in_case
CRA004775	ENSGALG00010023205	DNAJC17	case	control	-0.774393336	0.297352492	-2.604294084	0.009206372	0.194465171	down_in_case
CRA004775	ENSGALG00010023206	HSPB9	case	control	1.501486639	0.715339588	2.098984405	0.035818275	0.359120808	up_in_case
CRA004775	ENSGALG00010023207	MNS1	case	control	0.403992179	0.380701428	1.061178522	0.288608778	0.742851819	up_in_case
CRA004775	ENSGALG00010023208	BACE1	case	control	-0.211334519	0.199403804	-1.059831935	0.289221065	0.743485368	down_in_case
CRA004775	ENSGALG00010023209	PIK3C2A	case	control	0.019390407	0.387454381	0.050045652	0.960086009	0.991431439	up_in_case
CRA004775	ENSGALG00010023210	ZNHIT6	case	control	-0.028934018	0.253241708	-0.114254553	0.909036008	0.98075825	down_in_case
CRA004775	ENSGALG00010023211	TLK1	case	control	0.378011622	0.223058686	1.694673404	0.090137394	0.517978072	up_in_case
CRA004775	ENSGALG00010023212	RIMBP2	case	control	-0.275086154	0.472050171	-0.582747706	0.560063153	0.880918893	down_in_case
CRA004775	ENSGALG00010023213	FCSK	case	control	-0.762528623	0.276002517	-2.762759667	0.005731495	0.152739903	down_in_case
CRA004775	ENSGALG00010023214	SUPV3L1	case	control	-0.113605469	0.231364435	-0.491023909	0.623409537	0.906441769	down_in_case
CRA004775	ENSGALG00010023215	TRIP12	case	control	0.217203106	0.239250365	0.907848588	0.363958223	0.793608387	up_in_case
CRA004775	ENSGALG00010023216	SLC35D1	case	control	-0.471907357	0.255166639	-1.849408522	0.064398846	0.456696419	down_in_case
CRA004775	ENSGALG00010023217	CREB3L1	case	control	-0.448290221	0.345356978	-1.298048828	0.194270557	0.65892248	down_in_case
CRA004775	ENSGALG00010023218	SSH3	case	control	-0.380327481	0.228939135	-1.661260233	0.096661193	0.530629094	down_in_case
CRA004775	ENSGALG00010023219	TEX9	case	control	-0.729681396	0.418984643	-1.741546873	0.081587765	0.497302017	down_in_case
CRA004775	ENSGALG00010023220	ST3GAL2	case	control	-0.256832589	0.20641593	-1.244247905	0.213408337	0.682777582	down_in_case
CRA004775	ENSGALG00010023221	AMY1A	case	control	0.251431792	0.315759564	0.796276091	0.425871579	0.822541484	up_in_case
CRA004775	ENSGALG00010023223	CEP164	case	control	-0.063195686	0.388464409	-0.162680761	0.870769796	0.973918682	down_in_case
CRA004775	ENSGALG00010023225	PDPR	case	control	-0.613612368	0.244774213	-2.506850541	0.012181221	0.222250112	down_in_case
CRA004775	ENSGALG00010023226	ENSGALG00010023226	case	control	0.193911026	0.554132158	0.349936425	0.72638641	0.935238044	up_in_case
CRA004775	ENSGALG00010023227	RNPC3	case	control	0.312996628	0.207861078	1.505797197	0.132119243	0.588332808	up_in_case
CRA004775	ENSGALG00010023228	GORASP2	case	control	0.032302102	0.177834579	0.181641289	0.855864244	0.970703965	up_in_case
CRA004775	ENSGALG00010023229	HKDC1	case	control	1.993375528	0.630438643	3.161886648	0.001567506	0.080693373	up_in_case
CRA004775	ENSGALG00010023230	ENSGALG00010023230	case	control	0.064091636	0.27297279	0.234791299	0.814370722	0.958686054	up_in_case
CRA004775	ENSGALG00010023231	ASIC1	case	control	0.795976371	0.344380868	2.31132576	0.020814868	0.283953623	up_in_case
CRA004775	ENSGALG00010023234	ENSGALG00010023234	case	control	0.184245626	0.366756368	0.502365172	0.615410673	0.902802125	up_in_case
CRA004775	ENSGALG00010023235	ENSGALG00010023235	case	control	0.063729631	0.31167992	0.204471404	0.837985137	0.965490877	up_in_case
CRA004775	ENSGALG00010023238	NUCB2	case	control	-0.160465355	0.345587505	-0.464326264	0.64241401	0.913111066	down_in_case
CRA004775	ENSGALG00010023239	RBPMS2	case	control	0.119972117	0.245989627	0.487712098	0.625753782	0.907183337	up_in_case
CRA004775	ENSGALG00010023243	HK1	case	control	0.131419997	0.249316567	0.527120996	0.598109567	0.896610123	up_in_case
CRA004775	ENSGALG00010023244	PPIB	case	control	-0.3222375	0.194561492	-1.656224449	0.097676374	0.532395151	down_in_case
CRA004775	ENSGALG00010023245	TANC2	case	control	-0.95828116	0.398769452	-2.403095714	0.016256932	0.25344686	down_in_case
CRA004775	ENSGALG00010023246	SP5	case	control	0.218968587	0.638733567	0.342816784	0.731736299	0.93670693	up_in_case
CRA004775	ENSGALG00010023248	PRDM10	case	control	-0.04868029	0.293316929	-0.165964814	0.868184641	0.973370973	down_in_case
CRA004775	ENSGALG00010023249	IL12RB2	case	control	0.459271948	0.409045219	1.122790163	0.261526624	0.7223728	up_in_case
CRA004775	ENSGALG00010023250	FZD10	case	control	-0.951431134	1.002016761	-0.949516187	0.342358143	0.781891186	down_in_case
CRA004775	ENSGALG00010023251	ENSGALG00010023251	case	control	-0.822379192	0.566306206	-1.452181141	0.146451245	0.609922856	down_in_case
CRA004775	ENSGALG00010023252	GLG1	case	control	-0.119803536	0.20586616	-0.58194866	0.560601263	0.881008373	down_in_case
CRA004775	ENSGALG00010023253	TACR2	case	control	-0.180532943	0.667071843	-0.270634932	0.786671826	0.95041797	down_in_case
CRA004775	ENSGALG00010023255	SNX22	case	control	-0.762207132	0.375534357	-2.029660181	0.042391093	0.386934578	down_in_case
CRA004775	ENSGALG00010023257	PAK6	case	control	0.409748937	0.443688189	0.923506522	0.355743303	0.788778426	up_in_case
CRA004775	ENSGALG00010023258	ENSGALG00010023258	case	control	0.207472233	0.53723327	0.386186493	0.699358556	0.929347461	up_in_case
CRA004775	ENSGALG00010023259	TSPAN15	case	control	-0.243746946	0.429541087	-0.567458979	0.570402389	0.885244439	down_in_case
CRA004775	ENSGALG00010023260	ENSGALG00010023260	case	control	0.573198114	0.864130029	0.663323916	0.507123123	0.862623709	up_in_case
CRA004775	ENSGALG00010023261	SNX1	case	control	-0.266409087	0.198335709	-1.343223006	0.179199774	0.644990528	down_in_case
CRA004775	ENSGALG00010023262	POLD4	case	control	0.134802991	0.390885473	0.344865697	0.73019534	0.936393482	up_in_case
CRA004775	ENSGALG00010023263	SRP14	case	control	-0.067390598	0.199074044	-0.338520266	0.734971158	0.936931635	down_in_case
CRA004775	ENSGALG00010023264	RACGAP1	case	control	0.313586934	0.50275987	0.623731036	0.532804237	0.869976453	up_in_case
CRA004775	ENSGALG00010023265	RAB5C	case	control	-0.133910696	0.161956148	-0.82683305	0.408331695	0.814384687	down_in_case
CRA004775	ENSGALG00010023266	RFWD3	case	control	-0.628718997	0.285143101	-2.204924452	0.027459396	0.316243014	down_in_case
CRA004775	ENSGALG00010023268	KCNJ11	case	control	-0.45914369	0.541624004	-0.847716656	0.396595788	0.808998414	down_in_case
CRA004775	ENSGALG00010023271	PATL2	case	control	-0.996001411	0.509644973	-1.954304397	0.050665229	0.416238146	down_in_case
CRA004775	ENSGALG00010023272	MLKL	case	control	0.320862065	0.526452994	0.60947904	0.542206962	0.875128455	up_in_case
CRA004775	ENSGALG00010023273	HDAC7	case	control	-0.263765311	0.265421446	-0.993760357	0.32033955	0.767037518	down_in_case
CRA004775	ENSGALG00010023274	ZBTB38	case	control	0.495252571	0.258435355	1.916349911	0.055320572	0.430998971	up_in_case
CRA004775	ENSGALG00010023276	ABCC8	case	control	0.029218388	0.352639191	0.082856328	0.93396578	0.985192041	up_in_case
CRA004775	ENSGALG00010023277	KCNH4	case	control	-0.566280071	0.273775838	-2.068407769	0.038601695	0.370251617	down_in_case
CRA004775	ENSGALG00010023278	ENSGALG00010023278	case	control	-0.268189075	0.503554689	-0.532591754	0.594316211	0.894956597	down_in_case
CRA004775	ENSGALG00010023279	FA2H	case	control	1.942297932	0.301458825	6.442995751	1.17138E-10	1.78974E-07	up_in_case
CRA004775	ENSGALG00010023281	B2M	case	control	-0.399654594	0.179288699	-2.229112024	0.025806452	0.308336492	down_in_case
CRA004775	ENSGALG00010023282	SERBP1	case	control	-0.189224948	0.136755934	-1.383669016	0.166459825	0.630194031	down_in_case
CRA004775	ENSGALG00010023285	SLC48A1	case	control	-0.0358679	0.281263789	-0.127524058	0.898525633	0.978790377	down_in_case
CRA004775	ENSGALG00010023286	HCRT	case	control	-0.734383955	0.747807033	-0.982050078	0.326075178	0.770998457	down_in_case
CRA004775	ENSGALG00010023287	RGS19	case	control	-0.656098586	0.495543966	-1.32399672	0.185504134	0.6491093	down_in_case
CRA004775	ENSGALG00010023289	NFRKB	case	control	-0.235388845	0.207566008	-1.134043322	0.256776376	0.719236059	down_in_case
CRA004775	ENSGALG00010023293	COL11A1	case	control	-0.928986722	0.628995265	-1.476937545	0.13969238	0.599534929	down_in_case
CRA004775	ENSGALG00010023295	ENSGALG00010023295	case	control	2.520419214	0.736352106	3.422845122	0.000619694	0.051333788	up_in_case
CRA004775	ENSGALG00010023299	PASK	case	control	0.085667591	0.384525104	0.222788032	0.823700483	0.960898591	up_in_case
CRA004775	ENSGALG00010023304	ENSGALG00010023304	case	control	-0.758653488	0.359042713	-2.112989512	0.034601666	0.353831224	down_in_case
CRA004775	ENSGALG00010023305	PLCB2	case	control	-0.06544432	0.358924099	-0.182334707	0.855320063	0.970581465	down_in_case
CRA004775	ENSGALG00010023309	PIK3R3	case	control	-0.317787511	0.383406357	-0.828853004	0.407187591	0.813842524	down_in_case
CRA004775	ENSGALG00010023311	ETS1	case	control	-0.288677461	0.315139435	-0.916030904	0.359650686	0.791966047	down_in_case
CRA004775	ENSGALG00010023312	UBR3	case	control	0.572977258	0.346708211	1.652620964	0.098408029	0.534442656	up_in_case
CRA004775	ENSGALG00010023314	CYB561	case	control	2.311981859	0.864492879	2.674379299	0.007486772	0.172634743	up_in_case
CRA004775	ENSGALG00010023317	RAPGEF3	case	control	-0.080667755	0.368585452	-0.218857674	0.826760918	0.961878235	down_in_case
CRA004775	ENSGALG00010023319	EIF2AK4	case	control	-0.022024494	0.201985462	-0.109039998	0.913170766	0.980803023	down_in_case
CRA004775	ENSGALG00010023320	ENSGALG00010023320	case	control	-0.096125259	0.282370758	-0.340422144	0.733538645	0.936819725	down_in_case
CRA004775	ENSGALG00010023322	PRKAB1	case	control	-0.261538956	0.198726562	-1.316074475	0.188149047	0.652111233	down_in_case
CRA004775	ENSGALG00010023324	LAG3	case	control	-1.015655393	0.678392702	-1.497149645	0.134354324	0.592420453	down_in_case
CRA004775	ENSGALG00010023328	TCEA2	case	control	-0.138898052	0.271329206	-0.51191707	0.608709051	0.901007337	down_in_case
CRA004775	ENSGALG00010023330	TSPAN1	case	control	2.360146899	0.922331124	2.558893263	0.010500598	0.20539647	up_in_case
CRA004775	ENSGALG00010023331	CD4	case	control	-0.750956746	0.672125324	-1.117286791	0.263871722	0.723981064	down_in_case
CRA004775	ENSGALG00010023332	PPP1R7	case	control	0.076784033	0.217982618	0.352248422	0.724651966	0.935238044	up_in_case
CRA004775	ENSGALG00010023333	ENDOU	case	control	-0.324244092	0.767944186	-0.422223513	0.672861881	0.921952322	down_in_case
CRA004775	ENSGALG00010023334	RPS17	case	control	-0.318061504	0.24003418	-1.325067554	0.185148746	0.6491093	down_in_case
CRA004775	ENSGALG00010023335	WDR59	case	control	-0.545413669	0.141094619	-3.865588034	0.000110822	0.022744947	down_in_case
CRA004775	ENSGALG00010023336	GPR162	case	control	-0.075634191	0.233001911	-0.324607599	0.745478075	0.939421865	down_in_case
CRA004775	ENSGALG00010023337	FLI1	case	control	-0.241116449	0.416670097	-0.578674714	0.562808681	0.881532201	down_in_case
CRA004775	ENSGALG00010023338	HDLBP	case	control	0.024277625	0.152469238	0.159229661	0.873487936	0.974079175	up_in_case
CRA004775	ENSGALG00010023340	P3H3	case	control	-0.188957706	0.372786955	-0.506878535	0.612240048	0.901413878	down_in_case
CRA004775	ENSGALG00010023342	FITM2	case	control	-0.134628431	0.267754027	-0.502806372	0.615100413	0.902639483	down_in_case
CRA004775	ENSGALG00010023343	TMEM233	case	control	0.345977937	0.582278183	0.594179804	0.552391859	0.878258849	up_in_case
CRA004775	ENSGALG00010023347	STAT5A	case	control	-0.336386543	0.302603136	-1.111642618	0.266291841	0.727311193	down_in_case
CRA004775	ENSGALG00010023349	ENSGALG00010023349	case	control	2.190667065	0.787941492	2.780240774	0.005431861	0.14978291	up_in_case
CRA004775	ENSGALG00010023350	ZNRF1	case	control	0.597910619	0.330141032	1.811076362	0.070129032	0.469724266	up_in_case
CRA004775	ENSGALG00010023351	GNB3	case	control	-1.23433245	0.528443759	-2.335787731	0.019502307	0.27590147	down_in_case
CRA004775	ENSGALG00010023354	SEPTIN2	case	control	-0.486412814	0.517052991	-0.940740741	0.346837735	0.784552667	down_in_case
CRA004775	ENSGALG00010023356	ENSGALG00010023356	case	control	0.181457283	0.327059156	0.55481487	0.579021295	0.888378369	up_in_case
CRA004775	ENSGALG00010023359	CDCA3	case	control	0.489813103	0.464760222	1.05390496	0.291926432	0.746148767	up_in_case
CRA004775	ENSGALG00010023362	GADD45A	case	control	-0.13813412	0.359090045	-0.38467822	0.700475834	0.929842957	down_in_case
CRA004775	ENSGALG00010023363	CPEB1	case	control	-0.413568171	0.427991869	-0.96629913	0.333894521	0.774300343	down_in_case
CRA004775	ENSGALG00010023364	CTRB1	case	control	2.547378599	1.559288789	1.633679801	0.102326102	0.542513917	up_in_case
CRA004775	ENSGALG00010023366	GDAP1L1	case	control	-0.611254468	0.679426646	-0.899662196	0.368300047	0.794665838	down_in_case
CRA004775	ENSGALG00010023368	ENSGALG00010023368	case	control	-2.209569836	0.734399756	-3.008674522	0.002623901	0.106489884	down_in_case
CRA004775	ENSGALG00010023369	ACE	case	control	0.299141424	0.452008163	0.661805359	0.508095971	0.862971569	up_in_case
CRA004775	ENSGALG00010023371	ENSGALG00010023371	case	control	2.753120028	1.130389586	2.43554971	0.014869181	0.244420343	up_in_case
CRA004775	ENSGALG00010023374	ENSGALG00010023374	case	control	-0.030362182	0.535574702	-0.056690844	0.954791467	0.990670511	down_in_case
CRA004775	ENSGALG00010023375	ST14	case	control	0.424841877	0.276179049	1.538284235	0.123979133	0.576485043	up_in_case
CRA004775	ENSGALG00010023377	OSER1	case	control	0.176540932	0.302002545	0.584567697	0.558838433	0.880754991	up_in_case
CRA004775	ENSGALG00010023380	USP5	case	control	-0.447524099	0.235606887	-1.899452537	0.057505002	0.439306264	down_in_case
CRA004775	ENSGALG00010023381	HOXB7	case	control	0.030917063	0.303641824	0.10182083	0.918898891	0.98189977	up_in_case
CRA004775	ENSGALG00010023383	HOXB6	case	control	0.172957284	0.367359525	0.470812029	0.637774973	0.910511177	up_in_case
CRA004775	ENSGALG00010023384	AP3B2	case	control	-0.165627104	0.274661338	-0.603023001	0.546493388	0.877099134	down_in_case
CRA004775	ENSGALG00010023387	ENSGALG00010023387	case	control	-0.043833587	0.543676637	-0.080624372	0.935740683	0.986264424	down_in_case
CRA004775	ENSGALG00010023388	FARP2	case	control	-0.119884878	0.31476136	-0.380875461	0.703295662	0.930068722	down_in_case
CRA004775	ENSGALG00010023389	GNG12	case	control	-0.037228345	0.231732186	-0.160652455	0.872367138	0.97401729	down_in_case
CRA004775	ENSGALG00010023390	HSPB8	case	control	0.987163966	0.669502015	1.474474973	0.140353751	0.600007375	up_in_case
CRA004775	ENSGALG00010023392	TPI1	case	control	0.031458407	0.599021907	0.052516288	0.958117317	0.991431439	up_in_case
CRA004775	ENSGALG00010023394	HOXB5	case	control	0.262027194	0.404226473	0.648218787	0.516843453	0.864913871	up_in_case
CRA004775	ENSGALG00010023396	ENSGALG00010023396	case	control	0.318436078	0.422039731	0.754516825	0.450538947	0.835404673	up_in_case
CRA004775	ENSGALG00010023397	ENSGALG00010023397	case	control	0.186682641	0.421577637	0.442819125	0.657896569	0.917354438	up_in_case

CRA004775	ENSGALG00010023401	LRRC23	case	control	0.02805976	0.422920079	0.066347667	0.947101034	0.98933351	up_in_case
CRA004775	ENSGALG00010023405	DNAJA2	case	control	-0.107416801	0.226955884	-0.473293747	0.636003622	0.910080596	down_in_case
CRA004775	ENSGALG00010023408	ZBTB44	case	control	0.013219677	0.354977123	0.037240928	0.970292906	0.994414835	up_in_case
CRA004775	ENSGALG00010023411	ENSGALG00010023411	case	control	-0.074849696	0.508115496	-0.14730843	0.882888579	0.975577339	down_in_case
CRA004775	ENSGALG00010023412	GSS	case	control	-0.35434622	0.155786125	-2.274568551	0.022931824	0.294156267	down_in_case
CRA004775	ENSGALG00010023414	SUDS3	case	control	-0.130491951	0.131315053	-0.993731856	0.320353429	0.767037518	down_in_case
CRA004775	ENSGALG00010023415	HOXB4	case	control	0.023636603	0.374440445	0.063125133	0.949666861	0.990168609	up_in_case
CRA004775	ENSGALG00010023421	OLFM3	case	control	-0.147392311	0.405596453	-0.363396449	0.716308754	0.934006649	down_in_case
CRA004775	ENSGALG00010023422	WLS	case	control	-0.269959041	0.178689749	-1.510769607	0.130847161	0.586275434	down_in_case
CRA004775	ENSGALG00010023428	HOXB3	case	control	-0.16058628	0.361899213	-0.443732051	0.65723632	0.917108353	down_in_case
CRA004775	ENSGALG00010023429	BMF	case	control	-0.110520969	0.286041866	-0.386380395	0.699214967	0.929347461	down_in_case
CRA004775	ENSGALG00010023431	BCAR1	case	control	-0.347062713	0.349101019	-0.994161273	0.320144359	0.767037518	down_in_case
CRA004775	ENSGALG00010023432	TMOD3	case	control	0.329830276	0.228489306	1.443526094	0.148872327	0.612367147	up_in_case
CRA004775	ENSGALG00010023434	ATN1	case	control	-0.189788483	0.284566495	-0.666938962	0.504811123	0.861471523	down_in_case
CRA004775	ENSGALG00010023437	KCNJ5	case	control	0.054692217	0.402247297	0.135966648	0.891847647	0.977819591	up_in_case
CRA004775	ENSGALG00010023438	HOXB2	case	control	0.066801842	0.361971415	0.184550047	0.853581969	0.970050055	up_in_case
CRA004775	ENSGALG00010023439	ACSS2	case	control	-0.870163836	0.277335942	-3.137580476	0.001703485	0.083959221	down_in_case
CRA004775	ENSGALG00010023441	ENSGALG00010023441	case	control	-0.060416833	0.500680524	-0.120669429	0.903952874	0.979540372	down_in_case
CRA004775	ENSGALG00010023442	STK25	case	control	0.191953402	0.20966334	0.915531545	0.35991265	0.791975934	up_in_case
CRA004775	ENSGALG00010023443	ENSGALG00010023443	case	control	-0.324442569	0.23173104	-1.400082478	0.161488621	0.627296619	down_in_case
CRA004775	ENSGALG00010023447	C12orf57	case	control	-0.020923454	0.400806272	-0.05220341	0.958366616	0.991431439	down_in_case
CRA004775	ENSGALG00010023448	FAM219B	case	control	-0.323140954	0.21158818	-1.52721647	0.126707192	0.580923609	down_in_case
CRA004775	ENSGALG00010023453	ARHGAP32	case	control	-0.138544326	0.479855632	-0.28872085	0.772795006	0.946296525	down_in_case
CRA004775	ENSGALG00010023454	HOXB1	case	control	-0.3928031	0.446410737	-0.879914097	0.378905848	0.800112763	down_in_case
CRA004775	ENSGALG00010023455	CFDP1	case	control	-0.016537807	0.193529475	-0.085453687	0.931900713	0.985044263	down_in_case
CRA004775	ENSGALG00010023456	MPI	case	control	-0.244714054	0.413124518	-0.592349385	0.553616652	0.878871229	down_in_case
CRA004775	ENSGALG00010023466	CLCF1	case	control	-1.139985192	0.503308543	-2.264982799	0.023513735	0.297457904	down_in_case
CRA004775	ENSGALG00010023469	ENSGALG00010023469	case	control	-0.330313048	0.349262281	-0.945744975	0.344278677	0.783803988	down_in_case
CRA004775	ENSGALG00010023472	PTPN6	case	control	-0.77956526	0.50751542	-1.53604251	0.124527955	0.576754431	down_in_case
CRA004775	ENSGALG00010023473	BOK	case	control	-0.107597045	0.250502463	-0.429524899	0.667541278	0.919546651	down_in_case
CRA004775	ENSGALG00010023476	SCAMP2	case	control	-0.493521711	0.171950892	-2.870131735	0.004103008	0.131210386	down_in_case
CRA004775	ENSGALG00010023478	TAOK3	case	control	0.286818704	0.280051354	1.024164673	0.305757502	0.758032511	up_in_case
CRA004775	ENSGALG00010023479	MCCC2L	case	control	-1.099967932	0.431781713	-2.547509307	0.010849496	0.210425132	down_in_case
CRA004775	ENSGALG00010023482	MTCL2	case	control	-0.241680211	0.339831996	-0.711175564	0.476975449	0.846417525	down_in_case
CRA004775	ENSGALG00010023485	BARX2	case	control	0.476087378	0.547636347	0.86934949	0.384656001	0.803182776	up_in_case
CRA004775	ENSGALG00010023487	GRN	case	control	0.015311131	0.32509618	0.047097234	0.962435732	0.991860454	up_in_case
CRA004775	ENSGALG00010023489	ETV5	case	control	0.100228798	0.370530382	0.270500889	0.786774933	0.95041797	up_in_case
CRA004775	ENSGALG00010023490	PHB2	case	control	-0.080000937	0.20197337	-0.396096461	0.692033869	0.927867527	down_in_case
CRA004775	ENSGALG00010023495	ENSGALG00010023495	case	control	1.702936305	1.651731163	1.031000894	0.302540401	0.755164773	up_in_case
CRA004775	ENSGALG00010023496	PLEKHA3	case	control	-0.380536871	0.32748341	-1.162003505	0.245234041	0.712046495	down_in_case
CRA004775	ENSGALG00010023498	DISP2	case	control	0.254566878	0.600177418	0.42415271	0.671454447	0.921605102	up_in_case
CRA004775	ENSGALG00010023499	PEBP1	case	control	-0.323549903	0.42364585	-0.763727303	0.445029767	0.833012432	down_in_case
CRA004775	ENSGALG00010023501	ENSGALG00010023501	case	control	2.852392056	1.34257674	2.124565376	0.033622895	0.348565623	up_in_case
CRA004775	ENSGALG00010023507	ENSGALG00010023507	case	control	2.309928494	0.833739432	2.770564046	0.005595929	0.15147564	up_in_case
CRA004775	ENSGALG00010023510	ASPHD2	case	control	-0.616291959	0.5420926	-1.136875801	0.25559021	0.718871765	down_in_case
CRA004775	ENSGALG00010023513	CDCA9	case	control	0.263478256	0.41485726	0.63510581	0.525359408	0.867484846	up_in_case
CRA004775	ENSGALG00010023514	ULK3	case	control	-0.462527691	0.277618867	-1.666052802	0.095702898	0.529205137	down_in_case
CRA004775	ENSGALG00010023518	METTL5	case	control	0.039722471	0.280392017	0.141667625	0.887342548	0.977118922	up_in_case
CRA004775	ENSGALG00010023519	DGKG	case	control	0.181104384	0.310750788	0.582796217	0.560030492	0.880918893	up_in_case
CRA004775	ENSGALG00010023522	EMG1	case	control	-0.534625405	0.267886249	-1.995717987	0.045964628	0.399027525	down_in_case
CRA004775	ENSGALG00010023526	KNSTRN	case	control	0.157152008	0.428134334	0.367062382	0.713572487	0.933244709	up_in_case
CRA004775	ENSGALG00010023527	TMEM98	case	control	0.350591193	0.462617604	0.757842309	0.44854539	0.83429564	up_in_case
CRA004775	ENSGALG00010023531	CPLX3	case	control	0.579102802	0.727854799	0.795629572	0.426247373	0.822541484	up_in_case
CRA004775	ENSGALG00010023533	FKBP7	case	control	-0.1315835	0.399475153	-0.329390949	0.741860208	0.938877854	down_in_case
CRA004775	ENSGALG00010023536	ENSGALG00010023536	case	control	-0.574523184	0.279726795	-2.053872546	0.039988029	0.375888502	down_in_case
CRA004775	ENSGALG00010023541	PRSS2	case	control	3.438237849	0.895186416	3.840806547	0.000122631	0.023828766	up_in_case
CRA004775	ENSGALG00010023544	SSB	case	control	-0.049257633	0.299641076	-0.164388788	0.86942509	0.973496509	down_in_case
CRA004775	ENSGALG00010023545	PTPRT	case	control	-0.572190153	0.674433354	-0.848401329	0.396214503	0.808998414	down_in_case
CRA004775	ENSGALG00010023546	SLC11A2	case	control	-0.54424508	0.349933412	-1.555281839	0.119878961	0.572133279	down_in_case
CRA004775	ENSGALG00010023551	ENSGALG00010023551	case	control	-0.490680537	0.434480093	-1.129351022	0.258749785	0.720037471	down_in_case
CRA004775	ENSGALG00010023552	SGPP2	case	control	0.016256598	0.667118565	0.024368379	0.980558771	0.996980737	up_in_case
CRA004775	ENSGALG00010023555	HPS4	case	control	-0.519255403	0.209517025	-2.478344678	0.013199357	0.231510654	down_in_case
CRA004775	ENSGALG00010023556	IVD	case	control	-0.09221482	0.31654199	-0.291319391	0.770807058	0.945853152	down_in_case
CRA004775	ENSGALG00010023559	PJVK	case	control	1.357178374	2.001959382	0.67792503	0.497819229	0.857810713	up_in_case
CRA004775	ENSGALG00010023565	ENSGALG00010023565	case	control	-0.989880947	0.571897854	-1.73087019	0.08347492	0.502115892	down_in_case
CRA004775	ENSGALG00010023569	LPCAT3	case	control	-0.425411622	0.219151547	-1.941175531	0.05223699	0.420135271	down_in_case
CRA004775	ENSGALG00010023571	APLP2	case	control	0.344499452	0.229349902	1.502069326	0.133079206	0.589932998	up_in_case
CRA004775	ENSGALG00010023572	MYBL2	case	control	-0.093943514	0.473330556	-0.198473377	0.842674713	0.966478468	down_in_case
CRA004775	ENSGALG00010023574	DEPDC1	case	control	0.546380633	0.643752692	0.848743066	0.396024277	0.808998414	up_in_case
CRA004775	ENSGALG00010023578	SRRD	case	control	-0.240814146	0.260985143	-0.922712087	0.356157265	0.789127613	down_in_case
CRA004775	ENSGALG00010023580	RDM1	case	control	-0.484043981	0.358440393	-1.350416946	0.176882278	0.642787581	down_in_case
CRA004775	ENSGALG00010023585	TFIP11	case	control	-0.080076334	0.15831082	-0.505817187	0.612984992	0.901859934	down_in_case
CRA004775	ENSGALG00010023588	FARSB	case	control	-0.113648088	0.268429237	-0.423381927	0.672016629	0.921952322	down_in_case
CRA004775	ENSGALG00010023590	S1PR1	case	control	0.28038916	0.250344423	1.120013608	0.262707963	0.723367482	up_in_case
CRA004775	ENSGALG00010023592	BAHD1	case	control	-0.243890845	0.265492517	-0.918635476	0.358286269	0.790696227	down_in_case
CRA004775	ENSGALG00010023593	CSK	case	control	-0.010990763	0.182603336	-0.060189277	0.952004886	0.990168609	down_in_case
CRA004775	ENSGALG00010023597	MOGAT1	case	control	-1.215922946	0.546722664	-2.224021477	0.026146999	0.30942116	down_in_case
CRA004775	ENSGALG00010023598	IL10RA	case	control	-0.208871199	0.277760263	-0.751983733	0.452060841	0.836647191	down_in_case
CRA004775	ENSGALG00010023600	CHST14	case	control	0.105822956	0.136392958	0.775868174	0.437826826	0.827567928	up_in_case
CRA004775	ENSGALG00010023601	TPST2	case	control	-0.174274045	0.245503956	-0.709862473	0.477789423	0.846563638	down_in_case
CRA004775	ENSGALG00010023603	C1S	case	control	0.246118283	0.424848834	0.579307892	0.562381441	0.881532201	up_in_case
CRA004775	ENSGALG00010023604	ENSGALG00010023604	case	control	-0.916012968	0.410651367	-2.230634162	0.025705372	0.308173121	down_in_case
CRA004775	ENSGALG00010023605	INSYN1	case	control	0.464055848	0.579541588	0.80072916	0.423288458	0.820552247	up_in_case
CRA004775	ENSGALG00010023612	GPT2	case	control	0.714511424	0.403378461	1.771317742	0.076507879	0.487052593	up_in_case
CRA004775	ENSGALG00010023613	COPB1	case	control	-0.157402528	0.184294178	-0.854083019	0.393058992	0.807553294	down_in_case
CRA004775	ENSGALG00010023615	PRSS3	case	control	2.289336282	0.949506183	2.411080964	0.015905317	0.252039758	up_in_case
CRA004775	ENSGALG00010023617	ENSGALG00010023617	case	control	-0.354801687	0.385805996	-0.919637566	0.357762189	0.790416653	down_in_case
CRA004775	ENSGALG00010023620	IRS1	case	control	0.489067713	0.389398397	1.255957184	0.209131494	0.678704224	up_in_case
CRA004775	ENSGALG00010023621	CD79B	case	control	1.018639664	0.604383347	1.685419808	0.091907605	0.52187835	up_in_case
CRA004775	ENSGALG00010023623	OSBPL6	case	control	0.699184669	0.414566682	1.68654332	0.091691198	0.521441551	up_in_case
CRA004775	ENSGALG00010023624	PSMA1	case	control	-0.347522357	0.243924733	-1.424711433	0.154240633	0.619284425	down_in_case
CRA004775	ENSGALG00010023626	LOXL1	case	control	0.243071578	0.275490645	0.882322438	0.377602466	0.798955456	up_in_case
CRA004775	ENSGALG00010023627	IFT52	case	control	-0.162918362	0.219703486	-0.741537448	0.458367635	0.840058241	down_in_case
CRA004775	ENSGALG00010023631	ENSGALG00010023631	case	control	0.033079663	0.36885597	0.089681789	0.928540088	0.98473447	up_in_case
CRA004775	ENSGALG00010023635	SCN4A	case	control	-0.094213648	0.49745543	-0.189391135	0.849786272	0.968413719	down_in_case
CRA004775	ENSGALG00010023636	PDE3B	case	control	-0.139844213	0.211168625	-0.662239537	0.507817719	0.862748056	down_in_case
CRA004775	ENSGALG00010023637	RHBDD1	case	control	-0.047283021	0.176550369	-0.267816042	0.788840922	0.95041797	down_in_case
CRA004775	ENSGALG00010023638	DCTN2	case	control	-0.001612381	0.311253608	-0.005180279	0.995866754	0.999355158	down_in_case
CRA004775	ENSGALG00010023639	KLHL23	case	control	-0.529116056	0.521043475	-1.015493105	0.309870832	0.762123736	down_in_case
CRA004775	ENSGALG00010023641	LRRC40	case	control	-0.4946965	0.313153126	-1.579727167	0.114169361	0.564321671	down_in_case
CRA004775	ENSGALG00010023643	SCN4B	case	control	-0.142229847	0.351322107	-0.404841722	0.685593858	0.926686318	down_in_case
CRA004775	ENSGALG00010023646	DPH5	case	control	-0.164752606	0.292980711	-0.562332603	0.573889429	0.886897708	down_in_case
CRA004775	ENSGALG00010023648	SCN2B	case	control	-0.730700229	0.587584334	-1.243566561	0.21365913	0.683061827	down_in_case
CRA004775	ENSGALG00010023652	SLC30A7	case	control	-0.438881138	0.359411487	-1.221110492	0.222044188	0.689550502	down_in_case
CRA004775	ENSGALG00010023655	C1R	case	control	-0.078224142	0.335182138	-0.233378017	0.815467882	0.958870945	down_in_case
CRA004775	ENSGALG00010023663	PHOSPHO2	case	control	0.15311999	0.232293888	0.659164951	0.509789851	0.862971569	up_in_case
CRA004775	ENSGALG00010023664	SLC6A9	case	control	-1.086285243	0.419091943	-2.59199744	0.009542048	0.197609494	down_in_case
CRA004775	ENSGALG00010023665	C16orf87	case	control	-0.142669184	0.346902681	-0.411265729	0.680877696	0.923896221	down_in_case
CRA004775	ENSGALG00010023666	ENSGALG00010023666	case	control	-0.590784437	0.432444061	-1.366152272	0.171891174	0.636057362	down_in_case
CRA004775	ENSGALG00010023668	RCN1	case	control	0.061904542	0.251235057	0.24640089	0.805371917	0.954959838	up_in_case
CRA004775	ENSGALG00010023670	EXTL2	case	control	0.017240487	0.270609204	0.063709905	0.949201218	0.990168609	up_in_case
CRA004775	ENSGALG00010023671	ENSGALG00010023671	case	control	-0.562398617	0.453569915	-1.239938097	0.214998291	0.683649824	down_in_case
CRA004775	ENSGALG00010023676	ENSGALG00010023676	case	control	0.149170942	0.520872551	0.286386645	0.774582002	0.946319184	up_in_case
CRA004775	ENSGALG00010023677	MN1	case	control	0.429238145	0.320031422	1.3412375	0.179843355	0.644990528	up_in_case
CRA004775	ENSGALG00010023680	MPZL3	case	control	1.552791611	0.632755641	2.454014647	0.014127123	0.238037235	up_in_case
CRA004775	ENSGALG00010023686	MYLK3	case	control	0.242058696	0.334554508	0.723525435	0.469357133	0.8440023	up_in_case
CRA004775	ENSGALG00010023689	WT1	case	control	1.674068782	0.516861397	3.23891239	0.001199864	0.072815451	up_in_case
CRA004775	ENSGALG00010023693	SRSF11	case	control	-0.074098983	0.171640088	-0.431711404	0.665951178	0.918888163	down_in_case
CRA004775	ENSGALG00010023694	PPCDC	case	control	-0.045177643	0.191178063	-0.236311857	0.813190687	0.95819924	down_in_case
CRA004775	ENSGALG00010023695	CLSTN3	case	control	-0.297940166	0.275399571	-1.081846876	0.279320574	0.735529913	down_in_case
CRA004775	ENSGALG00010023696	LRRC4C	case	control	0.6741452	0.682552978	0.987681867	0.323308478	0.768640193	up_in_case
CRA004775	ENSGALG00010023698	ENSGALG00010023698	case	control	-0.608685818	0.502815485	-1.210555038	0.226065985	0.694166578	down_in_case
CRA004775	ENSGALG00010023699	PPIG	case	control	0.409106182	0.272740934	1.499980861	0.13361936	0.591183984	up_in_case
CRA004775	ENSGALG00010023702	COL4A4	case	control	1.10938729	0.640840817	1.731143305	0.083426208	0.502054175	up_in_case
CRA004775	ENSGALG00010023703	PITPNB	case	control	-0.228507254	0.235621302	-0.96980728	0.332142563	0.772935571	down_in_case
CRA004775	ENSGALG00010023704	ANKRD13C	case	control	0.038552829	0.284039947	0.135730305	0.89203449	0.977819591	up_in_case

CRA004775	ENSGALG00010023706	CDK5RAP3	case	control	-0.606041133	0.147474134	-4.109474096	3.96561E-05	0.013135644	down_in_case
CRA004775	ENSGALG00010023707	MPZL2	case	control	-0.912070648	0.599877991	-1.520426924	0.128403712	0.582924874	down_in_case
CRA004775	ENSGALG00010023710	CDC14A	case	control	0.119341828	0.246432446	0.484278064	0.628188542	0.908314135	up_in_case
CRA004775	ENSGALG00010023711	ENSGALG00010023711	case	control	-1.099798887	0.659024623	-1.668828217	0.095151429	0.527976505	down_in_case
CRA004775	ENSGALG00010023712	ARHGAP40	case	control	-0.022273244	0.493147368	-0.045165494	0.963975398	0.992633346	down_in_case
CRA004775	ENSGALG00010023714	EIF3M	case	control	-0.054293906	0.146693208	-0.370118742	0.711294018	0.932234469	down_in_case
CRA004775	ENSGALG00010023715	CD3E	case	control	-0.744843029	0.464930784	-1.602051435	0.109144236	0.556276325	down_in_case
CRA004775	ENSGALG00010023716	FASTKD1	case	control	-0.209960675	0.248249214	-0.845765717	0.397683454	0.809315551	down_in_case
CRA004775	ENSGALG00010023720	SCAMP5	case	control	-0.403084096	0.193389583	-2.084311317	0.037131859	0.363933342	down_in_case
CRA004775	ENSGALG00010023722	PRR15L	case	control	1.440682924	0.647496168	2.225006102	0.026080829	0.308903939	up_in_case
CRA004775	ENSGALG00010023724	CYP2R1	case	control	0.399716733	0.299862782	1.332998811	0.182532194	0.647378665	up_in_case
CRA004775	ENSGALG00010023726	PNPO	case	control	-0.317134402	0.418426629	-0.757921175	0.448498173	0.83429564	down_in_case
CRA004775	ENSGALG00010023730	SP2	case	control	-0.168934943	0.185013408	-0.913095676	0.361192224	0.792567091	down_in_case
CRA004775	ENSGALG00010023731	ACTR5	case	control	-0.205517139	0.190402699	-1.079381437	0.280417723	0.736726042	down_in_case
CRA004775	ENSGALG00010023732	TM2D1	case	control	-0.077631378	0.255190602	-0.304209392	0.76096837	0.942236668	down_in_case
CRA004775	ENSGALG00010023733	ORC6	case	control	-0.475640072	0.428053276	-1.111170267	0.266495067	0.727311193	down_in_case
CRA004775	ENSGALG00010023736	KIF5A	case	control	-0.749227233	0.605263919	-1.237852133	0.215770894	0.683655659	down_in_case
CRA004775	ENSGALG00010023738	ENSGALG00010023738	case	control	-0.327765632	0.356982624	-0.918155703	0.358537356	0.79097996	down_in_case
CRA004775	ENSGALG00010023740	PPP1R16B	case	control	-0.151039437	0.363457001	-0.415563427	0.677729494	0.923000026	down_in_case
CRA004775	ENSGALG00010023743	CD3D	case	control	-0.77243773	0.407102216	-1.897404877	0.057774523	0.440142637	down_in_case
CRA004775	ENSGALG00010023744	MT4	case	control	0.124693497	0.366345273	0.340371519	0.733576764	0.936819725	up_in_case
CRA004775	ENSGALG00010023746	TTC28	case	control	-0.064738735	0.395319234	-0.16376318	0.869917578	0.973806302	down_in_case
CRA004775	ENSGALG00010023748	ENSGALG00010023748	case	control	-0.69068752	0.268426394	-2.573098378	0.010079255	0.202058223	down_in_case
CRA004775	ENSGALG00010023749	RPP25	case	control	0.488386308	0.42877665	1.139022631	0.254693715	0.718419134	up_in_case
CRA004775	ENSGALG00010023750	ENSGALG00010023750	case	control	-0.013643123	0.239275643	-0.057018435	0.95453051	0.990670511	down_in_case
CRA004775	ENSGALG00010023751	PRRG4	case	control	-0.000113055	0.312024781	-0.000362328	0.999710904	0.999914248	down_in_case
CRA004775	ENSGALG00010023752	C1QL1	case	control	1.453124487	0.525513354	2.765152355	0.005689622	0.152268906	up_in_case
CRA004775	ENSGALG00010023759	CALCA	case	control	-1.065453803	0.774458758	-1.375739885	0.168902179	0.632337016	down_in_case
CRA004775	ENSGALG00010023760	ZW10	case	control	-0.139820981	0.202389654	-0.690850435	0.489659537	0.854639532	down_in_case
CRA004775	ENSGALG00010023762	BBS5	case	control	0.864453447	0.537011204	1.609749368	0.107452583	0.553800541	up_in_case
CRA004775	ENSGALG00010023764	COX5A	case	control	0.246889956	0.332472523	0.742587549	0.457731429	0.839704957	up_in_case
CRA004775	ENSGALG00010023766	ENSGALG00010023766	case	control	-0.021753564	0.521524312	-0.041711504	0.966728683	0.993094598	down_in_case
CRA004775	ENSGALG00010023767	ENSGALG00010023767	case	control	-0.638336044	0.393395016	-1.622633785	0.104667714	0.547517634	down_in_case
CRA004775	ENSGALG00010023770	SCRN2	case	control	-0.222687503	0.326761961	-0.681497633	0.495556664	0.856382445	down_in_case
CRA004775	ENSGALG00010023773	OVOA	case	control	1.464435561	1.156707813	1.26603758	0.205499628	0.674045742	up_in_case
CRA004775	ENSGALG00010023775	ENSGALG00010023775	case	control	0.117069487	0.302883219	0.386516913	0.699113879	0.929347461	up_in_case
CRA004775	ENSGALG00010023777	OAZ2	case	control	-0.241706844	0.298604128	-0.809455801	0.418253016	0.818943344	down_in_case
CRA004775	ENSGALG00010023778	FANCF	case	control	-0.502336244	0.550759839	-0.912078565	0.361727361	0.792757982	down_in_case
CRA004775	ENSGALG00010023781	COL4A3	case	control	-0.843110532	0.902830011	-0.93385302	0.350379716	0.785466132	down_in_case
CRA004775	ENSGALG00010023782	TMPRSS5	case	control	-0.254376859	0.582580533	-0.436638103	0.662373825	0.918173506	down_in_case
CRA004775	ENSGALG00010023783	VPS35	case	control	-0.133373545	0.159160475	-0.837981571	0.402041054	0.811868062	down_in_case
CRA004775	ENSGALG00010023784	LRRC46	case	control	0.215833221	0.612835779	0.352187697	0.724697503	0.935238044	up_in_case
CRA004775	ENSGALG00010023786	ENSGALG00010023786	case	control	-0.561642866	0.692025577	-0.811592642	0.417025415	0.818943344	down_in_case
CRA004775	ENSGALG00010023788	RTCA	case	control	0.015262349	0.23373119	0.065298728	0.947936155	0.989905078	up_in_case
CRA004775	ENSGALG00010023791	VTI1B	case	control	0.023714193	0.212126127	0.111792889	0.910987626	0.98075825	up_in_case
CRA004775	ENSGALG00010023792	ZNF609	case	control	0.128650077	0.268585547	0.478991063	0.631944987	0.909651573	up_in_case
CRA004775	ENSGALG00010023793	RBM45	case	control	-0.15275372	0.169006225	-0.903834874	0.366082967	0.793608387	down_in_case
CRA004775	ENSGALG00010023794	MT3	case	control	-0.18594338	0.492386279	-0.377637208	0.70570012	0.931025842	down_in_case
CRA004775	ENSGALG00010023798	ARCN1	case	control	-0.160687445	0.164097464	-0.97921955	0.327471511	0.771474966	down_in_case
CRA004775	ENSGALG00010023799	NMT1	case	control	-0.694887039	0.25520939	-2.722811406	0.0064729	0.159800439	down_in_case
CRA004775	ENSGALG00010023800	PEX5	case	control	-0.08842694	0.232878663	-0.379712505	0.704158837	0.930465147	down_in_case
CRA004775	ENSGALG00010023802	ENSGALG00010023802	case	control	0.176242764	0.356041533	0.495006193	0.620595749	0.905283281	up_in_case
CRA004775	ENSGALG00010023805	GALNT6	case	control	0.236338573	0.507284465	0.465889633	0.641294502	0.912315404	up_in_case
CRA004775	ENSGALG00010023806	PATJ	case	control	0.203494142	0.382087639	0.532584991	0.594320894	0.894956597	up_in_case
CRA004775	ENSGALG00010023807	MRPL10	case	control	-0.291367214	0.378885931	-0.76901038	0.441887149	0.829654585	down_in_case
CRA004775	ENSGALG00010023808	DHX35	case	control	-0.298919196	0.287123965	-1.041080624	0.297838131	0.750344577	down_in_case
CRA004775	ENSGALG00010023809	CDON	case	control	-0.057235224	0.323648988	-0.176843512	0.85963131	0.971325143	down_in_case
CRA004775	ENSGALG00010023811	MRPL44	case	control	0.081115331	0.36078476	0.224830258	0.822111326	0.960766569	up_in_case
CRA004775	ENSGALG00010023813	CELA1	case	control	2.37958363	1.410454479	1.687104168	0.091583324	0.521441551	up_in_case
CRA004775	ENSGALG00010023815	PLCD3	case	control	-0.087174364	0.355633082	-0.245124452	0.80636007	0.955397873	down_in_case
CRA004775	ENSGALG00010023817	BBS2	case	control	-0.291096292	0.337617557	-0.862207211	0.388573502	0.804467665	down_in_case
CRA004775	ENSGALG00010023818	ALOX5	case	control	-0.376460981	0.290076234	-1.297800156	0.194356016	0.65892248	down_in_case
CRA004775	ENSGALG00010023819	BIN2	case	control	-0.816935039	0.470264609	-1.73718163	0.082355116	0.499148629	down_in_case
CRA004775	ENSGALG00010023820	CYP1C1	case	control	-1.573512078	0.718421038	-2.190236636	0.02850708	0.321586952	down_in_case
CRA004775	ENSGALG00010023821	KANK4	case	control	0.459610644	0.320459101	1.434225591	0.151507896	0.615657531	up_in_case
CRA004775	ENSGALG00010023822	CHEK2	case	control	-0.462427871	0.314265698	-1.471455122	0.141168076	0.600619496	down_in_case
CRA004775	ENSGALG00010023823	MUC2	case	control	2.575427452	0.695424479	3.703389123	0.000212738	0.029549119	up_in_case
CRA004775	ENSGALG00010023826	DBT	case	control	0.181794174	0.344450539	0.5277802	0.597651901	0.896490702	up_in_case
CRA004775	ENSGALG00010023828	CHEK1	case	control	-0.859689717	0.566717332	-1.516963869	0.129275806	0.584172159	down_in_case
CRA004775	ENSGALG00010023829	USP1	case	control	0.237601005	0.246168404	0.965197002	0.334446148	0.774742828	up_in_case
CRA004775	ENSGALG00010023830	AGFG1	case	control	0.070750169	0.122089605	0.579493799	0.562256029	0.881532201	up_in_case
CRA004775	ENSGALG00010023831	SATB2	case	control	0.093540771	0.682163754	0.137123631	0.890933074	0.977517012	up_in_case
CRA004775	ENSGALG00010023832	ENSGALG00010023832	case	control	0.172702976	0.313479152	0.550923323	0.581686238	0.889219177	up_in_case
CRA004775	ENSGALG00010023833	PIP4K2C	case	control	-0.263817296	0.16966818	-1.554901432	0.119969546	0.572133279	down_in_case
CRA004775	ENSGALG00010023835	INAFM2	case	control	-0.217586255	0.453569824	-0.479719424	0.631426914	0.909395104	down_in_case
CRA004775	ENSGALG00010023836	NDUFS3	case	control	-0.015203093	0.403204972	-0.037705618	0.969922397	0.994366623	down_in_case
CRA004775	ENSGALG00010023837	STT3A	case	control	-0.194973378	0.156391436	-1.246701112	0.212507111	0.682275341	down_in_case
CRA004775	ENSGALG00010023840	SMAGP	case	control	0.028460874	0.288472531	0.098660602	0.921407752	0.982481112	up_in_case
CRA004775	ENSGALG00010023841	OSBPL7	case	control	-0.399783878	0.166275733	-2.404342906	0.016201568	0.25344686	down_in_case
CRA004775	ENSGALG00010023847	PIK3AP1	case	control	0.569022084	0.46642171	1.21997341	0.222474955	0.6901691	up_in_case
CRA004775	ENSGALG00010023850	DAZAP2	case	control	-0.307737042	0.254874533	-1.207406006	0.227275824	0.694715356	down_in_case
CRA004775	ENSGALG00010023851	HSCB	case	control	0.199692941	0.415061244	0.481116808	0.630433483	0.9090909	up_in_case
CRA004775	ENSGALG00010023852	LRRC39	case	control	-0.040080261	0.566559053	-0.070743307	0.943602053	0.987862092	down_in_case
CRA004775	ENSGALG00010023854	SHCBP1	case	control	0.251075295	0.478527677	0.524682912	0.599803638	0.897206546	up_in_case
CRA004775	ENSGALG00010023857	ACBD4	case	control	-0.092171019	0.231621518	-0.397938067	0.690675839	0.927867527	down_in_case
CRA004775	ENSGALG00010023859	ENSGALG00010023859	case	control	-0.077183762	0.686795598	-0.112382436	0.910520181	0.98075825	down_in_case
CRA004775	ENSGALG00010023860	TRIP4	case	control	-0.326205793	0.207632111	-1.571075836	0.116165035	0.566086328	down_in_case
CRA004775	ENSGALG00010023861	ENSGALG00010023861	case	control	-0.297094344	0.210433983	-1.411817331	0.158003754	0.623088507	down_in_case
CRA004775	ENSGALG00010023862	TRMT13	case	control	-0.329876618	0.356023038	-0.926559752	0.35415516	0.78730441	down_in_case
CRA004775	ENSGALG00010023863	EYA2	case	control	-0.50821539	0.669407596	-0.759201707	0.447731905	0.834136489	down_in_case
CRA004775	ENSGALG00010023864	EPHA1	case	control	1.353126153	0.494651051	2.735516584	0.006228245	0.1586011	up_in_case
CRA004775	ENSGALG00010023866	AMBRA1	case	control	-0.197762978	0.137021154	-1.443302529	0.148935268	0.612442843	down_in_case
CRA004775	ENSGALG00010023867	ENSGALG00010023867	case	control	-0.329162644	0.519010714	-0.634211656	0.525942702	0.867878941	down_in_case
CRA004775	ENSGALG00010023869	DOCK7	case	control	0.20780966	0.235622939	0.881958525	0.377799236	0.799125872	up_in_case
CRA004775	ENSGALG00010023871	UQCRFS1	case	control	0.097743143	0.405721525	0.240911898	0.8096234	0.956043914	up_in_case
CRA004775	ENSGALG00010023874	HEXIM1	case	control	0.286978439	0.338617697	0.847499825	0.396716585	0.808998414	up_in_case
CRA004775	ENSGALG00010023876	POU6F1	case	control	0.017129811	0.531179715	0.032248616	0.974273786	0.994947809	up_in_case
CRA004775	ENSGALG00010023878	ENSGALG00010023878	case	control	2.741779886	0.832458199	3.293594668	0.00098915	0.066028181	up_in_case
CRA004775	ENSGALG00010023879	EI24	case	control	-0.260185047	0.276043114	-0.942552209	0.345909994	0.784552667	down_in_case
CRA004775	ENSGALG00010023880	ENSGALG00010023880	case	control	-0.331137226	0.436851407	-0.758008834	0.448445694	0.83429564	down_in_case
CRA004775	ENSGALG00010023881	VSTM2B	case	control	0.395786592	0.432527327	0.915055691	0.360162395	0.791975934	up_in_case
CRA004775	ENSGALG00010023883	TBX21	case	control	-0.228970692	0.383019142	-0.597804826	0.54997017	0.877623839	down_in_case
CRA004775	ENSGALG00010023884	C2orf69	case	control	0.52222602	0.261328923	1.998347574	0.045678991	0.397803551	up_in_case
CRA004775	ENSGALG00010023887	RASA2	case	control	0.268124929	0.314171372	0.85343527	0.39341797	0.807808048	up_in_case
CRA004775	ENSGALG00010023888	CTH	case	control	-0.057688929	0.27440119	-0.21023571	0.83348371	0.964019688	down_in_case
CRA004775	ENSGALG00010023889	SAMD8	case	control	0.626499699	0.418876094	1.495668308	0.134740113	0.592420453	up_in_case
CRA004775	ENSGALG00010023890	SASS6	case	control	-0.138997405	0.226462546	-0.613776571	0.539362982	0.873987916	down_in_case
CRA004775	ENSGALG00010023891	ENSGALG00010023891	case	control	0.163615369	0.318383221	0.513894447	0.60732579	0.900943922	up_in_case
CRA004775	ENSGALG00010023894	ENSGALG00010023894	case	control	2.127463689	0.61336437	3.468515278	0.000523343	0.046175703	up_in_case
CRA004775	ENSGALG00010023896	POP4	case	control	-0.413639377	0.216766323	-1.908227122	0.056361868	0.434435	down_in_case
CRA004775	ENSGALG00010023902	PTGER3	case	control	0.348494217	0.351039781	0.992748503	0.320832532	0.767130611	up_in_case
CRA004775	ENSGALG00010023903	TBKBP1	case	control	-0.27732085	0.301351776	-0.920256232	0.357438875	0.790309937	down_in_case
CRA004775	ENSGALG00010023909	FOXRED1	case	control	-0.220461226	0.283375902	-0.777981557	0.436579886	0.827068479	down_in_case
CRA004775	ENSGALG00010023910	RNF7	case	control	0.332403588	0.256882258	1.293992009	0.195668181	0.661073909	up_in_case
CRA004775	ENSGALG00010023911	TFCP2	case	control	-0.664261399	0.151760353	-4.37704173	1.20301E-05	0.005510836	down_in_case
CRA004775	ENSGALG00010023913	ZMYND8	case	control	0.002756514	0.253781275	0.010861772	0.99133373	0.998065748	up_in_case
CRA004775	ENSGALG00010023914	CCDC117	case	control	-0.14202859	0.218874354	-0.64890467	0.516399996	0.864913871	down_in_case
CRA004775	ENSGALG00010023917	ZRANB2	case	control	-0.182040442	0.162855106	-1.117806171	0.263649786	0.723981064	down_in_case
CRA004775	ENSGALG00010023918	MFSD14A	case	control	-0.228669294	0.267352449	-0.855310266	0.392379406	0.80746892	down_in_case
CRA004775	ENSGALG00010023921	GRK7	case	control	0.477509527	0.393223893	1.214345147	0.224615952	0.692859731	up_in_case
CRA004775	ENSGALG00010023923	CSNK1G1	case	control	0.203920033	0.230847206	0.883354997	0.377044498	0.798523018	up_in_case
CRA004775	ENSGALG00010023925	NCOA3	case	control	0.111927553	0.35191678	0.318051196	0.750446103	0.941495694	up_in_case
CRA004775	ENSGALG00010023926	ZYX	case	control	-0.276614633	0.320794949	-0.862278644	0.388534202	0.804467665	down_in_case
CRA004775	ENSGALG00010023927	KPNB1	case	control	0.287005212	0.193746875	1.481341115	0.138515698	0.597959259	up_in_case
CRA004775	ENSGALG00010023930	SRPRA	case	control	-0.018840147	0.20781166	-0.09065972	0.927762978	0.984691935	down_in_case
CRA004775	ENSGALG00010023931	CSRNP2	case	control	0.124462572	0.236472224	0.52633062	0.598658512	0.8970998	up_in_case

CRA004775	ENSGALG00010023937	XBP1	case	control	-0.151628888	0.22320686	-0.679320018	0.496935115	0.857679291	down_in_case
CRA004775	ENSGALG00010023940	ATP1B3	case	control	0.15292333	0.269906446	0.566579021	0.571000232	0.885321726	up_in_case
CRA004775	ENSGALG00010023942	NEGR1	case	control	-0.007934453	0.390165712	-0.020336111	0.983775249	0.99751066	down_in_case
CRA004775	ENSGALG00010023945	LETMD1	case	control	-0.474819908	0.323687722	-1.46690738	0.142401253	0.602473513	down_in_case
CRA004775	ENSGALG00010023946	ZNRF3	case	control	-0.075094912	0.323842188	-0.231887365	0.816625498	0.959451233	down_in_case
CRA004775	ENSGALG00010023948	SULF2	case	control	0.213983145	0.389012375	0.550067707	0.582272935	0.889579727	up_in_case
CRA004775	ENSGALG00010023953	SLC35A3	case	control	-0.380875498	0.318758462	-1.194871803	0.232137121	0.698952825	down_in_case
CRA004775	ENSGALG00010023954	PLEKHF1	case	control	0.252490062	0.189032946	1.335693415	0.181649502	0.647378665	up_in_case
CRA004775	ENSGALG00010023955	C1QL4	case	control	0.910983329	0.831277531	1.095883499	0.273129761	0.731001618	up_in_case
CRA004775	ENSGALG00010023956	KREMEN1	case	control	0.071091607	0.316846347	0.224372499	0.822467467	0.960868728	up_in_case
CRA004775	ENSGALG00010023957	YBX3	case	control	0.216874371	0.329686489	0.657820013	0.510653793	0.863170205	up_in_case
CRA004775	ENSGALG00010023962	FPGT	case	control	0.037217705	0.278471393	0.133650011	0.893679336	0.977834811	up_in_case
CRA004775	ENSGALG00010023966	PEAK1	case	control	0.382193329	0.416046721	0.918630793	0.35828872	0.790696227	up_in_case
CRA004775	ENSGALG00010023967	PRPH	case	control	0.260488146	0.536966747	0.485110387	0.627598044	0.908049317	up_in_case
CRA004775	ENSGALG00010023968	TYW5	case	control	0.049955472	0.210371704	0.237462885	0.812297709	0.957639386	up_in_case
CRA004775	ENSGALG00010023972	ENSGALG00010023972	case	control	-0.596245575	0.460824838	-1.293865968	0.195711722	0.661073909	down_in_case
CRA004775	ENSGALG00010023974	PREX1	case	control	-0.144557981	0.418005625	-0.345827836	0.729472104	0.936339759	down_in_case
CRA004775	ENSGALG00010023978	TFDP2	case	control	0.672745693	0.272436516	2.469366825	0.013535239	0.232945019	up_in_case
CRA004775	ENSGALG00010023979	FAM118B	case	control	-0.108618324	0.146015616	-0.743881557	0.456948134	0.839688534	down_in_case
CRA004775	ENSGALG00010023981	ENSGALG00010023981	case	control	-0.376341072	0.274803118	-1.369493457	0.170845079	0.635139279	down_in_case
CRA004775	ENSGALG00010023984	FMNL1	case	control	-0.555897664	0.344788602	-1.6122855	0.106899827	0.552884225	down_in_case
CRA004775	ENSGALG00010023986	SYCE3	case	control	0.259832874	0.626983954	0.414417103	0.678568666	0.92338129	up_in_case
CRA004775	ENSGALG00010023987	TNNI3K	case	control	0.838916069	0.901808402	0.930259761	0.352236606	0.786426886	up_in_case
CRA004775	ENSGALG00010023989	ENSGALG00010023989	case	control	-0.467948969	0.196009079	-2.387384152	0.01696875	0.257853739	down_in_case
CRA004775	ENSGALG00010023991	TSPAN3	case	control	-0.202214433	0.171963881	-1.175912244	0.239629946	0.706254731	down_in_case
CRA004775	ENSGALG00010023992	FAM131B	case	control	-0.222265429	0.386736057	-0.574721247	0.565479836	0.883033678	down_in_case
CRA004775	ENSGALG00010023993	C19orf12	case	control	-0.348261569	0.220063607	-1.582549578	0.113524164	0.562886277	down_in_case
CRA004775	ENSGALG00010023996	MYO7B	case	control	1.815049917	0.422876353	4.292152786	1.76949E-05	0.007603835	up_in_case
CRA004775	ENSGALG00010023997	ARFGEF2	case	control	-0.120639628	0.354432492	-0.340374066	0.733574846	0.936819725	down_in_case
CRA004775	ENSGALG00010023999	DGCR14	case	control	-0.31246065	0.174522732	-1.790372216	0.073394094	0.478313831	down_in_case
CRA004775	ENSGALG00010024001	ENSGALG00010024001	case	control	1.251051078	0.478784491	2.612973272	0.008975832	0.191062954	up_in_case
CRA004775	ENSGALG00010024002	DGCR2	case	control	-0.063761318	0.131308834	-0.485582853	0.627262956	0.907733515	down_in_case
CRA004775	ENSGALG00010024003	ERICH3	case	control	-0.836603298	0.293955732	-2.846017973	0.00442697	0.136798335	down_in_case
CRA004775	ENSGALG00010024004	EIF4B	case	control	-0.381783191	0.263832784	-1.447065009	0.147878717	0.611192154	down_in_case
CRA004775	ENSGALG00010024005	STYK1	case	control	0.699395223	0.465675165	1.501895044	0.133124217	0.589942348	up_in_case
CRA004775	ENSGALG00010024008	CCNE1	case	control	0.15160758	0.200723844	0.755304285	0.450066428	0.835337561	up_in_case
CRA004775	ENSGALG00010024009	CSE1L	case	control	0.022066158	0.133778977	0.164944882	0.868987365	0.973370973	up_in_case
CRA004775	ENSGALG00010024010	ENSGALG00010024010	case	control	0.177507212	0.221763073	0.800436295	0.423458059	0.820552247	up_in_case
CRA004775	ENSGALG00010024011	AGPS	case	control	0.034015963	0.184707124	0.184161619	0.853886668	0.970073177	up_in_case
CRA004775	ENSGALG00010024013	PTPRJ	case	control	-0.071173645	0.461037915	-0.15437699	0.877312496	0.974479909	down_in_case
CRA004775	ENSGALG00010024016	CRYZ	case	control	-0.50580868	0.246078674	-2.055475475	0.039833102	0.375888502	down_in_case
CRA004775	ENSGALG00010024017	TYW3	case	control	-0.028776839	0.236561396	-0.121646387	0.903179075	0.979310476	down_in_case
CRA004775	ENSGALG00010024018	KRT18	case	control	2.623293764	0.75637239	3.468256904	0.000523846	0.046175703	up_in_case
CRA004775	ENSGALG00010024019	MAP3K14	case	control	-0.341916982	0.183052391	-1.867864054	0.061781022	0.446686038	down_in_case
CRA004775	ENSGALG00010024020	MAIP1	case	control	0.071899988	0.308248014	0.233253694	0.815564414	0.958870945	up_in_case
CRA004775	ENSGALG00010024021	IGLL1	case	control	0.947392436	0.753258273	1.257725895	0.208490904	0.677287602	up_in_case
CRA004775	ENSGALG00010024024	AGL	case	control	0.482620958	0.455413986	1.059741185	0.28926236	0.743485368	up_in_case
CRA004775	ENSGALG00010024025	CLCN1	case	control	0.233108016	0.520359022	0.447975351	0.654170986	0.916560183	up_in_case
CRA004775	ENSGALG00010024026	TIRAP	case	control	-0.494460584	0.260529255	-1.897908105	0.057708188	0.439880987	down_in_case
CRA004775	ENSGALG00010024029	SCAPER	case	control	-0.228878012	0.288249152	-0.794028396	0.427178901	0.822776983	down_in_case
CRA004775	ENSGALG00010024030	STAU1	case	control	0.03411121	0.21283443	0.16027111	0.872667515	0.974030114	up_in_case
CRA004775	ENSGALG00010024031	LIMS2	case	control	0.234254998	0.287673484	0.814308622	0.415468171	0.818943344	up_in_case
CRA004775	ENSGALG00010024034	LRP2	case	control	-0.442954334	0.620260181	-0.714142786	0.475138891	0.84579952	down_in_case
CRA004775	ENSGALG00010024035	OSBPL5	case	control	-0.183141627	0.288804263	-0.634137545	0.525991062	0.867878941	down_in_case
CRA004775	ENSGALG00010024038	NKD1	case	control	-0.573501178	0.642880356	-0.892080732	0.372349662	0.796100128	down_in_case
CRA004775	ENSGALG00010024039	MRPL45	case	control	-0.253270858	0.208672725	-1.213722862	0.224853571	0.692859731	down_in_case
CRA004775	ENSGALG00010024042	DCPS	case	control	-0.17067595	0.204793171	-0.83340645	0.404615538	0.81313407	down_in_case
CRA004775	ENSGALG00010024045	PSTPIP1	case	control	-1.177047105	0.562421406	-2.092820599	0.036365172	0.360628495	down_in_case
CRA004775	ENSGALG00010024049	RCN2	case	control	-0.05540251	0.258065762	-0.214683689	0.83001394	0.963232966	down_in_case
CRA004775	ENSGALG00010024051	ENSGALG00010024051	case	control	2.262001337	0.658868036	3.43316296	0.000596583	0.05063962	up_in_case
CRA004775	ENSGALG00010024052	ENSGALG00010024052	case	control	0.046221772	0.296713964	0.155778891	0.87620731	0.974421894	up_in_case
CRA004775	ENSGALG00010024053	ISL2	case	control	-0.228478586	0.657245871	-0.347630309	0.728117841	0.935650625	down_in_case
CRA004775	ENSGALG00010024056	ENSGALG00010024056	case	control	0.569919201	0.841568066	0.677211059	0.498272052	0.858076267	up_in_case
CRA004775	ENSGALG00010024058	SLC44A5	case	control	0.522513478	0.179766414	2.906624579	0.003653514	0.12600076	up_in_case
CRA004775	ENSGALG00010024060	FRRS1	case	control	0.340512757	0.275630953	1.235393754	0.216683996	0.684341211	up_in_case
CRA004775	ENSGALG00010024062	ST3GAL4	case	control	-0.120646889	0.289043953	-0.417399803	0.676385999	0.922924485	down_in_case
CRA004775	ENSGALG00010024063	BRD7	case	control	0.267425159	0.250401917	1.06798367	0.285527878	0.74152976	up_in_case
CRA004775	ENSGALG00010024064	GPR17	case	control	-0.100735375	0.393687317	-0.255876608	0.798046118	0.952835572	down_in_case
CRA004775	ENSGALG00010024066	ENSGALG00010024066	case	control	0.781197114	0.875113375	0.892681036	0.372028009	0.795917016	up_in_case
CRA004775	ENSGALG00010024068	CASP2	case	control	-0.591291129	0.407774775	-1.45004342	0.147046412	0.610295079	down_in_case
CRA004775	ENSGALG00010024069	ENSGALG00010024069	case	control	0.568185529	0.785383154	0.72345011	0.469403394	0.8440023	up_in_case
CRA004775	ENSGALG00010024073	RABGGTB	case	control	0.077009875	0.274760674	0.280279831	0.779262823	0.94795144	up_in_case
CRA004775	ENSGALG00010024074	ADCY7	case	control	-0.387102096	0.602983924	-0.641977474	0.520887809	0.866005109	down_in_case
CRA004775	ENSGALG00010024075	SPATS2L	case	control	0.178647103	0.259328615	0.688883112	0.490896828	0.855445733	up_in_case
CRA004775	ENSGALG00010024077	SFT2D3	case	control	-0.109888057	0.357838773	-0.307088178	0.758776256	0.942236668	down_in_case
CRA004775	ENSGALG00010024078	KIRREL3	case	control	-0.643144562	0.532421996	-1.207960165	0.227062585	0.694715356	down_in_case
CRA004775	ENSGALG00010024080	TENT4B	case	control	0.646813453	0.205145553	3.152948938	0.001616301	0.080802173	up_in_case
CRA004775	ENSGALG00010024081	CARS	case	control	0.087174956	0.189070278	0.461071706	0.644747161	0.913918935	up_in_case
CRA004775	ENSGALG00010024083	PALMD	case	control	0.65197203	0.394558445	1.652409262	0.098451149	0.53445976	up_in_case
CRA004775	ENSGALG00010024086	GABARAPL1	case	control	0.374630168	0.464930468	0.805776763	0.420371579	0.819388688	up_in_case
CRA004775	ENSGALG00010024089	ENSGALG00010024089	case	control	0.748051155	0.804340304	0.930018241	0.35236164	0.786426886	up_in_case
CRA004775	ENSGALG00010024091	ETFA	case	control	-0.524963382	0.271232243	-1.935475576	0.052931963	0.423142991	down_in_case
CRA004775	ENSGALG00010024095	RBM12	case	control	-0.16372361	0.229192138	-0.714350898	0.475010226	0.84579952	down_in_case
CRA004775	ENSGALG00010024096	MYO1D	case	control	-0.425609129	0.276460689	-1.539492395	0.123684133	0.576340398	down_in_case
CRA004775	ENSGALG00010024099	ENSGALG00010024099	case	control	1.320438347	0.750163472	1.760200803	0.078373766	0.490987542	up_in_case
CRA004775	ENSGALG00010024101	CNEP1R1	case	control	-0.062891021	0.190705933	-0.329780096	0.741566128	0.938877854	down_in_case
CRA004775	ENSGALG00010024102	SGF29	case	control	-0.433958533	0.208722251	-2.079119655	0.037606354	0.365717803	down_in_case
CRA004775	ENSGALG00010024104	PLPPR5	case	control	-0.276965628	0.477591575	-0.579921512	0.561967548	0.881532201	down_in_case
CRA004775	ENSGALG00010024106	WDR33	case	control	0.051186891	0.201698691	0.253778993	0.799666303	0.952847322	up_in_case
CRA004775	ENSGALG00010024107	NFE2L2	case	control	-0.102541327	0.255057887	-0.402031589	0.687660779	0.927111919	down_in_case
CRA004775	ENSGALG00010024110	CPNE1	case	control	-0.303108008	0.194414673	-1.559079895	0.118977472	0.570892125	down_in_case
CRA004775	ENSGALG00010024111	ENSGALG00010024111	case	control	0.872915174	0.83004638	1.051646263	0.292961874	0.746438942	up_in_case
CRA004775	ENSGALG00010024112	NAP1L4	case	control	0.137642959	0.305980442	0.449842342	0.652824125	0.91629933	up_in_case
CRA004775	ENSGALG00010024113	ENSGALG00010024113	case	control	0.392337868	0.364222918	1.077191603	0.281394676	0.738078244	up_in_case
CRA004775	ENSGALG00010024115	POLR2D	case	control	-0.422883918	0.216961714	-1.949117708	0.051281369	0.418250363	down_in_case
CRA004775	ENSGALG00010024116	MCAM	case	control	-0.176350007	0.50872859	-0.346648508	0.728855399	0.935981564	down_in_case
CRA004775	ENSGALG00010024117	ENSGALG00010024117	case	control	0.690790716	1.022213423	0.675779344	0.499180751	0.858888202	up_in_case
CRA004775	ENSGALG00010024118	TMEM266	case	control	-0.121684915	0.247988057	-0.49068861	0.623646704	0.906441769	down_in_case
CRA004775	ENSGALG00010024119	AMMECR1L	case	control	-0.081212011	0.270185283	-0.300578958	0.763735579	0.94239774	down_in_case
CRA004775	ENSGALG00010024121	NRG4	case	control	0.371571642	0.293999946	1.263849355	0.206284102	0.674837165	up_in_case
CRA004775	ENSGALG00010024122	SOCS7	case	control	-0.153768295	0.314278163	-0.489274514	0.624647362	0.90646861	down_in_case
CRA004775	ENSGALG00010024123	PLPPR4	case	control	-1.646582396	1.007834164	-1.633783071	0.102304408	0.542513917	down_in_case
CRA004775	ENSGALG00010024124	DIDO1	case	control	-0.09057922	0.239301115	-0.378515662	0.705047561	0.930925979	down_in_case
CRA004775	ENSGALG00010024126	CDK5R1	case	control	-0.623599439	0.346581794	-1.799285047	0.071973602	0.473280705	down_in_case
CRA004775	ENSGALG00010024128	PHLDA2	case	control	0.285138213	0.345688744	0.824840895	0.409461926	0.814953096	up_in_case
CRA004775	ENSGALG00010024129	GID8	case	control	0.41194493	0.198212112	2.078303518	0.037681413	0.365870038	up_in_case
CRA004775	ENSGALG00010024132	ENSGALG00010024132	case	control	-0.187357725	0.225669822	-0.830229417	0.406409086	0.813842524	down_in_case
CRA004775	ENSGALG00010024133	PSMD11	case	control	-0.402563947	0.3360373	-1.197973998	0.23092714	0.697294488	down_in_case
CRA004775	ENSGALG00010024135	FBXO22	case	control	0.08552578	0.276808495	0.308970938	0.757343634	0.941900693	up_in_case
CRA004775	ENSGALG00010024136	SAP130	case	control	-0.239349016	0.250671598	-0.954831013	0.339663161	0.780011378	down_in_case
CRA004775	ENSGALG00010024137	ENSGALG00010024137	case	control	0.739061901	0.399863998	1.848283181	0.064561388	0.457298868	up_in_case
CRA004775	ENSGALG00010024138	CBL	case	control	-0.611730976	0.420714538	-1.454028612	0.145938374	0.609044185	down_in_case
CRA004775	ENSGALG00010024139	SLC22A18	case	control	1.967520582	0.763758485	2.576103076	0.009992085	0.201425687	up_in_case
CRA004775	ENSGALG00010024141	YTHDF1	case	control	0.265214398	0.177066202	1.497826208	0.13417841	0.592323374	up_in_case
CRA004775	ENSGALG00010024142	DNAJC6	case	control	-0.711772278	0.412415723	-1.725861159	0.084372394	0.503614276	down_in_case
CRA004775	ENSGALG00010024143	UBE2Q2	case	control	0.494162927	0.282416319	1.749767607	0.080158422	0.493676892	up_in_case
CRA004775	ENSGALG00010024144	HNRNPA3	case	control	-0.27410248	0.237674873	-1.153266545	0.248800997	0.713881011	down_in_case
CRA004775	ENSGALG00010024145	RAP1GAP3	case	control	-0.295597598	0.255190635	-1.158340306	0.246725189	0.712046495	down_in_case
CRA004775	ENSGALG00010024146	CDKN1C	case	control	-0.146372902	0.495163253	-0.29560534	0.767531495	0.944590059	down_in_case
CRA004775	ENSGALG00010024147	ENSGALG00010024147	case	control	-0.539194486	0.269118634	-2.003556865	0.04511755	0.394914981	down_in_case
CRA004775	ENSGALG00010024148	UGGT1	case	control	-0.092951755	0.312543473	-0.297404244	0.766157904	0.943612838	down_in_case
CRA004775	ENSGALG00010024149	TCIRG1	case	control	-0.061248145	0.45136525	-0.135695303	0.892062161	0.977819591	down_in_case
CRA004775	ENSGALG00010024150	NHERF4	case	control	0.109028964	0.31983954	0.340894532	0.733182981	0.936819725	up_in_case
CRA004775	ENSGALG00010024151	LEPROT	case	control	-0.035764533	0.302268699	-0.118320332	0.905813848	0.979739788	down_in_case
CRA004775	ENSGALG00010024152	ZNF423	case	control	-0.192053958	0.179419646	-1.070417659	0.284431354	0.741035375	down_in_case
CRA004775	ENSGALG00010024153	KCNQ1	case	control	0.424030802	0.428517025	0.989530817	0.3224035	0.767880594	up_in_case

CRA004775	ENSGALG00010024154	VPS29L	case	control	0.244921834	0.477013373	0.51344857	0.607637578	0.900943922	up_in_case
CRA004775	ENSGALG00010024155	SNX7	case	control	-0.066617494	0.257001856	-0.259210167	0.795473087	0.952503519	down_in_case
CRA004775	ENSGALG00010024158	ENSGALG00010024158	case	control	-0.510238974	0.332902917	-1.532696017	0.125350771	0.578422299	down_in_case
CRA004775	ENSGALG00010024159	NLRX1	case	control	-0.330989856	0.256301601	-1.291407683	0.196562348	0.66285976	down_in_case
CRA004775	ENSGALG00010024160	VPREB3	case	control	3.273363545	0.855552743	3.826021916	0.000130231	0.024531539	up_in_case
CRA004775	ENSGALG00010024162	SRCIN1	case	control	1.140369132	0.629480777	1.811602789	0.070047594	0.469724266	up_in_case
CRA004775	ENSGALG00010024163	HS6ST1	case	control	0.208767806	0.199488003	1.046518101	0.295321892	0.747601499	up_in_case
CRA004775	ENSGALG00010024164	SNAP29	case	control	0.611057779	0.246674503	2.477182569	0.013242414	0.231674862	up_in_case
CRA004775	ENSGALG00010024165	LEPR	case	control	0.049790281	0.464103322	0.107282751	0.914564668	0.981303879	up_in_case
CRA004775	ENSGALG00010024169	FAM168B	case	control	-0.015004612	0.239452738	-0.062662103	0.950035576	0.990168609	down_in_case
CRA004775	ENSGALG00010024170	NUP160	case	control	-0.458058447	0.202405221	-2.263076244	0.02363099	0.297845774	down_in_case
CRA004775	ENSGALG00010024172	ABCG4	case	control	0.85769829	0.710944386	1.206421074	0.227655174	0.694718454	up_in_case
CRA004775	ENSGALG00010024173	CBLN1	case	control	-0.425565495	0.740138793	-0.574980665	0.565304374	0.882950982	down_in_case
CRA004775	ENSGALG00010024174	PLEKHB2	case	control	0.128356855	0.252721153	0.507899136	0.61152408	0.901413878	up_in_case
CRA004775	ENSGALG00010024175	DHRS9	case	control	0.226479793	0.549117345	0.412443342	0.680014501	0.923896221	up_in_case
CRA004775	ENSGALG00010024177	ENSGALG00010024177	case	control	-0.680705862	1.034193838	-0.658199495	0.510409949	0.863170205	down_in_case
CRA004775	ENSGALG00010024178	N4BP1	case	control	0.40320465	0.229700267	1.755351247	0.079199246	0.491399489	up_in_case
CRA004775	ENSGALG00010024179	ATG4C	case	control	-0.156705066	0.209093645	-0.749449203	0.453586504	0.83804959	down_in_case
CRA004775	ENSGALG00010024180	HINFP	case	control	-0.291198897	0.256079435	-1.137142847	0.255478575	0.718871765	down_in_case
CRA004775	ENSGALG00010024181	FOXD3	case	control	0.968654618	0.680702991	1.423020951	0.154730082	0.619284425	up_in_case
CRA004775	ENSGALG00010024183	MTX2	case	control	-0.08303872	0.283022327	-0.293399893	0.769216507	0.945357282	down_in_case
CRA004775	ENSGALG00010024184	ENSGALG00010024184	case	control	0.293636947	0.553381784	0.530622719	0.595680249	0.895305942	up_in_case
CRA004775	ENSGALG00010024185	CHRNA5	case	control	0.224828177	0.409153339	0.549496131	0.582665019	0.889712708	up_in_case
CRA004775	ENSGALG00010024187	ENSGALG00010024187	case	control	0.667693769	0.917322024	0.72787282	0.466691443	0.8434807	up_in_case
CRA004775	ENSGALG00010024188	TSSC4	case	control	-0.191996961	0.275757237	-0.696253571	0.486270044	0.851808837	down_in_case
CRA004775	ENSGALG00010024189	KAT7	case	control	-0.174790218	0.173880257	-1.005233263	0.314784542	0.765098415	down_in_case
CRA004775	ENSGALG00010024190	WARS2	case	control	-0.340112233	0.281740405	-1.207183018	0.227361669	0.694715356	down_in_case
CRA004775	ENSGALG00010024191	DPYD	case	control	0.042368552	0.41556288	0.101954612	0.918792702	0.98189977	up_in_case
CRA004775	ENSGALG00010024192	ALG6	case	control	-0.382365526	0.446848737	-0.855693425	0.392167378	0.80746892	down_in_case
CRA004775	ENSGALG00010024193	GSTK1	case	control	-0.279722841	0.289075869	-0.967645075	0.333221658	0.773910112	down_in_case
CRA004775	ENSGALG00010024194	NKAIN4	case	control	0.461124326	0.383839949	1.201345318	0.229617278	0.695782399	up_in_case
CRA004775	ENSGALG00010024195	PSMA4	case	control	-0.107619166	0.221331672	-0.486234822	0.626800685	0.90758663	down_in_case
CRA004775	ENSGALG00010024196	MRPL21	case	control	0.18911213	0.291754713	0.648188776	0.51686286	0.864913871	up_in_case
CRA004775	ENSGALG00010024197	KRT7	case	control	0.442078649	0.661330987	0.668468071	0.503834856	0.861452528	up_in_case
CRA004775	ENSGALG00010024198	PI4KA	case	control	0.066586669	0.244011893	0.272882883	0.784943245	0.950244571	up_in_case
CRA004775	ENSGALG00010024199	SLC35B1	case	control	-0.29247717	0.169340314	-1.727156182	0.084139618	0.503263978	down_in_case
CRA004775	ENSGALG00010024201	IREB2	case	control	0.129974286	0.177550535	0.732041085	0.464143492	0.842643327	up_in_case
CRA004775	ENSGALG00010024203	SPOP	case	control	-0.130324116	0.144026063	-0.904864812	0.365537011	0.793608387	down_in_case
CRA004775	ENSGALG00010024204	PTBP2	case	control	0.057655352	0.280135767	0.205812178	0.836937629	0.965269623	up_in_case
CRA004775	ENSGALG00010024205	ANKRD13D	case	control	-0.300792732	0.324642112	-0.926536395	0.354167292	0.78730441	down_in_case
CRA004775	ENSGALG00010024206	KRT80	case	control	-2.746075282	1.104392035	-2.486504063	0.01290051	0.228267513	down_in_case
CRA004775	ENSGALG00010024207	KCTD18	case	control	-0.18188471	0.277488338	-0.655467942	0.51216652	0.863263816	down_in_case
CRA004775	ENSGALG00010024208	SERPIND1	case	control	0.897184288	0.497934152	1.80181312	0.071574813	0.471598108	up_in_case
CRA004775	ENSGALG00010024209	RWDD3	case	control	0.005209542	0.40776787	0.012775754	0.989806701	0.998065748	up_in_case
CRA004775	ENSGALG00010024211	TRPM5	case	control	2.656053704	0.709026509	3.746056982	0.000179636	0.027735809	up_in_case
CRA004775	ENSGALG00010024212	RIMS4	case	control	-0.11240817	0.567412951	-0.198106458	0.842961772	0.966478468	down_in_case
CRA004775	ENSGALG00010024213	ENSGALG00010024213	case	control	0.624176662	0.424068278	1.471877749	0.141053893	0.600619496	up_in_case
CRA004775	ENSGALG00010024215	ENSGALG00010024215	case	control	-0.70057496	0.686165969	-1.020999279	0.30725478	0.758947455	down_in_case
CRA004775	ENSGALG00010024218	NGFR	case	control	-0.305556594	0.492638069	-0.620245598	0.535096106	0.870981055	down_in_case
CRA004775	ENSGALG00010024220	SIAH1	case	control	0.833215306	0.355850722	2.34147426	0.01920775	0.273705467	up_in_case
CRA004775	ENSGALG00010024222	ENSGALG00010024222	case	control	-0.48748362	0.404182906	-1.206096579	0.227780254	0.694718454	down_in_case
CRA004775	ENSGALG00010024223	ENSGALG00010024223	case	control	0.253398651	0.79365985	0.319278657	0.749515216	0.941495694	up_in_case
CRA004775	ENSGALG00010024225	BCDIN3D	case	control	0.026291649	0.298164571	0.088178313	0.929734954	0.98473447	up_in_case
CRA004775	ENSGALG00010024226	POU2AF1	case	control	1.270426607	0.782391451	1.623773631			up_in_case
CRA004775	ENSGALG00010024230	LONP2	case	control	-0.169764226	0.309155256	-0.549122885	0.582921121	0.889748955	down_in_case
CRA004775	ENSGALG00010024233	FAIM2	case	control	1.113342123	0.710926278	1.566044408	0.117338221	0.567740281	up_in_case
CRA004775	ENSGALG00010024234	ENSGALG00010024234	case	control	0.183375543	0.386933424	0.47392014	0.635556857	0.909837348	up_in_case
CRA004775	ENSGALG00010024238	ATF7	case	control	-0.129330192	0.254117391	-0.508938769	0.610795144	0.901413878	down_in_case
CRA004775	ENSGALG00010024240	WDR61	case	control	-0.354673844	0.180807415	-1.961611163	0.049807772	0.413341381	down_in_case
CRA004775	ENSGALG00010024242	KMT2A	case	control	0.043862072	0.414863095	0.105726618	0.915799261	0.981626545	up_in_case
CRA004775	ENSGALG00010024243	MPP3	case	control	-0.226697943	0.374859277	-0.60475479	0.545341937	0.876563448	down_in_case
CRA004775	ENSGALG00010024245	TARBP2	case	control	-0.146568465	0.366080037	-0.400372732	0.688882005	0.927420456	down_in_case
CRA004775	ENSGALG00010024250	AP2A2	case	control	-0.180209191	0.223442225	-0.806513588	0.419946776	0.819388688	down_in_case
CRA004775	ENSGALG00010024251	ENSGALG00010024251	case	control	0.281601563	0.467174041	0.602776564	0.54665734	0.877099134	up_in_case
CRA004775	ENSGALG00010024254	ENSGALG00010024254	case	control	-0.847195249	0.465302976	-1.820738942	0.06864655	0.46645716	down_in_case
CRA004775	ENSGALG00010024255	CFAP97D1	case	control	-1.675982247	0.7623675	-2.19839152	0.027921217	0.318626275	down_in_case
CRA004775	ENSGALG00010024258	DNAJA4	case	control	1.074732682	0.451023557	2.382874832	0.017178034	0.259292146	up_in_case
CRA004775	ENSGALG00010024259	ENSGALG00010024259	case	control	0.032634776	0.358660035	0.090990836	0.927499873	0.984564671	up_in_case
CRA004775	ENSGALG00010024263	PHKB	case	control	0.375560299	0.481785832	0.779517108	0.435675164	0.826905159	up_in_case
CRA004775	ENSGALG00010024264	AMER3	case	control	1.201681229	0.536557092	2.239614846	0.025115938	0.304882883	up_in_case
CRA004775	ENSGALG00010024266	ENSGALG00010024266	case	control	-0.145717625	0.536845125	-0.271433265	0.78605782	0.95041797	down_in_case
CRA004775	ENSGALG00010024267	MAP3K12	case	control	0.100746227	0.287119176	0.350886446	0.72567354	0.935238044	up_in_case
CRA004775	ENSGALG00010024270	RAB18L	case	control	-1.477258725	0.563606979	-2.621079547	0.008765181	0.189215071	down_in_case
CRA004775	ENSGALG00010024274	ACSBG1	case	control	0.567937398	0.303985017	1.86830721	0.061719262	0.446686038	up_in_case
CRA004775	ENSGALG00010024277	ITGB3BP	case	control	-0.325314436	0.586720776	-0.554462105	0.579262633	0.888378369	down_in_case
CRA004775	ENSGALG00010024280	ENSGALG00010024280	case	control	-0.111051088	0.385913772	-0.287761402	0.773529385	0.946296525	down_in_case
CRA004775	ENSGALG00010024282	HIC2	case	control	-0.353246865	0.418551644	-0.84397438	0.398683722	0.810039516	down_in_case
CRA004775	ENSGALG00010024283	UBE4A	case	control	-0.274421622	0.19009713	-1.443586353	0.148855366	0.612367147	down_in_case
CRA004775	ENSGALG00010024285	CCND2	case	control	-0.165958711	0.221923852	-0.747818271	0.45456978	0.838254622	down_in_case
CRA004775	ENSGALG00010024286	ADA	case	control	0.199577919	0.186804466	1.068378733	0.285349706	0.741413527	up_in_case
CRA004775	ENSGALG00010024288	CHID1	case	control	0.154272911	0.34991026	0.44089279	0.659290614	0.917907174	up_in_case
CRA004775	ENSGALG00010024290	SOST	case	control	-1.90636736	0.849204169	-2.244886953	0.024775391	0.302344474	down_in_case
CRA004775	ENSGALG00010024291	ENSGALG00010024291	case	control	-0.914259002	0.652644695	-1.400852577	0.161258162	0.627121149	down_in_case
CRA004775	ENSGALG00010024300	MEOX1	case	control	0.249545041	0.712719571	0.350130755	0.726240571	0.935238044	up_in_case
CRA004775	ENSGALG00010024304	PKIG	case	control	0.161464559	0.23373407	0.690804548	0.489688377	0.854639532	up_in_case
CRA004775	ENSGALG00010024305	ENSGALG00010024305	case	control	-0.948980836	0.760342153	-1.2480971	0.2119955	0.682275341	down_in_case
CRA004775	ENSGALG00010024309	ABCB11	case	control	0.729849664	0.458252169	1.592681306	0.111231703	0.560478986	up_in_case
CRA004775	ENSGALG00010024311	KLHL6	case	control	-0.115492409	0.502403103	-0.229879967	0.818185043	0.959889304	down_in_case
CRA004775	ENSGALG00010024313	SERINC3	case	control	-0.184187754	0.160918116	-1.144605464	0.252372628	0.717019836	down_in_case
CRA004775	ENSGALG00010024314	ITFG1	case	control	0.3794183	0.266811376	1.422046935	0.155012626	0.619284425	up_in_case
CRA004775	ENSGALG00010024321	UBE2L3	case	control	-0.01663699	0.198756082	-0.083705565	0.933290532	0.985090297	down_in_case
CRA004775	ENSGALG00010024322	IDH3A	case	control	0.120377198	0.330766873	0.363933658	0.71590755	0.933921336	up_in_case
CRA004775	ENSGALG00010024323	TTPAL	case	control	-0.533100827	0.256528541	-2.078134561	0.037696967	0.365870038	down_in_case
CRA004775	ENSGALG00010024325	PCBP2	case	control	-0.185555316	0.321121837	-0.577834624	0.563375777	0.881641096	down_in_case
CRA004775	ENSGALG00010024326	KLHL24	case	control	0.607558418	0.486762599	1.248161669	0.211971858	0.682275341	up_in_case
CRA004775	ENSGALG00010024327	PPP1R1B	case	control	-0.538337246	0.638553175	-0.843057818	0.399196108	0.810714176	down_in_case
CRA004775	ENSGALG00010024328	YDJC	case	control	-0.145561744	0.280464647	-0.519002111	0.603759269	0.898692785	down_in_case
CRA004775	ENSGALG00010024330	SDF2L1	case	control	-0.287077408	0.365949344	-0.784473077	0.432762574	0.825642959	down_in_case
CRA004775	ENSGALG00010024332	EFCAB7	case	control	0.151709318	0.264387259	0.573814784	0.566093143	0.883033678	up_in_case
CRA004775	ENSGALG00010024333	ETV4	case	control	0.28611244	0.445032498	0.642902353	0.520287463	0.865611895	up_in_case
CRA004775	ENSGALG00010024334	USP28	case	control	-0.154993831	0.383072763	-0.404606764	0.685766586	0.926686318	down_in_case
CRA004775	ENSGALG00010024335	AMHR2	case	control	-0.289424212	0.731016942	-0.395919979	0.692164062	0.927867527	down_in_case
CRA004775	ENSGALG00010024336	TSPAN4	case	control	0.065238795	0.312360296	0.208857513	0.834559472	0.96420999	up_in_case
CRA004775	ENSGALG00010024338	NDUFA9	case	control	-0.012355454	0.291013095	-0.042456694	0.966134634	0.993094598	down_in_case
CRA004775	ENSGALG00010024339	TOP3B	case	control	-0.065963585	0.196868422	-0.335064325	0.737576563	0.93763662	down_in_case
CRA004775	ENSGALG00010024340	NETO2	case	control	0.923615265	0.503872479	1.83303376	0.066797551	0.462498477	up_in_case
CRA004775	ENSGALG00010024342	HNF4A	case	control	2.531889739	0.738661826	3.427671025	0.000608783	0.051333788	up_in_case
CRA004775	ENSGALG00010024343	POLR2L	case	control	-0.228399462	0.342112545	-0.667614987	0.504379388	0.861471523	down_in_case
CRA004775	ENSGALG00010024344	YEATS2	case	control	-0.006204698	0.252390539	-0.02458372	0.980387005	0.996980737	down_in_case
CRA004775	ENSGALG00010024345	STARD3	case	control	0.030610171	0.246873912	0.123991111	0.901322313	0.979157637	up_in_case
CRA004775	ENSGALG00010024346	PPM1F	case	control	-0.388491948	0.454703827	-0.854384603	0.392891924	0.807553294	down_in_case
CRA004775	ENSGALG00010024347	OTOG	case	control	-0.14407959	1.036926233	-0.138948737	0.889490658	0.977162915	down_in_case
CRA004775	ENSGALG00010024349	R3HDML	case	control	-0.795240566	1.344018954	-0.591688506			down_in_case
CRA004775	ENSGALG00010024350	PGM1	case	control	0.07953891	0.600899339	0.132366446	0.894694454	0.977834811	up_in_case
CRA004775	ENSGALG00010024351	SP1	case	control	0.222123837	0.253965861	0.874620851	0.381780239	0.801753951	up_in_case
CRA004775	ENSGALG00010024352	MAP6D1	case	control	-0.153425185	0.765218591	-0.200498506	0.841090726	0.966461921	down_in_case
CRA004775	ENSGALG00010024354	C2CD2L	case	control	-0.236880182	0.224010951	-1.057449118	0.290306665	0.744282131	down_in_case
CRA004775	ENSGALG00010024356	PARL	case	control	-0.115849932	0.281859394	-0.411020298	0.681057651	0.923896221	down_in_case
CRA004775	ENSGALG00010024358	MAPK3	case	control	0.023000177	0.1967064	0.116926427	0.906918356	0.980273095	up_in_case
CRA004775	ENSGALG00010024360	FTL	case	control	-0.40756892	0.292488386	-1.393453344	0.163482744	0.629717526	down_in_case
CRA004775	ENSGALG00010024361	H2AX	case	control	-0.623852832	0.62307064	-1.001255383	0.316703357	0.765914152	down_in_case
CRA004775	ENSGALG00010024362	DHX8	case	control	-0.247060178	0.196019021	-1.260388792	0.207529143	0.676400389	down_in_case
CRA004775	ENSGALG00010024363	EDEM2	case	control	-0.34571847	0.18413888	-1.877487632	0.060451291	0.445397937	down_in_case
CRA004775	ENSGALG00010024365	ENSGALG00010024365	case	control	-0.31327195	0.253820647	-1.234225637	0.217118837	0.684537009	down_in_case
CRA004775	ENSGALG00010024366	PARD6A	case	control	-0.294448064	0.527675949	-0.558009257	0.576838063	0.88797455	down_in_case
CRA004775	ENSGALG00010024367	ENSGALG00010024367	case	control	-0.092055772	0.563141934	-0.163468154	0.870149845	0.973907742	down_in_case

CRA004775	ENSGALG00010024368	DPAGT1	case	control	0.099815258	0.129596445	0.770200581	0.441180919	0.829353567	up_in_case
CRA004775	ENSGALG00010024369	MYOD1	case	control	1.154753693	0.676459538	1.707055084	0.087811814	0.512706568	up_in_case
CRA004775	ENSGALG00010024370	HERPUD1	case	control	-0.550829156	0.300701791	-1.831812022	0.066979431	0.462498477	down_in_case
CRA004775	ENSGALG00010024371	YPEL1	case	control	0.123509389	0.307401257	0.40178557	0.687841843	0.927111919	up_in_case
CRA004775	ENSGALG00010024373	TCAP	case	control	0.493287338	0.800662963	0.616098609	0.53782944	0.872603995	up_in_case
CRA004775	ENSGALG00010024374	ENSGALG00010024374	case	control	0.433000265	0.703092171	0.615851353	0.537992631	0.872603995	up_in_case
CRA004775	ENSGALG00010024377	CIB2	case	control	-0.702464667	0.424762908	-1.653780625	0.098172094	0.533793778	down_in_case
CRA004775	ENSGALG00010024378	ZDHHC1	case	control	0.039779537	0.299580184	0.132784274	0.894363991	0.977834811	up_in_case
CRA004775	ENSGALG00010024380	PNMT	case	control	0.905993654	0.540987513	1.674703449	0.09399243	0.526235729	up_in_case
CRA004775	ENSGALG00010024382	ENSGALG00010024382	case	control	0.227987686	0.488269722	0.466929805	0.640550101	0.911724439	up_in_case
CRA004775	ENSGALG00010024383	DUSP28	case	control	0.06261961	0.533106664	0.11746169	0.906494201	0.980055425	up_in_case
CRA004775	ENSGALG00010024386	TPPP3	case	control	-0.343083165	0.636653792	-0.538884978	0.589966223	0.893756256	down_in_case
CRA004775	ENSGALG00010024387	PPIL2	case	control	-0.058252963	0.18612667	-0.312974829	0.754299786	0.941900693	down_in_case
CRA004775	ENSGALG00010024389	AAAS	case	control	-0.28357152	0.33492632	-0.846668365	0.397179996	0.808998414	down_in_case
CRA004775	ENSGALG00010024391	TBC1D2B	case	control	-0.707634341	0.443276188	-1.59637346	0.110405438	0.558567026	down_in_case
CRA004775	ENSGALG00010024392	CHMP4B	case	control	0.240175301	0.192632872	1.246803306	0.212469628	0.682275341	up_in_case
CRA004775	ENSGALG00010024393	OGFOD1	case	control	-0.379011709	0.228094329	-1.661644598	0.096584056	0.530629094	down_in_case
CRA004775	ENSGALG00010024396	ENSGALG00010024396	case	control	-0.468724836	0.805680858	-0.581774819	0.560718367	0.881008373	down_in_case
CRA004775	ENSGALG00010024398	PHB	case	control	-0.074326991	0.230706246	-0.322171558	0.74732273	0.940417372	down_in_case
CRA004775	ENSGALG00010024399	ROR1	case	control	0.965245619	0.371544747	2.597925629	0.00937888	0.195703177	up_in_case
CRA004775	ENSGALG00010024401	KCNC1	case	control	0.346194612	0.461400957	0.750311862	0.4530669	0.837607279	up_in_case
CRA004775	ENSGALG00010024402	GPC1	case	control	0.192243259	0.281665305	0.682523745	0.494907834	0.856382445	up_in_case
CRA004775	ENSGALG00010024403	HMBS	case	control	-0.553217761	0.293276933	-1.886332333	0.059250185	0.44414236	down_in_case
CRA004775	ENSGALG00010024406	NUDT21	case	control	-0.327982189	0.195709898	-1.675858972	0.09376582	0.525845753	down_in_case
CRA004775	ENSGALG00010024407	HIRA	case	control	-0.136183429	0.220774224	-0.616844786	0.537337108	0.872603995	down_in_case
CRA004775	ENSGALG00010024410	MYG1	case	control	-0.376583471	0.280639293	-1.341877208	0.179635813	0.644990528	down_in_case
CRA004775	ENSGALG00010024412	PIGU	case	control	-0.62691443	0.170551729	-3.675802261	0.000237103	0.031051477	down_in_case
CRA004775	ENSGALG00010024413	AMFR	case	control	-0.248139389	0.256770243	-0.966386861	0.333850636	0.774300343	down_in_case
CRA004775	ENSGALG00010024415	SIK2	case	control	-0.023884762	0.240320712	-0.099387032	0.920830979	0.982481112	down_in_case
CRA004775	ENSGALG00010024416	VDR	case	control	-0.315645041	0.360879645	-0.874654601	0.381761869	0.801753951	down_in_case
CRA004775	ENSGALG00010024419	PGAP3	case	control	-0.489964638	0.197080941	-2.486108685	0.012914852	0.228267513	down_in_case
CRA004775	ENSGALG00010024420	TMEM106C	case	control	-0.702040492	0.255126497	-2.751734929	0.005928048	0.154441997	down_in_case
CRA004775	ENSGALG00010024421	DAPK2	case	control	0.304428187	0.507992042	0.599277471	0.548987869	0.877623839	up_in_case
CRA004775	ENSGALG00010024423	SERGEF	case	control	-0.183261439	0.27572321	-0.664657282	0.506269722	0.8622397	down_in_case
CRA004775	ENSGALG00010024424	SAMHD1	case	control	0.089943825	0.271800928	0.330918018	0.740706412	0.938450627	up_in_case
CRA004775	ENSGALG00010024425	MRPL40	case	control	-0.055208324	0.236529016	-0.233410365	0.815442765	0.958870945	down_in_case
CRA004775	ENSGALG00010024426	PHAF1	case	control	-0.05310899	0.356100377	-0.149140506	0.881442764	0.975011509	down_in_case
CRA004775	ENSGALG00010024427	C22orf39	case	control	-0.350409538	0.320931307	-1.091852151	0.274898092	0.731001618	down_in_case
CRA004775	ENSGALG00010024428	SAAL1	case	control	-0.25021949	0.215787251	-1.159565674	0.246225683	0.712046495	down_in_case
CRA004775	ENSGALG00010024430	ZNF652	case	control	0.151645071	0.141804256	1.069397175	0.28489074	0.74120185	up_in_case
CRA004775	ENSGALG00010024431	FEZ1	case	control	-0.143798993	0.30600891	-0.469917665	0.638413843	0.91099914	down_in_case
CRA004775	ENSGALG00010024433	ENSGALG00010024433	case	control	-0.325231538	0.404727026	-0.803582456	0.421638158	0.819812626	down_in_case
CRA004775	ENSGALG00010024434	B3GNT9	case	control	-0.086235025	0.381195121	-0.226222792	0.821028145	0.960766569	down_in_case
CRA004775	ENSGALG00010024437	HERC1	case	control	0.053958254	0.326719807	0.165151461	0.868824769	0.973370973	up_in_case
CRA004775	ENSGALG00010024438	RBL1	case	control	-0.693759572	0.505079861	-1.373564112	0.169577045	0.633147466	down_in_case
CRA004775	ENSGALG00010024439	UFD1	case	control	-0.130070183	0.336789943	-0.386205663	0.69934436	0.929347461	down_in_case
CRA004775	ENSGALG00010024440	PKNOX2	case	control	-0.390845507	0.462872774	-0.84439079	0.398451067	0.809918791	down_in_case
CRA004775	ENSGALG00010024441	SAA	case	control	-1.267925395	0.526265897	-2.409286639	0.015983739	0.252634935	down_in_case
CRA004775	ENSGALG00010024446	GRM4	case	control	0.646393658	0.830236165	0.77856601	0.436235408	0.82694408	up_in_case
CRA004775	ENSGALG00010024449	CACHD1	case	control	0.099983548	0.209388343	0.477502932	0.633004034	0.909723668	up_in_case
CRA004775	ENSGALG00010024451	PHOSPHO1	case	control	-0.239754571	0.257286566	-0.931858102	0.351409863	0.786113067	down_in_case
CRA004775	ENSGALG00010024452	SLC16A1	case	control	0.067417579	0.302602927	0.222792225	0.823697219	0.960898591	up_in_case
CRA004775	ENSGALG00010024453	HTR3A	case	control	-1.207913949	0.701091083	-1.72290588	0.084905547	0.505426915	down_in_case
CRA004775	ENSGALG00010024454	HPS5	case	control	-0.082231521	0.29415057	-0.279555879	0.779818261	0.948291556	down_in_case
CRA004775	ENSGALG00010024457	CHD6	case	control	-0.088211499	0.26509845	-0.332749961	0.739323033	0.938064864	down_in_case
CRA004775	ENSGALG00010024459	AHCYL1	case	control	-0.102651342	0.188764232	-0.543807163	0.586574179	0.891564224	down_in_case
CRA004775	ENSGALG00010024460	IMP4	case	control	-0.274079789	0.260558273	-1.051894403	0.292848	0.746438942	down_in_case
CRA004775	ENSGALG00010024462	ZBTB32	case	control	-0.181111032	0.603894396	-0.299905137	0.764249516	0.94239774	down_in_case
CRA004775	ENSGALG00010024463	FBXL22	case	control	-0.426303623	0.434031322	-0.982195526	0.326003531	0.770998457	down_in_case
CRA004775	ENSGALG00010024465	SENP1	case	control	-0.198628834	0.203351497	-0.976775862	0.328680128	0.77220987	down_in_case
CRA004775	ENSGALG00010024466	ADGRG3	case	control	-1.332202481	0.47556162	-2.801324636	0.005089329	0.143998699	down_in_case
CRA004775	ENSGALG00010024467	ENSGALG00010024467	case	control	-0.105619599	0.349181724	-0.302477455	0.762288117	0.94239774	down_in_case
CRA004775	ENSGALG00010024469	GTF2H1	case	control	0.086667244	0.268730181	0.322506553	0.747068974	0.940401452	up_in_case
CRA004775	ENSGALG00010024471	CCDC61	case	control	-0.279623077	0.226409699	-1.235031358	0.216818834	0.684403275	down_in_case
CRA004775	ENSGALG00010024472	LDHA	case	control	0.204518071	0.652049558	0.313654182	0.753783703	0.941900693	up_in_case
CRA004775	ENSGALG00010024473	RAVER2	case	control	-0.177092842	0.188659161	-0.938691985	0.34788891	0.785270866	down_in_case
CRA004775	ENSGALG00010024474	STRIP1	case	control	-0.532118735	0.215033363	-2.474586861	0.013339038	0.232183685	down_in_case
CRA004775	ENSGALG00010024475	ADGRG1	case	control	0.227793849	0.353096043	0.645132828	0.518841111	0.865435399	up_in_case
CRA004775	ENSGALG00010024476	KCNB1	case	control	-0.386921924	0.736599779	-0.525281076	0.599387811	0.897206546	down_in_case
CRA004775	ENSGALG00010024479	ERBB2	case	control	-0.193240757	0.351612086	-0.549585081	0.582603994	0.889712708	down_in_case
CRA004775	ENSGALG00010024480	CDC45	case	control	0.673775961	0.431164794	1.562687795	0.118126047	0.569418751	up_in_case
CRA004775	ENSGALG00010024482	USP3	case	control	0.074195473	0.305232249	0.243078748	0.807944394	0.955397873	up_in_case
CRA004775	ENSGALG00010024486	GPR37L1	case	control	1.226646688	0.46819531	2.619946553	0.008794355	0.189418079	up_in_case
CRA004775	ENSGALG00010024488	ADGRG5	case	control	-0.61050747	0.331107606	-1.843834027	0.065207332	0.457949961	down_in_case
CRA004775	ENSGALG00010024490	TGM3	case	control	-0.045958692	0.614109847	-0.074837901	0.940343686	0.987076872	down_in_case
CRA004775	ENSGALG00010024493	JAK1	case	control	0.26209066	0.237270136	1.104608716	0.269329173	0.72765348	up_in_case
CRA004775	ENSGALG00010024494	DDAH1	case	control	0.380961056	0.538602998	0.707313285	0.479371812	0.847280435	up_in_case
CRA004775	ENSGALG00010024495	CLDN5	case	control	0.691698054	0.404400582	1.710427938	0.087186768	0.510824565	up_in_case
CRA004775	ENSGALG00010024496	UEVLD	case	control	1.172111778	0.357458851	3.279011768	0.001041713	0.067571071	up_in_case
CRA004775	ENSGALG00010024497	ABI3	case	control	0.480378034	0.391142673	1.228140182	0.219394349	0.687020074	up_in_case
CRA004775	ENSGALG00010024498	CCDC102A	case	control	-0.098394536	0.247687023	-0.397253498	0.691180535	0.927867527	down_in_case
CRA004775	ENSGALG00010024499	CA12	case	control	-1.788167569	0.854392428	-2.092911303	0.036357073	0.360628495	down_in_case
CRA004775	ENSGALG00010024500	GNGT2	case	control	-0.065180795	0.323492274	-0.201491042	0.840314633	0.966461921	down_in_case
CRA004775	ENSGALG00010024501	ARL8A	case	control	-0.085871629	0.210094106	-0.40872936	0.682738285	0.924685724	down_in_case
CRA004775	ENSGALG00010024502	VPS8	case	control	-0.119604948	0.23424461	-0.510598508	0.609632221	0.901246133	down_in_case
CRA004775	ENSGALG00010024504	CASC3	case	control	-0.05230723	0.254310052	-0.205682903	0.837038614	0.965269623	down_in_case
CRA004775	ENSGALG00010024505	DNASE2B	case	control	-0.999479524	0.5919531	-1.68844377	0.091326074	0.521441551	down_in_case
CRA004775	ENSGALG00010024506	RAB8B	case	control	-0.076185607	0.179164852	-0.425226299	0.670671714	0.921535334	down_in_case
CRA004775	ENSGALG00010024508	RPS27L	case	control	0.214975651	0.377601841	0.569318332	0.569140128	0.884809383	up_in_case
CRA004775	ENSGALG00010024509	SEPTIN5	case	control	-0.316331951	0.376917902	-0.839259557	0.401323673	0.811868062	down_in_case
CRA004775	ENSGALG00010024510	CNN3	case	control	0.303086464	0.222758061	1.36060829	0.17363751	0.63910316	up_in_case
CRA004775	ENSGALG00010024511	ENSGALG00010024511	case	control	-0.462647725	0.583520632	-0.792855813	0.427861834	0.822830776	down_in_case
CRA004775	ENSGALG00010024512	CCNDBP1	case	control	-0.366170253	0.270918786	-1.351586792	0.176507538	0.641934714	down_in_case
CRA004775	ENSGALG00010024513	SPTY2D1	case	control	0.171556941	0.285149634	0.6016383	0.547414929	0.877277621	up_in_case
CRA004775	ENSGALG00010024514	FAF1	case	control	-0.038509049	0.233274411	-0.165080471	0.868880644	0.973370973	down_in_case
CRA004775	ENSGALG00010024515	C3orf70	case	control	0.050876988	0.426565899	0.119271109	0.905060572	0.979544699	up_in_case
CRA004775	ENSGALG00010024516	EHHADH	case	control	0.034746297	0.326511136	0.106416881	0.9152516	0.981469218	up_in_case
CRA004775	ENSGALG00010024517	LACTB	case	control	0.20820332	0.185863382	1.120195481	0.262630469	0.723367482	up_in_case
CRA004775	ENSGALG00010024519	MIEN1	case	control	-0.239529233	0.261912646	-0.914538632	0.360433888	0.791975934	down_in_case
CRA004775	ENSGALG00010024520	MAP3K13	case	control	0.065557214	0.28939568	0.226531418	0.820788126	0.960766569	up_in_case
CRA004775	ENSGALG00010024521	HEPACAM	case	control	1.135981814	0.470067826	2.416633838	0.015664766	0.250472326	up_in_case
CRA004775	ENSGALG00010024522	DNTTIP2	case	control	0.071904673	0.196346754	0.366212689	0.714206374	0.93334033	up_in_case
CRA004775	ENSGALG00010024523	TOX3	case	control	1.055735403	0.696536024	1.515693899	0.129596771	0.584172159	up_in_case
CRA004775	ENSGALG00010024524	TMEM41A	case	control	-0.138598899	0.36245214	-0.382392276	0.702170419	0.930068722	down_in_case
CRA004775	ENSGALG00010024525	GP1BB	case	control	-0.307072782	0.446857751	-0.687182401	0.491967793	0.855746361	down_in_case
CRA004775	ENSGALG00010024527	ATP6V0B	case	control	-0.577555607	0.249662251	-2.313347756	0.020703527	0.283842672	down_in_case
CRA004775	ENSGALG00010024528	TBX1	case	control	-0.921500772	0.365512145	-2.521122169	0.011698123	0.217673733	down_in_case
CRA004775	ENSGALG00010024529	GRB7	case	control	2.384414175	0.864876215	2.756942709	0.005834457	0.154441997	up_in_case
CRA004775	ENSGALG00010024530	ENSGALG00010024530	case	control	-0.248098311	1.021855218	-0.242792038	0.808166504	0.955397873	down_in_case
CRA004775	ENSGALG00010024531	CEP63	case	control	-0.463158078	0.321117591	-1.442331693	0.149208823	0.61265447	down_in_case
CRA004775	ENSGALG00010024532	TMEM86A	case	control	0.395745988	0.723590475	0.546919842	0.584433803	0.89025771	up_in_case
CRA004775	ENSGALG00010024533	CDKN2C	case	control	-0.382484235	0.280306187	-1.364522984	0.172403026	0.63702643	down_in_case
CRA004775	ENSGALG00010024534	VWF	case	control	0.468903809	0.460946726	1.017262479	0.309028581	0.761414086	up_in_case
CRA004775	ENSGALG00010024535	ENSGALG00010024535	case	control	-0.031657205	0.371734591	-0.085160772	0.932133576	0.985044263	down_in_case
CRA004775	ENSGALG00010024536	URI1	case	control	0.130164228	0.123623884	1.052905187	0.292384449	0.746262643	up_in_case
CRA004775	ENSGALG00010024537	EPB42	case	control	-0.58379084	0.448019779	-1.303047025	0.192558708	0.656993806	down_in_case
CRA004775	ENSGALG00010024539	RAPGEFL1	case	control	-0.1178678	0.492037579	-0.239550402	0.810678819	0.956879351	down_in_case
CRA004775	ENSGALG00010024541	WIPF2	case	control	0.159744282	0.22230331	0.718587061	0.472395388	0.845425322	up_in_case
CRA004775	ENSGALG00010024542	ZNFX1	case	control	-0.322855209	0.445349495	-0.724947963	0.468483955	0.8440023	down_in_case
CRA004775	ENSGALG00010024544	ZNF536	case	control	1.424990984	0.633021773	2.251093159	0.024379638	0.30145607	up_in_case
CRA004775	ENSGALG00010024545	PHACTR3	case	control	-0.051969103	0.67394236	-0.077112089	0.938534376	0.986707289	down_in_case
CRA004775	ENSGALG00010024546	KY	case	control	0.377990803	1.045268212	0.361620872	0.717635365	0.934011578	up_in_case
CRA004775	ENSGALG00010024547	PFKM	case	control	0.064280631	0.714629557	0.089949584	0.928327279	0.98473447	up_in_case
CRA004775	ENSGALG00010024548	CDC6	case	control	-0.502002988	0.560820893	-0.895121767	0.370721999	0.795641647	down_in_case
CRA004775	ENSGALG00010024549	GNB1L	case	control	-0.012620594	0.172131351	-0.073319552	0.941551833	0.987577169	down_in_case
CRA004775	ENSGALG00010024551	RNF11	case	control	0.074461299	0.204317993	0.364438283	0.715530752	0.933870859	up_in_case

CRA004775	ENSGALG00010024552	CD81	case	control	-0.156219298	0.175622066	-0.889519758	0.373723809	0.797250404	down_in_case
CRA004775	ENSGALG00010024553	ASB8	case	control	-0.487204856	0.28187062	-1.728469808	0.083904029	0.503010645	down_in_case
CRA004775	ENSGALG00010024554	ST6GALNAC3	case	control	-0.440914147	0.703033611	-0.627159414	0.530554744	0.869211096	down_in_case
CRA004775	ENSGALG00010024555	EPHB1	case	control	0.222168501	0.335322344	0.662552034	0.507617499	0.862748056	up_in_case
CRA004775	ENSGALG00010024556	ENSGALG00010024556	case	control	0.08677827	0.413793194	0.209714108	0.833890814	0.964019688	up_in_case
CRA004775	ENSGALG00010024557	TSPAN32	case	control	-0.151497659	0.566103447	-0.267614797	0.78899584	0.95041797	down_in_case
CRA004775	ENSGALG00010024558	PFDN5	case	control	-0.349206292	0.346431081	-1.008010861	0.313449248	0.765098415	down_in_case
CRA004775	ENSGALG00010024561	ROBO3	case	control	-0.293075202	0.546958714	-0.535826918	0.592078176	0.893804699	down_in_case
CRA004775	ENSGALG00010024563	ESPL1	case	control	-0.513320776	0.454448434	-1.129546803	0.258667238	0.720037471	down_in_case
CRA004775	ENSGALG00010024564	PPP1R3D	case	control	0.045653174	0.447782278	0.101953955	0.918793223	0.98189977	up_in_case
CRA004775	ENSGALG00010024565	FAM217B	case	control	0.181317596	0.161091366	1.125557508	0.260352862	0.721313541	up_in_case
CRA004775	ENSGALG00010024566	MTSS2	case	control	-0.246523693	0.381696428	-0.645863243	0.518367926	0.865435399	down_in_case
CRA004775	ENSGALG00010024567	IKZF3	case	control	-0.072252553	0.516449258	-0.13990252	0.88873701	0.977118922	down_in_case
CRA004775	ENSGALG00010024568	DZIP1L	case	control	0.291811109	0.445179622	0.655490716	0.512151862	0.863263816	up_in_case
CRA004775	ENSGALG00010024569	ASCL2	case	control	-0.089301918	0.497297232	-0.179574532	0.8574866	0.970943344	down_in_case
CRA004775	ENSGALG00010024570	TPM1	case	control	0.039635996	0.639219304	0.062006883	0.950557351	0.990168609	up_in_case
CRA004775	ENSGALG00010024571	KCNA3	case	control	-0.400118734	0.496070303	-0.806576672	0.419910419	0.819388688	down_in_case
CRA004775	ENSGALG00010024573	ENSGALG00010024573	case	control	-0.658696032	0.422118142	-1.56045421	0.118652583	0.570664629	down_in_case
CRA004775	ENSGALG00010024575	TXNRD2	case	control	-0.516669381	0.270380232	-1.910899245	0.05601753	0.433172413	down_in_case
CRA004775	ENSGALG00010024576	CSAD	case	control	-0.298503145	0.312158179	-0.956256043	0.338942892	0.779398613	down_in_case
CRA004775	ENSGALG00010024578	ENSGALG00010024578	case	control	-0.985799542	0.6223806	-1.583917528	0.113212488	0.562886277	down_in_case
CRA004775	ENSGALG00010024579	ZNF740	case	control	-0.238316383	0.20848805	-1.143069749	0.253009633	0.717347518	down_in_case
CRA004775	ENSGALG00010024580	IGF2	case	control	-0.275095761	0.368762654	-0.745996802	0.455669347	0.839192403	down_in_case
CRA004775	ENSGALG00010024581	RARA	case	control	-0.089220782	0.268634772	-0.332126705	0.739793586	0.938064864	down_in_case
CRA004775	ENSGALG00010024582	TLN2	case	control	0.620019093	0.369800492	1.676631335	0.093614595	0.525355698	up_in_case
CRA004775	ENSGALG00010024583	ITGB7	case	control	-0.480737202	0.639103077	-0.752206051	0.451927155	0.836512358	down_in_case
CRA004775	ENSGALG00010024585	TBCCD1	case	control	-0.656852623	0.273333421	-2.403118585	0.016255915	0.25344686	down_in_case
CRA004775	ENSGALG00010024586	DLGAP4	case	control	0.226240683	0.198986013	1.136967769	0.25555176	0.718871765	up_in_case
CRA004775	ENSGALG00010024587	ENSGALG00010024587	case	control	0.022625873	0.257639138	0.087820015	0.930019729	0.984882657	up_in_case
CRA004775	ENSGALG00010024588	TTC39A	case	control	0.898732764	0.615490235	1.460190127	0.144237828	0.606297475	up_in_case
CRA004775	ENSGALG00010024589	ADORA1	case	control	0.735717241	0.401989466	1.83019035	0.06722148	0.462875601	up_in_case
CRA004775	ENSGALG00010024590	MRPL23	case	control	-0.114214745	0.241644462	-0.472656164	0.636458506	0.910143606	down_in_case
CRA004775	ENSGALG00010024592	COMT	case	control	-0.513388269	0.331107171	-1.550519935	0.121016772	0.573473028	down_in_case
CRA004775	ENSGALG00010024593	SRSF6	case	control	-0.299000133	0.166713952	-1.793491961	0.072894295	0.476411335	down_in_case
CRA004775	ENSGALG00010024594	TNNI2	case	control	0.083186438	0.757906266	0.109758214	0.912601131	0.980787663	up_in_case
CRA004775	ENSGALG00010024595	MYBPHL	case	control	-0.078468891	0.712301951	-0.110162398	0.912280583	0.98075825	down_in_case
CRA004775	ENSGALG00010024596	EPS15	case	control	-0.266511192	0.237775494	-1.120852226	0.262350769	0.723230258	down_in_case
CRA004775	ENSGALG00010024597	DNAJB11	case	control	-0.453458103	0.255026751	-1.778080535	0.075390629	0.483342345	down_in_case
CRA004775	ENSGALG00010024598	LRRC3C	case	control	-0.03201492	0.363890729	-0.087979487	0.92989298	0.984824274	down_in_case
CRA004775	ENSGALG00010024599	ENSGALG00010024599	case	control	-0.450752503	0.444779246	-1.013429712	0.310854954	0.762763182	down_in_case
CRA004775	ENSGALG00010024600	CD9	case	control	2.29025455	0.413763299	5.535180519	3.10908E-08	3.28869E-05	up_in_case
CRA004775	ENSGALG00010024601	ENSGALG00010024601	case	control	1.702782075	0.84744922	2.00930278	0.044505033	0.393308941	up_in_case
CRA004775	ENSGALG00010024602	PROCR	case	control	0.270054295	0.404161786	0.668183645	0.504016374	0.861471523	up_in_case
CRA004775	ENSGALG00010024604	MFSD5	case	control	-0.018251979	0.358493742	-0.050912964	0.959394875	0.991431439	down_in_case
CRA004775	ENSGALG00010024605	LSP1P1	case	control	-0.64631435	0.350510451	-1.843923193	0.065194334	0.457949961	down_in_case
CRA004775	ENSGALG00010024607	RARG	case	control	-0.346937497	0.212398844	-1.633424603	0.102379726	0.542513917	down_in_case
CRA004775	ENSGALG00010024608	DENND2D	case	control	-0.218392001	0.444763816	-0.491029156	0.623405826	0.906441769	down_in_case
CRA004775	ENSGALG00010024609	PRR33	case	control	0.290438131	0.718941185	0.403980377	0.686227149	0.926725143	up_in_case
CRA004775	ENSGALG00010024610	PFKL	case	control	-0.364389791	0.275221035	-1.323989612	0.185506495	0.6491093	down_in_case
CRA004775	ENSGALG00010024611	ENSGALG00010024611	case	control	-0.496789599	0.354713913	-1.400535985	0.161352875	0.627121149	down_in_case
CRA004775	ENSGALG00010024613	ENSGALG00010024613	case	control	-0.299040112	0.202947095	-1.473488016	0.140619493	0.600007375	down_in_case
CRA004775	ENSGALG00010024616	ARVCF	case	control	0.206753202	0.345783508	0.597926729	0.549888824	0.877623839	up_in_case
CRA004775	ENSGALG00010024617	ENSGALG00010024617	case	control	0.84883327	0.462734569	1.834384823	0.066596891	0.462498477	up_in_case
CRA004775	ENSGALG00010024618	VPS13C	case	control	0.010418441	0.349867311	0.029778263	0.976243895	0.995591879	up_in_case
CRA004775	ENSGALG00010024619	ENSGALG00010024619	case	control	-0.114949745	0.295698896	-0.388739173	0.697469101	0.929092636	down_in_case
CRA004775	ENSGALG00010024620	MSANTD2	case	control	-0.211907729	0.17683028	-1.198367886	0.230773829	0.697290908	down_in_case
CRA004775	ENSGALG00010024621	THRA	case	control	-0.124500001	0.254829373	-0.488562207	0.625151675	0.906591226	down_in_case
CRA004775	ENSGALG00010024622	PRMT8	case	control	0.40209735	0.588692813	0.683034243	0.494585205	0.856382445	up_in_case
CRA004775	ENSGALG00010024623	NRDC	case	control	0.046636407	0.217688226	0.214234863	0.830363911	0.963232966	up_in_case
CRA004775	ENSGALG00010024624	CTSD	case	control	-0.230335387	0.273755046	-0.841392296	0.4001282	0.811658978	down_in_case
CRA004775	ENSGALG00010024625	SPRYD3	case	control	-0.469828398	0.246655756	-1.904793974	0.056806857	0.435477592	down_in_case
CRA004775	ENSGALG00010024626	TSHZ3	case	control	0.015545732	0.369200137	0.042106518	0.966413784	0.993094598	up_in_case
CRA004775	ENSGALG00010024627	CEPT1	case	control	-0.168170433	0.253188596	-0.664210141	0.506555824	0.8622397	down_in_case
CRA004775	ENSGALG00010024628	TANGO2	case	control	-0.057900341	0.237692156	-0.243593822	0.807545414	0.955397873	down_in_case
CRA004775	ENSGALG00010024629	ENSGALG00010024629	case	control	0.053526451	0.2282043	0.234554964	0.814554168	0.958758519	up_in_case
CRA004775	ENSGALG00010024630	CFAP410	case	control	-0.255430067	0.216035791	-1.18235069	0.23706658	0.703871614	down_in_case
CRA004775	ENSGALG00010024631	EPB41L1	case	control	-0.059273146	0.297197675	-0.199440142	0.841918463	0.966478468	down_in_case
CRA004775	ENSGALG00010024632	IFITM10	case	control	-0.429390186	0.541642006	-0.792756434	0.427919743	0.822830776	down_in_case
CRA004775	ENSGALG00010024633	NRGN	case	control	0.387248453	0.612851368	0.631879886	0.527465367	0.86830001	up_in_case
CRA004775	ENSGALG00010024634	ENSGALG00010024634	case	control	0.106633332	0.285877277	0.373003876	0.709145559	0.931874404	up_in_case
CRA004775	ENSGALG00010024635	ENSGALG00010024635	case	control	0.067690772	0.512940267	0.131966189	0.895011035	0.977834811	up_in_case
CRA004775	ENSGALG00010024636	PANX3	case	control	-0.792756453	0.264836502	-2.993380618	0.002759054	0.10984643	down_in_case
CRA004775	ENSGALG00010024637	DUSP8	case	control	0.742689506	0.797160591	0.931668618	0.35150781	0.786204276	up_in_case
CRA004775	ENSGALG00010024638	ZNF507	case	control	0.395430401	0.393163414	1.005766016	0.314528138	0.765098415	up_in_case
CRA004775	ENSGALG00010024639	AAR2	case	control	-0.042230013	0.0878701	-0.480595937	0.630803704	0.909338686	down_in_case
CRA004775	ENSGALG00010024640	GSDMA	case	control	-0.509692276	0.213448672	-2.387891525	0.016945343	0.257853739	down_in_case
CRA004775	ENSGALG00010024641	TRA2B	case	control	-0.366583101	0.279370313	-1.312176293	0.189460659	0.654755848	down_in_case
CRA004775	ENSGALG00010024642	RAB3B	case	control	0.660852431	0.418159803	1.580382492	0.114019299	0.563985387	up_in_case
CRA004775	ENSGALG00010024643	ETV7	case	control	-0.27421645	0.507820053	-0.539987439	0.589205695	0.893412703	down_in_case
CRA004775	ENSGALG00010024644	EIF6	case	control	-0.336224275	0.131227052	-2.562156738	0.010402436	0.204640771	down_in_case
CRA004775	ENSGALG00010024645	PARP11	case	control	-0.047709023	0.27640674	-0.172604415	0.862962382	0.971910432	down_in_case
CRA004775	ENSGALG00010024646	RORA	case	control	0.850937585	0.533023869	1.596434296	0.110391864	0.558567026	up_in_case
CRA004775	ENSGALG00010024647	RPUSD4	case	control	-0.209791267	0.16095216	-1.303438661	0.192425045	0.656959697	down_in_case
CRA004775	ENSGALG00010024648	MMP24	case	control	0.481819697	0.298814006	1.612440139	0.106866196	0.552884225	up_in_case
CRA004775	ENSGALG00010024649	NCOR2	case	control	0.164530277	0.278738522	0.590267453	0.555011356	0.87981923	up_in_case
CRA004775	ENSGALG00010024650	PRKAG1	case	control	-0.082828754	0.33264057	-0.249003764	0.80335787	0.954058041	down_in_case
CRA004775	ENSGALG00010024651	PSMD3	case	control	-0.513222082	0.356901572	-1.437993338	0.150435945	0.614205663	down_in_case
CRA004775	ENSGALG00010024652	PUS3	case	control	-0.250095499	0.193573627	-1.291991591	0.196360057	0.662612795	down_in_case
CRA004775	ENSGALG00010024653	RFLNA	case	control	-0.119257254	0.389088052	-0.306504538	0.759220525	0.942236668	down_in_case
CRA004775	ENSGALG00010024654	CCDC92	case	control	-0.318152011	0.560412994	-0.56770991	0.570231961	0.88518436	down_in_case
CRA004775	ENSGALG00010024655	IGF2BP2	case	control	-0.055891973	0.250568458	-0.223060688	0.823488274	0.960898591	down_in_case
CRA004775	ENSGALG00010024656	HYLS1	case	control	-0.372023892	0.277472078	-1.340761546	0.179997885	0.644990528	down_in_case
CRA004775	ENSGALG00010024657	DPY19L3	case	control	0.288821836	0.34715659	0.831964145	0.405429183	0.813520604	up_in_case
CRA004775	ENSGALG00010024659	BEND5	case	control	0.367510727	0.290233255	1.266259885	0.205420054	0.674045742	up_in_case
CRA004775	ENSGALG00010024660	KMT2D	case	control	-0.043429618	0.369164601	-0.117642963	0.906350562	0.980044552	down_in_case
CRA004775	ENSGALG00010024662	MOB2	case	control	0.148178097	0.208668382	0.710112836	0.477634167	0.846563638	up_in_case
CRA004775	ENSGALG00010024663	ICE2	case	control	0.089103363	0.268179829	0.332252292	0.739698761	0.938064864	up_in_case
CRA004775	ENSGALG00010024665	MAP1LC3A	case	control	-0.130788608	0.401966221	-0.325372136	0.744899441	0.939218065	down_in_case
CRA004775	ENSGALG00010024666	MED24	case	control	-0.448779318	0.124516802	-3.604166743	0.000313156	0.035519918	down_in_case
CRA004775	ENSGALG00010024667	RBM7	case	control	0.018801223	0.296880302	0.063329306	0.94950428	0.990168609	up_in_case
CRA004775	ENSGALG00010024668	ENSGALG00010024668	case	control	0.764602134	0.373005897	2.049839267	0.040380118	0.376363211	up_in_case
CRA004775	ENSGALG00010024669	STIL	case	control	-0.1477226	0.428229102	-0.344961608	0.730123233	0.936393482	down_in_case
CRA004775	ENSGALG00010024670	SENP2	case	control	-0.039785212	0.208356268	-0.190947997	0.848566336	0.968223172	down_in_case
CRA004775	ENSGALG00010024671	KCTD20	case	control	0.369689435	0.514196167	0.718965756	0.47216202	0.845425322	up_in_case
CRA004775	ENSGALG00010024672	DYNLRB1	case	control	0.003310917	0.249719351	0.013258551	0.989421517	0.998065748	up_in_case
CRA004775	ENSGALG00010024673	REXO2	case	control	0.064233668	0.246159562	0.260943217	0.794136297	0.951843306	up_in_case
CRA004775	ENSGALG00010024674	DHH	case	control	0.687086466	0.450612195	1.524784444	0.127312871	0.581272645	up_in_case
CRA004775	ENSGALG00010024675	NR1D1	case	control	-0.350260717	0.54786317	-0.639321524	0.522613789	0.866638286	down_in_case
CRA004775	ENSGALG00010024676	ENSGALG00010024676	case	control	1.050781056	0.391883589	2.68136019	0.007332355	0.170964283	up_in_case
CRA004775	ENSGALG00010024677	LMBR1L	case	control	-1.080042625	0.548446137	-1.969277477	0.048921236	0.41021055	down_in_case
CRA004775	ENSGALG00010024678	AMOTL2	case	control	0.152415741	0.309370614	0.492663925	0.622250071	0.906040637	up_in_case
CRA004775	ENSGALG00010024679	MSL1	case	control	-0.022981523	0.216667564	-0.106068128	0.915528298	0.981478883	down_in_case
CRA004775	ENSGALG00010024680	BRSK1	case	control	0.89388339	0.671980989	1.330221248	0.183445385	0.647806087	up_in_case
CRA004775	ENSGALG00010024681	ATP6V0A2	case	control	-0.141430194	0.153624687	-0.920621531	0.357248057	0.790175009	down_in_case
CRA004775	ENSGALG00010024682	ENSGALG00010024682	case	control	-1.186822342	0.420982077	-2.819175463	0.004814719	0.142429514	down_in_case
CRA004775	ENSGALG00010024683	SHISA4	case	control	-0.074300486	0.449920677	-0.1651413	0.868832766	0.973370973	down_in_case
CRA004775	ENSGALG00010024684	ENSGALG00010024684	case	control	-0.038187318	0.762113084	-0.050107155	0.960036998	0.991431439	down_in_case
CRA004775	ENSGALG00010024685	ENSGALG00010024685	case	control	-0.42126702	0.218621107	-1.926927481	0.053988668	0.427648719	down_in_case
CRA004775	ENSGALG00010024686	CMPK1	case	control	-0.24493511	0.274234483	-0.893159413	0.37177181	0.795818272	down_in_case
CRA004775	ENSGALG00010024687	RYK	case	control	0.135588675	0.210507487	0.644103812	0.519508119	0.865611895	up_in_case
CRA004775	ENSGALG00010024688	CADM1	case	control	-0.005302123	0.152107499	-0.034857733	0.972193184	0.994947809	down_in_case
CRA004775	ENSGALG00010024689	FOXD2	case	control	-0.042890359	0.494159712	-0.086794527	0.930834838	0.985044263	down_in_case
CRA004775	ENSGALG00010024690	TUBA1C	case	control	0.971902336	0.648042364	1.499751236	0.133678853	0.591256967	up_in_case
CRA004775	ENSGALG00010024691	TCTN2	case	control	-0.299375218	0.257795013	-1.161291734	0.24552328	0.712046495	down_in_case
CRA004775	ENSGALG00010024692	DRAM2	case	control	-0.074376864	0.191514496	-0.388361539	0.697748502	0.929092636	down_in_case
CRA004775	ENSGALG00010024693	ANXA2	case	control	-0.406790362	0.389603711	-1.044113162	0.296433037	0.748897794	down_in_case

CRA004775	ENSGALG00010024694	GTF2H3	case	control	-0.29749136	0.192331631	-1.54676253	0.121920519	0.574153785	down_in_case
CRA004775	ENSGALG00010024695	TRABD2B	case	control	0.157737094	0.546558492	0.288600573	0.772887057	0.946296525	up_in_case
CRA004775	ENSGALG00010024696	TRADD	case	control	-0.722960357	0.362746056	-1.993020584	0.046259193	0.399566686	down_in_case
CRA004775	ENSGALG00010024697	IGF2BP1	case	control	0.28988421	0.659427674	0.439599704	0.660227055	0.917907174	up_in_case
CRA004775	ENSGALG00010024698	ENSGALG00010024698	case	control	-0.369096469	0.372934799	-0.989707773	0.322316975	0.767880594	down_in_case
CRA004775	ENSGALG00010024699	FBXL8	case	control	-0.660655029	0.205069899	-3.22160898	0.00127473	0.07547801	down_in_case
CRA004775	ENSGALG00010024700	TOLLIP	case	control	-0.160330181	0.290996289	-0.550969848	0.581654344	0.889219177	down_in_case
CRA004775	ENSGALG00010024701	EIF2B1	case	control	-0.249522392	0.20116999	-1.240355939	0.21484377	0.683649824	down_in_case
CRA004775	ENSGALG00010024702	INAVA	case	control	0.588517186	0.376372856	1.5636547	0.117898682	0.569050463	up_in_case
CRA004775	ENSGALG00010024705	SLCO2A1	case	control	0.284044592	0.317017777	0.895989475	0.370258384	0.795584784	up_in_case
CRA004775	ENSGALG00010024706	ENSGALG00010024706	case	control	0.721274838	0.59347566	1.215340217	0.224236359	0.692293259	up_in_case
CRA004775	ENSGALG00010024707	ENSGALG00010024707	case	control	0.157339367	0.331978509	0.473944435	0.635539532	0.909837348	up_in_case
CRA004775	ENSGALG00010024708	BNIP2	case	control	0.046103459	0.126759943	0.363706847	0.71607693	0.933921336	up_in_case
CRA004775	ENSGALG00010024709	TEAD4	case	control	0.007184782	0.373394182	0.019241814	0.984648201	0.997562105	up_in_case
CRA004775	ENSGALG00010024710	HSDL1	case	control	-0.447264928	0.355133649	-1.259427064	0.207876119	0.676506608	down_in_case
CRA004775	ENSGALG00010024711	DDX55	case	control	0.160082021	0.210525082	0.760394054	0.447019077	0.834117509	up_in_case
CRA004775	ENSGALG00010024712	LRIF1	case	control	0.230531315	0.221867863	1.039047801	0.298782506	0.752069968	up_in_case
CRA004775	ENSGALG00010024713	RND2	case	control	0.293041227	0.832354892	0.35206284	0.724791136	0.935238044	up_in_case
CRA004775	ENSGALG00010024714	SRPRB	case	control	-0.551367029	0.212823716	-2.590721751	0.00957749	0.197747835	down_in_case
CRA004775	ENSGALG00010024715	DNAAF1	case	control	-0.260934304	0.495813637	-0.526274963	0.598697176	0.8970998	down_in_case
CRA004775	ENSGALG00010024716	GTF2A2	case	control	-0.142603886	0.200867296	-0.709940785	0.477740857	0.846563638	down_in_case
CRA004775	ENSGALG00010024717	TF	case	control	-0.795778719	0.556545862	-1.429852908	0.15275924	0.617472961	down_in_case
CRA004775	ENSGALG00010024718	ENSGALG00010024718	case	control	-0.122806753	0.375400473	-0.327135318	0.74356554	0.939218065	down_in_case
CRA004775	ENSGALG00010024719	PYM1	case	control	-0.393444918	0.237365157	-1.657551272	0.097408072	0.531987176	down_in_case
CRA004775	ENSGALG00010024720	BUD13	case	control	-0.487792918	0.168494554	-2.895007037	0.0037915	0.127263805	down_in_case
CRA004775	ENSGALG00010024722	BRCA1	case	control	-0.066522478	0.721756305	-0.092167505	0.926564955	0.984182984	down_in_case
CRA004775	ENSGALG00010024723	ITCH	case	control	0.234870389	0.276619144	0.849074965	0.39583958	0.808998414	up_in_case
CRA004775	ENSGALG00010024724	HOXD3	case	control	0.175540013	0.418863797	0.419086144	0.675153177	0.92288678	up_in_case
CRA004775	ENSGALG00010024726	TMED2	case	control	-0.169656383	0.157298791	-1.078561268	0.280783356	0.737061584	down_in_case
CRA004775	ENSGALG00010024728	SLC5A9	case	control	2.102232595	0.612173616	3.434046386	0.000594642	0.05063962	up_in_case
CRA004775	ENSGALG00010024729	FOXM1	case	control	0.479132484	0.354475441	1.352174025	0.176319651	0.641590771	up_in_case
CRA004775	ENSGALG00010024730	AQP12A	case	control	-0.702259041	1.087628093	-0.645679388	0.518487012	0.865435399	down_in_case
CRA004775	ENSGALG00010024731	AOX1	case	control	0.424593319	0.591004353	0.718426719	0.472494217	0.845425322	up_in_case
CRA004775	ENSGALG00010024732	AHCY	case	control	-0.243463444	0.223457002	-1.089531508	0.275919568	0.731001618	down_in_case
CRA004775	ENSGALG00010024733	MBTPS1	case	control	-0.048495091	0.143943881	-0.336902759	0.736190205	0.937385622	down_in_case
CRA004775	ENSGALG00010024734	ZPR1	case	control	-0.380072478	0.16756413	-2.268220993	0.023315741	0.296313338	down_in_case
CRA004775	ENSGALG00010024735	OTUD7A	case	control	0.365639858	0.401292308	0.91115591	0.362213231	0.792867581	up_in_case
CRA004775	ENSGALG00010024736	DGKA	case	control	-0.320007808	0.249505324	-1.282569053	0.199643098	0.666980623	down_in_case
CRA004775	ENSGALG00010024737	STK38	case	control	-0.21210221	0.288552037	-0.735057053	0.462304748	0.842230074	down_in_case
CRA004775	ENSGALG00010024738	NBR1	case	control	0.125764833	0.175230181	0.717712169	0.472934778	0.845445211	up_in_case
CRA004775	ENSGALG00010024741	RILPL2	case	control	0.021953613	0.34254281	0.064090128	0.948898462	0.990168609	up_in_case
CRA004775	ENSGALG00010024742	PAK2	case	control	0.000516771	0.251387324	0.002055675	0.99835981	0.999914248	up_in_case
CRA004775	ENSGALG00010024744	KLF13	case	control	0.224783559	0.248400794	0.904922867	0.365506252	0.793608387	up_in_case
CRA004775	ENSGALG00010024745	PMEL	case	control	8.570088783	0.41988209	20.41070336	1.34338E-92	1.84728E-88	up_in_case
CRA004775	ENSGALG00010024747	TULP3	case	control	-0.060481941	0.2063586	-0.29309145	0.769452252	0.945407363	down_in_case
CRA004775	ENSGALG00010024749	APOA5	case	control	-0.561673413	0.339169112	-1.656027607	0.097716228	0.532395151	down_in_case
CRA004775	ENSGALG00010024750	APOA4	case	control	2.577281079	1.078484353	2.389725054	0.01686099	0.25704598	up_in_case
CRA004775	ENSGALG00010024751	MTMR10	case	control	0.016599272	0.29557436	0.056159377	0.955214843	0.990670511	up_in_case
CRA004775	ENSGALG00010024752	MPP2	case	control	-0.151311925	0.356016952	-0.425013259	0.670827009	0.921535334	down_in_case
CRA004775	ENSGALG00010024753	HOXD8	case	control	0.139259475	0.319982226	0.435210032	0.663409981	0.918295135	up_in_case
CRA004775	ENSGALG00010024754	EIF2S2	case	control	0.182026325	0.360747877	0.504580446	0.613853547	0.902363507	up_in_case
CRA004775	ENSGALG00010024755	PIGX	case	control	-0.593304086	0.324450564	-1.828642484	0.067453184	0.463542595	down_in_case
CRA004775	ENSGALG00010024756	CDK2	case	control	-0.340128768	0.35806315	-0.949912796	0.342156564	0.781616177	down_in_case
CRA004775	ENSGALG00010024757	PPFIA1	case	control	0.389784257	0.234836515	1.659811111	0.096952458	0.53104893	up_in_case
CRA004775	ENSGALG00010024758	SPATA6	case	control	-0.122804802	0.347904311	-0.352984422	0.72410012	0.935238044	down_in_case
CRA004775	ENSGALG00010024759	APOC3	case	control	1.203133649	0.661136073	1.819797313	0.068789877	0.466664825	up_in_case
CRA004775	ENSGALG00010024760	ENSGALG00010024760	case	control	-0.149382964	0.239297516	-0.624256224	0.532459328	0.869946559	down_in_case
CRA004775	ENSGALG00010024761	NECAB2	case	control	-0.493654046	0.591037589	-0.83523291	0.403586583	0.812550381	down_in_case
CRA004775	ENSGALG00010024762	FAN1	case	control	-0.012145997	0.192682202	-0.063036425	0.949737499	0.990168609	down_in_case
CRA004775	ENSGALG00010024763	HOXD9	case	control	-0.545827624	0.344336812	-1.585156176	0.112930855	0.562886277	down_in_case
CRA004775	ENSGALG00010024764	ENSGALG00010024764	case	control	-0.460149597	0.756803071	-0.608017613	0.543175792	0.875456081	down_in_case
CRA004775	ENSGALG00010024765	SNRNP35	case	control	-0.070519467	0.44591935	-0.158143993	0.874343335	0.974161012	down_in_case
CRA004775	ENSGALG00010024767	CTTN	case	control	0.234038708	0.15955783	1.466795504	0.142431693	0.602473513	up_in_case
CRA004775	ENSGALG00010024769	RALY	case	control	-0.136137731	0.524388634	-0.259612285	0.795162859	0.952360933	down_in_case
CRA004775	ENSGALG00010024770	CDC27	case	control	0.168652056	0.208350937	0.809461469	0.418249757	0.818943344	up_in_case
CRA004775	ENSGALG00010024771	AOX2	case	control	0.200259419	0.483467553	0.414214807	0.678716799	0.92338129	up_in_case
CRA004775	ENSGALG00010024772	ELAPOR1	case	control	-0.121331334	0.305642385	-0.396971559	0.691388434	0.927867527	down_in_case
CRA004775	ENSGALG00010024773	APOA1	case	control	-0.435479897	0.475906584	-0.915053314	0.360163642	0.791975934	down_in_case
CRA004775	ENSGALG00010024775	ITFG2	case	control	0.296363886	0.26216812	1.130434494	0.258293186	0.720037471	up_in_case
CRA004775	ENSGALG00010024776	MPHOSPH10	case	control	0.18595862	0.29015374	0.640896854	0.5215897	0.866314221	up_in_case
CRA004775	ENSGALG00010024778	RAB5B	case	control	-0.203559965	0.224073881	-0.908450211	0.363640408	0.793608387	down_in_case
CRA004775	ENSGALG00010024781	CEP19	case	control	-0.11047382	0.338842893	-0.326032573	0.744399711	0.939218065	down_in_case
CRA004775	ENSGALG00010024783	UBE2T	case	control	0.087463344	0.356939646	0.245036787	0.806427948	0.955397873	up_in_case
CRA004775	ENSGALG00010024784	ENSGALG00010024784	case	control	0.093295138	0.272326582	0.342585501	0.731910311	0.936755276	up_in_case
CRA004775	ENSGALG00010024785	MCEE	case	control	-0.287918521	0.377178411	-0.763348358	0.445255669	0.833012432	down_in_case
CRA004775	ENSGALG00010024786	PDCD5	case	control	0.464285489	0.267570648	1.735188419	0.082707437	0.499833536	up_in_case
CRA004775	ENSGALG00010024787	SARS1	case	control	-0.319383277	0.216570298	-1.474732595	0.140284449	0.600007375	down_in_case
CRA004775	ENSGALG00010024788	SIK3	case	control	0.250801464	0.305937913	0.81977896	0.412342127	0.817035725	up_in_case
CRA004775	ENSGALG00010024789	SUOX	case	control	-0.162013711	0.282355907	-0.573792533	0.566108202	0.883033678	down_in_case
CRA004775	ENSGALG00010024792	BZW1	case	control	-0.401517727	0.162959126	-2.463916796	0.013742801	0.234005339	down_in_case
CRA004775	ENSGALG00010024793	FKBP4	case	control	-0.026157966	0.168470956	-0.155266919	0.876610894	0.974479909	down_in_case
CRA004775	ENSGALG00010024794	DDX6	case	control	0.004125583	0.250237477	0.016486672	0.986846135	0.997656315	up_in_case
CRA004775	ENSGALG00010024795	NRROS	case	control	-0.515689223	0.325312656	-1.585211071	0.112918386	0.562886277	down_in_case
CRA004775	ENSGALG00010024797	IKZF4	case	control	-0.139500835	0.256304445	-0.54427786	0.58625028	0.891503646	down_in_case
CRA004775	ENSGALG00010024798	FBXO45	case	control	-0.141533665	0.194930792	-0.726071359	0.467795026	0.843870188	down_in_case
CRA004775	ENSGALG00010024799	ENSGALG00010024799	case	control	0.472304024	0.335207033	1.408991988	0.158837537	0.624764009	up_in_case
CRA004775	ENSGALG00010024800	ENSGALG00010024800	case	control	0.271011219	0.209622855	1.292851479	0.196062432	0.662095898	up_in_case
CRA004775	ENSGALG00010024801	CXCR5	case	control	0.627445062	0.777569891	0.806930758	0.419706377	0.819388688	up_in_case
CRA004775	ENSGALG00010024802	CD59	case	control	-0.297172991	0.339145296	-0.876240935	0.380899067	0.801753951	down_in_case
CRA004775	ENSGALG00010024803	CLK1	case	control	0.287162344	0.219089233	1.310709522	0.18995592	0.655361714	up_in_case
CRA004775	ENSGALG00010024804	WDR53	case	control	-0.130382973	0.224364306	-0.581121726	0.561158417	0.881179558	down_in_case
CRA004775	ENSGALG00010024805	ENSGALG00010024805	case	control	0.004562474	0.593521351	0.007687126	0.993866621	0.998951824	up_in_case
CRA004775	ENSGALG00010024806	ANKRD27	case	control	0.120778201	0.244500782	0.493978793	0.621321157	0.90544587	up_in_case
CRA004775	ENSGALG00010024807	PPIL3	case	control	-0.520230083	0.268616609	-1.9367011	0.052781891	0.422982632	down_in_case
CRA004775	ENSGALG00010024808	CELSR2	case	control	0.882330746	0.384271158	2.29611494	0.02166931	0.288534247	up_in_case
CRA004775	ENSGALG00010024809	KANSL1	case	control	-0.076939796	0.215868823	-0.356419212	0.721526644	0.934511903	down_in_case
CRA004775	ENSGALG00010024810	ADIPOR1	case	control	-0.334831428	0.241769056	-1.384922597	0.166076137	0.630194031	down_in_case
CRA004775	ENSGALG00010024811	NIF3L1	case	control	-0.186428978	0.275958114	-0.675569839	0.499313796	0.858902178	down_in_case
CRA004775	ENSGALG00010024812	SBNO1	case	control	0.274237334	0.192723515	1.422957305	0.154748532	0.619284425	up_in_case
CRA004775	ENSGALG00010024813	RNF168	case	control	0.271625407	0.194765703	1.394626481	0.163128505	0.629717526	up_in_case
CRA004775	ENSGALG00010024814	APBA2	case	control	-0.263376386	0.647418613	-0.406810031	0.684147519	0.925591552	down_in_case
CRA004775	ENSGALG00010024815	GBX1	case	control	-1.818805405	0.971661466	-1.871850915	0.061227232	0.445483389	down_in_case
CRA004775	ENSGALG00010024816	NFIA	case	control	-0.142246007	0.280159003	-0.507733129	0.611640512	0.901413878	down_in_case
CRA004775	ENSGALG00010024817	UCKL1	case	control	-0.119284279	0.374350649	-0.318643173	0.749997111	0.941495694	down_in_case
CRA004775	ENSGALG00010024818	FBXO3	case	control	2.26426E-05	0.210679681	0.000107474	0.999914248	0.999914248	up_in_case
CRA004775	ENSGALG00010024820	NUDT19	case	control	0.183580404	0.268424327	0.683918652	0.494026535	0.856233789	up_in_case
CRA004775	ENSGALG00010024821	NECAP1	case	control	-0.603068157	0.224922893	-2.681221772	0.007335388	0.170964283	down_in_case
CRA004775	ENSGALG00010024822	RPS26	case	control	-0.021767755	0.323401559	-0.067308751	0.94633591	0.989106556	down_in_case
CRA004775	ENSGALG00010024823	ASB10	case	control	-0.310815501	0.673149316	-0.461733367	0.64427254	0.913739303	down_in_case
CRA004775	ENSGALG00010024824	DNAJC5	case	control	0.159282878	0.29685173	0.536573857	0.591562007	0.893804699	up_in_case
CRA004775	ENSGALG00010024825	UBXN7	case	control	-0.071731003	0.283168693	-0.253315442	0.800024464	0.952993446	down_in_case
CRA004775	ENSGALG00010024826	RBM33	case	control	0.149564247	0.29230408	0.511673484	0.608879547	0.90115757	up_in_case
CRA004775	ENSGALG00010024828	TIMM17B	case	control	-0.184106155	0.301697571	-0.610234132	0.541706723	0.875015758	down_in_case
CRA004775	ENSGALG00010024829	ERBB3	case	control	1.105742146	0.42986305	2.572312616	0.010102163	0.202058223	up_in_case
CRA004775	ENSGALG00010024830	ORC2	case	control	-0.301948337	0.331153078	-0.91180894	0.361869303	0.792867581	down_in_case
CRA004775	ENSGALG00010024831	SLC7A9	case	control	2.315851119	0.846867546	2.734608416	0.006245452	0.158635228	up_in_case
CRA004775	ENSGALG00010024833	MAPT	case	control	0.018558544	0.368721032	0.05033221	0.959857657	0.991431439	up_in_case
CRA004775	ENSGALG00010024834	INSIG1	case	control	0.563577322	0.388820065	1.449455344	0.147210463	0.610295079	up_in_case
CRA004775	ENSGALG00010024835	ENTREP2	case	control	-0.106996567	0.319989167	-0.334375592	0.738096155	0.937863632	down_in_case
CRA004775	ENSGALG00010024836	TXNDC12	case	control	-0.284657755	0.188171455	-1.512757367	0.130341302	0.58515287	down_in_case
CRA004775	ENSGALG00010024837	TNFRSF1A	case	control	-0.18538592	0.223398863	-0.829842719	0.406627715	0.813842524	down_in_case
CRA004775	ENSGALG00010024838	EN2	case	control	-2.866599977	1.215933862	-2.357529522	0.018396992	0.269466431	down_in_case
CRA004775	ENSGALG00010024839	PA2G4	case	control	-0.046814119	0.164841621	-0.283994529	0.776414573	0.946831926	down_in_case
CRA004775	ENSGALG00010024840	BTF3L4	case	control	-0.249887923	0.155395666	-1.608075243	0.1078187	0.55406723	down_in_case
CRA004775	ENSGALG00010024841	ENSGALG00010024841	case	control	-0.080940585	0.362380808	-0.223357815	0.823257034	0.960898591	down_in_case
CRA004775	ENSGALG00010024842	ZFYVE9	case	control	0.087031769	0.255828035	0.34019637	0.733708652	0.936819725	up_in_case

CRA004775	ENSGALG00010024843	BCL9L	case	control	0.019311646	0.320589751	0.060237877	0.951966179	0.990168609	up_in_case
CRA004775	ENSGALG00010024844	TM4SF19	case	control	1.433488527	0.714464788	2.006380931	0.044815621	0.394279979	up_in_case
CRA004775	ENSGALG00010024845	HYCC2	case	control	0.064034703	0.249845797	0.256296898	0.797721592	0.952835572	up_in_case
CRA004775	ENSGALG00010024846	LMO2	case	control	-0.034204043	0.392376509	-0.087171484	0.930535205	0.985044263	down_in_case
CRA004775	ENSGALG00010024847	PPIL1	case	control	-0.088566849	0.337076071	-0.262750329	0.792743022	0.951284723	down_in_case
CRA004775	ENSGALG00010024848	ESYT1	case	control	-0.303282915	0.180509461	-1.680149691	0.092928195	0.524612711	down_in_case
CRA004775	ENSGALG00010024849	CENATAC	case	control	0.212668719	0.188812352	1.126349614	0.260017565	0.720822628	up_in_case
CRA004775	ENSGALG00010024850	CDK2AP1	case	control	-0.069847547	0.252217272	-0.276934036	0.781830757	0.948976497	down_in_case
CRA004775	ENSGALG00010024852	CEP89	case	control	0.196280239	0.433188508	0.453105832	0.650472536	0.915428088	up_in_case
CRA004775	ENSGALG00010024853	TJP1	case	control	0.51535914	0.279451445	1.844181341	0.065156717	0.457949961	up_in_case
CRA004775	ENSGALG00010024854	C12orf65	case	control	0.213308219	0.27690731	0.770323541	0.441107995	0.829353567	up_in_case
CRA004775	ENSGALG00010024855	CAPRIN1	case	control	0.11712986	0.180826916	0.647745713	0.517149432	0.864913871	up_in_case
CRA004775	ENSGALG00010024856	SORT1	case	control	0.050660062	0.378052834	0.134002599	0.893400519	0.977834811	up_in_case
CRA004775	ENSGALG00010024857	SLC37A4	case	control	-0.127682787	0.196370251	-0.650214514	0.515553667	0.864897625	down_in_case
CRA004775	ENSGALG00010024858	SLC25A39	case	control	-0.389947163	0.211739849	-1.841633334	0.065528802	0.45911339	down_in_case
CRA004775	ENSGALG00010024859	TARSL2	case	control	-0.226156662	0.171311019	-1.320152453	0.186784123	0.650080606	down_in_case
CRA004775	ENSGALG00010024861	ORC1	case	control	-0.054072139	0.188664981	-0.286604006	0.774415546	0.946319184	down_in_case
CRA004775	ENSGALG00010024862	METTL16	case	control	0.075915208	0.252161962	0.301057333	0.763370776	0.94239774	up_in_case
CRA004775	ENSGALG00010024863	LMOD1	case	control	0.438016684	0.401143691	1.091919663	0.274868414	0.731001618	up_in_case
CRA004775	ENSGALG00010024864	MPHOSPH9	case	control	-0.579646732	0.36146726	-1.603594007	0.108803573	0.555936379	down_in_case
CRA004775	ENSGALG00010024865	SNAP47	case	control	-0.137056982	0.474802718	-0.288660905	0.772840883	0.946296525	down_in_case
CRA004775	ENSGALG00010024866	TRAPPC4	case	control	-0.321854538	0.308092667	-1.044667961	0.296176457	0.748897794	down_in_case
CRA004775	ENSGALG00010024867	TM2D3	case	control	-0.258968801	0.204375087	-1.267125093	0.205110565	0.674045742	down_in_case
CRA004775	ENSGALG00010024868	FAAP24	case	control	0.218378271	0.513442789	0.425321528	0.670602302	0.921535334	up_in_case
CRA004775	ENSGALG00010024869	NDUFB3	case	control	0.120520292	0.35299602	0.341421109	0.732786586	0.936819725	up_in_case
CRA004775	ENSGALG00010024870	TPD52L2	case	control	-0.018055187	0.152024496	-0.118764986	0.90546155	0.979739788	down_in_case
CRA004775	ENSGALG00010024871	POLQ	case	control	-0.219731583	0.607399901	-0.361757687	0.717533114	0.934011578	down_in_case
CRA004775	ENSGALG00010024872	RUNDC3A	case	control	-0.523536569	0.350631521	-1.493124655	0.13540456	0.593544183	down_in_case
CRA004775	ENSGALG00010024873	HYOU1	case	control	-0.265339667	0.297150559	-0.892946887	0.371885616	0.795818272	down_in_case
CRA004775	ENSGALG00010024874	JMJD4	case	control	-0.141562943	0.251401826	-0.563094331	0.573370652	0.886730131	down_in_case
CRA004775	ENSGALG00010024875	GAL3ST2	case	control	2.950775066	0.842349735	3.503028425	0.00046	0.044470881	up_in_case
CRA004775	ENSGALG00010024876	CFLAR	case	control	0.17162907	0.245129231	0.7001575	0.48382895	0.850349167	up_in_case
CRA004775	ENSGALG00010024877	PRPF38A	case	control	-0.323573564	0.237974275	-1.359699757	0.173924955	0.639185614	down_in_case
CRA004775	ENSGALG00010024878	RHPN2	case	control	1.258522652	0.450326347	2.794690254	0.005194946	0.146685229	up_in_case
CRA004775	ENSGALG00010024879	D2HGDH	case	control	-0.509362741	0.221964131	-2.294797536	0.02174473	0.288534247	down_in_case
CRA004775	ENSGALG00010024880	NAT10	case	control	-0.440899225	0.267563315	-1.647831369	0.09938728	0.537098329	down_in_case
CRA004775	ENSGALG00010024881	CASP10	case	control	-0.243437964	0.259343422	-0.938670285	0.347900054	0.785270866	down_in_case
CRA004775	ENSGALG00010024882	MYL6	case	control	-0.136648147	0.250642118	-0.545192276	0.585621284	0.891199455	down_in_case
CRA004775	ENSGALG00010024883	ING5	case	control	-0.177667947	0.190212495	-0.93404982	0.350278195	0.785466132	down_in_case
CRA004775	ENSGALG00010024884	ZBTB46	case	control	-0.167667445	0.275994642	-0.607502535	0.543517461	0.875657091	down_in_case
CRA004775	ENSGALG00010024885	SLC4A1	case	control	-0.200806335	0.386622343	-0.519386266	0.603491407	0.898692785	down_in_case
CRA004775	ENSGALG00010024886	ABTB2	case	control	0.529846876	0.412103046	1.285714537	0.198542706	0.665405984	up_in_case
CRA004775	ENSGALG00010024887	CASP18	case	control	-0.458976987	0.564902536	-0.812488805	0.416511208	0.818943344	down_in_case
CRA004775	ENSGALG00010024888	PITPNM2	case	control	-0.264385216	0.434136048	-0.608991621	0.542529993	0.875137006	down_in_case
CRA004775	ENSGALG00010024889	SLC2A4RG	case	control	0.120735491	0.276128223	0.437244297	0.661934188	0.918136475	up_in_case
CRA004775	ENSGALG00010024890	SMARCC2	case	control	-0.175244149	0.139419906	-1.256952142	0.208770966	0.677877107	down_in_case
CRA004775	ENSGALG00010024891	PKP1	case	control	0.606288549	0.832567389	0.728215586	0.466481626	0.8434807	up_in_case
CRA004775	ENSGALG00010024892	ENSGALG00010024892	case	control	-1.036909073	0.540238052	-1.919355864	0.054939313	0.430875458	down_in_case
CRA004775	ENSGALG00010024893	ARL6IP4	case	control	0.136447121	0.190969649	0.71449637	0.4749203	0.84579952	up_in_case
CRA004775	ENSGALG00010024894	TUT4	case	control	0.296481828	0.246737849	1.201606598	0.229515984	0.695782399	up_in_case
CRA004775	ENSGALG00010024896	RNF41	case	control	-0.387779502	0.362010172	-1.071183995	0.284086707	0.740820744	down_in_case
CRA004775	ENSGALG00010024897	ADAL	case	control	0.005729074	0.382466617	0.014979277	0.988048713	0.998065748	up_in_case
CRA004775	ENSGALG00010024898	NABP2	case	control	0.025064933	0.221349476	0.113236922	0.909842724	0.98075825	up_in_case
CRA004775	ENSGALG00010024899	OGFOD2	case	control	-0.610489517	0.29529728	-2.067372637	0.038699055	0.370564421	down_in_case
CRA004775	ENSGALG00010024900	DTYMK	case	control	-0.490302869	0.331785143	-1.477772223	0.139468755	0.59914295	down_in_case
CRA004775	ENSGALG00010024901	ZGPAT	case	control	-0.27068204	0.141577078	-1.91190583	0.055888274	0.432968819	down_in_case
CRA004775	ENSGALG00010024902	LARP6	case	control	0.062428272	0.2555673	0.244273317	0.807019147	0.955397873	up_in_case
CRA004775	ENSGALG00010024903	CASP8	case	control	-0.344320789	0.402461248	-0.855537747	0.392253517	0.80746892	down_in_case
CRA004775	ENSGALG00010024904	ALS2CL	case	control	-0.390490434	0.44605768	-0.875425873	0.381342227	0.801753951	down_in_case
CRA004775	ENSGALG00010024905	GPX7	case	control	-0.457458994	0.42732975	-1.070505841	0.284391681	0.741035375	down_in_case
CRA004775	ENSGALG00010024907	PHLDB1	case	control	-0.253498435	0.37223109	-0.681024346	0.495856085	0.856382445	down_in_case
CRA004775	ENSGALG00010024908	ENSGALG00010024908	case	control	-0.0269365	0.289646472	-0.092997852	0.925905268	0.98417264	down_in_case
CRA004775	ENSGALG00010024909	SLC39A5	case	control	2.843543179	0.764849605	3.717780804	0.000200981	0.029202004	up_in_case
CRA004775	ENSGALG00010024910	UBTF	case	control	-0.264926739	0.153095088	-1.730471839	0.083546008	0.502115892	down_in_case
CRA004775	ENSGALG00010024911	CAT	case	control	0.272237144	0.368422755	0.738925975	0.459951948	0.840995542	up_in_case
CRA004775	ENSGALG00010024915	STXBP5L	case	control	0.735127204	0.618736875	1.188109573	0.234790243	0.702145501	up_in_case
CRA004775	ENSGALG00010024917	TRAK2	case	control	0.136976108	0.19782763	0.692401298	0.488685351	0.854054691	up_in_case
CRA004775	ENSGALG00010024918	ATG4B	case	control	-0.334520482	0.151210321	-2.212286047	0.026946907	0.31493941	down_in_case
CRA004775	ENSGALG00010024919	ANKRD52	case	control	0.021320687	0.301687161	0.070671508	0.943659197	0.987862092	up_in_case
CRA004775	ENSGALG00010024920	VIPR2	case	control	0.764870728	0.535492755	1.428349348	0.153191331	0.617472961	up_in_case
CRA004775	ENSGALG00010024921	STRADB	case	control	-0.041444204	0.222119245	-0.186585381	0.851985729	0.969430695	down_in_case
CRA004775	ENSGALG00010024924	MNX1	case	control	0.342057563	0.596862638	0.573092603	0.566581995	0.883033678	up_in_case
CRA004775	ENSGALG00010024925	GTF2E1	case	control	-0.4256034	0.154778271	-2.749761955	0.005963857	0.154441997	down_in_case
CRA004775	ENSGALG00010024927	NOM1	case	control	-0.090340295	0.216461884	-0.417349666	0.676422666	0.922924485	down_in_case
CRA004775	ENSGALG00010024928	THAP4	case	control	-0.216076411	0.322020256	-0.671002544	0.502218909	0.860091011	down_in_case
CRA004775	ENSGALG00010024930	TMEM237	case	control	0.145778653	0.23050991	0.632418161	0.52711367	0.86830001	up_in_case
CRA004775	ENSGALG00010024931	UBE3C	case	control	0.248332993	0.214756562	1.156346473	0.247539467	0.712046495	up_in_case
CRA004775	ENSGALG00010024933	CS	case	control	-0.181849587	0.358362539	-0.50744586	0.611842015	0.901413878	down_in_case
CRA004775	ENSGALG00010024934	G6PC3	case	control	-0.340237835	0.320027351	-1.063152364	0.287712861	0.74258854	down_in_case
CRA004775	ENSGALG00010024935	KLHL30	case	control	-0.459106431	0.688349643	-0.666966906	0.504793273	0.861471523	down_in_case
CRA004775	ENSGALG00010024936	EHF	case	control	1.689524317	0.618190443	2.73301591	0.00627573	0.158635228	up_in_case
CRA004775	ENSGALG00010024937	ILKAP	case	control	-0.406544851	0.236378155	-1.719891806	0.08545211	0.506269697	down_in_case
CRA004775	ENSGALG00010024938	ENSGALG00010024938	case	control	-0.263380881	0.327807949	-0.803460933	0.421708369	0.819812626	down_in_case
CRA004775	ENSGALG00010024939	COQ10A	case	control	-0.373120224	0.426013809	-0.87584068	0.381116652	0.801753951	down_in_case
CRA004775	ENSGALG00010024940	RABL3	case	control	-0.34973055	0.15858166	-2.205365675	0.027428445	0.316243014	down_in_case
CRA004775	ENSGALG00010024941	PDHX	case	control	-0.235070013	0.349552075	-0.672489252	0.50127228	0.860057564	down_in_case
CRA004775	ENSGALG00010024942	DNAJB6	case	control	0.135196204	0.145436273	0.929590681	0.352583055	0.786426886	up_in_case
CRA004775	ENSGALG00010024943	ABCB9	case	control	0.086165098	0.569162131	0.151389373	0.879668572	0.974996282	up_in_case
CRA004775	ENSGALG00010024944	ENSGALG00010024944	case	control	-0.671871656	0.327909888	-2.048952109	0.040466798	0.376363211	down_in_case
CRA004775	ENSGALG00010024945	APIP	case	control	-0.089736989	0.202019789	-0.444199004	0.656898712	0.91710603	down_in_case
CRA004775	ENSGALG00010024946	KIF1A	case	control	-0.292980067	0.6698301	-0.437394597	0.661825201	0.918136475	down_in_case
CRA004775	ENSGALG00010024947	MPP4	case	control	0.756537949	0.580144225	1.304051504	0.192216022	0.656959697	up_in_case
CRA004775	ENSGALG00010024949	PAN2	case	control	-0.604722535	0.231051087	-2.617267646	0.008863681	0.190031214	down_in_case
CRA004775	ENSGALG00010024950	NCAM1	case	control	-0.727431314	0.398348629	-1.826117276	0.067832598	0.463543284	down_in_case
CRA004775	ENSGALG00010024953	NCAPG2	case	control	0.02947283	0.822209868	0.035845873	0.971405255	0.994908384	up_in_case
CRA004775	ENSGALG00010024954	ENSGALG00010024954	case	control	-1.920315076	0.87937728	-2.183721503	0.028982719	0.323755716	down_in_case
CRA004775	ENSGALG00010024956	VPS37B	case	control	0.691727993	0.248407843	2.784646352	0.005358613	0.149162532	up_in_case
CRA004775	ENSGALG00010024960	ARFRP1	case	control	-0.147185552	0.179880959	-0.818238643	0.413220934	0.817112606	down_in_case
CRA004775	ENSGALG00010024961	LSM12	case	control	0.411587038	0.212366968	1.938093488	0.052611818	0.422249808	up_in_case
CRA004775	ENSGALG00010024963	TNFRSF6B	case	control	0.35207779	0.697469338	0.504793216	0.613704081	0.902282137	up_in_case
CRA004775	ENSGALG00010024964	ITM2C	case	control	0.913417813	0.324567249	2.814263653	0.004888911	0.142429514	up_in_case
CRA004775	ENSGALG00010024965	STAT2	case	control	-0.48500866	0.208962949	-2.321027064	0.020285383	0.281477603	down_in_case
CRA004775	ENSGALG00010024966	AGAP3	case	control	-0.32764353	0.245985973	-1.331960218	0.182873261	0.647378665	down_in_case
CRA004775	ENSGALG00010024968	TMEM101	case	control	-0.154891725	0.289233219	-0.535525365	0.592286623	0.893923098	down_in_case
CRA004775	ENSGALG00010024970	CAB39	case	control	0.308690568	0.184381603	1.674193967	0.094092485	0.52638965	up_in_case
CRA004775	ENSGALG00010024973	RTEL1	case	control	0.421390194	0.487959381	0.863576377	0.387820646	0.804270424	up_in_case
CRA004775	ENSGALG00010024974	CD44	case	control	-0.215892643	0.389037041	-0.554941098	0.57893495	0.888378369	down_in_case
CRA004775	ENSGALG00010024977	LRP3	case	control	-0.177444064	0.441808612	-0.401631065	0.687955564	0.927111919	down_in_case
CRA004775	ENSGALG00010024983	APOF	case	control	-0.409559584	0.511537776	-0.800643869	0.423337847	0.820552247	down_in_case
CRA004775	ENSGALG00010024986	PTS	case	control	0.194035855	0.312038767	0.621832528	0.534051995	0.870956093	up_in_case
CRA004775	ENSGALG00010024989	TIMELESS	case	control	-0.569430598	0.444263539	-1.281740563	0.199933671	0.667140962	down_in_case
CRA004775	ENSGALG00010024994	NDUFB4	case	control	0.115792381	0.367405753	0.315162134	0.752638554	0.94167946	up_in_case
CRA004775	ENSGALG00010024996	COA7	case	control	-0.024538318	0.228796702	-0.107249438	0.914591096	0.981303879	down_in_case
CRA004775	ENSGALG00010024999	ENSGALG00010024999	case	control	-0.153905449	0.735174751	-0.209345396	0.834178616	0.964019688	down_in_case
CRA004775	ENSGALG00010025003	ENSGALG00010025003	case	control	0.318832614	0.6723294	0.474220842	0.635342432	0.909837348	up_in_case
CRA004775	ENSGALG00010025005	HIP1R	case	control	-0.170688092	0.192603635	-0.886214281	0.375502069	0.798503808	down_in_case
CRA004775	ENSGALG00010025007	ZYG11B	case	control	-0.070187517	0.173187455	-0.405269058	0.685279748	0.926656387	down_in_case
CRA004775	ENSGALG00010025008	ENSGALG00010025008	case	control	0.337938855	1.165770494	0.28988455	0.771904562	0.946283287	up_in_case
CRA004775	ENSGALG00010025012	ENSGALG00010025012	case	control	0.155743579	0.400435604	0.388935394	0.697323939	0.929092636	up_in_case
CRA004775	ENSGALG00010025026	DENR	case	control	0.053520945	0.335033195	0.159748187	0.873079441	0.97407675	up_in_case
CRA004775	ENSGALG00010025028	HES6	case	control	0.003605833	0.394298346	0.009144935	0.992703499	0.998439571	up_in_case
CRA004775	ENSGALG00010025030	ENSGALG00010025030	case	control	-0.172486973	0.339917526	-0.507437715	0.611847728	0.901413878	down_in_case
CRA004775	ENSGALG00010025031	ECHDC2	case	control	0.065608698	0.340379473	0.192751628	0.847153488	0.967891337	up_in_case
CRA004775	ENSGALG00010025032	FSTL1	case	control	0.171912995	0.349106606	0.492436958	0.622410478	0.90616903	up_in_case
CRA004775	ENSGALG00010025039	CEBPA	case	control	-1.443043347	0.585583802	-2.464281531	0.013728823	0.234005339	down_in_case
CRA004775	ENSGALG00010025042	BCO2	case	control	-0.333510959	0.526884867	-0.632986407	0.52674252	0.86830001	down_in_case

CRA004775	ENSGALG00010025044	TMEM9	case	control	-1.288989187	0.367229736	-3.510034897	0.000448048	0.043695791	down_in_case
CRA004775	ENSGALG00010025047	PER2	case	control	0.426626874	0.29652248	1.438767391	0.150216438	0.613766096	up_in_case
CRA004775	ENSGALG00010025048	ESYT2	case	control	-0.075453243	0.410507727	-0.183804684	0.854166681	0.970073177	down_in_case
CRA004775	ENSGALG00010025055	ALS2	case	control	0.113764774	0.292297831	0.389208409	0.697121983	0.929092636	up_in_case
CRA004775	ENSGALG00010025056	RBMS2	case	control	-0.426195152	0.187539994	-2.272556072	0.023052945	0.295240109	down_in_case
CRA004775	ENSGALG00010025057	SCP2	case	control	0.103551836	0.205396728	0.504155237	0.614152293	0.902363507	up_in_case
CRA004775	ENSGALG00010025060	GPR156	case	control	0.11002235	0.361716364	0.304167465	0.76100031	0.942236668	up_in_case
CRA004775	ENSGALG00010025064	GPN3	case	control	-0.165100765	0.253137699	-0.652217216	0.514261055	0.864287921	down_in_case
CRA004775	ENSGALG00010025067	SLC1A2	case	control	-0.704534439	0.518358378	-1.359164757	0.174094386	0.639244835	down_in_case
CRA004775	ENSGALG00010025069	PODN	case	control	0.448803714	0.335685391	1.336977199	0.18123008	0.647297358	up_in_case
CRA004775	ENSGALG00010025071	CDK15	case	control	-0.052164912	0.434890037	-0.119949659	0.904523026	0.979540372	down_in_case
CRA004775	ENSGALG00010025072	ENSGALG00010025072	case	control	-1.82169422	0.817164295	-2.229287588	0.025794776	0.308336492	down_in_case
CRA004775	ENSGALG00010025073	IL18	case	control	-0.291608212	0.448525363	-0.650148767	0.515596131	0.864897625	down_in_case
CRA004775	ENSGALG00010025080	BAZ2A	case	control	-0.315275741	0.262413118	-1.201448096	0.229577429	0.695782399	down_in_case
CRA004775	ENSGALG00010025092	COL1A1	case	control	-0.705842714	0.700960029	-1.006965712	0.313951252	0.765098415	down_in_case
CRA004775	ENSGALG00010025097	LMBR1	case	control	-0.243439102	0.248061526	-0.981365817	0.326412377	0.770998457	down_in_case
CRA004775	ENSGALG00010025098	ATP5B	case	control	0.013767231	0.410772043	0.033515502	0.973263504	0.994947809	up_in_case
CRA004775	ENSGALG00010025102	ARPC3	case	control	-0.352434711	0.346081228	-1.018358356	0.308507685	0.760948731	down_in_case
CRA004775	ENSGALG00010025103	GSK3B	case	control	0.100086893	0.167028189	0.599221567	0.549025143	0.877623839	up_in_case
CRA004775	ENSGALG00010025113	SDHD	case	control	0.013643525	0.326854293	0.041741918	0.966704438	0.993094598	up_in_case
CRA004775	ENSGALG00010025114	CEBPG	case	control	0.195762912	0.248400627	0.78809347	0.430642035	0.824577397	up_in_case
CRA004775	ENSGALG00010025115	MGAT4F	case	control	-0.01862829	0.424607563	-0.043871782	0.965006608	0.992951652	down_in_case
CRA004775	ENSGALG00010025116	ANAPC7	case	control	-0.094935308	0.188870184	-0.502648463	0.61521145	0.902664601	down_in_case
CRA004775	ENSGALG00010025121	DYNC2I1	case	control	0.011997488	0.35327733	0.033960536	0.97290862	0.994947809	up_in_case
CRA004775	ENSGALG00010025125	PIK3CB	case	control	0.295134378	0.455476167	0.647968872	0.517005083	0.864913871	up_in_case
CRA004775	ENSGALG00010025126	ENSGALG00010025126	case	control	0.175593204	0.230005464	0.763430578	0.44520665	0.833012432	up_in_case
CRA004775	ENSGALG00010025127	PAMR1	case	control	-0.114443094	0.523262171	-0.218710812	0.826875327	0.961878235	down_in_case
CRA004775	ENSGALG00010025130	ATP2A2	case	control	-0.186334866	0.324635241	-0.573982249	0.565979814	0.883033678	down_in_case
CRA004775	ENSGALG00010025131	SUMO1	case	control	-0.35056201	0.194046124	-1.80659115	0.07082605	0.469911807	down_in_case
CRA004775	ENSGALG00010025138	CFAP91	case	control	-0.399352743	0.634071024	-0.629823361	0.52881016	0.86916155	down_in_case
CRA004775	ENSGALG00010025139	COPB2	case	control	-0.106674537	0.164713797	-0.647635714	0.517220592	0.864927687	down_in_case
CRA004775	ENSGALG00010025144	PIH1D2	case	control	0.143509166	0.293707629	0.488612321	0.625116188	0.906591226	up_in_case
CRA004775	ENSGALG00010025145	CPT2	case	control	-0.464906568	0.282973643	-1.642932404	0.10039692	0.538583505	down_in_case
CRA004775	ENSGALG00010025147	COX17	case	control	-0.161571647	0.326542742	-0.494794788	0.620744984	0.905373809	down_in_case
CRA004775	ENSGALG00010025149	IFT81	case	control	0.404066123	0.337111527	1.198612597	0.230678618	0.697177844	up_in_case
CRA004775	ENSGALG00010025154	TMEM183A	case	control	0.110145354	0.320581711	0.343579656	0.731162426	0.936495391	up_in_case
CRA004775	ENSGALG00010025155	CZIB	case	control	0.206190926	0.282310891	0.7303683	0.46516509	0.843383332	up_in_case
CRA004775	ENSGALG00010025156	DLAT	case	control	-0.097006323	0.214496705	-0.452250876	0.651088261	0.915732298	down_in_case
CRA004775	ENSGALG00010025157	NOP58	case	control	0.151988324	0.231188877	0.657420573	0.510910527	0.863170205	up_in_case
CRA004775	ENSGALG00010025160	MRPS22	case	control	0.001845982	0.315362168	0.005853531	0.995329585	0.999001196	up_in_case
CRA004775	ENSGALG00010025161	ENSGALG00010025161	case	control	-0.377767019	0.209089452	-1.806724422	0.070805258	0.469911807	down_in_case
CRA004775	ENSGALG00010025163	LDLRAD3	case	control	0.022773428	0.404608078	0.056285153	0.955114646	0.990670511	up_in_case
CRA004775	ENSGALG00010025164	C11orf52	case	control	-0.680877076	0.445286516	-1.52907634	0.12624552	0.580543965	down_in_case
CRA004775	ENSGALG00010025167	PTGES3	case	control	-0.060017612	0.226009298	-0.265553728	0.790582926	0.951054854	down_in_case
CRA004775	ENSGALG00010025168	POPDC2	case	control	-0.517849195	0.586194312	-0.883408768	0.377015455	0.798523018	down_in_case
CRA004775	ENSGALG00010025169	RBP2	case	control	2.420406891	1.127632766	2.146449593	0.031837126	0.339997388	up_in_case
CRA004775	ENSGALG00010025170	COMMD9	case	control	-0.223564525	0.192946258	-1.158688063	0.246583358	0.712046495	down_in_case
CRA004775	ENSGALG00010025182	PLA1A	case	control	-0.461454022	0.29488343	-1.564869285	0.117613562	0.568272695	down_in_case
CRA004775	ENSGALG00010025186	TMEM120B	case	control	-0.32833452	0.273318531	-1.20128891	0.229639151	0.695782399	down_in_case
CRA004775	ENSGALG00010025188	PPFIA4	case	control	-0.404256826	0.533323838	-0.757995044	0.44845395	0.83429564	down_in_case
CRA004775	ENSGALG00010025190	PRR5L	case	control	-0.688178278	0.548931154	-1.25366956	0.209962137	0.67982426	down_in_case
CRA004775	ENSGALG00010025192	CRYAB	case	control	0.189787961	0.620405322	0.305909628	0.759673455	0.942236668	up_in_case
CRA004775	ENSGALG00010025195	P2RX7	case	control	-0.346548233	0.54915835	-0.631053379	0.528005623	0.868597358	down_in_case
CRA004775	ENSGALG00010025200	PEPD	case	control	-0.309511535	0.172193563	-1.797462867	0.072262168	0.474308865	down_in_case
CRA004775	ENSGALG00010025204	TRAF6	case	control	0.416041559	0.184825178	2.251000449	0.024385509	0.30145607	up_in_case
CRA004775	ENSGALG00010025207	BMPR2	case	control	0.410360657	0.353696961	1.16020408	0.245965726	0.712046495	up_in_case
CRA004775	ENSGALG00010025210	HSPB2	case	control	-0.18957416	0.737995163	-0.256877239	0.797273542	0.952835572	down_in_case
CRA004775	ENSGALG00010025212	P2RX4	case	control	-0.134717652	0.340042782	-0.396178536	0.691973324	0.927867527	down_in_case
CRA004775	ENSGALG00010025214	LRP8	case	control	0.197198	0.64199344	0.307165133	0.758717684	0.942236668	up_in_case
CRA004775	ENSGALG00010025215	MYOG	case	control	-0.438918202	0.723610313	-0.60656709	0.544138246	0.875729696	down_in_case
CRA004775	ENSGALG00010025219	NACA	case	control	-0.201906316	0.205894994	-0.98062761	0.326776414	0.770998457	down_in_case
CRA004775	ENSGALG00010025222	FAM117B	case	control	-0.690768277	0.585639037	-1.179512009	0.238194358	0.704790868	down_in_case
CRA004775	ENSGALG00010025225	DMRTB1	case	control	1.829186795	1.261113325	1.450453943	0.146931974	0.610295079	up_in_case
CRA004775	ENSGALG00010025228	FDXACB1	case	control	-0.650616567	0.209953415	-3.098861564	0.001942658	0.087873309	down_in_case
CRA004775	ENSGALG00010025229	ENSGALG00010025229	case	control	-0.199021326	0.35397597	-0.562245302	0.5739489	0.886897708	down_in_case
CRA004775	ENSGALG00010025232	PRIM1	case	control	-0.279218686	0.180942516	-1.543134763	0.122798082	0.57525466	down_in_case
CRA004775	ENSGALG00010025233	WDR12	case	control	-0.415271428	0.191317627	-2.170586339	0.029962456	0.330139205	down_in_case
CRA004775	ENSGALG00010025236	ENSGALG00010025236	case	control	0.51969457	0.320491002	1.621557445	0.104898141	0.547815057	up_in_case
CRA004775	ENSGALG00010025238	IGSF9B	case	control	-0.08629456	0.704271282	-0.122530284	0.902479064	0.979310476	down_in_case
CRA004775	ENSGALG00010025242	CLSTN2	case	control	0.129167474	0.597538658	0.216165888	0.828858442	0.962881849	up_in_case
CRA004775	ENSGALG00010025243	PAXIP1	case	control	-0.008642204	0.249146915	-0.034687182	0.972329182	0.994947809	down_in_case
CRA004775	ENSGALG00010025245	ENSGALG00010025245	case	control	0.085656505	0.201801371	0.424459483	0.671230748	0.921535334	up_in_case
CRA004775	ENSGALG00010025247	SGCA	case	control	-0.148636905	0.581439305	-0.255636149	0.798231803	0.952835572	down_in_case
CRA004775	ENSGALG00010025248	C11orf74	case	control	-0.595607207	0.332286589	-1.792450331	0.073060859	0.47704647	down_in_case
CRA004775	ENSGALG00010025250	GLIS1	case	control	-1.533825726	0.849317963	-1.805949942	0.070926159	0.469911807	down_in_case
CRA004775	ENSGALG00010025252	PPP1R9B	case	control	-0.510158086	0.240517889	-2.121081672	0.03391493	0.349772852	down_in_case
CRA004775	ENSGALG00010025256	ANAPC5	case	control	-0.337276562	0.277332349	-1.216145769	0.223929398	0.69173716	down_in_case
CRA004775	ENSGALG00010025257	LPCAT2	case	control	-0.851188266	0.434392367	-1.959491766	0.050055223	0.414161886	down_in_case
CRA004775	ENSGALG00010025262	CARF	case	control	-0.25938914	0.303869789	-0.853619379	0.393315918	0.807807863	down_in_case
CRA004775	ENSGALG00010025263	SAMD14	case	control	0.218189726	0.601514266	0.362734084	0.716803536	0.934011578	up_in_case
CRA004775	ENSGALG00010025268	ZBTB39	case	control	-0.392541423	0.194063666	-2.022745579	0.043099385	0.389478281	down_in_case
CRA004775	ENSGALG00010025270	NDC1	case	control	-0.427726527	0.375594164	-1.138799716	0.2547867	0.718438877	down_in_case
CRA004775	ENSGALG00010025271	RNF34	case	control	0.166622739	0.150702469	1.105640403	0.268882195	0.727586876	up_in_case
CRA004775	ENSGALG00010025272	NCAPD3	case	control	0.239238313	0.425174596	0.56268252	0.573651089	0.886897708	up_in_case
CRA004775	ENSGALG00010025274	PDK2	case	control	-0.261872196	0.50208296	-0.521571566	0.601968665	0.898185892	down_in_case
CRA004775	ENSGALG00010025280	HSPB11	case	control	0.021308296	0.384375093	0.055436205	0.955790953	0.990926004	up_in_case
CRA004775	ENSGALG00010025283	ENSGALG00010025283	case	control	-0.465682744	0.764344225	-0.609257882	0.54235352	0.875135326	down_in_case
CRA004775	ENSGALG00010025286	ITGA3	case	control	-0.240283515	0.244171158	-0.984078205	0.325077062	0.770251058	down_in_case
CRA004775	ENSGALG00010025289	KDM2B	case	control	0.008455813	0.241318767	0.035040014	0.972047834	0.994947809	up_in_case
CRA004775	ENSGALG00010025291	DUS2	case	control	-0.250647382	0.280865002	-0.8924123	0.372171981	0.796004755	down_in_case
CRA004775	ENSGALG00010025292	ATP1A1	case	control	0.136189295	0.219367694	0.62082658	0.534713733	0.870981055	up_in_case
CRA004775	ENSGALG00010025297	SPSB4	case	control	1.042733135	0.426384508	2.445523031	0.014464221	0.240504965	up_in_case
CRA004775	ENSGALG00010025298	ENSGALG00010025298	case	control	-0.972621459	0.605665781	-1.605871569	0.108302132	0.555197961	down_in_case
CRA004775	ENSGALG00010025301	MORN3	case	control	0.583959039	0.469260627	1.244423686	0.21334367	0.682777582	up_in_case
CRA004775	ENSGALG00010025303	TMCC2	case	control	-0.685990325	0.340342824	-2.015586278	0.043843256	0.391981676	down_in_case
CRA004775	ENSGALG00010025304	DDX28	case	control	-0.245888632	0.234051233	-1.050576101	0.293453322	0.746438942	down_in_case
CRA004775	ENSGALG00010025305	GPR182	case	control	1.233438	0.382975672	3.220669329	0.001278916	0.07547801	up_in_case
CRA004775	ENSGALG00010025312	PLEKHM3	case	control	0.228546498	0.280884619	0.813666831	0.415835841	0.818943344	up_in_case
CRA004775	ENSGALG00010025315	LRRC42	case	control	0.171869975	0.242150482	0.709765158	0.477849779	0.846563638	up_in_case
CRA004775	ENSGALG00010025316	SLC25A36	case	control	0.210792368	0.187365588	1.125032457	0.260575281	0.721540613	up_in_case
CRA004775	ENSGALG00010025318	NUAK2	case	control	0.809788367	0.572131644	1.41538818	0.156954713	0.621625652	up_in_case
CRA004775	ENSGALG00010025319	LDLRAD1	case	control	-0.453980321	0.588591012	-0.771300125	0.440529058	0.829353567	down_in_case
CRA004775	ENSGALG00010025320	JAM3	case	control	0.317868453	0.323590647	0.982316563	0.325943917	0.770998457	up_in_case
CRA004775	ENSGALG00010025323	KLHDC8A	case	control	0.215162001	0.438572641	0.490596041	0.623712187	0.906441769	up_in_case
CRA004775	ENSGALG00010025325	DPEP2	case	control	0.092201658	0.409216089	0.225312887	0.821735875	0.960766569	up_in_case
CRA004775	ENSGALG00010025327	TMEM59	case	control	-0.063911563	0.153593879	-0.416107486	0.677331352	0.922924485	down_in_case
CRA004775	ENSGALG00010025329	MYO1A	case	control	0.613871258	0.55760359	1.100909802	0.270935915	0.728665115	up_in_case
CRA004775	ENSGALG00010025330	USH1C	case	control	2.415611209	0.714357905	3.381513935	0.000720876	0.056322515	up_in_case
CRA004775	ENSGALG00010025331	ENSGALG00010025331	case	control	-0.595518887	0.389267412	-1.529845213	0.126055047	0.580487602	down_in_case
CRA004775	ENSGALG00010025333	FZD5	case	control	0.364381944	0.269419841	1.352468857	0.176225375	0.641590771	up_in_case
CRA004775	ENSGALG00010025334	RHOF	case	control	-0.597235839	0.583173714	-1.024113097	0.305781859	0.758032511	down_in_case
CRA004775	ENSGALG00010025336	ADAM10	case	control	0.236761957	0.322932663	0.733161999	0.463459629	0.842456502	up_in_case
CRA004775	ENSGALG00010025337	NEMP1	case	control	-0.466036824	0.168201228	-2.770710003	0.005593422	0.15147564	down_in_case
CRA004775	ENSGALG00010025338	FADD	case	control	-0.121331951	0.454344944	-0.267048094	0.789432132	0.950475151	down_in_case
CRA004775	ENSGALG00010025340	METTL21A	case	control	-0.274241323	0.317854324	-0.862789341	0.388253301	0.804270424	down_in_case
CRA004775	ENSGALG00010025341	VPS26B	case	control	-0.205362734	0.236980757	-0.86657979	0.386172291	0.803610044	down_in_case
CRA004775	ENSGALG00010025342	TCEANC2	case	control	-0.371541167	0.250831103	-1.481240414	0.13854252	0.597959259	down_in_case
CRA004775	ENSGALG00010025343	NAB2	case	control	-0.276937143	0.421617695	-0.656844214	0.511281091	0.863170205	down_in_case
CRA004775	ENSGALG00010025345	CCM2	case	control	0.010093255	0.28542622	0.035362044	0.97179105	0.994947809	up_in_case
CRA004775	ENSGALG00010025346	PXYLP1	case	control	-0.422057337	0.469235601	-0.899457195	0.368409186	0.794665838	down_in_case
CRA004775	ENSGALG00010025348	HIPK1	case	control	-0.082177856	0.41587526	-0.197602175	0.843356333	0.966478468	down_in_case
CRA004775	ENSGALG00010025349	SETD1A	case	control	-0.336824635	0.327556459	-1.028294895	0.303811132	0.756198056	down_in_case
CRA004775	ENSGALG00010025351	CREB1	case	control	-0.007384419	0.319757477	-0.023093811	0.981575442	0.997069916	down_in_case
CRA004775	ENSGALG00010025353	SLC12A4	case	control	0.242976112	0.268900745	0.903590327	0.366212672	0.793608387	up_in_case
CRA004775	ENSGALG00010025355	STAT6	case	control	-0.307373048	0.247851284	-1.240151123	0.214919503	0.683649824	down_in_case
CRA004775	ENSGALG00010025357	OLFML3	case	control	0.222359857	0.386589881	0.575182816	0.565167663	0.882950982	up_in_case

CRA004775	ENSGALG00010025358	INCENP	case	control	0.211601945	0.507160493	0.417228762	0.67651109	0.922924485	up_in_case
CRA004775	ENSGALG00010025359	CDCP2	case	control	0.511452897	0.511545448	0.999819076	0.317398073	0.766246646	up_in_case
CRA004775	ENSGALG00010025360	THYN1	case	control	-0.142979433	0.181935418	-0.785880145	0.431937708	0.825399586	down_in_case
CRA004775	ENSGALG00010025362	G6PC2	case	control	-0.596942725	0.488639443	-1.221642531	0.221842838	0.689550502	down_in_case
CRA004775	ENSGALG00010025364	EPHA4	case	control	-0.585423076	0.496908192	-1.178131263	0.23874428	0.705560412	down_in_case
CRA004775	ENSGALG00010025365	SIKE1	case	control	-0.12752238	0.283911284	-0.449162776	0.653314238	0.916442743	down_in_case
CRA004775	ENSGALG00010025368	LRP1	case	control	-0.266510315	0.383468884	-0.694998542	0.487056215	0.852751466	down_in_case
CRA004775	ENSGALG00010025369	ENSGALG00010025369	case	control	1.425166501	0.590838442	2.412108624	0.015860555	0.252039758	up_in_case
CRA004775	ENSGALG00010025370	HPD	case	control	-1.050733009	0.437279264	-2.402887801	0.016266177	0.25344686	down_in_case
CRA004775	ENSGALG00010025373	CYB5RL	case	control	-0.354261727	0.224943913	-1.574889146	0.115282034	0.565776219	down_in_case
CRA004775	ENSGALG00010025377	MYO1G	case	control	-0.579936273	0.479484219	-1.209500231	0.226470722	0.694513583	down_in_case
CRA004775	ENSGALG00010025378	PAX3	case	control	0.760387502	0.540319317	1.407292833	0.159340574	0.624907945	up_in_case
CRA004775	ENSGALG00010025379	ACAD8	case	control	0.122282751	0.379283138	0.322404924	0.747145955	0.940412267	up_in_case
CRA004775	ENSGALG00010025380	SPC25	case	control	-0.172174626	0.521763869	-0.329985719	0.741410753	0.938778937	down_in_case
CRA004775	ENSGALG00010025381	MRPL37	case	control	0.111333776	0.342642581	0.324926854	0.745236432	0.939384561	up_in_case
CRA004775	ENSGALG00010025382	MAB21L3	case	control	0.030701462	0.497729083	0.061683078	0.950815216	0.990168609	up_in_case
CRA004775	ENSGALG00010025386	TPCN2	case	control	0.056957276	0.29749395	0.191456922	0.848167627	0.968199438	up_in_case
CRA004775	ENSGALG00010025387	PSMD9	case	control	-0.276868148	0.240994399	-1.148857188	0.250614873	0.715557647	down_in_case
CRA004775	ENSGALG00010025389	SNED1	case	control	-0.09791036	0.306441343	-0.319507673	0.749341574	0.941366343	down_in_case
CRA004775	ENSGALG00010025390	BCL7A	case	control	0.402440903	0.255597004	1.57451338	0.115368811	0.565776219	up_in_case
CRA004775	ENSGALG00010025392	SLC6A2	case	control	0.261393637	0.351404172	0.743854677	0.456964397	0.839688534	up_in_case
CRA004775	ENSGALG00010025398	TSPAN2	case	control	0.103399882	0.358424442	0.288484461	0.772975924	0.946296525	up_in_case
CRA004775	ENSGALG00010025400	SLC22A15	case	control	-0.655714471	0.483659166	-1.355736677	0.175182965	0.640706322	down_in_case
CRA004775	ENSGALG00010025403	ENSGALG00010025403	case	control	-0.154965701	0.324689011	-0.47727424	0.633166852	0.909723668	down_in_case
CRA004775	ENSGALG00010025404	CERS6	case	control	-0.993767481	0.680622261	-1.460086655	0.14426626	0.606297475	down_in_case
CRA004775	ENSGALG00010025405	TAMALIN	case	control	0.14089707	0.310729426	0.453439738	0.650232127	0.915428088	up_in_case
CRA004775	ENSGALG00010025406	ENSGALG00010025406	case	control	0.198858692	0.269156136	0.738822805	0.460014602	0.840995542	up_in_case
CRA004775	ENSGALG00010025407	MLXIP	case	control	0.471304148	0.351685007	1.340131478	0.180202604	0.644990528	up_in_case
CRA004775	ENSGALG00010025413	NECAB3	case	control	-0.371978663	0.271672153	-1.369218959	0.170930841	0.635171699	down_in_case
CRA004775	ENSGALG00010025414	MMP2	case	control	0.575769563	0.368997795	1.560360443	0.118674727	0.570664629	up_in_case
CRA004775	ENSGALG00010025415	ENSGALG00010025415	case	control	-0.370540928	0.43767741	-0.846607386	0.397213996	0.808998414	down_in_case
CRA004775	ENSGALG00010025416	ENSGALG00010025416	case	control	-0.849177025	0.610130506	-1.391795718	0.163984267	0.629717526	down_in_case
CRA004775	ENSGALG00010025417	B3GAT1	case	control	0.295374273	0.532368438	0.55483055	0.579010569	0.888378369	up_in_case
CRA004775	ENSGALG00010025418	CASQ2	case	control	0.113667536	0.728753977	0.155975184	0.876052582	0.974421894	up_in_case
CRA004775	ENSGALG00010025419	CCND1	case	control	-0.055195308	0.274372856	-0.201168983	0.840566444	0.966461921	down_in_case
CRA004775	ENSGALG00010025420	STK39	case	control	-0.439263153	0.468774208	-0.937046335	0.348734724	0.785311667	down_in_case
CRA004775	ENSGALG00010025421	NR4A1	case	control	-0.241022394	0.664661824	-0.362624098	0.716885706	0.934011578	down_in_case
CRA004775	ENSGALG00010025422	SSBP3	case	control	-0.166300587	0.163516917	-1.017023744	0.309142134	0.761557414	down_in_case
CRA004775	ENSGALG00010025423	NGF	case	control	0.441569524	0.466200612	0.947166333	0.343554028	0.783191551	up_in_case
CRA004775	ENSGALG00010025424	IRX6	case	control	0.030289093	0.589348115	0.051394231	0.959011382	0.991431439	up_in_case
CRA004775	ENSGALG00010025425	ENSGALG00010025425	case	control	-0.386027535	0.286490578	-1.347435358	0.177840059	0.643716414	down_in_case
CRA004775	ENSGALG00010025426	ENSGALG00010025426	case	control	-0.092833564	0.423935265	-0.218980519	0.826665223	0.961878235	down_in_case
CRA004775	ENSGALG00010025427	SLC35G2	case	control	-0.460652594	0.391785673	-1.175777028	0.239683988	0.706254731	down_in_case
CRA004775	ENSGALG00010025428	VANGL1	case	control	-0.06305055	0.331555075	-0.190166143	0.849178941	0.968407664	down_in_case
CRA004775	ENSGALG00010025432	IRX3	case	control	-0.158153826	0.59137431	-0.267434386	0.789134727	0.95041797	down_in_case
CRA004775	ENSGALG00010025433	CHPF2	case	control	-0.470796026	0.229233415	-2.053784459	0.039996558	0.375888502	down_in_case
CRA004775	ENSGALG00010025434	SYT6	case	control	0.054988225	0.604282744	0.09099751	0.92749457	0.984564671	up_in_case
CRA004775	ENSGALG00010025435	SCLY	case	control	-0.367995382	0.222890123	-1.651017	0.098735104	0.535162165	down_in_case
CRA004775	ENSGALG00010025436	ENSGALG00010025436	case	control	-0.225612606	0.680802814	-0.331391999	0.740348408	0.938450627	down_in_case
CRA004775	ENSGALG00010025438	EXOSC6	case	control	-0.308401174	0.410295938	-0.751655441	0.452258293	0.836784182	down_in_case
CRA004775	ENSGALG00010025442	ABCF2	case	control	0.123493019	0.366589413	0.336870118	0.736214812	0.937385622	up_in_case
CRA004775	ENSGALG00010025443	ENSGALG00010025443	case	control	-1.522267265	0.709239901	-2.146336187	0.031846167	0.339997388	down_in_case
CRA004775	ENSGALG00010025445	B3GNT4	case	control	0.33015125	0.435507118	0.758084625	0.448400323	0.83429564	up_in_case
CRA004775	ENSGALG00010025448	CHD1L	case	control	-0.419378853	0.313947353	-1.335825417	0.181606343	0.647378665	down_in_case
CRA004775	ENSGALG00010025449	ENSGALG00010025449	case	control	-0.236133809	0.280649683	-0.841382774	0.400133533	0.811658978	down_in_case
CRA004775	ENSGALG00010025451	LRRC43	case	control	-0.651481344	0.588802793	-1.106450838	0.268531431	0.727528758	down_in_case
CRA004775	ENSGALG00010025452	TRIM33	case	control	0.255527863	0.262384105	0.973869443	0.330121367	0.77237154	up_in_case
CRA004775	ENSGALG00010025458	BCAS2	case	control	-0.373647203	0.295163508	-1.265899045	0.205549228	0.674045742	down_in_case
CRA004775	ENSGALG00010025459	DIABLO	case	control	0.049250286	0.218795005	0.225097855	0.82190315	0.960766569	up_in_case
CRA004775	ENSGALG00010025464	VPS33A	case	control	-0.294325171	0.175267107	-1.679294969	0.093094571	0.524831789	down_in_case
CRA004775	ENSGALG00010025465	ENSGALG00010025465	case	control	-0.313288007	0.281244076	-1.11393638	0.265306481	0.726437726	down_in_case
CRA004775	ENSGALG00010025470	ORAOV1	case	control	0.148959094	0.255739528	0.5824641	0.560254116	0.880918893	up_in_case
CRA004775	ENSGALG00010025472	ZCCHC8	case	control	-0.07285435	0.221006881	-0.32964743	0.74166638	0.938877854	down_in_case
CRA004775	ENSGALG00010025473	PARS2	case	control	-0.14928621	0.209564453	-0.712364184	0.476239288	0.846367006	down_in_case
CRA004775	ENSGALG00010025477	ENSGALG00010025477	case	control	0.109741744	0.143371194	0.765437884	0.444010841	0.832130395	up_in_case
CRA004775	ENSGALG00010025481	TMEM39A	case	control	-0.314468298	0.119240475	-2.637261369	0.008357841	0.18329931	down_in_case
CRA004775	ENSGALG00010025483	FGF4	case	control	1.208064926	0.714664242	1.69039509	0.090952394	0.520902277	up_in_case
CRA004775	ENSGALG00010025485	DENND2C	case	control	0.074682108	0.237318237	0.314691821	0.752995655	0.941826747	up_in_case
CRA004775	ENSGALG00010025486	B3GALT1	case	control	0.757719079	0.630125117	1.202489885	0.229173783	0.695782399	up_in_case
CRA004775	ENSGALG00010025488	SLC4A8	case	control	-0.823928816	0.436787414	-1.886338274	0.059249385	0.44414236	down_in_case
CRA004775	ENSGALG00010025490	ENSGALG00010025490	case	control	-0.162334097	0.831527919	-0.195223868	0.845217695	0.966993336	down_in_case
CRA004775	ENSGALG00010025491	ZMYND11	case	control	0.265595422	0.173421292	1.531504118	0.125644849	0.57939045	up_in_case
CRA004775	ENSGALG00010025496	ATG101	case	control	-0.160357701	0.253922165	-0.631523053	0.527698581	0.86830001	down_in_case
CRA004775	ENSGALG00010025499	STAC3	case	control	-0.36669604	0.684628242	-0.53561337	0.592225786	0.893923098	down_in_case
CRA004775	ENSGALG00010025504	AMPD1	case	control	-0.205640995	0.792105725	-0.259613065	0.795162257	0.952360933	down_in_case
CRA004775	ENSGALG00010025505	ENSGALG00010025505	case	control	0.866183705	0.829464958	1.044267991	0.296361418	0.748897794	up_in_case
CRA004775	ENSGALG00010025506	R3HDM2	case	control	-0.132233664	0.249905804	-0.529134026	0.596712479	0.895687512	down_in_case
CRA004775	ENSGALG00010025508	ENSGALG00010025508	case	control	-0.242091091	0.333472066	-0.725971125	0.467856472	0.843870188	down_in_case
CRA004775	ENSGALG00010025512	NRAS	case	control	-0.12344339	0.155194483	-0.795410943	0.426374496	0.822541484	down_in_case
CRA004775	ENSGALG00010025513	RSRC2	case	control	0.240391991	0.179198307	1.341485838	0.179762765	0.644990528	up_in_case
CRA004775	ENSGALG00010025520	ACOT11	case	control	0.077340738	0.551246618	0.140301519	0.888421764	0.977118922	up_in_case
CRA004775	ENSGALG00010025522	SUMO3	case	control	-0.128368232	0.172461264	-0.744330811	0.456676365	0.839688534	down_in_case
CRA004775	ENSGALG00010025523	B4GALT4	case	control	0.385119657	0.325182388	1.184318926	0.23628684	0.703419274	up_in_case
CRA004775	ENSGALG00010025527	KNTC1	case	control	0.023194693	0.80255723	0.028900983	0.976943562	0.995689064	up_in_case
CRA004775	ENSGALG00010025531	ENSGALG00010025531	case	control	-0.09331396	0.184820168	-0.504890572	0.613635696	0.902282137	down_in_case
CRA004775	ENSGALG00010025533	INHBC	case	control	-0.204079123	0.386272311	-0.52832967	0.597270542	0.896329502	down_in_case
CRA004775	ENSGALG00010025538	ANO1	case	control	0.276446193	0.34166673	0.80911066	0.418451497	0.818943344	up_in_case
CRA004775	ENSGALG00010025541	TTC21B	case	control	-0.023085482	0.257458539	-0.089666794	0.928552004	0.98473447	down_in_case
CRA004775	ENSGALG00010025544	CSDE1	case	control	0.307591412	0.183906828	1.672539373	0.094418011	0.526933666	up_in_case
CRA004775	ENSGALG00010025548	ENSGALG00010025548	case	control	1.289914142	0.447390875	2.883192782	0.003936664	0.129085442	up_in_case
CRA004775	ENSGALG00010025549	DHCR24	case	control	-0.060763619	0.526400817	-0.115432228	0.908102535	0.980606464	down_in_case
CRA004775	ENSGALG00010025553	UBE2G2	case	control	-0.31507558	0.179772152	-1.752638417	0.079664093	0.492120816	down_in_case
CRA004775	ENSGALG00010025554	DIP2C	case	control	0.489917941	0.308609479	1.587501271	0.112399159	0.562828756	up_in_case
CRA004775	ENSGALG00010025557	VPS29	case	control	-0.232467575	0.195301582	-1.190300524	0.233928295	0.701251407	down_in_case
CRA004775	ENSGALG00010025558	GALNT3	case	control	-0.176684105	0.724194854	-0.243973158	0.807251609	0.955397873	down_in_case
CRA004775	ENSGALG00010025559	SLC6A17	case	control	-0.610998418	0.899522833	-0.679247259	0.496981208	0.857679291	down_in_case
CRA004775	ENSGALG00010025562	GRAMD1B	case	control	-0.290198894	0.464580151	-0.624647638	0.532202347	0.869886675	down_in_case
CRA004775	ENSGALG00010025563	TSPEAR	case	control	0.203876385	0.396235784	0.514532996	0.606879398	0.900898526	up_in_case
CRA004775	ENSGALG00010025565	ENSGALG00010025565	case	control	-0.445320847	0.438907039	-1.014613134	0.310290276	0.762244121	down_in_case
CRA004775	ENSGALG00010025569	PPTC7	case	control	0.822121797	0.408604991	2.012020937	0.04421773	0.392535833	up_in_case
CRA004775	ENSGALG00010025571	SRSF3	case	control	-0.552930376	0.229591739	-2.408319997	0.016026127	0.253014091	down_in_case
CRA004775	ENSGALG00010025572	ENSGALG00010025572	case	control	-0.323618059	0.161989116	-1.997776562	0.045740889	0.398090488	down_in_case
CRA004775	ENSGALG00010025573	CLMP	case	control	-0.194038349	0.464220085	-0.417987835	0.675956011	0.922924485	down_in_case
CRA004775	ENSGALG00010025574	ARHGAP31	case	control	0.120287791	0.392377424	0.306561448	0.759177202	0.942236668	up_in_case
CRA004775	ENSGALG00010025575	TRPM2	case	control	-0.798821629	0.793168805	-1.007126887	0.313873802	0.765098415	down_in_case
CRA004775	ENSGALG00010025577	PTH2R	case	control	0.002834331	0.653170118	0.004339345	0.996537714	0.999606847	up_in_case
CRA004775	ENSGALG00010025578	ORMDL2	case	control	-0.45324741	0.19638038	-2.308007605	0.020998714	0.284887644	down_in_case
CRA004775	ENSGALG00010025580	RASSF7	case	control	-0.610141267	0.462845777	-1.318238812	0.187423717	0.651317546	down_in_case
CRA004775	ENSGALG00010025582	DMAP1	case	control	-0.396806701	0.239694056	-1.655471595	0.097828874	0.532770234	down_in_case
CRA004775	ENSGALG00010025585	SARNP	case	control	-0.359964309	0.206566767	-1.742605135	0.081402613	0.496834143	down_in_case
CRA004775	ENSGALG00010025586	TCTN1	case	control	0.670957141	0.269534082	2.489322081	0.012798696	0.227866767	up_in_case
CRA004775	ENSGALG00010025588	ENSGALG00010025588	case	control	-0.313891785	0.415059102	-0.756258043	0.449494499	0.835036044	down_in_case
CRA004775	ENSGALG00010025590	HRAS	case	control	-0.000253616	0.311126903	-0.000815154	0.999349601	0.999914248	down_in_case
CRA004775	ENSGALG00010025591	OTOS	case	control	0.216158721	0.448676838	0.481769287	0.62996985	0.908943555	up_in_case
CRA004775	ENSGALG00010025592	HSPA8	case	control	0.120996913	0.30796138	0.392896384	0.694396012	0.928765642	up_in_case
CRA004775	ENSGALG00010025595	ERI3	case	control	-0.241947178	0.288692759	-0.838078443	0.401986649	0.811868062	down_in_case
CRA004775	ENSGALG00010025598	ENSGALG00010025598	case	control	-0.365999082	0.358685853	-1.020388954	0.30754403	0.759249237	down_in_case
CRA004775	ENSGALG00010025599	COPS9	case	control	0.0441934	0.327444107	0.134964712	0.892639774	0.977834811	up_in_case
CRA004775	ENSGALG00010025600	ENSGALG00010025600	case	control	-0.425981152	0.399452031	-1.066413784	0.286236633	0.741582427	down_in_case
CRA004775	ENSGALG00010025604	HVCN1	case	control	0.672835993	0.312360914	2.154033882	0.031237515	0.336670173	up_in_case
CRA004775	ENSGALG00010025606	CCDC12	case	control	-0.162165897	0.283816657	-0.571375544	0.567745103	0.884152085	down_in_case
CRA004775	ENSGALG00010025608	ENSGALG00010025608	case	control	0.371999016	0.499305545	0.745032815	0.456251883	0.839657339	up_in_case
CRA004775	ENSGALG00010025610	RNH1	case	control	-0.24790469	0.183698572	-1.349518874	0.177170362	0.643154607	down_in_case
CRA004775	ENSGALG00010025612	TMEM53	case	control	-0.517270629	0.295068127	-1.753054908	0.079592583	0.492032795	down_in_case
CRA004775	ENSGALG00010025613	CDKN1A	case	control	0.179033008	0.51255725	0.34929368	0.726868843	0.935238044	up_in_case
CRA004775	ENSGALG00010025615	AQP5	case	control	2.906472894	0.740893846	3.922927568	8.74795E-05	0.020740173	up_in_case

CRA004775	ENSGALG00010025618	ADIPOQ	case	control	0.325967305	0.430529386	0.757131373	0.44897116	0.834536953	up_in_case
CRA004775	ENSGALG00010025621	PPP1CC	case	control	-0.07305523	0.33039053	-0.22111781	0.825000703	0.961768508	down_in_case
CRA004775	ENSGALG00010025623	ENSGALG00010025623	case	control	-0.651047429	0.496779828	-1.310535156	0.190014859	0.655361714	down_in_case
CRA004775	ENSGALG00010025624	ARMH1	case	control	-0.396226962	0.398078412	-0.995349032	0.319566538	0.767037518	down_in_case
CRA004775	ENSGALG00010025629	ST6GAL1	case	control	0.385509636	0.234459304	1.644249679	0.100124639	0.538583505	up_in_case
CRA004775	ENSGALG00010025635	CHKA	case	control	0.286254046	0.212906276	1.344507316	0.178784392	0.644859234	up_in_case
CRA004775	ENSGALG00010025637	C6orf89	case	control	-0.422696083	0.239895279	-1.762002508	0.078068874	0.490500984	down_in_case
CRA004775	ENSGALG00010025638	ENSGALG00010025638	case	control	0.478764986	0.807029427	0.59324353	0.553018185	0.878546876	up_in_case
CRA004775	ENSGALG00010025642	RPS8	case	control	-0.302921129	0.29171943	-1.038398878	0.299084392	0.75239174	down_in_case
CRA004775	ENSGALG00010025643	ENSGALG00010025643	case	control	0.59869932	0.706916761	0.84691629	0.397041782	0.808998414	up_in_case
CRA004775	ENSGALG00010025644	ENSGALG00010025644	case	control	0.14522734	0.664899938	0.218419844	0.827102006	0.961897808	up_in_case
CRA004775	ENSGALG00010025645	NBEAL2	case	control	-0.653372827	0.38147914	-1.712735399	0.08676123	0.509281038	down_in_case
CRA004775	ENSGALG00010025648	ENSGALG00010025648	case	control	-0.377144769	0.657263523	-0.573810589	0.566095982	0.883033678	down_in_case
CRA004775	ENSGALG00010025650	PPP1R1A	case	control	0.083976806	0.660666684	0.127109188	0.898853979	0.978790377	up_in_case
CRA004775	ENSGALG00010025652	KMT5B	case	control	0.368347328	0.236169053	1.559676527	0.118836342	0.570892125	up_in_case
CRA004775	ENSGALG00010025653	IGSF11	case	control	-0.634511513	0.707513136	-0.896819409	0.369815289	0.795234047	down_in_case
CRA004775	ENSGALG00010025654	CRTAM	case	control	-0.031469465	0.376541505	-0.083575024	0.933394326	0.985090297	down_in_case
CRA004775	ENSGALG00010025657	ENSGALG00010025657	case	control	-0.26617596	0.561064201	-0.47441266	0.635205667	0.909837348	down_in_case
CRA004775	ENSGALG00010025658	PDE1B	case	control	0.365456969	0.412249074	0.886495549	0.375350552	0.798503808	up_in_case
CRA004775	ENSGALG00010025659	COBLL1	case	control	0.281286616	0.433243366	0.649257757	0.516171785	0.864913871	up_in_case
CRA004775	ENSGALG00010025660	KIF2C	case	control	0.934618068	0.533664664	1.751320877	0.079890654	0.493298778	up_in_case
CRA004775	ENSGALG00010025661	SNRPE	case	control	-0.27063944	0.274949407	-0.984324507	0.324955983	0.770251058	down_in_case
CRA004775	ENSGALG00010025664	GPR55	case	control	1.431605112	0.681965043	2.09923533	0.035796161	0.359120808	up_in_case
CRA004775	ENSGALG00010025671	ENSGALG00010025671	case	control	2.363530344	0.692788839	3.411617236	0.000645787	0.052858455	up_in_case
CRA004775	ENSGALG00010025685	NCKAP1L	case	control	-0.601336495	0.420601183	-1.429707095	0.152801103	0.617472961	down_in_case
CRA004775	ENSGALG00010025688	BEST4	case	control	-0.233450001	0.603824087	-0.386619226	0.699038122	0.929347461	down_in_case
CRA004775	ENSGALG00010025691	ENSGALG00010025691	case	control	0.097123142	0.468184593	0.207446258	0.83566136	0.965077632	up_in_case
CRA004775	ENSGALG00010025692	ENSGALG00010025692	case	control	0.1458863	0.555106379	0.262807825	0.792698704	0.951284723	up_in_case
CRA004775	ENSGALG00010025696	MTCH1	case	control	-0.289749592	0.271562292	-1.066972845	0.285984099	0.741582427	down_in_case
CRA004775	ENSGALG00010025702	GTSF2	case	control	-0.265885281	0.360552099	-0.737439282	0.460855255	0.841484611	down_in_case
CRA004775	ENSGALG00010025703	UBASH3B	case	control	-0.264607447	0.468438307	-0.564871495	0.572161173	0.885642749	down_in_case
CRA004775	ENSGALG00010025705	ACSL3	case	control	-0.066012126	0.237334118	-0.278140061	0.780904844	0.948690036	down_in_case
CRA004775	ENSGALG00010025708	HOXC8	case	control	0.248917961	0.580812478	0.428568548	0.668237238	0.919720775	up_in_case
CRA004775	ENSGALG00010025720	PHETA1	case	control	-0.320249656	0.46974356	-0.681754224	0.495394374	0.856382445	down_in_case
CRA004775	ENSGALG00010025723	SETD2	case	control	0.700632983	0.252798095	2.771512118	0.00557966	0.15147564	up_in_case
CRA004775	ENSGALG00010025727	GLI1	case	control	0.112399288	0.375195147	0.299575538	0.764500946	0.942416182	up_in_case
CRA004775	ENSGALG00010025734	PLK3	case	control	-0.103619294	0.345367393	-0.30002628	0.76415711	0.94239774	down_in_case
CRA004775	ENSGALG00010025736	KIF9	case	control	0.253620396	0.41118589	0.616802284	0.537365145	0.872603995	up_in_case
CRA004775	ENSGALG00010025737	ENSGALG00010025737	case	control	-0.183300536	0.25043508	-0.731928353	0.4642123	0.842643327	down_in_case
CRA004775	ENSGALG00010025738	SOX13	case	control	-0.169896444	0.324880939	-0.522949867	0.601009141	0.89775697	down_in_case
CRA004775	ENSGALG00010025743	SH2B3	case	control	-0.087847629	0.330833035	-0.265534636	0.790597632	0.951054854	down_in_case
CRA004775	ENSGALG00010025745	COX14	case	control	-0.371869711	0.426433128	-0.872046955	0.383182762	0.802000938	down_in_case
CRA004775	ENSGALG00010025748	ETNK2	case	control	0.101186684	0.372610047	0.271561879	0.785958915	0.95041797	up_in_case
CRA004775	ENSGALG00010025753	SORL1	case	control	-0.725918549	0.509041511	-1.426049808	0.153853965	0.619153022	down_in_case
CRA004775	ENSGALG00010025755	ENSGALG00010025755	case	control	0.943448022	0.413916132	2.279321699	0.022647949	0.292762917	up_in_case
CRA004775	ENSGALG00010025787	KCNE4	case	control	0.252319251	0.348533674	0.723945114	0.469099432	0.8440023	up_in_case
CRA004775	ENSGALG00010025790	LRP5	case	control	-0.044959699	0.247770163	-0.181457276	0.856008665	0.970703965	down_in_case
CRA004775	ENSGALG00010025791	FIGN	case	control	0.901080708	0.477485414	1.887137661	0.059141812	0.44414236	up_in_case
CRA004775	ENSGALG00010025799	SC5D	case	control	-0.204604681	0.280327792	-0.729876549	0.465465648	0.843383332	down_in_case
CRA004775	ENSGALG00010025800	ARL2BP	case	control	-0.622226456	0.272667346	-2.281998435	0.022489433	0.292594999	down_in_case
CRA004775	ENSGALG00010025801	PTPN7	case	control	-0.690527432	0.44559438	-1.549677158	0.121219023	0.573473028	down_in_case
CRA004775	ENSGALG00010025805	ATXN2	case	control	0.162154039	0.205205147	0.790204538	0.429408326	0.823991481	up_in_case
CRA004775	ENSGALG00010025809	ENSGALG00010025809	case	control	-0.050009146	0.349422142	-0.143119567	0.886195752	0.976819452	down_in_case
CRA004775	ENSGALG00010025822	KLF7	case	control	-0.243260412	0.47632004	-0.510707909	0.609555602	0.901246133	down_in_case
CRA004775	ENSGALG00010025829	ENSGALG00010025829	case	control	-0.813851515	0.532257554	-1.529055828	0.126250605	0.580543965	down_in_case
CRA004775	ENSGALG00010025830	TBCEL	case	control	-1.069004214	0.542845959	-1.969258859	0.048923373	0.41021055	down_in_case
CRA004775	ENSGALG00010025831	BRAP	case	control	-0.060934072	0.153870223	-0.396009513	0.69209801	0.927867527	down_in_case
CRA004775	ENSGALG00010025832	ENKD1	case	control	0.177130379	0.455320993	0.389023087	0.697259068	0.929092636	up_in_case
CRA004775	ENSGALG00010025835	PPP6R3	case	control	0.059022016	0.287175689	0.205525809	0.837161335	0.965269623	up_in_case
CRA004775	ENSGALG00010025836	ENSGALG00010025836	case	control	0.29336057	0.282420223	1.038737831	0.298926682	0.752295168	up_in_case
CRA004775	ENSGALG00010025837	ENSGALG00010025837	case	control	-0.409638802	0.318477446	-1.286241166	0.198358908	0.665114204	down_in_case
CRA004775	ENSGALG00010025846	GRIK4	case	control	0.084534367	0.455671258	0.185516127	0.852824233	0.969725992	up_in_case
CRA004775	ENSGALG00010025847	DEF6	case	control	-0.435950477	0.363853346	-1.198148874	0.230859065	0.697294488	down_in_case
CRA004775	ENSGALG00010025849	OBSCN	case	control	0.866922549	0.606608634	1.429129922	0.152966895	0.617472961	up_in_case
CRA004775	ENSGALG00010025852	BTBD19	case	control	0.581621173	0.340881347	1.70622763	0.087965706	0.512984066	up_in_case
CRA004775	ENSGALG00010025862	PTCH2	case	control	0.783647957	0.496897216	1.577082608	0.114776519	0.564684045	up_in_case
CRA004775	ENSGALG00010025869	FASTKD2	case	control	-0.259976071	0.191898388	-1.354759017	0.175494348	0.641031789	down_in_case
CRA004775	ENSGALG00010025870	ENSGALG00010025870	case	control	-0.344275367	0.415292821	-0.828994264	0.407107653	0.813842524	down_in_case
CRA004775	ENSGALG00010025881	GAL	case	control	-0.476287713	1.055261237	-0.451345786	0.65174035	0.915892481	down_in_case
CRA004775	ENSGALG00010025882	LDHD	case	control	-0.247844852	0.418987484	-0.59153283	0.554163465	0.879382329	down_in_case
CRA004775	ENSGALG00010025886	ALDH2	case	control	0.297143094	0.290803113	1.021801628	0.306874798	0.7585475	up_in_case
CRA004775	ENSGALG00010025887	ZNF76	case	control	-0.545963987	0.146890009	-3.716821802	0.000201745	0.029202004	down_in_case
CRA004775	ENSGALG00010025890	SCG2	case	control	0.279193883	0.483007128	0.578032636	0.563242086	0.881532201	up_in_case
CRA004775	ENSGALG00010025891	NUP93	case	control	-0.338426677	0.293055667	-1.154820451	0.248163961	0.712818804	down_in_case
CRA004775	ENSGALG00010025892	ZC3H11A	case	control	0.09051093	0.165122486	0.548144184	0.583592904	0.889906855	up_in_case
CRA004775	ENSGALG00010025897	MAPKAPK5	case	control	0.407363741	0.262430689	1.552271736	0.120597218	0.572850296	up_in_case
CRA004775	ENSGALG00010025899	ENSGALG00010025899	case	control	-0.153237054	0.339576587	-0.451259184	0.651802758	0.915892481	down_in_case
CRA004775	ENSGALG00010025900	FAM237A	case	control	0.21408958	0.959205568	0.22319468	0.823383992	0.960898591	up_in_case
CRA004775	ENSGALG00010025904	RPL10A	case	control	-0.315628915	0.24269679	-1.300507171	0.1934272	0.657919837	down_in_case
CRA004775	ENSGALG00010025905	CPT1A	case	control	0.775120152	0.48077254	1.612238817	0.106909981	0.552884225	up_in_case
CRA004775	ENSGALG00010025907	ARHGEF12	case	control	0.186879965	0.266882277	0.700233703	0.483781367	0.850349167	up_in_case
CRA004775	ENSGALG00010025910	HEATR3	case	control	-0.124629999	0.200160119	-0.622651501	0.53351356	0.870554975	down_in_case
CRA004775	ENSGALG00010025913	EIF2B3	case	control	-0.214679047	0.23277359	-0.922265481	0.356390115	0.789127613	down_in_case
CRA004775	ENSGALG00010025917	SCUBE3	case	control	1.766604551	0.433584431	4.074418787	4.61294E-05	0.013496287	up_in_case
CRA004775	ENSGALG00010025919	RGS9BP	case	control	0.188911974	0.60868054	0.310363092	0.756284858	0.941900693	up_in_case
CRA004775	ENSGALG00010025920	GTSF1	case	control	-0.885282116	0.623015963	-1.420962173	0.155327757	0.619284425	down_in_case
CRA004775	ENSGALG00010025924	TMEM116	case	control	0.119487244	0.371389719	0.321730081	0.747657187	0.94062525	up_in_case
CRA004775	ENSGALG00010025925	KLHL18	case	control	-0.217226475	0.268788276	-0.808169456	0.418993038	0.818943344	down_in_case
CRA004775	ENSGALG00010025929	NELFE	case	control	-0.370090485	0.237462498	-1.558521819	0.1191096	0.570892125	down_in_case
CRA004775	ENSGALG00010025931	TMEM136	case	control	-0.34539296	0.366626925	-0.942082909	0.346150194	0.784552667	down_in_case
CRA004775	ENSGALG00010025932	AP1S3	case	control	-0.587438447	0.451900385	-1.29992907	0.193625281	0.657919837	down_in_case
CRA004775	ENSGALG00010025933	SKIC2	case	control	-0.16044137	0.248810288	-0.644834149	0.519034669	0.865435399	down_in_case
CRA004775	ENSGALG00010025935	CATH3	case	control	0.565423355	0.44564302	1.268780907			up_in_case
CRA004775	ENSGALG00010025936	GOSR2	case	control	0.069918233	0.212755513	0.328631826	0.742433989	0.938877854	up_in_case
CRA004775	ENSGALG00010025938	ERP29	case	control	-0.092501375	0.276365173	-0.334707062	0.737846074	0.937854704	down_in_case
CRA004775	ENSGALG00010025939	TMEM136-1	case	control	0.396496264	0.528386409	0.750390732	0.453019411	0.837607279	up_in_case
CRA004775	ENSGALG00010025940	HECTD3	case	control	-0.423957283	0.16756504	-2.530105829	0.011402813	0.21639381	down_in_case
CRA004775	ENSGALG00010025943	CATH1	case	control	1.897279659	0.695143125	2.729336721			up_in_case
CRA004775	ENSGALG00010025947	CATHB1	case	control	2.285791311	1.13632788	2.011559649	0.044266377	0.392641326	up_in_case
CRA004775	ENSGALG00010025949	GPATCH1	case	control	0.220760205	0.242975603	0.908569428	0.36357745	0.793608387	up_in_case
CRA004775	ENSGALG00010025951	TBRG4	case	control	-0.23223697	0.259208679	-0.895945963	0.370281624	0.795584784	down_in_case
CRA004775	ENSGALG00010025954	TUBA1A	case	control	-0.035988052	0.323406214	-0.111278172	0.911395764	0.98075825	down_in_case
CRA004775	ENSGALG00010025956	ENSGALG00010025956	case	control	0.096922601	0.550561134	0.176043304	0.860259922	0.971325143	up_in_case
CRA004775	ENSGALG00010025957	ENSGALG00010025957	case	control	-0.135013834	0.402608034	-0.335348087	0.737362523	0.937597314	down_in_case
CRA004775	ENSGALG00010025959	UROD	case	control	-0.635148836	0.280914652	-2.261002872	0.023759079	0.2983663	down_in_case
CRA004775	ENSGALG00010025960	CMKLR2	case	control	0.170673542	0.235844472	0.723669884	0.469268427	0.8440023	up_in_case
CRA004775	ENSGALG00010025962	ENSGALG00010025962	case	control	0.691449602	0.428270757	1.614515096	0.106415745	0.552224847	up_in_case
CRA004775	ENSGALG00010025963	OAF	case	control	0.102597444	0.517615937	0.198211524	0.842879572	0.966478468	up_in_case
CRA004775	ENSGALG00010025968	FADS2	case	control	0.071884093	0.366848984	0.195950094	0.844649228	0.96676973	up_in_case
CRA004775	ENSGALG00010025970	KLHL12	case	control	-0.292436183	0.159649844	-1.83173485	0.066990934	0.462498477	down_in_case
CRA004775	ENSGALG00010025971	TRAFD1	case	control	-0.403078234	0.308015816	-1.308628364	0.190660268	0.656098436	down_in_case
CRA004775	ENSGALG00010025974	EEF1B2	case	control	-0.025990446	0.2619749	-0.09920968	0.92097179	0.982481112	down_in_case
CRA004775	ENSGALG00010025976	NAA25	case	control	0.016193305	0.267841596	0.060458515	0.951790455	0.990168609	up_in_case
CRA004775	ENSGALG00010025980	HECTD4	case	control	-0.222518527	0.315917872	-0.704355613	0.481211341	0.848786191	down_in_case
CRA004775	ENSGALG00010025982	SERPINE2	case	control	-0.049153387	0.351747062	-0.139740719	0.888864853	0.977118922	down_in_case
CRA004775	ENSGALG00010025983	RNF26	case	control	-0.587458634	0.237621072	-2.472249738	0.013426568	0.232695952	down_in_case
CRA004775	ENSGALG00010025984	NDUFS1	case	control	0.104651803	0.29568259	0.353932921	0.723389155	0.935238044	up_in_case
CRA004775	ENSGALG00010025985	CHCHD10	case	control	0.431382744	0.584346446	0.738231142	0.460374002	0.841265006	up_in_case
CRA004775	ENSGALG00010025986	RAB3IL1	case	control	-0.50243681	0.19328366	-2.599478975	0.00933654	0.195703177	down_in_case
CRA004775	ENSGALG00010025988	NSF	case	control	-0.241035929	0.166684779	-1.446058423	0.148160816	0.611192154	down_in_case
CRA004775	ENSGALG00010025990	FAM124B	case	control	0.360279034	0.468413622	0.769147216	0.441805921	0.829654585	up_in_case
CRA004775	ENSGALG00010025992	MMP11	case	control	0.253800408	0.459620582	0.55219548	0.580814431	0.888901418	up_in_case
CRA004775	ENSGALG00010025994	INO80D	case	control	-0.137315595	0.331276517	-0.414504463	0.6785047	0.92338129	down_in_case
CRA004775	ENSGALG00010025995	BEST1	case	control	-1.043972876	0.560347591	-1.863080867	0.06245089	0.448843826	down_in_case
CRA004775	ENSGALG00010025996	CUL3	case	control	0.450586039	0.269541768	1.671674271	0.09458857	0.526933666	up_in_case
CRA004775	ENSGALG00010025997	CCDC43	case	control	-0.178391668	0.248852829	-0.716856096	0.473462894	0.845445211	down_in_case

CRA004775	ENSGALG00010025998	SMARCB1	case	control	-0.075413049	0.161543988	-0.466826716	0.64062386	0.911724439	down_in_case
CRA004775	ENSGALG00010025999	DBF4B	case	control	-0.370510057	0.279024752	-1.327875231	0.184219329	0.648706783	down_in_case
CRA004775	ENSGALG00010026001	ANKS1A	case	control	0.026308109	0.23699195	0.11100845	0.911609646	0.98075825	up_in_case
CRA004775	ENSGALG00010026002	FTH1	case	control	0.323767711	0.288114121	1.123748153			up_in_case
CRA004775	ENSGALG00010026003	NECTIN1	case	control	-0.43769121	0.430908329	-1.015740893	0.309752789	0.762070812	down_in_case
CRA004775	ENSGALG00010026005	DERL3	case	control	-0.283169103	0.219534095	-1.289863893	0.197097919	0.662987644	down_in_case
CRA004775	ENSGALG00010026006	ITGA2B	case	control	-0.657107197	0.553521903	-1.187138564	0.235172969	0.702145501	down_in_case
CRA004775	ENSGALG00010026008	NRP2	case	control	-0.23894013	0.329182834	-0.725858415	0.467925572	0.843870188	down_in_case
CRA004775	ENSGALG00010026009	ENSGALG00010026009	case	control	-0.278654936	0.402150169	-0.692912642	0.488364376	0.853845967	down_in_case
CRA004775	ENSGALG00010026010	ZSWIM5	case	control	0.12895473	0.269221936	0.478990429	0.631945438	0.909651573	up_in_case
CRA004775	ENSGALG00010026011	GJC2	case	control	0.295344812	0.277434166	1.064558186	0.28707591	0.741909972	up_in_case
CRA004775	ENSGALG00010026013	RABIF	case	control	-0.14310182	0.309620038	-0.462185265	0.643948469	0.913727726	down_in_case
CRA004775	ENSGALG00010026016	SLC2A11	case	control	-0.837338708	0.673260033	-1.243707731	0.21360715	0.683061827	down_in_case
CRA004775	ENSGALG00010026017	PLEKHM1	case	control	-0.521794832	0.172024863	-3.033252421	0.002419331	0.100508235	down_in_case
CRA004775	ENSGALG00010026018	FOXL2	case	control	0.528898516	0.502674896	1.052168151	0.292722409	0.746438942	up_in_case
CRA004775	ENSGALG00010026019	ENSGALG00010026019	case	control	-0.54334008	0.75005981	-0.724395672	0.468822856	0.8440023	down_in_case
CRA004775	ENSGALG00010026023	THY1	case	control	0.145745233	0.359694945	0.405191219	0.685336959	0.926656387	up_in_case
CRA004775	ENSGALG00010026030	IBA57	case	control	-0.100620075	0.138882258	-0.724499128	0.468759362	0.8440023	down_in_case
CRA004775	ENSGALG00010026036	TUB5A	case	control	-0.225397407	0.535253176	-0.421104287	0.673678931	0.922501393	down_in_case
CRA004775	ENSGALG00010026037	ENSGALG00010026037	case	control	-0.154629062	0.618494912	-0.250008624	0.802580679	0.953859914	down_in_case
CRA004775	ENSGALG00010026040	GUK1	case	control	-0.202133929	0.301048952	-0.671432097	0.501945303	0.860091011	down_in_case
CRA004775	ENSGALG00010026043	ENSGALG00010026043	case	control	-0.025065078	0.429466185	-0.058363332	0.953459222	0.990670511	down_in_case
CRA004775	ENSGALG00010026044	CERKL	case	control	-0.205587174	0.652552113	-0.315050967	0.752722957	0.94167946	down_in_case
CRA004775	ENSGALG00010026051	TAF11	case	control	-0.434745321	0.252747017	-1.720080918	0.085417734	0.506269697	down_in_case
CRA004775	ENSGALG00010026061	MPEG1	case	control	-0.695754398	0.328049784	-2.12088052	0.033931858	0.349772852	down_in_case
CRA004775	ENSGALG00010026062	ITGA4	case	control	-0.249443586	0.525541733	-0.474640871	0.63504297	0.909837348	down_in_case
CRA004775	ENSGALG00010026064	ENSGALG00010026064	case	control	-0.126064966	0.320118058	-0.39380773	0.693722997	0.928407293	down_in_case
CRA004775	ENSGALG00010026066	SELENOP2	case	control	1.623257019	0.571737966	2.839162548	0.004523211	0.138149936	up_in_case
CRA004775	ENSGALG00010026068	BLTP3A	case	control	-0.105945361	0.269779148	-0.39271145	0.694532613	0.928767671	down_in_case
CRA004775	ENSGALG00010026073	HPDL	case	control	-0.691319529	0.447636904	-1.544375638	0.122497358	0.57525466	down_in_case
CRA004775	ENSGALG00010026076	C1orf35	case	control	-0.045477202	0.203541455	-0.223429678	0.823201108	0.960898591	down_in_case
CRA004775	ENSGALG00010026078	UBE2E3	case	control	0.054492791	0.199406358	0.273275094	0.784641759	0.950244571	up_in_case
CRA004775	ENSGALG00010026079	ENSGALG00010026079	case	control	0.188930981	0.579992733	0.325747152	0.744615666	0.939218065	up_in_case
CRA004775	ENSGALG00010026081	KPNA1	case	control	0.223683993	0.264163236	0.846764283	0.39712652	0.808998414	up_in_case
CRA004775	ENSGALG00010026083	MUTYH	case	control	-0.576106508	0.275134799	-2.093906368	0.036268322	0.360628495	down_in_case
CRA004775	ENSGALG00010026088	SNRPC	case	control	-0.20722433	0.266182437	-0.778504894	0.436271423	0.82694408	down_in_case
CRA004775	ENSGALG00010026090	USP2	case	control	-0.173571333	0.68252754	-0.25430671	0.799258617	0.952835572	down_in_case
CRA004775	ENSGALG00010026091	DTX4	case	control	-0.362964595	0.313907438	-1.15627905	0.247567035	0.712046495	down_in_case
CRA004775	ENSGALG00010026093	TOE1	case	control	-0.159529642	0.25006364	-0.637956171	0.523502212	0.866638286	down_in_case
CRA004775	ENSGALG00010026094	C6orf106	case	control	0.077624447	0.217603057	0.356724983	0.7212977	0.934511903	up_in_case
CRA004775	ENSGALG00010026095	MIF	case	control	-0.421213536	0.267756537	-1.573121387	0.115690714	0.566086328	down_in_case
CRA004775	ENSGALG00010026097	ARF1	case	control	-0.038900716	0.146889122	-0.264830472	0.791140062	0.951284723	down_in_case
CRA004775	ENSGALG00010026098	STAT3	case	control	-0.490357209	0.2293309	-2.138208193	0.03249985	0.343523087	down_in_case
CRA004775	ENSGALG00010026102	IPO9	case	control	-0.157627644	0.184371909	-0.854943927	0.392582191	0.807538924	down_in_case
CRA004775	ENSGALG00010026104	DDX51	case	control	-0.301598742	0.18870079	-1.598290823	0.109978269	0.558048403	down_in_case
CRA004775	ENSGALG00010026105	CNTF	case	control	-0.849408574	0.452000619	-1.879219934	0.060214469	0.445397937	down_in_case
CRA004775	ENSGALG00010026106	TESK2	case	control	0.127511234	0.333413025	0.382442268	0.702133343	0.930068722	up_in_case
CRA004775	ENSGALG00010026109	SPDEF	case	control	2.049732325	0.647798329	3.164151916	0.001555356	0.080693373	up_in_case
CRA004775	ENSGALG00010026111	ENSGALG00010026111	case	control	0.055300193	0.235456519	0.234863718	0.814314511	0.958686054	up_in_case
CRA004775	ENSGALG00010026112	ZFP91	case	control	0.081407455	0.209523723	0.38853574	0.697619611	0.929092636	up_in_case
CRA004775	ENSGALG00010026113	MFRP	case	control	-0.39898465	0.802985751	-0.496876377	0.619276231	0.904574831	down_in_case
CRA004775	ENSGALG00010026114	CWC22	case	control	0.269911258	0.204251879	1.321462788	0.186347102	0.6491093	up_in_case
CRA004775	ENSGALG00010026115	MMACHC	case	control	-0.250058267	0.302672367	-0.826168144	0.408708716	0.814384687	down_in_case
CRA004775	ENSGALG00010026116	DLX3	case	control	-0.3080536	0.69127756	-0.445629394	0.655864974	0.917054032	down_in_case
CRA004775	ENSGALG00010026117	LZTR1	case	control	-0.416129865	0.144720163	-2.875410435	0.004035026	0.130247989	down_in_case
CRA004775	ENSGALG00010026120	C1QTNF5	case	control	-0.122734899	0.387001053	-0.31714358	0.751134658	0.941495694	down_in_case
CRA004775	ENSGALG00010026121	ENSGALG00010026121	case	control	-1.048812123	0.365692946	-2.868013001	0.004130586	0.131785807	down_in_case
CRA004775	ENSGALG00010026124	PACSIN1	case	control	0.49448514	0.610570627	0.809873777	0.418012725	0.818943344	up_in_case
CRA004775	ENSGALG00010026127	ENSGALG00010026127	case	control	-0.053426457	0.683479326	-0.078168359	0.937694131	0.986707289	down_in_case
CRA004775	ENSGALG00010026128	PRDX1	case	control	0.011999681	0.182416186	0.06578189	0.947551475	0.989653678	up_in_case
CRA004775	ENSGALG00010026132	DDTL	case	control	-0.590739024	0.249399027	-2.368650074	0.017853136	0.265690984	down_in_case
CRA004775	ENSGALG00010026137	LPXN	case	control	-0.605240931	0.355868424	-1.700743564	0.088991151	0.515249396	down_in_case
CRA004775	ENSGALG00010026143	RNPEP	case	control	-0.155690648	0.146130321	-1.065423291	0.286684422	0.741851239	down_in_case
CRA004775	ENSGALG00010026148	RPS10	case	control	-0.350499111	0.305431267	-1.147554784	0.251152405	0.715759105	down_in_case
CRA004775	ENSGALG00010026149	LASP1	case	control	-0.156588692	0.271951674	-0.575796023	0.564753062	0.882585553	down_in_case
CRA004775	ENSGALG00010026154	ZNF385B	case	control	-1.029491045	0.46197393	-2.228461342	0.025849766	0.308336492	down_in_case
CRA004775	ENSGALG00010026162	MED1	case	control	-0.029946481	0.303006699	-0.098831085	0.921272387	0.982481112	down_in_case
CRA004775	ENSGALG00010026163	CTNND1	case	control	-0.036413273	0.23633192	-0.154076829	0.877549158	0.974479909	down_in_case
CRA004775	ENSGALG00010026166	WNT9A	case	control	-0.249075699	0.45910672	-0.542522443	0.587458651	0.892218236	down_in_case
CRA004775	ENSGALG00010026167	CABIN1	case	control	0.225121201	0.226961186	0.991892953	0.321249747	0.767549427	up_in_case
CRA004775	ENSGALG00010026173	CDK12	case	control	-0.013733204	0.192336633	-0.071401913	0.943077887	0.987862092	down_in_case
CRA004775	ENSGALG00010026183	SESTD1	case	control	0.126888979	0.195869539	0.647823951	0.517098822	0.864913871	up_in_case
CRA004775	ENSGALG00010026184	NUDT3	case	control	-0.140879883	0.294641768	-0.478139554	0.632550881	0.909651573	down_in_case
CRA004775	ENSGALG00010026192	SMIM29	case	control	-0.119815624	0.406102859	-0.295037629	0.767965135	0.94482317	down_in_case
CRA004775	ENSGALG00010026199	TBX6	case	control	0.597575983	0.743771115	0.803440696	0.421720061	0.819812626	up_in_case
CRA004775	ENSGALG00010026203	TMX2	case	control	-0.691738425	0.196811829	-3.514719761	0.000440218	0.043515325	down_in_case
CRA004775	ENSGALG00010026205	ENSGALG00010026205	case	control	1.606060394	0.572037374	2.807614442	0.004990994	0.142684328	up_in_case
CRA004775	ENSGALG00010026209	MED19	case	control	-0.209315562	0.344538719	-0.607524063	0.543503179	0.875657091	down_in_case
CRA004775	ENSGALG00010026213	AKR1A1	case	control	-0.476043043	0.240634825	-1.97827992	0.047897137	0.406815029	down_in_case
CRA004775	ENSGALG00010026218	FTSJ3	case	control	-0.392165245	0.28320401	-1.384744675	0.166130553	0.630194031	down_in_case
CRA004775	ENSGALG00010026220	ENSGALG00010026220	case	control	-0.665719545	0.270074026	-2.464952132	0.013703156	0.234005339	down_in_case
CRA004775	ENSGALG00010026221	CRKL	case	control	-0.069639719	0.2422977	-0.287413869	0.773795443	0.946296525	down_in_case
CRA004775	ENSGALG00010026229	CRHR2	case	control	0.107822417	0.500081142	0.215609844	0.829291882	0.96297907	up_in_case
CRA004775	ENSGALG00010026232	PSMC5	case	control	-0.23612791	0.296181817	-0.797239726	0.425311818	0.822283278	down_in_case
CRA004775	ENSGALG00010026234	ZDHHC5	case	control	-0.124437057	0.255376937	-0.487268188	0.626068289	0.907183337	down_in_case
CRA004775	ENSGALG00010026237	KLHL22	case	control	0.193865175	0.241325814	0.803333765	0.421781847	0.819812626	up_in_case
CRA004775	ENSGALG00010026239	GIP	case	control	2.642151907	0.789688345	3.345816011	0.000820408	0.060328515	up_in_case
CRA004775	ENSGALG00010026242	CCDC141	case	control	-0.517166174	0.55212634	-0.936680857	0.348922746	0.785311667	down_in_case
CRA004775	ENSGALG00010026244	CLP1	case	control	-0.327086718	0.172053977	-1.901070367	0.057292796	0.437928428	down_in_case
CRA004775	ENSGALG00010026249	YPEL4	case	control	-0.571205335	0.697905556	-0.818456494	0.413096575	0.817112606	down_in_case
CRA004775	ENSGALG00010026252	ENO2	case	control	-0.769981181	0.77041822	-0.999432725	0.317585114	0.766294595	down_in_case
CRA004775	ENSGALG00010026259	MED15	case	control	-0.016919706	0.191294833	-0.088448315	0.929520362	0.98473447	down_in_case
CRA004775	ENSGALG00010026260	ENSGALG00010026260	case	control	2.245433921	1.539923578	1.458146335	0.144800212	0.607612974	up_in_case
CRA004775	ENSGALG00010026261	IFT70A	case	control	-0.317582897	0.273102552	-1.162870486	0.244882054	0.711767728	down_in_case
CRA004775	ENSGALG00010026262	NAV1	case	control	-0.240051384	0.365891686	-0.656072258	0.511777633	0.863170205	down_in_case
CRA004775	ENSGALG00010026264	FZD7	case	control	-0.108178396	0.273978213	-0.394843058	0.692958715	0.927867527	down_in_case
CRA004775	ENSGALG00010026268	GARS1	case	control	-0.276255136	0.229048921	-1.206096651	0.227780226	0.694718454	down_in_case
CRA004775	ENSGALG00010026272	LRRC58	case	control	-0.011562767	0.23205174	-0.049828398	0.960259136	0.991431439	down_in_case
CRA004775	ENSGALG00010026277	SMARCD2	case	control	-0.312865297	0.169482282	-1.846005923	0.064891345	0.457570489	down_in_case
CRA004775	ENSGALG00010026287	NASP	case	control	-0.148495121	0.286158515	-0.518926097	0.603812278	0.898692785	down_in_case
CRA004775	ENSGALG00010026291	SERPING1	case	control	-0.545417767	0.318537259	-1.712257366	0.08684925	0.509281038	down_in_case
CRA004775	ENSGALG00010026295	SMPD4	case	control	-0.219173197	0.190532177	-1.150321172	0.250011614	0.714890768	down_in_case
CRA004775	ENSGALG00010026298	SNF8	case	control	-0.018554043	0.257055768	-0.072179057	0.942459412	0.987711255	down_in_case
CRA004775	ENSGALG00010026300	HMGA1	case	control	-0.467467796	0.209755562	-2.228631224	0.025838452	0.308336492	down_in_case
CRA004775	ENSGALG00010026305	ATP5MC1	case	control	0.036973702	0.423385852	0.087328619	0.930410306	0.985044263	up_in_case
CRA004775	ENSGALG00010026315	SYT2	case	control	0.906417306	0.701194876	1.292675312	0.19612338	0.66213911	up_in_case
CRA004775	ENSGALG00010026316	ENSGALG00010026316	case	control	-0.380533585	0.275977002	-1.378859769	0.167937994	0.63138062	down_in_case
CRA004775	ENSGALG00010026320	ENSGALG00010026320	case	control	-0.308746797	0.343936189	-0.897686278	0.369352827	0.794896739	down_in_case
CRA004775	ENSGALG00010026321	GPBP1L1	case	control	0.033582181	0.170235335	0.197269155	0.843616916	0.966478468	up_in_case
CRA004775	ENSGALG00010026325	TMEM69	case	control	-0.185179705	0.203783532	-0.908707894	0.363504336	0.793608387	down_in_case
CRA004775	ENSGALG00010026329	ENSGALG00010026329	case	control	0.077347864	0.579621601	0.133445448	0.893841104	0.977834811	up_in_case
CRA004775	ENSGALG00010026334	ENSGALG00010026334	case	control	0.104249612	0.148116309	0.70383615	0.481534818	0.848818238	up_in_case
CRA004775	ENSGALG00010026335	IPP	case	control	-0.490607231	0.25347175	-1.93554994	0.052922847	0.423142991	down_in_case
CRA004775	ENSGALG00010026344	TIMM10	case	control	-0.321113341	0.32317021	-0.993635337	0.320400434	0.767037518	down_in_case
CRA004775	ENSGALG00010026346	FZD2	case	control	0.188900963	0.438878586	0.430417361	0.666892069	0.919168997	up_in_case
CRA004775	ENSGALG00010026354	ENSGALG00010026354	case	control	0.082009827	0.502798018	0.163106902	0.870434265	0.973918682	up_in_case
CRA004775	ENSGALG00010026355	PPP1R12B	case	control	0.147084108	0.378635053	0.388458772	0.697676558	0.929092636	up_in_case
CRA004775	ENSGALG00010026360	CTDSPL	case	control	0.348220416	0.302588627	1.150804705	0.249812589	0.714767563	up_in_case
CRA004775	ENSGALG00010026362	ENSGALG00010026362	case	control	1.014883204	0.380240156	2.669058458	0.007606421	0.174326492	up_in_case
CRA004775	ENSGALG00010026367	RBP	case	control	1.100920707	0.688524714	1.598955978	0.109830385	0.557566828	up_in_case
CRA004775	ENSGALG00010026377	ENSGALG00010026377	case	control	-1.145787789	0.527113528	-2.173702112	0.029727514	0.328603733	down_in_case
CRA004775	ENSGALG00010026388	SNRPD3	case	control	-0.233937766	0.230668714	-1.014172065	0.310500656	0.762244121	down_in_case
CRA004775	ENSGALG00010026393	SLC43A3	case	control	-0.44648611	0.277757634	-1.607466562	0.107952059	0.554312457	down_in_case
CRA004775	ENSGALG00010026402	TFEB	case	control	-0.107769893	0.216400193	-0.498012002	0.618475583	0.904143308	down_in_case
CRA004775	ENSGALG00010026403	GUCD1	case	control	0.373176379	0.263052767	1.418636965	0.156004883	0.619442611	up_in_case
CRA004775	ENSGALG00010026409	VILL	case	control	-0.250980121	0.698821224	-0.359147823	0.719484507	0.93407508	down_in_case

CRA004775	ENSGALG00010026410	ENSGALG00010026410	case	control	-1.414269449	0.800649934	-1.766401755	0.077328471	0.488893703	down_in_case
CRA004775	ENSGALG00010026416	ENSGALG00010026416	case	control	2.582368007	1.025345521	2.518534438	0.011784436	0.217806153	up_in_case
CRA004775	ENSGALG00010026427	PGC	case	control	-0.177059757	0.489304543	-0.36186003	0.71745663	0.934011578	down_in_case
CRA004775	ENSGALG00010026433	VPS11	case	control	-0.338799349	0.206961256	-1.637018228	0.101626661	0.541444485	down_in_case
CRA004775	ENSGALG00010026434	ENSGALG00010026434	case	control	-0.543831076	0.193409762	-2.811807799	0.004926393	0.142429514	down_in_case
CRA004775	ENSGALG00010026436	PLCD1	case	control	0.1650475	0.331517261	0.497854921	0.618586303	0.904143308	up_in_case
CRA004775	ENSGALG00010026438	TOMM6	case	control	-0.278968815	0.33492293	-0.832934355	0.404881753	0.81313407	down_in_case
CRA004775	ENSGALG00010026443	UPB1	case	control	0.736542899	0.403539225	1.825207694	0.067969693	0.463992389	up_in_case
CRA004775	ENSGALG00010026448	ADORA2A	case	control	-0.105202338	0.339346878	-0.310014163	0.756550186	0.941900693	down_in_case
CRA004775	ENSGALG00010026457	SSRP1	case	control	-0.392540255	0.196907454	-1.993526643	0.046203809	0.399541954	down_in_case
CRA004775	ENSGALG00010026458	DLEC1	case	control	0.331623189	0.464077289	0.714586119	0.474864825	0.84579952	up_in_case
CRA004775	ENSGALG00010026480	USP49	case	control	-0.198011117	0.390030298	-0.507681374	0.611676813	0.901413878	down_in_case
CRA004775	ENSGALG00010026481	DIXDC1	case	control	-0.112698471	0.328137114	-0.343449327	0.731260456	0.936524599	down_in_case
CRA004775	ENSGALG00010026484	TNKS1BP1	case	control	-0.253296462	0.238177879	-1.063476019	0.287566135	0.742456237	down_in_case
CRA004775	ENSGALG00010026485	ACAA1	case	control	-0.239516041	0.1240003	-1.931576298	0.053411824	0.425777384	down_in_case
CRA004775	ENSGALG00010026492	SPECC1L	case	control	0.305147241	0.303001088	1.007082988	0.313894895	0.765098415	up_in_case
CRA004775	ENSGALG00010026498	ENSGALG00010026498	case	control	-0.244331018	0.282939954	-0.863543713	0.387838596	0.804270424	down_in_case
CRA004775	ENSGALG00010026507	APLNR	case	control	-0.766604228	0.700870124	-1.093789279	0.274047407	0.731001618	down_in_case
CRA004775	ENSGALG00010026508	CSRP1	case	control	0.309293668	0.295986155	1.044959918	0.296041495	0.74873397	up_in_case
CRA004775	ENSGALG00010026516	ENSGALG00010026516	case	control	0.291358548	0.320944673	0.907815496	0.36397571	0.793608387	up_in_case
CRA004775	ENSGALG00010026522	MED20	case	control	-0.414942712	0.29705585	-1.396850835	0.162458432	0.628577911	down_in_case
CRA004775	ENSGALG00010026536	BCR	case	control	-0.285361154	0.366584897	-0.778431288	0.436314799	0.82694408	down_in_case
CRA004775	ENSGALG00010026537	FJX1	case	control	-0.31225835	0.465406337	-0.670937039	0.502260639	0.860091011	down_in_case
CRA004775	ENSGALG00010026539	ENSGALG00010026539	case	control	-0.500701659	0.913969286	-0.547832041	0.583807236	0.889908634	down_in_case
CRA004775	ENSGALG00010026550	MYD88	case	control	-0.412550012	0.232932218	-1.771116143	0.07654139	0.487052593	down_in_case
CRA004775	ENSGALG00010026553	PLPP3	case	control	0.479340818	0.302562044	1.584272807	0.113131651	0.562886277	up_in_case
CRA004775	ENSGALG00010026560	TNNI1	case	control	0.437352383	0.676933789	0.646078523	0.518228503	0.865435399	up_in_case
CRA004775	ENSGALG00010026566	ENSGALG00010026566	case	control	-0.211588477	0.502810016	-0.420811977	0.673892385	0.922518087	down_in_case
CRA004775	ENSGALG00010026568	BYSL	case	control	-0.594791663	0.31324374	-1.898814205	0.057588909	0.439596503	down_in_case
CRA004775	ENSGALG00010026569	GNAZ	case	control	0.696753729	0.594952252	1.17110865	0.241555107	0.708952282	up_in_case
CRA004775	ENSGALG00010026581	DNER	case	control	0.458251483	0.804079206	0.569908386	0.568739837	0.884663439	up_in_case
CRA004775	ENSGALG00010026584	ENSGALG00010026584	case	control	0.428144879	0.208861419	2.049899309	0.040374258	0.376363211	up_in_case
CRA004775	ENSGALG00010026588	CCND3	case	control	-0.119032677	0.16811357	-0.708049191	0.47891471	0.847184661	down_in_case
CRA004775	ENSGALG00010026595	PRKAA2	case	control	0.041727278	0.650406409	0.064155699	0.948846252	0.990168609	up_in_case
CRA004775	ENSGALG00010026612	MFF	case	control	-0.113320616	0.208067443	-0.544634058	0.586005226	0.89145601	down_in_case
CRA004775	ENSGALG00010026625	ENSGALG00010026625	case	control	1.406197942	1.007891684	1.395187564	0.162959286	0.629717526	up_in_case
CRA004775	ENSGALG00010026628	FYB2	case	control	1.755087735	0.728051491	2.410664297	0.015923498	0.252039758	up_in_case
CRA004775	ENSGALG00010026630	XIRP1	case	control	1.929084702	0.942525258	2.04671937	0.040685646	0.378019137	up_in_case
CRA004775	ENSGALG00010026633	SLC5A1	case	control	2.243796095	0.891413348	2.517121938	0.011831787	0.21809505	up_in_case
CRA004775	ENSGALG00010026638	LAD1	case	control	2.434464072	0.783858876	3.105742816	0.001898017	0.08697473	up_in_case
CRA004775	ENSGALG00010026643	YWHAH	case	control	0.501039271	0.403207145	1.242634902	0.214002403	0.683502083	up_in_case
CRA004775	ENSGALG00010026652	ENSGALG00010026652	case	control	1.76155581	0.688970632	2.556793757	0.010564183	0.206054018	up_in_case
CRA004775	ENSGALG00010026659	LIPH	case	control	-0.477077758	0.420206419	-1.135341435	0.256232288	0.719236059	down_in_case
CRA004775	ENSGALG00010026671	TAF8	case	control	-0.693740772	0.253089505	-2.741088663	0.006123598	0.156225598	down_in_case
CRA004775	ENSGALG00010026673	DEPDC5	case	control	-0.380289749	0.155757436	-2.441551166	0.014624316	0.241689368	down_in_case
CRA004775	ENSGALG00010026681	PLXDC1	case	control	-0.657174045	0.498507829	-1.318282293	0.187409166	0.651317546	down_in_case
CRA004775	ENSGALG00010026686	ENSGALG00010026686	case	control	0.950161249	0.454164493	2.092108175	0.03642884	0.360628495	up_in_case
CRA004775	ENSGALG00010026690	KRT14	case	control	1.253751287	0.573998451	2.184241586	0.028944501	0.323755716	up_in_case
CRA004775	ENSGALG00010026692	NMNAT3	case	control	0.518753895	0.52325431	0.991399183	0.321490699	0.767650058	up_in_case
CRA004775	ENSGALG00010026696	CSRNP1	case	control	0.694886105	0.362228224	1.918365437	0.05506469	0.430875458	up_in_case
CRA004775	ENSGALG00010026697	CHN1	case	control	0.130204949	0.234679532	0.554820218	0.579017637	0.888378369	up_in_case
CRA004775	ENSGALG00010026704	WDFY1	case	control	-0.192952541	0.212588648	-0.907633325	0.364071981	0.793608387	down_in_case
CRA004775	ENSGALG00010026706	KRT24	case	control	2.20900689	0.659010596	3.352005115	0.000802285	0.060285388	up_in_case
CRA004775	ENSGALG00010026707	GORASP1	case	control	-0.032699429	0.240810146	-0.135789248	0.891987891	0.977819591	down_in_case
CRA004775	ENSGALG00010026715	WDR48	case	control	0.082176749	0.212640493	0.386458609	0.699157051	0.929347461	up_in_case
CRA004775	ENSGALG00010026719	ENSGALG00010026719	case	control	-0.495053277	0.754796824	-0.655876205	0.511903779	0.863170205	down_in_case
CRA004775	ENSGALG00010026724	OLA1	case	control	0.167172904	0.286999441	0.582485121	0.560239961	0.880918893	up_in_case
CRA004775	ENSGALG00010026740	DAB1	case	control	1.290436571	0.462520817	2.790007552	0.005270681	0.147770484	up_in_case
CRA004775	ENSGALG00010026743	TNNT2	case	control	-0.704573161	0.8598123	-0.819449967	0.412529736	0.817038226	down_in_case
CRA004775	ENSGALG00010026755	ENSGALG00010026755	case	control	-0.17927475	0.353121823	-0.507685276	0.611674076	0.901413878	down_in_case
CRA004775	ENSGALG00010026759	OMA1	case	control	-0.410091629	0.284729933	-1.440282814	0.149787402	0.61366707	down_in_case
CRA004775	ENSGALG00010026775	BTG2	case	control	0.613719292	0.616723681	0.995128468	0.319673786	0.767037518	up_in_case
CRA004775	ENSGALG00010026780	MYSM1	case	control	0.008393991	0.189732957	0.044241081	0.964712236	0.992871055	up_in_case
CRA004775	ENSGALG00010026782	HOXB9	case	control	-0.439066589	0.604354139	-0.726505473	0.467528954	0.843870188	down_in_case
CRA004775	ENSGALG00010026786	ENSGALG00010026786	case	control	-0.448995732	0.476805938	-0.941673951	0.346359596	0.784552667	down_in_case
CRA004775	ENSGALG00010026796	DCAKD	case	control	-0.302109752	0.308095185	-0.980572779	0.326803463	0.770998457	down_in_case
CRA004775	ENSGALG00010026801	FMOD	case	control	1.071679763	0.545990682	1.962816944			up_in_case
CRA004775	ENSGALG00010026822	PRELP	case	control	-0.175365655	0.354900008	-0.494126939	0.621216534	0.90544587	down_in_case
CRA004775	ENSGALG00010026825	ADAM23	case	control	0.481858601	0.384918206	1.251846741	0.210625714	0.680684888	up_in_case
CRA004775	ENSGALG00010026827	FAM117A	case	control	-0.327461432	0.315807046	-1.036903501	0.299780832	0.752512244	down_in_case
CRA004775	ENSGALG00010026836	ENSGALG00010026836	case	control	-0.286623839	0.663217504	-0.432171705	0.665616623	0.918888163	down_in_case
CRA004775	ENSGALG00010026839	FGGY	case	control	0.210941294	0.32580707	0.647442345	0.517345697	0.865031698	up_in_case
CRA004775	ENSGALG00010026846	MICAL1	case	control	-0.481015687	0.381354999	-1.261333111	0.207188857	0.675932139	down_in_case
CRA004775	ENSGALG00010026854	SMPD2	case	control	-0.312840895	0.256443299	-1.219922286	0.222494337	0.6901691	down_in_case
CRA004775	ENSGALG00010026856	UBE2Z	case	control	-0.200630399	0.158449278	-1.266212133	0.205437145	0.674045742	down_in_case
CRA004775	ENSGALG00010026874	TEAD3	case	control	-0.247005232	0.451004909	-0.547677478	0.583913379	0.889908634	down_in_case
CRA004775	ENSGALG00010026885	TULP1	case	control	0.14252246	0.721384166	0.197568046	0.843383038	0.966478468	up_in_case
CRA004775	ENSGALG00010026886	JUN	case	control	0.483089534	0.392806944	1.229839598	0.218757177	0.686309227	up_in_case
CRA004775	ENSGALG00010026889	ENSGALG00010026889	case	control	-0.209099976	0.671770737	-0.311266873	0.755597749	0.941900693	down_in_case
CRA004775	ENSGALG00010026893	FKBP5	case	control	-0.00208984	0.681780154	-0.00306527	0.997554272	0.999646385	down_in_case
CRA004775	ENSGALG00010026894	IP6K1	case	control	-0.216813612	0.134301969	-1.614374042	0.106446319	0.552224847	down_in_case
CRA004775	ENSGALG00010026897	ENSGALG00010026897	case	control	0.075494963	0.413006639	0.182793582	0.854959985	0.970530418	up_in_case
CRA004775	ENSGALG00010026898	PNPLA2	case	control	0.089477395	0.513908831	0.174111417	0.861777901	0.97165529	up_in_case
CRA004775	ENSGALG00010026899	HOOK1	case	control	0.066408447	0.32740756	0.202831134	0.839267022	0.966077417	up_in_case
CRA004775	ENSGALG00010026901	FAM212A	case	control	0.328334525	0.427977703	0.767176706	0.442976461	0.831586254	up_in_case
CRA004775	ENSGALG00010026906	CLPS	case	control	2.150248995	1.032541291	2.082482332	0.037298435	0.36426902	up_in_case
CRA004775	ENSGALG00010026907	ENSGALG00010026907	case	control	-0.143546426	0.21464909	-0.668749287	0.503655422	0.861306517	down_in_case
CRA004775	ENSGALG00010026909	ENSGALG00010026909	case	control	-0.079350248	0.292592426	-0.271197205	0.786239363	0.95041797	down_in_case
CRA004775	ENSGALG00010026916	ENSGALG00010026916	case	control	-0.163103168	0.388848921	-0.419451256	0.674886372	0.92288678	down_in_case
CRA004775	ENSGALG00010026917	AMT	case	control	-0.262967376	0.26545852	-0.990615694	0.321873271	0.767750105	down_in_case
CRA004775	ENSGALG00010026919	NICN1	case	control	-0.160041517	0.256291859	-0.624450255	0.53233193	0.869886675	down_in_case
CRA004775	ENSGALG00010026920	GATA2	case	control	-0.74945029	0.566352321	-1.323293403	0.185737825	0.6491093	down_in_case
CRA004775	ENSGALG00010026924	TPH1	case	control	-0.210845307	0.552544473	-0.38158975	0.702765688	0.930068722	down_in_case
CRA004775	ENSGALG00010026942	CDK18	case	control	-0.327594942	0.423991791	-0.77264454	0.439732777	0.828842585	down_in_case
CRA004775	ENSGALG00010026943	EEFSEC	case	control	0.156997325	0.199006426	0.788905808	0.430167061	0.824153229	up_in_case
CRA004775	ENSGALG00010026950	LHFPL5	case	control	0.231641018	0.422752629	0.547935134	0.583736443	0.889906855	up_in_case
CRA004775	ENSGALG00010026951	RUVBL1	case	control	-0.191984835	0.211320241	-0.908501877	0.363613123	0.793608387	down_in_case
CRA004775	ENSGALG00010026954	MFSD4A	case	control	0.439940373	0.247701409	1.776091521	0.075717833	0.484198762	up_in_case
CRA004775	ENSGALG00010026958	CLTCL1	case	control	0.086841305	0.260410621	0.333478353	0.738773227	0.938064864	up_in_case
CRA004775	ENSGALG00010026960	SEC61A1	case	control	0.100684621	0.197559655	0.509641612	0.610302566	0.901413878	up_in_case
CRA004775	ENSGALG00010026964	SRPK1	case	control	-0.074830774	0.237425552	-0.315175739	0.752628225	0.94167946	down_in_case
CRA004775	ENSGALG00010026972	TNNT3	case	control	0.215391821	0.760871646	0.28308562	0.777111205	0.947261429	up_in_case
CRA004775	ENSGALG00010026973	MGLL	case	control	0.002880448	0.189174695	0.015226394	0.987851565	0.998065748	up_in_case
CRA004775	ENSGALG00010026977	ELK4	case	control	-0.269111513	0.40929657	-0.657497603	0.510861012	0.863170205	down_in_case
CRA004775	ENSGALG00010026980	LIMK2	case	control	0.040487872	0.245254545	0.165085103	0.868876998	0.973370973	up_in_case
CRA004775	ENSGALG00010026982	ABTB1	case	control	-0.130502653	0.240396793	-0.542863536	0.587223764	0.892195484	down_in_case
CRA004775	ENSGALG00010026984	PGR2/3	case	control	2.067637364	1.468117359	1.408359728			up_in_case
CRA004775	ENSGALG00010026999	MLEC	case	control	-0.424342144	0.259578569	-1.634734891	0.102104635	0.542100708	down_in_case
CRA004775	ENSGALG00010027001	SLC26A8	case	control	-0.132533221	0.412293757	-0.321453379	0.747866837	0.940802935	down_in_case
CRA004775	ENSGALG00010027003	SEPTIN2L	case	control	-0.109718219	0.136512147	-0.803724949	0.421555842	0.819812626	down_in_case
CRA004775	ENSGALG00010027012	RILPL1	case	control	0.673745283	0.296081901	2.275536872	0.022873743	0.294061167	up_in_case
CRA004775	ENSGALG00010027025	ORAI1	case	control	-0.087424709	0.208659401	-0.418982841	0.675228672	0.92288678	down_in_case
CRA004775	ENSGALG00010027026	SLC45A3	case	control	-0.2954325	0.595428814	-0.496167624	0.619776151	0.905089892	down_in_case
CRA004775	ENSGALG00010027027	ARHGEF3	case	control	-0.292747848	0.356210898	-0.82183855	0.41116879	0.816695368	down_in_case
CRA004775	ENSGALG00010027036	NUCKS1	case	control	0.01062194	0.231646897	0.045854012	0.963426609	0.992220813	up_in_case
CRA004775	ENSGALG00010027040	CLIP1	case	control	0.114780489	0.278762336	0.411750347	0.680522419	0.923896221	up_in_case
CRA004775	ENSGALG00010027041	MAPK14	case	control	-0.430036887	0.240332989	-1.789337736	0.07356044	0.478490829	down_in_case
CRA004775	ENSGALG00010027043	IL17RD	case	control	0.276998547	0.473487735	0.585017365	0.55853604	0.880754991	up_in_case
CRA004775	ENSGALG00010027045	MAPK13	case	control	0.133136223	0.542014441	0.245632244	0.805966927	0.955171181	up_in_case
CRA004775	ENSGALG00010027050	GSTT1	case	control	-0.901425756	0.395152527	-2.281209646	0.022536044	0.292594999	down_in_case
CRA004775	ENSGALG00010027052	APPL1	case	control	0.263529453	0.221125415	1.19176465	0.23335354	0.700409935	up_in_case
CRA004775	ENSGALG00010027059	ASB14	case	control	0.071860633	0.507444079	0.141612911	0.887385768	0.977118922	up_in_case
CRA004775	ENSGALG00010027063	RAB7L1	case	control	-0.207583422	0.25284291	-0.820997596	0.411647637	0.817006008	down_in_case
CRA004775	ENSGALG00010027074	BRPF3	case	control	-0.096003639	0.163608393	-0.586789203	0.557345289	0.880622207	down_in_case
CRA004775	ENSGALG00010027075	SLC41A1	case	control	-0.369661798	0.269099961	-1.373696959	0.169535781	0.633147466	down_in_case
CRA004775	ENSGALG00010027076	VDHAP	case	control	0.193141854	0.322137488	0.599563421	0.548797231	0.877623839	up_in_case

CRA004775	ENSGALG00010027077	ARF4	case	control	-0.286775301	0.184990159	-1.550219226	0.121088907	0.573473028	down_in_case
CRA004775	ENSGALG00010027078	PDE12	case	control	0.302305028	0.323209691	0.935321669	0.349622551	0.785360568	up_in_case
CRA004775	ENSGALG00010027079	PM20D1	case	control	-0.469141291	0.203976535	-2.299976762	0.021449537	0.28832119	down_in_case
CRA004775	ENSGALG00010027080	TBC1D22B	case	control	-0.380328136	0.259643753	-1.464807575	0.142973425	0.603260989	down_in_case
CRA004775	ENSGALG00010027081	PDE4B	case	control	-0.15206301	0.556742364	-0.273129943	0.78475333	0.950244571	down_in_case
CRA004775	ENSGALG00010027082	SLC26A9	case	control	0.716118871	0.63466001	1.128350391	0.25917197	0.720037471	up_in_case
CRA004775	ENSGALG00010027083	DENND6A	case	control	0.271549985	0.250975796	1.081976785	0.279262844	0.735529913	up_in_case
CRA004775	ENSGALG00010027084	OSBPL9	case	control	-0.070906334	0.209588571	-0.338312024	0.735128063	0.937036151	down_in_case
CRA004775	ENSGALG00010027085	PIM1	case	control	-0.892673665	0.313923381	-2.843603625	0.00446065	0.137451483	down_in_case
CRA004775	ENSGALG00010027086	CACNA1S	case	control	-0.469664383	0.646966216	-0.725948854	0.467870126	0.843870188	down_in_case
CRA004775	ENSGALG00010027087	RAB7B	case	control	1.204561929	0.478217127	2.518859866	0.01177355	0.217806153	up_in_case
CRA004775	ENSGALG00010027088	AGBL4	case	control	0.214910907	0.637799818	0.336956677	0.736149559	0.937385622	up_in_case
CRA004775	ENSGALG00010027092	ENSGALG00010027092	case	control	0.174503288	0.330733769	0.527624647	0.597759883	0.896490702	up_in_case
CRA004775	ENSGALG00010027093	SLMAP	case	control	0.171426872	0.227580027	0.753259739	0.451293847	0.836054284	up_in_case
CRA004775	ENSGALG00010027095	SCN5A	case	control	-0.309883835	0.99762604	-0.310621238	0.75608858	0.941900693	down_in_case
CRA004775	ENSGALG00010027096	YIPF1	case	control	-0.402203459	0.204396935	-1.967756802	0.049096027	0.410291029	down_in_case
CRA004775	ENSGALG00010027097	ENSGALG00010027097	case	control	-0.15154172	0.254201322	-0.596148434	0.55107607	0.87787848	down_in_case
CRA004775	ENSGALG00010027098	EXOG	case	control	0.038248675	0.288674872	0.132497419	0.894590864	0.977834811	up_in_case
CRA004775	ENSGALG00010027099	GNA11	case	control	0.229454487	0.152156272	1.508018589	0.13154977	0.587352231	up_in_case
CRA004775	ENSGALG00010027100	MIER2	case	control	-0.431245052	0.179483822	-2.402695945	0.016274713	0.25344686	down_in_case
CRA004775	ENSGALG00010027101	RNF220	case	control	-0.031009625	0.19201302	-0.161497513	0.87170157	0.97397971	down_in_case
CRA004775	ENSGALG00010027102	DUS3L	case	control	-0.046898997	0.228056898	-0.205646038	0.837067413	0.965269623	down_in_case
CRA004775	ENSGALG00010027103	ACVR2B	case	control	0.207782956	0.482711165	0.430449866	0.666868428	0.919168997	up_in_case
CRA004775	ENSGALG00010027105	CHERP	case	control	-0.104224621	0.113634573	-0.917191117	0.359042501	0.791216895	down_in_case
CRA004775	ENSGALG00010027106	CYP2J23	case	control	0.102173347	0.415934859	0.245647473	0.805955137	0.955171181	up_in_case
CRA004775	ENSGALG00010027107	KIF21B	case	control	-0.6953943	0.585474666	-1.187744475	0.234934096	0.702145501	down_in_case
CRA004775	ENSGALG00010027108	ENSGALG00010027108	case	control	0.48060239	0.288246132	1.667333353	0.095448138	0.5283846	up_in_case
CRA004775	ENSGALG00010027109	C19orf44	case	control	0.149723635	0.288452954	0.519057382	0.603720727	0.898692785	up_in_case
CRA004775	ENSGALG00010027110	IKBKE	case	control	-1.204432013	0.571408989	-2.107828255	0.035045848	0.354683583	down_in_case
CRA004775	ENSGALG00010027112	XYLB	case	control	-0.367914129	0.345245122	-1.065660615	0.286577088	0.741851239	down_in_case
CRA004775	ENSGALG00010027113	PIAS4	case	control	-0.176294143	0.237553024	-0.742125443	0.458011336	0.839796467	down_in_case
CRA004775	ENSGALG00010027114	FLNB	case	control	-0.034001698	0.393018276	-0.086514291	0.931057596	0.985044263	down_in_case
CRA004775	ENSGALG00010027116	CCDC94	case	control	-0.621251467	0.227655059	-2.728915707	0.006354294	0.158952969	down_in_case
CRA004775	ENSGALG00010027117	RASSF5	case	control	-1.106800896	0.608966165	-1.817508033	0.069139359	0.467651418	down_in_case
CRA004775	ENSGALG00010027118	ABHD6	case	control	0.24466125	0.162108554	1.509243304	0.131236617	0.586873082	up_in_case
CRA004775	ENSGALG00010027119	NUB1	case	control	-0.208566268	0.315665688	-0.660718841	0.508792639	0.862971569	down_in_case
CRA004775	ENSGALG00010027120	ENSGALG00010027120	case	control	-0.063371638	0.87590301	-0.072350063	0.942323325	0.987651835	down_in_case
CRA004775	ENSGALG00010027121	ELF3	case	control	2.180440304	0.690584364	3.157384408	0.001591913	0.080776387	up_in_case
CRA004775	ENSGALG00010027122	DNASE1L3	case	control	0.559485751	0.403943544	1.385059273	0.166034344	0.630194031	up_in_case
CRA004775	ENSGALG00010027123	WDR86	case	control	-0.877810621	0.303857247	-2.88889151	0.003866024	0.128409904	down_in_case
CRA004775	ENSGALG00010027124	IP6K2	case	control	0.405238949	0.18428666	2.198959755	0.027880784	0.318626275	up_in_case
CRA004775	ENSGALG00010027125	CRYGN	case	control	0.680454185	0.477899218	1.423844525	0.154491484	0.619284425	up_in_case
CRA004775	ENSGALG00010027126	ENSGALG00010027126	case	control	-1.335792434	0.476720483	-2.802045398	0.005077973	0.143998699	down_in_case
CRA004775	ENSGALG00010027127	S1PR4	case	control	-0.923647198	0.606678126	-1.522466623	0.127892202	0.5821156	down_in_case
CRA004775	ENSGALG00010027128	EIF2D	case	control	-0.163755826	0.217403067	-0.753236046	0.451308082	0.836054284	down_in_case
CRA004775	ENSGALG00010027129	NCLN	case	control	-0.212044679	0.228508676	-0.927950233	0.353433386	0.78730441	down_in_case
CRA004775	ENSGALG00010027130	NCKIPSD	case	control	-0.180221406	0.189327648	-0.951902205	0.341146581	0.781070036	down_in_case
CRA004775	ENSGALG00010027131	UBA52	case	control	0.024785302	0.273254057	0.090704242	0.9277276	0.984691935	up_in_case
CRA004775	ENSGALG00010027133	CELF5	case	control	-0.512747677	0.585205517	-0.876183942	0.380930045	0.801753951	down_in_case
CRA004775	ENSGALG00010027134	DYRK3	case	control	0.029988845	0.314872269	0.095241302	0.924123165	0.983849734	up_in_case
CRA004775	ENSGALG00010027135	PRKAG2	case	control	0.062560863	0.217784906	0.287259866	0.77391335	0.946296525	up_in_case
CRA004775	ENSGALG00010027136	MAPKAPK2	case	control	0.000142765	0.230015625	0.000620677	0.999504771	0.999914248	up_in_case
CRA004775	ENSGALG00010027137	FKBP8	case	control	-0.367301554	0.28534084	-1.287237936	0.198011368	0.664272829	down_in_case
CRA004775	ENSGALG00010027139	HSD11B1L	case	control	-0.143790579	0.246898389	-0.582387678	0.56030558	0.880918893	down_in_case
CRA004775	ENSGALG00010027141	PIGR	case	control	2.928279018	0.717133499	4.083310877	4.43986E-05	0.013292084	up_in_case
CRA004775	ENSGALG00010027143	GALNT11	case	control	-0.181277037	0.329453516	-0.550235552	0.582157822	0.889571309	down_in_case
CRA004775	ENSGALG00010027145	CELSR3	case	control	-0.265080248	0.271440381	-0.97656895	0.328782597	0.77220987	down_in_case
CRA004775	ENSGALG00010027146	KXD1	case	control	-0.282464671	0.294545024	-0.958986393	0.3375656	0.778184725	down_in_case
CRA004775	ENSGALG00010027147	KMT2C	case	control	0.482147738	0.333227287	1.446903532	0.147923944	0.611192154	up_in_case
CRA004775	ENSGALG00010027148	ELL	case	control	0.613931682	0.386262472	1.589415814	0.111966547	0.56149297	up_in_case
CRA004775	ENSGALG00010027149	C1orf116	case	control	-0.175808346	0.420170682	-0.418421259	0.675639146	0.922924485	down_in_case
CRA004775	ENSGALG00010027150	YOD1	case	control	-0.324937234	0.224756243	-1.445731741	0.148252458	0.611192154	down_in_case
CRA004775	ENSGALG00010027151	MICOS13	case	control	0.095534185	0.410859201	0.23252293	0.81613188	0.959129938	up_in_case
CRA004775	ENSGALG00010027152	C4BPS	case	control	-1.030449822	0.671658391	-1.534187373	0.124983563	0.577503017	down_in_case
CRA004775	ENSGALG00010027153	ENSGALG00010027153	case	control	0.334900397	0.344764559	0.971388701	0.331354753	0.772629743	up_in_case
CRA004775	ENSGALG00010027154	PLPP2	case	control	-0.001891398	0.255483536	-0.007403208	0.994093149	0.998960959	down_in_case
CRA004775	ENSGALG00010027155	ENSGALG00010027155	case	control	0.388255729	0.497319775	0.780696342	0.434981112	0.826848946	up_in_case
CRA004775	ENSGALG00010027156	ISYNA1	case	control	0.383095254	0.310692811	1.233035465	0.217562533	0.684913551	up_in_case
CRA004775	ENSGALG00010027157	ENSGALG00010027157	case	control	0.153622432	0.243207162	0.631652583	0.527613919	0.86830001	up_in_case
CRA004775	ENSGALG00010027158	XRCC2	case	control	-0.291861726	0.248021369	-1.176760402	0.239291152	0.706254731	down_in_case
CRA004775	ENSGALG00010027159	TMUB1	case	control	-0.231191902	0.36422302	-0.63475368	0.525589077	0.867561466	down_in_case
CRA004775	ENSGALG00010027160	FASTK	case	control	-0.00118966	0.374991137	-0.003172502	0.997468713	0.999646385	down_in_case
CRA004775	ENSGALG00010027161	C4BPG	case	control	-0.534084661	0.314773619	-1.696726244	0.089748429	0.516522282	down_in_case
CRA004775	ENSGALG00010027162	SLC4A2	case	control	-0.245716145	0.210787031	-1.165708079	0.243732493	0.710981228	down_in_case
CRA004775	ENSGALG00010027163	ENSGALG00010027163	case	control	0.123867243	0.171971865	0.720276207	0.471354951	0.845200296	up_in_case
CRA004775	ENSGALG00010027164	ENSGALG00010027164	case	control	2.651800216	0.679274154	3.903873276			up_in_case
CRA004775	ENSGALG00010027165	ENSGALG00010027165	case	control	-0.310408684	0.289454954	-1.072390297	0.283544764	0.740414746	down_in_case
CRA004775	ENSGALG00010027166	CDK5	case	control	-0.330971058	0.21369664	-1.548789244	0.121432392	0.573556613	down_in_case
CRA004775	ENSGALG00010027167	ENSGALG00010027167	case	control	-1.073583816	0.618905039	-1.734650307	0.082802763	0.499833536	down_in_case
CRA004775	ENSGALG00010027168	SSBP4	case	control	-0.045894046	0.191377255	-0.2398093	0.810478097	0.956724552	down_in_case
CRA004775	ENSGALG00010027169	ABCB8	case	control	-0.321394013	0.38146659	-0.842522049	0.399495803	0.811083241	down_in_case
CRA004775	ENSGALG00010027170	TLE2	case	control	-0.447329441	0.318130431	-1.406119622	0.159688607	0.624907945	down_in_case
CRA004775	ENSGALG00010027171	ATG9B	case	control	-0.462084975	0.646478197	-0.714772713	0.474749499	0.84579952	down_in_case
CRA004775	ENSGALG00010027172	ENSGALG00010027172	case	control	-0.967506652	0.704564545	-1.37319804	0.169690789	0.633147466	down_in_case
CRA004775	ENSGALG00010027173	ENSGALG00010027173	case	control	0.320123908	0.247997794	1.290833693	0.196761352	0.66285976	up_in_case
CRA004775	ENSGALG00010027174	SHISA5	case	control	-0.442306402	0.29633818	-1.492573123	0.135548963	0.593592099	down_in_case
CRA004775	ENSGALG00010027175	NOS3	case	control	0.146393823	0.389738995	0.375620159	0.707199302	0.931617022	up_in_case
CRA004775	ENSGALG00010027176	ENSGALG00010027176	case	control	0.257693307	0.232267787	1.109466403	0.26722903	0.727315996	up_in_case
CRA004775	ENSGALG00010027178	C4BPM	case	control	-0.487161944	0.365474483	-1.332957476	0.182545759	0.647378665	down_in_case
CRA004775	ENSGALG00010027183	CD34	case	control	-0.046579782	0.508605328	-0.091583355	0.927029077	0.984216865	down_in_case
CRA004775	ENSGALG00010027184	MPND	case	control	0.771457088	0.431904109	1.786176775	0.07407064	0.479993107	up_in_case
CRA004775	ENSGALG00010027185	ATRIP	case	control	-0.241415436	0.270186274	-0.893514804	0.371581548	0.795818272	down_in_case
CRA004775	ENSGALG00010027186	PLXNA2	case	control	0.122531721	0.421279349	0.290856225	0.771161282	0.945853152	up_in_case
CRA004775	ENSGALG00010027187	ENSGALG00010027187	case	control	-0.15558861	0.251427623	-0.61882067	0.536034504	0.871588629	down_in_case
CRA004775	ENSGALG00010027188	KCNH2	case	control	-0.9692932	0.525670388	-1.84391821	0.065195061	0.457949961	down_in_case
CRA004775	ENSGALG00010027189	ENSGALG00010027189	case	control	1.132173485	0.610287527	1.855147672	0.063575142	0.453669836	up_in_case
CRA004775	ENSGALG00010027190	IPPK	case	control	0.607960658	0.168882603	3.599901035	0.000318338	0.035519918	up_in_case
CRA004775	ENSGALG00010027191	AOC1	case	control	2.568158265	0.8713514	2.94732787	0.003205331	0.121089322	up_in_case
CRA004775	ENSGALG00010027192	SH3GL1	case	control	-0.168646119	0.201744309	-0.83593991	0.403188709	0.812213331	down_in_case
CRA004775	ENSGALG00010027193	CAMK1G	case	control	0.150674191	0.783137419	0.19239815	0.847430341	0.967938751	up_in_case
CRA004775	ENSGALG00010027194	ENSGALG00010027194	case	control	-0.083176865	0.374272834	-0.222235913	0.824130238	0.961042648	down_in_case
CRA004775	ENSGALG00010027195	CENPP	case	control	0.929997566	0.548974084	1.694064607	0.090253007	0.518408145	up_in_case
CRA004775	ENSGALG00010027196	ENSGALG00010027196	case	control	-0.454999163	0.411794487	-1.104918053	0.269195099	0.72765348	down_in_case
CRA004775	ENSGALG00010027197	ENSGALG00010027197	case	control	-2.651924832	0.772236535	-3.434083615	0.000594561	0.05063962	down_in_case
CRA004775	ENSGALG00010027198	ECM2	case	control	0.282640406	0.435043612	0.649682924	0.515897056	0.864897625	up_in_case
CRA004775	ENSGALG00010027199	LAMB3	case	control	-1.130452889	0.556806291	-2.030244463	0.042331696	0.386779506	down_in_case
CRA004775	ENSGALG00010027200	G0S2	case	control	-0.439698213	0.378823512	-1.160694094	0.245766325	0.712046495	down_in_case
CRA004775	ENSGALG00010027201	ASPN	case	control	0.596569501	0.438783445	1.359598926	0.173956878	0.639185614	up_in_case
CRA004775	ENSGALG00010027202	CHAF1A	case	control	-0.062122335	0.296756229	-0.209337931	0.834184443	0.964019688	down_in_case
CRA004775	ENSGALG00010027203	ENSGALG00010027203	case	control	-0.433736665	0.490959315	-0.883447267	0.376994662	0.798523018	down_in_case
CRA004775	ENSGALG00010027205	OGN	case	control	0.656903794	0.541891164	1.212243044	0.225419359	0.693278343	up_in_case
CRA004775	ENSGALG00010027206	ENSGALG00010027206	case	control	1.378960108	0.569456659	2.421536543	0.015455047	0.249560894	up_in_case
CRA004775	ENSGALG00010027207	NOL8	case	control	0.264647626	0.248371062	1.065533256	0.286634685	0.741851239	up_in_case
CRA004775	ENSGALG00010027208	UBXN6	case	control	-0.181020907	0.197359079	-0.917216011	0.359029458	0.791216895	down_in_case
CRA004775	ENSGALG00010027209	TRAF3IP3	case	control	-0.423143873	0.4775928	-0.885992991	0.375621304	0.798523018	down_in_case
CRA004775	ENSGALG00010027210	ENSGALG00010027210	case	control	-0.534502645	0.489012337	-1.093024868	0.274382881	0.731001618	down_in_case
CRA004775	ENSGALG00010027211	HDGFRP2	case	control	0.111083333	0.139309859	0.797383142	0.425228547	0.822283278	up_in_case
CRA004775	ENSGALG00010027212	IRF6	case	control	0.411143585	0.529660512	0.776239829	0.437607393	0.827521409	up_in_case
CRA004775	ENSGALG00010027213	KCNC4	case	control	-0.087675214	0.512827965	-0.170964183	0.864251929	0.972350383	down_in_case
CRA004775	ENSGALG00010027214	PLIN4	case	control	-0.072461158	0.271062122	-0.267323066	0.789220428	0.95041797	down_in_case
CRA004775	ENSGALG00010027215	ENSGALG00010027215	case	control	0.176322652	0.353231867	0.499169721	0.617659824	0.903976249	up_in_case
CRA004775	ENSGALG00010027216	ENSGALG00010027216	case	control	-0.427698543	0.610749887	-0.700284277	0.483749789	0.850349167	down_in_case
CRA004775	ENSGALG00010027217	SEMA6B	case	control	-0.29534664	0.534464305	-0.552603115	0.580535208	0.888770836	down_in_case
CRA004775	ENSGALG00010027218	IARS1	case	control	-0.042947236	0.20512166	-0.209374458	0.834155931	0.964019688	down_in_case
CRA004775	ENSGALG00010027219	ENSGALG00010027219	case	control	-0.280284828	0.203163544	-1.379601979	0.167709228	0.63138062	down_in_case
CRA004775	ENSGALG00010027220	ANKRD24	case	control	-0.801264044	0.795979282	-1.006639321	0.314108131	0.765098415	down_in_case

CRA004775	ENSGALG00010027221	LRRC61	case	control	0.214799454	0.25999853	0.826156417	0.408715367	0.814384687	up_in_case
CRA004775	ENSGALG00010027223	SIRT6	case	control	-0.282590219	0.157815336	-1.790638514	0.073351322	0.478261752	down_in_case
CRA004775	ENSGALG00010027224	RARRES2	case	control	0.191831591	0.187725843	1.02187098	0.306841969	0.7585475	up_in_case
CRA004775	ENSGALG00010027225	CREB3L3	case	control	2.2640588	0.661216212	3.424082409	0.000616879	0.051333788	up_in_case
CRA004775	ENSGALG00010027227	ENSGALG00010027227	case	control	-0.219886307	0.370349321	-0.593726772	0.552694874	0.878345607	down_in_case
CRA004775	ENSGALG00010027228	SSPO	case	control	-0.710906091	0.610994833	-1.163522264	0.24461767	0.711765017	down_in_case
CRA004775	ENSGALG00010027229	MDM4	case	control	-0.284567075	0.240370446	-1.183868814	0.236464997	0.703419274	down_in_case
CRA004775	ENSGALG00010027230	MAP2K2	case	control	-0.250076154	0.202210189	-1.236713916	0.21619331	0.683774974	down_in_case
CRA004775	ENSGALG00010027231	ENSGALG00010027231	case	control	0.676513151	0.430120669	1.572845016	0.115754709	0.566086328	up_in_case
CRA004775	ENSGALG00010027232	ARRDC2	case	control	-0.086247607	0.590027876	-0.146175479	0.883782863	0.976136397	down_in_case
CRA004775	ENSGALG00010027233	ENSGALG00010027233	case	control	-0.708237474	0.470528549	-1.50519554	0.132273811	0.588449428	down_in_case
CRA004775	ENSGALG00010027235	ENSGALG00010027235	case	control	-0.427541333	0.362554192	-1.179248075	0.238299408	0.704840228	down_in_case
CRA004775	ENSGALG00010027236	PEX11G	case	control	0.018873131	0.612394501	0.030818583	0.97541422	0.995245303	up_in_case
CRA004775	ENSGALG00010027237	CTC-575C13.4	case	control	-0.407752289	0.431670068	-0.944592455	0.34486698	0.783972357	down_in_case
CRA004775	ENSGALG00010027239	ENSGALG00010027239	case	control	-0.347741035	0.151547083	-2.294607249	0.021755642	0.288534247	down_in_case
CRA004775	ENSGALG00010027240	SLC6A1	case	control	0.46713086	0.700598176	0.666760029	0.504925428	0.861471523	up_in_case
CRA004775	ENSGALG00010027241	LRRN2	case	control	-0.089906188	0.199043872	-0.45169031	0.6514921	0.915892481	down_in_case
CRA004775	ENSGALG00010027242	ENSGALG00010027242	case	control	0.299375383	0.287290479	1.042065101	0.297381497	0.749916187	up_in_case
CRA004775	ENSGALG00010027243	HRH1	case	control	0.086952609	0.494941816	0.175682487	0.860543395	0.971325143	up_in_case
CRA004775	ENSGALG00010027244	ARHGEF18	case	control	0.05933568	0.456239762	0.130053724	0.896523922	0.978653683	up_in_case
CRA004775	ENSGALG00010027245	LRIG2	case	control	0.001892961	0.250812033	0.007547329	0.99397816	0.998960959	up_in_case
CRA004775	ENSGALG00010027246	ZNF767	case	control	-0.30599207	0.181234343	-1.688377958	0.091338699	0.521441551	down_in_case
CRA004775	ENSGALG00010027247	ATG7	case	control	-0.307070868	0.270871744	-1.13363935	0.25694586	0.719236059	down_in_case
CRA004775	ENSGALG00010027250	ENSGALG00010027250	case	control	-0.149887148	0.26825049	-0.558758151	0.576326787	0.887937128	down_in_case
CRA004775	ENSGALG00010027251	ENSGALG00010027251	case	control	-0.016109211	0.263690063	-0.061091459	0.951286371	0.990168609	down_in_case
CRA004775	ENSGALG00010027252	MAGI3	case	control	0.175475509	0.237047223	0.740255494	0.459144981	0.840589413	up_in_case
CRA004775	ENSGALG00010027253	VGLL4	case	control	-0.143105789	0.315976522	-0.452900071	0.6506207	0.915542902	down_in_case
CRA004775	ENSGALG00010027254	INSR	case	control	-0.004567832	0.417467916	-0.010941756	0.991269916	0.998065748	down_in_case
CRA004775	ENSGALG00010027255	ENSGALG00010027255	case	control	-0.377491605	0.302859112	-1.24642644	0.21260788	0.682280269	down_in_case
CRA004775	ENSGALG00010027256	PHTF1	case	control	-0.903078535	0.38258832	-2.360444603	0.018253044	0.2691835	down_in_case
CRA004775	ENSGALG00010027257	TAFA3	case	control	-0.223146689	0.449963879	-0.495921338	0.619949911	0.905089892	down_in_case
CRA004775	ENSGALG00010027258	ENSGALG00010027258	case	control	-0.139162394	0.39818744	-0.349489663	0.72672173	0.935238044	down_in_case
CRA004775	ENSGALG00010027259	ENSGALG00010027259	case	control	-0.319976027	0.177608703	-1.801578541	0.07161174	0.471615439	down_in_case
CRA004775	ENSGALG00010027260	LEMD2	case	control	-0.209225049	0.289253117	-0.723328588	0.469478033	0.844004763	down_in_case
CRA004775	ENSGALG00010027261	ENSGALG00010027261	case	control	-0.402129069	0.289270416	-1.39014931	0.164483542	0.63006979	down_in_case
CRA004775	ENSGALG00010027262	USE1	case	control	-0.288798563	0.19703477	-1.46572386	0.142723531	0.602997185	down_in_case
CRA004775	ENSGALG00010027263	TAMM41	case	control	-0.025820678	0.214574711	-0.12033421	0.904218406	0.979540372	down_in_case
CRA004775	ENSGALG00010027264	ENSGALG00010027264	case	control	-0.387003124	0.262507509	-1.474252442	0.140412827	0.600007375	down_in_case
CRA004775	ENSGALG00010027265	IP6K3	case	control	-0.037298466	0.634976677	-0.0587399	0.953159279	0.990670511	down_in_case
CRA004775	ENSGALG00010027266	SLC26A6	case	control	2.444981269	0.970135827	2.520246341	0.011727273	0.217806153	up_in_case
CRA004775	ENSGALG00010027267	MYO9B	case	control	-0.153441683	0.38656342	-0.396937928	0.691413235	0.927867527	down_in_case
CRA004775	ENSGALG00010027268	UQCRC1	case	control	-0.028454968	0.338764704	-0.083996259	0.933059406	0.985090297	down_in_case
CRA004775	ENSGALG00010027269	CDC25A	case	control	-0.570262415	0.291053947	-1.959301432	0.050077496	0.414161886	down_in_case
CRA004775	ENSGALG00010027270	ENSGALG00010027270	case	control	0.256519214	0.624048143	0.411056769	0.681030908	0.923896221	up_in_case
CRA004775	ENSGALG00010027271	INKA2	case	control	0.775233844	0.370198738	2.094101798	0.036250913	0.360628495	up_in_case
CRA004775	ENSGALG00010027272	DDX20	case	control	0.137546366	0.211713642	0.649681167	0.515898191	0.864897625	up_in_case
CRA004775	ENSGALG00010027273	HAUS8	case	control	-0.278997679	0.37297798	-0.748027212	0.454443744	0.838254622	down_in_case
CRA004775	ENSGALG00010027274	COPG1	case	control	-0.152622678	0.113522989	-1.344420886	0.178812324	0.644859234	down_in_case
CRA004775	ENSGALG00010027275	CPAMD8	case	control	0.7232169	0.466741194	1.549503043	0.121260841	0.573473028	up_in_case
CRA004775	ENSGALG00010027276	ENSGALG00010027276	case	control	0.058424796	0.290604507	0.201045731	0.840662816	0.966461921	up_in_case
CRA004775	ENSGALG00010027277	KCND3	case	control	0.455111555	0.734175615	0.619894675	0.535327132	0.870981055	up_in_case
CRA004775	ENSGALG00010027278	H1-10	case	control	-0.269961844	0.265676944	-1.016128232	0.309568326	0.762002042	down_in_case
CRA004775	ENSGALG00010027279	F2RL3	case	control	0.703301475	0.517654562	1.358630882	0.174263584	0.639555285	up_in_case
CRA004775	ENSGALG00010027280	HMCES	case	control	-0.031681838	0.196547992	-0.16119136	0.871942686	0.97401729	down_in_case
CRA004775	ENSGALG00010027281	ENSGALG00010027281	case	control	0.215132049	0.232094862	0.926914311	0.353971026	0.78730441	up_in_case
CRA004775	ENSGALG00010027282	ENSGALG00010027282	case	control	-0.2827605	0.380291407	-0.743536389	0.457156999	0.839688534	down_in_case
CRA004775	ENSGALG00010027283	RAB7A	case	control	-0.177029531	0.271077999	-0.653057539	0.513719185	0.863954532	down_in_case
CRA004775	ENSGALG00010027284	CTTNBP2NL	case	control	0.236417525	0.282752211	0.836129711	0.403081936	0.812213331	up_in_case
CRA004775	ENSGALG00010027285	PTPRS	case	control	0.152488539	0.299701985	0.508800563	0.610892025	0.901413878	up_in_case
CRA004775	ENSGALG00010027286	RPN1	case	control	-0.122392686	0.156110622	-0.784012542	0.433032751	0.825787569	down_in_case
CRA004775	ENSGALG00010027287	DHX30	case	control	-0.202011429	0.137089951	-1.47356846	0.140597818	0.600007375	down_in_case
CRA004775	ENSGALG00010027288	DAG1	case	control	0.193404072	0.321861362	0.600892479	0.547911603	0.877369241	up_in_case
CRA004775	ENSGALG00010027289	SMARCC1	case	control	0.071983784	0.210814641	0.341455335	0.732760824	0.936819725	up_in_case
CRA004775	ENSGALG00010027290	CSPG5	case	control	0.875838614	0.6560297	1.335059395	0.181856908	0.647378665	up_in_case
CRA004775	ENSGALG00010027291	MST1	case	control	-0.529165471	0.368410492	-1.436347451	0.150903498	0.614921512	down_in_case
CRA004775	ENSGALG00010027292	KDM4B	case	control	0.021724381	0.197914448	0.109766523	0.912594541	0.980787663	up_in_case
CRA004775	ENSGALG00010027293	ELP6	case	control	-0.534111272	0.241026281	-2.215987686	0.026692345	0.314252091	down_in_case
CRA004775	ENSGALG00010027295	ENSGALG00010027295	case	control	-0.295841062	0.485265433	-0.609647921	0.54209506	0.875128455	down_in_case
CRA004775	ENSGALG00010027299	PGPEP1	case	control	-0.233762513	0.26119152	-0.894985079	0.370795064	0.795641647	down_in_case
CRA004775	ENSGALG00010027301	SCAP	case	control	-0.261943469	0.146942619	-1.782624203	0.074647497	0.481462352	down_in_case
CRA004775	ENSGALG00010027303	APEH	case	control	-0.471573445	0.292979566	-1.609577934	0.107490029	0.553800541	down_in_case
CRA004775	ENSGALG00010027304	PTPN23	case	control	-0.154076376	0.254785367	-0.604730083	0.545358356	0.876563448	down_in_case
CRA004775	ENSGALG00010027305	LSM4	case	control	-0.562395125	0.167998573	-3.347618459	0.000815091	0.060328515	down_in_case
CRA004775	ENSGALG00010027306	ENSGALG00010027306	case	control	0.142795589	0.425192552	0.335837465	0.736993436	0.937564762	up_in_case
CRA004775	ENSGALG00010027307	JUND	case	control	0.469383571	0.593704259	0.790601658	0.429176479	0.823883065	up_in_case
CRA004775	ENSGALG00010027309	ENSGALG00010027309	case	control	0.960657313	0.590174861	1.62775031	0.103577841	0.545480965	up_in_case
CRA004775	ENSGALG00010027310	ARHGEF2	case	control	-0.318140431	0.187413099	-1.697535725	0.089595423	0.516522282	down_in_case
CRA004775	ENSGALG00010027311	NFASC	case	control	-0.099779849	0.496252526	-0.201066683	0.840646433	0.966461921	down_in_case
CRA004775	ENSGALG00010027312	ENSGALG00010027312	case	control	-0.846474066	0.557689897	-1.517822127	0.129059245	0.584172159	down_in_case
CRA004775	ENSGALG00010027313	BSN	case	control	-0.740237776	0.334302082	-2.214278091	0.026809655	0.314824563	down_in_case
CRA004775	ENSGALG00010027314	ENSGALG00010027314	case	control	-0.003554682	0.52874143	-0.006722912	0.994635933	0.999001196	down_in_case
CRA004775	ENSGALG00010027315	RAB3A	case	control	0.207973535	0.35178357	0.591197409	0.554388158	0.879486798	up_in_case
CRA004775	ENSGALG00010027316	TRAIP	case	control	0.436881819	0.627085164	0.696686581	0.485998958	0.851645437	up_in_case
CRA004775	ENSGALG00010027317	MPV17L2	case	control	-0.128246296	0.271762934	-0.471905031	0.636994577	0.910342177	down_in_case
CRA004775	ENSGALG00010027319	MST1R	case	control	-0.696721975	0.576916236	-1.207665744	0.22717586	0.694715356	down_in_case
CRA004775	ENSGALG00010027320	MON1A	case	control	-0.355337242	0.312621937	-1.13663566	0.255690626	0.718871765	down_in_case
CRA004775	ENSGALG00010027321	IFI30	case	control	-0.454106997	0.394413891	-1.151346358	0.249589773	0.714467496	down_in_case
CRA004775	ENSGALG00010027322	CRABP2	case	control	0.824791789	0.712620487	1.157406789	0.247106202	0.712046495	up_in_case
CRA004775	ENSGALG00010027323	ISG20L2	case	control	-0.327717864	0.162820905	-2.012750545	0.044140879	0.392535833	down_in_case
CRA004775	ENSGALG00010027324	PIK3R2	case	control	-0.128071707	0.227792239	-0.562230335	0.573959096	0.886897708	down_in_case
CRA004775	ENSGALG00010027325	METTL25B	case	control	-0.666779635	0.267123283	-2.496149446	0.012554969	0.225973017	down_in_case
CRA004775	ENSGALG00010027326	MRPL24	case	control	-0.255286178	0.34801572	-0.733547833	0.463224365	0.842456502	down_in_case
CRA004775	ENSGALG00010027327	RBM6	case	control	-0.023532833	0.20656832	-0.113922759	0.909299024	0.98075825	down_in_case
CRA004775	ENSGALG00010027328	RBM15	case	control	0.00926498	0.251455872	0.036845353	0.970608312	0.994548055	up_in_case
CRA004775	ENSGALG00010027329	MINDY4	case	control	-0.209125408	0.347428066	-0.601924335	0.547224505	0.877128356	down_in_case
CRA004775	ENSGALG00010027330	ENSGALG00010027330	case	control	0.524666844	0.400823018	1.308973837	0.190543213	0.656023968	up_in_case
CRA004775	ENSGALG00010027331	SLC16A4	case	control	0.047166536	0.267926195	0.176043018	0.860260147	0.971325143	up_in_case
CRA004775	ENSGALG00010027332	HDGF	case	control	0.097838218	0.203169763	0.481558949	0.630119294	0.908943555	up_in_case
CRA004775	ENSGALG00010027333	PROK1	case	control	-0.409258883	0.51512131	-0.794490297	0.426910055	0.822776983	down_in_case
CRA004775	ENSGALG00010027334	PRCC	case	control	-0.130614142	0.152414611	-0.856966013	0.391463668	0.807170025	down_in_case
CRA004775	ENSGALG00010027335	MAST3	case	control	-0.968546325	0.323849211	-2.990732395	0.002783093	0.109874886	down_in_case
CRA004775	ENSGALG00010027336	RAD54L2	case	control	0.31552306	0.437055306	0.721929365	0.470337906	0.844782383	up_in_case
CRA004775	ENSGALG00010027337	AQP1	case	control	0.280236361	0.408955749	0.685248615	0.493187051	0.856001758	up_in_case
CRA004775	ENSGALG00010027339	LAMTOR5	case	control	-0.127533576	0.275584256	-0.462775261	0.643525466	0.913638565	down_in_case
CRA004775	ENSGALG00010027340	ENSGALG00010027340	case	control	-1.165902828	0.586955566	-1.9863562	0.046993786	0.402720701	down_in_case
CRA004775	ENSGALG00010027341	ENSGALG00010027341	case	control	-0.44525516	0.403181379	-1.104354475	0.269439401	0.72765348	down_in_case
CRA004775	ENSGALG00010027342	ENSGALG00010027342	case	control	0.311770505	0.62984571	0.494995043	0.620603619	0.905283281	up_in_case
CRA004775	ENSGALG00010027344	ENSGALG00010027344	case	control	-2.700038475	1.341317408	-2.012975049	0.044117254	0.392535833	down_in_case
CRA004775	ENSGALG00010027346	SIN3B	case	control	0.083926973	0.209771769	0.400087071	0.689092387	0.927420456	up_in_case
CRA004775	ENSGALG00010027348	ENSGALG00010027348	case	control	-0.013841938	0.249913688	-0.055386874	0.955830253	0.990926004	down_in_case
CRA004775	ENSGALG00010027350	INSRR	case	control	-0.35124327	0.351252063	-0.999974967	0.317322623	0.766199014	down_in_case
CRA004775	ENSGALG00010027351	TMEM38A	case	control	0.391754113	0.492481723	0.795469345	0.426340536	0.822541484	up_in_case
CRA004775	ENSGALG00010027353	ENSGALG00010027353	case	control	-0.030899052	0.423535432	-0.072955059	0.94184188	0.987590948	down_in_case
CRA004775	ENSGALG00010027355	ADCYAP1R1	case	control	-0.856643142	0.957226698	-0.894921907	0.370828835	0.795641647	down_in_case
CRA004775	ENSGALG00010027356	ENSGALG00010027356	case	control	-0.276050873	0.309955239	-0.890615283	0.373135598	0.796804495	down_in_case
CRA004775	ENSGALG00010027358	RNF126	case	control	-0.176868571	0.202678565	-0.872655532	0.382850863	0.801925538	down_in_case
CRA004775	ENSGALG00010027359	ENSGALG00010027359	case	control	-0.17065195	0.380860782	-0.448069106	0.654103324	0.916560183	down_in_case
CRA004775	ENSGALG00010027360	SMIM7	case	control	0.069671295	0.274593089	0.253725595	0.799707559	0.952847322	up_in_case
CRA004775	ENSGALG00010027361	FANCD2	case	control	0.209490474	0.369812352	0.566477763	0.571069045	0.885321726	up_in_case
CRA004775	ENSGALG00010027362	ENSGALG00010027362	case	control	-0.028048126	0.223998895	-0.125215463	0.900352975	0.978988423	down_in_case
CRA004775	ENSGALG00010027363	VIPR1	case	control	0.484164636	0.28679691	1.688179401	0.091376796	0.521441551	up_in_case
CRA004775	ENSGALG00010027364	FSTL3	case	control	0.156915711	0.463388894	0.338626396	0.734891195	0.936931635	up_in_case
CRA004775	ENSGALG00010027365	PLEKHA6	case	control	0.059917963	0.528503755	0.113372823	0.909734985	0.98075825	up_in_case
CRA004775	ENSGALG00010027366	USP4	case	control	-0.221384854	0.180617392	-1.22571172	0.220307178	0.688374653	down_in_case
CRA004775	ENSGALG00010027367	SEC22C	case	control	-0.013189159	0.180403972	-0.073109025	0.94171936	0.987577169	down_in_case
CRA004775	ENSGALG00010027368	EMC3	case	control	-0.13666634	0.199340753	-0.685591571	0.492970698	0.855820432	down_in_case

CRA004775	ENSGALG00010027369	ENSGALG00010027369	case	control	0.740495713	0.234932054	3.151956914	0.001621802	0.080802173	up_in_case
CRA004775	ENSGALG00010027370	NUP210L	case	control	-0.107219597	0.384289058	-0.279007675	0.780238935	0.94835413	down_in_case
CRA004775	ENSGALG00010027371	PRSS57	case	control	-0.00826984	0.594641468	-0.013907271	0.988903961	0.998065748	down_in_case
CRA004775	ENSGALG00010027372	PIK3C2B	case	control	-0.269310067	0.420243766	-0.640842503	0.521625015	0.866314221	down_in_case
CRA004775	ENSGALG00010027374	NKTR	case	control	0.40059677	0.277173213	1.445293956	0.148375335	0.611192154	up_in_case
CRA004775	ENSGALG00010027375	FUT7	case	control	-1.436722075	0.577590683	-2.487439838	0.012866621	0.228267513	down_in_case
CRA004775	ENSGALG00010027376	DSTYK	case	control	-0.136707857	0.222409688	-0.614666824	0.538774774	0.873460494	down_in_case
CRA004775	ENSGALG00010027378	ZBTB47	case	control	-0.044839713	0.361026245	-0.124200702	0.901156366	0.979157637	down_in_case
CRA004775	ENSGALG00010027379	OPTC	case	control	-2.702727549	0.744597823	-3.629781698	0.000283661	0.034015459	down_in_case
CRA004775	ENSGALG00010027380	GPX1	case	control	-0.639771381	0.382645778	-1.671967697	0.094530692	0.526933666	down_in_case
CRA004775	ENSGALG00010027381	KLHL40	case	control	1.318935015	0.932984807	1.413672554	0.157458067	0.622106365	up_in_case
CRA004775	ENSGALG00010027382	PALM	case	control	0.015665183	0.370214257	0.042313829	0.966248522	0.993094598	up_in_case
CRA004775	ENSGALG00010027383	ASS1	case	control	-0.99529766	0.39584921	-2.514335347	0.011925698	0.219531816	down_in_case
CRA004775	ENSGALG00010027384	HHATL	case	control	-0.271492582	0.578112822	-0.469618683	0.638627474	0.91099914	down_in_case
CRA004775	ENSGALG00010027385	CNTN2	case	control	2.087099969	0.881717448	2.3670848	0.017928824	0.266202063	up_in_case
CRA004775	ENSGALG00010027386	RHOA	case	control	-0.015491884	0.153758262	-0.10075481	0.919745101	0.982170916	down_in_case
CRA004775	ENSGALG00010027388	URM1	case	control	-0.235708379	0.280634125	-0.839913461	0.40095691	0.811868062	down_in_case
CRA004775	ENSGALG00010027391	TPM3	case	control	-0.346896704	0.290293736	-1.19498515	0.232092832	0.698952825	down_in_case
CRA004775	ENSGALG00010027392	RBBP5	case	control	-0.136423609	0.130651828	-1.044176814	0.296403592	0.748897794	down_in_case
CRA004775	ENSGALG00010027393	TEX264	case	control	-0.344238521	0.244773891	-1.406353101	0.159619299	0.624907945	down_in_case
CRA004775	ENSGALG00010027394	SLC27A4	case	control	-0.401615047	0.161152984	-2.492135341	0.012697764	0.227057161	down_in_case
CRA004775	ENSGALG00010027395	BCL2L15	case	control	0.296885752	0.239724341	1.238446418	0.215550578	0.683654538	up_in_case
CRA004775	ENSGALG00010027397	RFX5	case	control	-0.449226251	0.261935316	-1.715027424	0.086340199	0.508360843	down_in_case
CRA004775	ENSGALG00010027399	MAPKAP1	case	control	0.105834758	0.164042971	0.645164846	0.518820365	0.865435399	up_in_case
CRA004775	ENSGALG00010027400	ENSGALG00010027400	case	control	0.528232361	0.85028089	0.621244541	0.534438738	0.870981055	up_in_case
CRA004775	ENSGALG00010027401	ENSGALG00010027401	case	control	0.224566439	0.251204042	0.893960292	0.371343137	0.795818272	up_in_case
CRA004775	ENSGALG00010027402	AP4B1	case	control	-0.516119165	0.152938337	-3.374687967	0.000738994	0.057411924	down_in_case
CRA004775	ENSGALG00010027403	ACKR2	case	control	-0.653664769	0.541084776	-1.2080635	0.227022838	0.694715356	down_in_case
CRA004775	ENSGALG00010027405	DCLRE1B	case	control	-1.403234126	0.598054904	-2.346329941	0.018959316	0.272278915	down_in_case
CRA004775	ENSGALG00010027410	ALAS1	case	control	-0.251784528	0.188455567	-1.336041871	0.181535588	0.647378665	down_in_case
CRA004775	ENSGALG00010027412	ENSGALG00010027412	case	control	-0.092946412	0.36278642	-0.256201465	0.797795278	0.952835572	down_in_case
CRA004775	ENSGALG00010027414	PBX3	case	control	-0.232880956	0.147706608	-1.57664548	0.114877122	0.56497686	down_in_case
CRA004775	ENSGALG00010027417	WNT2B	case	control	0.217091161	0.566599373	0.383147548	0.701610366	0.930068722	up_in_case
CRA004775	ENSGALG00010027418	ENSGALG00010027418	case	control	0.658036722	0.657605833	1.000655239	0.316993515	0.765974554	up_in_case
CRA004775	ENSGALG00010027419	PTBP1	case	control	-0.377515446	0.412805849	-0.914510896	0.360448455	0.791975934	down_in_case
CRA004775	ENSGALG00010027421	PLPPR3	case	control	-0.111028785	0.422672133	-0.262683003	0.792794919	0.951284723	down_in_case
CRA004775	ENSGALG00010027423	PEX11B	case	control	-0.020454324	0.268569638	-0.076160223	0.939291628	0.986950867	down_in_case
CRA004775	ENSGALG00010027425	CFD	case	control	-0.020996225	0.406360558	-0.051668953	0.958792476	0.991431439	down_in_case
CRA004775	ENSGALG00010027427	UBAC1	case	control	0.49860126	0.356738624	1.397665479	0.162213545	0.628160648	up_in_case
CRA004775	ENSGALG00010027438	ST7L	case	control	-0.489751158	0.133703297	-3.662969953	0.000249308	0.032213474	down_in_case
CRA004775	ENSGALG00010027440	TWF2	case	control	-0.76496126	0.549867322	-1.391174251	0.164172593	0.629717526	down_in_case
CRA004775	ENSGALG00010027443	MED16	case	control	-0.367385351	0.172346502	-2.131666999	0.033034232	0.347288776	down_in_case
CRA004775	ENSGALG00010027444	SELENBP1	case	control	-0.001524711	0.211806748	-0.007198597	0.9942564	0.999001196	down_in_case
CRA004775	ENSGALG00010027445	PPM1M	case	control	-0.4964206	0.473085412	-1.049325528	0.294028321	0.746438942	down_in_case
CRA004775	ENSGALG00010027448	ADAMTSL5	case	control	0.358258487	0.584475302	0.612957443	0.539904479	0.874206929	up_in_case
CRA004775	ENSGALG00010027450	CAMSAP1	case	control	0.407197584	0.271841655	1.497921954	0.134153529	0.592323374	up_in_case
CRA004775	ENSGALG00010027455	R3HDM4	case	control	-0.410719512	0.283705192	-1.447698254	0.147701458	0.611023693	down_in_case
CRA004775	ENSGALG00010027457	CAPZA1	case	control	-0.033926179	0.211259903	-0.160589769	0.872416514	0.97401729	down_in_case
CRA004775	ENSGALG00010027460	WDR82	case	control	-0.217570149	0.151784684	-1.433413066	0.151739827	0.616199194	down_in_case
CRA004775	ENSGALG00010027467	GLYCTK	case	control	-0.129811521	0.284416144	-0.45641404	0.648092272	0.914511732	down_in_case
CRA004775	ENSGALG00010027468	BSG	case	control	0.113648174	0.152698718	0.7442641	0.456716715	0.839688534	up_in_case
CRA004775	ENSGALG00010027471	ENSGALG00010027471	case	control	0.080533883	0.367213241	0.219310945	0.826407837	0.961878235	up_in_case
CRA004775	ENSGALG00010027475	ARID3A	case	control	-0.340022351	0.402288461	-0.845220242	0.397987882	0.809455905	down_in_case
CRA004775	ENSGALG00010027479	HCN2	case	control	-0.233020792	0.339318474	-0.686731817	0.492251743	0.855746361	down_in_case
CRA004775	ENSGALG00010027487	MPZ	case	control	2.235941786	0.696851192	3.208635947	0.001333662	0.0764133	up_in_case
CRA004775	ENSGALG00010027490	ATP2B4	case	control	0.090365335	0.276621982	0.326674454	0.743914124	0.939218065	up_in_case
CRA004775	ENSGALG00010027492	PCP4L1	case	control	1.155867533	0.66597583	1.735599824	0.082634617	0.499833536	up_in_case
CRA004775	ENSGALG00010027496	NR1I3	case	control	2.159575302	0.694816462	3.108123399	0.001882794	0.086751567	up_in_case
CRA004775	ENSGALG00010027497	KCNT1	case	control	-0.262999507	0.54470757	-0.482826974	0.629218597	0.908586927	down_in_case
CRA004775	ENSGALG00010027499	MOV10	case	control	-0.642493677	0.393522357	-1.632673889	0.102537602	0.542853845	down_in_case
CRA004775	ENSGALG00010027501	BAP1	case	control	-0.281282418	0.113881707	-2.469952607	0.013513095	0.232945019	down_in_case
CRA004775	ENSGALG00010027502	THOP1	case	control	-0.358289724	0.237364543	-1.50944922	0.131184023	0.586828725	down_in_case
CRA004775	ENSGALG00010027510	RHOC	case	control	-0.323928897	0.370692649	-0.873847641	0.382201232	0.801753951	down_in_case
CRA004775	ENSGALG00010027511	POLRMT	case	control	-0.146876623	0.230248358	-0.637905192	0.523535398	0.866638286	down_in_case
CRA004775	ENSGALG00010027513	SEMA3G	case	control	0.392461053	0.365542423	1.073640235	0.282983957	0.739371536	up_in_case
CRA004775	ENSGALG00010027516	PPM1J	case	control	-0.584116957	0.534415762	-1.093000991	0.274393365	0.731001618	down_in_case
CRA004775	ENSGALG00010027520	FANCE	case	control	-0.421429339	0.49520576	-0.851018653	0.394758991	0.808974685	down_in_case
CRA004775	ENSGALG00010027521	B3GNT10	case	control	-0.790158767	0.810666679	-0.974702412	0.329707896	0.77237154	down_in_case
CRA004775	ENSGALG00010027522	PSMD5	case	control	-0.064796	0.235303126	-0.275372459	0.783030101	0.94936527	down_in_case
CRA004775	ENSGALG00010027524	ENSGALG00010027524	case	control	-0.19633418	0.413909168	-0.47434122	0.635256602	0.909837348	down_in_case
CRA004775	ENSGALG00010027525	ENSGALG00010027525	case	control	-0.253618711	0.528453533	-0.479926229	0.63127985	0.909357765	down_in_case
CRA004775	ENSGALG00010027527	ENSGALG00010027527	case	control	0.516580547	0.59535624	0.867683099	0.385567841	0.803446489	up_in_case
CRA004775	ENSGALG00010027528	ENSGALG00010027528	case	control	1.731617895	0.528306897	3.277674215	0.001046661	0.067571071	up_in_case
CRA004775	ENSGALG00010027529	ACY1	case	control	-0.296960904	0.555541191	-0.534543449	0.592965597	0.894422261	down_in_case
CRA004775	ENSGALG00010027530	PHF19	case	control	-0.774615638	0.370652561	-2.089869919	0.036629486	0.3613286	down_in_case
CRA004775	ENSGALG00010027531	UFC1	case	control	-0.163135268	0.310593381	-0.525237428	0.599418149	0.897206546	down_in_case
CRA004775	ENSGALG00010027533	WDR18	case	control	-0.303383403	0.172600899	-1.757716241	0.078795803	0.491399489	down_in_case
CRA004775	ENSGALG00010027534	KCNN3	case	control	0.664519193	0.346649299	1.916978323	0.055240686	0.430875458	up_in_case
CRA004775	ENSGALG00010027536	USP21	case	control	-0.179466452	0.290230794	-0.618357719	0.536339562	0.871773679	down_in_case
CRA004775	ENSGALG00010027537	SGTA	case	control	0.082369131	0.171587308	0.480042096	0.631197461	0.909357765	up_in_case
CRA004775	ENSGALG00010027538	ABHD14A	case	control	-0.056420377	0.304094477	-0.185535686	0.852808893	0.969725992	down_in_case
CRA004775	ENSGALG00010027539	DDR2L	case	control	0.11700652	0.346288145	0.337887742	0.735447785	0.937109578	up_in_case
CRA004775	ENSGALG00010027540	PPOX	case	control	-0.258225197	0.30979509	-0.833535473	0.4045428	0.81313407	down_in_case
CRA004775	ENSGALG00010027543	TUBB4B	case	control	-0.217824368	0.259520291	-0.839334632	0.401281554	0.811868062	down_in_case
CRA004775	ENSGALG00010027545	PPARD	case	control	0.327065574	0.276558499	1.182627094	0.23695697	0.703871614	up_in_case
CRA004775	ENSGALG00010027547	B4GALT3	case	control	-0.05876337	0.339136114	-0.173273702	0.862436294	0.9717602	down_in_case
CRA004775	ENSGALG00010027548	PCBP4	case	control	0.169706283	0.27895147	0.608372068	0.542940733	0.875261197	up_in_case
CRA004775	ENSGALG00010027553	FGF22	case	control	-0.284627386	0.581754369	-0.489256981	0.624659773	0.90646861	down_in_case
CRA004775	ENSGALG00010027554	ENSGALG00010027554	case	control	-0.377436504	0.260656407	-1.448023117	0.147610585	0.610831526	down_in_case
CRA004775	ENSGALG00010027555	ENSGALG00010027555	case	control	-0.554518551	0.306442775	-1.809533776	0.070368118	0.469724266	down_in_case
CRA004775	ENSGALG00010027557	C6orf132	case	control	-0.644996499	0.74228632	-0.868932219	0.384884206	0.803182776	down_in_case
CRA004775	ENSGALG00010027559	ENSGALG00010027559	case	control	-0.303851526	0.318895266	-0.952825452	0.340678516	0.780875343	down_in_case
CRA004775	ENSGALG00010027561	ADAMTS4	case	control	-0.475904137	0.448658966	-1.060725793	0.288814535	0.742889762	down_in_case
CRA004775	ENSGALG00010027562	SLC39A3	case	control	-0.145436619	0.190594092	-0.763069923	0.445421696	0.833012432	down_in_case
CRA004775	ENSGALG00010027563	WDR77	case	control	-0.302920168	0.214230359	-1.413992721	0.15736404	0.622106365	down_in_case
CRA004775	ENSGALG00010027567	PARP3	case	control	-0.413185832	0.272562436	-1.515930949	0.129536814	0.584172159	down_in_case
CRA004775	ENSGALG00010027568	NELFB	case	control	-0.43650044	0.151938944	-2.872867405	0.004067648	0.130993507	down_in_case
CRA004775	ENSGALG00010027569	ATP5PB	case	control	0.205525084	0.260290417	0.78959912	0.429761923	0.824104894	up_in_case
CRA004775	ENSGALG00010027570	NDUFS2	case	control	-0.025832507	0.337562158	-0.076526667	0.939000099	0.986946546	down_in_case
CRA004775	ENSGALG00010027571	RBM10	case	control	-0.325677774	0.125319797	-2.598773555	0.009355747	0.195703177	down_in_case
CRA004775	ENSGALG00010027574	GNG7	case	control	-0.050805833	0.178422699	-0.284749827	0.775835814	0.946517228	down_in_case
CRA004775	ENSGALG00010027575	FCER1G	case	control	-0.113200991	0.472826298	-0.239413484	0.810784975	0.956922513	down_in_case
CRA004775	ENSGALG00010027576	GADD45B	case	control	0.031490943	0.676114898	0.046576319	0.962850906	0.991860454	up_in_case
CRA004775	ENSGALG00010027577	ADORA3	case	control	-1.144792431	0.60727628	-1.885126208	0.0594128	0.444981165	down_in_case
CRA004775	ENSGALG00010027578	ENSGALG00010027578	case	control	-0.481110108	0.479952525	-1.002411869	0.316144712	0.765818284	down_in_case
CRA004775	ENSGALG00010027579	C26H6ORF125	case	control	-0.129215605	0.265775188	-0.486183852	0.626836819	0.90758663	down_in_case
CRA004775	ENSGALG00010027582	ENSGALG00010027582	case	control	1.074299329	0.572345586	1.877011646	0.060516498	0.445397937	up_in_case
CRA004775	ENSGALG00010027583	GLE1	case	control	-0.025238125	0.211187252	-0.119505911	0.904874558	0.979540372	down_in_case
CRA004775	ENSGALG00010027586	HJV	case	control	-0.034723697	0.561967786	-0.061789479	0.950730482	0.990168609	down_in_case
CRA004775	ENSGALG00010027590	SEMA3F	case	control	0.169917599	0.319502082	0.531820005	0.594850666	0.895328208	up_in_case
CRA004775	ENSGALG00010027592	MAP1S	case	control	0.023986769	0.217569265	0.110248886	0.912211993	0.98075825	up_in_case
CRA004775	ENSGALG00010027593	ENSGALG00010027593	case	control	0.587770206	0.669956906	0.877325393	0.380309924	0.801354851	up_in_case
CRA004775	ENSGALG00010027594	ITPR3	case	control	0.143153136	0.446505501	0.320607777	0.748507644	0.940917938	up_in_case
CRA004775	ENSGALG00010027595	GNAT1	case	control	-0.016589299	0.316584982	-0.052400776	0.958209356	0.991431439	down_in_case
CRA004775	ENSGALG00010027597	EXOSC2	case	control	-0.296529975	0.209775106	-1.413561317	0.157490746	0.622106365	down_in_case
CRA004775	ENSGALG00010027600	KIRREL1	case	control	-0.106996371	0.300110069	-0.356523764	0.721448359	0.934511903	down_in_case
CRA004775	ENSGALG00010027605	FCHO1	case	control	-0.467131751	0.344193366	-1.357178251	0.17472458	0.639850251	down_in_case
CRA004775	ENSGALG00010027607	ABL1	case	control	-0.084725344	0.267919207	-0.316234677	0.751824389	0.941495694	down_in_case
CRA004775	ENSGALG00010027608	GNAI2	case	control	-0.107904334	0.233811813	-0.461500777	0.644439364	0.913812299	down_in_case
CRA004775	ENSGALG00010027609	RPL36	case	control	-0.238196528	0.263401036	-0.904311282	0.365830366	0.793608387	down_in_case
CRA004775	ENSGALG00010027611	B3GNT3	case	control	1.479068849	0.484715306	3.051417668	0.002277635	0.096690159	up_in_case
CRA004775	ENSGALG00010027612	BAK1	case	control	-0.67919407	0.241417906	-2.813354163	0.004902762	0.142429514	down_in_case
CRA004775	ENSGALG00010027614	LONP1	case	control	0.199586833	0.202564752	0.985298925	0.32447726	0.770087471	up_in_case
CRA004775	ENSGALG00010027616	SLC38A3	case	control	0.194095876	0.483156939	0.401724286	0.68788695	0.927111919	up_in_case
CRA004775	ENSGALG00010027618	PPP1R26	case	control	0.164715821	0.227050128	0.725460158	0.468169779	0.843870188	up_in_case
CRA004775	ENSGALG00010027619	JAK3	case	control	-0.573399002	0.429806009	-1.334087916	0.182175046	0.647378665	down_in_case

CRA004775	ENSGALG00010027621	TSPO2	case	control	-0.529791577	0.507583911	-1.043751714	0.296600277	0.748968786	down_in_case
CRA004775	ENSGALG00010027623	APOBEC2	case	control	0.11546011	0.574199202	0.201080234	0.840635838	0.966461921	up_in_case
CRA004775	ENSGALG00010027624	ENSGALG00010027624	case	control	-0.401128178	0.373457774	-1.074092458	0.282781245	0.739013609	down_in_case
CRA004775	ENSGALG00010027626	SEMA3B	case	control	0.05604492	0.348442559	0.160844072	0.872216212	0.97401729	up_in_case
CRA004775	ENSGALG00010027627	OARD1	case	control	0.035471563	0.356729587	0.099435437	0.920792547	0.982481112	up_in_case
CRA004775	ENSGALG00010027628	PIERCE1	case	control	0.132145488	0.411128085	0.321421699			up_in_case
CRA004775	ENSGALG00010027632	LAMC3	case	control	0.383482866	0.492764516	0.778227437	0.436434945	0.826983178	up_in_case
CRA004775	ENSGALG00010027634	OASL	case	control	-0.396578702	0.448527642	-0.884178957	0.376599616	0.798523018	down_in_case
CRA004775	ENSGALG00010027635	CD48	case	control	-0.625347121	0.447392517	-1.397759457	0.162185313	0.628160648	down_in_case
CRA004775	ENSGALG00010027636	SLC1A6	case	control	-1.484122274	0.587458323	-2.526344793	0.01152563	0.216528087	down_in_case
CRA004775	ENSGALG00010027638	MRPS2	case	control	-0.316886238	0.289492883	-1.094625316	0.27368082	0.731001618	down_in_case
CRA004775	ENSGALG00010027641	HYAL3	case	control	-0.770689711	0.307294657	-2.507982792	0.012142258	0.222032167	down_in_case
CRA004775	ENSGALG00010027642	DIPK1B	case	control	-0.068789046	0.304775635	-0.225703889	0.821431733	0.960766569	down_in_case
CRA004775	ENSGALG00010027644	NAA80	case	control	-0.327349327	0.369214239	-0.886610787	0.375288484	0.798503808	down_in_case
CRA004775	ENSGALG00010027645	SLAMF1	case	control	-0.952840147	0.57501629	-1.657066353	0.097506061	0.532277031	down_in_case
CRA004775	ENSGALG00010027648	NFYA	case	control	-0.115438207	0.272889466	-0.423021851	0.672279319	0.921952322	down_in_case
CRA004775	ENSGALG00010027649	FBLN2	case	control	0.549870888	0.327228246	1.680389436	0.09288157	0.524612711	up_in_case
CRA004775	ENSGALG00010027650	CCDC124	case	control	-0.397355613	0.369954652	-1.074065729	0.282793223	0.739013609	down_in_case
CRA004775	ENSGALG00010027654	AIF1L	case	control	-0.240033268	0.21248034	-1.129672835	0.258614108	0.720037471	down_in_case
CRA004775	ENSGALG00010027663	COPA	case	control	-0.279798462	0.147915184	-1.891614188	0.058542404	0.443329681	down_in_case
CRA004775	ENSGALG00010027664	TREMB1	case	control	0.249053958	0.520375229	0.478604561	0.632219973	0.909651573	up_in_case
CRA004775	ENSGALG00010027665	TRAF1	case	control	-0.624055329	0.309535051	-2.016105531	0.043788942	0.391981676	down_in_case
CRA004775	ENSGALG00010027667	PTPDC1	case	control	-0.076231142	0.330214881	-0.230853139	0.817428897	0.959812855	down_in_case
CRA004775	ENSGALG00010027668	RANBP3	case	control	-0.13710212	0.1188883	-1.153201119	0.248827844	0.713881011	down_in_case
CRA004775	ENSGALG00010027671	BARX1	case	control	0.189928602	0.673321844	0.282076994	0.777884473	0.947617375	up_in_case
CRA004775	ENSGALG00010027672	CNTRL	case	control	-0.089304353	0.354285268	-0.252069057	0.800987687	0.953541667	down_in_case
CRA004775	ENSGALG00010027674	TREM2	case	control	-0.819775579	0.772160819	-1.061664304	0.28838811	0.742851819	down_in_case
CRA004775	ENSGALG00010027685	ENSGALG00010027685	case	control	-0.763094564	0.392321631	-1.945073899	0.051766085	0.419476344	down_in_case
CRA004775	ENSGALG00010027692	PHF2	case	control	0.338747447	0.190493814	1.778259565	0.075361234	0.483342345	up_in_case
CRA004775	ENSGALG00010027693	RAB14	case	control	0.122662061	0.155567043	0.788483592	0.430413892	0.824320534	up_in_case
CRA004775	ENSGALG00010027695	TREMB2	case	control	-1.022786416	0.833898447	-1.226511957	0.220006079	0.688038114	down_in_case
CRA004775	ENSGALG00010027697	CATH2	case	control	1.527686543	0.771257529	1.980773588			up_in_case
CRA004775	ENSGALG00010027698	FAM120A	case	control	0.004789702	0.260435619	0.018391119	0.985326837	0.997562105	up_in_case
CRA004775	ENSGALG00010027699	VANGL2	case	control	0.01729149	0.295896216	0.058437685	0.953399997	0.990670511	up_in_case
CRA004775	ENSGALG00010027700	TCF3	case	control	-0.084044869	0.275269018	-0.305319027	0.760123186	0.942236668	down_in_case
CRA004775	ENSGALG00010027701	NHLH1	case	control	-1.162188645	0.598667852	-1.94129122	0.052222964	0.420135271	down_in_case
CRA004775	ENSGALG00010027702	FOXP4	case	control	-1.238081257	0.550651467	-2.24839364	0.0245511	0.302160964	down_in_case
CRA004775	ENSGALG00010027703	NCSTN	case	control	-0.108543671	0.215892953	-0.50276616	0.615128688	0.902639483	down_in_case
CRA004775	ENSGALG00010027704	MDFI	case	control	-0.39080179	0.53644216	-0.728506854	0.466303375	0.843480361	down_in_case
CRA004775	ENSGALG00010027705	S100A11	case	control	-0.727416613	0.441675516	-1.646948012	0.099568732	0.537098329	down_in_case
CRA004775	ENSGALG00010027706	GSN	case	control	0.241730679	0.405905268	0.595534717	0.5514861	0.878124752	up_in_case
CRA004775	ENSGALG00010027707	HIGD1C	case	control	-0.372269121	0.324621475	-1.146779095	0.251472931	0.715942914	down_in_case
CRA004775	ENSGALG00010027708	ENSGALG00010027708	case	control	-0.946674499	0.594041419	-1.593616991	0.111021846	0.560403826	down_in_case
CRA004775	ENSGALG00010027709	PSMA5	case	control	-0.366958627	0.302351486	-1.213682233	0.224869091	0.692859731	down_in_case
CRA004775	ENSGALG00010027710	ENSGALG00010027710	case	control	-0.330393533	0.686119337	-0.481539457	0.630133144	0.908943555	down_in_case
CRA004775	ENSGALG00010027711	WNK2	case	control	0.254876826	0.47433078	0.537339842	0.59103289	0.893804699	up_in_case
CRA004775	ENSGALG00010027712	STOM	case	control	-0.220170668	0.202804857	-1.085628179	0.277643528	0.732797725	down_in_case
CRA004775	ENSGALG00010027713	ENSGALG00010027713	case	control	0.22777703	0.385488947	0.590878238	0.554602008	0.879521649	up_in_case
CRA004775	ENSGALG00010027714	SYPL2	case	control	-0.751782911	0.732059027	-1.026943023	0.304447294	0.757044256	down_in_case
CRA004775	ENSGALG00010027715	NINJ1	case	control	-0.983281657	0.502910897	-1.955180655	0.050561751	0.416238146	down_in_case
CRA004775	ENSGALG00010027717	ATXN7L2	case	control	0.001006139	0.337628184	0.002980021	0.997622291	0.999646385	up_in_case
CRA004775	ENSGALG00010027718	ENSGALG00010027718	case	control	-0.741366258	0.536680226	-1.381392909	0.167158185	0.631096819	down_in_case
CRA004775	ENSGALG00010027719	GPR61	case	control	-1.209825951	0.786893009	-1.537471977	0.124177772	0.576485043	down_in_case
CRA004775	ENSGALG00010027720	DAB2IP	case	control	0.2155604	0.299893133	0.718790717	0.472269878	0.845425322	up_in_case
CRA004775	ENSGALG00010027721	SUSD3	case	control	-0.068060938	0.359444089	-0.189350555	0.849818075	0.968413719	down_in_case
CRA004775	ENSGALG00010027722	GNAI3	case	control	-0.423837656	0.27199239	-1.558270275	0.119169192	0.570892125	down_in_case
CRA004775	ENSGALG00010027723	ENSGALG00010027723	case	control	-1.034428087	0.759536981	-1.361919317	0.173223349	0.63806494	down_in_case
CRA004775	ENSGALG00010027724	TTLL11	case	control	0.034919172	0.256065706	0.136368016	0.891530357	0.977782257	up_in_case
CRA004775	ENSGALG00010027725	BTN1A1	case	control	-0.884438307	0.456993906	-1.935339387	0.052948662	0.423142991	down_in_case
CRA004775	ENSGALG00010027727	GSTM2	case	control	-0.046750802	0.387479671	-0.120653561	0.903965443	0.979540372	down_in_case
CRA004775	ENSGALG00010027729	EPS8L3	case	control	1.312715996	0.425243876	3.086972136	0.002022065	0.089985187	up_in_case
CRA004775	ENSGALG00010027730	ENSGALG00010027730	case	control	-0.127505846	0.255264411	-0.499504984	0.617423676	0.903976249	down_in_case
CRA004775	ENSGALG00010027731	OLFML2A	case	control	0.21420534	0.255865096	0.837180777	0.402490961	0.811996248	up_in_case
CRA004775	ENSGALG00010027732	EEF2	case	control	-0.252807293	0.23829236	-1.060912288	0.288729764	0.742851819	down_in_case
CRA004775	ENSGALG00010027733	RPL35	case	control	-0.302170266	0.258366355	-1.169541855	0.242185388	0.709690398	down_in_case
CRA004775	ENSGALG00010027734	CSF1	case	control	0.128758844	0.303348061	0.424459095	0.671231031	0.921535334	up_in_case
CRA004775	ENSGALG00010027738	ENSGALG00010027738	case	control	-0.816976625	0.276731807	-2.95223247	0.003154853	0.119840845	down_in_case
CRA004775	ENSGALG00010027739	FGD3	case	control	-0.182691921	0.326886741	-0.558884463	0.576240574	0.887937128	down_in_case
CRA004775	ENSGALG00010027740	MARCHF2	case	control	-0.194622062	0.184344305	-1.055753047	0.291081055	0.744957302	down_in_case
CRA004775	ENSGALG00010027741	RAB11B	case	control	-0.182012188	0.103684465	-1.755443196	0.079183529	0.491399489	down_in_case
CRA004775	ENSGALG00010027743	C1orf74	case	control	-0.209265922	0.456160952	-0.458754572	0.646410421	0.914043203	down_in_case
CRA004775	ENSGALG00010027745	BICD2	case	control	0.333365605	0.314865379	1.058755985	0.289710924	0.743943027	up_in_case
CRA004775	ENSGALG00010027746	UQCR11	case	control	-0.03927556	0.498190843	-0.078836374	0.937162772	0.986688955	down_in_case
CRA004775	ENSGALG00010027751	ANGPTL4	case	control	0.309407273	0.4655957	0.664540659	0.506344335	0.8622397	up_in_case
CRA004775	ENSGALG00010027752	SCAI	case	control	0.185806941	0.195268927	0.951543822	0.341328384	0.781226133	up_in_case
CRA004775	ENSGALG00010027775	MVB12B	case	control	0.081301701	0.17442695	0.466107454	0.641138588	0.91228236	up_in_case
CRA004775	ENSGALG00010027776	TKT	case	control	0.176848572	0.359219218	0.492313782	0.622497539	0.906199836	up_in_case
CRA004775	ENSGALG00010027784	PEAR1	case	control	-0.128680417	0.308702834	-0.416842357	0.676793719	0.922924485	down_in_case
CRA004775	ENSGALG00010027787	DCP1A	case	control	0.815981114	0.348333577	2.342527875	0.019153603	0.273365569	up_in_case
CRA004775	ENSGALG00010027789	UAP1L1	case	control	-0.224287752	0.429553013	-0.522142193	0.601571331	0.898185892	down_in_case
CRA004775	ENSGALG00010027801	MBD3	case	control	-0.309332508	0.263400169	-1.174382344	0.240241914	0.70694164	down_in_case
CRA004775	ENSGALG00010027803	SAPCD2	case	control	0.14466901	0.419791241	0.344621316	0.730379079	0.936393482	up_in_case
CRA004775	ENSGALG00010027811	MEX3D	case	control	0.043632416	0.206458628	0.211337336	0.832624051	0.963988657	up_in_case
CRA004775	ENSGALG00010027813	LRRC71	case	control	-1.335570699	0.603587362	-2.212721445	0.026916856	0.31493941	down_in_case
CRA004775	ENSGALG00010027814	ENSGALG00010027814	case	control	-0.28005625	0.222197383	-1.260394005	0.207527263	0.676400389	down_in_case
CRA004775	ENSGALG00010027816	ZMYND19	case	control	-0.154220904	0.199389102	-0.773467065	0.439246013	0.828542102	down_in_case
CRA004775	ENSGALG00010027821	NMRK2	case	control	-0.82888079	0.629225169	-1.317303933	0.187736767	0.651911184	down_in_case
CRA004775	ENSGALG00010027822	DPH7	case	control	0.02497153	0.252828809	0.09876853	0.921322055	0.982481112	up_in_case
CRA004775	ENSGALG00010027826	ARHGEF11	case	control	-0.151469608	0.286131766	-0.529370122	0.59654872	0.895574418	down_in_case
CRA004775	ENSGALG00010027831	ATCAY	case	control	-0.212574164	0.509596637	-0.417142007	0.676574541	0.922924485	down_in_case
CRA004775	ENSGALG00010027838	ENSGALG00010027838	case	control	2.075313056	0.578817388	3.585436614	0.000336515	0.036436331	up_in_case
CRA004775	ENSGALG00010027842	SLAMF8	case	control	0.856469737	0.479935813	1.784550587	0.074334242	0.481085493	up_in_case
CRA004775	ENSGALG00010027847	NRTN	case	control	-0.291275099	0.864583472	-0.336896446	0.736194965	0.937385622	down_in_case
CRA004775	ENSGALG00010027860	ENSGALG00010027860	case	control	0.135701402	0.465849563	0.291298763	0.770822833	0.945853152	up_in_case
CRA004775	ENSGALG00010027861	PNPLA7	case	control	-0.147217362	0.191421274	-0.769075239	0.441848646	0.829654585	down_in_case
CRA004775	ENSGALG00010027862	ENSGALG00010027862	case	control	0.038879002	0.432775234	0.089836477	0.928417161	0.98473447	up_in_case
CRA004775	ENSGALG00010027863	CHDH	case	control	0.444975866	0.480244583	0.926560925	0.354154551	0.78730441	up_in_case
CRA004775	ENSGALG00010027864	ACTR8	case	control	-0.239146009	0.208355897	-1.147776529	0.251060829	0.715684776	down_in_case
CRA004775	ENSGALG00010027865	SELENOK	case	control	-0.495227094	0.269157454	-1.839915957	0.065780577	0.460330134	down_in_case
CRA004775	ENSGALG00010027866	KCNA2	case	control	0.422578644	0.481225492	0.878130214	0.379873058	0.800802457	up_in_case
CRA004775	ENSGALG00010027867	UBAP2L	case	control	-0.440715314	0.184733802	-2.385677714	0.017047683	0.258174775	down_in_case
CRA004775	ENSGALG00010027869	NSMF	case	control	0.334849652	0.336593166	0.994820114	0.319823762	0.767037518	up_in_case
CRA004775	ENSGALG00010027870	HAX1	case	control	0.009900185	0.414935192	0.023859594	0.980964604	0.997068414	up_in_case
CRA004775	ENSGALG00010027872	KDM5B	case	control	-0.164262786	0.172926386	-0.949900068	0.342163032	0.781616177	down_in_case
CRA004775	ENSGALG00010027873	RFX2	case	control	-0.563792223	0.364982021	-1.544712316	0.122415865	0.57525466	down_in_case
CRA004775	ENSGALG00010027874	FRS3	case	control	-0.160476838	0.262627387	-0.611043804	0.541170581	0.874673178	down_in_case
CRA004775	ENSGALG00010027875	WNT5A	case	control	0.233414729	0.317516284	0.735126795	0.462262277	0.842230074	up_in_case
CRA004775	ENSGALG00010027876	ANAPC2	case	control	-0.508391697	0.195260935	-2.603652882	0.009223612	0.194530505	down_in_case
CRA004775	ENSGALG00010027877	OAZ1	case	control	-0.338293078	0.224482857	-1.50698847	0.131813613	0.587352231	down_in_case
CRA004775	ENSGALG00010027879	ENSGALG00010027879	case	control	-0.037356047	0.500390965	-0.074653719	0.940490232	0.987076872	down_in_case
CRA004775	ENSGALG00010027880	AMH	case	control	0.024088949	0.477409667	0.050457606	0.959757732	0.991431439	up_in_case
CRA004775	ENSGALG00010027881	ENSGALG00010027881	case	control	-0.376647155	0.316383601	-1.190476226	0.233859269	0.701220847	down_in_case
CRA004775	ENSGALG00010027882	CHIA	case	control	1.559106252	0.682654849	2.283886585	0.022378197	0.292422177	up_in_case
CRA004775	ENSGALG00010027883	ATP8B2	case	control	-0.465790667	0.192435152	-2.420507184	0.015498873	0.249560894	down_in_case
CRA004775	ENSGALG00010027884	SF3A2	case	control	-0.170691781	0.14608814	-1.16841642	0.242638835	0.709690398	down_in_case
CRA004775	ENSGALG00010027885	PLEKHJ1	case	control	-0.104975369	0.118314522	-0.887256838	0.374940638	0.798503808	down_in_case
CRA004775	ENSGALG00010027886	IL6R	case	control	-0.143152915	0.256028546	-0.559128727	0.57607387	0.887937128	down_in_case
CRA004775	ENSGALG00010027888	PFKFB2	case	control	-0.604073261	0.304066898	-1.986645913	0.046961649	0.402720701	down_in_case
CRA004775	ENSGALG00010027889	RBM8A	case	control	-0.31127863	0.22258615	-1.398463608	0.161973894	0.627882156	down_in_case
CRA004775	ENSGALG00010027890	ENSGALG00010027890	case	control	0.335099408	0.66010487	0.507645714	0.611701825	0.901413878	up_in_case
CRA004775	ENSGALG00010027891	CCDC66	case	control	0.395654252	0.290262872	1.363089428	0.172854328	0.637223104	up_in_case
CRA004775	ENSGALG00010027892	SHE	case	control	0.085841779	0.318806349	0.269259941	0.787729657	0.95041797	up_in_case
CRA004775	ENSGALG00010027893	DOT1L	case	control	0.070122019	0.295864639	0.237007098	0.812651284	0.957891977	up_in_case
CRA004775	ENSGALG00010027894	RSBN1	case	control	0.256445604	0.152785055	1.6784731	0.093254778	0.524906446	up_in_case
CRA004775	ENSGALG00010027895	LIX1L	case	control	-0.487241171	0.214815736	-2.268181935	0.02331812	0.296313338	down_in_case
CRA004775	ENSGALG00010027896	TOR4A	case	control	-0.256295245	0.314851766	-0.814018763	0.415634202	0.818943344	down_in_case

CRA004775	ENSGALG00010027897	AP3D1	case	control	-0.035960985	0.180288217	-0.199463866	0.841899906	0.966478468	down_in_case
CRA004775	ENSGALG00010027898	TASOR	case	control	-0.265400191	0.250614137	-1.058999282	0.289600106	0.743883257	down_in_case
CRA004775	ENSGALG00010027899	PMVK	case	control	-0.43740871	0.274224746	-1.595073806	0.110695732	0.559623902	down_in_case
CRA004775	ENSGALG00010027901	MOB3A	case	control	-0.410350847	0.232503555	-1.764922893	0.077576726	0.489338327	down_in_case
CRA004775	ENSGALG00010027902	UBE2Q1	case	control	-0.019031005	0.122040727	-0.155939791	0.87608048	0.974421894	down_in_case
CRA004775	ENSGALG00010027903	NDUFA8	case	control	0.036899004	0.358278192	0.102989811	0.917971057	0.981688766	up_in_case
CRA004775	ENSGALG00010027905	SYN2	case	control	0.027001672	0.421979825	0.063988063	0.948979731	0.990168609	up_in_case
CRA004775	ENSGALG00010027906	LHX6	case	control	0.010321736	0.516495222	0.019984184	0.984055989	0.997534931	up_in_case
CRA004775	ENSGALG00010027907	MKNK2	case	control	-0.176972921	0.238065851	-0.743378023	0.457252846	0.839701374	down_in_case
CRA004775	ENSGALG00010027908	PBXIP1	case	control	-0.3384104	0.308932489	-1.095418616	0.273333282	0.731001618	down_in_case
CRA004775	ENSGALG00010027909	TIMP4	case	control	-0.223996077	0.270520549	-0.828018713	0.407659902	0.814105656	down_in_case
CRA004775	ENSGALG00010027910	CHRNB2	case	control	-0.22919949	0.468425655	-0.489297475	0.624631109	0.90646861	down_in_case
CRA004775	ENSGALG00010027911	PTPN22	case	control	-0.853979502	0.45620757	-1.871909977	0.061219059	0.445483389	down_in_case
CRA004775	ENSGALG00010027912	RGS3	case	control	-0.006499486	0.245409847	-0.026484211	0.978871127	0.996554147	down_in_case
CRA004775	ENSGALG00010027913	PYGO2	case	control	-0.000257577	0.208476786	-0.001235519	0.999014199	0.999914248	down_in_case
CRA004775	ENSGALG00010027914	BTBD2	case	control	-0.269695333	0.357605223	-0.754170566	0.450746809	0.835552293	down_in_case
CRA004775	ENSGALG00010027915	GSTM3	case	control	-0.175360317	0.332581151	-0.527270761	0.598005576	0.89655197	down_in_case
CRA004775	ENSGALG00010027916	ADAR	case	control	-0.555764937	0.210542612	-2.639679125	0.008298456	0.183215969	down_in_case
CRA004775	ENSGALG00010027917	PPARG	case	control	0.005299984	0.532758899	0.009948184	0.992062628	0.998065748	up_in_case
CRA004775	ENSGALG00010027918	CSNK1G2	case	control	-0.151268074	0.233518918	-0.647776529	0.517129498	0.864913871	down_in_case
CRA004775	ENSGALG00010027919	TSEN2	case	control	0.011140996	0.237080411	0.04699248	0.962519221	0.991860454	up_in_case
CRA004775	ENSGALG00010027920	SCAMP4	case	control	-0.421699841	0.155788261	-2.706878158	0.006791918	0.161712092	down_in_case
CRA004775	ENSGALG00010027921	MKRN2OS	case	control	0.080269645	0.231935949	0.346085396	0.729278538	0.936339759	up_in_case
CRA004775	ENSGALG00010027922	SHC1	case	control	-0.156495606	0.162809826	-0.961217202	0.336442969	0.776900397	down_in_case
CRA004775	ENSGALG00010027923	AMPD2	case	control	-0.3977559	0.252096695	-1.57779101	0.11461363	0.564488547	down_in_case
CRA004775	ENSGALG00010027924	ENSGALG00010027924	case	control	0.119882495	0.434437624	0.275948695	0.782587472	0.94936527	up_in_case
CRA004775	ENSGALG00010027925	MKRN2	case	control	0.002637297	0.193091857	0.013658251	0.989102631	0.998065748	up_in_case
CRA004775	ENSGALG00010027926	CKS1B	case	control	-1.12320719	0.620509197	-1.810137859	0.070274411	0.469724266	down_in_case
CRA004775	ENSGALG00010027927	ENSGALG00010027927	case	control	-0.066638454	0.334782882	-0.199049764	0.842223817	0.966478468	down_in_case
CRA004775	ENSGALG00010027928	FLAD1	case	control	-0.109975822	0.359046437	-0.306299716	0.759376455	0.942236668	down_in_case
CRA004775	ENSGALG00010027929	CDK5RAP2	case	control	0.321280038	0.351983526	0.912770098	0.361363468	0.792647799	up_in_case
CRA004775	ENSGALG00010027930	RAF1	case	control	-0.030470894	0.28034545	-0.108690526	0.913447957	0.980803023	down_in_case
CRA004775	ENSGALG00010027931	ABHD17A	case	control	-0.318635742	0.364405249	-0.874399428	0.381900769	0.801753951	down_in_case
CRA004775	ENSGALG00010027932	POLE3	case	control	-0.420385282	0.295244321	-1.423855608	0.154488275	0.619284425	down_in_case
CRA004775	ENSGALG00010027933	ENSGALG00010027933	case	control	0.407396848	0.283497012	1.437041063	0.150706326	0.614397477	up_in_case
CRA004775	ENSGALG00010027934	CNBP	case	control	-0.033671265	0.267245431	-0.125993791	0.899736839	0.978929349	down_in_case
CRA004775	ENSGALG00010027935	ISY1	case	control	-0.256249495	0.233162033	-1.099018961	0.271759795	0.729164673	down_in_case
CRA004775	ENSGALG00010027936	ENSGALG00010027936	case	control	0.019591462	0.253700771	0.077222714	0.938446372	0.986707289	up_in_case
CRA004775	ENSGALG00010027937	HDHD3	case	control	-0.520392013	0.269993296	-1.927425688	0.053926601	0.427596115	down_in_case
CRA004775	ENSGALG00010027938	ENSGALG00010027938	case	control	0.239798192	0.372321489	0.644062186	0.51953511	0.865611895	up_in_case
CRA004775	ENSGALG00010027939	BSPRY	case	control	0.395033709	0.590685687	0.668771425	0.503641297	0.861306517	up_in_case
CRA004775	ENSGALG00010027940	ZBTB7B	case	control	-0.531343169	0.531070996	-1.000512498	0.317062552	0.765974554	down_in_case
CRA004775	ENSGALG00010027941	KLF2	case	control	0.329127557	0.313622729	1.049437832	0.293976654	0.746438942	up_in_case
CRA004775	ENSGALG00010027942	KHDC4	case	control	-0.153645872	0.186510834	-0.823790597	0.410058551	0.815668326	down_in_case
CRA004775	ENSGALG00010027943	RAB43	case	control	0.263616953	0.255715701	1.030898582	0.302588382	0.755164773	up_in_case
CRA004775	ENSGALG00010027944	WDR31	case	control	-0.084476	0.385104738	-0.219358506	0.82637079	0.961878235	down_in_case
CRA004775	ENSGALG00010027946	AP1M1	case	control	-0.211458587	0.169239966	-1.249460108	0.211496835	0.682057454	down_in_case
CRA004775	ENSGALG00010027947	GP9	case	control	-1.417878396	0.521285303	-2.719966182	0.006528859	0.16028494	down_in_case
CRA004775	ENSGALG00010027948	PRPF4	case	control	-0.488640048	0.170170851	-2.871467378	0.004085709	0.131210386	down_in_case
CRA004775	ENSGALG00010027950	CDC26	case	control	-0.043087692	0.275405239	-0.156451968	0.875676777	0.974412785	down_in_case
CRA004775	ENSGALG00010027951	FAM32A	case	control	-0.170326075	0.220973654	-0.77079811	0.440826608	0.829353567	down_in_case
CRA004775	ENSGALG00010027952	CIB3	case	control	0.185884946	0.334992345	0.554893114	0.578967772	0.888378369	up_in_case
CRA004775	ENSGALG00010027953	SLC31A1	case	control	-0.513640345	0.378299058	-1.357762686	0.174538999	0.639597725	down_in_case
CRA004775	ENSGALG00010027954	CFAP92	case	control	0.237102078	0.566967311	0.418193559	0.675805605	0.922924485	up_in_case
CRA004775	ENSGALG00010027955	ENSGALG00010027955	case	control	-0.383502083	0.22541066	-1.701348473	0.088877571	0.515249396	down_in_case
CRA004775	ENSGALG00010027956	RAB8A	case	control	-0.399008063	0.257088551	-1.552025798	0.120656051	0.572850296	down_in_case
CRA004775	ENSGALG00010027957	ENTREP3	case	control	0.35981258	0.464096677	0.775296609	0.438164413	0.827821102	up_in_case
CRA004775	ENSGALG00010027958	ENSGALG00010027958	case	control	0.03622834	0.434741189	0.083333121	0.933586665	0.985093894	up_in_case
CRA004775	ENSGALG00010027959	TPM4	case	control	0.045109645	0.319923609	0.141001301	0.887868915	0.977118922	up_in_case
CRA004775	ENSGALG00010027960	PPP6C	case	control	-0.402351788	0.232889705	-1.727649526	0.084051078	0.503010645	down_in_case
CRA004775	ENSGALG00010027962	SCAMP3	case	control	-0.506218533	0.308151148	-1.642760499	0.100432496	0.538583505	down_in_case
CRA004775	ENSGALG00010027964	RABEPK	case	control	-0.610589288	0.209306001	-2.9172087	0.003531794	0.125102734	down_in_case
CRA004775	ENSGALG00010027965	ACAD9	case	control	0.015825646	0.300153048	0.052725254	0.957950818	0.991431439	up_in_case
CRA004775	ENSGALG00010027966	CLK2	case	control	-0.261535463	0.211914179	-1.234157451	0.217144239	0.684537009	down_in_case
CRA004775	ENSGALG00010027968	FKBP15	case	control	-0.304417298	0.266104438	-1.143976781	0.252633266	0.717153621	down_in_case
CRA004775	ENSGALG00010027969	HSPA5	case	control	-0.35345456	0.310277744	-1.139155376	0.254638354	0.718419134	down_in_case
CRA004775	ENSGALG00010027970	GRIN3B	case	control	-0.134720206	0.178393757	-0.755184532	0.450138268	0.835337561	down_in_case
CRA004775	ENSGALG00010027971	ENSGALG00010027971	case	control	0.007343291	0.451803111	0.016253299	0.987032315	0.99777118	up_in_case
CRA004775	ENSGALG00010027972	TMEM259	case	control	-0.289940501	0.229017237	-1.266020433	0.205505767	0.674045742	down_in_case
CRA004775	ENSGALG00010027973	GAPVD1	case	control	0.059483914	0.228062958	0.260822338	0.794229518	0.951843306	up_in_case
CRA004775	ENSGALG00010027974	ENSGALG00010027974	case	control	-1.334897778	0.447475781	-2.983173247	0.002852765	0.111128517	down_in_case
CRA004775	ENSGALG00010027975	IQSEC1	case	control	-0.08643159	0.431246979	-0.200422483	0.841150177	0.966461921	down_in_case
CRA004775	ENSGALG00010027976	LRSAM1	case	control	-0.332473899	0.147180296	-2.258956596	0.023886085	0.298869476	down_in_case
CRA004775	ENSGALG00010027977	BOLA1	case	control	0.257685576	0.383093468	0.67264414	0.501173713	0.860057373	up_in_case
CRA004775	ENSGALG00010027979	RPL12	case	control	-0.197613866	0.244165796	-0.809342953	0.418317906	0.818943344	down_in_case
CRA004775	ENSGALG00010027980	NUP210	case	control	-0.991477159	0.493312134	-2.009837365	0.044448405	0.393273296	down_in_case
CRA004775	ENSGALG00010027981	CNN2	case	control	-0.594499349	0.354561987	-1.676714851	0.093598255	0.525355698	down_in_case
CRA004775	ENSGALG00010027982	ENTPD2	case	control	0.264015489	0.463982114	0.569020832	0.569342003	0.884809383	up_in_case
CRA004775	ENSGALG00010027983	POGZ	case	control	-0.050441973	0.192675746	-0.261797211	0.793477789	0.951605885	down_in_case
CRA004775	ENSGALG00010027984	ARHGAP45	case	control	-0.782353766	0.442710599	-1.767190051	0.077196405	0.488700005	down_in_case
CRA004775	ENSGALG00010027985	PSMB4	case	control	-0.110267938	0.331877736	-0.332254699	0.739696943	0.938064864	down_in_case
CRA004775	ENSGALG00010027986	CGN	case	control	1.065282047	0.515973989	2.064604164	0.038960469	0.37127194	up_in_case
CRA004775	ENSGALG00010027987	TMEM141	case	control	0.024989558	0.350539788	0.071288792	0.943167915	0.987862092	up_in_case
CRA004775	ENSGALG00010027988	HDAC11	case	control	0.506715017	0.219134302	2.312349148	0.02075845	0.283942565	up_in_case
CRA004775	ENSGALG00010027989	ENSGALG00010027989	case	control	-0.195152674	0.275329249	-0.708797467	0.47845017	0.846957812	down_in_case
CRA004775	ENSGALG00010027990	ENSGALG00010027990	case	control	-0.441542168	0.359319732	-1.228828057	0.21913628	0.68672356	down_in_case
CRA004775	ENSGALG00010027991	ENSGALG00010027991	case	control	0.063740952	0.317191269	0.200954308	0.840734302	0.966461921	up_in_case
CRA004775	ENSGALG00010027993	CLIC3	case	control	0.206676047	0.334003753	0.618783609	0.536058922	0.871588629	up_in_case
CRA004775	ENSGALG00010027994	TUFT1	case	control	0.059829297	0.293195822	0.204059172	0.838307259	0.965677031	up_in_case
CRA004775	ENSGALG00010027995	POLR2E	case	control	-0.22417049	0.295046802	-0.759779427	0.447386443	0.834136489	down_in_case
CRA004775	ENSGALG00010027996	GPX4	case	control	-0.287186546	0.397137757	-0.723140877	0.469593338	0.844062961	down_in_case
CRA004775	ENSGALG00010027998	SNX27	case	control	-0.254154645	0.267961022	-0.948476172	0.342887098	0.782449465	down_in_case
CRA004775	ENSGALG00010027999	ENSGALG00010027999	case	control	-0.213906281	0.608937429	-0.351277933	0.725379849	0.935238044	down_in_case
CRA004775	ENSGALG00010028000	MRPL9	case	control	0.05530659	0.369760489	0.149574094	0.881100649	0.974996282	up_in_case
CRA004775	ENSGALG00010028001	SBNO2	case	control	-0.904146751	0.41959604	-2.15480287	0.031177264	0.336670173	down_in_case
CRA004775	ENSGALG00010028002	ABCA2	case	control	-0.326711731	0.208158824	-1.56953102	0.116524257	0.566086328	down_in_case
CRA004775	ENSGALG00010028004	ENSGALG00010028004	case	control	-0.804916282	0.425672812	-1.890927161	0.058634068	0.443329681	down_in_case
CRA004775	ENSGALG00010028005	IER5L	case	control	0.257367766	0.292966305	0.878489304	0.379678239	0.800637244	up_in_case
CRA004775	ENSGALG00010028006	ENSGALG00010028006	case	control	0.379873476	0.568924949	0.66770402	0.504322543	0.861471523	up_in_case
CRA004775	ENSGALG00010028007	STK11	case	control	0.091698044	0.242522095	0.378101816	0.70535496	0.930927733	up_in_case
CRA004775	ENSGALG00010028008	NTMT1	case	control	-0.186976249	0.334898048	-0.55830797	0.576634103	0.887937128	down_in_case
CRA004775	ENSGALG00010028009	RRP9	case	control	-0.604720067	0.215233133	-2.809604916	0.004960235	0.142684328	down_in_case
CRA004775	ENSGALG00010028010	ASB6	case	control	-0.436536562	0.203703917	-2.142995424	0.032113466	0.340736995	down_in_case
CRA004775	ENSGALG00010028011	ENSGALG00010028011	case	control	-0.235018212	0.367489509	-0.639523594	0.52248237	0.866638286	down_in_case
CRA004775	ENSGALG00010028012	PRRX2	case	control	-0.418646683	0.459896664	-0.910305979	0.362661166	0.793469164	down_in_case
CRA004775	ENSGALG00010028013	CBARP	case	control	0.090687573	0.437970079	0.207063399	0.835960346	0.965107526	up_in_case
CRA004775	ENSGALG00010028014	ENSGALG00010028014	case	control	-1.205715587	0.449623575	-2.68161114	0.007326857	0.170964283	down_in_case
CRA004775	ENSGALG00010028015	DUSP7	case	control	-0.221329368	0.385460447	-0.574194759	0.565836015	0.883033678	down_in_case
CRA004775	ENSGALG00010028016	PRF1	case	control	-0.367246714	0.373005687	-0.98456063	0.324839936	0.770185561	down_in_case
CRA004775	ENSGALG00010028017	ENSGALG00010028017	case	control	-0.237834943	0.578794281	-0.410914467	0.681135254	0.923896221	down_in_case
CRA004775	ENSGALG00010028019	RPL29	case	control	-0.303652365	0.266891831	-1.137735699	0.255230862	0.718871765	down_in_case
CRA004775	ENSGALG00010028020	ENSGALG00010028020	case	control	-0.215788449	0.358415779	-0.602061801	0.547133001	0.877099134	down_in_case
CRA004775	ENSGALG00010028021	ATP5F1D	case	control	-0.153871919	0.425521184	-0.361608128	0.71764489	0.934011578	down_in_case
CRA004775	ENSGALG00010028022	ENSGALG00010028022	case	control	-0.045406725	0.185983611	-0.244143691	0.807119536	0.955397873	down_in_case
CRA004775	ENSGALG00010028024	RBM3	case	control	-0.525862065	0.24074355	-2.184324623	0.028938403	0.323755716	down_in_case
CRA004775	ENSGALG00010028025	TNNC1	case	control	0.556853053	0.758403668	0.734243617	0.462800275	0.842456502	up_in_case
CRA004775	ENSGALG00010028026	ANKMY1	case	control	-0.024413298	0.274659926	-0.088885547	0.92917287	0.98473447	down_in_case
CRA004775	ENSGALG00010028027	ENSGALG00010028027	case	control	-0.540263768	0.402598082	-1.341943225	0.179614405	0.644990528	down_in_case
CRA004775	ENSGALG00010028028	FAM174C	case	control	-0.463789957	0.241850136	-1.917674985	0.055152235	0.430875458	down_in_case
CRA004775	ENSGALG00010028029	EFNA2	case	control	-0.552747719	0.48527567	-1.139038598	0.254687056	0.718419134	down_in_case
CRA004775	ENSGALG00010028030	PFDN2	case	control	-0.455298435	0.287711585	-1.582482108	0.113539554	0.562886277	down_in_case
CRA004775	ENSGALG00010028031	NISCH	case	control	0.242858588	0.161480882	1.503946379	0.132595174	0.589307122	up_in_case
CRA004775	ENSGALG00010028033	ENSGALG00010028033	case	control	-0.103291902	0.40925116	-0.252392449	0.800737736	0.953541667	down_in_case
CRA004775	ENSGALG00010028034	MUM1L1	case	control	-0.095736076	0.209465046	-0.457050366	0.647634845	0.914210065	down_in_case
CRA004775	ENSGALG00010028035	ENSGALG00010028035	case	control	-2.127619483	1.152468317	-1.84614141	0.064871676	0.457570489	down_in_case
CRA004775	ENSGALG00010028036	NDUFS7	case	control	0.159728974	0.419905965	0.380392248	0.703654268	0.930200908	up_in_case

CRA004775	ENSGALG00010028037	ENSGALG00010028037	case	control	-0.610160021	0.350485359	-1.740900171	0.081701079	0.497331356	down_in_case
CRA004775	ENSGALG00010028039	STAB1	case	control	-0.192554015	0.370913123	-0.519135084	0.603666544	0.898692785	down_in_case
CRA004775	ENSGALG00010028040	ENSGALG00010028040	case	control	1.011248829	0.817691727	1.236711581	0.216194178	0.683774974	up_in_case
CRA004775	ENSGALG00010028041	ENSGALG00010028041	case	control	-0.658282082	0.795033389	-0.827993001	0.407674463	0.814105656	down_in_case
CRA004775	ENSGALG00010028042	DPP7	case	control	0.29456927	0.367887021	0.800705797	0.423301986	0.820552247	up_in_case
CRA004775	ENSGALG00010028043	NT5DC2	case	control	0.149390842	0.373457835	0.40002064	0.689141315	0.927420456	up_in_case
CRA004775	ENSGALG00010028044	GAMT	case	control	-0.082132808	0.569915916	-0.144113905	0.885410525	0.976602443	down_in_case
CRA004775	ENSGALG00010028045	ENSGALG00010028045	case	control	-0.741826927	0.63454703	-1.169065319	0.242377315	0.709690398	down_in_case
CRA004775	ENSGALG00010028046	SMIM4	case	control	-0.111009751	0.459118875	-0.241788689	0.808943904	0.955623701	down_in_case
CRA004775	ENSGALG00010028047	ENSGALG00010028047	case	control	0.18545594	0.58154583	0.318901676	0.749801073	0.941495694	up_in_case
CRA004775	ENSGALG00010028048	S100A10	case	control	-0.088873553	0.275252542	-0.322880043	0.746786091	0.9402175	down_in_case
CRA004775	ENSGALG00010028049	MAN1B1	case	control	-0.303331178	0.307083366	-0.987781207	0.323259813	0.768640193	down_in_case
CRA004775	ENSGALG00010028050	PBRM1	case	control	0.048037844	0.280555316	0.171224145	0.864047523	0.97230094	up_in_case
CRA004775	ENSGALG00010028051	GNL3	case	control	-0.211696995	0.22709339	-0.932202363	0.351231954	0.786098731	down_in_case
CRA004775	ENSGALG00010028052	ENSGALG00010028052	case	control	-0.646710569	0.440581005	-1.467858491	0.142142666	0.602343234	down_in_case
CRA004775	ENSGALG00010028053	ORM1	case	control	1.804909503	0.627674124	2.875551873	0.004033219	0.130247989	up_in_case
CRA004775	ENSGALG00010028054	RPS15	case	control	-0.196024814	0.221872677	-0.883501373	0.376965441	0.798523018	down_in_case
CRA004775	ENSGALG00010028055	ENSGALG00010028055	case	control	-0.128621263	0.197811809	-0.650220345	0.515549901	0.864897625	down_in_case
CRA004775	ENSGALG00010028056	SLC25A25	case	control	-0.649341489	0.520699459	-1.2470562	0.212376891	0.682275341	down_in_case
CRA004775	ENSGALG00010028057	APC2	case	control	0.317579869	0.887593678	0.357798706	0.720493959	0.934406529	up_in_case
CRA004775	ENSGALG00010028058	GLT8D1	case	control	-0.579337711	0.297584894	-1.946798117	0.051558943	0.419270859	down_in_case
CRA004775	ENSGALG00010028059	GZMM	case	control	-0.585863629	0.501273791	-1.168749771	0.242504463	0.709690398	down_in_case
CRA004775	ENSGALG00010028060	ENSGALG00010028060	case	control	-0.119944938	0.395340538	-0.303396505	0.761587709	0.942236668	down_in_case
CRA004775	ENSGALG00010028061	NAIF1	case	control	-0.029908903	0.124665641	-0.239912961	0.810397733	0.956724552	down_in_case
CRA004775	ENSGALG00010028062	ENSGALG00010028062	case	control	2.393998229	0.854189604	2.802654374			up_in_case
CRA004775	ENSGALG00010028064	SPCS1	case	control	-0.306090634	0.207050034	-1.478341385	0.139316426	0.599104916	down_in_case
CRA004775	ENSGALG00010028065	REEP6	case	control	1.308406856	0.572998368	2.28343906	0.022404519	0.292422177	up_in_case
CRA004775	ENSGALG00010028066	ENSGALG00010028066	case	control	0.102474019	0.35962053	0.284950415	0.775682131	0.946517228	up_in_case
CRA004775	ENSGALG00010028067	FAM102A	case	control	-0.303227229	0.331353406	-0.915117284	0.360130062	0.791975934	down_in_case
CRA004775	ENSGALG00010028068	POLE4	case	control	-0.317228149	0.339564578	-0.934220381	0.350190224	0.785466132	down_in_case
CRA004775	ENSGALG00010028069	NEK4	case	control	0.318800761	0.186632768	1.708171422	0.08760454	0.512398989	up_in_case
CRA004775	ENSGALG00010028070	MEX3A	case	control	-0.009944624	0.438790053	-0.022663741	0.981918499	0.997069916	down_in_case
CRA004775	ENSGALG00010028071	DPM2	case	control	-0.287731139	0.247626197	-1.16195759	0.245252692	0.712046495	down_in_case
CRA004775	ENSGALG00010028073	ST6GALNAC4	case	control	-0.428420158	0.447144845	-0.958123889	0.338000291	0.778663427	down_in_case
CRA004775	ENSGALG00010028074	ENSGALG00010028074	case	control	-0.49472121	0.324343716	-1.525299201	0.127184487	0.581272645	down_in_case
CRA004775	ENSGALG00010028075	RAB25	case	control	0.183637923	0.584313797	0.314279629	0.753308669	0.941900693	up_in_case
CRA004775	ENSGALG00010028076	ST6GALNAC6	case	control	-0.327305853	0.433518924	-0.754997844	0.450250276	0.835404673	down_in_case
CRA004775	ENSGALG00010028078	LAMTOR2	case	control	-0.19648203	0.353668453	-0.5555543	0.57851558	0.888378369	down_in_case
CRA004775	ENSGALG00010028079	ITIH3	case	control	-0.32479164	0.670480396	-0.484416311	0.628090445	0.90828391	down_in_case
CRA004775	ENSGALG00010028080	MUSTN1	case	control	-0.758389815	0.714648384	-1.061206925	0.288595873	0.742851819	down_in_case
CRA004775	ENSGALG00010028081	UBQLN4	case	control	-0.222472108	0.188537924	-1.179985983	0.23800579	0.70473329	down_in_case
CRA004775	ENSGALG00010028082	AK1	case	control	0.202736581	0.650185842	0.311813281	0.75518243	0.941900693	up_in_case
CRA004775	ENSGALG00010028083	ELAVL1	case	control	-0.049384607	0.138543059	-0.356456741	0.721498543	0.934511903	down_in_case
CRA004775	ENSGALG00010028084	ENG	case	control	0.158734889	0.273014634	0.581415313	0.560960579	0.881008373	up_in_case
CRA004775	ENSGALG00010028085	ENSGALG00010028085	case	control	-0.053207264	0.220223038	-0.241606258	0.809085273	0.955623701	down_in_case
CRA004775	ENSGALG00010028086	MATK	case	control	0.200108545	0.468418777	0.427200092	0.669233595	0.920631368	up_in_case
CRA004775	ENSGALG00010028087	STIMATE	case	control	0.036561009	0.310654605	0.117690222	0.906313115	0.980044552	up_in_case
CRA004775	ENSGALG00010028088	NAXE	case	control	0.004330611	0.392156207	0.011043076	0.991189079	0.998065748	up_in_case
CRA004775	ENSGALG00010028089	ENSGALG00010028089	case	control	-0.104971595	0.784939889	-0.133732018	0.893614486	0.977834811	down_in_case
CRA004775	ENSGALG00010028090	ENSGALG00010028090	case	control	0.308201626	0.304537266	1.012032548	0.311522491	0.763050512	up_in_case
CRA004775	ENSGALG00010028091	FPGS	case	control	-0.450005895	0.208656097	-2.15668702	0.031030059	0.336379059	down_in_case
CRA004775	ENSGALG00010028092	RAX2	case	control	0.553975759	0.567350297	0.976426314	0.328853247	0.77220987	up_in_case
CRA004775	ENSGALG00010028093	SFMBT1	case	control	-0.323055596	0.48874625	-0.660988388	0.50861976	0.862971569	down_in_case
CRA004775	ENSGALG00010028095	CDK9	case	control	-0.414984169	0.22622761	-1.834365701	0.066599727	0.462498477	down_in_case
CRA004775	ENSGALG00010028096	TOR2A	case	control	-0.312821391	0.325568815	-0.960845684	0.336629764	0.777068305	down_in_case
CRA004775	ENSGALG00010028097	TTC24	case	control	-0.645102378	0.657329166	-0.981399293	0.326395875	0.770998457	down_in_case
CRA004775	ENSGALG00010028098	RFT1	case	control	-0.444262465	0.268600277	-1.653991089	0.098129323	0.533793778	down_in_case
CRA004775	ENSGALG00010028099	PCASP2	case	control	-0.237120932	0.337104421	-0.703404989	0.481803399	0.848909035	down_in_case
CRA004775	ENSGALG00010028100	ENSGALG00010028100	case	control	1.51559362	0.543290015	2.789658523	0.005276366	0.147770484	up_in_case
CRA004775	ENSGALG00010028101	MRPL54	case	control	0.018893391	0.373562513	0.050576251	0.959663188	0.991431439	up_in_case
CRA004775	ENSGALG00010028102	CERCAM	case	control	-0.306039984	0.424781647	-0.720464233	0.471239214	0.845200296	down_in_case
CRA004775	ENSGALG00010028103	APBA3	case	control	-0.661554429	0.247401297	-2.674013583	0.007494942	0.172634743	down_in_case
CRA004775	ENSGALG00010028104	GOLGA1	case	control	0.10833174	0.184181373	0.588179676	0.556411698	0.87981923	up_in_case
CRA004775	ENSGALG00010028105	PRKCD	case	control	-0.089725227	0.478901297	-0.187356408	0.851381199	0.969284796	down_in_case
CRA004775	ENSGALG00010028106	ARPC5L	case	control	-0.471235496	0.268212557	-1.75694793	0.078926685	0.491399489	down_in_case
CRA004775	ENSGALG00010028107	NOTCH1	case	control	-0.138549868	0.400201816	-0.346199999	0.729192415	0.936326912	down_in_case
CRA004775	ENSGALG00010028108	IQGAP3	case	control	0.520221453	0.432549979	1.202685188	0.229098168	0.695782399	up_in_case
CRA004775	ENSGALG00010028109	TJP3	case	control	0.109449054	0.296196773	0.369514674	0.711744138	0.932407723	up_in_case
CRA004775	ENSGALG00010028110	COQ4	case	control	-0.320265631	0.258872323	-1.237156705	0.216028911	0.683774974	down_in_case
CRA004775	ENSGALG00010028111	TRUB2	case	control	-0.096146202	0.234576203	-0.409871933	0.681899895	0.924278508	down_in_case
CRA004775	ENSGALG00010028112	SWI5	case	control	0.119020529	0.276097327	0.431081785	0.666408904	0.919168997	up_in_case
CRA004775	ENSGALG00010028113	PIP5K1C	case	control	-0.324875109	0.193675819	-1.677416987	0.09346097	0.525355698	down_in_case
CRA004775	ENSGALG00010028114	PFKFB4	case	control	-0.12122873	0.261610262	-0.463394399	0.643081693	0.913561273	down_in_case
CRA004775	ENSGALG00010028115	CACTIN	case	control	-0.009558948	0.219077943	-0.043632637	0.965197236	0.993058423	down_in_case
CRA004775	ENSGALG00010028116	HYAL1	case	control	-0.01195095	0.282150787	-0.042356606	0.966214421	0.993094598	down_in_case
CRA004775	ENSGALG00010028117	ENSGALG00010028117	case	control	-0.394751081	0.421327379	-0.936922453	0.348798449	0.785311667	down_in_case
CRA004775	ENSGALG00010028118	ENSGALG00010028118	case	control	0.085991327	0.152270402	0.564727784	0.572258933	0.885642749	up_in_case
CRA004775	ENSGALG00010028119	HYAL2	case	control	0.074469566	0.296751701	0.250949078	0.80185348	0.953543664	up_in_case
CRA004775	ENSGALG00010028120	TBXA2R	case	control	-0.728013764	0.353483207	-2.059542714	0.039442278	0.374034172	down_in_case
CRA004775	ENSGALG00010028121	TUSC2	case	control	-0.291193127	0.349496398	-0.833179193	0.404743675	0.81313407	down_in_case
CRA004775	ENSGALG00010028122	SEC16A	case	control	-0.080644865	0.282312151	-0.285658499	0.775139694	0.946319184	down_in_case
CRA004775	ENSGALG00010028123	RASSF1	case	control	0.023013142	0.303888225	0.075728968	0.939634729	0.987076872	up_in_case
CRA004775	ENSGALG00010028124	SUGP2	case	control	-0.257048497	0.189497144	-1.356476889	0.174947484	0.640495966	down_in_case
CRA004775	ENSGALG00010028125	HMG20B	case	control	-0.190834384	0.181133558	-1.053556204	0.292086149	0.746262643	down_in_case
CRA004775	ENSGALG00010028126	ZMYND10	case	control	-0.262929124	0.301496946	-0.872078898	0.383165337	0.802000938	down_in_case
CRA004775	ENSGALG00010028127	MFSD12	case	control	0.431369433	0.427994772	1.007884819	0.31350976	0.765098415	up_in_case
CRA004775	ENSGALG00010028128	DNM1	case	control	-0.568817207	0.437647026	-1.299716833	0.193698039	0.657919837	down_in_case
CRA004775	ENSGALG00010028129	CYB561D2	case	control	-0.440196327	0.25476418	-1.727858005	0.084013685	0.503010645	down_in_case
CRA004775	ENSGALG00010028130	TMEM115	case	control	-0.303195147	0.292091421	-1.038014559	0.299263277	0.75239174	down_in_case
CRA004775	ENSGALG00010028131	TMEM161A	case	control	-0.593532227	0.387207515	-1.532853064	0.125312063	0.578422299	down_in_case
CRA004775	ENSGALG00010028132	CACNA2D2	case	control	-0.077479006	0.288396078	-0.268654853	0.788195297	0.95041797	down_in_case
CRA004775	ENSGALG00010028133	ENSGALG00010028133	case	control	0.453020405	0.375040799	1.207922992	0.227076885	0.694715356	up_in_case
CRA004775	ENSGALG00010028134	INPP5E	case	control	-0.151336153	0.180438934	-0.838711188	0.401631399	0.811868062	down_in_case
CRA004775	ENSGALG00010028135	TEKTIP1	case	control	0.134238019	0.626548564	0.214249982	0.830352121	0.963232966	up_in_case
CRA004775	ENSGALG00010028136	C3orf18	case	control	-1.243941595	0.338115876	-3.679039296	0.000234114	0.030954849	down_in_case
CRA004775	ENSGALG00010028137	NUP214	case	control	-0.195465434	0.204314173	-0.956690531	0.338723479	0.779285019	down_in_case
CRA004775	ENSGALG00010028138	ENSGALG00010028138	case	control	-0.2143318	0.398925681	-0.537272506	0.591079394	0.893804699	down_in_case
CRA004775	ENSGALG00010028139	FZR1	case	control	-0.345978682	0.183182793	-1.88870732	0.058931057	0.44414236	down_in_case
CRA004775	ENSGALG00010028140	HEMK1	case	control	-0.539090024	0.310089211	-1.738499777	0.082122789	0.49879438	down_in_case
CRA004775	ENSGALG00010028141	CISH	case	control	-0.462571518	0.443562999	-1.042854158	0.297015844	0.749543929	down_in_case
CRA004775	ENSGALG00010028142	PMPCA	case	control	-0.01884965	0.234960149	-0.080224882	0.936058401	0.986264424	down_in_case
CRA004775	ENSGALG00010028144	FAM78A	case	control	0.253210806	0.315987754	0.801331072	0.422940007	0.820552247	up_in_case
CRA004775	ENSGALG00010028145	ENSGALG00010028145	case	control	-0.050615303	0.286988497	-0.176367009	0.860005621	0.971325143	down_in_case
CRA004775	ENSGALG00010028146	MAPKAPK3	case	control	0.049219068	0.245747493	0.200283092	0.841259185	0.966461921	up_in_case
CRA004775	ENSGALG00010028147	SLC25A42	case	control	0.090383369	0.33201807	0.272224248	0.785449599	0.95041797	up_in_case
CRA004775	ENSGALG00010028148	ENSGALG00010028148	case	control	-0.134311518	0.519713173	-0.25843393	0.796072033	0.952835572	down_in_case
CRA004775	ENSGALG00010028149	SNAPC4	case	control	-0.115878847	0.248218407	-0.466842281	0.640612723	0.911724439	down_in_case
CRA004775	ENSGALG00010028150	ARMC6	case	control	-0.289329845	0.229909837	-1.258449175	0.208229357	0.677077771	down_in_case
CRA004775	ENSGALG00010028151	DOCK3	case	control	0.432514045	0.682443788	0.633772411	0.52622936	0.868167959	up_in_case
CRA004775	ENSGALG00010028152	ENSGALG00010028152	case	control	-2.814397321	1.20006465	-2.345204754	0.019016633	0.272278915	down_in_case
CRA004775	ENSGALG00010028153	MANF	case	control	-0.177106738	0.275406906	-0.643072972	0.520176752	0.865611895	down_in_case
CRA004775	ENSGALG00010028154	ENSGALG00010028154	case	control	-0.050077319	0.14495758	-0.345461884	0.72974716	0.936339759	down_in_case
CRA004775	ENSGALG00010028155	CIZ1	case	control	-0.068638558	0.177744058	-0.386165134	0.699374374	0.929347461	down_in_case
CRA004775	ENSGALG00010028156	DOHH	case	control	-0.334137268	0.307593969	-1.086293302	0.277349249	0.732448686	down_in_case
CRA004775	ENSGALG00010028157	PRRC2B	case	control	0.437953196	0.333097911	1.314788182	0.1885811	0.652667439	up_in_case
CRA004775	ENSGALG00010028158	DDX49	case	control	-0.042443681	0.184554963	-0.229978542	0.818108444	0.959889304	down_in_case
CRA004775	ENSGALG00010028159	DCAF1	case	control	-0.042903298	0.204413219	-0.209885146	0.833757316	0.964019688	down_in_case
CRA004775	ENSGALG00010028160	ENSGALG00010028160	case	control	-0.490209205	0.471108596	-1.040543963	0.29808725	0.750732193	down_in_case
CRA004775	ENSGALG00010028161	ENSGALG00010028161	case	control	-1.590673266	0.541083512	-2.939792527	0.003284321	0.122856914	down_in_case
CRA004775	ENSGALG00010028162	CARD9	case	control	-0.765154173	0.575675311	-1.329141894	0.183801161	0.648064042	down_in_case
CRA004775	ENSGALG00010028163	GBP	case	control	0.32006898	0.610634066	0.524158408	0.600168367	0.897206546	up_in_case
CRA004775	ENSGALG00010028164	BBLN	case	control	-0.474206426	0.234735741	-2.020171381	0.043365614	0.389812978	down_in_case
CRA004775	ENSGALG00010028165	NFIC	case	control	0.147724843	0.252481892	0.585090843	0.558486636	0.880754991	up_in_case
CRA004775	ENSGALG00010028167	ENSGALG00010028167	case	control	0.757567512	0.19907773	3.805385532	0.000141583	0.02543383	up_in_case
CRA004775	ENSGALG00010028168	PTGES2	case	control	0.260649456	0.394252204	0.66112365	0.50853302	0.862971569	up_in_case
CRA004775	ENSGALG00010028169	DNLZ	case	control	-0.244089601	0.373334032	-0.653810207	0.51323409	0.863903061	down_in_case

CRA004775	ENSGALG00010028170	COPE	case	control	-0.513069238	0.219479757	-2.337660861	0.019404848	0.275088727	down_in_case
CRA004775	ENSGALG00010028171	RNF123	case	control	-0.257390245	0.349553696	-0.736339649	0.461524023	0.842010079	down_in_case
CRA004775	ENSGALG00010028172	CERS1	case	control	-0.463808293	0.357641214	-1.296853593	0.194681566	0.659287597	down_in_case
CRA004775	ENSGALG00010028173	GDF3	case	control	-0.104783391	0.359175811	-0.291732873	0.770490873	0.945754599	down_in_case
CRA004775	ENSGALG00010028174	GMPPB	case	control	-0.425877025	0.235847749	-1.805728595	0.070960744	0.469911807	down_in_case
CRA004775	ENSGALG00010028175	POMT1	case	control	-0.369052464	0.21698924	-1.700786937	0.088983003	0.515249396	down_in_case
CRA004775	ENSGALG00010028176	UPF1	case	control	-0.250788978	0.292380711	-0.857748025	0.391031615	0.806521034	down_in_case
CRA004775	ENSGALG00010028177	ENSGALG00010028177	case	control	-0.074088052	0.597075973	-0.124084799	0.901248134	0.979157637	down_in_case
CRA004775	ENSGALG00010028178	ENSGALG00010028178	case	control	-0.524928425	0.450903409	-1.164170452	0.244354942	0.711765017	down_in_case
CRA004775	ENSGALG00010028179	MCM2	case	control	-1.014457389	0.582468122	-1.741653063	0.08156917	0.497302017	down_in_case
CRA004775	ENSGALG00010028180	ODF2	case	control	-0.164325842	0.369858277	-0.444294076	0.656829984	0.91710603	down_in_case
CRA004775	ENSGALG00010028181	BGLAP	case	control	-0.116653326	0.387790334	-0.300815456	0.763555222	0.94239774	down_in_case
CRA004775	ENSGALG00010028182	SMG5	case	control	-0.262512083	0.255299749	-1.028250453	0.303832031	0.756198056	down_in_case
CRA004775	ENSGALG00010028183	CRTC1	case	control	-0.084213566	0.233325706	-0.360927082	0.718153958	0.934011578	down_in_case
CRA004775	ENSGALG00010028184	TMEM79	case	control	-0.703205632	0.301589397	-2.331665628	0.019718288	0.278098649	down_in_case
CRA004775	ENSGALG00010028185	GLMP	case	control	-0.246155104	0.165317963	-1.488979775	0.136492695	0.595518184	down_in_case
CRA004775	ENSGALG00010028186	CCT3	case	control	-0.239918249	0.237046571	-1.012114406	0.311483355	0.763050512	down_in_case
CRA004775	ENSGALG00010028187	TPRA1	case	control	-0.286567652	0.118276615	-2.422859767	0.01539887	0.249441914	down_in_case
CRA004775	ENSGALG00010028188	ENSGALG00010028188	case	control	-0.341536716	0.281340499	-1.213962145	0.22476218	0.692859731	down_in_case
CRA004775	ENSGALG00010028189	GPSM1	case	control	-0.343038797	0.329522994	-1.041016268	0.297867998	0.750344577	down_in_case
CRA004775	ENSGALG00010028190	ILF2	case	control	-0.22522826	0.259555633	-0.867745606	0.385533613	0.803446489	down_in_case
CRA004775	ENSGALG00010028191	KLHL26	case	control	-0.340699506	0.261511763	-1.302807574	0.192640464	0.656993806	down_in_case
CRA004775	ENSGALG00010028192	ENSGALG00010028192	case	control	-0.03452615	0.89972944	-0.038373925	0.969389551	0.994262379	down_in_case
CRA004775	ENSGALG00010028193	BRINP1	case	control	0.659370894	0.870783546	0.75721561	0.4489207	0.834536953	up_in_case
CRA004775	ENSGALG00010028194	ENSGALG00010028194	case	control	0.009141845	0.359887685	0.025401939	0.979734365	0.996768811	up_in_case
CRA004775	ENSGALG00010028195	ENSGALG00010028195	case	control	0.287038534	0.523524446	0.548281053	0.583498935	0.889906855	up_in_case
CRA004775	ENSGALG00010028196	ENSGALG00010028196	case	control	-0.433923179	0.261606289	-1.658687871	0.097178705	0.531393157	down_in_case
CRA004775	ENSGALG00010028197	INTS3	case	control	-0.273630506	0.137755109	-1.986354679	0.046993955	0.402720701	down_in_case
CRA004775	ENSGALG00010028198	UCK1	case	control	-0.170067914	0.161439266	-1.053448258	0.292135597	0.746262643	down_in_case
CRA004775	ENSGALG00010028199	DPP9	case	control	-0.349712736	0.244145062	-1.432397337	0.15203014	0.616583868	down_in_case
CRA004775	ENSGALG00010028201	ENSGALG00010028201	case	control	-0.332174882	0.381578057	-0.870529308	0.38401121	0.802757395	down_in_case
CRA004775	ENSGALG00010028202	QSOX2	case	control	-0.424957354	0.338012625	-1.257223318	0.208672782	0.677718334	down_in_case
CRA004775	ENSGALG00010028205	PRRT1B	case	control	0.611240909	0.44328267	1.37889647	0.167926677	0.63138062	up_in_case
CRA004775	ENSGALG00010028206	RBM15B	case	control	0.16802817	0.184653712	0.909963673	0.362841668	0.793608387	up_in_case
CRA004775	ENSGALG00010028207	ENSGALG00010028207	case	control	-1.374137729	0.607686071	-2.261262507	0.023743006	0.2983663	down_in_case
CRA004775	ENSGALG00010028208	AMIGO3	case	control	-0.247084899	0.344119402	-0.71802083	0.472744443	0.845425322	down_in_case
CRA004775	ENSGALG00010028211	GATAD2B	case	control	0.264482668	0.266143453	0.993759813	0.320339815	0.767037518	up_in_case
CRA004775	ENSGALG00010028221	TNFAIP8L1	case	control	-0.799964126	0.547004489	-1.462445267	0.143619232	0.605057618	down_in_case
CRA004775	ENSGALG00010028232	C9orf69	case	control	-0.003172257	0.323940135	-0.009792725	0.992186661	0.998065748	down_in_case
CRA004775	ENSGALG00010028242	ENSGALG00010028242	case	control	0.238232987	0.349790551	0.681073248	0.495825144	0.856382445	up_in_case
CRA004775	ENSGALG00010028247	MVP	case	control	-0.132523163	0.189119912	-0.700736171	0.483467678	0.850257583	down_in_case
CRA004775	ENSGALG00010028253	ENSGALG00010028253	case	control	-0.782382128	0.501431402	-1.560297428	0.118689611	0.570664629	down_in_case
CRA004775	ENSGALG00010028262	C1orf43	case	control	-0.260068096	0.342418154	-0.759504403	0.447550881	0.834136489	down_in_case
CRA004775	ENSGALG00010028270	RAPGEF1	case	control	0.196961988	0.291802528	0.674983829	0.499686039	0.859327504	up_in_case
CRA004775	ENSGALG00010028283	ENSGALG00010028283	case	control	0.114700408	0.408247559	0.280957975	0.778742633	0.94792594	up_in_case
CRA004775	ENSGALG00010028301	FBN3	case	control	0.450082878	0.645296347	0.697482451	0.485500918	0.851329141	up_in_case
CRA004775	ENSGALG00010028311	ENSGALG00010028311	case	control	0.840477241	0.558258741	1.505533508	0.132186969	0.588356927	up_in_case
CRA004775	ENSGALG00010028314	MEF2D	case	control	0.18402783	0.259284798	0.709751715	0.477858116	0.846563638	up_in_case
CRA004775	ENSGALG00010028316	TIMM44	case	control	-0.181875527	0.237564635	-0.765583342	0.443924259	0.832130395	down_in_case
CRA004775	ENSGALG00010028318	NACC2	case	control	-0.911112777	0.411992258	-2.211480337	0.027002592	0.31493941	down_in_case
CRA004775	ENSGALG00010028321	RHBG	case	control	-0.121603491	0.324724775	-0.374481717	0.708045956	0.931801673	down_in_case
CRA004775	ENSGALG00010028322	STRBP	case	control	0.128208401	0.211207344	0.607026244	0.543833496	0.875729696	up_in_case
CRA004775	ENSGALG00010028326	CRB2	case	control	-0.19847297	0.576878727	-0.344046264	0.730811494	0.936394787	down_in_case
CRA004775	ENSGALG00010028328	HNRNPM	case	control	-0.037111756	0.2184747	-0.169867523	0.865114322	0.972546358	down_in_case
CRA004775	ENSGALG00010028329	HAPLN2	case	control	0.725227874	0.38496972	1.883856926	0.059584331	0.445083352	up_in_case
CRA004775	ENSGALG00010028333	ENSGALG00010028333	case	control	0.133094566	0.569847264	0.233561823	0.815325169	0.958870945	up_in_case
CRA004775	ENSGALG00010028337	ENSGALG00010028337	case	control	-0.354428153	0.545984382	-0.64915438	0.516238595	0.864913871	down_in_case
CRA004775	ENSGALG00010028339	BCAN	case	control	-0.252438903	0.927804823	-0.272081904	0.785559043	0.95041797	down_in_case
CRA004775	ENSGALG00010028344	LSM7	case	control	-0.201397263	0.311884562	-0.64574297	0.518445827	0.865435399	down_in_case
CRA004775	ENSGALG00010028346	DENND1A	case	control	-0.144219264	0.319758122	-0.451026117	0.651970726	0.915892481	down_in_case
CRA004775	ENSGALG00010028348	NES	case	control	0.473844158	0.574554584	0.824715651	0.409533044	0.814976684	up_in_case
CRA004775	ENSGALG00010028355	RBM18	case	control	-0.105069496	0.17572989	-0.597903381	0.549904403	0.877623839	down_in_case
CRA004775	ENSGALG00010028357	SPPL2C	case	control	0.157619983	0.192095331	0.820530005	0.411914031	0.817006008	up_in_case
CRA004775	ENSGALG00010028362	TMPRSS9	case	control	0.239978414	0.821181917	0.292235385			up_in_case
CRA004775	ENSGALG00010028364	MHM2	case	control	-2.133183375	0.966906007	-2.206195184	0.027370336	0.316243014	down_in_case
CRA004775	ENSGALG00010028366	MRRF	case	control	-0.301714858	0.243725243	-1.237930281	0.215741913	0.683655659	down_in_case
CRA004775	ENSGALG00010028368	TIMM13	case	control	-0.139026413	0.376224158	-0.369530797	0.711732123	0.932407723	down_in_case
CRA004775	ENSGALG00010028370	S100A12	case	control	-1.970896389	0.615939959	-3.199819007	0.001375139	0.078138588	down_in_case
CRA004775	ENSGALG00010028372	PTGS1	case	control	-0.019779115	0.350144681	-0.056488405	0.954952732	0.990670511	down_in_case
CRA004775	ENSGALG00010028374	FDPS	case	control	-0.015826279	0.375603006	-0.042135656	0.966390556	0.993094598	down_in_case
CRA004775	ENSGALG00010028375	LMNB2	case	control	-0.077843789	0.419419673	-0.185598803	0.852759393	0.969725992	down_in_case
CRA004775	ENSGALG00010028377	PDCL	case	control	-0.180781249	0.185187095	-0.976208672	0.328961066	0.772331164	down_in_case
CRA004775	ENSGALG00010028380	RUSC1	case	control	-0.495888889	0.216126431	-2.2944389	0.021765301	0.288534247	down_in_case
CRA004775	ENSGALG00010028391	MED26	case	control	0.124316066	0.263177348	0.472366133	0.636665473	0.910250252	up_in_case
CRA004775	ENSGALG00010028392	ASH1L	case	control	0.51105218	0.271943172	1.879260938	0.060208872	0.445397937	up_in_case
CRA004775	ENSGALG00010028394	ZBTB26	case	control	0.208622744	0.181152457	1.15164181	0.249468293	0.714467496	up_in_case
CRA004775	ENSGALG00010028399	SLC35E1	case	control	-0.137423525	0.170381992	-0.806561325	0.419919263	0.819388688	down_in_case
CRA004775	ENSGALG00010028405	S100A6	case	control	-0.273807884	0.331465591	-0.826052211	0.408774475	0.814384687	down_in_case
CRA004775	ENSGALG00010028412	ENSGALG00010028412	case	control	0.489621027	0.470340344	1.040993046	0.297878776	0.750344577	up_in_case
CRA004775	ENSGALG00010028414	KBTBD12	case	control	-0.71403614	0.615317584	-1.16043513	0.245871691	0.712046495	down_in_case
CRA004775	ENSGALG00010028415	FSD1	case	control	-0.029688545	0.438358041	-0.067726704	0.946003191	0.988996418	down_in_case
CRA004775	ENSGALG00010028416	ENSGALG00010028416	case	control	-1.543887317	0.799282843	-1.931590715	0.053410043	0.425777384	down_in_case
CRA004775	ENSGALG00010028418	ENSGALG00010028418	case	control	0.192261291	0.281470658	0.683059799	0.494569057	0.856382445	up_in_case
CRA004775	ENSGALG00010028419	ENSGALG00010028419	case	control	-0.850689811	0.47775096	-1.780613504	0.074975612	0.482673054	down_in_case
CRA004775	ENSGALG00010028420	ENSGALG00010028420	case	control	-2.129781131	0.775787108	-2.745316478	0.006045257	0.155380051	down_in_case
CRA004775	ENSGALG00010028421	ZBTB7A	case	control	0.268268879	0.277502793	0.966724968	0.333681542	0.774292083	up_in_case
CRA004775	ENSGALG00010028422	RXRA	case	control	-0.154698897	0.447790284	-0.345471759	0.729739738	0.936339759	down_in_case
CRA004775	ENSGALG00010028425	DAP3	case	control	-0.044482535	0.316979341	-0.1403326	0.888397208	0.977118922	down_in_case
CRA004775	ENSGALG00010028426	GPR21	case	control	0.77599539	0.475994022	1.630262889	0.103045947	0.544156998	up_in_case
CRA004775	ENSGALG00010028427	ENSGALG00010028427	case	control	0.261529318	0.253264616	1.032632673	0.301775832	0.754631653	up_in_case
CRA004775	ENSGALG00010028428	RABGAP1	case	control	0.041593518	0.297358976	0.139876449	0.888757609	0.977118922	up_in_case
CRA004775	ENSGALG00010028429	REXO1	case	control	-0.033668388	0.187040411	-0.180005957	0.857147892	0.970913248	down_in_case
CRA004775	ENSGALG00010028430	NR2C2AP	case	control	-0.235764421	0.288307232	-0.817754099	0.413497613	0.817353502	down_in_case
CRA004775	ENSGALG00010028431	ENSGALG00010028431	case	control	-0.166777399	0.312558171	-0.533588351	0.59362637	0.894594166	down_in_case
CRA004775	ENSGALG00010028432	RFXANK	case	control	-0.39152427	0.274073682	-1.428536545	0.153137484	0.617472961	down_in_case
CRA004775	ENSGALG00010028433	MSTO1	case	control	-0.562095668	0.266544131	-2.10882778	0.03495945	0.354362166	down_in_case
CRA004775	ENSGALG00010028434	COL5A1	case	control	0.091484319	0.464272775	0.19704864	0.843789475	0.966478468	up_in_case
CRA004775	ENSGALG00010028435	ATP8B3	case	control	-0.199669513	0.534269614	-0.373724254	0.708609479	0.931801673	down_in_case
CRA004775	ENSGALG00010028436	S100A1	case	control	0.152252478	0.56912051	0.267522388	0.789066979	0.95041797	up_in_case
CRA004775	ENSGALG00010028437	ENSGALG00010028437	case	control	-0.324636928	0.266983267	-1.215944848	0.224005932	0.69173716	down_in_case
CRA004775	ENSGALG00010028438	MED27	case	control	-0.499702018	0.199658665	-2.502781516	0.012322159	0.223538261	down_in_case
CRA004775	ENSGALG00010028439	BORCS8	case	control	-0.265801315	0.20756828	-1.280548815	0.200352188	0.667403813	down_in_case
CRA004775	ENSGALG00010028440	PSMB7	case	control	-0.399590498	0.230344715	-1.734750012	0.082785094	0.499833536	down_in_case
CRA004775	ENSGALG00010028442	CHTOP	case	control	-0.125298549	0.212912917	-0.588496698	0.556198949	0.87981923	down_in_case
CRA004775	ENSGALG00010028443	ENSGALG00010028443	case	control	-0.251490029	0.396220406	-0.634722558	0.525609379	0.867561466	down_in_case
CRA004775	ENSGALG00010028444	MEF2B	case	control	-0.714920613	0.583893049	-1.224403363	0.220800101	0.688641913	down_in_case
CRA004775	ENSGALG00010028445	SYT11	case	control	-0.243577298	0.368567513	-0.660875659	0.508692057	0.862971569	down_in_case
CRA004775	ENSGALG00010028446	ENSGALG00010028446	case	control	-0.305208868	0.472368304	-0.646124784	0.518198546	0.865435399	down_in_case
CRA004775	ENSGALG00010028447	OLFM1	case	control	0.59544203	0.477205955	1.247767393	0.212116253	0.682275341	up_in_case
CRA004775	ENSGALG00010028448	RIT1	case	control	-0.174189487	0.175096177	-0.994821761	0.31982296	0.767037518	down_in_case
CRA004775	ENSGALG00010028449	EFCAB12	case	control	-0.894233595	0.557199008	-1.604872911	0.108521776	0.555578162	down_in_case
CRA004775	ENSGALG00010028450	PTGDS	case	control	0.247916078	0.321410479	0.771337881	0.440506685	0.829353567	up_in_case
CRA004775	ENSGALG00010028451	TMEM59L	case	control	0.047415585	0.530221832	0.089425939	0.928743409	0.98473447	up_in_case
CRA004775	ENSGALG00010028452	GOLPH3L	case	control	0.788013673	0.456712351	1.725404782	0.08445455	0.503614276	up_in_case
CRA004775	ENSGALG00010028453	ENSGALG00010028453	case	control	-0.158775346	0.162779211	-0.975403097	0.329360348	0.77237154	down_in_case
CRA004775	ENSGALG00010028454	C8G	case	control	-0.460523387	0.330749772	-1.392361919	0.16381283	0.629717526	down_in_case
CRA004775	ENSGALG00010028455	ENSA	case	control	-0.351701503	0.228560058	-1.538770626	0.123860303	0.576485043	down_in_case
CRA004775	ENSGALG00010028456	FBXW5	case	control	-0.254790717	0.274383163	-0.928594579	0.353099233	0.787065859	down_in_case
CRA004775	ENSGALG00010028457	KANK3	case	control	-0.110136348	0.347005431	-0.317390847	0.750947052	0.941495694	down_in_case
CRA004775	ENSGALG00010028458	TRAF2	case	control	-0.032327221	0.278085925	-0.116249036	0.907455175	0.980354087	down_in_case
CRA004775	ENSGALG00010028459	MCL1	case	control	0.16531057	0.45656936	0.362071099	0.7172989	0.934011578	up_in_case
CRA004775	ENSGALG00010028460	CRLF1	case	control	-0.521491896	0.532092414	-0.980077675	0.327047778	0.770998457	down_in_case
CRA004775	ENSGALG00010028461	ENSGALG00010028461	case	control	-0.557756046	0.51199018	-1.089388172	0.275982745	0.731001618	down_in_case
CRA004775	ENSGALG00010028462	EDF1	case	control	0.065508431	0.236234898	0.277302091	0.781548154	0.948910196	up_in_case
CRA004775	ENSGALG00010028463	RPS28	case	control	-0.273501359	0.351710114	-0.777632908	0.436785455	0.827138935	down_in_case
CRA004775	ENSGALG00010028464	ENSGALG00010028464	case	control	-0.263716611	0.292735104	-0.900871155	0.36765683	0.794321403	down_in_case

CRA004775	ENSGALG00010028465	ENSGALG00010028465	case	control	0.035840058	0.402434506	0.089058115	0.929035725	0.98473447	up_in_case
CRA004775	ENSGALG00010028466	MAMDC4	case	control	2.025006743	0.694860197	2.914264987	0.003565271	0.125102734	up_in_case
CRA004775	ENSGALG00010028467	ENSGALG00010028467	case	control	-0.540215517	0.393326686	-1.37345249	0.169611721	0.633147466	down_in_case
CRA004775	ENSGALG00010028468	PHPT1	case	control	-0.206578795	0.283890705	-0.727670161	0.466815521	0.8434807	down_in_case
CRA004775	ENSGALG00010028470	AJM1	case	control	-0.289626792	0.533265128	-0.543119693	0.587047394	0.892086277	down_in_case
CRA004775	ENSGALG00010028471	TPGS1	case	control	-0.627174462	0.280623243	-2.234934123	0.025421674	0.305924309	down_in_case
CRA004775	ENSGALG00010028472	RPRD2	case	control	0.12501953	0.253278324	0.493605327	0.621584938	0.905734289	up_in_case
CRA004775	ENSGALG00010028473	CDC34	case	control	-0.577104833	0.409206981	-1.410300556	0.158450954	0.624136085	down_in_case
CRA004775	ENSGALG00010028474	PEX19	case	control	-0.155528671	0.409806362	-0.379517464	0.704303638	0.930465147	down_in_case
CRA004775	ENSGALG00010028475	AMBP	case	control	1.164727753	0.477258372	2.440455363	0.014668759	0.241689368	up_in_case
CRA004775	ENSGALG00010028476	ENSGALG00010028476	case	control	-0.350685876	0.326080298	-1.075458648	0.282169437	0.738291085	down_in_case
CRA004775	ENSGALG00010028477	NRARP	case	control	0.224513932	0.35726793	0.628418935	0.529729536	0.869211096	up_in_case
CRA004775	ENSGALG00010028478	PEA15	case	control	0.152651061	0.256525514	0.59507165	0.551795578	0.878258849	up_in_case
CRA004775	ENSGALG00010028479	CASQ1	case	control	-0.821050384	0.944652407	-0.869156081	0.384761766	0.803182776	down_in_case
CRA004775	ENSGALG00010028480	F11R	case	control	0.147267222	0.219759831	0.670128024	0.502776182	0.860552063	up_in_case
CRA004775	ENSGALG00010028481	ENSGALG00010028481	case	control	0.24522452	0.32749296	0.748793258	0.453981824	0.838254622	up_in_case
CRA004775	ENSGALG00010028482	CD320	case	control	-0.268587915	0.33135047	-0.810585585	0.417603698	0.818943344	down_in_case
CRA004775	ENSGALG00010028483	EXD3	case	control	-0.169106197	0.214230514	-0.789365594	0.429898359	0.82413667	down_in_case
CRA004775	ENSGALG00010028484	NOXA1	case	control	0.383783273	0.352426985	1.088972439	0.27616604	0.731001618	up_in_case
CRA004775	ENSGALG00010028485	USF1	case	control	-0.139757621	0.240390043	-0.581378577	0.560985333	0.881008373	down_in_case
CRA004775	ENSGALG00010028486	ARHGAP30	case	control	-0.769501339	0.477233026	-1.612422648	0.10687	0.552884225	down_in_case
CRA004775	ENSGALG00010028487	ENTPD8	case	control	0.039645798	0.699237205	0.056698639	0.954785258	0.990670511	up_in_case
CRA004775	ENSGALG00010028488	PTGES	case	control	-0.520810629	0.403680701	-1.290154887	0.196996886	0.66285976	down_in_case
CRA004775	ENSGALG00010028489	ENSGALG00010028489	case	control	-0.212464189	0.596600699	-0.356124607	0.72174725	0.934709591	down_in_case
CRA004775	ENSGALG00010028490	NECTIN4	case	control	0.393905886	0.410170723	0.960346177	0.336881017	0.777256856	up_in_case
CRA004775	ENSGALG00010028491	TOR1B	case	control	-0.267536448	0.15156213	-1.765193242	0.077531294	0.489276192	down_in_case
CRA004775	ENSGALG00010028492	ENSGALG00010028492	case	control	-0.131235076	0.38504361	-0.34083172	0.73323027	0.936819725	down_in_case
CRA004775	ENSGALG00010028493	TOR1A	case	control	-0.163995719	0.154291678	-1.06289413	0.287829965	0.74258854	down_in_case
CRA004775	ENSGALG00010028494	ENSGALG00010028494	case	control	-0.076351702	0.340188211	-0.224439588	0.82241527	0.960868728	down_in_case
CRA004775	ENSGALG00010028495	LINGO3	case	control	-0.952864568	0.635870547	-1.498519741	0.133998268	0.59228871	down_in_case
CRA004775	ENSGALG00010028496	ENSGALG00010028496	case	control	-0.297145714	0.209407116	-1.418985755	0.155903169	0.619442611	down_in_case
CRA004775	ENSGALG00010028497	ADMP	case	control	1.112323978	0.627719049	1.772009277	0.076393017	0.486784235	up_in_case
CRA004775	ENSGALG00010028498	C9orf78	case	control	0.114360016	0.216303081	0.52870267	0.597011725	0.896038881	up_in_case
CRA004775	ENSGALG00010028500	VPS45	case	control	-0.135427857	0.190226004	-0.711931357	0.476507283	0.846367006	down_in_case
CRA004775	ENSGALG00010028501	USP20	case	control	-0.238733097	0.128150432	-1.862912932	0.062474517	0.448843826	down_in_case
CRA004775	ENSGALG00010028502	PLEKHO1	case	control	-0.331432755	0.365743516	-0.906189011	0.364835818	0.793608387	down_in_case
CRA004775	ENSGALG00010028503	NDOR1	case	control	-0.606193777	0.215426129	-2.813928751	0.004894007	0.142429514	down_in_case
CRA004775	ENSGALG00010028507	ANP32E	case	control	-0.121084607	0.263516501	-0.45949535	0.64587849	0.914043203	down_in_case
CRA004775	ENSGALG00010028508	ENSGALG00010028508	case	control	-3.456315358	1.175978628	-2.939097087	0.003291699	0.122856914	down_in_case
CRA004775	ENSGALG00010028510	CILP2	case	control	-0.955503979	0.778030131	-1.228106651	0.219406934	0.687020074	down_in_case
CRA004775	ENSGALG00010028511	YJEFN3	case	control	0.473055828	0.499998091	0.946115269	0.344089797	0.783503692	up_in_case
CRA004775	ENSGALG00010028512	NDUFA13	case	control	-0.282721819	0.392278727	-0.720716671	0.471083853	0.845198132	down_in_case
CRA004775	ENSGALG00010028514	FNBP1	case	control	-0.143692106	0.170042032	-0.845038752	0.398089203	0.80954224	down_in_case
CRA004775	ENSGALG00010028516	GATAD2A	case	control	-0.32675155	0.176833355	-1.847793647	0.0646322	0.457298868	down_in_case
CRA004775	ENSGALG00010028517	RNF208	case	control	-1.189338382	0.582595914	-2.041446488	0.041206468	0.379270506	down_in_case
CRA004775	ENSGALG00010028518	MAU2	case	control	-0.020564728	0.256626399	-0.080134891	0.936129973	0.986264424	down_in_case
CRA004775	ENSGALG00010028523	SUGP1	case	control	-0.666575252	0.166486973	-4.003768212	6.23414E-05	0.015875134	down_in_case
CRA004775	ENSGALG00010028525	TM6SF2	case	control	2.586192288	0.760225471	3.401875348	0.000669252	0.054134577	up_in_case
CRA004775	ENSGALG00010028528	UHRF1	case	control	0.222177274	0.534510794	0.415664709	0.677655368	0.923000026	up_in_case
CRA004775	ENSGALG00010028535	TICAM1	case	control	0.049857992	0.29818054	0.167207398	0.867206864	0.973120718	up_in_case
CRA004775	ENSGALG00010028536	GPR107	case	control	-0.219762396	0.261771581	-0.839519686	0.401177747	0.811868062	down_in_case
CRA004775	ENSGALG00010028538	FEM1A	case	control	0.229359331	0.371659442	0.617122303	0.537154059	0.872478793	up_in_case
CRA004775	ENSGALG00010028541	ENSGALG00010028541	case	control	0.198638557	0.77920547	0.25492449	0.798781421	0.952835572	up_in_case
CRA004775	ENSGALG00010028543	ANP32B	case	control	-0.340622853	0.283565012	-1.201216083	0.229667392	0.695782399	down_in_case
CRA004775	ENSGALG00010028544	ZNF414	case	control	-0.338532936	0.283160929	-1.195549601	0.231872369	0.698920857	down_in_case
CRA004775	ENSGALG00010028547	ZNF618	case	control	0.19394567	0.273463189	0.70922039	0.478187722	0.846814631	up_in_case
CRA004775	ENSGALG00010028548	MYO1F	case	control	-0.79938817	0.530168723	-1.507799565	0.131605834	0.587352231	down_in_case
CRA004775	ENSGALG00010028549	ADAMTS10	case	control	0.132737115	0.40666341	0.326405355	0.744117686	0.939218065	up_in_case
CRA004775	ENSGALG00010028550	ZAP70	case	control	-0.910302204	0.548071643	-1.660918268	0.096729863	0.530779068	down_in_case
CRA004775	ENSGALG00010028551	NCS1	case	control	-0.173197921	0.497678135	-0.348011915	0.727831235	0.935548472	down_in_case
CRA004775	ENSGALG00010028553	NFILZ	case	control	0.000534988	0.426116938	0.001255497	0.998998259	0.999914248	up_in_case
CRA004775	ENSGALG00010028556	SPTAN1	case	control	0.321203426	0.302162129	1.063016823	0.287774322	0.74258854	up_in_case
CRA004775	ENSGALG00010028559	DYNC2I2	case	control	-0.500016935	0.2059515	-2.427838281	0.015189114	0.247470987	down_in_case
CRA004775	ENSGALG00010028561	SET	case	control	-0.069794818	0.198876366	-0.350945764	0.725629038	0.935238044	down_in_case
CRA004775	ENSGALG00010028562	ACSBG2	case	control	-0.010543454	0.262485609	-0.040167741	0.967959396	0.993568693	down_in_case
CRA004775	ENSGALG00010028565	PKN1	case	control	0.27023832	0.363710893	0.743003097	0.457479805	0.839704957	up_in_case
CRA004775	ENSGALG00010028566	ZDHHC12	case	control	-0.746934926	0.279477574	-2.672611313	0.00752634	0.173068062	down_in_case
CRA004775	ENSGALG00010028568	MLLT1	case	control	-0.13883312	0.216898201	-0.640084242	0.522117833	0.866416245	down_in_case
CRA004775	ENSGALG00010028569	ZER1	case	control	-0.239486623	0.219479774	-1.091155775	0.275204344	0.731001618	down_in_case
CRA004775	ENSGALG00010028572	TBC1D13	case	control	-0.273287342	0.135604371	-2.015328416	0.043870249	0.391981676	down_in_case
CRA004775	ENSGALG00010028573	KCNN1	case	control	-0.244040154	0.535343788	-0.455856889	0.648492892	0.91479542	down_in_case
CRA004775	ENSGALG00010028574	ENDOG	case	control	-0.242161372	0.277513269	-0.87261187	0.382874669	0.801925538	down_in_case
CRA004775	ENSGALG00010028575	SETDB1	case	control	-0.466166129	0.169362285	-2.752478968	0.005914595	0.154441997	down_in_case
CRA004775	ENSGALG00010028577	CERS2	case	control	-0.016224976	0.149413347	-0.10859121	0.913526733	0.980803023	down_in_case
CRA004775	ENSGALG00010028578	SPOUT1	case	control	-0.380825091	0.219213773	-1.737231586	0.082346302	0.499148629	down_in_case
CRA004775	ENSGALG00010028579	OCLN	case	control	0.30010681	0.414815814	0.723470031	0.46939116	0.8440023	up_in_case
CRA004775	ENSGALG00010028580	PRUNE1	case	control	0.111374042	0.346937825	0.321020178	0.7481951	0.940917938	up_in_case
CRA004775	ENSGALG00010028581	NR2F6	case	control	-0.163789074	0.318926945	-0.513562985	0.607557564	0.900943922	down_in_case
CRA004775	ENSGALG00010028582	KYAT1	case	control	-0.226046866	0.390288577	-0.57917879	0.562468541	0.881532201	down_in_case
CRA004775	ENSGALG00010028583	USHBP1	case	control	0.0230246	0.376946452	0.061081885	0.951293996	0.990168609	up_in_case
CRA004775	ENSGALG00010028584	LRRC8A	case	control	0.079052307	0.204012755	0.387487082	0.698395646	0.929347461	up_in_case
CRA004775	ENSGALG00010028585	MINDY1	case	control	-0.410684938	0.304599672	-1.348277678	0.177569089	0.643283771	down_in_case
CRA004775	ENSGALG00010028586	BABAM1	case	control	-0.165719978	0.349275648	-0.474467601	0.635166497	0.909837348	down_in_case
CRA004775	ENSGALG00010028587	ANKLE1	case	control	-0.693784572	0.291547617	-2.379661267	0.017328559	0.260991251	down_in_case
CRA004775	ENSGALG00010028588	ABHD8	case	control	-0.720771961	0.541697876	-1.330579264	0.183327489	0.647806087	down_in_case
CRA004775	ENSGALG00010028589	PHYHD1	case	control	-0.378004764	0.445444712	-0.848600856	0.396103431	0.808998414	down_in_case
CRA004775	ENSGALG00010028590	ENSGALG00010028590	case	control	-0.035277634	0.278001559	-0.126897253	0.899021721	0.978790377	down_in_case
CRA004775	ENSGALG00010028591	GABPB2	case	control	-0.323504251	0.290478456	-1.113694471	0.265410282	0.726444424	down_in_case
CRA004775	ENSGALG00010028592	SEMA6C	case	control	0.600483087	0.353935555	1.696588768	0.089774435	0.516522282	up_in_case
CRA004775	ENSGALG00010028593	DOLK	case	control	-0.283541969	0.302862719	-0.936206245	0.349167008	0.785311667	down_in_case
CRA004775	ENSGALG00010028594	ENSGALG00010028594	case	control	0.035387965	0.404708288	0.087440673	0.930321241	0.985044263	up_in_case
CRA004775	ENSGALG00010028595	SCNM1	case	control	-0.545506725	0.327587158	-1.665226221	0.095867632	0.529205137	down_in_case
CRA004775	ENSGALG00010028596	DDA1	case	control	-0.178563505	0.309343833	-0.577233119	0.563781987	0.881964623	down_in_case
CRA004775	ENSGALG00010028597	NUP188	case	control	-0.680227428	0.299889704	-2.268258694	0.023313444	0.296313338	down_in_case
CRA004775	ENSGALG00010028598	TMOD4	case	control	-0.10350397	0.568131548	-0.182183107	0.85543903	0.970635695	down_in_case
CRA004775	ENSGALG00010028599	VPS72	case	control	-0.291847729	0.322707609	-0.904372008	0.365798176	0.793608387	down_in_case
CRA004775	ENSGALG00010028600	PSMD4	case	control	-0.202061896	0.317887164	-0.635640312	0.525010889	0.867302346	down_in_case
CRA004775	ENSGALG00010028601	ANO8	case	control	-0.082470254	0.558364188	-0.147699756	0.882579724	0.975434318	down_in_case
CRA004775	ENSGALG00010028602	ZNF687	case	control	-0.160098893	0.19652918	-0.814631664	0.41528318	0.818921904	down_in_case
CRA004775	ENSGALG00010028603	SH3GLB2	case	control	-0.581678983	0.293419953	-1.98241114	0.047433244	0.404519261	down_in_case
CRA004775	ENSGALG00010028604	PI4KB	case	control	-0.238578893	0.180045027	-1.325106819	0.185135725	0.6491093	down_in_case
CRA004775	ENSGALG00010028605	GTPBP3	case	control	0.149009147	0.443310749	0.336127981	0.736774359	0.937564762	up_in_case
CRA004775	ENSGALG00010028606	PRPF3	case	control	0.164593059	0.203444632	0.80903122	0.418497189	0.818943344	up_in_case
CRA004775	ENSGALG00010028607	ENSGALG00010028607	case	control	-0.127282131	0.211107801	-0.602924809	0.546558711	0.877099134	down_in_case
CRA004775	ENSGALG00010028608	MIGA2	case	control	-0.647529755	0.246952824	-2.622078762	0.008739523	0.188957834	down_in_case
CRA004775	ENSGALG00010028609	MRPS21	case	control	0.106082832	0.335220568	0.31645681	0.751655802	0.941495694	up_in_case
CRA004775	ENSGALG00010028610	ENSGALG00010028610	case	control	-0.373142995	0.376833936	-0.99020539	0.322073739	0.767876089	down_in_case
CRA004775	ENSGALG00010028611	ENSGALG00010028611	case	control	0.392335283	0.575400488	0.681847324	0.495335497	0.856382445	up_in_case
CRA004775	ENSGALG00010028612	DIRAS1	case	control	0.58345848	0.514608924	1.133790053	0.256882625	0.719236059	up_in_case
CRA004775	ENSGALG00010028613	ENSGALG00010028613	case	control	-0.447116036	0.537731839	-0.831485146	0.405699616	0.813707034	down_in_case
CRA004775	ENSGALG00010028618	DOLPP1	case	control	-0.657457971	0.31849656	-2.064254539	0.038993589	0.371330222	down_in_case
CRA004775	ENSGALG00010028626	CRAT	case	control	0.406651678	0.300326643	1.354031313	0.175726388	0.641298716	up_in_case
CRA004775	ENSGALG00010028642	APH1A	case	control	-0.404273347	0.305798125	-1.322026898	0.186159194	0.6491093	down_in_case
CRA004775	ENSGALG00010028649	ENSGALG00010028649	case	control	-0.243580506	0.310090975	-0.785513043	0.432152826	0.825415344	down_in_case
CRA004775	ENSGALG00010028658	PTPA	case	control	-0.506719595	0.190712745	-2.656978147	0.007884455	0.178614738	down_in_case
CRA004775	ENSGALG00010028662	FCRL4	case	control	-0.040165336	0.266108414	-0.150935985	0.880026214	0.974996282	down_in_case
CRA004775	ENSGALG00010028669	ZBTB34	case	control	0.702970203	0.213290901	3.295828364	0.000981319	0.066028181	up_in_case
CRA004775	ENSGALG00010028671	CADM3	case	control	-0.508183923	0.512765686	-0.991064607	0.321654034	0.767750105	down_in_case
CRA004775	ENSGALG00010028675	ZBTB43	case	control	0.157073056	0.237963836	0.66007112	0.509208191	0.862971569	up_in_case
CRA004775	ENSGALG00010028678	ENSGALG00010028678	case	control	-1.348450072	0.385319712	-3.499561614	0.000466024	0.044470881	down_in_case
CRA004775	ENSGALG00010028683	CRPL2	case	control	1.024295867	0.390137788	2.625472072	0.008652891	0.188268829	up_in_case
CRA004775	ENSGALG00010028686	TAGLN2	case	control	-0.021354274	0.269184438	-0.079329527	0.936770521	0.98646154	down_in_case
CRA004775	ENSGALG00010028689	CFAP45	case	control	0.845073534	0.757147745	1.116127651	0.264367498	0.724455452	up_in_case
CRA004775	ENSGALG00010028692	RALGPS1	case	control	0.364634265	0.292003028	1.248734534	0.211762186	0.682220401	up_in_case
CRA004775	ENSGALG00010028700	ANGPTL2	case	control	0.629236693	0.423086565	1.487252835	0.136948046	0.596242097	up_in_case
CRA004775	ENSGALG00010028712	FBXW2	case	control	-0.141724006	0.194983721	-0.726850451	0.467317574	0.843870188	down_in_case

CRA004775	ENSGALG00010028726	SLC2A8	case	control	-0.445689384	0.241323264	-1.846856276	0.064767973	0.457570489	down_in_case
CRA004775	ENSGALG00010028727	ENSGALG00010028727	case	control	-0.148018577	0.252871116	-0.585351857	0.558311153	0.880754991	down_in_case
CRA004775	ENSGALG00010028732	ENSGALG00010028732	case	control	-0.468407735	1.195607683	-0.391773775	0.695225374	0.928971346	down_in_case
CRA004775	ENSGALG00010028733	ENSGALG00010028733	case	control	-0.410366775	0.519648774	-0.789700266	0.429702836	0.824104894	down_in_case
CRA004775	ENSGALG00010028734	ENSGALG00010028734	case	control	0.804179092	0.647710109	1.241572551	0.214394314	0.683562661	up_in_case
CRA004775	ENSGALG00010028735	ENSGALG00010028735	case	control	0.249608418	0.425992793	0.585945165	0.557912365	0.880754991	up_in_case
CRA004775	ENSGALG00010028738	MIDN	case	control	0.493741662	0.605859356	0.814944355	0.415104163	0.818834793	up_in_case
CRA004775	ENSGALG00010028739	GARNL3	case	control	0.04382101	0.212990141	0.205741966	0.836992475	0.965269623	up_in_case
CRA004775	ENSGALG00010028740	DAZAP1	case	control	0.286934863	0.190166586	1.508860567	0.131334419	0.58691881	up_in_case
CRA004775	ENSGALG00010028742	ENSGALG00010028742	case	control	-0.259159493	0.218203549	-1.18769605	0.23495318	0.702145501	down_in_case
CRA004775	ENSGALG00010028743	ARRDC1	case	control	-0.195029219	0.238753212	-0.816865322	0.414005397	0.817589409	down_in_case
CRA004775	ENSGALG00010028744	HOMER3	case	control	0.440997062	0.40265503	1.095223029	0.273418939	0.731001618	up_in_case
CRA004775	ENSGALG00010028745	ENSGALG00010028745	case	control	-0.256229612	0.247420404	-1.03560421	0.300386827	0.752927916	down_in_case
CRA004775	ENSGALG00010028746	MYDGF	case	control	-0.366443788	0.273217762	-1.341215103	0.179850624	0.644990528	down_in_case
CRA004775	ENSGALG00010028748	PIAS3	case	control	0.120746696	0.266867324	0.452459648	0.650937885	0.915614449	up_in_case
CRA004775	ENSGALG00010028750	OTUD7B	case	control	0.304682729	0.506077521	0.602047545	0.547142489	0.877099134	up_in_case
CRA004775	ENSGALG00010028751	EHMT1	case	control	-0.416116873	0.255666032	-1.627579814	0.103614012	0.545480965	down_in_case
CRA004775	ENSGALG00010028752	MTMR11	case	control	0.086233021	0.305043673	0.282690736	0.777413918	0.947546427	up_in_case
CRA004775	ENSGALG00010028753	SV2A	case	control	-0.204947767	0.688042465	-0.29787081	0.765801767	0.943512239	down_in_case
CRA004775	ENSGALG00010028760	SSR2	case	control	-0.399476136	0.181556036	-2.200291128	0.027786246	0.318297866	down_in_case
CRA004775	ENSGALG00010028761	CTSS	case	control	-0.083478305	0.420515809	-0.198514072	0.842642876	0.966478468	down_in_case
CRA004775	ENSGALG00010028763	CTSK	case	control	0.78663133	0.688039957	1.143293093	0.252916922	0.717347518	up_in_case
CRA004775	ENSGALG00010028764	ARNT	case	control	-0.179516853	0.19276563	-0.931270027	0.351713902	0.786426886	down_in_case
CRA004775	ENSGALG00010028768	NTNG2	case	control	-0.177351167	0.424413733	-0.417873301	0.676039754	0.922924485	down_in_case
CRA004775	ENSGALG00010028771	SETX	case	control	0.237210728	0.262025962	0.90529475	0.365309257	0.793608387	up_in_case
CRA004775	ENSGALG00010028772	TTF1	case	control	0.19353661	0.170795056	1.133151128	0.257150794	0.719236059	up_in_case
CRA004775	ENSGALG00010028782	MLLT11	case	control	-0.439170533	0.484326534	-0.906765379	0.364530882	0.793608387	down_in_case
CRA004775	ENSGALG00010028783	DDX31	case	control	-0.274024431	0.22644676	-1.210105325	0.22623848	0.694420835	down_in_case
CRA004775	ENSGALG00010028786	GTF3C4	case	control	-0.321076214	0.292518725	-1.09762619	0.272367752	0.73036169	down_in_case
CRA004775	ENSGALG00010028806	SPACA9	case	control	0.785709048	0.719972244	1.091304635	0.275138859	0.731001618	up_in_case
CRA004775	ENSGALG00010028818	TSC1	case	control	-0.01186029	0.248619758	-0.047704534	0.961951721	0.991860454	down_in_case
CRA004775	ENSGALG00010028823	GFI1B	case	control	0.225590251	0.374582194	0.602244994	0.54701107	0.877099134	up_in_case
CRA004775	ENSGALG00010028826	CEL	case	control	2.492899915	1.310392223	1.902407441	0.057117909	0.436969285	up_in_case
CRA004775	ENSGALG00010028829	GTF3C5	case	control	-0.087326927	0.229286334	-0.380864073	0.703304112	0.930068722	down_in_case
CRA004775	ENSGALG00010028833	GBGT1	case	control	-0.505758002	0.369866329	-1.367407526	0.171497605	0.636057362	down_in_case
CRA004775	ENSGALG00010028849	ENSGALG00010028849	case	control	0.095878625	0.787895581	0.121689507	0.903144924	0.979310476	up_in_case
CRA004775	ENSGALG00010028854	RPL7A	case	control	-0.249807252	0.278594575	-0.896669477	0.369895313	0.795234047	down_in_case
CRA004775	ENSGALG00010028855	SURF6	case	control	-0.038759931	0.254114304	-0.152529512	0.878769316	0.974981284	down_in_case
CRA004775	ENSGALG00010028856	ETV3	case	control	0.195142505	0.254731014	0.766072814	0.443632978	0.832130395	up_in_case
CRA004775	ENSGALG00010028857	MED22	case	control	-0.078687407	0.179746522	-0.437768732	0.661553939	0.918136475	down_in_case
CRA004775	ENSGALG00010028858	ENSGALG00010028858	case	control	0.722390673	0.838979146	0.861035315	0.389218593	0.805197062	up_in_case
CRA004775	ENSGALG00010028859	SURF1	case	control	-0.066765028	0.222750815	-0.299729671	0.764383364	0.94239774	down_in_case
CRA004775	ENSGALG00010028860	SURF2	case	control	0.098419601	0.287818712	0.341949974	0.73238854	0.936819725	up_in_case
CRA004775	ENSGALG00010028861	SURF4	case	control	-0.619822617	0.302454738	-2.049307018	0.040432103	0.376363211	down_in_case
CRA004775	ENSGALG00010028865	STKLD1	case	control	0.128004048	0.278094972	0.46028897	0.645308818	0.914043203	up_in_case
CRA004775	ENSGALG00010028867	REXO4	case	control	-0.139313026	0.234767567	-0.593408315	0.552907926	0.878546876	down_in_case
CRA004775	ENSGALG00010028869	ADAMTS13	case	control	0.161332266	0.284082211	0.567906965	0.570098142	0.88518436	up_in_case
CRA004775	ENSGALG00010028870	CACFD1	case	control	-0.666625889	0.423202366	-1.575194147	0.115211638	0.565776219	down_in_case
CRA004775	ENSGALG00010028871	PIP5K1A	case	control	-0.262265827	0.219826443	-1.193058595	0.232846426	0.699803243	down_in_case
CRA004775	ENSGALG00010028872	SLC2A6	case	control	-0.111386187	0.488594558	-0.227972632	0.819667517	0.960465449	down_in_case
CRA004775	ENSGALG00010028873	TMEM8C	case	control	0.252179599	0.80468492	0.313389244	0.753984955	0.941900693	up_in_case
CRA004775	ENSGALG00010028874	CRPL1	case	control	5.706505057	0.482342826	11.83080737	2.70527E-32	1.86001E-28	up_in_case
CRA004775	ENSGALG00010028875	DUSP23	case	control	-0.320123067	0.375513784	-0.852493517	0.393940236	0.808366404	down_in_case
CRA004775	ENSGALG00010028876	ADAMTSL2	case	control	-0.687999691	0.332377837	-2.069932513	0.038458665	0.370251617	down_in_case
CRA004775	ENSGALG00010028878	IGSF9	case	control	-0.232166877	0.160632515	-1.445329279	0.148365418	0.611192154	down_in_case
CRA004775	ENSGALG00010028881	SARDH	case	control	-0.070199223	0.546880801	-0.128362931	0.897861765	0.978790377	down_in_case
CRA004775	ENSGALG00010028884	VAV2	case	control	0.118204667	0.351903205	0.335901082	0.736945461	0.937564762	up_in_case
CRA004775	ENSGALG00010028885	BRD3	case	control	0.13341083	0.282909546	0.471567087	0.637235824	0.910421454	up_in_case
CRA004775	ENSGALG00010028887	SF3B4	case	control	-0.192682788	0.135192971	-1.425242646	0.154087073	0.619284425	down_in_case
CRA004775	ENSGALG00010028888	WDR5B	case	control	-0.070606303	0.181055316	-0.389970888	0.696558074	0.929092636	down_in_case
CRA004775	ENSGALG00010028889	FUBP3	case	control	0.044466944	0.144000254	0.308797677	0.757475437	0.941900693	up_in_case
CRA004775	ENSGALG00010028891	SLC31A2	case	control	-0.590432163	0.323350475	-1.82598205	0.067852966	0.463543284	down_in_case
CRA004775	ENSGALG00010028892	NIBAN2	case	control	0.35511911	0.248604658	1.428449143	0.153162623	0.617472961	up_in_case
CRA004775	ENSGALG00010028893	STXBP1	case	control	-0.098199029	0.164595523	-0.596608143	0.550769033	0.877827378	down_in_case
CRA004775	ENSGALG00010028894	AKNA	case	control	-0.480907985	0.361795688	-1.329225309	0.183773647	0.648064042	down_in_case
CRA004775	ENSGALG00010028896	ATP6V1G1	case	control	0.081070909	0.267385909	0.303198136	0.761738869	0.942236668	up_in_case
CRA004775	ENSGALG00010028897	TMEM268	case	control	0.077478579	0.508976379	0.152224312	0.879010019	0.974981284	up_in_case
CRA004775	ENSGALG00010028898	TNFSF15	case	control	0.543112395	0.359315413	1.511519895	0.130656044	0.586275434	up_in_case
CRA004775	ENSGALG00010028899	TNFSF8	case	control	0.527810328	0.520311764	1.014411674	0.310386356	0.762244121	up_in_case
CRA004775	ENSGALG00010028900	TNC	case	control	-0.075550033	0.520623418	-0.14511455	0.884620431	0.976277331	down_in_case
CRA004775	ENSGALG00010028901	PAPPA	case	control	-0.225744487	0.709847092	-0.318018472	0.750470925	0.941495694	down_in_case
CRA004775	ENSGALG00010028903	ASTN2	case	control	-0.010110545	0.353867537	-0.028571553	0.9772063	0.995817685	down_in_case
CRA004775	ENSGALG00010028904	TRIM32	case	control	-0.088963068	0.160648837	-0.553773497	0.579733869	0.888438428	down_in_case
CRA004775	ENSGALG00010028905	COLGALT1	case	control	-0.160769348	0.19577152	-0.821209067	0.411527193	0.817006008	down_in_case
CRA004775	ENSGALG00010028909	ENSGALG00010028909	case	control	0.291063784	0.360890523	0.806515454	0.419945701	0.819388688	up_in_case
CRA004775	ENSGALG00010028912	EXFABP	case	control	-2.673557649	0.654807969	-4.082964437	4.44648E-05	0.013292084	down_in_case
CRA004775	ENSGALG00010028914	TLR4	case	control	-0.880375696	0.516159349	-1.705627725	0.088077414	0.513317894	down_in_case
CRA004775	ENSGALG00010028915	ADGRD2	case	control	0.178934616	0.532265317	0.336175606	0.736738447	0.937564762	up_in_case
CRA004775	ENSGALG00010028916	NR5A1	case	control	1.752238045	0.630294314	2.780031495	0.005435363	0.14978291	up_in_case
CRA004775	ENSGALG00010028917	NR6A1	case	control	-0.344970396	0.240727769	-1.43303117	0.15184893	0.616314827	down_in_case
CRA004775	ENSGALG00010028918	AGPAT2	case	control	0.12651219	0.421816289	0.299922485	0.764236283	0.94239774	up_in_case
CRA004775	ENSGALG00010028919	EGFL7	case	control	-0.264356976	0.284792966	-0.928242644	0.353281718	0.787099305	down_in_case
CRA004775	ENSGALG00010028921	MRPL41	case	control	-0.269929721	0.280854188	-0.961102707	0.336500529	0.776900397	down_in_case
CRA004775	ENSGALG00010028923	ENSGALG00010028923	case	control	-0.529384345	0.486306353	-1.088582005	0.276338256	0.731001618	down_in_case
CRA004775	ENSGALG00010028924	TMEM203	case	control	-0.046922787	0.362090687	-0.129588496	0.896892006	0.978790377	down_in_case
CRA004775	ENSGALG00010028925	ENSGALG00010028925	case	control	-1.293687029	0.572479345	-2.259796864	0.023833861	0.298798294	down_in_case
CRA004775	ENSGALG00010028926	ENSGALG00010028926	case	control	2.279902139	1.527988235	1.49209404	0.135674494	0.593592099	up_in_case
CRA004775	ENSGALG00010029140	NPDC1	case	control	0.011696728	0.362957566	0.032226158	0.974291696	0.994947809	up_in_case
CRA004775	ENSGALG00010029142	ENSGALG00010029142	case	control	-0.043454314	0.306955572	-0.141565483	0.887423233	0.977118922	down_in_case
CRA004775	ENSGALG00010029143	SH2D3C	case	control	0.166276415	0.282563429	0.588456957	0.556225617	0.87981923	up_in_case
CRA004775	ENSGALG00010029144	ENSGALG00010029144	case	control	-0.108236393	0.505213076	-0.214239096	0.830360609	0.963232966	down_in_case
CRA004775	ENSGALG00010029145	RC3H2	case	control	0.262356948	0.369447501	0.710133231	0.477621521	0.846563638	up_in_case
CRA004775	ENSGALG00010029146	NEK6	case	control	0.069076262	0.197643211	0.349499796	0.726714124	0.935238044	up_in_case
CRA004775	ENSGALG00010029147	RABL6	case	control	-0.262914142	0.173526262	-1.515125949	0.129740511	0.584172159	down_in_case
CRA004775	ENSGALG00010029148	RALGDS	case	control	-0.314399179	0.367603131	-0.855267956	0.392402823	0.80746892	down_in_case
CRA004775	ENSGALG00010029149	ENSGALG00010029149	case	control	0.266208542	0.415818712	0.640203373	0.52204039	0.866416245	up_in_case
CRA004775	ENSGALG00010029150	NARF	case	control	0.332688407	0.29158703	1.140957497	0.253887611	0.718204856	up_in_case
CRA004775	ENSGALG00010029151	CYBC1	case	control	-0.030918937	0.302808462	-0.102107241	0.918671554	0.98189977	down_in_case
CRA004775	ENSGALG00010029152	HEXD	case	control	-0.834210087	0.257864603	-3.235070181	0.001216128	0.073346407	down_in_case
CRA004775	ENSGALG00010029153	OGFOD3	case	control	0.194803032	0.283623107	0.686837662	0.492185034	0.855746361	up_in_case
CRA004775	ENSGALG00010029154	UTS2R	case	control	0.205076548	0.429012194	0.478020323	0.63263574	0.909651573	up_in_case
CRA004775	ENSGALG00010029155	ENSGALG00010029155	case	control	-0.269021549	0.965198184	-0.278721565	0.780458511	0.948399168	down_in_case
CRA004775	ENSGALG00010029158	CD7	case	control	-0.912875005	0.548656648	-1.663836587	0.09614509	0.529546782	down_in_case
CRA004775	ENSGALG00010029160	DUS1L	case	control	0.100196404	0.198637602	0.504418113	0.613967593	0.902363507	up_in_case
CRA004775	ENSGALG00010029161	HNF1B	case	control	1.829473106	0.785434615	2.329249399	0.019845857	0.279077963	up_in_case
CRA004775	ENSGALG00010029162	ELN	case	control	0.354046509	0.525324898	0.673957223	0.50033851	0.859740701	up_in_case
CRA004775	ENSGALG00010029163	ANKFY1	case	control	-0.292668386	0.29637053	-0.987508394	0.32339347	0.768709353	down_in_case
CRA004775	ENSGALG00010029164	CYB5D2	case	control	-0.321260997	0.305242564	-1.052477718	0.292580428	0.746432925	down_in_case
CRA004775	ENSGALG00010029165	RAC3	case	control	-0.260760913	0.34339242	-0.75936712	0.447632977	0.834136489	down_in_case
CRA004775	ENSGALG00010029166	ZZEF1	case	control	0.314205765	0.275447824	1.140708831	0.253991111	0.718204856	up_in_case
CRA004775	ENSGALG00010029167	ENSGALG00010029167	case	control	0.279361905	0.332556455	0.840043548	0.40088397	0.811868062	up_in_case
CRA004775	ENSGALG00010029168	RFNG	case	control	0.170210904	0.223387322	0.76195418	0.446087351	0.833494028	up_in_case
CRA004775	ENSGALG00010029169	SBDS	case	control	0.237518306	0.207966367	1.142099608	0.253412618	0.717862912	up_in_case
CRA004775	ENSGALG00010029170	DCXR	case	control	-0.315893735	0.359851317	-0.877845156	0.380027756	0.800906556	down_in_case
CRA004775	ENSGALG00010029171	ENSGALG00010029171	case	control	-0.236805828	0.214834943	-1.102268673	0.270344884	0.728665115	down_in_case
CRA004775	ENSGALG00010029172	LRRC45	case	control	-0.275144473	0.160077697	-1.71881829	0.085647464	0.507059728	down_in_case
CRA004775	ENSGALG00010029173	ZNHIT3	case	control	-0.420611038	0.307924173	-1.36595654	0.171952605	0.636057362	down_in_case
CRA004775	ENSGALG00010029175	CENPX	case	control	-0.433196301	0.384302768	-1.127226598	0.259646688	0.720274683	down_in_case
CRA004775	ENSGALG00010029177	ASPSCR1	case	control	-0.119653901	0.183192319	-0.653160032	0.513653114	0.863954532	down_in_case
CRA004775	ENSGALG00010029178	NOTUM	case	control	0.301351612	0.394347395	0.764178022	0.444761162	0.832663137	up_in_case
CRA004775	ENSGALG00010029179	PIGW	case	control	-0.590718291	0.241163901	-2.449447402	0.01430756	0.239125488	down_in_case
CRA004775	ENSGALG00010029180	CA4	case	control	1.107485529	0.522544891	2.119407437	0.034056049	0.35069902	up_in_case
CRA004775	ENSGALG00010029181	MYO19	case	control	-0.165382658	0.150464948	-1.099144083	0.271705224	0.729164673	down_in_case
CRA004775	ENSGALG00010029182	HEATR6	case	control	-0.481227525	0.218224674	-2.20519301	0.027440553	0.316243014	down_in_case
CRA004775	ENSGALG00010029183	ENSGALG00010029183	case	control	-0.051206547	0.35795479	-0.143053112	0.886248235	0.976819452	down_in_case
CRA004775	ENSGALG00010029184	DYNLL2	case	control	-0.299347103	0.383817944	-0.77991951	0.435438254	0.826905159	down_in_case

CRA004775	ENSGALG00010029186	ZNF302	case	control	0.267830075	0.36726754	0.729250602	0.465848383	0.843390341	up_in_case
CRA004775	ENSGALG00010029187	SRSF1	case	control	-0.391401341	0.221400377	-1.767844063	0.077086976	0.488700005	down_in_case
CRA004775	ENSGALG00010029188	ENSGALG00010029188	case	control	0.175619751	0.37555094	0.467632303	0.64004756	0.911484465	up_in_case
CRA004775	ENSGALG00010029189	ENSGALG00010029189	case	control	0.295980084	0.68981118	0.42907406	0.667869329	0.919688403	up_in_case
CRA004775	ENSGALG00010029190	DHRS7C	case	control	0.023460105	0.622246387	0.037702277	0.969925061	0.994366623	up_in_case
CRA004775	ENSGALG00010029191	GSG1L2	case	control	1.249160563	0.537564893	2.323739104	0.020139481	0.280632942	up_in_case
CRA004775	ENSGALG00010029192	DTX2	case	control	-0.343790805	0.258802324	-1.328391489	0.18404881	0.648604609	down_in_case
CRA004775	ENSGALG00010029194	DHRS11	case	control	0.461878172	0.259973851	1.77663319	0.075628611	0.48393161	up_in_case
CRA004775	ENSGALG00010029196	MRM1	case	control	-0.33436184	0.387419434	-0.863048703	0.38811069	0.804270424	down_in_case
CRA004775	ENSGALG00010029198	PPM1D	case	control	0.212732207	0.338409323	0.628623956	0.529595273	0.869211096	up_in_case
CRA004775	ENSGALG00010029199	APPBP2	case	control	0.185316065	0.237187571	0.781305969	0.43462256	0.82652173	up_in_case
CRA004775	ENSGALG00010029200	GAS7	case	control	-0.089919198	0.29794818	-0.301794755	0.76280853	0.94239774	down_in_case
CRA004775	ENSGALG00010029201	PIGS	case	control	-0.217901232	0.140647827	-1.549268386	0.121317216	0.573473028	down_in_case
CRA004775	ENSGALG00010029202	PSMD12	case	control	-0.106170842	0.333320155	-0.31852512	0.750086643	0.941495694	down_in_case
CRA004775	ENSGALG00010029203	ALDOC	case	control	-0.346788073	0.368568017	-0.940906584	0.346752734	0.784552667	down_in_case
CRA004775	ENSGALG00010029204	HELZ	case	control	0.323984604	0.292110107	1.10911809	0.267379242	0.72734559	up_in_case
CRA004775	ENSGALG00010029205	CACNG1	case	control	-0.564457346	0.786421182	-0.717754504	0.47290867	0.845445211	down_in_case
CRA004775	ENSGALG00010029206	CACNG4	case	control	0.691575702	0.716836569	0.964760634	0.334664718	0.775004131	up_in_case
CRA004775	ENSGALG00010029207	SPAG5	case	control	0.232750158	0.511135012	0.455359449	0.648850663	0.915047477	up_in_case
CRA004775	ENSGALG00010029209	PRKCA	case	control	0.719183362	0.293786764	2.447977411	0.014366066	0.23945185	up_in_case
CRA004775	ENSGALG00010029210	RSKR	case	control	-0.964376648	0.588481218	-1.638755185	0.101264256	0.540680709	down_in_case
CRA004775	ENSGALG00010029211	SDF2	case	control	-0.306138833	0.258322195	-1.185104645	0.235976075	0.703274167	down_in_case
CRA004775	ENSGALG00010029212	SUPT6H	case	control	-0.018461111	0.241246634	-0.076523805	0.939002376	0.986946546	down_in_case
CRA004775	ENSGALG00010029213	APOH	case	control	2.509489573	0.605465808	4.144725501	3.40221E-05	0.012311524	up_in_case
CRA004775	ENSGALG00010029214	ENSGALG00010029214	case	control	1.037368259	0.741845743	1.398361141	0.162004646	0.627882156	up_in_case
CRA004775	ENSGALG00010029215	ENSGALG00010029215	case	control	0.148326202	0.659791213	0.224807786	0.822128809	0.960766569	up_in_case
CRA004775	ENSGALG00010029216	RAB34	case	control	-0.208572946	0.262189704	-0.795503951	0.426320414	0.822541484	down_in_case
CRA004775	ENSGALG00010029217	CEP112	case	control	-0.178030091	0.291579718	-0.6105709	0.541483693	0.87495415	down_in_case
CRA004775	ENSGALG00010029218	RPL23A	case	control	-0.20532404	0.26669258	-0.769890334	0.441364948	0.829353567	down_in_case
CRA004775	ENSGALG00010029219	TLCD1	case	control	-0.703731483	0.340614361	-2.066065217	0.038822322	0.370839453	down_in_case
CRA004775	ENSGALG00010029220	NEK8	case	control	-0.111792262	0.253855332	-0.440377836	0.659663475	0.917907174	down_in_case
CRA004775	ENSGALG00010029221	AXIN2	case	control	0.214766918	0.381432683	0.563053266	0.573398613	0.886730131	up_in_case
CRA004775	ENSGALG00010029222	TRAF4	case	control	-0.021297873	0.226338991	-0.094097233	0.925031918	0.984140717	down_in_case
CRA004775	ENSGALG00010029223	NME2	case	control	-0.109453736	0.214755571	-0.509666573	0.610285075	0.901413878	down_in_case
CRA004775	ENSGALG00010029224	FAM222B	case	control	0.087813418	0.17864124	0.491562965	0.62302833	0.906441769	up_in_case
CRA004775	ENSGALG00010029225	ENSGALG00010029225	case	control	0.539560668	0.665373761	0.810913655	0.417415258	0.818943344	up_in_case
CRA004775	ENSGALG00010029226	MBTD1	case	control	0.163061859	0.267969751	0.608508455	0.5428503	0.875218017	up_in_case
CRA004775	ENSGALG00010029227	UTP18	case	control	-0.057712444	0.178238637	-0.323793117	0.746094668	0.939637341	down_in_case
CRA004775	ENSGALG00010029228	ERAL1	case	control	-0.231466648	0.2104087	-1.100081169	0.271296758	0.728872973	down_in_case
CRA004775	ENSGALG00010029229	CBX4	case	control	-0.075972507	0.25268307	-0.300663227	0.763671313	0.94239774	down_in_case
CRA004775	ENSGALG00010029230	PPM1E	case	control	0.801878271	0.404293643	1.983405587	0.047322144	0.404178134	up_in_case
CRA004775	ENSGALG00010029231	CBX8	case	control	-0.358054795	0.290023747	-1.23457061	0.216990353	0.684522675	down_in_case
CRA004775	ENSGALG00010029233	CBX2	case	control	-0.494082626	0.485460839	-1.017760004	0.308792024	0.76124043	down_in_case
CRA004775	ENSGALG00010029234	TOM1L1	case	control	0.588502896	0.608135721	0.967716376	0.333186037	0.773910112	up_in_case
CRA004775	ENSGALG00010029235	TRIM37	case	control	0.149288505	0.188138643	0.793502616	0.427485046	0.822776983	up_in_case
CRA004775	ENSGALG00010029237	COX11	case	control	0.17557244	0.262878712	0.667883825	0.504207753	0.861471523	up_in_case
CRA004775	ENSGALG00010029238	CAMKK1	case	control	-0.129319843	0.430552154	-0.300358137	0.763903992	0.94239774	down_in_case
CRA004775	ENSGALG00010029239	RBFOX3	case	control	-0.205077903	0.497396193	-0.412302921	0.680117408	0.923896221	down_in_case
CRA004775	ENSGALG00010029240	MIS12	case	control	0.378489914	0.401363904	0.943009349	0.345676121	0.784552667	up_in_case
CRA004775	ENSGALG00010029241	STXBP4	case	control	-0.0512233	0.360341218	-0.142152208	0.886959781	0.977118922	down_in_case
CRA004775	ENSGALG00010029242	DERL2	case	control	-0.244960916	0.217449779	-1.1265172	0.259946664	0.720816008	down_in_case
CRA004775	ENSGALG00010029243	NME1	case	control	-0.725495441	0.57282536	-1.266521162	0.205326558	0.674045742	down_in_case
CRA004775	ENSGALG00010029244	HLF	case	control	0.618950659	0.338705542	1.827400444	0.067639583	0.463543284	up_in_case
CRA004775	ENSGALG00010029245	SKA2	case	control	-0.173928144	0.269929591	-0.64434634	0.519350873	0.865611895	down_in_case
CRA004775	ENSGALG00010029246	DHX33	case	control	-0.216567532	0.217940548	-0.993700043	0.320368922	0.767037518	down_in_case
CRA004775	ENSGALG00010029247	CCDC92B	case	control	0.339552118	0.692925764	0.49002669	0.624115013	0.906441769	up_in_case
CRA004775	ENSGALG00010029248	C1QBP	case	control	-0.192620929	0.24316482	-0.792141431	0.428278215	0.823075979	down_in_case
CRA004775	ENSGALG00010029249	PRR11	case	control	0.21245406	0.473381855	0.4488006	0.653575506	0.916470479	up_in_case
CRA004775	ENSGALG00010029250	SPAG9	case	control	0.238177902	0.289937949	0.821478881	0.411373549	0.816866089	up_in_case
CRA004775	ENSGALG00010029251	ENGASE	case	control	-0.318521675	0.319958012	-0.995510857	0.319487866	0.767037518	down_in_case
CRA004775	ENSGALG00010029252	RPAIN	case	control	-0.114564916	0.322631013	-0.355095796	0.722517826	0.935185555	down_in_case
CRA004775	ENSGALG00010029253	NUP88	case	control	0.032496942	0.214467768	0.151523665	0.879562644	0.974996282	up_in_case
CRA004775	ENSGALG00010029254	MMD	case	control	0.46001446	0.237527267	1.936680641	0.052784394	0.422982632	up_in_case
CRA004775	ENSGALG00010029255	RAP1GAP2	case	control	-0.349542612	0.439206412	-0.795850432	0.426118975	0.822541484	down_in_case
CRA004775	ENSGALG00010029256	RABEP1	case	control	0.26562184	0.311610961	0.852414943	0.39398383	0.808366404	up_in_case
CRA004775	ENSGALG00010029257	YPEL2	case	control	-0.048396628	0.476997441	-0.101460981	0.91918453	0.982029871	down_in_case
CRA004775	ENSGALG00010029258	TMEM100	case	control	-0.173292233	0.441398121	-0.392598483	0.69461606	0.928788938	down_in_case
CRA004775	ENSGALG00010029259	PTRH2	case	control	-0.362982051	0.259102493	-1.400920721	0.161237782	0.627121149	down_in_case
CRA004775	ENSGALG00010029260	DHX40	case	control	-0.227817958	0.287399861	-0.792686387	0.427960564	0.822830776	down_in_case
CRA004775	ENSGALG00010029261	IFT22	case	control	-0.081428063	0.258245124	-0.31531307	0.752523962	0.94167946	down_in_case
CRA004775	ENSGALG00010029262	CLTC	case	control	-0.19604619	0.308369746	-0.635750403	0.524939119	0.867302346	down_in_case
CRA004775	ENSGALG00010029263	TOB1	case	control	0.672124155	0.549839058	1.22240162	0.221555787	0.689550502	up_in_case
CRA004775	ENSGALG00010029264	COL26A1	case	control	-0.629820265	0.527348303	-1.194315525	0.232354567	0.699289485	down_in_case
CRA004775	ENSGALG00010029266	CANT1	case	control	-0.018490039	0.176031675	-0.105038137	0.916345547	0.981626545	down_in_case
CRA004775	ENSGALG00010029267	ENSGALG00010029267	case	control	-0.490327922	0.232798961	-2.106228998	0.035184465	0.355489775	down_in_case
CRA004775	ENSGALG00010029268	PCTP	case	control	0.196065562	0.342198962	0.572957794	0.566673271	0.883033678	up_in_case
CRA004775	ENSGALG00010029269	WSCD1	case	control	-0.052494736	0.427344649	-0.122839344	0.902234318	0.979310476	down_in_case
CRA004775	ENSGALG00010029270	TIMP2	case	control	0.267573414	0.308593092	0.86707519	0.385900813	0.803541109	up_in_case
CRA004775	ENSGALG00010029271	MYL10	case	control	0.52343071	0.784364236	0.667331179	0.504560615	0.861471523	up_in_case
CRA004775	ENSGALG00010029273	SLC13A5	case	control	-0.280342173	0.643348982	-0.435754436	0.663014905	0.918295135	down_in_case
CRA004775	ENSGALG00010029274	VMP1	case	control	-0.446480715	0.229755781	-1.943283923	0.051981863	0.419978024	down_in_case
CRA004775	ENSGALG00010029275	PCYT2	case	control	-0.110853121	0.230629765	-0.48065401	0.630762422	0.909338686	down_in_case
CRA004775	ENSGALG00010029276	TUBD1	case	control	0.006113546	0.324363611	0.018847816	0.984962509	0.997562105	up_in_case
CRA004775	ENSGALG00010029277	WFIKKN2	case	control	-1.253484032	0.956368889	-1.310670021	0.189969271	0.655361714	down_in_case
CRA004775	ENSGALG00010029278	MED31	case	control	-0.401400239	0.224056984	-1.791509602	0.073211555	0.477576895	down_in_case
CRA004775	ENSGALG00010029279	ENSGALG00010029279	case	control	-0.260551527	0.30369556	-0.857936568	0.390927491	0.806479902	down_in_case
CRA004775	ENSGALG00010029280	USP36	case	control	-0.074080584	0.313689997	-0.236158579	0.813309619	0.958257268	down_in_case
CRA004775	ENSGALG00010029281	KIAA0753	case	control	-0.272570722	0.382679534	-0.712268877	0.476298293	0.846367006	down_in_case
CRA004775	ENSGALG00010029282	LUC7L3	case	control	0.077765443	0.186927414	0.416019466	0.677395759	0.922924485	up_in_case
CRA004775	ENSGALG00010029283	RPS6KB1	case	control	0.207322462	0.176309853	1.175898329	0.239635507	0.706254731	up_in_case
CRA004775	ENSGALG00010029284	CYTH1	case	control	-0.219161817	0.184639917	-1.186968785	0.235239933	0.702145501	down_in_case
CRA004775	ENSGALG00010029285	CUX1	case	control	0.042859442	0.181145398	0.236602432	0.812965233	0.958114791	up_in_case
CRA004775	ENSGALG00010029286	MAFG	case	control	-0.075950468	0.273886782	-0.277306074	0.781545096	0.948910196	down_in_case
CRA004775	ENSGALG00010029287	ANKRD40	case	control	0.080910627	0.306422341	0.264049372	0.791741875	0.951284723	up_in_case
CRA004775	ENSGALG00010029288	PYCR1	case	control	-0.963578798	0.407133424	-2.366739604	0.017945554	0.266202063	down_in_case
CRA004775	ENSGALG00010029289	PITPNM3	case	control	-0.139068629	0.334078495	-0.416275309	0.677208558	0.922924485	down_in_case
CRA004775	ENSGALG00010029290	ENSGALG00010029290	case	control	-0.520542569	0.600037702	-0.867516438	0.385659109	0.803514911	down_in_case
CRA004775	ENSGALG00010029291	RNFT1	case	control	-0.143446905	0.157984775	-0.907979296	0.363889161	0.793608387	down_in_case
CRA004775	ENSGALG00010029292	ENSGALG00010029292	case	control	-0.393097489	0.413726735	-0.950137991	0.342042141	0.781616177	down_in_case
CRA004775	ENSGALG00010029293	DNAH17	case	control	-0.701541995	0.325317078	-2.156486833	0.031045671	0.336379059	down_in_case
CRA004775	ENSGALG00010029294	PGS1	case	control	-0.295113312	0.154387423	-1.911511355	0.055938899	0.433117004	down_in_case
CRA004775	ENSGALG00010029295	SOCS3	case	control	-1.016901954	0.511020163	-1.989944874	0.046597008	0.401475853	down_in_case
CRA004775	ENSGALG00010029296	FBXO39	case	control	-0.13471657	0.291130353	-0.46273626	0.643553424	0.913638565	down_in_case
CRA004775	ENSGALG00010029297	SH2B2	case	control	0.100330884	0.633075963	0.158481588	0.874077328	0.974115968	up_in_case
CRA004775	ENSGALG00010029298	ENDOV	case	control	-0.378968665	0.174833841	-2.167593321	0.030189642	0.331325011	down_in_case
CRA004775	ENSGALG00010029299	PRKRIP1	case	control	0.083851978	0.237578979	0.352943592	0.72413073	0.935238044	up_in_case
CRA004775	ENSGALG00010029300	TEKT1	case	control	0.352778326	0.493630136	0.714661242	0.474818392	0.84579952	up_in_case
CRA004775	ENSGALG00010029301	RNF213	case	control	-0.707810009	0.42731779	-1.656401924	0.097640452	0.532395151	down_in_case
CRA004775	ENSGALG00010029303	SLC26A11	case	control	-0.055558129	0.37047283	-0.149965463	0.880791864	0.974996282	down_in_case
CRA004775	ENSGALG00010029304	ORAI3	case	control	-0.512925212	0.284230286	-1.804611389	0.071135515	0.47030027	down_in_case
CRA004775	ENSGALG00010029305	SMTNL2	case	control	0.070952442	0.460254151	0.154159265	0.87748416	0.974479909	up_in_case
CRA004775	ENSGALG00010029306	CEP95	case	control	-0.114951839	0.284118132	-0.404591704	0.685777658	0.926686318	down_in_case
CRA004775	ENSGALG00010029307	SGSH	case	control	0.169933164	0.287223417	0.591641049	0.55409098	0.879382329	up_in_case
CRA004775	ENSGALG00010029308	GAA	case	control	-0.03840075	0.291337153	-0.131808627	0.895135663	0.977836869	down_in_case
CRA004775	ENSGALG00010029309	ALKBH4	case	control	-0.213810559	0.344323412	-0.620958529	0.534626909	0.870981055	down_in_case
CRA004775	ENSGALG00010029310	LRWD1	case	control	-0.477285556	0.13110692	-3.640429924	0.000272183	0.033417776	down_in_case
CRA004775	ENSGALG00010029311	ENSGALG00010029311	case	control	-0.06414357	0.35008621	-0.183222212	0.854623667	0.97035175	down_in_case
CRA004775	ENSGALG00010029312	SMURF2	case	control	-0.052306917	0.297639086	-0.175739409	0.860498673	0.971325143	down_in_case
CRA004775	ENSGALG00010029313	CCDC40	case	control	0.433571213	0.70378013	0.616060605	0.537854521	0.872603995	up_in_case
CRA004775	ENSGALG00010029314	MED13	case	control	0.407678836	0.230517077	1.768540717	0.076970552	0.488700005	up_in_case
CRA004775	ENSGALG00010029315	TBC1D16	case	control	-0.070891579	0.281280792	-0.252031356	0.801016828	0.953541667	down_in_case
CRA004775	ENSGALG00010029316	INTS2	case	control	-0.626255358	0.244371125	-2.562722412	0.010385505	0.204600391	down_in_case
CRA004775	ENSGALG00010029317	KPNA2	case	control	0.142626282	0.521484788	0.273500368	0.784468609	0.950165405	up_in_case
CRA004775	ENSGALG00010029318	C17orf58	case	control	-0.346461148	0.537098494	-0.645060732	0.51888783	0.865435399	down_in_case
CRA004775	ENSGALG00010029319	BRIP1	case	control	-0.575617531	0.54074335	-1.064493037	0.287105407	0.741909972	down_in_case
CRA004775	ENSGALG00010029321	TBX2	case	control	0.205421304	0.299363414	0.686193751	0.492590938	0.855763086	up_in_case

CRA004775	ENSGALG00010029322	BPTF	case	control	0.29147892	0.311318329	0.936272919	0.349132688	0.785311667	up_in_case
CRA004775	ENSGALG00010029323	NOL11	case	control	0.267440768	0.209355477	1.277448157	0.201444072	0.669290466	up_in_case
CRA004775	ENSGALG00010029324	BCAS3	case	control	-0.024894957	0.268543895	-0.092703493	0.926139122	0.98417264	down_in_case
CRA004775	ENSGALG00010029325	LRRC75A	case	control	-0.505710965	0.491323041	-1.029284041	0.30334622	0.755679718	down_in_case
CRA004775	ENSGALG00010029326	NCBP3	case	control	-0.043086946	0.18128342	-0.237677255	0.812131426	0.957585764	down_in_case
CRA004775	ENSGALG00010029327	CRK	case	control	0.297489426	0.132144961	2.251235484	0.024370627	0.30145607	up_in_case
CRA004775	ENSGALG00010029328	ENSGALG00010029328	case	control	0.02239308	0.316907946	0.07066115	0.943667441	0.987862092	up_in_case
CRA004775	ENSGALG00010029329	ENSGALG00010029329	case	control	-0.482506035	0.32956569	-1.464066345	0.143175822	0.603744472	down_in_case
CRA004775	ENSGALG00010029330	AFMID	case	control	0.158642839	0.443385255	0.357799086	0.720493675	0.934406529	up_in_case
CRA004775	ENSGALG00010029331	TK1	case	control	0.174324779	0.507028475	0.343816547	0.730984256	0.936441634	up_in_case
CRA004775	ENSGALG00010029332	YWHAE	case	control	-0.157191773	0.199318762	-0.788645141	0.43031944	0.824254439	down_in_case
CRA004775	ENSGALG00010029333	ENSGALG00010029333	case	control	0.131992849	0.290153472	0.454907012	0.649176138	0.915047477	up_in_case
CRA004775	ENSGALG00010029336	SYNGR2	case	control	-0.634849219	0.363439408	-1.74678146	0.080675254	0.495252417	down_in_case
CRA004775	ENSGALG00010029337	MYO1C	case	control	0.250578813	0.276273002	0.906997105	0.364408328	0.793608387	up_in_case
CRA004775	ENSGALG00010029338	INPP5K	case	control	0.019297872	0.29713738	0.064945958	0.948217028	0.989972846	up_in_case
CRA004775	ENSGALG00010029339	PITPNA	case	control	0.279746418	0.198418134	1.40988332	0.158574139	0.624442434	up_in_case
CRA004775	ENSGALG00010029340	NDEL1	case	control	-0.066026327	0.260605134	-0.253357738	0.799991783	0.952993446	down_in_case
CRA004775	ENSGALG00010029341	SLC43A2	case	control	0.220097795	0.599913353	0.36688264	0.713706561	0.933244709	up_in_case
CRA004775	ENSGALG00010029342	TMC6	case	control	-0.40480422	0.37303697	-1.085158449	0.277851484	0.733205863	down_in_case
CRA004775	ENSGALG00010029343	SCARF1	case	control	-0.067926626	0.237445857	-0.286072063	0.774822928	0.946319184	down_in_case
CRA004775	ENSGALG00010029344	ENSGALG00010029344	case	control	-0.49319061	0.399123598	-1.235683916	0.216576079	0.684157514	down_in_case
CRA004775	ENSGALG00010029345	RILP	case	control	-0.550777149	0.558116527	-0.986849739	0.323716307	0.769171227	down_in_case
CRA004775	ENSGALG00010029346	ARL16	case	control	-0.165929773	0.419537345	-0.395506561	0.692469081	0.927867527	down_in_case
CRA004775	ENSGALG00010029347	PRPF8	case	control	-0.263228799	0.241507499	-1.089940476	0.275739365	0.731001618	down_in_case
CRA004775	ENSGALG00010029348	HGS	case	control	-0.323280386	0.219624627	-1.471967832	0.141029564	0.600619496	down_in_case
CRA004775	ENSGALG00010029349	MYH10	case	control	0.410507091	0.259836511	1.579866856	0.114137361	0.564321671	up_in_case
CRA004775	ENSGALG00010029350	MRPL12	case	control	-0.096098092	0.359280407	-0.26747379	0.789104392	0.95041797	down_in_case
CRA004775	ENSGALG00010029351	ENSGALG00010029351	case	control	0.041275612	0.285581526	0.144531802	0.885080546	0.976602443	up_in_case
CRA004775	ENSGALG00010029352	AATF	case	control	-0.410925166	0.20506186	-2.003908315	0.045079883	0.394836603	down_in_case
CRA004775	ENSGALG00010029353	GCGR	case	control	-1.3270909	0.556875081	-2.383103401	0.017167372	0.259292146	down_in_case
CRA004775	ENSGALG00010029354	TLCD2	case	control	0.683581344	0.344177396	1.986130851	0.047018797	0.402720701	up_in_case
CRA004775	ENSGALG00010029355	MCRIP1	case	control	0.393619202	0.445499712	0.883545358	0.376941687	0.798523018	up_in_case
CRA004775	ENSGALG00010029357	ARHGDIA	case	control	-0.187534084	0.124190016	-1.510057657	0.131028713	0.586487428	down_in_case
CRA004775	ENSGALG00010029359	ENSGALG00010029359	case	control	-0.873829687	0.571777809	-1.528267927	0.12644603	0.580747947	down_in_case
CRA004775	ENSGALG00010029360	ACACA	case	control	-0.057729812	0.215794551	-0.267522104	0.789067197	0.95041797	down_in_case
CRA004775	ENSGALG00010029361	ALYREF	case	control	-0.314543227	0.21700019	-1.449506688	0.147196134	0.610295079	down_in_case
CRA004775	ENSGALG00010029362	TADA2A	case	control	-0.867895701	0.27370992	-3.170859507	0.001519886	0.080693373	down_in_case
CRA004775	ENSGALG00010029363	PIK3R6	case	control	-0.944953786	0.481683581	-1.961772882	0.049788932	0.413341381	down_in_case
CRA004775	ENSGALG00010029364	ANAPC11	case	control	-0.265961074	0.375408837	-0.708457148	0.478661414	0.847113656	down_in_case
CRA004775	ENSGALG00010029365	PIK3R5	case	control	-0.979313164	0.370523318	-2.64305407	0.008216191	0.183215969	down_in_case
CRA004775	ENSGALG00010029366	MGAT5B	case	control	0.482518568	0.595677133	0.810033727	0.417920792	0.818943344	up_in_case
CRA004775	ENSGALG00010029367	DUSP14	case	control	0.085220723	0.261875674	0.325424358	0.744859923	0.939218065	up_in_case
CRA004775	ENSGALG00010029368	DDX52	case	control	-0.142768121	0.225601365	-0.63283359	0.526842319	0.86830001	down_in_case
CRA004775	ENSGALG00010029369	MFSD11	case	control	-0.268446177	0.23239352	-1.155136242	0.248034639	0.712818804	down_in_case
CRA004775	ENSGALG00010029370	SRSF2	case	control	-0.124712237	0.220478398	-0.56564379	0.571635954	0.885498028	down_in_case
CRA004775	ENSGALG00010029371	AUTS2	case	control	0.082285082	0.337867485	0.243542469	0.80758519	0.955397873	up_in_case
CRA004775	ENSGALG00010029372	METTL23	case	control	-0.188096722	0.320234706	-0.587371445	0.556954268	0.880409028	down_in_case
CRA004775	ENSGALG00010029373	GATSL2	case	control	0.314370203	0.567254575	0.554195976	0.579444732	0.888438428	up_in_case
CRA004775	ENSGALG00010029374	RCC1L	case	control	-0.273843848	0.194887496	-1.405138107	0.159980214	0.625090553	down_in_case
CRA004775	ENSGALG00010029375	JMJD6	case	control	0.170871135	0.347940418	0.491093089	0.62336061	0.906441769	up_in_case
CRA004775	ENSGALG00010029376	ENSGALG00010029376	case	control	-0.555940539	0.332935484	-1.669814621	0.094956047	0.527652809	down_in_case
CRA004775	ENSGALG00010029377	NCF1C	case	control	0.370274603	0.557674786	0.663961527	0.506714935	0.8622397	up_in_case
CRA004775	ENSGALG00010029378	ST6GALNAC1	case	control	-0.017265524	0.599068967	-0.028820595	0.977007675	0.995689064	down_in_case
CRA004775	ENSGALG00010029379	GTF2I	case	control	-0.02239153	0.376733093	-0.059436058	0.952604794	0.990418004	down_in_case
CRA004775	ENSGALG00010029380	RGS9	case	control	-0.391237855	0.337088596	-1.160638061	0.24578912	0.712046495	down_in_case
CRA004775	ENSGALG00010029382	GTF2IRD1	case	control	-0.262728414	0.422002902	-0.6225749	0.53356391	0.870554975	down_in_case
CRA004775	ENSGALG00010029383	GNA13	case	control	0.606278421	0.323131014	1.87626193	0.060619322	0.445397937	up_in_case
CRA004775	ENSGALG00010029384	WDR81	case	control	-0.405272205	0.240177076	-1.687389203	0.091528539	0.521441551	down_in_case
CRA004775	ENSGALG00010029385	CLIP2	case	control	0.196435448	0.289628801	0.678231748	0.497624766	0.857810713	up_in_case
CRA004775	ENSGALG00010029386	SLC16A6	case	control	0.236531172	0.428815772	0.551591586	0.581228203	0.888998848	up_in_case
CRA004775	ENSGALG00010029387	SERPINF1	case	control	-0.337091478	0.53964627	-0.624652661	0.532199049	0.869886675	down_in_case
CRA004775	ENSGALG00010029388	ST6GALNAC2	case	control	-0.368633603	0.362806083	-1.016062353	0.309599695	0.762002042	down_in_case
CRA004775	ENSGALG00010029389	SMYD4	case	control	-0.236598945	0.22510178	-1.051075409	0.293223957	0.746438942	down_in_case
CRA004775	ENSGALG00010029390	RPA1	case	control	-0.317854525	0.248136475	-1.280966551	0.200205415	0.667171435	down_in_case
CRA004775	ENSGALG00010029391	ARSG	case	control	-0.669298786	0.194556355	-3.440128119	0.000581439	0.05028532	down_in_case
CRA004775	ENSGALG00010029392	RTN4RL1	case	control	0.204044818	0.571760161	0.356871346	0.721188121	0.934511903	up_in_case
CRA004775	ENSGALG00010029394	CYGB	case	control	-0.523875069	0.355519885	-1.473546462	0.140603745	0.600007375	down_in_case
CRA004775	ENSGALG00010029395	DPH1	case	control	-0.341015152	0.168393876	-2.025104235	0.042856662	0.38854564	down_in_case
CRA004775	ENSGALG00010029396	WIPI1	case	control	0.240275741	0.309200985	0.777085949	0.437108064	0.827232725	up_in_case
CRA004775	ENSGALG00010029397	OVCA2	case	control	-0.489422364	0.267911467	-1.826806333	0.067728893	0.463543284	down_in_case
CRA004775	ENSGALG00010029398	RHBDF2	case	control	-0.262440997	0.207004272	-1.267804739	0.204867689	0.674045742	down_in_case
CRA004775	ENSGALG00010029399	PRKAR1A	case	control	0.079867421	0.18138723	0.440314467	0.659709364	0.917907174	up_in_case
CRA004775	ENSGALG00010029400	AANAT	case	control	0.910755251	0.632776671	1.439299665	0.150065638	0.61366707	up_in_case
CRA004775	ENSGALG00010029401	HIC1	case	control	0.204034562	0.457901235	0.445586399	0.655896037	0.917054032	up_in_case
CRA004775	ENSGALG00010029402	SMG6	case	control	-0.113743849	0.164615735	-0.690965835	0.489587012	0.854639532	down_in_case
CRA004775	ENSGALG00010029403	FAM20A	case	control	-0.202053958	0.314926389	-0.641591066	0.521138735	0.86621283	down_in_case
CRA004775	ENSGALG00010029404	SRR	case	control	-0.175996286	0.280611625	-0.627188152	0.530535909	0.869211096	down_in_case
CRA004775	ENSGALG00010029405	POLDIP2	case	control	-0.364902231	0.273586178	-1.333774366	0.182277814	0.647378665	down_in_case
CRA004775	ENSGALG00010029406	TMEM199	case	control	-0.371897347	0.209558773	-1.774668468	0.075952645	0.484761048	down_in_case
CRA004775	ENSGALG00010029407	UBE2O	case	control	0.181117027	0.286905877	0.631276811	0.527859546	0.86846095	up_in_case
CRA004775	ENSGALG00010029408	KIF12	case	control	0.176478892	0.431342767	0.409138406	0.682438093	0.924564673	up_in_case
CRA004775	ENSGALG00010029409	ENSGALG00010029409	case	control	0.246578618	0.422729455	0.583301248	0.559690522	0.880918893	up_in_case
CRA004775	ENSGALG00010029410	VTN	case	control	0.460065415	0.425955392	1.080078862	0.280107064	0.73661445	up_in_case
CRA004775	ENSGALG00010029411	SARM1	case	control	-1.252602597	0.629825771	-1.988808103	0.046722388	0.40180085	down_in_case
CRA004775	ENSGALG00010029412	SLC46A1	case	control	-0.49784195	0.285605783	-1.743108788	0.081314614	0.496834143	down_in_case
CRA004775	ENSGALG00010029413	B3GNTL1	case	control	0.10406878	0.360918489	0.288344275	0.773083219	0.946296525	up_in_case
CRA004775	ENSGALG00010029414	SLC13A2	case	control	2.467555317	0.608581658	4.054600208	5.02201E-05	0.013833034	up_in_case
CRA004775	ENSGALG00010029417	ENSGALG00010029417	case	control	-0.419525994	0.347679191	-1.206646831	0.227568183	0.694718454	down_in_case
CRA004775	ENSGALG00010029418	ABCA5	case	control	-0.140022822	0.258282272	-0.542130982	0.587728277	0.892529159	down_in_case
CRA004775	ENSGALG00010029419	RFC2	case	control	-0.389764317	0.290785101	-1.340386132	0.180119842	0.644990528	down_in_case
CRA004775	ENSGALG00010029420	LAT2	case	control	0.405050086	0.283077476	1.430880659	0.152464421	0.617173463	up_in_case
CRA004775	ENSGALG00010029421	TBCD	case	control	0.019433545	0.19322621	0.100574065	0.919888586	0.982171593	up_in_case
CRA004775	ENSGALG00010029422	EIF4H	case	control	0.088680673	0.154789973	0.572909673	0.566705855	0.883033678	up_in_case
CRA004775	ENSGALG00010029423	MYBBP1A	case	control	-0.09485224	0.240240253	-0.394822427	0.692973942	0.927867527	down_in_case
CRA004775	ENSGALG00010029424	MAP2K6	case	control	0.163554417	0.546947335	0.299031381	0.764916102	0.942843431	up_in_case
CRA004775	ENSGALG00010029425	SPNS2	case	control	0.002315049	0.383576894	0.006035423	0.995184458	0.999001196	up_in_case
CRA004775	ENSGALG00010029427	SPNS3	case	control	-1.301302011	0.427096998	-3.046853567	0.002312503	0.097543661	down_in_case
CRA004775	ENSGALG00010029428	FN3K	case	control	-0.096827294	0.420662816	-0.230177924	0.817953515	0.959889304	down_in_case
CRA004775	ENSGALG00010029429	UBE2G1	case	control	0.29751801	0.317275068	0.937728929	0.348383733	0.785270866	up_in_case
CRA004775	ENSGALG00010029430	FN3KRP	case	control	0.008740316	0.218248716	0.040047504	0.968055254	0.993568693	up_in_case
CRA004775	ENSGALG00010029431	ENSGALG00010029431	case	control	-0.080291196	1.872963713	-0.042868527	0.965806337	0.993094598	down_in_case
CRA004775	ENSGALG00010029432	ENSGALG00010029432	case	control	-0.313772997	1.817920492	-0.172599956	0.862965887	0.971910432	down_in_case
CRA004775	ENSGALG00010029434	ENSGALG00010029434	case	control	0.613561835	3.062555052	0.20034312			up_in_case
CRA004775	ENSGALG00010029436	WDR45B	case	control	-0.116897827	0.201261253	-0.580826292	0.561357533	0.881391577	down_in_case
CRA004775	ENSGALG00010029437	ENSGALG00010029437	case	control	0.252089024	1.409778651	0.178814613	0.858083271	0.971038554	up_in_case
CRA004775	ENSGALG00010029438	ENSGALG00010029438	case	control	-1.012793329	0.509208428	-1.988956338	0.046706022	0.40180085	down_in_case
CRA004775	ENSGALG00010029439	ENSGALG00010029439	case	control	-0.369116723	0.299816845	-1.231140711	0.218270241	0.685728601	down_in_case
CRA004775	ENSGALG00010029440	FOXK2	case	control	0.492122984	0.36622127	1.343785914	0.179017626	0.644923737	up_in_case
CRA004775	ENSGALG00010029442	ENSGALG00010029442	case	control	-0.606838893	0.566549298	-1.071114014	0.284118168	0.740820744	down_in_case
CRA004775	ENSGALG00010029443	ENSGALG00010029443	case	control	-2.02864226	0.614362571	-3.302027754	0.000959886	0.0656686	down_in_case
CRA004775	ENSGALG00010029445	SPHK1	case	control	-0.178141833	0.280491264	-0.63510653	0.525358938	0.867484846	down_in_case
CRA004775	ENSGALG00010029446	PRPSAP1	case	control	-0.280336046	0.195758477	-1.432050605	0.152129339	0.616604893	down_in_case
CRA004775	ENSGALG00010029447	ENSGALG00010029447	case	control	0.254020675	0.614300281	0.413512224	0.679231369	0.923483345	up_in_case
CRA004775	ENSGALG00010029449	UBALD2	case	control	0.222881876	0.272880153	0.816775694	0.414056625	0.817589409	up_in_case
CRA004775	ENSGALG00010029451	ENSGALG00010029451	case	control	-0.221986377	0.459157072	-0.483465006	0.628765601	0.908586927	down_in_case
CRA004775	ENSGALG00010029452	ENSGALG00010029452	case	control	-1.585676914	1.112819175	-1.424918756	0.154180687	0.619284425	down_in_case
CRA004775	ENSGALG00010029453	ENSGALG00010029453	case	control	-6.992643989	1.316142353	-5.312984552	1.07844E-07	9.26853E-05	down_in_case
CRA004775	ENSGALG00010029454	ENSGALG00010029454	case	control	-0.73006738	0.53990056	-1.352225639	0.176303144	0.641590771	down_in_case
CRA004775	ENSGALG00010029455	ENSGALG00010029455	case	control	-1.162584618	0.476196826	-2.441395146	0.014630636	0.241689368	down_in_case
CRA004775	ENSGALG00010029456	RNF157	case	control	1.382129754	0.631243657	2.189534482			up_in_case
CRA004775	ENSGALG00010029457	ENSGALG00010029457	case	control	-0.282370073	0.209717031	-1.346433677	0.178162696	0.644368088	down_in_case
CRA004775	ENSGALG00010029458	ENSGALG00010029458	case	control	-0.620648643	0.868166463	-0.714895898	0.474673372	0.84579952	down_in_case
CRA004775	ENSGALG00010029460	ENSGALG00010029460	case	control	-0.896988412	0.275952719	-3.250514857	0.001151962	0.071729642	down_in_case
CRA004775	ENSGALG00010029461	ENSGALG00010029461	case	control	-1.364328043	0.431918618	-3.158761824	0.001584409	0.080693373	down_in_case
CRA004775	ENSGALG00010029462	ENSGALG00010029462	case	control	0.821128999	0.824682548	0.99569101	0.319400299	0.767037518	up_in_case
CRA004775	ENSGALG00010029463	ENSGALG00010029463	case	control	-0.954929335	0.567183243	-1.683634605	0.092252307	0.522041757	down_in_case

CRA004775	ENSGALG00010029464	ENSGALG00010029464	case	control	-0.40518139	0.646303465	-0.626921272	0.530710843	0.869211096	down_in_case
CRA004775	ENSGALG00010029465	ENSGALG00010029465	case	control	-3.637914973	1.754315797	-2.073694474	0.038107695	0.368767707	down_in_case
CRA004775	ENSGALG00010029466	SLC39A11	case	control	-0.544132466	0.261127447	-2.083781202	0.037180074	0.363933342	down_in_case
CRA004775	ENSGALG00010029468	ENSGALG00010029468	case	control	-0.49145116	0.933258929	-0.526596795	0.598473618	0.897049692	down_in_case
CRA004775	ENSGALG00010029469	ENSGALG00010029469	case	control	-0.505351169	0.689316631	-0.733119072	0.463485809	0.842456502	down_in_case
CRA004775	ENSGALG00010029470	SSTR2	case	control	-3.144244979	0.747439037	-4.206690877	2.59137E-05	0.010181131	down_in_case
CRA004775	ENSGALG00010029472	ENSGALG00010029472	case	control	0.59119957	0.687527234	0.859892585	0.389848256	0.80601464	up_in_case
CRA004775	ENSGALG00010029473	COG1	case	control	-0.532826364	0.337646171	-1.578061325	0.114551522	0.564488547	down_in_case
CRA004775	ENSGALG00010029477	FAM104A	case	control	-0.155148083	0.164142737	-0.945202241	0.344555636	0.783972357	down_in_case
CRA004775	ENSGALG00010029479	ENSGALG00010029479	case	control	-1.037412761	0.767184776	-1.352233248	0.176300711	0.641590771	down_in_case
CRA004775	ENSGALG00010029480	ENSGALG00010029480	case	control	-0.074644699	0.252125363	-0.29606184	0.767182856	0.944367689	down_in_case
CRA004775	ENSGALG00010029481	ENSGALG00010029481	case	control	-0.500283155	0.870048517	-0.575006043	0.56528721	0.882950982	down_in_case
CRA004775	ENSGALG00010029482	DDX25	case	control	-0.99193969	0.363636085	-2.727836236	0.006375125	0.15910044	down_in_case
CRA004775	ENSGALG00010029485	ENSGALG00010029485	case	control	-0.810498472	0.854199673	-0.948839596	0.3427022	0.782225706	down_in_case
CRA004775	ENSGALG00010029487	CDC42EP4	case	control	-0.456466041	0.404712934	-1.127876089	0.259372253	0.720037471	down_in_case
CRA004775	ENSGALG00010029489	ENSGALG00010029489	case	control	2.811530978	0.860809491	3.266147746	0.001090214	0.068768468	up_in_case
CRA004775	ENSGALG00010029491	ENSGALG00010029491	case	control	-0.072835586	0.319275705	-0.228127556	0.819547078	0.960465449	down_in_case
CRA004775	ENSGALG00010029492	SDK2	case	control	-0.571563882	0.494045231	-1.156905981	0.247310775	0.712046495	down_in_case
CRA004775	ENSGALG00010029494	MYH7	case	control	2.521785272	1.023181914	2.464649968	0.013714716	0.234005339	up_in_case
CRA004775	ENSGALG00010029495	RPL38	case	control	-0.025523179	0.215054262	-0.118682509	0.905526894	0.979739788	down_in_case
CRA004775	ENSGALG00010029496	TTYH2	case	control	0.039841602	0.316037669	0.126065991	0.899679688	0.978929349	up_in_case
CRA004775	ENSGALG00010029497	ENSGALG00010029497	case	control	-0.522501848	0.724790567	-0.720900453	0.470970764	0.845198132	down_in_case
CRA004775	ENSGALG00010029498	ENSGALG00010029498	case	control	0.054164169	0.371592915	0.145762115	0.884109185	0.976208708	up_in_case
CRA004775	ENSGALG00010029500	ENSGALG00010029500	case	control	2.677706619	0.659240623	4.061804636	4.86948E-05	0.013833034	up_in_case
CRA004775	ENSGALG00010029501	ENSGALG00010029501	case	control	0.055951246	0.238656554	0.234442527	0.814641447	0.958758519	up_in_case
CRA004775	ENSGALG00010029505	VEZF1	case	control	0.121303688	0.132770105	0.913637053	0.36090759	0.792531424	up_in_case
CRA004775	ENSGALG00010029506	OXLD1	case	control	-0.432308266	0.29220923	-1.479447677	0.139020706	0.599082959	down_in_case
CRA004775	ENSGALG00010029507	VPS37D	case	control	-0.061155601	0.429884278	-0.142260613	0.886874156	0.977118922	down_in_case
CRA004775	ENSGALG00010029508	ENSGALG00010029508	case	control	-0.674991093	0.418144063	-1.614254879	0.106472153	0.552224847	down_in_case
CRA004775	ENSGALG00010029509	DNAJC30	case	control	-0.853506128	0.408519939	-2.089264309	0.036683937	0.361347294	down_in_case
CRA004775	ENSGALG00010029510	PDE6G	case	control	-0.25703242	0.612215123	-0.419840037	0.674602315	0.92284684	down_in_case
CRA004775	ENSGALG00010029511	CUEDC1	case	control	0.028942383	0.161239236	0.179499629	0.857545409	0.970943344	up_in_case
CRA004775	ENSGALG00010029512	TSPAN10	case	control	-0.654115248	0.865497388	-0.75576802	0.449788294	0.835138918	down_in_case
CRA004775	ENSGALG00010029513	WBSCR22	case	control	-0.374160344	0.243748525	-1.535026085	0.124777421	0.577326485	down_in_case
CRA004775	ENSGALG00010029514	MRPS23	case	control	-0.357399643	0.25413857	-1.406317991	0.15962972	0.624907945	down_in_case
CRA004775	ENSGALG00010029515	NPLOC4	case	control	-0.142606512	0.27804121	-0.512897034	0.608023348	0.900943922	down_in_case
CRA004775	ENSGALG00010029516	ENSGALG00010029516	case	control	-0.732828271	0.533380784	-1.373930769	0.169463176	0.633147466	down_in_case
CRA004775	ENSGALG00010029517	MSI2	case	control	0.141650385	0.339887436	0.416756755	0.676856337	0.922924485	up_in_case
CRA004775	ENSGALG00010029518	FAAP100	case	control	-0.73099051	0.304667618	-2.399304904	0.016426231	0.25465287	down_in_case
CRA004775	ENSGALG00010029519	STX1A	case	control	-0.264789657	0.255493016	-1.036387067	0.300021602	0.75270882	down_in_case
CRA004775	ENSGALG00010029520	MKS1	case	control	-0.519003666	0.178058203	-2.914797837	0.00355919	0.125102734	down_in_case
CRA004775	ENSGALG00010029521	ACTG1	case	control	-0.206337435	0.197807546	-1.043122162	0.296891718	0.749368211	down_in_case
CRA004775	ENSGALG00010029522	AKAP1	case	control	0.033716101	0.22200138	0.151873384	0.879286801	0.974996282	up_in_case
CRA004775	ENSGALG00010029523	FSCN2	case	control	-1.688781463	0.729224316	-2.315860053	0.020565911	0.282237368	down_in_case
CRA004775	ENSGALG00010029524	ENSGALG00010029524	case	control	0.318641019	0.335394664	0.950047968	0.34208788	0.781616177	up_in_case
CRA004775	ENSGALG00010029525	SLC38A10	case	control	-0.435994555	0.290089002	-1.502968233	0.132847236	0.589506365	down_in_case
CRA004775	ENSGALG00010029526	BCL7B	case	control	-0.409286934	0.24604172	-1.663485906	0.09621521	0.529546782	down_in_case
CRA004775	ENSGALG00010029527	NDUFAF8	case	control	-0.264327817	0.411103541	-0.642971393	0.520242663	0.865611895	down_in_case
CRA004775	ENSGALG00010029528	TBL2	case	control	-0.372700997	0.1983136	-1.87935168	0.060196489	0.445397937	down_in_case
CRA004775	ENSGALG00010029529	TEPSIN	case	control	-0.102047701	0.225772789	-0.451992916	0.651274086	0.915825397	down_in_case
CRA004775	ENSGALG00010029530	ENSGALG00010029530	case	control	-0.744894207	1.148816232	-0.648401534	0.516725279	0.864913871	down_in_case
CRA004775	ENSGALG00010029531	CEP131	case	control	-0.11488409	0.248961339	-0.461453534	0.644473251	0.913812299	down_in_case
CRA004775	ENSGALG00010029532	EVI2A	case	control	0.506824455	0.508458602	0.996786078	0.318868357	0.767037518	up_in_case
CRA004775	ENSGALG00010029533	MLXIPL	case	control	1.514239058	0.404506536	3.743422969	0.00018153	0.027735809	up_in_case
CRA004775	ENSGALG00010029534	NF1	case	control	0.161546121	0.339862511	0.475327863	0.634553302	0.909837348	up_in_case
CRA004775	ENSGALG00010029535	ANGPT1L	case	control	2.737054966	1.180223436	2.31909898	0.02038967	0.281976732	up_in_case
CRA004775	ENSGALG00010029536	BAZ1B	case	control	-0.081018306	0.254354051	-0.318525713	0.750086193	0.941495694	down_in_case
CRA004775	ENSGALG00010029537	MYH1B	case	control	0.76616537	0.760618967	1.007291961	0.313794492	0.765098415	up_in_case
CRA004775	ENSGALG00010029538	FZD9	case	control	-0.570198837	0.522415798	-1.091465532	0.275068091	0.731001618	down_in_case
CRA004775	ENSGALG00010029539	FKBP6	case	control	-0.718608646	0.646390067	-1.111726005	0.266255975	0.727311193	down_in_case
CRA004775	ENSGALG00010029541	ENSGALG00010029541	case	control	-0.247936395	0.265327105	-0.934455584	0.350068937	0.785466132	down_in_case
CRA004775	ENSGALG00010029542	AATK	case	control	0.422580796	0.329349742	1.283076141	0.199465402	0.666548904	up_in_case
CRA004775	ENSGALG00010029543	NSUN5	case	control	-0.332328409	0.258921355	-1.283511005	0.199313107	0.666525907	down_in_case
CRA004775	ENSGALG00010029544	ENSGALG00010029544	case	control	-0.159834743	0.255034439	-0.626718274	0.530843923	0.869211096	down_in_case
CRA004775	ENSGALG00010029545	OMG	case	control	-0.527046898	0.375195237	-1.404727047	0.16010246	0.625090553	down_in_case
CRA004775	ENSGALG00010029546	WSB1	case	control	-0.100828659	0.199707073	-0.504882764	0.613641181	0.902282137	down_in_case
CRA004775	ENSGALG00010029547	HIP1	case	control	0.147043929	0.142514135	1.031784875	0.302172906	0.754801023	up_in_case
CRA004775	ENSGALG00010029548	BAIAP2	case	control	0.336751234	0.260852163	1.290965847	0.196715521	0.66285976	up_in_case
CRA004775	ENSGALG00010029549	KSR1	case	control	0.450883587	0.279501414	1.61317104	0.106707353	0.552875967	up_in_case
CRA004775	ENSGALG00010029550	CHMP6	case	control	-0.092725231	0.390882986	-0.237219922	0.812486182	0.957779467	down_in_case
CRA004775	ENSGALG00010029552	NOS2	case	control	0.076585391	0.330191475	0.231942362	0.816582782	0.959451233	up_in_case
CRA004775	ENSGALG00010029553	MYH1C	case	control	0.714309618	0.785398827	0.909486484	0.363093389	0.793608387	up_in_case
CRA004775	ENSGALG00010029554	ENSGALG00010029554	case	control	0.87618042	0.745165018	1.175820656	0.23966655	0.706254731	up_in_case
CRA004775	ENSGALG00010029555	LYRM9	case	control	-0.029025075	0.281739399	-0.103021001	0.917946303	0.981688766	down_in_case
CRA004775	ENSGALG00010029556	NPTX1	case	control	0.256045593	0.790851764	0.323759274	0.746120291	0.939637341	up_in_case
CRA004775	ENSGALG00010029558	ENSGALG00010029558	case	control	1.72610613	0.577382409	2.989537094	0.002794005	0.109874886	up_in_case
CRA004775	ENSGALG00010029560	ENSGALG00010029560	case	control	-0.410961421	0.187998946	-2.185977264	0.028817269	0.323746947	down_in_case
CRA004775	ENSGALG00010029561	SCO1	case	control	-0.233719757	0.117921442	-1.98199542	0.047479753	0.404519261	down_in_case
CRA004775	ENSGALG00010029562	ENSGALG00010029562	case	control	0.041811069	0.450272855	0.092857184	0.926017021	0.98417264	up_in_case
CRA004775	ENSGALG00010029563	ADPRM	case	control	-0.179058931	0.25969685	-0.689492116	0.490513633	0.855184934	down_in_case
CRA004775	ENSGALG00010029564	NLK	case	control	-0.008629488	0.240391412	-0.035897653	0.971363967	0.994908384	down_in_case
CRA004775	ENSGALG00010029566	TMEM97	case	control	-0.227149712	0.270836836	-0.838695783	0.401640046	0.811868062	down_in_case
CRA004775	ENSGALG00010029567	IFT20	case	control	-0.02246359	0.35422439	-0.063416272	0.94943503	0.990168609	down_in_case
CRA004775	ENSGALG00010029569	TMEM220	case	control	-0.239266487	0.3750831	-0.637902607	0.523537081	0.866638286	down_in_case
CRA004775	ENSGALG00010029570	TNFAIP1	case	control	0.23690782	0.216960838	1.091938168	0.27486028	0.731001618	up_in_case
CRA004775	ENSGALG00010029571	EPX	case	control	-0.436148844	0.809328221	-0.538902305			down_in_case
CRA004775	ENSGALG00010029572	TSPOAP1	case	control	-0.480125532	0.223465975	-2.148539759	0.031670901	0.339179568	down_in_case
CRA004775	ENSGALG00010029573	MAP2K4	case	control	-0.049162917	0.252054243	-0.195048957	0.845354622	0.966993336	down_in_case
CRA004775	ENSGALG00010029574	SGSM2	case	control	-0.03305584	0.255466274	-0.129394146	0.89704578	0.978790377	down_in_case
CRA004775	ENSGALG00010029576	ATP2A3	case	control	-0.395104399	0.435765284	-0.906690856	0.3645703	0.793608387	down_in_case
CRA004775	ENSGALG00010029577	NOG	case	control	-0.44287378	0.998472332	-0.443551379	0.657366965	0.917152307	down_in_case
CRA004775	ENSGALG00010029579	GALNT17	case	control	-0.093648209	0.402268369	-0.232800329	0.815916458	0.959064856	down_in_case
CRA004775	ENSGALG00010029580	P2RX1	case	control	0.922652354	0.379277825	2.432655675	0.014988547	0.244783262	up_in_case
CRA004775	ENSGALG00010029581	DGKE	case	control	0.006650875	0.289409741	0.022980825	0.981665568	0.997069916	up_in_case
CRA004775	ENSGALG00010029582	SCPEP1	case	control	0.027228702	0.283347601	0.096096464	0.92344396	0.983371633	up_in_case
CRA004775	ENSGALG00010029583	LIMK1	case	control	0.090686994	0.361355883	0.250963104	0.801842635	0.953543664	up_in_case
CRA004775	ENSGALG00010029584	TRIM25	case	control	-0.538834136	0.340281295	-1.583496198	0.113308413	0.562886277	down_in_case
CRA004775	ENSGALG00010029585	ENSGALG00010029585	case	control	0.233294764	0.2991674	0.779813457	0.435500684	0.826905159	up_in_case
CRA004775	ENSGALG00010029586	COIL	case	control	-0.24994667	0.177894739	-1.405025646	0.160013652	0.625090553	down_in_case
CRA004775	ENSGALG00010029588	RAB11FIP4	case	control	0.322284595	0.327683886	0.983522868	0.325350166	0.770430538	up_in_case
CRA004775	ENSGALG00010029590	UTP6	case	control	-0.191471206	0.246741458	-0.77599933	0.437749381	0.827535296	down_in_case
CRA004775	ENSGALG00010029591	SUZ12	case	control	0.195640123	0.366036403	0.534482696	0.593007618	0.894422261	up_in_case
CRA004775	ENSGALG00010029592	MNT	case	control	-0.041617601	0.30824879	-0.135013023	0.892601578	0.977834811	down_in_case
CRA004775	ENSGALG00010029593	PAFAH1B1	case	control	0.146880217	0.192541693	0.762848892	0.445553519	0.833012432	up_in_case
CRA004775	ENSGALG00010029594	CRLF3	case	control	-0.669735949	0.405354227	-1.652223919	0.098488912	0.53445976	down_in_case
CRA004775	ENSGALG00010029595	CLUH	case	control	-0.109080994	0.225756083	-0.483180753	0.6289674	0.908586927	down_in_case
CRA004775	ENSGALG00010029596	ATAD5	case	control	0.498833847	0.449657215	1.109364714	0.267272878	0.727315996	up_in_case
CRA004775	ENSGALG00010029597	ADAP2	case	control	-0.588871146	0.429355375	-1.371523871	0.170211708	0.63378857	down_in_case
CRA004775	ENSGALG00010029598	SUPT4H1	case	control	-0.397690629	0.18002935	-2.209032185	0.027172402	0.31561562	down_in_case
CRA004775	ENSGALG00010029599	RNF43	case	control	0.449129031	0.750712305	0.598270507	0.549659452	0.877623839	up_in_case
CRA004775	ENSGALG00010029600	RHOT1	case	control	0.181140293	0.174606321	1.037421165	0.299539619	0.752512244	up_in_case
CRA004775	ENSGALG00010029602	MTMR4	case	control	-0.522126412	0.172771715	-3.02205956	0.002510611	0.103363527	down_in_case
CRA004775	ENSGALG00010029603	RHBDL3	case	control	0.227241175	0.658953268	0.344851731	0.73020584	0.936393482	up_in_case
CRA004775	ENSGALG00010029604	ENSGALG00010029604	case	control	-0.114371821	0.239940945	-0.476666543	0.633599591	0.909837348	down_in_case
CRA004775	ENSGALG00010029605	C17orf75	case	control	-0.363797301	0.272579784	-1.334645205	0.181992495	0.647378665	down_in_case
CRA004775	ENSGALG00010029606	ABHD11	case	control	-0.153855938	0.263794883	-0.583240798	0.55973121	0.880918893	down_in_case
CRA004775	ENSGALG00010029607	ZNF207	case	control	-0.015340514	0.175278297	-0.087520897	0.930257475	0.985044263	down_in_case
CRA004775	ENSGALG00010029608	ENSGALG00010029608	case	control	-0.34918989	0.337377163	-1.035013416	0.300662646	0.753217716	down_in_case
CRA004775	ENSGALG00010029609	METTL27	case	control	-0.235391555	0.424260318	-0.554828121	0.579012231	0.888378369	down_in_case
CRA004775	ENSGALG00010029610	ERN1	case	control	-0.193714147	0.283946999	-0.682219387	0.495100237	0.856382445	down_in_case
CRA004775	ENSGALG00010029611	YWHAG	case	control	-0.041210873	0.304531977	-0.135325273	0.892354704	0.977834811	down_in_case
CRA004775	ENSGALG00010029612	HSPB1	case	control	-0.124945118	0.594275912	-0.210247656	0.833474386	0.964019688	down_in_case
CRA004775	ENSGALG00010029614	MDH2	case	control	0.055846827	0.377624694	0.147889765	0.882429766	0.975352603	up_in_case
CRA004775	ENSGALG00010029615	ENSGALG00010029615	case	control	-0.328562249	0.61902997	-0.530769534	0.595578495	0.895305942	down_in_case
CRA004775	ENSGALG00010029616	TMEM120A	case	control	-0.460534129	0.185485736	-2.482854684	0.013033424	0.229478386	down_in_case
CRA004775	ENSGALG00010029617	POR	case	control	-0.018873665	0.139329611	-0.135460548	0.892247755	0.977834811	down_in_case

CRA004775	ENSGALG00010029618	TEX2	case	control	0.404652153	0.218602813	1.851083921	0.064157481	0.455932569	up_in_case
CRA004775	ENSGALG00010029619	ENSGALG00010029619	case	control	-0.132467191	0.248917583	-0.532172897	0.594606253	0.895102715	down_in_case
CRA004775	ENSGALG00010029620	ENSGALG00010029620	case	control	-0.190174365	0.391202615	-0.486127541	0.626876741	0.90758663	down_in_case
CRA004775	ENSGALG00010029621	TAF15	case	control	-0.358416636	0.16810582	-2.132089398	0.032999498	0.347189057	down_in_case
CRA004775	ENSGALG00010029622	MMP28	case	control	-0.078732641	0.318328603	-0.247331342	0.804651804	0.954576316	down_in_case
CRA004775	ENSGALG00010029623	ENSGALG00010029623	case	control	0.168918805	0.659182166	0.25625512	0.797753849	0.952835572	up_in_case
CRA004775	ENSGALG00010029624	RASL10B	case	control	-0.728490302	0.625154834	-1.1652958	0.243899279	0.71125478	down_in_case
CRA004775	ENSGALG00010029625	PECAM1	case	control	0.703924511	0.335844626	2.095982653	0.03608373	0.360339411	up_in_case
CRA004775	ENSGALG00010029626	AP2B1	case	control	-0.443873233	0.245426792	-1.808576927	0.070516756	0.469895261	down_in_case
CRA004775	ENSGALG00010029627	PEX12	case	control	-0.039356345	0.235946174	-0.166802217	0.867525674	0.973120718	down_in_case
CRA004775	ENSGALG00010029628	UNC45B	case	control	0.411742017	0.656809656	0.626881796	0.530736721	0.869211096	up_in_case
CRA004775	ENSGALG00010029629	ENSGALG00010029629	case	control	0.534285142	0.43474382	1.228965469	0.219084753	0.686718587	up_in_case
CRA004775	ENSGALG00010029630	RAD51D	case	control	-0.732883625	0.227735049	-3.218141559	0.001290241	0.075498328	down_in_case
CRA004775	ENSGALG00010029631	LIG3	case	control	-0.17416089	0.174298122	-0.999212656	0.317691686	0.76641726	down_in_case
CRA004775	ENSGALG00010029632	TMEM132E	case	control	0.059883646	0.522550248	0.114598828	0.908763109	0.98075825	up_in_case
CRA004775	ENSGALG00010029633	POLG2	case	control	-0.163850154	0.247379732	-0.662342677	0.507751632	0.862748056	down_in_case
CRA004775	ENSGALG00010029634	DDX5	case	control	0.002444682	0.18592352	0.013148856	0.989509033	0.998065748	up_in_case
CRA004775	ENSGALG00010029635	ENSGALG00010029635	case	control	-1.152578168	0.499803417	-2.306063001	0.021107112	0.285392234	down_in_case
CRA004775	ENSGALG00010029636	SPATA20	case	control	-0.179730487	0.203282672	-0.884140715	0.376620257	0.798523018	down_in_case
CRA004775	ENSGALG00010029638	EPN3	case	control	-0.190425392	0.461293376	-0.412807557	0.679747614	0.92381987	down_in_case
CRA004775	ENSGALG00010029639	ENSGALG00010029639	case	control	-2.222812819	1.025564857	-2.16740346	0.030204104	0.331325011	down_in_case
CRA004775	ENSGALG00010029641	ENSGALG00010029641	case	control	-0.496398489	0.505833991	-0.981346643	0.326421829	0.770998457	down_in_case
CRA004775	ENSGALG00010029642	NAT9	case	control	-0.082149289	0.451359879	-0.182003967	0.855579613	0.970635695	down_in_case
CRA004775	ENSGALG00010029644	MYCBPAP	case	control	-0.135663188	0.286314764	-0.473825332	0.635624469	0.909837348	down_in_case
CRA004775	ENSGALG00010029646	MRPS17	case	control	0.0051341	0.243592741	0.021076572	0.983184574	0.997398088	up_in_case
CRA004775	ENSGALG00010029647	RSAD1	case	control	-0.072919393	0.250035555	-0.291636096	0.770564874	0.945754599	down_in_case
CRA004775	ENSGALG00010029648	NIPSNAP2	case	control	0.05180266	0.384538783	0.134713745	0.892838205	0.977834811	up_in_case
CRA004775	ENSGALG00010029649	PSPH	case	control	0.524244355	0.534473327	0.980861585	0.326661003	0.770998457	up_in_case
CRA004775	ENSGALG00010029650	ACSF2	case	control	0.057691892	0.304538206	0.189440572	0.849747528	0.968413719	up_in_case
CRA004775	ENSGALG00010029651	ENSGALG00010029651	case	control	-0.036265574	0.583214494	-0.062182223	0.950417719	0.990168609	down_in_case
CRA004775	ENSGALG00010029652	LRRC59	case	control	-0.008195677	0.19287515	-0.042492136	0.966106381	0.993094598	down_in_case
CRA004775	ENSGALG00010029653	CCT6A	case	control	-0.108250755	0.138141793	-0.783620605	0.433262762	0.825828172	down_in_case
CRA004775	ENSGALG00010029654	EME1	case	control	-1.088599353	0.575719233	-1.890851116	0.058644222	0.443329681	down_in_case
CRA004775	ENSGALG00010029655	PHKG1	case	control	-0.438989906	0.605709266	-0.724753493	0.468603272	0.8440023	down_in_case
CRA004775	ENSGALG00010029656	MRPL27	case	control	-0.504161457	0.343601249	-1.467286449	0.142298148	0.602473513	down_in_case
CRA004775	ENSGALG00010029657	CHCHD2	case	control	0.150551183	0.440206129	0.34200156	0.732349719	0.936819725	up_in_case
CRA004775	ENSGALG00010029658	XYLT2	case	control	-0.340337931	0.127158275	-2.676490617	0.007439765	0.172229305	down_in_case
CRA004775	ENSGALG00010029659	VKORC1L1	case	control	-0.398629631	0.203438628	-1.959458908	0.050059068	0.414161886	down_in_case
CRA004775	ENSGALG00010029660	CD300LG	case	control	-0.352971219	0.30471544	-1.158363418	0.246715761	0.712046495	down_in_case
CRA004775	ENSGALG00010029661	KRABZFP	case	control	0.217324684	0.566536703	0.383602127	0.701273363	0.930068722	up_in_case
CRA004775	ENSGALG00010029663	GUSB	case	control	-0.099620784	0.447404208	-0.22266394	0.823797068	0.960898591	down_in_case
CRA004775	ENSGALG00010029664	RAB37	case	control	-0.941559823	0.464853238	-2.025499116	0.042816139	0.38854564	down_in_case
CRA004775	ENSGALG00010029665	ASL1	case	control	-0.422780125	0.303868765	-1.39132472	0.164126981	0.629717526	down_in_case
CRA004775	ENSGALG00010029666	SLC9A3R1	case	control	-0.414474113	0.407404472	-1.017352879	0.30898559	0.761414086	down_in_case
CRA004775	ENSGALG00010029667	TMEM104	case	control	-0.293485634	0.18724676	-1.567373629	0.117027384	0.56743426	down_in_case
CRA004775	ENSGALG00010029668	CRCP	case	control	-0.08264049	0.302014891	-0.273630516	0.784368581	0.950165405	down_in_case
CRA004775	ENSGALG00010029669	HID1	case	control	-0.368033577	0.16537918	-2.225392437	0.026054905	0.308862928	down_in_case
CRA004775	ENSGALG00010029670	TPST1	case	control	-0.126490408	0.323630026	-0.390848804	0.695908999	0.929092636	down_in_case
CRA004775	ENSGALG00010029671	GRIN2C	case	control	-0.189876197	0.573369289	-0.331158645	0.740524656	0.938450627	down_in_case
CRA004775	ENSGALG00010029672	KCTD7	case	control	-0.648278251	0.384900637	-1.684274301	0.09212867	0.52187835	down_in_case
CRA004775	ENSGALG00010029673	TMEM248	case	control	-0.148830492	0.264409628	-0.562878488	0.57351763	0.886814453	down_in_case
CRA004775	ENSGALG00010029674	FDXR	case	control	-0.350130144	0.165873699	-2.110823759	0.034787463	0.354006537	down_in_case
CRA004775	ENSGALG00010029675	DHRS13	case	control	0.04857216	0.23190725	0.209446491	0.834099703	0.964019688	up_in_case
CRA004775	ENSGALG00010029677	PHF12	case	control	0.100603595	0.180749161	0.556592321	0.577806001	0.888378369	up_in_case
CRA004775	ENSGALG00010029680	OTOP3	case	control	1.812441919	0.824356797	2.198613423	0.027905422	0.318626275	up_in_case
CRA004775	ENSGALG00010029682	CDR2L	case	control	0.708080766	0.343190359	2.063230353	0.039090748	0.371740574	up_in_case
CRA004775	ENSGALG00010029683	MRPL58	case	control	0.170165008	0.325865732	0.522193624	0.601535525	0.898185892	up_in_case
CRA004775	ENSGALG00010029684	ENSGALG00010029684	case	control	0.197039191	0.730303353	0.269804583	0.787310597	0.95041797	up_in_case
CRA004775	ENSGALG00010029685	ATP5PD	case	control	-0.045320679	0.323044903	-0.140292198	0.888429129	0.977118922	down_in_case
CRA004775	ENSGALG00010029686	KCTD2	case	control	-0.221881796	0.109763933	-2.021445384	0.043233681	0.389812978	down_in_case
CRA004775	ENSGALG00010029687	SLC16A5	case	control	0.621916832	0.580275796	1.071760767	0.283827499	0.740508942	up_in_case
CRA004775	ENSGALG00010029688	PIPOX	case	control	1.803605896	0.761756736	2.367692744	0.017899394	0.266091423	up_in_case
CRA004775	ENSGALG00010029689	ENSGALG00010029689	case	control	0.276020967	0.34842758	0.792190351	0.428249694	0.823075979	up_in_case
CRA004775	ENSGALG00010029690	ENSGALG00010029690	case	control	0.131241396	0.383495458	0.34222412	0.732182235	0.936819725	up_in_case
CRA004775	ENSGALG00010029691	NUFIP2	case	control	0.357715719	0.284717669	1.256387496	0.208975513	0.678381085	up_in_case
CRA004775	ENSGALG00010029692	CRYBA1	case	control	1.09745474	0.71964974	1.524984557	0.12726295	0.581272645	up_in_case
CRA004775	ENSGALG00010029693	HN1	case	control	-0.07161271	0.232118029	-0.308518517	0.757687813	0.941900693	down_in_case
CRA004775	ENSGALG00010029694	ARMC7	case	control	-0.132791696	0.219780162	-0.604202375	0.545709102	0.876735967	down_in_case
CRA004775	ENSGALG00010029695	TAOK1	case	control	0.147876696	0.298524828	0.495358113	0.620347356	0.905283281	up_in_case
CRA004775	ENSGALG00010029696	SUMO2	case	control	0.137537319	0.22529958	0.610464161	0.541554377	0.87495415	up_in_case
CRA004775	ENSGALG00010029697	GIT1	case	control	-0.231551574	0.445845119	-0.519354288	0.603513703	0.898692785	down_in_case
CRA004775	ENSGALG00010029698	NUP85	case	control	-0.437131371	0.125551776	-3.481682094	0.000498275	0.045678518	down_in_case
CRA004775	ENSGALG00010029699	ANKRD13B	case	control	-0.120951801	0.295773672	-0.40893363	0.682588369	0.924573745	down_in_case
CRA004775	ENSGALG00010029700	GGA3	case	control	-0.336847592	0.205525832	-1.638955013	0.101222629	0.540680709	down_in_case
CRA004775	ENSGALG00010029701	MRPS7	case	control	-0.14960857	0.186770614	-0.801028419	0.423115194	0.820552247	down_in_case
CRA004775	ENSGALG00010029702	CORO6	case	control	-0.314655618	0.592847486	-0.530753061	0.595589911	0.895305942	down_in_case
CRA004775	ENSGALG00010029703	MIF4GD	case	control	-0.214897279	0.135851048	-1.581859559	0.113681635	0.562992809	down_in_case
CRA004775	ENSGALG00010029704	SLC25A19	case	control	-0.314830324	0.275658361	-1.142103303	0.253411083	0.717862912	down_in_case
CRA004775	ENSGALG00010029705	GRB2	case	control	-0.149333289	0.215369034	-0.693383291	0.488069045	0.853655233	down_in_case
CRA004775	ENSGALG00010029706	SSH2	case	control	-0.645933122	0.413328839	-1.562758418	0.118109428	0.569418751	down_in_case
CRA004775	ENSGALG00010029707	NSRP1	case	control	0.037884824	0.267475358	0.141638559	0.887365508	0.977118922	up_in_case
CRA004775	ENSGALG00010029708	TMEM94	case	control	-0.316152634	0.168560716	-1.875600919	0.0607101	0.445397937	down_in_case
CRA004775	ENSGALG00010029709	SLC6A4	case	control	0.696056275	0.388020185	1.793866148	0.072834536	0.476411335	up_in_case
CRA004775	ENSGALG00010029710	BLMH	case	control	0.053028923	0.171665324	0.308908762	0.757390932	0.941900693	up_in_case
CRA004775	ENSGALG00010029711	CASKIN2	case	control	0.043907984	0.383941941	0.114361	0.908951628	0.98075825	up_in_case
CRA004775	ENSGALG00010029713	CPD	case	control	-0.10242518	0.224175946	-0.456896387	0.647745521	0.914210065	down_in_case
CRA004775	ENSGALG00010029716	GOSR1	case	control	-0.032356661	0.179861256	-0.179897891	0.857232731	0.970913248	down_in_case
CRA004775	ENSGALG00010029717	TRARG1	case	control	0.036415334	0.660264642	0.055152633	0.956016865	0.990926004	up_in_case
CRA004775	ENSGALG00010029718	ABR	case	control	0.041179213	0.227135911	0.181297675	0.856133931	0.970703965	up_in_case
CRA004775	ENSGALG00010029720	TIMM22	case	control	-0.252285326	0.228333225	-1.104899761	0.269203026	0.72765348	down_in_case
CRA004775	ENSGALG00010029721	EXOC7	case	control	-0.263320928	0.167378545	-1.573205981	0.115671131	0.566086328	down_in_case
CRA004775	ENSGALG00010029722	SPECC1	case	control	0.106949786	0.222621119	0.480411683	0.630934689	0.909357765	up_in_case
CRA004775	ENSGALG00010029723	ADORA2B	case	control	0.418105929	0.355301128	1.176764991	0.239289319	0.706254731	up_in_case
CRA004775	ENSGALG00010029724	CACNA1G	case	control	-0.122169113	0.398369786	-0.306672638	0.759092559	0.942236668	down_in_case
CRA004775	ENSGALG00010029725	TSEN54	case	control	-0.670618417	0.186305953	-3.599554432	0.000318763	0.035519918	down_in_case
CRA004775	ENSGALG00010029726	SEPTIN9	case	control	-0.232254724	0.246234993	-0.943223872	0.345566405	0.784552667	down_in_case
CRA004775	ENSGALG00010029727	ZSWIM7	case	control	-0.115756961	0.312608912	-0.37029322	0.711164025	0.932234469	down_in_case
CRA004775	ENSGALG00010029728	TNRC6C	case	control	0.245612649	0.312517262	0.785917065	0.431916076	0.825399586	up_in_case
CRA004775	ENSGALG00010029730	TTC19	case	control	-0.19506954	0.346951657	-0.562238387	0.573953611	0.886897708	down_in_case
CRA004775	ENSGALG00010029731	NCOR1	case	control	0.353698116	0.434567195	0.813908918	0.415697131	0.818943344	up_in_case
CRA004775	ENSGALG00010029732	PIGL	case	control	-0.413847496	0.299247262	-1.38296168	0.166676616	0.630220864	down_in_case
CRA004775	ENSGALG00010029733	ENSGALG00010029733	case	control	-0.275979834	0.511819804	-0.539212887	0.589739969	0.893511935	down_in_case
CRA004775	ENSGALG00010029734	SEC14L1	case	control	-0.279866877	0.309444515	-0.90441699	0.365774332	0.793608387	down_in_case
CRA004775	ENSGALG00010029735	SRP68	case	control	-0.261615531	0.169525661	-1.543220829	0.122777205	0.57525466	down_in_case
CRA004775	ENSGALG00010029736	ENSGALG00010029736	case	control	0.02516671	0.460937543	0.054598958	0.956457969	0.9909743	up_in_case
CRA004775	ENSGALG00010029737	ENSGALG00010029737	case	control	1.346954174	0.672750902	2.002158851	0.04526765	0.395473603	up_in_case
CRA004775	ENSGALG00010029738	TRPV2	case	control	0.075819307	0.210238638	0.360634505	0.718372691	0.934011578	up_in_case
CRA004775	ENSGALG00010029739	P2RX5	case	control	0.361640322	0.310696615	1.16396608	0.244437758	0.711765017	up_in_case
CRA004775	ENSGALG00010029740	HASPIN	case	control	-0.659108143	0.588161481	-1.120624461	0.262447748	0.723230258	down_in_case
CRA004775	ENSGALG00010029741	EMC6	case	control	-0.050833127	0.36625725	-0.138790774	0.889615485	0.977162915	down_in_case
CRA004775	ENSGALG00010029742	TAX1BP3	case	control	0.265001409	0.173552495	1.52692365	0.126779998	0.580923609	up_in_case
CRA004775	ENSGALG00010029743	MYOCD	case	control	0.855831707	0.549337722	1.557933621	0.119248984	0.57095849	up_in_case
CRA004775	ENSGALG00010029744	CTNS	case	control	0.1949235	0.212806405	0.915966319	0.35968456	0.791966047	up_in_case
CRA004775	ENSGALG00010029745	SHPK	case	control	-0.270294322	0.280646875	-0.96311182	0.335491411	0.776134318	down_in_case
CRA004775	ENSGALG00010029747	TRPV3	case	control	0.879932419	0.526314741	1.671874927	0.094548988	0.526933666	up_in_case
CRA004775	ENSGALG00010029749	ASPA	case	control	0.265200592	0.550060838	0.482129563	0.62971391	0.908911092	up_in_case
CRA004775	ENSGALG00010029750	TEN1	case	control	-1.332749171	0.716828857	-1.85922924	0.062994652	0.450462537	down_in_case
CRA004775	ENSGALG00010029752	ENSGALG00010029752	case	control	0.767932677	0.447044326	1.717799852	0.085833129	0.507503846	up_in_case
CRA004775	ENSGALG00010029753	AKAP10	case	control	-0.023688637	0.223289046	-0.106089563	0.915511292	0.981478883	down_in_case
CRA004775	ENSGALG00010029754	NLE1	case	control	-0.86652552	0.46356466	-1.869265704	0.061585857	0.446401278	down_in_case
CRA004775	ENSGALG00010029755	ACOX1	case	control	-0.005107241	0.21805586	-0.02342171	0.981313888	0.997069916	down_in_case
CRA004775	ENSGALG00010029756	ELAC2	case	control	-0.436133717	0.169261704	-2.576682764	0.009975345	0.201425687	down_in_case
CRA004775	ENSGALG00010029757	ULK2	case	control	0.499074123	0.44980528	1.109533716	0.267200008	0.727315996	up_in_case
CRA004775	ENSGALG00010029758	DNAH9	case	control	-0.787209059	0.538518699	-1.461804504	0.14379479	0.605426256	down_in_case
CRA004775	ENSGALG00010029759	ALDH3A2	case	control	-0.478223085	0.374758577	-1.276083095	0.201926147	0.670354119	down_in_case
CRA004775	ENSGALG00010029761	MRPL38	case	control	-0.327213038	0.214918421	-1.522498797	0.127884147	0.5821156	down_in_case

CRA004775	ENSGALG00010029762	SLC47A2	case	control	0.270193891	1.138708192	0.237281063			up_in_case
CRA004775	ENSGALG00010029763	GEMIN4	case	control	-0.349342528	0.259592649	-1.345733513	0.178388474	0.644368088	down_in_case
CRA004775	ENSGALG00010029764	NXN	case	control	-0.264857958	0.326968266	-0.810041785	0.417916161	0.818943344	down_in_case
CRA004775	ENSGALG00010029765	KCNJ16	case	control	1.727479594	0.661793696	2.61029926	0.009046305	0.191968729	up_in_case
CRA004775	ENSGALG00010029766	ENSGALG00010029766	case	control	-0.1277936	0.323887336	-0.394561892	0.693166242	0.928023463	down_in_case
CRA004775	ENSGALG00010029767	GLOD4	case	control	-0.434147207	0.234327665	-1.852735603	0.063920264	0.455187755	down_in_case
CRA004775	ENSGALG00010029768	KCNJ2	case	control	0.877068392	0.367478647	2.386719337	0.016999464	0.258012829	up_in_case
CRA004775	ENSGALG00010029770	TLCD3A	case	control	0.021884011	0.281834628	0.077648409	0.938107734	0.986707289	up_in_case
CRA004775	ENSGALG00010029771	SOX9	case	control	0.783205982	0.389133427	2.012692634	0.044146975	0.392535833	up_in_case
CRA004775	ENSGALG00010029772	VPS53	case	control	-0.407746365	0.198746281	-2.051592425	0.040209289	0.376363211	down_in_case
CRA004775	ENSGALG00010029773	TRIM65	case	control	-0.294990519	0.224041526	-1.316677867	0.187946626	0.652111233	down_in_case
CRA004775	ENSGALG00010029774	RFLNB	case	control	-0.076765822	0.294245661	-0.260890241	0.794177151	0.951843306	down_in_case
CRA004775	ENSGALG00010029775	ENSGALG00010029775	case	control	-0.40672458	0.307245803	-1.323775868	0.185577493	0.6491093	down_in_case
CRA004775	ENSGALG00010029776	WBP2	case	control	0.169633176	0.267090626	0.635114674	0.525353627	0.867484846	up_in_case
CRA004775	ENSGALG00010029777	ENSGALG00010029777	case	control	-0.206419305	0.26615455	-0.775561812	0.438007756	0.827682376	down_in_case
CRA004775	ENSGALG00010029778	DOC2B	case	control	-0.256742288	0.673217709	-0.381365916	0.702931749	0.930068722	down_in_case
CRA004775	ENSGALG00010029780	TEX14	case	control	-0.640159377	0.51791783	-1.236024983	0.216449278	0.684071252	down_in_case
CRA004775	ENSGALG00010029781	UNC13D	case	control	-1.202678382	0.558840175	-2.15209721	0.031389699	0.337301359	down_in_case
CRA004775	ENSGALG00010029782	UNK	case	control	-0.214565889	0.22372445	-0.95906321	0.337526903	0.778184725	down_in_case
CRA004775	ENSGALG00010029783	RAD51C	case	control	-0.735164939	0.257142507	-2.858978654	0.004250074	0.133620227	down_in_case
CRA004775	ENSGALG00010029784	CLDN3	case	control	2.493061059	0.736574806	3.384667842	0.000712644	0.055997557	up_in_case
CRA004775	ENSGALG00010029785	ENSGALG00010029785	case	control	-0.416680261	0.581557247	-0.716490532	0.473688512	0.845445211	down_in_case
CRA004775	ENSGALG00010029787	H3-3B	case	control	-0.176060978	0.228175818	-0.771602266	0.440350032	0.829353567	down_in_case
CRA004775	ENSGALG00010029791	GALK1	case	control	-0.22704299	0.174271343	-1.302813106	0.192638575	0.656993806	down_in_case
CRA004775	ENSGALG00010029804	ITGB4	case	control	0.153034139	0.437301735	0.349950908	0.726375541	0.935238044	up_in_case
CRA004775	ENSGALG00010029812	SAP30BP	case	control	-0.337301403	0.221241911	-1.524581853	0.127363427	0.581272645	down_in_case
CRA004775	ENSGALG00010029835	RECQL5	case	control	-0.306030234	0.318286496	-0.961492988	0.33630435	0.776900397	down_in_case
CRA004775	ENSGALG00010029841	SMIM5	case	control	0.390342386	0.372067051	1.049118392	0.294123632	0.746438942	up_in_case
CRA004775	ENSGALG00010029850	ENSGALG00010029850	case	control	2.423051921	0.72590197	3.337987802	0.000843875	0.06088574	up_in_case
CRA004775	ENSGALG00010029859	CSNK1D	case	control	0.077910098	0.183263227	0.425126736	0.670744289	0.921535334	up_in_case
CRA004775	ENSGALG00010029864	SLC16A3	case	control	-0.131601929	0.563557521	-0.233519958	0.815357673	0.958870945	down_in_case
CRA004775	ENSGALG00010029879	ENSGALG00010029879	case	control	0.022472879	0.594566365	0.037797091	0.969849464	0.994366623	up_in_case
CRA004775	ENSGALG00010029895	FASN	case	control	-0.265233253	0.389369087	-0.681187237	0.495753023	0.856382445	down_in_case
CRA004775	ENSGALG00010029907	NTN1	case	control	0.108710614	0.384865884	0.282463629	0.777588032	0.947570394	up_in_case
CRA004775	ENSGALG00010029921	STX8	case	control	-0.333202136	0.192018202	-1.735263285	0.082694182	0.499833536	down_in_case
CRA004775	ENSGALG00010029925	GGNBP2	case	control	0.368333807	0.219252961	1.679949063	0.092967227	0.524612711	up_in_case
CRA004775	ENSGALG00010029926	BLTP2	case	control	-0.212497448	0.234627678	-0.905679371	0.365105584	0.793608387	down_in_case
CRA004775	ENSGALG00010029927	ENSGALG00010029927	case	control	-0.147979049	0.606831137	-0.243855399	0.807342813	0.955397873	down_in_case
CRA004775	ENSGALG00010029928	USP43	case	control	0.237531132	0.320291743	0.741608666	0.458324472	0.840058241	up_in_case
CRA004775	ENSGALG00010029929	GDPD1	case	control	-0.029988636	0.173933381	-0.172414493	0.863111679	0.971910432	down_in_case
CRA004775	ENSGALG00010029930	ARHGAP44	case	control	1.096927142	0.634450954	1.728939226	0.083819973	0.503010645	up_in_case
CRA004775	ENSGALG00010029932	RASA4B	case	control	-0.585388135	0.436620622	-1.340724889	0.18000979	0.644990528	down_in_case
CRA004775	ENSGALG00010029933	TVP23B	case	control	-0.176445943	0.213003524	-0.828371001	0.407460424	0.814105656	down_in_case
CRA004775	ENSGALG00010029934	TEKT3	case	control	-0.047558834	0.614817256	-0.077354423	0.938341597	0.986707289	down_in_case
CRA004775	ENSGALG00010029935	SYNRG	case	control	-0.100121151	0.280467749	-0.356979192	0.721107383	0.934511903	down_in_case
CRA004775	ENSGALG00010029936	SERPINF2	case	control	-0.938144808	0.436082195	-2.151302708	0.031452315	0.337627463	down_in_case
CRA004775	ENSGALG00010029937	PMP22	case	control	0.431664585	0.378704557	1.139845237	0.254350783	0.718335926	up_in_case
CRA004775	ENSGALG00010029938	ENSGALG00010029938	case	control	0.086309606	0.381784767	0.226068752	0.821147948	0.960766569	up_in_case
CRA004775	ENSGALG00010029939	ENSGALG00010029939	case	control	-1.547101844	1.33641116	-1.157654089	0.247005227	0.712046495	down_in_case
CRA004775	ENSGALG00010029941	COX10	case	control	-0.174907534	0.272786384	-0.641188652	0.521400122	0.866299649	down_in_case
CRA004775	ENSGALG00010029943	HS3ST3A1	case	control	-0.030099666	0.427448118	-0.070417121	0.943861664	0.987976079	down_in_case
CRA004775	ENSGALG00010029945	CCL4	case	control	0.36657576	0.699428703	0.524107401	0.600203841	0.897206546	up_in_case
CRA004775	ENSGALG00010029946	METRNL	case	control	-0.226314903	0.250075439	-0.90498653	0.365472523	0.793608387	down_in_case
CRA004775	ENSGALG00010029947	CCL5	case	control	-0.181481306	0.470043815	-0.386094445	0.699426723	0.929347461	down_in_case
CRA004775	ENSGALG00010029948	MYADML2	case	control	-0.521746686	0.572584549	-0.911213353	0.36218297	0.792867581	down_in_case
CRA004775	ENSGALG00010029950	RFFL	case	control	-0.007332894	0.279937165	-0.026194786	0.979101975	0.996641591	down_in_case
CRA004775	ENSGALG00010029956	CCLI5	case	control	-0.341546038	0.696194239	-0.490590153	0.623716353	0.906441769	down_in_case
CRA004775	ENSGALG00010029962	ENSGALG00010029962	case	control	-0.55509267	0.904609153	-0.613627076	0.539461787	0.873987916	down_in_case
CRA004775	ENSGALG00010029966	RABGEF1	case	control	0.038252554	0.147345525	0.259611234	0.79516367	0.952360933	up_in_case
CRA004775	ENSGALG00010030004	FLOT2	case	control	-0.225916445	0.125646072	-1.798038269	0.072170944	0.473936316	down_in_case
CRA004775	ENSGALG00010030014	ENSGALG00010030014	case	control	-0.329397554	0.792673023	-0.415552875			down_in_case
CRA004775	ENSGALG00010030102	GPS1	case	control	-0.267286241	0.250799976	-1.065734712	0.286543582	0.741851239	down_in_case
CRA004775	ENSGALG00010030103	C1QTNF1	case	control	0.110382601	0.431973197	0.255531135	0.798312899	0.952835572	up_in_case
CRA004775	ENSGALG00010030104	P4HB	case	control	-0.042657151	0.237885107	-0.17931829	0.857687786	0.971024596	down_in_case
CRA004775	ENSGALG00010030105	RAB40B	case	control	0.185721509	0.595717968	0.311760798	0.755222319	0.941900693	up_in_case
CRA004775	ENSGALG00010030106	GPRC5C	case	control	0.22887723	0.356046696	0.642829248	0.520334903	0.865611895	up_in_case
CRA004775	ENSGALG00010030107	RPTOR	case	control	-0.334736008	0.312220543	-1.072113978	0.283668841	0.740508942	down_in_case
CRA004775	ENSGALG00010030108	LLGL2	case	control	-0.098150851	0.177999041	-0.551412249	0.581351105	0.888998848	down_in_case
CRA004775	ENSGALG00010030109	FBF1	case	control	-0.062260839	0.234361243	-0.265661841	0.790499654	0.951054854	down_in_case
PRJNA8274(	ENSGALG00010000007	ND1	case	control	-0.081423706	0.07474206	-1.089396074	0.275979262	0.701472589	down_in_case
PRJNA8274(	ENSGALG00010000011	ND2	case	control	-0.307373962	0.089156338	-3.44758396	0.000565625	0.034565946	down_in_case
PRJNA8274(	ENSGALG00010000017	COX1	case	control	-0.104102866	0.093134121	-1.117773642	0.263663681	0.68964455	down_in_case
PRJNA8274(	ENSGALG00010000020	COX2	case	control	-0.125210527	0.08041173	-1.557117692	0.119442545	0.516645008	down_in_case
PRJNA8274(	ENSGALG00010000022	ATP8	case	control	-0.21165993	0.108115058	-1.957728499	0.050261879	0.367061596	down_in_case
PRJNA8274(	ENSGALG00010000023	ATP6	case	control	-0.165539238	0.088439686	-1.871775507	0.061237668	0.402191009	down_in_case
PRJNA8274(	ENSGALG00010000024	COX3	case	control	-0.078020281	0.087751859	-0.889101181	0.373948704	0.774369566	down_in_case
PRJNA8274(	ENSGALG00010000026	ND3	case	control	-0.405335036	0.085542166	-4.738423829	2.15387E-06	0.000481255	down_in_case
PRJNA8274(	ENSGALG00010000028	ND4L	case	control	-0.261828678	0.088628237	-2.95423545	0.003134448	0.095164759	down_in_case
PRJNA8274(	ENSGALG00010000029	ND4	case	control	-0.242329089	0.088703548	-2.73189848	0.006297054	0.138110236	down_in_case
PRJNA8274(	ENSGALG00010000033	ND5	case	control	-0.200862368	0.086943755	-2.310256426	0.020873962	0.259355211	down_in_case
PRJNA8274(	ENSGALG00010000034	CYTB	case	control	-0.140968238	0.096063614	-1.46744676	0.142254562	0.543914503	down_in_case
PRJNA8274(	ENSGALG00010000037	ND6	case	control	-0.106326641	0.110896603	-0.958790785	0.337664153	0.750948271	down_in_case
PRJNA8274(	ENSGALG00010000039	PDIA3	case	control	0.052600017	0.046100475	1.140986445	0.253875564	0.679599506	up_in_case
PRJNA8274(	ENSGALG00010000042	CKMT1B	case	control	0.013197612	0.361420295	0.036515968	0.970870947	0.992761697	up_in_case
PRJNA8274(	ENSGALG00010000043	PPIP5K1	case	control	-0.002587299	0.091274393	-0.028346387	0.977385884	0.994661197	down_in_case
PRJNA8274(	ENSGALG00010000044	MAP1A	case	control	-0.132102972	0.100393934	-1.315846158	0.188225683	0.611129484	down_in_case
PRJNA8274(	ENSGALG00010000045	TP53BP1	case	control	-0.025012355	0.055505127	-0.450631439	0.652255204	0.905706359	down_in_case
PRJNA8274(	ENSGALG00010000046	TUBGCP4	case	control	0.044767735	0.066456223	0.673642486	0.500538635	0.840987941	up_in_case
PRJNA8274(	ENSGALG00010000047	IQGAP1	case	control	-0.009348574	0.072145689	-0.12957911	0.896899432	0.980176778	down_in_case
PRJNA8274(	ENSGALG00010000048	MAN2A2	case	control	0.05562277	0.08399062	0.662249784	0.507811153	0.843702533	up_in_case
PRJNA8274(	ENSGALG00010000049	FES	case	control	0.024394742	0.156530832	0.155846242	0.87615422	0.975666505	up_in_case
PRJNA8274(	ENSGALG00010000050	CIB1	case	control	-0.163682678	0.092232256	-1.774679326	0.075950851	0.442710866	down_in_case
PRJNA8274(	ENSGALG00010000051	GDPGP1	case	control	0.045540156	0.160393935	0.283926918	0.776466387	0.947228003	up_in_case
PRJNA8274(	ENSGALG00010000052	TTLL13	case	control	-0.008422909	0.114689985	-0.073440664	0.94145546	0.989551456	down_in_case
PRJNA8274(	ENSGALG00010000053	HDDC3	case	control	0.107070108	0.180745328	0.592381051	0.553595452	0.866337858	up_in_case
PRJNA8274(	ENSGALG00010000054	VPS33B	case	control	0.025345279	0.06139157	0.412846242	0.67971927	0.915381147	up_in_case
PRJNA8274(	ENSGALG00010000055	PRC1	case	control	-0.032267773	0.098010326	-0.3292283	0.741983134	0.937315893	down_in_case
PRJNA8274(	ENSGALG00010000056	UNC45A	case	control	0.085442206	0.073356318	1.164755917	0.244117808	0.668164293	up_in_case
PRJNA8274(	ENSGALG00010000058	FURIN	case	control	0.015529531	0.06389637	0.243042455	0.807972509	0.955875648	up_in_case
PRJNA8274(	ENSGALG00010000059	MFAP1	case	control	-0.021528996	0.0523095	-0.411569527	0.680654971	0.915564489	down_in_case
PRJNA8274(	ENSGALG00010000060	HYPK	case	control	-0.033998366	0.05966324	-0.569837731	0.568787762	0.874681686	down_in_case
PRJNA8274(	ENSGALG00010000061	SERINC4	case	control	-0.111286441	0.210499573	-0.528677754	0.597029012	0.884680373	down_in_case
PRJNA8274(	ENSGALG00010000063	SERF2	case	control	-0.07924411	0.095951537	-0.825876401	0.408874209	0.795170398	down_in_case
PRJNA8274(	ENSGALG00010000069	ENSGALG00010000069	case	control	-0.124529438	0.182653741	-0.681778745	0.495378866	0.838118945	down_in_case
PRJNA8274(	ENSGALG00010000071	ENSGALG00010000071	case	control	0.247655374	0.458097402	0.540617285	0.588771402	0.882817557	up_in_case
PRJNA8274(	ENSGALG00010000075	ENSGALG00010000075	case	control	-0.003995729	0.420139827	-0.009510475	0.992411853	0.997995042	down_in_case
PRJNA8274(	ENSGALG00010000076	ENSGALG00010000076	case	control	-0.323519699	0.12110236	-2.671456607	0.007552284	0.151682101	down_in_case
PRJNA8274(	ENSGALG00010000078	ENSGALG00010000078	case	control	2.089509745	0.401884874	5.19927442	2.00068E-07	6.50221E-05	up_in_case
PRJNA8274(	ENSGALG00010000081	ENSGALG00010000081	case	control	-0.739066454	0.19559878	-3.778481932	0.000157787	0.01474744	down_in_case
PRJNA8274(	ENSGALG00010000084	ENSGALG00010000084	case	control	-0.018487747	0.229145077	-0.080681405	0.935695326	0.988425668	down_in_case
PRJNA8274(	ENSGALG00010000085	ENSGALG00010000085	case	control	0.143149432	0.092276338	1.551312448	0.120826825	0.519499513	up_in_case
PRJNA8274(	ENSGALG00010000089	ENSGALG00010000089	case	control	0.193781202	0.162945156	1.189241871	0.234344502	0.659210368	up_in_case
PRJNA8274(	ENSGALG00010000091	ENSGALG00010000091	case	control	-0.633213264	0.96619858	-0.655365551	0.512232426	0.845834144	down_in_case
PRJNA8274(	ENSGALG00010000092	INPPL1	case	control	-0.135678967	0.083193751	-1.630879304	0.10291579	0.492370622	down_in_case
PRJNA8274(	ENSGALG00010000093	ENSGALG00010000093	case	control	-0.058535802	0.275745476	-0.212282003	0.831887035	0.962371901	down_in_case
PRJNA8274(	ENSGALG00010000098	ENSGALG00010000098	case	control	-0.070350473	0.108144294	-0.650524128	0.515353722	0.847750297	down_in_case
PRJNA8274(	ENSGALG00010000105	CACNA1F	case	control	-0.644074512	0.290932679	-2.213826624	0.026840708	0.291658148	down_in_case
PRJNA8274(	ENSGALG00010000107	ENSGALG00010000107	case	control	-0.941409283	0.437922575	-2.149716264	0.031577665	0.3098361	down_in_case
PRJNA8274(	ENSGALG00010000121	ENSGALG00010000121	case	control	-0.127987782	0.106499432	-1.201769621	0.229452799	0.653695091	down_in_case
PRJNA8274(	ENSGALG00010000123	ENSGALG00010000123	case	control	0.038456702	0.190820287	0.201533612	0.84028135	0.965297502	up_in_case
PRJNA8274(	ENSGALG00010000124	ENSGALG00010000124	case	control	0.013854719	0.193688795	0.071530823	0.942975294	0.990054824	up_in_case
PRJNA8274(	ENSGALG00010000126	ENSGALG00010000126	case	control	-0.106785318	0.202241562	-0.528008766	0.597493251	0.884680373	down_in_case
PRJNA8274(	ENSGALG00010000127	ENSGALG00010000127	case	control	-0.046466735	0.089966398	-0.516489894	0.605512295	0.88576746	down_in_case
PRJNA8274(	ENSGALG00010000133	USP39	case	control	0.014454904	0.068097358	0.212268202	0.831897801	0.962371901	up_in_case
PRJNA8274(	ENSGALG00010000134	ENSGALG00010000134	case	control	0.070989296	0.071577835	0.991777637	0.321306009	0.73942679	up_in_case
PRJNA8274(	ENSGALG00010000137	ENSGALG00010000137	case	control	-1.292741738	0.298176455	-4.335492351	1.45434E-05	0.002385485	down_in_case

PRJNA8274(	ENSGALG00010000138	ENSGALG00010000138	case	control	-0.265583402	0.397339123	-0.668404863	0.503875192	0.842675107	down_in_case
PRJNA8274(	ENSGALG00010000140	SEPHS3	case	control	-0.00987014	0.066897878	-0.147540409	0.882705487	0.976801393	down_in_case
PRJNA8274(	ENSGALG00010000141	ENSGALG00010000141	case	control	-0.067853118	0.064342833	-1.054555961	0.291628454	0.714417151	down_in_case
PRJNA8274(	ENSGALG00010000142	ENSGALG00010000142	case	control	-0.949342	0.356359197	-2.664003088	0.007721685	0.153361252	down_in_case
PRJNA8274(	ENSGALG00010000143	SCARF2	case	control	0.054562057	0.067299679	0.810732796	0.417519135	0.799987395	up_in_case
PRJNA8274(	ENSGALG00010000145	ENSGALG00010000145	case	control	0.196291275	0.094640374	2.074075442	0.038072305	0.330206289	up_in_case
PRJNA8274(	ENSGALG00010000146	ACADM	case	control	-0.512593218	0.098139213	-5.223123373	1.7593E-07	5.8507E-05	down_in_case
PRJNA8274(	ENSGALG00010000147	ENSGALG00010000147	case	control	0.027325886	0.074618822	0.366206341	0.714211111	0.926862121	up_in_case
PRJNA8274(	ENSGALG00010000148	ENSGALG00010000148	case	control	-0.167000242	0.128862333	-1.295958545	0.194989767	0.617865399	down_in_case
PRJNA8274(	ENSGALG00010000149	ENSGALG00010000149	case	control	-0.008210265	0.103929268	-0.078998581	0.937033752	0.988800485	down_in_case
PRJNA8274(	ENSGALG00010000150	B3GAT3	case	control	0.117305252	0.119621977	0.98063295	0.32677378	0.744561034	up_in_case
PRJNA8274(	ENSGALG00010000151	TBX5	case	control	0.118428979	0.157451739	0.752160501	0.451954544	0.82064437	up_in_case
PRJNA8274(	ENSGALG00010000153	NF2	case	control	0.022419417	0.050383575	0.444974725	0.65633802	0.907494354	up_in_case
PRJNA8274(	ENSGALG00010000154	ENSGALG00010000154	case	control	-0.389609807	2.093325385	-0.186120042	0.852350626	0.969582953	down_in_case
PRJNA8274(	ENSGALG00010000155	MAZ	case	control	-0.064084264	0.088603169	-0.72327282	0.469512288	0.829101076	down_in_case
PRJNA8274(	ENSGALG00010000156	ENSGALG00010000156	case	control	0.293705306	0.46193446	0.635815968	0.524896379	0.85237545	up_in_case
PRJNA8274(	ENSGALG00010000157	ENSGALG00010000157	case	control	-0.764546706	1.884591842	-0.405682911	0.6849756	0.916548243	down_in_case
PRJNA8274(	ENSGALG00010000161	CDC42EP2	case	control	0.021829963	0.131021611	0.166613457	0.867674204	0.973996477	up_in_case
PRJNA8274(	ENSGALG00010000166	PFDN6	case	control	0.027569549	0.091537339	0.301183637	0.763274467	0.943786458	up_in_case
PRJNA8274(	ENSGALG00010000171	DPF2	case	control	-0.166662112	0.071490113	-2.331261	0.019739601	0.252483273	down_in_case
PRJNA8274(	ENSGALG00010000172	ENSGALG00010000172	case	control	-0.260123251	0.940262321	-0.27664966	0.782049129	0.949394183	down_in_case
PRJNA8274(	ENSGALG00010000173	NIPSNAP1	case	control	0.167766403	0.134897146	1.243661618	0.213624128	0.638602829	up_in_case
PRJNA8274(	ENSGALG00010000174	ENSGALG00010000174	case	control	-0.049678021	0.186399692	-0.266513427	0.78984382	0.951843754	down_in_case
PRJNA8274(	ENSGALG00010000176	ENSGALG00010000176	case	control	0.336048408	0.341197365	0.984909155	0.324668696	0.742593544	up_in_case
PRJNA8274(	ENSGALG00010000177	ENSGALG00010000177	case	control	-0.128128516	0.099616799	-1.286213943	0.198368406	0.621920427	down_in_case
PRJNA8274(	ENSGALG00010000184	ENSGALG00010000184	case	control	1.417630445	2.945626134	0.48126625			up_in_case
PRJNA8274(	ENSGALG00010000186	THOC5	case	control	-0.101675696	0.044932509	-2.262853735	0.023644707	0.272018755	down_in_case
PRJNA8274(	ENSGALG00010000188	ENSGALG00010000188	case	control	-1.059721775	1.299639245	-0.815396872	0.414845174	0.798853486	down_in_case
PRJNA8274(	ENSGALG00010000189	ENSGALG00010000189	case	control	0.727774263	0.429695761	1.69369663	0.090322945	0.473926492	up_in_case
PRJNA8274(	ENSGALG00010000191	NEFH	case	control	-0.840193435	0.369046029	-2.276662986	0.022806358	0.267253668	down_in_case
PRJNA8274(	ENSGALG00010000192	ENSGALG00010000192	case	control	-0.045341425	0.504427957	-0.089886821	0.928377154	0.986776285	down_in_case
PRJNA8274(	ENSGALG00010000196	KSR2	case	control	-0.18308741	0.423757036	-0.43205751	0.665699615	0.912179427	down_in_case
PRJNA8274(	ENSGALG00010000197	ENSGALG00010000197	case	control	-0.267312289	0.538645212	-0.496267827	0.619705462	0.892425791	down_in_case
PRJNA8274(	ENSGALG00010000200	GAL3ST1	case	control	0.283416373	0.184676179	1.534666651	0.124865732	0.52390459	up_in_case
PRJNA8274(	ENSGALG00010000202	ENSGALG00010000202	case	control	0.872331724	0.351606044	2.480991832			up_in_case
PRJNA8274(	ENSGALG00010000205	RNF215	case	control	0.065168068	0.075704697	0.86081936	0.389337541	0.783669208	up_in_case
PRJNA8274(	ENSGALG00010000206	ENSGALG00010000206	case	control	3.048710251	0.633109779	4.815452791	1.46866E-06	0.000352447	up_in_case
PRJNA8274(	ENSGALG00010000210	CCDC157	case	control	-0.928064874	0.449000642	-2.066956675	0.038738237	0.334111449	down_in_case
PRJNA8274(	ENSGALG00010000215	SF3A1	case	control	-0.072853017	0.049559167	-1.470021011	0.141556064	0.543501954	down_in_case
PRJNA8274(	ENSGALG00010000221	TBC1D10A	case	control	0.117959836	0.057958835	2.035234757	0.041827257	0.341355095	up_in_case
PRJNA8274(	ENSGALG00010000223	CASTOR1	case	control	0.013245824	0.11773657	0.112503907	0.910423872	0.981829666	up_in_case
PRJNA8274(	ENSGALG00010000224	MYBPC2	case	control	-0.78515581	0.250418816	-3.135370663	0.001716371	0.066515188	down_in_case
PRJNA8274(	ENSGALG00010000225	LIF	case	control	0.451098218	0.370362284	1.217991782	0.223227096	0.647021328	up_in_case
PRJNA8274(	ENSGALG00010000226	ENSGALG00010000226	case	control	-0.028053703	0.263937296	-0.10628927	0.915352844	0.983445506	down_in_case
PRJNA8274(	ENSGALG00010000227	ENSGALG00010000227	case	control	-0.114004757	0.233507614	-0.488227152	0.625388955	0.895111806	down_in_case
PRJNA8274(	ENSGALG00010000230	ENSGALG00010000230	case	control	0.32174725	0.475651401	0.67643499	0.498764508	0.840142058	up_in_case
PRJNA8274(	ENSGALG00010000231	ENSGALG00010000231	case	control	0.389531894	0.445583715	0.874205858	0.382006157	0.77998936	up_in_case
PRJNA8274(	ENSGALG00010000232	ENSGALG00010000232	case	control	0.213773773	0.096117788	2.224081274	0.026142977	0.28691064	up_in_case
PRJNA8274(	ENSGALG00010000233	HORMAD2	case	control	0.306279787	0.276382414	1.108173933	0.267786706	0.694521603	up_in_case
PRJNA8274(	ENSGALG00010000234	ENSGALG00010000234	case	control	-0.257029925	1.829833503	-0.140466291	0.888291584	0.977948171	down_in_case
PRJNA8274(	ENSGALG00010000239	ENSGALG00010000239	case	control	-0.377052724	2.098082401	-0.17971302	0.857377872	0.971282862	down_in_case
PRJNA8274(	ENSGALG00010000241	ENSGALG00010000241	case	control	0.12401941	0.498248583	0.248910712	0.803429849	0.954891353	up_in_case
PRJNA8274(	ENSGALG00010000242	ENSGALG00010000242	case	control	-0.561701154	0.215652039	-2.604664234	0.009196433	0.171458928	down_in_case
PRJNA8274(	ENSGALG00010000246	ENSGALG00010000246	case	control	-0.814979634	1.55494843	-0.524120041	0.60019505	0.884946428	down_in_case
PRJNA8274(	ENSGALG00010000247	ENSGALG00010000247	case	control	0.376123959	0.284668576	1.321269684	0.186411459	0.609851564	up_in_case
PRJNA8274(	ENSGALG00010000249	ENSGALG00010000249	case	control	-0.358712998	2.013890586	-0.178119407	0.858629201	0.971828322	down_in_case
PRJNA8274(	ENSGALG00010000254	ENSGALG00010000254	case	control	0.393934988	0.34116851	1.154663975	0.248228057	0.672435338	up_in_case
PRJNA8274(	ENSGALG00010000255	ENSGALG00010000255	case	control	-0.100217113	0.138257896	-0.724856341	0.468540167	0.828596747	down_in_case
PRJNA8274(	ENSGALG00010000256	ENSGALG00010000256	case	control	-0.329237737	0.258053519	-1.275850602	0.202008336	0.625073547	down_in_case
PRJNA8274(	ENSGALG00010000258	ENSGALG00010000258	case	control	-0.188666344	0.6913548	-0.272893664	0.784934958	0.949518053	down_in_case
PRJNA8274(	ENSGALG00010000262	HNRNPKL	case	control	-0.256326137	1.614592473	-0.158755934	0.873861168	0.975062028	down_in_case
PRJNA8274(	ENSGALG00010000263	MTMR3	case	control	-0.071188456	0.071473631	-0.996010059	0.319245258	0.73861962	down_in_case
PRJNA8274(	ENSGALG00010000267	ENSGALG00010000267	case	control	0.021928856	0.091308303	0.240162779	0.810204067	0.955972758	up_in_case
PRJNA8274(	ENSGALG00010000269	ENSGALG00010000269	case	control	0.169387809	0.364889816	0.464216324	0.642492767	0.901845477	up_in_case
PRJNA8274(	ENSGALG00010000271	RTN4R	case	control	-0.001464823	0.227473613	-0.006439529	0.994862035	0.998586153	down_in_case
PRJNA8274(	ENSGALG00010000272	ENSGALG00010000272	case	control	-0.618992895	0.477218625	-1.297084528	0.194602104	0.617304812	down_in_case
PRJNA8274(	ENSGALG00010000278	ENSGALG00010000278	case	control	-0.716657163	2.359073105	-0.303787603	0.761289712	0.942985066	down_in_case
PRJNA8274(	ENSGALG00010000279	ENSGALG00010000279	case	control	-0.156080202	0.124957569	-1.249065605	0.211641079	0.636501957	down_in_case
PRJNA8274(	ENSGALG00010000281	OSBP2	case	control	-0.164605675	0.105678198	-1.557612435	0.119325149	0.516645008	down_in_case
PRJNA8274(	ENSGALG00010000282	ENSGALG00010000282	case	control	-0.773757365	1.986685212	-0.389471548	0.696927354	0.922031058	down_in_case
PRJNA8274(	ENSGALG00010000283	ENSGALG00010000283	case	control	-0.869199199	0.465156766	-1.868615621	0.061676311	0.404573966	down_in_case
PRJNA8274(	ENSGALG00010000284	ENSGALG00010000284	case	control	-0.110840209	0.122229813	-0.906818118	0.364502987	0.770152588	down_in_case
PRJNA8274(	ENSGALG00010000285	MYLPF	case	control	-0.260847122	0.124488333	-2.095353966	0.036139538	0.32592088	down_in_case
PRJNA8274(	ENSGALG00010000286	TCN2	case	control	-0.131806298	0.104674855	-1.259197322	0.207959069	0.63249038	down_in_case
PRJNA8274(	ENSGALG00010000287	IQCD	case	control	0.028209797	0.190265559	0.148265387	0.88213333	0.976801393	up_in_case
PRJNA8274(	ENSGALG00010000288	VARS2	case	control	-0.136872227	0.066609097	-2.05485786	0.039892736	0.336547209	down_in_case
PRJNA8274(	ENSGALG00010000289	QPRT	case	control	0.104412962	0.387351529	0.269556086	0.787501788	0.951121248	up_in_case
PRJNA8274(	ENSGALG00010000292	ABHD16A	case	control	0.113114502	0.112719867	1.00350103	0.31561918	0.736748426	up_in_case
PRJNA8274(	ENSGALG00010000293	ENSGALG00010000293	case	control	-0.243835695	0.223129763	-1.092797715	0.274482625	0.699970438	down_in_case
PRJNA8274(	ENSGALG00010000294	DUSP21	case	control	-0.244725824	0.266815341	-0.917210468	0.359032362	0.765374836	down_in_case
PRJNA8274(	ENSGALG00010000298	SLC35E4	case	control	0.259222057	0.20752001	1.249142468	0.21161297	0.636501957	up_in_case
PRJNA8274(	ENSGALG00010000301	UQCR10	case	control	-0.211850195	0.074267091	-2.852544678	0.004337072	0.113589968	down_in_case
PRJNA8274(	ENSGALG00010000302	ENSGALG00010000302	case	control	0.225338834	0.112402571	2.004748034	0.044989991	0.352144447	up_in_case
PRJNA8274(	ENSGALG00010000303	ENSGALG00010000303	case	control	-0.058827692	0.063870764	-0.921042555	0.35702821	0.763373775	down_in_case
PRJNA8274(	ENSGALG00010000306	ENSGALG00010000306	case	control	-0.177448244	0.094751207	-1.872780835	0.061098655	0.401706103	down_in_case
PRJNA8274(	ENSGALG00010000307	ZMAT5	case	control	0.20099551	0.078717195	2.553387584	0.010668071	0.184200521	up_in_case
PRJNA8274(	ENSGALG00010000308	ENSGALG00010000308	case	control	0.114794405	0.213132207	0.53860656	0.59015836	0.882949889	up_in_case
PRJNA8274(	ENSGALG00010000309	ENSGALG00010000309	case	control	-0.159758752	1.982300025	-0.08059262	0.935765936	0.988425668	down_in_case
PRJNA8274(	ENSGALG00010000312	CABP7	case	control	0.429859552	0.346996978	1.238799123	0.215419899	0.640470741	up_in_case
PRJNA8274(	ENSGALG00010000313	ENSGALG00010000313	case	control	-0.128774918	1.564707873	-0.082299655	0.934408429	0.988140189	down_in_case
PRJNA8274(	ENSGALG00010000318	ENSGALG00010000318	case	control	0.113382644	1.84849216	0.061337909	0.951090101	0.991802555	up_in_case
PRJNA8274(	ENSGALG00010000323	AP1B1	case	control	-0.082106874	0.050840952	-1.614975158	0.106316075	0.496267271	down_in_case
PRJNA8274(	ENSGALG00010000324	ENSGALG00010000324	case	control	-0.229093886	0.145711917	-1.572238499	0.11589525	0.512919984	down_in_case
PRJNA8274(	ENSGALG00010000326	ENSGALG00010000326	case	control	-0.041886548	1.260559479	-0.033228537	0.973492341	0.993553916	down_in_case
PRJNA8274(	ENSGALG00010000331	ENSGALG00010000331	case	control	-0.087768913	0.111469429	-0.787381025	0.431058851	0.806930928	down_in_case
PRJNA8274(	ENSGALG00010000338	ENSGALG00010000338	case	control	-0.095274292	0.116950594	-0.814654197	0.415270278	0.799221279	down_in_case
PRJNA8274(	ENSGALG00010000340	NAA10	case	control	-0.035218576	0.074634506	-0.471880602	0.637012014	0.899286272	down_in_case
PRJNA8274(	ENSGALG00010000341	EWSR1	case	control	-0.050168642	0.059918695	-0.837278623	0.402435973	0.792021921	down_in_case
PRJNA8274(	ENSGALG00010000346	ENSGALG00010000346	case	control	-0.125563506	0.065129693	-1.927899561	0.05386762	0.377972017	down_in_case
PRJNA8274(	ENSGALG00010000349	CHIR-IG1-5	case	control	1.199364689	0.304073355	3.944326823	8.00245E-05	0.009232611	up_in_case
PRJNA8274(	ENSGALG00010000350	ENSGALG00010000350	case	control	-0.170600817	0.061573595	-2.770681459	0.005593912	0.130142086	down_in_case
PRJNA8274(	ENSGALG00010000353	ENSGALG00010000353	case	control	-0.199196839	0.100560552	-1.980864602	0.04760646	0.359816265	down_in_case
PRJNA8274(	ENSGALG00010000356	ENSGALG00010000356	case	control	-0.148525602	0.159313551	-0.932284798	0.351189362	0.760831854	down_in_case
PRJNA8274(	ENSGALG00010000366	EMID1	case	control	0.182388151	0.209243041	0.871656951	0.383395552	0.780061953	up_in_case
PRJNA8274(	ENSGALG00010000367	HSD17B10	case	control	-0.135692877	0.066698211	-2.03443053	0.041908206	0.341355095	down_in_case
PRJNA8274(	ENSGALG00010000369	ENSGALG00010000369	case	control	-0.644427854	1.987274387	-0.32427724	0.745728149	0.938117773	down_in_case
PRJNA8274(	ENSGALG00010000373	ENSGALG00010000373	case	control	0.062859888	0.442351543	0.142103919	0.886997923	0.977630122	up_in_case
PRJNA8274(	ENSGALG00010000376	ASCC2	case	control	0.008521329	0.056495543	0.150831888	0.880108331	0.976380848	up_in_case
PRJNA8274(	ENSGALG00010000377	ENSGALG00010000377	case	control	-0.137175223	0.116312655	-1.179366277	0.238252358	0.663100178	down_in_case
PRJNA8274(	ENSGALG00010000381	ENSGALG00010000381	case	control	0.292581434	0.193026968	1.515754182	0.129581521	0.531989321	up_in_case
PRJNA8274(	ENSGALG00010000382	ENSGALG00010000382	case	control	-0.068009318	0.254076349	-0.267672761	0.788951218	0.951826746	down_in_case
PRJNA8274(	ENSGALG00010000384	ENSGALG00010000384	case	control	0.210101384	0.120594611	1.742212051	0.081471346	0.454383872	up_in_case
PRJNA8274(	ENSGALG00010000386	ENSGALG00010000386	case	control	-0.13502924	0.152362734	-0.886235347	0.37549072	0.774921593	down_in_case
PRJNA8274(	ENSGALG00010000387	DTX1	case	control	-0.235810736	0.312309074	-0.755055667	0.450215582	0.819302981	down_in_case
PRJNA8274(	ENSGALG00010000388	ENSGALG00010000388	case	control	-0.067980107	0.066604024	-1.020660657	0.307415241	0.730481545	down_in_case
PRJNA8274(	ENSGALG00010000402	PFKFB1	case	control	-0.040579018	0.343786626	-0.118035477	0.906039548	0.981730793	down_in_case
PRJNA8274(	ENSGALG00010000405	ENSGALG00010000405	case	control	-0.664026964	0.251609649	-2.639115658	0.008312262	0.160448204	down_in_case
PRJNA8274(	ENSGALG00010000406	NSMCE3	case	control	0.030287827	0.108556304	0.279005695	0.780240455	0.948875232	up_in_case
PRJNA8274(	ENSGALG00010000407	WSB2	case	control	-0.049397834	0.060085866	-0.822120701	0.411008205	0.796718771	down_in_case
PRJNA8274(	ENSGALG00010000408	ENSGALG00010000408	case	control	-0.445487926	0.213283395	-2.088713593	0.036733513	0.327994159	down_in_case
PRJNA8274(	ENSGALG00010000412	RFC5	case	control	0.011750075	0.074127137	0.158512467	0.874052997	0.975062028	up_in_case
PRJNA8274(	ENSGALG00010000422	ITIH6	case	control	0.460931471	0.494175417	0.932728451	0.350960191	0.760831854	up_in_case
PRJNA8274(	ENSGALG00010000423	ENSGALG00010000423	case	control	0.764809889	0.209068031	3.658186699	0.000254006	0.021415026	up_in_case
PRJNA8274(	ENSGALG00010000428	GNL3L	case	control	0.000327845	0.083785737	0.003912893	0.996877971	0.998921977	up_in_case
PRJNA8274(	ENSGALG00010000431	AIFM3	case	control	0.968248746	0.493612152	1.961557757	0.049813995	0.365302626	up_in_case
PRJNA8274(	ENSGALG00010000432	ENSGALG00010000432	case	control	-0.159767357	0.100146231	-1.595340687	0.110636072	0.503115856	down_in_case

PRJNA8274(	ENSGALG00010000434	TP53	case	control	-0.274449004	0.076395603	-3.592471181	0.000327557	0.024396164	down_in_case
PRJNA8274(	ENSGALG00010000435	P2RX6	case	control	0.342802332	0.243842626	1.405834319	0.159773329	0.572017525	up_in_case
PRJNA8274(	ENSGALG00010000436	ENSGALG00010000436	case	control	0.098787634	0.220453157	0.448111679	0.6540726	0.906478152	up_in_case
PRJNA8274(	ENSGALG00010000440	ENSGALG00010000440	case	control	1.13741319	0.388871938	2.924904263	0.003445622	0.100147146	up_in_case
PRJNA8274(	ENSGALG00010000441	SLC7A4	case	control	0.06640698	0.164323816	0.404122675	0.686122511	0.917034645	up_in_case
PRJNA8274(	ENSGALG00010000442	DGCR6	case	control	-0.098565972	0.061214338	-1.610177861	0.107359033	0.498776534	down_in_case
PRJNA8274(	ENSGALG00010000443	TBX3	case	control	-0.146129855	0.100547093	-1.453347388	0.146127326	0.551205689	down_in_case
PRJNA8274(	ENSGALG00010000444	RBM19	case	control	0.067871612	0.058140083	1.16738071	0.243056658	0.667473598	up_in_case
PRJNA8274(	ENSGALG00010000449	PLBD2	case	control	-0.060625463	0.065731836	-0.922315071	0.356364255	0.763373775	down_in_case
PRJNA8274(	ENSGALG00010000450	ENSGALG00010000450	case	control	-0.162297055	0.306588705	-0.529364102	0.596552895	0.884680373	down_in_case
PRJNA8274(	ENSGALG00010000452	SLC8B1	case	control	0.169334397	0.15680115	1.079930831	0.280172983	0.705762606	up_in_case
PRJNA8274(	ENSGALG00010000453	ACHE	case	control	0.013539268	0.165686616	0.081716122	0.934872458	0.988149615	up_in_case
PRJNA8274(	ENSGALG00010000457	TPCN1	case	control	0.100778238	0.056353393	1.788326014	0.073723425	0.437929428	up_in_case
PRJNA8274(	ENSGALG00010000458	ENSGALG00010000458	case	control	-0.168605472	0.10343366	-1.630083207	0.103083912	0.492515853	down_in_case
PRJNA8274(	ENSGALG00010000462	ENSGALG00010000462	case	control	-0.183003104	0.19663203	-0.930688169	0.352014891	0.760831854	down_in_case
PRJNA8274(	ENSGALG00010000465	ENSGALG00010000465	case	control	-0.085153366	0.149300263	-0.570349741	0.56844051	0.874500231	down_in_case
PRJNA8274(	ENSGALG00010000466	FBXO21	case	control	-0.087714517	0.055966372	-1.567271814	0.117051171	0.513845851	down_in_case
PRJNA8274(	ENSGALG00010000467	RNASEK	case	control	-0.025909936	0.151074486	-0.171504381	0.863827186	0.97295831	down_in_case
PRJNA8274(	ENSGALG00010000468	TESC	case	control	0.165837415	0.278561909	0.595334141	0.551620139	0.86521531	up_in_case
PRJNA8274(	ENSGALG00010000469	ENSGALG00010000469	case	control	0.022020714	0.207186323	0.106284591	0.915356557	0.983445506	up_in_case
PRJNA8274(	ENSGALG00010000470	FBXW8	case	control	-0.006915575	0.06723846	-0.102851482	0.918080845	0.98348611	down_in_case
PRJNA8274(	ENSGALG00010000471	ENSGALG00010000471	case	control	0.112363554	0.147606731	0.761235975	0.446516135	0.817679602	up_in_case
PRJNA8274(	ENSGALG00010000475	RNFT2	case	control	0.005195427	0.074882045	0.069381476	0.944685973	0.990313016	up_in_case
PRJNA8274(	ENSGALG00010000476	ENSGALG00010000476	case	control	-0.722947848	0.186261922	-3.881350728	0.000103878	0.011055819	down_in_case
PRJNA8274(	ENSGALG00010000479	C12orf49	case	control	0.195191583	0.076283604	2.558761936	0.010504565	0.183125942	up_in_case
PRJNA8274(	ENSGALG00010000483	ENSGALG00010000483	case	control	-0.160065562	0.150629121	-1.062646861	0.287942127	0.712013215	down_in_case
PRJNA8274(	ENSGALG00010000485	MED13L	case	control	-0.172889195	0.066809243	-2.587803547	0.009659005	0.175953849	down_in_case
PRJNA8274(	ENSGALG00010000486	ENSGALG00010000486	case	control	0.050238534	0.221912429	0.226389006	0.820898878	0.958669267	up_in_case
PRJNA8274(	ENSGALG00010000491	ENSGALG00010000491	case	control	0.02145976	0.088807778	0.241642805	0.809056952	0.955875648	up_in_case
PRJNA8274(	ENSGALG00010000495	ENSGALG00010000495	case	control	0.313182019	0.373285847	0.838987122	0.401476536	0.791223052	up_in_case
PRJNA8274(	ENSGALG00010000501	ENSGALG00010000501	case	control	0.058154584	0.075185082	0.773485678	0.439235001	0.812366461	up_in_case
PRJNA8274(	ENSGALG00010000507	TRPT1	case	control	-0.032484676	0.212322536	-0.152996837	0.87840077	0.976160112	down_in_case
PRJNA8274(	ENSGALG00010000509	ENSGALG00010000509	case	control	0.79128545	0.898376124	0.88079528			up_in_case
PRJNA8274(	ENSGALG00010000511	FERMT3	case	control	0.417193329	0.120914597	3.450314014	0.000559935	0.034365099	up_in_case
PRJNA8274(	ENSGALG00010000525	ENSGALG00010000525	case	control	-0.01124573	0.522602094	-0.021518723	0.982831868	0.996591771	down_in_case
PRJNA8274(	ENSGALG00010000526	POLD1	case	control	-0.170178942	0.097342953	-1.748241011	0.080422303	0.452237095	down_in_case
PRJNA8274(	ENSGALG00010000529	STIP1	case	control	-0.044707228	0.048528337	-0.921260255	0.356914566	0.763373775	down_in_case
PRJNA8274(	ENSGALG00010000532	ENSGALG00010000532	case	control	0.172498023	0.406753543	0.424084868	0.671503921	0.913676733	up_in_case
PRJNA8274(	ENSGALG00010000546	FLRT1	case	control	0.23651814	0.550478792	0.42965895	0.667443749	0.912432583	up_in_case
PRJNA8274(	ENSGALG00010000555	OTUB1	case	control	0.025639477	0.063821935	0.40173457	0.68787938	0.917290381	up_in_case
PRJNA8274(	ENSGALG00010000561	ENSGALG00010000561	case	control	0.009962071	0.077517738	0.128513435	0.897742666	0.980176778	up_in_case
PRJNA8274(	ENSGALG00010000564	NAA40	case	control	0.001013304	0.056957837	0.017790421	0.985806046	0.996595084	up_in_case
PRJNA8274(	ENSGALG00010000569	SIPA1	case	control	0.042901472	0.124078874	0.345759678	0.729523331	0.933556014	up_in_case
PRJNA8274(	ENSGALG00010000571	ENSGALG00010000571	case	control	1.823071422	1.614520736	1.129171884	0.258825332	0.686345498	up_in_case
PRJNA8274(	ENSGALG00010000574	ENSGALG00010000574	case	control	-0.101997069	0.095087679	-1.072663353	0.283422189	0.707725096	down_in_case
PRJNA8274(	ENSGALG00010000575	ENSGALG00010000575	case	control	-0.076815365	0.144477653	-0.531676445	0.594950109	0.884326039	down_in_case
PRJNA8274(	ENSGALG00010000576	NOS1	case	control	-0.354303102	0.384642665	-0.92112273	0.356986354	0.763373775	down_in_case
PRJNA8274(	ENSGALG00010000577	ENSGALG00010000577	case	control	0.353957893	0.165152025	2.143224663	0.032095063	0.3098361	up_in_case
PRJNA8274(	ENSGALG00010000579	ENSGALG00010000579	case	control	0.576864965	0.500623446	1.152293146	0.249200633	0.674421685	up_in_case
PRJNA8274(	ENSGALG00010000580	ENSGALG00010000580	case	control	0.242709448	0.316660995	0.766464618	0.443399897	0.814675386	up_in_case
PRJNA8274(	ENSGALG00010000581	ENSGALG00010000581	case	control	0.032871003	0.035192643	0.934030537	0.350288141	0.759878704	up_in_case
PRJNA8274(	ENSGALG00010000583	CCS	case	control	0.018068613	0.096294049	0.187639974	0.851158888	0.969234083	up_in_case
PRJNA8274(	ENSGALG00010000584	RBM14	case	control	-0.106983902	0.059211703	-1.806803324	0.07079295	0.4302334	down_in_case
PRJNA8274(	ENSGALG00010000585	SPTBN2	case	control	-0.151185253	0.271398727	-0.55705955	0.577486743	0.878143389	down_in_case
PRJNA8274(	ENSGALG00010000586	ENSGALG00010000586	case	control	-0.178844766	0.152922577	-1.169511846	0.242197471	0.666540259	down_in_case
PRJNA8274(	ENSGALG00010000589	ENSGALG00010000589	case	control	0.054000529	0.142953073	0.377750041	0.70561629	0.924320939	up_in_case
PRJNA8274(	ENSGALG00010000590	DNAH5	case	control	-0.819643942	0.422989072	-1.937742593	0.052654635	0.374713019	down_in_case
PRJNA8274(	ENSGALG00010000591	SBK2	case	control	0.159717392	0.24072912	0.663473502	0.507027345	0.843472666	up_in_case
PRJNA8274(	ENSGALG00010000592	CCT5	case	control	-0.020096533	0.056720922	-0.354305475	0.723109965	0.930215097	down_in_case
PRJNA8274(	ENSGALG00010000593	ATPSCKMT	case	control	0.095397657	0.171870179	0.555056483	0.578856027	0.879142433	up_in_case
PRJNA8274(	ENSGALG00010000595	MYO10	case	control	-0.128911933	0.143501138	-0.898333876	0.369007577	0.771834431	down_in_case
PRJNA8274(	ENSGALG00010000596	ENSGALG00010000596	case	control	0.012935233	0.687537949	0.018813846	0.984989609	0.996591771	up_in_case
PRJNA8274(	ENSGALG00010000598	ENSGALG00010000598	case	control	1.216501074	0.395845279	3.073173128	0.002117956	0.075874574	up_in_case
PRJNA8274(	ENSGALG00010000599	RETREG1	case	control	-0.366879532	0.155657765	-2.356962615	0.018425102	0.242205346	down_in_case
PRJNA8274(	ENSGALG00010000601	ENSGALG00010000601	case	control	-0.003415864	0.267566301	-0.012766423	0.989814145	0.997323462	down_in_case
PRJNA8274(	ENSGALG00010000602	ENSGALG00010000602	case	control	0.489756177	0.440615564	1.111527185	0.266341495	0.692362004	up_in_case
PRJNA8274(	ENSGALG00010000603	ZNF622	case	control	0.077530946	0.070408724	1.101155384	0.270829036	0.698323327	up_in_case
PRJNA8274(	ENSGALG00010000604	ENSGALG00010000604	case	control	0.109047466	0.136004044	0.801795764	0.422671109	0.803322284	up_in_case
PRJNA8274(	ENSGALG00010000605	MARCHF11	case	control	0.821382181	0.440868127	1.863101754	0.062447951	0.406465956	up_in_case
PRJNA8274(	ENSGALG00010000606	UBE2QL1	case	control	-0.306314528	0.125228663	-2.446041668	0.014443431	0.212076065	down_in_case
PRJNA8274(	ENSGALG00010000607	ENSGALG00010000607	case	control	-0.124181084	0.22145285	-0.560756313	0.574963673	0.877910508	down_in_case
PRJNA8274(	ENSGALG00010000608	ENSGALG00010000608	case	control	0.073988031	0.514443074	0.143821609	0.885641339	0.977630122	up_in_case
PRJNA8274(	ENSGALG00010000610	ENSGALG00010000610	case	control	-0.130470427	0.064803726	-2.013316761	0.044081316	0.350032868	down_in_case
PRJNA8274(	ENSGALG00010000611	C7orf57	case	control	-0.121096147	0.274655407	-0.440902105	0.65928387	0.908599575	down_in_case
PRJNA8274(	ENSGALG00010000612	ENSGALG00010000612	case	control	0.096403293	0.195842823	0.492248281	0.622543837	0.893944471	up_in_case
PRJNA8274(	ENSGALG00010000613	ENSGALG00010000613	case	control	-0.162352941	0.104648406	-1.551413406	0.120802645	0.519499513	down_in_case
PRJNA8274(	ENSGALG00010000614	KAT8	case	control	-0.330276137	0.138425578	-2.38594733	0.017035191	0.232224238	down_in_case
PRJNA8274(	ENSGALG00010000615	ENSGALG00010000615	case	control	0.093330856	0.60315288	0.154738307	0.877027629	0.975813349	up_in_case
PRJNA8274(	ENSGALG00010000616	PSENEN	case	control	-0.600244038	0.313091132	-1.917154393	0.05521832	0.383470355	down_in_case
PRJNA8274(	ENSGALG00010000619	ENSGALG00010000619	case	control	-0.012777314	0.246768295	-0.051778589	0.958705116	0.992056045	down_in_case
PRJNA8274(	ENSGALG00010000621	UPP1	case	control	-0.181219829	0.150111834	-1.207232131	0.227342759	0.65088213	down_in_case
PRJNA8274(	ENSGALG00010000623	ENSGALG00010000623	case	control	-0.120112156	0.100465597	-1.195555096	0.231870223	0.657233736	down_in_case
PRJNA8274(	ENSGALG00010000625	ENSGALG00010000625	case	control	-0.009643521	0.252676941	-0.038165418	0.969555793	0.992761697	down_in_case
PRJNA8274(	ENSGALG00010000627	ENSGALG00010000627	case	control	-0.07755556	0.157314337	-0.492997408	0.622014419	0.893715721	down_in_case
PRJNA8274(	ENSGALG00010000628	ENSGALG00010000628	case	control	0.288596187	0.407704139	0.707856897	0.479034129	0.831333501	up_in_case
PRJNA8274(	ENSGALG00010000630	ENSGALG00010000630	case	control	-0.554107158	0.273956146	-2.022612624	0.043113102	0.346358064	down_in_case
PRJNA8274(	ENSGALG00010000632	ENSGALG00010000632	case	control	0.781827113	0.452293748	1.728582621	0.083883822	0.458188945	up_in_case
PRJNA8274(	ENSGALG00010000633	VWC2	case	control	-0.409028002	0.369719097	-1.106321003	0.268587604	0.695419653	down_in_case
PRJNA8274(	ENSGALG00010000634	ENSGALG00010000634	case	control	-0.047345179	0.138385295	-0.342125797	0.732256224	0.933793462	down_in_case
PRJNA8274(	ENSGALG00010000635	ENSGALG00010000635	case	control	0.013614328	0.160958642	0.08458277	0.932593098	0.987717353	up_in_case
PRJNA8274(	ENSGALG00010000636	ENSGALG00010000636	case	control	-0.017507092	0.132186065	-0.132442795	0.894634068	0.980176778	down_in_case
PRJNA8274(	ENSGALG00010000639	SCN1B	case	control	-0.417463268	0.15392869	-2.712056258	0.006686724	0.141032681	down_in_case
PRJNA8274(	ENSGALG00010000641	ENSGALG00010000641	case	control	-0.985861195	0.273760545	-3.601180707	0.000316775	0.024072921	down_in_case
PRJNA8274(	ENSGALG00010000643	VKORC1	case	control	-0.265420934	0.167706391	-1.582652467	0.113500698	0.508062524	down_in_case
PRJNA8274(	ENSGALG00010000645	ENSGALG00010000645	case	control	0.594963031	0.228750676	2.600923599	0.009297316	0.172470753	up_in_case
PRJNA8274(	ENSGALG00010000646	ENSGALG00010000646	case	control	0.37015825	0.500436993	0.739670039	0.459500232	0.824480387	up_in_case
PRJNA8274(	ENSGALG00010000647	ENSGALG00010000647	case	control	0.147385685	0.600686929	0.245361898	0.806176229	0.9556344	up_in_case
PRJNA8274(	ENSGALG00010000649	PAPD7	case	control	-0.191081328	0.080332947	-2.378617176	0.017377713	0.234759681	down_in_case
PRJNA8274(	ENSGALG00010000650	BCKDK	case	control	-0.329096524	0.106914399	-3.078130974	0.002083033	0.075147345	down_in_case
PRJNA8274(	ENSGALG00010000651	ENSGALG00010000651	case	control	-0.130309934	0.247966634	-0.525513987	0.599225933	0.884854486	down_in_case
PRJNA8274(	ENSGALG00010000652	SRD5A1	case	control	0.228202188	0.139647415	1.634131137	0.102231318	0.491460867	up_in_case
PRJNA8274(	ENSGALG00010000653	NSUN2	case	control	0.062296307	0.047997836	1.297898226	0.19432231	0.61723879	up_in_case
PRJNA8274(	ENSGALG00010000654	ENSGALG00010000654	case	control	-0.024900328	0.080924285	-0.307699077	0.758311322	0.941903352	down_in_case
PRJNA8274(	ENSGALG00010000655	ENSGALG00010000655	case	control	0.941404857	0.57565161	1.635372577	0.101970968	0.491460867	up_in_case
PRJNA8274(	ENSGALG00010000656	ENSGALG00010000656	case	control	-0.451246632	0.175174396	-2.575985081	0.009995496	0.177559741	down_in_case
PRJNA8274(	ENSGALG00010000659	ENSGALG00010000659	case	control	0.033795634	0.405094547	0.083426535	0.93351239	0.988097743	up_in_case
PRJNA8274(	ENSGALG00010000660	ENSGALG00010000660	case	control	-0.162366132	0.13316617	-1.219274627	0.222739976	0.646365317	down_in_case
PRJNA8274(	ENSGALG00010000661	ENSGALG00010000661	case	control	0.391450088	0.26683343	1.467020411	0.142370503	0.54406686	up_in_case
PRJNA8274(	ENSGALG00010000670	ENSGALG00010000670	case	control	-0.124780547	0.243315601	-0.512834142	0.608067345	0.887502645	down_in_case
PRJNA8274(	ENSGALG00010000674	GRB10	case	control	-0.068521123	0.073262846	-0.935277928	0.349645087	0.759405338	down_in_case
PRJNA8274(	ENSGALG00010000684	COBL	case	control	-0.186453984	0.139311463	-1.338396567	0.180767197	0.60147482	down_in_case
PRJNA8274(	ENSGALG00010000685	ENSGALG00010000685	case	control	0.044774162	0.339278436	0.131968782	0.895008984	0.980176778	up_in_case
PRJNA8274(	ENSGALG00010000686	ENSGALG00010000686	case	control	0.134356198	0.122584001	1.096033707	0.273064024	0.698936152	up_in_case
PRJNA8274(	ENSGALG00010000687	IKZF1	case	control	-0.025562208	0.247194578	-0.103409259	0.917638164	0.983445506	down_in_case
PRJNA8274(	ENSGALG00010000688	ENSGALG00010000688	case	control	-0.453844824	0.25503596	-1.779532675	0.075152473	0.440984966	down_in_case
PRJNA8274(	ENSGALG00010000695	FIGNL1	case	control	0.020114798	0.127202583	0.158131994	0.87435279	0.975139986	up_in_case
PRJNA8274(	ENSGALG00010000697	ENSGALG00010000697	case	control	-0.01776488	0.170981743	-0.10389928	0.917249279	0.983445506	down_in_case
PRJNA8274(	ENSGALG00010000702	DDC	case	control	-0.190376134	0.480299084	-0.396369972	0.691832115	0.918929895	down_in_case
PRJNA8274(	ENSGALG00010000704	ENSGALG00010000704	case	control	-0.014334234	0.080897787	-0.177189444	0.859359587	0.971828322	down_in_case
PRJNA8274(	ENSGALG00010000708	ENSGALG00010000708	case	control	0.484546683	0.484507254	1.000081379	0.317271127	0.737768794	up_in_case
PRJNA8274(	ENSGALG00010000710	ENSGALG00010000710	case	control	-0.262570541	0.257906875	-1.018082749	0.308638633	0.731823162	down_in_case
PRJNA8274(	ENSGALG00010000711	SPRY2	case	control	-0.018989296	0.059106796	-0.321270937	0.748005079	0.939358271	down_in_case
PRJNA8274(	ENSGALG00010000713	ENSGALG00010000713	case	control	1.813331004	0.676754136	2.679453155	0.007374252	0.149577028	up_in_case
PRJNA8274(	ENSGALG00010000714	FBXL7	case	control	-0.1338589	0.062979826	-2.125425061	0.033551161	0.316061661	down_in_case
PRJNA8274(	ENSGALG00010000715	PCDH20	case	control	0.233587112	0.166994635	1.398770161	0.161881917	0.575570547	up_in_case
PRJNA8274(	ENSGALG00010000716	ENSGALG00010000716	case	control	0.917210909	0.513457632	1.786341952	0.074043908	0.438111241	up_in_case

PRJNA8274(	ENSGALG00010000717	NDFIP2	case	control	-0.105182344	0.056793364	-1.852018201	0.064023209	0.410839656	down_in_case
PRJNA8274(	ENSGALG00010000718	EEF1G	case	control	-0.054689831	0.063540812	-0.860703993	0.389401093	0.783669208	down_in_case
PRJNA8274(	ENSGALG00010000720	RBM26	case	control	-0.031052577	0.052800635	-0.588109913	0.556458519	0.867878646	down_in_case
PRJNA8274(	ENSGALG00010000721	ENSGALG00010000721	case	control	0.328818283	0.143624588	2.289428899	0.022054445	0.264801476	up_in_case
PRJNA8274(	ENSGALG00010000722	ANKH	case	control	0.292630457	0.095430646	3.066420165	0.002166387	0.076380185	up_in_case
PRJNA8274(	ENSGALG00010000724	OBI1	case	control	0.003912523	0.083650126	0.046772473	0.962694568	0.992068146	up_in_case
PRJNA8274(	ENSGALG00010000727	ENSGALG00010000727	case	control	-0.134935703	0.134513246	-1.003140637	0.31579301	0.736794815	down_in_case
PRJNA8274(	ENSGALG00010000728	OTULIN	case	control	0.152857196	0.215082984	0.710689395	0.477276733	0.830520323	up_in_case
PRJNA8274(	ENSGALG00010000729	EDNRB	case	control	-0.058390502	0.161113444	-0.362418555	0.717039276	0.926862121	down_in_case
PRJNA8274(	ENSGALG00010000730	INTS5	case	control	-0.317649686	0.107136293	-2.964912054	0.003027695	0.09371436	down_in_case
PRJNA8274(	ENSGALG00010000731	ENSGALG00010000731	case	control	0.31592632	0.31889991	0.990675476	0.32184407	0.739571618	up_in_case
PRJNA8274(	ENSGALG00010000732	ENSGALG00010000732	case	control	-0.065534264	0.119324142	-0.549212108	0.582859896	0.88115454	down_in_case
PRJNA8274(	ENSGALG00010000733	ENSGALG00010000733	case	control	-0.38023018	0.762557406	-0.498624992	0.618043595	0.891468975	down_in_case
PRJNA8274(	ENSGALG00010000734	SLAIN1	case	control	-0.333920302	0.285612316	-1.169138315	0.242347909	0.666713178	down_in_case
PRJNA8274(	ENSGALG00010000735	ENSGALG00010000735	case	control	0.058722586	0.539803146	0.108785186	0.913372874	0.982728035	up_in_case
PRJNA8274(	ENSGALG00010000737	ENSGALG00010000737	case	control	-0.310709485	0.701364106	-0.443007395	0.657760386	0.908307607	down_in_case
PRJNA8274(	ENSGALG00010000741	FAM105A	case	control	0.455794182	0.166909635	2.730784121	0.006318384	0.138365847	up_in_case
PRJNA8274(	ENSGALG00010000753	TRIO	case	control	0.025230149	0.061736202	0.408676726	0.682776916	0.915901011	up_in_case
PRJNA8274(	ENSGALG00010000757	SCEL	case	control	0.313506132	0.623557696	0.502770047	0.615125955	0.890945118	up_in_case
PRJNA8274(	ENSGALG00010000760	CDH10	case	control	0.198608782	0.168864765	1.176141046	0.239538518	0.664610169	up_in_case
PRJNA8274(	ENSGALG00010000762	ENSGALG00010000762	case	control	0.045362526	0.339819404	0.1334901	0.893805793	0.979888889	up_in_case
PRJNA8274(	ENSGALG00010000768	ENSGALG00010000768	case	control	0.656607679	0.337437358	1.9458654	0.05167091	0.371751242	up_in_case
PRJNA8274(	ENSGALG00010000770	ENSGALG00010000770	case	control	-0.430068359	0.562218922	-0.764948211	0.444302384	0.815565915	down_in_case
PRJNA8274(	ENSGALG00010000777	MYCBP2	case	control	-0.106455678	0.065350226	-1.629002429	0.103312505	0.492970761	down_in_case
PRJNA8274(	ENSGALG00010000783	DAP	case	control	-0.09277311	0.089540491	-1.036102315	0.300154412	0.723279006	down_in_case
PRJNA8274(	ENSGALG00010000784	FBXL3	case	control	-0.043981293	0.073510039	-0.598303216	0.549637631	0.864234145	down_in_case
PRJNA8274(	ENSGALG00010000785	CLN5	case	control	0.084459895	0.086705182	0.974104352	0.330004729	0.746250947	up_in_case
PRJNA8274(	ENSGALG00010000786	ANKRD33B	case	control	0.143530946	0.294556049	0.487278894	0.626060702	0.895445894	up_in_case
PRJNA8274(	ENSGALG00010000787	SNRNP48	case	control	-0.145520817	0.124954199	-1.164593249	0.244183678	0.668164293	down_in_case
PRJNA8274(	ENSGALG00010000788	GATD3AL1	case	control	-0.137989443	0.069461519	-1.986559528	0.04697123	0.357384486	down_in_case
PRJNA8274(	ENSGALG00010000789	ROPN1L	case	control	1.604872893	0.40819737	3.931610076	8.43788E-05	0.009576329	up_in_case
PRJNA8274(	ENSGALG00010000790	MARCHF6	case	control	0.069233234	0.054713638	1.265374358	0.205737163	0.628775684	up_in_case
PRJNA8274(	ENSGALG00010000791	ACOD1	case	control	-0.13004559	0.315763725	-0.411844616	0.680453318	0.915381147	down_in_case
PRJNA8274(	ENSGALG00010000792	ENSGALG00010000792	case	control	-0.139119827	1.44279389	-0.096423909	0.923183904	0.985482967	down_in_case
PRJNA8274(	ENSGALG00010000793	ENSGALG00010000793	case	control	0.524843606	1.054745211	0.497602266	0.618764407	0.891656847	up_in_case
PRJNA8274(	ENSGALG00010000795	ENSGALG00010000795	case	control	-0.278986228	0.20191214	-1.381720919	0.167057409	0.582805792	down_in_case
PRJNA8274(	ENSGALG00010000796	ENSGALG00010000796	case	control	-0.22263239	0.144345102	-1.542361932	0.122985667	0.522394484	down_in_case
PRJNA8274(	ENSGALG00010000797	ENSGALG00010000797	case	control	0.873735613	0.353679059	2.470419417	0.013495472	0.204001322	up_in_case
PRJNA8274(	ENSGALG00010000798	ENSGALG00010000798	case	control	-0.179092712	0.240134875	-0.745800512	0.455787931	0.822065071	down_in_case
PRJNA8274(	ENSGALG00010000799	ENSGALG00010000799	case	control	-0.215212969	0.099602135	-2.16072647	0.030716475	0.308259818	down_in_case
PRJNA8274(	ENSGALG00010000800	LMO7	case	control	-0.115139724	0.088305686	-1.303876673	0.192275635	0.615090794	down_in_case
PRJNA8274(	ENSGALG00010000802	SEMA5A	case	control	0.014705009	0.091969288	0.15989043	0.872967387	0.975062028	up_in_case
PRJNA8274(	ENSGALG00010000803	MTRR	case	control	0.089876881	0.090438354	0.993791644	0.320324315	0.738813491	up_in_case
PRJNA8274(	ENSGALG00010000805	FASTKD3	case	control	0.047246055	0.079699044	0.592805795	0.553311129	0.866252369	up_in_case
PRJNA8274(	ENSGALG00010000806	CFAP90	case	control	0.054533397	0.258583259	0.210892989	0.832970775	0.962544007	up_in_case
PRJNA8274(	ENSGALG00010000808	ADCY2	case	control	0.040852448	0.134953248	0.302715564	0.762106635	0.943297504	up_in_case
PRJNA8274(	ENSGALG00010000810	UCHL3	case	control	-0.057061356	0.073442523	-0.776952553	0.437186764	0.810720016	down_in_case
PRJNA8274(	ENSGALG00010000811	ENSGALG00010000811	case	control	-0.258448519	0.388624725	-0.665033649	0.506028971	0.843280685	down_in_case
PRJNA8274(	ENSGALG00010000812	ENSGALG00010000812	case	control	-0.054896831	0.063461915	-0.865035834	0.387019121	0.782604263	down_in_case
PRJNA8274(	ENSGALG00010000813	ENSGALG00010000813	case	control	-0.345513312	0.232176114	-1.488151843	0.136710854	0.539854583	down_in_case
PRJNA8274(	ENSGALG00010000814	CDH18	case	control	-0.232444372	0.223433673	-1.040328294	0.298187403	0.721487387	down_in_case
PRJNA8274(	ENSGALG00010000815	ENSGALG00010000815	case	control	0.045030202	0.103389191	0.435540717	0.663169991	0.911044469	up_in_case
PRJNA8274(	ENSGALG00010000816	TBC1D4	case	control	-0.153174131	0.08739907	-1.752583072	0.079673599	0.450131333	down_in_case
PRJNA8274(	ENSGALG00010000817	ENSGALG00010000817	case	control	-0.165958634	0.079712262	-2.081971198	0.037345101	0.328438691	down_in_case
PRJNA8274(	ENSGALG00010000818	KLF12	case	control	-0.068482481	0.096507649	-0.709606764	0.477948024	0.830520323	down_in_case
PRJNA8274(	ENSGALG00010000820	KLF5	case	control	-0.292381092	0.113309065	-2.580385714	0.009869002	0.177161817	down_in_case
PRJNA8274(	ENSGALG00010000821	ENSGALG00010000821	case	control	-0.025423811	0.174101422	-0.146028736	0.883898703	0.977325559	down_in_case
PRJNA8274(	ENSGALG00010000822	BASP1	case	control	-0.135386174	0.075699906	-1.788458958	0.073701991	0.437929428	down_in_case
PRJNA8274(	ENSGALG00010000823	PIBF1	case	control	-0.179308051	0.090907464	-1.972423858	0.048561241	0.363399983	down_in_case
PRJNA8274(	ENSGALG00010000843	DIS3	case	control	-0.042924127	0.064390401	-0.66662307	0.505012929	0.842675288	down_in_case
PRJNA8274(	ENSGALG00010000853	ENSGALG00010000853	case	control	-0.030613092	0.127850758	-0.239443962	0.810761345	0.956022547	down_in_case
PRJNA8274(	ENSGALG00010000857	ENSGALG00010000857	case	control	-0.136746128	0.397396615	-0.344104915	0.730767387	0.933770678	down_in_case
PRJNA8274(	ENSGALG00010000859	BORA	case	control	0.094675284	0.078060335	1.212847529	0.22518812	0.649074535	up_in_case
PRJNA8274(	ENSGALG00010000864	MZT1	case	control	0.077443402	0.072463928	1.068716596	0.285197391	0.709820465	up_in_case
PRJNA8274(	ENSGALG00010000870	DACH1	case	control	0.132658457	0.12078122	1.098336786	0.272057454	0.698334518	up_in_case
PRJNA8274(	ENSGALG00010000901	PCDH9	case	control	-0.451868622	0.246740338	-1.831352849	0.067047894	0.419231207	down_in_case
PRJNA8274(	ENSGALG00010001066	SLC33A1	case	control	0.044637788	0.066885076	0.667380399	0.504529183	0.842675107	up_in_case
PRJNA8274(	ENSGALG00010001070	C3orf33	case	control	-0.272058655	0.144353679	-1.884667277	0.059474773	0.397424884	down_in_case
PRJNA8274(	ENSGALG00010001082	P2RY1	case	control	-0.073996722	0.142931673	-0.517706962	0.60466274	0.885268908	down_in_case
PRJNA8274(	ENSGALG00010001104	MBNL1	case	control	-0.090622574	0.065725702	-1.378799633	0.16795654	0.584227328	down_in_case
PRJNA8274(	ENSGALG00010001111	SUCNR1	case	control	0.511351597	0.363607093	1.406330091	0.159626129	0.572017525	up_in_case
PRJNA8274(	ENSGALG00010001112	TSC22D2	case	control	0.004729357	0.07080825	0.066791046	0.946748052	0.990723333	up_in_case
PRJNA8274(	ENSGALG00010001113	IGSF10	case	control	0.06328789	0.246029168	0.257237347	0.796995555	0.95280257	up_in_case
PRJNA8274(	ENSGALG00010001114	MED12L	case	control	0.02813587	0.086081993	0.326849655	0.743781601	0.937448285	up_in_case
PRJNA8274(	ENSGALG00010001115	PFN2	case	control	-0.003956387	0.048816594	-0.081045947	0.935405412	0.988425668	down_in_case
PRJNA8274(	ENSGALG00010001116	RNF13	case	control	-0.015707799	0.032435732	-0.484274529	0.62819105	0.895894287	down_in_case
PRJNA8274(	ENSGALG00010001117	P2RY13	case	control	-0.002877819	0.229642623	-0.01253173	0.990001388	0.997323462	down_in_case
PRJNA8274(	ENSGALG00010001118	P2RY12	case	control	-0.378326909	0.220091312	-1.718954311	0.085622692	0.462737903	down_in_case
PRJNA8274(	ENSGALG00010001119	COMMD2	case	control	0.062706463	0.052334708	1.198181211	0.230846478	0.655335776	up_in_case
PRJNA8274(	ENSGALG00010001121	WWTR1	case	control	0.025431738	0.056721234	0.44836362	0.653890793	0.906478152	up_in_case
PRJNA8274(	ENSGALG00010001122	P2RY14	case	control	-1.266045629	0.283413606	-4.467130735	7.92757E-06	0.001417053	down_in_case
PRJNA8274(	ENSGALG00010001123	CP	case	control	0.041959194	0.096340456	0.435530365	0.663177503	0.911044469	up_in_case
PRJNA8274(	ENSGALG00010001125	CLRN1	case	control	-0.163584625	0.186865864	-0.875412029	0.381349757	0.779265722	down_in_case
PRJNA8274(	ENSGALG00010001127	SIAH2	case	control	-0.106039597	0.088284479	-1.201112562	0.229707541	0.653695091	down_in_case
PRJNA8274(	ENSGALG00010001129	TM4SF4	case	control	1.537637353	1.057516722	1.454007602	0.145944199	0.551107254	up_in_case
PRJNA8274(	ENSGALG00010001131	HPS3	case	control	0.022178126	0.08833459	0.251069551	0.801760337	0.954294696	up_in_case
PRJNA8274(	ENSGALG00010001132	SELENOT	case	control	0.079031697	0.063223977	1.250027288	0.211289579	0.636501957	up_in_case
PRJNA8274(	ENSGALG00010001133	EIF2A	case	control	0.05590138	0.049843365	1.121541054	0.262057627	0.68793423	up_in_case
PRJNA8274(	ENSGALG00010001134	ENSGALG00010001134	case	control	0.053392047	0.412979372	0.129285022	0.897132123	0.980176778	up_in_case
PRJNA8274(	ENSGALG00010001135	GYG1	case	control	-0.115947239	0.076749504	-1.510722978	0.130859045	0.533889972	down_in_case
PRJNA8274(	ENSGALG00010001136	SERP1	case	control	0.163822133	0.057688038	2.839793819	0.00451427	0.114389963	up_in_case
PRJNA8274(	ENSGALG00010001138	SPTSSB	case	control	1.30456091	0.850607537	1.533681343	0.125108066	0.523982046	up_in_case
PRJNA8274(	ENSGALG00010001140	NMD3	case	control	-0.191410442	0.084467373	-2.2660873	0.023446039	0.270324231	down_in_case
PRJNA8274(	ENSGALG00010001141	MTAP	case	control	0.142399652	0.191586888	0.743264083	0.457321813	0.823225317	up_in_case
PRJNA8274(	ENSGALG00010001142	PPM1L	case	control	-0.352609215	0.138539299	-2.545192714	0.010921744	0.185137791	down_in_case
PRJNA8274(	ENSGALG00010001143	CDKN2A	case	control	0.11384021	0.429502081	0.265051591	0.790969719	0.95239351	up_in_case
PRJNA8274(	ENSGALG00010001145	TRIM36	case	control	0.192847506	0.214479069	0.899143713	0.368576117	0.771770041	up_in_case
PRJNA8274(	ENSGALG00010001146	DIPK1C	case	control	-0.081527888	0.118145465	-0.690063625	0.490154177	0.836347578	down_in_case
PRJNA8274(	ENSGALG00010001147	PGGT1B	case	control	0.026798144	0.090365551	0.296552656	0.766808061	0.944312372	up_in_case
PRJNA8274(	ENSGALG00010001149	CIDEA	case	control	0.206718455	0.5305649	0.389619545	0.696817897	0.922031058	up_in_case
PRJNA8274(	ENSGALG00010001150	ENSGALG00010001150	case	control	0.024167075	0.483210241	0.050013583	0.960111564	0.992056045	up_in_case
PRJNA8274(	ENSGALG00010001151	CCDC112	case	control	0.035503963	0.24888827	0.142650205	0.886566445	0.977630122	up_in_case
PRJNA8274(	ENSGALG00010001152	FEM1C	case	control	0.000635597	0.127453123	0.004986905	0.996021042	0.998710499	up_in_case
PRJNA8274(	ENSGALG00010001153	TSHZ1	case	control	0.150944631	0.122673643	1.230456903	0.218526056	0.643443819	up_in_case
PRJNA8274(	ENSGALG00010001154	PTGR3	case	control	-0.037794313	0.052808592	-0.715684915	0.474185928	0.82979716	down_in_case
PRJNA8274(	ENSGALG00010001155	CYB5A	case	control	0.125568048	0.10130205	1.239541037	0.215145201	0.640404074	up_in_case
PRJNA8274(	ENSGALG00010001156	ALDH7A1	case	control	-0.125591268	0.193217561	-0.649999241	0.515692712	0.847810358	down_in_case
PRJNA8274(	ENSGALG00010001157	TIMM21	case	control	-0.085781879	0.091926106	-0.933161239	0.350736724	0.760621043	down_in_case
PRJNA8274(	ENSGALG00010001158	FRMPD1	case	control	0.418906708	0.283487255	1.477691504	0.13949037	0.54159986	up_in_case
PRJNA8274(	ENSGALG00010001159	RTTN	case	control	0.19055037	0.095829572	1.988429729	0.046764184	0.356907825	up_in_case
PRJNA8274(	ENSGALG00010001160	TRMT10B	case	control	0.136215085	0.166070513	0.82022439	0.4120882	0.797194435	up_in_case
PRJNA8274(	ENSGALG00010001161	B3GALNT1	case	control	-0.222428595	0.0978086	-2.274121047	0.02295871	0.267253668	down_in_case
PRJNA8274(	ENSGALG00010001162	ENSGALG00010001162	case	control	-0.141765794	0.138359815	-1.024616823	0.305544023	0.728862914	down_in_case
PRJNA8274(	ENSGALG00010001164	KPNA4	case	control	-0.054970469	0.03692446	-1.488727772	0.136559069	0.539848047	down_in_case
PRJNA8274(	ENSGALG00010001165	ENSGALG00010001165	case	control	0.006549101	0.173965028	0.037646078	0.969969869	0.992761697	up_in_case
PRJNA8274(	ENSGALG00010001166	TRIM59	case	control	0.017195799	0.060112972	0.286058047	0.774833663	0.947085948	up_in_case
PRJNA8274(	ENSGALG00010001167	SMC4	case	control	-0.076425683	0.089771539	-0.851335336	0.394583101	0.786978937	down_in_case
PRJNA8274(	ENSGALG00010001168	DOK6	case	control	-0.323548329	0.206048854	-1.570250562	0.116356831	0.5134816	down_in_case
PRJNA8274(	ENSGALG00010001169	ENSGALG00010001169	case	control	-0.301942257	0.090106324	-3.350955223	0.000805333	0.043457605	down_in_case
PRJNA8274(	ENSGALG00010001170	DCAF10	case	control	-0.065390696	0.128194627	-0.51008921	0.609988964	0.888273136	down_in_case
PRJNA8274(	ENSGALG00010001171	POLR1E	case	control	0.006071893	0.167601004	0.036228264	0.97110035	0.992761697	up_in_case
PRJNA8274(	ENSGALG00010001172	TMEM200C	case	control	-0.099073156	0.14317594	-0.691967906	0.488957484	0.836347578	down_in_case
PRJNA8274(	ENSGALG00010001173	IFT80	case	control	-0.0926384	0.092588503	-1.000538911	0.317049777	0.737684968	down_in_case
PRJNA8274(	ENSGALG00010001174	ENSGALG00010001174	case	control	-0.023853408	0.062936556	-0.379007193	0.704682522	0.923973441	down_in_case
PRJNA8274(	ENSGALG00010001177	SEH1L	case	control	0.042563491	0.059009306	0.721301325	0.470724142	0.829101076	up_in_case
PRJNA8274(	ENSGALG00010001178	ZBTB5	case	control	0.026350911	0.131815534	0.199907482	0.841552939	0.9661041	up_in_case

PRJNA8274(	ENSGALG00010001179	GRHPR	case	control	0.41438432	0.249625954	1.660020977	0.096910233	0.485229806	up_in_case
PRJNA8274(	ENSGALG00010001180	SPIRE1	case	control	0.180854212	0.139554059	1.295943755	0.194994863	0.617865399	up_in_case
PRJNA8274(	ENSGALG00010001181	MFSD1	case	control	0.072824633	0.050604212	1.439102208	0.150121567	0.557230917	up_in_case
PRJNA8274(	ENSGALG00010001182	ZCCHC7	case	control	0.196307073	0.161574645	1.214962121	0.224380539	0.648105158	up_in_case
PRJNA8274(	ENSGALG00010001183	RARRES1	case	control	-0.000813694	0.145029417	-0.005610544	0.995523457	0.998710499	down_in_case
PRJNA8274(	ENSGALG00010001185	PTPN2	case	control	-0.062429175	0.043066338	-1.449604916	0.147168724	0.552345978	down_in_case
PRJNA8274(	ENSGALG00010001187	GFM1	case	control	-0.062545518	0.081338566	-0.7689528	0.441921331	0.813507909	down_in_case
PRJNA8274(	ENSGALG00010001188	PSMG2	case	control	-0.044128394	0.080830524	-0.545937251	0.585109071	0.881307382	down_in_case
PRJNA8274(	ENSGALG00010001190	LXN	case	control	0.534957544	0.275623736	1.94089795	0.052270657	0.373200684	up_in_case
PRJNA8274(	ENSGALG00010001191	TOMM5	case	control	0.061589286	0.180842208	0.340569197	0.733427922	0.933846596	up_in_case
PRJNA8274(	ENSGALG00010001192	ENSGALG00010001192	case	control	0.0517368	0.098204666	0.526826298	0.598314219	0.884717127	up_in_case
PRJNA8274(	ENSGALG00010001193	PRELID3A	case	control	0.368301861	0.18232944	2.019980213	0.04338544	0.347864599	up_in_case
PRJNA8274(	ENSGALG00010001194	POT1	case	control	-0.110411187	0.066137207	-1.669426197	0.095032945	0.48148469	down_in_case
PRJNA8274(	ENSGALG00010001195	ZNF608	case	control	0.213288292	0.129560112	1.646249672	0.099712366	0.488707918	up_in_case
PRJNA8274(	ENSGALG00010001196	LSMEM1	case	control	-0.164966469	0.142418928	-1.158318433	0.246734112	0.671238151	down_in_case
PRJNA8274(	ENSGALG00010001197	CEP76	case	control	0.075210875	0.092485229	0.813220398	0.416091707	0.799987395	up_in_case
PRJNA8274(	ENSGALG00010001198	IFRD1	case	control	0.075385658	0.073225373	1.029501859	0.303243906	0.725925651	up_in_case
PRJNA8274(	ENSGALG00010001199	AFG3L2	case	control	-0.047679673	0.03753133	-1.27039656	0.203943408	0.626103636	down_in_case
PRJNA8274(	ENSGALG00010001200	CTTNBP2	case	control	-0.01390291	0.073342662	-0.189561019	0.849653135	0.968927451	down_in_case
PRJNA8274(	ENSGALG00010001201	TUBB6	case	control	0.023296877	0.08563705	0.272042028	0.785589704	0.949929982	up_in_case
PRJNA8274(	ENSGALG00010001202	CFTR	case	control	-0.454757272	0.178897146	-2.542004067	0.011021889	0.185137791	down_in_case
PRJNA8274(	ENSGALG00010001203	TMX3	case	control	0.042370032	0.05530434	0.766124912	0.443601981	0.814764409	up_in_case
PRJNA8274(	ENSGALG00010001204	SNX2	case	control	-0.079832712	0.114620189	-0.696497823	0.48611712	0.834635018	down_in_case
PRJNA8274(	ENSGALG00010001205	CEP192	case	control	-0.129963995	0.062107226	-2.092574459	0.036387159	0.32664727	down_in_case
PRJNA8274(	ENSGALG00010001206	RSRC1	case	control	-0.14448811	0.068927103	-2.096245228	0.036060443	0.325852945	down_in_case
PRJNA8274(	ENSGALG00010001208	MOV10L1	case	control	0.488802165	0.662376317	0.737952358	0.460543401	0.82454265	up_in_case
PRJNA8274(	ENSGALG00010001209	CDH7	case	control	0.005042441	0.142052098	0.035497122	0.971683341	0.993081005	up_in_case
PRJNA8274(	ENSGALG00010001210	PANX2	case	control	-0.123762869	0.205212536	-0.603096046	0.546444796	0.86246958	down_in_case
PRJNA8274(	ENSGALG00010001213	PPIC	case	control	0.153033123	0.204250476	0.749242431	0.453711099	0.821282604	up_in_case
PRJNA8274(	ENSGALG00010001214	TES	case	control	-0.163630399	0.082860478	-1.974770159	0.048294237	0.361953663	down_in_case
PRJNA8274(	ENSGALG00010001215	PRDM6	case	control	0.345925146	0.536236398	0.64509822	0.518863538	0.848941486	up_in_case
PRJNA8274(	ENSGALG00010001216	SHOX2	case	control	0.074133563	0.378297103	0.19596651	0.844636378	0.96721237	up_in_case
PRJNA8274(	ENSGALG00010001218	ENSGALG00010001218	case	control	0.087502581	0.107141098	0.816704165	0.414097511	0.798684741	up_in_case
PRJNA8274(	ENSGALG00010001219	TFEC	case	control	-0.032668685	0.337025364	-0.096932423	0.92278006	0.985482967	down_in_case
PRJNA8274(	ENSGALG00010001220	PLXNB2	case	control	-0.062487875	0.053612982	-1.165536271	0.243801987	0.667830028	down_in_case
PRJNA8274(	ENSGALG00010001221	GRAMD2B	case	control	0.179923206	0.163313663	1.101703326	0.27059067	0.698323327	up_in_case
PRJNA8274(	ENSGALG00010001222	ZNF800	case	control	0.0519388	0.084065981	0.617833745	0.536684937	0.857336818	up_in_case
PRJNA8274(	ENSGALG00010001223	GRM8	case	control	0.902048096	0.363215036	2.483509788	0.013009476	0.200161779	up_in_case
PRJNA8274(	ENSGALG00010001224	DNAJB9	case	control	0.087567192	0.071121204	1.231238884	0.218233532	0.643443819	up_in_case
PRJNA8274(	ENSGALG00010001225	SOCS6	case	control	0.00115642	0.071249732	0.016230518	0.987050488	0.996781748	up_in_case
PRJNA8274(	ENSGALG00010001226	VEPH1	case	control	-0.12647297	0.41221178	-0.306815515	0.758983798	0.942078947	down_in_case
PRJNA8274(	ENSGALG00010001227	ENSGALG00010001227	case	control	0.496251034	1.05816725	0.46897221	0.6390895	0.900303403	up_in_case
PRJNA8274(	ENSGALG00010001228	THAP5	case	control	-0.012971097	0.060565783	-0.214165433	0.830418051	0.962228709	down_in_case
PRJNA8274(	ENSGALG00010001229	ZNF407	case	control	-0.115841553	0.054112775	-2.140743174	0.032294755	0.309838273	down_in_case
PRJNA8274(	ENSGALG00010001230	PTX3	case	control	-0.028091335	0.204850685	-0.137130783	0.890927421	0.978917427	down_in_case
PRJNA8274(	ENSGALG00010001231	CNDP1	case	control	0.104028113	0.493191866	0.210928282	0.832943235	0.962544007	up_in_case
PRJNA8274(	ENSGALG00010001232	TSPAN12	case	control	0.122891462	0.093385343	1.315960915	0.188187162	0.611129484	up_in_case
PRJNA8274(	ENSGALG00010001233	CNDP2	case	control	0.052623989	0.084702011	0.621283828	0.534412892	0.856211151	up_in_case
PRJNA8274(	ENSGALG00010001234	AASS	case	control	-0.318071845	0.079637512	-3.993995253	6.49692E-05	0.008149641	down_in_case
PRJNA8274(	ENSGALG00010001235	CCDC102B	case	control	-0.437068845	0.245042547	-1.783644718	0.074481414	0.43920999	down_in_case
PRJNA8274(	ENSGALG00010001237	MELK	case	control	0.087616849	0.125935145	0.695729925	0.486597981	0.835095382	up_in_case
PRJNA8274(	ENSGALG00010001238	GOLIM4	case	control	0.052767949	0.081893803	0.644346056	0.519351057	0.8492533	up_in_case
PRJNA8274(	ENSGALG00010001239	ENSGALG00010001239	case	control	-0.070498017	0.309817064	-0.227547237	0.819998243	0.958669267	down_in_case
PRJNA8274(	ENSGALG00010001242	ENSGALG00010001242	case	control	0.031143183	0.626137929	0.049738534	0.960330749	0.992056045	up_in_case
PRJNA8274(	ENSGALG00010001244	ENSGALG00010001244	case	control	-0.192384945	0.129466433	-1.485983208	0.137283563	0.539854583	down_in_case
PRJNA8274(	ENSGALG00010001245	SERPINI1	case	control	-0.038408309	0.159998937	-0.240053526	0.810288762	0.955972758	down_in_case
PRJNA8274(	ENSGALG00010001248	PTPRZ1	case	control	0.155536308	0.180602014	0.861210263	0.389122249	0.783669208	up_in_case
PRJNA8274(	ENSGALG00010001249	PDCD10	case	control	0.032024119	0.062590302	0.511646658	0.608898325	0.8877909	up_in_case
PRJNA8274(	ENSGALG00010001250	ENSGALG00010001250	case	control	-0.063282295	0.540062692	-0.117175832	0.906720719	0.981730793	down_in_case
PRJNA8274(	ENSGALG00010001251	ZNF277	case	control	-0.095031095	0.083174998	-1.142543998	0.253227969	0.678882631	down_in_case
PRJNA8274(	ENSGALG00010001252	SNCAIP	case	control	-0.121993857	0.198590168	-0.614299578	0.539017381	0.858476757	down_in_case
PRJNA8274(	ENSGALG00010001254	NETO1	case	control	-0.120071387	0.28704705	-0.418298626	0.675728794	0.914247368	down_in_case
PRJNA8274(	ENSGALG00010001258	ENSGALG00010001258	case	control	-0.004804058	0.116645659	-0.041185057	0.967148367	0.992731535	down_in_case
PRJNA8274(	ENSGALG00010001259	ENSGALG00010001259	case	control	-0.207761954	0.311255151	-0.667497239	0.504454573	0.842675107	down_in_case
PRJNA8274(	ENSGALG00010001260	ENSGALG00010001260	case	control	-0.709842713	0.618093034	-1.148439916	0.250787003	0.676268933	down_in_case
PRJNA8274(	ENSGALG00010001262	ENSGALG00010001262	case	control	-0.010563125	0.101613609	-0.103953838	0.917205982	0.983445506	down_in_case
PRJNA8274(	ENSGALG00010001264	FAM3C	case	control	-0.058212488	0.05846037	-0.995759829	0.319366853	0.738739525	down_in_case
PRJNA8274(	ENSGALG00010001265	OPN1LW	case	control	-0.276614734	0.365011506	-0.757824698	0.448555935	0.818701429	down_in_case
PRJNA8274(	ENSGALG00010001267	BCHE	case	control	-0.329390875	0.093355957	-3.528332697	0.000418186	0.028760256	down_in_case
PRJNA8274(	ENSGALG00010001268	CBLN2	case	control	-0.597711832	0.183884443	-3.250475246	0.001152123	0.052995354	down_in_case
PRJNA8274(	ENSGALG00010001270	DRAP1	case	control	-0.004469912	0.053804671	-0.083076651	0.933790591	0.988101632	down_in_case
PRJNA8274(	ENSGALG00010001271	ENSGALG00010001271	case	control	-0.143673674	0.118231416	-1.215190334	0.224293506	0.648105158	down_in_case
PRJNA8274(	ENSGALG00010001273	ENSGALG00010001273	case	control	0.473575067	0.37242562	1.27159637	0.203516567	0.62581674	up_in_case
PRJNA8274(	ENSGALG00010001274	DLGAP1	case	control	0.065394242	0.321649627	0.203308933	0.838893574	0.965176451	up_in_case
PRJNA8274(	ENSGALG00010001276	ENSGALG00010001276	case	control	-0.47621333	0.288642844	-1.649835909	0.098976502	0.488407564	down_in_case
PRJNA8274(	ENSGALG00010001277	ENSGALG00010001277	case	control	-0.029159467	0.078851234	-0.369803566	0.711528858	0.926542436	down_in_case
PRJNA8274(	ENSGALG00010001279	LPIN2	case	control	0.189489577	0.122382277	1.548341645	0.121540063	0.519686058	up_in_case
PRJNA8274(	ENSGALG00010001281	LRRN3	case	control	0.092106277	0.106271269	0.866709107	0.386101414	0.78218735	up_in_case
PRJNA8274(	ENSGALG00010001283	ENSGALG00010001283	case	control	-0.095402761	0.085222168	-1.119459437	0.262944186	0.688674415	down_in_case
PRJNA8274(	ENSGALG00010001285	ENSGALG00010001285	case	control	-0.012391254	0.131171578	-0.094465994	0.924738994	0.985571367	down_in_case
PRJNA8274(	ENSGALG00010001286	SRFBP1	case	control	0.073301168	0.207799675	0.352749193	0.724276477	0.9309801	up_in_case
PRJNA8274(	ENSGALG00010001287	PLPP4	case	control	0.20977452	0.313524592	0.66908474	0.503441424	0.842604443	up_in_case
PRJNA8274(	ENSGALG00010001288	TGIF1	case	control	-0.035637496	0.091958854	-0.387537409	0.698358396	0.922288441	down_in_case
PRJNA8274(	ENSGALG00010001289	ENSGALG00010001289	case	control	0.456254461	0.227711534	2.003651079	0.04510745	0.352291176	up_in_case
PRJNA8274(	ENSGALG00010001291	ENSGALG00010001291	case	control	0.02595486	0.087296793	0.297317452	0.766224159	0.943992243	up_in_case
PRJNA8274(	ENSGALG00010001292	LOX	case	control	-0.185848666	0.165077916	-1.125823917	0.260240059	0.687282842	down_in_case
PRJNA8274(	ENSGALG00010001295	ENSGALG00010001295	case	control	-0.05333829	0.060408458	-0.882960624	0.377257547	0.775926253	down_in_case
PRJNA8274(	ENSGALG00010001297	MYL12A	case	control	0.162960699	0.078054619	2.087777771	0.036817886	0.328026329	up_in_case
PRJNA8274(	ENSGALG00010001298	DOCK4	case	control	0.029436897	0.095435325	0.308448644	0.757740973	0.941765572	up_in_case
PRJNA8274(	ENSGALG00010001300	MRPL49	case	control	0.065813663	0.151351627	0.434839484	0.663678943	0.911069299	up_in_case
PRJNA8274(	ENSGALG00010001301	GMPS	case	control	0.037831693	0.04466925	0.846929212	0.397034579	0.788531716	up_in_case
PRJNA8274(	ENSGALG00010001302	ENSGALG00010001302	case	control	0.162496133	0.222492887	0.730343048	0.465180521	0.826255378	up_in_case
PRJNA8274(	ENSGALG00010001304	SYVN1	case	control	-0.099026183	0.073177421	-1.35323412	0.175980848	0.594809383	down_in_case
PRJNA8274(	ENSGALG00010001305	WNT16	case	control	0.145125706	0.250123584	0.580216003	0.561768963	0.870629259	up_in_case
PRJNA8274(	ENSGALG00010001306	CEP120	case	control	0.045656064	0.134400175	0.339702415	0.734080643	0.933846596	up_in_case
PRJNA8274(	ENSGALG00010001307	CALU	case	control	0.116117103	0.079397309	1.462481587	0.143609287	0.546900878	up_in_case
PRJNA8274(	ENSGALG00010001308	MGMT	case	control	0.102039475	0.087612045	1.164674039	0.244150961	0.668164293	up_in_case
PRJNA8274(	ENSGALG00010001313	EMILIN2	case	control	-0.01597565	0.122656236	-0.130247349	0.896370735	0.980176778	down_in_case
PRJNA8274(	ENSGALG00010001320	CPED1	case	control	0.18453601	0.200001103	0.922674958	0.35617662	0.763373775	up_in_case
PRJNA8274(	ENSGALG00010001321	HOXA13	case	control	0.146482805	0.761578314	0.192341093	0.847475032	0.968194691	up_in_case
PRJNA8274(	ENSGALG00010001330	ENSGALG00010001330	case	control	-0.052474875	0.098250138	-0.534094668	0.593276038	0.883625114	down_in_case
PRJNA8274(	ENSGALG00010001333	PLCH1	case	control	-0.09499778	0.199807356	-0.47544686	0.634468501	0.898107199	down_in_case
PRJNA8274(	ENSGALG00010001334	EBF3	case	control	-0.052182525	0.099625133	-0.523788761	0.600425471	0.884954589	down_in_case
PRJNA8274(	ENSGALG00010001343	ENSGALG00010001343	case	control	-0.417349291	0.306843733	-1.360136272	0.173786805	0.592926641	down_in_case
PRJNA8274(	ENSGALG00010001347	MYOM1	case	control	-0.216519185	0.095843511	-2.259090711	0.023877743	0.273286623	down_in_case
PRJNA8274(	ENSGALG00010001349	ING3	case	control	-0.031203752	0.072804156	-0.428598506	0.668215433	0.912740538	down_in_case
PRJNA8274(	ENSGALG00010001351	CAPN1	case	control	-0.328148809	0.079207672	-4.14289173	3.42954E-05	0.004808079	down_in_case
PRJNA8274(	ENSGALG00010001353	GLRX3	case	control	-0.02678985	0.058718892	-0.456239022	0.648218108	0.903510177	down_in_case
PRJNA8274(	ENSGALG00010001358	TCERG1L	case	control	0.72835293	0.338329381	2.152792429	0.031334995	0.3098361	up_in_case
PRJNA8274(	ENSGALG00010001363	HIBADH	case	control	-0.006671381	0.068682688	-0.097133375	0.922620477	0.985482967	down_in_case
PRJNA8274(	ENSGALG00010001364	SMCHD1	case	control	0.012911712	0.055918724	0.230901415	0.817391391	0.958325563	up_in_case
PRJNA8274(	ENSGALG00010001365	ENSGALG00010001365	case	control	-0.531424272	0.110268201	-4.819379167	1.44006E-06	0.000352447	down_in_case
PRJNA8274(	ENSGALG00010001367	ENSGALG00010001367	case	control	-0.47991033	0.486263265	-0.986935194	0.323674409	0.741159977	down_in_case
PRJNA8274(	ENSGALG00010001368	WIPF3	case	control	-0.096472598	0.106627096	-0.904766253	0.365589233	0.771194282	down_in_case
PRJNA8274(	ENSGALG00010001369	PPP2R2D	case	control	0.044591858	0.051840524	0.860173754	0.38969327	0.783877029	up_in_case
PRJNA8274(	ENSGALG00010001372	ENSGALG00010001372	case	control	-0.313090702	0.233958557	-1.33823146	0.180820997	0.60147482	down_in_case
PRJNA8274(	ENSGALG00010001373	IMPA2	case	control	-0.166470466	0.055708053	-2.988265744	0.002805655	0.08899933	down_in_case
PRJNA8274(	ENSGALG00010001378	POLA2	case	control	0.123913907	0.058833404	2.106182863	0.035188471	0.323390191	up_in_case
PRJNA8274(	ENSGALG00010001380	SCRN1	case	control	0.104294043	0.116989366	0.891483098	0.372670055	0.774252766	up_in_case
PRJNA8274(	ENSGALG00010001381	MDFIC	case	control	0.05774245	0.060442032	0.955336014	0.339407801	0.751259351	up_in_case
PRJNA8274(	ENSGALG00010001382	BNIP3	case	control	-0.17724645	0.105933927	-1.673179263	0.094292011	0.479477093	down_in_case
PRJNA8274(	ENSGALG00010001385	MME	case	control	0.155380812	0.161583713	0.961611844	0.33624462	0.750473129	up_in_case
PRJNA8274(	ENSGALG00010001386	FKBP14	case	control	0.354146725	0.116423907	3.041872879	0.002351112	0.079482024	up_in_case
PRJNA8274(	ENSGALG00010001388	MPPE1	case	control	0.025497739	0.091577323	0.278428522	0.780683428	0.948875232	up_in_case
PRJNA8274(	ENSGALG00010001395	GNAL	case	control	-0.071092597	0.063746435	-1.115240357	0.264747436	0.690729491	down_in_case
PRJNA8274(	ENSGALG00010001397	KLHL7	case	control	0.028928004	0.04833301	0.598514426	0.549496736	0.864234145	up_in_case

PRJNA8274(	ENSGALG00010001398	WDR11	case	control	-0.006980214	0.057437858	-0.121526358	0.903274139	0.980997505	down_in_case
PRJNA8274(	ENSGALG00010001400	SNX24	case	control	0.113316083	0.158871233	0.713257405	0.47568649	0.830520323	up_in_case
PRJNA8274(	ENSGALG00010001402	NUP42	case	control	0.146737111	0.111959066	1.310631793	0.189982193	0.613281846	up_in_case
PRJNA8274(	ENSGALG00010001406	EML3	case	control	0.069117601	0.063060936	1.096044642	0.273059239	0.698936152	up_in_case
PRJNA8274(	ENSGALG00010001407	FOXP2	case	control	-0.071464853	0.073624592	-0.970665517	0.331714869	0.748120789	down_in_case
PRJNA8274(	ENSGALG00010001409	GPNMB	case	control	6.315247678	0.790963068	7.984251015	1.41378E-15	1.14363E-12	up_in_case
PRJNA8274(	ENSGALG00010001411	FGFR2	case	control	-0.050315352	0.240154285	-0.209512615	0.834048089	0.963078474	down_in_case
PRJNA8274(	ENSGALG00010001412	ENSGALG00010001412	case	control	-0.070168018	0.134172633	-0.522968185	0.600996393	0.884954589	down_in_case
PRJNA8274(	ENSGALG00010001414	CCDC126	case	control	-0.009081896	0.068990661	-0.131639497	0.895269443	0.980176778	down_in_case
PRJNA8274(	ENSGALG00010001415	MC5R	case	control	0.058662631	0.185691737	0.315914064	0.752067737	0.940427752	up_in_case
PRJNA8274(	ENSGALG00010001416	AP4M1	case	control	0.052659579	0.068032718	0.77403314	0.438911194	0.812213456	up_in_case
PRJNA8274(	ENSGALG00010001418	RNMT	case	control	-0.045226434	0.061254947	-0.738331128	0.460313255	0.82454265	down_in_case
PRJNA8274(	ENSGALG00010001419	KCND2	case	control	0.103893884	0.281660751	0.368861774	0.712230757	0.92680349	up_in_case
PRJNA8274(	ENSGALG00010001420	FAM221A	case	control	1.012479529	0.326617181	3.09989672	0.001935881	0.071904164	up_in_case
PRJNA8274(	ENSGALG00010001422	AP1S1	case	control	0.124805384	0.109507239	1.139699857	0.254411367	0.679971816	up_in_case
PRJNA8274(	ENSGALG00010001423	FAM210A	case	control	-0.064315928	0.067059609	-0.959085932	0.337515457	0.750948271	down_in_case
PRJNA8274(	ENSGALG00010001426	ATE1	case	control	-3.78196E-05	0.03671355	-0.001030125	0.999178079	0.99976345	down_in_case
PRJNA8274(	ENSGALG00010001427	ASB11	case	control	-1.333664352	0.333504849	-3.998935412	6.3628E-05	0.008052044	down_in_case
PRJNA8274(	ENSGALG00010001432	NSMCE4A	case	control	0.080731894	0.076161097	1.060014844	0.289137846	0.712801679	up_in_case
PRJNA8274(	ENSGALG00010001440	CDCA7L	case	control	-0.137757576	0.05764738	-2.389658935	0.016864025	0.230991919	down_in_case
PRJNA8274(	ENSGALG00010001441	HMGCLL1	case	control	0.261697106	0.175612432	1.490196922	0.136172465	0.539840644	up_in_case
PRJNA8274(	ENSGALG00010001442	LDLRAD4	case	control	-0.032356964	0.105203201	-0.307566344	0.758412333	0.941903352	down_in_case
PRJNA8274(	ENSGALG00010001443	ANKRD26	case	control	0.081086715	0.092887268	0.872958342	0.382685784	0.780035772	up_in_case
PRJNA8274(	ENSGALG00010001448	GPR37	case	control	0.48855025	0.208476261	2.343433477	0.019107169	0.246599748	up_in_case
PRJNA8274(	ENSGALG00010001450	GPR149	case	control	0.337969644	0.458731559	0.736748186	0.461275498	0.82454265	up_in_case
PRJNA8274(	ENSGALG00010001453	TAX1BP1	case	control	-0.068608959	0.032525787	-2.109371226	0.034912551	0.322721061	down_in_case
PRJNA8274(	ENSGALG00010001454	VEGFD	case	control	0.11580549	0.126235345	0.9173777	0.358944754	0.765374836	up_in_case
PRJNA8274(	ENSGALG00010001456	LSM8	case	control	0.13181824	0.132345188	0.996018382	0.319241214	0.73861962	up_in_case
PRJNA8274(	ENSGALG00010001457	NDC80	case	control	0.000534236	0.11204887	0.004767888	0.99619579	0.998710499	up_in_case
PRJNA8274(	ENSGALG00010001458	JAZF1	case	control	0.021119794	0.070282035	0.300500607	0.763795334	0.943786458	up_in_case
PRJNA8274(	ENSGALG00010001460	MALSU1	case	control	-0.075658087	0.047326353	-1.598646044	0.109899273	0.502095719	down_in_case
PRJNA8274(	ENSGALG00010001461	ASB9	case	control	0.512142809	0.243104081	2.106681249	0.035145219	0.323390191	up_in_case
PRJNA8274(	ENSGALG00010001462	DHX36	case	control	0.040787569	0.044511309	0.91634171	0.359487698	0.765436879	up_in_case
PRJNA8274(	ENSGALG00010001463	DSEL	case	control	-0.061980531	0.051013206	-1.214989922	0.224369935	0.648105158	down_in_case
PRJNA8274(	ENSGALG00010001465	TACC2	case	control	-0.283786393	0.079366345	-3.575651506	0.000349357	0.025231323	down_in_case
PRJNA8274(	ENSGALG00010001466	PRKX	case	control	-0.109289411	0.072046482	-1.51692918	0.129284566	0.53134802	down_in_case
PRJNA8274(	ENSGALG00010001468	ANOS1	case	control	-0.065791499	0.118149806	-0.556848136	0.577631192	0.878215701	down_in_case
PRJNA8274(	ENSGALG00010001469	ARHGEF26	case	control	0.135798673	0.087650549	1.54931914	0.121305021	0.519567065	up_in_case
PRJNA8274(	ENSGALG00010001470	GPR85	case	control	-0.427721754	0.254970055	-1.677537209	0.09343748	0.478523919	down_in_case
PRJNA8274(	ENSGALG00010001471	BMP5	case	control	0.196445739	0.178571916	1.100093137	0.271291544	0.698323327	up_in_case
PRJNA8274(	ENSGALG00010001472	ENSGALG00010001472	case	control	-0.081546972	0.132110973	-0.617261161	0.53706248	0.857483532	down_in_case
PRJNA8274(	ENSGALG00010001475	BMT2	case	control	0.133528892	0.089501839	1.491912278	0.135722144	0.539774365	up_in_case
PRJNA8274(	ENSGALG00010001476	IGF2BP3	case	control	-0.165752972	0.072308488	-2.292303114	0.021888158	0.26456604	down_in_case
PRJNA8274(	ENSGALG00010001477	NLGN4	case	control	-0.008366571	0.223310645	-0.037466064	0.970113398	0.992761697	down_in_case
PRJNA8274(	ENSGALG00010001479	TMEM168	case	control	-0.073617101	0.057542109	-1.279360501	0.200770136	0.623527532	down_in_case
PRJNA8274(	ENSGALG00010001480	JAKMIP3	case	control	0.096635877	0.191347071	0.5050293	0.613538257	0.890268602	up_in_case
PRJNA8274(	ENSGALG00010001482	TRA2A	case	control	-0.006649694	0.040841643	-0.162816504	0.870662913	0.974794532	down_in_case
PRJNA8274(	ENSGALG00010001483	ENSGALG00010001483	case	control	0.116485848	0.212174431	0.549009828	0.582998705	0.881183964	up_in_case
PRJNA8274(	ENSGALG00010001486	WNT2	case	control	-0.288886889	0.244545075	-1.181323685	0.237474162	0.662652947	down_in_case
PRJNA8274(	ENSGALG00010001487	CDH19	case	control	0.372583455	0.148159793	2.51474066	0.011911997	0.191395011	up_in_case
PRJNA8274(	ENSGALG00010001488	GRPR	case	control	0.37527334	0.294699152	1.273411672	0.202871998	0.625537942	up_in_case
PRJNA8274(	ENSGALG00010001491	PHF3	case	control	-0.032607236	0.067374188	-0.483972226	0.62840558	0.896021517	down_in_case
PRJNA8274(	ENSGALG00010001492	ENSGALG00010001492	case	control	-0.039227375	0.316256521	-0.124036573	0.901286317	0.980628041	down_in_case
PRJNA8274(	ENSGALG00010001495	CCNL1	case	control	0.065643249	0.083737956	0.783912732	0.433091319	0.808138728	up_in_case
PRJNA8274(	ENSGALG00010001496	PTP4A1	case	control	-0.053864862	0.088584706	-0.608060512	0.543147341	0.860419516	down_in_case
PRJNA8274(	ENSGALG00010001497	LEKR1	case	control	0.060960708	0.252018913	0.241889417	0.808865851	0.955875648	up_in_case
PRJNA8274(	ENSGALG00010001499	TIPARP	case	control	-0.108560991	0.101147524	-1.073293608	0.283139402	0.707353853	down_in_case
PRJNA8274(	ENSGALG00010001501	CAPZA2	case	control	-0.057074498	0.050159618	-1.137857508	0.255179987	0.68117861	down_in_case
PRJNA8274(	ENSGALG00010001504	RBMS3	case	control	-0.142251385	0.051160219	-2.780507756	0.005427396	0.127480206	down_in_case
PRJNA8274(	ENSGALG00010001505	SSR3	case	control	0.009908478	0.05230215	0.189446862	0.849742598	0.968927451	up_in_case
PRJNA8274(	ENSGALG00010001506	DPYSL4	case	control	0.178261926	0.221451998	0.804968696	0.420837745	0.801969583	up_in_case
PRJNA8274(	ENSGALG00010001507	METTL4	case	control	0.013711061	0.104107217	0.131701354	0.895220514	0.980176778	up_in_case
PRJNA8274(	ENSGALG00010001508	CD109	case	control	0.172040449	0.234120167	0.734838226	0.462438023	0.825020344	up_in_case
PRJNA8274(	ENSGALG00010001510	AP1S2	case	control	0.023733749	0.062357256	0.380609253	0.703493215	0.923694148	up_in_case
PRJNA8274(	ENSGALG00010001512	PLCL2	case	control	-0.052979118	0.101042758	-0.524323751	0.60005338	0.884888453	down_in_case
PRJNA8274(	ENSGALG00010001513	ZRSR2	case	control	0.017620171	0.045524663	0.387046708	0.698721629	0.922288441	up_in_case
PRJNA8274(	ENSGALG00010001514	MET	case	control	-0.040012312	0.103306449	-0.387316687	0.698521773	0.922288441	down_in_case
PRJNA8274(	ENSGALG00010001516	CLTRN	case	control	0.199184028	0.63961271	0.311413493	0.755486298	0.941559531	up_in_case
PRJNA8274(	ENSGALG00010001517	CAV2	case	control	-0.104365888	0.077902328	-1.33970179	0.180342315	0.601000958	down_in_case
PRJNA8274(	ENSGALG00010001518	SLC17A5	case	control	0.157185215	0.079228513	1.983947558	0.047261686	0.358156924	up_in_case
PRJNA8274(	ENSGALG00010001520	KCNAB1	case	control	0.15560863	0.113964045	1.365418626	0.172121513	0.590390414	up_in_case
PRJNA8274(	ENSGALG00010001521	TBC1D5	case	control	-0.184816321	0.072181268	-2.560447141	0.010453757	0.182972727	down_in_case
PRJNA8274(	ENSGALG00010001525	EEF1A1	case	control	0.032385385	0.058657039	0.552114217	0.580870102	0.879575141	up_in_case
PRJNA8274(	ENSGALG00010001526	PPP1R3A	case	control	-0.227406764	0.10889303	-2.088350049	0.03676627	0.327994159	down_in_case
PRJNA8274(	ENSGALG00010001527	STK32C	case	control	0.022087735	0.115093853	0.191910639	0.847812204	0.968313864	up_in_case
PRJNA8274(	ENSGALG00010001528	NR1D2	case	control	-0.012182043	0.118254261	-0.103015676	0.917950529	0.983445506	down_in_case
PRJNA8274(	ENSGALG00010001529	ENSGALG00010001529	case	control	1.610928922	1.269831437	1.268616349	0.20457793	0.627358178	up_in_case
PRJNA8274(	ENSGALG00010001532	ENSGALG00010001532	case	control	0.213738881	0.149564525	1.429074716	0.15298276	0.561193576	up_in_case
PRJNA8274(	ENSGALG00010001533	ENSGALG00010001533	case	control	0.255506868	0.124243338	2.056503565	0.039734003	0.336210795	up_in_case
PRJNA8274(	ENSGALG00010001534	SLITRK3	case	control	-0.218081381	0.311739399	-0.699563102	0.484200192	0.834033076	down_in_case
PRJNA8274(	ENSGALG00010001535	RAP2B	case	control	-0.000969908	0.046091322	-0.021043175	0.983211214	0.996591771	down_in_case
PRJNA8274(	ENSGALG00010001540	TRABD	case	control	-0.014467092	0.082631552	-0.175079508	0.86101716	0.97240131	down_in_case
PRJNA8274(	ENSGALG00010001542	MTO1	case	control	-0.074943044	0.048749626	-1.537304987	0.124218641	0.523029039	down_in_case
PRJNA8274(	ENSGALG00010001545	SELENOO	case	control	0.04064523	0.149154062	0.272505019	0.785233733	0.949745613	up_in_case
PRJNA8274(	ENSGALG00010001546	CGAS	case	control	-0.114333432	0.086808232	-1.317080531	0.187811632	0.610946377	down_in_case
PRJNA8274(	ENSGALG00010001547	PWWP2B	case	control	-0.188067128	0.082550216	-2.278214851	0.02271378	0.267253668	down_in_case
PRJNA8274(	ENSGALG00010001548	AKAP17A	case	control	0.108405505	0.056935488	1.904005883	0.056909418	0.388573099	up_in_case
PRJNA8274(	ENSGALG00010001551	FHIP1B	case	control	-0.083632347	0.068563415	-1.219780953	0.222547924	0.646365317	down_in_case
PRJNA8274(	ENSGALG00010001552	TUBGCP6	case	control	-0.215411884	0.064564979	-3.336358013	0.000848838	0.04446293	down_in_case
PRJNA8274(	ENSGALG00010001553	CNGA4	case	control	0.014892254	0.274321998	0.054287494	0.956706114	0.992056045	up_in_case
PRJNA8274(	ENSGALG00010001554	ENSGALG00010001554	case	control	0.063221598	0.320663931	0.197158432	0.843703559	0.96721237	up_in_case
PRJNA8274(	ENSGALG00010001556	EPB41L3	case	control	-0.108617588	0.056261356	-1.930589584	0.053533827	0.37782383	down_in_case
PRJNA8274(	ENSGALG00010001557	THRB	case	control	0.137288121	0.324730323	0.422775795	0.672458849	0.913676733	up_in_case
PRJNA8274(	ENSGALG00010001558	CCKBR	case	control	0.080661906	0.336361698	0.23980705	0.810479842	0.955972758	up_in_case
PRJNA8274(	ENSGALG00010001559	HDAC10	case	control	-0.198007759	0.109739806	-1.804338526	0.071178254	0.431840649	down_in_case
PRJNA8274(	ENSGALG00010001567	INPP5A	case	control	-0.044881243	0.057621536	-0.778897027	0.436040375	0.81000376	down_in_case
PRJNA8274(	ENSGALG00010001570	SMPD1	case	control	0.085803358	0.069639228	1.232112412	0.217907096	0.643019288	up_in_case
PRJNA8274(	ENSGALG00010001585	ENSGALG00010001585	case	control	0.125071714	0.085786124	1.457948076	0.144854856	0.548818979	up_in_case
PRJNA8274(	ENSGALG00010001586	ZBTB14	case	control	0.024774238	0.078811349	0.314348611	0.753256282	0.940830189	up_in_case
PRJNA8274(	ENSGALG00010001588	ENSGALG00010001588	case	control	0.965010029	0.40057936	2.409035827	0.015994728	0.224387437	up_in_case
PRJNA8274(	ENSGALG00010001595	AKAIN1	case	control	-0.005361666	0.341838139	-0.015684809	0.987485846	0.996908047	down_in_case
PRJNA8274(	ENSGALG00010001599	TBLX1	case	control	-0.109376789	0.048229632	-2.267833761	0.023339341	0.269590124	down_in_case
PRJNA8274(	ENSGALG00010001604	ENSGALG00010001604	case	control	-0.360337127	0.193333807	-1.863808165	0.062348649	0.406004406	down_in_case
PRJNA8274(	ENSGALG00010001605	APBB1	case	control	-0.009719508	0.077252032	-0.125815562	0.899877923	0.98028579	down_in_case
PRJNA8274(	ENSGALG00010001606	PIGA	case	control	0.073409342	0.077610821	0.945864783	0.344217558	0.755870938	up_in_case
PRJNA8274(	ENSGALG00010001608	ENSGALG00010001608	case	control	0.129766092	0.510736255	0.254076524	0.799436439	0.954053991	up_in_case
PRJNA8274(	ENSGALG00010001609	ENSGALG00010001609	case	control	-0.769633987	0.49769232	-1.54640519	0.122006742	0.520338922	down_in_case
PRJNA8274(	ENSGALG00010001611	MAPK12	case	control	-0.056917968	0.129337637	-0.440072736	0.659884427	0.90882667	down_in_case
PRJNA8274(	ENSGALG00010001612	HPX	case	control	1.235814307	0.611831729	2.01985979	0.043397933	0.347864599	up_in_case
PRJNA8274(	ENSGALG00010001616	OPNVA	case	control	0.070015194	0.414594989	0.168876122	0.865894081	0.973289701	up_in_case
PRJNA8274(	ENSGALG00010001617	TRIM3	case	control	0.009961475	0.048193432	0.206697762	0.836245906	0.964302593	up_in_case
PRJNA8274(	ENSGALG00010001620	MOSPD2	case	control	-0.005018405	0.097131908	-0.051665875	0.958794929	0.992056045	down_in_case
PRJNA8274(	ENSGALG00010001621	DLGAP2	case	control	-0.160078109	0.123258931	-1.298714085	0.194042068	0.617169773	down_in_case
PRJNA8274(	ENSGALG00010001627	ARFIP2	case	control	-0.058951929	0.052566729	-1.121468461	0.262088509	0.68793423	down_in_case
PRJNA8274(	ENSGALG00010001628	MAPK11	case	control	0.069297191	0.103507649	0.669488603	0.503183848	0.842485272	up_in_case
PRJNA8274(	ENSGALG00010001632	FANCB	case	control	-0.222900024	0.147126579	-1.515022133	0.129766799	0.532166684	down_in_case
PRJNA8274(	ENSGALG00010001633	B3GAT2	case	control	-0.185465629	0.274549767	-0.675526448	0.499341354	0.840142058	down_in_case
PRJNA8274(	ENSGALG00010001635	TIMM10B	case	control	0.104296841	0.094338657	1.10555783	0.268917951	0.695896977	up_in_case
PRJNA8274(	ENSGALG00010001639	ENSGALG00010001639	case	control	0.040537415	0.089129252	0.454816059	0.649241576	0.904011281	up_in_case
PRJNA8274(	ENSGALG00010001642	ENSGALG00010001642	case	control	-0.103194402	0.110399381	-0.934737144	0.34992378	0.759549188	down_in_case
PRJNA8274(	ENSGALG00010001647	ARL8BL	case	control	-0.210414661	0.157328736	-1.337420405	0.181085448	0.601788276	down_in_case
PRJNA8274(	ENSGALG00010001649	UBE2E2	case	control	-0.155267372	0.178274485	-0.870945565	0.383783876	0.780115057	down_in_case
PRJNA8274(	ENSGALG00010001654	RPS11	case	control	-0.0636883	0.064053861	-0.994292914	0.320080285	0.738813491	down_in_case
PRJNA8274(	ENSGALG00010001657	SMAP1	case	control	0.031557444	0.044897194	0.702882336	0.48212908	0.832931266	up_in_case
PRJNA8274(	ENSGALG00010001661	TCTN3	case	control	0.083628112	0.048841088	1.712249163	0.086850761	0.465853669	up_in_case
PRJNA8274(	ENSGALG00010001666	PIEZO2	case	control	-0.107298361	0.095836391	-1.119599344	0.262884535	0.688674415	down_in_case

PRJNA8274(	ENSGALG00010001668	UBE2E1	case	control	-0.122087594	0.062789685	-1.94438932	0.051848521	0.371953818	down_in_case
PRJNA8274(	ENSGALG00010001670	SHOX	case	control	0.284947487	0.235829618	1.20827693	0.226940759	0.650578187	up_in_case
PRJNA8274(	ENSGALG00010001674	ENSGALG00010001674	case	control	0.010416532	0.048634153	0.214181427	0.830405579	0.962228709	up_in_case
PRJNA8274(	ENSGALG00010001677	SDHAF4	case	control	0.070710119	0.093035808	0.76003122	0.447235924	0.817835513	up_in_case
PRJNA8274(	ENSGALG00010001678	SYAP1	case	control	-0.041762974	0.047036103	-0.887891892	0.374598906	0.774434633	down_in_case
PRJNA8274(	ENSGALG00010001681	NAPG	case	control	0.001297673	0.036765473	0.035295978	0.97184373	0.993165536	up_in_case
PRJNA8274(	ENSGALG00010001686	WASL	case	control	-0.073124572	0.040122255	-1.822543913	0.068372497	0.422366828	down_in_case
PRJNA8274(	ENSGALG00010001688	ENSGALG00010001688	case	control	0.013426249	0.089939956	0.149280137	0.881332588	0.97667824	up_in_case
PRJNA8274(	ENSGALG00010001689	ILK	case	control	0.003394138	0.052124867	0.065115523	0.948082021	0.991291133	up_in_case
PRJNA8274(	ENSGALG00010001691	CTPS2	case	control	-0.083788848	0.093890943	-0.892406084	0.372175311	0.774077746	down_in_case
PRJNA8274(	ENSGALG00010001692	ALDH18A1	case	control	0.228078585	0.124579673	1.83078491	0.067132653	0.419241238	up_in_case
PRJNA8274(	ENSGALG00010001693	NKIRAS1	case	control	-0.011286678	0.177171377	-0.063704864	0.949205232	0.99138853	down_in_case
PRJNA8274(	ENSGALG00010001694	ENSGALG00010001694	case	control	-0.070708234	0.112029756	-0.631155829	0.52793864	0.853386536	down_in_case
PRJNA8274(	ENSGALG00010001695	LMOD2	case	control	-0.265089937	0.147801475	-1.793554066	0.072884374	0.436712105	down_in_case
PRJNA8274(	ENSGALG00010001696	FAM135A	case	control	-0.118004709	0.050456746	-2.33873005	0.019349409	0.24882783	down_in_case
PRJNA8274(	ENSGALG00010001697	TAF10	case	control	0.041597457	0.073840241	0.563344003	0.57320066	0.877449175	up_in_case
PRJNA8274(	ENSGALG00010001698	RPL15	case	control	-0.034356853	0.061554763	-0.55815101	0.576741271	0.878085957	down_in_case
PRJNA8274(	ENSGALG00010001699	ASB15	case	control	-0.476243348	0.145830889	-3.265723404	0.001091848	0.051553287	down_in_case
PRJNA8274(	ENSGALG00010001700	ENSGALG00010001700	case	control	-0.266017331	0.19555104	-1.360347304	0.173720046	0.592926641	down_in_case
PRJNA8274(	ENSGALG00010001701	NDUFA5	case	control	-0.304138098	0.097450003	-3.120965522	0.001802591	0.068556002	down_in_case
PRJNA8274(	ENSGALG00010001702	PLEKHA1	case	control	0.123374121	0.06268463	1.968171785	0.049048275	0.36379167	up_in_case
PRJNA8274(	ENSGALG00010001704	ENSGALG00010001704	case	control	-0.095923743	0.114943922	-0.834526447	0.40398439	0.79260318	down_in_case
PRJNA8274(	ENSGALG00010001705	HTRA1	case	control	-0.067245421	0.131545587	-0.511194806	0.609214655	0.8877909	down_in_case
PRJNA8274(	ENSGALG00010001706	IQUB	case	control	0.366870318	0.225206822	1.629037323	0.103305118	0.492970761	up_in_case
PRJNA8274(	ENSGALG00010001708	AADAC	case	control	0.20402774	0.489913851	0.416456361	0.677076094	0.914726861	up_in_case
PRJNA8274(	ENSGALG00010001710	LCMT2	case	control	0.118812872	0.088053143	1.349331414	0.17723054	0.5959216	up_in_case
PRJNA8274(	ENSGALG00010001711	CREB5	case	control	-0.085056725	0.12433314	-0.684103412	0.493909867	0.838023719	down_in_case
PRJNA8274(	ENSGALG00010001712	ATP6AP1	case	control	-0.061248785	0.085962874	-0.712502756	0.476153505	0.830520323	down_in_case
PRJNA8274(	ENSGALG00010001713	PNPLA4	case	control	0.348468682	0.172996974	2.014305075	0.043977513	0.349803675	up_in_case
PRJNA8274(	ENSGALG00010001716	TRIL	case	control	-0.109966682	0.078183259	-1.406524654	0.159568389	0.572017525	down_in_case
PRJNA8274(	ENSGALG00010001717	STARD10	case	control	0.308127836	0.442179747	0.69683842	0.485903919	0.834635018	up_in_case
PRJNA8274(	ENSGALG00010001721	TM4SF1	case	control	-0.021942461	0.063452395	-0.345809812	0.729485651	0.933556014	down_in_case
PRJNA8274(	ENSGALG00010001725	MTA2	case	control	-0.07962662	0.06522998	-1.220705883	0.222197399	0.646080279	down_in_case
PRJNA8274(	ENSGALG00010001727	COL9A1	case	control	0.219934267	0.863624129	0.254664338			up_in_case
PRJNA8274(	ENSGALG00010001728	STS	case	control	-0.253768856	0.12727289	-1.993895598	0.046163465	0.356053762	down_in_case
PRJNA8274(	ENSGALG00010001729	ENSGALG00010001729	case	control	0.379961587	0.242333594	1.567927831	0.116897976	0.513560999	up_in_case
PRJNA8274(	ENSGALG00010001731	ENSGALG00010001731	case	control	-0.020267032	0.105633312	-0.19186213	0.847850202	0.968313864	down_in_case
PRJNA8274(	ENSGALG00010001738	PUDP	case	control	-0.150553689	0.090425164	-1.664953444	0.095922044	0.483185646	down_in_case
PRJNA8274(	ENSGALG00010001741	SATB1	case	control	-0.08420284	0.199126474	-0.422861101	0.672396605	0.913676733	down_in_case
PRJNA8274(	ENSGALG00010001745	CADPS2	case	control	-0.122078532	0.141603626	-0.862114447	0.388624542	0.783274271	down_in_case
PRJNA8274(	ENSGALG00010001749	VAPA	case	control	-0.083477699	0.060154261	-1.387727107	0.165220158	0.580618526	down_in_case
PRJNA8274(	ENSGALG00010001751	AVPR2C	case	control	-0.181287655	0.244771551	-0.740640216	0.458911617	0.824116052	down_in_case
PRJNA8274(	ENSGALG00010001752	ARRB1	case	control	-0.153601102	0.075188551	-2.042878866	0.041064431	0.338261153	down_in_case
PRJNA8274(	ENSGALG00010001753	PNPLA8	case	control	-0.128213722	0.068197734	-1.880029082	0.060104114	0.398649739	down_in_case
PRJNA8274(	ENSGALG00010001754	APCDD1	case	control	0.047147361	0.232105918	0.203128646	0.839034482	0.965260909	up_in_case
PRJNA8274(	ENSGALG00010001756	ENSGALG00010001756	case	control	0.222889377	0.365512387	0.609799788	0.541994442	0.85975913	up_in_case
PRJNA8274(	ENSGALG00010001758	COL19A1	case	control	-0.156691412	0.133298524	-1.175492476	0.239797746	0.664684583	down_in_case
PRJNA8274(	ENSGALG00010001759	C10orf88	case	control	-0.016243153	0.046626924	-0.348364253	0.727566645	0.932979874	down_in_case
PRJNA8274(	ENSGALG00010001760	RAB5A	case	control	0.00539027	0.055476338	0.0971634	0.922596633	0.985482967	up_in_case
PRJNA8274(	ENSGALG00010001761	ELOB	case	control	-0.009246511	0.089364278	-0.103469879	0.917590054	0.983445506	down_in_case
PRJNA8274(	ENSGALG00010001763	ENSGALG00010001763	case	control	0.025724621	0.417057807	0.061681189	0.95081672	0.991589783	up_in_case
PRJNA8274(	ENSGALG00010001767	ENSGALG00010001767	case	control	-0.126420335	0.119060341	-1.061817346	0.288318614	0.712697697	down_in_case
PRJNA8274(	ENSGALG00010001771	ENSGALG00010001771	case	control	-0.070957429	0.111850972	-0.634392606	0.525824634	0.85251736	down_in_case
PRJNA8274(	ENSGALG00010001773	NRCAM	case	control	-0.111269051	0.107548691	-1.034592338	0.300859334	0.72356012	down_in_case
PRJNA8274(	ENSGALG00010001776	LMBRD1	case	control	-0.047952306	0.069412071	-0.690835252	0.489669079	0.836347578	down_in_case
PRJNA8274(	ENSGALG00010001778	KAT2B	case	control	0.036458238	0.061975612	0.588267495	0.55635276	0.867878646	up_in_case
PRJNA8274(	ENSGALG00010001779	ENSGALG00010001779	case	control	0.407290207	0.552975079	0.736543513	0.461399997	0.82454265	up_in_case
PRJNA8274(	ENSGALG00010001780	ENSGALG00010001780	case	control	0.420897298	0.135960917	3.095722706	0.001963339	0.072174149	up_in_case
PRJNA8274(	ENSGALG00010001783	SGO1	case	control	0.127400397	0.109035817	1.168427037	0.242634554	0.667245024	up_in_case
PRJNA8274(	ENSGALG00010001785	CNTN1	case	control	-0.140777363	0.199146629	-0.706903066	0.479626718	0.831851816	down_in_case
PRJNA8274(	ENSGALG00010001786	P2RY8	case	control	0.086727762	0.154588546	0.561023211	0.574781714	0.877910508	up_in_case
PRJNA8274(	ENSGALG00010001788	HOXA1	case	control	0.234157576	0.262232297	0.892939497	0.371889574	0.77369739	up_in_case
PRJNA8274(	ENSGALG00010001790	HOXA2	case	control	-0.144621756	0.136448298	-1.059901503	0.289189412	0.712801679	down_in_case
PRJNA8274(	ENSGALG00010001793	IKZF5	case	control	-0.010745061	0.061781209	-0.17392118	0.861927407	0.972643856	down_in_case
PRJNA8274(	ENSGALG00010001795	ENSGALG00010001795	case	control	0.465077647	0.465060027	1.000037886	0.317292174	0.737768794	up_in_case
PRJNA8274(	ENSGALG00010001796	SLCO2B1	case	control	-0.065745282	0.099638693	-0.659836864	0.509358524	0.844521404	down_in_case
PRJNA8274(	ENSGALG00010001798	PSTK	case	control	0.187453354	0.097683526	1.918986358	0.05498606	0.38300081	up_in_case
PRJNA8274(	ENSGALG00010001800	ENSGALG00010001800	case	control	-0.01909763	0.100021489	-0.19093527	0.848576307	0.968692766	down_in_case
PRJNA8274(	ENSGALG00010001801	HOXA3	case	control	0.038929304	0.074877015	0.519909932	0.603126353	0.885268908	up_in_case
PRJNA8274(	ENSGALG00010001803	PPP4R1	case	control	-0.00742845	0.051495349	-0.144254785	0.885299281	0.977630122	down_in_case
PRJNA8274(	ENSGALG00010001805	ENSGALG00010001805	case	control	-0.126610545	0.102969057	-1.229597986	0.218847685	0.643443819	down_in_case
PRJNA8274(	ENSGALG00010001807	ASMTL	case	control	0.033767986	0.083615711	0.403847387	0.686324948	0.917152299	up_in_case
PRJNA8274(	ENSGALG00010001808	HOXA4	case	control	-0.042087004	0.131607773	-0.319791176	0.749126637	0.939503405	down_in_case
PRJNA8274(	ENSGALG00010001810	OPN2SW	case	control	0.423850998	0.363156599	1.167130099	0.243157834	0.667473598	up_in_case
PRJNA8274(	ENSGALG00010001811	ACADSB	case	control	-0.360401052	0.129940316	-2.773589165	0.005544164	0.129757041	down_in_case
PRJNA8274(	ENSGALG00010001813	HOXA5	case	control	-0.003797299	0.118134281	-0.032143917	0.97435728	0.993775355	down_in_case
PRJNA8274(	ENSGALG00010001817	SLC25A6	case	control	0.166860905	0.047384498	3.521423895	0.000429236	0.029368769	up_in_case
PRJNA8274(	ENSGALG00010001818	ADGRB3	case	control	-0.314974983	0.252586228	-1.246999827	0.212397561	0.63781712	down_in_case
PRJNA8274(	ENSGALG00010001819	HOXA6	case	control	0.064732612	0.071324017	0.907585063	0.364097489	0.770055025	up_in_case
PRJNA8274(	ENSGALG00010001820	PGM2L1	case	control	-0.140928768	0.15594956	-0.903681731	0.366164189	0.771724083	down_in_case
PRJNA8274(	ENSGALG00010001822	KCNE3	case	control	0.08328063	0.257729685	0.323131697	0.746595506	0.938412212	up_in_case
PRJNA8274(	ENSGALG00010001823	RALBP1	case	control	-0.002372085	0.05312781	-0.044648651	0.964387364	0.992068146	down_in_case
PRJNA8274(	ENSGALG00010001825	LIPT2	case	control	-0.269979247	0.123833239	-2.180183996	0.029243825	0.304235014	down_in_case
PRJNA8274(	ENSGALG00010001826	HOXA7	case	control	0.005738754	0.060045094	0.095574067	0.923858862	0.985541988	up_in_case
PRJNA8274(	ENSGALG00010001827	PDZRN4	case	control	0.097549436	0.18084143	0.539419731	0.58959727	0.882949889	up_in_case
PRJNA8274(	ENSGALG00010001828	ENSGALG00010001828	case	control	-0.992394229	1.111439964	-0.892890539	0.371915794	0.77369739	down_in_case
PRJNA8274(	ENSGALG00010001829	POLD3	case	control	-0.060307542	0.064670102	-0.932541312	0.351056847	0.760831854	down_in_case
PRJNA8274(	ENSGALG00010001831	CSF2RA	case	control	0.146669245	0.144135776	1.017576959	0.308879042	0.731823162	up_in_case
PRJNA8274(	ENSGALG00010001832	BUB3	case	control	0.102560833	0.049401155	2.076081659	0.037886399	0.330206289	up_in_case
PRJNA8274(	ENSGALG00010001834	ZCRB1	case	control	-0.069064243	0.071149415	-0.970693051	0.331701153	0.748120789	down_in_case
PRJNA8274(	ENSGALG00010001835	MECP2	case	control	-0.082633947	0.087511243	-0.944266634	0.345033412	0.755870938	down_in_case
PRJNA8274(	ENSGALG00010001836	TWSG1	case	control	0.104240552	0.069154812	1.507350664	0.131720798	0.535159035	up_in_case
PRJNA8274(	ENSGALG00010001837	TAB3	case	control	-0.05601555	0.048059129	-1.165554819	0.243794484	0.667830028	down_in_case
PRJNA8274(	ENSGALG00010001838	PPHLN1	case	control	-0.013365065	0.061421297	-0.217596591	0.827743441	0.961369482	down_in_case
PRJNA8274(	ENSGALG00010001840	HOXA9	case	control	0.144168066	0.118185875	1.219841762	0.222524866	0.646365317	up_in_case
PRJNA8274(	ENSGALG00010001843	PRICKLE1	case	control	0.094149697	0.076672912	1.227939485	0.219469686	0.643443819	up_in_case
PRJNA8274(	ENSGALG00010001844	HOXA10	case	control	0.254753317	0.142452631	1.788337043	0.073721647	0.437929428	up_in_case
PRJNA8274(	ENSGALG00010001845	ANKRD12	case	control	-0.135053724	0.114241227	-1.182180265	0.237134181	0.662437741	down_in_case
PRJNA8274(	ENSGALG00010001848	HOXA11	case	control	0.627522094	0.27866081	2.251920872	0.024327273	0.275772854	up_in_case
PRJNA8274(	ENSGALG00010001849	NDUFV2	case	control	-0.030754686	0.060999593	-0.504178548	0.614135914	0.890440426	down_in_case
PRJNA8274(	ENSGALG00010001850	MYOM2	case	control	-0.207957084	0.118522772	-1.754574931	0.079332043	0.44982086	down_in_case
PRJNA8274(	ENSGALG00010001852	CPXM2	case	control	0.218615243	0.170051171	1.285585053	0.198587916	0.622082628	up_in_case
PRJNA8274(	ENSGALG00010001853	ENSGALG00010001853	case	control	-0.216611094	0.134692161	-1.608193767	0.107792748	0.499655201	down_in_case
PRJNA8274(	ENSGALG00010001854	CHRDL2	case	control	0.054404971	0.203788141	0.266968289	0.789493577	0.951843754	up_in_case
PRJNA8274(	ENSGALG00010001856	CHST15	case	control	0.158072468	0.089350785	1.769122331	0.076873462	0.444876773	up_in_case
PRJNA8274(	ENSGALG00010001857	HNRNPA2B1	case	control	-0.040969846	0.043520728	-0.941386947	0.3465066	0.75695759	down_in_case
PRJNA8274(	ENSGALG00010001858	ADAMTS20	case	control	-0.167952394	0.109300468	-1.536611847	0.12438839	0.523029039	down_in_case
PRJNA8274(	ENSGALG00010001860	RNF169	case	control	0.10626735	0.074188151	1.432403268	0.152028444	0.560293917	up_in_case
PRJNA8274(	ENSGALG00010001861	CBX3	case	control	-0.008971874	0.042405517	-0.211573282	0.832439956	0.962371901	down_in_case
PRJNA8274(	ENSGALG00010001864	ENSGALG00010001864	case	control	-0.3757067	0.169690694	-2.214067789	0.026824116	0.291658148	down_in_case
PRJNA8274(	ENSGALG00010001865	CYBB	case	control	0.241053648	0.158408666	1.52172008	0.128079234	0.529650968	up_in_case
PRJNA8274(	ENSGALG00010001866	PUS7L	case	control	-0.018820761	0.082729639	-0.227497201	0.820037145	0.958669267	down_in_case
PRJNA8274(	ENSGALG00010001868	ENSGALG00010001868	case	control	0.046234745	0.09378946	0.492963127	0.622038641	0.893715721	up_in_case
PRJNA8274(	ENSGALG00010001869	CRLF2	case	control	0.240699362	0.705648585	0.341103726	0.733025496	0.933846596	up_in_case
PRJNA8274(	ENSGALG00010001870	PLP2	case	control	-0.066293874	0.149907554	-0.442231711	0.658321541	0.908367791	down_in_case
PRJNA8274(	ENSGALG00010001871	ENSGALG00010001871	case	control	-0.135540467	0.151892084	-0.892347141	0.372206894	0.774077746	down_in_case
PRJNA8274(	ENSGALG00010001873	ENSGALG00010001873	case	control	-0.132601205	0.182200675	-0.727775595	0.466750966	0.827292691	down_in_case
PRJNA8274(	ENSGALG00010001877	OAT	case	control	-0.092002103	0.058855305	-1.563191351	0.118007594	0.514604253	down_in_case
PRJNA8274(	ENSGALG00010001878	XK	case	control	-0.237997372	0.25521667	-0.932530668	0.351062345	0.760831854	down_in_case
PRJNA8274(	ENSGALG00010001879	IRAK4	case	control	0.222679493	0.194068546	1.14742702	0.251205179	0.677013581	up_in_case
PRJNA8274(	ENSGALG00010001880	MTCL1	case	control	-0.058140526	0.077619597	-0.749044426	0.453830429	0.821282604	down_in_case
PRJNA8274(	ENSGALG00010001883	KBTBD11	case	control	-0.032710636	0.089519457	-0.365402526	0.714810956	0.926862121	down_in_case
PRJNA8274(	ENSGALG00010001885	SMPX	case	control	-0.201150302	0.178254186	-1.128446444	0.259131423	0.686345498	down_in_case
PRJNA8274(	ENSGALG00010001886	NEU3	case	control	0.048295545	0.042559542	1.134775951	0.256469205	0.682835529	up_in_case
PRJNA8274(	ENSGALG00010001888	SNX10	case	control	0.369127977	0.139044843	2.654740511	0.007936944	0.1561187	up_in_case
PRJNA8274(	ENSGALG00010001890	KLHL34	case	control	-0.091401227	0.173405332	-0.527095826	0.598127045	0.884680373	down_in_case

PRJNA8274(	ENSGALG00010001892	RAB12	case	control	-0.044769829	0.055967959	-0.799918914	0.423757779	0.803723282	down_in_case
PRJNA8274(	ENSGALG00010001895	P4HA3	case	control	0.091361539	0.115411384	0.791616361	0.428584404	0.805355714	up_in_case
PRJNA8274(	ENSGALG00010001896	ARHGEF10	case	control	-0.100783258	0.110886853	-0.908883746	0.363411494	0.769779939	down_in_case
PRJNA8274(	ENSGALG00010001897	TLR7	case	control	0.495325268	0.18467194	2.682190202	0.007314186	0.148898802	up_in_case
PRJNA8274(	ENSGALG00010001898	LHPP	case	control	0.033344765	0.071564778	0.465938217	0.641259725	0.901516193	up_in_case
PRJNA8274(	ENSGALG00010001899	TWF1	case	control	0.12015386	0.050134964	2.396608061	0.016547613	0.22864029	up_in_case
PRJNA8274(	ENSGALG00010001901	PRPS2	case	control	0.332156788	0.18830262	1.763952023	0.077740057	0.447281736	up_in_case
PRJNA8274(	ENSGALG00010001902	WDR45	case	control	0.031787354	0.06156223	0.516345068	0.605613425	0.88576746	up_in_case
PRJNA8274(	ENSGALG00010001903	NARS2	case	control	0.139972766	0.090810094	1.541378942	0.123224586	0.522571644	up_in_case
PRJNA8274(	ENSGALG00010001906	SKAP2	case	control	2.99818E-05	0.081008235	0.000370108	0.999704697	0.999774611	up_in_case
PRJNA8274(	ENSGALG00010001908	FAM83B	case	control	-0.079418068	0.35011322	-0.226835388	0.820551745	0.958669267	down_in_case
PRJNA8274(	ENSGALG00010001910	FAM53B	case	control	0.038052604	0.051050712	0.74538832	0.456037003	0.822099797	up_in_case
PRJNA8274(	ENSGALG00010001911	USP35	case	control	-0.551539866	0.472333424	-1.167691801	0.242931105	0.667473598	down_in_case
PRJNA8274(	ENSGALG00010001912	TMEM117	case	control	-0.017294256	0.142814918	-0.121095587	0.903615324	0.980997505	down_in_case
PRJNA8274(	ENSGALG00010001914	ENSGALG00010001914	case	control	-0.345119509	0.197721628	-1.745481832	0.080901032	0.453075204	down_in_case
PRJNA8274(	ENSGALG00010001915	EEF1AKMT2	case	control	-0.486287906	0.133007288	-3.656099711	0.000256082	0.021415026	down_in_case
PRJNA8274(	ENSGALG00010001918	CNKSR2	case	control	-0.171542564	0.109695929	-1.563800632	0.117864396	0.514604253	down_in_case
PRJNA8274(	ENSGALG00010001921	ENSGALG00010001921	case	control	0.392950646	0.709088221	0.554163268	0.579467114	0.879142433	up_in_case
PRJNA8274(	ENSGALG00010001923	RAPGEF5	case	control	0.084450439	0.069428743	1.216361351	0.2238473	0.647995977	up_in_case
PRJNA8274(	ENSGALG00010001925	FRMPD4	case	control	-0.242371805	0.121438345	-1.995842453	0.045951074	0.355959026	down_in_case
PRJNA8274(	ENSGALG00010001928	KCTD21	case	control	0.089577892	0.095618011	0.936830734	0.348845633	0.759283494	up_in_case
PRJNA8274(	ENSGALG00010001929	POLR2I	case	control	0.160702943	0.077845651	2.064379198	0.038981777	0.335091843	up_in_case
PRJNA8274(	ENSGALG00010001935	ABRAXAS2	case	control	-0.064044262	0.076861153	-0.833246185	0.4047059	0.793319877	down_in_case
PRJNA8274(	ENSGALG00010001938	ALG8	case	control	0.121457994	0.075786897	1.602625232	0.10901742	0.501108679	up_in_case
PRJNA8274(	ENSGALG00010001940	PTPRM	case	control	-0.204059515	0.077751736	-2.624501092	0.008677601	0.165452934	down_in_case
PRJNA8274(	ENSGALG00010001947	TINAG	case	control	0.238117329	0.305496432	0.779443897	0.435718274	0.81000376	up_in_case
PRJNA8274(	ENSGALG00010001948	EMX2	case	control	-0.084709346	0.219945693	-0.385137554	0.700135506	0.922288441	down_in_case
PRJNA8274(	ENSGALG00010001952	ENSGALG00010001952	case	control	-0.05621267	0.044671302	-1.258362042	0.208260853	0.632880396	down_in_case
PRJNA8274(	ENSGALG00010001957	NDUFC2	case	control	-0.111993707	0.091717883	-1.221067291	0.222060543	0.645952056	down_in_case
PRJNA8274(	ENSGALG00010001958	RAB11FIP2	case	control	-0.087775034	0.064681453	-1.357035584	0.174769904	0.593777531	down_in_case
PRJNA8274(	ENSGALG00010001961	ENSGALG00010001961	case	control	0.202571482	0.246926098	0.820372914	0.412003551	0.797194435	up_in_case
PRJNA8274(	ENSGALG00010001966	ARHGAP6	case	control	-0.026876685	0.080059119	-0.335710473	0.737089208	0.935060877	down_in_case
PRJNA8274(	ENSGALG00010001967	LAMA1	case	control	-0.079486758	0.181430718	-0.438110804	0.661305963	0.909792535	down_in_case
PRJNA8274(	ENSGALG00010001969	PLEKHA8	case	control	0.259342862	0.119131422	2.176947588	0.029484478	0.305245958	up_in_case
PRJNA8274(	ENSGALG00010001971	MLIP	case	control	-0.308349959	0.127664051	-2.415323316	0.015721248	0.222636791	down_in_case
PRJNA8274(	ENSGALG00010001972	FAM204A	case	control	-0.253453988	0.085382145	-2.968465937	0.002992903	0.093242939	down_in_case
PRJNA8274(	ENSGALG00010001974	ENSGALG00010001974	case	control	-0.049194857	0.210201712	-0.234036423	0.814956702	0.957354871	down_in_case
PRJNA8274(	ENSGALG00010001976	THRSP	case	control	-1.117798699	1.011621089	-1.104957885	0.269177838	0.696212628	down_in_case
PRJNA8274(	ENSGALG00010001982	DMD	case	control	-0.321822324	0.086271243	-3.730354564	0.00019121	0.017196919	down_in_case
PRJNA8274(	ENSGALG00010001987	KCTD14	case	control	-0.266183215	0.463064089	-0.574830182	0.565406152	0.872906976	down_in_case
PRJNA8274(	ENSGALG00010001988	ENSGALG00010001988	case	control	-0.167285372	0.480444772	-0.348188558	0.72769858	0.932979874	down_in_case
PRJNA8274(	ENSGALG00010001991	TOMM7	case	control	0.053390755	0.092492058	0.577246916	0.563772668	0.871916948	up_in_case
PRJNA8274(	ENSGALG00010001995	LRRC1	case	control	0.043979387	0.10394738	0.423092787	0.672227565	0.913676733	up_in_case
PRJNA8274(	ENSGALG00010001996	ENSGALG00010001996	case	control	0.153894297	0.501947097	0.306594656	0.759151922	0.942078947	up_in_case
PRJNA8274(	ENSGALG00010001997	INTS4	case	control	-0.088941274	0.034157097	-2.603888564	0.009217272	0.171623685	down_in_case
PRJNA8274(	ENSGALG00010002005	COLQ	case	control	-0.378178715	0.137842493	-2.743556839	0.006077753	0.135166192	down_in_case
PRJNA8274(	ENSGALG00010002006	ENSGALG00010002006	case	control	-0.28839113	0.361263491	-0.798284735	0.424705274	0.804195634	down_in_case
PRJNA8274(	ENSGALG00010002012	MID1	case	control	-0.13177807	0.139563029	-0.944219043	0.345057726	0.755870938	down_in_case
PRJNA8274(	ENSGALG00010002017	ARHGAP28	case	control	-0.050560276	0.068575675	-0.737291699	0.460944979	0.82454265	down_in_case
PRJNA8274(	ENSGALG00010002022	ERNI	case	control	-0.756985952	0.843306858	-0.897639981	0.369377517	0.772123738	down_in_case
PRJNA8274(	ENSGALG00010002023	CLCN4	case	control	-0.001109123	0.174650662	-0.006350525	0.994933048	0.998586153	down_in_case
PRJNA8274(	ENSGALG00010002024	KLHL31	case	control	-0.167462489	0.10097845	-1.658398292	0.097237101	0.485691283	down_in_case
PRJNA8274(	ENSGALG00010002027	ENSGALG00010002027	case	control	0.188110539	0.362552564	0.518850388	0.603865077	0.885268908	up_in_case
PRJNA8274(	ENSGALG00010002034	WWC3	case	control	-0.049358329	0.051062754	-0.966620972	0.333733547	0.748508541	down_in_case
PRJNA8274(	ENSGALG00010002039	AAMDC	case	control	0.117465062	0.102643342	1.144400211	0.252457701	0.677706987	up_in_case
PRJNA8274(	ENSGALG00010002041	GCLC	case	control	0.104435303	0.113540749	0.919804594	0.357674882	0.763851675	up_in_case
PRJNA8274(	ENSGALG00010002046	FAM126A	case	control	0.029348291	0.070081881	0.418771452	0.675383171	0.914247368	up_in_case
PRJNA8274(	ENSGALG00010002050	ENSGALG00010002050	case	control	-0.274618871	0.327368623	-0.838867416	0.401543714	0.791246398	down_in_case
PRJNA8274(	ENSGALG00010002054	RSF1	case	control	-0.073731375	0.06178463	-1.193361113	0.232727978	0.657934961	down_in_case
PRJNA8274(	ENSGALG00010002056	PRLHR	case	control	0.22527864	0.566212841	0.397869183	0.690726617	0.918784485	up_in_case
PRJNA8274(	ENSGALG00010002062	GSTA4	case	control	0.324614955	0.227991018	1.423805895	0.154502669	0.563332015	up_in_case
PRJNA8274(	ENSGALG00010002063	ENSGALG00010002063	case	control	-1.017235386	0.890116046	-1.142812098	0.253116615	0.678711342	down_in_case
PRJNA8274(	ENSGALG00010002065	CLNS1A	case	control	0.009078853	0.066000218	0.137557928	0.890589808	0.978745332	up_in_case
PRJNA8274(	ENSGALG00010002072	NEURL4	case	control	-0.032093051	0.08246455	-0.389173911	0.697147501	0.922031058	down_in_case
PRJNA8274(	ENSGALG00010002079	ARX	case	control	-0.240198426	0.150616991	-1.594763145	0.110765212	0.503115856	down_in_case
PRJNA8274(	ENSGALG00010002084	HACL1	case	control	0.021523645	0.11450921	0.187964316	0.850904625	0.969234083	up_in_case
PRJNA8274(	ENSGALG00010002087	POLA1	case	control	-0.049138935	0.064365333	-0.763437915	0.445202276	0.816727715	down_in_case
PRJNA8274(	ENSGALG00010002088	OZFL1	case	control	0.020664961	0.134813141	0.153285956	0.878172775	0.976160112	up_in_case
PRJNA8274(	ENSGALG00010002090	AQP11	case	control	0.268249288	0.200165482	1.340137592	0.180200617	0.600999032	up_in_case
PRJNA8274(	ENSGALG00010002097	PCYT1B	case	control	-0.761959034	0.344509489	-2.211721473	0.026985916	0.292513391	down_in_case
PRJNA8274(	ENSGALG00010002098	ENSGALG00010002098	case	control	-0.063770182	0.479644822	-0.132952925	0.89423061	0.979959976	down_in_case
PRJNA8274(	ENSGALG00010002100	BTD	case	control	-0.248054356	0.099364081	-2.49641875	0.012545441	0.196926234	down_in_case
PRJNA8274(	ENSGALG00010002102	PAK1	case	control	-0.133850206	0.193222412	-0.692726092	0.488481462	0.835960376	down_in_case
PRJNA8274(	ENSGALG00010002113	GDPD4	case	control	0.171793851	0.188659136	0.910604463	0.362503818	0.7686543	up_in_case
PRJNA8274(	ENSGALG00010002118	ENSGALG00010002118	case	control	-0.158045502	0.33243633	-0.475415856	0.634490595	0.898107199	down_in_case
PRJNA8274(	ENSGALG00010002122	ENSGALG00010002122	case	control	-0.627083512	0.674606111	-0.929555043	0.352601514	0.76143184	down_in_case
PRJNA8274(	ENSGALG00010002126	ENSGALG00010002126	case	control	-0.163984993	0.145906509	-1.123904576	0.26105351	0.687516348	down_in_case
PRJNA8274(	ENSGALG00010002134	ANKRD28	case	control	0.039286423	0.048667755	0.807237198	0.419529839	0.80093491	up_in_case
PRJNA8274(	ENSGALG00010002135	ELOVL5	case	control	-0.072466898	0.067457063	-1.074267006	0.282703028	0.706698604	down_in_case
PRJNA8274(	ENSGALG00010002136	ACAP1	case	control	-0.004170773	0.21683477	-0.019234799	0.984653797	0.996591771	down_in_case
PRJNA8274(	ENSGALG00010002137	PDK3	case	control	-0.277229677	0.09044213	-3.065271415	0.002174725	0.076380185	down_in_case
PRJNA8274(	ENSGALG00010002138	MYO7A	case	control	0.099935165	0.139442801	0.716674971	0.473574673	0.82959752	up_in_case
PRJNA8274(	ENSGALG00010002140	ENSGALG00010002140	case	control	-0.388894874	0.398974462	-0.974736258	0.329691103	0.746174551	down_in_case
PRJNA8274(	ENSGALG00010002142	CLDN34	case	control	0.908192982	0.468266123	1.93948043	0.052442865	0.373844951	up_in_case
PRJNA8274(	ENSGALG00010002145	ENSGALG00010002145	case	control	-0.261184514	0.385032363	-0.678344312	0.497553409	0.839230872	down_in_case
PRJNA8274(	ENSGALG00010002146	CACUL1	case	control	0.021924745	0.073508614	0.298260896	0.765504047	0.943786458	up_in_case
PRJNA8274(	ENSGALG00010002147	ENSGALG00010002147	case	control	-0.005706174	0.646409373	-0.008827493	0.992956771	0.998196863	down_in_case
PRJNA8274(	ENSGALG00010002148	POLR2A	case	control	-0.294204955	0.133200545	-2.208736874	0.027192948	0.293158316	down_in_case
PRJNA8274(	ENSGALG00010002149	ENSGALG00010002149	case	control	0.021619581	0.220706175	0.097956394	0.921966919	0.985482967	up_in_case
PRJNA8274(	ENSGALG00010002150	EIF3A	case	control	-0.154894335	0.066533749	-2.328056617	0.019909098	0.253291899	down_in_case
PRJNA8274(	ENSGALG00010002159	GALNT15	case	control	-0.340921159	0.114091812	-2.988129934	0.002806902	0.08899933	down_in_case
PRJNA8274(	ENSGALG00010002160	CAPN5	case	control	0.001137111	0.075771296	0.015007149	0.988026477	0.996942188	up_in_case
PRJNA8274(	ENSGALG00010002161	CDKL5	case	control	-0.103532848	0.120227649	-0.861140089	0.389160892	0.783669208	down_in_case
PRJNA8274(	ENSGALG00010002174	ENSGALG00010002174	case	control	-0.439385725	0.423969679	-1.0363612	0.300033665	0.723279006	down_in_case
PRJNA8274(	ENSGALG00010002177	FBXO9	case	control	-0.189590176	0.074847494	-2.533019677	0.01130846	0.186343114	down_in_case
PRJNA8274(	ENSGALG00010002181	FIS1	case	control	0.206973134	0.150236033	1.377653086	0.168310422	0.58432606	up_in_case
PRJNA8274(	ENSGALG00010002185	ENSGALG00010002185	case	control	0.712395056	0.446390974	1.595899327	0.110511272	0.503054901	up_in_case
PRJNA8274(	ENSGALG00010002186	CILK1	case	control	0.053400695	0.051633217	1.034231415	0.301027991	0.723722305	up_in_case
PRJNA8274(	ENSGALG00010002192	ENSGALG00010002192	case	control	0.006785461	0.115058049	0.058974238	0.952972627	0.992056045	up_in_case
PRJNA8274(	ENSGALG00010002194	SHROOM2	case	control	-0.036007944	0.123713724	-0.291058603	0.7710065	0.94558971	down_in_case
PRJNA8274(	ENSGALG00010002196	ENSGALG00010002196	case	control	0.041777178	0.06997242	0.597052058	0.550472625	0.86456492	up_in_case
PRJNA8274(	ENSGALG00010002197	GSTA2	case	control	0.734442513	0.219550375	3.345211838	0.000822197	0.044035295	up_in_case
PRJNA8274(	ENSGALG00010002202	SFXN4	case	control	-0.020497536	0.080771342	-0.253772382	0.799671411	0.954053991	down_in_case
PRJNA8274(	ENSGALG00010002206	DPH3P1	case	control	-0.03505265	0.09190364	-0.381406542	0.702901607	0.923606	down_in_case
PRJNA8274(	ENSGALG00010002210	GPR143	case	control	3.752073335	0.596833359	6.28663475	3.24421E-10	1.85569E-07	up_in_case
PRJNA8274(	ENSGALG00010002211	ACER3	case	control	0.14674781	0.067505355	2.173869179	0.029714961	0.305970455	up_in_case
PRJNA8274(	ENSGALG00010002215	YLEC18	case	control	-0.268215398	0.385748988	-0.695310698	0.486860611	0.835185549	down_in_case
PRJNA8274(	ENSGALG00010002225	PRDX3	case	control	-0.246101934	0.059626552	-4.127388343	3.66906E-05	0.005093944	down_in_case
PRJNA8274(	ENSGALG00010002226	OXNAD1	case	control	0.242187705	0.108307197	2.236118299	0.025344023	0.282919227	up_in_case
PRJNA8274(	ENSGALG00010002227	ENSGALG00010002227	case	control	-1.626096748	1.462578421	-1.111801408	0.266223546	0.69218122	down_in_case
PRJNA8274(	ENSGALG00010002231	TMEM14A	case	control	-0.020572568	0.054002771	-0.380953931	0.703237433	0.923659131	down_in_case
PRJNA8274(	ENSGALG00010002235	MXRA5	case	control	-0.170784201	0.157613341	-1.083564375	0.278557993	0.703953892	down_in_case
PRJNA8274(	ENSGALG00010002238	ENSGALG00010002238	case	control	0.153924454	0.392704198	0.391960296	0.69508755	0.920773689	up_in_case
PRJNA8274(	ENSGALG00010002239	AGPAT5	case	control	-0.199241128	0.070734486	-2.816746651	0.004851278	0.119309498	down_in_case
PRJNA8274(	ENSGALG00010002243	ARSH	case	control	0.265171918	0.163573319	1.621119624	0.104991986	0.494770427	up_in_case
PRJNA8274(	ENSGALG00010002247	RFTN1	case	control	-0.171273118	0.11430049	-1.498446057	0.134017398	0.537883101	down_in_case
PRJNA8274(	ENSGALG00010002251	GRK5	case	control	-0.039625945	0.169479337	-0.23380989	0.815132571	0.957374769	down_in_case
PRJNA8274(	ENSGALG00010002253	ENSGALG00010002253	case	control	0.248988295	0.248824746	1.000657285	0.316992525	0.737671783	up_in_case
PRJNA8274(	ENSGALG00010002254	ENSGALG00010002254	case	control	0.618768408	0.515249727	1.200909724	0.229786222	0.653788892	up_in_case
PRJNA8274(	ENSGALG00010002261	NR0B1	case	control	-0.547280211	0.433437865	-1.262649748	0.206715081	0.630417074	down_in_case
PRJNA8274(	ENSGALG00010002269	MCPH1	case	control	-0.039420911	0.098936638	-0.398446036	0.690301429	0.918688733	down_in_case
PRJNA8274(	ENSGALG00010002270	IMMP2L	case	control	-0.457775563	0.204908782	-2.234045602	0.025480073	0.28355256	down_in_case
PRJNA8274(	ENSGALG00010002274	ENSGALG00010002274	case	control	1.112980938	0.959851298	1.159534753	0.246238279	0.670921661	up_in_case
PRJNA8274(	ENSGALG00010002275	IL1RAPL1	case	control	0.115459511	0.25668682	0.449806933	0.652849658	0.905885495	up_in_case
PRJNA8274(	ENSGALG00010002277	RGS10	case	control	-0.10727275	0.086788069	-1.236031071	0.216447015	0.641623613	down_in_case
PRJNA8274(	ENSGALG00010002279	ENSGALG00010002279	case	control	0.453721808	0.719287538	0.630793367	0.528175641	0.853519488	up_in_case

PRJNA8274(	ENSGALG00010002280	ST7	case	control	-0.145400047	0.146033553	-0.995661917	0.319414439	0.738739525	down_in_case
PRJNA8274(	ENSGALG00010002281	TGFBR2	case	control	-0.135866239	0.073661159	-1.844475989	0.065113802	0.414724068	down_in_case
PRJNA8274(	ENSGALG00010002282	TSKU	case	control	0.106165693	0.065427955	1.622635048	0.104667444	0.494770427	up_in_case
PRJNA8274(	ENSGALG00010002283	ENSGALG00010002283	case	control	0.074510173	0.839400669	0.08876592	0.929267942	0.986894287	up_in_case
PRJNA8274(	ENSGALG00010002285	CAV1	case	control	-0.164157223	0.083968049	-1.95499627	0.05058351	0.367719502	down_in_case
PRJNA8274(	ENSGALG00010002288	ANGPT2	case	control	-0.174281555	0.096934297	-1.797934902	0.072187324	0.434277972	down_in_case
PRJNA8274(	ENSGALG00010002289	ENSGALG00010002289	case	control	0.155652763	0.165649369	0.93965201	0.347396089	0.758205749	up_in_case
PRJNA8274(	ENSGALG00010002291	RNASEH1	case	control	-0.116383548	0.062825226	-1.852497085	0.063954476	0.410839656	down_in_case
PRJNA8274(	ENSGALG00010002293	GYG2	case	control	-0.17087687	0.176266405	-0.969423923	0.332333721	0.748293551	down_in_case
PRJNA8274(	ENSGALG00010002294	DAZL	case	control	-1.075911607	0.626817257	-1.716467749	0.086076466	0.463278022	down_in_case
PRJNA8274(	ENSGALG00010002295	YAF2	case	control	-0.098278938	0.079658141	-1.233758878	0.217292767	0.642778841	down_in_case
PRJNA8274(	ENSGALG00010002298	ENSGALG00010002298	case	control	0.164100031	0.373408363	0.439465334	0.660324395	0.909188753	up_in_case
PRJNA8274(	ENSGALG00010002308	GXYLT1	case	control	-0.0365834	0.073563504	-0.497303661	0.61897493	0.891822822	down_in_case
PRJNA8274(	ENSGALG00010002309	GADL1	case	control	0.104352121	0.165306111	0.631265962	0.527866639	0.853386536	up_in_case
PRJNA8274(	ENSGALG00010002310	PRRG1	case	control	0.156096685	0.072620161	2.149495167	0.031595169	0.3098361	up_in_case
PRJNA8274(	ENSGALG00010002312	ENSGALG00010002312	case	control	-0.021841962	0.134192574	-0.162765801	0.870702836	0.974794532	down_in_case
PRJNA8274(	ENSGALG00010002315	ENSGALG00010002315	case	control	0.068833503	0.099329347	0.692982538	0.48832051	0.835960376	up_in_case
PRJNA8274(	ENSGALG00010002324	SNTG2	case	control	0.262695989	0.640164905	0.410356749	0.681544269	0.915594727	up_in_case
PRJNA8274(	ENSGALG00010002328	ENSGALG00010002328	case	control	-0.188401647	0.265451299	-0.709740909	0.477864819	0.830520323	down_in_case
PRJNA8274(	ENSGALG00010002329	STT3B	case	control	0.004804449	0.033610088	0.142946625	0.886332335	0.977630122	up_in_case
PRJNA8274(	ENSGALG00010002330	KHDRBS2	case	control	0.173009575	0.216366457	0.79961366	0.423934671	0.803723282	up_in_case
PRJNA8274(	ENSGALG00010002331	ENSGALG00010002331	case	control	-0.084925227	0.279286199	-0.304079568	0.761067272	0.942985066	down_in_case
PRJNA8274(	ENSGALG00010002332	ENSGALG00010002332	case	control	0.280291202	0.723658831	0.387325062	0.698515574	0.922288441	up_in_case
PRJNA8274(	ENSGALG00010002333	ENSGALG00010002333	case	control	-0.193209195	0.693209826	-0.278716757	0.780462202	0.948875232	down_in_case
PRJNA8274(	ENSGALG00010002334	OSBPL10	case	control	-0.138022061	0.077006117	-1.792351928	0.07307661	0.436871039	down_in_case
PRJNA8274(	ENSGALG00010002335	TMEM47	case	control	-0.148946079	0.145124102	-1.026335925	0.304733269	0.728073294	down_in_case
PRJNA8274(	ENSGALG00010002336	PRIM2	case	control	-0.052431243	0.085117213	-0.615988717	0.537901967	0.857740557	down_in_case
PRJNA8274(	ENSGALG00010002338	CD99	case	control	-0.04520519	0.083349569	-0.542356617	0.58757286	0.882771329	down_in_case
PRJNA8274(	ENSGALG00010002339	ZNFY4	case	control	-0.548044146	0.570725793	-0.960258242	0.336925261	0.750735231	down_in_case
PRJNA8274(	ENSGALG00010002340	RAB23	case	control	0.130029733	0.055343941	2.349484541	0.018799425	0.244837681	up_in_case
PRJNA8274(	ENSGALG00010002341	FAM49A	case	control	0.053911652	0.074796297	0.720779691	0.471045072	0.829101076	up_in_case
PRJNA8274(	ENSGALG00010002342	BAG2	case	control	-0.166043484	0.084202297	-1.971959085	0.048614277	0.363399983	down_in_case
PRJNA8274(	ENSGALG00010002343	GPD1L	case	control	0.052502918	0.0567996	0.92435366	0.355302213	0.763161644	up_in_case
PRJNA8274(	ENSGALG00010002345	SCML2	case	control	-0.24353156	0.118730547	-2.051128082	0.040254475	0.33753444	down_in_case
PRJNA8274(	ENSGALG00010002346	FBXO15	case	control	-0.342068957	0.247191269	-1.383822972	0.166412667	0.58174452	down_in_case
PRJNA8274(	ENSGALG00010002347	ZNF451	case	control	0.003005096	0.052119192	0.057658147	0.954020932	0.992056045	up_in_case
PRJNA8274(	ENSGALG00010002348	THAP12	case	control	0.034624763	0.065946624	0.525042239	0.599553828	0.884888453	up_in_case
PRJNA8274(	ENSGALG00010002349	ZBED1	case	control	-0.014234364	0.049418203	-0.288038884	0.773316975	0.946459495	down_in_case
PRJNA8274(	ENSGALG00010002350	BEND6	case	control	0.438225854	0.12104793	3.620267218	0.000294299	0.023376995	up_in_case
PRJNA8274(	ENSGALG00010002352	ENSGALG00010002352	case	control	-0.363937747	0.086616439	-4.201716817	2.64898E-05	0.00382631	down_in_case
PRJNA8274(	ENSGALG00010002353	CMTM8	case	control	0.121391622	0.127363692	0.953110104	0.340534287	0.752068001	up_in_case
PRJNA8274(	ENSGALG00010002355	UFM1	case	control	0.073923939	0.064016822	1.154758012	0.248189536	0.672435338	up_in_case
PRJNA8274(	ENSGALG00010002356	WNT11	case	control	-0.008240616	0.192066267	-0.042905065	0.965777211	0.992340816	down_in_case
PRJNA8274(	ENSGALG00010002357	RAB31	case	control	0.040691231	0.091275198	0.445808195	0.655735801	0.907453191	up_in_case
PRJNA8274(	ENSGALG00010002358	FREM2	case	control	0.003339114	0.157868785	0.021151196	0.983125045	0.996591771	up_in_case
PRJNA8274(	ENSGALG00010002359	CMTM6	case	control	0.236252268	0.125682729	1.879751261	0.060141986	0.398715993	up_in_case
PRJNA8274(	ENSGALG00010002360	DHRSX	case	control	-0.069146852	0.068196987	-1.013928255	0.310616988	0.733587496	down_in_case
PRJNA8274(	ENSGALG00010002361	ENSGALG00010002361	case	control	0.049563946	0.055762299	0.888843304	0.374087299	0.774369566	up_in_case
PRJNA8274(	ENSGALG00010002362	TRPC4	case	control	-0.394040568	0.287174579	-1.372128998	0.170023284	0.586428595	down_in_case
PRJNA8274(	ENSGALG00010002364	VSNL1	case	control	-0.158187835	0.221925609	-0.712796669	0.475971587	0.830520323	down_in_case
PRJNA8274(	ENSGALG00010002365	RAI2	case	control	-0.199016093	0.078394035	-2.538663711	0.011127673	0.185137791	down_in_case
PRJNA8274(	ENSGALG00010002366	ENSGALG00010002366	case	control	0.125691138	0.151681555	0.82865143	0.407301677	0.794380997	up_in_case
PRJNA8274(	ENSGALG00010002367	STK31	case	control	-0.062127705	0.832846745	-0.074596804	0.940535517	0.989551456	down_in_case
PRJNA8274(	ENSGALG00010002368	UVRAG	case	control	-0.073499743	0.071420861	-1.029107484	0.30342917	0.726198683	down_in_case
PRJNA8274(	ENSGALG00010002369	GEMIN8	case	control	0.031430841	0.093078125	0.337682358	0.735602571	0.934202199	up_in_case
PRJNA8274(	ENSGALG00010002370	ENSGALG00010002370	case	control	-0.051343189	0.106647931	-0.481426961	0.630213078	0.89637578	down_in_case
PRJNA8274(	ENSGALG00010002371	BAG3	case	control	-0.107873869	0.08514424	-1.266954402	0.205171595	0.627986687	down_in_case
PRJNA8274(	ENSGALG00010002372	SMC6	case	control	-0.048447196	0.09558764	-0.506835368	0.612270338	0.889873548	down_in_case
PRJNA8274(	ENSGALG00010002373	POSTN	case	control	-0.151472628	0.110211077	-1.374386603	0.169321693	0.585146363	down_in_case
PRJNA8274(	ENSGALG00010002374	DST	case	control	-0.220259685	0.091573341	-2.40528175	0.016160001	0.225671894	down_in_case
PRJNA8274(	ENSGALG00010002375	NPY	case	control	0.579812144	0.370404819	1.565347194	0.117501523	0.51430076	up_in_case
PRJNA8274(	ENSGALG00010002376	GEN1	case	control	0.035816657	0.104848774	0.34160301	0.732649671	0.933846596	up_in_case
PRJNA8274(	ENSGALG00010002377	ART1A	case	control	0.53486245	0.260403889	2.05397259	0.039978345	0.336547209	up_in_case
PRJNA8274(	ENSGALG00010002378	ENSGALG00010002378	case	control	0.025646947	0.153136172	0.167478048	0.866993919	0.973570415	up_in_case
PRJNA8274(	ENSGALG00010002379	NHS	case	control	0.087953674	0.126017995	0.697945355	0.485211369	0.834423421	up_in_case
PRJNA8274(	ENSGALG00010002380	CMPK2	case	control	0.131727568	0.278050695	0.47375378	0.635675498	0.898681129	up_in_case
PRJNA8274(	ENSGALG00010002381	TDRD3	case	control	-0.082069331	0.076104482	-1.078377107	0.280865499	0.705762606	down_in_case
PRJNA8274(	ENSGALG00010002383	INPP5F	case	control	0.114130771	0.088682084	1.286965376	0.198106357	0.621485489	up_in_case
PRJNA8274(	ENSGALG00010002384	ENSGALG00010002384	case	control	-0.076055136	0.413187048	-0.184069507	0.853958928	0.969998446	down_in_case
PRJNA8274(	ENSGALG00010002385	PALS2	case	control	-0.03795161	0.095332366	-0.398097846	0.690558062	0.918784485	down_in_case
PRJNA8274(	ENSGALG00010002386	MHCY2B2	case	control	-0.245649331	0.191726262	-1.281250303	0.200105763	0.622902456	down_in_case
PRJNA8274(	ENSGALG00010002387	MCMBP	case	control	0.104014923	0.053094003	1.959071025	0.05010447	0.366754215	up_in_case
PRJNA8274(	ENSGALG00010002388	ENSGALG00010002388	case	control	0.64105757	0.427273464	1.500344916	0.13352508	0.537206146	up_in_case
PRJNA8274(	ENSGALG00010002389	COL21A1	case	control	0.078180088	0.153999263	0.507665337	0.611688061	0.889602558	up_in_case
PRJNA8274(	ENSGALG00010002391	REPS2	case	control	0.246438959	0.098613132	2.499048087	0.012452741	0.195901209	up_in_case
PRJNA8274(	ENSGALG00010002392	DFNA5	case	control	0.137019789	0.114336389	1.198391781	0.230764531	0.655335776	up_in_case
PRJNA8274(	ENSGALG00010002393	GPM6B	case	control	-0.035881967	0.055079738	-0.65145493	0.514752862	0.847493921	down_in_case
PRJNA8274(	ENSGALG00010002395	CLN8	case	control	0.030680581	0.05742905	0.534234522	0.593179288	0.883625114	up_in_case
PRJNA8274(	ENSGALG00010002396	SEC23IP	case	control	0.040161451	0.058732698	0.68380055	0.494101119	0.83803731	up_in_case
PRJNA8274(	ENSGALG00010002397	ENSGALG00010002397	case	control	0.379610758	0.293904416	1.291612977	0.196491208	0.619313262	up_in_case
PRJNA8274(	ENSGALG00010002398	ENSGALG00010002398	case	control	0.654154987	0.692996494	0.943951366	0.345194502	0.755935621	up_in_case
PRJNA8274(	ENSGALG00010002399	CSMD1	case	control	-0.677341206	0.222604835	-3.042796471	0.002343908	0.079426268	down_in_case
PRJNA8274(	ENSGALG00010002400	RBBP7	case	control	-0.104988531	0.078531103	-1.336903808	0.181254038	0.601795389	down_in_case
PRJNA8274(	ENSGALG00010002401	ZRANB1	case	control	-0.137717263	0.055360302	-2.487653757	0.012858886	0.199684901	down_in_case
PRJNA8274(	ENSGALG00010002402	TXLNG	case	control	0.0022453	0.085989511	0.026111323	0.979168546	0.995528632	up_in_case
PRJNA8274(	ENSGALG00010002403	ZNFY3	case	control	0.193772115	0.235088159	0.82425298	0.40979583	0.795863751	up_in_case
PRJNA8274(	ENSGALG00010002404	OSBPL3	case	control	0.432985406	0.171735347	2.521236384	0.011694326	0.189141894	up_in_case
PRJNA8274(	ENSGALG00010002405	KCNQ5	case	control	-0.217018111	0.204058582	-1.063508866	0.287551247	0.71132024	down_in_case
PRJNA8274(	ENSGALG00010002406	DIAPH3	case	control	-0.052520686	0.114800392	-0.457495699	0.647314793	0.903059048	down_in_case
PRJNA8274(	ENSGALG00010002408	OFD1	case	control	-0.005144979	0.103086277	-0.049909442	0.960194553	0.992056045	down_in_case
PRJNA8274(	ENSGALG00010002409	CYCS	case	control	0.111450569	0.081988834	1.359338381	0.174039387	0.593127559	up_in_case
PRJNA8274(	ENSGALG00010002410	RNF121	case	control	-0.003320468	0.065292385	-0.050855371	0.959440768	0.992056045	down_in_case
PRJNA8274(	ENSGALG00010002411	ENSGALG00010002411	case	control	0.186871422	0.121316091	1.540367972	0.123470684	0.522840029	up_in_case
PRJNA8274(	ENSGALG00010002413	TSSC1	case	control	-0.074356959	0.087904645	-0.845882028	0.397618559	0.788728117	down_in_case
PRJNA8274(	ENSGALG00010002414	ENSGALG00010002414	case	control	0.358328575	0.439225829	0.81581854	0.414603928	0.798853486	up_in_case
PRJNA8274(	ENSGALG00010002415	PCDH17	case	control	0.091313542	0.247624517	0.368758082	0.712308051	0.92680349	up_in_case
PRJNA8274(	ENSGALG00010002416	SH3BGRL2	case	control	0.576732847	0.242522386	2.378060252	0.017403982	0.234759681	up_in_case
PRJNA8274(	ENSGALG00010002418	TRAPPC2	case	control	0.100553597	0.067591468	1.487667011	0.136838732	0.539854583	up_in_case
PRJNA8274(	ENSGALG00010002419	CTBP2	case	control	-0.033821027	0.065334469	-0.51765979	0.604695657	0.885268908	down_in_case
PRJNA8274(	ENSGALG00010002420	TMEM272	case	control	0.19660324	0.368328495	0.533771464	0.593499659	0.883625114	up_in_case
PRJNA8274(	ENSGALG00010002421	ENSGALG00010002421	case	control	0.057468047	0.156645031	0.366867989	0.71371749	0.926862121	up_in_case
PRJNA8274(	ENSGALG00010002422	TRAPPC12	case	control	0.024609628	0.053726157	0.458056737	0.64691168	0.90295498	up_in_case
PRJNA8274(	ENSGALG00010002423	ENSGALG00010002423	case	control	-0.632918231	0.430822419	-1.469093072	0.141807547	0.543501954	down_in_case
PRJNA8274(	ENSGALG00010002424	CASK	case	control	0.046198553	0.046058654	1.003037409	0.315842812	0.736794815	up_in_case
PRJNA8274(	ENSGALG00010002425	FDX2	case	control	0.029370139	0.10179567	0.288520511	0.772948332	0.94645256	up_in_case
PRJNA8274(	ENSGALG00010002427	CYB5R4	case	control	-0.04299239	0.071060392	-0.605012009	0.545171017	0.861997112	down_in_case
PRJNA8274(	ENSGALG00010002428	IBSP	case	control	-0.083669293	0.816673752	-0.102451307	0.918398461	0.983678974	down_in_case
PRJNA8274(	ENSGALG00010002429	ENSGALG00010002429	case	control	-0.225214482	0.416374072	-0.540894587	0.588580242	0.882805047	down_in_case
PRJNA8274(	ENSGALG00010002430	NUMA1	case	control	-0.055501198	0.070726083	-0.784734514	0.432609242	0.807718	down_in_case
PRJNA8274(	ENSGALG00010002431	BCKDHB	case	control	-0.032002207	0.083186129	-0.384706047	0.700455215	0.922500421	down_in_case
PRJNA8274(	ENSGALG00010002432	RAVER1	case	control	0.037586762	0.087407355	0.430018291	0.667182336	0.912432583	up_in_case
PRJNA8274(	ENSGALG00010002433	GRHL1	case	control	-0.031381225	0.3930419	-0.079841934	0.936362972	0.988482984	down_in_case
PRJNA8274(	ENSGALG00010002434	GPR34	case	control	-0.024174515	0.14227282	-0.169916607	0.86507572	0.973289701	down_in_case
PRJNA8274(	ENSGALG00010002435	ATP7B	case	control	2.240042917	0.471323575	4.752664697	2.00753E-06	0.000463027	up_in_case
PRJNA8274(	ENSGALG00010002436	ENSGALG00010002436	case	control	0.528781674	0.623832619	0.847633898	0.39664189	0.788531716	up_in_case
PRJNA8274(	ENSGALG00010002437	TTK	case	control	0.004089205	0.089218003	0.045833854	0.963442676	0.992068146	up_in_case
PRJNA8274(	ENSGALG00010002438	RARB	case	control	0.147016507	0.068408642	2.149092624	0.031627058	0.3098361	up_in_case
PRJNA8274(	ENSGALG00010002440	LRRC51	case	control	-0.495078129	0.193735674	-2.555430906	0.010605641	0.183831118	down_in_case
PRJNA8274(	ENSGALG00010002441	USO1	case	control	-0.019248235	0.042126342	-0.45691684	0.647730819	0.903487194	down_in_case
PRJNA8274(	ENSGALG00010002442	ENSGALG00010002442	case	control	0.226043547	0.389198576	0.580792328	0.561380426	0.870498818	up_in_case
PRJNA8274(	ENSGALG00010002443	TYK2	case	control	-0.068522921	0.061332948	-1.117228556	0.263896614	0.689768156	down_in_case
PRJNA8274(	ENSGALG00010002444	KLF11	case	control	-0.156530182	0.081249328	-1.926541244	0.054036827	0.378787562	down_in_case
PRJNA8274(	ENSGALG00010002445	EDRF1	case	control	-0.032686447	0.052534839	-0.622186102	0.533819504	0.855817434	down_in_case
PRJNA8274(	ENSGALG00010002446	RIMS1	case	control	-0.434327135	0.221179935	-1.963682351	0.049566941	0.364833243	down_in_case
PRJNA8274(	ENSGALG00010002447	YLEC12	case	control	0.585767507	0.563116904	1.040223625	0.298236018	0.721487387	up_in_case
PRJNA8274(	ENSGALG00010002448	RAB9A	case	control	-0.026006336	0.081908586	-0.317504393	0.750860907	0.939972946	down_in_case

PRJNA8274(	ENSGALG00010002449	ALG11	case	control	-0.012812477	0.078599418	-0.163009827	0.870510697	0.974794532	down_in_case
PRJNA8274(	ENSGALG00010002450	ENSGALG00010002450	case	control	-0.05234285	0.204674642	-0.255736857	0.798154033	0.953278433	down_in_case
PRJNA8274(	ENSGALG00010002453	TOP2B	case	control	-0.031499796	0.037434172	-0.841471705	0.40008373	0.789900496	down_in_case
PRJNA8274(	ENSGALG00010002454	NEK5	case	control	0.362727295	0.307674574	1.178931658	0.23842539	0.663323557	up_in_case
PRJNA8274(	ENSGALG00010002455	NYX	case	control	0.398782626	0.171483513	2.325486681	0.020045951	0.254128638	up_in_case
PRJNA8274(	ENSGALG00010002456	ELOVL4	case	control	0.795052323	0.564789265	1.4076973	0.159220722	0.571787122	up_in_case
PRJNA8274(	ENSGALG00010002457	ENSGALG00010002457	case	control	-0.285463232	0.479384902	-0.595478144	0.551523904	0.865159262	down_in_case
PRJNA8274(	ENSGALG00010002458	ENSGALG00010002458	case	control	0.140445654	0.090568207	1.55071696	0.120969528	0.519499513	up_in_case
PRJNA8274(	ENSGALG00010002459	G3BP2	case	control	0.008116682	0.041797975	0.194188406	0.84602836	0.967563913	up_in_case
PRJNA8274(	ENSGALG00010002460	LAMTOR1	case	control	-0.240611471	0.07951616	-3.025944302	0.00247858	0.081856092	down_in_case
PRJNA8274(	ENSGALG00010002461	TOMT	case	control	-0.076742856	0.122157581	-0.628228359	0.529854355	0.854806007	down_in_case
PRJNA8274(	ENSGALG00010002462	RRM2	case	control	-0.103894245	0.115455774	-0.89986184	0.36819378	0.771770041	down_in_case
PRJNA8274(	ENSGALG00010002463	ASRGL1	case	control	0.007092856	0.104208773	0.068063904	0.945734764	0.990313016	up_in_case
PRJNA8274(	ENSGALG00010002464	NGLY1	case	control	0.003843724	0.07116761	0.054009456	0.956927631	0.992056045	up_in_case
PRJNA8274(	ENSGALG00010002466	UROS	case	control	0.032124316	0.095276149	0.337170601	0.735988298	0.934360144	up_in_case
PRJNA8274(	ENSGALG00010002467	NEK3	case	control	0.726564326	0.163159914	4.453081076	8.46468E-06	0.001494381	up_in_case
PRJNA8274(	ENSGALG00010002468	ENSGALG00010002468	case	control	-0.514149764	0.514351138	-0.999608489	0.317500013	0.737774213	down_in_case
PRJNA8274(	ENSGALG00010002470	OXSM	case	control	-0.009034662	0.126798896	-0.071251896	0.943197279	0.990069816	down_in_case
PRJNA8274(	ENSGALG00010002471	OGFRL1	case	control	0.150733818	0.09660207	1.560358053	0.118675292	0.515665959	up_in_case
PRJNA8274(	ENSGALG00010002472	CDC37	case	control	-0.031905186	0.066653979	-0.478668892	0.6321742	0.89724192	down_in_case
PRJNA8274(	ENSGALG00010002473	ENSGALG00010002473	case	control	0.268239646	0.69768589	0.384470505	0.700629753	0.922516526	up_in_case
PRJNA8274(	ENSGALG00010002474	HPCAL1	case	control	0.11071616	0.095501603	1.159312065	0.246329006	0.670921661	up_in_case
PRJNA8274(	ENSGALG00010002475	ENSGALG00010002475	case	control	0.200087018	0.211890207	0.944295731	0.345018547	0.755870938	up_in_case
PRJNA8274(	ENSGALG00010002476	DDX3X	case	control	0.030162774	0.049490972	0.609460122	0.542219498	0.85975913	up_in_case
PRJNA8274(	ENSGALG00010002478	PPME1	case	control	0.015452677	0.040429281	0.382214984	0.702301908	0.923231962	up_in_case
PRJNA8274(	ENSGALG00010002481	CKAP2	case	control	0.032881326	0.099833725	0.329360907	0.741882912	0.937315893	up_in_case
PRJNA8274(	ENSGALG00010002483	COQ2	case	control	-0.191130141	0.094460351	-2.023390122	0.043032942	0.345908414	down_in_case
PRJNA8274(	ENSGALG00010002487	BCCIP	case	control	-0.050379979	0.059123511	-0.852114133	0.394150748	0.786978937	down_in_case
PRJNA8274(	ENSGALG00010002492	RPL8	case	control	-0.066849157	0.061250568	-1.091404687	0.275094851	0.700894634	down_in_case
PRJNA8274(	ENSGALG00010002500	OSR1	case	control	0.049677896	0.089528749	0.554882047	0.578975342	0.879142433	up_in_case
PRJNA8274(	ENSGALG00010002501	TCEANC	case	control	-0.057547111	0.117432574	-0.490043849	0.624102871	0.894353977	down_in_case
PRJNA8274(	ENSGALG00010002504	ENSGALG00010002504	case	control	-0.074620482	0.034891197	-2.138662133	0.032463041	0.310633901	down_in_case
PRJNA8274(	ENSGALG00010002507	C2CD3	case	control	-0.014156955	0.074582906	-0.189815014	0.84945409	0.968927451	down_in_case
PRJNA8274(	ENSGALG00010002511	VPS36	case	control	-0.023783134	0.054790119	-0.434077067	0.664232478	0.911435195	down_in_case
PRJNA8274(	ENSGALG00010002512	DAXX	case	control	-0.140880571	0.087318052	-1.61341861	0.106653593	0.49679035	down_in_case
PRJNA8274(	ENSGALG00010002518	MAST1	case	control	-0.582149798	0.269497231	-2.160132761	0.030762394	0.308259818	down_in_case
PRJNA8274(	ENSGALG00010002520	TTC32	case	control	0.635008663	0.179829081	3.53117893	0.000413712	0.028718828	up_in_case
PRJNA8274(	ENSGALG00010002522	HPSE	case	control	-0.05728079	0.239937629	-0.238732001	0.811313401	0.956205737	down_in_case
PRJNA8274(	ENSGALG00010002523	DNAJB13	case	control	0.240177033	0.246426622	0.974639148	0.329739288	0.746174551	up_in_case
PRJNA8274(	ENSGALG00010002526	ZBTB22	case	control	-0.250624404	0.118836289	-2.108988818	0.034945547	0.322792221	down_in_case
PRJNA8274(	ENSGALG00010002527	LRRC3B	case	control	-0.179158645	0.162247797	-1.104228521	0.269494021	0.696612479	down_in_case
PRJNA8274(	ENSGALG00010002528	MED14	case	control	-0.035878639	0.056597373	-0.633927625	0.526128056	0.85251736	down_in_case
PRJNA8274(	ENSGALG00010002530	STAG3	case	control	0.006536271	0.10511424	0.062182547	0.950417461	0.991497374	up_in_case
PRJNA8274(	ENSGALG00010002533	LPAR2	case	control	-0.012753589	0.12331354	-0.103424078	0.917626403	0.983445506	down_in_case
PRJNA8274(	ENSGALG00010002536	RGL2	case	control	-0.196884431	0.15657102	-1.257476832	0.208581023	0.633434867	down_in_case
PRJNA8274(	ENSGALG00010002539	SNAP91	case	control	0.493024931	0.3009928	1.637995763	0.101422578	0.491460867	up_in_case
PRJNA8274(	ENSGALG00010002543	UCP3	case	control	-0.22170464	0.272172159	-0.814575015	0.415315617	0.799221279	down_in_case
PRJNA8274(	ENSGALG00010002544	THSD1	case	control	0.059331359	0.074403637	0.79742552	0.425203943	0.804500712	up_in_case
PRJNA8274(	ENSGALG00010002546	ATP6AP2	case	control	0.02764981	0.042445752	0.65141524	0.514778476	0.847493921	up_in_case
PRJNA8274(	ENSGALG00010002547	PBX4	case	control	-0.043047504	0.058375111	-0.737429087	0.460861453	0.82454265	down_in_case
PRJNA8274(	ENSGALG00010002551	WDR35	case	control	0.205890099	0.116532563	1.766803153	0.0772612	0.445884536	up_in_case
PRJNA8274(	ENSGALG00010002553	ENSGALG00010002553	case	control	0.114295951	0.256669758	0.445303536	0.656100413	0.907494354	up_in_case
PRJNA8274(	ENSGALG00010002554	EGFL6	case	control	-0.09869824	0.126816915	-0.778273462	0.436407817	0.810200135	down_in_case
PRJNA8274(	ENSGALG00010002556	ENSGALG00010002556	case	control	-0.329165251	0.141359034	-2.328575981	0.01988154	0.253209146	down_in_case
PRJNA8274(	ENSGALG00010002557	ENSGALG00010002557	case	control	0.243070569	0.249775817	0.973154938	0.330476303	0.746554834	up_in_case
PRJNA8274(	ENSGALG00010002561	PAAF1	case	control	-0.134089399	0.063519132	-2.11100805	0.03477162	0.322042853	down_in_case
PRJNA8274(	ENSGALG00010002565	MRPS18C	case	control	0.027200083	0.080615247	0.33740619	0.735810719	0.934357951	up_in_case
PRJNA8274(	ENSGALG00010002566	TMSB4X	case	control	0.021929121	0.063934836	0.34299174	0.731604674	0.933770678	up_in_case
PRJNA8274(	ENSGALG00010002567	COA4	case	control	0.165310616	0.201996487	0.818383622	0.413138171	0.797633779	up_in_case
PRJNA8274(	ENSGALG00010002569	GTPBP6	case	control	0.009264393	0.103091815	0.089865459	0.92839413	0.986776285	up_in_case
PRJNA8274(	ENSGALG00010002570	ADAM12	case	control	0.080158429	0.11253266	0.71231258	0.476271236	0.830520323	up_in_case
PRJNA8274(	ENSGALG00010002571	BCOR	case	control	0.025943084	0.051464059	0.504101009	0.614190398	0.890440426	up_in_case
PRJNA8274(	ENSGALG00010002573	MVB12A	case	control	0.040546258	0.062385138	0.649934565	0.51573449	0.847810358	up_in_case
PRJNA8274(	ENSGALG00010002575	BGV	case	control	0.190707836	0.317238037	0.601150601	0.547739683	0.863104956	up_in_case
PRJNA8274(	ENSGALG00010002576	HELQ	case	control	0.190638112	0.089338138	2.133893945	0.032851463	0.311991307	up_in_case
PRJNA8274(	ENSGALG00010002577	MRPL48	case	control	-0.028879027	0.072707963	-0.397192084	0.691225819	0.918812261	down_in_case
PRJNA8274(	ENSGALG00010002579	MATN3	case	control	-0.358746706	0.762277889	-0.470624573			down_in_case
PRJNA8274(	ENSGALG00010002581	ATP13A1	case	control	-0.015021271	0.052198597	-0.287771539	0.773521625	0.94652185	down_in_case
PRJNA8274(	ENSGALG00010002582	ENSGALG00010002582	case	control	0.191625564	0.297542889	0.644026697	0.519558123	0.849320777	up_in_case
PRJNA8274(	ENSGALG00010002584	ENSGALG00010002584	case	control	0.342230393	0.304289203	1.124687927	0.2607213	0.687359696	up_in_case
PRJNA8274(	ENSGALG00010002586	GPR63	case	control	0.253228464	0.085666312	2.955986519	0.003116707	0.095164759	up_in_case
PRJNA8274(	ENSGALG00010002587	DOCK1	case	control	-0.044406815	0.059784179	-0.742785395	0.457611619	0.823337749	down_in_case
PRJNA8274(	ENSGALG00010002588	FAM175A	case	control	-0.036388381	0.063554183	-0.572556823	0.566944801	0.87333105	down_in_case
PRJNA8274(	ENSGALG00010002589	SLC4A7	case	control	-0.022258992	0.058938304	-0.377665973	0.705678748	0.924320939	down_in_case
PRJNA8274(	ENSGALG00010002590	ATP6V1C2	case	control	0.290247663	0.149075381	1.946985882	0.051536427	0.371479245	up_in_case
PRJNA8274(	ENSGALG00010002591	ENSGALG00010002591	case	control	-0.353005377	0.118401771	-2.981419738	0.002869152	0.090571479	down_in_case
PRJNA8274(	ENSGALG00010002592	RPS6KA3	case	control	-0.242673261	0.081885762	-2.963558689	0.003041041	0.09392415	down_in_case
PRJNA8274(	ENSGALG00010002597	ENSGALG00010002597	case	control	0.040466621	0.098550331	0.410618824	0.681352059	0.915594727	up_in_case
PRJNA8274(	ENSGALG00010002598	NMT2	case	control	-0.058972288	0.052058034	-1.13281818	0.257290615	0.684130866	down_in_case
PRJNA8274(	ENSGALG00010002599	KIFC1	case	control	-0.256622112	0.33016508	-0.777253949	0.437008959	0.810535423	down_in_case
PRJNA8274(	ENSGALG00010002600	TRPA1	case	control	-1.388230426	0.544323794	-2.550376157	0.010760675	0.184728043	down_in_case
PRJNA8274(	ENSGALG00010002601	PLCXD1	case	control	-0.052107835	0.156100183	-0.333810207	0.73852278	0.935998915	down_in_case
PRJNA8274(	ENSGALG00010002603	INSYN2A	case	control	0.146290238	0.114179847	1.28122644	0.200114142	0.622902456	up_in_case
PRJNA8274(	ENSGALG00010002604	BLEC3	case	control	-0.294839579	0.468358362	-0.62951706	0.529010603	0.854302837	down_in_case
PRJNA8274(	ENSGALG00010002606	ENSGALG00010002606	case	control	0.484236416	0.189940035	2.54941733	0.010790309	0.184728043	up_in_case
PRJNA8274(	ENSGALG00010002608	RPP38	case	control	0.044464596	0.117588221	0.378138179	0.705327948	0.924182432	up_in_case
PRJNA8274(	ENSGALG00010002609	PDIA6	case	control	0.034399296	0.046840258	0.73439595	0.462707455	0.825091817	up_in_case
PRJNA8274(	ENSGALG00010002611	NPS	case	control	0.210162145	0.493965716	0.42545897	0.670502126	0.913465866	up_in_case
PRJNA8274(	ENSGALG00010002612	JPH1	case	control	-0.19008336	0.091302647	-2.08190414	0.037351227	0.328438691	down_in_case
PRJNA8274(	ENSGALG00010002613	ENSGALG00010002613	case	control	-0.032973107	0.064905544	-0.508016814	0.611441552	0.88947908	down_in_case
PRJNA8274(	ENSGALG00010002614	GPAT3	case	control	-0.028205797	0.118146915	-0.238734942	0.81131112	0.956205737	down_in_case
PRJNA8274(	ENSGALG00010002616	ZNFB3	case	control	0.089878896	0.132191462	0.679914528	0.496558582	0.83858637	up_in_case
PRJNA8274(	ENSGALG00010002619	RAB6A	case	control	-0.142536083	0.054858063	-2.598270423	0.009369467	0.172879234	down_in_case
PRJNA8274(	ENSGALG00010002621	SLC25A15	case	control	0.392727675	0.154631851	2.539759256	0.01109288	0.185137791	up_in_case
PRJNA8274(	ENSGALG00010002622	AZI2	case	control	0.034863011	0.045795079	0.761282919	0.446488101	0.817679602	up_in_case
PRJNA8274(	ENSGALG00010002623	MID1IP1	case	control	0.076950604	0.062161054	1.237923077	0.215744584	0.640470741	up_in_case
PRJNA8274(	ENSGALG00010002625	FHL5	case	control	-0.037272923	0.093712189	-0.39773826	0.690823132	0.918784485	down_in_case
PRJNA8274(	ENSGALG00010002626	CHD7	case	control	-0.054377335	0.060043469	-0.905632801	0.365130241	0.770843433	down_in_case
PRJNA8274(	ENSGALG00010002627	MRPS31	case	control	-0.115815156	0.061374883	-1.887012252	0.059158678	0.395497471	down_in_case
PRJNA8274(	ENSGALG00010002628	TRIM7.2	case	control	-0.047628962	0.058619438	-0.812511408	0.416498243	0.799987395	down_in_case
PRJNA8274(	ENSGALG00010002629	ENSGALG00010002629	case	control	0.669279687	0.90556705	0.739072481	0.459862986	0.824480387	up_in_case
PRJNA8274(	ENSGALG00010002632	HPGD	case	control	-0.087998465	0.172471808	-0.510219418	0.60989775	0.888273136	down_in_case
PRJNA8274(	ENSGALG00010002633	ENSGALG00010002633	case	control	0.138110129	0.084037677	1.643431067	0.100293777	0.489689587	up_in_case
PRJNA8274(	ENSGALG00010002634	RGN	case	control	0.200772552	0.188375897	1.065808073	0.286510411	0.710659943	up_in_case
PRJNA8274(	ENSGALG00010002638	FABP9	case	control	0.606088243	0.340929248	1.777753733	0.07544431	0.442021549	up_in_case
PRJNA8274(	ENSGALG00010002644	KCNF1	case	control	-0.48679116	0.245142837	-1.985744984	0.047061647	0.357398596	down_in_case
PRJNA8274(	ENSGALG00010002646	FOXO1	case	control	0.0660824	0.056656483	1.166369609	0.24346504	0.667780411	up_in_case
PRJNA8274(	ENSGALG00010002647	FAM168A	case	control	-0.047094442	0.050125	-0.939539984	0.347453574	0.758215489	down_in_case
PRJNA8274(	ENSGALG00010002649	UFL1	case	control	-0.013373529	0.04329027	-0.308926906	0.757377129	0.941659745	down_in_case
PRJNA8274(	ENSGALG00010002653	EIF1AX	case	control	-0.025669943	0.043414063	-0.591281753	0.554331654	0.866646786	down_in_case
PRJNA8274(	ENSGALG00010002655	FABP4	case	control	-0.408573209	0.774457085	-0.527560813	0.597804197	0.884680373	down_in_case
PRJNA8274(	ENSGALG00010002656	GLRA3	case	control	-0.600109146	0.544960523	-1.101197465	0.270810725	0.698323327	down_in_case
PRJNA8274(	ENSGALG00010002658	FBXL12	case	control	-0.133647692	0.088237757	-1.514631558	0.129865736	0.532363328	down_in_case
PRJNA8274(	ENSGALG00010002661	ACBD7	case	control	-0.028337255	0.190757163	-0.14855146	0.881907577	0.976801393	down_in_case
PRJNA8274(	ENSGALG00010002664	ZNFB1	case	control	0.110722227	0.117063206	0.94583286	0.344233842	0.755870938	up_in_case
PRJNA8274(	ENSGALG00010002667	ENSGALG00010002667	case	control	1.098819405	1.09239513	1.005880908	0.314472861	0.736432557	up_in_case
PRJNA8274(	ENSGALG00010002669	IL4I1	case	control	-0.528402413	0.54891738	-0.962626494	0.335734997	0.749888354	down_in_case
PRJNA8274(	ENSGALG00010002673	SLC66A3	case	control	-0.112917202	0.113110161	-0.998294064	0.318136785	0.738087569	down_in_case
PRJNA8274(	ENSGALG00010002678	CCNI	case	control	-0.107249764	0.084314421	-1.272021594	0.203365447	0.625537942	down_in_case
PRJNA8274(	ENSGALG00010002681	PLEKHB1	case	control	0.092244158	0.117150937	0.78739582	0.431050193	0.806930928	up_in_case
PRJNA8274(	ENSGALG00010002682	UBL5	case	control	-0.038549991	0.094661272	-0.407241421	0.683830682	0.916302357	down_in_case
PRJNA8274(	ENSGALG00010002685	CA8	case	control	0.006188659	0.137458215	0.045022113	0.964089683	0.992068146	up_in_case
PRJNA8274(	ENSGALG00010002689	COG6	case	control	-0.176652141	0.056038194	-3.152352532	0.001619606	0.064874977	down_in_case
PRJNA8274(	ENSGALG00010002693	MSC	case	control	-0.043077497	0.11181385	-0.38526083	0.700044179	0.922288441	down_in_case
PRJNA8274(	ENSGALG00010002700	LHFPL6	case	control	-0.042485518	0.070877905	-0.599418363	0.548893935	0.86406685	down_in_case
PRJNA8274(	ENSGALG00010002701	TMEM196	case	control	0.910857201	0.42479204	2.14424263	0.032013451	0.3098361	up_in_case

PRJNA8274(	ENSGALG00010002703	PIN1	case	control	-0.128897622	0.062657861	-2.057166009	0.03967026	0.336080244	down_in_case
PRJNA8274(	ENSGALG00010002704	KCNB2	case	control	-0.161547715	0.274764204	-0.587950366	0.556565608	0.867878646	down_in_case
PRJNA8274(	ENSGALG00010002705	JADE3	case	control	0.097517665	0.044658533	2.183628909	0.028989527	0.302591418	up_in_case
PRJNA8274(	ENSGALG00010002706	ROCK2	case	control	-0.010419053	0.063486127	-0.164115433	0.869640273	0.974718971	down_in_case
PRJNA8274(	ENSGALG00010002707	RELT	case	control	-0.003931715	0.155090478	-0.025351107	0.979774909	0.995636523	down_in_case
PRJNA8274(	ENSGALG00010002712	PTPRE	case	control	0.085365466	0.078483917	1.087681005	0.276735956	0.702359305	up_in_case
PRJNA8274(	ENSGALG00010002713	OLFM2	case	control	0.068872288	0.099360545	0.693155296	0.488212099	0.835899547	up_in_case
PRJNA8274(	ENSGALG00010002716	NHLRC3	case	control	0.131253866	0.078114639	1.680272334	0.092904341	0.478061202	up_in_case
PRJNA8274(	ENSGALG00010002722	TRIM7.1	case	control	-0.177255195	0.107401902	-1.650391589	0.09886287	0.488407564	down_in_case
PRJNA8274(	ENSGALG00010002725	TSPAN7	case	control	0.00212048	0.103465405	0.020494578	0.983648837	0.996591771	up_in_case
PRJNA8274(	ENSGALG00010002728	RP2	case	control	-0.081824581	0.061497321	-1.330538946	0.183340763	0.60491829	down_in_case
PRJNA8274(	ENSGALG00010002730	E2F6	case	control	-0.116648585	0.074037052	-1.575543344	0.115131082	0.510979041	down_in_case
PRJNA8274(	ENSGALG00010002733	RDH8	case	control	-0.222110819	0.21164259	-1.049461826	0.293965616	0.716378375	down_in_case
PRJNA8274(	ENSGALG00010002741	PDK4	case	control	-0.335955259	0.287914903	-1.166856091	0.243268491	0.667473598	down_in_case
PRJNA8274(	ENSGALG00010002742	PROSER1	case	control	0.026532457	0.065652998	0.404131681	0.686115889	0.917034645	up_in_case
PRJNA8274(	ENSGALG00010002747	FUT9	case	control	0.092144122	0.343569086	0.26819678	0.788547853	0.951738421	up_in_case
PRJNA8274(	ENSGALG00010002750	ENSGALG00010002750	case	control	-0.113977485	0.126201608	-0.903138137	0.366452586	0.771763177	down_in_case
PRJNA8274(	ENSGALG00010002752	NDUFA4	case	control	-0.102783979	0.068126898	-1.508713619	0.131371984	0.534908166	down_in_case
PRJNA8274(	ENSGALG00010002753	SLC9A7	case	control	0.127474632	0.09722739	1.311097963	0.189824669	0.613281846	up_in_case
PRJNA8274(	ENSGALG00010002758	GREB1	case	control	0.041011128	0.297839321	0.137695479	0.890481092	0.978745332	up_in_case
PRJNA8274(	ENSGALG00010002760	RAB2A	case	control	-0.021858187	0.048538034	-0.450331102	0.652471716	0.90585879	down_in_case
PRJNA8274(	ENSGALG00010002762	PPAN	case	control	-0.041155747	0.090187679	-0.456334474	0.648149477	0.903510177	down_in_case
PRJNA8274(	ENSGALG00010002763	SEPTIN11	case	control	0.064700455	0.054764869	1.181422617	0.237434878	0.662652947	up_in_case
PRJNA8274(	ENSGALG00010002765	MAP7D2	case	control	0.309545267	0.436411035	0.709297525	0.478139864	0.830520323	up_in_case
PRJNA8274(	ENSGALG00010002766	NANOS1	case	control	0.525920962	0.253895759	2.071405069	0.038320958	0.331512217	up_in_case
PRJNA8274(	ENSGALG00010002768	TRIM39.2	case	control	0.459512029	0.457855166	1.003618749	0.315562414	0.736748426	up_in_case
PRJNA8274(	ENSGALG00010002774	TBX18	case	control	0.090265	0.105239952	0.857706582	0.391054505	0.784275639	up_in_case
PRJNA8274(	ENSGALG00010002779	FBXO8	case	control	-0.017339717	0.071862358	-0.24129067	0.809329841	0.955875648	down_in_case
PRJNA8274(	ENSGALG00010002781	EIF3G	case	control	-0.052682759	0.058984275	-0.89316617	0.371768192	0.77369739	down_in_case
PRJNA8274(	ENSGALG00010002782	GSTA3	case	control	0.536541409	0.170914135	3.139245383	0.001693835	0.065999582	up_in_case
PRJNA8274(	ENSGALG00010002784	TRIM27.2	case	control	1.048028163	0.337188911	3.108133537	0.00188273	0.070664136	up_in_case
PRJNA8274(	ENSGALG00010002787	CEP44	case	control	0.00684716	0.09642183	0.071012554	0.943387764	0.990129937	up_in_case
PRJNA8274(	ENSGALG00010002788	ENSGALG00010002788	case	control	-0.341310107	0.409217172	-0.83405617	0.404249332	0.792645749	down_in_case
PRJNA8274(	ENSGALG00010002790	BCLAF3	case	control	-0.125271831	0.079461973	-1.576500381	0.114910531	0.510316955	down_in_case
PRJNA8274(	ENSGALG00010002792	ENSGALG00010002792	case	control	0.192070031	0.108824793	1.764947365	0.077572612	0.446933261	up_in_case
PRJNA8274(	ENSGALG00010002796	PRDM8	case	control	-0.139559328	0.266170024	-0.524323988	0.600053216	0.884888453	down_in_case
PRJNA8274(	ENSGALG00010002800	TUBGCP5	case	control	-0.015052176	0.059992329	-0.250901676	0.801890129	0.954294696	down_in_case
PRJNA8274(	ENSGALG00010002801	FGF5	case	control	-0.145411488	0.24098816	-0.603396813	0.546244742	0.862459954	down_in_case
PRJNA8274(	ENSGALG00010002805	FAM171A1	case	control	-0.072000095	0.036536571	-1.970630866	0.048766113	0.363542241	down_in_case
PRJNA8274(	ENSGALG00010002807	P2RY6	case	control	0.265050964	0.124697118	2.125558055	0.033540075	0.316061661	up_in_case
PRJNA8274(	ENSGALG00010002809	SH3KBP1	case	control	-0.131934957	0.063083438	-2.091435748	0.03648902	0.326967374	down_in_case
PRJNA8274(	ENSGALG00010002810	TRIM27.1	case	control	0.168833989	0.230346007	0.732958175	0.463583939	0.825091817	up_in_case
PRJNA8274(	ENSGALG00010002812	TRIM13	case	control	-0.080782949	0.088269933	-0.915180813	0.360096716	0.765754313	down_in_case
PRJNA8274(	ENSGALG00010002814	TERF1	case	control	-0.108803514	0.109902558	-0.989999834	0.3221742	0.739857245	down_in_case
PRJNA8274(	ENSGALG00010002817	SHFM1	case	control	-0.142434892	0.069427594	-2.051560249	0.040212419	0.337463373	down_in_case
PRJNA8274(	ENSGALG00010002819	BMP3	case	control	-0.222516221	0.138762689	-1.603573853	0.108808018	0.500951275	down_in_case
PRJNA8274(	ENSGALG00010002824	NXPH1	case	control	-0.295433909	0.393368841	-0.751035359	0.452631376	0.821045895	down_in_case
PRJNA8274(	ENSGALG00010002825	TRIM41	case	control	-0.234737772	0.075049191	-3.127785499	0.001761287	0.06788787	down_in_case
PRJNA8274(	ENSGALG00010002827	SBSPON	case	control	-0.049999191	0.216125578	-0.231343239	0.817048158	0.958001694	down_in_case
PRJNA8274(	ENSGALG00010002832	LPIN1	case	control	-0.316834755	0.177438139	-1.785606838	0.074162939	0.438111241	down_in_case
PRJNA8274(	ENSGALG00010002834	MRPL53	case	control	-0.020256873	0.078425352	-0.258294957	0.796179278	0.95280257	down_in_case
PRJNA8274(	ENSGALG00010002835	CYFIP1	case	control	0.033651064	0.041230809	0.816163068	0.414406876	0.798853486	up_in_case
PRJNA8274(	ENSGALG00010002837	DNMT1	case	control	-0.04422954	0.056074212	-0.788767923	0.430247661	0.806256264	down_in_case
PRJNA8274(	ENSGALG00010002838	ENSGALG00010002838	case	control	-0.120043138	0.169854078	-0.706742749	0.479726358	0.831851816	down_in_case
PRJNA8274(	ENSGALG00010002840	SRPX	case	control	-0.075538697	0.087297158	-0.865305342	0.38687122	0.782604263	down_in_case
PRJNA8274(	ENSGALG00010002848	TRIB2	case	control	0.168743262	0.077494434	2.177488808	0.029444116	0.305109318	up_in_case
PRJNA8274(	ENSGALG00010002853	CEP162	case	control	0.072352433	0.155121998	0.466422777	0.640912911	0.901451228	up_in_case
PRJNA8274(	ENSGALG00010002855	RACK1	case	control	-0.077991111	0.061416249	-1.269877472	0.204128279	0.62653668	down_in_case
PRJNA8274(	ENSGALG00010002857	DCAF15	case	control	-0.017442996	0.100858532	-0.172945168	0.862694528	0.972787494	down_in_case
PRJNA8274(	ENSGALG00010002860	NIPA2	case	control	-0.114752124	0.056145613	-2.04383063	0.040970282	0.337874876	down_in_case
PRJNA8274(	ENSGALG00010002864	RNASEH2B	case	control	-0.178567413	0.089501709	-1.995128522	0.046028864	0.355983104	down_in_case
PRJNA8274(	ENSGALG00010002866	ENSGALG00010002866	case	control	0.202554199	0.108899974	1.860002264	0.062885206	0.408011997	up_in_case
PRJNA8274(	ENSGALG00010002868	PPP1R9A	case	control	-0.081444848	0.067244657	-1.211172031	0.22582948	0.649498359	down_in_case
PRJNA8274(	ENSGALG00010002870	FABP5	case	control	-0.03848894	0.111669291	-0.344668972	0.730343247	0.933770678	down_in_case
PRJNA8274(	ENSGALG00010002878	P2RY2	case	control	-0.228582438	0.17842326	-1.281124657	0.200149884	0.622902456	down_in_case
PRJNA8274(	ENSGALG00010002880	RASGEF1B	case	control	-0.141723728	0.188683524	-0.751118725	0.452581208	0.821045895	down_in_case
PRJNA8274(	ENSGALG00010002881	ENSGALG00010002881	case	control	-0.042015618	0.326511696	-0.128680284	0.897610637	0.980176778	down_in_case
PRJNA8274(	ENSGALG00010002883	MAP3K15	case	control	0.057341845	0.227502026	0.252049821	0.801002555	0.954294696	up_in_case
PRJNA8274(	ENSGALG00010002884	ADHFE1	case	control	0.051855987	0.206136371	0.251561562	0.801379972	0.954294696	up_in_case
PRJNA8274(	ENSGALG00010002886	HERC2	case	control	-0.061927237	0.066793992	-0.927137837	0.353854973	0.762350539	down_in_case
PRJNA8274(	ENSGALG00010002888	ENSGALG00010002888	case	control	0.397702409	0.355337236	1.119225257	0.263044053	0.688798747	up_in_case
PRJNA8274(	ENSGALG00010002890	VXN	case	control	0.555006174	0.159903671	3.470878247	0.000518759	0.033449927	up_in_case
PRJNA8274(	ENSGALG00010002894	FCHSD2	case	control	0.049194913	0.049247535	0.998931491	0.31782788	0.738054349	up_in_case
PRJNA8274(	ENSGALG00010002896	OCA2	case	control	3.619670911	0.345570632	10.47447491	1.13164E-25	1.34854E-22	up_in_case
PRJNA8274(	ENSGALG00010002904	PON1	case	control	0.121432588	0.10550026	1.151016956	0.24972526	0.675060722	up_in_case
PRJNA8274(	ENSGALG00010002907	GABRG3	case	control	-0.653566792	0.293142424	-2.229519641	0.02577935	0.284977951	down_in_case
PRJNA8274(	ENSGALG00010002908	S1PR2	case	control	-0.086050527	0.128049031	-0.672012325	0.50157585	0.841254358	down_in_case
PRJNA8274(	ENSGALG00010002911	MYBL1	case	control	-0.156998593	0.145437805	-1.079489568	0.280369542	0.705762606	down_in_case
PRJNA8274(	ENSGALG00010002912	RPS3	case	control	-0.089733557	0.077423238	-1.159000301	0.246456062	0.670921661	down_in_case
PRJNA8274(	ENSGALG00010002913	NXNL1	case	control	-0.19682728	0.320801189	-0.613549096	0.53951333	0.858521589	down_in_case
PRJNA8274(	ENSGALG00010002914	SLC27A1	case	control	-0.136643932	0.116594078	-1.171962887	0.241211957	0.666227315	down_in_case
PRJNA8274(	ENSGALG00010002916	SDCBP	case	control	0.029085267	0.04471634	0.650439348	0.515408468	0.847750297	up_in_case
PRJNA8274(	ENSGALG00010002917	PGLS	case	control	0.044826268	0.055197154	0.81211194	0.416727403	0.799987395	up_in_case
PRJNA8274(	ENSGALG00010002918	LRATD1	case	control	0.241486863	0.304743812	0.792425812	0.428112434	0.805337183	up_in_case
PRJNA8274(	ENSGALG00010002919	ENSGALG00010002919	case	control	-0.183380128	0.077262813	-2.373459105	0.017622344	0.23617575	down_in_case
PRJNA8274(	ENSGALG00010002920	VCPIP1	case	control	-0.013609447	0.056575481	-0.240553796	0.809900964	0.955972758	down_in_case
PRJNA8274(	ENSGALG00010002922	TMEM106B	case	control	0.062403802	0.08970418	0.695662136	0.486640443	0.835095382	up_in_case
PRJNA8274(	ENSGALG00010002923	SERPINH1	case	control	0.062326435	0.065877245	0.946099598	0.344097789	0.755852285	up_in_case
PRJNA8274(	ENSGALG00010002925	INTS6	case	control	0.002780589	0.089761477	0.03097753	0.97528746	0.99399062	up_in_case
PRJNA8274(	ENSGALG00010002926	GALNTL6	case	control	0.204272943	0.39681032	0.514787374	0.606701609	0.886827457	up_in_case
PRJNA8274(	ENSGALG00010002927	GABRA5	case	control	0.769945786	0.633710731	1.214979878	0.224373766	0.648105158	up_in_case
PRJNA8274(	ENSGALG00010002928	ASB4	case	control	-0.283765128	0.31892689	-0.889749773	0.373600262	0.774369566	down_in_case
PRJNA8274(	ENSGALG00010002929	PDHA2	case	control	-0.032489143	0.086454703	-0.375793818	0.707070184	0.92461283	down_in_case
PRJNA8274(	ENSGALG00010002930	SERAF	case	control	0.276660712	0.158995394	1.740054881	0.081849382	0.454736389	up_in_case
PRJNA8274(	ENSGALG00010002934	VWDE	case	control	-0.362206256	0.349481191	-1.036411301	0.3000103	0.723279006	down_in_case
PRJNA8274(	ENSGALG00010002935	SYTL5	case	control	-0.548471215	0.677531651	-0.809513791	0.418219673	0.800393647	down_in_case
PRJNA8274(	ENSGALG00010002937	GALNT7	case	control	0.051497483	0.057202247	0.900270269	0.367976439	0.771770041	up_in_case
PRJNA8274(	ENSGALG00010002939	HMGB2	case	control	-0.059683381	0.094520642	-0.631432246	0.527757937	0.853386536	down_in_case
PRJNA8274(	ENSGALG00010002940	BG1	case	control	-0.434024005	0.261707751	-1.658430077	0.09723069	0.485691283	down_in_case
PRJNA8274(	ENSGALG00010002942	GABRB3	case	control	-0.058630783	0.411493218	-0.142482988	0.886698516	0.977630122	down_in_case
PRJNA8274(	ENSGALG00010002943	SAP30	case	control	0.0100894	0.092537388	0.109030528	0.913178277	0.982728035	up_in_case
PRJNA8274(	ENSGALG00010002946	GDPD5	case	control	0.025349125	0.05887448	0.430562183	0.666786743	0.912284937	up_in_case
PRJNA8274(	ENSGALG00010002947	ENSGALG00010002947	case	control	0.397919115	0.399193414	0.996807814	0.318857804	0.738427335	up_in_case
PRJNA8274(	ENSGALG00010002948	DLX6	case	control	-0.187847916	0.806737686	-0.232848817	0.815878804	0.957374769	down_in_case
PRJNA8274(	ENSGALG00010002949	ENSGALG00010002949	case	control	-0.808078407	0.60228841	-1.341680155	0.179699724	0.600679303	down_in_case
PRJNA8274(	ENSGALG00010002951	ENSGALG00010002951	case	control	-3.034728169	2.923239083	-1.038138887			down_in_case
PRJNA8274(	ENSGALG00010002952	NBAS	case	control	-0.018167536	0.050144126	-0.362306353	0.717123111	0.926862121	down_in_case
PRJNA8274(	ENSGALG00010002953	WDFY2	case	control	-0.277726432	0.120127255	-2.311935217	0.020781254	0.258860564	down_in_case
PRJNA8274(	ENSGALG00010002954	CHST7	case	control	0.022609769	0.132854971	0.170183841	0.86486556	0.973211953	up_in_case
PRJNA8274(	ENSGALG00010002955	BLEC1	case	control	-0.805116492	0.383080033	-2.101692653	0.035580208	0.324939791	down_in_case
PRJNA8274(	ENSGALG00010002956	SCRG1	case	control	0.323720727	0.202702053	1.597027372	0.110259606	0.502938553	up_in_case
PRJNA8274(	ENSGALG00010002957	ADGRG2	case	control	-0.03665006	0.191590351	-0.191293874	0.84829536	0.968692766	down_in_case
PRJNA8274(	ENSGALG00010002958	ENSGALG00010002958	case	control	-0.147485021	0.179269334	-0.822700782	0.410678171	0.796543221	down_in_case
PRJNA8274(	ENSGALG00010002959	DLX5	case	control	0.387773795	0.492267542	0.787729765	0.430854791	0.806930928	up_in_case
PRJNA8274(	ENSGALG00010002960	BLEC2	case	control	-3.88339505	1.042058802	-3.726656349	0.000194037	0.017342033	down_in_case
PRJNA8274(	ENSGALG00010002965	PRSS35	case	control	-0.092106327	0.103734961	-0.887900533	0.374594258	0.774434633	down_in_case
PRJNA8274(	ENSGALG00010002972	ME1	case	control	-0.054407567	0.122936091	-0.442567894	0.658078312	0.908367791	down_in_case
PRJNA8274(	ENSGALG00010002978	DYNLT3	case	control	0.008035956	0.077712551	0.103406149	0.917640632	0.983445506	up_in_case
PRJNA8274(	ENSGALG00010002981	KLHL35	case	control	-0.060594177	0.231694244	-0.261526465	0.793686544	0.95280257	down_in_case
PRJNA8274(	ENSGALG00010002982	HAND2	case	control	-0.030329797	0.213251832	-0.142225261	0.886902079	0.977630122	down_in_case
PRJNA8274(	ENSGALG00010002983	NSMAF	case	control	0.05181992	0.053260883	0.972945188	0.330580545	0.746554834	up_in_case
PRJNA8274(	ENSGALG00010002985	ENSGALG00010002985	case	control	0.05599321	0.109192113	0.512795369	0.60809447	0.887502645	up_in_case
PRJNA8274(	ENSGALG00010002987	PCDH8	case	control	-0.433720877	0.251341724	-1.72562227	0.08441539	0.459687768	down_in_case
PRJNA8274(	ENSGALG00010002991	BLB1	case	control	-0.427801071	0.221102444	-1.93485456	0.053008146	0.37618684	down_in_case
PRJNA8274(	ENSGALG00010002992	ENSGALG00010002992	case	control	0.003578408	0.258007858	0.013869375	0.988934194	0.997164011	up_in_case
PRJNA8274(	ENSGALG00010002994	DUSP4	case	control	0.070872329	0.127988323	0.553740584	0.579756397	0.879142433	up_in_case

PRJNA8274(	ENSGALG00010002995	RWDD2A	case	control	-0.039185037	0.125570451	-0.312056194	0.754997817	0.941559531	down_in_case
PRJNA8274(	ENSGALG00010002998	LECT1	case	control	0.267531809	0.332653872	0.804234767			up_in_case
PRJNA8274(	ENSGALG00010002999	CMTM7	case	control	0.102755798	0.19839159	0.517944325	0.604497115	0.885268908	up_in_case
PRJNA8274(	ENSGALG00010003001	MAP6	case	control	-0.40647004	0.298958734	-1.359619217	0.173950454	0.593015599	down_in_case
PRJNA8274(	ENSGALG00010003002	PHKA2	case	control	-0.179195437	0.189759402	-0.944329691	0.345001198	0.755870938	down_in_case
PRJNA8274(	ENSGALG00010003006	CYP7B1	case	control	-0.116034772	0.169511509	-0.684524447	0.493644058	0.837916219	down_in_case
PRJNA8274(	ENSGALG00010003009	TNKS	case	control	-0.104090585	0.046269083	-2.249679	0.02446933	0.276609812	down_in_case
PRJNA8274(	ENSGALG00010003017	PGM3	case	control	0.22342181	0.101860331	2.19341335	0.028277611	0.299627134	up_in_case
PRJNA8274(	ENSGALG00010003018	MOGAT2	case	control	-0.625193021	0.367675347	-1.700394181	0.089056806	0.471671229	down_in_case
PRJNA8274(	ENSGALG00010003019	TAPBP	case	control	-0.063728449	0.155559455	-0.409672618	0.682046119	0.915594727	down_in_case
PRJNA8274(	ENSGALG00010003022	BLB2	case	control	-0.668917762	0.31106936	-2.150381389	0.031525059	0.3098361	down_in_case
PRJNA8274(	ENSGALG00010003024	SUGT1	case	control	0.044166942	0.05771313	0.765284109	0.444102385	0.81533754	up_in_case
PRJNA8274(	ENSGALG00010003025	MFHAS1	case	control	-0.124145701	0.082524953	-1.504341372	0.132493492	0.535857167	down_in_case
PRJNA8274(	ENSGALG00010003027	SCIN	case	control	-0.01448202	0.305775202	-0.047361655	0.962224989	0.992056045	down_in_case
PRJNA8274(	ENSGALG00010003030	PPEF1	case	control	-0.420964348	0.509162413	-0.826778131	0.408362827	0.794775742	down_in_case
PRJNA8274(	ENSGALG00010003034	ERI1	case	control	-0.033012989	0.039184954	-0.842491462	0.399512917	0.789900496	down_in_case
PRJNA8274(	ENSGALG00010003035	DGAT2	case	control	-0.444357076	0.511074515	-0.869456533	0.384597473	0.780971676	down_in_case
PRJNA8274(	ENSGALG00010003042	TOX	case	control	0.388887741	0.174252064	2.231753994	0.025631226	0.283909015	up_in_case
PRJNA8274(	ENSGALG00010003052	ENSGALG00010003052	case	control	-0.048814992	0.039831133	-1.225548659	0.220368568	0.644563413	down_in_case
PRJNA8274(	ENSGALG00010003057	DDX1	case	control	0.003938943	0.02462291	0.159970653	0.872904192	0.975062028	up_in_case
PRJNA8274(	ENSGALG00010003061	ENSGALG00010003061	case	control	-0.299190435	0.385397413	-0.776316668	0.437562034	0.810720016	down_in_case
PRJNA8274(	ENSGALG00010003063	GGH	case	control	0.04242203	0.066528722	0.637649849	0.523701638	0.85188641	up_in_case
PRJNA8274(	ENSGALG00010003064	VAV1	case	control	0.238478259	0.22795041	1.046184824	0.295475708	0.718955697	up_in_case
PRJNA8274(	ENSGALG00010003068	BRD2	case	control	-0.032956685	0.059014252	-0.558452989	0.576535097	0.878085957	down_in_case
PRJNA8274(	ENSGALG00010003072	ELF1	case	control	-0.029399253	0.062615647	-0.469519279	0.638698508	0.900303403	down_in_case
PRJNA8274(	ENSGALG00010003074	TTPA	case	control	-0.226254061	0.268282824	-0.843341581	0.399037434	0.789900496	down_in_case
PRJNA8274(	ENSGALG00010003077	EIF2S3	case	control	-0.00822334	0.054122127	-0.15194044	0.879233912	0.976318821	down_in_case
PRJNA8274(	ENSGALG00010003081	DOP1A	case	control	0.236293293	0.084809684	2.786159334	0.005333665	0.126907498	up_in_case
PRJNA8274(	ENSGALG00010003087	TWISTNB	case	control	-0.113227043	0.092693163	-1.221525289	0.221887196	0.645834386	down_in_case
PRJNA8274(	ENSGALG00010003090	DMA	case	control	0.131399829	0.183331612	0.716733069	0.473538817	0.82959752	up_in_case
PRJNA8274(	ENSGALG00010003097	TFPI2	case	control	0.027166212	0.095105382	0.285643267	0.775151361	0.947087967	up_in_case
PRJNA8274(	ENSGALG00010003101	KLHL15	case	control	0.065171388	0.053226041	1.224426728	0.220791292	0.644744838	up_in_case
PRJNA8274(	ENSGALG00010003103	YTHDF3	case	control	-0.170063244	0.069938065	-2.431626374	0.015031204	0.21667965	down_in_case
PRJNA8274(	ENSGALG00010003107	GNG11	case	control	0.033539129	0.126687522	0.264739011	0.791210523	0.95239351	up_in_case
PRJNA8274(	ENSGALG00010003108	PPP1R3B	case	control	-0.01793093	0.171005907	-0.104855618	0.916490377	0.983445506	down_in_case
PRJNA8274(	ENSGALG00010003110	BF1	case	control	-0.477382941	0.194376006	-2.455976692	0.014050227	0.208540979	down_in_case
PRJNA8274(	ENSGALG00010003113	WBP4	case	control	0.078917827	0.094986482	0.830832185	0.406068438	0.7941993	up_in_case
PRJNA8274(	ENSGALG00010003115	UBE3D	case	control	0.168415985	0.137124087	1.228201317	0.219371404	0.643443819	up_in_case
PRJNA8274(	ENSGALG00010003117	ID2	case	control	0.081032906	0.074607263	1.086126242	0.277423144	0.702897051	up_in_case
PRJNA8274(	ENSGALG00010003121	ARMC1	case	control	0.02419989	0.044989245	0.537903897	0.590643398	0.882949889	up_in_case
PRJNA8274(	ENSGALG00010003125	APOO	case	control	0.038713734	0.073988173	0.52324219	0.600805725	0.884954589	up_in_case
PRJNA8274(	ENSGALG00010003126	ENSGALG00010003126	case	control	0.456136422	0.334526359	1.36352909	0.172715823	0.591719278	up_in_case
PRJNA8274(	ENSGALG00010003131	TAP1	case	control	0.046253025	0.311675222	0.148401354	0.882026032	0.976801393	up_in_case
PRJNA8274(	ENSGALG00010003132	TPBG	case	control	0.237701491	0.151696294	1.566956478	0.117124865	0.513845851	up_in_case
PRJNA8274(	ENSGALG00010003133	SAT1	case	control	-0.058941872	0.129902284	-0.453740073	0.650015921	0.904241824	down_in_case
PRJNA8274(	ENSGALG00010003136	NUP98	case	control	-0.069494109	0.056702435	-1.225593032	0.220351861	0.644563413	down_in_case
PRJNA8274(	ENSGALG00010003152	ENSGALG00010003152	case	control	0.029176771	0.170807614	0.170816572	0.864367998	0.97295831	up_in_case
PRJNA8274(	ENSGALG00010003154	MTFR1	case	control	0.049268702	0.056992283	0.864480226	0.387324139	0.782604263	up_in_case
PRJNA8274(	ENSGALG00010003159	TAP2	case	control	-0.156136963	0.22864396	-0.682882516	0.494681083	0.83803731	down_in_case
PRJNA8274(	ENSGALG00010003160	KIDINS220	case	control	-0.026154898	0.061148943	-0.427724447	0.668851749	0.913032039	down_in_case
PRJNA8274(	ENSGALG00010003164	C3	case	control	0.363045619	0.46291175	0.784265293			up_in_case
PRJNA8274(	ENSGALG00010003165	BMP2K	case	control	-0.054734451	0.06007403	-0.911116695	0.362233891	0.768423771	down_in_case
PRJNA8274(	ENSGALG00010003166	PGAP2	case	control	-0.039955404	0.062087357	-0.643535265	0.51987684	0.849611068	down_in_case
PRJNA8274(	ENSGALG00010003169	MTRF1	case	control	-0.115722874	0.100387884	-1.152757376	0.249009984	0.674274336	down_in_case
PRJNA8274(	ENSGALG00010003170	C4	case	control	0.592150796	0.373070404	1.587236056	0.112459191	0.506349634	up_in_case
PRJNA8274(	ENSGALG00010003171	PAQR3	case	control	0.037840881	0.12096874	0.312815367	0.75442094	0.941311206	up_in_case
PRJNA8274(	ENSGALG00010003176	PRAG1	case	control	-0.463165597	0.108099648	-4.284617085	1.83054E-05	0.002814704	down_in_case
PRJNA8274(	ENSGALG00010003178	CENPA	case	control	-0.401733708	0.391599357	-1.025879386	0.30494844	0.728073294	down_in_case
PRJNA8274(	ENSGALG00010003180	IBTK	case	control	-0.022618054	0.041437678	-0.545833044	0.585180707	0.881307382	down_in_case
PRJNA8274(	ENSGALG00010003183	NAA16	case	control	-0.142869505	0.051594025	-2.769109486	0.005620974	0.130251961	down_in_case
PRJNA8274(	ENSGALG00010003184	BET1	case	control	0.00625752	0.057241519	0.109317863	0.912950379	0.982728035	up_in_case
PRJNA8274(	ENSGALG00010003186	CYP21A1	case	control	-0.455128721	0.222125667	-2.04896952	0.040465095	0.33753444	down_in_case
PRJNA8274(	ENSGALG00010003187	RHOG	case	control	-0.01722626	0.106053381	-0.162430085	0.870967181	0.974937823	down_in_case
PRJNA8274(	ENSGALG00010003189	ANTXR2	case	control	-0.127760352	0.073354685	-1.741679511	0.08156454	0.454549071	down_in_case
PRJNA8274(	ENSGALG00010003191	CC2D1A	case	control	-0.221436278	0.133599175	-1.657467406	0.097425013	0.485860997	down_in_case
PRJNA8274(	ENSGALG00010003192	RGCC	case	control	-0.328513074	0.129152532	-2.543605378	0.010971496	0.185137791	down_in_case
PRJNA8274(	ENSGALG00010003193	ACOT9	case	control	-0.440108419	0.07668709	-5.739015797	9.52283E-09	4.53922E-06	down_in_case
PRJNA8274(	ENSGALG00010003198	PRKG2	case	control	0.105247995	0.249682365	0.421527546	0.673369901	0.91379937	up_in_case
PRJNA8274(	ENSGALG00010003201	MBOAT2	case	control	-0.275779929	0.097334099	-2.833333156	0.004606535	0.11597438	down_in_case
PRJNA8274(	ENSGALG00010003202	FAM46A	case	control	0.055364917	0.097673163	0.566838586	0.570823853	0.876116893	up_in_case
PRJNA8274(	ENSGALG00010003204	PKD2	case	control	-0.072700571	0.097951467	-0.74221013	0.457960032	0.823337749	down_in_case
PRJNA8274(	ENSGALG00010003205	ENSGALG00010003205	case	control	-0.432797063	0.210137463	-2.059590216	0.039437732	0.335938843	down_in_case
PRJNA8274(	ENSGALG00010003206	ENSGALG00010003206	case	control	1.19025936	0.447267617	2.661179384	0.007786746	0.154438929	up_in_case
PRJNA8274(	ENSGALG00010003208	ENSGALG00010003208	case	control	2.050397303	0.646933379	3.169410282	0.001527486	0.062408717	up_in_case
PRJNA8274(	ENSGALG00010003210	EYA1	case	control	-0.197991334	0.094337311	-2.098759581	0.0358381	0.324974524	down_in_case
PRJNA8274(	ENSGALG00010003214	PRDX4	case	control	0.114733406	0.066640778	1.721669659	0.085129377	0.461467053	up_in_case
PRJNA8274(	ENSGALG00010003218	MAP3K7	case	control	-0.072915345	0.03781507	-1.928208645	0.053829179	0.377891678	down_in_case
PRJNA8274(	ENSGALG00010003220	SPP1	case	control	-0.660797559	0.914489397	-0.722586353	0.469934055	0.829101076	down_in_case
PRJNA8274(	ENSGALG00010003222	ITGA8	case	control	0.172751017	0.066196618	2.609665311	0.009063084	0.169858595	up_in_case
PRJNA8274(	ENSGALG00010003223	AREG	case	control	0.50959071	0.685784818	0.743076686	0.457435254	0.823244158	up_in_case
PRJNA8274(	ENSGALG00010003229	STIM1	case	control	-0.092721935	0.084795234	-1.09348051	0.274182882	0.699970438	down_in_case
PRJNA8274(	ENSGALG00010003239	RRM1	case	control	0.009834672	0.047148266	0.208590322	0.834768066	0.963411173	up_in_case
PRJNA8274(	ENSGALG00010003242	ASAP2	case	control	0.026173129	0.107056146	0.244480395	0.806858784	0.955875648	up_in_case
PRJNA8274(	ENSGALG00010003243	VPS50	case	control	-0.069552696	0.071971409	-0.966393418	0.333847356	0.748508541	down_in_case
PRJNA8274(	ENSGALG00010003245	ENSGALG00010003245	case	control	0.350299473	0.20076454	1.744827411	0.081014916	0.453075204	up_in_case
PRJNA8274(	ENSGALG00010003246	VWA8	case	control	0.078521093	0.104417905	0.751988779	0.452057807	0.820671149	up_in_case
PRJNA8274(	ENSGALG00010003247	ENSGALG00010003247	case	control	-0.077584213	0.189446537	-0.409530914	0.682150085	0.915594727	down_in_case
PRJNA8274(	ENSGALG00010003248	MRI1	case	control	0.110491262	0.17224407	0.641480792	0.521210357	0.850251894	up_in_case
PRJNA8274(	ENSGALG00010003249	YJU2B	case	control	0.013839933	0.159008553	0.087038919	0.930640577	0.987258715	up_in_case
PRJNA8274(	ENSGALG00010003251	ELOF1	case	control	0.174441268	0.155058069	1.125006064	0.260586465	0.687359696	up_in_case
PRJNA8274(	ENSGALG00010003252	PHEX	case	control	-0.156422567	0.178187852	-0.877852025	0.380024027	0.77805894	down_in_case
PRJNA8274(	ENSGALG00010003253	ITGB1BP1	case	control	0.022573266	0.098374693	0.229462123	0.818509757	0.958536226	up_in_case
PRJNA8274(	ENSGALG00010003254	DGKH	case	control	-0.181669712	0.129160889	-1.406538107	0.159564397	0.572017525	down_in_case
PRJNA8274(	ENSGALG00010003255	CALCR	case	control	0.033859723	0.241797182	0.140033574	0.888633464	0.977948171	up_in_case
PRJNA8274(	ENSGALG00010003259	VEGFC	case	control	0.083864575	0.095985474	0.873721523	0.382269927	0.77998936	up_in_case
PRJNA8274(	ENSGALG00010003260	ACP5	case	control	0.387586555	0.215922926	1.795022709	0.072650082	0.435961465	up_in_case
PRJNA8274(	ENSGALG00010003261	PEX2	case	control	0.008137868	0.053222894	0.152901646	0.878475838	0.976160112	up_in_case
PRJNA8274(	ENSGALG00010003262	ENSGALG00010003262	case	control	-1.089836707	0.535025888	-2.03697939	0.041652107	0.340995489	down_in_case
PRJNA8274(	ENSGALG00010003263	ENSGALG00010003263	case	control	-0.972383041	0.606020385	-1.604538502	0.108595405	0.500951275	down_in_case
PRJNA8274(	ENSGALG00010003264	ENSGALG00010003264	case	control	-1.419214337	0.616278622	-2.302877767	0.021285721	0.261564457	down_in_case
PRJNA8274(	ENSGALG00010003266	ENSGALG00010003266	case	control	0.833644838	0.498281615	1.673039528	0.094319515	0.479477093	up_in_case
PRJNA8274(	ENSGALG00010003268	SMS	case	control	-0.0407706	0.066617107	-0.612013975	0.540528512	0.859221623	down_in_case
PRJNA8274(	ENSGALG00010003269	ENSGALG00010003269	case	control	0.01965154	0.310025513	0.063386847	0.949458461	0.991402409	up_in_case
PRJNA8274(	ENSGALG00010003271	AKAP11	case	control	-0.03210355	0.085878531	-0.373825095	0.708534448	0.92524028	down_in_case
PRJNA8274(	ENSGALG00010003272	CPSF3	case	control	0.096562368	0.04312188	2.239289399	0.025137092	0.281708792	up_in_case
PRJNA8274(	ENSGALG00010003273	NFIX	case	control	0.023139184	0.101431526	0.228126155	0.819548168	0.958669267	up_in_case
PRJNA8274(	ENSGALG00010003274	BACH2	case	control	-0.077130457	0.077656697	-0.993223503	0.320601047	0.738875399	down_in_case
PRJNA8274(	ENSGALG00010003275	MBTPS2	case	control	0.136681682	0.103617577	1.319097451	0.187136535	0.610664656	up_in_case
PRJNA8274(	ENSGALG00010003276	TNFSF11	case	control	0.017711913	0.395758384	0.04475436	0.964303104	0.992068146	up_in_case
PRJNA8274(	ENSGALG00010003278	ENSGALG00010003278	case	control	0.198785497	0.550360939	0.361191143	0.717956563	0.927322135	up_in_case
PRJNA8274(	ENSGALG00010003280	ENSGALG00010003280	case	control	0.112514209	0.06415207	1.75386715	0.079453274	0.450131333	up_in_case
PRJNA8274(	ENSGALG00010003287	ENSGALG00010003287	case	control	4.982843639	2.805340344	1.77619933	0.075700069	0.442474101	up_in_case
PRJNA8274(	ENSGALG00010003292	GADD45GIP1	case	control	0.071269332	0.063138698	1.128774172	0.258993111	0.686345498	up_in_case
PRJNA8274(	ENSGALG00010003295	IAH1	case	control	0.082045282	0.089304826	0.918710503	0.358247015	0.764616762	up_in_case
PRJNA8274(	ENSGALG00010003296	ENSGALG00010003296	case	control	-0.253789745	0.353373869	-0.718190471	0.472639853	0.829235491	down_in_case
PRJNA8274(	ENSGALG00010003299	RAD23A	case	control	-0.117972083	0.071903599	-1.640697886	0.100860144	0.490745171	down_in_case
PRJNA8274(	ENSGALG00010003300	CASP8AP2	case	control	0.03243411	0.0745703	0.434946751	0.663601079	0.911049869	up_in_case
PRJNA8274(	ENSGALG00010003303	MTHFD2L	case	control	-0.173378278	0.134048457	-1.293400026	0.195872741	0.619095929	down_in_case
PRJNA8274(	ENSGALG00010003305	CALR	case	control	0.128530875	0.045934173	2.7981537	0.005139565	0.123938918	up_in_case
PRJNA8274(	ENSGALG00010003307	COL1A2	case	control	-0.182914033	0.125773485	-1.454313149	0.145859507	0.551067621	down_in_case
PRJNA8274(	ENSGALG00010003308	MDN1	case	control	-0.103314637	0.065989088	-1.56563214	0.117434761	0.514181593	down_in_case
PRJNA8274(	ENSGALG00010003315	EPGN	case	control	0.015505267	0.311760824	0.049734495	0.960333967	0.992056045	up_in_case
PRJNA8274(	ENSGALG00010003317	LYRM2	case	control	-0.090348343	0.137697879	-0.6561346	0.511737524	0.845834144	down_in_case
PRJNA8274(	ENSGALG00010003320	OR51E2	case	control	0.145601249	0.238697511	0.609982266	0.541873555	0.85975913	up_in_case
PRJNA8274(	ENSGALG00010003323	STMN2	case	control	0.047167566	0.668044257	0.07060545	0.943711772	0.990183239	up_in_case
PRJNA8274(	ENSGALG00010003324	ENSGALG00010003324	case	control	-0.481410925	1.416165864	-0.339939648	0.733901978	0.933846596	down_in_case

PRJNA8274(	ENSGALG00010003330	HEY1	case	control	-0.025928866	0.076576188	-0.33860221	0.734909418	0.934096263	down_in_case
PRJNA8274(	ENSGALG00010003331	EPSTI1	case	control	-0.729183222	0.33797032	-2.157536262	0.030963905	0.308259818	down_in_case
PRJNA8274(	ENSGALG00010003333	ENSGALG00010003333	case	control	0.095596071	0.503506355	0.189860704	0.849418286	0.968927451	up_in_case
PRJNA8274(	ENSGALG00010003334	TAC1	case	control	-1.601972999	0.834460329	-1.919771309	0.054886792	0.382868844	down_in_case
PRJNA8274(	ENSGALG00010003339	ENSGALG00010003339	case	control	-1.880169084	0.66960107	-2.807894386	0.004986658	0.120658556	down_in_case
PRJNA8274(	ENSGALG00010003344	ZFAND1	case	control	0.160426772	0.111685697	1.436412859	0.150884896	0.558399072	up_in_case
PRJNA8274(	ENSGALG00010003347	GPM6A	case	control	0.070751758	0.294960836	0.239868313	0.810432346	0.955972758	up_in_case
PRJNA8274(	ENSGALG00010003348	ADAM17	case	control	-0.002775074	0.049520075	-0.056039379	0.955310437	0.992056045	down_in_case
PRJNA8274(	ENSGALG00010003352	FARSA	case	control	0.027622341	0.058072472	0.475652918	0.634321668	0.898107199	up_in_case
PRJNA8274(	ENSGALG00010003356	ENSGALG00010003356	case	control	0.165873944	0.185187761	0.895706841	0.370409356	0.772700698	up_in_case
PRJNA8274(	ENSGALG00010003358	DNAJC15	case	control	-0.196625646	0.088930581	-2.211001468	0.027035735	0.292515655	down_in_case
PRJNA8274(	ENSGALG00010003363	HBE1	case	control	-0.612794198	0.427146539	-1.43462288	0.151394591	0.559493525	down_in_case
PRJNA8274(	ENSGALG00010003364	ANKRD6	case	control	-0.03146004	0.111820882	-0.281343157	0.778447212	0.948329573	down_in_case
PRJNA8274(	ENSGALG00010003366	CASD1	case	control	-0.017644709	0.076932115	-0.229354263	0.818593581	0.958536226	down_in_case
PRJNA8274(	ENSGALG00010003371	ENSGALG00010003371	case	control	0.520239686	0.300197186	1.732993212	0.083096875	0.457082972	up_in_case
PRJNA8274(	ENSGALG00010003372	ENOX1	case	control	-0.095687406	0.072489054	-1.320025583	0.186826476	0.610613817	down_in_case
PRJNA8274(	ENSGALG00010003374	SYCE2	case	control	0.004907341	0.305518263	0.016062351	0.98718465	0.996796053	up_in_case
PRJNA8274(	ENSGALG00010003375	YWHAQ	case	control	0.066198188	0.03769871	1.755980179	0.079091793	0.449171025	up_in_case
PRJNA8274(	ENSGALG00010003379	ENSGALG00010003379	case	control	0.962967945	0.44068517	2.18516077	0.028877061	0.302078982	up_in_case
PRJNA8274(	ENSGALG00010003382	ASNS	case	control	0.051693113	0.142015356	0.363996647	0.715860513	0.926862121	up_in_case
PRJNA8274(	ENSGALG00010003383	UBE2J1	case	control	0.014071483	0.067145313	0.20956761	0.834005162	0.963078474	up_in_case
PRJNA8274(	ENSGALG00010003388	PXDN	case	control	-0.037504782	0.107877115	-0.347662078	0.728093979	0.933057319	down_in_case
PRJNA8274(	ENSGALG00010003389	ENSGALG00010003389	case	control	-0.608047665	0.379322477	-1.602983482	0.108938301	0.501108679	down_in_case
PRJNA8274(	ENSGALG00010003397	SPATA4	case	control	-0.424467729	0.29712822	-1.428567532	0.153128572	0.561471429	down_in_case
PRJNA8274(	ENSGALG00010003400	PKIA	case	control	-0.104057773	0.082752976	-1.257450522	0.208590545	0.633434867	down_in_case
PRJNA8274(	ENSGALG00010003401	ENSGALG00010003401	case	control	-0.119359651	0.787173545	-0.151630668	0.879478243	0.976318821	down_in_case
PRJNA8274(	ENSGALG00010003403	GCDH	case	control	0.067278397	0.096403111	0.697886154	0.485248394	0.834423421	up_in_case
PRJNA8274(	ENSGALG00010003405	GABRR2	case	control	-0.00695368	0.184880483	-0.03761176	0.969997231	0.992761697	down_in_case
PRJNA8274(	ENSGALG00010003406	C1GALT1	case	control	0.085978856	0.048258699	1.781623968	0.074810573	0.439711709	up_in_case
PRJNA8274(	ENSGALG00010003407	CHMP4C	case	control	0.169796218	0.163060607	1.041307409	0.297732899	0.721370179	up_in_case
PRJNA8274(	ENSGALG00010003408	ENSGALG00010003408	case	control	0.112256196	0.183023609	0.613342709	0.539649759	0.858521589	up_in_case
PRJNA8274(	ENSGALG00010003412	ASB5	case	control	-0.127505244	0.078664253	-1.620879111	0.105043568	0.494770427	down_in_case
PRJNA8274(	ENSGALG00010003419	ENSGALG00010003419	case	control	-0.544724548	1.043876364	-0.521828606	0.601789671	0.884954589	down_in_case
PRJNA8274(	ENSGALG00010003420	ENSGALG00010003420	case	control	-1.022504065	0.795064442	-1.286064388	0.198420591	0.621920427	down_in_case
PRJNA8274(	ENSGALG00010003422	ENSGALG00010003422	case	control	1.413783216	1.920822139	0.736030259	0.461712283	0.82479521	up_in_case
PRJNA8274(	ENSGALG00010003428	SNX16	case	control	0.110511897	0.087370007	1.264872243	0.205917129	0.62881082	up_in_case
PRJNA8274(	ENSGALG00010003431	PM20D2	case	control	-0.486834433	0.183747327	-2.649477636	0.008061631	0.157703584	down_in_case
PRJNA8274(	ENSGALG00010003432	LACC1	case	control	-0.200165542	0.279311951	-0.716637944	0.473597526	0.82959752	down_in_case
PRJNA8274(	ENSGALG00010003437	PNRC1	case	control	-0.039935425	0.103539985	-0.385700513	0.699718483	0.922288441	down_in_case
PRJNA8274(	ENSGALG00010003438	KCNS3	case	control	-0.433349393	0.189777296	-2.283462791	0.022403122	0.266593144	down_in_case
PRJNA8274(	ENSGALG00010003439	GMIP	case	control	-0.023833456	0.219463327	-0.108598811	0.913520704	0.982728035	down_in_case
PRJNA8274(	ENSGALG00010003442	SPCS3	case	control	0.079229937	0.045270717	1.750136574	0.08009475	0.451460358	up_in_case
PRJNA8274(	ENSGALG00010003447	ZC2HC1A	case	control	0.006365291	0.082837765	0.076840444	0.938750476	0.989551456	up_in_case
PRJNA8274(	ENSGALG00010003449	BORCS6	case	control	-0.001584166	0.083985192	-0.018862447	0.984950837	0.996591771	down_in_case
PRJNA8274(	ENSGALG00010003453	ENSGALG00010003453	case	control	-0.625911335	0.71513936	-0.875229879	0.381448839	0.779356821	down_in_case
PRJNA8274(	ENSGALG00010003454	BPNT2	case	control	0.121729764	0.082808432	1.47001653	0.141557277	0.543501954	up_in_case
PRJNA8274(	ENSGALG00010003456	CDS1	case	control	0.280879008	0.423875522	0.662645029	0.507557924	0.843702533	up_in_case
PRJNA8274(	ENSGALG00010003466	TSC22D1	case	control	-0.072403004	0.065401564	-1.107053094	0.268270974	0.694977341	down_in_case
PRJNA8274(	ENSGALG00010003468	ILVBL	case	control	-0.128175654	0.104619277	-1.22516288	0.220513857	0.644724628	down_in_case
PRJNA8274(	ENSGALG00010003470	METTL3	case	control	-0.11037925	0.080390149	-1.373044475	0.169738521	0.585894132	down_in_case
PRJNA8274(	ENSGALG00010003471	ADI1	case	control	0.059402399	0.063485861	0.935679186	0.34943839	0.759342183	up_in_case
PRJNA8274(	ENSGALG00010003472	FAM110B	case	control	0.130475686	0.066501146	1.962006565	0.04976172	0.365106513	up_in_case
PRJNA8274(	ENSGALG00010003473	ENSGALG00010003473	case	control	-0.193366291	0.128822578	-1.501027959	0.13334833	0.536998345	down_in_case
PRJNA8274(	ENSGALG00010003474	RPS7	case	control	-0.066668643	0.057499052	-1.159473786	0.246263116	0.670921661	down_in_case
PRJNA8274(	ENSGALG00010003476	ENSGALG00010003476	case	control	0.560227097	0.35813975	1.564269527	0.117754285	0.514604253	up_in_case
PRJNA8274(	ENSGALG00010003478	COLEC11	case	control	0.091303621	0.148219516	0.616002693	0.537892742	0.857740557	up_in_case
PRJNA8274(	ENSGALG00010003479	SYDE1	case	control	0.003502744	0.072682479	0.048192408	0.9615629	0.992056045	up_in_case
PRJNA8274(	ENSGALG00010003485	RNGTT	case	control	-0.020351948	0.055505505	-0.366665401	0.713868618	0.926862121	down_in_case
PRJNA8274(	ENSGALG00010003489	SGCE	case	control	0.056809499	0.05194874	1.093568359	0.274144333	0.699970438	up_in_case
PRJNA8274(	ENSGALG00010003495	ENSGALG00010003495	case	control	-0.30052178	0.192907977	-1.557850455	0.119268702	0.516645008	down_in_case
PRJNA8274(	ENSGALG00010003503	NUFIP1	case	control	-0.065634771	0.0533468	-1.230341291	0.218569328	0.643443819	down_in_case
PRJNA8274(	ENSGALG00010003506	DYNC1I1	case	control	0.160986965	0.196032361	0.821226476	0.411517279	0.796952476	up_in_case
PRJNA8274(	ENSGALG00010003507	WDFY3	case	control	-0.04804775	0.050355954	-0.954162242	0.340001524	0.751456625	down_in_case
PRJNA8274(	ENSGALG00010003509	GPALPP1	case	control	-0.037228781	0.06932412	-0.537024936	0.59125039	0.882949889	down_in_case
PRJNA8274(	ENSGALG00010003511	UBXN2B	case	control	-0.163969437	0.08594504	-1.907840612	0.05641182	0.386530439	down_in_case
PRJNA8274(	ENSGALG00010003512	ENSGALG00010003512	case	control	-0.913715531	0.351588959	-2.598817479	0.00935455	0.172879234	down_in_case
PRJNA8274(	ENSGALG00010003517	GTF2F2	case	control	-0.302556783	0.098561169	-3.069736136	0.00214248	0.076212584	down_in_case
PRJNA8274(	ENSGALG00010003519	KCTD4	case	control	-0.385871263	0.278687844	-1.384600265	0.16617473	0.581422731	down_in_case
PRJNA8274(	ENSGALG00010003522	ENSGALG00010003522	case	control	-0.835800399	0.513273811	-1.628371408	0.103446156	0.492970761	down_in_case
PRJNA8274(	ENSGALG00010003525	NOL10	case	control	-0.092280905	0.062162401	-1.484513199	0.137672824	0.539854583	down_in_case
PRJNA8274(	ENSGALG00010003528	GDAP1	case	control	-0.281392352	0.17466473	-1.611042773	0.1071704	0.498385925	down_in_case
PRJNA8274(	ENSGALG00010003531	PPM1K	case	control	-0.090349161	0.108239884	-0.834712278	0.403879727	0.79260318	down_in_case
PRJNA8274(	ENSGALG00010003539	ENSGALG00010003539	case	control	0.064799613	0.15704897	0.41260769	0.679894066	0.915381147	up_in_case
PRJNA8274(	ENSGALG00010003541	CNR1	case	control	0.091104463	0.103916598	0.87670752	0.380645521	0.778246195	up_in_case
PRJNA8274(	ENSGALG00010003543	SOX11	case	control	0.015475865	0.089469871	0.172972918	0.862672715	0.972787494	up_in_case
PRJNA8274(	ENSGALG00010003552	PI15	case	control	-0.172909703	0.136015555	-1.271249476	0.20363991	0.62581674	down_in_case
PRJNA8274(	ENSGALG00010003558	TPT1	case	control	0.038940888	0.074816199	0.520487389	0.602723915	0.885101354	up_in_case
PRJNA8274(	ENSGALG00010003560	ADGRL3	case	control	-0.163272786	0.085779375	-1.903403773	0.056987879	0.388573099	down_in_case
PRJNA8274(	ENSGALG00010003571	RPL7	case	control	-0.050440529	0.068554835	-0.735769089	0.461871236	0.82487307	down_in_case
PRJNA8274(	ENSGALG00010003582	RDH10	case	control	0.083209323	0.100518594	0.827800311	0.407783598	0.794388636	up_in_case
PRJNA8274(	ENSGALG00010003583	RSAD2	case	control	0.154929308	0.383436834	0.404054318	0.686172776	0.917034645	up_in_case
PRJNA8274(	ENSGALG00010003585	PNPLA6	case	control	0.045325816	0.076658693	0.59126779	0.554341008	0.866646786	up_in_case
PRJNA8274(	ENSGALG00010003589	SLC25A30	case	control	0.291980192	0.128487499	2.272440461	0.02305992	0.267659788	up_in_case
PRJNA8274(	ENSGALG00010003595	SNX13	case	control	-0.020136948	0.053309799	-0.37773445	0.705627873	0.924320939	down_in_case
PRJNA8274(	ENSGALG00010003596	CRISPLD1	case	control	-0.006826266	0.077315465	-0.088291076	0.929645331	0.987129367	down_in_case
PRJNA8274(	ENSGALG00010003597	RNF144A	case	control	-0.092470979	0.109823813	-0.841993885	0.399791377	0.789900496	down_in_case
PRJNA8274(	ENSGALG00010003604	ODC	case	control	-0.311954562	0.102975393	-3.029408794	0.002450329	0.081856092	down_in_case
PRJNA8274(	ENSGALG00010003617	COG3	case	control	0.086642357	0.06087454	1.423293813	0.154651	0.563441859	up_in_case
PRJNA8274(	ENSGALG00010003619	HNF4G	case	control	0.418537666	0.34650827	1.207872083	0.227096469	0.650797497	up_in_case
PRJNA8274(	ENSGALG00010003627	MCOLN1	case	control	-0.015559072	0.060643815	-0.256564854	0.797514709	0.95280257	down_in_case
PRJNA8274(	ENSGALG00010003632	TECRL	case	control	-0.808578186	0.412871726	-1.958424698	0.050180199	0.366770069	down_in_case
PRJNA8274(	ENSGALG00010003634	CYP4F11	case	control	0.568979198	0.267417299	2.127682839	0.033363389	0.314915154	up_in_case
PRJNA8274(	ENSGALG00010003639	ENSGALG00010003639	case	control	-1.1625331	0.714650088	-1.626716514	0.10379732	0.493779665	down_in_case
PRJNA8274(	ENSGALG00010003650	SPACA1	case	control	0.15592978	0.400686299	0.389156754	0.697160192	0.922031058	up_in_case
PRJNA8274(	ENSGALG00010003652	MYCN	case	control	0.075141095	0.079027348	0.950823959	0.341693748	0.753465011	up_in_case
PRJNA8274(	ENSGALG00010003660	IGFBP7	case	control	-0.052163681	0.091720007	-0.568727394	0.569541154	0.875087408	down_in_case
PRJNA8274(	ENSGALG00010003665	RDH14	case	control	-0.165161298	0.067557981	-2.444734051	0.014495899	0.212076065	down_in_case
PRJNA8274(	ENSGALG00010003666	SLC25A13	case	control	0.107195883	0.084117033	1.27436595	0.202533754	0.62541643	up_in_case
PRJNA8274(	ENSGALG00010003671	ENSGALG00010003671	case	control	0.321707989	0.490807262	0.655467051	0.512167093	0.845834144	up_in_case
PRJNA8274(	ENSGALG00010003674	AKIRIN2	case	control	-0.039195809	0.046790574	-0.837685999	0.402207079	0.792021921	down_in_case
PRJNA8274(	ENSGALG00010003676	POLR2B	case	control	-0.049336899	0.046391477	-1.063490589	0.287559531	0.71132024	down_in_case
PRJNA8274(	ENSGALG00010003678	ZNFY1	case	control	0.247179839	0.297135697	0.831875276	0.405479349	0.793782506	up_in_case
PRJNA8274(	ENSGALG00010003680	TRAPPC5	case	control	-0.046666023	0.067513397	-0.69121131	0.489432757	0.836347578	down_in_case
PRJNA8274(	ENSGALG00010003681	NT5C1B	case	control	-0.128063326	0.099069559	-1.292660704	0.196128434	0.619095929	down_in_case
PRJNA8274(	ENSGALG00010003690	ENSGALG00010003690	case	control	0.289767273	0.393361426	0.736643844	0.461338965	0.82454265	up_in_case
PRJNA8274(	ENSGALG00010003691	ERICH1	case	control	0.143415936	0.153519137	0.934189309	0.350206249	0.759878704	up_in_case
PRJNA8274(	ENSGALG00010003692	ZNFY2	case	control	-0.114017652	0.236836185	-0.481419895	0.630218099	0.89637578	down_in_case
PRJNA8274(	ENSGALG00010003695	NOA1	case	control	-0.110454584	0.048427108	-2.280842052	0.022557795	0.2670335	down_in_case
PRJNA8274(	ENSGALG00010003698	MACC1	case	control	0.262752502	0.827409187	0.317560533	0.750818316	0.939972946	up_in_case
PRJNA8274(	ENSGALG00010003700	REST	case	control	0.029142033	0.045377997	0.642206249	0.520739276	0.850168083	up_in_case
PRJNA8274(	ENSGALG00010003701	SARAF	case	control	-0.06456173	0.076466251	-0.844316675	0.398492471	0.789453932	down_in_case
PRJNA8274(	ENSGALG00010003703	TDRP	case	control	-0.084209858	0.177400262	-0.474688465	0.635009041	0.898182917	down_in_case
PRJNA8274(	ENSGALG00010003713	SPERT	case	control	0.559320032	0.35432506	1.578550589	0.114439176	0.509446069	up_in_case
PRJNA8274(	ENSGALG00010003714	ORC3	case	control	0.121125993	0.077871742	1.555455031	0.119837736	0.517634321	up_in_case
PRJNA8274(	ENSGALG00010003716	YLEC8	case	control	0.082978683	0.565627557	0.146701981	0.883367255	0.977115076	up_in_case
PRJNA8274(	ENSGALG00010003717	LEPROTL1	case	control	-0.0190161	0.069293748	-0.274427363	0.783756217	0.949518053	down_in_case
PRJNA8274(	ENSGALG00010003719	RPGR	case	control	-0.104583206	0.074491553	-1.403960614	0.160330578	0.572752253	down_in_case
PRJNA8274(	ENSGALG00010003722	MHCY11	case	control	0.760085774	0.467683507	1.625213982	0.104116972	0.493794905	up_in_case
PRJNA8274(	ENSGALG00010003724	ENSGALG00010003724	case	control	0.308504166	0.180205541	1.711957163	0.086904563	0.465967473	up_in_case
PRJNA8274(	ENSGALG00010003726	ENPEP	case	control	0.158651182	0.353286744	0.449071992	0.653379724	0.906151774	up_in_case
PRJNA8274(	ENSGALG00010003727	FBXO25	case	control	0.076741804	0.071247552	1.077114968	0.281428907	0.705762606	up_in_case
PRJNA8274(	ENSGALG00010003729	RARS2	case	control	0.014807566	0.097901516	0.151249612	0.879778816	0.976318821	up_in_case
PRJNA8274(	ENSGALG00010003731	ENSGALG00010003731	case	control	-0.068734883	0.542643913	-0.126666643	0.899204248	0.98028579	down_in_case
PRJNA8274(	ENSGALG00010003733	STAU2	case	control	-0.042483761	0.042885684	-0.990628037	0.321867242	0.739571618	down_in_case
PRJNA8274(	ENSGALG00010003734	ZC3H13	case	control	-0.062737462	0.045255722	-1.386287959	0.165658992	0.580618526	down_in_case
PRJNA8274(	ENSGALG00010003737	SRP72	case	control	-0.028983318	0.045635206	-0.635108742	0.525357496	0.85251736	down_in_case

PRJNA8274(	ENSGALG00010003740	PITX2	case	control	-0.184261282	0.10956986	-1.681678535	0.092631192	0.477342721	down_in_case
PRJNA8274(	ENSGALG00010003742	ZFHX4	case	control	-0.119015301	0.098281087	-1.210968507	0.225907475	0.649498359	down_in_case
PRJNA8274(	ENSGALG00010003743	ARL9	case	control	0.022270502	0.134604037	0.165451963	0.868588256	0.974483475	up_in_case
PRJNA8274(	ENSGALG00010003746	HOPX	case	control	-0.551178525	0.227194031	-2.426025552	0.015265195	0.219195419	down_in_case
PRJNA8274(	ENSGALG00010003749	MHCY15	case	control	0.34134003	1.076901914	0.316964828			up_in_case
PRJNA8274(	ENSGALG00010003750	CPB2	case	control	1.214034028	1.798884535	0.674881575	0.499751008	0.840559799	up_in_case
PRJNA8274(	ENSGALG00010003753	SLCO5A1	case	control	0.131343601	0.154308401	0.851175955	0.394671618	0.786978937	up_in_case
PRJNA8274(	ENSGALG00010003754	FAM110C	case	control	0.454387011	0.212253809	2.140771996	0.032292429	0.309838273	up_in_case
PRJNA8274(	ENSGALG00010003755	SLC35A1	case	control	0.143567247	0.081111471	1.769999313	0.076727255	0.444570401	up_in_case
PRJNA8274(	ENSGALG00010003756	MHCY7	case	control	-0.631888636	0.498536402	-1.267487458	0.204981046	0.627537776	down_in_case
PRJNA8274(	ENSGALG00010003761	UGT8	case	control	-0.008474704	0.2066373	-0.04101246	0.967285963	0.992731535	down_in_case
PRJNA8274(	ENSGALG00010003762	EVI5L	case	control	-0.102637987	0.07366062	-1.393390203	0.163501827	0.577587975	down_in_case
PRJNA8274(	ENSGALG00010003764	HDAC9	case	control	-0.2494249	0.093418837	-2.669963647	0.007585946	0.152061299	down_in_case
PRJNA8274(	ENSGALG00010003765	ENSGALG00010003765	case	control	0.719773819	0.463866028	1.551684701	0.120737685	0.519499513	up_in_case
PRJNA8274(	ENSGALG00010003766	CFAP206	case	control	0.371748341	0.519654841	0.715375499	0.474377048	0.82979716	up_in_case
PRJNA8274(	ENSGALG00010003767	ENSGALG00010003767	case	control	-1.98357408	1.593604551	-1.244709095	0.213238701	0.638582746	down_in_case
PRJNA8274(	ENSGALG00010003768	ARSJ	case	control	-0.176772647	0.064187714	-2.753995069	0.005887266	0.133681071	down_in_case
PRJNA8274(	ENSGALG00010003769	C6orf163	case	control	0.236346176	0.515883657	0.458138521	0.646852926	0.90295498	up_in_case
PRJNA8274(	ENSGALG00010003771	SPARCL1	case	control	-0.377428135	0.27496657	-1.372632807	0.169866528	0.586029275	down_in_case
PRJNA8274(	ENSGALG00010003772	ENSGALG00010003772	case	control	-0.428439046	0.454183527	-0.94331701	0.345518778	0.755935621	down_in_case
PRJNA8274(	ENSGALG00010003774	ENSGALG00010003774	case	control	0.241567933	0.613593115	0.39369401	0.693806965	0.920103713	up_in_case
PRJNA8274(	ENSGALG00010003775	NCOA2	case	control	-0.10358761	0.068730197	-1.507163012	0.131768879	0.535159035	down_in_case
PRJNA8274(	ENSGALG00010003777	ENSGALG00010003777	case	control	-0.493900642	0.717693824	-0.688177362	0.491341098	0.83694791	down_in_case
PRJNA8274(	ENSGALG00010003778	LCA5	case	control	-0.10893842	0.124578491	-0.874456091	0.381869922	0.77998936	down_in_case
PRJNA8274(	ENSGALG00010003786	ENSGALG00010003786	case	control	-0.514335824	0.637855192	-0.806352023	0.420039902	0.801303442	down_in_case
PRJNA8274(	ENSGALG00010003787	SMIM8	case	control	-0.272266697	0.182264022	-1.493803846	0.135226896	0.539774365	down_in_case
PRJNA8274(	ENSGALG00010003788	NUDT9	case	control	-0.08328186	0.131330143	-0.634141243	0.525988649	0.85251736	down_in_case
PRJNA8274(	ENSGALG00010003790	ELOVL6	case	control	0.11711625	0.107026103	1.094277443	0.273833315	0.699576755	up_in_case
PRJNA8274(	ENSGALG00010003791	ITGB8	case	control	-0.026829233	0.137951042	-0.194483725	0.845797138	0.967563913	down_in_case
PRJNA8274(	ENSGALG00010003793	TRAM1L1	case	control	-0.003418496	0.044800602	-0.076304692	0.939176693	0.989551456	down_in_case
PRJNA8274(	ENSGALG00010003795	LCP1	case	control	0.158693904	0.108812763	1.458412594	0.144726851	0.548818979	up_in_case
PRJNA8274(	ENSGALG00010003796	ENSGALG00010003796	case	control	0.309650571	0.366895215	0.843975494	0.3986831	0.789635503	up_in_case
PRJNA8274(	ENSGALG00010003797	ENSGALG00010003797	case	control	-0.033972162	0.176449166	-0.192532287	0.847325279	0.968178305	down_in_case
PRJNA8274(	ENSGALG00010003803	LACTB2	case	control	-0.162109909	0.079924647	-2.028284323	0.042531238	0.344353525	down_in_case
PRJNA8274(	ENSGALG00010003804	PSME1	case	control	-0.302493045	0.146636108	-2.062882398	0.039123803	0.335091843	down_in_case
PRJNA8274(	ENSGALG00010003806	EGF	case	control	-0.315899276	0.189900046	-1.663502891	0.096211813	0.483634708	down_in_case
PRJNA8274(	ENSGALG00010003808	TWIST1	case	control	-0.008932008	0.186582868	-0.047871536	0.961818624	0.992056045	down_in_case
PRJNA8274(	ENSGALG00010003810	HMGN2P46	case	control	-0.144394038	0.058371555	-2.473705544	0.013371986	0.203413453	down_in_case
PRJNA8274(	ENSGALG00010003811	PGLYRP2	case	control	1.157093286	1.406792731	0.822504453	0.410789853	0.796622579	up_in_case
PRJNA8274(	ENSGALG00010003812	BG8	case	control	-0.12511482	0.367342256	-0.340594685	0.733408731	0.933846596	down_in_case
PRJNA8274(	ENSGALG00010003813	LRIT3	case	control	-0.602236063	0.484596191	-1.242758559	0.213956818	0.638877113	down_in_case
PRJNA8274(	ENSGALG00010003814	TPP1	case	control	0.166335252	0.061625514	2.699129635	0.006952109	0.145020586	up_in_case
PRJNA8274(	ENSGALG00010003815	RASAL3	case	control	0.228012613	0.533059387	0.42774336	0.668837978	0.913032039	up_in_case
PRJNA8274(	ENSGALG00010003816	KLHL8	case	control	-0.138851246	0.094339009	-1.471832784	0.141066038	0.543438671	down_in_case
PRJNA8274(	ENSGALG00010003817	BF2	case	control	-0.272245639	0.243740032	-1.116950863	0.264015336	0.689826292	down_in_case
PRJNA8274(	ENSGALG00010003818	GAB2	case	control	-0.07674644	0.151070987	-0.508015748	0.6114423	0.88947908	down_in_case
PRJNA8274(	ENSGALG00010003819	PHIP	case	control	-0.005199043	0.065858872	-0.078942179	0.937078614	0.988800485	down_in_case
PRJNA8274(	ENSGALG00010003820	SH3YL1	case	control	-0.423230073	0.248705796	-1.701729836	0.088806025	0.470938334	down_in_case
PRJNA8274(	ENSGALG00010003821	RUBCNL	case	control	0.126696514	0.096622964	1.311246412	0.189774527	0.613281846	up_in_case
PRJNA8274(	ENSGALG00010003822	ENSGALG00010003822	case	control	0.110000633	0.086777129	1.267622408	0.204932826	0.627537776	up_in_case
PRJNA8274(	ENSGALG00010003823	PTPN13	case	control	-0.044829951	0.064100712	-0.699367451	0.484322424	0.834033076	down_in_case
PRJNA8274(	ENSGALG00010003824	SP4	case	control	-0.076261688	0.073546309	-1.036920678	0.299772826	0.723136203	down_in_case
PRJNA8274(	ENSGALG00010003826	ENSGALG00010003826	case	control	-0.074054027	0.064647167	-1.145510777	0.251997634	0.677148133	down_in_case
PRJNA8274(	ENSGALG00010003828	ENSGALG00010003828	case	control	0.443449598	0.358398541	1.237308603	0.215972535	0.640556775	up_in_case
PRJNA8274(	ENSGALG00010003829	ENSGALG00010003829	case	control	-0.00362377	0.088980569	-0.040725409	0.967514805	0.992731535	down_in_case
PRJNA8274(	ENSGALG00010003830	DMB2	case	control	-1.012102431	0.367939731	-2.750728843	0.005946284	0.134331538	down_in_case
PRJNA8274(	ENSGALG00010003831	IRAK1BP1	case	control	0.024222698	0.119999495	0.201856668	0.840028778	0.965286179	up_in_case
PRJNA8274(	ENSGALG00010003833	ACP1	case	control	-0.0139102	0.05754114	-0.241743557	0.808978877	0.955875648	down_in_case
PRJNA8274(	ENSGALG00010003834	MAPK10	case	control	-0.256078742	0.312396964	-0.819722249	0.412374463	0.797581687	down_in_case
PRJNA8274(	ENSGALG00010003835	ENSGALG00010003835	case	control	0.129016624	0.080847141	1.595809357	0.110531364	0.503054901	up_in_case
PRJNA8274(	ENSGALG00010003836	CD1F	case	control	0.228824056	0.227261026	1.006877689	0.313993555	0.73560089	up_in_case
PRJNA8274(	ENSGALG00010003837	ALKAL2	case	control	-0.232518991	0.30136886	-0.771542857	0.44038523	0.813001392	down_in_case
PRJNA8274(	ENSGALG00010003838	GAR1	case	control	-0.015913433	0.093128599	-0.170875902	0.864321345	0.97295831	down_in_case
PRJNA8274(	ENSGALG00010003839	ENSGALG00010003839	case	control	0.932400788	0.555025725	1.679923554	0.09297219	0.478066279	up_in_case
PRJNA8274(	ENSGALG00010003841	UBE2W	case	control	0.026637018	0.132588348	0.200900144	0.840776655	0.965711339	up_in_case
PRJNA8274(	ENSGALG00010003842	ENSGALG00010003842	case	control	-0.106398506	0.064206111	-1.657139853	0.097491203	0.485860997	down_in_case
PRJNA8274(	ENSGALG00010003843	LRCH1	case	control	0.01420999	0.078803347	0.180322164	0.856899656	0.971145061	up_in_case
PRJNA8274(	ENSGALG00010003844	TMEM18	case	control	-0.052431963	0.08610065	-0.60896129	0.542550097	0.85975913	down_in_case
PRJNA8274(	ENSGALG00010003845	CFI	case	control	1.166324311	0.634100836	1.839335709	0.065865824	0.416182558	up_in_case
PRJNA8274(	ENSGALG00010003848	ESD	case	control	0.342100725	0.12450889	2.747600801	0.006003305	0.134447684	up_in_case
PRJNA8274(	ENSGALG00010003850	PLA2G12A	case	control	-0.006210079	0.059884343	-0.103701219	0.917406459	0.983445506	down_in_case
PRJNA8274(	ENSGALG00010003851	PHF14	case	control	0.028819229	0.045940673	0.627314039	0.530453403	0.855473515	up_in_case
PRJNA8274(	ENSGALG00010003852	TCEB1	case	control	-0.013291675	0.058212407	-0.228330625	0.819389218	0.958669267	down_in_case
PRJNA8274(	ENSGALG00010003853	ENSGALG00010003853	case	control	0.030491379	0.091840171	0.332004817	0.739885622	0.9363983	up_in_case
PRJNA8274(	ENSGALG00010003858	SUCLA2	case	control	-0.05289381	0.084706051	-0.624439575	0.532338942	0.855755691	down_in_case
PRJNA8274(	ENSGALG00010003860	TMEM70	case	control	-0.142944272	0.045828299	-3.119126696	0.001813879	0.068802325	down_in_case
PRJNA8274(	ENSGALG00010003861	NUDT15	case	control	-0.057892617	0.080513124	-0.719045716	0.472112753	0.829235491	down_in_case
PRJNA8274(	ENSGALG00010003862	PCK2	case	control	0.459154551	0.271814692	1.689219036	0.091177462	0.476374757	up_in_case
PRJNA8274(	ENSGALG00010003863	HTR1B	case	control	-0.187593516	0.151444016	-1.238698769	0.215457075	0.640470741	down_in_case
PRJNA8274(	ENSGALG00010003865	AKAP8L	case	control	-0.022209459	0.069204182	-0.32092654	0.748266061	0.939503405	down_in_case
PRJNA8274(	ENSGALG00010003868	HBBA	case	control	0.379689193	0.179902586	2.110526597	0.034813023	0.322217621	up_in_case
PRJNA8274(	ENSGALG00010003869	LY96	case	control	-0.320240497	0.21763423	-1.471461989	0.14116622	0.543501954	down_in_case
PRJNA8274(	ENSGALG00010003870	MED4	case	control	0.085573902	0.088377999	0.96827155	0.33290877	0.748503677	up_in_case
PRJNA8274(	ENSGALG00010003871	ENSGALG00010003871	case	control	0.052946459	0.107195407	0.493924698	0.621359361	0.893175881	up_in_case
PRJNA8274(	ENSGALG00010003874	YES1	case	control	0.059816403	0.059056951	1.012859665	0.311127197	0.733935816	up_in_case
PRJNA8274(	ENSGALG00010003875	AKAP8	case	control	0.08103983	0.048430303	1.673329	0.094262547	0.479477093	up_in_case
PRJNA8274(	ENSGALG00010003878	ARHGAP24	case	control	0.079052469	0.084508557	0.935437442	0.349562908	0.759342183	up_in_case
PRJNA8274(	ENSGALG00010003879	CASP6	case	control	0.059171741	0.116311891	0.508733377	0.610939124	0.889056885	up_in_case
PRJNA8274(	ENSGALG00010003882	ENSGALG00010003882	case	control	-0.077344289	0.061886391	-1.249778628	0.211380425	0.636501957	down_in_case
PRJNA8274(	ENSGALG00010003884	TYMS	case	control	-0.051458842	0.071656975	-0.718127478	0.472678689	0.829235491	down_in_case
PRJNA8274(	ENSGALG00010003887	GIPC1	case	control	0.04351591	0.092344122	0.471236377	0.637471944	0.899378897	up_in_case
PRJNA8274(	ENSGALG00010003889	ITM2B	case	control	-0.068877048	0.052787474	-1.304799094	0.191961268	0.615090794	down_in_case
PRJNA8274(	ENSGALG00010003894	COPS4	case	control	-0.100608067	0.065363851	-1.539200415	0.123755376	0.522883756	down_in_case
PRJNA8274(	ENSGALG00010003895	THSD7A	case	control	-0.20682234	0.232876218	-0.888121346	0.374475481	0.774434633	down_in_case
PRJNA8274(	ENSGALG00010003896	MCUB	case	control	0.228287262	0.090979927	2.509204725	0.012100333	0.193549413	up_in_case
PRJNA8274(	ENSGALG00010003898	NDST4	case	control	0.093466892	0.450236211	0.207595235	0.835545025	0.963998106	up_in_case
PRJNA8274(	ENSGALG00010003899	LIN54	case	control	-0.119733405	0.073673582	-1.625187775	0.104122554	0.493794905	down_in_case
PRJNA8274(	ENSGALG00010003900	RB1	case	control	0.007694038	0.06079823	0.12655036	0.899296288	0.98028579	up_in_case
PRJNA8274(	ENSGALG00010003902	ENSGALG00010003902	case	control	-0.056571061	0.085630621	-0.660640554	0.508842855	0.844259682	down_in_case
PRJNA8274(	ENSGALG00010003903	COL28A1	case	control	0.036881519	0.199936456	0.184466203	0.853647739	0.969998446	up_in_case
PRJNA8274(	ENSGALG00010003909	DNAJB1	case	control	0.038195307	0.073200274	0.521791862	0.601815256	0.884954589	up_in_case
PRJNA8274(	ENSGALG00010003910	THAP9	case	control	-0.114415423	0.080618967	-1.419212215	0.155837156	0.565028227	down_in_case
PRJNA8274(	ENSGALG00010003913	PSMB5	case	control	0.050201513	0.079391833	0.632325912	0.527173935	0.852877845	up_in_case
PRJNA8274(	ENSGALG00010003914	CAMK2D	case	control	-0.232157335	0.061146175	-3.796759739	0.0001466	0.01379195	down_in_case
PRJNA8274(	ENSGALG00010003915	MIOS	case	control	-0.042311598	0.059234571	-0.714305818	0.475038096	0.830111175	down_in_case
PRJNA8274(	ENSGALG00010003918	HTR1E	case	control	-0.285005502	0.511367358	-0.557340036	0.577295127	0.878143389	down_in_case
PRJNA8274(	ENSGALG00010003920	LPAR6	case	control	0.039282078	0.08660206	0.453592881	0.650121879	0.904241824	up_in_case
PRJNA8274(	ENSGALG00010003921	COLEC12	case	control	-0.040880241	0.104017948	-0.393011413	0.694311052	0.920308089	down_in_case
PRJNA8274(	ENSGALG00010003923	ENSGALG00010003923	case	control	-0.030830929	0.057466633	-0.536501407	0.591612064	0.882949889	down_in_case
PRJNA8274(	ENSGALG00010003925	SEC31A	case	control	0.020647945	0.050063226	0.412437359	0.680018885	0.915381147	up_in_case
PRJNA8274(	ENSGALG00010003928	ENSGALG00010003928	case	control	-0.035825951	0.09326947	-0.384112308	0.700895209	0.922516526	down_in_case
PRJNA8274(	ENSGALG00010003929	RCBTB2	case	control	0.108087841	0.091607044	1.17990753	0.238036995	0.662913814	up_in_case
PRJNA8274(	ENSGALG00010003933	TECR	case	control	-0.353454972	0.102254189	-3.456630744	0.000546974	0.034007501	down_in_case
PRJNA8274(	ENSGALG00010003937	ENSGALG00010003937	case	control	-0.079004414	0.152324504	-0.518658598	0.603998838	0.885268908	down_in_case
PRJNA8274(	ENSGALG00010003948	SCD5	case	control	0.21834673	0.10190215	2.142709743	0.032136413	0.3098361	up_in_case
PRJNA8274(	ENSGALG00010003958	CYSLTR2	case	control	0.153172927	0.282652058	0.541913363	0.587878191	0.882771329	up_in_case
PRJNA8274(	ENSGALG00010003960	ASPH	case	control	-0.344167792	0.107047189	-3.215103491	0.001303975	0.057198904	down_in_case
PRJNA8274(	ENSGALG00010003962	UMAD1	case	control	-0.237700896	0.096249822	-2.469624275	0.013525503	0.204023937	down_in_case
PRJNA8274(	ENSGALG00010003965	SNTG1	case	control	0.225837912	0.302018447	0.747761979	0.454603739	0.821657174	up_in_case
PRJNA8274(	ENSGALG00010003968	DSN1	case	control	0.039961783	0.074400227	0.537119101	0.591185348	0.882949889	up_in_case
PRJNA8274(	ENSGALG00010003972	PENK	case	control	0.16256754	0.13712508	1.18554199	0.235803223	0.660526168	up_in_case
PRJNA8274(	ENSGALG00010003977	MYO6	case	control	0.09646788	0.101933743	0.946378274	0.343955683	0.755852285	up_in_case
PRJNA8274(	ENSGALG00010003978	ANK2	case	control	-0.122693624	0.073498532	-1.669334339	0.095051138	0.48148469	down_in_case
PRJNA8274(	ENSGALG00010003980	CHCHD7	case	control	-0.064244775	0.131394448	-0.488945888	0.624880007	0.894741574	down_in_case
PRJNA8274(	ENSGALG00010003981	TMEM150C	case	control	0.062734086	0.130537191	0.480584002	0.630812188	0.896844877	up_in_case
PRJNA8274(	ENSGALG00010003982	GLCCI1	case	control	0.065540105	0.074095109	0.884540234	0.376404653	0.775199984	up_in_case

PRJNA8274(	ENSGALG00010003985	PRMT5	case	control	-0.017806734	0.070173391	-0.253753361	0.799686107	0.954053991	down_in_case
PRJNA8274(	ENSGALG00010003986	FNDC3A	case	control	-0.077985498	0.056520303	-1.379778488	0.167654858	0.583750784	down_in_case
PRJNA8274(	ENSGALG00010003988	ENOPH1	case	control	0.030769594	0.062177275	0.494868806	0.620692731	0.893038138	up_in_case
PRJNA8274(	ENSGALG00010003990	ICA1	case	control	0.006299216	0.124512906	0.050590866	0.959651541	0.992056045	up_in_case
PRJNA8274(	ENSGALG00010003993	ENSGALG00010003993	case	control	0.002815106	0.0597047	0.0471505	0.962393279	0.992068146	up_in_case
PRJNA8274(	ENSGALG00010003994	SDR16C5	case	control	0.089250057	0.114134436	0.781973085	0.434230391	0.809152357	up_in_case
PRJNA8274(	ENSGALG00010003995	MLNR	case	control	-0.311220102	0.193106126	-1.611653183	0.107037431	0.497973773	down_in_case
PRJNA8274(	ENSGALG00010004000	ENSGALG00010004000	case	control	0.138424848	0.089611536	1.544721286	0.122413694	0.520832363	up_in_case
PRJNA8274(	ENSGALG00010004001	LARP7	case	control	-0.093011228	0.062812758	-1.480769686	0.138667957	0.540932258	down_in_case
PRJNA8274(	ENSGALG00010004002	CDADC1	case	control	-0.012100138	0.111425762	-0.108593723	0.913524739	0.982728035	down_in_case
PRJNA8274(	ENSGALG00010004003	HNRNPDL	case	control	0.078938745	0.045733484	1.726060167	0.084336589	0.459607696	up_in_case
PRJNA8274(	ENSGALG00010004005	ENSGALG00010004005	case	control	0.272690492	0.817614172	0.333519771			up_in_case
PRJNA8274(	ENSGALG00010004006	NKAIN3	case	control	-0.48911691	0.24781709	-1.973701292	0.048415718	0.362674055	down_in_case
PRJNA8274(	ENSGALG00010004007	ENSGALG00010004007	case	control	-0.075094337	0.118017497	-0.636298338	0.524581988	0.852356213	down_in_case
PRJNA8274(	ENSGALG00010004016	ENSGALG00010004016	case	control	-0.192764668	0.436844788	-0.441265807	0.659020577	0.908599575	down_in_case
PRJNA8274(	ENSGALG00010004018	HNRNPD	case	control	-0.095495436	0.05396213	-1.769675076	0.076781284	0.444703266	down_in_case
PRJNA8274(	ENSGALG00010004029	ARL4A	case	control	-0.066455324	0.049078531	-1.354060973	0.175716926	0.594727928	down_in_case
PRJNA8274(	ENSGALG00010004034	NEDD8	case	control	-0.203249191	0.144493515	-1.406631928	0.15953656	0.572017525	down_in_case
PRJNA8274(	ENSGALG00010004036	ADCYAP1	case	control	-0.551992398	0.451931799	-1.221406414	0.22193218	0.645834386	down_in_case
PRJNA8274(	ENSGALG00010004038	SENP6	case	control	-0.050494317	0.05064403	-0.99704381	0.318743245	0.738427335	down_in_case
PRJNA8274(	ENSGALG00010004039	ALPK1	case	control	0.127862958	0.260012065	0.491757786	0.622890582	0.894021703	up_in_case
PRJNA8274(	ENSGALG00010004040	LGALS1B	case	control	0.163677787	0.158839578	1.030459722	0.30279425	0.725470538	up_in_case
PRJNA8274(	ENSGALG00010004042	PLAG1	case	control	0.079675571	0.073754793	1.080276521	0.280019062	0.70572305	up_in_case
PRJNA8274(	ENSGALG00010004043	CAB39L	case	control	-0.052082791	0.101404987	-0.513611735	0.607523474	0.887328057	down_in_case
PRJNA8274(	ENSGALG00010004044	RNF138	case	control	-0.065907975	0.117410312	-0.561347409	0.574560728	0.877910508	down_in_case
PRJNA8274(	ENSGALG00010004046	ENSGALG00010004046	case	control	-0.124058693	0.10160903	-1.220941606	0.22210813	0.645952056	down_in_case
PRJNA8274(	ENSGALG00010004047	PDE7A	case	control	-0.022183219	0.068824026	-0.32231795	0.747211836	0.939021817	down_in_case
PRJNA8274(	ENSGALG00010004049	SETDB2	case	control	0.051806151	0.053652834	0.965580887	0.334253943	0.748686141	up_in_case
PRJNA8274(	ENSGALG00010004055	FILIP1	case	control	-0.25953547	0.078366485	-3.311817161	0.000926921	0.046346052	down_in_case
PRJNA8274(	ENSGALG00010004056	SHROOM3	case	control	0.12105159	0.127569593	0.948906298	0.342668271	0.75456672	up_in_case
PRJNA8274(	ENSGALG00010004057	ENSGALG00010004057	case	control	-0.455268784	0.230196432	-1.977740402	0.047958	0.360378034	down_in_case
PRJNA8274(	ENSGALG00010004058	TMEM30A	case	control	0.049344221	0.038763669	1.272950236	0.203035701	0.625537942	up_in_case
PRJNA8274(	ENSGALG00010004060	ETV1	case	control	-0.036076411	0.062835143	-0.574143861	0.565870455	0.872906976	down_in_case
PRJNA8274(	ENSGALG00010004063	ENSGALG00010004063	case	control	0.233703559	0.332548463	0.702765414	0.482201954	0.832931266	up_in_case
PRJNA8274(	ENSGALG00010004065	TIFA	case	control	-0.326288132	0.313706753	-1.040105539	0.298290871	0.721487387	down_in_case
PRJNA8274(	ENSGALG00010004070	ARL11	case	control	-0.234207465	0.236870864	-0.988755902	0.322782584	0.740689923	down_in_case
PRJNA8274(	ENSGALG00010004072	SGK3	case	control	0.23882662	0.126453698	1.888648755	0.058938909	0.395497471	up_in_case
PRJNA8274(	ENSGALG00010004076	COX7A2	case	control	0.131150877	0.087741419	1.494743071	0.134981509	0.539774365	up_in_case
PRJNA8274(	ENSGALG00010004079	AP1AR	case	control	0.015910131	0.137185105	0.115975645	0.907671844	0.981730793	up_in_case
PRJNA8274(	ENSGALG00010004081	RPS20	case	control	-0.056418981	0.085054303	-0.66332894	0.507119905	0.843483653	down_in_case
PRJNA8274(	ENSGALG00010004082	FAM241A	case	control	-0.144223549	0.119654575	-1.205332507	0.228074965	0.651681204	down_in_case
PRJNA8274(	ENSGALG00010004083	PHF11	case	control	0.099266171	0.100907446	0.983734843	0.325245903	0.743110335	up_in_case
PRJNA8274(	ENSGALG00010004085	RB1CC1	case	control	-0.084518561	0.071569775	-1.180925342	0.237632383	0.662652947	down_in_case
PRJNA8274(	ENSGALG00010004086	UTP3	case	control	-0.005490883	0.073122652	-0.075091404	0.940141987	0.989551456	down_in_case
PRJNA8274(	ENSGALG00010004087	NCOA7	case	control	0.087116908	0.095595846	0.911304327	0.362135047	0.768423771	up_in_case
PRJNA8274(	ENSGALG00010004094	DGKB	case	control	0.137841992	0.153397449	0.898593771	0.368869078	0.771834431	up_in_case
PRJNA8274(	ENSGALG00010004095	ANKRD50	case	control	0.087413449	0.071986687	1.214300206	0.224633107	0.648677995	up_in_case
PRJNA8274(	ENSGALG00010004096	COL12A1	case	control	-0.141845512	0.188064456	-0.754238814	0.450705835	0.819777848	down_in_case
PRJNA8274(	ENSGALG00010004097	ENSGALG00010004097	case	control	0.174074096	0.117143085	1.485995497	0.137280312	0.539854583	up_in_case
PRJNA8274(	ENSGALG00010004101	ARID1B	case	control	-0.118470804	0.054479712	-2.174585737	0.029661174	0.305970455	down_in_case
PRJNA8274(	ENSGALG00010004102	PCMTD1	case	control	0.013177451	0.088779298	0.148429318	0.882003964	0.976801393	up_in_case
PRJNA8274(	ENSGALG00010004104	SCARB2	case	control	0.021894679	0.101594526	0.215510422	0.829369388	0.962192296	up_in_case
PRJNA8274(	ENSGALG00010004108	PERPB	case	control	0.326686682	0.218978143	1.491868903	0.135733516	0.539774365	up_in_case
PRJNA8274(	ENSGALG00010004109	AGMO	case	control	-0.714315965	0.424205937	-1.6838896	0.092203007	0.476713154	down_in_case
PRJNA8274(	ENSGALG00010004113	ECHDC1	case	control	0.074986436	0.167980317	0.446400133	0.655308237	0.907302833	up_in_case
PRJNA8274(	ENSGALG00010004116	SPRY1	case	control	0.012419777	0.075930655	0.163567365	0.870071738	0.974794532	up_in_case
PRJNA8274(	ENSGALG00010004117	STBD1	case	control	0.086129607	0.154304262	0.55818035	0.576721238	0.878085957	up_in_case
PRJNA8274(	ENSGALG00010004122	TRIM55	case	control	-0.020921282	0.096985893	-0.215714691	0.829210149	0.962142561	down_in_case
PRJNA8274(	ENSGALG00010004123	NDUFAF4	case	control	0.177166768	0.066304326	2.67202426	0.00753952	0.151682101	up_in_case
PRJNA8274(	ENSGALG00010004125	MANEA	case	control	-0.002117141	0.073402426	-0.028842935	0.976989858	0.994586386	down_in_case
PRJNA8274(	ENSGALG00010004127	EPHA7	case	control	-0.381762481	0.164341071	-2.322988885	0.02017975	0.254471268	down_in_case
PRJNA8274(	ENSGALG00010004128	MCMDC2	case	control	0.970277206	0.881164188	1.101131003	0.270839645	0.698323327	up_in_case
PRJNA8274(	ENSGALG00010004131	NUDT6	case	control	0.533910561	0.149199106	3.578510451	0.000345558	0.025211633	up_in_case
PRJNA8274(	ENSGALG00010004135	NUP54	case	control	-0.05642747	0.054440431	-1.036499329	0.299969252	0.723279006	down_in_case
PRJNA8274(	ENSGALG00010004137	CROT	case	control	0.038569189	0.055635622	0.693246301	0.488154996	0.835899547	up_in_case
PRJNA8274(	ENSGALG00010004141	KPNA3	case	control	-0.12442729	0.052207945	-2.383301793	0.017158122	0.233233021	down_in_case
PRJNA8274(	ENSGALG00010004142	RBBP8	case	control	-0.100426154	0.071750692	-1.399654154	0.161616909	0.575192085	down_in_case
PRJNA8274(	ENSGALG00010004143	TCF24	case	control	0.023899861	0.30249344	0.07900952	0.937025051	0.988800485	up_in_case
PRJNA8274(	ENSGALG00010004145	PPP1R42	case	control	-0.464623108	0.720186849	-0.645142449	0.518834877	0.848941486	down_in_case
PRJNA8274(	ENSGALG00010004146	NAAA	case	control	-0.364982194	0.160013876	-2.280940896	0.022551945	0.2670335	down_in_case
PRJNA8274(	ENSGALG00010004147	SPRYD7	case	control	0.070474207	0.060651635	1.161950665	0.245255505	0.669759186	up_in_case
PRJNA8274(	ENSGALG00010004148	ENSGALG00010004148	case	control	0.076891382	0.121935013	0.630593134	0.528306589	0.853551489	up_in_case
PRJNA8274(	ENSGALG00010004149	SDAD1	case	control	-0.193922705	0.073662484	-2.632584398	0.008473797	0.162433368	down_in_case
PRJNA8274(	ENSGALG00010004154	COPS5	case	control	-0.002150938	0.063556421	-0.033842976	0.973002365	0.993497596	down_in_case
PRJNA8274(	ENSGALG00010004158	JCHAIN	case	control	-0.364061255	0.31433563	-1.158192776	0.246785376	0.671238151	down_in_case
PRJNA8274(	ENSGALG00010004172	ST8SIA6	case	control	-0.041612709	0.160203499	-0.259749066	0.795057342	0.95280257	down_in_case
PRJNA8274(	ENSGALG00010004177	CSPP1	case	control	0.163457418	0.081581971	2.003597321	0.045113213	0.352291176	up_in_case
PRJNA8274(	ENSGALG00010004181	BBS12	case	control	0.043638159	0.089958729	0.485090873	0.627611886	0.895865363	up_in_case
PRJNA8274(	ENSGALG00010004187	ARFGEF1	case	control	-0.093417337	0.058616006	-1.593717212	0.110999387	0.503480037	down_in_case
PRJNA8274(	ENSGALG00010004188	BMI1	case	control	-0.051460855	0.056669365	-0.908089499	0.363830938	0.770036689	down_in_case
PRJNA8274(	ENSGALG00010004191	CABLES1	case	control	-0.131021834	0.075433466	-1.736919188	0.082401436	0.456367364	down_in_case
PRJNA8274(	ENSGALG00010004200	ABCB1	case	control	0.392582283	0.268409708	1.462623263	0.143570494	0.546898792	up_in_case
PRJNA8274(	ENSGALG00010004201	CPA6	case	control	0.38886467	0.432455578	0.899201421	0.368545384	0.771770041	up_in_case
PRJNA8274(	ENSGALG00010004210	SYNCRIP	case	control	-0.052528801	0.042456394	-1.237241237	0.215997536	0.640556775	down_in_case
PRJNA8274(	ENSGALG00010004211	RNF146	case	control	0.056713021	0.044433107	1.276368557	0.201825266	0.6249678	up_in_case
PRJNA8274(	ENSGALG00010004212	RSPO3	case	control	-0.137470764	0.230544352	-0.59628771	0.55098304	0.864743359	down_in_case
PRJNA8274(	ENSGALG00010004214	REPS1	case	control	-0.146810664	0.046604774	-3.150120718	0.00163203	0.065055828	down_in_case
PRJNA8274(	ENSGALG00010004216	SNX14	case	control	0.105175661	0.047855783	2.197762826	0.027966012	0.298666145	up_in_case
PRJNA8274(	ENSGALG00010004217	CCDC28A	case	control	0.083042441	0.112520302	0.738021846	0.460501174	0.82454265	up_in_case
PRJNA8274(	ENSGALG00010004219	FGF2	case	control	0.08855385	0.118643689	0.746384836	0.455434977	0.822001789	up_in_case
PRJNA8274(	ENSGALG00010004222	SPAG6	case	control	-0.049702457	0.136811736	-0.363290887	0.7163876	0.926862121	down_in_case
PRJNA8274(	ENSGALG00010004224	NT5E	case	control	-0.275081271	0.171955394	-1.599724581	0.109659696	0.50180277	down_in_case
PRJNA8274(	ENSGALG00010004225	CETN4	case	control	0.004453624	0.073341316	0.060724626	0.95157852	0.991857576	up_in_case
PRJNA8274(	ENSGALG00010004226	RUNDC3B	case	control	-0.263576209	0.139953616	-1.883311174	0.05965821	0.397773519	down_in_case
PRJNA8274(	ENSGALG00010004230	TAF1B	case	control	0.02864043	0.080192581	0.357145628	0.720982788	0.92919613	up_in_case
PRJNA8274(	ENSGALG00010004231	ARMC3	case	control	-0.037495564	0.268644754	-0.139573038	0.888997345	0.978017246	down_in_case
PRJNA8274(	ENSGALG00010004233	NHSL1	case	control	0.110983995	0.057098631	1.943724253	0.051928712	0.371953818	up_in_case
PRJNA8274(	ENSGALG00010004235	ADAD1	case	control	0.292967269	0.715356994	0.409539952	0.682143454	0.915594727	up_in_case
PRJNA8274(	ENSGALG00010004236	DBF4	case	control	0.134687485	0.101061799	1.332723996	0.182622395	0.604094438	up_in_case
PRJNA8274(	ENSGALG00010004237	RUFY3	case	control	0.160726527	0.078025525	2.059922409	0.039405959	0.335091843	up_in_case
PRJNA8274(	ENSGALG00010004238	CCNC	case	control	-0.040913092	0.061683087	-0.663278944	0.507151919	0.843483653	down_in_case
PRJNA8274(	ENSGALG00010004240	ENSGALG00010004240	case	control	0.018325595	0.097259872	0.188418873	0.850548306	0.969234083	up_in_case
PRJNA8274(	ENSGALG00010004241	PREX2	case	control	-0.024701079	0.081460563	-0.303227451	0.76171653	0.943290152	down_in_case
PRJNA8274(	ENSGALG00010004242	ADAM22	case	control	0.081239053	0.121310138	0.669680659	0.503061384	0.842485272	up_in_case
PRJNA8274(	ENSGALG00010004243	BLTP1	case	control	-0.017682582	0.046925377	-0.376823438	0.706304822	0.924449984	down_in_case
PRJNA8274(	ENSGALG00010004244	TMEM241	case	control	-0.037695296	0.066791458	-0.56437301	0.572500303	0.876814097	down_in_case
PRJNA8274(	ENSGALG00010004245	ARFGEF3	case	control	-0.194340919	0.192881291	-1.007567498	0.313662139	0.735267739	down_in_case
PRJNA8274(	ENSGALG00010004246	C8orf34	case	control	0.086676675	0.258189242	0.335709861	0.737089669	0.935060877	up_in_case
PRJNA8274(	ENSGALG00010004247	MEOX2	case	control	0.095685068	0.077322162	1.237485689	0.215906824	0.640553441	up_in_case
PRJNA8274(	ENSGALG00010004248	GRSF1	case	control	-0.13655843	0.050288452	-2.715502753	0.006617523	0.140023533	down_in_case
PRJNA8274(	ENSGALG00010004249	USP45	case	control	-0.127360318	0.067810184	-1.878188639	0.060355364	0.399259348	down_in_case
PRJNA8274(	ENSGALG00010004250	MOB1B	case	control	-0.036404629	0.047962336	-0.759025341	0.447837398	0.818621346	down_in_case
PRJNA8274(	ENSGALG00010004251	CCN2	case	control	0.254999364	0.19384088	1.315508699	0.188338996	0.611129484	up_in_case
PRJNA8274(	ENSGALG00010004255	RPS12	case	control	-0.134255497	0.080645726	-1.664756503	0.095961345	0.483185646	down_in_case
PRJNA8274(	ENSGALG00010004256	ENSGALG00010004256	case	control	0.38074753	0.773674464	0.492128858	0.622628253	0.893944471	up_in_case
PRJNA8274(	ENSGALG00010004257	ENSGALG00010004257	case	control	0.494059803	0.458727983	1.077021288	0.281470755	0.705762606	up_in_case
PRJNA8274(	ENSGALG00010004265	PIP4K2A	case	control	-0.197823526	0.090117491	-2.195173471	0.028151157	0.299572314	down_in_case
PRJNA8274(	ENSGALG00010004275	ADARB2	case	control	-0.500175966	0.559360248	-0.894192907	0.371218686	0.773034399	down_in_case
PRJNA8274(	ENSGALG00010004281	VIM	case	control	-0.098929192	0.069718213	-1.41898634	0.155902999	0.565123671	down_in_case
PRJNA8274(	ENSGALG00010004283	DCK	case	control	0.068415216	0.084383605	0.810764312	0.417501033	0.799987395	up_in_case
PRJNA8274(	ENSGALG00010004285	SLC18B1	case	control	-0.025448105	0.111093689	-0.229068858	0.818815397	0.958580449	down_in_case
PRJNA8274(	ENSGALG00010004296	ENSGALG00010004296	case	control	0.887560596	0.985935708	0.900221575	0.368002347	0.771770041	up_in_case
PRJNA8274(	ENSGALG00010004297	SULF1	case	control	0.011471301	0.133276894	0.086071193	0.931409823	0.987258715	up_in_case
PRJNA8274(	ENSGALG00010004300	VNN2	case	control	-0.080994396	0.19904759	-0.406909705	0.684074307	0.916328843	down_in_case
PRJNA8274(	ENSGALG00010004302	CRPPA	case	control	-0.140510278	0.13565584	-1.035784952	0.300302479	0.723279006	down_in_case
PRJNA8274(	ENSGALG00010004306	PNISR	case	control	0.039309979	0.084002707	0.467960863	0.639812578	0.900806349	up_in_case

PRJNA8274(	ENSGALG00010004307	NPC2	case	control	0.205622277	0.159347491	1.290401726	0.196911213	0.620029043	up_in_case
PRJNA8274(	ENSGALG00010004308	DSG2	case	control	0.22226059	0.466036858	0.476916335			up_in_case
PRJNA8274(	ENSGALG00010004309	MRPS28	case	control	0.047790484	0.07403496	0.645512386	0.518595194	0.848941486	up_in_case
PRJNA8274(	ENSGALG00010004312	PDLIM5	case	control	-0.117174368	0.111167833	-1.05403124	0.291868615	0.714632191	down_in_case
PRJNA8274(	ENSGALG00010004319	TAAR1	case	control	0.399195171	0.688662559	0.579667307	0.562138994	0.870919569	up_in_case
PRJNA8274(	ENSGALG00010004323	TRDMT1	case	control	0.196394986	0.097823369	2.007648959	0.044680608	0.351254918	up_in_case
PRJNA8274(	ENSGALG00010004325	LTBP2	case	control	-0.035102162	0.27762091	-0.126439185	0.899384286	0.98028579	down_in_case
PRJNA8274(	ENSGALG00010004326	ENSGALG00010004326	case	control	-0.110643703	0.522105734	-0.211918191	0.832170859	0.962371901	down_in_case
PRJNA8274(	ENSGALG00010004328	ENSGALG00010004328	case	control	0.347422084	0.388037522	0.89533116	0.370610088	0.772791771	up_in_case
PRJNA8274(	ENSGALG00010004329	COQ3	case	control	-0.273343305	0.093315707	-2.929231471	0.003398013	0.099572914	down_in_case
PRJNA8274(	ENSGALG00010004330	STX7	case	control	0.068917664	0.059832988	1.151833906	0.249389332	0.674421685	up_in_case
PRJNA8274(	ENSGALG00010004332	ENSGALG00010004332	case	control	-0.349980774	0.626979577	-0.558201234	0.576706978	0.878085957	down_in_case
PRJNA8274(	ENSGALG00010004333	DIO2	case	control	0.08779476	0.354080853	0.247951164	0.804172193	0.954997212	up_in_case
PRJNA8274(	ENSGALG00010004334	ENSGALG00010004334	case	control	0.163986015	0.160287511	1.023074186	0.306272771	0.729293348	up_in_case
PRJNA8274(	ENSGALG00010004336	ENSGALG00010004336	case	control	-0.708876163	0.34774956	-2.038467459	0.041503206	0.340562528	down_in_case
PRJNA8274(	ENSGALG00010004337	BMPR1B	case	control	-0.163028126	0.101645443	-1.603890157	0.108738267	0.500951275	down_in_case
PRJNA8274(	ENSGALG00010004338	ENSGALG00010004338	case	control	0.153265211	0.178453652	0.858851634	0.390422376	0.783914628	up_in_case
PRJNA8274(	ENSGALG00010004339	B4GALT6	case	control	0.565098264	0.14775672	3.824518186	0.000131028	0.013103306	up_in_case
PRJNA8274(	ENSGALG00010004340	SLC4A4	case	control	-0.027116911	0.102508785	-0.264532561	0.791369578	0.95239351	down_in_case
PRJNA8274(	ENSGALG00010004341	ENSGALG00010004341	case	control	0.05576284	0.145251249	0.383906096	0.701048048	0.922516526	up_in_case
PRJNA8274(	ENSGALG00010004342	ZNF438	case	control	0.016849546	0.074673308	0.225643499	0.821478706	0.95897887	up_in_case
PRJNA8274(	ENSGALG00010004344	SOSTDC1	case	control	-0.144432419	0.247473914	-0.58362684	0.559471398	0.869613151	down_in_case
PRJNA8274(	ENSGALG00010004345	FAXC	case	control	-0.047444879	0.11565883	-0.410214069	0.681648921	0.915594727	down_in_case
PRJNA8274(	ENSGALG00010004346	CEP128	case	control	-0.043622087	0.144068258	-0.302787631	0.76205171	0.943297504	down_in_case
PRJNA8274(	ENSGALG00010004350	MOXD1	case	control	-0.221316171	0.136327473	-1.623415777	0.104500554	0.494612261	down_in_case
PRJNA8274(	ENSGALG00010004352	CHMP2A	case	control	-0.067633992	0.053078385	-1.274228519	0.202582441	0.62541643	down_in_case
PRJNA8274(	ENSGALG00010004355	ANKMY2	case	control	-0.007632523	0.066445644	-0.114868679	0.908549211	0.981730793	down_in_case
PRJNA8274(	ENSGALG00010004356	TRAPPC8	case	control	0.042016301	0.041876903	1.003328741	0.315702274	0.736748426	up_in_case
PRJNA8274(	ENSGALG00010004358	GC	case	control	0.059689052	1.65161422	0.036139827	0.971170866	0.992761697	up_in_case
PRJNA8274(	ENSGALG00010004362	FBXL4	case	control	-0.209486261	0.106497673	-1.967050116	0.049177433	0.364182961	down_in_case
PRJNA8274(	ENSGALG00010004364	TSHR	case	control	0.084977623	0.297811179	0.285340609	0.775383204	0.947087967	up_in_case
PRJNA8274(	ENSGALG00010004365	TPD52	case	control	0.185246658	0.10408366	1.779786162	0.075110963	0.440922321	up_in_case
PRJNA8274(	ENSGALG00010004366	UNC5C	case	control	0.150423233	0.307947676	0.488470102	0.625216898	0.89506388	up_in_case
PRJNA8274(	ENSGALG00010004367	TSPAN13	case	control	-0.349329399	0.173576175	-2.012542329	0.044162799	0.35007097	down_in_case
PRJNA8274(	ENSGALG00010004368	GJD4	case	control	0.483929099	0.253172494	1.911460012	0.055945491	0.38536634	up_in_case
PRJNA8274(	ENSGALG00010004369	UBE2M	case	control	-0.157983644	0.076128365	-2.075227064	0.037965496	0.330206289	down_in_case
PRJNA8274(	ENSGALG00010004370	ATP6V1H	case	control	0.143031512	0.068402489	2.091027879	0.036525565	0.327060475	up_in_case
PRJNA8274(	ENSGALG00010004372	SYNJ2	case	control	-0.348261557	0.151284685	-2.302027845	0.021333602	0.261638514	down_in_case
PRJNA8274(	ENSGALG00010004373	MMS22L	case	control	-0.016113351	0.119342676	-0.135017509	0.892598031	0.979446888	down_in_case
PRJNA8274(	ENSGALG00010004374	ILDR1	case	control	0.783054275	0.670428777	1.16799025	0.242810698	0.667472699	up_in_case
PRJNA8274(	ENSGALG00010004375	ENSGALG00010004375	case	control	0.435781874	0.258486624	1.685897189	0.091815603	0.476713154	up_in_case
PRJNA8274(	ENSGALG00010004377	AGR2	case	control	-0.241470808	0.457846506	-0.527405593	0.59791196	0.884680373	down_in_case
PRJNA8274(	ENSGALG00010004378	TRIM28	case	control	-0.052201875	0.064994085	-0.803178863	0.421871361	0.80254895	down_in_case
PRJNA8274(	ENSGALG00010004379	ZBTB10	case	control	-0.023400918	0.045596147	-0.513221394	0.607796462	0.887448737	down_in_case
PRJNA8274(	ENSGALG00010004380	ISCA2	case	control	-0.085241319	0.055457715	-1.537050682	0.124280899	0.523029039	down_in_case
PRJNA8274(	ENSGALG00010004381	ENSGALG00010004381	case	control	-0.468651172	0.289599718	-1.618272197	0.10560395	0.495266487	down_in_case
PRJNA8274(	ENSGALG00010004385	ADAMTS3	case	control	0.316026027	0.189738961	1.665583203	0.095796459	0.483185646	up_in_case
PRJNA8274(	ENSGALG00010004386	CFAP44	case	control	0.291583025	0.089807788	3.246745427	0.001167328	0.053331577	up_in_case
PRJNA8274(	ENSGALG00010004389	ENSGALG00010004389	case	control	-0.017064371	0.076121497	-0.224172827	0.822622827	0.959737817	down_in_case
PRJNA8274(	ENSGALG00010004390	PNLDC1	case	control	-1.049358061	0.376492706	-2.787193602	0.005316671	0.12671398	down_in_case
PRJNA8274(	ENSGALG00010004393	ZBTB45	case	control	0.021491247	0.114308776	0.188010473	0.850868442	0.969234083	up_in_case
PRJNA8274(	ENSGALG00010004394	CCNY	case	control	-0.004314329	0.067670538	-0.063754907	0.949165384	0.99138853	down_in_case
PRJNA8274(	ENSGALG00010004395	BOC	case	control	0.261316706	0.092426476	2.827292761	0.004694339	0.116746168	up_in_case
PRJNA8274(	ENSGALG00010004397	ENSGALG00010004397	case	control	0.119194522	0.112717095	1.05746623	0.290298859	0.714060795	up_in_case
PRJNA8274(	ENSGALG00010004398	COX18	case	control	0.139009014	0.091289061	1.522734633	0.127825109	0.529650968	up_in_case
PRJNA8274(	ENSGALG00010004404	RGS20	case	control	0.012651769	0.260171858	0.048628508	0.96121535	0.992056045	up_in_case
PRJNA8274(	ENSGALG00010004406	ENSGALG00010004406	case	control	0.159446587	0.097408263	1.636889758	0.101653506	0.491460867	up_in_case
PRJNA8274(	ENSGALG00010004408	SERAC1	case	control	-0.258951446	0.11602893	-2.231783452	0.025629279	0.283909015	down_in_case
PRJNA8274(	ENSGALG00010004416	STXBP6	case	control	0.046612044	0.167327535	0.278567686	0.780576614	0.948875232	up_in_case
PRJNA8274(	ENSGALG00010004420	ENSGALG00010004420	case	control	0.02945618	0.279281037	0.105471463	0.916001713	0.983445506	up_in_case
PRJNA8274(	ENSGALG00010004421	RAP1GDS1	case	control	0.014219133	0.062978538	0.225777445	0.821374521	0.95897887	up_in_case
PRJNA8274(	ENSGALG00010004424	MRPL18	case	control	0.107076288	0.081149358	1.31949643	0.187003203	0.610664656	up_in_case
PRJNA8274(	ENSGALG00010004425	CREM	case	control	0.051532142	0.06954201	0.741021752	0.458680252	0.824070529	up_in_case
PRJNA8274(	ENSGALG00010004429	ENSGALG00010004429	case	control	0.512487667	0.294055791	1.742824604	0.081364257	0.454140857	up_in_case
PRJNA8274(	ENSGALG00010004430	TCEA1	case	control	-0.044448295	0.058433025	-0.76067079	0.446853726	0.817833512	down_in_case
PRJNA8274(	ENSGALG00010004433	AHR1A	case	control	0.087875191	0.102474464	0.857532576	0.39115062	0.784275639	up_in_case
PRJNA8274(	ENSGALG00010004434	TCP1	case	control	0.027655605	0.06440769	0.429383596	0.66764409	0.912432583	up_in_case
PRJNA8274(	ENSGALG00010004436	NOVA1	case	control	-0.05622144	0.054075839	-1.039677618	0.298489703	0.721501478	down_in_case
PRJNA8274(	ENSGALG00010004440	GTF2H5	case	control	0.004474381	0.08213213	0.054477835	0.956554468	0.992056045	up_in_case
PRJNA8274(	ENSGALG00010004443	ANKRD17	case	control	-0.049896174	0.051603535	-0.966913892	0.333587082	0.748508541	down_in_case
PRJNA8274(	ENSGALG00010004445	EPHA6	case	control	0.05289775	0.334043551	0.15835585	0.874176402	0.975062028	up_in_case
PRJNA8274(	ENSGALG00010004446	ACAT2	case	control	0.170513271	0.106484722	1.601293286	0.109311975	0.501568961	up_in_case
PRJNA8274(	ENSGALG00010004451	CLDND1	case	control	-0.018263471	0.04724865	-0.386539524	0.699097137	0.922288441	down_in_case
PRJNA8274(	ENSGALG00010004454	SRI	case	control	0.18105713	0.054587969	3.316795485	0.000910562	0.046010752	up_in_case
PRJNA8274(	ENSGALG00010004457	AGA	case	control	0.116839521	0.084228215	1.38717793	0.165387514	0.580618526	up_in_case
PRJNA8274(	ENSGALG00010004460	ENSGALG00010004460	case	control	-0.687271671	0.414422722	-1.658383178	0.09724015	0.485691283	down_in_case
PRJNA8274(	ENSGALG00010004468	LYPLA1	case	control	0.066207355	0.045168313	1.465792088	0.142704937	0.544908038	up_in_case
PRJNA8274(	ENSGALG00010004471	C1H3ORF52	case	control	0.211361636	0.236578007	0.893412026	0.371636565	0.773566649	up_in_case
PRJNA8274(	ENSGALG00010004472	NEIL3	case	control	-0.271137636	0.178242395	-1.521173659	0.128216264	0.530035955	down_in_case
PRJNA8274(	ENSGALG00010004473	JDP2	case	control	-0.134111675	0.085222069	-1.57367307	0.115563051	0.512260269	down_in_case
PRJNA8274(	ENSGALG00010004477	TULP4	case	control	-0.096538912	0.076859753	-1.256039844	0.209101525	0.634076945	down_in_case
PRJNA8274(	ENSGALG00010004481	ZNF704	case	control	-0.039776219	0.07108592	-0.559551294	0.575785535	0.877910508	down_in_case
PRJNA8274(	ENSGALG00010004483	STEAP4	case	control	-0.13216665	0.425769298	-0.31041846	0.756242758	0.941659745	down_in_case
PRJNA8274(	ENSGALG00010004487	ABHD10	case	control	-0.067660542	0.102460696	-0.660356067	0.509025358	0.844438819	down_in_case
PRJNA8274(	ENSGALG00010004489	LIG1	case	control	-0.124954006	0.099811953	-1.25189421	0.210608415	0.636249596	down_in_case
PRJNA8274(	ENSGALG00010004494	WTAP	case	control	-0.00236363	0.025554701	-0.092492966	0.92630638	0.98618953	down_in_case
PRJNA8274(	ENSGALG00010004495	MRPL15	case	control	-0.320379717	0.173028601	-1.851599766	0.064083317	0.410842437	down_in_case
PRJNA8274(	ENSGALG00010004501	TAGLN3	case	control	0.062249041	0.330672293	0.188249944	0.850680723	0.969234083	up_in_case
PRJNA8274(	ENSGALG00010004503	ENSGALG00010004503	case	control	0.045024446	0.125899672	0.357621629	0.72062649	0.929128014	up_in_case
PRJNA8274(	ENSGALG00010004504	ZNF804B	case	control	0.212507536	0.544824071	0.390047994	0.696501058	0.921903481	up_in_case
PRJNA8274(	ENSGALG00010004505	ENSGALG00010004505	case	control	-0.097885287	0.081247826	-1.204774237	0.228290468	0.651867751	down_in_case
PRJNA8274(	ENSGALG00010004507	CUL2	case	control	-0.076939279	0.036359231	-2.11608651	0.034337451	0.319886347	down_in_case
PRJNA8274(	ENSGALG00010004515	ENSGALG00010004515	case	control	0.124781299	0.172678618	0.722621602	0.469912393	0.829101076	up_in_case
PRJNA8274(	ENSGALG00010004516	ENSGALG00010004516	case	control	-0.002748582	0.107947598	-0.025462189	0.979686307	0.995636523	down_in_case
PRJNA8274(	ENSGALG00010004522	TSPAN5	case	control	-0.031156497	0.058198393	-0.535349783	0.592408008	0.883604289	down_in_case
PRJNA8274(	ENSGALG00010004533	PAG1	case	control	0.028889998	0.075825526	0.381006239	0.703198619	0.923659131	up_in_case
PRJNA8274(	ENSGALG00010004534	CUBN	case	control	-0.320802782	0.198097876	-1.619415558	0.105357882	0.495041148	down_in_case
PRJNA8274(	ENSGALG00010004542	ERMARD	case	control	-0.073090455	0.117443799	-0.622344098	0.53371563	0.855817434	down_in_case
PRJNA8274(	ENSGALG00010004545	TMPRSS7	case	control	-0.747307479	0.379640785	-1.96845942	0.049015201	0.36379167	down_in_case
PRJNA8274(	ENSGALG00010004553	BATF	case	control	-0.147427691	0.271766557	-0.54247915	0.587488467	0.882771329	down_in_case
PRJNA8274(	ENSGALG00010004565	ENSGALG00010004565	case	control	-0.052361618	0.422500143	-0.123932782	0.901368497	0.980642841	down_in_case
PRJNA8274(	ENSGALG00010004568	RSU1	case	control	-0.11983301	0.068241126	-1.756023336	0.079084424	0.449171025	down_in_case
PRJNA8274(	ENSGALG00010004576	STEAP2	case	control	-0.173219269	0.105855778	-1.636370467	0.101762076	0.491460867	down_in_case
PRJNA8274(	ENSGALG00010004577	ZPLD1	case	control	-0.787427932	0.492178259	-1.599883614	0.109624405	0.501801854	down_in_case
PRJNA8274(	ENSGALG00010004583	XKR4	case	control	-0.199401132	0.14379393	-1.386714532	0.165528828	0.580618526	down_in_case
PRJNA8274(	ENSGALG00010004584	EIF4E	case	control	-0.067670232	0.047741982	-1.417415647	0.156361441	0.566211345	down_in_case
PRJNA8274(	ENSGALG00010004586	ENSGALG00010004586	case	control	0.418090392	0.458076723	0.912708222	0.361396018	0.767557265	up_in_case
PRJNA8274(	ENSGALG00010004588	MINDY3	case	control	-0.02893963	0.079275907	-0.365049503	0.715074454	0.926862121	down_in_case
PRJNA8274(	ENSGALG00010004591	RRS1	case	control	-0.028734805	0.082774939	-0.347143778	0.728483306	0.933057319	down_in_case
PRJNA8274(	ENSGALG00010004602	DYNLT2	case	control	-0.03858624	0.33555069	-0.114993775	0.908450056	0.981730793	down_in_case
PRJNA8274(	ENSGALG00010004609	CFAP69	case	control	-0.637386509	0.435985538	-1.461944155	0.143756514	0.546917268	down_in_case
PRJNA8274(	ENSGALG00010004610	TGS1	case	control	0.029910451	0.051220656	0.583952922	0.559251987	0.869461123	up_in_case
PRJNA8274(	ENSGALG00010004622	ENSGALG00010004622	case	control	-0.180471677	0.104260562	-1.730967811	0.083457506	0.45743286	down_in_case
PRJNA8274(	ENSGALG00010004623	MBIP	case	control	0.043477895	0.073091391	0.594842892	0.551948494	0.86554046	up_in_case
PRJNA8274(	ENSGALG00010004624	PHF10	case	control	-0.011579876	0.049037685	-0.236142397	0.813322176	0.956712425	down_in_case
PRJNA8274(	ENSGALG00010004630	ENSGALG00010004630	case	control	0.285845758	0.161083714	1.774516814	0.075977703	0.442710866	up_in_case
PRJNA8274(	ENSGALG00010004639	HBS1L	case	control	-0.043175686	0.040478994	-1.066619521	0.286143682	0.710557161	down_in_case
PRJNA8274(	ENSGALG00010004641	METAP1	case	control	0.114748914	0.054445259	2.107601591	0.035065466	0.323298621	up_in_case
PRJNA8274(	ENSGALG00010004650	GTPBP10	case	control	-0.478839159	0.111063952	-4.311382321	1.62237E-05	0.002576354	down_in_case
PRJNA8274(	ENSGALG00010004654	NKX2-1	case	control	-0.46879721	2.973453327	-0.157660861	0.874724043	0.975474836	down_in_case
PRJNA8274(	ENSGALG00010004666	C6orf120	case	control	-0.020559393	0.087175137	-0.235840097	0.813556753	0.956712425	down_in_case
PRJNA8274(	ENSGALG00010004669	ELMOD1	case	control	-0.020532752	0.272971695	-0.075219344	0.940040193	0.989551456	down_in_case
PRJNA8274(	ENSGALG00010004673	RAB18	case	control	0.030502049	0.043773144	0.696821067	0.48591478	0.834635018	up_in_case
PRJNA8274(	ENSGALG00010004689	BBX	case	control	0.058409901	0.06898916	0.846653314	0.397188388	0.788531716	up_in_case
PRJNA8274(	ENSGALG00010004705	ENSGALG00010004705	case	control	0.311134709	0.113049582	2.752196894	0.005919692	0.133942393	up_in_case
PRJNA8274(	ENSGALG00010004707	OTUD1	case	control	-0.004775443	0.159765982	-0.029890239	0.976154591	0.994231527	down_in_case

PRJNA8274(	ENSGALG00010004716	ADH6	case	control	1.86078775	0.728755979	2.553375622	0.010668437	0.184200521	up_in_case
PRJNA8274(	ENSGALG00010004717	ALKBH8	case	control	-0.158763249	0.146481948	-1.083841733	0.278434978	0.703953892	down_in_case
PRJNA8274(	ENSGALG00010004721	LAMA2	case	control	-0.254230222	0.122565392	-2.074241498	0.038056888	0.330206289	down_in_case
PRJNA8274(	ENSGALG00010004728	ENSGALG00010004728	case	control	-0.104349121	0.080441137	-1.297210919	0.194558625	0.617304812	down_in_case
PRJNA8274(	ENSGALG00010004729	ENSGALG00010004729	case	control	0.070486526	0.451104011	0.156253379	0.875833303	0.975666505	up_in_case
PRJNA8274(	ENSGALG00010004730	CLDN12	case	control	0.017371513	0.064210833	0.27053866	0.786745879	0.950529407	up_in_case
PRJNA8274(	ENSGALG00010004735	ENSGALG00010004735	case	control	-0.171333951	0.091190059	-1.878866542	0.060262718	0.399259348	down_in_case
PRJNA8274(	ENSGALG00010004736	CD47	case	control	0.14731865	0.061345682	2.401451001	0.016330196	0.226415743	up_in_case
PRJNA8274(	ENSGALG00010004739	ENSGALG00010004739	case	control	0.991557975	0.378604214	2.618982931	0.008819236	0.166598514	up_in_case
PRJNA8274(	ENSGALG00010004746	LYN	case	control	0.042552739	0.103272852	0.412041873	0.680308732	0.915381147	up_in_case
PRJNA8274(	ENSGALG00010004751	PTER	case	control	0.009155636	0.167904572	0.054528806	0.956513859	0.992056045	up_in_case
PRJNA8274(	ENSGALG00010004755	PAX9	case	control	-0.022241606	0.355118962	-0.062631424	0.950060006	0.991488793	down_in_case
PRJNA8274(	ENSGALG00010004757	TRMT10A	case	control	-0.018838565	0.123484954	-0.152557575	0.878747184	0.976234053	down_in_case
PRJNA8274(	ENSGALG00010004760	THBS2	case	control	0.034354506	0.165873117	0.207113164	0.835921481	0.964083973	up_in_case
PRJNA8274(	ENSGALG00010004762	IFT57	case	control	0.016251221	0.138549877	0.117295096	0.906626211	0.981730793	up_in_case
PRJNA8274(	ENSGALG00010004765	ENSGALG00010004765	case	control	0.511771926	0.260456439	1.964904104	0.049425338	0.364696764	up_in_case
PRJNA8274(	ENSGALG00010004769	HTR2A	case	control	0.119809784	0.206419371	0.580419288	0.561631901	0.870616281	up_in_case
PRJNA8274(	ENSGALG00010004777	CDK14	case	control	-0.05351204	0.075802041	-0.705944578	0.480222604	0.831851816	down_in_case
PRJNA8274(	ENSGALG00010004778	ENSGALG00010004778	case	control	0.497052246	0.459211156	1.082404553	0.279072806	0.704580001	up_in_case
PRJNA8274(	ENSGALG00010004780	ENSGALG00010004780	case	control	0.104766168	0.237039493	0.441977694	0.658505347	0.908367791	up_in_case
PRJNA8274(	ENSGALG00010004781	ENSGALG00010004781	case	control	0.137936089	0.096935637	1.422965721	0.154746093	0.563644709	up_in_case
PRJNA8274(	ENSGALG00010004787	RIOK3	case	control	0.043649614	0.048080808	0.907838611	0.363963495	0.770036689	up_in_case
PRJNA8274(	ENSGALG00010004791	FZD1	case	control	0.088532721	0.064665058	1.369096743	0.170969036	0.58841329	up_in_case
PRJNA8274(	ENSGALG00010004798	PTPRK	case	control	-0.106800852	0.097442945	-1.096034736	0.273063573	0.698936152	down_in_case
PRJNA8274(	ENSGALG00010004800	ZC3H12C	case	control	0.030028003	0.186906748	0.16065767	0.872363031	0.975062028	up_in_case
PRJNA8274(	ENSGALG00010004803	RMC1	case	control	0.124796331	0.067285407	1.854731026	0.063634646	0.409661205	up_in_case
PRJNA8274(	ENSGALG00010004824	ATM	case	control	-0.037708275	0.060853083	-0.619660875	0.53548108	0.857009451	down_in_case
PRJNA8274(	ENSGALG00010004835	HHLA2	case	control	1.302031334	0.223654128	5.82162891	5.82768E-09	2.97628E-06	up_in_case
PRJNA8274(	ENSGALG00010004837	CENPC	case	control	0.083264061	0.085181424	0.977490835	0.328326213	0.745445783	up_in_case
PRJNA8274(	ENSGALG00010004841	AKAP9	case	control	-0.057736999	0.091014693	-0.634370085	0.525839328	0.85251736	down_in_case
PRJNA8274(	ENSGALG00010004846	TTLL2	case	control	-0.057271789	0.268875145	-0.213005145	0.83132295	0.962371901	down_in_case
PRJNA8274(	ENSGALG00010004851	MTCL3	case	control	-0.090724914	0.340484644	-0.266458166	0.789886373	0.951843754	down_in_case
PRJNA8274(	ENSGALG00010004852	ENSGALG00010004852	case	control	0.276439228	0.146792595	1.883196002	0.059673811	0.397773519	up_in_case
PRJNA8274(	ENSGALG00010004855	ZEB1	case	control	-0.021060991	0.079596871	-0.264595716	0.791320921	0.95239351	down_in_case
PRJNA8274(	ENSGALG00010004859	NPC1	case	control	-0.112420279	0.066878921	-1.680952339	0.092772172	0.477724907	down_in_case
PRJNA8274(	ENSGALG00010004866	SLC25A21	case	control	0.302616331	0.147391721	2.05314335	0.040058677	0.336547209	up_in_case
PRJNA8274(	ENSGALG00010004868	NPAT	case	control	-0.086565518	0.050029838	-1.730277822	0.08358065	0.457756911	down_in_case
PRJNA8274(	ENSGALG00010004871	C11orf87	case	control	-0.202199128	0.182477735	-1.108075614	0.267829162	0.694521603	down_in_case
PRJNA8274(	ENSGALG00010004872	KIAA0408	case	control	-0.23744497	0.141924249	-1.673040178	0.094319387	0.479477093	down_in_case
PRJNA8274(	ENSGALG00010004879	ENSGALG00010004879	case	control	-0.04156546	0.611432193	-0.06798049	0.945801165	0.990313016	down_in_case
PRJNA8274(	ENSGALG00010004881	MIPOL1	case	control	-0.01643312	0.089063359	-0.184510449	0.853613031	0.969998446	down_in_case
PRJNA8274(	ENSGALG00010004883	KRIT1	case	control	0.012821837	0.051878295	0.247152252	0.804790397	0.955458919	up_in_case
PRJNA8274(	ENSGALG00010004887	MYH15	case	control	0.042655381	0.246603279	0.172971669	0.862673697	0.972787494	up_in_case
PRJNA8274(	ENSGALG00010004888	FOXA1	case	control	1.330903956	1.070340969	1.243439236	0.21370602	0.638602829	up_in_case
PRJNA8274(	ENSGALG00010004890	ACAT1	case	control	-0.014041246	0.111448912	-0.125988186	0.899741276	0.98028579	down_in_case
PRJNA8274(	ENSGALG00010004891	ANKIB1	case	control	0.014007889	0.054733266	0.255930076	0.798004831	0.953179843	up_in_case
PRJNA8274(	ENSGALG00010004892	HEY2	case	control	-0.057901573	0.183405269	-0.315702888	0.752228034	0.940427752	down_in_case
PRJNA8274(	ENSGALG00010004896	UBA6	case	control	-0.036406112	0.074181544	-0.490770476	0.623588794	0.89432552	down_in_case
PRJNA8274(	ENSGALG00010004899	GATAD1	case	control	-0.244747719	0.081061452	-3.019286163	0.002533711	0.082910904	down_in_case
PRJNA8274(	ENSGALG00010004901	CUL5	case	control	-0.144850567	0.045278962	-3.199069931	0.001378717	0.058654283	down_in_case
PRJNA8274(	ENSGALG00010004902	ANKRD29	case	control	0.152265497	0.277285988	0.549127988	0.582917619	0.88115454	up_in_case
PRJNA8274(	ENSGALG00010004903	ENSGALG00010004903	case	control	1.233907545	0.452929586	2.724281177	0.006444162	0.13904105	up_in_case
PRJNA8274(	ENSGALG00010004907	ENSGALG00010004907	case	control	-0.021416492	0.115400044	-0.185584784	0.852770387	0.969809672	down_in_case
PRJNA8274(	ENSGALG00010004917	ENSGALG00010004917	case	control	-0.172231515	0.081510923	-2.112986934	0.034601886	0.321094727	down_in_case
PRJNA8274(	ENSGALG00010004921	RRAGD	case	control	-0.122806622	0.09264677	-1.325535927	0.184993461	0.607859949	down_in_case
PRJNA8274(	ENSGALG00010004924	ENSGALG00010004924	case	control	1.085714173	0.242142903	4.483774501	7.33342E-06	0.00136192	up_in_case
PRJNA8274(	ENSGALG00010004927	KIF25	case	control	0.212426688	0.155602836	1.365185195	0.17219485	0.590500326	up_in_case
PRJNA8274(	ENSGALG00010004928	HDDC2	case	control	0.103068275	0.088035202	1.170762067	0.241694429	0.666243691	up_in_case
PRJNA8274(	ENSGALG00010004929	ENSGALG00010004929	case	control	0.568720489	0.14300123	3.977032141	6.97807E-05	0.00860228	up_in_case
PRJNA8274(	ENSGALG00010004933	SPTLC2	case	control	0.03569693	0.070592955	0.505672694	0.61308644	0.890154949	up_in_case
PRJNA8274(	ENSGALG00010004934	CIP2A	case	control	0.038650728	0.109414593	0.353250207	0.723900871	0.930580947	up_in_case
PRJNA8274(	ENSGALG00010004935	SVIL	case	control	-0.086652345	0.052345862	-1.655380985	0.097847241	0.487003223	down_in_case
PRJNA8274(	ENSGALG00010004939	DDX10	case	control	-0.048973292	0.063273715	-0.773991099	0.438936055	0.812213456	down_in_case
PRJNA8274(	ENSGALG00010004942	GABRR1	case	control	0.371543176	0.370240911	1.003517346	0.315611312	0.736748426	up_in_case
PRJNA8274(	ENSGALG00010004944	ALKBH1	case	control	-0.009382656	0.067427979	-0.139150788	0.889330995	0.978038394	down_in_case
PRJNA8274(	ENSGALG00010004946	SH2D1B	case	control	0.43671895	0.757868077	0.576246663	0.564448469	0.872237167	up_in_case
PRJNA8274(	ENSGALG00010004949	RAB39A	case	control	-0.379287955	0.160594004	-2.361781547	0.018187356	0.241260842	down_in_case
PRJNA8274(	ENSGALG00010004950	SLIRP	case	control	0.096002382	0.099534175	0.964516779	0.3347869	0.749092892	up_in_case
PRJNA8274(	ENSGALG00010004951	AFDN	case	control	-0.045384403	0.066758746	-0.679827065	0.496613967	0.83858637	down_in_case
PRJNA8274(	ENSGALG00010004952	PEX1	case	control	0.118015335	0.073759503	1.600001772	0.10959819	0.501801854	up_in_case
PRJNA8274(	ENSGALG00010004957	ENSGALG00010004957	case	control	-0.043031699	0.208335097	-0.206550409	0.836360993	0.964357539	down_in_case
PRJNA8274(	ENSGALG00010004958	TPD52L1	case	control	-0.179242449	0.280634692	-0.638703818	0.523015628	0.851254665	down_in_case
PRJNA8274(	ENSGALG00010004962	ENSGALG00010004962	case	control	0.006654152	0.061442064	0.108299618	0.913758025	0.982831122	up_in_case
PRJNA8274(	ENSGALG00010004963	ENSGALG00010004963	case	control	0.457370295	0.265819015	1.720607894	0.085322	0.461985839	up_in_case
PRJNA8274(	ENSGALG00010004965	JCAD	case	control	0.054948387	0.064548038	0.851278965	0.394614407	0.786978937	up_in_case
PRJNA8274(	ENSGALG00010004966	LAMA3	case	control	-0.090702711	0.204110033	-0.444381443	0.656766827	0.907508591	down_in_case
PRJNA8274(	ENSGALG00010004967	SLC35F2	case	control	-0.00332772	0.175403828	-0.018971766	0.984863629	0.996591771	down_in_case
PRJNA8274(	ENSGALG00010004969	YTHDC1	case	control	-0.086553793	0.054514513	-1.587720195	0.112349624	0.506149199	down_in_case
PRJNA8274(	ENSGALG00010004970	RBM48	case	control	0.013302523	0.097658693	0.136214426	0.891651771	0.978917427	up_in_case
PRJNA8274(	ENSGALG00010004973	SLN	case	control	-0.722521274	0.219846956	-3.286473864	0.001014502	0.049344819	down_in_case
PRJNA8274(	ENSGALG00010004974	MTPAP	case	control	-0.047453424	0.046651758	-1.017184062	0.309065877	0.731849981	down_in_case
PRJNA8274(	ENSGALG00010004975	SNW1	case	control	-0.007165356	0.041453516	-0.17285279	0.862767141	0.972787494	down_in_case
PRJNA8274(	ENSGALG00010004976	ENSGALG00010004976	case	control	-0.051042878	0.132484214	-0.385275169	0.700033557	0.922288441	down_in_case
PRJNA8274(	ENSGALG00010004977	RNF217	case	control	-0.369556302	0.136942385	-2.698626155	0.006962634	0.145020586	down_in_case
PRJNA8274(	ENSGALG00010004980	FNDC1	case	control	-0.140333075	0.196146284	-0.715451105	0.474330343	0.82979716	down_in_case
PRJNA8274(	ENSGALG00010004981	ENSGALG00010004981	case	control	0.28570946	0.207752582	1.375239038	0.169057349	0.585038804	up_in_case
PRJNA8274(	ENSGALG00010004983	CLXN	case	control	0.237812238	0.19980586	1.190216532	0.233961297	0.658462221	up_in_case
PRJNA8274(	ENSGALG00010004987	MAP3K8	case	control	-0.158884964	0.135606326	-1.171663367	0.241332236	0.666243691	down_in_case
PRJNA8274(	ENSGALG00010004988	HJURP	case	control	0.020400068	0.096452859	0.211502992	0.832494798	0.962371901	up_in_case
PRJNA8274(	ENSGALG00010004991	FAM133B	case	control	-0.032283009	0.052081874	-0.619851151	0.535355789	0.857009451	down_in_case
PRJNA8274(	ENSGALG00010004993	SULT1B	case	control	0.147043913	0.181241145	0.811316394	0.417183998	0.799987395	up_in_case
PRJNA8274(	ENSGALG00010004995	PIT54	case	control	-0.455553753	1.248267464	-0.364948832			down_in_case
PRJNA8274(	ENSGALG00010004996	GPR158	case	control	0.412334418	0.266774861	1.545626963	0.122194686	0.520784563	up_in_case
PRJNA8274(	ENSGALG00010004999	ADCK1	case	control	0.0128728	0.14114505	0.091202629	0.927331586	0.986531794	up_in_case
PRJNA8274(	ENSGALG00010005003	EXPH5	case	control	0.976965916	0.622475724	1.569484363	0.11653512	0.51350396	up_in_case
PRJNA8274(	ENSGALG00010005004	CDK6	case	control	-0.04302388	0.090339168	-0.476248351	0.633897457	0.898107199	down_in_case
PRJNA8274(	ENSGALG00010005008	ENSGALG00010005008	case	control	1.870903194	1.392305127	1.343745102	0.179030827	0.599333225	up_in_case
PRJNA8274(	ENSGALG00010005010	NKAIN2	case	control	-0.622175683	0.231775566	-2.684388586	0.00726626	0.148651665	down_in_case
PRJNA8274(	ENSGALG00010005014	SULT1B1	case	control	0.158875466	0.122736943	1.294438843	0.195513881	0.618824368	up_in_case
PRJNA8274(	ENSGALG00010005015	NRXN3	case	control	0.407096799	0.353747025	1.150813351	0.249809031	0.675159543	up_in_case
PRJNA8274(	ENSGALG00010005016	STX11	case	control	0.048634174	0.161501042	0.301138455	0.763308918	0.943786458	up_in_case
PRJNA8274(	ENSGALG00010005017	ENSGALG00010005017	case	control	-0.029349927	0.149664451	-0.196104867	0.844528087	0.96721237	down_in_case
PRJNA8274(	ENSGALG00010005023	POGLUT3	case	control	-0.144590655	0.114217164	-1.265927554	0.205539021	0.628438742	down_in_case
PRJNA8274(	ENSGALG00010005025	THOC1	case	control	0.025152643	0.045661001	0.550856149	0.58173229	0.880290776	up_in_case
PRJNA8274(	ENSGALG00010005029	FAT4	case	control	-0.18415025	0.117677873	-1.564867257	0.117614038	0.514336617	down_in_case
PRJNA8274(	ENSGALG00010005033	USP14	case	control	-0.04822035	0.071609453	-0.673379664	0.500705783	0.840987941	down_in_case
PRJNA8274(	ENSGALG00010005035	NECTIN3	case	control	-0.045832605	0.076121452	-0.602098405	0.547108636	0.862585833	down_in_case
PRJNA8274(	ENSGALG00010005037	THNSL1	case	control	-0.050960378	0.148112729	-0.344064813	0.730797545	0.933770678	down_in_case
PRJNA8274(	ENSGALG00010005042	SLC46A3	case	control	0.10994862	0.176860523	0.62166852	0.534159855	0.856190278	up_in_case
PRJNA8274(	ENSGALG00010005045	UTRN	case	control	-0.083149881	0.073154469	-1.136634325	0.255691185	0.681937229	down_in_case
PRJNA8274(	ENSGALG00010005048	ROCK1	case	control	-0.092606389	0.065195069	-1.420450812	0.155476479	0.564463894	down_in_case
PRJNA8274(	ENSGALG00010005057	FLVCR2	case	control	0.145705074	0.168659743	0.86389954	0.387643079	0.782619799	up_in_case
PRJNA8274(	ENSGALG00010005060	POMP	case	control	0.007530015	0.045939424	0.163911832	0.869800552	0.974776481	up_in_case
PRJNA8274(	ENSGALG00010005065	ENKUR	case	control	-0.094968778	0.277337512	-0.342430338	0.732027061	0.933770678	down_in_case
PRJNA8274(	ENSGALG00010005068	PLCXD2	case	control	-0.344783558	0.1709551	-2.016807681	0.043715587	0.349041259	down_in_case
PRJNA8274(	ENSGALG00010005070	ALB	case	control	1.072691693	1.121148914	0.956778961			up_in_case
PRJNA8274(	ENSGALG00010005075	TRDN	case	control	-0.375079598	0.148594169	-2.524187852	0.011596594	0.188230756	down_in_case
PRJNA8274(	ENSGALG00010005077	ERG28	case	control	-0.031247144	0.097755806	-0.31964489	0.749237541	0.939503405	down_in_case
PRJNA8274(	ENSGALG00010005081	GREB1L	case	control	0.186439587	0.141390435	1.318615273	0.187297765	0.610800009	up_in_case
PRJNA8274(	ENSGALG00010005082	ENSGALG00010005082	case	control	0.017345494	0.051922104	0.33406762	0.738328533	0.935918627	up_in_case
PRJNA8274(	ENSGALG00010005087	ENPP1	case	control	-0.320570799	0.123955963	-2.586166825	0.009704995	0.176297435	down_in_case
PRJNA8274(	ENSGALG00010005092	ESCO1	case	control	-0.02057037	0.053228116	-0.38645684	0.699158361	0.922288441	down_in_case
PRJNA8274(	ENSGALG00010005104	ENPP3	case	control	-0.384162838	0.220697485	-1.740676106	0.081740369	0.454736389	down_in_case
PRJNA8274(	ENSGALG00010005107	TTLL5	case	control	-0.003124109	0.056049143	-0.055738747	0.955549932	0.992056045	down_in_case
PRJNA8274(	ENSGALG00010005109	ABHD3	case	control	0.23283213	0.220628177	1.055314572	0.291281479	0.714222439	up_in_case
PRJNA8274(	ENSGALG00010005111	RASSF6	case	control	0.176755065	0.336304808	0.525579952	0.599180089	0.884854486	up_in_case

PRJNA8274(	ENSGALG00010005114	PHLDB2	case	control	-0.122581529	0.068345625	-1.793553404	0.07288448	0.436712105	down_in_case
PRJNA8274(	ENSGALG00010005118	PRTFDC1	case	control	-0.141064561	0.088610395	-1.591964032	0.111392787	0.50408761	down_in_case
PRJNA8274(	ENSGALG00010005119	MIB1	case	control	0.040527531	0.046860281	0.86485891	0.387116233	0.782604263	up_in_case
PRJNA8274(	ENSGALG00010005123	MTIF3	case	control	-0.039678981	0.072948591	-0.543930734	0.586489139	0.881932581	down_in_case
PRJNA8274(	ENSGALG00010005126	MED23	case	control	0.003478198	0.045372211	0.076659218	0.938894648	0.989551456	up_in_case
PRJNA8274(	ENSGALG00010005127	GATA6	case	control	-0.129542612	0.183318331	-0.70665389	0.479781591	0.831851816	down_in_case
PRJNA8274(	ENSGALG00010005131	IL8L2	case	control	0.165446294	0.543079448	0.304644734	0.760636746	0.942883622	up_in_case
PRJNA8274(	ENSGALG00010005132	LNX2	case	control	-0.032088847	0.045959162	-0.698203491	0.485049943	0.834423421	down_in_case
PRJNA8274(	ENSGALG00010005138	ENSGALG00010005138	case	control	0.054311104	0.306408898	0.177250413	0.859311699	0.971828322	up_in_case
PRJNA8274(	ENSGALG00010005140	DCT	case	control	7.636065224	0.615837066	12.39948949	2.62994E-35	6.26802E-32	up_in_case
PRJNA8274(	ENSGALG00010005141	CCDC170	case	control	0.215065584	0.221307716	0.971794331	0.331152877	0.747393645	up_in_case
PRJNA8274(	ENSGALG00010005143	GTF3A	case	control	0.063896111	0.075540199	0.845855741	0.397633225	0.788728117	up_in_case
PRJNA8274(	ENSGALG00010005145	AKAP7	case	control	-0.373163042	0.105121673	-3.549820219	0.000385494	0.027155511	down_in_case
PRJNA8274(	ENSGALG00010005146	TGFB3	case	control	-0.001180091	0.078774819	-0.014980561	0.988047689	0.996942188	down_in_case
PRJNA8274(	ENSGALG00010005148	TTC39C	case	control	0.044293555	0.067205222	0.659079062	0.509845	0.8447206	up_in_case
PRJNA8274(	ENSGALG00010005150	ALCAM	case	control	-0.046983812	0.109501023	-0.429071904	0.667870898	0.912456967	down_in_case
PRJNA8274(	ENSGALG00010005154	CWF19L2	case	control	-0.118059968	0.070770987	-1.668197285	0.09527657	0.482284936	down_in_case
PRJNA8274(	ENSGALG00010005157	IMPA1	case	control	-0.00830091	0.081419446	-0.101952427	0.918794436	0.983734684	down_in_case
PRJNA8274(	ENSGALG00010005158	IFT43	case	control	0.02655623	0.146345942	0.181462017	0.856004944	0.970727256	up_in_case
PRJNA8274(	ENSGALG00010005159	ARHGAP21	case	control	0.047341695	0.047497122	0.996727656	0.318896722	0.738427335	up_in_case
PRJNA8274(	ENSGALG00010005163	GPC6	case	control	-0.421037104	0.339621069	-1.239726101	0.21507672	0.640404074	down_in_case
PRJNA8274(	ENSGALG00010005165	METTL21C	case	control	0.47947659	0.653629362	0.73356036	0.463216728	0.825091817	up_in_case
PRJNA8274(	ENSGALG00010005166	CBLB	case	control	-0.025027513	0.056942373	-0.439523532	0.660282235	0.909188753	down_in_case
PRJNA8274(	ENSGALG00010005167	GUCY1A2	case	control	-0.135235945	0.12716309	-1.063484261	0.287562399	0.71132024	down_in_case
PRJNA8274(	ENSGALG00010005170	GPATCH2L	case	control	3.60163E-05	0.076351098	0.000471719	0.999623622	0.99976345	up_in_case
PRJNA8274(	ENSGALG00010005172	OSBPL1A	case	control	-0.146115538	0.098705036	-1.48032506	0.138786519	0.540932258	down_in_case
PRJNA8274(	ENSGALG00010005174	TPP2	case	control	-0.032937439	0.046554899	-0.707496741	0.479257837	0.831519907	down_in_case
PRJNA8274(	ENSGALG00010005176	BAMBI	case	control	0.450527468	0.462287389	0.974561451	0.329777843	0.746174551	up_in_case
PRJNA8274(	ENSGALG00010005177	AASDHPPT	case	control	0.015581786	0.144785107	0.107620091	0.91429706	0.982854306	up_in_case
PRJNA8274(	ENSGALG00010005178	EPHA5	case	control	0.050128606	0.104423485	0.480051077	0.631191074	0.897039706	up_in_case
PRJNA8274(	ENSGALG00010005180	WAC	case	control	-0.100911849	0.040805674	-2.472985734	0.013398949	0.203413453	down_in_case
PRJNA8274(	ENSGALG00010005181	IMPACT	case	control	-0.032085328	0.102602434	-0.312715082	0.754497136	0.941311206	down_in_case
PRJNA8274(	ENSGALG00010005182	NEPRO	case	control	0.182688409	0.095969948	1.90360016	0.056962278	0.388573099	up_in_case
PRJNA8274(	ENSGALG00010005184	FGF14	case	control	-0.360890652	0.240723085	-1.499194197	0.13382326	0.537548489	down_in_case
PRJNA8274(	ENSGALG00010005186	ESR1	case	control	-0.281304948	0.158587479	-1.773815632	0.076093652	0.442710866	down_in_case
PRJNA8274(	ENSGALG00010005187	KBTBD3	case	control	-0.154717504	0.229517522	-0.674098876	0.500248454	0.840855399	down_in_case
PRJNA8274(	ENSGALG00010005190	ENSGALG00010005190	case	control	-0.313546766	0.262787441	-1.193157348	0.232807755	0.657934961	down_in_case
PRJNA8274(	ENSGALG00010005191	MSANTD4	case	control	0.065975565	0.068351925	0.965233466	0.334427888	0.748686141	up_in_case
PRJNA8274(	ENSGALG00010005193	EPB41L2	case	control	-0.068377387	0.074795378	-0.914192684	0.360615608	0.766468964	down_in_case
PRJNA8274(	ENSGALG00010005194	ENSGALG00010005194	case	control	4.517715568	0.822586081	5.492088517	3.97208E-08	1.62288E-05	up_in_case
PRJNA8274(	ENSGALG00010005198	TRPC3	case	control	0.173511338	0.232646336	0.745815905	0.455778631	0.822065071	up_in_case
PRJNA8274(	ENSGALG00010005203	ENSGALG00010005203	case	control	-0.067740899	0.078870078	-0.858892248	0.390399966	0.783914628	down_in_case
PRJNA8274(	ENSGALG00010005204	ITGBL1	case	control	0.195899606	0.285877669	0.685256762	0.493181911	0.837887766	up_in_case
PRJNA8274(	ENSGALG00010005208	ENSGALG00010005208	case	control	0.024364336	0.093951464	0.259328962	0.795381435	0.95280257	up_in_case
PRJNA8274(	ENSGALG00010005211	ESRRB	case	control	-0.00247519	0.166964373	-0.014824658	0.988172068	0.996942188	down_in_case
PRJNA8274(	ENSGALG00010005212	TAF13	case	control	-0.040887315	0.073245637	-0.558221853	0.5766929	0.878085957	down_in_case
PRJNA8274(	ENSGALG00010005213	ENSGALG00010005213	case	control	-0.181693576	0.134222373	-1.353675782	0.175839838	0.594727928	down_in_case
PRJNA8274(	ENSGALG00010005214	BBS7	case	control	-0.150304166	0.063637949	-2.361863769	0.018183323	0.241260842	down_in_case
PRJNA8274(	ENSGALG00010005215	VASH1	case	control	0.026353503	0.063770713	0.413254009	0.679420522	0.915351915	up_in_case
PRJNA8274(	ENSGALG00010005216	ENSGALG00010005216	case	control	-0.084545159	0.153728974	-0.549962424	0.582345147	0.880661548	down_in_case
PRJNA8274(	ENSGALG00010005217	CCNA2	case	control	-0.033911164	0.101505303	-0.334082686	0.738317165	0.935918627	down_in_case
PRJNA8274(	ENSGALG00010005218	GTPBP8	case	control	-0.072640687	0.092453236	-0.78570194	0.432042126	0.807390038	down_in_case
PRJNA8274(	ENSGALG00010005219	NALCN	case	control	-0.314439057	0.167565277	-1.8765168	0.06058435	0.400164529	down_in_case
PRJNA8274(	ENSGALG00010005220	ANGEL1	case	control	-0.049119652	0.059794923	-0.821468606	0.411379399	0.796793365	down_in_case
PRJNA8274(	ENSGALG00010005221	ENSGALG00010005221	case	control	-0.679013068	0.389020076	-1.745444798	0.080907473	0.453075204	down_in_case
PRJNA8274(	ENSGALG00010005223	GRIA4	case	control	0.011878804	0.269132981	0.044137303	0.964794958	0.992291928	up_in_case
PRJNA8274(	ENSGALG00010005226	MYCT1	case	control	-0.009342573	0.070746032	-0.132057905	0.894938491	0.980176778	down_in_case
PRJNA8274(	ENSGALG00010005227	EXOSC9	case	control	-0.004940456	0.072689697	-0.067966385	0.945812393	0.990313016	down_in_case
PRJNA8274(	ENSGALG00010005228	ZNF521	case	control	-0.018629089	0.082161028	-0.226738752	0.820626892	0.958669267	down_in_case
PRJNA8274(	ENSGALG00010005229	CIPC	case	control	0.058519923	0.071514414	0.818295491	0.41318848	0.797633779	up_in_case
PRJNA8274(	ENSGALG00010005231	ENSGALG00010005231	case	control	-0.959434152	0.337368095	-2.843879334	0.004456792	0.114389963	down_in_case
PRJNA8274(	ENSGALG00010005232	GATA3	case	control	0.090770571	0.47697968	0.190302804	0.849071856	0.968833396	up_in_case
PRJNA8274(	ENSGALG00010005236	TMTC4	case	control	0.080009417	0.096460206	0.829455178	0.40684689	0.794253996	up_in_case
PRJNA8274(	ENSGALG00010005237	RAP2A	case	control	-0.009611994	0.074747172	-0.12859342	0.897679373	0.980176778	down_in_case
PRJNA8274(	ENSGALG00010005238	TMEM63C	case	control	-0.100552395	0.094386235	-1.065329026	0.286727062	0.710659943	down_in_case
PRJNA8274(	ENSGALG00010005239	ENSGALG00010005239	case	control	-0.496199255	0.228198468	-2.174419749	0.029673626	0.305970455	down_in_case
PRJNA8274(	ENSGALG00010005240	VIP	case	control	0.23164503	0.163624807	1.415708502	0.156860868	0.567595301	up_in_case
PRJNA8274(	ENSGALG00010005241	C4orf54	case	control	-0.064173867	0.114527411	-0.560336317	0.57525006	0.877910508	down_in_case
PRJNA8274(	ENSGALG00010005242	CCDC80	case	control	-0.03644756	0.106661298	-0.341713079	0.732566828	0.933846596	down_in_case
PRJNA8274(	ENSGALG00010005243	NGB	case	control	-0.008918476	0.472636519	-0.018869629	0.984945108	0.996591771	down_in_case
PRJNA8274(	ENSGALG00010005244	FBXO5	case	control	-0.064399983	0.089071769	-0.723012278	0.469672341	0.829101076	down_in_case
PRJNA8274(	ENSGALG00010005245	KIAA1217	case	control	-0.108991826	0.053469454	-2.038394224	0.041510523	0.340562528	down_in_case
PRJNA8274(	ENSGALG00010005246	ENSGALG00010005246	case	control	0.149621219	0.150491512	0.994216995	0.320117236	0.738813491	up_in_case
PRJNA8274(	ENSGALG00010005247	THAP7	case	control	-0.173125603	0.060530291	-2.860148217	0.004234431	0.112622544	down_in_case
PRJNA8274(	ENSGALG00010005248	TMEM200A	case	control	-0.001610274	0.093279391	-0.017262913	0.986226873	0.996597442	down_in_case
PRJNA8274(	ENSGALG00010005249	PDGFD	case	control	-0.097174157	0.121842943	-0.797536194	0.425139691	0.804485588	down_in_case
PRJNA8274(	ENSGALG00010005250	SLC35A5	case	control	0.162596449	0.060418667	2.691162475	0.007120351	0.146516526	up_in_case
PRJNA8274(	ENSGALG00010005251	MTRF1L	case	control	-0.11459897	0.093330517	-1.22788315	0.219490836	0.643443819	down_in_case
PRJNA8274(	ENSGALG00010005252	KLHL14	case	control	-0.060953016	0.144585043	-0.421572068	0.673337398	0.91379937	down_in_case
PRJNA8274(	ENSGALG00010005253	POMT2	case	control	-0.03142126	0.067502772	-0.465481024	0.641587023	0.901539099	down_in_case
PRJNA8274(	ENSGALG00010005254	ENSGALG00010005254	case	control	0.524275536	0.539390088	0.971978438	0.331061275	0.747305731	up_in_case
PRJNA8274(	ENSGALG00010005255	BZW2	case	control	-0.081742324	0.066447185	-1.230184903	0.218627872	0.643443819	down_in_case
PRJNA8274(	ENSGALG00010005256	OPRM1	case	control	0.563021992	0.441761475	1.274493191	0.202488684	0.62541643	up_in_case
PRJNA8274(	ENSGALG00010005257	SAMD3	case	control	0.421342561	0.491055358	0.858034748	0.390873276	0.78415935	up_in_case
PRJNA8274(	ENSGALG00010005258	ITIH5	case	control	-0.323941764	0.193117136	-1.677436666	0.093457124	0.478523919	down_in_case
PRJNA8274(	ENSGALG00010005259	SLC27A5	case	control	1.08717756	0.90182349	1.205532537	0.227997785	0.651681204	up_in_case
PRJNA8274(	ENSGALG00010005260	ANXA5	case	control	-0.061398893	0.057636754	-1.065273254	0.286752292	0.710659943	down_in_case
PRJNA8274(	ENSGALG00010005261	ATG3	case	control	-0.066380205	0.053261301	-1.246312111	0.212649833	0.638306594	down_in_case
PRJNA8274(	ENSGALG00010005262	ENSGALG00010005262	case	control	-0.006108664	0.088483756	-0.069037126	0.944960068	0.990313016	down_in_case
PRJNA8274(	ENSGALG00010005263	ENSGALG00010005263	case	control	-0.77711859	0.35310992	-2.200783794	0.027751333	0.297038973	down_in_case
PRJNA8274(	ENSGALG00010005264	ITIH2	case	control	0.987939341	0.813992148	1.213696402	0.224863679	0.648819735	up_in_case
PRJNA8274(	ENSGALG00010005265	DYNC2H1	case	control	-0.16434091	0.069900944	-2.35105422	0.018720306	0.244475226	down_in_case
PRJNA8274(	ENSGALG00010005266	SLC19A2	case	control	0.127559371	0.169440415	0.752827307	0.451553695	0.820302668	up_in_case
PRJNA8274(	ENSGALG00010005267	CYP51A1	case	control	0.254629296	0.129366273	1.96828192	0.049035609	0.36379167	up_in_case
PRJNA8274(	ENSGALG00010005268	MTTP	case	control	0.465826221	0.62375276	0.74681228	0.455176882	0.821701039	up_in_case
PRJNA8274(	ENSGALG00010005269	KIN	case	control	-0.016930033	0.064208791	-0.26367157	0.792033005	0.952571233	down_in_case
PRJNA8274(	ENSGALG00010005270	GSTZ1	case	control	0.070969159	0.144020236	0.492772132	0.622173604	0.893819825	up_in_case
PRJNA8274(	ENSGALG00010005272	MBNL2	case	control	-0.15932231	0.079493376	-2.004221218	0.045046369	0.352291176	down_in_case
PRJNA8274(	ENSGALG00010005273	ENSGALG00010005273	case	control	-0.903352997	0.381331687	-2.368942913	0.017839007	0.237964362	down_in_case
PRJNA8274(	ENSGALG00010005275	L3MBTL3	case	control	-0.003219774	0.072051321	-0.044687236	0.964356608	0.992068146	down_in_case
PRJNA8274(	ENSGALG00010005277	ENSGALG00010005277	case	control	0.029198253	0.188435717	0.154950735	0.876860156	0.975813349	up_in_case
PRJNA8274(	ENSGALG00010005278	DAPP1	case	control	-0.113329168	0.111865716	-1.013082228	0.311020886	0.733806083	down_in_case
PRJNA8274(	ENSGALG00010005279	CCDC181	case	control	-0.363891146	0.350775046	-1.03739177	0.299553312	0.722972551	down_in_case
PRJNA8274(	ENSGALG00010005280	TMEM244	case	control	0.145495681	0.426076918	0.3414775	0.73274414	0.933846596	up_in_case
PRJNA8274(	ENSGALG00010005281	TMED8	case	control	0.069129047	0.046396911	1.489949338	0.136237557	0.539840644	up_in_case
PRJNA8274(	ENSGALG00010005282	FLT1	case	control	-0.077299004	0.095481052	-0.809574281	0.418184895	0.800393647	down_in_case
PRJNA8274(	ENSGALG00010005283	ATP5C1	case	control	-0.072665602	0.073549047	-0.987988355	0.323158352	0.740689923	down_in_case
PRJNA8274(	ENSGALG00010005284	ENSGALG00010005284	case	control	-0.064404853	0.188583523	-0.341518984	0.732712916	0.933846596	down_in_case
PRJNA8274(	ENSGALG00010005285	MPP7	case	control	-0.065169535	0.110214057	-0.591299666	0.554319653	0.866646786	down_in_case
PRJNA8274(	ENSGALG00010005286	ENSGALG00010005286	case	control	1.411840463	0.538606527	2.621283614	0.008759935	0.166357336	up_in_case
PRJNA8274(	ENSGALG00010005287	ENSGALG00010005287	case	control	-0.005948504	0.339344225	-0.017529409	0.986014271	0.996597442	down_in_case
PRJNA8274(	ENSGALG00010005288	ENSGALG00010005288	case	control	0.154802982	0.067864351	2.281064809	0.022544612	0.2670335	up_in_case
PRJNA8274(	ENSGALG00010005291	IFNGR1	case	control	-0.123191968	0.094215296	-1.307558036	0.191023258	0.614227329	down_in_case
PRJNA8274(	ENSGALG00010005292	TAF3	case	control	-0.099668255	0.051208336	-1.946328724	0.051615265	0.371650702	down_in_case
PRJNA8274(	ENSGALG00010005293	BLZF1	case	control	-0.037195251	0.082285308	-0.452027851	0.651248918	0.904951854	down_in_case
PRJNA8274(	ENSGALG00010005295	ENSGALG00010005295	case	control	0.41070968	0.349386964	1.175515181	0.239788667	0.664684583	up_in_case
PRJNA8274(	ENSGALG00010005296	PAN3	case	control	-0.015319508	0.061793701	-0.24791374	0.804201149	0.954997212	down_in_case
PRJNA8274(	ENSGALG00010005297	IL22RA2	case	control	-0.514754078	0.391879576	-1.313551686	0.188997117	0.611783722	down_in_case
PRJNA8274(	ENSGALG00010005299	ODAD2	case	control	0.248305002	0.459797513	0.540031197	0.589175518	0.882949889	up_in_case
PRJNA8274(	ENSGALG00010005301	ENSGALG00010005301	case	control	-0.0808145	0.411790638	-0.196251426	0.844413378	0.96721237	down_in_case
PRJNA8274(	ENSGALG00010005302	PSPC1	case	control	-0.126522315	0.048627386	-2.601873681	0.0092716	0.172410759	down_in_case
PRJNA8274(	ENSGALG00010005303	ENSGALG00010005303	case	control	-0.509342758	0.309716532	-1.644544946	0.100063688	0.489464811	down_in_case
PRJNA8274(	ENSGALG00010005304	ENSGALG00010005304	case	control	0.246469784	0.15058504	1.636748139	0.101683106	0.491460867	up_in_case
PRJNA8274(	ENSGALG00010005305	SCAF8	case	control	-0.116614163	0.046347822	-2.516065627	0.011867308	0.190891461	down_in_case
PRJNA8274(	ENSGALG00010005307	TIAM2	case	control	0.103819835	0.089411367	1.161148049	0.245581698	0.670194327	up_in_case
PRJNA8274(	ENSGALG00010005309	ASXL3	case	control	0.01474768	0.072532824	0.203324223	0.838881624	0.965176451	up_in_case

PRJNA8274(	ENSGALG00010005310	LAMTOR3	case	control	-0.061570417	0.068108192	-0.904008975	0.365990642	0.771585756	down_in_case
PRJNA8274(	ENSGALG00010005311	YME1L1	case	control	-0.003217795	0.037672333	-0.085415327	0.931931208	0.98752251	down_in_case
PRJNA8274(	ENSGALG00010005313	MPHOSPH8	case	control	-0.033629565	0.058224553	-0.577583912	0.563545071	0.871916948	down_in_case
PRJNA8274(	ENSGALG00010005314	IL20RA	case	control	0.681009417	0.455696428	1.494436594	0.135061543	0.539774365	up_in_case
PRJNA8274(	ENSGALG00010005315	ENSGALG00010005315	case	control	-0.278798194	0.247036033	-1.128572989	0.25907801	0.686345498	down_in_case
PRJNA8274(	ENSGALG00010005316	PARP4	case	control	-0.125717498	0.066246419	-1.897725207	0.05773229	0.391451754	down_in_case
PRJNA8274(	ENSGALG00010005317	CHCHD3	case	control	-0.095090939	0.056449365	-1.684535129	0.092078296	0.476713154	down_in_case
PRJNA8274(	ENSGALG00010005318	SLC35D3	case	control	-0.128113511	0.350353984	-0.365668773	0.71461225	0.926862121	down_in_case
PRJNA8274(	ENSGALG00010005319	NOL4	case	control	-0.546727365	0.673419335	-0.811867637	0.416867586	0.799987395	down_in_case
PRJNA8274(	ENSGALG00010005320	ENSGALG00010005320	case	control	0.847520946	0.401165254	2.112647936	0.034630913	0.321155674	up_in_case
PRJNA8274(	ENSGALG00010005321	HS6ST3	case	control	-0.067438063	0.268484745	-0.25118024	0.801674761	0.954294696	down_in_case
PRJNA8274(	ENSGALG00010005322	TFB1M	case	control	0.079717495	0.120502528	0.661542094	0.508264729	0.843702533	up_in_case
PRJNA8274(	ENSGALG00010005323	DNAJB14	case	control	-0.035944278	0.059485735	-0.604250385	0.545677187	0.862133047	down_in_case
PRJNA8274(	ENSGALG00010005324	CENPJ	case	control	0.01594002	0.140916362	0.113116888	0.909937886	0.981730793	up_in_case
PRJNA8274(	ENSGALG00010005326	MASTL	case	control	-0.192360674	0.095120342	-2.022287457	0.043146664	0.346433066	down_in_case
PRJNA8274(	ENSGALG00010005329	VIPAS39	case	control	-0.019669782	0.064121893	-0.306756103	0.759029023	0.942078947	down_in_case
PRJNA8274(	ENSGALG00010005330	ENSGALG00010005330	case	control	-0.033661623	0.403897573	-0.083341978	0.933579623	0.988097743	down_in_case
PRJNA8274(	ENSGALG00010005332	ENSGALG00010005332	case	control	0.248026143	0.414443758	0.598455491	0.549536049	0.864234145	up_in_case
PRJNA8274(	ENSGALG00010005333	PLXNA4	case	control	-0.167608738	0.169032231	-0.991578568	0.321403148	0.73942679	down_in_case
PRJNA8274(	ENSGALG00010005334	NME7	case	control	0.177781633	0.106814713	1.664392745	0.096033969	0.483381118	up_in_case
PRJNA8274(	ENSGALG00010005335	AHSA1	case	control	0.012265558	0.073801721	0.166196096	0.868002631	0.974212199	up_in_case
PRJNA8274(	ENSGALG00010005336	PEX7	case	control	0.13734433	0.099955231	1.37405845	0.169423537	0.585206904	up_in_case
PRJNA8274(	ENSGALG00010005337	DCTN1	case	control	0.200106118	0.641382798	0.311991714	0.755046821	0.941559531	up_in_case
PRJNA8274(	ENSGALG00010005338	PCGF1	case	control	0.066261183	0.096943015	0.683506528	0.494286827	0.83803731	up_in_case
PRJNA8274(	ENSGALG00010005340	ENSGALG00010005340	case	control	-0.09267284	0.696151827	-0.133121593	0.894097219	0.979888889	down_in_case
PRJNA8274(	ENSGALG00010005341	UGGT2	case	control	-0.10972347	0.093466012	-1.173939788	0.240419144	0.66577879	down_in_case
PRJNA8274(	ENSGALG00010005342	MAP3K5	case	control	-0.015803012	0.098899028	-0.159789357	0.873047008	0.975062028	down_in_case
PRJNA8274(	ENSGALG00010005344	ENSGALG00010005344	case	control	1.230065308	0.466160796	2.638714619	0.0083221	0.160448204	up_in_case
PRJNA8274(	ENSGALG00010005345	NOX3	case	control	2.197689627	0.500987817	4.386712718	1.15077E-05	0.001959042	up_in_case
PRJNA8274(	ENSGALG00010005346	FBXO30	case	control	-0.022380314	0.082633489	-0.270838299	0.786515402	0.950331242	down_in_case
PRJNA8274(	ENSGALG00010005347	DOK1	case	control	0.17736125	0.098931442	1.792769274	0.073009825	0.436837025	up_in_case
PRJNA8274(	ENSGALG00010005349	ACBD5	case	control	-0.118176307	0.088565332	-1.334340473	0.182092299	0.603068611	down_in_case
PRJNA8274(	ENSGALG00010005350	ARHGAP20	case	control	-0.479843515	0.193415833	-2.480890568	0.01310546	0.200730816	down_in_case
PRJNA8274(	ENSGALG00010005351	DTNA	case	control	-0.429230597	0.109882473	-3.906269912	9.37318E-05	0.010422294	down_in_case
PRJNA8274(	ENSGALG00010005353	ENSGALG00010005353	case	control	-0.025323095	0.055300741	-0.457916012	0.647012783	0.90295498	down_in_case
PRJNA8274(	ENSGALG00010005354	M1AP	case	control	0.185571119	0.216242549	0.858161913	0.390803064	0.784128499	up_in_case
PRJNA8274(	ENSGALG00010005355	FDX1	case	control	-0.046864278	0.089011544	-0.526496628	0.598543194	0.88476024	down_in_case
PRJNA8274(	ENSGALG00010005356	DNAJC3	case	control	0.030355879	0.03943838	0.769703994	0.4414755	0.813334147	up_in_case
PRJNA8274(	ENSGALG00010005358	SHPRH	case	control	-0.049790645	0.06607113	-0.753591544	0.451094523	0.820067593	down_in_case
PRJNA8274(	ENSGALG00010005359	MEIS2	case	control	-0.228576566	0.127805866	-1.788466945	0.073700704	0.437929428	down_in_case
PRJNA8274(	ENSGALG00010005360	CAMK1D	case	control	0.015806705	0.160789333	0.098306927	0.921688578	0.985482967	up_in_case
PRJNA8274(	ENSGALG00010005361	ENSGALG00010005361	case	control	0.699755653	0.474023011	1.476206084	0.139888577	0.542025835	up_in_case
PRJNA8274(	ENSGALG00010005362	ATP1B1	case	control	-0.267822583	0.074047404	-3.61690714	0.000298144	0.023376995	down_in_case
PRJNA8274(	ENSGALG00010005363	RDX	case	control	-0.082692436	0.048236289	-1.714320008	0.08646997	0.464682667	down_in_case
PRJNA8274(	ENSGALG00010005364	MAP7	case	control	0.562495155	0.393637447	1.42896759	0.153013549	0.561193576	up_in_case
PRJNA8274(	ENSGALG00010005366	AUP1	case	control	0.036224417	0.053694908	0.674634114	0.499908254	0.840669773	up_in_case
PRJNA8274(	ENSGALG00010005367	H2AZ1	case	control	-0.08889939	0.051915554	-1.712384493	0.086825835	0.465853669	down_in_case
PRJNA8274(	ENSGALG00010005368	CCDC3	case	control	-0.076209793	0.145898675	-0.522347395	0.601428476	0.884954589	down_in_case
PRJNA8274(	ENSGALG00010005369	DPT	case	control	-0.382676142	0.170211147	-2.248243715	0.024560654	0.277422864	down_in_case
PRJNA8274(	ENSGALG00010005372	CDIN1	case	control	-0.16410225	0.099606083	-1.647512341	0.099452782	0.488584926	down_in_case
PRJNA8274(	ENSGALG00010005376	HTRA2	case	control	-0.008484642	0.057938187	-0.146443003	0.883571682	0.977115076	down_in_case
PRJNA8274(	ENSGALG00010005377	EMCN	case	control	-0.007574093	0.064894215	-0.116714457	0.907086333	0.981730793	down_in_case
PRJNA8274(	ENSGALG00010005378	ENSGALG00010005378	case	control	0.506167989	0.670978002	0.754373449	0.45062501	0.819735103	up_in_case
PRJNA8274(	ENSGALG00010005379	DZIP1	case	control	0.091826849	0.083799841	1.095787868	0.273171619	0.698936152	up_in_case
PRJNA8274(	ENSGALG00010005381	FGF9	case	control	0.570305383	0.188471084	3.025956927	0.002478476	0.081856092	up_in_case
PRJNA8274(	ENSGALG00010005382	RAB32	case	control	0.077357298	0.10389055	0.744603794	0.456511274	0.822283816	up_in_case
PRJNA8274(	ENSGALG00010005383	OPTN	case	control	-0.022928814	0.053435516	-0.429093162	0.667855429	0.912456967	down_in_case
PRJNA8274(	ENSGALG00010005384	MAPRE2	case	control	0.033242284	0.060602232	0.548532341	0.583326429	0.881306702	up_in_case
PRJNA8274(	ENSGALG00010005385	PDSS1	case	control	-0.02389429	0.085589205	-0.279174103	0.780111217	0.948864508	down_in_case
PRJNA8274(	ENSGALG00010005386	CPOX	case	control	0.262570656	0.066339618	3.95797658	7.55874E-05	0.009001807	up_in_case
PRJNA8274(	ENSGALG00010005387	CLDN10	case	control	-0.822628576	0.570369967	-1.4422719	0.149225684	0.555854983	down_in_case
PRJNA8274(	ENSGALG00010005388	ENSGALG00010005388	case	control	-0.259330589	0.442792293	-0.585670963	0.558096651	0.869085619	down_in_case
PRJNA8274(	ENSGALG00010005389	MCM10	case	control	0.082956478	0.115211445	0.720036787	0.471502346	0.829101076	up_in_case
PRJNA8274(	ENSGALG00010005391	ADGB	case	control	0.240118983	0.332479225	0.72220748	0.470166925	0.829101076	up_in_case
PRJNA8274(	ENSGALG00010005392	ENSGALG00010005392	case	control	-0.193425986	0.113332354	-1.706714626	0.087875107	0.46853618	down_in_case
PRJNA8274(	ENSGALG00010005394	PHYH	case	control	-0.07773662	0.095466504	-0.814281627	0.415483633	0.799372288	down_in_case
PRJNA8274(	ENSGALG00010005395	ENSGALG00010005395	case	control	0.455280713	0.306808714	1.483923671	0.137829171	0.539854583	up_in_case
PRJNA8274(	ENSGALG00010005396	MICU2	case	control	-0.140640245	0.071606219	-1.964078644	0.049520972	0.364833243	down_in_case
PRJNA8274(	ENSGALG00010005398	SEPHS1	case	control	-0.207155618	0.086560544	-2.39318756	0.0167027	0.229833552	down_in_case
PRJNA8274(	ENSGALG00010005399	NDNF	case	control	0.163060671	0.304717385	0.535120998	0.592566191	0.883604289	up_in_case
PRJNA8274(	ENSGALG00010005401	BCLAF1	case	control	-0.015160187	0.03992462	-0.379720257	0.704153081	0.923966697	down_in_case
PRJNA8274(	ENSGALG00010005402	ABCC4	case	control	0.183956066	0.124221047	1.480876791	0.138639409	0.540932258	up_in_case
PRJNA8274(	ENSGALG00010005403	ENSGALG00010005403	case	control	0.126443117	0.145405179	0.869591565	0.384523649	0.780971676	up_in_case
PRJNA8274(	ENSGALG00010005404	BEND7	case	control	-0.113932027	0.072185621	-1.57832024	0.114492058	0.509446069	down_in_case
PRJNA8274(	ENSGALG00010005405	CEBPD	case	control	-0.1503851	0.167708526	-0.896705155	0.369876269	0.772544067	down_in_case
PRJNA8274(	ENSGALG00010005407	DPH6	case	control	0.086549944	0.117978911	0.733605213	0.463189383	0.825091817	up_in_case
PRJNA8274(	ENSGALG00010005408	APBB1IP	case	control	-0.003114672	0.15562244	-0.020014286	0.984031976	0.996591771	down_in_case
PRJNA8274(	ENSGALG00010005409	PRKCQ	case	control	-0.052420525	0.158224671	-0.331304371	0.74041459	0.936901924	down_in_case
PRJNA8274(	ENSGALG00010005411	STXBP5	case	control	-0.00107362	0.076926437	-0.013956445	0.988864729	0.997164011	down_in_case
PRJNA8274(	ENSGALG00010005412	MTFR2	case	control	-0.143841972	0.094173868	-1.527408557	0.12665945	0.526979964	down_in_case
PRJNA8274(	ENSGALG00010005413	GAD2	case	control	0.279921937	0.293112048	0.954999765	0.339577816	0.751259351	up_in_case
PRJNA8274(	ENSGALG00010005414	ENSGALG00010005414	case	control	-0.455753167	0.176584802	-2.580930876	0.009853431	0.177161817	down_in_case
PRJNA8274(	ENSGALG00010005415	PRKDC	case	control	-0.065630529	0.064681556	-1.014671463	0.310262461	0.733226442	down_in_case
PRJNA8274(	ENSGALG00010005416	PPP3CA	case	control	-0.23061664	0.079932041	-2.885158913	0.003912161	0.107773305	down_in_case
PRJNA8274(	ENSGALG00010005417	LOXL3	case	control	0.188012783	0.152940555	1.229319344	0.218952098	0.643443819	up_in_case
PRJNA8274(	ENSGALG00010005419	ENSGALG00010005419	case	control	1.226330769	0.444274446	2.760300034	0.00577483	0.132339851	up_in_case
PRJNA8274(	ENSGALG00010005420	DHRS4	case	control	0.061665085	0.060491357	1.019403241	0.308011568	0.731290955	up_in_case
PRJNA8274(	ENSGALG00010005421	ENSGALG00010005421	case	control	-0.294026056	0.219853548	-1.337372352	0.181101125	0.601788276	down_in_case
PRJNA8274(	ENSGALG00010005423	ST3GAL6	case	control	-0.116553091	0.101732989	-1.14567646	0.251929047	0.677148133	down_in_case
PRJNA8274(	ENSGALG00010005424	ENSGALG00010005424	case	control	0.052228151	0.122954545	0.42477609	0.670999909	0.913676733	up_in_case
PRJNA8274(	ENSGALG00010005425	ELAPOR2	case	control	0.022913628	0.151045975	0.151699693	0.879423799	0.976318821	up_in_case
PRJNA8274(	ENSGALG00010005427	ENSGALG00010005427	case	control	0.04096531	0.098048515	0.417806533	0.676088574	0.914247368	up_in_case
PRJNA8274(	ENSGALG00010005428	ZNF770	case	control	0.06047199	0.064261874	0.941024367	0.346692372	0.757096367	up_in_case
PRJNA8274(	ENSGALG00010005429	ZDHHC20	case	control	-0.085785763	0.076346681	-1.123634467	0.261168128	0.687516348	down_in_case
PRJNA8274(	ENSGALG00010005430	MCM4	case	control	-0.041852806	0.067959993	-0.615844767	0.537996978	0.857740557	down_in_case
PRJNA8274(	ENSGALG00010005431	GRM3	case	control	-1.116491724	0.620038885	-1.800680168	0.071753305	0.432759285	down_in_case
PRJNA8274(	ENSGALG00010005433	GPC5	case	control	-0.18688057	0.241151177	-0.774951929	0.438368066	0.8116876	down_in_case
PRJNA8274(	ENSGALG00010005434	PRDM5	case	control	0.089652821	0.084512018	1.060829249	0.288767507	0.712801679	up_in_case
PRJNA8274(	ENSGALG00010005435	MAD2L1	case	control	0.024670226	0.071041912	0.347262975	0.728393764	0.933057319	up_in_case
PRJNA8274(	ENSGALG00010005436	AQR	case	control	-0.063725622	0.067873055	-0.938894256	0.347785038	0.758360178	down_in_case
PRJNA8274(	ENSGALG00010005437	PDE7B	case	control	-0.297511193	0.104193671	-2.85536723	0.004298709	0.113207259	down_in_case
PRJNA8274(	ENSGALG00010005438	GPR180	case	control	-0.00172427	0.092773786	-0.018585746	0.985171574	0.996591771	down_in_case
PRJNA8274(	ENSGALG00010005439	TBX19	case	control	-0.317971296	0.399056926	-0.796806859	0.425563211	0.804804048	down_in_case
PRJNA8274(	ENSGALG00010005441	UBE2V2	case	control	-0.125884795	0.048829657	-2.578039723	0.009936258	0.177161817	down_in_case
PRJNA8274(	ENSGALG00010005442	SASH1	case	control	-0.021585622	0.064052141	-0.337000793	0.736116302	0.934439691	down_in_case
PRJNA8274(	ENSGALG00010005443	BANK1	case	control	0.144104537	0.443092915	0.325224195	0.745011398	0.937849656	up_in_case
PRJNA8274(	ENSGALG00010005444	ACTC1	case	control	0.01998173	0.154132705	0.129639778	0.896851431	0.980176778	up_in_case
PRJNA8274(	ENSGALG00010005445	SFT2D2	case	control	0.113195067	0.092280272	1.226644271	0.219956322	0.644148148	up_in_case
PRJNA8274(	ENSGALG00010005446	ENSGALG00010005446	case	control	-0.466602386	0.385490556	-1.210411976	0.226120849	0.649563707	down_in_case
PRJNA8274(	ENSGALG00010005447	MYO3A	case	control	-0.154518152	0.19371382	-0.797661997	0.425066663	0.804485588	down_in_case
PRJNA8274(	ENSGALG00010005448	ENSGALG00010005448	case	control	-0.017548457	0.045292851	-0.387444301	0.698427313	0.922288441	down_in_case
PRJNA8274(	ENSGALG00010005449	TIPRL	case	control	-0.051922821	0.054948409	-0.944937668	0.344690699	0.755870938	down_in_case
PRJNA8274(	ENSGALG00010005450	ENSGALG00010005450	case	control	-0.371024887	0.205876013	-1.802176373	0.071517661	0.432432368	down_in_case
PRJNA8274(	ENSGALG00010005453	UST	case	control	-0.099992748	0.050478032	-1.98091611	0.047600682	0.359816265	down_in_case
PRJNA8274(	ENSGALG00010005454	GPR161	case	control	0.032093426	0.061940064	0.518136783	0.604362838	0.885268908	up_in_case
PRJNA8274(	ENSGALG00010005455	ENSGALG00010005455	case	control	-0.860459513	0.438457248	-1.962470724	0.049707706	0.36489743	down_in_case
PRJNA8274(	ENSGALG00010005456	RPL7L1	case	control	0.056784645	0.055249988	1.027776594	0.304054929	0.726844782	up_in_case
PRJNA8274(	ENSGALG00010005457	SEMA3D	case	control	-0.141323222	0.106962022	-1.321246732	0.186419109	0.609851564	down_in_case
PRJNA8274(	ENSGALG00010005458	PRKD1	case	control	0.025492484	0.070739326	0.36037216	0.718568843	0.927813495	up_in_case
PRJNA8274(	ENSGALG00010005459	ZC3H12D	case	control	0.534330384	0.326833384	1.634870888	0.102076116	0.491460867	up_in_case
PRJNA8274(	ENSGALG00010005460	TGDS	case	control	0.038505915	0.081541807	0.472222977	0.636767641	0.899158415	up_in_case
PRJNA8274(	ENSGALG00010005462	AHI1	case	control	-0.182313303	0.104957731	-1.737016422	0.082384272	0.456367364	down_in_case
PRJNA8274(	ENSGALG00010005463	SKA3	case	control	0.005172787	0.118491507	0.043655343	0.965179136	0.992291928	up_in_case
PRJNA8274(	ENSGALG00010005464	SLC39A8	case	control	0.571916275	0.501439969	1.140547844	0.254058132	0.679960937	up_in_case
PRJNA8274(	ENSGALG00010005465	ENSGALG00010005465	case	control	0.180921642	0.078522114	2.30408522	0.02121786	0.261114804	up_in_case
PRJNA8274(	ENSGALG00010005466	LATS2	case	control	-0.015436811	0.062067372	-0.248710568	0.803584674	0.954899521	down_in_case

PRJNA8274(	ENSGALG00010005467	ENSGALG00010005467	case	control	-0.175980837	0.183019303	-0.961542494	0.33627947	0.750473129	down_in_case
PRJNA8274(	ENSGALG00010005468	PPIL4	case	control	0.143579733	0.073036358	1.965866529	0.04931403	0.364571979	up_in_case
PRJNA8274(	ENSGALG00010005470	SEMA3A	case	control	-0.060182057	0.084678199	-0.710714888	0.477260932	0.830520323	down_in_case
PRJNA8274(	ENSGALG00010005472	PFKP	case	control	0.007297443	0.052337109	0.139431527	0.889109161	0.978017246	up_in_case
PRJNA8274(	ENSGALG00010005474	COL8A1	case	control	0.234731165	0.216952329	1.081948123	0.27927558	0.704960168	up_in_case
PRJNA8274(	ENSGALG00010005475	TMEM181	case	control	0.13692548	0.083861349	1.632760279	0.102519424	0.491460867	up_in_case
PRJNA8274(	ENSGALG00010005476	NFKB1	case	control	-0.098864388	0.051849278	-1.906765002	0.056551025	0.387277461	down_in_case
PRJNA8274(	ENSGALG00010005477	TMEM161B	case	control	0.042960722	0.150670253	0.285130747	0.775543975	0.947146475	up_in_case
PRJNA8274(	ENSGALG00010005478	ENSGALG00010005478	case	control	1.762182114	1.022195973	1.723918074	0.084722634	0.460659189	up_in_case
PRJNA8274(	ENSGALG00010005479	ENSGALG00010005479	case	control	0.757773182	0.372440295	2.034616532	0.041889473	0.341355095	up_in_case
PRJNA8274(	ENSGALG00010005481	ENSGALG00010005481	case	control	0.613794145	0.458641858	1.338286366	0.180803105	0.60147482	up_in_case
PRJNA8274(	ENSGALG00010005482	XPO4	case	control	-0.067699761	0.065646107	-1.031283724	0.302407789	0.725089098	down_in_case
PRJNA8274(	ENSGALG00010005484	GINM1	case	control	-0.045296241	0.084712135	-0.534707814	0.592851918	0.883625114	down_in_case
PRJNA8274(	ENSGALG00010005485	PITRM1	case	control	-0.024659189	0.08390828	-0.293882671	0.76884756	0.945375926	down_in_case
PRJNA8274(	ENSGALG00010005486	IRF5	case	control	-0.081230741	0.174614656	-0.465200019	0.641788225	0.901539099	down_in_case
PRJNA8274(	ENSGALG00010005487	ENSGALG00010005487	case	control	0.076376997	0.082810544	0.922310043	0.356366877	0.763373775	up_in_case
PRJNA8274(	ENSGALG00010005489	G2E3	case	control	0.063785493	0.076651238	0.832152159	0.405323064	0.793662853	up_in_case
PRJNA8274(	ENSGALG00010005490	DCBLD2	case	control	-0.031211461	0.063402741	-0.49227305	0.62252633	0.893944471	down_in_case
PRJNA8274(	ENSGALG00010005491	GGACT	case	control	0.311509685	0.131003322	2.377876232	0.017412669	0.234759681	up_in_case
PRJNA8274(	ENSGALG00010005492	TNPO3	case	control	-0.018596211	0.056523729	-0.328998305	0.742156969	0.937364834	down_in_case
PRJNA8274(	ENSGALG00010005493	KLF6	case	control	-0.004427622	0.059542637	-0.074360536	0.94072351	0.989551456	down_in_case
PRJNA8274(	ENSGALG00010005494	PPDPFL	case	control	0.210179151	0.49312388	0.426219779	0.669947707	0.913465866	up_in_case
PRJNA8274(	ENSGALG00010005495	TXN	case	control	0.048929813	0.183556255	0.266565759	0.789803522	0.951843754	up_in_case
PRJNA8274(	ENSGALG00010005497	MKX	case	control	0.267817165	0.184526043	1.451378686	0.146674441	0.55166873	up_in_case
PRJNA8274(	ENSGALG00010005498	SMO	case	control	-0.14769212	0.070253827	-2.102264416	0.03553012	0.324939791	down_in_case
PRJNA8274(	ENSGALG00010005499	PDE5A	case	control	0.026906293	0.079604734	0.337998653	0.735364203	0.934096263	up_in_case
PRJNA8274(	ENSGALG00010005500	KATNA1	case	control	-0.017743685	0.107261477	-0.165424586	0.868609803	0.974483475	down_in_case
PRJNA8274(	ENSGALG00010005502	SCFD1	case	control	0.132179698	0.044922688	2.942381759	0.003256981	0.097324927	up_in_case
PRJNA8274(	ENSGALG00010005503	DYNLT1	case	control	-0.009028296	0.10472467	-0.086209834	0.931299614	0.987258715	down_in_case
PRJNA8274(	ENSGALG00010005504	LATS1	case	control	-0.002875179	0.053700577	-0.053540941	0.957300911	0.992056045	down_in_case
PRJNA8274(	ENSGALG00010005505	FABP2	case	control	0.806845888	0.8675784	0.929997667	0.352372292	0.761166734	up_in_case
PRJNA8274(	ENSGALG00010005507	USP53	case	control	0.000476032	0.091877315	0.005181165	0.995866047	0.998710499	up_in_case
PRJNA8274(	ENSGALG00010005508	SS18	case	control	-0.025174774	0.056625377	-0.444584654	0.65661994	0.907494354	down_in_case
PRJNA8274(	ENSGALG00010005509	COCH	case	control	0.222637551	0.214071295	1.040015904	0.298332512	0.721487387	up_in_case
PRJNA8274(	ENSGALG00010005510	MYOZ2	case	control	0.042154244	0.115031202	0.366459219	0.714022438	0.926862121	up_in_case
PRJNA8274(	ENSGALG00010005511	AHCYL2	case	control	-0.064173089	0.056357364	-1.138681524	0.254836012	0.680514466	down_in_case
PRJNA8274(	ENSGALG00010005512	SMARCAD1	case	control	-0.153394436	0.10353708	-1.481541073	0.138462448	0.540932258	down_in_case
PRJNA8274(	ENSGALG00010005513	TMEM30CP	case	control	0.023167039	0.177992678	0.130157257	0.896442011	0.980176778	up_in_case
PRJNA8274(	ENSGALG00010005514	EEF1AKMT1	case	control	-0.08308545	0.10056074	-0.82622154	0.408678432	0.795170398	down_in_case
PRJNA8274(	ENSGALG00010005515	PCCA	case	control	-0.186818717	0.073447051	-2.543583637	0.010972179	0.185137791	down_in_case
PRJNA8274(	ENSGALG00010005516	LARP4B	case	control	-0.204025915	0.116031606	-1.758365005	0.078685423	0.449003015	down_in_case
PRJNA8274(	ENSGALG00010005518	HPGDS	case	control	3.977305942	0.566489963	7.02096454	2.20342E-12	1.57544E-09	up_in_case
PRJNA8274(	ENSGALG00010005519	PTBP3	case	control	0.570752991	0.193728092	2.946155018	0.003217511	0.096457878	up_in_case
PRJNA8274(	ENSGALG00010005520	ZIC2	case	control	-1.267057224	0.832631084	-1.521751047	0.128071472	0.529650968	down_in_case
PRJNA8274(	ENSGALG00010005521	SYTL3	case	control	-0.041905767	0.468709614	-0.089406672	0.928758721	0.986776285	down_in_case
PRJNA8274(	ENSGALG00010005522	SYNPO2	case	control	-0.203770132	0.09361254	-2.176739706	0.029499994	0.305245958	down_in_case
PRJNA8274(	ENSGALG00010005524	STRIP2	case	control	-0.304512155	0.169611451	-1.795351393	0.072597732	0.435830211	down_in_case
PRJNA8274(	ENSGALG00010005526	STRN3	case	control	-0.069612036	0.033776311	-2.060972138	0.039305698	0.335091843	down_in_case
PRJNA8274(	ENSGALG00010005527	NUP43	case	control	-0.030244867	0.060624261	-0.498890484	0.617856539	0.891419534	down_in_case
PRJNA8274(	ENSGALG00010005529	ENSGALG00010005529	case	control	0.282612604	0.256825108	1.100408783	0.271154053	0.698323327	up_in_case
PRJNA8274(	ENSGALG00010005530	IL17D	case	control	-0.21028641	0.129156566	-1.628151143	0.103492842	0.49298722	down_in_case
PRJNA8274(	ENSGALG00010005531	SUSD1	case	control	0.316082933	0.16700271	1.892681462	0.058400243	0.393617875	up_in_case
PRJNA8274(	ENSGALG00010005532	GTPBP4	case	control	-0.025672931	0.062591942	-0.410163515	0.681686003	0.915594727	down_in_case
PRJNA8274(	ENSGALG00010005533	OPN4-1	case	control	-0.414671271	0.539523478	-0.768588	0.442137932	0.813611173	down_in_case
PRJNA8274(	ENSGALG00010005534	ENSGALG00010005534	case	control	0.285903882	0.142583781	2.005164128	0.044945504	0.352144447	up_in_case
PRJNA8274(	ENSGALG00010005535	AP4S1	case	control	-0.074156309	0.108717735	-0.682099463	0.49517606	0.838118945	down_in_case
PRJNA8274(	ENSGALG00010005536	TAF4B	case	control	-0.09005798	0.153036824	-0.588472616	0.556215109	0.867878646	down_in_case
PRJNA8274(	ENSGALG00010005537	GRIN3A	case	control	-0.451719947	0.25609176	-1.763898794	0.07774902	0.447281736	down_in_case
PRJNA8274(	ENSGALG00010005538	ENSGALG00010005538	case	control	0.975760423	0.501475311	1.945779588	0.051681222	0.371751242	up_in_case
PRJNA8274(	ENSGALG00010005539	IFT88	case	control	-0.021432289	0.112940352	-0.189766446	0.84949215	0.968927451	down_in_case
PRJNA8274(	ENSGALG00010005540	PCMT1	case	control	-0.120868048	0.061049572	-1.979834474	0.047722131	0.360119697	down_in_case
PRJNA8274(	ENSGALG00010005542	KCTD1	case	control	-0.012738334	0.139339958	-0.091419105	0.927159582	0.986486757	down_in_case
PRJNA8274(	ENSGALG00010005543	SEC24D	case	control	0.042887082	0.077205689	0.555491207	0.578558723	0.879025684	up_in_case
PRJNA8274(	ENSGALG00010005544	AQP4	case	control	-0.690513756	0.340728743	-2.02657912	0.042705474	0.344511316	down_in_case
PRJNA8274(	ENSGALG00010005545	RPL34	case	control	-0.093034575	0.074299226	-1.252160751	0.210511294	0.636249596	down_in_case
PRJNA8274(	ENSGALG00010005546	NRF1	case	control	-0.079993787	0.077612757	-1.030678333	0.302691689	0.725404918	down_in_case
PRJNA8274(	ENSGALG00010005547	LRP11	case	control	0.109434539	0.133847766	0.817604529	0.413583041	0.798035013	up_in_case
PRJNA8274(	ENSGALG00010005548	EZR	case	control	-0.125499751	0.176718185	-0.710168853	0.477599433	0.830520323	down_in_case
PRJNA8274(	ENSGALG00010005549	RNF20	case	control	-0.018354374	0.157607029	-0.116456569	0.907290705	0.981730793	down_in_case
PRJNA8274(	ENSGALG00010005550	ENSGALG00010005550	case	control	0.602840476	0.299931941	2.009924231	0.044439209	0.350320113	up_in_case
PRJNA8274(	ENSGALG00010005551	METTL14	case	control	0.054225442	0.050274888	1.078579073	0.280775414	0.705762606	up_in_case
PRJNA8274(	ENSGALG00010005552	WDR37	case	control	-0.056515741	0.098232574	-0.575325872	0.565070927	0.87262573	down_in_case
PRJNA8274(	ENSGALG00010005553	PPP1R14C	case	control	-0.206003488	0.107939834	-1.908502924	0.056326246	0.386129105	down_in_case
PRJNA8274(	ENSGALG00010005554	CRYL1	case	control	-0.028548703	0.099855564	-0.285899975	0.774954733	0.947085948	down_in_case
PRJNA8274(	ENSGALG00010005555	PRSS12	case	control	-0.044293211	0.129766196	-0.341330887	0.732854498	0.933846596	down_in_case
PRJNA8274(	ENSGALG00010005556	IYD	case	control	0.324436152	0.736380793	0.44058204	0.659515608	0.908599575	up_in_case
PRJNA8274(	ENSGALG00010005557	CMSS1	case	control	-0.173481007	0.080296771	-2.160497928	0.030734144	0.308259818	down_in_case
PRJNA8274(	ENSGALG00010005558	TAGAP	case	control	-0.28078297	0.415186114	-0.67628218	0.498861504	0.840142058	down_in_case
PRJNA8274(	ENSGALG00010005559	FILIP1L	case	control	-0.193531212	0.07534911	-2.56846048	0.010215136	0.179675823	down_in_case
PRJNA8274(	ENSGALG00010005560	RSPH3	case	control	-0.317874654	0.22007547	-1.4443893	0.148629499	0.55519893	down_in_case
PRJNA8274(	ENSGALG00010005561	CLYBL	case	control	-0.506842136	0.154956114	-3.270875375	0.001072151	0.05127681	down_in_case
PRJNA8274(	ENSGALG00010005562	HECTD1	case	control	-0.076420627	0.044478054	-1.718164824	0.085766556	0.462815753	down_in_case
PRJNA8274(	ENSGALG00010005564	TM9SF2	case	control	-1.6852E-05	0.02948821	-0.000571483	0.999544023	0.99976345	down_in_case
PRJNA8274(	ENSGALG00010005565	PGAP4	case	control	0.042632519	0.189724184	0.22470788	0.822206534	0.959330405	up_in_case
PRJNA8274(	ENSGALG00010005567	NDST3	case	control	-0.366971388	0.120337589	-3.049515868	0.002292105	0.078791118	down_in_case
PRJNA8274(	ENSGALG00010005568	PLEKHG1	case	control	-0.038518856	0.115510295	-0.333466863	0.738781899	0.93607843	down_in_case
PRJNA8274(	ENSGALG00010005572	ENSGALG00010005572	case	control	-0.099034029	0.085659136	-1.156140872	0.24762354	0.672031667	down_in_case
PRJNA8274(	ENSGALG00010005573	CHST9	case	control	-0.163625055	0.149015838	-1.098038012	0.272187891	0.698418597	down_in_case
PRJNA8274(	ENSGALG00010005575	ENSGALG00010005575	case	control	1.533661713	0.485770996	3.157170203	0.001593083	0.064172088	up_in_case
PRJNA8274(	ENSGALG00010005578	HEATR5A	case	control	0.010804979	0.076921674	0.140467286	0.888290798	0.977948171	up_in_case
PRJNA8274(	ENSGALG00010005583	ALDOB	case	control	0.62926877	0.848324401	0.741778462	0.458221572	0.823633217	up_in_case
PRJNA8274(	ENSGALG00010005587	LEF1	case	control	-0.043121431	0.254330709	-0.169548661	0.865365101	0.973289701	down_in_case
PRJNA8274(	ENSGALG00010005588	PLAGL1	case	control	-0.212793724	0.068060509	-3.12653737	0.00176878	0.067952978	down_in_case
PRJNA8274(	ENSGALG00010005590	TBC1D23	case	control	0.030774815	0.044901705	0.685381893	0.493102967	0.837887766	up_in_case
PRJNA8274(	ENSGALG00010005594	MRPL50	case	control	0.113123687	0.195558144	0.578465744	0.562949718	0.871703408	up_in_case
PRJNA8274(	ENSGALG00010005596	PARD3	case	control	-0.053291506	0.042626975	-1.250182692	0.211232818	0.636501957	down_in_case
PRJNA8274(	ENSGALG00010005601	TMEM215	case	control	0.360962805	0.329399176	1.095821823	0.273156756	0.698936152	up_in_case
PRJNA8274(	ENSGALG00010005604	ENSGALG00010005604	case	control	0.385197349	0.473572541	0.813386157	0.415996694	0.799987395	up_in_case
PRJNA8274(	ENSGALG00010005609	GJB6	case	control	0.229217767	0.443885202	0.516389748	0.605582225	0.88576746	up_in_case
PRJNA8274(	ENSGALG00010005610	MTHFD1L	case	control	-0.0719054	0.083019356	-0.866128138	0.386419896	0.782470195	down_in_case
PRJNA8274(	ENSGALG00010005617	DTD2	case	control	-0.784127681	0.182966709	-4.285630346	1.82222E-05	0.002814704	down_in_case
PRJNA8274(	ENSGALG00010005618	PHACTR2	case	control	0.02952111	0.068077671	0.43363866	0.664550856	0.911721094	up_in_case
PRJNA8274(	ENSGALG00010005619	CELF2	case	control	-0.051945363	0.056166674	-0.924843139	0.355047507	0.763161644	down_in_case
PRJNA8274(	ENSGALG00010005621	CDH2	case	control	-0.162238085	0.060782397	-2.669162361	0.007604068	0.152061299	down_in_case
PRJNA8274(	ENSGALG00010005629	NRP1	case	control	-0.083247168	0.088661276	-0.938934919	0.34776416	0.758360178	down_in_case
PRJNA8274(	ENSGALG00010005630	NIT2	case	control	-0.249478334	0.112631846	-2.214989304	0.026760799	0.291454243	down_in_case
PRJNA8274(	ENSGALG00010005632	ITGB1	case	control	0.020319903	0.046999024	0.432347344	0.665488982	0.912007336	up_in_case
PRJNA8274(	ENSGALG00010005634	HADH	case	control	-0.236726269	0.068999139	-3.430858306	0.000601675	0.035999789	down_in_case
PRJNA8274(	ENSGALG00010005637	TOPORS	case	control	0.018827036	0.146472569	0.128536262	0.897724604	0.980176778	up_in_case
PRJNA8274(	ENSGALG00010005645	FUCA2	case	control	0.1008033	0.124187104	0.811705053	0.416960894	0.799987395	up_in_case
PRJNA8274(	ENSGALG00010005649	TOMM70	case	control	-0.076566333	0.048471315	-1.579621535	0.114193564	0.509446069	down_in_case
PRJNA8274(	ENSGALG00010005651	USP6NL	case	control	0.097656042	0.100255374	0.97407289	0.330020349	0.746250947	up_in_case
PRJNA8274(	ENSGALG00010005656	CYP2U1	case	control	0.619396554	0.217426188	2.848767024	0.004388901	0.114268826	up_in_case
PRJNA8274(	ENSGALG00010005660	UBAC2	case	control	0.011406523	0.052264056	0.218247941	0.827235934	0.961319258	up_in_case
PRJNA8274(	ENSGALG00010005668	LNP1	case	control	0.072485274	0.241581052	0.300045361	0.764142556	0.943786458	up_in_case
PRJNA8274(	ENSGALG00010005670	SGMS2	case	control	0.108907034	0.206430109	0.5275734	0.597795459	0.884680373	up_in_case
PRJNA8274(	ENSGALG00010005675	GJA3	case	control	0.114853793	0.271358416	0.423254952	0.672109258	0.913676733	up_in_case
PRJNA8274(	ENSGALG00010005679	TMEM45A	case	control	0.101982551	0.176160078	0.578919763	0.562643314	0.871417675	up_in_case
PRJNA8274(	ENSGALG00010005680	ENSGALG00010005680	case	control	-0.389785815	0.425156577	-0.916805328	0.35924466	0.765374836	down_in_case
PRJNA8274(	ENSGALG00010005681	EPC1	case	control	-0.041820245	0.047988469	-0.87146446	0.383500603	0.780061953	down_in_case
PRJNA8274(	ENSGALG00010005692	SEC24B	case	control	-0.001754882	0.039846306	-0.04404127	0.964871507	0.992291928	down_in_case
PRJNA8274(	ENSGALG00010005694	ENSGALG00010005694	case	control	-0.076478979	0.054394372	-1.40600904	0.15972144	0.572017525	down_in_case
PRJNA8274(	ENSGALG00010005695	KIF5B	case	control	0.058031813	0.040718825	1.425183873	0.154104057	0.562900869	up_in_case
PRJNA8274(	ENSGALG00010005696	NUBPL	case	control	-0.252275276	0.094249285	-2.67668105	0.007435538	0.150393485	down_in_case
PRJNA8274(	ENSGALG00010005699	PRPF18	case	control	-0.078107456	0.047837037	-1.63278208	0.102514837	0.491460867	down_in_case
PRJNA8274(	ENSGALG00010005704	AKAP12	case	control	-0.097040106	0.104824377	-0.925739881	0.354581177	0.763161644	down_in_case

PRJNA8274(	ENSGALG00010005705	ECHDC3	case	control	-0.074812478	0.090573847	-0.825983228	0.408813606	0.795170398	down_in_case
PRJNA8274(	ENSGALG00010005712	ENSGALG00010005712	case	control	-0.146630343	0.112213149	-1.306712665	0.191310316	0.614339451	down_in_case
PRJNA8274(	ENSGALG00010005714	ARHGAP5	case	control	-0.102997484	0.075383758	-1.366308704	0.17184209	0.58957339	down_in_case
PRJNA8274(	ENSGALG00010005716	ZBTB2	case	control	-0.001945884	0.063080934	-0.030847422	0.975391221	0.99399062	down_in_case
PRJNA8274(	ENSGALG00010005720	COL25A1	case	control	-0.060952981	0.153655885	-0.396684975	0.691599782	0.918877346	down_in_case
PRJNA8274(	ENSGALG00010005724	PROSER2	case	control	-0.002333682	0.092475748	-0.02523561	0.979867034	0.995636523	down_in_case
PRJNA8274(	ENSGALG00010005738	ELAVL2	case	control	-0.305178096	0.430917379	-0.708205588	0.478817597	0.83124975	down_in_case
PRJNA8274(	ENSGALG00010005740	ENSGALG00010005740	case	control	-0.125114634	0.29919214	-0.418174869	0.675819269	0.914247368	down_in_case
PRJNA8274(	ENSGALG00010005742	GPR183	case	control	-0.005703874	0.090527389	-0.063007165	0.949760799	0.991488793	down_in_case
PRJNA8274(	ENSGALG00010005743	ETNPPL	case	control	0.114248656	0.210648765	0.542365657	0.587566634	0.882771329	up_in_case
PRJNA8274(	ENSGALG00010005748	AIG1	case	control	-0.110662546	0.102638826	-1.078174314	0.280955973	0.705762606	down_in_case
PRJNA8274(	ENSGALG00010005752	RMND1	case	control	0.019505301	0.075860325	0.257121245	0.797085177	0.95280257	up_in_case
PRJNA8274(	ENSGALG00010005755	ZMYM2	case	control	-0.057558175	0.064212736	-0.896366963	0.370056806	0.772544067	down_in_case
PRJNA8274(	ENSGALG00010005756	TET2	case	control	0.068458656	0.07805062	0.87710586	0.380429142	0.778246195	up_in_case
PRJNA8274(	ENSGALG00010005757	ARHGAP12	case	control	0.02702763	0.079818874	0.338612024	0.734902024	0.934096263	up_in_case
PRJNA8274(	ENSGALG00010005758	HIVEP2	case	control	-0.008034536	0.085574867	-0.093888971	0.925197355	0.985571367	down_in_case
PRJNA8274(	ENSGALG00010005760	GPR18	case	control	0.160937282	0.278448081	0.5779795	0.56327796	0.871916948	up_in_case
PRJNA8274(	ENSGALG00010005763	AKAP6	case	control	-0.22416481	0.122679688	-1.827236549	0.067664211	0.42089154	down_in_case
PRJNA8274(	ENSGALG00010005768	CXXC4	case	control	0.329794379	0.303743286	1.085766808	0.277582175	0.70305085	up_in_case
PRJNA8274(	ENSGALG00010005769	TFG	case	control	-0.095614842	0.058132104	-1.644785517	0.10001405	0.489464811	down_in_case
PRJNA8274(	ENSGALG00010005772	SVEP1	case	control	-0.358333332	0.120458325	-2.974749421	0.00293228	0.091955261	down_in_case
PRJNA8274(	ENSGALG00010005777	ENSGALG00010005777	case	control	-1.937014471	1.177911231	-1.644448596	0.100083574	0.489464811	down_in_case
PRJNA8274(	ENSGALG00010005779	ARMT1	case	control	0.040153462	0.061919277	0.648480802	0.516674024	0.848054666	up_in_case
PRJNA8274(	ENSGALG00010005784	RASL11A	case	control	-0.080077242	0.357980898	-0.223691382	0.822997452	0.95978336	down_in_case
PRJNA8274(	ENSGALG00010005785	RPL21	case	control	-0.049013628	0.059883444	-0.818483783	0.413080998	0.797633779	down_in_case
PRJNA8274(	ENSGALG00010005789	RNASET2	case	control	0.035731155	0.086698059	0.412133272	0.680241743	0.915381147	up_in_case
PRJNA8274(	ENSGALG00010005790	MUSK	case	control	-0.17781519	0.159129986	-1.117421009	0.263814356	0.689768156	down_in_case
PRJNA8274(	ENSGALG00010005792	ADGRG6	case	control	-0.00250577	0.132697362	-0.018883346	0.984934165	0.996591771	down_in_case
PRJNA8274(	ENSGALG00010005799	UFSP2	case	control	-0.14666041	0.102907511	-1.425167203	0.154108874	0.562900869	down_in_case
PRJNA8274(	ENSGALG00010005800	FRMD4A	case	control	0.014031314	0.054619989	0.256889734	0.797263896	0.95280257	up_in_case
PRJNA8274(	ENSGALG00010005814	UPF2	case	control	-0.04057056	0.042457408	-0.955559033	0.339295067	0.751259351	down_in_case
PRJNA8274(	ENSGALG00010005817	VTA1	case	control	-0.051410469	0.037852795	-1.358168355	0.174410269	0.593194623	down_in_case
PRJNA8274(	ENSGALG00010005821	DOCK9	case	control	-0.068307555	0.071240196	-0.958834468	0.337642142	0.750948271	down_in_case
PRJNA8274(	ENSGALG00010005826	LRP2BP	case	control	0.205018378	0.228043085	0.899033521	0.368634806	0.771770041	up_in_case
PRJNA8274(	ENSGALG00010005828	LPAR1	case	control	0.023382566	0.093856737	0.24913039	0.803259922	0.954891353	up_in_case
PRJNA8274(	ENSGALG00010005829	NPAS3	case	control	-0.350499542	0.092266605	-3.798769256	0.000145416	0.01379195	down_in_case
PRJNA8274(	ENSGALG00010005830	USP12	case	control	-0.134863654	0.211952657	-0.636291406	0.524586506	0.852356213	down_in_case
PRJNA8274(	ENSGALG00010005831	SEC61A2	case	control	-0.052452736	0.060322937	-0.8695322	0.384556104	0.780971676	down_in_case
PRJNA8274(	ENSGALG00010005836	NEBL	case	control	0.009442164	0.193912736	0.04869285	0.961164074	0.992056045	up_in_case
PRJNA8274(	ENSGALG00010005839	RPS6KA2	case	control	0.081347143	0.113235951	0.718386189	0.4725192	0.829235491	up_in_case
PRJNA8274(	ENSGALG00010005841	ENSGALG00010005841	case	control	-0.383529347	0.241833416	-1.585923703	0.112756618	0.507003543	down_in_case
PRJNA8274(	ENSGALG00010005843	GPR12	case	control	-0.228884437	0.272776548	-0.839091331	0.40141806	0.791216852	down_in_case
PRJNA8274(	ENSGALG00010005847	ENSGALG00010005847	case	control	0.188577586	0.420873563	0.448062322	0.65410822	0.906478152	up_in_case
PRJNA8274(	ENSGALG00010005848	WASF3	case	control	-0.168823262	0.124897154	-1.351698229	0.176471872	0.595527443	down_in_case
PRJNA8274(	ENSGALG00010005849	SNX25	case	control	0.069419501	0.060774884	1.142239961	0.25335429	0.678954688	up_in_case
PRJNA8274(	ENSGALG00010005851	SLC15A1	case	control	0.820989815	0.739081855	1.110823935	0.266644142	0.692519293	up_in_case
PRJNA8274(	ENSGALG00010005853	COMMD3	case	control	0.047726615	0.093275232	0.511675112	0.608878407	0.8877909	up_in_case
PRJNA8274(	ENSGALG00010005858	CFAP97	case	control	0.096225744	0.091993983	1.04600041	0.295560843	0.719040499	up_in_case
PRJNA8274(	ENSGALG00010005859	ENSGALG00010005859	case	control	-0.408039617	0.556796798	-0.732833987	0.463659689	0.825091817	down_in_case
PRJNA8274(	ENSGALG00010005860	ABI3BP	case	control	-0.187808398	0.155114242	-1.210774688	0.225981769	0.649498359	down_in_case
PRJNA8274(	ENSGALG00010005861	MPC1L	case	control	-0.151620505	0.100258659	-1.51229337	0.130459247	0.533019209	down_in_case
PRJNA8274(	ENSGALG00010005864	ENSGALG00010005864	case	control	-0.337893767	0.422987068	-0.798827653	0.424390354	0.803723282	down_in_case
PRJNA8274(	ENSGALG00010005866	DNAJC1	case	control	0.103950604	0.078368545	1.326432743	0.184696399	0.607639686	up_in_case
PRJNA8274(	ENSGALG00010005871	EGLN3	case	control	-0.047890398	0.0957863	-0.499971269	0.617095308	0.891081782	down_in_case
PRJNA8274(	ENSGALG00010005872	STK24	case	control	0.069060294	0.067793692	1.018683177	0.308353402	0.731823162	up_in_case
PRJNA8274(	ENSGALG00010005875	CDK8	case	control	0.035116041	0.089528603	0.392232651	0.694886321	0.920623921	up_in_case
PRJNA8274(	ENSGALG00010005879	FAM107B	case	control	0.075807502	0.067938918	1.115818506	0.264499831	0.690350289	up_in_case
PRJNA8274(	ENSGALG00010005880	SLC25A4	case	control	-0.100362742	0.118531192	-0.846720092	0.397151157	0.788531716	down_in_case
PRJNA8274(	ENSGALG00010005881	CORO2A	case	control	0.628748998	0.408814574	1.537980882	0.124053289	0.523029039	up_in_case
PRJNA8274(	ENSGALG00010005884	MYB	case	control	-0.143433132	0.198380662	-0.723019723	0.469667767	0.829101076	down_in_case
PRJNA8274(	ENSGALG00010005885	SLITRK5	case	control	-0.215178027	0.193698726	-1.110890255	0.266615591	0.692519293	down_in_case
PRJNA8274(	ENSGALG00010005887	CCNH	case	control	0.070810467	0.174890665	0.404884197	0.685562635	0.917034645	up_in_case
PRJNA8274(	ENSGALG00010005888	IMPG2	case	control	0.300369871	0.193966595	1.548564957	0.121486336	0.519686058	up_in_case
PRJNA8274(	ENSGALG00010005890	SNX32	case	control	-0.064494459	0.058286528	-1.106507123	0.268507083	0.695337066	down_in_case
PRJNA8274(	ENSGALG00010005891	RNF6	case	control	0.069113012	0.085209756	0.811092711	0.417312431	0.799987395	up_in_case
PRJNA8274(	ENSGALG00010005892	ENSGALG00010005892	case	control	-1.697709534	0.684353642	-2.480748884	0.01311067	0.200730816	down_in_case
PRJNA8274(	ENSGALG00010005893	SFT2D1	case	control	-0.015391226	0.053795003	-0.286108845	0.774794758	0.947085948	down_in_case
PRJNA8274(	ENSGALG00010005895	MLLT10	case	control	0.026722744	0.043958708	0.60790559	0.543250092	0.860486964	up_in_case
PRJNA8274(	ENSGALG00010005899	ENSGALG00010005899	case	control	0.09205007	0.17536365	0.52490964	0.599646008	0.884888453	up_in_case
PRJNA8274(	ENSGALG00010005900	SPTSSA	case	control	-0.075793537	0.056268577	-1.346995811	0.177981582	0.59730458	down_in_case
PRJNA8274(	ENSGALG00010005901	ENSGALG00010005901	case	control	0.148335324	0.248834553	0.596120283	0.551094875	0.864743359	up_in_case
PRJNA8274(	ENSGALG00010005902	EAPP	case	control	-0.057949272	0.06475428	-0.894910298	0.370835041	0.772835133	down_in_case
PRJNA8274(	ENSGALG00010005903	PRR18	case	control	0.236217224	0.228805962	1.032391031	0.301888972	0.724695702	up_in_case
PRJNA8274(	ENSGALG00010005905	CITED2	case	control	0.131728376	0.121129244	1.087502673	0.276814718	0.702359305	up_in_case
PRJNA8274(	ENSGALG00010005909	BRMS1L	case	control	-0.017934574	0.056701793	-0.316296419	0.751777529	0.940427752	down_in_case
PRJNA8274(	ENSGALG00010005910	ENSGALG00010005910	case	control	0.371232626	0.451082317	0.822981995	0.410518232	0.796497837	up_in_case
PRJNA8274(	ENSGALG00010005911	TBXT	case	control	-0.355919142	0.213121281	-1.670030987	0.094913233	0.481468333	down_in_case
PRJNA8274(	ENSGALG00010005913	NFKBIA	case	control	-0.226912678	0.096633418	-2.348180182	0.018865393	0.245250105	down_in_case
PRJNA8274(	ENSGALG00010005915	PAPSS1	case	control	0.133311648	0.099488464	1.339970913	0.180254802	0.600999032	up_in_case
PRJNA8274(	ENSGALG00010005926	NUDT5	case	control	0.542607426	0.350659275	1.547392197	0.121768702	0.519686058	up_in_case
PRJNA8274(	ENSGALG00010005934	SLITRK6	case	control	-0.28825981	0.253964271	-1.135040804	0.256358222	0.682794296	down_in_case
PRJNA8274(	ENSGALG00010005935	ATP8A2	case	control	-0.10771396	0.283506789	-0.379934323	0.703994169	0.923942815	down_in_case
PRJNA8274(	ENSGALG00010005941	TXLNB	case	control	-0.400096529	0.175136556	-2.284483259	0.022343145	0.266477881	down_in_case
PRJNA8274(	ENSGALG00010005943	PROX2	case	control	0.215041474	0.483332488	0.444914173	0.65638178	0.907494354	up_in_case
PRJNA8274(	ENSGALG00010005949	SLITRK1	case	control	-0.492805771	0.312284588	-1.578066257	0.114550389	0.509446069	down_in_case
PRJNA8274(	ENSGALG00010005956	TXNL4B	case	control	0.087590783	0.088425705	0.99055793	0.321901489	0.739571618	up_in_case
PRJNA8274(	ENSGALG00010005963	UGCG	case	control	0.081552473	0.095827956	0.851030075	0.394752646	0.786978937	up_in_case
PRJNA8274(	ENSGALG00010005964	HSPA14	case	control	0.062684869	0.070305379	0.891608446	0.372602841	0.774225608	up_in_case
PRJNA8274(	ENSGALG00010005966	PLXDC2	case	control	-0.022636358	0.101330248	-0.223391915	0.823230496	0.959820309	down_in_case
PRJNA8274(	ENSGALG00010005967	GJD2	case	control	0.636186593	0.161442118	3.9406482	8.12617E-05	0.009296342	up_in_case
PRJNA8274(	ENSGALG00010005969	HECA	case	control	-0.036522561	0.056476609	-0.646684741	0.517836001	0.848425162	down_in_case
PRJNA8274(	ENSGALG00010005970	SHISA2	case	control	-0.007716848	0.139976531	-0.055129584	0.956035227	0.992056045	down_in_case
PRJNA8274(	ENSGALG00010005975	ABRACL	case	control	0.017869855	0.074943564	0.238444158	0.811536622	0.956205737	up_in_case
PRJNA8274(	ENSGALG00010005978	VPS16	case	control	-0.184517338	0.070664584	-2.611171353	0.009023267	0.16955679	down_in_case
PRJNA8274(	ENSGALG00010005979	SUV39H2	case	control	-0.029044673	0.069448794	-0.418217092	0.6757884	0.914247368	down_in_case
PRJNA8274(	ENSGALG00010005981	AREL1	case	control	0.085329608	0.078404071	1.088331342	0.276448859	0.702295022	up_in_case
PRJNA8274(	ENSGALG00010005984	IDH3B	case	control	0.042040648	0.076932245	0.546463291	0.584747515	0.881307382	up_in_case
PRJNA8274(	ENSGALG00010005985	ENSGALG00010005985	case	control	0.253593376	0.174586457	1.452537495	0.146352212	0.551598784	up_in_case
PRJNA8274(	ENSGALG00010005988	NUP58	case	control	-0.19861143	0.046091589	-4.309060129	1.6395E-05	0.002576354	down_in_case
PRJNA8274(	ENSGALG00010005989	FCF1	case	control	-0.018336856	0.068713766	-0.266858558	0.789578066	0.951843754	down_in_case
PRJNA8274(	ENSGALG00010005990	SLC2A12	case	control	-0.198324522	0.085781794	-2.311965204	0.020779601	0.258860564	down_in_case
PRJNA8274(	ENSGALG00010005991	FARP1	case	control	-0.077206676	0.044181903	-1.747472858	0.080555349	0.45280719	down_in_case
PRJNA8274(	ENSGALG00010005997	ENSGALG00010005997	case	control	-0.192934006	0.186236541	-1.035962141	0.300219805	0.723279006	down_in_case
PRJNA8274(	ENSGALG00010005998	YLPM1	case	control	-0.061340103	0.05440738	-1.127422472	0.259563902	0.686756482	down_in_case
PRJNA8274(	ENSGALG00010005999	PDE10A	case	control	0.024554948	0.094069797	0.261029028	0.794070122	0.95280257	up_in_case
PRJNA8274(	ENSGALG00010006001	NOP56	case	control	0.020808136	0.058503908	0.355670866	0.722087066	0.929484914	up_in_case
PRJNA8274(	ENSGALG00010006004	DCLRE1C	case	control	-0.018031462	0.124706223	-0.144591519	0.885033394	0.977630122	down_in_case
PRJNA8274(	ENSGALG00010006010	IPO5	case	control	0.076505059	0.052707196	1.451510705	0.146637704	0.55166873	up_in_case
PRJNA8274(	ENSGALG00010006013	TRMT10C	case	control	-0.033104146	0.065700864	-0.503861652	0.6143586	0.890555295	down_in_case
PRJNA8274(	ENSGALG00010006015	CDC123	case	control	0.077230212	0.064050762	1.205765703	0.227907843	0.651681204	up_in_case
PRJNA8274(	ENSGALG00010006016	MTMR6	case	control	0.308074122	0.056619474	5.441133605	5.29426E-08	2.103E-05	up_in_case
PRJNA8274(	ENSGALG00010006021	ENSGALG00010006021	case	control	-0.007864996	0.073108828	-0.107579286	0.914329429	0.982854306	down_in_case
PRJNA8274(	ENSGALG00010006022	AFG2A	case	control	0.086610621	0.06051125	1.431314348	0.152340144	0.560614325	up_in_case
PRJNA8274(	ENSGALG00010006024	AMER2	case	control	0.315618628	0.390091771	0.809088147	0.418464446	0.800647789	up_in_case
PRJNA8274(	ENSGALG00010006025	TNFAIP3	case	control	0.015119262	0.085447166	0.176942812	0.85955331	0.971828322	up_in_case
PRJNA8274(	ENSGALG00010006028	ARL5B	case	control	-0.181167954	0.055047319	-3.291131274	0.000997853	0.048700693	down_in_case
PRJNA8274(	ENSGALG00010006029	HSDL2	case	control	-0.139916627	0.161612377	-0.865754402	0.38662486	0.782604263	down_in_case
PRJNA8274(	ENSGALG00010006031	ERCC5	case	control	0.031967894	0.074077376	0.431547333	0.666070444	0.912284937	up_in_case
PRJNA8274(	ENSGALG00010006033	NSUN6	case	control	0.125348142	0.129994426	0.964257821	0.334916682	0.749266044	up_in_case
PRJNA8274(	ENSGALG00010006035	ADH1C	case	control	1.750555201	0.651460008	2.687126116	0.007206974	0.147992151	up_in_case
PRJNA8274(	ENSGALG00010006037	METTL21EP	case	control	0.657619597	0.506650833	1.297973978	0.194296277	0.61723879	up_in_case
PRJNA8274(	ENSGALG00010006038	ENSGALG00010006038	case	control	0.136486443	0.14023504	0.973269185	0.330419534	0.746554834	up_in_case
PRJNA8274(	ENSGALG00010006039	PCNP	case	control	-0.003646856	0.036958919	-0.098673236	0.92139772	0.985267883	down_in_case
PRJNA8274(	ENSGALG00010006041	QKI	case	control	-0.063418114	0.050722839	-1.250287152	0.21119467	0.636501957	down_in_case
PRJNA8274(	ENSGALG00010006047	ZBTB11	case	control	0.220721284	0.119863905	1.841432449	0.065558211	0.415550718	up_in_case
PRJNA8274(	ENSGALG00010006049	AIMP1	case	control	0.059799315	0.06485342	0.922068798	0.356492691	0.763373775	up_in_case

PRJNA8274(	ENSGALG00010006052	RALGAPA1	case	control	-0.041133041	0.050137723	-0.820401045	0.41198752	0.797194435	down_in_case
PRJNA8274(	ENSGALG00010006053	TMEM243	case	control	-0.420512315	0.192886472	-2.180102686	0.029249851	0.304235014	down_in_case
PRJNA8274(	ENSGALG00010006059	BIVM	case	control	0.045228976	0.047072309	0.960840394	0.336632425	0.750654067	up_in_case
PRJNA8274(	ENSGALG00010006061	RPL24	case	control	-0.080034446	0.060148417	-1.330616002	0.183315394	0.60491829	down_in_case
PRJNA8274(	ENSGALG00010006062	ENSGALG00010006062	case	control	-0.715521101	0.670992814	-1.066361793	0.286260126	0.710557161	down_in_case
PRJNA8274(	ENSGALG00010006063	POGLUT2	case	control	-0.053083141	0.062791669	-0.845385091	0.397895866	0.788896694	down_in_case
PRJNA8274(	ENSGALG00010006064	DMTF1	case	control	-0.032271161	0.075603226	-0.426849002	0.669489312	0.913446904	down_in_case
PRJNA8274(	ENSGALG00010006065	WDR54	case	control	0.03830378	0.146593326	0.261292795	0.793866723	0.95280257	up_in_case
PRJNA8274(	ENSGALG00010006066	ARHGAP18	case	control	0.083453887	0.132766611	0.628575866	0.529626764	0.854718737	up_in_case
PRJNA8274(	ENSGALG00010006068	PACRG	case	control	0.014891729	0.195541633	0.076156308	0.939294743	0.989551456	up_in_case
PRJNA8274(	ENSGALG00010006069	TTR	case	control	2.463294438	1.25314164	1.965695146	0.049333836	0.364571979	up_in_case
PRJNA8274(	ENSGALG00010006070	TMEM242	case	control	-0.167971596	0.104100834	-1.613547064	0.106625707	0.49679035	down_in_case
PRJNA8274(	ENSGALG00010006071	TEX30	case	control	-0.020791617	0.173439821	-0.119877987	0.904579802	0.981225151	down_in_case
PRJNA8274(	ENSGALG00010006072	ENSGALG00010006072	case	control	-0.139312404	0.08147786	-1.709819125	0.087299325	0.467042649	down_in_case
PRJNA8274(	ENSGALG00010006073	CEP97	case	control	-0.040907885	0.115142431	-0.355280712	0.722379304	0.929627793	down_in_case
PRJNA8274(	ENSGALG00010006075	SOX21	case	control	-0.743934055	0.428840016	-1.734758948	0.08278351	0.456624721	down_in_case
PRJNA8274(	ENSGALG00010006076	PODXL	case	control	-0.169696579	0.083011307	-2.044258622	0.040928005	0.337874876	down_in_case
PRJNA8274(	ENSGALG00010006077	TBCK	case	control	0.143472075	0.073870651	1.942206732	0.052112078	0.372764678	up_in_case
PRJNA8274(	ENSGALG00010006078	SPATA13	case	control	0.043548663	0.181924965	0.239377056	0.810813219	0.956022547	up_in_case
PRJNA8274(	ENSGALG00010006079	ENSGALG00010006079	case	control	0.833708385	0.579874124	1.437740281	0.150507759	0.558014249	up_in_case
PRJNA8274(	ENSGALG00010006080	ENSGALG00010006080	case	control	0.066656981	0.112386778	0.593103402	0.553111954	0.866252369	up_in_case
PRJNA8274(	ENSGALG00010006081	ZDHHC14	case	control	-0.010532442	0.068117366	-0.154621974	0.877119346	0.975813349	down_in_case
PRJNA8274(	ENSGALG00010006083	CACNB2	case	control	-0.026967101	0.127162589	-0.212067885	0.832054074	0.962371901	down_in_case
PRJNA8274(	ENSGALG00010006084	TMEM68	case	control	-0.10985659	0.085738544	-1.281297593	0.200089158	0.622902456	down_in_case
PRJNA8274(	ENSGALG00010006085	DLST	case	control	0.00270276	0.06756457	0.040002623	0.968091035	0.992761697	up_in_case
PRJNA8274(	ENSGALG00010006086	ENSGALG00010006086	case	control	0.513340581	0.349134874	1.470321699	0.141474647	0.543501954	up_in_case
PRJNA8274(	ENSGALG00010006087	ENSGALG00010006087	case	control	-0.087946006	0.325709466	-0.270013664	0.787149742	0.950937003	down_in_case
PRJNA8274(	ENSGALG00010006091	RTKN	case	control	-0.137681585	0.08204688	-1.678084358	0.093330632	0.478523919	down_in_case
PRJNA8274(	ENSGALG00010006096	EDIL3	case	control	-0.478432596	0.096887218	-4.938036278	7.89132E-07	0.000225692	down_in_case
PRJNA8274(	ENSGALG00010006100	RPS6KL1	case	control	-0.169195807	0.160332713	-1.055279384	0.291297567	0.714222439	down_in_case
PRJNA8274(	ENSGALG00010006101	ENSGALG00010006101	case	control	0.060379462	0.09669971	0.624401683	0.53236382	0.855755691	up_in_case
PRJNA8274(	ENSGALG00010006103	NXPE3	case	control	0.167976698	0.126554654	1.327305574	0.184407621	0.607190646	up_in_case
PRJNA8274(	ENSGALG00010006106	SHOC1	case	control	-0.657427456	0.354513127	-1.854451658	0.06367457	0.409661205	down_in_case
PRJNA8274(	ENSGALG00010006107	PGF	case	control	0.001074775	0.129117596	0.008324002	0.993358484	0.998196863	up_in_case
PRJNA8274(	ENSGALG00010006108	MKLN1	case	control	-0.032033859	0.056493913	-0.56703204	0.570692414	0.87600918	down_in_case
PRJNA8274(	ENSGALG00010006111	WBP1	case	control	0.012449082	0.060201784	0.206789249	0.836174452	0.964297957	up_in_case
PRJNA8274(	ENSGALG00010006113	EIF2B2	case	control	-0.070335196	0.045168615	-1.557169615	0.11943022	0.516645008	down_in_case
PRJNA8274(	ENSGALG00010006118	NPNT	case	control	-0.057789689	0.152234404	-0.379609917	0.704234998	0.923973441	down_in_case
PRJNA8274(	ENSGALG00010006120	MOGS	case	control	0.121991283	0.081882753	1.489828793	0.136269258	0.539840644	up_in_case
PRJNA8274(	ENSGALG00010006121	ENSGALG00010006121	case	control	0.237386896	0.211044978	1.124816609	0.260666755	0.687359696	up_in_case
PRJNA8274(	ENSGALG00010006123	MLH3	case	control	0.390773643	0.112040678	3.487783646	0.000487042	0.032243985	up_in_case
PRJNA8274(	ENSGALG00010006126	ENSGALG00010006126	case	control	0.194437105	0.209212587	0.929375749	0.352694391	0.761513292	up_in_case
PRJNA8274(	ENSGALG00010006128	ACYP1	case	control	-0.243217097	0.089179927	-2.727262783	0.006386216	0.13904105	down_in_case
PRJNA8274(	ENSGALG00010006131	ENSGALG00010006131	case	control	0.089984934	0.087253709	1.031302107	0.302399171	0.725089098	up_in_case
PRJNA8274(	ENSGALG00010006132	MIPEP	case	control	-0.076482267	0.073837645	-1.035816712	0.30028766	0.723279006	down_in_case
PRJNA8274(	ENSGALG00010006135	ZC2HC1C	case	control	0.245130849	0.1215248	2.017126132	0.043682352	0.349041259	up_in_case
PRJNA8274(	ENSGALG00010006139	GAREM1	case	control	-0.237465673	0.106231554	-2.235359118	0.025393782	0.283111687	down_in_case
PRJNA8274(	ENSGALG00010006146	CCDC142	case	control	0.092484357	0.153244164	0.603509814	0.546169589	0.862436519	up_in_case
PRJNA8274(	ENSGALG00010006147	ENSGALG00010006147	case	control	0.338006848	0.228620014	1.478465695	0.139283173	0.541505735	up_in_case
PRJNA8274(	ENSGALG00010006151	ENSGALG00010006151	case	control	0.035931945	0.112466866	0.319489165	0.749355606	0.939569063	up_in_case
PRJNA8274(	ENSGALG00010006155	TNFRSF19	case	control	0.073553895	0.082690285	0.889510726	0.373728661	0.774369566	up_in_case
PRJNA8274(	ENSGALG00010006156	NFKBIZ	case	control	0.085090519	0.213358147	0.398815419	0.690029215	0.918477347	up_in_case
PRJNA8274(	ENSGALG00010006157	SNX9	case	control	0.046524286	0.048055942	0.968127644	0.332980625	0.748503677	up_in_case
PRJNA8274(	ENSGALG00010006163	ENSGALG00010006163	case	control	0.517623253	0.436910165	1.184736117	0.236121798	0.660642087	up_in_case
PRJNA8274(	ENSGALG00010006172	ENSGALG00010006172	case	control	-0.065860629	0.081556708	-0.80754398	0.419353147	0.80093491	down_in_case
PRJNA8274(	ENSGALG00010006173	TBPL1	case	control	-0.049164345	0.055173849	-0.891080579	0.372885943	0.77436767	down_in_case
PRJNA8274(	ENSGALG00010006175	IL2RA	case	control	0.310957339	0.434205451	0.716152545	0.473897161	0.829769733	up_in_case
PRJNA8274(	ENSGALG00010006176	MMR1L3	case	control	0.422647089	0.200347961	2.109565209	0.034895824	0.322721061	up_in_case
PRJNA8274(	ENSGALG00010006177	NEK9	case	control	-0.056836159	0.066222048	-0.85826641	0.390745373	0.784128499	down_in_case
PRJNA8274(	ENSGALG00010006178	RPS23	case	control	0.045666408	0.195919606	0.233087483	0.815693474	0.957374769	up_in_case
PRJNA8274(	ENSGALG00010006180	CD200L	case	control	0.646033999	0.74075538	0.872128663	0.383138191	0.780061953	up_in_case
PRJNA8274(	ENSGALG00010006183	PRKN	case	control	-0.135722111	0.133088082	-1.019791627	0.307827295	0.731009321	down_in_case
PRJNA8274(	ENSGALG00010006184	LBX2	case	control	-0.407181884	0.267807793	-1.520425821	0.128403989	0.530380428	down_in_case
PRJNA8274(	ENSGALG00010006186	CHIR1AB3	case	control	-0.153104327	0.317593023	-0.482077112	0.629751168	0.896269608	down_in_case
PRJNA8274(	ENSGALG00010006187	TCF21	case	control	-0.034385772	0.515817125	-0.06666272	0.946850214	0.990723333	down_in_case
PRJNA8274(	ENSGALG00010006188	TMED10	case	control	0.00927883	0.037293467	0.248805774	0.803511024	0.954891353	up_in_case
PRJNA8274(	ENSGALG00010006193	RBM17	case	control	-0.058235124	0.037894377	-1.536774804	0.124348466	0.523029039	down_in_case
PRJNA8274(	ENSGALG00010006197	ENSGALG00010006197	case	control	-0.675196699	0.40881698	-1.65158673	0.098618827	0.488407564	down_in_case
PRJNA8274(	ENSGALG00010006198	ENSGALG00010006198	case	control	0.609876225	0.344071346	1.772528378	0.076306889	0.443212231	up_in_case
PRJNA8274(	ENSGALG00010006200	GSTCD	case	control	0.097361093	0.098201476	0.991442257	0.321469675	0.73942679	up_in_case
PRJNA8274(	ENSGALG00010006201	SSTR1	case	control	-0.49244923	0.401812563	-1.225569521	0.220360713	0.644563413	down_in_case
PRJNA8274(	ENSGALG00010006202	SACS	case	control	-0.004298484	0.10381086	-0.041406882	0.966971526	0.992726888	down_in_case
PRJNA8274(	ENSGALG00010006203	MMR1L4	case	control	-0.018216861	0.116938207	-0.155781942	0.876204905	0.975666505	down_in_case
PRJNA8274(	ENSGALG00010006205	CGGBP1	case	control	-0.043752148	0.042278114	-1.034865163	0.300731886	0.723511633	down_in_case
PRJNA8274(	ENSGALG00010006207	ENSGALG00010006207	case	control	-0.049088862	0.158551253	-0.309608796	0.756858467	0.941659745	down_in_case
PRJNA8274(	ENSGALG00010006212	FOS	case	control	-0.061107672	0.174693277	-0.349799792	0.726488955	0.932231879	down_in_case
PRJNA8274(	ENSGALG00010006219	CLEC14A	case	control	0.0204778	0.138810606	0.147523312	0.88271898	0.976801393	up_in_case
PRJNA8274(	ENSGALG00010006220	SGCG	case	control	-0.188194885	0.082152635	-2.290795492	0.021975245	0.264739677	down_in_case
PRJNA8274(	ENSGALG00010006223	ATG10	case	control	0.038608934	0.186096321	0.207467475	0.835644792	0.963998106	up_in_case
PRJNA8274(	ENSGALG00010006227	AGPAT4	case	control	0.117632465	0.078445307	1.499547512	0.133731653	0.537331451	up_in_case
PRJNA8274(	ENSGALG00010006229	INTS12	case	control	0.047942362	0.080352816	0.596648183	0.550742295	0.86457121	up_in_case
PRJNA8274(	ENSGALG00010006232	ZNF654	case	control	0.022842021	0.062957854	0.362814472	0.71674348	0.926862121	up_in_case
PRJNA8274(	ENSGALG00010006236	C3orf38	case	control	0.112252136	0.067350688	1.666681352	0.095577783	0.482930958	up_in_case
PRJNA8274(	ENSGALG00010006237	ENSGALG00010006237	case	control	0.342159732	0.297581631	1.149801251	0.25022574	0.675774897	up_in_case
PRJNA8274(	ENSGALG00010006239	EYA4	case	control	0.060719233	0.092967443	0.653123622	0.513676585	0.847240503	up_in_case
PRJNA8274(	ENSGALG00010006241	MRC1	case	control	0.231993537	0.292155075	0.794076697	0.427150783	0.805305429	up_in_case
PRJNA8274(	ENSGALG00010006242	ENSGALG00010006242	case	control	-0.633446797	0.420146852	-1.507679504	0.131636574	0.535159035	down_in_case
PRJNA8274(	ENSGALG00010006244	EPHA3	case	control	-0.216832659	0.106780511	-2.030638901	0.042291639	0.343424436	down_in_case
PRJNA8274(	ENSGALG00010006245	PFKFB3	case	control	-0.412752109	0.195312027	-2.113295917	0.034575448	0.321057731	down_in_case
PRJNA8274(	ENSGALG00010006246	SUPT20H	case	control	-0.059425237	0.03836442	-1.548967426	0.121389551	0.519631973	down_in_case
PRJNA8274(	ENSGALG00010006249	C9orf72	case	control	-0.120040765	0.170277676	-0.704970655	0.480828498	0.8322127	down_in_case
PRJNA8274(	ENSGALG00010006251	SEMA3E	case	control	-0.006135947	0.277473698	-0.022113615	0.982357326	0.996591771	down_in_case
PRJNA8274(	ENSGALG00010006252	COX7C	case	control	-0.040219521	0.18750283	-0.214500876	0.830156484	0.962228709	down_in_case
PRJNA8274(	ENSGALG00010006254	PROS1	case	control	0.000618964	0.08151767	0.007593007	0.993941715	0.998238292	up_in_case
PRJNA8274(	ENSGALG00010006259	PPA2	case	control	0.040252121	0.082422576	0.488362812	0.625292878	0.89506388	up_in_case
PRJNA8274(	ENSGALG00010006260	MAP3K4	case	control	0.026407692	0.054474884	0.484768218	0.62784077	0.895865363	up_in_case
PRJNA8274(	ENSGALG00010006261	PCLO	case	control	-0.189576754	0.322165957	-0.588444403	0.556234041	0.867878646	down_in_case
PRJNA8274(	ENSGALG00010006263	EXOSC8	case	control	-0.100036312	0.067053394	-1.491890356	0.135727891	0.539774365	down_in_case
PRJNA8274(	ENSGALG00010006266	CENPW	case	control	-0.029163421	0.09107227	-0.320222839	0.748799411	0.939503405	down_in_case
PRJNA8274(	ENSGALG00010006269	ENSGALG00010006269	case	control	0.859711709	1.255608868	0.684697067	0.4935351	0.837916219	up_in_case
PRJNA8274(	ENSGALG00010006270	STAM	case	control	-0.135269238	0.060582873	-2.232796671	0.025562356	0.283805659	down_in_case
PRJNA8274(	ENSGALG00010006273	ARL13B	case	control	-0.107743312	0.06828094	-1.577941257	0.114579106	0.509446069	down_in_case
PRJNA8274(	ENSGALG00010006277	GNG10	case	control	0.221420237	0.130632544	1.694985264	0.090078216	0.473572975	up_in_case
PRJNA8274(	ENSGALG00010006279	HACD1	case	control	-0.119769481	0.091384357	-1.310612507	0.189988712	0.613281846	down_in_case
PRJNA8274(	ENSGALG00010006280	VRTN	case	control	0.443240819	0.421856465	1.050691067	0.2934005	0.716170594	up_in_case
PRJNA8274(	ENSGALG00010006281	TRMT11	case	control	-0.020972218	0.076601007	-0.273785152	0.784249734	0.949518053	down_in_case
PRJNA8274(	ENSGALG00010006283	DNAJC25	case	control	0.096113574	0.071033217	1.353079289	0.176030301	0.594809383	up_in_case
PRJNA8274(	ENSGALG00010006285	FZD8	case	control	-0.003179725	0.106104264	-0.029967932	0.976092628	0.994231527	down_in_case
PRJNA8274(	ENSGALG00010006287	ECPAS	case	control	-0.1012611	0.073467891	-1.378304155	0.168109401	0.58432606	down_in_case
PRJNA8274(	ENSGALG00010006288	ARF5	case	control	0.209950967	0.097820835	2.146280656	0.031850594	0.3098361	up_in_case
PRJNA8274(	ENSGALG00010006289	SKIDA1	case	control	0.099061776	0.123033755	0.805159332	0.420727741	0.801969583	up_in_case
PRJNA8274(	ENSGALG00010006291	ENSGALG00010006291	case	control	0.63259442	0.152354587	4.152119282	3.2941E-05	0.004663931	up_in_case
PRJNA8274(	ENSGALG00010006293	SLC22A3	case	control	0.242871946	0.350787487	0.692362059	0.488709987	0.836251383	up_in_case
PRJNA8274(	ENSGALG00010006300	GCC1	case	control	0.109899453	0.089146694	1.232793362	0.217652869	0.642912219	up_in_case
PRJNA8274(	ENSGALG00010006301	ENSGALG00010006301	case	control	0.215978889	0.084439085	2.55780707	0.010533451	0.183125942	up_in_case
PRJNA8274(	ENSGALG00010006310	ALG5	case	control	0.105771736	0.082833915	1.276913399	0.201632823	0.624913171	up_in_case
PRJNA8274(	ENSGALG00010006311	ABCD4	case	control	0.350194186	0.120782066	2.899388933	0.003738908	0.105649139	up_in_case
PRJNA8274(	ENSGALG00010006323	ANAPC10	case	control	-0.111605064	0.071625222	-1.55818104	0.119190338	0.516645008	down_in_case
PRJNA8274(	ENSGALG00010006327	DENND6B	case	control	-0.052335543	0.058600831	-0.893085332	0.371811477	0.77369739	down_in_case
PRJNA8274(	ENSGALG00010006328	SYNDIG1L	case	control	0.057720314	0.193003005	0.299064328	0.764890964	0.943786458	up_in_case
PRJNA8274(	ENSGALG00010006330	STX19	case	control	-0.367163362	0.33170143	-1.106909192	0.268333191	0.695012613	down_in_case
PRJNA8274(	ENSGALG00010006331	SMAD9	case	control	0.080748369	0.116729748	0.691754842	0.4890913	0.836347578	up_in_case
PRJNA8274(	ENSGALG00010006336	ALDH8A1	case	control	1.926455866	1.063609205	1.81124407			up_in_case
PRJNA8274(	ENSGALG00010006341	HHIP	case	control	0.082361341	0.150659061	0.546673665	0.58460295	0.881307382	up_in_case
PRJNA8274(	ENSGALG00010006342	RFXAP	case	control	-0.027925577	0.071512496	-0.390499261	0.696167402	0.921691866	down_in_case
PRJNA8274(	ENSGALG00010006343	SEC23A	case	control	0.06378798	0.039169013	1.628531716	0.10341219	0.492970761	up_in_case

PRJNA8274(	ENSGALG00010006344	HINT3	case	control	-0.117483286	0.099306305	-1.18303955	0.236793473	0.661744512	down_in_case
PRJNA8274(	ENSGALG00010006345	SMC2	case	control	-0.024239146	0.213165255	-0.113710587	0.90946722	0.981730793	down_in_case
PRJNA8274(	ENSGALG00010006346	NSUN3	case	control	-0.091322964	0.147723544	-0.618201819	0.536442311	0.857336818	down_in_case
PRJNA8274(	ENSGALG00010006347	PPP6R2	case	control	-0.156412914	0.067972126	-2.301133172	0.021384105	0.261808818	down_in_case
PRJNA8274(	ENSGALG00010006351	IGF2R	case	control	-0.114021358	0.047491515	-2.400878489	0.016355767	0.226415743	down_in_case
PRJNA8274(	ENSGALG00010006352	GEMIN2	case	control	-0.087350772	0.075302444	-1.15999917	0.246049144	0.670869818	down_in_case
PRJNA8274(	ENSGALG00010006353	ABCE1	case	control	0.01452788	0.046530006	0.312226051	0.754868735	0.941559531	up_in_case
PRJNA8274(	ENSGALG00010006355	PTGR1	case	control	0.034032737	0.08440387	0.403212989	0.686791545	0.917290381	up_in_case
PRJNA8274(	ENSGALG00010006359	SGK1	case	control	0.179456026	0.075644062	2.372374278	0.017674177	0.23642725	up_in_case
PRJNA8274(	ENSGALG00010006361	SERTM1	case	control	-0.204731763	0.15161184	-1.35036791	0.176897999	0.595582794	down_in_case
PRJNA8274(	ENSGALG00010006363	TRAPPC6A	case	control	0.023997498	0.078720085	0.304845942	0.760483489	0.942883622	up_in_case
PRJNA8274(	ENSGALG00010006372	SBF1	case	control	-0.01302593	0.055830175	-0.233313443	0.81551802	0.957374769	down_in_case
PRJNA8274(	ENSGALG00010006378	CAAP1	case	control	0.055414066	0.189723421	0.292078151	0.770226872	0.945428692	up_in_case
PRJNA8274(	ENSGALG00010006381	SPART	case	control	-0.177520218	0.098219132	-1.807389401	0.070701585	0.430226668	down_in_case
PRJNA8274(	ENSGALG00010006382	PNN	case	control	0.032616369	0.054984726	0.593189622	0.553054257	0.866252369	up_in_case
PRJNA8274(	ENSGALG00010006387	OTUD4	case	control	-0.046384483	0.046524726	-0.996985636	0.318771482	0.738427335	down_in_case
PRJNA8274(	ENSGALG00010006404	SMAD1	case	control	-0.078142808	0.05893529	-1.325908614	0.184869969	0.607676259	down_in_case
PRJNA8274(	ENSGALG00010006408	ENSGALG00010006408	case	control	0.080952936	0.369236411	0.219244185	0.826459838	0.961207256	up_in_case
PRJNA8274(	ENSGALG00010006411	RGS17	case	control	-0.387883771	0.427484626	-0.90736309	0.364214821	0.770055025	down_in_case
PRJNA8274(	ENSGALG00010006421	LMF2	case	control	0.060060339	0.059559071	1.008416317	0.313254643	0.734712382	up_in_case
PRJNA8274(	ENSGALG00010006434	MMAA	case	control	-0.146514511	0.103630425	-1.413817521	0.157415488	0.568732055	down_in_case
PRJNA8274(	ENSGALG00010006446	LSAMP	case	control	-0.201029019	0.105330785	-1.908549514	0.05632023	0.386129105	down_in_case
PRJNA8274(	ENSGALG00010006448	MAS1	case	control	-0.60385161	0.483236331	-1.249598946	0.211446088	0.636501957	down_in_case
PRJNA8274(	ENSGALG00010006454	SOD2	case	control	-0.052676601	0.070251788	-0.749825765	0.453359649	0.821282604	down_in_case
PRJNA8274(	ENSGALG00010006457	NCAPH2	case	control	-0.061732942	0.096612171	-0.638976863	0.522837984	0.851159286	down_in_case
PRJNA8274(	ENSGALG00010006463	SCO2	case	control	0.06040483	0.078572495	0.76877831	0.442024927	0.813507909	up_in_case
PRJNA8274(	ENSGALG00010006465	PLAA	case	control	0.038009872	0.173745221	0.218767869	0.826830878	0.96126918	up_in_case
PRJNA8274(	ENSGALG00010006472	DCLK1	case	control	-0.303316337	0.115395102	-2.628502701	0.008576167	0.163956142	down_in_case
PRJNA8274(	ENSGALG00010006478	ENSGALG00010006478	case	control	0.035687031	0.062485204	0.571127702	0.567913082	0.874276786	up_in_case
PRJNA8274(	ENSGALG00010006480	IFT74	case	control	-0.065288708	0.177591209	-0.367634797	0.713145565	0.926862121	down_in_case
PRJNA8274(	ENSGALG00010006486	CEP43	case	control	0.061293125	0.075910598	0.807438313	0.419414001	0.80093491	up_in_case
PRJNA8274(	ENSGALG00010006492	GAP43	case	control	-0.361591823	0.282399625	-1.280426002	0.200395354	0.622968166	down_in_case
PRJNA8274(	ENSGALG00010006496	UBE2H	case	control	-0.156196566	0.048653187	-3.210407723	0.001325468	0.05778718	down_in_case
PRJNA8274(	ENSGALG00010006506	ZNF827	case	control	0.010761607	0.072223651	0.149003924	0.881550536	0.976768377	up_in_case
PRJNA8274(	ENSGALG00010006515	FBXO33	case	control	0.061404547	0.055978659	1.096927792	0.272672959	0.698931401	up_in_case
PRJNA8274(	ENSGALG00010006516	NBEA	case	control	-0.006467014	0.072697387	-0.088957994	0.929115294	0.986878757	down_in_case
PRJNA8274(	ENSGALG00010006517	ZBTB20	case	control	-0.121140218	0.226487561	-0.534864771	0.59274337	0.883625114	down_in_case
PRJNA8274(	ENSGALG00010006521	ZNF410	case	control	0.023701538	0.086452924	0.274155423	0.783965183	0.949518053	up_in_case
PRJNA8274(	ENSGALG00010006524	TAB2	case	control	-0.036395374	0.042392509	-0.858533156	0.39059813	0.784047342	down_in_case
PRJNA8274(	ENSGALG00010006526	LSM6	case	control	-0.072698643	0.095704198	-0.759618126	0.447482881	0.818077884	down_in_case
PRJNA8274(	ENSGALG00010006528	ZC3HC1	case	control	-0.05150042	0.072973808	-0.705738416	0.480350826	0.831902242	down_in_case
PRJNA8274(	ENSGALG00010006531	FAM161B	case	control	0.320598111	0.09576229	3.347853418	0.000814401	0.043781694	up_in_case
PRJNA8274(	ENSGALG00010006532	KLHDC10	case	control	-0.147950796	0.059504595	-2.486376007	0.012905153	0.199897239	down_in_case
PRJNA8274(	ENSGALG00010006537	DRD3	case	control	0.184984331	0.165913177	1.114946586	0.264873312	0.690855045	up_in_case
PRJNA8274(	ENSGALG00010006538	TEK	case	control	-0.059391458	0.081000735	-0.733221221	0.463423515	0.825091817	down_in_case
PRJNA8274(	ENSGALG00010006542	RFC3	case	control	-0.000438773	0.08423536	-0.00520889	0.995843926	0.998710499	down_in_case
PRJNA8274(	ENSGALG00010006544	MOB3B	case	control	0.216477933	0.226797759	0.954497672	0.339831787	0.751350877	up_in_case
PRJNA8274(	ENSGALG00010006546	SMOC2	case	control	-0.225440262	0.150961252	-1.493365078	0.135341649	0.539774365	down_in_case
PRJNA8274(	ENSGALG00010006548	TMEM209	case	control	-0.159387002	0.066311469	-2.40361137	0.016234021	0.226036602	down_in_case
PRJNA8274(	ENSGALG00010006549	ZDHHC23	case	control	0.247272397	0.138751143	1.782128719	0.074728244	0.439711709	up_in_case
PRJNA8274(	ENSGALG00010006557	COQ6	case	control	-0.072523552	0.093402307	-0.776464244	0.437474925	0.810720016	down_in_case
PRJNA8274(	ENSGALG00010006558	USP10	case	control	-0.06073251	0.050438437	-1.204091818	0.228554092	0.651938858	down_in_case
PRJNA8274(	ENSGALG00010006564	SLC10A7	case	control	0.14503142	0.082832002	1.750910486	0.07996133	0.451063915	up_in_case
PRJNA8274(	ENSGALG00010006568	ENTPD5	case	control	0.162158726	0.12039664	1.346870865	0.178021826	0.59730458	up_in_case
PRJNA8274(	ENSGALG00010006570	ENSGALG00010006570	case	control	-0.188229458	0.35172092	-0.53516708	0.592534328	0.883604289	down_in_case
PRJNA8274(	ENSGALG00010006575	SSBP2	case	control	-0.096850781	0.140336421	-0.690132902	0.490110614	0.836347578	down_in_case
PRJNA8274(	ENSGALG00010006576	CCDC191	case	control	0.064212767	0.070006332	0.917242268	0.359015702	0.765374836	up_in_case
PRJNA8274(	ENSGALG00010006580	ALDH6A1	case	control	-0.217857585	0.076397278	-2.851640661	0.004349424	0.113705229	down_in_case
PRJNA8274(	ENSGALG00010006581	CEP41	case	control	-0.079397549	0.112698152	-0.704515091	0.481112056	0.8322127	down_in_case
PRJNA8274(	ENSGALG00010006585	TTC29	case	control	0.293044298	0.197979555	1.48017455	0.13882667	0.540932258	up_in_case
PRJNA8274(	ENSGALG00010006592	STARD13	case	control	0.120658139	0.052571003	2.295146223	0.021724746	0.264012564	up_in_case
PRJNA8274(	ENSGALG00010006598	ENSGALG00010006598	case	control	0.267527422	0.103179817	2.592827057	0.009519062	0.174964773	up_in_case
PRJNA8274(	ENSGALG00010006602	EDNRA	case	control	0.05057201	0.064554484	0.783400423	0.433392008	0.808138728	up_in_case
PRJNA8274(	ENSGALG00010006603	MEST	case	control	-0.201968989	0.134180896	-1.505199291	0.132272847	0.535684428	down_in_case
PRJNA8274(	ENSGALG00010006606	SULT	case	control	2.024134597	1.103025462	1.835075133	0.066494558	0.417598673	up_in_case
PRJNA8274(	ENSGALG00010006608	KL	case	control	0.429955308	0.263254641	1.633229735	0.102420688	0.491460867	up_in_case
PRJNA8274(	ENSGALG00010006614	TMEM184C	case	control	0.000367759	0.048264829	0.007619602	0.993920496	0.998238292	up_in_case
PRJNA8274(	ENSGALG00010006615	GRAMD1C	case	control	-0.018465381	0.105914899	-0.174341675	0.861596949	0.972599205	down_in_case
PRJNA8274(	ENSGALG00010006616	COPG2	case	control	-0.032914273	0.053378153	-0.616624422	0.537482482	0.85762101	down_in_case
PRJNA8274(	ENSGALG00010006619	LIN52	case	control	-0.142098032	0.07146941	-1.988235696	0.046785629	0.356907825	down_in_case
PRJNA8274(	ENSGALG00010006621	DACT2	case	control	0.014519746	0.111157514	0.13062316	0.89607342	0.980176778	up_in_case
PRJNA8274(	ENSGALG00010006623	ENSGALG00010006623	case	control	0.075378889	0.156703257	0.481029494	0.630495537	0.89653616	up_in_case
PRJNA8274(	ENSGALG00010006627	SF3B5	case	control	-0.056719587	0.065727881	-0.862945611	0.388167371	0.783067156	down_in_case
PRJNA8274(	ENSGALG00010006633	FBXO18	case	control	0.061337372	0.064407176	0.95233756	0.340925815	0.752467843	up_in_case
PRJNA8274(	ENSGALG00010006634	PRMT9	case	control	0.058266244	0.063254924	0.921133729	0.356980613	0.763373775	up_in_case
PRJNA8274(	ENSGALG00010006635	ENSGALG00010006635	case	control	0.853668735	0.457747826	1.864932364	0.062190885	0.405537748	up_in_case
PRJNA8274(	ENSGALG00010006641	ENSGALG00010006641	case	control	0.421251373	0.390509269	1.07872311	0.280711181	0.705762606	up_in_case
PRJNA8274(	ENSGALG00010006651	ENSGALG00010006651	case	control	-0.051927477	0.060975245	-0.851615725	0.394427409	0.786978937	down_in_case
PRJNA8274(	ENSGALG00010006653	CFL2	case	control	-0.060847096	0.070389436	-0.864435055	0.387348943	0.782604263	down_in_case
PRJNA8274(	ENSGALG00010006657	PDS5B	case	control	-0.105506765	0.073804019	-1.429553108	0.152845322	0.561193576	down_in_case
PRJNA8274(	ENSGALG00010006670	ANKRD16	case	control	-0.110714855	0.170684775	-0.648651033	0.516563962	0.848054666	down_in_case
PRJNA8274(	ENSGALG00010006673	BAZ1A	case	control	0.02171996	0.074661166	0.290913745	0.771117289	0.94558971	up_in_case
PRJNA8274(	ENSGALG00010006678	ENSGALG00010006678	case	control	0.029500739	0.426435155	0.069179894	0.944846427	0.990313016	up_in_case
PRJNA8274(	ENSGALG00010006681	CRISPLD2	case	control	-0.130975176	0.217257212	-0.60285767	0.546603378	0.86246958	down_in_case
PRJNA8274(	ENSGALG00010006682	ARHGAP10	case	control	0.100820847	0.095366347	1.057195231	0.290422496	0.714060795	up_in_case
PRJNA8274(	ENSGALG00010006685	ENSGALG00010006685	case	control	0.076545076	0.101112844	0.757026241	0.449034141	0.818701429	up_in_case
PRJNA8274(	ENSGALG00010006701	LINGO2	case	control	-0.557917891	0.419353893	-1.330422589	0.183379076	0.60491829	down_in_case
PRJNA8274(	ENSGALG00010006715	IRF8	case	control	-0.05413843	0.063894055	-0.847315607	0.39681923	0.788531716	down_in_case
PRJNA8274(	ENSGALG00010006716	ENSGALG00010006716	case	control	-0.004899605	0.071862064	-0.068180689	0.945641799	0.990313016	down_in_case
PRJNA8274(	ENSGALG00010006720	SRP54	case	control	-0.011971623	0.057238088	-0.209154836	0.834327368	0.963099884	down_in_case
PRJNA8274(	ENSGALG00010006728	ENSGALG00010006728	case	control	0.206934725	0.119286451	1.734771416	0.082781301	0.456624721	up_in_case
PRJNA8274(	ENSGALG00010006730	HAPLN1	case	control	0.630116534	0.760476347	0.828581371			
PRJNA8274(	ENSGALG00010006735	NR3C2	case	control	-0.076357039	0.085062428	-0.897658823	0.369367468	0.772123738	down_in_case
PRJNA8274(	ENSGALG00010006739	VCAN	case	control	0.052059917	0.117010342	0.444917228	0.656379572	0.907494354	up_in_case
PRJNA8274(	ENSGALG00010006740	BRCA2	case	control	-0.0650038	0.081258393	-0.799964129	0.42373158	0.803723282	down_in_case
PRJNA8274(	ENSGALG00010006748	FAM177A1	case	control	-0.045485982	0.053328974	-0.852931883	0.39369708	0.786843919	down_in_case
PRJNA8274(	ENSGALG00010006760	TASOR2	case	control	-0.112488985	0.050001994	-2.249689982	0.024468632	0.276609812	down_in_case
PRJNA8274(	ENSGALG00010006761	PPP2R3C	case	control	-0.035652434	0.073193334	-0.487099461	0.626187849	0.895536157	down_in_case
PRJNA8274(	ENSGALG00010006767	ENSGALG00010006767	case	control	-0.062251178	0.321300171	-0.193747727	0.846373419	0.967631907	down_in_case
PRJNA8274(	ENSGALG00010006794	PERPA	case	control	1.050556774	0.735180332	1.428978345	0.153010458	0.561193576	up_in_case
PRJNA8274(	ENSGALG00010006803	COX4I1	case	control	-0.117440075	0.071858394	-1.634326466	0.102190319	0.491460867	down_in_case
PRJNA8274(	ENSGALG00010006804	PSMA6	case	control	-0.028575311	0.059973309	-0.476467133	0.633741617	0.898107199	down_in_case
PRJNA8274(	ENSGALG00010006814	ENSGALG00010006814	case	control	0.477882853	0.432962688	1.103750662	0.269701312	0.696915209	up_in_case
PRJNA8274(	ENSGALG00010006816	ENSGALG00010006816	case	control	0.118042085	0.481130023	0.245343421	0.806190534	0.9556344	up_in_case
PRJNA8274(	ENSGALG00010006819	EMC8	case	control	0.050333043	0.047634184	1.056658034	0.290667684	0.714060795	up_in_case
PRJNA8274(	ENSGALG00010006821	XRCC4	case	control	0.03288815	0.213693687	0.153903237	0.877686032	0.975889142	up_in_case
PRJNA8274(	ENSGALG00010006828	GTF2A1	case	control	0.002193283	0.036437271	0.060193396	0.952001606	0.992056045	up_in_case
PRJNA8274(	ENSGALG00010006833	LTV1	case	control	-0.109554656	0.0806504	-1.358389503	0.174340123	0.593194623	down_in_case
PRJNA8274(	ENSGALG00010006835	FRY	case	control	-0.245866811	0.101430125	-2.42400185	0.015350527	0.219732273	down_in_case
PRJNA8274(	ENSGALG00010006838	ASB13	case	control	-0.038576453	0.092228221	-0.418271683	0.675748491	0.914247368	down_in_case
PRJNA8274(	ENSGALG00010006840	ENSGALG00010006840	case	control	-0.097008009	0.104870729	-0.925024646	0.354953087	0.763161644	down_in_case
PRJNA8274(	ENSGALG00010006843	STON2	case	control	-0.190330266	0.143455504	-1.326754708	0.184589837	0.607639686	down_in_case
PRJNA8274(	ENSGALG00010006845	CALML3	case	control	1.03994572	0.946202696	1.099072877	0.271736279	0.698323327	up_in_case
PRJNA8274(	ENSGALG00010006847	SEL1L	case	control	0.123709427	0.047101918	2.626420169	0.008628823	0.164742549	up_in_case
PRJNA8274(	ENSGALG00010006850	IRF2BPL	case	control	-0.057805428	0.068217565	-0.847368677	0.396789658	0.788531716	down_in_case
PRJNA8274(	ENSGALG00010006851	TMEM167A	case	control	-0.06365149	0.127905613	-0.497644225	0.618734827	0.891656847	down_in_case
PRJNA8274(	ENSGALG00010006853	NET1	case	control	-0.028936365	0.096961165	-0.298432523	0.765373069	0.943786458	down_in_case
PRJNA8274(	ENSGALG00010006854	FOXG1	case	control	-0.406605486	2.236619589	-0.181794655	0.85574388	0.970656588	down_in_case
PRJNA8274(	ENSGALG00010006855	DCLK2	case	control	-0.061392551	0.112547416	-0.545481653	0.585422295	0.881307382	down_in_case
PRJNA8274(	ENSGALG00010006865	EFCAB2	case	control	0.037349401	0.310610865	0.120244993	0.904289077	0.981104465	up_in_case
PRJNA8274(	ENSGALG00010006877	GSE1	case	control	-0.077726759	0.083084151	-0.935518492	0.349521158	0.759342183	down_in_case
PRJNA8274(	ENSGALG00010006883	LRBA	case	control	0.020001723	0.104578231	0.191260865	0.84832122	0.968692766	up_in_case
PRJNA8274(	ENSGALG00010006885	RHOU	case	control	0.085810212	0.112853555	0.760367824	0.447034751	0.817833512	up_in_case
PRJNA8274(	ENSGALG00010006901	TUBAL3	case	control	0.051612159	0.093143312	0.554115667	0.579499757	0.879142433	up_in_case
PRJNA8274(	ENSGALG00010006903	B3GLCT	case	control	0.03890955	0.06306039	0.617020457	0.537221233	0.857491196	up_in_case
PRJNA8274(	ENSGALG00010006912	ENSGALG00010006912	case	control	0.162150516	0.118503197	1.36832187	0.171211348	0.588680519	up_in_case

PRJNA8274(	ENSGALG00010006913	HSPH1	case	control	0.18492666	0.112630922	1.641881793	0.100614504	0.490291708	up_in_case
PRJNA8274(	ENSGALG00010006922	SMYD3	case	control	-0.070321575	0.138217471	-0.508774863	0.610910041	0.889056885	down_in_case
PRJNA8274(	ENSGALG00010006923	ENSGALG00010006923	case	control	-0.252023508	0.215400097	-1.170025044	0.24199089	0.666243691	down_in_case
PRJNA8274(	ENSGALG00010006926	ID4	case	control	-0.061275252	0.169028288	-0.362514777	0.716967383	0.926862121	down_in_case
PRJNA8274(	ENSGALG00010006927	UCN3	case	control	0.14039781	0.537519702	0.261195654	0.79394163	0.95280257	up_in_case
PRJNA8274(	ENSGALG00010006939	FBXO31	case	control	0.000695342	0.0843906	0.008239561	0.993425856	0.998196863	up_in_case
PRJNA8274(	ENSGALG00010006942	ALOX5AP	case	control	0.145311257	0.164588569	0.882875756	0.377303404	0.775926253	up_in_case
PRJNA8274(	ENSGALG00010006944	MAB21L2	case	control	0.47408554	0.693147231	0.683960808			up_in_case
PRJNA8274(	ENSGALG00010006946	PLPPR1	case	control	0.184189825	0.319830555	0.57589815	0.564684026	0.872275547	up_in_case
PRJNA8274(	ENSGALG00010006948	ENSGALG00010006948	case	control	0.063032831	0.299507926	0.210454635	0.833312855	0.962628147	up_in_case
PRJNA8274(	ENSGALG00010006953	MAP1LC3B	case	control	0.104185176	0.061797862	1.6859026	0.091814561	0.476713154	up_in_case
PRJNA8274(	ENSGALG00010006957	PALM2AKAP2	case	control	-0.10732813	0.073907848	-1.452188534	0.14644919	0.55166873	down_in_case
PRJNA8274(	ENSGALG00010006958	KIF26B	case	control	0.093114687	0.177571065	0.52437984	0.600014376	0.884888453	up_in_case
PRJNA8274(	ENSGALG00010006961	ZCCHC14	case	control	-0.060391923	0.071132045	-0.84901149	0.395874899	0.787783337	down_in_case
PRJNA8274(	ENSGALG00010006963	ENSGALG00010006963	case	control	-0.094292137	0.412165906	-0.228772287	0.819045907	0.958669267	down_in_case
PRJNA8274(	ENSGALG00010006966	PGBD5	case	control	-0.188994634	0.275591233	-0.685778832	0.492852587	0.837825962	down_in_case
PRJNA8274(	ENSGALG00010006983	GTPBP2	case	control	0.065290285	0.073289743	0.890851602	0.373008788	0.774369566	up_in_case
PRJNA8274(	ENSGALG00010006988	USPL1	case	control	0.150510362	0.092926349	1.619673688	0.105302391	0.495041148	up_in_case
PRJNA8274(	ENSGALG00010006992	SLC35B4	case	control	0.03939662	0.072788685	0.541246486	0.5883377	0.882771329	up_in_case
PRJNA8274(	ENSGALG00010006998	POLH	case	control	0.043891672	0.063264558	0.693779802	0.487820311	0.835553062	up_in_case
PRJNA8274(	ENSGALG00010007000	LRGUK	case	control	0.072098013	0.46818973	0.153993153	0.877615134	0.975889142	up_in_case
PRJNA8274(	ENSGALG00010007001	SNX30	case	control	0.073158411	0.070834727	1.032804305	0.301695489	0.724474473	up_in_case
PRJNA8274(	ENSGALG00010007002	EXOC4	case	control	-0.064258132	0.058686324	-1.094942186	0.273541966	0.699383177	down_in_case
PRJNA8274(	ENSGALG00010007007	ACSL1	case	control	-0.038913616	0.255842208	-0.152100063	0.879108015	0.976318821	down_in_case
PRJNA8274(	ENSGALG00010007010	ENSGALG00010007010	case	control	1.301492461	0.899840083	1.446359731	0.14807633	0.553857883	up_in_case
PRJNA8274(	ENSGALG00010007011	HMGB1	case	control	-0.042489164	0.045708955	-0.929558843	0.352599545	0.76143184	down_in_case
PRJNA8274(	ENSGALG00010007014	ENSGALG00010007014	case	control	1.230474622	0.39119463	3.145428206	0.001658439	0.065655176	up_in_case
PRJNA8274(	ENSGALG00010007017	SRD5A2	case	control	-0.057652871	0.331616365	-0.173854119	0.861980111	0.972643856	down_in_case
PRJNA8274(	ENSGALG00010007021	SULT6B1	case	control	-0.018286429	0.211238862	-0.086567544	0.931015266	0.987258715	down_in_case
PRJNA8274(	ENSGALG00010007028	KATNAL1	case	control	0.008848294	0.105090051	0.084197261	0.932899596	0.987962988	up_in_case
PRJNA8274(	ENSGALG00010007033	CEBPZOS	case	control	-0.100431652	0.120753106	-0.831710713	0.405572252	0.793782506	down_in_case
PRJNA8274(	ENSGALG00010007038	CENPU	case	control	0.016732616	0.127484513	0.131252149	0.895575844	0.980176778	up_in_case
PRJNA8274(	ENSGALG00010007041	NAE1	case	control	-0.003550286	0.066803346	-0.053145327	0.957616117	0.992056045	down_in_case
PRJNA8274(	ENSGALG00010007049	ENSGALG00010007049	case	control	-0.478537504	0.603094463	-0.793470234	0.427503905	0.805305429	down_in_case
PRJNA8274(	ENSGALG00010007051	E2F3	case	control	0.073742505	0.126126189	0.584672426	0.558767998	0.869450004	up_in_case
PRJNA8274(	ENSGALG00010007066	INIP	case	control	-0.093224513	0.170899433	-0.545493398	0.58541422	0.881307382	down_in_case
PRJNA8274(	ENSGALG00010007067	CASP3	case	control	0.008221226	0.065727878	0.125079748	0.900460416	0.98028579	up_in_case
PRJNA8274(	ENSGALG00010007068	NCAM2	case	control	-0.044961643	0.290901164	-0.15455986	0.877168317	0.975813349	down_in_case
PRJNA8274(	ENSGALG00010007070	UBL3	case	control	0.067444911	0.052072432	1.295213381	0.19524663	0.618389105	up_in_case
PRJNA8274(	ENSGALG00010007085	PRIMPOL	case	control	-0.14630422	0.1137147	-1.286590214	0.198237156	0.621664767	down_in_case
PRJNA8274(	ENSGALG00010007090	CEBPZ	case	control	-0.066404914	0.05288805	-1.255575013	0.209270096	0.63414862	down_in_case
PRJNA8274(	ENSGALG00010007092	DYNC1LI2	case	control	-0.023384103	0.034134546	-0.685056815	0.49330807	0.837916219	down_in_case
PRJNA8274(	ENSGALG00010007097	ZCCHC9	case	control	0.056425563	0.176952257	0.318874503	0.749821679	0.939741456	up_in_case
PRJNA8274(	ENSGALG00010007098	NDUFAF7	case	control	0.049726823	0.083114206	0.598295111	0.549643038	0.864234145	up_in_case
PRJNA8274(	ENSGALG00010007102	CMTM3	case	control	0.048529191	0.063966848	0.758661606	0.44805501	0.818697952	up_in_case
PRJNA8274(	ENSGALG00010007106	SLC7A1	case	control	-0.183077328	0.083298777	-2.197839309	0.027960559	0.298666145	down_in_case
PRJNA8274(	ENSGALG00010007108	MBOAT1	case	control	0.218832092	0.139382731	1.57000864	0.116413101	0.5134816	up_in_case
PRJNA8274(	ENSGALG00010007110	ARL6	case	control	0.031664768	0.04768667	0.664017187	0.506679311	0.843472666	up_in_case
PRJNA8274(	ENSGALG00010007114	CKLF	case	control	0.176096105	0.173697766	1.013807539	0.310674597	0.733587496	up_in_case
PRJNA8274(	ENSGALG00010007115	ENSGALG00010007115	case	control	-0.081703732	0.162077921	-0.504101558	0.614190012	0.890440426	down_in_case
PRJNA8274(	ENSGALG00010007118	ENSGALG00010007118	case	control	0.093259886	0.129031721	0.722767121	0.46982297	0.829101076	up_in_case
PRJNA8274(	ENSGALG00010007123	ENSGALG00010007123	case	control	0.135441662	0.300580228	0.450600703	0.65227736	0.905706359	up_in_case
PRJNA8274(	ENSGALG00010007126	ENSGALG00010007126	case	control	1.965988153	1.040389471	1.889665561	0.058802703	0.395497471	up_in_case
PRJNA8274(	ENSGALG00010007130	IRF2	case	control	-0.067532733	0.044716042	-1.510257399	0.130977758	0.534010989	down_in_case
PRJNA8274(	ENSGALG00010007133	BLVRA	case	control	0.233206186	0.155025871	1.504304957	0.132502863	0.535857167	up_in_case
PRJNA8274(	ENSGALG00010007135	TK2	case	control	0.060393026	0.093548398	0.645580542	0.518551042	0.848941486	up_in_case
PRJNA8274(	ENSGALG00010007137	ENSGALG00010007137	case	control	1.056902501	0.292059276	3.618794501	0.000295979	0.023376995	up_in_case
PRJNA8274(	ENSGALG00010007141	PRKD3	case	control	0.101329302	0.067225876	1.507296123	0.131734771	0.535159035	up_in_case
PRJNA8274(	ENSGALG00010007143	CKMT2	case	control	-0.431156627	0.371296628	-1.161218807	0.245552928	0.670194327	down_in_case
PRJNA8274(	ENSGALG00010007146	ENSGALG00010007146	case	control	0.026587457	0.061043112	0.435552127	0.663161711	0.911044469	up_in_case
PRJNA8274(	ENSGALG00010007160	ENSGALG00010007160	case	control	0.181804652	0.125873752	1.444341245	0.148643009	0.55519893	up_in_case
PRJNA8274(	ENSGALG00010007162	ENSGALG00010007162	case	control	-0.093540966	0.185173127	-0.505154109	0.6134506	0.890252839	down_in_case
PRJNA8274(	ENSGALG00010007165	ENSGALG00010007165	case	control	-0.372728672	0.298452153	-1.24887245	0.21171173	0.636501957	down_in_case
PRJNA8274(	ENSGALG00010007166	RASGRF2	case	control	-0.014376089	0.149886128	-0.095913403	0.92358935	0.98553537	down_in_case
PRJNA8274(	ENSGALG00010007168	MINA	case	control	-0.103210439	0.065208466	-1.582776668	0.113472377	0.508062524	down_in_case
PRJNA8274(	ENSGALG00010007169	QPCT	case	control	0.038501619	0.080126506	0.480510387	0.630864519	0.896844877	up_in_case
PRJNA8274(	ENSGALG00010007175	FOXC2	case	control	0.351790051	0.349981641	1.005167155	0.314816368	0.736442673	up_in_case
PRJNA8274(	ENSGALG00010007181	GJB2	case	control	1.025395783	0.647899344	1.582646738	0.113502004	0.508062524	up_in_case
PRJNA8274(	ENSGALG00010007182	ENSGALG00010007182	case	control	0.503169191	0.256940509	1.958310085	0.050193638	0.366770069	up_in_case
PRJNA8274(	ENSGALG00010007183	VOPP1	case	control	0.052634457	0.221025651	0.238137324	0.811774587	0.956240049	up_in_case
PRJNA8274(	ENSGALG00010007184	VGLL3	case	control	-0.081826362	0.071385329	-1.146263001	0.251686347	0.677084435	down_in_case
PRJNA8274(	ENSGALG00010007187	ENPP6	case	control	-0.113706006	0.099567197	-1.142002683	0.253452904	0.678954688	down_in_case
PRJNA8274(	ENSGALG00010007188	MAB21L1	case	control	-0.096053679	0.120001809	-0.800435262	0.423458657	0.803723282	down_in_case
PRJNA8274(	ENSGALG00010007190	ENSGALG00010007190	case	control	-0.187266911	0.149074388	-1.256197755	0.209044281	0.634076945	down_in_case
PRJNA8274(	ENSGALG00010007201	ENSGALG00010007201	case	control	-0.5470921	0.376825516	-1.451844625	0.146544813	0.55166873	down_in_case
PRJNA8274(	ENSGALG00010007203	SLC30A6	case	control	0.120086159	0.063339801	1.895903625	0.05797279	0.39192679	up_in_case
PRJNA8274(	ENSGALG00010007204	LANCL2	case	control	-0.007335207	0.05155991	-0.142265712	0.886870129	0.977630122	down_in_case
PRJNA8274(	ENSGALG00010007207	EPM2A	case	control	-0.034366558	0.089105588	-0.385683533	0.69973106	0.922288441	down_in_case
PRJNA8274(	ENSGALG00010007214	MTHFSD	case	control	0.221762386	0.082348991	2.692958141	0.007082116	0.146516526	up_in_case
PRJNA8274(	ENSGALG00010007217	MSH3	case	control	0.081067268	0.161106917	0.503189246	0.614831225	0.890803484	up_in_case
PRJNA8274(	ENSGALG00010007223	FOXF1	case	control	-0.121716024	0.404040901	-0.30124679	0.763226313	0.943786458	down_in_case
PRJNA8274(	ENSGALG00010007224	DHFR	case	control	0.160460733	0.215331509	0.745179996	0.456162914	0.822099797	up_in_case
PRJNA8274(	ENSGALG00010007226	ENSGALG00010007226	case	control	1.599040856	1.135477122	1.408254579	0.1590557	0.571481535	up_in_case
PRJNA8274(	ENSGALG00010007230	ENSGALG00010007230	case	control	0.464398592	0.266845563	1.740327203	0.08180158	0.454736389	up_in_case
PRJNA8274(	ENSGALG00010007243	BEAN1	case	control	-0.14877545	0.112164531	-1.326403708	0.184706011	0.607639686	down_in_case
PRJNA8274(	ENSGALG00010007251	SPAST	case	control	-0.00986877	0.055384715	-0.178185796	0.858577065	0.971828322	down_in_case
PRJNA8274(	ENSGALG00010007252	NFATC1	case	control	-0.063925173	0.126155196	-0.506718515	0.612352338	0.889886045	down_in_case
PRJNA8274(	ENSGALG00010007259	ANKRD34B	case	control	0.124868814	0.135123739	0.924107156	0.355430528	0.763161644	up_in_case
PRJNA8274(	ENSGALG00010007265	CD200R1A	case	control	-0.149115826	0.206715328	-0.72135834	0.470689071	0.829101076	down_in_case
PRJNA8274(	ENSGALG00010007270	CDH5	case	control	-0.024196942	0.066525665	-0.363723412	0.716064559	0.926862121	down_in_case
PRJNA8274(	ENSGALG00010007271	CADM2	case	control	0.126605973	0.300913714	0.420738462	0.673946072	0.913846845	up_in_case
PRJNA8274(	ENSGALG00010007275	DPY30	case	control	-0.073049355	0.069317107	-1.053843099	0.291954758	0.714644477	down_in_case
PRJNA8274(	ENSGALG00010007277	FAM151B	case	control	0.3538988	0.386565237	0.915495669	0.359931475	0.765754313	up_in_case
PRJNA8274(	ENSGALG00010007283	STOX2	case	control	-0.068184655	0.086857911	-0.785013754	0.432445505	0.807707587	down_in_case
PRJNA8274(	ENSGALG00010007284	ENSGALG00010007284	case	control	0.084062885	0.120080248	0.700055897	0.483892397	0.833895068	up_in_case
PRJNA8274(	ENSGALG00010007287	ENSGALG00010007287	case	control	-0.25467226	0.434675297	-0.585890806	0.557948896	0.869041413	down_in_case
PRJNA8274(	ENSGALG00010007294	TRAPPC11	case	control	0.165245881	0.047654283	3.46759767	0.000525133	0.033449927	up_in_case
PRJNA8274(	ENSGALG00010007300	CCDC125	case	control	-0.024044125	0.324876193	-0.074010117	0.941002335	0.989551456	down_in_case
PRJNA8274(	ENSGALG00010007302	CDH11	case	control	-0.116842367	0.099376074	-1.175759539	0.239690979	0.664684583	down_in_case
PRJNA8274(	ENSGALG00010007304	F5	case	control	1.000720719	0.443076285	2.258574323	0.023909877	0.273286623	up_in_case
PRJNA8274(	ENSGALG00010007318	RMDN2	case	control	0.012564827	0.107645311	0.116724335	0.907078506	0.981730793	up_in_case
PRJNA8274(	ENSGALG00010007319	ATP9B	case	control	-0.004841332	0.073238798	-0.066103382	0.947295518	0.991025379	down_in_case
PRJNA8274(	ENSGALG00010007320	ENSGALG00010007320	case	control	-0.475776101	0.123344448	-3.857296459	0.000114648	0.011880199	down_in_case
PRJNA8274(	ENSGALG00010007321	CD200	case	control	0.129107748	0.144001271	0.896573671	0.369946452	0.772544067	up_in_case
PRJNA8274(	ENSGALG00010007322	AK6	case	control	0.028212176	0.206115779	0.136875381	0.891129299	0.978917427	up_in_case
PRJNA8274(	ENSGALG00010007324	ENSGALG00010007324	case	control	0.068790052	0.174282936	0.394703309	0.693061861	0.919538375	up_in_case
PRJNA8274(	ENSGALG00010007326	RWDD4	case	control	-0.054274875	0.066510044	-0.816040278	0.4144771	0.798853486	down_in_case
PRJNA8274(	ENSGALG00010007328	NOVA1	case	control	-0.037079746	0.111527063	-0.332473081	0.739532063	0.936365194	down_in_case
PRJNA8274(	ENSGALG00010007330	MMP13	case	control	-0.075961817	0.726779065	-0.104518444	0.916757932	0.983445506	down_in_case
PRJNA8274(	ENSGALG00010007333	QTRT2	case	control	0.112237846	0.060813079	1.845620182	0.064947374	0.414434381	up_in_case
PRJNA8274(	ENSGALG00010007334	SALL3	case	control	0.856002614	0.933460942	0.917020279	0.359132014	0.765374836	up_in_case
PRJNA8274(	ENSGALG00010007337	PRORP	case	control	0.147586327	0.073270766	2.01425938	0.043982308	0.349803675	up_in_case
PRJNA8274(	ENSGALG00010007339	ENSGALG00010007339	case	control	0.116103276	0.183389612	0.633096253	0.526670789	0.85251736	up_in_case
PRJNA8274(	ENSGALG00010007344	RAD17	case	control	0.054595966	0.09660321	0.565156849	0.571967084	0.876814097	up_in_case
PRJNA8274(	ENSGALG00010007353	ING2	case	control	-0.211288029	0.109108019	-1.936503212	0.0528061	0.375125297	down_in_case
PRJNA8274(	ENSGALG00010007357	NOX4	case	control	0.094015438	0.126822595	0.741314573	0.458502727	0.824002639	up_in_case
PRJNA8274(	ENSGALG00010007360	ROBO1	case	control	0.08327736	0.111289709	0.748293445	0.454283177	0.821331802	up_in_case
PRJNA8274(	ENSGALG00010007365	MEMO1	case	control	-0.024604938	0.069178316	-0.355674145	0.72208461	0.929484914	down_in_case
PRJNA8274(	ENSGALG00010007369	ENSGALG00010007369	case	control	0.212971244	0.268250033	0.793928118	0.427237279	0.805305429	up_in_case
PRJNA8274(	ENSGALG00010007374	CMIP	case	control	-0.082923222	0.099251962	-0.835481948	0.403446407	0.79260318	down_in_case
PRJNA8274(	ENSGALG00010007375	CHORDC1	case	control	0.156930486	0.051312446	3.058331837	0.00222573	0.077252265	up_in_case
PRJNA8274(	ENSGALG00010007376	CDKN2AIP	case	control	-0.082380806	0.050299662	-1.637800378	0.101463343	0.491460867	down_in_case
PRJNA8274(	ENSGALG00010007381	EGFR	case	control	-0.123364517	0.065826128	-1.874096516	0.060917123	0.401065776	down_in_case
PRJNA8274(	ENSGALG00010007382	EIF2AK2	case	control	-0.039071618	0.093556666	-0.41762516	0.676221198	0.914247368	down_in_case
PRJNA8274(	ENSGALG00010007387	ENSGALG00010007387	case	control	0.471976237	0.379405465	1.243989031	0.2135036	0.638582746	up_in_case
PRJNA8274(	ENSGALG00010007388	CYP1B1	case	control	0.118854034	0.162362936	0.73202688	0.464152162	0.825544268	up_in_case

PRJNA8274(	ENSGALG00010007391	NANP	case	control	-0.011905918	0.054366657	-0.218993015	0.826655489	0.961207256	down_in_case
PRJNA8274(	ENSGALG00010007392	NAALAD2	case	control	-0.024185413	0.139532018	-0.173332351	0.862390196	0.972787494	down_in_case
PRJNA8274(	ENSGALG00010007393	PLCG2	case	control	0.191622644	0.121101242	1.58233425	0.113573286	0.508165829	up_in_case
PRJNA8274(	ENSGALG00010007398	ENSGALG00010007398	case	control	1.867707693	0.925392702	2.018286602	0.043561421	0.348491091	up_in_case
PRJNA8274(	ENSGALG00010007399	WWC2	case	control	0.098404622	0.108688092	0.905385498	0.365261196	0.770843433	up_in_case
PRJNA8274(	ENSGALG00010007401	SEC61G	case	control	0.055206708	0.090534346	0.609787442	0.542002622	0.85975913	up_in_case
PRJNA8274(	ENSGALG00010007403	SDR42E1	case	control	0.165387821	0.169467381	0.975927168	0.329100558	0.745636104	up_in_case
PRJNA8274(	ENSGALG00010007404	FOLH1	case	control	-0.466977715	0.165755616	-2.81726633	0.004843434	0.119309498	down_in_case
PRJNA8274(	ENSGALG00010007408	HCLS1	case	control	0.014062796	0.149216739	0.09424409	0.924915262	0.985571367	up_in_case
PRJNA8274(	ENSGALG00010007411	ENSGALG00010007411	case	control	1.88315546	0.815206902	2.310033753	0.020886286	0.259355211	up_in_case
PRJNA8274(	ENSGALG00010007413	GPATCH11	case	control	-0.025910663	0.08349168	-0.310338269	0.756303732	0.941659745	down_in_case
PRJNA8274(	ENSGALG00010007414	ROBO2	case	control	0.113617402	0.164749735	0.689636324	0.490422919	0.836479931	up_in_case
PRJNA8274(	ENSGALG00010007416	DCTD	case	control	-0.090437976	0.077617449	-1.16517584	0.243947823	0.668030232	down_in_case
PRJNA8274(	ENSGALG00010007418	NME6	case	control	0.127606633	0.104341002	1.222976874	0.221338431	0.645683305	up_in_case
PRJNA8274(	ENSGALG00010007419	RABL2A	case	control	-0.038380605	0.118485492	-0.323926618	0.745993591	0.938117773	down_in_case
PRJNA8274(	ENSGALG00010007423	HEATR5B	case	control	-0.022658596	0.052334334	-0.432958529	0.665044898	0.9119814	down_in_case
PRJNA8274(	ENSGALG00010007425	ARSA	case	control	-0.110120927	0.059463551	-1.851906348	0.064039272	0.410839656	down_in_case
PRJNA8274(	ENSGALG00010007429	C21orf91	case	control	-0.107827059	0.094190989	-1.14477043	0.252304267	0.677422273	down_in_case
PRJNA8274(	ENSGALG00010007430	MEF2C	case	control	0.036347576	0.130830214	0.277822488	0.781148628	0.949220375	up_in_case
PRJNA8274(	ENSGALG00010007432	NAT	case	control	0.117483009	0.221926116	0.529378929	0.596542612	0.884680373	up_in_case
PRJNA8274(	ENSGALG00010007433	RNF144B	case	control	-0.060494487	0.233177738	-0.259435086	0.795299561	0.95280257	down_in_case
PRJNA8274(	ENSGALG00010007434	ENSGALG00010007434	case	control	0.103882991	0.225363123	0.460958251	0.644828558	0.902255223	up_in_case
PRJNA8274(	ENSGALG00010007436	PNAT3	case	control	-0.136223817	0.114659599	-1.188071633	0.234805189	0.659409702	down_in_case
PRJNA8274(	ENSGALG00010007437	TUSC1	case	control	0.175749905	0.208910733	0.841267955	0.400197838	0.789900496	up_in_case
PRJNA8274(	ENSGALG00010007439	HSD17B2	case	control	0.349896814	0.283725388	1.233223491	0.217492393	0.642877222	up_in_case
PRJNA8274(	ENSGALG00010007441	STRN	case	control	-0.051653824	0.099841959	-0.517355872	0.604907758	0.885268908	down_in_case
PRJNA8274(	ENSGALG00010007455	TRPC6	case	control	0.064856416	0.161191768	0.402355633	0.687422317	0.917290381	up_in_case
PRJNA8274(	ENSGALG00010007457	SHANK3	case	control	-0.205450706	0.089011682	-2.308131935	0.020991799	0.259673644	down_in_case
PRJNA8274(	ENSGALG00010007465	PNAT10	case	control	0.120549404	0.237204743	0.508208237	0.611307315	0.889468315	up_in_case
PRJNA8274(	ENSGALG00010007467	CHODL	case	control	-0.091995932	0.160060393	-0.574757626	0.565455228	0.872906976	down_in_case
PRJNA8274(	ENSGALG00010007479	NFX1	case	control	-0.01292302	0.065622171	-0.196930695	0.843881773	0.96721237	down_in_case
PRJNA8274(	ENSGALG00010007480	TENM3	case	control	-0.115451541	0.078429105	-1.472049708	0.141007455	0.54338031	down_in_case
PRJNA8274(	ENSGALG00010007485	CTSC	case	control	-0.114982482	0.074458267	-1.544254071	0.122526794	0.521003021	down_in_case
PRJNA8274(	ENSGALG00010007500	MPHOSPH6	case	control	-0.004155336	0.10208527	-0.04070456	0.967531427	0.992731535	down_in_case
PRJNA8274(	ENSGALG00010007506	RAB38	case	control	4.36019255	0.368210826	11.84156531	2.3797E-32	4.25371E-29	up_in_case
PRJNA8274(	ENSGALG00010007513	GALNT12	case	control	-0.041299414	0.085644515	-0.482219026	0.629650362	0.896269608	down_in_case
PRJNA8274(	ENSGALG00010007514	VIT	case	control	0.756978155	0.590951378	1.280948287	0.200211831	0.622902456	up_in_case
PRJNA8274(	ENSGALG00010007521	SLC12A7	case	control	-0.114712524	0.084163309	-1.362975451	0.172890248	0.59217498	down_in_case
PRJNA8274(	ENSGALG00010007529	CDH13	case	control	-0.047482581	0.09990081	-0.475297261	0.634575111	0.898107199	down_in_case
PRJNA8274(	ENSGALG00010007532	DCUN1D5	case	control	-0.033522406	0.057166902	-0.586395364	0.557609859	0.868797231	down_in_case
PRJNA8274(	ENSGALG00010007536	GABBR2	case	control	-0.098729232	0.147982888	-0.667166544	0.504665759	0.842675107	down_in_case
PRJNA8274(	ENSGALG00010007539	RPS3A	case	control	-0.065497313	0.073911518	-0.886158403	0.375532175	0.774921593	down_in_case
PRJNA8274(	ENSGALG00010007542	MRPL36	case	control	-0.107611943	0.10733156	-1.002612312	0.316047953	0.736866972	down_in_case
PRJNA8274(	ENSGALG00010007544	MLYCD	case	control	-0.02096632	0.091440202	-0.229289953	0.818643562	0.958536226	down_in_case
PRJNA8274(	ENSGALG00010007548	HSBP1	case	control	-0.053496121	0.048677084	-1.099000107	0.271768019	0.698323327	down_in_case
PRJNA8274(	ENSGALG00010007549	SH3D19	case	control	-0.162129199	0.078753959	-2.058679996	0.039524904	0.335234949	down_in_case
PRJNA8274(	ENSGALG00010007551	FEZ2	case	control	-3.92551E-05	0.050670755	-0.00077471	0.999381871	0.99976345	down_in_case
PRJNA8274(	ENSGALG00010007555	NDUFS6	case	control	-0.045689932	0.09148215	-0.499440947	0.617468779	0.891081782	down_in_case
PRJNA8274(	ENSGALG00010007564	IRX4	case	control	0.010844141	0.583060704	0.018598648	0.985161281	0.996591771	up_in_case
PRJNA8274(	ENSGALG00010007566	FHIP1A	case	control	-0.080549741	0.172884004	-0.465917834	0.641274316	0.901516193	down_in_case
PRJNA8274(	ENSGALG00010007567	TRIP13	case	control	-0.073169702	0.086800796	-0.842961189	0.39925015	0.789900496	down_in_case
PRJNA8274(	ENSGALG00010007569	GPR89A	case	control	-0.017697818	0.078150349	-0.226458598	0.820844757	0.958669267	down_in_case
PRJNA8274(	ENSGALG00010007572	PDZK1	case	control	0.047789837	0.225242425	0.212170675	0.831973884	0.962371901	up_in_case
PRJNA8274(	ENSGALG00010007574	ENSGALG00010007574	case	control	0.076571079	0.231260807	0.331102707	0.740566907	0.937011747	up_in_case
PRJNA8274(	ENSGALG00010007575	MMP27	case	control	0.197898401	0.194409164	1.017947904	0.308702714	0.731823162	up_in_case
PRJNA8274(	ENSGALG00010007577	WFDC1	case	control	0.332852442	0.09889346	3.365767979	0.000763309	0.041821136	up_in_case
PRJNA8274(	ENSGALG00010007579	GATB	case	control	-0.207221736	0.097263628	-2.130516217	0.033129018	0.313323388	down_in_case
PRJNA8274(	ENSGALG00010007580	BRD9	case	control	0.008805396	0.067176446	0.131078626	0.89571311	0.980176778	up_in_case
PRJNA8274(	ENSGALG00010007581	LPCAT1	case	control	-0.051720618	0.084053553	-0.615329348	0.53833724	0.857836253	down_in_case
PRJNA8274(	ENSGALG00010007584	ELP2	case	control	0.052561623	0.079775166	0.658872005	0.509977964	0.844843013	up_in_case
PRJNA8274(	ENSGALG00010007585	KCNG4	case	control	0.29711423	0.588471834	0.504891165	0.61363528	0.890319045	up_in_case
PRJNA8274(	ENSGALG00010007588	TTC27	case	control	-0.066195827	0.073100814	-0.905541588	0.365178538	0.770843433	down_in_case
PRJNA8274(	ENSGALG00010007590	FBXW7	case	control	-0.228825561	0.079343073	-2.884001738	0.003926566	0.107773305	down_in_case
PRJNA8274(	ENSGALG00010007593	SEC61B	case	control	0.10082536	0.089767018	1.123189366	0.261357078	0.687516348	up_in_case
PRJNA8274(	ENSGALG00010007595	ATP2C2	case	control	0.19347229	0.193176217	1.00153266	0.316569359	0.737286943	up_in_case
PRJNA8274(	ENSGALG00010007596	ENSGALG00010007596	case	control	0.351415915	0.511566559	0.686940749	0.492120067	0.83747673	up_in_case
PRJNA8274(	ENSGALG00010007601	TMEM154	case	control	0.194236536	0.346379404	0.560762373	0.57495954	0.877910508	up_in_case
PRJNA8274(	ENSGALG00010007603	ALG2	case	control	0.218169995	0.089248348	2.44452698	0.014504223	0.212076065	up_in_case
PRJNA8274(	ENSGALG00010007604	ENSGALG00010007604	case	control	-1.005288226	0.44200475	-2.274383309	0.02294295	0.267253668	down_in_case
PRJNA8274(	ENSGALG00010007610	TLDC1	case	control	0.012152812	0.057267736	0.212210441	0.831942861	0.962371901	up_in_case
PRJNA8274(	ENSGALG00010007614	BIRC6	case	control	-0.194350612	0.066256786	-2.933293699	0.003353865	0.099502622	down_in_case
PRJNA8274(	ENSGALG00010007624	TPPP	case	control	0.104679241	0.12963221	0.807509498	0.419373005	0.80093491	up_in_case
PRJNA8274(	ENSGALG00010007626	DLK2	case	control	0.010231701	0.123948717	0.082547861	0.934211061	0.988140189	up_in_case
PRJNA8274(	ENSGALG00010007633	ENSGALG00010007633	case	control	-0.000375402	0.106662034	-0.003519544	0.997191816	0.998921977	down_in_case
PRJNA8274(	ENSGALG00010007640	TMEM123	case	control	0.487553157	0.317717395	1.534549775	0.124894458	0.52390459	up_in_case
PRJNA8274(	ENSGALG00010007642	ZNF318	case	control	-0.011047871	0.043049557	-0.25663146	0.797463287	0.95280257	down_in_case
PRJNA8274(	ENSGALG00010007643	COTL1	case	control	0.059328263	0.071085134	0.83460859	0.403938124	0.79260318	up_in_case
PRJNA8274(	ENSGALG00010007650	KLHL36	case	control	-0.011786686	0.077751423	-0.151594471	0.879506794	0.976318821	down_in_case
PRJNA8274(	ENSGALG00010007651	CTNNAL1	case	control	0.069884384	0.081685864	0.855526045	0.392259992	0.785827491	up_in_case
PRJNA8274(	ENSGALG00010007654	HTR1F	case	control	1.316555156	0.653865499	2.013495372	0.044062541	0.350032868	up_in_case
PRJNA8274(	ENSGALG00010007658	BIRC2	case	control	0.163068097	0.07612086	2.142226161	0.032175287	0.3098361	up_in_case
PRJNA8274(	ENSGALG00010007660	FAM98A	case	control	0.017606214	0.068573021	0.256751326	0.797370747	0.95280257	up_in_case
PRJNA8274(	ENSGALG00010007663	ARFIP1	case	control	-0.026216758	0.04866854	-0.538679771	0.590107834	0.882949889	down_in_case
PRJNA8274(	ENSGALG00010007671	FAM206A	case	control	0.00646753	0.099656671	0.06489811	0.948255125	0.991291133	up_in_case
PRJNA8274(	ENSGALG00010007673	FHDC1	case	control	0.255027298	0.203779931	1.251483875	0.210757993	0.636310899	up_in_case
PRJNA8274(	ENSGALG00010007678	CDH8	case	control	-0.347253609	0.284673987	-1.219829085	0.222529673	0.646365317	down_in_case
PRJNA8274(	ENSGALG00010007679	TMEM135	case	control	-0.430539653	0.106679246	-4.035833298	5.44088E-05	0.006946843	down_in_case
PRJNA8274(	ENSGALG00010007680	CHMP2B	case	control	-0.0598225	0.054875132	-1.090156817	0.275644072	0.700894634	down_in_case
PRJNA8274(	ENSGALG00010007695	RASGRP3	case	control	0.187958989	0.092034704	2.042262125	0.041125537	0.338374671	up_in_case
PRJNA8274(	ENSGALG00010007698	ADAMTS18	case	control	-0.365007697	0.14469498	-2.522600975	0.01164905	0.188867818	down_in_case
PRJNA8274(	ENSGALG00010007699	SLC39A6	case	control	-0.170276021	0.066155604	-2.573871473	0.010056762	0.178205332	down_in_case
PRJNA8274(	ENSGALG00010007707	ENSGALG00010007707	case	control	0.946435199	0.698110334	1.355710054	0.175191439	0.594080527	up_in_case
PRJNA8274(	ENSGALG00010007713	SCLT1	case	control	0.009837298	0.107874352	0.091192182	0.927339887	0.986531794	up_in_case
PRJNA8274(	ENSGALG00010007719	LTBP1	case	control	0.028872371	0.122319585	0.236040457	0.813401277	0.956712425	up_in_case
PRJNA8274(	ENSGALG00010007724	RBM11	case	control	0.155260725	0.260208752	0.596677569	0.550722671	0.86457121	up_in_case
PRJNA8274(	ENSGALG00010007730	COG2	case	control	0.053236949	0.053519673	0.994717385	0.319873737	0.738813491	up_in_case
PRJNA8274(	ENSGALG00010007731	ENSGALG00010007731	case	control	0.073948685	0.18045084	0.409799618	0.681952946	0.915594727	up_in_case
PRJNA8274(	ENSGALG00010007741	ENSGALG00010007741	case	control	0.149076925	0.144190884	1.033885923	0.301189497	0.723867193	up_in_case
PRJNA8274(	ENSGALG00010007748	CSGALNACT1	case	control	0.234126589	0.231769169	1.010171413	0.312413173	0.734364975	up_in_case
PRJNA8274(	ENSGALG00010007751	HSPA13	case	control	0.174868098	0.101647707	1.720334905	0.085371582	0.462079343	up_in_case
PRJNA8274(	ENSGALG00010007753	PRSS23	case	control	-0.092123208	0.117614796	-0.783262069	0.433473233	0.808170434	down_in_case
PRJNA8274(	ENSGALG00010007758	DEK	case	control	-0.087644629	0.050336479	-1.741175224	0.081652869	0.454736389	down_in_case
PRJNA8274(	ENSGALG00010007759	AGT	case	control	1.095316963	1.212576803	0.903296979	0.366368299	0.771763177	up_in_case
PRJNA8274(	ENSGALG00010007761	ENSGALG00010007761	case	control	0.038354751	0.082977717	0.462229528	0.64391673	0.902038523	up_in_case
PRJNA8274(	ENSGALG00010007762	SH2D4A	case	control	0.44408502	0.341012017	1.302256217	0.192828813	0.615090794	up_in_case
PRJNA8274(	ENSGALG00010007763	FZD4	case	control	-0.062855546	0.081723786	-0.769121811	0.441821001	0.813507909	down_in_case
PRJNA8274(	ENSGALG00010007765	NUDT7	case	control	-0.021853491	0.069266816	-0.315497269	0.752384124	0.940427752	down_in_case
PRJNA8274(	ENSGALG00010007766	ENSGALG00010007766	case	control	0.045768538	0.123811987	0.369661602	0.711634645	0.926556991	up_in_case
PRJNA8274(	ENSGALG00010007767	VAT1L	case	control	1.101389008	0.160489025	6.862706074	6.75682E-12	4.60107E-09	up_in_case
PRJNA8274(	ENSGALG00010007770	ENSGALG00010007770	case	control	0.152969431	0.409665586	0.373400735	0.708850213	0.925521953	up_in_case
PRJNA8274(	ENSGALG00010007771	CAPN9	case	control	0.367442512	0.378365244	0.971131777	0.331482662	0.748019893	up_in_case
PRJNA8274(	ENSGALG00010007772	CLEC3A	case	control	0.545926447	0.842466697	0.648009529	0.516978787	0.848054666	up_in_case
PRJNA8274(	ENSGALG00010007774	ENSGALG00010007774	case	control	-0.029995113	0.351525687	-0.085328368	0.93200034	0.987522589	down_in_case
PRJNA8274(	ENSGALG00010007777	KIF13A	case	control	-0.067837171	0.050229906	-1.350533511	0.176844912	0.595582794	down_in_case
PRJNA8274(	ENSGALG00010007780	SIRT5	case	control	-0.105612302	0.084143963	-1.255138198	0.209428596	0.63414862	down_in_case
PRJNA8274(	ENSGALG00010007781	MBP	case	control	0.00047054	0.231818941	0.002029773	0.998380477	0.999428829	up_in_case
PRJNA8274(	ENSGALG00010007783	NOL7	case	control	-0.119814176	0.081846207	-1.463894061	0.143222896	0.546011042	down_in_case
PRJNA8274(	ENSGALG00010007784	INTS10	case	control	-0.114368929	0.065015228	-1.759109872	0.078558848	0.448818031	down_in_case
PRJNA8274(	ENSGALG00010007786	SAMSN1	case	control	-0.161658285	0.180771652	-0.894267893	0.371178574	0.773034399	down_in_case
PRJNA8274(	ENSGALG00010007787	C1orf198	case	control	0.072698913	0.077065739	0.943336359	0.345508884	0.755935621	up_in_case
PRJNA8274(	ENSGALG00010007788	PALM2AKAP2	case	control	-0.096000399	0.171404579	-0.560080713	0.575424385	0.877910508	down_in_case
PRJNA8274(	ENSGALG00010007789	WWOX	case	control	-0.12277465	0.083722462	-1.466448151	0.142526237	0.54451648	down_in_case
PRJNA8274(	ENSGALG00010007792	ENSGALG00010007792	case	control	-0.0738289	0.218450417	-0.337966395	0.735388512	0.934096263	down_in_case
PRJNA8274(	ENSGALG00010007795	ZNF236	case	control	0.023613284	0.065956734	0.35801172	0.720334543	0.92895404	up_in_case
PRJNA8274(	ENSGALG00010007796	MCUR1	case	control	0.018266481	0.088042498	0.207473455	0.835640121	0.963998106	up_in_case
PRJNA8274(	ENSGALG00010007798	HGSNAT	case	control	-0.119136265	0.062090075	-1.91876502	0.055014079	0.383009409	down_in_case

PRJNA8274(	ENSGALG00010007800	MAF	case	control	0.009356678	0.061810842	0.151375993	0.879679126	0.976318821	up_in_case
PRJNA8274(	ENSGALG00010007801	TTC13	case	control	0.048296074	0.088306313	0.546915301	0.584436923	0.881307382	up_in_case
PRJNA8274(	ENSGALG00010007802	ENSGALG00010007802	case	control	0.57390293	0.860440207	0.666987578	0.504780068	0.842675107	up_in_case
PRJNA8274(	ENSGALG00010007803	ME3	case	control	-0.064082328	0.08782394	-0.729668104	0.465593082	0.826565	down_in_case
PRJNA8274(	ENSGALG00010007805	ENSGALG00010007805	case	control	-0.212806929	0.908144454	-0.234331584	0.814727568	0.957275016	down_in_case
PRJNA8274(	ENSGALG00010007806	DYNLRB2	case	control	0.081027605	0.266554927	0.303980893	0.761142448	0.942985066	up_in_case
PRJNA8274(	ENSGALG00010007809	ARV1	case	control	0.043206067	0.090183955	0.479088182	0.631875897	0.89724192	up_in_case
PRJNA8274(	ENSGALG00010007811	PRCP	case	control	-0.159157736	0.106129675	-1.49965348	0.133704187	0.537331451	down_in_case
PRJNA8274(	ENSGALG00010007812	POMK	case	control	0.076767427	0.071847077	1.068483653	0.2853024	0.709820465	up_in_case
PRJNA8274(	ENSGALG00010007817	CDYL2	case	control	-0.00369156	0.120253389	-0.030698181	0.975510242	0.99399062	down_in_case
PRJNA8274(	ENSGALG00010007818	ENSGALG00010007818	case	control	0.137459625	0.200168338	0.68672012	0.492259116	0.837514022	up_in_case
PRJNA8274(	ENSGALG00010007819	NRIP1	case	control	0.116821941	0.087183021	1.339962055	0.180257682	0.600999032	up_in_case
PRJNA8274(	ENSGALG00010007821	FAM89A	case	control	0.019548292	0.053524689	0.365220096	0.714947118	0.926862121	up_in_case
PRJNA8274(	ENSGALG00010007826	ENSGALG00010007826	case	control	-2.163431386	1.984033546	-1.09042077	0.275527837	0.700894634	down_in_case
PRJNA8274(	ENSGALG00010007830	SMIM19	case	control	-0.02417828	0.06191627	-0.390499629	0.69616713	0.921691866	down_in_case
PRJNA8274(	ENSGALG00010007835	GUCY1A1	case	control	-0.035135593	0.090938994	-0.386364435	0.699226786	0.922288441	down_in_case
PRJNA8274(	ENSGALG00010007845	TFAP2B	case	control	2.572125203	0.623566559	4.124860718	3.70959E-05	0.005100689	up_in_case
PRJNA8274(	ENSGALG00010007846	ENSGALG00010007846	case	control	-0.091749103	0.1233212	-0.743984841	0.456885646	0.822647285	down_in_case
PRJNA8274(	ENSGALG00010007847	CMC2	case	control	-0.06181678	0.08994313	-0.687287404	0.491901635	0.837304294	down_in_case
PRJNA8274(	ENSGALG00010007849	CXADR	case	control	0.719296717	0.379427553	1.895741917	0.05799418	0.39192679	up_in_case
PRJNA8274(	ENSGALG00010007850	ENSGALG00010007850	case	control	0.039603235	0.406637276	0.097392043	0.922415063	0.985482967	up_in_case
PRJNA8274(	ENSGALG00010007851	AvBD7	case	control	0.38093325	0.53965221	0.705886574	0.480258677	0.831851816	up_in_case
PRJNA8274(	ENSGALG00010007852	SLC20A2	case	control	-0.171644391	0.099153962	-1.731089589	0.083435787	0.45743286	down_in_case
PRJNA8274(	ENSGALG00010007853	C1orf131	case	control	0.095712836	0.109109264	0.877220064	0.38036712	0.778246195	up_in_case
PRJNA8274(	ENSGALG00010007854	FAM181B	case	control	0.213264864	0.066032378	3.229701408	0.001239196	0.055677577	up_in_case
PRJNA8274(	ENSGALG00010007855	RNF182	case	control	0.215610309	0.158430498	1.360914171	0.173540815	0.592914702	up_in_case
PRJNA8274(	ENSGALG00010007856	PDCD2	case	control	-0.1164346	0.065307902	-1.782856225	0.074609711	0.439603981	down_in_case
PRJNA8274(	ENSGALG00010007857	ENSGALG00010007857	case	control	0.761898248	0.294263997	2.589165699	0.009620879	0.17593167	up_in_case
PRJNA8274(	ENSGALG00010007859	CENPN	case	control	0.014847971	0.105733342	0.14042847	0.888321465	0.977948171	up_in_case
PRJNA8274(	ENSGALG00010007860	DEFB4A	case	control	0.589903459	0.609823218	0.967335191	0.333376497	0.748508541	up_in_case
PRJNA8274(	ENSGALG00010007861	INTU	case	control	0.027997387	0.127462839	0.219651364	0.826142685	0.961207256	up_in_case
PRJNA8274(	ENSGALG00010007862	ATMIN	case	control	-0.043988172	0.060450348	-0.727674412	0.466812918	0.827292691	down_in_case
PRJNA8274(	ENSGALG00010007863	AvBD1	case	control	0.223077731	0.538119386	0.414550631	0.678470895	0.914942347	up_in_case
PRJNA8274(	ENSGALG00010007864	TYR	case	control	6.87768365	0.623253395	11.03513226	2.58671E-28	3.69899E-25	up_in_case
PRJNA8274(	ENSGALG00010007865	GABRR3	case	control	0.152284889	0.297719482	0.511504613	0.608997759	0.8877909	up_in_case
PRJNA8274(	ENSGALG00010007866	TBP	case	control	0.005049115	0.048245275	0.104655114	0.91664948	0.983445506	up_in_case
PRJNA8274(	ENSGALG00010007867	ZNF516	case	control	-0.097016043	0.077499026	-1.251835638	0.210629761	0.636249596	down_in_case
PRJNA8274(	ENSGALG00010007868	INHBA	case	control	-0.091550309	0.129133388	-0.70895924	0.478349771	0.830750756	down_in_case
PRJNA8274(	ENSGALG00010007869	GCSH	case	control	0.174460633	0.133649741	1.305357063	0.191771292	0.615090794	up_in_case
PRJNA8274(	ENSGALG00010007870	AvBD3	case	control	0.447647854	0.349171285	1.282029405	0.199832331	0.622902456	up_in_case
PRJNA8274(	ENSGALG00010007871	HSPA4L	case	control	0.009550926	0.083223101	0.11476292	0.90863304	0.981730793	up_in_case
PRJNA8274(	ENSGALG00010007872	KLHL2	case	control	0.103456369	0.079982714	1.2934841	0.19584368	0.619095929	up_in_case
PRJNA8274(	ENSGALG00010007873	ENSGALG00010007873	case	control	-1.171907044	0.429803556	-2.726610863	0.006398845	0.13904105	down_in_case
PRJNA8274(	ENSGALG00010007874	ENSGALG00010007874	case	control	1.425296982	0.54026885	2.638125412	0.008336574	0.160448204	up_in_case
PRJNA8274(	ENSGALG00010007876	PKD1L2	case	control	-0.01434088	0.692477298	-0.020709531	0.983477366	0.996591771	down_in_case
PRJNA8274(	ENSGALG00010007877	MSMO1	case	control	0.310926632	0.133298648	2.332556537	0.019671432	0.251933438	up_in_case
PRJNA8274(	ENSGALG00010007878	PSMB1	case	control	-0.000800038	0.050049153	-0.015985037	0.987246329	0.996796053	down_in_case
PRJNA8274(	ENSGALG00010007879	PLK4	case	control	-0.097962889	0.08939358	-1.095860448	0.27313985	0.698936152	down_in_case
PRJNA8274(	ENSGALG00010007880	BCO1	case	control	-0.470142959	0.29793006	-1.578031297	0.11455842	0.509446069	down_in_case
PRJNA8274(	ENSGALG00010007881	AvBD14	case	control	0.016828878	0.389945431	0.043157007	0.965576376	0.992291928	up_in_case
PRJNA8274(	ENSGALG00010007882	CPE	case	control	0.026682656	0.136529128	0.195435626	0.845051929	0.967281084	up_in_case
PRJNA8274(	ENSGALG00010007883	DHTKD1	case	control	-0.294245429	0.100526769	-2.927035561	0.003422097	0.099869376	down_in_case
PRJNA8274(	ENSGALG00010007884	GLI3	case	control	0.027217237	0.090290849	0.301439594	0.763079306	0.943786458	up_in_case
PRJNA8274(	ENSGALG00010007885	USP38	case	control	-0.035405049	0.055499629	-0.63793308	0.523517243	0.851780245	down_in_case
PRJNA8274(	ENSGALG00010007886	GRM5	case	control	0.621850946	0.426514226	1.457984067	0.144844935	0.548818979	up_in_case
PRJNA8274(	ENSGALG00010007887	CDT1	case	control	-0.034356832	0.088807458	-0.386868772	0.698853361	0.922288441	down_in_case
PRJNA8274(	ENSGALG00010007889	DCAF6	case	control	-0.109383911	0.069280444	-1.578856973	0.114368867	0.509446069	down_in_case
PRJNA8274(	ENSGALG00010007890	ENSGALG00010007890	case	control	0.025060068	0.087102021	0.287709374	0.773569214	0.94652185	up_in_case
PRJNA8274(	ENSGALG00010007891	GDI1	case	control	-0.035091582	0.044175271	-0.794371641	0.426979109	0.805305429	down_in_case
PRJNA8274(	ENSGALG00010007892	ENSGALG00010007892	case	control	0.656004274	0.501484633	1.30812438	0.190831126	0.613935676	up_in_case
PRJNA8274(	ENSGALG00010007893	MFSD8	case	control	0.04051272	0.095879278	0.422538849	0.672631751	0.913676733	up_in_case
PRJNA8274(	ENSGALG00010007894	CD88	case	control	0.004851189	0.147087259	0.03298171	0.973689173	0.993553916	up_in_case
PRJNA8274(	ENSGALG00010007895	GOLGB1	case	control	-0.030227124	0.074387441	-0.406347144	0.68448755	0.916433328	down_in_case
PRJNA8274(	ENSGALG00010007896	TIMMDC1	case	control	-0.262699174	0.068411165	-3.840004405	0.000123032	0.012477724	down_in_case
PRJNA8274(	ENSGALG00010007897	EFHC1	case	control	-0.140300424	0.240197915	-0.58410342	0.559150734	0.869461123	down_in_case
PRJNA8274(	ENSGALG00010007898	GAB1	case	control	-0.030423331	0.060107934	-0.506145003	0.61275486	0.890080771	down_in_case
PRJNA8274(	ENSGALG00010007899	APRT	case	control	0.03836306	0.127471502	0.300954016	0.76344956	0.943786458	up_in_case
PRJNA8274(	ENSGALG00010007900	FHOD3	case	control	-0.258192171	0.128790799	-2.004740816	0.044990763	0.352144447	down_in_case
PRJNA8274(	ENSGALG00010007901	FSTL5	case	control	-0.686922723	0.334462302	-2.053812096	0.039993882	0.336547209	down_in_case
PRJNA8274(	ENSGALG00010007902	C7orf25	case	control	-0.010195906	0.068253147	-0.149383669	0.881250898	0.97667824	down_in_case
PRJNA8274(	ENSGALG00010007903	PSMA2	case	control	-0.002714578	0.04940627	-0.054943994	0.956183083	0.992056045	down_in_case
PRJNA8274(	ENSGALG00010007904	ENSGALG00010007904	case	control	0.104951305	0.084806504	1.237538402	0.215887267	0.640553441	up_in_case
PRJNA8274(	ENSGALG00010007905	MRPL32	case	control	0.037340487	0.078998992	0.472670422	0.636448332	0.898973839	up_in_case
PRJNA8274(	ENSGALG00010007906	NAF1	case	control	-0.155376451	0.067959183	-2.286320184	0.022235534	0.26563754	down_in_case
PRJNA8274(	ENSGALG00010007907	IRX2	case	control	0.262524038	0.585044783	0.448724688	0.653630273	0.906323369	up_in_case
PRJNA8274(	ENSGALG00010007908	IRX1	case	control	0.293754555	0.548956899	0.53511406	0.592570988	0.883604289	up_in_case
PRJNA8274(	ENSGALG00010007909	GALNS	case	control	-0.189682325	0.136615018	-1.38844417	0.165001834	0.580021198	down_in_case
PRJNA8274(	ENSGALG00010007910	ANKS6	case	control	-0.005743819	0.075303219	-0.076275882	0.939199614	0.989551456	down_in_case
PRJNA8274(	ENSGALG00010007911	IFI6	case	control	-0.553724592	0.435187211	-1.272382503	0.203237248	0.625537942	down_in_case
PRJNA8274(	ENSGALG00010007912	TRAPPC2L	case	control	-0.032372429	0.079786778	-0.405736761	0.684936029	0.916548243	down_in_case
PRJNA8274(	ENSGALG00010007914	PAQR8	case	control	0.187979495	0.098465796	1.909084197	0.056251231	0.385984934	up_in_case
PRJNA8274(	ENSGALG00010007915	HECW1	case	control	-0.002568711	0.100099062	-0.025661684	0.979527185	0.995636523	down_in_case
PRJNA8274(	ENSGALG00010007916	MCM3	case	control	-0.052145472	0.067132664	-0.77675261	0.437304741	0.810720016	down_in_case
PRJNA8274(	ENSGALG00010007917	POGLUT1	case	control	-0.070084894	0.062291085	-1.125119188	0.260538531	0.687359696	down_in_case
PRJNA8274(	ENSGALG00010007920	FAT3	case	control	-0.075361311	0.110189755	-0.683923027	0.494023773	0.838023719	down_in_case
PRJNA8274(	ENSGALG00010007921	FAM120B	case	control	0.057060826	0.0640673	0.890638835	0.373122958	0.774369566	up_in_case
PRJNA8274(	ENSGALG00010007922	IL17A	case	control	0.339542054	0.220548241	1.539536441	0.123673389	0.522883756	up_in_case
PRJNA8274(	ENSGALG00010007923	YAP1	case	control	0.015113287	0.04572502	0.33052553	0.741002905	0.937158859	up_in_case
PRJNA8274(	ENSGALG00010007924	ABHD18	case	control	-0.052135018	0.081229096	-0.641826888	0.52098559	0.850168083	down_in_case
PRJNA8274(	ENSGALG00010007925	STK17A	case	control	-0.196410181	0.113230316	-1.734607719	0.082810311	0.456624721	down_in_case
PRJNA8274(	ENSGALG00010007926	EIF3E	case	control	-0.073161127	0.065418492	-1.118355455	0.263415211	0.689391933	down_in_case
PRJNA8274(	ENSGALG00010007927	DLL1	case	control	0.006657752	0.15336171	0.043412088	0.965373042	0.992291928	up_in_case
PRJNA8274(	ENSGALG00010007928	C11orf70	case	control	-0.488813179	0.370401567	-1.319684425	0.186940402	0.610664656	down_in_case
PRJNA8274(	ENSGALG00010007929	LRRC14B	case	control	-0.153106544	0.209454818	-0.730976472	0.464793524	0.826068531	down_in_case
PRJNA8274(	ENSGALG00010007930	CBFA2T3	case	control	0.067788743	0.081426417	0.832515357	0.405118114	0.793478842	up_in_case
PRJNA8274(	ENSGALG00010007931	GBE1	case	control	-0.060428207	0.097232274	-0.621483018	0.534281865	0.856211151	down_in_case
PRJNA8274(	ENSGALG00010007932	NPY5R	case	control	0.063377466	0.161400451	0.392672174	0.694561625	0.920418056	up_in_case
PRJNA8274(	ENSGALG00010007933	CEP126	case	control	0.215350607	0.383108861	0.562113354	0.57403879	0.877573415	up_in_case
PRJNA8274(	ENSGALG00010007934	PKHD1	case	control	0.558793602	0.28547782	1.95739761	0.050300739	0.367061596	up_in_case
PRJNA8274(	ENSGALG00010007935	LARP1B	case	control	-0.024470482	0.074125997	-0.330120111	0.741309208	0.937205003	down_in_case
PRJNA8274(	ENSGALG00010007936	ENSGALG00010007936	case	control	-0.002460682	0.118575191	-0.020752084	0.983443421	0.996591771	down_in_case
PRJNA8274(	ENSGALG00010007937	ANGPTL5	case	control	0.228870523	0.372892198	0.613771283	0.539366476	0.858476757	up_in_case
PRJNA8274(	ENSGALG00010007938	BCL9	case	control	-0.014516841	0.053417674	-0.271761007	0.785805789	0.94995543	down_in_case
PRJNA8274(	ENSGALG00010007940	GLIPR2	case	control	-0.112286138	0.114415273	-0.981391164	0.326399882	0.744287835	down_in_case
PRJNA8274(	ENSGALG00010007941	MRPL19	case	control	-0.098826267	0.056248977	-1.756943379	0.078927461	0.449171025	down_in_case
PRJNA8274(	ENSGALG00010007942	PGRMC2	case	control	0.022919094	0.042515287	0.539078887	0.589832423	0.882949889	up_in_case
PRJNA8274(	ENSGALG00010007943	BLOC1S4	case	control	-0.022596698	0.049464725	-0.456824491	0.647797201	0.903491658	down_in_case
PRJNA8274(	ENSGALG00010007944	ACSF3	case	control	-0.052451518	0.147419001	-0.3557989	0.721991173	0.929484914	down_in_case
PRJNA8274(	ENSGALG00010007945	ENSGALG00010007945	case	control	0.103757803	0.06949334	1.493061114	0.13542119	0.539774365	up_in_case
PRJNA8274(	ENSGALG00010007946	ENSGALG00010007946	case	control	-0.153852357	0.127856639	-1.203319266	0.228852796	0.652401326	down_in_case
PRJNA8274(	ENSGALG00010007948	ENSGALG00010007948	case	control	0.233470586	0.236924574	0.985421575	0.324417036	0.742384961	up_in_case
PRJNA8274(	ENSGALG00010007950	ARC	case	control	0.03593276	0.383419706	0.093716519	0.925334347	0.985571367	up_in_case
PRJNA8274(	ENSGALG00010007951	TGFBR1	case	control	0.029662688	0.068141631	0.435309337	0.663337908	0.911044469	up_in_case
PRJNA8274(	ENSGALG00010007952	EMC2	case	control	0.032545935	0.052298134	0.622315425	0.533734481	0.855817434	up_in_case
PRJNA8274(	ENSGALG00010007953	SCGN	case	control	-0.053764347	0.506490119	-0.106150831	0.915462682	0.983445506	down_in_case
PRJNA8274(	ENSGALG00010007954	PSCA	case	control	1.369921483	0.57710876	2.373766574	0.017607678	0.23617575	up_in_case
PRJNA8274(	ENSGALG00010007955	TMA16	case	control	-0.016490708	0.070527222	-0.233820465	0.815124361	0.957374769	down_in_case
PRJNA8274(	ENSGALG00010007956	HS1BP3	case	control	-0.146673425	0.123518734	-1.187458943	0.235046642	0.659686983	down_in_case
PRJNA8274(	ENSGALG00010007957	CDH15	case	control	-0.075659627	0.103130918	-0.73362701	0.463176095	0.825091817	down_in_case
PRJNA8274(	ENSGALG00010007960	TSNAX	case	control	0.104014594	0.045649805	2.278533143	0.022694832	0.267253668	up_in_case
PRJNA8274(	ENSGALG00010007962	ADCY8	case	control	0.159970823	0.200112504	0.799404433	0.424055941	0.803723282	up_in_case
PRJNA8274(	ENSGALG00010007963	LDAH	case	control	-0.021440453	0.06105744	-0.351152185	0.72547418	0.931921256	down_in_case
PRJNA8274(	ENSGALG00010007964	RECQL4	case	control	-0.221312853	0.10074214	-2.196825023	0.028032945	0.298971144	down_in_case
PRJNA8274(	ENSGALG00010007965	EGLN1	case	control	-0.143071691	0.102354479	-1.397805867	0.162171372	0.575677776	down_in_case
PRJNA8274(	ENSGALG00010007966	TMEM74	case	control	0.213589918	0.244523117	0.873495811	0.382392889	0.77998936	up_in_case
PRJNA8274(	ENSGALG00010007968	SLC22A31	case	control	-0.089317611	0.171074748	-0.522096988	0.601602804	0.884954589	down_in_case

PRJNA8274(	ENSGALG00010007970	SPRTN	case	control	0.240610323	0.069492606	3.462387383	0.000535406	0.033580284	up_in_case
PRJNA8274(	ENSGALG00010007972	EXOC8	case	control	0.079494262	0.05506971	1.443520611	0.148873871	0.555199933	up_in_case
PRJNA8274(	ENSGALG00010007973	CARMIL1	case	control	-0.05990763	0.076910273	-0.778928844	0.436021632	0.81000376	down_in_case
PRJNA8274(	ENSGALG00010007975	ANKRD11	case	control	-0.13965433	0.065666712	-2.126714208	0.033443836	0.315466265	down_in_case
PRJNA8274(	ENSGALG00010007976	ENSGALG00010007976	case	control	-0.518170806	0.245723111	-2.108758932	0.034965395	0.322792221	down_in_case
PRJNA8274(	ENSGALG00010007977	APOB	case	control	0.812113275	1.123473853	0.722859079	0.469766466	0.829101076	up_in_case
PRJNA8274(	ENSGALG00010007978	JADE1	case	control	0.035143286	0.045372492	0.774550502	0.438605315	0.811916636	up_in_case
PRJNA8274(	ENSGALG00010007979	RAB30	case	control	0.069222208	0.057051893	1.213320081	0.225007468	0.648879142	up_in_case
PRJNA8274(	ENSGALG00010007980	MARCHF1	case	control	0.041315528	0.17913018	0.230645266	0.817590399	0.95838887	up_in_case
PRJNA8274(	ENSGALG00010007981	TDRD15	case	control	-0.741858834	0.597256288	-1.242111384	0.214195469	0.639112236	down_in_case
PRJNA8274(	ENSGALG00010007982	RECK	case	control	0.077488027	0.062860797	1.232692406	0.217690546	0.642912219	up_in_case
PRJNA8274(	ENSGALG00010007983	RCHY1	case	control	-0.088496686	0.07787099	-1.136452554	0.255767212	0.68198231	down_in_case
PRJNA8274(	ENSGALG00010007984	EIF3H	case	control	-0.079508348	0.054695256	-1.453660776	0.146040378	0.551173819	down_in_case
PRJNA8274(	ENSGALG00010007985	APELA	case	control	0.272606071	0.187290325	1.455526713	0.1455235	0.550234282	up_in_case
PRJNA8274(	ENSGALG00010007986	MYC	case	control	-0.032105322	0.052160422	-0.615511156	0.538217204	0.857740557	down_in_case
PRJNA8274(	ENSGALG00010007987	ENSGALG00010007987	case	control	-0.183487941	0.155287025	-1.181605103	0.237362428	0.662652947	down_in_case
PRJNA8274(	ENSGALG00010007988	TARBP1	case	control	0.072091982	0.081472115	0.884866943	0.376228399	0.775113975	up_in_case
PRJNA8274(	ENSGALG00010007989	SPG7	case	control	-0.07533021	0.043488653	-1.732180796	0.083241377	0.457254339	down_in_case
PRJNA8274(	ENSGALG00010007991	PCF11	case	control	0.070676809	0.052559411	1.344703211	0.178721097	0.5988078	up_in_case
PRJNA8274(	ENSGALG00010007992	LAPTM4A	case	control	-0.107795465	0.07026715	-1.534080517	0.125009846	0.523982046	down_in_case
PRJNA8274(	ENSGALG00010007993	LRRC14	case	control	-0.059107263	0.11607297	-0.509225042	0.610594492	0.888923338	down_in_case
PRJNA8274(	ENSGALG00010007994	FAM65B	case	control	-0.10862012	0.105290366	-1.031624484	0.302248066	0.725070851	down_in_case
PRJNA8274(	ENSGALG00010007995	KIAA0319	case	control	-0.37461678	0.486039662	-0.77075352	0.440853042	0.813083204	down_in_case
PRJNA8274(	ENSGALG00010007996	NUDCD1	case	control	-0.081517021	0.062585021	-1.302500491	0.19274535	0.615090794	down_in_case
PRJNA8274(	ENSGALG00010007997	TMEM192	case	control	-0.085011312	0.078871237	-1.077849363	0.281100986	0.705762606	down_in_case
PRJNA8274(	ENSGALG00010007998	RPL13	case	control	-0.070484918	0.06526978	-1.079901272	0.280186147	0.705762606	down_in_case
PRJNA8274(	ENSGALG00010007999	SDC1	case	control	0.274270622	0.124326423	2.206052535	0.027380322	0.293762083	up_in_case
PRJNA8274(	ENSGALG00010008000	C8orf82	case	control	0.016257053	0.088791482	0.183092488	0.85472545	0.970313652	up_in_case
PRJNA8274(	ENSGALG00010008001	ALDH5A1	case	control	0.21140567	0.134754294	1.56882325	0.116689129	0.51350396	up_in_case
PRJNA8274(	ENSGALG00010008002	GJA8	case	control	0.414734816	0.141879057	2.923157416	0.003465013	0.100506459	up_in_case
PRJNA8274(	ENSGALG00010008004	ENY2	case	control	-0.083217836	0.080026517	-1.039878264	0.298396462	0.721501478	down_in_case
PRJNA8274(	ENSGALG00010008005	ANKRD42	case	control	0.450403758	0.287139231	1.568590107	0.116743479	0.51350396	up_in_case
PRJNA8274(	ENSGALG00010008007	CBR4	case	control	-0.129541518	0.07375934	-1.756272739	0.07904185	0.449171025	down_in_case
PRJNA8274(	ENSGALG00010008008	BTC	case	control	0.253533408	0.171659565	1.476954745	0.139687769	0.541778931	up_in_case
PRJNA8274(	ENSGALG00010008009	GPLD1	case	control	0.903293193	0.639067418	1.413455243	0.157521912	0.568972808	up_in_case
PRJNA8274(	ENSGALG00010008010	CERK	case	control	0.204955868	0.066461481	3.083829395	0.002043547	0.074169362	up_in_case
PRJNA8274(	ENSGALG00010008011	ADNP2	case	control	-0.030920257	0.052154953	-0.592853691	0.553279072	0.866252369	down_in_case
PRJNA8274(	ENSGALG00010008012	CCDC90B	case	control	0.032613944	0.078513177	0.415394529	0.677853111	0.914893769	up_in_case
PRJNA8274(	ENSGALG00010008013	ENSGALG00010008013	case	control	0.005110158	0.130580245	0.039134238	0.968783364	0.992761697	up_in_case
PRJNA8274(	ENSGALG00010008015	ARHGAP39	case	control	-0.005184851	0.105590421	-0.049103418	0.96083688	0.992056045	down_in_case
PRJNA8274(	ENSGALG00010008016	GSR	case	control	-0.010113095	0.076737983	-0.131787339	0.895152501	0.980176778	down_in_case
PRJNA8274(	ENSGALG00010008017	GMNN	case	control	0.008304099	0.096380233	0.086159773	0.931339409	0.987258715	up_in_case
PRJNA8274(	ENSGALG00010008018	ENSGALG00010008018	case	control	0.206095503	0.202770419	1.016398267	0.309439771	0.732384442	up_in_case
PRJNA8274(	ENSGALG00010008019	PPP2CB	case	control	-0.055633547	0.040634796	-1.369111011	0.170964577	0.58841329	down_in_case
PRJNA8274(	ENSGALG00010008020	COA6	case	control	-0.146774866	0.1109289	-1.32314362	0.185787621	0.60893032	down_in_case
PRJNA8274(	ENSGALG00010008022	SLC35F3	case	control	-0.130922382	0.234048916	-0.559380425	0.575902118	0.877910508	down_in_case
PRJNA8274(	ENSGALG00010008023	ATAD2	case	control	0.077189162	0.074730097	1.032905949	0.301647914	0.724474473	up_in_case
PRJNA8274(	ENSGALG00010008024	MRS2	case	control	-0.123901358	0.109975946	-1.126622341	0.259902189	0.687227573	down_in_case
PRJNA8274(	ENSGALG00010008025	TBC1D22A	case	control	-0.068169071	0.05177659	-1.316600248	0.187972656	0.611104467	down_in_case
PRJNA8274(	ENSGALG00010008026	PURG	case	control	0.228513305	0.092639477	2.466694687	0.013636658	0.205051738	up_in_case
PRJNA8274(	ENSGALG00010008027	C6orf62	case	control	-0.177233776	0.099167284	-1.787220222	0.073901902	0.437990779	down_in_case
PRJNA8274(	ENSGALG00010008028	KCNK1	case	control	0.210990105	0.28546565	0.739108557	0.459841081	0.824480387	up_in_case
PRJNA8274(	ENSGALG00010008029	LY6E	case	control	0.076346269	0.092032821	0.829554813	0.406790534	0.794253996	up_in_case
PRJNA8274(	ENSGALG00010008030	GJA5	case	control	0.543968101	0.124836822	4.357433124	1.31597E-05	0.002213921	up_in_case
PRJNA8274(	ENSGALG00010008031	MAP3K21	case	control	0.158002744	0.158080601	0.999507487	0.317548914	0.737774213	up_in_case
PRJNA8274(	ENSGALG00010008032	PUM2	case	control	0.047411768	0.035631882	1.330599601	0.183320794	0.60491829	up_in_case
PRJNA8274(	ENSGALG00010008033	ACOT13	case	control	-0.004160594	0.090790912	-0.045826103	0.963448854	0.992068146	down_in_case
PRJNA8274(	ENSGALG00010008034	DPEP1	case	control	-0.033015523	0.223842104	-0.147494695	0.882741566	0.976801393	down_in_case
PRJNA8274(	ENSGALG00010008035	TDP2	case	control	-0.066821051	0.103260572	-0.64711099	0.517560113	0.848372336	down_in_case
PRJNA8274(	ENSGALG00010008036	SPOCK3	case	control	0.00662531	0.147102984	0.045038581	0.964076557	0.992068146	up_in_case
PRJNA8274(	ENSGALG00010008037	ENSGALG00010008037	case	control	0.206677366	0.799466955	0.25851896	0.796006418	0.95280257	up_in_case
PRJNA8274(	ENSGALG00010008038	WRN	case	control	-0.053240419	0.073943666	-0.72001325	0.471516838	0.829101076	down_in_case
PRJNA8274(	ENSGALG00010008039	CHMP1A	case	control	0.07337674	0.062287103	1.17804066	0.238780397	0.663794649	up_in_case
PRJNA8274(	ENSGALG00010008041	ENSGALG00010008041	case	control	0.080860692	0.145975115	0.553934772	0.579623487	0.879142433	up_in_case
PRJNA8274(	ENSGALG00010008043	IL17REL	case	control	0.29795777	0.302304098	0.985622661	0.324318314	0.74227783	up_in_case
PRJNA8274(	ENSGALG00010008045	DCDC2	case	control	-1.483129366	0.665741046	-2.227787177	0.02589471	0.285280707	down_in_case
PRJNA8274(	ENSGALG00010008046	RHOB	case	control	0.004874017	0.052728028	0.092436926	0.926350902	0.98618953	up_in_case
PRJNA8274(	ENSGALG00010008047	ASAP1	case	control	-0.047716887	0.078758617	-0.605862427	0.544606111	0.861585064	down_in_case
PRJNA8274(	ENSGALG00010008048	ENSGALG00010008048	case	control	0.438901395	0.651863703	0.673302398	0.500754927	0.840987941	up_in_case
PRJNA8274(	ENSGALG00010008050	CDK10	case	control	0.033071689	0.072785961	0.454369059	0.649563219	0.904102981	up_in_case
PRJNA8274(	ENSGALG00010008051	ENSGALG00010008051	case	control	-0.087132841	0.066587998	-1.308536723	0.190691327	0.613935676	down_in_case
PRJNA8274(	ENSGALG00010008052	RBFA	case	control	-0.18898493	0.086588832	-2.182555489	0.029068561	0.302974066	down_in_case
PRJNA8274(	ENSGALG00010008054	CRISP3	case	control	1.530269506	0.713022973	2.14617139	0.031859307	0.3098361	up_in_case
PRJNA8274(	ENSGALG00010008055	MLC1	case	control	0.025216225	0.581604527	0.043356308	0.965417506	0.992291928	up_in_case
PRJNA8274(	ENSGALG00010008056	GTF2E2	case	control	-0.053061924	0.05678518	-0.934432623	0.350080776	0.75977464	down_in_case
PRJNA8274(	ENSGALG00010008057	BAG1	case	control	-0.061695713	0.055088846	-1.119931117	0.262743117	0.688674415	down_in_case
PRJNA8274(	ENSGALG00010008058	USP25	case	control	-0.127694154	0.054510066	-2.342579322	0.019150963	0.246942079	down_in_case
PRJNA8274(	ENSGALG00010008059	ENSGALG00010008059	case	control	-0.110894558	0.066548721	-1.666366486	0.095640442	0.482930958	down_in_case
PRJNA8274(	ENSGALG00010008062	VPS9D1	case	control	0.169048647	0.113526694	1.489065184	0.136470205	0.539840644	up_in_case
PRJNA8274(	ENSGALG00010008063	KCNV1	case	control	0.329368088	0.1672476	1.969344184	0.048913581	0.36379167	up_in_case
PRJNA8274(	ENSGALG00010008064	DDX60	case	control	-0.065875756	0.197191491	-0.334069973	0.738326758	0.935918627	down_in_case
PRJNA8274(	ENSGALG00010008065	CHMP5	case	control	-0.000907887	0.037159161	-0.024432376	0.980507724	0.99596963	down_in_case
PRJNA8274(	ENSGALG00010008066	PCNX2	case	control	0.223405569	0.14229401	1.570027918	0.116408616	0.5134816	up_in_case
PRJNA8274(	ENSGALG00010008067	MPC2	case	control	0.189767088	0.077596103	2.44557499	0.014462137	0.212076065	up_in_case
PRJNA8274(	ENSGALG00010008069	DLG2	case	control	-0.124633904	0.088297304	-1.411525599	0.158089693	0.570159548	down_in_case
PRJNA8274(	ENSGALG00010008071	SMIM18	case	control	0.161573474	0.225560943	0.716318488	0.473794714	0.829698758	up_in_case
PRJNA8274(	ENSGALG00010008072	NRSN1	case	control	-0.521418725	0.159045251	-3.278430008	0.001043862	0.050260045	down_in_case
PRJNA8274(	ENSGALG00010008073	ZNF276	case	control	-0.02530542	0.055413974	-0.45666134	0.647914483	0.903500828	down_in_case
PRJNA8274(	ENSGALG00010008074	RHAG	case	control	0.439865575	0.182043144	2.416271022	0.015680385	0.222636791	up_in_case
PRJNA8274(	ENSGALG00010008075	SDHA	case	control	-0.124830812	0.077753424	-1.605470288	0.108390347	0.500478515	down_in_case
PRJNA8274(	ENSGALG00010008078	GUCY1B1	case	control	0.05692765	0.063818327	0.892026665	0.37237864	0.774201112	up_in_case
PRJNA8274(	ENSGALG00010008079	CCDC127	case	control	0.011613942	0.063115825	0.184009981	0.854005625	0.969998446	up_in_case
PRJNA8274(	ENSGALG00010008081	TOP1MT	case	control	0.001230113	0.08436872	0.014580206	0.988367091	0.996942188	up_in_case
PRJNA8274(	ENSGALG00010008083	FANCA	case	control	0.116875157	0.158266063	0.738472638	0.460227288	0.82454265	up_in_case
PRJNA8274(	ENSGALG00010008084	CYP2AC1	case	control	1.002633224	0.89840464	1.116015189			up_in_case
PRJNA8274(	ENSGALG00010008086	RHPN1	case	control	0.072362237	0.100218158	0.722047161	0.470265483	0.829101076	up_in_case
PRJNA8274(	ENSGALG00010008087	CPNE8	case	control	0.089442344	0.141246263	0.633236887	0.526578961	0.85251736	up_in_case
PRJNA8274(	ENSGALG00010008088	SLC35A4	case	control	-0.116422496	0.068171694	-1.707783518	0.087676518	0.468000826	down_in_case
PRJNA8274(	ENSGALG00010008091	ENSGALG00010008091	case	control	0.592363001	0.228644269	2.590762513	0.009576355	0.175791886	up_in_case
PRJNA8274(	ENSGALG00010008092	SPIRE2	case	control	1.004791194	0.719411802	1.396684334	0.162508517	0.576163465	up_in_case
PRJNA8274(	ENSGALG00010008093	EFR3A	case	control	0.006372504	0.061790766	0.103130365	0.917859506	0.983445506	up_in_case
PRJNA8274(	ENSGALG00010008094	PIM3	case	control	0.26962507	0.092040013	2.929433207	0.003395808	0.099572914	up_in_case
PRJNA8274(	ENSGALG00010008096	CPSF1	case	control	-0.117164712	0.090304969	-1.297433727	0.194481994	0.617304812	down_in_case
PRJNA8274(	ENSGALG00010008098	ENSGALG00010008098	case	control	0.045489147	0.090768648	0.501154833	0.616262159	0.891081782	up_in_case
PRJNA8274(	ENSGALG00010008099	PANK3	case	control	0.031946627	0.048293862	0.661504903	0.508288572	0.843702533	up_in_case
PRJNA8274(	ENSGALG00010008100	ENSGALG00010008100	case	control	0.262633372	0.147506655	1.780484906	0.074996637	0.440431997	up_in_case
PRJNA8274(	ENSGALG00010008101	CENPQ	case	control	-0.020249779	0.123320737	-0.16420417	0.869570419	0.974718971	down_in_case
PRJNA8274(	ENSGALG00010008103	RBPMS	case	control	-0.015095925	0.081285554	-0.185714731	0.852668475	0.969809672	down_in_case
PRJNA8274(	ENSGALG00010008104	TCF25	case	control	-0.098947172	0.056291979	-1.757749019	0.078790223	0.449171025	down_in_case
PRJNA8274(	ENSGALG00010008105	MMUT	case	control	-0.322748889	0.055642236	-5.80042992	6.61451E-09	3.26164E-06	down_in_case
PRJNA8274(	ENSGALG00010008106	ENSGALG00010008106	case	control	-0.631931717	0.237745059	-2.658022506	0.007860064	0.155677176	down_in_case
PRJNA8274(	ENSGALG00010008107	TUBB3	case	control	-0.14889409	0.093017052	-1.600718233	0.109439341	0.501801854	down_in_case
PRJNA8274(	ENSGALG00010008108	ENSGALG00010008108	case	control	-0.197916513	0.205448047	-0.963340931	0.335376459	0.749356776	down_in_case
PRJNA8274(	ENSGALG00010008110	DEF8	case	control	0.215518592	0.073541736	2.930561664	0.003383498	0.099555609	up_in_case
PRJNA8274(	ENSGALG00010008111	SYTL2	case	control	0.222756387	0.322457994	0.690807457	0.489686549	0.836347578	up_in_case
PRJNA8274(	ENSGALG00010008113	DBNDD1	case	control	0.167662373	0.177531286	0.944410291	0.344960025	0.755870938	up_in_case
PRJNA8274(	ENSGALG00010008115	ENSGALG00010008115	case	control	0.014560629	0.092868391	0.15678778	0.875412104	0.975666505	up_in_case
PRJNA8274(	ENSGALG00010008116	URAH	case	control	-0.107142792	0.308791431	-0.346974628	0.72861038	0.933057319	down_in_case
PRJNA8274(	ENSGALG00010008117	ENSGALG00010008117	case	control	-0.034083356	0.108873262	-0.313055337	0.754238621	0.941235036	down_in_case
PRJNA8274(	ENSGALG00010008118	ENSGALG00010008118	case	control	-0.130140082	0.356523474	-0.365025282	0.715092534	0.926862121	down_in_case
PRJNA8274(	ENSGALG00010008119	CCDC83	case	control	0.361981668	0.277814476	1.302961864	0.192587782	0.615090794	up_in_case
PRJNA8274(	ENSGALG00010008120	CDC5L	case	control	-0.011394789	0.040209298	-0.283386931	0.776880246	0.947379834	down_in_case
PRJNA8274(	ENSGALG00010008121	MPZL1	case	control	-0.019302057	0.045253457	-0.426532199	0.669720091	0.913465866	down_in_case
PRJNA8274(	ENSGALG00010008123	ENSGALG00010008123	case	control	-0.227578872	0.084019958	-2.708628736	0.00675619	0.141870059	down_in_case
PRJNA8274(	ENSGALG00010008124	ENSGALG00010008124	case	control	0.008563475	0.148511358	0.057662088	0.954017793	0.992056045	up_in_case
PRJNA8274(	ENSGALG00010008126	CDH3	case	control	2.437006139	0.461152147	5.284603256	1.25977E-07	4.50369E-05	up_in_case

PRJNA8274(	ENSGALG00010008127	TBC1D2	case	control	0.236356936	0.143724765	1.644510854	0.100070724	0.489464811	up_in_case
PRJNA8274(	ENSGALG00010008128	RARS	case	control	-0.039812294	0.040692428	-0.978371078	0.327890827	0.745445783	down_in_case
PRJNA8274(	ENSGALG00010008129	TANGO6	case	control	0.033505106	0.058023987	0.57743543	0.563645346	0.871916948	up_in_case
PRJNA8274(	ENSGALG00010008130	RCSD1	case	control	-0.171133202	0.081587708	-2.097536581	0.035946102	0.325541016	down_in_case
PRJNA8274(	ENSGALG00010008131	OC90	case	control	0.320666269	0.40886205	0.784289636	0.432870179	0.8079942	up_in_case
PRJNA8274(	ENSGALG00010008132	TDO2	case	control	1.602927473	0.60344038	2.656314569	0.007899988	0.155766219	up_in_case
PRJNA8274(	ENSGALG00010008133	CREG1	case	control	-0.019068106	0.09661504	-0.197361675	0.843544519	0.967181416	down_in_case
PRJNA8274(	ENSGALG00010008134	SLC2A13	case	control	0.262126114	0.098654946	2.656999226	0.007883962	0.155766219	up_in_case
PRJNA8274(	ENSGALG00010008135	WWC1	case	control	0.104182154	0.090553369	1.150505551	0.249935709	0.675246674	up_in_case
PRJNA8274(	ENSGALG00010008136	ELP1	case	control	0.226542472	0.187119632	1.210682547	0.226017094	0.649498359	up_in_case
PRJNA8274(	ENSGALG00010008137	HHLA1	case	control	0.321533177	0.298321811	1.077806466	0.281120133	0.705762606	up_in_case
PRJNA8274(	ENSGALG00010008139	ENSGALG00010008139	case	control	0.738686037	0.341423576	2.163547248	0.030499114	0.307572163	up_in_case
PRJNA8274(	ENSGALG00010008141	APTX	case	control	0.138666454	0.166404122	0.83331141	0.404669123	0.793319877	up_in_case
PRJNA8274(	ENSGALG00010008142	CD247	case	control	0.155513556	0.231032523	0.673124088	0.500868351	0.840987941	up_in_case
PRJNA8274(	ENSGALG00010008143	DCTN6	case	control	0.097284556	0.060827212	1.599359113	0.109740832	0.501817242	up_in_case
PRJNA8274(	ENSGALG00010008144	NTPCR	case	control	0.044755926	0.136306373	0.328348004	0.742648552	0.937448285	up_in_case
PRJNA8274(	ENSGALG00010008145	ENSGALG00010008145	case	control	0.00912194	0.169316785	0.053874988	0.957034765	0.992056045	up_in_case
PRJNA8274(	ENSGALG00010008146	SOX4	case	control	-0.085026992	0.053583463	-1.586814058	0.112554764	0.506620435	down_in_case
PRJNA8274(	ENSGALG00010008147	CDKAL1	case	control	-0.103398031	0.054611908	-1.893323908	0.058314808	0.393535512	down_in_case
PRJNA8274(	ENSGALG00010008148	ABCD2	case	control	0.043261732	0.065566344	0.65981614	0.509371825	0.844521404	up_in_case
PRJNA8274(	ENSGALG00010008149	DNAJA1	case	control	0.080846007	0.134974771	0.598971247	0.549192058	0.864234145	up_in_case
PRJNA8274(	ENSGALG00010008150	TERT	case	control	-0.387429746	0.476758998	-0.812632268	0.416428925	0.799987395	down_in_case
PRJNA8274(	ENSGALG00010008151	SIPA1L2	case	control	-0.030473414	0.099984953	-0.304779997	0.760533717	0.942883622	down_in_case
PRJNA8274(	ENSGALG00010008152	ENSGALG00010008152	case	control	-0.409439435	0.127881054	-3.20172084	0.001366093	0.058654283	down_in_case
PRJNA8274(	ENSGALG00010008153	SMU1	case	control	0.028268495	0.039409381	0.7173037	0.473186724	0.82959752	up_in_case
PRJNA8274(	ENSGALG00010008154	KCNQ3	case	control	-0.169455503	0.18486841	-0.916627683	0.359337773	0.765374836	down_in_case
PRJNA8274(	ENSGALG00010008155	MAP10	case	control	-0.366885951	0.17607621	-2.083677009	0.037189557	0.328438691	down_in_case
PRJNA8274(	ENSGALG00010008157	CTSO	case	control	-0.033341209	0.073540406	-0.453372651	0.650280426	0.904245691	down_in_case
PRJNA8274(	ENSGALG00010008158	CLPTM1L	case	control	0.059309879	0.050465994	1.17524444	0.239896935	0.664830655	up_in_case
PRJNA8274(	ENSGALG00010008159	SUPT3H	case	control	0.198970989	0.179104536	1.110920994	0.266602358	0.692519293	up_in_case
PRJNA8274(	ENSGALG00010008160	TXNL4A	case	control	-0.057947095	0.070640747	-0.820306938	0.412041152	0.797194435	down_in_case
PRJNA8274(	ENSGALG00010008161	TMEFF1	case	control	-0.265037197	0.145231819	-1.824925136	0.068012327	0.421339236	down_in_case
PRJNA8274(	ENSGALG00010008162	HSBP1L1	case	control	-0.355968843	0.214253183	-1.661440165	0.096625077	0.48430176	down_in_case
PRJNA8274(	ENSGALG00010008163	CHTF8	case	control	-0.048413294	0.115255539	-0.420051777	0.67444763	0.914018759	down_in_case
PRJNA8274(	ENSGALG00010008164	TG	case	control	-0.493503943	0.476653398	-1.035351777	0.300504657	0.723437138	down_in_case
PRJNA8274(	ENSGALG00010008165	MRPL1	case	control	0.018388917	0.069525361	0.264492212	0.791400665	0.95239351	up_in_case
PRJNA8274(	ENSGALG00010008166	RUNX2	case	control	0.362365718	0.672341676	0.538960667	0.589913995	0.882949889	up_in_case
PRJNA8274(	ENSGALG00010008167	UTP4	case	control	-0.031866695	0.071386568	-0.446396236	0.655311052	0.907302833	down_in_case
PRJNA8274(	ENSGALG00010008168	CAVIN4	case	control	-0.296193721	0.117495401	-2.520896289	0.011705635	0.189141894	down_in_case
PRJNA8274(	ENSGALG00010008169	HTR4	case	control	0.145580242	0.437458745	0.33278622	0.739295661	0.936326594	up_in_case
PRJNA8274(	ENSGALG00010008170	PICALM	case	control	0.023962349	0.047347055	0.506100105	0.612786377	0.890080771	up_in_case
PRJNA8274(	ENSGALG00010008171	KIF21A	case	control	0.459865476	0.153657017	2.992804914	0.002764264	0.088234315	up_in_case
PRJNA8274(	ENSGALG00010008172	SNTB2	case	control	0.08620597	0.088224006	0.977125997	0.328506779	0.745445783	up_in_case
PRJNA8274(	ENSGALG00010008174	SLC26A2	case	control	0.273616386	0.157989219	1.731867449	0.083297166	0.457254339	up_in_case
PRJNA8274(	ENSGALG00010008175	ENSGALG00010008175	case	control	-0.023109554	0.184616517	-0.125175981	0.900384232	0.98028579	down_in_case
PRJNA8274(	ENSGALG00010008176	SLA	case	control	-0.128561889	0.258330826	-0.497663755	0.618721059	0.891656847	down_in_case
PRJNA8274(	ENSGALG00010008177	NREP	case	control	0.054289601	0.149152055	0.363988287	0.715866756	0.926862121	up_in_case
PRJNA8274(	ENSGALG00010008178	PGR	case	control	0.301882015	0.302407314	0.998262942	0.318151873	0.738087569	up_in_case
PRJNA8274(	ENSGALG00010008179	SEMA6A	case	control	-0.00619476	0.126089855	-0.049129729	0.960815911	0.992056045	down_in_case
PRJNA8274(	ENSGALG00010008180	POU2F1	case	control	-0.168161065	0.103412395	-1.626120974	0.103923923	0.493794905	down_in_case
PRJNA8274(	ENSGALG00010008181	DISC1	case	control	0.117363383	0.077333918	1.517618484	0.129110604	0.530998459	up_in_case
PRJNA8274(	ENSGALG00010008182	CLIC5	case	control	0.326787313	0.299568825	1.090858877	0.275334984	0.700894634	up_in_case
PRJNA8274(	ENSGALG00010008183	ALG10	case	control	-0.05844158	0.061035531	-0.957500967	0.338314459	0.751259351	down_in_case
PRJNA8274(	ENSGALG00010008184	SLC66A2	case	control	-0.051249169	0.051868086	-0.988067481	0.323119601	0.740689923	down_in_case
PRJNA8274(	ENSGALG00010008185	PDGFC	case	control	-0.046023732	0.105064739	-0.438051172	0.661349189	0.909792535	down_in_case
PRJNA8274(	ENSGALG00010008186	NOP53	case	control	-0.238288037	0.10511981	-2.266823318	0.023401021	0.270084424	down_in_case
PRJNA8274(	ENSGALG00010008187	ENSGALG00010008187	case	control	0.216107025	0.17490628	1.235558981	0.21662254	0.641771515	up_in_case
PRJNA8274(	ENSGALG00010008188	VPS4A	case	control	-0.063003956	0.050039095	-1.259094633	0.207996153	0.63249038	down_in_case
PRJNA8274(	ENSGALG00010008189	EXOC3	case	control	-0.040818566	0.051688279	-0.789706432	0.429699234	0.806015686	down_in_case
PRJNA8274(	ENSGALG00010008190	TSNARE1	case	control	-0.175172215	0.085692226	-2.044201948	0.040933601	0.337874876	down_in_case
PRJNA8274(	ENSGALG00010008191	ENSGALG00010008191	case	control	-0.117763427	0.255168212	-0.461512924	0.644430652	0.902239118	down_in_case
PRJNA8274(	ENSGALG00010008192	HNRNPU	case	control	-0.07007111	0.044100041	-1.588912602	0.112080126	0.505685634	down_in_case
PRJNA8274(	ENSGALG00010008193	CNOT6L	case	control	-0.047731286	0.066097393	-0.722135686	0.47021106	0.829101076	down_in_case
PRJNA8274(	ENSGALG00010008194	PDF	case	control	0.293272903	0.119049637	2.463450627	0.013760685	0.205985143	up_in_case
PRJNA8274(	ENSGALG00010008195	ENSGALG00010008195	case	control	0.023122105	0.312754778	0.07393046	0.941065719	0.989551456	up_in_case
PRJNA8274(	ENSGALG00010008196	PDCD6	case	control	-0.043510887	0.048079816	-0.904971982	0.365480231	0.77107809	down_in_case
PRJNA8274(	ENSGALG00010008197	STARD4	case	control	0.44879429	0.224732888	1.997012066	0.045823872	0.355358662	up_in_case
PRJNA8274(	ENSGALG00010008198	GLRB	case	control	-0.110008034	0.307354995	-0.357918484	0.720404318	0.92895404	down_in_case
PRJNA8274(	ENSGALG00010008199	TPRKB	case	control	0.044170109	0.087609237	0.504171827	0.614140636	0.890440426	up_in_case
PRJNA8274(	ENSGALG00010008200	COG8	case	control	0.059027103	0.074482159	0.792499886	0.428069258	0.805337183	up_in_case
PRJNA8274(	ENSGALG00010008201	ENPP4	case	control	-0.104537283	0.113692381	-0.919474839	0.35784726	0.764105692	down_in_case
PRJNA8274(	ENSGALG00010008202	KCNG2	case	control	-0.105872022	0.117668943	-0.899744822	0.368256064	0.771770041	down_in_case
PRJNA8274(	ENSGALG00010008204	ENSGALG00010008204	case	control	0.14121894	0.118262113	1.194118186	0.23243174	0.657912487	up_in_case
PRJNA8274(	ENSGALG00010008205	ENSGALG00010008205	case	control	0.136833541	0.177079068	0.772725668	0.439684752	0.812401646	up_in_case
PRJNA8274(	ENSGALG00010008207	CAMK4	case	control	0.06172555	0.146421307	0.421561256	0.673345291	0.91379937	up_in_case
PRJNA8274(	ENSGALG00010008208	ILDR2	case	control	0.026399984	0.094422621	0.279593851	0.779789125	0.948864508	up_in_case
PRJNA8274(	ENSGALG00010008209	PPARGC1B	case	control	-0.085070457	0.114165067	-0.745153131	0.456179153	0.822099797	down_in_case
PRJNA8274(	ENSGALG00010008210	CTDP1	case	control	0.009024755	0.070729725	0.127594937	0.898469538	0.98028579	up_in_case
PRJNA8274(	ENSGALG00010008211	ADGRB1	case	control	-0.114173506	0.139958775	-0.815765252	0.41463441	0.798853486	down_in_case
PRJNA8274(	ENSGALG00010008212	GTSE1	case	control	-0.013892377	0.132932754	-0.104506801	0.916767171	0.983445506	down_in_case
PRJNA8274(	ENSGALG00010008213	ISCA1	case	control	0.010543033	0.05528291	0.190710522	0.848752395	0.968741311	up_in_case
PRJNA8274(	ENSGALG00010008214	CCNG2	case	control	0.023144645	0.079095963	0.292614739	0.769816647	0.945428692	up_in_case
PRJNA8274(	ENSGALG00010008215	SELENOW	case	control	-0.069346365	0.064050035	-1.082690519	0.278945814	0.704440209	down_in_case
PRJNA8274(	ENSGALG00010008217	ARHGEF37	case	control	0.055215277	0.11013462	0.501343514	0.616129386	0.891081782	up_in_case
PRJNA8274(	ENSGALG00010008218	ARHGAP42	case	control	0.033742175	0.090608491	0.372395285	0.709598564	0.925630832	up_in_case
PRJNA8274(	ENSGALG00010008219	NIP7	case	control	0.126583281	0.075100293	1.685523125	0.091887687	0.476713154	up_in_case
PRJNA8274(	ENSGALG00010008221	ENSGALG00010008221	case	control	0.005469046	0.146138635	0.037423682	0.97014719	0.992761697	up_in_case
PRJNA8274(	ENSGALG00010008222	STYXL2	case	control	-0.02922335	0.127689964	-0.228861757	0.818976365	0.958669267	down_in_case
PRJNA8274(	ENSGALG00010008223	ENSGALG00010008223	case	control	0.045683152	0.085251312	0.535864497	0.592052203	0.883475581	up_in_case
PRJNA8274(	ENSGALG00010008224	ENSGALG00010008224	case	control	-0.046374852	0.111631497	-0.415428025	0.677828594	0.914893769	down_in_case
PRJNA8274(	ENSGALG00010008226	COX20	case	control	-0.067335721	0.089608511	-0.751443362	0.452385875	0.820911365	down_in_case
PRJNA8274(	ENSGALG00010008227	DUSP1	case	control	-0.097743086	0.120586435	-0.810564522	0.417615798	0.799987395	down_in_case
PRJNA8274(	ENSGALG00010008229	PIGY	case	control	0.133003905	0.069921006	1.902202385	0.057144701	0.388573099	up_in_case
PRJNA8274(	ENSGALG00010008230	TRMU	case	control	0.07929341	0.087338375	0.907887401	0.363937715	0.770036689	up_in_case
PRJNA8274(	ENSGALG00010008231	ZCCHC6	case	control	0.03931724	0.130116237	0.302170131	0.762522373	0.943503499	up_in_case
PRJNA8274(	ENSGALG00010008232	PYURF	case	control	0.112516778	0.065589908	1.715458685	0.086261164	0.464083764	up_in_case
PRJNA8274(	ENSGALG00010008233	CYRIB	case	control	-0.005260341	0.047099106	-0.111686646	0.911071868	0.982306244	down_in_case
PRJNA8274(	ENSGALG00010008234	COMMD10	case	control	-0.139313018	0.193452274	-0.720141535	0.471437856	0.829101076	down_in_case
PRJNA8274(	ENSGALG00010008236	ENSGALG00010008236	case	control	-0.250339715	0.382815629	-0.653943297	0.513148338	0.846564516	down_in_case
PRJNA8274(	ENSGALG00010008237	GRIA2	case	control	-0.341153862	0.292593966	-1.165963423	0.243629234	0.667780411	down_in_case
PRJNA8274(	ENSGALG00010008238	NTAQ1	case	control	-0.011426901	0.1248618	-0.091516391	0.927082283	0.986477911	down_in_case
PRJNA8274(	ENSGALG00010008240	RCAN2	case	control	0.095701192	0.159040168	0.601742273	0.547345706	0.862769356	up_in_case
PRJNA8274(	ENSGALG00010008241	HERC3	case	control	0.143870194	0.061914464	2.323692788	0.020141965	0.254443554	up_in_case
PRJNA8274(	ENSGALG00010008242	CNTN5	case	control	0.197789352	0.626474904	0.315717918	0.752216625	0.940427752	up_in_case
PRJNA8274(	ENSGALG00010008243	YTHDC2	case	control	0.252365616	0.181085785	1.393624659	0.163430978	0.577480352	up_in_case
PRJNA8274(	ENSGALG00010008245	FBXO32	case	control	-0.12368722	0.17603425	-0.702631561	0.482285388	0.832932494	down_in_case
PRJNA8274(	ENSGALG00010008246	TERF2	case	control	0.006228394	0.042521477	0.146476434	0.883545292	0.977115076	up_in_case
PRJNA8274(	ENSGALG00010008247	GASK1B	case	control	0.118672647	0.105381277	1.12612649	0.260111983	0.687282842	up_in_case
PRJNA8274(	ENSGALG00010008248	TPK1	case	control	-0.224975703	0.086533915	-2.599855832	0.009326293	0.172753877	down_in_case
PRJNA8274(	ENSGALG00010008250	KLHL38	case	control	0.210881807	0.237158401	0.889202347	0.373894342	0.774369566	up_in_case
PRJNA8274(	ENSGALG00010008251	AHRR	case	control	-0.066557467	0.361289346	-0.184222059	0.853839255	0.969998446	down_in_case
PRJNA8274(	ENSGALG00010008252	ZFP92	case	control	0.160801067	0.094726738	1.697525639	0.089597328	0.472813801	up_in_case
PRJNA8274(	ENSGALG00010008253	ENSGALG00010008253	case	control	1.041044398	0.59818179	1.74034786	0.081797955	0.454736389	up_in_case
PRJNA8274(	ENSGALG00010008255	MSANTD3	case	control	0.015056338	0.077315038	0.194740101	0.845596416	0.967517103	up_in_case
PRJNA8274(	ENSGALG00010008257	DESI2	case	control	-0.092620668	0.036492802	-2.538053081	0.011147108	0.185137791	down_in_case
PRJNA8274(	ENSGALG00010008258	CCDC82	case	control	0.072507826	0.103572437	0.700068745	0.483884374	0.833895068	up_in_case
PRJNA8274(	ENSGALG00010008259	MCC	case	control	0.103132678	0.079077685	1.304194443	0.192167294	0.615090794	up_in_case
PRJNA8274(	ENSGALG00010008261	CRX	case	control	0.067684621	0.442159192	0.153077494	0.878337163	0.976160112	up_in_case
PRJNA8274(	ENSGALG00010008262	CYB5B	case	control	-0.030468876	0.047456705	-0.642035209	0.520850322	0.850168083	down_in_case
PRJNA8274(	ENSGALG00010008263	C1orf101	case	control	0.097511469	0.315038405	0.309522483	0.756924112	0.941659745	up_in_case
PRJNA8274(	ENSGALG00010008264	CNTNAP2	case	control	-0.007218567	0.153964237	-0.046884698	0.962605124	0.992068146	down_in_case
PRJNA8274(	ENSGALG00010008265	ENSGALG00010008265	case	control	0.167552614	0.558922626	0.299777834	0.764346624	0.943786458	up_in_case
PRJNA8274(	ENSGALG00010008266	ECSCR	case	control	0.018574725	0.082210357	0.225941426	0.821246978	0.958936428	up_in_case
PRJNA8274(	ENSGALG00010008267	MAML2	case	control	-0.120022682	0.07025512	-1.708383423	0.087565221	0.467581277	down_in_case
PRJNA8274(	ENSGALG00010008268	ENSGALG00010008268	case	control	0.229555383	0.225107044	1.019760992	0.307841827	0.731009321	up_in_case
PRJNA8274(	ENSGALG00010008269	ENSGALG00010008269	case	control	-0.061457261	0.380476686	-0.161527008	0.871678341	0.975062028	down_in_case

PRJNA8274(	ENSGALG00010008270	ENSGALG00010008270	case	control	-0.065348762	0.132175625	-0.494408577	0.621017658	0.893114457	down_in_case
PRJNA8274(	ENSGALG00010008272	DCP2	case	control	0.102620559	0.102402111	1.002133239	0.316279246	0.736866972	up_in_case
PRJNA8274(	ENSGALG00010008273	TMEM144	case	control	0.024769598	0.150383955	0.164709046	0.869172997	0.974684273	up_in_case
PRJNA8274(	ENSGALG00010008274	NFAT5	case	control	0.050292825	0.074715724	0.673122369	0.500869444	0.840987941	up_in_case
PRJNA8274(	ENSGALG00010008275	CELSR1	case	control	-0.239603933	0.113027003	-2.119882211	0.03401598	0.318007143	down_in_case
PRJNA8274(	ENSGALG00010008276	DNAJC18	case	control	-0.0439352	0.058992811	-0.744755152	0.456419751	0.8222491	down_in_case
PRJNA8274(	ENSGALG00010008278	CYP39A1	case	control	0.199072818	0.175346355	1.135311984	0.256244623	0.682618872	up_in_case
PRJNA8274(	ENSGALG00010008279	MAPK15	case	control	-0.014576974	0.126678935	-0.115070226	0.90838946	0.981730793	down_in_case
PRJNA8274(	ENSGALG00010008282	FAM83H	case	control	0.154794818	0.242138964	0.639280911	0.522640205	0.851055244	up_in_case
PRJNA8274(	ENSGALG00010008283	NQO1	case	control	0.680215389	0.209868858	3.241144955	0.001190506	0.054045207	up_in_case
PRJNA8274(	ENSGALG00010008284	BCKDHA	case	control	-0.270156695	0.074466655	-3.627888173	0.000285749	0.023091909	down_in_case
PRJNA8274(	ENSGALG00010008285	ADSS2	case	control	0.085079275	0.062151751	1.368895853	0.171031832	0.58844277	up_in_case
PRJNA8274(	ENSGALG00010008289	MTMR2	case	control	-0.054421819	0.068945188	-0.789349053	0.429908024	0.806015686	down_in_case
PRJNA8274(	ENSGALG00010008290	FAM13A	case	control	-0.11343337	0.092464342	-1.226779619	0.219905433	0.644131031	down_in_case
PRJNA8274(	ENSGALG00010008291	PTGFRN	case	control	-0.027666812	0.0758756	-0.364633849	0.715384744	0.926862121	down_in_case
PRJNA8274(	ENSGALG00010008292	REEP5	case	control	0.035952711	0.080871236	0.444567342	0.656632453	0.907494354	up_in_case
PRJNA8274(	ENSGALG00010008293	PLA2G7	case	control	0.391776165	0.074939417	5.227905158	1.71441E-07	5.83717E-05	up_in_case
PRJNA8274(	ENSGALG00010008294	NOB1	case	control	0.043219643	0.070353955	0.61431718	0.539005752	0.858476757	up_in_case
PRJNA8274(	ENSGALG00010008295	DMAC2	case	control	-0.072053512	0.108123382	-0.666400834	0.50515493	0.842675288	down_in_case
PRJNA8274(	ENSGALG00010008296	PALLD	case	control	-0.192295993	0.070131222	-2.741945557	0.006107646	0.135266583	down_in_case
PRJNA8274(	ENSGALG00010008297	ENSGALG00010008297	case	control	0.241885775	0.248270534	0.974283059	0.329916014	0.746250947	up_in_case
PRJNA8274(	ENSGALG00010008298	ENSGALG00010008298	case	control	0.185406089	0.064535323	2.872939644	0.004066718	0.110559064	up_in_case
PRJNA8274(	ENSGALG00010008300	CUL1	case	control	-0.023262307	0.048960575	-0.475123235	0.634699138	0.898107199	down_in_case
PRJNA8274(	ENSGALG00010008301	FAM91A1	case	control	0.01936351	0.051290027	0.377529733	0.705779972	0.924320939	up_in_case
PRJNA8274(	ENSGALG00010008302	ENSGALG00010008302	case	control	-0.02559649	0.103067597	-0.248346625	0.803866227	0.954997212	down_in_case
PRJNA8274(	ENSGALG00010008303	TEX10	case	control	-0.035947466	0.043758241	-0.821501613	0.411360606	0.796793365	down_in_case
PRJNA8274(	ENSGALG00010008306	ENSGALG00010008306	case	control	-0.302073883	0.326702244	-0.924615269	0.355166068	0.763161644	down_in_case
PRJNA8274(	ENSGALG00010008308	WWP2	case	control	0.30952942	0.165378945	1.871637408	0.061256784	0.402191009	up_in_case
PRJNA8274(	ENSGALG00010008309	ENSGALG00010008309	case	control	-0.102426719	0.276480069	-0.370466917	0.711034622	0.926028697	down_in_case
PRJNA8274(	ENSGALG00010008310	EXOSC5	case	control	-0.052245336	0.0874306	-0.59756351	0.550131218	0.864491914	down_in_case
PRJNA8274(	ENSGALG00010008311	SRP19	case	control	0.08890997	0.212009716	0.419367431	0.674947623	0.914247368	up_in_case
PRJNA8274(	ENSGALG00010008312	ZBTB18	case	control	-0.175618982	0.081841514	-2.145842285	0.031885564	0.3098361	down_in_case
PRJNA8274(	ENSGALG00010008313	PSMD7	case	control	-0.017697945	0.051977572	-0.340491961	0.733486076	0.933846596	down_in_case
PRJNA8274(	ENSGALG00010008314	INVS	case	control	0.128667569	0.096688619	1.330741608	0.183274047	0.60491829	up_in_case
PRJNA8274(	ENSGALG00010008316	ADGRF5	case	control	0.068962723	0.082031149	0.840689462	0.400521926	0.790138851	up_in_case
PRJNA8274(	ENSGALG00010008317	CEP57	case	control	0.039196742	0.072818228	0.538282002	0.590382375	0.882949889	up_in_case
PRJNA8274(	ENSGALG00010008318	ADPRH	case	control	0.52160696	0.111279009	4.687379634	2.76725E-06	0.000590623	up_in_case
PRJNA8274(	ENSGALG00010008319	ERP44	case	control	0.0356067	0.033753977	1.054889025	0.291476082	0.714303573	up_in_case
PRJNA8274(	ENSGALG00010008320	GPRIN3	case	control	0.042029405	0.078160694	0.537730703	0.590762979	0.882949889	up_in_case
PRJNA8274(	ENSGALG00010008321	ZFHX3	case	control	-0.026715177	0.075447027	-0.354091852	0.723270048	0.930215097	down_in_case
PRJNA8274(	ENSGALG00010008323	AKT3	case	control	-0.022879322	0.066209865	-0.345557601	0.729675215	0.933556014	down_in_case
PRJNA8274(	ENSGALG00010008324	SCRIB	case	control	-0.028862313	0.062786517	-0.45968966	0.645738992	0.902645903	down_in_case
PRJNA8274(	ENSGALG00010008325	AADAT	case	control	0.185176127	0.120469322	1.537122671	0.124263272	0.523029039	up_in_case
PRJNA8274(	ENSGALG00010008327	TGFB1	case	control	0.073650752	0.099965645	0.73676063	0.461267929	0.82454265	up_in_case
PRJNA8274(	ENSGALG00010008330	EZH2	case	control	-0.051502659	0.053133135	-0.96931338	0.332388856	0.748293551	down_in_case
PRJNA8274(	ENSGALG00010008331	CCDC97	case	control	-0.164256424	0.102531246	-1.60201334	0.10915266	0.501407977	down_in_case
PRJNA8274(	ENSGALG00010008332	DHX38	case	control	-0.025529714	0.03960073	-0.644677867	0.519135962	0.849150363	down_in_case
PRJNA8274(	ENSGALG00010008333	APC	case	control	0.01866348	0.116696236	0.159932147	0.872934525	0.975062028	up_in_case
PRJNA8274(	ENSGALG00010008334	STX17	case	control	-0.019656909	0.06757995	-0.290868947	0.771151552	0.94558971	down_in_case
PRJNA8274(	ENSGALG00010008335	HNRNPUL1	case	control	-0.093260308	0.056247698	-1.658028889	0.097311636	0.485860997	down_in_case
PRJNA8274(	ENSGALG00010008336	PAIP2B	case	control	-0.120122634	0.064542797	-1.861131516	0.062725607	0.407531201	down_in_case
PRJNA8274(	ENSGALG00010008339	FAM76B	case	control	0.028086907	0.067055381	0.418861347	0.675317468	0.914247368	up_in_case
PRJNA8274(	ENSGALG00010008340	PDIA4	case	control	0.105080759	0.064069987	1.640093345	0.10098576	0.49118924	up_in_case
PRJNA8274(	ENSGALG00010008341	DHODH	case	control	0.150829863	0.091352005	1.651084324	0.098721358	0.488407564	up_in_case
PRJNA8274(	ENSGALG00010008342	PLD3	case	control	-0.042209876	0.10357026	-0.407548229	0.683605379	0.916172158	down_in_case
PRJNA8274(	ENSGALG00010008343	TNFRSF21	case	control	0.098106556	0.07425865	1.321146502	0.18645252	0.609851564	up_in_case
PRJNA8274(	ENSGALG00010008344	MFAP3L	case	control	-0.207060686	0.269469346	-0.768401636	0.442248609	0.813710127	down_in_case
PRJNA8274(	ENSGALG00010008346	NR4A3	case	control	0.036111509	0.207709603	0.17385575	0.861978829	0.972643856	up_in_case
PRJNA8274(	ENSGALG00010008347	PUF60	case	control	0.027797899	0.042642828	0.651877469	0.514480221	0.847493921	up_in_case
PRJNA8274(	ENSGALG00010008348	ENSGALG00010008348	case	control	-0.039062059	0.066350447	-0.588723373	0.556046856	0.867878646	down_in_case
PRJNA8274(	ENSGALG00010008350	LRRC6	case	control	0.145754617	0.206365701	0.70629284	0.480006045	0.831851816	up_in_case
PRJNA8274(	ENSGALG00010008351	IST1	case	control	-0.00349143	0.046161634	-0.075634878	0.939709587	0.989551456	down_in_case
PRJNA8274(	ENSGALG00010008352	ENSGALG00010008352	case	control	-0.121525792	0.090740417	-1.339268601	0.180483247	0.601050402	down_in_case
PRJNA8274(	ENSGALG00010008354	TMEM71	case	control	-0.376425997	0.173844345	-2.165304813	0.03036435	0.307296678	down_in_case
PRJNA8274(	ENSGALG00010008355	MAFA	case	control	-0.188502561	0.14381122	-1.310763936	0.18993753	0.613281846	down_in_case
PRJNA8274(	ENSGALG00010008356	ENSGALG00010008356	case	control	-0.045187448	0.186588442	-0.2421771	0.808642939	0.955875648	down_in_case
PRJNA8274(	ENSGALG00010008358	HPF1	case	control	-0.018290316	0.06683615	-0.273659037	0.78434666	0.949518053	down_in_case
PRJNA8274(	ENSGALG00010008360	ZC3H3	case	control	-0.108587475	0.041952823	-2.588323455	0.009644437	0.175953849	down_in_case
PRJNA8274(	ENSGALG00010008361	MATR3	case	control	-0.070046673	0.034096532	-2.054363553	0.039940518	0.336547209	down_in_case
PRJNA8274(	ENSGALG00010008362	RAMP3	case	control	0.1482636	0.164605077	0.900723127	0.367735549	0.771770041	up_in_case
PRJNA8274(	ENSGALG00010008364	ENSGALG00010008364	case	control	0.596175965	0.429019793	1.38962345	0.164643251	0.579922223	up_in_case
PRJNA8274(	ENSGALG00010008366	RPRD1A	case	control	-0.033657625	0.090650289	-0.371290874	0.710420894	0.925650915	down_in_case
PRJNA8274(	ENSGALG00010008367	ENSGALG00010008367	case	control	0.216444132	0.516289781	0.419229936	0.675048096	0.914247368	up_in_case
PRJNA8274(	ENSGALG00010008368	SNCA	case	control	-0.040982185	0.191520512	-0.213983267	0.830560106	0.962228709	down_in_case
PRJNA8274(	ENSGALG00010008372	CLCN3	case	control	0.013256494	0.058153665	0.227956302	0.819680212	0.958669267	up_in_case
PRJNA8274(	ENSGALG00010008373	MACIR	case	control	-0.01474458	0.151773323	-0.09714869	0.922608315	0.985482967	down_in_case
PRJNA8274(	ENSGALG00010008374	ZNF821	case	control	0.150441572	0.07853364	1.915632237	0.055411923	0.383724215	up_in_case
PRJNA8274(	ENSGALG00010008375	ENSGALG00010008375	case	control	0.033897029	0.045213515	0.749710096	0.453429326	0.821282604	up_in_case
PRJNA8274(	ENSGALG00010008376	MMRN1	case	control	-0.332139443	0.180094059	-1.844255417	0.065145926	0.414724068	down_in_case
PRJNA8274(	ENSGALG00010008377	AKT2	case	control	-0.076054718	0.050979349	-1.491873053	0.135732428	0.539774365	down_in_case
PRJNA8274(	ENSGALG00010008380	SIL1	case	control	0.104362763	0.077005824	1.355258045	0.175335357	0.594211832	up_in_case
PRJNA8274(	ENSGALG00010008381	C18orf21	case	control	0.106679987	0.09880631	1.079687995	0.280281142	0.705762606	up_in_case
PRJNA8274(	ENSGALG00010008382	ENSGALG00010008382	case	control	0.017113849	0.10091718	0.169583104	0.865338011	0.973289701	up_in_case
PRJNA8274(	ENSGALG00010008383	PLD5	case	control	-0.192757474	0.28161183	-0.68447932	0.493672543	0.837916219	down_in_case
PRJNA8274(	ENSGALG00010008385	CTNNA1	case	control	0.008751246	0.038130626	0.22950701	0.818474872	0.958536226	up_in_case
PRJNA8274(	ENSGALG00010008386	SESN3	case	control	-0.006973811	0.100781384	-0.069197411	0.944832484	0.990313016	down_in_case
PRJNA8274(	ENSGALG00010008387	PHF20L1	case	control	0.008507751	0.070419085	0.120815989	0.903836785	0.981088965	up_in_case
PRJNA8274(	ENSGALG00010008388	ADCY1	case	control	-0.263425021	0.327969981	-0.803198575	0.421859969	0.80254895	down_in_case
PRJNA8274(	ENSGALG00010008389	GALNT1	case	control	-0.0582162	0.066060271	-0.881258885	0.378177714	0.776556765	down_in_case
PRJNA8274(	ENSGALG00010008391	ENSGALG00010008391	case	control	-0.096021625	0.081045466	-1.184787129	0.236101623	0.660642087	down_in_case
PRJNA8274(	ENSGALG00010008392	CD36	case	control	-0.483088056	0.316180724	-1.527885855			down_in_case
PRJNA8274(	ENSGALG00010008394	CD2AP	case	control	-0.048743325	0.074794164	-0.651699572	0.514594999	0.847493921	down_in_case
PRJNA8274(	ENSGALG00010008395	ENDOD1	case	control	0.06401515	0.097859107	0.65415629	0.513011119	0.846564516	up_in_case
PRJNA8274(	ENSGALG00010008396	LRRTM2	case	control	-0.201570611	0.354349632	-0.568846678	0.569460194	0.875057036	down_in_case
PRJNA8274(	ENSGALG00010008397	ATXN1L	case	control	0.059788085	0.043330541	1.379813937	0.16764394	0.583750784	up_in_case
PRJNA8274(	ENSGALG00010008399	INO80C	case	control	-0.063750126	0.065738331	-0.969755774	0.332168242	0.748268094	down_in_case
PRJNA8274(	ENSGALG00010008400	MAP1LC3C	case	control	-0.320558966	0.193446922	-1.657090034	0.097501274	0.485860997	down_in_case
PRJNA8274(	ENSGALG00010008401	PSMD8	case	control	-0.020868897	0.065252386	-0.319818144	0.749106192	0.939503405	down_in_case
PRJNA8274(	ENSGALG00010008403	GNAT3	case	control	-0.104988916	0.406814545	-0.258075619	0.796348547	0.95280257	down_in_case
PRJNA8274(	ENSGALG00010008405	LRATD2	case	control	0.086058362	0.092374622	0.931623434	0.351531168	0.760831854	up_in_case
PRJNA8274(	ENSGALG00010008407	GNAI1	case	control	-0.07033345	0.083417039	-0.843154471	0.399142058	0.789900496	down_in_case
PRJNA8274(	ENSGALG00010008408	SMARCA4	case	control	-0.098125221	0.07651452	-1.282439204	0.199688619	0.622902456	down_in_case
PRJNA8274(	ENSGALG00010008409	CWC15	case	control	-0.04275236	0.046911609	-0.911338588	0.362117001	0.768423771	down_in_case
PRJNA8274(	ENSGALG00010008410	NAPRT	case	control	0.188414955	0.093598152	2.013020039	0.044112521	0.350032868	up_in_case
PRJNA8274(	ENSGALG00010008412	NEK1	case	control	0.072735307	0.055840013	1.302566086	0.192722942	0.615090794	up_in_case
PRJNA8274(	ENSGALG00010008416	MSX2	case	control	0.85666686	0.57958647	1.478065663	0.139390204	0.541505735	up_in_case
PRJNA8274(	ENSGALG00010008417	IGFBP1	case	control	1.016186822	0.739453967	1.374239463			up_in_case
PRJNA8274(	ENSGALG00010008418	TMEM245	case	control	-0.111728194	0.069503175	-1.607526478	0.107938926	0.499736588	down_in_case
PRJNA8274(	ENSGALG00010008422	EXO1	case	control	-0.120789016	0.132192944	-0.913732706	0.360857314	0.766754768	down_in_case
PRJNA8274(	ENSGALG00010008424	PPIP5K2	case	control	0.034005947	0.159045499	0.213812699	0.830693121	0.962228709	up_in_case
PRJNA8274(	ENSGALG00010008425	MAGI2	case	control	0.207578357	0.088931939	2.334126077	0.019589121	0.251232676	up_in_case
PRJNA8274(	ENSGALG00010008426	AP1G1	case	control	0.051568409	0.040262465	1.280806038	0.200261803	0.622902456	up_in_case
PRJNA8274(	ENSGALG00010008428	ENSGALG00010008428	case	control	0.019468857	0.207616069	0.093773363	0.925289191	0.985571367	up_in_case
PRJNA8274(	ENSGALG00010008429	IGFBP3	case	control	-0.124781349	0.075678227	-1.648840816	0.099180251	0.488407564	down_in_case
PRJNA8274(	ENSGALG00010008430	ENSGALG00010008430	case	control	0.215189648	0.309366759	0.695581025	0.486691253	0.835095382	up_in_case
PRJNA8274(	ENSGALG00010008431	SH3RF1	case	control	-0.047742814	0.076853901	-0.621215232	0.534458019	0.856211151	down_in_case
PRJNA8274(	ENSGALG00010008433	EBAG9	case	control	-0.12273781	0.045770487	-2.681592843	0.007327258	0.148898802	down_in_case
PRJNA8274(	ENSGALG00010008434	PTCHD4	case	control	0.151449211	0.517782813	0.292495632	0.769907699	0.945428692	up_in_case
PRJNA8274(	ENSGALG00010008437	SHKBP1	case	control	-0.075934012	0.072148301	-1.052471246	0.292583396	0.714959426	down_in_case
PRJNA8274(	ENSGALG00010008438	SYBU	case	control	-0.066941917	0.201496476	-0.332223762	0.739720302	0.9363983	down_in_case
PRJNA8274(	ENSGALG00010008441	PHLPP2	case	control	0.063197864	0.05931738	1.065418998	0.286686363	0.710659943	up_in_case
PRJNA8274(	ENSGALG00010008442	WDR64	case	control	0.425349898	0.253376459	1.678726981	0.093205265	0.478404627	up_in_case
PRJNA8274(	ENSGALG00010008443	RSFR	case	control	0.419436724	0.236894821	1.770560969	0.076633737	0.44430328	up_in_case
PRJNA8274(	ENSGALG00010008444	SLC7A11	case	control	0.72067352	0.372019637	1.937192148	0.05272186	0.374713019	up_in_case
PRJNA8274(	ENSGALG00010008447	FRRS1L	case	control	0.092174985	0.072511831	1.271171665	0.203667584	0.62581674	up_in_case
PRJNA8274(	ENSGALG00010008448	TNS3	case	control	0.041507219	0.069999742	0.59296246	0.553206276	0.866252369	up_in_case

PRJNA8274(	ENSGALG00010008449	SRA1	case	control	0.063497897	0.081033231	0.783603177	0.433272991	0.808138728	up_in_case
PRJNA8274(	ENSGALG00010008450	GIN1	case	control	-0.126065255	0.146205014	-0.862249879	0.388550027	0.783234479	down_in_case
PRJNA8274(	ENSGALG00010008451	OPN3	case	control	-0.412043868	0.216574615	-1.902549232	0.057099389	0.388573099	down_in_case
PRJNA8274(	ENSGALG00010008453	AMOTL1	case	control	-0.078210168	0.083881284	-0.932391169	0.351134407	0.760831854	down_in_case
PRJNA8274(	ENSGALG00010008455	FKBP1B	case	control	-0.108475442	0.08919013	-1.216226977	0.22389847	0.647995977	down_in_case
PRJNA8274(	ENSGALG00010008456	NHLRC1	case	control	-0.027368391	0.080574994	-0.339663585	0.734109888	0.933846596	down_in_case
PRJNA8274(	ENSGALG00010008457	MARVELD3	case	control	0.072700481	0.119371191	0.6090287	0.542505416	0.85975913	up_in_case
PRJNA8274(	ENSGALG00010008459	EPB41L4B	case	control	0.255193418	0.238963287	1.067918931	0.285557082	0.709921118	up_in_case
PRJNA8274(	ENSGALG00010008464	SF3B6	case	control	-0.078109157	0.058900385	-1.326123025	0.184798951	0.607639686	down_in_case
PRJNA8274(	ENSGALG00010008466	C11orf97	case	control	-0.721549547	0.336539051	-2.144029182	0.032030549	0.3098361	down_in_case
PRJNA8274(	ENSGALG00010008468	EPPK1	case	control	-0.09912113	0.29745177	-0.333234293	0.738957434	0.936217889	down_in_case
PRJNA8274(	ENSGALG00010008469	NOCT	case	control	-0.069191615	0.066130748	-1.04628508	0.295429432	0.718955697	down_in_case
PRJNA8274(	ENSGALG00010008473	CSMD3	case	control	-0.930988525	0.562177125	-1.656041279	0.09771346	0.486525931	down_in_case
PRJNA8274(	ENSGALG00010008474	ENSGALG00010008474	case	control	-0.209742147	0.113244679	-1.852114804	0.064009339	0.410839656	down_in_case
PRJNA8274(	ENSGALG00010008476	RNF166	case	control	-0.117743751	0.059154409	-1.990447585	0.046541652	0.356907825	down_in_case
PRJNA8274(	ENSGALG00010008486	CTU2	case	control	0.019633618	0.066729301	0.294227833	0.768583814	0.945375926	up_in_case
PRJNA8274(	ENSGALG00010008488	CCSER1	case	control	0.055426423	0.126824592	0.437032139	0.66208804	0.91054616	up_in_case
PRJNA8274(	ENSGALG00010008492	ANKRD49	case	control	-0.013309791	0.072065463	-0.184690282	0.853471969	0.969998446	down_in_case
PRJNA8274(	ENSGALG00010008497	PAM	case	control	-0.060260595	0.066810535	-0.901962459	0.36707681	0.771770041	down_in_case
PRJNA8274(	ENSGALG00010008498	ENSGALG00010008498	case	control	-0.074337229	0.08532806	-0.871193238	0.383648651	0.780061953	down_in_case
PRJNA8274(	ENSGALG00010008500	ENSGALG00010008500	case	control	-0.269421304	0.11282309	-2.387997906	0.016940438	0.231594902	down_in_case
PRJNA8274(	ENSGALG00010008501	ENSGALG00010008501	case	control	0.410145018	0.159984248	2.56365876	0.010357532	0.181733384	up_in_case
PRJNA8274(	ENSGALG00010008504	RNF139	case	control	-0.012012677	0.040998915	-0.292999867	0.769522254	0.945428692	down_in_case
PRJNA8274(	ENSGALG00010008505	PTPN3	case	control	0.026959959	0.172717451	0.156092851	0.875959833	0.975666505	up_in_case
PRJNA8274(	ENSGALG00010008509	MGARP	case	control	0.450384892	0.407260024	1.105890254	0.268774024	0.695650414	up_in_case
PRJNA8274(	ENSGALG00010008514	PIEZO1	case	control	0.029795815	0.10836184	0.274965938	0.783342408	0.949518053	up_in_case
PRJNA8274(	ENSGALG00010008515	TAFA5	case	control	0.12876111	0.162085737	0.794401234	0.426961886	0.805305429	up_in_case
PRJNA8274(	ENSGALG00010008516	MRE11	case	control	-0.096332171	0.061347424	-1.570272479	0.116351734	0.5134816	down_in_case
PRJNA8274(	ENSGALG00010008522	NUDT12	case	control	-0.144663396	0.176293889	-0.820580885	0.411885039	0.797194435	down_in_case
PRJNA8274(	ENSGALG00010008527	CHUK	case	control	0.002174675	0.040572978	0.053599087	0.957254584	0.992056045	up_in_case
PRJNA8274(	ENSGALG00010008529	GPR83	case	control	0.369185254	0.390653729	0.945044747	0.344636032	0.755870938	up_in_case
PRJNA8274(	ENSGALG00010008534	RAB33B	case	control	-0.032820842	0.04174995	-0.786128902	0.431791973	0.807276582	down_in_case
PRJNA8274(	ENSGALG00010008542	MOCOS	case	control	0.078915915	0.147002958	0.536832156	0.591383558	0.882949889	up_in_case
PRJNA8274(	ENSGALG00010008561	EEF1D	case	control	-0.075182761	0.074830412	-1.004708626	0.315037174	0.736508113	down_in_case
PRJNA8274(	ENSGALG00010008580	PANX1	case	control	-0.36766235	0.200123524	-1.837177066	0.06618376	0.416388539	down_in_case
PRJNA8274(	ENSGALG00010008588	PYCR3	case	control	-0.125758191	0.10181124	-1.2352093	0.216752619	0.641864248	down_in_case
PRJNA8274(	ENSGALG00010008592	KIAA0513	case	control	-0.118919048	0.093254158	-1.275214434	0.202233354	0.625148499	down_in_case
PRJNA8274(	ENSGALG00010008596	ENSGALG00010008596	case	control	-0.064911673	0.098131132	-0.661478902	0.50830524	0.843702533	down_in_case
PRJNA8274(	ENSGALG00010008618	EIF4EBP3	case	control	-0.038052334	0.054213441	-0.701898512	0.482742457	0.833419912	down_in_case
PRJNA8274(	ENSGALG00010008623	TRPS1	case	control	-0.067032949	0.100185117	-0.669090888	0.503437502	0.842604443	down_in_case
PRJNA8274(	ENSGALG00010008636	AGTPBP1	case	control	0.254105446	0.204643697	1.241696907	0.214348412	0.639112236	up_in_case
PRJNA8274(	ENSGALG00010008637	RGS7	case	control	-0.424829891	0.519855159	-0.817208185	0.413809466	0.798364188	down_in_case
PRJNA8274(	ENSGALG00010008653	ENSGALG00010008653	case	control	-0.103489305	0.106181195	-0.974648146	0.329734823	0.746174551	down_in_case
PRJNA8274(	ENSGALG00010008656	CCDC6	case	control	-0.131981494	0.048520287	-2.720130099	0.006525624	0.139695237	down_in_case
PRJNA8274(	ENSGALG00010008658	TSTA3	case	control	0.013345846	0.114103325	0.116962811	0.906889524	0.981730793	up_in_case
PRJNA8274(	ENSGALG00010008666	SLC16A9	case	control	-0.254369782	0.100748153	-2.524808396	0.011576139	0.188192574	down_in_case
PRJNA8274(	ENSGALG00010008668	ENSGALG00010008668	case	control	0.205149028	0.29371302	0.6984676	0.484884813	0.834423421	up_in_case
PRJNA8274(	ENSGALG00010008674	RXFP1	case	control	-0.244538459	0.164213133	-1.489152877	0.136447116	0.539840644	down_in_case
PRJNA8274(	ENSGALG00010008686	ENSGALG00010008686	case	control	-0.301363576	0.349479616	-0.862320899	0.388510955	0.783234479	down_in_case
PRJNA8274(	ENSGALG00010008688	HBEGF	case	control	-0.041967923	0.173582022	-0.241775744	0.808953935	0.955875648	down_in_case
PRJNA8274(	ENSGALG00010008690	YIF1B	case	control	-0.045521391	0.065828355	-0.691516471	0.489241034	0.836347578	down_in_case
PRJNA8274(	ENSGALG00010008693	C4orf46	case	control	-0.167510729	0.071682482	-2.336843319	0.019447332	0.249862403	down_in_case
PRJNA8274(	ENSGALG00010008696	PFN4	case	control	0.055567077	0.219451745	0.253208638	0.800106992	0.954053991	up_in_case
PRJNA8274(	ENSGALG00010008699	NAA35	case	control	0.070227351	0.137316078	0.511428464	0.609051068	0.8877909	up_in_case
PRJNA8274(	ENSGALG00010008700	ENSGALG00010008700	case	control	-0.265912175	0.126140881	-2.108057071	0.035026053	0.323143587	down_in_case
PRJNA8274(	ENSGALG00010008708	ZC3H4	case	control	-0.159925721	0.092534689	-1.728278594	0.083938289	0.45831139	down_in_case
PRJNA8274(	ENSGALG00010008716	TATDN1	case	control	-0.030194746	0.072031548	-0.419187792	0.675078893	0.914247368	down_in_case
PRJNA8274(	ENSGALG00010008729	TMEM254	case	control	0.297969498	0.139189347	2.140749302	0.03229426	0.309838273	up_in_case
PRJNA8274(	ENSGALG00010008731	NDUFB9	case	control	0.007635117	0.051821529	0.147334851	0.882867726	0.976865404	up_in_case
PRJNA8274(	ENSGALG00010008739	VSTM5	case	control	0.0349636	0.19648505	0.177945343	0.858765901	0.971828322	up_in_case
PRJNA8274(	ENSGALG00010008745	GRAMD4	case	control	-0.025032246	0.055449632	-0.45144116	0.651671624	0.905275327	down_in_case
PRJNA8274(	ENSGALG00010008750	PFDN1	case	control	-0.054564288	0.077815383	-0.701201819	0.483177075	0.833529046	down_in_case
PRJNA8274(	ENSGALG00010008752	GOLM1	case	control	-0.598778611	0.190531932	-3.142668032	0.001674156	0.065655176	down_in_case
PRJNA8274(	ENSGALG00010008756	ITSN2	case	control	-0.213196197	0.067234307	-3.170943606	0.001519446	0.062408717	down_in_case
PRJNA8274(	ENSGALG00010008762	SAE1	case	control	-0.055485624	0.044589092	-1.24437664	0.213360976	0.638582746	down_in_case
PRJNA8274(	ENSGALG00010008764	ETFDH	case	control	-0.207559329	0.079169161	-2.621719454	0.008748742	0.166357336	down_in_case
PRJNA8274(	ENSGALG00010008768	PRXL2C	case	control	0.032825141	0.150452903	0.218175522	0.827292356	0.961319258	up_in_case
PRJNA8274(	ENSGALG00010008775	MED17	case	control	0.022835604	0.049372999	0.462511978	0.643714215	0.902038523	up_in_case
PRJNA8274(	ENSGALG00010008780	KARS1	case	control	0.048923538	0.041951603	1.16618997	0.243537647	0.667780411	up_in_case
PRJNA8274(	ENSGALG00010008782	CCDC9	case	control	0.066186416	0.291196869	0.227290959	0.820197503	0.958669267	up_in_case
PRJNA8274(	ENSGALG00010008790	GPN1	case	control	0.024167429	0.07551842	0.320020327	0.748952922	0.939503405	up_in_case
PRJNA8274(	ENSGALG00010008793	C11orf54	case	control	-0.121273994	0.099029716	-1.224622254	0.220717579	0.644744838	down_in_case
PRJNA8274(	ENSGALG00010008799	ENSGALG00010008799	case	control	-0.044601439	0.112385057	-0.396862716	0.6914687	0.918812261	down_in_case
PRJNA8274(	ENSGALG00010008807	BRD1	case	control	-0.06008832	0.055365891	-1.08529491	0.27779106	0.703206258	down_in_case
PRJNA8274(	ENSGALG00010008811	TERF2IP	case	control	0.026664214	0.055547649	0.480024163	0.631210211	0.897039706	up_in_case
PRJNA8274(	ENSGALG00010008814	ENSGALG00010008814	case	control	0.013127218	0.176882271	0.074214438	0.940839758	0.989551456	up_in_case
PRJNA8274(	ENSGALG00010008821	ENSGALG00010008821	case	control	0.289305153	0.141421152	2.045699313	0.040785963	0.337767404	up_in_case
PRJNA8274(	ENSGALG00010008824	GREM2	case	control	-0.677532457	0.286072705	-2.368392532	0.01786557	0.238096598	down_in_case
PRJNA8274(	ENSGALG00010008825	PPID	case	control	-0.041372546	0.050189389	-0.824328551	0.4097529	0.795863751	down_in_case
PRJNA8274(	ENSGALG00010008830	DHX34	case	control	-0.107717207	0.081508334	-1.321548375	0.186318584	0.60983194	down_in_case
PRJNA8274(	ENSGALG00010008832	MTSS1	case	control	0.206525942	0.073493255	2.810134625	0.004952078	0.120231831	up_in_case
PRJNA8274(	ENSGALG00010008837	ENSGALG00010008837	case	control	-0.015892995	0.086807614	-0.183082965	0.854732922	0.970313652	down_in_case
PRJNA8274(	ENSGALG00010008839	ZNF512	case	control	-0.040026226	0.04053858	-0.987361337	0.323465531	0.740800303	down_in_case
PRJNA8274(	ENSGALG00010008846	SLC35D2	case	control	0.339958905	0.260366344	1.305694507	0.191656467	0.615055537	up_in_case
PRJNA8274(	ENSGALG00010008850	CYSTM1	case	control	-0.190728074	0.08878001	-2.148322281	0.031688162	0.3098361	down_in_case
PRJNA8274(	ENSGALG00010008852	KHDRBS3	case	control	-0.033700168	0.078134776	-0.431308179	0.666244304	0.912284937	down_in_case
PRJNA8274(	ENSGALG00010008859	ENSGALG00010008859	case	control	0.249860763	0.427053802	0.585080292	0.55849373	0.869321905	up_in_case
PRJNA8274(	ENSGALG00010008860	SQLE	case	control	0.243514093	0.149254433	1.631536749	0.102777112	0.492036392	up_in_case
PRJNA8274(	ENSGALG00010008862	ZNF367	case	control	0.040572413	0.152372492	0.266271246	0.790030316	0.951843754	up_in_case
PRJNA8274(	ENSGALG00010008864	ADAT1	case	control	0.328426143	0.101981974	3.220433264	0.00127997	0.056667398	up_in_case
PRJNA8274(	ENSGALG00010008866	WASHC5	case	control	-0.026960948	0.050480342	-0.534088071	0.593280603	0.883625114	down_in_case
PRJNA8274(	ENSGALG00010008871	PRR7	case	control	0.012510448	0.110272194	0.113450615	0.909673314	0.981730793	up_in_case
PRJNA8274(	ENSGALG00010008877	FNDC4	case	control	-0.399958381	0.152642005	-2.620237989	0.008786842	0.166436729	down_in_case
PRJNA8274(	ENSGALG00010008879	HABP4	case	control	-0.067527466	0.083816253	-0.805660751	0.420438486	0.801849874	down_in_case
PRJNA8274(	ENSGALG00010008880	FMN2	case	control	-0.287451353	0.167057045	-1.720677825	0.085309302	0.461985839	down_in_case
PRJNA8274(	ENSGALG00010008884	FAM135B	case	control	0.307991715	0.43313349	0.711078043	0.477035875	0.830520323	up_in_case
PRJNA8274(	ENSGALG00010008888	FBXO46	case	control	0.001425707	0.073441649	0.019412784	0.984511812	0.996591771	up_in_case
PRJNA8274(	ENSGALG00010008892	ENSGALG00010008892	case	control	-0.08212686	0.087519991	-0.938378297	0.348050035	0.758675915	down_in_case
PRJNA8274(	ENSGALG00010008893	ENSGALG00010008893	case	control	-0.46003869	0.125732562	-3.658866761	0.000253333	0.021415026	down_in_case
PRJNA8274(	ENSGALG00010008897	NSMCE2	case	control	-0.212666234	0.077841943	-2.73202626	0.006294612	0.138110236	down_in_case
PRJNA8274(	ENSGALG00010008903	ENSGALG00010008903	case	control	-0.126103272	0.269842318	-0.467322076	0.640269464	0.901077979	down_in_case
PRJNA8274(	ENSGALG00010008905	GABARAPL2	case	control	-0.016115185	0.078197885	-0.206082108	0.836726775	0.964701514	down_in_case
PRJNA8274(	ENSGALG00010008906	DBN1	case	control	-0.006967094	0.094325968	-0.073861884	0.941120285	0.989551456	down_in_case
PRJNA8274(	ENSGALG00010008910	TRIB1	case	control	0.124660795	0.084645574	1.472738488	0.140821564	0.543368104	up_in_case
PRJNA8274(	ENSGALG00010008918	PLAC9	case	control	0.018969575	0.106168044	0.178675	0.858192901	0.971828322	up_in_case
PRJNA8274(	ENSGALG00010008921	OPA3	case	control	0.113572617	0.165834483	0.684855253	0.493435265	0.837916219	up_in_case
PRJNA8274(	ENSGALG00010008928	CFC1	case	control	0.603674451	0.430497756	1.402270842	0.160834384	0.573265281	up_in_case
PRJNA8274(	ENSGALG00010008931	FNIP2	case	control	0.01439392	0.067949722	0.211831911	0.832238172	0.962371901	up_in_case
PRJNA8274(	ENSGALG00010008932	VASP	case	control	-0.104110772	0.084782175	-1.227979492	0.219454667	0.643443819	down_in_case
PRJNA8274(	ENSGALG00010008936	IFT172	case	control	0.09463919	0.070537427	1.341687586	0.179697314	0.600679303	up_in_case
PRJNA8274(	ENSGALG00010008938	ZBED4	case	control	0.041747588	0.057470905	0.726412567	0.467585889	0.828046838	up_in_case
PRJNA8274(	ENSGALG00010008939	RANBP9	case	control	0.000655539	0.047758408	0.013726147	0.989048463	0.997164011	up_in_case
PRJNA8274(	ENSGALG00010008943	ENSGALG00010008943	case	control	-0.189194518	0.196545267	-0.962600224	0.335748185	0.749888354	down_in_case
PRJNA8274(	ENSGALG00010008946	COL22A1	case	control	0.066103321	0.151703715	0.43573963	0.663025649	0.911044469	up_in_case
PRJNA8274(	ENSGALG00010008947	SPMIP2	case	control	0.638802539	0.286063445	2.233079938	0.025543673	0.283805659	up_in_case
PRJNA8274(	ENSGALG00010008955	KRTCAP3	case	control	-0.16665454	0.258491972	-0.644718436	0.519109667	0.849150363	down_in_case
PRJNA8274(	ENSGALG00010008956	LVRN	case	control	-0.468394541	0.27848864	-1.681916146	0.092585101	0.477342721	down_in_case
PRJNA8274(	ENSGALG00010008960	LRRC2	case	control	-0.589717238	0.314705084	-1.873872614	0.060947985	0.4010843	down_in_case
PRJNA8274(	ENSGALG00010008966	NRBP1	case	control	0.0481251	0.068985694	0.697609853	0.485421218	0.834423421	up_in_case
PRJNA8274(	ENSGALG00010008969	PPP1R37	case	control	0.055085927	0.063297671	0.870267836	0.384154052	0.780645579	up_in_case
PRJNA8274(	ENSGALG00010008971	ANXA11	case	control	-0.326284112	0.065108847	-5.011363681	5.40457E-07	0.000157725	down_in_case
PRJNA8274(	ENSGALG00010008974	SMCO4	case	control	-0.042940252	0.081628645	-0.526043911	0.598857698	0.884767547	down_in_case
PRJNA8274(	ENSGALG00010008980	ENSGALG00010008980	case	control	-0.627357615	0.342677134	-1.83075424	0.067137233	0.419241238	down_in_case
PRJNA8274(	ENSGALG00010008983	ENSGALG00010008983	case	control	0.008279963	0.369263746	0.022422896	0.982110617	0.996591771	up_in_case
PRJNA8274(	ENSGALG00010008988	PPM1G	case	control	-0.044088463	0.058232675	-0.757108671	0.44898476	0.818701429	down_in_case
PRJNA8274(	ENSGALG00010008991	ENSGALG00010008991	case	control	-0.013463293	0.076523942	-0.175935691	0.860344465	0.971871858	down_in_case

PRJNA8274(	ENSGALG00010008995	ECD	case	control	0.060139602	0.056695686	1.060743886	0.28880631	0.712801679	up_in_case
PRJNA8274(	ENSGALG00010008996	CHRM3	case	control	-0.249399533	0.145914228	-1.709220111	0.087410185	0.467276876	down_in_case
PRJNA8274(	ENSGALG00010008997	CHST6	case	control	-0.019098451	0.059423898	-0.321393443	0.747912252	0.939324187	down_in_case
PRJNA8274(	ENSGALG00010008999	TRAPPC9	case	control	-0.115046816	0.063182308	-1.820870744	0.068626507	0.422837898	down_in_case
PRJNA8274(	ENSGALG00010009000	FAM149B1	case	control	0.069189326	0.050324097	1.374874644	0.169170312	0.585038804	up_in_case
PRJNA8274(	ENSGALG00010009002	ENSGALG00010009002	case	control	-0.224375476	0.081655127	-2.747843088	0.005998871	0.134447684	down_in_case
PRJNA8274(	ENSGALG00010009003	ALG12	case	control	0.150373693	0.058500197	2.570481841	0.010155715	0.179292254	up_in_case
PRJNA8274(	ENSGALG00010009007	TMEM65	case	control	-0.097052479	0.061882002	-1.56834745	0.116800068	0.51350396	down_in_case
PRJNA8274(	ENSGALG00010009011	ACP6	case	control	0.176121315	0.13294418	1.324776418	0.185245318	0.60826821	up_in_case
PRJNA8274(	ENSGALG00010009013	LIPG	case	control	0.15398408	0.136470769	1.128330127	0.259180525	0.686348427	up_in_case
PRJNA8274(	ENSGALG00010009015	TMEM231	case	control	0.03775997	0.061889209	0.610122031	0.541780974	0.85975913	up_in_case
PRJNA8274(	ENSGALG00010009016	DNAJC9	case	control	0.01986617	0.066891555	0.296990697	0.766473612	0.944063105	up_in_case
PRJNA8274(	ENSGALG00010009019	ZNF513	case	control	-0.007614475	0.058001996	-0.13127954	0.895554177	0.980176778	down_in_case
PRJNA8274(	ENSGALG00010009020	ARHGAP35	case	control	-0.112470142	0.072423758	-1.552945404	0.120436178	0.51937194	down_in_case
PRJNA8274(	ENSGALG00010009021	HNF4beta	case	control	0.882343684	0.493649968	1.787387301	0.073874913	0.437990779	up_in_case
PRJNA8274(	ENSGALG00010009024	PMAIP1	case	control	-0.058148532	0.155088868	-0.374936852	0.707707431	0.924870864	down_in_case
PRJNA8274(	ENSGALG00010009025	UTP23	case	control	0.161809634	0.099601515	1.624570014	0.104254211	0.493800828	up_in_case
PRJNA8274(	ENSGALG00010009026	RAPGEF2	case	control	0.008366866	0.044947003	0.186149597	0.852327449	0.969582953	up_in_case
PRJNA8274(	ENSGALG00010009028	PDLIM7	case	control	-0.068889876	0.070030032	-0.983719036	0.325253677	0.743110335	down_in_case
PRJNA8274(	ENSGALG00010009029	IGSF3	case	control	-0.032085937	0.103764238	-0.309219605	0.757154482	0.941659745	down_in_case
PRJNA8274(	ENSGALG00010009030	ZDHHC7	case	control	0.079522574	0.052702991	1.508881598	0.131329043	0.534891859	up_in_case
PRJNA8274(	ENSGALG00010009031	SPIN1	case	control	0.064769797	0.173238105	0.373877309	0.708495599	0.92524028	up_in_case
PRJNA8274(	ENSGALG00010009034	SNX17	case	control	-0.091531472	0.042536019	-2.151857994	0.031408541	0.3098361	down_in_case
PRJNA8274(	ENSGALG00010009035	ENSGALG00010009035	case	control	-0.138069955	0.067449783	-2.047003705	0.040657721	0.337749022	down_in_case
PRJNA8274(	ENSGALG00010009037	ENSGALG00010009037	case	control	0.052702755	0.217177907	0.242670885	0.808260363	0.955875648	up_in_case
PRJNA8274(	ENSGALG00010009038	RAD21	case	control	-0.006669635	0.038434502	-0.173532504	0.862232881	0.972698817	down_in_case
PRJNA8274(	ENSGALG00010009039	SLC36A4	case	control	0.135842527	0.138226446	0.982753524	0.325728761	0.74363678	up_in_case
PRJNA8274(	ENSGALG00010009040	CRELD2	case	control	0.284653184	0.062511341	4.553624699	5.27294E-06	0.00101896	up_in_case
PRJNA8274(	ENSGALG00010009043	TFAM	case	control	-0.071422004	0.059278298	-1.204859222	0.228257653	0.651867751	down_in_case
PRJNA8274(	ENSGALG00010009047	SUPT5H	case	control	-0.027790213	0.059865525	-0.464210634	0.642496843	0.901845477	down_in_case
PRJNA8274(	ENSGALG00010009050	PIK3CG	case	control	0.120011819	0.082441521	1.455720592	0.145469875	0.550176993	up_in_case
PRJNA8274(	ENSGALG00010009051	TROJANZ	case	control	0.453016202	0.334234217	1.355385472	0.175294776	0.594211832	up_in_case
PRJNA8274(	ENSGALG00010009054	RYR2	case	control	-0.883645298	0.307826674	-2.870593654	0.004097018	0.110751151	down_in_case
PRJNA8274(	ENSGALG00010009057	NXNL2	case	control	0.360736343	0.418522378	0.861928447	0.388726894	0.783368493	up_in_case
PRJNA8274(	ENSGALG00010009059	MED29	case	control	0.101065502	0.091714179	1.101961583	0.270478374	0.698292245	up_in_case
PRJNA8274(	ENSGALG00010009065	EIF2B4	case	control	-0.104820123	0.062460115	-1.678192926	0.093309442	0.478523919	down_in_case
PRJNA8274(	ENSGALG00010009067	ENSGALG00010009067	case	control	-1.628707965	1.036105892	-1.571951262			down_in_case
PRJNA8274(	ENSGALG00010009068	AGO2	case	control	0.012166584	0.085065239	0.143026508	0.886269246	0.977630122	up_in_case
PRJNA8274(	ENSGALG00010009069	GRID2	case	control	0.518220274	0.268612287	1.929250069	0.053699825	0.377891678	up_in_case
PRJNA8274(	ENSGALG00010009070	ENSGALG00010009070	case	control	2.311548229	1.452209997	1.591745157	0.111441977	0.504150673	up_in_case
PRJNA8274(	ENSGALG00010009073	PRKAR2B	case	control	-0.611468145	0.345217692	-1.771253789	0.076518508	0.444080628	down_in_case
PRJNA8274(	ENSGALG00010009076	TMEM214	case	control	0.254421585	0.096385283	2.639631057	0.008299633	0.160448204	up_in_case
PRJNA8274(	ENSGALG00010009078	PTTG2	case	control	-0.041579248	0.105456694	-0.394277942	0.693375846	0.919776842	down_in_case
PRJNA8274(	ENSGALG00010009079	KCTD15	case	control	-0.140603903	0.071052986	-1.978859869	0.047831786	0.360228144	down_in_case
PRJNA8274(	ENSGALG00010009080	TLL1	case	control	0.10534089	0.162044581	0.650073511	0.515644739	0.847810358	up_in_case
PRJNA8274(	ENSGALG00010009081	HBP1	case	control	-0.012639003	0.053838172	-0.234759149	0.814395676	0.957063027	down_in_case
PRJNA8274(	ENSGALG00010009083	MED30	case	control	-0.062496928	0.07433396	-0.840758761	0.400483094	0.790138851	down_in_case
PRJNA8274(	ENSGALG00010009085	PAF1	case	control	0.01113055	0.051956915	0.21422654	0.830370401	0.962228709	up_in_case
PRJNA8274(	ENSGALG00010009086	UBE2D1	case	control	-0.134934665	0.073945393	-1.824787998	0.068033028	0.421339236	down_in_case
PRJNA8274(	ENSGALG00010009088	ENSGALG00010009088	case	control	-0.323210397	0.212746275	-1.51922941	0.128704761	0.530596961	down_in_case
PRJNA8274(	ENSGALG00010009089	AGBL5	case	control	0.047443317	0.068682465	0.690763169	0.489714385	0.836347578	up_in_case
PRJNA8274(	ENSGALG00010009091	EXT1	case	control	0.01124036	0.046327448	0.242628512	0.808293192	0.955875648	up_in_case
PRJNA8274(	ENSGALG00010009092	EED	case	control	-0.135132389	0.106541091	-1.268359342	0.204669654	0.627358178	down_in_case
PRJNA8274(	ENSGALG00010009093	SLU7	case	control	0.025335695	0.070385108	0.35995817	0.718878415	0.928045621	up_in_case
PRJNA8274(	ENSGALG00010009094	DARS	case	control	-0.122384982	0.059708846	-2.049696001	0.040394105	0.33753444	down_in_case
PRJNA8274(	ENSGALG00010009097	C1QTNF2	case	control	-0.135309955	0.144400468	-0.937046509	0.348734635	0.759193168	down_in_case
PRJNA8274(	ENSGALG00010009098	CAD	case	control	0.046225046	0.075181904	0.61484271	0.5386586	0.858157084	up_in_case
PRJNA8274(	ENSGALG00010009099	CXCR4	case	control	0.264417578	0.117050613	2.259002066	0.023883256	0.273286623	up_in_case
PRJNA8274(	ENSGALG00010009100	ENSGALG00010009100	case	control	-0.012978676	0.092830917	-0.139809848	0.888810232	0.978017246	down_in_case
PRJNA8274(	ENSGALG00010009101	HIKESHI	case	control	-0.045460479	0.063241296	-0.718841676	0.472238476	0.829235491	down_in_case
PRJNA8274(	ENSGALG00010009102	HTRA3	case	control	-0.143487767	0.198244501	-0.723791917	0.469193493	0.829101076	down_in_case
PRJNA8274(	ENSGALG00010009103	AP3S1	case	control	0.001487674	0.108995374	0.013648966	0.989110039	0.997164011	up_in_case
PRJNA8274(	ENSGALG00010009104	MTR	case	control	-0.018349953	0.086583752	-0.21193299	0.832159313	0.962371901	down_in_case
PRJNA8274(	ENSGALG00010009106	THSD7B	case	control	0.171603393	0.107147611	1.6015606	0.109252809	0.501658961	up_in_case
PRJNA8274(	ENSGALG00010009109	ENSGALG00010009109	case	control	0.12242967	0.186393108	0.656835819	0.51128649	0.845639232	up_in_case
PRJNA8274(	ENSGALG00010009111	LRFN1	case	control	-0.046838679	0.122734952	-0.381624615	0.702739823	0.923552934	down_in_case
PRJNA8274(	ENSGALG00010009112	ENSGALG00010009112	case	control	0.069934973	0.177764298	0.393414052	0.694013693	0.920203598	up_in_case
PRJNA8274(	ENSGALG00010009113	RPLP1	case	control	0.062206926	0.091591612	0.679177112	0.497025648	0.838837102	up_in_case
PRJNA8274(	ENSGALG00010009114	SETD7	case	control	-0.012004998	0.066233991	-0.181251311	0.856170321	0.970837807	down_in_case
PRJNA8274(	ENSGALG00010009115	COG5	case	control	0.038989694	0.049977848	0.780139524	0.435308755	0.81000376	up_in_case
PRJNA8274(	ENSGALG00010009116	S1PR3	case	control	0.38746789	0.11487833	3.372854478	0.000743933	0.04107427	up_in_case
PRJNA8274(	ENSGALG00010009117	ERGIC1	case	control	-0.009390853	0.049420884	-0.190017914	0.849295093	0.968927451	down_in_case
PRJNA8274(	ENSGALG00010009118	ACTN2	case	control	-0.173334456	0.105830536	-1.637849167	0.101453162	0.491460867	down_in_case
PRJNA8274(	ENSGALG00010009119	CISD1	case	control	-0.047069985	0.069850767	-0.673864971	0.500397164	0.840855399	down_in_case
PRJNA8274(	ENSGALG00010009120	ATG12	case	control	0.161038152	0.079839634	2.017020179	0.043693408	0.349041259	up_in_case
PRJNA8274(	ENSGALG00010009123	ENSGALG00010009123	case	control	-0.010762231	0.107275844	-0.100322965	0.920087926	0.984638357	down_in_case
PRJNA8274(	ENSGALG00010009126	JPH3	case	control	0.034139692	0.10543854	0.323787604	0.746098841	0.938117773	up_in_case
PRJNA8274(	ENSGALG00010009127	SCOC	case	control	-0.018509745	0.060380171	-0.306553365	0.759183355	0.942078947	down_in_case
PRJNA8274(	ENSGALG00010009129	ENSGALG00010009129	case	control	0.164008916	0.378274704	0.433570931	0.664600048	0.911721094	up_in_case
PRJNA8274(	ENSGALG00010009130	HEATR1	case	control	-0.060581306	0.050626217	-1.196639007	0.23144729	0.656555494	down_in_case
PRJNA8274(	ENSGALG00010009131	CDKL2	case	control	0.135366951	0.252227887	0.53668511	0.591485143	0.882949889	up_in_case
PRJNA8274(	ENSGALG00010009133	BOD1L1	case	control	-0.069182512	0.058605553	-1.180477084	0.237810519	0.662664656	down_in_case
PRJNA8274(	ENSGALG00010009135	TENM4	case	control	-0.184910782	0.110304947	-1.676359832	0.093667732	0.478545396	down_in_case
PRJNA8274(	ENSGALG00010009136	KHK	case	control	-0.08267955	0.372510901	-0.221952029	0.824351227	0.960422238	down_in_case
PRJNA8274(	ENSGALG00010009137	IPMK	case	control	-0.097300505	0.07129533	-1.364752858	0.17233074	0.590683026	down_in_case
PRJNA8274(	ENSGALG00010009138	ENSGALG00010009138	case	control	0.001041639	0.09069018	0.011485684	0.990835952	0.997673152	up_in_case
PRJNA8274(	ENSGALG00010009139	CKS2	case	control	-0.248334512	0.240422852	-1.032907273	0.301647295	0.724474473	down_in_case
PRJNA8274(	ENSGALG00010009141	MAML3	case	control	-0.089223739	0.08212698	-1.086412024	0.277296744	0.702814456	down_in_case
PRJNA8274(	ENSGALG00010009142	LGALS8	case	control	0.208710423	0.096650886	2.159425867	0.030817143	0.308259818	up_in_case
PRJNA8274(	ENSGALG00010009143	MCCC2	case	control	0.001466182	0.073819856	0.019861626	0.984153757	0.996591771	up_in_case
PRJNA8274(	ENSGALG00010009144	CREBZF	case	control	0.194472374	0.154253833	1.260729607	0.207406283	0.631716687	up_in_case
PRJNA8274(	ENSGALG00010009147	HNMT	case	control	-0.259517432	0.162761673	-1.594462797	0.110832417	0.503115856	down_in_case
PRJNA8274(	ENSGALG00010009149	FUT4	case	control	0.291721367	0.167015553	1.746671865	0.080694274	0.452876025	up_in_case
PRJNA8274(	ENSGALG00010009150	ENSGALG00010009150	case	control	-0.158314329	0.293634045	-0.539155223	0.589779754	0.882949889	down_in_case
PRJNA8274(	ENSGALG00010009151	CCNJL	case	control	0.448567334	0.213656729	2.099476745	0.035774896	0.324974524	up_in_case
PRJNA8274(	ENSGALG00010009152	RPP25L	case	control	0.225641625	0.171285841	1.31733962	0.18772481	0.610946377	up_in_case
PRJNA8274(	ENSGALG00010009153	SECISBP2	case	control	0.149765951	0.093168109	1.607480846	0.107948928	0.499736588	up_in_case
PRJNA8274(	ENSGALG00010009155	PAK4	case	control	-0.122195706	0.086520228	-1.412336848	0.157850802	0.56958528	down_in_case
PRJNA8274(	ENSGALG00010009157	ABHD1	case	control	-0.208526575	0.113127821	-1.843282869	0.065287721	0.414904609	down_in_case
PRJNA8274(	ENSGALG00010009159	PTK2	case	control	-0.047885799	0.034324658	-1.395084512	0.162990356	0.576993135	down_in_case
PRJNA8274(	ENSGALG00010009160	ENSGALG00010009160	case	control	-0.006877124	0.262364515	-0.026212098	0.979088166	0.995525769	down_in_case
PRJNA8274(	ENSGALG00010009163	SAMD12	case	control	-0.37047572	0.114757567	-3.228333694	0.001245136	0.055677577	down_in_case
PRJNA8274(	ENSGALG00010009164	TMEM128	case	control	-0.088095762	0.09953421	-0.885080241	0.376113355	0.7751003	down_in_case
PRJNA8274(	ENSGALG00010009166	CLGN	case	control	-0.250157912	0.186483761	-1.341446089	0.179775662	0.600773729	down_in_case
PRJNA8274(	ENSGALG00010009167	DCTN3	case	control	-0.01319888	0.192543746	-0.068550032	0.945347794	0.990313016	down_in_case
PRJNA8274(	ENSGALG00010009168	MRPS12	case	control	0.03202569	0.08527296	0.375566767	0.707239001	0.92461283	up_in_case
PRJNA8274(	ENSGALG00010009170	RPL26L1	case	control	-0.130079007	0.068276334	-1.90518442	0.056756103	0.387774615	down_in_case
PRJNA8274(	ENSGALG00010009172	SPOPL	case	control	-0.017759319	0.082317035	-0.21574294	0.829188128	0.962142561	down_in_case
PRJNA8274(	ENSGALG00010009173	LYAR	case	control	0.051310834	0.076905592	0.6671925	0.504649181	0.842675107	up_in_case
PRJNA8274(	ENSGALG00010009178	ENSGALG00010009178	case	control	-0.135728293	0.38159151	-0.355690022	0.722072718	0.929484914	down_in_case
PRJNA8274(	ENSGALG00010009180	NFKBIB	case	control	-0.059397679	0.061992582	-0.958141716	0.337991302	0.751259351	down_in_case
PRJNA8274(	ENSGALG00010009181	ARID3C	case	control	0.72895669	0.434940401	1.67599213	0.093739734	0.478571295	up_in_case
PRJNA8274(	ENSGALG00010009186	PREB	case	control	-0.000825342	0.075484207	-0.010933968	0.991276129	0.99790247	down_in_case
PRJNA8274(	ENSGALG00010009188	ENSGALG00010009188	case	control	-0.04346618	0.453107291	-0.095929112	0.923576873	0.98553537	down_in_case
PRJNA8274(	ENSGALG00010009189	ENSGALG00010009189	case	control	0.011889438	0.215661885	0.055129992	0.956034902	0.992056045	up_in_case
PRJNA8274(	ENSGALG00010009190	ZBTB49	case	control	0.032631906	0.071993746	0.45326029	0.650361324	0.904245691	up_in_case
PRJNA8274(	ENSGALG00010009192	LSM14A	case	control	-0.053664214	0.041817915	-1.283282865	0.199392994	0.622902456	down_in_case
PRJNA8274(	ENSGALG00010009193	EDARADD	case	control	-0.402674092	0.213371843	-1.887194139	0.059134218	0.395497471	down_in_case
PRJNA8274(	ENSGALG00010009197	FH	case	control	-0.136104521	0.062810234	-2.16691633	0.030241235	0.30654407	down_in_case
PRJNA8274(	ENSGALG00010009198	KCNN2	case	control	0.223123685	0.109112826	2.044889604	0.040865743	0.337874876	up_in_case
PRJNA8274(	ENSGALG00010009199	SEMA4D	case	control	0.644458049	0.152045771	4.238579254	2.24939E-05	0.003350651	up_in_case
PRJNA8274(	ENSGALG00010009200	ADGRA3	case	control	0.216083038	0.066342059	3.257104807	0.001125549	0.05223113	up_in_case
PRJNA8274(	ENSGALG00010009202	FABP6	case	control	-0.184206736	0.218926711	-0.84140823	0.400119276	0.789900496	down_in_case
PRJNA8274(	ENSGALG00010009205	SIRT2	case	control	-0.049888909	0.041118828	-1.213286255	0.225020396	0.648879142	down_in_case
PRJNA8274(	ENSGALG00010009208	SLC5A6	case	control	0.227266895	0.1724764	1.317669515	0.187614304	0.610946377	up_in_case
PRJNA8274(	ENSGALG00010009210	PCDH15	case	control	-0.362247578	0.293757641	-1.233151167	0.21751937	0.642877222	down_in_case

PRJNA8274(	ENSGALG00010009212	NXPH2	case	control	0.136468851	0.20001509	0.682292774	0.495053841	0.838118945	up_in_case
PRJNA8274(	ENSGALG00010009213	DUS4L	case	control	0.066346172	0.068437237	0.969445516	0.332322952	0.748293551	up_in_case
PRJNA8274(	ENSGALG00010009214	PCDH10	case	control	0.527768786	0.210638902	2.505561797	0.012225703	0.19425284	up_in_case
PRJNA8274(	ENSGALG00010009216	NSG1	case	control	-0.175875951	0.210784403	-0.834387882	0.404062443	0.79260318	down_in_case
PRJNA8274(	ENSGALG00010009219	TNFRSF11B	case	control	0.004813876	0.190739387	0.025237978	0.979865145	0.995636523	up_in_case
PRJNA8274(	ENSGALG00010009220	GADD45G	case	control	-0.111306563	0.190354998	-0.5847315	0.55872827	0.869450004	down_in_case
PRJNA8274(	ENSGALG00010009223	ENSGALG00010009223	case	control	0.13393675	0.112154579	1.194215619	0.232393635	0.657912487	up_in_case
PRJNA8274(	ENSGALG00010009224	ENSGALG00010009224	case	control	-0.908567439	0.322725656	-2.815293496	0.004873271	0.119457531	down_in_case
PRJNA8274(	ENSGALG00010009225	HNRNPL	case	control	-0.035220561	0.061795138	-0.569956832	0.568706977	0.874651514	down_in_case
PRJNA8274(	ENSGALG00010009227	ENSGALG00010009227	case	control	0.043801915	0.066969421	0.654058443	0.513074154	0.846564516	up_in_case
PRJNA8274(	ENSGALG00010009228	ATP6V0E1	case	control	-0.002744708	0.088566408	-0.030990393	0.975277201	0.99399062	down_in_case
PRJNA8274(	ENSGALG00010009232	ECH1	case	control	-0.128220833	0.14482268	-0.885364315	0.375960172	0.774921593	down_in_case
PRJNA8274(	ENSGALG00010009234	PCDH18	case	control	-0.129268201	0.082087581	-1.574759539	0.115311959	0.511623026	down_in_case
PRJNA8274(	ENSGALG00010009235	KLHDC4	case	control	-0.062078538	0.061415185	-1.010801123	0.312111625	0.734364975	down_in_case
PRJNA8274(	ENSGALG00010009236	DTWD2	case	control	0.113941924	0.171640516	0.66384049	0.506792408	0.843472666	up_in_case
PRJNA8274(	ENSGALG00010009237	DENND3	case	control	0.202414737	0.113329519	1.786072491	0.074087522	0.438111241	up_in_case
PRJNA8274(	ENSGALG00010009239	STX18	case	control	-0.011144512	0.051175658	-0.217769784	0.827608488	0.961319258	down_in_case
PRJNA8274(	ENSGALG00010009243	BCAP29	case	control	0.034610763	0.069308138	0.499375164	0.617515112	0.891081782	up_in_case
PRJNA8274(	ENSGALG00010009246	RUSC2	case	control	-0.144106379	0.161281153	-0.893510345	0.371583935	0.773566649	down_in_case
PRJNA8274(	ENSGALG00010009250	ERO1B	case	control	0.070918016	0.07669064	0.924728443	0.35510718	0.763161644	up_in_case
PRJNA8274(	ENSGALG00010009252	SIRT1	case	control	0.027619689	0.048236909	0.572584129	0.566926308	0.87333105	up_in_case
PRJNA8274(	ENSGALG00010009253	SLC45A4	case	control	-0.085522942	0.115640039	-0.739561686	0.459565997	0.824480387	down_in_case
PRJNA8274(	ENSGALG00010009254	SLC26A4	case	control	0.184341146	0.604952946	0.304719809	0.760579561	0.942883622	up_in_case
PRJNA8274(	ENSGALG00010009256	CREBRF	case	control	-0.013382443	0.10134153	-0.132052903	0.894942448	0.980176778	down_in_case
PRJNA8274(	ENSGALG00010009257	ENSGALG00010009257	case	control	-0.221198134	0.10994295	-2.011935587	0.044226727	0.350201601	down_in_case
PRJNA8274(	ENSGALG00010009258	DNAJC12	case	control	-0.173361622	0.154027094	-1.125526797	0.260365868	0.687323596	down_in_case
PRJNA8274(	ENSGALG00010009263	COLEC10	case	control	0.057443303	0.116017364	0.495126776	0.620510634	0.892955829	up_in_case
PRJNA8274(	ENSGALG00010009266	PCDH7	case	control	-0.037182709	0.170724208	-0.217794005	0.827589616	0.961319258	down_in_case
PRJNA8274(	ENSGALG00010009268	PWWP2A	case	control	-0.071478648	0.058211455	-1.227913771	0.219479339	0.643443819	down_in_case
PRJNA8274(	ENSGALG00010009269	ENSGALG00010009269	case	control	-0.021901858	0.641587115	-0.034136998	0.972767905	0.99340857	down_in_case
PRJNA8274(	ENSGALG00010009273	MAL2	case	control	0.941169809	0.563380629	1.670575381	0.094805579	0.481263676	up_in_case
PRJNA8274(	ENSGALG00010009275	CBLL1	case	control	-0.007388165	0.054480891	-0.135610213	0.892129431	0.979233257	down_in_case
PRJNA8274(	ENSGALG00010009276	ENSGALG00010009276	case	control	-0.072186591	0.123041439	-0.586685197	0.557415151	0.868661404	down_in_case
PRJNA8274(	ENSGALG00010009277	ACTN4	case	control	-0.052394429	0.05638195	-0.929276642	0.352745737	0.761513292	down_in_case
PRJNA8274(	ENSGALG00010009279	ELMOD2	case	control	0.00366938	0.0405741	0.090436506	0.927940348	0.986776285	up_in_case
PRJNA8274(	ENSGALG00010009280	HSD17B4	case	control	0.068848816	0.16341133	0.421322167	0.673519845	0.91379937	up_in_case
PRJNA8274(	ENSGALG00010009284	CCN3	case	control	0.634866273	0.415241628	1.528908062	0.126287237	0.525954642	up_in_case
PRJNA8274(	ENSGALG00010009286	ENSGALG00010009286	case	control	0.632357294	0.135082283	4.68127486	2.85096E-06	0.000599541	up_in_case
PRJNA8274(	ENSGALG00010009289	GPR137B	case	control	-0.042220292	0.089481658	-0.471831803	0.637046849	0.899286272	down_in_case
PRJNA8274(	ENSGALG00010009294	ACOX3	case	control	-0.083748675	0.088119379	-0.950400199	0.341908943	0.753707089	down_in_case
PRJNA8274(	ENSGALG00010009297	CTNNA3	case	control	-0.617873203	0.207889479	-2.972123482	0.002957477	0.0925425	down_in_case
PRJNA8274(	ENSGALG00010009299	MAP4K1	case	control	0.341143677	0.277667354	1.228605641	0.2192197	0.643443819	up_in_case
PRJNA8274(	ENSGALG00010009303	TTC1	case	control	0.026163659	0.054560124	0.479538114	0.631555861	0.897039706	up_in_case
PRJNA8274(	ENSGALG00010009306	TBC1D9	case	control	0.027078385	0.094296216	0.287163004	0.773987513	0.946671454	up_in_case
PRJNA8274(	ENSGALG00010009307	DIRAS2	case	control	0.466837191	0.271021441	1.722510181	0.08497714	0.461224852	up_in_case
PRJNA8274(	ENSGALG00010009309	TRIM54	case	control	-0.126999875	0.099541768	-1.275845083	0.202010287	0.625073547	down_in_case
PRJNA8274(	ENSGALG00010009311	BNIP1	case	control	-0.043194249	0.076805721	-0.562383221	0.573854949	0.877573415	down_in_case
PRJNA8274(	ENSGALG00010009313	UNC13B	case	control	-0.185832883	0.223477094	-0.831552263	0.405661717	0.793782506	down_in_case
PRJNA8274(	ENSGALG00010009317	LRRTM3	case	control	0.115438786	0.408089367	0.28287624	0.777271708	0.947649879	up_in_case
PRJNA8274(	ENSGALG00010009320	GPR20	case	control	-0.014706422	0.118399212	-0.124210474	0.90114863	0.980627456	down_in_case
PRJNA8274(	ENSGALG00010009322	ENSGALG00010009322	case	control	0.222241975	0.105614991	2.104265433	0.0353553	0.324492998	up_in_case
PRJNA8274(	ENSGALG00010009323	RNF150	case	control	-0.069547283	0.062551172	-1.111846204	0.266204282	0.69218122	down_in_case
PRJNA8274(	ENSGALG00010009324	NKX2-5	case	control	-0.445497087	1.311896843	-0.339582407	0.734171029	0.933846596	down_in_case
PRJNA8274(	ENSGALG00010009326	DLD	case	control	-0.071541356	0.067142554	-1.065514357	0.286643232	0.710659943	down_in_case
PRJNA8274(	ENSGALG00010009327	NID1	case	control	-0.166780937	0.083002309	-2.009352989	0.044499712	0.350410728	down_in_case
PRJNA8274(	ENSGALG00010009328	ENSGALG00010009328	case	control	0.072073135	0.133157799	0.541261084	0.58832764	0.882771329	up_in_case
PRJNA8274(	ENSGALG00010009330	EIF3K	case	control	0.084292748	0.05933358	1.420658385	0.155416096	0.564463894	up_in_case
PRJNA8274(	ENSGALG00010009332	ENSGALG00010009332	case	control	0.193928175	0.058153349	3.334772247	0.000853693	0.044554045	up_in_case
PRJNA8274(	ENSGALG00010009333	ADRA1B	case	control	0.294268531	0.225682985	1.303902155	0.192266945	0.615090794	up_in_case
PRJNA8274(	ENSGALG00010009334	SS18L2	case	control	-0.032644726	0.111926636	-0.291661815	0.770545207	0.945448224	down_in_case
PRJNA8274(	ENSGALG00010009335	PTP4A3	case	control	-0.126443056	0.09991321	-1.265528911	0.205681791	0.628740833	down_in_case
PRJNA8274(	ENSGALG00010009336	LAMB1	case	control	-0.163912738	0.09534112	-1.719223965	0.085573599	0.462647434	down_in_case
PRJNA8274(	ENSGALG00010009337	ZNF330	case	control	-0.118628754	0.0672726	-1.76340373	0.077832421	0.447528598	down_in_case
PRJNA8274(	ENSGALG00010009338	KYNU	case	control	1.489676572	0.479112228	3.109243484	0.001875671	0.070584453	up_in_case
PRJNA8274(	ENSGALG00010009340	SYK	case	control	0.084741119	0.112548608	0.752929076	0.451492535	0.820302668	up_in_case
PRJNA8274(	ENSGALG00010009344	ENPP2	case	control	0.008633424	0.179093706	0.048206181	0.961551924	0.992056045	up_in_case
PRJNA8274(	ENSGALG00010009345	ZFAT	case	control	-0.07961763	0.083384147	-0.954829341	0.339664007	0.751259351	down_in_case
PRJNA8274(	ENSGALG00010009346	STC2	case	control	-0.31355228	0.147858433	-2.120624927	0.033953379	0.317757405	down_in_case
PRJNA8274(	ENSGALG00010009347	TESK1	case	control	-0.052751806	0.131866204	-0.400040374	0.68912678	0.918180693	down_in_case
PRJNA8274(	ENSGALG00010009348	ERGIC2	case	control	-0.068038065	0.04126471	-1.648819682	0.099184581	0.488407564	down_in_case
PRJNA8274(	ENSGALG00010009351	GTF3C2	case	control	0.091314007	0.054980425	1.66084577	0.096744426	0.484569279	up_in_case
PRJNA8274(	ENSGALG00010009352	HSF1	case	control	-0.194666854	0.076125295	-2.557190139	0.010552152	0.183125942	down_in_case
PRJNA8274(	ENSGALG00010009355	GARRE1	case	control	-0.037849883	0.045777765	-0.826818053	0.408340196	0.794775742	down_in_case
PRJNA8274(	ENSGALG00010009357	LYST	case	control	0.069482025	0.074651348	0.930753787	0.35198094	0.760831854	up_in_case
PRJNA8274(	ENSGALG00010009359	REEP3	case	control	0.029614866	0.076286399	0.388206371	0.697863319	0.922288441	up_in_case
PRJNA8274(	ENSGALG00010009360	TNFAIP8	case	control	0.003875679	0.07696817	0.050354308	0.959840047	0.992056045	up_in_case
PRJNA8274(	ENSGALG00010009361	LRRK2	case	control	0.165316419	0.070335436	2.350400139	0.018753239	0.244681859	up_in_case
PRJNA8274(	ENSGALG00010009362	IL15	case	control	-0.499969282	0.284138034	-1.759599991	0.078475653	0.448818031	down_in_case
PRJNA8274(	ENSGALG00010009363	TAF2	case	control	-0.066643976	0.029186259	-2.283402439	0.022406674	0.266593144	down_in_case
PRJNA8274(	ENSGALG00010009365	CA5B	case	control	0.291410161	0.186565565	1.561971852	0.11829462	0.51463738	up_in_case
PRJNA8274(	ENSGALG00010009367	DMXL1	case	control	-0.002147054	0.13271817	-0.016177544	0.987092751	0.996781748	down_in_case
PRJNA8274(	ENSGALG00010009368	DSCC1	case	control	0.0334083	0.103756461	0.321987657	0.747462045	0.939105545	up_in_case
PRJNA8274(	ENSGALG00010009370	ENSGALG00010009370	case	control	0.091988757	0.570823593	0.161150937	0.871974522	0.975062028	up_in_case
PRJNA8274(	ENSGALG00010009371	PHTF2	case	control	0.043246711	0.104887985	0.412313301	0.680109801	0.915381147	up_in_case
PRJNA8274(	ENSGALG00010009372	RAB28	case	control	-0.076387729	0.090906746	-0.84028669	0.400747663	0.790340383	down_in_case
PRJNA8274(	ENSGALG00010009373	ARHGAP15	case	control	-0.121064014	0.153677595	-0.787779208	0.430825865	0.806930928	down_in_case
PRJNA8274(	ENSGALG00010009376	CDO1	case	control	-0.303930963	0.11213492	-2.71040425	0.006720125	0.141528402	down_in_case
PRJNA8274(	ENSGALG00010009378	ENSGALG00010009378	case	control	0.026433357	0.052735678	0.501242391	0.616200544	0.891081782	up_in_case
PRJNA8274(	ENSGALG00010009379	RYR1	case	control	-0.2136896	0.120168248	-1.778253441	0.075362239	0.441853228	down_in_case
PRJNA8274(	ENSGALG00010009381	INPP4B	case	control	0.004346013	0.095769669	0.045379846	0.963804545	0.992068146	up_in_case
PRJNA8274(	ENSGALG00010009383	ASXL2	case	control	-0.074957056	0.065038145	-1.152509136	0.249111918	0.674421685	down_in_case
PRJNA8274(	ENSGALG00010009385	GPI	case	control	-0.120688728	0.134611877	-0.896568202	0.369949372	0.772544067	down_in_case
PRJNA8274(	ENSGALG00010009386	TMEM60	case	control	0.053801612	0.078765517	0.683060485	0.494568623	0.83803731	up_in_case
PRJNA8274(	ENSGALG00010009387	DEPTOR	case	control	-0.121917828	0.091492658	-1.33254221	0.182682081	0.604152117	down_in_case
PRJNA8274(	ENSGALG00010009388	TMED7	case	control	-0.03108455	0.057666196	-0.539042842	0.589857294	0.882949889	down_in_case
PRJNA8274(	ENSGALG00010009389	CPEB4	case	control	-0.156282257	0.088061559	-1.774693286	0.075948545	0.442710866	down_in_case
PRJNA8274(	ENSGALG00010009391	RSBN1L	case	control	-0.09148651	0.07188491	-1.27268031	0.203131507	0.625537942	down_in_case
PRJNA8274(	ENSGALG00010009392	MAPRE3	case	control	0.012892871	0.061821338	0.208550507	0.83479915	0.963411173	up_in_case
PRJNA8274(	ENSGALG00010009393	MARVELD2	case	control	0.017005369	0.184084026	0.0923783	0.92639748	0.98618953	up_in_case
PRJNA8274(	ENSGALG00010009394	BANP	case	control	0.092132378	0.065553127	1.405461221	0.159884173	0.572055887	up_in_case
PRJNA8274(	ENSGALG00010009396	AUH	case	control	-0.214670405	0.099624779	-2.154789268	0.031178328	0.3098361	down_in_case
PRJNA8274(	ENSGALG00010009398	WTIP	case	control	-0.053629367	0.087467896	-0.613132005	0.539789059	0.858598814	down_in_case
PRJNA8274(	ENSGALG00010009400	CNTFR	case	control	-0.151098934	0.11186523	-1.350722957	0.176784196	0.595527443	down_in_case
PRJNA8274(	ENSGALG00010009404	GNG4	case	control	0.156117801	0.314078088	0.497066835	0.619141921	0.891973552	up_in_case
PRJNA8274(	ENSGALG00010009407	PTPN12	case	control	0.055192768	0.044794259	1.232139324	0.217897044	0.643019288	up_in_case
PRJNA8274(	ENSGALG00010009408	ENSGALG00010009408	case	control	0.138788406	0.082075771	1.690979003	0.090840813	0.475136658	up_in_case
PRJNA8274(	ENSGALG00010009410	JMJD1C	case	control	-0.052755953	0.055642893	-0.948116642	0.343070077	0.755102678	down_in_case
PRJNA8274(	ENSGALG00010009411	SMARCA5	case	control	0.047763656	0.075815809	0.629995994	0.528697205	0.853893159	up_in_case
PRJNA8274(	ENSGALG00010009415	HMP19	case	control	-0.277360114	0.216006107	-1.284038296	0.199128556	0.622902456	down_in_case
PRJNA8274(	ENSGALG00010009416	COL14A1	case	control	-0.210417327	0.135755945	-1.549967683	0.121149273	0.519499513	down_in_case
PRJNA8274(	ENSGALG00010009424	ENSGALG00010009424	case	control	0.903798333	0.832376993	1.085804077	0.277565682	0.70305085	up_in_case
PRJNA8274(	ENSGALG00010009431	SMN	case	control	-0.006951658	0.060081149	-0.115704474	0.90788676	0.981730793	down_in_case
PRJNA8274(	ENSGALG00010009432	BOP1	case	control	0.035939969	0.086695851	0.414552358	0.678469631	0.914942347	up_in_case
PRJNA8274(	ENSGALG00010009435	GTDC1	case	control	0.040333966	0.103626124	0.389225852	0.697109081	0.922031058	up_in_case
PRJNA8274(	ENSGALG00010009437	DPYSL5	case	control	0.391436141	0.261504539	1.496861748	0.134429235	0.539063179	up_in_case
PRJNA8274(	ENSGALG00010009439	B3GALNT2	case	control	-0.14520283	0.079430616	-1.828046124	0.06754263	0.42089154	down_in_case
PRJNA8274(	ENSGALG00010009440	DOK3	case	control	0.02278976	0.268186626	0.084977244	0.932279482	0.987669602	up_in_case
PRJNA8274(	ENSGALG00010009441	ZNF518B	case	control	0.054352761	0.05982448	0.90853713	0.363594506	0.769880166	up_in_case
PRJNA8274(	ENSGALG00010009444	NFIL3	case	control	-0.294152887	0.137160282	-2.144592324	0.031985457	0.3098361	down_in_case
PRJNA8274(	ENSGALG00010009446	ENSGALG00010009446	case	control	-0.151685416	0.091223555	-1.662787824	0.096354915	0.483634708	down_in_case
PRJNA8274(	ENSGALG00010009447	DDX41	case	control	0.035767212	0.052615711	0.679781985	0.496642515	0.83858637	up_in_case
PRJNA8274(	ENSGALG00010009451	ROR2	case	control	-0.035450308	0.102178585	-0.346944596	0.728632942	0.933057319	down_in_case
PRJNA8274(	ENSGALG00010009452	TBCE	case	control	0.223653164	0.082313721	2.717082407	0.006586021	0.140023533	up_in_case
PRJNA8274(	ENSGALG00010009453	GSAP	case	control	0.008141196	0.078534453	0.103664007	0.917435991	0.983445506	up_in_case
PRJNA8274(	ENSGALG00010009457	BDP1	case	control	-0.028373808	0.102980111	-0.275527073	0.782911329	0.949518053	down_in_case
PRJNA8274(	ENSGALG00010009458	SCX	case	control	0.686766952	0.293963486	2.336232166	0.019479145	0.250046472	up_in_case

PRJNA8274(	ENSGALG00010009461	ZEB2	case	control	-0.024488459	0.056268171	-0.435209793	0.663410155	0.911044469	down_in_case
PRJNA8274(	ENSGALG00010009462	NRBF2	case	control	-0.069716442	0.067934605	-1.026228704	0.304783794	0.728073294	down_in_case
PRJNA8274(	ENSGALG00010009463	MRPL13	case	control	0.019254904	0.053648556	0.358908145	0.719663806	0.928640356	up_in_case
PRJNA8274(	ENSGALG00010009464	SPTLC1	case	control	0.09371008	0.195172695	0.480139297	0.631128347	0.897039706	up_in_case
PRJNA8274(	ENSGALG00010009465	GGPS1	case	control	0.046235207	0.07896806	0.585492505	0.558216605	0.869085619	up_in_case
PRJNA8274(	ENSGALG00010009466	FAM193B	case	control	-0.001564085	0.065126422	-0.024016139	0.980839736	0.996165356	down_in_case
PRJNA8274(	ENSGALG00010009467	EGR2	case	control	0.085742381	0.188019498	0.4560292	0.648368981	0.903557349	up_in_case
PRJNA8274(	ENSGALG00010009469	DNMT3A	case	control	-0.094740484	0.089809177	-1.054908721	0.291467073	0.714303573	down_in_case
PRJNA8274(	ENSGALG00010009470	ZNF469	case	control	-0.056557631	0.129273155	-0.43750484	0.661745267	0.910162289	down_in_case
PRJNA8274(	ENSGALG00010009472	ADO	case	control	-0.020365786	0.064427651	-0.316103196	0.751924181	0.940427752	down_in_case
PRJNA8274(	ENSGALG00010009474	ACVR2A	case	control	-0.142471064	0.064553971	-2.207006976	0.027313572	0.2934516	down_in_case
PRJNA8274(	ENSGALG00010009477	ZNF365	case	control	0.01072519	0.065856032	0.162858129	0.870630139	0.974794532	up_in_case
PRJNA8274(	ENSGALG00010009479	MTBP	case	control	0.180356629	0.122462697	1.472747493	0.140819135	0.543368104	up_in_case
PRJNA8274(	ENSGALG00010009482	ARID4B	case	control	-0.031110856	0.070634379	-0.440449202	0.659611796	0.90862621	down_in_case
PRJNA8274(	ENSGALG00010009484	RTKN2	case	control	-0.062737745	0.087965702	-0.713206895	0.47571774	0.830520323	down_in_case
PRJNA8274(	ENSGALG00010009486	B4GALT1	case	control	0.196598249	0.071385759	2.754026154	0.005886706	0.133681071	up_in_case
PRJNA8274(	ENSGALG00010009489	MROH1	case	control	-0.055905743	0.081507537	-0.685896613	0.492778306	0.837799284	down_in_case
PRJNA8274(	ENSGALG00010009490	SNTB1	case	control	0.110330045	0.119701963	0.921706227	0.356681832	0.763373775	up_in_case
PRJNA8274(	ENSGALG00010009491	RBM34	case	control	0.150711563	0.100655563	1.497299891	0.134315243	0.538919186	up_in_case
PRJNA8274(	ENSGALG00010009492	CDC42SE2	case	control	-0.022351022	0.113655093	-0.196656581	0.84409629	0.96721237	down_in_case
PRJNA8274(	ENSGALG00010009493	ENSGALG00010009493	case	control	0.929170709	0.479551237	1.937583802	0.05267402	0.374713019	up_in_case
PRJNA8274(	ENSGALG00010009494	UBA2	case	control	-0.002120265	0.035386087	-0.059918027	0.952220922	0.992056045	down_in_case
PRJNA8274(	ENSGALG00010009497	HS3ST1	case	control	0.287775174	0.156890476	1.834242474	0.066618009	0.418006815	up_in_case
PRJNA8274(	ENSGALG00010009498	ORC4	case	control	0.053248522	0.102018709	0.521948601	0.601706118	0.884954589	up_in_case
PRJNA8274(	ENSGALG00010009499	MXD3	case	control	0.160695392	0.144619918	1.111156708	0.266500902	0.692519293	up_in_case
PRJNA8274(	ENSGALG00010009500	CCDC146	case	control	0.412415741	0.348776514	1.182464201	0.237021562	0.662252509	up_in_case
PRJNA8274(	ENSGALG00010009501	HGH1	case	control	0.047992902	0.09005261	0.532942933	0.594073084	0.883735816	up_in_case
PRJNA8274(	ENSGALG00010009502	ARID5B	case	control	-0.030296567	0.06569039	-0.461202427	0.644653381	0.902239118	down_in_case
PRJNA8274(	ENSGALG00010009503	IL11RA	case	control	0.025892693	0.120925422	0.214121172	0.830452566	0.962228709	up_in_case
PRJNA8274(	ENSGALG00010009506	HAS2	case	control	0.175798008	0.077867301	2.257661503	0.023966771	0.273286623	up_in_case
PRJNA8274(	ENSGALG00010009508	TOMM20	case	control	-0.101345817	0.06902796	-1.468185009	0.142053975	0.543501954	down_in_case
PRJNA8274(	ENSGALG00010009511	ZFPM1	case	control	0.242625838	0.173168773	1.401094624	0.161185779	0.574086336	up_in_case
PRJNA8274(	ENSGALG00010009514	PRELID1	case	control	0.001222352	0.085614158	0.014277454	0.988608627	0.997115486	up_in_case
PRJNA8274(	ENSGALG00010009515	MBD5	case	control	-0.110836309	0.055702107	-1.989804609	0.046612464	0.356907825	down_in_case
PRJNA8274(	ENSGALG00010009518	FGL2	case	control	-0.509916285	0.318814872	-1.599411852	0.109729121	0.501817242	down_in_case
PRJNA8274(	ENSGALG00010009522	TAF5L	case	control	-0.044998339	0.049670001	-0.905946008	0.364964431	0.770785905	down_in_case
PRJNA8274(	ENSGALG00010009524	FAM185A	case	control	-0.157143213	0.096234436	-1.632920813	0.102485652	0.491460867	down_in_case
PRJNA8274(	ENSGALG00010009525	ENSGALG00010009525	case	control	-0.001547013	0.205040048	-0.007544931	0.993980073	0.998238292	down_in_case
PRJNA8274(	ENSGALG00010009526	ENSGALG00010009526	case	control	0.317888911	0.152820007	2.080152439	0.037511553	0.32868579	up_in_case
PRJNA8274(	ENSGALG00010009527	ENSGALG00010009527	case	control	-0.417013159	0.541449276	-0.770179549	0.441193393	0.813083204	down_in_case
PRJNA8274(	ENSGALG00010009530	KIF3C	case	control	-0.091967439	0.126857751	-0.724965076	0.468473456	0.828596747	down_in_case
PRJNA8274(	ENSGALG00010009531	FBXL13	case	control	-0.297341109	0.324937054	-0.915072951	0.360153334	0.765754313	down_in_case
PRJNA8274(	ENSGALG00010009534	XPA	case	control	0.089239705	0.217801393	0.409729726	0.682004222	0.915594727	up_in_case
PRJNA8274(	ENSGALG00010009538	CRIM1	case	control	-0.032843686	0.082982643	-0.395789827	0.692260082	0.918988424	down_in_case
PRJNA8274(	ENSGALG00010009540	RAB10	case	control	-0.050418886	0.048545135	-1.03859812	0.298991681	0.722470604	down_in_case
PRJNA8274(	ENSGALG00010009541	PMPCB	case	control	0.041734755	0.064022802	0.651873294	0.514482914	0.847493921	up_in_case
PRJNA8274(	ENSGALG00010009542	WDR1	case	control	0.081519173	0.052816449	1.543442899	0.122723352	0.52168369	up_in_case
PRJNA8274(	ENSGALG00010009543	MAF1	case	control	-0.154754932	0.095458723	-1.6211712	0.104980927	0.494770427	down_in_case
PRJNA8274(	ENSGALG00010009546	GAREM2	case	control	-0.272485758	0.435286572	-0.625991647	0.53132042	0.855560567	down_in_case
PRJNA8274(	ENSGALG00010009548	PIK3C3	case	control	0.141416518	0.132661833	1.065992488	0.286427038	0.710659943	up_in_case
PRJNA8274(	ENSGALG00010009551	HADHA	case	control	-0.104359695	0.112422346	-0.928282489	0.353261055	0.761954708	down_in_case
PRJNA8274(	ENSGALG00010009554	ABCB10	case	control	-0.136127583	0.084935915	-1.602709329	0.108998843	0.501108679	down_in_case
PRJNA8274(	ENSGALG00010009555	EPC2	case	control	-0.042587947	0.044088653	-0.965961637	0.334063378	0.748645401	down_in_case
PRJNA8274(	ENSGALG00010009557	CIDEC	case	control	-0.127406806	0.723306944	-0.176144868	0.860180132	0.971871635	down_in_case
PRJNA8274(	ENSGALG00010009558	TMEM26	case	control	-0.207523105	0.126046491	-1.646401289	0.099681168	0.488707918	down_in_case
PRJNA8274(	ENSGALG00010009560	RAB24	case	control	-0.037847445	0.091090724	-0.415491762	0.677781944	0.914893769	down_in_case
PRJNA8274(	ENSGALG00010009562	HADHB	case	control	-0.058617255	0.102206901	-0.57351563	0.566295619	0.873008555	down_in_case
PRJNA8274(	ENSGALG00010009565	TMEM8B	case	control	-0.148938672	0.189363012	-0.786524628	0.431560197	0.807261374	down_in_case
PRJNA8274(	ENSGALG00010009567	NSD1	case	control	-0.071179123	0.050799903	-1.401166518	0.161164284	0.574086336	down_in_case
PRJNA8274(	ENSGALG00010009569	ZC3H18	case	control	-0.081200793	0.070240473	-1.156039949	0.247664817	0.672031667	down_in_case
PRJNA8274(	ENSGALG00010009570	SHARPIN	case	control	-0.164108133	0.108305841	-1.515228825	0.129714466	0.532166684	down_in_case
PRJNA8274(	ENSGALG00010009571	DNAJC2	case	control	0.07993225	0.075575409	1.057648919	0.290215531	0.714060795	up_in_case
PRJNA8274(	ENSGALG00010009574	TAF1C	case	control	-0.161534436	0.219715988	-0.735196548	0.462219801	0.824958777	down_in_case
PRJNA8274(	ENSGALG00010009575	RHOBTB1	case	control	-0.406741563	0.267116187	-1.522714022	0.127830268	0.529650968	down_in_case
PRJNA8274(	ENSGALG00010009578	KIF5C	case	control	-0.016525932	0.132938113	-0.124312971	0.901067478	0.98061377	down_in_case
PRJNA8274(	ENSGALG00010009582	TIGD5	case	control	-0.116117454	0.119676635	-0.97026001	0.331916905	0.748173352	down_in_case
PRJNA8274(	ENSGALG00010009583	LYPD6B	case	control	-0.053867593	0.272558944	-0.197636491	0.843329482	0.967012396	down_in_case
PRJNA8274(	ENSGALG00010009591	LYPD6	case	control	-0.110048603	0.319326477	-0.344627242	0.730374623	0.933770678	down_in_case
PRJNA8274(	ENSGALG00010009593	NCBP1	case	control	-0.022337189	0.050136578	-0.445526792	0.655939102	0.907494354	down_in_case
PRJNA8274(	ENSGALG00010009594	HINT2	case	control	-0.113188673	0.221619869	-0.510733418	0.609537737	0.888004876	down_in_case
PRJNA8274(	ENSGALG00010009595	ENSGALG00010009595	case	control	-0.167281711	0.178591213	-0.936673809	0.348926373	0.759342183	down_in_case
PRJNA8274(	ENSGALG00010009596	URB2	case	control	0.108222721	0.079865248	1.355066491	0.175396373	0.594211832	up_in_case
PRJNA8274(	ENSGALG00010009597	CDK1	case	control	-0.080768509	0.095277723	-0.847716616	0.39659581	0.788531716	down_in_case
PRJNA8274(	ENSGALG00010009600	LRRC17	case	control	-0.119850733	0.141379829	-0.847721588	0.396593041	0.788531716	down_in_case
PRJNA8274(	ENSGALG00010009601	ZHX2	case	control	-0.015433694	0.059753345	-0.258290035	0.796183076	0.95280257	down_in_case
PRJNA8274(	ENSGALG00010009602	TSTD2	case	control	-0.016735291	0.119256436	-0.140330296	0.888399029	0.977948171	down_in_case
PRJNA8274(	ENSGALG00010009603	HINT1	case	control	-0.13729582	0.17629281	-0.778794213	0.436100947	0.81000376	down_in_case
PRJNA8274(	ENSGALG00010009605	ENSGALG00010009605	case	control	0.130934508	0.133236523	0.982722343	0.325744111	0.74363678	up_in_case
PRJNA8274(	ENSGALG00010009606	CHSY3	case	control	0.23891408	0.169160793	1.412349021	0.157847219	0.56958528	up_in_case
PRJNA8274(	ENSGALG00010009608	AFAP1	case	control	0.03628438	0.082866506	0.437865456	0.661483818	0.909890207	up_in_case
PRJNA8274(	ENSGALG00010009609	MMADHC	case	control	-0.129220427	0.043317755	-2.983082259	0.002853613	0.090280231	down_in_case
PRJNA8274(	ENSGALG00010009612	DERL1	case	control	-0.004094121	0.046105572	-0.088798833	0.929241785	0.986894287	down_in_case
PRJNA8274(	ENSGALG00010009615	ENSGALG00010009615	case	control	-0.511227166	0.324790165	-1.574022929	0.115482149	0.512219208	down_in_case
PRJNA8274(	ENSGALG00010009616	ARMC10	case	control	-0.166702643	0.123339553	-1.351574892	0.176511347	0.595527443	down_in_case
PRJNA8274(	ENSGALG00010009617	SLC25A46	case	control	-0.116301598	0.078371546	-1.483977333	0.137814934	0.539854583	down_in_case
PRJNA8274(	ENSGALG00010009619	ENSGALG00010009619	case	control	0.025746445	0.219895874	0.117084712	0.906792924	0.981730793	up_in_case
PRJNA8274(	ENSGALG00010009620	TMOD1	case	control	-0.293013915	0.232284203	-1.261445728	0.207148302	0.631334339	down_in_case
PRJNA8274(	ENSGALG00010009623	XPO5	case	control	-0.038705072	0.05244812	-0.737968715	0.460533461	0.82454265	down_in_case
PRJNA8274(	ENSGALG00010009634	TBC1D31	case	control	0.033922186	0.073309945	0.462722841	0.643563044	0.902038523	up_in_case
PRJNA8274(	ENSGALG00010009635	TDRD7	case	control	0.249060112	0.127922545	1.946960269	0.051539498	0.371479245	up_in_case
PRJNA8274(	ENSGALG00010009637	CYBA	case	control	-0.131024706	0.119473521	-1.096684055	0.272779529	0.698933392	down_in_case
PRJNA8274(	ENSGALG00010009638	RND3	case	control	0.064991408	0.070110369	0.926987099	0.353933233	0.762350539	up_in_case
PRJNA8274(	ENSGALG00010009640	NPR2	case	control	-0.214437795	0.184445451	-1.162608203	0.244988501	0.669469819	down_in_case
PRJNA8274(	ENSGALG00010009643	PSMC2	case	control	-0.084211062	0.059285843	-1.420424462	0.155484145	0.564463894	down_in_case
PRJNA8274(	ENSGALG00010009644	SLC27A6	case	control	-0.100092079	0.216987233	-0.461280962	0.644597042	0.902239118	down_in_case
PRJNA8274(	ENSGALG00010009646	MVD	case	control	-0.11265215	0.1176395	-0.957604799	0.338262078	0.751259351	down_in_case
PRJNA8274(	ENSGALG00010009647	GALNT2	case	control	0.082731993	0.066761829	1.239211001	0.215267367	0.640470741	up_in_case
PRJNA8274(	ENSGALG00010009649	TENM2	case	control	-0.049177592	0.165327814	-0.297455041	0.766119128	0.943987552	down_in_case
PRJNA8274(	ENSGALG00010009650	KLHL29	case	control	-0.019126189	0.074025219	-0.258373953	0.796118317	0.95280257	down_in_case
PRJNA8274(	ENSGALG00010009652	CIAO2B	case	control	0.007668596	0.079804894	0.096091796	0.923447667	0.98553537	up_in_case
PRJNA8274(	ENSGALG00010009653	ABLIM2	case	control	-0.20843375	0.140740712	-1.480976946	0.138612717	0.540932258	down_in_case
PRJNA8274(	ENSGALG00010009654	NDUFA12	case	control	-0.121959112	0.069002819	-1.767451157	0.077152702	0.445884536	down_in_case
PRJNA8274(	ENSGALG00010009655	FAM83A	case	control	-0.469297627	0.714704372	-0.656631812	0.511417689	0.845660609	down_in_case
PRJNA8274(	ENSGALG00010009657	MSMP	case	control	-0.017780099	0.405294187	-0.043869612	0.965008338	0.992291928	down_in_case
PRJNA8274(	ENSGALG00010009658	NAPEPLD	case	control	0.095191584	0.104751114	0.908740539	0.3634871	0.769826056	up_in_case
PRJNA8274(	ENSGALG00010009659	APOLD1	case	control	-0.395828107	0.109940541	-3.600383457	0.000317748	0.024072921	down_in_case
PRJNA8274(	ENSGALG00010009663	FER	case	control	-0.052245575	0.123214057	-0.424022847	0.671549151	0.913676733	down_in_case
PRJNA8274(	ENSGALG00010009665	DGKQ	case	control	-0.014905317	0.192584087	-0.077396413	0.938308194	0.989367879	down_in_case
PRJNA8274(	ENSGALG00010009666	RRAD	case	control	0.032236006	0.177279213	0.181837484	0.855710267	0.970656588	up_in_case
PRJNA8274(	ENSGALG00010009668	TFAP2A	case	control	1.657613777	0.593822577	2.791429363	0.005247581	0.125485633	up_in_case
PRJNA8274(	ENSGALG00010009672	DDX47	case	control	-0.062096863	0.064881619	-0.957079441	0.33852716	0.751259351	down_in_case
PRJNA8274(	ENSGALG00010009676	ACO1	case	control	0.23025452	0.154417092	1.49112069	0.135929811	0.539774365	up_in_case
PRJNA8274(	ENSGALG00010009677	SLC26A5	case	control	0.402947175	0.159119948	2.532348594	0.011330129	0.186445159	up_in_case
PRJNA8274(	ENSGALG00010009678	JARID2	case	control	0.032208095	0.078745236	0.40901642	0.682527611	0.915874198	up_in_case
PRJNA8274(	ENSGALG00010009679	ANK3	case	control	-0.244216054	0.089612595	-2.725242524	0.006425427	0.13904105	down_in_case
PRJNA8274(	ENSGALG00010009683	ADAMTS19	case	control	-0.002278058	0.139086346	-0.016378731	0.986932248	0.996781748	down_in_case
PRJNA8274(	ENSGALG00010009686	MAT2B	case	control	-0.042436066	0.048692138	-0.871517819	0.383471481	0.780061953	down_in_case
PRJNA8274(	ENSGALG00010009689	ENSGALG00010009689	case	control	-0.479718928	0.49681536	-0.965587955	0.334250405	0.748686141	down_in_case
PRJNA8274(	ENSGALG00010009690	RBM43	case	control	0.09816411	0.121097832	0.810618228	0.417584946	0.799987395	up_in_case
PRJNA8274(	ENSGALG00010009695	ATAD2B	case	control	0.061027658	0.058548612	1.042341665	0.297253302	0.721027687	up_in_case
PRJNA8274(	ENSGALG00010009697	ENSGALG00010009697	case	control	-0.051536564	0.198967615	-0.259019863	0.795619915	0.95280257	down_in_case
PRJNA8274(	ENSGALG00010009699	POLR1C	case	control	0.046854021	0.068982472	0.679216315	0.497000811	0.838837102	up_in_case
PRJNA8274(	ENSGALG00010009700	SH3TC1	case	control	-0.09217695	0.131117756	-0.703008907	0.482050199	0.832928691	down_in_case
PRJNA8274(	ENSGALG00010009702	ENSGALG00010009702	case	control	0.569824936	0.133808181	4.258520912	2.05784E-05	0.003130543	up_in_case
PRJNA8274(	ENSGALG00010009704	RGP1	case	control	0.093082928	0.161163954	0.577566667	0.563556717	0.871916948	up_in_case
PRJNA8274(	ENSGALG00010009705	DTNBP1	case	control	0.052655874	0.061435769	0.857088223	0.391396129	0.784437932	up_in_case

PRJNA8274(	ENSGALG00010009710	ENSGALG00010009710	case	control	0.322723383	0.07195796	4.484887868	7.29523E-06	0.00136192	up_in_case
PRJNA8274(	ENSGALG00010009712	UBXN2A	case	control	-0.138227343	0.075769999	-1.824301768	0.068106463	0.421429002	down_in_case
PRJNA8274(	ENSGALG00010009715	NUDCD2	case	control	-0.186165588	0.054770666	-3.399001714	0.000676323	0.038540612	down_in_case
PRJNA8274(	ENSGALG00010009717	EDN1	case	control	-0.272381482	0.13352027	-2.040000983	0.041350228	0.339637139	down_in_case
PRJNA8274(	ENSGALG00010009718	ENSGALG00010009718	case	control	0.913030389	0.204027972	4.475025549	7.64022E-06	0.001400707	up_in_case
PRJNA8274(	ENSGALG00010009719	YIPF3	case	control	0.002218391	0.049631094	0.0446976	0.964348347	0.992068146	up_in_case
PRJNA8274(	ENSGALG00010009720	RELN	case	control	-0.138989854	0.1278547	-1.087092254	0.276996041	0.702359305	down_in_case
PRJNA8274(	ENSGALG00010009721	ZHX1	case	control	0.011403346	0.070982472	0.160650162	0.872368944	0.975062028	up_in_case
PRJNA8274(	ENSGALG00010009722	GBA2	case	control	0.025630652	0.099807147	0.256801768	0.797331805	0.95280257	up_in_case
PRJNA8274(	ENSGALG00010009724	ISOC1	case	control	0.128185833	0.093360343	1.373022308	0.169745412	0.585894132	up_in_case
PRJNA8274(	ENSGALG00010009726	PRR16	case	control	-0.104617506	0.167510497	-0.624542982	0.532271052	0.855755691	down_in_case
PRJNA8274(	ENSGALG00010009727	CDH20	case	control	-0.222773439	0.158377243	-1.406600056	0.159546016	0.572017525	down_in_case
PRJNA8274(	ENSGALG00010009728	CCDC149	case	control	0.030165625	0.079546021	0.379222303	0.704522791	0.923973441	up_in_case
PRJNA8274(	ENSGALG00010009730	C8orf76	case	control	-0.192707481	0.085511126	-2.253595397	0.024221633	0.275122308	down_in_case
PRJNA8274(	ENSGALG00010009731	NMI	case	control	0.084799505	0.1043134	0.812930118	0.416258125	0.799987395	up_in_case
PRJNA8274(	ENSGALG00010009732	HMMR	case	control	-0.013442086	0.134130438	-0.100216522	0.92017243	0.984638357	down_in_case
PRJNA8274(	ENSGALG00010009736	WISP1	case	control	0.09281944	0.158941984	0.583983147	0.559231651	0.869461123	up_in_case
PRJNA8274(	ENSGALG00010009738	ENSGALG00010009738	case	control	0.530989118	0.215933123	2.459044317	0.013930742	0.207988769	up_in_case
PRJNA8274(	ENSGALG00010009740	LRRC73	case	control	-0.198194671	0.189770378	-1.044392031	0.296304049	0.719868825	down_in_case
PRJNA8274(	ENSGALG00010009741	C2H6orf52	case	control	0.072196604	0.049143982	1.469083298	0.141810197	0.543501954	up_in_case
PRJNA8274(	ENSGALG00010009743	CCNG1	case	control	-0.009801403	0.073622754	-0.133130083	0.894090504	0.979888889	down_in_case
PRJNA8274(	ENSGALG00010009744	DAPK1	case	control	0.201889393	0.087482343	2.30777303	0.021011764	0.259695956	up_in_case
PRJNA8274(	ENSGALG00010009746	FOXE1	case	control	-0.538002004	0.953033716	-0.564515184	0.57240357	0.876814097	down_in_case
PRJNA8274(	ENSGALG00010009749	NDRG1	case	control	0.060899487	0.084134905	0.723831414	0.469169241	0.829101076	up_in_case
PRJNA8274(	ENSGALG00010009751	ENSGALG00010009751	case	control	0.093991919	0.249451662	0.376794118	0.706326612	0.924449984	up_in_case
PRJNA8274(	ENSGALG00010009761	PDP2	case	control	0.030569029	0.076368286	0.400284339	0.688947102	0.918082523	up_in_case
PRJNA8274(	ENSGALG00010009762	MAN2A1	case	control	0.074860434	0.072496925	1.032601499	0.301790426	0.724580775	up_in_case
PRJNA8274(	ENSGALG00010009765	STX10	case	control	0.078780986	0.057560061	1.368674478	0.171101052	0.58844277	up_in_case
PRJNA8274(	ENSGALG00010009769	TMCC3	case	control	0.042376479	0.193562774	0.218928869	0.826705458	0.961207256	up_in_case
PRJNA8274(	ENSGALG00010009772	ENSGALG00010009772	case	control	-1.100205259	0.747399316	-1.472044777	0.141008786	0.54338031	down_in_case
PRJNA8274(	ENSGALG00010009774	PIAS2	case	control	0.107212956	0.082698682	1.296428826	0.194827786	0.617640154	up_in_case
PRJNA8274(	ENSGALG00010009775	KIAA1614	case	control	0.087414177	0.129715076	0.673893737	0.500378874	0.840855399	up_in_case
PRJNA8274(	ENSGALG00010009777	ST3GAL1	case	control	-0.025654466	0.068011731	-0.377206485	0.706020161	0.924380911	down_in_case
PRJNA8274(	ENSGALG00010009778	ENSGALG00010009778	case	control	0.040344207	0.054110672	0.74558687	0.455917017	0.822065071	up_in_case
PRJNA8274(	ENSGALG00010009779	PPP1R3E	case	control	-0.438196543	0.242883072	-1.804146082	0.071208409	0.431840649	down_in_case
PRJNA8274(	ENSGALG00010009780	MFSD2B	case	control	-0.027431578	0.223651833	-0.122653041	0.902381851	0.980850383	down_in_case
PRJNA8274(	ENSGALG00010009784	ENSGALG00010009784	case	control	0.199582554	0.18948968	1.053263453	0.292220264	0.714677075	up_in_case
PRJNA8274(	ENSGALG00010009786	DIPK2B	case	control	-0.174832814	0.074710751	-2.340129245	0.019277068	0.248120672	down_in_case
PRJNA8274(	ENSGALG00010009788	PAK1IP1	case	control	0.062970921	0.052037807	1.210099432	0.226240741	0.649676349	up_in_case
PRJNA8274(	ENSGALG00010009790	MC1R	case	control	4.226813149	0.827639404	5.107070942	3.27191E-07	0.000101714	up_in_case
PRJNA8274(	ENSGALG00010009791	GABRG2	case	control	0.228685816	0.312925116	0.730800451	0.464901048	0.826156951	up_in_case
PRJNA8274(	ENSGALG00010009793	TNFAIP6	case	control	-0.113461447	0.163482033	-0.694030068	0.487663352	0.835553062	down_in_case
PRJNA8274(	ENSGALG00010009795	NDUFA6	case	control	-0.07801184	0.075956146	-1.027064226	0.304390222	0.727524683	down_in_case
PRJNA8274(	ENSGALG00010009796	ENSGALG00010009796	case	control	-0.63704301	0.303104825	-2.101725072	0.035577366	0.324939791	down_in_case
PRJNA8274(	ENSGALG00010009798	YIPF4	case	control	0.013169358	0.04533659	0.29047967	0.771449302	0.945630466	up_in_case
PRJNA8274(	ENSGALG00010009801	ENSGALG00010009801	case	control	0.508942379	0.476172459	1.068819435	0.28515104	0.709820465	up_in_case
PRJNA8274(	ENSGALG00010009804	XPR1	case	control	-0.025450938	0.050738117	-0.501613762	0.615939237	0.891081782	down_in_case
PRJNA8274(	ENSGALG00010009806	WDCP	case	control	0.089808429	0.082611253	1.087121008	0.276983334	0.702359305	up_in_case
PRJNA8274(	ENSGALG00010009809	SMDT1	case	control	-0.020165857	0.090838915	-0.221995794	0.824317157	0.960422238	down_in_case
PRJNA8274(	ENSGALG00010009811	ENSGALG00010009811	case	control	0.192070467	0.110230427	1.742445105	0.081430589	0.45433376	up_in_case
PRJNA8274(	ENSGALG00010009822	ENSGALG00010009822	case	control	-0.064678181	0.205556964	-0.314648455	0.753028584	0.940666434	down_in_case
PRJNA8274(	ENSGALG00010009830	RNASEL	case	control	0.067283952	0.145234329	0.463278567	0.643164707	0.902038523	up_in_case
PRJNA8274(	ENSGALG00010009831	N6AMT1	case	control	-0.09179141	0.108104954	-0.849095314	0.395828258	0.787783337	down_in_case
PRJNA8274(	ENSGALG00010009833	SOD3	case	control	0.02051328	0.147195754	0.139360543	0.88916525	0.978017246	up_in_case
PRJNA8274(	ENSGALG00010009840	ENSGALG00010009840	case	control	0.122971421	0.196090618	0.62711527	0.530583678	0.855495419	up_in_case
PRJNA8274(	ENSGALG00010009841	GABRA1	case	control	-0.362930085	0.3043042	-1.192655526	0.23300431	0.657982411	down_in_case
PRJNA8274(	ENSGALG00010009842	PHETA2	case	control	-0.149488426	0.072515228	-2.061476317	0.039257621	0.335091843	down_in_case
PRJNA8274(	ENSGALG00010009847	ENSGALG00010009847	case	control	0.070046575	0.083964942	0.834235964	0.404148029	0.79260318	up_in_case
PRJNA8274(	ENSGALG00010009850	RIF1	case	control	-0.035365064	0.076756102	-0.460745961	0.644980876	0.902291775	down_in_case
PRJNA8274(	ENSGALG00010009852	ENSGALG00010009852	case	control	-0.382843487	0.218750461	-1.75013797	0.080094509	0.451460358	down_in_case
PRJNA8274(	ENSGALG00010009857	ORC5	case	control	0.124450863	0.09507273	1.309006934	0.190532002	0.613935676	up_in_case
PRJNA8274(	ENSGALG00010009858	ST8SIA5	case	control	0.175326359	0.264933311	0.661775442	0.508115147	0.843702533	up_in_case
PRJNA8274(	ENSGALG00010009866	NAGA	case	control	-0.072747042	0.062971752	-1.15523293	0.247995053	0.672435338	down_in_case
PRJNA8274(	ENSGALG00010009868	LTN1	case	control	0.047407528	0.061710627	0.768223083	0.442354662	0.813800549	up_in_case
PRJNA8274(	ENSGALG00010009876	SELENOI	case	control	0.001049156	0.057232992	0.018331321	0.985374541	0.996591771	up_in_case
PRJNA8274(	ENSGALG00010009883	ENSGALG00010009883	case	control	0.168357074	0.202194026	0.832651077	0.405041544	0.793437545	up_in_case
PRJNA8274(	ENSGALG00010009884	GFOD1	case	control	0.115414315	0.105967693	1.089146249	0.276089398	0.701506466	up_in_case
PRJNA8274(	ENSGALG00010009910	ENSGALG00010009910	case	control	-0.062583688	0.167425458	-0.373800311	0.708552888	0.92524028	down_in_case
PRJNA8274(	ENSGALG00010009914	RWDD2B	case	control	0.163466272	0.104528731	1.563840588	0.11785501	0.514604253	up_in_case
PRJNA8274(	ENSGALG00010009941	CACNA1E	case	control	-0.491905026	0.291911215	-1.685118625	0.091965687	0.476713154	down_in_case
PRJNA8274(	ENSGALG00010009943	DHX15	case	control	0.003930948	0.037823099	0.103929828	0.917225036	0.983445506	up_in_case
PRJNA8274(	ENSGALG00010009948	WBP2NL	case	control	-0.020412011	0.046034633	-0.443405539	0.65747243	0.908128634	down_in_case
PRJNA8274(	ENSGALG00010009972	PTK2B	case	control	0.226090497	0.287824297	0.785515675	0.432151283	0.807390038	up_in_case
PRJNA8274(	ENSGALG00010009985	USP16	case	control	0.10287852	0.059861399	1.718612029	0.08568504	0.462802693	up_in_case
PRJNA8274(	ENSGALG00010009989	TBC1D7	case	control	0.056589952	0.084162859	0.672386286	0.501337811	0.841151085	up_in_case
PRJNA8274(	ENSGALG00010009991	LHFPL3	case	control	-0.276906273	0.298252568	-0.928428798	0.353185186	0.761954708	down_in_case
PRJNA8274(	ENSGALG00010009993	ENSGALG00010009993	case	control	0.786084001	0.389867987	2.016282506	0.043770443	0.349229229	up_in_case
PRJNA8274(	ENSGALG00010009995	SEPTIN3	case	control	-0.598804101	0.366611717	-1.633346871	0.102396064	0.491460867	down_in_case
PRJNA8274(	ENSGALG00010009999	GABRB2	case	control	-0.329279597	0.172055889	-1.913794397	0.055646432	0.384148446	down_in_case
PRJNA8274(	ENSGALG00010010006	PPARGC1A	case	control	-0.056087346	0.116351717	-0.48205001	0.629770421	0.896269608	down_in_case
PRJNA8274(	ENSGALG00010010007	CTIF	case	control	0.065590635	0.082958919	0.790639941	0.429154133	0.805896795	up_in_case
PRJNA8274(	ENSGALG00010010009	ELF2	case	control	0.018876952	0.051361338	0.367532332	0.713221979	0.926862121	up_in_case
PRJNA8274(	ENSGALG00010010011	EPB41L4A	case	control	0.056324157	0.124421545	0.45268813	0.650773328	0.904636952	up_in_case
PRJNA8274(	ENSGALG00010010014	GLUL	case	control	0.10317761	0.124601394	0.828061441	0.407635705	0.794388636	up_in_case
PRJNA8274(	ENSGALG00010010017	CCDC59	case	control	-0.084009599	0.092982604	-0.903498018	0.36626164	0.771763177	down_in_case
PRJNA8274(	ENSGALG00010010022	KMT2E	case	control	0.012224477	0.049729263	0.245820594	0.805821114	0.9556344	up_in_case
PRJNA8274(	ENSGALG00010010035	ATP10B	case	control	0.278725061	0.355448737	0.784149816	0.432952208	0.808041839	up_in_case
PRJNA8274(	ENSGALG00010010037	NAA15	case	control	-0.109566456	0.056158826	-1.951010426	0.051055805	0.369565336	down_in_case
PRJNA8274(	ENSGALG00010010042	METTL25	case	control	0.049936912	0.096886842	0.515414793	0.606263198	0.886458459	up_in_case
PRJNA8274(	ENSGALG00010010049	ZBTB7C	case	control	0.260330923	0.246711871	1.055202259	0.291332831	0.714222439	up_in_case
PRJNA8274(	ENSGALG00010010052	ZNF346	case	control	0.017948697	0.053378709	0.336251985	0.736680855	0.934907368	up_in_case
PRJNA8274(	ENSGALG00010010053	CHRNA2	case	control	0.22007435	0.733610404	0.299988043	0.764186276	0.943786458	up_in_case
PRJNA8274(	ENSGALG00010010059	ENSGALG00010010059	case	control	0.04708937	0.09839533	0.478573216	0.632242277	0.89724192	up_in_case
PRJNA8274(	ENSGALG00010010061	CYP2D6	case	control	-0.051850284	0.158187163	-0.327778077	0.743079464	0.937448285	down_in_case
PRJNA8274(	ENSGALG00010010062	RGS7BP	case	control	-0.08997121	0.108413858	-0.829886615	0.406602894	0.794253996	down_in_case
PRJNA8274(	ENSGALG00010010064	ENSGALG00010010064	case	control	0.007068307	0.172651997	0.040939617	0.967344034	0.992731535	up_in_case
PRJNA8274(	ENSGALG00010010071	GLT8D2	case	control	-0.000756386	0.102732033	-0.007362706	0.994125463	0.998301153	down_in_case
PRJNA8274(	ENSGALG00010010077	CCT8	case	control	0.047298482	0.068545862	0.690026795	0.490177337	0.836347578	up_in_case
PRJNA8274(	ENSGALG00010010080	CDC42EP3	case	control	-0.005202159	0.095751114	-0.054330007	0.956672243	0.992056045	down_in_case
PRJNA8274(	ENSGALG00010010084	HK3	case	control	-0.186090574	0.172798658	-1.076921409	0.281515378	0.705762606	down_in_case
PRJNA8274(	ENSGALG00010010085	TDG	case	control	0.184471605	0.049740526	3.708678169	0.000208344	0.018505091	up_in_case
PRJNA8274(	ENSGALG00010010094	SLC2A9	case	control	0.522124156	0.375866572	1.389121022	0.164795952	0.579922223	up_in_case
PRJNA8274(	ENSGALG00010010095	C12orf73	case	control	-0.090159343	0.081944718	-1.100245939	0.27122498	0.698323327	down_in_case
PRJNA8274(	ENSGALG00010010099	TNR	case	control	-0.430067339	0.490465491	-0.876855452	0.380565156	0.778246195	down_in_case
PRJNA8274(	ENSGALG00010010100	IRF2BP2	case	control	-0.110031229	0.079543445	-1.383284683	0.166577593	0.581841616	down_in_case
PRJNA8274(	ENSGALG00010010106	C7orf73	case	control	-0.110221481	0.074684797	-1.475822192	0.139991632	0.542025835	down_in_case
PRJNA8274(	ENSGALG00010010107	MGST2	case	control	0.514122092	0.317811188	1.617696645	0.105727989	0.495266487	up_in_case
PRJNA8274(	ENSGALG00010010111	CNOT4	case	control	-0.092220204	0.065951904	-1.398294789	0.162024562	0.575640059	down_in_case
PRJNA8274(	ENSGALG00010010115	NEB	case	control	-0.397509545	0.135638789	-2.930647998	0.003382558	0.099555609	down_in_case
PRJNA8274(	ENSGALG00010010116	DRD5	case	control	-0.427674092	0.335261412	-1.275643652	0.202081516	0.625073547	down_in_case
PRJNA8274(	ENSGALG00010010119	SRPK2	case	control	-0.051746542	0.070127847	-0.73788865	0.460582117	0.82454265	down_in_case
PRJNA8274(	ENSGALG00010010122	THBS4	case	control	0.510816564	0.177162896	2.883315726	0.003935128	0.107801401	up_in_case
PRJNA8274(	ENSGALG00010010124	ENSGALG00010010124	case	control	-0.271533923	0.201407629	-1.34818092	0.1776002	0.596589818	down_in_case
PRJNA8274(	ENSGALG00010010125	AHCTF1	case	control	0.024380724	0.058588414	0.416135592	0.677310787	0.914766174	up_in_case
PRJNA8274(	ENSGALG00010010127	FAM13C	case	control	0.084771756	0.202999974	0.417594913	0.676243317	0.914247368	up_in_case
PRJNA8274(	ENSGALG00010010136	EPHX2	case	control	0.051127755	0.077639801	0.658525063	0.510200797	0.844875756	up_in_case
PRJNA8274(	ENSGALG00010010142	ENSGALG00010010142	case	control	-0.119285537	0.190242152	-0.62701949	0.530646459	0.855495419	down_in_case
PRJNA8274(	ENSGALG00010010146	PHACTR1	case	control	-0.000943218	0.273214057	-0.003452305	0.997245464	0.998921977	down_in_case
PRJNA8274(	ENSGALG00010010150	NTRK2	case	control	-0.399370367	0.268799522	-1.485755498	0.137343805	0.539854583	down_in_case
PRJNA8274(	ENSGALG00010010153	ENSGALG00010010153	case	control	0.64366019	0.440287921	1.461907446	0.143766574	0.546917268	up_in_case
PRJNA8274(	ENSGALG00010010154	IQSEC3	case	control	0.144286014	0.190701483	0.75660667	0.449285544	0.818749597	up_in_case
PRJNA8274(	ENSGALG00010010155	BRINP3	case	control	-0.121096984	0.224700816	-0.538925432	0.589938308	0.882949889	down_in_case
PRJNA8274(	ENSGALG00010010156	ENSGALG00010010156	case	control	-0.002845088	0.055061592	-0.051671006	0.95879084	0.992056045	down_in_case
PRJNA8274(	ENSGALG00010010161	SEPSECS	case	control	-0.001422134	0.068641637	-0.020718235	0.983470422	0.996591771	down_in_case
PRJNA8274(	ENSGALG00010010167	PUS7	case	control	-0.073420542	0.075989889	-0.966188303	0.333949965	0.748508541	down_in_case
PRJNA8274(	ENSGALG00010010168	SNCB	case	control	-0.170039273	0.217712989	-0.781024933	0.434787831	0.809671309	down_in_case

PRJNA8274(	ENSGALG00010010171	ENSGALG00010010171	case	control	-0.134596119	0.515739892	-0.260976746	0.79411044	0.95280257	down_in_case
PRJNA8274(	ENSGALG00010010178	LGI2	case	control	0.385176537	0.135395987	2.844815022	0.004443722	0.114389963	up_in_case
PRJNA8274(	ENSGALG00010010183	NKX3-2	case	control	0.215016206	0.670417815	0.320719709	0.74842281	0.939503405	up_in_case
PRJNA8274(	ENSGALG00010010185	PHYHIPL	case	control	0.269970204	0.217464748	1.241443527	0.214441948	0.639124605	up_in_case
PRJNA8274(	ENSGALG00010010186	TNPO1	case	control	-0.066952256	0.072342109	-0.925494936	0.354708517	0.763161644	down_in_case
PRJNA8274(	ENSGALG00010010188	DSP	case	control	0.326254963	0.604103646	0.540064549	0.589152518	0.882949889	up_in_case
PRJNA8274(	ENSGALG00010010190	RNF44	case	control	-0.03474929	0.106366906	-0.326692681	0.743900337	0.937448285	down_in_case
PRJNA8274(	ENSGALG00010010191	RINT1	case	control	0.071984998	0.069516998	1.0355021	0.300434486	0.72338999	up_in_case
PRJNA8274(	ENSGALG00010010195	ENSGALG00010010195	case	control	-0.015701486	0.195376787	-0.080365157	0.935946838	0.988425668	down_in_case
PRJNA8274(	ENSGALG00010010196	EFCAB10	case	control	0.291536248	0.244281629	1.193443194	0.232695847	0.657934961	up_in_case
PRJNA8274(	ENSGALG00010010197	SCCPDH	case	control	0.009848061	0.068769919	0.14320304	0.886129829	0.977630122	up_in_case
PRJNA8274(	ENSGALG00010010201	RIOK1	case	control	0.118027251	0.06851067	1.722757208	0.08493244	0.461224852	up_in_case
PRJNA8274(	ENSGALG00010010205	ARL5A	case	control	0.060197691	0.068642067	0.876979573	0.380497733	0.778246195	up_in_case
PRJNA8274(	ENSGALG00010010206	SCARA5	case	control	-0.10670626	0.095062656	-1.122483473	0.261656931	0.687516348	down_in_case
PRJNA8274(	ENSGALG00010010214	FAF2	case	control	-0.060517804	0.059221013	-1.021897479	0.306829426	0.729504707	down_in_case
PRJNA8274(	ENSGALG00010010215	ENSGALG00010010215	case	control	-0.382012574	0.10748054	-3.554248733	0.000379061	0.027024726	down_in_case
PRJNA8274(	ENSGALG00010010216	ATXN7L1	case	control	-0.058740075	0.066790681	-0.879465137	0.379149128	0.777369321	down_in_case
PRJNA8274(	ENSGALG00010010218	PBK	case	control	0.03547529	0.106616171	0.332738367	0.739331786	0.936326594	up_in_case
PRJNA8274(	ENSGALG00010010220	LHX9	case	control	0.424227694	0.621514992	0.682570331	0.494878387	0.838081589	up_in_case
PRJNA8274(	ENSGALG00010010222	PAH	case	control	1.447186571	0.953375133	1.517961316	0.129024151	0.530795558	up_in_case
PRJNA8274(	ENSGALG00010010223	ENSGALG00010010223	case	control	-0.089967242	0.167684676	-0.53652632	0.591594851	0.882949889	down_in_case
PRJNA8274(	ENSGALG00010010226	BICC1	case	control	-0.033763969	0.090149725	-0.374532137	0.708008452	0.92490115	down_in_case
PRJNA8274(	ENSGALG00010010231	C1orf53	case	control	-0.106183547	0.165993089	-0.639686555	0.522376398	0.851040772	down_in_case
PRJNA8274(	ENSGALG00010010234	ENSGALG00010010234	case	control	0.289918807	0.145962553	1.986254698	0.04700505	0.357384486	up_in_case
PRJNA8274(	ENSGALG00010010243	ESCO2	case	control	0.059420731	0.086678992	0.685526321	0.493011857	0.837887766	up_in_case
PRJNA8274(	ENSGALG00010010246	CLTB	case	control	-0.011947754	0.049948077	-0.23920349	0.810947796	0.956022547	down_in_case
PRJNA8274(	ENSGALG00010010252	WDR7	case	control	0.027175645	0.124971085	0.217455462	0.827853413	0.961369482	up_in_case
PRJNA8274(	ENSGALG00010010253	IL10RB	case	control	0.004174617	0.072039465	0.057949026	0.953789232	0.992056045	up_in_case
PRJNA8274(	ENSGALG00010010254	WDR41	case	control	-0.130909332	0.178354872	-0.733982369	0.462959483	0.825091817	down_in_case
PRJNA8274(	ENSGALG00010010255	CACNB4	case	control	0.093731365	0.116520101	0.804422276	0.421153141	0.802114148	up_in_case
PRJNA8274(	ENSGALG00010010257	ENSGALG00010010257	case	control	0.145795383	0.156227899	0.933222455	0.350705123	0.760621043	up_in_case
PRJNA8274(	ENSGALG00010010259	IGF1	case	control	-0.000954921	0.188227549	-0.005073228	0.995952167	0.998710499	down_in_case
PRJNA8274(	ENSGALG00010010262	HSPA9	case	control	-0.147229522	0.060701659	-2.425461245	0.015288948	0.219195419	down_in_case
PRJNA8274(	ENSGALG00010010263	RAB4A	case	control	0.073131879	0.099804805	0.732749077	0.463711485	0.825091817	up_in_case
PRJNA8274(	ENSGALG00010010266	EFNA5	case	control	0.10671936	0.175326215	0.608690265	0.542729761	0.859948541	up_in_case
PRJNA8274(	ENSGALG00010010267	CCSER2	case	control	-0.035361755	0.104986421	-0.336822181	0.736250951	0.934527659	down_in_case
PRJNA8274(	ENSGALG00010010270	SSR1	case	control	0.006777381	0.038059168	0.178074865	0.858664181	0.971828322	up_in_case
PRJNA8274(	ENSGALG00010010271	HUNK	case	control	0.227757379	0.09568612	2.38025515	0.017300654	0.234501759	up_in_case
PRJNA8274(	ENSGALG00010010274	MIS18A	case	control	0.066337841	0.085197104	0.778639621	0.436192033	0.81000376	up_in_case
PRJNA8274(	ENSGALG00010010275	PDE8B	case	control	-0.581663799	0.540247674	-1.076661366	0.281631578	0.705775113	down_in_case
PRJNA8274(	ENSGALG00010010279	CCSAP	case	control	-0.06102412	0.126428732	-0.482676042	0.629325777	0.896269608	down_in_case
PRJNA8274(	ENSGALG00010010280	HSP90B1	case	control	0.192281137	0.055589197	3.458965888	0.000542254	0.033861249	up_in_case
PRJNA8274(	ENSGALG00010010284	CCDC25	case	control	0.002365144	0.046876327	0.050454971	0.959759831	0.992056045	up_in_case
PRJNA8274(	ENSGALG00010010285	ENSGALG00010010285	case	control	2.526030341	0.473897466	5.330330974	9.80339E-08	3.68917E-05	up_in_case
PRJNA8274(	ENSGALG00010010287	STAM2	case	control	0.063274948	0.053371958	1.185546694	0.235801364	0.660526168	up_in_case
PRJNA8274(	ENSGALG00010010288	ENSGALG00010010288	case	control	0.119544299	0.162324599	0.736452142	0.461455582	0.82454265	up_in_case
PRJNA8274(	ENSGALG00010010290	ENSGALG00010010290	case	control	0.034480095	0.11130286	0.309786244	0.756723513	0.941659745	up_in_case
PRJNA8274(	ENSGALG00010010291	TCF20	case	control	-0.102360368	0.059016992	-1.734421977	0.082843239	0.456624721	down_in_case
PRJNA8274(	ENSGALG00010010295	ENSGALG00010010295	case	control	-0.282074118	0.152778105	-1.84629936	0.064848751	0.413989792	down_in_case
PRJNA8274(	ENSGALG00010010298	KCNIP4	case	control	-0.224379222	0.214871813	-1.044246888	0.296371179	0.719909607	down_in_case
PRJNA8274(	ENSGALG00010010300	DENND1B	case	control	-0.194474229	0.134906539	-1.441547834	0.149429974	0.556014189	down_in_case
PRJNA8274(	ENSGALG00010010301	ENSGALG00010010301	case	control	0.03641103	0.318292861	0.114394742	0.908924882	0.981730793	up_in_case
PRJNA8274(	ENSGALG00010010302	WDR36	case	control	0.042829281	0.175848409	0.243557969	0.807573184	0.955875648	up_in_case
PRJNA8274(	ENSGALG00010010303	ETF1	case	control	-0.022198724	0.039560458	-0.56113414	0.574706096	0.877910508	down_in_case
PRJNA8274(	ENSGALG00010010305	URB1	case	control	0.06095814	0.086135851	0.707697662	0.47913303	0.831404238	up_in_case
PRJNA8274(	ENSGALG00010010308	PACRGL	case	control	-0.14880691	0.165405849	-0.899647201	0.36830803	0.771770041	down_in_case
PRJNA8274(	ENSGALG00010010310	NT5DC3	case	control	-0.388446622	0.107358579	-3.618216873	0.00029664	0.023376995	down_in_case
PRJNA8274(	ENSGALG00010010311	CTSV	case	control	0.205358336	0.06062883	3.387140025	0.000706253	0.039918666	up_in_case
PRJNA8274(	ENSGALG00010010313	GRID1	case	control	-0.15507075	0.22266199	-0.696440151	0.486153226	0.834635018	down_in_case
PRJNA8274(	ENSGALG00010010314	CRB1	case	control	-0.094178109	0.22686924	-0.41512066	0.678053576	0.914941113	down_in_case
PRJNA8274(	ENSGALG00010010317	ENSGALG00010010317	case	control	-0.022563288	0.052946133	-0.426155549	0.669994505	0.913465866	down_in_case
PRJNA8274(	ENSGALG00010010320	SYPL1	case	control	-0.545003722	0.611629347	-0.891068626	0.372892355	0.77436767	down_in_case
PRJNA8274(	ENSGALG00010010321	NUP133	case	control	0.018885659	0.06353695	0.297238995	0.766284053	0.943992243	up_in_case
PRJNA8274(	ENSGALG00010010322	ENSGALG00010010322	case	control	-0.122412845	0.11674522	-1.048546954	0.294386681	0.717160058	down_in_case
PRJNA8274(	ENSGALG00010010323	FBP2	case	control	-0.132602278	0.253991473	-0.522073738	0.601618991	0.884954589	down_in_case
PRJNA8274(	ENSGALG00010010327	FBP1	case	control	0.770463493	0.72896852	1.056922859	0.290546795	0.714060795	up_in_case
PRJNA8274(	ENSGALG00010010330	ALPK2	case	control	-0.263798871	0.129431069	-2.038141802	0.041535754	0.340574127	down_in_case
PRJNA8274(	ENSGALG00010010333	WAPL	case	control	-0.019172665	0.039733653	-0.482529632	0.629429753	0.896269608	down_in_case
PRJNA8274(	ENSGALG00010010334	ZBTB41	case	control	-0.080152819	0.068678837	-1.167067223	0.243183223	0.667473598	down_in_case
PRJNA8274(	ENSGALG00010010336	FMNL2	case	control	0.057951513	0.047528668	1.219295945	0.222731887	0.646365317	up_in_case
PRJNA8274(	ENSGALG00010010337	SCARA3	case	control	0.16853848	0.088131536	1.912351559	0.055831117	0.384949359	up_in_case
PRJNA8274(	ENSGALG00010010338	CNST	case	control	0.107192949	0.069619903	1.539688277	0.123636356	0.522883756	up_in_case
PRJNA8274(	ENSGALG00010010345	ENSGALG00010010345	case	control	0.255139812	0.218390164	1.168275195	0.242695777	0.667285064	up_in_case
PRJNA8274(	ENSGALG00010010346	TFB2M	case	control	-0.030786958	0.060463104	-0.509185858	0.610621955	0.888923338	down_in_case
PRJNA8274(	ENSGALG00010010347	FBXW11	case	control	-0.019552041	0.039380758	-0.496487164	0.619550742	0.892382716	down_in_case
PRJNA8274(	ENSGALG00010010349	RREB1	case	control	-0.201143661	0.085805308	-2.3441867	0.019068623	0.246324585	down_in_case
PRJNA8274(	ENSGALG00010010351	NAMPTP1	case	control	-0.15603368	0.105415415	-1.480178973	0.13882549	0.540932258	down_in_case
PRJNA8274(	ENSGALG00010010352	MALT1	case	control	0.219325758	0.102110728	2.147920812	0.031720047	0.3098361	up_in_case
PRJNA8274(	ENSGALG00010010356	STAB2	case	control	-0.017519217	0.271805509	-0.064454976	0.948607957	0.991294545	down_in_case
PRJNA8274(	ENSGALG00010010358	ASPM	case	control	0.026930358	0.077842296	0.345960472	0.729372421	0.933556014	up_in_case
PRJNA8274(	ENSGALG00010010361	PIGM	case	control	-0.660543826	0.16653786	-3.966328294	7.29883E-05	0.008784022	down_in_case
PRJNA8274(	ENSGALG00010010366	CLU	case	control	-0.174530869	0.087786463	-1.988129634	0.046797355	0.356907825	down_in_case
PRJNA8274(	ENSGALG00010010368	ENSGALG00010010368	case	control	0.119315718	0.127182698	0.938144257	0.34817028	0.758675915	up_in_case
PRJNA8274(	ENSGALG00010010369	ENSGALG00010010369	case	control	0.465996462	0.216702782	2.150394458	0.031524026	0.3098361	up_in_case
PRJNA8274(	ENSGALG00010010371	SOCS1L	case	control	0.226004422	0.121498346	1.860144019	0.062865153	0.408011997	up_in_case
PRJNA8274(	ENSGALG00010010374	CMTM4	case	control	0.069004137	0.109313742	0.631248515	0.527878045	0.853386536	up_in_case
PRJNA8274(	ENSGALG00010010379	SLC35F6	case	control	0.003634747	0.08031132	0.045258217	0.963901491	0.992068146	up_in_case
PRJNA8274(	ENSGALG00010010381	MAST4	case	control	-0.311031379	0.12927922	-2.405888426	0.016133191	0.225671894	down_in_case
PRJNA8274(	ENSGALG00010010382	SEC11C	case	control	0.183008427	0.07960972	2.29882013	0.021515155	0.262514266	up_in_case
PRJNA8274(	ENSGALG00010010383	SLIT2	case	control	-0.113073335	0.110459215	-1.02366593	0.305993093	0.728933908	down_in_case
PRJNA8274(	ENSGALG00010010386	PRPF40A	case	control	-0.070125039	0.058986656	-1.188828863	0.234507018	0.659210368	down_in_case
PRJNA8274(	ENSGALG00010010390	KCNK3	case	control	-0.089636325	0.220613194	-0.406305368	0.684518242	0.916433328	down_in_case
PRJNA8274(	ENSGALG00010010393	FANCC	case	control	0.078438623	0.162921891	0.481449253	0.630197239	0.89637578	up_in_case
PRJNA8274(	ENSGALG00010010394	MRPL42	case	control	0.0095575	0.091561736	0.104383125	0.916865314	0.983445506	up_in_case
PRJNA8274(	ENSGALG00010010397	PICK1	case	control	-0.056962232	0.054207162	-1.050824841	0.293339045	0.716170594	down_in_case
PRJNA8274(	ENSGALG00010010399	FGF18	case	control	-0.154411226	0.186768399	-0.826752421	0.408377402	0.794775742	down_in_case
PRJNA8274(	ENSGALG00010010403	ENSGALG00010010403	case	control	-0.181243576	0.467538478	-0.387654888	0.698271444	0.922288441	down_in_case
PRJNA8274(	ENSGALG00010010404	COP1	case	control	-0.042268377	0.059477442	-0.710662314	0.477293518	0.830520323	down_in_case
PRJNA8274(	ENSGALG00010010405	EVA1C	case	control	-0.196282053	0.194317402	-1.010110527	0.312442339	0.734364975	down_in_case
PRJNA8274(	ENSGALG00010010406	PTCH1	case	control	0.124347315	0.091872738	1.353473489	0.175904414	0.594805656	up_in_case
PRJNA8274(	ENSGALG00010010407	SOX10	case	control	0.515398846	0.11284879	4.567163264	4.94369E-06	0.000981872	up_in_case
PRJNA8274(	ENSGALG00010010408	GAS8	case	control	0.009940992	0.094525355	0.105167467	0.916242925	0.983445506	up_in_case
PRJNA8274(	ENSGALG00010010410	CACNA2D1	case	control	-0.210839756	0.089408005	-2.358175393	0.018365014	0.242045801	down_in_case
PRJNA8274(	ENSGALG00010010411	CD180	case	control	-0.047300729	0.404944099	-0.116808046	0.907012168	0.981730793	down_in_case
PRJNA8274(	ENSGALG00010010416	SLC35B3	case	control	0.038140565	0.07416767	0.514247853	0.607078715	0.887106645	up_in_case
PRJNA8274(	ENSGALG00010010417	OTOF	case	control	-2.468568926	0.72333169	-3.412775854	0.000643048	0.037533011	down_in_case
PRJNA8274(	ENSGALG00010010418	BRINP2	case	control	0.198075991	0.257084249	0.770471127	0.441020476	0.813083204	up_in_case
PRJNA8274(	ENSGALG00010010420	NPM1	case	control	0.016242176	0.076020939	0.21365398	0.830816901	0.962228709	up_in_case
PRJNA8274(	ENSGALG00010010421	POLR2F	case	control	-0.003528469	0.059994644	-0.058813071	0.953100998	0.992056045	down_in_case
PRJNA8274(	ENSGALG00010010422	CDH1	case	control	1.452035201	0.665061123	2.183310901	0.029012922	0.302614726	up_in_case
PRJNA8274(	ENSGALG00010010423	C21orf59	case	control	0.21791647	0.123711454	1.761489844	0.07815553	0.448561389	up_in_case
PRJNA8274(	ENSGALG00010010425	BLOC1S5	case	control	-0.045942123	0.063535913	-0.723089039	0.469625183	0.829101076	down_in_case
PRJNA8274(	ENSGALG00010010430	ASTN1	case	control	-0.147579011	0.462641728	-0.318992003	0.749732577	0.939712144	down_in_case
PRJNA8274(	ENSGALG00010010431	SOCS2	case	control	0.057156304	0.241058229	0.2371058	0.812574713	0.956599851	up_in_case
PRJNA8274(	ENSGALG00010010434	PROM1	case	control	0.277813866	0.09713954	2.85994627	0.004237128	0.112622544	up_in_case
PRJNA8274(	ENSGALG00010010435	GAS1	case	control	0.050773478	0.122675067	0.413885882	0.678957684	0.915132297	up_in_case
PRJNA8274(	ENSGALG00010010438	LDB3	case	control	-0.322476555	0.11882243	-2.713936706	0.006648887	0.140441775	down_in_case
PRJNA8274(	ENSGALG00010010439	HGF	case	control	-0.205289618	0.175911659	-1.167004049	0.243208734	0.667473598	down_in_case
PRJNA8274(	ENSGALG00010010444	TXNDC5	case	control	-0.04718379	0.057051066	-0.827044848	0.408211642	0.794775742	down_in_case
PRJNA8274(	ENSGALG00010010445	LMAN1L	case	control	0.122599895	0.077340894	1.585188483	0.112923516	0.507003543	up_in_case
PRJNA8274(	ENSGALG00010010446	C22orf23	case	control	-0.068015996	0.395578359	-0.17194064	0.863484196	0.972890026	down_in_case
PRJNA8274(	ENSGALG00010010447	BTF3	case	control	-0.044443047	0.138708487	-0.320406112	0.748660493	0.939503405	down_in_case
PRJNA8274(	ENSGALG00010010448	GALNT13	case	control	0.124184653	0.170637839	0.727767378	0.466755997	0.827292691	up_in_case
PRJNA8274(	ENSGALG00010010449	PAPPA2	case	control	-0.865247578	0.511637074	-1.691135419	0.090810942	0.475136658	down_in_case
PRJNA8274(	ENSGALG00010010451	RANBP17	case	control	-0.405949383	0.386391788	-1.050615968	0.293435004	0.716170594	down_in_case
PRJNA8274(	ENSGALG00010010453	DRC1	case	control	0.445789208	0.300124027	1.485349949	0.137451147	0.539854583	up_in_case

PRJNA8274(	ENSGALG00010010456	BMP6	case	control	0.197410108	0.113776735	1.735065684	0.082729172	0.456624721	up_in_case
PRJNA8274(	ENSGALG00010010457	AGGF1	case	control	0.024354083	0.124106698	0.196235044	0.8444262	0.96721237	up_in_case
PRJNA8274(	ENSGALG00010010460	SEMA3C	case	control	-0.232523316	0.08920328	-2.60666778	0.009142802	0.170681551	down_in_case
PRJNA8274(	ENSGALG00010010461	SLC7A5	case	control	0.519376652	0.128106362	4.054261201	5.0293E-05	0.006599787	up_in_case
PRJNA8274(	ENSGALG00010010463	ACAA2	case	control	-0.02345161	0.163942629	-0.143047666	0.886252536	0.977630122	down_in_case
PRJNA8274(	ENSGALG00010010465	BMPR1A	case	control	0.001547603	0.070559274	0.021933375	0.982501101	0.996591771	up_in_case
PRJNA8274(	ENSGALG00010010466	CRADD	case	control	0.258043406	0.092332237	2.794727124	0.005194354	0.124839094	up_in_case
PRJNA8274(	ENSGALG00010010467	FGFBP2	case	control	0.71530811	0.528320249	1.353929007	0.175759028	0.594727928	up_in_case
PRJNA8274(	ENSGALG00010010468	ENSGALG00010010468	case	control	0.049836272	0.775057384	0.064300106	0.948731269	0.991294545	up_in_case
PRJNA8274(	ENSGALG00010010471	KCNJ3	case	control	0.048577088	0.12039824	0.403470081	0.686602441	0.917265966	up_in_case
PRJNA8274(	ENSGALG00010010472	MMRN2	case	control	-0.156598521	0.088422217	-1.771031379	0.076555484	0.444114977	down_in_case
PRJNA8274(	ENSGALG00010010473	TRIM35	case	control	-0.015975596	0.132569621	-0.120507216	0.904081364	0.981104465	down_in_case
PRJNA8274(	ENSGALG00010010475	CCDC71L	case	control	0.065791063	0.079312953	0.829512209	0.406814631	0.794253996	up_in_case
PRJNA8274(	ENSGALG00010010478	ENSGALG00010010478	case	control	0.41892264	0.373196313	1.122526204	0.261638773	0.687516348	up_in_case
PRJNA8274(	ENSGALG00010010486	ENSGALG00010010486	case	control	-0.117976672	0.248938366	-0.473919203	0.635557525	0.898681129	down_in_case
PRJNA8274(	ENSGALG00010010494	ENSGALG00010010494	case	control	-0.262524615	0.172072421	-1.525663519	0.127093684	0.528018502	down_in_case
PRJNA8274(	ENSGALG00010010496	MRPL17	case	control	0.025205303	0.195683483	0.12880649	0.89751077	0.980176778	up_in_case
PRJNA8274(	ENSGALG00010010499	NR4A2	case	control	-0.128759734	0.165189852	-0.779465154	0.435705756	0.81000376	down_in_case
PRJNA8274(	ENSGALG00010010505	CD38	case	control	0.163311186	0.197356447	0.827493545	0.407957379	0.794577843	up_in_case
PRJNA8274(	ENSGALG00010010511	GABRP	case	control	-0.20887456	0.3648559	-0.572485082	0.566993389	0.87333105	down_in_case
PRJNA8274(	ENSGALG00010010512	PLXNC1	case	control	-0.169770952	0.111517327	-1.522372862	0.127915681	0.529650968	down_in_case
PRJNA8274(	ENSGALG00010010515	RXRG	case	control	0.720821995	0.195912538	3.679305069	0.00023387	0.020255986	up_in_case
PRJNA8274(	ENSGALG00010010517	STMN4	case	control	-0.212776535	0.322821942	-0.659114228	0.50982242	0.8447206	down_in_case
PRJNA8274(	ENSGALG00010010521	SNCG	case	control	-0.672347428	0.303305688	-2.216732015	0.02664141	0.290596612	down_in_case
PRJNA8274(	ENSGALG00010010526	ENSGALG00010010526	case	control	0.452405498	0.30801367	1.468783832	0.141891431	0.543501954	up_in_case
PRJNA8274(	ENSGALG00010010528	GPD2	case	control	-0.188753341	0.091447243	-2.064068135	0.039011257	0.335091843	down_in_case
PRJNA8274(	ENSGALG00010010534	SYNJ1	case	control	0.043433385	0.071012415	0.611630864	0.540782013	0.859433518	up_in_case
PRJNA8274(	ENSGALG00010010535	LMX1A	case	control	-0.013494313	0.32170926	-0.041945677	0.966542003	0.992570962	down_in_case
PRJNA8274(	ENSGALG00010010537	ENSGALG00010010537	case	control	0.758704481	0.266964059	2.841972374	0.004483538	0.114389963	up_in_case
PRJNA8274(	ENSGALG00010010544	ENSGALG00010010544	case	control	0.063400997	0.172636328	0.367251769	0.713431227	0.926862121	up_in_case
PRJNA8274(	ENSGALG00010010546	MICALL1	case	control	-0.081093353	0.110534824	-0.733645295	0.463164947	0.825091817	down_in_case
PRJNA8274(	ENSGALG00010010547	BST1	case	control	0.01641926	0.129940818	0.126359525	0.899447339	0.98028579	up_in_case
PRJNA8274(	ENSGALG00010010572	AQP3	case	control	-0.034369929	0.280561308	-0.122504165	0.902499748	0.980850383	down_in_case
PRJNA8274(	ENSGALG00010010573	CEP83	case	control	-0.007025167	0.093167423	-0.075403688	0.939893525	0.989551456	down_in_case
PRJNA8274(	ENSGALG00010010575	PAXBP1	case	control	0.099043373	0.054612777	1.813556818	0.069745984	0.425679712	up_in_case
PRJNA8274(	ENSGALG00010010577	PBX1	case	control	-0.129818159	0.080156753	-1.619553602	0.105328203	0.495041148	down_in_case
PRJNA8274(	ENSGALG00010010578	GLUD2	case	control	0.091139375	0.153611533	0.593310758	0.5529732	0.866252369	up_in_case
PRJNA8274(	ENSGALG00010010585	FBXL5	case	control	0.18973047	0.056149987	3.378993996	0.000727516	0.040323559	up_in_case
PRJNA8274(	ENSGALG00010010586	CYTIP	case	control	-0.034391796	0.362754481	-0.094807363	0.924467838	0.985571367	down_in_case
PRJNA8274(	ENSGALG00010010588	SLC25A3	case	control	-0.073222824	0.056091242	-1.305423461	0.191748694	0.615090794	down_in_case
PRJNA8274(	ENSGALG00010010593	EIF3L	case	control	-0.095994971	0.069663038	-1.377990012	0.168206372	0.58432606	down_in_case
PRJNA8274(	ENSGALG00010010596	SHLD2	case	control	-0.293171108	0.110611556	-2.650456409	0.00803831	0.157462784	down_in_case
PRJNA8274(	ENSGALG00010010599	F2RL1	case	control	-0.054618298	0.110701206	-0.49338485	0.621740685	0.893467169	down_in_case
PRJNA8274(	ENSGALG00010010604	HIVEP1	case	control	-0.012399605	0.063422378	-0.195508356	0.844994997	0.967281084	down_in_case
PRJNA8274(	ENSGALG00010010605	NUF2	case	control	-0.110556048	0.092997048	-1.188812442	0.234513481	0.659210368	down_in_case
PRJNA8274(	ENSGALG00010010614	IKBIP	case	control	0.111724016	0.115972309	0.963368035	0.335362862	0.749356776	up_in_case
PRJNA8274(	ENSGALG00010010618	APAF1	case	control	0.093550641	0.109360058	0.855437009	0.392309263	0.785827491	up_in_case
PRJNA8274(	ENSGALG00010010619	LRRC52	case	control	-0.001556142	0.192306326	-0.008091997	0.993543591	0.998196863	down_in_case
PRJNA8274(	ENSGALG00010010620	ANKRD54	case	control	-0.002776936	0.056781239	-0.04890587	0.960994311	0.992056045	down_in_case
PRJNA8274(	ENSGALG00010010623	SMAD2	case	control	0.095961074	0.17139404	0.559885714	0.575557393	0.877910508	up_in_case
PRJNA8274(	ENSGALG00010010625	FAM169A	case	control	0.18165823	0.235019884	0.772948344	0.439552952	0.812401646	up_in_case
PRJNA8274(	ENSGALG00010010626	ADTRP	case	control	-0.600433251	0.387308824	-1.550270002	0.121076724	0.519499513	down_in_case
PRJNA8274(	ENSGALG00010010633	EPCIP	case	control	0.29738601	0.324475867	0.916511952	0.359398442	0.765374836	up_in_case
PRJNA8274(	ENSGALG00010010635	ALDH9A1	case	control	0.014532665	0.079891607	0.181904774	0.855657458	0.970656588	up_in_case
PRJNA8274(	ENSGALG00010010638	ACVR1C	case	control	-0.268195038	0.389438848	-0.688670477	0.491030659	0.836917571	down_in_case
PRJNA8274(	ENSGALG00010010642	CC2D2A	case	control	-0.342080083	0.070016817	-4.885684601	1.0307E-06	0.000268555	down_in_case
PRJNA8274(	ENSGALG00010010645	FAM219A	case	control	0.063481207	0.146258798	0.434033426	0.664264168	0.911435195	up_in_case
PRJNA8274(	ENSGALG00010010646	MCM3AP	case	control	-0.01819023	0.043897036	-0.414384001	0.678592904	0.914942347	down_in_case
PRJNA8274(	ENSGALG00010010648	CENPM	case	control	-0.441405037	0.151485869	-2.913836378	0.00357017	0.102106855	down_in_case
PRJNA8274(	ENSGALG00010010651	NUAK1	case	control	0.020520837	0.067288933	0.304966011	0.760392039	0.942883622	up_in_case
PRJNA8274(	ENSGALG00010010655	C1QTNF7	case	control	-0.233683448	0.117359308	-1.991179494	0.046461157	0.356907825	down_in_case
PRJNA8274(	ENSGALG00010010657	TMEM170B	case	control	-0.030998354	0.109268497	-0.283689758	0.776648144	0.947241838	down_in_case
PRJNA8274(	ENSGALG00010010659	ADAMTS1	case	control	-0.1110655	0.094371867	-1.176892058	0.239238593	0.66429721	down_in_case
PRJNA8274(	ENSGALG00010010661	CPEB2	case	control	0.137387285	0.16272202	0.844306661	0.398498065	0.789453932	up_in_case
PRJNA8274(	ENSGALG00010010662	MGST3	case	control	0.048007346	0.077078809	0.622834559	0.533393245	0.855817434	up_in_case
PRJNA8274(	ENSGALG00010010663	EFR3B	case	control	0.125331802	0.090390938	1.386552742	0.165578187	0.580618526	up_in_case
PRJNA8274(	ENSGALG00010010666	C12orf75	case	control	-0.103272187	0.158760229	-0.650491545	0.515374761	0.847750297	down_in_case
PRJNA8274(	ENSGALG00010010671	SIGMAR1	case	control	0.029720379	0.123014809	0.241600007	0.809090117	0.955875648	up_in_case
PRJNA8274(	ENSGALG00010010678	KCNIP1	case	control	-0.576737239	0.279482786	-2.063587696	0.039056825	0.335091843	down_in_case
PRJNA8274(	ENSGALG00010010680	ENSGALG00010010680	case	control	0.217136493	0.348168913	0.623652729	0.532855673	0.855817434	up_in_case
PRJNA8274(	ENSGALG00010010682	SMIM13	case	control	0.087000885	0.072243307	1.204276071	0.228482892	0.651938858	up_in_case
PRJNA8274(	ENSGALG00010010685	STIM2	case	control	0.004373674	0.075120058	0.058222459	0.953571432	0.992056045	up_in_case
PRJNA8274(	ENSGALG00010010688	IQGAP2	case	control	0.089317673	0.117985992	0.757019303	0.449038298	0.818701429	up_in_case
PRJNA8274(	ENSGALG00010010689	POFUT2	case	control	-0.002634103	0.061538548	-0.042804107	0.96585769	0.992340816	down_in_case
PRJNA8274(	ENSGALG00010010690	MTPN	case	control	-0.034084138	0.052410758	-0.650327121	0.515480942	0.847771992	down_in_case
PRJNA8274(	ENSGALG00010010693	KCNMB1	case	control	-0.041586641	0.31945874	-0.130178443	0.89642525	0.980176778	down_in_case
PRJNA8274(	ENSGALG00010010697	NEDD9	case	control	-0.116066855	0.089034937	-1.303610233	0.19236651	0.615090794	down_in_case
PRJNA8274(	ENSGALG00010010698	GPRIN2	case	control	0.026937304	0.199197279	0.135229277	0.8924306	0.979422008	up_in_case
PRJNA8274(	ENSGALG00010010699	DNAJC27	case	control	-0.043570419	0.071491991	-0.609444756	0.54222968	0.85975913	down_in_case
PRJNA8274(	ENSGALG00010010706	SREBF2	case	control	0.026051762	0.083755707	0.311044623	0.755766698	0.941588405	up_in_case
PRJNA8274(	ENSGALG00010010710	UCK2	case	control	-0.048774918	0.078588914	-0.620633567	0.534840748	0.856463908	down_in_case
PRJNA8274(	ENSGALG00010010711	ENSGALG00010010711	case	control	-0.664202527	0.335832442	-1.977779525	0.047953584	0.360378034	down_in_case
PRJNA8274(	ENSGALG00010010712	ADAMTS5	case	control	-0.070341665	0.097605442	-0.720673599	0.471110359	0.829101076	down_in_case
PRJNA8274(	ENSGALG00010010713	NSA2	case	control	0.006205564	0.16115941	0.038505752	0.969284446	0.992761697	up_in_case
PRJNA8274(	ENSGALG00010010719	SH3BP4	case	control	0.007112879	0.101251151	0.070249858	0.943994792	0.990183239	up_in_case
PRJNA8274(	ENSGALG00010010722	TBC1D19	case	control	-0.116914763	0.086187364	-1.356518614	0.174934217	0.594053503	down_in_case
PRJNA8274(	ENSGALG00010010724	ADCY3	case	control	0.082345276	0.102374754	0.804351391	0.421194066	0.802114148	up_in_case
PRJNA8274(	ENSGALG00010010728	ELOVL2	case	control	-1.066578785	0.59651697	-1.788010801	0.073774265	0.437929428	down_in_case
PRJNA8274(	ENSGALG00010010732	CCKAR	case	control	-0.154307266	0.834606042	-0.184886351	0.853318177	0.96998807	down_in_case
PRJNA8274(	ENSGALG00010010733	GFM2	case	control	0.013234879	0.170168742	0.077775033	0.938007007	0.989228038	up_in_case
PRJNA8274(	ENSGALG00010010734	AMN1	case	control	0.152378041	0.110531442	1.378594524	0.168019806	0.584227328	up_in_case
PRJNA8274(	ENSGALG00010010740	CCDC134	case	control	-0.186468993	0.085122262	-2.190601944	0.02848061	0.300549329	down_in_case
PRJNA8274(	ENSGALG00010010742	LCP2	case	control	-0.008181938	0.163664539	-0.049992127	0.960128662	0.992056045	down_in_case
PRJNA8274(	ENSGALG00010010743	KLHL20	case	control	-0.052245521	0.076447446	-0.68341749	0.494343071	0.83803731	down_in_case
PRJNA8274(	ENSGALG00010010745	ATVR1	case	control	-0.03373032	0.029885197	-1.128663141	0.259039964	0.686345498	down_in_case
PRJNA8274(	ENSGALG00010010748	SF3B1	case	control	0.063881912	0.069182611	0.923381053	0.355808662	0.763373775	up_in_case
PRJNA8274(	ENSGALG00010010749	NCOA4	case	control	0.08170853	0.052837504	1.54641162	0.12200519	0.520338922	up_in_case
PRJNA8274(	ENSGALG00010010751	SLC30A5	case	control	0.2758172	0.127197167	2.168422516	0.030126554	0.306417012	up_in_case
PRJNA8274(	ENSGALG00010010752	ENSGALG00010010752	case	control	-0.269292217	0.188020264	-1.432251031	0.152071992	0.560293917	down_in_case
PRJNA8274(	ENSGALG00010010753	ENSGALG00010010753	case	control	0.270308809	0.215990896	1.251482416	0.210758525	0.636310899	up_in_case
PRJNA8274(	ENSGALG00010010754	DNAI1	case	control	0.580223475	0.213069204	2.723169114	0.006465895	0.13904105	up_in_case
PRJNA8274(	ENSGALG00010010756	CENPL	case	control	-0.12106018	0.119719744	-1.011196454	0.311922413	0.734358001	down_in_case
PRJNA8274(	ENSGALG00010010759	ENSGALG00010010759	case	control	0.221177574	0.227924502	0.970398406	0.331847943	0.748135832	up_in_case
PRJNA8274(	ENSGALG00010010761	MEI1	case	control	0.092214983	0.252128904	0.365745387	0.714555075	0.926862121	up_in_case
PRJNA8274(	ENSGALG00010010762	MRPL39	case	control	-0.086434315	0.070348153	-1.228665024	0.219197425	0.643443819	down_in_case
PRJNA8274(	ENSGALG00010010766	CENPO	case	control	0.02302262	0.112129546	0.205321625	0.83732085	0.964826648	up_in_case
PRJNA8274(	ENSGALG00010010768	ENSGALG00010010768	case	control	0.089995433	0.064976729	1.385040988	0.166039935	0.581099135	up_in_case
PRJNA8274(	ENSGALG00010010769	HEXB	case	control	0.124050833	0.09483195	1.308112217	0.19083525	0.613935676	up_in_case
PRJNA8274(	ENSGALG00010010771	ENSGALG00010010771	case	control	-0.06939476	0.058245243	-1.191423641	0.233487317	0.657982411	down_in_case
PRJNA8274(	ENSGALG00010010774	KPTN	case	control	-0.318842224	0.118702923	-2.686052005	0.007230183	0.148125534	down_in_case
PRJNA8274(	ENSGALG00010010776	GCAT	case	control	-0.135091523	0.101035362	-1.337071695	0.181199235	0.601788276	down_in_case
PRJNA8274(	ENSGALG00010010778	UPP2	case	control	-0.301456764	0.318952813	-0.945145339	0.344584681	0.755870938	down_in_case
PRJNA8274(	ENSGALG00010010779	DOCK2	case	control	0.095310728	0.158080783	0.602924187	0.546559125	0.86246958	up_in_case
PRJNA8274(	ENSGALG00010010780	PARG	case	control	-0.002002543	0.047657058	-0.042019867	0.966482861	0.992570962	down_in_case
PRJNA8274(	ENSGALG00010010781	NAPA	case	control	-0.087033902	0.05232096	-1.663461484	0.096220095	0.483634708	down_in_case
PRJNA8274(	ENSGALG00010010783	F2RL2	case	control	-0.007673172	0.208678508	-0.036770301	0.970668154	0.992761697	down_in_case
PRJNA8274(	ENSGALG00010010784	CCDC148	case	control	0.089866173	0.199202998	0.451128618	0.651896853	0.905500243	up_in_case
PRJNA8274(	ENSGALG00010010786	NABP1	case	control	0.245431608	0.101604174	2.415566198	0.015710766	0.222636791	up_in_case
PRJNA8274(	ENSGALG00010010789	ENSGALG00010010789	case	control	-0.189886796	0.168265269	-1.128496674	0.259110221	0.686345498	down_in_case
PRJNA8274(	ENSGALG00010010791	H-10	case	control	0.315365372	0.156473705	2.015452835	0.043857223	0.349476736	up_in_case
PRJNA8274(	ENSGALG00010010792	XRCC6	case	control	-0.069425709	0.064834737	-1.070810377	0.2842547	0.708901676	down_in_case
PRJNA8274(	ENSGALG00010010796	CAVIN2	case	control	-0.123185038	0.072756335	-1.693117698	0.090433066	0.474044299	down_in_case
PRJNA8274(	ENSGALG00010010799	DARS2	case	control	-0.150562508	0.082304406	-1.829337155	0.067349116	0.420197363	down_in_case
PRJNA8274(	ENSGALG00010010800	BICRA	case	control	-0.057029962	0.075005743	-0.760341268	0.44705062	0.817833512	down_in_case
PRJNA8274(	ENSGALG00010010805	PTRHD1	case	control	0.136024299	0.15576039	0.87329198	0.382503951	0.77998936	up_in_case
PRJNA8274(	ENSGALG00010010809	CENPH	case	control	0.066576042	0.174170048	0.382247367	0.702277891	0.923231962	up_in_case

PRJNA8274(	ENSGALG00010010810	GALT	case	control	0.142546175	0.20004728	0.712562426	0.476116569	0.830520323	up_in_case
PRJNA8274(	ENSGALG00010010813	EHD2	case	control	-0.120307164	0.089729231	-1.340780062	0.179991872	0.600808892	down_in_case
PRJNA8274(	ENSGALG00010010816	AP2S1	case	control	-0.034561778	0.082259663	-0.420154631	0.674372496	0.914018759	down_in_case
PRJNA8274(	ENSGALG00010010821	RGS8	case	control	-0.170622548	0.328924438	-0.518728708	0.60394994	0.885268908	down_in_case
PRJNA8274(	ENSGALG00010010822	OGDHL	case	control	-0.760580836	0.43916822	-1.731866747	0.083297291	0.457254339	down_in_case
PRJNA8274(	ENSGALG00010010823	TMEFF2	case	control	-0.356738499	0.17459182	-2.043271551	0.041025564	0.338135774	down_in_case
PRJNA8274(	ENSGALG00010010824	PKP4	case	control	-0.024863735	0.084246351	-0.295131303	0.767893578	0.945079453	down_in_case
PRJNA8274(	ENSGALG00010010825	MAK	case	control	0.035996927	0.30266027	0.118935093	0.905326779	0.981730793	up_in_case
PRJNA8274(	ENSGALG00010010826	NCOA1	case	control	-0.257682052	0.083889243	-3.071693613	0.002128481	0.075903429	down_in_case
PRJNA8274(	ENSGALG00010010828	SPDL1	case	control	0.184882184	0.072099806	2.564253565	0.010339798	0.181645093	up_in_case
PRJNA8274(	ENSGALG00010010829	LAMB4	case	control	-0.284400326	0.22500521	-1.26397218	0.206240012	0.62937093	down_in_case
PRJNA8274(	ENSGALG00010010831	DESI1	case	control	-0.020247836	0.067227537	-0.301183672	0.76327444	0.943786458	down_in_case
PRJNA8274(	ENSGALG00010010834	KGD4	case	control	-0.039752608	0.179015109	-0.222062866	0.824264945	0.960422238	down_in_case
PRJNA8274(	ENSGALG00010010836	DIP2A	case	control	0.035045874	0.059958123	0.58450585	0.55888003	0.869450004	up_in_case
PRJNA8274(	ENSGALG00010010837	RBPJ	case	control	-0.056022579	0.088096387	-0.635923685	0.524826164	0.852358222	down_in_case
PRJNA8274(	ENSGALG00010010838	JAM2	case	control	-0.20384959	0.090287774	-2.257776238	0.023959613	0.273286623	down_in_case
PRJNA8274(	ENSGALG00010010840	RGS16	case	control	-0.2672629	0.581148226	-0.459887664	0.645596855	0.902535441	down_in_case
PRJNA8274(	ENSGALG00010010841	PMM1	case	control	0.373116592	0.118453177	3.14990784	0.00163322	0.065055828	up_in_case
PRJNA8274(	ENSGALG00010010843	ZNF395	case	control	-0.14749143	0.076403941	-1.930416508	0.053555251	0.37782383	down_in_case
PRJNA8274(	ENSGALG00010010844	CDC14B	case	control	0.085619898	0.073936487	1.158019556	0.246856056	0.671238182	up_in_case
PRJNA8274(	ENSGALG00010010845	FKRP	case	control	-0.291293057	0.114329498	-2.547838165	0.010839274	0.184966135	down_in_case
PRJNA8274(	ENSGALG00010010848	RGR	case	control	0.06160883	0.352487995	0.174782774	0.861250324	0.972434239	up_in_case
PRJNA8274(	ENSGALG00010010849	ACBD6	case	control	-0.048258552	0.063995814	-0.754089193	0.450795666	0.819836962	down_in_case
PRJNA8274(	ENSGALG00010010850	ENSGALG00010010850	case	control	-0.127376976	0.148533381	-0.857564643	0.391132906	0.784275639	down_in_case
PRJNA8274(	ENSGALG00010010851	PNOC	case	control	-0.984138284	0.222396924	-4.425143409	9.63782E-06	0.001660493	down_in_case
PRJNA8274(	ENSGALG00010010853	SMIM20	case	control	-0.125466082	0.076714113	-1.635501962	0.101943864	0.491460867	down_in_case
PRJNA8274(	ENSGALG00010010854	LRIT1	case	control	0.428215587	0.467793013	0.915395431	0.359984076	0.765754313	up_in_case
PRJNA8274(	ENSGALG00010010855	ATP5PF	case	control	-0.141920325	0.067077212	-2.115775535	0.034363903	0.319924355	down_in_case
PRJNA8274(	ENSGALG00010010856	CSDC2	case	control	0.754557898	0.530105373	1.423411147	0.154617003	0.563441859	up_in_case
PRJNA8274(	ENSGALG00010010857	SEL1L3	case	control	0.09285719	0.073400805	1.265070456	0.205846072	0.62881082	up_in_case
PRJNA8274(	ENSGALG00010010859	ENSGALG00010010859	case	control	-0.10277054	0.112819697	-0.910927279	0.362333691	0.768450658	down_in_case
PRJNA8274(	ENSGALG00010010861	ENSGALG00010010861	case	control	0.056893827	0.219846868	0.258788434	0.795798482	0.95280257	up_in_case
PRJNA8274(	ENSGALG00010010862	STRN4	case	control	-0.055851937	0.076147826	-0.73346726	0.46327349	0.825091817	down_in_case
PRJNA8274(	ENSGALG00010010865	ENSGALG00010010865	case	control	-0.038136918	0.047702572	-0.799473001	0.424016196	0.803723282	down_in_case
PRJNA8274(	ENSGALG00010010867	QSOX1	case	control	-0.068806548	0.089636106	-0.767620898	0.442712443	0.814107419	down_in_case
PRJNA8274(	ENSGALG00010010868	SLC39A10	case	control	0.024462548	0.08948516	0.273369891	0.784568895	0.949518053	up_in_case
PRJNA8274(	ENSGALG00010010869	ENSGALG00010010869	case	control	-0.202254248	0.147168409	-1.374304787	0.169347081	0.585146363	down_in_case
PRJNA8274(	ENSGALG00010010870	NOL6	case	control	0.043599405	0.206867412	0.210760142	0.833074442	0.962550652	up_in_case
PRJNA8274(	ENSGALG00010010871	GCNT2	case	control	0.788351901	0.783983033	1.005572656	0.314621183	0.736432557	up_in_case
PRJNA8274(	ENSGALG00010010874	ENSGALG00010010874	case	control	0.417629982	0.214848144	1.943837979	0.051914992	0.371953818	up_in_case
PRJNA8274(	ENSGALG00010010876	ENSGALG00010010876	case	control	0.005886359	0.115459269	0.050982126	0.959339763	0.992056045	up_in_case
PRJNA8274(	ENSGALG00010010880	ELP3	case	control	-0.235779116	0.058404012	-4.037036274	5.41307E-05	0.006946843	down_in_case
PRJNA8274(	ENSGALG00010010882	CEP350	case	control	-0.116455536	0.08781448	-1.326154146	0.184788644	0.607639686	down_in_case
PRJNA8274(	ENSGALG00010010884	DNAH7	case	control	-0.014644951	0.252571318	-0.05798343	0.953761827	0.992056045	down_in_case
PRJNA8274(	ENSGALG00010010885	SNRPD2	case	control	-0.11315063	0.102032602	-1.108965446	0.267445089	0.694221232	down_in_case
PRJNA8274(	ENSGALG00010010887	GABPA	case	control	0.033686784	0.03940884	0.854802713	0.392660376	0.786090351	up_in_case
PRJNA8274(	ENSGALG00010010888	GNPAT	case	control	-0.03042065	0.057593964	-0.528191636	0.597366334	0.884680373	down_in_case
PRJNA8274(	ENSGALG00010010889	CCL19	case	control	0.097655585	0.343698887	0.284131223	0.77630982	0.947228003	up_in_case
PRJNA8274(	ENSGALG00010010890	NOL12	case	control	0.046796778	0.078268183	0.597902956	0.549904687	0.864351329	up_in_case
PRJNA8274(	ENSGALG00010010891	POLR3H	case	control	-0.092878548	0.069138562	-1.343368228	0.179152769	0.599551744	down_in_case
PRJNA8274(	ENSGALG00010010893	CCL21	case	control	-0.516285784	0.316891434	-1.629219756	0.103266506	0.492970761	down_in_case
PRJNA8274(	ENSGALG00010010894	SPAG1	case	control	0.343154949	0.111938796	3.065558689	0.002172637	0.076380185	up_in_case
PRJNA8274(	ENSGALG00010010896	LY86	case	control	0.268907555	0.140607869	1.912464479	0.055816645	0.384949359	up_in_case
PRJNA8274(	ENSGALG00010010897	CEP170	case	control	-0.042433592	0.056167762	-0.755479482	0.449961339	0.81905641	down_in_case
PRJNA8274(	ENSGALG00010010898	PHF24	case	control	-1.062778161	0.931961536	-1.140366979	0.254133444	0.679971816	down_in_case
PRJNA8274(	ENSGALG00010010900	SLC34A2	case	control	0.389515969	0.935269484	0.416474584	0.677062762	0.914726861	up_in_case
PRJNA8274(	ENSGALG00010010904	TOR1AIP1	case	control	-0.088740789	0.059741802	-1.485405283	0.137436497	0.539854583	down_in_case
PRJNA8274(	ENSGALG00010010905	ENC1	case	control	0.137441735	0.116498997	1.179767543	0.238092682	0.662913814	up_in_case
PRJNA8274(	ENSGALG00010010906	ATP6V1C1	case	control	0.048624856	0.058219171	0.835203501	0.403603139	0.79260318	up_in_case
PRJNA8274(	ENSGALG00010010908	STK17B	case	control	0.057949543	0.088409952	0.65546403	0.512169038	0.845834144	up_in_case
PRJNA8274(	ENSGALG00010010909	SLIT3	case	control	-0.117285338	0.168814812	-0.694757393	0.487207354	0.835461642	down_in_case
PRJNA8274(	ENSGALG00010010911	ENSGALG00010010911	case	control	-0.284977402	0.428763199	-0.664649864	0.506274468	0.843280685	down_in_case
PRJNA8274(	ENSGALG00010010912	BAALC	case	control	0.354090998	0.259091881	1.366661884	0.17173131	0.58957339	up_in_case
PRJNA8274(	ENSGALG00010010913	IFT56	case	control	0.108886064	0.087874923	1.239102812	0.215307426	0.640470741	up_in_case
PRJNA8274(	ENSGALG00010010915	APP	case	control	-0.032992849	0.05001688	-0.659634292	0.509488542	0.844521404	down_in_case
PRJNA8274(	ENSGALG00010010917	TANC1	case	control	0.006005654	0.050909007	0.117968391	0.906092703	0.981730793	up_in_case
PRJNA8274(	ENSGALG00010010918	TOR1AIP2	case	control	0.130103131	0.077282891	1.683466147	0.092284888	0.476713154	up_in_case
PRJNA8274(	ENSGALG00010010919	CDHR1	case	control	-0.007516492	0.53493349	-0.014051265	0.988789082	0.997164011	down_in_case
PRJNA8274(	ENSGALG00010010920	UBN2	case	control	0.029624906	0.076788276	0.385799857	0.699644901	0.922288441	up_in_case
PRJNA8274(	ENSGALG00010010921	ENSGALG00010010921	case	control	0.096999336	0.081282057	1.193367134	0.232725621	0.657934961	up_in_case
PRJNA8274(	ENSGALG00010010922	DYM	case	control	-0.139285631	0.059817419	-2.328512881	0.019884886	0.253209146	down_in_case
PRJNA8274(	ENSGALG00010010923	F13A1	case	control	0.058702521	0.143742902	0.40838553	0.682990655	0.915918327	up_in_case
PRJNA8274(	ENSGALG00010010924	ENSGALG00010010924	case	control	0.176965076	0.107303823	1.649196374	0.099107411	0.488407564	up_in_case
PRJNA8274(	ENSGALG00010010925	FBXO16	case	control	-0.181716336	0.381857983	-0.475874132	0.634164052	0.898107199	down_in_case
PRJNA8274(	ENSGALG00010010928	HECW2	case	control	0.02132376	0.083155232	0.25643317	0.797616378	0.95280257	up_in_case
PRJNA8274(	ENSGALG00010010929	FMC1	case	control	-0.071608689	0.122914163	-0.582591031	0.560168645	0.86984941	down_in_case
PRJNA8274(	ENSGALG00010010930	FAM163A	case	control	-0.0454247	0.244831268	-0.185534717	0.852809653	0.969809672	down_in_case
PRJNA8274(	ENSGALG00010010931	CYYR1	case	control	-0.17833424	0.105940619	-1.683341496	0.092309002	0.476713154	down_in_case
PRJNA8274(	ENSGALG00010010932	GHITM	case	control	-0.174318225	0.076220506	-2.2870253	0.022194346	0.26563754	down_in_case
PRJNA8274(	ENSGALG00010010933	ANAPC4	case	control	0.018315773	0.049623187	0.369097063	0.712055378	0.92680349	up_in_case
PRJNA8274(	ENSGALG00010010934	ENSGALG00010010934	case	control	0.07575261	0.095587162	0.792497737	0.428070511	0.805337183	up_in_case
PRJNA8274(	ENSGALG00010010935	LGALS1A	case	control	-0.084163042	0.137119537	-0.61379322	0.539351978	0.858476757	down_in_case
PRJNA8274(	ENSGALG00010010937	CDK7	case	control	0.239795846	0.147851036	1.621874647	0.104830191	0.494770427	up_in_case
PRJNA8274(	ENSGALG00010010938	FZD6	case	control	-0.079244112	0.095445916	-0.830251467	0.406396622	0.794253996	down_in_case
PRJNA8274(	ENSGALG00010010939	ZCCHC4	case	control	0.069105953	0.130838232	0.528178589	0.597375388	0.884680373	up_in_case
PRJNA8274(	ENSGALG00010010940	NRG3	case	control	-1.031507791	0.210587906	-4.898229001	9.67043E-07	0.000260919	down_in_case
PRJNA8274(	ENSGALG00010010941	RIPK4	case	control	0.798858783	0.734316924	1.087893738	0.276642021	0.702359305	up_in_case
PRJNA8274(	ENSGALG00010010942	NRN1	case	control	-0.886092789	0.262851524	-3.371077241	0.000748749	0.041181174	down_in_case
PRJNA8274(	ENSGALG00010010943	WDSUB1	case	control	0.230029208	0.088443403	2.600863388	0.009298948	0.172470753	up_in_case
PRJNA8274(	ENSGALG00010010945	PI4K2B	case	control	0.094410664	0.092552503	1.020076828	0.307692025	0.730896339	up_in_case
PRJNA8274(	ENSGALG00010010946	SH2D4B	case	control	-0.433564749	0.459449992	-0.943660369	0.345343232	0.755935621	down_in_case
PRJNA8274(	ENSGALG00010010947	LUC7L2	case	control	-0.041229014	0.039118077	-1.053963204	0.291899764	0.714632191	down_in_case
PRJNA8274(	ENSGALG00010010948	PRDM15	case	control	0.017898345	0.059201592	0.302328771	0.762401448	0.943435506	up_in_case
PRJNA8274(	ENSGALG00010010949	FZD3	case	control	0.043106253	0.164474543	0.26208465	0.793256181	0.95280257	up_in_case
PRJNA8274(	ENSGALG00010010951	ENSGALG00010010951	case	control	0.115998993	0.215250241	0.538902965	0.589953811	0.882949889	up_in_case
PRJNA8274(	ENSGALG00010010952	ENSGALG00010010952	case	control	0.580611379	0.373450803	1.554719859	0.120012803	0.518014815	up_in_case
PRJNA8274(	ENSGALG00010010953	EXTL3	case	control	-0.103511764	0.04513236	-2.293515421	0.021818349	0.264012564	down_in_case
PRJNA8274(	ENSGALG00010010954	MTERF2	case	control	-0.453141622	0.181147966	-2.501499916	0.012366847	0.195842652	down_in_case
PRJNA8274(	ENSGALG00010010955	GTF3C3	case	control	-0.033872794	0.087452162	-0.387329404	0.69851236	0.922288441	down_in_case
PRJNA8274(	ENSGALG00010010956	AXDND1	case	control	-0.458445708	0.348571778	-1.315211779	0.188438738	0.611314417	down_in_case
PRJNA8274(	ENSGALG00010010957	ZMAT2	case	control	-0.121142648	0.042772169	-2.832277394	0.004621773	0.116153533	down_in_case
PRJNA8274(	ENSGALG00010010958	LCORL	case	control	0.057847789	0.095685636	0.604560844	0.545470831	0.862111144	up_in_case
PRJNA8274(	ENSGALG00010010959	TMEM263	case	control	0.019633973	0.04834621	0.406111939	0.684660353	0.916433328	up_in_case
PRJNA8274(	ENSGALG00010010960	POLDIP3	case	control	0.000383434	0.036344842	0.010549896	0.991582557	0.99790247	up_in_case
PRJNA8274(	ENSGALG00010010961	C2orf66	case	control	0.531915193	0.354054813	1.502352665	0.133006054	0.536224013	up_in_case
PRJNA8274(	ENSGALG00010010962	ANKDD1B	case	control	0.646890428	0.330956057	1.95461124	0.050628974	0.367719502	up_in_case
PRJNA8274(	ENSGALG00010010963	TSPAN14	case	control	-0.071147083	0.074289043	-0.957706283	0.338210888	0.751259351	down_in_case
PRJNA8274(	ENSGALG00010010964	INTS9	case	control	-0.083895235	0.076890636	-1.091098207	0.275229672	0.700894634	down_in_case
PRJNA8274(	ENSGALG00010010965	BAZ2B	case	control	0.02004756	0.048044333	0.417272106	0.676479389	0.914247368	up_in_case
PRJNA8274(	ENSGALG00010010966	ENSGALG00010010966	case	control	-1.110476242	0.433830467	-2.55970091	0.010476228	0.183125942	down_in_case
PRJNA8274(	ENSGALG00010010967	PRXL2A	case	control	0.180139886	0.157482752	1.143870575	0.252677316	0.678169222	up_in_case
PRJNA8274(	ENSGALG00010010968	RRP7A	case	control	0.052377103	0.074614579	0.701968752	0.482698651	0.833419912	up_in_case
PRJNA8274(	ENSGALG00010010970	TPM2	case	control	-0.062599798	0.139174306	-0.449794215	0.652858829	0.905885495	down_in_case
PRJNA8274(	ENSGALG00010010971	LCORL	case	control	0.091207641	0.10364191	0.880026634	0.378844881	0.777229179	up_in_case
PRJNA8274(	ENSGALG00010010972	EXOSC3	case	control	-0.057000386	0.080705544	-0.706275968	0.480016535	0.831851816	down_in_case
PRJNA8274(	ENSGALG00010010973	PGAP1	case	control	-0.024691629	0.090278904	-0.273503868	0.784465919	0.949518053	down_in_case
PRJNA8274(	ENSGALG00010010974	NCAPG	case	control	0.151329622	0.086015098	1.759337907	0.078520132	0.448818031	up_in_case
PRJNA8274(	ENSGALG00010010975	ATP8B1	case	control	0.248806854	0.491388923	0.506333868	0.612622291	0.890080771	up_in_case
PRJNA8274(	ENSGALG00010010976	DYDC2	case	control	0.453740229	0.405864656	1.117959453	0.263584311	0.68964455	up_in_case
PRJNA8274(	ENSGALG00010010977	FARS2	case	control	0.054052821	0.071996609	0.750768982	0.4527917	0.821045895	up_in_case
PRJNA8274(	ENSGALG00010010978	SERHL	case	control	0.075483713	0.094759854	0.796579032	0.425695559	0.804804048	up_in_case
PRJNA8274(	ENSGALG00010010979	ENSGALG00010010979	case	control	0.148955617	0.26105355	0.570594106	0.568274815	0.874461133	up_in_case
PRJNA8274(	ENSGALG00010010980	POLK	case	control	0.018558357	0.152524993	0.121674205	0.903157043	0.980997505	up_in_case
PRJNA8274(	ENSGALG00010010981	RIC8B	case	control	0.12826245	0.065633381	1.954225845	0.050674514	0.367840382	up_in_case
PRJNA8274(	ENSGALG00010010982	ZBTB21	case	control	-0.059656827	0.04545534	-1.312427243	0.18937602	0.612687123	down_in_case
PRJNA8274(	ENSGALG00010010983	MED28	case	control	-0.192510522	0.087126496	-2.209551979	0.027136271	0.292866927	down_in_case

PRJNA8274(	ENSGALG00010010984	MAT1A	case	control	0.062926544	0.109080142	0.576883588	0.564018098	0.871916948	up_in_case
PRJNA8274(	ENSGALG00010010985	CERT1	case	control	0.066377915	0.143587479	0.462282059	0.643879064	0.902038523	up_in_case
PRJNA8274(	ENSGALG00010010986	NARS	case	control	-0.026156708	0.036312397	-0.720324462	0.471325247	0.829101076	down_in_case
PRJNA8274(	ENSGALG00010010987	MARCHF7	case	control	0.012419479	0.046317742	0.26813654	0.788594221	0.951738421	up_in_case
PRJNA8274(	ENSGALG00010010988	ANKRD44	case	control	0.217387466	0.072516896	2.99774919	0.002719814	0.087009711	up_in_case
PRJNA8274(	ENSGALG00010010989	TRIQK	case	control	0.155488036	0.088504952	1.75682866	0.078947018	0.449171025	up_in_case
PRJNA8274(	ENSGALG00010010990	MED18	case	control	-0.137953663	0.173766706	-0.793901582	0.427252728	0.805305429	down_in_case
PRJNA8274(	ENSGALG00010010991	C2CD2	case	control	0.09893752	0.084279291	1.173924441	0.240425292	0.66577879	up_in_case
PRJNA8274(	ENSGALG00010010992	PCDHA6	case	control	-0.032804064	0.234886507	-0.139659211	0.888929255	0.978017246	down_in_case
PRJNA8274(	ENSGALG00010010993	ENSGALG00010010993	case	control	-0.131297463	0.265688987	-0.494177289	0.621180978	0.893175881	down_in_case
PRJNA8274(	ENSGALG00010010994	NAA20	case	control	-0.020984976	0.065150826	-0.322098382	0.747378164	0.939105545	down_in_case
PRJNA8274(	ENSGALG00010010995	HEBP1	case	control	0.105470381	0.095516112	1.104215606	0.269499622	0.696612479	up_in_case
PRJNA8274(	ENSGALG00010010996	RFX4	case	control	0.216667772	0.316583107	0.684394611	0.493726018	0.837916219	up_in_case
PRJNA8274(	ENSGALG00010010997	LAP3	case	control	-0.101888894	0.103623921	-0.983256504	0.325481212	0.743511394	down_in_case
PRJNA8274(	ENSGALG00010010998	SOAT1	case	control	-0.111669895	0.075098072	-1.486987503	0.137018112	0.539854583	down_in_case
PRJNA8274(	ENSGALG00010011001	ANGPT1	case	control	0.132089153	0.161395047	0.818421353	0.413116633	0.797633779	up_in_case
PRJNA8274(	ENSGALG00010011003	FECH	case	control	0.090206868	0.110091278	0.819382509	0.412568211	0.797581687	up_in_case
PRJNA8274(	ENSGALG00010011004	UMODL1	case	control	0.154587176	0.222708818	0.694122386	0.487605461	0.835553062	up_in_case
PRJNA8274(	ENSGALG00010011006	TMEM64	case	control	-0.021325439	0.053477515	-0.39877393	0.690059788	0.918477347	down_in_case
PRJNA8274(	ENSGALG00010011007	ENSGALG00010011007	case	control	0.015172029	0.153054886	0.099128026	0.92103662	0.985102742	up_in_case
PRJNA8274(	ENSGALG00010011013	ENSGALG00010011013	case	control	0.162003109	0.422438983	0.383494694	0.701353003	0.922832899	up_in_case
PRJNA8274(	ENSGALG00010011015	LYRM4	case	control	-0.191144648	0.133003132	-1.437143963	0.150677092	0.558207879	down_in_case
PRJNA8274(	ENSGALG00010011018	QDPR	case	control	-0.019598263	0.109259018	-0.179374333	0.857643784	0.971507138	down_in_case
PRJNA8274(	ENSGALG00010011019	NECAB1	case	control	0.159997336	0.150630374	1.062185079	0.288151672	0.71240818	up_in_case
PRJNA8274(	ENSGALG00010011020	SFTPA2	case	control	0.806218016	0.489327255	1.647604967	0.099433761	0.488584926	up_in_case
PRJNA8274(	ENSGALG00010011021	PPP1R3G	case	control	0.04717754	0.18760366	0.251474517	0.801447262	0.954294696	up_in_case
PRJNA8274(	ENSGALG00010011022	C5orf22	case	control	0.08829166	0.119840874	0.736740789	0.461279997	0.82454265	up_in_case
PRJNA8274(	ENSGALG00010011025	POLR3B	case	control	-0.091867135	0.050588837	-1.815956668	0.069377019	0.425095479	down_in_case
PRJNA8274(	ENSGALG00010011027	FAM234B	case	control	-0.04651345	0.083471223	-0.557239349	0.577363908	0.878143389	down_in_case
PRJNA8274(	ENSGALG00010011028	HMBOX1	case	control	0.01167129	0.102848713	0.113480176	0.909649879	0.981730793	up_in_case
PRJNA8274(	ENSGALG00010011029	HMGCR	case	control	0.166802402	0.090767517	1.837688274	0.066108353	0.41627012	up_in_case
PRJNA8274(	ENSGALG00010011033	DROSHA	case	control	-0.023282994	0.060242177	-0.38648991	0.699133874	0.922288441	down_in_case
PRJNA8274(	ENSGALG00010011034	FAM180A	case	control	0.228556209	0.454397156	0.502987762	0.614972876	0.890903873	up_in_case
PRJNA8274(	ENSGALG00010011038	GSG1	case	control	0.080828526	0.40287521	0.200629186	0.840988535	0.965799554	up_in_case
PRJNA8274(	ENSGALG00010011040	CDH6	case	control	-0.273385382	0.194309526	-1.406958204	0.159439783	0.572017525	down_in_case
PRJNA8274(	ENSGALG00010011042	SLC13A4	case	control	0.320539154	0.577696294	0.554857557	0.578992095	0.879142433	up_in_case
PRJNA8274(	ENSGALG00010011044	MBL2	case	control	1.146731599	0.52402078	2.1883323	0.028645406	0.300624184	up_in_case
PRJNA8274(	ENSGALG00010011045	ENSGALG00010011045	case	control	0.35790396	0.170927427	2.09389427	0.0362694	0.326196489	up_in_case
PRJNA8274(	ENSGALG00010011049	ABL2	case	control	-0.021853049	0.062885022	-0.347508021	0.728209693	0.933057319	down_in_case
PRJNA8274(	ENSGALG00010011050	SYT10	case	control	-1.691671694	0.435978405	-3.880173134	0.000104382	0.011055819	down_in_case
PRJNA8274(	ENSGALG00010011055	TOR3A	case	control	0.175380366	0.132587216	1.322754719	0.18591696	0.608935531	up_in_case
PRJNA8274(	ENSGALG00010011056	ENSGALG00010011056	case	control	0.130913975	0.468683662	0.279322677	0.779997205	0.948864508	up_in_case
PRJNA8274(	ENSGALG00010011057	ASCL1	case	control	0.168747922	0.564188867	0.299098283	0.764865056	0.943786458	up_in_case
PRJNA8274(	ENSGALG00010011059	ENSGALG00010011059	case	control	0.052966241	0.131370527	0.403182067	0.686814291	0.917290381	up_in_case
PRJNA8274(	ENSGALG00010011061	KIF13B	case	control	-0.059991194	0.071256647	-0.841903131	0.399842178	0.789900496	down_in_case
PRJNA8274(	ENSGALG00010011062	TMEM55A	case	control	-0.165662489	0.076755568	-2.15831234	0.030903556	0.308259818	down_in_case
PRJNA8274(	ENSGALG00010011064	MYLIP	case	control	0.061224506	0.071804881	0.852651032	0.393852854	0.786935281	up_in_case
PRJNA8274(	ENSGALG00010011067	TMTC2	case	control	-0.00859604	0.09909067	-0.086749241	0.930870835	0.987258715	down_in_case
PRJNA8274(	ENSGALG00010011069	ENSGALG00010011069	case	control	-0.208667968	0.573121175	-0.364090488	0.71579044	0.926862121	down_in_case
PRJNA8274(	ENSGALG00010011076	PKP2	case	control	0.119113871	0.149004785	0.799396284	0.424060665	0.803723282	up_in_case
PRJNA8274(	ENSGALG00010011077	RASGEF1A	case	control	0.148367575	0.122892821	1.20729245	0.227319537	0.65088213	up_in_case
PRJNA8274(	ENSGALG00010011080	EMP1	case	control	0.155543203	0.142604035	1.09073494	0.275389532	0.700894634	up_in_case
PRJNA8274(	ENSGALG00010011085	UGT1A1	case	control	0.266768721	0.777006134	0.343328977	0.731350984	0.933770678	up_in_case
PRJNA8274(	ENSGALG00010011086	OTUD6A	case	control	-0.036278986	0.032358783	-1.121148039	0.262224853	0.688165792	down_in_case
PRJNA8274(	ENSGALG00010011090	GRIN2B	case	control	-0.021825995	0.272243282	-0.080170922	0.936101316	0.988425668	down_in_case
PRJNA8274(	ENSGALG00010011091	YARS2	case	control	0.116391005	0.065171339	1.785923166	0.074111699	0.438111241	up_in_case
PRJNA8274(	ENSGALG00010011094	CSGALNACT2	case	control	0.111712873	0.056968672	1.960952731	0.049884537	0.365445124	up_in_case
PRJNA8274(	ENSGALG00010011095	TNN	case	control	0.005153948	0.635682798	0.008107736	0.993531033	0.998196863	up_in_case
PRJNA8274(	ENSGALG00010011096	LDB2	case	control	-0.040529761	0.060972179	-0.664725487	0.506226089	0.843280685	down_in_case
PRJNA8274(	ENSGALG00010011099	CCBE1	case	control	-0.123050725	0.128845708	-0.955023856	0.339565633	0.751259351	down_in_case
PRJNA8274(	ENSGALG00010011101	ENSGALG00010011101	case	control	-1.293512868	0.508957692	-2.541493895	0.011037987	0.185137791	down_in_case
PRJNA8274(	ENSGALG00010011104	ABCG1	case	control	-0.096165971	0.196555029	-0.48925724	0.62465959	0.894741574	down_in_case
PRJNA8274(	ENSGALG00010011106	USP40	case	control	0.007223753	0.082917464	0.087119797	0.93057629	0.987258715	up_in_case
PRJNA8274(	ENSGALG00010011107	ENSGALG00010011107	case	control	-0.007822982	0.153419841	-0.050990682	0.959332946	0.992056045	down_in_case
PRJNA8274(	ENSGALG00010011109	ENSGALG00010011109	case	control	0.131837756	0.740849687	0.177954797	0.858758475	0.971828322	up_in_case
PRJNA8274(	ENSGALG00010011111	MRPS14	case	control	-0.006244098	0.076078026	-0.08207492	0.934587136	0.988140189	down_in_case
PRJNA8274(	ENSGALG00010011116	TAPT1	case	control	-0.046441511	0.079894288	-0.581287005	0.561047038	0.870480544	down_in_case
PRJNA8274(	ENSGALG00010011117	SLC6A15	case	control	3.124272299	0.63952985	4.885264225	1.0329E-06	0.000268555	up_in_case
PRJNA8274(	ENSGALG00010011118	GMPR2	case	control	0.040901755	0.098834211	0.413842076	0.678989767	0.915132297	up_in_case
PRJNA8274(	ENSGALG00010011119	ENSGALG00010011119	case	control	-0.439655014	0.154617891	-2.843493799	0.004462188	0.114389963	down_in_case
PRJNA8274(	ENSGALG00010011121	NUP35	case	control	0.021734899	0.073702031	0.294902307	0.76806851	0.945213398	up_in_case
PRJNA8274(	ENSGALG00010011124	CACYBP	case	control	-0.057434511	0.04921396	-1.167036965	0.243195442	0.667473598	down_in_case
PRJNA8274(	ENSGALG00010011126	ATXN1	case	control	0.022620187	0.107098556	0.211209079	0.832724126	0.962414532	up_in_case
PRJNA8274(	ENSGALG00010011128	GCNT4	case	control	0.314735882	0.259726024	1.211799563	0.225589116	0.649491335	up_in_case
PRJNA8274(	ENSGALG00010011129	ENSGALG00010011129	case	control	-0.316109475	0.111637718	-2.831565177	0.004632079	0.116208303	down_in_case
PRJNA8274(	ENSGALG00010011130	DNM1L	case	control	-0.030189941	0.039501032	-0.76428234	0.444699007	0.815952113	down_in_case
PRJNA8274(	ENSGALG00010011131	RET	case	control	-0.108508356	0.239068571	-0.453879635	0.649915462	0.904241824	down_in_case
PRJNA8274(	ENSGALG00010011133	CDC37L1	case	control	-0.077550319	0.097762755	-0.793250143	0.427632098	0.805305429	down_in_case
PRJNA8274(	ENSGALG00010011136	ENSGALG00010011136	case	control	-0.753342222	0.285773231	-2.636153919	0.008385169	0.160950217	down_in_case
PRJNA8274(	ENSGALG00010011141	MSX1	case	control	0.753930999	0.58431513	1.290281494	0.19695294	0.620029043	up_in_case
PRJNA8274(	ENSGALG00010011142	DUSP19	case	control	-0.52534885	0.104686867	-5.018288008	5.2134E-07	0.000155316	down_in_case
PRJNA8274(	ENSGALG00010011143	AK3	case	control	0.114708053	0.239900414	0.478148623	0.632544427	0.897270638	up_in_case
PRJNA8274(	ENSGALG00010011144	ATF7IP	case	control	-0.03198604	0.059196669	-0.540335124	0.58896594	0.882949889	down_in_case
PRJNA8274(	ENSGALG00010011146	BMS1	case	control	-0.036227931	0.066208267	-0.547181377	0.584254129	0.881307382	down_in_case
PRJNA8274(	ENSGALG00010011148	ENSGALG00010011148	case	control	-0.112394787	0.093706117	-1.199439163	0.230357227	0.654892316	down_in_case
PRJNA8274(	ENSGALG00010011150	FGD4	case	control	0.077717399	0.137566548	0.564944022	0.57211184	0.876814097	up_in_case
PRJNA8274(	ENSGALG00010011152	SLC18A3	case	control	-0.582642314	0.569537365	-1.023009813	0.306303206	0.729293348	down_in_case
PRJNA8274(	ENSGALG00010011154	SLC26A7	case	control	0.976742981	0.493670637	1.978531652	0.047868761	0.360228144	up_in_case
PRJNA8274(	ENSGALG00010011157	ENSGALG00010011157	case	control	-0.284188528	0.810512353	-0.35062825	0.72586726	0.932158578	down_in_case
PRJNA8274(	ENSGALG00010011161	ENSGALG00010011161	case	control	0.016425998	0.061420206	0.267436388	0.789133186	0.951843754	up_in_case
PRJNA8274(	ENSGALG00010011166	SOX7	case	control	0.014175036	0.083466196	0.169829665	0.865144097	0.973289701	up_in_case
PRJNA8274(	ENSGALG00010011170	RBM24	case	control	-0.046098136	0.089086505	-0.517453637	0.604839525	0.885268908	down_in_case
PRJNA8274(	ENSGALG00010011175	GPR52	case	control	-0.56025344	0.335555934	-1.669627576	0.094993071	0.48148469	down_in_case
PRJNA8274(	ENSGALG00010011178	RSPO2	case	control	-0.308312284	0.188858864	-1.632500996	0.102573989	0.491557654	down_in_case
PRJNA8274(	ENSGALG00010011181	ENSGALG00010011181	case	control	-0.376832679	0.196504023	-1.9176843	0.055151054	0.383470355	down_in_case
PRJNA8274(	ENSGALG00010011184	FAM8A1	case	control	-0.137906319	0.05729113	-2.407114676	0.01607912	0.225202171	down_in_case
PRJNA8274(	ENSGALG00010011187	STK32B	case	control	0.455805137	0.384357663	1.185887989	0.235666537	0.660526168	up_in_case
PRJNA8274(	ENSGALG00010011190	ENSGALG00010011190	case	control	-0.173360516	0.167584514	-1.034466207	0.300918267	0.723580161	down_in_case
PRJNA8274(	ENSGALG00010011192	RPL17	case	control	-0.081575981	0.178644139	-0.456639561	0.647930139	0.903500828	down_in_case
PRJNA8274(	ENSGALG00010011193	FAM20B	case	control	0.051857823	0.060659287	0.85490328	0.392604695	0.78608893	up_in_case
PRJNA8274(	ENSGALG00010011198	PINX1	case	control	-0.043063974	0.079567202	-0.541227709	0.588350641	0.882771329	down_in_case
PRJNA8274(	ENSGALG00010011200	PCDHB4	case	control	0.073584513	0.547465286	0.134409459	0.893078802	0.979748897	up_in_case
PRJNA8274(	ENSGALG00010011203	ENSGALG00010011203	case	control	0.126468697	0.159104692	0.794877228	0.426684922	0.805305429	up_in_case
PRJNA8274(	ENSGALG00010011205	CYTL1	case	control	1.170645009	0.875006405	1.337870217			up_in_case
PRJNA8274(	ENSGALG00010011210	NUP153	case	control	-0.026345816	0.050478083	-0.521925847	0.601721961	0.884954589	down_in_case
PRJNA8274(	ENSGALG00010011211	SLC37A1	case	control	-0.087216972	0.109404281	-0.797198899	0.425335525	0.804536774	down_in_case
PRJNA8274(	ENSGALG00010011212	NCKAP1	case	control	-0.020821969	0.028096932	-0.741076268	0.458647198	0.824070529	down_in_case
PRJNA8274(	ENSGALG00010011213	XKR6	case	control	-0.036934568	0.195909707	-0.188528523	0.850462358	0.969234083	down_in_case
PRJNA8274(	ENSGALG00010011218	ENSGALG00010011218	case	control	0.463329783	0.421482019	1.099287187	0.271642819	0.698323327	up_in_case
PRJNA8274(	ENSGALG00010011222	RCL1	case	control	0.041099306	0.068279128	0.601930738	0.547220243	0.862666682	up_in_case
PRJNA8274(	ENSGALG00010011231	PLBD1	case	control	-0.244190227	0.131479757	-1.857245797	0.063276192	0.409249003	down_in_case
PRJNA8274(	ENSGALG00010011235	ENSGALG00010011235	case	control	0.104992333	0.079529463	1.320169026	0.186778591	0.610613817	up_in_case
PRJNA8274(	ENSGALG00010011237	ENSGALG00010011237	case	control	-0.063249068	0.261851456	-0.241545603	0.809132277	0.955875648	down_in_case
PRJNA8274(	ENSGALG00010011239	RALGPS2	case	control	0.008099656	0.064528147	0.125521283	0.900110877	0.98028579	up_in_case
PRJNA8274(	ENSGALG00010011243	ALX1	case	control	0.923199829	0.407905221	2.263270438	0.023619023	0.271942058	up_in_case
PRJNA8274(	ENSGALG00010011244	PDE9A	case	control	0.177351368	0.12111915	1.464271896	0.143119673	0.545763019	up_in_case
PRJNA8274(	ENSGALG00010011246	DNAJB5	case	control	-0.124820995	0.115378029	-1.081843711	0.27932198	0.704960168	down_in_case
PRJNA8274(	ENSGALG00010011247	ENSGALG00010011247	case	control	0.005122602	0.066498308	0.077033562	0.938596845	0.989551456	up_in_case
PRJNA8274(	ENSGALG00010011250	ENSGALG00010011250	case	control	-0.016915666	0.073502234	-0.23013812	0.817984444	0.958536226	down_in_case
PRJNA8274(	ENSGALG00010011251	FRZB	case	control	0.101890749	0.089119491	1.14330488	0.25291203	0.678440497	up_in_case
PRJNA8274(	ENSGALG00010011253	KDM4C	case	control	0.132407034	0.068770057	1.925358781	0.054184489	0.37945063	up_in_case
PRJNA8274(	ENSGALG00010011262	ENSGALG00010011262	case	control	-0.280193231	0.244455884	-1.146191397	0.251715967	0.677084435	down_in_case
PRJNA8274(	ENSGALG00010011267	EVC2	case	control	0.049002942	0.088610943	0.553012304	0.580254985	0.879378502	up_in_case
PRJNA8274(	ENSGALG00010011271	ANGPTL1	case	control	0.003013789	0.064383524	0.046809946	0.962664702	0.992068146	up_in_case
PRJNA8274(	ENSGALG00010011273	RUNX1T1	case	control	-0.001803578	0.070109429	-0.02572518	0.97947654	0.995636523	down_in_case
PRJNA8274(	ENSGALG00010011274	ENSGALG00010011274	case	control	-1.884220441	0.589244858	-3.19768669	0.001385347	0.058654283	down_in_case

PRJNA8274(	ENSGALG00010011275	WDR4	case	control	0.020585948	0.075012883	0.274432166	0.783752527	0.949518053	up_in_case
PRJNA8274(	ENSGALG00010011278	ENSGALG00010011278	case	control	-0.204213732	0.193121305	-1.057437616	0.290311912	0.714060795	down_in_case
PRJNA8274(	ENSGALG00010011280	FAM199X	case	control	-0.015195238	0.050713208	-0.29963078	0.764458803	0.943786458	down_in_case
PRJNA8274(	ENSGALG00010011281	ENSGALG00010011281	case	control	0.800230322	0.545042358	1.468198409	0.142050336	0.543501954	up_in_case
PRJNA8274(	ENSGALG00010011282	CRY1	case	control	-0.112079583	0.079920201	-1.402393658	0.160797726	0.573265281	down_in_case
PRJNA8274(	ENSGALG00010011283	LONRF3	case	control	0.065301121	0.137323402	0.475527985	0.634410692	0.898107199	up_in_case
PRJNA8274(	ENSGALG00010011285	ENSGALG00010011285	case	control	-0.136977041	0.304719465	-0.449518513	0.653057657	0.906056514	down_in_case
PRJNA8274(	ENSGALG00010011287	ENSGALG00010011287	case	control	0.067883852	0.146244151	0.464181656	0.642517603	0.901845477	up_in_case
PRJNA8274(	ENSGALG00010011288	ENSGALG00010011288	case	control	-0.24292544	0.269366828	-0.901838738	0.367142538	0.771770041	down_in_case
PRJNA8274(	ENSGALG00010011293	NDUFV3	case	control	-0.131471649	0.090563101	-1.451713195	0.146581369	0.55166873	down_in_case
PRJNA8274(	ENSGALG00010011295	FAM167A	case	control	-0.241711437	0.392904782	-0.615190876	0.538428672	0.857886353	down_in_case
PRJNA8274(	ENSGALG00010011297	OSR2	case	control	-0.158369793	0.101385053	-1.562062529	0.118273259	0.51463738	down_in_case
PRJNA8274(	ENSGALG00010011300	EVC	case	control	-0.149068746	0.106668045	-1.397501437	0.162262835	0.575677776	down_in_case
PRJNA8274(	ENSGALG00010011304	COMMD5	case	control	-0.1429227	0.066685022	-2.143250416	0.032092996	0.3098361	down_in_case
PRJNA8274(	ENSGALG00010011306	ECI2	case	control	-0.173169131	0.111104537	-1.558614397	0.119087673	0.516645008	down_in_case
PRJNA8274(	ENSGALG00010011307	ENSGALG00010011307	case	control	0.301844513	0.428371668	0.704632297	0.481039094	0.8322127	up_in_case
PRJNA8274(	ENSGALG00010011311	DOCK8	case	control	-0.026441677	0.145705176	-0.181473834	0.85599567	0.970727256	down_in_case
PRJNA8274(	ENSGALG00010011312	VPS13B	case	control	-0.135637618	0.051169575	-2.650747432	0.008031388	0.157462784	down_in_case
PRJNA8274(	ENSGALG00010011313	RASSF9	case	control	-0.046204235	0.225593378	-0.204812021	0.837718995	0.964826648	down_in_case
PRJNA8274(	ENSGALG00010011316	PARPBP	case	control	-0.076004836	0.178041919	-0.426892926	0.669457318	0.913446904	down_in_case
PRJNA8274(	ENSGALG00010011317	BLK	case	control	0.053557985	0.533584217	0.100374006	0.920047406	0.984638357	up_in_case
PRJNA8274(	ENSGALG00010011318	PRP4K	case	control	-0.004815959	0.085453107	-0.056357921	0.955056678	0.992056045	down_in_case
PRJNA8274(	ENSGALG00010011321	MAP2	case	control	0.001482985	0.11401853	0.013006529	0.989622584	0.997323462	up_in_case
PRJNA8274(	ENSGALG00010011322	GATA4	case	control	0.310438126	1.235790068	0.251206199	0.801654692	0.954294696	up_in_case
PRJNA8274(	ENSGALG00010011326	COX6C	case	control	-0.143090861	0.064949166	-2.203120827	0.027586234	0.295714499	down_in_case
PRJNA8274(	ENSGALG00010011329	PCDHGC5	case	control	0.037082261	0.062003341	0.598068768	0.549794049	0.864342007	up_in_case
PRJNA8274(	ENSGALG00010011331	PMCH	case	control	0.030864483	0.300735356	0.102630043	0.918256598	0.9836007	up_in_case
PRJNA8274(	ENSGALG00010011333	ENSGALG00010011333	case	control	-0.201237352	0.121264081	-1.659496781	0.097015729	0.485418097	down_in_case
PRJNA8274(	ENSGALG00010011335	VCP	case	control	0.062731924	0.172067922	0.364576516	0.715427547	0.926862121	up_in_case
PRJNA8274(	ENSGALG00010011339	ENSGALG00010011339	case	control	-1.511276888	0.331286247	-4.561846149	5.07058E-06	0.000993278	down_in_case
PRJNA8274(	ENSGALG00010011340	PKNOX1	case	control	0.09260876	0.068738511	1.347261658	0.177895976	0.597302761	up_in_case
PRJNA8274(	ENSGALG00010011342	RASAL2	case	control	-0.036008326	0.085011037	-0.423572362	0.671877715	0.913676733	down_in_case
PRJNA8274(	ENSGALG00010011343	PXDC1	case	control	0.051490624	0.063086525	0.816190521	0.414391177	0.798853486	up_in_case
PRJNA8274(	ENSGALG00010011345	CRMP1	case	control	0.129007492	0.165570337	0.779170319	0.435879391	0.81000376	up_in_case
PRJNA8274(	ENSGALG00010011347	RGS22	case	control	0.096988974	0.367180833	0.264144981	0.791668205	0.95239351	up_in_case
PRJNA8274(	ENSGALG00010011348	SLC22A23	case	control	0.46268709	0.135062216	3.42573299	0.000613143	0.036533092	up_in_case
PRJNA8274(	ENSGALG00010011349	ENSGALG00010011349	case	control	-0.01236634	0.063652125	-0.194280076	0.845956585	0.967563913	down_in_case
PRJNA8274(	ENSGALG00010011351	KANK1	case	control	-0.008344832	0.094786233	-0.088038437	0.929846127	0.987215058	down_in_case
PRJNA8274(	ENSGALG00010011352	NTS	case	control	-0.699467779	0.528533313	-1.323412853	0.18569812	0.608776505	down_in_case
PRJNA8274(	ENSGALG00010011353	ENSGALG00010011353	case	control	-0.163644172	0.252127675	-0.649052794	0.516304253	0.848054666	down_in_case
PRJNA8274(	ENSGALG00010011357	UNC80	case	control	-0.186029706	0.285544055	-0.651492135	0.514728852	0.847493921	down_in_case
PRJNA8274(	ENSGALG00010011359	RNF19A	case	control	-0.052062286	0.058801436	-0.885391403	0.375945568	0.774921593	down_in_case
PRJNA8274(	ENSGALG00010011360	TCF4	case	control	0.061075878	0.207166861	0.294814903	0.768135281	0.945214226	up_in_case
PRJNA8274(	ENSGALG00010011361	NUP37	case	control	0.022626331	0.062002623	0.364925387	0.715167103	0.926862121	up_in_case
PRJNA8274(	ENSGALG00010011363	FDFT1	case	control	0.002591577	0.115779972	0.022383641	0.98214193	0.996591771	up_in_case
PRJNA8274(	ENSGALG00010011365	PSMG4	case	control	-0.521734192	0.134538011	-3.877968685	0.000105332	0.011055819	down_in_case
PRJNA8274(	ENSGALG00010011367	IER3IP1	case	control	0.022916982	0.088975692	0.257564529	0.796743008	0.95280257	up_in_case
PRJNA8274(	ENSGALG00010011368	ENSGALG00010011368	case	control	0.442819988	0.27104415	1.633755931	0.102310109	0.491460867	up_in_case
PRJNA8274(	ENSGALG00010011370	MGAT4C	case	control	-0.194702134	1.242107334	-0.156751456	0.875440732	0.975666505	down_in_case
PRJNA8274(	ENSGALG00010011371	ENSGALG00010011371	case	control	-0.084072	0.215893905	-0.389413494	0.696970292	0.922031058	down_in_case
PRJNA8274(	ENSGALG00010011372	VMA21	case	control	-0.00838888	0.042258062	-0.198515493	0.842641765	0.966519616	down_in_case
PRJNA8274(	ENSGALG00010011374	ENSGALG00010011374	case	control	0.102354701	0.084243927	1.214980181	0.22437365	0.648105158	up_in_case
PRJNA8274(	ENSGALG00010011375	C12orf50	case	control	-0.224465038	0.129006226	-1.739955076	0.081866907	0.454736389	down_in_case
PRJNA8274(	ENSGALG00010011377	SEC16B	case	control	0.173099813	0.268785354	0.644007609	0.5195705	0.849320777	up_in_case
PRJNA8274(	ENSGALG00010011378	TUBB2B	case	control	-0.085337709	0.198100191	-0.430780547	0.666627945	0.912284937	down_in_case
PRJNA8274(	ENSGALG00010011379	CELF4	case	control	-0.187384653	0.593279453	-0.315845512	0.752119771	0.940427752	down_in_case
PRJNA8274(	ENSGALG00010011381	RLIG1	case	control	0.152942744	0.089419112	1.710403297	0.087191322	0.467042649	up_in_case
PRJNA8274(	ENSGALG00010011382	ENSGALG00010011382	case	control	-0.185990598	0.189925932	-0.979279638	0.327441829	0.745061912	down_in_case
PRJNA8274(	ENSGALG00010011384	PUM3	case	control	-0.111233293	0.109941527	-1.011749573	0.311657806	0.734218555	down_in_case
PRJNA8274(	ENSGALG00010011386	CTSB	case	control	-0.001104172	0.081157391	-0.013605321	0.989144859	0.997164011	down_in_case
PRJNA8274(	ENSGALG00010011388	GPR50	case	control	0.082027752	0.147693249	0.555392701	0.578626084	0.879034633	up_in_case
PRJNA8274(	ENSGALG00010011389	RC3H1	case	control	-0.139259552	0.068989734	-2.018554684	0.043533525	0.348491091	down_in_case
PRJNA8274(	ENSGALG00010011394	SERPINC1	case	control	1.056119783	0.832374464	1.268803679	0.204511093	0.627307727	up_in_case
PRJNA8274(	ENSGALG00010011398	ENSGALG00010011398	case	control	0.311815195	0.152423571	2.045715071	0.040784412	0.337767404	up_in_case
PRJNA8274(	ENSGALG00010011399	JAKMIP1	case	control	0.249953307	0.273845215	0.912753971	0.361371952	0.767557265	up_in_case
PRJNA8274(	ENSGALG00010011400	BPHL	case	control	0.145541771	0.255819858	0.568922884	0.569408475	0.875057036	up_in_case
PRJNA8274(	ENSGALG00010011401	ANKRD46	case	control	0.037640759	0.039719058	0.94767501	0.343294927	0.755132664	up_in_case
PRJNA8274(	ENSGALG00010011403	SV2C	case	control	0.17596702	0.409609159	0.429597377	0.667488546	0.912432583	up_in_case
PRJNA8274(	ENSGALG00010011406	UBE2R2	case	control	-0.025106695	0.123975754	-0.202512944	0.83951574	0.965286179	down_in_case
PRJNA8274(	ENSGALG00010011407	CEP290	case	control	0.166439016	0.102409351	1.625232597	0.104113007	0.493794905	up_in_case
PRJNA8274(	ENSGALG00010011411	ENSGALG00010011411	case	control	-0.528584586	0.208886763	-2.530483875	0.011390532	0.186780127	down_in_case
PRJNA8274(	ENSGALG00010011412	KANSL1L	case	control	0.02481454	0.058540401	0.423887417	0.671647921	0.913676733	up_in_case
PRJNA8274(	ENSGALG00010011413	ZBTB37	case	control	0.028754439	0.098362284	0.292331959	0.770032826	0.945428692	up_in_case
PRJNA8274(	ENSGALG00010011414	PABPC1	case	control	-0.009302334	0.054290234	-0.171344521	0.863952875	0.97295831	down_in_case
PRJNA8274(	ENSGALG00010011416	ENSGALG00010011416	case	control	0.667463125	0.423115336	1.577496887	0.114681238	0.509615196	up_in_case
PRJNA8274(	ENSGALG00010011417	WFS1	case	control	0.226105163	0.078064166	2.896401457	0.003774692	0.105649139	up_in_case
PRJNA8274(	ENSGALG00010011418	RIPK1	case	control	0.011179575	0.071591454	0.156157948	0.875908523	0.975666505	up_in_case
PRJNA8274(	ENSGALG00010011419	RNF180	case	control	-0.026178371	0.135778635	-0.192801842	0.847114161	0.968026275	down_in_case
PRJNA8274(	ENSGALG00010011420	HMGB3	case	control	0.02429984	0.038970201	0.62354927	0.532923635	0.855817434	up_in_case
PRJNA8274(	ENSGALG00010011421	ENSGALG00010011421	case	control	-0.02589626	0.060849802	-0.425576743	0.67041629	0.913465866	down_in_case
PRJNA8274(	ENSGALG00010011422	KATNAL2	case	control	0.574390307	0.24873471	2.309248707	0.020929784	0.259355211	up_in_case
PRJNA8274(	ENSGALG00010011423	GDF6	case	control	0.157614737	0.422753549	0.372828891	0.709275799	0.925521953	up_in_case
PRJNA8274(	ENSGALG00010011424	ACADL	case	control	-0.109564638	0.076164958	-1.43851767	0.150287228	0.557485695	down_in_case
PRJNA8274(	ENSGALG00010011425	WASHC3	case	control	-0.039809984	0.049643192	-0.801922323	0.422597893	0.803289893	down_in_case
PRJNA8274(	ENSGALG00010011426	NQO2	case	control	-0.061488702	0.102844776	-0.59787871	0.549920867	0.864351329	down_in_case
PRJNA8274(	ENSGALG00010011427	TMTC3	case	control	-0.099924045	0.038764534	-2.577718231	0.009945506	0.177161817	down_in_case
PRJNA8274(	ENSGALG00010011428	UQCRB	case	control	-0.075718038	0.085370579	-0.886933642	0.375114628	0.774681835	down_in_case
PRJNA8274(	ENSGALG00010011429	UBAP2	case	control	-0.09216091	0.158372063	-0.581926563	0.560616148	0.870162912	down_in_case
PRJNA8274(	ENSGALG00010011430	ENSGALG00010011430	case	control	-0.442786041	0.244022758	-1.814527649	0.06959653	0.425209175	down_in_case
PRJNA8274(	ENSGALG00010011431	DRAM1	case	control	0.616782069	0.265338264	2.324512343	0.020098051	0.254401597	up_in_case
PRJNA8274(	ENSGALG00010011433	RNASE6	case	control	0.283910501	0.240684213	1.179597523	0.238160329	0.662973078	up_in_case
PRJNA8274(	ENSGALG00010011434	NPL	case	control	0.038233726	0.08873727	0.430864348	0.666567008	0.912284937	up_in_case
PRJNA8274(	ENSGALG00010011436	UBAP2L2	case	control	-0.456403968	1.657893357	-0.275291512			down_in_case
PRJNA8274(	ENSGALG00010011437	DMRT1	case	control	-0.140208713	0.432741988	-0.324000715	0.745937493	0.938117773	down_in_case
PRJNA8274(	ENSGALG00010011438	ENSGALG00010011438	case	control	0.326015561	0.128945051	2.528329383	0.011460678	0.187514518	up_in_case
PRJNA8274(	ENSGALG00010011439	ENSGALG00010011439	case	control	-0.636872879	0.344388296	-1.849287236	0.064416348	0.411965016	down_in_case
PRJNA8274(	ENSGALG00010011440	FUNDC1	case	control	-0.038707372	0.071573159	-0.540808492	0.588639589	0.882805047	down_in_case
PRJNA8274(	ENSGALG00010011441	CD99L2	case	control	-0.01602955	0.082186273	-0.195039256	0.845362216	0.967517103	down_in_case
PRJNA8274(	ENSGALG00010011442	ENSGALG00010011442	case	control	-0.285624153	1.692366237	-0.16877207	0.865975928	0.973289701	down_in_case
PRJNA8274(	ENSGALG00010011443	ENSGALG00010011443	case	control	0.049813018	0.353309498	0.14098975	0.88787804	0.977944694	up_in_case
PRJNA8274(	ENSGALG00010011445	MTERF3	case	control	-0.171853977	0.105525931	-1.628547366	0.103408874	0.492970761	down_in_case
PRJNA8274(	ENSGALG00010011446	ENSGALG00010011446	case	control	-0.317977759	0.200283956	-1.587634702	0.112368967	0.506149199	down_in_case
PRJNA8274(	ENSGALG00010011447	LAMC1	case	control	-0.236821947	0.113082148	-2.094246987	0.036237984	0.326119051	down_in_case
PRJNA8274(	ENSGALG00010011448	AvBD10	case	control	1.494430316	1.544338279	0.96768327	0.333202576	0.748508541	up_in_case
PRJNA8274(	ENSGALG00010011449	DUSP22	case	control	-0.041971286	0.080929858	-0.518613114	0.604030562	0.885268908	down_in_case
PRJNA8274(	ENSGALG00010011450	PTDSS1	case	control	-0.103729007	0.062382529	-1.662789397	0.0963546	0.483634708	down_in_case
PRJNA8274(	ENSGALG00010011451	CRHBP	case	control	0.133399025	0.298873251	0.446339794	0.655351816	0.907302833	up_in_case
PRJNA8274(	ENSGALG00010011452	KITLG	case	control	0.329070638	0.123309655	2.668652662	0.007615616	0.152061299	up_in_case
PRJNA8274(	ENSGALG00010011453	GNPTAB	case	control	0.021234577	0.071161427	0.298400107	0.765397808	0.943786458	up_in_case
PRJNA8274(	ENSGALG00010011456	KIAA1328	case	control	0.123948739	0.136670118	0.906919088	0.364449586	0.770152588	up_in_case
PRJNA8274(	ENSGALG00010011457	ENSGALG00010011457	case	control	-0.250724535	0.301826267	-0.830691566	0.406147892	0.7941993	down_in_case
PRJNA8274(	ENSGALG00010011458	TPGS2	case	control	0.148473901	0.192815876	0.77002944	0.44128243	0.813083204	up_in_case
PRJNA8274(	ENSGALG00010011459	ENSGALG00010011459	case	control	0.631085146	0.205394429	3.072552397	0.002122366	0.075874574	up_in_case
PRJNA8274(	ENSGALG00010011460	LAMC2	case	control	-0.257021193	0.412274221	-0.623422905	0.53300665	0.855817434	down_in_case
PRJNA8274(	ENSGALG00010011461	IRF4	case	control	0.12128994	0.277801242	0.436606902	0.662396456	0.910707559	up_in_case
PRJNA8274(	ENSGALG00010011463	IMP3	case	control	0.196603611	0.105930993	1.855959291	0.063459361	0.409507608	up_in_case
PRJNA8274(	ENSGALG00010011464	FANCG	case	control	0.091596592	0.187310446	0.489009522	0.624834955	0.894741574	up_in_case
PRJNA8274(	ENSGALG00010011465	PPP2R2C	case	control	0.061079916	0.21500387	0.28408752	0.77634331	0.947228003	up_in_case
PRJNA8274(	ENSGALG00010011466	MTMR1	case	control	-0.097400505	0.043220255	-2.253584701	0.024222307	0.275122308	down_in_case
PRJNA8274(	ENSGALG00010011467	ENSGALG00010011467	case	control	-0.200812247	1.617745975	-0.12413089			down_in_case
PRJNA8274(	ENSGALG00010011468	EFHC2	case	control	-0.011237293	0.208749113	-0.053831575	0.957069353	0.992056045	down_in_case
PRJNA8274(	ENSGALG00010011469	CPS1	case	control	0.065782552	0.197785189	0.332595946	0.739439304	0.936330652	up_in_case
PRJNA8274(	ENSGALG00010011471	NMNAT2	case	control	-0.460227767	0.32467658	-1.417496044	0.15633795	0.566211345	down_in_case
PRJNA8274(	ENSGALG00010011475	NDP	case	control	0.508264506	0.255572933	1.988725878	0.046731468	0.356907825	up_in_case
PRJNA8274(	ENSGALG00010011477	SDC2	case	control	0.002632457	0.074181297	0.035486805	0.971691568	0.993081005	up_in_case
PRJNA8274(	ENSGALG00010011478	HDAC3	case	control	-0.124404245	0.053868154	-2.309420994	0.020920231	0.259355211	down_in_case

PRJNA8274(	ENSGALG00010011480	BLTP3B	case	control	0.000824312	0.078825648	0.010457406	0.99165635	0.99790247	up_in_case
PRJNA8274(	ENSGALG00010011482	DMRT2	case	control	-0.143915823	0.109928551	-1.309176026	0.190474731	0.613935676	down_in_case
PRJNA8274(	ENSGALG00010011484	CHPT1	case	control	-0.190089925	0.059630814	-3.18778016	0.001433695	0.060122691	down_in_case
PRJNA8274(	ENSGALG00010011485	MTM1	case	control	0.107498926	0.066272483	1.622074826	0.104787328	0.494770427	up_in_case
PRJNA8274(	ENSGALG00010011487	MAN2B2	case	control	0.195606631	0.107383393	1.821572455	0.068519884	0.422606297	up_in_case
PRJNA8274(	ENSGALG00010011493	ENSGALG00010011493	case	control	-0.140260957	0.510833051	-0.274572988	0.783644322	0.949518053	down_in_case
PRJNA8274(	ENSGALG00010011496	ACTR6	case	control	0.075180722	0.060916171	1.234166907	0.217140716	0.642491319	up_in_case
PRJNA8274(	ENSGALG00010011497	CPQ	case	control	-0.069023349	0.100566395	-0.68634606	0.49249491	0.837671284	down_in_case
PRJNA8274(	ENSGALG00010011499	SMG7	case	control	-0.043980922	0.055536868	-0.791922972	0.428405591	0.805337183	down_in_case
PRJNA8274(	ENSGALG00010011503	NCF2	case	control	0.089309405	0.343913372	0.259685761	0.795106177	0.95280257	up_in_case
PRJNA8274(	ENSGALG00010011505	MYL1	case	control	-0.327512172	0.121895295	-2.686831935	0.007213324	0.147992151	down_in_case
PRJNA8274(	ENSGALG00010011509	EXOC2	case	control	-0.012448755	0.049646223	-0.250749292	0.802007948	0.954294696	down_in_case
PRJNA8274(	ENSGALG00010011510	ENSGALG00010011510	case	control	-0.627244875	0.484969421	-1.293369948	0.195883139	0.619095929	down_in_case
PRJNA8274(	ENSGALG00010011515	SERINC5	case	control	0.011815004	0.134859623	0.087609648	0.930186934	0.987258715	up_in_case
PRJNA8274(	ENSGALG00010011521	ENSGALG00010011521	case	control	-0.98634328	1.019796393	-0.967196282	0.33344592	0.748508541	down_in_case
PRJNA8274(	ENSGALG00010011523	PIGO	case	control	-0.010904958	0.084029793	-0.129774897	0.896744524	0.980176778	down_in_case
PRJNA8274(	ENSGALG00010011525	ENSGALG00010011525	case	control	0.279581331	0.226805642	1.232691253	0.217690976	0.642912219	up_in_case
PRJNA8274(	ENSGALG00010011529	SCYL2	case	control	0.044865374	0.064421078	0.69643936	0.486153721	0.834635018	up_in_case
PRJNA8274(	ENSGALG00010011530	ARPC5	case	control	0.05008678	0.04889037	1.024471279	0.30561273	0.728862914	up_in_case
PRJNA8274(	ENSGALG00010011531	MTDH	case	control	-0.10338005	0.047068575	-2.196370943	0.028065404	0.29905758	down_in_case
PRJNA8274(	ENSGALG00010011533	ENSGALG00010011533	case	control	-0.663443441	0.195999878	-3.384917633	0.000711996	0.039927628	down_in_case
PRJNA8274(	ENSGALG00010011536	FCHSD1	case	control	0.300722823	0.178258622	1.687002959	0.091602783	0.476713154	up_in_case
PRJNA8274(	ENSGALG00010011538	ENSGALG00010011538	case	control	-0.234846032	0.189617091	-1.238527766	0.215520433	0.640470741	down_in_case
PRJNA8274(	ENSGALG00010011539	LANCL1	case	control	-0.081741824	0.059673629	-1.369814869	0.170744699	0.5880658	down_in_case
PRJNA8274(	ENSGALG00010011544	STOML2	case	control	0.072002537	0.073367682	0.9813931	0.326398927	0.744287835	up_in_case
PRJNA8274(	ENSGALG00010011545	USP12-like	case	control	0.030705524	0.04604425	0.666869899	0.50485524	0.842675288	up_in_case
PRJNA8274(	ENSGALG00010011546	MAOA	case	control	0.016950849	0.09200061	0.184247136	0.853819583	0.969998446	up_in_case
PRJNA8274(	ENSGALG00010011549	KIAA0232	case	control	0.014916692	0.055808065	0.267285605	0.78924927	0.951843754	up_in_case
PRJNA8274(	ENSGALG00010011551	GMDS	case	control	0.045871795	0.077197122	0.594216381	0.552367398	0.866007432	up_in_case
PRJNA8274(	ENSGALG00010011555	ENSGALG00010011555	case	control	-0.08497375	1.670749181	-0.050859669	0.959437344	0.992056045	down_in_case
PRJNA8274(	ENSGALG00010011556	LAPTM4B	case	control	-0.104634136	0.119726498	-0.873943014	0.382149289	0.77998936	down_in_case
PRJNA8274(	ENSGALG00010011558	MYBPC1	case	control	-0.281040137	0.116556422	-2.411193929	0.015900391	0.223714308	down_in_case
PRJNA8274(	ENSGALG00010011559	MTX3	case	control	0.270351519	0.092219474	2.931609849	0.003372101	0.099555609	up_in_case
PRJNA8274(	ENSGALG00010011562	ENSGALG00010011562	case	control	0.059309992	0.482210418	0.122996081	0.902110201	0.980763983	up_in_case
PRJNA8274(	ENSGALG00010011564	ARAP3	case	control	0.01177655	0.073521856	0.160177532	0.872741228	0.975062028	up_in_case
PRJNA8274(	ENSGALG00010011566	NR1H4	case	control	2.225090504	1.139419934	1.952827432	0.050840047	0.368480827	up_in_case
PRJNA8274(	ENSGALG00010011570	RGL1	case	control	-0.026711669	0.065464964	-0.408029991	0.683251657	0.915968569	down_in_case
PRJNA8274(	ENSGALG00010011571	ATOSB	case	control	0.176575906	0.112368966	1.571393888	0.116091185	0.513328368	up_in_case
PRJNA8274(	ENSGALG00010011572	PTCD2	case	control	-0.046209301	0.1413117	-0.327002654	0.743665878	0.937448285	down_in_case
PRJNA8274(	ENSGALG00010011573	CMAS	case	control	0.096072032	0.109012653	0.881292487	0.378159531	0.776556765	up_in_case
PRJNA8274(	ENSGALG00010011574	GAS2L3	case	control	0.177777923	0.094372165	1.883796184	0.05959255	0.397773519	up_in_case
PRJNA8274(	ENSGALG00010011575	ENSGALG00010011575	case	control	0.088214394	0.333930924	0.264169586	0.791649246	0.95239351	up_in_case
PRJNA8274(	ENSGALG00010011577	ENSGALG00010011577	case	control	0.071089709	0.16215508	0.438405686	0.661092226	0.909646511	up_in_case
PRJNA8274(	ENSGALG00010011578	MYLK4	case	control	0.261994368	0.187713686	1.39571266	0.16280104	0.576616758	up_in_case
PRJNA8274(	ENSGALG00010011584	ENSGALG00010011584	case	control	-0.342972763	0.756065925	-0.453628118	0.650096512	0.904241824	down_in_case
PRJNA8274(	ENSGALG00010011586	ENSGALG00010011586	case	control	0.010342063	0.200898968	0.051478927	0.958943893	0.992056045	up_in_case
PRJNA8274(	ENSGALG00010011588	COLGALT2	case	control	0.314673738	0.120617555	2.608855224	0.009084567	0.170020898	up_in_case
PRJNA8274(	ENSGALG00010011589	RERG	case	control	-0.040685063	0.128958732	-0.315489014	0.752390391	0.940427752	down_in_case
PRJNA8274(	ENSGALG00010011593	WRNIP1	case	control	0.066552256	0.083617136	0.795916477	0.426080583	0.805094126	up_in_case
PRJNA8274(	ENSGALG00010011595	ENSGALG00010011595	case	control	0.185047205	0.174652459	1.059516746	0.289364507	0.712911094	up_in_case
PRJNA8274(	ENSGALG00010011597	TBC1D14	case	control	0.021595484	0.056232994	0.384035823	0.700951897	0.922516526	up_in_case
PRJNA8274(	ENSGALG00010011603	ENSGALG00010011603	case	control	-0.440415699	0.44200739	-0.99639895	0.319056343	0.73861962	down_in_case
PRJNA8274(	ENSGALG00010011605	APPL2	case	control	-0.002397859	0.069479435	-0.034511782	0.972469048	0.99340857	down_in_case
PRJNA8274(	ENSGALG00010011607	TSEN15	case	control	-0.171430146	0.114142016	-1.501902213	0.133122365	0.536390482	down_in_case
PRJNA8274(	ENSGALG00010011609	ENSGALG00010011609	case	control	-0.059118649	0.385195071	-0.153477168	0.878021994	0.976160112	down_in_case
PRJNA8274(	ENSGALG00010011612	ENSGALG00010011612	case	control	-0.414115552	0.35363653	-1.17102029	0.241590622	0.666243691	down_in_case
PRJNA8274(	ENSGALG00010011617	MAMLD1	case	control	-0.117941753	0.055781258	-2.114361657	0.03448439	0.320609886	down_in_case
PRJNA8274(	ENSGALG00010011619	PCDH1	case	control	0.000194093	0.079702216	0.002435223	0.998056975	0.999244889	up_in_case
PRJNA8274(	ENSGALG00010011621	WASHC4	case	control	0.080101192	0.060178091	1.331069001	0.183166308	0.60491829	up_in_case
PRJNA8274(	ENSGALG00010011622	BACH1	case	control	0.00799765	0.059715676	0.133928824	0.893458857	0.979888889	up_in_case
PRJNA8274(	ENSGALG00010011625	ERBB4	case	control	-0.199237689	0.132329279	-1.5056206	0.132164597	0.535549374	down_in_case
PRJNA8274(	ENSGALG00010011626	SERPINB1	case	control	0.128034603	0.560925812	0.228255858	0.819447339	0.958669267	up_in_case
PRJNA8274(	ENSGALG00010011628	MATN2	case	control	-0.129856373	0.121260028	-1.070891821	0.284218074	0.708901676	down_in_case
PRJNA8274(	ENSGALG00010011630	DELE1	case	control	-0.170512898	0.084814237	-2.010427794	0.044385931	0.350320113	down_in_case
PRJNA8274(	ENSGALG00010011633	TADA2B	case	control	-0.107092614	0.102483719	-1.044971973	0.296035923	0.719662167	down_in_case
PRJNA8274(	ENSGALG00010011634	AVD	case	control	-0.267185814	0.440060402	-0.607157138	0.543746634	0.861081181	down_in_case
PRJNA8274(	ENSGALG00010011635	PCDH12	case	control	0.001654544	0.101148849	0.016357515	0.986949173	0.996781748	up_in_case
PRJNA8274(	ENSGALG00010011636	GRIK1	case	control	-0.536034402	0.37010577	-1.4483276	0.147525452	0.552833848	down_in_case
PRJNA8274(	ENSGALG00010011637	CECR2	case	control	0.218887623	0.072467001	3.020514428	0.002523457	0.082910904	up_in_case
PRJNA8274(	ENSGALG00010011638	GRPEL1	case	control	-0.102650148	0.052256664	-1.964345603	0.049490027	0.364797618	down_in_case
PRJNA8274(	ENSGALG00010011639	ENSGALG00010011639	case	control	0.354431909	0.240467768	1.473926888	0.140501277	0.54301845	up_in_case
PRJNA8274(	ENSGALG00010011641	ENSGALG00010011641	case	control	-0.0979911	0.224289841	-0.436894954	0.662187532	0.910595413	down_in_case
PRJNA8274(	ENSGALG00010011643	C1orf21	case	control	-0.337541484	0.114315772	-2.952711405	0.003149963	0.095433201	down_in_case
PRJNA8274(	ENSGALG00010011647	FBXO38	case	control	-0.017047066	0.049795776	-0.342339589	0.732095346	0.933770678	down_in_case
PRJNA8274(	ENSGALG00010011648	ENSGALG00010011648	case	control	-0.226535443	0.156778951	-1.444935315	0.148476056	0.55494187	down_in_case
PRJNA8274(	ENSGALG00010011649	MRPS27	case	control	0.336498121	0.153688603	2.189479998	0.028561971	0.300549329	up_in_case
PRJNA8274(	ENSGALG00010011650	RPL30	case	control	-0.033108219	0.077107811	-0.429375682	0.667649848	0.912432583	down_in_case
PRJNA8274(	ENSGALG00010011651	SERPINB6	case	control	0.127735701	0.077564134	1.64683977	0.099590984	0.488667622	up_in_case
PRJNA8274(	ENSGALG00010011653	NR2C1	case	control	0.098462126	0.074355163	1.324213701	0.185432082	0.608601967	up_in_case
PRJNA8274(	ENSGALG00010011654	CLDN8	case	control	-0.065206855	1.174079689	-0.055538696	0.955709302	0.992056045	down_in_case
PRJNA8274(	ENSGALG00010011655	SORCS2	case	control	0.02165865	0.119792857	0.180800847	0.856523899	0.971059279	up_in_case
PRJNA8274(	ENSGALG00010011657	ALDH1L2	case	control	0.055888779	0.101294712	0.551744294	0.581123558	0.879651411	up_in_case
PRJNA8274(	ENSGALG00010011658	TENM1	case	control	-0.131063411	0.346631017	-0.378106415	0.705351543	0.924182432	down_in_case
PRJNA8274(	ENSGALG00010011659	CD14	case	control	0.099408587	0.133233995	0.74612029	0.455594754	0.822065071	up_in_case
PRJNA8274(	ENSGALG00010011660	ENSGALG00010011660	case	control	-0.076687586	0.080260724	-0.955480875	0.339334572	0.751259351	down_in_case
PRJNA8274(	ENSGALG00010011662	RIDA	case	control	0.101763742	0.14846038	0.685460605	0.493053312	0.837887766	up_in_case
PRJNA8274(	ENSGALG00010011663	UTP20	case	control	-0.071562907	0.07678745	-0.931960979	0.351356692	0.760831854	down_in_case
PRJNA8274(	ENSGALG00010011664	SERPINB10B	case	control	-0.570566027	0.524500508	-1.087827406	0.276671308	0.702359305	down_in_case
PRJNA8274(	ENSGALG00010011667	ENSGALG00010011667	case	control	-0.930033783	0.34064222	-2.730236383	0.006328893	0.138384046	down_in_case
PRJNA8274(	ENSGALG00010011669	ENSGALG00010011669	case	control	0.830606805	0.322618241	2.574581038	0.010036157	0.178060855	up_in_case
PRJNA8274(	ENSGALG00010011670	TMCO6	case	control	-0.230280061	0.101517273	-2.268383046	0.02330587	0.269590124	down_in_case
PRJNA8274(	ENSGALG00010011671	SLC41A2	case	control	0.073586827	0.131301834	0.560440205	0.575179214	0.877910508	up_in_case
PRJNA8274(	ENSGALG00010011673	POP1	case	control	-0.165995508	0.127512568	-1.301797233	0.19298571	0.615324823	down_in_case
PRJNA8274(	ENSGALG00010011674	ENSGALG00010011674	case	control	0.020014594	0.537352167	0.037246699	0.970288304	0.992761697	up_in_case
PRJNA8274(	ENSGALG00010011677	EDEM3	case	control	0.0238568	0.053951842	0.442186937	0.658353938	0.908367791	up_in_case
PRJNA8274(	ENSGALG00010011679	OVAL	case	control	-0.081661888	0.756771086	-0.107908308	0.914068427	0.982854306	down_in_case
PRJNA8274(	ENSGALG00010011681	MAP1B	case	control	-0.30832359	0.115397725	-2.671834208	0.007543791	0.151682101	down_in_case
PRJNA8274(	ENSGALG00010011682	USP15	case	control	-0.120109843	0.067564657	-1.777702254	0.075452769	0.442021549	down_in_case
PRJNA8274(	ENSGALG00010011683	NIPAL2	case	control	-0.622808972	0.307126335	-2.027859226	0.042574618	0.344353525	down_in_case
PRJNA8274(	ENSGALG00010011685	NDUFA2	case	control	-0.076468642	0.097275843	-0.786101046	0.431808292	0.807276582	down_in_case
PRJNA8274(	ENSGALG00010011688	PDE3A	case	control	-0.075522603	0.116939377	-0.645826968	0.518391421	0.848941486	down_in_case
PRJNA8274(	ENSGALG00010011692	ARL1	case	control	0.0817851	0.044810817	1.825119595	0.067982984	0.421339236	up_in_case
PRJNA8274(	ENSGALG00010011696	ZFC3H1	case	control	0.014687122	0.087702906	0.16746449	0.867004586	0.973570415	up_in_case
PRJNA8274(	ENSGALG00010011699	TIAM1	case	control	0.068258152	0.081568792	0.836817002	0.402695439	0.792021921	up_in_case
PRJNA8274(	ENSGALG00010011702	NUP205	case	control	-0.127531935	0.055772449	-2.286647567	0.022216402	0.26563754	down_in_case
PRJNA8274(	ENSGALG00010011703	YWHAZ	case	control	0.03161701	0.03616684	0.874198842	0.382009977	0.77998936	up_in_case
PRJNA8274(	ENSGALG00010011708	IK	case	control	-0.049379355	0.062443164	-0.790788808	0.429067242	0.805896795	down_in_case
PRJNA8274(	ENSGALG00010011712	DCAF12L2	case	control	0.034903041	0.049136382	0.710329894	0.477499586	0.830520323	up_in_case
PRJNA8274(	ENSGALG00010011713	AVPR1A	case	control	-1.044225724	0.467483966	-2.233714521	0.025501863	0.28357437	down_in_case
PRJNA8274(	ENSGALG00010011715	SLCO1B3	case	control	0.423813338	1.469132487	0.288478637	0.772980381	0.94645256	up_in_case
PRJNA8274(	ENSGALG00010011720	IKZF2	case	control	-0.091012264	0.205913979	-0.441991673	0.658495232	0.908367791	down_in_case
PRJNA8274(	ENSGALG00010011723	SPRY3	case	control	0.00359224	0.109178222	0.032902528	0.973752317	0.993553916	up_in_case
PRJNA8274(	ENSGALG00010011724	ARHGEF28	case	control	-0.093238573	0.186017052	-0.5012367	0.616204549	0.891081782	down_in_case
PRJNA8274(	ENSGALG00010011726	WDR55	case	control	-0.068498481	0.090681185	-0.755376995	0.450022812	0.81905641	down_in_case
PRJNA8274(	ENSGALG00010011729	FAM129A	case	control	0.06503105	0.091182581	0.713195974	0.475724497	0.830520323	up_in_case
PRJNA8274(	ENSGALG00010011734	ENSGALG00010011734	case	control	0.519333635	0.243526626	2.132553816	0.032961345	0.31242754	up_in_case
PRJNA8274(	ENSGALG00010011738	FGD6	case	control	-0.157234769	0.073554289	-2.137669615	0.032543567	0.310662892	down_in_case
PRJNA8274(	ENSGALG00010011745	AFAP1L1	case	control	-0.065926484	0.091656485	-0.719277898	0.471969711	0.829235491	down_in_case
PRJNA8274(	ENSGALG00010011746	SOD1	case	control	0.039524371	0.063652887	0.620936023	0.534641719	0.856336982	up_in_case
PRJNA8274(	ENSGALG00010011747	UTP15	case	control	0.153029919	0.15716055	0.973717126	0.330197012	0.746414364	up_in_case
PRJNA8274(	ENSGALG00010011751	ZNF706	case	control	0.011751911	0.082482746	0.142477204	0.886703084	0.977630122	up_in_case
PRJNA8274(	ENSGALG00010011752	RNF2	case	control	-0.005011386	0.131770049	-0.038031301	0.969662725	0.992761697	down_in_case
PRJNA8274(	ENSGALG00010011758	SERPINB11	case	control	-0.019957972	0.472944044	-0.042199436	0.966339713	0.992505774	down_in_case
PRJNA8274(	ENSGALG00010011759	CREB3	case	control	-0.001909307	0.093389493	-0.020444563	0.983688735	0.996591771	down_in_case
PRJNA8274(	ENSGALG00010011764	PCYOX1L	case	control	-0.018231512	0.106477507	-0.171224067	0.864047585	0.97295831	down_in_case
PRJNA8274(	ENSGALG00010011770	CHST11	case	control	0.426029	0.410220472	1.038536663			up_in_case

PRJNA8274(	ENSGALG00010011772	GRHL2	case	control	0.174316267	0.430357954	0.405049483	0.685441138	0.916999558	up_in_case
PRJNA8274(	ENSGALG00010011774	ANKRA2	case	control	0.129991462	0.146448883	0.887623448	0.374743337	0.774509281	up_in_case
PRJNA8274(	ENSGALG00010011775	VEZT	case	control	-0.009940015	0.05396834	-0.18418233	0.853870421	0.969998446	down_in_case
PRJNA8274(	ENSGALG00010011776	IL17RA	case	control	-0.065237333	0.086659181	-0.752803482	0.451568014	0.820302668	down_in_case
PRJNA8274(	ENSGALG00010011780	SERPINB5	case	control	0.514151724	0.589662661	0.871942142	0.383239942	0.780061953	up_in_case
PRJNA8274(	ENSGALG00010011784	GRPEL2	case	control	-0.146805703	0.070250456	-2.089747325	0.036640503	0.327474499	down_in_case
PRJNA8274(	ENSGALG00010011789	TXNRD1	case	control	0.194945666	0.142678295	1.366330216	0.171835341	0.58957339	up_in_case
PRJNA8274(	ENSGALG00010011790	AMY2A	case	control	-0.988798044	0.349366251	-2.830262059	0.004650989	0.116274729	down_in_case
PRJNA8274(	ENSGALG00010011792	HDHD5	case	control	-0.027363639	0.100293314	-0.272836124	0.78497919	0.949518053	down_in_case
PRJNA8274(	ENSGALG00010011793	TLN1	case	control	-0.082504573	0.097154416	-0.849210735	0.395764041	0.787783337	down_in_case
PRJNA8274(	ENSGALG00010011796	METAP2	case	control	-0.014330251	0.047622611	-0.300912746	0.763481031	0.943786458	down_in_case
PRJNA8274(	ENSGALG00010011797	SCAF4	case	control	-0.04108105	0.040906248	-1.004273224	0.315246936	0.736508113	down_in_case
PRJNA8274(	ENSGALG00010011802	VPS4B	case	control	0.042956531	0.049581194	0.866387587	0.386277649	0.78229293	up_in_case
PRJNA8274(	ENSGALG00010011808	SMARCA2	case	control	-0.148288578	0.08553612	-1.733636956	0.082982519	0.456931082	down_in_case
PRJNA8274(	ENSGALG00010011810	FGFR4	case	control	-0.08371181	0.090120956	-0.928882846	0.352949804	0.761723844	down_in_case
PRJNA8274(	ENSGALG00010011814	ENSGALG00010011814	case	control	-0.522605722	0.332233807	-1.57300585	0.115717464	0.512785785	down_in_case
PRJNA8274(	ENSGALG00010011817	KDSR	case	control	0.100731106	0.053491815	1.883112508	0.059685123	0.397773519	up_in_case
PRJNA8274(	ENSGALG00010011819	USP44	case	control	0.031594555	0.081016449	0.389977048	0.696553519	0.921903481	up_in_case
PRJNA8274(	ENSGALG00010011823	ENSGALG00010011823	case	control	0.101816365	0.386679092	0.263309723	0.792311866	0.952773914	up_in_case
PRJNA8274(	ENSGALG00010011825	TAFA2	case	control	-0.13442354	0.413214949	-0.325311416	0.744945391	0.937849656	down_in_case
PRJNA8274(	ENSGALG00010011837	VLDLR	case	control	0.082047847	0.10735832	0.764243024	0.444722432	0.815952113	up_in_case
PRJNA8274(	ENSGALG00010011839	CBS	case	control	-0.270268875	0.14374997	-1.880131699	0.060090131	0.398649739	down_in_case
PRJNA8274(	ENSGALG00010011841	KCNMB4	case	control	-0.230450814	0.136630715	-1.686669161	0.091666985	0.476713154	down_in_case
PRJNA8274(	ENSGALG00010011843	BCL2	case	control	-0.182414377	0.093198056	-1.957276633	0.050314953	0.367061596	down_in_case
PRJNA8274(	ENSGALG00010011846	UBAP1	case	control	0.022332648	0.100797137	0.221560343	0.824656157	0.960673484	up_in_case
PRJNA8274(	ENSGALG00010011850	SPAG16	case	control	0.423769182	0.275646125	1.537366731	0.124203528	0.523029039	up_in_case
PRJNA8274(	ENSGALG00010011856	NFYB	case	control	0.024234374	0.071759452	0.337716817	0.735576601	0.934202199	up_in_case
PRJNA8274(	ENSGALG00010011857	ENSGALG00010011857	case	control	-0.146016301	0.471869219	-0.30944231	0.75698509	0.941659745	down_in_case
PRJNA8274(	ENSGALG00010011859	NTN4	case	control	0.01160739	0.120333487	0.096460181	0.923155098	0.985482967	up_in_case
PRJNA8274(	ENSGALG00010011860	CECR1	case	control	0.111176691	0.105311707	1.055691662	0.291109108	0.714222439	up_in_case
PRJNA8274(	ENSGALG00010011861	ENSGALG00010011861	case	control	-0.046747378	0.332645642	-0.140532062	0.888239622	0.977948171	down_in_case
PRJNA8274(	ENSGALG00010011864	ENSGALG00010011864	case	control	0.027440417	0.26285207	0.104394907	0.916855965	0.983445506	up_in_case
PRJNA8274(	ENSGALG00010011866	KLF8	case	control	-0.190556384	0.082496025	-2.309885644	0.020894486	0.259355211	down_in_case
PRJNA8274(	ENSGALG00010011867	ENSGALG00010011867	case	control	0.259441284	0.499589679	0.519308734	0.603545464	0.885268908	up_in_case
PRJNA8274(	ENSGALG00010011868	SNRPF	case	control	0.005573207	0.078140626	0.071322783	0.943140863	0.990069816	up_in_case
PRJNA8274(	ENSGALG00010011870	AvBD6	case	control	0.096978832	0.627109332	0.154644217	0.877101809	0.975813349	up_in_case
PRJNA8274(	ENSGALG00010011871	CCT2	case	control	0.012785682	0.055791015	0.229170995	0.818736016	0.958565992	up_in_case
PRJNA8274(	ENSGALG00010011873	RFX3	case	control	0.058399878	0.111347116	0.52448487	0.59994134	0.884888453	up_in_case
PRJNA8274(	ENSGALG00010011874	KCNS2	case	control	-0.090947258	0.265481285	-0.342575028	0.731918191	0.933770678	down_in_case
PRJNA8274(	ENSGALG00010011875	PSD2	case	control	-0.074269109	0.143841581	-0.516325727	0.605626931	0.88576746	down_in_case
PRJNA8274(	ENSGALG00010011876	AMDHD1	case	control	0.891170079	0.45017451	1.979610263	0.047747339	0.360119697	up_in_case
PRJNA8274(	ENSGALG00010011879	CYP2AC2	case	control	1.656261685	0.887773581	1.865635249	0.062092414	0.405537748	up_in_case
PRJNA8274(	ENSGALG00010011880	U2AF1	case	control	-0.027657526	0.095387806	-0.289948235	0.771855839	0.945804499	down_in_case
PRJNA8274(	ENSGALG00010011881	RIOK2	case	control	0.207139101	0.126999299	1.631025551	0.102884928	0.492370622	up_in_case
PRJNA8274(	ENSGALG00010011882	ENSGALG00010011882	case	control	-0.012594208	0.409233302	-0.030775131	0.975448874	0.99399062	down_in_case
PRJNA8274(	ENSGALG00010011883	HCFC2	case	control	0.14150738	0.112282683	1.260277862	0.207569143	0.631809014	up_in_case
PRJNA8274(	ENSGALG00010011885	NCALD	case	control	-0.085323266	0.127888711	-0.66716808	0.504664778	0.842675107	down_in_case
PRJNA8274(	ENSGALG00010011886	NTNG1	case	control	-0.31154034	0.129993188	-2.396589742	0.016548441	0.22864029	down_in_case
PRJNA8274(	ENSGALG00010011887	PGM5	case	control	-0.106988181	0.091841375	-1.164923559	0.244049936	0.668164293	down_in_case
PRJNA8274(	ENSGALG00010011888	CAND1	case	control	0.00557852	0.041442637	0.134608219	0.892921643	0.979726808	up_in_case
PRJNA8274(	ENSGALG00010011889	CRYAA	case	control	0.252757481	0.440828906	0.573368664	0.566395103	0.873067799	up_in_case
PRJNA8274(	ENSGALG00010011891	HAL	case	control	1.685230448	0.511117909	3.297146157	0.000976727	0.048048938	up_in_case
PRJNA8274(	ENSGALG00010011892	ENSGALG00010011892	case	control	0.540395428	0.491746602	1.098930681	0.271798303	0.698323327	up_in_case
PRJNA8274(	ENSGALG00010011893	LIX1	case	control	0.173136055	0.543287935	0.318681943	0.749967709	0.939759747	up_in_case
PRJNA8274(	ENSGALG00010011894	VWC2L	case	control	1.480940271	0.272877218	5.427130489	5.72673E-08	2.2133E-05	up_in_case
PRJNA8274(	ENSGALG00010011895	PHF21B	case	control	0.735570088	0.449171155	1.637616487	0.101501722	0.491460867	up_in_case
PRJNA8274(	ENSGALG00010011896	SIK1	case	control	-0.233073983	0.085831231	-2.715491545	0.006617747	0.140023533	down_in_case
PRJNA8274(	ENSGALG00010011898	ENSGALG00010011898	case	control	-0.071289617	0.068621047	-1.0388885	0.298856595	0.722266235	down_in_case
PRJNA8274(	ENSGALG00010011899	PHLPP1	case	control	0.022400342	0.057031902	0.39276863	0.694490376	0.920408932	up_in_case
PRJNA8274(	ENSGALG00010011902	TP53I3	case	control	0.555801683	0.176769532	3.144216523	0.001665322	0.065655176	up_in_case
PRJNA8274(	ENSGALG00010011903	LTA4H	case	control	-0.029072636	0.056087456	-0.518344712	0.604217782	0.885268908	down_in_case
PRJNA8274(	ENSGALG00010011904	KIF24	case	control	0.512761078	0.203032758	2.52550898	0.011553083	0.188192574	up_in_case
PRJNA8274(	ENSGALG00010011906	ENSGALG00010011906	case	control	-1.794250072	2.91186223	-0.616186457			down_in_case
PRJNA8274(	ENSGALG00010011907	BIK	case	control	-0.068747898	0.192689086	-0.356781485	0.721255398	0.929292592	down_in_case
PRJNA8274(	ENSGALG00010011908	ENSGALG00010011908	case	control	0.048489555	0.120846911	0.401247782	0.688237704	0.917479181	up_in_case
PRJNA8274(	ENSGALG00010011909	C8orf48	case	control	0.173945624	0.199222669	0.873121643	0.382596778	0.7800305	up_in_case
PRJNA8274(	ENSGALG00010011911	NUDT2	case	control	0.035650549	0.185278318	0.192416197	0.847416206	0.968194691	up_in_case
PRJNA8274(	ENSGALG00010011912	TCP11X2	case	control	0.070473117	0.070928577	0.993578612	0.320428061	0.738813491	up_in_case
PRJNA8274(	ENSGALG00010011913	CXXC5	case	control	0.120698536	0.066468474	1.815876435	0.069389329	0.425095479	up_in_case
PRJNA8274(	ENSGALG00010011915	RRM2B	case	control	-0.193719195	0.106171591	-1.824585967	0.068063533	0.421345678	down_in_case
PRJNA8274(	ENSGALG00010011916	MCAT	case	control	-0.135871062	0.08053048	-1.687200453	0.091564814	0.476713154	down_in_case
PRJNA8274(	ENSGALG00010011917	HSF2BP	case	control	-0.622184757	0.223540379	-2.783321565	0.005380545	0.127387074	down_in_case
PRJNA8274(	ENSGALG00010011921	MEGF10	case	control	0.159843336	0.119227821	1.340654681	0.180032595	0.600808892	up_in_case
PRJNA8274(	ENSGALG00010011922	PIK3R1	case	control	-0.100218738	0.096352988	-1.040120707	0.298283824	0.721487387	down_in_case
PRJNA8274(	ENSGALG00010011923	BARD1	case	control	-0.043645006	0.097036929	-0.449777278	0.652871043	0.905885495	down_in_case
PRJNA8274(	ENSGALG00010011925	RRP1B	case	control	0.062193549	0.057352076	1.084416696	0.278180084	0.703443892	up_in_case
PRJNA8274(	ENSGALG00010011926	DYRK2	case	control	-0.182970007	0.078727706	-2.32408659	0.020120854	0.254401597	down_in_case
PRJNA8274(	ENSGALG00010011927	ENSGALG00010011927	case	control	0.923865847	0.573123973	1.611982557	0.106965735	0.497919928	up_in_case
PRJNA8274(	ENSGALG00010011929	ELK3	case	control	0.013158142	0.065059759	0.202247011	0.839723623	0.965286179	up_in_case
PRJNA8274(	ENSGALG00010011930	TSPO	case	control	-0.003962738	0.094796849	-0.041802427	0.9666562	0.992616951	down_in_case
PRJNA8274(	ENSGALG00010011931	ENSGALG00010011931	case	control	-0.442503418	1.186341364	-0.37299839	0.709149643	0.925521953	down_in_case
PRJNA8274(	ENSGALG00010011932	VAV3	case	control	0.256130352	0.183519659	1.395656211	0.162818047	0.576616758	up_in_case
PRJNA8274(	ENSGALG00010011935	ST8SIA4	case	control	0.143584045	0.108355356	1.325121806	0.185130755	0.608031647	up_in_case
PRJNA8274(	ENSGALG00010011936	CKAP4	case	control	0.06673786	0.084555671	0.789277165	0.42995003	0.806015686	up_in_case
PRJNA8274(	ENSGALG00010011937	SLC1A1	case	control	-0.421456388	0.490662784	-0.858953241	0.390366314	0.783914628	down_in_case
PRJNA8274(	ENSGALG00010011941	ENSGALG00010011941	case	control	0.005384516	0.14612554	0.036848565	0.970605751	0.992761697	up_in_case
PRJNA8274(	ENSGALG00010011943	TTLL12	case	control	-0.042860983	0.055618079	-0.770630423	0.440926023	0.813083204	down_in_case
PRJNA8274(	ENSGALG00010011947	THAP1	case	control	-0.006797797	0.141908131	-0.047902801	0.961793707	0.992056045	down_in_case
PRJNA8274(	ENSGALG00010011949	ABCA12	case	control	0.424277799	0.365844073	1.159723036	0.246161588	0.670921661	up_in_case
PRJNA8274(	ENSGALG00010011950	PDXK	case	control	-0.155019246	0.126590094	-1.224576432	0.220734852	0.644744838	down_in_case
PRJNA8274(	ENSGALG00010011952	UBE2D2	case	control	-0.008998605	0.067484686	-0.133342916	0.893922189	0.979888889	down_in_case
PRJNA8274(	ENSGALG00010011953	CDK17	case	control	-0.079250658	0.05004011	-1.583742664	0.113252292	0.507878574	down_in_case
PRJNA8274(	ENSGALG00010011956	ENSGALG00010011956	case	control	-0.039209551	0.516581756	-0.075901927	0.939497124	0.989551456	down_in_case
PRJNA8274(	ENSGALG00010011957	ENSGALG00010011957	case	control	-0.239189472	0.111603428	-2.143209014	0.032096319	0.3098361	down_in_case
PRJNA8274(	ENSGALG00010011960	CFAP54	case	control	0.056790656	0.224596786	0.252856051	0.800379452	0.954053991	up_in_case
PRJNA8274(	ENSGALG00010011961	AGPAT3	case	control	0.020334708	0.080702385	0.251971587	0.801063025	0.954294696	up_in_case
PRJNA8274(	ENSGALG00010011962	CPSF6	case	control	-0.039266553	0.048467204	-0.810167496	0.417843916	0.800243236	down_in_case
PRJNA8274(	ENSGALG00010011963	ZCCHC2	case	control	0.015919005	0.050040916	0.318119773	0.750394086	0.939972946	up_in_case
PRJNA8274(	ENSGALG00010011964	ENSGALG00010011964	case	control	-0.036883989	1.819180218	-0.02027506	0.983823951	0.996591771	down_in_case
PRJNA8274(	ENSGALG00010011965	ATIC	case	control	0.15502109	0.121113016	1.27997052	0.200555504	0.623059681	up_in_case
PRJNA8274(	ENSGALG00010011966	SLC25A24	case	control	0.243840452	0.063843921	3.819321348	0.000133819	0.013289004	up_in_case
PRJNA8274(	ENSGALG00010011967	MTMR9	case	control	-0.181025505	0.054908732	-3.296843668	0.000977779	0.048048938	down_in_case
PRJNA8274(	ENSGALG00010011968	ENSGALG00010011968	case	control	-0.208524255	0.264196578	-0.789276898	0.429950186	0.806015686	down_in_case
PRJNA8274(	ENSGALG00010011969	TMEM173	case	control	0.021577633	0.186330839	0.1158028	0.907808831	0.981730793	up_in_case
PRJNA8274(	ENSGALG00010011971	RNF170	case	control	-0.100994314	0.080127652	-1.260417736	0.207518707	0.631809014	down_in_case
PRJNA8274(	ENSGALG00010011972	IPO11	case	control	0.095857918	0.14534345	0.659526925	0.509557462	0.84453775	up_in_case
PRJNA8274(	ENSGALG00010011973	TRAPPC10	case	control	-0.07795932	0.053464392	-1.458154046	0.144798087	0.548818979	down_in_case
PRJNA8274(	ENSGALG00010011976	TNFRSF11A	case	control	0.029132349	0.090912719	0.320443053	0.748632493	0.939503405	up_in_case
PRJNA8274(	ENSGALG00010011977	GATD3A	case	control	0.043539321	0.05568515	0.781883878	0.434282821	0.809152357	up_in_case
PRJNA8274(	ENSGALG00010011978	ENSGALG00010011978	case	control	-0.337299067	1.909630784	-0.176630514	0.859798624	0.971828322	down_in_case
PRJNA8274(	ENSGALG00010011979	NEDD1	case	control	0.096833155	0.05641036	1.716584614	0.086055096	0.463278022	up_in_case
PRJNA8274(	ENSGALG00010011980	AvBD9	case	control	1.747992378	1.717865846	1.017537185	0.308897952	0.731823162	up_in_case
PRJNA8274(	ENSGALG00010011981	SCUBE1	case	control	-0.011535472	0.342457856	-0.033684356	0.973128854	0.99354775	down_in_case
PRJNA8274(	ENSGALG00010011982	STK3	case	control	-0.124211617	0.05994911	-2.071950991	0.038270012	0.331271895	down_in_case
PRJNA8274(	ENSGALG00010011983	LYZ	case	control	0.507963621	0.520675192	0.975586372	0.329269481	0.745732273	up_in_case
PRJNA8274(	ENSGALG00010011984	FN1	case	control	-0.347240122	0.139155975	-2.495330315	0.012583993	0.197314796	down_in_case
PRJNA8274(	ENSGALG00010011988	HINTW	case	control	-0.214482752	1.506322053	-0.142388377	0.886773243	0.977630122	down_in_case
PRJNA8274(	ENSGALG00010011989	H1-010	case	control	-0.141612943	0.171550986	-0.825486036	0.409095707	0.795170398	down_in_case
PRJNA8274(	ENSGALG00010011991	CSNK1A1	case	control	-0.066653508	0.03916796	-1.701735476	0.088804967	0.470938334	down_in_case
PRJNA8274(	ENSGALG00010011992	YEATS4	case	control	-0.130158403	0.072026499	-1.807090525	0.070748166	0.4302334	down_in_case
PRJNA8274(	ENSGALG00010011993	RELCH	case	control	-0.098714946	0.058989029	-1.67344585	0.094239558	0.479477093	down_in_case
PRJNA8274(	ENSGALG00010011994	TSPAN8	case	control	-0.279314265	0.738082093	-0.378432518	0.705109315	0.924182432	down_in_case
PRJNA8274(	ENSGALG00010011996	LRRC70	case	control	0.208560769	0.21585786	0.966194927	0.333946651	0.748508541	up_in_case
PRJNA8274(	ENSGALG00010011997	DENND5B	case	control	-0.232927515	0.108098223	-2.154776548	0.031179324	0.3098361	down_in_case
PRJNA8274(	ENSGALG00010011998	MPPED1	case	control	-0.020409556	0.101105947	-0.201863063	0.840023779	0.965286179	down_in_case
PRJNA8274(	ENSGALG00010012001	EEIG2	case	control	0.053306538	0.152155177	0.350343244	0.726081115	0.932223791	up_in_case

PRJNA8274(	ENSGALG00010012004	LGR5	case	control	-0.015096997	0.186322115	-0.08102633	0.935421013	0.988425668	down_in_case
PRJNA8274(	ENSGALG00010012007	MREG	case	control	-0.102813843	0.162300023	-0.633480149	0.52642014	0.85251736	down_in_case
PRJNA8274(	ENSGALG00010012008	ZNF366	case	control	0.015291738	0.120827857	0.126558048	0.899290202	0.98028579	up_in_case
PRJNA8274(	ENSGALG00010012010	HENMT1	case	control	-0.017785314	0.183736634	-0.096797867	0.922886918	0.985482967	down_in_case
PRJNA8274(	ENSGALG00010012011	HOOK3	case	control	-0.177140605	0.077868402	-2.27487145	0.022913642	0.267253668	down_in_case
PRJNA8274(	ENSGALG00010012013	FOXD1	case	control	-0.656274776	0.444343486	-1.476953747	0.139688036	0.541778931	down_in_case
PRJNA8274(	ENSGALG00010012015	ENSGALG00010012015	case	control	-0.364000357	0.242542146	-1.500771568	0.133414655	0.53711418	down_in_case
PRJNA8274(	ENSGALG00010012016	WDR91	case	control	-0.04132082	0.052024702	-0.794253863	0.427047657	0.805305429	down_in_case
PRJNA8274(	ENSGALG00010012017	N4BP3	case	control	-0.037893123	0.060649892	-0.62478467	0.532112393	0.855755691	down_in_case
PRJNA8274(	ENSGALG00010012019	ENSGALG00010012019	case	control	0.037612162	0.338159462	0.111226111	0.911437046	0.982477366	up_in_case
PRJNA8274(	ENSGALG00010012021	ENSGALG00010012021	case	control	-0.008509275	0.063893308	-0.133179445	0.894051467	0.979888889	down_in_case
PRJNA8274(	ENSGALG00010012024	UBR5	case	control	-0.007917257	0.046062585	-0.171880432	0.86353153	0.972890026	down_in_case
PRJNA8274(	ENSGALG00010012026	SYT1	case	control	0.635641772	0.216836849	2.931428738	0.003374068	0.099555609	up_in_case
PRJNA8274(	ENSGALG00010012028	TMEM140	case	control	-0.142714086	0.097123569	-1.469407351	0.141722335	0.543501954	down_in_case
PRJNA8274(	ENSGALG00010012031	GRK6	case	control	-0.019309447	0.06722797	-0.28722341	0.773941263	0.946671454	down_in_case
PRJNA8274(	ENSGALG00010012034	ARHGEF9	case	control	-0.078794607	0.071882599	-1.096156902	0.273010117	0.698936152	down_in_case
PRJNA8274(	ENSGALG00010012040	B4GALT7	case	control	0.1991056	0.072604557	2.742329265	0.006100516	0.135266583	up_in_case
PRJNA8274(	ENSGALG00010012041	ICOSLG	case	control	0.167473888	0.110588032	1.514394334	0.129925856	0.532363328	up_in_case
PRJNA8274(	ENSGALG00010012042	FNDC7	case	control	-0.231741	0.298320534	-0.776818803	0.437265682	0.810720016	down_in_case
PRJNA8274(	ENSGALG00010012043	NUP50	case	control	-0.002679744	0.062543247	-0.042846253	0.965824093	0.992340816	down_in_case
PRJNA8274(	ENSGALG00010012045	ENSGALG00010012045	case	control	-0.372657248	0.4211411	-0.884875041	0.376224031	0.775113975	down_in_case
PRJNA8274(	ENSGALG00010012046	LOXHD1	case	control	-0.124152194	0.237002637	-0.52384309	0.60038768	0.884954589	down_in_case
PRJNA8274(	ENSGALG00010012047	LMAN2	case	control	0.026464454	0.035004894	0.756021533	0.449636285	0.81887403	up_in_case
PRJNA8274(	ENSGALG00010012048	PDE6H	case	control	-0.329679151	0.356105045	-0.925791857	0.35455416	0.763161644	down_in_case
PRJNA8274(	ENSGALG00010012049	IFNAR1	case	control	0.074782544	0.074926827	0.998074343	0.31824331	0.738134246	up_in_case
PRJNA8274(	ENSGALG00010012051	ARK2C	case	control	-0.036102396	0.180765456	-0.199719552	0.841699922	0.966150978	down_in_case
PRJNA8274(	ENSGALG00010012052	KLF10	case	control	-0.100008311	0.087317892	-1.145335838	0.252070066	0.677148133	down_in_case
PRJNA8274(	ENSGALG00010012054	FAM60A	case	control	0.029558968	0.057530158	0.513799526	0.607392159	0.887328057	up_in_case
PRJNA8274(	ENSGALG00010012055	ENSGALG00010012055	case	control	-0.116136321	0.090807896	-1.278923158	0.200924117	0.623527532	down_in_case
PRJNA8274(	ENSGALG00010012058	ENSGALG00010012058	case	control	-0.048385315	0.318731159	-0.151806039	0.879339919	0.976318821	down_in_case
PRJNA8274(	ENSGALG00010012059	ARHGDIB	case	control	-0.00609434	0.081250681	-0.075006629	0.940209438	0.989551456	down_in_case
PRJNA8274(	ENSGALG00010012060	ENSGALG00010012060	case	control	0.034006683	0.178703747	0.190296418	0.84907686	0.968833396	up_in_case
PRJNA8274(	ENSGALG00010012061	AZIN1	case	control	-0.06984608	0.051768601	-1.349197741	0.17727346	0.5959216	down_in_case
PRJNA8274(	ENSGALG00010012062	HMGA2	case	control	-0.000143538	0.092785273	-0.001546993	0.998765678	0.999604507	down_in_case
PRJNA8274(	ENSGALG00010012063	PTPRD	case	control	-0.057443178	0.086012527	-0.667846645	0.504231488	0.842675107	down_in_case
PRJNA8274(	ENSGALG00010012064	RGS14	case	control	0.144083943	0.119256096	1.208189328	0.226974446	0.650578187	up_in_case
PRJNA8274(	ENSGALG00010012066	STXBP3	case	control	0.26072851	0.112406306	2.319518534	0.020366937	0.256379581	up_in_case
PRJNA8274(	ENSGALG00010012067	ENSGALG00010012067	case	control	0.004650592	0.128063807	0.03631465	0.971031468	0.992761697	up_in_case
PRJNA8274(	ENSGALG00010012070	DNAJC28	case	control	-0.430966107	0.12214758	-3.528241066	0.000418331	0.028760256	down_in_case
PRJNA8274(	ENSGALG00010012071	ENSGALG00010012071	case	control	0.081037868	0.094985494	0.853160462	0.393570326	0.786843919	up_in_case
PRJNA8274(	ENSGALG00010012072	CAPRIN2	case	control	0.115005927	0.095870796	1.199592898	0.230297486	0.654892316	up_in_case
PRJNA8274(	ENSGALG00010012074	KIAA0930	case	control	-0.002282377	0.081103953	-0.028141377	0.977549393	0.994661197	down_in_case
PRJNA8274(	ENSGALG00010012075	PIGN	case	control	0.008542321	0.108618956	0.078644845	0.937315117	0.988831145	up_in_case
PRJNA8274(	ENSGALG00010012077	IFNGR2	case	control	-0.038528424	0.039433196	-0.977055576	0.328541639	0.745445783	down_in_case
PRJNA8274(	ENSGALG00010012078	TMBIM4	case	control	0.08383592	0.068269894	1.22800718	0.219444273	0.643443819	up_in_case
PRJNA8274(	ENSGALG00010012079	ENSGALG00010012079	case	control	-0.553120449	0.261103463	-2.118395685	0.034141571	0.318892531	down_in_case
PRJNA8274(	ENSGALG00010012081	CHIC1	case	control	0.029234684	0.088930254	0.328737215	0.742354322	0.937444457	up_in_case
PRJNA8274(	ENSGALG00010012082	HAUS1	case	control	0.309211065	0.187011086	1.653437083	0.098241941	0.487629211	up_in_case
PRJNA8274(	ENSGALG00010012083	MGP	case	control	0.193702568	0.22418937	0.864013169	0.387580656	0.782604263	up_in_case
PRJNA8274(	ENSGALG00010012084	TMEM50B	case	control	-0.04267805	0.059127892	-0.721792185	0.470422255	0.829101076	down_in_case
PRJNA8274(	ENSGALG00010012085	IPO8	case	control	0.105074618	0.046328062	2.268055551	0.023325821	0.269590124	up_in_case
PRJNA8274(	ENSGALG00010012086	CIBAR1	case	control	-0.023529191	0.074920268	-0.314056418	0.75347819	0.940858857	down_in_case
PRJNA8274(	ENSGALG00010012087	TMEM171	case	control	0.253665371	0.151582304	1.673449765	0.094238788	0.479477093	up_in_case
PRJNA8274(	ENSGALG00010012088	ATP5F1AZ	case	control	-0.067516574	0.221010947	-0.305489725	0.759993194	0.942675708	down_in_case
PRJNA8274(	ENSGALG00010012089	ENSGALG00010012089	case	control	-0.099059907	0.178227259	-0.555806715	0.578342994	0.878884678	down_in_case
PRJNA8274(	ENSGALG00010012090	HELB	case	control	0.081206621	0.084282105	0.963509643	0.335291827	0.749356776	up_in_case
PRJNA8274(	ENSGALG00010012091	ENSGALG00010012091	case	control	-1.076307603	0.713945104	-1.507549525	0.13166986	0.535159035	down_in_case
PRJNA8274(	ENSGALG00010012092	OC3	case	control	0.457116925	0.537639611	0.850229252	0.395197641	0.787092796	up_in_case
PRJNA8274(	ENSGALG00010012093	ENSGALG00010012093	case	control	-0.04493553	0.041546689	-1.081567071	0.279444943	0.705021645	down_in_case
PRJNA8274(	ENSGALG00010012094	ENSGALG00010012094	case	control	0.176645717	0.318990551	0.553764731	0.579739868	0.879142433	up_in_case
PRJNA8274(	ENSGALG00010012095	RNF38	case	control	0.008098316	0.104632735	0.077397533	0.938307303	0.989367879	up_in_case
PRJNA8274(	ENSGALG00010012096	ENSGALG00010012096	case	control	0.067026096	0.084143591	0.796568039	0.425701946	0.804804048	up_in_case
PRJNA8274(	ENSGALG00010012097	GART	case	control	0.28488004	0.144436108	1.972360259	0.048568495	0.363399983	up_in_case
PRJNA8274(	ENSGALG00010012098	ART4	case	control	-0.441466067	0.40943457	-1.078233495	0.280929568	0.705762606	down_in_case
PRJNA8274(	ENSGALG00010012099	UPK3A	case	control	-0.463365355	0.348396886	-1.329992815	0.183520639	0.605106095	down_in_case
PRJNA8274(	ENSGALG00010012100	UBE2F	case	control	-0.05087242	0.078950842	-0.644355629	0.519344851	0.8492533	down_in_case
PRJNA8274(	ENSGALG00010012101	ENSGALG00010012101	case	control	0.027339436	0.058831146	0.464710237	0.642138976	0.901756591	up_in_case
PRJNA8274(	ENSGALG00010012102	SON	case	control	-0.042582383	0.043123708	-0.987447155	0.323423477	0.740800303	down_in_case
PRJNA8274(	ENSGALG00010012103	ENSGALG00010012103	case	control	-0.190735314	0.129825352	-1.469168476	0.141787098	0.543501954	down_in_case
PRJNA8274(	ENSGALG00010012105	DONSON	case	control	-0.031602071	0.080691345	-0.391641398	0.695323195	0.920915226	down_in_case
PRJNA8274(	ENSGALG00010012106	TMTC1	case	control	0.154071957	0.112042937	1.375115292	0.169095704	0.585038804	up_in_case
PRJNA8274(	ENSGALG00010012107	CLCC1	case	control	-0.192778544	0.081674292	-2.360333224	0.018258526	0.241560424	down_in_case
PRJNA8274(	ENSGALG00010012108	PSTPIP2	case	control	0.250504112	0.29943499	0.83658931	0.402823457	0.792021921	up_in_case
PRJNA8274(	ENSGALG00010012110	ENSGALG00010012110	case	control	-0.02012682	0.091529107	-0.219895293	0.825952703	0.961207256	down_in_case
PRJNA8274(	ENSGALG00010012111	DRD1	case	control	-0.775787397	0.304381502	-2.548733715	0.010811482	0.184728043	down_in_case
PRJNA8274(	ENSGALG00010012112	MBD2	case	control	-0.308503528	0.219027054	-1.408517906	0.158977769	0.571481535	down_in_case
PRJNA8274(	ENSGALG00010012113	WDR47	case	control	0.082763554	0.053397921	1.549939635	0.121156005	0.519499513	up_in_case
PRJNA8274(	ENSGALG00010012114	TAL2	case	control	-0.483197819	0.376866333	-1.282146416	0.199791289	0.622902456	down_in_case
PRJNA8274(	ENSGALG00010012116	RBM12B	case	control	-0.020396202	0.04881197	-0.417852467	0.676054987	0.914247368	down_in_case
PRJNA8274(	ENSGALG00010012118	ENSGALG00010012118	case	control	0.871724583	0.527477942	1.652627559	0.098406686	0.487941198	up_in_case
PRJNA8274(	ENSGALG00010012119	EPG5	case	control	-0.043819129	0.114774247	-0.381785378	0.702620566	0.923481074	down_in_case
PRJNA8274(	ENSGALG00010012120	ENSGALG00010012120	case	control	-0.024824903	0.118239188	-0.209954949	0.833702835	0.963000851	down_in_case
PRJNA8274(	ENSGALG00010012121	SREK1	case	control	0.16926534	0.11512521	1.470271711	0.141488179	0.543501954	up_in_case
PRJNA8274(	ENSGALG00010012122	GRIP1	case	control	-0.055085793	0.130285215	-0.422809244	0.672434443	0.913676733	down_in_case
PRJNA8274(	ENSGALG00010012123	ENSGALG00010012123	case	control	0.12496538	0.158322338	0.78930985	0.429930931	0.806015686	up_in_case
PRJNA8274(	ENSGALG00010012124	ERAP1	case	control	0.058386038	0.193812379	0.301250304	0.763223633	0.943786458	up_in_case
PRJNA8274(	ENSGALG00010012125	RNF152	case	control	-0.092293073	0.100318069	-0.920004485	0.357570416	0.763851675	down_in_case
PRJNA8274(	ENSGALG00010012127	ENSGALG00010012127	case	control	-0.06010011	0.056152778	-1.070296295	0.284485961	0.709107417	down_in_case
PRJNA8274(	ENSGALG00010012129	CRYZL1	case	control	0.181094173	0.104149131	1.738796765	0.082070518	0.455552638	up_in_case
PRJNA8274(	ENSGALG00010012130	TMEM67	case	control	0.040564345	0.080803709	0.502010929	0.615659833	0.891081782	up_in_case
PRJNA8274(	ENSGALG00010012131	SLC14A1	case	control	-0.216306778	0.432684084	-0.4999185	0.617132465	0.891081782	down_in_case
PRJNA8274(	ENSGALG00010012132	MC4R	case	control	-0.279497558	0.804873692	-0.347256421	0.728398687	0.933057319	down_in_case
PRJNA8274(	ENSGALG00010012133	RAB3IP	case	control	0.303271507	0.146618248	2.068443121	0.038598374	0.333106064	up_in_case
PRJNA8274(	ENSGALG00010012135	CAMSAP2	case	control	0.03215725	0.057974439	0.554679795	0.579113699	0.879142433	up_in_case
PRJNA8274(	ENSGALG00010012136	ENSGALG00010012136	case	control	-0.045697757	0.173768526	-0.26298063	0.792565509	0.95280257	down_in_case
PRJNA8274(	ENSGALG00010012138	DDX11	case	control	0.196944089	0.119802495	1.643906398	0.100195538	0.489677444	up_in_case
PRJNA8274(	ENSGALG00010012139	FOXQ1	case	control	-0.261581535	0.79182366	-0.33035327	0.741133047	0.937158859	down_in_case
PRJNA8274(	ENSGALG00010012140	GLOD5	case	control	-0.88519762	0.373249773	-2.371595867	0.017711451	0.236704443	down_in_case
PRJNA8274(	ENSGALG00010012141	ENSGALG00010012141	case	control	0.053621289	0.148477972	0.361139689	0.717995025	0.927322135	up_in_case
PRJNA8274(	ENSGALG00010012144	H1-01	case	control	-0.143832609	0.104150216	-1.381011143	0.167275534	0.583424425	down_in_case
PRJNA8274(	ENSGALG00010012147	ARFGAP3	case	control	0.16283462	0.074207158	2.194324977	0.028212055	0.299591493	up_in_case
PRJNA8274(	ENSGALG00010012148	SETBP1	case	control	-0.146089396	0.167631214	-0.871492795	0.383485138	0.780061953	down_in_case
PRJNA8274(	ENSGALG00010012149	SFXN1	case	control	0.058675225	0.081406284	0.720770223	0.471050899	0.829101076	up_in_case
PRJNA8274(	ENSGALG00010012154	CWC27	case	control	-0.004318918	0.200049264	-0.021589273	0.98277559	0.996591771	down_in_case
PRJNA8274(	ENSGALG00010012156	ENSGALG00010012156	case	control	0.040817176	0.503240842	0.081108632	0.935355561	0.988425668	up_in_case
PRJNA8274(	ENSGALG00010012157	H2BC32	case	control	-0.882375011	0.371030435	-2.378174209	0.017398604	0.234759681	down_in_case
PRJNA8274(	ENSGALG00010012159	WASHC1	case	control	-0.116110832	0.073536261	-1.578960241	0.114345177	0.509446069	down_in_case
PRJNA8274(	ENSGALG00010012160	SYT4	case	control	-0.84177126	0.368832782	-2.282257164	0.022474162	0.266705822	down_in_case
PRJNA8274(	ENSGALG00010012163	PDP1	case	control	0.115507326	0.070030229	1.64939238	0.099067275	0.488407564	up_in_case
PRJNA8274(	ENSGALG00010012168	ENSGALG00010012168	case	control	0.744804353	0.407444922	1.827987816	0.06755138	0.42089154	up_in_case
PRJNA8274(	ENSGALG00010012171	CDH17	case	control	0.258909529	0.261978602	0.988285023	0.323013078	0.740689923	up_in_case
PRJNA8274(	ENSGALG00010012172	CNOT2	case	control	0.012016381	0.044185047	0.271955836	0.785655977	0.949929982	up_in_case
PRJNA8274(	ENSGALG00010012175	PACSIN2	case	control	0.025495196	0.071653819	0.355810702	0.721982334	0.929484914	up_in_case
PRJNA8274(	ENSGALG00010012179	ENSGALG00010012179	case	control	-0.059259242	0.209210605	-0.283251615	0.776983965	0.947379834	down_in_case
PRJNA8274(	ENSGALG00010012180	DDX59	case	control	-0.104304583	0.085386387	-1.221559852	0.221874119	0.645834386	down_in_case
PRJNA8274(	ENSGALG00010012186	KIF20A	case	control	-0.124992026	0.100108951	-1.248559944	0.211826071	0.636501957	down_in_case
PRJNA8274(	ENSGALG00010012187	ENSGALG00010012187	case	control	0.376412503	0.434064355	0.867181327	0.385842666	0.78196572	up_in_case
PRJNA8274(	ENSGALG00010012188	ADAMTS6	case	control	0.082773042	0.196524871	0.421183547	0.673621058	0.91379937	up_in_case
PRJNA8274(	ENSGALG00010012190	CAST	case	control	-0.058183119	0.065437881	-0.889135135	0.373930458	0.774369566	down_in_case
PRJNA8274(	ENSGALG00010012191	BEST3	case	control	0.319896819	0.197629393	1.618670246	0.105518232	0.495266487	up_in_case
PRJNA8274(	ENSGALG00010012193	TTLL1	case	control	0.073294628	0.224311128	0.326754309	0.74385372	0.937448285	up_in_case
PRJNA8274(	ENSGALG00010012194	LRRFIP1	case	control	-0.075392086	0.078531497	-0.960023537	0.337043369	0.750735231	down_in_case
PRJNA8274(	ENSGALG00010012195	ITSN1	case	control	-0.160778272	0.070679386	-2.274754811	0.022920642	0.267253668	down_in_case
PRJNA8274(	ENSGALG00010012196	CENPK	case	control	0.062941649	0.169268622	0.371844752	0.710008441	0.925630832	up_in_case
PRJNA8274(	ENSGALG00010012198	TMEM5	case	control	-0.170482067	0.096906717	-1.759238905	0.078536939	0.448818031	down_in_case
PRJNA8274(	ENSGALG00010012199	ENSGALG00010012199	case	control	-0.231055715	0.496215289	-0.465636025	0.641476052	0.901539099	down_in_case

PRJNA8274(	ENSGALG00010012200	GEM	case	control	-0.187303465	0.113359908	-1.652290204	0.098475405	0.487941198	down_in_case
PRJNA8274(	ENSGALG00010012205	SLC6A13	case	control	0.461397151	0.231141583	1.996166785	0.045915771	0.355878337	up_in_case
PRJNA8274(	ENSGALG00010012206	RIBC2	case	control	0.413300223	0.530821529	0.778604861	0.436212515	0.81000376	up_in_case
PRJNA8274(	ENSGALG00010012207	GRIA3	case	control	0.006915287	0.121393407	0.056965917	0.954572346	0.992056045	up_in_case
PRJNA8274(	ENSGALG00010012209	KIF14	case	control	-0.023377641	0.100274074	-0.23313744	0.815654683	0.957374769	down_in_case
PRJNA8274(	ENSGALG00010012210	PPWD1	case	control	0.003474702	0.15522426	0.022385042	0.982140812	0.996591771	up_in_case
PRJNA8274(	ENSGALG00010012211	ENSGALG00010012211	case	control	-0.202567121	1.607516039	-0.126012504	0.899722027	0.98028579	down_in_case
PRJNA8274(	ENSGALG00010012212	RAD54B	case	control	-0.025560386	0.110020282	-0.232324314	0.816286129	0.957422215	down_in_case
PRJNA8274(	ENSGALG00010012215	RAB17	case	control	-1.245162907	0.606230864	-2.053941793	0.039981326	0.336547209	down_in_case
PRJNA8274(	ENSGALG00010012216	BRD8	case	control	-0.044262099	0.045721635	-0.968077778	0.333005527	0.748503677	down_in_case
PRJNA8274(	ENSGALG00010012219	KDM5A	case	control	-0.053334174	0.031464565	-1.695055157	0.090064958	0.473572975	down_in_case
PRJNA8274(	ENSGALG00010012226	SLCO4C1	case	control	0.262249015	0.523553301	0.500902228	0.616439935	0.891081782	up_in_case
PRJNA8274(	ENSGALG00010012228	NHP2	case	control	0.241291029	0.111993969	2.154500192	0.031200967	0.3098361	up_in_case
PRJNA8274(	ENSGALG00010012229	FSBP	case	control	-0.066279726	0.097801937	-0.677693394	0.497966116	0.839532593	down_in_case
PRJNA8274(	ENSGALG00010012230	PTPRQ	case	control	-0.232688165	0.137798003	-1.688617836	0.09129269	0.476454552	down_in_case
PRJNA8274(	ENSGALG00010012232	PCSK1	case	control	0.244488981	0.249806978	0.978711576	0.327722511	0.745416241	up_in_case
PRJNA8274(	ENSGALG00010012234	C1GALT1C1	case	control	0.150048936	0.053903101	2.783679108	0.005374618	0.127387074	up_in_case
PRJNA8274(	ENSGALG00010012240	TRIM14	case	control	-0.070868315	0.229434159	-0.308883016	0.757410517	0.941659745	down_in_case
PRJNA8274(	ENSGALG00010012242	CCDC77	case	control	-0.02686898	0.084920401	-0.31640195	0.751697437	0.940427752	down_in_case
PRJNA8274(	ENSGALG00010012243	ENSGALG00010012243	case	control	-0.051808872	1.414905406	-0.036616492	0.970790794	0.992761697	down_in_case
PRJNA8274(	ENSGALG00010012244	ENSGALG00010012244	case	control	-0.048280687	0.044256607	-1.090926085	0.275305408	0.700894634	down_in_case
PRJNA8274(	ENSGALG00010012245	ENSGALG00010012245	case	control	-0.027198027	0.054319002	-0.500709259	0.616575755	0.891081782	down_in_case
PRJNA8274(	ENSGALG00010012247	TRIM23	case	control	-0.023335398	0.136359169	-0.171131857	0.864120087	0.97295831	down_in_case
PRJNA8274(	ENSGALG00010012248	H2AC39	case	control	-0.036953922	0.233226781	-0.158446305	0.874105129	0.975062028	down_in_case
PRJNA8274(	ENSGALG00010012250	NANS	case	control	0.348455836	0.104623648	3.330564782	0.0008667	0.044742993	up_in_case
PRJNA8274(	ENSGALG00010012253	MYF6	case	control	-0.149916884	0.104818521	-1.430251861	0.152644746	0.561193576	down_in_case
PRJNA8274(	ENSGALG00010012259	IL1RAPL2	case	control	-0.08101059	0.214178204	-0.378239188	0.705252916	0.924182432	down_in_case
PRJNA8274(	ENSGALG00010012260	ENSGALG00010012260	case	control	0.655022761	0.386070469	1.696640418	0.089764664	0.472887198	up_in_case
PRJNA8274(	ENSGALG00010012263	SHLD3	case	control	0.274794635	0.199049085	1.380537043	0.167421352	0.583641076	up_in_case
PRJNA8274(	ENSGALG00010012264	MYF5	case	control	-0.047277533	0.097341911	-0.485685271	0.627190328	0.895838586	down_in_case
PRJNA8274(	ENSGALG00010012268	ENSGALG00010012268	case	control	-0.005659425	0.226099445	-0.025030691	0.980030483	0.995636523	down_in_case
PRJNA8274(	ENSGALG00010012271	ENSGALG00010012271	case	control	-0.000593368	0.166402082	-0.003565871	0.997154852	0.998921977	down_in_case
PRJNA8274(	ENSGALG00010012274	B4GALNT3	case	control	0.171386451	0.057989746	2.955461343	0.003122018	0.095164759	up_in_case
PRJNA8274(	ENSGALG00010012275	VIRMA	case	control	-0.091854707	0.04245437	-2.163610157	0.030494281	0.307572163	down_in_case
PRJNA8274(	ENSGALG00010012277	H3C33	case	control	-0.444945153	0.207384812	-2.145505008	0.031912491	0.3098361	down_in_case
PRJNA8274(	ENSGALG00010012282	ENSGALG00010012282	case	control	-0.381127455	1.023856346	-0.372247001	0.709708954	0.925630832	down_in_case
PRJNA8274(	ENSGALG00010012283	CUPIN1A	case	control	0.920674042	0.512446345	1.796625247	0.072395133	0.434979163	up_in_case
PRJNA8274(	ENSGALG00010012285	FBLN1	case	control	0.004154556	0.091280128	0.045514351	0.963697336	0.992068146	up_in_case
PRJNA8274(	ENSGALG00010012286	ENSGALG00010012286	case	control	-0.294344975	1.806444913	-0.162941573	0.870564438	0.974794532	down_in_case
PRJNA8274(	ENSGALG00010012288	MCTS1	case	control	-0.055657671	0.056992947	-0.976571199	0.328781483	0.745453498	down_in_case
PRJNA8274(	ENSGALG00010012290	ENSGALG00010012290	case	control	0.082320577	0.091332443	0.901328973	0.367413433	0.771770041	up_in_case
PRJNA8274(	ENSGALG00010012291	NR5A2	case	control	0.101969092	0.102447168	0.995333442	0.319574118	0.738813491	up_in_case
PRJNA8274(	ENSGALG00010012293	SRGAP1	case	control	-0.136739297	0.055323244	-2.471642801	0.013449382	0.203519755	down_in_case
PRJNA8274(	ENSGALG00010012294	ENSGALG00010012294	case	control	0.202671896	0.572575766	0.353965202	0.723364962	0.930215097	up_in_case
PRJNA8274(	ENSGALG00010012295	CUL4B	case	control	-0.028391975	0.043284951	-0.655931774	0.511868023	0.845834144	down_in_case
PRJNA8274(	ENSGALG00010012297	ABLIM3	case	control	0.042533669	0.098988018	0.429685027	0.667424776	0.912432583	up_in_case
PRJNA8274(	ENSGALG00010012300	NINJ2	case	control	0.049723657	0.193589651	0.256850802	0.797293951	0.95280257	up_in_case
PRJNA8274(	ENSGALG00010012303	CA9	case	control	-0.082340208	0.131390645	-0.626682427	0.530867425	0.855560567	down_in_case
PRJNA8274(	ENSGALG00010012304	ESRP1	case	control	0.541345455	0.68159844	0.794229304	0.427061952	0.805305429	up_in_case
PRJNA8274(	ENSGALG00010012307	ENSGALG00010012307	case	control	-0.057203416	0.174008458	-0.328739287	0.742352757	0.937444457	down_in_case
PRJNA8274(	ENSGALG00010012308	TRABD2A	case	control	-0.251516486	0.248951304	-1.010303952	0.312349688	0.734364975	down_in_case
PRJNA8274(	ENSGALG00010012309	TRAPPC13	case	control	0.06234129	0.167836052	0.371441588	0.710308655	0.925630832	up_in_case
PRJNA8274(	ENSGALG00010012311	ENSGALG00010012311	case	control	4.812685233	0.345482057	13.93034788	4.14332E-44	1.48124E-40	up_in_case
PRJNA8274(	ENSGALG00010012312	SH3TC2	case	control	0.320239708	0.141553575	2.262321581	0.023677542	0.272177533	up_in_case
PRJNA8274(	ENSGALG00010012314	ADRB2	case	control	0.174453485	0.223746253	0.779693436	0.435571344	0.81000376	up_in_case
PRJNA8274(	ENSGALG00010012315	ATP6V1E1	case	control	0.07741613	0.067789961	1.141999918	0.253454054	0.678954688	up_in_case
PRJNA8274(	ENSGALG00010012316	ENSGALG00010012316	case	control	1.042163508	0.715347797	1.456862678	0.14515429	0.549384008	up_in_case
PRJNA8274(	ENSGALG00010012318	POLR1D	case	control	0.039099034	0.129179114	0.30267303	0.762139053	0.943297504	up_in_case
PRJNA8274(	ENSGALG00010012320	DPY19L4	case	control	-0.03544746	0.057444771	-0.617070264	0.537188382	0.857491196	down_in_case
PRJNA8274(	ENSGALG00010012322	ENSGALG00010012322	case	control	0.376397675	0.155400928	2.422107003	0.015430806	0.220440088	up_in_case
PRJNA8274(	ENSGALG00010012323	SMAD7B	case	control	-0.444134847	1.6140505	-0.275167875	0.783187266	0.949518053	down_in_case
PRJNA8274(	ENSGALG00010012324	ENSGALG00010012324	case	control	-0.067641731	0.369549062	-0.183038567	0.854767758	0.970313652	down_in_case
PRJNA8274(	ENSGALG00010012325	MLPH	case	control	5.122146748	0.563422673	9.091126424	9.80182E-20	1.0782E-16	up_in_case
PRJNA8274(	ENSGALG00010012326	SGTB	case	control	0.076990466	0.07690569	1.001102338	0.316777335	0.737372905	up_in_case
PRJNA8274(	ENSGALG00010012327	UBTD2	case	control	-0.044067726	0.054877082	-0.803026042	0.421959682	0.802594118	down_in_case
PRJNA8274(	ENSGALG00010012329	ATXN10	case	control	-0.056235804	0.088340705	-0.636578616	0.524399358	0.852356213	down_in_case
PRJNA8274(	ENSGALG00010012331	ENSGALG00010012331	case	control	-0.30783264	0.549732284	-0.559968278	0.575501075	0.877910508	down_in_case
PRJNA8274(	ENSGALG00010012332	ENSGALG00010012332	case	control	-1.252927528	0.698425225	-1.793932238	0.072823985	0.436712105	down_in_case
PRJNA8274(	ENSGALG00010012334	ARHGEF39	case	control	-0.25500049	0.245739202	-1.037687466	0.299415582	0.722840853	down_in_case
PRJNA8274(	ENSGALG00010012336	ENSGALG00010012336	case	control	-0.158426461	1.635044979	-0.096894252	0.922810373	0.985482967	down_in_case
PRJNA8274(	ENSGALG00010012337	ACSS3	case	control	-0.155239685	0.105948411	-1.465238445	0.142855874	0.545197906	down_in_case
PRJNA8274(	ENSGALG00010012339	WNK1	case	control	-0.121536919	0.071232124	-1.706209389	0.087969101	0.468839159	down_in_case
PRJNA8274(	ENSGALG00010012340	ENHO	case	control	0.529377175	0.15331649	3.452839118	0.00055472	0.034195028	up_in_case
PRJNA8274(	ENSGALG00010012341	GOPC	case	control	0.05646474	0.053747011	1.05056522	0.293458322	0.716170594	up_in_case
PRJNA8274(	ENSGALG00010012342	ENSGALG00010012342	case	control	-0.098741842	1.616267901	-0.061092497	0.951285545	0.991857576	down_in_case
PRJNA8274(	ENSGALG00010012343	BCL2L13	case	control	-0.143304948	0.074152714	-1.932565118	0.053289793	0.376996967	down_in_case
PRJNA8274(	ENSGALG00010012346	NLN	case	control	-0.074987575	0.090825019	-0.825626858	0.409015795	0.795170398	down_in_case
PRJNA8274(	ENSGALG00010012347	ENSGALG00010012347	case	control	0.678533857	0.25684608	2.641791752	0.008246875	0.160013983	up_in_case
PRJNA8274(	ENSGALG00010012348	ENSGALG00010012348	case	control	0.431812021	0.734491734	0.587905896	0.556595459	0.867878646	up_in_case
PRJNA8274(	ENSGALG00010012350	H2AC32	case	control	-0.165299281	0.208117077	-0.794261019	0.427043492	0.805305429	down_in_case
PRJNA8274(	ENSGALG00010012352	PTPRC	case	control	0.066859732	0.127983063	0.52241078	0.601384352	0.884954589	up_in_case
PRJNA8274(	ENSGALG00010012353	PIGG	case	control	0.236384532	0.164057628	1.440862792	0.14962345	0.556322239	up_in_case
PRJNA8274(	ENSGALG00010012354	DCBLD1	case	control	-0.104789469	0.127882393	-0.819420616	0.412546477	0.797581687	down_in_case
PRJNA8274(	ENSGALG00010012358	STK10	case	control	-0.027475399	0.116619509	-0.235598647	0.813744124	0.956712425	down_in_case
PRJNA8274(	ENSGALG00010012360	ROS1	case	control	0.020987712	0.263512193	0.079646076	0.936518749	0.988574452	up_in_case
PRJNA8274(	ENSGALG00010012361	WIF1	case	control	-0.033372048	0.45614575	-0.073160932	0.941678055	0.989551456	down_in_case
PRJNA8274(	ENSGALG00010012362	COL6A3	case	control	-0.29706904	0.155018836	-1.916341574	0.055321632	0.383470355	down_in_case
PRJNA8274(	ENSGALG00010012366	INTS8	case	control	-0.023146456	0.057864701	-0.400009952	0.689149187	0.918180693	down_in_case
PRJNA8274(	ENSGALG00010012367	RAD52	case	control	0.069372085	0.085552332	0.810873102	0.417438549	0.799987395	up_in_case
PRJNA8274(	ENSGALG00010012371	ATP6V1G3	case	control	0.689247582	0.46227548	1.49098884	0.135964425	0.539774365	up_in_case
PRJNA8274(	ENSGALG00010012372	LEMD3	case	control	0.032575622	0.055021459	0.592053032	0.553815077	0.866377377	up_in_case
PRJNA8274(	ENSGALG00010012373	WNT7B	case	control	0.018872939	0.663339941	0.028451384	0.977302143	0.994661197	up_in_case
PRJNA8274(	ENSGALG00010012376	VGLL2	case	control	-0.190003195	0.110133931	-1.725201252	0.08449121	0.459925506	down_in_case
PRJNA8274(	ENSGALG00010012378	RPS19BP1	case	control	-0.035281308	0.06060712	-0.582131399	0.560478177	0.870138205	down_in_case
PRJNA8274(	ENSGALG00010012382	SH3PXD2B	case	control	0.172939688	0.075100254	2.302784324	0.021290981	0.261564457	up_in_case
PRJNA8274(	ENSGALG00010012384	NRK	case	control	0.034861422	0.081091481	0.429902395	0.667266643	0.912432583	up_in_case
PRJNA8274(	ENSGALG00010012387	ERBIN	case	control	-0.084601504	0.086228021	-0.98113702	0.326525176	0.744287835	down_in_case
PRJNA8274(	ENSGALG00010012390	MMP16	case	control	-0.108254197	0.118192322	-0.915915643	0.359711141	0.765570668	down_in_case
PRJNA8274(	ENSGALG00010012391	NEURL1B	case	control	0.065181053	0.065501781	0.995103524	0.319685917	0.738813491	up_in_case
PRJNA8274(	ENSGALG00010012393	ENSGALG00010012393	case	control	-0.240777846	1.339254016	-0.179785047	0.857321323	0.971282862	down_in_case
PRJNA8274(	ENSGALG00010012398	ENSGALG00010012398	case	control	0.396660917	0.45869493	0.864759758	0.387170663	0.782604263	up_in_case
PRJNA8274(	ENSGALG00010012399	ERC1	case	control	-0.068493655	0.095674159	-0.715905483	0.474049713	0.82979716	down_in_case
PRJNA8274(	ENSGALG00010012401	RIPK2	case	control	0.027282767	0.079331539	0.343908203	0.730915323	0.933770678	up_in_case
PRJNA8274(	ENSGALG00010012404	VAMP7	case	control	0.0592821	0.03640254	1.628515466	0.103415632	0.492970761	up_in_case
PRJNA8274(	ENSGALG00010012405	MSRB3	case	control	-0.193116072	0.102322054	-1.887335764	0.059115179	0.395497471	down_in_case
PRJNA8274(	ENSGALG00010012406	ENSGALG00010012406	case	control	-0.200628115	0.137861626	-1.455286153	0.145590058	0.550340427	down_in_case
PRJNA8274(	ENSGALG00010012407	ATF4	case	control	-0.020752006	0.057120314	-0.363303429	0.716378232	0.926862121	down_in_case
PRJNA8274(	ENSGALG00010012410	CACNA1I	case	control	0.242850571	0.268718354	0.903736449	0.366135167	0.771724083	up_in_case
PRJNA8274(	ENSGALG00010012412	PURA	case	control	0.022081668	0.07659979	0.288273217	0.773137607	0.946459495	up_in_case
PRJNA8274(	ENSGALG00010012413	NEK7	case	control	-0.094771376	0.068155096	-1.390525166	0.164369463	0.579452754	down_in_case
PRJNA8274(	ENSGALG00010012414	ENSGALG00010012414	case	control	-0.5204992	0.255830077	-2.034550456	0.041896127	0.341355095	down_in_case
PRJNA8274(	ENSGALG00010012415	BID	case	control	-0.0789393	0.079923719	-0.987683016	0.323307915	0.740795254	down_in_case
PRJNA8274(	ENSGALG00010012418	ENSGALG00010012418	case	control	0.527731018	0.409444109	1.28889635	0.197434123	0.62094535	up_in_case
PRJNA8274(	ENSGALG00010012419	HACE1	case	control	0.144693448	0.066945371	2.161365993	0.030667079	0.308259818	up_in_case
PRJNA8274(	ENSGALG00010012426	MIEF1	case	control	-0.038291806	0.099235413	-0.385868365	0.699594161	0.922288441	down_in_case
PRJNA8274(	ENSGALG00010012427	C7	case	control	0.663195881	0.321536828	2.0625814	0.039152416	0.335091843	up_in_case
PRJNA8274(	ENSGALG00010012428	CCNE2	case	control	0.008867905	0.103815615	0.085419757	0.931927687	0.98752251	up_in_case
PRJNA8274(	ENSGALG00010012429	CXCR7	case	control	-0.17238127	0.087129416	-1.978450879	0.047877865	0.360228144	down_in_case
PRJNA8274(	ENSGALG00010012430	F13B	case	control	1.224623015	0.674738299	1.814959988	0.069530059	0.425096829	up_in_case
PRJNA8274(	ENSGALG00010012431	LAMP2	case	control	-0.024486552	0.074960135	-0.326660986	0.743924311	0.937448285	down_in_case
PRJNA8274(	ENSGALG00010012436	ENSGALG00010012436	case	control	-0.195543506	0.252883424	-0.773255528	0.439371169	0.812366461	down_in_case
PRJNA8274(	ENSGALG00010012437	ENSGALG00010012437	case	control	-0.196919765	1.674422979	-0.117604553	0.906380997	0.981730793	down_in_case
PRJNA8274(	ENSGALG00010012439	TENT2	case	control	0.112024447	0.126991783	0.88213933	0.377701466	0.776247623	up_in_case
PRJNA8274(	ENSGALG00010012441	FST	case	control	-0.204275009	0.097673853	-2.0913991	0.036492303	0.326967374	down_in_case
PRJNA8274(	ENSGALG00010012442	CPLX1	case	control	-0.091222002	0.360028956	-0.25337407	0.799979163	0.954053991	down_in_case
PRJNA8274(	ENSGALG00010012445	SOBP	case	control	-0.171161431	0.083479467	-2.050341695	0.040331098	0.33753444	down_in_case

PRJNA8274(	ENSGALG00010012447	COPS8	case	control	-0.063499356	0.059032149	-1.07567413	0.282073021	0.706000864	down_in_case
PRJNA8274(	ENSGALG00010012449	SUB1	case	control	0.012036588	0.074967981	0.160556384	0.87244281	0.975062028	up_in_case
PRJNA8274(	ENSGALG00010012450	PPFIA2	case	control	-0.092343645	0.204908354	-0.450658275	0.652325859	0.905706359	down_in_case
PRJNA8274(	ENSGALG00010012454	IQCA1	case	control	-0.427620446	0.285818917	-1.496123667	0.134621432	0.539542172	down_in_case
PRJNA8274(	ENSGALG00010012456	ENSGALG00010012456	case	control	0.15451651	0.079053384	1.954584377	0.050632147	0.367719502	up_in_case
PRJNA8274(	ENSGALG00010012458	MRPS30	case	control	0.015088356	0.19176394	0.078681924	0.937285623	0.988831145	up_in_case
PRJNA8274(	ENSGALG00010012463	ENSGALG00010012463	case	control	0.884184811	0.55611458	1.589932799	0.111849954	0.504878263	up_in_case
PRJNA8274(	ENSGALG00010012464	ENSGALG00010012464	case	control	0.000845527	0.668205122	0.001265371	0.99899038	0.999759426	up_in_case
PRJNA8274(	ENSGALG00010012466	PDSS2	case	control	0.07861856	0.058419592	1.345756738	0.178380982	0.597948438	up_in_case
PRJNA8274(	ENSGALG00010012469	CETN2	case	control	0.118237278	0.126039643	0.938095943	0.348195107	0.758675915	up_in_case
PRJNA8274(	ENSGALG00010012470	CHD1	case	control	-0.034443367	0.176704369	-0.194920856	0.845454907	0.967517103	down_in_case
PRJNA8274(	ENSGALG00010012474	ASB18	case	control	-0.822237561	0.371900755	-2.210905866	0.027042356	0.292515655	down_in_case
PRJNA8274(	ENSGALG00010012477	ENSGALG00010012477	case	control	0.04663055	0.175035073	0.266406891	0.789925859	0.951843754	up_in_case
PRJNA8274(	ENSGALG00010012483	ENSGALG00010012483	case	control	0.327425391	0.211082016	1.551176157	0.120859474	0.519499513	up_in_case
PRJNA8274(	ENSGALG00010012486	RADX	case	control	0.082875441	0.052249202	1.586157047	0.112703689	0.50697161	up_in_case
PRJNA8274(	ENSGALG00010012489	GBX2	case	control	-0.053271939	0.482180976	-0.110481213	0.912027749	0.98266608	down_in_case
PRJNA8274(	ENSGALG00010012497	PLCXD3	case	control	0.199109929	0.340609456	0.584569588	0.558837162	0.869450004	up_in_case
PRJNA8274(	ENSGALG00010012498	KCNT2	case	control	-0.149680394	0.175463053	-0.853059327	0.393626405	0.786843919	down_in_case
PRJNA8274(	ENSGALG00010012506	TP53INP1	case	control	0.050118774	0.056779064	0.882698136	0.377399389	0.775926253	up_in_case
PRJNA8274(	ENSGALG00010012508	MDM1	case	control	-0.03058609	0.06813676	-0.44889264	0.653509106	0.906243232	down_in_case
PRJNA8274(	ENSGALG00010012509	MICAL3	case	control	-0.072900184	0.073493217	-0.99193078	0.321231293	0.73942679	down_in_case
PRJNA8274(	ENSGALG00010012511	OXCT1	case	control	-0.256278253	0.102141027	-2.509062829	0.012105195	0.193549413	down_in_case
PRJNA8274(	ENSGALG00010012517	MGAT3	case	control	-0.080978762	0.070074748	-1.155605467	0.247842571	0.67225887	down_in_case
PRJNA8274(	ENSGALG00010012519	ENSGALG00010012519	case	control	-0.213372597	1.309003463	-0.163003844	0.870515408	0.974794532	down_in_case
PRJNA8274(	ENSGALG00010012521	HOMER1	case	control	-0.238494529	0.129762204	-1.837935243	0.066071948	0.41627012	down_in_case
PRJNA8274(	ENSGALG00010012529	BEND3	case	control	0.038463999	0.048977092	0.785346732	0.432250303	0.807469541	up_in_case
PRJNA8274(	ENSGALG00010012532	FAM174A	case	control	0.120775922	0.195909231	0.616489181	0.53757171	0.857667685	up_in_case
PRJNA8274(	ENSGALG00010012535	JMY	case	control	0.087836197	0.098152908	0.894891438	0.370845124	0.772835133	up_in_case
PRJNA8274(	ENSGALG00010012536	FBXO4	case	control	0.084516673	0.160347891	0.52708316	0.598135841	0.884680373	up_in_case
PRJNA8274(	ENSGALG00010012538	RAP1B	case	control	-0.03181968	0.037745887	-0.842997273	0.399229969	0.789900496	down_in_case
PRJNA8274(	ENSGALG00010012547	ENSGALG00010012547	case	control	0.311608421	0.376446384	0.827763086	0.407804684	0.794388636	up_in_case
PRJNA8274(	ENSGALG00010012550	TAB1	case	control	-0.134648645	0.051620319	-2.60844271	0.009095524	0.170020898	down_in_case
PRJNA8274(	ENSGALG00010012551	PCGF3	case	control	0.04505129	0.127231159	0.35409007	0.723271384	0.930215097	up_in_case
PRJNA8274(	ENSGALG00010012553	ENSGALG00010012553	case	control	-0.100683774	0.384014549	-0.262187393	0.793176972	0.95280257	down_in_case
PRJNA8274(	ENSGALG00010012555	ENSGALG00010012555	case	control	0.167828133	0.156098885	1.075139861	0.282312115	0.706141927	up_in_case
PRJNA8274(	ENSGALG00010012561	OSGIN2	case	control	0.157896126	0.094574182	1.669547882	0.095008849	0.48148469	up_in_case
PRJNA8274(	ENSGALG00010012565	SYNGR1	case	control	-0.079111839	0.052939585	-1.494379662	0.135076414	0.539774365	down_in_case
PRJNA8274(	ENSGALG00010012569	C6orf203	case	control	-0.053169015	0.05632458	-0.94397535	0.345182245	0.755935621	down_in_case
PRJNA8274(	ENSGALG00010012571	AGAP1	case	control	-0.176725376	0.080575464	-2.193290194	0.028286478	0.299627134	down_in_case
PRJNA8274(	ENSGALG00010012574	PHAX	case	control	-0.055109692	0.156698039	-0.351693566	0.725068086	0.93183011	down_in_case
PRJNA8274(	ENSGALG00010012578	NDUFS4	case	control	-0.057646915	0.195996092	-0.294122776	0.768664087	0.945375926	down_in_case
PRJNA8274(	ENSGALG00010012582	CD24	case	control	-0.128205927	0.159994439	-0.801314891	0.422949372	0.803637526	down_in_case
PRJNA8274(	ENSGALG00010012583	NDUFAF6	case	control	-0.004609248	0.078425231	-0.058772513	0.953133302	0.992056045	down_in_case
PRJNA8274(	ENSGALG00010012586	CDC73	case	control	-0.070605991	0.05929536	-1.190750699	0.233751468	0.65800118	down_in_case
PRJNA8274(	ENSGALG00010012587	MBLAC2	case	control	0.092681772	0.181258635	0.511323346	0.60912466	0.8877909	up_in_case
PRJNA8274(	ENSGALG00010012588	PEX26	case	control	0.234383835	0.070209998	3.338325629	0.000842849	0.044416733	up_in_case
PRJNA8274(	ENSGALG00010012593	TUBA8B	case	control	0.666282438	1.17172579	0.568633416	0.569604943	0.875091393	up_in_case
PRJNA8274(	ENSGALG00010012599	DMGDH	case	control	0.18972358	0.172061099	1.10265238	0.270178154	0.697769117	up_in_case
PRJNA8274(	ENSGALG00010012600	NUP107	case	control	0.009129063	0.065115764	0.14019743	0.888504003	0.977948171	up_in_case
PRJNA8274(	ENSGALG00010012601	TUBA8A	case	control	-0.382031927	0.172603074	-2.213355293	0.02687316	0.291789061	down_in_case
PRJNA8274(	ENSGALG00010012604	ENSGALG00010012604	case	control	-0.197673734	1.530895568	-0.129122938	0.897260372	0.980176778	down_in_case
PRJNA8274(	ENSGALG00010012606	ATP5PO	case	control	-0.097924076	0.075445946	-1.297936887	0.194309023	0.61723879	down_in_case
PRJNA8274(	ENSGALG00010012607	LMNB1	case	control	0.060625038	0.108394484	0.559300027	0.575956977	0.877910508	up_in_case
PRJNA8274(	ENSGALG00010012610	RPL3	case	control	-0.047520888	0.06021887	-0.789136163	0.430032428	0.806064191	down_in_case
PRJNA8274(	ENSGALG00010012614	LHFPL2	case	control	-0.008728154	0.097002944	-0.089978237	0.928304509	0.986776285	down_in_case
PRJNA8274(	ENSGALG00010012618	B3GALT2	case	control	-0.830875089	0.208210922	-3.990545167	6.59216E-05	0.008197207	down_in_case
PRJNA8274(	ENSGALG00010012624	PDGFB	case	control	0.074725377	0.103990561	0.718578459	0.47240069	0.829235491	up_in_case
PRJNA8274(	ENSGALG00010012629	NUS1	case	control	0.00090998	0.053472139	0.017017831	0.986422391	0.996597442	up_in_case
PRJNA8274(	ENSGALG00010012631	GLRX2	case	control	-0.264439184	0.104708467	-2.525480427	0.011554022	0.188192574	down_in_case
PRJNA8274(	ENSGALG00010012633	USP18	case	control	0.410645373	0.132968504	3.088290532	0.002013116	0.073625454	up_in_case
PRJNA8274(	ENSGALG00010012640	PRRC1	case	control	0.119225144	0.057105695	2.087797792	0.036816079	0.328026329	up_in_case
PRJNA8274(	ENSGALG00010012648	MRPS6	case	control	-0.178267956	0.104272629	-1.70963327	0.087333709	0.467042649	down_in_case
PRJNA8274(	ENSGALG00010012650	DSE	case	control	0.003009536	0.091395772	0.032928612	0.973731516	0.993553916	up_in_case
PRJNA8274(	ENSGALG00010012653	CBX7	case	control	-0.044091279	0.152756114	-0.28863839	0.772858114	0.94645256	down_in_case
PRJNA8274(	ENSGALG00010012654	TENT5B	case	control	-0.052069649	0.143383756	-0.363148867	0.716493682	0.926862121	down_in_case
PRJNA8274(	ENSGALG00010012662	SLC35E3	case	control	0.112507418	0.078209027	1.438547718	0.150278708	0.557485695	up_in_case
PRJNA8274(	ENSGALG00010012667	SCAMP1	case	control	-0.034705183	0.050964456	-0.68096837	0.495891505	0.83840725	down_in_case
PRJNA8274(	ENSGALG00010012669	ERCC8	case	control	0.175438404	0.1759815	0.996913905	0.318806302	0.738427335	up_in_case
PRJNA8274(	ENSGALG00010012670	ENSGALG00010012670	case	control	0.43155664	0.294366589	1.466051705	0.142634201	0.544783408	up_in_case
PRJNA8274(	ENSGALG00010012671	NBN	case	control	0.158689398	0.137922183	1.150571974	0.249908368	0.675246674	up_in_case
PRJNA8274(	ENSGALG00010012672	RO60	case	control	-0.006581996	0.073556706	-0.089481926	0.928689917	0.986776285	down_in_case
PRJNA8274(	ENSGALG00010012673	ENSGALG00010012673	case	control	-0.062225791	0.337862377	-0.184174962	0.853876201	0.969998446	down_in_case
PRJNA8274(	ENSGALG00010012674	ENSGALG00010012674	case	control	-0.137260065	0.115697107	-1.186374223	0.235474547	0.660526168	down_in_case
PRJNA8274(	ENSGALG00010012676	ENSGALG00010012676	case	control	-0.037979662	0.162276763	-0.234042517	0.814951971	0.957354871	down_in_case
PRJNA8274(	ENSGALG00010012680	TRPM8	case	control	-0.646763035	0.21416969	-3.019862594	0.002528894	0.082910904	down_in_case
PRJNA8274(	ENSGALG00010012682	ENSGALG00010012682	case	control	0.073717277	0.121938406	0.604545192	0.545481233	0.862111144	up_in_case
PRJNA8274(	ENSGALG00010012683	MDM2	case	control	0.089022134	0.060140434	1.480237636	0.13880984	0.540932258	up_in_case
PRJNA8274(	ENSGALG00010012685	NPTXR	case	control	-0.207094145	0.180639596	-1.146449336	0.251609279	0.677081801	down_in_case
PRJNA8274(	ENSGALG00010012688	NDUFAF2	case	control	-0.031459987	0.141267834	-0.222697457	0.823770981	0.960215603	down_in_case
PRJNA8274(	ENSGALG00010012690	NT5DC1	case	control	-0.139288405	0.074172938	-1.877887116	0.060396609	0.399293348	down_in_case
PRJNA8274(	ENSGALG00010012693	UCHL5	case	control	0.038203639	0.053140158	0.718922201	0.472188857	0.829235491	up_in_case
PRJNA8274(	ENSGALG00010012696	DNAL4	case	control	-0.17235311	0.066411522	-2.595229035	0.00945279	0.173970275	down_in_case
PRJNA8274(	ENSGALG00010012699	RNF128	case	control	0.09198457	0.116457166	0.789857529	0.429610977	0.806015686	up_in_case
PRJNA8274(	ENSGALG00010012702	PTPRO	case	control	-0.965849461	0.351741966	-2.7459034	0.006034453	0.134449153	down_in_case
PRJNA8274(	ENSGALG00010012704	SMIM15	case	control	-0.019063173	0.07865473	-0.242365243	0.808497164	0.955875648	down_in_case
PRJNA8274(	ENSGALG00010012705	CPM	case	control	0.078273962	0.184501344	0.424246025	0.671386398	0.913676733	up_in_case
PRJNA8274(	ENSGALG00010012706	TBC1D8B	case	control	0.035676639	0.082385924	0.433042893	0.664983609	0.9119814	up_in_case
PRJNA8274(	ENSGALG00010012708	SPP2	case	control	1.243938918	0.872224383	1.426168475	0.153819717	0.56256316	up_in_case
PRJNA8274(	ENSGALG00010012709	PPP1R8	case	control	-0.027638168	0.08383569	-0.329670666	0.741648821	0.937315893	down_in_case
PRJNA8274(	ENSGALG00010012711	ELL2	case	control	0.208583532	0.136005987	1.533634933	0.125119489	0.523982046	up_in_case
PRJNA8274(	ENSGALG00010012712	EEF1E1	case	control	0.112118196	0.133883741	0.837429513	0.402351182	0.792021921	up_in_case
PRJNA8274(	ENSGALG00010012714	RGS2	case	control	0.054734125	0.102232911	0.53538655	0.592382589	0.883604289	up_in_case
PRJNA8274(	ENSGALG00010012719	COL10A1	case	control	0.521289201	0.885486019	0.588704045			up_in_case
PRJNA8274(	ENSGALG00010012720	ACO2	case	control	-0.180620366	0.081322353	-2.22104206	0.026348113	0.288056585	down_in_case
PRJNA8274(	ENSGALG00010012722	ENSGALG00010012722	case	control	0.229253202	0.161251501	1.421712044	0.155109862	0.563829072	up_in_case
PRJNA8274(	ENSGALG00010012725	ENSGALG00010012725	case	control	0.178493632	0.259569254	0.687653215	0.491671189	0.837145631	up_in_case
PRJNA8274(	ENSGALG00010012726	IL7R	case	control	1.196866353	0.423763737	2.824371813	0.004737339	0.117407198	up_in_case
PRJNA8274(	ENSGALG00010012730	CAP2	case	control	-0.161922349	0.081278854	-1.992182983	0.046350985	0.356443312	down_in_case
PRJNA8274(	ENSGALG00010012732	MGST1	case	control	0.167235348	0.115262367	1.450910238	0.146804857	0.551868941	up_in_case
PRJNA8274(	ENSGALG00010012733	ENSGALG00010012733	case	control	1.156025459	0.609596045	1.896379527	0.057909877	0.391912561	up_in_case
PRJNA8274(	ENSGALG00010012734	TMEM19	case	control	0.05465922	0.114814557	0.476065242	0.634027898	0.898107199	up_in_case
PRJNA8274(	ENSGALG00010012738	RAB21	case	control	-0.094050276	0.052316409	-1.797720398	0.072221327	0.434299097	down_in_case
PRJNA8274(	ENSGALG00010012741	ENSGALG00010012741	case	control	0.184340476	0.33641823	0.54795032	0.583726016	0.881307382	up_in_case
PRJNA8274(	ENSGALG00010012742	ENSGALG00010012742	case	control	-0.176127291	0.105668856	-1.666785251	0.095557114	0.482930958	down_in_case
PRJNA8274(	ENSGALG00010012743	ENSGALG00010012743	case	control	0.621656562	0.243021992	2.558025952	0.010526824	0.183125942	up_in_case
PRJNA8274(	ENSGALG00010012744	ARL4C	case	control	-0.013724591	0.109718927	-0.125088636	0.90045338	0.98028579	down_in_case
PRJNA8274(	ENSGALG00010012745	MARCHF3	case	control	0.458645677	0.283557455	1.617470001	0.105776866	0.495266487	up_in_case
PRJNA8274(	ENSGALG00010012747	TOB2	case	control	0.075600758	0.081228898	0.9307126	0.35200225	0.760831854	up_in_case
PRJNA8274(	ENSGALG00010012748	ENSGALG00010012748	case	control	0.057667521	0.370498289	0.155648548	0.876310055	0.975666505	up_in_case
PRJNA8274(	ENSGALG00010012749	RGS4	case	control	-0.011176933	0.100398885	-0.111325272	0.911358415	0.982467044	down_in_case
PRJNA8274(	ENSGALG00010012751	AP3B1	case	control	0.077039721	0.150096667	0.513267366	0.607764308	0.887448737	up_in_case
PRJNA8274(	ENSGALG00010012753	GTPBP1	case	control	0.028151793	0.042547099	0.661661869	0.508187948	0.843702533	up_in_case
PRJNA8274(	ENSGALG00010012755	CLVS2	case	control	0.165936858	0.331524321	0.500526954	0.616704082	0.891081782	up_in_case
PRJNA8274(	ENSGALG00010012756	RPA2	case	control	0.02817885	0.065425997	0.430698058	0.666687931	0.912284937	up_in_case
PRJNA8274(	ENSGALG00010012758	NAB1	case	control	0.015220628	0.037841625	0.402219196	0.687522716	0.917290381	up_in_case
PRJNA8274(	ENSGALG00010012759	ATP1B4	case	control	-0.12016607	0.149940529	-0.801424872	0.422885721	0.803623363	down_in_case
PRJNA8274(	ENSGALG00010012760	PLEKHF2	case	control	0.007900427	0.056395961	0.140088531	0.888590042	0.977948171	up_in_case
PRJNA8274(	ENSGALG00010012761	SMPDL3A	case	control	0.145709779	0.146865289	0.992132181	0.32113305	0.73942679	up_in_case
PRJNA8274(	ENSGALG00010012763	SMPDL3B	case	control	0.076140904	0.101893873	0.747256945	0.454908476	0.821657174	up_in_case
PRJNA8274(	ENSGALG00010012765	PHF5A	case	control	0.025925014	0.055951958	0.463344174	0.643117687	0.902038523	up_in_case
PRJNA8274(	ENSGALG00010012767	JOSD1	case	control	0.119639389	0.044207152	2.70633562	0.006803025	0.142644079	up_in_case
PRJNA8274(	ENSGALG00010012768	EPS8	case	control	-0.15158658	0.080707906	-1.878212274	0.060352132	0.399259348	down_in_case
PRJNA8274(	ENSGALG00010012769	NEMP2	case	control	0.003810953	0.082371501	0.046265427	0.963098695	0.992068146	up_in_case
PRJNA8274(	ENSGALG00010012771	ITGA2	case	control	0.004575342	0.140274956	0.032616956	0.973980048	0.993644481	up_in_case
PRJNA8274(	ENSGALG00010012772	TOMM22	case	control	-0.093815663	0.066752631	-1.405422699	0.159895621	0.572055887	down_in_case
PRJNA8274(	ENSGALG00010012773	XKR8	case	control	-0.213868309	0.17496148	-1.222373683	0.221566347	0.645689578	down_in_case

PRJNA8274(	ENSGALG00010012775	KCNE2	case	control	-0.130652667	0.083923479	-1.556807098	0.119516291	0.516807671	down_in_case
PRJNA8274(	ENSGALG00010012778	TBC1D15	case	control	0.035196026	0.065360795	0.538488343	0.590239951	0.882949889	up_in_case
PRJNA8274(	ENSGALG00010012780	FABP7	case	control	-0.021270061	0.250185217	-0.085017258	0.932247671	0.987669602	down_in_case
PRJNA8274(	ENSGALG00010012781	SPIN1L	case	control	-0.240568055	1.654807148	-0.145375282	0.88441458	0.977579768	down_in_case
PRJNA8274(	ENSGALG00010012786	TEF	case	control	-0.049022346	0.071676284	-0.68394095	0.494012455	0.838023719	down_in_case
PRJNA8274(	ENSGALG00010012790	PITX1	case	control	-0.213932912	0.430664428	-0.496750828	0.619364774	0.892204721	down_in_case
PRJNA8274(	ENSGALG00010012791	CFAP418	case	control	0.087157	0.137388206	0.634384874	0.525829679	0.85251736	up_in_case
PRJNA8274(	ENSGALG00010012792	CBY1	case	control	-0.002508564	0.099725015	-0.02515481	0.979931482	0.995636523	down_in_case
PRJNA8274(	ENSGALG00010012795	TBCA	case	control	0.04342624	0.147356025	0.294702844	0.768220891	0.945238232	up_in_case
PRJNA8274(	ENSGALG00010012796	MOCS2	case	control	-0.043540875	0.138000714	-0.315511954	0.752372976	0.940427752	down_in_case
PRJNA8274(	ENSGALG00010012797	ZC3H7B	case	control	-0.036780119	0.049667362	-0.740528938	0.458979109	0.82413376	down_in_case
PRJNA8274(	ENSGALG00010012798	MFSD6	case	control	0.082479792	0.08291748	0.994721405	0.319871781	0.738813491	up_in_case
PRJNA8274(	ENSGALG00010012801	DMC1	case	control	-0.040517868	0.291994615	-0.138762381	0.889637922	0.97815026	down_in_case
PRJNA8274(	ENSGALG00010012803	NUP155	case	control	-0.051043646	0.164072988	-0.311103289	0.755722101	0.941588405	down_in_case
PRJNA8274(	ENSGALG00010012804	ENSGALG00010012804	case	control	-0.014184736	0.128385676	-0.110485351	0.912024467	0.98266608	down_in_case
PRJNA8274(	ENSGALG00010012808	EYA3	case	control	-0.073580783	0.049428587	-1.488628088	0.136585331	0.539848047	down_in_case
PRJNA8274(	ENSGALG00010012809	RHOBTB3	case	control	0.744116648	0.195981213	3.796877439	0.00014653	0.01379195	up_in_case
PRJNA8274(	ENSGALG00010012812	DECR1	case	control	-0.038590146	0.104429125	-0.369534319	0.711729497	0.926596123	down_in_case
PRJNA8274(	ENSGALG00010012818	RGS18	case	control	-0.170225105	0.118283349	-1.439129913	0.150113718	0.557230917	down_in_case
PRJNA8274(	ENSGALG00010012819	RANGAP1	case	control	0.140073272	0.076494375	1.831157802	0.067076993	0.419231207	up_in_case
PRJNA8274(	ENSGALG00010012821	ZBTB33	case	control	0.008407051	0.047255153	0.177907594	0.858795547	0.971828322	up_in_case
PRJNA8274(	ENSGALG00010012824	HIBCH	case	control	-0.086580126	0.070547587	-1.2272585	0.219725448	0.643867604	down_in_case
PRJNA8274(	ENSGALG00010012827	ENSGALG00010012827	case	control	0.021871981	0.602706484	0.036289606	0.971051437	0.992761697	up_in_case
PRJNA8274(	ENSGALG00010012829	RGS5	case	control	0.061709512	0.150510928	0.410000213	0.681805791	0.915594727	up_in_case
PRJNA8274(	ENSGALG00010012830	ARRDC3	case	control	0.138602065	0.123682924	1.1206241	0.262447902	0.68849844	up_in_case
PRJNA8274(	ENSGALG00010012831	ENSGALG00010012831	case	control	-0.16016859	0.254542047	-0.629242168	0.529190526	0.85440042	down_in_case
PRJNA8274(	ENSGALG00010012832	CHADL	case	control	1.554607393	0.479454828	3.242448094	0.001185075	0.053969991	up_in_case
PRJNA8274(	ENSGALG00010012833	JAK2	case	control	-0.072644858	0.093357411	-0.77813702	0.436488241	0.810200135	down_in_case
PRJNA8274(	ENSGALG00010012834	TPH2	case	control	-0.223807467	0.817650351	-0.273720261	0.784299606	0.949518053	down_in_case
PRJNA8274(	ENSGALG00010012836	ENSGALG00010012836	case	control	-0.320159858	0.561102931	-0.570590243	0.568277434	0.874461133	down_in_case
PRJNA8274(	ENSGALG00010012837	PKIB	case	control	0.371472587	0.263674973	1.408827631	0.158886143	0.571302953	up_in_case
PRJNA8274(	ENSGALG00010012838	C2orf88	case	control	0.264746339	0.147902577	1.790004909	0.073453122	0.43784062	up_in_case
PRJNA8274(	ENSGALG00010012839	TGFBI	case	control	-0.016836881	0.116839215	-0.144102999	0.885419137	0.977630122	down_in_case
PRJNA8274(	ENSGALG00010012841	L3MBTL2	case	control	-0.028301263	0.077132803	-0.366916052	0.713681637	0.926862121	down_in_case
PRJNA8274(	ENSGALG00010012842	DDX17	case	control	0.02905361	0.048242136	0.602245517	0.547010722	0.862526554	up_in_case
PRJNA8274(	ENSGALG00010012843	ENSGALG00010012843	case	control	0.067097946	0.226660572	0.296028311	0.767208461	0.944480113	up_in_case
PRJNA8274(	ENSGALG00010012845	HDHD2	case	control	0.088803633	0.173298368	0.512432018	0.608348687	0.887692472	up_in_case
PRJNA8274(	ENSGALG00010012848	SERINC1	case	control	-0.0513783	0.052426591	-0.980004588	0.327083854	0.744857565	down_in_case
PRJNA8274(	ENSGALG00010012849	TRHDE	case	control	0.456695645	0.328653276	1.389597118	0.164651251	0.579922223	up_in_case
PRJNA8274(	ENSGALG00010012850	CALB1	case	control	-1.018630184	0.606497858	-1.679528081	0.093049171	0.478122824	down_in_case
PRJNA8274(	ENSGALG00010012851	ENSGALG00010012851	case	control	-0.262402444	0.350305015	-0.749068478	0.453815933	0.821282604	down_in_case
PRJNA8274(	ENSGALG00010012852	MSTN	case	control	-0.343331727	0.132125594	-2.598525507	0.009362509	0.172879234	down_in_case
PRJNA8274(	ENSGALG00010012854	LECT2	case	control	0.123912448	0.487326455	0.254269897	0.799287054	0.954053991	up_in_case
PRJNA8274(	ENSGALG00010012857	NSDHL	case	control	0.15654737	0.160298704	0.976597852	0.328768283	0.745453498	up_in_case
PRJNA8274(	ENSGALG00010012859	FBXL21	case	control	-0.004542781	0.089071666	-0.051001412	0.959324396	0.992056045	down_in_case
PRJNA8274(	ENSGALG00010012860	ENSGALG00010012860	case	control	-0.230456212	0.493378223	-0.467098468	0.64042943	0.901166378	down_in_case
PRJNA8274(	ENSGALG00010012861	WDR70	case	control	-0.172035781	0.205953437	-0.835313961	0.403540959	0.79260318	down_in_case
PRJNA8274(	ENSGALG00010012863	KCNE1	case	control	-0.273404886	0.378141917	-0.723021897	0.469666432	0.829101076	down_in_case
PRJNA8274(	ENSGALG00010012865	EP300	case	control	-0.112912943	0.072958069	-1.547641606	0.121708608	0.519686058	down_in_case
PRJNA8274(	ENSGALG00010012867	A4GALT	case	control	-0.029461909	0.071531108	-0.411875476	0.680430697	0.915381147	down_in_case
PRJNA8274(	ENSGALG00010012868	CTHRC1	case	control	0.271107717	0.307975535	0.880289782	0.378702347	0.777229179	up_in_case
PRJNA8274(	ENSGALG00010012869	ENSGALG00010012869	case	control	0.230290152	0.178762568	1.288245937	0.197660366	0.62094535	up_in_case
PRJNA8274(	ENSGALG00010012870	KCNC2	case	control	-0.457880111	0.165395578	-2.768393902	0.005633332	0.130251961	down_in_case
PRJNA8274(	ENSGALG00010012871	ENSGALG00010012871	case	control	-0.191573264	0.230792733	-0.830066274	0.406501314	0.794253996	down_in_case
PRJNA8274(	ENSGALG00010012873	PTAFR	case	control	0.00347674	0.108541297	0.032031498	0.974446932	0.993775355	up_in_case
PRJNA8274(	ENSGALG00010012875	HSF2	case	control	-0.056696733	0.046666327	-1.214938849	0.224389415	0.648105158	down_in_case
PRJNA8274(	ENSGALG00010012876	KDELR3	case	control	0.019506868	0.063260868	0.308355993	0.757811464	0.941765572	up_in_case
PRJNA8274(	ENSGALG00010012877	SLC25A32	case	control	0.023821993	0.055289771	0.430857149	0.666572243	0.912284937	up_in_case
PRJNA8274(	ENSGALG00010012878	GDNF	case	control	0.021015324	0.21739686	0.09666802	0.922990038	0.985482967	up_in_case
PRJNA8274(	ENSGALG00010012879	RCAN1	case	control	0.124075035	0.098724685	1.256778234	0.20883395	0.633836842	up_in_case
PRJNA8274(	ENSGALG00010012880	CLK4	case	control	0.105564633	0.092072895	1.146533225	0.251574588	0.677081801	up_in_case
PRJNA8274(	ENSGALG00010012882	ENSGALG00010012882	case	control	-1.538120346	0.344313516	-4.467208737	7.92468E-06	0.001417053	down_in_case
PRJNA8274(	ENSGALG00010012883	DCAF13	case	control	-0.022025601	0.042532602	-0.517852178	0.60456141	0.885268908	down_in_case
PRJNA8274(	ENSGALG00010012884	ENSGALG00010012884	case	control	-0.18141719	0.334872192	-0.541750539	0.587990369	0.882771329	down_in_case
PRJNA8274(	ENSGALG00010012885	PMS1	case	control	0.145499617	0.074065944	1.964460449	0.049476719	0.364797618	up_in_case
PRJNA8274(	ENSGALG00010012887	KCNJ4	case	control	-0.386214726	0.256794313	-1.503984734	0.132585298	0.535904608	down_in_case
PRJNA8274(	ENSGALG00010012888	ENSGALG00010012888	case	control	-0.306397461	1.573346992	-0.194742458			down_in_case
PRJNA8274(	ENSGALG00010012889	RIC1	case	control	-0.001044067	0.056110865	-0.018607218	0.985154445	0.996591771	down_in_case
PRJNA8274(	ENSGALG00010012890	MFAP3	case	control	-0.005766499	0.050158681	-0.11496512	0.908472769	0.981730793	down_in_case
PRJNA8274(	ENSGALG00010012891	CAPS2	case	control	0.458297502	0.246662964	1.857990734	0.06317033	0.409119439	up_in_case
PRJNA8274(	ENSGALG00010012892	ORMDL1	case	control	-0.1756325	0.139097294	-1.262659358	0.206711626	0.630417074	down_in_case
PRJNA8274(	ENSGALG00010012893	ENSGALG00010012893	case	control	0.338277691	0.193776466	1.745710914	0.080861198	0.453075204	up_in_case
PRJNA8274(	ENSGALG00010012894	CLIC6	case	control	0.020626425	0.308192977	0.06692698	0.946639834	0.990701817	up_in_case
PRJNA8274(	ENSGALG00010012896	STK38L	case	control	0.113096716	0.05744056	1.968934774	0.048960582	0.36379167	up_in_case
PRJNA8274(	ENSGALG00010012897	ENSGALG00010012897	case	control	-0.077692483	0.547597749	-0.141878747	0.887175782	0.977630122	down_in_case
PRJNA8274(	ENSGALG00010012898	FAM114A2	case	control	-0.029691566	0.049853466	-0.595576759	0.551458007	0.865150795	down_in_case
PRJNA8274(	ENSGALG00010012899	ENSGALG00010012899	case	control	-0.156040195	0.090043523	-1.732941906	0.083105995	0.457082972	down_in_case
PRJNA8274(	ENSGALG00010012900	RBX1	case	control	0.076867749	0.072955753	1.053621492	0.292056245	0.714648239	up_in_case
PRJNA8274(	ENSGALG00010012903	RGMB	case	control	-0.021238791	0.104318395	-0.203595838	0.838669346	0.96509516	down_in_case
PRJNA8274(	ENSGALG00010012904	ERMP1	case	control	-0.007020647	0.140443491	-0.049989125	0.960131054	0.992056045	down_in_case
PRJNA8274(	ENSGALG00010012905	NKAIN1	case	control	-0.090074832	0.147716107	-0.609783413	0.542005291	0.85975913	down_in_case
PRJNA8274(	ENSGALG00010012908	STRAP	case	control	-0.060801173	0.04302995	-1.412996603	0.157656723	0.569315943	down_in_case
PRJNA8274(	ENSGALG00010012910	XPNPEP3	case	control	-0.086731652	0.061269504	-1.415576206	0.156899622	0.567595301	down_in_case
PRJNA8274(	ENSGALG00010012911	CSNK1E	case	control	-0.084546669	0.056200297	-1.504381185	0.132483246	0.535857167	down_in_case
PRJNA8274(	ENSGALG00010012912	NKAPL	case	control	-0.015102426	0.083998371	-0.179794271	0.857314081	0.971282862	down_in_case
PRJNA8274(	ENSGALG00010012913	EGFLAM	case	control	-0.042231379	0.087175907	-0.484438655	0.628074591	0.895865363	down_in_case
PRJNA8274(	ENSGALG00010012915	ENSGALG00010012915	case	control	-0.257153937	0.23075691	-1.114393227	0.265110528	0.69125489	down_in_case
PRJNA8274(	ENSGALG00010012917	RUNX1	case	control	0.181572461	0.240089578	0.75626965	0.449487541	0.818749597	up_in_case
PRJNA8274(	ENSGALG00010012918	GLRX	case	control	0.75841742	0.299678924	2.530766625	0.011381355	0.186780127	up_in_case
PRJNA8274(	ENSGALG00010012921	EBF1	case	control	0.143269401	0.092717762	1.545220647	0.122292902	0.520832363	up_in_case
PRJNA8274(	ENSGALG00010012922	SNRNP40	case	control	-0.015840227	0.056132523	-0.282193398	0.77779522	0.947849555	down_in_case
PRJNA8274(	ENSGALG00010012923	MLANA	case	control	7.779636532	0.647585694	12.01329277	3.02554E-33	6.18074E-30	up_in_case
PRJNA8274(	ENSGALG00010012924	GLIPR1	case	control	-0.043999873	0.191612675	-0.229629241	0.818379883	0.958536226	down_in_case
PRJNA8274(	ENSGALG00010012925	SETD4	case	control	0.073620957	0.140311797	0.524695416	0.599794944	0.884888453	up_in_case
PRJNA8274(	ENSGALG00010012926	OSGEPL1	case	control	0.252418328	0.168890498	1.494567966	0.135027232	0.539774365	up_in_case
PRJNA8274(	ENSGALG00010012927	ENSGALG00010012927	case	control	-0.254537364	1.476666692	-0.172372929	0.863144353	0.972850732	down_in_case
PRJNA8274(	ENSGALG00010012930	IL6ST	case	control	-0.086141544	0.060260964	-1.429475042	0.152867743	0.561193576	down_in_case
PRJNA8274(	ENSGALG00010012932	ATF6	case	control	-0.036361255	0.055842426	-0.651140312	0.514955916	0.847688454	down_in_case
PRJNA8274(	ENSGALG00010012933	ZCCHC17	case	control	-0.144253569	0.075337712	-1.914759099	0.055523233	0.383937248	down_in_case
PRJNA8274(	ENSGALG00010012934	NDUFA1	case	control	-0.190783779	0.077453995	-2.463188359	0.013770755	0.205985143	down_in_case
PRJNA8274(	ENSGALG00010012938	UPF3B	case	control	-0.102145883	0.065931668	-1.549268898	0.121317093	0.519567065	down_in_case
PRJNA8274(	ENSGALG00010012939	GJA1	case	control	0.010975851	0.186940676	0.058713019	0.95318069	0.992056045	up_in_case
PRJNA8274(	ENSGALG00010012940	FBN2	case	control	-0.104369427	0.122395337	-0.852723886	0.393812442	0.786935281	down_in_case
PRJNA8274(	ENSGALG00010012943	ASNSD1	case	control	-0.011802555	0.086634177	-0.136234396	0.891635984	0.978917427	down_in_case
PRJNA8274(	ENSGALG00010012944	NCK1	case	control	0.057284556	0.05556634	1.030921873	0.302577458	0.725374293	up_in_case
PRJNA8274(	ENSGALG00010012945	CLINT1	case	control	-0.013793532	0.033832019	-0.407706458	0.683489196	0.916102305	down_in_case
PRJNA8274(	ENSGALG00010012948	ST13P5	case	control	-0.161301423	0.074302092	-2.170886697	0.029939738	0.305970455	down_in_case
PRJNA8274(	ENSGALG00010012949	ENSGALG00010012949	case	control	0.005270982	0.121945462	0.043224096	0.965522897	0.992291928	up_in_case
PRJNA8274(	ENSGALG00010012952	BRD10	case	control	0.072753312	0.069118315	1.052590947	0.292528508	0.714947473	up_in_case
PRJNA8274(	ENSGALG00010012954	RIMS2	case	control	-0.223464482	0.348575696	-0.641078782	0.521471499	0.850363352	down_in_case
PRJNA8274(	ENSGALG00010012955	RPL39L	case	control	-0.284117457	0.07290603	-3.897036447	9.7377E-05	0.010711467	down_in_case
PRJNA8274(	ENSGALG00010012956	FABP3	case	control	-0.138569458	0.111513101	-1.242629402	0.21400443	0.638885877	down_in_case
PRJNA8274(	ENSGALG00010012957	DERA	case	control	0.01017103	0.116841836	0.087049554	0.930632124	0.987258715	up_in_case
PRJNA8274(	ENSGALG00010012958	OLFML2B	case	control	-0.090313119	0.137155327	-0.658473285	0.510234058	0.844875756	down_in_case
PRJNA8274(	ENSGALG00010012959	TBC1D32	case	control	-0.132978905	0.094134919	-1.412641618	0.157761126	0.569549128	down_in_case
PRJNA8274(	ENSGALG00010012961	ANKRD55	case	control	1.191594267	0.544622154	2.18792838	0.02867482	0.300624184	up_in_case
PRJNA8274(	ENSGALG00010012962	SLC25A17	case	control	-0.092303886	0.059903446	-1.540877732	0.123346546	0.522778783	down_in_case
PRJNA8274(	ENSGALG00010012963	UIMC1	case	control	-0.033324828	0.062920792	-0.529631411	0.596367511	0.884680373	down_in_case
PRJNA8274(	ENSGALG00010012964	CBR3	case	control	-0.072063641	0.074924393	-0.961818147	0.336140961	0.750473129	down_in_case
PRJNA8274(	ENSGALG00010012965	ENSGALG00010012965	case	control	-0.013662925	0.065241783	-0.209419851	0.834120497	0.963078474	down_in_case
PRJNA8274(	ENSGALG00010012966	GLIPR1L	case	control	-0.111665771	0.208824431	-0.534735186	0.592832987	0.883625114	down_in_case
PRJNA8274(	ENSGALG00010012967	ECRG4	case	control	0.274375746	0.153668767	1.785501058	0.07418008	0.438111241	up_in_case
PRJNA8274(	ENSGALG00010012968	LSM11	case	control	-0.165357369	0.058450915	-2.828995391	0.004669437	0.116334528	down_in_case
PRJNA8274(	ENSGALG00010012969	SLC12A2	case	control	-0.092033659	0.094097054	-0.978071626	0.3280389	0.745445783	down_in_case
PRJNA8274(	ENSGALG00010012970	UNC5A	case	control	-0.160079194	0.106057319	-1.509364898	0.131205558	0.534541163	down_in_case
PRJNA8274(	ENSGALG00010012971	ENSGALG00010012971	case	control	0.279709888	0.273652463	1.022135466	0.306716789	0.729504707	up_in_case

PRJNA8274(	ENSGALG00010012973	GLDC	case	control	0.317081098	0.219821919	1.442445317	0.149176788	0.55581763	up_in_case
PRJNA8274(	ENSGALG00010012974	SLC49A3	case	control	0.590767731	0.14592507	4.048432045	5.15619E-05	0.006703047	up_in_case
PRJNA8274(	ENSGALG00010012975	KRR1	case	control	-0.069162914	0.077972405	-0.887017837	0.375069297	0.774681835	down_in_case
PRJNA8274(	ENSGALG00010012976	SLC9A2	case	control	-0.142771968	0.197682775	-0.722227659	0.470154521	0.829101076	down_in_case
PRJNA8274(	ENSGALG00010012977	NKRF	case	control	0.053672117	0.063742177	0.842018889	0.399777381	0.789900496	up_in_case
PRJNA8274(	ENSGALG00010012978	HSD17B7	case	control	0.342855695	0.218622709	1.568252884	0.116822127	0.51350396	up_in_case
PRJNA8274(	ENSGALG00010012979	HTR1A	case	control	-0.625419498	0.252416251	-2.477730713	0.013222089	0.201788559	down_in_case
PRJNA8274(	ENSGALG00010012980	KICS2	case	control	0.024608861	0.053194204	0.462622982	0.643634633	0.902038523	up_in_case
PRJNA8274(	ENSGALG00010012982	DDR2	case	control	0.03168444	0.085311172	0.371398488	0.710340752	0.925630832	up_in_case
PRJNA8274(	ENSGALG00010012985	ARNTL2	case	control	0.048933739	0.1012865	0.483122031	0.629009092	0.896269608	up_in_case
PRJNA8274(	ENSGALG00010012986	TMEM184B	case	control	-0.096757188	0.05705518	-1.695852812	0.089913758	0.473404543	down_in_case
PRJNA8274(	ENSGALG00010012988	RPL18A	case	control	-0.07314858	0.062906212	-1.162819652	0.244902682	0.669469819	down_in_case
PRJNA8274(	ENSGALG00010012991	MFSD9	case	control	0.098966368	0.077280641	1.280610082	0.200330657	0.622902456	up_in_case
PRJNA8274(	ENSGALG00010012992	HARS1	case	control	-0.080387021	0.038493321	-2.088336878	0.036767457	0.327994159	down_in_case
PRJNA8274(	ENSGALG00010012993	DOP1B	case	control	-0.009173028	0.084386468	-0.108702591	0.913438387	0.982728035	down_in_case
PRJNA8274(	ENSGALG00010012995	SERINC2	case	control	-0.10879792	0.098822673	-1.100940876	0.27092239	0.698323327	down_in_case
PRJNA8274(	ENSGALG00010012997	UBE2A	case	control	-0.026610552	0.028029369	-0.949381081	0.342426829	0.754383554	down_in_case
PRJNA8274(	ENSGALG00010012998	MAFF	case	control	0.035887451	0.090588026	0.396161087	0.691986196	0.918963837	up_in_case
PRJNA8274(	ENSGALG00010013001	ADGRV1	case	control	0.39456412	0.301626193	1.308122866	0.190831639	0.613935676	up_in_case
PRJNA8274(	ENSGALG00010013004	UAP1	case	control	0.098712994	0.102082508	0.966992246	0.33354791	0.748508541	up_in_case
PRJNA8274(	ENSGALG00010013005	SLC40A1	case	control	0.010544111	0.121629463	0.086690436	0.930917579	0.987258715	up_in_case
PRJNA8274(	ENSGALG00010013006	GRIK2	case	control	-0.570987374	0.804530452	-0.709715055	0.477880855	0.830520323	down_in_case
PRJNA8274(	ENSGALG00010013008	STEEP1	case	control	0.006866532	0.072721021	0.094422928	0.924773204	0.985571367	up_in_case
PRJNA8274(	ENSGALG00010013012	TMEM182	case	control	-0.074487487	0.085835239	-0.867796114	0.385505958	0.781541515	down_in_case
PRJNA8274(	ENSGALG00010013015	PLA2G6	case	control	0.019238162	0.087708079	0.219343103	0.826382788	0.961207256	up_in_case
PRJNA8274(	ENSGALG00010013016	QRSL1	case	control	-0.0071456	0.085968007	-0.083119297	0.933756682	0.988101632	down_in_case
PRJNA8274(	ENSGALG00010013017	SCMH1	case	control	-0.094134677	0.069613288	-1.352251564	0.176294853	0.595527443	down_in_case
PRJNA8274(	ENSGALG00010013018	ID1	case	control	-0.067197502	0.084121832	-0.798811682	0.424399616	0.803723282	down_in_case
PRJNA8274(	ENSGALG00010013019	DPYS	case	control	0.823491139	1.207252444	0.682120084	0.495163022	0.838118945	up_in_case
PRJNA8274(	ENSGALG00010013021	SLC25A5	case	control	0.083032882	0.08104332	1.024549365	0.305575867	0.728862914	up_in_case
PRJNA8274(	ENSGALG00010013027	UHMK1	case	control	0.013716708	0.049235861	0.278591806	0.780558101	0.948875232	up_in_case
PRJNA8274(	ENSGALG00010013029	MYLK2	case	control	-0.530132346	0.15951578	-3.323384978	0.000889321	0.045257255	down_in_case
PRJNA8274(	ENSGALG00010013032	BAIAP2L2	case	control	0.486574436	0.443468042	1.097202932	0.272552692	0.698931401	up_in_case
PRJNA8274(	ENSGALG00010013033	MAP3K1	case	control	0.04659998	0.096372933	0.483538046	0.628713753	0.896269608	up_in_case
PRJNA8274(	ENSGALG00010013037	MORC4	case	control	0.053560798	0.042071801	1.273080689	0.202989411	0.625537942	up_in_case
PRJNA8274(	ENSGALG00010013040	SLC25A43	case	control	-0.015009063	0.093232161	-0.1609859	0.872104506	0.975062028	down_in_case
PRJNA8274(	ENSGALG00010013041	LRP12	case	control	-0.022224839	0.047347535	-0.469398014	0.638785168	0.900303403	down_in_case
PRJNA8274(	ENSGALG00010013042	SLC25A48	case	control	0.189077494	0.188216699	1.004573421	0.315102301	0.736508113	up_in_case
PRJNA8274(	ENSGALG00010013044	ENSGALG00010013044	case	control	-0.047182116	0.080877195	-0.583379721	0.559637706	0.869682591	down_in_case
PRJNA8274(	ENSGALG00010013045	FSD1L	case	control	0.006022753	0.124118946	0.048524042	0.961298604	0.992056045	up_in_case
PRJNA8274(	ENSGALG00010013046	SLC12A5	case	control	0.091137	0.264451667	0.344626302	0.73037533	0.933770678	up_in_case
PRJNA8274(	ENSGALG00010013047	CTPS1	case	control	-0.047969646	0.049104254	-0.976893877	0.328621693	0.745445783	down_in_case
PRJNA8274(	ENSGALG00010013048	WDR75	case	control	0.049582178	0.084414074	0.587368623	0.556956162	0.868346393	up_in_case
PRJNA8274(	ENSGALG00010013051	XPOT	case	control	0.016327826	0.036475942	0.447632757	0.654418258	0.906800494	up_in_case
PRJNA8274(	ENSGALG00010013053	MRTFA	case	control	-0.170284927	0.066693969	-2.553228258	0.010672953	0.184200521	down_in_case
PRJNA8274(	ENSGALG00010013056	ENSGALG00010013056	case	control	-0.010334439	0.128237311	-0.080588394	0.935769296	0.988842668	down_in_case
PRJNA8274(	ENSGALG00010013058	ENSGALG00010013058	case	control	-0.017938995	0.566297944	-0.031677663	0.974729108	0.993775355	down_in_case
PRJNA8274(	ENSGALG00010013062	ENSGALG00010013062	case	control	0.135843053	0.11986177	1.133330943	0.257075302	0.683997349	up_in_case
PRJNA8274(	ENSGALG00010013063	PARP12	case	control	0.154237038	0.111036187	1.389070008	0.164811463	0.579922223	up_in_case
PRJNA8274(	ENSGALG00010013065	FKTN	case	control	0.002344347	0.12219702	0.019184978	0.984693541	0.996591771	up_in_case
PRJNA8274(	ENSGALG00010013069	ENSGALG00010013069	case	control	-2.847996037	2.601312998	-1.094830203	0.273591032	0.699383582	down_in_case
PRJNA8274(	ENSGALG00010013071	ENSGALG00010013071	case	control	-0.032101868	0.14137503	-0.227068868	0.820370192	0.958669267	down_in_case
PRJNA8274(	ENSGALG00010013074	ENSGALG00010013074	case	control	-0.033061688	0.595047148	-0.055561459	0.955691168	0.992056045	down_in_case
PRJNA8274(	ENSGALG00010013076	RTN4IP1	case	control	0.005947989	0.106140915	0.056038605	0.955311053	0.992056045	up_in_case
PRJNA8274(	ENSGALG00010013078	COL5A2	case	control	-0.091945816	0.092693888	-0.99192965	0.321231844	0.73942679	down_in_case
PRJNA8274(	ENSGALG00010013079	ENSGALG00010013079	case	control	0.032666054	0.365323408	0.08941681	0.928750665	0.986776285	up_in_case
PRJNA8274(	ENSGALG00010013080	ARHGEF7	case	control	-0.027595506	0.060686251	-0.45472418	0.649307683	0.904011281	down_in_case
PRJNA8274(	ENSGALG00010013081	NCOA5	case	control	-0.002263565	0.046900412	-0.048263221	0.961506466	0.992056045	down_in_case
PRJNA8274(	ENSGALG00010013082	CHAF1B	case	control	-0.145735556	0.069164961	-2.107072067	0.035111333	0.323358784	down_in_case
PRJNA8274(	ENSGALG00010013084	SGSM3	case	control	0.158321771	0.08475863	1.867913285	0.061774159	0.405030019	up_in_case
PRJNA8274(	ENSGALG00010013085	LYSMD3	case	control	0.171365542	0.144303846	1.187532745	0.235017548	0.659686983	up_in_case
PRJNA8274(	ENSGALG00010013086	CXCL14	case	control	0.007825091	0.259396899	0.030166478	0.975934283	0.994231527	up_in_case
PRJNA8274(	ENSGALG00010013087	GPN2	case	control	0.182923072	0.062717243	2.916631278	0.003538338	0.101807307	up_in_case
PRJNA8274(	ENSGALG00010013089	TBK1	case	control	0.047334645	0.057162682	0.828068988	0.407631431	0.794388636	up_in_case
PRJNA8274(	ENSGALG00010013093	GPATCH3	case	control	-0.035960233	0.095046626	-0.378343076	0.70517575	0.924182432	down_in_case
PRJNA8274(	ENSGALG00010013095	ADSL	case	control	-0.021577514	0.096370538	-0.223901566	0.822833898	0.959776917	down_in_case
PRJNA8274(	ENSGALG00010013097	ENSGALG00010013097	case	control	0.245377569	0.304664149	0.805403488	0.42058688	0.801969583	up_in_case
PRJNA8274(	ENSGALG00010013099	SIM2	case	control	-0.037358308	0.110240207	-0.33888097	0.7346994	0.934096263	down_in_case
PRJNA8274(	ENSGALG00010013100	ZFPM2	case	control	0.063647238	0.174929032	0.363846053	0.715972971	0.926862121	up_in_case
PRJNA8274(	ENSGALG00010013103	AIRIM	case	control	0.034456768	0.155559316	0.22150244	0.824701237	0.960673484	up_in_case
PRJNA8274(	ENSGALG00010013104	HLCS	case	control	-0.074946818	0.066657298	-1.124360281	0.260860215	0.687359696	down_in_case
PRJNA8274(	ENSGALG00010013106	SETD9	case	control	0.325639898	0.160759317	2.025636232	0.042802076	0.344633831	up_in_case
PRJNA8274(	ENSGALG00010013109	ENSGALG00010013109	case	control	-0.513278084	0.996549776	-0.515055139	0.60651449	0.886735222	down_in_case
PRJNA8274(	ENSGALG00010013110	ANKRD10	case	control	0.093470775	0.079572011	1.17466901	0.240127162	0.665210852	up_in_case
PRJNA8274(	ENSGALG00010013111	RFESD	case	control	0.223082012	0.29910423	0.745833693	0.455767884	0.822065071	up_in_case
PRJNA8274(	ENSGALG00010013112	CD40	case	control	0.148449171	0.115801007	1.281933332	0.199866034	0.622902456	up_in_case
PRJNA8274(	ENSGALG00010013114	PIGP	case	control	-0.067691671	0.109268115	-0.619500676	0.535586577	0.857079477	down_in_case
PRJNA8274(	ENSGALG00010013115	ENSGALG00010013115	case	control	0.677226407	0.207312743	3.266689722	0.001088129	0.051553287	up_in_case
PRJNA8274(	ENSGALG00010013116	TARS	case	control	0.131376845	0.164443073	0.798919905	0.424336856	0.803723282	up_in_case
PRJNA8274(	ENSGALG00010013117	ARSK	case	control	0.016623086	0.175581954	0.094674228	0.924573589	0.985571367	up_in_case
PRJNA8274(	ENSGALG00010013119	RNF114	case	control	-0.120734277	0.053875716	-2.240977671	0.025027521	0.280921158	down_in_case
PRJNA8274(	ENSGALG00010013121	PCBD2	case	control	0.008052948	0.110056958	0.073170732	0.941670257	0.989551456	up_in_case
PRJNA8274(	ENSGALG00010013123	PPFIBP1	case	control	-0.101604923	0.047988253	-2.11728738	0.034235465	0.319352351	down_in_case
PRJNA8274(	ENSGALG00010013125	POU3F3	case	control	1.044527527	0.589031903	1.773295335	0.076179782	0.44283369	up_in_case
PRJNA8274(	ENSGALG00010013129	RASSF3	case	control	-0.023892151	0.06962917	-0.343134223	0.731497486	0.933770678	down_in_case
PRJNA8274(	ENSGALG00010013131	SPATA2	case	control	-0.043632454	0.056852824	-0.767463277	0.442806119	0.814107419	down_in_case
PRJNA8274(	ENSGALG00010013132	SKIC3	case	control	-0.123078101	0.150453368	-0.818048158	0.41332969	0.797761447	down_in_case
PRJNA8274(	ENSGALG00010013133	TXNDC15	case	control	0.08330367	0.0720361	1.156415602	0.247511203	0.672031667	up_in_case
PRJNA8274(	ENSGALG00010013134	ENSGALG00010013134	case	control	-0.034214961	1.796509548	-0.019045243	0.984805013	0.996591771	down_in_case
PRJNA8274(	ENSGALG00010013135	TTC3	case	control	0.006711238	0.067224468	0.09983326	0.920476703	0.984866723	up_in_case
PRJNA8274(	ENSGALG00010013137	TMEM121	case	control	-0.095298152	0.133888638	-0.711771762	0.476606122	0.830520323	down_in_case
PRJNA8274(	ENSGALG00010013139	TMEM185A	case	control	0.057337817	0.057332853	1.000086574	0.317268613	0.737768794	up_in_case
PRJNA8274(	ENSGALG00010013143	ENSGALG00010013143	case	control	-0.257355236	0.70489839	-0.365095508	0.715040114	0.926862121	down_in_case
PRJNA8274(	ENSGALG00010013144	TEDC1	case	control	-0.120720876	0.099959004	-1.207703867	0.22716119	0.650852538	down_in_case
PRJNA8274(	ENSGALG00010013145	ENSGALG00010013145	case	control	0.251391084	0.267195804	0.940849671	0.346781902	0.757096367	up_in_case
PRJNA8274(	ENSGALG00010013146	TNRC6B	case	control	-0.041677697	0.098976733	-0.421085804	0.673692427	0.91379937	down_in_case
PRJNA8274(	ENSGALG00010013149	MCTP1	case	control	-0.050978194	0.128089695	-0.397988256	0.690638842	0.918784485	down_in_case
PRJNA8274(	ENSGALG00010013150	GNS	case	control	-0.142719399	0.077852888	-1.833193378	0.066773818	0.418617099	down_in_case
PRJNA8274(	ENSGALG00010013151	ENSGALG00010013151	case	control	-2.614107097	1.422901966	-1.837165988	0.066185395	0.416388539	down_in_case
PRJNA8274(	ENSGALG00010013152	TBXAS1	case	control	-0.037872813	0.157029428	-0.241182904	0.80941336	0.955875648	down_in_case
PRJNA8274(	ENSGALG00010013154	ENSGALG00010013154	case	control	-0.18958769	0.657974458	-0.288138373	0.773240821	0.946459495	down_in_case
PRJNA8274(	ENSGALG00010013156	ABCC9	case	control	-0.113310758	0.105119296	-1.077925383	0.281067057	0.705762606	down_in_case
PRJNA8274(	ENSGALG00010013158	CRIP1	case	control	0.159156006	0.129147007	1.232363103	0.217813477	0.643008408	up_in_case
PRJNA8274(	ENSGALG00010013159	ENSGALG00010013159	case	control	2.043833535	0.953122083	2.144356501	0.032004333	0.3098361	up_in_case
PRJNA8274(	ENSGALG00010013160	CRYBG1	case	control	0.398118965	0.540055759	0.737181223	0.461012151	0.82454265	up_in_case
PRJNA8274(	ENSGALG00010013162	IDS	case	control	0.119803614	0.074824905	1.601119487	0.109350457	0.501671971	up_in_case
PRJNA8274(	ENSGALG00010013163	ENSGALG00010013163	case	control	0.021533794	0.292177416	0.073701091	0.941248231	0.989551456	up_in_case
PRJNA8274(	ENSGALG00010013164	FAM83F	case	control	0.186425139	0.495894798	0.375936871	0.706963828	0.92461283	up_in_case
PRJNA8274(	ENSGALG00010013165	NUDC	case	control	0.038253195	0.043848224	0.872400102	0.382990145	0.780061953	up_in_case
PRJNA8274(	ENSGALG00010013167	ENSGALG00010013167	case	control	-0.345984857	0.42630625	-0.811587579	0.417028321	0.799987395	down_in_case
PRJNA8274(	ENSGALG00010013168	VPS26C	case	control	0.018659948	0.056176015	0.332169307	0.739761419	0.9363983	up_in_case
PRJNA8274(	ENSGALG00010013170	COL3A1	case	control	-0.28705819	0.129171934	-2.222295368	0.026263351	0.287789976	down_in_case
PRJNA8274(	ENSGALG00010013171	SLC9A8	case	control	0.138621481	0.092889757	1.492322565	0.135614604	0.539774365	up_in_case
PRJNA8274(	ENSGALG00010013173	KCNJ8	case	control	0.150149728	0.065778748	2.28264799	0.022451112	0.266705822	up_in_case
PRJNA8274(	ENSGALG00010013174	ADAMTS12	case	control	-0.023246732	0.180860941	-0.128533736	0.897726602	0.980176778	down_in_case
PRJNA8274(	ENSGALG00010013175	HIPK2	case	control	-0.188722832	0.070639006	-2.671651855	0.007547891	0.151682101	down_in_case
PRJNA8274(	ENSGALG00010013177	OXR1	case	control	-0.100112384	0.050812368	-1.970236555	0.048811266	0.363542241	down_in_case
PRJNA8274(	ENSGALG00010013179	TBC1D30	case	control	-0.16457866	0.174191192	-0.944816198	0.344752721	0.755870938	down_in_case
PRJNA8274(	ENSGALG00010013180	C5orf24	case	control	-0.033927086	0.034630394	-0.979691011	0.327238664	0.744857565	down_in_case
PRJNA8274(	ENSGALG00010013181	DYRK1A	case	control	-0.106064266	0.051826133	-2.046540221	0.04070325	0.337767404	down_in_case
PRJNA8274(	ENSGALG00010013183	CLTA	case	control	0.312807908	0.172473132	1.813661667	0.06972983	0.425679712	up_in_case
PRJNA8274(	ENSGALG00010013184	LDHB	case	control	0.229245898	0.149251833	1.535967053	0.124546461	0.523366845	up_in_case
PRJNA8274(	ENSGALG00010013185	ENSGALG00010013185	case	control	-0.190482077	0.338871878	-0.562106474	0.574043477	0.877573415	down_in_case
PRJNA8274(	ENSGALG00010013186	DDX46	case	control	-0.003279663	0.039217634	-0.083627242	0.933352807	0.988003934	down_in_case
PRJNA8274(	ENSGALG00010013187	KDF1	case	control	-0.126946982	0.230532074	-0.550669502	0.581860255	0.880300639	down_in_case
PRJNA8274(	ENSGALG00010013188	ENSGALG00010013188	case	control	-0.123945074	0.185507759	-0.668139571	0.504044504	0.842675107	down_in_case

PRJNA8274(	ENSGALG00010013190	SLC45A2	case	control	4.730446089	0.549530034	8.608166607	7.42383E-18	7.58291E-15	up_in_case
PRJNA8274(	ENSGALG00010013191	ABRA	case	control	-0.255327579	0.118767716	-2.149806254	0.031570543	0.3098361	down_in_case
PRJNA8274(	ENSGALG00010013192	B4GALT5	case	control	0.086003992	0.053183307	1.617123828	0.105851554	0.495266487	up_in_case
PRJNA8274(	ENSGALG00010013194	GRAP2	case	control	-0.365858149	0.670274044	-0.545833682	0.585180268	0.881307382	down_in_case
PRJNA8274(	ENSGALG00010013196	MRPS9	case	control	-0.038765068	0.060604717	-0.639637795	0.522408105	0.851040772	down_in_case
PRJNA8274(	ENSGALG00010013198	CAMLG	case	control	0.072492767	0.057589048	1.258794326	0.20810463	0.632630998	up_in_case
PRJNA8274(	ENSGALG00010013199	RALYL	case	control	-0.490336479	0.695948483	-0.704558586	0.481084979	0.8322127	down_in_case
PRJNA8274(	ENSGALG00010013201	GYS2	case	control	0.930401209	1.132109293	0.821829849	0.411173743	0.796718771	up_in_case
PRJNA8274(	ENSGALG00010013202	ENSGALG00010013202	case	control	0.384311553	0.45384268	0.84679465	0.397109591	0.788531716	up_in_case
PRJNA8274(	ENSGALG00010013203	AMACR	case	control	0.020232436	0.140965613	0.143527459	0.885873627	0.977630122	up_in_case
PRJNA8274(	ENSGALG00010013204	ENSGALG00010013204	case	control	0.160721309	0.326876046	0.491688855	0.622939318	0.894021703	up_in_case
PRJNA8274(	ENSGALG00010013205	KCNJ6	case	control	-0.134101373	0.232876151	-0.57584846	0.564717615	0.872275547	down_in_case
PRJNA8274(	ENSGALG00010013206	AFF2	case	control	-0.093035133	0.112283681	-0.828572171	0.407346541	0.794380997	down_in_case
PRJNA8274(	ENSGALG00010013207	C1QTNF3	case	control	0.135235634	0.116869953	1.157146296	0.247212595	0.671824421	up_in_case
PRJNA8274(	ENSGALG00010013208	ENSGALG00010013208	case	control	0.176530952	0.480774018	0.367180724	0.713484216	0.926862121	up_in_case
PRJNA8274(	ENSGALG00010013209	KIAA1549	case	control	-0.116182747	0.078651147	-1.477190763	0.139624509	0.541778931	down_in_case
PRJNA8274(	ENSGALG00010013212	ENSGALG00010013212	case	control	0.000713425	0.108720832	0.006561991	0.994764326	0.998586153	up_in_case
PRJNA8274(	ENSGALG00010013213	SH2D1A	case	control	0.662025945	0.417605493	1.585290318	0.112900388	0.507003543	up_in_case
PRJNA8274(	ENSGALG00010013214	RAI14	case	control	-0.053696514	0.087885537	-0.610982378	0.541211247	0.85975913	down_in_case
PRJNA8274(	ENSGALG00010013215	SF3A3	case	control	0.024132536	0.048265263	0.499998024	0.617076469	0.891081782	up_in_case
PRJNA8274(	ENSGALG00010013217	TGFBRAP1	case	control	0.02769762	0.09212378	0.300656575	0.763676386	0.943786458	up_in_case
PRJNA8274(	ENSGALG00010013220	CSTF1	case	control	0.061738099	0.038088008	1.62093275	0.105032062	0.494770427	up_in_case
PRJNA8274(	ENSGALG00010013221	C2orf49	case	control	-0.027960529	0.074435114	-0.37563628	0.707187315	0.92461283	down_in_case
PRJNA8274(	ENSGALG00010013225	GNE	case	control	0.153119792	0.083540437	1.832882347	0.06682007	0.418723486	up_in_case
PRJNA8274(	ENSGALG00010013226	DUSP6	case	control	-0.043717685	0.079671884	-0.548721623	0.583196505	0.881296631	down_in_case
PRJNA8274(	ENSGALG00010013227	CITED4	case	control	0.2407363	0.127456173	1.888777093	0.058921703	0.395497471	up_in_case
PRJNA8274(	ENSGALG00010013228	RTFDC1	case	control	0.058792347	0.047840447	1.228925535	0.219099727	0.643443819	up_in_case
PRJNA8274(	ENSGALG00010013230	ENSGALG00010013230	case	control	-0.065272461	0.121006173	-0.539414308	0.589601011	0.882949889	down_in_case
PRJNA8274(	ENSGALG00010013232	FHL2	case	control	-0.000512207	0.106049758	-0.00482987	0.996146336	0.998710499	down_in_case
PRJNA8274(	ENSGALG00010013233	LRRCC1	case	control	-0.193135635	0.125460577	-1.539412938	0.123703517	0.522883756	down_in_case
PRJNA8274(	ENSGALG00010013235	GCNT7	case	control	0.392999801	0.195359676	2.011673083	0.04425441	0.350201601	up_in_case
PRJNA8274(	ENSGALG00010013236	MON2	case	control	0.137554462	0.075810812	1.814443863	0.069609418	0.425209175	up_in_case
PRJNA8274(	ENSGALG00010013238	RAD1	case	control	0.260940882	0.167545331	1.557434519	0.119367356	0.516645008	up_in_case
PRJNA8274(	ENSGALG00010013241	SEC24A	case	control	-0.115890989	0.050912996	-2.276255523	0.022830721	0.267253668	down_in_case
PRJNA8274(	ENSGALG00010013242	SULT4A1	case	control	0.142639528	0.189378749	0.753197119	0.45133147	0.820289785	up_in_case
PRJNA8274(	ENSGALG00010013244	TRIM24	case	control	-0.061129778	0.061464923	-0.994547382	0.31995645	0.738813491	down_in_case
PRJNA8274(	ENSGALG00010013246	LNPEP	case	control	-0.173375314	0.091214179	-1.900749587	0.05733482	0.389495453	down_in_case
PRJNA8274(	ENSGALG00010013247	ATG5	case	control	-0.039243736	0.062620475	-0.626691766	0.530861302	0.855560567	down_in_case
PRJNA8274(	ENSGALG00010013248	ENSGALG00010013248	case	control	-0.330041034	0.942762866	-0.350078525	0.726279767	0.932226286	down_in_case
PRJNA8274(	ENSGALG00010013249	AURKA	case	control	-0.070200153	0.096928702	-0.724245263	0.468915175	0.828963655	down_in_case
PRJNA8274(	ENSGALG00010013250	E2F5	case	control	0.020676426	0.085408185	0.242089512	0.808710805	0.955875648	up_in_case
PRJNA8274(	ENSGALG00010013251	MTA1	case	control	-0.031490241	0.041577228	-0.757391559	0.448815311	0.818701429	down_in_case
PRJNA8274(	ENSGALG00010013252	PPM1H	case	control	1.004776019	0.203668809	4.933381923	8.08179E-07	0.00022607	up_in_case
PRJNA8274(	ENSGALG00010013253	SHB	case	control	0.013641594	0.06134867	0.22236169	0.824032332	0.960422238	up_in_case
PRJNA8274(	ENSGALG00010013254	SAR1B	case	control	-0.027770669	0.073014601	-0.38034405	0.703690041	0.923760915	down_in_case
PRJNA8274(	ENSGALG00010013256	BRIX1	case	control	0.172482194	0.170357298	1.012473169	0.311311869	0.734097745	up_in_case
PRJNA8274(	ENSGALG00010013257	STX12	case	control	0.094816948	0.057948112	1.636238787	0.101789622	0.491460867	up_in_case
PRJNA8274(	ENSGALG00010013258	RBIS	case	control	-0.081515688	0.084310719	-0.966848442	0.333619804	0.748508541	down_in_case
PRJNA8274(	ENSGALG00010013259	PTPRB	case	control	-0.020686926	0.090749899	-0.227955363	0.819680942	0.958669267	down_in_case
PRJNA8274(	ENSGALG00010013260	FAM210B	case	control	0.233829673	0.106535941	2.194843071	0.028174857	0.299572314	up_in_case
PRJNA8274(	ENSGALG00010013261	JADE2	case	control	-0.085114429	0.062639891	-1.358789542	0.174213288	0.593154767	down_in_case
PRJNA8274(	ENSGALG00010013262	CA13	case	control	0.261527507	0.205288338	1.273952085	0.202680398	0.625537942	up_in_case
PRJNA8274(	ENSGALG00010013263	ATP11A	case	control	0.061728923	0.089684318	0.688291159	0.491269448	0.836925555	up_in_case
PRJNA8274(	ENSGALG00010013264	DNAJC21	case	control	0.113684426	0.109528526	1.037943541	0.299296341	0.722718284	up_in_case
PRJNA8274(	ENSGALG00010013265	PACS2	case	control	-0.145434059	0.075203706	-1.933868251	0.053129329	0.376522532	down_in_case
PRJNA8274(	ENSGALG00010013266	KIAA1755	case	control	-0.012423868	0.101067864	-0.122926003	0.902165695	0.980763983	down_in_case
PRJNA8274(	ENSGALG00010013267	PNPLA3	case	control	0.03337501	0.119509307	0.27926704	0.780039898	0.948864508	up_in_case
PRJNA8274(	ENSGALG00010013268	PTPRR	case	control	-0.363232638	0.323288025	-1.123557355	0.261200856	0.687516348	down_in_case
PRJNA8274(	ENSGALG00010013269	INPP5B	case	control	-0.047481278	0.066130759	-0.717990817	0.472762949	0.829235491	down_in_case
PRJNA8274(	ENSGALG00010013271	GULP1	case	control	-0.134507061	0.072115336	-1.865165843	0.062158162	0.405537748	down_in_case
PRJNA8274(	ENSGALG00010013273	STAG2	case	control	-0.058393984	0.039156961	-1.491279788	0.135888053	0.539774365	down_in_case
PRJNA8274(	ENSGALG00010013274	MTF1	case	control	0.004078743	0.057959597	0.070372173	0.943897439	0.990183239	up_in_case
PRJNA8274(	ENSGALG00010013275	MAPRE1	case	control	-0.052670025	0.064022937	-0.822674309	0.410693229	0.796543221	down_in_case
PRJNA8274(	ENSGALG00010013276	POC1B	case	control	-0.088354299	0.082805163	-1.067014364	0.28596535	0.710420738	down_in_case
PRJNA8274(	ENSGALG00010013277	YRDC	case	control	-0.12333368	0.085416812	-1.443904038	0.148765971	0.55519893	down_in_case
PRJNA8274(	ENSGALG00010013278	SAMM50	case	control	-0.07212923	0.058821037	-1.226248877	0.220105033	0.644319749	down_in_case
PRJNA8274(	ENSGALG00010013280	FRS2	case	control	-0.024441807	0.082264389	-0.297112856	0.76638035	0.944029546	down_in_case
PRJNA8274(	ENSGALG00010013281	BIRC8	case	control	-0.179244686	0.089906595	-1.993676734	0.046187394	0.356053762	down_in_case
PRJNA8274(	ENSGALG00010013282	SEC22B	case	control	0.05747717	0.056304358	1.020829867	0.307335051	0.730412371	up_in_case
PRJNA8274(	ENSGALG00010013283	PAWR	case	control	0.025743822	0.219540528	0.117262277	0.906652217	0.981730793	up_in_case
PRJNA8274(	ENSGALG00010013284	MANEAL	case	control	0.130233216	0.187715959	0.69377807	0.487821398	0.835553062	up_in_case
PRJNA8274(	ENSGALG00010013285	ATP2B1	case	control	0.02223605	0.067220455	0.330792913	0.740800914	0.937059095	up_in_case
PRJNA8274(	ENSGALG00010013286	RNF14	case	control	-0.13808201	0.058579951	-2.357154739	0.018415571	0.242205346	down_in_case
PRJNA8274(	ENSGALG00010013288	CA3A	case	control	-0.371514897	0.380750379	-0.975743999	0.329191342	0.745673403	down_in_case
PRJNA8274(	ENSGALG00010013289	TM9SF4	case	control	0.011748269	0.05411809	0.217085808	0.828141474	0.96154783	up_in_case
PRJNA8274(	ENSGALG00010013290	AKR1D1	case	control	0.143053973	0.15533334	0.92094829	0.357077425	0.763373775	up_in_case
PRJNA8274(	ENSGALG00010013292	PRDM1	case	control	-0.064620254	0.26940932	-0.239859016	0.810439554	0.955972758	down_in_case
PRJNA8274(	ENSGALG00010013293	TUBGCP3	case	control	-0.053722377	0.054031551	-0.994277915	0.320087584	0.738813491	down_in_case
PRJNA8274(	ENSGALG00010013295	CA3B	case	control	-0.898107132	0.3813698	-2.35495084	0.018525156	0.242591332	down_in_case
PRJNA8274(	ENSGALG00010013296	AGXT2	case	control	0.647543828	0.500843148	1.292907432	0.196043077	0.619095929	up_in_case
PRJNA8274(	ENSGALG00010013297	FCHO2	case	control	0.011399695	0.120613834	0.09451399	0.92470087	0.985571367	up_in_case
PRJNA8274(	ENSGALG00010013298	EPYC	case	control	0.700475663	0.758317123	0.923723917	0.355630077	0.763360867	up_in_case
PRJNA8274(	ENSGALG00010013299	PLAGL2	case	control	-0.080461583	0.058324724	-1.379545026	0.167726774	0.583859022	down_in_case
PRJNA8274(	ENSGALG00010013300	GNPDA1	case	control	0.210903256	0.064660248	3.261714292	0.001107407	0.051921048	up_in_case
PRJNA8274(	ENSGALG00010013301	PARVB	case	control	-0.084814259	0.094614731	-0.896417051	0.370030064	0.772544067	down_in_case
PRJNA8274(	ENSGALG00010013303	TFPI	case	control	0.285229374	0.19149589	1.489480395	0.136360912	0.539840644	up_in_case
PRJNA8274(	ENSGALG00010013304	KCNJ15	case	control	0.066351244	0.138369071	0.479523665	0.631566137	0.897039706	up_in_case
PRJNA8274(	ENSGALG00010013305	THOC2	case	control	0.018339051	0.073460005	0.249646737	0.802860552	0.954891353	up_in_case
PRJNA8274(	ENSGALG00010013306	ARSB	case	control	0.15585599	0.157316365	0.990716955	0.32182381	0.739571618	up_in_case
PRJNA8274(	ENSGALG00010013307	POFUT1	case	control	0.080207004	0.050115262	1.60045066	0.109498644	0.501801854	up_in_case
PRJNA8274(	ENSGALG00010013309	ENSGALG00010013309	case	control	-0.06019911	0.205124709	-0.293475661	0.7691586	0.94539152	down_in_case
PRJNA8274(	ENSGALG00010013310	MYO16	case	control	-0.005212401	0.197621896	-0.026375626	0.978957735	0.995525769	down_in_case
PRJNA8274(	ENSGALG00010013311	RSPO1	case	control	-0.123941082	0.30748995	-0.403073603	0.686894079	0.917290381	down_in_case
PRJNA8274(	ENSGALG00010013312	ENSGALG00010013312	case	control	0.081561192	0.275145518	0.296429296	0.766902255	0.944347046	up_in_case
PRJNA8274(	ENSGALG00010013313	GAK	case	control	0.065607484	0.131841321	0.497624597	0.618748663	0.891656847	up_in_case
PRJNA8274(	ENSGALG00010013314	KIT	case	control	0.722356566	0.106577819	6.777738324	1.22072E-11	7.93466E-09	up_in_case
PRJNA8274(	ENSGALG00010013316	KDM7A	case	control	0.04540332	0.070122744	0.647483505	0.517319067	0.848258532	up_in_case
PRJNA8274(	ENSGALG00010013317	PREP	case	control	-0.071524908	0.045116921	-1.585323323	0.112892893	0.507003543	down_in_case
PRJNA8274(	ENSGALG00010013318	KERA	case	control	-0.100154126	0.121911306	-0.821532714	0.411342898	0.796793365	down_in_case
PRJNA8274(	ENSGALG00010013319	ENSGALG00010013319	case	control	-0.418008402	1.328558125	-0.31463313	0.753040221	0.940666434	down_in_case
PRJNA8274(	ENSGALG00010013320	CALCRL	case	control	0.055209615	0.086858425	0.635627632	0.525019155	0.852401131	up_in_case
PRJNA8274(	ENSGALG00010013322	IRS2	case	control	-0.418819903	0.134016558	-3.1251355	0.001777232	0.067952978	down_in_case
PRJNA8274(	ENSGALG00010013323	NOTCH2	case	control	-0.111583623	0.082106894	-1.359004309	0.174145223	0.593154767	down_in_case
PRJNA8274(	ENSGALG00010013324	PARVG	case	control	-0.102733673	0.089426272	-1.148808624	0.250634903	0.676241341	down_in_case
PRJNA8274(	ENSGALG00010013325	CA2	case	control	0.288670842	0.206469148	1.398130637	0.162073841	0.575672114	up_in_case
PRJNA8274(	ENSGALG00010013326	FGF20	case	control	-0.850773008	1.289639264	-0.659698438	0.509447369	0.844521404	down_in_case
PRJNA8274(	ENSGALG00010013327	SLF1	case	control	0.171060894	0.1787715	0.956868931	0.338633415	0.751259351	up_in_case
PRJNA8274(	ENSGALG00010013328	SLC37A3	case	control	-0.014887811	0.056951425	-0.26141244	0.793774465	0.95280257	down_in_case
PRJNA8274(	ENSGALG00010013329	LIFR	case	control	-0.066863528	0.095778161	-0.698108287	0.485109476	0.834423421	down_in_case
PRJNA8274(	ENSGALG00010013330	ERG	case	control	-0.001169936	0.10855985	-0.010776877	0.991401463	0.99790247	down_in_case
PRJNA8274(	ENSGALG00010013331	KIF3B	case	control	-0.06059666	0.053790067	-1.126539957	0.259937038	0.687227573	down_in_case
PRJNA8274(	ENSGALG00010013332	LUM	case	control	-0.120599	0.109021786	-1.106191748	0.268643534	0.695438547	down_in_case
PRJNA8274(	ENSGALG00010013333	PPP1R12A	case	control	-0.009292923	0.055413073	-0.167702718	0.866817158	0.973570415	down_in_case
PRJNA8274(	ENSGALG00010013334	SH3BGRL3	case	control	0.020829185	0.06244683	0.333550708	0.738718619	0.93607843	up_in_case
PRJNA8274(	ENSGALG00010013335	POPDC3	case	control	0.03189371	0.108482297	0.293999208	0.768758508	0.945375926	up_in_case
PRJNA8274(	ENSGALG00010013337	RAB19	case	control	0.14354817	0.164703914	0.871552877	0.383452348	0.780061953	up_in_case
PRJNA8274(	ENSGALG00010013339	ATP6V0D2	case	control	-0.071354115	0.736107216	-0.096934405	0.922778486	0.985482967	down_in_case
PRJNA8274(	ENSGALG00010013340	LIN28B	case	control	0.282009828	0.26479036	1.065030571	0.286862096	0.710659943	up_in_case
PRJNA8274(	ENSGALG00010013341	ENSGALG00010013341	case	control	0.482317733	1.133761375	0.425413799	0.670535049	0.913465866	up_in_case
PRJNA8274(	ENSGALG00010013343	SHISAL1	case	control	-0.249511054	0.228299937	-1.092908994	0.274433759	0.699970438	down_in_case
PRJNA8274(	ENSGALG00010013344	OSMR	case	control	-0.157288979	0.082932723	-1.896585246	0.057882699	0.391912561	down_in_case
PRJNA8274(	ENSGALG00010013345	DCN	case	control	-0.039187866	0.085037479	-0.460830518	0.644920205	0.902291775	down_in_case
PRJNA8274(	ENSGALG00010013346	HMGCS2	case	control	1.697869458	0.947708011	1.791553345	0.073204542	0.436961143	up_in_case
PRJNA8274(	ENSGALG00010013347	ENSGALG00010013347	case	control	-0.09517888	0.644149243	-0.147759049	0.882532929	0.976801393	down_in_case
PRJNA8274(	ENSGALG00010013348	MKRN4P	case	control	-0.000264748	0.042738647	-0.006194572	0.995057478	0.998618986	down_in_case
PRJNA8274(	ENSGALG00010013349	ETS2	case	control	-0.024723255	0.078042924	-0.316790472	0.751402594	0.940420766	down_in_case

PRJNA8274(	ENSGALG00010013351	EDAR	case	control	-0.065900071	0.410198873	-0.160653955	0.872365957	0.975062028	down_in_case
PRJNA8274(	ENSGALG00010013352	ASXL1	case	control	-0.010249008	0.052210529	-0.196301552	0.844374146	0.96721237	down_in_case
PRJNA8274(	ENSGALG00010013353	NDFIP1	case	control	-0.009543499	0.047122644	-0.2025247	0.83950655	0.965286179	down_in_case
PRJNA8274(	ENSGALG00010013354	WWP1	case	control	-0.1232962	0.083027631	-1.485002037	0.137543285	0.539854583	down_in_case
PRJNA8274(	ENSGALG00010013355	FAM171B	case	control	-0.062056169	0.093670096	-0.662497121	0.507652679	0.843702533	down_in_case
PRJNA8274(	ENSGALG00010013356	AMER1	case	control	0.035005751	0.052609144	0.665392906	0.505799221	0.843280685	up_in_case
PRJNA8274(	ENSGALG00010013357	RICTOR	case	control	-0.140428057	0.123885067	-1.133534983	0.256989659	0.683997349	down_in_case
PRJNA8274(	ENSGALG00010013358	RPS16	case	control	-0.186011452	0.070500867	-2.638427849	0.008329142	0.160448204	down_in_case
PRJNA8274(	ENSGALG00010013359	BTG1	case	control	-0.092732454	0.068432312	-1.355097498	0.175386496	0.594211832	down_in_case
PRJNA8274(	ENSGALG00010013360	PRR5	case	control	0.177502731	0.089270724	1.988364412	0.046771402	0.356907825	up_in_case
PRJNA8274(	ENSGALG00010013361	ENSGALG00010013361	case	control	-0.245573906	0.258077889	-0.951549577	0.341325464	0.752827754	down_in_case
PRJNA8274(	ENSGALG00010013362	ENSGALG00010013362	case	control	-0.447622311	0.228798394	-1.956404952	0.050417469	0.367061596	down_in_case
PRJNA8274(	ENSGALG00010013363	HS3ST5	case	control	-0.005306657	0.162548532	-0.0326466	0.973956408	0.993644481	down_in_case
PRJNA8274(	ENSGALG00010013364	OTOGL	case	control	0.223605617	0.330876561	0.675797694	0.499169099	0.840142058	up_in_case
PRJNA8274(	ENSGALG00010013365	CEP85	case	control	-0.179657978	0.102368079	-1.755019532	0.079255967	0.449567761	down_in_case
PRJNA8274(	ENSGALG00010013367	NOL4L	case	control	-0.064255172	0.078815384	-0.815261804	0.414922467	0.798894747	down_in_case
PRJNA8274(	ENSGALG00010013369	MTMR8	case	control	-0.068741041	0.058960698	-1.165879032	0.243663358	0.667780411	down_in_case
PRJNA8274(	ENSGALG00010013370	CNKSR1	case	control	-0.27822228	0.151585923	-1.835409746	0.066445001	0.417470787	down_in_case
PRJNA8274(	ENSGALG00010013371	FAT1	case	control	-0.353326471	0.094165258	-3.752195647	0.000175293	0.016102953	down_in_case
PRJNA8274(	ENSGALG00010013372	SPRY4	case	control	0.2230104	0.093351113	2.388942053	0.016896967	0.231221657	up_in_case
PRJNA8274(	ENSGALG00010013373	ENSGALG00010013373	case	control	-0.100342252	0.430863175	-0.232886582	0.815849478	0.957374769	down_in_case
PRJNA8274(	ENSGALG00010013374	RMDN1	case	control	-0.145337763	0.143179336	-1.015074995	0.31007008	0.73300948	down_in_case
PRJNA8274(	ENSGALG00010013375	PSMG1	case	control	-0.060947177	0.054060093	-1.127396826	0.25957474	0.686756482	down_in_case
PRJNA8274(	ENSGALG00010013376	ARHGAP8	case	control	-0.529405861	0.469057119	-1.128659687	0.259041421	0.686345498	down_in_case
PRJNA8274(	ENSGALG00010013378	SH3RF3	case	control	-0.161869433	0.099962046	-1.619308931	0.10538081	0.495041148	down_in_case
PRJNA8274(	ENSGALG00010013379	ZNF593	case	control	-0.108459232	0.077534737	-1.398846971	0.161858878	0.575570547	down_in_case
PRJNA8274(	ENSGALG00010013380	PHGDH	case	control	0.373699234	0.207272205	1.802939446	0.071397725	0.43228239	up_in_case
PRJNA8274(	ENSGALG00010013381	DENND2A	case	control	-0.007767026	0.050772458	-0.152977163	0.878416285	0.976160112	down_in_case
PRJNA8274(	ENSGALG00010013382	ASB12	case	control	-0.179868429	0.108751492	-1.653939883	0.098139728	0.487291011	down_in_case
PRJNA8274(	ENSGALG00010013383	SEPTIN10	case	control	0.034798528	0.061480742	0.566006963	0.571389047	0.876702079	up_in_case
PRJNA8274(	ENSGALG00010013385	PGRMC1	case	control	-0.184817551	0.111656579	-1.655232076	0.097877431	0.487003223	down_in_case
PRJNA8274(	ENSGALG00010013386	ITGAV	case	control	0.004055544	0.079217923	0.051194779	0.959170312	0.992056045	up_in_case
PRJNA8274(	ENSGALG00010013387	NDST1	case	control	-0.027771273	0.082736804	-0.335658028	0.73712876	0.935060877	down_in_case
PRJNA8274(	ENSGALG00010013388	SOWAHC	case	control	0.245977184	0.192777662	1.275963105	0.201968562	0.625073547	up_in_case
PRJNA8274(	ENSGALG00010013389	NAP1L1	case	control	0.029807896	0.05046451	0.590670469	0.554741239	0.867067408	up_in_case
PRJNA8274(	ENSGALG00010013390	SLC16A2	case	control	-0.024384585	0.12564004	-0.194082914	0.84611096	0.967563913	down_in_case
PRJNA8274(	ENSGALG00010013391	HMGN1	case	control	0.031850621	0.057570104	0.553249326	0.580092695	0.879378502	up_in_case
PRJNA8274(	ENSGALG00010013392	HDAC2	case	control	0.084172864	0.04583404	1.836470529	0.066288097	0.416668038	up_in_case
PRJNA8274(	ENSGALG00010013393	ADCK2	case	control	0.148696595	0.085028279	1.748789885	0.080327346	0.452049261	up_in_case
PRJNA8274(	ENSGALG00010013394	DNMT3B	case	control	0.179791842	0.105405635	1.705713764	0.088061386	0.469004774	up_in_case
PRJNA8274(	ENSGALG00010013396	FYB1	case	control	-0.17575966	0.126379148	-1.390733067	0.164306386	0.579452754	down_in_case
PRJNA8274(	ENSGALG00010013397	MATN1	case	control	1.395596209	0.840345835	1.660740318			up_in_case
PRJNA8274(	ENSGALG00010013398	CHAMP1	case	control	-0.108397659	0.057705556	-1.878461378	0.060318076	0.399259348	down_in_case
PRJNA8274(	ENSGALG00010013399	FGF1	case	control	0.00373149	0.209518874	0.017809802	0.985790585	0.996595084	up_in_case
PRJNA8274(	ENSGALG00010013400	BBS10	case	control	-0.160718785	0.145156255	-1.107212259	0.268202168	0.694924988	down_in_case
PRJNA8274(	ENSGALG00010013402	MARCKS	case	control	-0.002973421	0.088597007	-0.033561196	0.973227066	0.99354775	down_in_case
PRJNA8274(	ENSGALG00010013403	CPNE3	case	control	-0.085789793	0.087896307	-0.976034105	0.329047564	0.745636104	down_in_case
PRJNA8274(	ENSGALG00010013404	ENSGALG00010013404	case	control	-0.138243787	0.173792398	-0.79545359	0.426349698	0.805283407	down_in_case
PRJNA8274(	ENSGALG00010013405	RPS14	case	control	-0.056525855	0.061296537	-0.922170456	0.356439671	0.763373775	down_in_case
PRJNA8274(	ENSGALG00010013406	ENSGALG00010013406	case	control	-0.130224472	0.461147284	-0.282392364	0.777642669	0.947849555	down_in_case
PRJNA8274(	ENSGALG00010013407	ZC3H15	case	control	-0.032264741	0.053398071	-0.604230462	0.545690431	0.862133047	down_in_case
PRJNA8274(	ENSGALG00010013408	RLIM	case	control	-0.014013194	0.034500454	-0.406174182	0.684614622	0.916433328	down_in_case
PRJNA8274(	ENSGALG00010013409	DAB2	case	control	0.108961884	0.110642201	0.98481306	0.324715904	0.742593544	up_in_case
PRJNA8274(	ENSGALG00010013410	KIAA0825	case	control	0.122140704	0.19735792	0.618879163	0.535995967	0.857238203	up_in_case
PRJNA8274(	ENSGALG00010013411	ENSGALG00010013411	case	control	1.338681455	0.547856995	2.443487017	0.014546093	0.212471021	up_in_case
PRJNA8274(	ENSGALG00010013412	COMMD7	case	control	-0.054304293	0.068541436	-0.792284144	0.428195015	0.805337183	down_in_case
PRJNA8274(	ENSGALG00010013413	WRB	case	control	0.038006808	0.065048246	0.584286443	0.559027612	0.869461123	up_in_case
PRJNA8274(	ENSGALG00010013414	LAPTM5	case	control	0.103926246	0.085329198	1.217944718	0.223244981	0.647021328	up_in_case
PRJNA8274(	ENSGALG00010013416	NDUFB2	case	control	0.10393885	0.072618796	1.431294035	0.152345963	0.560614325	up_in_case
PRJNA8274(	ENSGALG00010013417	AKR1B1	case	control	0.043875417	0.090560984	0.484484768	0.628041872	0.895865363	up_in_case
PRJNA8274(	ENSGALG00010013418	HCN1	case	control	-0.96258032	0.502026573	-1.917389183	0.055188507	0.383470355	down_in_case
PRJNA8274(	ENSGALG00010013419	ENSGALG00010013419	case	control	-0.193885891	0.152550583	-1.27096132	0.20374241	0.62581674	down_in_case
PRJNA8274(	ENSGALG00010013420	ENSGALG00010013420	case	control	-0.19538562	2.176028479	-0.089790011	0.928454087	0.986776285	down_in_case
PRJNA8274(	ENSGALG00010013423	ZNF804A	case	control	-0.182436204	0.203072499	-0.898379666	0.368983173	0.771834431	down_in_case
PRJNA8274(	ENSGALG00010013425	ENSGALG00010013425	case	control	-0.114368551	0.351763608	-0.325129004	0.745083439	0.937849656	down_in_case
PRJNA8274(	ENSGALG00010013427	NEXMIF	case	control	0.125887892	0.136110954	0.924891702	0.355022243	0.763161644	up_in_case
PRJNA8274(	ENSGALG00010013428	BPGM	case	control	0.021496681	0.090101569	0.238582756	0.811429138	0.956205737	up_in_case
PRJNA8274(	ENSGALG00010013429	PDE4DIP	case	control	-0.155998448	0.090186696	-1.729727943	0.083678894	0.458084422	down_in_case
PRJNA8274(	ENSGALG00010013431	TMEM175	case	control	0.319880895	0.152084333	2.103312604	0.035438452	0.32485248	up_in_case
PRJNA8274(	ENSGALG00010013434	CORIN	case	control	0.414905844	0.291294446	1.424352061	0.154344584	0.56303838	up_in_case
PRJNA8274(	ENSGALG00010013435	SDC3	case	control	-0.045668251	0.124419375	-0.367050955	0.71358101	0.926862121	down_in_case
PRJNA8274(	ENSGALG00010013436	NPAS2	case	control	-0.045204755	0.185509744	-0.243678602	0.807479747	0.955875648	down_in_case
PRJNA8274(	ENSGALG00010013437	ENSGALG00010013437	case	control	0.23853185	0.130543448	1.827221921	0.067666409	0.42089154	up_in_case
PRJNA8274(	ENSGALG00010013438	RPL37	case	control	-0.122148453	0.066464739	-1.837793309	0.066092868	0.41627012	down_in_case
PRJNA8274(	ENSGALG00010013440	EEA1	case	control	-0.08915916	0.075565219	-1.179896809	0.238041259	0.662913814	down_in_case
PRJNA8274(	ENSGALG00010013442	ENSGALG00010013442	case	control	-0.057128647	0.081061852	-0.704753789	0.480963471	0.8322127	down_in_case
PRJNA8274(	ENSGALG00010013444	OSBPL8	case	control	0.098409417	0.079234163	1.242007403	0.214233831	0.639112236	up_in_case
PRJNA8274(	ENSGALG00010013446	ATP5I	case	control	-0.007079267	0.19013783	-0.037232289	0.970299794	0.992761697	down_in_case
PRJNA8274(	ENSGALG00010013449	LCA5L	case	control	0.292448092	0.246086843	1.188393851	0.234678278	0.659294991	up_in_case
PRJNA8274(	ENSGALG00010013450	CD74	case	control	-0.136636984	0.119306971	-1.145255656	0.252103269	0.677148133	down_in_case
PRJNA8274(	ENSGALG00010013451	PRKAB2	case	control	-0.21926942	0.091867222	-2.386807997	0.016995365	0.231902401	down_in_case
PRJNA8274(	ENSGALG00010013452	LSS	case	control	0.172041446	0.119677559	1.437541408	0.150564216	0.558039903	up_in_case
PRJNA8274(	ENSGALG00010013453	PUM1	case	control	-0.069714681	0.04449529	-1.566787899	0.117164277	0.513845851	down_in_case
PRJNA8274(	ENSGALG00010013457	SH3BGR	case	control	0.037841411	0.096590239	0.391772617	0.695226229	0.92087209	up_in_case
PRJNA8274(	ENSGALG00010013458	LAMA4	case	control	-0.109155687	0.091656373	-1.190923049	0.233683795	0.65800118	down_in_case
PRJNA8274(	ENSGALG00010013459	ATP10D	case	control	0.01323978	0.099290648	0.133343676	0.893921587	0.979888889	up_in_case
PRJNA8274(	ENSGALG00010013461	S100B	case	control	-0.120025364	0.436422653	-0.275020931	0.783300158	0.949518053	down_in_case
PRJNA8274(	ENSGALG00010013463	ENSGALG00010013463	case	control	-0.149690685	0.138158545	-1.083470335	0.278599711	0.703953892	down_in_case
PRJNA8274(	ENSGALG00010013464	ABCB7	case	control	0.03968679	0.053316713	0.744359263	0.456659157	0.822343024	up_in_case
PRJNA8274(	ENSGALG00010013465	CALD1	case	control	-0.027303533	0.093306464	-0.292622099	0.769811021	0.945428692	down_in_case
PRJNA8274(	ENSGALG00010013466	BRAF	case	control	-0.021613622	0.052895608	-0.408609006	0.682826621	0.915901011	down_in_case
PRJNA8274(	ENSGALG00010013468	DEPDC1B	case	control	-0.00866247	0.136371181	-0.063521269	0.949351423	0.99138853	down_in_case
PRJNA8274(	ENSGALG00010013470	RPL31	case	control	-0.028594938	0.060820504	-0.470152931	0.63824576	0.900090175	down_in_case
PRJNA8274(	ENSGALG00010013472	ENSGALG00010013472	case	control	0.172807852	0.208250492	0.829807655	0.406647542	0.794253996	up_in_case
PRJNA8274(	ENSGALG00010013476	ZDHHC17	case	control	0.056052284	0.062493133	0.896935095	0.369753552	0.772544067	up_in_case
PRJNA8274(	ENSGALG00010013477	RFC1	case	control	-0.076430277	0.064742071	-1.180534948	0.237787519	0.662664656	down_in_case
PRJNA8274(	ENSGALG00010013480	TBC1D8	case	control	-0.038425598	0.07464343	-0.514788752	0.606700646	0.886827457	down_in_case
PRJNA8274(	ENSGALG00010013482	ENSGALG00010013482	case	control	0.176800519	0.176618683	1.001029542	0.316812526	0.737372905	up_in_case
PRJNA8274(	ENSGALG00010013483	ENSGALG00010013483	case	control	0.10821052	0.439035935	0.246473036	0.805316075	0.9556344	up_in_case
PRJNA8274(	ENSGALG00010013484	TCOF1	case	control	0.075895495	0.057117896	1.328751597	0.183929936	0.605757274	up_in_case
PRJNA8274(	ENSGALG00010013486	PLPP6	case	control	0.015199938	0.083324048	0.182419585	0.855253457	0.970644797	up_in_case
PRJNA8274(	ENSGALG00010013487	ENSGALG00010013487	case	control	0.203856979	0.158185238	1.288723153	0.19749435	0.62094535	up_in_case
PRJNA8274(	ENSGALG00010013488	UPRT	case	control	-0.017039677	0.061171225	-0.278557065	0.780584766	0.948875232	down_in_case
PRJNA8274(	ENSGALG00010013491	CNOT11	case	control	-0.085799229	0.048741254	-1.760299973	0.078356959	0.448818031	down_in_case
PRJNA8274(	ENSGALG00010013492	B3GALT5	case	control	0.458555209	0.606231986	0.756402201	0.449408089	0.818749597	up_in_case
PRJNA8274(	ENSGALG00010013493	ENSGALG00010013493	case	control	-0.161302376	0.061334237	-2.629891291	0.008541218	0.163506575	down_in_case
PRJNA8274(	ENSGALG00010013494	ASB1	case	control	0.138037436	0.078384039	1.761040103	0.078231616	0.448561389	up_in_case
PRJNA8274(	ENSGALG00010013497	FAM172A	case	control	0.098411639	0.196553004	0.500687535	0.616591046	0.891081782	up_in_case
PRJNA8274(	ENSGALG00010013498	KDR	case	control	0.018509567	0.072938531	0.253769405	0.799673711	0.954053991	up_in_case
PRJNA8274(	ENSGALG00010013501	AKR1B10	case	control	-0.305308061	0.147428588	-2.070887782	0.038369284	0.331548912	down_in_case
PRJNA8274(	ENSGALG00010013502	PAFAH2	case	control	-0.122091287	0.210552399	-0.57986177	0.562007838	0.870810714	down_in_case
PRJNA8274(	ENSGALG00010013504	IGSF5	case	control	0.483475764	0.686073359	0.704699807	0.480997071	0.8322127	up_in_case
PRJNA8274(	ENSGALG00010013506	RNF149	case	control	0.014475686	0.072308355	0.200193816	0.841329004	0.96603539	up_in_case
PRJNA8274(	ENSGALG00010013508	TUBE1	case	control	0.199839967	0.155180615	1.287789503	0.197819247	0.621110634	up_in_case
PRJNA8274(	ENSGALG00010013509	ENSGALG00010013509	case	control	0.168470938	0.116140007	1.450584872	0.14689549	0.551919473	up_in_case
PRJNA8274(	ENSGALG00010013510	MRPS33	case	control	-0.270771744	0.078831801	-3.434803497	0.000592984	0.035779182	down_in_case
PRJNA8274(	ENSGALG00010013511	FAM78B	case	control	0.273326274	0.109337201	2.499846994	0.012424696	0.195901209	up_in_case
PRJNA8274(	ENSGALG00010013512	ENSGALG00010013512	case	control	0.181158649	1.449082404	0.125016112	0.900510795	0.98028579	up_in_case
PRJNA8274(	ENSGALG00010013514	LMBRD2	case	control	0.135152937	0.136000531	0.993767722	0.320335964	0.738813491	up_in_case
PRJNA8274(	ENSGALG00010013515	RBP4L	case	control	-0.435366012	0.404178344	-1.077163136	0.281407391	0.705762606	down_in_case
PRJNA8274(	ENSGALG00010013516	CSRP2	case	control	0.113205148	0.092920424	1.218302098	0.223109193	0.646996863	up_in_case
PRJNA8274(	ENSGALG00010013517	STMN1	case	control	0.094514978	0.07284394	1.29749953	0.194459367	0.617304812	up_in_case
PRJNA8274(	ENSGALG00010013520	CREG2	case	control	-0.418931776	0.123222856	-3.399789528	0.000674377	0.038540612	down_in_case
PRJNA8274(	ENSGALG00010013523	TMEM178B	case	control	0.114839522	0.218246396	0.526192067	0.598754766	0.88476024	up_in_case
PRJNA8274(	ENSGALG00010013524	ZDHHC15	case	control	0.031109506	0.112416742	0.276733748	0.781984556	0.949394183	up_in_case
PRJNA8274(	ENSGALG00010013526	SIRPA	case	control	-0.375500246	0.323030536	-1.162429567	0.245061019	0.66954004	down_in_case

PRJNA8274(	ENSGALG00010013527	ADARB1	case	control	-0.078911818	0.084652449	-0.932185884	0.351240469	0.760831854	down_in_case
PRJNA8274(	ENSGALG00010013528	PRDX6	case	control	-0.011008783	0.066640625	-0.165196274	0.868789498	0.974483475	down_in_case
PRJNA8274(	ENSGALG00010013531	ARSI	case	control	0.285738855	0.178184662	1.603610833	0.108799861	0.500951275	up_in_case
PRJNA8274(	ENSGALG00010013532	WDR19	case	control	0.046399576	0.09616741	0.482487523	0.62945966	0.896269608	up_in_case
PRJNA8274(	ENSGALG00010013533	SKP2	case	control	0.015635878	0.061727277	0.253305815	0.800031903	0.954053991	up_in_case
PRJNA8274(	ENSGALG00010013537	LMO3	case	control	-0.483862668	0.358833566	-1.348432014	0.177519473	0.596458756	down_in_case
PRJNA8274(	ENSGALG00010013538	E2F7	case	control	-0.112747742	0.110241639	-1.022732824	0.306434187	0.729466972	down_in_case
PRJNA8274(	ENSGALG00010013539	NSFL1C	case	control	-0.003829156	0.056453397	-0.067828619	0.945922062	0.990313016	down_in_case
PRJNA8274(	ENSGALG00010013544	ENSGALG00010013544	case	control	-0.989145678	0.296895213	-3.331632293	0.000863383	0.044742993	down_in_case
PRJNA8274(	ENSGALG00010013546	IDUA	case	control	0.139524043	0.133409703	1.045831294	0.29563893	0.71910813	up_in_case
PRJNA8274(	ENSGALG00010013549	PCP4	case	control	-0.029323494	0.207076649	-0.141606956	0.887390472	0.977633571	down_in_case
PRJNA8274(	ENSGALG00010013550	KLHL5	case	control	0.008641268	0.055555897	0.155541875	0.876394144	0.975666505	up_in_case
PRJNA8274(	ENSGALG00010013551	UBE2N	case	control	-0.039718961	0.053048605	-0.748727715	0.454021336	0.821331802	down_in_case
PRJNA8274(	ENSGALG00010013552	FGF16	case	control	0.120250339	0.123956645	0.97009998	0.331996659	0.748235181	up_in_case
PRJNA8274(	ENSGALG00010013553	TADA1	case	control	-0.022744947	0.081446526	-0.279262333	0.780043511	0.948864508	down_in_case
PRJNA8274(	ENSGALG00010013554	ENSGALG00010013554	case	control	-0.171376692	2.124826597	-0.080654436	0.935716773	0.988425668	down_in_case
PRJNA8274(	ENSGALG00010013556	RASL11B	case	control	0.082932005	0.15004551	0.55271234	0.580460402	0.879392282	up_in_case
PRJNA8274(	ENSGALG00010013558	SNAPC3	case	control	-0.046208771	0.068759848	-0.672031317	0.50156376	0.841254358	down_in_case
PRJNA8274(	ENSGALG00010013561	AGK	case	control	-0.196505244	0.098791232	-1.989096002	0.046690608	0.356907825	down_in_case
PRJNA8274(	ENSGALG00010013562	RERGL	case	control	-0.084377149	0.090126384	-0.936209186	0.349165495	0.759342183	down_in_case
PRJNA8274(	ENSGALG00010013563	FKBP1A	case	control	-0.228191739	0.066340746	-3.439692064	0.000582376	0.035288061	down_in_case
PRJNA8274(	ENSGALG00010013565	ENSGALG00010013565	case	control	-1.610917262	2.402944816	-0.670392949	0.502607328	0.84219414	down_in_case
PRJNA8274(	ENSGALG00010013569	ENSGALG00010013569	case	control	0.113840454	0.43911705	0.25924854	0.795443482	0.95280257	up_in_case
PRJNA8274(	ENSGALG00010013572	DENND11	case	control	0.248223954	0.073849378	3.361219269	0.000775992	0.042032898	up_in_case
PRJNA8274(	ENSGALG00010013573	ENSGALG00010013573	case	control	0.406616473	0.291148001	1.396597169	0.162534742	0.576163465	up_in_case
PRJNA8274(	ENSGALG00010013574	NR2F1	case	control	0.15648034	0.103393307	1.513447476	0.130166033	0.532650639	up_in_case
PRJNA8274(	ENSGALG00010013576	CAMK2A	case	control	-0.21946838	0.113283707	-1.937334025	0.052704526	0.374713019	down_in_case
PRJNA8274(	ENSGALG00010013580	RANBP3L	case	control	-0.355177703	0.235876669	-1.505777169	0.132124386	0.535549374	down_in_case
PRJNA8274(	ENSGALG00010013582	MAP4K4	case	control	-0.045693022	0.062323701	-0.733156423	0.46346303	0.825091817	down_in_case
PRJNA8274(	ENSGALG00010013583	FASLG	case	control	-0.155796423	0.507404228	-0.30704597	0.758808382	0.942078947	down_in_case
PRJNA8274(	ENSGALG00010013587	SLC26A1	case	control	0.291498456	0.507511052	0.574368686	0.565718339	0.872906976	up_in_case
PRJNA8274(	ENSGALG00010013588	ENSGALG00010013588	case	control	-0.921700555	0.463819215	-1.987197869	0.046900474	0.357313147	down_in_case
PRJNA8274(	ENSGALG00010013589	DSCAM	case	control	-0.114163441	0.363928757	-0.313697225	0.753751009	0.940872855	down_in_case
PRJNA8274(	ENSGALG00010013592	H3C33	case	control	0.151218481	0.447050548	0.338258126	0.735168676	0.934096263	up_in_case
PRJNA8274(	ENSGALG00010013595	WEE2	case	control	2.628769351	0.564193053	4.659343705	3.17219E-06	0.000657425	up_in_case
PRJNA8274(	ENSGALG00010013596	SDCBP2	case	control	-0.283330941	0.093097274	-3.043386017	0.00233932	0.079426268	down_in_case
PRJNA8274(	ENSGALG00010013598	PAQR7	case	control	0.372255454	0.210847755	1.765517753	0.077476789	0.446561099	up_in_case
PRJNA8274(	ENSGALG00010013604	FYN	case	control	0.101873983	0.055897329	1.822519694	0.068376168	0.422366828	up_in_case
PRJNA8274(	ENSGALG00010013609	PIGC	case	control	-0.19565854	0.113863824	-1.718355611	0.085731772	0.462802693	down_in_case
PRJNA8274(	ENSGALG00010013612	TWIST2	case	control	0.028785368	0.222839761	0.129175189	0.897219028	0.980176778	up_in_case
PRJNA8274(	ENSGALG00010013614	ATRX	case	control	-0.042854213	0.081031457	-0.528858977	0.596903282	0.884680373	down_in_case
PRJNA8274(	ENSGALG00010013617	PIK3C2G	case	control	0.079516356	0.157672533	0.504313302	0.614041231	0.890440426	up_in_case
PRJNA8274(	ENSGALG00010013619	IL1R2	case	control	0.216573713	0.126171183	1.716506955	0.086069296	0.463278022	up_in_case
PRJNA8274(	ENSGALG00010013621	PDGFRA	case	control	-0.062619909	0.088209591	-0.709899102	0.477766707	0.830520323	down_in_case
PRJNA8274(	ENSGALG00010013624	SST1	case	control	-0.939238801	0.754880716	-1.244221479	0.213418061	0.638582746	down_in_case
PRJNA8274(	ENSGALG00010013625	SSBP1	case	control	0.110108988	0.073228085	1.503644235	0.132672995	0.535938934	up_in_case
PRJNA8274(	ENSGALG00010013627	MYCBP	case	control	-0.009932514	0.114241268	-0.086943313	0.930716571	0.987258715	down_in_case
PRJNA8274(	ENSGALG00010013632	SLC1A3	case	control	-0.744473393	0.341823918	-2.177944123	0.029410197	0.305109318	down_in_case
PRJNA8274(	ENSGALG00010013635	BCL6	case	control	0.003517766	0.09007113	0.039055425	0.9688462	0.992761697	up_in_case
PRJNA8274(	ENSGALG00010013638	COMMD8	case	control	-0.107836121	0.067097336	-1.607159497	0.108019384	0.499736588	down_in_case
PRJNA8274(	ENSGALG00010013639	ENSGALG00010013639	case	control	-0.170216037	0.235469382	-0.722879703	0.469753795	0.829101076	down_in_case
PRJNA8274(	ENSGALG00010013640	SNPH	case	control	0.063630577	0.579611952	0.10978134	0.91258279	0.982717125	up_in_case
PRJNA8274(	ENSGALG00010013645	RRAGC	case	control	0.021580575	0.043577849	0.495218917	0.620445599	0.892955829	up_in_case
PRJNA8274(	ENSGALG00010013646	CLDN1	case	control	0.298555356	0.330710333	0.902769967	0.366647994	0.771770041	up_in_case
PRJNA8274(	ENSGALG00010013648	NAV3	case	control	-0.037443096	0.074216466	-0.504511979	0.613901647	0.890440426	down_in_case
PRJNA8274(	ENSGALG00010013650	BACE2	case	control	0.4786055	0.11055107	4.329270627	1.49604E-05	0.00240375	up_in_case
PRJNA8274(	ENSGALG00010013651	ENSGALG00010013651	case	control	0.078172091	0.088421333	0.884086325	0.376649615	0.775199984	up_in_case
PRJNA8274(	ENSGALG00010013654	SLC6A7	case	control	0.443235216	0.238269653	1.860225213	0.062853669	0.408011997	up_in_case
PRJNA8274(	ENSGALG00010013657	MAGT1	case	control	0.128133583	0.036968688	3.466002971	0.000528257	0.033449927	up_in_case
PRJNA8274(	ENSGALG00010013661	GABRA4	case	control	0.609275343	0.357424834	1.704625101	0.088264367	0.469561177	up_in_case
PRJNA8274(	ENSGALG00010013662	RAD21L1	case	control	0.036094943	0.527900961	0.068374461	0.945487552	0.990313016	up_in_case
PRJNA8274(	ENSGALG00010013663	OPA1	case	control	-0.088884824	0.044501576	-1.997341018	0.04578815	0.355274309	down_in_case
PRJNA8274(	ENSGALG00010013664	REV3L	case	control	-0.107231501	0.057931821	-1.850994823	0.064170298	0.410842437	down_in_case
PRJNA8274(	ENSGALG00010013666	COX7B	case	control	-0.114616107	0.078964417	-1.451490586	0.146643302	0.55166873	down_in_case
PRJNA8274(	ENSGALG00010013667	SOWAHA	case	control	0.756362872	0.416740303	1.81495014	0.069531572	0.425096829	up_in_case
PRJNA8274(	ENSGALG00010013669	NPR3	case	control	0.138149629	0.207260144	0.666551832	0.505058445	0.842675288	up_in_case
PRJNA8274(	ENSGALG00010013672	ENSGALG00010013672	case	control	1.315550418	0.395528478	3.326057389	0.000880838	0.04498565	up_in_case
PRJNA8274(	ENSGALG00010013675	CHRNA6	case	control	-1.267356733	0.470882309	-2.69145115	0.007114192	0.146516526	down_in_case
PRJNA8274(	ENSGALG00010013677	GABRG1	case	control	0.073956755	0.453497803	0.163080736	0.870454867	0.974794532	up_in_case
PRJNA8274(	ENSGALG00010013678	ATP7A	case	control	-0.021966538	0.047973697	-0.457887115	0.647033544	0.90295498	down_in_case
PRJNA8274(	ENSGALG00010013679	MFSD4B	case	control	-0.172527752	0.06797014	-2.538287443	0.011139645	0.185137791	down_in_case
PRJNA8274(	ENSGALG00010013686	UQCRQ	case	control	-0.148283082	0.11335085	-1.308177952	0.190812958	0.613935676	down_in_case
PRJNA8274(	ENSGALG00010013690	CREB3L2	case	control	0.018568124	0.079827372	0.232603478	0.816069327	0.957422215	up_in_case
PRJNA8274(	ENSGALG00010013696	CETN3	case	control	0.000954731	0.237368614	0.004022146	0.996790801	0.998921977	up_in_case
PRJNA8274(	ENSGALG00010013697	HDAC4	case	control	-0.058468186	0.049530057	-1.180458685	0.237817833	0.662664656	down_in_case
PRJNA8274(	ENSGALG00010013698	TFAP2E	case	control	-0.30659175	0.876118733	-0.349943151	0.726381362	0.932226286	down_in_case
PRJNA8274(	ENSGALG00010013704	LEAP2	case	control	0.497894328	0.867061023	0.574232164	0.565810706	0.872906976	up_in_case
PRJNA8274(	ENSGALG00010013709	PSMF1	case	control	-0.195363656	0.06455308	-3.02640333	0.002474819	0.081856092	down_in_case
PRJNA8274(	ENSGALG00010013715	IL1R1	case	control	0.067208525	0.173117924	0.388223954	0.697850308	0.922288441	up_in_case
PRJNA8274(	ENSGALG00010013719	NCDN	case	control	0.207163084	0.062193421	3.330948536	0.000865506	0.044742993	up_in_case
PRJNA8274(	ENSGALG00010013720	SLC16A10	case	control	0.103616215	0.102600444	1.009900267	0.312543075	0.734364975	up_in_case
PRJNA8274(	ENSGALG00010013732	FNTA	case	control	0.013327006	0.069804201	0.190919821	0.848588411	0.968692766	up_in_case
PRJNA8274(	ENSGALG00010013737	AFF4	case	control	-0.011903085	0.066776626	-0.178252267	0.858524864	0.971828322	down_in_case
PRJNA8274(	ENSGALG00010013740	GNPDA2	case	control	0.063566821	0.070603803	0.900331397	0.367943917	0.771770041	up_in_case
PRJNA8274(	ENSGALG00010013742	RSPO4	case	control	0.241464516	0.480898577	0.502111106	0.615589368	0.891081782	up_in_case
PRJNA8274(	ENSGALG00010013744	ENSGALG00010013744	case	control	0.118066358	0.251733653	0.469013009	0.639060338	0.900303403	up_in_case
PRJNA8274(	ENSGALG00010013746	ENSGALG00010013746	case	control	-0.044626173	0.184881202	-0.241377558	0.809262505	0.955875648	down_in_case
PRJNA8274(	ENSGALG00010013749	NELL2	case	control	0.035097862	0.185518809	0.189187619	0.849945771	0.969081847	up_in_case
PRJNA8274(	ENSGALG00010013751	FUT10	case	control	0.226867586	0.19455552	1.166081467	0.243581509	0.667780411	up_in_case
PRJNA8274(	ENSGALG00010013753	PLEKHA5	case	control	-0.375001115	0.112173004	-3.343060294	0.000828599	0.044212556	down_in_case
PRJNA8274(	ENSGALG00010013755	ANGPT4	case	control	0.050224015	0.109667368	0.457966814	0.646976284	0.90295498	up_in_case
PRJNA8274(	ENSGALG00010013757	DGKI	case	control	-0.203395512	0.23406067	-0.868986284	0.384854633	0.781070289	down_in_case
PRJNA8274(	ENSGALG00010013758	FAM3B	case	control	-0.83560475	0.543659565	-1.536999998	0.12429331	0.523029039	down_in_case
PRJNA8274(	ENSGALG00010013760	RPF2	case	control	-0.116314546	0.071234746	-1.632834445	0.102503821	0.491460867	down_in_case
PRJNA8274(	ENSGALG00010013766	DNM3	case	control	-0.006456135	0.104044106	-0.062051901	0.9505215	0.991497374	down_in_case
PRJNA8274(	ENSGALG00010013767	PGK2	case	control	-0.106559091	0.118444592	-0.899653489	0.368304682	0.771770041	down_in_case
PRJNA8274(	ENSGALG00010013768	FAM110A	case	control	-0.049255207	0.145000714	-0.339689413	0.734090436	0.933846596	down_in_case
PRJNA8274(	ENSGALG00010013771	PELO	case	control	0.187681416	0.185143827	1.013706044	0.310723039	0.733587496	up_in_case
PRJNA8274(	ENSGALG00010013772	KIAA0319L	case	control	0.062960206	0.061116036	1.030174906	0.302927907	0.725484689	up_in_case
PRJNA8274(	ENSGALG00010013775	DBX2	case	control	0.097478289	0.174195288	0.559591999	0.575757764	0.877910508	up_in_case
PRJNA8274(	ENSGALG00010013777	CPLANE1	case	control	-0.003872274	0.18365555	-0.02108444	0.983178297	0.996591771	down_in_case
PRJNA8274(	ENSGALG00010013778	ENSGALG00010013778	case	control	-1.869859747	0.663629079	-2.817627809	0.004837986	0.119309498	down_in_case
PRJNA8274(	ENSGALG00010013779	SLC52A3	case	control	0.219543576	0.16490475	1.331335674	0.183078585	0.604903827	up_in_case
PRJNA8274(	ENSGALG00010013783	NDUFA10	case	control	-0.151481906	0.055764698	-2.716448076	0.006598654	0.140023533	down_in_case
PRJNA8274(	ENSGALG00010013784	ANO6	case	control	-0.188418815	0.130280096	-1.446259418	0.148104454	0.553857883	down_in_case
PRJNA8274(	ENSGALG00010013787	ZCCHC10	case	control	-0.055623771	0.073533474	-0.756441488	0.449384541	0.818749597	down_in_case
PRJNA8274(	ENSGALG00010013791	AEBP2	case	control	-0.020577639	0.052001966	-0.395708871	0.69231981	0.918988424	down_in_case
PRJNA8274(	ENSGALG00010013792	FGF10	case	control	0.305795838	0.189219121	1.616093747	0.106074046	0.495705509	up_in_case
PRJNA8274(	ENSGALG00010013793	METTL13	case	control	0.005567358	0.079255028	0.070246116	0.943997769	0.990183239	up_in_case
PRJNA8274(	ENSGALG00010013794	GTF3C6	case	control	-0.194006274	0.084437166	-2.297640744	0.021582245	0.263108354	down_in_case
PRJNA8274(	ENSGALG00010013797	IL1RL1	case	control	0.020160333	0.197438613	0.102109374	0.918669861	0.983734684	up_in_case
PRJNA8274(	ENSGALG00010013799	SCRT2	case	control	-0.393516183	0.207922355	-1.892611227	0.05840959	0.393617875	down_in_case
PRJNA8274(	ENSGALG00010013802	PYROXD1	case	control	-0.08326066	0.113602192	-0.732914198	0.463610763	0.825091817	down_in_case
PRJNA8274(	ENSGALG00010013805	HSPA4	case	control	0.005181545	0.033104903	0.156518967	0.87562397	0.975666505	up_in_case
PRJNA8274(	ENSGALG00010013808	PLAAT1	case	control	-0.246141687	0.115851163	-2.124637164	0.0336169	0.316364727	down_in_case
PRJNA8274(	ENSGALG00010013814	AMD1	case	control	-0.015408218	0.070158247	-0.219620914	0.826166402	0.961207256	down_in_case
PRJNA8274(	ENSGALG00010013815	SRXN1	case	control	-0.058670357	0.086481146	-0.678417889	0.49750677	0.839230872	down_in_case
PRJNA8274(	ENSGALG00010013820	MASP1	case	control	0.568944933	0.62647737	0.908165179	0.363790958	0.770036689	up_in_case
PRJNA8274(	ENSGALG00010013823	GUF1	case	control	-0.044038108	0.101193745	-0.435186068	0.663427374	0.911044469	down_in_case
PRJNA8274(	ENSGALG00010013828	PTN	case	control	0.003321191	0.24868186	0.013355179	0.989344426	0.997294889	up_in_case
PRJNA8274(	ENSGALG00010013831	TCERG1	case	control	-0.047465614	0.039195327	-1.211001845	0.225894697	0.649498359	down_in_case
PRJNA8274(	ENSGALG00010013833	TMPRSS2	case	control	2.483410363	0.951509983	2.609967744	0.009055076	0.169858595	up_in_case
PRJNA8274(	ENSGALG00010013836	IL18R1	case	control	0.154716811	0.147374536	1.049820514	0.293800642	0.716378375	up_in_case
PRJNA8274(	ENSGALG00010013837	IFI27L2	case	control	-0.001001526	0.243741197	-0.004108973	0.996721523	0.998921977	down_in_case
PRJNA8274(	ENSGALG00010013842	CDK19	case	control	0.008939581	0.060565052	0.147602955	0.882656123	0.976801393	up_in_case
PRJNA8274(	ENSGALG00010013846	YIPF7	case	control	-0.035338715	0.356166587	-0.099219623	0.920963895	0.985098638	down_in_case

PRJNA8274(	ENSGALG00010013848	ITPA	case	control	0.013619911	0.106083986	0.128388003	0.897841925	0.980176778	up_in_case
PRJNA8274(	ENSGALG00010013859	MYOC	case	control	0.163387601	0.245072139	0.666691863	0.504968978	0.842675288	up_in_case
PRJNA8274(	ENSGALG00010013861	TCF15	case	control	-0.029533631	0.117121501	-0.252162334	0.800915591	0.954294696	down_in_case
PRJNA8274(	ENSGALG00010013866	KCTD8	case	control	-0.217340796	0.346975122	-0.626387259	0.531060964	0.855560567	down_in_case
PRJNA8274(	ENSGALG00010013867	RECQL	case	control	0.017772341	0.074670675	0.238009642	0.811873616	0.956240049	up_in_case
PRJNA8274(	ENSGALG00010013871	ENSGALG00010013871	case	control	0.115228769	0.211824307	0.543982748	0.586453344	0.881932581	up_in_case
PRJNA8274(	ENSGALG00010013872	GLS2	case	control	0.132490823	0.077247011	1.715157928	0.086316276	0.464183347	up_in_case
PRJNA8274(	ENSGALG00010013878	ARID2	case	control	-0.013259942	0.077201093	-0.171758473	0.863627414	0.972890026	down_in_case
PRJNA8274(	ENSGALG00010013880	ZMYM4	case	control	0.035102618	0.044562437	0.787717662	0.430861872	0.806930928	up_in_case
PRJNA8274(	ENSGALG00010013886	SLC9A4	case	control	-0.787210302	0.697338483	-1.128878331	0.258949163	0.686345498	down_in_case
PRJNA8274(	ENSGALG00010013889	ENSGALG00010013889	case	control	-0.041456708	0.113297049	-0.365911636	0.714431013	0.926862121	down_in_case
PRJNA8274(	ENSGALG00010013890	SFPQ	case	control	0.042734458	0.044858376	0.952652828	0.340766001	0.752236352	up_in_case
PRJNA8274(	ENSGALG00010013893	CSNK2A1	case	control	-0.038243699	0.038703362	-0.988123428	0.323092203	0.740689923	down_in_case
PRJNA8274(	ENSGALG00010013897	AMOT	case	control	0.044441157	0.062527595	0.710744706	0.477242451	0.830520323	up_in_case
PRJNA8274(	ENSGALG00010013898	EMB	case	control	0.032025309	0.251206372	0.127486053	0.898555711	0.98028579	up_in_case
PRJNA8274(	ENSGALG00010013899	WNT5B	case	control	-0.038148768	0.081163506	-0.47002366	0.638338114	0.900131647	down_in_case
PRJNA8274(	ENSGALG00010013901	NFXL1	case	control	0.073895271	0.076140126	0.970516802	0.331788954	0.748120789	up_in_case
PRJNA8274(	ENSGALG00010013908	TBC1D20	case	control	-0.046101797	0.06161388	-0.748237192	0.454317102	0.821331802	down_in_case
PRJNA8274(	ENSGALG00010013910	ADIPOR2	case	control	0.189042855	0.058975266	3.205459979	0.001348468	0.058257076	up_in_case
PRJNA8274(	ENSGALG00010013911	TNIK	case	control	-0.097599965	0.090149958	-1.082640158	0.278968175	0.704440209	down_in_case
PRJNA8274(	ENSGALG00010013914	NRG1	case	control	-0.206060605	0.153856831	-1.339300979	0.18047271	0.601050402	down_in_case
PRJNA8274(	ENSGALG00010013915	ENSGALG00010013915	case	control	0.16775594	0.221086616	0.758779266	0.447984611	0.818681142	up_in_case
PRJNA8274(	ENSGALG00010013917	TRPC5	case	control	-0.377954618	0.436037461	-0.866793914	0.386054937	0.78218735	down_in_case
PRJNA8274(	ENSGALG00010013921	PPP2R2B	case	control	-0.019369305	0.078759316	-0.245930333	0.805736163	0.9556344	down_in_case
PRJNA8274(	ENSGALG00010013922	ENSGALG00010013922	case	control	1.163964728	0.548331982	2.12273726	0.033775875	0.31692586	up_in_case
PRJNA8274(	ENSGALG00010013928	CCNO	case	control	-1.137097456	0.314457522	-3.616060598	0.00029912	0.023376995	down_in_case
PRJNA8274(	ENSGALG00010013930	SLC22A16	case	control	0.090817243	0.089366329	1.016235571	0.309517221	0.732384442	up_in_case
PRJNA8274(	ENSGALG00010013934	STAT1	case	control	-0.061435802	0.048911835	-1.25605188	0.209097161	0.634076945	down_in_case
PRJNA8274(	ENSGALG00010013936	DLGAP3	case	control	0.047463893	0.18419962	0.257676387	0.796656671	0.95280257	up_in_case
PRJNA8274(	ENSGALG00010013938	ENSGALG00010013938	case	control	0.866865608	0.338778632	2.558796587	0.010503518	0.183125942	up_in_case
PRJNA8274(	ENSGALG00010013940	UPF3A	case	control	-0.171246013	0.249981929	-0.685033567	0.49332274	0.837916219	down_in_case
PRJNA8274(	ENSGALG00010013942	CACNA2D4	case	control	0.54157618	0.44484156	1.217458595	0.223429781	0.647349714	up_in_case
PRJNA8274(	ENSGALG00010013944	METTL24	case	control	0.112205999	0.125250505	0.895852663	0.370331459	0.772700698	up_in_case
PRJNA8274(	ENSGALG00010013945	SCAF11	case	control	-0.017556058	0.035419473	-0.495661179	0.620133481	0.892952249	down_in_case
PRJNA8274(	ENSGALG00010013947	ENSGALG00010013947	case	control	-0.024735159	0.23335551	-0.10599775	0.915584137	0.983445506	down_in_case
PRJNA8274(	ENSGALG00010013949	PRRC2C	case	control	-0.122066165	0.065758828	-1.856270381	0.063415029	0.409498817	down_in_case
PRJNA8274(	ENSGALG00010013953	DHX29	case	control	-0.01276001	0.123900565	-0.102985891	0.917974169	0.983445506	down_in_case
PRJNA8274(	ENSGALG00010013954	LPL	case	control	-0.136862358	0.158570183	-0.863102736	0.388080984	0.783061671	down_in_case
PRJNA8274(	ENSGALG00010013958	TUSC3	case	control	-0.152468116	0.153394787	-0.993958913	0.320242871	0.738813491	down_in_case
PRJNA8274(	ENSGALG00010013959	STK32A	case	control	-0.035510794	0.37865182	-0.093782183	0.925282185	0.985571367	down_in_case
PRJNA8274(	ENSGALG00010013964	SLC38A1	case	control	0.14167126	0.079766888	1.77606604	0.075722033	0.442474101	up_in_case
PRJNA8274(	ENSGALG00010013965	SLC2A2	case	control	2.028975542	0.950375529	2.134919806	0.032767561	0.311760562	up_in_case
PRJNA8274(	ENSGALG00010013967	MLLT3	case	control	0.010646592	0.102058569	0.104318452	0.916916635	0.983445506	up_in_case
PRJNA8274(	ENSGALG00010013971	ENSGALG00010013971	case	control	-0.347721158	0.232529753	-1.495383511	0.134814381	0.539774365	down_in_case
PRJNA8274(	ENSGALG00010013973	ENSGALG00010013973	case	control	0.159748554	0.28806214	0.554562823	0.579193724	0.879142433	up_in_case
PRJNA8274(	ENSGALG00010013976	CDC40	case	control	-0.077569738	0.047938328	-1.618115213	0.105637771	0.495266487	down_in_case
PRJNA8274(	ENSGALG00010013977	SLC38A2	case	control	-0.004514991	0.063217731	-0.071419696	0.943063735	0.990069816	down_in_case
PRJNA8274(	ENSGALG00010013981	RLF	case	control	-0.157969346	0.050004514	-3.159101699	0.001582563	0.063928374	down_in_case
PRJNA8274(	ENSGALG00010013989	ENSGALG00010013989	case	control	0.047231669	0.2439299	0.193628042	0.846467139	0.967661691	up_in_case
PRJNA8274(	ENSGALG00010013994	CHRM2	case	control	0.288726797	0.419901594	0.687605861	0.491701017	0.837145631	up_in_case
PRJNA8274(	ENSGALG00010013995	REM1	case	control	-0.128688761	0.110746484	-1.162012151	0.245230529	0.669759186	down_in_case
PRJNA8274(	ENSGALG00010014001	CDC16	case	control	-0.018333291	0.057260269	-0.320174735	0.748835875	0.939503405	down_in_case
PRJNA8274(	ENSGALG00010014002	ZDHHC18	case	control	0.084523204	0.158462181	0.5333967	0.593759001	0.883625114	up_in_case
PRJNA8274(	ENSGALG00010014010	EIF5A2	case	control	-0.00328332	0.049269448	-0.06664008	0.946868237	0.990723333	down_in_case
PRJNA8274(	ENSGALG00010014011	SFN	case	control	1.291537201	0.578616476	2.232112727	0.025607514	0.283909015	up_in_case
PRJNA8274(	ENSGALG00010014014	PARP8	case	control	0.276500787	0.164406375	1.681813046	0.092605098	0.477342721	up_in_case
PRJNA8274(	ENSGALG00010014018	DPYSL3	case	control	0.005915508	0.116485475	0.050783223	0.95949826	0.992056045	up_in_case
PRJNA8274(	ENSGALG00010014019	IRAG2	case	control	-0.279355531	0.264278087	-1.057051436	0.290488114	0.714060795	down_in_case
PRJNA8274(	ENSGALG00010014020	PDXP	case	control	-0.021007718	0.071414275	-0.294166921	0.768630356	0.945375926	down_in_case
PRJNA8274(	ENSGALG00010014026	SH3BP1	case	control	-0.094888843	0.40181077	-0.236153061	0.813313901	0.956712425	down_in_case
PRJNA8274(	ENSGALG00010014030	WASF1	case	control	-0.009346624	0.110359828	-0.084692267	0.932506044	0.987717353	down_in_case
PRJNA8274(	ENSGALG00010014031	RPL22L1	case	control	-0.108518005	0.106197413	-1.021851679	0.306851106	0.729504707	down_in_case
PRJNA8274(	ENSGALG00010014032	PSD3	case	control	0.097092446	0.115475505	0.840805556	0.400456874	0.790138851	up_in_case
PRJNA8274(	ENSGALG00010014033	SLC38A4	case	control	0.890629681	0.490070648	1.817349569	0.069163605	0.424591509	up_in_case
PRJNA8274(	ENSGALG00010014035	PSIP1	case	control	0.04086401	0.078661772	0.519490076	0.603419032	0.885268908	up_in_case
PRJNA8274(	ENSGALG00010014041	SKIV2L2	case	control	-0.046494718	0.160603014	-0.289500907	0.772198086	0.945899659	down_in_case
PRJNA8274(	ENSGALG00010014042	SGCZ	case	control	-0.210734419	0.330734737	-0.637170503	0.524013789	0.852011056	down_in_case
PRJNA8274(	ENSGALG00010014045	GOLT1B	case	control	0.058034034	0.058330306	0.994920791	0.31977479	0.738813491	up_in_case
PRJNA8274(	ENSGALG00010014047	HM13	case	control	-0.021902555	0.043439434	-0.504209033	0.614114493	0.890440426	down_in_case
PRJNA8274(	ENSGALG00010014056	FMO3	case	control	0.223707401	0.345595982	0.647309032			up_in_case
PRJNA8274(	ENSGALG00010014062	GGA1	case	control	-0.000785054	0.053322728	-0.014722689	0.988253418	0.996942188	down_in_case
PRJNA8274(	ENSGALG00010014065	CHUNK-1	case	control	0.250095924	0.401698019	0.622596857	0.533549477	0.855817434	up_in_case
PRJNA8274(	ENSGALG00010014066	BCL2L1	case	control	0.134491085	0.084393794	1.593613443	0.111022642	0.503480037	up_in_case
PRJNA8274(	ENSGALG00010014076	PRRX1	case	control	-0.026870101	0.149419477	-0.179829973	0.857286052	0.971282862	down_in_case
PRJNA8274(	ENSGALG00010014077	SLITRK2	case	control	-0.235095137	0.082746203	-2.841159229	0.004494986	0.114389963	down_in_case
PRJNA8274(	ENSGALG00010014080	MYO1B	case	control	0.064896306	0.08000862	0.811116424	0.417298815	0.799987395	up_in_case
PRJNA8274(	ENSGALG00010014082	HNRNPR	case	control	-0.054101332	0.04580122	-1.181220334	0.237515205	0.662652947	down_in_case
PRJNA8274(	ENSGALG00010014083	ENSGALG00010014083	case	control	0.111541374	0.207879093	0.536568506	0.591565703	0.882949889	up_in_case
PRJNA8274(	ENSGALG00010014089	FIG4	case	control	0.226829361	0.078108592	2.904025723	0.003683979	0.104942029	up_in_case
PRJNA8274(	ENSGALG00010014091	CHIC2	case	control	0.010442411	0.055450247	0.188320377	0.850625513	0.969234083	up_in_case
PRJNA8274(	ENSGALG00010014092	TYRP1	case	control	8.809537202	0.439256684	20.05555641	1.80513E-89	8.60446E-86	up_in_case
PRJNA8274(	ENSGALG00010014093	PLD1	case	control	0.028750538	0.072243696	0.397966038	0.69065522	0.918784485	up_in_case
PRJNA8274(	ENSGALG00010014096	JAKMIP2	case	control	0.202424826	0.089031112	2.273641449	0.022987553	0.267253668	up_in_case
PRJNA8274(	ENSGALG00010014099	TPX2	case	control	-0.140728671	0.075403453	-1.866342522	0.061993459	0.405537748	down_in_case
PRJNA8274(	ENSGALG00010014102	TRIM2	case	control	-0.170737528	0.094272283	-1.811110567	0.070123738	0.427620237	down_in_case
PRJNA8274(	ENSGALG00010014104	ALDH1A1	case	control	-0.160133838	0.62240631	-0.257281835	0.796961214	0.95280257	down_in_case
PRJNA8274(	ENSGALG00010014105	AHR1B	case	control	-0.300535988	0.486136079	-0.618213708	0.536434475	0.857336818	down_in_case
PRJNA8274(	ENSGALG00010014109	AMIGO2	case	control	-0.170170611	0.163033823	-1.04377489	0.296589552	0.720288443	down_in_case
PRJNA8274(	ENSGALG00010014111	GORAB	case	control	0.088910854	0.062600796	1.420283134	0.155525269	0.564469886	up_in_case
PRJNA8274(	ENSGALG00010014114	ANXA1	case	control	-0.084454133	0.218590132	-0.386358395	0.699231258	0.922288441	down_in_case
PRJNA8274(	ENSGALG00010014120	SOX5	case	control	0.258584048	0.121869358	2.121813492	0.033853403	0.317237002	up_in_case
PRJNA8274(	ENSGALG00010014122	ABCA1	case	control	-0.144927067	0.089217476	-1.62442465	0.10428521	0.493800828	down_in_case
PRJNA8274(	ENSGALG00010014125	PLPP1	case	control	-0.146248431	0.113645845	-1.286878823	0.198136527	0.621485489	down_in_case
PRJNA8274(	ENSGALG00010014128	COL4A1	case	control	-0.052828388	0.100177541	-0.527347624	0.597952208	0.884680373	down_in_case
PRJNA8274(	ENSGALG00010014131	CDC23	case	control	0.061684617	0.054789126	1.125855094	0.260226859	0.687282842	up_in_case
PRJNA8274(	ENSGALG00010014134	RPAP3	case	control	0.018909359	0.058749626	0.321863472	0.747556127	0.939105545	up_in_case
PRJNA8274(	ENSGALG00010014135	ZBTB24	case	control	0.061203782	0.08853924	0.691261653	0.489401125	0.836347578	up_in_case
PRJNA8274(	ENSGALG00010014142	PPIL6	case	control	-0.589869295	0.402838684	-1.464281655	0.143117007	0.545763019	down_in_case
PRJNA8274(	ENSGALG00010014143	NFATC2	case	control	0.041348991	0.11220565	0.368510778	0.71249241	0.926808752	up_in_case
PRJNA8274(	ENSGALG00010014145	NTMT2	case	control	0.054836455	0.208564071	0.262923786	0.792609322	0.95280257	up_in_case
PRJNA8274(	ENSGALG00010014147	ENSGALG00010014147	case	control	-0.32588447	0.228456019	-1.426464803	0.15373422	0.562474506	down_in_case
PRJNA8274(	ENSGALG00010014148	KCNG1	case	control	-0.170611517	0.189315521	-0.901201951	0.367480954	0.771770041	down_in_case
PRJNA8274(	ENSGALG00010014149	LGALS2	case	control	0.392929924	0.496150727	0.791956763	0.428385888	0.805337183	up_in_case
PRJNA8274(	ENSGALG00010014151	ATP23	case	control	-0.074656018	0.065734813	-1.135715062	0.256075835	0.682403711	down_in_case
PRJNA8274(	ENSGALG00010014153	FOCAD	case	control	0.031796053	0.157509321	0.201867756	0.84002011	0.965286179	up_in_case
PRJNA8274(	ENSGALG00010014154	DNAI7	case	control	0.331533752	0.328740891	1.008495629	0.313216585	0.734712382	up_in_case
PRJNA8274(	ENSGALG00010014156	CD164	case	control	-0.027994585	0.051581547	-0.542724799	0.587319297	0.882769177	down_in_case
PRJNA8274(	ENSGALG00010014159	CDC42EP1	case	control	0.162000682	0.04958055	3.267424083	0.00108531	0.051553287	up_in_case
PRJNA8274(	ENSGALG00010014161	GFRA3	case	control	0.544282761	0.332148638	1.638672265	0.101281533	0.491460867	up_in_case
PRJNA8274(	ENSGALG00010014162	COL18A1	case	control	-0.019440226	0.098932273	-0.196500345	0.844218563	0.96721237	down_in_case
PRJNA8274(	ENSGALG00010014165	LUZP1	case	control	-0.001073637	0.073631251	-0.014581269	0.988366243	0.996942188	down_in_case
PRJNA8274(	ENSGALG00010014166	FGF12	case	control	-0.010024426	0.174453377	-0.057461922	0.954177238	0.992056045	down_in_case
PRJNA8274(	ENSGALG00010014167	SLC38A9	case	control	0.041107988	0.143767707	0.285933392	0.774929138	0.947085948	up_in_case
PRJNA8274(	ENSGALG00010014170	CEMIP2	case	control	0.101294663	0.080911699	1.251916157	0.210600417	0.636249596	up_in_case
PRJNA8274(	ENSGALG00010014171	DLC1	case	control	-0.013778149	0.071443828	-0.19285289	0.847074181	0.968026275	down_in_case
PRJNA8274(	ENSGALG00010014173	MND1	case	control	0.098751664	0.097445207	1.013407095	0.310865752	0.733682167	up_in_case
PRJNA8274(	ENSGALG00010014176	ABHD17B	case	control	0.064600668	0.124838901	0.517472256	0.604826532	0.885268908	up_in_case
PRJNA8274(	ENSGALG00010014178	LRTM2	case	control	-0.154339053	0.222172562	-0.694680978	0.487255252	0.835461642	down_in_case
PRJNA8274(	ENSGALG00010014179	CTBPL	case	control	0.025506486	0.037416518	0.681690543	0.495434649	0.838118945	up_in_case
PRJNA8274(	ENSGALG00010014180	SLC19A1	case	control	-0.031842405	0.076565495	-0.415884528	0.677494502	0.914864532	down_in_case
PRJNA8274(	ENSGALG00010014184	SLC44A1	case	control	-0.072364868	0.11682825	-0.619412414	0.535644705	0.857079477	down_in_case
PRJNA8274(	ENSGALG00010014186	MOCS3	case	control	-0.054936082	0.067997958	-0.807907818	0.41914365	0.80093491	down_in_case
PRJNA8274(	ENSGALG00010014189	DDX4	case	control	-0.244987261	0.613220068	-0.399509531	0.689517803	0.918335352	down_in_case
PRJNA8274(	ENSGALG00010014190	HACD4	case	control	-0.136798484	0.213354242	-0.641180052	0.521405708	0.850363352	down_in_case
PRJNA8274(	ENSGALG00010014192	KDM1A	case	control	0.011554571	0.046261694	0.249765408	0.802768772	0.954891353	up_in_case
PRJNA8274(	ENSGALG00010014193	CARD10	case	control	0.370809929	0.290288479	1.277384246	0.201466624	0.624725388	up_in_case

PRJNA8274(	ENSGALG00010014194	LRIG3	case	control	0.091407598	0.081294219	1.12440465	0.2608414	0.687359696	up_in_case
PRJNA8274(	ENSGALG00010014197	SH3GL2	case	control	-0.311092198	0.158214064	-1.96627399	0.04926697	0.364571979	down_in_case
PRJNA8274(	ENSGALG00010014198	KDM3B	case	control	-0.023272626	0.034172448	-0.681034799	0.495849472	0.83840725	down_in_case
PRJNA8274(	ENSGALG00010014199	LNX1	case	control	0.17013222	0.236076713	0.720664979	0.471115664	0.829101076	up_in_case
PRJNA8274(	ENSGALG00010014200	DCP1B	case	control	-0.046025275	0.07156169	-0.643155225	0.520123383	0.849641807	down_in_case
PRJNA8274(	ENSGALG00010014203	TMEM38B	case	control	-0.108233173	0.098654343	-1.097094866	0.272599924	0.698931401	down_in_case
PRJNA8274(	ENSGALG00010014204	KIFAP3	case	control	-0.049728725	0.074458529	-0.667871446	0.504215655	0.842675107	down_in_case
PRJNA8274(	ENSGALG00010014205	DPM1	case	control	0.051199191	0.058593365	0.87380527	0.38222431	0.77998936	up_in_case
PRJNA8274(	ENSGALG00010014209	CCDC50	case	control	-0.051583678	0.047731778	-1.080698842	0.279831099	0.705498009	down_in_case
PRJNA8274(	ENSGALG00010014210	ENSGALG00010014210	case	control	-0.167513466	0.18785193	-0.891731411	0.372536913	0.774201112	down_in_case
PRJNA8274(	ENSGALG00010014213	ETFRF1	case	control	-0.246111666	0.090068189	-2.732503768	0.006285495	0.138110236	down_in_case
PRJNA8274(	ENSGALG00010014214	REEP2	case	control	0.078728757	0.103300408	0.76213403	0.445980014	0.817315674	up_in_case
PRJNA8274(	ENSGALG00010014217	RASA3	case	control	-0.047159068	0.071945278	-0.655485247	0.512155382	0.845834144	down_in_case
PRJNA8274(	ENSGALG00010014218	IL31RA	case	control	0.230863551	0.147840633	1.56157036	0.118389237	0.514757029	up_in_case
PRJNA8274(	ENSGALG00010014220	C9orf85	case	control	0.106379445	0.194783146	0.546142965	0.584967668	0.881307382	up_in_case
PRJNA8274(	ENSGALG00010014221	SCYL3	case	control	-0.017541876	0.053701244	-0.326656782	0.743927492	0.937448285	down_in_case
PRJNA8274(	ENSGALG00010014222	TBX22	case	control	-0.442302927	0.281242404	-1.572675102	0.115794068	0.512807423	down_in_case
PRJNA8274(	ENSGALG00010014224	NMUR2	case	control	-0.697603104	0.443542527	-1.572798685	0.11576544	0.512807423	down_in_case
PRJNA8274(	ENSGALG00010014225	ZMPSTE24	case	control	0.093901239	0.038266558	2.453872119	0.014132723	0.209211118	up_in_case
PRJNA8274(	ENSGALG00010014226	SLC16A7	case	control	0.084735032	0.132327483	0.640343415	0.521949361	0.850840147	up_in_case
PRJNA8274(	ENSGALG00010014234	GLRA1	case	control	-0.032099994	0.510773288	-0.062845874	0.949889236	0.991488793	down_in_case
PRJNA8274(	ENSGALG00010014236	WDR44	case	control	0.023755591	0.048742288	0.487371268	0.625995251	0.895445894	up_in_case
PRJNA8274(	ENSGALG00010014238	PHLDA1	case	control	0.308308525	0.095532643	3.22725841	0.001249825	0.055677577	up_in_case
PRJNA8274(	ENSGALG00010014240	PIGV	case	control	0.193807452	0.115355444	1.680089334	0.092939936	0.478066279	up_in_case
PRJNA8274(	ENSGALG00010014241	ENSGALG00010014241	case	control	-1.533548825	0.927061599	-1.654203805	0.09808611	0.487193947	down_in_case
PRJNA8274(	ENSGALG00010014243	ENSGALG00010014243	case	control	0.123259095	0.092329044	1.334998066	0.18187698	0.602744104	up_in_case
PRJNA8274(	ENSGALG00010014244	SESN1	case	control	-0.333223477	0.099105943	-3.362295607	0.000772973	0.042028584	down_in_case
PRJNA8274(	ENSGALG00010014246	KRAS	case	control	0.007121803	0.071079235	0.10019527	0.920189301	0.984638357	up_in_case
PRJNA8274(	ENSGALG00010014249	ENSGALG00010014249	case	control	-0.086132027	0.05789056	-1.487842348	0.136792475	0.539854583	down_in_case
PRJNA8274(	ENSGALG00010014250	ENSGALG00010014250	case	control	-0.277587716	0.141899829	-1.956223056	0.050438884	0.367061596	down_in_case
PRJNA8274(	ENSGALG00010014257	NOP16	case	control	0.109917663	0.102606929	1.071249905	0.284057078	0.708692203	up_in_case
PRJNA8274(	ENSGALG00010014258	ENSGALG00010014258	case	control	-0.232325649	0.353805263	-0.65664837	0.51140704	0.845660609	down_in_case
PRJNA8274(	ENSGALG00010014260	ADNP	case	control	0.013701338	0.037513997	0.365232702	0.714937709	0.926862121	up_in_case
PRJNA8274(	ENSGALG00010014263	ZNF131	case	control	0.056162687	0.14617065	0.384226841	0.700810326	0.922516526	up_in_case
PRJNA8274(	ENSGALG00010014265	ZNF462	case	control	0.008129242	0.104573576	0.077737057	0.938037216	0.989228038	up_in_case
PRJNA8274(	ENSGALG00010014267	SCFD2	case	control	0.096991704	0.071310453	1.360133061	0.173787821	0.592926641	up_in_case
PRJNA8274(	ENSGALG00010014272	PARD6B	case	control	0.008487872	0.123612366	0.068665235	0.945256092	0.990313016	up_in_case
PRJNA8274(	ENSGALG00010014274	FIRRM	case	control	-0.131065335	0.105431765	-1.243129472	0.213820127	0.638602829	down_in_case
PRJNA8274(	ENSGALG00010014278	G3BP1	case	control	0.019891657	0.049156833	0.404657001	0.685729653	0.917034645	up_in_case
PRJNA8274(	ENSGALG00010014284	TMEM131L	case	control	0.092584896	0.053913678	1.717280282	0.085927973	0.463278022	up_in_case
PRJNA8274(	ENSGALG00010014288	ARMC2	case	control	0.063833903	0.187987718	0.339564219	0.734184728	0.933846596	up_in_case
PRJNA8274(	ENSGALG00010014291	OSTN	case	control	-0.236845788	0.407342597	-0.581441248	0.560943105	0.870480544	down_in_case
PRJNA8274(	ENSGALG00010014292	NIM1K	case	control	-0.529437197	0.293656842	-1.802911159	0.071402168	0.43228239	down_in_case
PRJNA8274(	ENSGALG00010014294	TMPO	case	control	-0.045355075	0.056197122	-0.807071133	0.419625502	0.80093491	down_in_case
PRJNA8274(	ENSGALG00010014295	CACNA1C	case	control	-0.378405688	0.175213897	-2.159678504	0.030797566	0.308259818	down_in_case
PRJNA8274(	ENSGALG00010014298	METTL18	case	control	-0.091020198	0.095159946	-0.956496943	0.338821228	0.751259351	down_in_case
PRJNA8274(	ENSGALG00010014300	ELFN2	case	control	0.302966256	0.381503616	0.794137311	0.427115499	0.805305429	up_in_case
PRJNA8274(	ENSGALG00010014301	ARID1A	case	control	-0.076673059	0.057507168	-1.3332783	0.182440492	0.603772052	down_in_case
PRJNA8274(	ENSGALG00010014302	FOXO3	case	control	-0.327116353	0.088429833	-3.699162871	0.000216312	0.019094186	down_in_case
PRJNA8274(	ENSGALG00010014303	GABRA2	case	control	-1.033927591	0.302500687	-3.417934687	0.000630982	0.03713189	down_in_case
PRJNA8274(	ENSGALG00010014305	ARL10	case	control	0.075284173	0.289238621	0.260283956	0.794644748	0.95280257	up_in_case
PRJNA8274(	ENSGALG00010014306	ENSGALG00010014306	case	control	0.336489499	0.311305954	1.080896445	0.27974318	0.705400718	up_in_case
PRJNA8274(	ENSGALG00010014308	ENSGALG00010014308	case	control	-0.061550616	0.133702975	-0.460353375	0.645262595	0.902421038	down_in_case
PRJNA8274(	ENSGALG00010014310	COL6A1	case	control	-0.15525993	0.078478955	-1.97836388	0.047887671	0.360228144	down_in_case
PRJNA8274(	ENSGALG00010014312	KIAA1191	case	control	0.14967751	0.06321867	2.367615596	0.017903126	0.23837496	up_in_case
PRJNA8274(	ENSGALG00010014314	ENSGALG00010014314	case	control	-0.39902124	0.19157234	-2.082875012	0.037262618	0.328438691	down_in_case
PRJNA8274(	ENSGALG00010014317	RAD23B	case	control	-0.003257097	0.059580955	-0.054666748	0.956403961	0.992056045	down_in_case
PRJNA8274(	ENSGALG00010014318	ENSGALG00010014318	case	control	0.038736849	0.288121112	0.134446408	0.893049586	0.979748897	up_in_case
PRJNA8274(	ENSGALG00010014319	ENSGALG00010014319	case	control	-0.058403763	0.08463298	-0.690082794	0.490142123	0.836347578	down_in_case
PRJNA8274(	ENSGALG00010014320	UTS2B	case	control	-0.663297895	0.733682126	-0.904067131	0.365959806	0.771585756	down_in_case
PRJNA8274(	ENSGALG00010014323	KLF4	case	control	0.133905129	0.154033736	0.869323389	0.384670274	0.780971676	up_in_case
PRJNA8274(	ENSGALG00010014324	COL4A2	case	control	-0.082444897	0.104322788	-0.790286561	0.429360434	0.806015686	down_in_case
PRJNA8274(	ENSGALG00010014328	RASSF8	case	control	-0.042436467	0.065667959	-0.646227892	0.518131779	0.848618078	down_in_case
PRJNA8274(	ENSGALG00010014329	IER5	case	control	0.140027796	0.107657804	1.300674829	0.193369782	0.616082781	up_in_case
PRJNA8274(	ENSGALG00010014334	HMGCS1	case	control	0.167160777	0.090153115	1.85418747	0.063712344	0.409661205	up_in_case
PRJNA8274(	ENSGALG00010014338	BHLHE41	case	control	-0.212156478	0.143470351	-1.478747883	0.13920771	0.541505735	down_in_case
PRJNA8274(	ENSGALG00010014340	EFNB1	case	control	-0.055855495	0.082112572	-0.680230737	0.496358373	0.83858637	down_in_case
PRJNA8274(	ENSGALG00010014344	LSG1	case	control	-0.02819933	0.056640613	-0.497864141	0.618579804	0.891656847	down_in_case
PRJNA8274(	ENSGALG00010014346	AFG1L	case	control	0.063217081	0.124775704	0.506645757	0.612403397	0.889886045	up_in_case
PRJNA8274(	ENSGALG00010014347	KIAA0040	case	control	0.066315432	0.3060806	0.216660029	0.828473301	0.961776928	up_in_case
PRJNA8274(	ENSGALG00010014350	PTGER4	case	control	0.077145629	0.157366357	0.490229491	0.623971515	0.894353977	up_in_case
PRJNA8274(	ENSGALG00010014355	RAB20	case	control	-0.019393165	0.072401912	-0.267854325	0.788811452	0.951738421	down_in_case
PRJNA8274(	ENSGALG00010014359	TMEM271	case	control	0.456817781	0.39788413	1.148117622	0.25092001	0.676372507	up_in_case
PRJNA8274(	ENSGALG00010014361	SSPN	case	control	-0.053630879	0.078156133	-0.686201801	0.492585862	0.837671284	down_in_case
PRJNA8274(	ENSGALG00010014362	NAXD	case	control	0.043348516	0.059817636	0.724677854	0.468649684	0.828596747	up_in_case
PRJNA8274(	ENSGALG00010014367	OPHN1	case	control	0.030720179	0.053710233	0.571961379	0.567348139	0.873689252	up_in_case
PRJNA8274(	ENSGALG00010014371	TTC33	case	control	0.021278632	0.133275834	0.159658592	0.873150021	0.975062028	up_in_case
PRJNA8274(	ENSGALG00010014372	CCDC171	case	control	-0.026053506	0.164575582	-0.15830724	0.874214703	0.975062028	down_in_case
PRJNA8274(	ENSGALG00010014375	H2BC35	case	control	0.062780698	0.388620445	0.161547594	0.871662129	0.975062028	up_in_case
PRJNA8274(	ENSGALG00010014379	CARS2	case	control	0.009376512	0.057147171	0.164076574	0.869670863	0.974718971	up_in_case
PRJNA8274(	ENSGALG00010014380	SNX3	case	control	-0.060718184	0.055490361	-1.094211372	0.273862285	0.699576755	down_in_case
PRJNA8274(	ENSGALG00010014388	MFNG	case	control	-0.06369744	0.196795328	-0.32367354	0.746185205	0.938143875	down_in_case
PRJNA8274(	ENSGALG00010014389	COL6A2	case	control	-0.173129682	0.07610051	-2.275013424	0.022905125	0.267253668	down_in_case
PRJNA8274(	ENSGALG00010014390	SPARC	case	control	-0.001274322	0.123516724	-0.010316999	0.991768372	0.99790247	down_in_case
PRJNA8274(	ENSGALG00010014393	ATP8A1	case	control	-0.032620373	0.093865844	-0.347521222	0.728199778	0.933057319	down_in_case
PRJNA8274(	ENSGALG00010014396	ING1	case	control	-0.029646015	0.048651822	-0.609350564	0.542292099	0.85975913	down_in_case
PRJNA8274(	ENSGALG00010014397	PRKAA1	case	control	0.100397388	0.092501705	1.085357157	0.2777635	0.703206258	up_in_case
PRJNA8274(	ENSGALG00010014399	GAS6	case	control	-0.076336433	0.107490521	-0.710168969	0.477599362	0.830520323	down_in_case
PRJNA8274(	ENSGALG00010014400	RIPOR3	case	control	0.144385184	0.086066008	1.677609865	0.093423286	0.478523919	up_in_case
PRJNA8274(	ENSGALG00010014407	YIPF6	case	control	-0.055318865	0.043689256	-1.266189201	0.205445353	0.628438742	down_in_case
PRJNA8274(	ENSGALG00010014408	ENSGALG00010014408	case	control	-0.525931916	0.236012516	-2.228406884	0.025853394	0.285045136	down_in_case
PRJNA8274(	ENSGALG00010014411	ATP13A3	case	control	0.10519207	0.078319083	1.343121819	0.179232531	0.599678334	up_in_case
PRJNA8274(	ENSGALG00010014414	TMEM255B	case	control	-0.218229487	0.385938615	-0.565451288	0.571766848	0.876814097	down_in_case
PRJNA8274(	ENSGALG00010014416	H2BC32	case	control	-0.885768349	0.404765332	-2.188350333	0.028644093	0.300624184	down_in_case
PRJNA8274(	ENSGALG00010014418	RAC2	case	control	0.617801224	0.158186292	3.90552947	9.40193E-05	0.010422294	up_in_case
PRJNA8274(	ENSGALG00010014427	TMEM267	case	control	0.082215507	0.106769264	0.770029729	0.441282258	0.813083204	up_in_case
PRJNA8274(	ENSGALG00010014431	ENSGALG00010014431	case	control	0.248290601	0.473482588	0.524392254	0.600005743	0.884888453	up_in_case
PRJNA8274(	ENSGALG00010014439	ENSGALG00010014439	case	control	-0.210208112	0.227600386	-0.923584166	0.355702861	0.763373775	down_in_case
PRJNA8274(	ENSGALG00010014442	SHISA3	case	control	0.541835277	0.813245107	0.666263187	0.505242892	0.842675288	up_in_case
PRJNA8274(	ENSGALG00010014449	ENSGALG00010014449	case	control	-0.196234982	0.488946208	-0.401342681	0.688167844	0.91747158	down_in_case
PRJNA8274(	ENSGALG00010014453	NR2E1	case	control	-0.138761874	0.200543439	-0.691929261	0.488981754	0.836347578	down_in_case
PRJNA8274(	ENSGALG00010014455	C4orf19	case	control	-0.265754526	0.229840257	-1.156257522	0.247575837	0.672031667	down_in_case
PRJNA8274(	ENSGALG00010014465	SIMC1	case	control	-0.006906682	0.080149741	-0.086172236	0.931329501	0.987258715	down_in_case
PRJNA8274(	ENSGALG00010014469	TMCO3	case	control	0.036659561	0.069560159	0.527019515	0.598180036	0.884680373	up_in_case
PRJNA8274(	ENSGALG00010014470	RPS6KA1	case	control	-0.039680622	0.056986567	-0.696315357	0.486231358	0.834635018	down_in_case
PRJNA8274(	ENSGALG00010014474	PTPN1	case	control	-0.082419948	0.048610526	-1.695516475	0.089977488	0.473565724	down_in_case
PRJNA8274(	ENSGALG00010014476	C5orf34	case	control	-0.087105238	0.15245363	-0.571355617	0.567758608	0.874133085	down_in_case
PRJNA8274(	ENSGALG00010014477	ITPR2	case	control	0.004345278	0.076968385	0.056455357	0.954979059	0.992056045	up_in_case
PRJNA8274(	ENSGALG00010014482	THOC3	case	control	-0.034091442	0.069852092	-0.488051834	0.625513127	0.895199931	down_in_case
PRJNA8274(	ENSGALG00010014487	OSTM1	case	control	0.096735352	0.091461093	1.057666697	0.290207423	0.714060795	up_in_case
PRJNA8274(	ENSGALG00010014488	ENSGALG00010014488	case	control	-0.112236352	0.101005757	-1.111187664	0.26648758	0.692519293	down_in_case
PRJNA8274(	ENSGALG00010014492	CPLX2	case	control	0.026496124	0.162411045	0.163142377	0.870406334	0.974794532	up_in_case
PRJNA8274(	ENSGALG00010014494	KLHL9	case	control	-0.026905431	0.05393921	-0.498810258	0.61791306	0.891419534	down_in_case
PRJNA8274(	ENSGALG00010014504	SEC63	case	control	-0.015653698	0.035086767	-0.446142506	0.65549431	0.907412259	down_in_case
PRJNA8274(	ENSGALG00010014505	CYTH4	case	control	0.166409555	0.133600867	1.245572416	0.212921414	0.638582746	up_in_case
PRJNA8274(	ENSGALG00010014512	HMGN4	case	control	-0.106644533	0.049166417	-2.169052347	0.03007871	0.306407857	down_in_case
PRJNA8274(	ENSGALG00010014515	ETNK1	case	control	0.097098561	0.081299774	1.194327563	0.23234986	0.657912487	up_in_case
PRJNA8274(	ENSGALG00010014517	DCUN1D2	case	control	0.148428374	0.095924783	1.547341259	0.121780978	0.519686058	up_in_case
PRJNA8274(	ENSGALG00010014522	FAT2	case	control	-0.463643712	0.392645859	-1.180819054	0.237674613	0.662652947	down_in_case
PRJNA8274(	ENSGALG00010014537	GKAP1	case	control	0.028108984	0.124421285	0.225917811	0.821265345	0.958936428	up_in_case
PRJNA8274(	ENSGALG00010014539	PAIP1	case	control	-0.031129412	0.048378239	-0.643458977	0.519926325	0.849611068	down_in_case
PRJNA8274(	ENSGALG00010014540	FTCD	case	control	1.08130873	1.938936681	0.557681301	0.577062029	0.878143389	up_in_case
PRJNA8274(	ENSGALG00010014547	DHDDS	case	control	0.160130082	0.059353574	2.697901269	0.006977813	0.145020586	up_in_case
PRJNA8274(	ENSGALG00010014550	SCML4	case	control	0.137699942	0.483147096	0.285006249	0.775639355	0.947146475	up_in_case
PRJNA8274(	ENSGALG00010014555	VGLL1	case	control	0.809540871	0.95211206	0.850257974	0.395181676	0.787092796	up_in_case
PRJNA8274(	ENSGALG00010014567	UTP11	case	control	-0.213044042	0.111785083	-1.905836054	0.05667148	0.387381529	down_in_case

PRJNA8274(	ENSGALG00010014570	KIF27	case	control	-0.083064797	0.120360817	-0.690131548	0.490111465	0.836347578	down_in_case
PRJNA8274(	ENSGALG00010014572	IL1RAP	case	control	0.242802335	0.140600403	1.726896433	0.084186265	0.459139432	up_in_case
PRJNA8274(	ENSGALG00010014573	CEBPB	case	control	0.127121075	0.163745443	0.776333512	0.437552091	0.810720016	up_in_case
PRJNA8274(	ENSGALG00010014576	HRH2	case	control	0.243600635	0.321535484	0.757616646	0.448680512	0.818701429	up_in_case
PRJNA8274(	ENSGALG00010014578	ENSGALG00010014578	case	control	-0.175219105	0.151466818	-1.156815118	0.247347904	0.671936745	down_in_case
PRJNA8274(	ENSGALG00010014579	NNT	case	control	-0.149259631	0.090219964	-1.654396935	0.098046889	0.487193947	down_in_case
PRJNA8274(	ENSGALG00010014592	QNG1	case	control	0.095305372	0.137302294	0.694128036	0.487601918	0.835553062	up_in_case
PRJNA8274(	ENSGALG00010014599	SSTR3	case	control	0.050704152	0.421464426	0.120304702	0.90424178	0.981104465	up_in_case
PRJNA8274(	ENSGALG00010014603	ENSGALG00010014603	case	control	-0.07485314	0.109627201	-0.682797147	0.494735033	0.83803731	down_in_case
PRJNA8274(	ENSGALG00010014611	TMEM189	case	control	-0.086149785	0.051508322	-1.672541097	0.094417671	0.47963506	down_in_case
PRJNA8274(	ENSGALG00010014614	GHR	case	control	0.064034242	0.120763112	0.530246706	0.595940892	0.884680373	up_in_case
PRJNA8274(	ENSGALG00010014617	GRTP1	case	control	0.042676868	0.079262529	0.538424258	0.590284183	0.882949889	up_in_case
PRJNA8274(	ENSGALG00010014626	C2CD5	case	control	0.007171597	0.066621566	0.107646781	0.914275887	0.982854306	up_in_case
PRJNA8274(	ENSGALG00010014632	NWD2	case	control	0.125459391	0.139508706	0.89929435	0.368495896	0.771770041	up_in_case
PRJNA8274(	ENSGALG00010014634	CCDC152	case	control	-1.148047841	0.376409956	-3.049993295	0.002288465	0.078791118	down_in_case
PRJNA8274(	ENSGALG00010014641	DOCK11	case	control	-0.065756166	0.070899447	-0.927456684	0.353689472	0.762284769	down_in_case
PRJNA8274(	ENSGALG00010014645	SLC36A1	case	control	-0.071086485	0.101347558	-0.701412907	0.483045369	0.833529046	down_in_case
PRJNA8274(	ENSGALG00010014648	C1QTNF6	case	control	0.386004147	0.118395614	3.260290928	0.00111298	0.052011805	up_in_case
PRJNA8274(	ENSGALG00010014653	FAM162B	case	control	0.157069442	0.161393244	0.973209527	0.330449177	0.746554834	up_in_case
PRJNA8274(	ENSGALG00010014654	LAMP1	case	control	0.020164056	0.049963425	0.403576334	0.686524292	0.917247255	up_in_case
PRJNA8274(	ENSGALG00010014655	FHL3	case	control	-0.121922399	0.087240669	-1.39754086	0.162250988	0.575677776	down_in_case
PRJNA8274(	ENSGALG00010014658	SELENOP1	case	control	-0.199946971	0.124614673	-1.60452189	0.108599063	0.500951275	down_in_case
PRJNA8274(	ENSGALG00010014668	GP5	case	control	0.0499042	0.13044504	0.38256878	0.702039522	0.923226519	up_in_case
PRJNA8274(	ENSGALG00010014672	ISL1	case	control	-0.635224284	0.665592559	-0.954374077	0.339894323	0.751350877	down_in_case
PRJNA8274(	ENSGALG00010014676	UBE2V1	case	control	-0.063638437	0.037276552	-1.707197512	0.087785347	0.468232177	down_in_case
PRJNA8274(	ENSGALG00010014677	ENSGALG00010014677	case	control	0.046209997	0.14787317	0.31249751	0.754662455	0.941435323	up_in_case
PRJNA8274(	ENSGALG00010014682	IL13RA1	case	control	-0.090451603	0.092025796	-0.982894004	0.325659609	0.74363678	down_in_case
PRJNA8274(	ENSGALG00010014687	CUL4A	case	control	-0.062729008	0.060602528	-1.035088971	0.300627363	0.723511633	down_in_case
PRJNA8274(	ENSGALG00010014690	CSF3R	case	control	-0.281618844	0.233902359	-1.204001726	0.228588912	0.651938858	down_in_case
PRJNA8274(	ENSGALG00010014691	PABIR2	case	control	-0.046042684	0.041017855	-1.122503447	0.261648443	0.687516348	down_in_case
PRJNA8274(	ENSGALG00010014696	SPINK5	case	control	0.914893745	0.550598028	1.661636437			up_in_case
PRJNA8274(	ENSGALG00010014697	ENSGALG00010014697	case	control	0.282781113	0.269933032	1.047597289	0.294824187	0.717981245	up_in_case
PRJNA8274(	ENSGALG00010014702	ENSGALG00010014702	case	control	-0.295656956	0.332093183	-0.890283123	0.373313881	0.774369566	down_in_case
PRJNA8274(	ENSGALG00010014703	PCID2	case	control	-0.027646553	0.060632657	-0.455968023	0.648412973	0.903557349	down_in_case
PRJNA8274(	ENSGALG00010014705	ARAP2	case	control	0.173010631	0.215610928	0.80242051	0.422309753	0.803062429	up_in_case
PRJNA8274(	ENSGALG00010014707	KPNA5	case	control	-0.006100329	0.048627061	-0.12545132	0.900166262	0.98028579	down_in_case
PRJNA8274(	ENSGALG00010014710	NPEPL1	case	control	-0.086185176	0.068238139	-1.263005947	0.206587043	0.630295436	down_in_case
PRJNA8274(	ENSGALG00010014713	ST8SIA1	case	control	-0.018704574	0.110735809	-0.168911705	0.865866093	0.973289701	down_in_case
PRJNA8274(	ENSGALG00010014716	TMEM35A	case	control	0.162540493	0.11393019	1.42666745	0.153675772	0.562468273	up_in_case
PRJNA8274(	ENSGALG00010014717	ELOVL7	case	control	-0.026933247	0.123830977	-0.217500077	0.827818647	0.961369482	down_in_case
PRJNA8274(	ENSGALG00010014719	ENSGALG00010014719	case	control	0.114840414	0.510845693	0.224804507	0.82213136	0.959320967	up_in_case
PRJNA8274(	ENSGALG00010014722	KCTD17	case	control	-0.10490224	0.223297135	-0.469787669	0.638506725	0.900280632	down_in_case
PRJNA8274(	ENSGALG00010014726	PWP2	case	control	-0.104569507	0.071509013	-1.462326247	0.143651829	0.546917241	down_in_case
PRJNA8274(	ENSGALG00010014728	INTS13	case	control	-0.044771772	0.043268598	-1.034740543	0.300790096	0.723515287	down_in_case
PRJNA8274(	ENSGALG00010014729	MRPS15	case	control	0.066453374	0.049676114	1.337732948	0.180983506	0.601788276	up_in_case
PRJNA8274(	ENSGALG00010014734	ZUP1	case	control	-0.035197744	0.083595377	-0.421048928	0.673719354	0.91379937	down_in_case
PRJNA8274(	ENSGALG00010014735	STX16	case	control	0.012297707	0.045844999	0.268245337	0.788510479	0.951738421	up_in_case
PRJNA8274(	ENSGALG00010014736	KIF2A	case	control	-0.014979068	0.057566744	-0.260203498	0.794706807	0.95280257	down_in_case
PRJNA8274(	ENSGALG00010014737	RMI1	case	control	0.08282664	0.162820143	0.508700205	0.610962378	0.889056885	up_in_case
PRJNA8274(	ENSGALG00010014738	BRWD1	case	control	0.058128787	0.054372785	1.069078714	0.285034202	0.709820465	up_in_case
PRJNA8274(	ENSGALG00010014739	TRMT2B	case	control	-0.115811948	0.094482712	-1.225747503	0.220293708	0.644563413	down_in_case
PRJNA8274(	ENSGALG00010014740	TRMT9B	case	control	-0.034030896	0.346446189	-0.09822852	0.921750836	0.985482967	down_in_case
PRJNA8274(	ENSGALG00010014742	PROZ	case	control	1.370321698	1.767770767	0.77516934			up_in_case
PRJNA8274(	ENSGALG00010014744	IL2RB	case	control	1.038595015	0.460088384	2.257381517	0.023984246	0.273286623	up_in_case
PRJNA8274(	ENSGALG00010014745	LSM10	case	control	-0.126671021	0.205348277	-0.616859431	0.537327447	0.857563269	down_in_case
PRJNA8274(	ENSGALG00010014748	FGFR1OP2	case	control	0.134169314	0.090217077	1.487183118	0.136966454	0.539854583	up_in_case
PRJNA8274(	ENSGALG00010014750	LONRF1	case	control	0.073250156	0.093918444	0.779933662	0.435429924	0.81000376	up_in_case
PRJNA8274(	ENSGALG00010014753	ENSGALG00010014753	case	control	-0.462244803	0.356125877	-1.297981507	0.19429369	0.61723879	down_in_case
PRJNA8274(	ENSGALG00010014754	DIMT1	case	control	0.044064161	0.154452528	0.285292583	0.775419995	0.947087967	up_in_case
PRJNA8274(	ENSGALG00010014758	FXN	case	control	0.163224617	0.138195975	1.181109774	0.237559118	0.662652947	up_in_case
PRJNA8274(	ENSGALG00010014762	STK40	case	control	-0.015878463	0.04932351	-0.321924845	0.747509631	0.939105545	down_in_case
PRJNA8274(	ENSGALG00010014764	ENSGALG00010014764	case	control	0.245876183	1.735219326	0.141697467	0.887318976	0.977630122	up_in_case
PRJNA8274(	ENSGALG00010014766	F10	case	control	0.461530499	0.338708241	1.362619633	0.173002418	0.592369563	up_in_case
PRJNA8274(	ENSGALG00010014767	LRRC15	case	control	-0.091129093	0.16545798	-0.550768797	0.581792177	0.880290776	down_in_case
PRJNA8274(	ENSGALG00010014769	CCDC69	case	control	-0.005397076	0.143255548	-0.037674461	0.969947238	0.992761697	down_in_case
PRJNA8274(	ENSGALG00010014777	APCDD1L	case	control	0.167303031	0.286743389	0.583459069	0.559584304	0.869682591	up_in_case
PRJNA8274(	ENSGALG00010014778	TMPRSS6	case	control	0.675837028	1.371747698	0.492683188	0.622236459	0.893820328	up_in_case
PRJNA8274(	ENSGALG00010014780	EVA1B	case	control	0.034450627	0.077729646	0.443210903	0.657613193	0.908235336	up_in_case
PRJNA8274(	ENSGALG00010014783	ENSGALG00010014783	case	control	0.001561191	0.057084675	0.027348681	0.978181629	0.995091221	up_in_case
PRJNA8274(	ENSGALG00010014789	F7	case	control	1.232709672	0.833959204	1.478141456	0.13936992	0.541505735	up_in_case
PRJNA8274(	ENSGALG00010014792	ENSGALG00010014792	case	control	0.017365216	0.182753816	0.095019716	0.924299167	0.985571367	up_in_case
PRJNA8274(	ENSGALG00010014793	TLR2A	case	control	-0.181165374	0.14632521	-1.238100892	0.215678653	0.640470741	down_in_case
PRJNA8274(	ENSGALG00010014795	THRAP3	case	control	-0.012452228	0.031646615	-0.393477414	0.693966902	0.920203598	down_in_case
PRJNA8274(	ENSGALG00010014796	PRLR	case	control	-0.091956581	0.242674235	-0.378930139	0.704739743	0.923973441	down_in_case
PRJNA8274(	ENSGALG00010014797	ZDHHC9	case	control	-0.179541499	0.056096759	-3.200568103	0.00137157	0.058654283	down_in_case
PRJNA8274(	ENSGALG00010014801	MPST	case	control	0.114600915	0.090081558	1.272190639	0.203305393	0.625537942	up_in_case
PRJNA8274(	ENSGALG00010014802	ENSGALG00010014802	case	control	0.324475768	0.185147672	1.752524155	0.07968372	0.450131333	up_in_case
PRJNA8274(	ENSGALG00010014805	SPEF2	case	control	-0.299545253	0.309240249	-0.968648984	0.332720354	0.748503677	down_in_case
PRJNA8274(	ENSGALG00010014806	MAP7D1	case	control	0.022310617	0.086606131	0.257610133	0.796707809	0.95280257	up_in_case
PRJNA8274(	ENSGALG00010014807	HES1	case	control	-0.030074099	0.069563808	-0.432323927	0.665505999	0.912007336	down_in_case
PRJNA8274(	ENSGALG00010014810	ENTREP1	case	control	-0.000984928	0.126896651	-0.007761657	0.993807156	0.998238292	down_in_case
PRJNA8274(	ENSGALG00010014812	RWDD1	case	control	0.04107593	0.063466324	0.647208279	0.517497154	0.848372336	up_in_case
PRJNA8274(	ENSGALG00010014813	SASH3	case	control	0.71501597	0.253148975	2.824486928	0.004735638	0.117407198	up_in_case
PRJNA8274(	ENSGALG00010014816	TST	case	control	0.156954694	0.100364549	1.563845966	0.117853746	0.514604253	up_in_case
PRJNA8274(	ENSGALG00010014818	ENSGALG00010014818	case	control	-0.308075609	0.366635169	-0.840278388	0.400752316	0.790340383	down_in_case
PRJNA8274(	ENSGALG00010014821	C3AR1L	case	control	0.045363312	0.154266046	0.294058952	0.768712856	0.945375926	up_in_case
PRJNA8274(	ENSGALG00010014824	ANXA6	case	control	0.019661578	0.077630572	0.25327107	0.80005875	0.954053991	up_in_case
PRJNA8274(	ENSGALG00010014829	ENSGALG00010014829	case	control	0.17776178	0.340690413	0.521769245	0.601831006	0.884954589	up_in_case
PRJNA8274(	ENSGALG00010014830	TRAPPC3L	case	control	0.055014105	0.656566269	0.083790636	0.933222893	0.987991895	up_in_case
PRJNA8274(	ENSGALG00010014831	VAPB	case	control	-0.072434202	0.0571141	-1.268236777	0.204713407	0.627358178	down_in_case
PRJNA8274(	ENSGALG00010014837	APBA1	case	control	0.04997102	0.120802369	0.413659269	0.67912366	0.915226495	up_in_case
PRJNA8274(	ENSGALG00010014839	TM7SF3	case	control	0.042652534	0.086830359	0.491216832	0.623273095	0.894052087	up_in_case
PRJNA8274(	ENSGALG00010014840	NCF4	case	control	-0.231108205	0.144413906	-1.60031822	0.109528007	0.501801854	down_in_case
PRJNA8274(	ENSGALG00010014847	PAICS	case	control	0.048634423	0.086728203	0.560768248	0.574955535	0.877910508	up_in_case
PRJNA8274(	ENSGALG00010014851	CALHM4	case	control	0.220351592	0.312310443	0.70555307	0.480466117	0.831953766	up_in_case
PRJNA8274(	ENSGALG00010014856	MMGT1	case	control	-0.016627719	0.057412961	-0.289616122	0.772109931	0.945872699	down_in_case
PRJNA8274(	ENSGALG00010014859	TRAPPC3	case	control	0.050613157	0.052712405	0.960175445	0.336966923	0.750735231	up_in_case
PRJNA8274(	ENSGALG00010014861	MED21	case	control	-0.023013044	0.078815042	-0.291987974	0.770295819	0.94543217	down_in_case
PRJNA8274(	ENSGALG00010014862	ENSGALG00010014862	case	control	-0.097402882	0.362271545	-0.268867053	0.788031992	0.951551316	down_in_case
PRJNA8274(	ENSGALG00010014865	TLR2	case	control	-0.471568973	0.25677381	-1.836515074	0.066281515	0.416668038	down_in_case
PRJNA8274(	ENSGALG00010014866	FAM26E	case	control	0.068882184	0.10699269	0.643802711	0.519703375	0.849440881	up_in_case
PRJNA8274(	ENSGALG00010014867	TP63	case	control	-0.074494111	0.194775286	-0.382461825	0.702118839	0.923231962	down_in_case
PRJNA8274(	ENSGALG00010014876	PCNT	case	control	-0.05508426	0.071339741	-0.772139888	0.44003158	0.812753079	down_in_case
PRJNA8274(	ENSGALG00010014877	ENSGALG00010014877	case	control	0.091853738	0.136103278	0.674882629	0.499750338	0.840559799	up_in_case
PRJNA8274(	ENSGALG00010014878	TMCO1	case	control	-0.090531285	0.050533933	-1.791494921	0.073213909	0.436961143	down_in_case
PRJNA8274(	ENSGALG00010014880	SFRP2	case	control	-0.106819612	0.08447287	-1.264543424	0.206035045	0.628879646	down_in_case
PRJNA8274(	ENSGALG00010014881	ENSGALG00010014881	case	control	0.188302467	0.29836507	0.631114315	0.527965782	0.853386536	up_in_case
PRJNA8274(	ENSGALG00010014882	XPNPEP2	case	control	1.268059922	0.555592473	2.282356195	0.022468319	0.266705822	up_in_case
PRJNA8274(	ENSGALG00010014883	PPAT	case	control	0.067069211	0.108487111	0.61822285	0.53642845	0.857336818	up_in_case
PRJNA8274(	ENSGALG00010014886	RABGAP1L	case	control	-0.059119433	0.070642261	-0.836884786	0.402657333	0.792021921	down_in_case
PRJNA8274(	ENSGALG00010014887	MTMR12	case	control	0.222930428	0.100095386	2.227179863	0.025935255	0.28550743	up_in_case
PRJNA8274(	ENSGALG00010014889	PVALB	case	control	-0.389989702	0.270469789	-1.441897461	0.149331303	0.555958768	down_in_case
PRJNA8274(	ENSGALG00010014893	SLC9A6	case	control	0.026465016	0.066344594	0.398902376	0.689965138	0.918477347	up_in_case
PRJNA8274(	ENSGALG00010014894	RAB22A	case	control	-0.018818527	0.12124493	-0.155210834	0.876655107	0.975805093	down_in_case
PRJNA8274(	ENSGALG00010014897	COL8A2	case	control	0.090201148	0.224001052	0.402681807	0.687182319	0.917290381	up_in_case
PRJNA8274(	ENSGALG00010014898	ENSGALG00010014898	case	control	0.081255514	0.266487799	0.3049127	0.760432643	0.942883622	up_in_case
PRJNA8274(	ENSGALG00010014900	UBLCP1	case	control	-0.020956754	0.05074177	-0.41300795	0.679600789	0.915351915	down_in_case
PRJNA8274(	ENSGALG00010014902	AASDH	case	control	0.175022762	0.092324159	1.895741749	0.057994202	0.39192679	up_in_case
PRJNA8274(	ENSGALG00010014903	MCF2L	case	control	0.008815558	0.142182143	0.062001864	0.950561348	0.991497374	up_in_case
PRJNA8274(	ENSGALG00010014906	IFT27	case	control	-0.174886098	0.09051077	-1.932213114	0.053333207	0.376996967	down_in_case
PRJNA8274(	ENSGALG00010014907	ENSGALG00010014907	case	control	-0.016269869	0.102345354	-0.158970278	0.873692291	0.975062028	down_in_case
PRJNA8274(	ENSGALG00010014908	ENSGALG00010014908	case	control	0.089457761	0.064098133	1.395637549	0.162823669	0.576616758	up_in_case
PRJNA8274(	ENSGALG00010014909	FBXL14	case	control	-0.050487636	0.071751577	-0.703644961	0.481653904	0.832529825	down_in_case
PRJNA8274(	ENSGALG00010014912	ENSGALG00010014912	case	control	-0.24154503	0.264334274	-0.913786271	0.360829162	0.766754768	down_in_case
PRJNA8274(	ENSGALG00010014914	PTAR1	case	control	0.128874409	0.112408822	1.146479489	0.251596809	0.677081801	up_in_case
PRJNA8274(	ENSGALG00010014916	DYNLT2B	case	control	0.111120399	0.140221079	0.792465724	0.42808917	0.805337183	up_in_case

PRJNA8274(	ENSGALG00010014917	EIF3D	case	control	0.028529114	0.074868688	0.38105535	0.703162178	0.923659131	up_in_case
PRJNA8274(	ENSGALG00010014918	PLRG1	case	control	-0.063920947	0.045956533	-1.39090013	0.164255714	0.579452754	down_in_case
PRJNA8274(	ENSGALG00010014922	MAN1A1	case	control	0.217718589	0.137958114	1.578149939	0.114531168	0.509446069	up_in_case
PRJNA8274(	ENSGALG00010014923	CIMIP1	case	control	0.439950996	0.294873947	1.49199684	0.135699974	0.539774365	up_in_case
PRJNA8274(	ENSGALG00010014928	SUCO	case	control	0.112427995	0.053037559	2.119780738	0.03402454	0.318007143	up_in_case
PRJNA8274(	ENSGALG00010014929	FHL1	case	control	-0.25935437	0.166019024	-1.562196691	0.11824166	0.51463738	down_in_case
PRJNA8274(	ENSGALG00010014931	RNF145	case	control	0.044980401	0.067519415	0.666184699	0.505293052	0.842675288	up_in_case
PRJNA8274(	ENSGALG00010014934	MAMDC2	case	control	0.058920112	0.140030448	0.420766436	0.673925643	0.913846845	up_in_case
PRJNA8274(	ENSGALG00010014935	PCYT1A	case	control	-0.111388931	0.095020747	-1.172259054	0.241093066	0.666204503	down_in_case
PRJNA8274(	ENSGALG00010014936	FOXRED2	case	control	0.089395225	0.096884439	0.922699511	0.356163821	0.763373775	up_in_case
PRJNA8274(	ENSGALG00010014940	VAMP4	case	control	0.05211934	0.100946635	0.516305869	0.605640798	0.88576746	up_in_case
PRJNA8274(	ENSGALG00010014942	PMEPA1	case	control	0.082182682	0.086201342	0.95338054	0.340397297	0.751881581	up_in_case
PRJNA8274(	ENSGALG00010014943	APLN	case	control	0.114741297	0.14237552	0.805906083	0.420297003	0.801686961	up_in_case
PRJNA8274(	ENSGALG00010014944	FGB	case	control	0.956068712	0.823667625	1.160745771	0.245745302	0.670512845	up_in_case
PRJNA8274(	ENSGALG00010014945	TXN2	case	control	0.090511222	0.10364473	0.873283401	0.382508626	0.77998936	up_in_case
PRJNA8274(	ENSGALG00010014946	PCK1	case	control	0.474408665	0.589313109	0.805019704	0.420808309	0.801969583	up_in_case
PRJNA8274(	ENSGALG00010014948	RBM38	case	control	-0.290777785	0.095484071	-3.045301506	0.002324472	0.079329425	down_in_case
PRJNA8274(	ENSGALG00010014949	AGO3	case	control	-0.000852924	0.088121256	-0.009678983	0.992277409	0.997995042	down_in_case
PRJNA8274(	ENSGALG00010014956	FGG	case	control	1.33107032	1.378936333	0.965287728	0.334400717	0.748686141	up_in_case
PRJNA8274(	ENSGALG00010014963	KMO	case	control	0.729197946	0.998560947	0.730248813	0.46523811	0.826255378	up_in_case
PRJNA8274(	ENSGALG00010014965	ABHD13	case	control	-0.031445207	0.056852177	-0.553104703	0.580191716	0.879378502	down_in_case
PRJNA8274(	ENSGALG00010014967	HNRNPH1	case	control	-0.06981799	0.045455421	-1.535966203	0.12454667	0.523366845	down_in_case
PRJNA8274(	ENSGALG00010014971	SMC5	case	control	0.070641816	0.185998595	0.379797577	0.704095681	0.923966697	up_in_case
PRJNA8274(	ENSGALG00010014972	FGA	case	control	1.546755514	1.452576106	1.064836127	0.286950094	0.710666148	up_in_case
PRJNA8274(	ENSGALG00010014974	LIG4	case	control	0.181643531	0.139406138	1.302980866	0.192581294	0.615090794	up_in_case
PRJNA8274(	ENSGALG00010014976	ITGB2	case	control	-0.088489786	0.132068557	-0.670029174	0.502839193	0.842443289	down_in_case
PRJNA8274(	ENSGALG00010014980	ENSGALG00010014980	case	control	-0.335352663	0.305756195	-1.09679761	0.272729875	0.698931401	down_in_case
PRJNA8274(	ENSGALG00010014982	ENSGALG00010014982	case	control	-0.35703562	0.336744704	-1.060256081	0.289028114	0.712801679	down_in_case
PRJNA8274(	ENSGALG00010014984	LPP	case	control	0.066188327	0.142309099	0.465102562	0.641858012	0.901539099	up_in_case
PRJNA8274(	ENSGALG00010014985	ENSGALG00010014985	case	control	0.017916426	0.059240489	0.302435482	0.76232011	0.943416492	up_in_case
PRJNA8274(	ENSGALG00010014987	KLF9	case	control	-0.159872465	0.191039325	-0.836856311	0.40267334	0.792021921	down_in_case
PRJNA8274(	ENSGALG00010014990	AGO1	case	control	-0.035998497	0.052455243	-0.686270714	0.492542413	0.837671284	down_in_case
PRJNA8274(	ENSGALG00010014992	ARL15	case	control	-0.085338336	0.048201147	-1.770462757	0.076650083	0.44430328	down_in_case
PRJNA8274(	ENSGALG00010014994	ENSGALG00010014994	case	control	-0.126594982	0.075532743	-1.676027863	0.093732735	0.478571295	down_in_case
PRJNA8274(	ENSGALG00010014995	LRAT	case	control	0.881269403	0.289069453	3.048642445	0.002298779	0.078831031	up_in_case
PRJNA8274(	ENSGALG00010014997	RAE1	case	control	0.029017588	0.056867889	0.510263144	0.60986712	0.888273136	up_in_case
PRJNA8274(	ENSGALG00010015002	ADAM19	case	control	0.11772741	0.090333467	1.303253537	0.192488218	0.615090794	up_in_case
PRJNA8274(	ENSGALG00010015006	HSPB3	case	control	-0.504067058	0.235888689	-2.136885243	0.032607327	0.311063889	down_in_case
PRJNA8274(	ENSGALG00010015009	AGO4	case	control	-0.153453168	0.042436963	-3.616026167	0.00029916	0.023376995	down_in_case
PRJNA8274(	ENSGALG00010015013	SNX18	case	control	0.037190585	0.091283003	0.407420704	0.683699023	0.916211792	up_in_case
PRJNA8274(	ENSGALG00010015016	ARGLU1	case	control	0.077606458	0.063519524	1.221773285	0.221793374	0.645834386	up_in_case
PRJNA8274(	ENSGALG00010015017	FAM184A	case	control	-0.004079739	0.128204524	-0.031822114	0.974613911	0.993775355	down_in_case
PRJNA8274(	ENSGALG00010015018	MAP7D3	case	control	0.016796822	0.044104697	0.380839755	0.703322158	0.923659131	up_in_case
PRJNA8274(	ENSGALG00010015020	NIPAL4	case	control	1.135893864	0.81483029	1.394025085	0.16331003	0.577197348	up_in_case
PRJNA8274(	ENSGALG00010015024	CRACD	case	control	-0.041081343	0.120530228	-0.340838504	0.733225162	0.933846596	down_in_case
PRJNA8274(	ENSGALG00010015025	TRAF3IP1	case	control	0.02613428	0.094925489	0.275313621	0.7830753	0.949518053	up_in_case
PRJNA8274(	ENSGALG00010015027	CLSPN	case	control	-0.077107622	0.07312366	-1.054482538	0.291662051	0.714417151	down_in_case
PRJNA8274(	ENSGALG00010015028	MCM9	case	control	-0.587134497	0.236956475	-2.477815799	0.013218937	0.201788559	down_in_case
PRJNA8274(	ENSGALG00010015029	TFRC	case	control	0.123968638	0.156287665	0.793208075	0.427656603	0.805305429	up_in_case
PRJNA8274(	ENSGALG00010015031	MYH9	case	control	-0.052586525	0.057485204	-0.914783655	0.360305217	0.765923087	down_in_case
PRJNA8274(	ENSGALG00010015032	C1orf216	case	control	1.035147612	0.218208726	4.743841515	2.09703E-06	0.000475992	up_in_case
PRJNA8274(	ENSGALG00010015033	BMP7	case	control	-0.012855394	0.201231807	-0.063883511	0.949062982	0.99138853	down_in_case
PRJNA8274(	ENSGALG00010015035	SLC35F1	case	control	0.126422614	0.09295449	1.360048493	0.173814579	0.592926641	up_in_case
PRJNA8274(	ENSGALG00010015037	CYFIP2	case	control	-0.006473761	0.165235637	-0.039178961	0.968747707	0.992761697	down_in_case
PRJNA8274(	ENSGALG00010015038	PCSK5	case	control	0.03233059	0.098726616	0.327475925	0.743307949	0.937448285	up_in_case
PRJNA8274(	ENSGALG00010015039	FAM114A1	case	control	0.112522399	0.063439703	1.773690502	0.076114358	0.442710866	up_in_case
PRJNA8274(	ENSGALG00010015040	ZC4H2	case	control	0.053958706	0.053940952	1.000329134	0.317151253	0.737768794	up_in_case
PRJNA8274(	ENSGALG00010015041	TFAP2C	case	control	0.068808313	0.497256798	0.138375812	0.889943412	0.978410917	up_in_case
PRJNA8274(	ENSGALG00010015042	EFNB2	case	control	-0.109171131	0.063006208	-1.732704355	0.08314823	0.457139442	down_in_case
PRJNA8274(	ENSGALG00010015043	COQ10B	case	control	0.174282652	0.093099454	1.872005093	0.061205899	0.402191009	up_in_case
PRJNA8274(	ENSGALG00010015044	ZC3H12B	case	control	0.041453582	0.094009622	0.44095042	0.659248891	0.908599575	up_in_case
PRJNA8274(	ENSGALG00010015046	PSMB2	case	control	-0.078375131	0.049959229	-1.568781831	0.116698783	0.51350396	down_in_case
PRJNA8274(	ENSGALG00010015047	NPY2R	case	control	0.152581794	0.228136423	0.668818209	0.503611449	0.842675107	up_in_case
PRJNA8274(	ENSGALG00010015049	DCK2	case	control	-0.044646901	0.085880704	-0.519871156	0.603153381	0.885268908	down_in_case
PRJNA8274(	ENSGALG00010015050	HIVEP3	case	control	-0.020538114	0.096157668	-0.213587895	0.83086844	0.962228709	down_in_case
PRJNA8274(	ENSGALG00010015052	ENSGALG00010015052	case	control	-0.095981639	0.083516522	-1.149253298	0.250451549	0.676001728	down_in_case
PRJNA8274(	ENSGALG00010015054	ENSGALG00010015054	case	control	-0.360850787	0.12416398	-2.906243729	0.003657964	0.104408952	down_in_case
PRJNA8274(	ENSGALG00010015055	ZNF335	case	control	-0.053876502	0.039113755	-1.377431065	0.168379014	0.584422305	down_in_case
PRJNA8274(	ENSGALG00010015056	ENSGALG00010015056	case	control	0.132040418	0.359780315	0.367002898	0.713616856	0.926862121	up_in_case
PRJNA8274(	ENSGALG00010015057	LAS1L	case	control	-0.124733625	0.084961256	-1.468133606	0.142070651	0.543501954	down_in_case
PRJNA8274(	ENSGALG00010015059	ENSGALG00010015059	case	control	-0.066421613	0.09250511	-0.718031821	0.472737666	0.829235491	down_in_case
PRJNA8274(	ENSGALG00010015061	MMP9	case	control	0.093958955	0.763586686	0.123049494	0.902067906	0.980763983	up_in_case
PRJNA8274(	ENSGALG00010015062	ENSGALG00010015062	case	control	-0.242272022	0.158682702	-1.526770211	0.126818161	0.527026942	down_in_case
PRJNA8274(	ENSGALG00010015063	EEF1AKMT4	case	control	-0.087606719	0.163193084	-0.536828628	0.591385995	0.882949889	down_in_case
PRJNA8274(	ENSGALG00010015064	RFK	case	control	-0.000394026	0.141524298	-0.002784155	0.997778568	0.999142415	down_in_case
PRJNA8274(	ENSGALG00010015066	RUFY1	case	control	-0.089594942	0.058602995	-1.528845805	0.126302674	0.525954642	down_in_case
PRJNA8274(	ENSGALG00010015068	ESM1	case	control	0.099782136	0.162108823	0.615525632	0.538207647	0.857740557	up_in_case
PRJNA8274(	ENSGALG00010015069	HSPD1	case	control	0.106629448	0.074596169	1.429422566	0.152882816	0.561193576	up_in_case
PRJNA8274(	ENSGALG00010015070	EDN2	case	control	0.162850099	0.323296897	0.503716866	0.614460355	0.890612515	up_in_case
PRJNA8274(	ENSGALG00010015071	SNAI1	case	control	0.137596634	0.07426994	1.852655776	0.063931712	0.410839656	up_in_case
PRJNA8274(	ENSGALG00010015072	MAP9	case	control	0.154303368	0.094010144	1.641348059	0.100725185	0.490422251	up_in_case
PRJNA8274(	ENSGALG00010015075	ENSGALG00010015075	case	control	-0.148358615	0.099235411	-1.495016885	0.134910035	0.539774365	down_in_case
PRJNA8274(	ENSGALG00010015078	GABRQ	case	control	-0.696376801	0.634710317	-1.097156896	0.272572812	0.698931401	down_in_case
PRJNA8274(	ENSGALG00010015080	ASF1A	case	control	-0.033751672	0.062653671	-0.538702219	0.590092343	0.882949889	down_in_case
PRJNA8274(	ENSGALG00010015083	CEP135	case	control	-0.061952632	0.113694572	-0.54490404	0.585819518	0.881576413	down_in_case
PRJNA8274(	ENSGALG00010015084	FOXO6	case	control	-0.082096088	0.1272524	-0.64514373	0.518834047	0.848941486	down_in_case
PRJNA8274(	ENSGALG00010015085	PSMD2	case	control	-0.004729527	0.067553991	-0.070011074	0.944184845	0.990306827	down_in_case
PRJNA8274(	ENSGALG00010015086	FOXN3	case	control	-0.094368963	0.064735825	-1.457754843	0.144908129	0.548818979	down_in_case
PRJNA8274(	ENSGALG00010015087	GABRA3	case	control	0.413670984	0.368572775	1.122359035	0.261709815	0.687516348	up_in_case
PRJNA8274(	ENSGALG00010015088	ENSGALG00010015088	case	control	-0.266315864	0.296278926	-0.898868737	0.368722582	0.771770041	down_in_case
PRJNA8274(	ENSGALG00010015090	GZMA	case	control	-0.388932244	0.75816016	-0.512994832	0.607954936	0.887502645	down_in_case
PRJNA8274(	ENSGALG00010015091	MSN	case	control	0.045635858	0.046502479	0.981363983	0.326413281	0.744287835	up_in_case
PRJNA8274(	ENSGALG00010015092	HSPE1	case	control	-0.015676771	0.069148568	-0.226711438	0.820648133	0.958669267	down_in_case
PRJNA8274(	ENSGALG00010015094	CDK5RAP1	case	control	0.129469867	0.083126318	1.557507547	0.11935003	0.516645008	up_in_case
PRJNA8274(	ENSGALG00010015095	RBFOX2	case	control	-0.131246169	0.074569948	-1.760041056	0.078400846	0.448818031	down_in_case
PRJNA8274(	ENSGALG00010015096	GABRE	case	control	-1.166982743	0.756820505	-1.541954446	0.123084664	0.522552563	down_in_case
PRJNA8274(	ENSGALG00010015097	MFSD2A	case	control	0.01156173	0.089779188	0.128779628	0.897532025	0.980176778	up_in_case
PRJNA8274(	ENSGALG00010015098	HAVCR1	case	control	0.059799013	0.146213107	0.408985309	0.682550443	0.915874198	up_in_case
PRJNA8274(	ENSGALG00010015100	MOB4	case	control	-0.035021983	0.043616991	-0.802943564	0.422007355	0.802594118	down_in_case
PRJNA8274(	ENSGALG00010015101	CAP1	case	control	-0.017747505	0.044906698	-0.395208405	0.69268909	0.91912907	down_in_case
PRJNA8274(	ENSGALG00010015103	ITPK1	case	control	0.154837627	0.213114164	0.7265478	0.467503015	0.828046838	up_in_case
PRJNA8274(	ENSGALG00010015104	CNGA2	case	control	0.130710381	0.287962562	0.453914495	0.649890371	0.904241824	up_in_case
PRJNA8274(	ENSGALG00010015107	MB	case	control	-0.047477817	0.441009919	-0.107657028	0.914267758	0.982854306	down_in_case
PRJNA8274(	ENSGALG00010015108	ENSGALG00010015108	case	control	0.045150119	0.092305823	0.489136202	0.624745273	0.894741574	up_in_case
PRJNA8274(	ENSGALG00010015109	PPT1	case	control	0.014679056	0.049147888	0.298671136	0.765190983	0.943786458	up_in_case
PRJNA8274(	ENSGALG00010015111	ADAMTS2	case	control	-0.106446636	0.144825759	-0.734997953	0.462340741	0.824990341	down_in_case
PRJNA8274(	ENSGALG00010015112	CBLN4	case	control	0.357816747	0.364714549	0.98108712	0.326549781	0.744287835	up_in_case
PRJNA8274(	ENSGALG00010015115	CLDN2	case	control	0.290867583	0.141261441	2.059072748	0.03948727	0.335234949	up_in_case
PRJNA8274(	ENSGALG00010015117	RASD2	case	control	-0.102300075	0.165864173	-0.616770178	0.537386325	0.857563269	down_in_case
PRJNA8274(	ENSGALG00010015118	GPX8	case	control	-0.182804619	0.149389298	-1.223679486	0.221073161	0.645172695	down_in_case
PRJNA8274(	ENSGALG00010015119	TLR1A	case	control	0.712538981	0.205591272	3.465803653	0.000528649	0.033449927	up_in_case
PRJNA8274(	ENSGALG00010015121	UNC79	case	control	0.347055213	0.243098773	1.427630456	0.153398253	0.562068571	up_in_case
PRJNA8274(	ENSGALG00010015122	ENSGALG00010015122	case	control	-0.027772617	0.086878861	-0.319670596	0.749218052	0.939503405	down_in_case
PRJNA8274(	ENSGALG00010015123	CEP85L	case	control	-0.097226646	0.090329276	-1.076358083	0.281767141	0.705775113	down_in_case
PRJNA8274(	ENSGALG00010015124	UXS1	case	control	-0.138923284	0.087234159	-1.592533093	0.111264974	0.503925506	down_in_case
PRJNA8274(	ENSGALG00010015126	MCM5	case	control	0.009692748	0.084076711	0.115284575	0.908219565	0.981730793	up_in_case
PRJNA8274(	ENSGALG00010015127	RPL11	case	control	-0.059628818	0.063742339	-0.935466436	0.349547973	0.759342183	down_in_case
PRJNA8274(	ENSGALG00010015128	OCRL	case	control	-0.09079641	0.053579782	-1.694602086	0.090150931	0.473607022	down_in_case
PRJNA8274(	ENSGALG00010015129	PRRG3	case	control	0.109690805	0.097380695	1.126412221	0.259991077	0.687227573	up_in_case
PRJNA8274(	ENSGALG00010015131	DOK5	case	control	-0.170369407	0.188766673	-0.902539646	0.366770271	0.771770041	down_in_case
PRJNA8274(	ENSGALG00010015132	GCNT1	case	control	-0.280125191	0.28700603	-0.976025454	0.32905185	0.745636104	down_in_case
PRJNA8274(	ENSGALG00010015133	RFTN2	case	control	0.155837081	0.079273233	1.965822185	0.049319154	0.364571979	up_in_case
PRJNA8274(	ENSGALG00010015134	PLN	case	control	0.587740121	0.426217332	1.37896814	0.167904577	0.584193542	up_in_case
PRJNA8274(	ENSGALG00010015136	TCEB3	case	control	-0.026060545	0.052767414	-0.493875719	0.621393954	0.893175881	down_in_case
PRJNA8274(	ENSGALG00010015137	AMN	case	control	0.13293865	0.752837703	0.176583412	0.859835625	0.971828322	up_in_case
PRJNA8274(	ENSGALG00010015138	ENSGALG00010015138	case	control	-0.893434072	0.502761873	-1.777052159	0.075559658	0.442285352	down_in_case

PRJNA8274(	ENSGALG00010015140	ASCC3	case	control	-0.006002748	0.044090076	-0.136147366	0.891704783	0.978917427	down_in_case
PRJNA8274(	ENSGALG00010015141	PFDN4	case	control	-0.134523013	0.081717578	-1.646194333	0.099723756	0.488707918	down_in_case
PRJNA8274(	ENSGALG00010015142	HNRNPAB	case	control	-0.024548341	0.037717408	-0.650849095	0.515143904	0.847750297	down_in_case
PRJNA8274(	ENSGALG00010015143	EXOC1	case	control	0.016134464	0.058837735	0.27421966	0.783915821	0.949518053	up_in_case
PRJNA8274(	ENSGALG00010015145	CLIC2	case	control	-0.019679512	0.075513169	-0.260610336	0.794393018	0.95280257	down_in_case
PRJNA8274(	ENSGALG00010015146	ENSGALG00010015146	case	control	-0.078316469	0.074399981	-1.052640975	0.29250557	0.714947473	down_in_case
PRJNA8274(	ENSGALG00010015148	ENSGALG00010015148	case	control	0.020281945	0.125204077	0.161991091	0.871312869	0.975062028	up_in_case
PRJNA8274(	ENSGALG00010015149	ENSGALG00010015149	case	control	0.111611519	0.083062854	1.343699553	0.179045562	0.599333225	up_in_case
PRJNA8274(	ENSGALG00010015151	ENSGALG00010015151	case	control	0.278561279	0.274452306	1.014971538	0.310119395	0.73300948	up_in_case
PRJNA8274(	ENSGALG00010015152	BOLL	case	control	-0.027136841	0.159630661	-0.169997673	0.865011967	0.973289701	down_in_case
PRJNA8274(	ENSGALG00010015153	ERCC6L	case	control	0.023524699	0.10104788	0.232807446	0.81591093	0.957374769	up_in_case
PRJNA8274(	ENSGALG00010015154	CYP24A1	case	control	0.401996215	0.624869288	0.643328489	0.520010974	0.849641807	up_in_case
PRJNA8274(	ENSGALG00010015155	HMOX1	case	control	0.287922323	0.148162797	1.943283524	0.051981911	0.372042707	up_in_case
PRJNA8274(	ENSGALG00010015156	CNOT6	case	control	0.019778036	0.055512677	0.356279625	0.721631167	0.929484914	up_in_case
PRJNA8274(	ENSGALG00010015157	CDC42BPB	case	control	-0.068303202	0.045137319	-1.513231256	0.130220927	0.532650639	down_in_case
PRJNA8274(	ENSGALG00010015158	PIN4	case	control	0.078337528	0.069801185	1.12229509	0.261736993	0.687516348	up_in_case
PRJNA8274(	ENSGALG00010015160	SIM1	case	control	-0.071502915	0.410333179	-0.174255749	0.861664474	0.972599205	down_in_case
PRJNA8274(	ENSGALG00010015161	GFPT2	case	control	-0.031007388	0.100562881	-0.3083383	0.757824926	0.941765572	down_in_case
PRJNA8274(	ENSGALG00010015163	PLK2	case	control	0.042851684	0.137579418	0.311468714	0.755444323	0.941559531	up_in_case
PRJNA8274(	ENSGALG00010015164	ENSGALG00010015164	case	control	0.7106537	0.446613894	1.591203743	0.111563731	0.504204693	up_in_case
PRJNA8274(	ENSGALG00010015165	KLF3	case	control	-0.034040924	0.064617626	-0.526805555	0.598328625	0.884717127	down_in_case
PRJNA8274(	ENSGALG00010015166	YTHDF2	case	control	0.00961637	0.045899497	0.209509273	0.834050697	0.963078474	up_in_case
PRJNA8274(	ENSGALG00010015167	RAB3C	case	control	-0.220453183	0.368324977	-0.598529008	0.549487009	0.864234145	down_in_case
PRJNA8274(	ENSGALG00010015170	EXOC1L	case	control	0.606427815	0.651617282	0.930650293	0.35203449	0.760831854	up_in_case
PRJNA8274(	ENSGALG00010015171	NHSL2	case	control	-0.011693678	0.095719057	-0.122166666	0.902767025	0.980895712	down_in_case
PRJNA8274(	ENSGALG00010015172	PPP4R3A	case	control	-0.055645228	0.050848038	-1.094343665	0.273804282	0.699576755	down_in_case
PRJNA8274(	ENSGALG00010015173	ENS-1	case	control	-0.466074626	0.542403511	-0.859276566	0.39018795	0.783914628	down_in_case
PRJNA8274(	ENSGALG00010015174	ST6GAL2	case	control	0.15704198	0.198271137	0.792056685	0.428327625	0.805337183	up_in_case
PRJNA8274(	ENSGALG00010015175	MAPK9	case	control	-0.08808137	0.06571831	-1.340286584	0.180152192	0.600999032	down_in_case
PRJNA8274(	ENSGALG00010015177	TC2N	case	control	0.100934288	0.10944256	0.922258108	0.35639396	0.763373775	up_in_case
PRJNA8274(	ENSGALG00010015180	PITHD1	case	control	-0.099646179	0.083644276	-1.191309006	0.2335323	0.657982411	down_in_case
PRJNA8274(	ENSGALG00010015181	VPS13A	case	control	-0.177341894	0.090237903	-1.965270555	0.049382932	0.364571979	down_in_case
PRJNA8274(	ENSGALG00010015182	PHYKPL	case	control	0.165948786	0.083439296	1.988856496	0.046717045	0.356907825	up_in_case
PRJNA8274(	ENSGALG00010015183	SLC5A7	case	control	-1.495815195	0.764887426	-1.955601757	0.050512086	0.367407338	down_in_case
PRJNA8274(	ENSGALG00010015186	ENSGALG00010015186	case	control	0.541720699	0.27864198	1.944146032	0.051877844	0.371953818	up_in_case
PRJNA8274(	ENSGALG00010015188	SULT1C3	case	control	1.480324765	0.853552362	1.734310431	0.082863018	0.456624721	up_in_case
PRJNA8274(	ENSGALG00010015190	FBLN5	case	control	0.127475697	0.278278075	0.458087461	0.646889607	0.90295498	up_in_case
PRJNA8274(	ENSGALG00010015191	TOM1	case	control	-0.128237274	0.112524348	-1.139640235	0.254436215	0.679971816	down_in_case
PRJNA8274(	ENSGALG00010015192	ENSGALG00010015192	case	control	-0.1246176	0.259817264	-0.479635564	0.631486553	0.897039706	down_in_case
PRJNA8274(	ENSGALG00010015193	LYPLA2	case	control	0.033364876	0.063303581	0.527061428	0.598150932	0.884680373	up_in_case
PRJNA8274(	ENSGALG00010015194	TMLHE	case	control	0.001788015	0.049361212	0.036223074	0.971104488	0.992761697	up_in_case
PRJNA8274(	ENSGALG00010015196	PDE4D	case	control	-0.377827263	0.152118346	-2.483771827	0.012999908	0.200161779	down_in_case
PRJNA8274(	ENSGALG00010015198	NME5	case	control	0.030036581	0.146230564	0.205405627	0.837255224	0.964826648	up_in_case
PRJNA8274(	ENSGALG00010015199	ENSGALG00010015199	case	control	-0.057822994	0.110088664	-0.525240223	0.599416207	0.884888453	down_in_case
PRJNA8274(	ENSGALG00010015200	TBC1D1	case	control	-0.046624909	0.061097376	-0.763124569	0.445389109	0.816860877	down_in_case
PRJNA8274(	ENSGALG00010015201	GCC2	case	control	0.046965888	0.090157804	0.520929807	0.60241567	0.885101354	up_in_case
PRJNA8274(	ENSGALG00010015203	HMGXB4	case	control	-0.003709511	0.050471539	-0.073497075	0.941410572	0.989551456	down_in_case
PRJNA8274(	ENSGALG00010015204	ENSGALG00010015204	case	control	0.194918387	0.098384997	1.981179981	0.047571093	0.359816265	up_in_case
PRJNA8274(	ENSGALG00010015207	AKR1E2	case	control	-0.231174627	0.11598868	-1.993079204	0.046252775	0.356365668	down_in_case
PRJNA8274(	ENSGALG00010015212	GNAQ	case	control	-0.015790838	0.079312913	-0.199095417	0.842188106	0.966478606	down_in_case
PRJNA8274(	ENSGALG00010015213	RUBCN	case	control	0.060250119	0.091070973	0.661573235	0.508244765	0.843702533	up_in_case
PRJNA8274(	ENSGALG00010015218	PLCL1	case	control	-0.03125347	0.111273935	-0.280869642	0.778810386	0.948479876	down_in_case
PRJNA8274(	ENSGALG00010015227	FYTTD1	case	control	-0.045046255	0.032747995	-1.375542361	0.168963362	0.585038804	down_in_case
PRJNA8274(	ENSGALG00010015233	ENSGALG00010015233	case	control	-0.173330356	0.531320187	-0.326225806	0.744253518	0.937611251	down_in_case
PRJNA8274(	ENSGALG00010015234	TRIP11	case	control	0.079035108	0.097279152	0.812456798	0.416529566	0.799987395	up_in_case
PRJNA8274(	ENSGALG00010015241	ENSGALG00010015241	case	control	-0.078506961	0.19673933	-0.399040503	0.68986336	0.918477347	down_in_case
PRJNA8274(	ENSGALG00010015248	ENSGALG00010015248	case	control	0.016412922	0.081185727	0.202165114	0.839787645	0.965286179	up_in_case
PRJNA8274(	ENSGALG00010015250	RORB	case	control	0.22911607	0.413686787	0.553839468	0.579688715	0.879142433	up_in_case
PRJNA8274(	ENSGALG00010015253	ENSGALG00010015253	case	control	0.104657427	0.056647538	1.847519433	0.064671894	0.413229709	up_in_case
PRJNA8274(	ENSGALG00010015260	RASGEF1C	case	control	-0.194866031	0.110804512	-1.75864708	0.078637471	0.449003015	down_in_case
PRJNA8274(	ENSGALG00010015261	IQCG	case	control	0.017976503	0.193637572	0.092835822	0.926033992	0.986137638	up_in_case
PRJNA8274(	ENSGALG00010015283	ENSGALG00010015283	case	control	0.893490852	0.606417998	1.473391052	0.140645622	0.543282655	up_in_case
PRJNA8274(	ENSGALG00010015285	ALG13	case	control	-0.061153679	0.313840615	-0.194855848	0.8455058	0.967517103	down_in_case
PRJNA8274(	ENSGALG00010015291	ZFP64	case	control	0.040452009	0.044657946	0.905818841	0.365031747	0.770814232	up_in_case
PRJNA8274(	ENSGALG00010015296	RPL35A	case	control	-0.082434013	0.076554507	-1.076801573	0.281568923	0.70577311	down_in_case
PRJNA8274(	ENSGALG00010015298	ENSGALG00010015298	case	control	0.157422965	0.212882864	0.739481618	0.459614598	0.824480387	up_in_case
PRJNA8274(	ENSGALG00010015299	GALE	case	control	0.055756276	0.069689513	0.800066943	0.423672013	0.803723282	up_in_case
PRJNA8274(	ENSGALG00010015300	ATXN3	case	control	-0.196045655	0.077227997	-2.538530864	0.011131898	0.185137791	down_in_case
PRJNA8274(	ENSGALG00010015306	BCAT1	case	control	-0.111386493	0.092147515	-1.208784554	0.226745627	0.650445465	down_in_case
PRJNA8274(	ENSGALG00010015310	PGM2	case	control	0.172845551	0.074172921	2.330305282	0.019790022	0.252676171	up_in_case
PRJNA8274(	ENSGALG00010015314	GMEB1	case	control	0.080160531	0.053389151	1.501438571	0.133242164	0.536721956	up_in_case
PRJNA8274(	ENSGALG00010015318	SALL4	case	control	0.274644107	0.159449634	1.722450535	0.084987936	0.461224852	up_in_case
PRJNA8274(	ENSGALG00010015320	TRPM6	case	control	0.882512508	0.363884622	2.425253649	0.015297694	0.219195419	up_in_case
PRJNA8274(	ENSGALG00010015327	LARGE1	case	control	-0.164152182	0.105547681	-1.555241969	0.119888452	0.517634321	down_in_case
PRJNA8274(	ENSGALG00010015333	MED7	case	control	-0.05800578	0.075259137	-0.770747348	0.440856701	0.813083204	down_in_case
PRJNA8274(	ENSGALG00010015339	ENSGALG00010015339	case	control	0.002786085	0.714619889	0.003898695	0.996889299	0.998921977	up_in_case
PRJNA8274(	ENSGALG00010015348	CPSF2	case	control	0.034445341	0.044681906	0.77090132	0.440765424	0.813083204	up_in_case
PRJNA8274(	ENSGALG00010015353	RELL1	case	control	0.048056167	0.061810737	0.77747281	0.43687987	0.810506246	up_in_case
PRJNA8274(	ENSGALG00010015356	ENSGALG00010015356	case	control	-0.185845492	0.099651101	-1.864961755	0.062186765	0.405537748	down_in_case
PRJNA8274(	ENSGALG00010015362	ATP9A	case	control	-0.066238963	0.193462758	-0.342386122	0.732060331	0.933770678	down_in_case
PRJNA8274(	ENSGALG00010015364	CLOCK	case	control	-0.083013528	0.068144132	-1.218205077	0.223146051	0.646996863	down_in_case
PRJNA8274(	ENSGALG00010015370	ENSGALG00010015370	case	control	-0.102054732	0.058722521	-1.737914684	0.082225848	0.455747917	down_in_case
PRJNA8274(	ENSGALG00010015374	LIMS1	case	control	0.017817689	0.111376958	0.159976434	0.872899638	0.975062028	up_in_case
PRJNA8274(	ENSGALG00010015384	TAF12	case	control	0.008638461	0.067927205	0.127172334	0.898804002	0.98028579	up_in_case
PRJNA8274(	ENSGALG00010015386	SLC24A4	case	control	1.253585023	0.258796537	4.843901849	1.27314E-06	0.000319401	up_in_case
PRJNA8274(	ENSGALG00010015388	PSMD1	case	control	0.00129325	0.079158008	0.016337571	0.986965084	0.996781748	up_in_case
PRJNA8274(	ENSGALG00010015390	CCNB3	case	control	-0.031245245	0.09954457	-0.313881961	0.753610691	0.940858857	down_in_case
PRJNA8274(	ENSGALG00010015391	SYN3	case	control	0.016197042	0.355120296	0.045610014	0.963621088	0.992068146	up_in_case
PRJNA8274(	ENSGALG00010015392	TMEM165	case	control	-0.027560319	0.060402884	-0.456274884	0.648192322	0.903510177	down_in_case
PRJNA8274(	ENSGALG00010015393	DCX	case	control	-0.067885302	0.153828989	-0.441303699	0.658993149	0.908599575	down_in_case
PRJNA8274(	ENSGALG00010015397	SRD5A3	case	control	0.122118251	0.088682408	1.377029026	0.168503274	0.584687698	up_in_case
PRJNA8274(	ENSGALG00010015404	BEND4	case	control	-0.212174174	0.191200439	-1.109695012	0.267130473	0.693530458	down_in_case
PRJNA8274(	ENSGALG00010015407	TIMP3	case	control	0.031345722	0.075271103	0.416437657	0.677089778	0.914726861	up_in_case
PRJNA8274(	ENSGALG00010015409	FAM13B	case	control	0.112602034	0.076585269	1.470283198	0.14148507	0.543501954	up_in_case
PRJNA8274(	ENSGALG00010015410	HTR2B	case	control	-0.142888626	0.167448106	-0.8533308	0.393475884	0.786843919	down_in_case
PRJNA8274(	ENSGALG00010015413	CARNMT1	case	control	0.092838471	0.075382998	1.231557164	0.218114551	0.64336594	up_in_case
PRJNA8274(	ENSGALG00010015416	CAPN6	case	control	-0.297876797	0.155858408	-1.911201333	0.055978713	0.385409528	down_in_case
PRJNA8274(	ENSGALG00010015422	ENSGALG00010015422	case	control	-0.151052721	0.329197563	-0.458851272	0.646340973	0.902693223	down_in_case
PRJNA8274(	ENSGALG00010015430	HMGCL	case	control	0.05763937	0.078951033	0.730064798	0.465350578	0.826255378	up_in_case
PRJNA8274(	ENSGALG00010015431	RIN3	case	control	-0.119786708	0.095009897	-1.260781368	0.207387628	0.631716687	down_in_case
PRJNA8274(	ENSGALG00010015436	SLC30A9	case	control	-0.069940518	0.05322233	-1.314119803	0.188805891	0.611783722	down_in_case
PRJNA8274(	ENSGALG00010015440	ENSGALG00010015440	case	control	0.028216219	0.399262086	0.070670921	0.943659664	0.990183239	up_in_case
PRJNA8274(	ENSGALG00010015443	PAK3	case	control	-0.065899639	0.05780697	-1.13999469	0.254288514	0.679971816	down_in_case
PRJNA8274(	ENSGALG00010015449	HDX	case	control	0.006826641	0.060504371	0.112828894	0.910166211	0.981730793	up_in_case
PRJNA8274(	ENSGALG00010015466	NMRK1	case	control	0.13096691	0.164912694	0.794159059	0.42710284	0.805305429	up_in_case
PRJNA8274(	ENSGALG00010015469	TMEM33	case	control	-0.120823894	0.067822561	-1.781470536	0.074835614	0.439711709	down_in_case
PRJNA8274(	ENSGALG00010015472	LGMN	case	control	-0.052408684	0.069472682	-0.754378308	0.450622093	0.819735103	down_in_case
PRJNA8274(	ENSGALG00010015477	ARMC9	case	control	0.06263612	0.093191286	0.672124216	0.501504621	0.841254358	up_in_case
PRJNA8274(	ENSGALG00010015478	FBXO7	case	control	-0.055402964	0.047396538	-1.168924286	0.242434138	0.666822114	down_in_case
PRJNA8274(	ENSGALG00010015481	GOLGA5	case	control	0.122186259	0.058269126	2.096929657	0.035999803	0.325820999	up_in_case
PRJNA8274(	ENSGALG00010015483	SGCD	case	control	-0.297602937	0.109262296	-2.7237478	0.006454577	0.13904105	down_in_case
PRJNA8274(	ENSGALG00010015485	BCAS1	case	control	0.025595436	0.214205817	0.119489921	0.904887225	0.981484171	up_in_case
PRJNA8274(	ENSGALG00010015486	MTFR1L	case	control	-0.021300507	0.0648112	-0.328654728	0.742416677	0.937444457	down_in_case
PRJNA8274(	ENSGALG00010015487	RPS6KA6	case	control	0.063196674	0.088826251	0.711463931	0.476796794	0.830520323	up_in_case
PRJNA8274(	ENSGALG00010015490	RANBP2	case	control	-0.066298457	0.058647748	-1.130451887	0.258285861	0.685757113	down_in_case
PRJNA8274(	ENSGALG00010015493	OSTF1	case	control	0.133648147	0.075266181	1.77567328	0.075786784	0.442474101	up_in_case
PRJNA8274(	ENSGALG00010015494	CHRDL1	case	control	-0.082802299	0.185733458	-0.445812508	0.655732686	0.907453191	down_in_case
PRJNA8274(	ENSGALG00010015498	MID2	case	control	-0.102279024	0.053023458	-1.92893914	0.053738418	0.377891678	down_in_case
PRJNA8274(	ENSGALG00010015505	CHGA	case	control	-0.398395667	0.409511211	-0.972856557	0.330624599	0.746554834	down_in_case
PRJNA8274(	ENSGALG00010015507	AMMECR1	case	control	0.060089704	0.055356548	1.085503088	0.277698897	0.703206258	up_in_case
PRJNA8274(	ENSGALG00010015508	FUCA1	case	control	-0.140996923	0.069352544	-2.033046151	0.042047861	0.342027535	down_in_case
PRJNA8274(	ENSGALG00010015511	BPIFB4	case	control	-0.952051076	0.418204687	-2.276519382	0.022814942	0.267253668	down_in_case
PRJNA8274(	ENSGALG00010015516	SELENON	case	control	0.117109169	0.057143781	2.049377319	0.040425233	0.33753444	up_in_case
PRJNA8274(	ENSGALG00010015520	UBE3A	case	control	-0.092190982	0.04028547	-2.288442492	0.022111765	0.265266985	down_in_case
PRJNA8274(	ENSGALG00010015521	DKC1	case	control	0.009057013	0.068863672	0.131520907	0.895363249	0.980176778	up_in_case
PRJNA8274(	ENSGALG00010015523	MRPL22	case	control	-0.060755186	0.07504081	-0.809628593	0.418153669	0.800393647	down_in_case

PRJNA8274(	ENSGALG00010015532	NXT2	case	control	0.08674005	0.065423065	1.325832869	0.184895063	0.607676259	up_in_case
PRJNA8274(	ENSGALG00010015533	PSAT1	case	control	0.26509045	0.135942503	1.950018894	0.051173867	0.370042007	up_in_case
PRJNA8274(	ENSGALG00010015540	ACSL4	case	control	0.02848615	0.064831919	0.439384641	0.660382854	0.909188753	up_in_case
PRJNA8274(	ENSGALG00010015541	RASEF	case	control	0.266877175	0.303893885	0.878191987	0.37983954	0.77805894	up_in_case
PRJNA8274(	ENSGALG00010015542	CNGA3	case	control	0.168925168	0.251937187	0.670505096	0.502535859	0.84219414	up_in_case
PRJNA8274(	ENSGALG00010015544	RTCB	case	control	-0.028318549	0.050124267	-0.564966858	0.572096307	0.876814097	down_in_case
PRJNA8274(	ENSGALG00010015553	ASCL4	case	control	-1.696226169	0.891691357	-1.90225705	0.057137558	0.388573099	down_in_case
PRJNA8274(	ENSGALG00010015555	PNRC2	case	control	0.085265128	0.053870035	1.582793264	0.113468593	0.508062524	up_in_case
PRJNA8274(	ENSGALG00010015557	IRS4	case	control	0.015209594	0.071087828	0.213954966	0.830582176	0.962228709	up_in_case
PRJNA8274(	ENSGALG00010015558	MPP1	case	control	0.040431357	0.103833585	0.389386119	0.696990539	0.922031058	up_in_case
PRJNA8274(	ENSGALG00010015562	IRF9	case	control	-0.356667027	0.191151236	-1.8658892	0.062056869	0.405537748	down_in_case
PRJNA8274(	ENSGALG00010015563	B3GNT7	case	control	0.271731299	0.275177479	0.987476517	0.323409089	0.740800303	up_in_case
PRJNA8274(	ENSGALG00010015564	PKD2L2	case	control	-0.236551522	0.175062453	-1.351240764	0.17661832	0.595527443	down_in_case
PRJNA8274(	ENSGALG00010015565	ENSGALG00010015565	case	control	1.950549827	1.99320236	0.978601002	0.327777164	0.745421985	up_in_case
PRJNA8274(	ENSGALG00010015567	ENSGALG00010015567	case	control	-0.379149166	0.366638664	-1.034122158	0.301079059	0.723723406	down_in_case
PRJNA8274(	ENSGALG00010015570	LIMCH1	case	control	-0.061451548	0.07295	-0.842378988	0.39957585	0.789900496	down_in_case
PRJNA8274(	ENSGALG00010015571	DUSP15	case	control	0.160955819	0.190814763	0.843518693	0.398938416	0.789900496	up_in_case
PRJNA8274(	ENSGALG00010015573	MAN1C1	case	control	-0.117833671	0.063527179	-1.85485445	0.063617014	0.409661205	down_in_case
PRJNA8274(	ENSGALG00010015574	C14orf132	case	control	0.086385717	0.214921029	0.40194167	0.687726955	0.917290381	up_in_case
PRJNA8274(	ENSGALG00010015580	GEMIN5	case	control	-0.0209342	0.040574621	-0.515943221	0.605894066	0.886009321	down_in_case
PRJNA8274(	ENSGALG00010015583	PRDM4	case	control	-0.100641021	0.06527806	-1.541728122	0.123139675	0.522552563	down_in_case
PRJNA8274(	ENSGALG00010015584	TTLL9	case	control	0.132232613	0.224662999	0.58858207	0.556141665	0.867878646	up_in_case
PRJNA8274(	ENSGALG00010015585	BDKRB1	case	control	-0.404469024	0.190437827	-2.123890149	0.03367933	0.316643277	down_in_case
PRJNA8274(	ENSGALG00010015588	BDKRB2	case	control	0.53910142	0.45461064	1.185853065	0.235680331	0.660526168	up_in_case
PRJNA8274(	ENSGALG00010015589	FRMD3	case	control	-0.122585968	0.183407805	-0.668379229	0.503891551	0.842675107	down_in_case
PRJNA8274(	ENSGALG00010015596	UCHL1	case	control	-0.337097079	0.200365396	-1.682411658	0.092489042	0.477342721	down_in_case
PRJNA8274(	ENSGALG00010015605	LDLRAP1	case	control	-0.031018226	0.053380232	-0.581080763	0.561186023	0.870480544	down_in_case
PRJNA8274(	ENSGALG00010015606	TMEM131	case	control	-0.153822818	0.061697519	-2.493176713	0.012660582	0.198081314	down_in_case
PRJNA8274(	ENSGALG00010015610	ENSGALG00010015610	case	control	-0.06792393	0.085377474	-0.795572036	0.426280826	0.805259684	down_in_case
PRJNA8274(	ENSGALG00010015613	PWP1	case	control	-0.113058542	0.072833951	-1.552278029	0.120595713	0.519499513	down_in_case
PRJNA8274(	ENSGALG00010015614	ATG2B	case	control	0.008504456	0.050538777	0.168275852	0.86636627	0.973368767	up_in_case
PRJNA8274(	ENSGALG00010015616	NADK2	case	control	0.199358186	0.146481667	1.360977042	0.173520945	0.592914702	up_in_case
PRJNA8274(	ENSGALG00010015617	SRSF10	case	control	0.025026989	0.043781994	0.571627439	0.567574402	0.873943572	up_in_case
PRJNA8274(	ENSGALG00010015618	NCL	case	control	-0.062842021	0.055789777	-1.126407465	0.259993089	0.687227573	down_in_case
PRJNA8274(	ENSGALG00010015620	PDRG1	case	control	-0.181821352	0.089023495	-2.042397383	0.041112129	0.338374671	down_in_case
PRJNA8274(	ENSGALG00010015621	IDNK	case	control	-0.055637654	0.142988223	-0.389106547	0.69719733	0.922031058	down_in_case
PRJNA8274(	ENSGALG00010015624	ITGA1	case	control	-0.100307287	0.093959045	-1.067563927	0.285717263	0.710150198	down_in_case
PRJNA8274(	ENSGALG00010015626	XKR7	case	control	-0.505868845	0.339218258	-1.491278353	0.13588843	0.539774365	down_in_case
PRJNA8274(	ENSGALG00010015628	UBQLN1	case	control	0.033799902	0.088659959	0.381230739	0.703032042	0.923659131	up_in_case
PRJNA8274(	ENSGALG00010015631	GSKIP	case	control	-0.054763215	0.071437849	-0.766585437	0.443328037	0.814648025	down_in_case
PRJNA8274(	ENSGALG00010015632	COL4A5	case	control	0.010121763	0.085442799	0.118462444	0.905701251	0.981730793	up_in_case
PRJNA8274(	ENSGALG00010015633	CCM2L	case	control	0.042138306	0.176116642	0.239263625	0.81090117	0.956022547	up_in_case
PRJNA8274(	ENSGALG00010015635	ABTB3	case	control	0.128345546	0.119264461	1.076142419	0.281863567	0.705892996	up_in_case
PRJNA8274(	ENSGALG00010015636	APBB2	case	control	-0.029591273	0.074070096	-0.399503636	0.689522145	0.918335352	down_in_case
PRJNA8274(	ENSGALG00010015643	ENSGALG00010015643	case	control	0.183163015	0.203094698	0.901860153	0.367131161	0.771770041	up_in_case
PRJNA8274(	ENSGALG00010015645	MACO1	case	control	-0.058312962	0.061451349	-0.948928924	0.342656763	0.75456672	down_in_case
PRJNA8274(	ENSGALG00010015647	PDZD2	case	control	-0.232825941	0.113032775	-2.059809129	0.039416792	0.335091843	down_in_case
PRJNA8274(	ENSGALG00010015653	HCK	case	control	0.646899718	0.415069435	1.558533739	0.119106777	0.516645008	up_in_case
PRJNA8274(	ENSGALG00010015655	ENSGALG00010015655	case	control	-0.256872675	0.288970428	-0.888923745	0.374044063	0.774369566	down_in_case
PRJNA8274(	ENSGALG00010015657	NMUR1	case	control	-0.303384939	0.46699757	-0.649649931	0.515918373	0.847910516	down_in_case
PRJNA8274(	ENSGALG00010015660	KLHL3	case	control	0.048206514	0.083551793	0.576965643	0.563962665	0.871916948	up_in_case
PRJNA8274(	ENSGALG00010015668	ZNF217	case	control	0.02364571	0.058204514	0.406252167	0.684557328	0.916433328	up_in_case
PRJNA8274(	ENSGALG00010015669	INPP4A	case	control	-0.077542038	0.054530396	-1.421996618	0.155027233	0.563829072	down_in_case
PRJNA8274(	ENSGALG00010015670	ENSGALG00010015670	case	control	-0.033547218	0.058168323	-0.576726589	0.564124168	0.871916948	down_in_case
PRJNA8274(	ENSGALG00010015674	RFKL	case	control	0.195498126	0.104948266	1.862804719	0.062489746	0.406552942	up_in_case
PRJNA8274(	ENSGALG00010015676	TGM2	case	control	-0.211817134	0.133139564	-1.59094057	0.111622951	0.504204693	down_in_case
PRJNA8274(	ENSGALG00010015677	GRHL3	case	control	0.02219656	0.165865556	0.133822598	0.893542857	0.979888889	up_in_case
PRJNA8274(	ENSGALG00010015680	ENSGALG00010015680	case	control	-0.042832677	0.060191386	-0.711608082	0.476707501	0.830520323	down_in_case
PRJNA8274(	ENSGALG00010015684	PDE6D	case	control	0.032706534	0.059985541	0.545240286	0.585588268	0.881464445	up_in_case
PRJNA8274(	ENSGALG00010015686	CNOT8	case	control	0.092930243	0.042428652	2.190270937	0.028504593	0.300549329	up_in_case
PRJNA8274(	ENSGALG00010015691	EXOC3L4	case	control	-0.021597595	0.99484819	-0.021709438	0.982679735	0.996591771	down_in_case
PRJNA8274(	ENSGALG00010015693	COA5	case	control	-0.079862453	0.073190424	-1.091159855	0.275202549	0.700894634	down_in_case
PRJNA8274(	ENSGALG00010015695	ASAH1	case	control	-0.073278898	0.089905532	-0.815065501	0.415034818	0.799003486	down_in_case
PRJNA8274(	ENSGALG00010015697	FAXDC2	case	control	-0.003226983	0.143271147	-0.02252361	0.982030279	0.996591771	down_in_case
PRJNA8274(	ENSGALG00010015698	TNFAIP2	case	control	0.024853653	0.093988396	0.264433205	0.791446128	0.95239351	up_in_case
PRJNA8274(	ENSGALG00010015701	UNC50	case	control	0.012015951	0.07368437	0.163073261	0.870460753	0.974794532	up_in_case
PRJNA8274(	ENSGALG00010015703	GK5	case	control	0.265584296	0.123874834	2.143972976	0.032035052	0.3098361	up_in_case
PRJNA8274(	ENSGALG00010015705	NPY6R	case	control	0.83968135	0.602343775	1.394023453	0.163310522	0.577197348	up_in_case
PRJNA8274(	ENSGALG00010015706	ENSGALG00010015706	case	control	0.462341018	0.164526775	2.810126302	0.004952206	0.120231831	up_in_case
PRJNA8274(	ENSGALG00010015709	HNRNPA0	case	control	0.011745176	0.04662364	0.251914613	0.801107064	0.954294696	up_in_case
PRJNA8274(	ENSGALG00010015711	LBFABP	case	control	0.510733073	0.263146625	1.940868797	0.052274194	0.373200684	up_in_case
PRJNA8274(	ENSGALG00010015716	MGAT4A	case	control	0.05060748	0.100953397	0.501295465	0.616163197	0.891081782	up_in_case
PRJNA8274(	ENSGALG00010015719	XRN1	case	control	-0.067086432	0.057615507	-1.164381532	0.244269429	0.668186525	down_in_case
PRJNA8274(	ENSGALG00010015729	RHCE	case	control	0.068394847	0.138074862	0.495346119	0.620355822	0.892955829	up_in_case
PRJNA8274(	ENSGALG00010015731	PHF6	case	control	0.090900696	0.043301543	2.099248435	0.035795006	0.324974524	up_in_case
PRJNA8274(	ENSGALG00010015733	COL4A6	case	control	-0.107722568	0.097820734	-1.101224286	0.270799054	0.698323327	down_in_case
PRJNA8274(	ENSGALG00010015734	TSHZ2	case	control	0.027072425	0.095336054	0.283968381	0.776434612	0.947228003	up_in_case
PRJNA8274(	ENSGALG00010015738	CRACDL	case	control	0.491162901	0.386860344	1.269612947	0.204222535	0.626691471	up_in_case
PRJNA8274(	ENSGALG00010015742	HPRT1	case	control	0.010139464	0.094169722	0.107672226	0.914255701	0.982854306	up_in_case
PRJNA8274(	ENSGALG00010015746	PAPOLB	case	control	-0.059462179	0.038078955	-1.561549659	0.118394117	0.514757029	down_in_case
PRJNA8274(	ENSGALG00010015749	ATR	case	control	-0.038859008	0.08157763	-0.476343924	0.633829378	0.898107199	down_in_case
PRJNA8274(	ENSGALG00010015751	MYOM3	case	control	0.185965005	0.147672111	1.259310263	0.207918288	0.63249038	up_in_case
PRJNA8274(	ENSGALG00010015753	RPRD1B	case	control	-0.025434102	0.034774565	-0.731399565	0.464535131	0.825814566	down_in_case
PRJNA8274(	ENSGALG00010015756	PCM1	case	control	-0.067250299	0.045503234	-1.477923483	0.13942826	0.541505735	down_in_case
PRJNA8274(	ENSGALG00010015758	PCDH19	case	control	-0.054474439	0.085906535	-0.634112861	0.52600717	0.85251736	down_in_case
PRJNA8274(	ENSGALG00010015759	EIF5	case	control	0.05472029	0.055094642	0.993205292	0.32060992	0.738875399	up_in_case
PRJNA8274(	ENSGALG00010015764	TTI1	case	control	0.001361264	0.06751388	0.020162728	0.98391356	0.996591771	up_in_case
PRJNA8274(	ENSGALG00010015766	TLE1	case	control	-0.03573305	0.091413013	-0.390896756	0.695873552	0.921558614	down_in_case
PRJNA8274(	ENSGALG00010015769	TMEM50A	case	control	0.047240575	0.047824943	0.987781101	0.323259865	0.740795254	up_in_case
PRJNA8274(	ENSGALG00010015774	PLS1	case	control	0.954045107	0.309139483	3.086131538	0.002027791	0.073972986	up_in_case
PRJNA8274(	ENSGALG00010015776	SPOCK1	case	control	0.050546031	0.212757539	0.237575746	0.812210164	0.956328147	up_in_case
PRJNA8274(	ENSGALG00010015778	PABIR3	case	control	-0.045571155	0.066757123	-0.682641094	0.494833661	0.838081589	down_in_case
PRJNA8274(	ENSGALG00010015782	ATG4A	case	control	-0.042711123	0.05857356	-0.729187754	0.465886821	0.826881164	down_in_case
PRJNA8274(	ENSGALG00010015785	FGL1	case	control	-0.208723652	0.148480819	-1.405728044	0.159804896	0.572017525	down_in_case
PRJNA8274(	ENSGALG00010015788	TRPC1	case	control	0.003467317	0.078752704	0.044027908	0.964882158	0.992291928	up_in_case
PRJNA8274(	ENSGALG00010015792	MARK3	case	control	-0.13255977	0.047676361	-2.780408713	0.005429052	0.127480206	down_in_case
PRJNA8274(	ENSGALG00010015793	RSRP1	case	control	0.21131194	0.110171679	1.918024145	0.05510795	0.383470355	up_in_case
PRJNA8274(	ENSGALG00010015795	PSMD10	case	control	0.114938454	0.077767842	1.477968919	0.139416098	0.541505735	up_in_case
PRJNA8274(	ENSGALG00010015796	LARP1	case	control	-0.026458076	0.073650965	-0.359235969	0.719418571	0.928491477	down_in_case
PRJNA8274(	ENSGALG00010015797	PIP5K1B	case	control	-0.065877973	0.108918885	-0.60483517	0.545288523	0.861997112	down_in_case
PRJNA8274(	ENSGALG00010015798	IL22RA1	case	control	-0.10526798	0.201468674	-0.522502965	0.601320183	0.884954589	down_in_case
PRJNA8274(	ENSGALG00010015800	BVES	case	control	-0.136310849	0.140559104	-0.969776025	0.332158146	0.748268094	down_in_case
PRJNA8274(	ENSGALG00010015801	VSTM2L	case	control	-0.264837811	0.19666083	-1.346672905	0.178085602	0.597378399	down_in_case
PRJNA8274(	ENSGALG00010015802	TRPC7	case	control	-0.336179893	0.506375685	-0.663894225	0.506758012	0.843472666	down_in_case
PRJNA8274(	ENSGALG00010015803	SYF2	case	control	0.052458963	0.069344318	0.756499802	0.449349591	0.818749597	up_in_case
PRJNA8274(	ENSGALG00010015804	CKB	case	control	0.130498029	0.143225577	0.911136347	0.362223537	0.768423771	up_in_case
PRJNA8274(	ENSGALG00010015807	ENSGALG00010015807	case	control	-0.042534222	0.893264448	-0.047616607	0.962021797	0.992056045	down_in_case
PRJNA8274(	ENSGALG00010015809	PCOLCE2	case	control	0.570682495	0.151667459	3.762722079	0.000168074	0.015606866	up_in_case
PRJNA8274(	ENSGALG00010015810	LIPT1	case	control	0.105742716	0.068150964	1.551595312	0.120759085	0.519499513	up_in_case
PRJNA8274(	ENSGALG00010015811	CTNNBL1	case	control	-0.02344382	0.05492392	-0.426841705	0.669494628	0.913446904	down_in_case
PRJNA8274(	ENSGALG00010015813	ENSGALG00010015813	case	control	-0.042990655	0.066839279	-0.643194464	0.520097925	0.849641807	down_in_case
PRJNA8274(	ENSGALG00010015814	PAQR9	case	control	0.551381691	0.446336101	1.23535087	0.216699949	0.641841192	up_in_case
PRJNA8274(	ENSGALG00010015815	VSIG1	case	control	-0.684911962	0.709611251	-0.965193211	0.334448047	0.748686141	down_in_case
PRJNA8274(	ENSGALG00010015816	MITD1	case	control	-0.050783982	0.088463979	-0.574063967	0.565924516	0.872906976	down_in_case
PRJNA8274(	ENSGALG00010015820	ENSGALG00010015820	case	control	0.094235538	0.197560897	0.476994888	0.633365762	0.898079365	up_in_case
PRJNA8274(	ENSGALG00010015825	RUNX3	case	control	0.109089125	0.689090304	0.158308896	0.874213399	0.975062028	up_in_case
PRJNA8274(	ENSGALG00010015827	ENSGALG00010015827	case	control	0.039412975	0.129023687	0.305470848	0.760007569	0.942675708	up_in_case
PRJNA8274(	ENSGALG00010015829	SMAD5	case	control	-0.005187864	0.042173604	-0.123012115	0.902097504	0.980763983	down_in_case
PRJNA8274(	ENSGALG00010015831	LYG2	case	control	0.359229781	0.436412942	0.823141906	0.4104273	0.796464701	up_in_case
PRJNA8274(	ENSGALG00010015832	RAB39B	case	control	0.013433861	0.044011458	0.305235539	0.760186767	0.942816198	up_in_case
PRJNA8274(	ENSGALG00010015834	STARD8	case	control	-0.124912772	0.084045598	-1.486250017	0.137213002	0.539854583	down_in_case
PRJNA8274(	ENSGALG00010015835	HAND1	case	control	-0.30377875	1.17960958	-0.257524824	0.796773654	0.95280257	down_in_case
PRJNA8274(	ENSGALG00010015837	GDA	case	control	-0.160019724	0.156799975	-1.020534114	0.307475219	0.73050268	down_in_case
PRJNA8274(	ENSGALG00010015838	IFNLR1	case	control	0.401275446	1.455016352	0.275787585	0.78271122	0.949518053	up_in_case
PRJNA8274(	ENSGALG00010015839	U2SURP	case	control	0.042475382	0.056919567	0.746235156	0.455525375	0.822061189	up_in_case
PRJNA8274(	ENSGALG00010015840	MTUS1	case	control	-0.226729019	0.089257068	-2.540180004	0.011079543	0.185137791	down_in_case

PRJNA8274(	ENSGALG00010015843	ENSGALG00010015843	case	control	0.216661032	0.266472452	0.813071032	0.416177334	0.799987395	up_in_case
PRJNA8274(	ENSGALG00010015845	SAP30L	case	control	-0.04761557	0.067393354	-0.706532129	0.47985728	0.831851816	down_in_case
PRJNA8274(	ENSGALG00010015848	UBE2B	case	control	-0.067818904	0.087714354	-0.773179082	0.439416404	0.812366461	down_in_case
PRJNA8274(	ENSGALG00010015852	ZFAND5	case	control	-0.027622993	0.15426203	-0.179065405	0.857886345	0.97170493	down_in_case
PRJNA8274(	ENSGALG00010015855	NIPAL3	case	control	0.013011487	0.054575745	0.238411539	0.811561918	0.956205737	up_in_case
PRJNA8274(	ENSGALG00010015856	TXNDC9	case	control	-0.019908862	0.055881186	-0.356271279	0.721637416	0.929484914	down_in_case
PRJNA8274(	ENSGALG00010015858	CHST2	case	control	0.071115386	0.07219788	0.985006564	0.324620847	0.742593544	up_in_case
PRJNA8274(	ENSGALG00010015859	ENSGALG00010015859	case	control	0.749579385	1.281076038	0.585117014	0.55846904	0.869321905	up_in_case
PRJNA8274(	ENSGALG00010015861	PPP2CA	case	control	-0.052421585	0.032807248	-1.597865961	0.110072811	0.502591904	down_in_case
PRJNA8274(	ENSGALG00010015862	PDGFRL	case	control	0.091074957	0.094887459	0.959820793	0.337145415	0.750845576	up_in_case
PRJNA8274(	ENSGALG00010015863	ENSGALG00010015863	case	control	0.322011313	0.23851641	1.350059363	0.176996942	0.595582794	up_in_case
PRJNA8274(	ENSGALG00010015865	VRK1	case	control	0.029724245	0.092782428	0.320365022	0.748691638	0.939503405	up_in_case
PRJNA8274(	ENSGALG00010015866	ARHGEF6	case	control	0.058707818	0.054804753	1.071217626	0.284071588	0.708692203	up_in_case
PRJNA8274(	ENSGALG00010015867	PICALML	case	control	-0.065985633	0.034017904	-1.939732466	0.052412212	0.373812782	down_in_case
PRJNA8274(	ENSGALG00010015869	SLC7A2	case	control	-0.062194831	0.119448538	-0.520683065	0.602587574	0.885101354	down_in_case
PRJNA8274(	ENSGALG00010015870	EIF5B	case	control	-0.144188453	0.045118147	-3.195797341	0.00139445	0.058654283	down_in_case
PRJNA8274(	ENSGALG00010015872	TMC1	case	control	-0.083850382	0.244198518	-0.343369739	0.731320322	0.933770678	down_in_case
PRJNA8274(	ENSGALG00010015874	CLIC4	case	control	0.056270812	0.059403829	0.947259001	0.343506817	0.755250228	up_in_case
PRJNA8274(	ENSGALG00010015875	DNTTIP1	case	control	-0.085189776	0.062913464	-1.354078626	0.175711294	0.594727928	down_in_case
PRJNA8274(	ENSGALG00010015876	SKP1	case	control	-0.019975789	0.047788586	-0.41800334	0.675944675	0.914247368	down_in_case
PRJNA8274(	ENSGALG00010015877	REV1	case	control	0.13036553	0.081757276	1.594543459	0.110814365	0.503115856	up_in_case
PRJNA8274(	ENSGALG00010015879	TRMT61A	case	control	-0.024301005	0.068259587	-0.356008672	0.721834071	0.929484914	down_in_case
PRJNA8274(	ENSGALG00010015881	GAB3	case	control	-0.057603779	0.140039612	-0.411339181	0.680823844	0.915594727	down_in_case
PRJNA8274(	ENSGALG00010015886	AFF3	case	control	0.036383493	0.140887873	0.258244324	0.796218352	0.95280257	up_in_case
PRJNA8274(	ENSGALG00010015887	SLC9A9	case	control	-0.076534491	0.123493064	-0.619747282	0.535424182	0.857009451	down_in_case
PRJNA8274(	ENSGALG00010015889	SH3BP5	case	control	-0.098677262	0.066012091	-1.494836183	0.134957201	0.539774365	down_in_case
PRJNA8274(	ENSGALG00010015892	UBE2C	case	control	-0.107511701	0.124510073	-0.863477935	0.387874746	0.782976971	down_in_case
PRJNA8274(	ENSGALG00010015893	TCF7	case	control	-0.191004271	0.271080579	-0.704603301	0.481057143	0.8322127	down_in_case
PRJNA8274(	ENSGALG00010015894	CEP78	case	control	0.229515044	0.166491481	1.378539266	0.168036853	0.584227328	up_in_case
PRJNA8274(	ENSGALG00010015895	RBMXL1	case	control	0.147039171	0.064072661	2.294881613	0.02173991	0.264012564	up_in_case
PRJNA8274(	ENSGALG00010015896	LONRF2	case	control	-0.222539465	0.070586319	-3.152728022	0.001617524	0.064874977	down_in_case
PRJNA8274(	ENSGALG00010015898	ENSGALG00010015898	case	control	0.221661931	0.182344631	1.215620825	0.224129397	0.648105158	up_in_case
PRJNA8274(	ENSGALG00010015899	SMARCA1	case	control	-0.05860129	0.062919603	-0.931367762	0.351663361	0.760831854	down_in_case
PRJNA8274(	ENSGALG00010015900	ENSGALG00010015900	case	control	0.044948472	0.122433811	0.36712467	0.713526026	0.926862121	up_in_case
PRJNA8274(	ENSGALG00010015904	MTMR7	case	control	-0.165303742	0.183784856	-0.899441586	0.368417497	0.771770041	down_in_case
PRJNA8274(	ENSGALG00010015905	ANPEP	case	control	0.089112028	0.116951233	0.761958858	0.44608456	0.817402512	up_in_case
PRJNA8274(	ENSGALG00010015906	BAG5	case	control	-0.063632464	0.075147878	-0.84676329	0.397127073	0.788531716	down_in_case
PRJNA8274(	ENSGALG00010015908	CHRD	case	control	0.076249155	0.103507221	0.736655417	0.461331925	0.82454265	up_in_case
PRJNA8274(	ENSGALG00010015909	TNNC2	case	control	-0.136247827	0.110934052	-1.228187601	0.219376552	0.643443819	down_in_case
PRJNA8274(	ENSGALG00010015910	C5orf15	case	control	0.034872914	0.041553521	0.839228857	0.401340897	0.791181795	up_in_case
PRJNA8274(	ENSGALG00010015911	TM9SF2L	case	control	0.010095072	0.049720918	0.203034708	0.839107904	0.96526772	up_in_case
PRJNA8274(	ENSGALG00010015912	ENSGALG00010015912	case	control	-0.155914491	0.285502612	-0.546105304	0.584993554	0.881307382	down_in_case
PRJNA8274(	ENSGALG00010015913	CHST10	case	control	-0.079676484	0.054588952	-1.459571609	0.144407847	0.548627048	down_in_case
PRJNA8274(	ENSGALG00010015914	SRRM1	case	control	-0.015465862	0.037641439	-0.410873299	0.681165442	0.915594727	down_in_case
PRJNA8274(	ENSGALG00010015916	COA8	case	control	0.021542326	0.135005016	0.15956686	0.873222287	0.975062028	up_in_case
PRJNA8274(	ENSGALG00010015917	TMEM63A	case	control	0.009650687	0.070682078	0.13653655	0.891397132	0.978917427	up_in_case
PRJNA8274(	ENSGALG00010015918	BICD1	case	control	0.006412828	0.16880468	0.037989634	0.969695947	0.992761697	up_in_case
PRJNA8274(	ENSGALG00010015919	ENSGALG00010015919	case	control	0.25638104	0.240493187	1.066063634	0.286394877	0.710659943	up_in_case
PRJNA8274(	ENSGALG00010015921	SNX21	case	control	0.080941195	0.084663832	0.956030377	0.339056888	0.751259351	up_in_case
PRJNA8274(	ENSGALG00010015923	RCAN3	case	control	0.13488624	0.071717704	1.880794172	0.059999923	0.398649739	up_in_case
PRJNA8274(	ENSGALG00010015927	GLA	case	control	-0.121172673	0.094872167	-1.277220471	0.201524422	0.624725388	down_in_case
PRJNA8274(	ENSGALG00010015928	CAPN7	case	control	0.07932273	0.060669095	1.3074652	0.191054766	0.614227329	up_in_case
PRJNA8274(	ENSGALG00010015929	SLC6A12	case	control	1.273400446	0.547513815	2.325786876	0.020029923	0.254128638	up_in_case
PRJNA8274(	ENSGALG00010015931	RBM47	case	control	0.16834869	0.214873154	0.783479399	0.433345647	0.808138728	up_in_case
PRJNA8274(	ENSGALG00010015932	ACOT8	case	control	0.193718087	0.123314062	1.570932658	0.116198292	0.5134816	up_in_case
PRJNA8274(	ENSGALG00010015933	ENSGALG00010015933	case	control	0.249521344	0.280123089	0.890756076	0.373060044	0.774369566	up_in_case
PRJNA8274(	ENSGALG00010015934	TMC2	case	control	0.158196953	0.648275483	0.244027358	0.807209632	0.955875648	up_in_case
PRJNA8274(	ENSGALG00010015935	EPHX1	case	control	0.105764684	0.103941244	1.01754299	0.308895192	0.731823162	up_in_case
PRJNA8274(	ENSGALG00010015936	RPL36AL	case	control	-0.018376831	0.071504318	-0.25700309	0.797176388	0.95280257	down_in_case
PRJNA8274(	ENSGALG00010015937	ENSGALG00010015937	case	control	-0.511301548	0.205334851	-2.490086539	0.012771199	0.199334349	down_in_case
PRJNA8274(	ENSGALG00010015939	SUN2	case	control	-0.008458381	0.05942655	-0.142333375	0.886816685	0.977630122	down_in_case
PRJNA8274(	ENSGALG00010015942	AP3S2	case	control	-0.019013649	0.083674533	-0.227233406	0.820242253	0.958669267	down_in_case
PRJNA8274(	ENSGALG00010015946	TLE4	case	control	0.147415313	0.115834358	1.272638925	0.203146199	0.625537942	up_in_case
PRJNA8274(	ENSGALG00010015954	CTSA	case	control	-0.017918435	0.054318605	-0.329876571	0.741493227	0.937271559	down_in_case
PRJNA8274(	ENSGALG00010015957	ENSGALG00010015957	case	control	-0.079531919	0.163306119	-0.487011263	0.626250349	0.895536157	down_in_case
PRJNA8274(	ENSGALG00010015959	TRNAU1AP	case	control	0.048292832	0.080364874	0.60091965	0.547893505	0.863252217	up_in_case
PRJNA8274(	ENSGALG00010015963	DIPK2A	case	control	-0.000830833	0.055986336	-0.014839922	0.98815989	0.996942188	down_in_case
PRJNA8274(	ENSGALG00010015964	KLC1	case	control	-0.108461067	0.086355377	-1.255985105	0.20912137	0.634076945	down_in_case
PRJNA8274(	ENSGALG00010015967	ZSWIM1	case	control	0.09501896	0.18575909	0.511517152	0.608988981	0.8877909	up_in_case
PRJNA8274(	ENSGALG00010015970	THPO	case	control	-0.156317205	0.268227569	-0.582778294	0.560042559	0.86984941	down_in_case
PRJNA8274(	ENSGALG00010015971	ENSGALG00010015971	case	control	-0.004742682	0.090049261	-0.052667641	0.957996722	0.992056045	down_in_case
PRJNA8274(	ENSGALG00010015972	FSTL4	case	control	-0.047756542	0.154088339	-0.309929631	0.756614469	0.941659745	down_in_case
PRJNA8274(	ENSGALG00010015974	VPS37A	case	control	-0.060195011	0.051298135	-1.173434689	0.240621533	0.665904651	down_in_case
PRJNA8274(	ENSGALG00010015975	BTK	case	control	-0.009306387	0.129465386	-0.071883206	0.942694855	0.990046805	down_in_case
PRJNA8274(	ENSGALG00010015979	ENSGALG00010015979	case	control	0.355145998	0.132377083	2.682835949	0.007300079	0.148898802	up_in_case
PRJNA8274(	ENSGALG00010015980	EXOSC7	case	control	0.103923823	0.083919638	1.238373102	0.215577749	0.640470741	up_in_case
PRJNA8274(	ENSGALG00010015983	CNOT7	case	control	-0.011453256	0.03495047	-0.327699636	0.743138778	0.937448285	down_in_case
PRJNA8274(	ENSGALG00010015984	PDCL3	case	control	0.031707692	0.074954201	0.423027551	0.67227516	0.913676733	up_in_case
PRJNA8274(	ENSGALG00010015985	TIMM8A	case	control	-0.058117489	0.087054452	-0.667599276	0.50438942	0.842675107	down_in_case
PRJNA8274(	ENSGALG00010015986	NEURL2	case	control	-0.182707722	0.149785264	-1.219797713	0.222541569	0.646365317	down_in_case
PRJNA8274(	ENSGALG00010015987	POLR2H	case	control	0.038354736	0.081173796	0.472501452	0.636568906	0.899055343	up_in_case
PRJNA8274(	ENSGALG00010015989	ENAH	case	control	-0.091774271	0.082367364	-1.114206722	0.265190512	0.69125489	down_in_case
PRJNA8274(	ENSGALG00010015992	ATP10A	case	control	0.253866339	0.155722523	1.630248045	0.103049083	0.492515853	up_in_case
PRJNA8274(	ENSGALG00010015994	ENSGALG00010015994	case	control	-0.036454673	0.100967191	-0.36105464	0.718058601	0.927322135	down_in_case
PRJNA8274(	ENSGALG00010016004	XRCC3	case	control	0.016596728	0.084888871	0.195511239	0.84499274	0.967281084	up_in_case
PRJNA8274(	ENSGALG00010016006	PLTP	case	control	0.546766076	0.110904698	4.930053348	8.22072E-07	0.00022607	up_in_case
PRJNA8274(	ENSGALG00010016008	TAF7	case	control	-0.031236886	0.056549422	-0.552382051	0.580686625	0.879548643	down_in_case
PRJNA8274(	ENSGALG00010016011	ADD1	case	control	-0.03195645	0.050234483	-0.636145691	0.524681467	0.852358222	down_in_case
PRJNA8274(	ENSGALG00010016015	ZDHHC3	case	control	-0.013214156	0.052697698	-0.250753959	0.80200434	0.954294696	down_in_case
PRJNA8274(	ENSGALG00010016016	ZDHHC2	case	control	-0.11585085	0.114554977	-1.01131224	0.311867009	0.734348466	down_in_case
PRJNA8274(	ENSGALG00010016017	RAPGEF6	case	control	-0.06754957	0.05007161	-1.349059266	0.177317931	0.5959216	down_in_case
PRJNA8274(	ENSGALG00010016021	ENSGALG00010016021	case	control	0.29810247	0.418252131	0.712733894	0.476010439	0.830520323	up_in_case
PRJNA8274(	ENSGALG00010016022	EPHB3	case	control	-0.035334667	0.07244022	-0.487776915	0.625707865	0.895389019	down_in_case
PRJNA8274(	ENSGALG00010016025	PCIF1	case	control	0.028815064	0.038389577	0.750596039	0.452895805	0.821045895	up_in_case
PRJNA8274(	ENSGALG00010016027	COPS2	case	control	-0.024519952	0.067486705	-0.363330118	0.716358297	0.926862121	down_in_case
PRJNA8274(	ENSGALG00010016030	TGM4	case	control	0.807565037	0.553414184	1.459241668	0.144498604	0.548736091	up_in_case
PRJNA8274(	ENSGALG00010016040	RPSA2	case	control	-0.047816313	0.05059038	-0.945166105	0.344574081	0.755870938	down_in_case
PRJNA8274(	ENSGALG00010016041	ENSGALG00010016041	case	control	0.112743561	0.077601472	1.452853386	0.146264466	0.551432074	up_in_case
PRJNA8274(	ENSGALG00010016043	ADAMTSL1	case	control	0.205178733	0.142466555	1.440188776	0.149813998	0.556819093	up_in_case
PRJNA8274(	ENSGALG00010016047	SRP9	case	control	-0.02718048	0.072987058	-0.37240136	0.709594041	0.925630832	down_in_case
PRJNA8274(	ENSGALG00010016049	ZFYVE21	case	control	-0.011512375	0.057902282	-0.198824199	0.842400266	0.966519616	down_in_case
PRJNA8274(	ENSGALG00010016053	DRP2	case	control	0.020327556	0.257971789	0.078797592	0.937193619	0.988831145	up_in_case
PRJNA8274(	ENSGALG00010016057	SOHO1	case	control	-0.072777778	0.294331273	-0.24726485	0.80470326	0.95543479	down_in_case
PRJNA8274(	ENSGALG00010016059	EPDR1	case	control	-0.097380514	0.090251182	-1.078994335	0.280590253	0.705762606	down_in_case
PRJNA8274(	ENSGALG00010016060	ENSGALG00010016060	case	control	0.010130906	0.809533312	0.012514502	0.990015133	0.997323462	up_in_case
PRJNA8274(	ENSGALG00010016066	C14orf2	case	control	-0.119487647	0.073526958	-1.625086235	0.104144185	0.493794905	down_in_case
PRJNA8274(	ENSGALG00010016067	AHSG	case	control	0.560507768	0.362594077	1.545827148	0.122146319	0.520778878	up_in_case
PRJNA8274(	ENSGALG00010016069	GALK2	case	control	-0.278435415	0.109117259	-2.551708305	0.010719622	0.184687469	down_in_case
PRJNA8274(	ENSGALG00010016076	RAB3GAP2	case	control	-0.06417724	0.046289616	-1.386428449	0.165616114	0.580618526	down_in_case
PRJNA8274(	ENSGALG00010016083	CDH22	case	control	0.190062275	0.386377189	0.491908635	0.622783934	0.894021703	up_in_case
PRJNA8274(	ENSGALG00010016086	TDRD9	case	control	0.648037095	0.247889472	2.614217897	0.008943198	0.168495036	up_in_case
PRJNA8274(	ENSGALG00010016087	IARS2	case	control	-0.06709282	0.046897871	-1.430615485	0.152540447	0.561185591	down_in_case
PRJNA8274(	ENSGALG00010016091	HMX1	case	control	-0.709664232	0.46069899	-1.540407614	0.123461027	0.522840029	down_in_case
PRJNA8274(	ENSGALG00010016094	FGF7	case	control	-0.222203076	0.127814835	-1.738476417	0.082126902	0.455552638	down_in_case
PRJNA8274(	ENSGALG00010016097	FNIP1	case	control	-0.093734566	0.090983678	-1.030234955	0.302899725	0.725484689	down_in_case
PRJNA8274(	ENSGALG00010016100	SFRP4	case	control	-0.445261716	0.301868187	-1.475020341	0.140207075	0.542467849	down_in_case
PRJNA8274(	ENSGALG00010016103	BPNT1	case	control	-0.01274375	0.126640346	-0.100629463	0.919844607	0.984637913	down_in_case
PRJNA8274(	ENSGALG00010016107	UVSSA	case	control	-0.023610682	0.070007447	-0.337259575	0.735921231	0.934357951	down_in_case
PRJNA8274(	ENSGALG00010016109	MLH1	case	control	-0.011613772	0.100616907	-0.115425652	0.908107747	0.981730793	down_in_case
PRJNA8274(	ENSGALG00010016111	EPB41	case	control	0.103512107	0.091546538	1.130704769	0.258179374	0.685601679	up_in_case
PRJNA8274(	ENSGALG00010016113	DTWD1	case	control	-0.03797374	0.072828406	-0.521413852	0.602078504	0.885002041	down_in_case
PRJNA8274(	ENSGALG00010016114	HAUS6	case	control	0.08920347	0.182006941	0.490110267	0.624055873	0.894353977	up_in_case
PRJNA8274(	ENSGALG00010016115	RD3L	case	control	-0.039953372	0.180930749	-0.220821346	0.825231542	0.960903617	down_in_case
PRJNA8274(	ENSGALG00010016117	ENSGALG00010016117	case	control	-0.309859753	0.211009846	-1.468461113	0.141979012	0.543501954	down_in_case
PRJNA8274(	ENSGALG00010016118	EPRS	case	control	-0.121833462	0.039987169	-3.046813905	0.002312808	0.079122395	down_in_case
PRJNA8274(	ENSGALG00010016119	FETUB	case	control	2.010518054	1.357993357	1.480506545	0.138738115	0.540932258	up_in_case

PRJNA8274(	ENSGALG00010016121	SLC35C2	case	control	0.149548917	0.072511016	2.062430317	0.039166785	0.335091843	up_in_case
PRJNA8274(	ENSGALG00010016122	AFG2B	case	control	-0.208529461	0.093919019	-2.220311321	0.026397642	0.288377601	down_in_case
PRJNA8274(	ENSGALG00010016127	ENSGALG00010016127	case	control	0.076030052	0.206945693	0.367391325	0.713327141	0.926862121	up_in_case
PRJNA8274(	ENSGALG00010016129	CENPI	case	control	-0.053571278	0.098336775	-0.544773587	0.585909247	0.881576413	down_in_case
PRJNA8274(	ENSGALG00010016131	SMYD1	case	control	-0.239450918	0.099371917	-2.40964374	0.015968105	0.224306381	down_in_case
PRJNA8274(	ENSGALG00010016137	STARD3NL	case	control	0.057793333	0.079298613	0.728806359	0.466120121	0.82702452	up_in_case
PRJNA8274(	ENSGALG00010016139	LURAP1L	case	control	0.081190937	0.196555857	0.413068009	0.679556787	0.915351915	up_in_case
PRJNA8274(	ENSGALG00010016141	RRP15	case	control	-0.007064657	0.068157247	-0.103652323	0.917445263	0.983445506	down_in_case
PRJNA8274(	ENSGALG00010016147	FABP1	case	control	-0.099079446	0.289455802	-0.342295594	0.732128451	0.933770678	down_in_case
PRJNA8274(	ENSGALG00010016150	POLG	case	control	-0.03097365	0.082714118	-0.374466306	0.70805742	0.92490115	down_in_case
PRJNA8274(	ENSGALG00010016159	ASPG	case	control	0.188819513	0.137298217	1.375251019	0.169053636	0.585038804	up_in_case
PRJNA8274(	ENSGALG00010016168	PLIN2	case	control	-0.009306712	0.18124539	-0.051348683	0.959047676	0.992056045	down_in_case
PRJNA8274(	ENSGALG00010016169	RHOH	case	control	0.02398459	0.112738172	0.21274595	0.831525124	0.962371901	up_in_case
PRJNA8274(	ENSGALG00010016173	ENSGALG00010016173	case	control	0.177412148	0.204093345	0.869269638	0.384699666	0.780971676	up_in_case
PRJNA8274(	ENSGALG00010016177	ENSGALG00010016177	case	control	-0.64883935	1.781564445	-0.364196396	0.715711358	0.926862121	down_in_case
PRJNA8274(	ENSGALG00010016181	FANCI	case	control	-0.041629883	0.10038455	-0.414704087	0.67835854	0.914942347	down_in_case
PRJNA8274(	ENSGALG00010016182	ELMO2	case	control	0.088379429	0.043034867	2.053670321	0.040007611	0.336547209	up_in_case
PRJNA8274(	ENSGALG00010016190	THNSL2	case	control	0.381450883	0.12157777	3.137505168	0.001703923	0.066212214	up_in_case
PRJNA8274(	ENSGALG00010016193	TCAIM	case	control	-0.065040576	0.082023569	-0.79294984	0.427807048	0.805337183	down_in_case
PRJNA8274(	ENSGALG00010016197	SNRPN	case	control	0.172196983	0.071629072	2.40400967	0.016216344	0.226017275	up_in_case
PRJNA8274(	ENSGALG00010016199	RLBP1	case	control	0.767710302	0.331739158	2.31419862	0.02065683	0.25845743	up_in_case
PRJNA8274(	ENSGALG00010016200	MICU3	case	control	-0.00313325	0.064026956	-0.048936412	0.960969971	0.992056045	down_in_case
PRJNA8274(	ENSGALG00010016206	KNG1	case	control	1.006297222	1.084421996	0.927957221	0.353429761	0.761954708	up_in_case
PRJNA8274(	ENSGALG00010016209	ADAMTS7	case	control	0.102906634	0.117499552	0.875804482	0.381136333	0.778940912	up_in_case
PRJNA8274(	ENSGALG00010016211	TACC3	case	control	-0.038728047	0.071750134	-0.539762714	0.589360684	0.882949889	down_in_case
PRJNA8274(	ENSGALG00010016215	SPATA17	case	control	0.046216301	0.28911666	0.15985347	0.872996503	0.975062028	up_in_case
PRJNA8274(	ENSGALG00010016218	N4BP2	case	control	-0.011481819	0.079482403	-0.144457365	0.885139321	0.977630122	down_in_case
PRJNA8274(	ENSGALG00010016221	TMEM200B	case	control	-0.063590673	0.116323678	-0.546670067	0.584605422	0.881307382	down_in_case
PRJNA8274(	ENSGALG00010016224	MOSPD1	case	control	-0.049320126	0.065084441	-0.757786739	0.448578662	0.818701429	down_in_case
PRJNA8274(	ENSGALG00010016225	P4HA2	case	control	-0.127531251	0.088997698	-1.432972469	0.151865705	0.560144335	down_in_case
PRJNA8274(	ENSGALG00010016229	ENSGALG00010016229	case	control	0.961880715	0.391781771	2.455144128	0.014082811	0.208688292	up_in_case
PRJNA8274(	ENSGALG00010016235	DDX27	case	control	0.026601996	0.054467412	0.488402063	0.625265081	0.89506388	up_in_case
PRJNA8274(	ENSGALG00010016236	GPATCH2	case	control	0.023536478	0.043734545	0.538166735	0.590461943	0.882949889	up_in_case
PRJNA8274(	ENSGALG00010016240	EIF4A2	case	control	-0.067288855	0.059029153	-1.13992582	0.254317207	0.679971816	down_in_case
PRJNA8274(	ENSGALG00010016241	TOPAZ1	case	control	1.720379847	0.650672654	2.644002072	0.008193215	0.159353687	up_in_case
PRJNA8274(	ENSGALG00010016242	PDS5A	case	control	-0.088972187	0.044653825	-1.992487476	0.046317598	0.356443312	down_in_case
PRJNA8274(	ENSGALG00010016245	FAM96A	case	control	0.051163723	0.086033665	0.594694217	0.552047888	0.865601403	up_in_case
PRJNA8274(	ENSGALG00010016247	KIF26A	case	control	0.071777593	0.090425433	0.7937766	0.427325497	0.805305429	up_in_case
PRJNA8274(	ENSGALG00010016251	MPDZ	case	control	-0.111604565	0.093687344	-1.191244846	0.233557479	0.657982411	down_in_case
PRJNA8274(	ENSGALG00010016253	EDN3	case	control	1.672965155	0.134530248	12.43560598	1.67466E-35	4.78953E-32	up_in_case
PRJNA8274(	ENSGALG00010016257	ENSGALG00010016257	case	control	0.104076912	0.071689009	1.45178337	0.146561849	0.55166873	up_in_case
PRJNA8274(	ENSGALG00010016258	CTNNB1	case	control	-0.065873644	0.068605256	-0.960183624	0.336962807	0.750735231	down_in_case
PRJNA8274(	ENSGALG00010016260	INTS6L	case	control	0.069665465	0.065757228	1.059434332	0.289402021	0.712911094	up_in_case
PRJNA8274(	ENSGALG00010016262	SPG11	case	control	0.005595272	0.041221778	0.135735834	0.892030118	0.979199408	up_in_case
PRJNA8274(	ENSGALG00010016263	HGFAC	case	control	1.677025912	1.12214329	1.494484642	0.135048993	0.539774365	up_in_case
PRJNA8274(	ENSGALG00010016266	PDLIM4	case	control	0.019126708	0.06281312	0.3045018	0.760745622	0.942936846	up_in_case
PRJNA8274(	ENSGALG00010016270	RFC4	case	control	-0.080612101	0.056661132	-1.422705446	0.154821561	0.563775993	down_in_case
PRJNA8274(	ENSGALG00010016274	ZNF831	case	control	1.27862999	0.384385863	3.32642304	0.000879683	0.04498565	up_in_case
PRJNA8274(	ENSGALG00010016276	ACTR2	case	control	0.068010949	0.057345756	1.18598051	0.235629996	0.660526168	up_in_case
PRJNA8274(	ENSGALG00010016279	PRELID3B	case	control	1.339309662	0.064319504	20.82276109	2.69217E-96	1.9249E-92	up_in_case
PRJNA8274(	ENSGALG00010016281	SLC22A4	case	control	-0.007934531	0.176766188	-0.044887154	0.964197256	0.992068146	down_in_case
PRJNA8274(	ENSGALG00010016286	ULK4	case	control	-0.01283806	0.108833024	-0.117961068	0.906098506	0.981730793	down_in_case
PRJNA8274(	ENSGALG00010016287	BCL11B	case	control	-0.022134901	0.416959295	-0.05308648	0.957663004	0.992056045	down_in_case
PRJNA8274(	ENSGALG00010016289	IRF1	case	control	-0.163464432	0.159954935	-1.02194053	0.306809048	0.729504707	down_in_case
PRJNA8274(	ENSGALG00010016292	PHKA1	case	control	0.04856214	0.101190917	0.479906113	0.631294154	0.897039706	up_in_case
PRJNA8274(	ENSGALG00010016295	UBE2K	case	control	-0.025660867	0.041888074	-0.612605571	0.540137174	0.858981493	down_in_case
PRJNA8274(	ENSGALG00010016299	SRSF4	case	control	-0.075824764	0.078774605	-0.96255341	0.335771688	0.749888354	down_in_case
PRJNA8274(	ENSGALG00010016303	ENSGALG00010016303	case	control	1.029164483	0.091783478	11.21296016	3.52213E-29	5.59628E-26	up_in_case
PRJNA8274(	ENSGALG00010016304	EIF3J	case	control	-0.076974284	0.057669099	-1.334757881	0.181955603	0.602864949	down_in_case
PRJNA8274(	ENSGALG00010016305	DENND4C	case	control	-0.026807704	0.112795518	-0.237666397	0.812139848	0.956328147	down_in_case
PRJNA8274(	ENSGALG00010016307	RAD50	case	control	0.001748343	0.073895448	0.023659686	0.981124063	0.99638336	up_in_case
PRJNA8274(	ENSGALG00010016308	RPS6	case	control	0.008901953	0.178080818	0.049988278	0.960131729	0.992056045	up_in_case
PRJNA8274(	ENSGALG00010016310	HDAC8	case	control	0.025067833	0.065067736	0.385257489	0.700046654	0.922288441	up_in_case
PRJNA8274(	ENSGALG00010016312	MECR	case	control	0.120760728	0.088148103	1.369975336	0.170694601	0.588034881	up_in_case
PRJNA8274(	ENSGALG00010016314	ACER2	case	control	0.181690495	0.082026101	2.215032684	0.026757821	0.291454243	up_in_case
PRJNA8274(	ENSGALG00010016315	RPS4Y1	case	control	-0.026653047	0.061042057	-0.436634156	0.662376688	0.910707559	down_in_case
PRJNA8274(	ENSGALG00010016317	CTDSPL2	case	control	-0.121355625	0.039143959	-3.10023896	0.001933646	0.071904164	down_in_case
PRJNA8274(	ENSGALG00010016319	RBM41	case	control	0.022278701	0.113612587	0.196093603	0.844536903	0.96721237	up_in_case
PRJNA8274(	ENSGALG00010016322	EBF4	case	control	-0.005754447	0.078482507	-0.073321402	0.941550361	0.989551456	down_in_case
PRJNA8274(	ENSGALG00010016324	TUBB1	case	control	0.526701899	0.187365608	2.811091671	0.004937372	0.120231831	up_in_case
PRJNA8274(	ENSGALG00010016328	USH2A	case	control	0.058308597	0.246759939	0.236296854	0.813202328	0.956712425	up_in_case
PRJNA8274(	ENSGALG00010016332	ENSGALG00010016332	case	control	0.055251067	0.134606538	0.410463477	0.68146599	0.915594727	up_in_case
PRJNA8274(	ENSGALG00010016335	ABHD4	case	control	-0.074570993	0.063908879	-1.166833069	0.24327779	0.667473598	down_in_case
PRJNA8274(	ENSGALG00010016336	SLC24A2	case	control	-0.533607646	0.326395089	-1.634851941	0.102080089	0.491460867	down_in_case
PRJNA8274(	ENSGALG00010016338	PTPRU	case	control	0.19211586	0.092815309	2.069872558	0.03846428	0.332149281	up_in_case
PRJNA8274(	ENSGALG00010016339	SETD3	case	control	-0.045845581	0.067971167	-0.674485713	0.500002566	0.840686266	down_in_case
PRJNA8274(	ENSGALG00010016340	RGS12	case	control	0.197563733	0.088058247	2.243557418	0.024860892	0.279489584	up_in_case
PRJNA8274(	ENSGALG00010016341	SLX4	case	control	0.075743146	0.049717693	1.523464616	0.127642507	0.529529401	up_in_case
PRJNA8274(	ENSGALG00010016344	SMIM14	case	control	0.182727131	0.063119046	2.894960264	0.003792065	0.105704745	up_in_case
PRJNA8274(	ENSGALG00010016345	CTSZ	case	control	0.286505714	0.099297501	2.885326533	0.003910079	0.107773305	up_in_case
PRJNA8274(	ENSGALG00010016346	TRAP1	case	control	-0.026891325	0.050978121	-0.527507183	0.59784143	0.884680373	down_in_case
PRJNA8274(	ENSGALG00010016347	ANO10	case	control	0.085016364	0.075737017	1.122520634	0.261641139	0.687516348	up_in_case
PRJNA8274(	ENSGALG00010016350	CASC4	case	control	0.000669789	0.038917767	0.017210378	0.986268783	0.996597442	up_in_case
PRJNA8274(	ENSGALG00010016353	C1orf232	case	control	0.18190291	0.168888057	1.077062009	0.281452564	0.705762606	up_in_case
PRJNA8274(	ENSGALG00010016355	MCF2L2	case	control	-0.153961557	0.150056611	-1.026023149	0.304880673	0.728073294	down_in_case
PRJNA8274(	ENSGALG00010016361	NELFCD	case	control	-0.003489435	0.038764034	-0.090017324	0.928273449	0.986776285	down_in_case
PRJNA8274(	ENSGALG00010016362	NUP62	case	control	-0.109398698	0.063275785	-1.728918873	0.083823616	0.458188945	down_in_case
PRJNA8274(	ENSGALG00010016366	KIF3A	case	control	0.032410745	0.053085428	0.610539384	0.541504563	0.85975913	up_in_case
PRJNA8274(	ENSGALG00010016368	CCNK	case	control	-0.002770432	0.055031287	-0.050342853	0.959849175	0.992056045	down_in_case
PRJNA8274(	ENSGALG00010016369	MSANTD1	case	control	0.413797996	0.247051912	1.674943507	0.093945316	0.479278638	up_in_case
PRJNA8274(	ENSGALG00010016370	TMEM255A	case	control	0.038754152	0.106989105	0.362225223	0.717183732	0.926862121	up_in_case
PRJNA8274(	ENSGALG00010016372	DNAAF6	case	control	0.359221325	0.134648467	2.66784564	0.007633932	0.152061299	up_in_case
PRJNA8274(	ENSGALG00010016373	SRL	case	control	-0.215833573	0.101068716	-2.135513164	0.032719116	0.311575029	down_in_case
PRJNA8274(	ENSGALG00010016374	FRMD5	case	control	0.147817134	0.20031793	0.737912648	0.460567532	0.82454265	up_in_case
PRJNA8274(	ENSGALG00010016375	CCNI2	case	control	0.313253926	0.110474555	2.835530092	0.00457497	0.11558671	up_in_case
PRJNA8274(	ENSGALG00010016377	FRMPD3	case	control	0.040178329	0.175143033	0.229402954	0.81855574	0.958536226	up_in_case
PRJNA8274(	ENSGALG00010016378	WDR76	case	control	-0.031679537	0.102718931	-0.308409919	0.757770435	0.941765572	down_in_case
PRJNA8274(	ENSGALG00010016379	TFAP4	case	control	0.005623981	0.093244955	0.060314054	0.951905509	0.992056045	up_in_case
PRJNA8274(	ENSGALG00010016382	UGDH	case	control	0.223319155	0.061081003	3.656114731	0.000256067	0.021415026	up_in_case
PRJNA8274(	ENSGALG00010016383	FAM110D	case	control	0.093411981	0.100759835	0.927075561	0.353887304	0.762350539	up_in_case
PRJNA8274(	ENSGALG00010016384	GNAS	case	control	0.018612745	0.054578501	0.34102705	0.733083218	0.933846596	up_in_case
PRJNA8274(	ENSGALG00010016385	HTT	case	control	-0.009957689	0.059439228	-0.167527228	0.866955226	0.973570415	down_in_case
PRJNA8274(	ENSGALG00010016387	CCDC85C	case	control	-0.118046933	0.130528918	-0.9043738	0.365797226	0.771519223	down_in_case
PRJNA8274(	ENSGALG00010016390	ENSGALG00010016390	case	control	-0.050584841	0.409776698	-0.123444894	0.901754809	0.980763983	down_in_case
PRJNA8274(	ENSGALG00010016392	STMN3	case	control	-0.679623477	0.408680073	-1.662971899	0.096318061	0.483634708	down_in_case
PRJNA8274(	ENSGALG00010016393	BLM	case	control	-0.026141927	0.084950547	-0.30773112	0.758286938	0.941903352	down_in_case
PRJNA8274(	ENSGALG00010016394	PDIK1L	case	control	0.088361309	0.050934868	1.734790169	0.082777978	0.456624721	up_in_case
PRJNA8274(	ENSGALG00010016395	ENSGALG00010016395	case	control	0.004431517	0.101734519	0.043559625	0.965255436	0.992291928	up_in_case
PRJNA8274(	ENSGALG00010016397	LIAS	case	control	-0.029483195	0.05637559	-0.522978037	0.600989537	0.884954589	down_in_case
PRJNA8274(	ENSGALG00010016398	SEPTIN6	case	control	-0.059594211	0.045295717	-1.315669884	0.188284867	0.611129484	down_in_case
PRJNA8274(	ENSGALG00010016399	ACSM5	case	control	-0.304677609	0.290303139	-1.049515378	0.293940981	0.716378375	down_in_case
PRJNA8274(	ENSGALG00010016400	ENSGALG00010016400	case	control	-0.065987331	0.056255656	-1.172990163	0.240799751	0.665967363	down_in_case
PRJNA8274(	ENSGALG00010016402	SEPTIN8	case	control	0.047524283	0.066492404	0.714732519	0.47477434	0.830093946	up_in_case
PRJNA8274(	ENSGALG00010016403	GRK4	case	control	-0.193253233	0.084848102	-2.277637661	0.022748175	0.267253668	down_in_case
PRJNA8274(	ENSGALG00010016404	ESRRG	case	control	-0.102576325	0.07947941	-1.290602495	0.19684155	0.620029043	down_in_case
PRJNA8274(	ENSGALG00010016407	RPL9	case	control	-0.133447225	0.064036279	-2.083931587	0.037166391	0.328438691	down_in_case
PRJNA8274(	ENSGALG00010016409	GMEB2	case	control	-0.030047793	0.074755241	-0.40194898	0.687721575	0.917290381	down_in_case
PRJNA8274(	ENSGALG00010016410	TRAK1	case	control	-0.094972843	0.068807014	-1.380278515	0.167500907	0.583641076	down_in_case
PRJNA8274(	ENSGALG00010016411	CTSH	case	control	0.033780733	0.137700155	0.245320951	0.80620793	0.9556344	up_in_case
PRJNA8274(	ENSGALG00010016412	TMSB15B	case	control	-0.141780101	0.127045999	-1.115974547	0.26443303	0.690350289	down_in_case
PRJNA8274(	ENSGALG00010016414	KLB	case	control	1.883781354	0.609702379	3.089673616	0.002003766	0.073471404	up_in_case
PRJNA8274(	ENSGALG00010016415	RASGRF1	case	control	0.054802676	0.339441042	0.161449763	0.871739175	0.975062028	up_in_case
PRJNA8274(	ENSGALG00010016416	CYP46A1	case	control	0.001425153	0.076582908	0.018609278	0.985152802	0.996591771	up_in_case
PRJNA8274(	ENSGALG00010016417	NOP14	case	control	0.11439332	0.084930079	1.346911736	0.178008661	0.59730458	up_in_case
PRJNA8274(	ENSGALG00010016418	NFIB	case	control	-0.140848102	0.109326254	-1.288328261	0.19763172	0.62094535	down_in_case
PRJNA8274(	ENSGALG00010016421	ENSGALG00010016421	case	control	0.158656687	0.304700481	0.520697199	0.602577726	0.885101354	up_in_case

PRJNA8274(	ENSGALG00010016422	KCTD3	case	control	-0.033169923	0.078641592	-0.421786003	0.673181223	0.91379937	down_in_case
PRJNA8274(	ENSGALG00010016423	TRMT44	case	control	-0.052253715	0.074707865	-0.699440613	0.484276714	0.834033076	down_in_case
PRJNA8274(	ENSGALG00010016425	TRIM63	case	control	-0.160534066	0.102704048	-1.563074378	0.118035101	0.514604253	down_in_case
PRJNA8274(	ENSGALG00010016426	HELZ2	case	control	0.186231749	0.1033747	1.801521546	0.071620714	0.432606532	up_in_case
PRJNA8274(	ENSGALG00010016428	ADRA1D	case	control	0.048006879	0.160845481	0.29846582	0.765347659	0.943786458	up_in_case
PRJNA8274(	ENSGALG00010016429	ENSGALG00010016429	case	control	1.025067799	0.359883994	2.848328392	0.004394955	0.114268826	up_in_case
PRJNA8274(	ENSGALG00010016431	B3GNT5	case	control	0.208935562	0.132466299	1.577273337	0.114732645	0.50968525	up_in_case
PRJNA8274(	ENSGALG00010016433	TMED3	case	control	0.045356479	0.068526851	0.661878939	0.50804881	0.843702533	up_in_case
PRJNA8274(	ENSGALG00010016434	GPR139	case	control	-0.036625068	0.331891341	-0.110352587	0.912129753	0.98266608	down_in_case
PRJNA8274(	ENSGALG00010016441	ENSGALG00010016441	case	control	-0.578259142	0.347707761	-1.663060785	0.096300269	0.483634708	down_in_case
PRJNA8274(	ENSGALG00010016445	ZDHHC21	case	control	0.159135079	0.154384882	1.03076854	0.302649374	0.725404918	up_in_case
PRJNA8274(	ENSGALG00010016447	ID3	case	control	0.101023067	0.083273501	1.213147836	0.225073304	0.648900855	up_in_case
PRJNA8274(	ENSGALG00010016449	FMR1	case	control	-0.091481049	0.062010398	-1.475253367	0.140144438	0.542372252	down_in_case
PRJNA8274(	ENSGALG00010016450	ENSGALG00010016450	case	control	0.319252018	0.245264536	1.301664004	0.19303127	0.615324823	up_in_case
PRJNA8274(	ENSGALG00010016451	USP46	case	control	-0.054151583	0.114017789	-0.474939775	0.6348299	0.898107199	down_in_case
PRJNA8274(	ENSGALG00010016452	GLCE	case	control	-0.053630176	0.055495457	-0.966388577	0.333849778	0.748508541	down_in_case
PRJNA8274(	ENSGALG00010016453	MYCL	case	control	0.020805134	0.097117756	0.214225848	0.83037094	0.962228709	up_in_case
PRJNA8274(	ENSGALG00010016454	ANKS3	case	control	0.046814546	0.050943458	0.918951074	0.358121163	0.764462254	up_in_case
PRJNA8274(	ENSGALG00010016455	RBM27	case	control	0.004703093	0.049925377	0.094202449	0.92494834	0.985571367	up_in_case
PRJNA8274(	ENSGALG00010016456	LAMP3	case	control	0.219970204	0.338151521	0.650507806	0.515364261	0.847750297	up_in_case
PRJNA8274(	ENSGALG00010016457	SMOX	case	control	0.058914188	0.082796059	0.711557878	0.476738598	0.830520323	up_in_case
PRJNA8274(	ENSGALG00010016458	ZC3H12A	case	control	-0.11732944	0.065555969	-1.78975984	0.073492528	0.437892979	down_in_case
PRJNA8274(	ENSGALG00010016459	PAQR5	case	control	-0.095753605	0.169950816	-0.563419504	0.573149259	0.877449175	down_in_case
PRJNA8274(	ENSGALG00010016460	LBR	case	control	0.10571741	0.042497994	2.487585889	0.012861339	0.199684901	up_in_case
PRJNA8274(	ENSGALG00010016461	HPCAL4	case	control	-0.000123207	0.218389398	-0.000564162	0.999549864	0.99976345	down_in_case
PRJNA8274(	ENSGALG00010016462	RNF24	case	control	0.003475656	0.083457271	0.04164593	0.966780958	0.992654885	up_in_case
PRJNA8274(	ENSGALG00010016463	FREM1	case	control	0.156866048	0.118937826	1.318891158	0.187205503	0.610664656	up_in_case
PRJNA8274(	ENSGALG00010016464	PANK2	case	control	0.01207207	0.068792588	0.175485034	0.860698529	0.972153185	up_in_case
PRJNA8274(	ENSGALG00010016465	STK35	case	control	-0.139266054	0.14061953	-0.990374904	0.321990907	0.739571618	down_in_case
PRJNA8274(	ENSGALG00010016466	TMEM164	case	control	0.224206554	0.123350829	1.817633135	0.069120224	0.424578693	up_in_case
PRJNA8274(	ENSGALG00010016468	TTC39B	case	control	-0.038430111	0.117371908	-0.327421714	0.743348945	0.937448285	down_in_case
PRJNA8274(	ENSGALG00010016469	MAVS	case	control	0.021023403	0.080333156	0.261702689	0.793550668	0.95280257	up_in_case
PRJNA8274(	ENSGALG00010016470	TNIP1	case	control	-0.0108884	0.054785364	-0.19874651	0.84246104	0.966519616	down_in_case
PRJNA8274(	ENSGALG00010016471	POMGNT2	case	control	-0.051163755	0.091608369	-0.558505243	0.576499425	0.878085957	down_in_case
PRJNA8274(	ENSGALG00010016472	TFDP1	case	control	0.037092376	0.047481538	0.781195761	0.434687367	0.809589652	up_in_case
PRJNA8274(	ENSGALG00010016473	RAB9B	case	control	0.05572555	0.068793585	0.810039913	0.417917237	0.800243236	up_in_case
PRJNA8274(	ENSGALG00010016474	MAPK8IP3	case	control	0.254965197	0.128762411	1.980121333	0.047689897	0.360119697	up_in_case
PRJNA8274(	ENSGALG00010016475	MCCC1	case	control	-0.120493115	0.067386141	-1.788099365	0.073759978	0.437929428	down_in_case
PRJNA8274(	ENSGALG00010016477	CNIH3	case	control	0.268653971	0.211154411	1.272310487	0.203262824	0.625537942	up_in_case
PRJNA8274(	ENSGALG00010016478	SOX12	case	control	-0.32164617	0.514308873	-0.625394947	0.531711878	0.855623068	down_in_case
PRJNA8274(	ENSGALG00010016479	ENSGALG00010016479	case	control	0.089052619	0.192644114	0.462264937	0.643891341	0.902038523	up_in_case
PRJNA8274(	ENSGALG00010016480	GPX3	case	control	-0.215487878	0.120386146	-1.790418563	0.073386648	0.437626802	down_in_case
PRJNA8274(	ENSGALG00010016482	EML1	case	control	-0.116531238	0.084790557	-1.374342173	0.169335479	0.585146363	down_in_case
PRJNA8274(	ENSGALG00010016483	WDR26	case	control	-0.09368914	0.055654097	-1.683418579	0.092294089	0.476713154	down_in_case
PRJNA8274(	ENSGALG00010016489	CCK	case	control	-1.071183783	0.386303165	-2.772909676	0.005555754	0.129767478	down_in_case
PRJNA8274(	ENSGALG00010016491	DNAJC8	case	control	-0.042566417	0.041804778	-1.018218949	0.308573916	0.731823162	down_in_case
PRJNA8274(	ENSGALG00010016496	PLP1	case	control	0.022945287	0.202781301	0.113152876	0.909909355	0.981730793	up_in_case
PRJNA8274(	ENSGALG00010016498	KIF23	case	control	0.014757584	0.090125823	0.163744231	0.869932496	0.974794532	up_in_case
PRJNA8274(	ENSGALG00010016500	ENSGALG00010016500	case	control	-0.085497816	0.062950155	-1.358182783	0.174405692	0.593194623	down_in_case
PRJNA8274(	ENSGALG00010016502	YIPF5	case	control	0.100375653	0.060953047	1.646770054	0.099605319	0.488667622	up_in_case
PRJNA8274(	ENSGALG00010016504	SNRK	case	control	-0.086704526	0.065660532	-1.320496856	0.186669185	0.610420614	down_in_case
PRJNA8274(	ENSGALG00010016505	KCTD16	case	control	0.205827182	0.315781181	0.651803194	0.514528141	0.847493921	up_in_case
PRJNA8274(	ENSGALG00010016507	SPATA18	case	control	-0.199494802	0.119254946	-1.672843	0.094358207	0.479503328	down_in_case
PRJNA8274(	ENSGALG00010016508	CREBBP	case	control	-0.158142528	0.066844906	-2.365812691	0.017990544	0.239316072	down_in_case
PRJNA8274(	ENSGALG00010016510	THG1L	case	control	-0.122634083	0.121976617	-1.005390099	0.314709046	0.736432557	down_in_case
PRJNA8274(	ENSGALG00010016514	GASK1A	case	control	0.127918475	0.152526789	0.83866235	0.401658812	0.791364151	up_in_case
PRJNA8274(	ENSGALG00010016518	TMEM42	case	control	-0.176293266	0.07660713	-2.301264481	0.021376686	0.261808818	down_in_case
PRJNA8274(	ENSGALG00010016519	MEX3B	case	control	0.049659098	0.090876179	0.546447902	0.58475809	0.881307382	up_in_case
PRJNA8274(	ENSGALG00010016520	CHMP1B	case	control	0.018318528	0.04594003	0.398748721	0.690078365	0.918877347	up_in_case
PRJNA8274(	ENSGALG00010016526	EIF1B	case	control	-0.032644376	0.073460911	-0.444377499	0.656769679	0.907508591	down_in_case
PRJNA8274(	ENSGALG00010016527	MRPS34	case	control	0.040730358	0.060502583	0.673200319	0.500819859	0.840987941	up_in_case
PRJNA8274(	ENSGALG00010016529	DCUN1D1	case	control	-0.056661734	0.053387858	-1.061322467	0.288543379	0.712801679	down_in_case
PRJNA8274(	ENSGALG00010016533	CNIH4	case	control	-0.035623642	0.076955229	-0.462913852	0.643426118	0.902038523	down_in_case
PRJNA8274(	ENSGALG00010016534	TRIT1	case	control	0.166993259	0.090723677	1.840680015	0.065668464	0.415733817	up_in_case
PRJNA8274(	ENSGALG00010016535	BNC2	case	control	-0.076055788	0.136275351	-0.558103774	0.576773524	0.878085957	down_in_case
PRJNA8274(	ENSGALG00010016541	UBE2J2	case	control	-0.030628054	0.037322315	-0.820636508	0.411853346	0.797194435	down_in_case
PRJNA8274(	ENSGALG00010016542	ENOX2	case	control	0.100432588	0.046535585	2.158189009	0.03091314	0.308259818	up_in_case
PRJNA8274(	ENSGALG00010016543	TCEA3	case	control	-0.408079122	0.177700805	-2.296439352	0.021650773	0.263494515	down_in_case
PRJNA8274(	ENSGALG00010016544	ADCY9	case	control	-0.065474861	0.056629778	-1.156191374	0.247602887	0.672031667	down_in_case
PRJNA8274(	ENSGALG00010016546	C1QTNF12	case	control	0.200077919	0.352487709	0.567616726	0.570295248	0.875775563	up_in_case
PRJNA8274(	ENSGALG00010016547	SGCB	case	control	-0.256200365	0.119694647	-2.140449655	0.032318445	0.309838273	down_in_case
PRJNA8274(	ENSGALG00010016548	EVL	case	control	-0.157364672	0.050788681	-3.098420138	0.001945554	0.072043631	down_in_case
PRJNA8274(	ENSGALG00010016549	PTPRA	case	control	-0.016440839	0.047928443	-0.343028866	0.731576744	0.933770678	down_in_case
PRJNA8274(	ENSGALG00010016550	FBLIM1	case	control	-0.277596515	0.123633445	-2.245318936	0.024747665	0.279094332	down_in_case
PRJNA8274(	ENSGALG00010016552	ENSGALG00010016552	case	control	-0.065240626	0.187167205	-0.348568681	0.727413144	0.932979874	down_in_case
PRJNA8274(	ENSGALG00010016553	SLC25A14	case	control	0.110956369	0.066647939	1.664813222	0.095950025	0.483185646	up_in_case
PRJNA8274(	ENSGALG00010016556	ASAP3	case	control	0.110265727	0.116252168	0.948504696	0.342872583	0.754784166	up_in_case
PRJNA8274(	ENSGALG00010016558	NR2F2	case	control	-0.039037164	0.048299219	-0.808235915	0.418954787	0.80093491	down_in_case
PRJNA8274(	ENSGALG00010016559	ROGDI	case	control	-0.051125901	0.075674309	-0.675604461	0.499291808	0.840142058	down_in_case
PRJNA8274(	ENSGALG00010016560	DCUN1D4	case	control	0.098313114	0.094445248	1.040953524	0.297897119	0.721487387	up_in_case
PRJNA8274(	ENSGALG00010016561	DEGS2	case	control	0.072204953	0.479929793	0.150448991	0.880410392	0.976399289	up_in_case
PRJNA8274(	ENSGALG00010016562	GLIS2	case	control	0.005979395	0.091487582	0.065357448	0.947889403	0.991291133	up_in_case
PRJNA8274(	ENSGALG00010016564	SPEN	case	control	-0.220855617	0.085591189	-2.580354601	0.009869891	0.177161817	down_in_case
PRJNA8274(	ENSGALG00010016565	NVL	case	control	-0.076305984	0.063659234	-1.198663238	0.230658918	0.655228949	down_in_case
PRJNA8274(	ENSGALG00010016566	CNTLN	case	control	0.012708895	0.204559119	0.062128224	0.950460721	0.991497374	up_in_case
PRJNA8274(	ENSGALG00010016567	E2F2	case	control	0.003679954	0.127574209	0.028845599	0.976987733	0.994586386	up_in_case
PRJNA8274(	ENSGALG00010016568	PAM16	case	control	-0.077293963	0.090782095	-0.851422995	0.394534422	0.786978937	down_in_case
PRJNA8274(	ENSGALG00010016569	YY1	case	control	-0.020050493	0.035753787	-0.560793548	0.574938285	0.877910508	down_in_case
PRJNA8274(	ENSGALG00010016570	ENSGALG00010016570	case	control	-0.296155963	0.283875376	-1.043260485	0.296827667	0.720505834	down_in_case
PRJNA8274(	ENSGALG00010016572	RAB33A	case	control	0.01084199	0.052687569	0.205778907	0.836963618	0.964784002	up_in_case
PRJNA8274(	ENSGALG00010016573	DEGS1	case	control	0.053623313	0.060454767	0.886998921	0.375079481	0.774681835	up_in_case
PRJNA8274(	ENSGALG00010016575	CWH43	case	control	-0.175088843	0.439011687	-0.398825015	0.690022144	0.918477347	down_in_case
PRJNA8274(	ENSGALG00010016576	MRPS26	case	control	-0.00834998	0.072039224	-0.115908802	0.90772482	0.981730793	down_in_case
PRJNA8274(	ENSGALG00010016577	ZBTB17	case	control	-0.052976288	0.059729741	-0.886933157	0.375114889	0.774681835	down_in_case
PRJNA8274(	ENSGALG00010016578	GRIK3	case	control	-0.625153703	0.403309146	-1.550060811	0.121126921	0.519499513	down_in_case
PRJNA8274(	ENSGALG00010016580	MARS2	case	control	-0.073560281	0.071418385	-1.029990828	0.303014311	0.725570102	down_in_case
PRJNA8274(	ENSGALG00010016581	H2AFYMACROH2A1	case	control	0.030981992	0.058317722	0.531262043	0.595237204	0.884461254	up_in_case
PRJNA8274(	ENSGALG00010016583	VPS41	case	control	-0.036834557	0.085914743	-0.428733837	0.668116933	0.912693174	down_in_case
PRJNA8274(	ENSGALG00010016584	EHBP1	case	control	-0.016046068	0.067123548	-0.239052741	0.811064686	0.956081528	down_in_case
PRJNA8274(	ENSGALG00010016588	ATP11B	case	control	-0.128164429	0.044230065	-2.897676693	0.003759379	0.105649139	down_in_case
PRJNA8274(	ENSGALG00010016589	FBXO28	case	control	-0.013067605	0.050908082	-0.256690194	0.797417942	0.95280257	down_in_case
PRJNA8274(	ENSGALG00010016591	HOMER2	case	control	0.041780427	0.205032799	0.203774359	0.838529831	0.965068538	up_in_case
PRJNA8274(	ENSGALG00010016592	TMEM129	case	control	0.029921685	0.062528369	0.47852975	0.632273205	0.89724192	up_in_case
PRJNA8274(	ENSGALG00010016597	FRYL	case	control	-0.030188766	0.048712681	-0.619731163	0.535434795	0.857009451	down_in_case
PRJNA8274(	ENSGALG00010016598	AIFM1	case	control	-0.114729361	0.054176461	-2.117697583	0.034200688	0.319236184	down_in_case
PRJNA8274(	ENSGALG00010016599	ENSGALG00010016599	case	control	-0.047715082	0.275866011	-0.172964702	0.862679173	0.972787494	down_in_case
PRJNA8274(	ENSGALG00010016600	GNL2	case	control	0.016111806	0.072114367	0.223420195	0.823208488	0.959820309	up_in_case
PRJNA8274(	ENSGALG00010016602	HSPB7	case	control	-0.036171451	0.29812272	-0.121330743	0.90342907	0.980997505	down_in_case
PRJNA8274(	ENSGALG00010016603	ENSGALG00010016603	case	control	0.139307613	0.075118134	1.854513765	0.063665693	0.409661205	up_in_case
PRJNA8274(	ENSGALG00010016604	TP53BP2	case	control	0.047447104	0.044428763	1.067936628	0.285549099	0.709921118	up_in_case
PRJNA8274(	ENSGALG00010016605	WDPCP	case	control	-0.145451935	0.111549927	-1.303917806	0.192261609	0.615090794	down_in_case
PRJNA8274(	ENSGALG00010016606	VASN	case	control	-0.007556355	0.075215445	-0.100462811	0.919976906	0.984638357	down_in_case
PRJNA8274(	ENSGALG00010016607	FAM103A1	case	control	0.073896469	0.076188591	0.969915153	0.332088786	0.748268094	up_in_case
PRJNA8274(	ENSGALG00010016609	SOX2	case	control	0.357374905	0.413979203	0.863267774	0.387990258	0.78298909	up_in_case
PRJNA8274(	ENSGALG00010016611	DNALI1	case	control	0.276041095	0.158573397	1.740778092	0.081722484	0.454736389	up_in_case
PRJNA8274(	ENSGALG00010016612	GRIA1	case	control	-0.39211563	0.224875261	-1.743702834	0.081210921	0.453461995	down_in_case
PRJNA8274(	ENSGALG00010016613	SNIP1	case	control	-0.040622484	0.046736831	-0.869174979	0.384751431	0.780971676	down_in_case
PRJNA8274(	ENSGALG00010016614	DNAJC19	case	control	-0.230954496	0.12297053	-1.878128809	0.060363546	0.399259348	down_in_case
PRJNA8274(	ENSGALG00010016615	BCORL1	case	control	-0.075478205	0.098342921	-0.767500134	0.442784213	0.814107419	down_in_case
PRJNA8274(	ENSGALG00010016616	SLBP	case	control	-0.045656266	0.072131969	-0.632954663	0.52676325	0.85251736	down_in_case
PRJNA8274(	ENSGALG00010016618	TRPM3	case	control	-0.220014011	0.53018447	-0.41497634	0.678159223	0.914941113	down_in_case
PRJNA8274(	ENSGALG00010016619	SLC25A47	case	control	-0.321301168	0.177826976	-1.806819049	0.070790498	0.4302334	down_in_case
PRJNA8274(	ENSGALG00010016620	ENSGALG00010016620	case	control	0.070117001	0.123427471	0.568082622	0.569978867	0.875477744	up_in_case
PRJNA8274(	ENSGALG00010016622	KIAA1143	case	control	0.04496284	0.107732365	0.417356848	0.676417413	0.914247368	up_in_case
PRJNA8274(	ENSGALG00010016623	DNAJA3	case	control	-0.119664213	0.055128732	-2.170632399	0.029958971	0.305970455	down_in_case
PRJNA8274(	ENSGALG00010016624	MDH1	case	control	-0.007037538	0.075846275	-0.09278686	0.926072891	0.986137638	down_in_case

PRJNA8274(	ENSGALG00010016626	CAPN2	case	control	-0.079123697	0.162981147	-0.485477606	0.627337594	0.895838586	down_in_case
PRJNA8274(	ENSGALG00010016627	SH3RF2	case	control	-0.915518531	0.594635887	-1.539628789	0.123650864	0.522883756	down_in_case
PRJNA8274(	ENSGALG00010016629	LARS1	case	control	-0.069530331	0.054123857	-1.284652195	0.198913851	0.622830756	down_in_case
PRJNA8274(	ENSGALG00010016630	ENSGALG00010016630	case	control	-0.07172942	0.363929289	-0.197097134	0.843751526	0.96721237	down_in_case
PRJNA8274(	ENSGALG00010016631	FAM53A	case	control	-0.098113786	0.06841443	-1.434109535	0.151541007	0.559523988	down_in_case
PRJNA8274(	ENSGALG00010016640	OCIAD1	case	control	-0.044628492	0.049646338	-0.898928171	0.368690921	0.771770041	down_in_case
PRJNA8274(	ENSGALG00010016643	BTBD1	case	control	0.004293814	0.09791595	0.043852041	0.965022344	0.992291928	up_in_case
PRJNA8274(	ENSGALG00010016644	NMRAL1	case	control	0.07698991	0.169825359	0.453347547	0.6502985	0.904245691	up_in_case
PRJNA8274(	ENSGALG00010016647	EGR1	case	control	-0.043417413	0.125810043	-0.34510292	0.730016998	0.933770678	down_in_case
PRJNA8274(	ENSGALG00010016648	ADRA2C	case	control	-0.097301439	0.1497761	-0.649645962	0.515920937	0.847910516	down_in_case
PRJNA8274(	ENSGALG00010016651	ENSGALG00010016651	case	control	-0.165926413	0.10308741	-1.60957009	0.107491742	0.498867454	down_in_case
PRJNA8274(	ENSGALG00010016652	FXR1	case	control	-0.134348982	0.055008134	-2.442347585	0.01459209	0.212925391	down_in_case
PRJNA8274(	ENSGALG00010016653	EPHA2	case	control	0.167279336	0.098451485	1.699104242	0.089299544	0.47258594	up_in_case
PRJNA8274(	ENSGALG00010016654	SMIM3	case	control	-0.281923485	0.126076559	-2.236129284	0.025343304	0.282919227	down_in_case
PRJNA8274(	ENSGALG00010016655	TRANK1	case	control	-0.138472476	0.072883844	-1.899906329	0.057445413	0.390061448	down_in_case
PRJNA8274(	ENSGALG00010016657	SLC25A29	case	control	0.036343658	0.09906211	0.366877484	0.713710407	0.926862121	up_in_case
PRJNA8274(	ENSGALG00010016658	ZAR1	case	control	-0.338643613	0.551520794	-0.614017851	0.539203531	0.858476757	down_in_case
PRJNA8274(	ENSGALG00010016661	UBE4B	case	control	0.051388406	0.050863869	1.010312556	0.312345567	0.734364975	up_in_case
PRJNA8274(	ENSGALG00010016664	TM6SF1	case	control	-0.360997666	0.142417331	-2.534787468	0.011251557	0.185840265	down_in_case
PRJNA8274(	ENSGALG00010016667	WARS1	case	control	-0.028241839	0.045194702	-0.624892704	0.532041481	0.855755691	down_in_case
PRJNA8274(	ENSGALG00010016671	CASS4	case	control	0.209334913	0.206964432	1.011453566	0.311799395	0.734310169	up_in_case
PRJNA8274(	ENSGALG00010016672	DCTN4	case	control	-0.060243035	0.03480777	-1.730735264	0.083498993	0.457484902	down_in_case
PRJNA8274(	ENSGALG00010016673	LRPAP1	case	control	0.014702537	0.069567314	0.211342603	0.832619941	0.962371901	up_in_case
PRJNA8274(	ENSGALG00010016674	SLC10A4	case	control	-0.088499172	0.309429145	-0.286007873	0.774872092	0.947085948	down_in_case
PRJNA8274(	ENSGALG00010016675	SKIL	case	control	0.075889028	0.086352877	0.878824544	0.379496416	0.777798094	up_in_case
PRJNA8274(	ENSGALG00010016677	MEAF6	case	control	0.033045991	0.052034832	0.635074425	0.525379876	0.85251736	up_in_case
PRJNA8274(	ENSGALG00010016678	NOX1	case	control	0.065652049	0.214357687	0.306273362	0.75939652	0.942244706	up_in_case
PRJNA8274(	ENSGALG00010016680	BPIFB3	case	control	0.312969887	0.350876684	0.891965472	0.37241144	0.774201112	up_in_case
PRJNA8274(	ENSGALG00010016681	SUSD4	case	control	-0.153597821	0.154759345	-0.99249464	0.320956293	0.73942679	down_in_case
PRJNA8274(	ENSGALG00010016682	GPR141	case	control	0.648183897	0.421015714	1.539571745	0.123664777	0.522883756	up_in_case
PRJNA8274(	ENSGALG00010016683	RBM22	case	control	-0.069052661	0.037201694	-1.856169779	0.063429362	0.409498817	down_in_case
PRJNA8274(	ENSGALG00010016691	ENSGALG00010016691	case	control	-0.033298559	0.2700905	-0.123286671	0.901880095	0.980763983	down_in_case
PRJNA8274(	ENSGALG00010016692	PRKCI	case	control	-0.059301119	0.064424797	-0.920470408	0.35732699	0.763549838	down_in_case
PRJNA8274(	ENSGALG00010016696	DOK7	case	control	-0.153435621	0.09169658	-1.673297092	0.094268825	0.479477093	down_in_case
PRJNA8274(	ENSGALG00010016699	SYNPO	case	control	0.015851283	0.070258912	0.225612422	0.821502878	0.95897887	up_in_case
PRJNA8274(	ENSGALG00010016700	CLUAP1	case	control	-0.096145128	0.120948282	-0.794927603	0.426655616	0.805305429	down_in_case
PRJNA8274(	ENSGALG00010016702	WDR25	case	control	-0.087781788	0.108209688	-0.8112193	0.417239744	0.799987395	down_in_case
PRJNA8274(	ENSGALG00010016704	CSTF2	case	control	-0.004338387	0.070230265	-0.061773754	0.950743005	0.991585221	down_in_case
PRJNA8274(	ENSGALG00010016706	RTN4	case	control	-0.181910084	0.063531328	-2.863313097	0.004192361	0.111848429	down_in_case
PRJNA8274(	ENSGALG00010016707	KIF1B	case	control	-0.101512613	0.068574078	-1.480335074	0.138783848	0.540932258	down_in_case
PRJNA8274(	ENSGALG00010016712	ENSGALG00010016712	case	control	0.234439166	0.097965179	2.393086688	0.016707293	0.229833552	up_in_case
PRJNA8274(	ENSGALG00010016720	INTS15	case	control	0.027442393	0.05889865	0.46592567	0.641268706	0.901516193	up_in_case
PRJNA8274(	ENSGALG00010016724	PGD	case	control	0.078699488	0.071381336	1.102521933	0.270234829	0.697789464	up_in_case
PRJNA8274(	ENSGALG00010016729	ELMO1	case	control	-0.050025409	0.068358182	-0.731813038	0.46428269	0.825629877	down_in_case
PRJNA8274(	ENSGALG00010016731	C4orf48	case	control	0.226772211	0.181036509	1.25263248	0.210339489	0.636098721	up_in_case
PRJNA8274(	ENSGALG00010016732	ARHGAP26	case	control	0.125177222	0.054056168	2.31568805	0.020575307	0.257989413	up_in_case
PRJNA8274(	ENSGALG00010016733	PHC3	case	control	0.099477436	0.104409543	0.952761916	0.340710715	0.752236352	up_in_case
PRJNA8274(	ENSGALG00010016735	ENSGALG00010016735	case	control	0.60455661	0.273504326	2.210409678	0.027076743	0.292666229	up_in_case
PRJNA8274(	ENSGALG00010016736	CENPS	case	control	0.155096369	0.101497341	1.528083066	0.126491915	0.526480127	up_in_case
PRJNA8274(	ENSGALG00010016738	NAA60	case	control	-0.107047603	0.070774578	-1.512514881	0.13040293	0.532941383	down_in_case
PRJNA8274(	ENSGALG00010016741	NELFA	case	control	0.037304247	0.040327044	0.925042925	0.354943579	0.763161644	up_in_case
PRJNA8274(	ENSGALG00010016742	TRMT12	case	control	-0.007724767	0.105854118	-0.072975593	0.94182554	0.989551456	down_in_case
PRJNA8274(	ENSGALG00010016744	CRYBG2	case	control	-0.114484451	0.677656857	-0.168941625	0.865842558	0.973289701	down_in_case
PRJNA8274(	ENSGALG00010016745	BEGAIN	case	control	0.141032188	0.338888801	0.416160663	0.677292443	0.914766174	up_in_case
PRJNA8274(	ENSGALG00010016746	HDGFL3	case	control	-0.036423261	0.068751025	-0.529784991	0.596261013	0.884680373	down_in_case
PRJNA8274(	ENSGALG00010016750	SYTL4	case	control	0.037379566	0.098839069	0.378186139	0.705292322	0.924182432	up_in_case
PRJNA8274(	ENSGALG00010016751	DFFA	case	control	-0.117334722	0.0801776	-1.463435206	0.143348331	0.546343586	down_in_case
PRJNA8274(	ENSGALG00010016752	ENSGALG00010016752	case	control	0.00239043	0.112684928	0.021213399	0.983075426	0.996591771	up_in_case
PRJNA8274(	ENSGALG00010016754	ENSGALG00010016754	case	control	-0.042023415	0.148029879	-0.283884683	0.776498755	0.947228003	down_in_case
PRJNA8274(	ENSGALG00010016756	NUDT16L1	case	control	0.02468723	0.053341725	0.462812738	0.6434986	0.902038523	up_in_case
PRJNA8274(	ENSGALG00010016757	SRPX2	case	control	0.123932126	0.107565823	1.152151519	0.249258816	0.674421685	up_in_case
PRJNA8274(	ENSGALG00010016758	NR3C1	case	control	-0.0736761	0.053123445	-1.386884826	0.165476886	0.580618526	down_in_case
PRJNA8274(	ENSGALG00010016760	DLK1	case	control	-0.422293929	0.12827461	-3.29210845	0.000994393	0.048697993	down_in_case
PRJNA8274(	ENSGALG00010016761	GPR160	case	control	0.021804012	0.088375458	0.246720214	0.805124761	0.9556344	up_in_case
PRJNA8274(	ENSGALG00010016762	EML6	case	control	-0.159425101	0.11466174	-1.390394922	0.164408988	0.579452754	down_in_case
PRJNA8274(	ENSGALG00010016763	UBALD1	case	control	0.056654186	0.07133479	0.794201335	0.427078231	0.805305429	up_in_case
PRJNA8274(	ENSGALG00010016766	AOAH	case	control	-0.16176372	0.263578498	-0.61372123	0.539399557	0.858476757	down_in_case
PRJNA8274(	ENSGALG00010016767	SLAIN2	case	control	0.00306411	0.049447772	0.061966587	0.95058944	0.991497374	up_in_case
PRJNA8274(	ENSGALG00010016768	GALNT10	case	control	0.059552492	0.085135371	0.699503522	0.484237412	0.834033076	up_in_case
PRJNA8274(	ENSGALG00010016769	BNC1	case	control	0.200264387	0.449274942	0.445750181	0.655777712	0.907453191	up_in_case
PRJNA8274(	ENSGALG00010016772	TSPAN6	case	control	0.044382573	0.052747493	0.841415779	0.400115049	0.789900496	up_in_case
PRJNA8274(	ENSGALG00010016773	RPS27A	case	control	-0.047419722	0.066154554	-0.716802079	0.473496229	0.82959752	down_in_case
PRJNA8274(	ENSGALG00010016774	PEX14	case	control	-0.049565964	0.051770354	-0.957419852	0.338355382	0.751259351	down_in_case
PRJNA8274(	ENSGALG00010016777	TNMD	case	control	0.174804819	0.239259033	0.730609068	0.465017971	0.826255378	up_in_case
PRJNA8274(	ENSGALG00010016778	MTIF2	case	control	-0.011447001	0.048569444	-0.235683177	0.813678525	0.956712425	down_in_case
PRJNA8274(	ENSGALG00010016779	CTNNA2	case	control	-0.072151612	0.106813487	-0.675491587	0.499363495	0.840142058	down_in_case
PRJNA8274(	ENSGALG00010016780	PRORSD1P	case	control	0.011981012	0.084953772	0.141029788	0.88784641	0.977944694	up_in_case
PRJNA8274(	ENSGALG00010016781	CDIP1	case	control	-0.056056344	0.092391243	-0.606727887	0.544031512	0.861114226	down_in_case
PRJNA8274(	ENSGALG00010016783	CLBA1	case	control	0.417475268	0.208744429	1.999934895	0.045507295	0.354248401	up_in_case
PRJNA8274(	ENSGALG00010016785	PDGFRB	case	control	-0.011585023	0.093880904	-0.123401273	0.901789349	0.980763983	down_in_case
PRJNA8274(	ENSGALG00010016786	DISP1	case	control	0.033259102	0.095192665	0.349387239	0.726798612	0.932420272	up_in_case
PRJNA8274(	ENSGALG00010016787	PPP2R5C	case	control	-0.047057415	0.052331979	-0.899209552	0.368541054	0.771770041	down_in_case
PRJNA8274(	ENSGALG00010016788	ANLN	case	control	-0.054809664	0.104912365	-0.522432832	0.601369001	0.884954589	down_in_case
PRJNA8274(	ENSGALG00010016789	DISP3	case	control	-0.070724944	0.075021332	-0.942731112	0.345818455	0.756227431	down_in_case
PRJNA8274(	ENSGALG00010016791	LRRTM1	case	control	0.296944981	0.414357218	0.716640059	0.47359622	0.82959752	up_in_case
PRJNA8274(	ENSGALG00010016792	HMOX2	case	control	0.080388417	0.075238426	1.068448955	0.285318044	0.709820465	up_in_case
PRJNA8274(	ENSGALG00010016793	SLC9A1	case	control	0.004128344	0.089078511	0.046345007	0.963035267	0.992068146	up_in_case
PRJNA8274(	ENSGALG00010016796	BROX	case	control	-0.002987935	0.073282831	-0.040772644	0.967477148	0.992731535	down_in_case
PRJNA8274(	ENSGALG00010016797	SEC62	case	control	-0.045410171	0.053942902	-0.841819203	0.399889161	0.789900496	down_in_case
PRJNA8274(	ENSGALG00010016799	WDTC1	case	control	-0.175085685	0.057982679	-3.019620488	0.002530916	0.082910904	down_in_case
PRJNA8274(	ENSGALG00010016800	RPL14	case	control	-0.014232814	0.066641877	-0.213571629	0.830881126	0.962228709	down_in_case
PRJNA8274(	ENSGALG00010016802	TMEM222	case	control	0.100752486	0.051831204	1.943857705	0.051912613	0.371953818	up_in_case
PRJNA8274(	ENSGALG00010016804	CSF1R	case	control	-0.076448976	0.076574857	-0.998356105	0.318106711	0.738087569	down_in_case
PRJNA8274(	ENSGALG00010016805	ENSGALG00010016805	case	control	-0.177788482	0.824424124	-0.215651722	0.829259236	0.962142561	down_in_case
PRJNA8274(	ENSGALG00010016807	VSIG4	case	control	0.217308448	0.226433626	0.95970043	0.337206007	0.750863577	up_in_case
PRJNA8274(	ENSGALG00010016808	ENTPD3	case	control	-0.491282913	0.310670005	-1.581365774	0.113794428	0.508641701	down_in_case
PRJNA8274(	ENSGALG00010016809	SYTL1	case	control	0.355933845	0.569793798	0.624671322	0.532186799	0.855755691	up_in_case
PRJNA8274(	ENSGALG00010016810	SH3GL3	case	control	0.138867381	0.126384352	1.098770371	0.271868239	0.698323327	up_in_case
PRJNA8274(	ENSGALG00010016812	LRRC31	case	control	0.071580392	0.124802887	0.573547569	0.566274001	0.873008555	up_in_case
PRJNA8274(	ENSGALG00010016813	ENSGALG00010016813	case	control	-0.008021193	0.375164692	-0.021380459	0.982942162	0.996591771	down_in_case
PRJNA8274(	ENSGALG00010016814	HMGXB3	case	control	0.007242708	0.062588121	0.115720172	0.907874319	0.981730793	up_in_case
PRJNA8274(	ENSGALG00010016816	MAP3K6	case	control	-0.32943919	0.285213869	-1.155060207	0.248065772	0.672435338	down_in_case
PRJNA8274(	ENSGALG00010016817	GGCT	case	control	0.036638112	0.111180182	0.329538153	0.741748961	0.937315893	up_in_case
PRJNA8274(	ENSGALG00010016820	EIF2AK3	case	control	0.136988331	0.071031547	1.928556199	0.053785981	0.377891678	up_in_case
PRJNA8274(	ENSGALG00010016823	HSF3	case	control	0.011488333	0.051488922	0.223122418	0.823440231	0.959986573	up_in_case
PRJNA8274(	ENSGALG00010016825	ENSGALG00010016825	case	control	-0.095697403	0.165637635	-0.577751568	0.563431858	0.871916948	down_in_case
PRJNA8274(	ENSGALG00010016828	LACTBL1	case	control	-0.331459311	0.286984937	-1.154971106	0.248102259	0.672435338	down_in_case
PRJNA8274(	ENSGALG00010016831	AIDA	case	control	-0.012279824	0.051953171	-0.236363319	0.813150757	0.956712425	down_in_case
PRJNA8274(	ENSGALG00010016832	DYNC1H1	case	control	-0.125997788	0.050968942	-2.472050314	0.013434061	0.203503251	down_in_case
PRJNA8274(	ENSGALG00010016834	ENSGALG00010016834	case	control	-0.085352253	0.072817027	-1.172146914	0.241138077	0.666204503	down_in_case
PRJNA8274(	ENSGALG00010016835	CCDC88A	case	control	0.010621635	0.102727347	0.103396368	0.917648395	0.983445506	up_in_case
PRJNA8274(	ENSGALG00010016836	HEPH	case	control	-0.11590614	0.130648386	-0.887160903	0.374992278	0.774681835	down_in_case
PRJNA8274(	ENSGALG00010016840	LRRC34	case	control	0.105865101	0.06083142	1.740302978	0.081805831	0.454736389	up_in_case
PRJNA8274(	ENSGALG00010016842	NPY7R	case	control	0.122709645	0.192526821	0.637363896	0.52388784	0.851995464	up_in_case
PRJNA8274(	ENSGALG00010016845	MIA3	case	control	-0.059348762	0.050612383	-1.172613475	0.240950843	0.666073277	down_in_case
PRJNA8274(	ENSGALG00010016847	EPHB2	case	control	-0.02455922	0.089781927	-0.27354303	0.78443582	0.949518053	down_in_case
PRJNA8274(	ENSGALG00010016848	ENSGALG00010016848	case	control	0.109396096	0.091848329	1.191051575	0.233633338	0.65800118	up_in_case
PRJNA8274(	ENSGALG00010016849	WASF2	case	control	-0.049113999	0.054411785	-0.902635329	0.36671947	0.771770041	down_in_case
PRJNA8274(	ENSGALG00010016851	TEC	case	control	0.01968738	0.102119005	0.1927886	0.847124532	0.968026275	up_in_case
PRJNA8274(	ENSGALG00010016852	AHDC1	case	control	-0.023488284	0.070181829	-0.334677571	0.737868323	0.935832995	down_in_case
PRJNA8274(	ENSGALG00010016854	TAF1A	case	control	0.106945863	0.089688707	1.192411693	0.233099857	0.657982411	up_in_case
PRJNA8274(	ENSGALG00010016856	NOD1	case	control	0.379090424	0.114553466	3.309288111	0.000935335	0.046603816	up_in_case
PRJNA8274(	ENSGALG00010016858	ENSGALG00010016858	case	control	0.047790929	0.124194127	0.384808285	0.700379461	0.922485612	up_in_case
PRJNA8274(	ENSGALG00010016859	RPIA	case	control	-0.120268302	0.078630793	-1.529531853	0.126132649	0.525939433	down_in_case
PRJNA8274(	ENSGALG00010016860	VSX1	case	control	-0.115073799	0.399998542	-0.287685546	0.773587455	0.94652185	down_in_case
PRJNA8274(	ENSGALG00010016861	FGR	case	control	0.023707894	0.057661835	0.411154002	0.680959614	0.915594727	up_in_case

PRJNA8274(	ENSGALG00010016863	MYNN	case	control	0.11127714	0.066882673	1.663766353	0.096159131	0.483634708	up_in_case
PRJNA8274(	ENSGALG00010016864	ENSGALG00010016864	case	control	0.177881523	0.224942685	0.790785986	0.429068889	0.805896795	up_in_case
PRJNA8274(	ENSGALG00010016865	CFAP36	case	control	0.028584186	0.056375594	0.507031214	0.612132917	0.889814948	up_in_case
PRJNA8274(	ENSGALG00010016866	ZNRF2	case	control	-0.15407231	0.095313926	-1.616472179	0.105992264	0.495485247	down_in_case
PRJNA8274(	ENSGALG00010016867	FLT4	case	control	0.043036617	0.063432656	0.678461529	0.497479108	0.839230872	up_in_case
PRJNA8274(	ENSGALG00010016869	HSP90AA1	case	control	0.181324351	0.091391812	1.984032787	0.047252185	0.358156924	up_in_case
PRJNA8274(	ENSGALG00010016872	EDA2R	case	control	-0.228567664	0.183754557	-1.243874807	0.213545643	0.638582746	down_in_case
PRJNA8274(	ENSGALG00010016873	ENTPD6	case	control	0.256022669	0.10417645	2.457586809	0.0139874	0.208137171	up_in_case
PRJNA8274(	ENSGALG00010016874	ADAMTSL3	case	control	-0.072535208	0.100440395	-0.722171668	0.47018894	0.829101076	down_in_case
PRJNA8274(	ENSGALG00010016875	SESN2	case	control	-0.024807498	0.089257808	-0.277930843	0.781065447	0.949199957	down_in_case
PRJNA8274(	ENSGALG00010016876	MTURN	case	control	0.140811233	0.094192513	1.494930217	0.134932655	0.539774365	up_in_case
PRJNA8274(	ENSGALG00010016877	UBE2D3	case	control	-0.105764407	0.045605244	-2.319128204	0.020388086	0.256420078	down_in_case
PRJNA8274(	ENSGALG00010016878	SUCLG1	case	control	-0.147092671	0.091119519	-1.61428279	0.106466101	0.496402103	down_in_case
PRJNA8274(	ENSGALG00010016879	RNF130	case	control	0.05664464	0.069463374	0.815460535	0.414808746	0.798853486	up_in_case
PRJNA8274(	ENSGALG00010016880	AR	case	control	-0.053451848	0.112584913	-0.474769194	0.634951493	0.898182917	down_in_case
PRJNA8274(	ENSGALG00010016881	C1QB	case	control	0.637848652	0.254221909	2.509023136	0.012106555	0.193549413	up_in_case
PRJNA8274(	ENSGALG00010016882	ENSGALG00010016882	case	control	0.007595445	0.041629791	0.182452156	0.855227898	0.970644797	up_in_case
PRJNA8274(	ENSGALG00010016883	TMEM251	case	control	0.035206146	0.045789614	0.76886751	0.441971967	0.813507909	up_in_case
PRJNA8274(	ENSGALG00010016884	SBK1	case	control	0.008218935	0.126973195	0.064729685	0.948389227	0.991291133	up_in_case
PRJNA8274(	ENSGALG00010016885	CDCA4	case	control	-0.044355361	0.051100921	-0.867995336	0.385396886	0.781541515	down_in_case
PRJNA8274(	ENSGALG00010016887	TJP2	case	control	-0.064375233	0.077267184	-0.83315101	0.404759568	0.793319877	down_in_case
PRJNA8274(	ENSGALG00010016888	ENSGALG00010016888	case	control	-0.035670495	0.088817756	-0.401614461	0.687967785	0.917290381	down_in_case
PRJNA8274(	ENSGALG00010016889	FUNDC2	case	control	-0.194854151	0.067445879	-2.889044593	0.003864143	0.107295613	down_in_case
PRJNA8274(	ENSGALG00010016890	UBR7	case	control	0.043143005	0.053414992	0.807694682	0.419266366	0.80093491	up_in_case
PRJNA8274(	ENSGALG00010016891	ENSGALG00010016891	case	control	-0.058235557	0.20083384	-0.289968845	0.771840073	0.945804499	down_in_case
PRJNA8274(	ENSGALG00010016892	MAL	case	control	0.040235113	0.221268141	0.181838711	0.855709304	0.970656588	up_in_case
PRJNA8274(	ENSGALG00010016893	TBC1D9B	case	control	0.004473763	0.035326498	0.126640446	0.899224984	0.98028579	up_in_case
PRJNA8274(	ENSGALG00010016894	BTBD7	case	control	-0.040541672	0.039803413	-1.018547631	0.308417777	0.731823162	down_in_case
PRJNA8274(	ENSGALG00010016895	ENSGALG00010016895	case	control	0.128631498	0.10630062	1.210072891	0.226250924	0.649676349	up_in_case
PRJNA8274(	ENSGALG00010016896	SPTBN1	case	control	-0.00220454	0.056752264	-0.038844979	0.969013984	0.992761697	down_in_case
PRJNA8274(	ENSGALG00010016897	TMEM179	case	control	-0.254537982	0.803534564	-0.31677291	0.751415921	0.940420766	down_in_case
PRJNA8274(	ENSGALG00010016899	SAXO2	case	control	-0.094999594	0.143486462	-0.66208054	0.507919607	0.843702533	down_in_case
PRJNA8274(	ENSGALG00010016900	CISD2	case	control	-0.087782952	0.082581997	-1.062979281	0.287791348	0.71176345	down_in_case
PRJNA8274(	ENSGALG00010016901	MECOM	case	control	8.651E-05	0.157335161	0.000549845	0.999561287	0.99976345	up_in_case
PRJNA8274(	ENSGALG00010016902	MRNIP	case	control	0.001831219	0.116920549	0.015662081	0.987503978	0.996908047	up_in_case
PRJNA8274(	ENSGALG00010016903	PPM1A	case	control	-0.410642093	0.172617062	-2.378919487	0.017363468	0.234759681	down_in_case
PRJNA8274(	ENSGALG00010016904	EAF1	case	control	-0.030971188	0.046344712	-0.668278775	0.503955659	0.842675107	down_in_case
PRJNA8274(	ENSGALG00010016905	C1QC	case	control	0.799085498	0.325390939	2.455770592	0.014058287	0.208540979	up_in_case
PRJNA8274(	ENSGALG00010016907	SQSTM1	case	control	0.044634033	0.136549342	0.326871093	0.743765386	0.937448285	up_in_case
PRJNA8274(	ENSGALG00010016908	CMC4	case	control	-0.102154223	0.07143949	-1.42994054	0.152734085	0.561193576	down_in_case
PRJNA8274(	ENSGALG00010016909	MRPS5	case	control	-0.078141997	0.055038903	-1.419759354	0.155677752	0.564716939	down_in_case
PRJNA8274(	ENSGALG00010016910	NSD2	case	control	0.119305653	0.077398451	1.541447564	0.123207895	0.522571644	up_in_case
PRJNA8274(	ENSGALG00010016911	AXIN1	case	control	-0.09830582	0.055399979	-1.774473943	0.075984788	0.442710866	down_in_case
PRJNA8274(	ENSGALG00010016912	CCDC88C	case	control	-0.222836845	0.061894076	-3.600293582	0.000317858	0.024072921	down_in_case
PRJNA8274(	ENSGALG00010016913	BRCC3	case	control	0.052113655	0.081268422	0.641253435	0.521358038	0.850363352	up_in_case
PRJNA8274(	ENSGALG00010016915	PLOD2	case	control	0.023551761	0.130473368	0.1805101	0.856752126	0.971145061	up_in_case
PRJNA8274(	ENSGALG00010016916	ACYP2	case	control	-0.358749796	0.147920132	-2.425293909	0.015295998	0.219195419	down_in_case
PRJNA8274(	ENSGALG00010016917	METTL6	case	control	0.008600787	0.081736342	0.10522598	0.916196496	0.983445506	up_in_case
PRJNA8274(	ENSGALG00010016918	RCOR1	case	control	0.022844093	0.051723937	0.441654176	0.658739474	0.908474731	up_in_case
PRJNA8274(	ENSGALG00010016919	ARHGDIG	case	control	-0.002488877	0.208645475	-0.011928735	0.990482473	0.997457701	down_in_case
PRJNA8274(	ENSGALG00010016920	C1QA	case	control	0.493945301	0.291109706	1.69676686	0.089740747	0.472887198	up_in_case
PRJNA8274(	ENSGALG00010016921	EFL1	case	control	-0.005981535	0.069395548	-0.086194797	0.931311567	0.987258715	down_in_case
PRJNA8274(	ENSGALG00010016922	ENSGALG00010016922	case	control	-0.033094377	0.100027129	-0.330854014	0.740754759	0.937059095	down_in_case
PRJNA8274(	ENSGALG00010016923	ENSGALG00010016923	case	control	0.102309717	0.102956852	0.993714509	0.320361877	0.738813491	up_in_case
PRJNA8274(	ENSGALG00010016924	LETM1	case	control	0.179124196	0.062674654	2.858000557	0.004263196	0.112687078	up_in_case
PRJNA8274(	ENSGALG00010016925	PHACTR4	case	control	0.030868954	0.039404461	0.783387297	0.433399713	0.808138728	up_in_case
PRJNA8274(	ENSGALG00010016926	ENSGALG00010016926	case	control	-0.156051265	0.181639448	-0.859126509	0.390270724	0.783914628	down_in_case
PRJNA8274(	ENSGALG00010016927	MGAT4B	case	control	-0.032045182	0.094177334	-0.34026427	0.733657522	0.933846596	down_in_case
PRJNA8274(	ENSGALG00010016928	RGS11	case	control	-0.035071743	0.324409109	-0.108109613	0.913908742	0.982854306	down_in_case
PRJNA8274(	ENSGALG00010016930	C10H15ORF48	case	control	0.224600046	0.552864425	0.406247963	0.684560416	0.916433328	up_in_case
PRJNA8274(	ENSGALG00010016931	SLC30A4	case	control	-0.025099883	0.035366741	-0.709703004	0.477888329	0.830520323	down_in_case
PRJNA8274(	ENSGALG00010016932	OGT	case	control	0.1555102	0.099452369	1.563665118	0.117896234	0.514604253	up_in_case
PRJNA8274(	ENSGALG00010016934	SLC25A38	case	control	-0.010155026	0.080989128	-0.125387525	0.900216763	0.98028579	down_in_case
PRJNA8274(	ENSGALG00010016935	PRIMA1	case	control	0.292642784	0.159006804	1.840441901	0.065703387	0.415733817	up_in_case
PRJNA8274(	ENSGALG00010016936	RCC1	case	control	-0.012528116	0.111505184	-0.112354559	0.910542284	0.981855322	down_in_case
PRJNA8274(	ENSGALG00010016937	DHRS7	case	control	0.312021385	0.229989705	1.356675441	0.174884359	0.594025258	up_in_case
PRJNA8274(	ENSGALG00010016938	PSME4	case	control	-0.005744842	0.058687604	-0.097888515	0.92202082	0.985482967	down_in_case
PRJNA8274(	ENSGALG00010016939	FAM234A	case	control	0.069580938	0.063190098	1.101136744	0.270837147	0.698323327	up_in_case
PRJNA8274(	ENSGALG00010016940	FGFR3	case	control	0.396781232	0.174315094	2.276229924	0.022832252	0.267253668	up_in_case
PRJNA8274(	ENSGALG00010016941	SLC8A1	case	control	-0.36336444	0.180046508	-2.018169885	0.043573571	0.348491091	down_in_case
PRJNA8274(	ENSGALG00010016942	ZBTB40	case	control	0.180443061	0.126994895	1.420868613	0.155354959	0.564424775	up_in_case
PRJNA8274(	ENSGALG00010016943	PCNX4	case	control	0.147723714	0.069083237	2.138343839	0.032488847	0.310633901	up_in_case
PRJNA8274(	ENSGALG00010016944	OPRD1	case	control	-0.872960124	0.316639349	-2.756954016	0.005834256	0.133274528	down_in_case
PRJNA8274(	ENSGALG00010016945	BDH2	case	control	0.050615344	0.074871181	0.676032391	0.49902008	0.840142058	up_in_case
PRJNA8274(	ENSGALG00010016946	TAF1	case	control	-0.160848479	0.034105346	-4.716224817	2.40261E-06	0.000520565	down_in_case
PRJNA8274(	ENSGALG00010016947	CANX	case	control	0.066714674	0.044032883	1.515110301	0.129744473	0.532166684	up_in_case
PRJNA8274(	ENSGALG00010016949	LUC7L	case	control	0.084932226	0.045801824	1.85434155	0.063690311	0.409661205	up_in_case
PRJNA8274(	ENSGALG00010016950	ENSGALG00010016950	case	control	0.055634701	0.823765873	0.067537031	0.946154183	0.99041101	up_in_case
PRJNA8274(	ENSGALG00010016952	GINS1	case	control	0.018722513	0.111030548	0.16862488	0.86609171	0.973289701	up_in_case
PRJNA8274(	ENSGALG00010016953	GJA4	case	control	-0.135523677	0.08903535	-1.522133368	0.127975666	0.529650968	down_in_case
PRJNA8274(	ENSGALG00010016955	ENSGALG00010016955	case	control	-0.209343957	0.181754834	-1.151793066	0.249406118	0.674421685	down_in_case
PRJNA8274(	ENSGALG00010016956	CENPE	case	control	-0.072864371	0.125763382	-0.579376679	0.562335037	0.871034556	down_in_case
PRJNA8274(	ENSGALG00010016957	WNT4	case	control	-0.179837829	0.171077284	-1.051208109	0.29316302	0.716008742	down_in_case
PRJNA8274(	ENSGALG00010016958	TLR15	case	control	0.763457745	0.39131293	1.951015891	0.051055155	0.369565336	up_in_case
PRJNA8274(	ENSGALG00010016959	PERCC1	case	control	0.943416096	0.724363561	1.302406894	0.192777327	0.615090794	up_in_case
PRJNA8274(	ENSGALG00010016960	GJB3	case	control	0.698228785	0.759052089	0.919869395	0.357641014	0.763851675	up_in_case
PRJNA8274(	ENSGALG00010016961	EIF4A3	case	control	-0.049229157	0.045673718	-1.077844303	0.281103245	0.705762606	down_in_case
PRJNA8274(	ENSGALG00010016962	SQOR	case	control	-0.072618411	0.085677917	-0.847574424	0.396675023	0.788531716	down_in_case
PRJNA8274(	ENSGALG00010016964	MAML1	case	control	0.072600524	0.048313737	1.502689072	0.132919242	0.536224013	up_in_case
PRJNA8274(	ENSGALG00010016965	SLC25A33	case	control	-0.085996154	0.103700915	-0.829270929	0.406951117	0.794349027	down_in_case
PRJNA8274(	ENSGALG00010016966	KDM3A	case	control	-0.023803407	0.042289772	-0.562864395	0.573527227	0.877542213	down_in_case
PRJNA8274(	ENSGALG00010016967	ERLEC1	case	control	0.05879495	0.04737538	1.241044393	0.21458935	0.639430653	up_in_case
PRJNA8274(	ENSGALG00010016968	C15orf61	case	control	-0.225477462	0.088046378	-2.560894231	0.010440314	0.182961378	down_in_case
PRJNA8274(	ENSGALG00010016969	CHMP3	case	control	-0.036217738	0.076863352	-0.471196445	0.637500457	0.899378897	down_in_case
PRJNA8274(	ENSGALG00010016970	SYNE3	case	control	0.067888026	0.107926949	0.629018298	0.529337077	0.85444409	up_in_case
PRJNA8274(	ENSGALG00010016972	ASB3	case	control	-0.073053062	0.09079095	-0.804629346	0.421033602	0.802114148	down_in_case
PRJNA8274(	ENSGALG00010016973	PLSCR1	case	control	0.078193291	0.104332355	0.749463491	0.453577896	0.821282604	up_in_case
PRJNA8274(	ENSGALG00010016975	MALL	case	control	-0.359444108	0.211315137	-1.700986086	0.0889456	0.47143146	down_in_case
PRJNA8274(	ENSGALG00010016977	CLCN7	case	control	0.17849847	0.054815866	3.256328547	0.001128631	0.05223113	up_in_case
PRJNA8274(	ENSGALG00010016978	FAM181A	case	control	-0.50162853	0.139768776	-3.588988497	0.000331963	0.024516337	down_in_case
PRJNA8274(	ENSGALG00010016979	PLSCR5	case	control	-0.390806805	0.301823672	-1.294818271	0.195382928	0.618546793	down_in_case
PRJNA8274(	ENSGALG00010016980	NPHP1	case	control	0.131197885	0.084747187	1.548109035	0.121596047	0.519686058	up_in_case
PRJNA8274(	ENSGALG00010016981	ENSGALG00010016981	case	control	0.14392822	0.492417907	0.292288762	0.770065851	0.945428692	up_in_case
PRJNA8274(	ENSGALG00010016982	GLRX5	case	control	0.063402505	0.078335849	0.809367694	0.418303679	0.800447292	up_in_case
PRJNA8274(	ENSGALG00010016983	ZFAND2A	case	control	0.42108026	0.120332807	3.499297237	0.000466486	0.03102676	up_in_case
PRJNA8274(	ENSGALG00010016984	RNF103	case	control	-0.047592568	0.057013615	-0.834757939	0.403854012	0.79260318	down_in_case
PRJNA8274(	ENSGALG00010016985	ENSGALG00010016985	case	control	-0.048840556	0.171123143	-0.285411751	0.775328706	0.947087967	down_in_case
PRJNA8274(	ENSGALG00010016986	GFER	case	control	-0.056806764	0.088889729	-0.639070052	0.522777361	0.851157494	down_in_case
PRJNA8274(	ENSGALG00010016987	ASB2	case	control	-0.451873394	0.120446299	-3.751658591	0.000175669	0.016102953	down_in_case
PRJNA8274(	ENSGALG00010016988	ECI1	case	control	-0.034703745	0.075615643	-0.458949277	0.646270591	0.902693223	down_in_case
PRJNA8274(	ENSGALG00010016989	RHOG2	case	control	0.056048106	0.060233858	0.930508326	0.352107955	0.760831854	up_in_case
PRJNA8274(	ENSGALG00010016991	PDLIM3	case	control	-0.37581643	0.135862667	-2.766149363	0.005672255	0.130407144	down_in_case
PRJNA8274(	ENSGALG00010016993	ENSGALG00010016993	case	control	-0.322581287	0.104186778	-3.096182569	0.001960296	0.072174149	down_in_case
PRJNA8274(	ENSGALG00010016994	ATL2	case	control	-0.079244504	0.053266056	-1.487711134	0.13682709	0.539854583	down_in_case
PRJNA8274(	ENSGALG00010016996	ZIC4	case	control	0.097676993	0.521701912	0.187227592	0.851482192	0.969234083	up_in_case
PRJNA8274(	ENSGALG00010016999	ITGB1BP2	case	control	-0.160987027	0.095597994	-1.684000055	0.092181658	0.476713154	down_in_case
PRJNA8274(	ENSGALG00010017001	TPCN3	case	control	-0.054715746	0.081301111	-0.673001206	0.500946523	0.840987941	down_in_case
PRJNA8274(	ENSGALG00010017003	CPZ	case	control	0.033115327	0.170605334	0.194104878	0.846093762	0.967563913	up_in_case
PRJNA8274(	ENSGALG00010017004	CX3CR1	case	control	0.479187692	0.458629182	1.044825998	0.296103397	0.719662167	up_in_case
PRJNA8274(	ENSGALG00010017006	DNAH3	case	control	0.030445006	0.172489237	0.176503799	0.859898165	0.971828322	up_in_case
PRJNA8274(	ENSGALG00010017008	RTN1	case	control	-0.172264252	0.237187068	-0.726280121	0.467667064	0.82808804	down_in_case
PRJNA8274(	ENSGALG00010017010	NONO	case	control	0.008567915	0.057454191	0.149126024	0.881454191	0.976737306	up_in_case
PRJNA8274(	ENSGALG00010017012	SYNGR3	case	control	0.142077563	0.210311777	0.675556857	0.499322041	0.840142058	up_in_case
PRJNA8274(	ENSGALG00010017016	ZIC1	case	control	0.008982044	0.460582067	0.019501506	0.984441035	0.996591771	up_in_case
PRJNA8274(	ENSGALG00010017017	ENSGALG00010017017	case	control	0.35276526	0.268899608	1.311884619	0.189559068	0.612863366	up_in_case
PRJNA8274(	ENSGALG00010017018	HP1BP3	case	control	-0.071453863	0.044276318	-1.613816716	0.106567188	0.496711468	down_in_case

PRJNA8274(	ENSGALG00010017020	GJB5	case	control	0.26106906	0.401320287	0.650525448	0.515352869	0.847750297	up_in_case
PRJNA8274(	ENSGALG00010017022	CCR4	case	control	-0.13745993	0.223288684	-0.615615298	0.538148452	0.857740557	down_in_case
PRJNA8274(	ENSGALG00010017024	BUB1	case	control	-0.048767396	0.101284831	-0.481487655	0.630169951	0.89637578	down_in_case
PRJNA8274(	ENSGALG00010017033	LDAF1	case	control	-0.117683754	0.062300118	-1.888981245	0.058894342	0.395497471	down_in_case
PRJNA8274(	ENSGALG00010017034	ZMYM3	case	control	-0.044706828	0.060911051	-0.733969081	0.462967582	0.825091817	down_in_case
PRJNA8274(	ENSGALG00010017035	PIK3CD	case	control	0.115559952	0.115678433	0.998975777	0.317806426	0.738054349	up_in_case
PRJNA8274(	ENSGALG00010017038	ZNF598	case	control	-0.000908901	0.040420069	-0.022486384	0.982059973	0.996591771	down_in_case
PRJNA8274(	ENSGALG00010017039	MAP2K5	case	control	0.091281539	0.040674133	2.244215949	0.02481851	0.279452518	up_in_case
PRJNA8274(	ENSGALG00010017041	JKAMP	case	control	0.014066171	0.06186159	0.227381337	0.820127231	0.958669267	up_in_case
PRJNA8274(	ENSGALG00010017042	HNRNPLL	case	control	-0.0587351	0.043453542	-1.351675772	0.176479059	0.595527443	down_in_case
PRJNA8274(	ENSGALG00010017045	GLB1	case	control	0.034102421	0.054553462	0.62511928	0.531892775	0.855755691	up_in_case
PRJNA8274(	ENSGALG00010017049	TRAF3	case	control	0.087757973	0.099790282	0.879424045	0.3791714	0.777369321	up_in_case
PRJNA8274(	ENSGALG00010017050	L3HYPDH	case	control	-0.22109545	0.17401471	-1.27055609	0.203886617	0.626063693	down_in_case
PRJNA8274(	ENSGALG00010017054	GALM	case	control	0.20319717	0.081267236	2.500357835	0.012406792	0.195901209	up_in_case
PRJNA8274(	ENSGALG00010017055	GPR135	case	control	0.184361479	0.238844187	0.771890163	0.440179484	0.81275826	up_in_case
PRJNA8274(	ENSGALG00010017056	AGTR1	case	control	-0.210193019	0.111248819	-1.889395508	0.058838852	0.395497471	down_in_case
PRJNA8274(	ENSGALG00010017057	NDUFAB1	case	control	-0.048976683	0.090498485	-0.541187879	0.588378091	0.882771329	down_in_case
PRJNA8274(	ENSGALG00010017060	CLSTN1	case	control	-0.051750337	0.120900579	-0.428040439	0.668621679	0.913032039	down_in_case
PRJNA8274(	ENSGALG00010017063	TMPPE	case	control	-0.089144623	0.104757558	-0.850961253	0.394790877	0.786978937	down_in_case
PRJNA8274(	ENSGALG00010017065	VRK2	case	control	-0.057299923	0.088421484	-0.648031678	0.516964462	0.848054666	down_in_case
PRJNA8274(	ENSGALG00010017068	ZP2	case	control	0.482768938	0.731334168	0.660120857	0.509176275	0.844493243	up_in_case
PRJNA8274(	ENSGALG00010017070	BLOC1S6	case	control	0.189699506	0.088334376	2.147516219	0.031752207	0.3098361	up_in_case
PRJNA8274(	ENSGALG00010017077	CTNNBIP1	case	control	-0.041798007	0.063820885	-0.654926787	0.512514893	0.846105168	down_in_case
PRJNA8274(	ENSGALG00010017079	ENSGALG00010017079	case	control	-0.356131022	0.791269089	-0.450075742	0.652655828	0.905885495	down_in_case
PRJNA8274(	ENSGALG00010017080	PALB2	case	control	0.009969307	0.093020265	0.107173491	0.914651345	0.983126446	up_in_case
PRJNA8274(	ENSGALG00010017083	ENSGALG00010017083	case	control	-0.214975114	0.375281537	-0.572836905	0.566755129	0.87333105	down_in_case
PRJNA8274(	ENSGALG00010017085	ENSGALG00010017085	case	control	0.002830928	0.072381364	0.039111289	0.968801661	0.992761697	up_in_case
PRJNA8274(	ENSGALG00010017089	CRTAP	case	control	0.194807137	0.09710828	2.006081638	0.044847539	0.351986719	up_in_case
PRJNA8274(	ENSGALG00010017092	ANKS4B	case	control	0.065097769	0.403168947	0.161465237	0.871726989	0.975062028	up_in_case
PRJNA8274(	ENSGALG00010017094	SORBS2	case	control	0.121411755	0.124280737	0.976915313	0.32861108	0.745445783	up_in_case
PRJNA8274(	ENSGALG00010017096	DCTN5	case	control	-0.02252836	0.05891701	-0.382374454	0.702183636	0.923231962	down_in_case
PRJNA8274(	ENSGALG00010017098	DAAM1	case	control	0.112653647	0.062560578	1.800713026	0.071748123	0.432759285	up_in_case
PRJNA8274(	ENSGALG00010017100	SUSD5	case	control	0.326359126	0.5264956	0.619870567	0.535343005	0.857009451	up_in_case
PRJNA8274(	ENSGALG00010017101	CSMD2	case	control	-0.304657999	0.156446333	-1.947364274	0.051491078	0.371479245	down_in_case
PRJNA8274(	ENSGALG00010017104	FANCL	case	control	-0.03155794	0.109262814	-0.288825985	0.772714546	0.946370161	down_in_case
PRJNA8274(	ENSGALG00010017105	PLK1	case	control	-0.10063378	0.103166927	-0.975446142	0.329339005	0.745771619	down_in_case
PRJNA8274(	ENSGALG00010017106	CRYM	case	control	-0.043477621	0.078191327	-0.556041475	0.578182502	0.878734167	down_in_case
PRJNA8274(	ENSGALG00010017109	ITGB1BP3	case	control	-0.082846324	0.069489837	-1.192207784	0.233179782	0.657982411	down_in_case
PRJNA8274(	ENSGALG00010017110	BRF1	case	control	-0.009439273	0.075488378	-0.125042729	0.900489723	0.98028579	down_in_case
PRJNA8274(	ENSGALG00010017111	GJB1	case	control	0.551464211	0.360649345	1.529086962	0.126242887	0.525954642	up_in_case
PRJNA8274(	ENSGALG00010017112	LZIC	case	control	-0.004182851	0.054659239	-0.076525969	0.939000654	0.989551456	down_in_case
PRJNA8274(	ENSGALG00010017115	LGALSL	case	control	-0.241524675	0.113769403	-2.122931735	0.033759573	0.31692586	down_in_case
PRJNA8274(	ENSGALG00010017116	ENSGALG00010017116	case	control	-0.038990432	0.214569865	-0.181714391	0.855586872	0.970656588	down_in_case
PRJNA8274(	ENSGALG00010017119	BTBD6	case	control	0.061631563	0.081020075	0.760694964	0.446839283	0.817833512	up_in_case
PRJNA8274(	ENSGALG00010017120	ERN2	case	control	0.033240626	0.078475162	0.423581497	0.671871052	0.913676733	up_in_case
PRJNA8274(	ENSGALG00010017122	NCBP2	case	control	-0.00156055	0.112847692	-0.013828819	0.98896655	0.997164011	down_in_case
PRJNA8274(	ENSGALG00010017127	SOX8	case	control	0.053479788	0.096606852	0.553581727	0.579865135	0.879142433	up_in_case
PRJNA8274(	ENSGALG00010017131	TLR3	case	control	0.161833368	0.334280747	0.484124106	0.628297794	0.895957166	up_in_case
PRJNA8274(	ENSGALG00010017132	TMEM108	case	control	-0.102007144	0.16742046	-0.609287202	0.542334088	0.85975913	down_in_case
PRJNA8274(	ENSGALG00010017133	WDR20	case	control	-0.119419499	0.103419225	-1.15471276	0.248208072	0.672435338	down_in_case
PRJNA8274(	ENSGALG00010017135	ENSGALG00010017135	case	control	-0.070502165	0.131104014	-0.537757482	0.590744489	0.882949889	down_in_case
PRJNA8274(	ENSGALG00010017136	ENSGALG00010017136	case	control	0.120190873	0.246513847	0.487562359	0.625859863	0.895445894	up_in_case
PRJNA8274(	ENSGALG00010017139	COL9A2	case	control	0.4473654	0.822526622	0.543891697	0.586516003	0.881932581	up_in_case
PRJNA8274(	ENSGALG00010017140	UQCRC2	case	control	-0.134054227	0.072928402	-1.838162134	0.066038518	0.41627012	down_in_case
PRJNA8274(	ENSGALG00010017141	AFTPH	case	control	-0.103301122	0.052460492	-1.969122244	0.048939055	0.36379167	down_in_case
PRJNA8274(	ENSGALG00010017142	RBP7	case	control	-0.431315884	0.541635955	-0.796320628	0.425845699	0.804969398	down_in_case
PRJNA8274(	ENSGALG00010017143	FAM149A	case	control	0.244777175	0.177905348	1.375884305	0.168857455	0.585038804	up_in_case
PRJNA8274(	ENSGALG00010017144	CPB1	case	control	0.213188046	0.460638007	0.462810368	0.643500299	0.902038523	up_in_case
PRJNA8274(	ENSGALG00010017147	SRSF7L	case	control	0.061647192	0.051871994	1.188448483	0.234656765	0.659294991	up_in_case
PRJNA8274(	ENSGALG00010017148	MOK	case	control	-0.059363859	0.31571709	-0.188028652	0.850854191	0.969234083	down_in_case
PRJNA8274(	ENSGALG00010017152	CYP4V2	case	control	0.428509096	0.332777969	1.287672669	0.197859931	0.621110634	up_in_case
PRJNA8274(	ENSGALG00010017153	SLC35F4	case	control	-0.537645798	0.398528948	-1.349075898	0.177312589	0.5959216	down_in_case
PRJNA8274(	ENSGALG00010017154	PIGZ	case	control	-0.176260796	0.207029153	-0.851381522	0.394557453	0.786978937	down_in_case
PRJNA8274(	ENSGALG00010017162	BCL11A	case	control	-0.134069828	0.092308114	-1.4524165	0.146385831	0.551598784	down_in_case
PRJNA8274(	ENSGALG00010017163	CDV3	case	control	0.002574018	0.050679132	0.050790499	0.959492462	0.992056045	up_in_case
PRJNA8274(	ENSGALG00010017167	SMAP2	case	control	0.021780372	0.053869763	0.404315352	0.685980838	0.917034645	up_in_case
PRJNA8274(	ENSGALG00010017169	CCDC198	case	control	0.251620599	0.277330048	0.907296562	0.364249992	0.770055025	up_in_case
PRJNA8274(	ENSGALG00010017174	DHX57	case	control	-0.010636185	0.056398102	-0.188591194	0.850413235	0.969234083	down_in_case
PRJNA8274(	ENSGALG00010017180	ENSGALG00010017180	case	control	-0.026306107	0.421229255	-0.062450807	0.950203835	0.991497374	down_in_case
PRJNA8274(	ENSGALG00010017183	NFYC	case	control	-0.055587498	0.043061993	-1.290871491	0.196748242	0.619986749	down_in_case
PRJNA8274(	ENSGALG00010017184	CARD11	case	control	0.038471468	0.156756646	0.24542161	0.806129998	0.9556344	up_in_case
PRJNA8274(	ENSGALG00010017185	NAA30	case	control	-0.073703968	0.05511774	-1.337209531	0.181154252	0.601788276	down_in_case
PRJNA8274(	ENSGALG00010017187	ZNF839	case	control	0.05515375	0.074875769	0.736603449	0.461363537	0.82454265	up_in_case
PRJNA8274(	ENSGALG00010017188	ENSGALG00010017188	case	control	0.050954555	0.096180302	0.529781613	0.596263355	0.884680373	up_in_case
PRJNA8274(	ENSGALG00010017190	STK26	case	control	0.05050217	0.058372477	0.865170932	0.386944977	0.782604263	up_in_case
PRJNA8274(	ENSGALG00010017198	TOPBP1	case	control	-0.036724926	0.056158184	-0.653955018	0.513140786	0.846564516	down_in_case
PRJNA8274(	ENSGALG00010017200	KCNQ4	case	control	-0.085889808	0.173669318	-0.494559479	0.620911111	0.893114457	down_in_case
PRJNA8274(	ENSGALG00010017201	AP5M1	case	control	0.206220447	0.10526902	1.958985141	0.050114527	0.366754215	up_in_case
PRJNA8274(	ENSGALG00010017207	ENSGALG00010017207	case	control	-0.250349084	0.194516582	-1.287032093	0.198083102	0.621485489	down_in_case
PRJNA8274(	ENSGALG00010017208	CINP	case	control	-0.044390429	0.093278001	-0.47589387	0.634149989	0.898107199	down_in_case
PRJNA8274(	ENSGALG00010017211	UBFD1	case	control	0.034974899	0.048826335	0.716312183	0.473798605	0.829698758	up_in_case
PRJNA8274(	ENSGALG00010017212	TINAGL1	case	control	-0.073791445	0.103078753	-0.715874446	0.474068879	0.82979716	down_in_case
PRJNA8274(	ENSGALG00010017218	ARHGEF33	case	control	-0.093323384	0.481342348	-0.193881517	0.846268657	0.967616222	down_in_case
PRJNA8274(	ENSGALG00010017219	EARS2	case	control	-0.130876753	0.100783763	-1.298589661	0.194084788	0.617169773	down_in_case
PRJNA8274(	ENSGALG00010017221	SPPL2A	case	control	-0.031628482	0.055085211	-0.574173737	0.56585024	0.872906976	down_in_case
PRJNA8274(	ENSGALG00010017223	NOC2L	case	control	0.018792359	0.065964894	0.284884237	0.775732833	0.947146475	up_in_case
PRJNA8274(	ENSGALG00010017226	PEF1	case	control	0.222061598	0.081594782	2.721517163	0.006498301	0.139528086	up_in_case
PRJNA8274(	ENSGALG00010017228	RAP2C	case	control	0.010332316	0.046519448	0.22210746	0.82423023	0.960422238	up_in_case
PRJNA8274(	ENSGALG00010017240	MELTF	case	control	0.479195404	0.293938764	1.630255897	0.103047425	0.492515853	up_in_case
PRJNA8274(	ENSGALG00010017246	GGA2	case	control	0.053287441	0.077365027	0.688779445	0.490962073	0.836900422	up_in_case
PRJNA8274(	ENSGALG00010017248	TECPR2	case	control	-0.090599851	0.041379809	-2.189470024	0.028562695	0.300549329	down_in_case
PRJNA8274(	ENSGALG00010017250	COL16A1	case	control	0.018524146	0.113214898	0.163619329	0.870030827	0.974794532	up_in_case
PRJNA8274(	ENSGALG00010017252	EXOC5	case	control	0.037709123	0.064063921	0.588617162	0.556118119	0.867878646	up_in_case
PRJNA8274(	ENSGALG00010017256	CARHSP1	case	control	0.094040521	0.057261463	1.642300348	0.100527775	0.490291708	up_in_case
PRJNA8274(	ENSGALG00010017263	COG7	case	control	-0.017726182	0.051816047	-0.342098303	0.732276915	0.933793462	down_in_case
PRJNA8274(	ENSGALG00010017264	PIAS1	case	control	-0.011892126	0.059432097	-0.200096011	0.841405493	0.966045648	down_in_case
PRJNA8274(	ENSGALG00010017266	PMM2	case	control	-0.053092853	0.059004052	-0.899817063	0.368217612	0.771770041	down_in_case
PRJNA8274(	ENSGALG00010017272	NRXN1	case	control	-0.12246258	0.192101882	-0.637487664	0.523807242	0.851961279	down_in_case
PRJNA8274(	ENSGALG00010017278	CCR5	case	control	0.205595529	0.142854498	1.43919535	0.150095183	0.557230917	up_in_case
PRJNA8274(	ENSGALG00010017280	SOS1	case	control	-0.075649935	0.049507809	-1.52804044	0.126502498	0.526480127	down_in_case
PRJNA8274(	ENSGALG00010017283	INF2	case	control	0.0799141	0.082486967	0.968808799	0.332640596	0.748503677	up_in_case
PRJNA8274(	ENSGALG00010017284	ENSGALG00010017284	case	control	0.044485482	0.161769796	0.274992509	0.783321993	0.949518053	up_in_case
PRJNA8274(	ENSGALG00010017289	ADGRB2	case	control	-0.109189244	0.204527019	-0.533862196	0.593436879	0.883625114	down_in_case
PRJNA8274(	ENSGALG00010017290	DACT1	case	control	0.149064187	0.058458953	2.549894918	0.010775539	0.184728043	up_in_case
PRJNA8274(	ENSGALG00010017296	SCNN1G	case	control	-0.115636876	0.103461731	-1.117677763	0.263704643	0.68964455	down_in_case
PRJNA8274(	ENSGALG00010017299	USP31	case	control	0.004268147	0.0725184	0.058856059	0.953066757	0.992056045	up_in_case
PRJNA8274(	ENSGALG00010017300	TRPM7	case	control	0.105740289	0.062046239	1.704217547	0.088340452	0.46961653	up_in_case
PRJNA8274(	ENSGALG00010017301	LHCGR	case	control	-0.354538811	0.275958564	-1.284753792	0.198878334	0.622830756	down_in_case
PRJNA8274(	ENSGALG00010017304	ANKRD9	case	control	-0.004684677	0.235532254	-0.019889748	0.984131324	0.996591771	down_in_case
PRJNA8274(	ENSGALG00010017305	HS3ST2	case	control	-0.442015047	0.506167322	-0.873258758	0.382522055	0.77998936	down_in_case
PRJNA8274(	ENSGALG00010017307	TMEM186	case	control	0.158159286	0.117931285	1.3411139	0.179883475	0.600808892	up_in_case
PRJNA8274(	ENSGALG00010017310	SAMD11	case	control	0.213677545	0.081756668	2.613579417	0.008959926	0.168588076	up_in_case
PRJNA8274(	ENSGALG00010017312	PTP4A2	case	control	-0.150858897	0.063915387	-2.360290754	0.018260617	0.241560424	down_in_case
PRJNA8274(	ENSGALG00010017313	MAP4K3	case	control	0.040867367	0.046365925	0.881409506	0.378096214	0.776556765	up_in_case
PRJNA8274(	ENSGALG00010017314	CALML4	case	control	0.068110028	0.086211178	0.790037083	0.429506112	0.806015686	up_in_case
PRJNA8274(	ENSGALG00010017315	HS3ST4	case	control	1.367287164	0.859377271	1.591020859	0.111604881	0.504204693	up_in_case
PRJNA8274(	ENSGALG00010017318	ADSS1	case	control	-0.373554902	0.11377896	-3.283163257	0.001026492	0.049590661	down_in_case
PRJNA8274(	ENSGALG00010017322	KHDRBS1	case	control	0.01419957	0.039542402	0.35909731	0.719522294	0.92854154	up_in_case
PRJNA8274(	ENSGALG00010017323	ENSGALG00010017323	case	control	0.048527272	0.055906298	0.868010833	0.385388402	0.781541515	up_in_case
PRJNA8274(	ENSGALG00010017326	STON1	case	control	0.119900438	0.106025691	1.130862126	0.258113126	0.685553066	up_in_case
PRJNA8274(	ENSGALG00010017327	CLN6	case	control	-0.00062613	0.087301993	-0.007171999	0.994277621	0.998301153	down_in_case
PRJNA8274(	ENSGALG00010017332	FGFR1	case	control	-0.024022304	0.052819566	-0.454799349	0.649253599	0.904011281	down_in_case
PRJNA8274(	ENSGALG00010017334	KDM8	case	control	-0.094205124	0.135963908	-0.692868611	0.48839201	0.835960376	down_in_case
PRJNA8274(	ENSGALG00010017335	THUMPD2	case	control	-0.067947557	0.090963667	-0.746974693	0.455078837	0.821670122	down_in_case
PRJNA8274(	ENSGALG00010017337	ABAT	case	control	0.089058383	0.076095779	1.170345908	0.241861794	0.666243691	up_in_case
PRJNA8274(	ENSGALG00010017338	MBNL3	case	control	-0.119106044	0.068074535	-1.749641668	0.080180165	0.451585805	down_in_case

PRJNA8274(	ENSGALG00010017340	SIVA1	case	control	0.047782879	0.106648001	0.448042893	0.654122241	0.906478152	up_in_case
PRJNA8274(	ENSGALG00010017341	TA3	case	control	0.008760785	0.059335875	0.147647364	0.882621074	0.976801393	up_in_case
PRJNA8274(	ENSGALG00010017343	DLG1	case	control	-0.07910478	0.041891358	-1.888331691	0.058981435	0.395497471	down_in_case
PRJNA8274(	ENSGALG00010017344	RERE	case	control	-0.240193497	0.078153143	-3.073369636	0.002116561	0.075874574	down_in_case
PRJNA8274(	ENSGALG00010017346	GM2A	case	control	0.091037923	0.168326179	0.540842331	0.588616263	0.882805047	up_in_case
PRJNA8274(	ENSGALG00010017347	TMEM39B	case	control	-0.065690082	0.05899206	-1.113541081	0.265476115	0.691495163	down_in_case
PRJNA8274(	ENSGALG00010017349	ENSGALG00010017349	case	control	0.015395163	0.082984388	0.185518793	0.852822142	0.969809672	up_in_case
PRJNA8274(	ENSGALG00010017351	CCR2	case	control	-0.070186987	0.109494466	-0.641009447	0.521516545	0.850363352	down_in_case
PRJNA8274(	ENSGALG00010017352	GPCPD1	case	control	0.116413343	0.075362577	1.544710219	0.122416372	0.520832363	up_in_case
PRJNA8274(	ENSGALG00010017354	FEM1B	case	control	-0.064154693	0.043968219	-1.459115127	0.144533423	0.548736091	down_in_case
PRJNA8274(	ENSGALG00010017356	NSMCE1	case	control	-0.217176162	0.078052067	-2.782452395	0.005394978	0.127480206	down_in_case
PRJNA8274(	ENSGALG00010017359	EHD3	case	control	0.002758104	0.051210992	0.053857665	0.957048566	0.992056045	up_in_case
PRJNA8274(	ENSGALG00010017361	METTL22	case	control	-0.146688715	0.062490872	-2.347362271	0.018906862	0.245565961	down_in_case
PRJNA8274(	ENSGALG00010017362	ENO1	case	control	-0.079750282	0.083483009	-0.955287591	0.339432281	0.751259351	down_in_case
PRJNA8274(	ENSGALG00010017363	TIMM9	case	control	-0.094541609	0.074896244	-1.262301059	0.206840476	0.63066499	down_in_case
PRJNA8274(	ENSGALG00010017364	AKT1	case	control	-0.000142928	0.051842735	-0.002756955	0.997800271	0.999142415	down_in_case
PRJNA8274(	ENSGALG00010017365	CD8BP	case	control	-0.521886352	0.534306568	-0.976754514	0.3286907	0.745453498	down_in_case
PRJNA8274(	ENSGALG00010017367	TIMD4	case	control	0.110706158	0.240608672	0.460108764	0.645438154	0.902490037	up_in_case
PRJNA8274(	ENSGALG00010017368	ERLIN2	case	control	0.018080242	0.030749582	0.587983328	0.556543483	0.867878646	up_in_case
PRJNA8274(	ENSGALG00010017371	TMEM114	case	control	0.640604337	0.570661282	1.122564921	0.261622321	0.687516348	up_in_case
PRJNA8274(	ENSGALG00010017372	ENSGALG00010017372	case	control	0.039344244	0.056347814	0.69823904	0.485027715	0.834423421	up_in_case
PRJNA8274(	ENSGALG00010017373	RP1-27O5.3	case	control	0.117133525	0.094076355	1.245089962	0.213098684	0.638582746	up_in_case
PRJNA8274(	ENSGALG00010017375	BDH1A	case	control	0.062454085	0.085054157	0.734286096	0.46277439	0.825091817	up_in_case
PRJNA8274(	ENSGALG00010017376	GALNT14	case	control	0.157094577	0.209291007	0.750603572	0.452891271	0.821045895	up_in_case
PRJNA8274(	ENSGALG00010017377	USP8	case	control	-0.08221113	0.054731315	-1.502085789	0.133074954	0.536350577	down_in_case
PRJNA8274(	ENSGALG00010017378	ENSGALG00010017378	case	control	-0.048774568	0.387288096	-0.125938724	0.89978043	0.98028579	down_in_case
PRJNA8274(	ENSGALG00010017380	MARCKSL1	case	control	0.123823355	0.060545294	2.04513591	0.040841461	0.337874876	up_in_case
PRJNA8274(	ENSGALG00010017381	CA6	case	control	-0.07420766	0.231546014	-0.320487747	0.748598617	0.939503405	down_in_case
PRJNA8274(	ENSGALG00010017383	ENSGALG00010017383	case	control	0.2522863	0.100922264	2.49980817	0.012426057	0.195901209	up_in_case
PRJNA8274(	ENSGALG00010017384	ARID4A	case	control	0.000361493	0.057170241	0.006323099	0.99495493	0.998586153	up_in_case
PRJNA8274(	ENSGALG00010017386	ENSGALG00010017386	case	control	0.169295281	0.648405022	0.261094956	0.794019281	0.95280257	up_in_case
PRJNA8274(	ENSGALG00010017387	HS6ST2	case	control	-0.094436678	0.057826387	-1.633107	0.102446495	0.491460867	down_in_case
PRJNA8274(	ENSGALG00010017388	PLPBP	case	control	-0.005367633	0.053910844	-0.099564993	0.920689687	0.984952684	down_in_case
PRJNA8274(	ENSGALG00010017390	DDX24	case	control	0.037181131	0.069661187	0.533742421	0.593519755	0.883625114	up_in_case
PRJNA8274(	ENSGALG00010017391	CRAMP1	case	control	-0.059394696	0.062059052	-0.957067394	0.33853324	0.751259351	down_in_case
PRJNA8274(	ENSGALG00010017393	VDAC1	case	control	-0.056542116	0.089302106	-0.633155459	0.526632129	0.85251736	down_in_case
PRJNA8274(	ENSGALG00010017395	BRF2	case	control	0.016370693	0.090796716	0.180300491	0.85691667	0.971145061	up_in_case
PRJNA8274(	ENSGALG00010017396	CCR8L	case	control	0.00081805	0.222339043	0.003679291	0.997064357	0.998921977	up_in_case
PRJNA8274(	ENSGALG00010017398	SLC22A5	case	control	0.145948923	0.116879843	1.248709104	0.21177149	0.636501957	up_in_case
PRJNA8274(	ENSGALG00010017399	CPXM1	case	control	-0.120223574	0.125577857	-0.957362839	0.338384148	0.751259351	down_in_case
PRJNA8274(	ENSGALG00010017400	GPC4	case	control	-0.012427322	0.059706478	-0.208140265	0.835119449	0.963703043	down_in_case
PRJNA8274(	ENSGALG00010017402	SLC2A5	case	control	-0.048395649	0.200666079	-0.241175039	0.809419456	0.955875648	down_in_case
PRJNA8274(	ENSGALG00010017403	ITGA11	case	control	0.261200101	0.188723694	1.384034482	0.166347897	0.58174452	up_in_case
PRJNA8274(	ENSGALG00010017406	IFT140	case	control	0.101189001	0.073812184	1.370898338	0.17040665	0.587325883	up_in_case
PRJNA8274(	ENSGALG00010017407	EPN2	case	control	0.034538599	0.036709138	0.940871993	0.346770462	0.757096367	up_in_case
PRJNA8274(	ENSGALG00010017409	GPC3	case	control	0.03949719	0.342214868	0.115416347	0.908115123	0.981730793	up_in_case
PRJNA8274(	ENSGALG00010017411	RAB11FIP1	case	control	0.050612671	0.076276791	0.663539601	0.506985026	0.843472666	up_in_case
PRJNA8274(	ENSGALG00010017413	ZIC3	case	control	1.467377715	0.68527554	2.141295917	0.032250182	0.309838273	up_in_case
PRJNA8274(	ENSGALG00010017414	ENSGALG00010017414	case	control	-0.013121	0.104771388	-0.125234577	0.900337844	0.98028579	down_in_case
PRJNA8274(	ENSGALG00010017415	HDAC1	case	control	-0.099959789	0.0561123	-1.781423833	0.074843238	0.439711709	down_in_case
PRJNA8274(	ENSGALG00010017418	CHGB	case	control	0.448417988	0.178806909	2.50783368	0.012147383	0.19369863	up_in_case
PRJNA8274(	ENSGALG00010017419	B9D1	case	control	0.100331656	0.076753847	1.307187324	0.1911491	0.61425441	up_in_case
PRJNA8274(	ENSGALG00010017421	GPR157	case	control	-0.346913652	0.113695585	-3.051249986	0.002278907	0.078715887	down_in_case
PRJNA8274(	ENSGALG00010017422	ENSGALG00010017422	case	control	0.290492093	0.208235489	1.395017216	0.163010648	0.576993135	up_in_case
PRJNA8274(	ENSGALG00010017423	TMEM204	case	control	0.04060874	0.056758536	0.715464906	0.474321818	0.82979716	up_in_case
PRJNA8274(	ENSGALG00010017424	APOD	case	control	0.263024859	0.403716981	0.651508039	0.51471859	0.847493921	up_in_case
PRJNA8274(	ENSGALG00010017425	H6PD	case	control	0.02005774	0.094239354	0.212838258	0.831453122	0.962371901	up_in_case
PRJNA8274(	ENSGALG00010017428	PSMA3	case	control	-0.019106102	0.062124156	-0.307547069	0.758427002	0.941903352	down_in_case
PRJNA8274(	ENSGALG00010017429	TELO2	case	control	0.041645965	0.081740624	0.509489199	0.610409367	0.888794823	up_in_case
PRJNA8274(	ENSGALG00010017430	CACNA1H	case	control	-0.084359066	0.125331287	-0.673088643	0.500890899	0.840987941	down_in_case
PRJNA8274(	ENSGALG00010017431	FYCO1	case	control	-0.068827837	0.078543867	-0.876298041	0.38086803	0.778503834	down_in_case
PRJNA8274(	ENSGALG00010017433	RNPS1	case	control	-0.000899613	0.039104951	-0.023005083	0.981646218	0.996591771	down_in_case
PRJNA8274(	ENSGALG00010017434	FGF13	case	control	-0.455054312	0.163745878	-2.779027588	0.005452189	0.127813621	down_in_case
PRJNA8274(	ENSGALG00010017435	ACTR10	case	control	0.013390023	0.076031689	0.176111079	0.860206677	0.971871635	up_in_case
PRJNA8274(	ENSGALG00010017437	DRG2	case	control	-0.071510695	0.059686973	-1.198095515	0.230879835	0.655335776	down_in_case
PRJNA8274(	ENSGALG00010017439	PPP4R4	case	control	0.128234102	0.302615904	0.423752025	0.671746669	0.913676733	up_in_case
PRJNA8274(	ENSGALG00010017441	SPSB1	case	control	-0.048538125	0.098897904	-0.490790234	0.623574818	0.89432552	down_in_case
PRJNA8274(	ENSGALG00010017442	LCK	case	control	-0.046709287	0.240513948	-0.194206145	0.846014472	0.967563913	down_in_case
PRJNA8274(	ENSGALG00010017445	F9	case	control	1.25772087	0.822395065	1.529339029	0.126180419	0.525939433	up_in_case
PRJNA8274(	ENSGALG00010017447	CAPN13	case	control	-0.276451641	0.393398826	-0.702726147	0.48222643	0.832931266	down_in_case
PRJNA8274(	ENSGALG00010017449	TRMT6	case	control	-0.063510175	0.066099534	-0.960826369	0.336639478	0.750654067	down_in_case
PRJNA8274(	ENSGALG00010017450	ENSGALG00010017450	case	control	-0.225763685	0.147156898	-1.534169909	0.124987858	0.523982046	down_in_case
PRJNA8274(	ENSGALG00010017451	GABPB1	case	control	0.006675736	0.054995523	0.121386895	0.903384597	0.980997505	up_in_case
PRJNA8274(	ENSGALG00010017452	ARMH4	case	control	-1.31785253	0.461901602	-2.853102313	0.004329468	0.113589968	down_in_case
PRJNA8274(	ENSGALG00010017453	PPP1R2	case	control	0.00646213	0.069760579	0.092632976	0.926195145	0.98618953	up_in_case
PRJNA8274(	ENSGALG00010017454	ENSGALG00010017454	case	control	0.09090024	0.084988062	1.069564801	0.284815246	0.709583124	up_in_case
PRJNA8274(	ENSGALG00010017455	CCR9	case	control	-0.031780911	0.109165129	-0.291126946	0.770954232	0.94558971	down_in_case
PRJNA8274(	ENSGALG00010017457	ZMAT4	case	control	-0.215124281	0.175981404	-1.222426211	0.221546492	0.645689578	down_in_case
PRJNA8274(	ENSGALG00010017458	ENSGALG00010017458	case	control	0.219122713	0.097059342	2.2576159	0.023969617	0.273286623	up_in_case
PRJNA8274(	ENSGALG00010017459	FAM167B	case	control	0.274806122	0.241113918	1.139735625	0.25439646	0.679971816	up_in_case
PRJNA8274(	ENSGALG00010017460	SERPINA10	case	control	1.827348862	1.711810862	1.067494606	0.285748548	0.710150198	up_in_case
PRJNA8274(	ENSGALG00010017462	MCM8	case	control	0.318008905	0.128831632	2.468407021	0.01357159	0.204288151	up_in_case
PRJNA8274(	ENSGALG00010017463	PPP1R36	case	control	-0.202029532	0.242100473	-0.834486316	0.404006995	0.79260318	down_in_case
PRJNA8274(	ENSGALG00010017464	SH3BP2	case	control	0.045137068	0.065264099	0.69160639	0.489184548	0.836347578	up_in_case
PRJNA8274(	ENSGALG00010017465	TMEM234	case	control	0.15568111	0.119494032	1.302835861	0.192630805	0.615090794	up_in_case
PRJNA8274(	ENSGALG00010017466	SPIA4	case	control	1.668704329	1.980314587	0.842646083	0.39942641	0.789900496	up_in_case
PRJNA8274(	ENSGALG00010017467	SFRP1	case	control	0.115903024	0.09109811	1.27228791	0.203270843	0.625537942	up_in_case
PRJNA8274(	ENSGALG00010017468	AMDHD2	case	control	0.232357681	0.057246948	4.05886586	4.93116E-05	0.006590246	up_in_case
PRJNA8274(	ENSGALG00010017470	HSPA2	case	control	-0.130443467	0.114865539	-1.135618807	0.256116134	0.682403711	down_in_case
PRJNA8274(	ENSGALG00010017471	CRLS1	case	control	-0.006739085	0.051127914	-0.131808335	0.895135894	0.980176778	down_in_case
PRJNA8274(	ENSGALG00010017472	LZTFL1	case	control	0.115429873	0.090230291	1.279280729	0.200798216	0.623527532	up_in_case
PRJNA8274(	ENSGALG00010017473	EIF3I	case	control	0.001731877	0.047193901	0.036697043	0.970726566	0.992761697	up_in_case
PRJNA8274(	ENSGALG00010017475	STUM	case	control	-0.100779672	0.120444397	-0.83673192	0.402743273	0.792021921	down_in_case
PRJNA8274(	ENSGALG00010017476	ATP6V0C	case	control	-0.056687066	0.059331704	-0.955426235	0.339362192	0.751259351	down_in_case
PRJNA8274(	ENSGALG00010017477	MCF2	case	control	-0.064233984	0.129833354	-0.494741776	0.620782408	0.893077308	down_in_case
PRJNA8274(	ENSGALG00010017479	SSTR5	case	control	0.185079057	0.373615647	0.495372875	0.620336938	0.892955829	up_in_case
PRJNA8274(	ENSGALG00010017480	DCDC2B	case	control	0.17110309	0.158035483	1.082687803	0.27894702	0.704440209	up_in_case
PRJNA8274(	ENSGALG00010017481	SPIA1	case	control	1.162924141	1.600869167	0.726432968	0.467573386	0.828046838	up_in_case
PRJNA8274(	ENSGALG00010017482	CORO2B	case	control	0.062530002	0.089154578	0.701366133	0.483074552	0.833529046	up_in_case
PRJNA8274(	ENSGALG00010017484	IQCC	case	control	0.291435508	0.12788768	2.278839591	0.022676602	0.267253668	up_in_case
PRJNA8274(	ENSGALG00010017485	LBH	case	control	-0.037725968	0.05437075	-0.693865132	0.487766792	0.835553062	down_in_case
PRJNA8274(	ENSGALG00010017487	C1QTNF8	case	control	0.093817905	0.500502383	0.18744747	0.851309807	0.969234083	up_in_case
PRJNA8274(	ENSGALG00010017489	TNFRSF1B	case	control	-0.014597618	0.098899828	-0.14760004	0.882658424	0.976801393	down_in_case
PRJNA8274(	ENSGALG00010017490	SCNN1B	case	control	-0.015256471	0.311791245	-0.048931686	0.960973738	0.992056045	down_in_case
PRJNA8274(	ENSGALG00010017491	CCDC28B	case	control	0.340213018	0.264223469	1.287595759	0.197886717	0.621110634	up_in_case
PRJNA8274(	ENSGALG00010017493	YPEL5	case	control	-0.006393938	0.055892744	-0.114396569	0.908923434	0.981730793	down_in_case
PRJNA8274(	ENSGALG00010017494	TBC1D24	case	control	-0.029247405	0.062910525	-0.464904795	0.641999636	0.90164946	down_in_case
PRJNA8274(	ENSGALG00010017495	NT5M	case	control	-0.098155833	0.060023725	-1.635283913	0.101989544	0.491460867	down_in_case
PRJNA8274(	ENSGALG00010017496	PARP1	case	control	-0.107295967	0.061318601	-1.749811077	0.080150919	0.451585805	down_in_case
PRJNA8274(	ENSGALG00010017499	SNRPD1	case	control	0.034720071	0.067580226	0.513760808	0.607419232	0.887328057	up_in_case
PRJNA8274(	ENSGALG00010017500	ACAP2	case	control	0.049249358	0.069650649	0.707091157	0.479509831	0.831821257	up_in_case
PRJNA8274(	ENSGALG00010017501	ENSGALG00010017501	case	control	1.17020983	2.196583404	0.532740905	0.594212946	0.883840767	up_in_case
PRJNA8274(	ENSGALG00010017502	NAT8B	case	control	-0.027816074	0.114562757	-0.242802069	0.808158733	0.955875648	down_in_case
PRJNA8274(	ENSGALG00010017503	MED9	case	control	-0.100424409	0.059983687	-1.674195338	0.094092215	0.479477093	down_in_case
PRJNA8274(	ENSGALG00010017505	TNFRSF8	case	control	-0.457365083	0.27322255	-1.673965357	0.094137408	0.479477093	down_in_case
PRJNA8274(	ENSGALG00010017509	RASD1	case	control	0.34928168	0.279720414	1.248681409	0.211781623	0.636501957	up_in_case
PRJNA8274(	ENSGALG00010017510	HDC	case	control	0.269691612	0.362948952	0.743056593	0.457447418	0.823244158	up_in_case
PRJNA8274(	ENSGALG00010017513	TNIP2	case	control	-0.083850543	0.109222434	-0.7677044	0.442662822	0.814107419	down_in_case
PRJNA8274(	ENSGALG00010017514	TXLNA	case	control	-0.016804131	0.059558727	-0.282143885	0.777833184	0.947849555	down_in_case
PRJNA8274(	ENSGALG00010017516	AAK1	case	control	-0.053131399	0.04916993	-1.080566893	0.279889816	0.705521658	down_in_case
PRJNA8274(	ENSGALG00010017517	LIN9	case	control	0.010900449	0.061837413	0.176275959	0.860077148	0.971871635	up_in_case
PRJNA8274(	ENSGALG00010017518	PEMT	case	control	0.031323183	0.063885432	0.490302438	0.623919902	0.894353977	up_in_case
PRJNA8274(	ENSGALG00010017519	NTN3	case	control	0.003895465	0.133493445	0.029180946	0.976720278	0.994586386	up_in_case
PRJNA8274(	ENSGALG00010017520	ZBTB1	case	control	-0.039690426	0.04099922	-0.968077588	0.333005622	0.748503677	down_in_case
PRJNA8274(	ENSGALG00010017521	MIIP	case	control	-0.03614205	0.079335913	-0.455557241	0.648708397	0.903708435	down_in_case
PRJNA8274(	ENSGALG00010017522	XXYLT1	case	control	-0.002110525	0.054391566	-0.038802424	0.969047912	0.992761697	down_in_case

PRJNA8274(	ENSGALG00010017524	ACBD3	case	control	-0.040966061	0.040478103	-1.012054852	0.311511827	0.734109585	down_in_case
PRJNA8274(	ENSGALG00010017525	ENSGALG00010017525	case	control	0.777789557	0.685371175	1.134844278	0.25644057	0.682835529	up_in_case
PRJNA8274(	ENSGALG00010017526	PLEKHA2	case	control	0.148256139	0.122951209	1.205812774	0.227889689	0.651681204	up_in_case
PRJNA8274(	ENSGALG00010017528	RAI1	case	control	-0.104165586	0.070802538	-1.471212609	0.141233628	0.543501954	down_in_case
PRJNA8274(	ENSGALG00010017529	MFN2	case	control	0.080472629	0.068718861	1.171041364	0.241582151	0.666243691	up_in_case
PRJNA8274(	ENSGALG00010017530	TLR1B	case	control	-0.02112289	0.117090621	-0.180397797	0.856840284	0.971145061	down_in_case
PRJNA8274(	ENSGALG00010017531	SACM1L	case	control	0.098723561	0.049100723	2.010633542	0.044364178	0.350320113	up_in_case
PRJNA8274(	ENSGALG00010017532	MIXL1	case	control	-0.982475337	0.381185915	-2.577417734	0.009954157	0.177161817	down_in_case
PRJNA8274(	ENSGALG00010017533	FAM193A	case	control	-0.016551557	0.042610074	-0.38844235	0.697688709	0.922288441	down_in_case
PRJNA8274(	ENSGALG00010017534	ATP11C	case	control	-0.000205855	0.06931281	-0.002969946	0.99763033	0.999142415	down_in_case
PRJNA8274(	ENSGALG00010017535	LIMD1	case	control	-0.072145468	0.074044119	-0.974357849	0.329878891	0.746250947	down_in_case
PRJNA8274(	ENSGALG00010017536	GSC	case	control	0.170901324	0.387824295	0.440666884	0.659454174	0.908599575	up_in_case
PRJNA8274(	ENSGALG00010017537	MANBA	case	control	0.154934575	0.091448044	1.694236068	0.090220434	0.473624156	up_in_case
PRJNA8274(	ENSGALG00010017538	H3-3A	case	control	-0.072469506	0.050789433	-1.426861873	0.153619712	0.56245196	down_in_case
PRJNA8274(	ENSGALG00010017539	KPNA6	case	control	-0.034890455	0.032371378	-1.077818035	0.281114969	0.705762606	down_in_case
PRJNA8274(	ENSGALG00010017540	EIF4G1	case	control	-0.122009293	0.092236528	-1.322787136	0.185906176	0.608935531	down_in_case
PRJNA8274(	ENSGALG00010017541	SREBF1	case	control	-0.143328066	0.079619387	-1.800165403	0.071834525	0.433066487	down_in_case
PRJNA8274(	ENSGALG00010017542	PLOD1	case	control	-0.025304139	0.063166626	-0.400593489	0.68871944	0.918035793	down_in_case
PRJNA8274(	ENSGALG00010017543	ZBTB25	case	control	0.046926245	0.118657443	0.395476623	0.69249117	0.918988424	up_in_case
PRJNA8274(	ENSGALG00010017544	SDE2	case	control	-0.107608768	0.044633042	-2.410966466	0.015910311	0.223714308	down_in_case
PRJNA8274(	ENSGALG00010017545	DICER1	case	control	-0.02676698	0.073092305	-0.366207909	0.714209941	0.926862121	down_in_case
PRJNA8274(	ENSGALG00010017546	NFU1	case	control	-0.045928566	0.086956277	-0.528180001	0.597374409	0.884680373	down_in_case
PRJNA8274(	ENSGALG00010017547	RNF4	case	control	0.092882801	0.046448546	1.999692319	0.045533498	0.354259534	up_in_case
PRJNA8274(	ENSGALG00010017550	TM2D2	case	control	-0.111525152	0.066977162	-1.665122094	0.0958884	0.483185646	down_in_case
PRJNA8274(	ENSGALG00010017551	NALF2	case	control	-0.1296233	0.163319096	-0.793681225	0.427381033	0.805305429	down_in_case
PRJNA8274(	ENSGALG00010017552	LARS2	case	control	0.019019497	0.069638688	0.273116827	0.784763412	0.949518053	up_in_case
PRJNA8274(	ENSGALG00010017553	EDA	case	control	-0.062168978	0.070173388	-0.885933819	0.375653191	0.774921593	down_in_case
PRJNA8274(	ENSGALG00010017554	FAM131A	case	control	0.200367619	0.276288453	0.725211702	0.468322165	0.828596747	up_in_case
PRJNA8274(	ENSGALG00010017556	TOM1L2	case	control	-0.047719768	0.055370254	-0.861830401	0.388780853	0.783368493	down_in_case
PRJNA8274(	ENSGALG00010017558	ENSGALG00010017558	case	control	-0.052091513	0.048888179	-1.065523693	0.28663901	0.710659943	down_in_case
PRJNA8274(	ENSGALG00010017559	DUSP10	case	control	0.027028035	0.110829403	0.243870616	0.807331027	0.955875648	up_in_case
PRJNA8274(	ENSGALG00010017560	ENSGALG00010017560	case	control	-0.690483144	0.745207412	-0.926565051	0.354152407	0.762707745	down_in_case
PRJNA8274(	ENSGALG00010017562	SLC30A10	case	control	1.28481519	0.465133855	2.762248281	0.005740481	0.131763843	up_in_case
PRJNA8274(	ENSGALG00010017563	ALK	case	control	-0.180873509	0.153025689	-1.181981336	0.237213107	0.662528794	down_in_case
PRJNA8274(	ENSGALG00010017565	ENSGALG00010017565	case	control	-0.065290574	0.086957138	-0.750836277	0.452751194	0.821045895	down_in_case
PRJNA8274(	ENSGALG00010017568	CDCP1	case	control	0.073891743	0.298023503	0.247939315	0.80418136	0.954997212	up_in_case
PRJNA8274(	ENSGALG00010017569	CLMN	case	control	0.100200266	0.127653949	0.784936673	0.432490699	0.807707587	up_in_case
PRJNA8274(	ENSGALG00010017570	DRC3	case	control	-0.416973659	0.437287403	-0.95354601	0.340313496	0.751812605	down_in_case
PRJNA8274(	ENSGALG00010017571	ADAM9	case	control	-0.069126013	0.046268607	-1.494015457	0.135171579	0.539774365	down_in_case
PRJNA8274(	ENSGALG00010017573	ENSGALG00010017573	case	control	-0.097654902	0.453975828	-0.215110357	0.829681279	0.962228709	down_in_case
PRJNA8274(	ENSGALG00010017574	DYNC1LI1	case	control	-0.016542661	0.044183253	-0.374410212	0.708099146	0.92490115	down_in_case
PRJNA8274(	ENSGALG00010017576	ENSGALG00010017576	case	control	-0.163831304	0.149570265	-1.095346752	0.273364753	0.699305181	down_in_case
PRJNA8274(	ENSGALG00010017577	CLPX	case	control	-0.15824191	0.063074412	-2.508813072	0.012113757	0.193549413	down_in_case
PRJNA8274(	ENSGALG00010017578	BSDC1	case	control	-0.026497736	0.049663125	-0.533549502	0.593653254	0.883625114	down_in_case
PRJNA8274(	ENSGALG00010017579	P3H2	case	control	-0.020005906	0.08886706	-0.225121729	0.821884578	0.959267831	down_in_case
PRJNA8274(	ENSGALG00010017580	LYPLAL1	case	control	-0.051533168	0.14077137	-0.366077054	0.714307579	0.926862121	down_in_case
PRJNA8274(	ENSGALG00010017581	EFCAB11	case	control	-0.662059213	0.256146887	-2.584685765	0.009746778	0.176652637	down_in_case
PRJNA8274(	ENSGALG00010017582	CLCN6	case	control	0.058429463	0.044363485	1.317062052	0.187817826	0.610946377	up_in_case
PRJNA8274(	ENSGALG00010017583	MTHFD1	case	control	0.095415742	0.143639292	0.664273266	0.506515429	0.843411729	up_in_case
PRJNA8274(	ENSGALG00010017585	ENSGALG00010017585	case	control	-0.065289294	0.081287228	-0.803192519	0.421863469	0.80254895	down_in_case
PRJNA8274(	ENSGALG00010017586	ZFYVE28	case	control	0.032537088	0.125479237	0.259302562	0.795401803	0.95280257	up_in_case
PRJNA8274(	ENSGALG00010017587	MB21D2	case	control	-0.037281046	0.110276872	-0.338067682	0.735312184	0.934096263	down_in_case
PRJNA8274(	ENSGALG00010017589	ATPAF2	case	control	-0.044128657	0.06445306	-0.684663495	0.49355629	0.837916219	down_in_case
PRJNA8274(	ENSGALG00010017590	ENSGALG00010017590	case	control	0.130905436	0.138782296	0.943243055	0.345556596	0.755935621	up_in_case
PRJNA8274(	ENSGALG00010017591	ESR2	case	control	0.644452239	0.311000939	2.072187439	0.038247964	0.331271895	up_in_case
PRJNA8274(	ENSGALG00010017592	GID4	case	control	-0.06694125	0.076988262	-0.869499434	0.384574018	0.780971676	down_in_case
PRJNA8274(	ENSGALG00010017593	MTHFR	case	control	0.035727011	0.049768164	0.717868787	0.472838195	0.829235491	up_in_case
PRJNA8274(	ENSGALG00010017594	CEP68	case	control	-0.126577707	0.096729544	-1.308573392	0.190678899	0.613935676	down_in_case
PRJNA8274(	ENSGALG00010017595	CCNF	case	control	0.024943424	0.08350241	0.298715018	0.765157497	0.943786458	up_in_case
PRJNA8274(	ENSGALG00010017596	TDP1	case	control	-0.007026804	0.047517984	-0.14787673	0.882440053	0.976801393	down_in_case
PRJNA8274(	ENSGALG00010017597	CNOT10	case	control	0.032882339	0.068672868	0.478825775	0.632062578	0.89724192	up_in_case
PRJNA8274(	ENSGALG00010017598	ENSGALG00010017598	case	control	-0.06676071	0.073619388	-0.90683599	0.364493534	0.770152588	down_in_case
PRJNA8274(	ENSGALG00010017599	KCNK13	case	control	-0.008827513	0.185286698	-0.047642455	0.962001196	0.992056045	down_in_case
PRJNA8274(	ENSGALG00010017600	MXD4	case	control	-0.000857283	0.058392095	-0.014681492	0.988286285	0.996942188	down_in_case
PRJNA8274(	ENSGALG00010017601	ENSGALG00010017601	case	control	-0.027348405	0.105458535	-0.259328513	0.795381782	0.95280257	down_in_case
PRJNA8274(	ENSGALG00010017603	CYP3A5	case	control	0.545841651	0.767025479	0.711634314	0.476691253	0.830520323	up_in_case
PRJNA8274(	ENSGALG00010017604	GATM	case	control	-0.166291145	0.142075751	-1.170440026	0.241823936	0.666243691	down_in_case
PRJNA8274(	ENSGALG00010017605	GFPT1	case	control	0.027635868	0.056705782	0.48735539	0.626006501	0.895445894	up_in_case
PRJNA8274(	ENSGALG00010017606	ENSGALG00010017606	case	control	-0.091396445	0.238521542	-0.383178994	0.701587052	0.922880366	down_in_case
PRJNA8274(	ENSGALG00010017608	RBBP4	case	control	-0.11567101	0.047837209	-2.418013361	0.015605504	0.221827738	down_in_case
PRJNA8274(	ENSGALG00010017609	TRIM71	case	control	-0.133815267	0.138881505	-0.963521147	0.335286057	0.749356776	down_in_case
PRJNA8274(	ENSGALG00010017610	PSMC1	case	control	0.063018151	0.045568419	1.382934783	0.166684864	0.582074127	up_in_case
PRJNA8274(	ENSGALG00010017612	CLDN11	case	control	-0.659424923	0.346492256	-1.903144764	0.057021658	0.388573099	down_in_case
PRJNA8274(	ENSGALG00010017613	HAUS3	case	control	-0.098622794	0.083783072	-1.177120766	0.239147308	0.66429721	down_in_case
PRJNA8274(	ENSGALG00010017615	AGTRAP	case	control	0.065704025	0.131040141	0.501403802	0.616086964	0.891081782	up_in_case
PRJNA8274(	ENSGALG00010017616	PDCD7	case	control	-0.098389085	0.072629187	-1.354676944	0.175520507	0.594350757	down_in_case
PRJNA8274(	ENSGALG00010017619	POLN	case	control	0.317334372	0.311557642	1.018541451	0.308420712	0.731823162	up_in_case
PRJNA8274(	ENSGALG00010017620	UBAP1L	case	control	-0.230276667	0.223153413	-1.031920883	0.302109182	0.724980921	down_in_case
PRJNA8274(	ENSGALG00010017621	SYNE2	case	control	-0.405487599	0.100016663	-4.054200452	5.03061E-05	0.006599787	down_in_case
PRJNA8274(	ENSGALG00010017622	RAB1B	case	control	-0.013931408	0.033475944	-0.416161784	0.677291622	0.914766174	down_in_case
PRJNA8274(	ENSGALG00010017625	YBX1	case	control	-0.043415884	0.05103973	-0.850629183	0.394975373	0.786978937	down_in_case
PRJNA8274(	ENSGALG00010017626	COPS7B	case	control	-0.01907202	0.057167754	-0.333614992	0.738670104	0.93607843	down_in_case
PRJNA8274(	ENSGALG00010017628	NRDE2	case	control	-0.021118705	0.067127068	-0.314607882	0.753059394	0.940666434	down_in_case
PRJNA8274(	ENSGALG00010017629	ENSGALG00010017629	case	control	0.073128391	0.069024278	1.059458979	0.289390802	0.712911094	up_in_case
PRJNA8274(	ENSGALG00010017631	NAT8L	case	control	0.172438282	0.159771258	1.079282246	0.280461925	0.705762606	up_in_case
PRJNA8274(	ENSGALG00010017632	AWAT1	case	control	1.18528802	0.516688163	2.294010401	0.021789901	0.264012564	up_in_case
PRJNA8274(	ENSGALG00010017633	HLX	case	control	-0.019695655	0.110989398	-0.177455279	0.85915079	0.971828322	down_in_case
PRJNA8274(	ENSGALG00010017634	PPIH	case	control	0.036810142	0.07993112	0.460523289	0.645140659	0.902338755	up_in_case
PRJNA8274(	ENSGALG00010017636	ZBTB8OS	case	control	-0.014936309	0.08857522	-0.168628523	0.866088844	0.973289701	down_in_case
PRJNA8274(	ENSGALG00010017638	GFRA2	case	control	0.347970815	0.168648308	2.063292654	0.039084832	0.335091843	up_in_case
PRJNA8274(	ENSGALG00010017639	ENSGALG00010017639	case	control	-0.036951884	0.108980798	-0.339067843	0.734558623	0.934037729	down_in_case
PRJNA8274(	ENSGALG00010017640	P2RY4	case	control	0.447158386	0.536773372	0.833048749	0.404817236	0.793324171	up_in_case
PRJNA8274(	ENSGALG00010017641	GPRC5B	case	control	-0.072399368	0.086938316	-0.832767084	0.404976102	0.793437545	down_in_case
PRJNA8274(	ENSGALG00010017642	MTARC1	case	control	-0.007049802	0.092170078	-0.076486883	0.939031749	0.989551456	down_in_case
PRJNA8274(	ENSGALG00010017643	CALM1	case	control	-0.075781913	0.051263959	-1.47826884	0.139335835	0.541505735	down_in_case
PRJNA8274(	ENSGALG00010017644	RBBP6	case	control	-0.091262369	0.067057269	-1.36096161	0.173525822	0.592914702	down_in_case
PRJNA8274(	ENSGALG00010017645	SYNC	case	control	-0.121632227	0.109220949	-1.113634589	0.265435981	0.691495163	down_in_case
PRJNA8274(	ENSGALG00010017646	MAEA	case	control	0.036653857	0.036498605	1.004253636	0.315256375	0.736508113	up_in_case
PRJNA8274(	ENSGALG00010017647	ITGA9	case	control	-0.117478019	0.089263764	-1.316077361	0.188148079	0.611129484	down_in_case
PRJNA8274(	ENSGALG00010017648	ENSGALG00010017648	case	control	0.13351688	0.124197435	1.075037343	0.28235801	0.706141927	up_in_case
PRJNA8274(	ENSGALG00010017649	ENSGALG00010017649	case	control	-0.135362489	0.101467616	-1.334046209	0.182188713	0.603078379	down_in_case
PRJNA8274(	ENSGALG00010017651	PPCS	case	control	0.129196921	0.071174689	1.815208787	0.06949183	0.425096829	up_in_case
PRJNA8274(	ENSGALG00010017652	C1orf115	case	control	0.016342144	0.143568998	0.113827806	0.909374296	0.981730793	up_in_case
PRJNA8274(	ENSGALG00010017653	ENSGALG00010017653	case	control	-0.053777245	0.112978396	-0.475995822	0.634077354	0.898107199	down_in_case
PRJNA8274(	ENSGALG00010017654	KBTBD13	case	control	0.14526931	0.184924086	0.785561868	0.432124212	0.807390038	up_in_case
PRJNA8274(	ENSGALG00010017655	ENSGALG00010017655	case	control	0.009808218	0.089961773	0.109026508	0.913181465	0.982728035	up_in_case
PRJNA8274(	ENSGALG00010017656	FSD2	case	control	-0.174385511	0.125958057	-1.384472853	0.166213715	0.581422731	down_in_case
PRJNA8274(	ENSGALG00010017657	DIS3L2	case	control	-0.011730584	0.075785205	-0.154787254	0.87698904	0.975813349	down_in_case
PRJNA8274(	ENSGALG00010017658	WHAMM	case	control	-0.014804434	0.099017031	-0.149514014	0.881148052	0.97667824	down_in_case
PRJNA8274(	ENSGALG00010017659	TNRC6A	case	control	-0.059532808	0.06789956	-0.876777524	0.38060749	0.778246195	down_in_case
PRJNA8274(	ENSGALG00010017660	CLIP4	case	control	-0.116125642	0.101898125	-1.139624916	0.2544426	0.679971816	down_in_case
PRJNA8274(	ENSGALG00010017661	NHSL3	case	control	-0.037646653	0.094839796	-0.396949958	0.691404363	0.918812261	down_in_case
PRJNA8274(	ENSGALG00010017662	GOLGA4	case	control	-0.126645387	0.088363248	-1.433235991	0.151790407	0.560075381	down_in_case
PRJNA8274(	ENSGALG00010017663	ZMYND12	case	control	-0.107640051	0.196556882	-0.547627993	0.583947364	0.881307382	down_in_case
PRJNA8274(	ENSGALG00010017664	ENSGALG00010017664	case	control	0.005894672	0.045060834	0.130815861	0.895920975	0.980176778	up_in_case
PRJNA8274(	ENSGALG00010017665	CTBP1	case	control	-0.022761213	0.048517975	-0.469129483	0.638977086	0.900303403	down_in_case
PRJNA8274(	ENSGALG00010017666	PCARE	case	control	0.259628027	0.592237786	0.438384773	0.661107383	0.909646511	up_in_case
PRJNA8274(	ENSGALG00010017667	SLC2A1	case	control	0.021598716	0.071324885	0.302821601	0.762025821	0.943297504	up_in_case
PRJNA8274(	ENSGALG00010017669	ENSGALG00010017669	case	control	-0.176811747	0.041658203	-4.244344057	2.19234E-05	0.003300045	down_in_case
PRJNA8274(	ENSGALG00010017670	FAM179A	case	control	-0.240338314	0.199421167	-1.205179554	0.228133993	0.651681204	down_in_case
PRJNA8274(	ENSGALG00010017671	ANTXR1	case	control	-0.077621271	0.112727269	-0.688575813	0.491090246	0.83691938	down_in_case
PRJNA8274(	ENSGALG00010017672	SPRED2	case	control	-0.009649075	0.045493051	-0.212099971	0.832029042	0.962371901	down_in_case
PRJNA8274(	ENSGALG00010017673	WDR89	case	control	-0.049612441	0.061736049	-0.803621902	0.42161537	0.802413627	down_in_case
PRJNA8274(	ENSGALG00010017676	SPON2	case	control	-0.2861749	0.139398517	-2.052926432	0.040079713	0.336547209	down_in_case
PRJNA8274(	ENSGALG00010017677	WDR43	case	control	0.020113059	0.041602144	0.483462064	0.628767689	0.896269608	up_in_case
PRJNA8274(	ENSGALG00010017678	YARS	case	control	0.042033563	0.06844732	0.614100925	0.539148637	0.858476757	up_in_case

PRJNA8274(	ENSGALG00010017679	RASL12	case	control	0.294903566	0.188229017	1.56672744	0.117178414	0.513845851	up_in_case
PRJNA8274(	ENSGALG00010017684	TRMT61B	case	control	-0.129659419	0.081730482	-1.586426694	0.112642549	0.506856026	down_in_case
PRJNA8274(	ENSGALG00010017685	PHC2	case	control	-0.056542303	0.066330788	-0.852429225	0.393975905	0.786978937	down_in_case
PRJNA8274(	ENSGALG00010017687	TTC7B	case	control	-0.114747291	0.077500776	-1.480595382	0.138714427	0.540932258	down_in_case
PRJNA8274(	ENSGALG00010017688	SPDYA	case	control	0.10504727	0.138769864	0.756989072	0.44905641	0.818701429	up_in_case
PRJNA8274(	ENSGALG00010017691	LZTS1	case	control	0.055715831	0.131639863	0.423244368	0.672116979	0.913676733	up_in_case
PRJNA8274(	ENSGALG00010017692	EPCAM	case	control	0.593808875	0.749174009	0.792618094	0.428000363	0.805337183	up_in_case
PRJNA8274(	ENSGALG00010017693	MTFMT	case	control	0.221304179	0.150619379	1.469294196	0.14175301	0.543501954	up_in_case
PRJNA8274(	ENSGALG00010017694	ATP6V1B2	case	control	0.010337676	0.042593986	0.242702713	0.808235705	0.955875648	up_in_case
PRJNA8274(	ENSGALG00010017695	FNDC5	case	control	-0.019846446	0.12852049	-0.154422426	0.877276672	0.975813349	down_in_case
PRJNA8274(	ENSGALG00010017696	SLC18A1	case	control	0.206796112	0.191997903	1.077074845	0.28144683	0.705762606	up_in_case
PRJNA8274(	ENSGALG00010017699	RNF19B	case	control	0.001444418	0.13681714	0.010557289	0.991576658	0.99790247	up_in_case
PRJNA8274(	ENSGALG00010017700	VPS35L	case	control	0.011066587	0.060760657	0.182134095	0.855477492	0.970656588	up_in_case
PRJNA8274(	ENSGALG00010017701	PPP2R5E	case	control	-0.053317212	0.039914305	-1.335792048	0.181617253	0.602709285	down_in_case
PRJNA8274(	ENSGALG00010017702	ECEL1	case	control	-0.573757214	0.657592642	-0.872511609	0.382929338	0.780061953	down_in_case
PRJNA8274(	ENSGALG00010017703	RPS6KA5	case	control	-0.070290569	0.071514491	-0.98288568	0.325663707	0.74363678	down_in_case
PRJNA8274(	ENSGALG00010017704	ZNF362	case	control	-0.090769302	0.068767954	-1.319936056	0.186856368	0.610613817	down_in_case
PRJNA8274(	ENSGALG00010017705	MAK16	case	control	-0.142287005	0.056745154	-2.507474112	0.012159749	0.19369863	down_in_case
PRJNA8274(	ENSGALG00010017706	FGFRL1	case	control	-0.063949376	0.105502741	-0.606139472	0.544422142	0.861579972	down_in_case
PRJNA8274(	ENSGALG00010017707	AK2	case	control	-0.006453612	0.05708564	-0.113051407	0.909989799	0.981730793	down_in_case
PRJNA8274(	ENSGALG00010017708	MEIS1	case	control	-0.247073054	0.082376298	-2.999322155	0.00270581	0.087009711	down_in_case
PRJNA8274(	ENSGALG00010017710	RAB41	case	control	0.125289621	0.119558162	1.047938663	0.294666868	0.717720356	up_in_case
PRJNA8274(	ENSGALG00010017711	LRRFIP2	case	control	0.037224845	0.055346134	0.67258257	0.501212894	0.841040176	up_in_case
PRJNA8274(	ENSGALG00010017712	MSH2	case	control	-0.122549216	0.056785978	-2.158089368	0.030920884	0.308259818	down_in_case
PRJNA8274(	ENSGALG00010017713	ALKBH5	case	control	0.029362387	0.078731623	0.372942741	0.70919106	0.925521953	up_in_case
PRJNA8274(	ENSGALG00010017714	ATAD3A	case	control	-0.012094753	0.078486222	-0.154100332	0.877530626	0.975889142	down_in_case
PRJNA8274(	ENSGALG00010017715	CILP	case	control	-0.181705115	0.170820532	-1.063719403	0.287455832	0.71132024	down_in_case
PRJNA8274(	ENSGALG00010017717	LLGL1	case	control	-0.069581538	0.068780845	-1.011641226	0.311709627	0.734219678	down_in_case
PRJNA8274(	ENSGALG00010017718	ATOH8	case	control	-0.131708089	0.138667052	-0.949815311	0.342206105	0.754129649	down_in_case
PRJNA8274(	ENSGALG00010017720	ENSGALG00010017720	case	control	-0.122143816	0.298602217	-0.40905194	0.682501545	0.915874198	down_in_case
PRJNA8274(	ENSGALG00010017721	VWA1	case	control	0.038400362	0.105252572	0.364840128	0.715230749	0.926862121	up_in_case
PRJNA8274(	ENSGALG00010017723	KCNK12	case	control	-0.138907917	0.214787604	-0.646722226	0.517811735	0.848425162	down_in_case
PRJNA8274(	ENSGALG00010017724	FLII	case	control	-0.142265234	0.077233451	-1.842015757	0.065472845	0.415377855	down_in_case
PRJNA8274(	ENSGALG00010017725	ENSGALG00010017725	case	control	0.708438626	0.545540517	1.298599469	0.19408142	0.617169773	up_in_case
PRJNA8274(	ENSGALG00010017726	TTI2	case	control	0.073602658	0.116187326	0.633482675	0.526418491	0.85251736	up_in_case
PRJNA8274(	ENSGALG00010017727	AZIN2	case	control	-0.022129463	0.130760446	-0.169236676	0.865610483	0.973289701	down_in_case
PRJNA8274(	ENSGALG00010017729	MIEF2	case	control	-0.230083641	0.099937697	-2.302270797	0.021319906	0.261638514	down_in_case
PRJNA8274(	ENSGALG00010017730	TARP	case	control	0.063491691	0.557735073	0.11383844	0.909365866	0.981730793	up_in_case
PRJNA8274(	ENSGALG00010017732	RHOJ	case	control	0.061586527	0.124580473	0.494351368	0.621058053	0.893114457	up_in_case
PRJNA8274(	ENSGALG00010017733	TRIM62	case	control	0.067777951	0.08543017	0.793372549	0.4275608	0.805305429	up_in_case
PRJNA8274(	ENSGALG00010017734	TOP3A	case	control	-0.215245768	0.118094254	-1.822660807	0.06835478	0.422366828	down_in_case
PRJNA8274(	ENSGALG00010017737	ENSGALG00010017737	case	control	-0.008054902	0.236634437	-0.034039431	0.972845707	0.99340857	down_in_case
PRJNA8274(	ENSGALG00010017738	NDUFS5	case	control	-0.016529445	0.066137849	-0.249924141	0.802646014	0.954891353	down_in_case
PRJNA8274(	ENSGALG00010017739	MRPL20	case	control	-0.031077265	0.06286067	-0.494383286	0.621035515	0.893114457	down_in_case
PRJNA8274(	ENSGALG00010017741	SMCR8	case	control	-0.092342923	0.059101538	-1.562445356	0.118183109	0.51463738	down_in_case
PRJNA8274(	ENSGALG00010017742	DGLUCY	case	control	-0.240764617	0.141780759	-1.698147328	0.089479959	0.472586803	down_in_case
PRJNA8274(	ENSGALG00010017743	KCNH5	case	control	0.134178217	0.263027723	0.510129564	0.609960694	0.888273136	up_in_case
PRJNA8274(	ENSGALG00010017744	ETAA1	case	control	-0.032118881	0.05915676	-0.542945229	0.587167514	0.882726603	down_in_case
PRJNA8274(	ENSGALG00010017745	SHMT1	case	control	0.439038493	0.222247819	1.975445673	0.048217594	0.361748551	up_in_case
PRJNA8274(	ENSGALG00010017746	MSH6	case	control	-0.104658132	0.052273905	-2.002110444	0.045272854	0.352807531	down_in_case
PRJNA8274(	ENSGALG00010017747	DUSP26	case	control	-0.096594084	0.117201408	-0.824171701	0.409842004	0.795863751	down_in_case
PRJNA8274(	ENSGALG00010017748	ENSGALG00010017748	case	control	-0.382308463	0.348030797	-1.098490325	0.27199044	0.698323327	down_in_case
PRJNA8274(	ENSGALG00010017749	TMEM88B	case	control	-0.503849964	0.358871857	-1.403982937	0.160323931	0.572752253	down_in_case
PRJNA8274(	ENSGALG00010017751	ST3GAL5	case	control	0.294563901	0.16074473	1.832494922	0.066877718	0.418849217	up_in_case
PRJNA8274(	ENSGALG00010017752	ENSGALG00010017752	case	control	1.424672769	0.959505587	1.484798827	0.137597123	0.539854583	up_in_case
PRJNA8274(	ENSGALG00010017753	GNRHR	case	control	0.155074679	0.356213139	0.435342389	0.663313921	0.911044469	up_in_case
PRJNA8274(	ENSGALG00010017754	PDZD11	case	control	0.054307417	0.090463487	0.600324166	0.548290216	0.86339387	up_in_case
PRJNA8274(	ENSGALG00010017755	C1D	case	control	-0.053433445	0.066205195	-0.807088392	0.41961556	0.80093491	down_in_case
PRJNA8274(	ENSGALG00010017756	EIF2B5	case	control	-0.022255222	0.058717258	-0.379023516	0.704670401	0.923973441	down_in_case
PRJNA8274(	ENSGALG00010017757	ENSGALG00010017757	case	control	-0.111040946	0.060551963	-1.833812495	0.066681832	0.418223773	down_in_case
PRJNA8274(	ENSGALG00010017759	ENSGALG00010017759	case	control	-0.738137783	0.34520774	-2.138242273	0.032497085	0.310633901	down_in_case
PRJNA8274(	ENSGALG00010017760	ENSGALG00010017760	case	control	0.177750605	0.492151874	0.361170229	0.717972196	0.927322135	up_in_case
PRJNA8274(	ENSGALG00010017761	PARP16	case	control	-0.049570129	0.093309255	-0.531245584	0.595248609	0.884461254	down_in_case
PRJNA8274(	ENSGALG00010017762	FBXO11	case	control	-0.023985768	0.032788286	-0.731534682	0.464452628	0.825770556	down_in_case
PRJNA8274(	ENSGALG00010017763	POLR1A	case	control	-0.066643167	0.076272782	-0.873747695	0.382255671	0.77998936	down_in_case
PRJNA8274(	ENSGALG00010017764	IGDCC3	case	control	-0.299602456	0.191781889	-1.562204112	0.118239912	0.51463738	down_in_case
PRJNA8274(	ENSGALG00010017765	SYT16	case	control	-0.026952821	0.152231295	-0.177051777	0.85946772	0.971828322	down_in_case
PRJNA8274(	ENSGALG00010017766	DNAAF10	case	control	-0.156847542	0.080812936	-1.940871727	0.052273838	0.373200684	down_in_case
PRJNA8274(	ENSGALG00010017767	KIF4B	case	control	-0.045979287	0.102610447	-0.448095571	0.654084224	0.906478152	down_in_case
PRJNA8274(	ENSGALG00010017768	FOXN2	case	control	-0.078939387	0.089938866	-0.877700493	0.380106277	0.77805894	down_in_case
PRJNA8274(	ENSGALG00010017770	PTCD3	case	control	0.008764028	0.054718818	0.160164798	0.872751259	0.975062028	up_in_case
PRJNA8274(	ENSGALG00010017771	PNO1	case	control	-0.062440113	0.07149708	-0.873323959	0.382486525	0.77998936	down_in_case
PRJNA8274(	ENSGALG00010017772	ENSGALG00010017772	case	control	-0.090511467	0.239837402	-0.37738679	0.705886182	0.924320939	down_in_case
PRJNA8274(	ENSGALG00010017773	DVL3	case	control	-0.05723405	0.062484544	-0.91597133	0.359681933	0.765570668	down_in_case
PRJNA8274(	ENSGALG00010017774	ASH2L	case	control	-0.0909827	0.044504811	-2.044334066	0.040920556	0.337874876	down_in_case
PRJNA8274(	ENSGALG00010017775	PRPSAP2	case	control	-0.073016683	0.062324658	-1.171553679	0.241376294	0.666243691	down_in_case
PRJNA8274(	ENSGALG00010017776	GPR68	case	control	0.065114354	0.192288716	0.338628054	0.734889946	0.934096263	up_in_case
PRJNA8274(	ENSGALG00010017778	PPP3R1	case	control	-0.167533058	0.056229374	-2.979458019	0.002887588	0.090752764	down_in_case
PRJNA8274(	ENSGALG00010017779	SNAPC1	case	control	-0.021902536	0.068601903	-0.319270101	0.749521703	0.93961255	down_in_case
PRJNA8274(	ENSGALG00010017780	TAF9	case	control	0.064704949	0.061315806	1.055273553	0.291300233	0.714222439	up_in_case
PRJNA8274(	ENSGALG00010017781	CYP26C1	case	control	0.013507085	0.276135099	0.048914771	0.960987217	0.992056045	up_in_case
PRJNA8274(	ENSGALG00010017782	IGDCC4	case	control	-0.057703928	0.102917057	-0.560683816	0.575013102	0.877910508	down_in_case
PRJNA8274(	ENSGALG00010017783	STAR	case	control	0.14211598	0.357451722	0.397580906	0.690939138	0.918812261	up_in_case
PRJNA8274(	ENSGALG00010017786	KAZALD1	case	control	-0.089104642	0.094525019	-0.942656702	0.345856527	0.756227431	down_in_case
PRJNA8274(	ENSGALG00010017787	CNRIP1	case	control	0.058196278	0.169760358	0.342814301	0.731738167	0.933770678	up_in_case
PRJNA8274(	ENSGALG00010017789	IMMT	case	control	-0.125206649	0.071739255	-1.745301776	0.080932353	0.453075204	down_in_case
PRJNA8274(	ENSGALG00010017790	R3HCC1L	case	control	0.149527452	0.09367526	1.596232043	0.110436996	0.503054901	up_in_case
PRJNA8274(	ENSGALG00010017791	CCNL2	case	control	0.162396499	0.085176865	1.906579915	0.056575008	0.387277461	up_in_case
PRJNA8274(	ENSGALG00010017792	LSM1	case	control	0.205597744	0.127742144	1.609474661	0.107512591	0.498867454	up_in_case
PRJNA8274(	ENSGALG00010017793	FAM83G	case	control	0.435052797	0.443220235	0.981572507	0.326310498	0.744287835	up_in_case
PRJNA8274(	ENSGALG00010017795	MACF1	case	control	0.06127388	0.061350515	0.998750865	0.317915394	0.738087569	up_in_case
PRJNA8274(	ENSGALG00010017796	MXI1	case	control	-0.099004881	0.056394987	-1.755561726	0.079163272	0.449249472	down_in_case
PRJNA8274(	ENSGALG00010017797	PPP1R21	case	control	0.073533753	0.058588474	1.255089067	0.209446429	0.63414862	up_in_case
PRJNA8274(	ENSGALG00010017798	BAG4	case	control	0.116889745	0.060296564	1.938580524	0.052552437	0.374252913	up_in_case
PRJNA8274(	ENSGALG00010017799	GRAPL	case	control	0.951097274	0.300888123	3.160966492	0.001572466	0.063700459	up_in_case
PRJNA8274(	ENSGALG00010017801	SMNDC1	case	control	0.051243323	0.04469318	1.146557993	0.251564346	0.677081801	up_in_case
PRJNA8274(	ENSGALG00010017803	ENSGALG00010017803	case	control	0.036991843	0.056735014	0.652010815	0.514394196	0.847493921	up_in_case
PRJNA8274(	ENSGALG00010017804	DUSP5	case	control	0.005497849	0.092488996	0.059443282	0.95259904	0.992056045	up_in_case
PRJNA8274(	ENSGALG00010017805	DPP8	case	control	0.01840336	0.067183855	0.273925329	0.784142006	0.949518053	up_in_case
PRJNA8274(	ENSGALG00010017806	ENSGALG00010017806	case	control	-0.311500774	0.304813628	-1.021938476	0.306810021	0.729504707	down_in_case
PRJNA8274(	ENSGALG00010017807	MRPL35	case	control	0.099375704	0.067441054	1.473519438	0.140611027	0.543282655	up_in_case
PRJNA8274(	ENSGALG00010017808	SFRP5	case	control	0.183193567	0.206872745	0.88553747	0.37586682	0.774921593	up_in_case
PRJNA8274(	ENSGALG00010017809	MOSMO	case	control	0.171861106	0.083855384	2.049494	0.040413834	0.33753444	up_in_case
PRJNA8274(	ENSGALG00010017810	HIF1A	case	control	0.025929256	0.051852181	0.500061045	0.617032094	0.891081782	up_in_case
PRJNA8274(	ENSGALG00010017811	HACD3	case	control	0.11079966	0.060053896	1.845003691	0.065037002	0.414658096	up_in_case
PRJNA8274(	ENSGALG00010017813	DDHD2	case	control	-0.052807637	0.067080541	-0.78722736	0.431148784	0.806993142	down_in_case
PRJNA8274(	ENSGALG00010017814	ENSGALG00010017814	case	control	0.256646349	0.268805089	0.954767449	0.339695312	0.751259351	up_in_case
PRJNA8274(	ENSGALG00010017815	VWA9	case	control	-0.038895463	0.06417956	-0.606041285	0.544487339	0.861585064	down_in_case
PRJNA8274(	ENSGALG00010017816	PRKCH	case	control	0.253002999	0.321774528	0.786274169	0.431706882	0.807276582	up_in_case
PRJNA8274(	ENSGALG00010017817	REEP1	case	control	-0.227790403	0.102692004	-2.218190265	0.026541862	0.289731779	down_in_case
PRJNA8274(	ENSGALG00010017818	SLC38A6	case	control	0.121348634	0.119865441	1.012373813	0.311359354	0.734097745	up_in_case
PRJNA8274(	ENSGALG00010017820	FLRT2	case	control	0.000743344	0.07481912	0.00993521	0.99207298	0.997934975	up_in_case
PRJNA8274(	ENSGALG00010017821	ENSGALG00010017821	case	control	0.026752328	0.509719566	0.052484405	0.958142721	0.992056045	up_in_case
PRJNA8274(	ENSGALG00010017822	VWA3A	case	control	-0.041331285	0.204871616	-0.201742368	0.840118139	0.965286179	down_in_case
PRJNA8274(	ENSGALG00010017823	PPIE	case	control	-0.052760602	0.058814333	-0.897070482	0.369681309	0.772544067	down_in_case
PRJNA8274(	ENSGALG00010017824	YAE1	case	control	0.026656836	0.109160633	0.244198255	0.807077279	0.955875648	up_in_case
PRJNA8274(	ENSGALG00010017825	PLPP5	case	control	0.015692998	0.099643594	0.157491285	0.874857676	0.975474836	up_in_case
PRJNA8274(	ENSGALG00010017826	PABPC4	case	control	-0.409867261	0.140419673	-2.9188735	0.003512987	0.101486305	down_in_case
PRJNA8274(	ENSGALG00010017828	ENSGALG00010017828	case	control	-0.012052747	0.068259732	-0.176571849	0.859844708	0.971828322	down_in_case
PRJNA8274(	ENSGALG00010017830	DENND4A	case	control	0.055865326	0.077763594	0.718399486	0.472511004	0.829235491	up_in_case
PRJNA8274(	ENSGALG00010017831	PLEK	case	control	-0.09674775	0.139454755	-0.693757274	0.487834442	0.835553062	down_in_case
PRJNA8274(	ENSGALG00010017832	POU3F1	case	control	0.057995401	0.322146649	0.180027951	0.857130625	0.971282862	up_in_case
PRJNA8274(	ENSGALG00010017833	ENSGALG00010017833	case	control	-0.122259452	0.199341921	-0.613315311	0.539667872	0.858521589	down_in_case
PRJNA8274(	ENSGALG00010017834	ENSGALG00010017834	case	control	-0.501750252	0.257744682	-1.946694873	0.051571327	0.371521396	down_in_case
PRJNA8274(	ENSGALG00010017838	B3GNT2	case	control	0.05056563	0.091641415	0.551777058	0.581101107	0.879651411	up_in_case
PRJNA8274(	ENSGALG00010017841	GALC	case	control	0.001809913	0.175625802	0.010305509	0.991777539	0.99790247	up_in_case

PRJNA8274(	ENSGALG00010017843	NSD3	case	control	-0.005199377	0.035984168	-0.144490671	0.885113022	0.977630122	down_in_case
PRJNA8274(	ENSGALG00010017848	CYP3A4	case	control	0.342374437	0.165038733	2.074509609	0.038032007	0.330206289	up_in_case
PRJNA8274(	ENSGALG00010017849	FBXO48	case	control	-0.037116987	0.150688196	-0.246316484	0.805437251	0.9556344	down_in_case
PRJNA8274(	ENSGALG00010017850	TRMT5	case	control	0.123552711	0.084994482	1.453655685	0.14604179	0.551173819	up_in_case
PRJNA8274(	ENSGALG00010017852	LETM2	case	control	0.060022098	0.076710776	0.782446759	0.433952063	0.808852255	up_in_case
PRJNA8274(	ENSGALG00010017855	SDR42E2	case	control	-0.524910365	0.206162051	-2.546105657	0.010893221	0.185137791	down_in_case
PRJNA8274(	ENSGALG00010017856	FASTKD5	case	control	-0.092044689	0.084422188	-1.090290258	0.275585305	0.700894634	down_in_case
PRJNA8274(	ENSGALG00010017858	GPR65	case	control	-0.054251941	0.175402303	-0.309300052	0.757093292	0.941659745	down_in_case
PRJNA8274(	ENSGALG00010017859	EXOC6	case	control	-0.122165868	0.086631329	-1.410181166	0.158486196	0.570582225	down_in_case
PRJNA8274(	ENSGALG00010017860	CYSLTR1	case	control	-0.089988261	0.164127784	-0.548281704	0.583498489	0.881307382	down_in_case
PRJNA8274(	ENSGALG00010017861	PRSS56	case	control	0.28677436	0.441700159	0.649251203	0.51617602	0.848054666	up_in_case
PRJNA8274(	ENSGALG00010017865	UBOX5	case	control	-0.026830141	0.076532725	-0.350570829	0.725910344	0.932158578	down_in_case
PRJNA8274(	ENSGALG00010017866	RALA	case	control	-0.032389371	0.060872148	-0.532088529	0.594664682	0.884223506	down_in_case
PRJNA8274(	ENSGALG00010017867	APLF	case	control	0.028292046	0.084162164	0.336161098	0.736749387	0.93491137	up_in_case
PRJNA8274(	ENSGALG00010017868	CHRND	case	control	-0.316795241	0.128070713	-2.473596297	0.013376075	0.203413453	down_in_case
PRJNA8274(	ENSGALG00010017870	RAB11A	case	control	0.020442021	0.036905889	0.553895912	0.579650083	0.879142433	up_in_case
PRJNA8274(	ENSGALG00010017873	HSPG2	case	control	-0.230362654	0.135354127	-1.701925596	0.088769317	0.470938334	down_in_case
PRJNA8274(	ENSGALG00010017876	COMMD1	case	control	0.00469973	0.079166216	0.059365355	0.952661107	0.992056045	up_in_case
PRJNA8274(	ENSGALG00010017878	CHRNG	case	control	-0.011955272	0.196355127	-0.06088597	0.951450023	0.991857576	down_in_case
PRJNA8274(	ENSGALG00010017880	CDK13	case	control	-0.024475932	0.047171529	-0.518870868	0.603850794	0.885268908	down_in_case
PRJNA8274(	ENSGALG00010017881	KCNK10	case	control	-0.12826381	0.362877457	-0.353463152	0.723741249	0.930459395	down_in_case
PRJNA8274(	ENSGALG00010017882	LZTS3	case	control	0.108637973	0.136114686	0.798135575	0.424791819	0.804253014	up_in_case
PRJNA8274(	ENSGALG00010017886	DDRGK1	case	control	-0.041009931	0.050810144	-0.807120944	0.419596806	0.80093491	down_in_case
PRJNA8274(	ENSGALG00010017887	PROKR1	case	control	-0.299782561	0.297022532	-1.009292324	0.31283446	0.734574752	down_in_case
PRJNA8274(	ENSGALG00010017888	MPLKIP	case	control	0.08415531	0.070314295	1.19684496	0.23136699	0.656457929	up_in_case
PRJNA8274(	ENSGALG00010017890	ANAPC1	case	control	0.04884931	0.051832099	0.942452872	0.345960829	0.756227431	up_in_case
PRJNA8274(	ENSGALG00010017891	GOLGA7B	case	control	-0.342542393	0.236799268	-1.446551739	0.148022511	0.553857883	down_in_case
PRJNA8274(	ENSGALG00010017894	KAT6A	case	control	-0.020396129	0.045409776	-0.449157231	0.653318237	0.906151774	down_in_case
PRJNA8274(	ENSGALG00010017895	MNAT1	case	control	0.006097887	0.055348281	0.110173015	0.912272163	0.98266608	up_in_case
PRJNA8274(	ENSGALG00010017898	NHERF2	case	control	-0.065830095	0.040209094	-1.637194168	0.101589905	0.491460867	down_in_case
PRJNA8274(	ENSGALG00010017901	HTR7L	case	control	-0.03779464	0.519571459	-0.072741949	0.942011467	0.989551456	down_in_case
PRJNA8274(	ENSGALG00010017903	ENSGALG00010017903	case	control	0.12435453	0.396287703	0.31379861	0.753674	0.940858857	up_in_case
PRJNA8274(	ENSGALG00010017904	MEGF11	case	control	-0.248241095	0.1884807	-1.317063739	0.18781726	0.610946377	down_in_case
PRJNA8274(	ENSGALG00010017905	EIF4E2	case	control	0.048508041	0.071647151	0.677040753	0.498380098	0.83983448	up_in_case
PRJNA8274(	ENSGALG00010017909	NTHL1	case	control	0.007079188	0.139312349	0.050815226	0.959472758	0.992056045	up_in_case
PRJNA8274(	ENSGALG00010017915	BFSP1	case	control	-0.272046979	0.443446482	-0.613483229	0.539556869	0.858521589	down_in_case
PRJNA8274(	ENSGALG00010017918	SIX4	case	control	-0.133450676	0.071847336	-1.857419951	0.06325143	0.409249003	down_in_case
PRJNA8274(	ENSGALG00010017920	AP3M2	case	control	0.166095347	0.059584729	2.787548922	0.005310843	0.12671398	up_in_case
PRJNA8274(	ENSGALG00010017921	SPATA7	case	control	-0.169819206	0.135936213	-1.249256559	0.211571251	0.636501957	down_in_case
PRJNA8274(	ENSGALG00010017922	MERTK	case	control	0.051682062	0.061912264	0.834762925	0.403851204	0.79260318	up_in_case
PRJNA8274(	ENSGALG00010017927	PCSK2	case	control	-0.292759747	0.147931094	-1.979027788	0.047812878	0.360228144	down_in_case
PRJNA8274(	ENSGALG00010017930	TSC2	case	control	-0.057841293	0.050050197	-1.155665633	0.247817951	0.67225887	down_in_case
PRJNA8274(	ENSGALG00010017933	SIX1	case	control	-0.073984075	0.088432544	-0.836615932	0.402808487	0.792021921	down_in_case
PRJNA8274(	ENSGALG00010017937	PTPN21	case	control	-0.200885991	0.091248645	-2.201522996	0.027699021	0.296701119	down_in_case
PRJNA8274(	ENSGALG00010017941	DIS3L	case	control	0.106829053	0.062939854	1.697319679	0.089636239	0.472813801	up_in_case
PRJNA8274(	ENSGALG00010017946	PTGDR	case	control	0.092699009	0.17882818	0.518369134	0.604200746	0.885268908	up_in_case
PRJNA8274(	ENSGALG00010017948	PLAT	case	control	-0.041332841	0.111170578	-0.371796586	0.710044306	0.925630832	down_in_case
PRJNA8274(	ENSGALG00010017949	SFXN3	case	control	-0.081151391	0.072956333	-1.112328265	0.265997035	0.69218122	down_in_case
PRJNA8274(	ENSGALG00010017950	FBLN7	case	control	0.332757038	0.298756112	1.113808303	0.265361434	0.691495163	up_in_case
PRJNA8274(	ENSGALG00010017954	PTGER2	case	control	0.10829069	0.156053501	0.693933097	0.487724166	0.835553062	up_in_case
PRJNA8274(	ENSGALG00010017955	IKBKB	case	control	-0.021990122	0.062946976	-0.349343583	0.726831383	0.932420272	down_in_case
PRJNA8274(	ENSGALG00010017956	TIPIN	case	control	0.00724533	0.086333248	0.083922818	0.933117797	0.987991895	up_in_case
PRJNA8274(	ENSGALG00010017957	THUMPD1	case	control	-0.094794047	0.061876346	-1.531991669	0.125524491	0.524979916	down_in_case
PRJNA8274(	ENSGALG00010017958	CAPN10	case	control	0.035085466	0.101905424	0.34429439	0.730624903	0.933770678	up_in_case
PRJNA8274(	ENSGALG00010017959	PDZD7	case	control	-0.387962964	0.17924284	-2.164454464	0.030429488	0.307572163	down_in_case
PRJNA8274(	ENSGALG00010017962	TXNDC16	case	control	0.06041163	0.05612376	1.076400267	0.281748283	0.705775113	up_in_case
PRJNA8274(	ENSGALG00010017963	LPAR4	case	control	0.003223998	0.065422453	0.04927968	0.960696413	0.992056045	up_in_case
PRJNA8274(	ENSGALG00010017967	ACSM3	case	control	1.127704448	0.681531271	1.654662811	0.097992915	0.487193947	up_in_case
PRJNA8274(	ENSGALG00010017970	AMPH	case	control	1.202786684	0.731890379	1.643397315	0.100300755	0.489689587	up_in_case
PRJNA8274(	ENSGALG00010017972	RNPEPL1	case	control	-0.102091055	0.053067123	-1.923809865	0.05437842	0.380622326	down_in_case
PRJNA8274(	ENSGALG00010017973	PKD1	case	control	-0.105045385	0.098232681	-1.069352717	0.284910764	0.709671474	down_in_case
PRJNA8274(	ENSGALG00010017974	MXRA8	case	control	0.090730198	0.080716648	1.124058061	0.260988395	0.687516348	up_in_case
PRJNA8274(	ENSGALG00010017976	GPR137C	case	control	0.01908285	0.077765428	0.245389892	0.806154555	0.9556344	up_in_case
PRJNA8274(	ENSGALG00010017979	ENSGALG00010017979	case	control	0.159234812	0.116429482	1.367650263	0.171421575	0.588978502	up_in_case
PRJNA8274(	ENSGALG00010017983	SLC4A11	case	control	0.056581718	0.12723644	0.44469743	0.656538427	0.907494354	up_in_case
PRJNA8274(	ENSGALG00010017985	ABCC5	case	control	0.294915723	0.147854923	1.994629045	0.046083353	0.356019424	up_in_case
PRJNA8274(	ENSGALG00010017987	ERI2	case	control	0.139888931	0.154495009	0.905459229	0.36522215	0.770843433	up_in_case
PRJNA8274(	ENSGALG00010017991	RAB26	case	control	-0.080577213	0.104725265	-0.769415218	0.441646857	0.813488561	down_in_case
PRJNA8274(	ENSGALG00010017993	REXO5	case	control	0.064658825	0.078082078	0.828087908	0.407620717	0.794388636	up_in_case
PRJNA8274(	ENSGALG00010017995	ENSGALG00010017995	case	control	-0.603687293	1.008477637	-0.598612474	0.549431336	0.864234145	down_in_case
PRJNA8274(	ENSGALG00010017996	ZC3H14	case	control	-0.005167529	0.037395745	-0.138184959	0.890094242	0.978501511	down_in_case
PRJNA8274(	ENSGALG00010017997	ENSGALG00010017997	case	control	1.904965297	0.542678868	3.510299382	0.000447602	0.03019205	up_in_case
PRJNA8274(	ENSGALG00010018000	MAP2K1	case	control	0.05246078	0.047513098	1.10413301	0.269535444	0.696612479	up_in_case
PRJNA8274(	ENSGALG00010018001	TRAF7	case	control	-0.037380059	0.054183075	-0.689884419	0.490266875	0.836347578	down_in_case
PRJNA8274(	ENSGALG00010018002	ZC3H8	case	control	0.038861759	0.050057761	0.776338343	0.437549239	0.810720016	up_in_case
PRJNA8274(	ENSGALG00010018004	CDC42BPA	case	control	-0.257485062	0.095450755	-2.697569666	0.006984767	0.145020586	down_in_case
PRJNA8274(	ENSGALG00010018006	DCUN1D3	case	control	-0.132494156	0.080365028	-1.648654388	0.09921846	0.488407564	down_in_case
PRJNA8274(	ENSGALG00010018007	DNAAF9	case	control	0.317824376	0.089923872	3.534371545	0.000408746	0.02851253	up_in_case
PRJNA8274(	ENSGALG00010018008	ABCF3	case	control	-0.059631811	0.057446177	-1.038046633	0.299248345	0.722718284	down_in_case
PRJNA8274(	ENSGALG00010018010	ENSGALG00010018010	case	control	-0.050632252	0.060481711	-0.837149797	0.402508372	0.792021921	down_in_case
PRJNA8274(	ENSGALG00010018011	LIPA	case	control	0.096551235	0.085772439	1.125667358	0.260306345	0.687293341	up_in_case
PRJNA8274(	ENSGALG00010018012	DVL1	case	control	-0.031696696	0.06568815	-0.482532938	0.629427406	0.896269608	down_in_case
PRJNA8274(	ENSGALG00010018014	LYRM1	case	control	0.093355797	0.05124362	1.82180333	0.068484832	0.422606297	up_in_case
PRJNA8274(	ENSGALG00010018016	IFIT5	case	control	-0.550211973	0.333554261	-1.649542632	0.099036517	0.488407564	down_in_case
PRJNA8274(	ENSGALG00010018019	NAPB	case	control	0.462194153	0.299910374	1.541107587	0.123290604	0.5226966	up_in_case
PRJNA8274(	ENSGALG00010018020	SNAPC5	case	control	0.093805765	0.077810402	1.205568448	0.227983931	0.651681204	up_in_case
PRJNA8274(	ENSGALG00010018021	ATRN	case	control	-0.103831138	0.081329957	-1.27666536	0.201720416	0.6249678	down_in_case
PRJNA8274(	ENSGALG00010018022	XPO6	case	control	0.068969853	0.061430138	1.122736416	0.261549457	0.687516348	up_in_case
PRJNA8274(	ENSGALG00010018025	CPTP	case	control	0.066941573	0.079696987	0.839951113	0.400935797	0.790593202	up_in_case
PRJNA8274(	ENSGALG00010018026	CCDC39	case	control	-0.665840119	0.385010993	-1.729405474	0.083736551	0.458084422	down_in_case
PRJNA8274(	ENSGALG00010018027	EML5	case	control	0.51708707	0.305621462	1.691920019	0.090661224	0.474544476	up_in_case
PRJNA8274(	ENSGALG00010018030	POLB	case	control	-0.042394246	0.054788936	-0.773773845	0.439064542	0.812346093	down_in_case
PRJNA8274(	ENSGALG00010018032	ERO1A	case	control	-0.0276547	0.072203448	-0.38301079	0.701711764	0.922880366	down_in_case
PRJNA8274(	ENSGALG00010018033	COQ8A	case	control	-0.164937988	0.105142101	-1.56871498	0.116714366	0.51350396	down_in_case
PRJNA8274(	ENSGALG00010018035	ITM2A	case	control	0.00726798	0.040771886	0.178259612	0.858519096	0.971828322	up_in_case
PRJNA8274(	ENSGALG00010018036	RPL4	case	control	0.014015805	0.055733481	0.251479088	0.801443728	0.954294696	up_in_case
PRJNA8274(	ENSGALG00010018037	GZF1	case	control	0.210294551	0.060662668	3.466622203	0.000527042	0.033449927	up_in_case
PRJNA8274(	ENSGALG00010018039	TTC14	case	control	0.072960385	0.083670732	0.871994103	0.383211594	0.780061953	up_in_case
PRJNA8274(	ENSGALG00010018042	ZWILCH	case	control	-0.233015815	0.106314742	-2.191754505	0.028397238	0.300549329	down_in_case
PRJNA8274(	ENSGALG00010018043	PSMC6	case	control	-0.088424841	0.048317547	-1.830077192	0.067238397	0.419689688	down_in_case
PRJNA8274(	ENSGALG00010018044	INTS11	case	control	0.103201896	0.066629004	1.548903479	0.121404925	0.519631973	up_in_case
PRJNA8274(	ENSGALG00010018045	PSEN2	case	control	0.124282505	0.060451835	2.055893026	0.039792828	0.33650943	up_in_case
PRJNA8274(	ENSGALG00010018046	CAPN15	case	control	0.114115884	0.076971832	1.482566816	0.138189539	0.540363798	up_in_case
PRJNA8274(	ENSGALG00010018048	GFRA4	case	control	0.2276457	0.14707075	1.547865224	0.121654749	0.519686058	up_in_case
PRJNA8274(	ENSGALG00010018049	SUGCT	case	control	-0.331619715	0.196732001	-1.685641951	0.091864784	0.476713154	down_in_case
PRJNA8274(	ENSGALG00010018051	GNPNAT1	case	control	0.00146692	0.058239428	0.025187752	0.979905206	0.995636523	up_in_case
PRJNA8274(	ENSGALG00010018052	PPP1RL	case	control	0.046184359	0.043050037	1.07280651	0.283357939	0.707725096	up_in_case
PRJNA8274(	ENSGALG00010018053	DIAPH2	case	control	-0.041080863	0.064872563	-0.633254822	0.526567251	0.85251736	down_in_case
PRJNA8274(	ENSGALG00010018054	SMAD6	case	control	0.017090347	0.157309435	0.108641588	0.913486774	0.982728035	up_in_case
PRJNA8274(	ENSGALG00010018055	PEX5L	case	control	-0.047618024	0.370410297	-0.128554807	0.897709929	0.980176778	down_in_case
PRJNA8274(	ENSGALG00010018057	SSTR4	case	control	0.138615186	0.110921494	1.249669296	0.211420378	0.636501957	up_in_case
PRJNA8274(	ENSGALG00010018058	ITPKB	case	control	-0.01814685	0.060404492	-0.300422197	0.763855134	0.943786458	down_in_case
PRJNA8274(	ENSGALG00010018060	CASKIN1	case	control	-0.287679563	0.141558415	-2.032232149	0.04213016	0.342502151	down_in_case
PRJNA8274(	ENSGALG00010018061	NDUFAF5	case	control	-0.079557558	0.104656329	-0.760179139	0.447147513	0.817833512	down_in_case
PRJNA8274(	ENSGALG00010018062	GOLGA7	case	control	0.063212795	0.052073799	1.213907873	0.224782906	0.648819735	up_in_case
PRJNA8274(	ENSGALG00010018063	DECR2	case	control	0.174450759	0.06967642	2.503727369	0.012289269	0.194829874	up_in_case
PRJNA8274(	ENSGALG00010018064	TTC8	case	control	0.226348177	0.095015412	2.382225904	0.017208337	0.233693469	up_in_case
PRJNA8274(	ENSGALG00010018067	ENSGALG00010018067	case	control	-0.15675171	0.134911427	-1.161886089	0.245281738	0.669759186	down_in_case
PRJNA8274(	ENSGALG00010018068	ADAM33	case	control	-0.019750135	0.087241589	-0.226384401	0.82090246	0.958669267	down_in_case
PRJNA8274(	ENSGALG00010018069	ENSGALG00010018069	case	control	0.060542587	0.329623065	0.18367218	0.854270634	0.970137355	up_in_case
PRJNA8274(	ENSGALG00010018071	GPAT4	case	control	-0.018892467	0.048742664	-0.387596103	0.698314953	0.922288441	down_in_case
PRJNA8274(	ENSGALG00010018075	MLST8	case	control	-0.018273923	0.062440943	-0.292659312	0.769782573	0.945428692	down_in_case
PRJNA8274(	ENSGALG00010018076	FOXA2	case	control	0.113415075	0.981325233	0.115573381	0.90799066	0.981730793	up_in_case
PRJNA8274(	ENSGALG00010018078	NKX6-3	case	control	0.237618266	0.469838076	0.505745018	0.613035661	0.890154949	up_in_case
PRJNA8274(	ENSGALG00010018080	BRICD5	case	control	-0.025011461	0.122405738	-0.204332421	0.838093736	0.964826648	down_in_case
PRJNA8274(	ENSGALG00010018081	DIO3	case	control	0.109231115	0.206084507	0.530030698	0.596090647	0.884680373	up_in_case

PRJNA8274(	ENSGALG00010018082	RAB11FIP3	case	control	0.26918496	0.067867819	3.966312198	7.29932E-05	0.008784022	up_in_case
PRJNA8274(	ENSGALG00010018083	PAX1	case	control	0.135854627	0.557658838	0.24361602	0.80752822	0.955875648	up_in_case
PRJNA8274(	ENSGALG00010018085	PGP	case	control	0.073029835	0.139821959	0.522305904	0.601457359	0.884954589	up_in_case
PRJNA8274(	ENSGALG00010018087	ENSGALG00010018087	case	control	0.028677159	0.059766894	0.479816779	0.631357681	0.897039706	up_in_case
PRJNA8274(	ENSGALG00010018089	TASP1	case	control	0.080440641	0.105539508	0.762185106	0.445949534	0.817315674	up_in_case
PRJNA8274(	ENSGALG00010018092	ENSGALG00010018092	case	control	-0.142630469	0.111149702	-1.283228525	0.199412025	0.622902456	down_in_case
PRJNA8274(	ENSGALG00010018095	E4F1	case	control	0.023828521	0.056179563	0.424149275	0.671456952	0.913676733	up_in_case
PRJNA8274(	ENSGALG00010018099	SMAD3	case	control	-0.010605398	0.062128902	-0.170699909	0.864459734	0.972984982	down_in_case
PRJNA8274(	ENSGALG00010018102	ZDHHC4	case	control	0.067873918	0.132156509	0.513587401	0.60754049	0.887328057	up_in_case
PRJNA8274(	ENSGALG00010018103	BTRC	case	control	0.003088686	0.078385175	0.03940396	0.968568323	0.992761697	up_in_case
PRJNA8274(	ENSGALG00010018111	NKX2-2	case	control	-0.200929127	0.321038323	-0.625872717	0.531398431	0.855560567	down_in_case
PRJNA8274(	ENSGALG00010018115	KLHL4	case	control	0.000482381	0.128480676	0.003754499	0.99700435	0.998921977	up_in_case
PRJNA8274(	ENSGALG00010018119	GRID2IP	case	control	-0.092119135	0.39967195	-0.230486866	0.817713469	0.95838887	down_in_case
PRJNA8274(	ENSGALG00010018121	FERMT2	case	control	-0.103468798	0.077805566	-1.329838002	0.183571652	0.605134769	down_in_case
PRJNA8274(	ENSGALG00010018123	USP13	case	control	-0.209006647	0.084032431	-2.487214109	0.012874789	0.199684901	down_in_case
PRJNA8274(	ENSGALG00010018124	FBXO41	case	control	-0.645173197	0.961561745	-0.670963878	0.502243541	0.842077927	down_in_case
PRJNA8274(	ENSGALG00010018128	SEPTIN12	case	control	-0.095824037	0.095861795	-0.999606121	0.31750116	0.737774213	down_in_case
PRJNA8274(	ENSGALG00010018137	NDUFB5	case	control	-0.086515869	0.079003783	-1.095085143	0.273479337	0.69934809	down_in_case
PRJNA8274(	ENSGALG00010018140	CHPF	case	control	-0.050635541	0.096776595	-0.523220935	0.600820515	0.884954589	down_in_case
PRJNA8274(	ENSGALG00010018142	DDHD1	case	control	0.155059482	0.072210951	2.14731255	0.031768407	0.3098361	up_in_case
PRJNA8274(	ENSGALG00010018143	MRPL47	case	control	-0.048873593	0.068881028	-0.709536347	0.477991704	0.830520323	down_in_case
PRJNA8274(	ENSGALG00010018145	ENSGALG00010018145	case	control	0.505631493	0.23671133	2.136067986	0.032673873	0.31149092	up_in_case
PRJNA8274(	ENSGALG00010018147	ISM1	case	control	-0.004803485	0.120247814	-0.03994655	0.968135739	0.992761697	down_in_case
PRJNA8274(	ENSGALG00010018148	XRN2	case	control	-0.072847765	0.04216791	-1.727564033	0.084066416	0.45883578	down_in_case
PRJNA8274(	ENSGALG00010018149	ENSGALG00010018149	case	control	-0.132246887	0.136670035	-0.967636301	0.333226041	0.748508541	down_in_case
PRJNA8274(	ENSGALG00010018150	AAGAB	case	control	-0.045873945	0.070282811	-0.652705044	0.51394645	0.847493921	down_in_case
PRJNA8274(	ENSGALG00010018154	SLC16A12	case	control	-0.2261956	0.240355486	-0.941087737	0.346659899	0.757096367	down_in_case
PRJNA8274(	ENSGALG00010018157	FBXL2	case	control	-0.025269387	0.147852066	-0.170909933	0.864294586	0.97295831	down_in_case
PRJNA8274(	ENSGALG00010018160	ENSGALG00010018160	case	control	0.149120693	0.143979303	1.035709232	0.300337814	0.723279006	up_in_case
PRJNA8274(	ENSGALG00010018162	ENSGALG00010018162	case	control	0.516889015	0.258627605	1.998584083	0.045653374	0.354999047	up_in_case
PRJNA8274(	ENSGALG00010018163	CCT7	case	control	0.038251747	0.051295194	0.745717962	0.455837807	0.822065071	up_in_case
PRJNA8274(	ENSGALG00010018165	DACH2	case	control	0.015587805	0.093093789	0.167441944	0.867022324	0.973570415	up_in_case
PRJNA8274(	ENSGALG00010018166	MSS51	case	control	-0.343721607	0.165153142	-2.081229602	0.037412895	0.328438691	down_in_case
PRJNA8274(	ENSGALG00010018168	ENSGALG00010018168	case	control	-0.305885292	0.174473013	-1.75319544	0.079568465	0.450131333	down_in_case
PRJNA8274(	ENSGALG00010018172	ACTL6A	case	control	0.033902294	0.078962514	0.429346684	0.667670948	0.912432583	up_in_case
PRJNA8274(	ENSGALG00010018174	BMP4	case	control	-0.05298978	0.137071273	-0.386585598	0.699063022	0.922288441	down_in_case
PRJNA8274(	ENSGALG00010018177	KIF20B	case	control	-0.076380432	0.109016873	-0.70062945	0.483534294	0.833691495	down_in_case
PRJNA8274(	ENSGALG00010018179	PRADC1	case	control	-0.040050491	0.084577818	-0.473534219	0.635832094	0.898691227	down_in_case
PRJNA8274(	ENSGALG00010018183	CYP17A1	case	control	-0.128662079	0.231948467	-0.55470114	0.579099097	0.879142433	down_in_case
PRJNA8274(	ENSGALG00010018185	GNB4	case	control	-0.026331286	0.03700915	-0.711480436	0.47678657	0.830520323	down_in_case
PRJNA8274(	ENSGALG00010018186	SPTLC3	case	control	0.485897175	0.477649131	1.017267997	0.309025957	0.731849981	up_in_case
PRJNA8274(	ENSGALG00010018188	UBP1	case	control	-0.047663535	0.03289986	-1.448745832	0.147408576	0.552558944	down_in_case
PRJNA8274(	ENSGALG00010018189	MIS18BP1	case	control	0.0563228	0.094368869	0.59683665	0.550616446	0.86457121	up_in_case
PRJNA8274(	ENSGALG00010018192	IQCH	case	control	-0.062975294	0.198155772	-0.317807011	0.750631332	0.939972946	down_in_case
PRJNA8274(	ENSGALG00010018193	ANK1	case	control	-0.09366867	0.108588782	-0.862599871	0.388357501	0.783067156	down_in_case
PRJNA8274(	ENSGALG00010018198	BTBD3	case	control	-0.038144391	0.061801359	-0.617209571	0.537096503	0.857483532	down_in_case
PRJNA8274(	ENSGALG00010018199	BORCS7	case	control	-0.020872448	0.057434285	-0.363414423	0.71629533	0.926862121	down_in_case
PRJNA8274(	ENSGALG00010018206	KIZ	case	control	0.034836948	0.107214607	0.32492726	0.745236125	0.937849656	up_in_case
PRJNA8274(	ENSGALG00010018209	GMPPA	case	control	0.103207963	0.053233264	1.938787067	0.052527272	0.374252913	up_in_case
PRJNA8274(	ENSGALG00010018215	CFAP70	case	control	-0.646451256	0.195148499	-3.312611974	0.000924291	0.046346052	down_in_case
PRJNA8274(	ENSGALG00010018216	FANCM	case	control	0.009493583	0.062820082	0.151123373	0.879878395	0.976318821	up_in_case
PRJNA8274(	ENSGALG00010018217	ENSGALG00010018217	case	control	0.363110802	0.327728026	1.107963839	0.267877433	0.694521603	up_in_case
PRJNA8274(	ENSGALG00010018219	SLC24A5	case	control	4.619915216	0.541026727	8.539162643	1.35198E-17	1.28889E-14	up_in_case
PRJNA8274(	ENSGALG00010018226	JAG1	case	control	0.001127235	0.061454224	0.018342672	0.985365486	0.996591771	up_in_case
PRJNA8274(	ENSGALG00010018229	AS3MT	case	control	-0.559424583	0.126053372	-4.437997765	9.07996E-06	0.001583456	down_in_case
PRJNA8274(	ENSGALG00010018231	SMYD5	case	control	-0.020413524	0.068437424	-0.298280134	0.765489365	0.943786458	down_in_case
PRJNA8274(	ENSGALG00010018233	RASSF2	case	control	0.350872832	0.090510085	3.87661586	0.000105919	0.011055819	up_in_case
PRJNA8274(	ENSGALG00010018240	SOUL	case	control	0.32503241	0.393744997	0.825489627	0.409093669	0.795170398	up_in_case
PRJNA8274(	ENSGALG00010018241	RALGAPA2	case	control	-0.027923077	0.067920116	-0.411116455	0.680987144	0.915594727	down_in_case
PRJNA8274(	ENSGALG00010018244	CHM	case	control	-0.121222778	0.064390493	-1.882619194	0.059751993	0.397790272	down_in_case
PRJNA8274(	ENSGALG00010018251	USP48	case	control	-0.039758813	0.03436912	-1.156817874	0.247346778	0.671936745	down_in_case
PRJNA8274(	ENSGALG00010018255	MFN1	case	control	-0.089970597	0.061568671	-1.461304834	0.143931804	0.547285923	down_in_case
PRJNA8274(	ENSGALG00010018258	OSCP1	case	control	0.164276692	0.131559574	1.248686709	0.211779684	0.636501957	up_in_case
PRJNA8274(	ENSGALG00010018259	WDR90	case	control	-0.221539716	0.080461741	-2.753354739	0.005898794	0.133681071	down_in_case
PRJNA8274(	ENSGALG00010018261	PRNP	case	control	-0.010860047	0.074142573	-0.14647519	0.883546274	0.977115076	down_in_case
PRJNA8274(	ENSGALG00010018263	SLX4IP	case	control	0.050193491	0.101637394	0.493848659	0.621413066	0.893175881	up_in_case
PRJNA8274(	ENSGALG00010018264	CFAP61	case	control	0.376961827	0.344960134	1.09276925	0.274495126	0.699970438	up_in_case
PRJNA8274(	ENSGALG00010018265	PDCD4	case	control	-0.029141609	0.068403344	-0.42602609	0.670088835	0.913465866	down_in_case
PRJNA8274(	ENSGALG00010018267	ADPRHL2	case	control	-0.053294225	0.074933435	-0.711220896	0.476947362	0.830520323	down_in_case
PRJNA8274(	ENSGALG00010018273	PROM2	case	control	0.670840394	0.808490805	0.829743999	0.406683539	0.794253996	up_in_case
PRJNA8274(	ENSGALG00010018274	ZNF711	case	control	0.035390718	0.05720028	0.618715824	0.536103584	0.857238203	up_in_case
PRJNA8274(	ENSGALG00010018275	ZNF639	case	control	-0.036644441	0.051540642	-0.710981462	0.477095724	0.830520323	down_in_case
PRJNA8274(	ENSGALG00010018276	ARHGAP17	case	control	-0.034742316	0.053531811	-0.649003181	0.51633632	0.848054666	down_in_case
PRJNA8274(	ENSGALG00010018278	PUSL1	case	control	-0.050074668	0.139059408	-0.360095507	0.718775713	0.927996812	down_in_case
PRJNA8274(	ENSGALG00010018279	INSM1	case	control	1.004292795	0.662470396	1.515981396	0.129524057	0.531933949	up_in_case
PRJNA8274(	ENSGALG00010018283	POF1B	case	control	0.02131775	0.142703074	0.149385358	0.881249565	0.97667824	up_in_case
PRJNA8274(	ENSGALG00010018284	ENSGALG00010018284	case	control	-0.08024987	0.099298835	-0.808165271	0.418995447	0.80093491	down_in_case
PRJNA8274(	ENSGALG00010018285	BBIP1	case	control	-0.023399319	0.083530939	-0.280127566	0.779379636	0.948763837	down_in_case
PRJNA8274(	ENSGALG00010018287	MKKS	case	control	0.030160143	0.050358917	0.598903719	0.549237092	0.864234145	up_in_case
PRJNA8274(	ENSGALG00010018288	CRNKL1	case	control	0.046905127	0.041388843	1.133279481	0.257096906	0.683997349	up_in_case
PRJNA8274(	ENSGALG00010018289	HEYL	case	control	-0.006438506	0.092604893	-0.069526624	0.944570441	0.990313016	down_in_case
PRJNA8274(	ENSGALG00010018290	RHOT2	case	control	0.033358784	0.06265091	0.532454901	0.59441097	0.883951421	up_in_case
PRJNA8274(	ENSGALG00010018294	FKBP3	case	control	-0.100294466	0.069459662	-1.443923908	0.148760381	0.55519893	down_in_case
PRJNA8274(	ENSGALG00010018296	PIK3CA	case	control	-0.09308386	0.054661364	-1.70291874	0.088583276	0.470732387	down_in_case
PRJNA8274(	ENSGALG00010018298	ENSGALG00010018298	case	control	0.159281405	0.140643268	1.132520643	0.257415611	0.684335979	up_in_case
PRJNA8274(	ENSGALG00010018299	SHOC2	case	control	0.039189692	0.05317292	0.7370235	0.461108059	0.82454265	up_in_case
PRJNA8274(	ENSGALG00010018301	ENSGALG00010018301	case	control	0.143605687	0.2870433	0.500292768	0.616868946	0.891081782	up_in_case
PRJNA8274(	ENSGALG00010018302	RAB11FIP5	case	control	0.042917775	0.054147425	0.792609724	0.428005242	0.805337183	up_in_case
PRJNA8274(	ENSGALG00010018306	DES	case	control	-0.160508771	0.143263267	-1.120376314	0.262553434	0.688522667	down_in_case
PRJNA8274(	ENSGALG00010018308	ACAP3	case	control	0.177169442	0.092425672	1.916885636	0.055252462	0.383470355	up_in_case
PRJNA8274(	ENSGALG00010018309	DPYSL2	case	control	0.051437012	0.08563926	0.600624196	0.548090318	0.863372059	up_in_case
PRJNA8274(	ENSGALG00010018310	APOOL	case	control	0.053423318	0.05322401	1.003744696	0.315501687	0.736748426	up_in_case
PRJNA8274(	ENSGALG00010018312	PRPF39	case	control	0.050598224	0.060006683	0.843209816	0.399111109	0.789900496	up_in_case
PRJNA8274(	ENSGALG00010018313	MYEF2	case	control	0.01565258	0.043181008	0.362487605	0.716987684	0.926862121	up_in_case
PRJNA8274(	ENSGALG00010018314	SLC24A3	case	control	-0.108811073	0.062933854	-1.728975222	0.08381353	0.458188945	down_in_case
PRJNA8274(	ENSGALG00010018315	CLASP2	case	control	-0.093723714	0.073638776	-1.272749472	0.203106956	0.625537942	down_in_case
PRJNA8274(	ENSGALG00010018317	ZMAT3	case	control	-0.107773072	0.141219835	-0.763158176	0.445369069	0.816860877	down_in_case
PRJNA8274(	ENSGALG00010018319	STUB1	case	control	0.000576696	0.052968082	0.010887613	0.991313113	0.99790247	up_in_case
PRJNA8274(	ENSGALG00010018322	NT5C1A	case	control	0.065307168	0.170489928	0.383055871	0.701678339	0.922880366	up_in_case
PRJNA8274(	ENSGALG00010018323	GOT1	case	control	-0.238538525	0.097833232	-2.438215722	0.014759963	0.214499461	down_in_case
PRJNA8274(	ENSGALG00010018325	ENSGALG00010018325	case	control	-0.020351217	0.207562861	-0.098048453	0.921893819	0.985482967	down_in_case
PRJNA8274(	ENSGALG00010018326	LCMT1	case	control	-0.053756179	0.066894756	-0.803593311	0.421631887	0.802413627	down_in_case
PRJNA8274(	ENSGALG00010018327	SNAP25	case	control	-0.221282052	0.55073391	-0.401794856	0.687835008	0.917290381	down_in_case
PRJNA8274(	ENSGALG00010018329	ENSGALG00010018329	case	control	0.11857711	0.194173753	0.610675276	0.541414577	0.85975913	up_in_case
PRJNA8274(	ENSGALG00010018333	ENSGALG00010018333	case	control	0.265893764	0.299283656	0.888433964	0.374307363	0.774434633	up_in_case
PRJNA8274(	ENSGALG00010018336	JMJD8	case	control	0.10921316	0.084207456	1.296953565	0.194647165	0.617310812	up_in_case
PRJNA8274(	ENSGALG00010018337	TMEM151B	case	control	-0.070562744	0.15030139	-0.46947499	0.638730157	0.900303403	down_in_case
PRJNA8274(	ENSGALG00010018338	PDCD6IP	case	control	0.016823575	0.059877114	0.280968374	0.778734657	0.948479876	up_in_case
PRJNA8274(	ENSGALG00010018339	FAM76A	case	control	0.02558815	0.05400822	0.473782506	0.635655011	0.898681129	up_in_case
PRJNA8274(	ENSGALG00010018340	DNPEP	case	control	-0.07132615	0.06239332	-1.143169644	0.252968163	0.678440497	down_in_case
PRJNA8274(	ENSGALG00010018342	BNIP3L	case	control	0.005930435	0.054705497	0.108406558	0.9136732	0.982813807	up_in_case
PRJNA8274(	ENSGALG00010018344	SFXN5	case	control	0.022229906	0.083974103	0.264723355	0.791222585	0.95239351	up_in_case
PRJNA8274(	ENSGALG00010018345	WDR24	case	control	-0.08830126	0.082034225	-1.076395364	0.281750475	0.705775113	down_in_case
PRJNA8274(	ENSGALG00010018347	CTXN2	case	control	-0.815314386	0.504838016	-1.615001961	0.10631027	0.496267271	down_in_case
PRJNA8274(	ENSGALG00010018348	TOGARAM1	case	control	0.053090327	0.056543556	0.938927987	0.347767719	0.758360178	up_in_case
PRJNA8274(	ENSGALG00010018349	KCNMB2	case	control	-0.255202511	0.128259498	-1.989735773	0.04662005	0.356907825	down_in_case
PRJNA8274(	ENSGALG00010018350	CNNM1	case	control	-0.07971257	0.341583301	-0.233362024	0.8154803	0.957374769	down_in_case
PRJNA8274(	ENSGALG00010018351	RIN2	case	control	-0.049643509	0.058086049	-0.854654589	0.392742397	0.786144496	down_in_case
PRJNA8274(	ENSGALG00010018354	AARS2	case	control	0.041261559	0.066631479	0.619250234	0.535751523	0.857154485	up_in_case
PRJNA8274(	ENSGALG00010018355	PRKCB	case	control	-0.074803825	0.132419543	-0.564900192	0.572141653	0.876814097	down_in_case
PRJNA8274(	ENSGALG00010018356	ADRA1A	case	control	0.335535426	0.437981006	0.766095838	0.443619279	0.814764409	up_in_case
PRJNA8274(	ENSGALG00010018358	FBXL16	case	control	-0.173361132	0.371444456	-0.46672155	0.64069911	0.901327818	down_in_case
PRJNA8274(	ENSGALG00010018359	CACNG3	case	control	0.01944023	0.409439295	0.047480128	0.962130568	0.992056045	up_in_case
PRJNA8274(	ENSGALG00010018361	ASIC4	case	control	-0.057357099	0.160591752	-0.357160927	0.720971335	0.92919613	down_in_case
PRJNA8274(	ENSGALG00010018363	TCTE1	case	control	-0.036204733	0.149844703	-0.241615035	0.809078471	0.955875648	down_in_case
PRJNA8274(	ENSGALG00010018365	HPSE2	case	control	-0.096606703	0.170960951	-0.565080517	0.572018999	0.876814097	down_in_case

PRJNA8274(	ENSGALG00010018367	PAK5	case	control	-0.096663821	0.129114286	-0.748668668	0.454056932	0.821331802	down_in_case
PRJNA8274(	ENSGALG00010018370	ZBTB8A	case	control	0.081778582	0.07301986	1.119949866	0.262735127	0.688674415	up_in_case
PRJNA8274(	ENSGALG00010018371	ENSGALG00010018371	case	control	0.027016454	0.137537954	0.19642908	0.844274337	0.96721237	up_in_case
PRJNA8274(	ENSGALG00010018372	NLGN3	case	control	-0.018963066	0.165907186	-0.114299243	0.909000583	0.981730793	down_in_case
PRJNA8274(	ENSGALG00010018374	HPS1	case	control	0.07122322	0.099513737	0.715712441	0.474168927	0.82979716	up_in_case
PRJNA8274(	ENSGALG00010018376	PYGB	case	control	-0.146536586	0.07886281	-1.858120277	0.063151936	0.409119439	down_in_case
PRJNA8274(	ENSGALG00010018377	RAP1GAP	case	control	-0.097016995	0.234069404	-0.414479608	0.678522898	0.914942347	down_in_case
PRJNA8274(	ENSGALG00010018378	ARPP21	case	control	-0.226389558	0.091082424	-2.485546025	0.012935286	0.199897239	down_in_case
PRJNA8274(	ENSGALG00010018379	SRBD1	case	control	0.05162258	0.095262033	0.541900883	0.587886788	0.882771329	up_in_case
PRJNA8274(	ENSGALG00010018380	SLC20A1	case	control	0.245859024	0.118161075	2.080710799	0.037460385	0.328438691	up_in_case
PRJNA8274(	ENSGALG00010018381	HAPSTR1	case	control	-0.086082355	0.050237724	-1.713500313	0.086620536	0.465142194	down_in_case
PRJNA8274(	ENSGALG00010018383	METRN	case	control	-0.040146763	0.101270086	-0.396432594	0.691785925	0.918929895	down_in_case
PRJNA8274(	ENSGALG00010018384	LAMP5	case	control	-0.109197797	0.160422565	-0.68068851	0.496068608	0.838518426	down_in_case
PRJNA8274(	ENSGALG00010018385	SPR	case	control	0.359305805	0.125130088	2.871458106	0.004085829	0.110657879	up_in_case
PRJNA8274(	ENSGALG00010018387	NT5DC4	case	control	0.734589311	0.239822817	3.063050131	0.002190934	0.076473225	up_in_case
PRJNA8274(	ENSGALG00010018388	SMC3	case	control	-0.081474502	0.052958332	-1.538464262	0.12393514	0.523029039	down_in_case
PRJNA8274(	ENSGALG00010018391	STAC	case	control	-0.03028876	0.183303723	-0.165238106	0.868756574	0.974483475	down_in_case
PRJNA8274(	ENSGALG00010018393	PRKCE	case	control	-0.0157205	0.084389292	-0.18628548	0.852220893	0.969582953	down_in_case
PRJNA8274(	ENSGALG00010018394	KLHL28	case	control	0.078840895	0.05002505	1.576028325	0.115019276	0.510641304	up_in_case
PRJNA8274(	ENSGALG00010018395	MED12	case	control	-0.042938717	0.048715519	-0.881417628	0.37809182	0.776556765	down_in_case
PRJNA8274(	ENSGALG00010018396	TBL1XR1	case	control	-0.096943579	0.045156264	-2.146846735	0.031805485	0.3098361	down_in_case
PRJNA8274(	ENSGALG00010018397	FAM173A	case	control	-0.246707879	0.110862472	-2.225350699	0.026057704	0.286194449	down_in_case
PRJNA8274(	ENSGALG00010018398	LRFN5	case	control	-0.155694707	0.175077644	-0.889289476	0.373847526	0.774369566	down_in_case
PRJNA8274(	ENSGALG00010018399	ABHD12	case	control	-0.067060004	0.056555196	-1.185744353	0.235723273	0.660526168	down_in_case
PRJNA8274(	ENSGALG00010018401	ENSGALG00010018401	case	control	0.019565476	0.053350328	0.366735806	0.713816095	0.926862121	up_in_case
PRJNA8274(	ENSGALG00010018404	IL2RG	case	control	-0.015234306	0.137774596	-0.110574131	0.911954062	0.98266608	down_in_case
PRJNA8274(	ENSGALG00010018405	DCLK3	case	control	0.243712122	0.124167734	1.96276532	0.049673449	0.364833243	up_in_case
PRJNA8274(	ENSGALG00010018406	TMEM260	case	control	0.15981497	0.113318335	1.410318731	0.15844559	0.570582225	up_in_case
PRJNA8274(	ENSGALG00010018410	EXOC6B	case	control	-0.081059136	0.079973773	-1.01357149	0.310787268	0.733618014	down_in_case
PRJNA8274(	ENSGALG00010018413	SLC15A2	case	control	-0.14732537	0.339161394	-0.434381306	0.66401157	0.911350941	down_in_case
PRJNA8274(	ENSGALG00010018414	EPAS1	case	control	0.048942438	0.090420454	0.541276176	0.588317239	0.882771329	up_in_case
PRJNA8274(	ENSGALG00010018415	PELI2	case	control	0.198762291	0.068904712	2.884596478	0.003919157	0.107773305	up_in_case
PRJNA8274(	ENSGALG00010018416	DUT	case	control	-0.191827186	0.064894537	-2.955983598	0.003116737	0.095164759	down_in_case
PRJNA8274(	ENSGALG00010018418	PLCB4	case	control	0.056058303	0.097791488	0.573243168	0.56648006	0.87310464	up_in_case
PRJNA8274(	ENSGALG00010018419	USP7	case	control	-0.131036905	0.042913493	-3.053512921	0.00226179	0.078313788	down_in_case
PRJNA8274(	ENSGALG00010018420	RHOQ	case	control	-0.06246849	0.071259802	-0.876630142	0.380687562	0.778246195	down_in_case
PRJNA8274(	ENSGALG00010018422	ENSGALG00010018422	case	control	-0.116565037	0.101461816	-1.148856214	0.250615275	0.676241341	down_in_case
PRJNA8274(	ENSGALG00010018423	IGFALS	case	control	-0.002013487	0.803537115	-0.002505779	0.99800068	0.999244889	down_in_case
PRJNA8274(	ENSGALG00010018425	CYP26B1	case	control	-0.068057278	0.143290647	-0.474959668	0.63481572	0.898107199	down_in_case
PRJNA8274(	ENSGALG00010018426	ENSGALG00010018426	case	control	0.350753402	0.244956199	1.431902532	0.152171717	0.560293917	up_in_case
PRJNA8274(	ENSGALG00010018427	PIGF	case	control	0.13646267	0.124492932	1.096147932	0.273014041	0.698936152	up_in_case
PRJNA8274(	ENSGALG00010018428	PPP2R5D	case	control	0.002536706	0.043759003	0.057969914	0.953772593	0.992056045	up_in_case
PRJNA8274(	ENSGALG00010018430	LOXL4	case	control	0.107324645	0.256627514	0.418211761	0.675792298	0.914247368	up_in_case
PRJNA8274(	ENSGALG00010018434	HSPBAP1	case	control	-0.005778727	0.11470517	-0.050378954	0.959820407	0.992056045	down_in_case
PRJNA8274(	ENSGALG00010018436	FOXO4	case	control	-0.055586599	0.062333264	-0.891764623	0.372519107	0.774201112	down_in_case
PRJNA8274(	ENSGALG00010018438	PPP2R2A	case	control	-0.084729846	0.0408174	-2.07582662	0.037909989	0.330206289	down_in_case
PRJNA8274(	ENSGALG00010018440	MEA1	case	control	0.071313698	0.092690908	0.769371016	0.44167309	0.813488561	up_in_case
PRJNA8274(	ENSGALG00010018441	CRIPT	case	control	0.134802176	0.078885187	1.708840165	0.087480561	0.467303704	up_in_case
PRJNA8274(	ENSGALG00010018443	PLCB1	case	control	-0.192431136	0.127161684	-1.513279232	0.130208746	0.532650639	down_in_case
PRJNA8274(	ENSGALG00010018444	NUBP2	case	control	-0.096994064	0.091438445	-1.060758017	0.288799886	0.712801679	down_in_case
PRJNA8274(	ENSGALG00010018446	FBN1	case	control	-0.025132175	0.150326897	-0.167183491	0.867225674	0.973675289	down_in_case
PRJNA8274(	ENSGALG00010018448	KLHDC3	case	control	-0.296604434	0.082654394	-3.588489596	0.000332599	0.024516337	down_in_case
PRJNA8274(	ENSGALG00010018451	PIK3R4	case	control	0.080723833	0.060603528	1.3319989	0.18286055	0.604462752	up_in_case
PRJNA8274(	ENSGALG00010018452	SPSB3	case	control	-0.06722671	0.075850394	-0.88630666	0.375452301	0.774921593	down_in_case
PRJNA8274(	ENSGALG00010018453	TMX4	case	control	-0.014953646	0.053930357	-0.277276979	0.781567435	0.949394183	down_in_case
PRJNA8274(	ENSGALG00010018455	RBM20	case	control	-0.279512166	0.120126729	-2.326810762	0.019975339	0.253908758	down_in_case
PRJNA8274(	ENSGALG00010018458	SEMA5B	case	control	-0.148798552	0.107106129	-1.389262722	0.164752875	0.579922223	down_in_case
PRJNA8274(	ENSGALG00010018459	SNX12	case	control	0.052454467	0.032757616	1.601290736	0.10931254	0.501658961	up_in_case
PRJNA8274(	ENSGALG00010018462	KTN1	case	control	-0.076142012	0.049988088	-1.52320314	0.127707891	0.529567933	down_in_case
PRJNA8274(	ENSGALG00010018464	ENSGALG00010018464	case	control	1.417374986	1.356607538	1.044793683	0.296118335	0.719662167	up_in_case
PRJNA8274(	ENSGALG00010018465	NAALADL2	case	control	0.217510232	0.108136677	2.011438101	0.044279202	0.350201601	up_in_case
PRJNA8274(	ENSGALG00010018466	DYSF	case	control	-0.2228207	0.105261041	-2.11683923	0.034273495	0.319498679	down_in_case
PRJNA8274(	ENSGALG00010018467	TLX1	case	control	-0.101122438	0.448593385	-0.225421152	0.821651658	0.959074256	down_in_case
PRJNA8274(	ENSGALG00010018469	KLHDC7A	case	control	0.054561925	0.130281009	0.41880183	0.675360967	0.914247368	up_in_case
PRJNA8274(	ENSGALG00010018470	IQCB1	case	control	-0.243122879	0.136794936	-1.777279815	0.075522213	0.442247193	down_in_case
PRJNA8274(	ENSGALG00010018471	HHEX	case	control	0.075372008	0.103488443	0.728313284	0.466421832	0.827112128	up_in_case
PRJNA8274(	ENSGALG00010018472	LMF1	case	control	-0.145936001	0.10036883	-1.453997227	0.145947075	0.551107254	down_in_case
PRJNA8274(	ENSGALG00010018476	ENSGALG00010018476	case	control	-0.224811861	0.141188617	-1.592280353	0.111321726	0.503925506	down_in_case
PRJNA8274(	ENSGALG00010018480	EME2	case	control	0.250068751	0.122238267	2.045748489	0.040781122	0.337767404	up_in_case
PRJNA8274(	ENSGALG00010018482	TWNK	case	control	-0.004353161	0.085481236	-0.050925336	0.959385016	0.992056045	down_in_case
PRJNA8274(	ENSGALG00010018484	NLGN1	case	control	-0.011658332	0.124395782	-0.093719672	0.925331843	0.985571367	down_in_case
PRJNA8274(	ENSGALG00010018485	ATP2C1	case	control	0.004222211	0.036677746	0.115116428	0.908352838	0.981730793	up_in_case
PRJNA8274(	ENSGALG00010018486	ENSGALG00010018486	case	control	-0.007414017	0.047554699	-0.155905029	0.876107881	0.975666505	down_in_case
PRJNA8274(	ENSGALG00010018487	ENSGALG00010018487	case	control	0.043868398	0.084521359	0.519021445	0.603745786	0.885268908	up_in_case
PRJNA8274(	ENSGALG00010018488	ENSGALG00010018488	case	control	-0.27321632	0.148539319	-1.839353523	0.065863205	0.416182558	down_in_case
PRJNA8274(	ENSGALG00010018489	SLC7A3	case	control	0.116328772	0.087508898	1.329336497	0.183736978	0.605261182	up_in_case
PRJNA8274(	ENSGALG00010018490	NME3	case	control	0.041088489	0.07706972	0.533134019	0.593940811	0.883711747	up_in_case
PRJNA8274(	ENSGALG00010018491	TIA1	case	control	-0.078333274	0.059614076	-1.314006331	0.188844074	0.611783722	down_in_case
PRJNA8274(	ENSGALG00010018492	ENSGALG00010018492	case	control	0.02683642	0.082818141	0.324040356	0.745907482	0.938117773	up_in_case
PRJNA8274(	ENSGALG00010018494	HAO1	case	control	1.236728428	0.640656089	1.93040923	0.053556152	0.37782383	up_in_case
PRJNA8274(	ENSGALG00010018495	ENSGALG00010018495	case	control	0.263431382	0.402695443	0.654170259	0.51300212	0.846564516	up_in_case
PRJNA8274(	ENSGALG00010018496	GNG13	case	control	0.137444393	0.423612825	0.324457583	0.745591631	0.938057392	up_in_case
PRJNA8274(	ENSGALG00010018497	ASTE1	case	control	-0.168433867	0.102080992	-1.65000225	0.098942476	0.488407564	down_in_case
PRJNA8274(	ENSGALG00010018498	ENSGALG00010018498	case	control	-0.023078797	0.165832776	-0.139169096	0.889316529	0.978038394	down_in_case
PRJNA8274(	ENSGALG00010018501	ATG14	case	control	0.064832755	0.067761233	0.956782402	0.338677096	0.751259351	up_in_case
PRJNA8274(	ENSGALG00010018502	ARHGEF10L	case	control	0.095284009	0.054376964	1.75228629	0.079724592	0.450131333	up_in_case
PRJNA8274(	ENSGALG00010018503	BMP2	case	control	0.149392446	0.1498895	0.996683857	0.318917988	0.738427335	up_in_case
PRJNA8274(	ENSGALG00010018504	SLCO3A1	case	control	-0.010431337	0.081259501	-0.128370675	0.897855637	0.980176778	down_in_case
PRJNA8274(	ENSGALG00010018505	CHTF18	case	control	-0.01425003	0.051022188	-0.279290843	0.780021633	0.948864508	down_in_case
PRJNA8274(	ENSGALG00010018506	ENSGALG00010018506	case	control	0.022219071	0.154925992	0.143417321	0.885960604	0.977630122	up_in_case
PRJNA8274(	ENSGALG00010018508	ENSGALG00010018508	case	control	0.020528739	0.057674246	0.355942913	0.721883318	0.929484914	up_in_case
PRJNA8274(	ENSGALG00010018509	ENSGALG00010018509	case	control	-0.007879058	0.072232643	-0.109078906	0.913139906	0.982728035	down_in_case
PRJNA8274(	ENSGALG00010018510	ENSGALG00010018510	case	control	-0.150294354	0.073001411	-2.058786981	0.039514649	0.335234949	down_in_case
PRJNA8274(	ENSGALG00010018511	SOCS5	case	control	0.13241583	0.057428706	2.305742896	0.021125003	0.260420292	up_in_case
PRJNA8274(	ENSGALG00010018512	FERMT1	case	control	0.029283949	0.111812499	0.261902286	0.793396777	0.95280257	up_in_case
PRJNA8274(	ENSGALG00010018513	RPUSD1	case	control	0.144475202	0.074317364	1.944030218	0.051891807	0.371953818	up_in_case
PRJNA8274(	ENSGALG00010018514	EAF2	case	control	0.238810925	0.156879354	1.522258468	0.12794433	0.529650968	up_in_case
PRJNA8274(	ENSGALG00010018515	SEMA4G	case	control	0.298041859	0.119462525	2.494856509	0.012600807	0.197362044	up_in_case
PRJNA8274(	ENSGALG00010018516	LRRN4	case	control	-0.208653219	0.123618185	-1.687884509	0.091433401	0.476493306	down_in_case
PRJNA8274(	ENSGALG00010018517	ECT2	case	control	-0.096996768	0.112094103	-0.865315526	0.386865631	0.782604263	down_in_case
PRJNA8274(	ENSGALG00010018518	ENSGALG00010018518	case	control	-0.014195487	0.655655332	-0.021650838	0.98272648	0.996591771	down_in_case
PRJNA8274(	ENSGALG00010018519	OGDH	case	control	-0.208828116	0.087825585	-2.377759477	0.017418183	0.234759681	down_in_case
PRJNA8274(	ENSGALG00010018520	ENSGALG00010018520	case	control	0.38084971	0.103102752	3.693885012	0.000220854	0.019375509	up_in_case
PRJNA8274(	ENSGALG00010018521	ENSGALG00010018521	case	control	-0.127613941	0.079420247	-1.606818736	0.108094137	0.499756275	down_in_case
PRJNA8274(	ENSGALG00010018522	NEK11	case	control	-0.123096495	0.206336141	-0.596582326	0.550786274	0.86457121	down_in_case
PRJNA8274(	ENSGALG00010018523	TEX11	case	control	-0.163190236	0.139120193	-1.173016173	0.24078932	0.665967363	down_in_case
PRJNA8274(	ENSGALG00010018524	SEC22A	case	control	0.021219011	0.044831748	0.473303216	0.635996868	0.898691227	up_in_case
PRJNA8274(	ENSGALG00010018525	ST8SIA2	case	control	-0.005047697	0.101697107	-0.049634616	0.960413561	0.992056045	down_in_case
PRJNA8274(	ENSGALG00010018526	ENSGALG00010018526	case	control	-0.032653826	0.074844134	-0.436291051	0.662625574	0.910934984	down_in_case
PRJNA8274(	ENSGALG00010018527	CCT4	case	control	-0.004054383	0.045954074	-0.08822684	0.929696386	0.987129367	down_in_case
PRJNA8274(	ENSGALG00010018528	ENSGALG00010018528	case	control	-0.874908967	0.558678682	-1.566032489	0.117341011	0.514085926	down_in_case
PRJNA8274(	ENSGALG00010018529	UBR2	case	control	0.060252651	0.041568311	1.449485186	0.147202134	0.552345978	up_in_case
PRJNA8274(	ENSGALG00010018530	FAM161A	case	control	0.122904395	0.165527046	0.742503403	0.457782391	0.823337749	up_in_case
PRJNA8274(	ENSGALG00010018531	ADCY5	case	control	0.088083955	0.106987948	0.823307262	0.410333285	0.796464701	up_in_case
PRJNA8274(	ENSGALG00010018532	MFSD13A	case	control	0.06805467	0.082429511	0.8256105	0.409025077	0.795170398	up_in_case
PRJNA8274(	ENSGALG00010018533	PCYOX1	case	control	-0.077674446	0.099112362	-0.783700886	0.433215643	0.808138728	down_in_case
PRJNA8274(	ENSGALG00010018534	NCEH1	case	control	0.067080327	0.101542022	0.660616421	0.508858335	0.844259682	up_in_case
PRJNA8274(	ENSGALG00010018535	FAM174B	case	control	0.187559938	0.061688517	3.040435164	0.002362365	0.079674119	up_in_case
PRJNA8274(	ENSGALG00010018536	ENSGALG00010018536	case	control	-0.005091664	0.20707432	-0.024588583	0.980383126	0.99591381	down_in_case
PRJNA8274(	ENSGALG00010018537	ENSGALG00010018537	case	control	0.202878106	0.544004453	0.372934643	0.709197088	0.925521953	up_in_case
PRJNA8274(	ENSGALG00010018539	CHD2	case	control	-0.123160592	0.051129531	-2.40879563	0.016005258	0.224387437	down_in_case
PRJNA8274(	ENSGALG00010018540	TNFSF10	case	control	-0.113922374	0.063313418	-1.79934014	0.071964892	0.433669599	down_in_case
PRJNA8274(	ENSGALG00010018542	ZMIZ2	case	control	-0.415330338	0.131965691	-3.147259986	0.001648083	0.065465539	down_in_case
PRJNA8274(	ENSGALG00010018543	MRPL3	case	control	0.036897919	0.056895694	0.648518652	0.516649551	0.848054666	up_in_case
PRJNA8274(	ENSGALG00010018544	HACD2	case	control	0.115042558	0.099159576	1.160175978	0.245977165	0.670869818	up_in_case
PRJNA8274(	ENSGALG00010018546	SNRPGP15	case	control	0.035682695	0.07670446	0.46519714	0.641790286	0.901539099	up_in_case

PRJNA8274(	ENSGALG00010018548	RGMA	case	control	-0.036517902	0.070369589	-0.51894437	0.603799535	0.885268908	down_in_case
PRJNA8274(	ENSGALG00010018549	GHSR	case	control	-0.154255079	0.207007028	-0.745168319	0.456169972	0.822099797	down_in_case
PRJNA8274(	ENSGALG00010018551	FBXO34	case	control	-0.04623705	0.069686243	-0.663503258	0.507008294	0.843472666	down_in_case
PRJNA8274(	ENSGALG00010018552	DTD1	case	control	-0.06332877	0.052273236	-1.211495111	0.225705707	0.649498359	down_in_case
PRJNA8274(	ENSGALG00010018554	PPIA	case	control	-0.109507485	0.057025063	-1.920339583	0.05481502	0.382741592	down_in_case
PRJNA8274(	ENSGALG00010018555	UBN1	case	control	0.028113051	0.050462881	0.557103566	0.577456671	0.878143389	up_in_case
PRJNA8274(	ENSGALG00010018556	MRPL2	case	control	-0.047368287	0.097901667	-0.483835349	0.628502726	0.896070685	down_in_case
PRJNA8274(	ENSGALG00010018557	TBCC	case	control	-0.016644535	0.088912396	-0.187201508	0.851502643	0.969234083	down_in_case
PRJNA8274(	ENSGALG00010018558	ENSGALG00010018558	case	control	0.291637605	0.207559725	1.405078011	0.159998082	0.572279283	up_in_case
PRJNA8274(	ENSGALG00010018559	DLG3	case	control	0.106236969	0.080168014	1.325179011	0.185111785	0.608031647	up_in_case
PRJNA8274(	ENSGALG00010018560	ENSGALG00010018560	case	control	-0.032055059	0.04976552	-0.644121856	0.519496418	0.849320777	down_in_case
PRJNA8274(	ENSGALG00010018561	ACTR1A	case	control	-0.067669328	0.044905122	-1.506940081	0.131826017	0.535212583	down_in_case
PRJNA8274(	ENSGALG00010018562	ENSGALG00010018562	case	control	-0.166296853	0.232811794	-0.714297376	0.475043314	0.830111175	down_in_case
PRJNA8274(	ENSGALG00010018564	DLGAP5	case	control	-0.085442486	0.071716059	-1.191399618	0.233496743	0.657982411	down_in_case
PRJNA8274(	ENSGALG00010018566	ENSGALG00010018566	case	control	0.16182345	0.097668216	1.656869121	0.097545939	0.485860997	up_in_case
PRJNA8274(	ENSGALG00010018568	FAM136A	case	control	-0.159589521	0.1292598	-1.234641561	0.216963934	0.642356989	down_in_case
PRJNA8274(	ENSGALG00010018573	SEC23B	case	control	0.160082057	0.065478136	2.444816957	0.014492567	0.212076065	up_in_case
PRJNA8274(	ENSGALG00010018574	ENSGALG00010018574	case	control	0.294395951	0.226850472	1.29775331	0.194372118	0.617259892	up_in_case
PRJNA8274(	ENSGALG00010018576	PTK7	case	control	-0.092577376	0.062148298	-1.489620451	0.136324062	0.539840644	down_in_case
PRJNA8274(	ENSGALG00010018577	NARFL	case	control	-0.060571405	0.07505661	-0.807009598	0.419660953	0.80093491	down_in_case
PRJNA8274(	ENSGALG00010018579	FNDC3B	case	control	-0.03372204	0.074274425	-0.454019541	0.649814762	0.904241824	down_in_case
PRJNA8274(	ENSGALG00010018581	SUFU	case	control	0.028905755	0.048810079	0.592208733	0.553710822	0.866337858	up_in_case
PRJNA8274(	ENSGALG00010018582	RCC2	case	control	0.002578364	0.041185638	0.062603462	0.950082273	0.991488793	up_in_case
PRJNA8274(	ENSGALG00010018583	PPL	case	control	-0.428704355	0.196792754	-2.178455996	0.029372105	0.305026217	down_in_case
PRJNA8274(	ENSGALG00010018585	GDPD2	case	control	0.230454519	0.353404151	0.652099074	0.514337261	0.847493921	up_in_case
PRJNA8274(	ENSGALG00010018587	SRF	case	control	-0.034845836	0.088119341	-0.395439138	0.692518829	0.918988424	down_in_case
PRJNA8274(	ENSGALG00010018588	POLR3F	case	control	-0.039175951	0.081785056	-0.479011113	0.631930723	0.89724192	down_in_case
PRJNA8274(	ENSGALG00010018590	TGFA	case	control	0.500120889	0.325296541	1.537430703	0.124187873	0.523029039	up_in_case
PRJNA8274(	ENSGALG00010018593	LGALS3	case	control	0.576559883	0.342285742	1.684440256	0.092096616	0.476713154	up_in_case
PRJNA8274(	ENSGALG00010018594	BICRAL	case	control	0.048210256	0.089840953	0.536617813	0.591531637	0.882949889	up_in_case
PRJNA8274(	ENSGALG00010018598	ENSGALG00010018598	case	control	-0.049531844	0.042069887	-1.177370509	0.239047656	0.664279338	down_in_case
PRJNA8274(	ENSGALG00010018601	CPNE4	case	control	0.353885277	0.411887147	0.859180189	0.390241112	0.783914628	up_in_case
PRJNA8274(	ENSGALG00010018602	MCTP2	case	control	-0.092245943	0.122843504	-0.750922436	0.452699337	0.821045895	down_in_case
PRJNA8274(	ENSGALG00010018604	FAM43A	case	control	-0.025084244	0.099196585	-0.252874064	0.800365532	0.954053991	down_in_case
PRJNA8274(	ENSGALG00010018606	DZANK1	case	control	0.077147969	0.227379062	0.339292318	0.734389529	0.933988817	up_in_case
PRJNA8274(	ENSGALG00010018607	HAGHL	case	control	-0.054072894	0.112081412	-0.482443013	0.629491272	0.896269608	down_in_case
PRJNA8274(	ENSGALG00010018608	TRIM8	case	control	-0.033139734	0.09168446	-0.361454214	0.717759927	0.927271384	down_in_case
PRJNA8274(	ENSGALG00010018609	ENSGALG00010018609	case	control	0.098766762	0.075518776	1.307843789	0.190926299	0.614090434	up_in_case
PRJNA8274(	ENSGALG00010018611	NUDCD3	case	control	0.002192424	0.044079408	0.049738057	0.960331129	0.992056045	up_in_case
PRJNA8274(	ENSGALG00010018613	PADI3	case	control	-0.17827453	0.255553114	-0.697602652	0.485425722	0.834423421	down_in_case
PRJNA8274(	ENSGALG00010018615	HTR2C	case	control	-0.045080264	0.200418358	-0.224930811	0.8220331	0.959284587	down_in_case
PRJNA8274(	ENSGALG00010018616	ENSGALG00010018616	case	control	-0.916514393	0.390613631	-2.346345136	0.018958543	0.245790719	down_in_case
PRJNA8274(	ENSGALG00010018618	FAHD1	case	control	0.007320957	0.089330148	0.081953933	0.934683347	0.988149615	up_in_case
PRJNA8274(	ENSGALG00010018619	MAPK1IP1L	case	control	-0.070051397	0.039649296	-1.766775306	0.077265866	0.445884536	down_in_case
PRJNA8274(	ENSGALG00010018623	ARRDC4	case	control	-0.071422666	0.083288467	-0.857533685	0.391150007	0.784275639	down_in_case
PRJNA8274(	ENSGALG00010018624	ENSGALG00010018624	case	control	-0.142574899	0.415516428	-0.343126985	0.731502931	0.933770678	down_in_case
PRJNA8274(	ENSGALG00010018627	MYLK	case	control	-0.096221682	0.150687902	-0.638549484	0.523116053	0.85132122	down_in_case
PRJNA8274(	ENSGALG00010018628	XDH	case	control	1.659676692	0.61366866	2.704515974	0.006840398	0.143217705	up_in_case
PRJNA8274(	ENSGALG00010018631	KAT14	case	control	-0.02728722	0.0581986	-0.468863855	0.639166954	0.900304115	down_in_case
PRJNA8274(	ENSGALG00010018633	ENSGALG00010018633	case	control	0.045318554	0.120691949	0.375489457	0.707296486	0.92461283	up_in_case
PRJNA8274(	ENSGALG00010018638	SEC14L5	case	control	-0.032288482	0.122328136	-0.26394976	0.791818632	0.95239351	down_in_case
PRJNA8274(	ENSGALG00010018641	ENSGALG00010018641	case	control	0.512029103	0.843994535	0.606673481	0.544067624	0.861114226	up_in_case
PRJNA8274(	ENSGALG00010018644	ESF1	case	control	-0.004336945	0.067166611	-0.064569952	0.948516409	0.991291133	down_in_case
PRJNA8274(	ENSGALG00010018649	HAGH	case	control	0.071571912	0.077693593	0.92120739	0.356942161	0.763373775	up_in_case
PRJNA8274(	ENSGALG00010018654	ENSGALG00010018654	case	control	0.40031601	0.219874797	1.82065437	0.068659412	0.422837898	up_in_case
PRJNA8274(	ENSGALG00010018655	BIRC5	case	control	-0.120545497	0.1198693	-1.005641116	0.314588238	0.736432557	down_in_case
PRJNA8274(	ENSGALG00010018665	IL13RA2	case	control	-0.084446563	0.179501271	-0.470451061	0.638032791	0.899878579	down_in_case
PRJNA8274(	ENSGALG00010018668	OVOL2	case	control	0.146042579	0.502495541	0.290634577	0.771330812	0.945630466	up_in_case
PRJNA8274(	ENSGALG00010018670	MRAS	case	control	-0.034961411	0.105302465	-0.332009427	0.739882141	0.9363983	down_in_case
PRJNA8274(	ENSGALG00010018672	PKDCC	case	control	0.032473878	0.067313595	0.482426731	0.629502836	0.896269608	up_in_case
PRJNA8274(	ENSGALG00010018673	NAGPA	case	control	0.114265501	0.067316846	1.697428014	0.08961577	0.472813801	up_in_case
PRJNA8274(	ENSGALG00010018684	PADI2	case	control	0.228242625	0.330549544	0.690494447	0.489883301	0.836347578	up_in_case
PRJNA8274(	ENSGALG00010018685	ARL3	case	control	0.061259867	0.078543241	0.779950845	0.435419809	0.81000376	up_in_case
PRJNA8274(	ENSGALG00010018693	ENSGALG00010018693	case	control	0.073278156	0.166303127	0.440630056	0.65948084	0.908599575	up_in_case
PRJNA8274(	ENSGALG00010018694	MGME1	case	control	-0.211017639	0.102210283	-2.06454412	0.038966155	0.335091843	down_in_case
PRJNA8274(	ENSGALG00010018698	MRPS16	case	control	0.062997216	0.07280212	0.865321171	0.386862534	0.782604263	up_in_case
PRJNA8274(	ENSGALG00010018701	SFXN2	case	control	0.013598568	0.08315826	0.163526367	0.870104014	0.974794532	up_in_case
PRJNA8274(	ENSGALG00010018704	PGPEP1L	case	control	-0.034061825	0.236963548	-0.143742889	0.885703502	0.977630122	down_in_case
PRJNA8274(	ENSGALG00010018707	SLC45A1	case	control	0.117746971	0.091262009	1.290207976	0.196978458	0.620029043	up_in_case
PRJNA8274(	ENSGALG00010018709	SNX5	case	control	0.056514898	0.075352226	0.750009668	0.453248882	0.821282604	up_in_case
PRJNA8274(	ENSGALG00010018713	ENSGALG00010018713	case	control	0.170998367	0.097875109	1.747107814	0.080618638	0.452876025	up_in_case
PRJNA8274(	ENSGALG00010018715	DNAJC13	case	control	-0.043115993	0.04302481	-1.002119301	0.316285977	0.736866972	down_in_case
PRJNA8274(	ENSGALG00010018717	MEIOB	case	control	-0.396839258	0.726878685	-0.54594978	0.585100458	0.881307382	down_in_case
PRJNA8274(	ENSGALG00010018719	ERRFI1	case	control	0.059873673	0.061353368	0.975882423	0.329122734	0.745636104	up_in_case
PRJNA8274(	ENSGALG00010018721	LRCH2	case	control	-0.065659305	0.076432907	-0.859044983	0.390315699	0.783914628	down_in_case
PRJNA8274(	ENSGALG00010018724	RRBP1	case	control	0.212629974	0.091531714	2.323019691	0.020178095	0.254471268	up_in_case
PRJNA8274(	ENSGALG00010018726	EBF2	case	control	-0.049026804	0.081536882	-0.601283774	0.547650995	0.863104956	down_in_case
PRJNA8274(	ENSGALG00010018727	PARK7	case	control	-0.081999943	0.063967401	-1.281902065	0.199877003	0.622902456	down_in_case
PRJNA8274(	ENSGALG00010018729	HS3ST6	case	control	0.156078245	0.60104771	0.259676964	0.795112963	0.95280257	up_in_case
PRJNA8274(	ENSGALG00010018730	IGF1R	case	control	-0.150294911	0.074929027	-2.005830284	0.044874358	0.352004018	down_in_case
PRJNA8274(	ENSGALG00010018732	ENSGALG00010018732	case	control	0.005730211	0.120023846	0.047742268	0.961921647	0.992056045	up_in_case
PRJNA8274(	ENSGALG00010018736	EEF2KMT	case	control	0.026473214	0.090134331	0.293708442	0.768980703	0.94539152	up_in_case
PRJNA8274(	ENSGALG00010018747	FLRT3	case	control	-0.001593703	0.078701637	-0.020249939	0.98384399	0.996591771	down_in_case
PRJNA8274(	ENSGALG00010018750	SOCS4	case	control	-0.063379103	0.071070186	-0.89178187	0.372509861	0.774201112	down_in_case
PRJNA8274(	ENSGALG00010018752	PLS3	case	control	-0.001513584	0.069585158	-0.021751535	0.982646155	0.996591771	down_in_case
PRJNA8274(	ENSGALG00010018753	SYNM	case	control	-0.185280144	0.101115917	-1.832353884	0.066898714	0.418849217	down_in_case
PRJNA8274(	ENSGALG00010018756	EML4	case	control	-0.050465271	0.066142315	-0.762980104	0.445475261	0.816914111	down_in_case
PRJNA8274(	ENSGALG00010018758	ENSGALG00010018758	case	control	0.183068792	0.089678231	2.041396122	0.04121147	0.338772367	up_in_case
PRJNA8274(	ENSGALG00010018764	TMEM177	case	control	-0.160547512	0.091941546	-1.746191118	0.080777748	0.453075204	down_in_case
PRJNA8274(	ENSGALG00010018767	NDUFB6	case	control	-0.030862566	0.078632171	-0.39249287	0.694694078	0.920508277	down_in_case
PRJNA8274(	ENSGALG00010018774	MSRB1	case	control	0.091374996	0.107953436	0.846429717	0.397313067	0.788560285	up_in_case
PRJNA8274(	ENSGALG00010018780	WDHD1	case	control	-0.138301342	0.101587363	-1.36140301	0.173386365	0.592914702	down_in_case
PRJNA8274(	ENSGALG00010018783	ENSGALG00010018783	case	control	-0.143064772	0.119629946	-1.195894316	0.231737803	0.657171791	down_in_case
PRJNA8274(	ENSGALG00010018786	DSTN	case	control	0.106713162	0.045515947	2.344522471	0.019051462	0.246324585	up_in_case
PRJNA8274(	ENSGALG00010018796	PER3	case	control	0.234357001	0.112506892	2.083045736	0.037247055	0.328438691	up_in_case
PRJNA8274(	ENSGALG00010018799	ENSGALG00010018799	case	control	-0.092517645	0.297512278	-0.310970847	0.755822784	0.941588405	down_in_case
PRJNA8274(	ENSGALG00010018806	GCH1	case	control	0.39440955	0.239166906	1.649097512	0.099127659	0.488407564	up_in_case
PRJNA8274(	ENSGALG00010018807	RD3	case	control	-0.181293243	0.17122687	-1.058789681	0.289695574	0.713388447	down_in_case
PRJNA8274(	ENSGALG00010018808	MATCAP2	case	control	0.155696607	0.067779728	2.297096956	0.021613239	0.263261774	up_in_case
PRJNA8274(	ENSGALG00010018811	VAMP3	case	control	-0.062194965	0.056896735	-1.093120105	0.27434107	0.699970438	down_in_case
PRJNA8274(	ENSGALG00010018815	ENSGALG00010018815	case	control	-0.061672393	0.156450129	-0.394198416	0.693434555	0.919776842	down_in_case
PRJNA8274(	ENSGALG00010018819	LRRC28	case	control	-0.086799296	0.059822082	-1.450957455	0.146791707	0.551868941	down_in_case
PRJNA8274(	ENSGALG00010018822	TRAF5	case	control	-0.128734444	0.157408673	-0.817835773	0.413450968	0.797887833	down_in_case
PRJNA8274(	ENSGALG00010018823	RAB40C	case	control	0.048366457	0.053353644	0.906525845	0.364657592	0.770251634	up_in_case
PRJNA8274(	ENSGALG00010018829	PTPN4	case	control	-0.200361448	0.092301351	-2.17073147	0.029951477	0.305970455	down_in_case
PRJNA8274(	ENSGALG00010018831	WBP1L	case	control	-0.058291122	0.061418572	-0.949079733	0.342580062	0.75456672	down_in_case
PRJNA8274(	ENSGALG00010018832	EEPD1	case	control	-0.270631594	0.104517057	-2.58935338	0.009615636	0.17593167	down_in_case
PRJNA8274(	ENSGALG00010018834	SAMD4A	case	control	-0.113187077	0.072315772	-1.565178298	0.117541109	0.51430076	down_in_case
PRJNA8274(	ENSGALG00010018835	CAMK2B	case	control	-0.051309259	0.105388735	-0.48685715	0.626359567	0.895536157	down_in_case
PRJNA8274(	ENSGALG00010018837	RCOR3	case	control	0.026997153	0.057599175	0.468707284	0.63927888	0.900304115	up_in_case
PRJNA8274(	ENSGALG00010018838	WFIKKN1	case	control	-0.048279564	0.222532842	-0.216954783	0.828243584	0.961588312	down_in_case
PRJNA8274(	ENSGALG00010018839	RPL3L	case	control	0.455586845	0.341243001	1.33508041	0.18185003	0.602744104	up_in_case
PRJNA8274(	ENSGALG00010018843	MCRIP2	case	control	-0.041182408	0.100472759	-0.409886309	0.681889349	0.915594727	down_in_case
PRJNA8274(	ENSGALG00010018847	CGRRF1	case	control	0.155919392	0.121742837	1.280727447	0.200289416	0.622902456	up_in_case
PRJNA8274(	ENSGALG00010018848	MEF2A	case	control	-0.176290096	0.061761245	-2.854380542	0.004312085	0.113350751	down_in_case
PRJNA8274(	ENSGALG00010018849	KCNH1	case	control	-0.161974766	0.395162774	-0.409893787	0.681883864	0.915594727	down_in_case
PRJNA8274(	ENSGALG00010018852	NDUFB10	case	control	-0.046400132	0.083137512	-0.558113073	0.576767174	0.878085957	down_in_case
PRJNA8274(	ENSGALG00010018854	LYSMD4	case	control	-0.00970714	0.069280969	-0.140112644	0.888570991	0.977948171	down_in_case
PRJNA8274(	ENSGALG00010018855	SYNDIG1	case	control	-0.455797465	0.328219011	-1.388699158	0.16492425	0.580021198	down_in_case
PRJNA8274(	ENSGALG00010018857	ENSGALG00010018857	case	control	-0.045721291	0.061088538	-0.748443031	0.454192976	0.821331802	down_in_case
PRJNA8274(	ENSGALG00010018858	EPB41L5	case	control	0.004662501	0.067095626	0.069490382	0.944599288	0.990313016	up_in_case
PRJNA8274(	ENSGALG00010018860	AGTR2	case	control	-0.139064197	0.118153378	-1.176980293	0.239203372	0.66429721	down_in_case
PRJNA8274(	ENSGALG00010018864	ABCA3	case	control	0.01849763	0.067992907	0.272052348	0.785581769	0.949929982	up_in_case
PRJNA8274(	ENSGALG00010018865	ENSGALG00010018865	case	control	0.038515209	0.107602734	0.357938944	0.720389006	0.92895404	up_in_case
PRJNA8274(	ENSGALG00010018867	RPS2	case	control	-0.071219112	0.074849534	-0.951497063	0.341352109	0.752827754	down_in_case
PRJNA8274(	ENSGALG00010018872	KCTD9	case	control	-0.017363144	0.066639784	-0.260552233	0.79443783	0.95280257	down_in_case

PRJNA8274(	ENSGALG00010018874	ENSGALG00010018874	case	control	-0.232918347	0.146270182	-1.592384338	0.111298373	0.503925506	down_in_case
PRJNA8274(	ENSGALG00010018876	GMFB	case	control	-0.013167203	0.070700013	-0.186240461	0.852256195	0.969582953	down_in_case
PRJNA8274(	ENSGALG00010018880	TMEM17	case	control	-0.002122985	0.165577682	-0.012821685	0.989770056	0.997323462	down_in_case
PRJNA8274(	ENSGALG00010018881	ENSGALG00010018881	case	control	-1.201565462	0.768622427	-1.563271407	0.117988771	0.514604253	down_in_case
PRJNA8274(	ENSGALG00010018882	HHAT	case	control	-0.100039352	0.099080746	-1.009674999	0.312651024	0.734380691	down_in_case
PRJNA8274(	ENSGALG00010018883	TBL3	case	control	-0.018716899	0.068493262	-0.273266281	0.784648533	0.949518053	down_in_case
PRJNA8274(	ENSGALG00010018885	EFEMP1	case	control	-0.160646673	0.13337699	-1.204455684	0.228413501	0.651938858	down_in_case
PRJNA8274(	ENSGALG00010018887	ADAMTS17	case	control	-0.183916301	0.142279428	-1.29264156	0.196135059	0.619095929	down_in_case
PRJNA8274(	ENSGALG00010018891	GNRH1	case	control	0.131144887	0.280562607	0.467435373	0.64018842	0.901077979	up_in_case
PRJNA8274(	ENSGALG00010018892	APMAP	case	control	-0.049716781	0.063665754	-0.780903043	0.434859522	0.809699371	down_in_case
PRJNA8274(	ENSGALG00010018894	SERTAD4	case	control	0.001973118	0.045623117	0.043248207	0.965503677	0.992291928	up_in_case
PRJNA8274(	ENSGALG00010018895	SEPTIN7	case	control	-0.007933835	0.040283445	-0.19695026	0.843866462	0.96721237	down_in_case
PRJNA8274(	ENSGALG00010018897	CCDC85A	case	control	0.034763578	0.075759482	0.458867689	0.646329183	0.902693223	up_in_case
PRJNA8274(	ENSGALG00010018899	ACSS1	case	control	0.11681386	0.087040321	1.34206605	0.179574581	0.600541745	up_in_case
PRJNA8274(	ENSGALG00010018901	TCF7L2	case	control	0.020316541	0.091099554	0.223014709	0.823524058	0.960006035	up_in_case
PRJNA8274(	ENSGALG00010018902	NOXO1	case	control	-0.305263404	0.577185668	-0.528882509	0.596886957	0.884680373	down_in_case
PRJNA8274(	ENSGALG00010018903	CAMTA1	case	control	-0.100927612	0.083740554	-1.205241758	0.228109986	0.651681204	down_in_case
PRJNA8274(	ENSGALG00010018904	UGP2	case	control	0.069513242	0.117190566	0.593164151	0.553071301	0.866252369	up_in_case
PRJNA8274(	ENSGALG00010018908	ENSGALG00010018908	case	control	0.116242427	0.132305305	0.878592338	0.379622352	0.777847776	up_in_case
PRJNA8274(	ENSGALG00010018909	TTBK1	case	control	0.043513672	0.098315171	0.442593663	0.658059669	0.908367791	up_in_case
PRJNA8274(	ENSGALG00010018911	CNIH1	case	control	-0.08184467	0.039241378	-2.085672644	0.037008286	0.32835414	down_in_case
PRJNA8274(	ENSGALG00010018913	ENSGALG00010018913	case	control	-0.152417156	0.20388037	-0.747581318	0.454712737	0.821657174	down_in_case
PRJNA8274(	ENSGALG00010018915	DOCK5	case	control	0.070669969	0.100368756	0.704103264	0.481368467	0.832354182	up_in_case
PRJNA8274(	ENSGALG00010018916	CERS3	case	control	0.22951122	0.304185435	0.754510881	0.450542514	0.819735103	up_in_case
PRJNA8274(	ENSGALG00010018917	DNAJC11	case	control	-0.07401705	0.072862258	-1.015848977	0.309701309	0.732384442	down_in_case
PRJNA8274(	ENSGALG00010018919	RALB	case	control	-0.011176482	0.038370737	-0.291276201	0.770840087	0.94558971	down_in_case
PRJNA8274(	ENSGALG00010018920	ACAD11	case	control	-0.038607894	0.145287796	-0.265733912	0.790444144	0.952262112	down_in_case
PRJNA8274(	ENSGALG00010018922	EEF2K	case	control	-0.189816795	0.063486952	-2.989855232	0.002791097	0.088892397	down_in_case
PRJNA8274(	ENSGALG00010018923	VPS54	case	control	0.023043785	0.065559186	0.351495896	0.725216351	0.93185316	up_in_case
PRJNA8274(	ENSGALG00010018925	LINS1	case	control	0.018519051	0.128818725	0.143760549	0.885689557	0.977630122	up_in_case
PRJNA8274(	ENSGALG00010018926	SLC22A7	case	control	-0.577226412	0.642889334	-0.897862791	0.369258704	0.772101106	down_in_case
PRJNA8274(	ENSGALG00010018932	CDKN3	case	control	-0.176403681	0.132667112	-1.329671525	0.183626521	0.605176134	down_in_case
PRJNA8274(	ENSGALG00010018933	PHF13	case	control	0.00571223	0.062320928	0.091658298	0.926969531	0.986471245	up_in_case
PRJNA8274(	ENSGALG00010018936	SYT14	case	control	-0.339171056	0.188112032	-1.803026913	0.071383988	0.43228239	down_in_case
PRJNA8274(	ENSGALG00010018937	INHBB	case	control	0.066199612	0.198112057	0.334152365	0.738264587	0.935918627	up_in_case
PRJNA8274(	ENSGALG00010018941	KLHL21	case	control	-0.329100513	0.086626447	-3.799076675	0.000145236	0.01379195	down_in_case
PRJNA8274(	ENSGALG00010018943	ACKR4	case	control	-0.070739733	0.279894443	-0.252737182	0.800471314	0.954053991	down_in_case
PRJNA8274(	ENSGALG00010018944	NEFL	case	control	-0.37277037	0.258417578	-1.442511662	0.149158084	0.55581763	down_in_case
PRJNA8274(	ENSGALG00010018945	HABP2	case	control	0.717001593	0.481405007	1.489393719	0.136383721	0.539840644	up_in_case
PRJNA8274(	ENSGALG00010018947	ENSGALG00010018947	case	control	-0.031092368	0.176141895	-0.176518869	0.859886326	0.971828322	down_in_case
PRJNA8274(	ENSGALG00010018948	POLR3E	case	control	-0.038221734	0.043203484	-0.884691005	0.376323309	0.775197827	down_in_case
PRJNA8274(	ENSGALG00010018950	TTL	case	control	-0.000783048	0.043802437	-0.017876824	0.985737118	0.996595084	down_in_case
PRJNA8274(	ENSGALG00010018951	YKT6	case	control	-0.020482261	0.054531846	-0.375601835	0.707212926	0.92461283	down_in_case
PRJNA8274(	ENSGALG00010018955	ZBTB48	case	control	-0.037698987	0.072556797	-0.51957898	0.603357052	0.885268908	down_in_case
PRJNA8274(	ENSGALG00010018958	PELI3	case	control	-0.003568115	0.042615325	-0.083728441	0.933272344	0.987991895	down_in_case
PRJNA8274(	ENSGALG00010018959	UBA5	case	control	0.091460894	0.056555214	1.617196516	0.105835868	0.495266487	up_in_case
PRJNA8274(	ENSGALG00010018960	RBFOX1	case	control	-0.365491479	0.168686781	-2.166687138	0.030258718	0.30654407	down_in_case
PRJNA8274(	ENSGALG00010018961	ASB7	case	control	0.00045388	0.044720923	0.010149163	0.991902279	0.99790247	up_in_case
PRJNA8274(	ENSGALG00010018968	GLI2	case	control	-0.065257742	0.080480686	-0.81084972	0.417451977	0.799987395	down_in_case
PRJNA8274(	ENSGALG00010018969	DIEXF	case	control	0.039290015	0.075159687	0.522753841	0.601145565	0.884954589	up_in_case
PRJNA8274(	ENSGALG00010018971	POLR1B	case	control	0.031644051	0.044853143	0.705503534	0.480496934	0.831953766	up_in_case
PRJNA8274(	ENSGALG00010018973	NEFM	case	control	-0.880943828	0.584709343	-1.506635456	0.131904124	0.535212583	down_in_case
PRJNA8274(	ENSGALG00010018974	TAS1R1	case	control	0.308358032	0.17837986	1.728659462	0.083870061	0.458188945	up_in_case
PRJNA8274(	ENSGALG00010018977	NPHP3	case	control	0.134710585	0.092327674	1.459048831	0.144551668	0.548736091	up_in_case
PRJNA8274(	ENSGALG00010018983	MDGA2	case	control	0.209710592	0.143443957	1.461968819	0.143749755	0.546917268	up_in_case
PRJNA8274(	ENSGALG00010018985	NOL9	case	control	0.069015491	0.05943382	1.161215805	0.245554149	0.670194327	up_in_case
PRJNA8274(	ENSGALG00010018986	CTDSP1	case	control	-0.110567588	0.073018285	-1.514245196	0.129963663	0.532363328	down_in_case
PRJNA8274(	ENSGALG00010018987	MRPS10	case	control	0.006508936	0.101826488	0.063921831	0.949032469	0.99138853	up_in_case
PRJNA8274(	ENSGALG00010018990	GCK	case	control	-0.035199997	0.244849378	-0.143761839	0.885688538	0.977630122	down_in_case
PRJNA8274(	ENSGALG00010018991	RYBP	case	control	-0.018082057	0.077799857	-0.232417611	0.816213672	0.957422215	down_in_case
PRJNA8274(	ENSGALG00010018993	ENSGALG00010018993	case	control	-0.043832248	0.385860547	-0.113596086	0.90955799	0.981730793	down_in_case
PRJNA8274(	ENSGALG00010018998	RPS29	case	control	-0.044190786	0.0805523	-0.548597447	0.583281738	0.881306702	down_in_case
PRJNA8274(	ENSGALG00010018999	THUMPD3	case	control	0.008682723	0.047106322	0.184321816	0.853761	0.969998446	up_in_case
PRJNA8274(	ENSGALG00010019000	TRERF1	case	control	-0.067706235	0.079097531	-0.855984172	0.392006535	0.785441144	down_in_case
PRJNA8274(	ENSGALG00010019001	ALDH1A3	case	control	-0.08883985	0.135085933	-0.657654341	0.510760268	0.845159897	down_in_case
PRJNA8274(	ENSGALG00010019004	ECE2	case	control	0.064023231	0.170136152	0.376305862	0.706689522	0.924581603	up_in_case
PRJNA8274(	ENSGALG00010019005	ENSGALG00010019005	case	control	0.173398683	0.084945321	2.041297625	0.041221253	0.338772367	up_in_case
PRJNA8274(	ENSGALG00010019006	ENSGALG00010019006	case	control	0.133056021	0.06824488	1.949685047	0.05121367	0.370042007	up_in_case
PRJNA8274(	ENSGALG00010019008	HERPUD2	case	control	-0.041934187	0.054073271	-0.775506749	0.438040279	0.811390476	down_in_case
PRJNA8274(	ENSGALG00010019009	NRAP	case	control	-0.151015519	0.152753802	-0.988620367	0.322848917	0.740689923	down_in_case
PRJNA8274(	ENSGALG00010019011	VIL1	case	control	0.178346077	0.363040936	0.491256109	0.623245319	0.894052087	up_in_case
PRJNA8274(	ENSGALG00010019012	ADAM28	case	control	0.022041701	0.194386874	0.113390891	0.909720661	0.981730793	up_in_case
PRJNA8274(	ENSGALG00010019013	LRRK1	case	control	0.146362526	0.072623839	2.015351007	0.043867884	0.349476736	up_in_case
PRJNA8274(	ENSGALG00010019015	GSG1L	case	control	0.653313999	0.442442006	1.476609339	0.139780387	0.541990111	up_in_case
PRJNA8274(	ENSGALG00010019016	PLEKHG5	case	control	0.065459296	0.075134624	0.871226764	0.383630349	0.780061953	up_in_case
PRJNA8274(	ENSGALG00010019017	IRAK2	case	control	0.095950139	0.065953088	1.454824065	0.145717974	0.550678391	up_in_case
PRJNA8274(	ENSGALG00010019018	SH3BGRL	case	control	0.003100543	0.071211695	0.043539795	0.965271243	0.992291928	up_in_case
PRJNA8274(	ENSGALG00010019019	TBX20	case	control	-0.114671446	0.252843344	-0.453527645	0.650168842	0.904241824	down_in_case
PRJNA8274(	ENSGALG00010019020	KCNK2	case	control	0.470800539	0.159358285	2.954352436	0.00313326	0.095164759	up_in_case
PRJNA8274(	ENSGALG00010019021	LRR1	case	control	-0.00079189	0.091766316	-0.008629416	0.993114807	0.998196863	down_in_case
PRJNA8274(	ENSGALG00010019023	AP2M1	case	control	-0.027444759	0.035849006	-0.765565389	0.443934945	0.815134786	down_in_case
PRJNA8274(	ENSGALG00010019024	CNPY3	case	control	0.100455873	0.062625216	1.60408027	0.10869636	0.500951275	up_in_case
PRJNA8274(	ENSGALG00010019025	DNAAF2	case	control	0.280783656	0.166340673	1.688003604	0.091410537	0.476493306	up_in_case
PRJNA8274(	ENSGALG00010019026	CHSY1	case	control	0.098296463	0.05708855	1.721824469	0.085101321	0.461467053	up_in_case
PRJNA8274(	ENSGALG00010019027	GNLY	case	control	0.247227475	0.213132007	1.159973477	0.246059605	0.670869818	up_in_case
PRJNA8274(	ENSGALG00010019028	TNFRSF25	case	control	0.643008804	0.36192614	1.776629909	0.075629151	0.442474101	up_in_case
PRJNA8274(	ENSGALG00010019029	CENPF	case	control	-0.129834054	0.155595283	-0.834434383	0.404036249	0.79260318	down_in_case
PRJNA8274(	ENSGALG00010019030	CDR2	case	control	0.175246805	0.076685508	2.285266266	0.02229722	0.266152121	up_in_case
PRJNA8274(	ENSGALG00010019032	GNMT	case	control	0.063575405	0.10873795	0.584666208	0.55877218	0.869450004	up_in_case
PRJNA8274(	ENSGALG00010019033	CYP2AB4	case	control	0.892151541	1.039501119	0.858249717	0.390754588	0.784128499	up_in_case
PRJNA8274(	ENSGALG00010019034	CASP7	case	control	0.108857696	0.152657873	0.713082755	0.47579455	0.830520323	up_in_case
PRJNA8274(	ENSGALG00010019035	ENSGALG00010019035	case	control	0.110045932	0.265129848	0.415064287	0.678094842	0.914941113	up_in_case
PRJNA8274(	ENSGALG00010019036	REL	case	control	-0.020545465	0.069983949	-0.293573959	0.769083477	0.94539152	down_in_case
PRJNA8274(	ENSGALG00010019038	PLEKHS1	case	control	0.035040137	0.479215032	0.073119862	0.941710736	0.989551456	up_in_case
PRJNA8274(	ENSGALG00010019040	HMGN5	case	control	0.012947802	0.064049623	0.202152666	0.839797376	0.965286179	up_in_case
PRJNA8274(	ENSGALG00010019041	KATNIP	case	control	0.103148164	0.071321376	1.446244738	0.14810857	0.553857883	up_in_case
PRJNA8274(	ENSGALG00010019042	PEX6	case	control	0.09991557	0.07089325	1.409380586	0.158722662	0.570858667	up_in_case
PRJNA8274(	ENSGALG00010019043	METTL9	case	control	-0.132366641	0.084879915	-1.559457737	0.11888808	0.516128861	down_in_case
PRJNA8274(	ENSGALG00010019045	LBX1	case	control	-0.061597515	0.113367829	-0.543342111	0.586894275	0.882408594	down_in_case
PRJNA8274(	ENSGALG00010019046	SELENOS	case	control	-0.03115999	0.055728051	-0.559143713	0.576063644	0.877910508	down_in_case
PRJNA8274(	ENSGALG00010019047	GHRL	case	control	-0.555317474	0.345020247	-1.609521411	0.107502377	0.498867454	down_in_case
PRJNA8274(	ENSGALG00010019049	POLD2	case	control	-0.031679852	0.070880115	-0.446949785	0.654911317	0.907132102	down_in_case
PRJNA8274(	ENSGALG00010019050	TNS1	case	control	-0.150904031	0.0856179	-1.762528987	0.077979963	0.448028441	down_in_case
PRJNA8274(	ENSGALG00010019051	IGSF6	case	control	-0.222392196	0.20215113	-1.100128385	0.271276188	0.698323327	down_in_case
PRJNA8274(	ENSGALG00010019059	BRWD3	case	control	0.016162102	0.073035464	0.221291159	0.824865733	0.960744547	up_in_case
PRJNA8274(	ENSGALG00010019062	PUS10	case	control	-0.011566576	0.094669237	-0.122178821	0.902757399	0.980895712	down_in_case
PRJNA8274(	ENSGALG00010019064	ENSGALG00010019064	case	control	-0.053118037	0.074241955	-0.715471957	0.474317463	0.82979716	down_in_case
PRJNA8274(	ENSGALG00010019065	CAND2	case	control	-0.190675098	0.084074731	-2.267923974	0.023333841	0.269590124	down_in_case
PRJNA8274(	ENSGALG00010019067	GTF3C1	case	control	-0.002610493	0.040544275	-0.06438622	0.948662702	0.991294545	down_in_case
PRJNA8274(	ENSGALG00010019068	SNRPA1	case	control	-0.157654568	0.081747963	-1.928544306	0.053787459	0.377891678	down_in_case
PRJNA8274(	ENSGALG00010019072	AEBP1	case	control	-0.239169254	0.129748885	-1.84332415	0.065281697	0.414904609	down_in_case
PRJNA8274(	ENSGALG00010019076	ESPN	case	control	0.088503467	0.319824263	0.276725307	0.781991038	0.949394183	up_in_case
PRJNA8274(	ENSGALG00010019079	CAPG	case	control	-0.27964113	0.175488239	-1.593503538	0.111047275	0.503480037	down_in_case
PRJNA8274(	ENSGALG00010019081	PCSK6	case	control	-0.12788126	0.093005722	-1.374982704	0.169136807	0.585038804	down_in_case
PRJNA8274(	ENSGALG00010019082	POLR3K	case	control	-0.242508304	0.121891386	-1.98954424	0.046641164	0.356907825	down_in_case
PRJNA8274(	ENSGALG00010019083	OTOA	case	control	0.056727782	0.169878541	0.333931418	0.738431311	0.935965942	up_in_case
PRJNA8274(	ENSGALG00010019087	PEX13	case	control	-0.046020538	0.055987811	-0.821974232	0.411091563	0.796718771	down_in_case
PRJNA8274(	ENSGALG00010019093	RPL32	case	control	-0.118107203	0.089241448	-1.323456821	0.185683507	0.608776505	down_in_case
PRJNA8274(	ENSGALG00010019094	SNRNP25	case	control	-0.125261216	0.096182356	-1.3023305	0.19280343	0.615090794	down_in_case
PRJNA8274(	ENSGALG00010019097	STC1	case	control	0.073477048	0.193033127	0.380644758	0.703466865	0.923694148	up_in_case
PRJNA8274(	ENSGALG00010019101	POLE2	case	control	0.04439469	0.103800743	0.427691445	0.668875779	0.913032039	up_in_case
PRJNA8274(	ENSGALG00010019104	POLL	case	control	-0.212420044	0.143192161	-1.483461408	0.137951862	0.539854583	down_in_case
PRJNA8274(	ENSGALG00010019108	KDELR2	case	control	0.101673466	0.061629656	1.64974905	0.098994273	0.488407564	up_in_case
PRJNA8274(	ENSGALG00010019110	MBD4	case	control	0.048962479	0.094319656	0.519112148	0.603682537	0.885268908	up_in_case
PRJNA8274(	ENSGALG00010019113	KLHDC1	case	control	-0.113788237	0.112416449	-1.012202733	0.31144113	0.734097745	down_in_case
PRJNA8274(	ENSGALG00010019114	TENT5D	case	control	0.837708821	0.317236475	2.640644715	0.008274844	0.160339127	up_in_case

PRJNA8274(	ENSGALG00010019115	DBNL	case	control	-0.005602687	0.053716063	-0.104301884	0.916929783	0.983445506	down_in_case
PRJNA8274(	ENSGALG00010019120	DAGLB	case	control	-0.190964802	0.075340214	-2.534699495	0.011254382	0.185840265	down_in_case
PRJNA8274(	ENSGALG00010019123	PLEKHO2	case	control	0.024116452	0.090410234	0.266744716	0.789665722	0.951843754	up_in_case
PRJNA8274(	ENSGALG00010019124	BMPER	case	control	-0.115931612	0.120339184	-0.963373755	0.335359992	0.749356776	down_in_case
PRJNA8274(	ENSGALG00010019125	ENSGALG00010019125	case	control	0.069961466	0.118880144	0.588504217	0.556193904	0.867878646	up_in_case
PRJNA8274(	ENSGALG00010019127	TMBIM1	case	control	-0.042482427	0.057960203	-0.732958567	0.4635837	0.825091817	down_in_case
PRJNA8274(	ENSGALG00010019130	RPS6KC1	case	control	-0.034292288	0.048386413	-0.708717295	0.47849993	0.830910626	down_in_case
PRJNA8274(	ENSGALG00010019132	ENSGALG00010019132	case	control	0.100419639	0.065929732	1.523131297	0.12772586	0.529567933	up_in_case
PRJNA8274(	ENSGALG00010019135	KIAA1841	case	control	0.016784365	0.186459835	0.090015979	0.928274517	0.986776285	up_in_case
PRJNA8274(	ENSGALG00010019139	DPCD	case	control	0.038036838	0.091548011	0.415485137	0.677786793	0.914893769	up_in_case
PRJNA8274(	ENSGALG00010019141	ACOT7	case	control	0.266489923	0.126944525	2.099262827	0.035793738	0.324974524	up_in_case
PRJNA8274(	ENSGALG00010019144	IFT122	case	control	-0.024117934	0.064859738	-0.371847543	0.710006364	0.925630832	down_in_case
PRJNA8274(	ENSGALG00010019145	RAC1	case	control	-0.004691683	0.03898409	-0.12034867	0.904206951	0.981104465	down_in_case
PRJNA8274(	ENSGALG00010019146	KLHDC2	case	control	-0.090608316	0.057402374	-1.578476795	0.114456115	0.509446069	down_in_case
PRJNA8274(	ENSGALG00010019148	ENSGALG00010019148	case	control	0.261354065	0.334770162	0.780697012	0.434980718	0.80981959	up_in_case
PRJNA8274(	ENSGALG00010019149	COX5B	case	control	-0.133865866	0.093127903	-1.437440999	0.150592726	0.558039903	down_in_case
PRJNA8274(	ENSGALG00010019157	ENSGALG00010019157	case	control	-0.078984899	0.370010041	-0.213466908	0.830962798	0.962245365	down_in_case
PRJNA8274(	ENSGALG00010019158	ENSGALG00010019158	case	control	0.236917973	0.190706165	1.242319425	0.214118731	0.639093687	up_in_case
PRJNA8274(	ENSGALG00010019166	PNPT1	case	control	-0.047162068	0.051315982	-0.919052241	0.358068248	0.764462254	down_in_case
PRJNA8274(	ENSGALG00010019169	GPR153	case	control	0.468006595	0.203553099	2.299186782	0.021494335	0.262484197	up_in_case
PRJNA8274(	ENSGALG00010019172	NEMF	case	control	-0.055712737	0.048371443	-1.151769179	0.249415936	0.674421685	down_in_case
PRJNA8274(	ENSGALG00010019173	RHO	case	control	-0.14597944	0.344166812	-0.424153159	0.671454119	0.913676733	down_in_case
PRJNA8274(	ENSGALG00010019174	ENSGALG00010019174	case	control	0.381655801	0.35771623	1.066923357	0.286006447	0.710420738	up_in_case
PRJNA8274(	ENSGALG00010019183	ANGEL2	case	control	0.127392216	0.054618216	2.332412587	0.019678997	0.251933438	up_in_case
PRJNA8274(	ENSGALG00010019184	PNKD	case	control	-0.022265056	0.132303185	-0.168288135	0.866356608	0.973368767	down_in_case
PRJNA8274(	ENSGALG00010019185	ANKDD1A	case	control	0.114084267	0.183366719	0.622164524	0.533833691	0.855817434	up_in_case
PRJNA8274(	ENSGALG00010019189	ICMT	case	control	-0.059075019	0.055076129	-1.072606592	0.283447666	0.707725096	down_in_case
PRJNA8274(	ENSGALG00010019194	ENSGALG00010019194	case	control	-0.129574205	0.156385792	-0.828554836	0.407356353	0.794380997	down_in_case
PRJNA8274(	ENSGALG00010019204	ARF6	case	control	0.024186599	0.060809867	0.397741351	0.690820853	0.918784485	up_in_case
PRJNA8274(	ENSGALG00010019205	ENSGALG00010019205	case	control	-0.271062282	0.42238449	-0.641742983	0.521040076	0.850168083	down_in_case
PRJNA8274(	ENSGALG00010019206	RNF207	case	control	-0.171199522	0.164221617	-1.042490786	0.297184195	0.721027687	down_in_case
PRJNA8274(	ENSGALG00010019208	PLXND1	case	control	-0.129205808	0.074080585	-1.744125096	0.081137279	0.453227768	down_in_case
PRJNA8274(	ENSGALG00010019209	VCPKMT	case	control	0.150742081	0.08182858	1.842169105	0.065450418	0.415377855	up_in_case
PRJNA8274(	ENSGALG00010019212	RPL22	case	control	0.035493131	0.060349743	0.588123969	0.556449086	0.867878646	up_in_case
PRJNA8274(	ENSGALG00010019213	RHBDF1	case	control	0.133175002	0.0638499	2.085751166	0.037001169	0.32835414	up_in_case
PRJNA8274(	ENSGALG00010019215	ENSGALG00010019215	case	control	0.054430406	0.056387837	0.965286293	0.334401435	0.748686141	up_in_case
PRJNA8274(	ENSGALG00010019218	RMND5A	case	control	0.008555517	0.056024629	0.152709922	0.878627036	0.976234053	up_in_case
PRJNA8274(	ENSGALG00010019221	VASH2	case	control	0.026728243	0.089131469	0.299874374	0.764272982	0.943786458	up_in_case
PRJNA8274(	ENSGALG00010019223	AAMP	case	control	0.075614657	0.050717857	1.490888264	0.135990833	0.539774365	up_in_case
PRJNA8274(	ENSGALG00010019225	PROK2	case	control	-0.008486005	0.397785927	-0.021333096	0.982979943	0.996591771	down_in_case
PRJNA8274(	ENSGALG00010019227	CLCN5	case	control	0.05889691	0.067393168	0.873929988	0.382156383	0.77998936	up_in_case
PRJNA8274(	ENSGALG00010019230	SPG21	case	control	0.142085052	0.050775227	2.798314483	0.005137007	0.123938918	up_in_case
PRJNA8274(	ENSGALG00010019231	ENSGALG00010019231	case	control	-0.103063886	0.178564997	-0.577178548	0.563818847	0.871916948	down_in_case
PRJNA8274(	ENSGALG00010019232	SOS2	case	control	-0.089826258	0.043553942	-2.062413973	0.03916834	0.335091843	down_in_case
PRJNA8274(	ENSGALG00010019233	ARPC2	case	control	0.060464334	0.0439362	1.376184872	0.168764404	0.585038804	up_in_case
PRJNA8274(	ENSGALG00010019236	MPG	case	control	0.095824199	0.110414354	0.867859985	0.385470987	0.781541515	up_in_case
PRJNA8274(	ENSGALG00010019237	SORCS3	case	control	-0.163916174	0.218088073	-0.751605402	0.452288394	0.820911365	down_in_case
PRJNA8274(	ENSGALG00010019239	CYTH3	case	control	-0.005033216	0.06006984	-0.083789399	0.933223877	0.987991895	down_in_case
PRJNA8274(	ENSGALG00010019240	ENSGALG00010019240	case	control	0.015670717	0.228965448	0.068441406	0.945434262	0.990313016	up_in_case
PRJNA8274(	ENSGALG00010019243	SERTAD2	case	control	-0.172305966	0.073422189	-2.346783291	0.018936265	0.245724672	down_in_case
PRJNA8274(	ENSGALG00010019246	IL17RC	case	control	0.158363475	0.078924446	2.006519945	0.044800803	0.351986719	up_in_case
PRJNA8274(	ENSGALG00010019247	HBZ	case	control	0.244525981	0.375231879	0.65166633	0.514616448	0.847493921	up_in_case
PRJNA8274(	ENSGALG00010019248	RMI2	case	control	0.128336011	0.176452643	0.727311354	0.467035244	0.827584138	up_in_case
PRJNA8274(	ENSGALG00010019250	CXCR1	case	control	-0.40293919	0.322394751	-1.249831729	0.211361023	0.636501957	down_in_case
PRJNA8274(	ENSGALG00010019252	TATDN3	case	control	0.12456335	0.099183515	1.255887642	0.20915671	0.634076945	up_in_case
PRJNA8274(	ENSGALG00010019253	HBAD	case	control	0.252285515	0.17432773	1.447190965	0.147843446	0.553590281	up_in_case
PRJNA8274(	ENSGALG00010019256	BBS9	case	control	0.026807179	0.102084098	0.262598969	0.792859695	0.95280257	up_in_case
PRJNA8274(	ENSGALG00010019257	SLC1A4	case	control	0.188643306	0.096765319	1.949492937	0.051236586	0.370042007	up_in_case
PRJNA8274(	ENSGALG00010019259	L2HGDH	case	control	0.079748852	0.068751192	1.159963198	0.24606379	0.670869818	up_in_case
PRJNA8274(	ENSGALG00010019260	TRAIL-LIKE	case	control	-0.077403741	0.0733029	-1.055943777	0.290993902	0.714222439	down_in_case
PRJNA8274(	ENSGALG00010019262	TMC3	case	control	0.261991242	0.240328604	1.090137574	0.275652547	0.700894634	up_in_case
PRJNA8274(	ENSGALG00010019263	SNRPB2	case	control	0.001682017	0.046510054	0.036164596	0.971151116	0.992761697	up_in_case
PRJNA8274(	ENSGALG00010019264	HK2	case	control	0.145342122	0.086217776	1.685755879	0.091842829	0.476713154	up_in_case
PRJNA8274(	ENSGALG00010019266	ATP5S	case	control	0.110524863	0.125776907	0.87873733	0.379543713	0.777798094	up_in_case
PRJNA8274(	ENSGALG00010019267	STARD5	case	control	0.069816592	0.227045742	0.307500117	0.758462734	0.941903352	up_in_case
PRJNA8274(	ENSGALG00010019268	NSL1	case	control	-0.044432342	0.059382446	-0.748240341	0.454315202	0.821331802	down_in_case
PRJNA8274(	ENSGALG00010019269	SOCS1	case	control	-0.010084471	0.161075555	-0.062607086	0.950079387	0.991488793	down_in_case
PRJNA8274(	ENSGALG00010019270	ENSGALG00010019270	case	control	0.729674376	0.284063707	2.568699755	0.010208086	0.179675823	up_in_case
PRJNA8274(	ENSGALG00010019271	CDKL1	case	control	0.321275247	0.149723168	2.14579514	0.031889327	0.3098361	up_in_case
PRJNA8274(	ENSGALG00010019275	CTAGE1	case	control	0.298518832	0.389554367	0.766308525	0.443492747	0.814741301	up_in_case
PRJNA8274(	ENSGALG00010019277	IGFBP5	case	control	-0.053643623	0.092034489	-0.582864354	0.559984618	0.86984941	down_in_case
PRJNA8274(	ENSGALG00010019278	JPT2	case	control	-0.024109632	0.058017188	-0.415560153	0.67773189	0.914893769	down_in_case
PRJNA8274(	ENSGALG00010019280	TLNRD1	case	control	0.031602981	0.048894094	0.646355787	0.518048967	0.848618078	up_in_case
PRJNA8274(	ENSGALG00010019282	IGFBP2	case	control	-0.055206162	0.071505222	-0.772057764	0.440080216	0.812753079	down_in_case
PRJNA8274(	ENSGALG00010019283	NT5C3A	case	control	-0.049646697	0.117581223	-0.422233207	0.672854806	0.913776846	down_in_case
PRJNA8274(	ENSGALG00010019284	CHD5	case	control	0.073449339	0.115275008	0.637166202	0.52401659	0.852011056	up_in_case
PRJNA8274(	ENSGALG00010019285	BATF3	case	control	-0.244271982	0.181772066	-1.343836749	0.179001183	0.599333225	down_in_case
PRJNA8274(	ENSGALG00010019287	AHSA2	case	control	0.04982595	0.0739402	0.673868209	0.500395105	0.840855399	up_in_case
PRJNA8274(	ENSGALG00010019288	RPL37A	case	control	0.028708386	0.083848881	0.342382459	0.732063088	0.933770678	up_in_case
PRJNA8274(	ENSGALG00010019289	FKBP9	case	control	0.045552668	0.08751894	0.520489255	0.602722615	0.885101354	up_in_case
PRJNA8274(	ENSGALG00010019291	ADD2	case	control	0.332957857	0.249280715	1.335674354	0.181655735	0.602709285	up_in_case
PRJNA8274(	ENSGALG00010019292	ENSGALG00010019292	case	control	0.078725039	0.109307637	0.720215359	0.471392409	0.829101076	up_in_case
PRJNA8274(	ENSGALG00010019294	SMARCAL1	case	control	0.043820648	0.095459059	0.45905175	0.646197004	0.902693223	up_in_case
PRJNA8274(	ENSGALG00010019295	MAP4K5	case	control	-0.072469133	0.108992246	-0.664901729	0.50611335	0.843280685	down_in_case
PRJNA8274(	ENSGALG00010019296	ENSGALG00010019296	case	control	-0.005602868	0.092188042	-0.06077652	0.95153719	0.991857576	down_in_case
PRJNA8274(	ENSGALG00010019297	SLC25A26	case	control	-0.150458094	0.131168501	-1.147059644	0.251356971	0.677081801	down_in_case
PRJNA8274(	ENSGALG00010019298	PGAP6	case	control	-0.110201557	0.070364918	-1.566143474	0.117315032	0.514085926	down_in_case
PRJNA8274(	ENSGALG00010019300	MESDC2	case	control	0.040769163	0.048030123	0.848824877	0.395978745	0.787880348	up_in_case
PRJNA8274(	ENSGALG00010019302	USP34	case	control	0.003154588	0.041827124	0.075419679	0.939880802	0.989551456	up_in_case
PRJNA8274(	ENSGALG00010019305	SNRNP27	case	control	0.001971925	0.062261227	0.031671798	0.974733785	0.993775355	up_in_case
PRJNA8274(	ENSGALG00010019307	ATF3	case	control	0.134901733	0.20159286	0.669179125	0.503381221	0.842604443	up_in_case
PRJNA8274(	ENSGALG00010019310	CRELD1	case	control	-0.066689463	0.079041449	-0.843727729	0.398821569	0.789800365	down_in_case
PRJNA8274(	ENSGALG00010019312	KBTBD2	case	control	0.050590172	0.035600617	1.421047627	0.155302914	0.564379078	up_in_case
PRJNA8274(	ENSGALG00010019313	MRPL28	case	control	0.092138938	0.071794124	1.28337714	0.199359979	0.622902456	up_in_case
PRJNA8274(	ENSGALG00010019314	GKN2	case	control	-1.203622624	0.710040104	-1.695147384			down_in_case
PRJNA8274(	ENSGALG00010019316	CLEC16A	case	control	0.131818497	0.076794462	1.716510445	0.086068658	0.463278022	up_in_case
PRJNA8274(	ENSGALG00010019318	NOG2	case	control	-0.193874419	0.18899447	-1.025820596	0.304976156	0.728073294	down_in_case
PRJNA8274(	ENSGALG00010019319	ACTR3	case	control	0.055357497	0.055532508	0.996848489	0.318838058	0.738427335	up_in_case
PRJNA8274(	ENSGALG00010019322	EGR3	case	control	0.234398587	0.153014721	1.531869516	0.125554637	0.524979916	up_in_case
PRJNA8274(	ENSGALG00010019323	LRIG1	case	control	-0.085240029	0.064725326	-1.316950163	0.18785533	0.610946377	down_in_case
PRJNA8274(	ENSGALG00010019325	SLITRK4	case	control	-0.684208636	0.189663034	-3.607496001	0.000309166	0.024027598	down_in_case
PRJNA8274(	ENSGALG00010019331	CEMIP	case	control	0.113108687	0.268061671	0.421950241	0.673061338	0.91379937	up_in_case
PRJNA8274(	ENSGALG00010019334	ATL1	case	control	0.090846704	0.062001501	1.465233944	0.142857102	0.545197906	up_in_case
PRJNA8274(	ENSGALG00010019335	HIPK3	case	control	-0.198592975	0.083653528	-2.373994012	0.017596836	0.23617575	down_in_case
PRJNA8274(	ENSGALG00010019336	DEXI	case	control	-0.05029569	0.204213151	-0.246290161	0.805457626	0.9556344	down_in_case
PRJNA8274(	ENSGALG00010019337	XPO1	case	control	0.005471972	0.04509621	0.121339964	0.903421767	0.980997505	up_in_case
PRJNA8274(	ENSGALG00010019344	DNAJC24	case	control	0.222075084	0.073138033	3.036383044	0.00239435	0.080562828	up_in_case
PRJNA8274(	ENSGALG00010019345	ENSGALG00010019345	case	control	0.061714457	0.099165301	0.622339228	0.533718832	0.855817434	up_in_case
PRJNA8274(	ENSGALG00010019346	POU3F4	case	control	0.202133162	0.243862747	0.828880855	0.407171829	0.794380997	up_in_case
PRJNA8274(	ENSGALG00010019350	SAV1	case	control	0.007111179	0.04014703	0.177128387	0.859407545	0.971828322	up_in_case
PRJNA8274(	ENSGALG00010019355	ENTPD4	case	control	0.02946547	0.076437977	0.38548207	0.699880288	0.922288441	up_in_case
PRJNA8274(	ENSGALG00010019356	AVL9	case	control	-0.044873584	0.062325468	-0.719987921	0.471532433	0.829101076	down_in_case
PRJNA8274(	ENSGALG00010019362	ENSGALG00010019362	case	control	-0.021825808	0.224481033	-0.097227849	0.922545453	0.985482967	down_in_case
PRJNA8274(	ENSGALG00010019365	ENSGALG00010019365	case	control	0.140191459	0.153094932	0.915715876	0.359815936	0.765679744	up_in_case
PRJNA8274(	ENSGALG00010019371	TMEM87A	case	control	-0.011089713	0.038757248	-0.286132622	0.774776547	0.947085948	down_in_case
PRJNA8274(	ENSGALG00010019373	DENND5A	case	control	-0.047353598	0.053304125	-0.888366481	0.37434365	0.774434633	down_in_case
PRJNA8274(	ENSGALG00010019376	ENSGALG00010019376	case	control	0.600495434	0.369642822	1.624528863	0.104262986	0.493800828	up_in_case
PRJNA8274(	ENSGALG00010019388	GPR75	case	control	-0.068241283	0.093154036	-0.732563893	0.46382446	0.82516668	down_in_case
PRJNA8274(	ENSGALG00010019390	TVP23A	case	control	0.239719755	0.328673081	0.729356218	0.465783792	0.826800922	up_in_case
PRJNA8274(	ENSGALG00010019391	GANC	case	control	0.096611179	0.106472058	0.907385291	0.364203085	0.770055025	up_in_case
PRJNA8274(	ENSGALG00010019392	NIN	case	control	0.276685872	0.076856304	3.60004135	0.000318167	0.024072921	up_in_case
PRJNA8274(	ENSGALG00010019398	SLC35F5	case	control	-0.07903702	0.078471925	-1.007201236	0.313838079	0.735477467	down_in_case
PRJNA8274(	ENSGALG00010019399	CD93	case	control	0.085729959	0.092731821	0.92449343	0.35522947	0.763161644	up_in_case
PRJNA8274(	ENSGALG00010019403	PACC1	case	control	0.053138597	0.086261382	0.616018384	0.537882387	0.857740557	up_in_case
PRJNA8274(	ENSGALG00010019411	LSM5	case	control	-0.014200789	0.117190533	-0.121176932	0.903550895	0.980997505	down_in_case
PRJNA8274(	ENSGALG00010019416	GPR39	case	control	0.079534389	0.521688178	0.152455801	0.878827448	0.976247379	up_in_case

PRJNA8274(	ENSGALG00010019418	ZNF703	case	control	-0.030425987	0.092611853	-0.328532325	0.742509208	0.937448285	down_in_case
PRJNA8274(	ENSGALG00010019422	IMMP1L	case	control	0.036160736	0.126273809	0.286367667	0.774596537	0.947085948	up_in_case
PRJNA8274(	ENSGALG00010019423	LYPD1	case	control	0.077299064	0.130434351	0.592628117	0.553430058	0.866337858	up_in_case
PRJNA8274(	ENSGALG00010019426	GRM7	case	control	-0.550121202	0.171980313	-3.198745207	0.001380271	0.058654283	down_in_case
PRJNA8274(	ENSGALG00010019430	ENSGALG00010019430	case	control	-0.049360694	0.238253702	-0.207177028	0.835871607	0.964083973	down_in_case
PRJNA8274(	ENSGALG00010019441	LMCD1	case	control	-0.122455669	0.154312952	-0.793554053	0.42745509	0.805305429	down_in_case
PRJNA8274(	ENSGALG00010019443	NCKAP5	case	control	-0.215444266	0.088039243	-2.447139021	0.014399529	0.212063088	down_in_case
PRJNA8274(	ENSGALG00010019451	CAV3	case	control	-0.199552168	0.148452005	-1.344220086	0.178877228	0.599190528	down_in_case
PRJNA8274(	ENSGALG00010019458	ELP4	case	control	-0.200948126	0.067941809	-2.957650529	0.003099934	0.095126715	down_in_case
PRJNA8274(	ENSGALG00010019461	MGAT5	case	control	0.00540968	0.076843478	0.070398687	0.943876336	0.990183239	up_in_case
PRJNA8274(	ENSGALG00010019470	TGFB2	case	control	-0.174825049	0.072974979	-2.395684822	0.016589348	0.228984248	down_in_case
PRJNA8274(	ENSGALG00010019471	NUBP1	case	control	-0.151400572	0.081164654	-1.865351048	0.062132214	0.405537748	down_in_case
PRJNA8274(	ENSGALG00010019472	CAPN3	case	control	-0.269294556	0.134806739	-1.997634226	0.04575633	0.35522015	down_in_case
PRJNA8274(	ENSGALG00010019473	OXTR	case	control	0.606118154	0.384069215	1.578148236	0.114531559	0.509446069	up_in_case
PRJNA8274(	ENSGALG00010019474	TMEM163	case	control	1.096766506	0.557443233	1.96749452	0.049126227	0.364180947	up_in_case
PRJNA8274(	ENSGALG00010019477	NCAPH	case	control	-0.002148856	0.076194215	-0.028202353	0.97750076	0.994661197	down_in_case
PRJNA8274(	ENSGALG00010019481	PYGL	case	control	-0.002446572	0.089370514	-0.027375605	0.978160155	0.995091221	down_in_case
PRJNA8274(	ENSGALG00010019483	SCUBE2	case	control	0.366317777	0.176319741	2.077576653	0.037748368	0.329750561	up_in_case
PRJNA8274(	ENSGALG00010019495	EMP2	case	control	0.05194805	0.083118896	0.624984845	0.531981005	0.855755691	up_in_case
PRJNA8274(	ENSGALG00010019498	ENSGALG00010019498	case	control	0.122050932	0.219070407	0.557131077	0.577437876	0.878143389	up_in_case
PRJNA8274(	ENSGALG00010019509	ZNF106	case	control	-0.166248369	0.110076682	-1.510295976	0.130967918	0.534010989	down_in_case
PRJNA8274(	ENSGALG00010019515	ENSGALG00010019515	case	control	-0.033561799	0.08715146	-0.385097383	0.700165267	0.922288441	down_in_case
PRJNA8274(	ENSGALG00010019524	HHIPL2	case	control	-0.811978364	0.389038848	-2.087139541	0.036875523	0.328139374	down_in_case
PRJNA8274(	ENSGALG00010019529	SNAP23	case	control	0.091423872	0.045649531	2.002734085	0.045205838	0.352477364	up_in_case
PRJNA8274(	ENSGALG00010019534	PPP2R5A	case	control	-0.172311364	0.047883216	-3.598575387	0.000319965	0.024081584	down_in_case
PRJNA8274(	ENSGALG00010019540	UNC5D	case	control	-0.046892265	0.218216448	-0.214888776	0.829854036	0.962228709	down_in_case
PRJNA8274(	ENSGALG00010019546	HSD17B12	case	control	-0.048761434	0.056878688	-0.857288305	0.39128557	0.784326276	down_in_case
PRJNA8274(	ENSGALG00010019550	ACMSD	case	control	0.674736748	0.400561676	1.684476543	0.092089609	0.476713154	up_in_case
PRJNA8274(	ENSGALG00010019557	TRIM9	case	control	0.041833006	0.137982897	0.303175301	0.761756271	0.943290152	up_in_case
PRJNA8274(	ENSGALG00010019572	NFKBIE	case	control	0.094758033	0.151425867	0.625771769	0.531464652	0.855560567	up_in_case
PRJNA8274(	ENSGALG00010019577	MXD1	case	control	-0.070601184	0.074493529	-0.947749219	0.343257138	0.755132664	down_in_case
PRJNA8274(	ENSGALG00010019581	SLC35B2	case	control	-0.02265347	0.07405861	-0.305885709	0.759691668	0.942529138	down_in_case
PRJNA8274(	ENSGALG00010019584	RAD18	case	control	0.068227379	0.084053949	0.811709377	0.416958413	0.799987395	up_in_case
PRJNA8274(	ENSGALG00010019585	PDE1C	case	control	-0.362186308	0.158194545	-2.28949935	0.022050356	0.264801476	down_in_case
PRJNA8274(	ENSGALG00010019586	GLYR1	case	control	0.048149211	0.030765176	1.5650556	0.117569873	0.51430076	up_in_case
PRJNA8274(	ENSGALG00010019587	CCNT2	case	control	0.137256655	0.097729974	1.404447889	0.160185519	0.572721131	up_in_case
PRJNA8274(	ENSGALG00010019588	CHMP7	case	control	-0.162924015	0.053129545	-3.066542656	0.002165499	0.076380185	down_in_case
PRJNA8274(	ENSGALG00010019591	ENSGALG00010019591	case	control	0.043360003	0.05716987	0.758441517	0.448186712	0.818697952	up_in_case
PRJNA8274(	ENSGALG00010019592	ALKBH3	case	control	-0.119039184	0.10888774	-1.093228526	0.274293475	0.699970438	down_in_case
PRJNA8274(	ENSGALG00010019594	TMX1	case	control	-0.647189821	0.323341726	-2.001566049	0.045331423	0.353071545	down_in_case
PRJNA8274(	ENSGALG00010019595	GRIN2A	case	control	-0.343087633	0.13958432	-2.457923869	0.013974279	0.208137171	down_in_case
PRJNA8274(	ENSGALG00010019598	KCNAB2	case	control	-0.086199862	0.097481474	-0.884269176	0.376550924	0.775199984	down_in_case
PRJNA8274(	ENSGALG00010019600	MAP3K19	case	control	-0.125809648	0.256966658	-0.489595225	0.624420356	0.894531266	down_in_case
PRJNA8274(	ENSGALG00010019602	SLC29A1	case	control	-0.060949741	0.081453775	-0.74827399	0.45429491	0.821331802	down_in_case
PRJNA8274(	ENSGALG00010019610	CORO7	case	control	0.177564615	0.097803069	1.815532146	0.069442171	0.425095479	up_in_case
PRJNA8274(	ENSGALG00010019613	FRMD6	case	control	0.035110408	0.051235335	0.685277212	0.493169009	0.837887766	up_in_case
PRJNA8274(	ENSGALG00010019614	MICALL2	case	control	-0.023025185	0.087164488	-0.264157861	0.79165828	0.95239351	down_in_case
PRJNA8274(	ENSGALG00010019617	ANO3	case	control	0.086816768	0.118919274	0.730047916	0.465360896	0.826255378	up_in_case
PRJNA8274(	ENSGALG00010019618	NPHP4	case	control	-0.070482598	0.09051794	-0.778658882	0.436180683	0.81000376	down_in_case
PRJNA8274(	ENSGALG00010019621	ENSGALG00010019621	case	control	0.031999844	0.116373605	0.274975098	0.78333537	0.949518053	up_in_case
PRJNA8274(	ENSGALG00010019622	C2orf42	case	control	0.075886998	0.061124452	1.241516207	0.214415115	0.639124605	up_in_case
PRJNA8274(	ENSGALG00010019624	SRGAP3	case	control	0.087711373	0.084757399	1.034852111	0.300737982	0.723511633	up_in_case
PRJNA8274(	ENSGALG00010019625	INTS1	case	control	-0.069008391	0.050837408	-1.35743333	0.174643564	0.593489298	down_in_case
PRJNA8274(	ENSGALG00010019630	AJAP1	case	control	-0.187401071	0.338487731	-0.553642139	0.579823781	0.879142433	down_in_case
PRJNA8274(	ENSGALG00010019634	GNG2	case	control	-0.116386564	0.103167698	-1.128129892	0.259265067	0.686374086	down_in_case
PRJNA8274(	ENSGALG00010019635	LRRC57	case	control	-0.030565664	0.062908183	-0.485877401	0.627054092	0.895838586	down_in_case
PRJNA8274(	ENSGALG00010019640	MGRN1	case	control	-0.048302477	0.056987557	-0.847596904	0.396662499	0.788531716	down_in_case
PRJNA8274(	ENSGALG00010019641	CAPN11	case	control	0.065593181	0.067742433	0.968273186	0.332907953	0.748503677	up_in_case
PRJNA8274(	ENSGALG00010019643	RAB3GAP1	case	control	-0.040563801	0.040084643	-1.011953652	0.311560215	0.734109585	down_in_case
PRJNA8274(	ENSGALG00010019645	ENSGALG00010019645	case	control	-0.01124626	0.113652621	-0.098952931	0.921175642	0.985177749	down_in_case
PRJNA8274(	ENSGALG00010019646	VPS37C	case	control	0.070994696	0.068041488	1.043403046	0.296761663	0.720490965	up_in_case
PRJNA8274(	ENSGALG00010019648	PRDM16	case	control	0.086283842	0.191817636	0.449822257	0.652838608	0.905885495	up_in_case
PRJNA8274(	ENSGALG00010019649	TMEM63B	case	control	0.137560281	0.053793342	2.557199021	0.010551883	0.183125942	up_in_case
PRJNA8274(	ENSGALG00010019650	KBTBD8	case	control	0.095109723	0.062030199	1.533280961	0.125206644	0.523984491	up_in_case
PRJNA8274(	ENSGALG00010019652	TNFRSF10B	case	control	0.003411343	0.356753394	0.009562187	0.992370595	0.997995042	up_in_case
PRJNA8274(	ENSGALG00010019655	RTRAF	case	control	-0.011992334	0.049468304	-0.242424603	0.808451172	0.955875648	down_in_case
PRJNA8274(	ENSGALG00010019657	PPP1R17	case	control	0.212470396	0.299946995	0.708359809	0.478721845	0.831195043	up_in_case
PRJNA8274(	ENSGALG00010019659	MRPL14	case	control	0.086689929	0.064629631	1.341334118	0.179811997	0.600773729	up_in_case
PRJNA8274(	ENSGALG00010019660	ENSGALG00010019660	case	control	-0.115125229	0.514860646	-0.223604639	0.823064954	0.959783808	down_in_case
PRJNA8274(	ENSGALG00010019670	ENSGALG00010019670	case	control	-0.012927899	0.090539358	-0.142787619	0.886457916	0.977630122	down_in_case
PRJNA8274(	ENSGALG00010019673	NID2	case	control	0.008399505	0.097076477	0.086524612	0.931049392	0.987258715	up_in_case
PRJNA8274(	ENSGALG00010019675	MAFK	case	control	-0.056061246	0.058449038	-0.959147446	0.337484471	0.750948271	down_in_case
PRJNA8274(	ENSGALG00010019679	RHOBTB2	case	control	0.047456663	0.099690252	0.476041161	0.634045054	0.898107199	up_in_case
PRJNA8274(	ENSGALG00010019680	CCDC34	case	control	-0.073203147	0.097222638	-0.752943431	0.451483908	0.820302668	down_in_case
PRJNA8274(	ENSGALG00010019682	TMEM158	case	control	-0.133221254	0.208399792	-0.639258093	0.522655046	0.851055244	down_in_case
PRJNA8274(	ENSGALG00010019683	TMEM184A	case	control	0.150712963	0.198272945	0.760128735	0.447177638	0.817833512	up_in_case
PRJNA8274(	ENSGALG00010019684	PIGQ	case	control	0.035862557	0.110034586	0.325920771	0.7444843	0.937654174	up_in_case
PRJNA8274(	ENSGALG00010019690	ENSGALG00010019690	case	control	-0.167054688	0.0618047	-2.702944736	0.006872817	0.143686091	down_in_case
PRJNA8274(	ENSGALG00010019692	MMEL1	case	control	0.297474726	0.192877058	1.542302277	0.123000156	0.522394484	up_in_case
PRJNA8274(	ENSGALG00010019693	HAUS2	case	control	0.01638864	0.059248537	0.276608343	0.782080858	0.949394183	up_in_case
PRJNA8274(	ENSGALG00010019696	ENSGALG00010019696	case	control	1.003542014	0.516006485	1.94482442	0.051796114	0.371953818	up_in_case
PRJNA8274(	ENSGALG00010019697	EIF4EBP1	case	control	-0.143334156	0.072929712	-1.965373955	0.049370972	0.364571979	down_in_case
PRJNA8274(	ENSGALG00010019700	HES5B	case	control	-0.022361175	0.154115476	-0.145093638	0.884636941	0.977612694	down_in_case
PRJNA8274(	ENSGALG00010019706	NPRL3	case	control	-0.198328032	0.077958344	-2.544025704	0.010958302	0.185137791	down_in_case
PRJNA8274(	ENSGALG00010019713	NUDT14	case	control	-0.20355668	0.096890907	-2.100885285	0.035651037	0.324939791	down_in_case
PRJNA8274(	ENSGALG00010019716	VEGFA	case	control	0.084892906	0.069107522	1.228417742	0.219290191	0.643443819	up_in_case
PRJNA8274(	ENSGALG00010019717	ZRANB3	case	control	0.025558064	0.095391902	0.26792698	0.788755526	0.951738421	up_in_case
PRJNA8274(	ENSGALG00010019724	HES5A	case	control	0.315990711	0.190701173	1.656994055	0.097520677	0.485860997	up_in_case
PRJNA8274(	ENSGALG00010019726	PEBP4	case	control	-0.172255257	0.204532037	-0.842192059	0.399680458	0.789900496	down_in_case
PRJNA8274(	ENSGALG00010019731	PANK4	case	control	0.025973103	0.063825196	0.406941212	0.684051166	0.916328843	up_in_case
PRJNA8274(	ENSGALG00010019732	HBA1	case	control	0.188483358	0.164115289	1.148481407	0.250769884	0.676268933	up_in_case
PRJNA8274(	ENSGALG00010019735	ENSGALG00010019735	case	control	-0.132958964	0.111890782	-1.188292383	0.234718238	0.659294991	down_in_case
PRJNA8274(	ENSGALG00010019738	ADRA2A	case	control	-0.221469617	0.205239034	-1.079081361	0.280551459	0.705762606	down_in_case
PRJNA8274(	ENSGALG00010019744	KBP	case	control	0.192899914	0.397758338	0.484967618	0.627699316	0.895865363	up_in_case
PRJNA8274(	ENSGALG00010019752	GPAM	case	control	0.198476774	0.108751697	1.825045307	0.067994193	0.421339236	up_in_case
PRJNA8274(	ENSGALG00010019761	MRPS18A	case	control	-0.006757276	0.072002352	-0.09384799	0.92522991	0.985571367	down_in_case
PRJNA8274(	ENSGALG00010019767	SUCLG2	case	control	-0.096955938	0.081138625	-1.19494184	0.232109754	0.657651969	down_in_case
PRJNA8274(	ENSGALG00010019771	VWCE	case	control	0.331270265	0.276359312	1.198694058	0.230646929	0.655228949	up_in_case
PRJNA8274(	ENSGALG00010019772	ENSGALG00010019772	case	control	-0.023389584	0.073706794	-0.317332812	0.750991082	0.940053613	down_in_case
PRJNA8274(	ENSGALG00010019778	ABHD17C	case	control	-0.057973373	0.06961958	-0.832716491	0.405004642	0.793437545	down_in_case
PRJNA8274(	ENSGALG00010019785	ACSL5	case	control	0.266023058	0.39126996	0.679896453	0.496570028	0.83858637	up_in_case
PRJNA8274(	ENSGALG00010019786	R3HDM1	case	control	-0.095225194	0.049241845	-1.933826703	0.053134438	0.376522532	down_in_case
PRJNA8274(	ENSGALG00010019789	ARNT2	case	control	0.042795344	0.061072015	0.700735745	0.483467945	0.833691495	up_in_case
PRJNA8274(	ENSGALG00010019794	RSPH9	case	control	0.060377124	0.155565665	0.38811343	0.697932094	0.922288441	up_in_case
PRJNA8274(	ENSGALG00010019799	DOK2	case	control	0.264354621	0.225764372	1.170931529	0.2416263	0.666243691	up_in_case
PRJNA8274(	ENSGALG00010019800	EXT2	case	control	0.031507711	0.048597801	0.648336143	0.516767562	0.848054666	up_in_case
PRJNA8274(	ENSGALG00010019801	FIBIN	case	control	0.095930207	0.189207801	0.507009788	0.612147951	0.889814948	up_in_case
PRJNA8274(	ENSGALG00010019803	FAH	case	control	1.07648843	0.672739508	1.600156402	0.109563891	0.501801854	up_in_case
PRJNA8274(	ENSGALG00010019804	ELFN1	case	control	-0.660427844	0.223032391	-2.961129737	0.003065128	0.094464072	down_in_case
PRJNA8274(	ENSGALG00010019807	ZDHHC6	case	control	-0.006389282	0.096540441	-0.066182444	0.947232574	0.991025379	down_in_case
PRJNA8274(	ENSGALG00010019810	ZFAND6	case	control	-0.080199144	0.068217233	-1.175643463	0.23973738	0.664684583	down_in_case
PRJNA8274(	ENSGALG00010019811	UBXN4	case	control	-0.028817501	0.057243387	-0.503420618	0.614668579	0.890733753	down_in_case
PRJNA8274(	ENSGALG00010019813	ENSGALG00010019813	case	control	0.187019606	0.319056097	0.586165278	0.557764453	0.868943422	up_in_case
PRJNA8274(	ENSGALG00010019816	ENSGALG00010019816	case	control	-0.772429419	0.913095315	-0.845946098	0.397582814	0.788728117	down_in_case
PRJNA8274(	ENSGALG00010019820	BBOX1	case	control	-0.074382035	0.142080243	-0.523521307	0.600611527	0.884954589	down_in_case
PRJNA8274(	ENSGALG00010019823	LCT	case	control	0.160316044	0.231974833	0.691092396	0.489507478	0.836347578	up_in_case
PRJNA8274(	ENSGALG00010019825	BCL2A1	case	control	0.1907314	0.268180269	0.711205939	0.476956629	0.830520323	up_in_case
PRJNA8274(	ENSGALG00010019830	JAG2	case	control	-0.123825852	0.073548579	-1.683592725	0.092260406	0.476713154	down_in_case
PRJNA8274(	ENSGALG00010019832	MAD2L1BP	case	control	-0.091940039	0.084507678	-1.087948944	0.276617648	0.702359305	down_in_case
PRJNA8274(	ENSGALG00010019833	MTHFS	case	control	0.044433288	0.115711019	0.384002216	0.700976805	0.922516526	up_in_case
PRJNA8274(	ENSGALG00010019834	PLCH2	case	control	0.14764074	0.162741359	0.907210936	0.364295262	0.770055025	up_in_case
PRJNA8274(	ENSGALG00010019836	MINAR1	case	control	-0.198633408	0.405418286	-0.48994684	0.624171517	0.894353977	down_in_case
PRJNA8274(	ENSGALG00010019838	ALX4	case	control	-0.144678757	0.193087798	-0.749290006	0.45368243	0.821282604	down_in_case
PRJNA8274(	ENSGALG00010019843	MCM6	case	control	-0.052124287	0.056473896	-0.922980183	0.356017532	0.763373775	down_in_case
PRJNA8274(	ENSGALG00010019846	ENSGALG00010019846	case	control	-0.053896745	0.079317577	-0.679505693	0.496817501	0.83878161	down_in_case
PRJNA8274(	ENSGALG00010019848	MAD1L1	case	control	-0.236641308	0.077725109	-3.044592828	0.002329955	0.079329425	down_in_case

PRJNA8274(	ENSGALG00010019854	CCDC14	case	control	0.109230247	0.074105383	1.473985327	0.140485542	0.54301845	up_in_case
PRJNA8274(	ENSGALG00010019855	TTC7A	case	control	-0.026041707	0.071893724	-0.362225036	0.717183872	0.926862121	down_in_case
PRJNA8274(	ENSGALG00010019859	BIN3	case	control	0.02381636	0.081834663	0.291030214	0.771028212	0.94558971	up_in_case
PRJNA8274(	ENSGALG00010019865	ENSGALG00010019865	case	control	-0.158915619	0.074181781	-2.142245919	0.032173698	0.3098361	down_in_case
PRJNA8274(	ENSGALG00010019867	PEX11A	case	control	0.31165425	0.331750737	0.939422933	0.347513643	0.758230866	up_in_case
PRJNA8274(	ENSGALG00010019871	TAFA1	case	control	-1.086167304	0.450239447	-2.412421457	0.015846951	0.223703263	down_in_case
PRJNA8274(	ENSGALG00010019872	VTI1A	case	control	0.07254372	0.07200407	1.007494715	0.313697096	0.735267739	up_in_case
PRJNA8274(	ENSGALG00010019873	LGR4	case	control	-0.032232026	0.064523353	-0.499540467	0.617398686	0.891081782	down_in_case
PRJNA8274(	ENSGALG00010019875	PLEKHH1	case	control	-0.293439748	0.223361277	-1.313744939	0.188932053	0.611783722	down_in_case
PRJNA8274(	ENSGALG00010019876	MRM2	case	control	-0.092590168	0.114736802	-0.806978811	0.419678691	0.80093491	down_in_case
PRJNA8274(	ENSGALG00010019878	MCFD2	case	control	0.034393739	0.053171251	0.646848414	0.517730055	0.848425162	up_in_case
PRJNA8274(	ENSGALG00010019891	PIGH	case	control	-0.098706052	0.104316398	-0.946217992	0.344037411	0.755852285	down_in_case
PRJNA8274(	ENSGALG00010019896	DDB1	case	control	-0.205776723	0.05904183	-3.485270072	0.000491641	0.032249816	down_in_case
PRJNA8274(	ENSGALG00010019898	NUDT1	case	control	-0.284063614	0.098923119	-2.871559432	0.00408452	0.110657879	down_in_case
PRJNA8274(	ENSGALG00010019899	ENSGALG00010019899	case	control	-0.140452173	0.260885305	-0.538367513	0.59032335	0.882949889	down_in_case
PRJNA8274(	ENSGALG00010019905	ENSGALG00010019905	case	control	-0.016079298	0.057364538	-0.280300322	0.779247104	0.948683261	down_in_case
PRJNA8274(	ENSGALG00010019906	ENSGALG00010019906	case	control	0.017439269	0.075338696	0.231478236	0.816943291	0.957957283	up_in_case
PRJNA8274(	ENSGALG00010019910	ENSGALG00010019910	case	control	0.229340345	0.130296628	1.760140296	0.078384022	0.448818031	up_in_case
PRJNA8274(	ENSGALG00010019912	ARG2	case	control	-0.105055598	0.165926039	-0.633147144	0.526637558	0.85251736	down_in_case
PRJNA8274(	ENSGALG00010019916	NOC3L	case	control	-0.02750938	0.054417572	-0.505523836	0.613190962	0.89021632	down_in_case
PRJNA8274(	ENSGALG00010019919	LIN7C	case	control	0.029785482	0.049162099	0.605862697	0.544605932	0.861585064	up_in_case
PRJNA8274(	ENSGALG00010019921	CALM2	case	control	-0.071541739	0.060451409	-1.183458585	0.236627451	0.661690689	down_in_case
PRJNA8274(	ENSGALG00010019925	RDH11	case	control	-0.044359371	0.114946216	-0.385914151	0.69956025	0.922288441	down_in_case
PRJNA8274(	ENSGALG00010019926	KIF7	case	control	0.021313257	0.057349628	0.371637228	0.710162967	0.925630832	up_in_case
PRJNA8274(	ENSGALG00010019931	ZFYVE26	case	control	0.073777885	0.07568584	0.974791126	0.32966388	0.746174551	up_in_case
PRJNA8274(	ENSGALG00010019932	KALRN	case	control	0.093909869	0.093397304	1.005488001	0.314661924	0.736432557	up_in_case
PRJNA8274(	ENSGALG00010019934	TKFC	case	control	0.297545649	0.147383258	2.018856505	0.043502136	0.348491091	up_in_case
PRJNA8274(	ENSGALG00010019935	ENSGALG00010019935	case	control	1.627866113	0.569788198	2.856967061	0.004277102	0.112846055	up_in_case
PRJNA8274(	ENSGALG00010019938	TAFA4	case	control	-0.298469868	0.404942881	-0.73706659	0.461081856	0.82454265	down_in_case
PRJNA8274(	ENSGALG00010019947	ENSGALG00010019947	case	control	0.166148764	0.284513181	0.583975631	0.559236708	0.869461123	up_in_case
PRJNA8274(	ENSGALG00010019948	TICRR	case	control	-0.023168839	0.076273938	-0.303758259	0.761312069	0.942985066	down_in_case
PRJNA8274(	ENSGALG00010019954	CYB561A3	case	control	-0.02239287	0.109419268	-0.204651977	0.837844043	0.964826648	down_in_case
PRJNA8274(	ENSGALG00010019956	SLC29A3	case	control	0.111034954	0.075061428	1.479254499	0.139072309	0.541505735	up_in_case
PRJNA8274(	ENSGALG00010019961	HTATSF1	case	control	-0.01196877	0.044380096	-0.269687788	0.787400457	0.95107919	down_in_case
PRJNA8274(	ENSGALG00010019962	SNX8	case	control	0.118649272	0.109514979	1.083406788	0.278627904	0.703953892	up_in_case
PRJNA8274(	ENSGALG00010019966	ENSGALG00010019966	case	control	-0.293241673	0.386641268	-0.758433456	0.448191535	0.818697952	down_in_case
PRJNA8274(	ENSGALG00010019967	CEP170B	case	control	0.130661315	0.124794256	1.047013848	0.295093191	0.718513985	up_in_case
PRJNA8274(	ENSGALG00010019968	RHCG	case	control	0.17232694	0.61906741	0.278365388	0.780731886	0.948875232	up_in_case
PRJNA8274(	ENSGALG00010019972	PPP1CB	case	control	-0.103807194	0.053386277	-1.94445464	0.05184065	0.371953818	down_in_case
PRJNA8274(	ENSGALG00010019975	ENSGALG00010019975	case	control	0.024892625	0.084561066	0.294374537	0.768471722	0.945375926	up_in_case
PRJNA8274(	ENSGALG00010019977	ZMIZ1	case	control	-0.144059113	0.074656709	-1.929620445	0.053653884	0.37782383	down_in_case
PRJNA8274(	ENSGALG00010019979	ENSGALG00010019979	case	control	0.380782331	0.383144982	0.993833531	0.320303919	0.738813491	up_in_case
PRJNA8274(	ENSGALG00010019982	PLCE1	case	control	-0.100467753	0.061470497	-1.634406063	0.102173616	0.491460867	down_in_case
PRJNA8274(	ENSGALG00010019985	UMPS	case	control	0.12111677	0.064319529	1.883048141	0.059693844	0.397773519	up_in_case
PRJNA8274(	ENSGALG00010019989	ENSGALG00010019989	case	control	-0.320371331	0.301016527	-1.064298145	0.287193658	0.711057292	down_in_case
PRJNA8274(	ENSGALG00010019990	MARCHF8	case	control	-0.0179874	0.071668148	-0.250981792	0.801828187	0.954294696	down_in_case
PRJNA8274(	ENSGALG00010019991	EIF3B	case	control	-0.043209174	0.053258819	-0.811305522	0.417190239	0.799987395	down_in_case
PRJNA8274(	ENSGALG00010019992	CHAC2	case	control	-0.068589748	0.101080923	-0.678562739	0.497414959	0.839230872	down_in_case
PRJNA8274(	ENSGALG00010019996	ITGB5	case	control	-0.157960251	0.085066226	-1.856909126	0.063324084	0.409373599	down_in_case
PRJNA8274(	ENSGALG00010019997	FSHR	case	control	-0.484669778	0.296669202	-1.633704391	0.102320936	0.491460867	down_in_case
PRJNA8274(	ENSGALG00010019998	PLEK2	case	control	0.046556224	0.56911263	0.08180494	0.934801828	0.988149615	up_in_case
PRJNA8274(	ENSGALG00010019999	ERLIN1	case	control	-0.159468197	0.046717389	-3.413465538	0.000641423	0.037533011	down_in_case
PRJNA8274(	ENSGALG00010020003	ENSGALG00010020003	case	control	-0.223757711	0.118847473	-1.882730072	0.059736958	0.397790272	down_in_case
PRJNA8274(	ENSGALG00010020005	EOGT	case	control	-0.068342145	0.060557601	-1.128547769	0.259088655	0.686345498	down_in_case
PRJNA8274(	ENSGALG00010020006	PLB1	case	control	-0.06238373	0.331905179	-0.187956483	0.850910765	0.969234083	down_in_case
PRJNA8274(	ENSGALG00010020008	EIF2S1	case	control	-0.022853466	0.047398251	-0.482158425	0.629693408	0.896269608	down_in_case
PRJNA8274(	ENSGALG00010020009	MACROD2	case	control	-0.145619241	0.070662529	-2.060770289	0.03932496	0.335091843	down_in_case
PRJNA8274(	ENSGALG00010020010	SORBS3	case	control	0.112890493	0.103850263	1.08705062	0.277014439	0.702359305	up_in_case
PRJNA8274(	ENSGALG00010020011	TLE3	case	control	-0.150943823	0.104735038	-1.441196998	0.149529036	0.556115792	down_in_case
PRJNA8274(	ENSGALG00010020013	ADAM8	case	control	0.427445685	0.594553588	0.718935507	0.472180658	0.829235491	up_in_case
PRJNA8274(	ENSGALG00010020015	ATP6V1D	case	control	-0.039244862	0.055638972	-0.705348447	0.480593418	0.832020081	down_in_case
PRJNA8274(	ENSGALG00010020017	ENSGALG00010020017	case	control	-0.23143718	0.142291553	-1.626499775	0.103843381	0.493794905	down_in_case
PRJNA8274(	ENSGALG00010020018	ENSGALG00010020018	case	control	-0.353857547	0.215475808	-1.642214734	0.10054551	0.490291708	down_in_case
PRJNA8274(	ENSGALG00010020019	PEX10	case	control	0.000635336	0.072993311	0.008704026	0.99305528	0.998196863	up_in_case
PRJNA8274(	ENSGALG00010020023	FOSL2	case	control	0.116406999	0.177187478	0.656970796	0.511199694	0.84559348	up_in_case
PRJNA8274(	ENSGALG00010020024	ENSGALG00010020024	case	control	4.410480351	1.439145879	3.064651342	0.002179239	0.076380185	up_in_case
PRJNA8274(	ENSGALG00010020025	LOXL2	case	control	-0.112781277	0.129358507	-0.871850482	0.383289951	0.780061953	down_in_case
PRJNA8274(	ENSGALG00010020027	RER1	case	control	0.00771183	0.052175788	0.14780476	0.882496852	0.976801393	up_in_case
PRJNA8274(	ENSGALG00010020030	MRPL33	case	control	0.024678204	0.067336705	0.366489633	0.713999747	0.926862121	up_in_case
PRJNA8274(	ENSGALG00010020032	CHST12	case	control	0.061047831	0.077387808	0.78885592	0.430196222	0.806256264	up_in_case
PRJNA8274(	ENSGALG00010020036	TMF1	case	control	0.005611254	0.053316355	0.105244517	0.916181787	0.983445506	up_in_case
PRJNA8274(	ENSGALG00010020037	MPP5	case	control	-0.053874037	0.070240256	-0.766996601	0.443083536	0.814303376	down_in_case
PRJNA8274(	ENSGALG00010020038	SKI	case	control	-0.131639041	0.073043123	-1.802209915	0.071512385	0.432432368	down_in_case
PRJNA8274(	ENSGALG00010020041	CDH23	case	control	-0.113290424	0.096741983	-1.171057497	0.241575667	0.666243691	down_in_case
PRJNA8274(	ENSGALG00010020046	BDNF	case	control	-0.168389959	0.210361528	-0.800478871	0.423433401	0.803723282	down_in_case
PRJNA8274(	ENSGALG00010020048	ENSGALG00010020048	case	control	-0.587985974	0.171987507	-3.418771428	0.000629045	0.03713189	down_in_case
PRJNA8274(	ENSGALG00010020053	LFNG	case	control	0.110036366	0.085849455	1.28173633	0.199935156	0.622902456	up_in_case
PRJNA8274(	ENSGALG00010020055	R3HCC1	case	control	-0.062706543	0.053738631	-1.166880166	0.243258767	0.667473598	down_in_case
PRJNA8274(	ENSGALG00010020056	UACA	case	control	0.067115396	0.130473722	0.5143978	0.606973897	0.887044121	up_in_case
PRJNA8274(	ENSGALG00010020057	RBKS	case	control	0.17812567	0.1190101	1.496727334	0.134464221	0.539063179	up_in_case
PRJNA8274(	ENSGALG00010020059	PRKCZ	case	control	-0.004376086	0.122045204	-0.035856272	0.971396964	0.992921843	down_in_case
PRJNA8274(	ENSGALG00010020061	UBA3	case	control	-0.022249502	0.041199588	-0.540041865	0.589168161	0.882949889	down_in_case
PRJNA8274(	ENSGALG00010020063	ENSGALG00010020063	case	control	-0.055945728	0.073142797	-0.764883631	0.444340842	0.815565915	down_in_case
PRJNA8274(	ENSGALG00010020064	TMEM138	case	control	-0.020446487	0.085209524	-0.239955414	0.810364821	0.955972758	down_in_case
PRJNA8274(	ENSGALG00010020065	TTYH3	case	control	0.054101111	0.086491516	0.625507721	0.531637883	0.855623068	up_in_case
PRJNA8274(	ENSGALG00010020066	ENSGALG00010020066	case	control	0.618868639	0.389003337	1.590908303	0.111630214	0.504204693	up_in_case
PRJNA8274(	ENSGALG00010020069	TMEM216	case	control	0.099115233	0.214880424	0.461257623	0.644613785	0.902239118	up_in_case
PRJNA8274(	ENSGALG00010020070	ARL6IP5	case	control	-0.138454596	0.058679167	-2.359518783	0.018298654	0.241839888	down_in_case
PRJNA8274(	ENSGALG00010020071	ENSGALG00010020071	case	control	0.352733743	0.063758166	5.532369635	3.15933E-08	1.32878E-05	up_in_case
PRJNA8274(	ENSGALG00010020074	ENSGALG00010020074	case	control	0.086069398	0.113715955	0.756880579	0.449121412	0.818701429	up_in_case
PRJNA8274(	ENSGALG00010020075	CPSF7	case	control	-0.022954489	0.061742628	-0.371776995	0.710058893	0.925630832	down_in_case
PRJNA8274(	ENSGALG00010020080	SLC4A1AP	case	control	0.113237817	0.047785632	2.369704288	0.017802317	0.237696674	up_in_case
PRJNA8274(	ENSGALG00010020083	CFAP74	case	control	-0.138307098	0.319766508	-0.432525279	0.665359683	0.912007336	down_in_case
PRJNA8274(	ENSGALG00010020084	ENSGALG00010020084	case	control	0.120312537	0.08194051	1.468291287	0.142025117	0.543501954	up_in_case
PRJNA8274(	ENSGALG00010020087	ENSGALG00010020087	case	control	0.108880845	0.474213516	0.229602998	0.818400277	0.958536226	up_in_case
PRJNA8274(	ENSGALG00010020088	LMOD3	case	control	-0.228592902	0.106332886	-2.149785541	0.031572182	0.3098361	down_in_case
PRJNA8274(	ENSGALG00010020090	MORF4L2	case	control	-0.062701957	0.046243323	-1.355913719	0.175126622	0.594080527	down_in_case
PRJNA8274(	ENSGALG00010020091	SUPT7L	case	control	-0.020002953	0.047584549	-0.42036655	0.6742177	0.913965794	down_in_case
PRJNA8274(	ENSGALG00010020092	SDHAF2	case	control	0.033113999	0.081033107	0.408647772	0.682798167	0.915901011	up_in_case
PRJNA8274(	ENSGALG00010020094	CLEC3B	case	control	-0.181591337	0.103331471	-1.757367202	0.07885524	0.449171025	down_in_case
PRJNA8274(	ENSGALG00010020095	LRFN2	case	control	-0.233111106	0.252002792	-0.925033824	0.354948313	0.763161644	down_in_case
PRJNA8274(	ENSGALG00010020096	GPHN	case	control	0.021732319	0.06567677	0.330898105	0.740721453	0.937059095	up_in_case
PRJNA8274(	ENSGALG00010020097	RETSAT	case	control	0.064339445	0.156156775	0.412018276	0.680326027	0.915381147	up_in_case
PRJNA8274(	ENSGALG00010020098	GABRD	case	control	0.081981503	0.321496533	0.254999649	0.79872337	0.953639826	up_in_case
PRJNA8274(	ENSGALG00010020100	MOCS1	case	control	-0.241084994	0.085843691	-2.808418306	0.004978551	0.120658556	down_in_case
PRJNA8274(	ENSGALG00010020101	KIF15	case	control	0.098867815	0.104573706	0.945436651	0.344435998	0.755870938	up_in_case
PRJNA8274(	ENSGALG00010020102	TMEM127	case	control	-0.025713125	0.121307379	-0.211966704	0.83213301	0.962371901	down_in_case
PRJNA8274(	ENSGALG00010020103	PSAP	case	control	0.006912704	0.063305839	0.109195361	0.91304754	0.982728035	up_in_case
PRJNA8274(	ENSGALG00010020104	BRAT1	case	control	-0.041622983	0.09044166	-0.460219143	0.645358932	0.902467507	down_in_case
PRJNA8274(	ENSGALG00010020106	ENSGALG00010020106	case	control	-0.072331308	0.096116656	-0.752536663	0.45172839	0.820489772	down_in_case
PRJNA8274(	ENSGALG00010020108	RPAP1	case	control	-0.079248492	0.060785467	-1.303740778	0.192321981	0.615090794	down_in_case
PRJNA8274(	ENSGALG00010020109	WDFY4	case	control	-0.017318076	0.164579287	-0.105226339	0.916196211	0.983445506	down_in_case
PRJNA8274(	ENSGALG00010020111	ADRA2B	case	control	-0.015561402	0.080987407	-0.192145942	0.847627889	0.968291964	down_in_case
PRJNA8274(	ENSGALG00010020113	CIAO1	case	control	-0.099609609	0.073377914	-1.357487604	0.17462633	0.593489298	down_in_case
PRJNA8274(	ENSGALG00010020116	GNA12	case	control	-0.103141446	0.058356533	-1.767436153	0.077155213	0.445884536	down_in_case
PRJNA8274(	ENSGALG00010020117	AKAP13	case	control	-0.007337551	0.065878633	-0.111379835	0.91131515	0.982467044	down_in_case
PRJNA8274(	ENSGALG00010020118	GNB1	case	control	0.0017287	0.037939168	0.045565036	0.963656937	0.992068146	up_in_case
PRJNA8274(	ENSGALG00010020120	DAAM2	case	control	0.078302869	0.110929629	0.705878759	0.480263538	0.831851816	up_in_case
PRJNA8274(	ENSGALG00010020121	ENSGALG00010020121	case	control	-0.05619597	0.220220954	-0.255179943	0.798584122	0.953626437	down_in_case
PRJNA8274(	ENSGALG00010020123	ENSGALG00010020123	case	control	-0.273218596	0.112851524	-2.421044807	0.01547597	0.220644431	down_in_case
PRJNA8274(	ENSGALG00010020125	NAV2	case	control	-0.103446419	0.080461494	-1.285663661	0.198560469	0.622082628	down_in_case
PRJNA8274(	ENSGALG00010020126	NADK	case	control	0.270509319	0.069575829	3.887978359	0.000101083	0.010950619	up_in_case
PRJNA8274(	ENSGALG00010020127	FRMD4B	case	control	-0.042115584	0.101854096	-0.413489351	0.679248123	0.915258927	down_in_case
PRJNA8274(	ENSGALG00010020128	KIF6	case	control	0.62797583	0.426900391	1.471012544	0.141287725	0.543501954	up_in_case
PRJNA8274(	ENSGALG00010020129	MGAT2	case	control	0.046863645	0.050122615	0.934980052	0.34979858	0.759427074	up_in_case
PRJNA8274(	ENSGALG00010020130	PDXDC1	case	control	0.154641011	0.071375995	2.166568912	0.03026774	0.30654407	up_in_case

PRJNA8274(ENSGALG00010020131	SNRNP200	case	control	-0.095564317	0.067535059	-1.415032705	0.157058907	0.568017798	down_in_case
PRJNA8274(ENSGALG00010020132	SHC4	case	control	0.110716353	0.118244652	0.936332859	0.349101835	0.759342183	up_in_case
PRJNA8274(ENSGALG00010020136	KCNK17	case	control	1.677855234	0.255200216	6.574662292	4.87637E-11	3.03183E-08	up_in_case
PRJNA8274(ENSGALG00010020137	NTAN1	case	control	-0.005716701	0.059814833	-0.095573308	0.923859465	0.985541988	down_in_case
PRJNA8274(ENSGALG00010020138	ENSGALG00010020138	case	control	0.119872027	0.055167504	2.172873838	0.029789814	0.305970455	up_in_case
PRJNA8274(ENSGALG00010020140	SECISBP2L	case	control	0.039672918	0.059981788	0.661416065	0.508345526	0.843702533	up_in_case
PRJNA8274(ENSGALG00010020141	THOC7	case	control	-0.138220969	0.074409745	-1.857565419	0.063230753	0.409249003	down_in_case
PRJNA8274(ENSGALG00010020142	SLC35E2B	case	control	0.088865657	0.059834765	1.485184375	0.13749499	0.539854583	up_in_case
PRJNA8274(ENSGALG00010020143	KCNK5	case	control	0.36461567	0.178056757	2.04774969	0.040584532	0.337631189	up_in_case
PRJNA8274(ENSGALG00010020147	ABHD2	case	control	0.072672124	0.103648057	0.701143138	0.483213692	0.833529046	up_in_case
PRJNA8274(ENSGALG00010020149	FAHD2A	case	control	0.113001073	0.095057437	1.188766251	0.234531662	0.659210368	up_in_case
PRJNA8274(ENSGALG00010020152	ATXN7	case	control	-0.045289961	0.067986882	-0.66615735	0.505310531	0.842675288	down_in_case
PRJNA8274(ENSGALG00010020153	AXL	case	control	0.04048937	0.057530133	0.703794141	0.481560983	0.832529825	up_in_case
PRJNA8274(ENSGALG00010020155	MFGE8	case	control	0.007036129	0.10246613	0.068667856	0.945254005	0.990313016	up_in_case
PRJNA8274(ENSGALG00010020160	SYT7	case	control	-0.170605497	0.247301039	-0.689869716	0.490276122	0.836347578	down_in_case
PRJNA8274(ENSGALG00010020163	CDC2L1	case	control	0.09589546	0.07713976	1.243139208	0.21381654	0.638602829	up_in_case
PRJNA8274(ENSGALG00010020164	C10orf71	case	control	-0.196127011	0.100709963	-1.947443974	0.05148153	0.371479245	down_in_case
PRJNA8274(ENSGALG00010020166	HAPLN3	case	control	-0.040543395	0.147892839	-0.274140352	0.783976765	0.949518053	down_in_case
PRJNA8274(ENSGALG00010020168	COX7A2L	case	control	-0.081088894	0.072018454	-1.125946043	0.260188359	0.687282842	down_in_case
PRJNA8274(ENSGALG00010020170	SORCS1	case	control	-0.347636602	0.160674697	-2.163605153	0.030494666	0.307572163	down_in_case
PRJNA8274(ENSGALG00010020171	RRN3	case	control	0.07685305	0.075067494	1.023786002	0.305936363	0.728933908	up_in_case
PRJNA8274(ENSGALG00010020175	ACAN	case	control	0.108282744	0.790837401	0.136921627	0.891092744	0.978917427	up_in_case
PRJNA8274(ENSGALG00010020176	ENSGALG00010020176	case	control	0.00891793	0.182073183	0.048979918	0.9609353	0.992056045	up_in_case
PRJNA8274(ENSGALG00010020177	KCNG3	case	control	0.124754098	0.146708739	0.850352193	0.395129307	0.787092796	up_in_case
PRJNA8274(ENSGALG00010020179	PSMD6	case	control	-0.046030427	0.047604555	-0.966933264	0.333577396	0.748508541	down_in_case
PRJNA8274(ENSGALG00010020180	TCF7L1	case	control	-0.028326005	0.084708795	-0.334392726	0.738083227	0.935918627	down_in_case
PRJNA8274(ENSGALG00010020181	LEUTX	case	control	0.039698138	0.151813557	0.261492711	0.79371257	0.95280257	up_in_case
PRJNA8274(ENSGALG00010020183	XPNPEP1	case	control	-0.019616305	0.062957909	-0.311578083	0.755361193	0.941559531	down_in_case
PRJNA8274(ENSGALG00010020184	RSL1D1	case	control	-0.112915291	0.059356725	-1.902316726	0.05712976	0.388573099	down_in_case
PRJNA8274(ENSGALG00010020186	MGA	case	control	-0.10001404	0.062670753	-1.595864655	0.110519014	0.503054901	down_in_case
PRJNA8274(ENSGALG00010020187	MMP23B	case	control	-0.006631837	0.112122309	-0.059148238	0.952834037	0.992056045	down_in_case
PRJNA8274(ENSGALG00010020193	VSTM4	case	control	0.065183364	0.085615685	0.761348389	0.446449005	0.817679602	up_in_case
PRJNA8274(ENSGALG00010020194	PRICKLE2	case	control	0.042301644	0.099315055	0.425933853	0.670156047	0.913465866	up_in_case
PRJNA8274(ENSGALG00010020199	DET1	case	control	-0.037388831	0.05151742	-0.72575122	0.467991296	0.828456986	down_in_case
PRJNA8274(ENSGALG00010020202	MAPKBP1	case	control	-0.082004592	0.067263908	-1.219146991	0.222788407	0.646365317	down_in_case
PRJNA8274(ENSGALG00010020204	GSPT2	case	control	-0.08880037	0.043220715	-2.054578926	0.039919693	0.336547209	down_in_case
PRJNA8274(ENSGALG00010020206	MRPS11	case	control	-0.049384703	0.067283355	-0.733980979	0.462960331	0.825091817	down_in_case
PRJNA8274(ENSGALG00010020207	DRGX	case	control	-1.682621981	0.894951286	-1.880126893	0.060090786	0.398649739	down_in_case
PRJNA8274(ENSGALG00010020211	ADD3	case	control	-0.107942379	0.06828209	-1.580830045	0.113916903	0.508907126	down_in_case
PRJNA8274(ENSGALG00010020212	JMJD7	case	control	-0.137911252	0.102855842	-1.340820797	0.179978642	0.600808892	down_in_case
PRJNA8274(ENSGALG00010020215	PRMT3	case	control	0.030158228	0.054444066	0.553930476	0.579626427	0.879142433	up_in_case
PRJNA8274(ENSGALG00010020224	MRPL46	case	control	-0.056433066	0.075508062	-0.747378024	0.454835407	0.821657174	down_in_case
PRJNA8274(ENSGALG00010020226	TCIM	case	control	0.060683906	0.109520841	0.554085462	0.579520359	0.879142433	up_in_case
PRJNA8274(ENSGALG00010020228	TM9SF3	case	control	-0.03016925	0.042880467	-0.703566273	0.481702921	0.832529825	down_in_case
PRJNA8274(ENSGALG00010020230	ENSGALG00010020230	case	control	0.058510598	0.137531091	0.425435425	0.670519286	0.913465866	up_in_case
PRJNA8274(ENSGALG00010020231	BMP10	case	control	-0.222627039	1.030626476	-0.216011372	0.828978883	0.962129538	down_in_case
PRJNA8274(ENSGALG00010020236	SLC35G1	case	control	0.494089978	0.207956092	2.375934135	0.017504587	0.235259018	up_in_case
PRJNA8274(ENSGALG00010020237	MIB2	case	control	0.004256724	0.051457837	0.082722559	0.934072147	0.988127868	up_in_case
PRJNA8274(ENSGALG00010020240	SV2B	case	control	0.263769258	0.286586833	0.920381637	0.357373361	0.763549838	up_in_case
PRJNA8274(ENSGALG00010020244	SLC25A37	case	control	0.225701551	0.120366706	1.875116122	0.06077675	0.400562592	up_in_case
PRJNA8274(ENSGALG00010020246	ADAMTS9	case	control	-0.003302908	0.080426074	-0.041067631	0.96724198	0.992731535	down_in_case
PRJNA8274(ENSGALG00010020248	NOLC1	case	control	-0.034110018	0.056633411	-0.602294964	0.546977813	0.862526554	down_in_case
PRJNA8274(ENSGALG00010020250	ENSGALG00010020250	case	control	-0.087269921	0.196746908	-0.443564383	0.657357561	0.908057682	down_in_case
PRJNA8274(ENSGALG00010020252	DMTN	case	control	0.219233831	0.133621392	1.640709076	0.10085782	0.490745171	up_in_case
PRJNA8274(ENSGALG00010020254	ENSGALG00010020254	case	control	0.154159714	0.074274212	2.075548286	0.037935749	0.330206289	up_in_case
PRJNA8274(ENSGALG00010020257	ZNF710	case	control	0.044497823	0.065617647	0.678138044	0.497684171	0.839255147	up_in_case
PRJNA8274(ENSGALG00010020259	ENSGALG00010020259	case	control	-0.006697742	0.179173459	-0.037381329	0.97018096	0.992761697	down_in_case
PRJNA8274(ENSGALG00010020261	SSU72	case	control	0.069379907	0.052601136	1.318981141	0.187175417	0.610664656	up_in_case
PRJNA8274(ENSGALG00010020265	ENSGALG00010020265	case	control	-0.224173508	0.157293132	-1.425195781	0.154100616	0.562900869	down_in_case
PRJNA8274(ENSGALG00010020268	CPLANE2	case	control	-0.088384756	0.064315056	-1.374246753	0.169365091	0.585146363	down_in_case
PRJNA8274(ENSGALG00010020269	ENSGALG00010020269	case	control	0.051740604	0.616071214	0.083984777	0.933068535	0.987991895	up_in_case
PRJNA8274(ENSGALG00010020272	ENSGALG00010020272	case	control	-0.027309434	0.059382065	-0.459893634	0.64559257	0.902535441	down_in_case
PRJNA8274(ENSGALG00010020277	IDH2	case	control	-0.209545909	0.077838071	-2.692074798	0.007100902	0.146516526	down_in_case
PRJNA8274(ENSGALG00010020282	MAGI1	case	control	0.022523587	0.082213813	0.273963532	0.784112647	0.949518053	up_in_case
PRJNA8274(ENSGALG00010020283	FBXO42	case	control	-0.021554222	0.06570259	-0.328057418	0.742868249	0.937448285	down_in_case
PRJNA8274(ENSGALG00010020284	HEG1	case	control	-0.151961427	0.09835257	-1.545068189	0.122329771	0.520832363	down_in_case
PRJNA8274(ENSGALG00010020285	ADAMTS14	case	control	0.071901531	0.103185686	0.696816917	0.485917378	0.834635018	up_in_case
PRJNA8274(ENSGALG00010020287	GALNT16	case	control	0.093456979	0.129420948	0.722116324	0.470222963	0.829101076	up_in_case
PRJNA8274(ENSGALG00010020290	NELL1	case	control	0.037436119	0.234423162	0.159694626	0.873121635	0.975062028	up_in_case
PRJNA8274(ENSGALG00010020291	SNX29	case	control	-0.073899987	0.098057886	-0.753636345	0.451067614	0.820067593	down_in_case
PRJNA8274(ENSGALG00010020292	NPM2	case	control	-0.15660817	0.284275296	-0.5509032	0.581700033	0.880290776	down_in_case
PRJNA8274(ENSGALG00010020293	SEMA4B	case	control	0.10267828	0.086353717	1.189042977	0.234422755	0.659210368	up_in_case
PRJNA8274(ENSGALG00010020295	LGI1	case	control	0.151716843	0.10336238	1.467814914	0.142154505	0.5436773	up_in_case
PRJNA8274(ENSGALG00010020298	ENSGALG00010020298	case	control	-0.106844618	0.081331696	-1.31368979	0.188950618	0.611783722	down_in_case
PRJNA8274(ENSGALG00010020300	EHD4	case	control	0.003724528	0.069083479	0.053913443	0.957004126	0.992056045	up_in_case
PRJNA8274(ENSGALG00010020302	HAAO	case	control	1.114935244	1.351336939	0.82506088	0.409337028	0.795423223	up_in_case
PRJNA8274(ENSGALG00010020303	EXD2	case	control	-0.006865466	0.045597189	-0.150567749	0.880316704	0.976399289	down_in_case
PRJNA8274(ENSGALG00010020304	ZFP36L2	case	control	-0.097584185	0.06929096	-1.408324913	0.159034882	0.571481535	down_in_case
PRJNA8274(ENSGALG00010020305	XPO7	case	control	-0.031819134	0.044079756	-0.721853689	0.470384436	0.829101076	down_in_case
PRJNA8274(ENSGALG00010020307	SHQ1	case	control	0.031442993	0.180446925	0.174250646	0.861668485	0.972599205	up_in_case
PRJNA8274(ENSGALG00010020309	GXYLT2	case	control	-0.02196687	0.083843313	-0.261999064	0.793322164	0.95280257	down_in_case
PRJNA8274(ENSGALG00010020311	SGPL1	case	control	-0.034962632	0.066722544	-0.524000278	0.600278346	0.884946428	down_in_case
PRJNA8274(ENSGALG00010020313	FRA10AC1	case	control	0.015269477	0.087962978	0.173589818	0.862187834	0.972698817	up_in_case
PRJNA8274(ENSGALG00010020314	THADA	case	control	-0.036283675	0.088821209	-0.408502381	0.682904883	0.915918327	down_in_case
PRJNA8274(ENSGALG00010020315	ANO5	case	control	0.008343656	0.078287617	0.106576952	0.915124604	0.983445506	up_in_case
PRJNA8274(ENSGALG00010020316	PDPK1	case	control	-0.038741119	0.047717741	-0.811880826	0.416860017	0.799987395	down_in_case
PRJNA8274(ENSGALG00010020319	NTRK3	case	control	-0.044645068	0.104870319	-0.425716908	0.67031414	0.913465866	down_in_case
PRJNA8274(ENSGALG00010020320	PDE6C	case	control	0.285053413	0.299482486	0.95181998	0.341188287	0.752814768	up_in_case
PRJNA8274(ENSGALG00010020321	SZRD1	case	control	-0.00131068	0.056134539	-0.023348907	0.98137196	0.99656434	down_in_case
PRJNA8274(ENSGALG00010020323	ARHGAP25	case	control	-0.064899358	0.102980896	-0.630207745	0.528558673	0.853862293	down_in_case
PRJNA8274(ENSGALG00010020324	PLEKHH2	case	control	0.053708891	0.110617169	0.485538466	0.627294433	0.895838586	up_in_case
PRJNA8274(ENSGALG00010020325	ENSGALG00010020325	case	control	0.373657405	0.796457016	0.469149493	0.638962784	0.900303403	up_in_case
PRJNA8274(ENSGALG00010020327	MTTPL	case	control	1.832896963	1.050361991	1.745014556	0.080982335	0.453075204	up_in_case
PRJNA8274(ENSGALG00010020328	SLC17A6	case	control	0.207094564	0.595716763	0.347639309	0.728111081	0.933057319	up_in_case
PRJNA8274(ENSGALG00010020329	PPP4R2	case	control	-0.012684848	0.040680152	-0.3118191	0.755178008	0.941559531	down_in_case
PRJNA8274(ENSGALG00010020336	OSBP	case	control	-0.002297333	0.040411498	-0.056848507	0.954665873	0.992056045	down_in_case
PRJNA8274(ENSGALG00010020338	NECAP2	case	control	0.053933813	0.063162411	0.853890974	0.393165401	0.786660869	up_in_case
PRJNA8274(ENSGALG00010020340	DCAF5	case	control	-0.014470618	0.044473181	-0.325378531	0.744894602	0.937849656	down_in_case
PRJNA8274(ENSGALG00010020341	DYNC2LI1	case	control	0.051938684	0.113377101	0.458105595	0.64687658	0.90295498	up_in_case
PRJNA8274(ENSGALG00010020342	CPPED1	case	control	-0.08642508	0.143293366	-0.603133858	0.546419644	0.86246958	down_in_case
PRJNA8274(ENSGALG00010020343	PATL1	case	control	-0.124499847	0.068380963	-1.820679942	0.068655523	0.422837898	down_in_case
PRJNA8274(ENSGALG00010020345	AGBL1	case	control	0.033368244	0.15190268	0.219668566	0.826129287	0.961207256	up_in_case
PRJNA8274(ENSGALG00010020346	PCBD1	case	control	0.543690087	0.278283993	1.953723899	0.050733878	0.368084455	up_in_case
PRJNA8274(ENSGALG00010020350	PDZRN3	case	control	-0.200933439	0.074893303	-2.682929341	0.007298041	0.148898802	down_in_case
PRJNA8274(ENSGALG00010020351	CROCC	case	control	0.126039878	0.092732627	1.359175099	0.17409111	0.593154767	up_in_case
PRJNA8274(ENSGALG00010020356	STX3	case	control	0.234763682	0.287374894	0.816924815	0.413971396	0.79856886	up_in_case
PRJNA8274(ENSGALG00010020357	RBP4	case	control	1.077287655	0.792231218	1.359814698	0.17388857	0.593015599	up_in_case
PRJNA8274(ENSGALG00010020362	CDS2	case	control	0.13223935	0.070450314	1.877058346	0.060510098	0.399858778	up_in_case
PRJNA8274(ENSGALG00010020364	MRPL16	case	control	0.176404174	0.079766755	2.211499939	0.027001236	0.292513391	up_in_case
PRJNA8274(ENSGALG00010020365	UNC5B	case	control	-0.14505085	0.101845809	-1.424220123	0.154382761	0.56303838	down_in_case
PRJNA8274(ENSGALG00010020369	CNTN3	case	control	-0.08034748	0.235024326	-0.341868783	0.732449643	0.933846596	down_in_case
PRJNA8274(ENSGALG00010020370	CEP55	case	control	-0.020646539	0.135783941	-0.152054349	0.87914407	0.976318821	down_in_case
PRJNA8274(ENSGALG00010020373	MS4A4A	case	control	0.02761607	0.07203585	0.383365639	0.701448677	0.922873869	up_in_case
PRJNA8274(ENSGALG00010020374	PCNA	case	control	0.015561879	0.04774534	0.325935028	0.744473513	0.937654174	up_in_case
PRJNA8274(ENSGALG00010020375	LRPPRC	case	control	-0.188113727	0.052555635	-3.579325574	0.000344482	0.025211633	down_in_case
PRJNA8274(ENSGALG00010020377	TMEM230	case	control	-0.14099388	0.097310511	-1.448906995	0.147363557	0.552558944	down_in_case
PRJNA8274(ENSGALG00010020379	COL13A1	case	control	-0.082440882	0.120966937	-0.681515832	0.495545153	0.838118945	down_in_case
PRJNA8274(ENSGALG00010020382	MFAP2	case	control	0.003225734	0.06695262	0.048179352	0.961573306	0.992056045	up_in_case
PRJNA8274(ENSGALG00010020383	BAAT	case	control	0.107906485	0.068864108	1.566948132	0.117126816	0.513845851	up_in_case
PRJNA8274(ENSGALG00010020385	SLC12A8	case	control	-0.065458237	0.309364946	-0.211589058	0.832427647	0.962371901	down_in_case
PRJNA8274(ENSGALG00010020386	PPM1B	case	control	-0.071299802	0.04002281	-1.781479171	0.074834205	0.439711709	down_in_case
PRJNA8274(ENSGALG00010020388	MYOF	case	control	-0.115321407	0.146804125	-0.785546098	0.432133454	0.807390038	down_in_case
PRJNA8274(ENSGALG00010020389	ACTN1	case	control	0.027110875	0.086973169	0.311715392	0.755256829	0.941559531	up_in_case
PRJNA8274(ENSGALG00010020390	KLHL25	case	control	0.140298314	0.128193823	1.094423353	0.273769346	0.699576755	up_in_case
PRJNA8274(ENSGALG00010020392	SLC23A2	case	control	0.189987353	0.116734735	1.627513463	0.103628092	0.493302833	up_in_case
PRJNA8274(ENSGALG00010020393	SHISA9	case	control	-0.398344492	0.172376712	-2.310895051	0.020838653	0.259349635	down_in_case

PRJNA8274(	ENSGALG00010020396	CHL1	case	control	0.487632447	0.218806402	2.22860228	0.025840379	0.285045136	up_in_case
PRJNA8274(	ENSGALG00010020399	ATP13A2	case	control	0.026855543	0.098176861	0.273542487	0.784436237	0.949518053	up_in_case
PRJNA8274(	ENSGALG00010020402	SLC3A1	case	control	0.294133938	0.207925505	1.414612113	0.157182255	0.568233173	up_in_case
PRJNA8274(	ENSGALG00010020405	UBE2I	case	control	0.013564205	0.045872613	0.295692869	0.767464644	0.944714161	up_in_case
PRJNA8274(	ENSGALG00010020413	CYP26A1	case	control	0.301101702	0.290901445	1.035064306	0.300638881	0.723511633	up_in_case
PRJNA8274(	ENSGALG00010020415	TSR3	case	control	0.059603322	0.070505937	0.845366002	0.39790652	0.788896694	up_in_case
PRJNA8274(	ENSGALG00010020416	ENSGALG00010020416	case	control	-0.198945059	0.941070792	-0.211402862	0.832572924	0.962371901	down_in_case
PRJNA8274(	ENSGALG00010020417	ENSGALG00010020417	case	control	0.844019056	0.55738083	1.51425921	0.12996011	0.532363328	up_in_case
PRJNA8274(	ENSGALG00010020422	ERCC4	case	control	0.161867564	0.067698394	2.391010391	0.016802077	0.230806634	up_in_case
PRJNA8274(	ENSGALG00010020424	ENSGALG00010020424	case	control	0.590563602	0.878019386	0.672608842	0.501196175	0.841040176	up_in_case
PRJNA8274(	ENSGALG00010020428	SEMA4C	case	control	0.100547517	0.070937069	1.417418548	0.156360593	0.566211345	up_in_case
PRJNA8274(	ENSGALG00010020429	ZNF148	case	control	0.095012503	0.057417604	1.654762574	0.097972669	0.487193947	up_in_case
PRJNA8274(	ENSGALG00010020433	GNPTG	case	control	0.02758753	0.069619588	0.396261029	0.691912474	0.918951275	up_in_case
PRJNA8274(	ENSGALG00010020436	CNTN4	case	control	-0.211400347	0.135306002	-1.562387064	0.118196833	0.51463738	down_in_case
PRJNA8274(	ENSGALG00010020437	SNX4	case	control	-0.079159095	0.041364884	-1.913678644	0.055661229	0.384148446	down_in_case
PRJNA8274(	ENSGALG00010020438	HTR7	case	control	0.081899148	0.200614024	0.408242388	0.683095731	0.915918327	up_in_case
PRJNA8274(	ENSGALG00010020440	PAX7	case	control	-0.015209427	0.102572785	-0.14827936	0.882122304	0.976801393	down_in_case
PRJNA8274(	ENSGALG00010020443	PREPL	case	control	-0.306622677	0.132251821	-2.318476028	0.020423464	0.256639314	down_in_case
PRJNA8274(	ENSGALG00010020444	ENSGALG00010020444	case	control	0.921661094	0.831891829	1.107909782	0.267900781	0.694521603	up_in_case
PRJNA8274(	ENSGALG00010020449	PRPF19	case	control	-0.099913886	0.046112899	-2.166723144	0.030255971	0.30654407	down_in_case
PRJNA8274(	ENSGALG00010020450	ANKRD39	case	control	-0.03430768	0.167733528	-0.204536805	0.837934034	0.964826648	down_in_case
PRJNA8274(	ENSGALG00010020453	ENSGALG00010020453	case	control	0.01005544	0.111671691	0.090044663	0.928251723	0.986776285	up_in_case
PRJNA8274(	ENSGALG00010020455	ENSGALG00010020455	case	control	0.596819364	0.913258022	0.653505745			up_in_case
PRJNA8274(	ENSGALG00010020460	MAPK8	case	control	-0.053442888	0.061849515	-0.86407934	0.387544307	0.782604263	down_in_case
PRJNA8274(	ENSGALG00010020462	CNNM4	case	control	-0.013372196	0.063058591	-0.21205986	0.832060335	0.962371901	down_in_case
PRJNA8274(	ENSGALG00010020467	ALDH4A1	case	control	0.077239816	0.075105072	1.02842343	0.303750692	0.726603361	up_in_case
PRJNA8274(	ENSGALG00010020468	ENSGALG00010020468	case	control	-0.236722958	0.25019289	-0.946161813	0.34406606	0.755852285	down_in_case
PRJNA8274(	ENSGALG00010020473	TMEM109	case	control	0.158237095	0.191286938	0.827223731	0.408110263	0.794767365	up_in_case
PRJNA8274(	ENSGALG00010020476	TMEM132A	case	control	0.06277121	0.078501783	0.799615085	0.423933845	0.803723282	up_in_case
PRJNA8274(	ENSGALG00010020481	RPP30	case	control	-0.134791343	0.101332189	-1.330192746	0.183454774	0.605028428	down_in_case
PRJNA8274(	ENSGALG00010020484	TRNT1	case	control	-0.214582365	0.068652412	-3.125634738	0.001774218	0.067952978	down_in_case
PRJNA8274(	ENSGALG00010020485	LMAN2L	case	control	-0.214261933	0.050609049	-4.233668403	2.2991E-05	0.003389393	down_in_case
PRJNA8274(	ENSGALG00010020486	MRTFB	case	control	-0.025328189	0.067307734	-0.376304285	0.706690694	0.924581603	down_in_case
PRJNA8274(	ENSGALG00010020489	IFFO2	case	control	0.145480107	0.112567443	1.292381726	0.196224982	0.619095929	up_in_case
PRJNA8274(	ENSGALG00010020493	DEPDC7	case	control	0.08337273	0.099785775	0.83551719	0.403426573	0.79260318	up_in_case
PRJNA8274(	ENSGALG00010020497	OSBPL11	case	control	-0.128411026	0.059888895	-2.144154208	0.032020533	0.3098361	down_in_case
PRJNA8274(	ENSGALG00010020507	ANKRD1	case	control	0.178083912	0.342159256	0.52047083	0.602735454	0.885101354	up_in_case
PRJNA8274(	ENSGALG00010020509	CRBN	case	control	0.029331389	0.098340977	0.298262127	0.765503108	0.943786458	up_in_case
PRJNA8274(	ENSGALG00010020514	CAMKMT	case	control	0.023913728	0.091984493	0.259975648	0.794882558	0.95280257	up_in_case
PRJNA8274(	ENSGALG00010020517	UBR4	case	control	-0.139495669	0.068191736	-2.045638917	0.04079191	0.337767404	down_in_case
PRJNA8274(	ENSGALG00010020520	ARHGAP22	case	control	0.087390602	0.084268251	1.03705251	0.299711386	0.723109974	up_in_case
PRJNA8274(	ENSGALG00010020521	ENSGALG00010020521	case	control	-0.211794629	0.666419936	-0.317809564	0.750629396	0.939972946	down_in_case
PRJNA8274(	ENSGALG00010020524	ENSGALG00010020524	case	control	0.023651182	0.270312157	0.087495812	0.930277414	0.987258715	up_in_case
PRJNA8274(	ENSGALG00010020525	ENSGALG00010020525	case	control	0.329301434	0.665360423	0.494921885	0.620655262	0.893038138	up_in_case
PRJNA8274(	ENSGALG00010020530	SRSF5A	case	control	-0.006781829	0.032001567	-0.211921789	0.832168051	0.962371901	down_in_case
PRJNA8274(	ENSGALG00010020533	ENSGALG00010020533	case	control	0.070654271	0.075852476	0.931469536	0.351610736	0.760831854	up_in_case
PRJNA8274(	ENSGALG00010020535	LMLN	case	control	0.300471118	0.148036722	2.029706642	0.042386367	0.343873176	up_in_case
PRJNA8274(	ENSGALG00010020537	CCDC167	case	control	0.040077231	0.087851899	0.456190833	0.648252757	0.903510177	up_in_case
PRJNA8274(	ENSGALG00010020540	PCGF5	case	control	-0.013038471	0.12312942	-0.105892409	0.915667717	0.983445506	down_in_case
PRJNA8274(	ENSGALG00010020544	BMERB1	case	control	-0.049026981	0.062318746	-0.786713208	0.43144977	0.807261374	down_in_case
PRJNA8274(	ENSGALG00010020545	STYX	case	control	-0.091639326	0.103196077	-0.888011728	0.374534443	0.774434633	down_in_case
PRJNA8274(	ENSGALG00010020546	LRRC20	case	control	-0.417733277	0.096397673	-4.333437352	1.46799E-05	0.002385485	down_in_case
PRJNA8274(	ENSGALG00010020548	CMTR1	case	control	0.041104842	0.087264908	0.47103518	0.637615612	0.899378897	up_in_case
PRJNA8274(	ENSGALG00010020550	HECTD2	case	control	0.327000331	0.13655285	2.394679638	0.016634893	0.229391479	up_in_case
PRJNA8274(	ENSGALG00010020554	ZFP36L1	case	control	-0.048562492	0.081956074	-0.592542934	0.55348708	0.866337858	down_in_case
PRJNA8274(	ENSGALG00010020556	EMC1	case	control	0.032683123	0.065387684	0.499836062	0.617190516	0.891081782	up_in_case
PRJNA8274(	ENSGALG00010020557	ENSGALG00010020557	case	control	0.105005385	0.145896499	0.719725185	0.471694217	0.829161315	up_in_case
PRJNA8274(	ENSGALG00010020558	EIF4EBP2	case	control	-0.141334843	0.063375854	-2.230105538	0.025740437	0.284898029	down_in_case
PRJNA8274(	ENSGALG00010020562	LRRN1	case	control	-0.036046735	0.149285394	-0.2414619	0.809197143	0.955875648	down_in_case
PRJNA8274(	ENSGALG00010020568	PLA2G4F	case	control	-0.227027434	0.45463695	-0.499359839	0.617525906	0.891081782	down_in_case
PRJNA8274(	ENSGALG00010020569	DTX3L	case	control	-0.033454928	0.129841595	-0.257659562	0.796669658	0.95280257	down_in_case
PRJNA8274(	ENSGALG00010020571	MRTO4	case	control	0.121349593	0.075398211	1.609449237	0.107518146	0.498867454	up_in_case
PRJNA8274(	ENSGALG00010020572	PPP1R3C	case	control	0.184560162	0.128856434	1.432292949	0.15206	0.560293917	up_in_case
PRJNA8274(	ENSGALG00010020574	DTL	case	control	-0.032552252	0.085243259	-0.381874799	0.702554234	0.923478771	down_in_case
PRJNA8274(	ENSGALG00010020576	AKR7L	case	control	-0.037290906	0.086661825	-0.430303718	0.666974723	0.912284937	down_in_case
PRJNA8274(	ENSGALG00010020578	TNKS2	case	control	-0.012075717	0.068078156	-0.177380201	0.859209758	0.971828322	down_in_case
PRJNA8274(	ENSGALG00010020580	ENSGALG00010020580	case	control	0.043240675	0.07085759	0.610247606	0.541697799	0.85975913	up_in_case
PRJNA8274(	ENSGALG00010020582	KANSL3	case	control	0.033601592	0.053962497	0.622684153	0.533492098	0.855817434	up_in_case
PRJNA8274(	ENSGALG00010020584	PARP9	case	control	-0.046477063	0.081520743	-0.570125609	0.568592507	0.874569523	down_in_case
PRJNA8274(	ENSGALG00010020586	C11orf16	case	control	0.136827692	0.07166665	1.90922405	0.056233195	0.385984934	up_in_case
PRJNA8274(	ENSGALG00010020588	PALD1	case	control	0.101731273	0.054273909	1.874404746	0.06087466	0.400970813	up_in_case
PRJNA8274(	ENSGALG00010020590	INTS7	case	control	-0.031099051	0.065850398	-0.472268228	0.636735345	0.899158415	down_in_case
PRJNA8274(	ENSGALG00010020594	PQLC2	case	control	-0.006953694	0.098361445	-0.070695323	0.943640243	0.990183239	down_in_case
PRJNA8274(	ENSGALG00010020595	ZFYVE27	case	control	0.031870315	0.091133003	0.349712111	0.726554764	0.932232671	up_in_case
PRJNA8274(	ENSGALG00010020596	SUMF1	case	control	-0.069173321	0.080035329	-0.864284836	0.387431438	0.782604263	down_in_case
PRJNA8274(	ENSGALG00010020597	ARID5A	case	control	0.087127892	0.096104285	0.906597369	0.364619753	0.770251634	up_in_case
PRJNA8274(	ENSGALG00010020599	PARN	case	control	-0.015281477	0.05531802	-0.276247727	0.782357802	0.949459648	down_in_case
PRJNA8274(	ENSGALG00010020601	AKIP1	case	control	-0.135563521	0.120203983	-1.127778943	0.259413288	0.686583384	down_in_case
PRJNA8274(	ENSGALG00010020602	DENND2B	case	control	0.01280034	0.052654539	0.243100404	0.807927618	0.955875648	up_in_case
PRJNA8274(	ENSGALG00010020603	VPS39	case	control	0.058789494	0.089311042	0.658255605	0.5103739	0.844911643	up_in_case
PRJNA8274(	ENSGALG00010020605	STAMBP	case	control	-0.036680604	0.048419298	-0.757561655	0.448713443	0.818701429	down_in_case
PRJNA8274(	ENSGALG00010020607	ENSGALG00010020607	case	control	-0.20577804	0.109228056	-1.883930251	0.059574411	0.397773519	down_in_case
PRJNA8274(	ENSGALG00010020608	CAPZB	case	control	-0.064274698	0.060095555	-1.069541646	0.284825674	0.709583124	down_in_case
PRJNA8274(	ENSGALG00010020609	AVPI1	case	control	0.092660285	0.1381571	0.670687827	0.502419419	0.84219414	up_in_case
PRJNA8274(	ENSGALG00010020611	LPGAT1	case	control	-0.114520592	0.07952598	-1.44004	0.149856083	0.556819093	down_in_case
PRJNA8274(	ENSGALG00010020612	RPL27A	case	control	-0.016515259	0.071236303	-0.231837681	0.816664089	0.957784679	down_in_case
PRJNA8274(	ENSGALG00010020613	ACTG2	case	control	-0.450380694	0.258006632	-1.745616729	0.080877573	0.453075204	down_in_case
PRJNA8274(	ENSGALG00010020614	FAIM	case	control	-0.207778773	0.09351985	-2.221761193	0.026299449	0.287964866	down_in_case
PRJNA8274(	ENSGALG00010020616	PI4K2A	case	control	-0.0301518	0.066187155	-0.455553642	0.648710985	0.903708435	down_in_case
PRJNA8274(	ENSGALG00010020617	DGUOK	case	control	-0.038584984	0.093113297	-0.414387477	0.678590359	0.914942347	down_in_case
PRJNA8274(	ENSGALG00010020618	ENSGALG00010020618	case	control	-0.395643708	0.39395771	-1.004279641	0.315243844	0.736508113	down_in_case
PRJNA8274(	ENSGALG00010020619	QARS	case	control	-0.010356789	0.064450561	-0.160693535	0.872334782	0.975062028	down_in_case
PRJNA8274(	ENSGALG00010020620	MARF1	case	control	-0.067026822	0.034761269	-1.928204098	0.053829745	0.377891678	down_in_case
PRJNA8274(	ENSGALG00010020622	ABCG2	case	control	0.143784068	0.092184171	1.559747913	0.118819465	0.516128861	up_in_case
PRJNA8274(	ENSGALG00010020623	NEK2	case	control	-0.069164119	0.128840961	-0.536817783	0.591393487	0.882949889	down_in_case
PRJNA8274(	ENSGALG00010020624	MINOS1	case	control	-0.191396281	0.106737667	-1.793146565	0.072949492	0.436712105	down_in_case
PRJNA8274(	ENSGALG00010020625	SLC30A1	case	control	0.06080723	0.06121571	0.993327197	0.320550528	0.738875399	up_in_case
PRJNA8274(	ENSGALG00010020626	MORN4	case	control	0.061039579	0.07103622	0.859274026	0.390189351	0.783914628	up_in_case
PRJNA8274(	ENSGALG00010020629	TRIM66	case	control	-0.218790989	0.329965262	-0.663072798	0.507283931	0.843605095	down_in_case
PRJNA8274(	ENSGALG00010020630	QRICH1	case	control	-0.018463139	0.045734673	-0.403701119	0.686432517	0.917210333	down_in_case
PRJNA8274(	ENSGALG00010020632	BLOC1S2	case	control	-0.114722092	0.074806675	-1.53358096	0.125132775	0.523982046	down_in_case
PRJNA8274(	ENSGALG00010020633	HOGA1	case	control	0.244484863	0.099427002	2.458938289	0.013934857	0.207988769	up_in_case
PRJNA8274(	ENSGALG00010020635	NDE1	case	control	0.049854206	0.068620347	0.726522202	0.467518702	0.828046838	up_in_case
PRJNA8274(	ENSGALG00010020636	NBL1	case	control	-0.178242996	0.118636833	-1.50242544	0.13298727	0.536224013	down_in_case
PRJNA8274(	ENSGALG00010020638	IMPDH2	case	control	-0.104584688	0.086391521	-1.210589734	0.226052681	0.649498359	down_in_case
PRJNA8274(	ENSGALG00010020640	PTPN14	case	control	-0.021417212	0.077327792	-0.276966558	0.781805785	0.949394183	down_in_case
PRJNA8274(	ENSGALG00010020642	ANKRD2	case	control	-0.152530272	0.425214077	-0.358714069	0.719809002	0.928743908	down_in_case
PRJNA8274(	ENSGALG00010020646	CEP70	case	control	-0.180216891	0.100990444	-1.784494479	0.074343351	0.438757705	down_in_case
PRJNA8274(	ENSGALG00010020648	TET3	case	control	-0.104759489	0.082209757	-1.274295077	0.202558861	0.62541643	down_in_case
PRJNA8274(	ENSGALG00010020649	MYH11	case	control	-0.375218081	0.165014309	-2.273851789	0.022974899	0.267253668	down_in_case
PRJNA8274(	ENSGALG00010020651	LMO1	case	control	0.233727775	0.163859794	1.426388806	0.153756143	0.562474506	up_in_case
PRJNA8274(	ENSGALG00010020653	NDUFAF3	case	control	-0.150115543	0.097880629	-1.533659365	0.125113475	0.523982046	down_in_case
PRJNA8274(	ENSGALG00010020654	SAYSD1	case	control	-0.086384897	0.089379355	-0.966497203	0.333795446	0.748508541	down_in_case
PRJNA8274(	ENSGALG00010020655	RIC3	case	control	-0.107129167	0.124580167	-0.859921525	0.389832302	0.783877029	down_in_case
PRJNA8274(	ENSGALG00010020657	DALRD3	case	control	0.042975932	0.075305274	0.570689535	0.568210114	0.874461133	up_in_case
PRJNA8274(	ENSGALG00010020659	RAD51B	case	control	-0.029800804	0.125181562	-0.238060646	0.811834057	0.956240049	down_in_case
PRJNA8274(	ENSGALG00010020662	BFAR	case	control	-0.051900605	0.06184374	-0.839221638	0.401344947	0.791181795	down_in_case
PRJNA8274(	ENSGALG00010020664	ESYT3	case	control	0.278998299	0.411647893	0.67775957	0.497924149	0.839532593	up_in_case
PRJNA8274(	ENSGALG00010020666	TMCO4	case	control	0.03727689	0.149747355	0.248931878	0.803413476	0.954891353	up_in_case
PRJNA8274(	ENSGALG00010020667	UBTD1	case	control	0.098796673	0.133756216	0.738632387	0.460130252	0.82454265	up_in_case
PRJNA8274(	ENSGALG00010020670	ENSGALG00010020670	case	control	-0.039810793	0.090221284	-0.441257219	0.659026793	0.908599575	down_in_case
PRJNA8274(	ENSGALG00010020673	MMS19	case	control	-0.131818065	0.068775719	-1.91663667	0.055284106	0.383470355	down_in_case
PRJNA8274(	ENSGALG00010020674	TRPV4	case	control	0.294000669	0.852457531	0.344886001	0.730180075	0.933770678	up_in_case
PRJNA8274(	ENSGALG00010020675	SMYD2	case	control	0.021792965	0.142498676	0.152934511	0.87844992	0.976160112	up_in_case
PRJNA8274(	ENSGALG00010020676	CYP2C23a	case	control	1.266253838	0.713941558	1.773609932	0.076127694	0.442710866	up_in_case
PRJNA8274(	ENSGALG00010020677	CYR61	case	control	0.294507754	0.201541655	1.461274865	0.143940025	0.547285923	up_in_case

PRJNA8274(	ENSGALG00010020679	OTUD3	case	control	-0.024677816	0.071846554	-0.343479464	0.731237787	0.933770678	down_in_case
PRJNA8274(	ENSGALG00010020681	BCL10	case	control	-0.008109672	0.062611815	-0.129523024	0.896943808	0.980176778	down_in_case
PRJNA8274(	ENSGALG00010020682	ZDHHC16	case	control	0.11482933	0.074482376	1.541697982	0.123147003	0.522552563	up_in_case
PRJNA8274(	ENSGALG00010020684	P4HTM	case	control	0.047669732	0.148136239	0.321796561	0.747606819	0.939105545	up_in_case
PRJNA8274(	ENSGALG00010020685	RPL6	case	control	0.010693544	0.062740084	0.170441982	0.864662559	0.973093769	up_in_case
PRJNA8274(	ENSGALG00010020686	C1orf52	case	control	-0.111821787	0.075774001	-1.475727638	0.140017023	0.542025835	down_in_case
PRJNA8274(	ENSGALG00010020690	ENSGALG00010020690	case	control	0.323684473	0.637746023	0.507544479	0.611772836	0.889602558	up_in_case
PRJNA8274(	ENSGALG00010020691	ENSGALG00010020691	case	control	0.333309731	0.105044589	3.17303094	0.001508564	0.062168507	up_in_case
PRJNA8274(	ENSGALG00010020692	TUB	case	control	0.049930154	0.093689523	0.532932095	0.594080587	0.883735816	up_in_case
PRJNA8274(	ENSGALG00010020693	PTGFR	case	control	0.052580482	0.150146719	0.350194015	0.726193098	0.932226286	up_in_case
PRJNA8274(	ENSGALG00010020695	ENSGALG00010020695	case	control	-0.202415373	0.585712508	-0.345588271	0.729652162	0.933556014	down_in_case
PRJNA8274(	ENSGALG00010020696	MOB1A	case	control	0.014942657	0.062319418	0.2397753	0.810504456	0.955972758	up_in_case
PRJNA8274(	ENSGALG00010020699	PLA2G10	case	control	0.203452119	0.167342334	1.215783922	0.224067245	0.648105158	up_in_case
PRJNA8274(	ENSGALG00010020700	MTHFD2	case	control	-0.151516491	0.095023288	-1.594519555	0.110819714	0.503115856	down_in_case
PRJNA8274(	ENSGALG00010020701	PTPN11	case	control	-0.059571812	0.033290886	-1.789433052	0.0735451	0.437929428	down_in_case
PRJNA8274(	ENSGALG00010020704	PROX1	case	control	0.482070218	0.284831565	1.6924747	0.0905555	0.474422807	up_in_case
PRJNA8274(	ENSGALG00010020705	EXOSC1	case	control	0.083632755	0.126784485	0.659645026	0.509481652	0.844521404	up_in_case
PRJNA8274(	ENSGALG00010020707	SLC4A5	case	control	-0.054082122	0.272526746	-0.198447023	0.84269533	0.966519616	down_in_case
PRJNA8274(	ENSGALG00010020708	FAM214A	case	control	0.037157057	0.058536192	0.634770654	0.525578005	0.85251736	up_in_case
PRJNA8274(	ENSGALG00010020711	PLA2G2E	case	control	0.216233618	0.193674293	1.116480738	0.264216411	0.690099483	up_in_case
PRJNA8274(	ENSGALG00010020712	ZC3H7A	case	control	0.027559098	0.038448827	0.716773439	0.473513903	0.82959752	up_in_case
PRJNA8274(	ENSGALG00010020713	SCTR	case	control	-1.072569659	0.915244402	-1.171894257	0.241239513	0.666227315	down_in_case
PRJNA8274(	ENSGALG00010020714	PGAM1	case	control	-0.069536927	0.09958195	-0.698288466	0.484996811	0.834423421	down_in_case
PRJNA8274(	ENSGALG00010020715	ENSGALG00010020715	case	control	0.006914379	0.854128937	0.008095241	0.993541003	0.998196863	up_in_case
PRJNA8274(	ENSGALG00010020716	RRAS	case	control	-0.017075822	0.055006192	-0.310434537	0.756230534	0.941659745	down_in_case
PRJNA8274(	ENSGALG00010020719	RPH3A	case	control	-0.410664258	0.438880589	-0.935708409	0.34942334	0.759342183	down_in_case
PRJNA8274(	ENSGALG00010020720	ENSGALG00010020720	case	control	0.16908066	0.307206491	0.550381144	0.582057979	0.88034085	up_in_case
PRJNA8274(	ENSGALG00010020721	PTDSS2	case	control	0.041519041	0.067067347	0.619064905	0.535873602	0.857238203	up_in_case
PRJNA8274(	ENSGALG00010020723	RPS24	case	control	-0.147274343	0.069996586	-2.104021812	0.035376544	0.324492998	down_in_case
PRJNA8274(	ENSGALG00010020724	TACR1	case	control	0.097319544	0.174933119	0.556324295	0.577989181	0.878627118	up_in_case
PRJNA8274(	ENSGALG00010020725	FOPNL	case	control	0.06015279	0.077716196	0.774005846	0.438927334	0.812213456	up_in_case
PRJNA8274(	ENSGALG00010020728	TMEM37	case	control	0.013767439	0.159840654	0.086132272	0.931361269	0.987258715	up_in_case
PRJNA8274(	ENSGALG00010020730	RRP12	case	control	-0.294895719	0.102564072	-2.875234114	0.00403728	0.110177685	down_in_case
PRJNA8274(	ENSGALG00010020732	ARIH2	case	control	-0.041382668	0.049727562	-0.832187737	0.405302985	0.793662853	down_in_case
PRJNA8274(	ENSGALG00010020733	TMEM229B	case	control	0.315039531	0.11467019	2.747353345	0.006007837	0.134447684	up_in_case
PRJNA8274(	ENSGALG00010020734	SIGIRR	case	control	0.759819686	1.064306283	0.71391074			up_in_case
PRJNA8274(	ENSGALG00010020736	LRRTM4	case	control	-0.18946365	0.202341393	-0.936356357	0.349089741	0.759342183	down_in_case
PRJNA8274(	ENSGALG00010020737	POLR3A	case	control	-0.002377535	0.056124377	-0.042361897	0.966210203	0.992505774	down_in_case
PRJNA8274(	ENSGALG00010020739	PKP3	case	control	-0.056179094	0.751459898	-0.074759936	0.940405719	0.989551456	down_in_case
PRJNA8274(	ENSGALG00010020742	THAP10	case	control	-0.250829343	0.18005523	-1.393068913	0.163598953	0.577788348	down_in_case
PRJNA8274(	ENSGALG00010020743	PURB	case	control	-0.128351816	0.123039915	-1.043172176	0.296868558	0.720505834	down_in_case
PRJNA8274(	ENSGALG00010020744	CHST3	case	control	0.075282685	0.074105234	1.015888901	0.309682295	0.732384442	up_in_case
PRJNA8274(	ENSGALG00010020745	ABCC1	case	control	0.164524555	0.087095665	1.889009682	0.058890531	0.395497471	up_in_case
PRJNA8274(	ENSGALG00010020747	DBI	case	control	-0.052887531	0.082707659	-0.639451434	0.522529298	0.851055244	down_in_case
PRJNA8274(	ENSGALG00010020750	AIFM2	case	control	0.826686411	0.261405332	3.162469578	0.001564371	0.063552564	up_in_case
PRJNA8274(	ENSGALG00010020753	SYDE2	case	control	0.070117223	0.089933619	0.779655304	0.435593794	0.81000376	up_in_case
PRJNA8274(	ENSGALG00010020754	SLC25A20	case	control	0.033527076	0.061588753	0.544370106	0.586186813	0.881808482	up_in_case
PRJNA8274(	ENSGALG00010020756	MACROH2A2	case	control	-0.026631399	0.084129493	-0.316552472	0.751583204	0.940427752	down_in_case
PRJNA8274(	ENSGALG00010020759	ARHGAP19	case	control	-0.158946754	0.078434508	-2.026490113	0.042714585	0.344511316	down_in_case
PRJNA8274(	ENSGALG00010020760	TXNDC11	case	control	0.1323217	0.077155355	1.715003449	0.086344595	0.464183347	up_in_case
PRJNA8274(	ENSGALG00010020762	TTBK2	case	control	-0.063312979	0.139260949	-0.454635553	0.649371453	0.904012049	down_in_case
PRJNA8274(	ENSGALG00010020764	PRKAR2A	case	control	-0.105674271	0.063677435	-1.659524628	0.097010122	0.485418097	down_in_case
PRJNA8274(	ENSGALG00010020765	GLT1D1	case	control	0.274169773	0.205860484	1.331823221	0.182918286	0.604513863	up_in_case
PRJNA8274(	ENSGALG00010020766	C2orf76	case	control	-0.257039621	0.126342672	-2.034464031	0.041904832	0.341355095	down_in_case
PRJNA8274(	ENSGALG00010020767	UBXN10	case	control	-0.058032259	0.065938192	-0.88010085	0.378804679	0.777229179	down_in_case
PRJNA8274(	ENSGALG00010020770	B4GALNT4	case	control	0.075866514	0.065478678	1.15864456	0.246601098	0.671183041	up_in_case
PRJNA8274(	ENSGALG00010020771	LINGO1	case	control	0.019619322	0.114175585	0.171834655	0.86356752	0.972890026	up_in_case
PRJNA8274(	ENSGALG00010020773	ZCCHC24	case	control	-0.117945941	0.076012796	-1.551659035	0.12074383	0.519499513	down_in_case
PRJNA8274(	ENSGALG00010020775	UBR1	case	control	-0.017065887	0.03862379	-0.441849094	0.65859841	0.908367791	down_in_case
PRJNA8274(	ENSGALG00010020776	MCOLN3	case	control	0.583927085	0.392044167	1.489442094	0.136370991	0.539840644	up_in_case
PRJNA8274(	ENSGALG00010020778	SLIT1	case	control	-0.25380757	0.153044429	-1.658391429	0.097238486	0.485691283	down_in_case
PRJNA8274(	ENSGALG00010020779	SLC15A4	case	control	0.051986861	0.048791035	1.065500291	0.286649594	0.710659943	up_in_case
PRJNA8274(	ENSGALG00010020781	SNN	case	control	-0.020774259	0.067224738	-0.309026998	0.75730099	0.941659745	down_in_case
PRJNA8274(	ENSGALG00010020782	PPIF	case	control	0.073631279	0.08210941	0.896745931	0.369854505	0.772544067	up_in_case
PRJNA8274(	ENSGALG00010020786	DDX19	case	control	-0.0978233	0.051114994	-1.913788751	0.055647153	0.384148446	down_in_case
PRJNA8274(	ENSGALG00010020787	TMEM132C	case	control	0.15905426	0.231861222	0.685989054	0.492720011	0.837799284	up_in_case
PRJNA8274(	ENSGALG00010020790	PRPH2L	case	control	0.164747398	0.334771684	0.492118677	0.622635449	0.893944471	up_in_case
PRJNA8274(	ENSGALG00010020794	STEAP3	case	control	-0.01199119	0.087117842	-0.137643335	0.890522305	0.978745332	down_in_case
PRJNA8274(	ENSGALG00010020797	DGCR8	case	control	0.036260483	0.054522126	0.665059953	0.506012148	0.843280685	up_in_case
PRJNA8274(	ENSGALG00010020798	C1QL2	case	control	0.427294375	0.523861093	0.815663505	0.414692617	0.798853486	up_in_case
PRJNA8274(	ENSGALG00010020803	ABCC6	case	control	0.246947255	0.172421334	1.432231435	0.152077598	0.560293917	up_in_case
PRJNA8274(	ENSGALG00010020806	HTD2	case	control	-0.051472913	0.091562068	-0.562164162	0.574004176	0.877573415	down_in_case
PRJNA8274(	ENSGALG00010020809	ENSGALG00010020809	case	control	-0.032580835	0.117587454	-0.277077479	0.781720614	0.949394183	down_in_case
PRJNA8274(	ENSGALG00010020810	IFITM5	case	control	0.070707672	0.108541957	0.651431705	0.51476785	0.847493921	up_in_case
PRJNA8274(	ENSGALG00010020811	MCOLN2	case	control	1.296780766	0.719052402	1.803457943	0.071316325	0.43228239	up_in_case
PRJNA8274(	ENSGALG00010020813	NPY4R	case	control	0.216944888	0.366348804	0.592181238	0.553729232	0.866337858	up_in_case
PRJNA8274(	ENSGALG00010020816	TRMT2A	case	control	-0.109820574	0.05292586	-2.074988937	0.03798756	0.330206289	down_in_case
PRJNA8274(	ENSGALG00010020818	TMEM62	case	control	0.277176017	0.126646507	2.188580041	0.028627378	0.300624184	up_in_case
PRJNA8274(	ENSGALG00010020820	VWA5B1	case	control	-0.105268843	0.35246698	-0.298662992	0.765197197	0.943786458	down_in_case
PRJNA8274(	ENSGALG00010020827	ENSGALG00010020827	case	control	-0.075489143	0.052950439	-1.4256566	0.15396749	0.562900869	down_in_case
PRJNA8274(	ENSGALG00010020828	CAMK2N1	case	control	-0.084604845	0.133688714	-0.632849572	0.526831882	0.85251736	down_in_case
PRJNA8274(	ENSGALG00010020831	MUL1	case	control	-0.005822992	0.077994084	-0.074659405	0.940485708	0.989551456	down_in_case
PRJNA8274(	ENSGALG00010020833	RANBP1	case	control	0.051527502	0.056512812	0.911784417	0.361882215	0.768247576	up_in_case
PRJNA8274(	ENSGALG00010020835	PTGR2	case	control	-0.262316459	0.108785548	-2.411317173	0.015895019	0.223714308	down_in_case
PRJNA8274(	ENSGALG00010020839	PGGHG	case	control	-0.057668307	0.119466555	-0.482715076	0.629298057	0.896269608	down_in_case
PRJNA8274(	ENSGALG00010020840	LPAR3	case	control	0.271271959	0.181619124	1.493631023	0.135272086	0.539774365	up_in_case
PRJNA8274(	ENSGALG00010020843	HMG20A	case	control	-0.084514498	0.061089662	-1.383450092	0.1665269	0.581806664	down_in_case
PRJNA8274(	ENSGALG00010020845	RAB35	case	control	-0.044254308	0.039395436	-1.123335912	0.261294857	0.687516348	down_in_case
PRJNA8274(	ENSGALG00010020847	MIDEAS	case	control	-0.08129718	0.072397373	-1.122929974	0.261467236	0.687516348	down_in_case
PRJNA8274(	ENSGALG00010020854	ENSGALG00010020854	case	control	-0.197441407	0.134954214	-1.463025137	0.143460501	0.546625409	down_in_case
PRJNA8274(	ENSGALG00010020855	DNAL1	case	control	0.180444624	0.093862655	1.922432564	0.054551351	0.381464567	up_in_case
PRJNA8274(	ENSGALG00010020856	VTG2	case	control	0.161371703	0.383542385	0.420740207	0.673944798	0.913846845	up_in_case
PRJNA8274(	ENSGALG00010020861	CDA	case	control	0.369976094	0.148720256	2.487731692	0.012856068	0.199684901	up_in_case
PRJNA8274(	ENSGALG00010020865	BICDL1	case	control	-0.994837601	0.55559915	-1.790567174	0.073362779	0.437626802	down_in_case
PRJNA8274(	ENSGALG00010020866	LCOR	case	control	-0.044797533	0.068560303	-0.653403379	0.513496261	0.847040782	down_in_case
PRJNA8274(	ENSGALG00010020870	RIOX1	case	control	-0.013603696	0.055622156	-0.244573325	0.80678682	0.955875648	down_in_case
PRJNA8274(	ENSGALG00010020873	PINK1	case	control	-0.242173409	0.086751881	-2.791563776	0.005245402	0.125485633	down_in_case
PRJNA8274(	ENSGALG00010020875	ENSGALG00010020875	case	control	-0.07352079	0.068937447	-1.066485543	0.28620421	0.710557161	down_in_case
PRJNA8274(	ENSGALG00010020878	XYLT1	case	control	0.055514302	0.118859522	0.467058094	0.640458315	0.901166378	up_in_case
PRJNA8274(	ENSGALG00010020880	PXK	case	control	-0.090368557	0.067677396	-1.335284199	0.181783348	0.602744104	down_in_case
PRJNA8274(	ENSGALG00010020886	GBF1	case	control	0.002586322	0.05148939	0.050230188	0.959938956	0.992056045	up_in_case
PRJNA8274(	ENSGALG00010020887	ARPP19	case	control	-0.053724888	0.040267978	-1.334183915	0.18214359	0.603068611	down_in_case
PRJNA8274(	ENSGALG00010020889	PPRC1	case	control	-0.116858015	0.086256627	-1.354771452	0.175490385	0.594350757	down_in_case
PRJNA8274(	ENSGALG00010020890	RPS15A	case	control	-0.032520794	0.06536114	-0.497555492	0.618797381	0.891656847	down_in_case
PRJNA8274(	ENSGALG00010020894	PSMD13	case	control	-0.01942608	0.049767707	-0.390335052	0.696288807	0.92176726	down_in_case
PRJNA8274(	ENSGALG00010020896	MARCO	case	control	0.791005033	0.421694079	1.875779321	0.060685589	0.400562592	up_in_case
PRJNA8274(	ENSGALG00010020898	ENSGALG00010020898	case	control	0.146108655	0.147369514	0.991444235	0.32146871	0.73942679	up_in_case
PRJNA8274(	ENSGALG00010020900	PDHB	case	control	-0.005321144	0.066895146	-0.079544549	0.936599499	0.988586717	down_in_case
PRJNA8274(	ENSGALG00010020903	AGMAT	case	control	0.214341074	0.557496206	0.38447091	0.700629452	0.922516526	up_in_case
PRJNA8274(	ENSGALG00010020905	KCTD6	case	control	-0.030527459	0.061507186	-0.496323459	0.619666218	0.892425791	down_in_case
PRJNA8274(	ENSGALG00010020906	SIRT3	case	control	0.124906206	0.089006874	1.403332128	0.160517822	0.572925686	up_in_case
PRJNA8274(	ENSGALG00010020912	PDE8A	case	control	0.125178227	0.142150938	0.880600784	0.378533934	0.777176635	up_in_case
PRJNA8274(	ENSGALG00010020913	TMEM132B	case	control	-0.072027208	0.158075068	-0.455651918	0.648640303	0.903708435	down_in_case
PRJNA8274(	ENSGALG00010020915	EN1	case	control	-0.13661186	0.285304297	-0.47882861	0.632060562	0.89724192	down_in_case
PRJNA8274(	ENSGALG00010020916	ARL6IP1	case	control	0.145527421	0.092642112	1.570856034	0.116216093	0.5134816	up_in_case
PRJNA8274(	ENSGALG00010020918	ENSGALG00010020918	case	control	0.051854961	0.292404139	0.177340038	0.859241303	0.971828322	up_in_case
PRJNA8274(	ENSGALG00010020920	ACOX2	case	control	-0.190007632	0.190755921	-0.996077243	0.319212616	0.73861962	down_in_case
PRJNA8274(	ENSGALG00010020921	LDB1	case	control	0.036234333	0.076597408	0.473049075	0.636178167	0.89885859	up_in_case
PRJNA8274(	ENSGALG00010020922	INSIG2	case	control	0.078133333	0.054712964	1.428058856	0.153274919	0.561863967	up_in_case
PRJNA8274(	ENSGALG00010020924	DNAJC16	case	control	-0.053011604	0.062731355	-0.845057519	0.398078725	0.789064551	down_in_case
PRJNA8274(	ENSGALG00010020925	RIC8A	case	control	-0.046070129	0.047130837	-0.977494393	0.328324453	0.745445783	down_in_case
PRJNA8274(	ENSGALG00010020927	ENSGALG00010020927	case	control	0.140765946	0.096528333	1.458286296	0.144761646	0.548818979	up_in_case
PRJNA8274(	ENSGALG00010020928	AACS	case	control	0.248263813	0.137545993	1.804951263	0.071082309	0.43162506	up_in_case
PRJNA8274(	ENSGALG00010020932	CASP9	case	control	0.071044034	0.102706119	0.691721527	0.489112225	0.836347578	up_in_case
PRJNA8274(	ENSGALG00010020934	ABCD3	case	control	0.010167279	0.065406782	0.155446863	0.876469042	0.975673929	up_in_case
PRJNA8274(	ENSGALG00010020935	BRI3BP	case	control	0.034125778	0.047622206	0.716593808	0.473624766	0.82959752	up_in_case

PRJNA8274(ENSGALG00010020936	BET1L	case	control	0.063348768	0.040333652	1.570618209	0.116271358	0.5134816	up_in_case
PRJNA8274(ENSGALG00010020938	SMG1	case	control	-0.113987734	0.051002026	-2.234964833	0.025419658	0.283111687	down_in_case
PRJNA8274(ENSGALG00010020940	ENSGALG00010020940	case	control	0.151143829	0.097415811	1.551532835	0.120774045	0.519499513	up_in_case
PRJNA8274(ENSGALG00010020941	ENSGALG00010020941	case	control	0.262991613	0.132597688	1.983380078	0.047324991	0.358446699	up_in_case
PRJNA8274(ENSGALG00010020943	FAM107A	case	control	0.538861771	0.427571394	1.260284899	0.207566606	0.631809014	up_in_case
PRJNA8274(ENSGALG00010020944	MYO5A	case	control	0.220818077	0.085644802	2.578300974	0.009928748	0.177161817	up_in_case
PRJNA8274(ENSGALG00010020945	NUMB	case	control	0.060743977	0.064448049	0.942526229	0.345923289	0.756227431	up_in_case
PRJNA8274(ENSGALG00010020946	CTBS	case	control	-0.063009713	0.094047881	-0.669974823	0.50287384	0.842443289	down_in_case
PRJNA8274(ENSGALG00010020949	HPS6	case	control	0.084019664	0.063705938	1.318867073	0.187213556	0.610664656	up_in_case
PRJNA8274(ENSGALG00010020950	EFHD2	case	control	-0.241534528	0.159768988	-1.511773533	0.130591485	0.533148629	down_in_case
PRJNA8274(ENSGALG00010020951	ENSGALG00010020951	case	control	0.903831143	0.449393416	2.011224709	0.044301727	0.350201601	up_in_case
PRJNA8274(ENSGALG00010020952	GNG5	case	control	-0.003992317	0.054132721	-0.073750533	0.941208889	0.989551456	down_in_case
PRJNA8274(ENSGALG00010020954	PLIN1	case	control	-0.449691679	0.916690525	-0.490559973	0.623737703	0.894353977	down_in_case
PRJNA8274(ENSGALG00010020957	RPF1	case	control	0.06534313	0.063243438	1.033200169	0.301510234	0.724474473	up_in_case
PRJNA8274(ENSGALG00010020959	ENSGALG00010020959	case	control	0.124527799	0.169741686	0.733631216	0.46317353	0.825091817	up_in_case
PRJNA8274(ENSGALG00010020960	CLEC19A	case	control	0.050057174	0.158122418	0.31657228	0.751568172	0.940427752	up_in_case
PRJNA8274(ENSGALG00010020962	MYO5C	case	control	0.178766201	0.270804651	0.66012973	0.509170582	0.844493243	up_in_case
PRJNA8274(ENSGALG00010020963	CCDC93	case	control	-0.034692453	0.081141205	-0.427556541	0.668974012	0.91307897	down_in_case
PRJNA8274(ENSGALG00010020965	DHX37	case	control	0.011229131	0.059630905	0.188310593	0.850633182	0.969234083	up_in_case
PRJNA8274(ENSGALG00010020966	PAPLN	case	control	-0.045635691	0.157247577	-0.290215541	0.771651347	0.945715998	down_in_case
PRJNA8274(ENSGALG00010020967	VPS51	case	control	0.10474402	0.059722817	1.753835874	0.079458634	0.450131333	up_in_case
PRJNA8274(ENSGALG00010020969	RBP3	case	control	0.445441287	0.321447589	1.385735347	0.165827731	0.580782892	up_in_case
PRJNA8274(ENSGALG00010020970	ARMH3	case	control	-0.05703667	0.044144897	-1.292033154	0.196345663	0.619264002	down_in_case
PRJNA8274(ENSGALG00010020971	TMEM51	case	control	0.412695561	0.221353896	1.86441517	0.062263424	0.405741851	up_in_case
PRJNA8274(ENSGALG00010020973	GNB5	case	control	0.197078936	0.132603185	1.486230788	0.137218086	0.539854583	up_in_case
PRJNA8274(ENSGALG00010020975	HCCS	case	control	-0.281106502	0.075099732	-3.74310925	0.000181757	0.016489578	down_in_case
PRJNA8274(ENSGALG00010020976	GDF10	case	control	-0.173659354	0.278966172	-0.622510438	0.533606282	0.855817434	down_in_case
PRJNA8274(ENSGALG00010020978	BCL2L10	case	control	-0.080712321	0.127359953	-0.633733905	0.526254494	0.85251736	down_in_case
PRJNA8274(ENSGALG00010020979	ENSGALG00010020979	case	control	0.077883487	0.192742603	0.40408029	0.686153679	0.917034645	up_in_case
PRJNA8274(ENSGALG00010020981	CIT	case	control	-0.038417167	0.095245959	-0.403346951	0.686693007	0.917290381	down_in_case
PRJNA8274(ENSGALG00010020982	TLCD4	case	control	-0.040849383	0.109965127	-0.371475799	0.710283178	0.925630832	down_in_case
PRJNA8274(ENSGALG00010020984	PSEN1	case	control	0.103775476	0.065511263	1.584086021	0.113174145	0.507809939	up_in_case
PRJNA8274(ENSGALG00010020986	CFAP20DC	case	control	-0.269812738	0.210346242	-1.28270767	0.199594512	0.622902456	down_in_case
PRJNA8274(ENSGALG00010020987	NADSYN1	case	control	-0.144866326	0.084188885	-1.72072983	0.085299861	0.461985839	down_in_case
PRJNA8274(ENSGALG00010020989	MAPK4	case	control	0.084726456	0.037237468	2.275301173	0.022887869	0.267253668	up_in_case
PRJNA8274(ENSGALG00010020990	UBB	case	control	0.06712243	0.053472133	1.255278714	0.2093776	0.63414862	up_in_case
PRJNA8274(ENSGALG00010020991	ALG14	case	control	-0.009529587	0.106770438	-0.089253046	0.928880809	0.986776285	down_in_case
PRJNA8274(ENSGALG00010020992	KCNH7	case	control	-0.23884069	0.384463194	-0.621231613	0.534447242	0.856211151	down_in_case
PRJNA8274(ENSGALG00010020994	DHCR7	case	control	0.014366605	0.099147833	0.144900851	0.884789155	0.977630122	up_in_case
PRJNA8274(ENSGALG00010020998	SYT17	case	control	-0.08933724	0.142012898	-0.629078351	0.529297763	0.85444409	down_in_case
PRJNA8274(ENSGALG00010020999	IFIH1	case	control	-0.259903141	0.161360346	-1.610700195	0.107245083	0.498409064	down_in_case
PRJNA8274(ENSGALG00010021000	NFKB2	case	control	-0.060382933	0.079930379	-0.755444105	0.449982558	0.81905641	down_in_case
PRJNA8274(ENSGALG00010021002	SLC44A3	case	control	-0.028521389	0.142432075	-0.200245549	0.841288546	0.96603539	down_in_case
PRJNA8274(ENSGALG00010021003	KAZN	case	control	0.039575552	0.084325188	0.469320642	0.638840463	0.900303403	up_in_case
PRJNA8274(ENSGALG00010021004	PSD	case	control	0.155790761	0.132388183	1.176772407	0.239286359	0.66429721	up_in_case
PRJNA8274(ENSGALG00010021010	LEO1	case	control	-0.028374456	0.05933109	-0.478239254	0.632479926	0.89726815	down_in_case
PRJNA8274(ENSGALG00010021011	SCARB1	case	control	-0.131267238	0.061606788	-2.130726845	0.033111652	0.313323388	down_in_case
PRJNA8274(ENSGALG00010021012	KCNIP2	case	control	0.319989959	0.521138498	0.614020958	0.539201478	0.858476757	up_in_case
PRJNA8274(ENSGALG00010021013	FAP	case	control	0.015348246	0.134140344	0.11441931	0.908905407	0.981730793	up_in_case
PRJNA8274(ENSGALG00010021014	FBXL15	case	control	-0.030031244	0.072539897	-0.413996233	0.678876866	0.915132297	down_in_case
PRJNA8274(ENSGALG00010021015	RBM25	case	control	-0.003996131	0.050873214	-0.07855078	0.937389939	0.988837129	down_in_case
PRJNA8274(ENSGALG00010021016	FHIT	case	control	0.011799051	0.14267661	0.082697862	0.934091785	0.988127868	up_in_case
PRJNA8274(ENSGALG00010021017	COQ7	case	control	0.115111461	0.089840746	1.281283453	0.200094123	0.622902456	up_in_case
PRJNA8274(ENSGALG00010021018	F3	case	control	-0.40280874	0.125752662	-3.203182612	0.001359178	0.058542893	down_in_case
PRJNA8274(ENSGALG00010021019	VSIG10	case	control	0.147068864	0.065804107	2.2349496	0.025420658	0.283111687	up_in_case
PRJNA8274(ENSGALG00010021020	ZDHHC13	case	control	0.019706252	0.084347285	0.233632325	0.81527043	0.957374769	up_in_case
PRJNA8274(ENSGALG00010021021	GCG	case	control	-0.326150533	0.352090147	-0.926326781	0.354276182	0.76285942	down_in_case
PRJNA8274(ENSGALG00010021022	ZFYVE1	case	control	0.08026613	0.058068959	1.382255357	0.166893308	0.582409284	up_in_case
PRJNA8274(ENSGALG00010021023	MGEA5	case	control	-0.036550677	0.061146041	-0.597760315	0.549999874	0.864380503	down_in_case
PRJNA8274(ENSGALG00010021024	ENSGALG00010021024	case	control	0.031239505	0.083080489	0.376014945	0.706905786	0.92461283	up_in_case
PRJNA8274(ENSGALG00010021025	CUEDC2	case	control	0.111429854	0.076269759	1.46099655	0.144016388	0.547430716	up_in_case
PRJNA8274(ENSGALG00010021026	ADGRA2	case	control	-0.018313407	0.066309069	-0.276182541	0.782407866	0.949459648	down_in_case
PRJNA8274(ENSGALG00010021027	PLA2G4A	case	control	-0.090715886	0.109076525	-0.831671952	0.405594137	0.793782506	down_in_case
PRJNA8274(ENSGALG00010021028	ENSGALG00010021028	case	control	-0.046987767	0.282435211	-0.166366532	0.867868511	0.974138124	down_in_case
PRJNA8274(ENSGALG00010021029	PRDM2	case	control	0.067018787	0.053260426	1.258322396	0.208275185	0.632880396	up_in_case
PRJNA8274(ENSGALG00010021030	CSRP3	case	control	-0.40319692	0.331489864	-1.216317493	0.223864	0.647995977	down_in_case
PRJNA8274(ENSGALG00010021031	DDX54	case	control	-0.034199495	0.073515088	-0.465203751	0.641785553	0.901539099	down_in_case
PRJNA8274(ENSGALG00010021032	TMC7	case	control	-0.178923274	0.075297529	-2.376217065	0.01749117	0.235259018	down_in_case
PRJNA8274(ENSGALG00010021033	RNF122	case	control	0.104356539	0.099397177	1.049894398	0.293766668	0.716378375	up_in_case
PRJNA8274(ENSGALG00010021034	NPM3	case	control	0.05366099	0.118062031	0.454515218	0.649458041	0.904044582	up_in_case
PRJNA8274(ENSGALG00010021036	E2F8	case	control	-0.205143246	0.097174883	-2.111072735	0.034766061	0.322042853	down_in_case
PRJNA8274(ENSGALG00010021038	PTGS2	case	control	-0.147737127	0.18531308	-0.797229894	0.425317528	0.804536774	down_in_case
PRJNA8274(ENSGALG00010021040	FGF8	case	control	0.728576909	0.479154884	1.520545724	0.128373876	0.530380428	up_in_case
PRJNA8274(ENSGALG00010021041	GLTP	case	control	0.445800977	0.163523885	2.726213217	0.00640656	0.13904105	up_in_case
PRJNA8274(ENSGALG00010021042	LRRC38	case	control	-0.238064481	0.087599225	-2.717655094	0.006574633	0.140023533	down_in_case
PRJNA8274(ENSGALG00010021043	PTPRG	case	control	-0.110043351	0.074137876	-1.484306778	0.137727553	0.539854583	down_in_case
PRJNA8274(ENSGALG00010021044	EIF4G2	case	control	-0.035186277	0.025456283	-1.382223704	0.166903024	0.582409284	down_in_case
PRJNA8274(ENSGALG00010021045	DPP4	case	control	-0.096295689	0.097371555	-0.988950919	0.322687156	0.740689923	down_in_case
PRJNA8274(ENSGALG00010021046	ENSGALG00010021046	case	control	0.319601456	0.143549145	2.226425356	0.025985704	0.28584274	up_in_case
PRJNA8274(ENSGALG00010021047	TMC5	case	control	-0.131373101	0.477558133	-0.275093423	0.783244465	0.949518053	down_in_case
PRJNA8274(ENSGALG00010021048	API5	case	control	0.002361213	0.030736705	0.07682064	0.938766231	0.989551456	up_in_case
PRJNA8274(ENSGALG00010021050	CTR9	case	control	-0.007279293	0.058315648	-0.124825717	0.900661527	0.980321194	down_in_case
PRJNA8274(ENSGALG00010021051	GDE1	case	control	0.028078349	0.094809784	0.296154546	0.76711206	0.944442742	up_in_case
PRJNA8274(ENSGALG00010021052	CEP15	case	control	-0.030035586	0.11532965	-0.260432471	0.794530198	0.95280257	down_in_case
PRJNA8274(ENSGALG00010021053	ENSGALG00010021053	case	control	0.019605293	0.354567791	0.055293496	0.955904644	0.992056045	up_in_case
PRJNA8274(ENSGALG00010021055	IRAG1	case	control	0.159726015	0.114378894	1.396464061	0.162574796	0.576163465	up_in_case
PRJNA8274(ENSGALG00010021057	ZDHHC8	case	control	-0.098767202	0.052499284	-1.881305701	0.059930346	0.398421175	down_in_case
PRJNA8274(ENSGALG00010021058	CCP110	case	control	0.020952176	0.050498173	0.414909579	0.678208097	0.914941113	up_in_case
PRJNA8274(ENSGALG00010021060	PAX2	case	control	-0.028123625	1.170049182	-0.024036276	0.980823673	0.996165356	down_in_case
PRJNA8274(ENSGALG00010021062	MYO9A	case	control	0.025381708	0.06543985	0.387863178	0.698117289	0.922288441	up_in_case
PRJNA8274(ENSGALG00010021064	LYVE1	case	control	0.174391799	0.218271924	0.798965784	0.424310252	0.803723282	up_in_case
PRJNA8274(ENSGALG00010021065	CNNM2	case	control	0.071649842	0.070415409	1.017530729	0.308901022	0.731823162	up_in_case
PRJNA8274(ENSGALG00010021066	ENSGALG00010021066	case	control	0.198439289	0.338924755	0.585496594	0.558213856	0.869085619	up_in_case
PRJNA8274(ENSGALG00010021067	SLC4A10	case	control	-0.056201308	0.170608243	-0.329417307	0.741840288	0.937315893	down_in_case
PRJNA8274(ENSGALG00010021069	PGAM5	case	control	-0.040170749	0.049458123	-0.812217407	0.416666893	0.799987395	down_in_case
PRJNA8274(ENSGALG00010021070	RNF141	case	control	-0.075120273	0.054683619	-1.373725332	0.169526969	0.585422763	down_in_case
PRJNA8274(ENSGALG00010021071	HIF1AN	case	control	-0.073751979	0.057900939	-1.273761352	0.202748006	0.625537942	down_in_case
PRJNA8274(ENSGALG00010021072	ENSGALG00010021072	case	control	-0.106737502	0.050834337	-2.099712691	0.035754123	0.324974524	down_in_case
PRJNA8274(ENSGALG00010021073	NDUFB8	case	control	-0.015772154	0.072146438	-0.218613071	0.82695147	0.96126918	down_in_case
PRJNA8274(ENSGALG00010021074	TTC17	case	control	0.002436676	0.038899705	0.062639961	0.950053208	0.991488793	up_in_case
PRJNA8274(ENSGALG00010021075	DHRS3	case	control	0.072236006	0.099669566	0.724754895	0.468602411	0.828596747	up_in_case
PRJNA8274(ENSGALG00010021077	SEC31B	case	control	-0.08730972	0.086164848	-1.013286995	0.310923097	0.733696418	down_in_case
PRJNA8274(ENSGALG00010021078	ENSGALG00010021078	case	control	-1.469169309	0.307651817	-4.775428673	1.79325E-06	0.000420384	down_in_case
PRJNA8274(ENSGALG00010021080	TTLL7	case	control	-0.030757547	0.102645873	-0.299647189	0.764446286	0.943786458	down_in_case
PRJNA8274(ENSGALG00010021081	KCNH6	case	control	0.010609054	0.095456743	0.111139912	0.9115054	0.982477366	up_in_case
PRJNA8274(ENSGALG00010021082	SENP8	case	control	-0.079851481	0.087729642	-0.910199553	0.36271728	0.768925833	down_in_case
PRJNA8274(ENSGALG00010021083	SCD	case	control	1.172758578	0.304488196	3.851573208	0.000117361	0.011987632	up_in_case
PRJNA8274(ENSGALG00010021085	AMPD3	case	control	-0.10929597	0.111263222	-0.982318941	0.325942746	0.743971472	down_in_case
PRJNA8274(ENSGALG00010021086	PSMD14	case	control	-0.014748524	0.062799543	-0.234850814	0.814324527	0.957063027	down_in_case
PRJNA8274(ENSGALG00010021087	THBS1	case	control	0.34754422	0.335549744	1.035745746	0.300320774	0.723279006	up_in_case
PRJNA8274(ENSGALG00010021088	ANKLE2	case	control	0.010502775	0.03863093	0.271874772	0.78571831	0.949929982	up_in_case
PRJNA8274(ENSGALG00010021090	ADM	case	control	-0.464302619	0.191409187	-2.425707076	0.015278597	0.219195419	down_in_case
PRJNA8274(ENSGALG00010021091	TANK	case	control	-0.02305031	0.059496666	-0.387421884	0.698443905	0.922288441	down_in_case
PRJNA8274(ENSGALG00010021092	VPS13D	case	control	-0.11271013	0.073935448	-1.524439679	0.127398914	0.528825682	down_in_case
PRJNA8274(ENSGALG00010021093	GRAMD2	case	control	0.099003	0.136589149	0.724823315	0.468560431	0.828596747	up_in_case
PRJNA8274(ENSGALG00010021094	ENSGALG00010021094	case	control	-0.43784913	0.212842341	-2.057152385	0.03967157	0.336080244	down_in_case
PRJNA8274(ENSGALG00010021095	NT5C2	case	control	-0.190831081	0.073930857	-2.581210186	0.009845462	0.177161817	down_in_case
PRJNA8274(ENSGALG00010021096	ZFAND4	case	control	0.125787517	0.078896625	1.5943333	0.110861403	0.503115856	up_in_case
PRJNA8274(ENSGALG00010021097	KATNBL1	case	control	-0.043313498	0.070364963	-0.615554903	0.538188323	0.857740557	down_in_case
PRJNA8274(ENSGALG00010021098	SDK1	case	control	-0.198004088	0.087385623	-2.265865715	0.023459606	0.270324231	down_in_case
PRJNA8274(ENSGALG00010021099	HMCN1	case	control	-0.006598197	0.08104254	-0.081416461	0.935110759	0.988328444	down_in_case
PRJNA8274(ENSGALG00010021100	EMC7	case	control	-0.085711934	0.061080589	-1.403259783	0.160539386	0.572925686	down_in_case
PRJNA8274(ENSGALG00010021101	TMEM240	case	control	0.203412061	0.262921107	0.773661971	0.439130715	0.812363418	up_in_case
PRJNA8274(ENSGALG00010021102	GOLGA3	case	control	-0.02886542	0.053692677	-0.537604414	0.590850182	0.882949889	down_in_case
PRJNA8274(ENSGALG00010021103	RBMS1	case	control	0.046692499	0.032890324	1.419642415	0.15571181	0.564716939	up_in_case
PRJNA8274(ENSGALG00010021104	INA	case	control	-0.084886012	0.211318612	-0.401696809	0.687907173	0.917290381	down_in_case

PRJNA8274(	ENSGALG00010021106	ITGB6	case	control	0.388767722	0.126245661	3.079454139	0.002073803	0.075076917	up_in_case
PRJNA8274(	ENSGALG00010021108	PCGF6	case	control	0.090881847	0.066054599	1.375859488	0.16886514	0.585038804	up_in_case
PRJNA8274(	ENSGALG00010021110	PKLR	case	control	-0.306455136	0.138918521	-2.206006331	0.027383556	0.293762083	down_in_case
PRJNA8274(	ENSGALG00010021111	AVEN	case	control	-0.152717547	0.074517998	-2.049404847	0.040422544	0.33753444	down_in_case
PRJNA8274(	ENSGALG00010021112	TAF5	case	control	-0.077043186	0.051017088	-1.510144734	0.131006497	0.534010989	down_in_case
PRJNA8274(	ENSGALG00010021113	SYP	case	control	0.777487415	0.354812387	2.191263451	0.028432733	0.300549329	up_in_case
PRJNA8274(	ENSGALG00010021114	SBF2	case	control	-0.049492882	0.073928067	-0.669473505	0.503193476	0.842485272	down_in_case
PRJNA8274(	ENSGALG00010021115	ALPL	case	control	0.169516636	0.257702171	0.657800575	0.510666285	0.845108488	up_in_case
PRJNA8274(	ENSGALG00010021116	FAM21C	case	control	0.060931059	0.052046867	1.170696001	0.241720993	0.666243691	up_in_case
PRJNA8274(	ENSGALG00010021117	CYP1A2	case	control	1.426479583	0.965514644	1.477429257	0.139560608	0.541725489	up_in_case
PRJNA8274(	ENSGALG00010021118	USMG5	case	control	-0.059415659	0.094894616	-0.626122548	0.531234563	0.855560567	down_in_case
PRJNA8274(	ENSGALG00010021120	PDCD11	case	control	0.002292743	0.071905435	0.031885527	0.974563341	0.993775355	up_in_case
PRJNA8274(	ENSGALG00010021121	PLA2R1	case	control	-0.302363518	0.097308204	-3.107276748	0.001888195	0.070683756	down_in_case
PRJNA8274(	ENSGALG00010021122	CYP1A1	case	control	1.090974327	0.774964897	1.407772573	0.159198424	0.571787122	up_in_case
PRJNA8274(	ENSGALG00010021123	RYR3	case	control	-0.176298342	0.145731977	-1.209743705	0.226377254	0.649908598	down_in_case
PRJNA8274(	ENSGALG00010021124	CALHM2	case	control	-0.037560051	0.08933089	-0.420459827	0.674149571	0.913965794	down_in_case
PRJNA8274(	ENSGALG00010021125	LY75	case	control	-0.104972528	0.099315172	-1.056963661	0.290528172	0.714060795	down_in_case
PRJNA8274(	ENSGALG00010021126	ECE1	case	control	-0.007612677	0.048424682	-0.157206553	0.875082064	0.975572894	down_in_case
PRJNA8274(	ENSGALG00010021127	SWAP70	case	control	-0.139127561	0.06576847	-2.115414283	0.034394653	0.320002306	down_in_case
PRJNA8274(	ENSGALG00010021129	TXNRD3	case	control	-0.002862141	0.07746803	-0.036946095	0.970527986	0.992761697	down_in_case
PRJNA8274(	ENSGALG00010021130	ENSGALG00010021130	case	control	0.471187469	1.220738358	0.385985634			up_in_case
PRJNA8274(	ENSGALG00010021131	EDC3	case	control	-0.106748107	0.054043265	-1.975234235	0.048241572	0.361748551	down_in_case
PRJNA8274(	ENSGALG00010021133	ENSGALG00010021133	case	control	0.125330363	0.043601827	2.874429192	0.004047585	0.11024851	up_in_case
PRJNA8274(	ENSGALG00010021134	CHFR	case	control	0.01118898	0.059048363	0.189488396	0.849710049	0.968927451	up_in_case
PRJNA8274(	ENSGALG00010021135	CLK3	case	control	-0.051000738	0.072723604	-0.701295521	0.483118608	0.833529046	down_in_case
PRJNA8274(	ENSGALG00010021136	WEE1	case	control	0.072763821	0.053478232	1.360625031	0.173632217	0.592914702	up_in_case
PRJNA8274(	ENSGALG00010021137	PLAU	case	control	0.277505577	0.1564078	1.774243853	0.076022823	0.442710866	up_in_case
PRJNA8274(	ENSGALG00010021138	DDX18	case	control	0.125704898	0.063900415	1.967200039	0.049160153	0.364182961	up_in_case
PRJNA8274(	ENSGALG00010021139	ARHGAP29	case	control	-0.038481496	0.058708596	-0.655466138	0.512167682	0.845834144	down_in_case
PRJNA8274(	ENSGALG00010021140	SLC11A1	case	control	-0.085092636	0.291051585	-0.292362731	0.770009301	0.945428692	down_in_case
PRJNA8274(	ENSGALG00010021141	SGSM1	case	control	0.063307876	0.154408699	0.410002005	0.681804477	0.915594727	up_in_case
PRJNA8274(	ENSGALG00010021142	WNT6	case	control	-0.074798918	0.587276631	-0.127365732	0.898650937	0.98028579	down_in_case
PRJNA8274(	ENSGALG00010021143	ABCA4	case	control	0.437350201	0.294785676	1.48362094	0.137909511	0.539854583	up_in_case
PRJNA8274(	ENSGALG00010021144	FOXK1	case	control	-0.208373381	0.073207982	-2.846320523	0.004422765	0.114389963	down_in_case
PRJNA8274(	ENSGALG00010021145	EIF4G3	case	control	-0.114861522	0.064394864	-1.783706259	0.074471409	0.43920999	down_in_case
PRJNA8274(	ENSGALG00010021146	AP5Z1	case	control	-0.19368244	0.060262894	-3.213958504	0.001309186	0.057251861	down_in_case
PRJNA8274(	ENSGALG00010021147	ARID3B	case	control	-0.052388859	0.0754959	-0.693929856	0.487726199	0.835553062	down_in_case
PRJNA8274(	ENSGALG00010021148	GPATCH8	case	control	0.048295845	0.058592598	0.824265302	0.40978883	0.795863751	up_in_case
PRJNA8274(	ENSGALG00010021149	ENSGALG00010021149	case	control	0.257471364	0.877757587	0.293328555	0.76927103	0.94539152	up_in_case
PRJNA8274(	ENSGALG00010021150	ENSGALG00010021150	case	control	0.036206204	0.218757763	0.165508204	0.868543993	0.974483475	up_in_case
PRJNA8274(	ENSGALG00010021151	WNT10A	case	control	0.41216409	0.790806621	0.521194537	0.60223126	0.885002041	up_in_case
PRJNA8274(	ENSGALG00010021152	STK11IP	case	control	0.006964161	0.065653363	0.106074706	0.91552308	0.983445506	up_in_case
PRJNA8274(	ENSGALG00010021153	PTPRF	case	control	0.047063838	0.114655186	0.410481547	0.681452737	0.915594727	up_in_case
PRJNA8274(	ENSGALG00010021154	CHST13	case	control	0.147913409	0.121979147	1.212612255	0.225278101	0.6490987	up_in_case
PRJNA8274(	ENSGALG00010021155	RADIL	case	control	0.198011658	0.185676575	1.06643317	0.286227874	0.710557161	up_in_case
PRJNA8274(	ENSGALG00010021156	DDOST	case	control	0.085785342	0.049409598	1.736208061	0.082527052	0.456372384	up_in_case
PRJNA8274(	ENSGALG00010021158	ACTG1L	case	control	0.112270746	0.05448479	2.060588752	0.039342291	0.335091843	up_in_case
PRJNA8274(	ENSGALG00010021159	MMD2	case	control	-0.203159462	0.221585422	-0.916844891	0.359223925	0.765374836	down_in_case
PRJNA8274(	ENSGALG00010021160	ZNF143	case	control	0.028048748	0.054158071	0.517905237	0.604524388	0.885268908	up_in_case
PRJNA8274(	ENSGALG00010021161	ENSGALG00010021161	case	control	-0.022052254	0.06334046	-0.348154306	0.727724302	0.932979874	down_in_case
PRJNA8274(	ENSGALG00010021162	WIPI2	case	control	-0.127529119	0.045855967	-2.781080158	0.005417836	0.127480206	down_in_case
PRJNA8274(	ENSGALG00010021163	INHA	case	control	-0.078256725	0.29382439	-0.266338423	0.789978584	0.951843754	down_in_case
PRJNA8274(	ENSGALG00010021164	SLC41A3	case	control	0.082203669	0.130932114	0.627834277	0.530112509	0.855116625	up_in_case
PRJNA8274(	ENSGALG00010021165	PTRF	case	control	-0.126784351	0.090510231	-1.400773704	0.161281754	0.574285132	down_in_case
PRJNA8274(	ENSGALG00010021166	NEURL1	case	control	0.508719388	0.163764525	3.106407736	0.001893754	0.07070674	up_in_case
PRJNA8274(	ENSGALG00010021167	KIAA1671	case	control	0.064405489	0.076288276	0.844238366	0.398536219	0.789453932	up_in_case
PRJNA8274(	ENSGALG00010021168	IVNS1ABP	case	control	-0.108319307	0.072921494	-1.48542359	0.13743165	0.539854583	down_in_case
PRJNA8274(	ENSGALG00010021169	CAMK2G	case	control	-0.130838087	0.053069101	-2.465428736	0.013684939	0.205561591	down_in_case
PRJNA8274(	ENSGALG00010021170	UBL7	case	control	-0.009889656	0.093141723	-0.106178578	0.915440667	0.983445506	down_in_case
PRJNA8274(	ENSGALG00010021171	ENSGALG00010021171	case	control	-0.062417843	0.098937283	-0.630882933	0.528117072	0.853519488	down_in_case
PRJNA8274(	ENSGALG00010021172	SLC29A4	case	control	-0.459619017	0.170789939	-2.691136366	0.007120908	0.146516526	down_in_case
PRJNA8274(	ENSGALG00010021173	KLF15	case	control	-0.186666431	0.111271534	-1.677575775	0.093429945	0.478523919	down_in_case
PRJNA8274(	ENSGALG00010021175	IPO7	case	control	-0.059169236	0.057569636	-1.027785474	0.304050751	0.726844782	down_in_case
PRJNA8274(	ENSGALG00010021176	ENSGALG00010021176	case	control	-0.078749639	0.065670716	-1.199159142	0.230466071	0.654941338	down_in_case
PRJNA8274(	ENSGALG00010021177	OBSL1	case	control	0.064221265	0.19764954	0.324924943	0.745237878	0.937849656	up_in_case
PRJNA8274(	ENSGALG00010021178	NDST2	case	control	0.001365642	0.045618026	0.029936465	0.976117724	0.994231527	up_in_case
PRJNA8274(	ENSGALG00010021180	TMEM198	case	control	-0.052649123	0.098544857	-0.534265564	0.593157813	0.883625114	down_in_case
PRJNA8274(	ENSGALG00010021181	DAGLA	case	control	0.081803335	0.085935216	0.951918653	0.341138239	0.752814768	up_in_case
PRJNA8274(	ENSGALG00010021182	ZSWIM8	case	control	-0.027336485	0.075334053	-0.362870225	0.71670183	0.926862121	down_in_case
PRJNA8274(	ENSGALG00010021183	CFAP100	case	control	0.282297934	0.26880213	1.050207208	0.293622856	0.716275477	up_in_case
PRJNA8274(	ENSGALG00010021184	DRAXIN	case	control	0.154702071	0.324022657	0.477442142	0.633047312	0.897716834	up_in_case
PRJNA8274(	ENSGALG00010021186	SWT1	case	control	0.018197015	0.078123224	0.232927086	0.815818025	0.957374769	up_in_case
PRJNA8274(	ENSGALG00010021187	SEMA7A	case	control	-0.142520015	0.096782537	-1.472579872	0.140864356	0.543368104	down_in_case
PRJNA8274(	ENSGALG00010021188	TNRC18	case	control	-0.147252852	0.070246669	-2.096225406	0.0360622	0.325852945	down_in_case
PRJNA8274(	ENSGALG00010021189	ENSGALG00010021189	case	control	-0.111678519	0.471373481	-0.236921515	0.812717678	0.956624009	down_in_case
PRJNA8274(	ENSGALG00010021190	ENSGALG00010021190	case	control	-0.068819855	0.061476515	-1.119449524	0.262948413	0.688674415	down_in_case
PRJNA8274(	ENSGALG00010021191	MYRF	case	control	-0.084604853	0.111234581	-0.760598478	0.446896929	0.817833512	down_in_case
PRJNA8274(	ENSGALG00010021192	ENSGALG00010021192	case	control	-0.136016277	0.094457253	-1.439977043	0.149873894	0.556819093	down_in_case
PRJNA8274(	ENSGALG00010021193	USP37	case	control	-0.019636852	0.055488534	-0.353890261	0.723421126	0.930215097	down_in_case
PRJNA8274(	ENSGALG00010021194	CYP11A1	case	control	0.341696477	0.218841929	1.56138487	0.11843297	0.514769443	up_in_case
PRJNA8274(	ENSGALG00010021195	GCLM	case	control	0.112882101	0.085220097	1.32459484	0.185305568	0.608326361	up_in_case
PRJNA8274(	ENSGALG00010021196	ATP6V0A1	case	control	0.23076351	0.106392304	2.168986873	0.030083681	0.306407857	up_in_case
PRJNA8274(	ENSGALG00010021197	CHCHD1	case	control	0.295598645	0.080999207	3.649401712	0.000262852	0.021602187	up_in_case
PRJNA8274(	ENSGALG00010021199	TMEM258	case	control	-0.06171519	0.088341271	-0.69859975	0.4848022	0.834423421	down_in_case
PRJNA8274(	ENSGALG00010021200	FTO	case	control	-0.031165918	0.201982958	-0.154299742	0.877373401	0.975845037	down_in_case
PRJNA8274(	ENSGALG00010021201	TRMT1L	case	control	-0.035718117	0.04718047	-0.757053007	0.449018106	0.818701429	down_in_case
PRJNA8274(	ENSGALG00010021202	UROC1	case	control	0.705441411	0.409373761	1.723220876	0.08484859	0.461061346	up_in_case
PRJNA8274(	ENSGALG00010021203	FUT11	case	control	-0.126055803	0.069858507	-1.804444558	0.071161643	0.431840649	down_in_case
PRJNA8274(	ENSGALG00010021204	NAGLU	case	control	0.007290173	0.063203989	0.115343564	0.908172809	0.981730793	up_in_case
PRJNA8274(	ENSGALG00010021205	CDK5R2	case	control	-1.857789887	1.060349703	-1.752053951	0.079764531	0.450131333	down_in_case
PRJNA8274(	ENSGALG00010021207	MAD2L2	case	control	-0.035228139	0.068101457	-0.517289061	0.604954389	0.885268908	down_in_case
PRJNA8274(	ENSGALG00010021210	ENSGALG00010021210	case	control	0.004552434	0.042900388	0.10611638	0.915490016	0.983445506	up_in_case
PRJNA8274(	ENSGALG00010021211	FEN1	case	control	0.037390528	0.081925591	0.456396202	0.648105097	0.903510177	up_in_case
PRJNA8274(	ENSGALG00010021212	MYO18B	case	control	-0.211293811	0.083865628	-2.51943275	0.011754409	0.189715633	down_in_case
PRJNA8274(	ENSGALG00010021213	FBXO44	case	control	0.286704058	0.163040167	1.758487268	0.078664636	0.449003015	up_in_case
PRJNA8274(	ENSGALG00010021214	FBXL18	case	control	0.027255282	0.090055995	0.302648174	0.762157997	0.943297504	up_in_case
PRJNA8274(	ENSGALG00010021215	SH3PXD2A	case	control	0.088931451	0.086490387	1.028223535	0.30384469	0.726706651	up_in_case
PRJNA8274(	ENSGALG00010021217	FBXO2	case	control	-0.062540216	0.199138937	-0.314053178	0.75348065	0.940858857	down_in_case
PRJNA8274(	ENSGALG00010021218	DPF3	case	control	-0.166102945	0.152309057	-1.090565123	0.275464283	0.700894634	down_in_case
PRJNA8274(	ENSGALG00010021219	STRA6	case	control	0.062303084	0.218128612	0.285625455	0.775165005	0.947087967	up_in_case
PRJNA8274(	ENSGALG00010021220	SYNPO2L	case	control	-0.227303538	0.140183306	-1.621473658	0.104916095	0.494770427	down_in_case
PRJNA8274(	ENSGALG00010021222	ENSGALG00010021222	case	control	-0.186825012	0.276484697	-0.675715561	0.499221255	0.840142058	down_in_case
PRJNA8274(	ENSGALG00010021223	DFFB	case	control	-0.086600638	0.090077091	-0.961405805	0.336348167	0.750473129	down_in_case
PRJNA8274(	ENSGALG00010021224	MYOZ1	case	control	-0.311598071	0.124684358	-2.499095113	0.012451089	0.195901209	down_in_case
PRJNA8274(	ENSGALG00010021227	CHCHD4	case	control	0.012786361	0.062191692	0.20559597	0.837106526	0.964784002	up_in_case
PRJNA8274(	ENSGALG00010021228	ESRP2	case	control	0.124057766	0.946533512	0.131065371	0.895723595	0.980176778	up_in_case
PRJNA8274(	ENSGALG00010021229	GLO1	case	control	-0.046193145	0.095118654	-0.485637072	0.627224507	0.895838586	down_in_case
PRJNA8274(	ENSGALG00010021230	C1orf174	case	control	0.111736788	0.043178036	2.587815443	0.009658671	0.175953849	up_in_case
PRJNA8274(	ENSGALG00010021231	STN1	case	control	0.274574339	0.127265057	2.157499829	0.03096674	0.308259818	up_in_case
PRJNA8274(	ENSGALG00010021232	ACTB	case	control	0.037367836	0.071067662	0.525806464	0.599022683	0.884828142	up_in_case
PRJNA8274(	ENSGALG00010021233	COASY	case	control	-0.000286545	0.039775456	-0.007204077	0.994252028	0.998301153	down_in_case
PRJNA8274(	ENSGALG00010021234	ISLR2	case	control	-0.550104044	0.244060367	-2.25396713	0.024198236	0.275122308	down_in_case
PRJNA8274(	ENSGALG00010021235	XRCC5	case	control	-0.058535957	0.086069022	-0.680104823	0.496438091	0.83858637	down_in_case
PRJNA8274(	ENSGALG00010021236	BTBD9	case	control	-0.031585224	0.062393552	-0.506225765	0.61269817	0.890080771	down_in_case
PRJNA8274(	ENSGALG00010021237	FSCN1	case	control	0.021959162	0.081833757	0.268338683	0.788438633	0.951738421	up_in_case
PRJNA8274(	ENSGALG00010021238	NDUFS8	case	control	-0.199438152	0.068978519	-2.891308117	0.003836418	0.106733027	down_in_case
PRJNA8274(	ENSGALG00010021239	USP54	case	control	-0.039490355	0.049880705	-0.791696013	0.428537948	0.805355714	down_in_case
PRJNA8274(	ENSGALG00010021240	SLK	case	control	-0.004451019	0.061129438	-0.072813023	0.941954907	0.989551456	down_in_case
PRJNA8274(	ENSGALG00010021241	TMEM43	case	control	-0.135293436	0.089823939	-1.506206891	0.132014071	0.535394559	down_in_case
PRJNA8274(	ENSGALG00010021242	BCAR3	case	control	0.191886897	0.170565804	1.125002152	0.260588122	0.687359696	up_in_case
PRJNA8274(	ENSGALG00010021243	DYL1	case	control	-0.157793418	0.063546747	-2.483107721	0.01302417	0.200161779	down_in_case
PRJNA8274(	ENSGALG00010021244	PPP3CB	case	control	0.01409036	0.040751915	0.345759464	0.729523491	0.933556014	up_in_case
PRJNA8274(	ENSGALG00010021245	ZFAND3	case	control	-0.110652555	0.052795739	-2.095861486	0.03609448	0.325852945	down_in_case
PRJNA8274(	ENSGALG00010021246	PECR	case	control	-0.010580781	0.101197724	-0.104555526	0.916728507	0.983445506	down_in_case
PRJNA8274(	ENSGALG00010021247	GNAO1	case	control	0.185105864	0.150192808	1.232454911	0.2177792	0.643008408	up_in_case
PRJNA8274(	ENSGALG00010021248	ANXA7	case	control	-0.018895908	0.083268315	-0.226927946	0.820479771	0.958669267	down_in_case

PRJNA8274(	ENSGALG00010021249	ENSGALG00010021249	case	control	-0.118600311	0.404236164	-0.293393619	0.769221303	0.94539152	down_in_case
PRJNA8274(	ENSGALG00010021250	ISLR	case	control	-0.08342343	0.128721653	-0.648091662	0.516925667	0.848054666	down_in_case
PRJNA8274(	ENSGALG00010021251	RNF216	case	control	-0.016273208	0.052333853	-0.310949921	0.755838693	0.941588405	down_in_case
PRJNA8274(	ENSGALG00010021252	XPC	case	control	0.032017279	0.06223539	0.514454535	0.606934239	0.887044121	up_in_case
PRJNA8274(	ENSGALG00010021253	TMEM169	case	control	-0.002779335	0.080281348	-0.034619939	0.972382802	0.99340857	down_in_case
PRJNA8274(	ENSGALG00010021254	FNBP1L	case	control	-0.104703173	0.043866883	-2.386838648	0.016993948	0.231902401	down_in_case
PRJNA8274(	ENSGALG00010021255	EIF3F	case	control	-0.065605501	0.060227888	-1.089287755	0.276027011	0.701472589	down_in_case
PRJNA8274(	ENSGALG00010021256	ENSGALG00010021256	case	control	-0.010362118	0.126174578	-0.082125248	0.934547116	0.988140189	down_in_case
PRJNA8274(	ENSGALG00010021257	RGS6	case	control	0.222780666	0.269962252	0.825228951	0.40924162	0.795345904	up_in_case
PRJNA8274(	ENSGALG00010021258	LSM3	case	control	-0.120414533	0.075700481	-1.590670637	0.111683718	0.504287077	down_in_case
PRJNA8274(	ENSGALG00010021259	CEP104	case	control	0.079799784	0.062412256	1.278591574	0.201040919	0.62375464	up_in_case
PRJNA8274(	ENSGALG00010021260	KDM4A	case	control	-0.009541915	0.045603383	-0.209236996	0.834263233	0.963099884	down_in_case
PRJNA8274(	ENSGALG00010021261	JUP	case	control	-0.016297846	0.061648339	-0.264367968	0.791496391	0.95239351	down_in_case
PRJNA8274(	ENSGALG00010021262	MDGA1	case	control	0.104677888	0.204249634	0.512499757	0.608301291	0.887692472	up_in_case
PRJNA8274(	ENSGALG00010021263	STOML1	case	control	0.0387649	0.112477475	0.344645897	0.730360597	0.933770678	up_in_case
PRJNA8274(	ENSGALG00010021264	CBFB	case	control	-0.178202088	0.089699109	-1.986665087	0.046959523	0.357384486	down_in_case
PRJNA8274(	ENSGALG00010021265	SIPA1L1	case	control	-0.075148722	0.071542295	-1.050409717	0.29352978	0.716170594	down_in_case
PRJNA8274(	ENSGALG00010021266	COQ5	case	control	0.108195384	0.075788733	1.42759194	0.153409345	0.562068571	up_in_case
PRJNA8274(	ENSGALG00010021267	LRRC47	case	control	-0.091266728	0.058289591	-1.56574658	0.117407957	0.514181593	down_in_case
PRJNA8274(	ENSGALG00010021269	NUDT13	case	control	-0.030651117	0.092738045	-0.330512863	0.741012475	0.937158859	down_in_case
PRJNA8274(	ENSGALG00010021271	ENSGALG00010021271	case	control	0.12722219	0.089277105	1.425025942	0.154149702	0.562906216	up_in_case
PRJNA8274(	ENSGALG00010021272	ENSGALG00010021272	case	control	0.090968865	0.127331587	0.714424966	0.474964438	0.830111175	up_in_case
PRJNA8274(	ENSGALG00010021275	MAP3K2	case	control	-0.108473832	0.061428354	-1.765859335	0.077419451	0.446500211	down_in_case
PRJNA8274(	ENSGALG00010021277	ENSGALG00010021277	case	control	0.060377732	0.156638465	0.385459167	0.699897254	0.922288441	up_in_case
PRJNA8274(	ENSGALG00010021287	COL17A1	case	control	0.084271768	0.560233755	0.15042251	0.880431283	0.976399289	up_in_case
PRJNA8274(	ENSGALG00010021290	ERCC3	case	control	-0.010953258	0.034864778	-0.314163987	0.753396493	0.940858857	down_in_case
PRJNA8274(	ENSGALG00010021293	PCNX1	case	control	-0.008629843	0.046093114	-0.187226286	0.851483216	0.969234083	down_in_case
PRJNA8274(	ENSGALG00010021296	GATC	case	control	-0.056917607	0.069147167	-0.823137235	0.410429957	0.796464701	down_in_case
PRJNA8274(	ENSGALG00010021298	RPGRIP1L	case	control	0.115727765	0.138075824	0.838146472	0.401948445	0.791716635	up_in_case
PRJNA8274(	ENSGALG00010021299	CYP27C1	case	control	-0.125076814	0.132678961	-0.942702692	0.345832996	0.756227431	down_in_case
PRJNA8274(	ENSGALG00010021301	SMURF1	case	control	0.12923604	0.053071213	2.435143892	0.014885869	0.215235515	up_in_case
PRJNA8274(	ENSGALG00010021302	P4HA1	case	control	0.003042796	0.044818233	0.067891932	0.945871661	0.990313016	up_in_case
PRJNA8274(	ENSGALG00010021307	MAP3K9	case	control	-0.137915506	0.179323793	-0.769086488	0.441841969	0.813507909	down_in_case
PRJNA8274(	ENSGALG00010021309	ST3GAL3	case	control	0.005990136	0.06920577	0.086555446	0.931024882	0.987258715	up_in_case
PRJNA8274(	ENSGALG00010021311	TRIAP1	case	control	-0.441385061	0.087558378	-5.04103745	4.63015E-07	0.000140875	down_in_case
PRJNA8274(	ENSGALG00010021314	ZP3	case	control	-0.211477044	0.215815671	-0.979896607	0.327137158	0.744857565	down_in_case
PRJNA8274(	ENSGALG00010021315	COX6A1	case	control	-0.130232065	0.101825555	-1.27897231	0.200906807	0.623527532	down_in_case
PRJNA8274(	ENSGALG00010021316	PLA2G12B	case	control	1.236600259	0.690158768	1.791762006	0.073171097	0.436961143	up_in_case
PRJNA8274(	ENSGALG00010021318	TP73	case	control	0.342938772	0.221571281	1.547758226	0.121680518	0.519686058	up_in_case
PRJNA8274(	ENSGALG00010021320	SLC6A6	case	control	-0.006115766	0.108014666	-0.056619775	0.954848082	0.992056045	down_in_case
PRJNA8274(	ENSGALG00010021325	ARPC1A	case	control	-0.074142949	0.041236906	-1.797975576	0.072180878	0.434277972	down_in_case
PRJNA8274(	ENSGALG00010021329	PSMC3IP	case	control	-0.000504314	0.086347952	-0.005840491	0.995339989	0.998710499	down_in_case
PRJNA8274(	ENSGALG00010021330	COMMD4	case	control	-0.015177033	0.101302214	-0.149819362	0.880907133	0.97667824	down_in_case
PRJNA8274(	ENSGALG00010021332	OIT3	case	control	0.770883524	0.138457819	5.567641674	2.5821E-08	1.15388E-05	up_in_case
PRJNA8274(	ENSGALG00010021335	NFATC3	case	control	-0.049580849	0.052309291	-0.947840203	0.34321081	0.755132664	down_in_case
PRJNA8274(	ENSGALG00010021338	PDIA5	case	control	-0.046963148	0.078428393	-0.598802887	0.549304337	0.864234145	down_in_case
PRJNA8274(	ENSGALG00010021341	L3MBTL1	case	control	0.254966159	0.118767976	2.146758477	0.031812515	0.3098361	up_in_case
PRJNA8274(	ENSGALG00010021342	SFR1	case	control	-0.201307456	0.12992269	-1.549440326	0.121275906	0.519567065	down_in_case
PRJNA8274(	ENSGALG00010021345	NEIL1	case	control	-0.054875183	0.150760073	-0.363990157	0.715865359	0.926862121	down_in_case
PRJNA8274(	ENSGALG00010021346	RPS21	case	control	-0.222016079	0.091622672	-2.423156574	0.015386293	0.220023997	down_in_case
PRJNA8274(	ENSGALG00010021348	SLC4A3	case	control	-0.149795782	0.088267052	-1.697074715	0.089682536	0.472883577	down_in_case
PRJNA8274(	ENSGALG00010021350	CABLES2	case	control	0.015911254	0.085893721	0.1852435	0.853038051	0.969900941	up_in_case
PRJNA8274(	ENSGALG00010021352	CFAP43	case	control	-0.2967005	0.181071636	-1.638580768	0.1013006	0.491460867	down_in_case
PRJNA8274(	ENSGALG00010021353	IPO13	case	control	-0.151721838	0.079029264	-1.919818431	0.054880838	0.382868844	down_in_case
PRJNA8274(	ENSGALG00010021354	ENSGALG00010021354	case	control	-0.024231915	0.05143741	-0.471095166	0.637572777	0.899378897	down_in_case
PRJNA8274(	ENSGALG00010021358	MMP15	case	control	-0.012425419	0.051481499	-0.241356981	0.809278452	0.955875648	down_in_case
PRJNA8274(	ENSGALG00010021360	PTPRN	case	control	-0.176121397	0.210827271	-0.835382423	0.403502423	0.79260318	down_in_case
PRJNA8274(	ENSGALG00010021361	WRAP73	case	control	0.064943754	0.097709822	0.664659425	0.506268352	0.843280685	up_in_case
PRJNA8274(	ENSGALG00010021362	ARPC1B	case	control	0.101803094	0.063743109	1.597083916	0.110247003	0.502938553	up_in_case
PRJNA8274(	ENSGALG00010021363	ENSGALG00010021363	case	control	0.064713183	0.051174159	1.26456759	0.206026377	0.628879646	up_in_case
PRJNA8274(	ENSGALG00010021367	GRIP2	case	control	-0.067119628	0.08923478	-0.752168917	0.451949483	0.82064437	down_in_case
PRJNA8274(	ENSGALG00010021373	CEP250	case	control	-0.089316297	0.074062071	-1.205965422	0.227830823	0.651681204	down_in_case
PRJNA8274(	ENSGALG00010021375	TUBG1	case	control	0.055359007	0.051211205	1.080994036	0.279699767	0.705400718	up_in_case
PRJNA8274(	ENSGALG00010021378	KIF11	case	control	-0.080914695	0.144518998	-0.559889675	0.575554692	0.877910508	down_in_case
PRJNA8274(	ENSGALG00010021381	DNAJB2	case	control	0.059297617	0.066274727	0.894724414	0.370934425	0.772892652	up_in_case
PRJNA8274(	ENSGALG00010021382	TPRG1L	case	control	0.062185999	0.056453294	1.101547756	0.270658332	0.698323327	up_in_case
PRJNA8274(	ENSGALG00010021383	MSI1	case	control	0.061892969	0.066438018	0.931589629	0.351548645	0.760831854	up_in_case
PRJNA8274(	ENSGALG00010021384	ARTN	case	control	-0.255409039	0.320576268	-0.796718488	0.425614544	0.804804048	down_in_case
PRJNA8274(	ENSGALG00010021385	GDF5	case	control	-0.072060878	0.588552924	-0.122437381	0.902552636	0.980850383	down_in_case
PRJNA8274(	ENSGALG00010021387	SLC2A14	case	control	-0.002558906	0.048573055	-0.0526816	0.9579856	0.992056045	down_in_case
PRJNA8274(	ENSGALG00010021388	DPH2	case	control	0.166007146	0.120497612	1.377679967	0.168302119	0.58432606	up_in_case
PRJNA8274(	ENSGALG00010021390	GSTO2	case	control	1.401469256	0.225812177	6.20634935	5.42296E-10	2.98263E-07	up_in_case
PRJNA8274(	ENSGALG00010021391	ENSGALG00010021391	case	control	0.214028052	0.316104309	0.677080463	0.498354904	0.83983448	up_in_case
PRJNA8274(	ENSGALG00010021393	CCDC174	case	control	0.03527223	0.062192289	0.567147963	0.570613659	0.875982321	up_in_case
PRJNA8274(	ENSGALG00010021394	MAN2C1	case	control	0.047950658	0.088017028	0.544788423	0.585899042	0.881576413	up_in_case
PRJNA8274(	ENSGALG00010021396	MED6	case	control	0.087682731	0.058001613	1.511729179	0.130602773	0.533148629	up_in_case
PRJNA8274(	ENSGALG00010021400	B4GALT2	case	control	-0.07105088	0.067264288	-1.056294237	0.290833808	0.714222439	down_in_case
PRJNA8274(	ENSGALG00010021401	PDAP1	case	control	-0.095632053	0.058073819	-1.646732625	0.099613015	0.488667622	down_in_case
PRJNA8274(	ENSGALG00010021402	ENSGALG00010021402	case	control	-0.10816805	0.094945159	-1.139268719	0.254591091	0.680241518	down_in_case
PRJNA8274(	ENSGALG00010021404	UQCC1	case	control	-0.045264475	0.068219164	-0.663515531	0.507000436	0.843472666	down_in_case
PRJNA8274(	ENSGALG00010021405	IDE	case	control	-0.045583348	0.040798106	-1.117290794	0.26387001	0.689768156	down_in_case
PRJNA8274(	ENSGALG00010021407	SIN3A	case	control	-0.060872426	0.059076288	-1.030403706	0.302820534	0.725470538	down_in_case
PRJNA8274(	ENSGALG00010021408	TTC9	case	control	0.058660583	0.189437568	0.309656547	0.75682215	0.941659745	up_in_case
PRJNA8274(	ENSGALG00010021409	MEGF6	case	control	0.256675855	0.212261914	1.209241215	0.226570188	0.650201422	up_in_case
PRJNA8274(	ENSGALG00010021411	MCU	case	control	-0.190507015	0.089738139	-2.122921402	0.033760439	0.31692586	down_in_case
PRJNA8274(	ENSGALG00010021412	ZNF319	case	control	0.013408017	0.061564303	0.217788825	0.827593652	0.961319258	up_in_case
PRJNA8274(	ENSGALG00010021413	SYNJ2BP	case	control	0.094788414	0.166786539	0.568321728	0.569816528	0.875322414	up_in_case
PRJNA8274(	ENSGALG00010021414	BUD31	case	control	-0.080316945	0.057353559	-1.400382913	0.161398684	0.574558423	down_in_case
PRJNA8274(	ENSGALG00010021415	SIRT4	case	control	-0.156833159	0.107659081	-1.456757366	0.145183368	0.549384008	down_in_case
PRJNA8274(	ENSGALG00010021416	PLEKHH3	case	control	0.00405354	0.097483198	0.041581935	0.966831975	0.992654885	up_in_case
PRJNA8274(	ENSGALG00010021417	STK16	case	control	0.024376754	0.086486072	0.281857572	0.778052724	0.948036294	up_in_case
PRJNA8274(	ENSGALG00010021419	MANSC1	case	control	0.219218792	0.128773799	1.702355555	0.088688736	0.470938334	up_in_case
PRJNA8274(	ENSGALG00010021420	PLXNB3	case	control	-0.105102514	0.068831705	-1.526949159	0.126773654	0.527026942	down_in_case
PRJNA8274(	ENSGALG00010021421	GLB1L	case	control	0.478449224	0.147934529	3.234195736	0.001219858	0.055104058	up_in_case
PRJNA8274(	ENSGALG00010021424	PTCD1	case	control	-0.030259624	0.057197719	-0.529035493	0.596780829	0.884680373	down_in_case
PRJNA8274(	ENSGALG00010021425	PTPN9	case	control	0.1610885	0.077400172	2.08124213	0.037411749	0.328438691	up_in_case
PRJNA8274(	ENSGALG00010021426	SLC38A7	case	control	0.032213858	0.069154115	0.465827058	0.641339296	0.901518916	up_in_case
PRJNA8274(	ENSGALG00010021427	RPLP0	case	control	0.13550522	0.072659874	1.864925071	0.062191908	0.405537748	up_in_case
PRJNA8274(	ENSGALG00010021429	MED8	case	control	-0.151337584	0.080043166	-1.890699637	0.058664451	0.394963111	down_in_case
PRJNA8274(	ENSGALG00010021431	CPSF4	case	control	0.004995065	0.060353794	0.082763061	0.934039941	0.988127868	up_in_case
PRJNA8274(	ENSGALG00010021432	SYT9	case	control	-0.467805006	0.239787541	-1.950914562	0.05106721	0.369565336	down_in_case
PRJNA8274(	ENSGALG00010021433	ENSGALG00010021433	case	control	-0.014323917	0.129527358	-0.110586032	0.911944624	0.98266608	down_in_case
PRJNA8274(	ENSGALG00010021434	ANKZF1	case	control	0.043584399	0.073121318	0.59605598	0.55113783	0.864743359	up_in_case
PRJNA8274(	ENSGALG00010021435	GCN1	case	control	-0.094701473	0.045203418	-2.095006889	0.03617038	0.32592088	down_in_case
PRJNA8274(	ENSGALG00010021436	ENSGALG00010021436	case	control	-0.739098643	0.528512923	-1.398449519	0.161978122	0.575618076	down_in_case
PRJNA8274(	ENSGALG00010021438	GOT2	case	control	-0.15023306	0.072808682	-2.063394847	0.039075129	0.335091843	down_in_case
PRJNA8274(	ENSGALG00010021439	ITPRIP	case	control	0.007495541	0.086204998	0.086950188	0.930711107	0.987258715	up_in_case
PRJNA8274(	ENSGALG00010021441	ENSGALG00010021441	case	control	0.200956181	0.214088915	0.938657573	0.347906583	0.75850955	up_in_case
PRJNA8274(	ENSGALG00010021443	SNUPN	case	control	-0.086868913	0.067443334	-1.288028155	0.197736162	0.621047029	down_in_case
PRJNA8274(	ENSGALG00010021444	ATG9A	case	control	-0.075611651	0.055762731	-1.355953168	0.175114069	0.594080527	down_in_case
PRJNA8274(	ENSGALG00010021446	HOXB8	case	control	-0.350779786	0.314219107	-1.116354094	0.264270595	0.690114958	down_in_case
PRJNA8274(	ENSGALG00010021448	SZT2	case	control	-0.000543219	0.057014993	-0.009527656	0.992398145	0.997995042	down_in_case
PRJNA8274(	ENSGALG00010021450	OLFML1	case	control	-0.384906658	0.166229743	-2.315510156	0.020585029	0.257989413	down_in_case
PRJNA8274(	ENSGALG00010021452	SLC25A1	case	control	0.327042656	0.153460325	2.131121884	0.033079102	0.31326567	up_in_case
PRJNA8274(	ENSGALG00010021454	ATP5J2	case	control	-0.126730634	0.091262242	-1.388642579	0.164941463	0.580021198	down_in_case
PRJNA8274(	ENSGALG00010021455	CFAP58	case	control	-0.049423872	0.287411925	-0.1719618	0.863467561	0.972890026	down_in_case
PRJNA8274(	ENSGALG00010021457	USB1	case	control	-0.019374678	0.071461073	-0.271122124	0.786297106	0.950252288	down_in_case
PRJNA8274(	ENSGALG00010021458	USP30	case	control	0.051842007	0.071943196	0.720596388	0.471157877	0.829101076	up_in_case
PRJNA8274(	ENSGALG00010021459	ARHGEF16	case	control	0.086740161	0.081208353	1.068118709	0.285466967	0.709921118	up_in_case
PRJNA8274(	ENSGALG00010021461	SNX33	case	control	0.074281828	0.047479516	1.564502632	0.117699575	0.514553326	up_in_case
PRJNA8274(	ENSGALG00010021462	SETD5	case	control	-0.014508598	0.050531324	-0.28712088	0.774019765	0.946671454	down_in_case
PRJNA8274(	ENSGALG00010021465	ABCB6	case	control	0.161224552	0.090132992	1.788740719	0.073656582	0.437929428	up_in_case
PRJNA8274(	ENSGALG00010021466	HES4	case	control	0.018579923	0.106857552	0.173875618	0.861963215	0.972643856	up_in_case
PRJNA8274(	ENSGALG00010021467	LHFPL4	case	control	-0.062495656	0.20041444	-0.311832099	0.755168128	0.941559531	down_in_case
PRJNA8274(	ENSGALG00010021469	CSPG4	case	control	0.122139547	0.074844725	1.631905881	0.102699315	0.491993368	up_in_case
PRJNA8274(	ENSGALG00010021470	ENSGALG00010021470	case	control	0.787410547	0.401541877	1.960967443	0.049882821	0.365445124	up_in_case

PRJNA8274(	ENSGALG00010021473	SLC8A3	case	control	-0.053711369	0.077231792	-0.695456722	0.486769124	0.835128792	down_in_case
PRJNA8274(	ENSGALG00010021474	PPFIBP2	case	control	-0.003840221	0.112681659	-0.034080269	0.972813142	0.99340857	down_in_case
PRJNA8274(	ENSGALG00010021476	ALKBH2	case	control	-0.274307388	0.115448264	-2.376020029	0.017500513	0.235259018	down_in_case
PRJNA8274(	ENSGALG00010021477	ZFAND2B	case	control	0.097792405	0.090131489	1.084997112	0.277922935	0.703415569	up_in_case
PRJNA8274(	ENSGALG00010021478	SLC13A3	case	control	0.303858924	0.213334497	1.424330938	0.154350696	0.56303838	up_in_case
PRJNA8274(	ENSGALG00010021479	SKAP1	case	control	0.080509913	0.329428968	0.244392331	0.806926981	0.955875648	up_in_case
PRJNA8274(	ENSGALG00010021480	ENSGALG00010021480	case	control	0.158954164	0.050596931	3.141577167	0.001680405	0.065655176	up_in_case
PRJNA8274(	ENSGALG00010021481	MICU1	case	control	0.195196613	0.101161278	1.929558603	0.053661552	0.37782383	up_in_case
PRJNA8274(	ENSGALG00010021483	UNG	case	control	0.052403054	0.080883154	0.647885894	0.517058755	0.848054666	up_in_case
PRJNA8274(	ENSGALG00010021484	MARCHF5	case	control	-0.103286016	0.068845966	-1.500247902	0.133550199	0.537206146	down_in_case
PRJNA8274(	ENSGALG00010021485	SMOC1	case	control	0.392489004	0.210275077	1.866550283	0.061964416	0.405537748	up_in_case
PRJNA8274(	ENSGALG00010021487	KLHL17	case	control	0.19471089	0.077899173	2.49952447	0.012436011	0.195901209	up_in_case
PRJNA8274(	ENSGALG00010021488	HYI	case	control	-0.189864069	0.116131251	-1.634909357	0.102068051	0.491460867	down_in_case
PRJNA8274(	ENSGALG00010021489	CNPPD1	case	control	0.006929359	0.061002554	0.113591286	0.909561796	0.981730793	up_in_case
PRJNA8274(	ENSGALG00010021490	ACACB	case	control	-0.305464879	0.200929997	-1.520255235	0.12844684	0.53040422	down_in_case
PRJNA8274(	ENSGALG00010021492	PARP6	case	control	-0.094545336	0.080203955	-1.178811394	0.238473286	0.663327756	down_in_case
PRJNA8274(	ENSGALG00010021493	UNC93B1	case	control	-0.022349277	0.093061906	-0.240154941	0.810210143	0.955972758	down_in_case
PRJNA8274(	ENSGALG00010021494	SLC2A10	case	control	0.123518524	0.123693067	0.998588905	0.317993877	0.738087569	up_in_case
PRJNA8274(	ENSGALG00010021496	MTMR14	case	control	0.165487732	0.076648209	2.159055439	0.030845866	0.308259818	up_in_case
PRJNA8274(	ENSGALG00010021498	B3GALT6	case	control	0.048103215	0.077103192	0.623880974	0.532705756	0.855817434	up_in_case
PRJNA8274(	ENSGALG00010021500	FOXN4	case	control	0.429945279	0.614951351	0.69915332	0.484456219	0.834163027	up_in_case
PRJNA8274(	ENSGALG00010021502	DNAJB12	case	control	0.038288206	0.048338787	0.792080404	0.428313795	0.805337183	up_in_case
PRJNA8274(	ENSGALG00010021503	KCNS1	case	control	-0.013587242	0.292526596	-0.046447885	0.962953271	0.992068146	down_in_case
PRJNA8274(	ENSGALG00010021504	ODR4	case	control	0.057319195	0.057407247	0.998466173	0.318053359	0.738087569	up_in_case
PRJNA8274(	ENSGALG00010021505	IHH	case	control	1.266347451	0.619461453	2.044271591			up_in_case
PRJNA8274(	ENSGALG00010021506	MAX	case	control	0.034548054	0.091790775	0.376378276	0.706635694	0.924581603	up_in_case
PRJNA8274(	ENSGALG00010021509	DDIT4	case	control	-0.17535392	0.081629935	-2.148157038	0.031701282	0.3098361	down_in_case
PRJNA8274(	ENSGALG00010021510	NTSR1	case	control	0.064566816	0.316230811	0.204176234	0.838215783	0.96484215	up_in_case
PRJNA8274(	ENSGALG00010021512	CPNE9	case	control	-0.480896703	0.291891193	-1.647520425	0.099451121	0.488584926	down_in_case
PRJNA8274(	ENSGALG00010021513	SDF4	case	control	-0.003451219	0.052474301	-0.065769702	0.947561178	0.99115828	down_in_case
PRJNA8274(	ENSGALG00010021514	TPR	case	control	-0.103128021	0.069171954	-1.490893547	0.135989446	0.539774365	down_in_case
PRJNA8274(	ENSGALG00010021516	SRSF5	case	control	0.142503064	0.078383561	1.818022319	0.069060722	0.424577953	up_in_case
PRJNA8274(	ENSGALG00010021518	CPEB3	case	control	-0.358923463	0.114220398	-3.142376224	0.001675826	0.065655176	down_in_case
PRJNA8274(	ENSGALG00010021519	FNTB	case	control	0.060077757	0.057745061	1.040396469	0.298155742	0.721487387	up_in_case
PRJNA8274(	ENSGALG00010021521	ENSGALG00010021521	case	control	-0.01160204	0.193118047	-0.060077449	0.952093951	0.992056045	down_in_case
PRJNA8274(	ENSGALG00010021523	CELF6	case	control	-0.129085997	0.095211817	-1.355777067	0.17517011	0.594080527	down_in_case
PRJNA8274(	ENSGALG00010021525	ENSGALG00010021525	case	control	-0.182770379	0.108462002	-1.685109774	0.091967394	0.476713154	down_in_case
PRJNA8274(	ENSGALG00010021526	KCNJ12	case	control	0.087037747	0.126345188	0.688888497	0.490893439	0.836883187	up_in_case
PRJNA8274(	ENSGALG00010021528	BRPF1	case	control	-0.046219201	0.059135542	-0.781580749	0.434461004	0.80937889	down_in_case
PRJNA8274(	ENSGALG00010021529	BTAF1	case	control	-0.031512981	0.047290355	-0.666372264	0.505173186	0.842675288	down_in_case
PRJNA8274(	ENSGALG00010021530	MLF2	case	control	0.01335532	0.047001173	0.284148647	0.776296468	0.947228003	up_in_case
PRJNA8274(	ENSGALG00010021531	RAB15	case	control	0.075210567	0.310085213	0.242548061	0.808355521	0.955875648	up_in_case
PRJNA8274(	ENSGALG00010021532	DPP10	case	control	-0.053051788	0.161829491	-0.327825215	0.743043821	0.937448285	down_in_case
PRJNA8274(	ENSGALG00010021533	SSX2IP	case	control	0.453928028	0.159700551	2.842369835	0.004477951	0.114389963	up_in_case
PRJNA8274(	ENSGALG00010021534	KIFC3	case	control	-0.119122181	0.101808284	-1.170063742	0.241975318	0.666243691	down_in_case
PRJNA8274(	ENSGALG00010021535	SUSD6	case	control	0.112650676	0.051871469	2.171727103	0.029876253	0.305970455	up_in_case
PRJNA8274(	ENSGALG00010021538	TNFRSF18	case	control	0.619809024	0.336767534	1.840465489	0.065699926	0.415733817	up_in_case
PRJNA8274(	ENSGALG00010021539	HEXA	case	control	0.151833616	0.089370787	1.698917749	0.089334682	0.47258594	up_in_case
PRJNA8274(	ENSGALG00010021540	STK4	case	control	0.122587093	0.063123561	1.942018011	0.05213492	0.372764678	up_in_case
PRJNA8274(	ENSGALG00010021541	CYP27A1	case	control	0.270573718	0.270737894	0.999393598	0.31760406	0.737774213	up_in_case
PRJNA8274(	ENSGALG00010021543	PLEKHD1	case	control	0.072090665	0.272149741	0.264893382	0.791091597	0.95239351	up_in_case
PRJNA8274(	ENSGALG00010021545	CYB5R2	case	control	0.037658743	0.142082916	0.265047654	0.790972752	0.95239351	up_in_case
PRJNA8274(	ENSGALG00010021546	OGG1	case	control	0.463912844	0.163383126	2.83941712	0.004519603	0.114389963	up_in_case
PRJNA8274(	ENSGALG00010021549	ANAPC16	case	control	-0.005474307	0.079019099	-0.069278271	0.944768121	0.990313016	down_in_case
PRJNA8274(	ENSGALG00010021550	MAP2K3	case	control	0.098167892	0.080857845	1.214079991	0.22471718	0.648789758	up_in_case
PRJNA8274(	ENSGALG00010021552	ENSGALG00010021552	case	control	-0.28086709	0.102283646	-2.745962846	0.00603336	0.134449153	down_in_case
PRJNA8274(	ENSGALG00010021553	SLC39A9	case	control	0.003188539	0.035722881	0.089257612	0.928877181	0.986776285	up_in_case
PRJNA8274(	ENSGALG00010021554	SNX11	case	control	0.009079744	0.079873898	0.113675982	0.909494653	0.981730793	up_in_case
PRJNA8274(	ENSGALG00010021556	CAMK1	case	control	-0.089089453	0.056259479	-1.583545636	0.113297154	0.507883792	down_in_case
PRJNA8274(	ENSGALG00010021558	CHURC1	case	control	-0.148205231	0.102222464	-1.449830352	0.147105833	0.55227446	down_in_case
PRJNA8274(	ENSGALG00010021561	TGIF2	case	control	-0.003182414	0.063581884	-0.05005222	0.960080775	0.992056045	down_in_case
PRJNA8274(	ENSGALG00010021562	LRP6	case	control	-0.031165929	0.100550579	-0.30995276	0.75659688	0.941659745	down_in_case
PRJNA8274(	ENSGALG00010021564	PRKAG3	case	control	0.015108818	0.157780878	0.095758235	0.923712589	0.98553537	up_in_case
PRJNA8274(	ENSGALG00010021565	CBX1	case	control	-0.004335901	0.063336666	-0.068457999	0.945421053	0.990313016	down_in_case
PRJNA8274(	ENSGALG00010021566	ENSGALG00010021566	case	control	-0.109885771	0.066874892	-1.643154363	0.100351	0.489767677	down_in_case
PRJNA8274(	ENSGALG00010021568	TADA3	case	control	-0.07222498	0.069353221	-1.041407727	0.297686358	0.721370179	down_in_case
PRJNA8274(	ENSGALG00010021570	KATNB1	case	control	0.034984812	0.090585512	0.386207583	0.699342938	0.922288441	up_in_case
PRJNA8274(	ENSGALG00010021572	MYL9	case	control	0.069753744	0.097108	0.718310993	0.472565553	0.829235491	up_in_case
PRJNA8274(	ENSGALG00010021573	NATD1	case	control	0.030412416	0.108944551	0.279155001	0.780125875	0.948864508	up_in_case
PRJNA8274(	ENSGALG00010021574	TTLL4	case	control	0.054216754	0.075456703	0.718514741	0.472439962	0.829235491	up_in_case
PRJNA8274(	ENSGALG00010021575	ADGRL2	case	control	-0.163256045	0.044701118	-3.652169169	0.000260034	0.02149418	down_in_case
PRJNA8274(	ENSGALG00010021576	ERH	case	control	-0.047492663	0.045425188	-1.045513858	0.295785539	0.719342382	down_in_case
PRJNA8274(	ENSGALG00010021577	ARIH1	case	control	-0.046027004	0.048678848	-0.945523682	0.344391586	0.755870938	down_in_case
PRJNA8274(	ENSGALG00010021579	C20H20ORF24	case	control	0.011501689	0.045922052	0.250461132	0.802230759	0.954480394	up_in_case
PRJNA8274(	ENSGALG00010021580	KCTD10	case	control	0.033412499	0.051581018	0.647767355	0.517135433	0.848054666	up_in_case
PRJNA8274(	ENSGALG00010021582	STK36	case	control	0.032918568	0.095806319	0.34359496	0.731150916	0.933770678	up_in_case
PRJNA8274(	ENSGALG00010021583	NFE2L1	case	control	-0.044373038	0.056458609	-0.785939274	0.431903064	0.807348212	down_in_case
PRJNA8274(	ENSGALG00010021584	OVCH2	case	control	0.054478228	0.449788813	0.121119571	0.903596328	0.980997505	up_in_case
PRJNA8274(	ENSGALG00010021586	BBS4	case	control	0.000674679	0.077197602	0.008739638	0.993026867	0.998196863	up_in_case
PRJNA8274(	ENSGALG00010021588	RNF25	case	control	0.000138697	0.065991115	0.002101754	0.998323044	0.999428829	up_in_case
PRJNA8274(	ENSGALG00010021593	DCAF7	case	control	0.030365421	0.045012906	0.67459365	0.499933968	0.840669773	up_in_case
PRJNA8274(	ENSGALG00010021594	ADPGK	case	control	0.046127272	0.047777662	0.965456867	0.33431603	0.748686141	up_in_case
PRJNA8274(	ENSGALG00010021595	UBE3B	case	control	0.06801583	0.076589724	0.888054251	0.37451157	0.774434633	up_in_case
PRJNA8274(	ENSGALG00010021597	GGT7	case	control	0.118605839	0.098897986	1.199274565	0.230421202	0.654941338	up_in_case
PRJNA8274(	ENSGALG00010021599	ARPC4	case	control	0.142372189	0.052780361	2.697446293	0.006987355	0.145020586	up_in_case
PRJNA8274(	ENSGALG00010021600	FMN1	case	control	-0.060210596	0.07435725	-0.809747471	0.418085329	0.800393647	down_in_case
PRJNA8274(	ENSGALG00010021602	PLEKHG4	case	control	0.019012247	0.076667925	0.247981757	0.804148522	0.954997212	up_in_case
PRJNA8274(	ENSGALG00010021604	SPTB	case	control	-0.208327439	0.090101832	-2.312133231	0.020770342	0.258860564	down_in_case
PRJNA8274(	ENSGALG00010021605	MAP3K3	case	control	0.014641387	0.053318903	0.274600303	0.783623334	0.949518053	up_in_case
PRJNA8274(	ENSGALG00010021606	RNF8	case	control	-0.091189189	0.073638129	-1.238342011	0.215589273	0.640470741	down_in_case
PRJNA8274(	ENSGALG00010021607	SLC9A5	case	control	0.466810291	0.133368821	3.500145585	0.000465004	0.03102676	up_in_case
PRJNA8274(	ENSGALG00010021609	ENSGALG00010021609	case	control	-0.327696953	0.089146487	-3.675937926	0.000236977	0.020292055	down_in_case
PRJNA8274(	ENSGALG00010021610	TMEM11	case	control	0.135358961	0.0701486	1.929603163	0.053656027	0.37782383	up_in_case
PRJNA8274(	ENSGALG00010021611	GREM1	case	control	0.039581299	0.361635207	0.109450901	0.912844863	0.982728035	up_in_case
PRJNA8274(	ENSGALG00010021612	BCS1L	case	control	0.186102217	0.08570236	2.171494654	0.029893801	0.305970455	up_in_case
PRJNA8274(	ENSGALG00010021613	ADGRL4	case	control	-0.009170494	0.070294542	-0.130458129	0.896203979	0.980176778	down_in_case
PRJNA8274(	ENSGALG00010021615	FLVCR1	case	control	0.135963013	0.128283876	1.059860505	0.289208066	0.712801679	up_in_case
PRJNA8274(	ENSGALG00010021616	ENSGALG00010021616	case	control	2.644195257	0.69546145	3.80207308	0.00014349	0.01379195	up_in_case
PRJNA8274(	ENSGALG00010021617	SORBS1	case	control	0.157075856	0.071586494	2.194210757	0.028220262	0.299591493	up_in_case
PRJNA8274(	ENSGALG00010021618	NEO1	case	control	-0.110007356	0.078638967	-1.398891163	0.161845623	0.575570547	down_in_case
PRJNA8274(	ENSGALG00010021619	FHOD1	case	control	-0.123629575	0.106041246	-1.165863093	0.243669803	0.667780411	down_in_case
PRJNA8274(	ENSGALG00010021621	ZNF518A	case	control	-0.074989017	0.086698139	-0.864943796	0.387069638	0.782604263	down_in_case
PRJNA8274(	ENSGALG00010021622	PTHLH	case	control	-0.240691701	0.131401667	-1.831724866	0.066992422	0.419068957	down_in_case
PRJNA8274(	ENSGALG00010021623	FAR1	case	control	0.090528383	0.058166828	1.556357567	0.119623089	0.517113113	up_in_case
PRJNA8274(	ENSGALG00010021624	HSD11B2	case	control	-0.384175813	0.297243622	-1.29246108	0.196197516	0.619095929	down_in_case
PRJNA8274(	ENSGALG00010021625	HCN4	case	control	-0.297146976	0.550253811	-0.540018024	0.589184602	0.882949889	down_in_case
PRJNA8274(	ENSGALG00010021626	ZNF142	case	control	-0.06530813	0.073753661	-0.885490009	0.375892406	0.774921593	down_in_case
PRJNA8274(	ENSGALG00010021627	NCOA6	case	control	0.011992297	0.057225161	0.209563361	0.834008478	0.963078474	up_in_case
PRJNA8274(	ENSGALG00010021628	REC114	case	control	-0.159871977	0.226463306	-0.705950912	0.480218665	0.831851816	down_in_case
PRJNA8274(	ENSGALG00010021629	DHRS7B	case	control	-0.115907754	0.083579877	-1.386790205	0.165505745	0.580618526	down_in_case
PRJNA8274(	ENSGALG00010021630	VTG1	case	control	-0.072100606	0.320523	-0.224946746	0.822020703	0.959284587	down_in_case
PRJNA8274(	ENSGALG00010021632	LIMD2	case	control	0.045042161	0.110174079	0.408827203	0.682666475	0.915901011	up_in_case
PRJNA8274(	ENSGALG00010021633	CCNJ	case	control	0.125678802	0.07985872	1.573764293	0.115541952	0.512260269	up_in_case
PRJNA8274(	ENSGALG00010021634	PLCD4	case	control	-0.021166553	0.150516463	-0.140626162	0.888165279	0.977948171	down_in_case
PRJNA8274(	ENSGALG00010021635	FOXJ2	case	control	-0.052693139	0.052577428	-1.002200768	0.316246637	0.736866972	down_in_case
PRJNA8274(	ENSGALG00010021636	C1orf159	case	control	0.108135452	0.087975803	1.229149935	0.219015596	0.643443819	up_in_case
PRJNA8274(	ENSGALG00010021637	ATP6V0D1	case	control	0.096954745	0.064269164	1.508573309	0.131407859	0.534908166	up_in_case
PRJNA8274(	ENSGALG00010021638	SRC	case	control	0.052481405	0.072742063	0.721472595	0.470618796	0.829101076	up_in_case
PRJNA8274(	ENSGALG00010021639	SCG5	case	control	-0.171225895	0.237664972	-0.720450698	0.471247545	0.829101076	down_in_case
PRJNA8274(	ENSGALG00010021640	PLEKHG3	case	control	-0.042901816	0.091024345	-0.471322431	0.6374105	0.899378897	down_in_case
PRJNA8274(	ENSGALG00010021642	NPTN	case	control	-0.128948817	0.042521042	-3.032588358	0.002424661	0.081391203	down_in_case
PRJNA8274(	ENSGALG00010021643	ENSGALG00010021643	case	control	-0.119623023	0.091714521	-1.304297532	0.192132156	0.615090794	down_in_case
PRJNA8274(	ENSGALG00010021644	TARDBP	case	control	-0.005217789	0.042631584	-0.122392567	0.902588125	0.980850383	down_in_case
PRJNA8274(	ENSGALG00010021645	ASCC1	case	control	0.223802088	0.070508769	3.174102881	0.001503004	0.062118376	up_in_case
PRJNA8274(	ENSGALG00010021646	RPUSD3	case	control	-0.338312495	0.13302363	-2.543251109	0.010982627	0.185137791	down_in_case
PRJNA8274(	ENSGALG00010021647	ARHGAP11B	case	control	-0.108657896	0.110848906	-0.98023427	0.326970492	0.744857565	down_in_case

PRJNA8274(	ENSGALG00010021648	BRE	case	control	-0.094041691	0.060588091	-1.552148108	0.120626789	0.519499513	down_in_case
PRJNA8274(	ENSGALG00010021649	PXN	case	control	-0.036148596	0.057719721	-0.62627809	0.531132555	0.855560567	down_in_case
PRJNA8274(	ENSGALG00010021650	CD276	case	control	0.015347324	0.06390189	0.240170104	0.810198389	0.955972758	up_in_case
PRJNA8274(	ENSGALG00010021651	RNF113B	case	control	-0.048524701	0.079324196	-0.611726343	0.54071883	0.859428617	down_in_case
PRJNA8274(	ENSGALG00010021652	BORCS5	case	control	0.218863879	0.063087131	3.469231754	0.000521949	0.033449927	up_in_case
PRJNA8274(	ENSGALG00010021653	PTH	case	control	0.511976822	1.083443274	0.472546034			up_in_case
PRJNA8274(	ENSGALG00010021655	USP22	case	control	-0.028113585	0.041379154	-0.679414198	0.496875456	0.83878161	down_in_case
PRJNA8274(	ENSGALG00010021656	SETD6	case	control	0.232979166	0.065472362	3.558435339	0.000373071	0.026808591	up_in_case
PRJNA8274(	ENSGALG00010021657	JAGN1	case	control	0.054363609	0.106829496	0.508882013	0.610834929	0.889056885	up_in_case
PRJNA8274(	ENSGALG00010021658	STRADA	case	control	0.044273722	0.069929094	0.633123055	0.526653288	0.85251736	up_in_case
PRJNA8274(	ENSGALG00010021659	SPOCK2	case	control	-0.060997434	0.117134561	-0.520746685	0.602543248	0.885101354	down_in_case
PRJNA8274(	ENSGALG00010021660	RNF223	case	control	0.428483415	0.286789956	1.494067022	0.135158102	0.539774365	up_in_case
PRJNA8274(	ENSGALG00010021661	DUSP16	case	control	-0.072317435	0.077349043	-0.934949326	0.349814415	0.759427074	down_in_case
PRJNA8274(	ENSGALG00010021662	RASGRP1	case	control	-0.050576102	0.125708428	-0.402328647	0.687442174	0.917290381	down_in_case
PRJNA8274(	ENSGALG00010021663	CNOT9	case	control	-0.012266477	0.053350271	-0.229923425	0.818151273	0.958536226	down_in_case
PRJNA8274(	ENSGALG00010021664	TYSND1	case	control	0.003031063	0.135551221	0.022361012	0.98215998	0.996591771	up_in_case
PRJNA8274(	ENSGALG00010021665	CNOT1	case	control	-0.051685998	0.054057305	-0.956133453	0.339004815	0.751259351	down_in_case
PRJNA8274(	ENSGALG00010021666	CCDC47	case	control	-0.246199473	0.072731506	-3.385045707	0.000711664	0.039927628	down_in_case
PRJNA8274(	ENSGALG00010021667	TBX15	case	control	-0.145542032	0.114013409	-1.276534337	0.201766697	0.6249678	down_in_case
PRJNA8274(	ENSGALG00010021668	PYGO1	case	control	-0.132349761	0.110687956	-1.195701553	0.231813045	0.657201932	down_in_case
PRJNA8274(	ENSGALG00010021669	ENSGALG00010021669	case	control	0.064711316	0.047143801	1.372636795	0.169865287	0.586029275	up_in_case
PRJNA8274(	ENSGALG00010021670	BLCAP	case	control	-0.057680126	0.062479884	-0.923179153	0.355913851	0.763373775	down_in_case
PRJNA8274(	ENSGALG00010021671	TNFRSF13B	case	control	-0.342853027	0.363962213	-0.942001711	0.346191764	0.756500951	down_in_case
PRJNA8274(	ENSGALG00010021674	ING4	case	control	0.070937873	0.066838016	1.061340196	0.288535325	0.712801679	up_in_case
PRJNA8274(	ENSGALG00010021675	DDX42	case	control	-0.130283766	0.06026806	-2.161738155	0.030638365	0.308259818	down_in_case
PRJNA8274(	ENSGALG00010021676	TP53INP2	case	control	0.141718539	0.157560344	0.899455629	0.36841002	0.771770041	up_in_case
PRJNA8274(	ENSGALG00010021677	ENSGALG00010021677	case	control	-0.008067121	0.051196311	-0.157572302	0.874793831	0.975474836	down_in_case
PRJNA8274(	ENSGALG00010021678	DNAAF4	case	control	-0.11238379	0.124627323	-0.90175884	0.367184989	0.771770041	down_in_case
PRJNA8274(	ENSGALG00010021679	MASP2	case	control	1.658170486	0.593476404	2.793995641	0.005206118	0.124911893	up_in_case
PRJNA8274(	ENSGALG00010021680	PIGT	case	control	-0.046708071	0.050895701	-0.917721339	0.358764773	0.765374836	down_in_case
PRJNA8274(	ENSGALG00010021681	BTBD10	case	control	0.021111405	0.061138371	0.345305332	0.729864838	0.933715082	up_in_case
PRJNA8274(	ENSGALG00010021682	MYL4	case	control	0.145305723	0.098457885	1.475816005	0.139993293	0.542025835	up_in_case
PRJNA8274(	ENSGALG00010021683	HFM1	case	control	-0.100867822	0.276428211	-0.364896989	0.715188302	0.926862121	down_in_case
PRJNA8274(	ENSGALG00010021684	BIN1	case	control	-0.226665372	0.095219422	-2.380453137	0.01729136	0.234501759	down_in_case
PRJNA8274(	ENSGALG00010021685	DBNDD2	case	control	-0.121144627	0.145890115	-0.830382694	0.406322447	0.794253996	down_in_case
PRJNA8274(	ENSGALG00010021686	CCPG1	case	control	-0.066949872	0.086939623	-0.770073183	0.441256483	0.813083204	down_in_case
PRJNA8274(	ENSGALG00010021687	ZNF384	case	control	-0.083182527	0.044949386	-1.850582041	0.064229705	0.410955161	down_in_case
PRJNA8274(	ENSGALG00010021688	ITPR1	case	control	0.047543146	0.079759421	0.596081892	0.55112052	0.864743359	up_in_case
PRJNA8274(	ENSGALG00010021689	MPRIP	case	control	-0.079903884	0.054187775	-1.474573983	0.140327114	0.54263865	down_in_case
PRJNA8274(	ENSGALG00010021691	PIGB	case	control	-0.056263108	0.185114795	-0.303936312	0.761176413	0.942985066	down_in_case
PRJNA8274(	ENSGALG00010021693	PPA1	case	control	-0.078195418	0.051212513	-1.526881093	0.126790582	0.527026942	down_in_case
PRJNA8274(	ENSGALG00010021695	FNBP4	case	control	0.085828205	0.06275138	1.367750076	0.171390319	0.588978502	up_in_case
PRJNA8274(	ENSGALG00010021697	PIANP	case	control	0.025382036	0.15060273	0.168536358	0.866161343	0.973291467	up_in_case
PRJNA8274(	ENSGALG00010021698	GYPC	case	control	-0.154891641	0.095655229	-1.619269981	0.105389187	0.495041148	down_in_case
PRJNA8274(	ENSGALG00010021699	SYS1	case	control	-0.026532197	0.093986924	-0.282296686	0.777716026	0.947849555	down_in_case
PRJNA8274(	ENSGALG00010021700	FLCN	case	control	-0.00869415	0.058731854	-0.148031248	0.882318108	0.976801393	down_in_case
PRJNA8274(	ENSGALG00010021701	DR1	case	control	0.043593622	0.050450908	0.864080029	0.387543929	0.782604263	up_in_case
PRJNA8274(	ENSGALG00010021702	PLD6	case	control	0.005381055	0.314121156	0.017130507	0.986332502	0.996597442	up_in_case
PRJNA8274(	ENSGALG00010021703	CD82	case	control	-0.073923463	0.071190636	-1.038387455	0.299089708	0.722585372	down_in_case
PRJNA8274(	ENSGALG00010021704	COPS7A	case	control	-0.002100173	0.056237131	-0.037344959	0.970209959	0.992761697	down_in_case
PRJNA8274(	ENSGALG00010021705	CCDC18	case	control	0.288360074	0.141083907	2.043890627	0.040964353	0.337874876	up_in_case
PRJNA8274(	ENSGALG00010021706	ENSGALG00010021706	case	control	-0.122248723	0.141715226	-0.862636473	0.38833737	0.783067156	down_in_case
PRJNA8274(	ENSGALG00010021707	DLX2	case	control	2.488440194	0.676588143	3.677924629	0.000235139	0.020255986	up_in_case
PRJNA8274(	ENSGALG00010021708	CNTNAP5	case	control	-0.369046705	0.251300118	-1.468549668	0.141954975	0.543501954	down_in_case
PRJNA8274(	ENSGALG00010021709	DLX1	case	control	0.674044281	0.56435196	1.194368637	0.232333799	0.657912487	up_in_case
PRJNA8274(	ENSGALG00010021710	ENSGALG00010021710	case	control	0.668805554	0.233918476	2.859139493	0.004247919	0.112636686	up_in_case
PRJNA8274(	ENSGALG00010021711	ENSGALG00010021711	case	control	0.238612095	0.189756129	1.257467126	0.208584536	0.633434867	up_in_case
PRJNA8274(	ENSGALG00010021713	RAB27A	case	control	0.322914251	0.071651235	4.506750667	6.58279E-06	0.001255119	up_in_case
PRJNA8274(	ENSGALG00010021714	TSPAN18	case	control	-0.043890346	0.092144672	-0.476319954	0.633846452	0.898107199	down_in_case
PRJNA8274(	ENSGALG00010021715	MMAB	case	control	-0.097013246	0.105134135	-0.922756876	0.356133918	0.763373775	down_in_case
PRJNA8274(	ENSGALG00010021716	TFCP2L1	case	control	0.343591476	0.285982161	1.201443735	0.22957912	0.653695091	up_in_case
PRJNA8274(	ENSGALG00010021717	SDC4	case	control	-0.256330854	0.101198778	-2.532944169	0.011310897	0.186343114	down_in_case
PRJNA8274(	ENSGALG00010021718	SST7	case	control	0.00387698	0.38526405	0.010063177	0.991970882	0.99790247	up_in_case
PRJNA8274(	ENSGALG00010021719	BHLHE40	case	control	0.022898935	0.089028066	0.257210299	0.797016434	0.95280257	up_in_case
PRJNA8274(	ENSGALG00010021720	CACNB1	case	control	-0.187305659	0.090324737	-2.073691726	0.03810795	0.330268901	down_in_case
PRJNA8274(	ENSGALG00010021721	TP53I11	case	control	-0.05085986	0.138062079	-0.368383996	0.712586929	0.926808752	down_in_case
PRJNA8274(	ENSGALG00010021722	AARS1	case	control	-0.091589179	0.056348194	-1.625414644	0.104074238	0.493794905	down_in_case
PRJNA8274(	ENSGALG00010021723	COPS3	case	control	0.130750002	0.050540001	2.587059725	0.009679881	0.176109798	up_in_case
PRJNA8274(	ENSGALG00010021725	SH2D6	case	control	-0.090306152	0.10958722	-0.82405733	0.409906984	0.795881856	down_in_case
PRJNA8274(	ENSGALG00010021726	ARNTL	case	control	-0.006795518	0.051978889	-0.130736114	0.895984063	0.980176778	down_in_case
PRJNA8274(	ENSGALG00010021727	MVK	case	control	0.024300842	0.062924549	0.386190167	0.699355835	0.922288441	up_in_case
PRJNA8274(	ENSGALG00010021728	TMED5	case	control	-0.122214325	0.067581919	-1.808387899	0.07054615	0.429463578	down_in_case
PRJNA8274(	ENSGALG00010021729	RBPJL	case	control	0.458756759	0.460607861	0.995981175	0.319259292	0.73861962	up_in_case
PRJNA8274(	ENSGALG00010021730	ARL8B	case	control	-0.074689604	0.042623318	-1.752317911	0.079719158	0.450131333	down_in_case
PRJNA8274(	ENSGALG00010021731	SRM	case	control	0.169073286	0.068383052	2.472444268	0.013419264	0.203494665	up_in_case
PRJNA8274(	ENSGALG00010021732	CASR	case	control	0.162138265	0.232266468	0.69807005	0.485133387	0.834423421	up_in_case
PRJNA8274(	ENSGALG00010021733	ENSGALG00010021733	case	control	-0.020044841	0.150485983	-0.133200719	0.894034642	0.979888889	down_in_case
PRJNA8274(	ENSGALG00010021734	PARD3B	case	control	-0.108406094	0.090502489	-1.197824454	0.230985365	0.655505203	down_in_case
PRJNA8274(	ENSGALG00010021735	PIGBOS1	case	control	-0.114672017	0.105817337	-1.083678918	0.278507186	0.703953892	down_in_case
PRJNA8274(	ENSGALG00010021736	EDEM1	case	control	0.088076686	0.052516198	1.6771337	0.09351634	0.478523919	up_in_case
PRJNA8274(	ENSGALG00010021738	PRDM11	case	control	-0.030086795	0.039650404	-0.758801731	0.44797117	0.818681142	down_in_case
PRJNA8274(	ENSGALG00010021739	EXOSC10	case	control	-0.026112636	0.051694533	-0.505133408	0.613465138	0.890252839	down_in_case
PRJNA8274(	ENSGALG00010021740	PDLIM1	case	control	0.00379809	0.124028948	0.030622606	0.975570514	0.99399062	up_in_case
PRJNA8274(	ENSGALG00010021741	RPL19	case	control	-0.05858459	0.069173608	-0.846921126	0.397039086	0.788531716	down_in_case
PRJNA8274(	ENSGALG00010021742	MATN4	case	control	0.437243731	0.44441013	0.983874356	0.325177294	0.743110335	up_in_case
PRJNA8274(	ENSGALG00010021743	SYT13	case	control	-0.685517469	0.228624547	-2.998442108	0.002713637	0.087009711	down_in_case
PRJNA8274(	ENSGALG00010021744	LITAF	case	control	0.165511478	0.239922933	0.689852681	0.490286835	0.836347578	up_in_case
PRJNA8274(	ENSGALG00010021745	RSL24D1	case	control	-0.122465151	0.065312709	-1.875058521	0.060784673	0.400562592	down_in_case
PRJNA8274(	ENSGALG00010021746	MTOR	case	control	-0.12047921	0.070110757	-1.71841262	0.08572138	0.462802693	down_in_case
PRJNA8274(	ENSGALG00010021747	AKTIP	case	control	0.10335367	0.066102643	1.563533087	0.11792726	0.514604253	up_in_case
PRJNA8274(	ENSGALG00010021748	CDCA7	case	control	-0.046317826	0.084255683	-0.549729399	0.582504989	0.880810124	down_in_case
PRJNA8274(	ENSGALG00010021749	CLASP1	case	control	-0.121681307	0.059823816	-2.033994412	0.041952159	0.341443296	down_in_case
PRJNA8274(	ENSGALG00010021750	HELLS	case	control	-0.114176602	0.084700579	-1.348002619	0.17765754	0.59664228	down_in_case
PRJNA8274(	ENSGALG00010021751	MTCH2	case	control	-0.030380374	0.063947997	-0.475079367	0.634730405	0.898107199	down_in_case
PRJNA8274(	ENSGALG00010021752	CSTB	case	control	-0.23769653	0.084548398	-2.81136646	0.004933156	0.120231831	down_in_case
PRJNA8274(	ENSGALG00010021753	MTF2	case	control	0.072219459	0.056804392	1.271371045	0.203596678	0.62581674	up_in_case
PRJNA8274(	ENSGALG00010021754	SLC17A9	case	control	0.709509072	0.476772484	1.488150211	0.136711284	0.539854583	up_in_case
PRJNA8274(	ENSGALG00010021755	ENSGALG00010021755	case	control	-0.155552928	0.094400466	-1.647798301	0.099394068	0.488584926	down_in_case
PRJNA8274(	ENSGALG00010021756	USP42	case	control	0.172341557	0.086242741	1.998331175	0.045680768	0.355019011	up_in_case
PRJNA8274(	ENSGALG00010021757	ZP4	case	control	0.399946589	0.330041513	1.21180692	0.225586299	0.649491335	up_in_case
PRJNA8274(	ENSGALG00010021758	RASSF10	case	control	0.24949923	0.38065309	0.655450425	0.512177795	0.845834144	up_in_case
PRJNA8274(	ENSGALG00010021759	NIFK	case	control	0.037848105	0.050370983	0.751387052	0.452419753	0.820911365	up_in_case
PRJNA8274(	ENSGALG00010021760	CSTA	case	control	-0.315059243	0.283730411	-1.110417605	0.266819115	0.692847894	down_in_case
PRJNA8274(	ENSGALG00010021761	MAFB	case	control	-0.065717304	0.087169298	-0.753904243	0.450906722	0.819934655	down_in_case
PRJNA8274(	ENSGALG00010021762	LAMB2	case	control	-0.215759341	0.124607677	-1.731509212	0.083360983	0.457254339	down_in_case
PRJNA8274(	ENSGALG00010021764	FAM222A	case	control	0.122889258	0.461485894	0.266290388	0.790015575	0.951843754	up_in_case
PRJNA8274(	ENSGALG00010021765	EIF2AK1	case	control	0.091194546	0.061102671	1.492480503	0.135573225	0.539774365	up_in_case
PRJNA8274(	ENSGALG00010021766	AGRN	case	control	-0.106796139	0.090915897	-1.174669584	0.240126933	0.665210852	down_in_case
PRJNA8274(	ENSGALG00010021767	CCDC58	case	control	-0.049428451	0.099102258	-0.498762112	0.617946982	0.891419534	down_in_case
PRJNA8274(	ENSGALG00010021768	DIPK1A	case	control	0.023765089	0.109003514	0.218021312	0.827412506	0.961319258	up_in_case
PRJNA8274(	ENSGALG00010021769	ENSGALG00010021769	case	control	-0.050484157	0.08282969	-0.60949349	0.542197386	0.85975913	down_in_case
PRJNA8274(	ENSGALG00010021770	RALGAPB	case	control	-0.015389996	0.039609771	-0.388540391	0.69761617	0.922288441	down_in_case
PRJNA8274(	ENSGALG00010021771	ANKRD61	case	control	0.098828255	0.070383346	1.404142617	0.160274385	0.572752253	up_in_case
PRJNA8274(	ENSGALG00010021772	TSN	case	control	-0.04143165	0.049284792	-0.84065789	0.400539618	0.790138851	down_in_case
PRJNA8274(	ENSGALG00010021773	ENSGALG00010021773	case	control	0.107945073	0.776632728	0.138991146	0.889457146	0.978101906	up_in_case
PRJNA8274(	ENSGALG00010021774	FBXL20	case	control	-0.154713	0.062124721	-2.490361269	0.012761331	0.199334349	down_in_case
PRJNA8274(	ENSGALG00010021775	CCDC36	case	control	0.209741894	0.283289273	0.740380643	0.459069061	0.824191785	up_in_case
PRJNA8274(	ENSGALG00010021776	PMS2	case	control	0.015483697	0.084481081	0.183280058	0.854578281	0.970313652	up_in_case
PRJNA8274(	ENSGALG00010021778	RBL2	case	control	-0.118167004	0.040807217	-2.895737853	0.003782682	0.105649139	down_in_case
PRJNA8274(	ENSGALG00010021779	UNC13C	case	control	0.090131697	0.202169767	0.445821839	0.655725945	0.907453191	up_in_case
PRJNA8274(	ENSGALG00010021781	RAPGEF4	case	control	0.038057531	0.246343624	0.154489614	0.8772237	0.975813349	up_in_case
PRJNA8274(	ENSGALG00010021782	ADAM11	case	control	-0.299881489	0.213854493	-1.402268824	0.160834986	0.573265281	down_in_case
PRJNA8274(	ENSGALG00010021783	GIT2	case	control	-0.066660092	0.041278239	-1.614896681	0.106333071	0.496267271	down_in_case
PRJNA8274(	ENSGALG00010021784	ANGPTL7	case	control	1.128711104	0.478610083	2.35831033	0.018358339	0.242045801	up_in_case
PRJNA8274(	ENSGALG00010021785	CCDC71	case	control	0.014989333	0.041970292	0.357141504	0.720985875	0.92919613	up_in_case
PRJNA8274(	ENSGALG00010021786	FAM162A	case	control	0.060974845	0.12141329	0.502208983	0.615520525	0.891081782	up_in_case

PRJNA8274(	ENSGALG00010021788	RPL5	case	control	-0.041262392	0.06103522	-0.676042328	0.499013771	0.840142058	down_in_case
PRJNA8274(	ENSGALG00010021789	CHD9	case	control	-0.021193864	0.050802069	-0.41718506	0.676543052	0.914247368	down_in_case
PRJNA8274(	ENSGALG00010021790	PHF21A	case	control	-0.080551093	0.049967588	-1.612066879	0.106947387	0.497919928	down_in_case
PRJNA8274(	ENSGALG00010021791	PLEKHM2	case	control	-0.141120691	0.084879535	-1.662599712	0.096392589	0.483654042	down_in_case
PRJNA8274(	ENSGALG00010021792	KLHDC8B	case	control	0.03934811	0.175811204	0.223808885	0.822906016	0.959776917	up_in_case
PRJNA8274(	ENSGALG00010021793	ENSGALG00010021793	case	control	-0.321812893	0.134655852	-2.389891624	0.016853345	0.230991919	down_in_case
PRJNA8274(	ENSGALG00010021794	ENSGALG00010021794	case	control	-0.045111477	0.128479414	-0.351118329	0.725499578	0.931921256	down_in_case
PRJNA8274(	ENSGALG00010021795	NDRG3	case	control	-0.112308313	0.063762565	-1.761351874	0.078178865	0.448561389	down_in_case
PRJNA8274(	ENSGALG00010021797	SLC25A34	case	control	0.087073178	0.133624297	0.65162684	0.514641929	0.847493921	up_in_case
PRJNA8274(	ENSGALG00010021798	RANBP10	case	control	-0.082629553	0.069233105	-1.193497724	0.232674503	0.657934961	down_in_case
PRJNA8274(	ENSGALG00010021799	ENSGALG00010021799	case	control	0.367797983	0.145368471	2.53010835	0.011402731	0.186780127	up_in_case
PRJNA8274(	ENSGALG00010021800	TMEM82	case	control	0.126216894	0.298748321	0.422485702	0.672670535	0.913676733	up_in_case
PRJNA8274(	ENSGALG00010021801	VAMP1	case	control	0.003094059	0.202415611	0.015285675	0.987804271	0.996942188	up_in_case
PRJNA8274(	ENSGALG00010021803	TLL2	case	control	0.002975636	0.118939387	0.025018085	0.980040538	0.995636523	up_in_case
PRJNA8274(	ENSGALG00010021805	FUT8	case	control	0.040026646	0.09130725	0.438373136	0.661115817	0.909646511	up_in_case
PRJNA8274(	ENSGALG00010021807	CDC42	case	control	-0.100040163	0.031439869	-3.181952335	0.001462859	0.060810705	down_in_case
PRJNA8274(	ENSGALG00010021810	ANKRD13A	case	control	0.020642031	0.048499908	0.4256097	0.670392271	0.913465866	up_in_case
PRJNA8274(	ENSGALG00010021812	TEAD1	case	control	-0.209732394	0.059039791	-3.552390498	0.000381748	0.027024726	down_in_case
PRJNA8274(	ENSGALG00010021817	EVI5	case	control	-0.00766467	0.06795358	-0.112792726	0.910194885	0.981730793	down_in_case
PRJNA8274(	ENSGALG00010021821	PPP1R19L	case	control	-0.025596794	0.072718742	-0.351997208	0.724840356	0.931621166	down_in_case
PRJNA8274(	ENSGALG00010021823	ENSGALG00010021823	case	control	-0.071086465	0.196179198	-0.362354753	0.717086947	0.926862121	down_in_case
PRJNA8274(	ENSGALG00010021826	FBXO40	case	control	-0.151293696	0.174205164	-0.868479973	0.385131631	0.781521545	down_in_case
PRJNA8274(	ENSGALG00010021827	CCZ1	case	control	0.014606518	0.046408263	0.314739592	0.752959381	0.940666434	up_in_case
PRJNA8274(	ENSGALG00010021829	ERCC6	case	control	0.05175765	0.05151365	1.00473661	0.315023695	0.736508113	up_in_case
PRJNA8274(	ENSGALG00010021830	ENSGALG00010021830	case	control	1.998596976	1.855818047	1.076935845			up_in_case
PRJNA8274(	ENSGALG00010021831	ENSGALG00010021831	case	control	-0.117742892	0.337639618	-0.348723567	0.72729685	0.932933706	down_in_case
PRJNA8274(	ENSGALG00010021835	NUDT8	case	control	-0.142342041	0.120119338	-1.185005201	0.236015391	0.660642087	down_in_case
PRJNA8274(	ENSGALG00010021837	ENSGALG00010021837	case	control	-0.144367341	0.321479343	-0.449071906	0.653379786	0.906151774	down_in_case
PRJNA8274(	ENSGALG00010021839	C1orf50	case	control	-0.007878758	0.091870385	-0.085759501	0.931657602	0.987378915	down_in_case
PRJNA8274(	ENSGALG00010021840	ENSGALG00010021840	case	control	-0.076482181	0.301424629	-0.253735671	0.799699774	0.954053991	down_in_case
PRJNA8274(	ENSGALG00010021841	CENPT	case	control	-0.081232116	0.093227744	-0.871329845	0.383574079	0.780061953	down_in_case
PRJNA8274(	ENSGALG00010021842	ENSGALG00010021842	case	control	0.099672728	0.142888151	0.697557685	0.485453852	0.834423421	up_in_case
PRJNA8274(	ENSGALG00010021843	ENSGALG00010021843	case	control	-0.089771548	0.290593019	-0.308925345	0.757378317	0.941659745	down_in_case
PRJNA8274(	ENSGALG00010021847	P3H1	case	control	0.197737273	0.079419816	2.489772481	0.012782489	0.199334349	up_in_case
PRJNA8274(	ENSGALG00010021848	LARGE2	case	control	-0.108322865	0.104395764	-1.037617437	0.299448197	0.722840853	down_in_case
PRJNA8274(	ENSGALG00010021851	ENSGALG00010021851	case	control	-0.061460519	0.250158183	-0.245686624	0.805924828	0.9556344	down_in_case
PRJNA8274(	ENSGALG00010021854	TAPBPL	case	control	0.051462605	0.10858028	0.473959035	0.63552912	0.898681129	up_in_case
PRJNA8274(	ENSGALG00010021860	ENSGALG00010021860	case	control	0.362015864	0.190894245	1.896421045	0.057904391	0.391912561	up_in_case
PRJNA8274(	ENSGALG00010021862	PDK1	case	control	-0.050198318	0.050433581	-0.995335193	0.319573267	0.738813491	down_in_case
PRJNA8274(	ENSGALG00010021863	CLDN19	case	control	-0.052734248	0.228779494	-0.230502514	0.81770131	0.95838887	down_in_case
PRJNA8274(	ENSGALG00010021866	SCNN1A	case	control	0.012651545	0.222418237	0.056881778	0.95463937	0.992056045	up_in_case
PRJNA8274(	ENSGALG00010021867	CXCL12	case	control	0.01137037	0.119158967	0.095421856	0.923979757	0.985571367	up_in_case
PRJNA8274(	ENSGALG00010021869	GJC3	case	control	-0.04128185	0.074064537	-0.557376733	0.577270059	0.878143389	down_in_case
PRJNA8274(	ENSGALG00010021870	ENSGALG00010021870	case	control	-0.125193592	0.209594512	-0.597313309	0.55029822	0.86456492	down_in_case
PRJNA8274(	ENSGALG00010021871	ENSGALG00010021871	case	control	-0.178449496	0.135391097	-1.318029765	0.187493684	0.610946377	down_in_case
PRJNA8274(	ENSGALG00010021874	TMCC1	case	control	-0.039603609	0.069118724	-0.572979455	0.566658605	0.873285704	down_in_case
PRJNA8274(	ENSGALG00010021877	PLEKHN1	case	control	-0.270783441	0.122634337	-2.20805565	0.027240394	0.293158316	down_in_case
PRJNA8274(	ENSGALG00010021879	CREBL2	case	control	-0.026003584	0.088961867	-0.292300343	0.770056997	0.945428692	down_in_case
PRJNA8274(	ENSGALG00010021881	PHF20	case	control	-0.032399534	0.037139923	-0.872364082	0.383009789	0.780061953	down_in_case
PRJNA8274(	ENSGALG00010021886	ENSGALG00010021886	case	control	0.668693031	0.882395217	0.757815793	0.448561266	0.818701429	up_in_case
PRJNA8274(	ENSGALG00010021887	NUTF2	case	control	0.001987001	0.044149897	0.045005784	0.964102699	0.992068146	up_in_case
PRJNA8274(	ENSGALG00010021892	NDUFV1	case	control	-0.043665976	0.070568769	-0.618771968	0.536066592	0.857238203	down_in_case
PRJNA8274(	ENSGALG00010021893	FAM43B	case	control	0.384365297	0.27035532	1.421704209	0.155112138	0.563829072	up_in_case
PRJNA8274(	ENSGALG00010021897	ENSGALG00010021897	case	control	0.132016626	0.140883478	0.937062512	0.348726404	0.759193168	up_in_case
PRJNA8274(	ENSGALG00010021902	LMTK2	case	control	0.0154936	0.04338522	0.357117006	0.721004214	0.92919613	up_in_case
PRJNA8274(	ENSGALG00010021903	MORC2	case	control	-0.033728875	0.045813076	-0.736228128	0.461591877	0.824683138	down_in_case
PRJNA8274(	ENSGALG00010021905	RBSN	case	control	-0.001911819	0.042658759	-0.044816565	0.964253522	0.992068146	down_in_case
PRJNA8274(	ENSGALG00010021908	EDC4	case	control	-0.049202101	0.06013003	-0.818261711	0.413207765	0.797633779	down_in_case
PRJNA8274(	ENSGALG00010021913	ENSGALG00010021913	case	control	-0.122643838	0.121395863	-1.010280213	0.312361058	0.734364975	down_in_case
PRJNA8274(	ENSGALG00010021916	MRPS25	case	control	-0.184538198	0.063571304	-2.90285377	0.003697793	0.105126109	down_in_case
PRJNA8274(	ENSGALG00010021919	PARVA	case	control	-0.011265385	0.085651955	-0.131525135	0.895359904	0.980176778	down_in_case
PRJNA8274(	ENSGALG00010021922	MRPL51	case	control	-0.035238394	0.06942525	-0.507573172	0.611752709	0.889602558	down_in_case
PRJNA8274(	ENSGALG00010021924	ENSGALG00010021924	case	control	0.842931391	0.610491326	1.380742617	0.167358113	0.583570108	up_in_case
PRJNA8274(	ENSGALG00010021927	ENSGALG00010021927	case	control	0.339146057	0.535026039	0.633887012	0.526154563	0.85251736	up_in_case
PRJNA8274(	ENSGALG00010021931	ITGA6	case	control	-0.015130422	0.078854795	-0.191877011	0.847838545	0.968313864	down_in_case
PRJNA8274(	ENSGALG00010021932	RPAP2	case	control	-0.107087466	0.05397692	-1.983949174	0.047261506	0.358156924	down_in_case
PRJNA8274(	ENSGALG00010021933	PRR14L	case	control	0.004136094	0.061478052	0.067277578	0.946360727	0.990554706	up_in_case
PRJNA8274(	ENSGALG00010021934	EFTUD2	case	control	0.031293164	0.051905602	0.602886053	0.546584495	0.86246958	up_in_case
PRJNA8274(	ENSGALG00010021935	CETP	case	control	0.13912369	0.207783763	0.66955997	0.503138339	0.842485272	up_in_case
PRJNA8274(	ENSGALG00010021938	CABP2	case	control	0.443962021	0.1384736	3.206113086	0.001345411	0.058257076	up_in_case
PRJNA8274(	ENSGALG00010021943	OCM3	case	control	-0.240702541	0.240399934	-1.001258766	0.316701722	0.737355036	down_in_case
PRJNA8274(	ENSGALG00010021945	C1QTNF4	case	control	-0.024456618	0.133668741	-0.182964375	0.854825972	0.970313652	down_in_case
PRJNA8274(	ENSGALG00010021947	CDK2AP2	case	control	-0.055872226	0.108869632	-0.513203045	0.607809296	0.887448737	down_in_case
PRJNA8274(	ENSGALG00010021949	ENSGALG00010021949	case	control	-0.172087557	0.216244296	-0.795801603	0.42614736	0.805113918	down_in_case
PRJNA8274(	ENSGALG00010021951	PSKH1	case	control	-0.044626249	0.062903769	-0.709436799	0.478053459	0.830520323	down_in_case
PRJNA8274(	ENSGALG00010021953	PITPNM1	case	control	0.036109186	0.100766112	0.358346525	0.720084004	0.92895404	up_in_case
PRJNA8274(	ENSGALG00010021954	ENSGALG00010021954	case	control	0.172134474	1.783261292	0.096527903	0.923101314	0.985482967	up_in_case
PRJNA8274(	ENSGALG00010021958	BHLHA15	case	control	-0.39710927	0.324105968	-1.225245163	0.220482863	0.644724628	down_in_case
PRJNA8274(	ENSGALG00010021960	CDKN1B	case	control	0.087701984	0.062191629	1.41018953	0.158483727	0.570582225	up_in_case
PRJNA8274(	ENSGALG00010021965	PEX16	case	control	-0.083799136	0.064825321	-1.292691405	0.196117811	0.619095929	down_in_case
PRJNA8274(	ENSGALG00010021966	ADGRA1	case	control	-0.038422098	0.090552896	-0.424305568	0.671342979	0.913676733	down_in_case
PRJNA8274(	ENSGALG00010021969	CCDC103	case	control	-0.154704104	0.278725631	-0.555040826	0.578866736	0.879142433	down_in_case
PRJNA8274(	ENSGALG00010021970	ERGIC3	case	control	0.018011084	0.0485771	0.37077314	0.710806509	0.925900262	up_in_case
PRJNA8274(	ENSGALG00010021971	ICOS	case	control	0.447885441	0.428074908	1.046278193	0.295432611	0.718955697	up_in_case
PRJNA8274(	ENSGALG00010021972	PISD	case	control	-0.02442622	0.048571951	-0.502887368	0.615043463	0.890915875	down_in_case
PRJNA8274(	ENSGALG00010021974	GFAP	case	control	-0.69990854	0.399345446	-1.752639342	0.079663934	0.450131333	down_in_case
PRJNA8274(	ENSGALG00010021978	NR2C2	case	control	-0.027469803	0.059549219	-0.461295771	0.644586419	0.902239118	down_in_case
PRJNA8274(	ENSGALG00010021980	GLMN	case	control	0.001966275	0.099572608	0.019747152	0.984245076	0.996591771	up_in_case
PRJNA8274(	ENSGALG00010021983	CTLA4	case	control	1.064082589	0.741713608	1.434627296	0.151393332	0.559493525	up_in_case
PRJNA8274(	ENSGALG00010021988	FAM180B	case	control	-0.223143734	0.201951165	-1.10493908	0.269185987	0.696212628	down_in_case
PRJNA8274(	ENSGALG00010021989	NCAPD2	case	control	0.011408068	0.076994055	0.148168165	0.882210055	0.976801393	up_in_case
PRJNA8274(	ENSGALG00010021994	ENSGALG00010021994	case	control	0.465189196	0.175637916	2.648569326	0.008083327	0.157792878	up_in_case
PRJNA8274(	ENSGALG00010021999	ANKRD22	case	control	0.105894552	0.416413648	0.254301347	0.799262759	0.954053991	up_in_case
PRJNA8274(	ENSGALG00010022002	KIF18B	case	control	-0.143608434	0.095235701	-1.507926467	0.131573348	0.535159035	down_in_case
PRJNA8274(	ENSGALG00010022005	NLRC5	case	control	0.034953668	0.143692527	0.243253207	0.807809251	0.955875648	up_in_case
PRJNA8274(	ENSGALG00010022006	SFI1	case	control	0.043147478	0.103018018	0.418834282	0.675337249	0.914247368	up_in_case
PRJNA8274(	ENSGALG00010022019	BTBD8	case	control	0.033539795	0.129826655	0.258342902	0.796142278	0.95280257	up_in_case
PRJNA8274(	ENSGALG00010022021	TECPR1	case	control	-0.075136414	0.05411378	-1.388489489	0.164988043	0.580021198	down_in_case
PRJNA8274(	ENSGALG00010022022	STAMBPL1	case	control	0.248429426	0.194237649	1.278997288	0.200898011	0.623527532	up_in_case
PRJNA8274(	ENSGALG00010022024	MICAL2	case	control	-0.133130875	0.128039976	-1.03976023	0.29845131	0.721501478	down_in_case
PRJNA8274(	ENSGALG00010022027	EIF4ENIF1	case	control	-0.021930385	0.041744316	-0.525350201	0.599339765	0.884888453	down_in_case
PRJNA8274(	ENSGALG00010022028	FGD5	case	control	0.09857457	0.095119453	1.036323988	0.300051019	0.723279006	up_in_case
PRJNA8274(	ENSGALG00010022029	ENSGALG00010022029	case	control	1.488377514	0.473527621	3.143169367	0.001671291	0.065655176	up_in_case
PRJNA8274(	ENSGALG00010022030	ZNF341	case	control	0.083629945	0.08168396	1.023823343	0.305918723	0.728933908	up_in_case
PRJNA8274(	ENSGALG00010022031	ENSGALG00010022031	case	control	0.846543939	1.195861834	0.707894437	0.479010815	0.831333501	up_in_case
PRJNA8274(	ENSGALG00010022032	KBTBD4	case	control	-0.033494805	0.04820894	-0.694784103	0.487190612	0.835461642	down_in_case
PRJNA8274(	ENSGALG00010022033	RAPH1	case	control	-0.092997398	0.071159355	-1.30688928	0.191250317	0.61430358	down_in_case
PRJNA8274(	ENSGALG00010022034	ENSGALG00010022034	case	control	-0.099317508	0.080786898	-1.229376415	0.218930709	0.643443819	down_in_case
PRJNA8274(	ENSGALG00010022037	DRG1	case	control	0.013846079	0.058079313	0.238399493	0.811571261	0.956205737	up_in_case
PRJNA8274(	ENSGALG00010022038	GAPDH	case	control	-0.22904723	0.112938801	-2.028064994	0.042553615	0.344353525	down_in_case
PRJNA8274(	ENSGALG00010022041	MAPK8IP1	case	control	-0.098551625	0.100406546	-0.981525905	0.326333466	0.744287835	down_in_case
PRJNA8274(	ENSGALG00010022042	AIP	case	control	0.110027485	0.068529449	1.605550405	0.10837273	0.500478515	up_in_case
PRJNA8274(	ENSGALG00010022044	PXMP4	case	control	0.036625216	0.055110716	0.664575202	0.506322235	0.843280685	up_in_case
PRJNA8274(	ENSGALG00010022045	ENSGALG00010022045	case	control	0.963566722	1.356491826	0.710337286	0.477495003	0.830520323	up_in_case
PRJNA8274(	ENSGALG00010022046	CRY2	case	control	-0.116967613	0.06944391	-1.684346579	0.092114708	0.476713154	down_in_case
PRJNA8274(	ENSGALG00010022049	EPHX4	case	control	1.356164253	0.403132798	3.364063302	0.000768039	0.041919699	up_in_case
PRJNA8274(	ENSGALG00010022050	ENSGALG00010022050	case	control	0.269688127	0.210646188	1.280289618	0.200443298	0.622981777	up_in_case
PRJNA8274(	ENSGALG00010022051	BAIAP2L1	case	control	0.520891111	0.128250929	4.061499712	4.87585E-05	0.006577795	up_in_case
PRJNA8274(	ENSGALG00010022052	IFFO1	case	control	0.021639721	0.059436916	0.364078796	0.71579917	0.926862121	up_in_case
PRJNA8274(	ENSGALG00010022054	ENSGALG00010022054	case	control	0.329710012	0.383969543	0.858687927	0.390512713	0.783985932	up_in_case
PRJNA8274(	ENSGALG00010022055	E2F1	case	control	0.043882065	0.101795113	0.431082234	0.666408578	0.912284937	up_in_case
PRJNA8274(	ENSGALG00010022058	NLRP3	case	control	0.067677717	0.090773672	0.745565491	0.455929936	0.822065071	up_in_case
PRJNA8274(	ENSGALG00010022059	NPTX2	case	control	-0.192260187	0.116525505	-1.649940818	0.098955041	0.488407564	down_in_case
PRJNA8274(	ENSGALG00010022062	MITF	case	control	0.139362926	0.087275505	1.596816038	0.11030672	0.502993014	up_in_case
PRJNA8274(	ENSGALG00010022065	PABPC1L	case	control	0.060588729	0.139188178	0.435300827	0.663344085	0.911044469	up_in_case
PRJNA8274(	ENSGALG00010022066	DAD1	case	control	0.081096112	0.051368062	1.578726326	0.114398844	0.509446069	up_in_case

PRJNA8274(	ENSGALG00010022067	TMEM130	case	control	-0.018973112	0.1334693	-0.142153376	0.886958858	0.977630122	down_in_case
PRJNA8274(	ENSGALG00010022069	NOP2	case	control	-0.005726582	0.063699332	-0.089900192	0.928366529	0.986776285	down_in_case
PRJNA8274(	ENSGALG00010022071	ACTA2	case	control	0.067190957	0.114240237	0.588154916	0.556428315	0.867878646	up_in_case
PRJNA8274(	ENSGALG00010022072	SMTN	case	control	-0.177938738	0.104764201	-1.698468907	0.089419296	0.47258594	down_in_case
PRJNA8274(	ENSGALG00010022073	PTPMT1	case	control	-0.152941077	0.095356209	-1.603892159	0.108737825	0.500951275	down_in_case
PRJNA8274(	ENSGALG00010022075	ENSGALG00010022075	case	control	0.332057231	2.816538364	0.117895511	0.90615045	0.981730793	up_in_case
PRJNA8274(	ENSGALG00010022076	YWHAB	case	control	-0.056022948	0.038402653	-1.458830123	0.144611869	0.548818931	down_in_case
PRJNA8274(	ENSGALG00010022079	ABI2	case	control	0.002725912	0.055533627	0.049085782	0.960850934	0.992056045	up_in_case
PRJNA8274(	ENSGALG00010022081	FAS	case	control	-0.213845804	0.174151578	-1.227929179	0.219473555	0.643443819	down_in_case
PRJNA8274(	ENSGALG00010022082	TGFBR3	case	control	-0.139067063	0.099580339	-1.396531324	0.162554555	0.576163465	down_in_case
PRJNA8274(	ENSGALG00010022084	SELENOM	case	control	0.203132864	0.093017039	2.183824242	0.028975166	0.302591418	up_in_case
PRJNA8274(	ENSGALG00010022085	MLLT6	case	control	0.065551975	0.063676177	1.029458387	0.303264325	0.725925651	up_in_case
PRJNA8274(	ENSGALG00010022087	SLC35C1	case	control	0.009793343	0.059343941	0.165026832	0.868922863	0.974556622	up_in_case
PRJNA8274(	ENSGALG00010022088	DKK3	case	control	-0.142128095	0.131814595	-1.078242479	0.280925559	0.705762606	down_in_case
PRJNA8274(	ENSGALG00010022090	TRRAP	case	control	-0.092672277	0.048995139	-1.891458575	0.058563156	0.394466853	down_in_case
PRJNA8274(	ENSGALG00010022093	CHD4	case	control	-0.042240904	0.042728424	-0.988590259	0.322863654	0.740689923	down_in_case
PRJNA8274(	ENSGALG00010022097	INPP5J	case	control	-0.249609503	0.165013638	-1.512659837	0.130366086	0.532941383	down_in_case
PRJNA8274(	ENSGALG00010022098	CDC7	case	control	0.028770331	0.103801538	0.277166713	0.781652098	0.949394183	up_in_case
PRJNA8274(	ENSGALG00010022100	ENTPD1	case	control	-0.015650039	0.126497168	-0.123718489	0.901538172	0.980678218	down_in_case
PRJNA8274(	ENSGALG00010022101	ENSGALG00010022101	case	control	-0.170384772	0.149300726	-1.141218647	0.253778946	0.679468065	down_in_case
PRJNA8274(	ENSGALG00010022104	EXOC3L1	case	control	0.053599043	0.110203588	0.486363862	0.626709208	0.895656773	up_in_case
PRJNA8274(	ENSGALG00010022105	DLG5	case	control	0.091275399	0.044931266	2.031445089	0.042209864	0.342955148	up_in_case
PRJNA8274(	ENSGALG00010022108	USP47	case	control	-0.042403342	0.042765643	-0.991528235	0.321427712	0.73942679	down_in_case
PRJNA8274(	ENSGALG00010022111	PCGF2	case	control	-0.075047441	0.088211651	-0.850765641	0.394899551	0.786978937	down_in_case
PRJNA8274(	ENSGALG00010022115	E2F4	case	control	-0.050596284	0.052342354	-0.966641355	0.333723353	0.748508541	down_in_case
PRJNA8274(	ENSGALG00010022116	CYP20A1	case	control	-0.033654178	0.068361226	-0.492299219	0.622507832	0.893944471	down_in_case
PRJNA8274(	ENSGALG00010022117	FAM20C	case	control	-0.150020687	0.07858197	-1.909098061	0.056249443	0.385984934	down_in_case
PRJNA8274(	ENSGALG00010022120	FOXL3	case	control	-0.152935819	0.33019725	-0.463165028	0.643246081	0.902038523	down_in_case
PRJNA8274(	ENSGALG00010022122	PSMB3	case	control	0.050601529	0.058037104	0.871882393	0.38327254	0.780061953	up_in_case
PRJNA8274(	ENSGALG00010022124	ENSGALG00010022124	case	control	0.025616367	0.110142383	0.232575018	0.816091428	0.957422215	up_in_case
PRJNA8274(	ENSGALG00010022125	CBFA2T2	case	control	-0.167065185	0.088785963	-1.881662132	0.059881904	0.398284294	down_in_case
PRJNA8274(	ENSGALG00010022129	ELMO3	case	control	0.080429636	0.130262131	0.6174445	0.536941578	0.857433785	up_in_case
PRJNA8274(	ENSGALG00010022131	ENSGALG00010022131	case	control	1.68878397	1.281679955	1.317633129	0.18762649	0.610946377	up_in_case
PRJNA8274(	ENSGALG00010022134	CH25H	case	control	-0.276738211	0.168841594	-1.639040504	0.101204824	0.491460867	down_in_case
PRJNA8274(	ENSGALG00010022135	CHST1	case	control	0.124535769	0.180691498	0.689217646	0.490686314	0.836729584	up_in_case
PRJNA8274(	ENSGALG00010022137	PDDC1	case	control	0.049669816	0.059980985	0.828092702	0.407618002	0.794388636	up_in_case
PRJNA8274(	ENSGALG00010022138	SPAG17	case	control	0.123769564	0.155495403	0.795969279	0.426049892	0.805094126	up_in_case
PRJNA8274(	ENSGALG00010022142	TMEM208	case	control	0.078045235	0.080382413	0.970924259	0.331585997	0.748120789	up_in_case
PRJNA8274(	ENSGALG00010022143	MATCAP1	case	control	-0.00976936	0.086551706	-0.112873102	0.910131161	0.981730793	down_in_case
PRJNA8274(	ENSGALG00010022144	SNTA1	case	control	-0.140353866	0.090309176	-1.554148451	0.12014901	0.518289848	down_in_case
PRJNA8274(	ENSGALG00010022146	NPPA	case	control	-0.419076416	0.749071388	-0.559461252	0.575846969	0.877910508	down_in_case
PRJNA8274(	ENSGALG00010022148	TALDO1	case	control	0.146834733	0.076889	1.909697528	0.056172169	0.385984934	up_in_case
PRJNA8274(	ENSGALG00010022149	NBEAL1	case	control	0.078510488	0.07434645	1.056008559	0.290964304	0.714222439	up_in_case
PRJNA8274(	ENSGALG00010022150	CPNE2	case	control	-0.032103847	0.079362868	-0.404519744	0.685830562	0.917034645	down_in_case
PRJNA8274(	ENSGALG00010022151	PIP4K2B	case	control	-0.100468169	0.076218373	-1.318162082	0.187449396	0.610946377	down_in_case
PRJNA8274(	ENSGALG00010022152	ZNF644	case	control	0.027322615	0.063680306	0.429059102	0.667880215	0.912456967	up_in_case
PRJNA8274(	ENSGALG00010022154	CPN1	case	control	0.699791659	0.300236336	2.330802686	0.019763766	0.252566452	up_in_case
PRJNA8274(	ENSGALG00010022157	EPS8L2	case	control	0.097108774	0.203021192	0.47831841	0.632423595	0.89726815	up_in_case
PRJNA8274(	ENSGALG00010022158	CWC25	case	control	0.036641595	0.068806818	0.532528544	0.594359977	0.883951421	up_in_case
PRJNA8274(	ENSGALG00010022159	KIAA2013	case	control	0.020434658	0.036642913	0.557670128	0.57706966	0.878143389	up_in_case
PRJNA8274(	ENSGALG00010022161	CELF1	case	control	-0.040007548	0.064197144	-0.623198252	0.53315425	0.855817434	down_in_case
PRJNA8274(	ENSGALG00010022162	POLE	case	control	0.073986811	0.090341161	0.818971217	0.412802836	0.797581687	up_in_case
PRJNA8274(	ENSGALG00010022164	PDGFA	case	control	-0.090358865	0.070264457	-1.285982543	0.198449154	0.621920427	down_in_case
PRJNA8274(	ENSGALG00010022165	FAM192A	case	control	-0.039141766	0.04375726	-0.894520514	0.371043459	0.772959157	down_in_case
PRJNA8274(	ENSGALG00010022166	SDHB	case	control	-0.100263458	0.07174717	-1.397455227	0.162276721	0.575677776	down_in_case
PRJNA8274(	ENSGALG00010022167	WDR3	case	control	-0.009576335	0.072505992	-0.132076464	0.894923812	0.980176778	down_in_case
PRJNA8274(	ENSGALG00010022169	TOX2	case	control	-0.202635786	0.118521679	-1.709693849	0.087322501	0.467042649	down_in_case
PRJNA8274(	ENSGALG00010022170	RSPRY1	case	control	0.074814197	0.052851056	1.415566746	0.156902393	0.567595301	up_in_case
PRJNA8274(	ENSGALG00010022171	FOXP1	case	control	-0.007121702	0.077259264	-0.092179263	0.926555614	0.986284448	down_in_case
PRJNA8274(	ENSGALG00010022172	GIPC2	case	control	0.181096129	0.108617034	1.667290311	0.095456692	0.482930958	up_in_case
PRJNA8274(	ENSGALG00010022173	RPL23	case	control	-0.057898521	0.059728299	-0.969364973	0.332363122	0.748293551	down_in_case
PRJNA8274(	ENSGALG00010022174	KCNMA1	case	control	-0.09137041	0.095912012	-0.952648238	0.340768328	0.752236352	down_in_case
PRJNA8274(	ENSGALG00010022176	ARL2BPL	case	control	0.190385067	0.240344967	0.792132533	0.428283402	0.805337183	up_in_case
PRJNA8274(	ENSGALG00010022178	ENSGALG00010022178	case	control	-0.172073552	0.233268524	-0.737662966	0.460719282	0.82454265	down_in_case
PRJNA8274(	ENSGALG00010022180	PLLP	case	control	-0.022571566	0.19978252	-0.112980686	0.910045867	0.981730793	down_in_case
PRJNA8274(	ENSGALG00010022181	ENSGALG00010022181	case	control	0.068232862	0.110440487	0.617824707	0.536690895	0.857336818	up_in_case
PRJNA8274(	ENSGALG00010022182	CNTNAP1	case	control	-0.289985533	0.151829794	-1.909938258	0.056141163	0.385984934	down_in_case
PRJNA8274(	ENSGALG00010022184	PRKAR1B	case	control	-0.011302121	0.064188713	-0.176076446	0.860233885	0.971871635	down_in_case
PRJNA8274(	ENSGALG00010022186	DNAJB4	case	control	-0.038643107	0.050954727	-0.758381189	0.448222816	0.818697952	down_in_case
PRJNA8274(	ENSGALG00010022190	ACVR1B	case	control	-0.0716023	0.073091129	-0.979630513	0.327268537	0.744857565	down_in_case
PRJNA8274(	ENSGALG00010022193	GDAP2	case	control	-0.000436934	0.061397476	-0.007116489	0.994321911	0.998301153	down_in_case
PRJNA8274(	ENSGALG00010022194	ENSGALG00010022194	case	control	0.069028773	0.087287442	0.790821351	0.429048249	0.805896795	up_in_case
PRJNA8274(	ENSGALG00010022196	PLXNA1	case	control	-0.023142695	0.082866526	-0.27927676	0.78003244	0.948864508	down_in_case
PRJNA8274(	ENSGALG00010022199	RBM39	case	control	0.025248282	0.044737351	0.564366958	0.572504421	0.876814097	up_in_case
PRJNA8274(	ENSGALG00010022200	DNMBP	case	control	-0.05800122	0.054677147	-1.060794564	0.288783273	0.712801679	down_in_case
PRJNA8274(	ENSGALG00010022202	CX3CL1	case	control	-0.002460254	0.134817122	-0.018248826	0.985440352	0.996591771	down_in_case
PRJNA8274(	ENSGALG00010022203	DNAAF5	case	control	0.048023513	0.092750776	0.517769392	0.604619176	0.885268908	up_in_case
PRJNA8274(	ENSGALG00010022204	ENSGALG00010022204	case	control	0.026464141	0.368574133	0.071801406	0.942759954	0.990046805	up_in_case
PRJNA8274(	ENSGALG00010022205	GALNT18	case	control	-0.196928968	0.093954229	-2.096009618	0.036081338	0.325852945	down_in_case
PRJNA8274(	ENSGALG00010022207	ENSGALG00010022207	case	control	-0.044031978	0.151561134	-0.290522887	0.771416244	0.945630466	down_in_case
PRJNA8274(	ENSGALG00010022209	ZFYVE19	case	control	-0.116550566	0.072163073	-1.615099822	0.106289079	0.496267271	down_in_case
PRJNA8274(	ENSGALG00010022210	EZH1	case	control	0.121862273	0.078326232	1.55582962	0.119748613	0.517499293	up_in_case
PRJNA8274(	ENSGALG00010022211	ENSGALG00010022211	case	control	0.765162334	0.402198974	1.902447256	0.057112708	0.388573099	up_in_case
PRJNA8274(	ENSGALG00010022213	IDH1	case	control	0.014208576	0.097784796	0.14530455	0.884470422	0.977579768	up_in_case
PRJNA8274(	ENSGALG00010022214	ENSGALG00010022214	case	control	0.259140916	0.181785388	1.425532159	0.154003431	0.562900869	up_in_case
PRJNA8274(	ENSGALG00010022215	ENSGALG00010022215	case	control	0.097451518	0.081701008	1.19278233	0.232954632	0.657982411	up_in_case
PRJNA8274(	ENSGALG00010022216	ROMO1	case	control	-0.041040006	0.085323623	-0.480992303	0.630521969	0.89653616	down_in_case
PRJNA8274(	ENSGALG00010022218	ENSGALG00010022218	case	control	-0.196681458	0.178995958	-1.098803907	0.271853607	0.698323327	down_in_case
PRJNA8274(	ENSGALG00010022219	EIF4E3	case	control	-0.144076348	0.093087923	-1.54774479	0.121683754	0.519686058	down_in_case
PRJNA8274(	ENSGALG00010022220	PPP1R14D	case	control	0.112544862	0.336208704	0.334747022	0.737815927	0.935832995	up_in_case
PRJNA8274(	ENSGALG00010022221	CUTC	case	control	0.080554409	0.08739841	0.921691928	0.356689292	0.763373775	up_in_case
PRJNA8274(	ENSGALG00010022222	ENSGALG00010022222	case	control	0.036880361	0.140453665	0.262580268	0.792874111	0.95280257	up_in_case
PRJNA8274(	ENSGALG00010022223	DOK4	case	control	0.144225333	0.126150184	1.143282773	0.252921205	0.678440497	up_in_case
PRJNA8274(	ENSGALG00010022224	NHEJ1	case	control	-0.171371889	0.177792888	-0.963884946	0.335103612	0.749356776	down_in_case
PRJNA8274(	ENSGALG00010022226	FBRSL1	case	control	-0.054719965	0.054282285	-1.008063035	0.313424202	0.734989519	down_in_case
PRJNA8274(	ENSGALG00010022227	TMEM80	case	control	-0.030142905	0.09200016	-0.327639703	0.743184098	0.937448285	down_in_case
PRJNA8274(	ENSGALG00010022228	FUBP1	case	control	0.038128393	0.04166277	0.915167021	0.360103955	0.765754313	up_in_case
PRJNA8274(	ENSGALG00010022229	RAPSN	case	control	0.072288718	0.081319015	0.888952219	0.374028759	0.774369566	up_in_case
PRJNA8274(	ENSGALG00010022230	NFS1	case	control	-0.039934051	0.058623424	-0.681196159	0.495747378	0.838361815	down_in_case
PRJNA8274(	ENSGALG00010022231	ZNF503	case	control	-0.123791331	0.105822747	-1.169798876	0.242081916	0.666366006	down_in_case
PRJNA8274(	ENSGALG00010022232	GPR27	case	control	0.036351107	0.068079171	0.533953441	0.593373747	0.883625114	up_in_case
PRJNA8274(	ENSGALG00010022233	FAM46C	case	control	-0.273234048	0.107127715	-2.550544916	0.010755466	0.184728043	down_in_case
PRJNA8274(	ENSGALG00010022234	COX15	case	control	-0.19141353	0.092907476	-2.060259704	0.03937372	0.335091843	down_in_case
PRJNA8274(	ENSGALG00010022235	RAMP2	case	control	-0.002450139	0.057431331	-0.042662064	0.96597092	0.992340816	down_in_case
PRJNA8274(	ENSGALG00010022236	SHF	case	control	-0.193981345	0.085799074	-2.260879224	0.023766737	0.272786248	down_in_case
PRJNA8274(	ENSGALG00010022237	SUN1	case	control	0.068441985	0.053972665	1.268086061	0.204767219	0.627358178	up_in_case
PRJNA8274(	ENSGALG00010022238	COMTD1	case	control	0.132669123	0.103316991	1.284097819	0.199107731	0.622902456	up_in_case
PRJNA8274(	ENSGALG00010022239	DEAF1	case	control	-0.000839668	0.078047942	-0.010758357	0.991416239	0.99790247	down_in_case
PRJNA8274(	ENSGALG00010022240	ENSGALG00010022240	case	control	0.245128585	0.14786655	1.657769013	0.097364098	0.485860997	up_in_case
PRJNA8274(	ENSGALG00010022241	POLR2C	case	control	-0.064933999	0.060448178	-1.074209354	0.282728861	0.706698604	down_in_case
PRJNA8274(	ENSGALG00010022242	USP19	case	control	-0.001001025	0.052104975	-0.019211703	0.984672221	0.996591771	down_in_case
PRJNA8274(	ENSGALG00010022243	ENSGALG00010022243	case	control	0.810247353	0.492373988	1.645593336	0.099847512	0.48914677	up_in_case
PRJNA8274(	ENSGALG00010022244	ENTPD7	case	control	0.220483051	0.105962734	2.080760308	0.037455851	0.328438691	up_in_case
PRJNA8274(	ENSGALG00010022245	PIKFYVE	case	control	0.072248629	0.062572285	1.15464265	0.248236794	0.672435338	up_in_case
PRJNA8274(	ENSGALG00010022246	RHOV	case	control	-0.683509775	0.431115399	-1.585445048	0.112865253	0.507003543	down_in_case
PRJNA8274(	ENSGALG00010022247	GALNT9	case	control	0.44941281	0.187169035	2.401106622	0.016345573	0.226415743	up_in_case
PRJNA8274(	ENSGALG00010022248	FIGNL2	case	control	0.06104521	0.107718649	0.566709761	0.570911388	0.876157207	up_in_case
PRJNA8274(	ENSGALG00010022249	DRD4	case	control	-0.701017348	0.571176939	-1.227320818	0.219702034	0.643867604	down_in_case
PRJNA8274(	ENSGALG00010022250	SLC25A28	case	control	0.030449146	0.068481987	0.444630003	0.656587161	0.907494354	up_in_case
PRJNA8274(	ENSGALG00010022251	WNK4	case	control	0.129778667	0.139826195	0.928142735	0.353333534	0.761954708	up_in_case
PRJNA8274(	ENSGALG00010022252	COQ9	case	control	-0.136586912	0.086915312	-1.571494234	0.116067893	0.513328368	down_in_case
PRJNA8274(	ENSGALG00010022253	ALPK3	case	control	-0.168292588	0.130539785	-1.289205345	0.197326707	0.62094535	down_in_case
PRJNA8274(	ENSGALG00010022255	NOC4L	case	control	0.001118271	0.089629902	0.012476539	0.990045421	0.997323462	up_in_case
PRJNA8274(	ENSGALG00010022256	PSMC3	case	control	0.079063815	0.047451687	1.666196093	0.095674364	0.482930958	up_in_case
PRJNA8274(	ENSGALG00010022257	KCNK18	case	control	-0.325490906	0.506775774	-0.642277951	0.520692728	0.850168083	down_in_case
PRJNA8274(	ENSGALG00010022258	ENSGALG00010022258	case	control	0.772020724	0.769487397	1.003292228	0.315719885	0.736748426	up_in_case

PRJNA8274(	ENSGALG00010022259	GET4	case	control	-0.057807246	0.047060522	-1.228359644	0.21931199	0.643443819	down_in_case
PRJNA8274(	ENSGALG00010022264	SCN8A	case	control	0.358040546	0.163643737	2.187926973	0.028674922	0.300624184	up_in_case
PRJNA8274(	ENSGALG00010022266	COA3	case	control	0.00749261	0.086934474	0.086186861	0.931317875	0.987258715	up_in_case
PRJNA8274(	ENSGALG00010022269	CNTD1	case	control	-0.035436749	0.391175489	-0.090590413	0.92781805	0.986776285	down_in_case
PRJNA8274(	ENSGALG00010022271	SLC18A2	case	control	-0.233656499	0.271484101	-0.860663658	0.389423314	0.783669208	down_in_case
PRJNA8274(	ENSGALG00010022274	CCDC91	case	control	0.138073292	0.111400256	1.239434246	0.215184726	0.640404074	up_in_case
PRJNA8274(	ENSGALG00010022275	VDAC2	case	control	-0.129412737	0.068176746	-1.898194682	0.057670441	0.391217888	down_in_case
PRJNA8274(	ENSGALG00010022276	BECN1	case	control	0.040910617	0.051484995	0.794612433	0.426838984	0.805305429	up_in_case
PRJNA8274(	ENSGALG00010022279	EP400	case	control	-0.143561262	0.058839587	-2.439875398	0.014692329	0.213930485	down_in_case
PRJNA8274(	ENSGALG00010022280	PDZD8	case	control	-0.034573482	0.065324221	-0.529259771	0.596625258	0.884680373	down_in_case
PRJNA8274(	ENSGALG00010022282	TMT1A	case	control	0.025180045	0.167475825	0.150350323	0.880488232	0.976399289	up_in_case
PRJNA8274(	ENSGALG00010022290	NEXN	case	control	-0.107424143	0.116562302	-0.921602795	0.3567358	0.763373775	down_in_case
PRJNA8274(	ENSGALG00010022293	DNAJC10	case	control	0.022935537	0.050684242	0.452518112	0.650895776	0.904636952	up_in_case
PRJNA8274(	ENSGALG00010022294	ENSGALG00010022294	case	control	-1.352251915	0.728843456	-1.85533931	0.063547788	0.409661205	down_in_case
PRJNA8274(	ENSGALG00010022296	CIAPIN1	case	control	-0.127818352	0.050150333	-2.548703975	0.010812404	0.184728043	down_in_case
PRJNA8274(	ENSGALG00010022297	VPS18	case	control	-0.019150515	0.069904918	-0.273950897	0.784122357	0.949518053	down_in_case
PRJNA8274(	ENSGALG00010022299	MRGBP	case	control	0.007006692	0.051825692	0.135197264	0.89245591	0.979422008	up_in_case
PRJNA8274(	ENSGALG00010022302	ENSGALG00010022302	case	control	-0.057198483	0.130470303	-0.43840232	0.661094665	0.909646511	down_in_case
PRJNA8274(	ENSGALG00010022303	DUSP13A	case	control	0.154567152	0.186490171	0.828821974	0.407205152	0.794380997	up_in_case
PRJNA8274(	ENSGALG00010022304	ADAP1	case	control	1.127314607	0.214180641	5.263382351	1.41429E-07	4.93277E-05	up_in_case
PRJNA8274(	ENSGALG00010022307	ENSGALG00010022307	case	control	-0.859200218	0.315341519	-2.724665689	0.006436663	0.13904105	down_in_case
PRJNA8274(	ENSGALG00010022308	OGFR	case	control	-0.050022836	0.096434102	-0.518725588	0.603952115	0.885268908	down_in_case
PRJNA8274(	ENSGALG00010022309	WSCD2	case	control	0.075616495	0.272420512	0.277572693	0.781340397	0.949372731	up_in_case
PRJNA8274(	ENSGALG00010022310	DCLRE1A	case	control	0.20552452	0.072351038	2.840657512	0.004502063	0.114389963	up_in_case
PRJNA8274(	ENSGALG00010022311	DLL4	case	control	0.106929857	0.081052096	1.31927319	0.187077797	0.610664656	up_in_case
PRJNA8274(	ENSGALG00010022312	ZNF592	case	control	-0.076252523	0.05139446	-1.48367202	0.137895953	0.539854583	down_in_case
PRJNA8274(	ENSGALG00010022313	ENSGALG00010022313	case	control	0.076497558	0.308334963	0.248098877	0.804057904	0.954997212	up_in_case
PRJNA8274(	ENSGALG00010022314	DUSP13B	case	control	0.014496013	0.299843728	0.048345228	0.96144111	0.992056045	up_in_case
PRJNA8274(	ENSGALG00010022316	INO80	case	control	0.02817087	0.043408704	0.648968232	0.51635891	0.848054666	up_in_case
PRJNA8274(	ENSGALG00010022317	PSME3	case	control	0.129854914	0.040962765	3.170072005	0.001524012	0.062408717	up_in_case
PRJNA8274(	ENSGALG00010022320	COL9A3	case	control	0.562692181	0.646907219	0.869818986			up_in_case
PRJNA8274(	ENSGALG00010022322	ENSGALG00010022322	case	control	-0.094442434	0.345213409	-0.273576956	0.784409745	0.949518053	down_in_case
PRJNA8274(	ENSGALG00010022323	DUSP29	case	control	0.47745175	0.343414003	1.3903095	0.164434914	0.579452754	up_in_case
PRJNA8274(	ENSGALG00010022325	MIGA1	case	control	-0.050050498	0.082745051	-0.60487603	0.545261371	0.861997112	down_in_case
PRJNA8274(	ENSGALG00010022326	COX19	case	control	0.004936897	0.063055345	0.078294668	0.937593659	0.988906131	up_in_case
PRJNA8274(	ENSGALG00010022328	ENSGALG00010022328	case	control	-0.023532094	0.195788076	-0.120191659	0.904331325	0.981104465	down_in_case
PRJNA8274(	ENSGALG00010022329	CYP2AC7	case	control	-0.365969153	0.323400856	-1.131627039	0.257791267	0.685079934	down_in_case
PRJNA8274(	ENSGALG00010022332	KAT6B	case	control	-0.041230085	0.078328476	-0.526374159	0.598628266	0.88476024	down_in_case
PRJNA8274(	ENSGALG00010022333	ENSGALG00010022333	case	control	-0.025509812	0.110804801	-0.23022298	0.817918506	0.958536226	down_in_case
PRJNA8274(	ENSGALG00010022334	PDE1A	case	control	0.006393234	0.075591697	0.084575873	0.932598581	0.987717353	up_in_case
PRJNA8274(	ENSGALG00010022340	GFOD2	case	control	0.029982256	0.056633789	0.529405788	0.596523984	0.884680373	up_in_case
PRJNA8274(	ENSGALG00010022343	GATA5	case	control	0.383432986	0.497753584	0.770326922	0.44110599	0.813083204	up_in_case
PRJNA8274(	ENSGALG00010022345	USP33	case	control	0.103201459	0.059580621	1.732131325	0.083250183	0.457254339	up_in_case
PRJNA8274(	ENSGALG00010022347	SEC11A	case	control	-0.025403846	0.064772607	-0.39220045	0.694910112	0.920623921	down_in_case
PRJNA8274(	ENSGALG00010022348	G6PC1	case	control	1.210637663	0.581430179	2.082172042	0.037326758	0.328438691	up_in_case
PRJNA8274(	ENSGALG00010022353	SLC39A13	case	control	0.120035226	0.059783053	2.007847032	0.044659549	0.351254918	up_in_case
PRJNA8274(	ENSGALG00010022354	FAR2	case	control	-0.096799707	0.062431002	-1.550507036	0.121019866	0.519499513	down_in_case
PRJNA8274(	ENSGALG00010022356	TCFL5	case	control	-0.08865685	0.203360471	-0.435959113	0.662866395	0.911044469	down_in_case
PRJNA8274(	ENSGALG00010022357	ENSGALG00010022357	case	control	-0.026552418	0.409635937	-0.064819551	0.948317675	0.991291133	down_in_case
PRJNA8274(	ENSGALG00010022359	ZZZ3	case	control	-0.063228437	0.057582338	-1.098052615	0.272181515	0.698418597	down_in_case
PRJNA8274(	ENSGALG00010022363	KIF18A	case	control	-0.030019573	0.126169319	-0.237930844	0.811934732	0.956240049	down_in_case
PRJNA8274(	ENSGALG00010022365	PLCG1	case	control	0.142685435	0.065152289	2.190029496	0.028522098	0.300549329	up_in_case
PRJNA8274(	ENSGALG00010022366	AARSD1	case	control	-0.007819896	0.06127018	-0.127629715	0.898442014	0.98028579	down_in_case
PRJNA8274(	ENSGALG00010022373	AK5	case	control	0.040442419	0.200049377	0.202162187	0.839789933	0.965286179	up_in_case
PRJNA8274(	ENSGALG00010022375	NMB	case	control	-0.230737079	0.24652565	-0.935955667	0.349296014	0.759342183	down_in_case
PRJNA8274(	ENSGALG00010022377	ZHX3	case	control	-0.044357956	0.061646896	-0.719548893	0.471802788	0.829235491	down_in_case
PRJNA8274(	ENSGALG00010022379	METTL15	case	control	-0.112068445	0.097846412	-1.145350586	0.252063959	0.677148133	down_in_case
PRJNA8274(	ENSGALG00010022382	CALB2	case	control	0.040735248	0.578316023	0.070437696	0.943845289	0.990183239	up_in_case
PRJNA8274(	ENSGALG00010022385	ENSGALG00010022385	case	control	-0.404568323	0.443333506	-0.912559772	0.36147412	0.767609135	down_in_case
PRJNA8274(	ENSGALG00010022386	SPI1	case	control	0.031410689	0.104656226	0.300132061	0.764076425	0.943786458	up_in_case
PRJNA8274(	ENSGALG00010022388	CYP2W1	case	control	0.258541776	0.34756756	0.743860493	0.456960879	0.82267916	up_in_case
PRJNA8274(	ENSGALG00010022391	ENSGALG00010022391	case	control	0.004692973	0.088488917	0.053034588	0.95770435	0.992056045	up_in_case
PRJNA8274(	ENSGALG00010022392	EMILIN3	case	control	0.193277309	0.117152592	1.649791142	0.098985661	0.488407564	up_in_case
PRJNA8274(	ENSGALG00010022395	PTGES3L	case	control	-0.017022053	0.08929204	-0.190633483	0.848812756	0.968741311	down_in_case
PRJNA8274(	ENSGALG00010022396	PPP1R1C	case	control	0.118033022	0.20911946	0.564428685	0.572462422	0.876814097	up_in_case
PRJNA8274(	ENSGALG00010022397	ATF1	case	control	-0.041260473	0.038427324	-1.073727477	0.282944843	0.706991307	down_in_case
PRJNA8274(	ENSGALG00010022400	NHLRC2	case	control	0.12338733	0.077458076	1.592956292	0.111169997	0.503876688	up_in_case
PRJNA8274(	ENSGALG00010022401	LCAT	case	control	0.836737032	0.354434151	2.360768654	0.018237104	0.241560424	up_in_case
PRJNA8274(	ENSGALG00010022406	ITPRID2	case	control	0.124245101	0.075694334	1.641405559	0.100713257	0.490422251	up_in_case
PRJNA8274(	ENSGALG00010022407	RUNDC1	case	control	0.006280263	0.043090179	0.145746967	0.884121143	0.977420359	up_in_case
PRJNA8274(	ENSGALG00010022408	RAD51AP1	case	control	-0.039695722	0.074160955	-0.535264433	0.592467017	0.883604289	down_in_case
PRJNA8274(	ENSGALG00010022409	CARNS1	case	control	-0.286014685	0.259222649	-1.103355303	0.269872899	0.697106657	down_in_case
PRJNA8274(	ENSGALG00010022411	ADRB1	case	control	0.063729372	0.223771872	0.284796171	0.775800306	0.947147987	up_in_case
PRJNA8274(	ENSGALG00010022412	CHAC1	case	control	1.076730893	0.281534426	3.824508819	0.000131033	0.013103306	up_in_case
PRJNA8274(	ENSGALG00010022415	DIP2B	case	control	0.066644534	0.05352077	1.245208804	0.213055007	0.638582746	up_in_case
PRJNA8274(	ENSGALG00010022416	CCDC186	case	control	-0.033965787	0.056561016	-0.600515854	0.548162499	0.863390652	down_in_case
PRJNA8274(	ENSGALG00010022419	DUOX2	case	control	0.031150985	0.448146492	0.069510719	0.944583101	0.990313016	up_in_case
PRJNA8274(	ENSGALG00010022421	CMKLR1	case	control	0.064135967	0.128385824	0.499556453	0.617387427	0.891081782	up_in_case
PRJNA8274(	ENSGALG00010022422	RPS6KB2	case	control	0.041296937	0.058192534	0.709660413	0.477914746	0.830520323	up_in_case
PRJNA8274(	ENSGALG00010022425	VWA2	case	control	0.448432344	0.251577588	1.782481293	0.074670779	0.439711709	up_in_case
PRJNA8274(	ENSGALG00010022426	EXD1	case	control	-0.57377172	0.359091676	-1.597841883	0.110078171	0.502591904	down_in_case
PRJNA8274(	ENSGALG00010022427	RPL27	case	control	-0.063154023	0.06419716	-0.983751052	0.325237931	0.743110335	down_in_case
PRJNA8274(	ENSGALG00010022429	ADK	case	control	-0.008605165	0.064174255	-0.134090605	0.893330928	0.979875146	down_in_case
PRJNA8274(	ENSGALG00010022431	LARP4	case	control	-0.01272673	0.064950734	-0.195944365	0.844653712	0.96721237	down_in_case
PRJNA8274(	ENSGALG00010022433	LNPK	case	control	-0.234898592	0.073466049	-3.197376157	0.001386839	0.058654283	down_in_case
PRJNA8274(	ENSGALG00010022435	PIGK	case	control	-0.060521789	0.084643628	-0.715018844	0.474597399	0.830081068	down_in_case
PRJNA8274(	ENSGALG00010022436	AFAP1L2	case	control	0.059031561	0.063784768	0.925480529	0.354716007	0.763161644	up_in_case
PRJNA8274(	ENSGALG00010022438	RPN2	case	control	0.105429419	0.04821866	2.186485889	0.028780076	0.301284836	up_in_case
PRJNA8274(	ENSGALG00010022439	IFI35	case	control	-0.070008982	0.088698849	-0.789288507	0.429943403	0.806015686	down_in_case
PRJNA8274(	ENSGALG00010022440	SORD	case	control	-0.014652567	0.113683343	-0.128889306	0.897445239	0.980176778	down_in_case
PRJNA8274(	ENSGALG00010022441	FICD	case	control	0.209632907	0.107857947	1.943601865	0.051943481	0.371953818	up_in_case
PRJNA8274(	ENSGALG00010022448	FERRY3	case	control	0.094573268	0.138166892	0.684485743	0.493668489	0.837916219	up_in_case
PRJNA8274(	ENSGALG00010022449	ENSGALG00010022449	case	control	-0.048330544	0.062499347	-0.773296779	0.439346762	0.812366461	down_in_case
PRJNA8274(	ENSGALG00010022451	CHP1	case	control	0.228344571	0.076144792	2.998820586	0.002710269	0.087009711	up_in_case
PRJNA8274(	ENSGALG00010022452	AP3M1	case	control	-0.048389466	0.093509571	-0.517481421	0.604820135	0.885268908	down_in_case
PRJNA8274(	ENSGALG00010022453	ENSGALG00010022453	case	control	-0.110743229	0.126167287	-0.877749149	0.380079866	0.77805894	down_in_case
PRJNA8274(	ENSGALG00010022454	ABLIM1	case	control	-0.052681345	0.099288912	-0.530586384	0.595705433	0.884680373	down_in_case
PRJNA8274(	ENSGALG00010022455	C7orf50	case	control	0.04842316	0.169226941	0.286143328	0.774768348	0.947085948	up_in_case
PRJNA8274(	ENSGALG00010022456	ATP5G3	case	control	-0.00489346	0.09492741	-0.051549497	0.958887661	0.992056045	down_in_case
PRJNA8274(	ENSGALG00010022460	VAT1	case	control	0.156032329	0.085506031	1.82481079	0.068029587	0.421339236	up_in_case
PRJNA8274(	ENSGALG00010022462	ITGA5	case	control	0.085224776	0.118673066	0.718147602	0.472666282	0.829235491	up_in_case
PRJNA8274(	ENSGALG00010022463	DUSP12	case	control	0.009447087	0.087504079	0.107961679	0.91402609	0.982854306	up_in_case
PRJNA8274(	ENSGALG00010022466	NEDD4	case	control	0.038800607	0.063604698	0.610027379	0.541843671	0.85975913	up_in_case
PRJNA8274(	ENSGALG00010022467	SART3	case	control	0.005173569	0.041699671	0.124067379	0.901261926	0.980628041	up_in_case
PRJNA8274(	ENSGALG00010022468	MYBPC3	case	control	-0.210259222	0.151328339	-1.389423972	0.164703865	0.579922223	down_in_case
PRJNA8274(	ENSGALG00010022470	ENSGALG00010022470	case	control	0.722632455	0.190079084	3.801746313	0.00014368	0.01379195	up_in_case
PRJNA8274(	ENSGALG00010022471	ST6GALNAC5	case	control	-0.213514735	0.134914648	-1.582591208	0.113514669	0.508062524	down_in_case
PRJNA8274(	ENSGALG00010022472	QSER1	case	control	-0.159742338	0.069027985	-2.314167783	0.02065852	0.25845743	down_in_case
PRJNA8274(	ENSGALG00010022473	ZNF385A	case	control	-0.182856222	0.146885727	-1.244887619	0.213173062	0.638582746	down_in_case
PRJNA8274(	ENSGALG00010022474	VCL	case	control	-0.065365833	0.054325372	-1.203228436	0.228887934	0.652401326	down_in_case
PRJNA8274(	ENSGALG00010022476	OIP5	case	control	-0.120589097	0.100148959	-1.204097356	0.228551952	0.651938858	down_in_case
PRJNA8274(	ENSGALG00010022478	ATP6V1A	case	control	0.095835045	0.056706412	1.690021322	0.091023876	0.475746133	up_in_case
PRJNA8274(	ENSGALG00010022479	ENSGALG00010022479	case	control	0.880829186	0.613051189	1.436795494	0.150776111	0.558399072	up_in_case
PRJNA8274(	ENSGALG00010022480	PIF1	case	control	-0.027069568	0.155212536	-0.174403235	0.861548572	0.972599205	down_in_case
PRJNA8274(	ENSGALG00010022481	CORO1B	case	control	-0.099709633	0.091920504	-1.084737667	0.27803786	0.703443892	down_in_case
PRJNA8274(	ENSGALG00010022482	PRTG	case	control	1.342021893	0.284548753	4.716316204	2.40153E-06	0.000520565	up_in_case
PRJNA8274(	ENSGALG00010022483	TOP2A	case	control	-0.156025548	0.111556127	-1.398628227	0.161924497	0.575570547	down_in_case
PRJNA8274(	ENSGALG00010022485	ATF2	case	control	0.011301735	0.03719553	0.303846598	0.761244764	0.942985066	up_in_case
PRJNA8274(	ENSGALG00010022486	FAM183A	case	control	0.995310822	0.40301037	2.469690348	0.013523005	0.204023937	up_in_case
PRJNA8274(	ENSGALG00010022489	NUSAP1	case	control	-0.110538851	0.074760736	-1.478568262	0.13925574	0.541505735	down_in_case
PRJNA8274(	ENSGALG00010022493	ENSGALG00010022493	case	control	0.040164819	0.144770095	0.277438649	0.781443308	0.949394183	up_in_case
PRJNA8274(	ENSGALG00010022494	FHIP2A	case	control	0.117109896	0.069203929	1.692243442	0.090599567	0.474422807	up_in_case
PRJNA8274(	ENSGALG00010022496	TUBGCP2	case	control	0.016512741	0.051716854	0.319291296	0.749505632	0.93961255	up_in_case
PRJNA8274(	ENSGALG00010022497	CORO1C	case	control	0.005142559	0.056134674	0.091611097	0.927007034	0.986471245	up_in_case
PRJNA8274(	ENSGALG00010022498	NAA50	case	control	-0.163552841	0.048206823	-3.392732192	0.000691993	0.039267832	down_in_case
PRJNA8274(	ENSGALG00010022500	ENSGALG00010022500	case	control	0.732559509	0.477753702	1.533341355	0.12519177	0.523984491	up_in_case

PRJNA8274(	ENSGALG00010022502	ENSGALG00010022502	case	control	0.248532046	0.388355952	0.639959412	0.522198987	0.850893974	up_in_case
PRJNA8274(	ENSGALG00010022504	RAD9A	case	control	-0.133102178	0.096802681	-1.374984417	0.169136276	0.585038804	down_in_case
PRJNA8274(	ENSGALG00010022505	ZNF511	case	control	0.060944669	0.092836482	0.656473275	0.511519657	0.845731426	up_in_case
PRJNA8274(	ENSGALG00010022506	EBNA1BP2	case	control	0.094206775	0.071668581	1.314478029	0.188685387	0.611698262	up_in_case
PRJNA8274(	ENSGALG00010022507	ENSGALG00010022507	case	control	0.755917234	0.237391956	3.184257996	0.001451256	0.060509682	up_in_case
PRJNA8274(	ENSGALG00010022508	USF3	case	control	0.03387308	0.074840306	0.452604779	0.650833357	0.904636952	up_in_case
PRJNA8274(	ENSGALG00010022511	IGFBP4	case	control	0.210667697	0.108043461	1.949842166	0.051194934	0.370042007	up_in_case
PRJNA8274(	ENSGALG00010022513	NDUFAF1	case	control	0.027530927	0.046360923	0.593839074	0.552619752	0.866213137	up_in_case
PRJNA8274(	ENSGALG00010022515	CHRNA1	case	control	-0.082576775	0.166744249	-0.495230122	0.62043769	0.892955829	down_in_case
PRJNA8274(	ENSGALG00010022516	TRUB1	case	control	0.103279516	0.09816635	1.052086749	0.292759751	0.715268142	up_in_case
PRJNA8274(	ENSGALG00010022517	HNRNPA1	case	control	-0.055190489	0.062420116	-0.884177925	0.376600173	0.775199984	down_in_case
PRJNA8274(	ENSGALG00010022518	ECHS1	case	control	-0.072554318	0.067362319	-1.077075714	0.281446442	0.705762606	down_in_case
PRJNA8274(	ENSGALG00010022520	TMOD2	case	control	0.10555279	0.18242007	0.578624874	0.562842317	0.871631486	up_in_case
PRJNA8274(	ENSGALG00010022521	RTF1	case	control	-0.01573005	0.033757393	-0.465973501	0.641234469	0.901516193	down_in_case
PRJNA8274(	ENSGALG00010022522	TNS4	case	control	0.01225914	0.255324114	0.04801403	0.961705061	0.992056045	up_in_case
PRJNA8274(	ENSGALG00010022524	SOX18	case	control	-0.160207767	0.099230426	-1.614502455	0.106418485	0.496402103	down_in_case
PRJNA8274(	ENSGALG00010022525	ISCU	case	control	-0.037198871	0.050222994	-0.740674086	0.458891075	0.824116052	down_in_case
PRJNA8274(	ENSGALG00010022526	PPP1CA	case	control	-0.101201603	0.058000621	-1.744836533	0.081013328	0.453075204	down_in_case
PRJNA8274(	ENSGALG00010022528	SIDT1	case	control	0.373385424	0.28199691	1.324076293	0.185477708	0.608612029	up_in_case
PRJNA8274(	ENSGALG00010022529	CCR7	case	control	0.520368757	0.253994025	2.04874409	0.040487145	0.33753444	up_in_case
PRJNA8274(	ENSGALG00010022531	ENSGALG00010022531	case	control	-0.173903543	0.295138487	-0.589226924	0.555709058	0.867878646	down_in_case
PRJNA8274(	ENSGALG00010022532	CBX5	case	control	0.041846925	0.057887726	0.722898056	0.469742518	0.829101076	up_in_case
PRJNA8274(	ENSGALG00010022533	TMEM119	case	control	0.010332157	0.102001193	0.101294472	0.919316704	0.984146494	up_in_case
PRJNA8274(	ENSGALG00010022534	CFAP57	case	control	0.688688077	0.374603934	1.838443255	0.065997116	0.41627012	up_in_case
PRJNA8274(	ENSGALG00010022536	SMUG1	case	control	0.017508127	0.056488752	0.309940063	0.756606536	0.941659745	up_in_case
PRJNA8274(	ENSGALG00010022538	WIPF1	case	control	0.147794077	0.129063007	1.145131203	0.252154812	0.677148133	up_in_case
PRJNA8274(	ENSGALG00010022539	LYSMD2	case	control	0.052589306	0.094258727	0.557925058	0.57689556	0.878143389	up_in_case
PRJNA8274(	ENSGALG00010022540	GPR146	case	control	0.186536885	0.20057252	0.930022146	0.352359618	0.761166734	up_in_case
PRJNA8274(	ENSGALG00010022541	ITPKA	case	control	0.033040766	0.103526666	0.319152228	0.74961108	0.93964222	up_in_case
PRJNA8274(	ENSGALG00010022542	ENSGALG00010022542	case	control	1.069136076	0.658619787	1.623297836	0.104525752	0.494612261	up_in_case
PRJNA8274(	ENSGALG00010022544	SPICE1	case	control	-0.005762238	0.064300388	-0.089614357	0.928593675	0.986776285	down_in_case
PRJNA8274(	ENSGALG00010022545	CSTF3	case	control	-0.042340339	0.064367109	-0.657794637	0.510670101	0.845108488	down_in_case
PRJNA8274(	ENSGALG00010022546	MTG1	case	control	0.13820933	0.101487157	1.361840588	0.173248199	0.592691208	up_in_case
PRJNA8274(	ENSGALG00010022547	SMARCE1	case	control	-0.049043352	0.042759099	-1.146968799	0.251394517	0.677081801	down_in_case
PRJNA8274(	ENSGALG00010022548	ENSGALG00010022548	case	control	-0.055295656	0.08831865	-0.626092628	0.531254187	0.855560567	down_in_case
PRJNA8274(	ENSGALG00010022550	TRIM45	case	control	-0.141552947	0.139211651	-1.016818244	0.309239902	0.732140827	down_in_case
PRJNA8274(	ENSGALG00010022551	GPER1	case	control	-0.063696684	0.400384701	-0.159088707	0.873598985	0.975062028	down_in_case
PRJNA8274(	ENSGALG00010022552	GPR155	case	control	0.180034086	0.061719588	2.916968361	0.003534516	0.101807307	up_in_case
PRJNA8274(	ENSGALG00010022553	PRPF6	case	control	-0.017070924	0.042452002	-0.402122945	0.687593547	0.917290381	down_in_case
PRJNA8274(	ENSGALG00010022554	SCG3	case	control	-0.21794017	0.133250565	-1.63556658	0.10193033	0.491460867	down_in_case
PRJNA8274(	ENSGALG00010022557	WNT1	case	control	0.198076768	0.236951786	0.835937015	0.403190338	0.792416414	up_in_case
PRJNA8274(	ENSGALG00010022558	NYAP2	case	control	-0.430965198	0.438043889	-0.983840224	0.325194078	0.743110335	down_in_case
PRJNA8274(	ENSGALG00010022559	SSH1	case	control	0.053436531	0.07007655	0.762545125	0.445734722	0.817180323	up_in_case
PRJNA8274(	ENSGALG00010022560	SAMD10	case	control	0.705222009	0.563554008	1.251383184	0.21079471	0.636310899	up_in_case
PRJNA8274(	ENSGALG00010022562	GHRH	case	control	-0.442471515	0.610459727	-0.72481688	0.468564379	0.828596747	down_in_case
PRJNA8274(	ENSGALG00010022564	VTCN1	case	control	-0.576821594	0.321802258	-1.792472179	0.073057362	0.436871039	down_in_case
PRJNA8274(	ENSGALG00010022565	ARF3	case	control	-0.10238551	0.089400039	-1.145251288	0.252105078	0.677148133	down_in_case
PRJNA8274(	ENSGALG00010022566	ENSGALG00010022566	case	control	0.514790258	0.455210073	1.130885032	0.258103484	0.685553066	up_in_case
PRJNA8274(	ENSGALG00010022567	PAOX	case	control	-0.545667889	0.141558565	-3.85471476	0.000115865	0.011919887	down_in_case
PRJNA8274(	ENSGALG00010022568	SCRN3	case	control	-0.29988216	0.123120134	-2.435687399	0.014863523	0.215129943	down_in_case
PRJNA8274(	ENSGALG00010022569	KIAA1549L	case	control	0.024890643	0.135534851	0.183647548	0.854289959	0.970137355	up_in_case
PRJNA8274(	ENSGALG00010022571	RIPOR1	case	control	0.053689502	0.057845893	0.928147166	0.353331236	0.761954708	up_in_case
PRJNA8274(	ENSGALG00010022572	ENSGALG00010022572	case	control	0.749963282	0.265962289	2.819810603	0.0048052	0.11888298	up_in_case
PRJNA8274(	ENSGALG00010022573	AP4E1	case	control	-0.020802719	0.073595468	-0.282663037	0.777435153	0.947687554	down_in_case
PRJNA8274(	ENSGALG00010022574	ITPRIPL2	case	control	-0.01638642	0.101187337	-0.161941413	0.87135199	0.975062028	down_in_case
PRJNA8274(	ENSGALG00010022575	TMEM41B	case	control	0.138571012	0.063161552	2.193913989	0.028241594	0.29959554	up_in_case
PRJNA8274(	ENSGALG00010022578	ZNF512B	case	control	0.023659928	0.058298194	0.405843252	0.684857777	0.916548243	up_in_case
PRJNA8274(	ENSGALG00010022579	TNFAIP8L3	case	control	-0.131950389	0.115545959	-1.141973201	0.253465159	0.678954688	down_in_case
PRJNA8274(	ENSGALG00010022581	TMEM125	case	control	0.025942634	0.513975485	0.050474457	0.959744304	0.992056045	up_in_case
PRJNA8274(	ENSGALG00010022582	ENSGALG00010022582	case	control	-0.252894488	0.207121021	-1.220998654	0.222086529	0.645952056	down_in_case
PRJNA8274(	ENSGALG00010022583	FKBP11	case	control	-0.026258742	0.114279027	-0.229777437	0.818264718	0.958536226	down_in_case
PRJNA8274(	ENSGALG00010022585	CIR1	case	control	-0.029498699	0.057671698	-0.511493505	0.609005535	0.8877909	down_in_case
PRJNA8274(	ENSGALG00010022587	ENSGALG00010022587	case	control	0.326336729	1.050603977	0.310618212	0.756090881	0.941659745	up_in_case
PRJNA8274(	ENSGALG00010022588	TCP11L1	case	control	-0.115734472	0.076360439	-1.515633926	0.129611944	0.531989321	down_in_case
PRJNA8274(	ENSGALG00010022590	ENSGALG00010022590	case	control	0.030187375	0.089493103	0.337315102	0.735879376	0.934357951	up_in_case
PRJNA8274(	ENSGALG00010022593	MANBAL	case	control	0.049016923	0.092560147	0.529568333	0.596411255	0.884680373	up_in_case
PRJNA8274(	ENSGALG00010022597	SLCO4A1	case	control	0.156300497	0.271012533	0.57672793	0.564123262	0.871916948	up_in_case
PRJNA8274(	ENSGALG00010022599	RNLS	case	control	0.090653897	0.193585798	0.468287956	0.63957868	0.900637629	up_in_case
PRJNA8274(	ENSGALG00010022603	ATRNL1	case	control	-0.047466035	0.128006851	-0.37080855	0.710780133	0.925900262	down_in_case
PRJNA8274(	ENSGALG00010022608	P3H4	case	control	0.202889394	0.075387326	2.691293168	0.007117562	0.146516526	up_in_case
PRJNA8274(	ENSGALG00010022609	RBBP8NL	case	control	0.347832861	0.781418817	0.445129877	0.656225899	0.907494354	up_in_case
PRJNA8274(	ENSGALG00010022614	DAO	case	control	-0.406938267	0.106750498	-3.812050285	0.000137819	0.013591788	down_in_case
PRJNA8274(	ENSGALG00010022616	GLDN	case	control	-0.000985967	0.198113017	-0.00497679	0.996029113	0.998710499	down_in_case
PRJNA8274(	ENSGALG00010022619	MADD	case	control	0.064838545	0.081729932	0.793326794	0.42758745	0.805305429	up_in_case
PRJNA8274(	ENSGALG00010022622	MPPED2	case	control	0.02120731	0.072898324	0.290916295	0.771115338	0.94558971	up_in_case
PRJNA8274(	ENSGALG00010022623	TOP1	case	control	-0.051629556	0.058066168	-0.889150399	0.373922256	0.774369566	down_in_case
PRJNA8274(	ENSGALG00010022625	ENSGALG00010022625	case	control	-0.262120854	0.1272405	-2.060042622	0.039394466	0.335091843	down_in_case
PRJNA8274(	ENSGALG00010022627	CTCF	case	control	-0.003549074	0.044214601	-0.080269271	0.936023098	0.988425668	down_in_case
PRJNA8274(	ENSGALG00010022628	SVOP	case	control	0.23415767	0.531495664	0.440563652	0.659528922	0.908599575	up_in_case
PRJNA8274(	ENSGALG00010022630	ENSGALG00010022630	case	control	0.119242291	0.190651718	0.625445664	0.5316786	0.855623068	up_in_case
PRJNA8274(	ENSGALG00010022637	DMXL2	case	control	0.013363778	0.092783545	0.14403177	0.885475382	0.977630122	up_in_case
PRJNA8274(	ENSGALG00010022642	SEC13	case	control	0.06897661	0.043447394	1.587589119	0.11237928	0.506149199	up_in_case
PRJNA8274(	ENSGALG00010022643	SP3	case	control	-0.024480643	0.038318768	-0.638868207	0.522908672	0.851177463	down_in_case
PRJNA8274(	ENSGALG00010022644	ARMC8	case	control	-0.007925587	0.043766318	-0.181088731	0.85629793	0.970905518	down_in_case
PRJNA8274(	ENSGALG00010022645	ENSGALG00010022645	case	control	0.877363241	0.570159804	1.53880234	0.123852558	0.522908647	up_in_case
PRJNA8274(	ENSGALG00010022647	SVIP	case	control	-0.090385062	0.063011891	-1.434412769	0.151454506	0.559493525	down_in_case
PRJNA8274(	ENSGALG00010022649	PATZ1	case	control	-0.025920092	0.056486765	-0.458870171	0.6463274	0.902693223	down_in_case
PRJNA8274(	ENSGALG00010022650	NR1H3	case	control	-0.06440333	0.041942151	-1.535527581	0.124654288	0.523589042	down_in_case
PRJNA8274(	ENSGALG00010022652	CARMIL2	case	control	0.119014438	0.212874738	0.559082014	0.576105749	0.877910508	up_in_case
PRJNA8274(	ENSGALG00010022655	NRIP3	case	control	0.761514393	0.494873609	1.538805827	0.123851707	0.522908647	up_in_case
PRJNA8274(	ENSGALG00010022656	FKBP10	case	control	-0.041307966	0.088275406	-0.467944217	0.639824482	0.900806349	down_in_case
PRJNA8274(	ENSGALG00010022657	MRPS35	case	control	0.01933988	0.061156465	0.316236066	0.751823334	0.940427752	up_in_case
PRJNA8274(	ENSGALG00010022659	TIE1	case	control	0.001791291	0.06893806	0.025984059	0.979270053	0.995561052	up_in_case
PRJNA8274(	ENSGALG00010022660	METAP1D	case	control	0.290653355	0.17434534	1.667112842	0.095491969	0.482930958	up_in_case
PRJNA8274(	ENSGALG00010022661	ENSGALG00010022661	case	control	0.081751656	0.399922445	0.204418774	0.83802626	0.964826648	up_in_case
PRJNA8274(	ENSGALG00010022662	GFRA1	case	control	0.168361299	0.19775596	0.851358915	0.394570007	0.786978937	up_in_case
PRJNA8274(	ENSGALG00010022664	TRPC4AP	case	control	-0.09812742	0.04577426	-2.143724893	0.032054936	0.3098361	down_in_case
PRJNA8274(	ENSGALG00010022666	ENSGALG00010022666	case	control	0.029791165	0.064909331	0.45896583	0.646258704	0.902693223	up_in_case
PRJNA8274(	ENSGALG00010022667	PTEN	case	control	-0.056083646	0.051033915	-1.09894853	0.271790517	0.698323327	down_in_case
PRJNA8274(	ENSGALG00010022669	MANSC4	case	control	-0.081287315	0.635497135	-0.127911378	0.898219106	0.98028579	down_in_case
PRJNA8274(	ENSGALG00010022671	DBR1	case	control	0.020640296	0.048586662	0.424814029	0.67097225	0.913676733	up_in_case
PRJNA8274(	ENSGALG00010022673	KLHL42	case	control	0.153853939	0.125232086	1.228550479	0.219240392	0.643443819	up_in_case
PRJNA8274(	ENSGALG00010022674	ENSGALG00010022674	case	control	-0.191032689	0.304973252	-0.62639162	0.531058105	0.855560567	down_in_case
PRJNA8274(	ENSGALG00010022681	PPP2R3A	case	control	-0.143600382	0.100642898	-1.426830758	0.153628683	0.56245196	down_in_case
PRJNA8274(	ENSGALG00010022682	EIF1	case	control	-0.124568428	0.050943831	-2.445211238	0.014476732	0.212076065	down_in_case
PRJNA8274(	ENSGALG00010022684	ENSGALG00010022684	case	control	-0.116369984	0.30309824	-0.383934873	0.701026719	0.922516526	down_in_case
PRJNA8274(	ENSGALG00010022685	ATAD1	case	control	-0.053500224	0.068959142	-0.775824962	0.437852343	0.811152806	down_in_case
PRJNA8274(	ENSGALG00010022689	MPL	case	control	-0.014743477	0.256790976	-0.057414313	0.954215162	0.992056045	down_in_case
PRJNA8274(	ENSGALG00010022691	ASIC2	case	control	0.432929385	0.368179442	1.175865177	0.239648756	0.664684583	up_in_case
PRJNA8274(	ENSGALG00010022695	PIK3IP1	case	control	0.026091922	0.109810872	0.237607825	0.812185281	0.956328147	up_in_case
PRJNA8274(	ENSGALG00010022698	SEMA6D	case	control	-0.075557908	0.068721561	-1.099478916	0.271559225	0.698323327	down_in_case
PRJNA8274(	ENSGALG00010022702	CDC20	case	control	0.009428047	0.115265928	0.081793875	0.934810627	0.988149615	up_in_case
PRJNA8274(	ENSGALG00010022705	CRHR1	case	control	0.389712377	0.923568121	0.421963869	0.67305139	0.91379937	up_in_case
PRJNA8274(	ENSGALG00010022707	SALL1	case	control	1.174687182	0.288362596	4.073646157	4.62828E-05	0.006303274	up_in_case
PRJNA8274(	ENSGALG00010022708	SPRED1	case	control	-0.032638146	0.053578179	-0.609168625	0.542412674	0.85975913	down_in_case
PRJNA8274(	ENSGALG00010022709	RNF5	case	control	0.031913744	0.050004053	0.638223145	0.523328433	0.851569935	up_in_case
PRJNA8274(	ENSGALG00010022711	LRRC49	case	control	0.140510959	0.104025022	1.350741932	0.176778115	0.595527443	up_in_case
PRJNA8274(	ENSGALG00010022712	DDX23	case	control	-0.035469584	0.04840761	-0.732727446	0.463724681	0.825091817	down_in_case
PRJNA8274(	ENSGALG00010022717	ELOVL1	case	control	0.105021587	0.070994056	1.479301139	0.139059849	0.541505735	up_in_case
PRJNA8274(	ENSGALG00010022718	C12orf43	case	control	-0.055010884	0.116007912	-0.474199419	0.635357707	0.898587204	down_in_case
PRJNA8274(	ENSGALG00010022719	MSL2	case	control	0.011909953	0.055614256	0.214152879	0.830427841	0.962228709	up_in_case
PRJNA8274(	ENSGALG00010022724	LUZP2	case	control	0.360673307	0.243119623	1.483521988	0.137935779	0.539854583	up_in_case
PRJNA8274(	ENSGALG00010022725	KRT222	case	control	0.256524146	0.60221836	0.425965335	0.670133106	0.913465866	up_in_case
PRJNA8274(	ENSGALG00010022727	ACP2	case	control	0.211840364	0.100823582	2.101099372	0.035632244	0.324939791	up_in_case
PRJNA8274(	ENSGALG00010022729	ENSGALG00010022729	case	control	-0.076094164	0.084646011	-0.898969286	0.368669021	0.771770041	down_in_case
PRJNA8274(	ENSGALG00010022731	LRRC8B	case	control	0.002332648	0.131642047	0.017719628	0.985862522	0.996595084	up_in_case

PRJNA8274(	ENSGALG00010022733	PCCB	case	control	0.070321067	0.066944445	1.050439163	0.293516248	0.716170594	up_in_case
PRJNA8274(	ENSGALG00010022734	ENSGALG00010022734	case	control	-0.087008519	0.066340134	-1.311551749	0.189671423	0.613088007	down_in_case
PRJNA8274(	ENSGALG00010022737	CYLD	case	control	-0.040242327	0.066778386	-0.602625041	0.546758158	0.862508733	down_in_case
PRJNA8274(	ENSGALG00010022741	HNF1A	case	control	1.025472866	0.435168793	2.356494497	0.018448341	0.242205346	up_in_case
PRJNA8274(	ENSGALG00010022742	IFT46	case	control	0.103092296	0.088078021	1.170465631	0.241813637	0.666243691	up_in_case
PRJNA8274(	ENSGALG00010022743	ZNF326	case	control	0.045240978	0.060549277	0.747176185	0.454957217	0.821657174	up_in_case
PRJNA8274(	ENSGALG00010022745	PAPSS2	case	control	0.359186686	0.189697215	1.893473688	0.058294904	0.393535512	up_in_case
PRJNA8274(	ENSGALG00010022747	NT5C3B	case	control	0.044877396	0.089789317	0.499807749	0.617210454	0.891081782	up_in_case
PRJNA8274(	ENSGALG00010022748	ENSGALG00010022748	case	control	-0.266487313	0.282228238	-0.944226258	0.34505404	0.755870938	down_in_case
PRJNA8274(	ENSGALG00010022749	HSPA12A	case	control	-0.194484326	0.106548996	-1.825304161	0.067955143	0.421339236	down_in_case
PRJNA8274(	ENSGALG00010022751	SLC25A12	case	control	-0.046138624	0.091947926	-0.501790811	0.615814678	0.891081782	down_in_case
PRJNA8274(	ENSGALG00010022754	SNX20	case	control	-0.089839069	0.200780963	-0.447448146	0.65455152	0.906850514	down_in_case
PRJNA8274(	ENSGALG00010022755	MINPP1	case	control	0.110660438	0.082688769	1.338276515	0.180806315	0.60147482	up_in_case
PRJNA8274(	ENSGALG00010022759	THSD4	case	control	-0.06752602	0.108246437	-0.623817483	0.532747457	0.855817434	down_in_case
PRJNA8274(	ENSGALG00010022760	ELAVL4	case	control	-0.478009459	0.234700791	-2.036675956	0.041682526	0.340995489	down_in_case
PRJNA8274(	ENSGALG00010022764	MFAP5	case	control	-0.054666136	0.146619495	-0.372843575	0.709264869	0.925521953	down_in_case
PRJNA8274(	ENSGALG00010022765	KRT12	case	control	0.136792527	0.454823983	0.300759266	0.763598072	0.943786458	up_in_case
PRJNA8274(	ENSGALG00010022767	CACNB3	case	control	0.129758883	0.230505408	0.562932055	0.573481152	0.877542213	up_in_case
PRJNA8274(	ENSGALG00010022769	FXYD6	case	control	-0.063689099	0.094027076	-0.677348503	0.498184864	0.839703389	down_in_case
PRJNA8274(	ENSGALG00010022770	HAT1	case	control	0.043459095	0.062861889	0.691342492	0.489350334	0.836347578	up_in_case
PRJNA8274(	ENSGALG00010022771	SGMS1	case	control	0.057513886	0.071985598	0.798963792	0.424311407	0.803723282	up_in_case
PRJNA8274(	ENSGALG00010022774	A1CF	case	control	1.170996174	1.159636238	1.00979612	0.312592979	0.734364975	up_in_case
PRJNA8274(	ENSGALG00010022776	CDH4	case	control	-0.264760073	0.173938882	-1.522144273	0.127972935	0.529650968	down_in_case
PRJNA8274(	ENSGALG00010022778	GDF11	case	control	0.260743007	0.199492402	1.30703227	0.191201752	0.614285566	up_in_case
PRJNA8274(	ENSGALG00010022780	DYNC1I2	case	control	-0.037071524	0.038822059	-0.954908748	0.339623845	0.751259351	down_in_case
PRJNA8274(	ENSGALG00010022782	TMPRSS13	case	control	0.260676421	0.234140976	1.113331058	0.265566272	0.691591307	up_in_case
PRJNA8274(	ENSGALG00010022783	STAG1	case	control	-0.031679586	0.048625231	-0.651505098	0.514720488	0.847493921	down_in_case
PRJNA8274(	ENSGALG00010022786	KRT40	case	control	-0.277637076	0.32384537	-0.857313712	0.391271533	0.784326276	down_in_case
PRJNA8274(	ENSGALG00010022787	ENO4	case	control	0.241076281	0.290395873	0.83016428	0.406445908	0.794253996	up_in_case
PRJNA8274(	ENSGALG00010022788	CYBRD1	case	control	0.00395717	0.125578057	0.031511632	0.974861516	0.993834724	up_in_case
PRJNA8274(	ENSGALG00010022789	ENSGALG00010022789	case	control	0.244615408	0.22266335	1.098588551	0.271947574	0.698323327	up_in_case
PRJNA8274(	ENSGALG00010022792	DDB2	case	control	-0.276775009	0.168579401	-1.641807995	0.100629802	0.490291708	down_in_case
PRJNA8274(	ENSGALG00010022795	TAF4	case	control	-0.025522156	0.062515497	-0.408253275	0.683087739	0.915918327	down_in_case
PRJNA8274(	ENSGALG00010022796	RIMKLB	case	control	0.158109894	0.054483805	2.901961292	0.003708344	0.105216891	up_in_case
PRJNA8274(	ENSGALG00010022797	EFHD1	case	control	0.063235513	0.038584894	1.638867112	0.101240938	0.491460867	up_in_case
PRJNA8274(	ENSGALG00010022799	DCAF17	case	control	0.174688947	0.085039645	2.054205982	0.03995576	0.336547209	up_in_case
PRJNA8274(	ENSGALG00010022800	KLHL10	case	control	-0.086060612	0.29874098	-0.28807769	0.77328727	0.946459495	down_in_case
PRJNA8274(	ENSGALG00010022801	LSM14B	case	control	-0.057286384	0.07364045	-0.777920078	0.436616131	0.810227183	down_in_case
PRJNA8274(	ENSGALG00010022803	ENSGALG00010022803	case	control	-0.066455571	0.175124559	-0.379476023	0.704334406	0.923973441	down_in_case
PRJNA8274(	ENSGALG00010022804	SNX19	case	control	0.061140996	0.085893447	0.711823763	0.476573916	0.830520323	up_in_case
PRJNA8274(	ENSGALG00010022805	ENSGALG00010022805	case	control	-0.016260111	0.13964467	-0.116439182	0.907304483	0.981730793	down_in_case
PRJNA8274(	ENSGALG00010022806	SPPL3	case	control	-0.024966149	0.044363144	-0.562767791	0.573593016	0.877542213	down_in_case
PRJNA8274(	ENSGALG00010022807	ADCY6	case	control	0.046300167	0.073946327	0.626132071	0.531228318	0.855560567	up_in_case
PRJNA8274(	ENSGALG00010022808	PSMA7	case	control	0.027742131	0.056735241	0.488975295	0.624859187	0.894741574	up_in_case
PRJNA8274(	ENSGALG00010022809	MYO1E	case	control	0.003830303	0.064960413	0.058963648	0.952981063	0.992056045	up_in_case
PRJNA8274(	ENSGALG00010022811	GIGYF2	case	control	-0.06135204	0.04681725	-1.310458028	0.190040934	0.613311975	down_in_case
PRJNA8274(	ENSGALG00010022813	USP24	case	control	-0.170699003	0.075857595	-2.250255931	0.024432704	0.276609812	down_in_case
PRJNA8274(	ENSGALG00010022815	LRRC8D	case	control	0.114714462	0.122342207	0.937652383	0.348423082	0.758941366	up_in_case
PRJNA8274(	ENSGALG00010022816	METTL8	case	control	0.277496741	0.176513284	1.572101171	0.11592709	0.512919984	up_in_case
PRJNA8274(	ENSGALG00010022817	KRT23	case	control	0.260820836	0.691169685	0.37736151	0.705904966	0.924320939	up_in_case
PRJNA8274(	ENSGALG00010022819	PACSIN3	case	control	-0.263045421	0.107341556	-2.450546006	0.014263973	0.210935697	down_in_case
PRJNA8274(	ENSGALG00010022820	SHTN1	case	control	0.167628928	0.156914892	1.068279279	0.285394552	0.709887302	up_in_case
PRJNA8274(	ENSGALG00010022821	ENSGALG00010022821	case	control	2.194189199	0.981643301	2.235220468			up_in_case
PRJNA8274(	ENSGALG00010022822	KCNJ13	case	control	3.610347545	0.567462718	6.362263866	1.98802E-10	1.18453E-07	up_in_case
PRJNA8274(	ENSGALG00010022823	IGHMBP2	case	control	-0.052763947	0.055872871	-0.944357183	0.344987154	0.755870938	down_in_case
PRJNA8274(	ENSGALG00010022824	PUS1	case	control	0.008720152	0.056643559	0.153947818	0.87765088	0.975889142	up_in_case
PRJNA8274(	ENSGALG00010022825	SS18L1	case	control	0.010999427	0.189916415	0.057917201	0.953814581	0.992056045	up_in_case
PRJNA8274(	ENSGALG00010022826	SNORC	case	control	0.190609063	1.005036073	0.189653952	0.849580307	0.968927451	up_in_case
PRJNA8274(	ENSGALG00010022827	CMTR2	case	control	-0.04683902	0.060735658	-0.77119474	0.440591512	0.813083204	down_in_case
PRJNA8274(	ENSGALG00010022828	PRKG1	case	control	-0.301367192	0.106461401	-2.830764868	0.004643684	0.116274729	down_in_case
PRJNA8274(	ENSGALG00010022830	MTG2	case	control	0.084208326	0.105793453	0.795969165	0.426049958	0.805094126	up_in_case
PRJNA8274(	ENSGALG00010022831	LRRC8C	case	control	-0.077396055	0.066778582	-1.15899519	0.246458146	0.670921661	down_in_case
PRJNA8274(	ENSGALG00010022833	HRH3	case	control	0.113924053	0.3325386	0.342588958	0.73190771	0.933770678	up_in_case
PRJNA8274(	ENSGALG00010022834	CHRNA7	case	control	0.267285314	0.234069034	1.141908047	0.253492243	0.678954688	up_in_case
PRJNA8274(	ENSGALG00010022837	ITGB3	case	control	0.171402928	0.115795342	1.480223002	0.138813744	0.540932258	up_in_case
PRJNA8274(	ENSGALG00010022838	ARFGAP2	case	control	-0.077812762	0.044008357	-1.76813603	0.077038165	0.445649581	down_in_case
PRJNA8274(	ENSGALG00010022839	SYT12	case	control	0.473876825	0.214030195	2.214065283	0.026824288	0.291658148	up_in_case
PRJNA8274(	ENSGALG00010022840	MARVELD1	case	control	-0.025839952	0.115467895	-0.223784733	0.82292481	0.959776917	down_in_case
PRJNA8274(	ENSGALG00010022841	ENSGALG00010022841	case	control	-0.085831177	0.328433764	-0.261334815	0.793834321	0.95280257	down_in_case
PRJNA8274(	ENSGALG00010022842	PHC1	case	control	0.073551743	0.044580423	1.649866423	0.09897026	0.488407564	up_in_case
PRJNA8274(	ENSGALG00010022845	HYDIN	case	control	-0.264265651	0.294178592	-0.89831707	0.369016535	0.771834431	down_in_case
PRJNA8274(	ENSGALG00010022848	SGIP1	case	control	0.313464856	0.359969282	0.870810016	0.383857896	0.78015462	up_in_case
PRJNA8274(	ENSGALG00010022850	ULK1	case	control	-0.024253865	0.073420579	-0.330341518	0.741141926	0.937158859	down_in_case
PRJNA8274(	ENSGALG00010022858	HERC4	case	control	-0.133907511	0.052054432	-2.572451659	0.010098106	0.178716475	down_in_case
PRJNA8274(	ENSGALG00010022860	MOB3C	case	control	0.03818563	0.074311029	0.513862214	0.607348327	0.887328057	up_in_case
PRJNA8274(	ENSGALG00010022862	ACADS	case	control	-0.091837316	0.055858766	-1.644098553	0.100155847	0.489650807	down_in_case
PRJNA8274(	ENSGALG00010022863	FAM81A	case	control	-0.624140276	0.249007283	-2.506514142	0.012192818	0.193945826	down_in_case
PRJNA8274(	ENSGALG00010022864	CSTPP1	case	control	-0.108323658	0.112428605	-0.963488414	0.335302476	0.749356776	down_in_case
PRJNA8274(	ENSGALG00010022866	KLHL11	case	control	0.025319631	0.060442795	0.418902377	0.675287479	0.914247368	up_in_case
PRJNA8274(	ENSGALG00010022867	ATPAF1	case	control	0.153750914	0.085006882	1.808687848	0.070499512	0.429362445	up_in_case
PRJNA8274(	ENSGALG00010022868	VAC14	case	control	0.103160175	0.049650431	2.077729678	0.037734264	0.329750561	up_in_case
PRJNA8274(	ENSGALG00010022872	KRT13	case	control	0.525194086	0.405888992	1.293935279	0.195687777	0.619095929	up_in_case
PRJNA8274(	ENSGALG00010022873	NGEF	case	control	0.051212664	0.326356776	0.156922325	0.875306065	0.975666505	up_in_case
PRJNA8274(	ENSGALG00010022874	EFCAB14	case	control	-0.070245995	0.067731502	-1.037124423	0.299677874	0.723109974	down_in_case
PRJNA8274(	ENSGALG00010022875	SPON1	case	control	-0.154194288	0.304112213	-0.507030897	0.61213314	0.889814948	down_in_case
PRJNA8274(	ENSGALG00010022877	KYAT3	case	control	-0.041867859	0.120521279	-0.34738977	0.728298518	0.933057319	down_in_case
PRJNA8274(	ENSGALG00010022879	ENSGALG00010022879	case	control	-0.316720678	0.236843335	-1.337258144	0.181138389	0.601788276	down_in_case
PRJNA8274(	ENSGALG00010022881	CCNB1	case	control	0.008655607	0.063391033	0.136543076	0.891391974	0.978917427	up_in_case
PRJNA8274(	ENSGALG00010022885	M6PR	case	control	-0.005296738	0.038010803	-0.13934823	0.88917498	0.978017246	down_in_case
PRJNA8274(	ENSGALG00010022886	PLA2G15	case	control	-0.250330414	0.13061673	-1.91652642	0.055298124	0.383470355	down_in_case
PRJNA8274(	ENSGALG00010022888	OSBPL2	case	control	-0.049685221	0.065748526	-0.755685708	0.449837655	0.819037259	down_in_case
PRJNA8274(	ENSGALG00010022890	MYPN	case	control	-0.409276566	0.126806052	-3.227579123	0.001248425	0.055677577	down_in_case
PRJNA8274(	ENSGALG00010022891	NTM	case	control	-0.244615961	0.162667295	-1.503780839	0.132637806	0.535938934	down_in_case
PRJNA8274(	ENSGALG00010022900	MMP17	case	control	0.105370999	0.087009186	1.211033048	0.225882739	0.649498359	up_in_case
PRJNA8274(	ENSGALG00010022911	SMPD3	case	control	-0.028349425	0.539435268	-0.052553896	0.958087352	0.992056045	down_in_case
PRJNA8274(	ENSGALG00010022917	CYP4B7	case	control	0.573468847	0.413629712	1.386430499	0.165615489	0.580618526	up_in_case
PRJNA8274(	ENSGALG00010022919	ACLY	case	control	0.054857582	0.072540773	0.756231007	0.449510705	0.818749597	up_in_case
PRJNA8274(	ENSGALG00010022921	ENSGALG00010022921	case	control	0.002213512	0.110977182	0.019945654	0.984086726	0.996591771	up_in_case
PRJNA8274(	ENSGALG00010022923	CD63	case	control	0.048944808	0.063369404	0.772372851	0.439893629	0.812618382	up_in_case
PRJNA8274(	ENSGALG00010022928	RNF111	case	control	-0.008723665	0.042438804	-0.20555868	0.837135657	0.964784002	down_in_case
PRJNA8274(	ENSGALG00010022929	LRP4	case	control	-0.323363876	0.109010334	-2.966359836	0.003013477	0.093476614	down_in_case
PRJNA8274(	ENSGALG00010022930	GTF2B	case	control	0.088208439	0.066635611	1.323743229	0.185588336	0.608776505	up_in_case
PRJNA8274(	ENSGALG00010022931	RDH5	case	control	-0.076184314	0.131002085	-0.581550394	0.560869565	0.870461773	down_in_case
PRJNA8274(	ENSGALG00010022932	OPCML	case	control	-0.041363218	0.115770749	-0.357285567	0.720878034	0.92919613	down_in_case
PRJNA8274(	ENSGALG00010022933	UNC119B	case	control	0.092670762	0.061012188	1.518889338	0.128790353	0.530596961	up_in_case
PRJNA8274(	ENSGALG00010022937	BLOC1S1	case	control	-0.087870594	0.117590765	-0.74725761	0.454908075	0.821657174	down_in_case
PRJNA8274(	ENSGALG00010022942	NEU2	case	control	0.389002719	0.238619923	1.630218947	0.103055231	0.492515853	up_in_case
PRJNA8274(	ENSGALG00010022945	KCNA4	case	control	0.480220923	0.323608131	1.483958148	0.137820024	0.539854583	up_in_case
PRJNA8274(	ENSGALG00010022946	KRT17	case	control	0.152665905	0.410521843	0.371882539	0.709980306	0.925630832	up_in_case
PRJNA8274(	ENSGALG00010022948	PDZK1IP1	case	control	1.654146974	0.641841195	2.577190412	0.009960706	0.177161817	up_in_case
PRJNA8274(	ENSGALG00010022949	INSC	case	control	0.335021052	0.160120263	2.092308906	0.036410892	0.32664727	up_in_case
PRJNA8274(	ENSGALG00010022950	ITGA7	case	control	0.134555016	0.127471417	1.055570098	0.291164668	0.714222439	up_in_case
PRJNA8274(	ENSGALG00010022953	ENSGALG00010022953	case	control	0.352489342	1.025457712	0.343738545	0.731042921	0.933770678	up_in_case
PRJNA8274(	ENSGALG00010022954	ADRM1	case	control	0.003747798	0.066435044	0.056412975	0.955012821	0.992056045	up_in_case
PRJNA8274(	ENSGALG00010022956	PBLD	case	control	0.050458797	0.247867027	0.203572045	0.83868794	0.96509516	up_in_case
PRJNA8274(	ENSGALG00010022957	SLTM	case	control	-0.041254328	0.060990404	-0.676406873	0.498782355	0.840142058	down_in_case
PRJNA8274(	ENSGALG00010022958	SFSWAP	case	control	0.052651776	0.076948995	0.684242544	0.493822021	0.837979697	up_in_case
PRJNA8274(	ENSGALG00010022959	ADAMTS8	case	control	0.127188192	0.077108208	1.649476694	0.099050014	0.488407564	up_in_case
PRJNA8274(	ENSGALG00010022967	ADAMTS15	case	control	0.064238413	0.08947854	0.717919773	0.472806755	0.829235491	up_in_case
PRJNA8274(	ENSGALG00010022969	TTN	case	control	-0.348235888	0.116840718	-2.980432628	0.002878415	0.090663747	down_in_case
PRJNA8274(	ENSGALG00010022971	CKAP5	case	control	-0.009031261	0.041138498	-0.219533083	0.826234812	0.961207256	down_in_case
PRJNA8274(	ENSGALG00010022972	ENSGALG00010022972	case	control	0.72172519	0.889288111	0.81157634	0.417034772	0.799987395	up_in_case
PRJNA8274(	ENSGALG00010022974	INPP5D	case	control	-0.040412569	0.090329649	-0.447389855	0.6545936	0.906850514	down_in_case
PRJNA8274(	ENSGALG00010022976	MINDY2	case	control	-0.002231509	0.04394561	-0.050778893	0.95950171	0.992056045	down_in_case
PRJNA8274(	ENSGALG00010022978	ARL14EP	case	control	-0.081882624	0.057894592	-1.414339785	0.157262161	0.568321683	down_in_case
PRJNA8274(	ENSGALG00010022979	LAMA5	case	control	-0.177003534	0.115580904	-1.531425414	0.125664287	0.525056024	down_in_case

PRJNA8274(	ENSGALG00010022981	HNRNPH3	case	control	-0.072142148	0.0486045	-1.484268921	0.137737592	0.539854583	down_in_case
PRJNA8274(	ENSGALG00010022983	PKN2	case	control	-0.074086548	0.044041362	-1.682203821	0.092529323	0.477342721	down_in_case
PRJNA8274(	ENSGALG00010022985	ENSGALG00010022985	case	control	0.215823372	0.345706933	0.624295758	0.532433369	0.855771291	up_in_case
PRJNA8274(	ENSGALG00010022986	ALG9	case	control	-0.130059398	0.074031018	-1.756823044	0.078947976	0.449171025	down_in_case
PRJNA8274(	ENSGALG00010022987	F2	case	control	1.246223836	0.754227855	1.652317437	0.098469857	0.487941198	up_in_case
PRJNA8274(	ENSGALG00010022990	SLC25A22	case	control	0.11319272	0.09170025	1.234377445	0.21706229	0.642491319	up_in_case
PRJNA8274(	ENSGALG00010022991	TAL1	case	control	0.17589609	0.149198413	1.178940758	0.238421767	0.663323557	up_in_case
PRJNA8274(	ENSGALG00010022992	SLC7A6	case	control	0.108306047	0.080509707	1.345254507	0.178543059	0.598351476	up_in_case
PRJNA8274(	ENSGALG00010022994	LIPC	case	control	0.317107858	1.013845922	0.312777169	0.754449962	0.941311206	up_in_case
PRJNA8274(	ENSGALG00010022997	ARHGAP1	case	control	-0.103965682	0.06224131	-1.67036461	0.094847247	0.481304341	down_in_case
PRJNA8274(	ENSGALG00010022999	ENSGALG00010022999	case	control	0.597365795	0.332219568	1.798105389	0.072160308	0.434277972	up_in_case
PRJNA8274(	ENSGALG00010023002	ATG16L1	case	control	0.013154686	0.053032579	0.248049151	0.804096378	0.954997212	up_in_case
PRJNA8274(	ENSGALG00010023003	ENSGALG00010023003	case	control	0.485218795	0.640571593	0.757477853	0.448763629	0.818701429	up_in_case
PRJNA8274(	ENSGALG00010023005	IRF7	case	control	0.006371837	0.161937025	0.039347621	0.96861324	0.992761697	up_in_case
PRJNA8274(	ENSGALG00010023007	LMO4	case	control	-0.113891801	0.087267384	-1.305090131	0.191862159	0.615090794	down_in_case
PRJNA8274(	ENSGALG00010023013	ENSGALG00010023013	case	control	-0.262206631	0.554577087	-0.472804659	0.63635255	0.898973839	down_in_case
PRJNA8274(	ENSGALG00010023015	METTL2B	case	control	-0.047060242	0.064832816	-0.725870704	0.467918038	0.828429855	down_in_case
PRJNA8274(	ENSGALG00010023017	BUB1B	case	control	-0.064925149	0.106454795	-0.609884679	0.541938202	0.85975913	down_in_case
PRJNA8274(	ENSGALG00010023019	CABP1	case	control	-0.150276193	0.247644585	-0.606822046	0.543969016	0.861114226	down_in_case
PRJNA8274(	ENSGALG00010023024	PRMT7	case	control	-0.007993409	0.072575486	-0.110139247	0.912298943	0.98266608	down_in_case
PRJNA8274(	ENSGALG00010023026	PHRF1	case	control	-0.058996465	0.045086736	-1.308510456	0.19070023	0.613935676	down_in_case
PRJNA8274(	ENSGALG00010023028	TMEM61	case	control	-0.187943814	0.196602163	-0.95596005	0.339092419	0.751259351	down_in_case
PRJNA8274(	ENSGALG00010023030	HS2ST1	case	control	-0.023018948	0.042186689	-0.545644817	0.585310111	0.881307382	down_in_case
PRJNA8274(	ENSGALG00010023041	CD151	case	control	0.133178273	0.06804922	1.957087418	0.050337191	0.367061596	up_in_case
PRJNA8274(	ENSGALG00010023042	ENSGALG00010023042	case	control	-0.380624321	0.630570089	-0.603619372	0.54609673	0.862416703	down_in_case
PRJNA8274(	ENSGALG00010023043	CNP	case	control	-0.175529966	0.095480422	-1.838387002	0.066005399	0.41627012	down_in_case
PRJNA8274(	ENSGALG00010023046	SLC7A6OS	case	control	0.031195841	0.061004034	0.511373412	0.609089609	0.8877909	up_in_case
PRJNA8274(	ENSGALG00010023048	ARFGAP1	case	control	0.052248321	0.055821681	0.935986165	0.349280311	0.759342183	up_in_case
PRJNA8274(	ENSGALG00010023050	RUFY2	case	control	0.051795329	0.071115225	0.728329677	0.4664118	0.827112128	up_in_case
PRJNA8274(	ENSGALG00010023052	RAD54L	case	control	0.052620752	0.092182514	0.57083225	0.56811336	0.874461133	up_in_case
PRJNA8274(	ENSGALG00010023053	MAN1A2	case	control	-0.017800169	0.047512645	-0.374640665	0.707927726	0.92490115	down_in_case
PRJNA8274(	ENSGALG00010023054	AQP9	case	control	0.472596124	0.294414323	1.60520765	0.108448114	0.500583614	up_in_case
PRJNA8274(	ENSGALG00010023056	SAG	case	control	0.054931474	0.418685841	0.131199742	0.8956173	0.980176778	up_in_case
PRJNA8274(	ENSGALG00010023057	PAFAH1B2	case	control	0.0402081	0.041060611	0.979237739	0.327462526	0.745061912	up_in_case
PRJNA8274(	ENSGALG00010023058	PPARA	case	control	0.081405861	0.139660079	0.582885681	0.55997026	0.86984941	up_in_case
PRJNA8274(	ENSGALG00010023059	SELENOF	case	control	0.133627896	0.053986201	2.475223192	0.013315294	0.202994347	up_in_case
PRJNA8274(	ENSGALG00010023060	ALDH1A2	case	control	-0.119084534	0.112944633	-1.054362039	0.291717195	0.71442985	down_in_case
PRJNA8274(	ENSGALG00010023062	STX2	case	control	-0.044504655	0.071110653	-0.625850743	0.531412845	0.855560567	down_in_case
PRJNA8274(	ENSGALG00010023063	ENSGALG00010023063	case	control	-0.017500533	0.043449946	-0.402774567	0.687114072	0.917290381	down_in_case
PRJNA8274(	ENSGALG00010023064	ATG13	case	control	-0.031884401	0.043036312	-0.740872068	0.458771013	0.824070529	down_in_case
PRJNA8274(	ENSGALG00010023065	DNA2	case	control	-0.071494381	0.106900258	-0.668795211	0.503626122	0.842675107	down_in_case
PRJNA8274(	ENSGALG00010023066	TLR21	case	control	-0.294815186	0.184347497	-1.599236175	0.109768135	0.501817242	down_in_case
PRJNA8274(	ENSGALG00010023067	C15orf62	case	control	0.900242046	0.431450143	2.086549422	0.036928883	0.32835414	up_in_case
PRJNA8274(	ENSGALG00010023068	CDPF1	case	control	0.062660475	0.068700875	0.912076818	0.361728281	0.768034805	up_in_case
PRJNA8274(	ENSGALG00010023069	ENSGALG00010023069	case	control	0.303791458	0.273231145	1.111847839	0.266203579	0.69218122	up_in_case
PRJNA8274(	ENSGALG00010023070	SIDT2	case	control	-5.39135E-05	0.058210719	-0.000926178	0.999261017	0.99976345	down_in_case
PRJNA8274(	ENSGALG00010023071	POLR2M	case	control	-0.077061565	0.064040411	-1.203327139	0.228849751	0.652401326	down_in_case
PRJNA8274(	ENSGALG00010023073	POMGNT1	case	control	-0.103002665	0.065684722	-1.568137344	0.116849083	0.51350396	down_in_case
PRJNA8274(	ENSGALG00010023074	CCNT1	case	control	-0.005517918	0.05008074	-0.110180451	0.912266266	0.98266608	down_in_case
PRJNA8274(	ENSGALG00010023075	PKDREJ	case	control	0.096752173	0.1988828	0.486478332	0.626628064	0.895656773	up_in_case
PRJNA8274(	ENSGALG00010023076	DNAJC7	case	control	-0.147624853	0.065539009	-2.252473072	0.024292393	0.275699376	down_in_case
PRJNA8274(	ENSGALG00010023077	KANSL2	case	control	-0.036511055	0.074293687	-0.491442226	0.623113705	0.894052087	down_in_case
PRJNA8274(	ENSGALG00010023078	TAGLN	case	control	-0.107441066	0.208110735	-0.516268736	0.605666729	0.88576746	down_in_case
PRJNA8274(	ENSGALG00010023079	SLC25A16	case	control	0.145531439	0.087481941	1.66355979	0.096200433	0.483634708	up_in_case
PRJNA8274(	ENSGALG00010023080	MCRS1	case	control	-0.018535577	0.040045836	-0.462859026	0.643465419	0.902038523	down_in_case
PRJNA8274(	ENSGALG00010023081	PRLHR2	case	control	0.529325511	0.194276337	2.72460105	0.006437923	0.13904105	up_in_case
PRJNA8274(	ENSGALG00010023083	HARBI1	case	control	0.023485204	0.113371521	0.207152593	0.83589069	0.964083973	up_in_case
PRJNA8274(	ENSGALG00010023084	ENSGALG00010023084	case	control	-0.260029666	0.171474907	-1.516429835	0.1294107	0.531621088	down_in_case
PRJNA8274(	ENSGALG00010023086	PCSK7	case	control	0.096461672	0.075670301	1.274762635	0.20239327	0.625372462	up_in_case
PRJNA8274(	ENSGALG00010023087	SH3GLB1	case	control	-0.053044764	0.058334589	-0.909319242	0.363181637	0.769407023	down_in_case
PRJNA8274(	ENSGALG00010023089	ENSGALG00010023089	case	control	0.463231181	0.743682032	0.622888763	0.533357623	0.855817434	up_in_case
PRJNA8274(	ENSGALG00010023090	LRRC41	case	control	0.036874972	0.065316541	0.564557935	0.572374483	0.876814097	up_in_case
PRJNA8274(	ENSGALG00010023091	ENSGALG00010023091	case	control	-0.090621586	0.052709102	-1.719277742	0.085563811	0.462647434	down_in_case
PRJNA8274(	ENSGALG00010023093	POP5	case	control	-0.132583213	0.112955696	-1.173762967	0.240489981	0.66582899	down_in_case
PRJNA8274(	ENSGALG00010023094	CCDC32	case	control	-0.219210601	0.088307784	-2.482347436	0.013051995	0.20026129	down_in_case
PRJNA8274(	ENSGALG00010023096	SPATS2	case	control	-0.015568047	0.068273731	-0.228023964	0.819627611	0.958669267	down_in_case
PRJNA8274(	ENSGALG00010023097	LURAP1	case	control	0.042033433	0.122441759	0.343293276	0.731377838	0.933770678	up_in_case
PRJNA8274(	ENSGALG00010023099	DNAJC22	case	control	0.673576991	1.200685762	0.560993569	0.574801921	0.877910508	up_in_case
PRJNA8274(	ENSGALG00010023102	RNF10	case	control	-0.031549253	0.059839547	-0.527230809	0.598033316	0.884680373	down_in_case
PRJNA8274(	ENSGALG00010023103	DGKD	case	control	0.021154133	0.069010414	0.306535378	0.759197048	0.942078947	up_in_case
PRJNA8274(	ENSGALG00010023104	ENSGALG00010023104	case	control	0.200855863	0.129509206	1.550900269	0.120925586	0.519499513	up_in_case
PRJNA8274(	ENSGALG00010023107	ZC3HAV1	case	control	-0.016846781	0.134556539	-0.125202243	0.900363441	0.98028579	down_in_case
PRJNA8274(	ENSGALG00010023108	TET1	case	control	-0.100510459	0.106508683	-0.943683244	0.345331539	0.755935621	down_in_case
PRJNA8274(	ENSGALG00010023110	LIMA1	case	control	0.154886516	0.04967378	3.118073847	0.001820372	0.068865919	up_in_case
PRJNA8274(	ENSGALG00010023112	PCMT1L	case	control	-0.099202304	0.0983298	-1.00887324	0.313035431	0.734574752	down_in_case
PRJNA8274(	ENSGALG00010023113	MYZAP	case	control	0.032873485	0.095110631	0.345634183	0.729617653	0.933556014	up_in_case
PRJNA8274(	ENSGALG00010023114	UQCRHL	case	control	-0.045468555	0.082357568	-0.55208715	0.580888645	0.879575141	down_in_case
PRJNA8274(	ENSGALG00010023115	RPLP2	case	control	-0.005078083	0.061753938	-0.082230919	0.934463087	0.988140189	down_in_case
PRJNA8274(	ENSGALG00010023116	PROC	case	control	1.177238296	0.803886926	1.464432693	0.143075761	0.545763019	up_in_case
PRJNA8274(	ENSGALG00010023117	RPUSD2	case	control	-0.072857009	0.060810604	-1.198097117	0.230879211	0.655335776	down_in_case
PRJNA8274(	ENSGALG00010023118	PIDD1	case	control	0.087954145	0.123641967	0.711361583	0.476860199	0.830520323	up_in_case
PRJNA8274(	ENSGALG00010023119	NDRG4	case	control	0.000472036	0.122455128	0.003854769	0.996924347	0.998921977	up_in_case
PRJNA8274(	ENSGALG00010023120	CHRM4	case	control	-0.265675197	0.160963327	-1.650532468	0.098834078	0.488407564	down_in_case
PRJNA8274(	ENSGALG00010023122	ENSGALG00010023122	case	control	0.244627967	0.175705813	1.392258809	0.16384404	0.578511055	up_in_case
PRJNA8274(	ENSGALG00010023123	RNF214	case	control	-0.037405705	0.059053996	-0.633415316	0.526462466	0.85251736	down_in_case
PRJNA8274(	ENSGALG00010023124	NSUN4	case	control	0.126653998	0.098744411	1.282644728	0.199616572	0.622902456	up_in_case
PRJNA8274(	ENSGALG00010023125	COL20A1	case	control	0.015705451	0.130534975	0.120316038	0.9042328	0.981104465	up_in_case
PRJNA8274(	ENSGALG00010023126	CGNL1	case	control	-0.049322049	0.114484371	-0.43081906	0.66659994	0.912284937	down_in_case
PRJNA8274(	ENSGALG00010023127	CERS5	case	control	0.14105827	0.048312488	2.919706183	0.003503615	0.101420446	up_in_case
PRJNA8274(	ENSGALG00010023128	IWS1	case	control	0.001480032	0.043241017	0.034227496	0.972695741	0.99340857	up_in_case
PRJNA8274(	ENSGALG00010023130	DYNLT5	case	control	-0.458847677	0.387227829	-1.184955321	0.236035113	0.660642087	down_in_case
PRJNA8274(	ENSGALG00010023131	SOX6	case	control	-0.048496921	0.084440748	-0.574330781	0.565743983	0.872906976	down_in_case
PRJNA8274(	ENSGALG00010023134	KNL1	case	control	-0.079001447	0.094704032	-0.834193071	0.404172195	0.79260318	down_in_case
PRJNA8274(	ENSGALG00010023135	GINS3	case	control	0.039040701	0.073161345	0.533624711	0.593601208	0.883625114	up_in_case
PRJNA8274(	ENSGALG00010023136	MDK	case	control	-0.20852758	0.122126364	-1.70747391	0.087734003	0.468132927	down_in_case
PRJNA8274(	ENSGALG00010023137	ETV6	case	control	0.010806256	0.044719424	0.241645695	0.809054711	0.955875648	up_in_case
PRJNA8274(	ENSGALG00010023140	CCAR1	case	control	-0.018255877	0.071028504	-0.257021841	0.797161912	0.95280257	down_in_case
PRJNA8274(	ENSGALG00010023142	TLK2	case	control	-0.097693391	0.042361479	-2.3061846	0.02110032	0.260340442	down_in_case
PRJNA8274(	ENSGALG00010023144	C11orf58	case	control	-0.029431546	0.047420131	-0.620655093	0.534826582	0.856463908	down_in_case
PRJNA8274(	ENSGALG00010023147	RAD51	case	control	-0.118291071	0.087050239	-1.35888279	0.174183733	0.593154767	down_in_case
PRJNA8274(	ENSGALG00010023149	ADGRD1	case	control	-0.282559647	0.13232007	-2.135425467	0.032726272	0.311575029	down_in_case
PRJNA8274(	ENSGALG00010023151	ENSGALG00010023151	case	control	0.144577308	0.397800877	0.363441401	0.716275179	0.926862121	up_in_case
PRJNA8274(	ENSGALG00010023155	CFAP263	case	control	0.151791016	0.189961379	0.799062508	0.424254168	0.803723282	up_in_case
PRJNA8274(	ENSGALG00010023157	STOX1	case	control	0.403868042	0.268473048	1.504315032	0.13250027	0.535857167	up_in_case
PRJNA8274(	ENSGALG00010023159	DAW1	case	control	-0.465937638	0.342454181	-1.36058388	0.173645229	0.592914702	down_in_case
PRJNA8274(	ENSGALG00010023160	ENSGALG00010023160	case	control	-0.028694555	0.05341321	-0.537218328	0.591116812	0.882949889	down_in_case
PRJNA8274(	ENSGALG00010023161	ODF2L	case	control	0.236453814	0.300516793	0.786823964	0.431384923	0.807261374	up_in_case
PRJNA8274(	ENSGALG00010023162	CHRNA4	case	control	-0.247995654	0.199493098	-1.243128991	0.213820304	0.638602829	down_in_case
PRJNA8274(	ENSGALG00010023163	CRTAC1	case	control	0.02688337	0.12006305	0.223910439	0.822826993	0.959776917	up_in_case
PRJNA8274(	ENSGALG00010023164	NKIRAS2	case	control	0.008082493	0.06702933	0.120581441	0.90402257	0.981104465	up_in_case
PRJNA8274(	ENSGALG00010023165	CSNK2A2	case	control	-0.022592278	0.042805221	-0.527792577	0.59764331	0.884680373	down_in_case
PRJNA8274(	ENSGALG00010023166	KDM2A	case	control	-0.021980244	0.051774366	-0.424539133	0.671172672	0.913676733	down_in_case
PRJNA8274(	ENSGALG00010023167	GPD1	case	control	-0.609673286	0.284800381	-2.140703899	0.032297924	0.309838273	down_in_case
PRJNA8274(	ENSGALG00010023168	SPHKAP	case	control	0.360003272	0.379162337	0.949470021	0.342381612	0.754383554	up_in_case
PRJNA8274(	ENSGALG00010023169	RAN	case	control	0.010666697	0.048710389	0.218981979	0.826664086	0.961207256	up_in_case
PRJNA8274(	ENSGALG00010023170	TCF12	case	control	-0.048414812	0.055520618	-0.872015005	0.383200192	0.780061953	down_in_case
PRJNA8274(	ENSGALG00010023171	ENSGALG00010023171	case	control	0.139224058	0.066331986	2.098897769	0.035825914	0.324974524	up_in_case
PRJNA8274(	ENSGALG00010023172	DHX58	case	control	-0.044652988	0.181976575	-0.245377672	0.806164016	0.9556344	down_in_case
PRJNA8274(	ENSGALG00010023174	KCNQ2	case	control	0.127247413	0.196642216	0.647101195	0.517566452	0.848372336	up_in_case
PRJNA8274(	ENSGALG00010023175	GRK2	case	control	0.000408705	0.067561712	0.006049357	0.995173341	0.998665177	up_in_case
PRJNA8274(	ENSGALG00010023177	LZTS2	case	control	-0.095257402	0.070064202	-1.359573068	0.173965066	0.593015599	down_in_case
PRJNA8274(	ENSGALG00010023178	CFAP20	case	control	0.032192958	0.049674188	0.648082227	0.516931769	0.848054666	up_in_case
PRJNA8274(	ENSGALG00010023179	KAT2A	case	control	0.103232625	0.081050014	1.273690397	0.202773161	0.625537942	up_in_case
PRJNA8274(	ENSGALG00010023180	EEF1A2	case	control	-0.220708084	0.12761026	-1.729548108	0.083711044	0.458084422	down_in_case
PRJNA8274(	ENSGALG00010023181	PLEKHA7	case	control	-0.055025143	0.086810881	-0.633850764	0.52617822	0.85251736	down_in_case
PRJNA8274(	ENSGALG00010023182	EPHB6	case	control	-0.047405149	0.073784519	-0.64248097	0.520560941	0.850162341	down_in_case

PRJNA8274(	ENSGALG00010023183	ENSGALG00010023183	case	control	0.091019415	0.530192246	0.171672475	0.863695026	0.972890026	up_in_case
PRJNA8274(	ENSGALG00010023184	SMARCD1	case	control	0.055675391	0.047310472	1.176809039	0.239271734	0.66429721	up_in_case
PRJNA8274(	ENSGALG00010023185	FBXW4	case	control	0.008122833	0.094414854	0.086033425	0.931439846	0.987258715	up_in_case
PRJNA8274(	ENSGALG00010023186	MIER1	case	control	0.032889137	0.040670081	0.808681379	0.418698442	0.800881182	up_in_case
PRJNA8274(	ENSGALG00010023187	ENSGALG00010023187	case	control	-0.262331777	0.217534791	-1.205930214	0.2278444	0.651681204	down_in_case
PRJNA8274(	ENSGALG00010023188	KIF1BP	case	control	-0.056713514	0.046304091	-1.224805688	0.220648441	0.644744838	down_in_case
PRJNA8274(	ENSGALG00010023189	RMDN3	case	control	0.019661027	0.063592643	0.309171415	0.757191137	0.941659745	up_in_case
PRJNA8274(	ENSGALG00010023190	SF3B3	case	control	-0.024178939	0.052292624	-0.462377619	0.643810547	0.902038523	down_in_case
PRJNA8274(	ENSGALG00010023193	PPDPF	case	control	-0.159735049	0.115446342	-1.383630233	0.166471706	0.581755962	down_in_case
PRJNA8274(	ENSGALG00010023194	MRC2	case	control	0.007472978	0.08690619	0.085989019	0.931475146	0.987258715	up_in_case
PRJNA8274(	ENSGALG00010023195	ENSGALG00010023195	case	control	0.243926271	0.165577353	1.473186195	0.140700836	0.543349166	up_in_case
PRJNA8274(	ENSGALG00010023196	DGKZ	case	control	-0.142771312	0.053516495	-2.667800145	0.007634966	0.152061299	down_in_case
PRJNA8274(	ENSGALG00010023198	PTK6	case	control	-0.300544093	0.349525805	-0.859862389	0.389864903	0.783877029	down_in_case
PRJNA8274(	ENSGALG00010023199	RPS13	case	control	-0.088261405	0.070543108	-1.251169777	0.210872542	0.636310899	down_in_case
PRJNA8274(	ENSGALG00010023200	VPS26A	case	control	2.98542E-06	0.035307001	8.45561E-05	0.999932534	0.999932534	up_in_case
PRJNA8274(	ENSGALG00010023201	ZNF280D	case	control	-0.002820686	0.059206847	-0.047641205	0.962002192	0.992056045	down_in_case
PRJNA8274(	ENSGALG00010023202	PID1	case	control	-0.077392361	0.063311853	-1.222399245	0.221556685	0.645689578	down_in_case
PRJNA8274(	ENSGALG00010023203	COG4	case	control	0.036932449	0.042400235	0.871043492	0.383730406	0.780115057	up_in_case
PRJNA8274(	ENSGALG00010023204	SRGN	case	control	0.504725366	0.39503356	1.277677184	0.201363273	0.624619264	up_in_case
PRJNA8274(	ENSGALG00010023205	DNAJC17	case	control	0.005459165	0.08278504	0.065943858	0.947422523	0.991085741	up_in_case
PRJNA8274(	ENSGALG00010023206	HSPB9	case	control	-0.224945931	0.182863944	-1.230127305	0.218649437	0.643443819	down_in_case
PRJNA8274(	ENSGALG00010023207	MNS1	case	control	0.072184681	0.082530131	0.874646386	0.38176634	0.779894095	up_in_case
PRJNA8274(	ENSGALG00010023208	BACE1	case	control	-0.031961867	0.050258473	-0.635949823	0.524809127	0.852358222	down_in_case
PRJNA8274(	ENSGALG00010023209	PIK3C2A	case	control	0.034671621	0.050315189	0.689088556	0.490767541	0.836768313	up_in_case
PRJNA8274(	ENSGALG00010023210	ZNHIT6	case	control	-0.178155874	0.105547835	-1.687915941	0.091427366	0.476493306	down_in_case
PRJNA8274(	ENSGALG00010023211	TLK1	case	control	-0.01089187	0.04735218	-0.230018345	0.818077515	0.958536226	down_in_case
PRJNA8274(	ENSGALG00010023212	RIMBP2	case	control	-0.133001487	0.160244177	-0.829992638	0.406542946	0.794253996	down_in_case
PRJNA8274(	ENSGALG00010023213	FCSK	case	control	0.296343084	0.080563042	3.678399885	0.000234702	0.020255986	up_in_case
PRJNA8274(	ENSGALG00010023214	SUPV3L1	case	control	-0.059982393	0.056582478	-1.060087768	0.289104672	0.712801679	down_in_case
PRJNA8274(	ENSGALG00010023215	TRIP12	case	control	-0.078260613	0.035982286	-2.174976117	0.029631906	0.305970455	down_in_case
PRJNA8274(	ENSGALG00010023216	SLC35D1	case	control	0.168638231	0.085185063	1.979669031	0.047740731	0.360119697	up_in_case
PRJNA8274(	ENSGALG00010023217	CREB3L1	case	control	-0.029044807	0.105470082	-0.275384317	0.783020992	0.949518053	down_in_case
PRJNA8274(	ENSGALG00010023218	SSH3	case	control	0.107361593	0.101929042	1.053297379	0.29220472	0.714677075	up_in_case
PRJNA8274(	ENSGALG00010023219	TEX9	case	control	0.05240632	0.122919445	0.42634687	0.66985511	0.913465866	up_in_case
PRJNA8274(	ENSGALG00010023220	ST3GAL2	case	control	0.007677639	0.061398489	0.125046065	0.900487082	0.98028579	up_in_case
PRJNA8274(	ENSGALG00010023221	AMY1A	case	control	-0.224447073	0.142207059	-1.578311758	0.114494006	0.509446069	down_in_case
PRJNA8274(	ENSGALG00010023222	ENSGALG00010023222	case	control	-0.70585692	0.420999711	-1.676620912	0.093616635	0.478523919	down_in_case
PRJNA8274(	ENSGALG00010023223	CEP164	case	control	-0.018825698	0.094907425	-0.198358541	0.842764552	0.966519616	down_in_case
PRJNA8274(	ENSGALG00010023225	PDPR	case	control	-0.080380037	0.077144834	-1.041936737	0.29744101	0.721038557	down_in_case
PRJNA8274(	ENSGALG00010023226	ENSGALG00010023226	case	control	-0.152210711	0.237131931	-0.641881968	0.520949823	0.850168083	down_in_case
PRJNA8274(	ENSGALG00010023227	RNPC3	case	control	0.055349655	0.101074266	0.547613719	0.583957168	0.881307382	up_in_case
PRJNA8274(	ENSGALG00010023228	GORASP2	case	control	0.002056669	0.033760125	0.06092006	0.951422874	0.991857576	up_in_case
PRJNA8274(	ENSGALG00010023229	HKDC1	case	control	0.071085414	0.372110358	0.191033151	0.848499621	0.968692766	up_in_case
PRJNA8274(	ENSGALG00010023230	ENSGALG00010023230	case	control	-0.04398519	0.08245459	-0.533447438	0.593723887	0.883625114	down_in_case
PRJNA8274(	ENSGALG00010023231	ASIC1	case	control	-0.036675486	0.126152745	-0.290722858	0.771263288	0.945630466	down_in_case
PRJNA8274(	ENSGALG00010023233	SEZ6L	case	control	-0.839568092	0.672291077	-1.248816355	0.211732251	0.636501957	down_in_case
PRJNA8274(	ENSGALG00010023234	ENSGALG00010023234	case	control	-0.060808432	0.063421636	-0.958796329	0.337661359	0.750948271	down_in_case
PRJNA8274(	ENSGALG00010023235	ENSGALG00010023235	case	control	0.107053333	0.248758652	0.430350189	0.666940923	0.912284937	up_in_case
PRJNA8274(	ENSGALG00010023237	GAD1	case	control	0.962792681	0.684753864	1.406041984	0.159711658	0.572017525	up_in_case
PRJNA8274(	ENSGALG00010023238	NUCB2	case	control	0.042290215	0.067399966	0.62745157	0.530363273	0.855424633	up_in_case
PRJNA8274(	ENSGALG00010023239	RBPMS2	case	control	-0.151249625	0.123791713	-1.221807353	0.221780487	0.645834386	down_in_case
PRJNA8274(	ENSGALG00010023240	PIWIL1	case	control	-0.083604705	0.342763908	-0.243913386	0.807297901	0.955875648	down_in_case
PRJNA8274(	ENSGALG00010023242	MYT1	case	control	-0.176368817	0.518871717	-0.339908327	0.733925566	0.933846596	down_in_case
PRJNA8274(	ENSGALG00010023243	HK1	case	control	-0.191545404	0.070292927	-2.724959849	0.006430931	0.13904105	down_in_case
PRJNA8274(	ENSGALG00010023244	PPIB	case	control	0.127179365	0.056022905	2.270131589	0.0231996	0.269062679	up_in_case
PRJNA8274(	ENSGALG00010023245	TANC2	case	control	-0.044852365	0.079808541	-0.561999565	0.574116315	0.877590947	down_in_case
PRJNA8274(	ENSGALG00010023246	SP5	case	control	0.336926627	0.544437708	0.618852482	0.536013545	0.857238203	up_in_case
PRJNA8274(	ENSGALG00010023248	PRDM10	case	control	-0.013143344	0.051013108	-0.257646414	0.796679805	0.95280257	down_in_case
PRJNA8274(	ENSGALG00010023249	IL12RB2	case	control	-0.131242871	0.187965055	-0.69823016	0.485033268	0.834423421	down_in_case
PRJNA8274(	ENSGALG00010023250	FZD10	case	control	-0.095912822	0.449059178	-0.213586152	0.830869799	0.962228709	down_in_case
PRJNA8274(	ENSGALG00010023252	GLG1	case	control	-0.117314332	0.048447075	-2.421494647	0.015456829	0.220591467	down_in_case
PRJNA8274(	ENSGALG00010023253	TACR2	case	control	-0.362279701	0.675310842	-0.536463623	0.591638171	0.882949889	down_in_case
PRJNA8274(	ENSGALG00010023254	MYO3B	case	control	-0.110562054	0.220601774	-0.501183885	0.616241715	0.891081782	down_in_case
PRJNA8274(	ENSGALG00010023255	SNX22	case	control	-0.113059223	0.232947845	-0.485341356	0.627434224	0.895865363	down_in_case
PRJNA8274(	ENSGALG00010023257	PAK6	case	control	0.085325963	0.267861613	0.318544945	0.750071608	0.939807587	up_in_case
PRJNA8274(	ENSGALG00010023258	ENSGALG00010023258	case	control	-0.146754318	0.271429075	-0.540672798	0.588733131	0.882817557	down_in_case
PRJNA8274(	ENSGALG00010023259	TSPAN15	case	control	-0.18718395	0.195651627	-0.956720642	0.338708276	0.751259351	down_in_case
PRJNA8274(	ENSGALG00010023260	ENSGALG00010023260	case	control	-0.25631324	0.284225318	-0.901795949	0.367165272	0.771770041	down_in_case
PRJNA8274(	ENSGALG00010023261	SNX1	case	control	0.113110981	0.052109748	2.170629984	0.029959154	0.305970455	up_in_case
PRJNA8274(	ENSGALG00010023262	POLD4	case	control	-0.069725786	0.326069909	-0.213836922	0.830674231	0.962228709	down_in_case
PRJNA8274(	ENSGALG00010023263	SRP14	case	control	0.201997032	0.06148223	3.285453879	0.001018182	0.049355946	up_in_case
PRJNA8274(	ENSGALG00010023264	RACGAP1	case	control	0.069381757	0.083510103	0.830818727	0.406076042	0.7941993	up_in_case
PRJNA8274(	ENSGALG00010023265	RAB5C	case	control	0.043737496	0.033420757	1.308692548	0.190638517	0.613935676	up_in_case
PRJNA8274(	ENSGALG00010023266	RFWD3	case	control	0.196511955	0.085462318	2.299398839	0.021482302	0.262484197	up_in_case
PRJNA8274(	ENSGALG00010023267	DSCAML1	case	control	0.510397745	0.397870944	1.282822363	0.199554318	0.622902456	up_in_case
PRJNA8274(	ENSGALG00010023268	KCNJ11	case	control	-0.11299051	0.091097228	-1.240328746	0.214853824	0.640085351	down_in_case
PRJNA8274(	ENSGALG00010023269	OPRL1	case	control	-0.293892609	0.261329301	-1.124606419	0.260755852	0.687359696	down_in_case
PRJNA8274(	ENSGALG00010023270	ENSGALG00010023270	case	control	-0.396161601	0.589837571	-0.671645247	0.501809566	0.841514615	down_in_case
PRJNA8274(	ENSGALG00010023271	PATL2	case	control	-0.403641515	0.184749137	-2.184808657	0.028902879	0.302128048	down_in_case
PRJNA8274(	ENSGALG00010023273	HDAC7	case	control	-0.070444527	0.10739322	-0.655949476	0.511856633	0.845834144	down_in_case
PRJNA8274(	ENSGALG00010023274	ZBTB38	case	control	-0.192649709	0.089193926	-2.159897188	0.030780629	0.308259818	down_in_case
PRJNA8274(	ENSGALG00010023275	TWIST3	case	control	-0.121916512	0.628916516	-0.193851662	0.846292034	0.967616222	down_in_case
PRJNA8274(	ENSGALG00010023276	ABCC8	case	control	0.010380199	0.08881766	0.116870887	0.906962369	0.981730793	up_in_case
PRJNA8274(	ENSGALG00010023277	KCNH4	case	control	-0.196317958	0.317635528	-0.618060454	0.536535489	0.857336818	down_in_case
PRJNA8274(	ENSGALG00010023278	ENSGALG00010023278	case	control	-0.216158858	0.182526148	-1.184262425	0.236309198	0.661037075	down_in_case
PRJNA8274(	ENSGALG00010023279	FA2H	case	control	0.444075775	0.373410973	1.189241363	0.234344702	0.659210368	up_in_case
PRJNA8274(	ENSGALG00010023281	B2M	case	control	0.111075417	0.067901031	1.635842864	0.101872478	0.491460867	up_in_case
PRJNA8274(	ENSGALG00010023282	SERBP1	case	control	-0.074630418	0.049671513	-1.502479258	0.132973381	0.536224013	down_in_case
PRJNA8274(	ENSGALG00010023285	SLC48A1	case	control	0.042953219	0.07927899	0.541798268	0.587957485	0.882771329	up_in_case
PRJNA8274(	ENSGALG00010023287	RGS19	case	control	-0.066598567	0.114404802	-0.582130872	0.560478532	0.870138205	down_in_case
PRJNA8274(	ENSGALG00010023289	NFRKB	case	control	-0.092848654	0.057277814	-1.621023009	0.105012704	0.494770427	down_in_case
PRJNA8274(	ENSGALG00010023290	SLC16A14	case	control	-0.274548318	0.179280868	-1.531386595	0.125673875	0.525056024	down_in_case
PRJNA8274(	ENSGALG00010023293	COL11A1	case	control	0.219966028	0.317496296	0.692814469	0.488425991	0.835960376	up_in_case
PRJNA8274(	ENSGALG00010023295	ENSGALG00010023295	case	control	-0.170648745	0.536622261	-0.318005341	0.750480886	0.939972946	down_in_case
PRJNA8274(	ENSGALG00010023299	PASK	case	control	-0.134094838	0.09909849	-1.353147139	0.176008629	0.594809383	down_in_case
PRJNA8274(	ENSGALG00010023304	ENSGALG00010023304	case	control	0.041157147	0.092008451	0.447319201	0.654644606	0.906850514	up_in_case
PRJNA8274(	ENSGALG00010023305	PLCB2	case	control	-0.480795316	0.123547517	-3.891582176	9.95926E-05	0.010871564	down_in_case
PRJNA8274(	ENSGALG00010023309	PIK3R3	case	control	0.031188102	0.096429952	0.323427542	0.746371474	0.938295567	up_in_case
PRJNA8274(	ENSGALG00010023311	ETS1	case	control	0.07390702	0.077389823	0.954996636	0.339579398	0.751259351	up_in_case
PRJNA8274(	ENSGALG00010023312	UBR3	case	control	-0.090073725	0.048393224	-1.861287933	0.062703526	0.407531201	down_in_case
PRJNA8274(	ENSGALG00010023314	CYB561	case	control	-0.865784269	0.76158875	-1.136813364	0.255616315	0.681937229	down_in_case
PRJNA8274(	ENSGALG00010023317	RAPGEF3	case	control	0.221467125	0.080021724	2.76758753	0.005647288	0.130251961	up_in_case
PRJNA8274(	ENSGALG00010023319	EIF2AK4	case	control	0.060861581	0.068308878	0.890976145	0.372941968	0.77436767	up_in_case
PRJNA8274(	ENSGALG00010023320	ENSGALG00010023320	case	control	-0.020242959	0.059110151	-0.34246164	0.732003507	0.933770678	down_in_case
PRJNA8274(	ENSGALG00010023322	PRKAB1	case	control	0.005977666	0.061260097	0.097578459	0.92226703	0.985482967	up_in_case
PRJNA8274(	ENSGALG00010023328	TCEA2	case	control	0.221618573	0.06310327	3.511998239	0.000444751	0.030141889	up_in_case
PRJNA8274(	ENSGALG00010023330	TSPAN1	case	control	1.063077869	0.903708018	1.176351042	0.239454627	0.664610169	up_in_case
PRJNA8274(	ENSGALG00010023331	CD4	case	control	-0.07925766	0.398781337	-0.198749671	0.842458567	0.966519616	down_in_case
PRJNA8274(	ENSGALG00010023332	PPP1R7	case	control	-0.102170748	0.055033422	-1.856521808	0.063379218	0.409498817	down_in_case
PRJNA8274(	ENSGALG00010023333	ENDOU	case	control	0.805419724	0.441559787	1.824033231	0.068147049	0.421497749	up_in_case
PRJNA8274(	ENSGALG00010023334	RPS17	case	control	-0.059566004	0.064477711	-0.923823169	0.35557839	0.763360867	down_in_case
PRJNA8274(	ENSGALG00010023335	WDR59	case	control	-0.124164619	0.055712964	-2.228648602	0.025837295	0.285045136	down_in_case
PRJNA8274(	ENSGALG00010023336	GPR162	case	control	0.042984997	0.091184967	0.471404427	0.637351955	0.899378897	up_in_case
PRJNA8274(	ENSGALG00010023337	FLI1	case	control	0.029630467	0.066312017	0.446834052	0.654994883	0.907159983	up_in_case
PRJNA8274(	ENSGALG00010023338	HDLBP	case	control	-0.038732831	0.04891753	-0.791798576	0.428478133	0.805355714	down_in_case
PRJNA8274(	ENSGALG00010023340	P3H3	case	control	0.080448539	0.084162016	0.955877047	0.339134357	0.751259351	up_in_case
PRJNA8274(	ENSGALG00010023342	FITM2	case	control	-0.166633249	0.089336748	-1.865226267	0.062149695	0.405537748	down_in_case
PRJNA8274(	ENSGALG00010023343	TMEM233	case	control	-0.423587952	0.312445164	-1.355719344	0.175188482	0.594080527	down_in_case
PRJNA8274(	ENSGALG00010023347	STAT5A	case	control	-0.159794117	0.07558413	-2.11412259	0.034504798	0.320609886	down_in_case
PRJNA8274(	ENSGALG00010023349	ENSGALG00010023349	case	control	-0.098540296	0.368654601	-0.267297073	0.78924044	0.951843754	down_in_case
PRJNA8274(	ENSGALG00010023350	ZNRF1	case	control	0.038281376	0.082852957	0.46203995	0.644052672	0.90210829	up_in_case
PRJNA8274(	ENSGALG00010023351	GNB3	case	control	0.197374241	0.259265595	0.761282039	0.446488626	0.817679602	up_in_case
PRJNA8274(	ENSGALG00010023354	SEPTIN2	case	control	0.061089708	0.102679488	0.594955329	0.551873331	0.865517508	up_in_case
PRJNA8274(	ENSGALG00010023356	ENSGALG00010023356	case	control	-0.004521792	0.155857573	-0.029012332	0.976854755	0.994586386	down_in_case
PRJNA8274(	ENSGALG00010023359	CDCA3	case	control	-0.028304463	0.081654752	-0.346635837	0.728864919	0.933270804	down_in_case
PRJNA8274(	ENSGALG00010023362	GADD45A	case	control	0.162125693	0.112865751	1.43644721	0.150875128	0.558399072	up_in_case

PRJNA8274(	ENSGALG00010023363	CPEB1	case	control	0.185475982	0.138171346	1.34236213	0.179478607	0.600361189	up_in_case
PRJNA8274(	ENSGALG00010023366	GDAP1L1	case	control	0.180121617	0.222344081	0.810103045	0.417880955	0.800243236	up_in_case
PRJNA8274(	ENSGALG00010023368	ENSGALG00010023368	case	control	0.46824512	0.326504268	1.434116385	0.151539053	0.559523988	up_in_case
PRJNA8274(	ENSGALG00010023369	ACE	case	control	-0.015783736	0.143599678	-0.109914845	0.912476909	0.982717125	down_in_case
PRJNA8274(	ENSGALG00010023374	ENSGALG00010023374	case	control	-0.063815191	0.21223101	-0.300687403	0.763652875	0.943786458	down_in_case
PRJNA8274(	ENSGALG00010023375	ST14	case	control	0.077891687	0.114841585	0.678253323	0.497611089	0.839230872	up_in_case
PRJNA8274(	ENSGALG00010023377	OSER1	case	control	0.023806304	0.058489588	0.407017807	0.68399491	0.916328843	up_in_case
PRJNA8274(	ENSGALG00010023380	USP5	case	control	0.067368295	0.060488228	1.113742241	0.265389782	0.691495163	up_in_case
PRJNA8274(	ENSGALG00010023381	HOXB7	case	control	0.075244478	0.104525602	0.719866486	0.471607204	0.82911028	up_in_case
PRJNA8274(	ENSGALG00010023383	HOXB6	case	control	0.106310281	0.101999068	1.042267179	0.297287824	0.721027687	up_in_case
PRJNA8274(	ENSGALG00010023384	AP3B2	case	control	0.106270917	0.141697711	0.749983296	0.453264765	0.821282604	up_in_case
PRJNA8274(	ENSGALG00010023387	ENSGALG00010023387	case	control	-0.212341874	0.094767573	-2.240659612	0.025048132	0.280931992	down_in_case
PRJNA8274(	ENSGALG00010023388	FARP2	case	control	0.003523339	0.075162703	0.046876165	0.962611925	0.992068146	up_in_case
PRJNA8274(	ENSGALG00010023389	GNG12	case	control	0.124528483	0.042092259	2.958465192	0.003091752	0.095079672	up_in_case
PRJNA8274(	ENSGALG00010023390	HSPB8	case	control	-0.170941007	0.133109963	-1.284208962	0.19906885	0.622902456	down_in_case
PRJNA8274(	ENSGALG00010023392	TPI1	case	control	-0.150245196	0.1146054	-1.310978335	0.189865083	0.613281846	down_in_case
PRJNA8274(	ENSGALG00010023394	HOXB5	case	control	-0.065669498	0.152092753	-0.431772693	0.665906628	0.912284937	down_in_case
PRJNA8274(	ENSGALG00010023396	ENSGALG00010023396	case	control	0.201011955	0.180785704	1.111879702	0.266189877	0.69218122	up_in_case
PRJNA8274(	ENSGALG00010023397	ENSGALG00010023397	case	control	-0.309363611	0.219987123	-1.406280544	0.159640835	0.572017525	down_in_case
PRJNA8274(	ENSGALG00010023399	SRRM4	case	control	-0.728198727	0.505191374	-1.441431433	0.149462835	0.556014189	down_in_case
PRJNA8274(	ENSGALG00010023401	LRRC23	case	control	-0.021260173	0.295745324	-0.07188676	0.942692027	0.990046805	down_in_case
PRJNA8274(	ENSGALG00010023405	DNAJA2	case	control	-0.022519521	0.045905107	-0.490566794	0.623732877	0.894353977	down_in_case
PRJNA8274(	ENSGALG00010023408	ZBTB44	case	control	-0.043122779	0.072077568	-0.598282935	0.549651161	0.864234145	down_in_case
PRJNA8274(	ENSGALG00010023411	ENSGALG00010023411	case	control	-0.117071854	0.123399308	-0.948723747	0.342761133	0.754654997	down_in_case
PRJNA8274(	ENSGALG00010023412	GSS	case	control	0.100155303	0.059115191	1.69423969	0.090219746	0.473624156	up_in_case
PRJNA8274(	ENSGALG00010023414	SUDS3	case	control	0.027143227	0.040416741	0.671583766	0.501848716	0.841514615	up_in_case
PRJNA8274(	ENSGALG00010023415	HOXB4	case	control	-0.165799079	0.106992754	-1.549629051	0.121230576	0.519567065	down_in_case
PRJNA8274(	ENSGALG00010023421	OLFM3	case	control	-0.210204483	0.09090965	-2.312235089	0.020764732	0.258860564	down_in_case
PRJNA8274(	ENSGALG00010023422	WLS	case	control	-0.074419428	0.09145717	-0.81370797	0.415812267	0.799746526	down_in_case
PRJNA8274(	ENSGALG00010023428	HOXB3	case	control	0.01295376	0.136219779	0.09509456	0.924239719	0.985571367	up_in_case
PRJNA8274(	ENSGALG00010023429	BMF	case	control	0.240272906	0.098799518	2.431923861	0.015018864	0.21667965	up_in_case
PRJNA8274(	ENSGALG00010023431	BCAR1	case	control	0.019359554	0.076559773	0.252868486	0.800369842	0.954053991	up_in_case
PRJNA8274(	ENSGALG00010023432	TMOD3	case	control	-0.033928534	0.069212206	-0.490210271	0.623985113	0.894353977	down_in_case
PRJNA8274(	ENSGALG00010023434	ATN1	case	control	-0.179587088	0.093691282	-1.91679614	0.055263835	0.383470355	down_in_case
PRJNA8274(	ENSGALG00010023437	KCNJ5	case	control	0.080788944	0.148521992	0.543952734	0.586473999	0.881932581	up_in_case
PRJNA8274(	ENSGALG00010023438	HOXB2	case	control	0.088966455	0.142602258	0.623878308	0.532707507	0.855817434	up_in_case
PRJNA8274(	ENSGALG00010023439	ACSS2	case	control	-0.134147926	0.07967435	-1.683702799	0.09223912	0.476713154	down_in_case
PRJNA8274(	ENSGALG00010023441	ENSGALG00010023441	case	control	-0.167798208	0.143004964	-1.173373309	0.240646136	0.665904651	down_in_case
PRJNA8274(	ENSGALG00010023442	STK25	case	control	-0.031522297	0.032283097	-0.976433509	0.328849683	0.745489928	down_in_case
PRJNA8274(	ENSGALG00010023443	ENSGALG00010023443	case	control	-0.34578917	0.105909682	-3.264943894	0.001094858	0.051553287	down_in_case
PRJNA8274(	ENSGALG00010023447	C12orf57	case	control	-0.081299054	0.115460757	-0.704127152	0.481353592	0.832354182	down_in_case
PRJNA8274(	ENSGALG00010023448	FAM219B	case	control	-0.212607277	0.088465963	-2.403266413	0.016249345	0.226036602	down_in_case
PRJNA8274(	ENSGALG00010023453	ARHGAP32	case	control	-0.010896811	0.096572752	-0.112835255	0.910161168	0.981730793	down_in_case
PRJNA8274(	ENSGALG00010023454	HOXB1	case	control	0.132218671	0.227788606	0.580444622	0.561614821	0.870616281	up_in_case
PRJNA8274(	ENSGALG00010023455	CFDP1	case	control	0.039099559	0.058774334	0.665248854	0.505891338	0.843280685	up_in_case
PRJNA8274(	ENSGALG00010023456	MPI	case	control	-0.022762677	0.076734397	-0.29664242	0.766739522	0.944309289	down_in_case
PRJNA8274(	ENSGALG00010023466	CLCF1	case	control	-0.011312649	0.2829195	-0.039985399	0.968104767	0.992761697	down_in_case
PRJNA8274(	ENSGALG00010023469	ENSGALG00010023469	case	control	-0.268114435	0.263282522	-1.018352576	0.308510431	0.731823162	down_in_case
PRJNA8274(	ENSGALG00010023472	PTPN6	case	control	0.103545289	0.097785692	1.058900209	0.289645229	0.71338732	up_in_case
PRJNA8274(	ENSGALG00010023473	BOK	case	control	0.375543085	0.095655426	3.925998769	8.63706E-05	0.009725193	up_in_case
PRJNA8274(	ENSGALG00010023475	ENSGALG00010023475	case	control	-0.406796985	0.433573202	-0.938242914	0.348119589	0.758675915	down_in_case
PRJNA8274(	ENSGALG00010023476	SCAMP2	case	control	-0.022449318	0.059943997	-0.374504855	0.708028746	0.92490115	down_in_case
PRJNA8274(	ENSGALG00010023478	TAOK3	case	control	-0.217437114	0.065339424	-3.32780886	0.000875319	0.04498565	down_in_case
PRJNA8274(	ENSGALG00010023479	MCCC2L	case	control	-0.263549382	0.115782116	-2.276252931	0.022830876	0.267253668	down_in_case
PRJNA8274(	ENSGALG00010023482	MTCL2	case	control	0.051767108	0.087171511	0.593853505	0.552610099	0.866213137	up_in_case
PRJNA8274(	ENSGALG00010023485	BARX2	case	control	0.269304402	0.142706255	1.887124029	0.059143645	0.395497471	up_in_case
PRJNA8274(	ENSGALG00010023487	GRN	case	control	0.155150652	0.071911231	2.157530187	0.030964378	0.308259818	up_in_case
PRJNA8274(	ENSGALG00010023489	ETV5	case	control	-0.25695963	0.084872571	-3.027593337	0.002465096	0.081856092	down_in_case
PRJNA8274(	ENSGALG00010023490	PHB2	case	control	-0.037242245	0.047374116	-0.786130654	0.431790947	0.807276582	down_in_case
PRJNA8274(	ENSGALG00010023496	PLEKHA3	case	control	0.127795783	0.100783719	1.268020114	0.204790768	0.627358178	up_in_case
PRJNA8274(	ENSGALG00010023498	DISP2	case	control	-0.455895244	0.315069593	-1.446966808	0.14790622	0.553680353	down_in_case
PRJNA8274(	ENSGALG00010023499	PEBP1	case	control	-0.011287407	0.060148225	-0.187659844	0.85114331	0.969234083	down_in_case
PRJNA8274(	ENSGALG00010023506	RPE65	case	control	0.202915096	0.295457393	0.686782936	0.492219524	0.837514022	up_in_case
PRJNA8274(	ENSGALG00010023510	ASPHD2	case	control	-0.120274092	0.19303927	-0.623055047	0.533248349	0.855817434	down_in_case
PRJNA8274(	ENSGALG00010023513	CDCA9	case	control	-0.292696055	0.134838772	-2.170711357	0.029952998	0.305970455	down_in_case
PRJNA8274(	ENSGALG00010023514	ULK3	case	control	0.077479049	0.10098613	0.767224662	0.442947952	0.814158832	up_in_case
PRJNA8274(	ENSGALG00010023518	METTL5	case	control	-0.084332335	0.052579546	-1.603900024	0.108736092	0.500951275	down_in_case
PRJNA8274(	ENSGALG00010023519	DGKG	case	control	-0.139589937	0.14600042	-0.956092709	0.339025398	0.751259351	down_in_case
PRJNA8274(	ENSGALG00010023522	EMG1	case	control	-0.118620226	0.09097658	-1.303854533	0.192283185	0.615090794	down_in_case
PRJNA8274(	ENSGALG00010023526	KNSTRN	case	control	0.100201122	0.095898049	1.044871329	0.296082443	0.719662167	up_in_case
PRJNA8274(	ENSGALG00010023527	TMEM98	case	control	-0.061280633	0.090040918	-0.680586494	0.496133175	0.838518426	down_in_case
PRJNA8274(	ENSGALG00010023533	FKBP7	case	control	0.08387559	0.053864528	1.557158177	0.119432935	0.516645008	up_in_case
PRJNA8274(	ENSGALG00010023536	ENSGALG00010023536	case	control	0.275838614	0.168799379	1.634121025	0.102233441	0.491460867	up_in_case
PRJNA8274(	ENSGALG00010023543	ENSGALG00010023543	case	control	1.14096786	0.776217452	1.469907508	0.141586806	0.543501954	up_in_case
PRJNA8274(	ENSGALG00010023544	SSB	case	control	-0.07979915	0.065747722	-1.213717334	0.224855683	0.648819735	down_in_case
PRJNA8274(	ENSGALG00010023545	PTPRT	case	control	-0.036250891	0.105706023	-0.342940642	0.731643116	0.933770678	down_in_case
PRJNA8274(	ENSGALG00010023546	SLC11A2	case	control	-0.197512067	0.090963373	-2.171336223	0.029905766	0.305970455	down_in_case
PRJNA8274(	ENSGALG00010023551	ENSGALG00010023551	case	control	-0.042299647	0.087244858	-0.484838279	0.627791067	0.895865363	down_in_case
PRJNA8274(	ENSGALG00010023552	SGPP2	case	control	-0.018762521	0.253994506	-0.073869789	0.941113995	0.989551456	down_in_case
PRJNA8274(	ENSGALG00010023555	HPS4	case	control	0.074832792	0.072752482	1.028594349	0.303670335	0.726591644	up_in_case
PRJNA8274(	ENSGALG00010023556	IVD	case	control	-0.010890083	0.079471814	-0.137030756	0.891006485	0.978917427	down_in_case
PRJNA8274(	ENSGALG00010023569	LPCAT3	case	control	-0.033275686	0.080730733	-0.412181147	0.680206654	0.915381147	down_in_case
PRJNA8274(	ENSGALG00010023571	APLP2	case	control	0.027649294	0.066936596	0.41306693	0.679557577	0.915351915	up_in_case
PRJNA8274(	ENSGALG00010023572	MYBL2	case	control	0.048902997	0.086814119	0.563306948	0.573225888	0.877449175	up_in_case
PRJNA8274(	ENSGALG00010023574	DEPDC1	case	control	-0.072274813	0.090285255	-0.800516234	0.423411761	0.803723282	down_in_case
PRJNA8274(	ENSGALG00010023578	SRRD	case	control	-0.137385967	0.083584668	-1.643674266	0.100243504	0.489689587	down_in_case
PRJNA8274(	ENSGALG00010023580	RDM1	case	control	-0.091494462	0.103606984	-0.883091651	0.377186755	0.775926253	down_in_case
PRJNA8274(	ENSGALG00010023585	TFIP11	case	control	-0.101600243	0.050122932	-2.027021151	0.04266025	0.344511316	down_in_case
PRJNA8274(	ENSGALG00010023588	FARSB	case	control	-0.052994062	0.058993427	-0.898304532	0.369023217	0.771834431	down_in_case
PRJNA8274(	ENSGALG00010023590	S1PR1	case	control	-0.036600899	0.055259024	-0.662351537	0.507745955	0.843702533	down_in_case
PRJNA8274(	ENSGALG00010023592	BAHD1	case	control	0.064389203	0.049507627	1.30059157	0.193398294	0.616082781	up_in_case
PRJNA8274(	ENSGALG00010023593	CSK	case	control	0.082324661	0.048848709	1.685298621	0.091930972	0.476713154	up_in_case
PRJNA8274(	ENSGALG00010023597	MOGAT1	case	control	0.497889719	0.326468909	1.525075451	0.12724028	0.528320557	up_in_case
PRJNA8274(	ENSGALG00010023600	CHST14	case	control	-0.027211644	0.063170312	-0.430766348	0.66663827	0.912284937	down_in_case
PRJNA8274(	ENSGALG00010023601	TPST2	case	control	0.09793491	0.061911487	1.581853613	0.113682993	0.50849759	up_in_case
PRJNA8274(	ENSGALG00010023603	C1S	case	control	0.109839384	0.188434686	0.582904276	0.559957742	0.86984941	up_in_case
PRJNA8274(	ENSGALG00010023604	ENSGALG00010023604	case	control	0.615807835	0.255397777	2.411171473	0.01590137	0.223714308	up_in_case
PRJNA8274(	ENSGALG00010023605	INSYN1	case	control	-0.10814384	0.255834779	-0.42270969	0.672507085	0.913676733	down_in_case
PRJNA8274(	ENSGALG00010023606	LRRC7	case	control	-0.19500681	0.206659116	-0.943615815	0.345366008	0.755935621	down_in_case
PRJNA8274(	ENSGALG00010023612	GPT2	case	control	0.319030212	0.210005938	1.519148529	0.128725114	0.530596961	up_in_case
PRJNA8274(	ENSGALG00010023613	COPB1	case	control	0.027761717	0.039861006	0.696463032	0.486138901	0.834635018	up_in_case
PRJNA8274(	ENSGALG00010023617	ENSGALG00010023617	case	control	-0.477670285	0.240184962	-1.98876017	0.046727681	0.356907825	down_in_case
PRJNA8274(	ENSGALG00010023620	IRS1	case	control	-0.114571413	0.079402321	-1.442922722	0.149042242	0.555605854	down_in_case
PRJNA8274(	ENSGALG00010023621	CD79B	case	control	0.29672531	0.41513262	0.714772329	0.474749736	0.830093946	up_in_case
PRJNA8274(	ENSGALG00010023623	OSBPL6	case	control	0.008645442	0.15871363	0.054471959	0.956559149	0.992056045	up_in_case
PRJNA8274(	ENSGALG00010023624	PSMA1	case	control	-0.065646835	0.044084624	-1.489109556	0.136458522	0.539840644	down_in_case
PRJNA8274(	ENSGALG00010023626	LOXL1	case	control	-0.072989441	0.083012298	-0.879260583	0.379260003	0.777439512	down_in_case
PRJNA8274(	ENSGALG00010023627	IFT52	case	control	0.064599685	0.057942001	1.114902553	0.264892183	0.690855045	up_in_case
PRJNA8274(	ENSGALG00010023631	ENSGALG00010023631	case	control	-0.215255385	0.125862813	-1.710238155	0.087221843	0.467042649	down_in_case
PRJNA8274(	ENSGALG00010023635	SCN4A	case	control	-0.577922252	0.187779152	-3.077669942	0.002086258	0.075147345	down_in_case
PRJNA8274(	ENSGALG00010023636	PDE3B	case	control	-0.075246745	0.069964857	-1.075493446	0.282153865	0.706000864	down_in_case
PRJNA8274(	ENSGALG00010023637	RHBDD1	case	control	-0.077798268	0.095401883	-0.815479371	0.414797968	0.798853486	down_in_case
PRJNA8274(	ENSGALG00010023638	DCTN2	case	control	-0.112476259	0.054687065	-2.056725095	0.039712677	0.336210795	down_in_case
PRJNA8274(	ENSGALG00010023639	KLHL23	case	control	0.122819087	0.198917064	0.617438669	0.536945423	0.857433785	up_in_case
PRJNA8274(	ENSGALG00010023641	LRRC40	case	control	-0.146099455	0.062003837	-2.356296988	0.018458154	0.242205346	down_in_case
PRJNA8274(	ENSGALG00010023642	PAX6	case	control	-0.530543497	0.435179963	-1.219135856	0.222792633	0.646365317	down_in_case
PRJNA8274(	ENSGALG00010023643	SCN4B	case	control	-0.042678102	0.142983132	-0.298483473	0.765334188	0.943786458	down_in_case
PRJNA8274(	ENSGALG00010023646	DPH5	case	control	-0.043862063	0.080998567	-0.541516527	0.58815161	0.882771329	down_in_case
PRJNA8274(	ENSGALG00010023648	SCN2B	case	control	-0.034257586	0.127657404	-0.268355652	0.788425572	0.951738421	down_in_case
PRJNA8274(	ENSGALG00010023652	SLC30A7	case	control	0.089790929	0.093815231	0.957103956	0.338514787	0.751259351	up_in_case
PRJNA8274(	ENSGALG00010023655	C1R	case	control	0.04475875	0.076116846	0.588026869	0.556514258	0.867878646	up_in_case
PRJNA8274(	ENSGALG00010023658	ENSGALG00010023658	case	control	-0.109232405	0.240719183	-0.453775239	0.649990607	0.904241824	down_in_case
PRJNA8274(	ENSGALG00010023660	CRYBB1	case	control	-0.071572183	0.236357797	-0.30281287	0.762032475	0.943297504	down_in_case
PRJNA8274(	ENSGALG00010023663	PHOSPHO2	case	control	-0.038909768	0.095363141	-0.408016843	0.68326131	0.915968569	down_in_case
PRJNA8274(	ENSGALG00010023664	SLC6A9	case	control	0.042993232	0.089641339	0.479613893	0.631501966	0.897039706	up_in_case
PRJNA8274(	ENSGALG00010023665	C16orf87	case	control	-0.137799114	0.117487386	-1.172884327	0.240842196	0.665967363	down_in_case

PRJNA8274(	ENSGALG00010023666	ENSGALG00010023666	case	control	0.021570758	0.158146781	0.136397073	0.891507387	0.978917427	up_in_case
PRJNA8274(	ENSGALG00010023668	RCN1	case	control	0.020101111	0.063297143	0.317567435	0.75081308	0.939972946	up_in_case
PRJNA8274(	ENSGALG00010023670	EXTL2	case	control	-0.194677952	0.06351793	-3.064929089	0.002177216	0.076380185	down_in_case
PRJNA8274(	ENSGALG00010023671	ENSGALG00010023671	case	control	0.273142489	0.294492986	0.92750083	0.353666561	0.762284769	up_in_case
PRJNA8274(	ENSGALG00010023673	SGK2	case	control	0.719315204	1.389174929	0.517800306	0.604597605	0.885268908	up_in_case
PRJNA8274(	ENSGALG00010023676	ENSGALG00010023676	case	control	0.256337675	0.17162497	1.493591961	0.135282302	0.539774365	up_in_case
PRJNA8274(	ENSGALG00010023677	MN1	case	control	-0.120039381	0.094100337	-1.27565304	0.202078196	0.625073547	down_in_case
PRJNA8274(	ENSGALG00010023680	MPZL3	case	control	0.326308413	0.624451461	0.522552085	0.601285992	0.884954589	up_in_case
PRJNA8274(	ENSGALG00010023686	MYLK3	case	control	-0.28034484	0.193429077	-1.44934177	0.147242162	0.552351236	down_in_case
PRJNA8274(	ENSGALG00010023689	WT1	case	control	-0.156785371	0.18411051	-0.851582949	0.394445607	0.786978937	down_in_case
PRJNA8274(	ENSGALG00010023693	SRSF11	case	control	0.071138521	0.057465795	1.237928057	0.215742738	0.640470741	up_in_case
PRJNA8274(	ENSGALG00010023694	PPCDC	case	control	-0.139613086	0.116206488	-1.201422469	0.229587364	0.653695091	down_in_case
PRJNA8274(	ENSGALG00010023695	CLSTN3	case	control	0.012862892	0.072846262	0.176575869	0.85984155	0.971828322	up_in_case
PRJNA8274(	ENSGALG00010023696	LRRC4C	case	control	-0.022488766	0.164998827	-0.136296523	0.891586873	0.978917427	down_in_case
PRJNA8274(	ENSGALG00010023698	ENSGALG00010023698	case	control	-0.095307602	0.148449552	-0.642020142	0.520860105	0.850168083	down_in_case
PRJNA8274(	ENSGALG00010023699	PPIG	case	control	-0.118887278	0.082877598	-1.434492325	0.151431818	0.559493525	down_in_case
PRJNA8274(	ENSGALG00010023702	COL4A4	case	control	-0.183192839	0.215767155	-0.849030235	0.395864468	0.787783337	down_in_case
PRJNA8274(	ENSGALG00010023703	PITPNB	case	control	-0.024611603	0.042620573	-0.577458277	0.563629916	0.871916948	down_in_case
PRJNA8274(	ENSGALG00010023704	ANKRD13C	case	control	-0.029271646	0.038735452	-0.755681025	0.449840464	0.819037259	down_in_case
PRJNA8274(	ENSGALG00010023706	CDK5RAP3	case	control	-0.009502653	0.06634166	-0.143238088	0.88610215	0.977630122	down_in_case
PRJNA8274(	ENSGALG00010023707	MPZL2	case	control	0.209632456	0.367594239	0.570282211	0.568486304	0.874500231	up_in_case
PRJNA8274(	ENSGALG00010023710	CDC14A	case	control	0.015507467	0.065800595	0.235673664	0.813685907	0.956712425	up_in_case
PRJNA8274(	ENSGALG00010023711	ENSGALG00010023711	case	control	-0.736811819	0.313773023	-2.348231887	0.018862774	0.245250105	down_in_case
PRJNA8274(	ENSGALG00010023712	ARHGAP40	case	control	0.226624382	0.078486228	2.887441388	0.00388389	0.107634925	up_in_case
PRJNA8274(	ENSGALG00010023714	EIF3M	case	control	-0.010120535	0.077787557	-0.130104812	0.896483503	0.980176778	down_in_case
PRJNA8274(	ENSGALG00010023715	CD3E	case	control	0.169377709	0.295024663	0.574113729	0.565890843	0.872906976	up_in_case
PRJNA8274(	ENSGALG00010023716	FASTKD1	case	control	-0.12994129	0.091281421	-1.423523967	0.154584319	0.563441859	down_in_case
PRJNA8274(	ENSGALG00010023720	SCAMP5	case	control	0.055616703	0.08928965	0.622879612	0.533363637	0.855817434	up_in_case
PRJNA8274(	ENSGALG00010023724	CYP2R1	case	control	0.007377939	0.103134264	0.071537225	0.942970199	0.990054824	up_in_case
PRJNA8274(	ENSGALG00010023726	PNPO	case	control	0.13383807	0.141368632	0.946731026	0.343775857	0.755725557	up_in_case
PRJNA8274(	ENSGALG00010023730	SP2	case	control	-0.052994852	0.070935813	-0.747081764	0.455014207	0.821657174	down_in_case
PRJNA8274(	ENSGALG00010023731	ACTR5	case	control	-0.200690824	0.078967692	-2.541429527	0.01104002	0.185137791	down_in_case
PRJNA8274(	ENSGALG00010023732	TM2D1	case	control	-0.051133318	0.097449362	-0.524716804	0.599780074	0.884888453	down_in_case
PRJNA8274(	ENSGALG00010023733	ORC6	case	control	-0.030966984	0.107663889	-0.287626465	0.773632684	0.94652185	down_in_case
PRJNA8274(	ENSGALG00010023736	KIF5A	case	control	-0.991211282	0.542926334	-1.825682824	0.067898052	0.421339236	down_in_case
PRJNA8274(	ENSGALG00010023738	ENSGALG00010023738	case	control	-0.044737708	0.1315219	-0.34015406	0.733740513	0.933846596	down_in_case
PRJNA8274(	ENSGALG00010023740	PPP1R16B	case	control	0.087144199	0.093609174	0.93093652	0.351886402	0.760831854	up_in_case
PRJNA8274(	ENSGALG00010023742	SP6	case	control	1.052159175	1.090684796	0.964677585	0.334706326	0.749087957	up_in_case
PRJNA8274(	ENSGALG00010023743	CD3D	case	control	0.346955821	0.16681747	2.079853029	0.037539015	0.328724998	up_in_case
PRJNA8274(	ENSGALG00010023744	MT4	case	control	0.167590832	0.20425729	0.820488866	0.411937474	0.797194435	up_in_case
PRJNA8274(	ENSGALG00010023746	TTC28	case	control	0.020847032	0.075369265	0.27659858	0.782088355	0.949394183	up_in_case
PRJNA8274(	ENSGALG00010023748	ENSGALG00010023748	case	control	-0.23449825	0.117510279	-1.995555211	0.045982359	0.355983104	down_in_case
PRJNA8274(	ENSGALG00010023749	RPP25	case	control	0.195631789	0.154169272	1.268941509	0.204461926	0.627291471	up_in_case
PRJNA8274(	ENSGALG00010023750	ENSGALG00010023750	case	control	-0.038880799	0.097222739	-0.399914664	0.689219371	0.918188654	down_in_case
PRJNA8274(	ENSGALG00010023751	PRRG4	case	control	-0.238845014	0.237598316	-1.00524708	0.31477789	0.736442673	down_in_case
PRJNA8274(	ENSGALG00010023752	C1QL1	case	control	-0.152565296	0.222018483	-0.687173851	0.49197318	0.837326408	down_in_case
PRJNA8274(	ENSGALG00010023759	CALCA	case	control	0.253582965	0.265276164	0.955920655	0.339112323	0.751259351	up_in_case
PRJNA8274(	ENSGALG00010023760	ZW10	case	control	0.078420926	0.060856921	1.288611473	0.197533193	0.62094535	up_in_case
PRJNA8274(	ENSGALG00010023762	BBS5	case	control	-0.194601399	0.11455974	-1.698689254	0.089377749	0.47258594	down_in_case
PRJNA8274(	ENSGALG00010023764	COX5A	case	control	-0.038935531	0.067505011	-0.576779859	0.564088177	0.871916948	down_in_case
PRJNA8274(	ENSGALG00010023766	ENSGALG00010023766	case	control	-0.494599814	0.347867885	-1.421803608	0.155083272	0.563829072	down_in_case
PRJNA8274(	ENSGALG00010023767	ENSGALG00010023767	case	control	0.395268262	0.228053529	1.733225809	0.083055541	0.457082972	up_in_case
PRJNA8274(	ENSGALG00010023770	SCRN2	case	control	-0.192131685	0.121471861	-1.581697056	0.113718745	0.508498454	down_in_case
PRJNA8274(	ENSGALG00010023773	OVOA	case	control	0.196484329	0.265832905	0.739127196	0.459829764	0.824480387	up_in_case
PRJNA8274(	ENSGALG00010023775	ENSGALG00010023775	case	control	-0.016771065	0.133229209	-0.125881289	0.899825894	0.98028579	down_in_case
PRJNA8274(	ENSGALG00010023777	OAZ2	case	control	-0.11272924	0.055413209	-2.03433877	0.041917451	0.341355095	down_in_case
PRJNA8274(	ENSGALG00010023778	FANCF	case	control	-0.047686127	0.158727425	-0.300427772	0.763850883	0.943786458	down_in_case
PRJNA8274(	ENSGALG00010023781	COL4A3	case	control	0.064915557	0.178778405	0.363106251	0.716525515	0.926862121	up_in_case
PRJNA8274(	ENSGALG00010023782	TMPRSS5	case	control	1.3672304	0.455405246	3.00222804	0.002680113	0.086709547	up_in_case
PRJNA8274(	ENSGALG00010023783	VPS35	case	control	-0.037054475	0.036179133	-1.02419466	0.305743341	0.728933908	down_in_case
PRJNA8274(	ENSGALG00010023786	ENSGALG00010023786	case	control	0.030604674	0.151503988	0.202005733	0.839912242	0.965286179	up_in_case
PRJNA8274(	ENSGALG00010023788	RTCA	case	control	-0.007629276	0.078705813	-0.096934088	0.922778738	0.985482967	down_in_case
PRJNA8274(	ENSGALG00010023791	VTI1B	case	control	0.037159584	0.056076445	0.662659407	0.507548713	0.843702533	up_in_case
PRJNA8274(	ENSGALG00010023792	ZNF609	case	control	-0.03415753	0.056516882	-0.604377467	0.545592713	0.862133047	down_in_case
PRJNA8274(	ENSGALG00010023793	RBM45	case	control	0.047113713	0.055999932	0.84131733	0.400170185	0.789900496	up_in_case
PRJNA8274(	ENSGALG00010023794	MT3	case	control	0.716224822	0.475793739	1.505326288	0.13224021	0.535684428	up_in_case
PRJNA8274(	ENSGALG00010023798	ARCN1	case	control	0.06237575	0.036137137	1.726084454	0.08433222	0.459607696	up_in_case
PRJNA8274(	ENSGALG00010023799	NMT1	case	control	-0.066365187	0.067316581	-0.985866869	0.324198447	0.742122265	down_in_case
PRJNA8274(	ENSGALG00010023800	PEX5	case	control	-0.105195941	0.047886493	-2.196776882	0.028036385	0.298971144	down_in_case
PRJNA8274(	ENSGALG00010023802	ENSGALG00010023802	case	control	-0.272378001	0.231130207	-1.178461287	0.238612757	0.663457598	down_in_case
PRJNA8274(	ENSGALG00010023805	GALNT6	case	control	-0.05101937	0.296068953	-0.172322594	0.863183922	0.972850732	down_in_case
PRJNA8274(	ENSGALG00010023806	PATJ	case	control	0.038026665	0.095270455	0.399144365	0.689786834	0.918477347	up_in_case
PRJNA8274(	ENSGALG00010023807	MRPL10	case	control	0.284781613	0.080288106	3.546996299	0.00038965	0.027313706	up_in_case
PRJNA8274(	ENSGALG00010023808	DHX35	case	control	0.059258901	0.076904147	0.770555345	0.440970538	0.813083204	up_in_case
PRJNA8274(	ENSGALG00010023809	CDON	case	control	-0.000535927	0.060123538	-0.008913767	0.992887937	0.998196863	down_in_case
PRJNA8274(	ENSGALG00010023811	MRPL44	case	control	-0.076191673	0.061219875	-1.244557809	0.213294337	0.638582746	down_in_case
PRJNA8274(	ENSGALG00010023815	PLCD3	case	control	-0.187389924	0.090120826	-2.079318764	0.037588062	0.328953048	down_in_case
PRJNA8274(	ENSGALG00010023817	BBS2	case	control	0.009736309	0.076433642	0.127382514	0.898637656	0.98028579	up_in_case
PRJNA8274(	ENSGALG00010023818	ALOX5	case	control	-0.180949803	0.16740706	-1.080897085	0.279742896	0.705400718	down_in_case
PRJNA8274(	ENSGALG00010023819	BIN2	case	control	-0.023481428	0.187835083	-0.125010875	0.900514941	0.98028579	down_in_case
PRJNA8274(	ENSGALG00010023821	KANK4	case	control	0.219840178	0.075912509	2.895967746	0.003779912	0.105649139	up_in_case
PRJNA8274(	ENSGALG00010023822	CHEK2	case	control	0.111255638	0.106759719	1.042112508	0.29735952	0.721027687	up_in_case
PRJNA8274(	ENSGALG00010023823	MUC2	case	control	-0.199215502	0.379918567	-0.524363692	0.600025605	0.884888453	down_in_case
PRJNA8274(	ENSGALG00010023826	DBT	case	control	-0.013921478	0.055768073	-0.249631691	0.802872189	0.954891353	down_in_case
PRJNA8274(	ENSGALG00010023828	CHEK1	case	control	-0.068455377	0.091456559	-0.748501555	0.454157688	0.821331802	down_in_case
PRJNA8274(	ENSGALG00010023829	USP1	case	control	-0.021757315	0.044772201	-0.485955885	0.626998444	0.895838586	down_in_case
PRJNA8274(	ENSGALG00010023830	AGFG1	case	control	0.013407769	0.037463513	0.357888717	0.720426595	0.92895404	up_in_case
PRJNA8274(	ENSGALG00010023831	SATB2	case	control	0.094798915	0.577278573	0.164216931	0.869560373	0.974718971	up_in_case
PRJNA8274(	ENSGALG00010023832	ENSGALG00010023832	case	control	-0.00741301	0.126035222	-0.058816974	0.953097889	0.992056045	down_in_case
PRJNA8274(	ENSGALG00010023833	PIP4K2C	case	control	0.141122431	0.067189911	2.100351513	0.035697931	0.324939791	up_in_case
PRJNA8274(	ENSGALG00010023835	INAFM2	case	control	0.01286309	0.089728322	0.143355963	0.88600906	0.977630122	up_in_case
PRJNA8274(	ENSGALG00010023836	NDUFS3	case	control	-0.104665436	0.073482392	-1.424360773	0.154342064	0.56303838	down_in_case
PRJNA8274(	ENSGALG00010023837	STT3A	case	control	-0.039190365	0.069030426	-0.567725971	0.570221054	0.875755673	down_in_case
PRJNA8274(	ENSGALG00010023840	SMAGP	case	control	-0.026477891	0.160239998	-0.165238961	0.8687559	0.974483475	down_in_case
PRJNA8274(	ENSGALG00010023841	OSBPL7	case	control	-0.057093742	0.089825281	-0.635608834	0.52503141	0.852401131	down_in_case
PRJNA8274(	ENSGALG00010023847	PIK3AP1	case	control	0.066637886	0.136879439	0.486836347	0.62637431	0.895536157	up_in_case
PRJNA8274(	ENSGALG00010023850	DAZAP2	case	control	-0.090023076	0.104563222	-0.860943976	0.3892689	0.783669208	down_in_case
PRJNA8274(	ENSGALG00010023851	HSCB	case	control	-0.100465294	0.074986143	-1.33978479	0.180315322	0.601000958	down_in_case
PRJNA8274(	ENSGALG00010023852	LRRC39	case	control	-0.231197132	0.106548907	-2.169868632	0.030016799	0.306162788	down_in_case
PRJNA8274(	ENSGALG00010023854	SHCBP1	case	control	-0.590163054	0.17144491	-3.442289737	0.000576812	0.035099629	down_in_case
PRJNA8274(	ENSGALG00010023857	ACBD4	case	control	0.246958031	0.143618726	1.719539218	0.085516233	0.462647434	up_in_case
PRJNA8274(	ENSGALG00010023859	ENSGALG00010023859	case	control	0.559846222	0.407151405	1.375032029	0.169121515	0.585038804	up_in_case
PRJNA8274(	ENSGALG00010023860	TRIP4	case	control	0.074083531	0.056909049	1.301788256	0.192988779	0.615324823	up_in_case
PRJNA8274(	ENSGALG00010023861	ENSGALG00010023861	case	control	0.510237189	0.117743709	4.333456071	1.46787E-05	0.002385485	up_in_case
PRJNA8274(	ENSGALG00010023862	TRMT13	case	control	-0.264026855	0.12164893	-2.170400142	0.029976546	0.305970455	down_in_case
PRJNA8274(	ENSGALG00010023863	EYA2	case	control	0.019964864	0.117161994	0.170403927	0.864692484	0.973093769	up_in_case
PRJNA8274(	ENSGALG00010023864	EPHA1	case	control	0.766671554	0.692405184	1.107258542	0.268182163	0.694924988	up_in_case
PRJNA8274(	ENSGALG00010023866	AMBRA1	case	control	-0.033746766	0.055354932	-0.609643349	0.54209809	0.85975913	down_in_case
PRJNA8274(	ENSGALG00010023869	DOCK7	case	control	-0.011602606	0.052765554	-0.219889781	0.825956995	0.961207256	down_in_case
PRJNA8274(	ENSGALG00010023871	UQCRFS1	case	control	0.000637122	0.070400048	0.009050028	0.992779221	0.998196863	up_in_case
PRJNA8274(	ENSGALG00010023874	HEXIM1	case	control	-0.039777943	0.054875893	-0.724870986	0.468531182	0.828596747	down_in_case
PRJNA8274(	ENSGALG00010023876	POU6F1	case	control	0.152521416	0.327635334	0.465521877	0.641557774	0.901539099	up_in_case
PRJNA8274(	ENSGALG00010023879	EI24	case	control	-0.086562484	0.055056419	-1.572250531	0.115892461	0.512919984	down_in_case
PRJNA8274(	ENSGALG00010023880	ENSGALG00010023880	case	control	-0.083472551	0.117350999	-0.711306693	0.476894205	0.830520323	down_in_case
PRJNA8274(	ENSGALG00010023881	VSTM2B	case	control	-0.00714315	0.120523071	-0.059267903	0.952738726	0.992056045	down_in_case
PRJNA8274(	ENSGALG00010023883	TBX21	case	control	-0.315476563	0.165186453	-1.909821035	0.05615626	0.385984934	down_in_case
PRJNA8274(	ENSGALG00010023884	C2orf69	case	control	-0.034243745	0.06968948	-0.491376097	0.623160467	0.894052087	down_in_case
PRJNA8274(	ENSGALG00010023885	ANGPTL3	case	control	0.81758757	1.395380797	0.585924338	0.557926361	0.869041413	up_in_case
PRJNA8274(	ENSGALG00010023886	CYP2C18	case	control	-0.059155367	0.482585692	-0.122580026	0.902439672	0.980850383	down_in_case
PRJNA8274(	ENSGALG00010023887	RASA2	case	control	0.01947086	0.080388906	0.242208295	0.808618768	0.955875648	up_in_case
PRJNA8274(	ENSGALG00010023888	CTH	case	control	0.23355487	0.161622983	1.445059767	0.148441099	0.55494187	up_in_case
PRJNA8274(	ENSGALG00010023889	SAMD8	case	control	-0.057720907	0.079557026	-0.725528712	0.468127737	0.828595945	down_in_case
PRJNA8274(	ENSGALG00010023890	SASS6	case	control	-0.047547182	0.075386199	-0.630714685	0.528227095	0.853519488	down_in_case
PRJNA8274(	ENSGALG00010023891	ENSGALG00010023891	case	control	-0.411135918	0.376241497	-1.092744742	0.274505889	0.699970438	down_in_case
PRJNA8274(	ENSGALG00010023896	POP4	case	control	0.082258901	0.096855556	0.849294603	0.395717383	0.787783337	up_in_case
PRJNA8274(	ENSGALG00010023902	PTGER3	case	control	-0.058396222	0.148330339	-0.393690341	0.693809674	0.920103713	down_in_case

PRJNA8274(	ENSGALG00010023903	TBKBP1	case	control	0.578507357	0.271075666	2.13411763	0.032833153	0.311991307	up_in_case
PRJNA8274(	ENSGALG00010023909	FOXRED1	case	control	-0.100985916	0.109164727	-0.925078263	0.354925199	0.763161644	down_in_case
PRJNA8274(	ENSGALG00010023910	RNF7	case	control	-0.016813476	0.064451016	-0.260872159	0.794191095	0.95280257	down_in_case
PRJNA8274(	ENSGALG00010023911	TFCP2	case	control	-0.15719255	0.060710551	-2.589213009	0.009619557	0.17593167	down_in_case
PRJNA8274(	ENSGALG00010023913	ZMYND8	case	control	-0.049962586	0.05319424	-0.939248051	0.347603404	0.758311011	down_in_case
PRJNA8274(	ENSGALG00010023914	CCDC117	case	control	0.098973864	0.050540219	1.95831888	0.050192607	0.366770069	up_in_case
PRJNA8274(	ENSGALG00010023917	ZRANB2	case	control	-0.094408869	0.05945254	-1.587970308	0.112293053	0.506149199	down_in_case
PRJNA8274(	ENSGALG00010023918	MFSD14A	case	control	-0.02070616	0.05044873	-0.410439675	0.681483447	0.915594727	down_in_case
PRJNA8274(	ENSGALG00010023920	ENSGALG00010023920	case	control	-0.751870282	0.347391408	-2.16433183	0.030438892	0.307572163	down_in_case
PRJNA8274(	ENSGALG00010023921	GRK7	case	control	0.151163065	0.187404423	0.806614182	0.419888801	0.801159345	up_in_case
PRJNA8274(	ENSGALG00010023923	CSNK1G1	case	control	0.002323469	0.068201409	0.034067758	0.972823119	0.99340857	up_in_case
PRJNA8274(	ENSGALG00010023925	NCOA3	case	control	-0.037466165	0.064650389	-0.579519554	0.562238657	0.870979611	down_in_case
PRJNA8274(	ENSGALG00010023926	ZYX	case	control	-0.003146502	0.067795414	-0.046411718	0.962982097	0.992068146	down_in_case
PRJNA8274(	ENSGALG00010023927	KPNB1	case	control	-0.050198953	0.062911346	-0.797931639	0.424910162	0.804370574	down_in_case
PRJNA8274(	ENSGALG00010023930	SRPRA	case	control	-0.010863937	0.036179677	-0.300277345	0.763965613	0.943786458	down_in_case
PRJNA8274(	ENSGALG00010023931	CSRNP2	case	control	0.086599763	0.119466169	0.72488943	0.468519866	0.828596747	up_in_case
PRJNA8274(	ENSGALG00010023937	XBP1	case	control	0.362330519	0.069866116	5.18606931	2.14779E-07	6.8252E-05	up_in_case
PRJNA8274(	ENSGALG00010023940	ATP1B3	case	control	0.104825432	0.042636383	2.458591115	0.013948338	0.207988769	up_in_case
PRJNA8274(	ENSGALG00010023942	NEGR1	case	control	0.044990442	0.105081749	0.428147057	0.668544059	0.913032039	up_in_case
PRJNA8274(	ENSGALG00010023945	LETMD1	case	control	-0.124453802	0.099580406	-1.249782034	0.211379181	0.636501957	down_in_case
PRJNA8274(	ENSGALG00010023946	ZNRF3	case	control	-0.060283415	0.081213211	-0.742285825	0.457914178	0.823337749	down_in_case
PRJNA8274(	ENSGALG00010023947	ENSGALG00010023947	case	control	0.321663097	0.676412141	0.475543057	0.634399952	0.898107199	up_in_case
PRJNA8274(	ENSGALG00010023948	SULF2	case	control	-0.097490439	0.135355046	-0.720257145	0.471366686	0.829101076	down_in_case
PRJNA8274(	ENSGALG00010023953	SLC35A3	case	control	0.167455747	0.127644663	1.311889928	0.189557276	0.612863366	up_in_case
PRJNA8274(	ENSGALG00010023954	PLEKHF1	case	control	0.004863028	0.065845221	0.07385544	0.941125413	0.989551456	up_in_case
PRJNA8274(	ENSGALG00010023955	C1QL4	case	control	0.422731562	0.411218627	1.027997115	0.303951185	0.726839789	up_in_case
PRJNA8274(	ENSGALG00010023956	KREMEN1	case	control	0.013180983	0.137384832	0.095942052	0.923566596	0.98553537	up_in_case
PRJNA8274(	ENSGALG00010023957	YBX3	case	control	-0.17679779	0.073244902	-2.413789701	0.015787572	0.22330591	down_in_case
PRJNA8274(	ENSGALG00010023962	FPGT	case	control	-0.176747666	0.083988381	-2.104429965	0.035340958	0.324492998	down_in_case
PRJNA8274(	ENSGALG00010023966	PEAK1	case	control	0.079073405	0.070682687	1.118709659	0.263264023	0.689122374	up_in_case
PRJNA8274(	ENSGALG00010023967	PRPH	case	control	0.595421499	0.288963161	2.06054466	0.039346501	0.335091843	up_in_case
PRJNA8274(	ENSGALG00010023968	TYW5	case	control	-0.002036092	0.114093033	-0.017845895	0.985761792	0.996595084	down_in_case
PRJNA8274(	ENSGALG00010023972	ENSGALG00010023972	case	control	0.189232686	0.280683332	0.674185689	0.500193267	0.840855399	up_in_case
PRJNA8274(	ENSGALG00010023974	PREX1	case	control	0.061072845	0.066610226	0.916868909	0.359211338	0.765374836	up_in_case
PRJNA8274(	ENSGALG00010023978	TFDP2	case	control	-0.08394544	0.080557578	-1.042055162	0.297386105	0.721027687	down_in_case
PRJNA8274(	ENSGALG00010023979	FAM118B	case	control	0.008495397	0.067988525	0.124953392	0.900560449	0.98028579	up_in_case
PRJNA8274(	ENSGALG00010023981	ENSGALG00010023981	case	control	-0.166353025	0.203098458	-0.819075766	0.412743188	0.797581687	down_in_case
PRJNA8274(	ENSGALG00010023984	FMNL1	case	control	0.12724935	0.137120465	0.928011365	0.353401674	0.761954708	up_in_case
PRJNA8274(	ENSGALG00010023986	SYCE3	case	control	0.194963793	0.329143527	0.592336706	0.553625141	0.866337858	up_in_case
PRJNA8274(	ENSGALG00010023987	TNNI3K	case	control	0.071305361	0.286327574	0.249034208	0.803334321	0.954891353	up_in_case
PRJNA8274(	ENSGALG00010023989	ENSGALG00010023989	case	control	0.046631089	0.065033743	0.717029139	0.473356117	0.82959752	up_in_case
PRJNA8274(	ENSGALG00010023991	TSPAN3	case	control	-0.109589168	0.055674442	-1.968392751	0.049022865	0.36379167	down_in_case
PRJNA8274(	ENSGALG00010023992	FAM131B	case	control	0.164532249	0.108320129	1.518944359	0.128776502	0.530596961	up_in_case
PRJNA8274(	ENSGALG00010023993	C19orf12	case	control	0.031250704	0.078059214	0.400346134	0.688901593	0.918082523	up_in_case
PRJNA8274(	ENSGALG00010023996	MYO7B	case	control	0.195748674	0.371399597	0.527056777	0.598154161	0.884680373	up_in_case
PRJNA8274(	ENSGALG00010023997	ARFGEF2	case	control	0.036062519	0.072465005	0.497654264	0.618727749	0.891656847	up_in_case
PRJNA8274(	ENSGALG00010023999	DGCR14	case	control	0.024440903	0.056417487	0.433215018	0.664858569	0.911900781	up_in_case
PRJNA8274(	ENSGALG00010024001	ENSGALG00010024001	case	control	0.39149791	0.545069913	0.71825265	0.47260152	0.829235491	up_in_case
PRJNA8274(	ENSGALG00010024002	DGCR2	case	control	-0.050366292	0.040829188	-1.233585451	0.217357417	0.642778841	down_in_case
PRJNA8274(	ENSGALG00010024003	ERICH3	case	control	0.12458608	0.159650595	0.780367156	0.435174795	0.81000376	up_in_case
PRJNA8274(	ENSGALG00010024004	EIF4B	case	control	-0.06323548	0.055015632	-1.149409312	0.250387242	0.675955742	down_in_case
PRJNA8274(	ENSGALG00010024005	STYK1	case	control	0.053192528	0.17739192	0.299858798	0.764284863	0.943786458	up_in_case
PRJNA8274(	ENSGALG00010024008	CCNE1	case	control	0.029632068	0.090440211	0.327642621	0.743181892	0.937448285	up_in_case
PRJNA8274(	ENSGALG00010024009	CSE1L	case	control	-0.070469245	0.042289645	-1.666347534	0.095644214	0.482930958	down_in_case
PRJNA8274(	ENSGALG00010024010	ENSGALG00010024010	case	control	-0.256049038	0.068416426	-3.742508259	0.000182193	0.016489578	down_in_case
PRJNA8274(	ENSGALG00010024011	AGPS	case	control	-0.053562136	0.060198778	-0.889754545	0.373597699	0.774369566	down_in_case
PRJNA8274(	ENSGALG00010024013	PTPRJ	case	control	0.080567209	0.080287079	1.003489095	0.315624936	0.736748426	up_in_case
PRJNA8274(	ENSGALG00010024016	CRYZ	case	control	-0.041227805	0.066732872	-0.617803556	0.536704839	0.857336818	down_in_case
PRJNA8274(	ENSGALG00010024017	TYW3	case	control	-0.165124925	0.154536064	-1.06852032	0.285285869	0.709820465	down_in_case
PRJNA8274(	ENSGALG00010024018	KRT18	case	control	0.271790981	1.063786602	0.255493894	0.798341659	0.953422893	up_in_case
PRJNA8274(	ENSGALG00010024019	MAP3K14	case	control	-0.04494781	0.069499823	-0.646732733	0.517804935	0.848425162	down_in_case
PRJNA8274(	ENSGALG00010024020	MAIP1	case	control	-0.005774681	0.03936784	-0.146685232	0.883380476	0.977115076	down_in_case
PRJNA8274(	ENSGALG00010024023	LHX8	case	control	0.025505246	0.338674285	0.075309071	0.939968804	0.989551456	up_in_case
PRJNA8274(	ENSGALG00010024024	AGL	case	control	-0.20684895	0.100251989	-2.063290245	0.03908506	0.335091843	down_in_case
PRJNA8274(	ENSGALG00010024025	CLCN1	case	control	-0.315844496	0.227164309	-1.390379047	0.164413806	0.579452754	down_in_case
PRJNA8274(	ENSGALG00010024026	TIRAP	case	control	0.039929415	0.074283769	0.537525429	0.590904725	0.882949889	up_in_case
PRJNA8274(	ENSGALG00010024029	SCAPER	case	control	-0.116903906	0.086079102	-1.358098583	0.174432405	0.593194623	down_in_case
PRJNA8274(	ENSGALG00010024030	STAU1	case	control	0.00086383	0.051395633	0.016807468	0.986590212	0.996696576	up_in_case
PRJNA8274(	ENSGALG00010024031	LIMS2	case	control	-0.007484935	0.059775285	-0.125217885	0.900351059	0.98028579	down_in_case
PRJNA8274(	ENSGALG00010024034	LRP2	case	control	-0.307202736	0.180164644	-1.70512221	0.088171634	0.469417114	down_in_case
PRJNA8274(	ENSGALG00010024035	OSBPL5	case	control	0.072149346	0.068935719	1.046617732	0.29527592	0.71883651	up_in_case
PRJNA8274(	ENSGALG00010024038	NKD1	case	control	0.056975174	0.140975643	0.404149066	0.686103106	0.917034645	up_in_case
PRJNA8274(	ENSGALG00010024039	MRPL45	case	control	-0.062418704	0.064116972	-0.973512977	0.330298414	0.746525575	down_in_case
PRJNA8274(	ENSGALG00010024040	ENSGALG00010024040	case	control	-0.145238384	0.596439586	-0.243508962	0.807611143	0.955875648	down_in_case
PRJNA8274(	ENSGALG00010024042	DCPS	case	control	-0.228909951	0.075592901	-3.028193772	0.002460203	0.081856092	down_in_case
PRJNA8274(	ENSGALG00010024045	PSTPIP1	case	control	-0.219685445	0.151745187	-1.447725952	0.147693709	0.553319371	down_in_case
PRJNA8274(	ENSGALG00010024046	ENSGALG00010024046	case	control	0.109320257	0.769089739	0.142142394	0.886967532	0.977630122	up_in_case
PRJNA8274(	ENSGALG00010024049	RCN2	case	control	-0.038748208	0.064451955	-0.601195231	0.54770996	0.863104956	down_in_case
PRJNA8274(	ENSGALG00010024051	ENSGALG00010024051	case	control	0.391807756	0.662494255	0.591413061	0.554243691	0.866646786	up_in_case
PRJNA8274(	ENSGALG00010024052	ENSGALG00010024052	case	control	0.233386836	0.132088697	1.766894831	0.077245843	0.445884536	up_in_case
PRJNA8274(	ENSGALG00010024053	ISL2	case	control	-1.041365245	0.408359465	-2.550119034	0.010768614	0.184728043	down_in_case
PRJNA8274(	ENSGALG00010024054	ENSGALG00010024054	case	control	-0.156242405	0.874582477	-0.178647993	0.858214107	0.971828322	down_in_case
PRJNA8274(	ENSGALG00010024057	HSD3B1	case	control	1.562593431	0.596513815	2.619542737	0.008804774	0.166545327	up_in_case
PRJNA8274(	ENSGALG00010024058	SLC44A5	case	control	-0.068623376	0.060392087	-1.136297474	0.255832088	0.682028123	down_in_case
PRJNA8274(	ENSGALG00010024060	FRRS1	case	control	0.108526323	0.106807383	1.016093826	0.309584709	0.732384442	up_in_case
PRJNA8274(	ENSGALG00010024061	ENSGALG00010024061	case	control	1.775348392	1.029769744	1.72402462	0.084703399	0.460659189	up_in_case
PRJNA8274(	ENSGALG00010024062	ST3GAL4	case	control	0.160661169	0.089671008	1.791673505	0.073185281	0.436961143	up_in_case
PRJNA8274(	ENSGALG00010024063	BRD7	case	control	-0.067756714	0.044605477	-1.519022303	0.128756882	0.530596961	down_in_case
PRJNA8274(	ENSGALG00010024064	GPR17	case	control	0.028399396	0.103494166	0.274405766	0.783772813	0.949518053	up_in_case
PRJNA8274(	ENSGALG00010024068	CASP2	case	control	0.083137449	0.073419869	1.132356283	0.257484677	0.684392356	up_in_case
PRJNA8274(	ENSGALG00010024073	RABGGTB	case	control	-0.006167014	0.076856307	-0.080240837	0.936045711	0.988425668	down_in_case
PRJNA8274(	ENSGALG00010024074	ADCY7	case	control	-0.001603451	0.132304934	-0.012119356	0.99033039	0.997374785	down_in_case
PRJNA8274(	ENSGALG00010024075	SPATS2L	case	control	-0.006572875	0.069955436	-0.093958035	0.925142493	0.985571367	down_in_case
PRJNA8274(	ENSGALG00010024077	SFT2D3	case	control	0.120414469	0.073949668	1.628330088	0.103454913	0.492970761	up_in_case
PRJNA8274(	ENSGALG00010024078	KIRREL3	case	control	0.040411466	0.160795119	0.251322715	0.801564615	0.954294696	up_in_case
PRJNA8274(	ENSGALG00010024080	TENT4B	case	control	-0.073224912	0.068393115	-1.070647407	0.284327999	0.708960835	down_in_case
PRJNA8274(	ENSGALG00010024081	CARS	case	control	-0.006771014	0.039371564	-0.171977265	0.863455403	0.972890026	down_in_case
PRJNA8274(	ENSGALG00010024083	PALMD	case	control	0.052217584	0.092343255	0.565472634	0.571752333	0.876814097	up_in_case
PRJNA8274(	ENSGALG00010024086	GABARAPL1	case	control	-0.02629193	0.048198256	-0.545495462	0.585412801	0.881307382	down_in_case
PRJNA8274(	ENSGALG00010024091	ETFA	case	control	-0.068352394	0.076857598	-0.889338153	0.373821372	0.774369566	down_in_case
PRJNA8274(	ENSGALG00010024095	RBM12	case	control	-0.058199939	0.038117903	-1.526840012	0.126800799	0.527026942	down_in_case
PRJNA8274(	ENSGALG00010024096	MYO1D	case	control	0.112124989	0.145265758	0.771861109	0.440196694	0.81275826	up_in_case
PRJNA8274(	ENSGALG00010024101	CNEP1R1	case	control	0.04155916	0.056533712	0.735121728	0.462265362	0.824958777	up_in_case
PRJNA8274(	ENSGALG00010024102	SGF29	case	control	0.087245025	0.057911333	1.506527656	0.131931773	0.535212583	up_in_case
PRJNA8274(	ENSGALG00010024104	PLPPR5	case	control	0.011644867	0.094118049	0.123726179	0.901532083	0.980678218	up_in_case
PRJNA8274(	ENSGALG00010024106	WDR33	case	control	-0.074732929	0.037515071	-1.992077487	0.046362557	0.356443312	down_in_case
PRJNA8274(	ENSGALG00010024107	NFE2L2	case	control	-0.012469527	0.041865743	-0.297845582	0.765821023	0.943860474	down_in_case
PRJNA8274(	ENSGALG00010024110	CPNE1	case	control	0.092222168	0.068775921	1.340907783	0.179950395	0.600808892	up_in_case
PRJNA8274(	ENSGALG00010024112	NAP1L4	case	control	-0.103041812	0.038613902	-2.668515902	0.007618717	0.152061299	down_in_case
PRJNA8274(	ENSGALG00010024113	ENSGALG00010024113	case	control	-0.36824887	0.161697139	-2.277398804	0.022762421	0.267253668	down_in_case
PRJNA8274(	ENSGALG00010024115	POLR2D	case	control	-0.026035828	0.056256353	-0.462806887	0.643502795	0.902038523	down_in_case
PRJNA8274(	ENSGALG00010024116	MCAM	case	control	-0.080130035	0.146850125	-0.545658609	0.585300629	0.881307382	down_in_case
PRJNA8274(	ENSGALG00010024118	TMEM266	case	control	0.08618119	0.086736176	0.993601457	0.320416935	0.738813491	up_in_case
PRJNA8274(	ENSGALG00010024119	AMMECR1L	case	control	0.01043721	0.050998005	0.204659179	0.837838416	0.964826648	up_in_case
PRJNA8274(	ENSGALG00010024121	NRG4	case	control	0.096851473	0.132907565	0.728713021	0.466177226	0.82702452	up_in_case
PRJNA8274(	ENSGALG00010024122	SOCS7	case	control	-0.025136095	0.066359569	-0.378786288	0.70484657	0.924028782	down_in_case
PRJNA8274(	ENSGALG00010024123	PLPPR4	case	control	-0.735573853	0.256691972	-2.865589633	0.004162334	0.111582498	down_in_case
PRJNA8274(	ENSGALG00010024124	DIDO1	case	control	-0.021490907	0.047597847	-0.451510073	0.651621967	0.905275327	down_in_case
PRJNA8274(	ENSGALG00010024126	CDK5R1	case	control	-0.178576577	0.161841696	-1.103402778	0.269852291	0.697106657	down_in_case
PRJNA8274(	ENSGALG00010024128	PHLDA2	case	control	0.224115823	0.131634501	1.702561421	0.088650175	0.47091289	up_in_case
PRJNA8274(	ENSGALG00010024129	GID8	case	control	-0.013780842	0.050441764	-0.273203008	0.784697168	0.949518053	down_in_case
PRJNA8274(	ENSGALG00010024131	ENSGALG00010024131	case	control	1.02973665	1.392341678	0.739571807	0.459559854	0.824480387	up_in_case
PRJNA8274(	ENSGALG00010024132	ENSGALG00010024132	case	control	0.202595894	0.122150727	1.658572969	0.097201872	0.485691283	up_in_case
PRJNA8274(	ENSGALG00010024133	PSMD11	case	control	-0.067847484	0.076597716	-0.885763797	0.375744822	0.774921593	down_in_case
PRJNA8274(	ENSGALG00010024135	FBXO22	case	control	0.07195117	0.058050404	1.239460277	0.215175091	0.640404074	up_in_case
PRJNA8274(	ENSGALG00010024136	SAP130	case	control	-0.089840192	0.07351899	-1.221999808	0.2217077	0.645834386	down_in_case

PRJNA8274(	ENSGALG00010024137	ENSGALG00010024137	case	control	0.348220499	0.314200405	1.108275145	0.267743006	0.694521603	up_in_case
PRJNA8274(	ENSGALG00010024138	CBL	case	control	-0.057412682	0.067598426	-0.849319806	0.395703363	0.787783337	down_in_case
PRJNA8274(	ENSGALG00010024140	KRT6A	case	control	0.818275355	0.760871368	1.075445061	0.282175517	0.706000864	up_in_case
PRJNA8274(	ENSGALG00010024141	YTHDF1	case	control	-0.021478302	0.041084882	-0.52277872	0.60112825	0.884954589	down_in_case
PRJNA8274(	ENSGALG00010024142	DNAJC6	case	control	-0.175623293	0.20846063	-0.842477034	0.39952099	0.789900496	down_in_case
PRJNA8274(	ENSGALG00010024143	UBE2Q2	case	control	-0.037488675	0.079586311	-0.471044266	0.637609124	0.899378897	down_in_case
PRJNA8274(	ENSGALG00010024144	HNRNPA3	case	control	-0.089800596	0.041956201	-2.140341455	0.032327182	0.309838273	down_in_case
PRJNA8274(	ENSGALG00010024145	RAP1GAP3	case	control	-0.044634358	0.131308067	-0.339920916	0.733916085	0.933846596	down_in_case
PRJNA8274(	ENSGALG00010024146	CDKN1C	case	control	-0.043663562	0.111725877	-0.390809753	0.695937866	0.921558614	down_in_case
PRJNA8274(	ENSGALG00010024147	ENSGALG00010024147	case	control	-0.207681996	0.144910427	-1.433174957	0.151807845	0.560075381	down_in_case
PRJNA8274(	ENSGALG00010024148	UGGT1	case	control	0.045211857	0.055756958	0.810873805	0.417438145	0.799987395	up_in_case
PRJNA8274(	ENSGALG00010024149	TCIRG1	case	control	-0.058395792	0.094482091	-0.618062025	0.536534454	0.857336818	down_in_case
PRJNA8274(	ENSGALG00010024150	NHERF4	case	control	0.036670634	0.278383565	0.131727009	0.895200222	0.980176778	up_in_case
PRJNA8274(	ENSGALG00010024151	LEPROT	case	control	-0.037320805	0.050791746	-0.734780898	0.462472943	0.825020344	down_in_case
PRJNA8274(	ENSGALG00010024152	ZNF423	case	control	-0.084712181	0.055802656	-1.518067176	0.128997465	0.530795558	down_in_case
PRJNA8274(	ENSGALG00010024153	KCNQ1	case	control	-0.052296742	0.201660355	-0.259330805	0.795380013	0.95280257	down_in_case
PRJNA8274(	ENSGALG00010024155	SNX7	case	control	-0.021224213	0.056244668	-0.377355115	0.705909719	0.924320939	down_in_case
PRJNA8274(	ENSGALG00010024157	CHRNB4	case	control	-0.273448912	0.236229594	-1.157555699	0.247045397	0.671497658	down_in_case
PRJNA8274(	ENSGALG00010024158	ENSGALG00010024158	case	control	0.20805566	0.257947865	0.806580274	0.419908342	0.801159345	up_in_case
PRJNA8274(	ENSGALG00010024159	NLRX1	case	control	0.08825136	0.096962727	0.910157569	0.362739418	0.768925833	up_in_case
PRJNA8274(	ENSGALG00010024162	SRCIN1	case	control	0.048729791	0.163467824	0.298100202	0.765626687	0.943786458	up_in_case
PRJNA8274(	ENSGALG00010024163	HS6ST1	case	control	-0.030522216	0.060605365	-0.503622351	0.614526783	0.890618526	down_in_case
PRJNA8274(	ENSGALG00010024164	SNAP29	case	control	-0.010698909	0.048834825	-0.219083589	0.826584934	0.961207256	down_in_case
PRJNA8274(	ENSGALG00010024165	LEPR	case	control	-0.04384854	0.276409711	-0.158636032	0.873955639	0.975062028	down_in_case
PRJNA8274(	ENSGALG00010024167	CHRNA3	case	control	-0.339394537	0.349179361	-0.971977656	0.331061665	0.747305731	down_in_case
PRJNA8274(	ENSGALG00010024168	BIRC7	case	control	0.042920431	0.371669648	0.115480053	0.90806463	0.981730793	up_in_case
PRJNA8274(	ENSGALG00010024169	FAM168B	case	control	-0.066556406	0.043806874	-1.519314202	0.128683427	0.530596961	down_in_case
PRJNA8274(	ENSGALG00010024170	NUP160	case	control	-0.137311944	0.060521088	-2.268828082	0.023278783	0.269590124	down_in_case
PRJNA8274(	ENSGALG00010024172	ABCG4	case	control	-0.439238069	0.196297632	-2.237612672	0.025246324	0.282673383	down_in_case
PRJNA8274(	ENSGALG00010024173	CBLN1	case	control	-0.462907034	0.372748377	-1.241875386	0.214282543	0.639112236	down_in_case
PRJNA8274(	ENSGALG00010024174	PLEKHB2	case	control	0.123451412	0.059317256	2.081205723	0.03741508	0.328438691	up_in_case
PRJNA8274(	ENSGALG00010024175	DHRS9	case	control	-0.276570108	0.385342585	-0.717725262	0.472926704	0.829289009	down_in_case
PRJNA8274(	ENSGALG00010024178	N4BP1	case	control	-0.081841662	0.06777379	-1.207570976	0.227212329	0.65086865	down_in_case
PRJNA8274(	ENSGALG00010024179	ATG4C	case	control	0.097734749	0.059653504	1.638373995	0.1013437	0.491460867	up_in_case
PRJNA8274(	ENSGALG00010024180	HINFP	case	control	0.025409273	0.093671886	0.271258264	0.786192404	0.950252288	up_in_case
PRJNA8274(	ENSGALG00010024181	FOXD3	case	control	-0.150714521	0.141229636	-1.06715931	0.285899903	0.710402887	down_in_case
PRJNA8274(	ENSGALG00010024183	MTX2	case	control	-0.15812687	0.075860665	-2.084438241	0.037120322	0.328438691	down_in_case
PRJNA8274(	ENSGALG00010024184	ENSGALG00010024184	case	control	0.111504974	0.111570819	0.999409841	0.317596195	0.737774213	up_in_case
PRJNA8274(	ENSGALG00010024185	CHRNA5	case	control	-0.207010286	0.107073654	-1.933344735	0.053193744	0.376756089	down_in_case
PRJNA8274(	ENSGALG00010024186	HAO2	case	control	0.917088385	0.635947811	1.442081203	0.149279467	0.555910516	up_in_case
PRJNA8274(	ENSGALG00010024188	TSSC4	case	control	0.112256953	0.048442032	2.317346069	0.020484888	0.257185156	up_in_case
PRJNA8274(	ENSGALG00010024189	KAT7	case	control	-0.023223268	0.044289722	-0.524348921	0.600035877	0.884888453	down_in_case
PRJNA8274(	ENSGALG00010024190	WARS2	case	control	-0.124123046	0.081671037	-1.519792707	0.128563083	0.530577801	down_in_case
PRJNA8274(	ENSGALG00010024191	DPYD	case	control	-0.012800493	0.382871522	-0.033432867	0.9733294	0.99354775	down_in_case
PRJNA8274(	ENSGALG00010024192	ALG6	case	control	-0.146487194	0.146131295	-1.002435479	0.316133314	0.736866972	down_in_case
PRJNA8274(	ENSGALG00010024193	GSTK1	case	control	-0.038768173	0.103899221	-0.373132469	0.709049854	0.925521953	down_in_case
PRJNA8274(	ENSGALG00010024194	NKAIN4	case	control	0.002269043	0.080633189	0.028140313	0.977550242	0.994661197	up_in_case
PRJNA8274(	ENSGALG00010024195	PSMA4	case	control	-0.03206266	0.059394609	-0.539824417	0.589318126	0.882949889	down_in_case
PRJNA8274(	ENSGALG00010024196	MRPL21	case	control	0.046060191	0.074153514	0.62114643	0.534503283	0.856211151	up_in_case
PRJNA8274(	ENSGALG00010024197	KRT7	case	control	-0.183584047	0.304805504	-0.602298991	0.546975132	0.862526554	down_in_case
PRJNA8274(	ENSGALG00010024198	PI4KA	case	control	-0.018298593	0.048668767	-0.375982251	0.706930091	0.92461283	down_in_case
PRJNA8274(	ENSGALG00010024199	SLC35B1	case	control	0.092101447	0.072622013	1.268230434	0.204715671	0.627358178	up_in_case
PRJNA8274(	ENSGALG00010024200	GCHFR	case	control	-0.209530418	0.629562478	-0.332819101	0.739270839	0.936326594	down_in_case
PRJNA8274(	ENSGALG00010024201	IREB2	case	control	-0.052746289	0.048014152	-1.098557131	0.271961285	0.698323327	down_in_case
PRJNA8274(	ENSGALG00010024203	SPOP	case	control	-0.019707353	0.048995489	-0.402227901	0.68751631	0.917290381	down_in_case
PRJNA8274(	ENSGALG00010024204	PTBP2	case	control	-0.132446984	0.081681105	-1.621513124	0.104907638	0.494770427	down_in_case
PRJNA8274(	ENSGALG00010024205	ANKRD13D	case	control	-0.041515093	0.089247493	-0.465168164	0.641811035	0.901539099	down_in_case
PRJNA8274(	ENSGALG00010024206	KRT80	case	control	0.034121827	0.35634637	0.095754664	0.923715425	0.98553537	up_in_case
PRJNA8274(	ENSGALG00010024207	KCTD18	case	control	-0.045665341	0.08311976	-0.549392117	0.582736383	0.881066851	down_in_case
PRJNA8274(	ENSGALG00010024208	SERPIND1	case	control	1.072179249	0.775564116	1.382450822	0.166833321	0.582409284	up_in_case
PRJNA8274(	ENSGALG00010024209	RWDD3	case	control	-0.11732312	0.135482779	-0.865963337	0.386510268	0.78254238	down_in_case
PRJNA8274(	ENSGALG00010024211	TRPM5	case	control	0.541415305	0.352705256	1.535036115	0.124774957	0.523864324	up_in_case
PRJNA8274(	ENSGALG00010024212	RIMS4	case	control	-0.006283119	0.173031427	-0.036312011	0.971033573	0.992761697	down_in_case
PRJNA8274(	ENSGALG00010024213	ENSGALG00010024213	case	control	-0.051300321	0.155399135	-0.33011973	0.741309496	0.937205003	down_in_case
PRJNA8274(	ENSGALG00010024214	CRABP1	case	control	0.565057916	0.230685138	2.449476897	0.014306389	0.21093806	up_in_case
PRJNA8274(	ENSGALG00010024215	ENSGALG00010024215	case	control	-0.020367306	0.254700857	-0.079965595	0.936264619	0.988452127	down_in_case
PRJNA8274(	ENSGALG00010024218	NGFR	case	control	0.0094036	0.138247573	0.068019997	0.945769715	0.990313016	up_in_case
PRJNA8274(	ENSGALG00010024220	SIAH1	case	control	-0.146985101	0.052188139	-2.816446489	0.004855813	0.119309498	down_in_case
PRJNA8274(	ENSGALG00010024222	ENSGALG00010024222	case	control	0.005870838	0.151158268	0.038839017	0.969018737	0.992761697	up_in_case
PRJNA8274(	ENSGALG00010024223	ENSGALG00010024223	case	control	-0.03135268	0.237596928	-0.131957431	0.895017962	0.980176778	down_in_case
PRJNA8274(	ENSGALG00010024225	BCDIN3D	case	control	-0.079037872	0.106793966	-0.740096799	0.459241261	0.824397443	down_in_case
PRJNA8274(	ENSGALG00010024227	MUC6	case	control	-0.095198169	0.36767783	-0.258917348	0.795699013	0.95280257	down_in_case
PRJNA8274(	ENSGALG00010024228	ENSGALG00010024228	case	control	0.119763592	0.302510129	0.395899445	0.69217921	0.918988424	up_in_case
PRJNA8274(	ENSGALG00010024229	TTC36	case	control	0.630759003	1.259390715	0.500844571	0.616480515	0.891081782	up_in_case
PRJNA8274(	ENSGALG00010024230	LONP2	case	control	-0.062405926	0.065294695	-0.955757974	0.339194525	0.751259351	down_in_case
PRJNA8274(	ENSGALG00010024233	FAIM2	case	control	0.344303782	0.197098681	1.746859896	0.080661644	0.452876025	up_in_case
PRJNA8274(	ENSGALG00010024234	ENSGALG00010024234	case	control	-0.277990556	0.134034781	-2.074018052	0.038077634	0.330206289	down_in_case
PRJNA8274(	ENSGALG00010024238	ATF7	case	control	-0.13096226	0.08262874	-1.584948047	0.112978139	0.507089574	down_in_case
PRJNA8274(	ENSGALG00010024240	WDR61	case	control	0.07330379	0.07599195	0.964625723	0.334732311	0.749087957	up_in_case
PRJNA8274(	ENSGALG00010024242	KMT2A	case	control	-0.162744187	0.072500237	-2.24474006	0.024784825	0.279293139	down_in_case
PRJNA8274(	ENSGALG00010024243	MPP3	case	control	-0.019993171	0.217562414	-0.091896255	0.926780467	0.986376948	down_in_case
PRJNA8274(	ENSGALG00010024245	TARBP2	case	control	0.01343795	0.106949142	0.125648041	0.900010533	0.98028579	up_in_case
PRJNA8274(	ENSGALG00010024250	AP2A2	case	control	-0.057012517	0.035159647	-1.621532687	0.104903446	0.494770427	down_in_case
PRJNA8274(	ENSGALG00010024251	ENSGALG00010024251	case	control	-0.229845074	0.179333026	-1.28166618	0.199959774	0.622902456	down_in_case
PRJNA8274(	ENSGALG00010024254	ENSGALG00010024254	case	control	-0.39980047	0.190534914	-2.098327606	0.035876215	0.325113993	down_in_case
PRJNA8274(	ENSGALG00010024255	CFAP97D1	case	control	0.629441965	0.365285271	1.723151779	0.084861081	0.461061346	up_in_case
PRJNA8274(	ENSGALG00010024258	DNAJA4	case	control	-0.064656415	0.122210176	-0.529059176	0.5967644	0.884680373	down_in_case
PRJNA8274(	ENSGALG00010024259	ENSGALG00010024259	case	control	-0.062223987	0.063676521	-0.977188853	0.328475666	0.745445783	down_in_case
PRJNA8274(	ENSGALG00010024263	PHKB	case	control	-0.224452027	0.086963688	-2.580985609	0.009851869	0.177161817	down_in_case
PRJNA8274(	ENSGALG00010024264	AMER3	case	control	-0.080556469	0.085205647	-0.9454358	0.344436432	0.755870938	down_in_case
PRJNA8274(	ENSGALG00010024266	ENSGALG00010024266	case	control	-0.275622364	0.279930023	-0.984611655	0.324814862	0.742701076	down_in_case
PRJNA8274(	ENSGALG00010024267	MAP3K12	case	control	-0.122025252	0.148991191	-0.819009843	0.412780798	0.797581687	down_in_case
PRJNA8274(	ENSGALG00010024270	RAB18L	case	control	0.022656863	0.434552125	0.052138425	0.958418396	0.992056045	up_in_case
PRJNA8274(	ENSGALG00010024274	ACSBG1	case	control	-0.000988253	0.129502328	-0.007631162	0.993911273	0.998238292	down_in_case
PRJNA8274(	ENSGALG00010024277	ITGB3BP	case	control	-0.37383555	0.160451964	-2.329890774	0.019811925	0.252730173	down_in_case
PRJNA8274(	ENSGALG00010024280	ENSGALG00010024280	case	control	-0.081848789	0.079827597	-1.02531947	0.305212472	0.728515832	down_in_case
PRJNA8274(	ENSGALG00010024282	HIC2	case	control	0.03243248	0.095522399	0.339527483	0.734212397	0.933846596	up_in_case
PRJNA8274(	ENSGALG00010024283	UBE4A	case	control	0.004693995	0.05529294	0.084893209	0.932346291	0.987669602	up_in_case
PRJNA8274(	ENSGALG00010024285	CCND2	case	control	0.011775002	0.059865339	0.196691473	0.844068984	0.96721237	up_in_case
PRJNA8274(	ENSGALG00010024286	ADA	case	control	-0.127389107	0.101492175	-1.25516185	0.209420011	0.63414862	down_in_case
PRJNA8274(	ENSGALG00010024288	CHID1	case	control	0.05373915	0.089533021	0.600215983	0.548362303	0.86339387	up_in_case
PRJNA8274(	ENSGALG00010024290	SOST	case	control	0.281009543	0.404513426	0.694685331	0.487252523	0.835461642	up_in_case
PRJNA8274(	ENSGALG00010024291	ENSGALG00010024291	case	control	0.275099069	0.273554007	1.005648106	0.314584874	0.736432557	up_in_case
PRJNA8274(	ENSGALG00010024300	MEOX1	case	control	0.18921509	0.147811575	1.280110105	0.200506415	0.623042533	up_in_case
PRJNA8274(	ENSGALG00010024304	PKIG	case	control	-0.025573337	0.104747687	-0.244142257	0.807120646	0.955875648	down_in_case
PRJNA8274(	ENSGALG00010024305	ENSGALG00010024305	case	control	-0.858884817	0.349648475	-2.456423743	0.014032758	0.208540979	down_in_case
PRJNA8274(	ENSGALG00010024309	ABCB11	case	control	0.224940507	0.295661435	0.760804354	0.446773933	0.817833512	up_in_case
PRJNA8274(	ENSGALG00010024311	KLHL6	case	control	0.118191548	0.108686462	1.087454184	0.276836136	0.702359305	up_in_case
PRJNA8274(	ENSGALG00010024313	SERINC3	case	control	-0.082885636	0.066135256	-1.253274586	0.210105794	0.635741188	down_in_case
PRJNA8274(	ENSGALG00010024314	ITFG1	case	control	-0.017801901	0.062201172	-0.286198809	0.774725856	0.947085948	down_in_case
PRJNA8274(	ENSGALG00010024321	UBE2L3	case	control	-0.001518906	0.037943442	-0.040030787	0.968068582	0.992761697	down_in_case
PRJNA8274(	ENSGALG00010024322	IDH3A	case	control	0.027459918	0.09671757	0.28391861	0.776472754	0.947228003	up_in_case
PRJNA8274(	ENSGALG00010024323	TTPAL	case	control	0.037584367	0.065701592	0.572046522	0.567290457	0.873689252	up_in_case
PRJNA8274(	ENSGALG00010024325	PCBP2	case	control	-0.103136215	0.063677554	-1.61966358	0.105304564	0.495041148	down_in_case
PRJNA8274(	ENSGALG00010024326	KLHL24	case	control	-0.102273433	0.085770535	-1.192407544	0.233101484	0.657982411	down_in_case
PRJNA8274(	ENSGALG00010024327	PPP1R1B	case	control	-0.215132267	0.310281381	-0.69334572	0.488092617	0.835895141	down_in_case
PRJNA8274(	ENSGALG00010024328	YDJC	case	control	0.00672832	0.067449837	0.099752949	0.920540463	0.984866723	up_in_case
PRJNA8274(	ENSGALG00010024330	SDF2L1	case	control	0.175989285	0.056576563	3.110639382	0.001866828	0.070437034	up_in_case
PRJNA8274(	ENSGALG00010024332	EFCAB7	case	control	-0.097477111	0.132836269	-0.733813975	0.463062122	0.825091817	down_in_case
PRJNA8274(	ENSGALG00010024333	ETV4	case	control	-0.022810045	0.096416011	-0.236579427	0.812983082	0.956712425	down_in_case
PRJNA8274(	ENSGALG00010024334	USP28	case	control	-0.225809366	0.075315787	-2.998167793	0.002716081	0.087009711	down_in_case
PRJNA8274(	ENSGALG00010024335	AMHR2	case	control	0.242474438	0.409777429	0.591722288	0.554036569	0.866629068	up_in_case
PRJNA8274(	ENSGALG00010024336	TSPAN4	case	control	-0.152250517	0.079324101	-1.919347532	0.054940366	0.38300081	down_in_case
PRJNA8274(	ENSGALG00010024338	NDUFA9	case	control	-0.058608117	0.06096576	-0.961328404	0.336387071	0.750473129	down_in_case
PRJNA8274(	ENSGALG00010024339	TOP3B	case	control	0.000282256	0.056786875	0.004970444	0.996034175	0.998710499	up_in_case
PRJNA8274(	ENSGALG00010024340	NETO2	case	control	-0.084011336	0.183630926	-0.457501022	0.647310968	0.903059048	down_in_case

PRJNA8274(	ENSGALG00010024342	HNF4A	case	control	1.424236446	0.886277339	1.60698732	0.10805715	0.499746844	up_in_case
PRJNA8274(	ENSGALG00010024343	POLR2L	case	control	0.128649063	0.074137443	1.735277848	0.082691604	0.456624721	up_in_case
PRJNA8274(	ENSGALG00010024344	YEATS2	case	control	-0.098270444	0.045723476	-2.149233879	0.031615865	0.3098361	down_in_case
PRJNA8274(	ENSGALG00010024345	STARD3	case	control	0.033138724	0.051276619	0.646273573	0.518102199	0.848618078	up_in_case
PRJNA8274(	ENSGALG00010024346	PPM1F	case	control	0.021313365	0.081584253	0.261243613	0.793904647	0.95280257	up_in_case
PRJNA8274(	ENSGALG00010024347	OTOG	case	control	0.170677322	0.262611266	0.649923837	0.51574142	0.847810358	up_in_case
PRJNA8274(	ENSGALG00010024349	R3HDML	case	control	-0.052642395	0.414484517	-0.127006903	0.898934935	0.98028579	down_in_case
PRJNA8274(	ENSGALG00010024350	PGM1	case	control	-0.256473478	0.144784453	-1.771415868	0.076491572	0.444080628	down_in_case
PRJNA8274(	ENSGALG00010024351	SP1	case	control	-0.04554485	0.078470265	-0.580409019	0.561638824	0.870616281	down_in_case
PRJNA8274(	ENSGALG00010024352	MAP6D1	case	control	0.147588338	0.34850888	0.423485156	0.671941327	0.913676733	up_in_case
PRJNA8274(	ENSGALG00010024354	C2CD2L	case	control	0.040474065	0.07472769	0.541620718	0.588079817	0.882771329	up_in_case
PRJNA8274(	ENSGALG00010024356	PARL	case	control	-0.081955169	0.062342811	-1.314588926	0.188648094	0.611698262	down_in_case
PRJNA8274(	ENSGALG00010024358	MAPK3	case	control	-0.074080961	0.02618645	-2.828980687	0.004669652	0.116334528	down_in_case
PRJNA8274(	ENSGALG00010024360	FTL	case	control	0.32840027	0.170151932	1.930041385	0.05360171	0.37782383	up_in_case
PRJNA8274(	ENSGALG00010024361	H2AX	case	control	0.106928702	0.202268564	0.528647162	0.597050238	0.884680373	up_in_case
PRJNA8274(	ENSGALG00010024362	DHX8	case	control	-0.10118926	0.039227236	-2.579566371	0.009892445	0.177161817	down_in_case
PRJNA8274(	ENSGALG00010024363	EDEM2	case	control	0.033920122	0.092017969	0.368624985	0.71240727	0.92680349	up_in_case
PRJNA8274(	ENSGALG00010024365	ENSGALG00010024365	case	control	0.066525536	0.093106001	0.71451394	0.474909439	0.830111175	up_in_case
PRJNA8274(	ENSGALG00010024366	PARD6A	case	control	0.466992011	0.331880461	1.407109083	0.159395045	0.572017525	up_in_case
PRJNA8274(	ENSGALG00010024367	ENSGALG00010024367	case	control	0.04242421	0.112660626	0.376566434	0.706495836	0.924580439	up_in_case
PRJNA8274(	ENSGALG00010024368	DPAGT1	case	control	0.017519532	0.049423543	0.354477461	0.722981092	0.930215097	up_in_case
PRJNA8274(	ENSGALG00010024369	MYOD1	case	control	-0.152448323	0.104513214	-1.458651176	0.144661141	0.548818979	down_in_case
PRJNA8274(	ENSGALG00010024370	HERPUD1	case	control	0.267985006	0.04747843	5.64435273	1.65804E-08	7.64837E-06	up_in_case
PRJNA8274(	ENSGALG00010024371	YPEL1	case	control	-0.002087798	0.074775354	-0.027920943	0.977725205	0.99476844	down_in_case
PRJNA8274(	ENSGALG00010024373	TCAP	case	control	0.245545442	0.376591801	0.652020148	0.514388175	0.847493921	up_in_case
PRJNA8274(	ENSGALG00010024374	ENSGALG00010024374	case	control	0.263339599	0.81067541	0.324839752	0.745302357	0.937849656	up_in_case
PRJNA8274(	ENSGALG00010024377	CIB2	case	control	-0.119957133	0.162613139	-0.737684138	0.460706413	0.82454265	down_in_case
PRJNA8274(	ENSGALG00010024378	ZDHHC1	case	control	-0.067047798	0.070033964	-0.957361182	0.338384984	0.751259351	down_in_case
PRJNA8274(	ENSGALG00010024383	DUSP28	case	control	0.117325724	0.171642436	0.683547301	0.494261071	0.83803731	up_in_case
PRJNA8274(	ENSGALG00010024386	TPPP3	case	control	0.268177105	0.240351159	1.115772051	0.26451972	0.690350289	up_in_case
PRJNA8274(	ENSGALG00010024387	PPIL2	case	control	0.036985798	0.046842394	0.789579589	0.429773332	0.806015686	up_in_case
PRJNA8274(	ENSGALG00010024389	AAAS	case	control	-0.011713522	0.067915051	-0.172473136	0.86306558	0.972850732	down_in_case
PRJNA8274(	ENSGALG00010024391	TBC1D2B	case	control	0.025518277	0.086836972	0.293864194	0.76886168	0.945375926	up_in_case
PRJNA8274(	ENSGALG00010024392	CHMP4B	case	control	-0.069173419	0.044699661	-1.547515519	0.121738985	0.519686058	down_in_case
PRJNA8274(	ENSGALG00010024393	OGFOD1	case	control	0.022361195	0.077619195	0.28808847	0.773279018	0.946459495	up_in_case
PRJNA8274(	ENSGALG00010024396	ENSGALG00010024396	case	control	-0.058566494	0.232898824	-0.251467538	0.801452657	0.954294696	down_in_case
PRJNA8274(	ENSGALG00010024398	PHB	case	control	-0.039339767	0.059168448	-0.664877453	0.506128878	0.843280685	down_in_case
PRJNA8274(	ENSGALG00010024399	ROR1	case	control	-0.056497933	0.083985578	-0.672709933	0.501131847	0.841040176	down_in_case
PRJNA8274(	ENSGALG00010024400	KCNA1	case	control	0.062337577	0.285165194	0.218601632	0.826960381	0.96126918	up_in_case
PRJNA8274(	ENSGALG00010024401	KCNC1	case	control	-0.36795972	0.129334185	-2.845030649	0.004440715	0.114389963	down_in_case
PRJNA8274(	ENSGALG00010024402	GPC1	case	control	-0.000256616	0.093702121	-0.002738641	0.997814883	0.999142415	down_in_case
PRJNA8274(	ENSGALG00010024403	HMBS	case	control	0.057375578	0.063589038	0.902287253	0.366904295	0.771770041	up_in_case
PRJNA8274(	ENSGALG00010024406	NUDT21	case	control	-0.067611176	0.062344715	-1.084473251	0.278155021	0.703443892	down_in_case
PRJNA8274(	ENSGALG00010024407	HIRA	case	control	-0.059259725	0.070889706	-0.835942595	0.403187198	0.792416414	down_in_case
PRJNA8274(	ENSGALG00010024410	MYG1	case	control	0.058882363	0.066472647	0.885813427	0.375718073	0.774921593	up_in_case
PRJNA8274(	ENSGALG00010024412	PIGU	case	control	-0.049864222	0.073462663	-0.678769602	0.497283857	0.839173844	down_in_case
PRJNA8274(	ENSGALG00010024413	AMFR	case	control	-0.046115175	0.051055928	-0.903228608	0.366404577	0.771763177	down_in_case
PRJNA8274(	ENSGALG00010024415	SIK2	case	control	-0.011662936	0.041873748	-0.278526203	0.780608454	0.948875232	down_in_case
PRJNA8274(	ENSGALG00010024416	VDR	case	control	0.154289294	0.309771424	0.498074651	0.618431427	0.891656847	up_in_case
PRJNA8274(	ENSGALG00010024419	PGAP3	case	control	-0.018955412	0.07883211	-0.240452926	0.809979152	0.955972758	down_in_case
PRJNA8274(	ENSGALG00010024420	TMEM106C	case	control	0.027084464	0.109424703	0.247516905	0.804508211	0.955282522	up_in_case
PRJNA8274(	ENSGALG00010024421	DAPK2	case	control	0.125201088	0.096575901	1.296400924	0.194837394	0.617640154	up_in_case
PRJNA8274(	ENSGALG00010024423	SERGEF	case	control	-0.073933492	0.107243347	-0.689399333	0.490572003	0.836634424	down_in_case
PRJNA8274(	ENSGALG00010024424	SAMHD1	case	control	-0.070103182	0.077613383	-0.903235748	0.366400789	0.771763177	down_in_case
PRJNA8274(	ENSGALG00010024425	MRPL40	case	control	-0.164215162	0.085705902	-1.916030955	0.055361156	0.383558394	down_in_case
PRJNA8274(	ENSGALG00010024426	PHAF1	case	control	-0.142840831	0.080982679	-1.763844225	0.077758209	0.447281736	down_in_case
PRJNA8274(	ENSGALG00010024427	C22orf39	case	control	-0.143920191	0.094004627	-1.530990509	0.125771738	0.525273324	down_in_case
PRJNA8274(	ENSGALG00010024428	SAAL1	case	control	0.037473157	0.040419134	0.927114287	0.353867199	0.762350539	up_in_case
PRJNA8274(	ENSGALG00010024430	ZNF652	case	control	-0.026395411	0.030656091	-0.861016859	0.389228758	0.783669208	down_in_case
PRJNA8274(	ENSGALG00010024431	FEZ1	case	control	0.118716674	0.075984581	1.562378477	0.118198854	0.51463738	up_in_case
PRJNA8274(	ENSGALG00010024433	ENSGALG00010024433	case	control	-0.174833388	0.083751152	-2.087534122	0.03683988	0.328026329	down_in_case
PRJNA8274(	ENSGALG00010024434	B3GNT9	case	control	0.138847536	0.083210184	1.668636327	0.095189475	0.482014693	up_in_case
PRJNA8274(	ENSGALG00010024437	HERC1	case	control	-0.099326214	0.038992206	-2.547335066	0.010854915	0.185012262	down_in_case
PRJNA8274(	ENSGALG00010024438	RBL1	case	control	0.001491841	0.052276778	0.028537347	0.977233581	0.994661197	up_in_case
PRJNA8274(	ENSGALG00010024439	UFD1	case	control	-0.109485431	0.044053285	-2.48529547	0.012944395	0.199897239	down_in_case
PRJNA8274(	ENSGALG00010024440	PKNOX2	case	control	-0.098927932	0.096766184	-1.022339907	0.306620051	0.729504707	down_in_case
PRJNA8274(	ENSGALG00010024441	SAA	case	control	0.391475124	0.369244825	1.060204769	0.289051452	0.712801679	up_in_case
PRJNA8274(	ENSGALG00010024446	GRM4	case	control	-0.228499683	0.385365764	-0.592942353	0.553219733	0.866252369	down_in_case
PRJNA8274(	ENSGALG00010024449	CACHD1	case	control	-0.139568791	0.082137703	-1.699204933	0.089280577	0.47258594	down_in_case
PRJNA8274(	ENSGALG00010024451	PHOSPHO1	case	control	0.343418952	0.274656245	1.250359161	0.211168376	0.636501957	up_in_case
PRJNA8274(	ENSGALG00010024452	SLC16A1	case	control	0.147655978	0.10643333	1.387309567	0.165347387	0.580618526	up_in_case
PRJNA8274(	ENSGALG00010024453	HTR3A	case	control	-0.319330273	0.487123802	-0.655542333	0.51211864	0.845834144	down_in_case
PRJNA8274(	ENSGALG00010024454	HPS5	case	control	-0.002396425	0.060918874	-0.039337977	0.968620929	0.992761697	down_in_case
PRJNA8274(	ENSGALG00010024457	CHD6	case	control	-0.032442557	0.049293537	-0.658150309	0.510441552	0.844925823	down_in_case
PRJNA8274(	ENSGALG00010024459	AHCYL1	case	control	-0.014800208	0.063861728	-0.231753961	0.816729118	0.957784679	down_in_case
PRJNA8274(	ENSGALG00010024460	IMP4	case	control	-0.052144668	0.092370795	-0.564514661	0.572403926	0.876814097	down_in_case
PRJNA8274(	ENSGALG00010024462	ZBTB32	case	control	-0.983142564	0.184625275	-5.325070266	1.00914E-07	3.70018E-05	down_in_case
PRJNA8274(	ENSGALG00010024463	FBXL22	case	control	-0.002411906	0.15714412	-0.015348372	0.987754252	0.996942188	down_in_case
PRJNA8274(	ENSGALG00010024465	SENP1	case	control	0.095971547	0.081437265	1.178472123	0.23860844	0.663457598	up_in_case
PRJNA8274(	ENSGALG00010024467	ENSGALG00010024467	case	control	-0.151764933	0.228469474	-0.664267878	0.506518876	0.843411729	down_in_case
PRJNA8274(	ENSGALG00010024469	GTF2H1	case	control	-0.132292609	0.068549588	-1.929881882	0.053621475	0.37782383	down_in_case
PRJNA8274(	ENSGALG00010024471	CCDC61	case	control	-0.163111445	0.09817984	-1.661353755	0.09664242	0.48430176	down_in_case
PRJNA8274(	ENSGALG00010024472	LDHA	case	control	-0.372356818	0.265574204	-1.402082026	0.160890754	0.573321151	down_in_case
PRJNA8274(	ENSGALG00010024473	RAVER2	case	control	-0.100504949	0.054300464	-1.850904057	0.064183357	0.410842437	down_in_case
PRJNA8274(	ENSGALG00010024474	STRIP1	case	control	0.085351609	0.061680322	1.383773726	0.166427751	0.58174452	up_in_case
PRJNA8274(	ENSGALG00010024475	ADGRG1	case	control	-0.223103996	0.123204944	-1.810836388	0.070166182	0.427696675	down_in_case
PRJNA8274(	ENSGALG00010024476	KCNB1	case	control	-0.306731048	0.157024045	-1.95340178	0.050772005	0.368174279	down_in_case
PRJNA8274(	ENSGALG00010024479	ERBB2	case	control	-0.126550806	0.0629903	-2.009052278	0.04453159	0.350468759	down_in_case
PRJNA8274(	ENSGALG00010024480	CDC45	case	control	0.086641273	0.084236988	1.028541919	0.303694983	0.726591644	up_in_case
PRJNA8274(	ENSGALG00010024482	USP3	case	control	0.057836712	0.049673283	1.164342461	0.244285255	0.668186525	up_in_case
PRJNA8274(	ENSGALG00010024486	GPR37L1	case	control	0.011683717	0.198259614	0.0589314	0.953006748	0.992056045	up_in_case
PRJNA8274(	ENSGALG00010024488	ADGRG5	case	control	0.320057558	0.341473502	0.937283733	0.348612628	0.759193168	up_in_case
PRJNA8274(	ENSGALG00010024490	TGM3	case	control	0.889112119	0.584537122	1.521053302	0.128246462	0.530035955	up_in_case
PRJNA8274(	ENSGALG00010024492	NTF3	case	control	-0.014888729	0.156591512	-0.095080053	0.924251242	0.985571367	down_in_case
PRJNA8274(	ENSGALG00010024493	JAK1	case	control	-0.123041434	0.060407426	-2.036859407	0.041664133	0.340995489	down_in_case
PRJNA8274(	ENSGALG00010024494	DDAH1	case	control	-0.386720429	0.263018232	-1.470317954	0.141475661	0.543501954	down_in_case
PRJNA8274(	ENSGALG00010024495	CLDN5	case	control	0.049366776	0.113616243	0.434504561	0.663922084	0.911315588	up_in_case
PRJNA8274(	ENSGALG00010024496	UEVLD	case	control	-0.107074745	0.084175345	-1.272044036	0.203357474	0.625537942	down_in_case
PRJNA8274(	ENSGALG00010024497	ABI3	case	control	-0.16795611	0.245965153	-0.682845145	0.494704699	0.83803731	down_in_case
PRJNA8274(	ENSGALG00010024498	CCDC102A	case	control	0.119230541	0.060496915	1.970853235	0.048740665	0.363542241	up_in_case
PRJNA8274(	ENSGALG00010024499	CA12	case	control	-0.044155698	0.683891113	-0.064565392	0.94852004	0.991291133	down_in_case
PRJNA8274(	ENSGALG00010024500	GNGT2	case	control	-0.543760556	0.263959333	-2.060016404	0.039396973	0.335091843	down_in_case
PRJNA8274(	ENSGALG00010024501	ARL8A	case	control	0.011574501	0.067630157	0.171144076	0.864110479	0.97295831	up_in_case
PRJNA8274(	ENSGALG00010024502	VPS8	case	control	-0.09469253	0.048803814	-1.940269046	0.052347001	0.373533991	down_in_case
PRJNA8274(	ENSGALG00010024504	CASC3	case	control	-0.026745865	0.056584373	-0.472672283	0.636447004	0.898973839	down_in_case
PRJNA8274(	ENSGALG00010024505	DNASE2B	case	control	-0.205689015	0.111565978	-1.843653581	0.065233642	0.414904609	down_in_case
PRJNA8274(	ENSGALG00010024506	RAB8B	case	control	-0.017484289	0.055469032	-0.315208119	0.752603641	0.940590113	down_in_case
PRJNA8274(	ENSGALG00010024508	RPS27L	case	control	-0.201966266	0.100175975	-2.016114802	0.043787973	0.349229229	down_in_case
PRJNA8274(	ENSGALG00010024509	SEPTIN5	case	control	-0.131479819	0.090682371	-1.449893924	0.147088102	0.55227446	down_in_case
PRJNA8274(	ENSGALG00010024510	CNN3	case	control	-0.022222747	0.041380835	-0.537029944	0.59124693	0.882949889	down_in_case
PRJNA8274(	ENSGALG00010024511	ENSGALG00010024511	case	control	-0.036132926	0.080913517	-0.446562288	0.65519113	0.907302833	down_in_case
PRJNA8274(	ENSGALG00010024512	CCNDBP1	case	control	0.068374172	0.064420231	1.061377318	0.288518461	0.712801679	up_in_case
PRJNA8274(	ENSGALG00010024513	SPTY2D1	case	control	0.053419563	0.062525908	0.854358855	0.392906186	0.786252233	up_in_case
PRJNA8274(	ENSGALG00010024514	FAF1	case	control	0.025459462	0.047991231	0.530502381	0.595763659	0.884680373	up_in_case
PRJNA8274(	ENSGALG00010024515	C3orf70	case	control	-0.023983966	0.088875689	-0.269859686	0.787268203	0.950999772	down_in_case
PRJNA8274(	ENSGALG00010024516	EHHADH	case	control	0.39830387	0.247831376	1.607156756	0.108019986	0.499736588	up_in_case
PRJNA8274(	ENSGALG00010024517	LACTB	case	control	-0.153737035	0.061565394	-2.497133943	0.012520166	0.196745458	down_in_case
PRJNA8274(	ENSGALG00010024518	ENSGALG00010024518	case	control	1.242563458	0.753278719	1.649540106	0.099037034	0.488407564	up_in_case
PRJNA8274(	ENSGALG00010024519	MIEN1	case	control	0.029129623	0.098563187	0.295542621	0.767579398	0.944774092	up_in_case
PRJNA8274(	ENSGALG00010024520	MAP3K13	case	control	-0.096391292	0.148636953	-0.648501531	0.516660621	0.848054666	down_in_case
PRJNA8274(	ENSGALG00010024521	HEPACAM	case	control	0.293213202	0.245800843	1.192889325	0.232912721	0.657982411	up_in_case
PRJNA8274(	ENSGALG00010024522	DNTTIP2	case	control	-0.129896894	0.066168059	-1.963135939	0.049630381	0.364833243	down_in_case
PRJNA8274(	ENSGALG00010024523	TOX3	case	control	0.128757951	0.154982268	0.830791499	0.406091426	0.7941993	up_in_case
PRJNA8274(	ENSGALG00010024524	TMEM41A	case	control	0.106190309	0.104879421	1.012498994	0.311299527	0.734097745	up_in_case
PRJNA8274(	ENSGALG00010024525	GP1BB	case	control	0.299092738	0.224763364	1.330700581	0.183287552	0.60491829	up_in_case
PRJNA8274(	ENSGALG00010024526	TGM6	case	control	-0.034547362	0.549161355	-0.06290931	0.949838721	0.991488793	down_in_case

PRJNA8274(	ENSGALG00010024527	ATP6V0B	case	control	0.03936036	0.066571097	0.591252994	0.55435092	0.866646786	up_in_case
PRJNA8274(	ENSGALG00010024528	TBX1	case	control	-0.069863179	0.17719067	-0.394282492	0.693372488	0.919776842	down_in_case
PRJNA8274(	ENSGALG00010024529	GRB7	case	control	0.662283409	1.007632761	0.657266649	0.511009477	0.845376622	up_in_case
PRJNA8274(	ENSGALG00010024531	CEP63	case	control	0.058225647	0.077617871	0.750157741	0.453159707	0.821282604	up_in_case
PRJNA8274(	ENSGALG00010024532	TMEM86A	case	control	0.095303737	0.080409647	1.185227655	0.235927449	0.660642087	up_in_case
PRJNA8274(	ENSGALG00010024533	CDKN2C	case	control	0.031482381	0.121652797	0.258788796	0.795798203	0.95280257	up_in_case
PRJNA8274(	ENSGALG00010024534	VWF	case	control	-0.194162854	0.093569218	-2.075071899	0.037979872	0.330206289	down_in_case
PRJNA8274(	ENSGALG00010024535	ENSGALG00010024535	case	control	-0.157771935	0.107296035	-1.470435833	0.141443752	0.543501954	down_in_case
PRJNA8274(	ENSGALG00010024536	URI1	case	control	-0.096433982	0.047909876	-2.012820517	0.044133515	0.350032868	down_in_case
PRJNA8274(	ENSGALG00010024537	EPB42	case	control	0.123978198	0.158236191	0.783500901	0.433333025	0.808138728	up_in_case
PRJNA8274(	ENSGALG00010024539	RAPGEFL1	case	control	-0.051554341	0.474269058	-0.10870273	0.913438277	0.982728035	down_in_case
PRJNA8274(	ENSGALG00010024540	PTPN5	case	control	0.456594412	0.242613587	1.881982035	0.059838455	0.398180505	up_in_case
PRJNA8274(	ENSGALG00010024541	WIPF2	case	control	-0.082714291	0.094265851	-0.877457635	0.38023812	0.77821742	down_in_case
PRJNA8274(	ENSGALG00010024542	ZNFX1	case	control	0.01853425	0.129013492	0.143661332	0.885767907	0.977630122	up_in_case
PRJNA8274(	ENSGALG00010024544	ZNF536	case	control	0.168836392	0.167529778	1.007799298	0.313550823	0.735165891	up_in_case
PRJNA8274(	ENSGALG00010024545	PHACTR3	case	control	0.121476259	0.156659549	0.775415606	0.438094116	0.811390476	up_in_case
PRJNA8274(	ENSGALG00010024546	KY	case	control	-0.068126142	0.162836863	-0.418370513	0.675676242	0.914247368	down_in_case
PRJNA8274(	ENSGALG00010024547	PFKM	case	control	-0.350803661	0.162257139	-2.162022971	0.030616406	0.308259818	down_in_case
PRJNA8274(	ENSGALG00010024548	CDC6	case	control	-0.052640428	0.104154349	-0.505407879	0.613272386	0.89024415	down_in_case
PRJNA8274(	ENSGALG00010024549	GNB1L	case	control	-0.027059659	0.070151445	-0.385732028	0.69969514	0.922288441	down_in_case
PRJNA8274(	ENSGALG00010024551	RNF11	case	control	-0.137211918	0.081181586	-1.690185233	0.090992523	0.475746133	down_in_case
PRJNA8274(	ENSGALG00010024552	CD81	case	control	0.050660556	0.068662292	0.737822091	0.460622567	0.82454265	up_in_case
PRJNA8274(	ENSGALG00010024553	ASB8	case	control	0.100625783	0.078084325	1.288680957	0.197509025	0.62094535	up_in_case
PRJNA8274(	ENSGALG00010024554	ST6GALNAC3	case	control	-0.070650596	0.084274597	-0.838337988	0.401840906	0.791613853	down_in_case
PRJNA8274(	ENSGALG00010024555	EPHB1	case	control	-0.007036864	0.092141525	-0.076370173	0.939124599	0.989551456	down_in_case
PRJNA8274(	ENSGALG00010024556	ENSGALG00010024556	case	control	0.028732727	0.143133116	0.200741297	0.840900866	0.965776435	up_in_case
PRJNA8274(	ENSGALG00010024557	TSPAN32	case	control	0.150043591	0.233848482	0.641627387	0.521115146	0.850193564	up_in_case
PRJNA8274(	ENSGALG00010024558	PFDN5	case	control	-0.147555573	0.045629118	-3.23380284	0.001221538	0.055104058	down_in_case
PRJNA8274(	ENSGALG00010024559	ENSGALG00010024559	case	control	-0.036154207	0.256138918	-0.141150775	0.887750832	0.977944694	down_in_case
PRJNA8274(	ENSGALG00010024560	A4GNT	case	control	-0.152828058	0.414463972	-0.368736653	0.712324026	0.92680349	down_in_case
PRJNA8274(	ENSGALG00010024561	ROBO3	case	control	0.394634407	0.330532726	1.193934445	0.232503612	0.657934961	up_in_case
PRJNA8274(	ENSGALG00010024563	ESPL1	case	control	-0.036190252	0.111770842	-0.323789745	0.746097221	0.938117773	down_in_case
PRJNA8274(	ENSGALG00010024564	PPP1R3D	case	control	-0.160080038	0.117884944	-1.357934549	0.174484454	0.593230548	down_in_case
PRJNA8274(	ENSGALG00010024565	FAM217B	case	control	-0.156539148	0.061996348	-2.524973693	0.011570695	0.188192574	down_in_case
PRJNA8274(	ENSGALG00010024566	MTSS2	case	control	0.077305993	0.111886197	0.690934137	0.489606932	0.836347578	up_in_case
PRJNA8274(	ENSGALG00010024567	IKZF3	case	control	0.138130673	0.404907998	0.341140885	0.732997524	0.933846596	up_in_case
PRJNA8274(	ENSGALG00010024568	DZIP1L	case	control	0.058933214	0.188785989	0.312169425	0.754911767	0.941559531	up_in_case
PRJNA8274(	ENSGALG00010024569	ASCL2	case	control	0.034393244	0.214503764	0.160338652	0.872614313	0.975062028	up_in_case
PRJNA8274(	ENSGALG00010024570	TPM1	case	control	-0.140780471	0.120226152	-1.170963791	0.241613332	0.666243691	down_in_case
PRJNA8274(	ENSGALG00010024571	KCNA3	case	control	0.201448314	0.51263415	0.39296702	0.69434384	0.920308089	up_in_case
PRJNA8274(	ENSGALG00010024572	CLDN18	case	control	-0.434964692	0.865850103	-0.502355651	0.615417369	0.891081782	down_in_case
PRJNA8274(	ENSGALG00010024573	ENSGALG00010024573	case	control	-0.206897862	0.220805375	-0.937014609	0.348751043	0.759193168	down_in_case
PRJNA8274(	ENSGALG00010024575	TXNRD2	case	control	0.116596305	0.078270648	1.489655542	0.13631483	0.539840644	up_in_case
PRJNA8274(	ENSGALG00010024576	CSAD	case	control	0.467326032	0.163101249	2.865251094	0.004166787	0.111582498	up_in_case
PRJNA8274(	ENSGALG00010024578	ENSGALG00010024578	case	control	0.396635594	0.821318065	0.48292569	0.6291485	0.896269608	up_in_case
PRJNA8274(	ENSGALG00010024579	ZNF740	case	control	-0.062339748	0.064360106	-0.968608537	0.332740542	0.748503677	down_in_case
PRJNA8274(	ENSGALG00010024580	IGF2	case	control	0.060238395	0.119838997	0.50266104	0.615202606	0.890965897	up_in_case
PRJNA8274(	ENSGALG00010024581	RARA	case	control	-0.009936432	0.065771143	-0.151075864	0.879915872	0.976318821	down_in_case
PRJNA8274(	ENSGALG00010024582	TLN2	case	control	0.002784043	0.072235155	0.038541392	0.96925603	0.992761697	up_in_case
PRJNA8274(	ENSGALG00010024583	ITGB7	case	control	1.795473898	0.647601152	2.772499545	0.00556276	0.129767478	up_in_case
PRJNA8274(	ENSGALG00010024585	TBCCD1	case	control	0.007686383	0.101773466	0.075524429	0.939797462	0.989551456	up_in_case
PRJNA8274(	ENSGALG00010024586	DLGAP4	case	control	0.003573343	0.049078416	0.072808839	0.941958237	0.989551456	up_in_case
PRJNA8274(	ENSGALG00010024587	ENSGALG00010024587	case	control	0.075744204	0.093223069	0.812504936	0.416501955	0.799987395	up_in_case
PRJNA8274(	ENSGALG00010024588	TTC39A	case	control	0.603837943	0.406114842	1.486864996	0.137050471	0.539854583	up_in_case
PRJNA8274(	ENSGALG00010024589	ADORA1	case	control	0.015971585	0.282170781	0.056602547	0.954861805	0.992056045	up_in_case
PRJNA8274(	ENSGALG00010024590	MRPL23	case	control	0.052759207	0.09812879	0.537652681	0.590816853	0.882949889	up_in_case
PRJNA8274(	ENSGALG00010024592	COMT	case	control	-0.03449156	0.094247401	-0.365968283	0.714388743	0.926862121	down_in_case
PRJNA8274(	ENSGALG00010024593	SRSF6	case	control	-0.007052007	0.039924146	-0.176635132	0.859794996	0.971828322	down_in_case
PRJNA8274(	ENSGALG00010024594	TNNI2	case	control	-0.189355789	0.130560871	-1.450325719	0.14696771	0.552045773	down_in_case
PRJNA8274(	ENSGALG00010024595	MYBPHL	case	control	-0.167961628	0.173457446	-0.968316043	0.332886555	0.748503677	down_in_case
PRJNA8274(	ENSGALG00010024596	EPS15	case	control	-0.053123066	0.047526832	-1.117748926	0.263674241	0.68964455	down_in_case
PRJNA8274(	ENSGALG00010024597	DNAJB11	case	control	0.252345713	0.085610629	2.947597933	0.003202533	0.096413097	up_in_case
PRJNA8274(	ENSGALG00010024598	LRRC3C	case	control	-0.043730286	0.091350497	-0.478708795	0.632145808	0.89724192	down_in_case
PRJNA8274(	ENSGALG00010024599	ENSGALG00010024599	case	control	-0.050457045	0.292648583	-0.172415134	0.863111175	0.972850732	down_in_case
PRJNA8274(	ENSGALG00010024600	CD9	case	control	0.270431193	0.133551865	2.024915132	0.04287608	0.345035417	up_in_case
PRJNA8274(	ENSGALG00010024601	ENSGALG00010024601	case	control	0.018219995	0.179601672	0.101446688	0.919195876	0.984090816	up_in_case
PRJNA8274(	ENSGALG00010024602	PROCR	case	control	-0.145021172	0.146348052	-0.990933389	0.321718107	0.739571618	down_in_case
PRJNA8274(	ENSGALG00010024603	ENSGALG00010024603	case	control	1.193433639	1.108945124	1.076188183			up_in_case
PRJNA8274(	ENSGALG00010024604	MFSD5	case	control	0.046638036	0.061621379	0.756848306	0.449140749	0.818701429	up_in_case
PRJNA8274(	ENSGALG00010024605	LSP1P1	case	control	0.050283818	0.105113419	0.478376772	0.632382063	0.89726815	up_in_case
PRJNA8274(	ENSGALG00010024607	RARG	case	control	-0.000688941	0.065660693	-0.010492449	0.991628391	0.99790247	down_in_case
PRJNA8274(	ENSGALG00010024608	DENND2D	case	control	0.043452074	0.293821471	0.147885971	0.88243276	0.976801393	up_in_case
PRJNA8274(	ENSGALG00010024609	PRR33	case	control	-0.417135659	0.37354724	-1.116687835	0.264127821	0.689994125	down_in_case
PRJNA8274(	ENSGALG00010024610	PFKL	case	control	0.158013183	0.098379055	1.606166908	0.108237242	0.500094527	up_in_case
PRJNA8274(	ENSGALG00010024611	ENSGALG00010024611	case	control	-0.482692031	0.237033645	-2.036386151	0.041711595	0.341038198	down_in_case
PRJNA8274(	ENSGALG00010024613	ENSGALG00010024613	case	control	-0.02285359	0.060914819	-0.375172905	0.707531879	0.924751474	down_in_case
PRJNA8274(	ENSGALG00010024614	ENSGALG00010024614	case	control	0.23716761	0.622782591	0.380819267	0.703337362	0.923659131	up_in_case
PRJNA8274(	ENSGALG00010024615	SYT8	case	control	0.146706006	0.572119546	0.256425439	0.797622347	0.95280257	up_in_case
PRJNA8274(	ENSGALG00010024616	ARVCF	case	control	-0.094575943	0.06677603	-1.416315753	0.156683078	0.567232408	down_in_case
PRJNA8274(	ENSGALG00010024618	VPS13C	case	control	-0.214219577	0.093549231	-2.289912746	0.022026375	0.264801476	down_in_case
PRJNA8274(	ENSGALG00010024619	ENSGALG00010024619	case	control	-0.248103752	0.270342648	-0.917738115	0.358755988	0.765374836	down_in_case
PRJNA8274(	ENSGALG00010024620	MSANTD2	case	control	-0.006955584	0.0637703	-0.109072471	0.913145009	0.982728035	down_in_case
PRJNA8274(	ENSGALG00010024621	THRA	case	control	-0.088492789	0.069813673	-1.267556698	0.204956304	0.627537776	down_in_case
PRJNA8274(	ENSGALG00010024622	PRMT8	case	control	0.181431093	0.133012941	1.364010837	0.172564155	0.591341341	up_in_case
PRJNA8274(	ENSGALG00010024623	NRDC	case	control	-0.204829429	0.048409647	-4.231169634	2.32479E-05	0.003392299	down_in_case
PRJNA8274(	ENSGALG00010024624	CTSD	case	control	0.058118651	0.069445169	0.836899851	0.402648864	0.792021921	up_in_case
PRJNA8274(	ENSGALG00010024625	SPRYD3	case	control	0.020051277	0.063103022	0.317754623	0.750671074	0.939972946	up_in_case
PRJNA8274(	ENSGALG00010024626	TSHZ3	case	control	0.079086273	0.091980518	0.859815473	0.389890768	0.783877029	up_in_case
PRJNA8274(	ENSGALG00010024627	CEPT1	case	control	-0.004750132	0.052932777	-0.089738949	0.928494664	0.986776285	down_in_case
PRJNA8274(	ENSGALG00010024628	TANGO2	case	control	-0.041971795	0.077098932	-0.544388796	0.586173954	0.881808482	down_in_case
PRJNA8274(	ENSGALG00010024629	ENSGALG00010024629	case	control	0.04129006	0.075447707	0.547267266	0.584195129	0.881307382	up_in_case
PRJNA8274(	ENSGALG00010024630	CFAP410	case	control	0.082566083	0.066328696	1.244801844	0.213204598	0.638582746	up_in_case
PRJNA8274(	ENSGALG00010024631	EPB41L1	case	control	-0.085267658	0.056169101	-1.518052743	0.129001103	0.530795558	down_in_case
PRJNA8274(	ENSGALG00010024632	IFITM10	case	control	-0.30083514	0.335006237	-0.897998624	0.369186283	0.772062569	down_in_case
PRJNA8274(	ENSGALG00010024633	NRGN	case	control	-0.683539405	0.316321628	-2.160899994	0.030703065	0.308259818	down_in_case
PRJNA8274(	ENSGALG00010024634	ENSGALG00010024634	case	control	0.081799635	0.054544613	1.499683121	0.133696505	0.537331451	up_in_case
PRJNA8274(	ENSGALG00010024635	ENSGALG00010024635	case	control	-0.23530981	0.197952237	-1.188720135	0.234549815	0.659210368	down_in_case
PRJNA8274(	ENSGALG00010024636	PANX3	case	control	0.269059287	0.227841489	1.180905583	0.237640233	0.662652947	up_in_case
PRJNA8274(	ENSGALG00010024637	DUSP8	case	control	-0.16639711	0.134435396	-1.237747758	0.215809605	0.640530791	down_in_case
PRJNA8274(	ENSGALG00010024638	ZNF507	case	control	-0.000955751	0.078253136	-0.012213586	0.990255211	0.997369314	down_in_case
PRJNA8274(	ENSGALG00010024639	AAR2	case	control	0.002996481	0.046239153	0.064803976	0.948330076	0.991291133	up_in_case
PRJNA8274(	ENSGALG00010024640	GSDMA	case	control	-0.309588532	0.184331167	-1.679523528	0.093050057	0.478122824	down_in_case
PRJNA8274(	ENSGALG00010024641	TRA2B	case	control	0.066091557	0.050237853	1.315572883	0.18831744	0.611129484	up_in_case
PRJNA8274(	ENSGALG00010024642	RAB3B	case	control	0.259312467	0.185945491	1.394561737	0.16314804	0.577197348	up_in_case
PRJNA8274(	ENSGALG00010024643	ETV7	case	control	0.30704592	0.39538951	0.77656567	0.437415063	0.810720016	up_in_case
PRJNA8274(	ENSGALG00010024644	EIF6	case	control	-0.000368416	0.045856668	-0.00803408	0.993589801	0.998196863	down_in_case
PRJNA8274(	ENSGALG00010024645	PARP11	case	control	-0.035929919	0.081194052	-0.442519097	0.658113614	0.908367791	down_in_case
PRJNA8274(	ENSGALG00010024646	RORA	case	control	-0.059843861	0.139831073	-0.427972551	0.668671105	0.913032039	down_in_case
PRJNA8274(	ENSGALG00010024647	RPUSD4	case	control	0.124461087	0.118115504	1.053723544	0.292009506	0.714648239	up_in_case
PRJNA8274(	ENSGALG00010024648	MMP24	case	control	-0.066899777	0.058268285	-1.148133626	0.250913404	0.676372507	down_in_case
PRJNA8274(	ENSGALG00010024649	NCOR2	case	control	-0.086165395	0.093458428	-0.921964948	0.356546859	0.763373775	down_in_case
PRJNA8274(	ENSGALG00010024650	PRKAG1	case	control	-0.209998301	0.061766676	-3.399864074	0.000674194	0.038540612	down_in_case
PRJNA8274(	ENSGALG00010024651	PSMD3	case	control	0.077858626	0.061949638	1.25680518	0.20882419	0.633836842	up_in_case
PRJNA8274(	ENSGALG00010024652	PUS3	case	control	-0.030215187	0.079222596	-0.381396069	0.702909377	0.923606	down_in_case
PRJNA8274(	ENSGALG00010024653	RFLNA	case	control	0.149829935	0.12661809	1.183321716	0.23668167	0.661690689	up_in_case
PRJNA8274(	ENSGALG00010024654	CCDC92	case	control	0.106625678	0.170048165	0.627032216	0.530638118	0.855495419	up_in_case
PRJNA8274(	ENSGALG00010024655	IGF2BP2	case	control	-0.014458051	0.071678468	-0.201707026	0.840145769	0.965286179	down_in_case
PRJNA8274(	ENSGALG00010024656	HYLS1	case	control	-0.027641497	0.104713313	-0.263973091	0.791800654	0.95239351	down_in_case
PRJNA8274(	ENSGALG00010024657	DPY19L3	case	control	0.086450368	0.058274543	1.483501417	0.13794124	0.539854583	up_in_case
PRJNA8274(	ENSGALG00010024659	BEND5	case	control	-0.150084806	0.066461153	-2.258233546	0.023931103	0.273286623	down_in_case
PRJNA8274(	ENSGALG00010024660	KMT2D	case	control	-0.13328539	0.08116625	-1.642128231	0.100563432	0.490291708	down_in_case
PRJNA8274(	ENSGALG00010024662	MOB2	case	control	-0.024944275	0.042067334	-0.592960675	0.55320747	0.866252369	down_in_case
PRJNA8274(	ENSGALG00010024663	ICE2	case	control	-0.092688397	0.085695441	-1.081602425	0.279429226	0.705021645	down_in_case
PRJNA8274(	ENSGALG00010024664	DNAH10	case	control	-0.235205326	0.189417914	-1.241726939	0.214337327	0.639112236	down_in_case
PRJNA8274(	ENSGALG00010024665	MAP1LC3A	case	control	-0.323950337	0.090095249	-3.595642838	0.000323592	0.024227009	down_in_case
PRJNA8274(	ENSGALG00010024666	MED24	case	control	0.056403038	0.061093051	0.923231656	0.355886495	0.763373775	up_in_case

PRJNA8274(	ENSGALG00010024667	RBM7	case	control	0.028281198	0.077933203	0.362890235	0.716686882	0.926862121	up_in_case
PRJNA8274(	ENSGALG00010024668	ENSGALG00010024668	case	control	-0.054523956	0.131875045	-0.413451658	0.679275734	0.915258927	down_in_case
PRJNA8274(	ENSGALG00010024669	STIL	case	control	0.037654986	0.090547004	0.415861209	0.677511566	0.914864532	up_in_case
PRJNA8274(	ENSGALG00010024670	SENP2	case	control	-0.1318807	0.068195717	-1.933856064	0.053130827	0.376522532	down_in_case
PRJNA8274(	ENSGALG00010024671	KCTD20	case	control	-0.040262622	0.055135159	-0.730253129	0.465235473	0.826255378	down_in_case
PRJNA8274(	ENSGALG00010024672	DYNLRB1	case	control	-0.083531005	0.045017017	-1.855542871	0.063518743	0.409661205	down_in_case
PRJNA8274(	ENSGALG00010024673	REXO2	case	control	-0.005831226	0.041075434	-0.141963831	0.887108575	0.977630122	down_in_case
PRJNA8274(	ENSGALG00010024674	DHH	case	control	0.110923329	0.196492548	0.564516721	0.572402524	0.876814097	up_in_case
PRJNA8274(	ENSGALG00010024675	NR1D1	case	control	0.165164102	0.426162897	0.387560962	0.698340963	0.922288441	up_in_case
PRJNA8274(	ENSGALG00010024676	ENSGALG00010024676	case	control	0.107871955	0.084585379	1.275302616	0.202202152	0.625148499	up_in_case
PRJNA8274(	ENSGALG00010024677	LMBR1L	case	control	-0.06898204	0.178357064	-0.386763712	0.698931144	0.922288441	down_in_case
PRJNA8274(	ENSGALG00010024678	AMOTL2	case	control	-0.092530666	0.094353375	-0.980682091	0.326749538	0.744561034	down_in_case
PRJNA8274(	ENSGALG00010024679	MSL1	case	control	-0.112580018	0.038277994	-2.941115935	0.003270321	0.097324927	down_in_case
PRJNA8274(	ENSGALG00010024680	BRSK1	case	control	-0.559671246	0.229735652	-2.436153209	0.014844396	0.215129943	down_in_case
PRJNA8274(	ENSGALG00010024681	ATP6V0A2	case	control	0.136211445	0.063294277	2.152034155	0.031394664	0.3098361	up_in_case
PRJNA8274(	ENSGALG00010024682	ENSGALG00010024682	case	control	0.02822738	0.179884048	0.15691986	0.875308008	0.975666505	up_in_case
PRJNA8274(	ENSGALG00010024683	SHISA4	case	control	-0.148314803	0.090025271	-1.647479673	0.099459491	0.488584926	down_in_case
PRJNA8274(	ENSGALG00010024684	ENSGALG00010024684	case	control	-0.491783768	0.35693515	-1.377795849	0.168266328	0.58432606	down_in_case
PRJNA8274(	ENSGALG00010024685	ENSGALG00010024685	case	control	-0.249861789	0.149025085	-1.676642478	0.093612415	0.478523919	down_in_case
PRJNA8274(	ENSGALG00010024686	CMPK1	case	control	0.13766919	0.085146586	1.616849213	0.105910833	0.495266487	up_in_case
PRJNA8274(	ENSGALG00010024687	RYK	case	control	-0.181715539	0.055775259	-3.2579954	0.001122022	0.05223113	down_in_case
PRJNA8274(	ENSGALG00010024688	CADM1	case	control	-0.137327064	0.106312455	-1.29173073	0.196450411	0.619313262	down_in_case
PRJNA8274(	ENSGALG00010024689	FOXD2	case	control	0.002371856	0.137433835	0.017258166	0.986230659	0.996597442	up_in_case
PRJNA8274(	ENSGALG00010024690	TUBA1C	case	control	-0.257135418	0.327823718	-0.784371001	0.432822449	0.8079942	down_in_case
PRJNA8274(	ENSGALG00010024691	TCTN2	case	control	0.110662951	0.084459302	1.310251779	0.190110674	0.613398611	up_in_case
PRJNA8274(	ENSGALG00010024692	DRAM2	case	control	-0.114429954	0.066956554	-1.709017963	0.087447622	0.467302316	down_in_case
PRJNA8274(	ENSGALG00010024693	ANXA2	case	control	-0.057576697	0.124670032	-0.461832701	0.644201298	0.90210829	down_in_case
PRJNA8274(	ENSGALG00010024694	GTF2H3	case	control	-0.023606741	0.075203835	-0.313903424	0.75359439	0.940858857	down_in_case
PRJNA8274(	ENSGALG00010024695	TRABD2B	case	control	0.175998853	0.1413852	1.244818079	0.213198629	0.638582746	up_in_case
PRJNA8274(	ENSGALG00010024696	TRADD	case	control	0.119035092	0.065230133	1.82484823	0.068023935	0.421339236	up_in_case
PRJNA8274(	ENSGALG00010024697	IGF2BP1	case	control	-0.202098376	0.069008944	-2.928582335	0.003405116	0.099577022	down_in_case
PRJNA8274(	ENSGALG00010024698	ENSGALG00010024698	case	control	-0.102675559	0.142357725	-0.721250352	0.470755497	0.829101076	down_in_case
PRJNA8274(	ENSGALG00010024699	FBXL8	case	control	0.086533125	0.069156015	1.251274016	0.210834522	0.636310899	up_in_case
PRJNA8274(	ENSGALG00010024700	TOLLIP	case	control	-0.033742778	0.0592388	-0.569606033	0.568944936	0.874829309	down_in_case
PRJNA8274(	ENSGALG00010024701	EIF2B1	case	control	0.032694831	0.071311365	0.45847995	0.646607664	0.90295498	up_in_case
PRJNA8274(	ENSGALG00010024702	INAVA	case	control	0.24900012	0.315408332	0.789453209	0.429847167	0.806015686	up_in_case
PRJNA8274(	ENSGALG00010024705	SLCO2A1	case	control	-0.058038176	0.247214597	-0.234768402	0.814388494	0.957063027	down_in_case
PRJNA8274(	ENSGALG00010024706	ENSGALG00010024706	case	control	-0.305883584	0.246966457	-1.238563276	0.215507275	0.640470741	down_in_case
PRJNA8274(	ENSGALG00010024707	ENSGALG00010024707	case	control	-0.43896721	0.175271099	-2.504504246	0.012262313	0.194618291	down_in_case
PRJNA8274(	ENSGALG00010024708	BNIP2	case	control	-0.058796298	0.040726744	-1.443677834	0.148829619	0.555199933	down_in_case
PRJNA8274(	ENSGALG00010024709	TEAD4	case	control	0.014761933	0.077447304	0.190606153	0.848834169	0.968741311	up_in_case
PRJNA8274(	ENSGALG00010024710	HSDL1	case	control	0.084178193	0.119058878	0.707029954	0.479547863	0.831821257	up_in_case
PRJNA8274(	ENSGALG00010024711	DDX55	case	control	0.101850975	0.1020651	0.997902075	0.318326846	0.738134246	up_in_case
PRJNA8274(	ENSGALG00010024712	LRIF1	case	control	0.002791292	0.046013848	0.06066201	0.951628388	0.991857576	up_in_case
PRJNA8274(	ENSGALG00010024713	RND2	case	control	-0.065795121	0.311027298	-0.211541306	0.832464904	0.962371901	down_in_case
PRJNA8274(	ENSGALG00010024714	SRPRB	case	control	0.09731697	0.040510999	2.402235746	0.016295203	0.226234374	up_in_case
PRJNA8274(	ENSGALG00010024715	DNAAF1	case	control	0.07201258	0.110685854	0.650603281	0.515302612	0.847750297	up_in_case
PRJNA8274(	ENSGALG00010024716	GTF2A2	case	control	-0.050205105	0.057877362	-0.867439405	0.385701299	0.781790018	down_in_case
PRJNA8274(	ENSGALG00010024717	TF	case	control	0.851841708	0.806009067	1.056863679	0.290573807	0.714060795	up_in_case
PRJNA8274(	ENSGALG00010024718	ENSGALG00010024718	case	control	0.265383835	0.174004556	1.525154517	0.127220562	0.528320557	up_in_case
PRJNA8274(	ENSGALG00010024719	PYM1	case	control	0.102909553	0.102630833	1.002715749	0.315998029	0.736866972	up_in_case
PRJNA8274(	ENSGALG00010024720	BUD13	case	control	0.099576291	0.079077313	1.259227039	0.207948338	0.63249038	up_in_case
PRJNA8274(	ENSGALG00010024721	ENSGALG00010024721	case	control	-0.221084478	0.381021959	-0.580240779	0.561752257	0.870629259	down_in_case
PRJNA8274(	ENSGALG00010024722	BRCA1	case	control	0.02922955	0.102845939	0.284207143	0.776251642	0.947228003	up_in_case
PRJNA8274(	ENSGALG00010024723	ITCH	case	control	0.035326131	0.045858322	0.770331969	0.441102997	0.813083204	up_in_case
PRJNA8274(	ENSGALG00010024724	HOXD3	case	control	-0.165765911	0.133231031	-1.244198966	0.213426344	0.638582746	down_in_case
PRJNA8274(	ENSGALG00010024726	TMED2	case	control	0.04670954	0.0346211	1.349163955	0.17728431	0.5959216	up_in_case
PRJNA8274(	ENSGALG00010024728	SLC5A9	case	control	0.427459972	0.300083067	1.424472153	0.154309841	0.56303838	up_in_case
PRJNA8274(	ENSGALG00010024729	FOXM1	case	control	0.023027565	0.049989126	0.460651482	0.645048669	0.902298344	up_in_case
PRJNA8274(	ENSGALG00010024731	AOX1	case	control	-0.359162669	0.182255287	-1.970657062	0.048763115	0.363542241	down_in_case
PRJNA8274(	ENSGALG00010024732	AHCY	case	control	0.028527799	0.130170902	0.219156498	0.826528142	0.961207256	up_in_case
PRJNA8274(	ENSGALG00010024733	MBTPS1	case	control	-0.026382912	0.043383554	-0.608131657	0.543100158	0.860419516	down_in_case
PRJNA8274(	ENSGALG00010024734	ZPR1	case	control	-0.054476733	0.066271347	-0.822025439	0.411062419	0.796718771	down_in_case
PRJNA8274(	ENSGALG00010024735	OTUD7A	case	control	-0.269019585	0.11470832	-2.345249115	0.019014371	0.246291212	down_in_case
PRJNA8274(	ENSGALG00010024736	DGKA	case	control	-0.036694651	0.088433533	-0.414940457	0.678185492	0.914941113	down_in_case
PRJNA8274(	ENSGALG00010024737	STK38	case	control	-0.097506879	0.050915011	-1.915090995	0.0554809	0.383830126	down_in_case
PRJNA8274(	ENSGALG00010024738	NBR1	case	control	0.058545755	0.048351591	1.210834112	0.225958989	0.649498359	up_in_case
PRJNA8274(	ENSGALG00010024739	ASIP	case	control	0.114766606	0.39345032	0.29169275	0.770521553	0.945448224	up_in_case
PRJNA8274(	ENSGALG00010024741	RILPL2	case	control	0.047963435	0.084760699	0.565868807	0.571482968	0.876752112	up_in_case
PRJNA8274(	ENSGALG00010024742	PAK2	case	control	-0.013980591	0.048626235	-0.287511276	0.773720869	0.946548757	down_in_case
PRJNA8274(	ENSGALG00010024744	KLF13	case	control	-0.108954209	0.103487835	-1.052821417	0.292422847	0.714933615	down_in_case
PRJNA8274(	ENSGALG00010024745	PMEL	case	control	9.173565345	0.385890617	23.77244987	6.439E-125	9.2077E-121	up_in_case
PRJNA8274(	ENSGALG00010024746	HOXD4	case	control	-0.265833883	0.239838656	-1.108386308	0.267695016	0.694521603	down_in_case
PRJNA8274(	ENSGALG00010024747	TULP3	case	control	0.028359476	0.048885507	0.580120323	0.56183348	0.870634889	up_in_case
PRJNA8274(	ENSGALG00010024748	TRPM1	case	control	2.288320237	0.28399816	8.057517818	7.78592E-16	6.95867E-13	up_in_case
PRJNA8274(	ENSGALG00010024749	APOA5	case	control	0.607571114	0.446029897	1.36217576	0.173142424	0.592471084	up_in_case
PRJNA8274(	ENSGALG00010024750	APOA4	case	control	0.905651818	1.195030373	0.757848368	0.448541763	0.818701429	up_in_case
PRJNA8274(	ENSGALG00010024751	MTMR10	case	control	0.022339254	0.056333334	0.396554805	0.691695786	0.918919522	up_in_case
PRJNA8274(	ENSGALG00010024752	MPP2	case	control	-0.083993974	0.155081428	-0.541612077	0.58808577	0.882771329	down_in_case
PRJNA8274(	ENSGALG00010024753	HOXD8	case	control	-0.180373757	0.084746168	-2.128400145	0.033303921	0.314561474	down_in_case
PRJNA8274(	ENSGALG00010024754	EIF2S2	case	control	-0.032650579	0.041905507	-0.779147684	0.435892724	0.81000376	down_in_case
PRJNA8274(	ENSGALG00010024755	PIGX	case	control	-0.02837763	0.108545539	-0.261435244	0.793756881	0.95280257	down_in_case
PRJNA8274(	ENSGALG00010024756	CDK2	case	control	0.057246012	0.080707449	0.709302701	0.478136653	0.830520323	up_in_case
PRJNA8274(	ENSGALG00010024757	PPFIA1	case	control	0.005752291	0.036164124	0.159060715	0.873621039	0.975062028	up_in_case
PRJNA8274(	ENSGALG00010024758	SPATA6	case	control	-0.011805778	0.066724163	-0.176934078	0.859560171	0.971828322	down_in_case
PRJNA8274(	ENSGALG00010024759	APOC3	case	control	1.055159587	0.869578886	1.213414452	0.224971404	0.648879142	up_in_case
PRJNA8274(	ENSGALG00010024760	ENSGALG00010024760	case	control	-0.043598756	0.138376861	-0.315072589	0.75270654	0.940632484	down_in_case
PRJNA8274(	ENSGALG00010024761	NECAB2	case	control	0.091128307	0.126388213	0.721019027	0.470897808	0.829101076	up_in_case
PRJNA8274(	ENSGALG00010024762	FAN1	case	control	0.092678662	0.079936287	1.159406634	0.246290474	0.670921661	up_in_case
PRJNA8274(	ENSGALG00010024763	HOXD9	case	control	0.038570212	0.163752375	0.235539862	0.813789743	0.956712425	up_in_case
PRJNA8274(	ENSGALG00010024764	ENSGALG00010024764	case	control	0.077927622	0.191040202	0.407912164	0.683338162	0.915985725	up_in_case
PRJNA8274(	ENSGALG00010024765	SNRNP35	case	control	0.141339748	0.059279585	2.384290429	0.017112092	0.232828648	up_in_case
PRJNA8274(	ENSGALG00010024767	CTTN	case	control	0.021621713	0.051713958	0.418102073	0.67587249	0.914247368	up_in_case
PRJNA8274(	ENSGALG00010024769	RALY	case	control	-0.317373062	0.113449526	-2.797482488	0.005150256	0.123987642	down_in_case
PRJNA8274(	ENSGALG00010024770	CDC27	case	control	-0.140049267	0.036093998	-3.880126213	0.000104402	0.011055819	down_in_case
PRJNA8274(	ENSGALG00010024771	AOX2	case	control	-0.368490626	0.233544524	-1.577817455	0.114607553	0.509446069	down_in_case
PRJNA8274(	ENSGALG00010024772	ELAPOR1	case	control	0.147789297	0.135951674	1.087072286	0.277004865	0.702359305	up_in_case
PRJNA8274(	ENSGALG00010024773	APOA1	case	control	0.261015772	0.427666247	0.610325865	0.541645967	0.85975913	up_in_case
PRJNA8274(	ENSGALG00010024775	ITFG2	case	control	-0.050323539	0.05656186	-0.889707997	0.373622699	0.774369566	down_in_case
PRJNA8274(	ENSGALG00010024776	MPHOSPH10	case	control	-0.011397028	0.06305656	-0.18074294	0.856569352	0.971059279	down_in_case
PRJNA8274(	ENSGALG00010024778	RAB5B	case	control	-0.041916497	0.053182493	-0.788163445	0.430601108	0.806812873	down_in_case
PRJNA8274(	ENSGALG00010024779	ENSGALG00010024779	case	control	0.091787417	0.879190691	0.104399897	0.916852005	0.983445506	up_in_case
PRJNA8274(	ENSGALG00010024780	HOXD11	case	control	0.707752902	0.757735759	0.93403656	0.350285034	0.759878704	up_in_case
PRJNA8274(	ENSGALG00010024781	CEP19	case	control	-0.019278702	0.1274896	-0.151217841	0.879803877	0.976318821	down_in_case
PRJNA8274(	ENSGALG00010024782	HOXD12	case	control	1.985931099	1.221765171	1.625460559	0.104064461	0.493794905	up_in_case
PRJNA8274(	ENSGALG00010024783	UBE2T	case	control	-0.053089315	0.094592785	-0.561240638	0.574633503	0.877910508	down_in_case
PRJNA8274(	ENSGALG00010024784	ENSGALG00010024784	case	control	-0.016143463	0.101335259	-0.15930746	0.873426643	0.975062028	down_in_case
PRJNA8274(	ENSGALG00010024785	MCEE	case	control	-0.020537208	0.075416197	-0.272318264	0.785377314	0.949838937	down_in_case
PRJNA8274(	ENSGALG00010024786	PDCD5	case	control	-0.146869515	0.06366047	-2.307075571	0.021050608	0.259951371	down_in_case
PRJNA8274(	ENSGALG00010024787	SARS1	case	control	-0.020938317	0.07032685	-0.29772863	0.76591029	0.943860474	down_in_case
PRJNA8274(	ENSGALG00010024788	SIK3	case	control	0.119453032	0.080653123	1.481071374	0.138587555	0.540932258	up_in_case
PRJNA8274(	ENSGALG00010024789	SUOX	case	control	-0.004708986	0.083563473	-0.056352204	0.955061232	0.992056045	down_in_case
PRJNA8274(	ENSGALG00010024791	SHANK2	case	control	1.190806372	0.416550911	2.858729484	0.004253413	0.112636686	up_in_case
PRJNA8274(	ENSGALG00010024792	BZW1	case	control	-0.044284019	0.044646477	-0.991881598	0.321255287	0.73942679	down_in_case
PRJNA8274(	ENSGALG00010024793	FKBP4	case	control	-0.003582816	0.04323063	-0.082876784	0.933949514	0.988127868	down_in_case
PRJNA8274(	ENSGALG00010024794	DDX6	case	control	-0.100669967	0.041158235	-2.445925256	0.014448095	0.212076065	down_in_case
PRJNA8274(	ENSGALG00010024795	NRROS	case	control	0.059770885	0.129612624	0.461150183	0.64469086	0.902239118	up_in_case
PRJNA8274(	ENSGALG00010024797	IKZF4	case	control	-0.081790497	0.078586305	-1.040772897	0.297980962	0.721487387	down_in_case
PRJNA8274(	ENSGALG00010024798	FBXO45	case	control	-0.102464931	0.051839443	-1.976582409	0.048088852	0.360981935	down_in_case
PRJNA8274(	ENSGALG00010024799	ENSGALG00010024799	case	control	-0.088539857	0.102639557	-0.862628983	0.38834149	0.783067156	down_in_case
PRJNA8274(	ENSGALG00010024800	ENSGALG00010024800	case	control	0.066161003	0.074691824	0.885786414	0.375732632	0.774921593	up_in_case
PRJNA8274(	ENSGALG00010024802	CD59	case	control	0.02215546	0.094005206	0.235683335	0.813678403	0.956712425	up_in_case
PRJNA8274(	ENSGALG00010024803	CLK1	case	control	0.061592771	0.098310326	0.626513755	0.530978018	0.855560567	up_in_case
PRJNA8274(	ENSGALG00010024804	WDR53	case	control	-0.064671933	0.070822767	-0.913151748	0.361162737	0.767289726	down_in_case
PRJNA8274(	ENSGALG00010024805	ENSGALG00010024805	case	control	-0.086844666	0.235761105	-0.368358751	0.71260575	0.926808752	down_in_case
PRJNA8274(	ENSGALG00010024806	ANKRD27	case	control	-0.055634744	0.077218532	-0.720484354	0.471226829	0.829101076	down_in_case

PRJNA8274(	ENSGALG00010024807	PPIL3	case	control	0.147537572	0.108634276	1.358112532	0.174427979	0.593194623	up_in_case
PRJNA8274(	ENSGALG00010024808	CELSR2	case	control	-0.182708918	0.225867088	-0.80892227	0.418559858	0.800723207	down_in_case
PRJNA8274(	ENSGALG00010024809	KANSL1	case	control	-0.112458861	0.056395793	-1.994100193	0.046141106	0.356053762	down_in_case
PRJNA8274(	ENSGALG00010024810	ADIPOR1	case	control	0.032803518	0.052691041	0.622563481	0.533571415	0.855817434	up_in_case
PRJNA8274(	ENSGALG00010024811	NIF3L1	case	control	-0.110569546	0.07713364	-1.43348021	0.15172065	0.560042669	down_in_case
PRJNA8274(	ENSGALG00010024812	SBNO1	case	control	0.020843962	0.034703342	0.600632699	0.548084654	0.863372059	up_in_case
PRJNA8274(	ENSGALG00010024813	RNF168	case	control	-0.213899654	0.121545755	-1.759828259	0.07843693	0.448818031	down_in_case
PRJNA8274(	ENSGALG00010024814	APBA2	case	control	-0.034894266	0.099683213	-0.350051575	0.726299992	0.932226286	down_in_case
PRJNA8274(	ENSGALG00010024816	NFIA	case	control	0.022024237	0.069920389	0.314990191	0.752769101	0.940632484	up_in_case
PRJNA8274(	ENSGALG00010024817	UCKL1	case	control	-0.044088601	0.091018546	-0.484391399	0.628108121	0.895865363	down_in_case
PRJNA8274(	ENSGALG00010024818	FBXO3	case	control	0.024903379	0.052092752	0.478058431	0.632608618	0.897272687	up_in_case
PRJNA8274(	ENSGALG00010024820	NUDT19	case	control	0.156319979	0.08609515	1.815665329	0.069421726	0.425095479	up_in_case
PRJNA8274(	ENSGALG00010024821	NECAP1	case	control	-0.012444105	0.045908649	-0.271062333	0.786343091	0.950252288	down_in_case
PRJNA8274(	ENSGALG00010024822	RPS26	case	control	-0.079168611	0.052387917	-1.511199828	0.130737546	0.533546493	down_in_case
PRJNA8274(	ENSGALG00010024823	ASB10	case	control	-0.590336745	0.369786844	-1.596424412	0.110394069	0.503054901	down_in_case
PRJNA8274(	ENSGALG00010024824	DNAJC5	case	control	-0.140952657	0.086595998	-1.627704044	0.103587655	0.493274548	down_in_case
PRJNA8274(	ENSGALG00010024825	UBXN7	case	control	0.173945143	0.05914752	2.940869571	0.003272923	0.097324927	up_in_case
PRJNA8274(	ENSGALG00010024826	RBM33	case	control	-0.043222958	0.075035716	-0.576031791	0.564593694	0.872275547	down_in_case
PRJNA8274(	ENSGALG00010024827	ENSGALG00010024827	case	control	0.140879081	0.401288102	0.351067178	0.725537951	0.931921256	up_in_case
PRJNA8274(	ENSGALG00010024828	TIMM17B	case	control	-0.121685307	0.084802637	-1.434923619	0.151308864	0.559493525	down_in_case
PRJNA8274(	ENSGALG00010024829	ERBB3	case	control	-0.022785782	0.102985889	-0.221251499	0.824896612	0.960744547	down_in_case
PRJNA8274(	ENSGALG00010024830	ORC2	case	control	0.125482837	0.086349043	1.453204718	0.146166923	0.551209651	up_in_case
PRJNA8274(	ENSGALG00010024831	SLC7A9	case	control	0.31974539	0.4324271	0.739420333	0.459651799	0.824480387	up_in_case
PRJNA8274(	ENSGALG00010024833	MAPT	case	control	-0.333200526	0.183100002	-1.819773475	0.068793509	0.423481349	down_in_case
PRJNA8274(	ENSGALG00010024834	INSIG1	case	control	0.267093475	0.202994087	1.315769731	0.188251342	0.611129484	up_in_case
PRJNA8274(	ENSGALG00010024835	ENTREP2	case	control	-0.024391592	0.098123584	-0.248580324	0.80368543	0.954939896	down_in_case
PRJNA8274(	ENSGALG00010024836	TXNDC12	case	control	-0.016899695	0.035999048	-0.469448376	0.638749177	0.900303403	down_in_case
PRJNA8274(	ENSGALG00010024837	TNFRSF1A	case	control	-0.232880573	0.084126231	-2.768227797	0.005636205	0.130251961	down_in_case
PRJNA8274(	ENSGALG00010024838	EN2	case	control	0.690583211	0.562908372	1.226812827	0.219892949	0.644131031	up_in_case
PRJNA8274(	ENSGALG00010024839	PA2G4	case	control	-0.021948995	0.058391904	-0.37589106	0.706997887	0.92461283	down_in_case
PRJNA8274(	ENSGALG00010024840	BTF3L4	case	control	-0.040925949	0.048106643	-0.85073385	0.394917215	0.786978937	down_in_case
PRJNA8274(	ENSGALG00010024841	ENSGALG00010024841	case	control	-0.158386602	0.129391432	-1.224088796	0.220918732	0.644950086	down_in_case
PRJNA8274(	ENSGALG00010024842	ZFYVE9	case	control	0.047281922	0.049900722	0.947519804	0.34337397	0.755145216	up_in_case
PRJNA8274(	ENSGALG00010024843	BCL9L	case	control	-0.011129087	0.07764752	-0.143328296	0.886030909	0.977630122	down_in_case
PRJNA8274(	ENSGALG00010024845	HYCC2	case	control	0.004164524	0.113590002	0.036662772	0.970753892	0.992761697	up_in_case
PRJNA8274(	ENSGALG00010024846	LMO2	case	control	-0.0657937	0.064735516	-1.016346273	0.309464521	0.732384442	down_in_case
PRJNA8274(	ENSGALG00010024847	PPIL1	case	control	0.068918899	0.089148547	0.773079324	0.439475437	0.812370572	up_in_case
PRJNA8274(	ENSGALG00010024848	ESYT1	case	control	-0.040585368	0.065709975	-0.617643949	0.536810067	0.857409132	down_in_case
PRJNA8274(	ENSGALG00010024849	CENATAC	case	control	0.097822838	0.118874096	0.82291131	0.410558431	0.796497837	up_in_case
PRJNA8274(	ENSGALG00010024850	CDK2AP1	case	control	-0.138810732	0.086335143	-1.607812613	0.107876225	0.499736588	down_in_case
PRJNA8274(	ENSGALG00010024851	CPNE5	case	control	-0.485198256	0.424240652	-1.143686382	0.252753723	0.678246996	down_in_case
PRJNA8274(	ENSGALG00010024852	CEP89	case	control	-0.084234418	0.072732838	-1.158134618	0.246809105	0.671238151	down_in_case
PRJNA8274(	ENSGALG00010024853	TJP1	case	control	-0.039945237	0.053240139	-0.750284247	0.453083528	0.821282096	down_in_case
PRJNA8274(	ENSGALG00010024854	C12orf65	case	control	-0.093266851	0.086960375	-1.072521263	0.283485969	0.707725096	down_in_case
PRJNA8274(	ENSGALG00010024855	CAPRIN1	case	control	-0.102254084	0.03902505	-2.620216587	0.008787394	0.166436729	down_in_case
PRJNA8274(	ENSGALG00010024856	SORT1	case	control	-0.075790007	0.074751896	-1.01388742	0.310636475	0.733587496	down_in_case
PRJNA8274(	ENSGALG00010024857	SLC37A4	case	control	-0.08610426	0.065526924	-1.31402871	0.188836543	0.611783722	down_in_case
PRJNA8274(	ENSGALG00010024858	SLC25A39	case	control	-0.035619469	0.065105401	-0.547104673	0.584306822	0.881307382	down_in_case
PRJNA8274(	ENSGALG00010024859	TARSL2	case	control	-0.001414112	0.065965989	-0.021436989	0.982897068	0.996591771	down_in_case
PRJNA8274(	ENSGALG00010024860	NEU4	case	control	0.867076792	0.344438723	2.517361535	0.011823743	0.190619538	up_in_case
PRJNA8274(	ENSGALG00010024861	ORC1	case	control	-0.011000016	0.052225136	-0.21062685	0.833178459	0.962550652	down_in_case
PRJNA8274(	ENSGALG00010024862	METTL16	case	control	-0.212460237	0.066034215	-3.217426576	0.001293461	0.056912297	down_in_case
PRJNA8274(	ENSGALG00010024863	LMOD1	case	control	-0.290302217	0.171261744	-1.695079178	0.090060402	0.473572975	down_in_case
PRJNA8274(	ENSGALG00010024864	MPHOSPH9	case	control	-0.033443699	0.084178225	-0.397296325	0.691148957	0.918812261	down_in_case
PRJNA8274(	ENSGALG00010024865	SNAP47	case	control	0.08741452	0.083691043	1.044490746	0.296258398	0.719868825	up_in_case
PRJNA8274(	ENSGALG00010024866	TRAPPC4	case	control	-0.01221075	0.053867789	-0.226679997	0.820672582	0.958669267	down_in_case
PRJNA8274(	ENSGALG00010024867	TM2D3	case	control	0.113869523	0.080691426	1.411172513	0.158193753	0.570246977	up_in_case
PRJNA8274(	ENSGALG00010024868	FAAP24	case	control	-0.087109826	0.141906716	-0.613852735	0.539312646	0.858476757	down_in_case
PRJNA8274(	ENSGALG00010024869	NDUFB3	case	control	0.022454143	0.094367272	0.237944182	0.811924388	0.956240049	up_in_case
PRJNA8274(	ENSGALG00010024870	TPD52L2	case	control	-0.016836679	0.039550046	-0.425705668	0.670322331	0.913465866	down_in_case
PRJNA8274(	ENSGALG00010024871	POLQ	case	control	-0.230579739	0.137611107	-1.675589597	0.093818608	0.478803031	down_in_case
PRJNA8274(	ENSGALG00010024872	RUNDC3A	case	control	0.02529704	0.222568923	0.113659355	0.909507834	0.981730793	up_in_case
PRJNA8274(	ENSGALG00010024873	HYOU1	case	control	0.117078651	0.055953225	2.092437946	0.036399357	0.32664727	up_in_case
PRJNA8274(	ENSGALG00010024874	JMJD4	case	control	-0.075417701	0.082264204	-0.916774225	0.359260962	0.765374836	down_in_case
PRJNA8274(	ENSGALG00010024876	CFLAR	case	control	0.004614812	0.061933868	0.074511923	0.940603054	0.989551456	up_in_case
PRJNA8274(	ENSGALG00010024877	PRPF38A	case	control	0.027805816	0.052742188	0.527202556	0.598052934	0.884680373	up_in_case
PRJNA8274(	ENSGALG00010024878	RHPN2	case	control	0.02580486	0.209407642	0.123227883	0.901926647	0.980763983	up_in_case
PRJNA8274(	ENSGALG00010024879	D2HGDH	case	control	-0.165511064	0.072167106	-2.293441896	0.021822577	0.264012564	down_in_case
PRJNA8274(	ENSGALG00010024880	NAT10	case	control	0.017706262	0.08870974	0.199597725	0.841795208	0.966164004	up_in_case
PRJNA8274(	ENSGALG00010024881	CASP10	case	control	-0.047150811	0.161064242	-0.292745372	0.769716786	0.945428692	down_in_case
PRJNA8274(	ENSGALG00010024882	MYL6	case	control	0.253706213	0.076591218	3.312471327	0.000924756	0.046346052	up_in_case
PRJNA8274(	ENSGALG00010024883	ING5	case	control	-0.012842249	0.049920015	-0.257256514	0.79698076	0.95280257	down_in_case
PRJNA8274(	ENSGALG00010024884	ZBTB46	case	control	0.100426225	0.064071067	1.567419274	0.117016722	0.513845851	up_in_case
PRJNA8274(	ENSGALG00010024885	SLC4A1	case	control	0.357864812	0.166308959	2.151807179	0.031412544	0.3098361	up_in_case
PRJNA8274(	ENSGALG00010024886	ABTB2	case	control	0.375801851	0.104275119	3.603945543	0.000313423	0.024072921	up_in_case
PRJNA8274(	ENSGALG00010024887	CASP18	case	control	0.049482105	0.226982669	0.217999486	0.827429512	0.961319258	up_in_case
PRJNA8274(	ENSGALG00010024888	PITPNM2	case	control	-0.139297923	0.085663587	-1.626104255	0.103927479	0.493794905	down_in_case
PRJNA8274(	ENSGALG00010024889	SLC2A4RG	case	control	-0.447559261	0.214573237	-2.085811199	0.036995729	0.32835414	down_in_case
PRJNA8274(	ENSGALG00010024890	SMARCC2	case	control	-0.127517143	0.064002409	-1.992380382	0.046329338	0.356443312	down_in_case
PRJNA8274(	ENSGALG00010024891	PKP1	case	control	-0.105177841	0.455754109	-0.230777603	0.817487582	0.958359765	down_in_case
PRJNA8274(	ENSGALG00010024893	ARL6IP4	case	control	-0.009618746	0.056832412	-0.169247538	0.865601939	0.973289701	down_in_case
PRJNA8274(	ENSGALG00010024894	TUT4	case	control	-0.026734559	0.055533218	-0.481415637	0.630221125	0.89637578	down_in_case
PRJNA8274(	ENSGALG00010024896	RNF41	case	control	0.04084376	0.046312332	0.88191974	0.377820211	0.776380086	up_in_case
PRJNA8274(	ENSGALG00010024897	ADAL	case	control	-0.091705841	0.065778791	-1.394155174	0.163270751	0.577197348	down_in_case
PRJNA8274(	ENSGALG00010024898	NABP2	case	control	0.089951664	0.089171171	1.008752754	0.313093225	0.734574752	up_in_case
PRJNA8274(	ENSGALG00010024899	OGFOD2	case	control	0.066340741	0.087149783	0.76122669	0.446521679	0.817679602	up_in_case
PRJNA8274(	ENSGALG00010024900	DTYMK	case	control	-0.003596409	0.07208484	-0.049891337	0.960208981	0.992056045	down_in_case
PRJNA8274(	ENSGALG00010024901	ZGPAT	case	control	-0.006127919	0.052343352	-0.117071589	0.906803323	0.981730793	down_in_case
PRJNA8274(	ENSGALG00010024902	LARP6	case	control	-0.133276306	0.089042785	-1.496767035	0.134453887	0.539063179	down_in_case
PRJNA8274(	ENSGALG00010024903	CASP8	case	control	-0.011581563	0.12291923	-0.094220923	0.924933664	0.985571367	down_in_case
PRJNA8274(	ENSGALG00010024904	ALS2CL	case	control	0.194744148	0.094460367	2.061649295	0.039241137	0.335091843	up_in_case
PRJNA8274(	ENSGALG00010024905	GPX7	case	control	0.014582291	0.067160271	0.217126752	0.828109566	0.96154783	up_in_case
PRJNA8274(	ENSGALG00010024907	PHLDB1	case	control	-0.037622002	0.060522378	-0.62162134	0.534190886	0.856190278	down_in_case
PRJNA8274(	ENSGALG00010024908	ENSGALG00010024908	case	control	-0.233989528	0.058999307	-3.965970807	7.30978E-05	0.008784022	down_in_case
PRJNA8274(	ENSGALG00010024909	SLC39A5	case	control	1.836266495	1.13192454	1.622251687	0.104749469	0.494770427	up_in_case
PRJNA8274(	ENSGALG00010024910	UBTF	case	control	0.06023042	0.04615082	1.305078013	0.191866285	0.615090794	up_in_case
PRJNA8274(	ENSGALG00010024911	CAT	case	control	-0.157236775	0.079817941	-1.969942761	0.048844931	0.363603599	down_in_case
PRJNA8274(	ENSGALG00010024915	STXBP5L	case	control	-0.330438449	0.165414511	-1.997638822	0.045755831	0.35522015	down_in_case
PRJNA8274(	ENSGALG00010024917	TRAK2	case	control	0.049494825	0.059781857	0.827923845	0.40771363	0.794388636	up_in_case
PRJNA8274(	ENSGALG00010024918	ATG4B	case	control	0.048778728	0.060388964	0.807742413	0.419238882	0.80093491	up_in_case
PRJNA8274(	ENSGALG00010024919	ANKRD52	case	control	0.008878911	0.077668922	0.114317421	0.908986173	0.981730793	up_in_case
PRJNA8274(	ENSGALG00010024920	VIPR2	case	control	0.031028078	0.204156992	0.151981461	0.879201558	0.976318821	up_in_case
PRJNA8274(	ENSGALG00010024921	STRADB	case	control	-0.150623335	0.074537731	-2.020766304	0.043303962	0.347500933	down_in_case
PRJNA8274(	ENSGALG00010024924	MNX1	case	control	0.533770741	0.538172938	0.991820107	0.321285288	0.73942679	up_in_case
PRJNA8274(	ENSGALG00010024925	GTF2E1	case	control	0.006687998	0.060377217	0.110770229	0.911798553	0.98266608	up_in_case
PRJNA8274(	ENSGALG00010024926	ELF5	case	control	0.27461645	0.629881775	0.435980943	0.662850556	0.911044469	up_in_case
PRJNA8274(	ENSGALG00010024927	NOM1	case	control	0.032040268	0.093215432	0.343722782	0.731054776	0.933770678	up_in_case
PRJNA8274(	ENSGALG00010024928	THAP4	case	control	0.00760732	0.065291398	0.116513354	0.907245704	0.981730793	up_in_case
PRJNA8274(	ENSGALG00010024930	TMEM237	case	control	0.102017656	0.068415424	1.491149957	0.135922129	0.539774365	up_in_case
PRJNA8274(	ENSGALG00010024931	UBE3C	case	control	-0.036449671	0.048655541	-0.749137095	0.453774579	0.821282604	down_in_case
PRJNA8274(	ENSGALG00010024932	ANKK1	case	control	1.420139512	0.876726731	1.619820021	0.105270944	0.495041148	up_in_case
PRJNA8274(	ENSGALG00010024933	CS	case	control	-0.168406877	0.078560752	-2.14365154	0.032060818	0.3098361	down_in_case
PRJNA8274(	ENSGALG00010024934	G6PC3	case	control	0.029485251	0.114016067	0.258606104	0.795939173	0.95280257	up_in_case
PRJNA8274(	ENSGALG00010024935	KLHL30	case	control	-0.065124888	0.141269269	-0.460998268	0.644799848	0.902255223	down_in_case
PRJNA8274(	ENSGALG00010024936	EHF	case	control	0.908807703	0.541268406	1.679033345	0.093145545	0.478269765	up_in_case
PRJNA8274(	ENSGALG00010024937	ILKAP	case	control	-0.011381115	0.061140404	-0.186147204	0.852329326	0.969582953	down_in_case
PRJNA8274(	ENSGALG00010024938	ENSGALG00010024938	case	control	0.119037458	0.153940201	0.773270774	0.439362148	0.812366461	up_in_case
PRJNA8274(	ENSGALG00010024939	COQ10A	case	control	-0.004885883	0.148405824	-0.03292245	0.97373643	0.993553916	down_in_case
PRJNA8274(	ENSGALG00010024940	RABL3	case	control	-0.032037238	0.075349249	-0.425183238	0.670703103	0.913520751	down_in_case
PRJNA8274(	ENSGALG00010024941	PDHX	case	control	-0.009674128	0.050851219	-0.190243777	0.849118108	0.968833396	down_in_case
PRJNA8274(	ENSGALG00010024942	DNAJB6	case	control	0.013973379	0.039323218	0.355346782	0.722329812	0.929627793	up_in_case
PRJNA8274(	ENSGALG00010024943	ABCB9	case	control	-0.027302142	0.145375342	-0.187804492	0.851029914	0.969234083	down_in_case
PRJNA8274(	ENSGALG00010024944	ENSGALG00010024944	case	control	-0.022687946	0.067043567	-0.338406014	0.735057243	0.934096263	down_in_case
PRJNA8274(	ENSGALG00010024945	APIP	case	control	0.036659715	0.084728622	0.432672147	0.665252967	0.912007336	up_in_case
PRJNA8274(	ENSGALG00010024946	KIF1A	case	control	-0.060778232	0.228905826	-0.265516318	0.790611741	0.952383783	down_in_case
PRJNA8274(	ENSGALG00010024947	MPP4	case	control	0.005383362	0.185033997	0.029093909	0.976789694	0.994586386	up_in_case

PRJNA8274(	ENSGALG00010024948	HGD	case	control	0.879765679	1.180862356	0.745019667	0.456259831	0.82214158	up_in_case
PRJNA8274(	ENSGALG00010024949	PAN2	case	control	0.010312925	0.048510206	0.212592884	0.831644522	0.962371901	up_in_case
PRJNA8274(	ENSGALG00010024950	NCAM1	case	control	-0.160824606	0.08060707	-1.9951675	0.046024614	0.355983104	down_in_case
PRJNA8274(	ENSGALG00010024953	NCAPG2	case	control	-0.19654212	0.148629045	-1.322366841	0.186046025	0.609079249	down_in_case
PRJNA8274(	ENSGALG00010024956	VPS37B	case	control	-0.066041036	0.045975324	-1.436445239	0.150875688	0.558399072	down_in_case
PRJNA8274(	ENSGALG00010024959	AGXT	case	control	1.743274114	1.290258152	1.351104902	0.176661831	0.595527443	up_in_case
PRJNA8274(	ENSGALG00010024960	ARFRP1	case	control	0.049751903	0.050913564	0.977183665	0.328478233	0.745445783	up_in_case
PRJNA8274(	ENSGALG00010024961	LSM12	case	control	-0.164724599	0.081196433	-2.028717175	0.042487106	0.344353525	down_in_case
PRJNA8274(	ENSGALG00010024963	TNFRSF6B	case	control	-0.226408577	0.291275841	-0.77729954	0.436982066	0.810535423	down_in_case
PRJNA8274(	ENSGALG00010024964	ITM2C	case	control	0.111695171	0.190406086	0.586615549	0.557461937	0.868661404	up_in_case
PRJNA8274(	ENSGALG00010024965	STAT2	case	control	0.018848386	0.089009344	0.211757391	0.832296311	0.962371901	up_in_case
PRJNA8274(	ENSGALG00010024966	AGAP3	case	control	0.0574768	0.040189115	1.430158373	0.15267157	0.561193576	up_in_case
PRJNA8274(	ENSGALG00010024968	TMEM101	case	control	-0.047136991	0.073642207	-0.640081182	0.522119822	0.850861933	down_in_case
PRJNA8274(	ENSGALG00010024970	CAB39	case	control	1.86262E-05	0.034511188	0.000539716	0.999569369	0.99976345	up_in_case
PRJNA8274(	ENSGALG00010024973	RTEL1	case	control	0.07539345	0.076823835	0.98138097	0.326404907	0.744287835	up_in_case
PRJNA8274(	ENSGALG00010024974	CD44	case	control	0.042589758	0.114602427	0.371630511	0.710167969	0.925630832	up_in_case
PRJNA8274(	ENSGALG00010024977	LRP3	case	control	0.026958493	0.071548262	0.376787535	0.706331505	0.924449984	up_in_case
PRJNA8274(	ENSGALG00010024984	SHISAL2A	case	control	0.04470876	0.326890888	0.136769674	0.891212855	0.978917427	up_in_case
PRJNA8274(	ENSGALG00010024986	PTS	case	control	0.134445222	0.10472045	1.283848779	0.199194872	0.622902456	up_in_case
PRJNA8274(	ENSGALG00010024989	TIMELESS	case	control	-0.05674098	0.119076052	-0.476510425	0.633710782	0.898107199	down_in_case
PRJNA8274(	ENSGALG00010024994	NDUFB4	case	control	-0.174899622	0.081070773	-2.157369596	0.030976878	0.308259818	down_in_case
PRJNA8274(	ENSGALG00010024996	COA7	case	control	-0.057298882	0.096531695	-0.593575834	0.552795848	0.866252369	down_in_case
PRJNA8274(	ENSGALG00010024997	HES6	case	control	-0.031362581	0.299830049	-0.104601192	0.916692269	0.983445506	down_in_case
PRJNA8274(	ENSGALG00010025003	ENSGALG00010025003	case	control	-0.216037758	0.160022369	-1.350047244	0.177000829	0.595582794	down_in_case
PRJNA8274(	ENSGALG00010025005	HIP1R	case	control	0.087163563	0.093810406	0.929145995	0.352813431	0.761544462	up_in_case
PRJNA8274(	ENSGALG00010025006	ENSGALG00010025006	case	control	-0.060740006	0.324155848	-0.187379022	0.851363469	0.969234083	down_in_case
PRJNA8274(	ENSGALG00010025007	ZYG11B	case	control	0.052826592	0.065714111	0.803885064	0.421463357	0.802413627	up_in_case
PRJNA8274(	ENSGALG00010025008	ENSGALG00010025008	case	control	0.488526855	0.484244785	1.008842781	0.31305004	0.734574752	up_in_case
PRJNA8274(	ENSGALG00010025026	DENR	case	control	-0.055449179	0.06791621	-0.816435111	0.414251322	0.798785586	down_in_case
PRJNA8274(	ENSGALG00010025028	HES6	case	control	-0.009562341	0.084957271	-0.112554708	0.910383595	0.981829666	down_in_case
PRJNA8274(	ENSGALG00010025031	ECHDC2	case	control	-0.123576824	0.108278725	-1.14128444	0.253751575	0.679468065	down_in_case
PRJNA8274(	ENSGALG00010025032	FSTL1	case	control	0.023227673	0.08783528	0.264445823	0.791436406	0.95239351	up_in_case
PRJNA8274(	ENSGALG00010025039	CEBPA	case	control	0.000860621	0.220966199	0.00389481	0.996892399	0.998921977	up_in_case
PRJNA8274(	ENSGALG00010025042	BCO2	case	control	0.650337147	0.785510884	0.827916151	0.407717988	0.794388636	up_in_case
PRJNA8274(	ENSGALG00010025044	TMEM9	case	control	0.460859099	0.182432355	2.526191688	0.011530655	0.188192574	up_in_case
PRJNA8274(	ENSGALG00010025047	PER2	case	control	0.153939024	0.078395264	1.963626583	0.049573412	0.364833243	up_in_case
PRJNA8274(	ENSGALG00010025048	ESYT2	case	control	-0.110489458	0.089738698	-1.231235357	0.218234851	0.643443819	down_in_case
PRJNA8274(	ENSGALG00010025055	ALS2	case	control	-0.068810736	0.048996765	-1.404393468	0.160201715	0.572721131	down_in_case
PRJNA8274(	ENSGALG00010025056	RBMS2	case	control	-0.047140028	0.086965756	-0.54205276	0.587782161	0.882771329	down_in_case
PRJNA8274(	ENSGALG00010025057	SCP2	case	control	-0.01348614	0.086368256	-0.156146958	0.875917186	0.975666505	down_in_case
PRJNA8274(	ENSGALG00010025060	GPR156	case	control	-0.045071888	0.135364619	-0.332966537	0.739159543	0.936326594	down_in_case
PRJNA8274(	ENSGALG00010025064	GPN3	case	control	-0.023209827	0.064568655	-0.359459664	0.719251248	0.928443116	down_in_case
PRJNA8274(	ENSGALG00010025067	SLC1A2	case	control	0.14894132	0.169670996	0.877824278	0.380039087	0.77805894	up_in_case
PRJNA8274(	ENSGALG00010025069	PODN	case	control	0.109863171	0.180973463	0.607067851	0.543805885	0.861081181	up_in_case
PRJNA8274(	ENSGALG00010025071	CDK15	case	control	1.252462442	0.257337582	4.867001682	1.13304E-06	0.00028933	up_in_case
PRJNA8274(	ENSGALG00010025073	IL18	case	control	0.311026598	0.244724155	1.270927256	0.203754529	0.62581674	up_in_case
PRJNA8274(	ENSGALG00010025080	BAZ2A	case	control	0.104119278	0.131033146	0.794602594	0.426844709	0.805305429	up_in_case
PRJNA8274(	ENSGALG00010025092	COL1A1	case	control	-0.350462731	0.155463225	-2.254312763	0.024176499	0.275122308	down_in_case
PRJNA8274(	ENSGALG00010025097	LMBR1	case	control	0.187981492	0.072812424	2.581722751	0.009830853	0.177161817	up_in_case
PRJNA8274(	ENSGALG00010025098	ATP5B	case	control	-0.163553712	0.086395538	-1.893080537	0.05834716	0.393568108	down_in_case
PRJNA8274(	ENSGALG00010025102	ARPC3	case	control	0.08164607	0.069583957	1.173346183	0.240657009	0.665904651	up_in_case
PRJNA8274(	ENSGALG00010025103	GSK3B	case	control	-0.134454822	0.056188744	-2.392913804	0.016715167	0.229833552	down_in_case
PRJNA8274(	ENSGALG00010025113	SDHD	case	control	-0.073828965	0.061874375	-1.193207447	0.232788139	0.657934961	down_in_case
PRJNA8274(	ENSGALG00010025114	CEBPG	case	control	-0.111698334	0.061650903	-1.811787482	0.07001904	0.427163939	down_in_case
PRJNA8274(	ENSGALG00010025115	MGAT4F	case	control	0.411278692	0.136302258	3.017401909	0.002549515	0.083237603	up_in_case
PRJNA8274(	ENSGALG00010025116	ANAPC7	case	control	-0.124756121	0.052986153	-2.354504231	0.018547433	0.242660829	down_in_case
PRJNA8274(	ENSGALG00010025121	DYNC2I1	case	control	-0.027504275	0.081287442	-0.338358233	0.735093245	0.934096263	down_in_case
PRJNA8274(	ENSGALG00010025125	PIK3CB	case	control	0.146595424	0.073148258	2.004086317	0.045060815	0.352291176	up_in_case
PRJNA8274(	ENSGALG00010025126	ENSGALG00010025126	case	control	0.142874608	0.080545649	1.773833973	0.076090617	0.442710866	up_in_case
PRJNA8274(	ENSGALG00010025127	PAMR1	case	control	-0.040620445	0.139289997	-0.291624998	0.77057336	0.945448224	down_in_case
PRJNA8274(	ENSGALG00010025130	ATP2A2	case	control	0.189647303	0.121066757	1.566468842	0.117238897	0.513953474	up_in_case
PRJNA8274(	ENSGALG00010025131	SUMO1	case	control	-0.015969713	0.050957224	-0.313394497	0.753980965	0.941077751	down_in_case
PRJNA8274(	ENSGALG00010025138	CFAP91	case	control	0.949865598	0.20604932	4.609894359	4.02874E-06	0.000811422	up_in_case
PRJNA8274(	ENSGALG00010025139	COPB2	case	control	0.047644537	0.044926343	1.060503342	0.28891567	0.712801679	up_in_case
PRJNA8274(	ENSGALG00010025144	PIH1D2	case	control	0.229209951	0.12003485	1.909528369	0.056193966	0.385984934	up_in_case
PRJNA8274(	ENSGALG00010025145	CPT2	case	control	0.071173382	0.078989207	0.901051981	0.367560683	0.771770041	up_in_case
PRJNA8274(	ENSGALG00010025147	COX17	case	control	-0.016857238	0.080521354	-0.20935115	0.834174125	0.963078474	down_in_case
PRJNA8274(	ENSGALG00010025149	IFT81	case	control	-0.031469509	0.071774388	-0.438450397	0.66105982	0.909646511	down_in_case
PRJNA8274(	ENSGALG00010025154	TMEM183A	case	control	-0.012979329	0.053194266	-0.243998651	0.807231865	0.955875648	down_in_case
PRJNA8274(	ENSGALG00010025155	CZIB	case	control	-0.103489003	0.09344707	-1.10746119	0.268094583	0.694898049	down_in_case
PRJNA8274(	ENSGALG00010025156	DLAT	case	control	-0.169112411	0.08478128	-1.994690464	0.04607665	0.356019424	down_in_case
PRJNA8274(	ENSGALG00010025157	NOP58	case	control	0.065935723	0.055350657	1.191236512	0.233560749	0.657982411	up_in_case
PRJNA8274(	ENSGALG00010025160	MRPS22	case	control	-0.134052372	0.066821618	-2.006122808	0.044843147	0.351986719	down_in_case
PRJNA8274(	ENSGALG00010025161	ENSGALG00010025161	case	control	0.035916085	0.076614398	0.468790286	0.639219544	0.900304115	up_in_case
PRJNA8274(	ENSGALG00010025163	LDLRAD3	case	control	0.00758989	0.08111279	0.093572053	0.925449111	0.98562019	up_in_case
PRJNA8274(	ENSGALG00010025164	C11orf52	case	control	-0.234077648	0.068867891	-3.398937362	0.000676482	0.038540612	down_in_case
PRJNA8274(	ENSGALG00010025167	PTGES3	case	control	0.077283659	0.060209517	1.283578792	0.199289374	0.622902456	up_in_case
PRJNA8274(	ENSGALG00010025168	POPDC2	case	control	-0.184447239	0.145674519	-1.266159933	0.205455829	0.628438742	down_in_case
PRJNA8274(	ENSGALG00010025170	COMMD9	case	control	0.14518355	0.05429169	2.674139454	0.007492129	0.151324073	up_in_case
PRJNA8274(	ENSGALG00010025182	PLA1A	case	control	0.426306081	0.244732959	1.741923453	0.081521839	0.454488228	up_in_case
PRJNA8274(	ENSGALG00010025186	TMEM120B	case	control	-0.125195888	0.091622696	-1.366428778	0.171804421	0.58957339	down_in_case
PRJNA8274(	ENSGALG00010025188	PPFIA4	case	control	0.098033168	0.301150608	0.325528707	0.744780959	0.937849656	up_in_case
PRJNA8274(	ENSGALG00010025190	PRR5L	case	control	0.064334171	0.173347761	0.371127786	0.710542356	0.925724826	up_in_case
PRJNA8274(	ENSGALG00010025192	CRYAB	case	control	0.049989802	0.091577844	0.545872236	0.585153764	0.881307382	up_in_case
PRJNA8274(	ENSGALG00010025195	P2RX7	case	control	0.156559551	0.234175111	0.668557605	0.503777724	0.842675107	up_in_case
PRJNA8274(	ENSGALG00010025200	PEPD	case	control	0.076670201	0.069707594	1.099883045	0.271383083	0.698323327	up_in_case
PRJNA8274(	ENSGALG00010025204	TRAF6	case	control	-0.014215285	0.057047589	-0.249182925	0.803219287	0.954891353	down_in_case
PRJNA8274(	ENSGALG00010025207	BMPR2	case	control	-0.107345451	0.061697903	-1.739855753	0.08188435	0.454736389	down_in_case
PRJNA8274(	ENSGALG00010025210	HSPB2	case	control	0.218466915	0.153588618	1.422416047	0.154905507	0.563829072	up_in_case
PRJNA8274(	ENSGALG00010025211	RAG1	case	control	0.017894104	1.046770639	0.01709458	0.986361163	0.996597442	up_in_case
PRJNA8274(	ENSGALG00010025212	P2RX4	case	control	-0.258722472	0.159470969	-1.622379762	0.10472206	0.494770427	down_in_case
PRJNA8274(	ENSGALG00010025214	LRP8	case	control	0.215790147	0.140434205	1.536592501	0.12439313	0.523029039	up_in_case
PRJNA8274(	ENSGALG00010025215	MYOG	case	control	0.141834503	0.130776415	1.084557201	0.278117819	0.703443892	up_in_case
PRJNA8274(	ENSGALG00010025216	DPP6	case	control	0.079645613	0.218781156	0.364042381	0.715826362	0.926862121	up_in_case
PRJNA8274(	ENSGALG00010025219	NACA	case	control	-0.039948598	0.058611699	-0.681580617	0.495504175	0.838118945	down_in_case
PRJNA8274(	ENSGALG00010025220	NHLH2	case	control	-0.751442557	2.973166172	-0.252741527	0.800467956	0.954053991	down_in_case
PRJNA8274(	ENSGALG00010025222	FAM117B	case	control	0.050606414	0.133076849	0.380279621	0.703737861	0.923760915	up_in_case
PRJNA8274(	ENSGALG00010025228	FDXACB1	case	control	-0.075909254	0.099846272	-0.760261272	0.447098427	0.817833512	down_in_case
PRJNA8274(	ENSGALG00010025229	ENSGALG00010025229	case	control	0.054431317	0.074754602	0.72813333	0.466531973	0.827204862	up_in_case
PRJNA8274(	ENSGALG00010025230	CHST8	case	control	0.512369379	0.17578194	2.914801033	0.003559154	0.101995788	up_in_case
PRJNA8274(	ENSGALG00010025232	PRIM1	case	control	-0.066284219	0.068129744	-0.972911608	0.330597235	0.746554834	down_in_case
PRJNA8274(	ENSGALG00010025233	WDR12	case	control	0.010494171	0.069296494	0.151438706	0.879629659	0.976318821	up_in_case
PRJNA8274(	ENSGALG00010025236	ENSGALG00010025236	case	control	0.308903652	0.275591103	1.120876721	0.262340341	0.688342547	up_in_case
PRJNA8274(	ENSGALG00010025238	IGSF9B	case	control	-0.126671866	0.215892009	-0.586737169	0.55738024	0.868661404	down_in_case
PRJNA8274(	ENSGALG00010025239	RAG2	case	control	-0.379170176	0.515754966	-0.735175036	0.462232901	0.824958777	down_in_case
PRJNA8274(	ENSGALG00010025242	CLSTN2	case	control	0.211044839	0.160201033	1.317375021	0.18771295	0.610946377	up_in_case
PRJNA8274(	ENSGALG00010025243	PAXIP1	case	control	0.02818924	0.067060424	0.420355823	0.674225536	0.913965794	up_in_case
PRJNA8274(	ENSGALG00010025245	ENSGALG00010025245	case	control	-0.007777392	0.049416344	-0.157385004	0.874941431	0.975492161	down_in_case
PRJNA8274(	ENSGALG00010025247	SGCA	case	control	-0.115377126	0.101468467	-1.137073712	0.255507472	0.681925504	down_in_case
PRJNA8274(	ENSGALG00010025248	C11orf74	case	control	0.065946152	0.128934775	0.511469087	0.609022629	0.8877909	up_in_case
PRJNA8274(	ENSGALG00010025250	GLIS1	case	control	-0.056069667	0.375298914	-0.149400024	0.881237993	0.97667824	down_in_case
PRJNA8274(	ENSGALG00010025252	PPP1R9B	case	control	0.082017696	0.063990808	1.281710593	0.199944188	0.622902456	up_in_case
PRJNA8274(	ENSGALG00010025256	ANAPC5	case	control	0.249061742	0.09061494	2.748572624	0.005985537	0.134447684	up_in_case
PRJNA8274(	ENSGALG00010025257	LPCAT2	case	control	0.137174948	0.153141167	0.895741827	0.370390666	0.772700698	up_in_case
PRJNA8274(	ENSGALG00010025259	SHH	case	control	-0.209940546	0.683016588	-0.307372544	0.758559824	0.941942122	down_in_case
PRJNA8274(	ENSGALG00010025262	CARF	case	control	0.158348466	0.10729789	1.475783598	0.140001995	0.542025835	up_in_case
PRJNA8274(	ENSGALG00010025263	SAMD14	case	control	-0.151874752	0.303709584	-0.500065721	0.617028802	0.891081782	down_in_case
PRJNA8274(	ENSGALG00010025268	ZBTB39	case	control	0.282281994	0.101410142	2.783567685	0.005376464	0.127387074	up_in_case
PRJNA8274(	ENSGALG00010025270	NDC1	case	control	0.101857106	0.095313556	1.068652874	0.285226114	0.709820465	up_in_case
PRJNA8274(	ENSGALG00010025271	RNF34	case	control	0.027267928	0.048640937	0.560596269	0.575072795	0.877910508	up_in_case
PRJNA8274(	ENSGALG00010025272	NCAPD3	case	control	-0.052840729	0.091575607	-0.577017511	0.563927626	0.871916948	down_in_case
PRJNA8274(	ENSGALG00010025274	PDK2	case	control	-0.376305444	0.138390222	-2.719162082	0.006544753	0.139826638	down_in_case
PRJNA8274(	ENSGALG00010025280	HSPB11	case	control	0.107963999	0.104638602	1.03177983	0.30217527	0.725017847	up_in_case
PRJNA8274(	ENSGALG00010025286	ITGA3	case	control	-0.124203037	0.138137042	-0.899129119	0.368583889	0.771770041	down_in_case
PRJNA8274(	ENSGALG00010025289	KDM2B	case	control	-0.020035466	0.041202061	-0.486273402	0.626773335	0.895658907	down_in_case
PRJNA8274(	ENSGALG00010025291	DUS2	case	control	0.118282793	0.072424871	1.633179189	0.102431316	0.491460867	up_in_case

PRJNA8274(	ENSGALG00010025292	ATP1A1	case	control	0.019837894	0.052624506	0.376970656	0.706195412	0.924449984	up_in_case
PRJNA8274(	ENSGALG00010025297	SPSB4	case	control	0.136769421	0.091757274	1.490556718	0.136077915	0.539840644	up_in_case
PRJNA8274(	ENSGALG00010025298	ENSGALG00010025298	case	control	0.021794585	0.130797626	0.166628294	0.867662529	0.973996477	up_in_case
PRJNA8274(	ENSGALG00010025301	MORN3	case	control	-0.958633194	0.251729167	-3.808192766	0.000139986	0.01371097	down_in_case
PRJNA8274(	ENSGALG00010025303	TMCC2	case	control	-0.048805458	0.098076267	-0.497627604	0.618746543	0.891656847	down_in_case
PRJNA8274(	ENSGALG00010025304	DDX28	case	control	-0.034154082	0.077268308	-0.442019283	0.658475252	0.908367791	down_in_case
PRJNA8274(	ENSGALG00010025305	GPR182	case	control	-0.176298518	0.32269474	-0.54633217	0.584837626	0.881307382	down_in_case
PRJNA8274(	ENSGALG00010025312	PLEKHM3	case	control	0.093879757	0.081730231	1.148654002	0.25069868	0.676268933	up_in_case
PRJNA8274(	ENSGALG00010025313	DIO1	case	control	0.833298943	0.560461413	1.486808769	0.137065325	0.539854583	up_in_case
PRJNA8274(	ENSGALG00010025315	LRRC42	case	control	0.152610647	0.08952329	1.704703298	0.088249774	0.469561177	up_in_case
PRJNA8274(	ENSGALG00010025316	SLC25A36	case	control	0.072379572	0.064121302	1.12879136	0.258985858	0.686345498	up_in_case
PRJNA8274(	ENSGALG00010025318	NUAK2	case	control	0.347717938	0.378770704	0.918016979	0.358609977	0.765277223	up_in_case
PRJNA8274(	ENSGALG00010025319	LDLRAD1	case	control	-0.097430135	0.247828512	-0.393135295	0.694219557	0.920308089	down_in_case
PRJNA8274(	ENSGALG00010025320	JAM3	case	control	0.126028484	0.065556981	1.922426604	0.054552101	0.381464567	up_in_case
PRJNA8274(	ENSGALG00010025321	ENSGALG00010025321	case	control	1.815587915	2.255056831	0.805118474	0.420751316	0.801969583	up_in_case
PRJNA8274(	ENSGALG00010025323	KLHDC8A	case	control	0.337824653	0.153001289	2.20798566	0.027245273	0.293158316	up_in_case
PRJNA8274(	ENSGALG00010025325	DPEP2	case	control	0.146129067	0.16321426	0.895320468	0.370615802	0.772791771	up_in_case
PRJNA8274(	ENSGALG00010025327	TMEM59	case	control	0.048583381	0.058895618	0.824906549	0.409424648	0.795485389	up_in_case
PRJNA8274(	ENSGALG00010025329	MYO1A	case	control	0.112733713	0.104831128	1.075383954	0.282202863	0.706000864	up_in_case
PRJNA8274(	ENSGALG00010025330	USH1C	case	control	0.56690069	0.229239163	2.472966151	0.013399683	0.203413453	up_in_case
PRJNA8274(	ENSGALG00010025331	ENSGALG00010025331	case	control	-0.060366514	0.590720203	-0.102191382	0.918604768	0.983734684	down_in_case
PRJNA8274(	ENSGALG00010025333	FZD5	case	control	-0.081536367	0.058846595	-1.385574946	0.165876733	0.580812263	down_in_case
PRJNA8274(	ENSGALG00010025334	RHOF	case	control	0.222415332	0.217276153	1.023652755	0.305999318	0.728933908	up_in_case
PRJNA8274(	ENSGALG00010025336	ADAM10	case	control	0.066625868	0.052838914	1.260924244	0.207336143	0.631716687	up_in_case
PRJNA8274(	ENSGALG00010025337	NEMP1	case	control	0.04560166	0.107122568	0.425696102	0.670329303	0.913465866	up_in_case
PRJNA8274(	ENSGALG00010025338	FADD	case	control	-0.049239232	0.070610631	-0.697334545	0.485593454	0.834513447	down_in_case
PRJNA8274(	ENSGALG00010025340	METTL21A	case	control	-0.125794271	0.081677925	-1.540125692	0.123529718	0.522883756	down_in_case
PRJNA8274(	ENSGALG00010025341	VPS26B	case	control	-0.003815163	0.068823031	-0.055434401	0.95579239	0.992056045	down_in_case
PRJNA8274(	ENSGALG00010025342	TCEANC2	case	control	0.088054616	0.084474414	1.042382093	0.297234565	0.721027687	up_in_case
PRJNA8274(	ENSGALG00010025343	NAB2	case	control	0.145435398	0.138564444	1.049586706	0.293908172	0.716378375	up_in_case
PRJNA8274(	ENSGALG00010025344	ENSGALG00010025344	case	control	0.006359938	0.182805447	0.034790746	0.9722466	0.99340857	up_in_case
PRJNA8274(	ENSGALG00010025345	CCM2	case	control	0.011231783	0.060347372	0.18611885	0.85235156	0.969582953	up_in_case
PRJNA8274(	ENSGALG00010025346	PXYLP1	case	control	0.288094936	0.181690219	1.585638111	0.112821426	0.507003543	up_in_case
PRJNA8274(	ENSGALG00010025348	HIPK1	case	control	-0.031715751	0.06797637	-0.46657023	0.64080739	0.901391469	down_in_case
PRJNA8274(	ENSGALG00010025349	SETD1A	case	control	-0.002211737	0.060396086	-0.036620535	0.97078757	0.992761697	down_in_case
PRJNA8274(	ENSGALG00010025351	CREB1	case	control	0.013602627	0.043026261	0.316147097	0.75189086	0.940427752	up_in_case
PRJNA8274(	ENSGALG00010025353	SLC12A4	case	control	0.066351409	0.046336737	1.431939613	0.152161104	0.560293917	up_in_case
PRJNA8274(	ENSGALG00010025354	ENSGALG00010025354	case	control	2.554886149	2.075796703	1.230797864			up_in_case
PRJNA8274(	ENSGALG00010025355	STAT6	case	control	-0.022694685	0.052843974	-0.429465905	0.667584201	0.912432583	down_in_case
PRJNA8274(	ENSGALG00010025357	OLFML3	case	control	0.001549684	0.05712876	0.027126157	0.978359112	0.995130187	up_in_case
PRJNA8274(	ENSGALG00010025358	INCENP	case	control	-0.047066115	0.094171936	-0.499789178	0.617223532	0.891081782	down_in_case
PRJNA8274(	ENSGALG00010025359	CDCP2	case	control	0.511963285	0.421339926	1.215083721	0.224334162	0.648105158	up_in_case
PRJNA8274(	ENSGALG00010025360	THYN1	case	control	0.160075065	0.0657092	2.436113454	0.014846027	0.215129943	up_in_case
PRJNA8274(	ENSGALG00010025362	G6PC2	case	control	-0.060496743	0.142808097	-0.423622637	0.671841044	0.913676733	down_in_case
PRJNA8274(	ENSGALG00010025364	EPHA4	case	control	0.02663899	0.107039448	0.248870769	0.803460747	0.954891353	up_in_case
PRJNA8274(	ENSGALG00010025365	SIKE1	case	control	-0.079251623	0.049462956	-1.60224196	0.109102115	0.501336838	down_in_case
PRJNA8274(	ENSGALG00010025367	ENSGALG00010025367	case	control	-1.966245318	0.935825223	-2.101081773	0.035633789	0.324939791	down_in_case
PRJNA8274(	ENSGALG00010025368	LRP1	case	control	-0.195348153	0.118729371	-1.645322907	0.099903238	0.489252161	down_in_case
PRJNA8274(	ENSGALG00010025369	ENSGALG00010025369	case	control	0.954109416	0.660803721	1.443862051	0.148777783	0.55519893	up_in_case
PRJNA8274(	ENSGALG00010025370	HPD	case	control	0.594608234	0.564594714	1.053159407	0.292267939	0.714677075	up_in_case
PRJNA8274(	ENSGALG00010025373	CYB5RL	case	control	-0.19129928	0.075464877	-2.534944562	0.011246512	0.185840265	down_in_case
PRJNA8274(	ENSGALG00010025377	MYO1G	case	control	0.002522108	0.200652813	0.012569514	0.989971243	0.997323462	up_in_case
PRJNA8274(	ENSGALG00010025378	PAX3	case	control	0.192753079	0.212313735	0.907869097	0.363947387	0.770036689	up_in_case
PRJNA8274(	ENSGALG00010025379	ACAD8	case	control	-0.169515721	0.078085527	-2.170898096	0.029938876	0.305970455	down_in_case
PRJNA8274(	ENSGALG00010025380	SPC25	case	control	-0.15046407	0.101779266	-1.478337157	0.139317557	0.541505735	down_in_case
PRJNA8274(	ENSGALG00010025381	MRPL37	case	control	-0.119166335	0.077226628	-1.54307313	0.122813033	0.52190977	down_in_case
PRJNA8274(	ENSGALG00010025382	MAB21L3	case	control	0.955022949	0.273933178	3.486335449	0.000489687	0.032249816	up_in_case
PRJNA8274(	ENSGALG00010025386	TPCN2	case	control	0.157430131	0.106947668	1.472029579	0.14101289	0.54338031	up_in_case
PRJNA8274(	ENSGALG00010025387	PSMD9	case	control	-0.086462014	0.059899671	-1.443447231	0.148894527	0.555199933	down_in_case
PRJNA8274(	ENSGALG00010025389	SNED1	case	control	0.138043775	0.067227037	2.053396695	0.04003412	0.336547209	up_in_case
PRJNA8274(	ENSGALG00010025390	BCL7A	case	control	-0.133055958	0.066038892	-2.014812087	0.043924342	0.349731677	down_in_case
PRJNA8274(	ENSGALG00010025392	SLC6A2	case	control	-0.220796129	0.206048754	-1.071572256	0.2839122	0.708665468	down_in_case
PRJNA8274(	ENSGALG00010025398	TSPAN2	case	control	0.126243505	0.124979547	1.010113316	0.312441003	0.734364975	up_in_case
PRJNA8274(	ENSGALG00010025400	SLC22A15	case	control	0.477053972	0.202794755	2.352397986	0.018652805	0.243816378	up_in_case
PRJNA8274(	ENSGALG00010025403	ENSGALG00010025403	case	control	0.100476886	0.062689804	1.602762797	0.108987033	0.501108679	up_in_case
PRJNA8274(	ENSGALG00010025404	CERS6	case	control	0.105833257	0.124031696	0.853275898	0.393506322	0.786843919	up_in_case
PRJNA8274(	ENSGALG00010025405	TAMALIN	case	control	-0.065966894	0.131101311	-0.503174935	0.614841286	0.890803484	down_in_case
PRJNA8274(	ENSGALG00010025406	ENSGALG00010025406	case	control	-0.078369266	0.07144983	-1.096843279	0.272709907	0.698931401	down_in_case
PRJNA8274(	ENSGALG00010025407	MLXIP	case	control	-0.215401167	0.083571478	-2.577448324	0.009953276	0.177161817	down_in_case
PRJNA8274(	ENSGALG00010025413	NECAB3	case	control	-0.000953618	0.07662362	-0.012445479	0.9900702	0.997323462	down_in_case
PRJNA8274(	ENSGALG00010025414	MMP2	case	control	-0.048267327	0.076020122	-0.634928303	0.525475177	0.85251736	down_in_case
PRJNA8274(	ENSGALG00010025415	ENSGALG00010025415	case	control	0.442531891	0.228999624	1.932456847	0.053303143	0.376996967	up_in_case
PRJNA8274(	ENSGALG00010025417	B3GAT1	case	control	-0.096513451	0.097561145	-0.989261163	0.32253538	0.740567749	down_in_case
PRJNA8274(	ENSGALG00010025418	CASQ2	case	control	-0.165405558	0.105392073	-1.569430727	0.116547609	0.51350396	down_in_case
PRJNA8274(	ENSGALG00010025419	CCND1	case	control	-0.018612364	0.051436055	-0.361854435	0.717460811	0.927052463	down_in_case
PRJNA8274(	ENSGALG00010025420	STK39	case	control	-0.047515284	0.112539565	-0.422209591	0.672872041	0.913776846	down_in_case
PRJNA8274(	ENSGALG00010025421	NR4A1	case	control	0.008323829	0.18003872	0.046233552	0.963124101	0.992068146	up_in_case
PRJNA8274(	ENSGALG00010025422	SSBP3	case	control	-0.007957253	0.055743807	-0.142746847	0.886490117	0.977630122	down_in_case
PRJNA8274(	ENSGALG00010025423	NGF	case	control	-0.180192484	0.197756647	-0.911182943	0.36219899	0.768423771	down_in_case
PRJNA8274(	ENSGALG00010025424	IRX6	case	control	0.278063659	0.441290214	0.63011517	0.528619235	0.853863669	up_in_case
PRJNA8274(	ENSGALG00010025425	ENSGALG00010025425	case	control	-0.012930913	0.065097935	-0.198637842	0.84254605	0.966519616	down_in_case
PRJNA8274(	ENSGALG00010025426	ENSGALG00010025426	case	control	-0.074467935	0.070522852	-1.055940485	0.290995406	0.714222439	down_in_case
PRJNA8274(	ENSGALG00010025427	SLC35G2	case	control	0.24161795	0.079842948	3.026165212	0.002476769	0.081856092	up_in_case
PRJNA8274(	ENSGALG00010025428	VANGL1	case	control	-0.09943664	0.08942425	-1.111965048	0.266153178	0.69218122	down_in_case
PRJNA8274(	ENSGALG00010025429	SCN3B	case	control	-0.258150873	0.268543253	-0.961300907	0.336400892	0.750473129	down_in_case
PRJNA8274(	ENSGALG00010025430	IRX5	case	control	-0.131547456	0.591919476	-0.222238769	0.824128015	0.960422238	down_in_case
PRJNA8274(	ENSGALG00010025432	IRX3	case	control	-0.298277632	0.467724792	-0.63772038	0.523655716	0.85188641	down_in_case
PRJNA8274(	ENSGALG00010025433	CHPF2	case	control	-0.017179577	0.057420853	-0.299187077	0.764797309	0.943786458	down_in_case
PRJNA8274(	ENSGALG00010025434	SYT6	case	control	0.079170818	0.115152816	0.687528288	0.491749881	0.837145631	up_in_case
PRJNA8274(	ENSGALG00010025435	SCLY	case	control	-0.072555638	0.067326546	-1.077667619	0.281182114	0.705762606	down_in_case
PRJNA8274(	ENSGALG00010025438	EXOSC6	case	control	0.06218041	0.111121256	0.559572599	0.575770999	0.877910508	up_in_case
PRJNA8274(	ENSGALG00010025442	ABCF2	case	control	0.032370801	0.057497588	0.562994073	0.57343892	0.877542213	up_in_case
PRJNA8274(	ENSGALG00010025445	B3GNT4	case	control	0.225807797	0.16649901	1.356211051	0.175032027	0.594080527	up_in_case
PRJNA8274(	ENSGALG00010025448	CHD1L	case	control	0.023748105	0.117349305	0.202371075	0.839626639	0.965286179	up_in_case
PRJNA8274(	ENSGALG00010025449	ENSGALG00010025449	case	control	-0.012475313	0.080167916	-0.155614783	0.876336672	0.975666505	down_in_case
PRJNA8274(	ENSGALG00010025451	LRRC43	case	control	0.138937775	0.320542781	0.43344534	0.664691268	0.911758765	up_in_case
PRJNA8274(	ENSGALG00010025452	TRIM33	case	control	0.015415604	0.046824198	0.329223018	0.741987126	0.937315893	up_in_case
PRJNA8274(	ENSGALG00010025458	BCAS2	case	control	0.064380902	0.07139762	0.901723359	0.367203841	0.771770041	up_in_case
PRJNA8274(	ENSGALG00010025459	DIABLO	case	control	0.013971718	0.05549527	0.25176413	0.801223385	0.954294696	up_in_case
PRJNA8274(	ENSGALG00010025464	VPS33A	case	control	0.025844257	0.055625436	0.464612221	0.642209179	0.90176662	up_in_case
PRJNA8274(	ENSGALG00010025465	ENSGALG00010025465	case	control	0.097631776	0.056312212	1.733758494	0.082960943	0.456931082	up_in_case
PRJNA8274(	ENSGALG00010025470	ORAOV1	case	control	0.004389138	0.147530555	0.029750704	0.976265874	0.994274054	up_in_case
PRJNA8274(	ENSGALG00010025472	ZCCHC8	case	control	-0.006831275	0.078864362	-0.086620556	0.930973126	0.987258715	down_in_case
PRJNA8274(	ENSGALG00010025473	PARS2	case	control	0.003208776	0.069315439	0.046292374	0.963077217	0.992068146	up_in_case
PRJNA8274(	ENSGALG00010025477	ENSGALG00010025477	case	control	0.022725752	0.032909291	0.690557317	0.489843778	0.836347578	up_in_case
PRJNA8274(	ENSGALG00010025480	FGF19	case	control	0.515532372	0.309632387	1.664982068	0.095916333	0.483185646	up_in_case
PRJNA8274(	ENSGALG00010025481	TMEM39A	case	control	0.075107978	0.052640146	1.426819313	0.153631983	0.56245196	up_in_case
PRJNA8274(	ENSGALG00010025483	FGF4	case	control	-0.041455403	0.152966516	-0.271009654	0.786383607	0.950252288	down_in_case
PRJNA8274(	ENSGALG00010025485	DENND2C	case	control	0.263059728	0.089136792	2.951191325	0.003165508	0.095701395	up_in_case
PRJNA8274(	ENSGALG00010025486	B3GALT1	case	control	-0.273096747	0.132995979	-2.053421075	0.040031757	0.336547209	down_in_case
PRJNA8274(	ENSGALG00010025487	FGF3	case	control	-0.180737006	0.327184567	-0.552400767	0.580673805	0.879548643	down_in_case
PRJNA8274(	ENSGALG00010025488	SLC4A8	case	control	0.186502463	0.143776498	1.297169321	0.194572934	0.617304812	up_in_case
PRJNA8274(	ENSGALG00010025490	ENSGALG00010025490	case	control	-0.588503981	0.625742316	-0.940489346	0.346966612	0.757383995	down_in_case
PRJNA8274(	ENSGALG00010025491	ZMYND11	case	control	-0.049287637	0.057637915	-0.855125261	0.392481807	0.786063002	down_in_case
PRJNA8274(	ENSGALG00010025496	ATG101	case	control	0.005745375	0.071763631	0.080059698	0.936189776	0.988446087	up_in_case
PRJNA8274(	ENSGALG00010025499	STAC3	case	control	-0.018799139	0.156730636	-0.119945527	0.904526299	0.981225151	down_in_case
PRJNA8274(	ENSGALG00010025504	AMPD1	case	control	-0.242940386	0.129551773	-1.875237828	0.060760012	0.400562592	down_in_case
PRJNA8274(	ENSGALG00010025505	ENSGALG00010025505	case	control	-0.545029016	0.329686592	-1.653173133	0.098295633	0.487726422	down_in_case
PRJNA8274(	ENSGALG00010025506	R3HDM2	case	control	-0.012206135	0.085460624	-0.142827595	0.886426343	0.977630122	down_in_case
PRJNA8274(	ENSGALG00010025508	ENSGALG00010025508	case	control	-0.05460466	0.064470789	-0.846967464	0.397013257	0.788531716	down_in_case
PRJNA8274(	ENSGALG00010025512	NRAS	case	control	0.081678015	0.04511735	1.810346032	0.070242143	0.427977266	up_in_case
PRJNA8274(	ENSGALG00010025513	RSRC2	case	control	0.037449616	0.043332182	0.864244875	0.387453385	0.782604263	up_in_case
PRJNA8274(	ENSGALG00010025520	ACOT11	case	control	0.110065172	0.108327466	1.016041228	0.309609754	0.732384442	up_in_case
PRJNA8274(	ENSGALG00010025522	SUMO3	case	control	-0.081600084	0.057877349	-1.409879444	0.158575283	0.570759263	down_in_case
PRJNA8274(	ENSGALG00010025523	B4GALT4	case	control	0.013827172	0.121581401	0.11372769	0.909453661	0.981730793	up_in_case
PRJNA8274(	ENSGALG00010025527	KNTC1	case	control	-0.067929014	0.129108229	-0.526140082	0.598790882	0.88476024	down_in_case

PRJNA8274(	ENSGALG00010025531	ENSGALG00010025531	case	control	0.15149352	0.081226531	1.865074354	0.062170983	0.405537748	up_in_case
PRJNA8274(	ENSGALG00010025533	INHBC	case	control	0.789391995	0.50276892	1.570089087	0.116394387	0.5134816	up_in_case
PRJNA8274(	ENSGALG00010025538	ANO1	case	control	-0.156199936	0.195444795	-0.799202332	0.4241731	0.803723282	down_in_case
PRJNA8274(	ENSGALG00010025541	TTC21B	case	control	0.010762169	0.073756647	0.14591456	0.883988837	0.97734965	up_in_case
PRJNA8274(	ENSGALG00010025544	CSDE1	case	control	-0.066698292	0.035827421	-1.861654856	0.062651756	0.407421604	down_in_case
PRJNA8274(	ENSGALG00010025546	ENSGALG00010025546	case	control	0.49903833	0.73039798	0.683241663	0.49445415	0.83803731	up_in_case
PRJNA8274(	ENSGALG00010025548	ENSGALG00010025548	case	control	0.11771337	0.142385009	0.826725864	0.408392458	0.794775742	up_in_case
PRJNA8274(	ENSGALG00010025549	DHCR24	case	control	0.279979167	0.209848082	1.334199315	0.182138544	0.603068611	up_in_case
PRJNA8274(	ENSGALG00010025553	UBE2G2	case	control	-0.009701655	0.06632197	-0.146281172	0.883699429	0.977180779	down_in_case
PRJNA8274(	ENSGALG00010025554	DIP2C	case	control	0.080956459	0.065111143	1.243357973	0.21373595	0.638602829	up_in_case
PRJNA8274(	ENSGALG00010025557	VPS29	case	control	-0.007329021	0.056054861	-0.13074729	0.895975221	0.980176778	down_in_case
PRJNA8274(	ENSGALG00010025558	GALNT3	case	control	0.156440542	0.231606785	0.675457508	0.49938514	0.840142058	up_in_case
PRJNA8274(	ENSGALG00010025559	SLC6A17	case	control	1.659757287	0.762156675	2.177711411	0.029427529	0.305109318	up_in_case
PRJNA8274(	ENSGALG00010025562	GRAMD1B	case	control	-0.126596669	0.098262505	-1.288351729	0.197623554	0.62094535	down_in_case
PRJNA8274(	ENSGALG00010025563	TSPEAR	case	control	0.250218324	0.23382325	1.070117379	0.284566478	0.709184495	up_in_case
PRJNA8274(	ENSGALG00010025565	ENSGALG00010025565	case	control	0.207205934	0.123293407	1.680592167	0.092842158	0.477913198	up_in_case
PRJNA8274(	ENSGALG00010025568	MYL3	case	control	0.204772826	0.306934617	0.667154548	0.50467342	0.842675107	up_in_case
PRJNA8274(	ENSGALG00010025569	PPTC7	case	control	0.114604189	0.057662736	1.987491357	0.046867973	0.357255869	up_in_case
PRJNA8274(	ENSGALG00010025571	SRSF3	case	control	0.059815766	0.049044419	1.219624322	0.222607322	0.646365317	up_in_case
PRJNA8274(	ENSGALG00010025572	ENSGALG00010025572	case	control	0.060429849	0.046779298	1.291807524	0.196423809	0.619313262	up_in_case
PRJNA8274(	ENSGALG00010025573	CLMP	case	control	-0.077129174	0.051271578	-1.50432611	0.132497419	0.535857167	down_in_case
PRJNA8274(	ENSGALG00010025574	ARHGAP31	case	control	0.019385508	0.080563525	0.240623887	0.809846635	0.955972758	up_in_case
PRJNA8274(	ENSGALG00010025575	TRPM2	case	control	0.464527801	0.394935903	1.176210615	0.239510724	0.664610169	up_in_case
PRJNA8274(	ENSGALG00010025577	PTH2R	case	control	0.290930294	0.23643384	1.23049346	0.218512374	0.643443819	up_in_case
PRJNA8274(	ENSGALG00010025578	ORMDL2	case	control	0.042911066	0.078508791	0.546576577	0.584669665	0.881307382	up_in_case
PRJNA8274(	ENSGALG00010025579	CSRNP3	case	control	-0.026855188	0.16709952	-0.160713736	0.87231887	0.975062028	down_in_case
PRJNA8274(	ENSGALG00010025580	RASSF7	case	control	-0.172327642	0.142862105	-1.206251594	0.227720496	0.651681204	down_in_case
PRJNA8274(	ENSGALG00010025582	DMAP1	case	control	-0.114156542	0.055742352	-2.04793192	0.040566671	0.337631189	down_in_case
PRJNA8274(	ENSGALG00010025585	SARNP	case	control	-0.078587222	0.064286562	-1.222451776	0.22153683	0.645689578	down_in_case
PRJNA8274(	ENSGALG00010025586	TCTN1	case	control	-0.061667538	0.052704188	-1.170069034	0.241973189	0.666243691	down_in_case
PRJNA8274(	ENSGALG00010025587	LRRC56	case	control	0.268985295	0.166020563	1.620192645	0.105190901	0.495041148	up_in_case
PRJNA8274(	ENSGALG00010025588	ENSGALG00010025588	case	control	-0.235773259	0.263120486	-0.896065762	0.370217642	0.772700698	down_in_case
PRJNA8274(	ENSGALG00010025590	HRAS	case	control	-0.133600505	0.089038302	-1.500483524	0.133489198	0.537206146	down_in_case
PRJNA8274(	ENSGALG00010025591	OTOS	case	control	-0.169204614	0.274520725	-0.616363715	0.537654496	0.857704071	down_in_case
PRJNA8274(	ENSGALG00010025592	HSPA8	case	control	0.341044461	0.149128964	2.286909606	0.0222011	0.26563754	up_in_case
PRJNA8274(	ENSGALG00010025594	UPK1B	case	control	0.12756175	0.322563015	0.39546304	0.692501193	0.918988424	up_in_case
PRJNA8274(	ENSGALG00010025595	ERI3	case	control	-0.066530507	0.086094454	-0.772761827	0.439663348	0.812401646	down_in_case
PRJNA8274(	ENSGALG00010025597	SCN2A	case	control	0.617304312	0.593410584	1.040265086	0.29821676	0.721487387	up_in_case
PRJNA8274(	ENSGALG00010025598	ENSGALG00010025598	case	control	0.224421773	0.329164923	0.681791277	0.495370941	0.838118945	up_in_case
PRJNA8274(	ENSGALG00010025599	COPS9	case	control	-0.122334599	0.082395572	-1.484722982	0.137617221	0.539854583	down_in_case
PRJNA8274(	ENSGALG00010025600	ENSGALG00010025600	case	control	0.090277831	0.049202198	1.834833277	0.066530396	0.417640328	up_in_case
PRJNA8274(	ENSGALG00010025604	HVCN1	case	control	-0.008802561	0.105835785	-0.083171875	0.933714876	0.988101632	down_in_case
PRJNA8274(	ENSGALG00010025606	CCDC12	case	control	-0.055703002	0.058792152	-0.947456431	0.343406248	0.755145216	down_in_case
PRJNA8274(	ENSGALG00010025608	ENSGALG00010025608	case	control	-1.033264862	0.514078592	-2.009935595	0.044438006	0.350320113	down_in_case
PRJNA8274(	ENSGALG00010025610	RNH1	case	control	0.033203089	0.071898006	0.461808199	0.644218871	0.90210829	up_in_case
PRJNA8274(	ENSGALG00010025612	TMEM53	case	control	0.11468388	0.091568157	1.252442812	0.210408555	0.636118886	up_in_case
PRJNA8274(	ENSGALG00010025613	CDKN1A	case	control	0.444812942	0.158142172	2.812740824	0.004912123	0.120074107	up_in_case
PRJNA8274(	ENSGALG00010025615	AQP5	case	control	0.196221181	0.650503384	0.301645134	0.762922598	0.943786458	up_in_case
PRJNA8274(	ENSGALG00010025618	ADIPOQ	case	control	-0.083010614	0.138266864	-0.600365207	0.54826287	0.86339387	down_in_case
PRJNA8274(	ENSGALG00010025621	PPP1CC	case	control	-0.082983134	0.030292454	-2.739399522	0.006155153	0.136041239	down_in_case
PRJNA8274(	ENSGALG00010025623	ENSGALG00010025623	case	control	0.168130594	0.379009366	0.443605382	0.657327914	0.908057682	up_in_case
PRJNA8274(	ENSGALG00010025624	ARMH1	case	control	0.326036441	0.2615066	1.246761806	0.212484849	0.637945273	up_in_case
PRJNA8274(	ENSGALG00010025628	ENSGALG00010025628	case	control	-0.313356404	0.16441123	-1.90593066	0.056659203	0.387381529	down_in_case
PRJNA8274(	ENSGALG00010025629	ST6GAL1	case	control	-0.018960836	0.076158177	-0.248966517	0.803386682	0.954891353	down_in_case
PRJNA8274(	ENSGALG00010025635	CHKA	case	control	0.105459641	0.086691559	1.216492609	0.223797325	0.647995977	up_in_case
PRJNA8274(	ENSGALG00010025637	C6orf89	case	control	-0.003486101	0.055700632	-0.062586388	0.950095869	0.991488793	down_in_case
PRJNA8274(	ENSGALG00010025639	MYL2	case	control	0.239333017	0.251743088	0.950703429	0.341754947	0.753483771	up_in_case
PRJNA8274(	ENSGALG00010025640	SLC38A11	case	control	4.98799647	0.467597745	10.66728086	1.44802E-26	1.88242E-23	up_in_case
PRJNA8274(	ENSGALG00010025642	RPS8	case	control	-0.113091761	0.06226543	-1.816284904	0.06932668	0.425095479	down_in_case
PRJNA8274(	ENSGALG00010025644	ENSGALG00010025644	case	control	0.193650165	0.263323142	0.735408834	0.462090543	0.824955651	up_in_case
PRJNA8274(	ENSGALG00010025645	NBEAL2	case	control	0.047621464	0.082759751	0.575418162	0.565008524	0.872623598	up_in_case
PRJNA8274(	ENSGALG00010025647	JHY	case	control	-0.295750411	0.277919684	-1.064157843	0.287257201	0.711057292	down_in_case
PRJNA8274(	ENSGALG00010025648	ENSGALG00010025648	case	control	0.643609153	0.450250993	1.429445271	0.152876294	0.561193576	up_in_case
PRJNA8274(	ENSGALG00010025650	PPP1R1A	case	control	-0.252538745	0.25075977	-1.007094343	0.313889439	0.735477467	down_in_case
PRJNA8274(	ENSGALG00010025652	KMT5B	case	control	-0.007828973	0.062321978	-0.125621384	0.900031635	0.98028579	down_in_case
PRJNA8274(	ENSGALG00010025653	IGSF11	case	control	0.047479114	0.123543606	0.384310572	0.700748274	0.922516526	up_in_case
PRJNA8274(	ENSGALG00010025658	PDE1B	case	control	0.080783676	0.328903199	0.245615356	0.805980001	0.9556344	up_in_case
PRJNA8274(	ENSGALG00010025659	COBLL1	case	control	0.007895826	0.091171431	0.086604165	0.930986155	0.987258715	up_in_case
PRJNA8274(	ENSGALG00010025660	KIF2C	case	control	-0.057566621	0.114205205	-0.504063026	0.614217088	0.890440426	down_in_case
PRJNA8274(	ENSGALG00010025661	SNRPE	case	control	-0.309271916	0.086485609	-3.575992802	0.000348901	0.025231323	down_in_case
PRJNA8274(	ENSGALG00010025666	CUX2	case	control	0.1062744	0.216967343	0.489817492	0.624263051	0.894395515	up_in_case
PRJNA8274(	ENSGALG00010025674	GRB14	case	control	-0.693060826	0.326735954	-2.121164865	0.033907931	0.31753989	down_in_case
PRJNA8274(	ENSGALG00010025679	ENSGALG00010025679	case	control	-0.257174782	1.724004969	-0.149172877			down_in_case
PRJNA8274(	ENSGALG00010025685	NCKAP1L	case	control	0.074219602	0.109107758	0.680241287	0.496351694	0.83858637	up_in_case
PRJNA8274(	ENSGALG00010025688	BEST4	case	control	0.700586546	0.388087141	1.805229993	0.071038699	0.431543501	up_in_case
PRJNA8274(	ENSGALG00010025691	ENSGALG00010025691	case	control	0.033952304	0.12106984	0.280435688	0.77914326	0.948637601	up_in_case
PRJNA8274(	ENSGALG00010025695	ENSGALG00010025695	case	control	-0.226671767	1.405812353	-0.161238992	0.871905171	0.975062028	down_in_case
PRJNA8274(	ENSGALG00010025696	MTCH1	case	control	-0.010770475	0.072144243	-0.149290841	0.881324142	0.97667824	down_in_case
PRJNA8274(	ENSGALG00010025701	ENSGALG00010025701	case	control	-0.24784563	2.61747917	-0.094688673	0.924562115	0.985571367	down_in_case
PRJNA8274(	ENSGALG00010025702	GTSF2	case	control	0.205489741	0.197258425	1.041728591	0.297537529	0.721150283	up_in_case
PRJNA8274(	ENSGALG00010025703	UBASH3B	case	control	-0.176740724	0.077749491	-2.273207491	0.023013679	0.267340058	down_in_case
PRJNA8274(	ENSGALG00010025705	ACSL3	case	control	0.0089158	0.079392205	0.112300703	0.910584984	0.981855322	up_in_case
PRJNA8274(	ENSGALG00010025706	ENSGALG00010025706	case	control	-0.732606713	1.43449561	-0.510706835	0.609556354	0.888004876	down_in_case
PRJNA8274(	ENSGALG00010025708	HOXC8	case	control	0.419432692	0.23292291	1.800736099	0.071744485	0.432759285	up_in_case
PRJNA8274(	ENSGALG00010025710	ENSGALG00010025710	case	control	-1.960516215	1.813066801	-1.081325969			down_in_case
PRJNA8274(	ENSGALG00010025719	HOXC11	case	control	-0.038586933	1.044381531	-0.036947161	0.970527136	0.992761697	down_in_case
PRJNA8274(	ENSGALG00010025720	PHETA1	case	control	0.222039054	0.191990012	1.156513571	0.247471152	0.672031667	up_in_case
PRJNA8274(	ENSGALG00010025723	SETD2	case	control	-0.082509633	0.05308396	-1.554323256	0.120107329	0.518266386	down_in_case
PRJNA8274(	ENSGALG00010025727	GLI1	case	control	0.106091691	0.114583735	0.92588788	0.354504251	0.763161644	up_in_case
PRJNA8274(	ENSGALG00010025734	PLK3	case	control	0.183269569	0.11480511	1.596353761	0.110409833	0.503054901	up_in_case
PRJNA8274(	ENSGALG00010025736	KIF9	case	control	-0.264701138	0.260202039	-1.017290791	0.309015116	0.731849981	down_in_case
PRJNA8274(	ENSGALG00010025737	ENSGALG00010025737	case	control	0.139613118	0.114680582	1.217408516	0.223448824	0.647349714	up_in_case
PRJNA8274(	ENSGALG00010025738	SOX13	case	control	-0.17224516	0.07794892	-2.209718361	0.027124715	0.292866927	down_in_case
PRJNA8274(	ENSGALG00010025743	SH2B3	case	control	0.079712843	0.06223544	1.280827178	0.200254376	0.622902456	up_in_case
PRJNA8274(	ENSGALG00010025745	COX14	case	control	-0.151669704	0.204295082	-0.742405066	0.457841951	0.823337749	down_in_case
PRJNA8274(	ENSGALG00010025748	ETNK2	case	control	-0.187932345	0.128650739	-1.460794917	0.144071731	0.547495551	down_in_case
PRJNA8274(	ENSGALG00010025753	SORL1	case	control	0.11094409	0.117621399	0.943230497	0.345563018	0.755935621	up_in_case
PRJNA8274(	ENSGALG00010025755	ENSGALG00010025755	case	control	0.138301617	0.266269271	0.519405101	0.603478275	0.885268908	up_in_case
PRJNA8274(	ENSGALG00010025762	ENSGALG00010025762	case	control	0.166940476	2.300339361	0.072572108			up_in_case
PRJNA8274(	ENSGALG00010025782	ENSGALG00010025782	case	control	-0.286850033	2.944325656	-0.097424697			down_in_case
PRJNA8274(	ENSGALG00010025784	ENSGALG00010025784	case	control	-2.233559192	2.946416024	-0.758059681			down_in_case
PRJNA8274(	ENSGALG00010025787	KCNE4	case	control	-0.074005551	0.116509687	-0.635187969	0.525305829	0.85251736	down_in_case
PRJNA8274(	ENSGALG00010025790	LRP5	case	control	0.03988416	0.068994299	0.578079073	0.563210735	0.871916948	up_in_case
PRJNA8274(	ENSGALG00010025791	FIGN	case	control	-0.255319834	0.124715793	-2.047213335	0.040637143	0.337749022	down_in_case
PRJNA8274(	ENSGALG00010025794	ENSGALG00010025794	case	control	-0.263221576	2.948353109	-0.089277494			down_in_case
PRJNA8274(	ENSGALG00010025799	SC5D	case	control	0.089580926	0.089180326	1.004492028	0.315141512	0.736508113	up_in_case
PRJNA8274(	ENSGALG00010025800	ARL2BP	case	control	0.161041946	0.157316453	1.023681518	0.305985728	0.728933908	up_in_case
PRJNA8274(	ENSGALG00010025801	PTPN7	case	control	0.246258167	0.453651411	0.542835668	0.587242953	0.882747212	up_in_case
PRJNA8274(	ENSGALG00010025804	ENSGALG00010025804	case	control	-0.217499181	2.395392331	-0.09079898			down_in_case
PRJNA8274(	ENSGALG00010025805	ATXN2	case	control	-0.138984065	0.050616719	-2.74581338	0.006036109	0.134449153	down_in_case
PRJNA8274(	ENSGALG00010025809	ENSGALG00010025809	case	control	-0.017606738	0.128964736	-0.136523664	0.891407319	0.978917427	down_in_case
PRJNA8274(	ENSGALG00010025822	KLF7	case	control	0.101000381	0.09258288	1.090918543	0.275308727	0.700894634	up_in_case
PRJNA8274(	ENSGALG00010025829	ENSGALG00010025829	case	control	0.184667904	0.087918715	2.100439073	0.035690235	0.324939791	up_in_case
PRJNA8274(	ENSGALG00010025830	TBCEL	case	control	0.179263068	0.12926502	1.386787151	0.165506676	0.580618526	up_in_case
PRJNA8274(	ENSGALG00010025831	BRAP	case	control	-0.033873566	0.043439752	-0.779782675	0.435518806	0.81000376	down_in_case
PRJNA8274(	ENSGALG00010025832	ENKD1	case	control	-0.14274823	0.122773897	-1.162692021	0.24495448	0.669469819	down_in_case
PRJNA8274(	ENSGALG00010025835	PPP6R3	case	control	0.09318659	0.038740926	2.405378472	0.016155724	0.225671894	up_in_case
PRJNA8274(	ENSGALG00010025836	ENSGALG00010025836	case	control	0.08431865	0.072484687	1.163261559	0.244723397	0.669128983	up_in_case
PRJNA8274(	ENSGALG00010025837	ENSGALG00010025837	case	control	-0.132482163	0.174229613	-0.760388321	0.447022502	0.817833512	down_in_case
PRJNA8274(	ENSGALG00010025846	GRIK4	case	control	-0.069400871	0.227067782	-0.305639444	0.759879185	0.942675708	down_in_case
PRJNA8274(	ENSGALG00010025847	DEF6	case	control	-0.128701456	0.084087401	-1.530567662	0.125876279	0.525491114	down_in_case
PRJNA8274(	ENSGALG00010025849	OBSCN	case	control	-0.269800164	0.129998371	-2.075411887	0.037948377	0.330206289	down_in_case
PRJNA8274(	ENSGALG00010025852	BTBD19	case	control	-0.115691386	0.13741435	-0.841916339	0.399834784	0.789900496	down_in_case
PRJNA8274(	ENSGALG00010025858	ENSGALG00010025858	case	control	-0.170824127	1.639093688	-0.104218647	0.916995836	0.983445506	down_in_case
PRJNA8274(	ENSGALG00010025862	PTCH2	case	control	0.11133752	0.235899557	0.471970025	0.636948184	0.899286272	up_in_case
PRJNA8274(	ENSGALG00010025865	LGR6	case	control	0.247686685	0.410875392	0.602826768	0.546623937	0.86246958	up_in_case

PRJNA8274(	ENSGALG00010025869	FASTKD2	case	control	0.077935794	0.059805767	1.303148481	0.192524075	0.615090794	up_in_case
PRJNA8274(	ENSGALG00010025870	ENSGALG00010025870	case	control	-0.302497308	0.132763289	-2.278471033	0.022698528	0.267253668	down_in_case
PRJNA8274(	ENSGALG00010025871	ENSGALG00010025871	case	control	-0.145952837	2.092548016	-0.069748859			down_in_case
PRJNA8274(	ENSGALG00010025874	MDH1B	case	control	-0.143195486	0.226367373	-0.632580056	0.527007915	0.852705723	down_in_case
PRJNA8274(	ENSGALG00010025876	ENSGALG00010025876	case	control	-0.184804022	1.569298014	-0.117762223	0.906256063	0.981730793	down_in_case
PRJNA8274(	ENSGALG00010025877	ENSGALG00010025877	case	control	-0.306965939	2.705854818	-0.113445088	0.909677695	0.981730793	down_in_case
PRJNA8274(	ENSGALG00010025881	GAL	case	control	-0.161790421	0.74976713	-0.215787562	0.829153344	0.962142561	down_in_case
PRJNA8274(	ENSGALG00010025882	LDHD	case	control	-0.156124857	0.107112628	-1.457576567	0.144957292	0.548818979	down_in_case
PRJNA8274(	ENSGALG00010025886	ALDH2	case	control	0.051820503	0.064717168	0.800722661	0.423292221	0.803723282	up_in_case
PRJNA8274(	ENSGALG00010025887	ZNF76	case	control	-0.295588916	0.118812101	-2.487868772	0.012851114	0.199684901	down_in_case
PRJNA8274(	ENSGALG00010025890	SCG2	case	control	-1.197425706	0.371459226	-3.223572392	0.001266023	0.056224	down_in_case
PRJNA8274(	ENSGALG00010025891	NUP93	case	control	-0.152946484	0.082278055	-1.858897657	0.063041646	0.40876339	down_in_case
PRJNA8274(	ENSGALG00010025892	ZC3H11A	case	control	0.026690089	0.060539721	0.440869048	0.659307802	0.908599575	up_in_case
PRJNA8274(	ENSGALG00010025893	ENSGALG00010025893	case	control	0.77382917	1.554859925	0.497684169	0.618706668	0.891656847	up_in_case
PRJNA8274(	ENSGALG00010025897	MAPKAPK5	case	control	0.010338036	0.068496618	0.150927681	0.880032764	0.976372762	up_in_case
PRJNA8274(	ENSGALG00010025898	ENSGALG00010025898	case	control	-0.550283538	1.370027852	-0.40165865	0.68793526	0.917290381	down_in_case
PRJNA8274(	ENSGALG00010025899	ENSGALG00010025899	case	control	-0.045079367	0.140966925	-0.319786838	0.749129926	0.939503405	down_in_case
PRJNA8274(	ENSGALG00010025900	FAM237A	case	control	-1.025506222	0.522407904	-1.96303734	0.049641835	0.364833243	down_in_case
PRJNA8274(	ENSGALG00010025902	ENSGALG00010025902	case	control	-0.078128374	2.277717906	-0.034301163	0.972636997	0.99340857	down_in_case
PRJNA8274(	ENSGALG00010025904	RPL10A	case	control	-0.081094356	0.066517437	-1.219144328	0.222789418	0.646365317	down_in_case
PRJNA8274(	ENSGALG00010025905	CPT1A	case	control	-0.032684906	0.111978483	-0.291885593	0.770374099	0.945447101	down_in_case
PRJNA8274(	ENSGALG00010025907	ARHGEF12	case	control	-0.104796358	0.05145284	-2.036745845	0.041675518	0.340995489	down_in_case
PRJNA8274(	ENSGALG00010025910	HEATR3	case	control	-0.019459912	0.068557944	-0.283846206	0.776528243	0.947228003	down_in_case
PRJNA8274(	ENSGALG00010025913	EIF2B3	case	control	-0.049336737	0.053861776	-0.91598793	0.359673226	0.765570668	down_in_case
PRJNA8274(	ENSGALG00010025914	ENSGALG00010025914	case	control	1.680531503	0.829509442	2.025934145	0.042771534	0.344633831	up_in_case
PRJNA8274(	ENSGALG00010025917	SCUBE3	case	control	0.19004618	0.236132598	0.804828227	0.420918811	0.802017188	up_in_case
PRJNA8274(	ENSGALG00010025919	RGS9BP	case	control	0.525732228	0.308953058	1.701657304	0.088819628	0.470938334	up_in_case
PRJNA8274(	ENSGALG00010025920	GTSF1	case	control	0.625963504	0.433488576	1.444013843	0.148735081	0.55519893	up_in_case
PRJNA8274(	ENSGALG00010025924	TMEM116	case	control	0.398056186	0.121928858	3.264659342	0.001095958	0.051553287	up_in_case
PRJNA8274(	ENSGALG00010025925	KLHL18	case	control	-0.057063297	0.056701456	-1.006381524	0.314232078	0.736039101	down_in_case
PRJNA8274(	ENSGALG00010025926	ENSGALG00010025926	case	control	-0.214639281	0.462904226	-0.463679675	0.642877261	0.902038523	down_in_case
PRJNA8274(	ENSGALG00010025927	ENSGALG00010025927	case	control	0.279909118	1.368399351	0.204552215	0.837921993	0.964826648	up_in_case
PRJNA8274(	ENSGALG00010025929	NELFE	case	control	0.012542885	0.062392663	0.201031402	0.84067402	0.965671017	up_in_case
PRJNA8274(	ENSGALG00010025931	TMEM136	case	control	0.043118671	0.129607352	0.332686922	0.739370622	0.936326594	up_in_case
PRJNA8274(	ENSGALG00010025932	AP1S3	case	control	0.323426049	0.330837873	0.977596808	0.328273777	0.745445783	up_in_case
PRJNA8274(	ENSGALG00010025933	SKIC2	case	control	-0.052056252	0.086364154	-0.602752987	0.546673026	0.86246958	down_in_case
PRJNA8274(	ENSGALG00010025935	CATH3	case	control	0.16973094	0.307584914	0.551818157	0.581072946	0.879651411	up_in_case
PRJNA8274(	ENSGALG00010025936	GOSR2	case	control	0.11420327	0.05700428	2.003415699	0.045132688	0.352291176	up_in_case
PRJNA8274(	ENSGALG00010025938	ERP29	case	control	0.12541953	0.055093312	2.276492836	0.022816529	0.267253668	up_in_case
PRJNA8274(	ENSGALG00010025939	TMEM136-1	case	control	0.076719363	0.595595795	0.128811122	0.897507105	0.980176778	up_in_case
PRJNA8274(	ENSGALG00010025940	HECTD3	case	control	-0.098876714	0.048306478	-2.046862413	0.040671596	0.337749022	down_in_case
PRJNA8274(	ENSGALG00010025941	ENSGALG00010025941	case	control	0.659929588	0.423181207	1.559449186	0.118890103	0.516128861	up_in_case
PRJNA8274(	ENSGALG00010025942	ENSGALG00010025942	case	control	0.819908799	1.164093763	0.704332267	0.481225876	0.832308906	up_in_case
PRJNA8274(	ENSGALG00010025943	CATH1	case	control	0.070334422	0.402194673	0.174876564	0.861176626	0.972434239	up_in_case
PRJNA8274(	ENSGALG00010025946	KRT5	case	control	0.807188212	0.876647966	0.920766651	0.35717227	0.763410721	up_in_case
PRJNA8274(	ENSGALG00010025948	WNT9B	case	control	-0.868834509	0.723087137	-1.201562667	0.229533013	0.653695091	down_in_case
PRJNA8274(	ENSGALG00010025949	GPATCH1	case	control	0.025614558	0.079456391	0.322372528	0.747170493	0.939021817	up_in_case
PRJNA8274(	ENSGALG00010025951	TBRG4	case	control	-0.047942599	0.06363248	-0.753429679	0.451191753	0.820140088	down_in_case
PRJNA8274(	ENSGALG00010025953	POU2F3	case	control	-0.077846571	0.763062117	-0.102018655	0.918741867	0.983734684	down_in_case
PRJNA8274(	ENSGALG00010025954	TUBA1A	case	control	-0.024963215	0.086147241	-0.289773812	0.771989284	0.945872699	down_in_case
PRJNA8274(	ENSGALG00010025957	ENSGALG00010025957	case	control	0.100088625	0.068866862	1.453364107	0.146122687	0.551205689	up_in_case
PRJNA8274(	ENSGALG00010025958	HOXC9	case	control	0.512968233	0.347497408	1.476178588	0.139895956	0.542025835	up_in_case
PRJNA8274(	ENSGALG00010025959	UROD	case	control	-0.144082243	0.066311072	-2.172823289	0.02979362	0.305970455	down_in_case
PRJNA8274(	ENSGALG00010025960	CMKLR2	case	control	-0.118098246	0.06929007	-1.704403606	0.088305711	0.46960642	down_in_case
PRJNA8274(	ENSGALG00010025962	ENSGALG00010025962	case	control	0.087046593	0.13596924	0.640193275	0.522046955	0.850840147	up_in_case
PRJNA8274(	ENSGALG00010025963	OAF	case	control	0.272742763	0.069149928	3.944223374	8.0059E-05	0.009232611	up_in_case
PRJNA8274(	ENSGALG00010025964	HOXC10	case	control	1.040069345	1.075084236	0.96743056	0.333328839	0.748508541	up_in_case
PRJNA8274(	ENSGALG00010025968	FADS2	case	control	0.103118729	0.119713421	0.861379855	0.389028868	0.783669208	up_in_case
PRJNA8274(	ENSGALG00010025970	KLHL12	case	control	0.000321259	0.057930425	0.005545596	0.995575277	0.998710499	up_in_case
PRJNA8274(	ENSGALG00010025971	TRAFD1	case	control	0.153719082	0.080017556	1.921066958	0.054723267	0.382287597	up_in_case
PRJNA8274(	ENSGALG00010025972	WNT3	case	control	-0.049361219	1.341060582	-0.036807598	0.970638416	0.992761697	down_in_case
PRJNA8274(	ENSGALG00010025974	EEF1B2	case	control	-0.051229973	0.067436845	-0.759673337	0.44744987	0.818077884	down_in_case
PRJNA8274(	ENSGALG00010025976	NAA25	case	control	0.087125573	0.054356758	1.602847134	0.108968408	0.501108679	up_in_case
PRJNA8274(	ENSGALG00010025978	TRIM29	case	control	0.435872905	0.825356559	0.528102553	0.597428159	0.884680373	up_in_case
PRJNA8274(	ENSGALG00010025980	HECTD4	case	control	-0.146014795	0.048518149	-3.009488159	0.002616883	0.085048691	down_in_case
PRJNA8274(	ENSGALG00010025982	SERPINE2	case	control	0.177482177	0.080686425	2.199653513	0.027831487	0.297674098	up_in_case
PRJNA8274(	ENSGALG00010025983	RNF26	case	control	-0.055883057	0.061814076	-0.904050675	0.365968531	0.771585756	down_in_case
PRJNA8274(	ENSGALG00010025984	NDUFS1	case	control	-0.202432449	0.079662977	-2.541110769	0.011050091	0.185137791	down_in_case
PRJNA8274(	ENSGALG00010025985	CHCHD10	case	control	0.077845219	0.172770052	0.450571256	0.652298587	0.905706359	up_in_case
PRJNA8274(	ENSGALG00010025986	RAB3IL1	case	control	0.061960117	0.052372258	1.183071343	0.236780874	0.661744512	up_in_case
PRJNA8274(	ENSGALG00010025988	NSF	case	control	-0.031546036	0.046236125	-0.68228113	0.495061202	0.838118945	down_in_case
PRJNA8274(	ENSGALG00010025990	FAM124B	case	control	0.064782654	0.217592267	0.297724985	0.765913073	0.943860474	up_in_case
PRJNA8274(	ENSGALG00010025992	MMP11	case	control	-0.22155298	0.186518042	-1.187836724	0.234897743	0.659540098	down_in_case
PRJNA8274(	ENSGALG00010025994	INO80D	case	control	-0.011569089	0.089888331	-0.128705125	0.89759098	0.980176778	down_in_case
PRJNA8274(	ENSGALG00010025996	CUL3	case	control	-0.175158937	0.065699964	-2.666043138	0.007674984	0.152645726	down_in_case
PRJNA8274(	ENSGALG00010025997	CCDC43	case	control	0.002260605	0.067600554	0.033440634	0.973323207	0.99354775	up_in_case
PRJNA8274(	ENSGALG00010025998	SMARCB1	case	control	0.024179266	0.042989444	0.562446596	0.573811779	0.877573415	up_in_case
PRJNA8274(	ENSGALG00010025999	DBF4B	case	control	-0.217699643	0.133789752	-1.62717726	0.103699456	0.493478276	down_in_case
PRJNA8274(	ENSGALG00010026001	ANKS1A	case	control	0.014049064	0.068766047	0.204302334	0.838117246	0.964826648	up_in_case
PRJNA8274(	ENSGALG00010026002	FTH1	case	control	0.086420588	0.08034294	1.075646328	0.28208546	0.706000864	up_in_case
PRJNA8274(	ENSGALG00010026003	NECTIN1	case	control	0.15013564	0.08778394	1.710285951	0.087213008	0.467042649	up_in_case
PRJNA8274(	ENSGALG00010026005	DERL3	case	control	0.147254702	0.091069819	1.616942965	0.105890593	0.495266487	up_in_case
PRJNA8274(	ENSGALG00010026006	ITGA2B	case	control	0.327035735	0.185567572	1.762353911	0.07800952	0.448028441	up_in_case
PRJNA8274(	ENSGALG00010026008	NRP2	case	control	-0.167586062	0.09823006	-1.706056806	0.087997504	0.468839159	down_in_case
PRJNA8274(	ENSGALG00010026009	ENSGALG00010026009	case	control	0.047858062	0.170478529	0.2807278	0.778919184	0.948479876	up_in_case
PRJNA8274(	ENSGALG00010026010	ZSWIM5	case	control	-0.160946645	0.082270554	-1.95630924	0.050428736	0.367061596	down_in_case
PRJNA8274(	ENSGALG00010026011	GJC2	case	control	0.002817682	0.075158418	0.03748991	0.970094385	0.992761697	up_in_case
PRJNA8274(	ENSGALG00010026013	RABIF	case	control	-0.048088634	0.063992675	-0.751470916	0.452369298	0.820911365	down_in_case
PRJNA8274(	ENSGALG00010026014	SOX14	case	control	-0.487419958	0.716103195	-0.68065603	0.496089164	0.838518426	down_in_case
PRJNA8274(	ENSGALG00010026016	SLC2A11	case	control	0.615207625	0.354343207	1.736191391	0.082529999	0.456372384	up_in_case
PRJNA8274(	ENSGALG00010026017	PLEKHM1	case	control	-0.090807364	0.074260075	-1.222828884	0.221394334	0.645689578	down_in_case
PRJNA8274(	ENSGALG00010026018	FOXL2	case	control	-0.364566146	0.618674858	-0.589269374	0.555680586	0.867878646	down_in_case
PRJNA8274(	ENSGALG00010026023	THY1	case	control	-0.024317418	0.188140847	-0.129251133	0.897158937	0.980176778	down_in_case
PRJNA8274(	ENSGALG00010026030	IBA57	case	control	-0.061264832	0.102600039	-0.597122892	0.550425335	0.86456492	down_in_case
PRJNA8274(	ENSGALG00010026036	TUB5A	case	control	0.040541364	0.069243845	0.585486891	0.558220378	0.869085619	up_in_case
PRJNA8274(	ENSGALG00010026040	GUK1	case	control	-0.00496086	0.101463043	-0.048893266	0.961004355	0.992056045	down_in_case
PRJNA8274(	ENSGALG00010026044	CERKL	case	control	0.028986728	0.192819783	0.150330673	0.880503734	0.976399289	up_in_case
PRJNA8274(	ENSGALG00010026051	TAF11	case	control	0.014468354	0.063863139	0.226552508	0.820771725	0.958669267	up_in_case
PRJNA8274(	ENSGALG00010026059	ENSGALG00010026059	case	control	1.090331383	0.676863958	1.6108575	0.107210785	0.498409064	up_in_case
PRJNA8274(	ENSGALG00010026061	MPEG1	case	control	-0.076297511	0.137670748	-0.554202778	0.579440077	0.879142433	down_in_case
PRJNA8274(	ENSGALG00010026062	ITGA4	case	control	0.056010089	0.100166279	0.559171103	0.576044952	0.877910508	up_in_case
PRJNA8274(	ENSGALG00010026064	ENSGALG00010026064	case	control	0.068318622	0.077616807	0.880203963	0.378748827	0.777229179	up_in_case
PRJNA8274(	ENSGALG00010026066	SELENOP2	case	control	0.798785064	0.509365495	1.56819626	0.116835337	0.51350396	up_in_case
PRJNA8274(	ENSGALG00010026068	BLTP3A	case	control	-0.144021717	0.050544003	-2.849432359	0.004379732	0.114268826	down_in_case
PRJNA8274(	ENSGALG00010026073	HPDL	case	control	0.009472562	0.210196435	0.045065285	0.964055272	0.992068146	up_in_case
PRJNA8274(	ENSGALG00010026076	C1orf35	case	control	-0.045271434	0.114068874	-0.396878066	0.69145738	0.918812261	down_in_case
PRJNA8274(	ENSGALG00010026078	UBE2E3	case	control	-0.078224285	0.052203874	-1.498438327	0.134019405	0.537883101	down_in_case
PRJNA8274(	ENSGALG00010026081	KPNA1	case	control	0.025387877	0.048047611	0.528389994	0.597228681	0.884680373	up_in_case
PRJNA8274(	ENSGALG00010026083	MUTYH	case	control	0.159952249	0.150779582	1.06083494	0.28876492	0.712801679	up_in_case
PRJNA8274(	ENSGALG00010026088	SNRPC	case	control	-0.077437184	0.062549464	-1.238015154	0.215710442	0.640470741	down_in_case
PRJNA8274(	ENSGALG00010026090	USP2	case	control	-0.200134647	0.104577969	-1.91373621	0.05565387	0.384148446	down_in_case
PRJNA8274(	ENSGALG00010026091	DTX4	case	control	-0.103700951	0.062566708	-1.657446172	0.097429303	0.485860997	down_in_case
PRJNA8274(	ENSGALG00010026093	TOE1	case	control	0.050683644	0.065911463	0.768965553	0.44191376	0.813507909	up_in_case
PRJNA8274(	ENSGALG00010026094	C6orf106	case	control	-0.099359663	0.047285592	-2.101267204	0.035617517	0.324939791	down_in_case
PRJNA8274(	ENSGALG00010026095	MIF	case	control	0.351256921	0.126951749	2.766853741	0.005660014	0.13033527	up_in_case
PRJNA8274(	ENSGALG00010026097	ARF1	case	control	0.028861269	0.027311227	1.056754767	0.290623523	0.714060795	up_in_case
PRJNA8274(	ENSGALG00010026098	STAT3	case	control	-0.015242457	0.038531014	-0.395589294	0.692408036	0.918988424	down_in_case
PRJNA8274(	ENSGALG00010026102	IPO9	case	control	-0.01908162	0.041151713	-0.463689559	0.642870179	0.902038523	down_in_case
PRJNA8274(	ENSGALG00010026104	DDX51	case	control	0.097118436	0.096956721	1.001667915	0.316504008	0.737254816	up_in_case
PRJNA8274(	ENSGALG00010026105	CNTF	case	control	0.002509948	0.203924498	0.012308222	0.990179708	0.997363515	up_in_case
PRJNA8274(	ENSGALG00010026106	TESK2	case	control	0.0131828	0.09356596	0.140893122	0.887954376	0.977948171	up_in_case
PRJNA8274(	ENSGALG00010026109	SPDEF	case	control	-1.089786564	0.667061485	-1.633712317	0.102319271	0.491460867	down_in_case
PRJNA8274(	ENSGALG00010026111	ENSGALG00010026111	case	control	0.221366532	0.091493526	2.419477556	0.01554282	0.221376816	up_in_case
PRJNA8274(	ENSGALG00010026112	ZFP91	case	control	0.016749331	0.038580861	0.434135746	0.664189869	0.911435195	up_in_case
PRJNA8274(	ENSGALG00010026113	MFRP	case	control	-0.460246442	0.340731664	-1.350759233	0.176772571	0.595527443	down_in_case
PRJNA8274(	ENSGALG00010026114	CWC22	case	control	0.054144012	0.051396004	1.053467349	0.292126851	0.714677075	up_in_case

PRJNA8274(	ENSGALG00010026115	MMACHC	case	control	0.410939498	0.145984868	2.814945852	0.004878545	0.119457531	up_in_case
PRJNA8274(	ENSGALG00010026116	DLX3	case	control	0.359489554	0.51538868	0.697511543	0.485482718	0.834423421	up_in_case
PRJNA8274(	ENSGALG00010026117	LZTR1	case	control	-0.061623581	0.044659758	-1.37984584	0.167634115	0.583750784	down_in_case
PRJNA8274(	ENSGALG00010026120	C1QTNF5	case	control	0.062573881	0.118722124	0.527061674	0.59815076	0.884680373	up_in_case
PRJNA8274(	ENSGALG00010026121	ENSGALG00010026121	case	control	0.578619607	0.202051995	2.863716376	0.004187027	0.111848429	up_in_case
PRJNA8274(	ENSGALG00010026124	PACSIN1	case	control	0.277592281	0.145924512	1.902300556	0.057131873	0.388573099	up_in_case
PRJNA8274(	ENSGALG00010026127	ENSGALG00010026127	case	control	-0.888276454	0.382833958	-2.320265577	0.020326515	0.256096181	down_in_case
PRJNA8274(	ENSGALG00010026128	PRDX1	case	control	0.080583256	0.046403975	1.736559334	0.082464983	0.456372384	up_in_case
PRJNA8274(	ENSGALG00010026132	DDTL	case	control	0.199912722	0.111490574	1.793090797	0.072958407	0.436712105	up_in_case
PRJNA8274(	ENSGALG00010026137	LPXN	case	control	0.666036965	0.310052218	2.148144497	0.031702278	0.3098361	up_in_case
PRJNA8274(	ENSGALG00010026141	WNT3A	case	control	-0.200064931	1.741297818	-0.114894149	0.908529023	0.981730793	down_in_case
PRJNA8274(	ENSGALG00010026143	RNPEP	case	control	-0.015073804	0.06297266	-0.239370604	0.810818222	0.956022547	down_in_case
PRJNA8274(	ENSGALG00010026148	RPS10	case	control	-0.043385114	0.069446527	-0.624726912	0.532150307	0.855755691	down_in_case
PRJNA8274(	ENSGALG00010026149	LASP1	case	control	-0.030932482	0.046958648	-0.658717467	0.510077215	0.844850541	down_in_case
PRJNA8274(	ENSGALG00010026154	ZNF385B	case	control	0.072335204	0.136289299	0.530747491	0.595593772	0.884680373	up_in_case
PRJNA8274(	ENSGALG00010026162	MED1	case	control	-0.127884045	0.038746027	-3.300571784	0.00096488	0.04774321	down_in_case
PRJNA8274(	ENSGALG00010026163	CTNND1	case	control	-0.007310173	0.038270721	-0.191012162	0.848516065	0.968692766	down_in_case
PRJNA8274(	ENSGALG00010026166	WNT9A	case	control	-0.240663349	0.134967771	-1.783117157	0.074567234	0.43953481	down_in_case
PRJNA8274(	ENSGALG00010026167	CABIN1	case	control	-0.059801355	0.052953576	-1.129316644	0.258764282	0.686345498	down_in_case
PRJNA8274(	ENSGALG00010026173	CDK12	case	control	-0.059785986	0.047690642	-1.253620906	0.209979829	0.635494508	down_in_case
PRJNA8274(	ENSGALG00010026183	SESTD1	case	control	-0.071881178	0.038075651	-1.887851592	0.059045877	0.395497471	down_in_case
PRJNA8274(	ENSGALG00010026184	NUDT3	case	control	-0.109627528	0.071521674	-1.532787501	0.125328221	0.524339838	down_in_case
PRJNA8274(	ENSGALG00010026192	SMIM29	case	control	-0.182457068	0.170457034	-1.070399173	0.284439671	0.709107417	down_in_case
PRJNA8274(	ENSGALG00010026199	TBX6	case	control	0.224519464	0.42651341	0.526406575	0.598605748	0.88476024	up_in_case
PRJNA8274(	ENSGALG00010026203	TMX2	case	control	0.056753819	0.068702685	0.826078613	0.408759499	0.795170398	up_in_case
PRJNA8274(	ENSGALG00010026205	ENSGALG00010026205	case	control	0.630092859	0.730513084	0.862534666			up_in_case
PRJNA8274(	ENSGALG00010026209	MED19	case	control	-0.09979466	0.081074	-1.230908299	0.218357163	0.643443819	down_in_case
PRJNA8274(	ENSGALG00010026213	AKR1A1	case	control	0.163677008	0.061913227	2.643651707	0.0082017	0.159353687	up_in_case
PRJNA8274(	ENSGALG00010026218	FTSJ3	case	control	0.20486469	0.078134005	2.621965825	0.00874242	0.166357336	up_in_case
PRJNA8274(	ENSGALG00010026220	ENSGALG00010026220	case	control	-0.030916923	0.078677267	-0.392958777	0.694349928	0.920308089	down_in_case
PRJNA8274(	ENSGALG00010026221	CRKL	case	control	-0.025257475	0.063419513	-0.398260321	0.690438305	0.918784485	down_in_case
PRJNA8274(	ENSGALG00010026229	CRHR2	case	control	-0.056621744	0.120117387	-0.471386746	0.637364579	0.899378897	down_in_case
PRJNA8274(	ENSGALG00010026232	PSMC5	case	control	0.027493979	0.061182567	0.449376021	0.653160426	0.906111185	up_in_case
PRJNA8274(	ENSGALG00010026234	ZDHHC5	case	control	-0.096008692	0.079257772	-1.21134735	0.225762309	0.649498359	down_in_case
PRJNA8274(	ENSGALG00010026237	KLHL22	case	control	0.005589149	0.063286388	0.088315177	0.929626177	0.987129367	up_in_case
PRJNA8274(	ENSGALG00010026242	CCDC141	case	control	0.043371351	0.106940538	0.405565107	0.685062171	0.916578316	up_in_case
PRJNA8274(	ENSGALG00010026244	CLP1	case	control	0.061913584	0.072765766	0.850861433	0.394846331	0.786978937	up_in_case
PRJNA8274(	ENSGALG00010026252	ENO2	case	control	0.057761494	0.226426413	0.255100512	0.798645469	0.953626437	up_in_case
PRJNA8274(	ENSGALG00010026254	ENSGALG00010026254	case	control	0.163062329	0.445002915	0.366429799	0.714044387	0.926862121	up_in_case
PRJNA8274(	ENSGALG00010026259	MED15	case	control	0.007027884	0.052630451	0.133532661	0.893772135	0.979888889	up_in_case
PRJNA8274(	ENSGALG00010026261	IFT70A	case	control	-0.028945134	0.08369447	-0.345842846	0.729460823	0.933556014	down_in_case
PRJNA8274(	ENSGALG00010026262	NAV1	case	control	-0.069677431	0.098056791	-0.710582418	0.477343041	0.830520323	down_in_case
PRJNA8274(	ENSGALG00010026264	FZD7	case	control	-0.126469185	0.075544316	-1.674105907	0.094109787	0.479477093	down_in_case
PRJNA8274(	ENSGALG00010026266	ENSGALG00010026266	case	control	0.1249598	0.222128035	0.562557538	0.573736213	0.877573415	up_in_case
PRJNA8274(	ENSGALG00010026268	GARS1	case	control	-0.033646325	0.039171053	-0.858958919	0.390363181	0.783914628	down_in_case
PRJNA8274(	ENSGALG00010026272	LRRC58	case	control	-0.038772195	0.068339288	-0.567348541	0.570477404	0.875913068	down_in_case
PRJNA8274(	ENSGALG00010026277	SMARCD2	case	control	0.025579551	0.073543153	0.347816895	0.727977701	0.933057319	up_in_case
PRJNA8274(	ENSGALG00010026287	NASP	case	control	-0.003922124	0.068244286	-0.057471831	0.954169345	0.992056045	down_in_case
PRJNA8274(	ENSGALG00010026291	SERPING1	case	control	0.011998003	0.095756873	0.125296526	0.900288803	0.98028579	up_in_case
PRJNA8274(	ENSGALG00010026295	SMPD4	case	control	0.021465759	0.067488678	0.318064599	0.750435936	0.939972946	up_in_case
PRJNA8274(	ENSGALG00010026298	SNF8	case	control	-0.063502147	0.066123267	-0.960360093	0.336874015	0.750735231	down_in_case
PRJNA8274(	ENSGALG00010026300	HMGA1	case	control	0.006898576	0.071515931	0.096462091	0.92315358	0.985482967	up_in_case
PRJNA8274(	ENSGALG00010026305	ATP5MC1	case	control	-0.033029095	0.09258159	-0.356756613	0.721274019	0.929292592	down_in_case
PRJNA8274(	ENSGALG00010026315	SYT2	case	control	-0.153661952	0.239783576	-0.640836018	0.521629229	0.850450117	down_in_case
PRJNA8274(	ENSGALG00010026316	ENSGALG00010026316	case	control	-0.02437987	0.153902512	-0.158411122	0.87413285	0.975062028	down_in_case
PRJNA8274(	ENSGALG00010026320	ENSGALG00010026320	case	control	-0.008907198	0.06517181	-0.136672561	0.891289619	0.978917427	down_in_case
PRJNA8274(	ENSGALG00010026321	GPBP1L1	case	control	-0.034906003	0.038942295	-0.896351961	0.370064815	0.772544067	down_in_case
PRJNA8274(	ENSGALG00010026325	TMEM69	case	control	-0.024046854	0.067586146	-0.355795612	0.721993635	0.929484914	down_in_case
PRJNA8274(	ENSGALG00010026329	ENSGALG00010026329	case	control	-0.759966811	0.216217106	-3.514832042	0.000440032	0.029964097	down_in_case
PRJNA8274(	ENSGALG00010026334	ENSGALG00010026334	case	control	0.027308794	0.10796415	0.252943163	0.800312134	0.954053991	up_in_case
PRJNA8274(	ENSGALG00010026335	IPP	case	control	0.097376097	0.072913271	1.335505816	0.181710853	0.602744104	up_in_case
PRJNA8274(	ENSGALG00010026344	TIMM10	case	control	-0.005898078	0.120490336	-0.04895063	0.96095864	0.992056045	down_in_case
PRJNA8274(	ENSGALG00010026346	FZD2	case	control	-0.130472521	0.0909535	-1.434496984	0.151430489	0.559493525	down_in_case
PRJNA8274(	ENSGALG00010026354	ENSGALG00010026354	case	control	-0.509301867	0.17763081	-2.867193292	0.0041413	0.111526534	down_in_case
PRJNA8274(	ENSGALG00010026355	PPP1R12B	case	control	-0.20059027	0.089675456	-2.236846938	0.025296346	0.28282857	down_in_case
PRJNA8274(	ENSGALG00010026360	CTDSPL	case	control	-0.026183604	0.057502772	-0.455345073	0.648861004	0.903741342	down_in_case
PRJNA8274(	ENSGALG00010026362	ENSGALG00010026362	case	control	0.055399717	0.259378357	0.213586505	0.830869524	0.962228709	up_in_case
PRJNA8274(	ENSGALG00010026367	RBP	case	control	0.650810908	0.515681739	1.262039856	0.206934445	0.630817004	up_in_case
PRJNA8274(	ENSGALG00010026377	ENSGALG00010026377	case	control	-0.161567373	0.132789653	-1.216716586	0.223712066	0.647981071	down_in_case
PRJNA8274(	ENSGALG00010026388	SNRPD3	case	control	-0.117983362	0.059879178	-1.970357081	0.048797461	0.363542241	down_in_case
PRJNA8274(	ENSGALG00010026393	SLC43A3	case	control	-0.020895374	0.123480788	-0.169219637	0.865623884	0.973289701	down_in_case
PRJNA8274(	ENSGALG00010026402	TFEB	case	control	-0.181614206	0.090280176	-2.01167314	0.044254404	0.350201601	down_in_case
PRJNA8274(	ENSGALG00010026403	GUCD1	case	control	-0.195651115	0.074196757	-2.63692273	0.008366189	0.160801744	down_in_case
PRJNA8274(	ENSGALG00010026409	VILL	case	control	0.123480192	0.20405777	0.605123696	0.54509681	0.861997112	up_in_case
PRJNA8274(	ENSGALG00010026410	ENSGALG00010026410	case	control	-0.914019833	0.153789736	-5.943308406	2.79326E-09	1.47939E-06	down_in_case
PRJNA8274(	ENSGALG00010026416	ENSGALG00010026416	case	control	0.259905813	0.63068978	0.412097709	0.680267808	0.915381147	up_in_case
PRJNA8274(	ENSGALG00010026422	P2RX3	case	control	-0.054393177	0.383678164	-0.141767716	0.887263485	0.977630122	down_in_case
PRJNA8274(	ENSGALG00010026427	PGC	case	control	0.180628842	0.329555649	0.548098151	0.58362451	0.881307382	up_in_case
PRJNA8274(	ENSGALG00010026433	VPS11	case	control	-0.044674005	0.054541252	-0.819086533	0.412737045	0.797581687	down_in_case
PRJNA8274(	ENSGALG00010026434	ENSGALG00010026434	case	control	0.297766667	0.117310677	2.53827421	0.011140066	0.185137791	up_in_case
PRJNA8274(	ENSGALG00010026436	PLCD1	case	control	-0.025734814	0.064849587	-0.396838521	0.691486542	0.918812261	down_in_case
PRJNA8274(	ENSGALG00010026438	TOMM6	case	control	-0.137391194	0.058467241	-2.349883329	0.018779296	0.244798485	down_in_case
PRJNA8274(	ENSGALG00010026443	UPB1	case	control	0.546588841	0.398409221	1.371928188	0.170085794	0.586502739	up_in_case
PRJNA8274(	ENSGALG00010026448	ADORA2A	case	control	0.177852271	0.075856141	2.344599508	0.019047527	0.246324585	up_in_case
PRJNA8274(	ENSGALG00010026457	SSRP1	case	control	0.009390681	0.048655682	0.19300276	0.846956806	0.968026275	up_in_case
PRJNA8274(	ENSGALG00010026458	DLEC1	case	control	0.178213443	0.115970938	1.536707784	0.124364884	0.523029039	up_in_case
PRJNA8274(	ENSGALG00010026480	USP49	case	control	-0.058761994	0.182806402	-0.321443851	0.747874057	0.939324187	down_in_case
PRJNA8274(	ENSGALG00010026481	DIXDC1	case	control	-0.14075041	0.094635214	-1.487294254	0.136937111	0.539854583	down_in_case
PRJNA8274(	ENSGALG00010026484	TNKS1BP1	case	control	-0.005245133	0.071681822	-0.073172424	0.94166891	0.989551456	down_in_case
PRJNA8274(	ENSGALG00010026485	ACAA1	case	control	0.101473892	0.100076097	1.013967321	0.310598346	0.733587496	up_in_case
PRJNA8274(	ENSGALG00010026492	SPECC1L	case	control	-0.028723752	0.071511342	-0.401667079	0.687929056	0.917290381	down_in_case
PRJNA8274(	ENSGALG00010026498	ENSGALG00010026498	case	control	-0.084377007	0.053361105	-1.581245485	0.113821919	0.508641701	down_in_case
PRJNA8274(	ENSGALG00010026507	APLNR	case	control	0.006149746	0.099111139	0.062048993	0.950523816	0.991497374	up_in_case
PRJNA8274(	ENSGALG00010026508	CSRP1	case	control	-0.009231081	0.142311165	-0.064865474	0.948281111	0.991291133	down_in_case
PRJNA8274(	ENSGALG00010026516	ENSGALG00010026516	case	control	-0.063313669	0.070263392	-0.901090411	0.367540251	0.771770041	down_in_case
PRJNA8274(	ENSGALG00010026522	MED20	case	control	-0.140841954	0.090861262	-1.550077008	0.121123034	0.519499513	down_in_case
PRJNA8274(	ENSGALG00010026523	LRRC55	case	control	0.031885749	0.437967338	0.072803942	0.941962134	0.989551456	up_in_case
PRJNA8274(	ENSGALG00010026536	BCR	case	control	-0.036798889	0.052292051	-0.703718609	0.481608029	0.832529825	down_in_case
PRJNA8274(	ENSGALG00010026537	FJX1	case	control	0.059359719	0.101676276	0.583810903	0.559347542	0.869515149	up_in_case
PRJNA8274(	ENSGALG00010026550	MYD88	case	control	0.078619528	0.046602866	1.687010565	0.09160132	0.476713154	up_in_case
PRJNA8274(	ENSGALG00010026553	PLPP3	case	control	-0.165936807	0.065416442	-2.53662234	0.011192763	0.185465245	down_in_case
PRJNA8274(	ENSGALG00010026559	RAB36	case	control	0.001747237	0.3277347	0.005331255	0.995746294	0.998710499	up_in_case
PRJNA8274(	ENSGALG00010026560	TNNI1	case	control	0.164837272	0.142288058	1.158475799	0.246669922	0.671238151	up_in_case
PRJNA8274(	ENSGALG00010026565	RSPH14	case	control	-0.065955781	0.304852832	-0.216352856	0.828712711	0.961976765	down_in_case
PRJNA8274(	ENSGALG00010026566	ENSGALG00010026566	case	control	0.007448321	0.106966915	0.069632003	0.944486564	0.990313016	up_in_case
PRJNA8274(	ENSGALG00010026568	BYSL	case	control	0.000650607	0.072750385	0.008943001	0.992864613	0.998196863	up_in_case
PRJNA8274(	ENSGALG00010026569	GNAZ	case	control	-0.224825802	0.107930684	-2.08305732	0.037245999	0.328438691	down_in_case
PRJNA8274(	ENSGALG00010026581	DNER	case	control	-0.577794541	0.166783856	-3.46433135	0.000531551	0.033485392	down_in_case
PRJNA8274(	ENSGALG00010026584	ENSGALG00010026584	case	control	-0.033077507	0.062902984	-0.525849576	0.598992726	0.884828142	down_in_case
PRJNA8274(	ENSGALG00010026588	CCND3	case	control	0.028749058	0.084491022	0.34026169	0.733659464	0.933846596	up_in_case
PRJNA8274(	ENSGALG00010026595	PRKAA2	case	control	0.053964482	0.127639984	0.422786659	0.672450922	0.913676733	up_in_case
PRJNA8274(	ENSGALG00010026612	MFF	case	control	-0.059015472	0.066633582	-0.88567161	0.375794511	0.774921593	down_in_case
PRJNA8274(	ENSGALG00010026628	FYB2	case	control	2.222164572	0.806845169	2.754140021	0.005884659	0.133681071	up_in_case
PRJNA8274(	ENSGALG00010026630	XIRP1	case	control	-0.244565427	0.112183133	-2.180055245	0.029253367	0.304235014	down_in_case
PRJNA8274(	ENSGALG00010026633	SLC5A1	case	control	0.627584522	0.345362853	1.817174364	0.069190419	0.424591509	up_in_case
PRJNA8274(	ENSGALG00010026635	C8A	case	control	-0.094105595	1.959185533	-0.048033019	0.961689928	0.992056045	down_in_case
PRJNA8274(	ENSGALG00010026638	LAD1	case	control	0.593018168	0.337019401	1.759596527	0.078476241	0.448818031	up_in_case
PRJNA8274(	ENSGALG00010026643	YWHAH	case	control	0.06361405	0.071576506	0.888756016	0.374134219	0.774369566	up_in_case
PRJNA8274(	ENSGALG00010026659	LIPH	case	control	0.623628893	0.179187479	3.480315122	0.000500824	0.032553582	up_in_case
PRJNA8274(	ENSGALG00010026666	ENSGALG00010026666	case	control	-0.327686625	0.213508048	-1.534774114	0.124839323	0.52390459	down_in_case
PRJNA8274(	ENSGALG00010026671	TAF8	case	control	-0.017108815	0.069556912	-0.245968589	0.805706548	0.9556344	down_in_case
PRJNA8274(	ENSGALG00010026673	DEPDC5	case	control	-0.037280281	0.059342766	-0.62821948	0.529860171	0.854806007	down_in_case
PRJNA8274(	ENSGALG00010026681	PLXDC1	case	control	0.166435093	0.124579589	1.335974007	0.181557769	0.602663905	up_in_case
PRJNA8274(	ENSGALG00010026686	ENSGALG00010026686	case	control	0.592553302	0.328937382	1.801416728	0.071637222	0.432606532	up_in_case
PRJNA8274(	ENSGALG00010026690	KRT14	case	control	0.2123179	0.397825386	0.533696208	0.593551733	0.883625114	up_in_case
PRJNA8274(	ENSGALG00010026696	CSRNP1	case	control	-0.013767648	0.09970177	-0.138088301	0.890170632	0.978510265	down_in_case

PRJNA8274(	ENSGALG00010026697	CHN1	case	control	0.069865393	0.062263757	1.122087653	0.261825173	0.687529163	up_in_case
PRJNA8274(	ENSGALG00010026700	C8B	case	control	0.323120851	0.757470225	0.426578948			up_in_case
PRJNA8274(	ENSGALG00010026704	WDFY1	case	control	0.158335143	0.050005578	3.166349631	0.001543651	0.062889498	up_in_case
PRJNA8274(	ENSGALG00010026706	KRT24	case	control	-0.246107058	0.375675277	-0.655105812	0.51239963	0.846012552	down_in_case
PRJNA8274(	ENSGALG00010026707	GORASP1	case	control	-0.031872735	0.075065012	-0.424601735	0.671127028	0.913676733	down_in_case
PRJNA8274(	ENSGALG00010026715	WDR48	case	control	0.036287617	0.046644931	0.777954137	0.436596051	0.810227183	up_in_case
PRJNA8274(	ENSGALG00010026719	ENSGALG00010026719	case	control	0.204365358	0.608576522	0.33580881	0.737015046	0.935060877	up_in_case
PRJNA8274(	ENSGALG00010026724	OLA1	case	control	-0.208465036	0.066734245	-3.123809036	0.001785263	0.068078022	down_in_case
PRJNA8274(	ENSGALG00010026731	ENSGALG00010026731	case	control	0.414967018	0.603115321	0.688039258	0.49142806	0.836996338	up_in_case
PRJNA8274(	ENSGALG00010026737	SCN11A	case	control	0.345778091	1.066511533	0.324214113	0.745775938	0.938117773	up_in_case
PRJNA8274(	ENSGALG00010026740	DAB1	case	control	-0.026862776	0.100447337	-0.267431438	0.789136997	0.951843754	down_in_case
PRJNA8274(	ENSGALG00010026743	TNNT2	case	control	0.445723734	0.181970641	2.449426626	0.014308386	0.21093806	up_in_case
PRJNA8274(	ENSGALG00010026746	HOXD10	case	control	0.447693168	0.327499286	1.36700502	0.171623732	0.589389858	up_in_case
PRJNA8274(	ENSGALG00010026755	ENSGALG00010026755	case	control	0.329627401	0.405837069	0.812216099	0.416667644	0.799987395	up_in_case
PRJNA8274(	ENSGALG00010026759	OMA1	case	control	0.025529993	0.068479044	0.372814689	0.70928637	0.925521953	up_in_case
PRJNA8274(	ENSGALG00010026775	BTG2	case	control	0.016978494	0.098466012	0.172429994	0.863099493	0.972850732	up_in_case
PRJNA8274(	ENSGALG00010026780	MYSM1	case	control	0.075144223	0.084383522	0.890508256	0.373193037	0.774369566	up_in_case
PRJNA8274(	ENSGALG00010026782	HOXB9	case	control	0.627394369	0.448551986	1.398710492	0.161899817	0.575570547	up_in_case
PRJNA8274(	ENSGALG00010026796	DCAKD	case	control	0.07501768	0.184379549	0.406865516	0.684106764	0.916328843	up_in_case
PRJNA8274(	ENSGALG00010026801	FMOD	case	control	0.192112101	0.313752799	0.612304025	0.540336627	0.859012092	up_in_case
PRJNA8274(	ENSGALG00010026822	PRELP	case	control	-0.030171194	0.103291136	-0.292098579	0.770211253	0.945428692	down_in_case
PRJNA8274(	ENSGALG00010026825	ADAM23	case	control	-0.26921568	0.112054293	-2.402546774	0.016281352	0.226234374	down_in_case
PRJNA8274(	ENSGALG00010026827	FAM117A	case	control	-0.037191563	0.083577221	-0.444996405	0.656322352	0.907494354	down_in_case
PRJNA8274(	ENSGALG00010026834	ENSGALG00010026834	case	control	0.682774508	1.500585514	0.455005397	0.649105356	0.90399363	up_in_case
PRJNA8274(	ENSGALG00010026839	FGGY	case	control	-0.320752706	0.117771431	-2.723518804	0.006459054	0.13904105	down_in_case
PRJNA8274(	ENSGALG00010026845	ENSGALG00010026845	case	control	-0.29553992	2.538031721	-0.116444533	0.907300243	0.981730793	down_in_case
PRJNA8274(	ENSGALG00010026846	MICAL1	case	control	0.197094381	0.16326518	1.207204018	0.227353583	0.65088213	up_in_case
PRJNA8274(	ENSGALG00010026851	ENSGALG00010026851	case	control	0.008614618	1.807024141	0.004767296	0.996196263	0.998710499	up_in_case
PRJNA8274(	ENSGALG00010026854	SMPD2	case	control	0.181711192	0.089529581	2.029621835	0.042394994	0.343873176	up_in_case
PRJNA8274(	ENSGALG00010026856	UBE2Z	case	control	0.057012272	0.045827249	1.244069248	0.213474078	0.638582746	up_in_case
PRJNA8274(	ENSGALG00010026874	TEAD3	case	control	-0.046558252	0.062837241	-0.74093407	0.458733416	0.824070529	down_in_case
PRJNA8274(	ENSGALG00010026885	TULP1	case	control	-0.32922123	0.39454225	-0.834438466	0.404033949	0.79260318	down_in_case
PRJNA8274(	ENSGALG00010026886	JUN	case	control	-0.039638974	0.075844376	-0.522635641	0.601227833	0.884954589	down_in_case
PRJNA8274(	ENSGALG00010026889	ENSGALG00010026889	case	control	-0.068846643	0.238060845	-0.289197674	0.772430112	0.946102835	down_in_case
PRJNA8274(	ENSGALG00010026893	FKBP5	case	control	-0.336161717	0.162001305	-2.075055615	0.037981381	0.330206289	down_in_case
PRJNA8274(	ENSGALG00010026894	IP6K1	case	control	0.117655904	0.045592463	2.580599849	0.009862883	0.177161817	up_in_case
PRJNA8274(	ENSGALG00010026897	ENSGALG00010026897	case	control	-0.002827241	0.216811652	-0.013040078	0.989595818	0.997323462	down_in_case
PRJNA8274(	ENSGALG00010026898	PNPLA2	case	control	-0.078928513	0.13042871	-0.605146776	0.545081476	0.861997112	down_in_case
PRJNA8274(	ENSGALG00010026899	HOOK1	case	control	0.100950795	0.135185791	0.746755961	0.455210883	0.821701039	up_in_case
PRJNA8274(	ENSGALG00010026901	FAM212A	case	control	0.105126386	0.097874254	1.074096424	0.282779467	0.706701569	up_in_case
PRJNA8274(	ENSGALG00010026907	ENSGALG00010026907	case	control	0.232562949	0.096289652	2.415243431	0.015724696	0.222636791	up_in_case
PRJNA8274(	ENSGALG00010026909	ENSGALG00010026909	case	control	-0.243580557	0.089893208	-2.709665876	0.006735102	0.141635229	down_in_case
PRJNA8274(	ENSGALG00010026916	ENSGALG00010026916	case	control	-0.144320553	0.177270322	-0.814126991	0.415572205	0.799392323	down_in_case
PRJNA8274(	ENSGALG00010026917	AMT	case	control	0.42078882	0.18360047	2.291872241	0.021913016	0.26456604	up_in_case
PRJNA8274(	ENSGALG00010026919	NICN1	case	control	0.083827425	0.102362221	0.818929332	0.412826734	0.797581687	up_in_case
PRJNA8274(	ENSGALG00010026920	GATA2	case	control	0.001702584	0.134307385	0.012676773	0.98988567	0.997323462	up_in_case
PRJNA8274(	ENSGALG00010026924	TPH1	case	control	-0.054400107	0.21650541	-0.251264422	0.80160968	0.954294696	down_in_case
PRJNA8274(	ENSGALG00010026940	TH	case	control	-0.765728629	1.163131144	-0.658333871	0.510323618	0.844911643	down_in_case
PRJNA8274(	ENSGALG00010026942	CDK18	case	control	0.07046117	0.075281169	0.935973381	0.349286893	0.759342183	up_in_case
PRJNA8274(	ENSGALG00010026943	EEFSEC	case	control	0.019634783	0.060053695	0.326953781	0.743702843	0.937448285	up_in_case
PRJNA8274(	ENSGALG00010026950	LHFPL5	case	control	0.036156772	0.171653124	0.210638591	0.833169296	0.962550652	up_in_case
PRJNA8274(	ENSGALG00010026951	RUVBL1	case	control	-0.095415844	0.056103744	-1.700703687	0.088998643	0.471537824	down_in_case
PRJNA8274(	ENSGALG00010026954	MFSD4A	case	control	-0.03822644	0.071604685	-0.533853895	0.593442623	0.883625114	down_in_case
PRJNA8274(	ENSGALG00010026958	CLTCL1	case	control	-0.014598428	0.054763856	-0.266570491	0.789799879	0.951843754	down_in_case
PRJNA8274(	ENSGALG00010026960	SEC61A1	case	control	0.077849033	0.071193588	1.09348377	0.274181451	0.699970438	up_in_case
PRJNA8274(	ENSGALG00010026964	SRPK1	case	control	0.060979739	0.044287088	1.376919133	0.168537251	0.584687698	up_in_case
PRJNA8274(	ENSGALG00010026972	TNNT3	case	control	-0.225047304	0.109192491	-2.061014482	0.039301658	0.335091843	down_in_case
PRJNA8274(	ENSGALG00010026973	MGLL	case	control	0.017362631	0.105769285	0.164155697	0.869608576	0.974718971	up_in_case
PRJNA8274(	ENSGALG00010026977	ELK4	case	control	-0.032890336	0.087664136	-0.375185761	0.707522319	0.924751474	down_in_case
PRJNA8274(	ENSGALG00010026980	LIMK2	case	control	-0.024769848	0.070554019	-0.351076358	0.725531064	0.931921256	down_in_case
PRJNA8274(	ENSGALG00010026982	ABTB1	case	control	-0.010932001	0.058717419	-0.186179864	0.852303714	0.969582953	down_in_case
PRJNA8274(	ENSGALG00010026984	PGR2/3	case	control	0.15656255	0.799435428	0.195841396	0.844734308	0.967227209	up_in_case
PRJNA8274(	ENSGALG00010026999	MLEC	case	control	-0.064877915	0.0577956	-1.122540728	0.261632601	0.687516348	down_in_case
PRJNA8274(	ENSGALG00010027001	SLC26A8	case	control	-0.254708204	0.168283298	-1.51356794	0.130135458	0.532650639	down_in_case
PRJNA8274(	ENSGALG00010027003	SEPTIN2L	case	control	0.015961279	0.035892204	0.444700433	0.656536257	0.907494354	up_in_case
PRJNA8274(	ENSGALG00010027012	RILPL1	case	control	-0.118220468	0.091607202	-1.290514987	0.196871912	0.620029043	down_in_case
PRJNA8274(	ENSGALG00010027015	ENSGALG00010027015	case	control	1.018310986	0.555904394	1.831809566	0.066979798	0.419068957	up_in_case
PRJNA8274(	ENSGALG00010027025	ORAI1	case	control	-0.084432105	0.081798806	-1.032192388	0.301982	0.724797349	down_in_case
PRJNA8274(	ENSGALG00010027026	SLC45A3	case	control	0.428547381	0.231814027	1.84866889	0.064505639	0.412351645	up_in_case
PRJNA8274(	ENSGALG00010027027	ARHGEF3	case	control	-0.035712271	0.100966502	-0.353704156	0.723560607	0.930310797	down_in_case
PRJNA8274(	ENSGALG00010027036	NUCKS1	case	control	-0.099232543	0.040734985	-2.436052036	0.014848548	0.215129943	down_in_case
PRJNA8274(	ENSGALG00010027040	CLIP1	case	control	-0.142566721	0.057419276	-2.482906969	0.013031512	0.200161779	down_in_case
PRJNA8274(	ENSGALG00010027041	MAPK14	case	control	-0.174617542	0.0638761	-2.733691364	0.006262872	0.137995482	down_in_case
PRJNA8274(	ENSGALG00010027043	IL17RD	case	control	0.020262411	0.078622787	0.257716768	0.796625504	0.95280257	up_in_case
PRJNA8274(	ENSGALG00010027045	MAPK13	case	control	0.014757696	0.142903337	0.103270482	0.917748303	0.983445506	up_in_case
PRJNA8274(	ENSGALG00010027046	HESX1	case	control	0.307186508	0.237402423	1.293948495	0.195683212	0.619095929	up_in_case
PRJNA8274(	ENSGALG00010027050	GSTT1	case	control	-0.147499275	0.149222414	-0.988452545	0.322931065	0.740689923	down_in_case
PRJNA8274(	ENSGALG00010027052	APPL1	case	control	0.058537948	0.084071338	0.696289008	0.486247856	0.834635018	up_in_case
PRJNA8274(	ENSGALG00010027059	ASB14	case	control	-0.213875563	0.102737421	-2.081768842	0.037363589	0.328438691	down_in_case
PRJNA8274(	ENSGALG00010027063	RAB7L1	case	control	0.121401417	0.067358689	1.802312648	0.07149623	0.432432368	up_in_case
PRJNA8274(	ENSGALG00010027073	DNAH12	case	control	-0.029058601	0.193348256	-0.150291509	0.880534632	0.976399289	down_in_case
PRJNA8274(	ENSGALG00010027074	BRPF3	case	control	0.063261159	0.041518866	1.523672611	0.127590515	0.529467313	up_in_case
PRJNA8274(	ENSGALG00010027075	SLC41A1	case	control	-0.020395427	0.039898518	-0.511182568	0.609223224	0.8877909	down_in_case
PRJNA8274(	ENSGALG00010027076	VDHAP	case	control	0.230316466	0.095461873	2.412653953	0.015836848	0.223703263	up_in_case
PRJNA8274(	ENSGALG00010027077	ARF4	case	control	0.064612674	0.051083208	1.264851543	0.20592455	0.62881082	up_in_case
PRJNA8274(	ENSGALG00010027078	PDE12	case	control	0.047020662	0.10169866	0.46235282	0.643828327	0.902038523	up_in_case
PRJNA8274(	ENSGALG00010027079	PM20D1	case	control	-0.001350474	0.075086621	-0.017985549	0.985650382	0.996595084	down_in_case
PRJNA8274(	ENSGALG00010027080	TBC1D22B	case	control	-0.03904177	0.052932189	-0.737580866	0.460769186	0.82454265	down_in_case
PRJNA8274(	ENSGALG00010027081	PDE4B	case	control	0.295507802	0.155769868	1.897079366	0.057817464	0.391843477	up_in_case
PRJNA8274(	ENSGALG00010027082	SLC26A9	case	control	-0.503104584	0.446890154	-1.125790262	0.260254307	0.687282842	down_in_case
PRJNA8274(	ENSGALG00010027083	DENND6A	case	control	-0.041910126	0.05918491	-0.708121819	0.478869611	0.83124975	down_in_case
PRJNA8274(	ENSGALG00010027084	OSBPL9	case	control	-0.083101394	0.03626642	-2.291414332	0.021939461	0.26456604	down_in_case
PRJNA8274(	ENSGALG00010027085	PIM1	case	control	0.229796332	0.114591881	2.005345662	0.044926106	0.352144447	up_in_case
PRJNA8274(	ENSGALG00010027086	CACNA1S	case	control	-0.306356054	0.137653636	-2.22555729	0.02604385	0.286194449	down_in_case
PRJNA8274(	ENSGALG00010027087	RAB7B	case	control	-0.146518332	0.163702009	-0.895030751	0.37077065	0.772835133	down_in_case
PRJNA8274(	ENSGALG00010027088	AGBL4	case	control	0.039338263	0.224219083	0.175445649	0.860729474	0.972153185	up_in_case
PRJNA8274(	ENSGALG00010027092	ENSGALG00010027092	case	control	0.048817368	0.077641813	0.62875101	0.529512077	0.854630102	up_in_case
PRJNA8274(	ENSGALG00010027093	SLMAP	case	control	-0.195977112	0.06890421	-2.8441965	0.004452358	0.114389963	down_in_case
PRJNA8274(	ENSGALG00010027095	SCN5A	case	control	0.100758175	0.150267186	0.670526798	0.50252203	0.84219414	up_in_case
PRJNA8274(	ENSGALG00010027096	YIPF1	case	control	0.09582278	0.05853476	1.637023536	0.101625552	0.491460867	up_in_case
PRJNA8274(	ENSGALG00010027097	ENSGALG00010027097	case	control	-0.090607108	0.056798769	-1.595230133	0.110660783	0.503115856	down_in_case
PRJNA8274(	ENSGALG00010027098	EXOG	case	control	-0.122522878	0.155023971	-0.790347955	0.429324588	0.806015686	down_in_case
PRJNA8274(	ENSGALG00010027099	GNA11	case	control	-0.085647719	0.064713787	-1.323484886	0.185674179	0.608776505	down_in_case
PRJNA8274(	ENSGALG00010027100	MIER2	case	control	0.103427095	0.064064985	1.614409106	0.106438718	0.496402103	up_in_case
PRJNA8274(	ENSGALG00010027101	RNF220	case	control	-0.070501648	0.05280518	-1.335127484	0.181834626	0.602744104	down_in_case
PRJNA8274(	ENSGALG00010027102	DUS3L	case	control	0.095903049	0.083152749	1.153335873	0.248772551	0.673758993	up_in_case
PRJNA8274(	ENSGALG00010027103	ACVR2B	case	control	-0.172491359	0.146477156	-1.177599049	0.238956489	0.664155062	down_in_case
PRJNA8274(	ENSGALG00010027105	CHERP	case	control	-0.053815729	0.043627231	-1.233535295	0.217376117	0.642778841	down_in_case
PRJNA8274(	ENSGALG00010027106	CYP2J23	case	control	0.027816864	0.109895108	0.253121949	0.800173978	0.954053991	up_in_case
PRJNA8274(	ENSGALG00010027107	KIF21B	case	control	0.098710378	0.109477489	0.901649998	0.367242823	0.771770041	up_in_case
PRJNA8274(	ENSGALG00010027108	ENSGALG00010027108	case	control	0.11416888	0.14236181	0.801962833	0.422574458	0.803289893	up_in_case
PRJNA8274(	ENSGALG00010027109	C19orf44	case	control	-0.128705366	0.106211698	-1.211781463	0.225596046	0.649491335	down_in_case
PRJNA8274(	ENSGALG00010027110	IKBKE	case	control	0.142156284	0.213019624	0.667338911	0.504555677	0.842675107	up_in_case
PRJNA8274(	ENSGALG00010027111	CALR3	case	control	0.385293226	0.739230824	0.52120828	0.602221687	0.885002041	up_in_case
PRJNA8274(	ENSGALG00010027112	XYLB	case	control	0.374578079	0.193520398	1.935599979	0.052916713	0.375724427	up_in_case
PRJNA8274(	ENSGALG00010027113	PIAS4	case	control	0.120661659	0.062806604	1.921161961	0.054711292	0.382287597	up_in_case
PRJNA8274(	ENSGALG00010027114	FLNB	case	control	-0.064246398	0.069505539	-0.924334939	0.355311957	0.763161644	down_in_case
PRJNA8274(	ENSGALG00010027115	C9ORF152	case	control	0.574236107	0.623337194	0.921228691	0.356931042	0.763373775	up_in_case
PRJNA8274(	ENSGALG00010027116	CCDC94	case	control	-0.075595966	0.065612685	-1.152154741	0.249257492	0.674421685	down_in_case
PRJNA8274(	ENSGALG00010027117	RASSF5	case	control	-0.019954965	0.217076484	-0.091925965	0.926756862	0.986376948	down_in_case
PRJNA8274(	ENSGALG00010027118	ABHD6	case	control	-0.070651545	0.133823257	-0.527946684	0.597536341	0.884680373	down_in_case
PRJNA8274(	ENSGALG00010027119	NUB1	case	control	-0.145968771	0.065058606	-2.243650448	0.024854901	0.279489584	down_in_case
PRJNA8274(	ENSGALG00010027120	ENSGALG00010027120	case	control	-0.56454545	0.597248875	-0.94524322	0.344534719	0.755870938	down_in_case
PRJNA8274(	ENSGALG00010027121	ELF3	case	control	-0.125259425	0.745523492	-0.168015396	0.866571167	0.973522483	down_in_case
PRJNA8274(	ENSGALG00010027122	DNASE1L3	case	control	0.035560585	0.173472307	0.204992865	0.8375777	0.964826648	up_in_case
PRJNA8274(	ENSGALG00010027123	WDR86	case	control	-0.165401058	0.077094149	-2.145442434	0.031917489	0.3098361	down_in_case

PRJNA8274(	ENSGALG00010027124	IP6K2	case	control	-0.01558365	0.05501685	-0.283252321	0.776983423	0.947379834	down_in_case
PRJNA8274(	ENSGALG00010027126	ENSGALG00010027126	case	control	-0.074125693	0.360054167	-0.205873727	0.836889549	0.964784002	down_in_case
PRJNA8274(	ENSGALG00010027128	EIF2D	case	control	-0.024854112	0.068742396	-0.361554349	0.717685084	0.927258466	down_in_case
PRJNA8274(	ENSGALG00010027129	NCLN	case	control	0.09239123	0.087474466	1.056207984	0.290873204	0.714222439	up_in_case
PRJNA8274(	ENSGALG00010027130	NCKIPSD	case	control	-0.069593688	0.10273556	-0.677406033	0.498148372	0.839703389	down_in_case
PRJNA8274(	ENSGALG00010027131	UBA52	case	control	-0.061921956	0.060483152	-1.023788513	0.305935177	0.728933908	down_in_case
PRJNA8274(	ENSGALG00010027133	CELF5	case	control	-0.136480178	0.143996439	-0.947802447	0.343230035	0.755132664	down_in_case
PRJNA8274(	ENSGALG00010027134	DYRK3	case	control	-0.115432861	0.087284398	-1.322491339	0.186004592	0.609079249	down_in_case
PRJNA8274(	ENSGALG00010027135	PRKAG2	case	control	0.081364336	0.048081532	1.692215967	0.090604803	0.474422807	up_in_case
PRJNA8274(	ENSGALG00010027136	MAPKAPK2	case	control	-0.178997387	0.07816375	-2.290030696	0.022019537	0.264801476	down_in_case
PRJNA8274(	ENSGALG00010027137	FKBP8	case	control	-0.082118191	0.054644923	-1.502759755	0.132901007	0.536224013	down_in_case
PRJNA8274(	ENSGALG00010027139	HSD11B1L	case	control	-0.070122061	0.142594736	-0.491757713	0.622890634	0.894021703	down_in_case
PRJNA8274(	ENSGALG00010027143	GALNT11	case	control	0.13113033	0.080037399	1.638363217	0.101345947	0.491460867	up_in_case
PRJNA8274(	ENSGALG00010027144	ENSGALG00010027144	case	control	-0.927493056	0.507574717	-1.827303498	0.06765415	0.42089154	down_in_case
PRJNA8274(	ENSGALG00010027145	CELSR3	case	control	0.084533062	0.102362071	0.825824074	0.408903896	0.795170398	up_in_case
PRJNA8274(	ENSGALG00010027146	KXD1	case	control	-0.065629817	0.081653137	-0.803763568	0.421533534	0.802413627	down_in_case
PRJNA8274(	ENSGALG00010027147	KMT2C	case	control	-0.043294292	0.058832863	-0.735886197	0.461799958	0.824848788	down_in_case
PRJNA8274(	ENSGALG00010027148	ELL	case	control	-0.171440356	0.08019081	-2.137905279	0.032524432	0.310662892	down_in_case
PRJNA8274(	ENSGALG00010027149	C1orf116	case	control	0.2814725	0.225598321	1.247671077	0.212151537	0.637212137	up_in_case
PRJNA8274(	ENSGALG00010027150	YOD1	case	control	0.03408541	0.072937664	0.467322483	0.640269173	0.901077979	up_in_case
PRJNA8274(	ENSGALG00010027151	MICOS13	case	control	-0.146038607	0.101483708	-1.439034988	0.15014061	0.557230917	down_in_case
PRJNA8274(	ENSGALG00010027152	C4BPS	case	control	0.562003983	0.230827772	2.434732949	0.014902784	0.215262433	up_in_case
PRJNA8274(	ENSGALG00010027153	ENSGALG00010027153	case	control	0.010843832	0.144041802	0.075282532	0.939989919	0.989551456	up_in_case
PRJNA8274(	ENSGALG00010027154	PLPP2	case	control	-0.054549055	0.076909092	-0.709266669	0.478159008	0.830520323	down_in_case
PRJNA8274(	ENSGALG00010027155	ENSGALG00010027155	case	control	0.292392844	0.176004513	1.661280381	0.096657148	0.48430176	up_in_case
PRJNA8274(	ENSGALG00010027156	ISYNA1	case	control	-0.040828415	0.075453107	-0.541109792	0.58843191	0.882771329	down_in_case
PRJNA8274(	ENSGALG00010027157	ENSGALG00010027157	case	control	0.219595531	0.168074523	1.30653669	0.191370111	0.614339451	up_in_case
PRJNA8274(	ENSGALG00010027158	XRCC2	case	control	0.143623303	0.088568042	1.62161542	0.104885719	0.494770427	up_in_case
PRJNA8274(	ENSGALG00010027159	TMUB1	case	control	-0.061461156	0.067473841	-0.91088866	0.362354041	0.768450658	down_in_case
PRJNA8274(	ENSGALG00010027160	FASTK	case	control	-0.057452119	0.061633764	-0.932153336	0.351257287	0.760831854	down_in_case
PRJNA8274(	ENSGALG00010027161	C4BPG	case	control	0.237909266	0.118186114	2.013005228	0.044114079	0.350032868	up_in_case
PRJNA8274(	ENSGALG00010027162	SLC4A2	case	control	0.106670559	0.092028154	1.159107887	0.246412212	0.670921661	up_in_case
PRJNA8274(	ENSGALG00010027163	ENSGALG00010027163	case	control	-0.02760149	0.043731685	-0.63115543	0.527938901	0.853386536	down_in_case
PRJNA8274(	ENSGALG00010027164	ENSGALG00010027164	case	control	0.015430419	0.410695056	0.037571474	0.970029352	0.992761697	up_in_case
PRJNA8274(	ENSGALG00010027165	ENSGALG00010027165	case	control	0.249965147	0.107482427	2.325637344	0.020037906	0.254128638	up_in_case
PRJNA8274(	ENSGALG00010027166	CDK5	case	control	-0.133705499	0.076257837	-1.753334517	0.079544604	0.450131333	down_in_case
PRJNA8274(	ENSGALG00010027167	ENSGALG00010027167	case	control	-0.114277952	0.307577102	-0.371542458	0.710233539	0.925630832	down_in_case
PRJNA8274(	ENSGALG00010027168	SSBP4	case	control	-0.041922594	0.084882643	-0.493888882	0.621384657	0.893175881	down_in_case
PRJNA8274(	ENSGALG00010027169	ABCB8	case	control	-0.111223553	0.085329184	-1.303464395	0.192416264	0.615090794	down_in_case
PRJNA8274(	ENSGALG00010027170	TLE2	case	control	0.161535367	0.115853682	1.394304998	0.163225522	0.577197348	up_in_case
PRJNA8274(	ENSGALG00010027171	ATG9B	case	control	-0.37820344	0.173965356	-2.174015836	0.029703946	0.305970455	down_in_case
PRJNA8274(	ENSGALG00010027172	ENSGALG00010027172	case	control	-0.768990188	0.358925523	-2.142478421	0.032155003	0.3098361	down_in_case
PRJNA8274(	ENSGALG00010027173	ENSGALG00010027173	case	control	-0.109946216	0.066530357	-1.652572164	0.098417967	0.487941198	down_in_case
PRJNA8274(	ENSGALG00010027174	SHISA5	case	control	0.106934092	0.059814749	1.787754589	0.07381561	0.437990779	up_in_case
PRJNA8274(	ENSGALG00010027175	NOS3	case	control	0.05255809	0.096999066	0.541841196	0.587927909	0.882771329	up_in_case
PRJNA8274(	ENSGALG00010027176	ENSGALG00010027176	case	control	0.034436048	0.051361924	0.67045868	0.502565438	0.84219414	up_in_case
PRJNA8274(	ENSGALG00010027178	C4BPM	case	control	0.223921441	0.066214597	3.381753424	0.000720248	0.040076036	up_in_case
PRJNA8274(	ENSGALG00010027183	CD34	case	control	0.024280322	0.083106669	0.292158528	0.770165419	0.945428692	up_in_case
PRJNA8274(	ENSGALG00010027184	MPND	case	control	0.188432242	0.176908498	1.065139568	0.286812776	0.710659943	up_in_case
PRJNA8274(	ENSGALG00010027185	ATRIP	case	control	0.105833285	0.064808531	1.633014723	0.1024659	0.491460867	up_in_case
PRJNA8274(	ENSGALG00010027186	PLXNA2	case	control	-0.034776974	0.113381014	-0.306726612	0.759051472	0.942078947	down_in_case
PRJNA8274(	ENSGALG00010027187	ENSGALG00010027187	case	control	0.167932791	0.098875669	1.698423813	0.089427801	0.47258594	up_in_case
PRJNA8274(	ENSGALG00010027188	KCNH2	case	control	-0.149869863	0.177498896	-0.844342503	0.398478042	0.789453932	down_in_case
PRJNA8274(	ENSGALG00010027189	ENSGALG00010027189	case	control	0.132907064	0.465485321	0.285523641	0.775242995	0.947087967	up_in_case
PRJNA8274(	ENSGALG00010027190	IPPK	case	control	0.063936807	0.072002009	0.887986437	0.374548047	0.774434633	up_in_case
PRJNA8274(	ENSGALG00010027191	AOC1	case	control	0.779297553	0.919190525	0.847808515	0.39654462	0.788531716	up_in_case
PRJNA8274(	ENSGALG00010027192	SH3GL1	case	control	-0.05582337	0.055753055	-1.001261187	0.316700552	0.737355036	down_in_case
PRJNA8274(	ENSGALG00010027193	CAMK1G	case	control	-0.382881771	0.371254184	-1.031319747	0.302390901	0.725089098	down_in_case
PRJNA8274(	ENSGALG00010027194	ENSGALG00010027194	case	control	0.297971859	0.302166254	0.986118919	0.324074761	0.741957906	up_in_case
PRJNA8274(	ENSGALG00010027195	CENPP	case	control	-0.086958541	0.156004868	-0.557409152	0.577247914	0.878143389	down_in_case
PRJNA8274(	ENSGALG00010027196	ENSGALG00010027196	case	control	-0.42639657	0.387406258	-1.100644506	0.271051407	0.698323327	down_in_case
PRJNA8274(	ENSGALG00010027197	ENSGALG00010027197	case	control	0.043287322	0.355574474	0.121739115	0.903105634	0.980997505	up_in_case
PRJNA8274(	ENSGALG00010027198	ECM2	case	control	-0.29697633	0.100676895	-2.94979629	0.003179835	0.095931735	down_in_case
PRJNA8274(	ENSGALG00010027199	LAMB3	case	control	-0.859936012	0.302761324	-2.840309986	0.004506971	0.114389963	down_in_case
PRJNA8274(	ENSGALG00010027200	G0S2	case	control	-0.380444605	0.189923238	-2.00314932	0.045161264	0.352321914	down_in_case
PRJNA8274(	ENSGALG00010027201	ASPN	case	control	-0.280355711	0.281783451	-0.994933203	0.319768753	0.738813491	down_in_case
PRJNA8274(	ENSGALG00010027202	CHAF1A	case	control	0.049872114	0.060673282	0.821978186	0.411089312	0.796718771	up_in_case
PRJNA8274(	ENSGALG00010027203	ENSGALG00010027203	case	control	-0.266108941	0.271514584	-0.980090782	0.327041309	0.744857565	down_in_case
PRJNA8274(	ENSGALG00010027204	OMD	case	control	-0.922965778	0.944746186	-0.976945757	0.328596007	0.745445783	down_in_case
PRJNA8274(	ENSGALG00010027205	OGN	case	control	-0.005164031	0.104231089	-0.049544055	0.96048573	0.992056045	down_in_case
PRJNA8274(	ENSGALG00010027206	ENSGALG00010027206	case	control	0.780719874	0.600736638	1.299604226	0.19373665	0.616682237	up_in_case
PRJNA8274(	ENSGALG00010027207	NOL8	case	control	0.000924853	0.055819057	0.016568766	0.986780642	0.996781748	up_in_case
PRJNA8274(	ENSGALG00010027208	UBXN6	case	control	-0.089854787	0.082392059	-1.090575816	0.275459576	0.700894634	down_in_case
PRJNA8274(	ENSGALG00010027209	TRAF3IP3	case	control	0.312797767	0.231698245	1.350022168	0.177008872	0.595582794	up_in_case
PRJNA8274(	ENSGALG00010027210	ENSGALG00010027210	case	control	-0.213970479	0.210230025	-1.017792199	0.30877672	0.731823162	down_in_case
PRJNA8274(	ENSGALG00010027211	HDGFRP2	case	control	-0.066129033	0.048264837	-1.370128596	0.170646763	0.588011737	down_in_case
PRJNA8274(	ENSGALG00010027212	IRF6	case	control	0.356976626	0.460521274	0.775157731	0.438246461	0.811567521	up_in_case
PRJNA8274(	ENSGALG00010027213	KCNC4	case	control	0.236045307	0.21635317	1.091018478	0.275264752	0.700894634	up_in_case
PRJNA8274(	ENSGALG00010027214	PLIN4	case	control	-0.018481028	0.098121207	-0.188348969	0.850603101	0.969234083	down_in_case
PRJNA8274(	ENSGALG00010027215	ENSGALG00010027215	case	control	0.210990828	0.170768831	1.235534767	0.216631546	0.641771515	up_in_case
PRJNA8274(	ENSGALG00010027216	ENSGALG00010027216	case	control	-0.098772381	0.274886628	-0.359320431	0.719355392	0.928491477	down_in_case
PRJNA8274(	ENSGALG00010027217	SEMA6B	case	control	0.002046371	0.10785901	0.01897265	0.984862923	0.996591771	up_in_case
PRJNA8274(	ENSGALG00010027218	IARS1	case	control	-0.106069987	0.051220177	-2.070863331	0.03837157	0.331548912	down_in_case
PRJNA8274(	ENSGALG00010027219	ENSGALG00010027219	case	control	-0.100113067	0.05968477	-1.677363712	0.093471381	0.478523919	down_in_case
PRJNA8274(	ENSGALG00010027221	LRRC61	case	control	-0.03740383	0.150095034	-0.249200981	0.803205321	0.954891353	down_in_case
PRJNA8274(	ENSGALG00010027222	GUCA1B	case	control	-0.566696358	0.554150464	-1.022639869	0.306478152	0.729466972	down_in_case
PRJNA8274(	ENSGALG00010027223	SIRT6	case	control	-0.007788647	0.060150581	-0.12948582	0.896973244	0.980176778	down_in_case
PRJNA8274(	ENSGALG00010027224	RARRES2	case	control	0.130941587	0.09285153	1.410225411	0.158473135	0.570582225	up_in_case
PRJNA8274(	ENSGALG00010027225	CREB3L3	case	control	-0.00103601	0.594713376	-0.001742033	0.998610059	0.999518713	down_in_case
PRJNA8274(	ENSGALG00010027227	ENSGALG00010027227	case	control	0.03816177	0.061160412	0.623961946	0.532652576	0.855817434	up_in_case
PRJNA8274(	ENSGALG00010027229	MDM4	case	control	0.034961464	0.089951147	0.388671681	0.697519033	0.922288441	up_in_case
PRJNA8274(	ENSGALG00010027230	MAP2K2	case	control	0.052232062	0.07929797	0.658680949	0.510100669	0.844850541	up_in_case
PRJNA8274(	ENSGALG00010027231	ENSGALG00010027231	case	control	-0.047420598	0.128538738	-0.368920676	0.712186851	0.92680349	down_in_case
PRJNA8274(	ENSGALG00010027232	ARRDC2	case	control	0.131581968	0.10649713	1.235544737	0.216627837	0.641771515	up_in_case
PRJNA8274(	ENSGALG00010027233	ENSGALG00010027233	case	control	-0.245430717	0.173832691	-1.411878951	0.157985606	0.569927894	down_in_case
PRJNA8274(	ENSGALG00010027234	SLC6A11	case	control	0.117554474	0.400468514	0.293542363	0.769107624	0.94539152	up_in_case
PRJNA8274(	ENSGALG00010027236	PEX11G	case	control	0.098848681	0.082328176	1.200666476	0.229880603	0.653927318	up_in_case
PRJNA8274(	ENSGALG00010027237	CTC-575C13.4	case	control	0.116493883	0.126953925	0.917607578	0.358824349	0.765374836	up_in_case
PRJNA8274(	ENSGALG00010027239	ENSGALG00010027239	case	control	0.027086215	0.043045376	0.62924797	0.529186729	0.85440042	up_in_case
PRJNA8274(	ENSGALG00010027240	SLC6A1	case	control	0.064953509	0.303455845	0.214045996	0.830511189	0.962228709	up_in_case
PRJNA8274(	ENSGALG00010027241	LRRN2	case	control	0.057736799	0.078245769	0.737890361	0.460581077	0.82454265	up_in_case
PRJNA8274(	ENSGALG00010027242	ENSGALG00010027242	case	control	-0.068687259	0.115003032	-0.597264763	0.550330626	0.86456492	down_in_case
PRJNA8274(	ENSGALG00010027243	HRH1	case	control	0.054636489	0.128190285	0.426213957	0.669951948	0.913465866	up_in_case
PRJNA8274(	ENSGALG00010027244	ARHGEF18	case	control	-0.030952418	0.044964806	-0.688369887	0.491219882	0.836925555	down_in_case
PRJNA8274(	ENSGALG00010027245	LRIG2	case	control	-0.08857822	0.052609385	-1.683696176	0.092240401	0.476713154	down_in_case
PRJNA8274(	ENSGALG00010027246	ZNF767	case	control	-0.050311451	0.073782318	-0.681890362	0.49530828	0.838118945	down_in_case
PRJNA8274(	ENSGALG00010027247	ATG7	case	control	0.088046563	0.072080381	1.221505237	0.221894784	0.645834386	up_in_case
PRJNA8274(	ENSGALG00010027248	ENSGALG00010027248	case	control	-0.246768121	0.314456861	-0.784743956	0.432603705	0.807718	down_in_case
PRJNA8274(	ENSGALG00010027249	C2CD4C	case	control	-0.626619643	0.293697204	-2.133556721	0.032879084	0.311991307	down_in_case
PRJNA8274(	ENSGALG00010027250	ENSGALG00010027250	case	control	0.076850312	0.089891969	0.854918555	0.392596238	0.78608893	up_in_case
PRJNA8274(	ENSGALG00010027251	ENSGALG00010027251	case	control	-0.00595046	0.09160609	-0.064957034	0.94820821	0.991291133	down_in_case
PRJNA8274(	ENSGALG00010027252	MAGI3	case	control	-0.019820384	0.060620223	-0.326959928	0.743698193	0.937448285	down_in_case
PRJNA8274(	ENSGALG00010027253	VGLL4	case	control	0.042531867	0.052151179	0.81554947	0.41475786	0.798853486	up_in_case
PRJNA8274(	ENSGALG00010027254	INSR	case	control	-0.243435601	0.078583701	-3.097787427	0.001949712	0.072043631	down_in_case
PRJNA8274(	ENSGALG00010027255	ENSGALG00010027255	case	control	0.06039015	0.144696868	0.417356301	0.676417813	0.914247368	up_in_case
PRJNA8274(	ENSGALG00010027256	PHTF1	case	control	0.017192656	0.066463763	0.258677137	0.795884361	0.95280257	up_in_case
PRJNA8274(	ENSGALG00010027257	TAFA3	case	control	-0.403932397	0.253187795	-1.595386529	0.110625827	0.503115856	down_in_case
PRJNA8274(	ENSGALG00010027258	ENSGALG00010027258	case	control	0.146121827	0.142991647	1.02189065	0.306832658	0.729504707	up_in_case
PRJNA8274(	ENSGALG00010027259	ENSGALG00010027259	case	control	-0.042195254	0.050925035	-0.828575845	0.407344461	0.794380997	down_in_case
PRJNA8274(	ENSGALG00010027260	LEMD2	case	control	-0.008038561	0.051317408	-0.156643936	0.875525474	0.975666505	down_in_case
PRJNA8274(	ENSGALG00010027261	ENSGALG00010027261	case	control	-0.086185788	0.077999138	-1.104958211	0.269177697	0.696212628	down_in_case
PRJNA8274(	ENSGALG00010027262	USE1	case	control	0.029640303	0.07103411	0.417268594	0.676481957	0.914247368	up_in_case
PRJNA8274(	ENSGALG00010027263	TAMM41	case	control	0.197746525	0.107511697	1.839302416	0.065870718	0.416182558	up_in_case
PRJNA8274(	ENSGALG00010027264	ENSGALG00010027264	case	control	0.126758834	0.063952764	1.982069664	0.047471444	0.359365618	up_in_case
PRJNA8274(	ENSGALG00010027265	IP6K3	case	control	0.049738361	0.104137207	0.477623347	0.632918311	0.897622914	up_in_case
PRJNA8274(	ENSGALG00010027267	MYO9B	case	control	0.097163822	0.053333749	1.821807477	0.068484203	0.422606297	up_in_case

PRJNA8274(	ENSGALG00010027268	UQCRC1	case	control	-0.148736693	0.079781635	-1.864297373	0.062279955	0.405741851	down_in_case
PRJNA8274(	ENSGALG00010027269	CDC25A	case	control	-0.231886192	0.086477933	-2.681449281	0.007330403	0.148898802	down_in_case
PRJNA8274(	ENSGALG00010027270	ENSGALG00010027270	case	control	-0.249430091	0.261437421	-0.954071878	0.34004726	0.751456625	down_in_case
PRJNA8274(	ENSGALG00010027271	INKA2	case	control	-0.214403425	0.120510993	-1.77911922	0.075220218	0.441201444	down_in_case
PRJNA8274(	ENSGALG00010027272	DDX20	case	control	-0.022089274	0.060346486	-0.366040768	0.714334655	0.926862121	down_in_case
PRJNA8274(	ENSGALG00010027273	HAUS8	case	control	0.05815163	0.089713276	0.648194254	0.516859318	0.848054666	up_in_case
PRJNA8274(	ENSGALG00010027274	COPG1	case	control	-0.033968118	0.038502468	-0.882232227	0.377651238	0.776247623	down_in_case
PRJNA8274(	ENSGALG00010027275	CPAMD8	case	control	-0.182608691	0.175911836	-1.038069381	0.299237755	0.722718284	down_in_case
PRJNA8274(	ENSGALG00010027276	ENSGALG00010027276	case	control	-0.081497548	0.069691161	-1.1694101	0.242238442	0.666540259	down_in_case
PRJNA8274(	ENSGALG00010027277	KCND3	case	control	-0.752037519	0.274287624	-2.741784364	0.006110644	0.135266583	down_in_case
PRJNA8274(	ENSGALG00010027278	H1-10	case	control	-0.156883029	0.070633517	-2.221084769	0.02634522	0.288056585	down_in_case
PRJNA8274(	ENSGALG00010027279	F2RL3	case	control	-0.121865648	0.164890364	-0.739070769	0.459864025	0.824480387	down_in_case
PRJNA8274(	ENSGALG00010027280	HMCES	case	control	0.179477345	0.07219398	2.486043081	0.012917233	0.199897239	up_in_case
PRJNA8274(	ENSGALG00010027281	ENSGALG00010027281	case	control	-0.324288432	0.192027403	-1.688761227	0.091265197	0.476454552	down_in_case
PRJNA8274(	ENSGALG00010027282	ENSGALG00010027282	case	control	0.095740185	0.072013082	1.329483224	0.183688597	0.605241229	up_in_case
PRJNA8274(	ENSGALG00010027283	RAB7A	case	control	-0.016481804	0.034819211	-0.473353759	0.635960814	0.898691227	down_in_case
PRJNA8274(	ENSGALG00010027284	CTTNBP2NL	case	control	-0.075783266	0.05223877	-1.450709254	0.146860838	0.551919473	down_in_case
PRJNA8274(	ENSGALG00010027285	PTPRS	case	control	-0.065332646	0.05696393	-1.146912546	0.251417767	0.677081801	down_in_case
PRJNA8274(	ENSGALG00010027286	RPN1	case	control	-0.016424622	0.044317616	-0.370611581	0.710926855	0.925972678	down_in_case
PRJNA8274(	ENSGALG00010027287	DHX30	case	control	0.038351654	0.04787372	0.801100357	0.42307355	0.803723282	up_in_case
PRJNA8274(	ENSGALG00010027288	DAG1	case	control	-0.080184875	0.088950053	-0.901459553	0.36734403	0.771770041	down_in_case
PRJNA8274(	ENSGALG00010027289	SMARCC1	case	control	-0.022335741	0.066017431	-0.33833096	0.735113795	0.934096263	down_in_case
PRJNA8274(	ENSGALG00010027290	CSPG5	case	control	0.02395252	0.160447095	0.149286096	0.881327886	0.97667824	up_in_case
PRJNA8274(	ENSGALG00010027291	MST1	case	control	-0.109310783	0.166936964	-0.654802752	0.512594759	0.846139334	down_in_case
PRJNA8274(	ENSGALG00010027292	KDM4B	case	control	-0.007339604	0.04627436	-0.158610611	0.873975668	0.975062028	down_in_case
PRJNA8274(	ENSGALG00010027293	ELP6	case	control	0.236499633	0.176620205	1.339029315	0.18056113	0.601169768	up_in_case
PRJNA8274(	ENSGALG00010027295	ENSGALG00010027295	case	control	0.10424391	0.164721302	0.632850205	0.526831468	0.85251736	up_in_case
PRJNA8274(	ENSGALG00010027299	PGPEP1	case	control	-0.050174993	0.085093925	-0.589642481	0.555430365	0.867878646	down_in_case
PRJNA8274(	ENSGALG00010027301	SCAP	case	control	-0.042964334	0.065543865	-0.655505038	0.512142644	0.845834144	down_in_case
PRJNA8274(	ENSGALG00010027303	APEH	case	control	0.006604153	0.073324116	0.090067954	0.928233214	0.986776285	up_in_case
PRJNA8274(	ENSGALG00010027304	PTPN23	case	control	-0.032513717	0.07029966	-0.462501761	0.64372154	0.902038523	down_in_case
PRJNA8274(	ENSGALG00010027305	LSM4	case	control	0.077338853	0.070267387	1.100636537	0.271054877	0.698323327	up_in_case
PRJNA8274(	ENSGALG00010027306	ENSGALG00010027306	case	control	-0.234522185	0.300630056	-0.780102257	0.435330688	0.81000376	down_in_case
PRJNA8274(	ENSGALG00010027307	JUND	case	control	0.093712515	0.097726589	0.958925468	0.337596293	0.750948271	up_in_case
PRJNA8274(	ENSGALG00010027310	ARHGEF2	case	control	-0.028346973	0.065159471	-0.435039941	0.663533437	0.911044469	down_in_case
PRJNA8274(	ENSGALG00010027311	NFASC	case	control	-0.061234717	0.154199524	-0.39711353	0.691283743	0.918812261	down_in_case
PRJNA8274(	ENSGALG00010027313	BSN	case	control	-0.323298458	0.154355844	-2.094500928	0.036215381	0.326119051	down_in_case
PRJNA8274(	ENSGALG00010027314	ENSGALG00010027314	case	control	0.121655866	0.178792662	0.680429859	0.49623232	0.83858637	up_in_case
PRJNA8274(	ENSGALG00010027315	RAB3A	case	control	-0.264782825	0.143038089	-1.851135091	0.064150121	0.410842437	down_in_case
PRJNA8274(	ENSGALG00010027316	TRAIP	case	control	0.025174369	0.123335804	0.204112412	0.838265655	0.96484215	up_in_case
PRJNA8274(	ENSGALG00010027317	MPV17L2	case	control	0.004424115	0.108113729	0.04092094	0.967358924	0.992731535	up_in_case
PRJNA8274(	ENSGALG00010027319	MST1R	case	control	-0.267669514	0.308843877	-0.866682277	0.386116119	0.78218735	down_in_case
PRJNA8274(	ENSGALG00010027320	MON1A	case	control	0.036579509	0.081594332	0.448309443	0.653929886	0.906478152	up_in_case
PRJNA8274(	ENSGALG00010027321	IFI30	case	control	0.359844002	0.107678592	3.341834202	0.000832268	0.044243221	up_in_case
PRJNA8274(	ENSGALG00010027322	CRABP2	case	control	0.076654425	0.125181303	0.61234724	0.540308041	0.859012092	up_in_case
PRJNA8274(	ENSGALG00010027323	ISG20L2	case	control	0.017095843	0.077989976	0.219205647	0.826489857	0.961207256	up_in_case
PRJNA8274(	ENSGALG00010027324	PIK3R2	case	control	-0.025712944	0.052992307	-0.485220305	0.62752008	0.895865363	down_in_case
PRJNA8274(	ENSGALG00010027325	METTL25B	case	control	0.091623043	0.159637619	0.573943933	0.566005742	0.872938105	up_in_case
PRJNA8274(	ENSGALG00010027326	MRPL24	case	control	-0.061096301	0.067179306	-0.909451211	0.363112	0.769373478	down_in_case
PRJNA8274(	ENSGALG00010027327	RBM6	case	control	-0.036307432	0.056488113	-0.642744645	0.520389807	0.849979924	down_in_case
PRJNA8274(	ENSGALG00010027328	RBM15	case	control	0.192546355	0.072495758	2.655967177	0.007908131	0.155766219	up_in_case
PRJNA8274(	ENSGALG00010027329	MINDY4	case	control	0.101826836	0.090349656	1.1270307	0.259729501	0.687038822	up_in_case
PRJNA8274(	ENSGALG00010027330	ENSGALG00010027330	case	control	0.037718814	0.171046535	0.220517851	0.825467872	0.961096773	up_in_case
PRJNA8274(	ENSGALG00010027331	SLC16A4	case	control	0.08747961	0.117464746	0.744730759	0.4564345	0.8222491	up_in_case
PRJNA8274(	ENSGALG00010027332	HDGF	case	control	-0.058901942	0.050539134	-1.16547194	0.243828012	0.667830028	down_in_case
PRJNA8274(	ENSGALG00010027333	PROK1	case	control	0.667503517	0.336140539	1.985787014	0.047056978	0.357398596	up_in_case
PRJNA8274(	ENSGALG00010027334	PRCC	case	control	-0.02450605	0.049867249	-0.491425756	0.623125351	0.894052087	down_in_case
PRJNA8274(	ENSGALG00010027335	MAST3	case	control	0.087295796	0.072491563	1.204220094	0.228504522	0.651938858	up_in_case
PRJNA8274(	ENSGALG00010027336	RAD54L2	case	control	-0.040562755	0.056339573	-0.719969151	0.47154399	0.829101076	down_in_case
PRJNA8274(	ENSGALG00010027337	AQP1	case	control	0.123514945	0.097053848	1.272643471	0.203144586	0.625537942	up_in_case
PRJNA8274(	ENSGALG00010027339	LAMTOR5	case	control	0.041712636	0.069052556	0.604070853	0.545796537	0.862133047	up_in_case
PRJNA8274(	ENSGALG00010027346	SIN3B	case	control	-0.047743816	0.049346004	-0.967531567	0.333278369	0.748508541	down_in_case
PRJNA8274(	ENSGALG00010027348	ENSGALG00010027348	case	control	0.160448044	0.109087467	1.470820141	0.141339764	0.543501954	up_in_case
PRJNA8274(	ENSGALG00010027350	INSRR	case	control	0.124590672	0.236767515	0.526215229	0.598738674	0.88476024	up_in_case
PRJNA8274(	ENSGALG00010027351	TMEM38A	case	control	-0.260875721	0.122793401	-2.124509298	0.033627579	0.316364727	down_in_case
PRJNA8274(	ENSGALG00010027353	ENSGALG00010027353	case	control	0.280825556	0.378281865	0.742371184	0.457862473	0.823337749	up_in_case
PRJNA8274(	ENSGALG00010027355	ADCYAP1R1	case	control	0.044589852	0.123167307	0.362026685	0.717332089	0.926969897	up_in_case
PRJNA8274(	ENSGALG00010027356	ENSGALG00010027356	case	control	-0.068389861	0.121316336	-0.563731676	0.572936757	0.87728832	down_in_case
PRJNA8274(	ENSGALG00010027358	RNF126	case	control	0.014697311	0.046589228	0.315465864	0.752407966	0.940427752	up_in_case
PRJNA8274(	ENSGALG00010027359	ENSGALG00010027359	case	control	-0.060030205	0.069283726	-0.86644019	0.386248812	0.78229293	down_in_case
PRJNA8274(	ENSGALG00010027360	SMIM7	case	control	-0.121891419	0.081316842	-1.498968911	0.133881697	0.537632203	down_in_case
PRJNA8274(	ENSGALG00010027361	FANCD2	case	control	-0.027395004	0.063540529	-0.431142215	0.666364967	0.912284937	down_in_case
PRJNA8274(	ENSGALG00010027362	ENSGALG00010027362	case	control	-0.06193304	0.070141509	-0.882972731	0.377251005	0.775926253	down_in_case
PRJNA8274(	ENSGALG00010027363	VIPR1	case	control	-0.057907544	0.190937472	-0.303280145	0.761676376	0.943290152	down_in_case
PRJNA8274(	ENSGALG00010027364	FSTL3	case	control	-0.09919796	0.087555314	-1.132974756	0.257224855	0.68408321	down_in_case
PRJNA8274(	ENSGALG00010027365	PLEKHA6	case	control	0.227539468	0.103890914	2.190176786	0.028511418	0.300549329	up_in_case
PRJNA8274(	ENSGALG00010027366	USP4	case	control	-0.051777934	0.046511452	-1.113229788	0.265609752	0.691591307	down_in_case
PRJNA8274(	ENSGALG00010027367	SEC22C	case	control	0.027473221	0.070740561	0.388365886	0.697745286	0.922288441	up_in_case
PRJNA8274(	ENSGALG00010027368	EMC3	case	control	0.066206605	0.057190898	1.157642347	0.24701002	0.671497658	up_in_case
PRJNA8274(	ENSGALG00010027369	ENSGALG00010027369	case	control	0.005877762	0.059096967	0.099459624	0.920773345	0.984968494	up_in_case
PRJNA8274(	ENSGALG00010027370	NUP210L	case	control	0.659444566	0.306901986	2.148713912	0.031657085	0.3098361	up_in_case
PRJNA8274(	ENSGALG00010027372	PIK3C2B	case	control	-0.121147325	0.078078483	-1.55160961	0.120755662	0.519499513	down_in_case
PRJNA8274(	ENSGALG00010027373	MLN	case	control	-1.423512468	0.683167089	-2.08369591	0.037187837	0.328438691	down_in_case
PRJNA8274(	ENSGALG00010027374	NKTR	case	control	0.046006521	0.08432854	0.545562876	0.585366449	0.881307382	up_in_case
PRJNA8274(	ENSGALG00010027375	FUT7	case	control	0.082343844	0.343296252	0.239862345	0.810436973	0.955972758	up_in_case
PRJNA8274(	ENSGALG00010027376	DSTYK	case	control	-0.101195164	0.046667891	-2.168410929	0.030127435	0.306417012	down_in_case
PRJNA8274(	ENSGALG00010027378	ZBTB47	case	control	0.079329978	0.076269449	1.040127851	0.298280506	0.721387387	up_in_case
PRJNA8274(	ENSGALG00010027379	OPTC	case	control	-0.031336381	0.350584522	-0.089383242	0.928777341	0.986776285	down_in_case
PRJNA8274(	ENSGALG00010027380	GPX1	case	control	-0.07853895	0.063345362	-1.239853205	0.215029695	0.640404074	down_in_case
PRJNA8274(	ENSGALG00010027381	KLHL40	case	control	-0.13729609	0.088118489	-1.558084929	0.119213117	0.516645008	down_in_case
PRJNA8274(	ENSGALG00010027382	PALM	case	control	-0.019742812	0.081282923	-0.24289003	0.808090589	0.955875648	down_in_case
PRJNA8274(	ENSGALG00010027383	ASS1	case	control	-0.02912909	0.198713279	-0.146588541	0.883456799	0.977115076	down_in_case
PRJNA8274(	ENSGALG00010027384	HHATL	case	control	-0.14008849	0.117592798	-1.191301607	0.233535203	0.657982411	down_in_case
PRJNA8274(	ENSGALG00010027385	CNTN2	case	control	-1.077442818	0.764463925	-1.409409629	0.158714078	0.570858667	down_in_case
PRJNA8274(	ENSGALG00010027386	RHOA	case	control	-0.00608184	0.035888277	-0.169465921	0.865430176	0.973289701	down_in_case
PRJNA8274(	ENSGALG00010027387	ENSGALG00010027387	case	control	0.160169078	0.216496436	0.739823164	0.459407301	0.824480387	up_in_case
PRJNA8274(	ENSGALG00010027388	URM1	case	control	-0.22712595	0.088402584	-2.569222981	0.010192685	0.179675823	down_in_case
PRJNA8274(	ENSGALG00010027391	TPM3	case	control	0.025500541	0.072881081	0.34989246	0.726419406	0.932226286	up_in_case
PRJNA8274(	ENSGALG00010027392	RBBP5	case	control	-0.017384606	0.044583422	-0.389934323	0.696585112	0.921903481	down_in_case
PRJNA8274(	ENSGALG00010027393	TEX264	case	control	-0.058371927	0.066020602	-0.884147157	0.37661678	0.775199984	down_in_case
PRJNA8274(	ENSGALG00010027394	SLC27A4	case	control	0.002371797	0.090519233	0.026202136	0.979096113	0.995525769	up_in_case
PRJNA8274(	ENSGALG00010027395	BCL2L15	case	control	0.33485563	0.429368077	0.779880127	0.435461437	0.81000376	up_in_case
PRJNA8274(	ENSGALG00010027397	RFX5	case	control	0.062933509	0.104170709	0.604138236	0.54575174	0.862133047	up_in_case
PRJNA8274(	ENSGALG00010027399	MAPKAP1	case	control	-0.078945182	0.051581896	-1.53048234	0.125897381	0.525491114	down_in_case
PRJNA8274(	ENSGALG00010027401	ENSGALG00010027401	case	control	-0.0229953	0.209557052	-0.109732887	0.912621218	0.982717125	down_in_case
PRJNA8274(	ENSGALG00010027402	AP4B1	case	control	-0.034455682	0.089180809	-0.386357592	0.699231853	0.922288441	down_in_case
PRJNA8274(	ENSGALG00010027403	ACKR2	case	control	0.809518027	0.425726069	1.901499783	0.057236581	0.389012883	up_in_case
PRJNA8274(	ENSGALG00010027405	DCLRE1B	case	control	-0.063488747	0.086488072	-0.734075178	0.462902921	0.825091817	down_in_case
PRJNA8274(	ENSGALG00010027407	ENSGALG00010027407	case	control	0.177421891	1.171378248	0.151464219	0.879609534	0.976318821	up_in_case
PRJNA8274(	ENSGALG00010027410	ALAS1	case	control	-0.056565193	0.068020011	-0.831596352	0.405636822	0.793782506	down_in_case
PRJNA8274(	ENSGALG00010027412	ENSGALG00010027412	case	control	0.034306979	0.2091078	0.164063604	0.869681073	0.974718971	up_in_case
PRJNA8274(	ENSGALG00010027414	PBX3	case	control	0.030747567	0.060534858	0.507931594	0.611501317	0.88947908	up_in_case
PRJNA8274(	ENSGALG00010027417	WNT2B	case	control	-0.129716571	0.314642036	-0.412267135	0.680143634	0.915381147	down_in_case
PRJNA8274(	ENSGALG00010027418	ENSGALG00010027418	case	control	-0.482664286	0.992239622	-0.486439239	0.626655776	0.895656773	down_in_case
PRJNA8274(	ENSGALG00010027419	PTBP1	case	control	-0.007462212	0.059943694	-0.124487019	0.900929678	0.980538427	down_in_case
PRJNA8274(	ENSGALG00010027421	PLPPR3	case	control	-0.052906597	0.1140564	-0.463863466	0.642745569	0.902038523	down_in_case
PRJNA8274(	ENSGALG00010027423	PEX11B	case	control	0.041715941	0.125813863	0.331568716	0.740214945	0.936732187	up_in_case
PRJNA8274(	ENSGALG00010027425	CFD	case	control	0.54360117	0.117774426	4.615612992	3.91937E-06	0.000800671	up_in_case
PRJNA8274(	ENSGALG00010027427	UBAC1	case	control	-0.096303596	0.057152495	-1.685028744	0.091983026	0.476713154	down_in_case
PRJNA8274(	ENSGALG00010027438	ST7L	case	control	-0.002048344	0.053406657	-0.038353717	0.969405662	0.992761697	down_in_case
PRJNA8274(	ENSGALG00010027440	TWF2	case	control	0.025236802	0.058526177	0.431205371	0.666319049	0.912284937	up_in_case
PRJNA8274(	ENSGALG00010027443	MED16	case	control	0.089844407	0.074351092	1.20838046	0.226900952	0.650578187	up_in_case
PRJNA8274(	ENSGALG00010027444	SELENBP1	case	control	-0.062183459	0.087119943	-0.713768362	0.475370426	0.830316001	down_in_case
PRJNA8274(	ENSGALG00010027445	PPM1M	case	control	0.025719234	0.095975318	0.267977586	0.788716572	0.951738421	up_in_case
PRJNA8274(	ENSGALG00010027448	ADAMTSL5	case	control	-0.335718479	0.132252079	-2.53847412	0.011133704	0.185137791	down_in_case
PRJNA8274(	ENSGALG00010027450	CAMSAP1	case	control	-0.111617579	0.038515367	-2.89800117	0.003755492	0.105649139	down_in_case

PRJNA8274(	ENSGALG00010027455	R3HDM4	case	control	0.043402808	0.093116409	0.466113422	0.641134316	0.901516193	up_in_case
PRJNA8274(	ENSGALG00010027457	CAPZA1	case	control	-0.002166467	0.041138176	-0.052663175	0.958000281	0.992056045	down_in_case
PRJNA8274(	ENSGALG00010027460	WDR82	case	control	-0.003582536	0.04574395	-0.078317158	0.937575769	0.988906131	down_in_case
PRJNA8274(	ENSGALG00010027467	GLYCTK	case	control	0.251951353	0.133489273	1.887427716	0.05910282	0.395497471	up_in_case
PRJNA8274(	ENSGALG00010027468	BSG	case	control	0.012818371	0.065586964	0.19544083	0.845047855	0.967281084	up_in_case
PRJNA8274(	ENSGALG00010027471	ENSGALG00010027471	case	control	-0.146609766	0.081508446	-1.798706437	0.07206513	0.434090717	down_in_case
PRJNA8274(	ENSGALG00010027473	DNAH1	case	control	0.090587017	0.280298951	0.323180007	0.746558921	0.938412212	up_in_case
PRJNA8274(	ENSGALG00010027475	ARID3A	case	control	0.164023723	0.091838901	1.78599397	0.074100234	0.438111241	up_in_case
PRJNA8274(	ENSGALG00010027479	HCN2	case	control	-0.24135941	0.178667205	-1.350888149	0.176731265	0.595527443	down_in_case
PRJNA8274(	ENSGALG00010027487	MPZ	case	control	-0.244867216	0.196233253	-1.247837521	0.212090565	0.637162832	down_in_case
PRJNA8274(	ENSGALG00010027490	ATP2B4	case	control	-0.01602374	0.057518744	-0.278582927	0.780564916	0.948875232	down_in_case
PRJNA8274(	ENSGALG00010027492	PCP4L1	case	control	0.770949714	0.644606709	1.19600014			up_in_case
PRJNA8274(	ENSGALG00010027497	KCNT1	case	control	-0.052179673	0.159899816	-0.326327287	0.744176745	0.937597133	down_in_case
PRJNA8274(	ENSGALG00010027499	MOV10	case	control	0.128223147	0.21765549	0.589110558	0.555787112	0.867878646	up_in_case
PRJNA8274(	ENSGALG00010027501	BAP1	case	control	-0.024307784	0.046032854	-0.528052945	0.597462589	0.884680373	down_in_case
PRJNA8274(	ENSGALG00010027502	THOP1	case	control	-0.010590263	0.06279984	-0.168635193	0.866083597	0.973289701	down_in_case
PRJNA8274(	ENSGALG00010027510	RHOC	case	control	-0.170709762	0.150657422	-1.133098922	0.257172714	0.684071765	down_in_case
PRJNA8274(	ENSGALG00010027511	POLRMT	case	control	-0.024424775	0.05407577	-0.451676882	0.651501775	0.905215253	down_in_case
PRJNA8274(	ENSGALG00010027513	SEMA3G	case	control	0.059065443	0.086485048	0.68295554	0.494634937	0.83803731	up_in_case
PRJNA8274(	ENSGALG00010027516	PPM1J	case	control	-0.232399962	0.211521515	-1.098706023	0.271896314	0.698323327	down_in_case
PRJNA8274(	ENSGALG00010027520	FANCE	case	control	-0.149189864	0.132664546	-1.124564689	0.260773544	0.687359696	down_in_case
PRJNA8274(	ENSGALG00010027522	PSMD5	case	control	-0.07900864	0.063247226	-1.24920323	0.211590751	0.636501957	down_in_case
PRJNA8274(	ENSGALG00010027524	ENSGALG00010027524	case	control	-0.182833553	0.073721789	-2.480047688	0.013136481	0.20091089	down_in_case
PRJNA8274(	ENSGALG00010027525	ENSGALG00010027525	case	control	0.014284142	0.18026332	0.079240424	0.936841392	0.988769053	up_in_case
PRJNA8274(	ENSGALG00010027527	ENSGALG00010027527	case	control	-0.226179696	0.452346821	-0.500013894	0.617065294	0.891081782	down_in_case
PRJNA8274(	ENSGALG00010027528	ENSGALG00010027528	case	control	0.019588003	1.663578117	0.011774622	0.990605428	0.997511276	up_in_case
PRJNA8274(	ENSGALG00010027529	ACY1	case	control	-0.05815472	0.090961044	-0.639336547	0.522604018	0.851055244	down_in_case
PRJNA8274(	ENSGALG00010027530	PHF19	case	control	0.018460035	0.098771958	0.186895506	0.851742563	0.96943001	up_in_case
PRJNA8274(	ENSGALG00010027531	UFC1	case	control	0.026638765	0.094904227	0.28069103	0.778947389	0.948479876	up_in_case
PRJNA8274(	ENSGALG00010027533	WDR18	case	control	-0.063942591	0.062298191	-1.026395631	0.304705136	0.728073294	down_in_case
PRJNA8274(	ENSGALG00010027534	KCNN3	case	control	-0.198345046	0.109112566	-1.817802053	0.069094393	0.424578693	down_in_case
PRJNA8274(	ENSGALG00010027536	USP21	case	control	0.072123559	0.137627548	0.52404886	0.600244556	0.884946428	up_in_case
PRJNA8274(	ENSGALG00010027537	SGTA	case	control	-0.128172902	0.04260811	-3.008180909	0.002628166	0.085221721	down_in_case
PRJNA8274(	ENSGALG00010027538	ABHD14A	case	control	0.091435336	0.125483066	0.728666734	0.466205546	0.82702452	up_in_case
PRJNA8274(	ENSGALG00010027539	DDR2L	case	control	-0.14205823	0.084731255	-1.676574134	0.093625788	0.478523919	down_in_case
PRJNA8274(	ENSGALG00010027540	PPOX	case	control	0.076502544	0.108946625	0.70220206	0.482553161	0.833294313	up_in_case
PRJNA8274(	ENSGALG00010027543	TUBB4B	case	control	0.084137775	0.066203038	1.270905051	0.203762429	0.62581674	up_in_case
PRJNA8274(	ENSGALG00010027545	PPARD	case	control	-0.023639204	0.075874586	-0.31155628	0.755377765	0.941559531	down_in_case
PRJNA8274(	ENSGALG00010027547	B4GALT3	case	control	-0.076892175	0.086388756	-0.890071563	0.373427461	0.774369566	down_in_case
PRJNA8274(	ENSGALG00010027548	PCBP4	case	control	-0.030460086	0.080175243	-0.379918845	0.704005658	0.923942815	down_in_case
PRJNA8274(	ENSGALG00010027553	FGF22	case	control	0.275666581	0.235158648	1.172257893	0.241093531	0.666204503	up_in_case
PRJNA8274(	ENSGALG00010027554	ENSGALG00010027554	case	control	0.037977617	0.126867042	0.299349752	0.764673197	0.943786458	up_in_case
PRJNA8274(	ENSGALG00010027555	ENSGALG00010027555	case	control	-0.098867481	0.15261876	-0.647806872	0.51710987	0.848054666	down_in_case
PRJNA8274(	ENSGALG00010027557	C6orf132	case	control	1.19778946	0.791619135	1.513088058	0.130257293	0.532650639	up_in_case
PRJNA8274(	ENSGALG00010027559	ENSGALG00010027559	case	control	0.241674311	0.199277462	1.212752856	0.225224325	0.649074535	up_in_case
PRJNA8274(	ENSGALG00010027561	ADAMTS4	case	control	0.059210923	0.110313853	0.536749659	0.591440549	0.882949889	up_in_case
PRJNA8274(	ENSGALG00010027562	SLC39A3	case	control	0.062863093	0.098191674	0.640207971	0.522037401	0.850840147	up_in_case
PRJNA8274(	ENSGALG00010027563	WDR77	case	control	-0.018581835	0.070578227	-0.26327999	0.792334782	0.952773914	down_in_case
PRJNA8274(	ENSGALG00010027567	PARP3	case	control	0.277079922	0.100839807	2.747723647	0.006001057	0.134447684	up_in_case
PRJNA8274(	ENSGALG00010027568	NELFB	case	control	0.03466687	0.067790242	0.511384375	0.609081934	0.8877909	up_in_case
PRJNA8274(	ENSGALG00010027569	ATP5PB	case	control	-0.054847927	0.057379205	-0.9558851	0.339130287	0.751259351	down_in_case
PRJNA8274(	ENSGALG00010027570	NDUFS2	case	control	-0.176763295	0.077413428	-2.283367357	0.022408738	0.266593144	down_in_case
PRJNA8274(	ENSGALG00010027571	RBM10	case	control	0.092160627	0.072774065	1.26639382	0.205372122	0.628438742	up_in_case
PRJNA8274(	ENSGALG00010027574	GNG7	case	control	-0.014807497	0.052950001	-0.27965055	0.77974562	0.948864508	down_in_case
PRJNA8274(	ENSGALG00010027575	FCER1G	case	control	-0.57872104	0.252566099	-2.291364685	0.02194233	0.26456604	down_in_case
PRJNA8274(	ENSGALG00010027576	GADD45B	case	control	-0.116324539	0.110280899	-1.05480224	0.29151578	0.714303573	down_in_case
PRJNA8274(	ENSGALG00010027578	ENSGALG00010027578	case	control	0.304290427	0.22254977	1.367291579	0.17153393	0.589222964	up_in_case
PRJNA8274(	ENSGALG00010027579	C26H6ORF125	case	control	-0.046529614	0.081724233	-0.569349047	0.569119288	0.875003314	down_in_case
PRJNA8274(	ENSGALG00010027583	GLE1	case	control	0.096525581	0.054364021	1.775541619	0.0758085	0.442474101	up_in_case
PRJNA8274(	ENSGALG00010027586	HJV	case	control	-0.389880167	0.111176682	-3.506851967	0.000453441	0.030442305	down_in_case
PRJNA8274(	ENSGALG00010027590	SEMA3F	case	control	-0.213585041	0.133699541	-1.597500175	0.110154259	0.502778774	down_in_case
PRJNA8274(	ENSGALG00010027592	MAP1S	case	control	-0.051942008	0.08153782	-0.637029639	0.524105538	0.852058799	down_in_case
PRJNA8274(	ENSGALG00010027593	ENSGALG00010027593	case	control	0.020258775	0.274485024	0.073806487	0.941164365	0.989551456	up_in_case
PRJNA8274(	ENSGALG00010027594	ITPR3	case	control	-0.039959936	0.099381394	-0.402086696	0.687620223	0.917290381	down_in_case
PRJNA8274(	ENSGALG00010027595	GNAT1	case	control	0.156985742	0.196829741	0.797571248	0.425119342	0.804485588	up_in_case
PRJNA8274(	ENSGALG00010027597	EXOSC2	case	control	0.068321776	0.080042206	0.85357188	0.393342245	0.786843919	up_in_case
PRJNA8274(	ENSGALG00010027600	KIRREL1	case	control	-0.078318833	0.07641349	-1.024934651	0.305394023	0.728705913	down_in_case
PRJNA8274(	ENSGALG00010027605	FCHO1	case	control	0.01774907	0.230989558	0.076839274	0.938751407	0.989551456	up_in_case
PRJNA8274(	ENSGALG00010027607	ABL1	case	control	-0.038540919	0.04819138	-0.799747155	0.423857306	0.803723282	down_in_case
PRJNA8274(	ENSGALG00010027608	GNAI2	case	control	0.027700202	0.043688389	0.634040374	0.526054474	0.85251736	up_in_case
PRJNA8274(	ENSGALG00010027609	RPL36	case	control	0.088887814	0.068608506	1.295580084	0.195120194	0.61812556	up_in_case
PRJNA8274(	ENSGALG00010027612	BAK1	case	control	0.09204963	0.067112834	1.371565232	0.170198824	0.586751007	up_in_case
PRJNA8274(	ENSGALG00010027614	LONP1	case	control	-0.057758445	0.046111139	-1.252592032	0.210354216	0.636098721	down_in_case
PRJNA8274(	ENSGALG00010027616	SLC38A3	case	control	-0.181340844	0.313630794	-0.578198464	0.563130136	0.871888365	down_in_case
PRJNA8274(	ENSGALG00010027618	PPP1R26	case	control	-0.047198107	0.064780683	-0.728583032	0.466256761	0.82702452	down_in_case
PRJNA8274(	ENSGALG00010027619	JAK3	case	control	0.283802817	0.320908095	0.884374131	0.376494283	0.775199984	up_in_case
PRJNA8274(	ENSGALG00010027621	TSPO2	case	control	0.540148119	0.148890496	3.627821337	0.000285823	0.023091909	up_in_case
PRJNA8274(	ENSGALG00010027622	FIBCD1	case	control	-0.023120302	0.668860792	-0.034566688	0.972425265	0.99340857	down_in_case
PRJNA8274(	ENSGALG00010027623	APOBEC2	case	control	-0.156066727	0.122354407	-1.275530081	0.202121684	0.625073547	down_in_case
PRJNA8274(	ENSGALG00010027626	SEMA3B	case	control	-0.165182753	0.093359887	-1.769311846	0.076841848	0.444873854	down_in_case
PRJNA8274(	ENSGALG00010027627	OARD1	case	control	-0.05976457	0.073967087	-0.807988688	0.419097094	0.80093491	down_in_case
PRJNA8274(	ENSGALG00010027628	PIERCE1	case	control	-0.310773256	0.156465311	-1.986211851	0.047009806	0.357384486	down_in_case
PRJNA8274(	ENSGALG00010027632	LAMC3	case	control	-0.014794284	0.542806023	-0.027255194	0.978256193	0.995096284	down_in_case
PRJNA8274(	ENSGALG00010027634	OASL	case	control	0.036015471	0.180205167	0.199858149	0.841591522	0.9661041	up_in_case
PRJNA8274(	ENSGALG00010027635	CD48	case	control	-0.052776387	0.125219361	-0.42147146	0.673410847	0.91379937	down_in_case
PRJNA8274(	ENSGALG00010027636	SLC1A6	case	control	-0.478264004	0.446434089	-1.071298128	0.284035402	0.708692203	down_in_case
PRJNA8274(	ENSGALG00010027638	MRPS2	case	control	-0.112739451	0.077890444	-1.44741055	0.147781973	0.553505031	down_in_case
PRJNA8274(	ENSGALG00010027641	HYAL3	case	control	-0.068123278	0.093165508	-0.731207066	0.464652685	0.825920869	down_in_case
PRJNA8274(	ENSGALG00010027642	DIPK1B	case	control	0.044699563	0.096784109	0.461848167	0.644190207	0.90210829	up_in_case
PRJNA8274(	ENSGALG00010027644	NAA80	case	control	0.000850725	0.163196058	0.005212904	0.995840723	0.998710499	up_in_case
PRJNA8274(	ENSGALG00010027648	NFYA	case	control	0.053877323	0.04730798	1.138863321	0.254760166	0.680438994	up_in_case
PRJNA8274(	ENSGALG00010027649	FBLN2	case	control	0.031757465	0.115871628	0.274074553	0.784027329	0.949518053	up_in_case
PRJNA8274(	ENSGALG00010027650	CCDC124	case	control	0.021176469	0.056489241	0.374876149	0.707752578	0.924870864	up_in_case
PRJNA8274(	ENSGALG00010027653	WNT7A	case	control	-0.165871131	0.62821272	-0.264036568	0.791751741	0.95239351	down_in_case
PRJNA8274(	ENSGALG00010027654	AIF1L	case	control	-0.078983156	0.063497664	-1.243874991	0.213545575	0.638582746	down_in_case
PRJNA8274(	ENSGALG00010027663	COPA	case	control	-0.019007696	0.035733934	-0.531922846	0.594779434	0.884223506	down_in_case
PRJNA8274(	ENSGALG00010027664	TREMB1	case	control	0.386714443	0.244696825	1.580381938	0.114019425	0.509206053	up_in_case
PRJNA8274(	ENSGALG00010027665	TRAF1	case	control	0.342115641	0.134790556	2.538127672	0.011144732	0.185137791	up_in_case
PRJNA8274(	ENSGALG00010027667	PTPDC1	case	control	0.042569766	0.107505279	0.395978377	0.69212098	0.918988424	up_in_case
PRJNA8274(	ENSGALG00010027668	RANBP3	case	control	-0.049605201	0.043071043	-1.151706504	0.249441699	0.674421685	down_in_case
PRJNA8274(	ENSGALG00010027671	BARX1	case	control	0.110278197	0.391456254	0.281712697	0.778163818	0.948090874	up_in_case
PRJNA8274(	ENSGALG00010027672	CNTRL	case	control	0.202939058	0.10299987	1.970284605	0.048805762	0.363542241	up_in_case
PRJNA8274(	ENSGALG00010027674	TREM2	case	control	0.281882663	0.157819181	1.786111558	0.074081197	0.438111241	up_in_case
PRJNA8274(	ENSGALG00010027685	ENSGALG00010027685	case	control	0.013183692	0.312021781	0.042252474	0.966297432	0.992505774	up_in_case
PRJNA8274(	ENSGALG00010027692	PHF2	case	control	-0.145865103	0.056553861	-2.579224461	0.009902242	0.177161817	down_in_case
PRJNA8274(	ENSGALG00010027693	RAB14	case	control	0.112004085	0.038413098	2.915778486	0.003548023	0.101880984	up_in_case
PRJNA8274(	ENSGALG00010027695	TREMB2	case	control	0.199115679	0.233648796	0.852200752	0.394102679	0.786978937	up_in_case
PRJNA8274(	ENSGALG00010027697	CATH2	case	control	-0.395153582	0.639357268	-0.618048159	0.536543593	0.857336818	down_in_case
PRJNA8274(	ENSGALG00010027698	FAM120A	case	control	-0.03455567	0.034242272	-1.009152368	0.312901566	0.734574752	down_in_case
PRJNA8274(	ENSGALG00010027699	VANGL2	case	control	-0.036923921	0.065417116	-0.564438218	0.572455935	0.876814097	down_in_case
PRJNA8274(	ENSGALG00010027700	TCF3	case	control	-0.046618112	0.060484029	-0.770750759	0.440854679	0.813083204	down_in_case
PRJNA8274(	ENSGALG00010027701	NHLH1	case	control	-0.01663364	0.332312507	-0.050054211	0.960079188	0.992056045	down_in_case
PRJNA8274(	ENSGALG00010027702	FOXP4	case	control	-0.016914408	0.139385521	-0.121349822	0.90341396	0.980997505	down_in_case
PRJNA8274(	ENSGALG00010027703	NCSTN	case	control	-0.017649793	0.067305307	-0.262234798	0.793140427	0.95280257	down_in_case
PRJNA8274(	ENSGALG00010027704	MDFI	case	control	0.079251331	0.072954546	1.086311057	0.277341397	0.702814456	up_in_case
PRJNA8274(	ENSGALG00010027705	S100A11	case	control	0.253776279	0.144494028	1.756309813	0.079035523	0.449171025	up_in_case
PRJNA8274(	ENSGALG00010027706	GSN	case	control	0.174461025	0.099757164	1.748857106	0.080315723	0.452049261	up_in_case
PRJNA8274(	ENSGALG00010027707	HIGD1C	case	control	0.398484949	0.124179045	3.208954859	0.001332184	0.057903441	up_in_case
PRJNA8274(	ENSGALG00010027708	ENSGALG00010027708	case	control	-0.11040831	0.371224022	-0.297416932	0.766148218	0.943987552	down_in_case
PRJNA8274(	ENSGALG00010027709	PSMA5	case	control	-0.094399726	0.073271203	-1.288360537	0.197620489	0.62094535	down_in_case
PRJNA8274(	ENSGALG00010027710	ENSGALG00010027710	case	control	0.435348207	0.260320892	1.672352162	0.094454899	0.479653786	up_in_case
PRJNA8274(	ENSGALG00010027711	WNK2	case	control	-0.129390304	0.0995663	-1.299539137	0.193758971	0.616682237	down_in_case
PRJNA8274(	ENSGALG00010027712	STOM	case	control	-0.363260204	0.176192026	-2.061728969	0.039233547	0.335091843	down_in_case
PRJNA8274(	ENSGALG00010027713	ENSGALG00010027713	case	control	0.033217499	0.096052161	0.345827716	0.729472195	0.933556014	up_in_case
PRJNA8274(	ENSGALG00010027714	SYPL2	case	control	-0.117331193	0.12007853	-0.977120494	0.328509503	0.745445783	down_in_case
PRJNA8274(	ENSGALG00010027715	NINJ1	case	control	0.075543757	0.095909986	0.787652676	0.430899894	0.806930928	up_in_case

PRJNA8274(	ENSGALG00010027717	ATXN7L2	case	control	-0.044554616	0.134099146	-0.332251303	0.739699507	0.9363983	down_in_case
PRJNA8274(	ENSGALG00010027718	ENSGALG00010027718	case	control	0.297946303	0.243564573	1.223274384	0.221226079	0.645487233	up_in_case
PRJNA8274(	ENSGALG00010027719	GPR61	case	control	-0.203501457	0.412164066	-0.49373896	0.621490546	0.893197469	down_in_case
PRJNA8274(	ENSGALG00010027720	DAB2IP	case	control	-0.137354751	0.05970389	-2.300599707	0.021414268	0.26195383	down_in_case
PRJNA8274(	ENSGALG00010027721	SUSD3	case	control	-0.024301504	0.1529238	-0.158912502	0.87373781	0.975062028	down_in_case
PRJNA8274(	ENSGALG00010027722	GNAI3	case	control	-0.018486579	0.042963623	-0.430284442	0.666988743	0.912284937	down_in_case
PRJNA8274(	ENSGALG00010027723	ENSGALG00010027723	case	control	1.355294882	0.726668083	1.865081065	0.062170042	0.405537748	up_in_case
PRJNA8274(	ENSGALG00010027724	TTLL11	case	control	-0.088664943	0.094171	-0.941531294	0.34643266	0.756911694	down_in_case
PRJNA8274(	ENSGALG00010027725	BTN1A1	case	control	-0.148608293	0.343691335	-0.432388826	0.665458838	0.912007336	down_in_case
PRJNA8274(	ENSGALG00010027727	GSTM2	case	control	0.199697512	0.179211204	1.114313766	0.265144603	0.69125489	up_in_case
PRJNA8274(	ENSGALG00010027728	CRNN	case	control	2.168794367	1.249136248	1.736235235	0.082522249	0.456372384	up_in_case
PRJNA8274(	ENSGALG00010027729	EPS8L3	case	control	0.066533374	0.204759544	0.324934177	0.745230889	0.937849656	up_in_case
PRJNA8274(	ENSGALG00010027730	ENSGALG00010027730	case	control	0.131066863	0.094010861	1.394167245	0.163267107	0.577197348	up_in_case
PRJNA8274(	ENSGALG00010027731	OLFML2A	case	control	-0.237928964	0.113686886	-2.092844406	0.036363046	0.32664727	down_in_case
PRJNA8274(	ENSGALG00010027732	EEF2	case	control	-0.11871029	0.064461434	-1.841570732	0.065537966	0.415550718	down_in_case
PRJNA8274(	ENSGALG00010027733	RPL35	case	control	-0.042350364	0.073827611	-0.573638558	0.566212414	0.873008555	down_in_case
PRJNA8274(	ENSGALG00010027734	CSF1	case	control	0.02039253	0.092540589	0.220363094	0.825588386	0.961158831	up_in_case
PRJNA8274(	ENSGALG00010027736	ENSGALG00010027736	case	control	-0.215105165	1.870034806	-0.115027359	0.908423437	0.981730793	down_in_case
PRJNA8274(	ENSGALG00010027738	ENSGALG00010027738	case	control	0.570524999	0.242135349	2.3562235	0.018461806	0.242205346	up_in_case
PRJNA8274(	ENSGALG00010027739	FGD3	case	control	0.079707451	0.053033435	1.502966022	0.132847806	0.536224013	up_in_case
PRJNA8274(	ENSGALG00010027740	MARCHF2	case	control	0.065361426	0.070916711	0.921664655	0.356703522	0.763373775	up_in_case
PRJNA8274(	ENSGALG00010027741	RAB11B	case	control	0.039203253	0.042540354	0.921554464	0.35676102	0.763373775	up_in_case
PRJNA8274(	ENSGALG00010027743	C1orf74	case	control	-0.178455785	0.186761393	-0.955528241	0.339310631	0.751259351	down_in_case
PRJNA8274(	ENSGALG00010027744	ENSGALG00010027744	case	control	0.730366444	1.325501131	0.551011558	0.581625751	0.880290776	up_in_case
PRJNA8274(	ENSGALG00010027745	BICD2	case	control	-0.011333988	0.052415618	-0.216233038	0.828806102	0.962007082	down_in_case
PRJNA8274(	ENSGALG00010027746	UQCR11	case	control	-0.105276851	0.119276402	-0.882629333	0.377436575	0.775926253	down_in_case
PRJNA8274(	ENSGALG00010027751	ANGPTL4	case	control	0.230617704	0.13844538	1.6657667	0.095759891	0.483185646	up_in_case
PRJNA8274(	ENSGALG00010027752	SCAI	case	control	0.032331759	0.066977527	0.482725474	0.629290673	0.896269608	up_in_case
PRJNA8274(	ENSGALG00010027759	ENSGALG00010027759	case	control	1.037233701	0.715961471	1.448728377	0.147413452	0.552558944	up_in_case
PRJNA8274(	ENSGALG00010027766	ENSGALG00010027766	case	control	0.211250782	0.694221425	0.304298852	0.76090022	0.942965001	up_in_case
PRJNA8274(	ENSGALG00010027767	ENSGALG00010027767	case	control	0.242423374	0.557137508	0.435123053	0.663473111	0.911044469	up_in_case
PRJNA8274(	ENSGALG00010027772	ENSGALG00010027772	case	control	1.770951702	0.794363617	2.229396796	0.025787515	0.284977951	up_in_case
PRJNA8274(	ENSGALG00010027775	MVB12B	case	control	-0.11075495	0.044959874	-2.463417751	0.013761947	0.205985143	down_in_case
PRJNA8274(	ENSGALG00010027776	TKT	case	control	0.209655096	0.107164514	1.956385463	0.050419763	0.367061596	up_in_case
PRJNA8274(	ENSGALG00010027784	PEAR1	case	control	0.108213632	0.100574289	1.075957207	0.281946395	0.705976791	up_in_case
PRJNA8274(	ENSGALG00010027787	DCP1A	case	control	-0.044346565	0.082922981	-0.534792221	0.592793543	0.883625114	down_in_case
PRJNA8274(	ENSGALG00010027789	UAP1L1	case	control	0.083595615	0.073753856	1.1334406	0.257029272	0.683997349	up_in_case
PRJNA8274(	ENSGALG00010027801	MBD3	case	control	-0.02354573	0.050441597	-0.466791914	0.640648761	0.901327818	down_in_case
PRJNA8274(	ENSGALG00010027803	SAPCD2	case	control	-0.135208659	0.156606735	-0.863364271	0.387937218	0.78298909	down_in_case
PRJNA8274(	ENSGALG00010027811	MEX3D	case	control	-0.025203907	0.07180787	-0.350990876	0.725595193	0.931921256	down_in_case
PRJNA8274(	ENSGALG00010027813	LRRC71	case	control	0.440205744	0.270914751	1.624886582	0.104186728	0.493800828	up_in_case
PRJNA8274(	ENSGALG00010027814	ENSGALG00010027814	case	control	-0.05621002	0.071467009	-0.786517038	0.431564641	0.807261374	down_in_case
PRJNA8274(	ENSGALG00010027816	ZMYND19	case	control	-0.027648557	0.067457305	-0.409867503	0.681903145	0.915594727	down_in_case
PRJNA8274(	ENSGALG00010027821	NMRK2	case	control	0.035240207	0.131085426	0.268833908	0.788057499	0.951551316	up_in_case
PRJNA8274(	ENSGALG00010027822	DPH7	case	control	0.248104141	0.098950411	2.507358358	0.012163732	0.19369863	up_in_case
PRJNA8274(	ENSGALG00010027826	ARHGEF11	case	control	-0.045619136	0.061811437	-0.738037135	0.460491883	0.82454265	down_in_case
PRJNA8274(	ENSGALG00010027830	CACNA1D	case	control	0.105804022	0.166657441	0.634859274	0.5255202	0.85251736	up_in_case
PRJNA8274(	ENSGALG00010027831	ATCAY	case	control	-0.04550525	0.111942485	-0.406505627	0.684371124	0.916433328	down_in_case
PRJNA8274(	ENSGALG00010027838	ENSGALG00010027838	case	control	0.195392981	0.350555011	0.557381792	0.577266603	0.878143389	up_in_case
PRJNA8274(	ENSGALG00010027842	SLAMF8	case	control	-0.238208181	0.176274252	-1.351349838	0.176583394	0.595527443	down_in_case
PRJNA8274(	ENSGALG00010027847	NRTN	case	control	1.03105093	0.572322653	1.80152039	0.071620896	0.432606532	up_in_case
PRJNA8274(	ENSGALG00010027860	ENSGALG00010027860	case	control	0.146650466	0.349368428	0.419758782	0.674661678	0.914210367	up_in_case
PRJNA8274(	ENSGALG00010027861	PNPLA7	case	control	-0.016280235	0.065290601	-0.249350362	0.803089778	0.954891353	down_in_case
PRJNA8274(	ENSGALG00010027862	ENSGALG00010027862	case	control	-0.010511999	0.323964459	-0.032448	0.974114784	0.993711044	down_in_case
PRJNA8274(	ENSGALG00010027863	CHDH	case	control	-0.056555005	0.07415977	-0.762610305	0.445695837	0.817180323	down_in_case
PRJNA8274(	ENSGALG00010027864	ACTR8	case	control	0.04358201	0.062238264	0.700244618	0.483774552	0.833892971	up_in_case
PRJNA8274(	ENSGALG00010027865	SELENOK	case	control	0.037839653	0.088473905	0.427692806	0.668874788	0.913032039	up_in_case
PRJNA8274(	ENSGALG00010027866	KCNA2	case	control	0.03081339	0.102983249	0.299207785	0.76478151	0.943786458	up_in_case
PRJNA8274(	ENSGALG00010027867	UBAP2L	case	control	-0.073221435	0.07573311	-0.966835172	0.333626439	0.748508541	down_in_case
PRJNA8274(	ENSGALG00010027868	CACNA2D3	case	control	0.019506622	0.336117762	0.058035083	0.953720683	0.992056045	up_in_case
PRJNA8274(	ENSGALG00010027869	NSMF	case	control	0.007923336	0.08226241	0.096317815	0.923268162	0.985499345	up_in_case
PRJNA8274(	ENSGALG00010027870	HAX1	case	control	-0.018480015	0.061288463	-0.301525187	0.763014047	0.943786458	down_in_case
PRJNA8274(	ENSGALG00010027872	KDM5B	case	control	-0.118827179	0.047066637	-2.524658357	0.011581081	0.188192574	down_in_case
PRJNA8274(	ENSGALG00010027873	RFX2	case	control	0.140301825	0.125593758	1.117108267	0.263948036	0.689776483	up_in_case
PRJNA8274(	ENSGALG00010027874	FRS3	case	control	0.032991324	0.055625991	0.593091885	0.553119661	0.866252369	up_in_case
PRJNA8274(	ENSGALG00010027875	WNT5A	case	control	-0.312707565	0.152620386	-2.048923955	0.040469551	0.33753444	down_in_case
PRJNA8274(	ENSGALG00010027876	ANAPC2	case	control	0.101778099	0.082796536	1.229255521	0.218976018	0.643443819	up_in_case
PRJNA8274(	ENSGALG00010027877	OAZ1	case	control	-0.114793712	0.047055416	-2.439543039	0.014705851	0.213930485	down_in_case
PRJNA8274(	ENSGALG00010027879	ENSGALG00010027879	case	control	-0.273016168	0.100416769	-2.718830451	0.006551318	0.139826638	down_in_case
PRJNA8274(	ENSGALG00010027880	AMH	case	control	-0.2825251	0.112263788	-2.516618273	0.011848712	0.190806969	down_in_case
PRJNA8274(	ENSGALG00010027881	ENSGALG00010027881	case	control	-0.041727074	0.233210419	-0.178924569	0.857996931	0.971753216	down_in_case
PRJNA8274(	ENSGALG00010027883	ATP8B2	case	control	-0.019834788	0.049024113	-0.404592489	0.68577708	0.917034645	down_in_case
PRJNA8274(	ENSGALG00010027884	SF3A2	case	control	-0.055617253	0.058450162	-0.951532899	0.341333926	0.752827754	down_in_case
PRJNA8274(	ENSGALG00010027885	PLEKHJ1	case	control	0.043702373	0.051648478	0.846150264	0.397468924	0.788728117	up_in_case
PRJNA8274(	ENSGALG00010027886	IL6R	case	control	-0.23814585	0.123679425	-1.925509044	0.054165706	0.37945063	down_in_case
PRJNA8274(	ENSGALG00010027887	ERC2	case	control	-0.309124744	0.262004671	-1.179844403	0.238062106	0.662913814	down_in_case
PRJNA8274(	ENSGALG00010027888	PFKFB2	case	control	-0.062617391	0.120577429	-0.519312704	0.603542695	0.885268908	down_in_case
PRJNA8274(	ENSGALG00010027889	RBM8A	case	control	0.122158313	0.093737852	1.303190875	0.192509605	0.615090794	up_in_case
PRJNA8274(	ENSGALG00010027890	ENSGALG00010027890	case	control	0.282080362	0.35090694	0.803860882	0.421477324	0.802413627	up_in_case
PRJNA8274(	ENSGALG00010027891	CCDC66	case	control	0.147379948	0.099688693	1.478401838	0.139300254	0.541505735	up_in_case
PRJNA8274(	ENSGALG00010027892	SHE	case	control	-0.054451359	0.063803972	-0.853416445	0.393428405	0.786843919	down_in_case
PRJNA8274(	ENSGALG00010027893	DOT1L	case	control	-0.076959384	0.089445663	-0.860403752	0.389566518	0.783772245	down_in_case
PRJNA8274(	ENSGALG00010027894	RSBN1	case	control	-0.006683746	0.041588927	-0.160709747	0.872322012	0.975062028	down_in_case
PRJNA8274(	ENSGALG00010027895	LIX1L	case	control	0.168018594	0.111136666	1.511819628	0.130579755	0.533148629	up_in_case
PRJNA8274(	ENSGALG00010027896	TOR4A	case	control	0.105829928	0.109038214	0.970576498	0.331759214	0.748120789	up_in_case
PRJNA8274(	ENSGALG00010027897	AP3D1	case	control	-0.012327031	0.040504283	-0.30433895	0.760869674	0.942965001	down_in_case
PRJNA8274(	ENSGALG00010027898	TASOR	case	control	0.003322893	0.046797394	0.071005931	0.943393035	0.990129937	up_in_case
PRJNA8274(	ENSGALG00010027899	PMVK	case	control	0.178986624	0.142537555	1.255715544	0.209219121	0.634131716	up_in_case
PRJNA8274(	ENSGALG00010027901	MOB3A	case	control	0.084401416	0.061699901	1.367934394	0.171332613	0.588955857	up_in_case
PRJNA8274(	ENSGALG00010027902	UBE2Q1	case	control	0.020445188	0.039191506	0.521673957	0.60189736	0.884961161	up_in_case
PRJNA8274(	ENSGALG00010027903	NDUFA8	case	control	-0.029917108	0.082168222	-0.36409584	0.715786443	0.926862121	down_in_case
PRJNA8274(	ENSGALG00010027904	MORN5	case	control	0.654190095	0.483268522	1.353678267	0.175839045	0.594727928	up_in_case
PRJNA8274(	ENSGALG00010027905	SYN2	case	control	-0.252275317	0.23895505	-1.055743819	0.291085272	0.714222439	down_in_case
PRJNA8274(	ENSGALG00010027906	LHX6	case	control	-0.125127615	0.127924148	-0.978139131	0.328005517	0.745445783	down_in_case
PRJNA8274(	ENSGALG00010027907	MKNK2	case	control	-0.150994118	0.088313146	-1.709758112	0.087310612	0.467042649	down_in_case
PRJNA8274(	ENSGALG00010027908	PBXIP1	case	control	-0.208820879	0.103082747	-2.025759747	0.042789411	0.344633831	down_in_case
PRJNA8274(	ENSGALG00010027909	TIMP4	case	control	-0.132965868	0.365778572	-0.363514644	0.716220476	0.926862121	down_in_case
PRJNA8274(	ENSGALG00010027910	CHRNB2	case	control	-0.094249722	0.122823816	-0.767357059	0.442869252	0.81411882	down_in_case
PRJNA8274(	ENSGALG00010027911	PTPN22	case	control	0.439052561	0.47981953	0.915036872	0.360172274	0.765754313	up_in_case
PRJNA8274(	ENSGALG00010027912	RGS3	case	control	-0.003921897	0.086271916	-0.045459717	0.963740883	0.992068146	down_in_case
PRJNA8274(	ENSGALG00010027913	PYGO2	case	control	0.0630463	0.094455205	0.667473009	0.504470045	0.842675107	up_in_case
PRJNA8274(	ENSGALG00010027914	BTBD2	case	control	-0.240345649	0.089766938	-2.677440647	0.0074187	0.150265444	down_in_case
PRJNA8274(	ENSGALG00010027915	GSTM3	case	control	0.065117002	0.077745136	0.83757011	0.402272186	0.792021921	up_in_case
PRJNA8274(	ENSGALG00010027916	ADAR	case	control	0.111046146	0.043771624	2.536943679	0.011182494	0.185465245	up_in_case
PRJNA8274(	ENSGALG00010027917	PPARG	case	control	-0.176762702	0.238305356	-0.741748759	0.458239572	0.823633217	down_in_case
PRJNA8274(	ENSGALG00010027918	CSNK1G2	case	control	-0.01621203	0.047718606	-0.339742324	0.734050586	0.933846596	down_in_case
PRJNA8274(	ENSGALG00010027919	TSEN2	case	control	0.106768866	0.117663414	0.907409211	0.364190441	0.770055025	up_in_case
PRJNA8274(	ENSGALG00010027920	SCAMP4	case	control	0.032699391	0.064007509	0.510868038	0.609443463	0.888004876	up_in_case
PRJNA8274(	ENSGALG00010027921	MKRN2OS	case	control	-0.258757546	0.15528144	-1.666377815	0.095638187	0.482930958	down_in_case
PRJNA8274(	ENSGALG00010027922	SHC1	case	control	-0.04378361	0.049761986	-0.87986059	0.378934836	0.777229179	down_in_case
PRJNA8274(	ENSGALG00010027923	AMPD2	case	control	-0.128091127	0.092795562	-1.380358331	0.167476343	0.583641076	down_in_case
PRJNA8274(	ENSGALG00010027924	ENSGALG00010027924	case	control	1.002839147	0.482632102	2.077854212	0.037722789	0.329750561	up_in_case
PRJNA8274(	ENSGALG00010027925	MKRN2	case	control	0.014273609	0.038848457	0.367417656	0.713307504	0.926862121	up_in_case
PRJNA8274(	ENSGALG00010027926	CKS1B	case	control	0.121516112	0.088732673	1.369463001	0.170854593	0.588302594	up_in_case
PRJNA8274(	ENSGALG00010027927	ENSGALG00010027927	case	control	-0.24359823	0.126065922	-1.932308323	0.053321462	0.376996967	down_in_case
PRJNA8274(	ENSGALG00010027928	FLAD1	case	control	0.13160779	0.081385174	1.617097857	0.105857159	0.495266487	up_in_case
PRJNA8274(	ENSGALG00010027929	CDK5RAP2	case	control	0.06006474	0.08573101	0.7006186	0.483541067	0.833691495	up_in_case
PRJNA8274(	ENSGALG00010027930	RAF1	case	control	-0.064907479	0.05907007	-1.098821776	0.271845812	0.698323327	down_in_case
PRJNA8274(	ENSGALG00010027931	ABHD17A	case	control	-0.061271723	0.060625783	-1.010654533	0.312181805	0.734364975	down_in_case
PRJNA8274(	ENSGALG00010027932	POLE3	case	control	-0.013622603	0.058491779	-0.232897737	0.815840816	0.957374769	down_in_case
PRJNA8274(	ENSGALG00010027933	ENSGALG00010027933	case	control	0.180071542	0.163865461	1.098898704	0.271812252	0.698323327	up_in_case
PRJNA8274(	ENSGALG00010027934	CNBP	case	control	-0.06751252	0.044144597	-1.529349584	0.126177804	0.525939433	down_in_case
PRJNA8274(	ENSGALG00010027935	ISY1	case	control	0.011901185	0.073430966	0.162073114	0.871248278	0.975062028	up_in_case
PRJNA8274(	ENSGALG00010027936	ENSGALG00010027936	case	control	0.022186589	0.049848237	0.445082712	0.656259982	0.907494354	up_in_case
PRJNA8274(	ENSGALG00010027937	HDHD3	case	control	-0.064743299	0.050864704	-1.272853161	0.203070153	0.625537942	down_in_case
PRJNA8274(	ENSGALG00010027938	ENSGALG00010027938	case	control	0.14873796	0.106952887	1.390686726	0.164320445	0.579452754	up_in_case

PRJNA8274(	ENSGALG00010027939	BSPRY	case	control	0.144716475	0.598727172	0.241706877	0.809007301	0.955875648	up_in_case
PRJNA8274(	ENSGALG00010027940	ZBTB7B	case	control	0.609385956	0.351929774	1.731555559	0.083352725	0.457254339	up_in_case
PRJNA8274(	ENSGALG00010027941	KLF2	case	control	0.0377253	0.09030903	0.417735632	0.676140417	0.914247368	up_in_case
PRJNA8274(	ENSGALG00010027942	KHDC4	case	control	0.021996407	0.053409717	0.411842788	0.680454657	0.915381147	up_in_case
PRJNA8274(	ENSGALG00010027943	RAB43	case	control	0.037573191	0.061678059	0.609182459	0.542403505	0.85975913	up_in_case
PRJNA8274(	ENSGALG00010027944	WDR31	case	control	-0.051549764	0.226211237	-0.227883303	0.819736963	0.958669267	down_in_case
PRJNA8274(	ENSGALG00010027946	AP1M1	case	control	-0.038940646	0.061651718	-0.631623045	0.527633224	0.853386536	down_in_case
PRJNA8274(	ENSGALG00010027947	GP9	case	control	-0.622384146	0.349237911	-1.782120802	0.074729534	0.439711709	down_in_case
PRJNA8274(	ENSGALG00010027948	PRPF4	case	control	0.032989696	0.051867629	0.636036319	0.52475275	0.852358222	up_in_case
PRJNA8274(	ENSGALG00010027949	ENSGALG00010027949	case	control	-2.464320387	0.786820423	-3.131998502	0.001736208	0.067102078	down_in_case
PRJNA8274(	ENSGALG00010027950	CDC26	case	control	-0.042009751	0.080603567	-0.521189733	0.602234606	0.885002041	down_in_case
PRJNA8274(	ENSGALG00010027951	FAM32A	case	control	-0.084549672	0.071340315	-1.185159781	0.235954278	0.660642087	down_in_case
PRJNA8274(	ENSGALG00010027952	CIB3	case	control	-0.020760313	0.067166721	-0.309086299	0.757255881	0.941659745	down_in_case
PRJNA8274(	ENSGALG00010027953	SLC31A1	case	control	0.285994187	0.107647922	2.6567553	0.007889669	0.155766219	up_in_case
PRJNA8274(	ENSGALG00010027954	CFAP92	case	control	0.083432555	0.293571296	0.284198611	0.776258179	0.947228003	up_in_case
PRJNA8274(	ENSGALG00010027955	ENSGALG00010027955	case	control	0.638049965	0.132538302	4.814079826	1.4788E-06	0.000352447	up_in_case
PRJNA8274(	ENSGALG00010027956	RAB8A	case	control	-0.047101149	0.052227727	-0.901841836	0.367140892	0.771770041	down_in_case
PRJNA8274(	ENSGALG00010027957	ENTREP3	case	control	-0.010266163	0.272072095	-0.037733246	0.969900369	0.992761697	down_in_case
PRJNA8274(	ENSGALG00010027958	ENSGALG00010027958	case	control	-0.212511033	0.132114006	-1.608542798	0.107716351	0.499602493	down_in_case
PRJNA8274(	ENSGALG00010027959	TPM4	case	control	0.113846296	0.082553306	1.379064038	0.167875011	0.584193542	up_in_case
PRJNA8274(	ENSGALG00010027960	PPP6C	case	control	-0.041699835	0.051648599	-0.807375914	0.419449939	0.80093491	down_in_case
PRJNA8274(	ENSGALG00010027961	TINCR	case	control	1.105346667	0.65743197	1.681309576	0.092702799	0.477539634	up_in_case
PRJNA8274(	ENSGALG00010027962	SCAMP3	case	control	-0.071590132	0.107126888	-0.668274174	0.503958596	0.842675107	down_in_case
PRJNA8274(	ENSGALG00010027964	RABEPK	case	control	-0.034495936	0.077920805	-0.44270508	0.657979068	0.908367791	down_in_case
PRJNA8274(	ENSGALG00010027965	ACAD9	case	control	-0.125637089	0.110371646	-1.138309469	0.254991281	0.680801964	down_in_case
PRJNA8274(	ENSGALG00010027966	CLK2	case	control	0.013526126	0.052094788	0.259644519	0.795137993	0.95280257	up_in_case
PRJNA8274(	ENSGALG00010027968	FKBP15	case	control	0.002558087	0.050535809	0.050619285	0.959628895	0.992056045	up_in_case
PRJNA8274(	ENSGALG00010027969	HSPA5	case	control	0.253876969	0.087551885	2.899731603	0.003734823	0.105649139	up_in_case
PRJNA8274(	ENSGALG00010027970	GRIN3B	case	control	-0.155703921	0.096292994	-1.616980791	0.105882427	0.495266487	down_in_case
PRJNA8274(	ENSGALG00010027971	ENSGALG00010027971	case	control	-0.427850391	0.223402105	-1.915158283	0.055472321	0.383830126	down_in_case
PRJNA8274(	ENSGALG00010027972	TMEM259	case	control	-0.038321174	0.055837027	-0.686303979	0.49252144	0.837671284	down_in_case
PRJNA8274(	ENSGALG00010027973	GAPVD1	case	control	0.042803621	0.04079625	1.049204794	0.294083873	0.71654445	up_in_case
PRJNA8274(	ENSGALG00010027974	ENSGALG00010027974	case	control	-0.186784976	0.513637519	-0.363651347	0.716118379	0.926862121	down_in_case
PRJNA8274(	ENSGALG00010027975	IQSEC1	case	control	-0.034690713	0.095321544	-0.363933604	0.715907591	0.926862121	down_in_case
PRJNA8274(	ENSGALG00010027976	LRSAM1	case	control	0.075122061	0.046770261	1.60619289	0.108231535	0.500094527	up_in_case
PRJNA8274(	ENSGALG00010027977	BOLA1	case	control	-0.059138261	0.121587603	-0.48638397	0.626694954	0.895656773	down_in_case
PRJNA8274(	ENSGALG00010027979	RPL12	case	control	-0.096359731	0.07522688	-1.280921541	0.200221226	0.622902456	down_in_case
PRJNA8274(	ENSGALG00010027980	NUP210	case	control	-0.050541895	0.087770463	-0.575841723	0.564722169	0.872275547	down_in_case
PRJNA8274(	ENSGALG00010027981	CNN2	case	control	0.233760717	0.084888859	2.753726683	0.005892095	0.133681071	up_in_case
PRJNA8274(	ENSGALG00010027982	ENTPD2	case	control	0.116794809	0.147715279	0.790675208	0.429133547	0.805896795	up_in_case
PRJNA8274(	ENSGALG00010027983	POGZ	case	control	-0.100265313	0.055180654	-1.817037411	0.069211385	0.424591509	down_in_case
PRJNA8274(	ENSGALG00010027984	ARHGAP45	case	control	0.143855296	0.12328325	1.166868138	0.243263625	0.667473598	up_in_case
PRJNA8274(	ENSGALG00010027985	PSMB4	case	control	-0.045727689	0.047597254	-0.960721162	0.336692388	0.750654996	down_in_case
PRJNA8274(	ENSGALG00010027986	CGN	case	control	0.280983843	0.225670843	1.245104768	0.213093242	0.638582746	up_in_case
PRJNA8274(	ENSGALG00010027987	TMEM141	case	control	-0.067606683	0.096158909	-0.703072483	0.48201058	0.832928691	down_in_case
PRJNA8274(	ENSGALG00010027988	HDAC11	case	control	0.093313505	0.103746437	0.899438164	0.368419319	0.771770041	up_in_case
PRJNA8274(	ENSGALG00010027989	ENSGALG00010027989	case	control	-0.097300696	0.101774968	-0.956037596	0.33905324	0.751259351	down_in_case
PRJNA8274(	ENSGALG00010027990	ENSGALG00010027990	case	control	-0.063666794	0.182820619	-0.348247338	0.72765444	0.932979874	down_in_case
PRJNA8274(	ENSGALG00010027991	ENSGALG00010027991	case	control	-0.137909499	0.206629489	-0.667424088	0.504501284	0.842675107	down_in_case
PRJNA8274(	ENSGALG00010027993	CLIC3	case	control	-0.144339885	0.123350346	-1.170161981	0.241935789	0.666243691	down_in_case
PRJNA8274(	ENSGALG00010027994	TUFT1	case	control	0.167286628	0.091643972	1.825396959	0.067941148	0.421339236	up_in_case
PRJNA8274(	ENSGALG00010027995	POLR2E	case	control	0.035406728	0.049866213	0.710034415	0.477682795	0.830520323	up_in_case
PRJNA8274(	ENSGALG00010027996	GPX4	case	control	0.074191339	0.075733741	0.979633887	0.327266871	0.744857565	up_in_case
PRJNA8274(	ENSGALG00010027998	SNX27	case	control	-0.04136022	0.049860987	-0.829510652	0.406815512	0.794253996	down_in_case
PRJNA8274(	ENSGALG00010027999	ENSGALG00010027999	case	control	-0.907045445	0.342647028	-2.647171496	0.008116818	0.158134189	down_in_case
PRJNA8274(	ENSGALG00010028000	MRPL9	case	control	-0.079157369	0.070692207	-1.119746738	0.262821702	0.688674415	down_in_case
PRJNA8274(	ENSGALG00010028001	SBNO2	case	control	0.070475397	0.057550605	1.224581348	0.220732999	0.644744838	up_in_case
PRJNA8274(	ENSGALG00010028002	ABCA2	case	control	-0.122345069	0.07224032	-1.693584271	0.090344309	0.473926492	down_in_case
PRJNA8274(	ENSGALG00010028004	ENSGALG00010028004	case	control	-0.951285774	0.22814135	-4.169720978	3.04973E-05	0.004361111	down_in_case
PRJNA8274(	ENSGALG00010028005	IER5L	case	control	-0.072945036	0.094406824	-0.772666981	0.439719492	0.812401646	down_in_case
PRJNA8274(	ENSGALG00010028007	STK11	case	control	-0.108979471	0.078677939	-1.385133778	0.166011565	0.581099135	down_in_case
PRJNA8274(	ENSGALG00010028008	NTMT1	case	control	-0.085086372	0.063833696	-1.3329382	0.182552085	0.604001577	down_in_case
PRJNA8274(	ENSGALG00010028009	RRP9	case	control	0.024027321	0.067567922	0.355602489	0.72213828	0.929484914	up_in_case
PRJNA8274(	ENSGALG00010028010	ASB6	case	control	-0.001588104	0.05498808	-0.02888087	0.976959603	0.994586386	down_in_case
PRJNA8274(	ENSGALG00010028011	ENSGALG00010028011	case	control	-0.104685901	0.0846359	-1.23689713	0.216125276	0.640802704	down_in_case
PRJNA8274(	ENSGALG00010028012	PRRX2	case	control	0.03647228	0.170647257	0.213729073	0.830758338	0.962228709	up_in_case
PRJNA8274(	ENSGALG00010028013	CBARP	case	control	-0.085493834	0.090755122	-0.942027648	0.346178485	0.756500951	down_in_case
PRJNA8274(	ENSGALG00010028014	ENSGALG00010028014	case	control	-0.271677004	0.362593457	-0.749260636	0.453700129	0.821282604	down_in_case
PRJNA8274(	ENSGALG00010028015	DUSP7	case	control	-0.015609977	0.075132313	-0.207766496	0.835411294	0.963962035	down_in_case
PRJNA8274(	ENSGALG00010028019	RPL29	case	control	-0.000159841	0.084627817	-0.001888752	0.998492995	0.999471498	down_in_case
PRJNA8274(	ENSGALG00010028020	ENSGALG00010028020	case	control	-0.143920225	0.131419911	-1.095117356	0.273465226	0.69934809	down_in_case
PRJNA8274(	ENSGALG00010028021	ATP5F1D	case	control	-0.171321185	0.113663636	-1.507264687	0.131742825	0.535159035	down_in_case
PRJNA8274(	ENSGALG00010028022	ENSGALG00010028022	case	control	-0.153516722	0.081016676	-1.894878069	0.058108557	0.392514109	down_in_case
PRJNA8274(	ENSGALG00010028024	RBM3	case	control	0.09132998	0.086320953	1.058027934	0.290042708	0.714060795	up_in_case
PRJNA8274(	ENSGALG00010028025	TNNC1	case	control	0.177434924	0.164731298	1.077117257	0.281427884	0.705762606	up_in_case
PRJNA8274(	ENSGALG00010028026	ANKMY1	case	control	0.024782579	0.136248445	0.181892568	0.855667037	0.970656588	up_in_case
PRJNA8274(	ENSGALG00010028027	ENSGALG00010028027	case	control	0.038857337	0.151424771	0.256611494	0.797478701	0.95280257	up_in_case
PRJNA8274(	ENSGALG00010028028	FAM174C	case	control	0.048765637	0.141974443	0.343481798	0.731236032	0.933770678	up_in_case
PRJNA8274(	ENSGALG00010028029	EFNA2	case	control	-0.068516972	0.112490133	-0.609093173	0.542462682	0.85975913	down_in_case
PRJNA8274(	ENSGALG00010028030	PFDN2	case	control	0.024494268	0.121479727	0.201632558	0.840203991	0.965286179	up_in_case
PRJNA8274(	ENSGALG00010028031	NISCH	case	control	0.033373198	0.055271423	0.603805664	0.545972854	0.862316303	up_in_case
PRJNA8274(	ENSGALG00010028032	ENSGALG00010028032	case	control	-0.066910645	0.16477961	-0.406061437	0.68469746	0.916433328	down_in_case
PRJNA8274(	ENSGALG00010028033	ENSGALG00010028033	case	control	0.046741544	0.211675295	0.220817191	0.825234777	0.960903617	up_in_case
PRJNA8274(	ENSGALG00010028034	MUM1L1	case	control	-0.023756509	0.044684933	-0.53164472	0.594972085	0.884326039	down_in_case
PRJNA8274(	ENSGALG00010028036	NDUFS7	case	control	0.055450745	0.07539898	0.735430973	0.462077064	0.824955651	up_in_case
PRJNA8274(	ENSGALG00010028037	ENSGALG00010028037	case	control	-0.152360729	0.201130926	-0.75752015	0.448738299	0.818701429	down_in_case
PRJNA8274(	ENSGALG00010028039	STAB1	case	control	0.052449768	0.09319978	0.562767078	0.573593501	0.877542213	up_in_case
PRJNA8274(	ENSGALG00010028042	DPP7	case	control	0.232009181	0.070861045	3.274142797	0.00105983	0.050857633	up_in_case
PRJNA8274(	ENSGALG00010028043	NT5DC2	case	control	0.078780096	0.065173064	1.208783067	0.226746199	0.650445465	up_in_case
PRJNA8274(	ENSGALG00010028044	GAMT	case	control	-0.602916712	0.185659073	-3.24744006	0.001164482	0.053331577	down_in_case
PRJNA8274(	ENSGALG00010028045	ENSGALG00010028045	case	control	0.038290692	0.250924281	0.152598591	0.878714836	0.976234053	up_in_case
PRJNA8274(	ENSGALG00010028046	SMIM4	case	control	-0.254291882	0.108570529	-2.342181487	0.01917139	0.246982771	down_in_case
PRJNA8274(	ENSGALG00010028047	ENSGALG00010028047	case	control	0.28783015	0.290576657	0.990548081	0.3219063	0.739571618	up_in_case
PRJNA8274(	ENSGALG00010028048	S100A10	case	control	0.241447546	0.102510101	2.355353695	0.018505083	0.242550577	up_in_case
PRJNA8274(	ENSGALG00010028049	MAN1B1	case	control	0.081192767	0.056812585	1.42913348	0.152965872	0.561193576	up_in_case
PRJNA8274(	ENSGALG00010028050	PBRM1	case	control	-0.061972112	0.042547496	-1.456539582	0.145243516	0.54946621	down_in_case
PRJNA8274(	ENSGALG00010028051	GNL3	case	control	-0.028913613	0.057754932	-0.500625876	0.616634448	0.891081782	down_in_case
PRJNA8274(	ENSGALG00010028052	ENSGALG00010028052	case	control	0.277458302	0.18272731	1.518428209	0.128906485	0.530769575	up_in_case
PRJNA8274(	ENSGALG00010028053	ORM1	case	control	1.27279773	1.891596878	0.672869439	0.501030356	0.841029944	up_in_case
PRJNA8274(	ENSGALG00010028054	RPS15	case	control	-0.088538605	0.067208139	-1.317379206	0.187711548	0.610946377	down_in_case
PRJNA8274(	ENSGALG00010028055	ENSGALG00010028055	case	control	0.154002962	0.075664307	2.035344892	0.041816182	0.341355095	up_in_case
PRJNA8274(	ENSGALG00010028056	SLC25A25	case	control	0.295393391	0.106620945	2.770500597	0.00559702	0.130142086	up_in_case
PRJNA8274(	ENSGALG00010028057	APC2	case	control	-0.76236445	0.340068453	-2.241797037	0.024974493	0.280546149	down_in_case
PRJNA8274(	ENSGALG00010028058	GLT8D1	case	control	0.152838708	0.062969772	2.427175828	0.015216879	0.219135318	up_in_case
PRJNA8274(	ENSGALG00010028060	ENSGALG00010028060	case	control	-0.089151045	0.074826346	-1.191439256	0.23348119	0.657982411	down_in_case
PRJNA8274(	ENSGALG00010028061	NAIF1	case	control	-0.105819289	0.056929401	-1.858781015	0.063058185	0.40876339	down_in_case
PRJNA8274(	ENSGALG00010028062	ENSGALG00010028062	case	control	0.147018742	0.532116709	0.276290406	0.782325024	0.949459648	up_in_case
PRJNA8274(	ENSGALG00010028064	SPCS1	case	control	-0.028076892	0.063285655	-0.443653342	0.657293234	0.908057682	down_in_case
PRJNA8274(	ENSGALG00010028065	REEP6	case	control	0.175354365	0.363708248	0.482129196	0.629714171	0.896269608	up_in_case
PRJNA8274(	ENSGALG00010028066	ENSGALG00010028066	case	control	-0.130216883	0.087224219	-1.492898234	0.135463828	0.539774365	down_in_case
PRJNA8274(	ENSGALG00010028067	FAM102A	case	control	-0.008966923	0.108859887	-0.082371229	0.934351514	0.988140189	down_in_case
PRJNA8274(	ENSGALG00010028068	POLE4	case	control	-0.025141203	0.107119986	-0.234701327	0.814440558	0.957063027	down_in_case
PRJNA8274(	ENSGALG00010028069	NEK4	case	control	0.149456321	0.088491454	1.688935089	0.09123187	0.476454552	up_in_case
PRJNA8274(	ENSGALG00010028070	MEX3A	case	control	0.065648254	0.120205539	0.546133355	0.584974274	0.881307382	up_in_case
PRJNA8274(	ENSGALG00010028071	DPM2	case	control	-0.038413116	0.09080201	-0.423042572	0.672264201	0.913676733	down_in_case
PRJNA8274(	ENSGALG00010028072	ENSGALG00010028072	case	control	0.227384191	0.298276359	0.762327229	0.445864726	0.817313881	up_in_case
PRJNA8274(	ENSGALG00010028073	ST6GALNAC4	case	control	-0.234305009	0.130489196	-1.795589335	0.072559853	0.435785763	down_in_case
PRJNA8274(	ENSGALG00010028074	ENSGALG00010028074	case	control	-0.695930846	0.41218796	-1.688382278	0.09133787	0.476493306	down_in_case
PRJNA8274(	ENSGALG00010028075	RAB25	case	control	-0.145900631	0.4469574	-0.326430731	0.74409849	0.937581144	down_in_case
PRJNA8274(	ENSGALG00010028076	ST6GALNAC6	case	control	-0.374076559	0.122163603	-3.062095012	0.002197937	0.076473225	down_in_case
PRJNA8274(	ENSGALG00010028078	LAMTOR2	case	control	-0.199616791	0.108347327	-1.842378556	0.065419796	0.415377855	down_in_case
PRJNA8274(	ENSGALG00010028079	ITIH3	case	control	1.470776759	1.496613698	0.982736401			up_in_case
PRJNA8274(	ENSGALG00010028080	MUSTN1	case	control	-0.18403802	0.197469362	-0.931982656	0.351345489	0.760831854	down_in_case
PRJNA8274(	ENSGALG00010028081	UBQLN4	case	control	-0.081187817	0.058584681	-1.385819893	0.165801906	0.580782892	down_in_case
PRJNA8274(	ENSGALG00010028082	AK1	case	control	-0.187804305	0.147133586	-1.27642036	0.201806963	0.6249678	down_in_case
PRJNA8274(	ENSGALG00010028083	ELAVL1	case	control	-0.012801569	0.030289387	-0.422642078	0.672556421	0.913676733	down_in_case

PRJNA8274(	ENSGALG00010028084	ENG	case	control	-0.015197163	0.112460934	-0.135132815	0.892506866	0.979422008	down_in_case
PRJNA8274(	ENSGALG00010028085	ENSGALG00010028085	case	control	-0.033257852	0.100778338	-0.330009925	0.741392463	0.937227035	down_in_case
PRJNA8274(	ENSGALG00010028086	MATK	case	control	0.313019636	0.356400449	0.878280701	0.379791406	0.77805894	up_in_case
PRJNA8274(	ENSGALG00010028087	STIMATE	case	control	0.09124797	0.060559087	1.50675935	0.131872353	0.535212583	up_in_case
PRJNA8274(	ENSGALG00010028088	NAXE	case	control	-0.190437225	0.104550525	-1.821485116	0.068533147	0.422606297	down_in_case
PRJNA8274(	ENSGALG00010028090	ENSGALG00010028090	case	control	0.02691403	0.112168303	0.239943276	0.810374231	0.955972758	up_in_case
PRJNA8274(	ENSGALG00010028091	FPGS	case	control	-0.078343576	0.068151561	-1.149549258	0.250329568	0.675927647	down_in_case
PRJNA8274(	ENSGALG00010028093	SFMBT1	case	control	-0.059343378	0.14128036	-0.420039827	0.67445636	0.914018759	down_in_case
PRJNA8274(	ENSGALG00010028095	CDK9	case	control	0.040945863	0.057636468	0.710415891	0.477446272	0.830520323	up_in_case
PRJNA8274(	ENSGALG00010028096	TOR2A	case	control	0.045992931	0.096798757	0.475139689	0.634687411	0.898107199	up_in_case
PRJNA8274(	ENSGALG00010028097	TTC24	case	control	0.086018596	0.155610376	0.552781878	0.580412779	0.879392282	up_in_case
PRJNA8274(	ENSGALG00010028098	RFT1	case	control	-0.114145574	0.155897308	-0.732184381	0.464056036	0.825475969	down_in_case
PRJNA8274(	ENSGALG00010028099	PCASP2	case	control	-0.368437867	0.170591874	-2.159762116	0.03079109	0.308259818	down_in_case
PRJNA8274(	ENSGALG00010028101	MRPL54	case	control	-0.07469618	0.101546282	-0.735587539	0.461981748	0.824955651	down_in_case
PRJNA8274(	ENSGALG00010028102	CERCAM	case	control	-0.066165391	0.066809423	-0.990360174	0.321998104	0.739571618	down_in_case
PRJNA8274(	ENSGALG00010028103	APBA3	case	control	-0.034858014	0.079444686	-0.438770864	0.660827574	0.909646511	down_in_case
PRJNA8274(	ENSGALG00010028104	GOLGA1	case	control	-0.090016632	0.088125266	-1.021462237	0.307035492	0.729821731	down_in_case
PRJNA8274(	ENSGALG00010028105	PRKCD	case	control	0.141940635	0.09871987	1.437812215	0.150487343	0.558014249	up_in_case
PRJNA8274(	ENSGALG00010028106	ARPC5L	case	control	-0.009077796	0.095521732	-0.095033833	0.924287954	0.985571367	down_in_case
PRJNA8274(	ENSGALG00010028107	NOTCH1	case	control	-0.077331083	0.082705432	-0.935018189	0.349778926	0.759427074	down_in_case
PRJNA8274(	ENSGALG00010028108	IQGAP3	case	control	0.183062709	0.090324805	2.026715799	0.042691486	0.344511316	up_in_case
PRJNA8274(	ENSGALG00010028109	TJP3	case	control	0.193503743	0.192612277	1.004628297	0.315075866	0.736508113	up_in_case
PRJNA8274(	ENSGALG00010028110	COQ4	case	control	0.047417862	0.082288468	0.576239457	0.564453339	0.872237167	up_in_case
PRJNA8274(	ENSGALG00010028111	TRUB2	case	control	-0.019032888	0.071025586	-0.26797228	0.788720656	0.951738421	down_in_case
PRJNA8274(	ENSGALG00010028112	SWI5	case	control	0.077655891	0.060090662	1.292312109	0.19624908	0.619095929	up_in_case
PRJNA8274(	ENSGALG00010028113	PIP5K1C	case	control	0.015957527	0.056835905	0.2807649	0.778890727	0.948479876	up_in_case
PRJNA8274(	ENSGALG00010028114	PFKFB4	case	control	-0.072738986	0.062498011	-1.163860819	0.24448042	0.668592467	down_in_case
PRJNA8274(	ENSGALG00010028115	CACTIN	case	control	-0.088493333	0.06364582	-1.390402918	0.164406561	0.579452754	down_in_case
PRJNA8274(	ENSGALG00010028116	HYAL1	case	control	-0.064514723	0.086916018	-0.742265056	0.45792676	0.823337749	down_in_case
PRJNA8274(	ENSGALG00010028117	ENSGALG00010028117	case	control	0.658338218	0.411304828	1.600609021	0.109463543	0.501801854	up_in_case
PRJNA8274(	ENSGALG00010028118	ENSGALG00010028118	case	control	0.01651613	0.025124276	0.657377354	0.510938309	0.845356684	up_in_case
PRJNA8274(	ENSGALG00010028119	HYAL2	case	control	0.020574855	0.072045005	0.285583369	0.775197242	0.947087967	up_in_case
PRJNA8274(	ENSGALG00010028120	TBXA2R	case	control	0.005213717	0.114799036	0.045416032	0.963775703	0.992068146	up_in_case
PRJNA8274(	ENSGALG00010028121	TUSC2	case	control	-0.060625414	0.067747193	-0.894877127	0.370852775	0.772835133	down_in_case
PRJNA8274(	ENSGALG00010028122	SEC16A	case	control	-0.017250149	0.052926281	-0.325927854	0.744478941	0.937654174	down_in_case
PRJNA8274(	ENSGALG00010028123	RASSF1	case	control	0.210431416	0.092324225	2.279265454	0.02265129	0.267253668	up_in_case
PRJNA8274(	ENSGALG00010028124	SUGP2	case	control	0.063497746	0.071264386	0.891016533	0.372920301	0.77436767	up_in_case
PRJNA8274(	ENSGALG00010028125	HMG20B	case	control	0.029743417	0.07055319	0.421574378	0.673335711	0.91379937	up_in_case
PRJNA8274(	ENSGALG00010028126	ZMYND10	case	control	-0.043406833	0.060812405	-0.713782539	0.475361658	0.830316001	down_in_case
PRJNA8274(	ENSGALG00010028127	MFSD12	case	control	0.254217619	0.116228461	2.187223479	0.028726214	0.300941286	up_in_case
PRJNA8274(	ENSGALG00010028128	DNM1	case	control	0.065131937	0.077080537	0.844985504	0.398118933	0.789064551	up_in_case
PRJNA8274(	ENSGALG00010028129	CYB561D2	case	control	0.030739847	0.058844434	0.52239175	0.601397599	0.884954589	up_in_case
PRJNA8274(	ENSGALG00010028130	TMEM115	case	control	0.033276862	0.062110943	0.535764884	0.592121054	0.883486131	up_in_case
PRJNA8274(	ENSGALG00010028131	TMEM161A	case	control	-0.260205984	0.113441233	-2.293751374	0.021804784	0.264012564	down_in_case
PRJNA8274(	ENSGALG00010028132	CACNA2D2	case	control	-0.132883	0.102251973	-1.299564161	0.19375039	0.616682237	down_in_case
PRJNA8274(	ENSGALG00010028133	ENSGALG00010028133	case	control	0.44150199	0.270592704	1.631610845	0.102761492	0.492036392	up_in_case
PRJNA8274(	ENSGALG00010028134	INPP5E	case	control	-0.132158745	0.065277361	-2.024572417	0.042911289	0.34512454	down_in_case
PRJNA8274(	ENSGALG00010028136	C3orf18	case	control	0.001519597	0.065351987	0.023252494	0.981448866	0.996571667	up_in_case
PRJNA8274(	ENSGALG00010028137	NUP214	case	control	-0.101055655	0.057196209	-1.766824373	0.077257646	0.445884536	down_in_case
PRJNA8274(	ENSGALG00010028138	ENSGALG00010028138	case	control	-0.405824702	0.264303042	-1.535452259	0.124672775	0.523589042	down_in_case
PRJNA8274(	ENSGALG00010028139	FZR1	case	control	-0.022624223	0.072896663	-0.310360198	0.756287058	0.941659745	down_in_case
PRJNA8274(	ENSGALG00010028140	HEMK1	case	control	-0.003156661	0.152311708	-0.020725008	0.98346502	0.996591771	down_in_case
PRJNA8274(	ENSGALG00010028141	CISH	case	control	-0.02704786	0.077612862	-0.348497131	0.727466868	0.932979874	down_in_case
PRJNA8274(	ENSGALG00010028142	PMPCA	case	control	-0.067141267	0.054610499	-1.229457112	0.218900468	0.643443819	down_in_case
PRJNA8274(	ENSGALG00010028144	FAM78A	case	control	-0.109541476	0.102929171	-1.064241312	0.287219397	0.711057292	down_in_case
PRJNA8274(	ENSGALG00010028145	ENSGALG00010028145	case	control	-0.221074093	0.149037936	-1.483341087	0.137983811	0.539854583	down_in_case
PRJNA8274(	ENSGALG00010028146	MAPKAPK3	case	control	-0.032674233	0.102049404	-0.320180537	0.748831477	0.939503405	down_in_case
PRJNA8274(	ENSGALG00010028147	SLC25A42	case	control	-0.187460603	0.11449403	-1.637295874	0.101568663	0.491460867	down_in_case
PRJNA8274(	ENSGALG00010028148	ENSGALG00010028148	case	control	-0.27852838	0.150461861	-1.851156018	0.064147111	0.410842437	down_in_case
PRJNA8274(	ENSGALG00010028149	SNAPC4	case	control	0.123156364	0.067741736	1.818027873	0.069059873	0.424577953	up_in_case
PRJNA8274(	ENSGALG00010028150	ARMC6	case	control	-0.061492432	0.064102207	-0.959287283	0.33741404	0.750948271	down_in_case
PRJNA8274(	ENSGALG00010028151	DOCK3	case	control	0.089562915	0.236161501	0.37924435	0.704506421	0.923973441	up_in_case
PRJNA8274(	ENSGALG00010028152	ENSGALG00010028152	case	control	-0.162988298	0.298787595	-0.545498878	0.585410452	0.881307382	down_in_case
PRJNA8274(	ENSGALG00010028153	MANF	case	control	0.166298883	0.056852096	2.92511439	0.003443296	0.100147146	up_in_case
PRJNA8274(	ENSGALG00010028154	ENSGALG00010028154	case	control	0.122925774	0.139259847	0.88270795	0.377394086	0.775926253	up_in_case
PRJNA8274(	ENSGALG00010028155	CIZ1	case	control	0.048943985	0.042704679	1.146103562	0.251752304	0.677084435	up_in_case
PRJNA8274(	ENSGALG00010028156	DOHH	case	control	0.040520898	0.067909191	0.596692395	0.550712771	0.86457121	up_in_case
PRJNA8274(	ENSGALG00010028157	PRRC2B	case	control	-0.083409546	0.063391251	-1.315789561	0.188244684	0.611129484	down_in_case
PRJNA8274(	ENSGALG00010028158	DDX49	case	control	-0.030819754	0.069576374	-0.442962916	0.657792558	0.908307607	down_in_case
PRJNA8274(	ENSGALG00010028159	DCAF1	case	control	-0.054117928	0.044194293	-1.224545614	0.22074647	0.644744838	down_in_case
PRJNA8274(	ENSGALG00010028160	ENSGALG00010028160	case	control	0.415978062	0.211024253	1.971233429	0.048697182	0.363542241	up_in_case
PRJNA8274(	ENSGALG00010028161	ENSGALG00010028161	case	control	1.34707124	0.403411498	3.339198926	0.000840204	0.044416733	up_in_case
PRJNA8274(	ENSGALG00010028162	CARD9	case	control	0.076765196	0.118243898	0.649210637	0.516202237	0.848054666	up_in_case
PRJNA8274(	ENSGALG00010028163	GBP	case	control	-0.022052732	0.414337143	-0.053224125	0.957553334	0.992056045	down_in_case
PRJNA8274(	ENSGALG00010028164	BBLN	case	control	-0.17984806	0.086269611	-2.084720899	0.037094643	0.328438691	down_in_case
PRJNA8274(	ENSGALG00010028165	NFIC	case	control	-0.067518851	0.119016941	-0.567304537	0.570507295	0.875913068	down_in_case
PRJNA8274(	ENSGALG00010028167	ENSGALG00010028167	case	control	-0.092837376	0.091440622	-1.015274975	0.30997477	0.732909922	down_in_case
PRJNA8274(	ENSGALG00010028168	PTGES2	case	control	-0.064901017	0.075221303	-0.862801028	0.388246874	0.783067156	down_in_case
PRJNA8274(	ENSGALG00010028169	DNLZ	case	control	0.030248852	0.119637172	0.252838244	0.800393213	0.954053991	up_in_case
PRJNA8274(	ENSGALG00010028170	COPE	case	control	0.163805386	0.090048157	1.819086499	0.068898234	0.423943525	up_in_case
PRJNA8274(	ENSGALG00010028171	RNF123	case	control	-0.16246922	0.074023959	-2.194819379	0.028176557	0.299572314	down_in_case
PRJNA8274(	ENSGALG00010028172	CERS1	case	control	-0.044586512	0.117596127	-0.379149492	0.704576856	0.923973441	down_in_case
PRJNA8274(	ENSGALG00010028173	GDF3	case	control	-0.017122909	0.107743773	-0.158922497	0.873729936	0.975062028	down_in_case
PRJNA8274(	ENSGALG00010028174	GMPPB	case	control	0.159291712	0.066249756	2.404412071	0.016198503	0.225988865	up_in_case
PRJNA8274(	ENSGALG00010028175	POMT1	case	control	-0.123564331	0.08688869	-1.422099141	0.154997472	0.563829072	down_in_case
PRJNA8274(	ENSGALG00010028176	UPF1	case	control	-0.096801994	0.043097628	-2.246109556	0.024696991	0.278742681	down_in_case
PRJNA8274(	ENSGALG00010028177	ENSGALG00010028177	case	control	0.169474056	0.29162832	0.581130311	0.561152631	0.870480544	up_in_case
PRJNA8274(	ENSGALG00010028178	ENSGALG00010028178	case	control	0.102213715	0.11060288	0.924150569	0.355407928	0.763161644	up_in_case
PRJNA8274(	ENSGALG00010028179	MCM2	case	control	-0.094136449	0.075153844	-1.252583282	0.210357402	0.636098721	down_in_case
PRJNA8274(	ENSGALG00010028180	ODF2	case	control	0.119341079	0.058214182	2.050034462	0.040361068	0.33753444	up_in_case
PRJNA8274(	ENSGALG00010028181	BGLAP	case	control	-0.176323064	0.330685411	-0.533204848	0.593891786	0.883711747	down_in_case
PRJNA8274(	ENSGALG00010028182	SMG5	case	control	0.11352505	0.073491869	1.544729401	0.12241173	0.520832363	up_in_case
PRJNA8274(	ENSGALG00010028183	CRTC1	case	control	-0.018958651	0.071620742	-0.264708942	0.791233689	0.95239351	down_in_case
PRJNA8274(	ENSGALG00010028184	TMEM79	case	control	0.084228253	0.12329643	0.683136186	0.494520791	0.83803731	up_in_case
PRJNA8274(	ENSGALG00010028185	GLMP	case	control	0.047619209	0.08080236	0.589329427	0.555640308	0.867878646	up_in_case
PRJNA8274(	ENSGALG00010028186	CCT3	case	control	-0.047982249	0.053249415	-0.90108501	0.367543122	0.771770041	down_in_case
PRJNA8274(	ENSGALG00010028187	TPRA1	case	control	0.028466752	0.049584133	0.574110115	0.565893289	0.872906976	up_in_case
PRJNA8274(	ENSGALG00010028188	ENSGALG00010028188	case	control	-0.09890862	0.08433365	-1.172825081	0.240865958	0.665967363	down_in_case
PRJNA8274(	ENSGALG00010028189	GPSM1	case	control	-0.077442934	0.092021184	-0.841577244	0.400024631	0.789900496	down_in_case
PRJNA8274(	ENSGALG00010028190	ILF2	case	control	0.018957449	0.045373147	0.417812082	0.676084517	0.914247368	up_in_case
PRJNA8274(	ENSGALG00010028191	KLHL26	case	control	-0.013934542	0.061220547	-0.227612182	0.819947749	0.958669267	down_in_case
PRJNA8274(	ENSGALG00010028193	BRINP1	case	control	-0.142715302	0.157698416	-0.904988812	0.365471314	0.77107809	down_in_case
PRJNA8274(	ENSGALG00010028194	ENSGALG00010028194	case	control	0.135862263	0.104531957	1.29971988	0.193696994	0.616682237	up_in_case
PRJNA8274(	ENSGALG00010028195	ENSGALG00010028195	case	control	-0.016539214	0.300862563	-0.054972654	0.95616025	0.992056045	down_in_case
PRJNA8274(	ENSGALG00010028196	ENSGALG00010028196	case	control	0.146765537	0.102499081	1.431871746	0.15218053	0.560293917	up_in_case
PRJNA8274(	ENSGALG00010028197	INTS3	case	control	-0.057998428	0.074224663	-0.781390253	0.434573002	0.809482081	down_in_case
PRJNA8274(	ENSGALG00010028198	UCK1	case	control	-0.033041031	0.0681886	-0.484553589	0.627993042	0.895865363	down_in_case
PRJNA8274(	ENSGALG00010028199	DPP9	case	control	-0.08994279	0.060659372	-1.482751745	0.138140381	0.540319324	down_in_case
PRJNA8274(	ENSGALG00010028201	ENSGALG00010028201	case	control	-0.10517413	0.106404264	-0.988439053	0.322937669	0.740689923	down_in_case
PRJNA8274(	ENSGALG00010028202	QSOX2	case	control	0.026306489	0.044801008	0.58718521	0.557079325	0.868443731	up_in_case
PRJNA8274(	ENSGALG00010028205	PRRT1B	case	control	0.356358522	0.28874631	1.23415784	0.217144094	0.642491319	up_in_case
PRJNA8274(	ENSGALG00010028206	RBM15B	case	control	-0.050948159	0.049706079	-1.024988504	0.305368612	0.728705913	down_in_case
PRJNA8274(	ENSGALG00010028207	ENSGALG00010028207	case	control	0.913602788	0.353313021	2.585816921	0.009714852	0.176297435	up_in_case
PRJNA8274(	ENSGALG00010028208	AMIGO3	case	control	-0.035136232	0.253075736	-0.138836828	0.889579091	0.97815026	down_in_case
PRJNA8274(	ENSGALG00010028211	GATAD2B	case	control	-0.048643858	0.082536981	-0.589358336	0.555620919	0.867878646	down_in_case
PRJNA8274(	ENSGALG00010028216	NKX2-3	case	control	1.049408928	0.870670598	1.20528812	0.228092094	0.651681204	up_in_case
PRJNA8274(	ENSGALG00010028219	ENSGALG00010028219	case	control	0.36455299	1.285182471	0.283658545	0.776672067	0.947241838	up_in_case
PRJNA8274(	ENSGALG00010028221	TNFAIP8L1	case	control	0.027751696	0.058210134	0.476750258	0.63353997	0.898107199	up_in_case
PRJNA8274(	ENSGALG00010028232	C9orf69	case	control	-0.11074684	0.069736777	-1.588069368	0.112270654	0.506149199	down_in_case
PRJNA8274(	ENSGALG00010028234	EDCH3	case	control	1.203140696	1.266108061	0.95026699	0.341976607	0.753740056	up_in_case
PRJNA8274(	ENSGALG00010028239	EDMPN2	case	control	-0.231485215	2.163910743	-0.106975399	0.914808496	0.983221458	down_in_case
PRJNA8274(	ENSGALG00010028242	ENSGALG00010028242	case	control	0.131356356	0.23867291	0.550361398	0.58207152	0.88034085	up_in_case
PRJNA8274(	ENSGALG00010028245	JAC1	case	control	0.705843533	1.166805186	0.604936918	0.545220912	0.861997112	up_in_case
PRJNA8274(	ENSGALG00010028247	MVP	case	control	-0.002831362	0.085537175	-0.033100951	0.973594084	0.993553916	down_in_case
PRJNA8274(	ENSGALG00010028262	C1orf43	case	control	-0.111180271	0.069124536	-1.608405309	0.10774644	0.499602493	down_in_case
PRJNA8274(	ENSGALG00010028270	RAPGEF1	case	control	-0.139959605	0.078817553	-1.77574158	0.075775521	0.442474101	down_in_case

PRJNA8274(	ENSGALG00010028283	ENSGALG00010028283	case	control	-0.016123201	0.09282201	-0.173700192	0.862101086	0.972698817	down_in_case
PRJNA8274(	ENSGALG00010028286	KCTD12A	case	control	0.7389576	1.350916795	0.547004525	0.584375623	0.881307382	up_in_case
PRJNA8274(	ENSGALG00010028292	ENSGALG00010028292	case	control	-0.528574871	0.401484754	-1.316550295	0.187989409	0.611104467	down_in_case
PRJNA8274(	ENSGALG00010028297	ENSGALG00010028297	case	control	-0.308049984	0.64020135	-0.481176718	0.630390906	0.896528092	down_in_case
PRJNA8274(	ENSGALG00010028301	FBN3	case	control	-0.099866493	0.113754081	-0.877915688	0.379989475	0.77805894	down_in_case
PRJNA8274(	ENSGALG00010028311	ENSGALG00010028311	case	control	0.280338784	0.227836869	1.230436429	0.218533719	0.643443819	up_in_case
PRJNA8274(	ENSGALG00010028314	MEF2D	case	control	-0.063943837	0.073676543	-0.867899525	0.385449339	0.781541515	down_in_case
PRJNA8274(	ENSGALG00010028316	TIMM44	case	control	-0.178940883	0.060229458	-2.970986104	0.002968452	0.092683112	down_in_case
PRJNA8274(	ENSGALG00010028318	NACC2	case	control	-0.125383154	0.134747756	-0.930502725	0.352110854	0.760831854	down_in_case
PRJNA8274(	ENSGALG00010028321	RHBG	case	control	-0.175884938	0.163179792	-1.077859799	0.281096328	0.705762606	down_in_case
PRJNA8274(	ENSGALG00010028322	STRBP	case	control	0.061344348	0.095557889	0.641960059	0.520899117	0.850168083	up_in_case
PRJNA8274(	ENSGALG00010028326	CRB2	case	control	0.198771573	0.167407326	1.187352895	0.235088452	0.659686983	up_in_case
PRJNA8274(	ENSGALG00010028328	HNRNPM	case	control	-0.010774317	0.048967198	-0.220031308	0.825846772	0.961207256	down_in_case
PRJNA8274(	ENSGALG00010028329	HAPLN2	case	control	-0.122609	0.377534514	-0.324762361	0.745360933	0.937849656	down_in_case
PRJNA8274(	ENSGALG00010028333	ENSGALG00010028333	case	control	-0.717659902	0.258112123	-2.780419196	0.005428877	0.127480206	down_in_case
PRJNA8274(	ENSGALG00010028337	ENSGALG00010028337	case	control	-0.33208391	0.199774868	-1.662290722	0.096454497	0.483794918	down_in_case
PRJNA8274(	ENSGALG00010028339	BCAN	case	control	0.224115405	0.26793087	0.836467276	0.402892079	0.792047942	up_in_case
PRJNA8274(	ENSGALG00010028344	LSM7	case	control	-0.071984579	0.05952295	-1.209358388	0.226525188	0.650201422	down_in_case
PRJNA8274(	ENSGALG00010028346	DENND1A	case	control	-0.013255366	0.044756385	-0.296167033	0.767102524	0.944442742	down_in_case
PRJNA8274(	ENSGALG00010028348	NES	case	control	0.072408722	0.143333319	0.505177177	0.613434399	0.890252839	up_in_case
PRJNA8274(	ENSGALG00010028353	ENSGALG00010028353	case	control	0.742945654	1.263527867	0.587993089	0.556536932	0.867878646	up_in_case
PRJNA8274(	ENSGALG00010028355	RBM18	case	control	-0.14707857	0.04025961	-3.653253675	0.000258938	0.02149418	down_in_case
PRJNA8274(	ENSGALG00010028357	SPPL2C	case	control	-0.05712503	0.057398661	-0.995232801	0.319623052	0.738813491	down_in_case
PRJNA8274(	ENSGALG00010028360	ENSGALG00010028360	case	control	1.476762626	0.645836941	2.286587422	0.022219916	0.26563754	up_in_case
PRJNA8274(	ENSGALG00010028362	TMPRSS9	case	control	-0.575022099	0.311697734	-1.8448068	0.065065648	0.414658096	down_in_case
PRJNA8274(	ENSGALG00010028364	MHM2	case	control	-0.407667609	0.462524876	-0.881396074	0.378103482	0.776556765	down_in_case
PRJNA8274(	ENSGALG00010028366	MRRF	case	control	-0.037154789	0.066345885	-0.560016484	0.575468194	0.877910508	down_in_case
PRJNA8274(	ENSGALG00010028368	TIMM13	case	control	-0.079004886	0.086202881	-0.916499372	0.359405037	0.765374836	down_in_case
PRJNA8274(	ENSGALG00010028370	S100A12	case	control	-0.258886725	0.40682432	-0.636360003	0.524541804	0.852356213	down_in_case
PRJNA8274(	ENSGALG00010028372	PTGS1	case	control	-0.123254356	0.10850061	-1.135978463	0.255965578	0.682256807	down_in_case
PRJNA8274(	ENSGALG00010028374	FDPS	case	control	0.14629403	0.116194458	1.259044819	0.208014144	0.63249038	up_in_case
PRJNA8274(	ENSGALG00010028375	LMNB2	case	control	-0.00176083	0.089484025	-0.019677593	0.984300565	0.996591771	down_in_case
PRJNA8274(	ENSGALG00010028377	PDCL	case	control	-0.010790528	0.0474692	-0.227316403	0.820177719	0.958669267	down_in_case
PRJNA8274(	ENSGALG00010028380	RUSC1	case	control	-0.188269401	0.086897915	-2.166558334	0.030268547	0.30654407	down_in_case
PRJNA8274(	ENSGALG00010028384	ENSGALG00010028384	case	control	0.731621398	0.999812158	0.731758853	0.464315767	0.825629877	up_in_case
PRJNA8274(	ENSGALG00010028386	NCAN	case	control	-0.607410401	0.304578443	-1.994265894	0.046123004	0.356053762	down_in_case
PRJNA8274(	ENSGALG00010028391	MED26	case	control	-0.038525036	0.051890481	-0.742429736	0.457827009	0.823337749	down_in_case
PRJNA8274(	ENSGALG00010028392	ASH1L	case	control	-0.143128809	0.060545249	-2.363997347	0.018078942	0.240268468	down_in_case
PRJNA8274(	ENSGALG00010028394	ZBTB26	case	control	-0.005076263	0.067706694	-0.07497431	0.940235152	0.989551456	down_in_case
PRJNA8274(	ENSGALG00010028399	SLC35E1	case	control	-0.036027403	0.041093758	-0.876712278	0.380642936	0.778246195	down_in_case
PRJNA8274(	ENSGALG00010028401	ENSGALG00010028401	case	control	0.784883368	1.09816115	0.714725127	0.474778908	0.830093946	up_in_case
PRJNA8274(	ENSGALG00010028405	S100A6	case	control	-0.057989558	0.144834689	-0.400384455	0.688873372	0.918082523	down_in_case
PRJNA8274(	ENSGALG00010028411	LHX2	case	control	1.064026561	0.538143516	1.977217096	0.048017096	0.360632601	up_in_case
PRJNA8274(	ENSGALG00010028412	ENSGALG00010028412	case	control	-0.012163051	0.269309231	-0.045163883	0.963976682	0.992068146	down_in_case
PRJNA8274(	ENSGALG00010028414	KBTBD12	case	control	0.192923498	0.236797974	0.814717688	0.415233926	0.799221279	up_in_case
PRJNA8274(	ENSGALG00010028415	FSD1	case	control	-0.01825914	0.099453543	-0.183594665	0.854331449	0.970137355	down_in_case
PRJNA8274(	ENSGALG00010028418	ENSGALG00010028418	case	control	0.073614163	0.08281813	0.888865319	0.374075466	0.774369566	up_in_case
PRJNA8274(	ENSGALG00010028419	ENSGALG00010028419	case	control	-0.233108545	0.263297872	-0.885341547	0.375972448	0.774921593	down_in_case
PRJNA8274(	ENSGALG00010028420	ENSGALG00010028420	case	control	1.012241529	0.48057596	2.106309125	0.035177509	0.323390191	up_in_case
PRJNA8274(	ENSGALG00010028421	ZBTB7A	case	control	0.010029539	0.068366543	0.146702441	0.883366892	0.977115076	up_in_case
PRJNA8274(	ENSGALG00010028422	RXRA	case	control	-0.272228025	0.100252858	-2.715414113	0.006619294	0.140023533	down_in_case
PRJNA8274(	ENSGALG00010028425	DAP3	case	control	-0.0136615	0.066806022	-0.204495045	0.837966664	0.964826648	down_in_case
PRJNA8274(	ENSGALG00010028426	GPR21	case	control	0.14745	0.108223644	1.362456436	0.173053884	0.592369563	up_in_case
PRJNA8274(	ENSGALG00010028427	ENSGALG00010028427	case	control	-0.028452711	0.276157205	-0.103030849	0.917938487	0.983445506	down_in_case
PRJNA8274(	ENSGALG00010028428	RABGAP1	case	control	-0.037378707	0.045906096	-0.814242767	0.41550589	0.799372288	down_in_case
PRJNA8274(	ENSGALG00010028429	REXO1	case	control	0.010926964	0.039469886	0.276843063	0.781900614	0.949394183	up_in_case
PRJNA8274(	ENSGALG00010028430	NR2C2AP	case	control	0.057802325	0.08264649	0.699392371	0.484306854	0.834033076	up_in_case
PRJNA8274(	ENSGALG00010028431	ENSGALG00010028431	case	control	0.470370165	0.358512299	1.31200566	0.189518225	0.612863366	up_in_case
PRJNA8274(	ENSGALG00010028432	RFXANK	case	control	0.009367075	0.090710186	0.103263767	0.917753632	0.983445506	up_in_case
PRJNA8274(	ENSGALG00010028433	MSTO1	case	control	0.157405325	0.080192729	1.962837872	0.049665016	0.364833243	up_in_case
PRJNA8274(	ENSGALG00010028434	COL5A1	case	control	-0.217350413	0.121722645	-1.785620192	0.074160775	0.438111241	down_in_case
PRJNA8274(	ENSGALG00010028435	ATP8B3	case	control	-0.175653973	0.301794172	-0.582032357	0.560544886	0.870146751	down_in_case
PRJNA8274(	ENSGALG00010028436	S100A1	case	control	-0.056726623	0.181197869	-0.313064514	0.754231649	0.941235036	down_in_case
PRJNA8274(	ENSGALG00010028437	ENSGALG00010028437	case	control	-0.002185384	0.040598835	-0.053828726	0.957071623	0.992056045	down_in_case
PRJNA8274(	ENSGALG00010028438	MED27	case	control	-0.12288759	0.058928965	-2.08535125	0.037037429	0.32835414	down_in_case
PRJNA8274(	ENSGALG00010028439	BORCS8	case	control	-0.242299724	0.099266655	-2.440897445	0.014650815	0.213564373	down_in_case
PRJNA8274(	ENSGALG00010028440	PSMB7	case	control	0.047160798	0.048059764	0.981294816	0.326447378	0.744287835	up_in_case
PRJNA8274(	ENSGALG00010028442	CHTOP	case	control	-0.048011083	0.05438797	-0.882751873	0.377370348	0.775926253	down_in_case
PRJNA8274(	ENSGALG00010028443	ENSGALG00010028443	case	control	0.320998753	0.186421916	1.72189386	0.085088748	0.461467053	up_in_case
PRJNA8274(	ENSGALG00010028444	MEF2B	case	control	0.052338782	0.202521881	0.258435195	0.796071057	0.95280257	up_in_case
PRJNA8274(	ENSGALG00010028445	SYT11	case	control	0.033830751	0.047479714	0.71253064	0.476136245	0.830520323	up_in_case
PRJNA8274(	ENSGALG00010028446	ENSGALG00010028446	case	control	0.031058511	0.175096721	0.177379172	0.859210567	0.971828322	up_in_case
PRJNA8274(	ENSGALG00010028447	OLFM1	case	control	-0.388128767	0.098181786	-3.953164646	7.71243E-05	0.009039983	down_in_case
PRJNA8274(	ENSGALG00010028448	RIT1	case	control	-0.042271384	0.050887469	-0.830683568	0.406152411	0.7941993	down_in_case
PRJNA8274(	ENSGALG00010028449	EFCAB12	case	control	-0.109352401	0.189590441	-0.576782252	0.564086561	0.871916948	down_in_case
PRJNA8274(	ENSGALG00010028450	PTGDS	case	control	0.227880551	0.372106369	0.612407015	0.540268502	0.859012092	up_in_case
PRJNA8274(	ENSGALG00010028451	TMEM59L	case	control	0.259555481	0.164295548	1.579808361	0.11415076	0.509446069	up_in_case
PRJNA8274(	ENSGALG00010028452	GOLPH3L	case	control	-0.049840935	0.693704113	-0.071847542	0.942723238	0.990046805	down_in_case
PRJNA8274(	ENSGALG00010028453	ENSGALG00010028453	case	control	0.022579391	0.073401498	0.307614859	0.758375412	0.941903352	up_in_case
PRJNA8274(	ENSGALG00010028454	C8G	case	control	0.392971498	0.19372363	2.028516075	0.042507604	0.344353525	up_in_case
PRJNA8274(	ENSGALG00010028455	ENSA	case	control	-0.149947428	0.057286378	-2.617505804	0.008857498	0.16710056	down_in_case
PRJNA8274(	ENSGALG00010028456	FBXW5	case	control	-0.051834435	0.054039849	-0.959189115	0.337463483	0.750948271	down_in_case
PRJNA8274(	ENSGALG00010028457	KANK3	case	control	0.055865536	0.077434006	0.721459977	0.470626556	0.829101076	up_in_case
PRJNA8274(	ENSGALG00010028458	TRAF2	case	control	0.041301802	0.07087596	0.582733582	0.560072662	0.86984941	up_in_case
PRJNA8274(	ENSGALG00010028459	MCL1	case	control	-0.019268108	0.058473961	-0.329516029	0.74176568	0.937315893	down_in_case
PRJNA8274(	ENSGALG00010028460	CRLF1	case	control	0.202937175	0.11581671	1.752227067	0.079734771	0.450131333	up_in_case
PRJNA8274(	ENSGALG00010028461	ENSGALG00010028461	case	control	-0.009421985	0.138059781	-0.068245693	0.945590054	0.990313016	down_in_case
PRJNA8274(	ENSGALG00010028462	EDF1	case	control	-0.034039486	0.040405411	-0.842448703	0.399536841	0.789900496	down_in_case
PRJNA8274(	ENSGALG00010028463	RPS28	case	control	0.016700094	0.099692353	0.167516299	0.866963824	0.973570415	up_in_case
PRJNA8274(	ENSGALG00010028464	ENSGALG00010028464	case	control	-0.045212088	0.079440862	-0.569128862	0.569268693	0.875057036	down_in_case
PRJNA8274(	ENSGALG00010028465	ENSGALG00010028465	case	control	-0.052873219	0.109068478	-0.484770849	0.627838903	0.895865363	down_in_case
PRJNA8274(	ENSGALG00010028466	MAMDC4	case	control	1.892002285	1.008841945	1.875419925	0.060734976	0.400562592	up_in_case
PRJNA8274(	ENSGALG00010028467	ENSGALG00010028467	case	control	-0.07355957	0.216930219	-0.339093236	0.734539494	0.934037729	down_in_case
PRJNA8274(	ENSGALG00010028468	PHPT1	case	control	0.055585	0.07801137	0.71252435	0.476140138	0.830520323	up_in_case
PRJNA8274(	ENSGALG00010028470	AJM1	case	control	-0.584937326	0.348892983	-1.676552281	0.093630065	0.478523919	down_in_case
PRJNA8274(	ENSGALG00010028471	TPGS1	case	control	-0.00609296	0.083294649	-0.073149476	0.941687171	0.989551456	down_in_case
PRJNA8274(	ENSGALG00010028472	RPRD2	case	control	-0.085305619	0.060775238	-1.403624602	0.160430665	0.572925686	down_in_case
PRJNA8274(	ENSGALG00010028473	CDC34	case	control	-0.151339723	0.06554829	-2.308827927	0.020953132	0.259419725	down_in_case
PRJNA8274(	ENSGALG00010028474	PEX19	case	control	-0.122470005	0.106796498	-1.146760493	0.251480621	0.677081801	down_in_case
PRJNA8274(	ENSGALG00010028475	AMBP	case	control	1.549970493	1.300643134	1.191695441	0.233380686	0.657982411	up_in_case
PRJNA8274(	ENSGALG00010028476	ENSGALG00010028476	case	control	-0.088234967	0.052113799	-1.693120973	0.090432443	0.474044299	down_in_case
PRJNA8274(	ENSGALG00010028477	NRARP	case	control	-0.033018176	0.068545381	-0.481698052	0.63002046	0.89637578	down_in_case
PRJNA8274(	ENSGALG00010028478	PEA15	case	control	0.316825892	0.09313608	3.401752489	0.000669552	0.038540612	up_in_case
PRJNA8274(	ENSGALG00010028479	CASQ1	case	control	-0.047526848	0.245593381	-0.193518442	0.846552964	0.967682445	down_in_case
PRJNA8274(	ENSGALG00010028480	F11R	case	control	-0.045333755	0.138741136	-0.32675064	0.743856496	0.937448285	down_in_case
PRJNA8274(	ENSGALG00010028481	ENSGALG00010028481	case	control	0.332146084	0.152454965	2.178650492	0.029357642	0.305026217	up_in_case
PRJNA8274(	ENSGALG00010028482	CD320	case	control	0.227035797	0.12805177	1.77300007	0.076228695	0.442937969	up_in_case
PRJNA8274(	ENSGALG00010028483	EXD3	case	control	0.028276832	0.096677841	0.292485139	0.769915721	0.945428692	up_in_case
PRJNA8274(	ENSGALG00010028484	NOXA1	case	control	0.248734912	0.408243008	0.609281498	0.542337868	0.85975913	up_in_case
PRJNA8274(	ENSGALG00010028485	USF1	case	control	-0.116043208	0.074612016	-1.555288477	0.11987738	0.517634321	down_in_case
PRJNA8274(	ENSGALG00010028486	ARHGAP30	case	control	0.178916354	0.164984041	1.084446432	0.278166906	0.703443892	up_in_case
PRJNA8274(	ENSGALG00010028487	ENTPD8	case	control	1.829484594	0.719237119	2.543645964	0.010970221	0.185137791	up_in_case
PRJNA8274(	ENSGALG00010028488	PTGES	case	control	-0.106186828	0.078903827	-1.345775386	0.178374966	0.597948438	down_in_case
PRJNA8274(	ENSGALG00010028490	NECTIN4	case	control	0.004853426	0.311439518	0.015583849	0.987566391	0.996908047	up_in_case
PRJNA8274(	ENSGALG00010028491	TOR1B	case	control	0.072459885	0.05601809	1.293508662	0.19583519	0.619095929	up_in_case
PRJNA8274(	ENSGALG00010028492	ENSGALG00010028492	case	control	0.155574737	0.118095167	1.317367522	0.187715462	0.610946377	up_in_case
PRJNA8274(	ENSGALG00010028493	TOR1A	case	control	0.192556706	0.067932805	2.834517234	0.004589498	0.11574925	up_in_case
PRJNA8274(	ENSGALG00010028494	ENSGALG00010028494	case	control	0.203573481	0.14505786	1.403395041	0.160499071	0.572925686	up_in_case
PRJNA8274(	ENSGALG00010028495	LINGO3	case	control	-0.209640307	0.212773582	-0.985274135	0.324489434	0.742431825	down_in_case
PRJNA8274(	ENSGALG00010028496	ENSGALG00010028496	case	control	0.020694341	0.14589174	0.141847245	0.887200665	0.977630122	up_in_case
PRJNA8274(	ENSGALG00010028498	C9orf78	case	control	-0.046239977	0.052138718	-0.886864479	0.375151867	0.774681835	down_in_case
PRJNA8274(	ENSGALG00010028500	VPS45	case	control	-0.019137469	0.056350723	-0.33961354	0.73414758	0.933846596	down_in_case
PRJNA8274(	ENSGALG00010028501	USP20	case	control	0.111788514	0.051477776	2.171587895	0.029886761	0.305970455	up_in_case
PRJNA8274(	ENSGALG00010028502	PLEKHO1	case	control	0.000673333	0.082297892	0.008181655	0.993472057	0.998196863	up_in_case
PRJNA8274(	ENSGALG00010028503	NDOR1	case	control	0.014736604	0.12424776	0.118606601	0.905587036	0.981730793	up_in_case
PRJNA8274(	ENSGALG00010028505	ENSGALG00010028505	case	control	0.293488477	1.293957376	0.226814641	0.820567879	0.958669267	up_in_case

PRJNA8274(	ENSGALG00010028506	SHD	case	control	-0.101840123	0.378637754	-0.268964524	0.787956983	0.951551316	down_in_case
PRJNA8274(	ENSGALG00010028507	ANP32E	case	control	0.025038008	0.051095002	0.490028524	0.624113715	0.894353977	up_in_case
PRJNA8274(	ENSGALG00010028508	ENSGALG00010028508	case	control	1.525995068	0.870417043	1.753176918	0.079571644	0.450131333	up_in_case
PRJNA8274(	ENSGALG00010028510	CILP2	case	control	0.235876307	0.391590478	0.602354554	0.546938154	0.862526554	up_in_case
PRJNA8274(	ENSGALG00010028511	YJEFN3	case	control	-0.151451211	0.412625251	-0.367043003	0.713586942	0.926862121	down_in_case
PRJNA8274(	ENSGALG00010028512	NDUFA13	case	control	-0.060621159	0.0658417	-0.92071072	0.357201478	0.763410721	down_in_case
PRJNA8274(	ENSGALG00010028513	ENSGALG00010028513	case	control	0.29520443	1.47948209	0.199532277	0.841846398	0.966164004	up_in_case
PRJNA8274(	ENSGALG00010028514	FNBP1	case	control	-0.183236227	0.052594284	-3.483957054	0.000494059	0.032260458	down_in_case
PRJNA8274(	ENSGALG00010028516	GATAD2A	case	control	0.002238816	0.042700353	0.052430854	0.95818539	0.992056045	up_in_case
PRJNA8274(	ENSGALG00010028517	RNF208	case	control	0.134044534	0.126718366	1.057814577	0.290139986	0.714060795	up_in_case
PRJNA8274(	ENSGALG00010028518	MAU2	case	control	0.026719597	0.06283446	0.425237943	0.670663227	0.913520751	up_in_case
PRJNA8274(	ENSGALG00010028519	ENSGALG00010028519	case	control	0.437466333	1.640984482	0.266587733	0.789786602	0.951843754	up_in_case
PRJNA8274(	ENSGALG00010028523	SUGP1	case	control	-0.002815793	0.047586639	-0.059171923	0.952815172	0.992056045	down_in_case
PRJNA8274(	ENSGALG00010028528	UHRF1	case	control	-0.097042197	0.087531564	-1.108653762	0.267579576	0.694444271	down_in_case
PRJNA8274(	ENSGALG00010028534	ENSGALG00010028534	case	control	-0.418456077	2.421247195	-0.172826665	0.862787677	0.972787494	down_in_case
PRJNA8274(	ENSGALG00010028535	TICAM1	case	control	-0.077094585	0.048407868	-1.592604415	0.111248963	0.503925506	down_in_case
PRJNA8274(	ENSGALG00010028536	GPR107	case	control	0.063307644	0.050825048	1.24559929	0.212911543	0.638582746	up_in_case
PRJNA8274(	ENSGALG00010028537	ENSGALG00010028537	case	control	0.245113051	1.348535299	0.181762429	0.855769171	0.970656588	up_in_case
PRJNA8274(	ENSGALG00010028538	FEM1A	case	control	-0.150691282	0.099794167	-1.51002094	0.131038081	0.534010989	down_in_case
PRJNA8274(	ENSGALG00010028539	ENSGALG00010028539	case	control	0.802409241	1.416955713	0.566290981	0.57119599	0.87649991	up_in_case
PRJNA8274(	ENSGALG00010028541	ENSGALG00010028541	case	control	0.503650298	0.443117368	1.13660699	0.255702617	0.681937229	up_in_case
PRJNA8274(	ENSGALG00010028542	ENSGALG00010028542	case	control	0.533761847	1.727430501	0.308991792	0.75732777	0.941659745	up_in_case
PRJNA8274(	ENSGALG00010028543	ANP32B	case	control	0.048350746	0.053982242	0.89567872	0.370424379	0.772700698	up_in_case
PRJNA8274(	ENSGALG00010028544	ZNF414	case	control	0.043835973	0.118551361	0.369763554	0.711558673	0.926542436	up_in_case
PRJNA8274(	ENSGALG00010028545	ENSGALG00010028545	case	control	0.926392211	1.272524569	0.72799554	0.466616316	0.827251838	up_in_case
PRJNA8274(	ENSGALG00010028546	ENSGALG00010028546	case	control	1.105316333	1.47913069	0.747274288	0.45489801	0.821657174	up_in_case
PRJNA8274(	ENSGALG00010028547	ZNF618	case	control	-0.016732897	0.073329552	-0.228187637	0.819500372	0.958669267	down_in_case
PRJNA8274(	ENSGALG00010028548	MYO1F	case	control	-0.079718122	0.131373142	-0.606806846	0.543979104	0.861114226	down_in_case
PRJNA8274(	ENSGALG00010028549	ADAMTS10	case	control	0.065668986	0.094994821	0.691290174	0.489383206	0.836347578	up_in_case
PRJNA8274(	ENSGALG00010028551	NCS1	case	control	-0.238128169	0.162165675	-1.468425234	0.141988751	0.543501954	down_in_case
PRJNA8274(	ENSGALG00010028552	ENSGALG00010028552	case	control	1.169313983	0.938377466	1.246101942	0.212726972	0.638404134	up_in_case
PRJNA8274(	ENSGALG00010028554	ENSGALG00010028554	case	control	0.615008371	1.058867801	0.580816954	0.561363827	0.870498818	up_in_case
PRJNA8274(	ENSGALG00010028555	ENSGALG00010028555	case	control	1.03570348	1.424751854	0.726936047	0.467265134	0.827888913	up_in_case
PRJNA8274(	ENSGALG00010028556	SPTAN1	case	control	-0.173109383	0.069679354	-2.484371253	0.012978043	0.200161779	down_in_case
PRJNA8274(	ENSGALG00010028558	ENSGALG00010028558	case	control	0.276166594	1.491767601	0.185127089	0.853129355	0.969924886	up_in_case
PRJNA8274(	ENSGALG00010028559	DYNC2I2	case	control	0.02478412	0.090833591	0.272851921	0.784967046	0.949518053	up_in_case
PRJNA8274(	ENSGALG00010028561	SET	case	control	0.006152268	0.039419517	0.156071628	0.875976562	0.975666505	up_in_case
PRJNA8274(	ENSGALG00010028562	ACSBG2	case	control	-0.027063027	0.109670255	-0.246767251	0.805088357	0.9556344	down_in_case
PRJNA8274(	ENSGALG00010028563	ENSGALG00010028563	case	control	0.453109524	1.610859754	0.281284278	0.778492368	0.948329573	up_in_case
PRJNA8274(	ENSGALG00010028564	ENSGALG00010028564	case	control	1.006589421	2.303277081	0.437024894			up_in_case
PRJNA8274(	ENSGALG00010028565	PKN1	case	control	-0.056535072	0.089536107	-0.631422044	0.527764606	0.853386536	down_in_case
PRJNA8274(	ENSGALG00010028566	ZDHHC12	case	control	-0.051211165	0.089059221	-0.575023724	0.565275252	0.872847005	down_in_case
PRJNA8274(	ENSGALG00010028567	ENSGALG00010028567	case	control	0.635874583	1.204778183	0.527793906	0.597642387	0.884680373	up_in_case
PRJNA8274(	ENSGALG00010028568	MLLT1	case	control	-0.017661648	0.047415739	-0.372484933	0.709531828	0.925630832	down_in_case
PRJNA8274(	ENSGALG00010028569	ZER1	case	control	-0.042270559	0.040597661	-1.041206775	0.297779592	0.721370179	down_in_case
PRJNA8274(	ENSGALG00010028572	TBC1D13	case	control	0.040569246	0.043596422	0.930563673	0.352079313	0.760831854	up_in_case
PRJNA8274(	ENSGALG00010028573	KCNN1	case	control	0.073513162	0.085352078	0.861293176	0.389076593	0.783669208	up_in_case
PRJNA8274(	ENSGALG00010028574	ENDOG	case	control	0.029656586	0.082809162	0.358131698	0.720244758	0.92895404	up_in_case
PRJNA8274(	ENSGALG00010028575	SETDB1	case	control	-0.057700269	0.04422135	-1.304805688	0.191959022	0.615090794	down_in_case
PRJNA8274(	ENSGALG00010028577	CERS2	case	control	-0.009702142	0.05725212	-0.169463453	0.865432117	0.973289701	down_in_case
PRJNA8274(	ENSGALG00010028578	SPOUT1	case	control	0.034238463	0.066058459	0.51830551	0.604245129	0.885268908	up_in_case
PRJNA8274(	ENSGALG00010028579	OCLN	case	control	-0.250914906	0.122482925	-2.048570489	0.040504133	0.33753444	down_in_case
PRJNA8274(	ENSGALG00010028580	PRUNE1	case	control	0.12796215	0.098962621	1.293035179	0.195998892	0.619095929	up_in_case
PRJNA8274(	ENSGALG00010028581	NR2F6	case	control	-0.019888955	0.043337085	-0.458936162	0.646280009	0.902693223	down_in_case
PRJNA8274(	ENSGALG00010028582	KYAT1	case	control	0.044942945	0.092546858	0.485623678	0.627234005	0.895838586	up_in_case
PRJNA8274(	ENSGALG00010028583	USHBP1	case	control	0.033214034	0.140553156	0.236309413	0.813192583	0.956712425	up_in_case
PRJNA8274(	ENSGALG00010028584	LRRC8A	case	control	0.016090237	0.046826906	0.343610926	0.731138907	0.933770678	up_in_case
PRJNA8274(	ENSGALG00010028585	MINDY1	case	control	-0.06266846	0.079357853	-0.789694502	0.429706203	0.806015686	down_in_case
PRJNA8274(	ENSGALG00010028586	BABAM1	case	control	-0.042798796	0.049778773	-0.859780045	0.389910301	0.783877029	down_in_case
PRJNA8274(	ENSGALG00010028587	ANKLE1	case	control	-0.138140317	0.131315174	-1.051975277	0.292810893	0.715270885	down_in_case
PRJNA8274(	ENSGALG00010028588	ABHD8	case	control	-0.328098862	0.304413957	-1.077804924	0.281120822	0.705762606	down_in_case
PRJNA8274(	ENSGALG00010028589	PHYHD1	case	control	0.351730775	0.162302274	2.167133989	0.030224639	0.30654407	up_in_case
PRJNA8274(	ENSGALG00010028590	ENSGALG00010028590	case	control	-0.054980698	0.068697591	-0.800329347	0.423520004	0.803723282	down_in_case
PRJNA8274(	ENSGALG00010028591	GABPB2	case	control	-0.044672406	0.103731352	-0.430654811	0.666719381	0.912284937	down_in_case
PRJNA8274(	ENSGALG00010028592	SEMA6C	case	control	-0.250679151	0.145129545	-1.727278561	0.084117648	0.458940237	down_in_case
PRJNA8274(	ENSGALG00010028593	DOLK	case	control	-0.022468172	0.089068802	-0.252256364	0.800842914	0.954294696	down_in_case
PRJNA8274(	ENSGALG00010028594	ENSGALG00010028594	case	control	-0.089041604	0.091094952	-0.977459253	0.328341841	0.745445783	down_in_case
PRJNA8274(	ENSGALG00010028595	SCNM1	case	control	-0.011988564	0.070086102	-0.1710548	0.864180677	0.97295831	down_in_case
PRJNA8274(	ENSGALG00010028596	DDA1	case	control	0.063846054	0.03490963	1.828895146	0.067415318	0.42042697	up_in_case
PRJNA8274(	ENSGALG00010028597	NUP188	case	control	-0.030241641	0.041810198	-0.723307752	0.469490831	0.829101076	down_in_case
PRJNA8274(	ENSGALG00010028598	TMOD4	case	control	-0.273606472	0.185791424	-1.472653938	0.140844373	0.543368104	down_in_case
PRJNA8274(	ENSGALG00010028599	VPS72	case	control	-0.21049063	0.070945267	-2.966943954	0.003007758	0.093476614	down_in_case
PRJNA8274(	ENSGALG00010028600	PSMD4	case	control	-0.016399287	0.066751353	-0.245677225	0.805932104	0.9556344	down_in_case
PRJNA8274(	ENSGALG00010028601	ANO8	case	control	-0.298713519	0.135053438	-2.211817218	0.026979297	0.292513391	down_in_case
PRJNA8274(	ENSGALG00010028602	ZNF687	case	control	0.013059703	0.067057276	0.194754448	0.845585185	0.967517103	up_in_case
PRJNA8274(	ENSGALG00010028603	SH3GLB2	case	control	0.107103725	0.162486237	0.659155673	0.509795808	0.8447206	up_in_case
PRJNA8274(	ENSGALG00010028604	PI4KB	case	control	-0.043057757	0.05468481	-0.787380573	0.431059116	0.806930928	down_in_case
PRJNA8274(	ENSGALG00010028605	GTPBP3	case	control	0.005519205	0.149201712	0.036991569	0.970491728	0.992761697	up_in_case
PRJNA8274(	ENSGALG00010028606	PRPF3	case	control	-0.057534637	0.045047167	-1.277208784	0.201528547	0.624725388	down_in_case
PRJNA8274(	ENSGALG00010028607	ENSGALG00010028607	case	control	0.030574958	0.05536713	0.552222198	0.580796127	0.879575141	up_in_case
PRJNA8274(	ENSGALG00010028608	MIGA2	case	control	0.106768821	0.097091064	1.099677118	0.271472828	0.698323327	up_in_case
PRJNA8274(	ENSGALG00010028609	MRPS21	case	control	-0.022204972	0.090796196	-0.244558391	0.806798385	0.955875648	down_in_case
PRJNA8274(	ENSGALG00010028610	ENSGALG00010028610	case	control	0.114567684	0.173112058	0.66181227	0.508091542	0.843702533	up_in_case
PRJNA8274(	ENSGALG00010028612	DIRAS1	case	control	-0.66266773	0.375286124	-1.765766675	0.077435002	0.446500211	down_in_case
PRJNA8274(	ENSGALG00010028613	ENSGALG00010028613	case	control	0.189172244	0.197040065	0.960069945	0.337020014	0.750735231	up_in_case
PRJNA8274(	ENSGALG00010028618	DOLPP1	case	control	0.048782889	0.091699314	0.531987506	0.594734649	0.884223506	up_in_case
PRJNA8274(	ENSGALG00010028626	CRAT	case	control	-0.179774069	0.094694395	-1.898465798	0.057634749	0.391161326	down_in_case
PRJNA8274(	ENSGALG00010028642	APH1A	case	control	-0.045029572	0.064552068	-0.697569791	0.485446279	0.834423421	down_in_case
PRJNA8274(	ENSGALG00010028649	ENSGALG00010028649	case	control	0.001624352	0.094664505	0.017159037	0.986309741	0.996597442	up_in_case
PRJNA8274(	ENSGALG00010028658	PTPA	case	control	-0.071948034	0.055070261	-1.306477083	0.191390368	0.614339451	down_in_case
PRJNA8274(	ENSGALG00010028662	FCRL4	case	control	-0.179542293	0.131172883	-1.368745496	0.171078843	0.58844277	down_in_case
PRJNA8274(	ENSGALG00010028669	ZBTB34	case	control	0.005817449	0.056963039	0.102126734	0.918656082	0.983734684	up_in_case
PRJNA8274(	ENSGALG00010028671	CADM3	case	control	0.142159471	0.194701664	0.73013999	0.465304619	0.826255378	up_in_case
PRJNA8274(	ENSGALG00010028675	ZBTB43	case	control	-0.072027616	0.063734888	-1.13011285	0.258428676	0.685881601	down_in_case
PRJNA8274(	ENSGALG00010028678	ENSGALG00010028678	case	control	0.166801191	0.266733473	0.625347804	0.531742812	0.855623068	up_in_case
PRJNA8274(	ENSGALG00010028683	CRPL2	case	control	0.778327302	0.326276358	2.385484829	0.017056626	0.232295	up_in_case
PRJNA8274(	ENSGALG00010028686	TAGLN2	case	control	0.109397963	0.081248817	1.346456073	0.178155478	0.597472638	up_in_case
PRJNA8274(	ENSGALG00010028689	CFAP45	case	control	-2.412173379	0.704653914	-3.423202982	0.000618878	0.036721833	down_in_case
PRJNA8274(	ENSGALG00010028692	RALGPS1	case	control	-0.051273162	0.116028076	-0.44190306	0.658559356	0.908367791	down_in_case
PRJNA8274(	ENSGALG00010028695	VSIG8	case	control	-1.412660691	1.212581726	-1.165002458			down_in_case
PRJNA8274(	ENSGALG00010028700	ANGPTL2	case	control	-0.078503906	0.137974158	-0.56897543	0.569372814	0.875057036	down_in_case
PRJNA8274(	ENSGALG00010028707	ENSGALG00010028707	case	control	0.179750567	0.824436789	0.218028319	0.827407047	0.961319258	up_in_case
PRJNA8274(	ENSGALG00010028712	FBXW2	case	control	-0.027503881	0.063574463	-0.432624663	0.665287469	0.912007336	down_in_case
PRJNA8274(	ENSGALG00010028726	SLC2A8	case	control	0.136853786	0.074098351	1.846920805	0.064758619	0.41359904	up_in_case
PRJNA8274(	ENSGALG00010028727	ENSGALG00010028727	case	control	0.092205854	0.118594759	0.777486754	0.436871646	0.810506246	up_in_case
PRJNA8274(	ENSGALG00010028734	ENSGALG00010028734	case	control	-0.246543706	0.141315797	-1.744629481	0.081049386	0.453090779	down_in_case
PRJNA8274(	ENSGALG00010028735	ENSGALG00010028735	case	control	-0.197550922	0.334661929	-0.590299955	0.55498957	0.867360748	down_in_case
PRJNA8274(	ENSGALG00010028736	NTRK1	case	control	1.017848357	0.933791748	1.09001644	0.275705902	0.700905672	up_in_case
PRJNA8274(	ENSGALG00010028738	MIDN	case	control	0.048819804	0.090435566	0.539829696	0.589314486	0.882949889	up_in_case
PRJNA8274(	ENSGALG00010028739	GARNL3	case	control	-0.022084707	0.039106249	-0.564736013	0.572253335	0.876814097	down_in_case
PRJNA8274(	ENSGALG00010028740	DAZAP1	case	control	0.018901866	0.047611661	0.397000756	0.691366903	0.918812261	up_in_case
PRJNA8274(	ENSGALG00010028742	ENSGALG00010028742	case	control	0.019872588	0.148167329	0.134122605	0.893305625	0.979875146	up_in_case
PRJNA8274(	ENSGALG00010028743	ARRDC1	case	control	0.017210685	0.08022595	0.21452765	0.830135607	0.962228709	up_in_case
PRJNA8274(	ENSGALG00010028744	HOMER3	case	control	-0.038335839	0.083806679	-0.457431789	0.64736072	0.903059048	down_in_case
PRJNA8274(	ENSGALG00010028745	ENSGALG00010028745	case	control	0.053300826	0.066693321	0.799192864	0.424178589	0.803723282	up_in_case
PRJNA8274(	ENSGALG00010028746	MYDGF	case	control	0.14910924	0.062849843	2.372467962	0.017669695	0.23642725	up_in_case
PRJNA8274(	ENSGALG00010028747	ENSGALG00010028747	case	control	-0.083201188	0.172404231	-0.482593657	0.629384284	0.896269608	down_in_case
PRJNA8274(	ENSGALG00010028748	PIAS3	case	control	0.176095217	0.10379628	1.696546512	0.08978243	0.472887198	up_in_case
PRJNA8274(	ENSGALG00010028750	OTUD7B	case	control	0.058988811	0.065398015	0.901996964	0.36705848	0.771770041	up_in_case
PRJNA8274(	ENSGALG00010028751	EHMT1	case	control	-0.092982438	0.044648178	-2.082558426	0.037291492	0.328438691	down_in_case
PRJNA8274(	ENSGALG00010028752	MTMR11	case	control	0.10917371	0.14089574	0.774854583	0.438425592	0.811689017	up_in_case
PRJNA8274(	ENSGALG00010028753	SV2A	case	control	-0.027951493	0.459767147	-0.06079489	0.95152256	0.991857576	down_in_case
PRJNA8274(	ENSGALG00010028756	ENSGALG00010028756	case	control	2.068744938	0.759151429	2.725075472			up_in_case
PRJNA8274(	ENSGALG00010028757	MAT2A	case	control	1.887740628	0.875246464	2.156810345			up_in_case
PRJNA8274(	ENSGALG00010028760	SSR2	case	control	-0.038141551	0.04736829	-0.80521275	0.42069692	0.801969583	down_in_case
PRJNA8274(	ENSGALG00010028761	CTSS	case	control	0.008955359	0.12495762	0.071667167	0.942866786	0.990054824	up_in_case

PRJNA8274(	ENSGALG00010028762	CACNA1B	case	control	0.548814745	0.41047017	1.337039289	0.181209812	0.601788276	up_in_case
PRJNA8274(	ENSGALG00010028763	CTSK	case	control	0.343562843	0.105699665	3.250368323	0.001152556	0.052995354	up_in_case
PRJNA8274(	ENSGALG00010028764	ARNT	case	control	-0.085781806	0.049056951	-1.748616754	0.080357288	0.452049261	down_in_case
PRJNA8274(	ENSGALG00010028768	NTNG2	case	control	0.070336139	0.132509256	0.530801705	0.595556199	0.884680373	up_in_case
PRJNA8274(	ENSGALG00010028771	SETX	case	control	0.113434758	0.056446986	2.009580422	0.044475615	0.350410728	up_in_case
PRJNA8274(	ENSGALG00010028772	TTF1	case	control	0.022739463	0.049516391	0.459231026	0.646068272	0.902693223	up_in_case
PRJNA8274(	ENSGALG00010028782	MLLT11	case	control	-0.258456927	0.232306922	-1.112566618	0.265894604	0.69218122	down_in_case
PRJNA8274(	ENSGALG00010028783	DDX31	case	control	-0.11647632	0.067121262	-1.735311839	0.082685586	0.456624721	down_in_case
PRJNA8274(	ENSGALG00010028786	GTF3C4	case	control	-0.032037088	0.045353819	-0.706381267	0.479951067	0.831851816	down_in_case
PRJNA8274(	ENSGALG00010028800	ENSGALG00010028800	case	control	0.219146512	0.1824498	1.201133201	0.229699536	0.653695091	up_in_case
PRJNA8274(	ENSGALG00010028806	SPACA9	case	control	0.273846224	0.254780068	1.074833782	0.282449153	0.706246352	up_in_case
PRJNA8274(	ENSGALG00010028818	TSC1	case	control	-0.060894086	0.077424095	-0.78650046	0.43157435	0.807261374	down_in_case
PRJNA8274(	ENSGALG00010028823	GFI1B	case	control	0.43551796	0.203226636	2.143016139	0.032111802	0.3098361	up_in_case
PRJNA8274(	ENSGALG00010028829	GTF3C5	case	control	-0.053699298	0.066104848	-0.81233525	0.416599289	0.799987395	down_in_case
PRJNA8274(	ENSGALG00010028833	GBGT1	case	control	-0.062238377	0.099069887	-0.628226996	0.529855248	0.854806007	down_in_case
PRJNA8274(	ENSGALG00010028849	ENSGALG00010028849	case	control	0.273207111	0.295586695	0.924287581	0.355336607	0.763161644	up_in_case
PRJNA8274(	ENSGALG00010028853	ENSGALG00010028853	case	control	0.470836398	1.355326611	0.347397	0.728293087	0.933057319	up_in_case
PRJNA8274(	ENSGALG00010028854	RPL7A	case	control	-0.106363049	0.068250209	-1.558428191	0.119131778	0.516645008	down_in_case
PRJNA8274(	ENSGALG00010028855	SURF6	case	control	-0.001107319	0.060682015	-0.018247895	0.985441094	0.996591771	down_in_case
PRJNA8274(	ENSGALG00010028856	ETV3	case	control	-0.05602644	0.063684446	-0.879750768	0.37899434	0.777229179	down_in_case
PRJNA8274(	ENSGALG00010028857	MED22	case	control	-0.044650016	0.076871612	-0.580838817	0.561349091	0.870498818	down_in_case
PRJNA8274(	ENSGALG00010028858	ENSGALG00010028858	case	control	-0.437910435	0.534865447	-0.81873009	0.412940425	0.797633779	down_in_case
PRJNA8274(	ENSGALG00010028859	SURF1	case	control	-0.041147233	0.077740226	-0.5292914	0.596603321	0.884680373	down_in_case
PRJNA8274(	ENSGALG00010028860	SURF2	case	control	0.00018976	0.06363867	0.002981833	0.997620845	0.999142415	up_in_case
PRJNA8274(	ENSGALG00010028861	SURF4	case	control	-0.016275517	0.033988507	-0.47885354	0.632042825	0.89724192	down_in_case
PRJNA8274(	ENSGALG00010028862	ENSGALG00010028862	case	control	1.616850851	1.235213529	1.308964655	0.190546324	0.613935676	up_in_case
PRJNA8274(	ENSGALG00010028865	STKLD1	case	control	0.112285642	0.177071757	0.634125083	0.525999195	0.85251736	up_in_case
PRJNA8274(	ENSGALG00010028866	HORMAD1	case	control	-0.079364139	0.328877124	-0.241318515	0.809308262	0.955875648	down_in_case
PRJNA8274(	ENSGALG00010028867	REXO4	case	control	-0.089169136	0.072383469	-1.23189918	0.217986748	0.64312162	down_in_case
PRJNA8274(	ENSGALG00010028868	ENSGALG00010028868	case	control	0.656014286	1.730848498	0.379013118	0.704678123	0.923973441	up_in_case
PRJNA8274(	ENSGALG00010028869	ADAMTS13	case	control	0.086975224	0.241292667	0.360455314	0.718506668	0.927813495	up_in_case
PRJNA8274(	ENSGALG00010028870	CACFD1	case	control	0.31284745	0.193361994	1.617936616	0.105676258	0.495266487	up_in_case
PRJNA8274(	ENSGALG00010028871	PIP5K1A	case	control	-0.103728467	0.059468566	-1.744257063	0.081114275	0.453227768	down_in_case
PRJNA8274(	ENSGALG00010028872	SLC2A6	case	control	-0.340193202	0.224000022	-1.518719503	0.128833115	0.530620261	down_in_case
PRJNA8274(	ENSGALG00010028873	TMEM8C	case	control	0.191498753	0.207925036	0.920999013	0.357050942	0.763373775	up_in_case
PRJNA8274(	ENSGALG00010028874	CRPL1	case	control	4.761646986	0.651337081	7.310572552	2.66006E-13	2.00205E-10	up_in_case
PRJNA8274(	ENSGALG00010028875	DUSP23	case	control	0.023800563	0.104890007	0.226909732	0.820493935	0.958669267	up_in_case
PRJNA8274(	ENSGALG00010028876	ADAMTSL2	case	control	-0.186580321	0.16490294	-1.131455395	0.257863467	0.685144479	down_in_case
PRJNA8274(	ENSGALG00010028877	FAM163B	case	control	-0.289763201	0.413228625	-0.70121764	0.483167203	0.833529046	down_in_case
PRJNA8274(	ENSGALG00010028878	IGSF9	case	control	0.046313811	0.089529692	0.517301136	0.604945961	0.885268908	up_in_case
PRJNA8274(	ENSGALG00010028881	SARDH	case	control	-0.071553618	0.107268021	-0.66705452	0.504737309	0.842675107	down_in_case
PRJNA8274(	ENSGALG00010028884	VAV2	case	control	0.082240331	0.059783433	1.375637487	0.168933895	0.585038804	up_in_case
PRJNA8274(	ENSGALG00010028885	BRD3	case	control	-0.077356344	0.061845867	-1.250792465	0.211010205	0.636501957	down_in_case
PRJNA8274(	ENSGALG00010028887	SF3B4	case	control	-0.03792465	0.071012748	-0.534054114	0.593304095	0.883625114	down_in_case
PRJNA8274(	ENSGALG00010028888	WDR5B	case	control	-0.130478437	0.051547515	-2.531226522	0.011366442	0.186780127	down_in_case
PRJNA8274(	ENSGALG00010028889	FUBP3	case	control	-0.002986754	0.04329907	-0.068979636	0.945005829	0.990313016	down_in_case
PRJNA8274(	ENSGALG00010028890	ENSGALG00010028890	case	control	-0.050329509	0.150430322	-0.334570242	0.737949296	0.93585269	down_in_case
PRJNA8274(	ENSGALG00010028891	SLC31A2	case	control	0.479646769	0.363662683	1.318933154	0.187191461	0.610664656	up_in_case
PRJNA8274(	ENSGALG00010028892	NIBAN2	case	control	0.148150955	0.07370898	2.009944441	0.04443707	0.350320113	up_in_case
PRJNA8274(	ENSGALG00010028893	STXBP1	case	control	-0.154863528	0.086652065	-1.787187955	0.073907115	0.437990779	down_in_case
PRJNA8274(	ENSGALG00010028894	AKNA	case	control	-0.003832203	0.066396618	-0.057716844	0.953974176	0.992056045	down_in_case
PRJNA8274(	ENSGALG00010028895	WHRN	case	control	-0.150614759	0.539472271	-0.279189065	0.780099735	0.948864508	down_in_case
PRJNA8274(	ENSGALG00010028896	ATP6V1G1	case	control	-0.011171418	0.094061253	-0.118767478	0.905459575	0.981730793	down_in_case
PRJNA8274(	ENSGALG00010028897	TMEM268	case	control	0.086405211	0.154769329	0.558283808	0.5766506	0.878085957	up_in_case
PRJNA8274(	ENSGALG00010028898	TNFSF15	case	control	-0.036974986	0.211461166	-0.174854733	0.86119378	0.972434239	down_in_case
PRJNA8274(	ENSGALG00010028899	TNFSF8	case	control	1.309757907	0.699305658	1.872940527	0.061076598	0.401706103	up_in_case
PRJNA8274(	ENSGALG00010028900	TNC	case	control	-0.001587586	0.391438178	-0.004055777			down_in_case
PRJNA8274(	ENSGALG00010028901	PAPPA	case	control	-0.135725237	0.107999784	-1.256717668	0.208855888	0.633836842	down_in_case
PRJNA8274(	ENSGALG00010028903	ASTN2	case	control	0.127903032	0.252283427	0.506981507	0.612167795	0.889814948	up_in_case
PRJNA8274(	ENSGALG00010028904	TRIM32	case	control	0.004151219	0.051455016	0.080676659	0.9356991	0.988425668	up_in_case
PRJNA8274(	ENSGALG00010028905	COLGALT1	case	control	-0.035018653	0.079429883	-0.440875038	0.659303466	0.908599575	down_in_case
PRJNA8274(	ENSGALG00010028909	ENSGALG00010028909	case	control	0.061441178	0.095231361	0.645177986	0.51881185	0.848941486	up_in_case
PRJNA8274(	ENSGALG00010028911	LMX1B	case	control	1.012465329	0.749572256	1.350724125	0.176783821	0.595527443	up_in_case
PRJNA8274(	ENSGALG00010028912	EXFABP	case	control	-0.233006119	0.260498457	-0.894462569	0.371074448	0.772959157	down_in_case
PRJNA8274(	ENSGALG00010028914	TLR4	case	control	0.006097256	0.109949967	0.055454823	0.955776121	0.992056045	up_in_case
PRJNA8274(	ENSGALG00010028915	ADGRD2	case	control	0.252582576	0.364894852	0.692206467	0.488807678	0.836318473	up_in_case
PRJNA8274(	ENSGALG00010028917	NR6A1	case	control	0.169847997	0.081063793	2.095238694	0.036149779	0.32592088	up_in_case
PRJNA8274(	ENSGALG00010028918	AGPAT2	case	control	-0.14198459	0.082267818	-1.725882544	0.084368546	0.459607696	down_in_case
PRJNA8274(	ENSGALG00010028919	EGFL7	case	control	0.002502011	0.052808831	0.047378647	0.962211447	0.992056045	up_in_case
PRJNA8274(	ENSGALG00010028921	MRPL41	case	control	-0.081997982	0.073747971	-1.111867637	0.266195066	0.69218122	down_in_case
PRJNA8274(	ENSGALG00010028923	ENSGALG00010028923	case	control	-0.20891727	0.134686099	-1.551142033	0.12086765	0.519499513	down_in_case
PRJNA8274(	ENSGALG00010028924	TMEM203	case	control	0.026431304	0.063063906	0.419119358	0.675128904	0.914247368	up_in_case
PRJNA8274(	ENSGALG00010028925	ENSGALG00010028925	case	control	-0.061383712	0.241430815	-0.2542497	0.799302657	0.954053991	down_in_case
PRJNA8274(	ENSGALG00010028926	ENSGALG00010028926	case	control	0.720619411	0.450689735	1.598925724	0.109837107	0.501972079	up_in_case
PRJNA8274(	ENSGALG00010029140	NPDC1	case	control	-0.122831748	0.081473352	-1.507630965	0.131649003	0.535159035	down_in_case
PRJNA8274(	ENSGALG00010029141	C5	case	control	0.403439283	0.625313859	0.64517886	0.518811284	0.848941486	up_in_case
PRJNA8274(	ENSGALG00010029142	ENSGALG00010029142	case	control	0.014958084	0.122970165	0.121639943	0.903184178	0.980997505	up_in_case
PRJNA8274(	ENSGALG00010029143	SH2D3C	case	control	-0.005430711	0.078334305	-0.069327363	0.944729045	0.990313016	down_in_case
PRJNA8274(	ENSGALG00010029144	ENSGALG00010029144	case	control	-0.35657889	0.124297884	-2.868744662	0.004121043	0.111190415	down_in_case
PRJNA8274(	ENSGALG00010029145	RC3H2	case	control	-0.061071278	0.054738454	-1.115692418	0.264553817	0.690350289	down_in_case
PRJNA8274(	ENSGALG00010029146	NEK6	case	control	0.094465371	0.083111238	1.136613684	0.255699817	0.681937229	up_in_case
PRJNA8274(	ENSGALG00010029147	RABL6	case	control	0.001278034	0.048314034	0.026452644	0.978896305	0.995525769	up_in_case
PRJNA8274(	ENSGALG00010029148	RALGDS	case	control	-0.043052288	0.083950029	-0.512832314	0.608068624	0.887502645	down_in_case
PRJNA8274(	ENSGALG00010029149	ENSGALG00010029149	case	control	0.162198905	0.19691962	0.823680773	0.410120967	0.796189224	up_in_case
PRJNA8274(	ENSGALG00010029150	NARF	case	control	0.108554169	0.06179138	1.756785005	0.078954462	0.449171025	up_in_case
PRJNA8274(	ENSGALG00010029151	CYBC1	case	control	-0.020549817	0.075815243	-0.271051261	0.786351607	0.950252288	down_in_case
PRJNA8274(	ENSGALG00010029152	HEXD	case	control	0.215687829	0.105254611	2.049200756	0.040442488	0.33753444	up_in_case
PRJNA8274(	ENSGALG00010029153	OGFOD3	case	control	0.160481689	0.093496068	1.71645389	0.086079	0.463278022	up_in_case
PRJNA8274(	ENSGALG00010029154	UTS2R	case	control	0.195854777	0.124857097	1.568631517	0.116733824	0.51350396	up_in_case
PRJNA8274(	ENSGALG00010029155	ENSGALG00010029155	case	control	-0.084156669	0.130551081	-0.644626366	0.519169344	0.849150363	down_in_case
PRJNA8274(	ENSGALG00010029156	ENSGALG00010029156	case	control	-0.040517495	0.538052046	-0.075304044	0.939972804	0.989551456	down_in_case
PRJNA8274(	ENSGALG00010029158	CD7	case	control	0.021715413	0.381568214	0.056910958	0.954616125	0.992056045	up_in_case
PRJNA8274(	ENSGALG00010029159	SSC4D	case	control	-0.021364115	0.526684901	-0.04056337	0.967643987	0.992761697	down_in_case
PRJNA8274(	ENSGALG00010029160	DUS1L	case	control	0.014346885	0.056674498	0.253145335	0.800155907	0.954053991	up_in_case
PRJNA8274(	ENSGALG00010029161	HNF1B	case	control	0.604280821	0.949682354	0.63629783	0.524582319	0.852356213	up_in_case
PRJNA8274(	ENSGALG00010029162	ELN	case	control	0.007383347	0.447958617	0.016482207			up_in_case
PRJNA8274(	ENSGALG00010029163	ANKFY1	case	control	0.001439087	0.053720367	0.026788472	0.978628448	0.995333344	up_in_case
PRJNA8274(	ENSGALG00010029164	CYB5D2	case	control	-0.002197525	0.071382977	-0.030785006	0.975440998	0.99399062	down_in_case
PRJNA8274(	ENSGALG00010029165	RAC3	case	control	-0.012335222	0.076245447	-0.161783062	0.871476692	0.975062028	down_in_case
PRJNA8274(	ENSGALG00010029166	ZZEF1	case	control	0.015264067	0.064866745	0.235314209	0.813964868	0.956818546	up_in_case
PRJNA8274(	ENSGALG00010029167	ENSGALG00010029167	case	control	-0.098216827	0.061100649	-1.607459618	0.107953581	0.499736588	down_in_case
PRJNA8274(	ENSGALG00010029168	RFNG	case	control	-0.043142008	0.042047852	-1.026021675	0.304881367	0.728073294	down_in_case
PRJNA8274(	ENSGALG00010029169	SBDS	case	control	-0.045058701	0.052915059	-0.851528873	0.394475632	0.786978937	down_in_case
PRJNA8274(	ENSGALG00010029170	DCXR	case	control	-0.00443137	0.093421845	-0.047433983	0.962167345	0.992056045	down_in_case
PRJNA8274(	ENSGALG00010029171	ENSGALG00010029171	case	control	-0.064229439	0.05282034	-1.215998213	0.223985603	0.648105158	down_in_case
PRJNA8274(	ENSGALG00010029172	LRRC45	case	control	0.020919274	0.059500832	0.351579521	0.725153626	0.93185316	up_in_case
PRJNA8274(	ENSGALG00010029173	ZNHIT3	case	control	-0.060966747	0.10072063	-0.605305452	0.54497606	0.861997112	down_in_case
PRJNA8274(	ENSGALG00010029174	CALN1	case	control	-0.217924198	0.208801238	-1.043692077	0.296627877	0.720288443	down_in_case
PRJNA8274(	ENSGALG00010029175	CENPX	case	control	0.010647852	0.145480948	0.073190697	0.941654369	0.989551456	up_in_case
PRJNA8274(	ENSGALG00010029177	ASPSCR1	case	control	-0.020613293	0.064475294	-0.319708403	0.749189389	0.939503405	down_in_case
PRJNA8274(	ENSGALG00010029178	NOTUM	case	control	0.174246764	0.098872878	1.762331268	0.078013344	0.448028441	up_in_case
PRJNA8274(	ENSGALG00010029179	PIGW	case	control	0.06776159	0.116311388	0.58258775	0.560170854	0.86984941	up_in_case
PRJNA8274(	ENSGALG00010029180	CA4	case	control	-0.757628804	0.325947652	-2.324387977	0.020104709	0.254401597	down_in_case
PRJNA8274(	ENSGALG00010029181	MYO19	case	control	0.121297149	0.08109989	1.495651219	0.134744569	0.539774365	up_in_case
PRJNA8274(	ENSGALG00010029182	HEATR6	case	control	0.061524959	0.072781761	0.845334855	0.397923906	0.788896694	up_in_case
PRJNA8274(	ENSGALG00010029183	ENSGALG00010029183	case	control	0.022874419	0.06703436	0.341234246	0.732927245	0.933846596	up_in_case
PRJNA8274(	ENSGALG00010029184	DYNLL2	case	control	0.039955327	0.071063227	0.562250387	0.573945436	0.877573415	up_in_case
PRJNA8274(	ENSGALG00010029186	ZNF302	case	control	0.154674547	0.086635005	1.785358555	0.074203177	0.438111241	up_in_case
PRJNA8274(	ENSGALG00010029187	SRSF1	case	control	0.062062297	0.045472084	1.364843904	0.172302116	0.590683026	up_in_case
PRJNA8274(	ENSGALG00010029188	ENSGALG00010029188	case	control	0.09804263	0.596531194	0.164354574	0.869452022	0.974718971	up_in_case
PRJNA8274(	ENSGALG00010029189	ENSGALG00010029189	case	control	0.690126422	0.545592044	1.264912913	0.205902548	0.62881082	up_in_case
PRJNA8274(	ENSGALG00010029190	DHRS7C	case	control	-0.563483624	0.176960602	-3.18423206	0.001451386	0.060509682	down_in_case
PRJNA8274(	ENSGALG00010029191	GSG1L2	case	control	-0.371099766	0.359243966	-1.033002085	0.301602923	0.724474473	down_in_case
PRJNA8274(	ENSGALG00010029192	DTX2	case	control	-0.041227161	0.074128217	-0.556160159	0.578101372	0.878704254	down_in_case
PRJNA8274(	ENSGALG00010029194	DHRS11	case	control	-0.026357307	0.091003067	-0.289630969	0.772098572	0.945872699	down_in_case
PRJNA8274(	ENSGALG00010029196	MRM1	case	control	-0.235800039	0.107692535	-2.189567176	0.028555642	0.300549329	down_in_case

PRJNA8274(	ENSGALG00010029197	LHX1	case	control	-0.547174911	0.385333594	-1.420003133	0.155606769	0.564622379	down_in_case
PRJNA8274(	ENSGALG00010029198	PPM1D	case	control	-0.054995057	0.075468566	-0.728714752	0.466176167	0.82702452	down_in_case
PRJNA8274(	ENSGALG00010029199	APPBP2	case	control	-0.137192893	0.053736903	-2.553048004	0.010678478	0.184200521	down_in_case
PRJNA8274(	ENSGALG00010029200	GAS7	case	control	0.025109654	0.10753639	0.233499135	0.815373841	0.957374769	up_in_case
PRJNA8274(	ENSGALG00010029201	PIGS	case	control	-0.027858949	0.067895928	-0.410318403	0.681572394	0.915594727	down_in_case
PRJNA8274(	ENSGALG00010029202	PSMD12	case	control	-0.058946698	0.045589825	-1.292979264	0.196018231	0.619095929	down_in_case
PRJNA8274(	ENSGALG00010029203	ALDOC	case	control	-0.023769899	0.101452235	-0.234296451	0.814754841	0.957275016	down_in_case
PRJNA8274(	ENSGALG00010029204	HELZ	case	control	-0.054068258	0.07043632	-0.76761901	0.442713565	0.814107419	down_in_case
PRJNA8274(	ENSGALG00010029205	CACNG1	case	control	-0.077765703	0.107507068	-0.72335433	0.469462222	0.829101076	down_in_case
PRJNA8274(	ENSGALG00010029206	CACNG4	case	control	0.255793381	0.182411336	1.402288842	0.160829011	0.573265281	up_in_case
PRJNA8274(	ENSGALG00010029207	SPAG5	case	control	-0.167130745	0.101490073	-1.64676939	0.099605455	0.488667622	down_in_case
PRJNA8274(	ENSGALG00010029208	CACNG5	case	control	0.414730256	0.198854502	2.085596508	0.037015188	0.32835414	up_in_case
PRJNA8274(	ENSGALG00010029209	PRKCA	case	control	0.058186852	0.072087348	0.807171489	0.419567689	0.80093491	up_in_case
PRJNA8274(	ENSGALG00010029210	RSKR	case	control	-0.347563835	0.213875106	-1.625078494	0.104145835	0.493794905	down_in_case
PRJNA8274(	ENSGALG00010029211	SDF2	case	control	0.065078907	0.08089094	0.804526531	0.421092954	0.802114148	up_in_case
PRJNA8274(	ENSGALG00010029212	SUPT6H	case	control	-0.148605467	0.044982384	-3.303636992	0.000954393	0.047388277	down_in_case
PRJNA8274(	ENSGALG00010029213	APOH	case	control	3.494233009	0.983351019	3.553393387	0.000380295	0.027024726	up_in_case
PRJNA8274(	ENSGALG00010029214	ENSGALG00010029214	case	control	-0.366440505	0.246151808	-1.488676877	0.136572477	0.539848047	down_in_case
PRJNA8274(	ENSGALG00010029215	ENSGALG00010029215	case	control	-0.280827707	0.152365235	-1.843121944	0.065311208	0.414904609	down_in_case
PRJNA8274(	ENSGALG00010029216	RAB34	case	control	-0.121984197	0.086944039	-1.40301967	0.160610974	0.573038154	down_in_case
PRJNA8274(	ENSGALG00010029217	CEP112	case	control	0.005723172	0.130992215	0.04369093	0.965150769	0.992291928	up_in_case
PRJNA8274(	ENSGALG00010029218	RPL23A	case	control	-0.098770529	0.068521867	-1.441445393	0.149458894	0.556014189	down_in_case
PRJNA8274(	ENSGALG00010029219	TLCD1	case	control	0.045067434	0.122232713	0.368701906	0.712349928	0.92680349	up_in_case
PRJNA8274(	ENSGALG00010029220	NEK8	case	control	-0.07965516	0.102217678	-0.779269908	0.435820737	0.81000376	down_in_case
PRJNA8274(	ENSGALG00010029221	AXIN2	case	control	-0.024495286	0.130576065	-0.187593999	0.851194931	0.969234083	down_in_case
PRJNA8274(	ENSGALG00010029222	TRAF4	case	control	0.166129808	0.109297841	1.519973383	0.128517665	0.53054348	up_in_case
PRJNA8274(	ENSGALG00010029223	NME2	case	control	0.017799434	0.050149569	0.354926968	0.722644305	0.929885139	up_in_case
PRJNA8274(	ENSGALG00010029224	FAM222B	case	control	-0.065923945	0.068059337	-0.96862456	0.332732545	0.748503677	down_in_case
PRJNA8274(	ENSGALG00010029225	ENSGALG00010029225	case	control	1.816136325	1.395703705	1.301233434	0.193178564	0.615657114	up_in_case
PRJNA8274(	ENSGALG00010029226	MBTD1	case	control	-0.016642854	0.057321177	-0.290343905	0.771553154	0.945676703	down_in_case
PRJNA8274(	ENSGALG00010029227	UTP18	case	control	0.033161685	0.044360105	0.747556505	0.454727708	0.821657174	up_in_case
PRJNA8274(	ENSGALG00010029228	ERAL1	case	control	0.097990411	0.0599394	1.634824682	0.102085805	0.491460867	up_in_case
PRJNA8274(	ENSGALG00010029229	CBX4	case	control	0.010177621	0.087846763	0.115856526	0.907766251	0.981730793	up_in_case
PRJNA8274(	ENSGALG00010029230	PPM1E	case	control	-0.162707263	0.077374157	-2.102863151	0.035477734	0.324939791	down_in_case
PRJNA8274(	ENSGALG00010029231	CBX8	case	control	0.159396199	0.083379383	1.911697981	0.055914943	0.385341538	up_in_case
PRJNA8274(	ENSGALG00010029232	CA10	case	control	0.394942039	0.268218541	1.472463601	0.140895729	0.543368104	up_in_case
PRJNA8274(	ENSGALG00010029233	CBX2	case	control	0.000700655	0.109597104	0.006393007	0.994899153	0.998586153	up_in_case
PRJNA8274(	ENSGALG00010029234	TOM1L1	case	control	0.028091886	0.193103277	0.145475968	0.884335089	0.977579768	up_in_case
PRJNA8274(	ENSGALG00010029235	TRIM37	case	control	-0.108771729	0.051103978	-2.128439571	0.033300655	0.314561474	down_in_case
PRJNA8274(	ENSGALG00010029237	COX11	case	control	0.038994415	0.088753029	0.439358698	0.660401649	0.909188753	up_in_case
PRJNA8274(	ENSGALG00010029238	CAMKK1	case	control	0.397751824	0.188776	2.107004192	0.035117216	0.323358784	up_in_case
PRJNA8274(	ENSGALG00010029239	RBFOX3	case	control	-0.429884071	0.200368164	-2.145470934	0.031915213	0.3098361	down_in_case
PRJNA8274(	ENSGALG00010029240	MIS12	case	control	-0.202884546	0.101937071	-1.990292088	0.046558769	0.356907825	down_in_case
PRJNA8274(	ENSGALG00010029241	STXBP4	case	control	-0.122743589	0.082643147	-1.485224042	0.137484485	0.539854583	down_in_case
PRJNA8274(	ENSGALG00010029242	DERL2	case	control	0.043612691	0.080023659	0.544997457	0.585755266	0.881576413	up_in_case
PRJNA8274(	ENSGALG00010029243	NME1	case	control	0.334287379	0.237791248	1.405801859	0.15978297	0.572017525	up_in_case
PRJNA8274(	ENSGALG00010029244	HLF	case	control	0.035759132	0.079432309	0.450183711	0.652577981	0.905885495	up_in_case
PRJNA8274(	ENSGALG00010029245	SKA2	case	control	-0.09144689	0.053262473	-1.716910317	0.085995559	0.463278022	down_in_case
PRJNA8274(	ENSGALG00010029246	DHX33	case	control	0.062035263	0.083324247	0.744504327	0.456571425	0.822288586	up_in_case
PRJNA8274(	ENSGALG00010029247	CCDC92B	case	control	-0.548062372	0.367617015	-1.490851482	0.136000492	0.539774365	down_in_case
PRJNA8274(	ENSGALG00010029248	C1QBP	case	control	-0.100153963	0.062928274	-1.591557437	0.11148418	0.504182092	down_in_case
PRJNA8274(	ENSGALG00010029249	PRR11	case	control	-0.242429906	0.088529	-2.738423639	0.00617345	0.136235074	down_in_case
PRJNA8274(	ENSGALG00010029250	SPAG9	case	control	-0.004318939	0.053720679	-0.080396202	0.935922147	0.988425668	down_in_case
PRJNA8274(	ENSGALG00010029251	ENGASE	case	control	0.055144149	0.161259735	0.341958576	0.732382066	0.933844276	up_in_case
PRJNA8274(	ENSGALG00010029252	RPAIN	case	control	0.121071329	0.126430519	0.957611581	0.338258657	0.751259351	up_in_case
PRJNA8274(	ENSGALG00010029253	NUP88	case	control	-0.048910749	0.050729251	-0.964152792	0.334969328	0.749266604	down_in_case
PRJNA8274(	ENSGALG00010029254	MMD	case	control	-0.034556347	0.056366967	-0.613060255	0.539836499	0.858598814	down_in_case
PRJNA8274(	ENSGALG00010029255	RAP1GAP2	case	control	-0.105505584	0.094001983	-1.122376157	0.261702538	0.687516348	down_in_case
PRJNA8274(	ENSGALG00010029256	RABEP1	case	control	-0.066097842	0.04297744	-1.537966003	0.124056928	0.523029039	down_in_case
PRJNA8274(	ENSGALG00010029257	YPEL2	case	control	-0.131044607	0.121319967	-1.08015696	0.280072291	0.705732821	down_in_case
PRJNA8274(	ENSGALG00010029258	TMEM100	case	control	0.033604438	0.102746276	0.327062343	0.743620733	0.937448285	up_in_case
PRJNA8274(	ENSGALG00010029259	PTRH2	case	control	0.015393728	0.106081741	0.145111949	0.884622484	0.977612694	up_in_case
PRJNA8274(	ENSGALG00010029260	DHX40	case	control	0.052121668	0.077147142	0.675613726	0.499285925	0.840142058	up_in_case
PRJNA8274(	ENSGALG00010029261	IFT22	case	control	-0.067768638	0.132301273	-0.512229672	0.608490279	0.8877909	down_in_case
PRJNA8274(	ENSGALG00010029262	CLTC	case	control	-0.040825516	0.045327026	-0.900688173	0.367754139	0.771770041	down_in_case
PRJNA8274(	ENSGALG00010029263	TOB1	case	control	0.071949781	0.06620305	1.086804623	0.277123164	0.702510414	up_in_case
PRJNA8274(	ENSGALG00010029264	COL26A1	case	control	0.057656178	0.144260209	0.399667924	0.68940112	0.918335352	up_in_case
PRJNA8274(	ENSGALG00010029266	CANT1	case	control	0.118360633	0.076630137	1.544570286	0.122450238	0.520832363	up_in_case
PRJNA8274(	ENSGALG00010029267	ENSGALG00010029267	case	control	0.216616027	0.153684258	1.409487412	0.158691093	0.570858667	up_in_case
PRJNA8274(	ENSGALG00010029268	PCTP	case	control	-0.138879437	0.161417935	-0.860371785	0.389584134	0.783772245	down_in_case
PRJNA8274(	ENSGALG00010029269	WSCD1	case	control	0.008760293	0.088772331	0.098682694	0.92139021	0.985267883	up_in_case
PRJNA8274(	ENSGALG00010029270	TIMP2	case	control	0.08870388	0.068058528	1.303347032	0.192456311	0.615090794	up_in_case
PRJNA8274(	ENSGALG00010029271	MYL10	case	control	0.116892735	0.251745931	0.464328199	0.642412624	0.901845477	up_in_case
PRJNA8274(	ENSGALG00010029273	SLC13A5	case	control	0.322563058	0.272576413	1.183385804	0.236656281	0.661690689	up_in_case
PRJNA8274(	ENSGALG00010029274	VMP1	case	control	0.109671864	0.05690756	1.927193211	0.053955556	0.378403358	up_in_case
PRJNA8274(	ENSGALG00010029275	PCYT2	case	control	0.056788243	0.050495401	1.124622069	0.260749218	0.687359696	up_in_case
PRJNA8274(	ENSGALG00010029276	TUBD1	case	control	-0.317575537	0.099920614	-3.178278485	0.001481524	0.061408087	down_in_case
PRJNA8274(	ENSGALG00010029277	WFIKKN2	case	control	0.295719678	0.30984392	0.954414979	0.339873627	0.751350877	up_in_case
PRJNA8274(	ENSGALG00010029278	MED31	case	control	-0.096987766	0.06301483	-1.539126026	0.123773533	0.522883756	down_in_case
PRJNA8274(	ENSGALG00010029279	ENSGALG00010029279	case	control	0.066865994	0.078988161	0.846531843	0.397256118	0.788556702	up_in_case
PRJNA8274(	ENSGALG00010029280	USP36	case	control	0.010523318	0.068768208	0.153025914	0.878377839	0.976160112	up_in_case
PRJNA8274(	ENSGALG00010029281	KIAA0753	case	control	-0.078439662	0.09605355	-0.816624287	0.414143171	0.798684741	down_in_case
PRJNA8274(	ENSGALG00010029282	LUC7L3	case	control	0.022444922	0.068482581	0.327746442	0.743103386	0.937448285	up_in_case
PRJNA8274(	ENSGALG00010029283	RPS6KB1	case	control	-0.041826117	0.038314241	-1.091659801	0.274982661	0.700894634	down_in_case
PRJNA8274(	ENSGALG00010029284	CYTH1	case	control	0.183107406	0.072826963	2.514280405	0.011927556	0.191429907	up_in_case
PRJNA8274(	ENSGALG00010029285	CUX1	case	control	-0.109319805	0.057919768	-1.887435126	0.059101824	0.395497471	down_in_case
PRJNA8274(	ENSGALG00010029286	MAFG	case	control	0.008626689	0.076452119	0.112837797	0.910159152	0.981730793	up_in_case
PRJNA8274(	ENSGALG00010029287	ANKRD40	case	control	0.088260364	0.087337348	1.010568404	0.312223045	0.734364975	up_in_case
PRJNA8274(	ENSGALG00010029288	PYCR1	case	control	0.133497979	0.07310899	1.826013188	0.067848275	0.421339236	up_in_case
PRJNA8274(	ENSGALG00010029289	PITPNM3	case	control	0.23788362	0.159474558	1.49167129	0.135785339	0.539774365	up_in_case
PRJNA8274(	ENSGALG00010029290	ENSGALG00010029290	case	control	0.143311953	0.109487893	1.308929681	0.190558172	0.613935676	up_in_case
PRJNA8274(	ENSGALG00010029291	RNFT1	case	control	0.078550166	0.058642479	1.339475528	0.180415916	0.601050402	up_in_case
PRJNA8274(	ENSGALG00010029292	ENSGALG00010029292	case	control	0.194748663	0.131134165	1.485110023	0.137514682	0.539854583	up_in_case
PRJNA8274(	ENSGALG00010029293	DNAH17	case	control	-0.112836176	0.129263388	-0.872916742	0.38270846	0.780035772	down_in_case
PRJNA8274(	ENSGALG00010029294	PGS1	case	control	-0.045994489	0.057341812	-0.802110832	0.42248885	0.803289893	down_in_case
PRJNA8274(	ENSGALG00010029295	SOCS3	case	control	0.29688247	0.128785827	2.305241781	0.021153036	0.260541271	up_in_case
PRJNA8274(	ENSGALG00010029296	FBXO39	case	control	0.186709547	0.115852848	1.611609466	0.10704695	0.497973773	up_in_case
PRJNA8274(	ENSGALG00010029297	SH2B2	case	control	-0.098744953	0.187204387	-0.527471365	0.597866296	0.884680373	down_in_case
PRJNA8274(	ENSGALG00010029298	ENDOV	case	control	-0.012741095	0.098751331	-0.129022011	0.897340232	0.980176778	down_in_case
PRJNA8274(	ENSGALG00010029299	PRKRIP1	case	control	-0.039057921	0.069271232	-0.563840426	0.572862737	0.877268916	down_in_case
PRJNA8274(	ENSGALG00010029300	TEKT1	case	control	0.117895065	0.21592058	0.546011246	0.585058207	0.881307382	up_in_case
PRJNA8274(	ENSGALG00010029301	RNF213	case	control	0.286676233	0.10803187	2.653626501	0.007963192	0.156419835	up_in_case
PRJNA8274(	ENSGALG00010029303	SLC26A11	case	control	-0.018912842	0.107445622	-0.176022451	0.860276304	0.971871635	down_in_case
PRJNA8274(	ENSGALG00010029304	ORAI3	case	control	0.184304355	0.061179771	3.012504818	0.002591013	0.084399748	up_in_case
PRJNA8274(	ENSGALG00010029305	SMTNL2	case	control	0.068814304	0.103996163	0.661700412	0.508163241	0.843702533	up_in_case
PRJNA8274(	ENSGALG00010029306	CEP95	case	control	0.049231085	0.0565938	0.869902451	0.384353716	0.780940343	up_in_case
PRJNA8274(	ENSGALG00010029307	SGSH	case	control	-0.026800887	0.131062274	-0.204489715	0.837970829	0.964826648	down_in_case
PRJNA8274(	ENSGALG00010029308	GAA	case	control	0.042219067	0.135723731	0.311066215	0.755750285	0.941588405	up_in_case
PRJNA8274(	ENSGALG00010029309	ALKBH4	case	control	0.006072383	0.202346472	0.030009827	0.976059216	0.994231527	up_in_case
PRJNA8274(	ENSGALG00010029310	LRWD1	case	control	-0.039146336	0.042049519	-0.930957994	0.351875294	0.760831854	down_in_case
PRJNA8274(	ENSGALG00010029311	ENSGALG00010029311	case	control	0.022968902	0.14139488	0.162445077	0.870955376	0.974937823	up_in_case
PRJNA8274(	ENSGALG00010029312	SMURF2	case	control	-0.033298259	0.058383778	-0.570334094	0.568451121	0.874500231	down_in_case
PRJNA8274(	ENSGALG00010029313	CCDC40	case	control	0.204861453	0.2030617	1.008863085	0.313040301	0.734574752	up_in_case
PRJNA8274(	ENSGALG00010029314	MED13	case	control	-0.17263272	0.067127227	-2.571724288	0.010119344	0.178870984	down_in_case
PRJNA8274(	ENSGALG00010029315	TBC1D16	case	control	0.060155245	0.092855414	0.647837776	0.51708988	0.848054666	up_in_case
PRJNA8274(	ENSGALG00010029316	INTS2	case	control	-0.099529976	0.065616658	-1.516840058	0.129307071	0.53134802	down_in_case
PRJNA8274(	ENSGALG00010029317	KPNA2	case	control	-0.086483877	0.095195373	-0.908488239	0.363620325	0.769880166	down_in_case
PRJNA8274(	ENSGALG00010029318	C17orf58	case	control	0.027345337	0.134989096	0.202574416	0.839467688	0.965286179	up_in_case
PRJNA8274(	ENSGALG00010029319	BRIP1	case	control	0.093933356	0.083887646	1.119751962	0.262819475	0.688674415	up_in_case
PRJNA8274(	ENSGALG00010029320	TBX4	case	control	0.728601496	0.525562391	1.38632731	0.165646981	0.580618526	up_in_case
PRJNA8274(	ENSGALG00010029321	TBX2	case	control	0.1454394	0.089866918	1.618386413	0.105579348	0.495266487	up_in_case
PRJNA8274(	ENSGALG00010029322	BPTF	case	control	-0.133507241	0.074272623	-1.79752964	0.072251577	0.434299097	down_in_case
PRJNA8274(	ENSGALG00010029323	NOL11	case	control	-0.070310504	0.053528725	-1.313509795	0.189011223	0.611783722	down_in_case
PRJNA8274(	ENSGALG00010029324	BCAS3	case	control	-0.108845498	0.06722339	-1.619161087	0.105412608	0.495041148	down_in_case
PRJNA8274(	ENSGALG00010029325	LRRC75A	case	control	0.0776953	0.086751802	0.895604456	0.370464055	0.772700698	up_in_case
PRJNA8274(	ENSGALG00010029326	NCBP3	case	control	-0.014083229	0.04396202	-0.320349907	0.748703095	0.939503405	down_in_case

PRJNA8274(	ENSGALG00010029327	CRK	case	control	-0.030960045	0.036141608	-0.856631639	0.391648494	0.784833726	down_in_case
PRJNA8274(	ENSGALG00010029328	ENSGALG00010029328	case	control	0.100241273	0.073659747	1.360869085	0.173555065	0.592914702	up_in_case
PRJNA8274(	ENSGALG00010029329	ENSGALG00010029329	case	control	-0.168363133	0.095809877	-1.7572628	0.078873025	0.449171025	down_in_case
PRJNA8274(	ENSGALG00010029330	AFMID	case	control	0.174008097	0.12556662	1.385783068	0.165813154	0.580782892	up_in_case
PRJNA8274(	ENSGALG00010029331	TK1	case	control	-0.034897682	0.07887789	-0.442426665	0.658180487	0.908367791	down_in_case
PRJNA8274(	ENSGALG00010029332	YWHAE	case	control	-0.08149896	0.038398264	-2.122464693	0.033798735	0.316932396	down_in_case
PRJNA8274(	ENSGALG00010029333	ENSGALG00010029333	case	control	-0.157327171	0.057217445	-2.749636433	0.005966142	0.134447684	down_in_case
PRJNA8274(	ENSGALG00010029334	PIRT	case	control	-1.146612932	0.798438835	-1.43606859	0.150982825	0.55861692	down_in_case
PRJNA8274(	ENSGALG00010029336	SYNGR2	case	control	-0.057988481	0.050635899	-1.145204945	0.25212427	0.677148133	down_in_case
PRJNA8274(	ENSGALG00010029337	MYO1C	case	control	-0.098467809	0.080814055	-1.218449057	0.223053372	0.646990512	down_in_case
PRJNA8274(	ENSGALG00010029338	INPP5K	case	control	0.158477153	0.102539684	1.54552019	0.122220489	0.520784563	up_in_case
PRJNA8274(	ENSGALG00010029339	PITPNA	case	control	-0.10511151	0.085242751	-1.233084453	0.217544257	0.642877222	down_in_case
PRJNA8274(	ENSGALG00010029340	NDEL1	case	control	0.134493592	0.048590756	2.767884328	0.005642148	0.130251961	up_in_case
PRJNA8274(	ENSGALG00010029341	SLC43A2	case	control	-0.325272992	0.101048878	-3.218966894	0.001286533	0.056782183	down_in_case
PRJNA8274(	ENSGALG00010029342	TMC6	case	control	-0.042688156	0.103068588	-0.414172321	0.678747911	0.915065064	down_in_case
PRJNA8274(	ENSGALG00010029343	SCARF1	case	control	-0.069596685	0.074727375	-0.931341231	0.35167708	0.760831854	down_in_case
PRJNA8274(	ENSGALG00010029344	ENSGALG00010029344	case	control	0.2099513	0.117065493	1.793451637	0.072900737	0.436712105	up_in_case
PRJNA8274(	ENSGALG00010029345	RILP	case	control	-0.051945277	0.121698564	-0.426835578	0.669499091	0.913446904	down_in_case
PRJNA8274(	ENSGALG00010029346	ARL16	case	control	-0.241908853	0.115158164	-2.100666113	0.035670286	0.324939791	down_in_case
PRJNA8274(	ENSGALG00010029347	PRPF8	case	control	-0.155116322	0.057014681	-2.720638233	0.006515602	0.139689826	down_in_case
PRJNA8274(	ENSGALG00010029348	HGS	case	control	-0.120852547	0.047925458	-2.521677447	0.011679675	0.189141894	down_in_case
PRJNA8274(	ENSGALG00010029349	MYH10	case	control	-0.003403425	0.079638758	-0.042735787	0.965912151	0.992340816	down_in_case
PRJNA8274(	ENSGALG00010029350	MRPL12	case	control	-0.10222647	0.069313001	-1.474852751	0.140252136	0.542495412	down_in_case
PRJNA8274(	ENSGALG00010029351	ENSGALG00010029351	case	control	0.084439336	0.144563339	0.584099237	0.559153548	0.869461123	up_in_case
PRJNA8274(	ENSGALG00010029352	AATF	case	control	0.012488245	0.099406058	0.125628607	0.900025917	0.98028579	up_in_case
PRJNA8274(	ENSGALG00010029353	GCGR	case	control	0.332029528	0.596306492	0.556810184	0.577657125	0.878215701	up_in_case
PRJNA8274(	ENSGALG00010029354	TLCD2	case	control	0.180343435	0.189074746	0.953820848	0.340174334	0.751621289	up_in_case
PRJNA8274(	ENSGALG00010029355	MCRIP1	case	control	-0.175437477	0.114563955	-1.53134969	0.125682991	0.525056024	down_in_case
PRJNA8274(	ENSGALG00010029357	ARHGDIA	case	control	0.009865315	0.059020675	0.16715016	0.867251899	0.973675289	up_in_case
PRJNA8274(	ENSGALG00010029359	ENSGALG00010029359	case	control	-0.091194519	0.286409138	-0.318406458	0.75017664	0.939856839	down_in_case
PRJNA8274(	ENSGALG00010029360	ACACA	case	control	-0.062391991	0.05886171	-1.059975846	0.289155588	0.712801679	down_in_case
PRJNA8274(	ENSGALG00010029361	ALYREF	case	control	0.004972276	0.055876634	0.088986671	0.929092503	0.986878757	up_in_case
PRJNA8274(	ENSGALG00010029362	TADA2A	case	control	0.039611439	0.07055898	0.56139472	0.574528482	0.877910508	up_in_case
PRJNA8274(	ENSGALG00010029363	PIK3R6	case	control	0.112122983	0.12969072	0.864541297	0.387290604	0.782604263	up_in_case
PRJNA8274(	ENSGALG00010029364	ANAPC11	case	control	0.042480391	0.096477568	0.440313655	0.659709952	0.908673889	up_in_case
PRJNA8274(	ENSGALG00010029365	PIK3R5	case	control	0.056757606	0.112231372	0.505719611	0.613053499	0.890154949	up_in_case
PRJNA8274(	ENSGALG00010029366	MGAT5B	case	control	-0.161571365	0.375417305	-0.430378043	0.666920665	0.912284937	down_in_case
PRJNA8274(	ENSGALG00010029367	DUSP14	case	control	0.017248598	0.079114901	0.218019588	0.82741385	0.961319258	up_in_case
PRJNA8274(	ENSGALG00010029368	DDX52	case	control	-0.001781464	0.062131634	-0.028672413	0.977125858	0.994654027	down_in_case
PRJNA8274(	ENSGALG00010029369	MFSD11	case	control	-0.015584747	0.070526448	-0.220977339	0.825110078	0.960903617	down_in_case
PRJNA8274(	ENSGALG00010029370	SRSF2	case	control	0.058003486	0.05092794	1.138932506	0.254731306	0.680438994	up_in_case
PRJNA8274(	ENSGALG00010029371	AUTS2	case	control	-0.086139425	0.065598086	-1.313139306	0.189136011	0.612049097	down_in_case
PRJNA8274(	ENSGALG00010029372	METTL23	case	control	0.101060808	0.118772395	0.850877917	0.394837173	0.786978937	up_in_case
PRJNA8274(	ENSGALG00010029373	GATSL2	case	control	-0.13371457	0.112064927	-1.193188389	0.232795601	0.657934961	down_in_case
PRJNA8274(	ENSGALG00010029374	RCC1L	case	control	-0.072757934	0.073463433	-0.990396597	0.321980308	0.739571618	down_in_case
PRJNA8274(	ENSGALG00010029375	JMJD6	case	control	0.123977009	0.097244726	1.274896991	0.202345704	0.625360616	up_in_case
PRJNA8274(	ENSGALG00010029376	ENSGALG00010029376	case	control	0.144072746	0.08201937	1.756569773	0.078991169	0.449171025	up_in_case
PRJNA8274(	ENSGALG00010029377	NCF1C	case	control	-0.091209594	0.192698642	-0.473327642	0.635979444	0.898691227	down_in_case
PRJNA8274(	ENSGALG00010029378	ST6GALNAC1	case	control	0.431430634	0.363839062	1.185773268	0.235711851	0.660526168	up_in_case
PRJNA8274(	ENSGALG00010029379	GTF2I	case	control	0.036453175	0.072654343	0.501734289	0.615854441	0.891081782	up_in_case
PRJNA8274(	ENSGALG00010029380	RGS9	case	control	0.040721949	0.125147792	0.32539087	0.744885265	0.937849656	up_in_case
PRJNA8274(	ENSGALG00010029382	GTF2IRD1	case	control	-0.175686039	0.079598763	-2.207145343	0.027303907	0.2934516	down_in_case
PRJNA8274(	ENSGALG00010029383	GNA13	case	control	0.004515673	0.041138436	0.109767727	0.912593586	0.982717125	up_in_case
PRJNA8274(	ENSGALG00010029384	WDR81	case	control	0.135407256	0.059961028	2.25825441	0.023929803	0.273286623	up_in_case
PRJNA8274(	ENSGALG00010029385	CLIP2	case	control	-0.092974071	0.077394533	-1.201300245	0.229634756	0.653695091	down_in_case
PRJNA8274(	ENSGALG00010029386	SLC16A6	case	control	-0.338596996	0.117648858	-2.878030446	0.004001665	0.109414554	down_in_case
PRJNA8274(	ENSGALG00010029387	SERPINF1	case	control	-0.023252184	0.084187685	-0.276194595	0.782398609	0.949459648	down_in_case
PRJNA8274(	ENSGALG00010029388	ST6GALNAC2	case	control	1.526419768	0.191232176	7.982023751	1.43953E-15	1.14363E-12	up_in_case
PRJNA8274(	ENSGALG00010029389	SMYD4	case	control	0.071838108	0.071124933	1.010027082	0.312482315	0.734364975	up_in_case
PRJNA8274(	ENSGALG00010029390	RPA1	case	control	0.194161529	0.06704015	2.896197728	0.003777143	0.105649139	up_in_case
PRJNA8274(	ENSGALG00010029391	ARSG	case	control	-0.037663412	0.093524373	-0.402712262	0.687159912	0.917290381	down_in_case
PRJNA8274(	ENSGALG00010029392	RTN4RL1	case	control	-0.857011375	0.399999513	-2.142531047	0.032150773	0.3098361	down_in_case
PRJNA8274(	ENSGALG00010029394	CYGB	case	control	0.099986356	0.094318311	1.060094856	0.289101448	0.712801679	up_in_case
PRJNA8274(	ENSGALG00010029395	DPH1	case	control	0.188332956	0.090097496	2.090323974	0.036588707	0.32721608	up_in_case
PRJNA8274(	ENSGALG00010029396	WIPI1	case	control	-0.015058208	0.045758007	-0.329083571	0.742092522	0.937364834	down_in_case
PRJNA8274(	ENSGALG00010029397	OVCA2	case	control	0.240662914	0.159830418	1.505739124	0.132134156	0.535549374	up_in_case
PRJNA8274(	ENSGALG00010029398	RHBDF2	case	control	0.061342074	0.074891415	0.819080187	0.412740665	0.797581687	up_in_case
PRJNA8274(	ENSGALG00010029399	PRKAR1A	case	control	-0.031973615	0.037418983	-0.854475778	0.392841425	0.786232662	down_in_case
PRJNA8274(	ENSGALG00010029400	AANAT	case	control	0.112014309	0.605339784	0.185043693	0.853194765	0.969924886	up_in_case
PRJNA8274(	ENSGALG00010029401	HIC1	case	control	0.037764239	0.083432051	0.452634673	0.650811827	0.904636952	up_in_case
PRJNA8274(	ENSGALG00010029402	SMG6	case	control	-0.052962828	0.044092882	-1.201165034	0.22968719	0.653695091	down_in_case
PRJNA8274(	ENSGALG00010029403	FAM20A	case	control	0.059984611	0.123071449	0.487396647	0.625977269	0.895445894	up_in_case
PRJNA8274(	ENSGALG00010029404	SRR	case	control	-0.195269743	0.129836348	-1.503968236	0.132589546	0.535904608	down_in_case
PRJNA8274(	ENSGALG00010029405	POLDIP2	case	control	-0.031042614	0.039654065	-0.782835593	0.433723663	0.808531923	down_in_case
PRJNA8274(	ENSGALG00010029406	TMEM199	case	control	0.133692849	0.079475737	1.682184454	0.092533078	0.477342721	up_in_case
PRJNA8274(	ENSGALG00010029407	UBE2O	case	control	-0.065009073	0.055010077	-1.181766629	0.237298312	0.662637349	down_in_case
PRJNA8274(	ENSGALG00010029408	KIF12	case	control	0.013911022	0.369609873	0.037637041	0.969977074	0.992761697	up_in_case
PRJNA8274(	ENSGALG00010029409	ENSGALG00010029409	case	control	-0.142896298	0.204193029	-0.699809873	0.484046048	0.834033076	down_in_case
PRJNA8274(	ENSGALG00010029410	VTN	case	control	0.80755421	0.789151748	1.023319294	0.306156904	0.72918783	up_in_case
PRJNA8274(	ENSGALG00010029411	SARM1	case	control	0.101553143	0.153509355	0.661543677	0.508263714	0.843702533	up_in_case
PRJNA8274(	ENSGALG00010029412	SLC46A1	case	control	-0.179228674	0.080644491	-2.222454025	0.026252638	0.287789976	down_in_case
PRJNA8274(	ENSGALG00010029413	B3GNTL1	case	control	0.198492877	0.180935687	1.097035527	0.272625862	0.698931401	up_in_case
PRJNA8274(	ENSGALG00010029416	UNC119	case	control	0.043420848	0.168096851	0.258308518	0.796168813	0.95280257	up_in_case
PRJNA8274(	ENSGALG00010029417	ENSGALG00010029417	case	control	-0.122967742	0.124432031	-0.988232219	0.323038933	0.740689923	down_in_case
PRJNA8274(	ENSGALG00010029418	ABCA5	case	control	-0.212685069	0.086340663	-2.463324482	0.013765527	0.205985143	down_in_case
PRJNA8274(	ENSGALG00010029419	RFC2	case	control	-0.086735486	0.068509726	-1.266031715	0.205501728	0.628438742	down_in_case
PRJNA8274(	ENSGALG00010029420	LAT2	case	control	0.351247546	0.399142026	0.880006421	0.378855831	0.777229179	up_in_case
PRJNA8274(	ENSGALG00010029421	TBCD	case	control	0.036651558	0.058910454	0.622157107	0.533838567	0.855817434	up_in_case
PRJNA8274(	ENSGALG00010029422	EIF4H	case	control	-0.048971114	0.035534943	-1.378111498	0.168168866	0.58432606	down_in_case
PRJNA8274(	ENSGALG00010029423	MYBBP1A	case	control	-0.030023768	0.074230762	-0.404465311	0.685870582	0.917034645	down_in_case
PRJNA8274(	ENSGALG00010029424	MAP2K6	case	control	-0.114177445	0.085701619	-1.332267069	0.182772444	0.604311204	down_in_case
PRJNA8274(	ENSGALG00010029425	SPNS2	case	control	0.032600654	0.135860456	0.239956903	0.810363667	0.955972758	up_in_case
PRJNA8274(	ENSGALG00010029426	ZNF750	case	control	0.938741121	0.976860166	0.960977992	0.336563234	0.750654067	up_in_case
PRJNA8274(	ENSGALG00010029427	SPNS3	case	control	-0.06409737	0.248287109	-0.258158267	0.796284765	0.95280257	down_in_case
PRJNA8274(	ENSGALG00010029428	FN3K	case	control	0.307094691	0.269369689	1.140049172	0.254265816	0.679971816	up_in_case
PRJNA8274(	ENSGALG00010029429	UBE2G1	case	control	-0.069764995	0.047863177	-1.457592232	0.144952972	0.548818979	down_in_case
PRJNA8274(	ENSGALG00010029430	FN3KRP	case	control	-0.28470394	0.08293141	-3.433004912	0.000596931	0.035866039	down_in_case
PRJNA8274(	ENSGALG00010029431	ENSGALG00010029431	case	control	0.825140504	2.009334709	0.410653586	0.681326565	0.915594727	up_in_case
PRJNA8274(	ENSGALG00010029432	ENSGALG00010029432	case	control	-1.044387734	2.914276963	-0.358369416			down_in_case
PRJNA8274(	ENSGALG00010029436	WDR45B	case	control	0.011107197	0.047577132	0.233456632	0.815406842	0.957374769	up_in_case
PRJNA8274(	ENSGALG00010029437	ENSGALG00010029437	case	control	1.574146708	0.918517604	1.713790461	0.086567216	0.465030498	up_in_case
PRJNA8274(	ENSGALG00010029438	ENSGALG00010029438	case	control	-0.094195965	0.156953949	-0.600150334	0.54840605	0.86339387	down_in_case
PRJNA8274(	ENSGALG00010029439	ENSGALG00010029439	case	control	0.363015633	0.227600278	1.594970076	0.110718928	0.503115856	up_in_case
PRJNA8274(	ENSGALG00010029440	FOXK2	case	control	-0.175470629	0.071068141	-2.469047683	0.013547317	0.204137646	down_in_case
PRJNA8274(	ENSGALG00010029441	ENSGALG00010029441	case	control	-5.010762145	2.904508587	-1.725166924			down_in_case
PRJNA8274(	ENSGALG00010029442	ENSGALG00010029442	case	control	-0.009449288	0.447251785	-0.021127447	0.98314399	0.996591771	down_in_case
PRJNA8274(	ENSGALG00010029443	ENSGALG00010029443	case	control	-0.201451252	0.233077516	-0.864310106	0.38741756	0.782604263	down_in_case
PRJNA8274(	ENSGALG00010029445	SPHK1	case	control	0.267976748	0.11080682	2.418413847	0.015588336	0.221804191	up_in_case
PRJNA8274(	ENSGALG00010029446	PRPSAP1	case	control	0.009364545	0.060058675	0.155923269	0.876093503	0.975666505	up_in_case
PRJNA8274(	ENSGALG00010029449	UBALD2	case	control	-0.295505885	0.111580585	-2.648362924	0.008088264	0.157792878	down_in_case
PRJNA8274(	ENSGALG00010029451	ENSGALG00010029451	case	control	0.45094895	0.132526688	3.402702937	0.000667228	0.038540612	up_in_case
PRJNA8274(	ENSGALG00010029452	ENSGALG00010029452	case	control	-4.992409138	1.618479717	-3.084628795	0.002038063	0.074158543	down_in_case
PRJNA8274(	ENSGALG00010029454	ENSGALG00010029454	case	control	-0.234636409	0.222011067	-1.056868075	0.2905718	0.714060795	down_in_case
PRJNA8274(	ENSGALG00010029455	ENSGALG00010029455	case	control	-0.190380109	0.271315262	-0.701693326	0.482870436	0.833529046	down_in_case
PRJNA8274(	ENSGALG00010029456	RNF157	case	control	0.00938124	0.302649675	0.030997027	0.97527191	0.99399062	up_in_case
PRJNA8274(	ENSGALG00010029457	ENSGALG00010029457	case	control	0.185830421	0.15594548	1.191637107	0.233403569	0.657982411	up_in_case
PRJNA8274(	ENSGALG00010029458	ENSGALG00010029458	case	control	-0.752973568	1.444086155	-0.52141873	0.602075107	0.885002041	down_in_case
PRJNA8274(	ENSGALG00010029459	FOXJ1	case	control	0.544673441	0.356157341	1.529305669	0.126188685	0.525939433	up_in_case
PRJNA8274(	ENSGALG00010029460	ENSGALG00010029460	case	control	0.007626938	0.134907642	0.056534515	0.954916	0.992056045	up_in_case
PRJNA8274(	ENSGALG00010029461	ENSGALG00010029461	case	control	-0.148905648	0.152299574	-0.977715463	0.328215072	0.745445783	down_in_case
PRJNA8274(	ENSGALG00010029462	ENSGALG00010029462	case	control	0.047383564	0.486731652	0.097350488	0.922448062	0.985482967	up_in_case
PRJNA8274(	ENSGALG00010029464	ENSGALG00010029464	case	control	0.828630661	0.442866648	1.871061331	0.061336581	0.402530107	up_in_case
PRJNA8274(	ENSGALG00010029465	ENSGALG00010029465	case	control	2.9739974	1.560230029	1.906127522	0.056633663	0.387381529	up_in_case
PRJNA8274(	ENSGALG00010029466	SLC39A11	case	control	0.120766758	0.085587208	1.411037467	0.158233567	0.570246977	up_in_case
PRJNA8274(	ENSGALG00010029468	ENSGALG00010029468	case	control	0.217834952	0.279927402	0.778183738	0.436460703	0.810200135	up_in_case
PRJNA8274(	ENSGALG00010029469	ENSGALG00010029469	case	control	0.19030327	0.318744027	0.597041053	0.550479972	0.86456492	up_in_case

PRJNA8274(	ENSGALG00010029470	SSTR2	case	control	-0.202722795	0.41839997	-0.484519143	0.628017482	0.895865363	down_in_case
PRJNA8274(	ENSGALG00010029472	ENSGALG00010029472	case	control	0.972854708	0.434822242	2.237361876	0.025262698	0.282673383	up_in_case
PRJNA8274(	ENSGALG00010029473	COG1	case	control	-0.165045615	0.051645006	-3.19577103	0.001394577	0.058654283	down_in_case
PRJNA8274(	ENSGALG00010029474	ENSGALG00010029474	case	control	1.313663148	0.875955383	1.499691849	0.133694243	0.537331451	up_in_case
PRJNA8274(	ENSGALG00010029477	FAM104A	case	control	0.07026452	0.051101088	1.375010255	0.169128266	0.585038804	up_in_case
PRJNA8274(	ENSGALG00010029479	ENSGALG00010029479	case	control	0.036722574	0.57732199	0.06360848	0.94928198	0.99138853	up_in_case
PRJNA8274(	ENSGALG00010029480	ENSGALG00010029480	case	control	-0.064450901	0.092504734	-0.696730843	0.485971253	0.834635018	down_in_case
PRJNA8274(	ENSGALG00010029481	ENSGALG00010029481	case	control	0.31911529	0.277517209	1.149893702	0.250187655	0.675774897	up_in_case
PRJNA8274(	ENSGALG00010029482	DDX25	case	control	-0.135415468	0.12327771	-1.098458657	0.272004261	0.698323327	down_in_case
PRJNA8274(	ENSGALG00010029484	ENSGALG00010029484	case	control	0.131780781	0.359312614	0.366758014	0.713799528	0.926862121	up_in_case
PRJNA8274(	ENSGALG00010029487	CDC42EP4	case	control	-0.071830013	0.084785934	-0.847192568	0.396887795	0.788531716	down_in_case
PRJNA8274(	ENSGALG00010029489	ENSGALG00010029489	case	control	-0.950984483	0.732466762	-1.298331244	0.194173534	0.61723879	down_in_case
PRJNA8274(	ENSGALG00010029491	ENSGALG00010029491	case	control	0.052301603	0.214757397	0.243538075	0.807588593	0.955875648	up_in_case
PRJNA8274(	ENSGALG00010029492	SDK2	case	control	-0.074104412	0.137978952	-0.537070411	0.591218979	0.882949889	down_in_case
PRJNA8274(	ENSGALG00010029494	MYH7	case	control	0.205231485	0.301016075	0.681795767	0.495368101	0.838118945	up_in_case
PRJNA8274(	ENSGALG00010029495	RPL38	case	control	-0.205454825	0.080685595	-2.546363135	0.010885189	0.185137791	down_in_case
PRJNA8274(	ENSGALG00010029496	TTYH2	case	control	0.08834116	0.088526389	0.997907635	0.31832415	0.738134246	up_in_case
PRJNA8274(	ENSGALG00010029498	ENSGALG00010029498	case	control	0.013759099	0.08345613	0.164866253	0.869049255	0.974621939	up_in_case
PRJNA8274(	ENSGALG00010029501	ENSGALG00010029501	case	control	-0.597327605	0.195052021	-3.062401524	0.002195687	0.076473225	down_in_case
PRJNA8274(	ENSGALG00010029502	BTBD17	case	control	0.0807568	0.340879917	0.236906887	0.812729027	0.956624009	up_in_case
PRJNA8274(	ENSGALG00010029504	GPR142	case	control	0.032952753	0.212639862	0.154969783	0.876845139	0.975813349	up_in_case
PRJNA8274(	ENSGALG00010029505	VEZF1	case	control	-0.081162983	0.033962201	-2.389803426	0.016857393	0.230991919	down_in_case
PRJNA8274(	ENSGALG00010029506	OXLD1	case	control	0.119351189	0.117736793	1.013711909	0.31072024	0.733587496	up_in_case
PRJNA8274(	ENSGALG00010029507	VPS37D	case	control	0.054669117	0.154469909	0.353914349	0.723403073	0.930215097	up_in_case
PRJNA8274(	ENSGALG00010029508	ENSGALG00010029508	case	control	0.04255811	0.172525129	0.246677746	0.80515763	0.9556344	up_in_case
PRJNA8274(	ENSGALG00010029509	DNAJC30	case	control	-0.066422581	0.115809615	-0.573549789	0.566272498	0.873008555	down_in_case
PRJNA8274(	ENSGALG00010029510	PDE6G	case	control	-0.142148957	0.287073597	-0.495165553	0.620483264	0.892955829	down_in_case
PRJNA8274(	ENSGALG00010029511	CUEDC1	case	control	0.024139617	0.063021543	0.383037545	0.701691926	0.922880366	up_in_case
PRJNA8274(	ENSGALG00010029512	TSPAN10	case	control	3.32399427	0.599993539	5.54005011	3.02385E-08	1.31034E-05	up_in_case
PRJNA8274(	ENSGALG00010029513	WBSCR22	case	control	-0.051263916	0.054800454	-0.935465177	0.349548622	0.759342183	down_in_case
PRJNA8274(	ENSGALG00010029514	MRPS23	case	control	-0.211491105	0.063731063	-3.318493275	0.000905045	0.045894127	down_in_case
PRJNA8274(	ENSGALG00010029515	NPLOC4	case	control	-0.075791781	0.063900846	-1.186084152	0.235589068	0.660526168	down_in_case
PRJNA8274(	ENSGALG00010029516	ENSGALG00010029516	case	control	0.525865949	0.152300712	3.452813466	0.000554772	0.034195028	up_in_case
PRJNA8274(	ENSGALG00010029517	MSI2	case	control	-0.036078032	0.062082682	-0.581128754	0.561153681	0.870480544	down_in_case
PRJNA8274(	ENSGALG00010029518	FAAP100	case	control	0.136552147	0.079728203	1.712720739	0.086763928	0.4657373	up_in_case
PRJNA8274(	ENSGALG00010029519	STX1A	case	control	0.076484933	0.113017056	0.676755671	0.498560988	0.840040312	up_in_case
PRJNA8274(	ENSGALG00010029520	MKS1	case	control	0.138081216	0.091212143	1.513846848	0.130064688	0.532624579	up_in_case
PRJNA8274(	ENSGALG00010029521	ACTG1	case	control	0.00637837	0.055560252	0.114800959	0.908602889	0.981730793	up_in_case
PRJNA8274(	ENSGALG00010029522	AKAP1	case	control	0.066839327	0.059141935	1.130151164	0.258412534	0.685881601	up_in_case
PRJNA8274(	ENSGALG00010029524	ENSGALG00010029524	case	control	-0.124544387	0.076175084	-1.634975367	0.102054212	0.491460867	down_in_case
PRJNA8274(	ENSGALG00010029525	SLC38A10	case	control	0.047955727	0.070167484	0.683446574	0.494324699	0.83803731	up_in_case
PRJNA8274(	ENSGALG00010029526	BCL7B	case	control	-0.000263341	0.061775473	-0.00426287	0.996598732	0.998921977	down_in_case
PRJNA8274(	ENSGALG00010029527	NDUFAF8	case	control	-0.109436368	0.097531879	-1.122057413	0.26183803	0.687529163	down_in_case
PRJNA8274(	ENSGALG00010029528	TBL2	case	control	-0.025479951	0.072731411	-0.350329392	0.726091509	0.932223791	down_in_case
PRJNA8274(	ENSGALG00010029529	TEPSIN	case	control	0.127386324	0.09729659	1.309257856	0.19044702	0.613935676	up_in_case
PRJNA8274(	ENSGALG00010029530	ENSGALG00010029530	case	control	-0.04331056	0.172404889	-0.251214225	0.801648487	0.954294696	down_in_case
PRJNA8274(	ENSGALG00010029531	CEP131	case	control	0.00229425	0.066781878	0.034354379	0.972594562	0.99340857	up_in_case
PRJNA8274(	ENSGALG00010029532	EVI2A	case	control	-0.017453628	0.272367852	-0.064081086	0.948905662	0.99138853	down_in_case
PRJNA8274(	ENSGALG00010029533	MLXIPL	case	control	0.050536532	0.314192153	0.16084594	0.872214741	0.975062028	up_in_case
PRJNA8274(	ENSGALG00010029534	NF1	case	control	-0.021738849	0.063149241	-0.344245617	0.730661579	0.933770678	down_in_case
PRJNA8274(	ENSGALG00010029536	BAZ1B	case	control	-0.01168242	0.047502112	-0.245934752	0.805732741	0.9556344	down_in_case
PRJNA8274(	ENSGALG00010029537	MYH1B	case	control	-0.411648204	0.139978299	-2.940800156	0.003273657	0.097324927	down_in_case
PRJNA8274(	ENSGALG00010029538	FZD9	case	control	0.125600659	0.092928305	1.351586682	0.176507573	0.595527443	up_in_case
PRJNA8274(	ENSGALG00010029539	FKBP6	case	control	0.382064148	0.375417049	1.017705906	0.30881774	0.731823162	up_in_case
PRJNA8274(	ENSGALG00010029540	TRIM50	case	control	-0.017941664	0.324166389	-0.055347083	0.955861953	0.992056045	down_in_case
PRJNA8274(	ENSGALG00010029541	ENSGALG00010029541	case	control	-0.154585836	0.174128147	-0.887770522	0.374664202	0.77445766	down_in_case
PRJNA8274(	ENSGALG00010029542	AATK	case	control	0.038897634	0.109778136	0.354329516	0.72309195	0.930215097	up_in_case
PRJNA8274(	ENSGALG00010029543	NSUN5	case	control	-0.208883015	0.085271506	-2.449622692	0.014300598	0.21093806	down_in_case
PRJNA8274(	ENSGALG00010029544	ENSGALG00010029544	case	control	-0.083542353	0.07455946	-1.12047958	0.262509449	0.688522667	down_in_case
PRJNA8274(	ENSGALG00010029545	OMG	case	control	-0.062553466	0.264382146	-0.23660246	0.812965212	0.956712425	down_in_case
PRJNA8274(	ENSGALG00010029546	WSB1	case	control	0.075812302	0.087579575	0.865639076	0.38668812	0.782604263	up_in_case
PRJNA8274(	ENSGALG00010029547	HIP1	case	control	-0.114473932	0.080493496	-1.422151321	0.154982327	0.563829072	down_in_case
PRJNA8274(	ENSGALG00010029548	BAIAP2	case	control	-0.163919291	0.161946953	-1.012178909	0.311452519	0.734097745	down_in_case
PRJNA8274(	ENSGALG00010029549	KSR1	case	control	-0.11131323	0.100805644	-1.104236086	0.26949074	0.696612479	down_in_case
PRJNA8274(	ENSGALG00010029550	CHMP6	case	control	-0.062568861	0.063915506	-0.978930859	0.327614144	0.7452883	down_in_case
PRJNA8274(	ENSGALG00010029552	NOS2	case	control	0.198306694	0.111632686	1.776421417	0.075663484	0.442474101	up_in_case
PRJNA8274(	ENSGALG00010029553	MYH1C	case	control	-0.54154547	0.162252703	-3.337666866	0.00084485	0.044416733	down_in_case
PRJNA8274(	ENSGALG00010029554	ENSGALG00010029554	case	control	-1.083908037	0.817257656	-1.326274559	0.184748771	0.607639686	down_in_case
PRJNA8274(	ENSGALG00010029555	LYRM9	case	control	0.074406772	0.195605168	0.380392668	0.703653956	0.923760915	up_in_case
PRJNA8274(	ENSGALG00010029556	NPTX1	case	control	0.123026523	0.363806167	0.338165029	0.735238828	0.934096263	up_in_case
PRJNA8274(	ENSGALG00010029558	ENSGALG00010029558	case	control	-0.260324306	0.310897995	-0.837330282	0.402406942	0.792021921	down_in_case
PRJNA8274(	ENSGALG00010029560	ENSGALG00010029560	case	control	-0.131133289	0.212908927	-0.615912594	0.537952209	0.857740557	down_in_case
PRJNA8274(	ENSGALG00010029561	SCO1	case	control	0.180277178	0.049528337	3.639879456	0.000272766	0.022288855	up_in_case
PRJNA8274(	ENSGALG00010029563	ADPRM	case	control	-0.063917538	0.115030673	-0.55565647	0.57844572	0.87894738	down_in_case
PRJNA8274(	ENSGALG00010029564	NLK	case	control	0.036377177	0.045449276	0.800390686	0.423484476	0.803723282	up_in_case
PRJNA8274(	ENSGALG00010029566	TMEM97	case	control	0.014987551	0.068444139	0.218974942	0.826669567	0.961207256	up_in_case
PRJNA8274(	ENSGALG00010029567	IFT20	case	control	-0.090872665	0.064241001	-1.414558676	0.157197932	0.568233173	down_in_case
PRJNA8274(	ENSGALG00010029568	ENSGALG00010029568	case	control	0.559487286	0.743891482	0.75210874	0.451985668	0.82064437	up_in_case
PRJNA8274(	ENSGALG00010029569	TMEM220	case	control	-0.256062402	0.155361987	-1.648166367	0.099318536	0.488584926	down_in_case
PRJNA8274(	ENSGALG00010029570	TNFAIP1	case	control	-0.00418515	0.061847516	-0.067668839	0.946049256	0.990373672	down_in_case
PRJNA8274(	ENSGALG00010029571	EPX	case	control	0.231299238	0.442183037	0.523084827	0.600915224	0.884954589	up_in_case
PRJNA8274(	ENSGALG00010029572	TSPOAP1	case	control	0.043062799	0.102266956	0.42108224	0.67369503	0.91379937	up_in_case
PRJNA8274(	ENSGALG00010029573	MAP2K4	case	control	-0.001502123	0.046756369	-0.032126599	0.974371091	0.993775355	down_in_case
PRJNA8274(	ENSGALG00010029574	SGSM2	case	control	0.165537142	0.090036884	1.838548098	0.065981681	0.41627012	up_in_case
PRJNA8274(	ENSGALG00010029575	ANKFN1	case	control	0.090949347	0.48150343	0.188886187	0.85018202	0.969234083	up_in_case
PRJNA8274(	ENSGALG00010029576	ATP2A3	case	control	-0.034942602	0.083573581	-0.418105841	0.675869735	0.914247368	down_in_case
PRJNA8274(	ENSGALG00010029577	NOG	case	control	0.402453303	0.56372735	0.713914808	0.47527986	0.830316001	up_in_case
PRJNA8274(	ENSGALG00010029578	C17orf67	case	control	-0.263347667	0.552632504	-0.476533076	0.633694649	0.898107199	down_in_case
PRJNA8274(	ENSGALG00010029579	GALNT17	case	control	0.087449816	0.102804513	0.850641797	0.394968364	0.786978937	up_in_case
PRJNA8274(	ENSGALG00010029580	P2RX1	case	control	0.337701006	0.230186642	1.467074733	0.142355727	0.54406686	up_in_case
PRJNA8274(	ENSGALG00010029581	DGKE	case	control	-0.010301776	0.0519343	-0.198361705	0.842762077	0.966519616	down_in_case
PRJNA8274(	ENSGALG00010029582	SCPEP1	case	control	-0.142652861	0.092607828	-1.540397442	0.123463505	0.522840029	down_in_case
PRJNA8274(	ENSGALG00010029583	LIMK1	case	control	-0.046693049	0.087859156	-0.531453424	0.595104609	0.884431086	down_in_case
PRJNA8274(	ENSGALG00010029584	TRIM25	case	control	0.170305633	0.075328197	2.260848374	0.023768648	0.272786248	up_in_case
PRJNA8274(	ENSGALG00010029585	ENSGALG00010029585	case	control	0.207077298	0.25195944	0.821867591	0.41115226	0.796718771	up_in_case
PRJNA8274(	ENSGALG00010029586	COIL	case	control	0.008870866	0.063590173	0.139500583	0.889054595	0.978017246	up_in_case
PRJNA8274(	ENSGALG00010029588	RAB11FIP4	case	control	0.385938032	0.180395	2.139405365	0.032402853	0.310355523	up_in_case
PRJNA8274(	ENSGALG00010029590	UTP6	case	control	0.000681957	0.066779413	0.010212092	0.991852071	0.99790247	up_in_case
PRJNA8274(	ENSGALG00010029591	SUZ12	case	control	-0.010993406	0.048328462	-0.22747271	0.820056187	0.958669267	down_in_case
PRJNA8274(	ENSGALG00010029592	MNT	case	control	-0.081321726	0.066642683	-1.220264877	0.22236448	0.646365317	down_in_case
PRJNA8274(	ENSGALG00010029593	PAFAH1B1	case	control	-0.032944445	0.026808223	-1.228893308	0.219111811	0.643443819	down_in_case
PRJNA8274(	ENSGALG00010029594	CRLF3	case	control	-0.135593919	0.059121009	-2.293498067	0.021819347	0.264012564	down_in_case
PRJNA8274(	ENSGALG00010029595	CLUH	case	control	-0.124491146	0.080948839	-1.5378991	0.124073287	0.523029039	down_in_case
PRJNA8274(	ENSGALG00010029596	ATAD5	case	control	-0.02743365	0.096296084	-0.284888526	0.775729547	0.947146475	down_in_case
PRJNA8274(	ENSGALG00010029597	ADAP2	case	control	-0.031852984	0.157375685	-0.202400925	0.839603305	0.965286179	down_in_case
PRJNA8274(	ENSGALG00010029598	SUPT4H1	case	control	0.04121381	0.113639352	0.362671991	0.716849925	0.926862121	up_in_case
PRJNA8274(	ENSGALG00010029599	RNF43	case	control	0.468925559	0.387988732	1.208606128	0.226814201	0.650510044	up_in_case
PRJNA8274(	ENSGALG00010029600	RHOT1	case	control	-0.033564028	0.061288262	-0.547642022	0.583937729	0.881307382	down_in_case
PRJNA8274(	ENSGALG00010029601	HSF5	case	control	-0.236972779	0.217787077	-1.088093849	0.276553679	0.702359305	down_in_case
PRJNA8274(	ENSGALG00010029602	MTMR4	case	control	0.080760424	0.067601203	1.194659564	0.232220067	0.657834184	up_in_case
PRJNA8274(	ENSGALG00010029603	RHBDL3	case	control	0.380063292	0.313597479	1.21194626	0.225532952	0.649491335	up_in_case
PRJNA8274(	ENSGALG00010029604	ENSGALG00010029604	case	control	-0.115458077	0.081428918	-1.417900167	0.156219914	0.566128932	down_in_case
PRJNA8274(	ENSGALG00010029605	C17orf75	case	control	0.122688807	0.070571317	1.738508106	0.082121323	0.455552638	up_in_case
PRJNA8274(	ENSGALG00010029606	ABHD11	case	control	0.292134131	0.163353912	1.788350991	0.073719398	0.437929428	up_in_case
PRJNA8274(	ENSGALG00010029607	ZNF207	case	control	0.025116954	0.050079076	0.501545867	0.615987006	0.891081782	up_in_case
PRJNA8274(	ENSGALG00010029608	ENSGALG00010029608	case	control	-0.028006175	0.141324929	-0.198168686	0.842913087	0.966612441	down_in_case
PRJNA8274(	ENSGALG00010029609	METTL27	case	control	-0.015272912	0.128363794	-0.118981462	0.905290043	0.981730793	down_in_case
PRJNA8274(	ENSGALG00010029610	ERN1	case	control	-0.003689177	0.058105866	-0.063490604	0.949375841	0.99138853	down_in_case
PRJNA8274(	ENSGALG00010029611	YWHAG	case	control	-0.068563245	0.064381568	-1.06495147	0.286897892	0.710659943	down_in_case
PRJNA8274(	ENSGALG00010029612	HSPB1	case	control	-0.167675778	0.128265729	-1.30725315	0.19112675	0.61425441	down_in_case
PRJNA8274(	ENSGALG00010029614	MDH2	case	control	-0.1127523	0.085240247	-1.322758948	0.185915553	0.608935531	down_in_case
PRJNA8274(	ENSGALG00010029615	ENSGALG00010029615	case	control	0.081098974	0.271945727	0.298217498	0.765537167	0.943786458	up_in_case
PRJNA8274(	ENSGALG00010029616	TMEM120A	case	control	-0.096316294	0.074161513	-1.298736901	0.194034236	0.617169773	down_in_case
PRJNA8274(	ENSGALG00010029617	POR	case	control	-0.070416686	0.051685723	-1.362401118	0.173071331	0.592369563	down_in_case
PRJNA8274(	ENSGALG00010029618	TEX2	case	control	-0.15909702	0.068771731	-2.313407254	0.020700259	0.258753233	down_in_case
PRJNA8274(	ENSGALG00010029619	ENSGALG00010029619	case	control	-0.090993001	0.08344989	-1.090390902	0.275540988	0.700894634	down_in_case

PRJNA8274(	ENSGALG00010029620	ENSGALG00010029620	case	control	0.163776378	0.279187787	0.586617273	0.557460779	0.868661404	up_in_case
PRJNA8274(	ENSGALG00010029621	TAF15	case	control	-0.06720658	0.060071631	-1.118774016	0.26323656	0.689122374	down_in_case
PRJNA8274(	ENSGALG00010029622	MMP28	case	control	0.099326338	0.111689199	0.88931015	0.373836418	0.774369566	up_in_case
PRJNA8274(	ENSGALG00010029623	ENSGALG00010029623	case	control	-0.669177015	0.394877426	-1.694644895	0.090142805	0.473607022	down_in_case
PRJNA8274(	ENSGALG00010029624	RASL10B	case	control	0.042195532	0.177729891	0.237413816	0.812335772	0.956397295	up_in_case
PRJNA8274(	ENSGALG00010029625	PECAM1	case	control	0.164486853	0.093696327	1.755531494	0.079168438	0.449249472	up_in_case
PRJNA8274(	ENSGALG00010029626	AP2B1	case	control	-0.077182176	0.054690954	-1.411242069	0.158173249	0.570246977	down_in_case
PRJNA8274(	ENSGALG00010029627	PEX12	case	control	-0.130757781	0.10682815	-1.224001183	0.220951781	0.644950086	down_in_case
PRJNA8274(	ENSGALG00010029628	UNC45B	case	control	-0.226795526	0.120603721	-1.880501902	0.060039707	0.398649739	down_in_case
PRJNA8274(	ENSGALG00010029629	ENSGALG00010029629	case	control	0.14578046	0.167995693	0.867763081	0.385524045	0.781541515	up_in_case
PRJNA8274(	ENSGALG00010029630	RAD51D	case	control	0.011567771	0.095708888	0.120864125	0.903798658	0.981088965	up_in_case
PRJNA8274(	ENSGALG00010029631	LIG3	case	control	0.022769196	0.061645202	0.369358761	0.711860332	0.926682089	up_in_case
PRJNA8274(	ENSGALG00010029632	TMEM132E	case	control	-0.147769053	0.167953687	-0.879820237	0.3789567	0.777229179	down_in_case
PRJNA8274(	ENSGALG00010029633	POLG2	case	control	0.228325271	0.153126499	1.491089213	0.135938074	0.539774365	up_in_case
PRJNA8274(	ENSGALG00010029634	DDX5	case	control	0.186692225	0.055204568	3.381825697	0.000720058	0.040076036	up_in_case
PRJNA8274(	ENSGALG00010029635	ENSGALG00010029635	case	control	0.28222962	0.281615227	1.002181673	0.316255858	0.736866972	up_in_case
PRJNA8274(	ENSGALG00010029636	SPATA20	case	control	-0.25061689	0.063348772	-3.956144378	7.61691E-05	0.009001807	down_in_case
PRJNA8274(	ENSGALG00010029638	EPN3	case	control	-0.213001637	0.120744402	-1.7640705	0.07772011	0.447281736	down_in_case
PRJNA8274(	ENSGALG00010029639	ENSGALG00010029639	case	control	0.150278488	0.44635999	0.336675533	0.736361509	0.934585034	up_in_case
PRJNA8274(	ENSGALG00010029641	ENSGALG00010029641	case	control	-0.120701843	0.233082925	-0.517849355	0.60456338	0.885268908	down_in_case
PRJNA8274(	ENSGALG00010029642	NAT9	case	control	-0.205973797	0.101592286	-2.027455091	0.042615893	0.344492519	down_in_case
PRJNA8274(	ENSGALG00010029644	MYCBPAP	case	control	-0.064841395	0.118162879	-0.548745895	0.583179845	0.881296631	down_in_case
PRJNA8274(	ENSGALG00010029645	ENSGALG00010029645	case	control	0.20605486	0.291904979	0.70589704	0.480252169	0.831851816	up_in_case
PRJNA8274(	ENSGALG00010029646	MRPS17	case	control	0.003204415	0.067193128	0.047689622	0.961963605	0.992056045	up_in_case
PRJNA8274(	ENSGALG00010029647	RSAD1	case	control	-0.033164595	0.168945234	-0.196303822	0.84437237	0.96721237	down_in_case
PRJNA8274(	ENSGALG00010029648	NIPSNAP2	case	control	-0.103154284	0.061428217	-1.679265468	0.093100318	0.478209247	down_in_case
PRJNA8274(	ENSGALG00010029649	PSPH	case	control	-0.015995804	0.125483137	-0.127473736	0.898565459	0.98028579	down_in_case
PRJNA8274(	ENSGALG00010029650	ACSF2	case	control	0.049286459	0.109014571	0.452108915	0.651190521	0.904951854	up_in_case
PRJNA8274(	ENSGALG00010029651	ENSGALG00010029651	case	control	0.202443715	0.222568231	0.909580465	0.363043805	0.769342977	up_in_case
PRJNA8274(	ENSGALG00010029652	LRRC59	case	control	0.017080931	0.053582813	0.318776306	0.749896147	0.939752423	up_in_case
PRJNA8274(	ENSGALG00010029653	CCT6A	case	control	0.011800113	0.054996561	0.214560927	0.83010966	0.962228709	up_in_case
PRJNA8274(	ENSGALG00010029654	EME1	case	control	0.161757993	0.088523289	1.827293068	0.067655717	0.42089154	up_in_case
PRJNA8274(	ENSGALG00010029655	PHKG1	case	control	-0.020808606	0.089542915	-0.232386963	0.816237474	0.957422215	down_in_case
PRJNA8274(	ENSGALG00010029656	MRPL27	case	control	0.034840997	0.080591118	0.43231808	0.665510249	0.912007336	up_in_case
PRJNA8274(	ENSGALG00010029657	CHCHD2	case	control	-0.087435904	0.087484123	-0.99944882	0.31757732	0.737774213	down_in_case
PRJNA8274(	ENSGALG00010029658	XYLT2	case	control	0.036992896	0.060593959	0.610504695	0.541527534	0.85975913	up_in_case
PRJNA8274(	ENSGALG00010029659	VKORC1L1	case	control	0.11021888	0.055887986	1.972139052	0.048593735	0.363399983	up_in_case
PRJNA8274(	ENSGALG00010029660	CD300LG	case	control	-0.280585185	0.200755562	-1.39764589	0.162219431	0.575677776	down_in_case
PRJNA8274(	ENSGALG00010029663	GUSB	case	control	0.043395319	0.069573238	0.623735795	0.532801111	0.855817434	up_in_case
PRJNA8274(	ENSGALG00010029665	ASL1	case	control	-0.111956867	0.202535148	-0.552777473	0.580415796	0.879392282	down_in_case
PRJNA8274(	ENSGALG00010029666	SLC9A3R1	case	control	0.173895401	0.113688528	1.529577386	0.12612137	0.525939433	up_in_case
PRJNA8274(	ENSGALG00010029667	TMEM104	case	control	0.19280371	0.075735027	2.545766717	0.010903803	0.185137791	up_in_case
PRJNA8274(	ENSGALG00010029668	CRCP	case	control	-0.013503537	0.101428424	-0.133133661	0.894087674	0.979888889	down_in_case
PRJNA8274(	ENSGALG00010029669	HID1	case	control	0.063520658	0.081206565	0.782210872	0.434090656	0.809005133	up_in_case
PRJNA8274(	ENSGALG00010029670	TPST1	case	control	0.04532356	0.074195207	0.610869105	0.54128624	0.85975913	up_in_case
PRJNA8274(	ENSGALG00010029671	GRIN2C	case	control	-0.007619675	0.116225856	-0.065559206	0.947728768	0.991261073	down_in_case
PRJNA8274(	ENSGALG00010029672	KCTD7	case	control	-0.045142545	0.159637772	-0.282781102	0.777344642	0.947658003	down_in_case
PRJNA8274(	ENSGALG00010029673	TMEM248	case	control	-3.79938E-05	0.054570941	-0.000696227	0.999444491	0.99976345	down_in_case
PRJNA8274(	ENSGALG00010029674	FDXR	case	control	0.040713132	0.136084331	0.299175753	0.764805949	0.943786458	up_in_case
PRJNA8274(	ENSGALG00010029675	DHRS13	case	control	0.31958454	0.254808637	1.25421392	0.209764262	0.634976491	up_in_case
PRJNA8274(	ENSGALG00010029676	FADS6	case	control	1.946220072	0.749170669	2.597832713	0.009381419	0.172879234	up_in_case
PRJNA8274(	ENSGALG00010029677	PHF12	case	control	-0.095390795	0.043901594	-2.172832184	0.02979295	0.305970455	down_in_case
PRJNA8274(	ENSGALG00010029681	SEZ6	case	control	-0.539085171	0.810197702	-0.665374845	0.50581077	0.843280685	down_in_case
PRJNA8274(	ENSGALG00010029682	CDR2L	case	control	0.081046411	0.063200335	1.282373107	0.199711793	0.622902456	up_in_case
PRJNA8274(	ENSGALG00010029683	MRPL58	case	control	-0.0936883	0.083051126	-1.12807982	0.259286211	0.686374086	down_in_case
PRJNA8274(	ENSGALG00010029684	ENSGALG00010029684	case	control	0.201435861	0.36795666	0.547444531	0.584073369	0.881307382	up_in_case
PRJNA8274(	ENSGALG00010029685	ATP5PD	case	control	-0.02990212	0.065094389	-0.459365548	0.645971684	0.902693223	down_in_case
PRJNA8274(	ENSGALG00010029686	KCTD2	case	control	-0.008850037	0.04323265	-0.204707265	0.837800844	0.964826648	down_in_case
PRJNA8274(	ENSGALG00010029687	SLC16A5	case	control	0.273650971	0.201098025	1.360783981	0.173581966	0.592914702	up_in_case
PRJNA8274(	ENSGALG00010029688	PIPOX	case	control	1.833921889	0.644394593	2.845961013	0.004427762	0.114389963	up_in_case
PRJNA8274(	ENSGALG00010029689	ENSGALG00010029689	case	control	-0.215445205	0.095678578	-2.251760099	0.024337436	0.275772854	down_in_case
PRJNA8274(	ENSGALG00010029690	ENSGALG00010029690	case	control	-0.193589432	0.08766256	-2.208347931	0.027220028	0.293158316	down_in_case
PRJNA8274(	ENSGALG00010029691	NUFIP2	case	control	-0.019029435	0.06384365	-0.298063088	0.765655014	0.943786458	down_in_case
PRJNA8274(	ENSGALG00010029692	CRYBA1	case	control	-0.015528922	0.264934318	-0.058614233	0.953259374	0.992056045	down_in_case
PRJNA8274(	ENSGALG00010029693	HN1	case	control	0.055714876	0.053079225	1.049655038	0.293876743	0.716378375	up_in_case
PRJNA8274(	ENSGALG00010029694	ARMC7	case	control	-0.025440914	0.071298107	-0.356824534	0.721223168	0.929292592	down_in_case
PRJNA8274(	ENSGALG00010029695	TAOK1	case	control	-0.034062782	0.037843416	-0.900097962	0.368068121	0.771770041	down_in_case
PRJNA8274(	ENSGALG00010029696	SUMO2	case	control	0.01446331	0.044222366	0.327058705	0.743623485	0.937448285	up_in_case
PRJNA8274(	ENSGALG00010029697	GIT1	case	control	-0.005808404	0.070733899	-0.082116272	0.934554254	0.988140189	down_in_case
PRJNA8274(	ENSGALG00010029698	NUP85	case	control	0.081651758	0.049359886	1.654212873	0.098084268	0.487193947	up_in_case
PRJNA8274(	ENSGALG00010029699	ANKRD13B	case	control	0.082868865	0.088999367	0.931117473	0.351792801	0.760831854	up_in_case
PRJNA8274(	ENSGALG00010029700	GGA3	case	control	-0.053673567	0.05670297	-0.94657418	0.343855806	0.755785126	down_in_case
PRJNA8274(	ENSGALG00010029701	MRPS7	case	control	-0.095071919	0.053985012	-1.761079907	0.078224879	0.448561389	down_in_case
PRJNA8274(	ENSGALG00010029702	CORO6	case	control	-0.204095156	0.166420453	-1.226382656	0.22005471	0.644304331	down_in_case
PRJNA8274(	ENSGALG00010029703	MIF4GD	case	control	0.066081153	0.068678507	0.962180977	0.335958703	0.750188859	up_in_case
PRJNA8274(	ENSGALG00010029704	SLC25A19	case	control	-0.003521111	0.064113399	-0.054920055	0.956202154	0.992056045	down_in_case
PRJNA8274(	ENSGALG00010029705	GRB2	case	control	0.005439841	0.03290149	0.165337222	0.868678563	0.974483475	up_in_case
PRJNA8274(	ENSGALG00010029706	SSH2	case	control	-0.149981706	0.085863188	-1.746752131	0.080680343	0.452876025	down_in_case
PRJNA8274(	ENSGALG00010029707	NSRP1	case	control	-0.096527478	0.073430644	-1.314539442	0.188664734	0.611698262	down_in_case
PRJNA8274(	ENSGALG00010029708	TMEM94	case	control	-0.024609478	0.046723232	-0.526707541	0.598396698	0.884726301	down_in_case
PRJNA8274(	ENSGALG00010029709	SLC6A4	case	control	-0.00280504	0.190181645	-0.014749266	0.988232215	0.996942188	down_in_case
PRJNA8274(	ENSGALG00010029710	BLMH	case	control	0.046525263	0.047777107	0.973798254	0.33015672	0.746414364	up_in_case
PRJNA8274(	ENSGALG00010029711	CASKIN2	case	control	0.04978346	0.049936475	0.996935819	0.318795664	0.738427335	up_in_case
PRJNA8274(	ENSGALG00010029713	CPD	case	control	-0.008603494	0.075627083	-0.113762073	0.909426405	0.981730793	down_in_case
PRJNA8274(	ENSGALG00010029715	GALR2	case	control	-0.123420985	0.665973731	-0.185324104	0.852974833	0.969900941	down_in_case
PRJNA8274(	ENSGALG00010029716	GOSR1	case	control	-0.023816256	0.040305604	-0.590891921	0.55459284	0.866930215	down_in_case
PRJNA8274(	ENSGALG00010029717	TRARG1	case	control	0.025932481	0.400025298	0.064827104	0.948311661	0.991291133	up_in_case
PRJNA8274(	ENSGALG00010029718	ABR	case	control	0.057412786	0.075080131	0.764686814	0.44445806	0.815676367	up_in_case
PRJNA8274(	ENSGALG00010029720	TIMM22	case	control	-0.066295072	0.100341108	-0.660697034	0.508806626	0.844259682	down_in_case
PRJNA8274(	ENSGALG00010029721	EXOC7	case	control	-0.014881206	0.042879824	-0.347044483	0.7285579	0.933057319	down_in_case
PRJNA8274(	ENSGALG00010029722	SPECC1	case	control	-0.109373348	0.085270733	-1.282659877	0.199611263	0.622902456	down_in_case
PRJNA8274(	ENSGALG00010029723	ADORA2B	case	control	0.083589227	0.139720057	0.598262187	0.549665003	0.864234145	up_in_case
PRJNA8274(	ENSGALG00010029724	CACNA1G	case	control	0.197225312	0.107150251	1.840642563	0.065673956	0.415733817	up_in_case
PRJNA8274(	ENSGALG00010029725	TSEN54	case	control	0.076531434	0.064022414	1.195385008	0.23193664	0.657291707	up_in_case
PRJNA8274(	ENSGALG00010029726	SEPTIN9	case	control	0.080960033	0.053358868	1.517274184	0.129197473	0.531202952	up_in_case
PRJNA8274(	ENSGALG00010029727	ZSWIM7	case	control	0.077197236	0.127130315	0.607229174	0.543698834	0.861081181	up_in_case
PRJNA8274(	ENSGALG00010029728	TNRC6C	case	control	-0.11512147	0.072456912	-1.588826615	0.112099543	0.505685634	down_in_case
PRJNA8274(	ENSGALG00010029729	RASL10A	case	control	-1.167075827	0.588205476	-1.984129483	0.047241407	0.358156924	down_in_case
PRJNA8274(	ENSGALG00010029730	TTC19	case	control	-0.133650276	0.072447779	-1.844780849	0.065069424	0.414658096	down_in_case
PRJNA8274(	ENSGALG00010029731	NCOR1	case	control	-0.112780697	0.061157176	-1.844112258	0.065166782	0.414724068	down_in_case
PRJNA8274(	ENSGALG00010029732	PIGL	case	control	-0.132071805	0.110103267	-1.199526665	0.230323223	0.654892316	down_in_case
PRJNA8274(	ENSGALG00010029733	ENSGALG00010029733	case	control	-0.007969282	0.119004245	-0.066966364	0.94660848	0.990701817	down_in_case
PRJNA8274(	ENSGALG00010029734	SEC14L1	case	control	0.124256841	0.060680319	2.04772886	0.040586574	0.337631189	up_in_case
PRJNA8274(	ENSGALG00010029735	SRP68	case	control	-0.034407582	0.039639587	-0.868010614	0.385388522	0.781541515	down_in_case
PRJNA8274(	ENSGALG00010029736	ENSGALG00010029736	case	control	-0.138652518	0.08165288	-1.698072599	0.08949406	0.472586803	down_in_case
PRJNA8274(	ENSGALG00010029737	ENSGALG00010029737	case	control	0.226747353	0.823687732	0.27528315	0.783098709	0.949518053	up_in_case
PRJNA8274(	ENSGALG00010029738	TRPV2	case	control	0.033762899	0.078407434	0.430608398	0.666753134	0.912284937	up_in_case
PRJNA8274(	ENSGALG00010029739	P2RX5	case	control	-0.196052193	0.102154868	-1.919166421	0.054963276	0.38300081	down_in_case
PRJNA8274(	ENSGALG00010029740	HASPIN	case	control	0.050669642	0.100184431	0.50576363	0.613022594	0.890154949	up_in_case
PRJNA8274(	ENSGALG00010029741	EMC6	case	control	-0.104501405	0.056819985	-1.839166359	0.065890721	0.416182558	down_in_case
PRJNA8274(	ENSGALG00010029742	TAX1BP3	case	control	-0.041111917	0.050204117	-0.81889534	0.412846129	0.797581687	down_in_case
PRJNA8274(	ENSGALG00010029743	MYOCD	case	control	-0.353862618	0.169272791	-2.090487292	0.036574049	0.32721608	down_in_case
PRJNA8274(	ENSGALG00010029744	CTNS	case	control	0.015073456	0.07758321	0.1942876	0.845950694	0.967563913	up_in_case
PRJNA8274(	ENSGALG00010029745	SHPK	case	control	-0.101862865	0.153432499	-0.663893672	0.506758367	0.843472666	down_in_case
PRJNA8274(	ENSGALG00010029746	TRPV1	case	control	0.082453541	0.267630694	0.308087014	0.758016122	0.941903352	up_in_case
PRJNA8274(	ENSGALG00010029747	TRPV3	case	control	0.267540884	0.223725101	1.195846522	0.231756458	0.657171791	up_in_case
PRJNA8274(	ENSGALG00010029749	ASPA	case	control	0.15795496	0.27756745	0.569068742	0.56930949	0.875057036	up_in_case
PRJNA8274(	ENSGALG00010029750	TEN1	case	control	0.097914208	0.215006849	0.455400413	0.648821198	0.903741342	up_in_case
PRJNA8274(	ENSGALG00010029752	ENSGALG00010029752	case	control	-0.059088851	0.112396534	-0.525717733	0.599084341	0.884828142	down_in_case
PRJNA8274(	ENSGALG00010029753	AKAP10	case	control	0.014374604	0.046300437	0.310463691	0.756208366	0.941659745	up_in_case
PRJNA8274(	ENSGALG00010029754	NLE1	case	control	-0.206196337	0.135480914	-1.521958553	0.128019466	0.529650968	down_in_case
PRJNA8274(	ENSGALG00010029755	ACOX1	case	control	0.096829065	0.106414674	0.909922114	0.362863586	0.769075039	up_in_case
PRJNA8274(	ENSGALG00010029756	ELAC2	case	control	0.025081446	0.065080068	0.385393659	0.69994578	0.922288441	up_in_case
PRJNA8274(	ENSGALG00010029757	ULK2	case	control	0.13683363	0.048124301	2.843337506	0.004464377	0.114389963	up_in_case
PRJNA8274(	ENSGALG00010029758	DNAH9	case	control	-0.330223024	0.27689223	-1.192604875	0.233024156	0.657982411	down_in_case
PRJNA8274(	ENSGALG00010029759	ALDH3A2	case	control	-0.08286337	0.083111776	-0.997011178	0.318759084	0.738427335	down_in_case

PRJNA8274(	ENSGALG00010029760	SHISA6	case	control	-0.175656587	0.177100944	-0.991844443	0.321273414	0.73942679	down_in_case
PRJNA8274(	ENSGALG00010029761	MRPL38	case	control	-0.009139892	0.076625282	-0.119280367	0.905053237	0.981589783	down_in_case
PRJNA8274(	ENSGALG00010029762	SLC47A2	case	control	0.228902547	0.278933011	0.820636274	0.411853479	0.797194435	up_in_case
PRJNA8274(	ENSGALG00010029763	GEMIN4	case	control	0.047749353	0.056455749	0.845783723	0.397673407	0.788728117	up_in_case
PRJNA8274(	ENSGALG00010029764	NXN	case	control	-0.104602721	0.070420852	-1.48539413	0.13743945	0.539854583	down_in_case
PRJNA8274(	ENSGALG00010029765	KCNJ16	case	control	-0.224245691	0.292821389	-0.765810488	0.443789073	0.814971586	down_in_case
PRJNA8274(	ENSGALG00010029766	ENSGALG00010029766	case	control	0.113058066	0.087312225	1.294870964	0.195364746	0.618546793	up_in_case
PRJNA8274(	ENSGALG00010029767	GLOD4	case	control	-0.215377583	0.074615366	-2.886504408	0.003895473	0.107747123	down_in_case
PRJNA8274(	ENSGALG00010029768	KCNJ2	case	control	-0.066920846	0.080742081	-0.828822403	0.407204909	0.794380997	down_in_case
PRJNA8274(	ENSGALG00010029770	TLCD3A	case	control	0.028839147	0.138203363	0.208671817	0.834704443	0.963411173	up_in_case
PRJNA8274(	ENSGALG00010029771	SOX9	case	control	0.075737114	0.24673182	0.306961275	0.758872848	0.942078947	up_in_case
PRJNA8274(	ENSGALG00010029772	VPS53	case	control	0.025148789	0.045686481	0.550464568	0.582000773	0.88034085	up_in_case
PRJNA8274(	ENSGALG00010029773	TRIM65	case	control	0.057781254	0.187383242	0.308358705	0.757809401	0.941765572	up_in_case
PRJNA8274(	ENSGALG00010029774	RFLNB	case	control	-0.042227006	0.061387843	-0.687872444	0.491533111	0.837075561	down_in_case
PRJNA8274(	ENSGALG00010029775	ENSGALG00010029775	case	control	-0.023983825	0.144793168	-0.165641969	0.868438717	0.974483475	down_in_case
PRJNA8274(	ENSGALG00010029776	WBP2	case	control	-0.01804628	0.076624195	-0.235516739	0.813807688	0.956712425	down_in_case
PRJNA8274(	ENSGALG00010029777	ENSGALG00010029777	case	control	0.192659839	0.12165121	1.583706726	0.113260473	0.507878574	up_in_case
PRJNA8274(	ENSGALG00010029778	DOC2B	case	control	-0.085940379	0.216274682	-0.397366803	0.691096992	0.918812261	down_in_case
PRJNA8274(	ENSGALG00010029780	TEX14	case	control	0.188173804	0.202624879	0.92868065	0.353054612	0.76183506	up_in_case
PRJNA8274(	ENSGALG00010029781	UNC13D	case	control	0.161656917	0.209688256	0.770939298	0.440742912	0.813083204	up_in_case
PRJNA8274(	ENSGALG00010029782	UNK	case	control	0.096152926	0.048327249	1.989621329	0.046632665	0.356907825	up_in_case
PRJNA8274(	ENSGALG00010029783	RAD51C	case	control	-0.277025478	0.100401209	-2.759184695	0.005794578	0.132579933	down_in_case
PRJNA8274(	ENSGALG00010029784	CLDN3	case	control	-0.34232422	0.42842814	-0.799023659	0.424276694	0.803723282	down_in_case
PRJNA8274(	ENSGALG00010029785	ENSGALG00010029785	case	control	0.348097626	0.760711304	0.457594917	0.647243496	0.903059048	up_in_case
PRJNA8274(	ENSGALG00010029787	H3-3B	case	control	0.00149454	0.046874827	0.03188363	0.974564853	0.993775355	up_in_case
PRJNA8274(	ENSGALG00010029791	GALK1	case	control	0.215901434	0.091541905	2.358498379	0.01834904	0.242045801	up_in_case
PRJNA8274(	ENSGALG00010029804	ITGB4	case	control	0.10699822	0.152660446	0.700890259	0.483371504	0.833691495	up_in_case
PRJNA8274(	ENSGALG00010029812	SAP30BP	case	control	0.01759915	0.049120263	0.358286961	0.720128575	0.92895404	up_in_case
PRJNA8274(	ENSGALG00010029835	RECQL5	case	control	-0.106098887	0.105153042	-1.008994931	0.312977065	0.734574752	down_in_case
PRJNA8274(	ENSGALG00010029841	SMIM5	case	control	0.098979371	0.09053927	1.093220336	0.27429707	0.699970438	up_in_case
PRJNA8274(	ENSGALG00010029859	CSNK1D	case	control	0.030185811	0.046787681	0.64516579	0.518819753	0.848941486	up_in_case
PRJNA8274(	ENSGALG00010029864	SLC16A3	case	control	0.039424968	0.164584295	0.239542711	0.810684781	0.956022547	up_in_case
PRJNA8274(	ENSGALG00010029879	ENSGALG00010029879	case	control	0.172352833	0.246090789	0.700362797	0.483700764	0.833866295	up_in_case
PRJNA8274(	ENSGALG00010029895	FASN	case	control	0.050806525	0.09082175	0.55940923	0.575882464	0.877910508	up_in_case
PRJNA8274(	ENSGALG00010029907	NTN1	case	control	-0.126815739	0.184198121	-0.688474658	0.491153924	0.836925555	down_in_case
PRJNA8274(	ENSGALG00010029921	STX8	case	control	-0.020654072	0.056215552	-0.367408506	0.713314328	0.926862121	down_in_case
PRJNA8274(	ENSGALG00010029925	GGNBP2	case	control	-0.067637464	0.056798925	-1.190822948	0.233723098	0.65800118	down_in_case
PRJNA8274(	ENSGALG00010029926	BLTP2	case	control	0.034754433	0.051906259	0.6695615	0.503137364	0.842485272	up_in_case
PRJNA8274(	ENSGALG00010029927	ENSGALG00010029927	case	control	0.421215289	0.562213562	0.749208696	0.453731429	0.821282604	up_in_case
PRJNA8274(	ENSGALG00010029928	USP43	case	control	-0.038691108	0.125809012	-0.30753845	0.758433561	0.941903352	down_in_case
PRJNA8274(	ENSGALG00010029929	GDPD1	case	control	0.027560286	0.03858689	0.714239637	0.475079011	0.830111175	up_in_case
PRJNA8274(	ENSGALG00010029930	ARHGAP44	case	control	-0.032631875	0.158649282	-0.205685615	0.837036496	0.964784002	down_in_case
PRJNA8274(	ENSGALG00010029932	RASA4B	case	control	0.051534648	0.188818195	0.272932635	0.784905	0.949518053	up_in_case
PRJNA8274(	ENSGALG00010029933	TVP23B	case	control	-0.064041597	0.044788846	-1.429855928	0.152758373	0.561193576	down_in_case
PRJNA8274(	ENSGALG00010029934	TEKT3	case	control	0.329738363	0.328855212	1.002685529	0.316012614	0.736866972	up_in_case
PRJNA8274(	ENSGALG00010029935	SYNRG	case	control	-0.037972342	0.080205495	-0.473438159	0.635900611	0.898691227	down_in_case
PRJNA8274(	ENSGALG00010029936	SERPINF2	case	control	1.140331105	0.661138804	1.724798329	0.084563823	0.460145612	up_in_case
PRJNA8274(	ENSGALG00010029937	PMP22	case	control	-0.134901296	0.077618473	-1.738005019	0.082209929	0.455747917	down_in_case
PRJNA8274(	ENSGALG00010029938	ENSGALG00010029938	case	control	0.548869573	0.290354463	1.890343159	0.05871208	0.395097763	up_in_case
PRJNA8274(	ENSGALG00010029941	COX10	case	control	-0.042055064	0.089971979	-0.467424025	0.640196537	0.901077979	down_in_case
PRJNA8274(	ENSGALG00010029943	HS3ST3A1	case	control	0.346942601	0.152566053	2.274048473	0.022963072	0.267253668	up_in_case
PRJNA8274(	ENSGALG00010029946	METRNL	case	control	0.052065921	0.061827889	0.842110613	0.399726041	0.789900496	up_in_case
PRJNA8274(	ENSGALG00010029948	MYADML2	case	control	0.085389488	0.091057859	0.937749785	0.348373013	0.758941366	up_in_case
PRJNA8274(	ENSGALG00010029950	RFFL	case	control	0.011639746	0.119784548	0.097172348	0.922589527	0.985482967	up_in_case
PRJNA8274(	ENSGALG00010029956	CCLI5	case	control	0.33211444	0.155648385	2.133748069	0.032863409	0.311991307	up_in_case
PRJNA8274(	ENSGALG00010029962	ENSGALG00010029962	case	control	0.330763066	0.577727169	0.572524686	0.566966567	0.87333105	up_in_case
PRJNA8274(	ENSGALG00010029966	RABGEF1	case	control	0.165143902	0.05761882	2.866145134	0.004155037	0.111582498	up_in_case
PRJNA8274(	ENSGALG00010030004	FLOT2	case	control	-0.04849473	0.087694695	-0.552995018	0.580266822	0.879378502	down_in_case
PRJNA8274(	ENSGALG00010030014	ENSGALG00010030014	case	control	0.033677402	0.131215829	0.25665655	0.797443916	0.95280257	up_in_case
PRJNA8274(	ENSGALG00010030102	GPS1	case	control	-0.087806184	0.055947513	-1.569438556	0.116545786	0.51350396	down_in_case
PRJNA8274(	ENSGALG00010030103	C1QTNF1	case	control	0.198050252	0.134741131	1.469857428	0.141600372	0.543501954	up_in_case
PRJNA8274(	ENSGALG00010030104	P4HB	case	control	0.163253048	0.055407943	2.946383502	0.003215135	0.096457878	up_in_case
PRJNA8274(	ENSGALG00010030105	RAB40B	case	control	-0.120656122	0.119483406	-1.00981489	0.312583985	0.734364975	down_in_case
PRJNA8274(	ENSGALG00010030106	GPRC5C	case	control	0.202725281	0.134839576	1.503455339	0.132721665	0.535984133	up_in_case
PRJNA8274(	ENSGALG00010030107	RPTOR	case	control	-0.079928726	0.093709028	-0.852945841	0.393689339	0.786843919	down_in_case
PRJNA8274(	ENSGALG00010030108	LLGL2	case	control	0.085628246	0.095775706	0.894049757	0.371295269	0.773081298	up_in_case
PRJNA8274(	ENSGALG00010030109	FBF1	case	control	0.140317623	0.065800711	2.132463618	0.032968752	0.31242754	up_in_case
Yang2025_S	ENSGALG00010000007	ND1	case	control	1.318376355	0.428402565	3.077424048	0.00208798	0.012354933	up_in_case
Yang2025_S	ENSGALG00010000011	ND2	case	control	1.304189856	0.551999176	2.36266631	0.018143998	0.063161783	up_in_case
Yang2025_S	ENSGALG00010000017	COX1	case	control	1.44498147	0.928056862	1.556996698	0.119471269	0.248774437	up_in_case
Yang2025_S	ENSGALG00010000020	COX2	case	control	0.938463678	0.396390792	2.36752139	0.017907685	0.062652169	up_in_case
Yang2025_S	ENSGALG00010000022	ATP8	case	control	0.728973258	0.465731282	1.565222879	0.117530659	0.245869148	up_in_case
Yang2025_S	ENSGALG00010000023	ATP6	case	control	0.854706537	0.410179073	2.083739987	0.037183825	0.107323239	up_in_case
Yang2025_S	ENSGALG00010000024	COX3	case	control	0.95739899	0.226340763	4.229900865	2.33794E-05	0.000331427	up_in_case
Yang2025_S	ENSGALG00010000026	ND3	case	control	0.889231002	0.394784196	2.252448326	0.024293955	0.07850281	up_in_case
Yang2025_S	ENSGALG00010000028	ND4L	case	control	2.686861784	0.683555021	3.93071765	8.46927E-05	0.000935176	up_in_case
Yang2025_S	ENSGALG00010000029	ND4	case	control	1.453257868	0.541603805	2.683249001	0.007291068	0.031869836	up_in_case
Yang2025_S	ENSGALG00010000033	ND5	case	control	1.33446566	0.539551733	2.473285839	0.013387702	0.050285074	up_in_case
Yang2025_S	ENSGALG00010000034	CYTB	case	control	1.26824586	0.402872291	3.148009652	0.001643863	0.010326855	up_in_case
Yang2025_S	ENSGALG00010000037	ND6	case	control	1.211516123	0.421547011	2.873976309	0.004053394	0.020347277	up_in_case
Yang2025_S	ENSGALG00010000039	PDIA3	case	control	0.145380176	0.095774659	1.517939894	0.129029552	0.262600672	up_in_case
Yang2025_S	ENSGALG00010000042	CKMT1B	case	control	0.729651697	0.399045509	1.828492442	0.067475679	0.165519018	up_in_case
Yang2025_S	ENSGALG00010000043	PPIP5K1	case	control	-0.142643615	0.112583813	-1.266999321	0.205155533	0.363396765	down_in_case
Yang2025_S	ENSGALG00010000044	MAP1A	case	control	0.10921461	0.191974577	0.568901422	0.56942304	0.713937399	up_in_case
Yang2025_S	ENSGALG00010000045	TP53BP1	case	control	0.262649665	0.101901719	2.577480211	0.009952358	0.04038617	up_in_case
Yang2025_S	ENSGALG00010000046	TUBGCP4	case	control	0.065397966	0.121345308	0.538941032	0.589927544	0.730853514	up_in_case
Yang2025_S	ENSGALG00010000047	IQGAP1	case	control	0.223173957	0.111227761	2.006459145	0.044807283	0.123445926	up_in_case
Yang2025_S	ENSGALG00010000048	MAN2A2	case	control	0.488135089	0.084530473	5.774664094	7.71068E-09	3.62751E-07	up_in_case
Yang2025_S	ENSGALG00010000049	FES	case	control	0.385714705	0.191063786	2.018774525	0.04351066	0.1206129	up_in_case
Yang2025_S	ENSGALG00010000050	CIB1	case	control	-0.2409931	0.14936547	-1.613445866	0.106647675	0.229271113	down_in_case
Yang2025_S	ENSGALG00010000051	GDPGP1	case	control	0.159253186	0.206194663	0.772343878	0.439910784	0.603302507	up_in_case
Yang2025_S	ENSGALG00010000052	TTLL13	case	control	0.048420683	0.202016354	0.239686946	0.810572955	0.885991329	up_in_case
Yang2025_S	ENSGALG00010000053	HDDC3	case	control	0.829253929	0.247564074	3.349653757	0.000809126	0.005905426	up_in_case
Yang2025_S	ENSGALG00010000054	VPS33B	case	control	-0.000602803	0.143134524	-0.004211446	0.996639762	0.997570398	down_in_case
Yang2025_S	ENSGALG00010000055	PRC1	case	control	0.354830744	0.144652961	2.452979472	0.014167842	0.05254961	up_in_case
Yang2025_S	ENSGALG00010000056	UNC45A	case	control	0.507728821	0.12928697	3.92714612	8.59598E-05	0.000943932	up_in_case
Yang2025_S	ENSGALG00010000058	FURIN	case	control	0.221813582	0.060751697	3.651150413	0.000261068	0.002324592	up_in_case
Yang2025_S	ENSGALG00010000059	MFAP1	case	control	0.524087627	0.173875229	3.014159226	0.002576925	0.014522991	up_in_case
Yang2025_S	ENSGALG00010000060	HYPK	case	control	0.719693619	0.18550993	3.879542287	0.000104653	0.001104805	up_in_case
Yang2025_S	ENSGALG00010000061	SERINC4	case	control	0.210815019	0.314595521	0.670114497	0.502784804	0.6591689	up_in_case
Yang2025_S	ENSGALG00010000062	ENSGALG00010000062	case	control	0.830551495	0.57010074	1.456850407	0.145157678	0.286308881	up_in_case
Yang2025_S	ENSGALG00010000063	SERF2	case	control	-0.30872651	0.305228995	-1.011458659	0.311796958	0.481428323	down_in_case
Yang2025_S	ENSGALG00010000069	ENSGALG00010000069	case	control	1.043489617	0.2071804	5.036623241	4.73816E-07	1.28206E-05	up_in_case
Yang2025_S	ENSGALG00010000075	ENSGALG00010000075	case	control	0.710931976	0.491274876	1.447116494	0.147864299	0.289923879	up_in_case
Yang2025_S	ENSGALG00010000076	ENSGALG00010000076	case	control	0.138231581	0.165836395	0.833541881	0.404539188	0.570814573	up_in_case
Yang2025_S	ENSGALG00010000078	ENSGALG00010000078	case	control	1.182604257	0.508064828	2.327664091	0.019929947	0.067605603	up_in_case
Yang2025_S	ENSGALG00010000081	ENSGALG00010000081	case	control	-0.092746925	0.26262419	-0.353154541	0.723972586	0.830470694	down_in_case
Yang2025_S	ENSGALG00010000084	ENSGALG00010000084	case	control	0.643651292	0.257061738	2.503878241	0.01228403	0.047221506	up_in_case
Yang2025_S	ENSGALG00010000085	ENSGALG00010000085	case	control	0.054637462	0.130702203	0.41803015	0.675925074	0.796197456	up_in_case
Yang2025_S	ENSGALG00010000089	ENSGALG00010000089	case	control	0.347094199	0.305371332	1.136629941	0.255693018	0.422007703	up_in_case
Yang2025_S	ENSGALG00010000091	ENSGALG00010000091	case	control	0.646708929	0.924443779	0.69956545	0.484198726	0.642111652	up_in_case
Yang2025_S	ENSGALG00010000092	INPPL1	case	control	0.390376615	0.164309831	2.375856718	0.01750826	0.061548336	up_in_case
Yang2025_S	ENSGALG00010000093	ENSGALG00010000093	case	control	0.160727884	0.351328211	0.457486416	0.647321464	0.775872645	up_in_case
Yang2025_S	ENSGALG00010000098	ENSGALG00010000098	case	control	0.737190891	0.183322953	4.021268902	5.78855E-05	0.000687668	up_in_case
Yang2025_S	ENSGALG00010000105	CACNA1F	case	control	-0.787677582	0.403642354	-1.951424505	0.051006569	0.135180018	down_in_case
Yang2025_S	ENSGALG00010000107	ENSGALG00010000107	case	control	-0.130508093	0.350320129	-0.372539522	0.709491192	0.820546083	down_in_case
Yang2025_S	ENSGALG00010000121	ENSGALG00010000121	case	control	0.106528953	0.287859207	0.370073113	0.711328015	0.821704942	up_in_case
Yang2025_S	ENSGALG00010000123	ENSGALG00010000123	case	control	0.085359096	0.145514415	0.586602337	0.557470813	0.704548864	up_in_case
Yang2025_S	ENSGALG00010000124	ENSGALG00010000124	case	control	-0.220681479	0.310594157	-0.710513943	0.477385487	0.636346544	down_in_case
Yang2025_S	ENSGALG00010000126	ENSGALG00010000126	case	control	1.43953253	0.360908872	3.9886316	6.64555E-05	0.00076979	up_in_case
Yang2025_S	ENSGALG00010000127	ENSGALG00010000127	case	control	0.366520549	0.184688971	1.984528615	0.047196941	0.128029857	up_in_case
Yang2025_S	ENSGALG00010000133	USP39	case	control	0.415344427	0.148369857	2.79938551	0.005119997	0.024417234	up_in_case
Yang2025_S	ENSGALG00010000134	ENSGALG00010000134	case	control	0.999619573	0.116705718	8.565300744	1.07794E-17	3.85157E-15	up_in_case
Yang2025_S	ENSGALG00010000137	ENSGALG00010000137	case	control	-0.581093332	0.418981905	-1.386917491	0.165466924	0.313072856	down_in_case
Yang2025_S	ENSGALG00010000138	ENSGALG00010000138	case	control	1.751350537	0.760264658	2.303606407	0.021244748	0.070852393	up_in_case
Yang2025_S	ENSGALG00010000140	SEPHS3	case	control	0.362266656	0.112402996	3.222927037	0.001268879	0.008435985	up_in_case

Yang2025_S	ENSGALG00010000141	ENSGALG00010000141	case	control	0.089330314	0.100819235	0.886044353	0.375593627	0.543743133	up_in_case
Yang2025_S	ENSGALG00010000142	ENSGALG00010000142	case	control	-1.427685544	0.530772132	-2.689827625	0.007148894	0.03143408	down_in_case
Yang2025_S	ENSGALG00010000143	SCARF2	case	control	0.056352033	0.14327943	0.39330163	0.694096715	0.810002321	up_in_case
Yang2025_S	ENSGALG00010000145	ENSGALG00010000145	case	control	0.221546852	0.164669342	1.345404368	0.178494685	0.330015051	up_in_case
Yang2025_S	ENSGALG00010000146	ACADM	case	control	0.067444459	0.153401849	0.439658708	0.660184313	0.785356702	up_in_case
Yang2025_S	ENSGALG00010000147	ENSGALG00010000147	case	control	0.702927475	0.223356698	3.14710721	0.001648945	0.010341155	up_in_case
Yang2025_S	ENSGALG00010000148	ENSGALG00010000148	case	control	0.605268857	0.198856829	3.043741868	0.002336555	0.013471233	up_in_case
Yang2025_S	ENSGALG00010000149	ENSGALG00010000149	case	control	0.537583891	0.119148762	4.511871389	6.42581E-06	0.000115031	up_in_case
Yang2025_S	ENSGALG00010000150	B3GAT3	case	control	0.240626191	0.235800656	1.020464467	0.307508233	0.477132527	up_in_case
Yang2025_S	ENSGALG00010000151	TBX5	case	control	0.758347317	0.317057794	2.391826765	0.016764753	0.059626553	up_in_case
Yang2025_S	ENSGALG00010000153	NF2	case	control	0.218396003	0.101070877	2.160820295	0.030709224	0.093312916	up_in_case
Yang2025_S	ENSGALG00010000154	ENSGALG00010000154	case	control	-11.40862362	2.949550013	-3.867920045	0.000109768	0.001144063	down_in_case
Yang2025_S	ENSGALG00010000155	MAZ	case	control	0.001564309	0.0812847	0.019244819	0.984645804	0.990868839	up_in_case
Yang2025_S	ENSGALG00010000156	ENSGALG00010000156	case	control	1.140713162	0.556403096	2.050156029	0.040349207	0.113911305	up_in_case
Yang2025_S	ENSGALG00010000157	ENSGALG00010000157	case	control	-6.108102965	2.588804729	-2.359429778	0.018303045	0.063477582	down_in_case
Yang2025_S	ENSGALG00010000161	CDC42EP2	case	control	0.161097857	0.17124514	0.940744111	0.346836008	0.515317174	up_in_case
Yang2025_S	ENSGALG00010000166	PFDN6	case	control	1.118301148	0.189149143	5.912271826	3.37421E-09	1.74794E-07	up_in_case
Yang2025_S	ENSGALG00010000171	DPF2	case	control	0.08576513	0.096517058	0.888600748	0.374217689	0.542240147	up_in_case
Yang2025_S	ENSGALG00010000172	ENSGALG00010000172	case	control	0.280522477	0.904378826	0.310182491	0.756422184	0.852063222	up_in_case
Yang2025_S	ENSGALG00010000173	NIPSNAP1	case	control	0.099351539	0.270233531	0.367650669	0.713133729	0.822771185	up_in_case
Yang2025_S	ENSGALG00010000174	ENSGALG00010000174	case	control	-0.056257598	0.258941674	-0.217259728	0.82800594	0.897011799	down_in_case
Yang2025_S	ENSGALG00010000176	ENSGALG00010000176	case	control	1.89353715	0.515398741	3.673926609	0.000238851	0.002161295	up_in_case
Yang2025_S	ENSGALG00010000177	ENSGALG00010000177	case	control	0.167651072	0.188158911	0.891007879	0.372924943	0.541179069	up_in_case
Yang2025_S	ENSGALG00010000178	ENSGALG00010000178	case	control	2.036501144	0.990920212	2.055161575	0.039863401	0.113089677	up_in_case
Yang2025_S	ENSGALG00010000184	ENSGALG00010000184	case	control	1.242885904	2.980778434	0.416966887	0.676702628	0.796843935	up_in_case
Yang2025_S	ENSGALG00010000186	THOC5	case	control	-0.026318183	0.080944866	-0.32513715	0.745077274	0.844536167	down_in_case
Yang2025_S	ENSGALG00010000188	ENSGALG00010000188	case	control	-1.040291047	2.219552004	-0.468694153	0.639288267	0.769251546	down_in_case
Yang2025_S	ENSGALG00010000189	ENSGALG00010000189	case	control	1.059336782	0.79127171	1.338777526	0.180643109	0.33292709	up_in_case
Yang2025_S	ENSGALG00010000191	NEFH	case	control	0.431883947	0.3667267	1.177672495	0.238927196	0.403141511	up_in_case
Yang2025_S	ENSGALG00010000192	ENSGALG00010000192	case	control	-0.305747747	0.575484177	-0.531287843	0.595219329	0.735121984	down_in_case
Yang2025_S	ENSGALG00010000196	KSR2	case	control	0.662193002	0.891222568	0.74301642			up_in_case
Yang2025_S	ENSGALG00010000197	ENSGALG00010000197	case	control	0.006805684	0.716750716	0.00949519	0.992424049	0.995042536	up_in_case
Yang2025_S	ENSGALG00010000200	GAL3ST1	case	control	0.481948334	0.298950584	1.612133776	0.106932832	0.229742371	up_in_case
Yang2025_S	ENSGALG00010000202	ENSGALG00010000202	case	control	0.233215787	0.305844424	0.76253078	0.44574328	0.608665615	up_in_case
Yang2025_S	ENSGALG00010000205	RNF215	case	control	0.604588502	0.166062586	3.64072676	0.00027187	0.002399305	up_in_case
Yang2025_S	ENSGALG00010000210	CCDC157	case	control	-0.563178251	0.409640486	-1.374811013	0.169190043	0.317830041	down_in_case
Yang2025_S	ENSGALG00010000215	SF3A1	case	control	0.024341552	0.131428291	0.18520786	0.853066005	0.913297079	up_in_case
Yang2025_S	ENSGALG00010000221	TBC1D10A	case	control	0.122671556	0.153197638	0.800740514	0.423281883	0.588714652	up_in_case
Yang2025_S	ENSGALG00010000223	CASTOR1	case	control	0.211979724	0.158822776	1.33469348	0.181976688	0.334676673	up_in_case
Yang2025_S	ENSGALG00010000224	MYBPC2	case	control	-0.640568658	0.233538198	-2.742886025	0.006090182	0.027759119	down_in_case
Yang2025_S	ENSGALG00010000225	LIF	case	control	-0.189457219	0.44286387	-0.427800125	0.668796646	0.791103443	down_in_case
Yang2025_S	ENSGALG00010000226	ENSGALG00010000226	case	control	0.402557212	0.373838126	1.076822252	0.281559682	0.449482664	up_in_case
Yang2025_S	ENSGALG00010000227	ENSGALG00010000227	case	control	0.294948706	0.370260054	0.796598776	0.425684089	0.590379098	up_in_case
Yang2025_S	ENSGALG00010000230	ENSGALG00010000230	case	control	0.830701337	0.67381302	1.232836577	0.217636742	0.378575459	up_in_case
Yang2025_S	ENSGALG00010000231	ENSGALG00010000231	case	control	0.338263245	0.232973219	1.45194047	0.146518158	0.288176505	up_in_case
Yang2025_S	ENSGALG00010000232	ENSGALG00010000232	case	control	-0.180552626	0.350012592	-0.515846088	0.60596191	0.743766699	down_in_case
Yang2025_S	ENSGALG00010000233	HORMAD2	case	control	0.882609791	0.395456353	2.231876626	0.025623118	0.08139005	up_in_case
Yang2025_S	ENSGALG00010000234	ENSGALG00010000234	case	control	-10.48913441	2.950148921	-3.555459296	0.000377319	0.003153859	down_in_case
Yang2025_S	ENSGALG00010000239	ENSGALG00010000239	case	control	-13.05928198	2.948966818	-4.428426222			down_in_case
Yang2025_S	ENSGALG00010000241	ENSGALG00010000241	case	control	0.006884932	0.542961305	0.012680337	0.989882826	0.993843973	up_in_case
Yang2025_S	ENSGALG00010000242	ENSGALG00010000242	case	control	0.522100316	0.332290446	1.571216755	0.116132309	0.243793873	up_in_case
Yang2025_S	ENSGALG00010000246	ENSGALG00010000246	case	control	-6.340068357	2.681165428	-2.364668845	0.018046199	0.062915633	down_in_case
Yang2025_S	ENSGALG00010000247	ENSGALG00010000247	case	control	0.594692176	0.246530797	2.412242947	0.015854713	0.057356029	up_in_case
Yang2025_S	ENSGALG00010000249	ENSGALG00010000249	case	control	-12.18903791	2.949226929	-4.132960334	3.58121E-05	0.000468583	down_in_case
Yang2025_S	ENSGALG00010000254	ENSGALG00010000254	case	control	0.610232216	0.551140316	1.107217524	0.268199893	0.435291995	up_in_case
Yang2025_S	ENSGALG00010000255	ENSGALG00010000255	case	control	3.131638189	0.254332444	12.31316831	7.69423E-35	1.5317E-31	up_in_case
Yang2025_S	ENSGALG00010000256	ENSGALG00010000256	case	control	-0.051830662	0.351968433	-0.147259404	0.882927274	0.931020944	down_in_case
Yang2025_S	ENSGALG00010000262	HNRNPKL	case	control	-11.49829687	2.948772813	-3.899349865			down_in_case
Yang2025_S	ENSGALG00010000263	MTMR3	case	control	-0.161485251	0.078897856	-2.046763501	0.040681311	0.114570345	down_in_case
Yang2025_S	ENSGALG00010000267	ENSGALG00010000267	case	control	0.767937592	0.167436992	4.586427295	4.50895E-06	8.47939E-05	up_in_case
Yang2025_S	ENSGALG00010000269	ENSGALG00010000269	case	control	-0.931856917	0.419843796	-2.219532423	0.026450524	0.083409833	down_in_case
Yang2025_S	ENSGALG00010000271	RTN4R	case	control	0.133753734	0.379133756	0.352787721	0.72424759	0.830512686	up_in_case
Yang2025_S	ENSGALG00010000272	ENSGALG00010000272	case	control	0.179584009	0.655908901	0.273794134	0.784242832	0.869543592	up_in_case
Yang2025_S	ENSGALG00010000279	ENSGALG00010000279	case	control	-0.342996106	0.170183862	-2.015444359	0.043858111	0.121394834	down_in_case
Yang2025_S	ENSGALG00010000281	OSBP2	case	control	0.075023073	0.112588903	0.666345178	0.505190495	0.660768683	up_in_case
Yang2025_S	ENSGALG00010000282	ENSGALG00010000282	case	control	-12.23875969	2.949270364	-4.149758477	3.32826E-05	0.000442246	down_in_case
Yang2025_S	ENSGALG00010000283	ENSGALG00010000283	case	control	-0.313343173	0.56342677	-0.556138241	0.578116354	0.72096397	down_in_case
Yang2025_S	ENSGALG00010000284	ENSGALG00010000284	case	control	0.391062634	0.287989272	1.357906951	0.174493212	0.32490151	up_in_case
Yang2025_S	ENSGALG00010000285	MYLPF	case	control	-0.20371243	0.30989636	-0.65735664	0.510951625	0.666302723	down_in_case
Yang2025_S	ENSGALG00010000286	TCN2	case	control	0.233723254	0.190635752	1.226020045	0.220191132	0.381637241	up_in_case
Yang2025_S	ENSGALG00010000287	IQCD	case	control	-0.200257982	0.253804311	-0.789025139	0.430097314	0.59424881	down_in_case
Yang2025_S	ENSGALG00010000288	VARS2	case	control	0.007682396	0.116465057	0.0659631	0.947407204	0.967543554	up_in_case
Yang2025_S	ENSGALG00010000289	QPRT	case	control	1.267900911	0.460409943	2.753852152	0.005889837	0.027008535	up_in_case
Yang2025_S	ENSGALG00010000292	ABHD16A	case	control	0.421056012	0.263924246	1.595366922	0.110630208	0.235327729	up_in_case
Yang2025_S	ENSGALG00010000293	ENSGALG00010000293	case	control	0.348653956	0.254183107	1.371664546	0.17016789	0.319107731	up_in_case
Yang2025_S	ENSGALG00010000294	DUSP21	case	control	-0.003095111	0.332690829	-0.009303266	0.992577175	0.99507647	down_in_case
Yang2025_S	ENSGALG00010000298	SLC35E4	case	control	0.915496823	0.240037311	3.813977158	0.000136748	0.001374881	up_in_case
Yang2025_S	ENSGALG00010000301	UQCR10	case	control	0.270590588	0.275929461	0.98065131	0.326764722	0.496633126	up_in_case
Yang2025_S	ENSGALG00010000302	ENSGALG00010000302	case	control	-0.427847763	0.15251874	-2.805214383	0.005028312	0.024078877	down_in_case
Yang2025_S	ENSGALG00010000303	ENSGALG00010000303	case	control	-0.04649623	0.102615256	-0.453112259	0.650467908	0.778316186	down_in_case
Yang2025_S	ENSGALG00010000306	ENSGALG00010000306	case	control	0.753257399	0.111620962	6.748350718	1.49535E-11	1.51794E-09	up_in_case
Yang2025_S	ENSGALG00010000307	ZMAT5	case	control	0.261580604	0.227425978	1.150179086	0.250070118	0.415888185	up_in_case
Yang2025_S	ENSGALG00010000308	ENSGALG00010000308	case	control	1.538278303	0.323184626	4.759750871	1.93832E-06	4.22699E-05	up_in_case
Yang2025_S	ENSGALG00010000309	ENSGALG00010000309	case	control	-12.77069971	2.949120537	-4.330341725	1.48878E-05	0.000228491	down_in_case
Yang2025_S	ENSGALG00010000312	CABP7	case	control	1.771115227	0.504732258	3.509019285	0.000449762	0.003626989	up_in_case
Yang2025_S	ENSGALG00010000313	ENSGALG00010000313	case	control	-7.965904443	2.47052119	-3.224382157	0.001262448	0.008397238	down_in_case
Yang2025_S	ENSGALG00010000318	ENSGALG00010000318	case	control	-12.6259732	2.949177224	-4.281184968	1.85901E-05	0.000273262	down_in_case
Yang2025_S	ENSGALG00010000323	AP1B1	case	control	-0.19605781	0.100930068	-1.942511428	0.052075218	0.137411127	down_in_case
Yang2025_S	ENSGALG00010000324	ENSGALG00010000324	case	control	-0.241336887	0.164010032	-1.471476377	0.141162332	0.280475777	down_in_case
Yang2025_S	ENSGALG00010000326	ENSGALG00010000326	case	control	-5.577937673	1.367435508	-4.07912303	4.52059E-05	0.000564466	down_in_case
Yang2025_S	ENSGALG00010000331	ENSGALG00010000331	case	control	0.423765703	0.164745858	2.572238894	0.010104314	0.040812643	up_in_case
Yang2025_S	ENSGALG00010000338	ENSGALG00010000338	case	control	1.067642041	0.511709472	2.086422277	0.036940389	0.106886279	up_in_case
Yang2025_S	ENSGALG00010000340	NAA10	case	control	-0.03152822	0.15092544	-0.208899308	0.834526844	0.90197251	down_in_case
Yang2025_S	ENSGALG00010000341	EWSR1	case	control	0.898010628	0.171922394	5.223348795	1.75716E-07	5.53982E-06	up_in_case
Yang2025_S	ENSGALG00010000346	ENSGALG00010000346	case	control	-0.187718224	0.126387754	-1.485256435	0.137475907	0.275169027	down_in_case
Yang2025_S	ENSGALG00010000349	CHIR-IG1-5	case	control	-0.028080553	0.344642572	-0.081477318	0.935062362	0.960213266	down_in_case
Yang2025_S	ENSGALG00010000350	ENSGALG00010000350	case	control	-0.172841466	0.145979095	-1.184015184	0.236407052	0.400088933	down_in_case
Yang2025_S	ENSGALG00010000353	ENSGALG00010000353	case	control	-1.088366051	0.336813727	-3.231358949	0.001232031	0.008236367	down_in_case
Yang2025_S	ENSGALG00010000356	ENSGALG00010000356	case	control	0.617856799	0.145007993	4.260846494	2.03654E-05	0.000295309	up_in_case
Yang2025_S	ENSGALG00010000366	EMID1	case	control	0.35763596	0.341369677	1.047650054	0.294799867	0.463504022	up_in_case
Yang2025_S	ENSGALG00010000367	HSD17B10	case	control	-0.599937341	0.274139111	-2.18844126	0.028637476	0.088386096	down_in_case
Yang2025_S	ENSGALG00010000376	ASCC2	case	control	0.266081163	0.132305193	2.011116554	0.044313147	0.122341497	up_in_case
Yang2025_S	ENSGALG00010000377	ENSGALG00010000377	case	control	1.015515621	0.31716791	3.201823353	0.001365607	0.008950957	up_in_case
Yang2025_S	ENSGALG00010000381	ENSGALG00010000381	case	control	0.665872989	0.267910797	2.485427968	0.012939577	0.049118226	up_in_case
Yang2025_S	ENSGALG00010000382	ENSGALG00010000382	case	control	2.852188526	0.346237333	8.237668942	1.75598E-16	5.09784E-14	up_in_case
Yang2025_S	ENSGALG00010000384	ENSGALG00010000384	case	control	0.150331147	0.171550466	0.876308588	0.380862298	0.548726844	up_in_case
Yang2025_S	ENSGALG00010000386	ENSGALG00010000386	case	control	-1.782291765	0.234259462	-7.608195409	2.77949E-14	5.09635E-12	down_in_case
Yang2025_S	ENSGALG00010000387	DTX1	case	control	0.039848794	0.517987929	0.076929966	0.938679258	0.962878892	up_in_case
Yang2025_S	ENSGALG00010000388	ENSGALG00010000388	case	control	0.232746725	0.078080317	2.98086295	0.002874374	0.015769449	up_in_case
Yang2025_S	ENSGALG00010000402	PFKFB1	case	control	0.165428127	0.224705288	0.736200417	0.461608738	0.622582053	up_in_case
Yang2025_S	ENSGALG00010000405	ENSGALG00010000405	case	control	1.135985201	0.299264073	3.7959291	0.000147092	0.001457838	up_in_case
Yang2025_S	ENSGALG00010000406	NSMCE3	case	control	-0.209966094	0.212050515	-0.990170164	0.322090953	0.491441742	down_in_case
Yang2025_S	ENSGALG00010000407	WSB2	case	control	-0.402486803	0.228309825	-1.762897429	0.077917791	0.18392589	down_in_case
Yang2025_S	ENSGALG00010000408	ENSGALG00010000408	case	control	1.0025497	0.162613284	6.165238631	7.03769E-10	4.37814E-08	up_in_case
Yang2025_S	ENSGALG00010000412	RFC5	case	control	0.15123509	0.198445319	0.762099557	0.446000587	0.608957298	up_in_case
Yang2025_S	ENSGALG00010000422	ITIH6	case	control	1.44766189	0.511639814	2.829455116	0.004662734	0.022734499	up_in_case
Yang2025_S	ENSGALG00010000423	ENSGALG00010000423	case	control	-0.21267746	0.200540855	-1.060519366	0.288908384	0.45775308	down_in_case
Yang2025_S	ENSGALG00010000428	GNL3L	case	control	0.156900096	0.174259325	0.900382785	0.367916579	0.536512927	up_in_case
Yang2025_S	ENSGALG00010000431	AIFM3	case	control	2.130044709	0.738817921	2.883044184	0.003938522	0.019935817	up_in_case
Yang2025_S	ENSGALG00010000432	ENSGALG00010000432	case	control	0.141534062	0.09156346	1.545748291	0.12216537	0.252990701	up_in_case
Yang2025_S	ENSGALG00010000434	TP53	case	control	0.000639229	0.227332795	0.002811866	0.997756458	0.998289914	up_in_case
Yang2025_S	ENSGALG00010000435	P2RX6	case	control	0.818067553	0.390453828	2.095171043	0.03615579	0.105227849	up_in_case
Yang2025_S	ENSGALG00010000436	ENSGALG00010000436	case	control	0.216450238	0.237668367	0.910723797	0.362440922	0.53114042	up_in_case
Yang2025_S	ENSGALG00010000440	ENSGALG00010000440	case	control	1.192768705	0.468140568	2.5478858	0.010837794	0.042977992	up_in_case
Yang2025_S	ENSGALG00010000441	SLC7A4	case	control	-0.187668559	0.237464238	-0.79030241	0.42935118	0.59361134	down_in_case
Yang2025_S	ENSGALG00010000442	DGCR6	case	control	0.125089605	0.182479825	0.685498274	0.493029549	0.650479717	up_in_case

Yang2025_S	ENSGALG00010000443	TBX3	case	control	0.425804585	0.183239602	2.323758515	0.02013844	0.068031313	up_in_case
Yang2025_S	ENSGALG00010000444	RBM19	case	control	0.198670766	0.094930741	2.092796959	0.036367283	0.105622779	up_in_case
Yang2025_S	ENSGALG00010000449	PLBD2	case	control	-0.139319024	0.110626042	-1.259369151	0.207897026	0.366668151	down_in_case
Yang2025_S	ENSGALG00010000450	ENSGALG00010000450	case	control	-0.490728197	0.291142777	-1.685524202	0.091887479	0.206891586	down_in_case
Yang2025_S	ENSGALG00010000452	SLC8B1	case	control	0.189295208	0.156895668	1.206503727	0.227623323	0.389873884	up_in_case
Yang2025_S	ENSGALG00010000453	ACHE	case	control	0.182238274	0.221738879	0.821859815	0.411156686	0.576579292	up_in_case
Yang2025_S	ENSGALG00010000454	ENSGALG00010000454	case	control	1.541770605	0.899866828	1.713331969	0.086651484	0.198306525	up_in_case
Yang2025_S	ENSGALG00010000457	TPCN1	case	control	0.017930539	0.106590912	0.168218275	0.866411564	0.921496348	up_in_case
Yang2025_S	ENSGALG00010000458	ENSGALG00010000458	case	control	0.759073422	0.099042608	7.664109812	1.80076E-14	3.48522E-12	up_in_case
Yang2025_S	ENSGALG00010000462	ENSGALG00010000462	case	control	0.290771765	0.379379211	0.766440956	0.443413971	0.606673902	up_in_case
Yang2025_S	ENSGALG00010000465	ENSGALG00010000465	case	control	-0.04242066	0.264125575	-0.160607925	0.872402213	0.925114134	down_in_case
Yang2025_S	ENSGALG00010000466	FBXO21	case	control	-0.060066351	0.100063514	-0.600282249	0.548318147	0.697154505	down_in_case
Yang2025_S	ENSGALG00010000467	RNASEK	case	control	-0.219350735	0.207569373	-1.056758675	0.290621739	0.459190859	down_in_case
Yang2025_S	ENSGALG00010000468	TESC	case	control	0.093466509	0.341205379	0.273930349	0.784138148	0.869496705	up_in_case
Yang2025_S	ENSGALG00010000469	ENSGALG00010000469	case	control	-0.136728825	0.192674999	-0.709634495	0.477930823	0.636951608	down_in_case
Yang2025_S	ENSGALG00010000470	FBXW8	case	control	0.176686835	0.106837181	1.653795362	0.098169099	0.216214065	up_in_case
Yang2025_S	ENSGALG00010000471	ENSGALG00010000471	case	control	0.824549984	0.184888585	4.45971277	8.20696E-06	0.000139129	up_in_case
Yang2025_S	ENSGALG00010000475	RNFT2	case	control	0.11241588	0.139289269	0.807067771	0.419627439	0.585277587	up_in_case
Yang2025_S	ENSGALG00010000476	ENSGALG00010000476	case	control	-0.232162789	0.265764234	-0.873566717	0.382354258	0.55019688	down_in_case
Yang2025_S	ENSGALG00010000479	C12orf49	case	control	-0.441668799	0.192667932	-2.292383552	0.02188352	0.072269491	down_in_case
Yang2025_S	ENSGALG00010000483	ENSGALG00010000483	case	control	0.457185921	0.183644303	2.48951867	0.01279162	0.048609551	up_in_case
Yang2025_S	ENSGALG00010000485	MED13L	case	control	-0.053623323	0.182135028	-0.29441521	0.768440646	0.858994096	down_in_case
Yang2025_S	ENSGALG00010000486	ENSGALG00010000486	case	control	1.111572359	0.393102299	2.827692341	0.004688484	0.022828276	up_in_case
Yang2025_S	ENSGALG00010000491	ENSGALG00010000491	case	control	0.337857965	0.248233846	1.361047137	0.173498794	0.323482164	up_in_case
Yang2025_S	ENSGALG00010000495	ENSGALG00010000495	case	control	-0.212229445	0.479365586	-0.442729832	0.657961162	0.784251885	down_in_case
Yang2025_S	ENSGALG00010000501	ENSGALG00010000501	case	control	0.453129221	0.117906396	3.843126714	0.000121477	0.001242862	up_in_case
Yang2025_S	ENSGALG00010000507	TRPT1	case	control	0.567142053	0.314025697	1.806037083	0.070912547	0.171526878	up_in_case
Yang2025_S	ENSGALG00010000511	FERMT3	case	control	0.708970257	0.230694445	3.073200381	0.002117762	0.01249408	up_in_case
Yang2025_S	ENSGALG00010000525	ENSGALG00010000525	case	control	-2.032053707	0.584176686	-3.478491619	0.000504244	0.003983358	down_in_case
Yang2025_S	ENSGALG00010000526	POLD1	case	control	0.140616017	0.152785856	0.920347081	0.357391413	0.526564743	up_in_case
Yang2025_S	ENSGALG00010000529	STIP1	case	control	0.324430058	0.077250031	4.199740169	2.67221E-05	0.000369785	up_in_case
Yang2025_S	ENSGALG00010000532	ENSGALG00010000532	case	control	-0.405221177	0.5010364	-0.808765945	0.418649788	0.584264877	down_in_case
Yang2025_S	ENSGALG00010000555	OTUB1	case	control	-0.005184732	0.198134024	-0.026167802	0.979123497	0.987413948	down_in_case
Yang2025_S	ENSGALG00010000561	ENSGALG00010000561	case	control	-0.263086846	0.220723328	-1.191930408	0.233288534	0.396422324	down_in_case
Yang2025_S	ENSGALG00010000564	NAA40	case	control	0.037767071	0.191050605	0.197680982	0.84329467	0.907018562	up_in_case
Yang2025_S	ENSGALG00010000569	SIPA1	case	control	0.638856509	0.184604603	3.460674864	0.000538823	0.004206443	up_in_case
Yang2025_S	ENSGALG00010000574	ENSGALG00010000574	case	control	0.467047426	0.192081025	2.431512568	0.015035927	0.05515284	up_in_case
Yang2025_S	ENSGALG00010000575	ENSGALG00010000575	case	control	-0.636953118	0.225255648	-2.827689891	0.00468852	0.022828276	down_in_case
Yang2025_S	ENSGALG00010000576	NOS1	case	control	-0.21400699	0.284417268	-0.752440213	0.451786371	0.613616804	down_in_case
Yang2025_S	ENSGALG00010000577	ENSGALG00010000577	case	control	1.186629785	0.36901307	3.215684973	0.001301336	0.008614782	up_in_case
Yang2025_S	ENSGALG00010000579	ENSGALG00010000579	case	control	1.617943174	0.598103086	2.705124272	0.006827884	0.030378852	up_in_case
Yang2025_S	ENSGALG00010000580	ENSGALG00010000580	case	control	-0.073370399	0.528693476	-0.138776818	0.889626513	0.935481114	down_in_case
Yang2025_S	ENSGALG00010000581	ENSGALG00010000581	case	control	0.558919785	0.067734194	8.251663598	1.56205E-16	4.6313E-14	up_in_case
Yang2025_S	ENSGALG00010000583	CCS	case	control	1.499638792	0.191784754	7.819384826	5.30821E-15	1.19306E-12	up_in_case
Yang2025_S	ENSGALG00010000584	RBM14	case	control	0.198655924	0.12097333	1.642146444	0.100559659	0.219880565	up_in_case
Yang2025_S	ENSGALG00010000585	SPTBN2	case	control	1.758659534	0.573674438	3.065605539	0.002172297	0.012734942	up_in_case
Yang2025_S	ENSGALG00010000586	ENSGALG00010000586	case	control	1.148081352	0.623064861	1.84263537	0.065382266	0.16177235	up_in_case
Yang2025_S	ENSGALG00010000589	ENSGALG00010000589	case	control	1.233937755	0.333845449	3.696134718	0.000218907	0.002008207	up_in_case
Yang2025_S	ENSGALG00010000590	DNAH5	case	control	-0.690392464	0.47688631	-1.44770871	0.147698533	0.289842325	down_in_case
Yang2025_S	ENSGALG00010000591	SBK2	case	control	-0.370499367	0.334643904	-1.107145124	0.268231189	0.435291995	down_in_case
Yang2025_S	ENSGALG00010000592	CCT5	case	control	0.126227111	0.10297022	1.225860362	0.220251227	0.381644323	up_in_case
Yang2025_S	ENSGALG00010000593	ATPSCKMT	case	control	-0.461920161	0.248247466	-1.860724577	0.062783081	0.157183298	down_in_case
Yang2025_S	ENSGALG00010000595	MYO10	case	control	0.035386592	0.214820081	0.164726649	0.869159141	0.922939315	up_in_case
Yang2025_S	ENSGALG00010000596	ENSGALG00010000596	case	control	1.479487536	0.981278438	1.507714302	0.131627663	0.266216472	up_in_case
Yang2025_S	ENSGALG00010000598	ENSGALG00010000598	case	control	1.948227201	0.647801927	3.007442737	0.002634558	0.014732167	up_in_case
Yang2025_S	ENSGALG00010000599	RETREG1	case	control	-0.446850209	0.145322758	-3.07488116	0.002105864	0.012439687	down_in_case
Yang2025_S	ENSGALG00010000601	ENSGALG00010000601	case	control	0.136673965	0.316900609	0.431283378	0.666262335	0.789218432	up_in_case
Yang2025_S	ENSGALG00010000602	ENSGALG00010000602	case	control	0.213302125	0.513994333	0.414989255	0.678149769	0.797865799	up_in_case
Yang2025_S	ENSGALG00010000603	ZNF622	case	control	0.232734105	0.137438973	1.693363242	0.090386347	0.204303933	up_in_case
Yang2025_S	ENSGALG00010000604	ENSGALG00010000604	case	control	-0.126973199	0.16305824	-0.778698449	0.43615737	0.600044718	down_in_case
Yang2025_S	ENSGALG00010000605	MARCHF11	case	control	1.148527992	0.819981946	1.400674731	0.161311362	0.307128547	up_in_case
Yang2025_S	ENSGALG00010000606	UBE2QL1	case	control	-0.161508847	0.104645715	-1.543387104	0.122736881	0.253654235	down_in_case
Yang2025_S	ENSGALG00010000607	ENSGALG00010000607	case	control	-0.015279266	0.262298522	-0.058251438	0.953548348	0.971460828	down_in_case
Yang2025_S	ENSGALG00010000608	ENSGALG00010000608	case	control	0.096957615	0.844442458	0.114818498	0.908588987	0.945305771	up_in_case
Yang2025_S	ENSGALG00010000609	ENSGALG00010000609	case	control	3.175786991	1.178441529	2.694904171	0.007040888	0.031108044	up_in_case
Yang2025_S	ENSGALG00010000610	ENSGALG00010000610	case	control	-0.606283034	0.204930259	-2.958484688	0.003091556	0.016691528	down_in_case
Yang2025_S	ENSGALG00010000611	C7orf57	case	control	0.084598185	0.332292334	0.254589639	0.799040063	0.878430916	up_in_case
Yang2025_S	ENSGALG00010000612	ENSGALG00010000612	case	control	0.10401446	0.356207576	0.292005188	0.770282657	0.859931005	up_in_case
Yang2025_S	ENSGALG00010000613	ENSGALG00010000613	case	control	0.222305142	0.150554619	1.476574705	0.139789676	0.278559866	up_in_case
Yang2025_S	ENSGALG00010000614	KAT8	case	control	-0.393845942	0.177184314	-2.222803666	0.026229042	0.082899001	down_in_case
Yang2025_S	ENSGALG00010000616	PSENEN	case	control	-0.428545752	0.297429161	-1.440833002	0.149631867	0.292088658	down_in_case
Yang2025_S	ENSGALG00010000619	ENSGALG00010000619	case	control	1.42158921	0.254822932	5.578733428	2.42276E-08	1.0048E-06	up_in_case
Yang2025_S	ENSGALG00010000621	UPP1	case	control	-0.462050559	0.267506451	-1.727250159	0.084122746	0.194162473	down_in_case
Yang2025_S	ENSGALG00010000623	ENSGALG00010000623	case	control	-0.132588188	0.164620323	-0.805418098	0.420578451	0.586310596	down_in_case
Yang2025_S	ENSGALG00010000625	ENSGALG00010000625	case	control	0.627299443	0.242632903	2.585384897	0.009727034	0.039737973	up_in_case
Yang2025_S	ENSGALG00010000626	ABCA13	case	control	1.100509507	0.633344912	1.737614822	0.082278706	0.190964985	up_in_case
Yang2025_S	ENSGALG00010000627	ENSGALG00010000627	case	control	1.1924692	0.397182309	3.002322043	0.002679286	0.014934339	up_in_case
Yang2025_S	ENSGALG00010000628	ENSGALG00010000628	case	control	0.219660184	0.501416115	0.438079626	0.661328563	0.786223072	up_in_case
Yang2025_S	ENSGALG00010000630	ENSGALG00010000630	case	control	0.665816908	0.382315203	1.741539189	0.08158911	0.189892397	up_in_case
Yang2025_S	ENSGALG00010000632	ENSGALG00010000632	case	control	0.452947772	0.61898925	0.731753858	0.464318817	0.625329344	up_in_case
Yang2025_S	ENSGALG00010000633	VWC2	case	control	0.148202155	0.573097064	0.258598699	0.795944887	0.876587085	up_in_case
Yang2025_S	ENSGALG00010000634	ENSGALG00010000634	case	control	0.858775435	0.342387133	2.508200081	0.012134793	0.046789802	up_in_case
Yang2025_S	ENSGALG00010000635	ENSGALG00010000635	case	control	0.200576129	0.201129807	0.997247162	0.318644554	0.488175677	up_in_case
Yang2025_S	ENSGALG00010000636	ENSGALG00010000636	case	control	0.65186114	0.146126286	4.460943744	8.15995E-06	0.000138669	up_in_case
Yang2025_S	ENSGALG00010000639	SCN1B	case	control	0.021062662	0.197412966	0.106693406	0.915032214	0.950120376	up_in_case
Yang2025_S	ENSGALG00010000641	ENSGALG00010000641	case	control	0.34841578	0.324601467	1.073364771	0.283107484	0.451384759	up_in_case
Yang2025_S	ENSGALG00010000643	VKORC1	case	control	-0.201463717	0.287765869	-0.70009594	0.483867391	0.641978329	down_in_case
Yang2025_S	ENSGALG00010000645	ENSGALG00010000645	case	control	1.033280427	0.550239232	1.87787487	0.060398285	0.153208018	up_in_case
Yang2025_S	ENSGALG00010000646	ENSGALG00010000646	case	control	-1.044081179	0.55618921	-1.877205024	0.060489999	0.153371204	down_in_case
Yang2025_S	ENSGALG00010000647	ENSGALG00010000647	case	control	0.418273969	0.843530796	0.495860934	0.61999253	0.755341485	up_in_case
Yang2025_S	ENSGALG00010000649	PAPD7	case	control	-0.161150334	0.087927567	-1.832762342	0.066837922	0.164468733	down_in_case
Yang2025_S	ENSGALG00010000650	BCKDK	case	control	0.360827522	0.216953468	1.66315628	0.096281157	0.213622349	up_in_case
Yang2025_S	ENSGALG00010000651	ENSGALG00010000651	case	control	0.718362757	0.324136501	2.216235307	0.026675391	0.083853276	up_in_case
Yang2025_S	ENSGALG00010000652	SRD5A1	case	control	0.305899026	0.164362632	1.861122706	0.06272685	0.157105699	up_in_case
Yang2025_S	ENSGALG00010000653	NSUN2	case	control	0.316097089	0.079179342	3.992166155	6.54725E-05	0.000760933	up_in_case
Yang2025_S	ENSGALG00010000654	ENSGALG00010000654	case	control	1.54194633	0.145507833	10.5969988	3.07696E-26	3.29827E-23	up_in_case
Yang2025_S	ENSGALG00010000655	ENSGALG00010000655	case	control	0.446878149	0.751413005	0.59471708	0.552032603	0.700088671	up_in_case
Yang2025_S	ENSGALG00010000656	ENSGALG00010000656	case	control	0.762995219	0.209424034	3.64330303	0.000269162	0.002384865	up_in_case
Yang2025_S	ENSGALG00010000659	ENSGALG00010000659	case	control	0.021220826	0.483554918	0.043885037	0.964996042	0.979253751	up_in_case
Yang2025_S	ENSGALG00010000660	ENSGALG00010000660	case	control	0.261434994	0.164335282	1.590863452	0.111640309	0.236969948	up_in_case
Yang2025_S	ENSGALG00010000661	ENSGALG00010000661	case	control	0.201632042	0.414039613	0.48698732	0.626267317	0.759929342	up_in_case
Yang2025_S	ENSGALG00010000670	ENSGALG00010000670	case	control	0.059862399	0.335710015	0.178315798	0.858474973	0.91669339	up_in_case
Yang2025_S	ENSGALG00010000674	GRB10	case	control	-0.299956281	0.107218823	-2.797608405	0.005148249	0.024518403	down_in_case
Yang2025_S	ENSGALG00010000684	COBL	case	control	-0.257926499	0.178981169	-1.441081764	0.149561585	0.292019151	down_in_case
Yang2025_S	ENSGALG00010000685	ENSGALG00010000685	case	control	-0.031533437	0.4849328	-0.065026405	0.948152976	0.967921209	down_in_case
Yang2025_S	ENSGALG00010000686	ENSGALG00010000686	case	control	0.31720685	0.166678364	1.903107529	0.057026516	0.147051165	up_in_case
Yang2025_S	ENSGALG00010000687	IKZF1	case	control	-0.426730794	0.311519257	-1.369837612	0.170737598	0.319726284	down_in_case
Yang2025_S	ENSGALG00010000688	ENSGALG00010000688	case	control	0.786180914	0.35321517	2.22578468	0.026028607	0.082377616	up_in_case
Yang2025_S	ENSGALG00010000695	FIGNL1	case	control	-0.154878935	0.1977832	-0.783074272	0.433583498	0.597624733	down_in_case
Yang2025_S	ENSGALG00010000697	ENSGALG00010000697	case	control	0.897721184	0.281647721	3.187390193	0.00143563	0.009296235	up_in_case
Yang2025_S	ENSGALG00010000702	DDC	case	control	0.446168456	0.367913251	1.212700153	0.225244481	0.386978405	up_in_case
Yang2025_S	ENSGALG00010000704	ENSGALG00010000704	case	control	-0.073351779	0.131925202	-0.556010364	0.57820377	0.72100846	down_in_case
Yang2025_S	ENSGALG00010000708	ENSGALG00010000708	case	control	0.570110974	0.561224832	1.015833479	0.30970869	0.479585576	up_in_case
Yang2025_S	ENSGALG00010000710	ENSGALG00010000710	case	control	0.06119072	0.376416146	0.162561358	0.870863814	0.924115691	up_in_case
Yang2025_S	ENSGALG00010000711	SPRY2	case	control	-0.637662497	0.113497822	-5.618279611	1.92868E-08	8.34664E-07	down_in_case
Yang2025_S	ENSGALG00010000714	FBXL7	case	control	-0.164542224	0.102530702	-1.604809299	0.108535779	0.232183924	down_in_case
Yang2025_S	ENSGALG00010000715	PCDH20	case	control	-0.050360682	0.213765258	-0.235588713	0.813751833	0.88798996	down_in_case
Yang2025_S	ENSGALG00010000717	NDFIP2	case	control	-0.09420609	0.164519171	-0.572614664	0.566905629	0.711703646	down_in_case
Yang2025_S	ENSGALG00010000718	EEF1G	case	control	-0.093456696	0.122445942	-0.763248618	0.445315139	0.608402971	down_in_case
Yang2025_S	ENSGALG00010000720	RBM26	case	control	0.29790003	0.143395676	2.077468716	0.03775832	0.108506737	up_in_case
Yang2025_S	ENSGALG00010000721	ENSGALG00010000721	case	control	0.417854403	0.175098814	2.386391968	0.017014606	0.060223147	up_in_case
Yang2025_S	ENSGALG00010000722	ANKH	case	control	-0.220744019	0.141597842	-1.558950453	0.119008108	0.248111607	down_in_case
Yang2025_S	ENSGALG00010000724	OBI1	case	control	0.053274448	0.11834523	0.450161347	0.652594105	0.780123432	up_in_case
Yang2025_S	ENSGALG00010000727	ENSGALG00010000727	case	control	0.179875169	0.21774501	0.826081705	0.408757745	0.574475118	up_in_case
Yang2025_S	ENSGALG00010000728	OTULIN	case	control	0.563108026	0.265998749	2.116957426	0.034263461	0.101000311	up_in_case
Yang2025_S	ENSGALG00010000729	EDNRB	case	control	-0.565972673	0.137724146	-4.109465846	3.96575E-05	0.000509955	down_in_case

Yang2025_S	ENSGALG00010000730	INTS5	case	control	-0.27870449	0.152126499	-1.832057478	0.066942858	0.164668794	down_in_case
Yang2025_S	ENSGALG00010000731	ENSGALG00010000731	case	control	1.045493923	0.58014039	1.802139517	0.071523458	0.172525426	up_in_case
Yang2025_S	ENSGALG00010000732	ENSGALG00010000732	case	control	-0.096623301	0.302594216	-0.319316416	0.749486585	0.847783798	down_in_case
Yang2025_S	ENSGALG00010000734	SLAIN1	case	control	-0.595201885	0.342526977	-1.737678857	0.082267416	0.190964985	down_in_case
Yang2025_S	ENSGALG00010000735	ENSGALG00010000735	case	control	-1.740339819	0.932853651	-1.865608627			down_in_case
Yang2025_S	ENSGALG00010000737	ENSGALG00010000737	case	control	0.490763433	0.867343013	0.565823932	0.571513476	0.715439066	up_in_case
Yang2025_S	ENSGALG00010000741	FAM105A	case	control	0.034726492	0.141374515	0.245634736	0.805964998	0.883489148	up_in_case
Yang2025_S	ENSGALG00010000753	TRIO	case	control	0.126708812	0.195758972	0.647269499	0.517457538	0.671957021	up_in_case
Yang2025_S	ENSGALG00010000757	SCEL	case	control	-0.386499553	0.747058911	-0.517361546	0.604903798	0.74299995	down_in_case
Yang2025_S	ENSGALG00010000760	CDH10	case	control	0.185907353	0.228847027	0.812365163	0.416582129	0.581743963	up_in_case
Yang2025_S	ENSGALG00010000762	ENSGALG00010000762	case	control	-0.096194823	0.412079461	-0.233437557	0.815421652	0.889255026	down_in_case
Yang2025_S	ENSGALG00010000768	ENSGALG00010000768	case	control	-0.333131168	0.445027469	-0.748563158	0.454120545	0.615885683	down_in_case
Yang2025_S	ENSGALG00010000777	MYCBP2	case	control	-0.015633025	0.167805334	-0.093161667	0.925775128	0.95539335	down_in_case
Yang2025_S	ENSGALG00010000783	DAP	case	control	0.338058201	0.192941699	1.752126175	0.079752114	0.186969333	up_in_case
Yang2025_S	ENSGALG00010000784	FBXL3	case	control	-0.289686847	0.103699537	-2.793521134	0.005213762	0.024728991	down_in_case
Yang2025_S	ENSGALG00010000785	CLN5	case	control	-0.295037717	0.122458713	-2.409283187	0.01598389	0.057643765	down_in_case
Yang2025_S	ENSGALG00010000786	ANKRD33B	case	control	0.213054431	0.332739333	0.640304315	0.521974776	0.675243084	up_in_case
Yang2025_S	ENSGALG00010000787	SNRNP48	case	control	0.061781352	0.183275843	0.337094898	0.736045363	0.839001402	up_in_case
Yang2025_S	ENSGALG00010000788	GATD3AL1	case	control	-0.448287326	0.294365054	-1.522895871	0.127784759	0.260752763	down_in_case
Yang2025_S	ENSGALG00010000789	ROPN1L	case	control	1.952482377	0.374517966	5.213320993	1.8549E-07	5.83475E-06	up_in_case
Yang2025_S	ENSGALG00010000790	MARCHF6	case	control	-0.218751011	0.069195671	-3.161339529	0.001570453	0.009960977	down_in_case
Yang2025_S	ENSGALG00010000791	ACOD1	case	control	2.112222825	0.368769982	5.727751526	1.0177E-08	4.69593E-07	up_in_case
Yang2025_S	ENSGALG00010000793	ENSGALG00010000793	case	control	-0.765843493	1.183504241	-0.647098225	0.517568374	0.672033636	down_in_case
Yang2025_S	ENSGALG00010000795	ENSGALG00010000795	case	control	0.991380863	0.252426334	3.927406658	8.58667E-05	0.000943932	up_in_case
Yang2025_S	ENSGALG00010000796	ENSGALG00010000796	case	control	0.074942287	0.241968044	0.309719771	0.756774067	0.852119509	up_in_case
Yang2025_S	ENSGALG00010000797	ENSGALG00010000797	case	control	-1.174718773	0.462981775	-2.537289447	0.011171455	0.043975768	down_in_case
Yang2025_S	ENSGALG00010000798	ENSGALG00010000798	case	control	0.143344762	0.435091163	0.329459144	0.74180867	0.842126584	up_in_case
Yang2025_S	ENSGALG00010000799	ENSGALG00010000799	case	control	-0.229715138	0.29907789	-0.768077968	0.442440865	0.605818361	down_in_case
Yang2025_S	ENSGALG00010000800	LMO7	case	control	0.441488758	0.160221495	2.755490191	0.005860426	0.026943266	up_in_case
Yang2025_S	ENSGALG00010000802	SEMA5A	case	control	0.015104687	0.155508315	0.097131055	0.922622319	0.953661386	up_in_case
Yang2025_S	ENSGALG00010000803	MTRR	case	control	-0.192226022	0.10002835	-1.921715409	0.054641577	0.142350043	down_in_case
Yang2025_S	ENSGALG00010000805	FASTKD3	case	control	-0.099737664	0.128206293	-0.777946712	0.436600429	0.60053568	down_in_case
Yang2025_S	ENSGALG00010000806	CFAP90	case	control	0.83611519	0.272140526	3.072365602	0.002123694	0.012519769	up_in_case
Yang2025_S	ENSGALG00010000807	ENSGALG00010000807	case	control	1.075580126	0.822769405	1.307268015	0.191121703	0.346240371	up_in_case
Yang2025_S	ENSGALG00010000808	ADCY2	case	control	0.330538236	0.147669893	2.238359021	0.025197652	0.080371427	up_in_case
Yang2025_S	ENSGALG00010000810	UCHL3	case	control	-0.005141423	0.157522444	-0.032639302	0.973962228	0.984417469	down_in_case
Yang2025_S	ENSGALG00010000811	ENSGALG00010000811	case	control	-0.787203416	0.474070383	-1.660520136	0.09680986	0.214372383	down_in_case
Yang2025_S	ENSGALG00010000812	ENSGALG00010000812	case	control	-0.618518213	0.134645603	-4.593675557	4.35506E-06	8.27937E-05	down_in_case
Yang2025_S	ENSGALG00010000813	ENSGALG00010000813	case	control	-0.468078231	0.27428194	-1.706558698	0.087904107	0.200580275	down_in_case
Yang2025_S	ENSGALG00010000814	CDH18	case	control	-0.033035452	0.244510051	-0.13510877	0.892525877	0.93692327	down_in_case
Yang2025_S	ENSGALG00010000815	ENSGALG00010000815	case	control	0.524928829	0.158626535	3.30921198	0.00093559	0.006644976	up_in_case
Yang2025_S	ENSGALG00010000816	TBC1D4	case	control	0.037839626	0.141911743	0.266641965	0.789744842	0.873209532	up_in_case
Yang2025_S	ENSGALG00010000817	ENSGALG00010000817	case	control	-0.135589422	0.274042844	-0.494774541	0.620759278	0.755766438	down_in_case
Yang2025_S	ENSGALG00010000818	KLF12	case	control	0.030626858	0.224216673	0.136594919	0.891350993	0.936336879	up_in_case
Yang2025_S	ENSGALG00010000820	KLF5	case	control	-0.002716527	0.219051749	-0.012401301	0.990105447	0.993885564	down_in_case
Yang2025_S	ENSGALG00010000821	ENSGALG00010000821	case	control	-0.244537312	0.16858114	-1.45056151	0.146902	0.288727696	down_in_case
Yang2025_S	ENSGALG00010000822	BASP1	case	control	0.180091686	0.179220609	1.004860365	0.314964092	0.4841726	up_in_case
Yang2025_S	ENSGALG00010000823	PIBF1	case	control	-0.673552657	0.130470331	-5.162496729	2.43678E-07	7.20944E-06	down_in_case
Yang2025_S	ENSGALG00010000843	DIS3	case	control	0.113456825	0.097100285	1.16844996	0.242625313	0.407592976	up_in_case
Yang2025_S	ENSGALG00010000853	ENSGALG00010000853	case	control	0.018899528	0.212591297	0.088900763	0.929160777	0.95718603	up_in_case
Yang2025_S	ENSGALG00010000857	ENSGALG00010000857	case	control	-1.195124865	0.443354991	-2.695638684	0.007025383	0.031059234	down_in_case
Yang2025_S	ENSGALG00010000859	BORA	case	control	-0.014473976	0.117006748	-0.123702058	0.901551182	0.941198361	down_in_case
Yang2025_S	ENSGALG00010000864	MZT1	case	control	-0.959632436	0.1202567	-7.979866668	1.46491E-15	3.51958E-13	down_in_case
Yang2025_S	ENSGALG00010000870	DACH1	case	control	0.036526783	0.208556167	0.175141228	0.860968664	0.918138454	up_in_case
Yang2025_S	ENSGALG00010000901	PCDH9	case	control	-0.778382386	0.343978571	-2.262880456	0.02364306	0.076972615	down_in_case
Yang2025_S	ENSGALG00010001066	SLC33A1	case	control	-0.422448021	0.161776511	-2.611306297	0.009019707	0.03769228	down_in_case
Yang2025_S	ENSGALG00010001070	C3orf33	case	control	0.402318658	0.170011806	2.366416	0.01796125	0.062808034	up_in_case
Yang2025_S	ENSGALG00010001082	P2RY1	case	control	0.078829159	0.150043758	0.525374462	0.599322903	0.738096744	up_in_case
Yang2025_S	ENSGALG00010001104	MBNL1	case	control	0.027334185	0.111308169	0.24557214	0.806013458	0.883489148	up_in_case
Yang2025_S	ENSGALG00010001111	SUCNR1	case	control	0.758651573	0.403570634	1.879848307	0.060128755	0.152733175	up_in_case
Yang2025_S	ENSGALG00010001112	TSC22D2	case	control	-0.155189834	0.122037089	-1.271661221	0.203493515	0.361601903	down_in_case
Yang2025_S	ENSGALG00010001113	IGSF10	case	control	1.093630088	0.385745479	2.83510799	0.00458102	0.022422378	up_in_case
Yang2025_S	ENSGALG00010001114	MED12L	case	control	0.802886516	0.128171052	6.264179828	3.74793E-10	2.5321E-08	up_in_case
Yang2025_S	ENSGALG00010001115	PFN2	case	control	-0.295764289	0.171958769	-1.719972122	0.085437509	0.19638851	down_in_case
Yang2025_S	ENSGALG00010001116	RNF13	case	control	-0.383354882	0.058574774	-6.544709566	5.96111E-11	5.35923E-09	down_in_case
Yang2025_S	ENSGALG00010001117	P2RY13	case	control	-0.165934815	0.284407317	-0.583440738	0.55959664	0.706210757	down_in_case
Yang2025_S	ENSGALG00010001118	P2RY12	case	control	-0.172093367	0.327832773	-0.524942534	0.59962314	0.738401242	down_in_case
Yang2025_S	ENSGALG00010001119	COMMD2	case	control	-0.268488576	0.08349989	-3.215436285	0.001302464	0.008618156	down_in_case
Yang2025_S	ENSGALG00010001121	WWTR1	case	control	0.05930171	0.149189328	0.397492977	0.691003965	0.80731145	up_in_case
Yang2025_S	ENSGALG00010001122	P2RY14	case	control	0.196450133	0.28101198	0.699080988	0.484501419	0.642336579	up_in_case
Yang2025_S	ENSGALG00010001123	CP	case	control	0.71363738	0.162798387	4.383565413	1.16753E-05	0.000188086	up_in_case
Yang2025_S	ENSGALG00010001124	GPR171	case	control	0.818111939	0.475758035	1.719596684	0.085505779	0.196458868	up_in_case
Yang2025_S	ENSGALG00010001125	CLRN1	case	control	-0.109097786	0.247811118	-0.440245727	0.659759144	0.785118189	down_in_case
Yang2025_S	ENSGALG00010001127	SIAH2	case	control	-0.13298735	0.09699099	-1.371130976	0.17033413	0.319247895	down_in_case
Yang2025_S	ENSGALG00010001131	HPS3	case	control	0.245401863	0.103865064	2.362698823	0.018142407	0.063161783	up_in_case
Yang2025_S	ENSGALG00010001132	SELENOT	case	control	-0.098882003	0.143323383	-0.689922331	0.490243032	0.647635541	down_in_case
Yang2025_S	ENSGALG00010001133	EIF2A	case	control	-0.114929064	0.079225857	-1.450650939	0.146877083	0.288719446	down_in_case
Yang2025_S	ENSGALG00010001134	ENSGALG00010001134	case	control	0.905900796	0.347406049	2.607613766	0.009117577	0.037937722	up_in_case
Yang2025_S	ENSGALG00010001135	GYG1	case	control	0.009799049	0.112492549	0.087108428	0.930585326	0.958016143	up_in_case
Yang2025_S	ENSGALG00010001136	SERP1	case	control	-0.261882238	0.182312359	-1.436448085	0.150874879	0.293760156	down_in_case
Yang2025_S	ENSGALG00010001140	NMD3	case	control	-0.394067047	0.098280877	-4.009600417	6.08216E-05	0.00071644	down_in_case
Yang2025_S	ENSGALG00010001141	MTAP	case	control	0.132934693	0.161229919	0.824503873	0.409653316	0.575105568	up_in_case
Yang2025_S	ENSGALG00010001142	PPM1L	case	control	-0.46174616	0.163770941	-2.819463306	0.004810403	0.023299606	down_in_case
Yang2025_S	ENSGALG00010001143	CDKN2A	case	control	-0.492666137	0.478782384	-1.028998045	0.303480594	0.473069799	down_in_case
Yang2025_S	ENSGALG00010001145	TRIM36	case	control	-0.271016372	0.262105664	-1.033996624	0.301137742	0.470759976	down_in_case
Yang2025_S	ENSGALG00010001146	DIPK1C	case	control	-0.455781776	0.143971549	-3.165776702	0.001546695	0.00983266	down_in_case
Yang2025_S	ENSGALG00010001147	PGGT1B	case	control	0.141145765	0.116260191	1.214050694	0.224728367	0.386329853	up_in_case
Yang2025_S	ENSGALG00010001149	CIDEA	case	control	-1.169463012	0.405496492	-2.884027444	0.003926245	0.019907242	down_in_case
Yang2025_S	ENSGALG00010001150	ENSGALG00010001150	case	control	-0.905499767	0.690679673	-1.311027097	0.189848609	0.344628806	down_in_case
Yang2025_S	ENSGALG00010001151	CCDC112	case	control	1.587006287	0.289846885	5.47532635	4.36706E-08	1.67184E-06	up_in_case
Yang2025_S	ENSGALG00010001152	FEM1C	case	control	0.142583501	0.157381053	0.905976278	0.364948409	0.533730925	up_in_case
Yang2025_S	ENSGALG00010001153	TSHZ1	case	control	0.160730107	0.144486752	1.112421068	0.26595715	0.433159525	up_in_case
Yang2025_S	ENSGALG00010001154	PTGR3	case	control	-0.710240203	0.139390883	-5.09531319	3.48165E-07	9.74233E-06	down_in_case
Yang2025_S	ENSGALG00010001155	CYB5A	case	control	-0.522236214	0.084518369	-6.17896698	6.45224E-10	4.07395E-08	down_in_case
Yang2025_S	ENSGALG00010001156	ALDH7A1	case	control	-0.230357042	0.16491342	-1.396836245	0.16246282	0.308688219	down_in_case
Yang2025_S	ENSGALG00010001157	TIMM21	case	control	0.208355374	0.144079585	1.446113089	0.148145485	0.290270997	up_in_case
Yang2025_S	ENSGALG00010001158	FRMPD1	case	control	0.126920712	0.415816827	0.305232265	0.76018926	0.854569001	up_in_case
Yang2025_S	ENSGALG00010001159	RTTN	case	control	0.146512814	0.162512756	0.901546544	0.367297798	0.535890987	up_in_case
Yang2025_S	ENSGALG00010001160	TRMT10B	case	control	0.050261514	0.166102898	0.302592636	0.762200327	0.855286959	up_in_case
Yang2025_S	ENSGALG00010001161	B3GALNT1	case	control	-0.031022844	0.175131789	-0.177139991	0.85939843	0.916995034	down_in_case
Yang2025_S	ENSGALG00010001162	ENSGALG00010001162	case	control	0.506780585	0.348587862	1.453810189	0.145998938	0.287439277	up_in_case
Yang2025_S	ENSGALG00010001164	KPNA4	case	control	-0.111040971	0.074435158	-1.491781226	0.135756507	0.272289272	down_in_case
Yang2025_S	ENSGALG00010001165	ENSGALG00010001165	case	control	0.665602859	0.219395489	3.033803749	0.002414915	0.013831416	up_in_case
Yang2025_S	ENSGALG00010001166	TRIM59	case	control	-0.834042917	0.12533725	-6.654389795	2.84478E-11	2.7152E-09	down_in_case
Yang2025_S	ENSGALG00010001167	SMC4	case	control	0.436752629	0.188889245	2.312215442	0.020765814	0.06957721	up_in_case
Yang2025_S	ENSGALG00010001168	DOK6	case	control	0.726689144	0.27374884	2.654583461	0.007940639	0.034077632	up_in_case
Yang2025_S	ENSGALG00010001169	ENSGALG00010001169	case	control	-0.161884146	0.123313553	-1.312784704	0.189255506	0.344174897	down_in_case
Yang2025_S	ENSGALG00010001170	DCAF10	case	control	-0.01182417	0.151682029	-0.077953664	0.937864912	0.962240285	down_in_case
Yang2025_S	ENSGALG00010001171	POLR1E	case	control	0.183938543	0.180100986	1.021307808	0.307108628	0.476754313	up_in_case
Yang2025_S	ENSGALG00010001172	TMEM200C	case	control	-0.29564753	0.266150291	-1.110829259	0.26664185	0.433808313	down_in_case
Yang2025_S	ENSGALG00010001173	IFT80	case	control	-0.298030838	0.108815156	-2.738872496	0.006165028	0.028004247	down_in_case
Yang2025_S	ENSGALG00010001174	ENSGALG00010001174	case	control	-0.112456704	0.080425955	-1.398263832	0.162033855	0.308167294	down_in_case
Yang2025_S	ENSGALG00010001177	SEH1L	case	control	0.099713656	0.078768678	1.265904914	0.205547127	0.363951616	up_in_case
Yang2025_S	ENSGALG00010001178	ZBTB5	case	control	-0.14231284	0.134298325	-1.059676954	0.289291591	0.458047758	down_in_case
Yang2025_S	ENSGALG00010001179	GRHPR	case	control	-0.558342811	0.299702339	-1.862991168	0.062463509	0.156777557	down_in_case
Yang2025_S	ENSGALG00010001180	SPIRE1	case	control	0.711045631	0.268582033	2.647405794	0.008111196	0.034578127	up_in_case
Yang2025_S	ENSGALG00010001181	MFSD1	case	control	-0.175259922	0.079303794	-2.209981547	0.027106443	0.084920927	down_in_case
Yang2025_S	ENSGALG00010001182	ZCCHC7	case	control	0.30403415	0.145300923	2.092444726	0.036398751	0.105670125	up_in_case
Yang2025_S	ENSGALG00010001183	RARRES1	case	control	0.04578442	0.16386947	0.279395668	0.779941195	0.86656678	up_in_case
Yang2025_S	ENSGALG00010001185	PTPN2	case	control	-0.076760957	0.106820299	-0.718598968	0.472388049	0.632182768	down_in_case
Yang2025_S	ENSGALG00010001186	ENSGALG00010001186	case	control	-0.132669558	0.565279696	-0.234697193	0.814443767	0.888605848	down_in_case
Yang2025_S	ENSGALG00010001187	GFM1	case	control	0.41027345	0.104440636	3.928293302	8.55508E-05	0.000941667	up_in_case
Yang2025_S	ENSGALG00010001188	PSMG2	case	control	0.379078586	0.125780669	3.013806403	0.002579924	0.014526088	up_in_case
Yang2025_S	ENSGALG00010001190	LXN	case	control	0.279020539	0.393790745	0.708550271	0.478603606	0.637401307	up_in_case
Yang2025_S	ENSGALG00010001191	TOMM5	case	control	-0.227238206	0.316938431	-0.716979022	0.473387041	0.632929363	down_in_case
Yang2025_S	ENSGALG00010001192	ENSGALG00010001192	case	control	0.279227969	0.149997234	1.861554119	0.062665966	0.157059394	up_in_case

Yang2025_S	ENSGALG00010001193	PRELID3A	case	control	0.092842964	0.226381954	0.410116455	0.681720523	0.800520391	up_in_case
Yang2025_S	ENSGALG00010001194	POT1	case	control	-0.496520695	0.131063172	-3.788407435	0.000151616	0.00149418	down_in_case
Yang2025_S	ENSGALG00010001195	ZNF608	case	control	1.022762237	0.251296401	4.069943827	4.70245E-05	0.000579386	up_in_case
Yang2025_S	ENSGALG00010001196	LSMEM1	case	control	-0.185277959	0.173122644	-1.07021216	0.284523822	0.452733274	down_in_case
Yang2025_S	ENSGALG00010001197	CEP76	case	control	-0.391555281	0.155457298	-2.518732057	0.011777824	0.045752858	down_in_case
Yang2025_S	ENSGALG00010001198	IFRD1	case	control	0.211114016	0.172424474	1.224385446	0.220806857	0.382260259	up_in_case
Yang2025_S	ENSGALG00010001199	AFG3L2	case	control	0.1962106	0.067631151	2.901186763	0.003717522	0.019122803	up_in_case
Yang2025_S	ENSGALG00010001200	CTTNBP2	case	control	0.244120027	0.155357537	1.571343314	0.116102925	0.243793873	up_in_case
Yang2025_S	ENSGALG00010001201	TUBB6	case	control	-0.129325086	0.12214463	-1.058786497	0.289697024	0.458459371	down_in_case
Yang2025_S	ENSGALG00010001202	CFTR	case	control	-0.362891291	0.166324927	-2.181821437	0.029122714	0.089507062	down_in_case
Yang2025_S	ENSGALG00010001203	TMX3	case	control	-0.406258483	0.074489097	-5.453932166	4.9268E-08	1.85054E-06	down_in_case
Yang2025_S	ENSGALG00010001204	SNX2	case	control	-0.054821535	0.131773411	-0.416028806	0.677388924	0.797237762	down_in_case
Yang2025_S	ENSGALG00010001205	CEP192	case	control	0.089467424	0.099878112	0.895766073	0.370377713	0.538973834	up_in_case
Yang2025_S	ENSGALG00010001206	RSRC1	case	control	0.31584228	0.102973492	3.067219264	0.002160603	0.012682394	up_in_case
Yang2025_S	ENSGALG00010001208	MOV10L1	case	control	1.779782412	1.053715966	1.689053283	0.091209219	0.205730086	up_in_case
Yang2025_S	ENSGALG00010001209	CDH7	case	control	0.1240941	0.173125598	0.716786546	0.473505814	0.632930794	up_in_case
Yang2025_S	ENSGALG00010001210	PANX2	case	control	0.683646716	0.301407185	2.268183204	0.023318043	0.076026421	up_in_case
Yang2025_S	ENSGALG00010001213	PPIC	case	control	0.167873884	0.202894024	0.827396888	0.408012144	0.573879823	up_in_case
Yang2025_S	ENSGALG00010001214	TES	case	control	-0.220110238	0.091158764	-2.414581196	0.015753311	0.057048439	down_in_case
Yang2025_S	ENSGALG00010001215	PRDM6	case	control	1.771219722	0.605085097	2.927224173	0.003420023	0.017962982	up_in_case
Yang2025_S	ENSGALG00010001216	SHOX2	case	control	0.441620007	0.417944582	1.056647285	0.290672592	0.459190859	up_in_case
Yang2025_S	ENSGALG00010001218	ENSGALG00010001218	case	control	0.276395847	0.127785799	2.162962147	0.030544091	0.09293361	up_in_case
Yang2025_S	ENSGALG00010001220	PLXNB2	case	control	0.1832109	0.161969797	1.13114237	0.257995174	0.424275547	up_in_case
Yang2025_S	ENSGALG00010001221	GRAMD2B	case	control	-0.155553289	0.178625017	-0.870837088	0.383843112	0.55159882	down_in_case
Yang2025_S	ENSGALG00010001222	ZNF800	case	control	-0.076166448	0.164242002	-0.463745249	0.642830274	0.772360741	down_in_case
Yang2025_S	ENSGALG00010001223	GRM8	case	control	0.077978212	0.542860997	0.143643056	0.88578234	0.933054419	up_in_case
Yang2025_S	ENSGALG00010001224	DNAJB9	case	control	-0.704081133	0.160671378	-4.382119207	1.1753E-05	0.000188685	down_in_case
Yang2025_S	ENSGALG00010001225	SOCS6	case	control	-0.325546506	0.096962817	-3.357436563	0.000786688	0.005772773	down_in_case
Yang2025_S	ENSGALG00010001226	VEPH1	case	control	-1.097394668	0.49151203	-2.23269137	0.025569304	0.081256157	down_in_case
Yang2025_S	ENSGALG00010001227	ENSGALG00010001227	case	control	-0.419319331	1.824161085	-0.229869683	0.818193035	0.89137049	down_in_case
Yang2025_S	ENSGALG00010001228	THAP5	case	control	-0.298007478	0.146736075	-2.030908072	0.042264321	0.117767109	down_in_case
Yang2025_S	ENSGALG00010001229	ZNF407	case	control	0.161137085	0.131372901	1.22656258	0.219987041	0.381330939	up_in_case
Yang2025_S	ENSGALG00010001230	PTX3	case	control	-0.692218901	0.267755239	-2.585267438	0.009730349	0.039739863	down_in_case
Yang2025_S	ENSGALG00010001231	CNDP1	case	control	0.336074904	0.633699229	0.530338193	0.595877471	0.735484972	up_in_case
Yang2025_S	ENSGALG00010001232	TSPAN12	case	control	0.012318092	0.105528484	0.116727647	0.907075881	0.944261228	up_in_case
Yang2025_S	ENSGALG00010001233	CNDP2	case	control	-0.020861004	0.117420697	-0.177660362	0.858989716	0.916888279	down_in_case
Yang2025_S	ENSGALG00010001234	AASS	case	control	-0.122113135	0.109990048	-1.11021986	0.266904297	0.433992275	down_in_case
Yang2025_S	ENSGALG00010001235	CCDC102B	case	control	-0.297660166	0.29804401	-0.998712123	0.317934166	0.487365231	down_in_case
Yang2025_S	ENSGALG00010001237	MELK	case	control	0.343809583	0.174560694	1.969570435	0.048887623	0.131160767	up_in_case
Yang2025_S	ENSGALG00010001238	GOLIM4	case	control	0.833920165	0.292429761	2.8516939	0.004348695	0.021484507	up_in_case
Yang2025_S	ENSGALG00010001239	ENSGALG00010001239	case	control	0.104556288	0.339409018	0.308053949	0.758041282	0.85295517	up_in_case
Yang2025_S	ENSGALG00010001242	ENSGALG00010001242	case	control	-0.596635191	0.853193073	-0.699296806	0.484366563	0.642273104	down_in_case
Yang2025_S	ENSGALG00010001244	ENSGALG00010001244	case	control	-0.354838234	0.343876363	-1.031877362	0.302129572	0.471677263	down_in_case
Yang2025_S	ENSGALG00010001245	SERPINI1	case	control	-0.387364625	0.259553031	-1.492429594	0.135586562	0.272130021	down_in_case
Yang2025_S	ENSGALG00010001248	PTPRZ1	case	control	0.631844855	0.32261478	1.958511806	0.050169987	0.133539564	up_in_case
Yang2025_S	ENSGALG00010001249	PDCD10	case	control	0.058327095	0.121992315	0.47812106	0.632564043	0.764176848	up_in_case
Yang2025_S	ENSGALG00010001250	ENSGALG00010001250	case	control	-0.235447696	0.644631442	-0.36524389	0.714929358	0.824031481	down_in_case
Yang2025_S	ENSGALG00010001251	ZNF277	case	control	0.039324745	0.113353183	0.346922282	0.728649706	0.833097929	up_in_case
Yang2025_S	ENSGALG00010001252	SNCAIP	case	control	0.326659729	0.207516181	1.574141006	0.115454854	0.242883967	up_in_case
Yang2025_S	ENSGALG00010001254	NETO1	case	control	0.056632183	0.36715322	0.15424673	0.877415198	0.928025294	up_in_case
Yang2025_S	ENSGALG00010001257	ENSGALG00010001257	case	control	1.939868863	0.769577042	2.520694819	0.011712338	0.045538905	up_in_case
Yang2025_S	ENSGALG00010001258	ENSGALG00010001258	case	control	0.005015368	0.157261198	0.031891962	0.974558209	0.984805557	up_in_case
Yang2025_S	ENSGALG00010001259	ENSGALG00010001259	case	control	0.582661097	0.318673689	1.828394114	0.067490424	0.165519018	up_in_case
Yang2025_S	ENSGALG00010001262	ENSGALG00010001262	case	control	1.132286222	0.11540123	9.811734396	1.00231E-22	7.75953E-20	up_in_case
Yang2025_S	ENSGALG00010001264	FAM3C	case	control	-0.18655608	0.102103477	-1.827127589	0.067680588	0.165810301	down_in_case
Yang2025_S	ENSGALG00010001265	OPN1LW	case	control	1.663162567	0.392065863	4.242048905	2.21488E-05	0.000316234	up_in_case
Yang2025_S	ENSGALG00010001267	BCHE	case	control	-0.218104843	0.099212436	-2.198361935	0.027923324	0.086700428	down_in_case
Yang2025_S	ENSGALG00010001268	CBLN2	case	control	-0.469594329	0.300485754	-1.562783998	0.118103409	0.246668316	down_in_case
Yang2025_S	ENSGALG00010001270	DRAP1	case	control	0.29824014	0.175743256	1.697021813	0.089692537	0.203337778	up_in_case
Yang2025_S	ENSGALG00010001271	ENSGALG00010001271	case	control	0.54808495	0.304336875	1.800915353	0.071716223	0.172870708	up_in_case
Yang2025_S	ENSGALG00010001273	ENSGALG00010001273	case	control	1.03007906	0.479983579	2.146071457	0.031867278	0.095870147	up_in_case
Yang2025_S	ENSGALG00010001274	DLGAP1	case	control	1.155348753	0.461157255	2.505324896	0.012233896	0.047106753	up_in_case
Yang2025_S	ENSGALG00010001276	ENSGALG00010001276	case	control	-0.289110301	0.314496208	-0.919280723	0.357948758	0.527051558	down_in_case
Yang2025_S	ENSGALG00010001277	ENSGALG00010001277	case	control	0.185003986	0.136323458	1.357095748	0.174750789	0.325207298	up_in_case
Yang2025_S	ENSGALG00010001279	LPIN2	case	control	-0.023983236	0.113141825	-0.211974984	0.832126551	0.900143771	down_in_case
Yang2025_S	ENSGALG00010001281	LRRN3	case	control	0.57712543	0.183533713	3.144519998	0.001663595	0.01040961	up_in_case
Yang2025_S	ENSGALG00010001283	ENSGALG00010001283	case	control	0.235324605	0.135752175	1.733486805	0.083009181	0.192308052	up_in_case
Yang2025_S	ENSGALG00010001285	ENSGALG00010001285	case	control	0.09731707	0.148766808	0.654158487	0.513009704	0.667986379	up_in_case
Yang2025_S	ENSGALG00010001286	SRFBP1	case	control	0.745909914	0.247855415	3.00945579	0.002617162	0.014670105	up_in_case
Yang2025_S	ENSGALG00010001287	PLPP4	case	control	-0.036902995	0.429821672	-0.085856525	0.931580472	0.958515861	down_in_case
Yang2025_S	ENSGALG00010001288	TGIF1	case	control	-0.51614542	0.153180037	-3.369534496	0.000752953	0.005574122	down_in_case
Yang2025_S	ENSGALG00010001289	ENSGALG00010001289	case	control	1.719399143	0.428897901	4.008877494	6.10081E-05	0.00071803	up_in_case
Yang2025_S	ENSGALG00010001291	ENSGALG00010001291	case	control	0.248863111	0.210783174	1.180659287	0.237738101	0.401719796	up_in_case
Yang2025_S	ENSGALG00010001292	LOX	case	control	0.069829149	0.193636613	0.360619555	0.718383869	0.826485326	up_in_case
Yang2025_S	ENSGALG00010001295	ENSGALG00010001295	case	control	0.419571428	0.078736441	5.328808636	9.88591E-08	3.40148E-06	up_in_case
Yang2025_S	ENSGALG00010001297	MYL12A	case	control	0.524089009	0.20904282	2.507089258	0.012172997	0.046911148	up_in_case
Yang2025_S	ENSGALG00010001298	DOCK4	case	control	0.278777138	0.172398464	1.617051169	0.105867236	0.228262407	up_in_case
Yang2025_S	ENSGALG00010001300	MRPL49	case	control	0.458440018	0.249882919	1.834619267	0.066562122	0.163992781	up_in_case
Yang2025_S	ENSGALG00010001301	GMPS	case	control	-0.32302019	0.07119854	-4.536893465	5.70889E-06	0.000104264	down_in_case
Yang2025_S	ENSGALG00010001302	ENSGALG00010001302	case	control	0.508562298	0.367828446	1.382607309	0.166785307	0.314542326	up_in_case
Yang2025_S	ENSGALG00010001304	SYVN1	case	control	0.629174191	0.201808658	3.117676897	0.001822825	0.011155498	up_in_case
Yang2025_S	ENSGALG00010001305	WNT16	case	control	-0.366650844	0.37457058	-0.978856493	0.327650892	0.49747387	down_in_case
Yang2025_S	ENSGALG00010001306	CEP120	case	control	0.111541929	0.140189885	0.79564891	0.42623613	0.590854461	up_in_case
Yang2025_S	ENSGALG00010001307	CALU	case	control	0.476858161	0.197204968	2.418083914	0.015602478	0.056678971	up_in_case
Yang2025_S	ENSGALG00010001308	MGMT	case	control	-0.284952437	0.152099463	-1.873461167	0.06100473	0.154255293	down_in_case
Yang2025_S	ENSGALG00010001313	EMILIN2	case	control	-0.304647518	0.150213218	-2.0281006	0.042549982	0.118412422	down_in_case
Yang2025_S	ENSGALG00010001320	CPED1	case	control	0.070941835	0.171461573	0.413747721	0.679058874	0.798606246	up_in_case
Yang2025_S	ENSGALG00010001322	ENSGALG00010001322	case	control	3.36117388	0.730376408	4.601974878	4.18504E-06	8.01078E-05	up_in_case
Yang2025_S	ENSGALG00010001330	ENSGALG00010001330	case	control	0.76871133	0.196990301	3.9022801	9.52908E-05	0.00102661	up_in_case
Yang2025_S	ENSGALG00010001333	PLCH1	case	control	0.038880888	0.281415395	0.13816191	0.890112458	0.935635294	up_in_case
Yang2025_S	ENSGALG00010001334	EBF3	case	control	0.44739692	0.237004178	1.887717436	0.059063894	0.150991629	up_in_case
Yang2025_S	ENSGALG00010001343	ENSGALG00010001343	case	control	-0.162908848	0.414350369	-0.393166894	0.694196219	0.810027114	down_in_case
Yang2025_S	ENSGALG00010001347	MYOM1	case	control	0.059946206	0.058177529	1.030401376	0.302821627	0.472491253	up_in_case
Yang2025_S	ENSGALG00010001349	ING3	case	control	0.247089031	0.145842759	1.694215285	0.090224381	0.204070241	up_in_case
Yang2025_S	ENSGALG00010001351	CAPN1	case	control	-0.31554913	0.105779886	-2.983073071	0.002853699	0.015683828	down_in_case
Yang2025_S	ENSGALG00010001353	GLRX3	case	control	-0.31973204	0.173844732	-1.839181638	0.065888475	0.162694563	down_in_case
Yang2025_S	ENSGALG00010001354	ENSGALG00010001354	case	control	0.981890241	0.760055692	1.291866177	0.196403493	0.352504202	up_in_case
Yang2025_S	ENSGALG00010001358	TCERG1L	case	control	1.354809337	0.412530036	3.284147141	0.001022915	0.007137867	up_in_case
Yang2025_S	ENSGALG00010001363	HIBADH	case	control	0.445172261	0.11388583	3.908934592	9.27041E-05	0.001003754	up_in_case
Yang2025_S	ENSGALG00010001364	SMCHD1	case	control	0.133580526	0.146841754	0.909690345	0.362985837	0.531715299	up_in_case
Yang2025_S	ENSGALG00010001365	ENSGALG00010001365	case	control	-0.780200939	0.159712619	-4.885030007	1.03413E-06	2.48888E-05	down_in_case
Yang2025_S	ENSGALG00010001367	ENSGALG00010001367	case	control	0.548628855	0.389991252	1.406772209	0.159494946	0.304920026	up_in_case
Yang2025_S	ENSGALG00010001368	WIPF3	case	control	-0.087446896	0.230996084	-0.378564409	0.705011356	0.817808478	down_in_case
Yang2025_S	ENSGALG00010001369	PPP2R2D	case	control	-0.142481422	0.077012999	-1.850095761	0.064299749	0.159803281	down_in_case
Yang2025_S	ENSGALG00010001372	ENSGALG00010001372	case	control	0.263633726	0.243620922	1.082147315	0.279187074	0.44703789	up_in_case
Yang2025_S	ENSGALG00010001373	IMPA2	case	control	0.063668496	0.104546085	0.608999329	0.542524884	0.69269601	up_in_case
Yang2025_S	ENSGALG00010001378	POLA2	case	control	0.525487925	0.114524538	4.588430872	4.4659E-06	8.43257E-05	up_in_case
Yang2025_S	ENSGALG00010001380	SCRN1	case	control	-0.002100128	0.179221161	-0.011718078	0.99065054	0.994000591	down_in_case
Yang2025_S	ENSGALG00010001381	MDFIC	case	control	-0.721562168	0.096150459	-7.50451087	6.16584E-14	1.04292E-11	down_in_case
Yang2025_S	ENSGALG00010001382	BNIP3	case	control	-0.271233833	0.189398914	-1.432077026	0.152121779	0.295609676	down_in_case
Yang2025_S	ENSGALG00010001385	MME	case	control	0.543366452	0.216817779	2.506097312	0.012207202	0.047024216	up_in_case
Yang2025_S	ENSGALG00010001386	FKBP14	case	control	-0.271778672	0.123489783	-2.200819094	0.027748833	0.086408937	down_in_case
Yang2025_S	ENSGALG00010001388	MPPE1	case	control	0.465880558	0.108119155	4.308954839	1.64028E-05	0.000246839	up_in_case
Yang2025_S	ENSGALG00010001395	GNAL	case	control	0.062341314	0.195366633	0.319099084	0.749651379	0.847783798	up_in_case
Yang2025_S	ENSGALG00010001397	KLHL7	case	control	0.033252996	0.078084156	0.425860984	0.670209146	0.792192467	up_in_case
Yang2025_S	ENSGALG00010001398	WDR11	case	control	-0.134284501	0.060831408	-2.207486329	0.027280101	0.085235024	down_in_case
Yang2025_S	ENSGALG00010001400	SNX24	case	control	-0.111314413	0.169717659	-0.655879968	0.511901358	0.667166613	down_in_case
Yang2025_S	ENSGALG00010001402	NUP42	case	control	-0.052775296	0.180470418	-0.292431839	0.769956467	0.859723027	down_in_case
Yang2025_S	ENSGALG00010001406	EML3	case	control	0.414591374	0.156077606	2.656315558	0.007899965	0.033945734	up_in_case
Yang2025_S	ENSGALG00010001407	FOXP2	case	control	0.460648235	0.21058612	2.18745772	0.028709126	0.088490253	up_in_case
Yang2025_S	ENSGALG00010001409	GPNMB	case	control	7.10704146	1.002655038	7.088221961	1.35846E-12	1.80287E-10	up_in_case
Yang2025_S	ENSGALG00010001411	FGFR2	case	control	0.27376908	0.291083056	0.940518776	0.346951523	0.515375905	up_in_case
Yang2025_S	ENSGALG00010001412	ENSGALG00010001412	case	control	0.56414749	0.195837297	2.880694829	0.003967996	0.020055868	up_in_case
Yang2025_S	ENSGALG00010001414	CCDC126	case	control	-0.065075841	0.101523949	-0.640990046	0.52152915	0.675105315	down_in_case
Yang2025_S	ENSGALG00010001415	MC5R	case	control	-0.523454892	0.227879812	-2.297065671	0.021615024	0.071630287	down_in_case
Yang2025_S	ENSGALG00010001416	AP4M1	case	control	0.570400234	0.268732603	2.122556874	0.033791002	0.100058991	up_in_case

Yang2025_S	ENSGALG00010001418	RNMT	case	control	-0.271968254	0.075262454	-3.613597987	0.000301977	0.002620207	down_in_case
Yang2025_S	ENSGALG00010001419	KCND2	case	control	0.437110504	0.368776621	1.185298847	0.235899309	0.399375152	up_in_case
Yang2025_S	ENSGALG00010001420	FAM221A	case	control	0.65869485	0.495920382	1.328227018	0.184103122	0.337473954	up_in_case
Yang2025_S	ENSGALG00010001422	AP1S1	case	control	0.470831579	0.260774958	1.805509178	0.070995041	0.171607266	up_in_case
Yang2025_S	ENSGALG00010001423	FAM210A	case	control	0.049553704	0.13535196	0.366109985	0.714283007	0.823558969	up_in_case
Yang2025_S	ENSGALG00010001426	ATE1	case	control	-0.288235822	0.099991991	-2.882589077	0.003944216	0.019957389	down_in_case
Yang2025_S	ENSGALG00010001427	ASB11	case	control	0.02983706	0.309207981	0.096495116	0.923127353	0.953733233	up_in_case
Yang2025_S	ENSGALG00010001432	NSMCE4A	case	control	-0.071499449	0.159257262	-0.448955655	0.653463646	0.780694094	down_in_case
Yang2025_S	ENSGALG00010001440	CDCA7L	case	control	-0.135275756	0.128879243	-1.049631834	0.293887415	0.462434635	down_in_case
Yang2025_S	ENSGALG00010001441	HMGCLL1	case	control	0.345923157	0.26010941	1.329914042	0.183546594	0.336893752	up_in_case
Yang2025_S	ENSGALG00010001442	LDLRAD4	case	control	0.397801282	0.158955352	2.502597602	0.012328563	0.047353506	up_in_case
Yang2025_S	ENSGALG00010001443	ANKRD26	case	control	1.259148749	0.195265027	6.448408957	1.1303E-10	9.48003E-09	up_in_case
Yang2025_S	ENSGALG00010001448	GPR37	case	control	-0.237441555	0.226664698	-1.047545371	0.294848118	0.463527586	down_in_case
Yang2025_S	ENSGALG00010001453	TAX1BP1	case	control	0.395179692	0.119866192	3.296840306	0.000977791	0.006881573	up_in_case
Yang2025_S	ENSGALG00010001454	VEGFD	case	control	-0.463498395	0.208446143	-2.223588253	0.02617616	0.082750632	down_in_case
Yang2025_S	ENSGALG00010001456	LSM8	case	control	-0.391210661	0.273072491	-1.43262567	0.151964842	0.295469523	down_in_case
Yang2025_S	ENSGALG00010001457	NDC80	case	control	-0.198283805	0.130510848	-1.519289835	0.128689557	0.262061812	down_in_case
Yang2025_S	ENSGALG00010001458	JAZF1	case	control	-0.176991352	0.159864046	-1.107136699	0.268234831	0.435291995	down_in_case
Yang2025_S	ENSGALG00010001460	MALSU1	case	control	0.056743793	0.11004062	0.515662248	0.606090326	0.743766699	up_in_case
Yang2025_S	ENSGALG00010001461	ASB9	case	control	0.092067231	0.203810135	0.451730386	0.651463225	0.779239488	up_in_case
Yang2025_S	ENSGALG00010001462	DHX36	case	control	0.261349559	0.111168869	2.350923982	0.018726859	0.064577774	up_in_case
Yang2025_S	ENSGALG00010001463	DSEL	case	control	-0.292340108	0.086806982	-3.36770271	0.000757973	0.005600398	down_in_case
Yang2025_S	ENSGALG00010001465	TACC2	case	control	0.270493026	0.192789478	1.403048701	0.160602317	0.306244432	up_in_case
Yang2025_S	ENSGALG00010001466	PRKX	case	control	-0.228831748	0.120198593	-1.903780589	0.056938766	0.146929537	down_in_case
Yang2025_S	ENSGALG00010001468	ANOS1	case	control	-0.056732547	0.180969945	-0.313491539	0.753907248	0.850181881	down_in_case
Yang2025_S	ENSGALG00010001469	ARHGEF26	case	control	0.111146459	0.174877498	0.635567527	0.525058341	0.678350452	up_in_case
Yang2025_S	ENSGALG00010001470	GPR85	case	control	0.14268482	0.350109778	0.407543089	0.683609154	0.801927229	up_in_case
Yang2025_S	ENSGALG00010001471	BMP5	case	control	0.780574454	0.29996841	2.602188852	0.009263083	0.038382711	up_in_case
Yang2025_S	ENSGALG00010001472	ENSGALG00010001472	case	control	0.483863173	0.191814409	2.522559051	0.011650439	0.045374193	up_in_case
Yang2025_S	ENSGALG00010001475	BMT2	case	control	-0.125616923	0.110203198	-1.139866404	0.254341964	0.420747898	down_in_case
Yang2025_S	ENSGALG00010001476	IGF2BP3	case	control	-0.233570941	0.116078013	-2.012189345	0.044199981	0.122140677	down_in_case
Yang2025_S	ENSGALG00010001477	NLGN4	case	control	0.233749628	0.350692797	0.666536723	0.505068099	0.6607165	up_in_case
Yang2025_S	ENSGALG00010001479	TMEM168	case	control	-0.540661158	0.0846971	-6.383467146	1.73123E-10	1.29702E-08	down_in_case
Yang2025_S	ENSGALG00010001480	JAKMIP3	case	control	0.762746161	0.323070131	2.360930607	0.018229141	0.063284276	up_in_case
Yang2025_S	ENSGALG00010001482	TRA2A	case	control	0.254963221	0.130453252	1.954441286	0.050649052	0.134539562	up_in_case
Yang2025_S	ENSGALG00010001483	ENSGALG00010001483	case	control	0.055053171	0.308512254	0.178447276	0.858371724	0.916653381	up_in_case
Yang2025_S	ENSGALG00010001486	WNT2	case	control	-0.235099735	0.289420061	-0.812313195	0.416611941	0.581743963	down_in_case
Yang2025_S	ENSGALG00010001487	CDH19	case	control	0.477083777	0.171945248	2.774626115	0.00552652	0.025806197	up_in_case
Yang2025_S	ENSGALG00010001491	PHF3	case	control	0.45758824	0.211471096	2.163833496	0.03047713	0.092800143	up_in_case
Yang2025_S	ENSGALG00010001492	ENSGALG00010001492	case	control	1.165061516	0.358936564	3.245870251	0.001170922	0.007913093	up_in_case
Yang2025_S	ENSGALG00010001495	CCNL1	case	control	0.736368296	0.17746327	4.14941242	3.3333E-05	0.000442246	up_in_case
Yang2025_S	ENSGALG00010001496	PTP4A1	case	control	-0.557522901	0.15303604	-3.64308239	0.000269393	0.002384997	down_in_case
Yang2025_S	ENSGALG00010001497	LEKR1	case	control	1.010740439	0.309999227	3.260461156	0.001112312	0.007627986	up_in_case
Yang2025_S	ENSGALG00010001499	TIPARP	case	control	-0.168589327	0.119157844	-1.414840361	0.157115307	0.302320051	down_in_case
Yang2025_S	ENSGALG00010001501	CAPZA2	case	control	-0.42104512	0.113714646	-3.702646356	0.000213362	0.001969008	down_in_case
Yang2025_S	ENSGALG00010001504	RBMS3	case	control	0.370188504	0.26327504	1.406090394	0.159697285	0.305181248	up_in_case
Yang2025_S	ENSGALG00010001505	SSR3	case	control	-0.309319149	0.147502132	-2.097048671	0.035989267	0.104896557	down_in_case
Yang2025_S	ENSGALG00010001506	DPYSL4	case	control	-0.032027612	0.228530755	-0.140145739	0.888544843	0.93504549	down_in_case
Yang2025_S	ENSGALG00010001507	METTL4	case	control	-0.089252101	0.184615669	-0.483448138	0.628777575	0.761384733	down_in_case
Yang2025_S	ENSGALG00010001508	CD109	case	control	0.749132159	0.331545816	2.259513234	0.023851478	0.077367399	up_in_case
Yang2025_S	ENSGALG00010001510	AP1S2	case	control	-0.074089488	0.075427342	-0.982263012	0.325970292	0.496003059	down_in_case
Yang2025_S	ENSGALG00010001512	PLCL2	case	control	0.018186625	0.140383345	0.129549737	0.896922672	0.938898545	up_in_case
Yang2025_S	ENSGALG00010001513	ZRSR2	case	control	0.594327427	0.163778917	3.628839655	0.000284698	0.002496706	up_in_case
Yang2025_S	ENSGALG00010001514	MET	case	control	-0.296051079	0.132128133	-2.240636202	0.02504965	0.08004285	down_in_case
Yang2025_S	ENSGALG00010001517	CAV2	case	control	-0.42202763	0.160236848	-2.633773918	0.008444169	0.035754938	down_in_case
Yang2025_S	ENSGALG00010001518	SLC17A5	case	control	-0.756239735	0.136033083	-5.559233965	2.70961E-08	1.10083E-06	down_in_case
Yang2025_S	ENSGALG00010001520	KCNAB1	case	control	0.094011338	0.169847471	0.553504495	0.579918004	0.722693381	up_in_case
Yang2025_S	ENSGALG00010001521	TBC1D5	case	control	-0.043286419	0.148613813	-0.291267805	0.770846508	0.860165446	down_in_case
Yang2025_S	ENSGALG00010001525	EEF1A1	case	control	-0.70783115	0.199756105	-3.543476935	0.000394888	0.003265734	down_in_case
Yang2025_S	ENSGALG00010001526	PPP1R3A	case	control	0.044921039	0.135858183	0.330646545	0.740911484	0.841450817	up_in_case
Yang2025_S	ENSGALG00010001527	STK32C	case	control	-0.680775426	0.1842608	-3.694629709	0.000220207	0.002014833	down_in_case
Yang2025_S	ENSGALG00010001528	NR1D2	case	control	0.061438537	0.187149739	0.328285456	0.74269584	0.842447418	up_in_case
Yang2025_S	ENSGALG00010001532	ENSGALG00010001532	case	control	0.304445993	0.163502951	1.862021396	0.062600074	0.157007206	up_in_case
Yang2025_S	ENSGALG00010001533	ENSGALG00010001533	case	control	0.612535094	0.157260308	3.895039391	9.81828E-05	0.001051635	up_in_case
Yang2025_S	ENSGALG00010001534	SLITRK3	case	control	1.308536579	0.491285185	2.663496925	0.007733312	0.033342673	up_in_case
Yang2025_S	ENSGALG00010001535	RAP2B	case	control	-0.219117663	0.11558429	-1.895739152	0.057994545	0.148831306	down_in_case
Yang2025_S	ENSGALG00010001540	TRABD	case	control	-0.180541679	0.116469285	-1.550122669	0.121112076	0.251369792	down_in_case
Yang2025_S	ENSGALG00010001542	MTO1	case	control	-0.166000835	0.110728652	-1.499167845	0.133830095	0.269731323	down_in_case
Yang2025_S	ENSGALG00010001545	SELENOO	case	control	-0.200872722	0.169738107	-1.183427369	0.236639816	0.400239815	down_in_case
Yang2025_S	ENSGALG00010001546	CGAS	case	control	0.018371728	0.122174941	0.150372309	0.880470887	0.929497106	up_in_case
Yang2025_S	ENSGALG00010001547	PWWP2B	case	control	-0.184328181	0.090864562	-2.02860365	0.042498677	0.118349132	down_in_case
Yang2025_S	ENSGALG00010001548	AKAP17A	case	control	0.437804064	0.122823347	3.564501992	0.000364548	0.003062069	up_in_case
Yang2025_S	ENSGALG00010001551	FHIP1B	case	control	0.231475619	0.10153848	2.279683715	0.022626454	0.074118391	up_in_case
Yang2025_S	ENSGALG00010001552	TUBGCP6	case	control	-0.031681768	0.094515186	-0.335202937	0.737472007	0.839871887	down_in_case
Yang2025_S	ENSGALG00010001553	CNGA4	case	control	0.81039214	0.331579421	2.444036293	0.014523965	0.05357673	up_in_case
Yang2025_S	ENSGALG00010001554	ENSGALG00010001554	case	control	0.535808461	0.27312436	1.961774705	0.04978872	0.132912991	up_in_case
Yang2025_S	ENSGALG00010001556	EPB41L3	case	control	0.096747177	0.154202261	0.627404399	0.530394185	0.682775331	up_in_case
Yang2025_S	ENSGALG00010001557	THRB	case	control	0.673133522	0.492489298	1.366798272	0.171688545	0.320879811	up_in_case
Yang2025_S	ENSGALG00010001558	CCKBR	case	control	0.768175906	0.401030055	1.915507072	0.055427868	0.144021507	up_in_case
Yang2025_S	ENSGALG00010001559	HDAC10	case	control	-0.026948575	0.164114113	-0.164206323	0.869568724	0.9231632	down_in_case
Yang2025_S	ENSGALG00010001567	INPP5A	case	control	-0.34198215	0.072902898	-4.690926702	2.7197E-06	5.60637E-05	down_in_case
Yang2025_S	ENSGALG00010001570	SMPD1	case	control	0.151104197	0.210420894	0.71810453	0.472692837	0.632390043	up_in_case
Yang2025_S	ENSGALG00010001585	ENSGALG00010001585	case	control	-0.099619155	0.137578382	-0.724090175	0.469010376	0.629639652	down_in_case
Yang2025_S	ENSGALG00010001586	ZBTB14	case	control	-0.124940579	0.135478976	-0.922213789	0.356417072	0.525804601	down_in_case
Yang2025_S	ENSGALG00010001588	ENSGALG00010001588	case	control	0.180795884	0.539585976	0.335064089	0.737576741	0.839922521	up_in_case
Yang2025_S	ENSGALG00010001595	AKAIN1	case	control	0.298485312	0.289034576	1.0326976	0.301745437	0.471339925	up_in_case
Yang2025_S	ENSGALG00010001599	TBLX1	case	control	-0.165119991	0.06259272	-2.638006335	0.008339502	0.035376245	down_in_case
Yang2025_S	ENSGALG00010001604	ENSGALG00010001604	case	control	-0.379273386	0.339592087	-1.116849891	0.264058513	0.431074903	down_in_case
Yang2025_S	ENSGALG00010001605	APBB1	case	control	0.045992499	0.194173887	0.236862429	0.812763518	0.887252024	up_in_case
Yang2025_S	ENSGALG00010001606	PIGA	case	control	-0.395865597	0.12316972	-3.213984715	0.001309066	0.008652403	down_in_case
Yang2025_S	ENSGALG00010001608	ENSGALG00010001608	case	control	-0.150469973	0.576104329	-0.261185284	0.793949626	0.875638151	down_in_case
Yang2025_S	ENSGALG00010001609	ENSGALG00010001609	case	control	-0.330381558	0.560911538	-0.589008312	0.555855698	0.703011282	down_in_case
Yang2025_S	ENSGALG00010001611	MAPK12	case	control	-0.183894336	0.143375	-1.28261089	0.199628433	0.356964226	down_in_case
Yang2025_S	ENSGALG00010001612	HPX	case	control	0.538454513	0.52214402	1.031237537	0.302429442	0.472037889	up_in_case
Yang2025_S	ENSGALG00010001616	OPNVA	case	control	-0.275867606	0.514343745	-0.536348714	0.591717569	0.732289905	down_in_case
Yang2025_S	ENSGALG00010001617	TRIM3	case	control	0.474091777	0.117746877	4.026363924	5.6646E-05	0.000675824	up_in_case
Yang2025_S	ENSGALG00010001620	MOSPD2	case	control	-0.222876899	0.156207196	-1.426803021	0.15363668	0.297618863	down_in_case
Yang2025_S	ENSGALG00010001621	DLGAP2	case	control	0.179941665	0.21185297	0.84937051	0.395675157	0.562855586	up_in_case
Yang2025_S	ENSGALG00010001627	ARFIP2	case	control	0.196886644	0.095916403	2.05269003	0.04010265	0.113560338	up_in_case
Yang2025_S	ENSGALG00010001628	MAPK11	case	control	-0.40535968	0.154023296	-2.631807597	0.008493195	0.035892169	down_in_case
Yang2025_S	ENSGALG00010001632	FANCB	case	control	-0.949273982	0.22843663	-4.15552436	3.24542E-05	0.000433605	down_in_case
Yang2025_S	ENSGALG00010001633	B3GAT2	case	control	0.052130147	0.455670849	0.114403076	0.908918275	0.945489412	up_in_case
Yang2025_S	ENSGALG00010001635	TIMM10B	case	control	0.347320265	0.307855821	1.128191321	0.259239128	0.425611561	up_in_case
Yang2025_S	ENSGALG00010001639	ENSGALG00010001639	case	control	0.713953046	0.237189203	3.010057099	0.002611986	0.014652988	up_in_case
Yang2025_S	ENSGALG00010001642	ENSGALG00010001642	case	control	0.86256032	0.272098632	3.170028143	0.001524242	0.00971651	up_in_case
Yang2025_S	ENSGALG00010001647	ARL8BL	case	control	0.127887793	0.196820323	0.64976925	0.515841285	0.67029544	up_in_case
Yang2025_S	ENSGALG00010001649	UBE2E2	case	control	0.15590183	0.21661981	0.71970255	0.471708156	0.631861675	up_in_case
Yang2025_S	ENSGALG00010001654	RPS11	case	control	-0.40117224	0.285050108	-1.407374454	0.159316382	0.304707129	down_in_case
Yang2025_S	ENSGALG00010001657	SMAP1	case	control	0.216504866	0.090158619	2.401377352	0.016333483	0.058540919	up_in_case
Yang2025_S	ENSGALG00010001661	TCTN3	case	control	-0.087986719	0.09212642	-0.955064995	0.33954483	0.507560552	down_in_case
Yang2025_S	ENSGALG00010001666	PIEZO2	case	control	-0.175777451	0.193944328	-0.90632942	0.364761518	0.533643229	down_in_case
Yang2025_S	ENSGALG00010001668	UBE2E1	case	control	-0.29864685	0.10856754	-2.7507932	0.005945116	0.027215898	down_in_case
Yang2025_S	ENSGALG00010001670	SHOX	case	control	0.358609721	0.242835	1.47676291	0.139739202	0.278518044	up_in_case
Yang2025_S	ENSGALG00010001672	ENSGALG00010001672	case	control	2.183757327	0.872945133	2.501597459	0.012363441	0.047461308	up_in_case
Yang2025_S	ENSGALG00010001674	ENSGALG00010001674	case	control	0.203444207	0.108190074	1.8804332	0.060049062	0.152614205	up_in_case
Yang2025_S	ENSGALG00010001677	SDHAF4	case	control	-0.367770151	0.215984186	-1.702764258	0.088612194	0.201826494	down_in_case
Yang2025_S	ENSGALG00010001678	SYAP1	case	control	-0.25192505	0.069174304	-3.641887737	0.000270646	0.002391537	down_in_case
Yang2025_S	ENSGALG00010001681	NAPG	case	control	-0.307918306	0.086260785	-3.569620945	0.000357498	0.003015579	down_in_case
Yang2025_S	ENSGALG00010001686	WASL	case	control	0.051611341	0.187229132	0.275658711	0.782810211	0.868623543	up_in_case
Yang2025_S	ENSGALG00010001688	ENSGALG00010001688	case	control	0.420571621	0.105799777	3.975165462	7.03303E-05	0.000804641	up_in_case
Yang2025_S	ENSGALG00010001689	ILK	case	control	0.306727646	0.096768412	3.169708369	0.00152592	0.009718325	up_in_case
Yang2025_S	ENSGALG00010001691	CTPS2	case	control	-0.076580917	0.114779972	-0.667197552	0.504645955	0.660366361	down_in_case
Yang2025_S	ENSGALG00010001692	ALDH18A1	case	control	0.08960671	0.125729663	0.712693471	0.476035458	0.63550578	up_in_case
Yang2025_S	ENSGALG00010001693	NKIRAS1	case	control	-0.339661806	0.204487961	-1.661035712	0.096706274	0.214238979	down_in_case
Yang2025_S	ENSGALG00010001694	ENSGALG00010001694	case	control	0.1481786	0.114654857	1.292388331	0.196222696	0.352344836	up_in_case
Yang2025_S	ENSGALG00010001695	LMOD2	case	control	0.800926133	0.261851389	3.058704925	0.00222296	0.012970202	up_in_case

Yang2025_S	ENSGALG00010001696	FAM135A	case	control	-0.300978673	0.097117621	-3.099114966	0.001940997	0.011724227	down_in_case
Yang2025_S	ENSGALG00010001697	TAF10	case	control	-0.223335456	0.298757807	-0.747546844	0.454733537	0.616411658	down_in_case
Yang2025_S	ENSGALG00010001698	RPL15	case	control	-1.068513796	0.3346676	-3.192761405	0.001409193	0.009189101	down_in_case
Yang2025_S	ENSGALG00010001699	ASB15	case	control	-0.551106912	0.198353526	-2.778407447	0.005462607	0.025621485	down_in_case
Yang2025_S	ENSGALG00010001700	ENSGALG00010001700	case	control	0.229553101	0.253823253	0.904381684	0.365793047	0.534505864	up_in_case
Yang2025_S	ENSGALG00010001701	NDUFA5	case	control	-0.019032749	0.241400303	-0.078843105	0.937157418	0.961656011	down_in_case
Yang2025_S	ENSGALG00010001702	PLEKHA1	case	control	0.023363559	0.090149751	0.259163874	0.795508803	0.876525276	up_in_case
Yang2025_S	ENSGALG00010001704	ENSGALG00010001704	case	control	-0.017786235	0.15459831	-0.115048053	0.908407034	0.945239847	down_in_case
Yang2025_S	ENSGALG00010001705	HTRA1	case	control	-0.262694196	0.141744072	-1.853299346	0.063839465	0.158971219	down_in_case
Yang2025_S	ENSGALG00010001706	IQUB	case	control	0.662321847	0.249510213	2.654487922	0.007942888	0.034077632	up_in_case
Yang2025_S	ENSGALG00010001708	AADAC	case	control	0.026322633	0.354210019	0.074313633	0.940760829	0.963650556	up_in_case
Yang2025_S	ENSGALG00010001710	LCMT2	case	control	0.079932687	0.118828491	0.672672743	0.501155512	0.657837421	up_in_case
Yang2025_S	ENSGALG00010001711	CREB5	case	control	0.67958271	0.367143382	1.851000845	0.064169432	0.159621747	up_in_case
Yang2025_S	ENSGALG00010001712	ATP6AP1	case	control	-0.383266522	0.132802183	-2.885995642	0.003901776	0.019840098	down_in_case
Yang2025_S	ENSGALG00010001713	PNPLA4	case	control	-0.110323643	0.217513712	-0.507203164	0.612012275	0.748892787	down_in_case
Yang2025_S	ENSGALG00010001716	TRIL	case	control	-0.353078584	0.087853874	-4.018930143	5.8463E-05	0.000691581	down_in_case
Yang2025_S	ENSGALG00010001717	STARD10	case	control	2.024515698	0.420488384	4.814676877	1.47438E-06	3.36261E-05	up_in_case
Yang2025_S	ENSGALG00010001721	TM4SF1	case	control	-1.07483944	0.248118085	-4.33196733	1.47783E-05	0.000227805	down_in_case
Yang2025_S	ENSGALG00010001725	MTA2	case	control	0.019925407	0.100796765	0.197679033	0.843296196	0.907018562	up_in_case
Yang2025_S	ENSGALG00010001727	COL9A1	case	control	2.316778548	1.023345855	2.263925278			up_in_case
Yang2025_S	ENSGALG00010001728	STS	case	control	-0.168455014	0.148928007	-1.131117091	0.258005812	0.424275547	down_in_case
Yang2025_S	ENSGALG00010001729	ENSGALG00010001729	case	control	-0.494383241	0.209263978	-2.362486112	0.018152822	0.063176715	down_in_case
Yang2025_S	ENSGALG00010001731	ENSGALG00010001731	case	control	-0.037042299	0.313677997	-0.118090204	0.905996185	0.943647271	down_in_case
Yang2025_S	ENSGALG00010001737	ENSGALG00010001737	case	control	1.292084821	0.849240151	1.521459883	0.128144471	0.261257236	up_in_case
Yang2025_S	ENSGALG00010001738	PUDP	case	control	0.049046784	0.171371216	0.286201999	0.774723413	0.862902307	up_in_case
Yang2025_S	ENSGALG00010001741	SATB1	case	control	0.232407635	0.242656407	0.957764264	0.338181643	0.50635796	up_in_case
Yang2025_S	ENSGALG00010001744	ENSGALG00010001744	case	control	3.368311237	1.100229341	3.061462833	0.002202583	0.012879983	up_in_case
Yang2025_S	ENSGALG00010001745	CADPS2	case	control	-0.362227423	0.151305239	-2.394017724	0.016664943	0.059392836	down_in_case
Yang2025_S	ENSGALG00010001749	VAPA	case	control	-0.089756193	0.105082879	-0.854146688	0.393023718	0.560013816	down_in_case
Yang2025_S	ENSGALG00010001751	AVPR2C	case	control	0.64176318	0.302454932	2.121847295	0.033850564	0.100128975	up_in_case
Yang2025_S	ENSGALG00010001752	ARRB1	case	control	0.459746866	0.266809633	1.72312694	0.084865572	0.195452919	up_in_case
Yang2025_S	ENSGALG00010001753	PNPLA8	case	control	-0.150092313	0.089733315	-1.672648693	0.094396475	0.210466381	down_in_case
Yang2025_S	ENSGALG00010001754	APCDD1	case	control	-0.424004984	0.170684912	-2.484138627	0.012986524	0.049202614	down_in_case
Yang2025_S	ENSGALG00010001756	ENSGALG00010001756	case	control	0.954902634	0.329050751	2.901991957	0.003707981	0.019096181	up_in_case
Yang2025_S	ENSGALG00010001758	COL19A1	case	control	0.039459916	0.187833247	0.210079509	0.833605618	0.901326373	up_in_case
Yang2025_S	ENSGALG00010001759	C10orf88	case	control	0.102732438	0.092932399	1.10545341	0.268963172	0.436169184	up_in_case
Yang2025_S	ENSGALG00010001760	RAB5A	case	control	-0.24535618	0.145253347	-1.689160252	0.091188724	0.205730086	down_in_case
Yang2025_S	ENSGALG00010001761	ELOB	case	control	0.567341235	0.199415741	2.845017315	0.004440901	0.021828555	up_in_case
Yang2025_S	ENSGALG00010001763	ENSGALG00010001763	case	control	0.633072904	0.398841502	1.587279408	0.112449377	0.238251797	up_in_case
Yang2025_S	ENSGALG00010001767	ENSGALG00010001767	case	control	0.845156811	0.279098791	3.028163642	0.002460448	0.01402512	up_in_case
Yang2025_S	ENSGALG00010001771	ENSGALG00010001771	case	control	0.385457581	0.193070483	1.996460438	0.045883828	0.125346234	up_in_case
Yang2025_S	ENSGALG00010001773	NRCAM	case	control	0.759832649	0.22410178	3.390569455	0.000697476	0.005236705	up_in_case
Yang2025_S	ENSGALG00010001776	LMBRD1	case	control	-0.69872815	0.210753615	-3.315379187	0.000915189	0.006520019	down_in_case
Yang2025_S	ENSGALG00010001778	KAT2B	case	control	0.065829476	0.124408855	0.529138183	0.596709596	0.735984087	up_in_case
Yang2025_S	ENSGALG00010001779	ENSGALG00010001779	case	control	3.323977037	0.562725613	5.906923304	3.48556E-09	1.79894E-07	up_in_case
Yang2025_S	ENSGALG00010001780	ENSGALG00010001780	case	control	0.264640557	0.163163849	1.621931319	0.104818055	0.226913095	up_in_case
Yang2025_S	ENSGALG00010001783	SGO1	case	control	-0.126372844	0.162047078	-0.779852656	0.435477608	0.599346219	down_in_case
Yang2025_S	ENSGALG00010001785	CNTN1	case	control	-0.270231731	0.245745292	-1.099641539	0.271488336	0.438681581	down_in_case
Yang2025_S	ENSGALG00010001786	P2RY8	case	control	-0.63969716	0.225079295	-2.842096869	0.004481787	0.022013995	down_in_case
Yang2025_S	ENSGALG00010001788	HOXA1	case	control	0.566153923	0.305575868	1.852744222	0.063919028	0.159140908	up_in_case
Yang2025_S	ENSGALG00010001790	HOXA2	case	control	0.660985823	0.219641471	3.009385334	0.002617769	0.014670105	up_in_case
Yang2025_S	ENSGALG00010001793	IKZF5	case	control	-0.39594765	0.124886056	-3.17047124	0.001521919	0.009710488	down_in_case
Yang2025_S	ENSGALG00010001795	ENSGALG00010001795	case	control	1.044588842	0.542650866	1.924974062	0.054232603	0.141628809	up_in_case
Yang2025_S	ENSGALG00010001796	SLCO2B1	case	control	-0.390465665	0.096320654	-4.053810353	5.03901E-05	0.00061166	down_in_case
Yang2025_S	ENSGALG00010001798	PSTK	case	control	0.191595484	0.199020778	0.962690861	0.335702684	0.504042334	up_in_case
Yang2025_S	ENSGALG00010001800	ENSGALG00010001800	case	control	0.194104274	0.127327354	1.524450697	0.127396164	0.260245964	up_in_case
Yang2025_S	ENSGALG00010001801	HOXA3	case	control	0.713408342	0.275051408	2.593727289	0.009494176	0.039084592	up_in_case
Yang2025_S	ENSGALG00010001803	PPP4R1	case	control	-0.074736377	0.055878723	-1.337474673	0.181067744	0.333489164	down_in_case
Yang2025_S	ENSGALG00010001805	ENSGALG00010001805	case	control	-0.288666155	0.153564962	-1.879765745	0.060140011	0.152733926	down_in_case
Yang2025_S	ENSGALG00010001807	ASMTL	case	control	-0.24986843	0.097540303	-2.561694214	0.010416298	0.04174608	down_in_case
Yang2025_S	ENSGALG00010001808	HOXA4	case	control	0.341960888	0.163137347	2.096153293	0.036068595	0.105039889	up_in_case
Yang2025_S	ENSGALG00010001810	OPN2SW	case	control	1.77694341	0.446818289	3.976881551	6.98249E-05	0.000800173	up_in_case
Yang2025_S	ENSGALG00010001811	ACADSB	case	control	-0.727812684	0.190529592	-3.819945648	0.000133481	0.001347869	down_in_case
Yang2025_S	ENSGALG00010001813	HOXA5	case	control	0.546336986	0.167941698	3.253134822	0.001141393	0.007781465	up_in_case
Yang2025_S	ENSGALG00010001817	SLC25A6	case	control	0.117998137	0.144208682	0.818245723	0.413216892	0.578711295	up_in_case
Yang2025_S	ENSGALG00010001818	ADGRB3	case	control	1.043205847	0.352380132	2.960455921	0.003071841	0.016623729	up_in_case
Yang2025_S	ENSGALG00010001819	HOXA6	case	control	0.249410311	0.092886801	2.685099573	0.00725082	0.031743694	up_in_case
Yang2025_S	ENSGALG00010001820	PGM2L1	case	control	-0.098935173	0.206072328	-0.480099263	0.631156812	0.763452777	down_in_case
Yang2025_S	ENSGALG00010001822	KCNE3	case	control	-0.727844779	0.387268891	-1.87943002	0.0601858	0.152822363	down_in_case
Yang2025_S	ENSGALG00010001823	RALBP1	case	control	0.233377141	0.17574664	1.327918084	0.18420517	0.337527816	up_in_case
Yang2025_S	ENSGALG00010001825	LIPT2	case	control	0.595496183	0.238138476	2.50062986	0.012397267	0.047546994	up_in_case
Yang2025_S	ENSGALG00010001826	HOXA7	case	control	0.14024819	0.137582002	1.019378898	0.30802312	0.477663229	up_in_case
Yang2025_S	ENSGALG00010001827	PDZRN4	case	control	0.578386026	0.301285646	1.919726457	0.05489246	0.142855525	up_in_case
Yang2025_S	ENSGALG00010001829	POLD3	case	control	-0.079167577	0.116058926	-0.682132601	0.495155108	0.652480986	down_in_case
Yang2025_S	ENSGALG00010001831	CSF2RA	case	control	-0.13849632	0.219560794	-0.630788027	0.528179133	0.680974322	down_in_case
Yang2025_S	ENSGALG00010001832	BUB3	case	control	-0.148609049	0.132873997	-1.11842085	0.263387293	0.430332036	down_in_case
Yang2025_S	ENSGALG00010001834	ZCRB1	case	control	0.128905392	0.160999136	0.800658903	0.423329141	0.588714652	up_in_case
Yang2025_S	ENSGALG00010001835	MECP2	case	control	-0.137810772	0.226692244	-0.607920101	0.543240467	0.693228563	down_in_case
Yang2025_S	ENSGALG00010001836	TWSG1	case	control	-0.451607957	0.10058498	-4.489815063	7.1285E-06	0.000125266	down_in_case
Yang2025_S	ENSGALG00010001837	TAB3	case	control	0.63843186	0.279617734	2.283230937	0.022416769	0.073552548	up_in_case
Yang2025_S	ENSGALG00010001838	PPHLN1	case	control	0.061936929	0.089511116	0.691946786	0.488970748	0.646287335	up_in_case
Yang2025_S	ENSGALG00010001840	HOXA9	case	control	0.393107983	0.14779956	2.659737162	0.007820165	0.033685936	up_in_case
Yang2025_S	ENSGALG00010001843	PRICKLE1	case	control	0.001809607	0.108625248	0.016659178	0.986708514	0.991904714	up_in_case
Yang2025_S	ENSGALG00010001844	HOXA10	case	control	0.194485684	0.143571809	1.354623068	0.17553768	0.32606206	up_in_case
Yang2025_S	ENSGALG00010001845	ANKRD12	case	control	0.688038141	0.267423977	2.572836399	0.010086888	0.040789547	up_in_case
Yang2025_S	ENSGALG00010001848	HOXA11	case	control	0.995149143	0.262854803	3.785927176	0.000153137	0.001501729	up_in_case
Yang2025_S	ENSGALG00010001849	NDUFV2	case	control	-0.297802222	0.192997417	-1.543037345	0.122821715	0.253709134	down_in_case
Yang2025_S	ENSGALG00010001850	MYOM2	case	control	0.065444073	0.085675202	0.763862488	0.444949194	0.608117597	up_in_case
Yang2025_S	ENSGALG00010001852	CPXM2	case	control	0.681082308	0.3454675	1.971480122	0.048668985	0.130775607	up_in_case
Yang2025_S	ENSGALG00010001853	ENSGALG00010001853	case	control	0.184562722	0.137731735	1.340015951	0.180240159	0.332388433	up_in_case
Yang2025_S	ENSGALG00010001854	CHRDL2	case	control	0.092571128	0.344648518	0.268595751	0.788240782	0.872340982	up_in_case
Yang2025_S	ENSGALG00010001856	CHST15	case	control	-0.089890173	0.112301763	-0.800434204	0.42345927	0.588714652	down_in_case
Yang2025_S	ENSGALG00010001857	HNRNPA2B1	case	control	0.446098848	0.116925443	3.815241882	0.00013605	0.001368846	up_in_case
Yang2025_S	ENSGALG00010001858	ADAMTS20	case	control	0.264117548	0.181292334	1.45685999	0.145155032	0.286308881	up_in_case
Yang2025_S	ENSGALG00010001860	RNF169	case	control	0.257468638	0.193833993	1.328294555	0.184080818	0.337473954	up_in_case
Yang2025_S	ENSGALG00010001861	CBX3	case	control	-0.104144782	0.07216591	-1.44312989	0.148983885	0.291381114	down_in_case
Yang2025_S	ENSGALG00010001864	ENSGALG00010001864	case	control	0.176387811	0.203090755	0.868517186	0.385111268	0.55265265	up_in_case
Yang2025_S	ENSGALG00010001865	CYBB	case	control	-0.329838842	0.242218124	-1.361743029	0.173278997	0.323158836	down_in_case
Yang2025_S	ENSGALG00010001866	PUS7L	case	control	0.025013643	0.109878812	0.227647557	0.819920245	0.892345253	up_in_case
Yang2025_S	ENSGALG00010001868	ENSGALG00010001868	case	control	0.288598004	0.171670987	1.681111112	0.092741334	0.208007484	up_in_case
Yang2025_S	ENSGALG00010001870	PLP2	case	control	0.302125612	0.248791614	1.214372169	0.224605638	0.386166511	up_in_case
Yang2025_S	ENSGALG00010001871	ENSGALG00010001871	case	control	0.237945585	0.216065496	1.101266001	0.270780904	0.438097295	up_in_case
Yang2025_S	ENSGALG00010001873	ENSGALG00010001873	case	control	0.608004528	0.287833437	2.112348497	0.034656569	0.101970765	up_in_case
Yang2025_S	ENSGALG00010001877	OAT	case	control	-0.451315232	0.097544028	-4.626784883	3.71386E-06	7.27885E-05	down_in_case
Yang2025_S	ENSGALG00010001878	XK	case	control	0.232759528	0.382367747	0.608732118	0.542702014	0.692728656	up_in_case
Yang2025_S	ENSGALG00010001879	IRAK4	case	control	0.501305108	0.172469686	2.906627359	0.003653481	0.018884	up_in_case
Yang2025_S	ENSGALG00010001880	MTCL1	case	control	0.416920727	0.214902305	1.940047722	0.05237389	0.137942373	up_in_case
Yang2025_S	ENSGALG00010001883	KBTBD11	case	control	-0.582957805	0.142832228	-4.081416438	4.47621E-05	0.000560431	down_in_case
Yang2025_S	ENSGALG00010001885	SMPX	case	control	-0.173673864	0.237322195	-0.731806241	0.464286839	0.625329344	down_in_case
Yang2025_S	ENSGALG00010001886	NEU3	case	control	-0.009140023	0.121864121	-0.075001751	0.940213319	0.963543774	down_in_case
Yang2025_S	ENSGALG00010001888	SNX10	case	control	-0.133770093	0.20186987	-0.662655069	0.507551492	0.663109886	down_in_case
Yang2025_S	ENSGALG00010001890	KLHL34	case	control	0.782532098	0.181553755	4.310195068	1.63111E-05	0.00024599	up_in_case
Yang2025_S	ENSGALG00010001892	RAB12	case	control	-0.365686005	0.066067633	-5.53502506	3.11184E-08	1.23192E-06	down_in_case
Yang2025_S	ENSGALG00010001895	P4HA3	case	control	-0.192406262	0.216757674	-0.887656055	0.374725791	0.542637836	down_in_case
Yang2025_S	ENSGALG00010001896	ARHGEF10	case	control	-0.428628715	0.143246059	-2.992254844	0.00276925	0.015313292	down_in_case
Yang2025_S	ENSGALG00010001897	TLR7	case	control	0.359112552	0.26083989	1.37675473	0.168588091	0.316912863	up_in_case
Yang2025_S	ENSGALG00010001898	LHPP	case	control	0.096676641	0.093045692	1.039023292	0.298793904	0.468198926	up_in_case
Yang2025_S	ENSGALG00010001899	TWF1	case	control	-0.126769927	0.100777734	-1.257916027	0.208422127	0.367316858	down_in_case
Yang2025_S	ENSGALG00010001901	PRPS2	case	control	-0.8070653	0.130765566	-6.171848778	6.7496E-10	4.23674E-08	down_in_case
Yang2025_S	ENSGALG00010001902	WDR45	case	control	0.089715973	0.112012294	0.80094755	0.423162011	0.588714652	up_in_case
Yang2025_S	ENSGALG00010001903	NARS2	case	control	-0.263967288	0.164728852	-1.60243506	0.109059437	0.232910844	down_in_case
Yang2025_S	ENSGALG00010001906	SKAP2	case	control	-0.214980139	0.119151739	-1.804255156	0.071191316	0.171990455	down_in_case
Yang2025_S	ENSGALG00010001908	FAM83B	case	control	-0.012374608	0.414467373	-0.029856651	0.976181378	0.985873859	down_in_case
Yang2025_S	ENSGALG00010001910	FAM53B	case	control	-0.080499872	0.119940028	-0.6711677	0.502113703	0.658677885	down_in_case
Yang2025_S	ENSGALG00010001911	USP35	case	control	0.291314959	0.783244255	0.371933732	0.709942189	0.820924745	up_in_case
Yang2025_S	ENSGALG00010001912	TMEM117	case	control	-0.354874555	0.117425792	-3.022117615	0.00251013	0.014237733	down_in_case

Yang2025_S	ENSGALG00010001914	ENSGALG00010001914	case	control	0.270680982	0.217962043	1.241872107	0.214283753	0.374801568	up_in_case
Yang2025_S	ENSGALG00010001915	EEF1AKMT2	case	control	0.428264282	0.169429102	2.527690214	0.011481561	0.044879538	up_in_case
Yang2025_S	ENSGALG00010001918	CNKSR2	case	control	0.243476937	0.195174499	1.247483348	0.212220322	0.372462529	up_in_case
Yang2025_S	ENSGALG00010001921	ENSGALG00010001921	case	control	-1.060216489	1.44962392	-0.731373478	0.46455106	0.625521212	down_in_case
Yang2025_S	ENSGALG00010001923	RAPGEF5	case	control	-0.172221815	0.111740807	-1.541261601	0.12325313	0.254336202	down_in_case
Yang2025_S	ENSGALG00010001925	FRMPD4	case	control	0.160835229	0.214266205	0.750632741	0.452873711	0.614667884	up_in_case
Yang2025_S	ENSGALG00010001928	KCTD21	case	control	-0.610118805	0.158574529	-3.847520839	0.000119319	0.001225285	down_in_case
Yang2025_S	ENSGALG00010001929	POLR2I	case	control	0.268755109	0.179468124	1.497508879	0.134260897	0.270364971	up_in_case
Yang2025_S	ENSGALG00010001935	ABRAXAS2	case	control	0.189103205	0.156247526	1.210279677	0.226171593	0.388236161	up_in_case
Yang2025_S	ENSGALG00010001938	ALG8	case	control	-0.499241848	0.145387867	-3.433861828	0.000595047	0.004586275	down_in_case
Yang2025_S	ENSGALG00010001940	PTPRM	case	control	0.12430787	0.13135933	0.946319308	0.343985748	0.51250309	up_in_case
Yang2025_S	ENSGALG00010001947	TINAG	case	control	-0.289240994	0.290439866	-0.995872219	0.319312235	0.488753953	down_in_case
Yang2025_S	ENSGALG00010001948	EMX2	case	control	-0.157920061	0.321910648	-0.4905711	0.623729831	0.757906801	down_in_case
Yang2025_S	ENSGALG00010001952	ENSGALG00010001952	case	control	0.295154095	0.167766873	1.759310944	0.078524709	0.184869372	up_in_case
Yang2025_S	ENSGALG00010001957	NDUFC2	case	control	-0.263654661	0.454248344	-0.580419643	0.561631662	0.708132954	down_in_case
Yang2025_S	ENSGALG00010001958	RAB11FIP2	case	control	-0.271408638	0.148809731	-1.82386351	0.06817271	0.166752099	down_in_case
Yang2025_S	ENSGALG00010001961	ENSGALG00010001961	case	control	1.225801036	0.265973681	4.608730572	4.05135E-06	7.79773E-05	up_in_case
Yang2025_S	ENSGALG00010001966	ARHGAP6	case	control	0.423230639	0.235380172	1.798072607	0.072165502	0.17359335	up_in_case
Yang2025_S	ENSGALG00010001967	LAMA1	case	control	-0.363671351	0.199092287	-1.826647113	0.067752845	0.165928979	down_in_case
Yang2025_S	ENSGALG00010001969	PLEKHA8	case	control	0.028553632	0.149501912	0.190991751	0.848532056	0.910118044	up_in_case
Yang2025_S	ENSGALG00010001971	MLIP	case	control	0.282383688	0.242624099	1.163873204	0.2444754	0.40976554	up_in_case
Yang2025_S	ENSGALG00010001972	FAM204A	case	control	-0.045759088	0.152780416	-0.29950886	0.764551813	0.856649925	down_in_case
Yang2025_S	ENSGALG00010001974	ENSGALG00010001974	case	control	0.465355413	0.261769008	1.777733031	0.075447712	0.179566843	up_in_case
Yang2025_S	ENSGALG00010001976	THRSP	case	control	-1.964279773	0.963874528	-2.037899868	0.041559948	0.116409623	down_in_case
Yang2025_S	ENSGALG00010001982	DMD	case	control	0.186446617	0.19372287	0.962439883	0.335828688	0.504122887	up_in_case
Yang2025_S	ENSGALG00010001988	ENSGALG00010001988	case	control	1.116852227	0.433089812	2.578800508	0.009914402	0.040279066	up_in_case
Yang2025_S	ENSGALG00010001991	TOMM7	case	control	0.675169972	0.332258151	2.032064434	0.042147133	0.117511065	up_in_case
Yang2025_S	ENSGALG00010001995	LRRC1	case	control	-0.160656581	0.159394533	-1.007917764	0.313493943	0.48287146	down_in_case
Yang2025_S	ENSGALG00010001996	ENSGALG00010001996	case	control	-0.682303978	0.652653252	-1.045431056	0.29582379	0.464432684	down_in_case
Yang2025_S	ENSGALG00010001997	INTS4	case	control	-0.349494625	0.08160096	-4.282971978	1.84413E-05	0.000271649	down_in_case
Yang2025_S	ENSGALG00010002005	COLQ	case	control	0.094984283	0.155732826	0.609918189	0.541916003	0.692235723	up_in_case
Yang2025_S	ENSGALG00010002006	ENSGALG00010002006	case	control	0.412065453	0.314199564	1.311476847	0.189696711	0.344497335	up_in_case
Yang2025_S	ENSGALG00010002012	MID1	case	control	-0.492165557	0.147026286	-3.347466429	0.000815539	0.005937582	down_in_case
Yang2025_S	ENSGALG00010002017	ARHGAP28	case	control	-0.137545617	0.124104321	-1.108306432	0.267729499	0.434826406	down_in_case
Yang2025_S	ENSGALG00010002022	ERNI	case	control	-1.892216442	0.957829235	-1.975525879	0.048208501	0.129955637	down_in_case
Yang2025_S	ENSGALG00010002023	CLCN4	case	control	-0.384701984	0.236108983	-1.629340735	0.103240907	0.224510307	down_in_case
Yang2025_S	ENSGALG00010002024	KLHL31	case	control	-0.521192157	0.117941448	-4.419075428	9.91241E-06	0.000162697	down_in_case
Yang2025_S	ENSGALG00010002027	ENSGALG00010002027	case	control	-0.597819015	0.481118589	-1.242560625	0.214029787	0.374498378	down_in_case
Yang2025_S	ENSGALG00010002034	WWC3	case	control	0.288127666	0.128039307	2.250306362	0.024429504	0.078747431	up_in_case
Yang2025_S	ENSGALG00010002039	AAMDC	case	control	0.203297739	0.146626703	1.386498739	0.165594665	0.313144478	up_in_case
Yang2025_S	ENSGALG00010002041	GCLC	case	control	0.423229313	0.160208647	2.641738254	0.008248177	0.035052866	up_in_case
Yang2025_S	ENSGALG00010002046	FAM126A	case	control	0.029357643	0.080971805	0.362566237	0.716928935	0.825653282	up_in_case
Yang2025_S	ENSGALG00010002050	ENSGALG00010002050	case	control	2.199813029	0.955573156	2.302087512	0.021330238	0.071011945	up_in_case
Yang2025_S	ENSGALG00010002054	RSF1	case	control	0.006525603	0.155130523	0.042065245	0.966446686	0.980027659	up_in_case
Yang2025_S	ENSGALG00010002062	GSTA4	case	control	0.003395001	0.343445252	0.009885129	0.992112937	0.994997748	up_in_case
Yang2025_S	ENSGALG00010002065	CLNS1A	case	control	-0.014655177	0.109833785	-0.133430502	0.893852924	0.937586789	down_in_case
Yang2025_S	ENSGALG00010002072	NEURL4	case	control	0.499963021	0.08821097	5.667810049	1.44634E-08	6.43922E-07	up_in_case
Yang2025_S	ENSGALG00010002079	ARX	case	control	0.132473164	0.258007951	0.513446052	0.607639339	0.744981013	up_in_case
Yang2025_S	ENSGALG00010002084	HACL1	case	control	0.056867252	0.105936631	0.536804424	0.591402715	0.732083986	up_in_case
Yang2025_S	ENSGALG00010002087	POLA1	case	control	-0.001879965	0.071478227	-0.026301221	0.979017081	0.987378087	down_in_case
Yang2025_S	ENSGALG00010002088	OZFL1	case	control	0.407028154	0.165969468	2.452427894	0.014189581	0.052560292	up_in_case
Yang2025_S	ENSGALG00010002090	AQP11	case	control	0.409556305	0.229244711	1.786546366	0.074010837	0.176997941	up_in_case
Yang2025_S	ENSGALG00010002097	PCYT1B	case	control	0.991813699	0.451681942	2.195823224	0.028104599	0.08715331	up_in_case
Yang2025_S	ENSGALG00010002098	ENSGALG00010002098	case	control	1.643791533	0.443508665	3.706334653	0.00021028	0.001952204	up_in_case
Yang2025_S	ENSGALG00010002100	BTD	case	control	-0.345927128	0.134321641	-2.575364071	0.010013462	0.040548951	down_in_case
Yang2025_S	ENSGALG00010002102	PAK1	case	control	0.107644937	0.277755236	0.387553223	0.698346691	0.813439491	up_in_case
Yang2025_S	ENSGALG00010002113	GDPD4	case	control	-0.155172209	0.312298925	-0.496870776	0.619280181	0.754901631	down_in_case
Yang2025_S	ENSGALG00010002118	ENSGALG00010002118	case	control	-0.192484221	0.479257213	-0.401630306	0.687956123	0.804790847	down_in_case
Yang2025_S	ENSGALG00010002122	ENSGALG00010002122	case	control	-1.119267345	0.73892771	-1.514718329	0.129843751	0.263680074	down_in_case
Yang2025_S	ENSGALG00010002126	ENSGALG00010002126	case	control	0.258173988	0.212773426	1.21337515	0.224986423	0.386582713	up_in_case
Yang2025_S	ENSGALG00010002134	ANKRD28	case	control	0.498085363	0.11682964	4.263347597	2.01387E-05	0.000292326	up_in_case
Yang2025_S	ENSGALG00010002135	ELOVL5	case	control	-0.598276235	0.099911124	-5.988084309	2.12327E-09	1.18826E-07	down_in_case
Yang2025_S	ENSGALG00010002136	ACAP1	case	control	0.415236005	0.290985186	1.427000496	0.153579752	0.297612827	up_in_case
Yang2025_S	ENSGALG00010002137	PDK3	case	control	-0.454667342	0.11310177	-4.019984321	5.8202E-05	0.000690145	down_in_case
Yang2025_S	ENSGALG00010002138	MYO7A	case	control	-0.587890246	0.236576131	-2.484993921	0.012955365	0.049151377	down_in_case
Yang2025_S	ENSGALG00010002140	ENSGALG00010002140	case	control	-0.184467664	0.532552734	-0.346383846	0.729054264	0.833418472	down_in_case
Yang2025_S	ENSGALG00010002142	CLDN34	case	control	1.444933169	0.502743137	2.874098249	0.004051829	0.020346752	up_in_case
Yang2025_S	ENSGALG00010002145	ENSGALG00010002145	case	control	-0.277702635	0.39795731	-0.697820163	0.485289668	0.642701722	down_in_case
Yang2025_S	ENSGALG00010002146	CACUL1	case	control	-0.385651557	0.092502965	-4.169072375	3.05842E-05	0.000412576	down_in_case
Yang2025_S	ENSGALG00010002147	ENSGALG00010002147	case	control	1.256074369	0.492453898	2.550643572	0.010752423	0.04273674	up_in_case
Yang2025_S	ENSGALG00010002148	POLR2A	case	control	0.670585248	0.421281768	1.591773722	0.111435557	0.236643475	up_in_case
Yang2025_S	ENSGALG00010002149	ENSGALG00010002149	case	control	0.224068052	0.453393162	0.49420254	0.621163146	0.7558516	up_in_case
Yang2025_S	ENSGALG00010002150	EIF3A	case	control	0.252572187	0.138606024	1.82223095	0.06841995	0.167180783	up_in_case
Yang2025_S	ENSGALG00010002159	GALNT15	case	control	-0.191694108	0.185225216	-1.03492447	0.300704186	0.470346035	down_in_case
Yang2025_S	ENSGALG00010002160	CAPN5	case	control	0.056362799	0.131336471	0.429148114	0.66781544	0.79018495	up_in_case
Yang2025_S	ENSGALG00010002161	CDKL5	case	control	0.144688322	0.232332042	0.622765248	0.533438798	0.685355779	up_in_case
Yang2025_S	ENSGALG00010002174	ENSGALG00010002174	case	control	-0.362870628	0.45118623	-0.804259093	0.421247357	0.586890814	down_in_case
Yang2025_S	ENSGALG00010002177	FBXO9	case	control	-0.470893505	0.154776943	-3.04240087	0.002346991	0.013520182	down_in_case
Yang2025_S	ENSGALG00010002181	FIS1	case	control	1.325393014	0.157361665	8.422591456	3.68247E-17	1.22179E-14	up_in_case
Yang2025_S	ENSGALG00010002185	ENSGALG00010002185	case	control	0.777816614	0.61910799	1.25635047	0.208988931	0.368221109	up_in_case
Yang2025_S	ENSGALG00010002186	CILK1	case	control	0.091536653	0.078381712	1.167831756	0.242874636	0.407804071	up_in_case
Yang2025_S	ENSGALG00010002192	ENSGALG00010002192	case	control	-0.955244432	0.170745754	-5.594542814	2.21204E-08	9.28457E-07	down_in_case
Yang2025_S	ENSGALG00010002194	SHROOM2	case	control	0.097787666	0.201643281	0.484953757	0.627709148	0.760884393	up_in_case
Yang2025_S	ENSGALG00010002196	ENSGALG00010002196	case	control	-0.493975332	0.071328012	-6.925404426	4.34732E-12	5.2224E-10	down_in_case
Yang2025_S	ENSGALG00010002197	GSTA2	case	control	0.344283179	0.260551539	1.321363058	0.186380338	0.340056735	up_in_case
Yang2025_S	ENSGALG00010002202	SFXN4	case	control	-0.292209079	0.181172404	-1.61287852	0.106770903	0.229500622	down_in_case
Yang2025_S	ENSGALG00010002206	DPH3P1	case	control	0.289695505	0.170710159	1.697002141	0.089696256	0.203337778	up_in_case
Yang2025_S	ENSGALG00010002210	GPR143	case	control	2.744875352	0.80594124	3.405800841	0.000659703	0.005009787	up_in_case
Yang2025_S	ENSGALG00010002211	ACER3	case	control	-0.378288133	0.122362113	-3.091546257	0.00199117	0.01192904	down_in_case
Yang2025_S	ENSGALG00010002215	YLEC18	case	control	-0.850011207	0.414644598	-2.049975355	0.040366836	0.113914916	down_in_case
Yang2025_S	ENSGALG00010002225	PRDX3	case	control	-0.683635798	0.195472312	-3.497353614	0.000469899	0.003761077	down_in_case
Yang2025_S	ENSGALG00010002226	OXNAD1	case	control	0.02001145	0.192573914	0.103915685	0.91723626	0.951236618	up_in_case
Yang2025_S	ENSGALG00010002231	TMEM14A	case	control	-0.747451277	0.131917021	-5.666071534	1.46109E-08	6.46629E-07	down_in_case
Yang2025_S	ENSGALG00010002235	MXRA5	case	control	-0.049570419	0.282816314	-0.175274255	0.86086414	0.918118919	down_in_case
Yang2025_S	ENSGALG00010002238	ENSGALG00010002238	case	control	0.71536149	0.575961212	1.242030671	0.214225246	0.374746272	up_in_case
Yang2025_S	ENSGALG00010002239	AGPAT5	case	control	-0.319010884	0.113208451	-2.817906968	0.004833781	0.023380334	down_in_case
Yang2025_S	ENSGALG00010002243	ARSH	case	control	0.484209194	0.174490773	2.774984523	0.005520433	0.025797197	up_in_case
Yang2025_S	ENSGALG00010002247	RFTN1	case	control	-0.5384472	0.122901495	-4.38112815	1.18066E-05	0.00018911	down_in_case
Yang2025_S	ENSGALG00010002251	GRK5	case	control	0.074571557	0.199248628	0.374263843	0.708208029	0.820014864	up_in_case
Yang2025_S	ENSGALG00010002253	ENSGALG00010002253	case	control	0.294492884	0.215417305	1.367080902	0.171599949	0.320757248	up_in_case
Yang2025_S	ENSGALG00010002254	ENSGALG00010002254	case	control	-1.086453207	0.492473436	-2.206115352	0.027375924	0.085457774	down_in_case
Yang2025_S	ENSGALG00010002261	NR0B1	case	control	-0.697020357	0.518130446	-1.345260372	0.178541166	0.330057196	down_in_case
Yang2025_S	ENSGALG00010002269	MCPH1	case	control	0.103831808	0.143357655	0.72428506	0.468890747	0.629539701	up_in_case
Yang2025_S	ENSGALG00010002270	IMMP2L	case	control	-0.757387521	0.308809974	-2.452600579	0.014182772	0.052560292	down_in_case
Yang2025_S	ENSGALG00010002274	ENSGALG00010002274	case	control	2.147730259	1.228128421	1.748783126	0.080328515	0.187878122	up_in_case
Yang2025_S	ENSGALG00010002275	IL1RAPL1	case	control	0.551815725	0.355009421	1.554369241	0.120096366	0.249707977	up_in_case
Yang2025_S	ENSGALG00010002277	RGS10	case	control	-0.378733579	0.102271829	-3.703205302	0.000212892	0.001967279	down_in_case
Yang2025_S	ENSGALG00010002279	ENSGALG00010002279	case	control	1.025678487	0.901748597	1.137432861	0.255357377	0.421711904	up_in_case
Yang2025_S	ENSGALG00010002280	ST7	case	control	0.375464946	0.164322305	2.284929886	0.022316939	0.073276755	up_in_case
Yang2025_S	ENSGALG00010002281	TGFBR2	case	control	-0.042505152	0.134949088	-0.31497176	0.752783095	0.84953131	down_in_case
Yang2025_S	ENSGALG00010002282	TSKU	case	control	-0.240782254	0.069277575	-3.475616098	0.000509681	0.004012661	down_in_case
Yang2025_S	ENSGALG00010002283	ENSGALG00010002283	case	control	1.543100121	1.145622588	1.346953296	0.177995275	0.329404418	up_in_case
Yang2025_S	ENSGALG00010002285	CAV1	case	control	-0.372576393	0.093112246	-4.001368347	6.29772E-05	0.000735614	down_in_case
Yang2025_S	ENSGALG00010002288	ANGPT2	case	control	0.276572765	0.175515252	1.575776248	0.115077378	0.24227274	up_in_case
Yang2025_S	ENSGALG00010002289	ENSGALG00010002289	case	control	1.491478739	0.408747459	3.648900331	0.000263365	0.002340558	up_in_case
Yang2025_S	ENSGALG00010002291	RNASEH1	case	control	0.254617919	0.11826504	2.152943235	0.03132314	0.094559782	up_in_case
Yang2025_S	ENSGALG00010002293	GYG2	case	control	-0.645870272	0.270575615	-2.387023209	0.016985419	0.060165687	down_in_case
Yang2025_S	ENSGALG00010002294	DAZL	case	control	-0.856254766	0.844184913	-1.014297641	0.310440749	0.48013228	down_in_case
Yang2025_S	ENSGALG00010002295	YAF2	case	control	0.23225305	0.122020221	1.903398044	0.056988626	0.14698066	up_in_case
Yang2025_S	ENSGALG00010002298	ENSGALG00010002298	case	control	0.609611325	0.485145132	1.256554554	0.20891498	0.36813736	up_in_case
Yang2025_S	ENSGALG00010002308	GXYLT1	case	control	-0.676108705	0.121294173	-5.574123539	2.48779E-08	1.02871E-06	down_in_case
Yang2025_S	ENSGALG00010002309	GADL1	case	control	-0.247532799	0.206335452	-1.199661992	0.23027064	0.392708526	down_in_case
Yang2025_S	ENSGALG00010002310	PRRG1	case	control	-0.312292175	0.119687071	-2.609239023	0.009074383	0.037837083	down_in_case
Yang2025_S	ENSGALG00010002312	ENSGALG00010002312	case	control	-0.199049343	0.158115234	-1.258887824	0.208070852	0.366928287	down_in_case
Yang2025_S	ENSGALG00010002315	ENSGALG00010002315	case	control	-0.259946937	0.17916664	-1.450866841	0.146816943	0.288641944	down_in_case
Yang2025_S	ENSGALG00010002328	ENSGALG00010002328	case	control	0.162430025	0.349690184	0.464496951	0.642291743	0.77178024	up_in_case

Yang2025_S	ENSGALG00010002329	STT3B	case	control	-0.151835494	0.070680417	-2.148197482	0.031698071	0.095484785	down_in_case
Yang2025_S	ENSGALG00010002330	KHDRBS2	case	control	0.541234537	0.313170389	1.728243016	0.083944665	0.193895061	up_in_case
Yang2025_S	ENSGALG00010002331	ENSGALG00010002331	case	control	0.10546274	0.367310496	0.287121497	0.774019292	0.862393767	up_in_case
Yang2025_S	ENSGALG00010002332	ENSGALG00010002332	case	control	-0.301296398	0.749784184	-0.401844164	0.687798717	0.804674261	down_in_case
Yang2025_S	ENSGALG00010002333	ENSGALG00010002333	case	control	0.642701291	0.812191665	0.79131727	0.428758873	0.593030686	up_in_case
Yang2025_S	ENSGALG00010002334	OSBPL10	case	control	-0.073518083	0.165130291	-0.445212581	0.656166136	0.782848896	down_in_case
Yang2025_S	ENSGALG00010002335	TMEM47	case	control	-0.208233623	0.156525677	-1.33034801	0.183403636	0.336767646	down_in_case
Yang2025_S	ENSGALG00010002336	PRIM2	case	control	-0.542117515	0.1396917	-3.880814058	0.000104107	0.001102175	down_in_case
Yang2025_S	ENSGALG00010002338	CD99	case	control	0.143953361	0.096279829	1.495155966	0.134873743	0.271246299	up_in_case
Yang2025_S	ENSGALG00010002339	ZNFY4	case	control	0.937577249	0.583225416	1.607572686	0.107928798	0.231169352	up_in_case
Yang2025_S	ENSGALG00010002340	RAB23	case	control	-0.183164368	0.121023086	-1.513466353	0.130161242	0.264209308	down_in_case
Yang2025_S	ENSGALG00010002341	FAM49A	case	control	0.121790855	0.145770516	0.835497177	0.403437836	0.569826296	up_in_case
Yang2025_S	ENSGALG00010002342	BAG2	case	control	-0.143488946	0.147413918	-0.97337448	0.330367218	0.499777142	down_in_case
Yang2025_S	ENSGALG00010002343	GPD1L	case	control	-0.507093624	0.150671012	-3.365568572	0.000763861	0.005628979	down_in_case
Yang2025_S	ENSGALG00010002345	SCML2	case	control	-0.277990124	0.153636503	-1.809401532	0.070388645	0.170674399	down_in_case
Yang2025_S	ENSGALG00010002346	FBXO15	case	control	-1.374174978	0.411847134	-3.336614161	0.000848056	0.006123139	down_in_case
Yang2025_S	ENSGALG00010002347	ZNF451	case	control	-0.194810386	0.079435087	-2.452447572	0.014188805	0.052560292	down_in_case
Yang2025_S	ENSGALG00010002348	THAP12	case	control	-0.03880386	0.077902335	-0.498109077	0.618407163	0.754267293	down_in_case
Yang2025_S	ENSGALG00010002349	ZBED1	case	control	-0.392782725	0.082283224	-4.773545644	1.81011E-06	3.99111E-05	down_in_case
Yang2025_S	ENSGALG00010002350	BEND6	case	control	0.544970253	0.184448484	2.954593288	0.003130815	0.016856206	up_in_case
Yang2025_S	ENSGALG00010002352	ENSGALG00010002352	case	control	-0.47621481	0.184211085	-2.585158272	0.009733431	0.039740801	down_in_case
Yang2025_S	ENSGALG00010002353	CMTM8	case	control	0.036934429	0.2043292	0.180759425	0.856556413	0.915205767	up_in_case
Yang2025_S	ENSGALG00010002355	UFM1	case	control	-0.704195531	0.158016004	-4.456482333	8.33155E-06	0.000140218	down_in_case
Yang2025_S	ENSGALG00010002356	WNT11	case	control	-0.721189658	0.241049593	-2.991872537	0.00277272	0.015320323	down_in_case
Yang2025_S	ENSGALG00010002357	RAB31	case	control	-0.203496503	0.13143051	-1.548320123	0.121545242	0.252006092	down_in_case
Yang2025_S	ENSGALG00010002358	FREM2	case	control	-0.113425641	0.19617888	-0.578174575	0.563146263	0.709277221	down_in_case
Yang2025_S	ENSGALG00010002359	CMTM6	case	control	-0.800607431	0.166592038	-4.805796488	1.54137E-06	3.48684E-05	down_in_case
Yang2025_S	ENSGALG00010002360	DHRSX	case	control	-0.464953742	0.198986374	-2.336610961	0.019459422	0.066462511	down_in_case
Yang2025_S	ENSGALG00010002361	ENSGALG00010002361	case	control	0.337949892	0.100225238	3.371904104	0.000746504	0.005539158	up_in_case
Yang2025_S	ENSGALG00010002362	TRPC4	case	control	0.002830225	0.451349602	0.006270582	0.994996832	0.996498552	up_in_case
Yang2025_S	ENSGALG00010002364	VSNL1	case	control	0.229908054	0.36841388	0.624048296	0.532595868	0.684787177	up_in_case
Yang2025_S	ENSGALG00010002365	RAI2	case	control	0.355702818	0.219655493	1.619366827	0.10536836	0.227679965	up_in_case
Yang2025_S	ENSGALG00010002366	ENSGALG00010002366	case	control	2.069536854	0.284097542	7.284599641	3.22627E-13	4.68313E-11	up_in_case
Yang2025_S	ENSGALG00010002368	UVRAG	case	control	-0.278607163	0.119985579	-2.322005413	0.020232644	0.068283336	down_in_case
Yang2025_S	ENSGALG00010002369	GEMIN8	case	control	0.318164645	0.119069645	2.67208863	0.007538074	0.032723693	up_in_case
Yang2025_S	ENSGALG00010002370	ENSGALG00010002370	case	control	-0.099874667	0.187134188	-0.533706152	0.593544852	0.733899514	down_in_case
Yang2025_S	ENSGALG00010002371	BAG3	case	control	0.416179328	0.222695724	1.868824961	0.061647171	0.155428501	up_in_case
Yang2025_S	ENSGALG00010002372	SMC6	case	control	0.09705187	0.111758453	0.86840742	0.385171334	0.55265265	up_in_case
Yang2025_S	ENSGALG00010002373	POSTN	case	control	-0.342019922	0.183772633	-1.861103672	0.062729538	0.157105699	down_in_case
Yang2025_S	ENSGALG00010002374	DST	case	control	0.786285171	0.219739632	3.578258338	0.000345891	0.002935443	up_in_case
Yang2025_S	ENSGALG00010002375	NPY	case	control	0.2146478	0.453661118	0.473145685	0.636109245	0.766761145	up_in_case
Yang2025_S	ENSGALG00010002376	GEN1	case	control	-0.034272477	0.147689528	-0.232057596	0.816493279	0.890075401	down_in_case
Yang2025_S	ENSGALG00010002377	ART1A	case	control	0.711944316	0.24813415	2.869191187	0.00411523	0.020590925	up_in_case
Yang2025_S	ENSGALG00010002378	ENSGALG00010002378	case	control	0.22267763	0.183807263	1.211473515	0.225713979	0.387641644	up_in_case
Yang2025_S	ENSGALG00010002379	NHS	case	control	0.709026431	0.278774002	2.543373573	0.010978778	0.043401212	up_in_case
Yang2025_S	ENSGALG00010002380	CMPK2	case	control	0.3811326	0.379164342	1.005191041	0.314804868	0.484045478	up_in_case
Yang2025_S	ENSGALG00010002381	TDRD3	case	control	0.524853416	0.21414366	2.450940714	0.014248342	0.052735895	up_in_case
Yang2025_S	ENSGALG00010002383	INPP5F	case	control	-0.056690074	0.118446247	-0.478614351	0.632213007	0.763944976	down_in_case
Yang2025_S	ENSGALG00010002384	ENSGALG00010002384	case	control	0.97624736	0.465857763	2.09559105	0.036118484	0.105141231	up_in_case
Yang2025_S	ENSGALG00010002385	PALS2	case	control	-0.047674571	0.136986185	-0.348024668	0.727821658	0.832760884	down_in_case
Yang2025_S	ENSGALG00010002386	MHCY2B2	case	control	0.15979186	0.269459059	0.59300979	0.5531746	0.701250496	up_in_case
Yang2025_S	ENSGALG00010002387	MCMBP	case	control	0.169913504	0.094262637	1.802554123	0.071458267	0.172427871	up_in_case
Yang2025_S	ENSGALG00010002388	ENSGALG00010002388	case	control	-0.54919239	0.590307485	-0.930349697	0.352190054	0.520994523	down_in_case
Yang2025_S	ENSGALG00010002389	COL21A1	case	control	0.195256199	0.239310617	0.815911143	0.414550959	0.579996748	up_in_case
Yang2025_S	ENSGALG00010002391	REPS2	case	control	-0.018239945	0.106012987	-0.172053873	0.863395176	0.919290108	down_in_case
Yang2025_S	ENSGALG00010002392	DFNA5	case	control	0.035602863	0.166258953	0.214141026	0.830437084	0.898876865	up_in_case
Yang2025_S	ENSGALG00010002393	GPM6B	case	control	-0.004721717	0.112508833	-0.041967525	0.966524587	0.980027659	down_in_case
Yang2025_S	ENSGALG00010002395	CLN8	case	control	-0.460299545	0.07940353	-5.796965795	6.75255E-09	3.21149E-07	down_in_case
Yang2025_S	ENSGALG00010002396	SEC23IP	case	control	-0.119741508	0.124205484	-0.96405975	0.335015971	0.503553829	down_in_case
Yang2025_S	ENSGALG00010002397	ENSGALG00010002397	case	control	-1.164406414	0.540171922	-2.15562188	0.031113203	0.094109503	down_in_case
Yang2025_S	ENSGALG00010002399	CSMD1	case	control	-0.637454472	0.312158446	-2.042086258	0.041142976	0.115543605	down_in_case
Yang2025_S	ENSGALG00010002400	RBBP7	case	control	-0.012073007	0.212960302	-0.056691352	0.954791063	0.972132325	down_in_case
Yang2025_S	ENSGALG00010002401	ZRANB1	case	control	0.06625299	0.101179581	0.654805929	0.512592713	0.667572691	up_in_case
Yang2025_S	ENSGALG00010002402	TXLNG	case	control	-0.169507101	0.121981535	-1.389612784	0.164646492	0.311901694	down_in_case
Yang2025_S	ENSGALG00010002403	ZNFY3	case	control	-1.12878189	0.321202105	-3.514241883	0.000441011	0.003568809	down_in_case
Yang2025_S	ENSGALG00010002404	OSBPL3	case	control	0.577037334	0.18413314	3.133804896	0.001725555	0.010715515	up_in_case
Yang2025_S	ENSGALG00010002405	KCNQ5	case	control	0.517730198	0.282983617	1.829541243	0.067318567	0.165272063	up_in_case
Yang2025_S	ENSGALG00010002406	DIAPH3	case	control	0.111776127	0.185500996	0.602563487	0.546799117	0.695855204	up_in_case
Yang2025_S	ENSGALG00010002408	OFD1	case	control	0.311764486	0.202590585	1.53888931	0.123831321	0.255057172	up_in_case
Yang2025_S	ENSGALG00010002409	CYCS	case	control	-0.396091757	0.22259197	-1.77945214	0.075165665	0.179109702	down_in_case
Yang2025_S	ENSGALG00010002410	RNF121	case	control	-0.083575759	0.248518498	-0.336295928	0.73664772	0.8392761	down_in_case
Yang2025_S	ENSGALG00010002411	ENSGALG00010002411	case	control	0.411318229	0.27429037	1.499572258	0.133725238	0.269597974	up_in_case
Yang2025_S	ENSGALG00010002413	TSSC1	case	control	-0.115894815	0.122348041	-0.947255182	0.343508763	0.512044604	down_in_case
Yang2025_S	ENSGALG00010002414	ENSGALG00010002414	case	control	1.496498449	0.634544385	2.35838262	0.018354764	0.063593642	up_in_case
Yang2025_S	ENSGALG00010002415	PCDH17	case	control	1.289010476	0.521532928	2.471580234	0.013451736	0.050484768	up_in_case
Yang2025_S	ENSGALG00010002416	SH3BGRL2	case	control	-0.680197511	0.293197395	-2.319930271	0.02034465	0.068611494	down_in_case
Yang2025_S	ENSGALG00010002418	TRAPPC2	case	control	-0.504889318	0.103202696	-4.89221053	9.97097E-07	2.41644E-05	down_in_case
Yang2025_S	ENSGALG00010002419	CTBP2	case	control	0.237329962	0.118648095	2.000284642	0.045469536	0.124826239	up_in_case
Yang2025_S	ENSGALG00010002420	TMEM272	case	control	-0.145463776	0.556923721	-0.261191561	0.793944785	0.875638151	down_in_case
Yang2025_S	ENSGALG00010002421	ENSGALG00010002421	case	control	-0.781978575	0.237287862	-3.29548494	0.00098252	0.006911364	down_in_case
Yang2025_S	ENSGALG00010002422	TRAPPC12	case	control	-0.056960103	0.129331851	-0.440418213	0.659634236	0.785037414	down_in_case
Yang2025_S	ENSGALG00010002423	ENSGALG00010002423	case	control	-0.444007696	0.55470685	-0.800436655	0.423457851	0.588714652	down_in_case
Yang2025_S	ENSGALG00010002424	CASK	case	control	0.086403712	0.113467292	0.761485626	0.446367061	0.609235587	up_in_case
Yang2025_S	ENSGALG00010002425	FDX2	case	control	-0.039330735	0.249122001	-0.157877405	0.874553403	0.92642396	down_in_case
Yang2025_S	ENSGALG00010002427	CYB5R4	case	control	0.120013619	0.106513452	1.126746121	0.259849836	0.426332672	up_in_case
Yang2025_S	ENSGALG00010002428	IBSP	case	control	0.444462781	0.864018277	0.514413634	0.606962829	0.744282561	up_in_case
Yang2025_S	ENSGALG00010002429	ENSGALG00010002429	case	control	0.151703705	0.498426378	0.304365323	0.760849584	0.85475967	up_in_case
Yang2025_S	ENSGALG00010002430	NUMA1	case	control	0.793396249	0.144845435	5.477537139	4.31287E-08	1.65564E-06	up_in_case
Yang2025_S	ENSGALG00010002431	BCKDHB	case	control	-0.353764127	0.130049243	-2.720232104	0.006523611	0.029315226	down_in_case
Yang2025_S	ENSGALG00010002432	RAVER1	case	control	0.85695695	0.142849622	5.999014475	1.98519E-09	1.11998E-07	up_in_case
Yang2025_S	ENSGALG00010002433	GRHL1	case	control	-0.037486249	0.497674652	-0.075322801	0.939957881	0.96346547	down_in_case
Yang2025_S	ENSGALG00010002434	GPR34	case	control	-0.566820614	0.147241354	-3.849602027	0.00011831	0.001216715	down_in_case
Yang2025_S	ENSGALG00010002435	ATP7B	case	control	1.161655478	0.645205118	1.800443682	0.071790609	0.172960258	up_in_case
Yang2025_S	ENSGALG00010002436	ENSGALG00010002436	case	control	0.680987392	0.833503842	0.817017701	0.413918313	0.57930146	up_in_case
Yang2025_S	ENSGALG00010002437	TTK	case	control	-0.41745785	0.162326455	-2.571717903	0.010119531	0.040862262	down_in_case
Yang2025_S	ENSGALG00010002438	RARB	case	control	0.13512795	0.151758183	0.890416233	0.373242429	0.541255415	up_in_case
Yang2025_S	ENSGALG00010002440	LRRC51	case	control	-0.591275796	0.348386999	-1.697180999	0.089662446	0.203337778	down_in_case
Yang2025_S	ENSGALG00010002441	USO1	case	control	0.371642415	0.06455413	5.757066472	8.55882E-09	3.96236E-07	up_in_case
Yang2025_S	ENSGALG00010002442	ENSGALG00010002442	case	control	1.272524884	0.376305764	3.38162475	0.000720585	0.005384103	up_in_case
Yang2025_S	ENSGALG00010002443	TYK2	case	control	0.215793134	0.083075021	2.597569414	0.009388614	0.038741979	up_in_case
Yang2025_S	ENSGALG00010002444	KLF11	case	control	0.108580289	0.16412392	0.661575042	0.508243607	0.663827413	up_in_case
Yang2025_S	ENSGALG00010002445	EDRF1	case	control	0.03079842	0.069760722	0.441486547	0.658860799	0.784658933	up_in_case
Yang2025_S	ENSGALG00010002446	RIMS1	case	control	0.240674165	0.444787847	0.541098787	0.588439494	0.729658689	up_in_case
Yang2025_S	ENSGALG00010002447	YLEC12	case	control	-0.035533401	0.520091833	-0.068321399	0.94552979	0.966310395	down_in_case
Yang2025_S	ENSGALG00010002448	RAB9A	case	control	-0.361095843	0.142489076	-2.534200184	0.011270433	0.044240417	down_in_case
Yang2025_S	ENSGALG00010002449	ALG11	case	control	-0.525610139	0.16660899	-3.154752558	0.001606343	0.010137856	down_in_case
Yang2025_S	ENSGALG00010002450	ENSGALG00010002450	case	control	0.72465168	0.459086757	1.578463481	0.114459171	0.24137236	up_in_case
Yang2025_S	ENSGALG00010002453	TOP2B	case	control	0.170736008	0.103426396	1.65079723	0.098779986	0.217216207	up_in_case
Yang2025_S	ENSGALG00010002454	NEK5	case	control	0.337692701	0.388299847	0.869669931	0.384480809	0.552002893	up_in_case
Yang2025_S	ENSGALG00010002455	NYX	case	control	0.392210509	0.167166091	2.346232464	0.018964275	0.065191228	up_in_case
Yang2025_S	ENSGALG00010002457	ENSGALG00010002457	case	control	-0.118139395	0.440740577	-0.268047467	0.788662782	0.872500466	down_in_case
Yang2025_S	ENSGALG00010002458	ENSGALG00010002458	case	control	0.219838164	0.122085724	1.800686908	0.071752242	0.17292762	up_in_case
Yang2025_S	ENSGALG00010002459	G3BP2	case	control	-0.046675981	0.079899786	-0.584181554	0.559098171	0.705950663	down_in_case
Yang2025_S	ENSGALG00010002460	LAMTOR1	case	control	0.26617408	0.256218972	1.038853908	0.298872686	0.468269718	up_in_case
Yang2025_S	ENSGALG00010002461	TOMT	case	control	0.791529923	0.187964001	4.211071896	2.54162E-05	0.00035524	up_in_case
Yang2025_S	ENSGALG00010002462	RRM2	case	control	0.123444496	0.173604731	0.711066426	0.477043074	0.636194395	up_in_case
Yang2025_S	ENSGALG00010002463	ASRGL1	case	control	0.086779337	0.168268362	0.515719865	0.606050078	0.743766699	up_in_case
Yang2025_S	ENSGALG00010002464	NGLY1	case	control	0.294348692	0.131752713	2.234099664	0.025476516	0.081016717	up_in_case
Yang2025_S	ENSGALG00010002465	ENSGALG00010002465	case	control	4.031967515	0.832447415	4.843510165	1.27565E-06	2.9822E-05	up_in_case
Yang2025_S	ENSGALG00010002466	UROS	case	control	0.138918871	0.132447944	1.048856385	0.294244222	0.462839285	up_in_case
Yang2025_S	ENSGALG00010002467	NEK3	case	control	0.102565006	0.196971713	0.520709318	0.602569283	0.740872548	up_in_case
Yang2025_S	ENSGALG00010002468	ENSGALG00010002468	case	control	0.004119788	0.43060581	0.009567423	0.992366417	0.995042536	up_in_case
Yang2025_S	ENSGALG00010002470	OXSM	case	control	-0.018330823	0.155164085	-0.118138312	0.905958067	0.943647271	down_in_case
Yang2025_S	ENSGALG00010002471	OGFRL1	case	control	-0.267408958	0.154672846	-1.728868155	0.083832695	0.19373277	down_in_case
Yang2025_S	ENSGALG00010002472	CDC37	case	control	0.548729598	0.10005516	5.484270824	4.15178E-08	1.59969E-06	up_in_case
Yang2025_S	ENSGALG00010002473	ENSGALG00010002473	case	control	1.876642381	1.184425825	1.584432171	0.113095406	0.239329458	up_in_case

Yang2025_S	ENSGALG00010002474	HPCAL1	case	control	-0.0131319	0.115277173	-0.113915876	0.90930448	0.945749958	down_in_case
Yang2025_S	ENSGALG00010002475	ENSGALG00010002475	case	control	0.212129828	0.255693913	0.829624084	0.406751355	0.572822652	up_in_case
Yang2025_S	ENSGALG00010002476	DDX3X	case	control	0.166994024	0.092196304	1.811287613	0.070096342	0.170172914	up_in_case
Yang2025_S	ENSGALG00010002478	PPME1	case	control	0.202704886	0.120507984	1.682086782	0.092552013	0.207783519	up_in_case
Yang2025_S	ENSGALG00010002481	CKAP2	case	control	0.010699471	0.128784334	0.08308053	0.933787507	0.959611277	up_in_case
Yang2025_S	ENSGALG00010002483	COQ2	case	control	-0.291627522	0.229879905	-1.268608154	0.204580855	0.362809824	down_in_case
Yang2025_S	ENSGALG00010002487	BCCIP	case	control	0.065407535	0.116221962	0.562781198	0.573583885	0.716529936	up_in_case
Yang2025_S	ENSGALG00010002492	RPL8	case	control	0.238453284	0.169930145	1.403242986	0.160544393	0.306244432	up_in_case
Yang2025_S	ENSGALG00010002500	OSR1	case	control	0.585398509	0.335636474	1.744144495	0.081133897	0.189158584	up_in_case
Yang2025_S	ENSGALG00010002501	TCEANC	case	control	-0.190688852	0.188924407	-1.009339421	0.31281188	0.482247323	down_in_case
Yang2025_S	ENSGALG00010002504	ENSGALG00010002504	case	control	0.061582111	0.127438363	0.48323056	0.628932039	0.761505601	up_in_case
Yang2025_S	ENSGALG00010002507	C2CD3	case	control	-0.054759205	0.129090653	-0.424191864	0.671425894	0.792975662	down_in_case
Yang2025_S	ENSGALG00010002511	VPS36	case	control	0.091302201	0.084520553	1.080236667	0.280036804	0.447821077	up_in_case
Yang2025_S	ENSGALG00010002512	DAXX	case	control	0.542161658	0.136818725	3.96262762	7.41294E-05	0.000837788	up_in_case
Yang2025_S	ENSGALG00010002518	MAST1	case	control	0.978061694	0.326981348	2.991184973	0.002778971	0.015342695	up_in_case
Yang2025_S	ENSGALG00010002520	TTC32	case	control	0.152039195	0.279798983	0.543387234	0.586863213	0.728487644	up_in_case
Yang2025_S	ENSGALG00010002522	HPSE	case	control	-0.298731506	0.296931098	-1.006063386	0.31438508	0.483656004	down_in_case
Yang2025_S	ENSGALG00010002523	DNAJB13	case	control	0.644970842	0.278623858	2.314844273	0.020621456	0.069210017	up_in_case
Yang2025_S	ENSGALG00010002526	ZBTB22	case	control	0.268162772	0.237376446	1.129694104	0.258605143	0.424910112	up_in_case
Yang2025_S	ENSGALG00010002527	LRRC3B	case	control	-0.34341946	0.253765995	-1.35329188	0.175962402	0.326667209	down_in_case
Yang2025_S	ENSGALG00010002528	MED14	case	control	0.353421422	0.16592096	2.130058933	0.033166748	0.098608627	up_in_case
Yang2025_S	ENSGALG00010002530	STAG3	case	control	0.219091554	0.115756937	1.892686164	0.058399617	0.149683111	up_in_case
Yang2025_S	ENSGALG00010002533	LPAR2	case	control	-0.400518788	0.167340808	-2.393431661	0.01669159	0.059447396	down_in_case
Yang2025_S	ENSGALG00010002536	RGL2	case	control	0.146029523	0.243140297	0.60059778	0.548107917	0.69703773	up_in_case
Yang2025_S	ENSGALG00010002539	SNAP91	case	control	1.029285035	0.333843777	3.08313381	0.00204833	0.012192767	up_in_case
Yang2025_S	ENSGALG00010002543	UCP3	case	control	-0.198220966	0.375890801	-0.52733657	0.597959883	0.737134729	down_in_case
Yang2025_S	ENSGALG00010002544	THSD1	case	control	-0.507223943	0.134729523	-3.764757205	0.000166711	0.001611039	down_in_case
Yang2025_S	ENSGALG00010002546	ATP6AP2	case	control	-0.381656337	0.08507101	-4.486326643	7.24616E-06	0.000126853	down_in_case
Yang2025_S	ENSGALG00010002547	PBX4	case	control	0.106204974	0.12838804	0.827218593	0.408113175	0.573928459	up_in_case
Yang2025_S	ENSGALG00010002551	WDR35	case	control	-0.256799666	0.114673618	-2.239396212	0.025130148	0.080226485	down_in_case
Yang2025_S	ENSGALG00010002553	ENSGALG00010002553	case	control	1.027567912	0.293653416	3.49925408	0.000466562	0.003740816	up_in_case
Yang2025_S	ENSGALG00010002554	EGFL6	case	control	-0.378203425	0.150223089	-2.517611819	0.011815346	0.045837095	down_in_case
Yang2025_S	ENSGALG00010002556	ENSGALG00010002556	case	control	-0.424190657	0.191997957	-2.209349852	0.027150316	0.08496623	down_in_case
Yang2025_S	ENSGALG00010002557	ENSGALG00010002557	case	control	0.016411914	0.229428058	0.071534033	0.94297274	0.965065006	up_in_case
Yang2025_S	ENSGALG00010002561	PAAF1	case	control	0.026752836	0.12100157	0.221094952	0.825018501	0.895244953	up_in_case
Yang2025_S	ENSGALG00010002565	MRPS18C	case	control	-0.144183752	0.205799884	-0.700601714	0.483551608	0.641803187	down_in_case
Yang2025_S	ENSGALG00010002566	TMSB4X	case	control	-0.247177819	0.242863769	-1.017763249	0.308790481	0.478429548	down_in_case
Yang2025_S	ENSGALG00010002567	COA4	case	control	-0.491415093	0.304457253	-1.614069256	0.106512405	0.22909836	down_in_case
Yang2025_S	ENSGALG00010002569	GTPBP6	case	control	-0.212863513	0.140152016	-1.518804499	0.128811713	0.262272242	down_in_case
Yang2025_S	ENSGALG00010002570	ADAM12	case	control	-0.050197688	0.21033035	-0.238661171	0.811368328	0.886368766	down_in_case
Yang2025_S	ENSGALG00010002571	BCOR	case	control	0.317510963	0.159911575	1.985540838	0.047084331	0.127823773	up_in_case
Yang2025_S	ENSGALG00010002573	MVB12A	case	control	0.178731226	0.138476642	1.290695844	0.196809166	0.352919281	up_in_case
Yang2025_S	ENSGALG00010002575	BGV	case	control	0.393213595	0.323062905	1.217142511	0.223549999	0.384921442	up_in_case
Yang2025_S	ENSGALG00010002576	HELQ	case	control	0.086960209	0.15009252	0.579377369	0.562334571	0.708639198	up_in_case
Yang2025_S	ENSGALG00010002577	MRPL48	case	control	0.111207811	0.160318372	0.693668538	0.487890101	0.645103763	up_in_case
Yang2025_S	ENSGALG00010002579	MATN3	case	control	1.28764556	0.973204891	1.323098118			up_in_case
Yang2025_S	ENSGALG00010002581	ATP13A1	case	control	-0.117176827	0.121849703	-0.961650494	0.336225198	0.504367781	down_in_case
Yang2025_S	ENSGALG00010002582	ENSGALG00010002582	case	control	-0.298573041	0.31329527	-0.953008455	0.340585787	0.508852036	down_in_case
Yang2025_S	ENSGALG00010002584	ENSGALG00010002584	case	control	0.828398177	0.414677494	1.997692637	0.045749993	0.125216666	up_in_case
Yang2025_S	ENSGALG00010002586	GPR63	case	control	0.10269762	0.208566799	0.492396777	0.622438877	0.756868433	up_in_case
Yang2025_S	ENSGALG00010002587	DOCK1	case	control	0.007138303	0.179379618	0.039794393	0.968257046	0.981213144	up_in_case
Yang2025_S	ENSGALG00010002588	FAM175A	case	control	0.068759842	0.117283037	0.586272696	0.557692277	0.704700932	up_in_case
Yang2025_S	ENSGALG00010002589	SLC4A7	case	control	0.383427778	0.093938921	4.081671099	4.4713E-05	0.00056032	up_in_case
Yang2025_S	ENSGALG00010002590	ATP6V1C2	case	control	0.564890675	0.256690197	2.200671008	0.027759323	0.086422288	up_in_case
Yang2025_S	ENSGALG00010002591	ENSGALG00010002591	case	control	-0.703642732	0.235517028	-2.987651204	0.002811302	0.015496635	down_in_case
Yang2025_S	ENSGALG00010002592	RPS6KA3	case	control	0.025701272	0.169184143	0.151913008	0.879255548	0.928681389	up_in_case
Yang2025_S	ENSGALG00010002597	ENSGALG00010002597	case	control	0.50835852	0.181733511	2.797274512	0.005153573	0.024535372	up_in_case
Yang2025_S	ENSGALG00010002598	NMT2	case	control	-0.289513636	0.079106502	-3.659795687	0.000252416	0.002260555	down_in_case
Yang2025_S	ENSGALG00010002599	KIFC1	case	control	-0.217484869	0.29137235	-0.746415607	0.455416394	0.61692729	down_in_case
Yang2025_S	ENSGALG00010002600	TRPA1	case	control	-0.354855837	0.71976618	-0.493015436	0.622001681	0.756554591	down_in_case
Yang2025_S	ENSGALG00010002601	PLCXD1	case	control	-0.575155273	0.129010373	-4.458209525	8.26471E-06	0.000139653	down_in_case
Yang2025_S	ENSGALG00010002603	INSYN2A	case	control	0.116040345	0.157497523	0.736775681	0.461258774	0.622307717	up_in_case
Yang2025_S	ENSGALG00010002604	BLEC3	case	control	-1.219928516	0.687217763	-1.775170233	0.075869783	0.1802635	down_in_case
Yang2025_S	ENSGALG00010002606	ENSGALG00010002606	case	control	-0.125777346	0.292112441	-0.430578532	0.666774854	0.789228274	down_in_case
Yang2025_S	ENSGALG00010002608	RPP38	case	control	-0.253422305	0.167280351	-1.514955602	0.129783648	0.263673296	down_in_case
Yang2025_S	ENSGALG00010002609	PDIA6	case	control	0.176108368	0.093777618	1.877936036	0.060389916	0.153208018	up_in_case
Yang2025_S	ENSGALG00010002611	NPS	case	control	-0.051999085	0.534844763	-0.097222761	0.922549493	0.953661386	down_in_case
Yang2025_S	ENSGALG00010002612	JPH1	case	control	0.024395616	0.156498221	0.155884305	0.876124217	0.927040476	up_in_case
Yang2025_S	ENSGALG00010002613	ENSGALG00010002613	case	control	0.103740848	0.116230459	0.89254442	0.372101195	0.540465932	up_in_case
Yang2025_S	ENSGALG00010002614	GPAT3	case	control	-0.407221605	0.16183629	-2.516256427	0.011860885	0.045926844	down_in_case
Yang2025_S	ENSGALG00010002616	ZNFB3	case	control	-0.079328936	0.181023302	-0.438224995	0.661223191	0.786189861	down_in_case
Yang2025_S	ENSGALG00010002619	RAB6A	case	control	0.419159057	0.133053325	3.150308763	0.00163098	0.0102526	up_in_case
Yang2025_S	ENSGALG00010002621	SLC25A15	case	control	0.205714785	0.191479356	1.074344459	0.282668326	0.450839318	up_in_case
Yang2025_S	ENSGALG00010002622	AZI2	case	control	0.095132605	0.084056873	1.13176474	0.257733355	0.424027663	up_in_case
Yang2025_S	ENSGALG00010002623	MID1IP1	case	control	0.018594521	0.137282844	0.135446792	0.89225863	0.936760643	up_in_case
Yang2025_S	ENSGALG00010002625	FHL5	case	control	-0.527434775	0.082129443	-6.421993795	1.34501E-10	1.06745E-08	down_in_case
Yang2025_S	ENSGALG00010002626	CHD7	case	control	0.442246706	0.213066642	2.075626202	0.037928536	0.108796655	up_in_case
Yang2025_S	ENSGALG00010002627	MRPS31	case	control	-0.083647378	0.18286704	-0.457421842	0.647367868	0.775872645	down_in_case
Yang2025_S	ENSGALG00010002628	TRIM7.2	case	control	-0.151444444	0.112631327	-1.344603213	0.178753405	0.330401254	down_in_case
Yang2025_S	ENSGALG00010002632	HPGD	case	control	0.351448429	0.254149667	1.382840411	0.166713805	0.314529759	up_in_case
Yang2025_S	ENSGALG00010002633	ENSGALG00010002633	case	control	0.607036093	0.168783314	3.596540914	0.000322477	0.002770478	up_in_case
Yang2025_S	ENSGALG00010002634	RGN	case	control	-0.056534801	0.25992007	-0.217508408	0.827812156	0.89687159	down_in_case
Yang2025_S	ENSGALG00010002638	FABP9	case	control	0.496144688	0.435665785	1.138819493	0.25477845	0.421184398	up_in_case
Yang2025_S	ENSGALG00010002644	KCNF1	case	control	-0.468030776	0.288005645	-1.625075006	0.104146578	0.225845403	down_in_case
Yang2025_S	ENSGALG00010002646	FOXO1	case	control	-0.549266646	0.076348479	-7.194205492	6.28255E-13	8.58307E-11	down_in_case
Yang2025_S	ENSGALG00010002647	FAM168A	case	control	0.123157398	0.132318527	0.930764576	0.351975358	0.520794648	up_in_case
Yang2025_S	ENSGALG00010002649	UFL1	case	control	0.141255279	0.092526409	1.526648234	0.126848506	0.25949538	up_in_case
Yang2025_S	ENSGALG00010002653	EIF1AX	case	control	-0.249683857	0.091713343	-2.722437646	0.006480226	0.029148467	down_in_case
Yang2025_S	ENSGALG00010002655	FABP4	case	control	-1.479103422	0.677094101	-2.184487237	0.028926464	0.089061042	down_in_case
Yang2025_S	ENSGALG00010002656	GLRA3	case	control	-1.129884058	0.605247197	-1.866814192	0.06192754	0.155994264	down_in_case
Yang2025_S	ENSGALG00010002658	FBXL12	case	control	0.427002094	0.237306479	1.799369723	0.071960216	0.173219141	up_in_case
Yang2025_S	ENSGALG00010002661	ACBD7	case	control	0.035192103	0.265587897	0.132506427	0.89458374	0.937909687	up_in_case
Yang2025_S	ENSGALG00010002664	ZNFB1	case	control	0.156222599	0.215558393	0.724734475	0.468614941	0.629230026	up_in_case
Yang2025_S	ENSGALG00010002667	ENSGALG00010002667	case	control	-2.296068563	0.885504927	-2.592948376	0.009515705	0.03915009	down_in_case
Yang2025_S	ENSGALG00010002669	IL4I1	case	control	-0.642499053	0.60452476	-1.062816771	0.287865053	0.456764433	down_in_case
Yang2025_S	ENSGALG00010002673	SLC66A3	case	control	-0.399883295	0.169040721	-2.365603343	0.018000719	0.062866639	down_in_case
Yang2025_S	ENSGALG00010002678	CCNI	case	control	0.065156334	0.144831208	0.449877721	0.652798612	0.780167124	up_in_case
Yang2025_S	ENSGALG00010002681	PLEKHB1	case	control	0.253944486	0.130415728	1.947192183	0.051511698	0.136130385	up_in_case
Yang2025_S	ENSGALG00010002682	UBL5	case	control	0.602404964	0.158829125	3.792786524	0.000148966	0.001472412	up_in_case
Yang2025_S	ENSGALG00010002685	CA8	case	control	-0.623845004	0.165267974	-3.774748291	0.000160169	0.001562997	down_in_case
Yang2025_S	ENSGALG00010002689	COG6	case	control	-0.227149334	0.110795641	-2.0501649	0.040348342	0.113911305	down_in_case
Yang2025_S	ENSGALG00010002693	MSC	case	control	-0.721514195	0.181097808	-3.98411335	6.77325E-05	0.000780044	down_in_case
Yang2025_S	ENSGALG00010002700	LHFPL6	case	control	-0.604269212	0.144817436	-4.172627468	3.01107E-05	0.000406976	down_in_case
Yang2025_S	ENSGALG00010002701	TMEM196	case	control	0.967405379	0.430577195	2.246764088	0.024655108	0.079199846	up_in_case
Yang2025_S	ENSGALG00010002703	PIN1	case	control	0.182033914	0.1309308	1.390306278	0.164435892	0.311545093	up_in_case
Yang2025_S	ENSGALG00010002704	KCNB2	case	control	0.132177167	0.258885968	0.510561341	0.609658252	0.747105724	up_in_case
Yang2025_S	ENSGALG00010002705	JADE3	case	control	0.101877393	0.085071155	1.197555081	0.231090272	0.393961701	up_in_case
Yang2025_S	ENSGALG00010002706	ROCK2	case	control	0.563063164	0.223232703	2.522314861	0.01165853	0.04538034	up_in_case
Yang2025_S	ENSGALG00010002707	RELT	case	control	0.105070654	0.21872951	0.480367986	0.630965754	0.763304782	up_in_case
Yang2025_S	ENSGALG00010002712	PTPRE	case	control	-0.089253703	0.08817577	-1.012224819	0.311430572	0.481091883	down_in_case
Yang2025_S	ENSGALG00010002713	OLFM2	case	control	0.299561565	0.157943142	1.896641793	0.057875231	0.148552466	up_in_case
Yang2025_S	ENSGALG00010002716	NHLRC3	case	control	-0.384648813	0.151141907	-2.544951429	0.010929294	0.043254675	down_in_case
Yang2025_S	ENSGALG00010002722	TRIM7.1	case	control	0.418588388	0.153704018	2.723340578	0.00646254	0.029101065	up_in_case
Yang2025_S	ENSGALG00010002725	TSPAN7	case	control	0.339497432	0.157969014	2.149139398	0.031623352	0.095300909	up_in_case
Yang2025_S	ENSGALG00010002728	RP2	case	control	-0.78591952	0.10294953	-7.634027252	2.27532E-14	4.28467E-12	down_in_case
Yang2025_S	ENSGALG00010002730	E2F6	case	control	-0.083449643	0.116380452	-0.717041754	0.473348334	0.632929363	down_in_case
Yang2025_S	ENSGALG00010002733	RDH8	case	control	0.055911309	0.293773913	0.190320878	0.849057694	0.910351313	up_in_case
Yang2025_S	ENSGALG00010002741	PDK4	case	control	-0.229282634	0.251791206	-0.910606202	0.362502902	0.531175387	down_in_case
Yang2025_S	ENSGALG00010002742	PROSER1	case	control	1.271027871	0.313289222	4.057043078	4.96979E-05	0.000606959	up_in_case
Yang2025_S	ENSGALG00010002747	FUT9	case	control	1.139167204	0.476807026	2.389157754	0.016887049	0.059908614	up_in_case
Yang2025_S	ENSGALG00010002750	ENSGALG00010002750	case	control	0.03926838	0.130035456	0.301982099	0.762665709	0.855408866	up_in_case
Yang2025_S	ENSGALG00010002752	NDUFA4	case	control	0.204658592	0.16774669	1.220045488	0.222447632	0.38403203	up_in_case
Yang2025_S	ENSGALG00010002753	SLC9A7	case	control	0.092447966	0.161728366	0.57162493	0.567576102	0.712288575	up_in_case
Yang2025_S	ENSGALG00010002758	GREB1	case	control	0.138473187	0.492742354	0.281025541	0.77869081	0.865798806	up_in_case
Yang2025_S	ENSGALG00010002760	RAB2A	case	control	0.334122738	0.09121504	3.663022445	0.000249257	0.002239453	up_in_case
Yang2025_S	ENSGALG00010002762	PPAN	case	control	0.809090537	0.095786144	8.446843201	2.99285E-17	1.0172E-14	up_in_case

Yang2025_S	ENSGALG00010002763	SEPTIN11	case	control	0.262776229	0.163064178	1.611489615	0.107073049	0.229937269	up_in_case
Yang2025_S	ENSGALG00010002765	MAP7D2	case	control	0.376862526	0.636347067	0.592227961	0.553697948	0.70174227	up_in_case
Yang2025_S	ENSGALG00010002766	NANOS1	case	control	0.905526376	0.376370808	2.405942113	0.01613082	0.057978587	up_in_case
Yang2025_S	ENSGALG00010002768	TRIM39.2	case	control	0.916505893	0.545532155	1.680021762	0.092953082	0.20834827	up_in_case
Yang2025_S	ENSGALG00010002774	TBX18	case	control	0.259370701	0.139849741	1.854638414	0.063647879	0.158664256	up_in_case
Yang2025_S	ENSGALG00010002779	FBXO8	case	control	-0.226496099	0.114577451	-1.976794705	0.04806484	0.129777863	down_in_case
Yang2025_S	ENSGALG00010002781	EIF3G	case	control	0.010912931	0.172604209	0.063225174	0.949587199	0.968633161	up_in_case
Yang2025_S	ENSGALG00010002782	GSTA3	case	control	0.122786135	0.116108512	1.057511921	0.290278017	0.458879656	up_in_case
Yang2025_S	ENSGALG00010002784	TRIM27.2	case	control	1.49285517	0.507269229	2.94292475	0.003251274	0.01729256	up_in_case
Yang2025_S	ENSGALG00010002787	CEP44	case	control	-0.218211569	0.095062156	-2.295462023	0.02170666	0.071831468	down_in_case
Yang2025_S	ENSGALG00010002788	ENSGALG00010002788	case	control	-1.107146242	0.414150497	-2.673294494	0.007511028	0.032616446	down_in_case
Yang2025_S	ENSGALG00010002790	BCLAF3	case	control	0.013897902	0.114214743	0.121682211	0.903150702	0.94209185	up_in_case
Yang2025_S	ENSGALG00010002792	ENSGALG00010002792	case	control	-0.302463027	0.23419824	-1.29148292	0.196536274	0.352573025	down_in_case
Yang2025_S	ENSGALG00010002796	PRDM8	case	control	0.264801887	0.234061773	1.131333335	0.257914819	0.424275547	up_in_case
Yang2025_S	ENSGALG00010002800	TUBGCP5	case	control	-0.042216515	0.09544021	-0.442334683	0.658247037	0.784458433	down_in_case
Yang2025_S	ENSGALG00010002801	FGF5	case	control	0.039462671	0.310696315	0.127013644	0.8989296	0.940010804	up_in_case
Yang2025_S	ENSGALG00010002805	FAM171A1	case	control	-0.017743584	0.116445057	-0.152377305	0.878889356	0.92862622	down_in_case
Yang2025_S	ENSGALG00010002807	P2RY6	case	control	-0.090961531	0.208891482	-0.43544873	0.663236746	0.786972416	down_in_case
Yang2025_S	ENSGALG00010002809	SH3KBP1	case	control	-0.043227804	0.146625528	-0.294817719	0.76813313	0.858788123	down_in_case
Yang2025_S	ENSGALG00010002810	TRIM27.1	case	control	-0.459024131	0.321461117	-1.42793049	0.153311867	0.29727739	down_in_case
Yang2025_S	ENSGALG00010002812	TRIM13	case	control	-0.478726319	0.082885657	-5.775743788	7.66139E-09	3.61903E-07	down_in_case
Yang2025_S	ENSGALG00010002814	TERF1	case	control	-0.226271923	0.145740204	-1.552570374	0.120525808	0.250376734	down_in_case
Yang2025_S	ENSGALG00010002817	SHFM1	case	control	-0.414744292	0.193813429	-2.139915141	0.032361625	0.096959632	down_in_case
Yang2025_S	ENSGALG00010002819	BMP3	case	control	-0.3672053	0.184785933	-1.987192937	0.04690102	0.127425564	down_in_case
Yang2025_S	ENSGALG00010002824	NXPH1	case	control	-0.315335971	0.860481831	-0.366464415			down_in_case
Yang2025_S	ENSGALG00010002825	TRIM41	case	control	0.137141574	0.108848473	1.259931076	0.207694227	0.366449615	up_in_case
Yang2025_S	ENSGALG00010002827	SBSPON	case	control	-1.349848162	0.520166421	-2.595031337	0.00945823	0.03899421	down_in_case
Yang2025_S	ENSGALG00010002832	LPIN1	case	control	-0.199193804	0.203903066	-0.976904409	0.328616479	0.498180008	down_in_case
Yang2025_S	ENSGALG00010002834	MRPL53	case	control	-0.171145307	0.238236249	-0.718384829	0.472520038	0.632219562	down_in_case
Yang2025_S	ENSGALG00010002835	CYFIP1	case	control	-0.28599525	0.070522258	-4.055389873	5.00507E-05	0.000609665	down_in_case
Yang2025_S	ENSGALG00010002837	DNMT1	case	control	0.033738081	0.079295528	0.425472688	0.670492127	0.79227574	up_in_case
Yang2025_S	ENSGALG00010002838	ENSGALG00010002838	case	control	0.405475588	0.26047424	1.556682104	0.11954598	0.248860655	up_in_case
Yang2025_S	ENSGALG00010002840	SRPX	case	control	-0.393255941	0.141522795	-2.778746288	0.005456913	0.025603394	down_in_case
Yang2025_S	ENSGALG00010002848	TRIB2	case	control	-0.489195119	0.131164142	-3.729640664	0.000191753	0.001800592	down_in_case
Yang2025_S	ENSGALG00010002853	CEP162	case	control	1.142204255	0.211957882	5.388826523	7.09192E-08	2.54706E-06	up_in_case
Yang2025_S	ENSGALG00010002855	RACK1	case	control	-0.40962381	0.181944247	-2.251369951	0.024362116	0.078639352	down_in_case
Yang2025_S	ENSGALG00010002857	DCAF15	case	control	0.549214696	0.187185006	2.934074193	0.003345442	0.017672001	up_in_case
Yang2025_S	ENSGALG00010002860	NIPA2	case	control	-0.871629413	0.153073917	-5.694173308	1.23971E-08	5.59074E-07	down_in_case
Yang2025_S	ENSGALG00010002864	RNASEH2B	case	control	-0.403170152	0.124248505	-3.244869253	0.001175046	0.007929424	down_in_case
Yang2025_S	ENSGALG00010002866	ENSGALG00010002866	case	control	1.211511386	0.139180086	8.704631679	3.18608E-18	1.30582E-15	up_in_case
Yang2025_S	ENSGALG00010002868	PPP1R9A	case	control	0.047136794	0.141576842	0.332941414	0.739178507	0.840406621	up_in_case
Yang2025_S	ENSGALG00010002870	FABP5	case	control	-0.087716484	0.184296338	-0.475953482	0.634107519	0.765245369	down_in_case
Yang2025_S	ENSGALG00010002878	P2RY2	case	control	-0.27013273	0.190029135	-1.421533233	0.155161799	0.299719944	down_in_case
Yang2025_S	ENSGALG00010002880	RASGEF1B	case	control	0.309192537	0.156671073	1.973513876	0.048437045	0.13040383	up_in_case
Yang2025_S	ENSGALG00010002881	ENSGALG00010002881	case	control	1.042317255	0.293811275	3.54757405	0.000388796	0.003228771	up_in_case
Yang2025_S	ENSGALG00010002883	MAP3K15	case	control	0.569319039	0.256980339	2.215418661	0.026731342	0.083953402	up_in_case
Yang2025_S	ENSGALG00010002884	ADHFE1	case	control	0.246662841	0.175316584	1.406956687	0.159440233	0.304857251	up_in_case
Yang2025_S	ENSGALG00010002886	HERC2	case	control	0.193801483	0.112823204	1.717744901	0.085843156	0.19694178	up_in_case
Yang2025_S	ENSGALG00010002888	ENSGALG00010002888	case	control	0.384814168	0.497605762	0.773331414	0.439326269	0.602975628	up_in_case
Yang2025_S	ENSGALG00010002890	VXN	case	control	1.026825506	0.279731514	3.670753759	0.000241836	0.002184049	up_in_case
Yang2025_S	ENSGALG00010002894	FCHSD2	case	control	0.021981742	0.091908418	0.23917006	0.810973718	0.886207556	up_in_case
Yang2025_S	ENSGALG00010002896	OCA2	case	control	3.346969738	0.306321879	10.92631629	8.62802E-28	1.20232E-24	up_in_case
Yang2025_S	ENSGALG00010002904	PON1	case	control	-0.136199795	0.105451669	-1.291585012	0.196500897	0.352573025	down_in_case
Yang2025_S	ENSGALG00010002907	GABRG3	case	control	-0.543711877	0.337702556	-1.610031867	0.1073909	0.230300429	down_in_case
Yang2025_S	ENSGALG00010002908	S1PR2	case	control	0.076493839	0.219255478	0.348879943	0.727179444	0.832465302	up_in_case
Yang2025_S	ENSGALG00010002911	MYBL1	case	control	-0.129918757	0.168525492	-0.770914568	0.440757572	0.604107088	down_in_case
Yang2025_S	ENSGALG00010002912	RPS3	case	control	-1.047713655	0.241969169	-4.329946911	1.49145E-05	0.00022864	down_in_case
Yang2025_S	ENSGALG00010002913	NXNL1	case	control	-0.087917045	0.413432148	-0.212651691	0.83159865	0.899784703	down_in_case
Yang2025_S	ENSGALG00010002914	SLC27A1	case	control	-0.003833244	0.190162807	-0.020157698	0.983917573	0.990242047	down_in_case
Yang2025_S	ENSGALG00010002916	SDCBP	case	control	-0.747862511	0.20456911	-3.655793925	0.000256387	0.002291311	down_in_case
Yang2025_S	ENSGALG00010002917	PGLS	case	control	0.277879557	0.125277523	2.218111841	0.026547208	0.083639009	up_in_case
Yang2025_S	ENSGALG00010002918	LRATD1	case	control	0.562251034	0.231665521	2.42699488	0.015224471	0.055645469	up_in_case
Yang2025_S	ENSGALG00010002919	ENSGALG00010002919	case	control	0.930422085	0.311157339	2.990198109	0.002787966	0.015386258	up_in_case
Yang2025_S	ENSGALG00010002920	VCPIP1	case	control	-0.181349258	0.118280127	-1.533218323	0.125222071	0.257142582	down_in_case
Yang2025_S	ENSGALG00010002922	TMEM106B	case	control	-0.17280835	0.104011166	-1.661440372	0.096625035	0.214135549	down_in_case
Yang2025_S	ENSGALG00010002923	SERPINH1	case	control	-0.329976728	0.120031536	-2.74908361	0.005976214	0.027331323	down_in_case
Yang2025_S	ENSGALG00010002925	INTS6	case	control	0.350085242	0.138561971	2.526560778	0.011518546	0.04499886	up_in_case
Yang2025_S	ENSGALG00010002926	GALNTL6	case	control	0.852942344	0.43064538	1.980614175	0.047634558	0.128940864	up_in_case
Yang2025_S	ENSGALG00010002928	ASB4	case	control	-1.078447643	0.355129146	-3.036775931	0.002391231	0.013723974	down_in_case
Yang2025_S	ENSGALG00010002929	PDHA2	case	control	0.133952957	0.167700995	0.798760657	0.424429208	0.589438011	up_in_case
Yang2025_S	ENSGALG00010002930	SERAF	case	control	0.102606787	0.251885883	0.407354259	0.683747817	0.801954872	up_in_case
Yang2025_S	ENSGALG00010002937	GALNT7	case	control	-0.077702346	0.087248213	-0.89058955	0.373149408	0.541255415	down_in_case
Yang2025_S	ENSGALG00010002939	HMGB2	case	control	-0.293013346	0.112432722	-2.606121606	0.009157394	0.038046598	down_in_case
Yang2025_S	ENSGALG00010002940	BG1	case	control	-0.122477165	0.529918985	-0.231124321	0.817218221	0.890656649	down_in_case
Yang2025_S	ENSGALG00010002942	GABRB3	case	control	-0.325528628	0.733706943	-0.443676635	0.65727639	0.783900793	down_in_case
Yang2025_S	ENSGALG00010002943	SAP30	case	control	-0.604711636	0.126962106	-4.76293009	1.90802E-06	4.17398E-05	down_in_case
Yang2025_S	ENSGALG00010002946	GDPD5	case	control	-0.221813609	0.065013365	-3.411815553	0.000645318	0.004927398	down_in_case
Yang2025_S	ENSGALG00010002947	ENSGALG00010002947	case	control	-0.73782818	0.689564588	-1.069991401	0.284623179	0.452816988	down_in_case
Yang2025_S	ENSGALG00010002949	ENSGALG00010002949	case	control	-0.673755955	0.619730348	-1.08717599	0.27695904	0.444326988	down_in_case
Yang2025_S	ENSGALG00010002951	ENSGALG00010002951	case	control	1.153903145	2.166080322	0.532714846	0.594230988	0.734509126	up_in_case
Yang2025_S	ENSGALG00010002952	NBAS	case	control	-0.230187619	0.084907809	-2.711030024	0.006707455	0.029929039	down_in_case
Yang2025_S	ENSGALG00010002953	WDFY2	case	control	-0.290487294	0.115761474	-2.509360702	0.012094991	0.046662153	down_in_case
Yang2025_S	ENSGALG00010002954	CHST7	case	control	0.003091129	0.265413722	0.011646457	0.990707682	0.994000591	up_in_case
Yang2025_S	ENSGALG00010002955	BLEC1	case	control	-0.305711153	0.532695809	-0.573894421	0.566039249	0.711190394	down_in_case
Yang2025_S	ENSGALG00010002956	SCRG1	case	control	0.556171769	0.307991393	1.80580296	0.070949123	0.171555792	up_in_case
Yang2025_S	ENSGALG00010002957	ADGRG2	case	control	-0.00986676	0.271910276	-0.036286824	0.971053656	0.98240189	down_in_case
Yang2025_S	ENSGALG00010002958	ENSGALG00010002958	case	control	0.098824983	0.226096586	0.437091885	0.662044713	0.786355857	up_in_case
Yang2025_S	ENSGALG00010002959	DLX5	case	control	0.329131394	0.476655586	0.690501492	0.489878872	0.647262492	up_in_case
Yang2025_S	ENSGALG00010002960	BLEC2	case	control	0.360783536	0.986544419	0.3657043	0.714585737	0.823703552	up_in_case
Yang2025_S	ENSGALG00010002965	PRSS35	case	control	-0.103794593	0.159519178	-0.650671561	0.515258525	0.669913001	down_in_case
Yang2025_S	ENSGALG00010002972	ME1	case	control	0.131511537	0.144092096	0.912690849	0.361405158	0.530012721	up_in_case
Yang2025_S	ENSGALG00010002978	DYNLT3	case	control	0.113646953	0.112519583	1.010019329	0.312486029	0.482171722	up_in_case
Yang2025_S	ENSGALG00010002981	KLHL35	case	control	-0.200698992	0.498778296	-0.402381166	0.687403528	0.804482084	down_in_case
Yang2025_S	ENSGALG00010002982	HAND2	case	control	-0.253345735	0.258426741	-0.980338701	0.326918957	0.496633126	down_in_case
Yang2025_S	ENSGALG00010002983	NSMAF	case	control	-0.072130965	0.068741832	-1.049302343	0.294038988	0.462568672	down_in_case
Yang2025_S	ENSGALG00010002985	ENSGALG00010002985	case	control	0.048442216	0.252971263	0.19149296	0.848139395	0.909910114	up_in_case
Yang2025_S	ENSGALG00010002987	PCDH8	case	control	-0.414507787	0.321913375	-1.287637667	0.197872121	0.354414911	down_in_case
Yang2025_S	ENSGALG00010002991	BLB1	case	control	-1.011159365	0.318943363	-3.170341456	0.001522599	0.009710488	down_in_case
Yang2025_S	ENSGALG00010002992	ENSGALG00010002992	case	control	0.837113282	0.301456248	2.776898102	0.005488038	0.025678306	up_in_case
Yang2025_S	ENSGALG00010002994	DUSP4	case	control	-0.043632654	0.234582972	-0.186000945	0.85244402	0.912771433	down_in_case
Yang2025_S	ENSGALG00010002995	RWDD2A	case	control	-0.590527033	0.151693732	-3.892890147	9.9075E-05	0.001058558	down_in_case
Yang2025_S	ENSGALG00010002998	LECT1	case	control	0.489714929	0.291822865	1.678123912	0.093322911	0.208774245	up_in_case
Yang2025_S	ENSGALG00010002999	CMTM7	case	control	0.143382679	0.261407025	0.548503544	0.583346197	0.725538134	up_in_case
Yang2025_S	ENSGALG00010003001	MAP6	case	control	0.33119366	0.501249352	0.660736335	0.508781418	0.664188525	up_in_case
Yang2025_S	ENSGALG00010003002	PHKA2	case	control	0.085378231	0.141560785	0.603120638	0.546428438	0.695513362	up_in_case
Yang2025_S	ENSGALG00010003006	CYP7B1	case	control	-0.400881315	0.214548518	-1.868487926	0.061694091	0.155518662	down_in_case
Yang2025_S	ENSGALG00010003009	TNKS	case	control	-0.125461461	0.088931073	-1.410771918	0.158311877	0.303699891	down_in_case
Yang2025_S	ENSGALG00010003017	PGM3	case	control	0.509621204	0.16457268	3.096633079	0.00195732	0.011793634	up_in_case
Yang2025_S	ENSGALG00010003018	MOGAT2	case	control	-0.540372293	0.632095646	-0.854890072	0.392612007	0.559583545	down_in_case
Yang2025_S	ENSGALG00010003019	TAPBP	case	control	-0.301042931	0.200319157	-1.502816479	0.132886375	0.268100715	down_in_case
Yang2025_S	ENSGALG00010003022	BLB2	case	control	0.160036652	0.321889745	0.497178474	0.6190632	0.754737155	up_in_case
Yang2025_S	ENSGALG00010003024	SUGT1	case	control	-0.369174271	0.136745152	-2.699724742	0.006939687	0.030768226	down_in_case
Yang2025_S	ENSGALG00010003025	MFHAS1	case	control	-0.226798132	0.09895401	-2.291954944	0.021908243	0.072282553	down_in_case
Yang2025_S	ENSGALG00010003027	SCIN	case	control	-0.215325511	0.197982072	-1.087601059	0.276771263	0.444076863	down_in_case
Yang2025_S	ENSGALG00010003030	PPEF1	case	control	0.110072084	0.521736988	0.21097236	0.832908839	0.900782668	up_in_case
Yang2025_S	ENSGALG00010003034	ERI1	case	control	-0.280477519	0.076634736	-3.659926745	0.000252287	0.002260555	down_in_case
Yang2025_S	ENSGALG00010003035	DGAT2	case	control	-1.053785239	0.444867257	-2.368763317	0.017847671	0.062520687	down_in_case
Yang2025_S	ENSGALG00010003042	TOX	case	control	0.089493875	0.346177103	0.258520491	0.796005237	0.876587085	up_in_case
Yang2025_S	ENSGALG00010003052	ENSGALG00010003052	case	control	0.131024041	0.15590541	0.840407275	0.400680071	0.56739488	up_in_case
Yang2025_S	ENSGALG00010003057	DDX1	case	control	0.066701903	0.058507491	1.140057482	0.254262354	0.420701247	up_in_case
Yang2025_S	ENSGALG00010003061	ENSGALG00010003061	case	control	-0.22835382	0.429689676	-0.531438926	0.595114653	0.735096401	down_in_case
Yang2025_S	ENSGALG00010003063	GGH	case	control	-0.673078021	0.079264844	-8.491507564	2.03974E-17	7.10593E-15	down_in_case
Yang2025_S	ENSGALG00010003064	VAV1	case	control	0.48529013	0.284869667	1.703551431	0.088464922	0.201562898	up_in_case
Yang2025_S	ENSGALG00010003068	BRD2	case	control	0.346981625	0.173923798	1.995020975	0.046040592	0.125626719	up_in_case
Yang2025_S	ENSGALG00010003072	ELF1	case	control	0.240575064	0.225495218	1.066874348	0.28602858	0.454533956	up_in_case
Yang2025_S	ENSGALG00010003074	TTPA	case	control	-0.059024703	0.240635879	-0.245286376	0.8062347	0.88359265	down_in_case

Yang2025_S	ENSGALG00010003077	EIF2S3	case	control	-0.050768312	0.128520397	-0.395021437	0.692827067	0.80892712	down_in_case
Yang2025_S	ENSGALG00010003081	DOP1A	case	control	0.591732842	0.142531359	4.151597553	3.30162E-05	0.000439848	up_in_case
Yang2025_S	ENSGALG00010003087	TWISTNB	case	control	0.318904999	0.169077125	1.886151062	0.059274601	0.151280506	up_in_case
Yang2025_S	ENSGALG00010003090	DMA	case	control	0.022567786	0.274394354	0.082245812	0.934451244	0.959939409	up_in_case
Yang2025_S	ENSGALG00010003097	TFPI2	case	control	-0.333274649	0.16091575	-2.071112676	0.038348268	0.109797228	down_in_case
Yang2025_S	ENSGALG00010003101	KLHL15	case	control	-0.073744563	0.115098688	-0.640707246	0.521712906	0.675243084	down_in_case
Yang2025_S	ENSGALG00010003103	YTHDF3	case	control	-0.248632158	0.097787841	-2.542567203	0.011004144	0.043476488	down_in_case
Yang2025_S	ENSGALG00010003107	GNG11	case	control	-0.449023699	0.345006914	-1.301491883	0.193090141	0.348492568	down_in_case
Yang2025_S	ENSGALG00010003108	PPP1R3B	case	control	-0.338209492	0.205696413	-1.64421677	0.100131434	0.219184973	down_in_case
Yang2025_S	ENSGALG00010003110	BF1	case	control	-0.056580272	0.186360521	-0.303606536	0.761427671	0.854874912	down_in_case
Yang2025_S	ENSGALG00010003113	WBP4	case	control	0.405422464	0.228517912	1.774138666	0.076040216	0.180555417	up_in_case
Yang2025_S	ENSGALG00010003115	UBE3D	case	control	-0.344889497	0.214057353	-1.61120135	0.107135844	0.229965801	down_in_case
Yang2025_S	ENSGALG00010003117	ID2	case	control	0.373566583	0.120360914	3.103720049	0.001911041	0.011583554	up_in_case
Yang2025_S	ENSGALG00010003121	ARMC1	case	control	-0.099182442	0.093679279	-1.058744722	0.289716055	0.458459371	down_in_case
Yang2025_S	ENSGALG00010003125	APOO	case	control	0.303419715	0.219159259	1.384471348	0.166214175	0.31397513	up_in_case
Yang2025_S	ENSGALG00010003126	ENSGALG00010003126	case	control	1.152487799	0.3503194	3.289820083	0.001002515	0.00703426	up_in_case
Yang2025_S	ENSGALG00010003131	TAP1	case	control	0.780136524	0.330323516	2.361734744	0.018189652	0.063241716	up_in_case
Yang2025_S	ENSGALG00010003132	TPBG	case	control	-0.3952872	0.185424957	-2.1317907	0.033024057	0.098310239	down_in_case
Yang2025_S	ENSGALG00010003133	SAT1	case	control	-0.266414877	0.256883082	-1.037105578	0.299686656	0.469333957	down_in_case
Yang2025_S	ENSGALG00010003136	NUP98	case	control	-0.261555497	0.068814333	-3.800886905	0.000144179	0.00143305	down_in_case
Yang2025_S	ENSGALG00010003152	ENSGALG00010003152	case	control	0.308566999	0.170115618	1.813866368	0.069698302	0.169459845	up_in_case
Yang2025_S	ENSGALG00010003154	MTFR1	case	control	0.102034076	0.105183019	0.970062253	0.332015463	0.501369253	up_in_case
Yang2025_S	ENSGALG00010003159	TAP2	case	control	0.519698743	0.279565706	1.858950266	0.063034188	0.157528948	up_in_case
Yang2025_S	ENSGALG00010003160	KIDINS220	case	control	-0.01430585	0.093873066	-0.152395683	0.878874861	0.92862622	down_in_case
Yang2025_S	ENSGALG00010003164	C3	case	control	-0.797191958	0.226258729	-3.523364438	0.000426105	0.003478987	down_in_case
Yang2025_S	ENSGALG00010003165	BMP2K	case	control	-0.027324261	0.134785274	-0.20272438	0.839350467	0.904799934	down_in_case
Yang2025_S	ENSGALG00010003166	PGAP2	case	control	0.077551439	0.101910765	0.760973966	0.446672617	0.609397192	up_in_case
Yang2025_S	ENSGALG00010003169	MTRF1	case	control	0.127137713	0.134749378	0.94351243	0.34541886	0.514128976	up_in_case
Yang2025_S	ENSGALG00010003170	C4	case	control	0.252933374	0.383608662	0.659352614	0.509669363	0.665048933	up_in_case
Yang2025_S	ENSGALG00010003171	PAQR3	case	control	0.179949522	0.12611002	1.426924856	0.153601556	0.297613692	up_in_case
Yang2025_S	ENSGALG00010003176	PRAG1	case	control	-0.385558644	0.131262222	-2.937316138	0.003310664	0.017528156	down_in_case
Yang2025_S	ENSGALG00010003178	CENPA	case	control	0.919887041	0.490874089	1.873977587	0.060933514	0.154121954	up_in_case
Yang2025_S	ENSGALG00010003180	IBTK	case	control	-0.08143638	0.115376437	-0.70583199	0.480292625	0.638755271	down_in_case
Yang2025_S	ENSGALG00010003183	NAA16	case	control	-0.013733225	0.103598451	-0.132562066	0.894539735	0.937909687	down_in_case
Yang2025_S	ENSGALG00010003184	BET1	case	control	-0.827174769	0.160820867	-5.143454221	2.69732E-07	7.76595E-06	down_in_case
Yang2025_S	ENSGALG00010003186	CYP21A1	case	control	0.185311312	0.478220346	0.387501941	0.698384648	0.813439491	up_in_case
Yang2025_S	ENSGALG00010003187	RHOG	case	control	0.646351166	0.241033572	2.681581496	0.007327506	0.031968966	up_in_case
Yang2025_S	ENSGALG00010003189	ANTXR2	case	control	-0.132657982	0.107061853	-1.239077961	0.215316628	0.376183201	down_in_case
Yang2025_S	ENSGALG00010003191	CC2D1A	case	control	0.644051163	0.207230805	3.107892975	0.001884263	0.011466028	up_in_case
Yang2025_S	ENSGALG00010003192	RGCC	case	control	0.095395305	0.217110244	0.439386477	0.660381524	0.785514721	up_in_case
Yang2025_S	ENSGALG00010003193	ACOT9	case	control	-0.397328257	0.15776047	-2.518553964	0.011783782	0.045752858	down_in_case
Yang2025_S	ENSGALG00010003198	PRKG2	case	control	0.314459022	0.358061172	0.878227092	0.379820492	0.547738649	up_in_case
Yang2025_S	ENSGALG00010003201	MBOAT2	case	control	-0.665690477	0.193240053	-3.444888709	0.000571295	0.004425231	down_in_case
Yang2025_S	ENSGALG00010003202	FAM46A	case	control	0.513802455	0.150384357	3.416595087	0.000634095	0.004844362	up_in_case
Yang2025_S	ENSGALG00010003204	PKD2	case	control	0.242210621	0.144889973	1.671686558	0.094586146	0.210788093	up_in_case
Yang2025_S	ENSGALG00010003205	ENSGALG00010003205	case	control	-0.330554355	0.204052517	-1.619947451	0.105243566	0.227516147	down_in_case
Yang2025_S	ENSGALG00010003206	ENSGALG00010003206	case	control	-0.35617003	0.7109811	-0.500955693	0.616402306	0.752546533	down_in_case
Yang2025_S	ENSGALG00010003210	EYA1	case	control	0.709085389	0.304539812	2.32838322	0.019891764	0.067545131	up_in_case
Yang2025_S	ENSGALG00010003214	PRDX4	case	control	-0.228515431	0.172015654	-1.328457183	0.184027119	0.337427658	down_in_case
Yang2025_S	ENSGALG00010003218	MAP3K7	case	control	-0.289843815	0.073981484	-3.917788591	8.9365E-05	0.000976707	down_in_case
Yang2025_S	ENSGALG00010003220	SPP1	case	control	-0.313725076	0.997861303	-0.314397477	0.753219172	0.849770657	down_in_case
Yang2025_S	ENSGALG00010003222	ITGA8	case	control	0.365524601	0.144615349	2.527564356	0.011485677	0.044883037	up_in_case
Yang2025_S	ENSGALG00010003223	AREG	case	control	-0.323679639	0.884694197	-0.365866127	0.714464973	0.823693931	down_in_case
Yang2025_S	ENSGALG00010003229	STIM1	case	control	0.276257028	0.122724304	2.251037653	0.024383153	0.078670811	up_in_case
Yang2025_S	ENSGALG00010003239	RRM1	case	control	-0.09980319	0.097343124	-1.025272116	0.305234809	0.474807136	down_in_case
Yang2025_S	ENSGALG00010003242	ASAP2	case	control	0.428165602	0.221362424	1.934228921	0.053084988	0.139363095	up_in_case
Yang2025_S	ENSGALG00010003243	VPS50	case	control	-0.192713142	0.086096296	-2.238344173	0.02519862	0.080371427	down_in_case
Yang2025_S	ENSGALG00010003245	ENSGALG00010003245	case	control	2.468589011	0.548718947	4.49882226	6.8331E-06	0.000120905	up_in_case
Yang2025_S	ENSGALG00010003246	VWA8	case	control	0.040541225	0.09395572	0.43149289	0.666110022	0.789172177	up_in_case
Yang2025_S	ENSGALG00010003247	ENSGALG00010003247	case	control	0.198597479	0.260430998	0.762572353	0.445718478	0.608665615	up_in_case
Yang2025_S	ENSGALG00010003248	MRI1	case	control	1.218759854	0.359800881	3.38731759	0.000705796	0.005290625	up_in_case
Yang2025_S	ENSGALG00010003249	YJU2B	case	control	1.560413962	0.264667087	5.895761277	3.72958E-09	1.91072E-07	up_in_case
Yang2025_S	ENSGALG00010003251	ELOF1	case	control	0.499744447	0.200381555	2.493964319	0.012632524	0.048162579	up_in_case
Yang2025_S	ENSGALG00010003252	PHEX	case	control	-0.384485033	0.208545576	-1.843649917	0.065234176	0.161520655	down_in_case
Yang2025_S	ENSGALG00010003253	ITGB1BP1	case	control	0.747107639	0.130969276	5.704449635	1.1672E-08	5.29801E-07	up_in_case
Yang2025_S	ENSGALG00010003254	DGKH	case	control	1.03238114	0.261856249	3.942549175	8.06201E-05	0.000899722	up_in_case
Yang2025_S	ENSGALG00010003255	CALCR	case	control	-0.348372335	0.33134834	-1.05137794	0.293085044	0.461705274	down_in_case
Yang2025_S	ENSGALG00010003256	ENSGALG00010003256	case	control	2.003595024	0.870852998	2.300727022	0.021407066	0.071127196	up_in_case
Yang2025_S	ENSGALG00010003257	ENSGALG00010003257	case	control	4.004106798	1.0968334	3.650606189	0.000261622	0.002328036	up_in_case
Yang2025_S	ENSGALG00010003259	VEGFC	case	control	-0.244526522	0.115875339	-2.110255079	0.034836391	0.1023498	down_in_case
Yang2025_S	ENSGALG00010003260	ACP5	case	control	0.079167969	0.214072065	0.369819241	0.711517177	0.82171568	up_in_case
Yang2025_S	ENSGALG00010003261	PEX2	case	control	-0.614480554	0.088439177	-6.948058296	3.70348E-12	4.52702E-10	down_in_case
Yang2025_S	ENSGALG00010003262	ENSGALG00010003262	case	control	0.775443064	0.292938264	2.647121114	0.008118027	0.034594713	up_in_case
Yang2025_S	ENSGALG00010003263	ENSGALG00010003263	case	control	3.087387529	0.909368544	3.395089428	0.000686062	0.005162134	up_in_case
Yang2025_S	ENSGALG00010003264	ENSGALG00010003264	case	control	-0.23033354	0.287389014	-0.801469538	0.422859872	0.588714652	down_in_case
Yang2025_S	ENSGALG00010003266	ENSGALG00010003266	case	control	2.005503124	0.723993624	2.770056334	0.00560466	0.026077107	up_in_case
Yang2025_S	ENSGALG00010003268	SMS	case	control	-0.318020138	0.076885694	-4.136271943	3.52994E-05	0.000463098	down_in_case
Yang2025_S	ENSGALG00010003269	ENSGALG00010003269	case	control	1.128833239	0.262656949	4.297747478	1.72543E-05	0.000256939	up_in_case
Yang2025_S	ENSGALG00010003271	AKAP11	case	control	0.110587516	0.15371056	0.71945295	0.471861882	0.631861675	up_in_case
Yang2025_S	ENSGALG00010003272	CPSF3	case	control	0.371555328	0.084713049	4.386045962	1.1543E-05	0.000186386	up_in_case
Yang2025_S	ENSGALG00010003273	NFIX	case	control	-0.005053735	0.129840942	-0.038922508	0.968952171	0.981620757	down_in_case
Yang2025_S	ENSGALG00010003274	BACH2	case	control	0.06312597	0.160578708	0.393115444	0.694234218	0.810027114	up_in_case
Yang2025_S	ENSGALG00010003275	MBTPS2	case	control	-0.7278167	0.148198702	-4.911086862	9.0573E-07	2.21816E-05	down_in_case
Yang2025_S	ENSGALG00010003276	TNFSF11	case	control	-0.470732333	0.573708097	-0.820508436	0.411926322	0.577291884	down_in_case
Yang2025_S	ENSGALG00010003278	ENSGALG00010003278	case	control	0.41021862	0.513983199	0.798116787	0.424802721	0.589604174	up_in_case
Yang2025_S	ENSGALG00010003280	ENSGALG00010003280	case	control	-0.257868981	0.116437455	-2.214656629	0.026783642	0.084079759	down_in_case
Yang2025_S	ENSGALG00010003292	GADD45GIP1	case	control	0.390089955	0.157949064	2.469719948	0.013521886	0.050632769	up_in_case
Yang2025_S	ENSGALG00010003295	IAH1	case	control	0.428222751	0.25238542	1.696701618	0.089753087	0.203433517	up_in_case
Yang2025_S	ENSGALG00010003296	ENSGALG00010003296	case	control	-0.540351923	0.387603773	-1.394083241	0.163292469	0.309968746	down_in_case
Yang2025_S	ENSGALG00010003299	RAD23A	case	control	0.377788727	0.167839718	2.250889906	0.024392511	0.078682788	up_in_case
Yang2025_S	ENSGALG00010003300	CASP8AP2	case	control	0.187130917	0.186680914	1.002410548	0.31614535	0.485376928	up_in_case
Yang2025_S	ENSGALG00010003303	MTHFD2L	case	control	-0.075121829	0.201399439	-0.372999198	0.709149041	0.820422738	down_in_case
Yang2025_S	ENSGALG00010003305	CALR	case	control	0.113143752	0.066312759	1.706213915	0.087968259	0.200693793	up_in_case
Yang2025_S	ENSGALG00010003307	COL1A2	case	control	-0.306209479	0.181856262	-1.683799479	0.092220428	0.207373191	down_in_case
Yang2025_S	ENSGALG00010003308	MDN1	case	control	0.450231989	0.18020233	2.4984804	0.012472704	0.04772299	up_in_case
Yang2025_S	ENSGALG00010003315	EPGN	case	control	0.884863894	0.424806661	2.082980274	0.037253022	0.107415843	up_in_case
Yang2025_S	ENSGALG00010003317	LYRM2	case	control	0.367476204	0.241262141	1.523140771	0.127723491	0.260704093	up_in_case
Yang2025_S	ENSGALG00010003320	OR51E2	case	control	-0.510510775	0.314877445	-1.621299917	0.104953333	0.227022366	down_in_case
Yang2025_S	ENSGALG00010003323	STMN2	case	control	1.436204047	0.781711941	1.837254839			up_in_case
Yang2025_S	ENSGALG00010003324	ENSGALG00010003324	case	control	0.950876319	0.856279262	1.110474539	0.266794594	0.433965527	up_in_case
Yang2025_S	ENSGALG00010003330	HEY1	case	control	-0.059449393	0.140428819	-0.423341832	0.672045878	0.793640619	down_in_case
Yang2025_S	ENSGALG00010003331	EPSTI1	case	control	-0.062270026	0.422836147	-0.147267509	0.882920877	0.931020944	down_in_case
Yang2025_S	ENSGALG00010003333	ENSGALG00010003333	case	control	2.156842973	1.198463609	1.799673312			up_in_case
Yang2025_S	ENSGALG00010003339	ENSGALG00010003339	case	control	-1.804152685	0.845659677	-2.133426405	0.032889763	0.098055073	down_in_case
Yang2025_S	ENSGALG00010003344	ZFAND1	case	control	0.237885417	0.218034794	1.091043371	0.275253799	0.442336123	up_in_case
Yang2025_S	ENSGALG00010003347	GPM6A	case	control	0.311884274	0.320247167	0.97388613	0.330113081	0.499633517	up_in_case
Yang2025_S	ENSGALG00010003348	ADAM17	case	control	0.142776883	0.098463386	1.450050505	0.147044436	0.288895163	up_in_case
Yang2025_S	ENSGALG00010003352	FARSA	case	control	0.255201901	0.131697613	1.937786844	0.052649234	0.138480005	up_in_case
Yang2025_S	ENSGALG00010003356	ENSGALG00010003356	case	control	1.2225637	0.418066237	2.92433015	0.003451984	0.018076495	up_in_case
Yang2025_S	ENSGALG00010003358	DNAJC15	case	control	0.175367803	0.195525151	0.896906624	0.369768745	0.538462416	up_in_case
Yang2025_S	ENSGALG00010003363	HBE1	case	control	-1.339848245	0.356959121	-3.753506119	0.000174378	0.00167468	down_in_case
Yang2025_S	ENSGALG00010003364	ANKRD6	case	control	-0.143034202	0.142585636	-1.00314594	0.315790452	0.485014873	down_in_case
Yang2025_S	ENSGALG00010003366	CASD1	case	control	-0.438856454	0.095560347	-4.592453543	4.38065E-06	8.31667E-05	down_in_case
Yang2025_S	ENSGALG00010003371	ENSGALG00010003371	case	control	-0.447947311	0.338334937	-1.323975922	0.185511041	0.339029031	down_in_case
Yang2025_S	ENSGALG00010003372	ENOX1	case	control	0.361466804	0.115931089	3.117945374	0.001821166	0.011150238	up_in_case
Yang2025_S	ENSGALG00010003374	SYCE2	case	control	0.671852064	0.389234314	1.72608642	0.084331866	0.194563669	up_in_case
Yang2025_S	ENSGALG00010003375	YWHAQ	case	control	-0.094541604	0.087046036	-1.086110388	0.277430157	0.444826745	down_in_case
Yang2025_S	ENSGALG00010003382	ASNS	case	control	-0.217303777	0.192615242	-1.1281754	0.259245851	0.425611561	down_in_case
Yang2025_S	ENSGALG00010003383	UBE2J1	case	control	-0.057304523	0.093819593	-0.610794832	0.541335415	0.6917183	down_in_case
Yang2025_S	ENSGALG00010003388	PXDN	case	control	-0.439568547	0.132326011	-3.321860487	0.000894194	0.006393326	down_in_case
Yang2025_S	ENSGALG00010003389	ENSGALG00010003389	case	control	-1.919518912	0.399378907	-4.806260114	1.5378E-06	3.48443E-05	down_in_case
Yang2025_S	ENSGALG00010003397	SPATA4	case	control	0.100393173	0.378938365	0.264932723	0.791061289	0.873985268	up_in_case
Yang2025_S	ENSGALG00010003400	PKIA	case	control	-0.335575379	0.119730303	-2.802760618	0.005066726	0.02422121	down_in_case
Yang2025_S	ENSGALG00010003401	ENSGALG00010003401	case	control	0.690426962	1.230452977	0.561116089	0.574718401	0.717496947	up_in_case
Yang2025_S	ENSGALG00010003403	GCDH	case	control	0.50761398	0.226381546	2.242293986	0.024942378	0.079773248	up_in_case
Yang2025_S	ENSGALG00010003405	GABRR2	case	control	0.079153593	0.286862097	0.275929074	0.782602543	0.868542931	up_in_case

Yang2025_S	ENSGALG00010003406	C1GALT1	case	control	-0.326086704	0.092520345	-3.524486477	0.000424304	0.003467849	down_in_case
Yang2025_S	ENSGALG00010003407	CHMP4C	case	control	-0.38128053	0.189484588	-2.012198117	0.044199057	0.122140677	down_in_case
Yang2025_S	ENSGALG00010003408	ENSGALG00010003408	case	control	1.34364321	0.481750183	2.789087074	0.005285685	0.024968143	up_in_case
Yang2025_S	ENSGALG00010003412	ASB5	case	control	-0.125739411	0.124063989	-1.013504497	0.310819249	0.480557665	down_in_case
Yang2025_S	ENSGALG00010003419	ENSGALG00010003419	case	control	-0.816023814	1.160151452	-0.703376971	0.481820854	0.640138932	down_in_case
Yang2025_S	ENSGALG00010003420	ENSGALG00010003420	case	control	0.166304684	0.395778818	0.420196019	0.674342263	0.795238135	up_in_case
Yang2025_S	ENSGALG00010003428	SNX16	case	control	-0.018765029	0.106046656	-0.176950691	0.859547121	0.916995034	down_in_case
Yang2025_S	ENSGALG00010003431	PM20D2	case	control	-0.252363087	0.210306963	-1.199974944	0.230149071	0.392597296	down_in_case
Yang2025_S	ENSGALG00010003432	LACC1	case	control	-0.65842217	0.33820922	-1.946789533	0.051559972	0.136206296	down_in_case
Yang2025_S	ENSGALG00010003437	PNRC1	case	control	0.306682105	0.127437981	2.406520435	0.016105303	0.057911329	up_in_case
Yang2025_S	ENSGALG00010003438	KCNS3	case	control	-0.02476838	0.237713612	-0.104194203	0.917015234	0.951213882	down_in_case
Yang2025_S	ENSGALG00010003439	GMIP	case	control	0.022691143	0.224652336	0.101005596	0.919546018	0.952562724	up_in_case
Yang2025_S	ENSGALG00010003442	SPCS3	case	control	-0.204865142	0.12543756	-1.633204132	0.102426071	0.223088276	down_in_case
Yang2025_S	ENSGALG00010003447	ZC2HC1A	case	control	0.223836989	0.17161036	1.304332606	0.192120203	0.347306047	up_in_case
Yang2025_S	ENSGALG00010003449	BORCS6	case	control	0.318815658	0.178240977	1.788677688	0.073666738	0.176443107	up_in_case
Yang2025_S	ENSGALG00010003453	ENSGALG00010003453	case	control	0.951594349	0.675395725	1.4089434	0.158851905	0.304385172	up_in_case
Yang2025_S	ENSGALG00010003454	BPNT2	case	control	-0.243291944	0.088920232	-2.736069592	0.006217787	0.028213894	down_in_case
Yang2025_S	ENSGALG00010003456	CDS1	case	control	0.077707091	0.360494277	0.215557071	0.829333021	0.898030589	up_in_case
Yang2025_S	ENSGALG00010003466	TSC22D1	case	control	-0.301937384	0.115091916	-2.623445636	0.008704534	0.036623694	down_in_case
Yang2025_S	ENSGALG00010003468	ILVBL	case	control	-0.041729476	0.240627115	-0.173419674	0.862321562	0.918905584	down_in_case
Yang2025_S	ENSGALG00010003470	METTL3	case	control	0.096117196	0.191254534	0.502561659	0.615272491	0.751430514	up_in_case
Yang2025_S	ENSGALG00010003471	ADI1	case	control	-0.12775392	0.131295286	-0.973027469	0.33053965	0.499899069	down_in_case
Yang2025_S	ENSGALG00010003472	FAM110B	case	control	-0.548911705	0.100874079	-5.441553575	5.28179E-08	1.95749E-06	down_in_case
Yang2025_S	ENSGALG00010003473	ENSGALG00010003473	case	control	1.057117816	0.618430166	1.709356809	0.087384876	0.199657034	up_in_case
Yang2025_S	ENSGALG00010003474	RPS7	case	control	-0.750709993	0.27253275	-2.754568004	0.005876968	0.026974816	down_in_case
Yang2025_S	ENSGALG00010003476	ENSGALG00010003476	case	control	-2.435033748	0.614197488	-3.964577834	7.3526E-05	0.000832319	down_in_case
Yang2025_S	ENSGALG00010003478	COLEC11	case	control	-0.362093962	0.117122306	-3.091588401	0.001990887	0.01192904	down_in_case
Yang2025_S	ENSGALG00010003479	SYDE1	case	control	0.016342034	0.097593525	0.167449978	0.867016003	0.921998474	up_in_case
Yang2025_S	ENSGALG00010003485	RNGTT	case	control	-0.105083308	0.104434386	-1.006213686	0.31431279	0.483598182	down_in_case
Yang2025_S	ENSGALG00010003489	SGCE	case	control	-0.07490047	0.109650257	-0.683085224	0.494552992	0.651987937	down_in_case
Yang2025_S	ENSGALG00010003495	ENSGALG00010003495	case	control	-0.599770366	0.380615253	-1.575791723	0.115073811	0.24227274	down_in_case
Yang2025_S	ENSGALG00010003503	NUFIP1	case	control	-0.129709596	0.075289207	-1.722817923	0.084921456	0.195535442	down_in_case
Yang2025_S	ENSGALG00010003506	DYNC1I1	case	control	0.528682975	0.250028983	2.114486765	0.034473714	0.101514399	up_in_case
Yang2025_S	ENSGALG00010003507	WDFY3	case	control	0.430474617	0.202326838	2.127619945	0.033368607	0.099060831	up_in_case
Yang2025_S	ENSGALG00010003509	GPALPP1	case	control	0.051275771	0.101823569	0.503574678	0.61456029	0.750823921	up_in_case
Yang2025_S	ENSGALG00010003511	UBXN2B	case	control	-0.274383189	0.112275301	-2.443842832	0.014531756	0.05357673	down_in_case
Yang2025_S	ENSGALG00010003512	ENSGALG00010003512	case	control	-1.117089162	0.489178202	-2.283603721	0.022394831	0.073497873	down_in_case
Yang2025_S	ENSGALG00010003517	GTF2F2	case	control	0.612191887	0.195053407	3.138585976	0.001697651	0.010589423	up_in_case
Yang2025_S	ENSGALG00010003519	KCTD4	case	control	-0.220172309	0.376842271	-0.584255869	0.559048178	0.705950663	down_in_case
Yang2025_S	ENSGALG00010003522	ENSGALG00010003522	case	control	-0.693119042	0.562426843	-1.232371909	0.217810189	0.378744266	down_in_case
Yang2025_S	ENSGALG00010003525	NOL10	case	control	-0.198546231	0.089575394	-2.216526457	0.026655468	0.083853276	down_in_case
Yang2025_S	ENSGALG00010003528	GDAP1	case	control	0.482769182	0.288094254	1.67573346	0.093790413	0.209423787	up_in_case
Yang2025_S	ENSGALG00010003531	PPM1K	case	control	-0.087930667	0.152694033	-0.57586184	0.564708571	0.710666841	down_in_case
Yang2025_S	ENSGALG00010003535	ENSGALG00010003535	case	control	2.370409014	0.827311321	2.86519591	0.004167513	0.020761542	up_in_case
Yang2025_S	ENSGALG00010003539	ENSGALG00010003539	case	control	0.481317993	0.215256762	2.236017994	0.025350592	0.08068522	up_in_case
Yang2025_S	ENSGALG00010003541	CNR1	case	control	-0.230233561	0.123666178	-1.86173426	0.062640557	0.157059394	down_in_case
Yang2025_S	ENSGALG00010003543	SOX11	case	control	0.409867727	0.091485911	4.480118563	7.46016E-06	0.000129699	up_in_case
Yang2025_S	ENSGALG00010003552	PI15	case	control	-1.00050814	0.207710418	-4.81684141	1.45849E-06	3.33973E-05	down_in_case
Yang2025_S	ENSGALG00010003558	TPT1	case	control	-0.532620626	0.218763715	-2.434684494	0.014904779	0.05471499	down_in_case
Yang2025_S	ENSGALG00010003560	ADGRL3	case	control	0.018892027	0.194326433	0.097217998	0.922553275	0.953661386	up_in_case
Yang2025_S	ENSGALG00010003571	RPL7	case	control	-0.826025276	0.26904773	-3.070181175	0.00213929	0.012578481	down_in_case
Yang2025_S	ENSGALG00010003582	RDH10	case	control	-0.148986544	0.09617779	-1.549074307	0.121363858	0.251817357	down_in_case
Yang2025_S	ENSGALG00010003583	RSAD2	case	control	-0.316832764	0.534918201	-0.592301334	0.553648823	0.70174227	down_in_case
Yang2025_S	ENSGALG00010003585	PNPLA6	case	control	0.330248189	0.089368824	3.695339993	0.000219593	0.002011851	up_in_case
Yang2025_S	ENSGALG00010003589	SLC25A30	case	control	0.440806126	0.162301042	2.715978409	0.006608023	0.029599099	up_in_case
Yang2025_S	ENSGALG00010003595	SNX13	case	control	0.0102796	0.147967533	0.069471997	0.944613922	0.966035154	up_in_case
Yang2025_S	ENSGALG00010003596	CRISPLD1	case	control	0.27686647	0.147567149	1.876206676	0.060626906	0.153606533	up_in_case
Yang2025_S	ENSGALG00010003597	RNF144A	case	control	-0.68581358	0.148941988	-4.604568453	4.13322E-06	7.92248E-05	down_in_case
Yang2025_S	ENSGALG00010003604	ODC	case	control	0.605687232	0.245239101	2.469782466	0.013519524	0.050632769	up_in_case
Yang2025_S	ENSGALG00010003617	COG3	case	control	0.088755787	0.090699731	0.97856726	0.327793843	0.497564348	up_in_case
Yang2025_S	ENSGALG00010003619	HNF4G	case	control	0.230109886	0.490908371	0.468743047	0.639253313	0.769251546	up_in_case
Yang2025_S	ENSGALG00010003627	MCOLN1	case	control	0.190091909	0.124266806	1.529707854	0.126089059	0.258509193	up_in_case
Yang2025_S	ENSGALG00010003632	TECRL	case	control	-1.315834429	0.378093426	-3.480183304	0.000501071	0.003962782	down_in_case
Yang2025_S	ENSGALG00010003634	CYP4F11	case	control	0.39982011	0.334610317	1.194882792	0.232132827	0.395281426	up_in_case
Yang2025_S	ENSGALG00010003639	ENSGALG00010003639	case	control	-0.528333996	0.705411581	-0.748972671	0.453873678	0.615725243	down_in_case
Yang2025_S	ENSGALG00010003650	SPACA1	case	control	0.889275181	0.42474723	2.0936574	0.03629051	0.105465748	up_in_case
Yang2025_S	ENSGALG00010003652	MYCN	case	control	-0.335777418	0.123664914	-2.715219753	0.006623181	0.029638414	down_in_case
Yang2025_S	ENSGALG00010003660	IGFBP7	case	control	-0.271286511	0.206266371	-1.315224141	0.188434584	0.343111973	down_in_case
Yang2025_S	ENSGALG00010003665	RDH14	case	control	0.276170412	0.099970784	2.762511206	0.005735859	0.026528111	up_in_case
Yang2025_S	ENSGALG00010003666	SLC25A13	case	control	-0.138424644	0.146345287	-0.945877022	0.344211314	0.51273153	down_in_case
Yang2025_S	ENSGALG00010003671	ENSGALG00010003671	case	control	1.086578184	0.495981824	2.190762105	0.028469012	0.087963567	up_in_case
Yang2025_S	ENSGALG00010003674	AKIRIN2	case	control	-0.193263889	0.117973994	-1.638190613	0.101381937	0.221331239	down_in_case
Yang2025_S	ENSGALG00010003676	POLR2B	case	control	-0.242316447	0.081739084	-2.964511424	0.00303164	0.016473049	down_in_case
Yang2025_S	ENSGALG00010003678	ZNFY1	case	control	0.659654329	0.363352143	1.81546839	0.069451959	0.169079849	up_in_case
Yang2025_S	ENSGALG00010003680	TRAPPC5	case	control	-0.249698651	0.291831025	-0.8556275	0.392203854	0.559402324	down_in_case
Yang2025_S	ENSGALG00010003681	NT5C1B	case	control	-0.513377515	0.144959742	-3.541517867	0.000397832	0.00328813	down_in_case
Yang2025_S	ENSGALG00010003690	ENSGALG00010003690	case	control	0.941065974	0.471088487	1.99764163	0.045755527	0.125216666	up_in_case
Yang2025_S	ENSGALG00010003691	ERICH1	case	control	0.707883193	0.172239549	4.109876025	3.95872E-05	0.000509955	up_in_case
Yang2025_S	ENSGALG00010003692	ZNFY2	case	control	0.442613472	0.232390032	1.904614704	0.056830174	0.1467787	up_in_case
Yang2025_S	ENSGALG00010003695	NOA1	case	control	-0.167799433	0.147212517	-1.13984487	0.254350936	0.420747898	down_in_case
Yang2025_S	ENSGALG00010003700	REST	case	control	0.537011592	0.190965695	2.812084095	0.004922163	0.023709072	up_in_case
Yang2025_S	ENSGALG00010003701	SARAF	case	control	-0.424298501	0.136881459	-3.099751447	0.001936831	0.011704137	down_in_case
Yang2025_S	ENSGALG00010003703	TDRP	case	control	-0.447810242	0.172264718	-2.599547061	0.009334688	0.038621994	down_in_case
Yang2025_S	ENSGALG00010003713	SPERT	case	control	0.023467974	0.330147172	0.071083371	0.943331403	0.965219406	up_in_case
Yang2025_S	ENSGALG00010003714	ORC3	case	control	-0.300888586	0.149943774	-2.006676095	0.044784163	0.123406627	down_in_case
Yang2025_S	ENSGALG00010003716	YLEC8	case	control	-0.236800512	0.559691283	-0.423091299	0.67222865	0.793789191	down_in_case
Yang2025_S	ENSGALG00010003717	LEPROTL1	case	control	-0.927197	0.157619084	-5.882517358	4.04073E-09	2.03572E-07	down_in_case
Yang2025_S	ENSGALG00010003719	RPGR	case	control	0.718069185	0.219200319	3.2758583	0.001053414	0.007295887	up_in_case
Yang2025_S	ENSGALG00010003722	MHCY11	case	control	-0.400111	0.475251583	-0.84189304	0.399847827	0.566799497	down_in_case
Yang2025_S	ENSGALG00010003724	ENSGALG00010003724	case	control	0.259177217	0.182606306	1.419322386	0.155805049	0.300587478	up_in_case
Yang2025_S	ENSGALG00010003726	ENPEP	case	control	-0.249182349	0.499015655	-0.499347759	0.617534415	0.753400637	down_in_case
Yang2025_S	ENSGALG00010003727	FBXO25	case	control	-0.161436126	0.125414786	-1.287217642	0.19801844	0.354594051	down_in_case
Yang2025_S	ENSGALG00010003729	RARS2	case	control	0.199095061	0.12447141	1.599524431	0.109704124	0.233714566	up_in_case
Yang2025_S	ENSGALG00010003731	ENSGALG00010003731	case	control	0.553635838	0.867833183	0.637951911	0.523504984	0.676845607	up_in_case
Yang2025_S	ENSGALG00010003733	STAU2	case	control	0.410718972	0.128749594	3.190060331	0.001422431	0.009244427	up_in_case
Yang2025_S	ENSGALG00010003734	ZC3H13	case	control	0.688314284	0.24103102	2.855708295	0.004294095	0.021256912	up_in_case
Yang2025_S	ENSGALG00010003737	SRP72	case	control	-0.039337797	0.082977936	-0.474075383	0.635446152	0.766393522	down_in_case
Yang2025_S	ENSGALG00010003740	PITX2	case	control	0.168310717	0.124998465	1.346502278	0.178140586	0.329504084	up_in_case
Yang2025_S	ENSGALG00010003742	ZFHX4	case	control	0.52991139	0.331200717	1.59997054	0.109605119	0.233617782	up_in_case
Yang2025_S	ENSGALG00010003743	ARL9	case	control	0.727220457	0.214505098	3.390224582	0.000698354	0.005240475	up_in_case
Yang2025_S	ENSGALG00010003746	HOPX	case	control	0.140282805	0.293774019	0.47751944	0.632992281	0.764561623	up_in_case
Yang2025_S	ENSGALG00010003749	MHCY15	case	control	0.931976652	1.123898766	0.829235408			up_in_case
Yang2025_S	ENSGALG00010003753	SLCO5A1	case	control	0.021841554	0.176468751	0.123770096	0.901497309	0.941198361	up_in_case
Yang2025_S	ENSGALG00010003754	FAM110C	case	control	-0.514335094	0.192954725	-2.665573972	0.007685702	0.033209383	down_in_case
Yang2025_S	ENSGALG00010003755	SLC35A1	case	control	-0.05266958	0.138971918	-0.378994408	0.704692016	0.817642236	down_in_case
Yang2025_S	ENSGALG00010003756	MHCY7	case	control	-0.598172916	0.551168911	-1.085280581	0.277797404	0.445261885	down_in_case
Yang2025_S	ENSGALG00010003761	UGT8	case	control	0.292386953	0.266482102	1.097210472			up_in_case
Yang2025_S	ENSGALG00010003762	EVI5L	case	control	0.55367284	0.085973366	6.440050721	1.19434E-10	9.79004E-09	up_in_case
Yang2025_S	ENSGALG00010003764	HDAC9	case	control	-0.513530316	0.138056082	-3.719722522	0.000199442	0.001865249	down_in_case
Yang2025_S	ENSGALG00010003765	ENSGALG00010003765	case	control	-0.564164552	0.701296418	-0.80445948	0.421131662	0.586788292	down_in_case
Yang2025_S	ENSGALG00010003767	ENSGALG00010003767	case	control	-0.932076943	1.94259319	-0.479810672	0.631362024	0.763452777	down_in_case
Yang2025_S	ENSGALG00010003768	ARSJ	case	control	-0.105995077	0.119687952	-0.885595207	0.375835695	0.543962445	down_in_case
Yang2025_S	ENSGALG00010003769	C6orf163	case	control	-0.448125592	0.555952011	-0.806050851	0.420213529	0.585916689	down_in_case
Yang2025_S	ENSGALG00010003771	SPARCL1	case	control	0.831193992	0.397211953	2.092570441	0.036387518	0.105659524	up_in_case
Yang2025_S	ENSGALG00010003772	ENSGALG00010003772	case	control	1.064082451	0.724197867	1.46932558	0.141744502	0.281168631	up_in_case
Yang2025_S	ENSGALG00010003774	ENSGALG00010003774	case	control	1.577172134	0.677854438	2.326712116	0.019980593	0.067665852	up_in_case
Yang2025_S	ENSGALG00010003775	NCOA2	case	control	0.127119928	0.174910707	0.726770418	0.467366608	0.628370374	up_in_case
Yang2025_S	ENSGALG00010003777	ENSGALG00010003777	case	control	1.210173843	0.575130288	2.104173382	0.035363326	0.103636732	up_in_case
Yang2025_S	ENSGALG00010003778	LCA5	case	control	0.601945714	0.184753577	3.258100465	0.001121607	0.007673503	up_in_case
Yang2025_S	ENSGALG00010003786	ENSGALG00010003786	case	control	-0.470481859	0.638905029	-0.736387785	0.461494736	0.622488544	down_in_case
Yang2025_S	ENSGALG00010003787	SMIM8	case	control	-0.300260482	0.288410671	-1.041086592	0.297835362	0.46695947	down_in_case
Yang2025_S	ENSGALG00010003788	NUDT9	case	control	0.681767774	0.160210155	4.255459174	2.0862E-05	0.000301661	up_in_case
Yang2025_S	ENSGALG00010003790	ELOVL6	case	control	0.884085287	0.15016188	5.887548081	3.91967E-09	1.9877E-07	up_in_case
Yang2025_S	ENSGALG00010003791	ITGB8	case	control	0.402780249	0.249041812	1.617319781	0.105809272	0.228172733	up_in_case
Yang2025_S	ENSGALG00010003793	TRAM1L1	case	control	-0.427586463	0.0598516	-7.144110812	9.05803E-13	1.22547E-10	down_in_case
Yang2025_S	ENSGALG00010003795	LCP1	case	control	-0.265193584	0.132980731	-1.994225637	0.046127402	0.125782072	down_in_case

Yang2025_S	ENSGALG00010003796	ENSGALG00010003796	case	control	-0.516357042	0.48134143	-1.072745893	0.283385143	0.45167242	down_in_case
Yang2025_S	ENSGALG00010003797	ENSGALG00010003797	case	control	0.459228561	0.173860568	2.64136121	0.008257363	0.035081206	up_in_case
Yang2025_S	ENSGALG00010003803	LACTB2	case	control	-0.295993285	0.106058128	-2.790859048	0.005256836	0.024882476	down_in_case
Yang2025_S	ENSGALG00010003804	PSME1	case	control	0.205776094	0.172220077	1.194843815	0.232148058	0.395281426	up_in_case
Yang2025_S	ENSGALG00010003806	EGF	case	control	0.01279971	0.199841057	0.064049449	0.948930853	0.968176265	up_in_case
Yang2025_S	ENSGALG00010003808	TWIST1	case	control	-0.295971252	0.151096787	-1.95881897	0.050133991	0.133491833	down_in_case
Yang2025_S	ENSGALG00010003810	HMGN2P46	case	control	0.241991513	0.1297152	1.865560193	0.062102923	0.156266947	up_in_case
Yang2025_S	ENSGALG00010003812	BG8	case	control	-0.626036791	0.343149749	-1.824383648	0.068094092	0.166589041	down_in_case
Yang2025_S	ENSGALG00010003814	TPP1	case	control	0.113349462	0.21278722	0.532689237	0.594248718	0.734509126	up_in_case
Yang2025_S	ENSGALG00010003816	KLHL8	case	control	-0.24669886	0.152896915	-1.613497964	0.106636365	0.229271113	down_in_case
Yang2025_S	ENSGALG00010003817	BF2	case	control	0.466581171	0.348468736	1.338947006	0.180587925	0.33286941	up_in_case
Yang2025_S	ENSGALG00010003818	GAB2	case	control	0.233918585	0.283123541	0.826206762	0.408686813	0.574475118	up_in_case
Yang2025_S	ENSGALG00010003819	PHIP	case	control	0.507766497	0.202718113	2.504790959	0.012252378	0.047133539	up_in_case
Yang2025_S	ENSGALG00010003820	SH3YL1	case	control	-0.460967596	0.302390302	-1.524412631	0.127405667	0.260245964	down_in_case
Yang2025_S	ENSGALG00010003821	RUBCNL	case	control	-0.179844748	0.191441133	-0.939425847	0.347512147	0.515991665	down_in_case
Yang2025_S	ENSGALG00010003822	ENSGALG00010003822	case	control	0.917530375	0.33284815	2.756603499	0.005840513	0.026878317	up_in_case
Yang2025_S	ENSGALG00010003823	PTPN13	case	control	0.141588429	0.150365694	0.941627207	0.346383536	0.514864487	up_in_case
Yang2025_S	ENSGALG00010003824	SP4	case	control	-0.087942852	0.16716699	-0.526077861	0.59883411	0.737810006	down_in_case
Yang2025_S	ENSGALG00010003826	ENSGALG00010003826	case	control	0.180305119	0.183366719	0.983303405	0.325458135	0.495331926	up_in_case
Yang2025_S	ENSGALG00010003828	ENSGALG00010003828	case	control	0.370978795	0.464799164	0.798148585	0.42478427	0.589604174	up_in_case
Yang2025_S	ENSGALG00010003829	ENSGALG00010003829	case	control	0.636512127	0.24343183	2.614744859	0.008929413	0.037400472	up_in_case
Yang2025_S	ENSGALG00010003830	DMB2	case	control	-0.553666494	0.458191445	-1.208373704	0.22690355	0.388923131	down_in_case
Yang2025_S	ENSGALG00010003831	IRAK1BP1	case	control	-0.049784292	0.261017432	-0.190731677	0.84873582	0.910199604	down_in_case
Yang2025_S	ENSGALG00010003833	ACP1	case	control	-0.300373304	0.223424373	-1.344407059	0.178816792	0.330435221	down_in_case
Yang2025_S	ENSGALG00010003834	MAPK10	case	control	0.286378758	0.370641219	0.772657609	0.439725041	0.603302507	up_in_case
Yang2025_S	ENSGALG00010003835	ENSGALG00010003835	case	control	0.507109368	0.267882136	1.89303167	0.058353658	0.149615129	up_in_case
Yang2025_S	ENSGALG00010003836	CD1F	case	control	0.739417169	0.320856885	2.304507723	0.021194159	0.070740266	up_in_case
Yang2025_S	ENSGALG00010003837	ALKAL2	case	control	-0.083012003	0.351557586	-0.236126331	0.813334642	0.887604217	down_in_case
Yang2025_S	ENSGALG00010003838	GAR1	case	control	0.304116023	0.111010898	2.739515038	0.00615299	0.027974524	up_in_case
Yang2025_S	ENSGALG00010003841	UBE2W	case	control	-0.219288232	0.170640118	-1.285091893	0.198760174	0.355614291	down_in_case
Yang2025_S	ENSGALG00010003842	ENSGALG00010003842	case	control	0.052074516	0.101689264	0.512094531	0.608584852	0.746008965	up_in_case
Yang2025_S	ENSGALG00010003843	LRCH1	case	control	0.647807222	0.233515081	2.77415583	0.005534516	0.02582836	up_in_case
Yang2025_S	ENSGALG00010003844	TMEM18	case	control	0.096383435	0.217349937	0.443448183	0.657441591	0.783900793	up_in_case
Yang2025_S	ENSGALG00010003845	CFI	case	control	0.165441861	0.41611306	0.397588727	0.690933372	0.80731145	up_in_case
Yang2025_S	ENSGALG00010003848	ESD	case	control	-0.244916076	0.216779544	-1.129793301	0.258563332	0.424891513	down_in_case
Yang2025_S	ENSGALG00010003850	PLA2G12A	case	control	-0.311019864	0.105472785	-2.948816274	0.003189936	0.017083686	down_in_case
Yang2025_S	ENSGALG00010003851	PHF14	case	control	0.543984484	0.158391836	3.43442248	0.000593818	0.004579332	up_in_case
Yang2025_S	ENSGALG00010003852	TCEB1	case	control	-0.245296104	0.10216256	-2.401037185	0.016348675	0.0585803	down_in_case
Yang2025_S	ENSGALG00010003853	ENSGALG00010003853	case	control	-0.220546135	0.138321535	-1.594445401	0.110836311	0.235694184	down_in_case
Yang2025_S	ENSGALG00010003858	SUCLA2	case	control	0.203640671	0.115231603	1.767229357	0.077189825	0.18252846	up_in_case
Yang2025_S	ENSGALG00010003860	TMEM70	case	control	-0.520151935	0.109348771	-4.756815572	1.96671E-06	4.26886E-05	down_in_case
Yang2025_S	ENSGALG00010003861	NUDT15	case	control	-0.360647364	0.134323049	-2.684925382	0.0072546	0.031750268	down_in_case
Yang2025_S	ENSGALG00010003862	PCK2	case	control	-0.213484686	0.155675872	-1.371340872	0.17026872	0.319247895	down_in_case
Yang2025_S	ENSGALG00010003863	HTR1B	case	control	0.254474354	0.267177482	0.952454349	0.340866607	0.509107843	up_in_case
Yang2025_S	ENSGALG00010003865	AKAP8L	case	control	0.644950802	0.235970109	2.733188552	0.006272441	0.028397164	up_in_case
Yang2025_S	ENSGALG00010003868	HBBA	case	control	-0.636718066	0.225859219	-2.81909266	0.004815961	0.023318423	down_in_case
Yang2025_S	ENSGALG00010003869	LY96	case	control	-0.653676977	0.31297534	-2.0885894	0.0367447	0.106430554	down_in_case
Yang2025_S	ENSGALG00010003870	MED4	case	control	-0.08853613	0.099726683	-0.887787779	0.374654918	0.542601301	down_in_case
Yang2025_S	ENSGALG00010003871	ENSGALG00010003871	case	control	1.45253521	0.18885872	7.691120699	1.45852E-14	2.90349E-12	up_in_case
Yang2025_S	ENSGALG00010003874	YES1	case	control	0.062450528	0.120459415	0.518436253	0.604153926	0.742376155	up_in_case
Yang2025_S	ENSGALG00010003875	AKAP8	case	control	0.558207024	0.111909836	4.988006794	6.10054E-07	1.58013E-05	up_in_case
Yang2025_S	ENSGALG00010003877	CLVS1	case	control	2.558077084	0.68403202	3.739703709	0.000184237	0.001746494	up_in_case
Yang2025_S	ENSGALG00010003878	ARHGAP24	case	control	0.096397469	0.110194132	0.874796756	0.381684503	0.54968722	up_in_case
Yang2025_S	ENSGALG00010003879	CASP6	case	control	-0.523556512	0.136607469	-3.832561399	0.000126816	0.001288972	down_in_case
Yang2025_S	ENSGALG00010003882	ENSGALG00010003882	case	control	-0.330832646	0.217586593	-1.520464294	0.128394326	0.261676189	down_in_case
Yang2025_S	ENSGALG00010003884	TYMS	case	control	-0.282767976	0.174422825	-1.621163837	0.104982506	0.227022366	down_in_case
Yang2025_S	ENSGALG00010003887	GIPC1	case	control	0.325109873	0.138946846	2.339814702	0.019293309	0.066027067	up_in_case
Yang2025_S	ENSGALG00010003889	ITM2B	case	control	-0.224114447	0.095447014	-2.348050887	0.018871943	0.065013726	down_in_case
Yang2025_S	ENSGALG00010003894	COPS4	case	control	-0.049434795	0.11200332	-0.441369014	0.65894587	0.784658933	down_in_case
Yang2025_S	ENSGALG00010003895	THSD7A	case	control	0.349620918	0.198429991	1.761935865	0.078080134	0.184164974	up_in_case
Yang2025_S	ENSGALG00010003896	MCUB	case	control	-0.283473363	0.1483276	-1.911130252	0.055987844	0.145016842	down_in_case
Yang2025_S	ENSGALG00010003899	LIN54	case	control	0.391647984	0.208328148	1.87995711	0.060113924	0.152733175	up_in_case
Yang2025_S	ENSGALG00010003900	RB1	case	control	0.009333858	0.133582855	0.069873172	0.944294607	0.965988078	up_in_case
Yang2025_S	ENSGALG00010003902	ENSGALG00010003902	case	control	0.629075766	0.242844415	2.590447738	0.009585117	0.039389151	up_in_case
Yang2025_S	ENSGALG00010003903	COL28A1	case	control	0.017844265	0.256347086	0.069609782	0.944504251	0.965993889	up_in_case
Yang2025_S	ENSGALG00010003909	DNAJB1	case	control	-0.227575475	0.137019663	-1.660896468	0.096734242	0.214238979	down_in_case
Yang2025_S	ENSGALG00010003910	THAP9	case	control	0.112708459	0.12207494	0.923272702	0.35586511	0.525204439	up_in_case
Yang2025_S	ENSGALG00010003913	PSMB5	case	control	0.275563764	0.284729613	0.967808586	0.333139975	0.502359653	up_in_case
Yang2025_S	ENSGALG00010003914	CAMK2D	case	control	0.058140541	0.166743165	0.348683203	0.727327156	0.832465302	up_in_case
Yang2025_S	ENSGALG00010003915	MIOS	case	control	0.139979556	0.140035478	0.99960066	0.317503804	0.487055868	up_in_case
Yang2025_S	ENSGALG00010003918	HTR1E	case	control	2.089521829	0.685096633	3.049966572	0.002288668	0.013249935	up_in_case
Yang2025_S	ENSGALG00010003920	LPAR6	case	control	-0.409700274	0.225697167	-1.815265471	0.069483123	0.169096632	down_in_case
Yang2025_S	ENSGALG00010003921	COLEC12	case	control	-0.030987715	0.175522051	-0.176545994	0.859865018	0.917263953	down_in_case
Yang2025_S	ENSGALG00010003923	ENSGALG00010003923	case	control	-0.589920808	0.234246356	-2.518377742	0.011789681	0.045759371	down_in_case
Yang2025_S	ENSGALG00010003925	SEC31A	case	control	0.072219586	0.09135675	0.790522708	0.429222566	0.593551301	up_in_case
Yang2025_S	ENSGALG00010003928	ENSGALG00010003928	case	control	-0.399528106	0.337469435	-1.183894196	0.236454948	0.400121397	down_in_case
Yang2025_S	ENSGALG00010003929	RCBTB2	case	control	-0.161552861	0.114620433	-1.409459517	0.158699336	0.304233766	down_in_case
Yang2025_S	ENSGALG00010003933	TECR	case	control	0.027486026	0.192882554	0.142501361	0.886684005	0.933580779	up_in_case
Yang2025_S	ENSGALG00010003937	ENSGALG00010003937	case	control	0.67901972	0.358762709	1.892670846	0.058401656	0.149683111	up_in_case
Yang2025_S	ENSGALG00010003948	SCD5	case	control	0.287229398	0.183472165	1.565520296	0.117460962	0.245805453	up_in_case
Yang2025_S	ENSGALG00010003958	CYSLTR2	case	control	-0.374842859	0.356160562	-1.052454704	0.292590982	0.461384557	down_in_case
Yang2025_S	ENSGALG00010003960	ASPH	case	control	0.192445009	0.142862961	1.347060203	0.177960844	0.329404418	up_in_case
Yang2025_S	ENSGALG00010003962	UMAD1	case	control	-0.638571431	0.146189745	-4.36810003	1.25332E-05	0.000198692	down_in_case
Yang2025_S	ENSGALG00010003965	SNTG1	case	control	0.199463155	0.495006227	0.402950799	0.68698442	0.804329347	up_in_case
Yang2025_S	ENSGALG00010003968	DSN1	case	control	-0.197896914	0.240638808	-0.822381542	0.410859781	0.576288952	down_in_case
Yang2025_S	ENSGALG00010003972	PENK	case	control	0.060735278	0.144618536	0.419968835	0.67450822	0.795335256	up_in_case
Yang2025_S	ENSGALG00010003977	MYO6	case	control	0.217689782	0.189991417	1.145787458	0.251883106	0.4177069	up_in_case
Yang2025_S	ENSGALG00010003978	ANK2	case	control	0.300687026	0.158903322	1.892263935	0.058455824	0.14973932	up_in_case
Yang2025_S	ENSGALG00010003980	CHCHD7	case	control	0.552127408	0.1738115	3.176587325	0.001490189	0.009573899	up_in_case
Yang2025_S	ENSGALG00010003981	TMEM150C	case	control	-0.398744325	0.239623267	-1.664046773	0.096103083	0.213349762	down_in_case
Yang2025_S	ENSGALG00010003982	GLCCI1	case	control	0.357832164	0.128645806	2.781529959	0.005410334	0.02545341	up_in_case
Yang2025_S	ENSGALG00010003985	PRMT5	case	control	0.172278458	0.126140618	1.365765137	0.172012692	0.321326207	up_in_case
Yang2025_S	ENSGALG00010003986	FNDC3A	case	control	-0.135355579	0.099602586	-1.358956479	0.17416038	0.324386541	down_in_case
Yang2025_S	ENSGALG00010003988	ENOPH1	case	control	-0.014839629	0.11740931	-0.126392265	0.899421425	0.940222773	down_in_case
Yang2025_S	ENSGALG00010003990	ICA1	case	control	0.54582303	0.190374142	2.867106972	0.00414243	0.020689877	up_in_case
Yang2025_S	ENSGALG00010003993	ENSGALG00010003993	case	control	-0.209678603	0.295849158	-0.70873483	0.478489046	0.637375795	down_in_case
Yang2025_S	ENSGALG00010003994	SDR16C5	case	control	-0.666155514	0.12884318	-5.170281539	2.33742E-07	7.01717E-06	down_in_case
Yang2025_S	ENSGALG00010003995	MLNR	case	control	-0.503875423	0.227301086	-2.216775252	0.026638453	0.083850655	down_in_case
Yang2025_S	ENSGALG00010004000	ENSGALG00010004000	case	control	0.488901935	0.177522048	2.75403501	0.005886547	0.027008535	up_in_case
Yang2025_S	ENSGALG00010004001	LARP7	case	control	0.481681557	0.186333474	2.585051128	0.009736456	0.039741509	up_in_case
Yang2025_S	ENSGALG00010004002	CDADC1	case	control	-0.10466309	0.120318908	-0.869880649	0.384365631	0.551947636	down_in_case
Yang2025_S	ENSGALG00010004003	HNRNPDL	case	control	0.376639779	0.105041281	3.585635795	0.000336258	0.002874696	up_in_case
Yang2025_S	ENSGALG00010004006	NKAIN3	case	control	-0.400521069	0.325617656	-1.230034863	0.21868405	0.379780936	down_in_case
Yang2025_S	ENSGALG00010004007	ENSGALG00010004007	case	control	0.030817091	0.1300807	0.236907478	0.812728568	0.887252024	up_in_case
Yang2025_S	ENSGALG00010004016	ENSGALG00010004016	case	control	1.11701105	0.411534706	2.714257229	0.006642457	0.029705595	up_in_case
Yang2025_S	ENSGALG00010004018	HNRNPD	case	control	0.110720939	0.122994622	0.900209596	0.36800872	0.536591139	up_in_case
Yang2025_S	ENSGALG00010004029	ARL4A	case	control	0.442649455	0.137092386	3.228840552	0.001242932	0.00829117	up_in_case
Yang2025_S	ENSGALG00010004034	NEDD8	case	control	-0.350617984	0.197715756	-1.773343674	0.076171776	0.180739826	down_in_case
Yang2025_S	ENSGALG00010004036	ADCYAP1	case	control	0.556584287	0.396345558	1.404290463	0.160232373	0.305868236	up_in_case
Yang2025_S	ENSGALG00010004038	SENP6	case	control	0.240941269	0.163439151	1.474195544	0.140428949	0.279443115	up_in_case
Yang2025_S	ENSGALG00010004039	ALPK1	case	control	0.373423427	0.317681413	1.175465144	0.239808674	0.404176811	up_in_case
Yang2025_S	ENSGALG00010004040	LGALS1B	case	control	0.318174247	0.228306008	1.393630637	0.163429172	0.310143744	up_in_case
Yang2025_S	ENSGALG00010004042	PLAG1	case	control	0.261064967	0.203158695	1.285029748	0.198781889	0.355614291	up_in_case
Yang2025_S	ENSGALG00010004043	CAB39L	case	control	-0.43366288	0.128458628	-3.375895318	0.000735759	0.005476925	down_in_case
Yang2025_S	ENSGALG00010004044	RNF138	case	control	-0.520488131	0.097940606	-5.314324205	1.07054E-07	3.64742E-06	down_in_case
Yang2025_S	ENSGALG00010004046	ENSGALG00010004046	case	control	0.461759009	0.267175695	1.728297212	0.083934953	0.193895061	up_in_case
Yang2025_S	ENSGALG00010004047	PDE7A	case	control	0.173663019	0.17185528	1.01051896	0.31224672	0.481855819	up_in_case
Yang2025_S	ENSGALG00010004049	SETDB2	case	control	0.213379102	0.086547398	2.465459459	0.013683766	0.051162672	up_in_case
Yang2025_S	ENSGALG00010004055	FILIP1	case	control	0.844015198	0.274798003	3.071402226	0.002130559	0.012543027	up_in_case
Yang2025_S	ENSGALG00010004056	SHROOM3	case	control	0.119729983	0.143762295	0.832832998	0.404938921	0.571251657	up_in_case
Yang2025_S	ENSGALG00010004057	ENSGALG00010004057	case	control	0.724090594	0.351741081	2.058589777	0.039533553	0.11234062	up_in_case
Yang2025_S	ENSGALG00010004058	TMEM30A	case	control	-0.51474601	0.058191735	-8.84568932	9.0965E-19	3.96124E-16	down_in_case
Yang2025_S	ENSGALG00010004060	ETV1	case	control	-0.193660263	0.099832642	-1.939849119	0.052398029	0.137975536	down_in_case
Yang2025_S	ENSGALG00010004063	ENSGALG00010004063	case	control	1.849345484	0.446166473	4.144967398	3.39862E-05	0.000448058	up_in_case
Yang2025_S	ENSGALG00010004065	TIFA	case	control	-0.386707766	0.379697832	-1.018461876	0.30845851	0.478021501	down_in_case
Yang2025_S	ENSGALG00010004070	ARL11	case	control	0.038998324	0.260302136	0.149819454	0.880907061	0.929747771	up_in_case

Yang2025_S	ENSGALG00010004072	SGK3	case	control	-0.400992696	0.143371381	-2.796881032	0.005159853	0.024548498	down_in_case
Yang2025_S	ENSGALG00010004076	COX7A2	case	control	-0.844138685	0.502608559	-1.679515141	0.093051691	0.208394777	down_in_case
Yang2025_S	ENSGALG00010004079	AP1AR	case	control	-0.175752491	0.131480151	-1.336722611	0.181313199	0.333852062	down_in_case
Yang2025_S	ENSGALG00010004081	RPS20	case	control	-0.641966135	0.267758554	-2.397556029	0.016504857	0.05900338	down_in_case
Yang2025_S	ENSGALG00010004082	FAM241A	case	control	-0.435268271	0.139792053	-3.113683952	0.001847673	0.011287734	down_in_case
Yang2025_S	ENSGALG00010004083	PHF11	case	control	0.19835224	0.158579326	1.250807686	0.21100465	0.370945904	up_in_case
Yang2025_S	ENSGALG00010004085	RB1CC1	case	control	0.168129207	0.138495533	1.213968444	0.224759774	0.386336186	up_in_case
Yang2025_S	ENSGALG00010004086	UTP3	case	control	0.51174112	0.119612445	4.278326742	1.88303E-05	0.000275921	up_in_case
Yang2025_S	ENSGALG00010004087	NCOA7	case	control	0.353259386	0.159980386	2.20814185	0.027234386	0.085149467	up_in_case
Yang2025_S	ENSGALG00010004094	DGKB	case	control	0.193519308	0.197970857	0.977514122	0.32831469	0.497993382	up_in_case
Yang2025_S	ENSGALG00010004095	ANKRD50	case	control	0.083235725	0.131515804	0.632895232	0.526802062	0.679783936	up_in_case
Yang2025_S	ENSGALG00010004096	COL12A1	case	control	0.618953488	0.291642879	2.122299336	0.033812609	0.10008044	up_in_case
Yang2025_S	ENSGALG00010004097	ENSGALG00010004097	case	control	0.621526695	0.15088332	4.119253826	3.80101E-05	0.000491802	up_in_case
Yang2025_S	ENSGALG00010004101	ARID1B	case	control	0.192958803	0.15690446	1.229785328	0.218777504	0.379842671	up_in_case
Yang2025_S	ENSGALG00010004102	PCMTD1	case	control	-0.188389258	0.166548938	-1.13113455	0.257998465	0.424275547	down_in_case
Yang2025_S	ENSGALG00010004104	SCARB2	case	control	-0.427449056	0.104766789	-4.080005324	4.50347E-05	0.000562832	down_in_case
Yang2025_S	ENSGALG00010004108	PERPB	case	control	-0.453195124	0.29099375	-1.55740501	0.119374357	0.248614807	down_in_case
Yang2025_S	ENSGALG00010004109	AGMO	case	control	0.264158087	0.415443279	0.635846336	0.524876583	0.678241393	up_in_case
Yang2025_S	ENSGALG00010004113	ECHDC1	case	control	-0.188699328	0.195093184	-0.967226653	0.333430741	0.502561032	down_in_case
Yang2025_S	ENSGALG00010004116	SPRY1	case	control	-0.451071859	0.118982494	-3.791077514	0.000149995	0.001479251	down_in_case
Yang2025_S	ENSGALG00010004117	STBD1	case	control	0.024581144	0.208082602	0.118131662	0.905963336	0.943647271	up_in_case
Yang2025_S	ENSGALG00010004122	TRIM55	case	control	0.597622926	0.160993424	3.712095255	0.000205551	0.001914671	up_in_case
Yang2025_S	ENSGALG00010004123	NDUFAF4	case	control	-0.310293064	0.153202486	-2.025378781	0.042828485	0.119005968	down_in_case
Yang2025_S	ENSGALG00010004125	MANEA	case	control	-0.195177955	0.130768138	-1.49254977	0.13555508	0.27212214	down_in_case
Yang2025_S	ENSGALG00010004127	EPHA7	case	control	-0.171446092	0.232365667	-0.737828846	0.460618462	0.621607424	down_in_case
Yang2025_S	ENSGALG00010004128	MCMDC2	case	control	0.410493805	0.941851699	0.435836985	0.662955008	0.786952362	up_in_case
Yang2025_S	ENSGALG00010004131	NUDT6	case	control	0.689028644	0.154474387	4.460471792	8.17794E-06	0.000138806	up_in_case
Yang2025_S	ENSGALG00010004133	CRH	case	control	1.90738299	0.786514222	2.425109346	0.015303777	0.055812124	up_in_case
Yang2025_S	ENSGALG00010004135	NUP54	case	control	-0.175879457	0.122581455	-1.434796624	0.15134506	0.294372915	down_in_case
Yang2025_S	ENSGALG00010004137	CROT	case	control	-0.107195331	0.110864723	-0.966902074	0.33359299	0.502561032	down_in_case
Yang2025_S	ENSGALG00010004139	PPEF2	case	control	0.979494895	0.414940731	2.360565791	0.018247081	0.06331501	up_in_case
Yang2025_S	ENSGALG00010004141	KPNA3	case	control	-0.288550293	0.096203867	-2.999362726	0.00270545	0.015050079	down_in_case
Yang2025_S	ENSGALG00010004142	RBBP8	case	control	0.247733913	0.111858524	2.214707511	0.026780147	0.084079759	up_in_case
Yang2025_S	ENSGALG00010004143	TCF24	case	control	0.449735202	0.310484042	1.448496995	0.147478106	0.289574102	up_in_case
Yang2025_S	ENSGALG00010004146	NAAA	case	control	-0.327065706	0.172096967	-1.900473396	0.057371023	0.147693554	down_in_case
Yang2025_S	ENSGALG00010004147	SPRYD7	case	control	-0.108777599	0.12125045	-0.897131512	0.369648745	0.538362799	down_in_case
Yang2025_S	ENSGALG00010004148	ENSGALG00010004148	case	control	0.409909062	0.155100593	2.642859422	0.008220916	0.034947671	up_in_case
Yang2025_S	ENSGALG00010004149	SDAD1	case	control	0.102003368	0.10169871	1.002995695	0.315862939	0.485072741	up_in_case
Yang2025_S	ENSGALG00010004154	COPS5	case	control	-0.073690005	0.157698496	-0.467284132	0.640296608	0.770046883	down_in_case
Yang2025_S	ENSGALG00010004158	JCHAIN	case	control	-0.786453157	0.411099032	-1.913050372	0.055741604	0.144620974	down_in_case
Yang2025_S	ENSGALG00010004172	ST8SIA6	case	control	-0.036676563	0.150366667	-0.243914188	0.807297281	0.88385352	down_in_case
Yang2025_S	ENSGALG00010004177	CSPP1	case	control	0.904186684	0.194643936	4.645337032	3.39522E-06	6.74927E-05	up_in_case
Yang2025_S	ENSGALG00010004181	BBS12	case	control	-0.070933915	0.144516943	-0.490834593	0.623543441	0.75777015	down_in_case
Yang2025_S	ENSGALG00010004187	ARFGEF1	case	control	0.055747155	0.096929255	0.575132402	0.565201755	0.71088981	up_in_case
Yang2025_S	ENSGALG00010004188	BMI1	case	control	0.041799248	0.13070915	0.319788234	0.749128868	0.847674443	up_in_case
Yang2025_S	ENSGALG00010004191	CABLES1	case	control	-0.727284751	0.122036576	-5.95956373	2.52912E-09	1.37669E-07	down_in_case
Yang2025_S	ENSGALG00010004200	ABCB1	case	control	0.032812705	0.161162837	0.2035997	0.838666327	0.904482259	up_in_case
Yang2025_S	ENSGALG00010004201	CPA6	case	control	0.835647984	0.568363832	1.470269458			up_in_case
Yang2025_S	ENSGALG00010004210	SYNCRIP	case	control	0.412360619	0.140683137	2.931130392	0.00337731	0.017799854	up_in_case
Yang2025_S	ENSGALG00010004211	RNF146	case	control	0.080049555	0.113369406	0.70609486	0.480129148	0.638659763	up_in_case
Yang2025_S	ENSGALG00010004212	RSPO3	case	control	0.475581772	0.19861626	2.394475521	0.016644154	0.05933392	up_in_case
Yang2025_S	ENSGALG00010004214	REPS1	case	control	0.1359321	0.199812437	0.680298496	0.496315477	0.653401432	up_in_case
Yang2025_S	ENSGALG00010004216	SNX14	case	control	0.243538405	0.138930865	1.752946731	0.079611151	0.186733107	up_in_case
Yang2025_S	ENSGALG00010004217	CCDC28A	case	control	0.026994194	0.134226148	0.201109802	0.840612718	0.905354913	up_in_case
Yang2025_S	ENSGALG00010004219	FGF2	case	control	-0.304922187	0.097176704	-3.13781157	0.001702143	0.010607942	down_in_case
Yang2025_S	ENSGALG00010004222	SPAG6	case	control	-0.770123179	0.311651057	-2.471107227	0.013469542	0.050537984	down_in_case
Yang2025_S	ENSGALG00010004224	NT5E	case	control	-0.282295413	0.219682945	-1.285012872	0.198787786	0.355614291	down_in_case
Yang2025_S	ENSGALG00010004225	CETN4	case	control	-0.330253882	0.103473077	-3.191688996	0.001414435	0.009207045	down_in_case
Yang2025_S	ENSGALG00010004226	RUNDC3B	case	control	-0.456221339	0.248464534	-1.836162816	0.06633358	0.163518822	down_in_case
Yang2025_S	ENSGALG00010004230	TAF1B	case	control	-0.007296349	0.144325156	-0.050554939	0.959680171	0.975643334	down_in_case
Yang2025_S	ENSGALG00010004231	ARMC3	case	control	-0.579561778	0.353433328	-1.639805114	0.101045695	0.220715292	down_in_case
Yang2025_S	ENSGALG00010004233	NHSL1	case	control	0.075806651	0.21306463	0.355791813	0.721996481	0.828817939	up_in_case
Yang2025_S	ENSGALG00010004236	DBF4	case	control	0.324986231	0.135033579	2.406706781	0.016097088	0.057911329	up_in_case
Yang2025_S	ENSGALG00010004237	RUFY3	case	control	0.153079428	0.095027192	1.61090131	0.107201235	0.230035295	up_in_case
Yang2025_S	ENSGALG00010004238	CCNC	case	control	-0.364756381	0.105590865	-3.454431219	0.000551455	0.00429303	down_in_case
Yang2025_S	ENSGALG00010004240	ENSGALG00010004240	case	control	-0.427516057	0.166470227	-2.568123223	0.01022508	0.041204885	down_in_case
Yang2025_S	ENSGALG00010004241	PREX2	case	control	0.140418758	0.169009072	0.830835625	0.406066494	0.572031601	up_in_case
Yang2025_S	ENSGALG00010004242	ADAM22	case	control	0.192482352	0.242366044	0.794180359	0.427090441	0.591483333	up_in_case
Yang2025_S	ENSGALG00010004243	BLTP1	case	control	0.003102953	0.149957668	0.020692195	0.983491195	0.990027436	up_in_case
Yang2025_S	ENSGALG00010004244	TMEM241	case	control	-0.061071232	0.117496663	-0.519769933	0.603223938	0.741408796	down_in_case
Yang2025_S	ENSGALG00010004245	ARFGEF3	case	control	-0.133144731	0.298037912	-0.446737566	0.655064555	0.781986044	down_in_case
Yang2025_S	ENSGALG00010004246	C8orf34	case	control	0.448083152	0.440309748	1.0176544	0.308842225	0.478456521	up_in_case
Yang2025_S	ENSGALG00010004247	MEOX2	case	control	0.473253542	0.213858103	2.21293248	0.026902301	0.084411785	up_in_case
Yang2025_S	ENSGALG00010004248	GRSF1	case	control	0.13207857	0.118718475	1.112535946	0.265907784	0.433129745	up_in_case
Yang2025_S	ENSGALG00010004249	USP45	case	control	0.08475225	0.106278338	0.797455544	0.425186512	0.590019323	up_in_case
Yang2025_S	ENSGALG00010004250	MOB1B	case	control	-0.196179316	0.0929441	-2.110723718	0.034796066	0.102252885	down_in_case
Yang2025_S	ENSGALG00010004251	CCN2	case	control	0.389082753	0.212101188	1.834420432	0.066591609	0.164036428	up_in_case
Yang2025_S	ENSGALG00010004255	RPS12	case	control	-0.167804755	0.276783632	-0.606266901	0.544337534	0.69418354	down_in_case
Yang2025_S	ENSGALG00010004256	ENSGALG00010004256	case	control	0.269196958	1.043415637	0.257995901	0.79641007	0.876731298	up_in_case
Yang2025_S	ENSGALG00010004257	ENSGALG00010004257	case	control	-0.991728702	0.560721674	-1.768664826	0.076949825	0.182084534	down_in_case
Yang2025_S	ENSGALG00010004265	PIP4K2A	case	control	-0.350945482	0.088650271	-3.958763787	7.53387E-05	0.000849389	down_in_case
Yang2025_S	ENSGALG00010004275	ADARB2	case	control	0.302317486	0.940541401	0.321429216	0.747885145	0.847161397	up_in_case
Yang2025_S	ENSGALG00010004281	VIM	case	control	0.068374064	0.081487064	0.839078747	0.401425122	0.567962135	up_in_case
Yang2025_S	ENSGALG00010004283	DCK	case	control	-0.627854861	0.18883115	-3.324953867	0.000884332	0.006342851	down_in_case
Yang2025_S	ENSGALG00010004285	SLC18B1	case	control	-0.921970791	0.174791836	-5.274678793	1.32989E-07	4.37075E-06	down_in_case
Yang2025_S	ENSGALG00010004296	ENSGALG00010004296	case	control	-1.371245343	1.43411499	-0.956161362	0.338990717	0.507121379	down_in_case
Yang2025_S	ENSGALG00010004297	SULF1	case	control	0.161219327	0.177989112	0.905781963	0.36505127	0.533730925	up_in_case
Yang2025_S	ENSGALG00010004300	VNN2	case	control	-0.191645027	0.232161756	-0.825480607	0.409098789	0.574651492	down_in_case
Yang2025_S	ENSGALG00010004302	CRPPA	case	control	-0.298993472	0.158320202	-1.888536447	0.05895397	0.150765933	down_in_case
Yang2025_S	ENSGALG00010004306	PNISR	case	control	1.600078831	0.248745315	6.432598858	1.2544E-10	1.01041E-08	up_in_case
Yang2025_S	ENSGALG00010004307	NPC2	case	control	-0.477221949	0.164449058	-2.901943943	0.003708549	0.019096181	down_in_case
Yang2025_S	ENSGALG00010004308	DSG2	case	control	-0.213251501	0.492157896	-0.433298954	0.664797598	0.788220414	down_in_case
Yang2025_S	ENSGALG00010004309	MRPS28	case	control	-0.032803096	0.200812184	-0.163352122	0.870241197	0.923654015	down_in_case
Yang2025_S	ENSGALG00010004312	PDLIM5	case	control	-0.004238894	0.102427649	-0.041384276	0.966989548	0.980427776	down_in_case
Yang2025_S	ENSGALG00010004323	TRDMT1	case	control	0.071014288	0.13739104	0.516877144	0.605241924	0.743151928	up_in_case
Yang2025_S	ENSGALG00010004325	LTBP2	case	control	-0.256384262	0.116076485	-2.208752812	0.027191839	0.085055629	down_in_case
Yang2025_S	ENSGALG00010004326	ENSGALG00010004326	case	control	-1.505384525	0.676873765	-2.224025517	0.026146727	0.082676344	down_in_case
Yang2025_S	ENSGALG00010004328	ENSGALG00010004328	case	control	-0.901645853	0.542406235	-1.662307317	0.096451171	0.213849972	down_in_case
Yang2025_S	ENSGALG00010004329	COQ3	case	control	-0.372738323	0.237043182	-1.572449038	0.115846449	0.243413792	down_in_case
Yang2025_S	ENSGALG00010004330	STX7	case	control	-0.428661791	0.120849237	-3.547079004	0.000389528	0.003230994	down_in_case
Yang2025_S	ENSGALG00010004332	ENSGALG00010004332	case	control	-1.053295323	0.717633797	-1.467733721	0.142176567	0.281865196	down_in_case
Yang2025_S	ENSGALG00010004333	DIO2	case	control	-0.314188943	0.487937459	-0.643912324	0.51963229	0.673525808	down_in_case
Yang2025_S	ENSGALG00010004334	ENSGALG00010004334	case	control	0.473964487	0.166966643	2.838677703	0.004530089	0.022219918	up_in_case
Yang2025_S	ENSGALG00010004336	ENSGALG00010004336	case	control	-0.029449887	0.395238914	-0.074511608	0.940603305	0.963601685	down_in_case
Yang2025_S	ENSGALG00010004337	BMPR1B	case	control	0.302424255	0.13752096	2.199113896	0.027869825	0.086592198	up_in_case
Yang2025_S	ENSGALG00010004338	ENSGALG00010004338	case	control	0.437237077	0.194282503	2.250522148	0.024415819	0.078739746	up_in_case
Yang2025_S	ENSGALG00010004339	B4GALT6	case	control	0.339020463	0.188679918	1.796802045	0.072367052	0.173958058	up_in_case
Yang2025_S	ENSGALG00010004340	SLC4A4	case	control	0.372917892	0.175747882	2.12189124	0.033846872	0.100128975	up_in_case
Yang2025_S	ENSGALG00010004341	ENSGALG00010004341	case	control	0.184722042	0.235467655	0.784490093	0.432752593	0.596774604	up_in_case
Yang2025_S	ENSGALG00010004342	ZNF438	case	control	0.2882049	0.160334367	1.797524167	0.072252445	0.173742506	up_in_case
Yang2025_S	ENSGALG00010004344	SOSTDC1	case	control	-0.089789643	0.334860932	-0.268140099	0.788591481	0.872490852	down_in_case
Yang2025_S	ENSGALG00010004345	FAXC	case	control	-0.211309187	0.206122912	-1.025161075	0.305287191	0.474807136	down_in_case
Yang2025_S	ENSGALG00010004346	CEP128	case	control	0.468791462	0.197841493	2.369530551	0.017810684	0.062438208	up_in_case
Yang2025_S	ENSGALG00010004350	MOXD1	case	control	-0.405210856	0.195119998	-2.076726428	0.037826816	0.108616666	down_in_case
Yang2025_S	ENSGALG00010004352	CHMP2A	case	control	-0.393986193	0.162967388	-2.417576905	0.015624232	0.056713642	down_in_case
Yang2025_S	ENSGALG00010004355	ANKMY2	case	control	-0.302714839	0.11266704	-2.686809193	0.007213815	0.031611481	down_in_case
Yang2025_S	ENSGALG00010004356	TRAPPC8	case	control	-0.046266463	0.072787831	-0.635634593	0.525014617	0.678350452	down_in_case
Yang2025_S	ENSGALG00010004358	GC	case	control	-7.582145245	2.238317946	-3.387429949			down_in_case
Yang2025_S	ENSGALG00010004362	FBXL4	case	control	-0.326509089	0.150875069	-2.164102339	0.030456496	0.092776908	down_in_case
Yang2025_S	ENSGALG00010004364	TSHR	case	control	-0.318823471	0.23056551	-1.382789087	0.166729546	0.314529759	down_in_case
Yang2025_S	ENSGALG00010004365	TPD52	case	control	0.3228483	0.180351977	1.790101255	0.073437635	0.176019526	up_in_case
Yang2025_S	ENSGALG00010004366	UNC5C	case	control	0.248185194	0.39173078	0.633560617	0.52636761	0.679475002	up_in_case
Yang2025_S	ENSGALG00010004367	TSPAN13	case	control	-1.421686732	0.180078834	-7.894801981	2.90777E-15	6.64258E-13	down_in_case
Yang2025_S	ENSGALG00010004368	GJD4	case	control	0.426307835	0.236800827	1.800280179	0.071816409	0.172991034	up_in_case
Yang2025_S	ENSGALG00010004369	UBE2M	case	control	-0.672620974	0.12818235	-5.247375897	1.54281E-07	4.94231E-06	down_in_case
Yang2025_S	ENSGALG00010004370	ATP6V1H	case	control	-0.154008896	0.153408001	-1.003916972	0.315418635	0.484623994	down_in_case

Yang2025_S	ENSGALG00010004372	SYNJ2	case	control	0.15444101	0.207618867	0.743867899	0.456956397	0.618522331	up_in_case
Yang2025_S	ENSGALG00010004373	MMS22L	case	control	-0.368747725	0.140036008	-2.633235063	0.008457579	0.03580084	down_in_case
Yang2025_S	ENSGALG00010004375	ENSGALG00010004375	case	control	2.061156057	0.364750842	5.650860309	1.59647E-08	7.01791E-07	up_in_case
Yang2025_S	ENSGALG00010004378	TRIM28	case	control	-0.103341684	0.088603164	-1.166343041	0.243475778	0.408529195	down_in_case
Yang2025_S	ENSGALG00010004379	ZBTB10	case	control	-0.108120559	0.095491688	-1.132251	0.257528924	0.423791398	down_in_case
Yang2025_S	ENSGALG00010004380	ISCA2	case	control	-0.798644246	0.238860282	-3.343562347	0.000827101	0.005996698	down_in_case
Yang2025_S	ENSGALG00010004381	ENSGALG00010004381	case	control	0.738010326	0.322644001	2.287382762	0.022173492	0.072934492	up_in_case
Yang2025_S	ENSGALG00010004385	ADAMTS3	case	control	0.856063734	0.254591301	3.362501908	0.000772396	0.00568286	up_in_case
Yang2025_S	ENSGALG00010004386	CFAP44	case	control	0.253226742	0.164116504	1.542969389	0.122838203	0.253709134	up_in_case
Yang2025_S	ENSGALG00010004389	ENSGALG00010004389	case	control	-0.155248619	0.106620606	-1.456084562	0.145369245	0.28656393	down_in_case
Yang2025_S	ENSGALG00010004390	PNLDC1	case	control	0.050887696	0.36715093	0.138601571	0.889765002	0.935552351	up_in_case
Yang2025_S	ENSGALG00010004393	ZBTB45	case	control	0.612213472	0.122808782	4.985095218	6.19313E-07	1.59817E-05	up_in_case
Yang2025_S	ENSGALG00010004394	CCNY	case	control	-0.101544617	0.108214146	-0.938367313	0.348055678	0.516513541	down_in_case
Yang2025_S	ENSGALG00010004395	BOC	case	control	0.113555295	0.138332497	0.820886613	0.411710857	0.577073869	up_in_case
Yang2025_S	ENSGALG00010004397	ENSGALG00010004397	case	control	0.237899819	0.12508509	1.901903884	0.057183721	0.147307033	up_in_case
Yang2025_S	ENSGALG00010004398	COX18	case	control	-0.354793798	0.229506952	-1.545895649	0.122129771	0.252954573	down_in_case
Yang2025_S	ENSGALG00010004404	RGS20	case	control	0.233962369	0.443531022	0.527499447	0.5978468	0.737060529	up_in_case
Yang2025_S	ENSGALG00010004406	ENSGALG00010004406	case	control	0.535295741	0.201852782	2.651911626	0.008003749	0.034254373	up_in_case
Yang2025_S	ENSGALG00010004408	SERAC1	case	control	-0.58542333	0.190094613	-3.079641868	0.002072496	0.012284235	down_in_case
Yang2025_S	ENSGALG00010004416	STXBP6	case	control	-0.03081074	0.245601011	-0.125450378	0.900167007	0.940592827	down_in_case
Yang2025_S	ENSGALG00010004420	ENSGALG00010004420	case	control	-0.133742304	0.400419158	-0.334005757	0.738375214	0.84021347	down_in_case
Yang2025_S	ENSGALG00010004421	RAP1GDS1	case	control	0.304336617	0.119269711	2.551667277	0.010720885	0.042660059	up_in_case
Yang2025_S	ENSGALG00010004424	MRPL18	case	control	0.311174409	0.221013631	1.40794216	0.159148198	0.304591421	up_in_case
Yang2025_S	ENSGALG00010004425	CREM	case	control	-0.297509875	0.145106687	-2.050283695	0.040336755	0.113911305	down_in_case
Yang2025_S	ENSGALG00010004429	ENSGALG00010004429	case	control	1.450649035	0.524782899	2.764284123			up_in_case
Yang2025_S	ENSGALG00010004430	TCEA1	case	control	-0.270708057	0.093476894	-2.895989011	0.003779656	0.019385172	down_in_case
Yang2025_S	ENSGALG00010004433	AHR1A	case	control	-0.190194063	0.116709406	-1.629637832	0.103178064	0.224443696	down_in_case
Yang2025_S	ENSGALG00010004434	TCP1	case	control	0.031709634	0.130781507	0.242462677	0.808421673	0.884598038	up_in_case
Yang2025_S	ENSGALG00010004436	NOVA1	case	control	-0.076144974	0.144660633	-0.5263697	0.598631364	0.737701455	down_in_case
Yang2025_S	ENSGALG00010004440	GTF2H5	case	control	-0.386395772	0.131136766	-2.946509839	0.003213822	0.017145715	down_in_case
Yang2025_S	ENSGALG00010004443	ANKRD17	case	control	0.342344894	0.171672994	1.994168599	0.046133632	0.125782072	up_in_case
Yang2025_S	ENSGALG00010004445	EPHA6	case	control	0.357734053	0.518545304	0.689880035	0.490269632	0.647635541	up_in_case
Yang2025_S	ENSGALG00010004446	ACAT2	case	control	0.13130811	0.181920662	0.721787772	0.470424969	0.630688083	up_in_case
Yang2025_S	ENSGALG00010004451	CLDND1	case	control	-0.781344703	0.15712145	-4.972871006	6.59686E-07	1.68674E-05	down_in_case
Yang2025_S	ENSGALG00010004454	SRI	case	control	-0.440264948	0.156629811	-2.810863054	0.004940881	0.023769828	down_in_case
Yang2025_S	ENSGALG00010004457	AGA	case	control	0.328127526	0.138928358	2.361847	0.018184145	0.063238349	up_in_case
Yang2025_S	ENSGALG00010004468	LYPLA1	case	control	-0.451851968	0.145180015	-3.112356525	0.001856002	0.011328685	down_in_case
Yang2025_S	ENSGALG00010004471	C1H3ORF52	case	control	0.24562781	0.424737401	0.578305111	0.563058144	0.709231449	up_in_case
Yang2025_S	ENSGALG00010004472	NEIL3	case	control	-0.144817309	0.213440514	-0.678490256	0.4974609	0.654382168	down_in_case
Yang2025_S	ENSGALG00010004473	JDP2	case	control	0.40592038	0.119428906	3.398845335	0.00067671	0.005111084	up_in_case
Yang2025_S	ENSGALG00010004477	TULP4	case	control	0.059545583	0.192375878	0.309527282	0.756920463	0.852200585	up_in_case
Yang2025_S	ENSGALG00010004481	ZNF704	case	control	-0.048183756	0.126190132	-0.381834578	0.702584069	0.81634023	down_in_case
Yang2025_S	ENSGALG00010004487	ABHD10	case	control	-0.384001339	0.136832944	-2.806351501	0.0050106	0.024010562	down_in_case
Yang2025_S	ENSGALG00010004489	LIG1	case	control	-0.011689593	0.169362858	-0.069020995	0.944972908	0.966047793	down_in_case
Yang2025_S	ENSGALG00010004494	WTAP	case	control	-0.418943794	0.055677705	-7.524444322	5.2945E-14	9.1085E-12	down_in_case
Yang2025_S	ENSGALG00010004495	MRPL15	case	control	-0.346888102	0.281273813	-1.233275499	0.217472996	0.378385091	down_in_case
Yang2025_S	ENSGALG00010004501	TAGLN3	case	control	0.980650422	0.419399987	2.338222348	0.019375717	0.066241564	up_in_case
Yang2025_S	ENSGALG00010004503	ENSGALG00010004503	case	control	-0.087056292	0.332191964	-0.262066218	0.793270391	0.875443328	down_in_case
Yang2025_S	ENSGALG00010004504	ZNF804B	case	control	1.722132834	0.718023838	2.398434068	0.01646534	0.05890745	up_in_case
Yang2025_S	ENSGALG00010004505	ENSGALG00010004505	case	control	-0.065499518	0.221721384	-0.295413628	0.767677924	0.858737931	down_in_case
Yang2025_S	ENSGALG00010004507	CUL2	case	control	0.151556812	0.111625258	1.357728655	0.174549801	0.324963457	up_in_case
Yang2025_S	ENSGALG00010004515	ENSGALG00010004515	case	control	-0.69358937	0.197111166	-3.518772596	0.000433548	0.003524792	down_in_case
Yang2025_S	ENSGALG00010004516	ENSGALG00010004516	case	control	0.568935976	0.239949131	2.371069123	0.017736714	0.062210195	up_in_case
Yang2025_S	ENSGALG00010004522	TSPAN5	case	control	-0.714816337	0.112546398	-6.351303545	2.13498E-10	1.56584E-08	down_in_case
Yang2025_S	ENSGALG00010004533	PAG1	case	control	-0.195957295	0.204405537	-0.95866921	0.337725414	0.505926181	down_in_case
Yang2025_S	ENSGALG00010004534	CUBN	case	control	-0.221431384	0.203396816	-1.088666914	0.276300797	0.443628484	down_in_case
Yang2025_S	ENSGALG00010004542	ERMARD	case	control	-0.593521114	0.187152977	-3.171315369	0.001517503	0.009691294	down_in_case
Yang2025_S	ENSGALG00010004545	TMPRSS7	case	control	0.681497556	0.521243139	1.307446573	0.191061089	0.346175565	up_in_case
Yang2025_S	ENSGALG00010004553	BATF	case	control	1.438503926	0.36864122	3.902178725	9.53307E-05	0.00102661	up_in_case
Yang2025_S	ENSGALG00010004565	ENSGALG00010004565	case	control	0.451939211	0.448497926	1.007672913	0.313611513	0.482999163	up_in_case
Yang2025_S	ENSGALG00010004568	RSU1	case	control	-0.079174176	0.101984397	-0.77633617	0.437550522	0.601464454	down_in_case
Yang2025_S	ENSGALG00010004576	STEAP2	case	control	-0.359548773	0.144671773	-2.485272469	0.012945231	0.049126306	down_in_case
Yang2025_S	ENSGALG00010004577	ZPLD1	case	control	-0.572982403	0.659346846	-0.869015157	0.38483884	0.552346198	down_in_case
Yang2025_S	ENSGALG00010004583	XKR4	case	control	-0.021438057	0.256108583	-0.083706904	0.933289468	0.959399477	down_in_case
Yang2025_S	ENSGALG00010004584	EIF4E	case	control	-0.478812452	0.061439423	-7.793244612	6.531E-15	1.40015E-12	down_in_case
Yang2025_S	ENSGALG00010004586	ENSGALG00010004586	case	control	-0.458309317	0.45756078	-1.001635928	0.316519462	0.485759769	down_in_case
Yang2025_S	ENSGALG00010004588	MINDY3	case	control	-0.305751574	0.110014694	-2.779188524	0.005449489	0.025593554	down_in_case
Yang2025_S	ENSGALG00010004591	RRS1	case	control	0.80402548	0.217847213	3.690776989	0.00022357	0.002036241	up_in_case
Yang2025_S	ENSGALG00010004602	DYNLT2	case	control	-0.006804224	0.429636047	-0.015837182	0.987364285	0.992277608	down_in_case
Yang2025_S	ENSGALG00010004610	TGS1	case	control	0.119226464	0.099375011	1.199763021	0.23023139	0.392689647	up_in_case
Yang2025_S	ENSGALG00010004622	ENSGALG00010004622	case	control	1.005581187	0.344302387	2.920633791	0.003493202	0.01824504	up_in_case
Yang2025_S	ENSGALG00010004623	MBIP	case	control	0.100899542	0.120516073	0.83722892	0.402463905	0.569027446	up_in_case
Yang2025_S	ENSGALG00010004624	PHF10	case	control	-0.208685178	0.074576336	-2.798276102	0.005137617	0.024484507	down_in_case
Yang2025_S	ENSGALG00010004630	ENSGALG00010004630	case	control	0.447430104	0.289631092	1.544827593	0.122387971	0.253414023	up_in_case
Yang2025_S	ENSGALG00010004639	HBS1L	case	control	-0.014136022	0.073190825	-0.193139258	0.846849908	0.909366839	down_in_case
Yang2025_S	ENSGALG00010004641	METAP1	case	control	-0.317012477	0.084866769	-3.73541351	0.000187407	0.001772923	down_in_case
Yang2025_S	ENSGALG00010004650	GTPBP10	case	control	0.24349264	0.121039201	2.011684139	0.044253244	0.122233687	up_in_case
Yang2025_S	ENSGALG00010004666	C6orf120	case	control	-0.094067995	0.090908404	-1.034755771	0.300782983	0.470416484	down_in_case
Yang2025_S	ENSGALG00010004669	ELMOD1	case	control	0.585206117	0.684367058	0.855105621	0.392492678	0.559491893	up_in_case
Yang2025_S	ENSGALG00010004673	RAB18	case	control	-0.114947669	0.088176717	-1.303605681	0.192368063	0.3476396	down_in_case
Yang2025_S	ENSGALG00010004689	BBX	case	control	0.110888272	0.144502225	0.767381074	0.442854978	0.606087617	up_in_case
Yang2025_S	ENSGALG00010004705	ENSGALG00010004705	case	control	1.116804446	0.33336451	3.350100007	0.000807824	0.005903003	up_in_case
Yang2025_S	ENSGALG00010004707	OTUD1	case	control	-0.577188935	0.186833049	-3.089329962	0.002006085	0.011992619	down_in_case
Yang2025_S	ENSGALG00010004716	ADH6	case	control	0.095759504	0.579981034	0.165107992	0.868858982	0.92285873	up_in_case
Yang2025_S	ENSGALG00010004717	ALKBH8	case	control	-0.199617298	0.158630406	-1.258379795	0.208254435	0.367159104	down_in_case
Yang2025_S	ENSGALG00010004721	LAMA2	case	control	0.077883944	0.178794165	0.43560674	0.66312208	0.786970378	up_in_case
Yang2025_S	ENSGALG00010004728	ENSGALG00010004728	case	control	0.504569956	0.188941106	2.670514454	0.007573511	0.032826963	up_in_case
Yang2025_S	ENSGALG00010004729	ENSGALG00010004729	case	control	0.629253627	0.491038384	1.281475434	0.200026723	0.357523193	up_in_case
Yang2025_S	ENSGALG00010004730	CLDN12	case	control	-0.527231992	0.112138517	-4.701613734	2.58113E-06	5.40648E-05	down_in_case
Yang2025_S	ENSGALG00010004735	ENSGALG00010004735	case	control	-1.154107099	0.224553357	-5.139567333	2.75372E-07	7.91197E-06	down_in_case
Yang2025_S	ENSGALG00010004736	CD47	case	control	0.033132009	0.097787265	0.338817221	0.734747427	0.838207564	up_in_case
Yang2025_S	ENSGALG00010004739	ENSGALG00010004739	case	control	0.714197553	0.368696839	1.937086182	0.052734809	0.138626593	up_in_case
Yang2025_S	ENSGALG00010004746	LYN	case	control	-0.303360237	0.131966093	-2.29877411	0.02151777	0.071375892	down_in_case
Yang2025_S	ENSGALG00010004751	PTER	case	control	-0.505495111	0.150681786	-3.354719393	0.000794455	0.005817517	down_in_case
Yang2025_S	ENSGALG00010004755	PAX9	case	control	0.943161705	0.398264121	2.368181451	0.017875767	0.06258764	up_in_case
Yang2025_S	ENSGALG00010004757	TRMT10A	case	control	0.102724739	0.107326609	0.95712275	0.338505302	0.506667173	up_in_case
Yang2025_S	ENSGALG00010004760	THBS2	case	control	-0.145220084	0.225830133	-0.643050075	0.520191608	0.673937343	down_in_case
Yang2025_S	ENSGALG00010004762	IFT57	case	control	0.545496418	0.128139658	4.257045998	2.07146E-05	0.00030006	up_in_case
Yang2025_S	ENSGALG00010004765	ENSGALG00010004765	case	control	-0.261293261	0.150998554	-1.730435526	0.083552491	0.193213403	down_in_case
Yang2025_S	ENSGALG00010004769	HTR2A	case	control	-0.845465606	0.27244792	-3.103219158	0.001914278	0.011587953	down_in_case
Yang2025_S	ENSGALG00010004777	CDK14	case	control	-0.358419575	0.143466405	-2.498282263	0.012479678	0.047736567	down_in_case
Yang2025_S	ENSGALG00010004778	ENSGALG00010004778	case	control	-1.919532079	0.523974568	-3.663406957	0.000248883	0.002237535	down_in_case
Yang2025_S	ENSGALG00010004780	ENSGALG00010004780	case	control	0.565503558	0.239422142	2.361951796	0.018179006	0.063236257	up_in_case
Yang2025_S	ENSGALG00010004781	ENSGALG00010004781	case	control	-0.18781727	0.172548261	-1.088491233	0.276378305	0.443661617	down_in_case
Yang2025_S	ENSGALG00010004787	RIOK3	case	control	-0.261585346	0.08853786	-2.954502682	0.003131734	0.016856206	down_in_case
Yang2025_S	ENSGALG00010004791	FZD1	case	control	-0.252394754	0.093109439	-2.710732182	0.006713483	0.029946345	down_in_case
Yang2025_S	ENSGALG00010004798	PTPRK	case	control	0.132058231	0.173415191	0.761514783	0.446349653	0.609235587	up_in_case
Yang2025_S	ENSGALG00010004800	ZC3H12C	case	control	-0.150470993	0.217412615	-0.692098724	0.488875333	0.646222516	down_in_case
Yang2025_S	ENSGALG00010004803	RMC1	case	control	-0.375772105	0.129407311	-2.903793469	0.003686713	0.019020489	down_in_case
Yang2025_S	ENSGALG00010004824	ATM	case	control	0.175484089	0.114517544	1.532377344	0.125429345	0.2574544	up_in_case
Yang2025_S	ENSGALG00010004835	HHLA2	case	control	0.640074495	0.288917889	2.215420085	0.026731244	0.083953402	up_in_case
Yang2025_S	ENSGALG00010004837	CENPC	case	control	0.073387074	0.116580208	0.629498569	0.529022705	0.681677541	up_in_case
Yang2025_S	ENSGALG00010004841	AKAP9	case	control	1.172287905	0.225686846	5.19431205	2.05478E-07	6.27925E-06	up_in_case
Yang2025_S	ENSGALG00010004846	TTLL2	case	control	-0.100299882	0.306118558	-0.327650447	0.743175974	0.842897046	down_in_case
Yang2025_S	ENSGALG00010004851	MTCL3	case	control	2.057279172	0.482393686	4.26473072			up_in_case
Yang2025_S	ENSGALG00010004852	ENSGALG00010004852	case	control	-0.253767657	0.155051924	-1.636662413	0.101701027	0.221784633	down_in_case
Yang2025_S	ENSGALG00010004855	ZEB1	case	control	0.473796375	0.256799421	1.845005618	0.065036721	0.161223277	up_in_case
Yang2025_S	ENSGALG00010004859	NPC1	case	control	-0.370760085	0.120835726	-3.068298572	0.002152814	0.012647329	down_in_case
Yang2025_S	ENSGALG00010004866	SLC25A21	case	control	-0.04249679	0.190723833	-0.222818457	0.823676803	0.894465038	down_in_case
Yang2025_S	ENSGALG00010004868	NPAT	case	control	0.247850011	0.196684966	1.260137043	0.207619929	0.366449615	up_in_case
Yang2025_S	ENSGALG00010004871	C11orf87	case	control	-0.55962006	0.236691879	-2.36433993	0.018062231	0.062955775	down_in_case
Yang2025_S	ENSGALG00010004872	KIAA0408	case	control	0.473793445	0.221567744	2.138368323	0.032486861	0.09720788	up_in_case
Yang2025_S	ENSGALG00010004881	MIPOL1	case	control	-0.198561052	0.153747895	-1.29147168	0.196540169	0.352573025	down_in_case
Yang2025_S	ENSGALG00010004883	KRIT1	case	control	-0.167816368	0.072830496	-2.304204663	0.021211158	0.070780048	down_in_case
Yang2025_S	ENSGALG00010004887	MYH15	case	control	0.840525301	0.258909676	3.24640358	0.00116873	0.00790595	up_in_case
Yang2025_S	ENSGALG00010004890	ACAT1	case	control	0.230144205	0.142828604	1.611331332	0.107107525	0.229940435	up_in_case

Yang2025_S	ENSGALG00010004891	ANKIB1	case	control	0.043538781	0.098707079	0.441090765	0.659147289	0.784658933	up_in_case
Yang2025_S	ENSGALG00010004892	HEY2	case	control	0.684820764	0.285281059	2.400512559	0.016372129	0.058649259	up_in_case
Yang2025_S	ENSGALG00010004896	UBA6	case	control	-0.234465756	0.096075513	-2.440431985	0.014669708	0.05399429	down_in_case
Yang2025_S	ENSGALG00010004899	GATAD1	case	control	-0.230324027	0.197189874	-1.168031719	0.242793971	0.407745411	down_in_case
Yang2025_S	ENSGALG00010004901	CUL5	case	control	-0.016693545	0.100488333	-0.166124208	0.868059204	0.922544616	down_in_case
Yang2025_S	ENSGALG00010004902	ANKRD29	case	control	0.298549389	0.312588178	0.955088547	0.33953292	0.507560552	up_in_case
Yang2025_S	ENSGALG00010004903	ENSGALG00010004903	case	control	0.836852981	0.513961278	1.628241302	0.10347373	0.224841171	up_in_case
Yang2025_S	ENSGALG00010004907	ENSGALG00010004907	case	control	-0.501102372	0.120912879	-4.144325852	3.40815E-05	0.00044889	down_in_case
Yang2025_S	ENSGALG00010004917	ENSGALG00010004917	case	control	-0.186758729	0.112494389	-1.660160387	0.096882191	0.214430327	down_in_case
Yang2025_S	ENSGALG00010004921	RRAGD	case	control	-0.408227819	0.112656903	-3.623637864	0.000290488	0.002537902	down_in_case
Yang2025_S	ENSGALG00010004924	ENSGALG00010004924	case	control	0.386719713	0.244967462	1.578657468	0.114414645	0.24137236	up_in_case
Yang2025_S	ENSGALG00010004927	KIF25	case	control	-0.213953698	0.244673655	-0.874445179	0.381875862	0.549788009	down_in_case
Yang2025_S	ENSGALG00010004928	HDDC2	case	control	0.244258052	0.184554344	1.323502051	0.185668475	0.339183297	up_in_case
Yang2025_S	ENSGALG00010004929	ENSGALG00010004929	case	control	0.485046186	0.181124743	2.677967566	0.007407039	0.032265423	up_in_case
Yang2025_S	ENSGALG00010004933	SPTLC2	case	control	-0.338624154	0.112332745	-3.014474123	0.002574252	0.014517279	down_in_case
Yang2025_S	ENSGALG00010004934	CIP2A	case	control	-0.467608936	0.127439624	-3.66925861	0.000243255	0.002194018	down_in_case
Yang2025_S	ENSGALG00010004935	SVIL	case	control	0.363827541	0.121488839	2.994740451	0.002746785	0.015225316	up_in_case
Yang2025_S	ENSGALG00010004939	DDX10	case	control	0.456695969	0.155994028	2.927650349	0.003415339	0.017945983	up_in_case
Yang2025_S	ENSGALG00010004942	GABRR1	case	control	-0.171773418	0.755945769	-0.227229816	0.820245044	0.892629027	down_in_case
Yang2025_S	ENSGALG00010004944	ALKBH1	case	control	0.090783168	0.114796363	0.790819205	0.429049501	0.593370861	up_in_case
Yang2025_S	ENSGALG00010004946	SH2D1B	case	control	2.085586167	1.015234528	2.054290029	0.039947629	0.113259453	up_in_case
Yang2025_S	ENSGALG00010004949	RAB39A	case	control	-0.504136076	0.198802132	-2.535868554	0.011216883	0.044092317	down_in_case
Yang2025_S	ENSGALG00010004950	SLIRP	case	control	-0.336824726	0.283676443	-1.187355294	0.235087506	0.398339543	down_in_case
Yang2025_S	ENSGALG00010004951	AFDN	case	control	0.198093862	0.193758184	1.022376743	0.306602623	0.47625767	up_in_case
Yang2025_S	ENSGALG00010004952	PEX1	case	control	0.047384037	0.08641502	0.548331024	0.583464629	0.725620669	up_in_case
Yang2025_S	ENSGALG00010004957	ENSGALG00010004957	case	control	1.090218668	0.217469093	5.013211997	5.35289E-07	1.41542E-05	up_in_case
Yang2025_S	ENSGALG00010004958	TPD52L1	case	control	-0.641742427	0.416989682	-1.538988743	0.123807044	0.255057172	down_in_case
Yang2025_S	ENSGALG00010004962	ENSGALG00010004962	case	control	-0.119289282	0.149899021	-0.795797607	0.426149683	0.590826369	down_in_case
Yang2025_S	ENSGALG00010004963	ENSGALG00010004963	case	control	0.823986953	0.246183068	3.34704966	0.000816766	0.005940296	up_in_case
Yang2025_S	ENSGALG00010004965	JCAD	case	control	-0.082325348	0.134185335	-0.613519713	0.539532752	0.690457572	down_in_case
Yang2025_S	ENSGALG00010004966	LAMA3	case	control	0.629602194	0.196016441	3.21198667	0.001318205	0.008701651	up_in_case
Yang2025_S	ENSGALG00010004967	SLC35F2	case	control	-0.218675245	0.330928136	-0.660793754	0.508744589	0.664188525	down_in_case
Yang2025_S	ENSGALG00010004969	YTHDC1	case	control	0.399964557	0.137121833	2.916855377	0.003535797	0.018391686	up_in_case
Yang2025_S	ENSGALG00010004970	RBM48	case	control	0.085530577	0.157303391	0.543730028	0.586627266	0.728364313	up_in_case
Yang2025_S	ENSGALG00010004973	SLN	case	control	-0.605904088	0.237309924	-2.553218503	0.010673252	0.042520619	down_in_case
Yang2025_S	ENSGALG00010004974	MTPAP	case	control	-0.138523253	0.094254575	-1.469671402	0.141650772	0.281045237	down_in_case
Yang2025_S	ENSGALG00010004975	SNW1	case	control	0.505800016	0.152802778	3.310149345	0.000932462	0.006626139	up_in_case
Yang2025_S	ENSGALG00010004976	ENSGALG00010004976	case	control	0.326316059	0.182429674	1.788722481	0.073659521	0.176443107	up_in_case
Yang2025_S	ENSGALG00010004977	RNF217	case	control	-0.34072164	0.198335853	-1.717902409	0.085814417	0.196908267	down_in_case
Yang2025_S	ENSGALG00010004980	FNDC1	case	control	0.844947677	0.326323047	2.589298194	0.009617177	0.039457687	up_in_case
Yang2025_S	ENSGALG00010004981	ENSGALG00010004981	case	control	-0.014163817	0.207325592	-0.068316781	0.945533466	0.966310395	down_in_case
Yang2025_S	ENSGALG00010004983	CLXN	case	control	-0.28444751	0.270259947	-1.052495987	0.292572051	0.461384557	down_in_case
Yang2025_S	ENSGALG00010004987	MAP3K8	case	control	0.383488027	0.207354788	1.849429333	0.064395843	0.159956519	up_in_case
Yang2025_S	ENSGALG00010004988	HJURP	case	control	-0.225971078	0.091772479	-2.462296768	0.013805039	0.051546949	down_in_case
Yang2025_S	ENSGALG00010004991	FAM133B	case	control	0.086786236	0.10226831	0.848613185	0.396096568	0.563249104	up_in_case
Yang2025_S	ENSGALG00010004993	SULT1B	case	control	-0.316599888	0.366708535	-0.863355655	0.387941953	0.555027835	down_in_case
Yang2025_S	ENSGALG00010004995	PIT54	case	control	-2.807995115	1.443723559	-1.944967301			down_in_case
Yang2025_S	ENSGALG00010004996	GPR158	case	control	0.304030139	0.314785169	0.965833749	0.334127378	0.502977748	up_in_case
Yang2025_S	ENSGALG00010004999	ADCK1	case	control	0.371032996	0.173958511	2.132882116	0.032934398	0.098148168	up_in_case
Yang2025_S	ENSGALG00010005003	EXPH5	case	control	-0.012865952	0.692941725	-0.018567148	0.98518641	0.991089563	down_in_case
Yang2025_S	ENSGALG00010005004	CDK6	case	control	0.118806254	0.179634551	0.661377517	0.508370241	0.663930582	up_in_case
Yang2025_S	ENSGALG00010005010	NKAIN2	case	control	-0.83918895	0.356359999	-2.354890987	0.01852814	0.064051013	down_in_case
Yang2025_S	ENSGALG00010005014	SULT1B1	case	control	0.32917413	0.21318513	1.544076408	0.122569823	0.253623053	up_in_case
Yang2025_S	ENSGALG00010005015	NRXN3	case	control	0.884574853	0.34793293	2.542371757	0.0110103	0.043476488	up_in_case
Yang2025_S	ENSGALG00010005016	STX11	case	control	0.17134245	0.134779692	1.27127795	0.203629784	0.36171162	up_in_case
Yang2025_S	ENSGALG00010005017	ENSGALG00010005017	case	control	-0.330434279	0.230115825	-1.435947651	0.151017238	0.293977709	down_in_case
Yang2025_S	ENSGALG00010005023	POGLUT3	case	control	-0.679469368	0.126667343	-5.364203211	8.13073E-08	2.86116E-06	down_in_case
Yang2025_S	ENSGALG00010005025	THOC1	case	control	0.453544605	0.117492832	3.860189576	0.000113299	0.00117647	up_in_case
Yang2025_S	ENSGALG00010005029	FAT4	case	control	-0.166723136	0.229397662	-0.726786554	0.467356721	0.628370374	down_in_case
Yang2025_S	ENSGALG00010005033	USP14	case	control	-0.15004961	0.124709826	-1.203189953	0.228902822	0.391167275	down_in_case
Yang2025_S	ENSGALG00010005035	NECTIN3	case	control	-0.137090477	0.098855039	-1.386782893	0.165507975	0.313096942	down_in_case
Yang2025_S	ENSGALG00010005037	THNSL1	case	control	0.302780087	0.174345136	1.736670687	0.082445315	0.191224605	up_in_case
Yang2025_S	ENSGALG00010005042	SLC46A3	case	control	-0.548755252	0.299751848	-1.830698478	0.06714556	0.164963573	down_in_case
Yang2025_S	ENSGALG00010005045	UTRN	case	control	-0.193310589	0.171013073	-1.130384861	0.25831409	0.424682262	down_in_case
Yang2025_S	ENSGALG00010005048	ROCK1	case	control	0.602937935	0.22738205	2.651651411	0.008009919	0.034259737	up_in_case
Yang2025_S	ENSGALG00010005057	FLVCR2	case	control	-0.021637015	0.141758863	-0.152632537	0.878688065	0.92862622	down_in_case
Yang2025_S	ENSGALG00010005060	POMP	case	control	-0.488342935	0.176823252	-2.761757468	0.005749117	0.026562978	down_in_case
Yang2025_S	ENSGALG00010005065	ENKUR	case	control	0.140515774	0.427084644	0.329011535	0.742146969	0.842167591	up_in_case
Yang2025_S	ENSGALG00010005068	PLCXD2	case	control	-0.595059226	0.176718386	-3.367274	0.000759152	0.00560614	down_in_case
Yang2025_S	ENSGALG00010005070	ALB	case	control	-3.780800625	1.056925742	-3.5771677			down_in_case
Yang2025_S	ENSGALG00010005075	TRDN	case	control	0.733954664	0.260907133	2.813087764	0.004906826	0.023667918	up_in_case
Yang2025_S	ENSGALG00010005077	ERG28	case	control	0.2546115	0.185684232	1.371206901	0.170310468	0.319247895	up_in_case
Yang2025_S	ENSGALG00010005081	GREB1L	case	control	0.36242334	0.20001369	1.811992675	0.069987328	0.169974707	up_in_case
Yang2025_S	ENSGALG00010005082	ENSGALG00010005082	case	control	0.163551263	0.148838685	1.098849159	0.271833866	0.438985389	up_in_case
Yang2025_S	ENSGALG00010005087	ENPP1	case	control	0.129083365	0.224948969	0.57383399	0.566080145	0.711190394	up_in_case
Yang2025_S	ENSGALG00010005092	ESCO1	case	control	0.01224692	0.097750755	0.125287219	0.90029617	0.940592827	up_in_case
Yang2025_S	ENSGALG00010005104	ENPP3	case	control	-0.483360045	0.236188733	-2.046499163	0.040707285	0.114602541	down_in_case
Yang2025_S	ENSGALG00010005107	TTLL5	case	control	0.277846698	0.118065697	2.353322817	0.018606473	0.064273971	up_in_case
Yang2025_S	ENSGALG00010005109	ABHD3	case	control	0.657997499	0.373581502	1.761322482	0.078183837	0.184347169	up_in_case
Yang2025_S	ENSGALG00010005111	RASSF6	case	control	-0.367188739	0.330856572	-1.109812439	0.267079858	0.434215655	down_in_case
Yang2025_S	ENSGALG00010005114	PHLDB2	case	control	-0.076972894	0.123258055	-0.624485713	0.53230865	0.684607387	down_in_case
Yang2025_S	ENSGALG00010005118	PRTFDC1	case	control	-0.720548772	0.136184999	-5.29095551	1.21679E-07	4.04677E-06	down_in_case
Yang2025_S	ENSGALG00010005119	MIB1	case	control	-0.128783661	0.093790388	-1.373100841	0.169721	0.318569791	down_in_case
Yang2025_S	ENSGALG00010005123	MTIF3	case	control	0.170684845	0.136110226	1.25401926	0.209835005	0.369105124	up_in_case
Yang2025_S	ENSGALG00010005126	MED23	case	control	-0.253529781	0.059194714	-4.282980058	1.84407E-05	0.000271649	down_in_case
Yang2025_S	ENSGALG00010005127	GATA6	case	control	-0.292708301	0.180421431	-1.622358826	0.10472654	0.226785444	down_in_case
Yang2025_S	ENSGALG00010005132	LNX2	case	control	-0.027400904	0.15801521	-0.173406753	0.862331717	0.918905584	down_in_case
Yang2025_S	ENSGALG00010005138	ENSGALG00010005138	case	control	-1.100580678	0.342871119	-3.209896135	0.001327829	0.008748607	down_in_case
Yang2025_S	ENSGALG00010005140	DCT	case	control	7.851882981	0.695683666	11.28657084	1.52886E-29	2.36718E-26	up_in_case
Yang2025_S	ENSGALG00010005141	CCDC170	case	control	-0.197411991	0.220904521	-0.893653014	0.371507572	0.539941387	down_in_case
Yang2025_S	ENSGALG00010005143	GTF3A	case	control	0.196811141	0.150245036	1.309934397	0.190218029	0.345096763	up_in_case
Yang2025_S	ENSGALG00010005145	AKAP7	case	control	-0.531637758	0.145209005	-3.66119	0.000251046	0.002252484	down_in_case
Yang2025_S	ENSGALG00010005146	TGFB3	case	control	0.12347864	0.104324822	1.183597893	0.236572275	0.400192246	up_in_case
Yang2025_S	ENSGALG00010005148	TTC39C	case	control	-0.295038293	0.107006053	-2.757211241	0.005829668	0.026855015	down_in_case
Yang2025_S	ENSGALG00010005150	ALCAM	case	control	0.359386902	0.216708075	1.658391834	0.097238404	0.214715409	up_in_case
Yang2025_S	ENSGALG00010005154	CWF19L2	case	control	0.589617549	0.169638089	3.475737975	0.00050945	0.004012661	up_in_case
Yang2025_S	ENSGALG00010005157	IMPA1	case	control	-0.387496673	0.102950309	-3.763919464	0.000167271	0.001614209	down_in_case
Yang2025_S	ENSGALG00010005158	IFT43	case	control	-0.281549498	0.312165543	-0.901923687	0.367097408	0.53582302	down_in_case
Yang2025_S	ENSGALG00010005159	ARHGAP21	case	control	0.423130698	0.168540167	2.510562948	0.012053883	0.046542216	up_in_case
Yang2025_S	ENSGALG00010005163	GPC6	case	control	-0.237651987	0.402516938	-0.590414873	0.554912541	0.702590066	down_in_case
Yang2025_S	ENSGALG00010005165	METTL21C	case	control	0.086074361	0.734603184	0.117171234	0.906724362	0.944055033	up_in_case
Yang2025_S	ENSGALG00010005166	CBLB	case	control	0.075854824	0.246766278	0.307395421	0.758542413	0.85303826	up_in_case
Yang2025_S	ENSGALG00010005167	GUCY1A2	case	control	-0.235399287	0.159987617	-1.47135942	0.141193942	0.280475777	down_in_case
Yang2025_S	ENSGALG00010005170	GPATCH2L	case	control	-0.176811055	0.143551163	-1.231693646	0.218063544	0.379047223	down_in_case
Yang2025_S	ENSGALG00010005172	OSBPL1A	case	control	0.456498549	0.150985929	3.023450941	0.002499095	0.014196859	up_in_case
Yang2025_S	ENSGALG00010005174	TPP2	case	control	-0.185345312	0.093856095	-1.97478183	0.048292912	0.130116343	down_in_case
Yang2025_S	ENSGALG00010005176	BAMBI	case	control	0.12986017	0.404343508	0.32116299	0.748086877	0.847252165	up_in_case
Yang2025_S	ENSGALG00010005177	AASDHPPT	case	control	-0.053648921	0.187889432	-0.285534533	0.775234651	0.863270828	down_in_case
Yang2025_S	ENSGALG00010005178	EPHA5	case	control	0.036242897	0.165914237	0.218443564	0.827083526	0.896430655	up_in_case
Yang2025_S	ENSGALG00010005180	WAC	case	control	0.347643431	0.201870345	1.722112434	0.085049153	0.195764775	up_in_case
Yang2025_S	ENSGALG00010005181	IMPACT	case	control	-0.014141553	0.187426768	-0.075451087	0.939855813	0.96346547	down_in_case
Yang2025_S	ENSGALG00010005182	NEPRO	case	control	-0.076729146	0.189224678	-0.40549228	0.685115692	0.803221199	down_in_case
Yang2025_S	ENSGALG00010005184	FGF14	case	control	0.059721265	0.363344775	0.164365279	0.869443595	0.9231632	up_in_case
Yang2025_S	ENSGALG00010005186	ESR1	case	control	0.075690246	0.251943798	0.30042512	0.763852905	0.856269828	up_in_case
Yang2025_S	ENSGALG00010005187	KBTBD3	case	control	-0.050289494	0.225484062	-0.22302904	0.823512905	0.894369288	down_in_case
Yang2025_S	ENSGALG00010005190	ENSGALG00010005190	case	control	-0.766942182	0.20411683	-3.757368661	0.000171709	0.001653078	down_in_case
Yang2025_S	ENSGALG00010005191	MSANTD4	case	control	0.520502178	0.094576129	5.503525917	3.7227E-08	1.4531E-06	up_in_case
Yang2025_S	ENSGALG00010005193	EPB41L2	case	control	0.057910914	0.132355576	0.437540413	0.661719474	0.786315287	up_in_case
Yang2025_S	ENSGALG00010005198	TRPC3	case	control	0.171204302	0.342093325	0.50046081	0.616750645	0.752839894	up_in_case
Yang2025_S	ENSGALG00010005203	ENSGALG00010005203	case	control	-0.123712059	0.123301004	-1.003333745	0.31569986	0.484929182	down_in_case
Yang2025_S	ENSGALG00010005204	ITGBL1	case	control	0.788924446	0.400596436	1.969374604	0.04891009	0.131184087	up_in_case
Yang2025_S	ENSGALG00010005208	ENSGALG00010005208	case	control	-0.72493345	0.192456658	-3.766736144	0.000165396	0.001600547	down_in_case
Yang2025_S	ENSGALG00010005211	ESRRB	case	control	0.587390182	0.192649	3.049017551	0.002295911	0.013280828	up_in_case
Yang2025_S	ENSGALG00010005212	TAF13	case	control	-0.342431942	0.20132406	-1.700899246	0.088961908	0.202420299	down_in_case
Yang2025_S	ENSGALG00010005213	ENSGALG00010005213	case	control	-0.345875013	0.261612447	-1.322089284	0.186138422	0.339741637	down_in_case
Yang2025_S	ENSGALG00010005214	BBS7	case	control	0.101262108	0.103390795	0.979411255	0.327376818	0.497111906	up_in_case

Yang2025_S	ENSGALG00010005215	VASH1	case	control	-0.529036986	0.119085019	-4.442515036	8.89134E-06	0.000148384	down_in_case
Yang2025_S	ENSGALG00010005216	ENSGALG00010005216	case	control	0.423935277	0.254428326	1.666226729	0.095668264	0.212583268	up_in_case
Yang2025_S	ENSGALG00010005217	CCNA2	case	control	-0.107661882	0.11727926	-0.917995919	0.358621002	0.527612204	down_in_case
Yang2025_S	ENSGALG00010005218	GTPBP8	case	control	-0.177706636	0.188805039	-0.941217656	0.346593331	0.515066446	down_in_case
Yang2025_S	ENSGALG00010005219	NALCN	case	control	0.044688057	0.256509414	0.174216051	0.861695671	0.918583934	up_in_case
Yang2025_S	ENSGALG00010005220	ANGEL1	case	control	-0.506037652	0.099874105	-5.066755295	4.04654E-07	1.1166E-05	down_in_case
Yang2025_S	ENSGALG00010005221	ENSGALG00010005221	case	control	-0.403133836	0.436390913	-0.92379063	0.355595335	0.524861878	down_in_case
Yang2025_S	ENSGALG00010005223	GRIA4	case	control	0.62189525	0.444725438	1.398380207	0.161998924	0.308142916	up_in_case
Yang2025_S	ENSGALG00010005226	MYCT1	case	control	-0.06257202	0.18790208	-0.333003339	0.739131762	0.840406621	down_in_case
Yang2025_S	ENSGALG00010005227	EXOSC9	case	control	0.154207731	0.103788766	1.485784418	0.137336153	0.275047326	up_in_case
Yang2025_S	ENSGALG00010005228	ZNF521	case	control	0.017778165	0.145866589	0.121879624	0.902994353	0.942069799	up_in_case
Yang2025_S	ENSGALG00010005229	CIPC	case	control	0.086267957	0.137075212	0.629347611	0.529121508	0.681703792	up_in_case
Yang2025_S	ENSGALG00010005231	ENSGALG00010005231	case	control	-0.224073625	0.397189684	-0.564147644	0.572653656	0.715881286	down_in_case
Yang2025_S	ENSGALG00010005232	GATA3	case	control	0.414831378	0.783209206	0.529655901	0.596350529	0.735736575	up_in_case
Yang2025_S	ENSGALG00010005236	TMTC4	case	control	0.081608493	0.158365618	0.515316985	0.606331532	0.743766699	up_in_case
Yang2025_S	ENSGALG00010005237	RAP2A	case	control	-0.110063221	0.157698337	-0.697935208	0.485217715	0.642701722	down_in_case
Yang2025_S	ENSGALG00010005238	TMEM63C	case	control	-0.138169311	0.105949922	-1.304100174	0.19219943	0.347379903	down_in_case
Yang2025_S	ENSGALG00010005239	ENSGALG00010005239	case	control	0.682493078	0.251142364	2.717554565	0.006576631	0.029515412	up_in_case
Yang2025_S	ENSGALG00010005240	VIP	case	control	0.480242574	0.251083758	1.912678772	0.055789188	0.144690553	up_in_case
Yang2025_S	ENSGALG00010005241	C4orf54	case	control	-0.270518551	0.192179541	-1.40763449	0.159239329	0.304640315	down_in_case
Yang2025_S	ENSGALG00010005242	CCDC80	case	control	-0.088895103	0.188486424	-0.471626026	0.637193747	0.767673023	down_in_case
Yang2025_S	ENSGALG00010005244	FBXO5	case	control	-0.284720643	0.140031728	-2.033258076	0.042026456	0.117247155	down_in_case
Yang2025_S	ENSGALG00010005245	KIAA1217	case	control	0.449326478	0.227665073	1.97362939	0.048423899	0.130393631	up_in_case
Yang2025_S	ENSGALG00010005246	ENSGALG00010005246	case	control	0.158214942	0.189069421	0.83680873	0.40270009	0.569188128	up_in_case
Yang2025_S	ENSGALG00010005247	THAP7	case	control	-0.053873609	0.132174119	-0.407595746	0.683570488	0.801927229	down_in_case
Yang2025_S	ENSGALG00010005248	TMEM200A	case	control	-0.497556781	0.126302723	-3.939398684	8.16861E-05	0.000906286	down_in_case
Yang2025_S	ENSGALG00010005249	PDGFD	case	control	-0.215334953	0.180173269	-1.195154831	0.232026543	0.395198069	down_in_case
Yang2025_S	ENSGALG00010005250	SLC35A5	case	control	-0.122096175	0.128226675	-0.952190137	0.341000562	0.50919876	down_in_case
Yang2025_S	ENSGALG00010005251	MTRF1L	case	control	0.094508991	0.100915058	0.936520213	0.349005411	0.517355519	up_in_case
Yang2025_S	ENSGALG00010005252	KLHL14	case	control	-0.162192988	0.150401717	-1.078398512	0.280855951	0.448821981	down_in_case
Yang2025_S	ENSGALG00010005253	POMT2	case	control	-0.463914056	0.1430919	-3.242070702	0.001186646	0.007996088	down_in_case
Yang2025_S	ENSGALG00010005254	ENSGALG00010005254	case	control	1.053399927	0.5148093	2.046194441	0.040737244	0.114635197	up_in_case
Yang2025_S	ENSGALG00010005255	BZW2	case	control	-0.253386702	0.103197107	-2.455366341	0.014074108	0.052271507	down_in_case
Yang2025_S	ENSGALG00010005256	OPRM1	case	control	0.674287056	0.632648053	1.06581701	0.28650637	0.455085634	up_in_case
Yang2025_S	ENSGALG00010005258	ITIH5	case	control	-0.434026794	0.126117488	-3.441448148	0.000578609	0.004476914	down_in_case
Yang2025_S	ENSGALG00010005259	SLC27A5	case	control	-1.037271278	0.692887836	-1.497026249	0.134386428	0.27047092	down_in_case
Yang2025_S	ENSGALG00010005260	ANXA5	case	control	-0.343999217	0.110088948	-3.124738875	0.00177963	0.01098279	down_in_case
Yang2025_S	ENSGALG00010005261	ATG3	case	control	0.430873602	0.106030513	4.063675533	4.8306E-05	0.000592556	up_in_case
Yang2025_S	ENSGALG00010005262	ENSGALG00010005262	case	control	-0.147081363	0.133598758	-1.100918642	0.270932068	0.438189225	down_in_case
Yang2025_S	ENSGALG00010005263	ENSGALG00010005263	case	control	-0.935548251	0.3942284	-2.373112268	0.017638902	0.061960699	down_in_case
Yang2025_S	ENSGALG00010005264	ITIH2	case	control	-1.800025863	0.507811415	-3.544673892	0.000393099	0.003254805	down_in_case
Yang2025_S	ENSGALG00010005265	DYNC2H1	case	control	-0.233150975	0.147473827	-1.580965116	0.113886014	0.240576877	down_in_case
Yang2025_S	ENSGALG00010005266	SLC19A2	case	control	-0.033315593	0.266912366	-0.124818469	0.900667266	0.940909989	down_in_case
Yang2025_S	ENSGALG00010005267	CYP51A1	case	control	0.101107222	0.20767285	0.486858164	0.626358848	0.759929342	up_in_case
Yang2025_S	ENSGALG00010005268	MTTP	case	control	0.058247769	0.323233683	0.180203278	0.856992985	0.915602027	up_in_case
Yang2025_S	ENSGALG00010005269	KIN	case	control	-0.092190328	0.144575883	-0.637660488	0.523694711	0.676965288	down_in_case
Yang2025_S	ENSGALG00010005270	GSTZ1	case	control	-0.135745829	0.177182214	-0.766136886	0.443594857	0.606818349	down_in_case
Yang2025_S	ENSGALG00010005272	MBNL2	case	control	0.48836658	0.208560834	2.34160254	0.019201151	0.065822395	up_in_case
Yang2025_S	ENSGALG00010005273	ENSGALG00010005273	case	control	-0.031669495	0.467683548	-0.067715648	0.946011992	0.96654279	down_in_case
Yang2025_S	ENSGALG00010005275	L3MBTL3	case	control	0.141298782	0.10694528	1.321225038	0.18642634	0.340077373	up_in_case
Yang2025_S	ENSGALG00010005277	ENSGALG00010005277	case	control	0.130970343	0.269203766	0.486510069	0.626605568	0.760075609	up_in_case
Yang2025_S	ENSGALG00010005278	DAPP1	case	control	-0.069028429	0.167212528	-0.412818526	0.679739577	0.799069598	down_in_case
Yang2025_S	ENSGALG00010005279	CCDC181	case	control	0.475407834	0.4333583	1.097031795	0.272627494	0.439910158	up_in_case
Yang2025_S	ENSGALG00010005280	TMEM244	case	control	-0.709055617	0.406345088	-1.744959243	0.080991964	0.188954129	down_in_case
Yang2025_S	ENSGALG00010005281	TMED8	case	control	-0.306523079	0.093023926	-3.295099348	0.000983869	0.006917363	down_in_case
Yang2025_S	ENSGALG00010005282	FLT1	case	control	0.077066499	0.121788156	0.632791407	0.526869869	0.679808483	up_in_case
Yang2025_S	ENSGALG00010005283	ATP5C1	case	control	0.073654063	0.199757264	0.368717821	0.712338064	0.822473355	up_in_case
Yang2025_S	ENSGALG00010005284	ENSGALG00010005284	case	control	0.726900871	0.308187955	2.358628428	0.018342612	0.063583157	up_in_case
Yang2025_S	ENSGALG00010005285	MPP7	case	control	-0.146096131	0.126312334	-1.156626012	0.24742519	0.413116466	down_in_case
Yang2025_S	ENSGALG00010005286	ENSGALG00010005286	case	control	-0.997250587	0.620446895	-1.607310142	0.107986351	0.231214263	down_in_case
Yang2025_S	ENSGALG00010005287	ENSGALG00010005287	case	control	0.135109577	0.526544588	0.256596649	0.797490162	0.87739029	up_in_case
Yang2025_S	ENSGALG00010005288	ENSGALG00010005288	case	control	0.026457981	0.068344655	0.387125822	0.698663062	0.81355977	up_in_case
Yang2025_S	ENSGALG00010005291	IFNGR1	case	control	-0.627855846	0.13964431	-4.496107613	6.92087E-06	0.000122079	down_in_case
Yang2025_S	ENSGALG00010005292	TAF3	case	control	-0.005437821	0.127632494	-0.042605304	0.966016168	0.979939965	down_in_case
Yang2025_S	ENSGALG00010005293	BLZF1	case	control	-0.272004808	0.114306733	-2.379604431	0.017331231	0.061080099	down_in_case
Yang2025_S	ENSGALG00010005295	ENSGALG00010005295	case	control	0.464107532	0.584206533	0.794423728	0.426948795	0.591345936	up_in_case
Yang2025_S	ENSGALG00010005296	PAN3	case	control	-0.020945119	0.129540276	-0.161688081	0.871551491	0.924500068	down_in_case
Yang2025_S	ENSGALG00010005301	ENSGALG00010005301	case	control	-0.802164157	0.488647645	-1.641600375	0.10067285	0.22009353	down_in_case
Yang2025_S	ENSGALG00010005302	PSPC1	case	control	-0.000748406	0.094497122	-0.007919885	0.993680912	0.995968029	down_in_case
Yang2025_S	ENSGALG00010005303	ENSGALG00010005303	case	control	0.098269656	0.354907245	0.276888278	0.781865893	0.868151492	up_in_case
Yang2025_S	ENSGALG00010005304	ENSGALG00010005304	case	control	-0.323260952	0.209653384	-1.541882826	0.12310207	0.254137385	down_in_case
Yang2025_S	ENSGALG00010005305	SCAF8	case	control	-0.171120028	0.123339711	-1.387387943	0.1653235	0.312928955	down_in_case
Yang2025_S	ENSGALG00010005307	TIAM2	case	control	0.128830501	0.114657929	1.123607427	0.261179604	0.427929193	up_in_case
Yang2025_S	ENSGALG00010005309	ASXL3	case	control	0.156548991	0.209700301	0.746536798	0.455343211	0.61692729	up_in_case
Yang2025_S	ENSGALG00010005310	LAMTOR3	case	control	-0.40613911	0.188484985	-2.154755771	0.031180951	0.094273917	down_in_case
Yang2025_S	ENSGALG00010005311	YME1L1	case	control	-0.296322958	0.055688205	-5.321108131	1.03137E-07	3.53994E-06	down_in_case
Yang2025_S	ENSGALG00010005313	MPHOSPH8	case	control	0.98016936	0.261504582	3.74819192	0.000178114	0.001706696	up_in_case
Yang2025_S	ENSGALG00010005314	IL20RA	case	control	-0.901926666	0.400786081	-2.250394187	0.024423934	0.078747431	down_in_case
Yang2025_S	ENSGALG00010005315	ENSGALG00010005315	case	control	0.186158187	0.442219017	0.420963775	0.673781534	0.794881957	up_in_case
Yang2025_S	ENSGALG00010005316	PARP4	case	control	-0.034019431	0.100481232	-0.338565022	0.734937437	0.838287074	down_in_case
Yang2025_S	ENSGALG00010005317	CHCHD3	case	control	-0.10752536	0.164959584	-0.651828511	0.514511807	0.669254366	down_in_case
Yang2025_S	ENSGALG00010005318	SLC35D3	case	control	-0.498606533	0.402999637	-1.237238169	0.215998675	0.376949472	down_in_case
Yang2025_S	ENSGALG00010005320	ENSGALG00010005320	case	control	1.625003133	0.577731668	2.812729894	0.00491229	0.023686074	up_in_case
Yang2025_S	ENSGALG00010005321	HS6ST3	case	control	0.525767773	0.529889567	0.992221409	0.321089531	0.490397042	up_in_case
Yang2025_S	ENSGALG00010005322	TFB1M	case	control	0.428744592	0.120592244	3.555324778	0.000377512	0.003153859	up_in_case
Yang2025_S	ENSGALG00010005323	DNAJB14	case	control	0.289903162	0.14730208	1.968086013	0.049058142	0.131441109	up_in_case
Yang2025_S	ENSGALG00010005324	CENPJ	case	control	0.099102917	0.139735061	0.709220123	0.478187888	0.637205529	up_in_case
Yang2025_S	ENSGALG00010005326	MASTL	case	control	-0.312018379	0.133352535	-2.339800882	0.019294023	0.066027067	down_in_case
Yang2025_S	ENSGALG00010005329	VIPAS39	case	control	0.401359768	0.118561807	3.385236586	0.000711169	0.005328035	up_in_case
Yang2025_S	ENSGALG00010005330	ENSGALG00010005330	case	control	0.16837724	0.53573369	0.314292798	0.753298667	0.849770657	up_in_case
Yang2025_S	ENSGALG00010005332	ENSGALG00010005332	case	control	-0.978909442	0.425523862	-2.300480723	0.021421	0.071156529	down_in_case
Yang2025_S	ENSGALG00010005333	PLXNA4	case	control	-0.298526999	0.162827207	-1.833397526	0.066743475	0.164294352	down_in_case
Yang2025_S	ENSGALG00010005334	NME7	case	control	-0.250243477	0.2064128	-1.21234476	0.225380437	0.387164249	down_in_case
Yang2025_S	ENSGALG00010005335	AHSA1	case	control	0.268083888	0.154498049	1.735192703	0.082706678	0.191797506	up_in_case
Yang2025_S	ENSGALG00010005336	PEX7	case	control	-0.55663796	0.142352698	-3.910273325	9.21917E-05	0.00099976	down_in_case
Yang2025_S	ENSGALG00010005337	DCTN1	case	control	1.83852398	0.757921817	2.425743577	0.01527706	0.055758469	up_in_case
Yang2025_S	ENSGALG00010005338	PCGF1	case	control	0.434605551	0.19365306	2.244248297	0.02481643	0.079534718	up_in_case
Yang2025_S	ENSGALG00010005341	UGGT2	case	control	-0.295785794	0.123746759	-2.390250842	0.016836869	0.059821971	down_in_case
Yang2025_S	ENSGALG00010005342	MAP3K5	case	control	0.079525111	0.095628414	0.831605459	0.405631679	0.571829321	up_in_case
Yang2025_S	ENSGALG00010005345	NOX3	case	control	3.727312443	0.62518482	5.961936896	2.49265E-09	1.36216E-07	up_in_case
Yang2025_S	ENSGALG00010005346	FBXO30	case	control	-0.589273414	0.165839319	-3.553279272	0.00038046	0.00317278	down_in_case
Yang2025_S	ENSGALG00010005347	DOK1	case	control	-0.13176417	0.19497421	-0.675803072	0.499165684	0.655992704	down_in_case
Yang2025_S	ENSGALG00010005349	ACBD5	case	control	0.266796997	0.113714177	2.346206991	0.018965572	0.065191228	up_in_case
Yang2025_S	ENSGALG00010005350	ARHGAP20	case	control	-0.697981176	0.270824217	-2.577248011	0.009959047	0.040401547	down_in_case
Yang2025_S	ENSGALG00010005351	DTNA	case	control	0.195436649	0.19309425	1.012130856	0.311475491	0.481091883	up_in_case
Yang2025_S	ENSGALG00010005353	ENSGALG00010005353	case	control	-0.963743373	0.128463513	-7.502078611	6.28137E-14	1.04292E-11	down_in_case
Yang2025_S	ENSGALG00010005354	M1AP	case	control	0.444193011	0.260740842	1.703580491	0.088459489	0.201562898	up_in_case
Yang2025_S	ENSGALG00010005355	FDX1	case	control	-0.133985716	0.098122647	-1.365492272	0.17209838	0.321430228	down_in_case
Yang2025_S	ENSGALG00010005356	DNAJC3	case	control	0.088513235	0.076751796	1.153239916	0.248811924	0.4145378	up_in_case
Yang2025_S	ENSGALG00010005358	SHPRH	case	control	-0.077872537	0.157149718	-0.495530874	0.620225434	0.755559177	down_in_case
Yang2025_S	ENSGALG00010005359	MEIS2	case	control	0.138048231	0.193464752	0.713557532	0.475500826	0.635049263	up_in_case
Yang2025_S	ENSGALG00010005360	CAMK1D	case	control	-0.00121137	0.17570409	-0.006894375	0.994499128	0.99628651	down_in_case
Yang2025_S	ENSGALG00010005361	ENSGALG00010005361	case	control	1.012212855	0.496267142	2.03965318	0.041384881	0.116012537	up_in_case
Yang2025_S	ENSGALG00010005362	ATP1B1	case	control	-0.10608464	0.077535172	-1.368213119	0.171245376	0.320309304	down_in_case
Yang2025_S	ENSGALG00010005363	RDX	case	control	-0.043159121	0.134890028	-0.319957834	0.749000295	0.847674443	down_in_case
Yang2025_S	ENSGALG00010005364	MAP7	case	control	1.720833514	0.414148408	4.155113188	3.25127E-05	0.000433969	up_in_case
Yang2025_S	ENSGALG00010005366	AUP1	case	control	-0.338968221	0.271345288	-1.249213587	0.211586964	0.371764511	down_in_case
Yang2025_S	ENSGALG00010005367	H2AZ1	case	control	-0.300403319	0.204320925	-1.470252344	0.141493423	0.280950534	down_in_case
Yang2025_S	ENSGALG00010005368	CCDC3	case	control	-0.201388214	0.205167424	-0.981579873	0.326306867	0.496244264	down_in_case
Yang2025_S	ENSGALG00010005369	DPT	case	control	-1.269556779	0.139920783	-9.073396749	1.15363E-19	5.95403E-17	down_in_case
Yang2025_S	ENSGALG00010005372	CDIN1	case	control	-0.45114383	0.122918535	-3.670266906	0.000242297	0.002186796	down_in_case
Yang2025_S	ENSGALG00010005376	HTRA2	case	control	-0.193152263	0.28412299	-0.679819198	0.496618949	0.653729931	down_in_case
Yang2025_S	ENSGALG00010005377	EMCN	case	control	-0.176883948	0.087757196	-2.015606195	0.043841172	0.121384209	down_in_case
Yang2025_S	ENSGALG00010005379	DZIP1	case	control	0.448797086	0.148112291	3.030113716	0.002444617	0.013967091	up_in_case
Yang2025_S	ENSGALG00010005381	FGF9	case	control	1.250429344	0.287174659	4.354246815	1.33525E-05	0.0002112	up_in_case
Yang2025_S	ENSGALG00010005382	RAB32	case	control	-0.476246705	0.16150421	-2.948819126	0.003189906	0.017083686	down_in_case

Yang2025_S	ENSGALG00010005383	OPTN	case	control	0.335686492	0.110687257	3.032747422	0.002423383	0.013868521	up_in_case
Yang2025_S	ENSGALG00010005384	MAPRE2	case	control	0.232779631	0.224536883	1.036709997	0.299871031	0.469517171	up_in_case
Yang2025_S	ENSGALG00010005385	PDSS1	case	control	-0.124430663	0.108096393	-1.151108367	0.249687656	0.415351258	down_in_case
Yang2025_S	ENSGALG00010005386	CPOX	case	control	0.038817051	0.144297281	0.269007504	0.787923908	0.87222991	up_in_case
Yang2025_S	ENSGALG00010005388	ENSGALG00010005388	case	control	-0.491585188	0.388858211	-1.264175923	0.206166891	0.364674332	down_in_case
Yang2025_S	ENSGALG00010005389	MCM10	case	control	-0.008985139	0.179401331	-0.050084018	0.960055436	0.975838719	down_in_case
Yang2025_S	ENSGALG00010005391	ADGB	case	control	1.38997492	0.579949934	2.396715371	0.016542768	0.059078288	up_in_case
Yang2025_S	ENSGALG00010005392	ENSGALG00010005392	case	control	0.003175489	0.1343148	0.023642139	0.981138059	0.988594337	up_in_case
Yang2025_S	ENSGALG00010005394	PHYH	case	control	-0.232491865	0.139622049	-1.665151502	0.095882534	0.212927986	down_in_case
Yang2025_S	ENSGALG00010005395	ENSGALG00010005395	case	control	0.415896609	0.384893168	1.080550771	0.279896991	0.44780305	up_in_case
Yang2025_S	ENSGALG00010005396	MICU2	case	control	-0.022674839	0.10996123	-0.206207579	0.836628768	0.903156104	down_in_case
Yang2025_S	ENSGALG00010005398	SEPHS1	case	control	-0.487440474	0.136015836	-3.583703836	0.000338756	0.002890733	down_in_case
Yang2025_S	ENSGALG00010005399	NDNF	case	control	0.200176502	0.32135018	0.622923262	0.533334951	0.685302401	up_in_case
Yang2025_S	ENSGALG00010005401	BCLAF1	case	control	0.487311941	0.166957257	2.918782632	0.003514012	0.018326254	up_in_case
Yang2025_S	ENSGALG00010005402	ABCC4	case	control	0.226571921	0.238260675	0.950941321	0.341634164	0.509762509	up_in_case
Yang2025_S	ENSGALG00010005403	ENSGALG00010005403	case	control	1.047881115	0.16917071	6.194223063	5.85732E-10	3.82672E-08	up_in_case
Yang2025_S	ENSGALG00010005404	BEND7	case	control	0.195844152	0.158238047	1.237655265	0.215843913	0.376773761	up_in_case
Yang2025_S	ENSGALG00010005405	CEBPD	case	control	-0.128668529	0.26508075	-0.485393709	0.627397094	0.760683246	down_in_case
Yang2025_S	ENSGALG00010005407	DPH6	case	control	0.152325031	0.203752703	0.747597596	0.454702915	0.616411658	up_in_case
Yang2025_S	ENSGALG00010005408	APBB1IP	case	control	-0.23556171	0.27240904	-0.864735288	0.387184096	0.554455902	down_in_case
Yang2025_S	ENSGALG00010005409	PRKCQ	case	control	0.094531388	0.182580963	0.517750516	0.604632348	0.742797476	up_in_case
Yang2025_S	ENSGALG00010005411	STXBP5	case	control	0.060247878	0.12172497	0.494950855	0.620634811	0.755748583	up_in_case
Yang2025_S	ENSGALG00010005412	MTFR2	case	control	0.033677998	0.183208509	0.183823327	0.854152056	0.913827938	up_in_case
Yang2025_S	ENSGALG00010005413	GAD2	case	control	-0.396024553	0.525526819	-0.753576295	0.451103683	0.613101513	down_in_case
Yang2025_S	ENSGALG00010005414	ENSGALG00010005414	case	control	0.486741065	0.18023663	2.700566831	0.006922143	0.030724674	up_in_case
Yang2025_S	ENSGALG00010005415	PRKDC	case	control	0.269335703	0.136966094	1.966440707	0.049247725	0.13182233	up_in_case
Yang2025_S	ENSGALG00010005416	PPP3CA	case	control	0.048743848	0.092152137	0.528949731	0.596840322	0.736080174	up_in_case
Yang2025_S	ENSGALG00010005417	LOXL3	case	control	0.687376836	0.170603291	4.029094825	5.5992E-05	0.000669742	up_in_case
Yang2025_S	ENSGALG00010005420	DHRS4	case	control	0.396277986	0.145004943	2.732858469	0.006278731	0.028416405	up_in_case
Yang2025_S	ENSGALG00010005421	ENSGALG00010005421	case	control	-1.026326039	0.261842491	-3.919631367	8.86845E-05	0.00097003	down_in_case
Yang2025_S	ENSGALG00010005423	ST3GAL6	case	control	-0.089378017	0.115709063	-0.772437478	0.439855364	0.603302507	down_in_case
Yang2025_S	ENSGALG00010005424	ENSGALG00010005424	case	control	-0.116884937	0.280998841	-0.415962347	0.677437556	0.797237762	down_in_case
Yang2025_S	ENSGALG00010005425	ELAPOR2	case	control	-0.011541637	0.376655998	-0.030642381	0.975554743	0.985455262	down_in_case
Yang2025_S	ENSGALG00010005427	ENSGALG00010005427	case	control	0.309155793	0.162981464	1.896877017	0.057844171	0.148527461	up_in_case
Yang2025_S	ENSGALG00010005428	ZNF770	case	control	0.044760075	0.073857065	0.606036472	0.544490534	0.694251587	up_in_case
Yang2025_S	ENSGALG00010005429	ZDHHC20	case	control	-0.189932313	0.073101386	-2.59820399	0.00937128	0.038704444	down_in_case
Yang2025_S	ENSGALG00010005430	MCM4	case	control	-0.222140196	0.105662883	-2.102348435	0.035522765	0.103971797	down_in_case
Yang2025_S	ENSGALG00010005431	GRM3	case	control	-0.279109734	1.128288643	-0.247374407	0.804618479	0.882654373	down_in_case
Yang2025_S	ENSGALG00010005433	GPC5	case	control	-0.474337206	0.337763315	-1.404347911	0.160215274	0.305868236	down_in_case
Yang2025_S	ENSGALG00010005434	PRDM5	case	control	-0.216537697	0.156049752	-1.387619624	0.165252903	0.312837821	down_in_case
Yang2025_S	ENSGALG00010005435	MAD2L1	case	control	-0.547930259	0.22723802	-2.411261372	0.015897451	0.057465884	down_in_case
Yang2025_S	ENSGALG00010005436	AQR	case	control	-0.346608528	0.093699958	-3.699132172	0.000216338	0.001988568	down_in_case
Yang2025_S	ENSGALG00010005437	PDE7B	case	control	0.182096127	0.117236994	1.553230954	0.120367969	0.250123419	up_in_case
Yang2025_S	ENSGALG00010005438	GPR180	case	control	0.024991067	0.116784653	0.213992729	0.830552727	0.898932214	up_in_case
Yang2025_S	ENSGALG00010005439	TBX19	case	control	0.170772831	0.396619494	0.430570946	0.666780371	0.789228274	up_in_case
Yang2025_S	ENSGALG00010005441	UBE2V2	case	control	0.159570472	0.162607641	0.981322098	0.326433929	0.496383326	up_in_case
Yang2025_S	ENSGALG00010005442	SASH1	case	control	-0.125823926	0.113613949	-1.107468998	0.268091209	0.435210973	down_in_case
Yang2025_S	ENSGALG00010005443	BANK1	case	control	-1.040290248	0.586789064	-1.772852139	0.076253211	0.180866127	down_in_case
Yang2025_S	ENSGALG00010005444	ACTC1	case	control	-0.217003134	0.204652161	-1.06035105	0.288984923	0.457822294	down_in_case
Yang2025_S	ENSGALG00010005445	SFT2D2	case	control	-0.327609883	0.121761832	-2.690579438	0.007132805	0.031418573	down_in_case
Yang2025_S	ENSGALG00010005446	ENSGALG00010005446	case	control	-0.11227144	0.489268298	-0.229468044	0.818505155	0.89157112	down_in_case
Yang2025_S	ENSGALG00010005447	MYO3A	case	control	0.43044833	0.309424423	1.391125904	0.164187251	0.311218637	up_in_case
Yang2025_S	ENSGALG00010005448	ENSGALG00010005448	case	control	1.063574809	0.134681052	7.896989168	2.85721E-15	6.63587E-13	up_in_case
Yang2025_S	ENSGALG00010005449	TIPRL	case	control	-0.052656464	0.093660527	-0.562205505	0.573976011	0.716798455	down_in_case
Yang2025_S	ENSGALG00010005450	ENSGALG00010005450	case	control	0.398809299	0.350459792	1.13796021	0.255137098	0.42149798	up_in_case
Yang2025_S	ENSGALG00010005453	UST	case	control	-0.511111741	0.093390515	-5.472844252	4.42869E-08	1.68617E-06	down_in_case
Yang2025_S	ENSGALG00010005454	GPR161	case	control	-0.268948927	0.094392304	-2.849267516	0.004382002	0.02161033	down_in_case
Yang2025_S	ENSGALG00010005455	ENSGALG00010005455	case	control	-0.298304617	0.500226422	-0.596339185	0.550948659	0.699095753	down_in_case
Yang2025_S	ENSGALG00010005456	RPL7L1	case	control	-0.052695492	0.122149513	-0.431401572	0.666176407	0.789183732	down_in_case
Yang2025_S	ENSGALG00010005457	SEMA3D	case	control	-0.514341503	0.160399231	-3.20663323	0.001342981	0.008826552	down_in_case
Yang2025_S	ENSGALG00010005458	PRKD1	case	control	-0.033753657	0.094173282	-0.358420733	0.720028478	0.827513142	down_in_case
Yang2025_S	ENSGALG00010005459	ZC3H12D	case	control	0.190701646	0.398387013	0.478684395	0.632163169	0.763944976	up_in_case
Yang2025_S	ENSGALG00010005460	TGDS	case	control	-0.787560872	0.129570244	-6.07825412	1.21498E-09	7.26642E-08	down_in_case
Yang2025_S	ENSGALG00010005462	AHI1	case	control	0.235712856	0.16765361	1.405951569	0.159738507	0.305218167	up_in_case
Yang2025_S	ENSGALG00010005463	SKA3	case	control	-0.209261892	0.164990971	-1.268323295	0.204682522	0.362835636	down_in_case
Yang2025_S	ENSGALG00010005464	SLC39A8	case	control	-0.395752196	0.656939797	-0.602417752	0.546896096	0.695855204	down_in_case
Yang2025_S	ENSGALG00010005465	ENSGALG00010005465	case	control	0.081161078	0.082820313	0.979965851	0.327102976	0.496804356	up_in_case
Yang2025_S	ENSGALG00010005466	LATS2	case	control	0.207747265	0.155830422	1.333162437	0.182478503	0.335334029	up_in_case
Yang2025_S	ENSGALG00010005467	ENSGALG00010005467	case	control	0.295696951	0.206768679	1.430085799	0.152692396	0.296305324	up_in_case
Yang2025_S	ENSGALG00010005468	PPIL4	case	control	0.556843286	0.10701371	5.203476123	1.95595E-07	6.08396E-06	up_in_case
Yang2025_S	ENSGALG00010005470	SEMA3A	case	control	-0.35192983	0.146380295	-2.404215883	0.016207199	0.0581631	down_in_case
Yang2025_S	ENSGALG00010005472	PFKP	case	control	0.090033735	0.066379248	1.356353636	0.174986678	0.325428981	up_in_case
Yang2025_S	ENSGALG00010005474	COL8A1	case	control	0.884873576	0.312306296	2.833351704	0.004606268	0.022514324	up_in_case
Yang2025_S	ENSGALG00010005475	TMEM181	case	control	-0.32808048	0.10208208	-3.213889057	0.001309503	0.008652403	down_in_case
Yang2025_S	ENSGALG00010005476	NFKB1	case	control	0.157732229	0.108793803	1.449827326	0.147106677	0.288926222	up_in_case
Yang2025_S	ENSGALG00010005477	TMEM161B	case	control	0.066430392	0.147368375	0.450777802	0.652149701	0.779725962	up_in_case
Yang2025_S	ENSGALG00010005479	ENSGALG00010005479	case	control	-1.100633684	0.688081582	-1.599568587	0.109694322	0.233714566	down_in_case
Yang2025_S	ENSGALG00010005481	ENSGALG00010005481	case	control	-1.808578111	0.778701938	-2.32255504	0.020203068	0.068216564	down_in_case
Yang2025_S	ENSGALG00010005482	XPO4	case	control	-0.197967588	0.09771809	-2.025905217	0.042774499	0.118879664	down_in_case
Yang2025_S	ENSGALG00010005484	GINM1	case	control	-0.70738362	0.118858511	-5.951476366	2.65734E-09	1.41878E-07	down_in_case
Yang2025_S	ENSGALG00010005485	PITRM1	case	control	0.097464855	0.084542051	1.152856522	0.24896928	0.414727702	up_in_case
Yang2025_S	ENSGALG00010005486	IRF5	case	control	0.37849629	0.227857509	1.661109575	0.096691442	0.214238979	up_in_case
Yang2025_S	ENSGALG00010005487	ENSGALG00010005487	case	control	0.711966639	0.227416654	3.130670629	0.001744076	0.010801647	up_in_case
Yang2025_S	ENSGALG00010005489	G2E3	case	control	-0.258906103	0.105585962	-2.452088315	0.01420298	0.052595941	down_in_case
Yang2025_S	ENSGALG00010005490	DCBLD2	case	control	-0.585763817	0.099581133	-5.882277111	4.0466E-09	2.03572E-07	down_in_case
Yang2025_S	ENSGALG00010005491	GGACT	case	control	0.161653732	0.132867468	1.216653965	0.223735901	0.385077218	up_in_case
Yang2025_S	ENSGALG00010005492	TNPO3	case	control	-0.013854901	0.077585183	-0.178576636	0.858270142	0.91663576	down_in_case
Yang2025_S	ENSGALG00010005493	KLF6	case	control	-0.018014571	0.111176228	-0.162036181	0.871277362	0.924369153	down_in_case
Yang2025_S	ENSGALG00010005494	PPDPFL	case	control	-0.838180043	0.423175989	-1.980689041	0.047626157	0.128940864	down_in_case
Yang2025_S	ENSGALG00010005495	TXN	case	control	-0.059922577	0.241595812	-0.24802821	0.80411258	0.882249311	down_in_case
Yang2025_S	ENSGALG00010005497	MKX	case	control	0.490106871	0.281666936	1.74002273	0.081855027	0.190330352	up_in_case
Yang2025_S	ENSGALG00010005498	SMO	case	control	-0.456285846	0.093328333	-4.889038827	1.0133E-06	2.45144E-05	down_in_case
Yang2025_S	ENSGALG00010005499	PDE5A	case	control	-0.175490178	0.105757334	-1.659366506	0.097041961	0.214637295	down_in_case
Yang2025_S	ENSGALG00010005500	KATNA1	case	control	0.068007351	0.078178322	0.869900368	0.384354854	0.551947636	up_in_case
Yang2025_S	ENSGALG00010005502	SCFD1	case	control	-0.024115337	0.069189048	-0.348542685	0.727432663	0.832465302	down_in_case
Yang2025_S	ENSGALG00010005503	DYNLT1	case	control	-0.935529151	0.292980754	-3.193142003	0.001407337	0.009189101	down_in_case
Yang2025_S	ENSGALG00010005504	LATS1	case	control	0.360411787	0.202727867	1.777810781	0.075434937	0.179566843	up_in_case
Yang2025_S	ENSGALG00010005507	USP53	case	control	0.066969023	0.162209687	0.412854648	0.67971311	0.799069598	up_in_case
Yang2025_S	ENSGALG00010005508	SS18	case	control	0.083600379	0.097470446	0.857699769	0.391058268	0.558397065	up_in_case
Yang2025_S	ENSGALG00010005509	COCH	case	control	0.528367072	0.212856604	2.4822677	0.013054916	0.049367775	up_in_case
Yang2025_S	ENSGALG00010005510	MYOZ2	case	control	0.571369265	0.215412275	2.652445245	0.007991109	0.034219624	up_in_case
Yang2025_S	ENSGALG00010005511	AHCYL2	case	control	-0.022688465	0.088307612	-0.256925362	0.797236392	0.877249614	down_in_case
Yang2025_S	ENSGALG00010005512	SMARCAD1	case	control	-0.068841053	0.097120258	-0.708822795	0.47843445	0.637375795	down_in_case
Yang2025_S	ENSGALG00010005513	TMEM30CP	case	control	0.113695632	0.250348425	0.454149578	0.649721171	0.777689789	up_in_case
Yang2025_S	ENSGALG00010005514	EEF1AKMT1	case	control	-0.538003033	0.133017748	-4.044595856	5.24135E-05	0.000631818	down_in_case
Yang2025_S	ENSGALG00010005515	PCCA	case	control	-0.067853193	0.090639348	-0.748606372	0.454094491	0.615885683	down_in_case
Yang2025_S	ENSGALG00010005516	LARP4B	case	control	0.395078459	0.167332965	2.361031851	0.018224165	0.063284276	up_in_case
Yang2025_S	ENSGALG00010005518	HPGDS	case	control	2.658886532	0.650089322	4.090032619	4.31313E-05	0.00054343	up_in_case
Yang2025_S	ENSGALG00010005519	PTBP3	case	control	0.597430389	0.204197773	2.925743901	0.003436337	0.018011468	up_in_case
Yang2025_S	ENSGALG00010005522	SYNPO2	case	control	0.398306779	0.275689888	1.444763833	0.148524233	0.290686122	up_in_case
Yang2025_S	ENSGALG00010005524	STRIP2	case	control	0.125781465	0.168764877	0.745305937	0.456086793	0.617705264	up_in_case
Yang2025_S	ENSGALG00010005526	STRN3	case	control	-0.242366357	0.084432308	-2.870540465	0.004097707	0.020525361	down_in_case
Yang2025_S	ENSGALG00010005527	NUP43	case	control	-0.231741317	0.091670418	-2.527983642	0.01147197	0.044854629	down_in_case
Yang2025_S	ENSGALG00010005529	ENSGALG00010005529	case	control	-0.490408521	0.384825702	-1.2743653	0.202533984	0.360448412	down_in_case
Yang2025_S	ENSGALG00010005530	IL17D	case	control	-0.377148582	0.168650645	-2.236271217	0.025334011	0.080660274	down_in_case
Yang2025_S	ENSGALG00010005531	SUSD1	case	control	0.200611887	0.147109652	1.363689495	0.172665312	0.322359474	up_in_case
Yang2025_S	ENSGALG00010005532	GTPBP4	case	control	0.004316506	0.116364579	0.037094671	0.970409521	0.982321994	up_in_case
Yang2025_S	ENSGALG00010005534	ENSGALG00010005534	case	control	0.165711292	0.206902902	0.800913328	0.423181824	0.588714652	up_in_case
Yang2025_S	ENSGALG00010005535	AP4S1	case	control	0.234568119	0.175037439	1.340102553	0.180212006	0.332388433	up_in_case
Yang2025_S	ENSGALG00010005536	TAF4B	case	control	0.183926597	0.250233532	0.735019786	0.462327444	0.623249776	up_in_case
Yang2025_S	ENSGALG00010005537	GRIN3A	case	control	-0.224573286	0.30658022	-0.732510681	0.463856926	0.624888463	down_in_case
Yang2025_S	ENSGALG00010005538	ENSGALG00010005538	case	control	0.063388294	0.595901935	0.106373701	0.915285858	0.950120376	up_in_case
Yang2025_S	ENSGALG00010005539	IFT88	case	control	0.16453645	0.136683657	1.203775588	0.228676328	0.391042413	up_in_case
Yang2025_S	ENSGALG00010005540	PCMT1	case	control	-0.128963038	0.086429425	-1.492119592	0.135667797	0.27221465	down_in_case
Yang2025_S	ENSGALG00010005542	KCTD1	case	control	0.18838143	0.156948461	1.20027574	0.230032269	0.39244609	up_in_case
Yang2025_S	ENSGALG00010005543	SEC24D	case	control	0.15105325	0.17665315	0.855083822	0.392504745	0.559491893	up_in_case

Yang2025_S	ENSGALG00010005544	AQP4	case	control	-0.480666763	0.144717348	-3.321417722	0.000895614	0.006400194	down_in_case
Yang2025_S	ENSGALG00010005545	RPL34	case	control	-0.642591127	0.352838582	-1.821204257	0.068575814	0.167385525	down_in_case
Yang2025_S	ENSGALG00010005546	NRF1	case	control	0.311371936	0.147215388	2.11507738	0.034423353	0.101414254	up_in_case
Yang2025_S	ENSGALG00010005547	LRP11	case	control	-0.07573327	0.106529397	-0.710914284	0.477137354	0.636259237	down_in_case
Yang2025_S	ENSGALG00010005548	EZR	case	control	0.081383441	0.158239337	0.514306003	0.607038066	0.744309322	up_in_case
Yang2025_S	ENSGALG00010005549	RNF20	case	control	0.634840038	0.221555417	2.865378087	0.004165116	0.020761542	up_in_case
Yang2025_S	ENSGALG00010005550	ENSGALG00010005550	case	control	1.125489815	0.479476035	2.347332782	0.018908358	0.065123078	up_in_case
Yang2025_S	ENSGALG00010005551	METTL14	case	control	-0.070013701	0.120341289	-0.581792847	0.560706223	0.707226757	down_in_case
Yang2025_S	ENSGALG00010005552	WDR37	case	control	0.001273049	0.166886183	0.007628245	0.9939136	0.995970515	up_in_case
Yang2025_S	ENSGALG00010005553	PPP1R14C	case	control	-0.520590441	0.132259546	-3.936127544	8.2807E-05	0.000916533	down_in_case
Yang2025_S	ENSGALG00010005554	CRYL1	case	control	-0.518695412	0.125947647	-4.118341433	3.81609E-05	0.000493295	down_in_case
Yang2025_S	ENSGALG00010005555	PRSS12	case	control	0.209163831	0.177038767	1.181457789	0.237420912	0.401366058	up_in_case
Yang2025_S	ENSGALG00010005556	IYD	case	control	0.28141975	0.680138621	0.413768225	0.679043856	0.798606246	up_in_case
Yang2025_S	ENSGALG00010005557	CMSS1	case	control	-0.353714933	0.236045427	-1.498503647	0.134002446	0.269922533	down_in_case
Yang2025_S	ENSGALG00010005558	TAGAP	case	control	-0.431545306	0.551335929	-0.782726617	0.433787669	0.597669682	down_in_case
Yang2025_S	ENSGALG00010005559	FILIP1L	case	control	0.653005622	0.186016056	3.510479878	0.000447299	0.003615491	up_in_case
Yang2025_S	ENSGALG00010005560	RSPH3	case	control	-0.100779492	0.388742896	-0.259244587	0.795446531	0.876525276	down_in_case
Yang2025_S	ENSGALG00010005561	CLYBL	case	control	-0.957377715	0.249616432	-3.8353954	0.000125362	0.001277768	down_in_case
Yang2025_S	ENSGALG00010005562	HECTD1	case	control	0.327101409	0.099191344	3.297680998	0.000974868	0.006871416	up_in_case
Yang2025_S	ENSGALG00010005564	TM9SF2	case	control	-0.712065258	0.110915094	-6.419913048	1.36352E-10	1.06745E-08	down_in_case
Yang2025_S	ENSGALG00010005565	PGAP4	case	control	-0.170404257	0.181827868	-0.937173483	0.348669328	0.517048748	down_in_case
Yang2025_S	ENSGALG00010005567	NDST3	case	control	-0.053656637	0.188134295	-0.285203911	0.775487924	0.863270828	down_in_case
Yang2025_S	ENSGALG00010005568	PLEKHG1	case	control	0.389768628	0.133549111	2.918541539	0.00351673	0.018333571	up_in_case
Yang2025_S	ENSGALG00010005572	ENSGALG00010005572	case	control	-0.197215227	0.375320707	-0.52545789	0.59926492	0.738096744	down_in_case
Yang2025_S	ENSGALG00010005573	CHST9	case	control	0.041858613	0.237813626	0.176014358	0.860282662	0.917628392	up_in_case
Yang2025_S	ENSGALG00010005575	ENSGALG00010005575	case	control	1.493654641	0.517604224	2.885707984	0.003905343	0.019847177	up_in_case
Yang2025_S	ENSGALG00010005578	HEATR5A	case	control	0.09363986	0.099370644	0.942329209	0.346024118	0.514727257	up_in_case
Yang2025_S	ENSGALG00010005583	ALDOB	case	control	-2.32401664	0.771508432	-3.012302319			down_in_case
Yang2025_S	ENSGALG00010005585	ENSGALG00010005585	case	control	1.066220596	0.513757198	2.075339481	0.037955083	0.108828	up_in_case
Yang2025_S	ENSGALG00010005587	LEF1	case	control	0.437565573	0.260442791	1.680083257	0.092941118	0.20834827	up_in_case
Yang2025_S	ENSGALG00010005588	PLAGL1	case	control	-0.158121456	0.152624841	-1.036013896	0.30019566	0.469814299	down_in_case
Yang2025_S	ENSGALG00010005590	TBC1D23	case	control	0.013382992	0.11630641	0.115066679	0.908392271	0.945239847	up_in_case
Yang2025_S	ENSGALG00010005594	MRPL50	case	control	0.033189416	0.289114079	0.114796954	0.908606063	0.945305771	up_in_case
Yang2025_S	ENSGALG00010005596	PARD3	case	control	0.189232381	0.149851594	1.262798582	0.206661574	0.365367805	up_in_case
Yang2025_S	ENSGALG00010005597	ENSGALG00010005597	case	control	0.588073414	0.582452785	1.009649932	0.312663038	0.482229123	up_in_case
Yang2025_S	ENSGALG00010005601	TMEM215	case	control	0.451773982	0.399766043	1.130095939	0.258435801	0.424782128	up_in_case
Yang2025_S	ENSGALG00010005604	ENSGALG00010005604	case	control	0.987961363	0.608506722	1.623583319	0.104464768	0.226333312	up_in_case
Yang2025_S	ENSGALG00010005609	GJB6	case	control	-0.541654948	0.590054712	-0.917974108	0.358632421	0.527612204	down_in_case
Yang2025_S	ENSGALG00010005610	MTHFD1L	case	control	-0.125176183	0.108444529	-1.154287679	0.248382246	0.414119	down_in_case
Yang2025_S	ENSGALG00010005617	DTD2	case	control	-0.36035271	0.254596483	-1.415387622	0.156954877	0.302178255	down_in_case
Yang2025_S	ENSGALG00010005618	PHACTR2	case	control	0.046954584	0.140424597	0.334375777	0.738096016	0.840126326	up_in_case
Yang2025_S	ENSGALG00010005619	CELF2	case	control	-0.066728568	0.151132912	-0.441522416	0.658834837	0.784658933	down_in_case
Yang2025_S	ENSGALG00010005621	CDH2	case	control	-0.326295109	0.136353331	-2.39301165	0.01671071	0.059479885	down_in_case
Yang2025_S	ENSGALG00010005629	NRP1	case	control	-0.233385958	0.115801132	-2.01540308	0.043862432	0.121394834	down_in_case
Yang2025_S	ENSGALG00010005630	NIT2	case	control	-0.159933245	0.10836857	-1.47582685	0.139990381	0.278840188	down_in_case
Yang2025_S	ENSGALG00010005632	ITGB1	case	control	0.046493338	0.109239151	0.425610573	0.670391635	0.792224172	up_in_case
Yang2025_S	ENSGALG00010005634	HADH	case	control	-0.352670485	0.181942409	-1.938363283	0.052578917	0.13837341	down_in_case
Yang2025_S	ENSGALG00010005637	TOPORS	case	control	0.1485645	0.164475672	0.90326124	0.366387263	0.535122787	up_in_case
Yang2025_S	ENSGALG00010005645	FUCA2	case	control	-0.114313803	0.174870966	-0.653703731	0.513302699	0.668305438	down_in_case
Yang2025_S	ENSGALG00010005649	TOMM70	case	control	-0.166876683	0.067344919	-2.477940206	0.013214329	0.049794936	down_in_case
Yang2025_S	ENSGALG00010005651	USP6NL	case	control	0.703113111	0.228990019	3.070496758	0.00213703	0.0125705	up_in_case
Yang2025_S	ENSGALG00010005656	CYP2U1	case	control	-0.370452466	0.138322309	-2.678183065	0.007402275	0.032254753	down_in_case
Yang2025_S	ENSGALG00010005660	UBAC2	case	control	-0.411875514	0.181510766	-2.269151983	0.023259085	0.075851943	down_in_case
Yang2025_S	ENSGALG00010005668	LNP1	case	control	0.269573872	0.254858845	1.05773795	0.290174929	0.458768735	up_in_case
Yang2025_S	ENSGALG00010005670	SGMS2	case	control	-0.182487811	0.241277894	-0.756338708	0.449446146	0.611683958	down_in_case
Yang2025_S	ENSGALG00010005675	GJA3	case	control	-0.416825179	0.401704991	-1.037640031	0.299437674	0.468996738	down_in_case
Yang2025_S	ENSGALG00010005679	TMEM45A	case	control	-0.355210341	0.293328678	-1.210963562	0.22590937	0.387881601	down_in_case
Yang2025_S	ENSGALG00010005680	ENSGALG00010005680	case	control	0.397638029	0.641094653	0.62024855	0.535094162	0.686479207	up_in_case
Yang2025_S	ENSGALG00010005681	EPC1	case	control	0.1903125	0.108734397	1.75025111	0.080074993	0.187473963	up_in_case
Yang2025_S	ENSGALG00010005692	SEC24B	case	control	0.331183505	0.130088328	2.545835657	0.01090165	0.04316979	up_in_case
Yang2025_S	ENSGALG00010005694	ENSGALG00010005694	case	control	0.125223695	0.111190246	1.126211154	0.260076153	0.42642607	up_in_case
Yang2025_S	ENSGALG00010005695	KIF5B	case	control	0.168890193	0.123323318	1.369491152	0.170845799	0.319821671	up_in_case
Yang2025_S	ENSGALG00010005696	NUBPL	case	control	-0.129911293	0.13446768	-0.966115381	0.333986449	0.502819918	down_in_case
Yang2025_S	ENSGALG00010005699	PRPF18	case	control	0.075645087	0.086488613	0.87462482	0.381778078	0.549730208	up_in_case
Yang2025_S	ENSGALG00010005704	AKAP12	case	control	0.108214784	0.235141778	0.460210793	0.645364925	0.774738131	up_in_case
Yang2025_S	ENSGALG00010005705	ECHDC3	case	control	-0.311150842	0.186413486	-1.669143409	0.095088962	0.211671675	down_in_case
Yang2025_S	ENSGALG00010005712	ENSGALG00010005712	case	control	0.268733191	0.151347024	1.775609343	0.075797329	0.180152785	up_in_case
Yang2025_S	ENSGALG00010005714	ARHGAP5	case	control	-0.197986185	0.129564148	-1.528093909	0.126489224	0.259094403	down_in_case
Yang2025_S	ENSGALG00010005716	ZBTB2	case	control	0.234526652	0.176106467	1.331732197	0.182948205	0.336064229	up_in_case
Yang2025_S	ENSGALG00010005720	COL25A1	case	control	0.611195297	0.210119157	2.908803298	0.003628151	0.018794899	up_in_case
Yang2025_S	ENSGALG00010005724	PROSER2	case	control	-0.306227443	0.126163914	-2.427218943	0.015215071	0.055645469	down_in_case
Yang2025_S	ENSGALG00010005738	ELAVL2	case	control	0.821829659	0.624291017	1.316420765	0.188032857	0.342449985	up_in_case
Yang2025_S	ENSGALG00010005740	ENSGALG00010005740	case	control	0.279426107	0.373641968	0.747844543	0.454553931	0.616348062	up_in_case
Yang2025_S	ENSGALG00010005742	GPR183	case	control	-0.232249521	0.093248056	-2.49066341	0.012750485	0.048506144	down_in_case
Yang2025_S	ENSGALG00010005743	ETNPPL	case	control	0.011514618	0.150234309	0.076644396	0.93890644	0.962932392	up_in_case
Yang2025_S	ENSGALG00010005748	AIG1	case	control	-0.431525152	0.132195365	-3.264298651	0.001097354	0.007540252	down_in_case
Yang2025_S	ENSGALG00010005752	RMND1	case	control	-0.047119302	0.149846628	-0.314450198	0.753179136	0.849770657	down_in_case
Yang2025_S	ENSGALG00010005755	ZMYM2	case	control	0.406756584	0.150404582	2.704416166	0.006842453	0.030433957	up_in_case
Yang2025_S	ENSGALG00010005756	TET2	case	control	0.572179135	0.232742555	2.458420781	0.013954956	0.051925851	up_in_case
Yang2025_S	ENSGALG00010005757	ARHGAP12	case	control	0.231938124	0.139864186	1.658309615	0.097254989	0.214715409	up_in_case
Yang2025_S	ENSGALG00010005758	HIVEP2	case	control	0.1898405	0.197398986	0.961709601	0.336195498	0.504367781	up_in_case
Yang2025_S	ENSGALG00010005760	GPR18	case	control	-0.089429554	0.367536118	-0.243321811	0.807756109	0.88400859	down_in_case
Yang2025_S	ENSGALG00010005763	AKAP6	case	control	0.009807297	0.151869326	0.064577206	0.948510633	0.967992436	up_in_case
Yang2025_S	ENSGALG00010005769	TFG	case	control	0.335964454	0.112934048	2.974873027	0.002931098	0.016016709	up_in_case
Yang2025_S	ENSGALG00010005772	SVEP1	case	control	-0.008811099	0.155561123	-0.056640754	0.954831369	0.972132325	down_in_case
Yang2025_S	ENSGALG00010005777	ENSGALG00010005777	case	control	0.958506922	1.125663284	0.851504118	0.394489377	0.561570075	up_in_case
Yang2025_S	ENSGALG00010005779	ARMT1	case	control	-0.330359105	0.097892575	-3.374710543	0.000738934	0.005494686	down_in_case
Yang2025_S	ENSGALG00010005784	RASL11A	case	control	0.279296197	0.478207467	0.584048173	0.559187902	0.705950663	up_in_case
Yang2025_S	ENSGALG00010005785	RPL21	case	control	0.35730505	0.151651137	2.35609873	0.018468008	0.063877051	up_in_case
Yang2025_S	ENSGALG00010005789	RNASET2	case	control	0.142410178	0.121787434	1.169333924	0.24226912	0.407289535	up_in_case
Yang2025_S	ENSGALG00010005790	MUSK	case	control	0.534141291	0.16465451	3.244012526	0.001178586	0.007949302	up_in_case
Yang2025_S	ENSGALG00010005792	ADGRG6	case	control	0.220764806	0.183829505	1.200921502	0.229781653	0.392162563	up_in_case
Yang2025_S	ENSGALG00010005799	UFSP2	case	control	0.065881578	0.115252667	0.571627365	0.567574452	0.712288575	up_in_case
Yang2025_S	ENSGALG00010005800	FRMD4A	case	control	0.074703195	0.178461219	0.418596236	0.675511241	0.796044678	up_in_case
Yang2025_S	ENSGALG00010005814	UPF2	case	control	0.279886318	0.1685864	1.660195116	0.096875207	0.214430327	up_in_case
Yang2025_S	ENSGALG00010005817	VTA1	case	control	-0.457616428	0.078831221	-5.80501508	6.43602E-09	3.08199E-07	down_in_case
Yang2025_S	ENSGALG00010005821	DOCK9	case	control	-0.024022106	0.151735852	-0.158315295	0.874208357	0.926381944	down_in_case
Yang2025_S	ENSGALG00010005826	LRP2BP	case	control	-0.164625461	0.267153251	-0.616221065	0.537748627	0.688996609	down_in_case
Yang2025_S	ENSGALG00010005828	LPAR1	case	control	-0.09650967	0.129865308	-0.743152051	0.457389629	0.618928382	down_in_case
Yang2025_S	ENSGALG00010005829	NPAS3	case	control	0.080800914	0.25055283	0.322490529	0.747081112	0.846388235	up_in_case
Yang2025_S	ENSGALG00010005830	USP12	case	control	-0.253278505	0.25127186	-1.007985948	0.313461208	0.48287146	down_in_case
Yang2025_S	ENSGALG00010005831	SEC61A2	case	control	-0.548542864	0.118798731	-4.617413512	3.88553E-06	7.52011E-05	down_in_case
Yang2025_S	ENSGALG00010005836	NEBL	case	control	0.013006106	0.221848755	0.058626003	0.953249999	0.971338769	up_in_case
Yang2025_S	ENSGALG00010005839	RPS6KA2	case	control	0.037864822	0.122840111	0.308244773	0.757896086	0.85295517	up_in_case
Yang2025_S	ENSGALG00010005841	ENSGALG00010005841	case	control	-0.747533417	0.375190157	-1.992412119	0.046325859	0.126256765	down_in_case
Yang2025_S	ENSGALG00010005843	GPR12	case	control	-1.0994959	0.289566159	-3.797045571	0.000146431	0.001453358	down_in_case
Yang2025_S	ENSGALG00010005847	ENSGALG00010005847	case	control	-1.142299564	0.794926535	-1.436987587	0.150721521	0.29355375	down_in_case
Yang2025_S	ENSGALG00010005848	WASF3	case	control	-0.120371661	0.214311636	-0.561666471	0.574343282	0.717157136	down_in_case
Yang2025_S	ENSGALG00010005849	SNX25	case	control	0.026066712	0.139233091	0.187216357	0.851491001	0.911961194	up_in_case
Yang2025_S	ENSGALG00010005853	COMMD3	case	control	0.606713874	0.165694743	3.661636225	0.00025061	0.002250157	up_in_case
Yang2025_S	ENSGALG00010005858	CFAP97	case	control	-0.163325517	0.119900283	-1.362177919	0.173141743	0.323075815	down_in_case
Yang2025_S	ENSGALG00010005859	ENSGALG00010005859	case	control	-0.673813555	0.775856189	-0.868477385	0.385133047	0.55265265	down_in_case
Yang2025_S	ENSGALG00010005860	ABI3BP	case	control	0.628700023	0.313705344	2.004110018	0.045058277	0.12394139	up_in_case
Yang2025_S	ENSGALG00010005861	MPC1L	case	control	-0.889081101	0.176295454	-5.043131173	4.57975E-07	1.2489E-05	down_in_case
Yang2025_S	ENSGALG00010005864	ENSGALG00010005864	case	control	0.667152444	0.498593826	1.338068	0.180874271	0.33326497	up_in_case
Yang2025_S	ENSGALG00010005866	DNAJC1	case	control	0.94868277	0.163776595	5.792541774	6.93291E-09	3.28606E-07	up_in_case
Yang2025_S	ENSGALG00010005871	EGLN3	case	control	-0.267107247	0.121919687	-2.190845898	0.028462946	0.087963567	down_in_case
Yang2025_S	ENSGALG00010005872	STK24	case	control	-0.037479157	0.11040594	-0.339466855	0.734258062	0.837882547	down_in_case
Yang2025_S	ENSGALG00010005875	CDK8	case	control	0.398595401	0.148253489	2.688607227	0.007175079	0.031491251	up_in_case
Yang2025_S	ENSGALG00010005879	FAM107B	case	control	-0.085397491	0.139447325	-0.612399637	0.540273382	0.690770674	down_in_case
Yang2025_S	ENSGALG00010005880	SLC25A4	case	control	-0.340675338	0.250552567	-1.359696058	0.173926126	0.324105452	down_in_case
Yang2025_S	ENSGALG00010005881	CORO2A	case	control	0.981372648	0.350181019	2.802472418	0.005071255	0.024234548	up_in_case
Yang2025_S	ENSGALG00010005884	MYB	case	control	-0.028031522	0.290803943	-0.096393198	0.923208294	0.953733233	down_in_case
Yang2025_S	ENSGALG00010005885	SLITRK5	case	control	0.204981831	0.328346974	0.624284207	0.532440954	0.684714349	up_in_case
Yang2025_S	ENSGALG00010005887	CCNH	case	control	0.404397989	0.206351526	1.959752839	0.050024686	0.133287571	up_in_case
Yang2025_S	ENSGALG00010005888	IMPG2	case	control	1.026044426	0.246671538	4.159557429	3.18865E-05	0.000428484	up_in_case

Yang2025_S	ENSGALG00010005890	SNX32	case	control	-0.371918029	0.069152152	-5.37825676	7.52105E-08	2.66682E-06	down_in_case
Yang2025_S	ENSGALG00010005891	RNF6	case	control	0.661243947	0.117640009	5.620910379	1.89954E-08	8.24613E-07	up_in_case
Yang2025_S	ENSGALG00010005892	ENSGALG00010005892	case	control	-2.407782049	0.799215773	-3.012680842	0.002589511	0.014556206	down_in_case
Yang2025_S	ENSGALG00010005893	SFT2D1	case	control	-0.121289294	0.146484666	-0.827999938	0.407670535	0.573653327	down_in_case
Yang2025_S	ENSGALG00010005895	MLLT10	case	control	0.604019639	0.201522432	2.997282398	0.002723982	0.015110946	up_in_case
Yang2025_S	ENSGALG00010005899	ENSGALG00010005899	case	control	0.252287055	0.190664883	1.32319623	0.185770129	0.339280046	up_in_case
Yang2025_S	ENSGALG00010005900	SPTSSA	case	control	-1.155062805	0.190668601	-6.057960257	1.37858E-09	8.0379E-08	down_in_case
Yang2025_S	ENSGALG00010005901	ENSGALG00010005901	case	control	0.795675782	0.27183191	2.927087487	0.003421526	0.017962982	up_in_case
Yang2025_S	ENSGALG00010005902	EAPP	case	control	0.463210919	0.158497747	2.922507908	0.003472248	0.018156014	up_in_case
Yang2025_S	ENSGALG00010005903	PRR18	case	control	0.414463563	0.35959495	1.152584492	0.249080972	0.414727702	up_in_case
Yang2025_S	ENSGALG00010005905	CITED2	case	control	0.438448884	0.22449178	1.953073223	0.050810919	0.134807785	up_in_case
Yang2025_S	ENSGALG00010005909	BRMS1L	case	control	-0.080629411	0.096976664	-0.831431071	0.405730152	0.571846836	down_in_case
Yang2025_S	ENSGALG00010005910	ENSGALG00010005910	case	control	-0.092825432	0.560787659	-0.16552688	0.868529294	0.922805694	down_in_case
Yang2025_S	ENSGALG00010005911	TBXT	case	control	0.595659401	0.319528592	1.864181845	0.062296172	0.156526714	up_in_case
Yang2025_S	ENSGALG00010005913	NFKBIA	case	control	-0.019574234	0.11196759	-0.174820531	0.861220655	0.918270777	down_in_case
Yang2025_S	ENSGALG00010005915	PAPSS1	case	control	0.095203026	0.112491499	0.846313071	0.397378118	0.564259453	up_in_case
Yang2025_S	ENSGALG00010005926	NUDT5	case	control	0.252327107	0.170388949	1.48088892	0.138636176	0.276736157	up_in_case
Yang2025_S	ENSGALG00010005934	SLITRK6	case	control	-0.465800013	0.332948878	-1.399013613	0.161808901	0.307865516	down_in_case
Yang2025_S	ENSGALG00010005935	ATP8A2	case	control	-0.56768543	0.326648803	-1.737907577	0.0822271	0.190908805	down_in_case
Yang2025_S	ENSGALG00010005941	TXLNB	case	control	0.264584234	0.157826942	1.676419949	0.093655964	0.2093177	up_in_case
Yang2025_S	ENSGALG00010005943	PROX2	case	control	1.010263554	0.760236108	1.328881309	0.183887131	0.337299878	up_in_case
Yang2025_S	ENSGALG00010005949	SLITRK1	case	control	-0.113872603	0.358941952	-0.317245178	0.751057572	0.848540188	down_in_case
Yang2025_S	ENSGALG00010005956	TXNL4B	case	control	0.136556204	0.129100403	1.057751952	0.290168543	0.458768735	up_in_case
Yang2025_S	ENSGALG00010005963	UGCG	case	control	-0.053821209	0.120762909	-0.44567665	0.655830834	0.782537285	down_in_case
Yang2025_S	ENSGALG00010005964	HSPA14	case	control	-0.084226599	0.144202647	-0.58408497	0.559163146	0.705950663	down_in_case
Yang2025_S	ENSGALG00010005966	PLXDC2	case	control	-0.187235334	0.186827729	-1.002181718	0.316255836	0.485462114	down_in_case
Yang2025_S	ENSGALG00010005967	GJD2	case	control	0.298592728	0.212552729	1.40479367	0.160082642	0.305749947	up_in_case
Yang2025_S	ENSGALG00010005969	HECA	case	control	-0.565556818	0.107488834	-5.261540196	1.42854E-07	4.65109E-06	down_in_case
Yang2025_S	ENSGALG00010005970	SHISA2	case	control	-0.009546058	0.158661582	-0.060166159	0.952023298	0.970549759	down_in_case
Yang2025_S	ENSGALG00010005975	ABRACL	case	control	-0.524174551	0.199905215	-2.622115436	0.008738583	0.036722945	down_in_case
Yang2025_S	ENSGALG00010005978	VPS16	case	control	-0.251252973	0.120478038	-2.085466998	0.037026931	0.106996968	down_in_case
Yang2025_S	ENSGALG00010005979	SUV39H2	case	control	-0.662949488	0.099617245	-6.654967094	2.83363E-11	2.7152E-09	down_in_case
Yang2025_S	ENSGALG00010005981	AREL1	case	control	-0.11461161	0.117455818	-0.975784866	0.329171086	0.498695269	down_in_case
Yang2025_S	ENSGALG00010005984	IDH3B	case	control	0.302411748	0.182484997	1.657186913	0.097481692	0.215005915	up_in_case
Yang2025_S	ENSGALG00010005985	ENSGALG00010005985	case	control	-0.040905601	0.237781238	-0.172030399	0.86341363	0.919290108	down_in_case
Yang2025_S	ENSGALG00010005988	NUP58	case	control	0.237552553	0.071993701	3.2996297	0.000968125	0.00682876	up_in_case
Yang2025_S	ENSGALG00010005989	FCF1	case	control	-0.296495842	0.177483803	-1.670551555	0.094810288	0.211153957	down_in_case
Yang2025_S	ENSGALG00010005990	SLC2A12	case	control	-0.493173999	0.138899941	-3.550570266	0.000384398	0.003200836	down_in_case
Yang2025_S	ENSGALG00010005991	FARP1	case	control	-0.074670771	0.10701699	-0.697746884	0.485335502	0.642701722	down_in_case
Yang2025_S	ENSGALG00010005997	ENSGALG00010005997	case	control	0.034035038	0.28892386	0.117799332	0.906226659	0.943746244	up_in_case
Yang2025_S	ENSGALG00010005998	YLPM1	case	control	0.206751137	0.166052039	1.245098454	0.213095562	0.373430215	up_in_case
Yang2025_S	ENSGALG00010005999	PDE10A	case	control	0.058679373	0.189446001	0.309741945	0.756757203	0.852119509	up_in_case
Yang2025_S	ENSGALG00010006001	NOP56	case	control	0.35447286	0.123234684	2.876404995	0.004022333	0.020264355	up_in_case
Yang2025_S	ENSGALG00010006004	DCLRE1C	case	control	0.166167195	0.172668365	0.962348806	0.33587442	0.504123419	up_in_case
Yang2025_S	ENSGALG00010006010	IPO5	case	control	-0.304704993	0.118864636	-2.563462135	0.0103634	0.041605579	down_in_case
Yang2025_S	ENSGALG00010006013	TRMT10C	case	control	-0.29407557	0.159931244	-1.838762476	0.06595013	0.162801604	down_in_case
Yang2025_S	ENSGALG00010006015	CDC123	case	control	-0.545087237	0.109827269	-4.963132017	6.93654E-07	1.75428E-05	down_in_case
Yang2025_S	ENSGALG00010006016	MTMR6	case	control	-0.633310171	0.108869104	-5.817170781	5.9852E-09	2.88594E-07	down_in_case
Yang2025_S	ENSGALG00010006021	ENSGALG00010006021	case	control	0.602175376	0.111843552	5.384086641	7.28134E-08	2.59503E-06	up_in_case
Yang2025_S	ENSGALG00010006022	AFG2A	case	control	0.2092606	0.07717742	2.711422574	0.006699519	0.0299032	up_in_case
Yang2025_S	ENSGALG00010006024	AMER2	case	control	1.321807427	0.584269865	2.262323465	0.023677426	0.077022565	up_in_case
Yang2025_S	ENSGALG00010006025	TNFAIP3	case	control	-0.360966118	0.120595823	-2.99318922	0.002760785	0.015284681	down_in_case
Yang2025_S	ENSGALG00010006028	ARL5B	case	control	-0.441471064	0.069442886	-6.357326029	2.05296E-10	1.51365E-08	down_in_case
Yang2025_S	ENSGALG00010006029	HSDL2	case	control	0.34328508	0.181871068	1.887518917	0.059090564	0.151004404	up_in_case
Yang2025_S	ENSGALG00010006031	ERCC5	case	control	0.628725543	0.13120859	4.791801682	1.6529E-06	3.70309E-05	up_in_case
Yang2025_S	ENSGALG00010006033	NSUN6	case	control	0.172627347	0.169956922	1.015712362	0.309766379	0.479621611	up_in_case
Yang2025_S	ENSGALG00010006035	ADH1C	case	control	0.376018446	0.334891385	1.122807163	0.261519403	0.428334847	up_in_case
Yang2025_S	ENSGALG00010006037	METTL21EP	case	control	-0.643786005	0.70485288	-0.91336224	0.361052058	0.529606361	down_in_case
Yang2025_S	ENSGALG00010006038	ENSGALG00010006038	case	control	0.166476118	0.342124798	0.486594714	0.62654557	0.760068993	up_in_case
Yang2025_S	ENSGALG00010006039	PCNP	case	control	-0.046817756	0.069354766	-0.675047415	0.499645641	0.656527654	down_in_case
Yang2025_S	ENSGALG00010006041	QKI	case	control	0.133112	0.174448067	0.763046574	0.44543562	0.608484008	up_in_case
Yang2025_S	ENSGALG00010006047	ZBTB11	case	control	0.10973612	0.113762729	0.964605202	0.334742593	0.503363766	up_in_case
Yang2025_S	ENSGALG00010006049	AIMP1	case	control	0.115196708	0.152473901	0.755517549	0.449938507	0.612226971	up_in_case
Yang2025_S	ENSGALG00010006052	RALGAPA1	case	control	0.470699286	0.127119258	3.702816509	0.000213219	0.001968991	up_in_case
Yang2025_S	ENSGALG00010006053	TMEM243	case	control	-0.390114954	0.297439684	-1.311576683	0.189663004	0.344497335	down_in_case
Yang2025_S	ENSGALG00010006059	BIVM	case	control	-0.333753883	0.061438378	-5.432335516	5.56212E-08	2.03969E-06	down_in_case
Yang2025_S	ENSGALG00010006060	ENSGALG00010006060	case	control	-0.379909093	0.498918554	-0.761465152	0.446379286	0.609235587	down_in_case
Yang2025_S	ENSGALG00010006061	RPL24	case	control	0.160350237	0.19449739	0.824433875	0.409693073	0.575105568	up_in_case
Yang2025_S	ENSGALG00010006062	ENSGALG00010006062	case	control	-2.283976514	0.793789999	-2.87730573	0.004010868	0.020213904	down_in_case
Yang2025_S	ENSGALG00010006063	POGLUT2	case	control	-0.166233454	0.072461014	-2.294108855	0.021784247	0.072053995	down_in_case
Yang2025_S	ENSGALG00010006064	DMTF1	case	control	0.105732603	0.143973445	0.734389613	0.462711316	0.623646599	up_in_case
Yang2025_S	ENSGALG00010006065	WDR54	case	control	0.093855543	0.228673731	0.410434303	0.681487387	0.800413294	up_in_case
Yang2025_S	ENSGALG00010006066	ARHGAP18	case	control	-0.578058218	0.144956366	-3.987808436	6.66865E-05	0.000771824	down_in_case
Yang2025_S	ENSGALG00010006068	PACRG	case	control	0.022254169	0.261112108	0.085228406	0.932079808	0.95863854	up_in_case
Yang2025_S	ENSGALG00010006069	TTR	case	control	-2.514681378	1.547221159	-1.625288902			down_in_case
Yang2025_S	ENSGALG00010006070	TMEM242	case	control	-0.18556599	0.121940443	-1.521775586	0.128065321	0.261171122	down_in_case
Yang2025_S	ENSGALG00010006071	TEX30	case	control	-0.215161902	0.268498992	-0.801350874	0.422928547	0.588714652	down_in_case
Yang2025_S	ENSGALG00010006072	ENSGALG00010006072	case	control	0.14026539	0.122935771	1.140964819	0.253884563	0.420423574	up_in_case
Yang2025_S	ENSGALG00010006073	CEP97	case	control	-0.523702509	0.1693652	-3.092149438	0.001987128	0.011915072	down_in_case
Yang2025_S	ENSGALG00010006075	SOX21	case	control	-0.271995614	0.527779348	-0.515358576	0.606302473	0.743766699	down_in_case
Yang2025_S	ENSGALG00010006076	PODXL	case	control	0.855456441	0.415727334	2.057734415	0.039615635	0.11252423	up_in_case
Yang2025_S	ENSGALG00010006077	TBCK	case	control	-0.097644296	0.104929253	-0.930572679	0.352074652	0.520879104	down_in_case
Yang2025_S	ENSGALG00010006078	SPATA13	case	control	0.079795016	0.206286053	0.386817307	0.698891463	0.813564352	up_in_case
Yang2025_S	ENSGALG00010006079	ENSGALG00010006079	case	control	-0.197582069	0.646719076	-0.305514521	0.759974312	0.854396292	down_in_case
Yang2025_S	ENSGALG00010006080	ENSGALG00010006080	case	control	0.930059745	0.170583619	5.452221914	4.97443E-08	1.86341E-06	up_in_case
Yang2025_S	ENSGALG00010006081	ZDHHC14	case	control	-0.145898837	0.096537982	-1.511310204	0.130709435	0.264980266	down_in_case
Yang2025_S	ENSGALG00010006083	CACNB2	case	control	0.433805359	0.220841518	1.964328821	0.049491971	0.13229822	up_in_case
Yang2025_S	ENSGALG00010006084	TMEM68	case	control	-0.168977142	0.136672331	-1.236366869	0.216322225	0.377277874	down_in_case
Yang2025_S	ENSGALG00010006085	DLST	case	control	0.046433197	0.113734	0.408261354	0.683081809	0.801622642	up_in_case
Yang2025_S	ENSGALG00010006086	ENSGALG00010006086	case	control	0.406889136	0.560230388	0.726288941	0.467661657	0.628604892	up_in_case
Yang2025_S	ENSGALG00010006087	ENSGALG00010006087	case	control	-0.283542464	0.480392679	-0.590230611	0.555036052	0.702682601	down_in_case
Yang2025_S	ENSGALG00010006091	RTKN	case	control	0.294329974	0.19806323	1.486040462	0.137268419	0.274951188	up_in_case
Yang2025_S	ENSGALG00010006096	EDIL3	case	control	-0.429399707	0.186606229	-2.3011006	0.021385945	0.071105219	down_in_case
Yang2025_S	ENSGALG00010006100	RPS6KL1	case	control	-0.118561639	0.242147929	-0.489624831	0.624399402	0.758588114	down_in_case
Yang2025_S	ENSGALG00010006101	ENSGALG00010006101	case	control	0.078610272	0.222223999	0.353743395	0.723531198	0.83016939	up_in_case
Yang2025_S	ENSGALG00010006103	NXPE3	case	control	0.453544529	0.143736384	3.155391244	0.00160283	0.01012945	up_in_case
Yang2025_S	ENSGALG00010006106	SHOC1	case	control	0.593140951	0.380017434	1.560825632	0.118564898	0.247372638	up_in_case
Yang2025_S	ENSGALG00010006107	PGF	case	control	0.475648325	0.206236054	2.306329648	0.02109222	0.070535177	up_in_case
Yang2025_S	ENSGALG00010006108	MKLN1	case	control	-0.349067958	0.069591753	-5.01593856	5.27752E-07	1.40589E-05	down_in_case
Yang2025_S	ENSGALG00010006111	WBP1	case	control	0.188139267	0.244457174	0.76962056	0.441525006	0.604900879	up_in_case
Yang2025_S	ENSGALG00010006113	EIF2B2	case	control	-0.310504817	0.118422461	-2.622009497	0.0087413	0.036722945	down_in_case
Yang2025_S	ENSGALG00010006118	NPNT	case	control	-0.341669853	0.265301844	-1.287853291	0.197797037	0.35432597	down_in_case
Yang2025_S	ENSGALG00010006120	MOGS	case	control	0.708567992	0.184975408	3.830606459	0.000127828	0.001296232	up_in_case
Yang2025_S	ENSGALG00010006121	ENSGALG00010006121	case	control	0.357194672	0.292604201	1.220743486	0.222183157	0.383753383	up_in_case
Yang2025_S	ENSGALG00010006123	MLH3	case	control	0.502150668	0.117681542	4.267029992	1.98093E-05	0.000287844	up_in_case
Yang2025_S	ENSGALG00010006126	ENSGALG00010006126	case	control	0.194982605	0.27046595	0.720913686	0.470962622	0.63116087	up_in_case
Yang2025_S	ENSGALG00010006128	ACYP1	case	control	0.002028868	0.188569295	0.010759269	0.991415511	0.99462744	up_in_case
Yang2025_S	ENSGALG00010006131	ENSGALG00010006131	case	control	0.372557034	0.244670703	1.522687552	0.127836894	0.260782772	up_in_case
Yang2025_S	ENSGALG00010006132	MIPEP	case	control	-0.225958612	0.097432865	-2.31912108	0.020388472	0.068709397	down_in_case
Yang2025_S	ENSGALG00010006135	ZC2HC1C	case	control	0.069978605	0.159971617	0.43744388	0.661789467	0.786315287	up_in_case
Yang2025_S	ENSGALG00010006139	GAREM1	case	control	-0.143078907	0.178761874	-0.800388269	0.423485875	0.588714652	down_in_case
Yang2025_S	ENSGALG00010006146	CCDC142	case	control	0.654898764	0.282834789	2.315481647	0.020586588	0.06914295	up_in_case
Yang2025_S	ENSGALG00010006147	ENSGALG00010006147	case	control	-0.236731034	0.307613851	-0.769572089	0.441553767	0.604900879	down_in_case
Yang2025_S	ENSGALG00010006151	ENSGALG00010006151	case	control	0.635181554	0.225928806	2.811423502	0.004932282	0.023749601	up_in_case
Yang2025_S	ENSGALG00010006155	TNFRSF19	case	control	-0.085647694	0.107058193	-0.800010648	0.423704628	0.588783805	down_in_case
Yang2025_S	ENSGALG00010006156	NFKBIZ	case	control	0.343414686	0.29430804	1.166854584	0.243269099	0.40828073	up_in_case
Yang2025_S	ENSGALG00010006157	SNX9	case	control	0.150460922	0.099220512	1.516429612	0.129410756	0.263040705	up_in_case
Yang2025_S	ENSGALG00010006163	ENSGALG00010006163	case	control	0.474568603	0.574979069	0.825366745	0.409163409	0.574651492	up_in_case
Yang2025_S	ENSGALG00010006172	ENSGALG00010006172	case	control	0.580683583	0.137160986	4.233591494	2.29989E-05	0.000326696	up_in_case
Yang2025_S	ENSGALG00010006173	TBPL1	case	control	-0.424534932	0.069868467	-6.0762022	1.23062E-09	7.29733E-08	down_in_case
Yang2025_S	ENSGALG00010006175	IL2RA	case	control	0.18918913	0.53160465	0.355883137	0.721928085	0.8288077	up_in_case
Yang2025_S	ENSGALG00010006176	MMR1L3	case	control	-0.394925196	0.346785757	-1.138816081	0.254779873	0.421184398	down_in_case
Yang2025_S	ENSGALG00010006177	NEK9	case	control	-0.371867637	0.142761884	-2.60481037	0.009192512	0.038135652	down_in_case
Yang2025_S	ENSGALG00010006178	RPS23	case	control	-0.008182435	0.313964927	-0.026061621	0.979208189	0.987427897	down_in_case
Yang2025_S	ENSGALG00010006180	CD200L	case	control	0.970531787	0.68765864	1.411356929	0.158139396	0.303452559	up_in_case
Yang2025_S	ENSGALG00010006183	PRKN	case	control	-0.01508557	0.153378078	-0.098355453	0.921650047	0.953337584	down_in_case

Yang2025_S	ENSGALG00010006184	LBX2	case	control	0.649256166	0.269319426	2.410729056	0.015920671	0.057505067	up_in_case
Yang2025_S	ENSGALG00010006186	CHIR1AB3	case	control	-0.382236952	0.41038477	-0.931411152	0.351640924	0.520458447	down_in_case
Yang2025_S	ENSGALG00010006187	TCF21	case	control	-0.805110465	0.546305925	-1.473735553	0.140552806	0.279640685	down_in_case
Yang2025_S	ENSGALG00010006188	TMED10	case	control	-0.2748618	0.089242282	-3.079950381	0.002070351	0.012280972	down_in_case
Yang2025_S	ENSGALG00010006193	RBM17	case	control	0.409133164	0.141826569	2.88474273	0.003917336	0.019893616	up_in_case
Yang2025_S	ENSGALG00010006197	ENSGALG00010006197	case	control	-0.180389574	0.39940458	-0.451646233	0.651523858	0.779245126	down_in_case
Yang2025_S	ENSGALG00010006198	ENSGALG00010006198	case	control	-0.977593015	0.496751868	-1.967970485	0.049071434	0.131451447	down_in_case
Yang2025_S	ENSGALG00010006200	GSTCD	case	control	-0.292012186	0.15315474	-1.906648044	0.056566179	0.146188744	down_in_case
Yang2025_S	ENSGALG00010006202	SACS	case	control	0.041157584	0.13996543	0.294055355	0.768715605	0.859094711	up_in_case
Yang2025_S	ENSGALG00010006203	MMR1L4	case	control	-0.708676369	0.157720286	-4.493248063	7.0145E-06	0.000123574	down_in_case
Yang2025_S	ENSGALG00010006205	CGGBP1	case	control	-0.373650708	0.070033752	-5.335294723	9.53895E-08	3.29023E-06	down_in_case
Yang2025_S	ENSGALG00010006207	ENSGALG00010006207	case	control	0.062418111	0.411437153	0.151707522	0.879417624	0.928681389	up_in_case
Yang2025_S	ENSGALG00010006212	FOS	case	control	-0.033394911	0.314168641	-0.10629613	0.915347402	0.950120376	down_in_case
Yang2025_S	ENSGALG00010006219	CLEC14A	case	control	-0.263786916	0.286230857	-0.921587974	0.356743534	0.525999486	down_in_case
Yang2025_S	ENSGALG00010006220	SGCG	case	control	-0.202618576	0.155579209	-1.302349955	0.192796782	0.348098362	down_in_case
Yang2025_S	ENSGALG00010006223	ATG10	case	control	0.045062263	0.18633185	0.241838759	0.808905105	0.884782737	up_in_case
Yang2025_S	ENSGALG00010006227	AGPAT4	case	control	0.121119601	0.12516546	0.967675919	0.333206248	0.502405223	up_in_case
Yang2025_S	ENSGALG00010006228	ENSGALG00010006228	case	control	1.264932107	0.835032563	1.514829677	0.129815543	0.263680074	up_in_case
Yang2025_S	ENSGALG00010006229	INTS12	case	control	-0.31998154	0.152499386	-2.09824805	0.035883239	0.104697013	down_in_case
Yang2025_S	ENSGALG00010006232	ZNF654	case	control	-0.204659834	0.120432279	-1.699376912	0.089248189	0.20264139	down_in_case
Yang2025_S	ENSGALG00010006236	C3orf38	case	control	0.250465943	0.09826003	2.549011456	0.010802875	0.042925026	up_in_case
Yang2025_S	ENSGALG00010006237	ENSGALG00010006237	case	control	0.540654269	0.338044318	1.599359137	0.109740826	0.233728642	up_in_case
Yang2025_S	ENSGALG00010006239	EYA4	case	control	0.513974896	0.198368948	2.591004792	0.009569616	0.039343276	up_in_case
Yang2025_S	ENSGALG00010006241	MRC1	case	control	-0.443950415	0.197436742	-2.248570405	0.024539841	0.078956981	down_in_case
Yang2025_S	ENSGALG00010006242	ENSGALG00010006242	case	control	0.187848591	0.671228667	0.279857819	0.77958659	0.866380025	up_in_case
Yang2025_S	ENSGALG00010006244	EPHA3	case	control	-0.607621418	0.157522118	-3.857372077	0.000114613	0.001188339	down_in_case
Yang2025_S	ENSGALG00010006245	PFKFB3	case	control	-0.268088286	0.274882503	-0.975283196	0.329419804	0.498963583	down_in_case
Yang2025_S	ENSGALG00010006246	SUPT20H	case	control	-0.009139378	0.110943264	-0.082378847	0.934345457	0.959922758	down_in_case
Yang2025_S	ENSGALG00010006249	C9orf72	case	control	-0.323846445	0.194809197	-1.662377601	0.096437087	0.213849972	down_in_case
Yang2025_S	ENSGALG00010006251	SEMA3E	case	control	-0.087808785	0.38737679	-0.226675391	0.820676164	0.892958721	down_in_case
Yang2025_S	ENSGALG00010006252	COX7C	case	control	-0.969562859	0.33037784	-2.934709112	0.003338605	0.017649265	down_in_case
Yang2025_S	ENSGALG00010006254	PROS1	case	control	-0.443958595	0.12106794	-3.667020325	0.000245393	0.002209015	down_in_case
Yang2025_S	ENSGALG00010006259	PPA2	case	control	0.100082804	0.163096317	0.613642327	0.539451707	0.690457572	up_in_case
Yang2025_S	ENSGALG00010006260	MAP3K4	case	control	0.22510236	0.103296969	2.179176807	0.029318536	0.089974191	up_in_case
Yang2025_S	ENSGALG00010006261	PCLO	case	control	1.811603233	0.552339567	3.279872274	0.001038541	0.007225196	up_in_case
Yang2025_S	ENSGALG00010006263	EXOSC8	case	control	-0.789623915	0.207234475	-3.810292259	0.000138803	0.001389522	down_in_case
Yang2025_S	ENSGALG00010006266	CENPW	case	control	-0.530173487	0.162404248	-3.264529672	0.00109646	0.007537823	down_in_case
Yang2025_S	ENSGALG00010006269	ENSGALG00010006269	case	control	-1.836768849	0.962518749	-1.908294099	0.056353215	0.145692404	down_in_case
Yang2025_S	ENSGALG00010006270	STAM	case	control	-0.127954837	0.143179573	-0.893666847	0.371500169	0.539941387	down_in_case
Yang2025_S	ENSGALG00010006273	ARL13B	case	control	0.397599494	0.212888823	1.867639119	0.06181239	0.155732355	up_in_case
Yang2025_S	ENSGALG00010006277	GNG10	case	control	0.006738221	0.183909796	0.036638729	0.970773063	0.982321994	up_in_case
Yang2025_S	ENSGALG00010006279	HACD1	case	control	-0.197099645	0.092266486	-2.136199761	0.032663135	0.097632086	down_in_case
Yang2025_S	ENSGALG00010006280	VRTN	case	control	1.997282919	0.428832312	4.657491663	3.20085E-06	6.39024E-05	up_in_case
Yang2025_S	ENSGALG00010006281	TRMT11	case	control	0.119600472	0.136092576	0.878817016	0.379500498	0.547561561	up_in_case
Yang2025_S	ENSGALG00010006283	DNAJC25	case	control	-0.176587394	0.112571376	-1.568670483	0.11672474	0.244640269	down_in_case
Yang2025_S	ENSGALG00010006285	FZD8	case	control	0.121211766	0.099111314	1.22298617	0.22133492	0.382773511	up_in_case
Yang2025_S	ENSGALG00010006287	ECPAS	case	control	0.244567138	0.074393156	3.28749512	0.001010829	0.007074163	up_in_case
Yang2025_S	ENSGALG00010006288	ARF5	case	control	-0.115109914	0.30828452	-0.373388562	0.708859271	0.820223694	down_in_case
Yang2025_S	ENSGALG00010006289	SKIDA1	case	control	0.213967655	0.157708458	1.356729102	0.174867302	0.32533137	up_in_case
Yang2025_S	ENSGALG00010006291	ENSGALG00010006291	case	control	1.11438687	0.193133758	5.770026345	7.92591E-09	3.7063E-07	up_in_case
Yang2025_S	ENSGALG00010006293	SLC22A3	case	control	0.352953277	0.389114685	0.907067478	0.364371115	0.533240021	up_in_case
Yang2025_S	ENSGALG00010006300	GCC1	case	control	0.379746245	0.102163222	3.717054316	0.000201559	0.001882524	up_in_case
Yang2025_S	ENSGALG00010006301	ENSGALG00010006301	case	control	-0.086509194	0.110087241	-0.785823982	0.431970615	0.596029566	down_in_case
Yang2025_S	ENSGALG00010006310	ALG5	case	control	0.607743262	0.114928455	5.288013863	1.23652E-07	4.08881E-06	up_in_case
Yang2025_S	ENSGALG00010006311	ABCD4	case	control	0.381531082	0.148459294	2.569937334	0.010171691	0.041025331	up_in_case
Yang2025_S	ENSGALG00010006323	ANAPC10	case	control	-0.012925246	0.126024477	-0.102561397	0.918311082	0.951977476	down_in_case
Yang2025_S	ENSGALG00010006327	DENND6B	case	control	0.130164637	0.078494743	1.658259288	0.097265143	0.214715409	up_in_case
Yang2025_S	ENSGALG00010006328	SYNDIG1L	case	control	0.45588861	0.261415268	1.743924958	0.081172176	0.189216171	up_in_case
Yang2025_S	ENSGALG00010006330	STX19	case	control	0.377792874	0.454086025	0.831985248	0.405417271	0.571694968	up_in_case
Yang2025_S	ENSGALG00010006331	SMAD9	case	control	0.548793021	0.230546622	2.380399317	0.017293886	0.061025653	up_in_case
Yang2025_S	ENSGALG00010006341	HHIP	case	control	0.106134986	0.172512863	0.615229401	0.538403234	0.6897085	up_in_case
Yang2025_S	ENSGALG00010006342	RFXAP	case	control	-0.869917189	0.131662015	-6.607199412	3.91658E-11	3.63851E-09	down_in_case
Yang2025_S	ENSGALG00010006343	SEC23A	case	control	-0.244442268	0.059619063	-4.100068973	4.13027E-05	0.00052803	down_in_case
Yang2025_S	ENSGALG00010006344	HINT3	case	control	-0.288576379	0.129417453	-2.229810381	0.025760034	0.081750415	down_in_case
Yang2025_S	ENSGALG00010006345	SMC2	case	control	0.550663049	0.211117156	2.608329224	0.00909854	0.037915118	up_in_case
Yang2025_S	ENSGALG00010006346	NSUN3	case	control	-0.203561671	0.249472929	-0.815966972	0.414519026	0.579996748	down_in_case
Yang2025_S	ENSGALG00010006347	PPP6R2	case	control	0.223650878	0.121972112	1.833623064	0.066709966	0.164269902	up_in_case
Yang2025_S	ENSGALG00010006351	IGF2R	case	control	0.008604404	0.107927323	0.079724055	0.936456727	0.961149333	up_in_case
Yang2025_S	ENSGALG00010006352	GEMIN2	case	control	-0.179378011	0.164212572	-1.092352487	0.274678198	0.442092942	down_in_case
Yang2025_S	ENSGALG00010006353	ABCE1	case	control	-0.318849424	0.116253907	-2.742698565	0.00609366	0.027759119	down_in_case
Yang2025_S	ENSGALG00010006355	PTGR1	case	control	-0.124333585	0.104430791	-1.190583572	0.233817104	0.396959228	down_in_case
Yang2025_S	ENSGALG00010006359	SGK1	case	control	0.026463396	0.117309801	0.225585551	0.82152378	0.893392686	up_in_case
Yang2025_S	ENSGALG00010006361	SERTM1	case	control	-0.817045348	0.160379841	-5.094439203	3.49775E-07	9.76777E-06	down_in_case
Yang2025_S	ENSGALG00010006363	TRAPPC6A	case	control	0.334825845	0.151067825	2.216394166	0.026664519	0.083853276	up_in_case
Yang2025_S	ENSGALG00010006372	SBF1	case	control	0.130331339	0.070933199	1.837381372	0.066153615	0.163188285	up_in_case
Yang2025_S	ENSGALG00010006378	CAAP1	case	control	0.386692863	0.194207454	1.991132963	0.046466271	0.126466307	up_in_case
Yang2025_S	ENSGALG00010006381	SPART	case	control	-0.135245844	0.110593674	-1.222907602	0.221364597	0.382773511	down_in_case
Yang2025_S	ENSGALG00010006382	PNN	case	control	1.510342046	0.250873335	6.020337105	1.74054E-09	9.94035E-08	up_in_case
Yang2025_S	ENSGALG00010006387	OTUD4	case	control	0.101582989	0.074752415	1.358925863	0.174170083	0.324386541	up_in_case
Yang2025_S	ENSGALG00010006404	SMAD1	case	control	-0.085839888	0.151620106	-0.566151091	0.571291075	0.715397298	down_in_case
Yang2025_S	ENSGALG00010006408	ENSGALG00010006408	case	control	0.295886101	0.550087835	0.537888828	0.590653802	0.731363136	up_in_case
Yang2025_S	ENSGALG00010006411	RGS17	case	control	0.035731326	0.484038326	0.073819208	0.941154243	0.963855699	up_in_case
Yang2025_S	ENSGALG00010006421	LMF2	case	control	-0.124749943	0.10138819	-1.230418881	0.218540286	0.37962589	down_in_case
Yang2025_S	ENSGALG00010006434	MMAA	case	control	0.369295053	0.118318277	3.121200391	0.001801154	0.011065404	up_in_case
Yang2025_S	ENSGALG00010006446	LSAMP	case	control	-0.238741102	0.174957942	-1.364562816	0.172390498	0.321932672	down_in_case
Yang2025_S	ENSGALG00010006448	MAS1	case	control	-0.136627467	0.488493694	-0.279691363	0.779714305	0.866452858	down_in_case
Yang2025_S	ENSGALG00010006454	SOD2	case	control	0.236347075	0.172880943	1.367108897	0.171591175	0.320757248	up_in_case
Yang2025_S	ENSGALG00010006457	NCAPH2	case	control	0.649995049	0.175952679	3.69414692	0.000220626	0.00201734	up_in_case
Yang2025_S	ENSGALG00010006463	SCO2	case	control	0.07018058	0.194537287	0.360756447	0.718281523	0.826459667	up_in_case
Yang2025_S	ENSGALG00010006465	PLAA	case	control	0.456843466	0.160330739	2.849381659	0.00438043	0.02161033	up_in_case
Yang2025_S	ENSGALG00010006472	DCLK1	case	control	-0.123606886	0.178771993	-0.691421986	0.489300391	0.646661699	down_in_case
Yang2025_S	ENSGALG00010006478	ENSGALG00010006478	case	control	0.171037518	0.186604246	0.916578916	0.359363337	0.528174132	up_in_case
Yang2025_S	ENSGALG00010006480	IFT74	case	control	0.879242909	0.222919469	3.944217669	8.00609E-05	0.000895384	up_in_case
Yang2025_S	ENSGALG00010006486	CEP43	case	control	-0.1490012	0.114694862	-1.29910963	0.193906309	0.349377348	down_in_case
Yang2025_S	ENSGALG00010006492	GAP43	case	control	0.572380259	0.49854189	1.148108656	0.250923711	0.416666144	up_in_case
Yang2025_S	ENSGALG00010006496	UBE2H	case	control	-0.331363032	0.084991573	-3.898775153	9.66805E-05	0.001039539	down_in_case
Yang2025_S	ENSGALG00010006506	ZNF827	case	control	0.634742998	0.204364608	3.105934064	0.00189679	0.011527156	up_in_case
Yang2025_S	ENSGALG00010006515	FBXO33	case	control	-0.205779953	0.113270944	-1.816705553	0.069262211	0.168757837	down_in_case
Yang2025_S	ENSGALG00010006516	NBEA	case	control	0.284261554	0.182868943	1.554455064	0.120075907	0.249707977	up_in_case
Yang2025_S	ENSGALG00010006517	ZBTB20	case	control	0.564506191	0.341844564	1.651353425	0.098666429	0.21700074	up_in_case
Yang2025_S	ENSGALG00010006520	ENSGALG00010006520	case	control	0.558854232	0.56962029	0.981099588	0.326543633	0.496491259	up_in_case
Yang2025_S	ENSGALG00010006521	ZNF410	case	control	-0.68801252	0.162340248	-4.238089634	2.2543E-05	0.000320875	down_in_case
Yang2025_S	ENSGALG00010006524	TAB2	case	control	0.3826802	0.218849244	1.748601883	0.080359861	0.187892871	up_in_case
Yang2025_S	ENSGALG00010006526	LSM6	case	control	0.170958256	0.227229577	0.75235917	0.451835093	0.613616804	up_in_case
Yang2025_S	ENSGALG00010006528	ZC3HC1	case	control	-0.206492871	0.171324447	-1.205273824	0.228097611	0.390244348	down_in_case
Yang2025_S	ENSGALG00010006531	FAM161B	case	control	0.240756201	0.151392196	1.590281456	0.111771377	0.2371759	up_in_case
Yang2025_S	ENSGALG00010006532	KLHDC10	case	control	-0.017593667	0.059520052	-0.295592267	0.76754148	0.858737931	down_in_case
Yang2025_S	ENSGALG00010006537	DRD3	case	control	0.354470305	0.245325028	1.444900704	0.148485778	0.290651682	up_in_case
Yang2025_S	ENSGALG00010006538	TEK	case	control	-0.019215557	0.13053554	-0.147205558	0.882969774	0.931020944	down_in_case
Yang2025_S	ENSGALG00010006542	RFC3	case	control	-0.468331364	0.125545802	-3.730362586	0.000191204	0.001799587	down_in_case
Yang2025_S	ENSGALG00010006544	MOB3B	case	control	-0.245771766	0.244376507	-1.005709466	0.314555348	0.483757728	down_in_case
Yang2025_S	ENSGALG00010006546	SMOC2	case	control	-0.248125856	0.167315814	-1.482979101	0.138079963	0.275884866	down_in_case
Yang2025_S	ENSGALG00010006548	TMEM209	case	control	-0.164610284	0.110033982	-1.495994976	0.134654965	0.270962734	down_in_case
Yang2025_S	ENSGALG00010006549	ZDHHC23	case	control	0.56804402	0.178073141	3.189977776	0.001422985	0.009244427	up_in_case
Yang2025_S	ENSGALG00010006557	COQ6	case	control	-0.153686732	0.188945043	-0.813393826	0.415992298	0.581409577	down_in_case
Yang2025_S	ENSGALG00010006558	USP10	case	control	0.057620897	0.071241803	0.808807392	0.418625944	0.584264877	up_in_case
Yang2025_S	ENSGALG00010006564	SLC10A7	case	control	-0.424757416	0.079992855	-5.30994194	1.0966E-07	3.71804E-06	down_in_case
Yang2025_S	ENSGALG00010006568	ENTPD5	case	control	0.041842357	0.127081008	0.329257359	0.741961172	0.842162493	up_in_case
Yang2025_S	ENSGALG00010006570	ENSGALG00010006570	case	control	-0.727214173	0.403389987	-1.802757123	0.071426367	0.172394495	down_in_case
Yang2025_S	ENSGALG00010006575	SSBP2	case	control	-0.080335964	0.154970623	-0.518394792	0.604182847	0.742376155	down_in_case
Yang2025_S	ENSGALG00010006576	CCDC191	case	control	0.334192062	0.217468445	1.536738182	0.124357437	0.25578168	up_in_case
Yang2025_S	ENSGALG00010006580	ALDH6A1	case	control	-0.435572604	0.084218134	-5.171957449	2.31654E-07	6.97215E-06	down_in_case
Yang2025_S	ENSGALG00010006581	CEP41	case	control	0.206251118	0.178586269	1.154910276	0.248127171	0.413842265	up_in_case
Yang2025_S	ENSGALG00010006585	TTC29	case	control	0.361014138	0.218267524	1.653998416	0.098127835	0.216157347	up_in_case
Yang2025_S	ENSGALG00010006592	STARD13	case	control	0.045079681	0.09063708	0.497364663	0.61893192	0.754737155	up_in_case
Yang2025_S	ENSGALG00010006598	ENSGALG00010006598	case	control	0.981972492	0.240792963	4.078078025	4.54095E-05	0.000565994	up_in_case

Yang2025_S	ENSGALG00010006602	EDNRA	case	control	-0.173108545	0.088894347	-1.947351549	0.051492603	0.136105731	down_in_case
Yang2025_S	ENSGALG00010006603	MEST	case	control	-0.436505472	0.149970973	-2.910599709	0.003607358	0.018701094	down_in_case
Yang2025_S	ENSGALG00010006606	SULT	case	control	-2.916009394	1.09568232	-2.661363921			down_in_case
Yang2025_S	ENSGALG00010006608	KL	case	control	0.389284494	0.306928355	1.268323659	0.204682392	0.362835636	up_in_case
Yang2025_S	ENSGALG00010006614	TMEM184C	case	control	-0.086249055	0.078033282	-1.10528549	0.269035903	0.436202327	down_in_case
Yang2025_S	ENSGALG00010006615	GRAMD1C	case	control	0.055627281	0.103874942	0.535521657	0.592289186	0.732671976	up_in_case
Yang2025_S	ENSGALG00010006616	COPG2	case	control	-0.175350722	0.137025684	-1.279692371	0.200653349	0.358337103	down_in_case
Yang2025_S	ENSGALG00010006619	LIN52	case	control	-0.59497376	0.086412531	-6.885271727	5.76773E-12	6.48172E-10	down_in_case
Yang2025_S	ENSGALG00010006621	DACT2	case	control	-0.211507111	0.169100106	-1.250780474	0.211014581	0.370945904	down_in_case
Yang2025_S	ENSGALG00010006623	ENSGALG00010006623	case	control	0.89658321	0.289815525	3.09363417	0.001977211	0.011872499	up_in_case
Yang2025_S	ENSGALG00010006627	SF3B5	case	control	-0.080818558	0.152730362	-0.529158428	0.596695553	0.735984087	down_in_case
Yang2025_S	ENSGALG00010006633	FBXO18	case	control	-0.107215725	0.104825201	-1.02280486	0.306400119	0.476093797	down_in_case
Yang2025_S	ENSGALG00010006634	PRMT9	case	control	-0.274556078	0.159793066	-1.718197692	0.085760562	0.19684952	down_in_case
Yang2025_S	ENSGALG00010006635	ENSGALG00010006635	case	control	-0.779623517	0.459847843	-1.695394529	0.090000603	0.203696346	down_in_case
Yang2025_S	ENSGALG00010006641	ENSGALG00010006641	case	control	1.399611086	0.486697896	2.875728655	0.004030961	0.020278497	up_in_case
Yang2025_S	ENSGALG00010006651	ENSGALG00010006651	case	control	0.318115187	0.188169247	1.690580112	0.090917026	0.205270352	up_in_case
Yang2025_S	ENSGALG00010006653	CFL2	case	control	0.053342259	0.134985637	0.395169891	0.692717512	0.80892712	up_in_case
Yang2025_S	ENSGALG00010006657	PDS5B	case	control	0.122894048	0.179934382	0.682993694	0.494610827	0.651987937	up_in_case
Yang2025_S	ENSGALG00010006670	ANKRD16	case	control	0.330707109	0.216579231	1.526956707	0.126771777	0.259407447	up_in_case
Yang2025_S	ENSGALG00010006673	BAZ1A	case	control	0.397645689	0.138296905	2.875304335	0.004036382	0.020283804	up_in_case
Yang2025_S	ENSGALG00010006678	ENSGALG00010006678	case	control	1.016183813	0.734803365	1.382932988	0.166685414	0.314529759	up_in_case
Yang2025_S	ENSGALG00010006681	CRISPLD2	case	control	0.351764471	0.317515998	1.107863772	0.267920654	0.435035459	up_in_case
Yang2025_S	ENSGALG00010006682	ARHGAP10	case	control	0.197098105	0.140539942	1.402434789	0.160785451	0.306485172	up_in_case
Yang2025_S	ENSGALG00010006685	ENSGALG00010006685	case	control	0.098073497	0.180389323	0.543676841	0.586663872	0.728364313	up_in_case
Yang2025_S	ENSGALG00010006701	LINGO2	case	control	1.395790089	0.636770648	2.191982458			up_in_case
Yang2025_S	ENSGALG00010006715	IRF8	case	control	-0.327193399	0.182056539	-1.79720762	0.072302665	0.173833271	down_in_case
Yang2025_S	ENSGALG00010006716	ENSGALG00010006716	case	control	0.105129889	0.106207915	0.989849857	0.322247512	0.491626787	up_in_case
Yang2025_S	ENSGALG00010006720	SRP54	case	control	-0.078607152	0.080181007	-0.980371229	0.326902906	0.496633126	down_in_case
Yang2025_S	ENSGALG00010006728	ENSGALG00010006728	case	control	-0.147303764	0.110784283	-1.329644965	0.183635276	0.336970973	down_in_case
Yang2025_S	ENSGALG00010006730	HAPLN1	case	control	2.468121221	0.837176827	2.948148041	0.003196839	0.017097578	up_in_case
Yang2025_S	ENSGALG00010006735	NR3C2	case	control	-0.092436507	0.142324086	-0.649479016	0.516028805	0.67047052	down_in_case
Yang2025_S	ENSGALG00010006739	VCAN	case	control	0.051305264	0.169635111	0.302444841	0.762312976	0.855300428	up_in_case
Yang2025_S	ENSGALG00010006740	BRCA2	case	control	0.30611462	0.156209905	1.959636425	0.050038301	0.13329836	up_in_case
Yang2025_S	ENSGALG00010006748	FAM177A1	case	control	-0.358418016	0.127189487	-2.817984605	0.004832613	0.023380334	down_in_case
Yang2025_S	ENSGALG00010006760	TASOR2	case	control	-0.332614791	0.086798789	-3.832021107	0.000127095	0.001290865	down_in_case
Yang2025_S	ENSGALG00010006761	PPP2R3C	case	control	-0.312360173	0.144843032	-2.156542634	0.031041318	0.093962617	down_in_case
Yang2025_S	ENSGALG00010006767	ENSGALG00010006767	case	control	-0.443345249	0.306635112	-1.4458398	0.14822214	0.290298738	down_in_case
Yang2025_S	ENSGALG00010006794	PERPA	case	control	-0.701957946	0.804243573	-0.8728176	0.382762505	0.550500104	down_in_case
Yang2025_S	ENSGALG00010006803	COX4I1	case	control	0.005917121	0.261524925	0.022625456	0.981949038	0.98883219	up_in_case
Yang2025_S	ENSGALG00010006804	PSMA6	case	control	-0.234232299	0.226988077	-1.031914549	0.30211215	0.471677263	down_in_case
Yang2025_S	ENSGALG00010006816	ENSGALG00010006816	case	control	0.352729518	0.313744851	1.124255958	0.260904456	0.427578925	up_in_case
Yang2025_S	ENSGALG00010006819	EMC8	case	control	-0.407916163	0.178823531	-2.281110099	0.022541933	0.073904015	down_in_case
Yang2025_S	ENSGALG00010006821	XRCC4	case	control	0.483494338	0.227091361	2.129074116	0.033248129	0.098787351	up_in_case
Yang2025_S	ENSGALG00010006828	GTF2A1	case	control	0.038026265	0.159995763	0.23767045	0.812136705	0.887130593	up_in_case
Yang2025_S	ENSGALG00010006833	LTV1	case	control	-0.054644656	0.102636428	-0.532409955	0.594442092	0.734617821	down_in_case
Yang2025_S	ENSGALG00010006835	FRY	case	control	0.066673788	0.200800263	0.332040344	0.739858795	0.840734919	up_in_case
Yang2025_S	ENSGALG00010006838	ASB13	case	control	-0.060979367	0.147558677	-0.413255042	0.679419765	0.798841721	down_in_case
Yang2025_S	ENSGALG00010006840	ENSGALG00010006840	case	control	-0.151019721	0.242780692	-0.622041725	0.533914432	0.685508673	down_in_case
Yang2025_S	ENSGALG00010006843	STON2	case	control	0.057223388	0.172094563	0.332511309	0.739503202	0.840536469	up_in_case
Yang2025_S	ENSGALG00010006845	CALML3	case	control	0.67865929	1.146186989	0.592101722	0.553782474	0.70174227	up_in_case
Yang2025_S	ENSGALG00010006847	SEL1L	case	control	-0.087614246	0.095912487	-0.91348112	0.360989559	0.529570429	down_in_case
Yang2025_S	ENSGALG00010006850	IRF2BPL	case	control	0.073309774	0.104391127	0.702260585	0.482516669	0.640795748	up_in_case
Yang2025_S	ENSGALG00010006851	TMEM167A	case	control	-0.550791083	0.125225543	-4.398392451	1.09056E-05	0.000177609	down_in_case
Yang2025_S	ENSGALG00010006853	NET1	case	control	0.038550079	0.096640486	0.398901956	0.689965448	0.806464395	up_in_case
Yang2025_S	ENSGALG00010006855	DCLK2	case	control	0.247229493	0.126354324	1.956636579	0.050390211	0.133954138	up_in_case
Yang2025_S	ENSGALG00010006865	EFCAB2	case	control	0.438481939	0.390196738	1.123745785	0.261120887	0.427883298	up_in_case
Yang2025_S	ENSGALG00010006877	GSE1	case	control	0.612138718	0.208830208	2.931274769	0.003375741	0.017798314	up_in_case
Yang2025_S	ENSGALG00010006883	LRBA	case	control	-0.182304579	0.141851657	-1.285177648	0.198730213	0.355614291	down_in_case
Yang2025_S	ENSGALG00010006885	RHOU	case	control	-0.212298676	0.123207857	-1.723093652	0.084871591	0.195452919	down_in_case
Yang2025_S	ENSGALG00010006901	TUBAL3	case	control	0.044425667	0.124147423	0.357846069	0.720458512	0.827802553	up_in_case
Yang2025_S	ENSGALG00010006903	B3GLCT	case	control	-0.795378322	0.09634241	-8.255744482	1.50958E-16	4.57305E-14	down_in_case
Yang2025_S	ENSGALG00010006912	ENSGALG00010006912	case	control	0.209855074	0.20319766	1.032763245	0.301714708	0.471339925	up_in_case
Yang2025_S	ENSGALG00010006913	HSPH1	case	control	0.179489962	0.174951694	1.02594012	0.30491981	0.474542948	up_in_case
Yang2025_S	ENSGALG00010006922	SMYD3	case	control	-0.386918317	0.159364225	-2.427886925	0.015187077	0.05557561	down_in_case
Yang2025_S	ENSGALG00010006923	ENSGALG00010006923	case	control	0.563183264	0.275867952	2.041495792	0.041201572	0.115684848	up_in_case
Yang2025_S	ENSGALG00010006926	ID4	case	control	0.661255698	0.309528293	2.136333619	0.032652231	0.097620432	up_in_case
Yang2025_S	ENSGALG00010006927	UCN3	case	control	0.14975471	0.666880322	0.224560097	0.82232151	0.893806762	up_in_case
Yang2025_S	ENSGALG00010006939	FBXO31	case	control	-0.124438444	0.119111292	-1.044724154	0.296150478	0.464788479	down_in_case
Yang2025_S	ENSGALG00010006942	ALOX5AP	case	control	-0.498267104	0.208234396	-2.392818443	0.016719512	0.059496017	down_in_case
Yang2025_S	ENSGALG00010006944	MAB21L2	case	control	1.180838988	0.608262323	1.941331797	0.052218045	0.137704076	up_in_case
Yang2025_S	ENSGALG00010006946	PLPPR1	case	control	0.53839876	0.375849993	1.432483092	0.152005614	0.295507565	up_in_case
Yang2025_S	ENSGALG00010006948	ENSGALG00010006948	case	control	1.355931517	0.288483238	4.700209011	2.59895E-06	5.41352E-05	up_in_case
Yang2025_S	ENSGALG00010006953	MAP1LC3B	case	control	-0.258531178	0.113041419	-2.287048232	0.022193008	0.072950644	down_in_case
Yang2025_S	ENSGALG00010006957	PALM2AKAP2	case	control	0.067773933	0.133931584	0.506033985	0.612832792	0.749304637	up_in_case
Yang2025_S	ENSGALG00010006958	KIF26B	case	control	0.06406469	0.341162708	0.18778339	0.851046457	0.911625212	up_in_case
Yang2025_S	ENSGALG00010006961	ZCCHC14	case	control	0.256970736	0.222105827	1.156974309	0.247282857	0.412977781	up_in_case
Yang2025_S	ENSGALG00010006963	ENSGALG00010006963	case	control	-0.607870573	0.692146768	-0.878239415	0.379813806	0.547738649	down_in_case
Yang2025_S	ENSGALG00010006966	PGBD5	case	control	-0.274111189	0.328557372	-0.834287137	0.404119198	0.570373994	down_in_case
Yang2025_S	ENSGALG00010006983	GTPBP2	case	control	-0.318503519	0.178900658	-1.780337328	0.075020772	0.178825599	down_in_case
Yang2025_S	ENSGALG00010006988	USPL1	case	control	0.422526744	0.117775743	3.587553213	0.000333796	0.002857151	up_in_case
Yang2025_S	ENSGALG00010006992	SLC35B4	case	control	-0.393971456	0.101569225	-3.878846722	0.000104953	0.001106292	down_in_case
Yang2025_S	ENSGALG00010006998	POLH	case	control	0.286467675	0.12019845	2.38328926	0.017158706	0.060610029	up_in_case
Yang2025_S	ENSGALG00010007000	LRGUK	case	control	2.043834274	0.567406721	3.602062151	0.000315703	0.002721721	up_in_case
Yang2025_S	ENSGALG00010007001	SNX30	case	control	0.484770942	0.112999335	4.290033596	1.78646E-05	0.000265098	up_in_case
Yang2025_S	ENSGALG00010007002	EXOC4	case	control	-0.01625638	0.123195723	-0.131955715	0.89501932	0.938174682	down_in_case
Yang2025_S	ENSGALG00010007007	ACSL1	case	control	0.015660184	0.226667791	0.069088703	0.944919013	0.966047793	up_in_case
Yang2025_S	ENSGALG00010007010	ENSGALG00010007010	case	control	2.133719202	0.986557861	2.162791749	0.030557201	0.092951816	up_in_case
Yang2025_S	ENSGALG00010007011	HMGB1	case	control	-0.277840424	0.087883358	-3.161467996	0.001569761	0.009960977	down_in_case
Yang2025_S	ENSGALG00010007014	ENSGALG00010007014	case	control	1.57403071	0.482575534	3.261729202	0.001107349	0.007605177	up_in_case
Yang2025_S	ENSGALG00010007017	SRD5A2	case	control	0.084389407	0.505066443	0.167085752	0.867302577	0.922021773	up_in_case
Yang2025_S	ENSGALG00010007021	SULT6B1	case	control	-0.499308568	0.32434574	-1.539433099	0.123698599	0.254915701	down_in_case
Yang2025_S	ENSGALG00010007028	KATNAL1	case	control	-0.435274794	0.179350152	-2.426955254	0.015226134	0.055645469	down_in_case
Yang2025_S	ENSGALG00010007033	CEBPZOS	case	control	-0.657506359	0.185228182	-3.54971015	0.000385656	0.003206509	down_in_case
Yang2025_S	ENSGALG00010007038	CENPU	case	control	-0.146597048	0.142592334	-1.028085058	0.303909818	0.473499924	down_in_case
Yang2025_S	ENSGALG00010007041	NAE1	case	control	-0.444129409	0.123881896	-3.585103403	0.000336945	0.002878801	down_in_case
Yang2025_S	ENSGALG00010007051	E2F3	case	control	0.003719139	0.197604361	0.018821137	0.984983792	0.991028819	up_in_case
Yang2025_S	ENSGALG00010007066	INIP	case	control	-0.121289592	0.221096938	-0.548581057	0.583292989	0.725536713	down_in_case
Yang2025_S	ENSGALG00010007067	CASP3	case	control	-0.406164833	0.100020889	-4.060800067	4.89048E-05	0.000599375	down_in_case
Yang2025_S	ENSGALG00010007068	NCAM2	case	control	1.153805858	0.402266149	2.868264858	0.004127299	0.020636493	up_in_case
Yang2025_S	ENSGALG00010007070	UBL3	case	control	-0.230314958	0.073481075	-3.134343901	0.001722389	0.010700618	down_in_case
Yang2025_S	ENSGALG00010007085	PRIMPOL	case	control	-0.25301959	0.140354281	-1.802720862	0.071432064	0.172394495	down_in_case
Yang2025_S	ENSGALG00010007090	CEBPZ	case	control	0.275342244	0.10446181	2.635817286	0.008393491	0.035572781	up_in_case
Yang2025_S	ENSGALG00010007092	DYNC1LI2	case	control	0.138018721	0.118460975	1.165098645	0.243979065	0.409242106	up_in_case
Yang2025_S	ENSGALG00010007097	ZCCHC9	case	control	-0.116041882	0.243330086	-0.476890809	0.633439877	0.764858952	down_in_case
Yang2025_S	ENSGALG00010007098	NDUFAF7	case	control	0.201310418	0.128620438	1.565151081	0.117547489	0.245869148	up_in_case
Yang2025_S	ENSGALG00010007102	CMTM3	case	control	-0.482049742	0.08822013	-5.464169505	4.65079E-08	1.75633E-06	down_in_case
Yang2025_S	ENSGALG00010007106	SLC7A1	case	control	-0.486640008	0.11707946	-4.156493441	3.23169E-05	0.0004326	down_in_case
Yang2025_S	ENSGALG00010007108	MBOAT1	case	control	0.033899745	0.168464489	0.201227839	0.840520424	0.905354913	up_in_case
Yang2025_S	ENSGALG00010007110	ARL6	case	control	-0.29047189	0.111283421	-2.610199145	0.009048953	0.037764947	down_in_case
Yang2025_S	ENSGALG00010007114	CKLF	case	control	-0.280057305	0.221502817	-1.264350986	0.206104077	0.364639678	down_in_case
Yang2025_S	ENSGALG00010007115	ENSGALG00010007115	case	control	0.524972483	0.356833428	1.471197598	0.141237687	0.280522686	up_in_case
Yang2025_S	ENSGALG00010007118	ENSGALG00010007118	case	control	-0.014506599	0.19239544	-0.075399911	0.93989653	0.96346547	down_in_case
Yang2025_S	ENSGALG00010007123	ENSGALG00010007123	case	control	0.500005901	0.268355951	1.863218982	0.062431463	0.156753593	up_in_case
Yang2025_S	ENSGALG00010007126	ENSGALG00010007126	case	control	1.255430741	0.939749491	1.335920639	0.181575214	0.334158823	up_in_case
Yang2025_S	ENSGALG00010007130	IRF2	case	control	-0.086155161	0.113512251	-0.75899438	0.447855918	0.610420332	down_in_case
Yang2025_S	ENSGALG00010007133	BLVRA	case	control	-0.235687607	0.233681517	-1.008584717	0.31317384	0.482591779	down_in_case
Yang2025_S	ENSGALG00010007135	TK2	case	control	-0.280290226	0.094996919	-2.950519116	0.003172404	0.017029065	down_in_case
Yang2025_S	ENSGALG00010007137	ENSGALG00010007137	case	control	0.154904514	0.412286099	0.375720923	0.707124381	0.819577331	up_in_case
Yang2025_S	ENSGALG00010007141	PRKD3	case	control	-0.110749267	0.093144485	-1.189005093	0.234437663	0.397625223	down_in_case
Yang2025_S	ENSGALG00010007143	CKMT2	case	control	0.002996858	0.360173086	0.008320604	0.993361195	0.995719195	up_in_case
Yang2025_S	ENSGALG00010007146	ENSGALG00010007146	case	control	-0.29809167	0.104807656	-2.844178402	0.004452611	0.021878397	down_in_case
Yang2025_S	ENSGALG00010007160	ENSGALG00010007160	case	control	0.344385534	0.256294346	1.343711006	0.179041857	0.330719549	up_in_case
Yang2025_S	ENSGALG00010007162	ENSGALG00010007162	case	control	-0.149041372	0.220377272	-0.676301015	0.498849549	0.655737049	down_in_case
Yang2025_S	ENSGALG00010007165	ENSGALG00010007165	case	control	-0.354671577	0.254958538	-1.391095117	0.164196585	0.311218637	down_in_case
Yang2025_S	ENSGALG00010007166	RASGRF2	case	control	0.511075288	0.259569978	1.968930665	0.048961054	0.131206208	up_in_case
Yang2025_S	ENSGALG00010007168	MINA	case	control	0.432451907	0.149660052	2.889561379	0.003857797	0.019662911	up_in_case

Yang2025_S	ENSGALG00010007169	QPCT	case	control	0.028810265	0.120403567	0.239280828	0.810887831	0.886194088	up_in_case
Yang2025_S	ENSGALG00010007175	FOXC2	case	control	1.59626124	0.459103631	3.476908332	0.000507231	0.004002416	up_in_case
Yang2025_S	ENSGALG00010007182	ENSGALG00010007182	case	control	-0.368147306	0.32667505	-1.12695263	0.25976251	0.426264927	down_in_case
Yang2025_S	ENSGALG00010007183	VOPP1	case	control	0.578907541	0.45592845	1.269733312	0.204179643	0.362359057	up_in_case
Yang2025_S	ENSGALG00010007184	VGLL3	case	control	0.14657101	0.188809616	0.77628996	0.4375778	0.601464454	up_in_case
Yang2025_S	ENSGALG00010007187	ENPP6	case	control	-0.381978968	0.16412224	-2.32740528	0.019943705	0.06761935	down_in_case
Yang2025_S	ENSGALG00010007188	MAB21L1	case	control	-0.147054348	0.189306529	-0.776805473	0.437273547	0.601224162	down_in_case
Yang2025_S	ENSGALG00010007190	ENSGALG00010007190	case	control	0.31495446	0.241305609	1.30520986	0.191821398	0.347056762	up_in_case
Yang2025_S	ENSGALG00010007201	ENSGALG00010007201	case	control	0.105815064	0.400018133	0.264525669	0.791374888	0.873985268	up_in_case
Yang2025_S	ENSGALG00010007203	SLC30A6	case	control	-0.162045554	0.102868515	-1.57526872	0.115194431	0.242445914	down_in_case
Yang2025_S	ENSGALG00010007204	LANCL2	case	control	-0.103810539	0.169755268	-0.61153059	0.540848373	0.691378964	down_in_case
Yang2025_S	ENSGALG00010007207	EPM2A	case	control	-0.074628856	0.09769742	-0.763877453	0.444940275	0.608117597	down_in_case
Yang2025_S	ENSGALG00010007214	MTHFSD	case	control	0.457649521	0.158161871	2.893551502	0.003809118	0.019478922	up_in_case
Yang2025_S	ENSGALG00010007217	MSH3	case	control	0.217205127	0.158218801	1.372814897	0.169809899	0.318650813	up_in_case
Yang2025_S	ENSGALG00010007223	FOXF1	case	control	0.410789224	0.406964635	1.009397841	0.312783874	0.482247323	up_in_case
Yang2025_S	ENSGALG00010007224	DHFR	case	control	0.274562257	0.230822504	1.189495186	0.234244864	0.397443344	up_in_case
Yang2025_S	ENSGALG00010007226	ENSGALG00010007226	case	control	-2.541796703	1.164393373	-2.182936422			down_in_case
Yang2025_S	ENSGALG00010007230	ENSGALG00010007230	case	control	-0.111974897	0.361284015	-0.309935929	0.75660968	0.852119509	down_in_case
Yang2025_S	ENSGALG00010007243	BEAN1	case	control	0.57439531	0.210441128	2.729482185	0.006343388	0.028662486	up_in_case
Yang2025_S	ENSGALG00010007251	SPAST	case	control	-0.50614523	0.062436625	-8.106543697	5.208E-16	1.39564E-13	down_in_case
Yang2025_S	ENSGALG00010007252	NFATC1	case	control	0.412615411	0.225251475	1.831798927	0.066981383	0.164734482	up_in_case
Yang2025_S	ENSGALG00010007259	ANKRD34B	case	control	0.038333821	0.155064557	0.247212013	0.804744149	0.882663441	up_in_case
Yang2025_S	ENSGALG00010007265	CD200R1A	case	control	-0.747670345	0.262566818	-2.847543155	0.004405812	0.021679022	down_in_case
Yang2025_S	ENSGALG00010007270	CDH5	case	control	-0.507330519	0.100230881	-5.061618888	4.15711E-07	1.14485E-05	down_in_case
Yang2025_S	ENSGALG00010007271	CADM2	case	control	0.845723064	0.43421943	1.947685905	0.051452558	0.136051497	up_in_case
Yang2025_S	ENSGALG00010007275	DPY30	case	control	0.173736942	0.171889041	1.01075055	0.312135836	0.481746511	up_in_case
Yang2025_S	ENSGALG00010007277	FAM151B	case	control	0.716849563	0.321438548	2.230129424	0.025738852	0.0817018	up_in_case
Yang2025_S	ENSGALG00010007283	STOX2	case	control	0.238607748	0.200572676	1.189632369	0.234190918	0.397418426	up_in_case
Yang2025_S	ENSGALG00010007284	ENSGALG00010007284	case	control	0.30943064	0.132386492	2.337327893	0.019422141	0.066364362	up_in_case
Yang2025_S	ENSGALG00010007287	ENSGALG00010007287	case	control	1.59918953	0.796520409	2.007719466	0.044673111	0.123149318	up_in_case
Yang2025_S	ENSGALG00010007294	TRAPPC11	case	control	-0.331810782	0.105641774	-3.140905065	0.001684266	0.010520058	down_in_case
Yang2025_S	ENSGALG00010007300	CCDC125	case	control	0.878905278	0.338762664	2.594457332	0.009474037	0.039024744	up_in_case
Yang2025_S	ENSGALG00010007302	CDH11	case	control	0.147455413	0.146121148	1.009131224	0.312911705	0.482294504	up_in_case
Yang2025_S	ENSGALG00010007304	F5	case	control	0.259480398	0.222156493	1.168007267	0.242803834	0.407745411	up_in_case
Yang2025_S	ENSGALG00010007318	RMDN2	case	control	0.345815784	0.160090256	2.160130114	0.030762598	0.093434352	up_in_case
Yang2025_S	ENSGALG00010007319	ATP9B	case	control	-0.028550849	0.092592985	-0.308347857	0.757817654	0.85295517	down_in_case
Yang2025_S	ENSGALG00010007320	ENSGALG00010007320	case	control	-0.285577405	0.218309949	-1.308128218	0.190829824	0.345801508	down_in_case
Yang2025_S	ENSGALG00010007321	CD200	case	control	-0.034878648	0.119548951	-0.291752017	0.770476234	0.860027742	down_in_case
Yang2025_S	ENSGALG00010007322	AK6	case	control	0.224112283	0.221943479	1.009771875	0.312604598	0.482229123	up_in_case
Yang2025_S	ENSGALG00010007324	ENSGALG00010007324	case	control	-0.164702914	0.199278131	-0.826497688	0.408521829	0.574387215	down_in_case
Yang2025_S	ENSGALG00010007326	RWDD4	case	control	0.376353872	0.173505495	2.169117887	0.030073735	0.091782194	up_in_case
Yang2025_S	ENSGALG00010007328	NOVA1	case	control	0.185758869	0.174322448	1.065604977	0.286602248	0.455186042	up_in_case
Yang2025_S	ENSGALG00010007330	MMP13	case	control	-0.388953384	0.73237458	-0.531085314			down_in_case
Yang2025_S	ENSGALG00010007333	QTRT2	case	control	0.19915164	0.113164829	1.759836881	0.078435468	0.184690477	up_in_case
Yang2025_S	ENSGALG00010007337	PRORP	case	control	-0.153610744	0.109318971	-1.405160902	0.159973437	0.305583256	down_in_case
Yang2025_S	ENSGALG00010007339	ENSGALG00010007339	case	control	-0.188541831	0.217274625	-0.867758167	0.385526736	0.553048699	down_in_case
Yang2025_S	ENSGALG00010007344	RAD17	case	control	0.077122282	0.127547768	0.604654106	0.545408848	0.694850275	up_in_case
Yang2025_S	ENSGALG00010007353	ING2	case	control	-0.38327953	0.175677255	-2.1817254	0.029129805	0.089509115	down_in_case
Yang2025_S	ENSGALG00010007357	NOX4	case	control	0.055929241	0.154609053	0.361746223	0.717541682	0.826085868	up_in_case
Yang2025_S	ENSGALG00010007360	ROBO1	case	control	0.057504972	0.177413505	0.324129618	0.745839904	0.845197377	up_in_case
Yang2025_S	ENSGALG00010007365	MEMO1	case	control	-0.173883998	0.132591751	-1.311423952	0.189714571	0.344497335	down_in_case
Yang2025_S	ENSGALG00010007369	ENSGALG00010007369	case	control	0.267241549	0.327802201	0.815252455	0.414927818	0.580407463	up_in_case
Yang2025_S	ENSGALG00010007374	CMIP	case	control	0.109489156	0.168597427	0.649411784	0.51607225	0.67047052	up_in_case
Yang2025_S	ENSGALG00010007375	CHORDC1	case	control	-0.067404466	0.13287618	-0.507272753	0.611963454	0.748892787	down_in_case
Yang2025_S	ENSGALG00010007376	CDKN2AIP	case	control	-0.200953968	0.101950011	-1.971102957	0.048712101	0.130841003	down_in_case
Yang2025_S	ENSGALG00010007381	EGFR	case	control	-0.162802313	0.152199915	-1.069661002	0.284771926	0.45295021	down_in_case
Yang2025_S	ENSGALG00010007382	EIF2AK2	case	control	-0.102247208	0.130885482	-0.781195948	0.434687256	0.598435775	down_in_case
Yang2025_S	ENSGALG00010007387	ENSGALG00010007387	case	control	-0.169477245	0.502524814	-0.337251495	0.735927322	0.839001402	down_in_case
Yang2025_S	ENSGALG00010007388	CYP1B1	case	control	0.434343733	0.124523286	3.488052291	0.000486553	0.003863313	up_in_case
Yang2025_S	ENSGALG00010007391	NANP	case	control	-0.273571722	0.100539025	-2.721050087	0.00650749	0.029252217	down_in_case
Yang2025_S	ENSGALG00010007392	NAALAD2	case	control	-0.091170253	0.160972778	-0.566370626	0.571141858	0.715397298	down_in_case
Yang2025_S	ENSGALG00010007393	PLCG2	case	control	0.268002562	0.167795511	1.597197448	0.110221701	0.234566189	up_in_case
Yang2025_S	ENSGALG00010007399	WWC2	case	control	0.571377535	0.156871399	3.642330841	0.000270181	0.002388938	up_in_case
Yang2025_S	ENSGALG00010007401	SEC61G	case	control	-1.643703173	0.290920248	-5.650012968	1.60436E-08	7.03041E-07	down_in_case
Yang2025_S	ENSGALG00010007403	SDR42E1	case	control	0.063855905	0.217963445	0.29296612	0.769548049	0.859542486	up_in_case
Yang2025_S	ENSGALG00010007404	FOLH1	case	control	-0.575771322	0.196203551	-2.934561164	0.003340197	0.017650985	down_in_case
Yang2025_S	ENSGALG00010007408	HCLS1	case	control	0.46854813	0.160555348	2.918296622	0.003519494	0.018341117	up_in_case
Yang2025_S	ENSGALG00010007411	ENSGALG00010007411	case	control	-0.55669283	1.160134941	-0.479851792	0.631332783	0.763452777	down_in_case
Yang2025_S	ENSGALG00010007413	GPATCH11	case	control	0.579589224	0.095988093	6.038136683	1.55904E-09	8.97736E-08	up_in_case
Yang2025_S	ENSGALG00010007414	ROBO2	case	control	0.597038282	0.264031679	2.261237303	0.023744566	0.077128329	up_in_case
Yang2025_S	ENSGALG00010007416	DCTD	case	control	-0.821677641	0.165364575	-4.968885504	6.73388E-07	1.71235E-05	down_in_case
Yang2025_S	ENSGALG00010007418	NME6	case	control	-0.238926612	0.136066705	-1.755952071	0.079096593	0.185838985	down_in_case
Yang2025_S	ENSGALG00010007419	RABL2A	case	control	-0.147549761	0.241622826	-0.610661516	0.541423688	0.691733666	down_in_case
Yang2025_S	ENSGALG00010007423	HEATR5B	case	control	-0.012257539	0.1226205	-0.099963209	0.920373534	0.952963458	down_in_case
Yang2025_S	ENSGALG00010007425	ARSA	case	control	-0.060930964	0.093555055	-0.651284577	0.514862802	0.669585922	down_in_case
Yang2025_S	ENSGALG00010007429	C21orf91	case	control	-0.050185219	0.148747516	-0.337385256	0.735826497	0.838957801	down_in_case
Yang2025_S	ENSGALG00010007430	MEF2C	case	control	0.940397291	0.275801834	3.409684694	0.00065038	0.004952486	up_in_case
Yang2025_S	ENSGALG00010007432	NAT	case	control	-0.830913413	0.301547029	-2.755501907	0.005860216	0.026943266	down_in_case
Yang2025_S	ENSGALG00010007433	RNF144B	case	control	0.237093084	0.244579044	0.969392471	0.332349407	0.501656087	up_in_case
Yang2025_S	ENSGALG00010007434	ENSGALG00010007434	case	control	-0.34133491	0.32364612	-1.054654727	0.291583265	0.460107892	down_in_case
Yang2025_S	ENSGALG00010007436	PNAT3	case	control	-0.399385091	0.182211547	-2.191875852	0.028388472	0.087831563	down_in_case
Yang2025_S	ENSGALG00010007437	TUSC1	case	control	0.433909128	0.281719284	1.540218054	0.123507211	0.254558938	up_in_case
Yang2025_S	ENSGALG00010007439	HSD17B2	case	control	0.410067568	0.314649625	1.30325141	0.192488944	0.347722768	up_in_case
Yang2025_S	ENSGALG00010007441	STRN	case	control	0.485576025	0.177843095	2.730361973	0.006326482	0.02859537	up_in_case
Yang2025_S	ENSGALG00010007455	TRPC6	case	control	0.035113994	0.211148279	0.166300167	0.867920735	0.922467808	up_in_case
Yang2025_S	ENSGALG00010007457	SHANK3	case	control	-0.109439982	0.130560494	-0.838231987	0.401900426	0.568495974	down_in_case
Yang2025_S	ENSGALG00010007465	PNAT10	case	control	-0.384606555	0.244847021	-1.570803486	0.116228302	0.243848448	down_in_case
Yang2025_S	ENSGALG00010007467	CHODL	case	control	-0.153395152	0.251877596	-0.609006732	0.542519977	0.69269601	down_in_case
Yang2025_S	ENSGALG00010007479	NFX1	case	control	0.015007941	0.108509106	0.138310425	0.889995087	0.935608284	up_in_case
Yang2025_S	ENSGALG00010007480	TENM3	case	control	0.053085631	0.173572966	0.305840431	0.759726143	0.854255128	up_in_case
Yang2025_S	ENSGALG00010007485	CTSC	case	control	-0.646438071	0.138143655	-4.679462624	2.87628E-06	5.85123E-05	down_in_case
Yang2025_S	ENSGALG00010007500	MPHOSPH6	case	control	0.444085171	0.181068378	2.452582694	0.014183477	0.052560292	up_in_case
Yang2025_S	ENSGALG00010007506	RAB38	case	control	4.531711822	0.431240597	10.50854639	7.89003E-26	7.85339E-23	up_in_case
Yang2025_S	ENSGALG00010007513	GALNT12	case	control	-0.243965546	0.11433164	-2.133841035	0.032855796	0.097976784	down_in_case
Yang2025_S	ENSGALG00010007514	VIT	case	control	1.659244873	0.758365151	2.187923418	0.028675181	0.088463283	up_in_case
Yang2025_S	ENSGALG00010007521	SLC12A7	case	control	0.076914377	0.127522001	0.603145938	0.546411608	0.695513362	up_in_case
Yang2025_S	ENSGALG00010007529	CDH13	case	control	0.120209792	0.193777122	0.620350799	0.535026857	0.686479207	up_in_case
Yang2025_S	ENSGALG00010007532	DCUN1D5	case	control	-0.227945018	0.211395832	-1.078285296	0.280906457	0.448851218	down_in_case
Yang2025_S	ENSGALG00010007536	GABBR2	case	control	0.565150283	0.281883671	2.004906073	0.044973089	0.123755924	up_in_case
Yang2025_S	ENSGALG00010007539	RPS3A	case	control	0.412916784	0.192354716	2.146642374	0.031821764	0.095753893	up_in_case
Yang2025_S	ENSGALG00010007542	MRPL36	case	control	-0.33917318	0.210061035	-1.614641102	0.106388439	0.229031809	down_in_case
Yang2025_S	ENSGALG00010007544	MLYCD	case	control	0.040340813	0.199983347	0.201720859	0.840134954	0.905085473	up_in_case
Yang2025_S	ENSGALG00010007548	HSBP1	case	control	-0.360643321	0.100777976	-3.578592611	0.000345449	0.002934027	down_in_case
Yang2025_S	ENSGALG00010007549	SH3D19	case	control	-0.035416238	0.229319931	-0.154440295	0.877262584	0.927937916	down_in_case
Yang2025_S	ENSGALG00010007551	FEZ2	case	control	0.079578125	0.146321256	0.543858953	0.586538537	0.728338488	up_in_case
Yang2025_S	ENSGALG00010007555	NDUFS6	case	control	0.143774436	0.294020475	0.488994639	0.624845492	0.75873804	up_in_case
Yang2025_S	ENSGALG00010007566	FHIP1A	case	control	0.017847705	0.193581587	0.092197325	0.926541263	0.955608219	up_in_case
Yang2025_S	ENSGALG00010007567	TRIP13	case	control	0.020396916	0.1297641	0.157184585	0.875099377	0.926564077	up_in_case
Yang2025_S	ENSGALG00010007569	GPR89A	case	control	0.366989762	0.124574459	2.945947059	0.003219675	0.017157236	up_in_case
Yang2025_S	ENSGALG00010007572	PDZK1	case	control	-0.746020184	0.43517181	-1.714311835	0.086471471	0.19805719	down_in_case
Yang2025_S	ENSGALG00010007574	ENSGALG00010007574	case	control	1.273016457	0.259294348	4.909541872	9.12894E-07	2.22398E-05	up_in_case
Yang2025_S	ENSGALG00010007575	MMP27	case	control	-0.365853936	0.263469676	-1.388599786	0.164954482	0.312400206	down_in_case
Yang2025_S	ENSGALG00010007577	WFDC1	case	control	-0.249639827	0.095770521	-2.606645806	0.009143389	0.037999738	down_in_case
Yang2025_S	ENSGALG00010007579	GATB	case	control	-0.137079388	0.101058184	-1.356440248	0.174959135	0.325421188	down_in_case
Yang2025_S	ENSGALG00010007580	BRD9	case	control	0.480750745	0.112087714	4.289058341	1.79432E-05	0.000265716	up_in_case
Yang2025_S	ENSGALG00010007581	LPCAT1	case	control	-0.227004899	0.106597162	-2.129558574	0.033208074	0.098710434	down_in_case
Yang2025_S	ENSGALG00010007584	ELP2	case	control	-0.154000272	0.137510795	-1.11991406	0.262750386	0.429591972	down_in_case
Yang2025_S	ENSGALG00010007588	TTC27	case	control	-0.095000826	0.097833781	-0.97104318	0.331526776	0.500794106	down_in_case
Yang2025_S	ENSGALG00010007590	FBXW7	case	control	0.160396043	0.129959222	1.234202858	0.217127323	0.377963586	up_in_case
Yang2025_S	ENSGALG00010007593	SEC61B	case	control	-0.641346636	0.344019471	-1.864274234	0.062283203	0.156526714	down_in_case
Yang2025_S	ENSGALG00010007595	ATP2C2	case	control	0.008247086	0.212077687	0.038887098	0.968980403	0.981620757	up_in_case
Yang2025_S	ENSGALG00010007596	ENSGALG00010007596	case	control	-0.90567141	0.494139261	-1.832826253	0.066828414	0.164468733	down_in_case
Yang2025_S	ENSGALG00010007601	TMEM154	case	control	0.378847173	0.370253547	1.02321011	0.306208514	0.475910734	up_in_case
Yang2025_S	ENSGALG00010007603	ALG2	case	control	-0.297500663	0.174761708	-1.702321783	0.088695063	0.201889204	down_in_case
Yang2025_S	ENSGALG00010007604	ENSGALG00010007604	case	control	-1.034384634	0.413915481	-2.49902379	0.012453595	0.047689158	down_in_case
Yang2025_S	ENSGALG00010007610	TLDC1	case	control	-0.352427007	0.086465224	-4.075939346	4.5829E-05	0.000570202	down_in_case

Yang2025_S	ENSGALG00010007614	BIRC6	case	control	-0.106488677	0.142573371	-0.746904392	0.455121275	0.616697293	down_in_case
Yang2025_S	ENSGALG00010007624	TPPP	case	control	0.664047687	0.272344084	2.438267346	0.014757855	0.054261401	up_in_case
Yang2025_S	ENSGALG00010007626	DLK2	case	control	-0.168723502	0.154428047	-1.09257033	0.274582496	0.442015967	down_in_case
Yang2025_S	ENSGALG00010007633	ENSGALG00010007633	case	control	0.651123036	0.146424006	4.446832562	8.71457E-06	0.000145783	up_in_case
Yang2025_S	ENSGALG00010007640	TMEM123	case	control	-0.453977408	0.145709682	-3.115629668	0.001835526	0.011223369	down_in_case
Yang2025_S	ENSGALG00010007642	ZNF318	case	control	0.559557755	0.197914796	2.827265901	0.004694733	0.022850542	up_in_case
Yang2025_S	ENSGALG00010007643	COTL1	case	control	-0.730260257	0.107801406	-6.77412551	1.25161E-11	1.31376E-09	down_in_case
Yang2025_S	ENSGALG00010007650	KLHL36	case	control	-0.079095189	0.100141473	-0.789834488	0.429624435	0.593753496	down_in_case
Yang2025_S	ENSGALG00010007651	CTNNAL1	case	control	0.379383347	0.124055738	3.058168471	0.002226943	0.012973435	up_in_case
Yang2025_S	ENSGALG00010007654	HTR1F	case	control	1.436837873	0.562125902	2.556078395	0.010585927	0.042255769	up_in_case
Yang2025_S	ENSGALG00010007658	BIRC2	case	control	-0.034844196	0.081777901	-0.426083274	0.670047168	0.792192467	down_in_case
Yang2025_S	ENSGALG00010007660	FAM98A	case	control	0.019931639	0.09103973	0.218933416	0.826701916	0.896430655	up_in_case
Yang2025_S	ENSGALG00010007663	ARFIP1	case	control	-0.410546072	0.077961446	-5.266014084	1.39418E-07	4.56053E-06	down_in_case
Yang2025_S	ENSGALG00010007671	FAM206A	case	control	-0.452763985	0.217430585	-2.082338068	0.037311601	0.107525867	down_in_case
Yang2025_S	ENSGALG00010007673	FHDC1	case	control	-0.08693568	0.162445173	-0.535169362	0.59253275	0.732778122	down_in_case
Yang2025_S	ENSGALG00010007678	CDH8	case	control	0.201558743	0.353273482	0.570545918	0.568307487	0.712821901	up_in_case
Yang2025_S	ENSGALG00010007679	TMEM135	case	control	-0.53181718	0.11929165	-4.458125794	8.26794E-06	0.000139653	down_in_case
Yang2025_S	ENSGALG00010007680	CHMP2B	case	control	-0.12622867	0.1058104	-1.19297035	0.232880985	0.395997136	down_in_case
Yang2025_S	ENSGALG00010007695	RASGRP3	case	control	0.24561884	0.092554776	2.65376731	0.00795987	0.034118974	up_in_case
Yang2025_S	ENSGALG00010007698	ADAMTS18	case	control	-0.203962444	0.274820473	-0.742166117	0.457986695	0.619195168	down_in_case
Yang2025_S	ENSGALG00010007699	SLC39A6	case	control	-0.294115052	0.098504519	-2.985802634	0.002828351	0.015565985	down_in_case
Yang2025_S	ENSGALG00010007713	SCLT1	case	control	0.805616717	0.206515493	3.900998933	9.57966E-05	0.00103083	up_in_case
Yang2025_S	ENSGALG00010007719	LTBP1	case	control	0.389389154	0.187747612	2.074003224	0.038079011	0.109115983	up_in_case
Yang2025_S	ENSGALG00010007724	RBM11	case	control	-0.235702662	0.429564005	-0.548702077	0.583209921	0.725536713	down_in_case
Yang2025_S	ENSGALG00010007730	COG2	case	control	-0.514181702	0.129002112	-3.985839419	6.7242E-05	0.000775035	down_in_case
Yang2025_S	ENSGALG00010007731	ENSGALG00010007731	case	control	-0.318673161	0.17141686	-1.859053773	0.063019517	0.157520532	down_in_case
Yang2025_S	ENSGALG00010007741	ENSGALG00010007741	case	control	0.110864033	0.17279901	0.641577938	0.521147262	0.674736327	up_in_case
Yang2025_S	ENSGALG00010007748	CSGALNACT1	case	control	1.026780989	0.297176664	3.45511985	0.000550048	0.004284473	up_in_case
Yang2025_S	ENSGALG00010007751	HSPA13	case	control	-0.262418016	0.115562424	-2.270790173	0.023159683	0.075563143	down_in_case
Yang2025_S	ENSGALG00010007753	PRSS23	case	control	-0.435902417	0.13266628	-3.28570619	0.001017271	0.007105597	down_in_case
Yang2025_S	ENSGALG00010007758	DEK	case	control	0.169267033	0.155174733	1.090815686	0.275353993	0.442336123	up_in_case
Yang2025_S	ENSGALG00010007759	AGT	case	control	-2.516360625	1.224380233	-2.055211737			down_in_case
Yang2025_S	ENSGALG00010007761	ENSGALG00010007761	case	control	-0.130860261	0.189521562	-0.690476901	0.489894331	0.647262492	down_in_case
Yang2025_S	ENSGALG00010007762	SH2D4A	case	control	0.810963895	0.437167151	1.855043072	0.063590076	0.158633675	up_in_case
Yang2025_S	ENSGALG00010007763	FZD4	case	control	-0.728835268	0.100651187	-7.241198921	4.44736E-13	6.32387E-11	down_in_case
Yang2025_S	ENSGALG00010007765	NUDT7	case	control	-0.002403832	0.134785832	-0.017834455	0.985770918	0.991427886	down_in_case
Yang2025_S	ENSGALG00010007766	ENSGALG00010007766	case	control	-0.04893255	0.171498408	-0.285323641	0.775396202	0.863270828	down_in_case
Yang2025_S	ENSGALG00010007767	VAT1L	case	control	0.578453692	0.223622905	2.586737217	0.009688945	0.03961721	up_in_case
Yang2025_S	ENSGALG00010007770	ENSGALG00010007770	case	control	1.4809648	0.417915759	3.543692162	0.000394566	0.003265007	up_in_case
Yang2025_S	ENSGALG00010007772	CLEC3A	case	control	0.529977532	0.792365803	0.668854625	0.503588217	0.659663641	up_in_case
Yang2025_S	ENSGALG00010007774	ENSGALG00010007774	case	control	0.377044834	0.369471233	1.020498488	0.307492106	0.477132527	up_in_case
Yang2025_S	ENSGALG00010007777	KIF13A	case	control	0.498069091	0.167339355	2.976401409	0.002916528	0.01595048	up_in_case
Yang2025_S	ENSGALG00010007780	SIRT5	case	control	-0.486635383	0.244380662	-1.991300694	0.046447839	0.126466307	down_in_case
Yang2025_S	ENSGALG00010007781	MBP	case	control	0.495511773	0.313517157	1.580493323			up_in_case
Yang2025_S	ENSGALG00010007783	NOL7	case	control	0.318770753	0.181846414	1.752966941	0.079607682	0.186733107	up_in_case
Yang2025_S	ENSGALG00010007784	INTS10	case	control	-0.053262893	0.11511072	-0.4627101	0.643572177	0.772918926	down_in_case
Yang2025_S	ENSGALG00010007786	SAMSN1	case	control	-0.04152637	0.302867241	-0.137110802	0.890943214	0.936155459	down_in_case
Yang2025_S	ENSGALG00010007787	C1orf198	case	control	0.01628479	0.097844904	0.16643473	0.867814845	0.922425619	up_in_case
Yang2025_S	ENSGALG00010007788	PALM2AKAP2	case	control	0.396631741	0.227260436	1.74527405	0.080937177	0.188952849	up_in_case
Yang2025_S	ENSGALG00010007789	WWOX	case	control	-0.256939841	0.100839399	-2.548010422	0.010833923	0.042976682	down_in_case
Yang2025_S	ENSGALG00010007792	ENSGALG00010007792	case	control	0.466548281	0.237281455	1.966223109	0.049272844	0.131838918	up_in_case
Yang2025_S	ENSGALG00010007795	ZNF236	case	control	0.134602706	0.142862067	0.942186469	0.34609718	0.514727257	up_in_case
Yang2025_S	ENSGALG00010007796	MCUR1	case	control	-0.373181879	0.107962843	-3.456577001	0.000547083	0.004263758	down_in_case
Yang2025_S	ENSGALG00010007798	HGSNAT	case	control	-0.178716182	0.104048536	-1.717623229	0.085865361	0.196960296	down_in_case
Yang2025_S	ENSGALG00010007800	MAF	case	control	0.012280609	0.11390303	0.107816349	0.914141374	0.949666936	up_in_case
Yang2025_S	ENSGALG00010007801	TTC13	case	control	0.384782662	0.108211845	3.555827556	0.000376791	0.003151612	up_in_case
Yang2025_S	ENSGALG00010007802	ENSGALG00010007802	case	control	0.373669128	1.215039482	0.307536614	0.758434959	0.85303826	up_in_case
Yang2025_S	ENSGALG00010007803	ME3	case	control	-0.000343755	0.133451382	-0.002575883	0.997944745	0.998374616	down_in_case
Yang2025_S	ENSGALG00010007805	ENSGALG00010007805	case	control	-0.119635805	0.899753367	-0.132965109	0.894220974	0.937885741	down_in_case
Yang2025_S	ENSGALG00010007806	DYNLRB2	case	control	-0.401487935	0.376579296	-1.066144473	0.286358338	0.454902354	down_in_case
Yang2025_S	ENSGALG00010007809	ARV1	case	control	-0.302458169	0.109801674	-2.754586148	0.005876642	0.026974816	down_in_case
Yang2025_S	ENSGALG00010007811	PRCP	case	control	-0.441962925	0.12728982	-3.472099527	0.000516405	0.004058764	down_in_case
Yang2025_S	ENSGALG00010007812	POMK	case	control	-0.293621183	0.133009994	-2.207512192	0.027278296	0.085235024	down_in_case
Yang2025_S	ENSGALG00010007817	CDYL2	case	control	0.167198327	0.170242846	0.982116616	0.326042401	0.49600446	up_in_case
Yang2025_S	ENSGALG00010007818	ENSGALG00010007818	case	control	0.631362318	0.228255322	2.766035478	0.005674236	0.026327475	up_in_case
Yang2025_S	ENSGALG00010007819	NRIP1	case	control	-0.173985336	0.151291257	-1.150002583	0.250142808	0.415924106	down_in_case
Yang2025_S	ENSGALG00010007821	FAM89A	case	control	-0.887471563	0.113832582	-7.796287711	6.37549E-15	1.40015E-12	down_in_case
Yang2025_S	ENSGALG00010007826	ENSGALG00010007826	case	control	-2.127341921	1.878463625	-1.132490346	0.257428341	0.423769667	down_in_case
Yang2025_S	ENSGALG00010007830	SMIM19	case	control	0.078666188	0.144263804	0.545294008	0.585551324	0.727566447	up_in_case
Yang2025_S	ENSGALG00010007835	GUCY1A1	case	control	-0.176697073	0.089288631	-1.978942567	0.047822473	0.12932392	down_in_case
Yang2025_S	ENSGALG00010007845	TFAP2B	case	control	2.946288296	0.719540661	4.094679366	4.22753E-05	0.000535065	up_in_case
Yang2025_S	ENSGALG00010007846	ENSGALG00010007846	case	control	0.183533316	0.187648255	0.978070997	0.328039211	0.497738067	up_in_case
Yang2025_S	ENSGALG00010007847	CMC2	case	control	-0.592862085	0.234846796	-2.524463162	0.011587515	0.045179636	down_in_case
Yang2025_S	ENSGALG00010007849	CXADR	case	control	-0.142204339	0.476582151	-0.298383686	0.765410339	0.857247499	down_in_case
Yang2025_S	ENSGALG00010007850	ENSGALG00010007850	case	control	0.032338086	0.497670687	0.064978884	0.948190813	0.967921209	up_in_case
Yang2025_S	ENSGALG00010007851	AvBD7	case	control	0.210279737	0.464535264	0.452666899	0.650788618	0.778633073	up_in_case
Yang2025_S	ENSGALG00010007852	SLC20A2	case	control	-0.334191322	0.125124403	-2.670872466	0.007565438	0.032811822	down_in_case
Yang2025_S	ENSGALG00010007853	C1orf131	case	control	0.936673369	0.238833191	3.921872696	8.78634E-05	0.000961804	up_in_case
Yang2025_S	ENSGALG00010007854	FAM181B	case	control	0.614560597	0.155905589	3.941876635	8.08466E-05	0.000900069	up_in_case
Yang2025_S	ENSGALG00010007855	RNF182	case	control	0.868037979	0.208517369	4.162904917	3.14224E-05	0.000423473	up_in_case
Yang2025_S	ENSGALG00010007856	PDCD2	case	control	0.26198541	0.14112952	1.856347353	0.063404064	0.158311347	up_in_case
Yang2025_S	ENSGALG00010007857	ENSGALG00010007857	case	control	0.581865973	0.290828232	2.000720388	0.045422531	0.124746348	up_in_case
Yang2025_S	ENSGALG00010007859	CENPN	case	control	-0.051645077	0.131790559	-0.391872354	0.695152531	0.810827029	down_in_case
Yang2025_S	ENSGALG00010007860	DEFB4A	case	control	-1.923117027	0.663124689	-2.900083588	0.003730632	0.019183156	down_in_case
Yang2025_S	ENSGALG00010007861	INTU	case	control	-0.620337573	0.141909842	-4.371349899	1.23481E-05	0.000196427	down_in_case
Yang2025_S	ENSGALG00010007862	ATMIN	case	control	-0.514434173	0.086710741	-5.932761817	2.97881E-09	1.57234E-07	down_in_case
Yang2025_S	ENSGALG00010007863	AvBD1	case	control	0.366094779	0.447058306	0.818897165	0.412845088	0.578248698	up_in_case
Yang2025_S	ENSGALG00010007864	TYR	case	control	6.562613686	0.797805553	8.225831049	1.93842E-16	5.50881E-14	up_in_case
Yang2025_S	ENSGALG00010007865	GABRR3	case	control	0.380616357	0.411208395	0.925604539	0.354651534	0.523883775	up_in_case
Yang2025_S	ENSGALG00010007866	TBP	case	control	-0.015577366	0.138787222	-0.112239193	0.910633754	0.946905746	down_in_case
Yang2025_S	ENSGALG00010007867	ZNF516	case	control	-0.12322887	0.1563739	-0.788039884	0.430673377	0.594672365	down_in_case
Yang2025_S	ENSGALG00010007868	INHBA	case	control	0.598498303	0.2087547	2.866993187	0.004143919	0.020689902	up_in_case
Yang2025_S	ENSGALG00010007869	GCSH	case	control	-0.407829845	0.199212065	-2.047214584	0.04063702	0.114468744	down_in_case
Yang2025_S	ENSGALG00010007870	AvBD3	case	control	0.593360972	0.436708005	1.358713293	0.174237458	0.324468659	up_in_case
Yang2025_S	ENSGALG00010007871	HSPA4L	case	control	-0.038409856	0.141687311	-0.271088887	0.786322668	0.871087239	down_in_case
Yang2025_S	ENSGALG00010007872	KLHL2	case	control	0.800131702	0.145054938	5.516059742	3.46684E-08	1.3647E-06	up_in_case
Yang2025_S	ENSGALG00010007873	ENSGALG00010007873	case	control	-1.090246143	0.322586956	-3.379696929	0.000725658	0.0054133	down_in_case
Yang2025_S	ENSGALG00010007874	ENSGALG00010007874	case	control	1.899487849	0.583996939	3.252564733	0.001143685	0.007793279	up_in_case
Yang2025_S	ENSGALG00010007877	MSMO1	case	control	-0.133626911	0.190553369	-0.701257142	0.483142555	0.641382443	down_in_case
Yang2025_S	ENSGALG00010007878	PSMB1	case	control	-0.132736067	0.154011539	-0.861857935	0.388765699	0.555749899	down_in_case
Yang2025_S	ENSGALG00010007879	PLK4	case	control	-0.095728302	0.125338185	-0.763760081	0.445010229	0.60814137	down_in_case
Yang2025_S	ENSGALG00010007880	BCO1	case	control	-1.746342815	0.402808579	-4.335416142	1.45485E-05	0.00022451	down_in_case
Yang2025_S	ENSGALG00010007882	CPE	case	control	0.201465237	0.123793733	1.627426784	0.103646487	0.225041102	up_in_case
Yang2025_S	ENSGALG00010007883	DHTKD1	case	control	-0.134141184	0.076662648	-1.749759333	0.080159851	0.187609593	down_in_case
Yang2025_S	ENSGALG00010007884	GLI3	case	control	0.333081796	0.245515037	1.356665565	0.174887499	0.32533137	up_in_case
Yang2025_S	ENSGALG00010007885	USP38	case	control	-0.235072838	0.082180155	-2.860457452	0.004230303	0.020985858	down_in_case
Yang2025_S	ENSGALG00010007887	CDT1	case	control	0.306322894	0.209780179	1.460208948	0.144232657	0.284927995	up_in_case
Yang2025_S	ENSGALG00010007889	DCAF6	case	control	-0.163549132	0.063881075	-2.560212563	0.010460816	0.041864293	down_in_case
Yang2025_S	ENSGALG00010007890	ENSGALG00010007890	case	control	-0.51519642	0.129107324	-3.990450751	6.59478E-05	0.000764545	down_in_case
Yang2025_S	ENSGALG00010007891	GDI1	case	control	-0.174295356	0.09297506	-1.874646348	0.060841392	0.153954023	down_in_case
Yang2025_S	ENSGALG00010007892	ENSGALG00010007892	case	control	0.68519677	0.519680097	1.318497233	0.187337251	0.341426182	up_in_case
Yang2025_S	ENSGALG00010007893	MFSD8	case	control	0.008804739	0.112758188	0.078085144	0.937760324	0.962203823	up_in_case
Yang2025_S	ENSGALG00010007894	CD88	case	control	-0.06326358	0.236177842	-0.267864162	0.78880388	0.872587288	down_in_case
Yang2025_S	ENSGALG00010007895	GOLGB1	case	control	0.40010298	0.19879406	2.012650582	0.044151402	0.122097595	up_in_case
Yang2025_S	ENSGALG00010007896	TIMMDC1	case	control	-0.158580066	0.15528865	-1.021195469	0.307161839	0.476754313	down_in_case
Yang2025_S	ENSGALG00010007897	EFHC1	case	control	0.468989754	0.336956496	1.391840667	0.163970652	0.310874971	up_in_case
Yang2025_S	ENSGALG00010007898	GAB1	case	control	0.235999763	0.23790504	0.991991439	0.321201702	0.490476309	up_in_case
Yang2025_S	ENSGALG00010007899	APRT	case	control	-0.223162369	0.262667641	-0.849599778	0.395547635	0.562731628	down_in_case
Yang2025_S	ENSGALG00010007900	FHOD3	case	control	0.063455367	0.273578683	0.231945582	0.816580281	0.890100611	up_in_case
Yang2025_S	ENSGALG00010007901	FSTL5	case	control	-0.838053571	0.319302259	-2.624640284	0.008674055	0.036539589	down_in_case
Yang2025_S	ENSGALG00010007902	C7orf25	case	control	0.185756763	0.128110669	1.449971064	0.147066589	0.288895163	up_in_case
Yang2025_S	ENSGALG00010007903	PSMA2	case	control	-0.608483077	0.148071119	-4.109397422	3.96693E-05	0.000509955	down_in_case
Yang2025_S	ENSGALG00010007904	ENSGALG00010007904	case	control	0.730487342	0.171971423	4.247725165	2.15952E-05	0.000309917	up_in_case
Yang2025_S	ENSGALG00010007905	MRPL32	case	control	0.281029123	0.169326447	1.659688294	0.096977176	0.214581514	up_in_case
Yang2025_S	ENSGALG00010007906	NAF1	case	control	0.152232432	0.159769094	0.952827789	0.340677332	0.508934243	up_in_case
Yang2025_S	ENSGALG00010007907	IRX2	case	control	0.612757134	0.71752913	0.853982241	0.39311483	0.560013816	up_in_case

Yang2025_S	ENSGALG00010007908	IRX1	case	control	0.680007844	0.587413717	1.157630175	0.24701499	0.412678801	up_in_case
Yang2025_S	ENSGALG00010007909	GALNS	case	control	-0.020379073	0.093573075	-0.217787785	0.827594463	0.896775182	down_in_case
Yang2025_S	ENSGALG00010007910	ANKS6	case	control	0.200957922	0.101291958	1.983947453	0.047261698	0.128180569	up_in_case
Yang2025_S	ENSGALG00010007911	IFI6	case	control	-0.678500177	0.525019238	-1.292333933	0.196241525	0.352344836	down_in_case
Yang2025_S	ENSGALG00010007912	TRAPPC2L	case	control	-0.263133903	0.284377201	-0.92529887	0.354810466	0.523980908	down_in_case
Yang2025_S	ENSGALG00010007914	PAQR8	case	control	-0.279388625	0.18820086	-1.484523637	0.137670057	0.275399404	down_in_case
Yang2025_S	ENSGALG00010007915	HECW1	case	control	-0.084308896	0.138426983	-0.60904958	0.542491576	0.69269601	down_in_case
Yang2025_S	ENSGALG00010007916	MCM3	case	control	-0.099441287	0.173636884	-0.572696796	0.566850007	0.711703646	down_in_case
Yang2025_S	ENSGALG00010007917	POGLUT1	case	control	-0.497857258	0.141168831	-3.526679741	0.000420805	0.003447339	down_in_case
Yang2025_S	ENSGALG00010007920	FAT3	case	control	0.158268827	0.244805071	0.646509595	0.517949385	0.672345103	up_in_case
Yang2025_S	ENSGALG00010007921	FAM120B	case	control	-0.534273517	0.08971873	-5.954983068	2.60099E-09	1.40569E-07	down_in_case
Yang2025_S	ENSGALG00010007922	IL17A	case	control	0.086424941	0.317305857	0.272371087	0.785336702	0.870271725	up_in_case
Yang2025_S	ENSGALG00010007923	YAP1	case	control	-0.00789266	0.141732808	-0.055686894	0.955591241	0.972763821	down_in_case
Yang2025_S	ENSGALG00010007924	ABHD18	case	control	0.133864395	0.127362733	1.051048392	0.293236365	0.461705274	up_in_case
Yang2025_S	ENSGALG00010007925	STK17A	case	control	-0.283244903	0.171847367	-1.648235339	0.099304388	0.217864637	down_in_case
Yang2025_S	ENSGALG00010007926	EIF3E	case	control	-0.616474564	0.147255778	-4.186420206	2.83388E-05	0.000388301	down_in_case
Yang2025_S	ENSGALG00010007927	DLL1	case	control	0.133316278	0.120863449	1.103032215	0.270013176	0.437362967	up_in_case
Yang2025_S	ENSGALG00010007928	C11orf70	case	control	1.289711014	0.409873739	3.146605629	0.001651776	0.01035425	up_in_case
Yang2025_S	ENSGALG00010007929	LRRC14B	case	control	-0.629010168	0.253836985	-2.478008349	0.013211805	0.049794936	down_in_case
Yang2025_S	ENSGALG00010007930	CBFA2T3	case	control	0.221357332	0.13139923	1.684616659	0.092062555	0.207185353	up_in_case
Yang2025_S	ENSGALG00010007931	GBE1	case	control	-0.241631747	0.130934945	-1.845433593	0.06497449	0.161135348	down_in_case
Yang2025_S	ENSGALG00010007932	NPY5R	case	control	0.574838125	0.224101443	2.565079985	0.010315202	0.041496058	up_in_case
Yang2025_S	ENSGALG00010007933	CEP126	case	control	1.087536032	0.44821301	2.426382116	0.015250204	0.055704218	up_in_case
Yang2025_S	ENSGALG00010007934	PKHD1	case	control	0.321244319	0.381021773	0.843112761	0.399165383	0.566086872	up_in_case
Yang2025_S	ENSGALG00010007935	LARP1B	case	control	0.759140142	0.210698661	3.602966137	0.000314607	0.002714577	up_in_case
Yang2025_S	ENSGALG00010007936	ENSGALG00010007936	case	control	-0.790230002	0.28012715	-2.820968986	0.004787883	0.023222816	down_in_case
Yang2025_S	ENSGALG00010007937	ANGPTL5	case	control	0.366278159	0.367221555	0.99743099	0.318555355	0.488175677	up_in_case
Yang2025_S	ENSGALG00010007938	BCL9	case	control	0.079839594	0.168871507	0.472783094	0.636367937	0.766976924	up_in_case
Yang2025_S	ENSGALG00010007940	GLIPR2	case	control	-0.620364246	0.122777088	-5.05276885	4.35451E-07	1.18981E-05	down_in_case
Yang2025_S	ENSGALG00010007941	MRPL19	case	control	0.27626367	0.130451322	2.117752933	0.034195997	0.100872401	up_in_case
Yang2025_S	ENSGALG00010007942	PGRMC2	case	control	-0.148554123	0.077576062	-1.91494798	0.055499138	0.144152934	down_in_case
Yang2025_S	ENSGALG00010007943	BLOC1S4	case	control	-0.373922817	0.108353035	-3.450967631	0.000558581	0.004331564	down_in_case
Yang2025_S	ENSGALG00010007944	ACSF3	case	control	0.075242799	0.204697762	0.367579977	0.713186447	0.822771185	up_in_case
Yang2025_S	ENSGALG00010007945	ENSGALG00010007945	case	control	-0.121288959	0.105600724	-1.148561819	0.250736709	0.416450064	down_in_case
Yang2025_S	ENSGALG00010007946	ENSGALG00010007946	case	control	0.606871954	0.215921393	2.810615225	0.004944688	0.023769828	up_in_case
Yang2025_S	ENSGALG00010007948	ENSGALG00010007948	case	control	-0.485045068	0.341496058	-1.420353343	0.155504839	0.300229259	down_in_case
Yang2025_S	ENSGALG00010007950	ARC	case	control	-0.32904928	0.522753928	-0.629453482	0.529052214	0.681677541	down_in_case
Yang2025_S	ENSGALG00010007951	TGFBR1	case	control	-0.209828509	0.103043958	-2.036300943	0.041720145	0.116694144	down_in_case
Yang2025_S	ENSGALG00010007952	EMC2	case	control	0.257427987	0.117850759	2.184355784	0.028936115	0.089071075	up_in_case
Yang2025_S	ENSGALG00010007954	PSCA	case	control	1.464912817	0.810658513	1.807065236	0.070752108	0.171227966	up_in_case
Yang2025_S	ENSGALG00010007955	TMA16	case	control	0.56822749	0.149275967	3.806557099	0.000140915	0.001407632	up_in_case
Yang2025_S	ENSGALG00010007956	HS1BP3	case	control	-0.276723902	0.173157507	-1.598105134	0.110019581	0.234171814	down_in_case
Yang2025_S	ENSGALG00010007957	CDH15	case	control	0.269444803	0.143938441	1.871944704	0.061214254	0.154616754	up_in_case
Yang2025_S	ENSGALG00010007960	TSNAX	case	control	-0.142314712	0.085339314	-1.667633653	0.095388473	0.212270581	down_in_case
Yang2025_S	ENSGALG00010007962	ADCY8	case	control	0.399609514	0.34591112	1.155237549	0.247993162	0.413816874	up_in_case
Yang2025_S	ENSGALG00010007963	LDAH	case	control	-0.344214827	0.077580114	-4.436895071	9.12658E-06	0.000151223	down_in_case
Yang2025_S	ENSGALG00010007964	RECQL4	case	control	0.932585654	0.280261536	3.327554925	0.000876117	0.006296386	up_in_case
Yang2025_S	ENSGALG00010007965	EGLN1	case	control	-0.244237354	0.099108666	-2.464339041	0.01372662	0.051284722	down_in_case
Yang2025_S	ENSGALG00010007966	TMEM74	case	control	0.226537686	0.251257789	0.901614582	0.367261642	0.535890987	up_in_case
Yang2025_S	ENSGALG00010007968	SLC22A31	case	control	-0.080597737	0.270277473	-0.29820368	0.765547713	0.857273175	down_in_case
Yang2025_S	ENSGALG00010007970	SPRTN	case	control	0.229150935	0.091759873	2.497289154	0.012514686	0.04783109	up_in_case
Yang2025_S	ENSGALG00010007972	EXOC8	case	control	-0.070158032	0.084316097	-0.832083489	0.405361821	0.571694968	down_in_case
Yang2025_S	ENSGALG00010007973	CARMIL1	case	control	0.057823143	0.147273325	0.392624683	0.694596706	0.810314366	up_in_case
Yang2025_S	ENSGALG00010007975	ANKRD11	case	control	0.310359873	0.233711913	1.32795915	0.184191602	0.337527816	up_in_case
Yang2025_S	ENSGALG00010007976	ENSGALG00010007976	case	control	0.896150805	0.477350901	1.877341813	0.060471261	0.153351596	up_in_case
Yang2025_S	ENSGALG00010007977	APOB	case	control	-4.645924103	1.083567445	-4.287618757			down_in_case
Yang2025_S	ENSGALG00010007978	JADE1	case	control	0.061462441	0.140549457	0.43730116	0.661892954	0.786315287	up_in_case
Yang2025_S	ENSGALG00010007979	RAB30	case	control	-0.116498852	0.086720479	-1.343383391	0.179147862	0.330739992	down_in_case
Yang2025_S	ENSGALG00010007980	MARCHF1	case	control	0.110628996	0.316623861	0.349401955	0.726787566	0.832329449	up_in_case
Yang2025_S	ENSGALG00010007982	RECK	case	control	-0.334275701	0.098838358	-3.382044269	0.000719485	0.005378772	down_in_case
Yang2025_S	ENSGALG00010007983	RCHY1	case	control	0.044084345	0.225351019	0.19562523	0.844903512	0.907836412	up_in_case
Yang2025_S	ENSGALG00010007984	EIF3H	case	control	-0.270066558	0.138972103	-1.943314895	0.051978123	0.137180898	down_in_case
Yang2025_S	ENSGALG00010007985	APELA	case	control	0.410108992	0.23641982	1.734664172	0.082800306	0.191887953	up_in_case
Yang2025_S	ENSGALG00010007986	MYC	case	control	-0.10645211	0.132732698	-0.80200366	0.422550841	0.588421804	down_in_case
Yang2025_S	ENSGALG00010007987	ENSGALG00010007987	case	control	-0.154576869	0.212241749	-0.728305668	0.466426494	0.627621976	down_in_case
Yang2025_S	ENSGALG00010007988	TARBP1	case	control	0.248441789	0.082307605	3.01845484	0.002540673	0.014368617	up_in_case
Yang2025_S	ENSGALG00010007989	SPG7	case	control	0.048059259	0.088296819	0.54429207	0.586240503	0.728098165	up_in_case
Yang2025_S	ENSGALG00010007991	PCF11	case	control	0.823993862	0.161695307	5.095966477	3.46967E-07	9.72833E-06	up_in_case
Yang2025_S	ENSGALG00010007992	LAPTM4A	case	control	-0.884243148	0.131045819	-6.747587612	1.50323E-11	1.51794E-09	down_in_case
Yang2025_S	ENSGALG00010007993	LRRC14	case	control	0.225524573	0.107119925	2.105346621	0.035261147	0.103423296	up_in_case
Yang2025_S	ENSGALG00010007994	FAM65B	case	control	-0.295689252	0.135689652	-2.179158449	0.029319899	0.089974191	down_in_case
Yang2025_S	ENSGALG00010007995	KIAA0319	case	control	1.436770355	0.830284077	1.730456352	0.083548773	0.193213403	up_in_case
Yang2025_S	ENSGALG00010007996	NUDCD1	case	control	-0.321568605	0.08380728	-3.837000862	0.000124546	0.001270534	down_in_case
Yang2025_S	ENSGALG00010007997	TMEM192	case	control	-0.308249757	0.110930103	-2.778774635	0.005456437	0.025603394	down_in_case
Yang2025_S	ENSGALG00010007998	RPL13	case	control	-0.475355073	0.30488146	-1.559147197	0.118961545	0.248088766	down_in_case
Yang2025_S	ENSGALG00010007999	SDC1	case	control	0.229239432	0.1309279	1.750882978	0.07996607	0.18731336	up_in_case
Yang2025_S	ENSGALG00010008000	C8orf82	case	control	0.147285827	0.211734993	0.695614005	0.486670593	0.643918982	up_in_case
Yang2025_S	ENSGALG00010008001	ALDH5A1	case	control	-0.12834072	0.16293472	-0.787681841	0.43088283	0.594800446	down_in_case
Yang2025_S	ENSGALG00010008002	GJA8	case	control	0.900151873	0.170191282	5.289059839	1.22947E-07	4.07919E-06	up_in_case
Yang2025_S	ENSGALG00010008004	ENY2	case	control	-0.757992202	0.187035815	-4.052658059	5.0639E-05	0.000614146	down_in_case
Yang2025_S	ENSGALG00010008005	ANKRD42	case	control	0.526319931	0.45279463	1.162381124	0.245080687	0.410530037	up_in_case
Yang2025_S	ENSGALG00010008006	CPNE7	case	control	-0.492933613	0.443567182	-1.111294148	0.266441757	0.433641132	down_in_case
Yang2025_S	ENSGALG00010008007	CBR4	case	control	-0.2755917	0.132760425	-2.075857315	0.03790715	0.108757696	down_in_case
Yang2025_S	ENSGALG00010008008	BTC	case	control	0.171855268	0.304516446	0.564354635	0.572512806	0.715833643	up_in_case
Yang2025_S	ENSGALG00010008009	GPLD1	case	control	-0.057730498	0.476910145	-0.1210511	0.90365056	0.942331105	down_in_case
Yang2025_S	ENSGALG00010008010	CERK	case	control	-0.192933445	0.077494345	-2.489645481	0.012787057	0.048605468	down_in_case
Yang2025_S	ENSGALG00010008011	ADNP2	case	control	-0.129118759	0.091349192	-1.413463611	0.157519454	0.302792466	down_in_case
Yang2025_S	ENSGALG00010008012	CCDC90B	case	control	-0.158518425	0.096395378	-1.644460853	0.100081044	0.219143518	down_in_case
Yang2025_S	ENSGALG00010008013	ENSGALG00010008013	case	control	0.352311167	0.171095225	2.059152535	0.039479629	0.112247805	up_in_case
Yang2025_S	ENSGALG00010008015	ARHGAP39	case	control	0.38100282	0.193905111	1.964893131	0.049426608	0.132174205	up_in_case
Yang2025_S	ENSGALG00010008016	GSR	case	control	-0.420051354	0.248015831	-1.693647343	0.090332316	0.204214929	down_in_case
Yang2025_S	ENSGALG00010008017	GMNN	case	control	-0.26170808	0.199244296	-1.313503502	0.189013342	0.343806411	down_in_case
Yang2025_S	ENSGALG00010008018	ENSGALG00010008018	case	control	0.554065641	0.243967157	2.271066514	0.023142952	0.075526237	up_in_case
Yang2025_S	ENSGALG00010008019	PPP2CB	case	control	-0.263047745	0.082274587	-3.197193126	0.00138772	0.009083079	down_in_case
Yang2025_S	ENSGALG00010008020	COA6	case	control	0.072893439	0.278532367	0.261705452	0.793548537	0.875535478	up_in_case
Yang2025_S	ENSGALG00010008022	SLC35F3	case	control	-0.683688138	0.417787827	-1.6364482	0.101745819	0.221812888	down_in_case
Yang2025_S	ENSGALG00010008023	ATAD2	case	control	-0.085439885	0.093680424	-0.912035634	0.36174996	0.530312397	down_in_case
Yang2025_S	ENSGALG00010008024	MRS2	case	control	-0.155465879	0.114387149	-1.359120144	0.17410852	0.324358587	down_in_case
Yang2025_S	ENSGALG00010008025	TBC1D22A	case	control	-0.023812914	0.105977609	-0.224697596	0.822214535	0.893795112	down_in_case
Yang2025_S	ENSGALG00010008026	PURG	case	control	0.194194672	0.16604895	1.169502562	0.242201209	0.407289535	up_in_case
Yang2025_S	ENSGALG00010008027	C6orf62	case	control	-0.468871301	0.155616393	-3.012994269	0.002586838	0.014552922	down_in_case
Yang2025_S	ENSGALG00010008028	KCNK1	case	control	-0.200260005	0.22425528	-0.893000178	0.371857077	0.540223975	down_in_case
Yang2025_S	ENSGALG00010008029	LY6E	case	control	-0.741481396	0.147935673	-5.012187936	5.38146E-07	1.42028E-05	down_in_case
Yang2025_S	ENSGALG00010008030	GJA5	case	control	0.233063359	0.139053991	1.676063795	0.093725697	0.209383792	up_in_case
Yang2025_S	ENSGALG00010008031	MAP3K21	case	control	-0.274201478	0.208498461	-1.31512471	0.188467993	0.343127971	down_in_case
Yang2025_S	ENSGALG00010008032	PUM2	case	control	-0.010599225	0.121225053	-0.087434281	0.930326322	0.958009922	down_in_case
Yang2025_S	ENSGALG00010008033	ACOT13	case	control	0.486745066	0.180114149	2.702425482	0.006883561	0.030607028	up_in_case
Yang2025_S	ENSGALG00010008034	DPEP1	case	control	-0.074073101	0.309909486	-0.239015274	0.811093739	0.886269211	down_in_case
Yang2025_S	ENSGALG00010008035	TDP2	case	control	-0.081389063	0.171781904	-0.473792997	0.635647529	0.766393522	down_in_case
Yang2025_S	ENSGALG00010008036	SPOCK3	case	control	-0.179842604	0.186538626	-0.964103832	0.334993872	0.503553829	down_in_case
Yang2025_S	ENSGALG00010008037	ENSGALG00010008037	case	control	-1.235797437	1.001900606	-1.233453128	0.217406754	0.378317072	down_in_case
Yang2025_S	ENSGALG00010008038	WRN	case	control	-0.014699582	0.096021269	-0.153086729	0.878329882	0.928362174	down_in_case
Yang2025_S	ENSGALG00010008039	CHMP1A	case	control	0.101337828	0.157526053	0.643308369	0.520024027	0.673908194	up_in_case
Yang2025_S	ENSGALG00010008040	ENSGALG00010008040	case	control	0.947582422	0.540677172	1.752584481	0.079673357	0.186847565	up_in_case
Yang2025_S	ENSGALG00010008041	ENSGALG00010008041	case	control	1.021837844	0.266446741	3.835054765	0.000125536	0.00127783	up_in_case
Yang2025_S	ENSGALG00010008043	IL17REL	case	control	-0.33957857	0.396057335	-0.857397503	0.391225239	0.55852861	down_in_case
Yang2025_S	ENSGALG00010008045	DCDC2	case	control	0.947151292	0.75467998	1.255036993	0.209465331	0.368659389	up_in_case
Yang2025_S	ENSGALG00010008046	RHOB	case	control	0.020873978	0.169868738	0.122882988	0.902199757	0.941381776	up_in_case
Yang2025_S	ENSGALG00010008047	ASAP1	case	control	0.560259232	0.265616273	2.109280525	0.034920375	0.102531695	up_in_case
Yang2025_S	ENSGALG00010008048	ENSGALG00010008048	case	control	-1.266858894	0.71032805	-1.783484256	0.074507509	0.177967455	down_in_case
Yang2025_S	ENSGALG00010008050	CDK10	case	control	-0.089108264	0.175944675	-0.506456157	0.61253646	0.749205264	down_in_case
Yang2025_S	ENSGALG00010008051	ENSGALG00010008051	case	control	0.236649621	0.100370757	2.357754672	0.018385839	0.063653831	up_in_case
Yang2025_S	ENSGALG00010008052	RBFA	case	control	-0.237887493	0.144778391	-1.64311463	0.100359218	0.219597126	down_in_case
Yang2025_S	ENSGALG00010008056	GTF2E2	case	control	-0.290545516	0.116936194	-2.484650017	0.012967886	0.049185489	down_in_case
Yang2025_S	ENSGALG00010008057	BAG1	case	control	-0.401499682	0.096958084	-4.140961376	3.45853E-05	0.000454238	down_in_case
Yang2025_S	ENSGALG00010008058	USP25	case	control	-0.010229737	0.098055625	-0.104325858	0.916910758	0.951194779	down_in_case

Yang2025_S	ENSGALG00010008059	ENSGALG00010008059	case	control	-0.219412702	0.095039181	-2.308655229	0.020962721	0.070169472	down_in_case
Yang2025_S	ENSGALG00010008062	VPS9D1	case	control	0.871769894	0.248003172	3.515156223	0.000439495	0.003560679	up_in_case
Yang2025_S	ENSGALG00010008063	KCNV1	case	control	-0.329472664	0.343412716	-0.959407292	0.337353603	0.50545355	down_in_case
Yang2025_S	ENSGALG00010008064	DDX60	case	control	0.741866006	0.267595215	2.772344068	0.005565418	0.025937824	up_in_case
Yang2025_S	ENSGALG00010008065	CHMP5	case	control	0.229016753	0.113214777	2.022852131	0.043088395	0.119632753	up_in_case
Yang2025_S	ENSGALG00010008066	PCNX2	case	control	0.239279903	0.24472565	0.977747543	0.328199201	0.497872401	up_in_case
Yang2025_S	ENSGALG00010008067	MPC2	case	control	0.125598828	0.157617544	0.796858169	0.425533407	0.590379098	up_in_case
Yang2025_S	ENSGALG00010008069	DLG2	case	control	0.018949698	0.144346198	0.13127951	0.8955542	0.938334765	up_in_case
Yang2025_S	ENSGALG00010008071	SMIM18	case	control	0.867739238	0.33841901	2.564097205	0.010344457	0.041565746	up_in_case
Yang2025_S	ENSGALG00010008072	NRSN1	case	control	-0.243607262	0.26007268	-0.936689166	0.348918471	0.517328203	down_in_case
Yang2025_S	ENSGALG00010008073	ZNF276	case	control	-0.106549313	0.126835057	-0.840062006	0.400873621	0.567527574	down_in_case
Yang2025_S	ENSGALG00010008074	RHAG	case	control	-0.154725894	0.272001423	-0.568842223	0.569463218	0.713937399	down_in_case
Yang2025_S	ENSGALG00010008075	SDHA	case	control	0.082906891	0.152757565	0.542735093	0.587312208	0.728714773	up_in_case
Yang2025_S	ENSGALG00010008078	GUCY1B1	case	control	-0.151956375	0.08347601	-1.82035983	0.068704226	0.167640235	down_in_case
Yang2025_S	ENSGALG00010008079	CCDC127	case	control	-0.104371215	0.116579832	-0.895276764	0.370639158	0.539185371	down_in_case
Yang2025_S	ENSGALG00010008081	TOP1MT	case	control	0.018161848	0.140477898	0.12928616	0.897131222	0.93902049	up_in_case
Yang2025_S	ENSGALG00010008083	FANCA	case	control	0.300498749	0.277777812	1.081795362	0.279343468	0.447174179	up_in_case
Yang2025_S	ENSGALG00010008084	CYP2AC1	case	control	-2.335949882	0.722215881	-3.234420543	0.001218898	0.008166033	down_in_case
Yang2025_S	ENSGALG00010008086	RHPN1	case	control	-0.32885303	0.164744948	-1.996134231	0.045919314	0.125394011	down_in_case
Yang2025_S	ENSGALG00010008087	CPNE8	case	control	-0.04297849	0.19861858	-0.216387057	0.828686054	0.897524675	down_in_case
Yang2025_S	ENSGALG00010008088	SLC35A4	case	control	-0.141869538	0.203534094	-0.697030829	0.485783501	0.643050544	down_in_case
Yang2025_S	ENSGALG00010008091	ENSGALG00010008091	case	control	-0.492755754	0.317206085	-1.553424657	0.120321716	0.250064596	down_in_case
Yang2025_S	ENSGALG00010008092	SPIRE2	case	control	1.58266391	0.934682773	1.693263165			up_in_case
Yang2025_S	ENSGALG00010008093	EFR3A	case	control	-0.443554969	0.07108304	-6.239954945	4.37697E-10	2.91833E-08	down_in_case
Yang2025_S	ENSGALG00010008094	PIM3	case	control	0.227906011	0.096360899	2.365129576	0.018023764	0.062876037	up_in_case
Yang2025_S	ENSGALG00010008096	CPSF1	case	control	-0.210519865	0.134701376	-1.562863503	0.118084704	0.246666219	down_in_case
Yang2025_S	ENSGALG00010008098	ENSGALG00010008098	case	control	-0.131616437	0.229613881	-0.573207669	0.566504093	0.711512801	down_in_case
Yang2025_S	ENSGALG00010008099	PANK3	case	control	-0.286418209	0.103354045	-2.771233664	0.005584434	0.026005182	down_in_case
Yang2025_S	ENSGALG00010008100	ENSGALG00010008100	case	control	0.544613841	0.22608697	2.408868764	0.016002051	0.057679405	up_in_case
Yang2025_S	ENSGALG00010008101	CENPQ	case	control	-0.385430075	0.187494993	-2.055681964	0.039813182	0.112993215	down_in_case
Yang2025_S	ENSGALG00010008103	RBPMS	case	control	0.440034284	0.104729791	4.20161523	2.65017E-05	0.000367464	up_in_case
Yang2025_S	ENSGALG00010008104	TCF25	case	control	-0.089467546	0.106398399	-0.840873047	0.400419059	0.567352044	down_in_case
Yang2025_S	ENSGALG00010008105	MMUT	case	control	0.301993295	0.095108084	3.175264202	0.001497001	0.00959996	up_in_case
Yang2025_S	ENSGALG00010008106	ENSGALG00010008106	case	control	-0.067253363	0.279919778	-0.240259419	0.810129152	0.885631893	down_in_case
Yang2025_S	ENSGALG00010008107	TUBB3	case	control	0.149935461	0.123247285	1.216541697	0.223778637	0.385077218	up_in_case
Yang2025_S	ENSGALG00010008108	ENSGALG00010008108	case	control	1.002077425	0.292342005	3.427757247	0.000608589	0.004659722	up_in_case
Yang2025_S	ENSGALG00010008110	DEF8	case	control	0.059568523	0.234059982	0.254501102	0.799108453	0.878430916	up_in_case
Yang2025_S	ENSGALG00010008111	SYTL2	case	control	0.204817462	0.369660184	0.554069576	0.57953123	0.722340581	up_in_case
Yang2025_S	ENSGALG00010008113	DBNDD1	case	control	0.167225653	0.216116778	0.773774506	0.439064151	0.602798321	up_in_case
Yang2025_S	ENSGALG00010008115	ENSGALG00010008115	case	control	0.371325289	0.183614866	2.022305147	0.043144837	0.119717903	up_in_case
Yang2025_S	ENSGALG00010008116	URAH	case	control	0.458483657	0.376248909	1.218564748	0.223009436	0.384474516	up_in_case
Yang2025_S	ENSGALG00010008117	ENSGALG00010008117	case	control	0.073201942	0.14925555	0.490447037	0.623817599	0.757947358	up_in_case
Yang2025_S	ENSGALG00010008118	ENSGALG00010008118	case	control	-1.003227997	0.369695554	-2.713659891	0.006654445	0.029744609	down_in_case
Yang2025_S	ENSGALG00010008119	CCDC83	case	control	1.053733091	0.417313772	2.525037899	0.011568581	0.045156353	up_in_case
Yang2025_S	ENSGALG00010008120	CDC5L	case	control	0.010419584	0.086020412	0.121129205	0.903588697	0.942331105	up_in_case
Yang2025_S	ENSGALG00010008121	MPZL1	case	control	-0.264918379	0.108403962	-2.443807156	0.014533193	0.05357673	down_in_case
Yang2025_S	ENSGALG00010008123	ENSGALG00010008123	case	control	-0.094155752	0.111144306	-0.847148677	0.396912256	0.563924581	down_in_case
Yang2025_S	ENSGALG00010008124	ENSGALG00010008124	case	control	0.520972906	0.209936464	2.481574174	0.013080348	0.049426538	up_in_case
Yang2025_S	ENSGALG00010008126	CDH3	case	control	2.157559535	0.359126766	6.007793738	1.88065E-09	1.06967E-07	up_in_case
Yang2025_S	ENSGALG00010008127	TBC1D2	case	control	0.664676018	0.160866377	4.131851718	3.59853E-05	0.000469967	up_in_case
Yang2025_S	ENSGALG00010008128	RARS	case	control	0.176071179	0.083407106	2.110985348	0.034773571	0.102208335	up_in_case
Yang2025_S	ENSGALG00010008129	TANGO6	case	control	0.196059906	0.200320882	0.97872925	0.327713776	0.497515139	up_in_case
Yang2025_S	ENSGALG00010008130	RCSD1	case	control	-0.016526097	0.099909287	-0.165411019	0.868620481	0.922805694	down_in_case
Yang2025_S	ENSGALG00010008131	OC90	case	control	-0.33849326	0.513813862	-0.658785768	0.510033348	0.665292024	down_in_case
Yang2025_S	ENSGALG00010008132	TDO2	case	control	1.047760519	0.440236611	2.37999406	0.017312917	0.061046432	up_in_case
Yang2025_S	ENSGALG00010008133	CREG1	case	control	-0.895045355	0.152934378	-5.852479791	4.84297E-09	2.40167E-07	down_in_case
Yang2025_S	ENSGALG00010008134	SLC2A13	case	control	-0.166845638	0.144435	-1.155160715	0.248024619	0.413819811	down_in_case
Yang2025_S	ENSGALG00010008135	WWC1	case	control	0.769325258	0.116672518	6.593885759	4.28461E-11	3.95405E-09	up_in_case
Yang2025_S	ENSGALG00010008136	ELP1	case	control	0.260742131	0.164675232	1.583371873	0.11333673	0.239730925	up_in_case
Yang2025_S	ENSGALG00010008137	HHLA1	case	control	0.027936032	0.395155787	0.07069625	0.943639505	0.965463767	up_in_case
Yang2025_S	ENSGALG00010008139	ENSGALG00010008139	case	control	1.373410802	0.364010082	3.773002095	0.000161295	0.00157178	up_in_case
Yang2025_S	ENSGALG00010008141	APTX	case	control	0.917965812	0.164926544	5.56590705	2.60792E-08	1.06573E-06	up_in_case
Yang2025_S	ENSGALG00010008142	CD247	case	control	0.565994779	0.292948556	1.93206202	0.053351851	0.139905542	up_in_case
Yang2025_S	ENSGALG00010008143	DCTN6	case	control	-0.411303765	0.181814943	-2.262211004	0.02368437	0.077022565	down_in_case
Yang2025_S	ENSGALG00010008144	NTPCR	case	control	-0.107987283	0.241397599	-0.447341994	0.65462815	0.781616252	down_in_case
Yang2025_S	ENSGALG00010008145	ENSGALG00010008145	case	control	0.464898577	0.158630031	2.930709747	0.003381886	0.017817233	up_in_case
Yang2025_S	ENSGALG00010008146	SOX4	case	control	-0.234376289	0.096177306	-2.436918851	0.014813003	0.054406747	down_in_case
Yang2025_S	ENSGALG00010008147	CDKAL1	case	control	0.222569157	0.12780421	1.741485336	0.081598541	0.189892397	up_in_case
Yang2025_S	ENSGALG00010008148	ABCD2	case	control	-0.082608788	0.085315278	-0.96827661	0.332906243	0.502061526	down_in_case
Yang2025_S	ENSGALG00010008149	DNAJA1	case	control	0.378404099	0.147021411	2.573802661	0.010058763	0.040697324	up_in_case
Yang2025_S	ENSGALG00010008151	SIPA1L2	case	control	0.316324214	0.147703361	2.141618245	0.032224214	0.096631036	up_in_case
Yang2025_S	ENSGALG00010008152	ENSGALG00010008152	case	control	0.039844677	0.221487382	0.179895924	0.857234275	0.915649212	up_in_case
Yang2025_S	ENSGALG00010008153	SMU1	case	control	-0.15042653	0.06068896	-2.478647355	0.013188162	0.049723226	down_in_case
Yang2025_S	ENSGALG00010008154	KCNQ3	case	control	-0.13467934	0.224062316	-0.601079836	0.547786813	0.696733227	down_in_case
Yang2025_S	ENSGALG00010008155	MAP10	case	control	-0.152929587	0.210157762	-0.727689455	0.466803707	0.627886539	down_in_case
Yang2025_S	ENSGALG00010008157	CTSO	case	control	-0.046078602	0.0946495	-0.486834078	0.626375918	0.759929342	down_in_case
Yang2025_S	ENSGALG00010008158	CLPTM1L	case	control	-0.20918675	0.060783823	-3.441487213	0.000578526	0.004476914	down_in_case
Yang2025_S	ENSGALG00010008159	SUPT3H	case	control	-0.119380857	0.198169876	-0.602416772	0.546896749	0.695855204	down_in_case
Yang2025_S	ENSGALG00010008160	TXNL4A	case	control	-0.13673686	0.153587583	-0.890285905	0.373312387	0.541255415	down_in_case
Yang2025_S	ENSGALG00010008161	TMEFF1	case	control	0.105879732	0.330812693	0.320059459	0.748923257	0.847674443	up_in_case
Yang2025_S	ENSGALG00010008162	HSBP1L1	case	control	-0.549009052	0.346901577	-1.582607543	0.113510943	0.239917335	down_in_case
Yang2025_S	ENSGALG00010008163	CHTF8	case	control	-0.31631893	0.211550394	-1.495241504	0.134851425	0.271240562	down_in_case
Yang2025_S	ENSGALG00010008164	TG	case	control	1.038723115	0.569983786	1.822373094	0.068398394	0.167180783	up_in_case
Yang2025_S	ENSGALG00010008165	MRPL1	case	control	-0.158061855	0.10576909	-1.494404971	0.135069803	0.271483875	down_in_case
Yang2025_S	ENSGALG00010008166	RUNX2	case	control	1.428570768	0.626275907	2.281056563	0.0225451	0.073904015	up_in_case
Yang2025_S	ENSGALG00010008167	UTP4	case	control	0.321383166	0.143018878	2.247138075	0.024631204	0.079141303	up_in_case
Yang2025_S	ENSGALG00010008168	CAVIN4	case	control	0.280066456	0.150040895	1.866600811	0.061957354	0.156041159	up_in_case
Yang2025_S	ENSGALG00010008169	HTR4	case	control	-0.190986768	0.490371622	-0.389473533	0.696925885	0.812283558	down_in_case
Yang2025_S	ENSGALG00010008170	PICALM	case	control	0.039843688	0.133948207	0.29745593	0.766118449	0.857636616	up_in_case
Yang2025_S	ENSGALG00010008171	KIF21A	case	control	0.908850209	0.251066	3.619965307	0.000294643	0.002567757	up_in_case
Yang2025_S	ENSGALG00010008172	SNTB2	case	control	-0.144388586	0.235196624	-0.61390586	0.539277538	0.690384243	down_in_case
Yang2025_S	ENSGALG00010008174	SLC26A2	case	control	-0.256297055	0.195124657	-1.313504192	0.189013109	0.343806411	down_in_case
Yang2025_S	ENSGALG00010008175	ENSGALG00010008175	case	control	0.095270336	0.223627993	0.426021515	0.670092169	0.792192467	up_in_case
Yang2025_S	ENSGALG00010008176	SLA	case	control	-0.303917817	0.364017683	-0.834898498	0.40377486	0.570126816	down_in_case
Yang2025_S	ENSGALG00010008177	NREP	case	control	-0.671161285	0.134011108	-5.008251155	5.49268E-07	1.44416E-05	down_in_case
Yang2025_S	ENSGALG00010008178	PGR	case	control	-0.825579142	0.437206731	-1.888303823	0.058985174	0.150818056	down_in_case
Yang2025_S	ENSGALG00010008179	SEMA6A	case	control	0.57752636	0.244610627	2.361002741	0.018225596	0.063284276	up_in_case
Yang2025_S	ENSGALG00010008180	POU2F1	case	control	0.589057264	0.285586791	2.062620827	0.039148667	0.111489649	up_in_case
Yang2025_S	ENSGALG00010008181	DISC1	case	control	-0.021587206	0.125773352	-0.171635773	0.863723882	0.919409694	down_in_case
Yang2025_S	ENSGALG00010008182	CLIC5	case	control	-0.507555962	0.277449891	-1.82936083	0.067345571	0.165309237	down_in_case
Yang2025_S	ENSGALG00010008183	ALG10	case	control	-0.670866234	0.097207664	-6.90137183	5.15027E-12	5.93133E-10	down_in_case
Yang2025_S	ENSGALG00010008184	SLC66A2	case	control	-0.646492356	0.130517041	-4.953317589	7.29588E-07	1.83848E-05	down_in_case
Yang2025_S	ENSGALG00010008185	PDGFC	case	control	-0.095663014	0.101138514	-0.945861377	0.344219295	0.51273153	down_in_case
Yang2025_S	ENSGALG00010008186	NOP53	case	control	0.138731668	0.161834744	0.857242796	0.391310716	0.55852861	up_in_case
Yang2025_S	ENSGALG00010008187	ENSGALG00010008187	case	control	0.136423264	0.193158099	0.706277732	0.480015438	0.638593662	up_in_case
Yang2025_S	ENSGALG00010008188	VPS4A	case	control	0.021680572	0.109595957	0.197822738	0.843183756	0.907018562	up_in_case
Yang2025_S	ENSGALG00010008189	EXOC3	case	control	0.03771234	0.072186214	0.522431328	0.601370048	0.73996394	up_in_case
Yang2025_S	ENSGALG00010008190	TSNARE1	case	control	-0.397316424	0.141536334	-2.807169106	0.0049979	0.023970981	down_in_case
Yang2025_S	ENSGALG00010008191	ENSGALG00010008191	case	control	1.153904151	0.328330463	3.514459611	0.00044065	0.003567957	up_in_case
Yang2025_S	ENSGALG00010008192	HNRNPU	case	control	0.078576267	0.069770814	1.126205393	0.260078591	0.42642607	up_in_case
Yang2025_S	ENSGALG00010008193	CNOT6L	case	control	-0.692460061	0.095189046	-7.27457713	3.47507E-13	4.99228E-11	down_in_case
Yang2025_S	ENSGALG00010008194	PDF	case	control	0.751244464	0.23558814	3.188804262	0.001428626	0.00926811	up_in_case
Yang2025_S	ENSGALG00010008195	ENSGALG00010008195	case	control	0.0853584	0.339615004	0.251338719	0.801552242	0.880210032	up_in_case
Yang2025_S	ENSGALG00010008196	PDCD6	case	control	-0.323688327	0.124541778	-2.599034105	0.009348648	0.038664719	down_in_case
Yang2025_S	ENSGALG00010008197	STARD4	case	control	0.079394058	0.202105562	0.3928346	0.694441648	0.810201303	up_in_case
Yang2025_S	ENSGALG00010008198	GLRB	case	control	0.650869134	0.46709003	1.393455421	0.163482117	0.310159741	up_in_case
Yang2025_S	ENSGALG00010008199	TPRKB	case	control	0.009194801	0.144590922	0.063591825	0.949295241	0.968477135	up_in_case
Yang2025_S	ENSGALG00010008200	COG8	case	control	0.151744217	0.232595436	0.652395505	0.514146063	0.6689035	up_in_case
Yang2025_S	ENSGALG00010008201	ENPP4	case	control	0.152734843	0.147502122	1.035475565	0.300446872	0.470049081	up_in_case
Yang2025_S	ENSGALG00010008202	KCNG2	case	control	0.103280107	0.093136258	1.10891407	0.267467253	0.434500554	up_in_case
Yang2025_S	ENSGALG00010008204	ENSGALG00010008204	case	control	0.634303124	0.228336143	2.7779357	0.005470544	0.025624213	up_in_case
Yang2025_S	ENSGALG00010008205	ENSGALG00010008205	case	control	0.123532387	0.266903178	0.462835953	0.643481959	0.772918926	up_in_case
Yang2025_S	ENSGALG00010008207	CAMK4	case	control	0.021851564	0.228649007	0.095568158	0.923863556	0.954127225	up_in_case
Yang2025_S	ENSGALG00010008208	ILDR2	case	control	-0.158428721	0.138050999	-1.147610098	0.251129559	0.416804479	down_in_case
Yang2025_S	ENSGALG00010008209	PPARGC1B	case	control	0.485002711	0.168498664	2.878377195	0.003997269	0.020174553	up_in_case
Yang2025_S	ENSGALG00010008210	CTDP1	case	control	0.263378625	0.147724361	1.78290584	0.074601632	0.178131211	up_in_case

Yang2025_S	ENSGALG00010008211	ADGRB1	case	control	-0.283855168	0.16704007	-1.699323813	0.089258188	0.20264139	down_in_case
Yang2025_S	ENSGALG00010008212	GTSE1	case	control	-0.109944907	0.125633035	-0.875127367	0.381504608	0.549484931	down_in_case
Yang2025_S	ENSGALG00010008213	ISCA1	case	control	-0.618957745	0.135463565	-4.56918246	4.8963E-06	9.04258E-05	down_in_case
Yang2025_S	ENSGALG00010008214	CCNG2	case	control	-0.679392131	0.158678933	-4.281552169	1.85594E-05	0.0002731	down_in_case
Yang2025_S	ENSGALG00010008215	SELENOW	case	control	0.288946938	0.223569205	1.292427269	0.196209218	0.352344836	up_in_case
Yang2025_S	ENSGALG00010008217	ARHGEF37	case	control	-0.102669687	0.172849779	-0.593982171	0.552524038	0.700584392	down_in_case
Yang2025_S	ENSGALG00010008218	ARHGAP42	case	control	0.198080851	0.225909086	0.876816666	0.380586225	0.54844561	up_in_case
Yang2025_S	ENSGALG00010008219	NIP7	case	control	0.412377053	0.260057628	1.585714117	0.112804175	0.238858256	up_in_case
Yang2025_S	ENSGALG00010008220	ENSGALG00010008220	case	control	1.209091135	0.62075448	1.947776737	0.051441683	0.136048559	up_in_case
Yang2025_S	ENSGALG00010008221	ENSGALG00010008221	case	control	-0.162883223	0.154412713	-1.0548563	0.291491051	0.460050187	down_in_case
Yang2025_S	ENSGALG00010008222	STYXL2	case	control	0.106993302	0.182353142	0.586736816	0.557380478	0.70449859	up_in_case
Yang2025_S	ENSGALG00010008223	ENSGALG00010008223	case	control	-0.315962768	0.102536295	-3.081472456	0.002059795	0.01222976	down_in_case
Yang2025_S	ENSGALG00010008224	ENSGALG00010008224	case	control	0.228510736	0.211250221	1.081706493	0.279382967	0.447186038	up_in_case
Yang2025_S	ENSGALG00010008225	ENSGALG00010008225	case	control	4.807423274	1.066427661	4.50796941	6.5451E-06	0.000116781	up_in_case
Yang2025_S	ENSGALG00010008226	COX20	case	control	-0.103977284	0.170829606	-0.608660795	0.542749298	0.692728656	down_in_case
Yang2025_S	ENSGALG00010008227	DUSP1	case	control	-0.303215691	0.13144225	-2.306835829	0.021063974	0.070457628	down_in_case
Yang2025_S	ENSGALG00010008229	PIGY	case	control	-0.194660414	0.125707984	-1.548512737	0.121498898	0.251947491	down_in_case
Yang2025_S	ENSGALG00010008230	TRMU	case	control	0.426194841	0.113629652	3.750736123	0.000176316	0.001690961	up_in_case
Yang2025_S	ENSGALG00010008231	ZCCHC6	case	control	0.723951227	0.145710017	4.968438298	6.74943E-07	1.71317E-05	up_in_case
Yang2025_S	ENSGALG00010008232	PYURF	case	control	-0.055975195	0.150206581	-0.372654743	0.709405424	0.820514988	down_in_case
Yang2025_S	ENSGALG00010008233	CYRIB	case	control	-0.138014723	0.081515715	-1.693105706	0.090435348	0.204381541	down_in_case
Yang2025_S	ENSGALG00010008234	COMMD10	case	control	-0.13953214	0.282109376	-0.494602988	0.620880392	0.755766438	down_in_case
Yang2025_S	ENSGALG00010008236	ENSGALG00010008236	case	control	1.463319788	0.373691277	3.915852147	9.00854E-05	0.000981612	up_in_case
Yang2025_S	ENSGALG00010008237	GRIA2	case	control	1.155657651	0.509515818	2.268148721			up_in_case
Yang2025_S	ENSGALG00010008238	NTAQ1	case	control	0.121017017	0.180429693	0.670715639	0.502401698	0.658914604	up_in_case
Yang2025_S	ENSGALG00010008240	RCAN2	case	control	0.206542393	0.094013089	2.196953593	0.028023761	0.086960076	up_in_case
Yang2025_S	ENSGALG00010008241	HERC3	case	control	0.011196491	0.086257561	0.129803007	0.896722284	0.938786882	up_in_case
Yang2025_S	ENSGALG00010008243	YTHDC2	case	control	0.289128766	0.176350747	1.639509734	0.101107145	0.220800512	up_in_case
Yang2025_S	ENSGALG00010008245	FBXO32	case	control	-0.378110978	0.229326185	-1.64879112	0.099190435	0.21780944	down_in_case
Yang2025_S	ENSGALG00010008246	TERF2	case	control	0.238006943	0.093447344	2.54696318	0.01086649	0.043067274	up_in_case
Yang2025_S	ENSGALG00010008247	GASK1B	case	control	-0.118664544	0.123191952	-0.963249162	0.335422499	0.503994298	down_in_case
Yang2025_S	ENSGALG00010008248	TPK1	case	control	0.145110337	0.105934507	1.36981179	0.170745661	0.319726284	up_in_case
Yang2025_S	ENSGALG00010008250	KLHL38	case	control	0.171522151	0.30063295	0.570536765	0.568313694	0.712821901	up_in_case
Yang2025_S	ENSGALG00010008251	AHRR	case	control	-0.367821177	0.399708191	-0.920224267	0.357455576	0.5266036	down_in_case
Yang2025_S	ENSGALG00010008252	ZFP92	case	control	0.322010165	0.153550585	2.097095001	0.035985166	0.104896557	up_in_case
Yang2025_S	ENSGALG00010008255	MSANTD3	case	control	0.156994989	0.138513	1.133431442	0.257033116	0.42337547	up_in_case
Yang2025_S	ENSGALG00010008257	DESI2	case	control	-0.523468924	0.058378458	-8.966816517	3.05208E-19	1.41769E-16	down_in_case
Yang2025_S	ENSGALG00010008258	CCDC82	case	control	0.449626139	0.14260615	3.152922489	0.001616447	0.010183499	up_in_case
Yang2025_S	ENSGALG00010008259	MCC	case	control	-0.039418045	0.142748522	-0.276136273	0.782443401	0.868509576	down_in_case
Yang2025_S	ENSGALG00010008262	CYB5B	case	control	0.089105674	0.167961515	0.530512444	0.595756683	0.735459725	up_in_case
Yang2025_S	ENSGALG00010008263	C1orf101	case	control	-0.905038258	0.432615851	-2.092013633	0.036437296	0.105737968	down_in_case
Yang2025_S	ENSGALG00010008264	CNTNAP2	case	control	0.046606485	0.243967203	0.191035861	0.848497497	0.910118044	up_in_case
Yang2025_S	ENSGALG00010008266	ECSCR	case	control	0.296144137	0.185202866	1.599025675	0.109814898	0.233771861	up_in_case
Yang2025_S	ENSGALG00010008267	MAML2	case	control	0.092502963	0.193694187	0.477572221	0.632954706	0.764561623	up_in_case
Yang2025_S	ENSGALG00010008268	ENSGALG00010008268	case	control	-1.164459046	0.278943743	-4.174530085	2.98602E-05	0.000404768	down_in_case
Yang2025_S	ENSGALG00010008269	ENSGALG00010008269	case	control	0.739968068	0.41628205	1.777564199	0.075475458	0.17957154	up_in_case
Yang2025_S	ENSGALG00010008270	ENSGALG00010008270	case	control	0.618276082	0.205219439	3.012755932	0.00258887	0.014556206	up_in_case
Yang2025_S	ENSGALG00010008272	DCP2	case	control	0.065995405	0.121563813	0.542886931	0.587207655	0.728714773	up_in_case
Yang2025_S	ENSGALG00010008273	TMEM144	case	control	-0.714466812	0.166737658	-4.284975686	1.82759E-05	0.000270069	down_in_case
Yang2025_S	ENSGALG00010008274	NFAT5	case	control	0.601865105	0.240663115	2.500861448	0.012389164	0.047533865	up_in_case
Yang2025_S	ENSGALG00010008275	CELSR1	case	control	-0.166582871	0.152392299	-1.093118691	0.27434169	0.441737989	down_in_case
Yang2025_S	ENSGALG00010008276	DNAJC18	case	control	0.473293475	0.134570579	3.517065	0.000436347	0.003545478	up_in_case
Yang2025_S	ENSGALG00010008278	CYP39A1	case	control	0.072925898	0.228465871	0.319198212	0.749576213	0.847783798	up_in_case
Yang2025_S	ENSGALG00010008279	MAPK15	case	control	0.36123327	0.184294839	1.960083484	0.049986033	0.133235534	up_in_case
Yang2025_S	ENSGALG00010008282	FAM83H	case	control	0.104713465	0.250872514	0.417397118	0.676387963	0.796540713	up_in_case
Yang2025_S	ENSGALG00010008283	NQO1	case	control	-0.062827509	0.219033876	-0.286839231	0.774235422	0.862565607	down_in_case
Yang2025_S	ENSGALG00010008284	BCKDHA	case	control	-0.212333616	0.151573197	-1.400865196	0.161254388	0.307103991	down_in_case
Yang2025_S	ENSGALG00010008285	ADSS2	case	control	-0.018967452	0.086638356	-0.218926732	0.826707123	0.896430655	down_in_case
Yang2025_S	ENSGALG00010008289	MTMR2	case	control	-0.184754597	0.08944946	-2.06546353	0.038879163	0.11090709	down_in_case
Yang2025_S	ENSGALG00010008290	FAM13A	case	control	-0.401713174	0.095458841	-4.208234362	2.57374E-05	0.000359009	down_in_case
Yang2025_S	ENSGALG00010008291	PTGFRN	case	control	-0.378269972	0.09281966	-4.075321696	4.59508E-05	0.000570234	down_in_case
Yang2025_S	ENSGALG00010008292	REEP5	case	control	-0.197109807	0.119655339	-1.647313095	0.099493708	0.218131658	down_in_case
Yang2025_S	ENSGALG00010008293	PLA2G7	case	control	0.000615835	0.134064606	0.004593572	0.996334872	0.997408509	up_in_case
Yang2025_S	ENSGALG00010008294	NOB1	case	control	0.556718676	0.163902009	3.396655589	0.000682148	0.00514101	up_in_case
Yang2025_S	ENSGALG00010008295	DMAC2	case	control	0.904188191	0.325725064	2.775924514	0.005504499	0.025731362	up_in_case
Yang2025_S	ENSGALG00010008296	PALLD	case	control	-0.01774713	0.173469268	-0.102307055	0.918512956	0.951982818	down_in_case
Yang2025_S	ENSGALG00010008297	ENSGALG00010008297	case	control	0.80900676	0.226960319	3.564529527	0.000364509	0.003062069	up_in_case
Yang2025_S	ENSGALG00010008298	ENSGALG00010008298	case	control	0.262685986	0.13396655	1.960832648	0.049898548	0.13309234	up_in_case
Yang2025_S	ENSGALG00010008300	CUL1	case	control	0.508624998	0.12858726	3.955485165	7.63795E-05	0.000859732	up_in_case
Yang2025_S	ENSGALG00010008301	FAM91A1	case	control	-0.03314189	0.077086716	-0.429929974	0.667246581	0.789645953	down_in_case
Yang2025_S	ENSGALG00010008302	ENSGALG00010008302	case	control	0.261554267	0.230016325	1.137111757	0.25549157	0.421783559	up_in_case
Yang2025_S	ENSGALG00010008303	TEX10	case	control	-0.375540342	0.095445081	-3.934622258	8.33276E-05	0.000921564	down_in_case
Yang2025_S	ENSGALG00010008306	ENSGALG00010008306	case	control	0.482091253	0.394732847	1.221310203	0.221968592	0.383500961	up_in_case
Yang2025_S	ENSGALG00010008308	WWP2	case	control	0.407044247	0.212225798	1.917977226	0.055113899	0.14330692	up_in_case
Yang2025_S	ENSGALG00010008309	ENSGALG00010008309	case	control	0.048071733	0.399861462	0.120220969	0.904308107	0.942875681	up_in_case
Yang2025_S	ENSGALG00010008310	EXOSC5	case	control	0.329164374	0.145773017	2.258061065	0.023941853	0.077570266	up_in_case
Yang2025_S	ENSGALG00010008311	SRP19	case	control	0.710002054	0.2274395	3.121718325	0.001797989	0.011065404	up_in_case
Yang2025_S	ENSGALG00010008312	ZBTB18	case	control	0.077629857	0.137850713	0.563144398	0.573336561	0.716341214	up_in_case
Yang2025_S	ENSGALG00010008313	PSMD7	case	control	0.273971536	0.12206555	2.244462386	0.024802666	0.079527187	up_in_case
Yang2025_S	ENSGALG00010008314	INVS	case	control	0.519649985	0.1821918	2.852213907	0.004341587	0.021469134	up_in_case
Yang2025_S	ENSGALG00010008316	ADGRF5	case	control	-0.127383201	0.13440928	-0.947726233	0.343268842	0.5118728	down_in_case
Yang2025_S	ENSGALG00010008317	CEP57	case	control	0.097374774	0.095519651	1.019421379	0.308002961	0.477663229	up_in_case
Yang2025_S	ENSGALG00010008318	ADPRH	case	control	-0.056116152	0.146936006	-0.381908788	0.702529022	0.81634023	down_in_case
Yang2025_S	ENSGALG00010008319	ERP44	case	control	0.094899466	0.084198051	1.127098131	0.259700994	0.426258344	up_in_case
Yang2025_S	ENSGALG00010008320	GPRIN3	case	control	-0.211279547	0.124933008	-1.691142727	0.090809547	0.205060935	down_in_case
Yang2025_S	ENSGALG00010008321	ZFHX3	case	control	0.37152184	0.263004085	1.412608626	0.157770831	0.303037427	up_in_case
Yang2025_S	ENSGALG00010008323	AKT3	case	control	0.124531352	0.11615047	1.072155379	0.283650248	0.451836558	up_in_case
Yang2025_S	ENSGALG00010008324	SCRIB	case	control	-0.060645972	0.093816789	-0.646429835	0.518001024	0.672349503	down_in_case
Yang2025_S	ENSGALG00010008325	AADAT	case	control	0.188865261	0.179374647	1.05290945	0.292382495	0.461160165	up_in_case
Yang2025_S	ENSGALG00010008327	TGFB1	case	control	0.306798847	0.147393064	2.081501245	0.03738805	0.107667386	up_in_case
Yang2025_S	ENSGALG00010008330	EZH2	case	control	0.187305326	0.093920068	1.994305699	0.046118657	0.125782072	up_in_case
Yang2025_S	ENSGALG00010008331	CCDC97	case	control	0.043476674	0.130362017	0.333507222	0.738751439	0.840267568	up_in_case
Yang2025_S	ENSGALG00010008332	DHX38	case	control	0.012232321	0.060872476	0.200949949	0.840737711	0.905384853	up_in_case
Yang2025_S	ENSGALG00010008333	APC	case	control	0.717641125	0.243531707	2.946807757	0.003210727	0.017135767	up_in_case
Yang2025_S	ENSGALG00010008334	STX17	case	control	0.039314594	0.097731479	0.402271553	0.687484188	0.804508915	up_in_case
Yang2025_S	ENSGALG00010008335	HNRNPUL1	case	control	-0.102828702	0.093708652	-1.097323448	0.272500024	0.439857273	down_in_case
Yang2025_S	ENSGALG00010008336	PAIP2B	case	control	-0.382068182	0.098251058	-3.888692794	0.000100786	0.001073736	down_in_case
Yang2025_S	ENSGALG00010008339	FAM76B	case	control	1.319117736	0.295038959	4.470995087	7.78565E-06	0.000133707	up_in_case
Yang2025_S	ENSGALG00010008340	PDIA4	case	control	-0.435122267	0.091940184	-4.732666887	2.21589E-06	4.74276E-05	down_in_case
Yang2025_S	ENSGALG00010008341	DHODH	case	control	0.334252999	0.223280687	1.497008108	0.134391148	0.27047092	up_in_case
Yang2025_S	ENSGALG00010008342	PLD3	case	control	0.147343032	0.138909867	1.060709613	0.28882189	0.457704426	up_in_case
Yang2025_S	ENSGALG00010008343	TNFRSF21	case	control	-0.007257457	0.10118683	-0.071723338	0.942822083	0.965052573	down_in_case
Yang2025_S	ENSGALG00010008344	MFAP3L	case	control	0.050778453	0.3262265	0.15565398	0.876305774	0.927136964	up_in_case
Yang2025_S	ENSGALG00010008346	NR4A3	case	control	0.296916935	0.262524916	1.131004781	0.258053079	0.424303204	up_in_case
Yang2025_S	ENSGALG00010008347	PUF60	case	control	0.128426486	0.088738915	1.447239759	0.147829785	0.289923879	up_in_case
Yang2025_S	ENSGALG00010008348	ENSGALG00010008348	case	control	0.287397	0.099336296	2.893172105	0.003813723	0.019495313	up_in_case
Yang2025_S	ENSGALG00010008350	LRRC6	case	control	0.607067529	0.307652952	1.973221856	0.048470291	0.13040913	up_in_case
Yang2025_S	ENSGALG00010008351	IST1	case	control	0.030279744	0.151749096	0.199538216	0.841841752	0.906153624	up_in_case
Yang2025_S	ENSGALG00010008352	ENSGALG00010008352	case	control	0.443003468	0.15438822	2.869412371	0.004112353	0.02058392	up_in_case
Yang2025_S	ENSGALG00010008354	TMEM71	case	control	-0.203718371	0.229256432	-0.888604823	0.374215498	0.542240147	down_in_case
Yang2025_S	ENSGALG00010008355	MAFA	case	control	-0.384067841	0.099279409	-3.868554875	0.000109482	0.00114261	down_in_case
Yang2025_S	ENSGALG00010008356	ENSGALG00010008356	case	control	0.465250173	0.252867539	1.839896787	0.065783392	0.162505152	up_in_case
Yang2025_S	ENSGALG00010008358	HPF1	case	control	0.268520868	0.108715376	2.469943799	0.013513428	0.050632769	up_in_case
Yang2025_S	ENSGALG00010008360	ZC3H3	case	control	-0.501985599	0.144045063	-3.484920547	0.000492283	0.003904363	down_in_case
Yang2025_S	ENSGALG00010008361	MATR3	case	control	0.295929215	0.121981005	2.426027029	0.015265133	0.055744139	up_in_case
Yang2025_S	ENSGALG00010008362	RAMP3	case	control	1.333107301	0.137997977	9.660339451	4.44389E-22	3.09628E-19	up_in_case
Yang2025_S	ENSGALG00010008364	ENSGALG00010008364	case	control	-1.768752411	0.499704473	-3.539596916	0.000400739	0.003300409	down_in_case
Yang2025_S	ENSGALG00010008366	RPRD1A	case	control	0.418542758	0.113346305	3.692601683	0.000221972	0.002024328	up_in_case
Yang2025_S	ENSGALG00010008367	ENSGALG00010008367	case	control	-2.46647171	0.738794874	-3.338506801	0.0008423	0.006084731	down_in_case
Yang2025_S	ENSGALG00010008368	SNCA	case	control	-0.347230425	0.246673924	-1.4076495	0.159234882	0.304640315	down_in_case
Yang2025_S	ENSGALG00010008372	CLCN3	case	control	-0.022294651	0.078982824	-0.282272147	0.777734841	0.864942938	down_in_case
Yang2025_S	ENSGALG00010008373	MACIR	case	control	-0.247659723	0.183700759	-1.348169298	0.177603937	0.328891809	down_in_case
Yang2025_S	ENSGALG00010008374	ZNF821	case	control	0.494789495	0.104986769	4.712874784	2.44246E-06	5.15442E-05	up_in_case
Yang2025_S	ENSGALG00010008375	ENSGALG00010008375	case	control	0.013110741	0.077073374	0.170107268	0.864925778	0.920197031	up_in_case
Yang2025_S	ENSGALG00010008376	MMRN1	case	control	-0.612353997	0.357190174	-1.71436406	0.086461885	0.19805719	down_in_case
Yang2025_S	ENSGALG00010008377	AKT2	case	control	-0.190120811	0.133266625	-1.426619837	0.153689503	0.297659934	down_in_case

Yang2025_S	ENSGALG00010008380	SIL1	case	control	0.163246322	0.105004366	1.554662227	0.120026535	0.249674543	up_in_case
Yang2025_S	ENSGALG00010008381	C18orf21	case	control	0.073734314	0.129682971	0.568573599	0.569645546	0.714042518	up_in_case
Yang2025_S	ENSGALG00010008382	ENSGALG00010008382	case	control	0.021587846	0.165936629	0.130096928	0.89648974	0.938786882	up_in_case
Yang2025_S	ENSGALG00010008383	PLD5	case	control	0.320662532	0.311751777	1.028582853	0.303675739	0.473273194	up_in_case
Yang2025_S	ENSGALG00010008385	CTNNA1	case	control	-0.073225165	0.042482431	-1.72365759	0.084769675	0.19534735	down_in_case
Yang2025_S	ENSGALG00010008386	SESN3	case	control	-0.105825177	0.124679798	-0.84877565	0.396006142	0.56321143	down_in_case
Yang2025_S	ENSGALG00010008387	PHF20L1	case	control	0.601262236	0.166493907	3.611316761	0.000304646	0.002640078	up_in_case
Yang2025_S	ENSGALG00010008388	ADCY1	case	control	0.073724544	0.468652226	0.15731184	0.874999089	0.926528291	up_in_case
Yang2025_S	ENSGALG00010008389	GALNT1	case	control	-0.445948242	0.065436287	-6.814999161	9.42643E-12	1.01044E-09	down_in_case
Yang2025_S	ENSGALG00010008391	ENSGALG00010008391	case	control	0.112695193	0.101150491	1.114133917	0.26522174	0.432416631	up_in_case
Yang2025_S	ENSGALG00010008392	CD36	case	control	-0.875891548	0.269750971	-3.247037609	0.00116613	0.007897142	down_in_case
Yang2025_S	ENSGALG00010008394	CD2AP	case	control	0.17406045	0.093960215	1.852490978	0.063955352	0.159202899	up_in_case
Yang2025_S	ENSGALG00010008395	ENDOD1	case	control	-0.017999431	0.238013195	-0.075623669	0.939718505	0.96346547	down_in_case
Yang2025_S	ENSGALG00010008396	LRRTM2	case	control	0.715415627	0.522846058	1.368310263	0.17121498	0.320295441	up_in_case
Yang2025_S	ENSGALG00010008397	ATXN1L	case	control	-0.250786406	0.110100912	-2.277786818	0.022739282	0.074418012	down_in_case
Yang2025_S	ENSGALG00010008399	INO80C	case	control	0.136604062	0.122938843	1.11115461	0.266501805	0.433641132	up_in_case
Yang2025_S	ENSGALG00010008400	MAP1LC3C	case	control	-0.35914089	0.338601605	-1.06065915	0.288844831	0.457704426	down_in_case
Yang2025_S	ENSGALG00010008401	PSMD8	case	control	0.189529576	0.123044646	1.540331762	0.123479505	0.254539484	up_in_case
Yang2025_S	ENSGALG00010008403	GNAT3	case	control	-1.494746131	0.49475234	-3.021200729	0.002517744	0.014267898	down_in_case
Yang2025_S	ENSGALG00010008405	LRATD2	case	control	-0.61957039	0.155171484	-3.992810893	6.52946E-05	0.0007595	down_in_case
Yang2025_S	ENSGALG00010008407	GNAI1	case	control	-0.432956059	0.083257541	-5.200202318	1.99072E-07	6.15092E-06	down_in_case
Yang2025_S	ENSGALG00010008408	SMARCA4	case	control	0.32155698	0.094611991	3.398691609	0.00067709	0.005111187	up_in_case
Yang2025_S	ENSGALG00010008409	CWC15	case	control	-0.311737582	0.106053466	-2.93943795	0.003288081	0.017461665	down_in_case
Yang2025_S	ENSGALG00010008410	NAPRT	case	control	-0.179116685	0.216130859	-0.828741833	0.407250509	0.573235943	down_in_case
Yang2025_S	ENSGALG00010008412	NEK1	case	control	0.935972133	0.202072217	4.631869464	3.62379E-06	7.13241E-05	up_in_case
Yang2025_S	ENSGALG00010008415	ENSGALG00010008415	case	control	0.863811398	0.481787731	1.792929423	0.07298421	0.175199822	up_in_case
Yang2025_S	ENSGALG00010008416	MSX2	case	control	1.094949307	0.79935123	1.369797489	0.170750126	0.319726284	up_in_case
Yang2025_S	ENSGALG00010008418	TMEM245	case	control	-0.150897538	0.111224916	-1.356688268	0.174880282	0.32533137	down_in_case
Yang2025_S	ENSGALG00010008422	EXO1	case	control	-0.181822862	0.157195583	-1.156666479	0.24740865	0.413116466	down_in_case
Yang2025_S	ENSGALG00010008424	PPIP5K2	case	control	0.336555463	0.164391435	2.04728101	0.040630501	0.114468744	up_in_case
Yang2025_S	ENSGALG00010008425	MAGI2	case	control	0.439410549	0.164744598	2.667222805	0.007648095	0.033087924	up_in_case
Yang2025_S	ENSGALG00010008426	AP1G1	case	control	-0.184121837	0.071568168	-2.572677787	0.010091511	0.040796404	down_in_case
Yang2025_S	ENSGALG00010008428	ENSGALG00010008428	case	control	1.374734382	0.531954882	2.584306354	0.009757508	0.039792471	up_in_case
Yang2025_S	ENSGALG00010008429	IGFBP3	case	control	-0.443703637	0.108407037	-4.092941293	4.25936E-05	0.000538115	down_in_case
Yang2025_S	ENSGALG00010008430	ENSGALG00010008430	case	control	0.552271353	0.389637145	1.417399137	0.156366265	0.301419823	up_in_case
Yang2025_S	ENSGALG00010008431	SH3RF1	case	control	-0.245492669	0.152223831	-1.612708524	0.106807848	0.229544627	down_in_case
Yang2025_S	ENSGALG00010008433	EBAG9	case	control	0.117801035	0.159376903	0.739134925	0.459825072	0.620973105	up_in_case
Yang2025_S	ENSGALG00010008437	SHKBP1	case	control	0.077029157	0.119304713	0.645650581	0.518505672	0.672691234	up_in_case
Yang2025_S	ENSGALG00010008438	SYBU	case	control	0.084790754	0.178101072	0.476082218	0.634015804	0.765245369	up_in_case
Yang2025_S	ENSGALG00010008440	ENSGALG00010008440	case	control	1.624474556	0.594753317	2.731341733	0.006307703	0.028528997	up_in_case
Yang2025_S	ENSGALG00010008441	PHLPP2	case	control	0.028986974	0.112042852	0.25871328	0.795856472	0.876582922	up_in_case
Yang2025_S	ENSGALG00010008442	WDR64	case	control	-1.441591657	0.480004949	-3.003284988	0.002670822	0.014893119	down_in_case
Yang2025_S	ENSGALG00010008443	RSFR	case	control	-0.772781677	0.31023348	-2.490968016	0.012739559	0.048477813	down_in_case
Yang2025_S	ENSGALG00010008444	SLC7A11	case	control	0.392508921	0.519478875	0.755582065	0.449899812	0.612226971	up_in_case
Yang2025_S	ENSGALG00010008447	FRRS1L	case	control	-0.019203922	0.116112019	-0.165391335	0.868635974	0.922805694	down_in_case
Yang2025_S	ENSGALG00010008448	TNS3	case	control	-0.103048887	0.124271839	-0.829221549	0.406979054	0.573027494	down_in_case
Yang2025_S	ENSGALG00010008449	SRA1	case	control	-0.042546244	0.304673243	-0.13964549	0.888940097	0.935249547	down_in_case
Yang2025_S	ENSGALG00010008450	GIN1	case	control	-0.233505872	0.159855443	-1.460731437	0.144089158	0.284684874	down_in_case
Yang2025_S	ENSGALG00010008451	OPN3	case	control	-0.17894456	0.243252423	-0.735633204	0.46195395	0.622866791	down_in_case
Yang2025_S	ENSGALG00010008452	ENSGALG00010008452	case	control	0.30328119	0.364951921	0.831016833	0.40596412	0.572003035	up_in_case
Yang2025_S	ENSGALG00010008453	AMOTL1	case	control	0.071534651	0.108501083	0.659298959	0.509703811	0.665048933	up_in_case
Yang2025_S	ENSGALG00010008455	FKBP1B	case	control	-0.073610028	0.139021895	-0.529485146	0.596468946	0.735817525	down_in_case
Yang2025_S	ENSGALG00010008456	NHLRC1	case	control	-0.069806118	0.140400823	-0.49719166	0.619053902	0.754737155	down_in_case
Yang2025_S	ENSGALG00010008457	MARVELD3	case	control	-0.064440531	0.237287209	-0.271571871	0.78595123	0.870744983	down_in_case
Yang2025_S	ENSGALG00010008459	EPB41L4B	case	control	0.462111656	0.282328863	1.636785028	0.101675395	0.221763442	up_in_case
Yang2025_S	ENSGALG00010008464	SF3B6	case	control	0.301274939	0.13728439	2.194531656	0.028197211	0.087395047	up_in_case
Yang2025_S	ENSGALG00010008466	C11orf97	case	control	-1.446498866	0.375683366	-3.850313844	0.000117967	0.001214977	down_in_case
Yang2025_S	ENSGALG00010008468	EPPK1	case	control	-0.388517116	0.406512825	-0.955731508	0.3392079	0.507391808	down_in_case
Yang2025_S	ENSGALG00010008469	NOCT	case	control	-0.006355949	0.172393839	-0.036868773	0.970589638	0.982321994	down_in_case
Yang2025_S	ENSGALG00010008473	CSMD3	case	control	0.845031344	0.872438846	0.968585189	0.332752196	0.50193785	up_in_case
Yang2025_S	ENSGALG00010008474	ENSGALG00010008474	case	control	0.178876728	0.114051231	1.568389275	0.116790312	0.244725149	up_in_case
Yang2025_S	ENSGALG00010008476	RNF166	case	control	-0.466836819	0.079816899	-5.848846857	4.94993E-09	2.42878E-07	down_in_case
Yang2025_S	ENSGALG00010008486	CTU2	case	control	-0.119928183	0.232365409	-0.516118918	0.605771356	0.743606752	down_in_case
Yang2025_S	ENSGALG00010008488	CCSER1	case	control	-0.067250558	0.186694115	-0.36021788	0.718684205	0.826504753	down_in_case
Yang2025_S	ENSGALG00010008492	ANKRD49	case	control	-0.181631974	0.172818113	-1.051000792	0.293258226	0.461705274	down_in_case
Yang2025_S	ENSGALG00010008497	PAM	case	control	-0.336213044	0.116919697	-2.875589424	0.004032739	0.020280122	down_in_case
Yang2025_S	ENSGALG00010008498	ENSGALG00010008498	case	control	0.271415717	0.150769305	1.800205402	0.071828211	0.172991034	up_in_case
Yang2025_S	ENSGALG00010008500	ENSGALG00010008500	case	control	0.071171654	0.207163723	0.343552688	0.73118271	0.835577421	up_in_case
Yang2025_S	ENSGALG00010008501	ENSGALG00010008501	case	control	-0.334869965	0.2817253	-1.188640015	0.234581355	0.397820516	down_in_case
Yang2025_S	ENSGALG00010008504	RNF139	case	control	-0.304146391	0.120774121	-2.518307645	0.011792027	0.045759371	down_in_case
Yang2025_S	ENSGALG00010008505	PTPN3	case	control	0.018011633	0.162336776	0.110952266	0.911654199	0.947628022	up_in_case
Yang2025_S	ENSGALG00010008509	MGARP	case	control	-0.021814365	0.291451091	-0.074847429	0.940336104	0.963543774	down_in_case
Yang2025_S	ENSGALG00010008514	PIEZO1	case	control	0.021754483	0.112356137	0.193620777	0.846472828	0.909031971	up_in_case
Yang2025_S	ENSGALG00010008515	TAFA5	case	control	0.526926976	0.160638558	3.280202347	0.001037327	0.007220353	up_in_case
Yang2025_S	ENSGALG00010008516	MRE11	case	control	0.195660002	0.092110606	2.124185373	0.033654646	0.099782445	up_in_case
Yang2025_S	ENSGALG00010008522	NUDT12	case	control	-0.001366082	0.177337635	-0.007703282	0.993853731	0.995970515	down_in_case
Yang2025_S	ENSGALG00010008527	CHUK	case	control	-0.101689515	0.071755159	-1.417173569	0.156432187	0.30147648	down_in_case
Yang2025_S	ENSGALG00010008529	GPR83	case	control	0.982852714	0.447802678	2.194834382	0.02817548	0.087347123	up_in_case
Yang2025_S	ENSGALG00010008534	RAB33B	case	control	-0.286035686	0.056183687	-5.091080693	3.56028E-07	9.92251E-06	down_in_case
Yang2025_S	ENSGALG00010008542	MOCOS	case	control	0.126026505	0.192811991	0.653623793	0.513354211	0.668310065	up_in_case
Yang2025_S	ENSGALG00010008561	EEF1D	case	control	-0.37867327	0.235372446	-1.608825826	0.107654432	0.230688069	down_in_case
Yang2025_S	ENSGALG00010008580	PANX1	case	control	0.069216791	0.331100838	0.209050484	0.834408828	0.90191491	up_in_case
Yang2025_S	ENSGALG00010008588	PYCR3	case	control	0.100748158	0.238374171	0.422647124	0.672552739	0.793835543	up_in_case
Yang2025_S	ENSGALG00010008592	KIAA0513	case	control	-0.163188618	0.109956532	-1.48411937	0.137777255	0.275534738	down_in_case
Yang2025_S	ENSGALG00010008596	ENSGALG00010008596	case	control	0.583630339	0.169752783	3.438119423	0.000585769	0.004527285	up_in_case
Yang2025_S	ENSGALG00010008618	EIF4EBP3	case	control	0.426131158	0.157215795	2.710485661	0.006718475	0.029959025	up_in_case
Yang2025_S	ENSGALG00010008623	TRPS1	case	control	0.55382194	0.227093562	2.438739056	0.014738607	0.054204931	up_in_case
Yang2025_S	ENSGALG00010008636	AGTPBP1	case	control	-0.026996282	0.266255369	-0.101392443	0.919238935	0.952418183	down_in_case
Yang2025_S	ENSGALG00010008637	RGS7	case	control	1.964214118	0.734201218	2.675307628	0.007466071	0.032471815	up_in_case
Yang2025_S	ENSGALG00010008653	ENSGALG00010008653	case	control	-0.214322853	0.196398894	-1.091263033	0.275157159	0.442336123	down_in_case
Yang2025_S	ENSGALG00010008656	CCDC6	case	control	0.415828498	0.202342862	2.055068778	0.039872362	0.113092075	up_in_case
Yang2025_S	ENSGALG00010008658	TSTA3	case	control	0.153827162	0.163158682	0.942807085	0.345779587	0.514515595	up_in_case
Yang2025_S	ENSGALG00010008666	SLC16A9	case	control	-0.629412517	0.108465924	-5.802859493	6.51934E-09	3.1112E-07	down_in_case
Yang2025_S	ENSGALG00010008668	ENSGALG00010008668	case	control	0.166577147	0.392497804	0.42440275	0.671272115	0.792861242	up_in_case
Yang2025_S	ENSGALG00010008674	RXFP1	case	control	-0.32921522	0.20335723	-1.61890099	0.105468568	0.227769128	down_in_case
Yang2025_S	ENSGALG00010008686	ENSGALG00010008686	case	control	0.171980036	0.445891491	0.3856993	0.699719381	0.814245476	up_in_case
Yang2025_S	ENSGALG00010008688	HBEGF	case	control	0.603097101	0.245770882	2.453899733	0.014131638	0.052443242	up_in_case
Yang2025_S	ENSGALG00010008690	YIF1B	case	control	0.121833716	0.177817239	0.685162566	0.493241343	0.65069754	up_in_case
Yang2025_S	ENSGALG00010008693	C4orf46	case	control	-0.105399467	0.253075613	-0.416474215	0.677063032	0.796968504	down_in_case
Yang2025_S	ENSGALG00010008696	PFN4	case	control	0.845632118	0.374041825	2.260795616	0.023771916	0.077163208	up_in_case
Yang2025_S	ENSGALG00010008699	NAA35	case	control	0.282357852	0.16134791	1.749993857	0.080119374	0.187546359	up_in_case
Yang2025_S	ENSGALG00010008700	ENSGALG00010008700	case	control	0.147449087	0.151613687	0.972531502	0.330786201	0.500109115	up_in_case
Yang2025_S	ENSGALG00010008708	ZC3H4	case	control	0.100633879	0.198310071	0.507457229	0.61183404	0.748892787	up_in_case
Yang2025_S	ENSGALG00010008716	TATDN1	case	control	0.469735009	0.097792899	4.803365221	1.56021E-06	3.52375E-05	up_in_case
Yang2025_S	ENSGALG00010008729	TMEM254	case	control	-0.449751205	0.151720127	-2.964347662	0.003033254	0.016473049	down_in_case
Yang2025_S	ENSGALG00010008731	NDUFB9	case	control	0.128682967	0.206336611	0.623655525	0.532853837	0.684831801	up_in_case
Yang2025_S	ENSGALG00010008739	VSTM5	case	control	0.043712934	0.318101606	0.13741815	0.890700286	0.936115589	up_in_case
Yang2025_S	ENSGALG00010008745	GRAMD4	case	control	-0.189378819	0.123192918	-1.537254106	0.124231095	0.255592714	down_in_case
Yang2025_S	ENSGALG00010008750	PFDN1	case	control	0.793945721	0.182613913	4.347673787	1.37589E-05	0.000215186	up_in_case
Yang2025_S	ENSGALG00010008752	GOLM1	case	control	-0.885011388	0.203370133	-4.351727438	1.35069E-05	0.000212569	down_in_case
Yang2025_S	ENSGALG00010008756	ITSN2	case	control	0.232109572	0.188832919	1.229179601	0.219004475	0.380028927	up_in_case
Yang2025_S	ENSGALG00010008762	SAE1	case	control	0.144539003	0.153217468	0.943358515	0.345497555	0.514150836	up_in_case
Yang2025_S	ENSGALG00010008764	ETFDH	case	control	0.071650845	0.088133571	0.812980158	0.416229434	0.581527689	up_in_case
Yang2025_S	ENSGALG00010008768	PRXL2C	case	control	0.065773593	0.186216296	0.353210724	0.723930469	0.830470694	up_in_case
Yang2025_S	ENSGALG00010008775	MED17	case	control	-0.08254896	0.097624084	-0.845579868	0.39778716	0.564591982	down_in_case
Yang2025_S	ENSGALG00010008780	KARS1	case	control	-0.201638026	0.111177991	-1.813650559	0.069731541	0.169493987	down_in_case
Yang2025_S	ENSGALG00010008782	CCDC9	case	control	1.171793361	0.279578845	4.191280494	2.77384E-05	0.000381952	up_in_case
Yang2025_S	ENSGALG00010008790	GPN1	case	control	-0.248155214	0.097175062	-2.553692365	0.010658738	0.04248556	down_in_case
Yang2025_S	ENSGALG00010008793	C11orf54	case	control	0.187932376	0.16406097	1.145503264	0.252000744	0.417802542	up_in_case
Yang2025_S	ENSGALG00010008799	ENSGALG00010008799	case	control	0.264501286	0.305946302	0.864534999	0.387294063	0.554556388	up_in_case
Yang2025_S	ENSGALG00010008807	BRD1	case	control	-0.100202462	0.132651688	-0.755380229	0.450020873	0.612226971	down_in_case
Yang2025_S	ENSGALG00010008811	TERF2IP	case	control	-0.383387165	0.130837498	-2.930254486	0.003386845	0.017836616	down_in_case
Yang2025_S	ENSGALG00010008814	ENSGALG00010008814	case	control	0.460863522	0.425232157	1.083792733	0.278456708	0.446062102	up_in_case
Yang2025_S	ENSGALG00010008821	ENSGALG00010008821	case	control	0.575237021	0.145775759	3.946040319	7.94542E-05	0.000890744	up_in_case
Yang2025_S	ENSGALG00010008824	GREM2	case	control	-0.85693203	0.254324919	-3.369437937	0.000753217	0.005574122	down_in_case
Yang2025_S	ENSGALG00010008825	PPID	case	control	0.06873705	0.150165129	0.457743085	0.64713703	0.77583924	up_in_case

Yang2025_S	ENSGALG00010008830	DHX34	case	control	0.349403799	0.141740828	2.46508931	0.01369791	0.051201819	up_in_case
Yang2025_S	ENSGALG00010008832	MTSS1	case	control	0.278991312	0.105120847	2.65400556	0.007954252	0.034115881	up_in_case
Yang2025_S	ENSGALG00010008837	ENSGALG00010008837	case	control	0.503234749	0.136168167	3.69568571	0.000219294	0.002010437	up_in_case
Yang2025_S	ENSGALG00010008839	ZNF512	case	control	0.073536985	0.073547256	0.999860349	0.317378095	0.48693384	up_in_case
Yang2025_S	ENSGALG00010008846	SLC35D2	case	control	0.928400545	0.280176556	3.313626799	0.000920943	0.006550968	up_in_case
Yang2025_S	ENSGALG00010008850	CYSTM1	case	control	-0.286004225	0.1961775	-1.457884949	0.144872258	0.285867306	down_in_case
Yang2025_S	ENSGALG00010008852	KHDRBS3	case	control	0.21382683	0.113101419	1.890576008	0.058680965	0.15023319	up_in_case
Yang2025_S	ENSGALG00010008859	ENSGALG00010008859	case	control	0.928934893	0.446500433	2.080479267	0.037481595	0.107846664	up_in_case
Yang2025_S	ENSGALG00010008860	SQLE	case	control	0.063919971	0.256387487	0.249310024	0.803120978	0.881427961	up_in_case
Yang2025_S	ENSGALG00010008862	ZNF367	case	control	0.042281443	0.155608076	0.271717536	0.785839216	0.870744983	up_in_case
Yang2025_S	ENSGALG00010008864	ADAT1	case	control	0.473839623	0.159915795	2.963057051	0.003046001	0.016520401	up_in_case
Yang2025_S	ENSGALG00010008866	WASHC5	case	control	0.088511964	0.069564824	1.272366682	0.203242866	0.361294724	up_in_case
Yang2025_S	ENSGALG00010008871	PRR7	case	control	0.985056182	0.291145617	3.383379744	0.000715996	0.005355555	up_in_case
Yang2025_S	ENSGALG00010008877	FNDC4	case	control	-0.073821121	0.18732479	-0.394080896	0.693521315	0.809492909	down_in_case
Yang2025_S	ENSGALG00010008879	HABP4	case	control	0.346864066	0.154296352	2.248038015	0.024573766	0.079011405	up_in_case
Yang2025_S	ENSGALG00010008880	FMN2	case	control	0.08956854	0.3309116	0.270672108	0.786643231	0.871165336	up_in_case
Yang2025_S	ENSGALG00010008884	FAM135B	case	control	0.393422112	0.871310888	0.451528975	0.651608347	0.779279293	up_in_case
Yang2025_S	ENSGALG00010008888	FBXO46	case	control	0.112366538	0.131168157	0.856660188	0.391632712	0.558759275	up_in_case
Yang2025_S	ENSGALG00010008892	ENSGALG00010008892	case	control	0.090229006	0.184446382	0.489188269	0.624708414	0.75873804	up_in_case
Yang2025_S	ENSGALG00010008893	ENSGALG00010008893	case	control	0.004496806	0.138546432	0.032457034	0.97410758	0.984421577	up_in_case
Yang2025_S	ENSGALG00010008897	NSMCE2	case	control	-0.210335858	0.139199468	-1.511039236	0.130778455	0.264980266	down_in_case
Yang2025_S	ENSGALG00010008903	ENSGALG00010008903	case	control	0.256013914	0.261241507	0.979989422	0.32709134	0.496804356	up_in_case
Yang2025_S	ENSGALG00010008905	GABARAPL2	case	control	-1.110315894	0.178004756	-6.237563062	4.4444E-10	2.94918E-08	down_in_case
Yang2025_S	ENSGALG00010008906	DBN1	case	control	0.155375555	0.135963785	1.14277162	0.253133425	0.419331226	up_in_case
Yang2025_S	ENSGALG00010008910	TRIB1	case	control	-0.273774239	0.167508271	-1.634392368	0.10217649	0.222647284	down_in_case
Yang2025_S	ENSGALG00010008918	PLAC9	case	control	-0.309375801	0.127161105	-2.432943627	0.014976632	0.05496428	down_in_case
Yang2025_S	ENSGALG00010008921	OPA3	case	control	1.819325271	0.377186351	4.823412259	1.41123E-06	3.23978E-05	up_in_case
Yang2025_S	ENSGALG00010008922	ENSGALG00010008922	case	control	0.731413801	0.720573338	1.015044218	0.31008475	0.47981832	up_in_case
Yang2025_S	ENSGALG00010008931	FNIP2	case	control	0.000588341	0.119825022	0.004909997	0.996082405	0.997299059	up_in_case
Yang2025_S	ENSGALG00010008932	VASP	case	control	0.203684096	0.174905435	1.164538406	0.244205888	0.409458435	up_in_case
Yang2025_S	ENSGALG00010008936	IFT172	case	control	0.320863156	0.097816875	3.280243366	0.001037176	0.007220353	up_in_case
Yang2025_S	ENSGALG00010008938	ZBED4	case	control	-0.358010129	0.09152254	-3.911715388	9.16429E-05	0.000995357	down_in_case
Yang2025_S	ENSGALG00010008939	RANBP9	case	control	-0.163715992	0.098570217	-1.660907281	0.09673207	0.214238979	down_in_case
Yang2025_S	ENSGALG00010008943	ENSGALG00010008943	case	control	0.309144316	0.283321559	1.09114293	0.275209995	0.442336123	up_in_case
Yang2025_S	ENSGALG00010008945	FER1L6	case	control	0.714948333	0.644555348	1.1092117	0.267338867	0.434394485	up_in_case
Yang2025_S	ENSGALG00010008946	COL22A1	case	control	0.230943364	0.319792705	0.72216583	0.470192529	0.630487082	up_in_case
Yang2025_S	ENSGALG00010008947	SPMIP2	case	control	0.275606604	0.411692024	0.669448491	0.503209428	0.659353397	up_in_case
Yang2025_S	ENSGALG00010008955	KRTCAP3	case	control	-0.576956543	0.353823487	-1.630633819	0.102967609	0.224100145	down_in_case
Yang2025_S	ENSGALG00010008956	LVRN	case	control	-0.164756586	0.233662091	-0.705106186	0.480744157	0.639233762	down_in_case
Yang2025_S	ENSGALG00010008960	LRRC2	case	control	0.232662657	0.274401476	0.847891421	0.396498443	0.563738986	up_in_case
Yang2025_S	ENSGALG00010008966	NRBP1	case	control	-0.106014905	0.107556229	-0.985669598	0.324295273	0.493885752	down_in_case
Yang2025_S	ENSGALG00010008969	PPP1R37	case	control	0.353965048	0.09107174	3.886661757	0.000101632	0.001081103	up_in_case
Yang2025_S	ENSGALG00010008971	ANXA11	case	control	0.043726599	0.097171423	0.449994425	0.652714461	0.780167124	up_in_case
Yang2025_S	ENSGALG00010008973	KCNK9	case	control	0.757504497	0.459112832	1.649931006	0.098957048	0.217434006	up_in_case
Yang2025_S	ENSGALG00010008974	SMCO4	case	control	-0.415580907	0.213438481	-1.947075825	0.051525645	0.136141422	down_in_case
Yang2025_S	ENSGALG00010008980	ENSGALG00010008980	case	control	0.420649258	0.459663748	0.915123849	0.360126616	0.528805521	up_in_case
Yang2025_S	ENSGALG00010008983	ENSGALG00010008983	case	control	0.862271203	0.503765487	1.711652	0.086960819	0.198851168	up_in_case
Yang2025_S	ENSGALG00010008988	PPM1G	case	control	-0.05912043	0.072814631	-0.811930636	0.416831434	0.581960328	down_in_case
Yang2025_S	ENSGALG00010008991	ENSGALG00010008991	case	control	0.291895701	0.205521834	1.420266134	0.155530217	0.300229259	up_in_case
Yang2025_S	ENSGALG00010008995	ECD	case	control	0.279845567	0.094008458	2.976812651	0.002912619	0.015941615	up_in_case
Yang2025_S	ENSGALG00010008996	CHRM3	case	control	0.093185402	0.241367661	0.386072442	0.699443018	0.81405984	up_in_case
Yang2025_S	ENSGALG00010008997	CHST6	case	control	-0.298860121	0.097840548	-3.054563015	0.002253887	0.013097555	down_in_case
Yang2025_S	ENSGALG00010008999	TRAPPC9	case	control	-0.274831028	0.111639596	-2.46177017	0.013825323	0.051595038	down_in_case
Yang2025_S	ENSGALG00010009000	FAM149B1	case	control	0.359480379	0.135252522	2.657846032	0.007864181	0.033833263	up_in_case
Yang2025_S	ENSGALG00010009002	ENSGALG00010009002	case	control	-0.043572869	0.210082297	-0.207408569	0.835690791	0.902740401	down_in_case
Yang2025_S	ENSGALG00010009003	ALG12	case	control	-0.452442934	0.084101031	-5.379754917	7.45873E-08	2.65146E-06	down_in_case
Yang2025_S	ENSGALG00010009007	TMEM65	case	control	-0.540707584	0.094539597	-5.719376884	1.06915E-08	4.91705E-07	down_in_case
Yang2025_S	ENSGALG00010009011	ACP6	case	control	-0.124389052	0.194573347	-0.639291322	0.522633433	0.676032385	down_in_case
Yang2025_S	ENSGALG00010009013	LIPG	case	control	0.083555605	0.201575344	0.414513022	0.678498433	0.798126416	up_in_case
Yang2025_S	ENSGALG00010009015	TMEM231	case	control	0.022591777	0.080493364	0.280666329	0.778966336	0.866036054	up_in_case
Yang2025_S	ENSGALG00010009016	DNAJC9	case	control	0.096070179	0.103394511	0.929161302	0.352805499	0.521628078	up_in_case
Yang2025_S	ENSGALG00010009019	ZNF513	case	control	0.317739924	0.128455476	2.473541298	0.013378134	0.050277397	up_in_case
Yang2025_S	ENSGALG00010009020	ARHGAP35	case	control	-0.010943939	0.098847548	-0.110715333	0.911842086	0.947752627	down_in_case
Yang2025_S	ENSGALG00010009021	HNF4beta	case	control	1.003507665	0.415360632	2.415991279	0.015692437	0.056887126	up_in_case
Yang2025_S	ENSGALG00010009024	PMAIP1	case	control	-0.44892246	0.21253102	-2.112267937	0.034663474	0.101970765	down_in_case
Yang2025_S	ENSGALG00010009025	UTP23	case	control	0.610555656	0.130409863	4.68182117	2.84337E-06	5.80974E-05	up_in_case
Yang2025_S	ENSGALG00010009026	RAPGEF2	case	control	0.101664433	0.150305992	0.676383105	0.498797441	0.65573041	up_in_case
Yang2025_S	ENSGALG00010009028	PDLIM7	case	control	0.543232025	0.109576362	4.95756579	7.13819E-07	1.802E-05	up_in_case
Yang2025_S	ENSGALG00010009029	IGSF3	case	control	-0.319785862	0.124686379	-2.564721688	0.010325859	0.041502985	down_in_case
Yang2025_S	ENSGALG00010009030	ZDHHC7	case	control	0.085312971	0.071439937	1.194191584	0.232403035	0.395516098	up_in_case
Yang2025_S	ENSGALG00010009031	SPIN1	case	control	-0.373426929	0.169751973	-2.199838521	0.027818354	0.086532428	down_in_case
Yang2025_S	ENSGALG00010009034	SNX17	case	control	-0.254233443	0.155665816	-1.633200212	0.102426895	0.223088276	down_in_case
Yang2025_S	ENSGALG00010009035	ENSGALG00010009035	case	control	-0.132455673	0.137237057	-0.965159671	0.334464843	0.503200046	down_in_case
Yang2025_S	ENSGALG00010009037	ENSGALG00010009037	case	control	0.37677205	0.25019699	1.505901611	0.132092433	0.267001458	up_in_case
Yang2025_S	ENSGALG00010009038	RAD21	case	control	-0.229064762	0.050649474	-4.522549696	6.10991E-06	0.000110002	down_in_case
Yang2025_S	ENSGALG00010009039	SLC36A4	case	control	-0.310080817	0.198365125	-1.563182118	0.118009765	0.246546638	down_in_case
Yang2025_S	ENSGALG00010009040	CRELD2	case	control	0.274005428	0.132582974	2.066671306	0.038765137	0.110660959	up_in_case
Yang2025_S	ENSGALG00010009043	TFAM	case	control	0.112054555	0.139887756	0.801031894	0.423113182	0.588714652	up_in_case
Yang2025_S	ENSGALG00010009047	SUPT5H	case	control	0.415815285	0.11109795	3.742780896	0.000181995	0.001732308	up_in_case
Yang2025_S	ENSGALG00010009050	PIK3CG	case	control	-0.010749211	0.143017258	-0.075160234	0.940087224	0.963527174	down_in_case
Yang2025_S	ENSGALG00010009051	TROJANZ	case	control	0.267291718	0.283685528	0.942211328	0.346084455	0.514727257	up_in_case
Yang2025_S	ENSGALG00010009054	RYR2	case	control	0.520447651	0.589962573	0.882170625	0.377684545	0.546068426	up_in_case
Yang2025_S	ENSGALG00010009057	NXNL2	case	control	1.037036465	0.355750183	2.915069378	0.003556095	0.018476578	up_in_case
Yang2025_S	ENSGALG00010009059	MED29	case	control	-0.967786726	0.319731669	-3.026871656	0.002470988	0.014060116	down_in_case
Yang2025_S	ENSGALG00010009065	EIF2B4	case	control	-0.170591792	0.152047443	-1.121964227	0.26187765	0.428730305	down_in_case
Yang2025_S	ENSGALG00010009067	ENSGALG00010009067	case	control	0.912541277	0.850846748	1.072509567	0.283491219	0.451738152	up_in_case
Yang2025_S	ENSGALG00010009068	AGO2	case	control	0.202465178	0.194261598	1.042229549	0.297305266	0.46628575	up_in_case
Yang2025_S	ENSGALG00010009069	GRID2	case	control	1.06551825	0.528043807	2.01785957	0.043605889	0.120780771	up_in_case
Yang2025_S	ENSGALG00010009070	ENSGALG00010009070	case	control	4.71638185	1.45918586	3.232200899	0.001228407	0.008221828	up_in_case
Yang2025_S	ENSGALG00010009073	PRKAR2B	case	control	-0.470959367	0.346268992	-1.360096854	0.173799277	0.323955714	down_in_case
Yang2025_S	ENSGALG00010009076	TMEM214	case	control	0.690054021	0.137335259	5.02459475	5.04497E-07	1.35456E-05	up_in_case
Yang2025_S	ENSGALG00010009078	PTTG2	case	control	-0.530341137	0.274876008	-1.929383144	0.053683315	0.140563132	down_in_case
Yang2025_S	ENSGALG00010009079	KCTD15	case	control	0.121420548	0.176833935	0.686636013	0.492312129	0.649902379	up_in_case
Yang2025_S	ENSGALG00010009080	TLL1	case	control	0.260563961	0.272079384	0.957676239	0.338226042	0.50635796	up_in_case
Yang2025_S	ENSGALG00010009081	HBP1	case	control	0.069950936	0.121905437	0.573813098	0.566094284	0.711190394	up_in_case
Yang2025_S	ENSGALG00010009083	MED30	case	control	-0.35592972	0.186204568	-1.911498323	0.055940572	0.144948284	down_in_case
Yang2025_S	ENSGALG00010009085	PAF1	case	control	0.824499748	0.174402221	4.727575968	2.27216E-06	4.84137E-05	up_in_case
Yang2025_S	ENSGALG00010009086	UBE2D1	case	control	0.011518524	0.11023901	0.104486825	0.916783023	0.951185424	up_in_case
Yang2025_S	ENSGALG00010009088	ENSGALG00010009088	case	control	0.93317219	0.298987968	3.121102823	0.001801751	0.011065404	up_in_case
Yang2025_S	ENSGALG00010009089	AGBL5	case	control	0.441076228	0.116784583	3.776836082	0.000158833	0.001551044	up_in_case
Yang2025_S	ENSGALG00010009091	EXT1	case	control	-0.183748878	0.080365014	-2.286428756	0.022229188	0.073040257	down_in_case
Yang2025_S	ENSGALG00010009092	EED	case	control	-0.651501356	0.194002699	-3.358207685	0.000784496	0.005759725	down_in_case
Yang2025_S	ENSGALG00010009093	SLU7	case	control	0.386289339	0.122339083	3.157530107	0.001591118	0.010069133	up_in_case
Yang2025_S	ENSGALG00010009094	DARS	case	control	-0.094345021	0.105690549	-0.892653328	0.372042852	0.540437521	down_in_case
Yang2025_S	ENSGALG00010009096	ENSGALG00010009096	case	control	1.262154894	1.440421275	0.876240108	0.380899517	0.548726844	up_in_case
Yang2025_S	ENSGALG00010009097	C1QTNF2	case	control	-0.202696003	0.185694393	-1.091556939	0.275027892	0.442234588	down_in_case
Yang2025_S	ENSGALG00010009098	CAD	case	control	0.120769329	0.108654509	1.111498554	0.266353812	0.433602847	up_in_case
Yang2025_S	ENSGALG00010009099	CXCR4	case	control	-0.534025035	0.192253139	-2.777718159	0.005474208	0.025632757	down_in_case
Yang2025_S	ENSGALG00010009100	ENSGALG00010009100	case	control	-0.156495201	0.107509053	-1.455646732	0.145490302	0.286762003	down_in_case
Yang2025_S	ENSGALG00010009101	HIKESHI	case	control	-0.729211123	0.305524921	-2.386748422	0.016998119	0.060195371	down_in_case
Yang2025_S	ENSGALG00010009102	HTRA3	case	control	-0.024255902	0.242033151	-0.100217271	0.920171835	0.952943376	down_in_case
Yang2025_S	ENSGALG00010009103	AP3S1	case	control	-0.813286562	0.198403364	-4.099157115	4.14657E-05	0.000529144	down_in_case
Yang2025_S	ENSGALG00010009104	MTR	case	control	-0.074026914	0.083993034	-0.881345869	0.378130646	0.546430012	down_in_case
Yang2025_S	ENSGALG00010009106	THSD7B	case	control	0.449996929	0.1362215	3.303420741	0.00095513	0.006763075	up_in_case
Yang2025_S	ENSGALG00010009109	ENSGALG00010009109	case	control	-0.039560136	0.290655575	-0.136106581	0.891737025	0.936424676	down_in_case
Yang2025_S	ENSGALG00010009111	LRFN1	case	control	0.215114163	0.144155462	1.492237342	0.135636936	0.272191922	up_in_case
Yang2025_S	ENSGALG00010009112	ENSGALG00010009112	case	control	0.244249917	0.208104592	1.17368826	0.240519914	0.40522851	up_in_case
Yang2025_S	ENSGALG00010009113	RPLP1	case	control	0.245621733	0.269318153	0.912013283	0.361761725	0.530312397	up_in_case
Yang2025_S	ENSGALG00010009114	SETD7	case	control	0.265965104	0.135347908	1.965047763	0.04940871	0.132151702	up_in_case
Yang2025_S	ENSGALG00010009115	COG5	case	control	-0.255818021	0.103322266	-2.475923452	0.013289207	0.050050026	down_in_case
Yang2025_S	ENSGALG00010009116	S1PR3	case	control	0.225814229	0.139890255	1.614224153	0.106478815	0.229084806	up_in_case
Yang2025_S	ENSGALG00010009117	ERGIC1	case	control	-0.085431864	0.055526825	-1.538569241	0.123909493	0.255086243	down_in_case
Yang2025_S	ENSGALG00010009118	ACTN2	case	control	0.410036945	0.116322454	3.525002538	0.000423479	0.003463132	up_in_case
Yang2025_S	ENSGALG00010009119	CISD1	case	control	-0.288835964	0.133757657	-2.159397605	0.030819334	0.093484417	down_in_case
Yang2025_S	ENSGALG00010009120	ATG12	case	control	-0.336457152	0.167442792	-2.009385709	0.044496245	0.122734594	down_in_case
Yang2025_S	ENSGALG00010009123	ENSGALG00010009123	case	control	1.539054166	0.165797829	9.282716013	1.65212E-20	1.00097E-17	up_in_case

Yang2025_S	ENSGALG00010009126	JPH3	case	control	0.284541	0.188000756	1.513509874	0.130150195	0.264209308	up_in_case
Yang2025_S	ENSGALG00010009127	SCOC	case	control	-0.303291101	0.073193975	-4.143662113	3.41803E-05	0.000449767	down_in_case
Yang2025_S	ENSGALG00010009129	ENSGALG00010009129	case	control	1.271390582	0.266428959	4.771968433	1.82434E-06	4.01614E-05	up_in_case
Yang2025_S	ENSGALG00010009130	HEATR1	case	control	-0.078777953	0.071126781	-1.1075709	0.268047177	0.435190191	down_in_case
Yang2025_S	ENSGALG00010009131	CDKL2	case	control	1.019262121	0.30831653	3.305895152	0.000946735	0.006718674	up_in_case
Yang2025_S	ENSGALG00010009132	SHC3	case	control	1.653735063	0.897081618	1.843461097	0.065261717	0.161560139	up_in_case
Yang2025_S	ENSGALG00010009133	BOD1L1	case	control	0.748783185	0.201312988	3.719497641	0.000199619	0.001865658	up_in_case
Yang2025_S	ENSGALG00010009135	TENM4	case	control	-0.401452901	0.222254719	-1.806273912	0.070875565	0.171467186	down_in_case
Yang2025_S	ENSGALG00010009136	KHK	case	control	0.425096604	0.486592473	0.873619358	0.38232558	0.55019688	up_in_case
Yang2025_S	ENSGALG00010009137	IPMK	case	control	-0.150533359	0.088572469	-1.699550221	0.08921556	0.20264139	down_in_case
Yang2025_S	ENSGALG00010009138	ENSGALG00010009138	case	control	-0.101063244	0.14468549	-0.698502968	0.484862702	0.64250302	down_in_case
Yang2025_S	ENSGALG00010009139	CKS2	case	control	-0.978372694	0.444768569	-2.19973434	0.027825749	0.086532428	down_in_case
Yang2025_S	ENSGALG00010009141	MAML3	case	control	0.192602184	0.108666419	1.772416775	0.076325399	0.180975742	up_in_case
Yang2025_S	ENSGALG00010009142	LGALS8	case	control	-0.13633782	0.137482293	-0.991675488	0.321355852	0.490588716	down_in_case
Yang2025_S	ENSGALG00010009143	MCCC2	case	control	0.215781461	0.121165733	1.780878592	0.074932287	0.178675807	up_in_case
Yang2025_S	ENSGALG00010009144	CREBZF	case	control	1.084958848	0.25852241	4.196769053	2.7075E-05	0.000373924	up_in_case
Yang2025_S	ENSGALG00010009147	HNMT	case	control	0.234578678	0.165528415	1.417150517	0.156438925	0.30147648	up_in_case
Yang2025_S	ENSGALG00010009149	FUT4	case	control	0.271521062	0.222939985	1.217911012	0.223257791	0.384728306	up_in_case
Yang2025_S	ENSGALG00010009150	ENSGALG00010009150	case	control	0.691185317	0.315950957	2.18763483	0.028696213	0.088490253	up_in_case
Yang2025_S	ENSGALG00010009151	CCNJL	case	control	0.571861211	0.242527764	2.357920605	0.018377623	0.063641197	up_in_case
Yang2025_S	ENSGALG00010009152	RPP25L	case	control	-0.207482959	0.241689562	-0.858468844	0.390633627	0.557995045	down_in_case
Yang2025_S	ENSGALG00010009153	SECISBP2	case	control	0.206700343	0.110988229	1.862362738	0.062551978	0.15694307	up_in_case
Yang2025_S	ENSGALG00010009155	PAK4	case	control	-0.046967039	0.120447443	-0.389938033	0.696582369	0.811951093	down_in_case
Yang2025_S	ENSGALG00010009157	ABHD1	case	control	0.298730007	0.198474747	1.505128541	0.132291033	0.26729118	up_in_case
Yang2025_S	ENSGALG00010009159	PTK2	case	control	0.040444584	0.102638163	0.39405015	0.693544014	0.809492909	up_in_case
Yang2025_S	ENSGALG00010009160	ENSGALG00010009160	case	control	0.182155071	0.297324563	0.612647233	0.54010962	0.690770674	up_in_case
Yang2025_S	ENSGALG00010009163	SAMD12	case	control	-0.518584632	0.184139391	-2.816261253	0.004858614	0.023484143	down_in_case
Yang2025_S	ENSGALG00010009164	TMEM128	case	control	-0.249335225	0.105702472	-2.358840055	0.018332155	0.063562723	down_in_case
Yang2025_S	ENSGALG00010009166	CLGN	case	control	-0.034255575	0.222392536	-0.154032037	0.877584475	0.928025294	down_in_case
Yang2025_S	ENSGALG00010009167	DCTN3	case	control	0.077054687	0.233181135	0.330449919	0.741060028	0.841482357	up_in_case
Yang2025_S	ENSGALG00010009168	MRPS12	case	control	-0.713880076	0.417800606	-1.708662138	0.087513551	0.199852727	down_in_case
Yang2025_S	ENSGALG00010009170	RPL26L1	case	control	-0.469460082	0.341529025	-1.374583265	0.16926068	0.317919878	down_in_case
Yang2025_S	ENSGALG00010009172	SPOPL	case	control	0.086233742	0.123354476	0.699072662	0.484506622	0.642336579	up_in_case
Yang2025_S	ENSGALG00010009173	LYAR	case	control	0.365973722	0.182122634	2.009490605	0.04448513	0.12272823	up_in_case
Yang2025_S	ENSGALG00010009178	ENSGALG00010009178	case	control	-0.63335821	0.389682526	-1.625318475	0.104094717	0.225768075	down_in_case
Yang2025_S	ENSGALG00010009180	NFKBIB	case	control	-0.16106631	0.205673564	-0.78311625	0.433558849	0.597624733	down_in_case
Yang2025_S	ENSGALG00010009181	ARID3C	case	control	0.198215905	0.396432788	0.499998766	0.617075947	0.753105037	up_in_case
Yang2025_S	ENSGALG00010009186	PREB	case	control	0.369732485	0.236494787	1.563385348	0.117961984	0.246483768	up_in_case
Yang2025_S	ENSGALG00010009188	ENSGALG00010009188	case	control	0.023445606	0.664320844	0.035292594	0.971846429	0.982991942	up_in_case
Yang2025_S	ENSGALG00010009189	ENSGALG00010009189	case	control	0.782190133	0.28474956	2.746940623	0.006015402	0.027474478	up_in_case
Yang2025_S	ENSGALG00010009190	ZBTB49	case	control	0.025949067	0.121078554	0.214315963	0.830300671	0.898799025	up_in_case
Yang2025_S	ENSGALG00010009192	LSM14A	case	control	0.043947922	0.082646002	0.531761023	0.594891521	0.734977689	up_in_case
Yang2025_S	ENSGALG00010009193	EDARADD	case	control	-0.659341207	0.249172879	-2.64611947	0.008142105	0.034665518	down_in_case
Yang2025_S	ENSGALG00010009197	FH	case	control	-0.149480045	0.102744319	-1.454874067	0.145704128	0.287102238	down_in_case
Yang2025_S	ENSGALG00010009198	KCNN2	case	control	0.018954648	0.135725961	0.139653811	0.888933522	0.935249547	up_in_case
Yang2025_S	ENSGALG00010009199	SEMA4D	case	control	-0.169453781	0.111357978	-1.521703098	0.128083491	0.261171122	down_in_case
Yang2025_S	ENSGALG00010009200	ADGRA3	case	control	0.07457284	0.115254196	0.647029285	0.51761299	0.672033636	up_in_case
Yang2025_S	ENSGALG00010009202	FABP6	case	control	0.720776872	0.23799152	3.028582163	0.002457043	0.014020839	up_in_case
Yang2025_S	ENSGALG00010009205	SIRT2	case	control	0.146394723	0.104927668	1.395196572	0.162956571	0.30949977	up_in_case
Yang2025_S	ENSGALG00010009208	SLC5A6	case	control	0.890978935	0.211219623	4.218258331	2.46197E-05	0.000346191	up_in_case
Yang2025_S	ENSGALG00010009210	PCDH15	case	control	1.193577556	0.462505643	2.58067674	0.009860687	0.040107609	up_in_case
Yang2025_S	ENSGALG00010009212	NXPH2	case	control	-0.197447217	0.228850135	-0.862779552	0.388258684	0.555254111	down_in_case
Yang2025_S	ENSGALG00010009213	DUS4L	case	control	0.153250602	0.116322667	1.317461215	0.187684074	0.341968824	up_in_case
Yang2025_S	ENSGALG00010009214	PCDH10	case	control	0.856195863	0.290405644	2.948275565	0.003195521	0.017097578	up_in_case
Yang2025_S	ENSGALG00010009216	NSG1	case	control	-0.509568784	0.305404993	-1.668501812			down_in_case
Yang2025_S	ENSGALG00010009219	TNFRSF11B	case	control	0.135266454	0.217364471	0.6223025	0.533742978	0.685501506	up_in_case
Yang2025_S	ENSGALG00010009220	GADD45G	case	control	-0.064371335	0.211008451	-0.305065199	0.760316496	0.854595062	down_in_case
Yang2025_S	ENSGALG00010009223	ENSGALG00010009223	case	control	-0.0272829	0.119353435	-0.228589152	0.819188257	0.892036287	down_in_case
Yang2025_S	ENSGALG00010009224	ENSGALG00010009224	case	control	0.433931129	0.324640612	1.336650788	0.181336653	0.333852062	up_in_case
Yang2025_S	ENSGALG00010009225	HNRNPL	case	control	0.370701617	0.083418401	4.443883062	8.83496E-06	0.00014762	up_in_case
Yang2025_S	ENSGALG00010009227	ENSGALG00010009227	case	control	-0.20969584	0.393068606	-0.533484071	0.593698535	0.734024406	down_in_case
Yang2025_S	ENSGALG00010009228	ATP6V0E1	case	control	-0.803027258	0.275875667	-2.910830324	0.003604697	0.018694252	down_in_case
Yang2025_S	ENSGALG00010009232	ECH1	case	control	0.046625699	0.263376781	0.177030408	0.859484505	0.916995034	up_in_case
Yang2025_S	ENSGALG00010009234	PCDH18	case	control	-0.021168912	0.152544267	-0.138772254	0.88963012	0.935481114	down_in_case
Yang2025_S	ENSGALG00010009235	KLHDC4	case	control	0.077990421	0.123696122	0.630500129	0.528367419	0.681088748	up_in_case
Yang2025_S	ENSGALG00010009236	DTWD2	case	control	0.046449732	0.181733869	0.25559205	0.798265858	0.877966435	up_in_case
Yang2025_S	ENSGALG00010009237	DENND3	case	control	0.35443531	0.188033944	1.884953864	0.059436067	0.151609297	up_in_case
Yang2025_S	ENSGALG00010009239	STX18	case	control	-0.400297324	0.113734235	-3.519585133	0.000432222	0.003516064	down_in_case
Yang2025_S	ENSGALG00010009243	BCAP29	case	control	-0.829691015	0.139361774	-5.953504997	2.6246E-09	1.41211E-07	down_in_case
Yang2025_S	ENSGALG00010009246	RUSC2	case	control	0.154651183	0.155917143	0.991880559	0.321255794	0.490489699	up_in_case
Yang2025_S	ENSGALG00010009250	ERO1B	case	control	-0.241835954	0.121604493	-1.988709039	0.046733328	0.127044269	down_in_case
Yang2025_S	ENSGALG00010009252	SIRT1	case	control	-0.234119445	0.114996569	-2.035881995	0.041762206	0.116741493	down_in_case
Yang2025_S	ENSGALG00010009253	SLC45A4	case	control	-0.207932009	0.158778945	-1.309569155	0.190341629	0.345212552	down_in_case
Yang2025_S	ENSGALG00010009256	CREBRF	case	control	-0.114342559	0.173494432	-0.659056073	0.509859762	0.665142963	down_in_case
Yang2025_S	ENSGALG00010009257	ENSGALG00010009257	case	control	0.295874593	0.131234895	2.254542082	0.024162086	0.078192911	up_in_case
Yang2025_S	ENSGALG00010009258	DNAJC12	case	control	0.111497228	0.143865292	0.775011311	0.438332976	0.602205464	up_in_case
Yang2025_S	ENSGALG00010009259	MGAT4D	case	control	1.805582573	0.481875193	3.746992165	0.000178968	0.001711678	up_in_case
Yang2025_S	ENSGALG00010009263	COLEC10	case	control	-0.354539366	0.1129553	-3.138758117	0.001696654	0.010587944	down_in_case
Yang2025_S	ENSGALG00010009266	PCDH7	case	control	0.398120242	0.299931028	1.327372644	0.184385444	0.337724917	up_in_case
Yang2025_S	ENSGALG00010009268	PWWP2A	case	control	-0.15882157	0.105305019	-1.508205131	0.131502034	0.266039612	down_in_case
Yang2025_S	ENSGALG00010009273	MAL2	case	control	-0.505009281	0.715029885	-0.706277167	0.48001579	0.638593662	down_in_case
Yang2025_S	ENSGALG00010009275	CBLL1	case	control	0.763842268	0.325486027	2.346774375	0.018936718	0.065168987	up_in_case
Yang2025_S	ENSGALG00010009276	ENSGALG00010009276	case	control	-0.110935566	0.183591388	-0.60425256	0.545675742	0.695063205	down_in_case
Yang2025_S	ENSGALG00010009277	ACTN4	case	control	0.242365556	0.076326743	3.175368756	0.001496462	0.00959996	up_in_case
Yang2025_S	ENSGALG00010009279	ELMOD2	case	control	-0.40079207	0.081307583	-4.929331987	8.25113E-07	2.06426E-05	down_in_case
Yang2025_S	ENSGALG00010009280	HSD17B4	case	control	0.053021377	0.140499002	0.377379032	0.705891947	0.818625523	up_in_case
Yang2025_S	ENSGALG00010009284	CCN3	case	control	0.732786641	0.387083259	1.893098251	0.058344805	0.149615129	up_in_case
Yang2025_S	ENSGALG00010009286	ENSGALG00010009286	case	control	0.670541376	0.344457079	1.94666162	0.051575316	0.136221006	up_in_case
Yang2025_S	ENSGALG00010009289	GPR137B	case	control	-0.061015557	0.20623021	-0.29586139	0.767335938	0.858585699	down_in_case
Yang2025_S	ENSGALG00010009294	ACOX3	case	control	-0.289488107	0.128728317	-2.248830046	0.024523311	0.078956682	down_in_case
Yang2025_S	ENSGALG00010009297	CTNNA3	case	control	-0.071754752	0.24961936	-0.287456677	0.77376267	0.862350567	down_in_case
Yang2025_S	ENSGALG00010009299	MAP4K1	case	control	0.235841889	0.432874376	0.544827557	0.585872124	0.727770373	up_in_case
Yang2025_S	ENSGALG00010009303	TTC1	case	control	0.084089318	0.113351732	0.741844145	0.45818177	0.61933873	up_in_case
Yang2025_S	ENSGALG00010009306	TBC1D9	case	control	-0.166664798	0.116644529	-1.428826533	0.153054097	0.296841872	down_in_case
Yang2025_S	ENSGALG00010009307	DIRAS2	case	control	1.17902107	0.243093962	4.850063161	1.23422E-06	2.89543E-05	up_in_case
Yang2025_S	ENSGALG00010009309	TRIM54	case	control	0.202333005	0.153875592	1.314912928	0.188539167	0.34321271	up_in_case
Yang2025_S	ENSGALG00010009311	BNIP1	case	control	-0.084266876	0.130131686	-0.647550789	0.517275535	0.671845892	down_in_case
Yang2025_S	ENSGALG00010009313	UNC13B	case	control	-0.243652694	0.243578559	-1.000304357	0.31716324	0.486694168	down_in_case
Yang2025_S	ENSGALG00010009317	LRRTM3	case	control	0.841340256	0.571430539	1.472340378	0.140928985	0.28014913	up_in_case
Yang2025_S	ENSGALG00010009320	GPR20	case	control	-0.278652028	0.178042257	-1.565089284	0.117561976	0.245869148	down_in_case
Yang2025_S	ENSGALG00010009322	ENSGALG00010009322	case	control	0.065239093	0.279200729	0.233663764	0.815246021	0.889255026	up_in_case
Yang2025_S	ENSGALG00010009323	RNF150	case	control	0.034081552	0.14923556	0.228374204	0.819355342	0.89209692	up_in_case
Yang2025_S	ENSGALG00010009326	DLD	case	control	0.195839095	0.076599716	2.556655636	0.010568378	0.042197808	up_in_case
Yang2025_S	ENSGALG00010009327	NID1	case	control	-0.607502605	0.094234261	-6.446727515	1.14291E-10	9.48003E-09	down_in_case
Yang2025_S	ENSGALG00010009328	ENSGALG00010009328	case	control	-0.169265364	0.183648337	-0.921681987	0.356694479	0.525982811	down_in_case
Yang2025_S	ENSGALG00010009330	EIF3K	case	control	-0.344881722	0.215374389	-1.601312596	0.109307701	0.233245566	down_in_case
Yang2025_S	ENSGALG00010009332	ENSGALG00010009332	case	control	0.42186654	0.107733757	3.915824998	9.00956E-05	0.000981612	up_in_case
Yang2025_S	ENSGALG00010009333	ADRA1B	case	control	0.015888269	0.199561238	0.079616006	0.936542665	0.961166743	up_in_case
Yang2025_S	ENSGALG00010009334	SS18L2	case	control	0.369721548	0.199321219	1.854903106	0.063610064	0.158655136	up_in_case
Yang2025_S	ENSGALG00010009335	PTP4A3	case	control	-0.071311495	0.14665397	-0.48625683	0.626785083	0.760227185	down_in_case
Yang2025_S	ENSGALG00010009336	LAMB1	case	control	-0.150739917	0.10569857	-1.426130146	0.153830778	0.297809377	down_in_case
Yang2025_S	ENSGALG00010009337	ZNF330	case	control	0.543928141	0.127394853	4.269624166	1.95803E-05	0.000285409	up_in_case
Yang2025_S	ENSGALG00010009338	KYNU	case	control	0.082586482	0.479631741	0.172187274	0.863290303	0.919290108	up_in_case
Yang2025_S	ENSGALG00010009340	SYK	case	control	0.413957611	0.256291647	1.615181828	0.106271325	0.228921148	up_in_case
Yang2025_S	ENSGALG00010009344	ENPP2	case	control	0.896168726	0.342462288	2.61683916	0.008874815	0.037205338	up_in_case
Yang2025_S	ENSGALG00010009345	ZFAT	case	control	0.010063017	0.136836603	0.073540386	0.941376108	0.963855699	up_in_case
Yang2025_S	ENSGALG00010009346	STC2	case	control	0.569967621	0.227966118	2.500229532	0.012411286	0.047578612	up_in_case
Yang2025_S	ENSGALG00010009347	TESK1	case	control	0.6686817	0.145319438	4.601460807	4.19538E-06	8.01957E-05	up_in_case
Yang2025_S	ENSGALG00010009348	ERGIC2	case	control	-0.230165021	0.074818207	-3.076323653	0.002095702	0.012390162	down_in_case
Yang2025_S	ENSGALG00010009351	GTF3C2	case	control	0.031923547	0.16273683	0.196166703	0.844479689	0.907479967	up_in_case
Yang2025_S	ENSGALG00010009352	HSF1	case	control	0.428436327	0.232220968	1.844951084	0.065044655	0.161223277	up_in_case
Yang2025_S	ENSGALG00010009355	GARRE1	case	control	0.024709098	0.128743035	0.191925704	0.847800402	0.909738128	up_in_case
Yang2025_S	ENSGALG00010009357	LYST	case	control	0.315910008	0.153281934	2.060973525	0.039305566	0.111848695	up_in_case
Yang2025_S	ENSGALG00010009359	REEP3	case	control	-0.261394557	0.125332139	-2.085614735	0.037013536	0.106996968	down_in_case
Yang2025_S	ENSGALG00010009360	TNFAIP8	case	control	-0.566901604	0.096257782	-5.88941062	3.87575E-09	1.97834E-07	down_in_case
Yang2025_S	ENSGALG00010009361	LRRK2	case	control	-0.088007214	0.115387496	-0.76271015	0.445636276	0.608638783	down_in_case

Yang2025_S	ENSGALG00010009362	IL15	case	control	0.069526647	0.27462496	0.253169438	0.800137282	0.879261338	up_in_case
Yang2025_S	ENSGALG00010009363	TAF2	case	control	0.138169767	0.164167248	0.841640269	0.399989342	0.566909223	up_in_case
Yang2025_S	ENSGALG00010009365	CA5B	case	control	-0.304205814	0.135003982	-2.253309932	0.024239614	0.078407386	down_in_case
Yang2025_S	ENSGALG00010009367	DMXL1	case	control	0.323692418	0.177557346	1.823030284	0.068298804	0.167001902	up_in_case
Yang2025_S	ENSGALG00010009368	DSCC1	case	control	-0.05315786	0.140095804	-0.379439346	0.704361638	0.81756109	down_in_case
Yang2025_S	ENSGALG00010009370	ENSGALG00010009370	case	control	0.946923109	0.604588748	1.566226814	0.117295527	0.245545061	up_in_case
Yang2025_S	ENSGALG00010009371	PHTF2	case	control	0.310431654	0.127358576	2.437461723	0.01479078	0.054368114	up_in_case
Yang2025_S	ENSGALG00010009372	RAB28	case	control	0.43925558	0.088027911	4.989958019	6.03924E-07	1.56717E-05	up_in_case
Yang2025_S	ENSGALG00010009373	ARHGAP15	case	control	-0.362165751	0.233819679	-1.548910482	0.121403241	0.251824079	down_in_case
Yang2025_S	ENSGALG00010009374	IL12B	case	control	1.168707655	0.65450791	1.785628008	0.074159509	0.177257763	up_in_case
Yang2025_S	ENSGALG00010009376	CDO1	case	control	-0.202426629	0.158152466	-1.279946082	0.200564099	0.358223625	down_in_case
Yang2025_S	ENSGALG00010009378	ENSGALG00010009378	case	control	-0.496179647	0.163154312	-3.041167846	0.002356624	0.01356446	down_in_case
Yang2025_S	ENSGALG00010009379	RYR1	case	control	0.165327892	0.169427252	0.975804597	0.329161306	0.498695269	up_in_case
Yang2025_S	ENSGALG00010009381	INPP4B	case	control	-0.055352416	0.164535979	-0.336415272	0.736557735	0.8392761	down_in_case
Yang2025_S	ENSGALG00010009383	ASXL2	case	control	-0.186829009	0.130507604	-1.431556502	0.152270786	0.295847655	down_in_case
Yang2025_S	ENSGALG00010009385	GPI	case	control	-0.466578466	0.183812966	-2.538332727	0.011138203	0.043869662	down_in_case
Yang2025_S	ENSGALG00010009386	TMEM60	case	control	-0.136023541	0.119299823	-1.140182259	0.254210377	0.420665195	down_in_case
Yang2025_S	ENSGALG00010009387	DEPTOR	case	control	-0.393864455	0.129578024	-3.039593008	0.00236898	0.013624326	down_in_case
Yang2025_S	ENSGALG00010009388	TMED7	case	control	-0.07825768	0.086627107	-0.903385586	0.366321287	0.535122787	down_in_case
Yang2025_S	ENSGALG00010009389	CPEB4	case	control	0.020616963	0.164107399	0.125630914	0.900024091	0.940590648	up_in_case
Yang2025_S	ENSGALG00010009391	RSBN1L	case	control	0.160778233	0.133558305	1.203805586	0.22866473	0.391042413	up_in_case
Yang2025_S	ENSGALG00010009392	MAPRE3	case	control	0.382649084	0.099584544	3.842454534	0.00012181	0.001245357	up_in_case
Yang2025_S	ENSGALG00010009393	MARVELD2	case	control	-0.433957155	0.205437261	-2.112358544	0.034655708	0.101970765	down_in_case
Yang2025_S	ENSGALG00010009394	BANP	case	control	0.035942544	0.097112537	0.370112293	0.711298823	0.821704942	up_in_case
Yang2025_S	ENSGALG00010009396	AUH	case	control	0.123811957	0.120783384	1.025074416	0.305328076	0.474807136	up_in_case
Yang2025_S	ENSGALG00010009398	WTIP	case	control	-0.212347509	0.158348622	-1.341012677	0.179916337	0.332009299	down_in_case
Yang2025_S	ENSGALG00010009400	CNTFR	case	control	-0.125575839	0.133870571	-0.938039164	0.348224284	0.516663692	down_in_case
Yang2025_S	ENSGALG00010009404	GNG4	case	control	-0.368430714	0.385633459	-0.955390942	0.339380033	0.507560552	down_in_case
Yang2025_S	ENSGALG00010009407	PTPN12	case	control	0.283198285	0.149315317	1.896645904	0.057874688	0.148552466	up_in_case
Yang2025_S	ENSGALG00010009408	ENSGALG00010009408	case	control	0.281715773	0.104344581	2.699860121	0.006936863	0.030765497	up_in_case
Yang2025_S	ENSGALG00010009410	JMJD1C	case	control	0.309583906	0.204884485	1.51101683	0.130784163	0.264980266	up_in_case
Yang2025_S	ENSGALG00010009411	SMARCA5	case	control	0.221860452	0.113676902	1.951675745	0.050976714	0.135170959	up_in_case
Yang2025_S	ENSGALG00010009415	HMP19	case	control	0.525446492	0.411708993	1.276257017	0.201864679	0.359808686	up_in_case
Yang2025_S	ENSGALG00010009416	COL14A1	case	control	-0.296240729	0.18692276	-1.584829629	0.113005049	0.239174567	down_in_case
Yang2025_S	ENSGALG00010009424	ENSGALG00010009424	case	control	-1.027741064	1.179232734	-0.871533696	0.383462815	0.551109152	down_in_case
Yang2025_S	ENSGALG00010009431	SMN	case	control	0.072462612	0.102556751	0.706561118	0.479839259	0.63857894	up_in_case
Yang2025_S	ENSGALG00010009432	BOP1	case	control	0.677025412	0.291512378	2.322458537			up_in_case
Yang2025_S	ENSGALG00010009435	GTDC1	case	control	0.126473241	0.137994418	0.916509833	0.359399552	0.528174132	up_in_case
Yang2025_S	ENSGALG00010009437	DPYSL5	case	control	0.588744106	0.324411839	1.814804624	0.06955394	0.169239418	up_in_case
Yang2025_S	ENSGALG00010009439	B3GALNT2	case	control	-0.119611783	0.100753775	-1.187169243	0.23516087	0.398366973	down_in_case
Yang2025_S	ENSGALG00010009440	DOK3	case	control	-0.60797559	0.369604199	-1.644936914	0.099982821	0.21896285	down_in_case
Yang2025_S	ENSGALG00010009441	ZNF518B	case	control	-0.41465486	0.099849332	-4.152805547	3.28424E-05	0.000437951	down_in_case
Yang2025_S	ENSGALG00010009444	NFIL3	case	control	-0.196696284	0.139280893	-1.412227332	0.157883035	0.303127597	down_in_case
Yang2025_S	ENSGALG00010009446	ENSGALG00010009446	case	control	-0.556097824	0.287064491	-1.937187784	0.052722393	0.138620103	down_in_case
Yang2025_S	ENSGALG00010009447	DDX41	case	control	0.032875178	0.083167539	0.395288574	0.692629931	0.80892712	up_in_case
Yang2025_S	ENSGALG00010009451	ROR2	case	control	-0.213566481	0.239156101	-0.893000345	0.371856988	0.540223975	down_in_case
Yang2025_S	ENSGALG00010009452	TBCE	case	control	-0.107023749	0.149828199	-0.714309784	0.475035643	0.634610458	down_in_case
Yang2025_S	ENSGALG00010009453	GSAP	case	control	-0.08588056	0.123696618	-0.694283816	0.487504239	0.644715912	down_in_case
Yang2025_S	ENSGALG00010009457	BDP1	case	control	0.50306368	0.143079769	3.515966529	0.000438156	0.003552804	up_in_case
Yang2025_S	ENSGALG00010009458	SCX	case	control	1.737854831	0.405720521	4.283379172	1.84076E-05	0.000271649	up_in_case
Yang2025_S	ENSGALG00010009461	ZEB2	case	control	0.311564671	0.22243812	1.4006802	0.161309726	0.307128547	up_in_case
Yang2025_S	ENSGALG00010009462	NRBF2	case	control	-0.027712554	0.108999489	-0.254244803	0.79930644	0.878556179	down_in_case
Yang2025_S	ENSGALG00010009463	MRPL13	case	control	-0.126043532	0.166908731	-0.755164402	0.450150345	0.612282169	down_in_case
Yang2025_S	ENSGALG00010009464	SPTLC1	case	control	0.074642735	0.183526367	0.406713956	0.684218089	0.80230386	up_in_case
Yang2025_S	ENSGALG00010009465	GGPS1	case	control	-0.195475463	0.118973645	-1.643014821	0.100379867	0.219597126	down_in_case
Yang2025_S	ENSGALG00010009466	FAM193B	case	control	0.138161073	0.121800704	1.134320804	0.256660006	0.423060939	up_in_case
Yang2025_S	ENSGALG00010009467	EGR2	case	control	-0.013659368	0.179883091	-0.075934699	0.93947105	0.963391647	down_in_case
Yang2025_S	ENSGALG00010009469	DNMT3A	case	control	0.260945581	0.110385019	2.363958288	0.018080848	0.062989155	up_in_case
Yang2025_S	ENSGALG00010009470	ZNF469	case	control	0.091198653	0.234401693	0.389069944	0.697224407	0.812563528	up_in_case
Yang2025_S	ENSGALG00010009472	ADO	case	control	-0.139238288	0.16589281	-0.839326838	0.401285927	0.567822846	down_in_case
Yang2025_S	ENSGALG00010009474	ACVR2A	case	control	0.009667982	0.121717812	0.079429478	0.936691023	0.961248207	up_in_case
Yang2025_S	ENSGALG00010009477	ZNF365	case	control	-0.130596604	0.114212197	-1.143455845	0.252849378	0.418960295	down_in_case
Yang2025_S	ENSGALG00010009479	MTBP	case	control	0.097197732	0.151598595	0.641151929	0.521423978	0.675031878	up_in_case
Yang2025_S	ENSGALG00010009482	ARID4B	case	control	0.598635962	0.170582818	3.509356744	0.000449192	0.003626588	up_in_case
Yang2025_S	ENSGALG00010009484	RTKN2	case	control	-0.044978744	0.101728216	-0.442146201	0.658383413	0.784553862	down_in_case
Yang2025_S	ENSGALG00010009486	B4GALT1	case	control	-0.323151712	0.089664629	-3.604004334	0.000313352	0.002707103	down_in_case
Yang2025_S	ENSGALG00010009489	MROH1	case	control	0.114450692	0.071390286	1.603168985	0.108897351	0.232671663	up_in_case
Yang2025_S	ENSGALG00010009490	SNTB1	case	control	0.096958318	0.133526339	0.726136277	0.467755232	0.628604892	up_in_case
Yang2025_S	ENSGALG00010009491	RBM34	case	control	0.409137886	0.160572438	2.547995725	0.01083438	0.042976682	up_in_case
Yang2025_S	ENSGALG00010009492	CDC42SE2	case	control	-0.237222165	0.131015954	-1.81063572	0.070197259	0.170328888	down_in_case
Yang2025_S	ENSGALG00010009493	ENSGALG00010009493	case	control	0.733699756	0.608703375	1.205348593	0.228068758	0.390244348	up_in_case
Yang2025_S	ENSGALG00010009494	UBA2	case	control	-0.282279378	0.05704351	-4.948492429	7.47905E-07	1.88124E-05	down_in_case
Yang2025_S	ENSGALG00010009497	HS3ST1	case	control	-0.433243306	0.220781582	-1.962316341	0.049725666	0.132775186	down_in_case
Yang2025_S	ENSGALG00010009498	ORC4	case	control	-0.13004141	0.162321862	-0.801133061	0.423054618	0.588714652	down_in_case
Yang2025_S	ENSGALG00010009499	MXD3	case	control	-0.022656959	0.344395798	-0.065787561	0.94754696	0.967543554	down_in_case
Yang2025_S	ENSGALG00010009500	CCDC146	case	control	1.176896941	0.373498634	3.151007347	0.001627084	0.010240928	up_in_case
Yang2025_S	ENSGALG00010009501	HGH1	case	control	0.457727455	0.283290443	1.615753251	0.106147673	0.228760875	up_in_case
Yang2025_S	ENSGALG00010009502	ARID5B	case	control	0.148877885	0.226970494	0.655934975	0.511865964	0.667166613	up_in_case
Yang2025_S	ENSGALG00010009503	IL11RA	case	control	-0.036141912	0.157209233	-0.229896877	0.818171903	0.89137049	down_in_case
Yang2025_S	ENSGALG00010009506	HAS2	case	control	0.004026715	0.109693453	0.036708801	0.970717192	0.982321994	up_in_case
Yang2025_S	ENSGALG00010009508	TOMM20	case	control	-0.235302239	0.164984296	-1.426209915	0.153807759	0.297806185	down_in_case
Yang2025_S	ENSGALG00010009511	ZFPM1	case	control	0.652277086	0.240896445	2.7077074	0.006774972	0.030191634	up_in_case
Yang2025_S	ENSGALG00010009514	PRELID1	case	control	-0.039721235	0.191124773	-0.207828816	0.835362632	0.902525839	down_in_case
Yang2025_S	ENSGALG00010009515	MBD5	case	control	0.496262657	0.236633889	2.097174919	0.035978094	0.104896557	up_in_case
Yang2025_S	ENSGALG00010009518	FGL2	case	control	-0.583973759	0.335747329	-1.739325106	0.081977594	0.190519975	down_in_case
Yang2025_S	ENSGALG00010009522	TAF5L	case	control	-0.259101917	0.078644379	-3.294601859	0.000985612	0.006926125	down_in_case
Yang2025_S	ENSGALG00010009524	FAM185A	case	control	-0.229673961	0.181588593	-1.264803901	0.205941633	0.364464904	down_in_case
Yang2025_S	ENSGALG00010009525	ENSGALG00010009525	case	control	-0.782503886	0.23603403	-3.315216393	0.000915722	0.006520484	down_in_case
Yang2025_S	ENSGALG00010009526	ENSGALG00010009526	case	control	0.470245159	0.242696112	1.937588348	0.052673465	0.138517595	up_in_case
Yang2025_S	ENSGALG00010009530	KIF3C	case	control	0.554248063	0.13352362	4.150936467	3.31118E-05	0.0004407	up_in_case
Yang2025_S	ENSGALG00010009531	FBXL13	case	control	0.588020086	0.404340593	1.454269237	0.145871676	0.287310502	up_in_case
Yang2025_S	ENSGALG00010009534	XPA	case	control	0.626589294	0.273788537	2.288588483	0.022103274	0.072815394	up_in_case
Yang2025_S	ENSGALG00010009538	CRIM1	case	control	0.208455715	0.141563925	1.472520029	0.140880503	0.28009271	up_in_case
Yang2025_S	ENSGALG00010009540	RAB10	case	control	-0.026880084	0.105262717	-0.255361871	0.798443617	0.878092637	down_in_case
Yang2025_S	ENSGALG00010009541	PMPCB	case	control	0.013478219	0.120157395	0.112171362	0.910687535	0.946905746	up_in_case
Yang2025_S	ENSGALG00010009542	WDR1	case	control	-0.477094213	0.173495907	-2.749887429	0.005961574	0.027273321	down_in_case
Yang2025_S	ENSGALG00010009543	MAF1	case	control	-0.106174227	0.096418185	-1.101184664	0.270816295	0.438097295	down_in_case
Yang2025_S	ENSGALG00010009548	PIK3C3	case	control	-0.047655742	0.129312314	-0.368532125	0.712476496	0.822496891	down_in_case
Yang2025_S	ENSGALG00010009551	HADHA	case	control	0.013899198	0.160559789	0.086567116	0.931015606	0.958317511	up_in_case
Yang2025_S	ENSGALG00010009554	ABCB10	case	control	-0.298429409	0.120491206	-2.476773357	0.013257606	0.049944509	down_in_case
Yang2025_S	ENSGALG00010009555	EPC2	case	control	0.058478197	0.157786355	0.370616314	0.710923329	0.821452454	up_in_case
Yang2025_S	ENSGALG00010009557	CIDEC	case	control	-0.839975452	0.698059933	-1.203299906	0.228860285	0.391167275	down_in_case
Yang2025_S	ENSGALG00010009558	TMEM26	case	control	-0.345646324	0.132533753	-2.60798715	0.009107637	0.03791901	down_in_case
Yang2025_S	ENSGALG00010009560	RAB24	case	control	0.451135817	0.191287555	2.35841698	0.018353064	0.063593642	up_in_case
Yang2025_S	ENSGALG00010009562	HADHB	case	control	-0.045318295	0.151318221	-0.299490004	0.764566198	0.856649925	down_in_case
Yang2025_S	ENSGALG00010009565	TMEM8B	case	control	0.201811021	0.209273146	0.964342655	0.334874162	0.503503609	up_in_case
Yang2025_S	ENSGALG00010009567	NSD1	case	control	-0.045193592	0.158514177	-0.285107572	0.775561729	0.863284023	down_in_case
Yang2025_S	ENSGALG00010009569	ZC3H18	case	control	0.014396193	0.110849965	0.129870978	0.896668506	0.938786882	up_in_case
Yang2025_S	ENSGALG00010009570	SHARPIN	case	control	0.144266211	0.097211971	1.484037509	0.13779897	0.27553862	up_in_case
Yang2025_S	ENSGALG00010009571	DNAJC2	case	control	1.219659945	0.270331721	4.511715982	6.43053E-06	0.000115031	up_in_case
Yang2025_S	ENSGALG00010009574	TAF1C	case	control	0.263013123	0.17952723	1.465031923	0.142912208	0.282880913	up_in_case
Yang2025_S	ENSGALG00010009575	RHOBTB1	case	control	-0.498535303	0.258249116	-1.930443404	0.053551921	0.140271809	down_in_case
Yang2025_S	ENSGALG00010009578	KIF5C	case	control	0.210443838	0.265367072	0.793029205	0.427760807	0.592176321	up_in_case
Yang2025_S	ENSGALG00010009582	TIGD5	case	control	-0.331190028	0.177760751	-1.863122347	0.062445055	0.156759474	down_in_case
Yang2025_S	ENSGALG00010009583	LYPD6B	case	control	-0.146204475	0.440657451	-0.331787138	0.740049997	0.840883619	down_in_case
Yang2025_S	ENSGALG00010009591	LYPD6	case	control	-0.114328142	0.413667693	-0.276376773	0.782258694	0.868380061	down_in_case
Yang2025_S	ENSGALG00010009593	NCBP1	case	control	-0.20467549	0.072172205	-2.83593233	0.004569213	0.022382981	down_in_case
Yang2025_S	ENSGALG00010009594	HINT2	case	control	0.587523407	0.321310261	1.828523638	0.067471001	0.165519018	up_in_case
Yang2025_S	ENSGALG00010009595	ENSGALG00010009595	case	control	-0.188132041	0.228458539	-0.823484391	0.41023259	0.575626801	down_in_case
Yang2025_S	ENSGALG00010009596	URB2	case	control	-0.176664843	0.106229877	-1.663042899	0.096303849	0.213625299	down_in_case
Yang2025_S	ENSGALG00010009597	CDK1	case	control	-0.595408255	0.197415893	-3.016009735	0.00256125	0.014461517	down_in_case
Yang2025_S	ENSGALG00010009600	LRRC17	case	control	-0.695017862	0.195994102	-3.546116202	0.000390954	0.003240893	down_in_case
Yang2025_S	ENSGALG00010009601	ZHX2	case	control	-0.147671826	0.113865489	-1.296897127	0.194666585	0.350475305	down_in_case
Yang2025_S	ENSGALG00010009602	TSTD2	case	control	-0.208930044	0.147894146	-1.412699892	0.157743983	0.303027627	down_in_case
Yang2025_S	ENSGALG00010009603	HINT1	case	control	0.161187041	0.256158185	0.629248061	0.529186669	0.681724713	up_in_case
Yang2025_S	ENSGALG00010009605	ENSGALG00010009605	case	control	0.070984579	0.139105192	0.510294245	0.609845334	0.747225422	up_in_case

Yang2025_S	ENSGALG00010009606	CHSY3	case	control	0.059020932	0.202533599	0.29141304	0.770735443	0.860110387	up_in_case
Yang2025_S	ENSGALG00010009608	AFAP1	case	control	-0.037495912	0.12312754	-0.30452904	0.760724872	0.85468847	down_in_case
Yang2025_S	ENSGALG00010009609	MMADHC	case	control	-0.474549995	0.06292802	-7.541155733	4.65824E-14	8.21679E-12	down_in_case
Yang2025_S	ENSGALG00010009612	DERL1	case	control	-0.69932309	0.195421138	-3.578543741	0.000345514	0.002934027	down_in_case
Yang2025_S	ENSGALG00010009615	ENSGALG00010009615	case	control	-1.097313844	0.406532154	-2.699205546	0.006950523	0.03080647	down_in_case
Yang2025_S	ENSGALG00010009616	ARMC10	case	control	-0.288237029	0.182292655	-1.581177421	0.113837476	0.240571009	down_in_case
Yang2025_S	ENSGALG00010009617	SLC25A46	case	control	-0.159947281	0.119484049	-1.338649655	0.180684753	0.332959804	down_in_case
Yang2025_S	ENSGALG00010009619	ENSGALG00010009619	case	control	-1.239617575	0.252860687	-4.902373677	9.46855E-07	2.30269E-05	down_in_case
Yang2025_S	ENSGALG00010009620	TMOD1	case	control	0.11585996	0.198796119	0.582807959	0.560022586	0.706620313	up_in_case
Yang2025_S	ENSGALG00010009623	XPO5	case	control	-0.170298095	0.071062886	-2.396442156	0.016555106	0.059092061	down_in_case
Yang2025_S	ENSGALG00010009634	TBC1D31	case	control	0.491435423	0.171839547	2.859850546	0.004238407	0.0210111	up_in_case
Yang2025_S	ENSGALG00010009635	TDRD7	case	control	0.460370882	0.140175582	3.284244493	0.001022562	0.007137867	up_in_case
Yang2025_S	ENSGALG00010009637	CYBA	case	control	0.135876276	0.237879344	0.571198295	0.567865234	0.712451791	up_in_case
Yang2025_S	ENSGALG00010009638	RND3	case	control	0.187828897	0.160611087	1.16946408	0.242216705	0.407289535	up_in_case
Yang2025_S	ENSGALG00010009640	NPR2	case	control	0.488550979	0.160715827	3.039843601	0.00236701	0.013618616	up_in_case
Yang2025_S	ENSGALG00010009643	PSMC2	case	control	-0.215317586	0.139403901	-1.54455926	0.122452907	0.253510809	down_in_case
Yang2025_S	ENSGALG00010009644	SLC27A6	case	control	-0.448980265	0.337224627	-1.331398213	0.183058017	0.336221625	down_in_case
Yang2025_S	ENSGALG00010009646	MVD	case	control	-0.106618046	0.234275329	-0.455097198	0.649039313	0.777007116	down_in_case
Yang2025_S	ENSGALG00010009647	GALNT2	case	control	0.030929486	0.104833375	0.295034725	0.767967353	0.858788123	up_in_case
Yang2025_S	ENSGALG00010009649	TENM2	case	control	0.745612489	0.245783952	3.033609331	0.002416471	0.013834646	up_in_case
Yang2025_S	ENSGALG00010009650	KLHL29	case	control	-0.166139859	0.147892839	-1.123380016	0.261276133	0.428037023	down_in_case
Yang2025_S	ENSGALG00010009652	CIAO2B	case	control	0.352947696	0.158960216	2.220352385	0.026394857	0.083309136	up_in_case
Yang2025_S	ENSGALG00010009653	ABLIM2	case	control	-0.031223731	0.137030303	-0.227860047	0.819755044	0.892304838	down_in_case
Yang2025_S	ENSGALG00010009654	NDUFA12	case	control	0.020125981	0.203488599	0.09890471	0.921213929	0.953298389	up_in_case
Yang2025_S	ENSGALG00010009655	FAM83A	case	control	0.029854374	0.654747937	0.045596744	0.963631664	0.978304476	up_in_case
Yang2025_S	ENSGALG00010009657	MSMP	case	control	0.696432363	0.357407498	1.948566742	0.051347187	0.135824421	up_in_case
Yang2025_S	ENSGALG00010009658	NAPEPLD	case	control	-0.271305148	0.130264708	-2.082721809	0.037276588	0.107457439	down_in_case
Yang2025_S	ENSGALG00010009659	APOLD1	case	control	-0.907879833	0.198635159	-4.570589812	4.86353E-06	9.02441E-05	down_in_case
Yang2025_S	ENSGALG00010009663	FER	case	control	-0.019740211	0.151599457	-0.130212942	0.896397956	0.938786882	down_in_case
Yang2025_S	ENSGALG00010009665	DGKQ	case	control	0.442086457	0.197945714	2.233372206	0.025524409	0.08113199	up_in_case
Yang2025_S	ENSGALG00010009666	RRAD	case	control	-0.159739638	0.207087732	-0.771362149	0.440492304	0.603981133	down_in_case
Yang2025_S	ENSGALG00010009668	TFAP2A	case	control	3.304222188	0.785730834	4.205285123	2.60753E-05	0.000363278	up_in_case
Yang2025_S	ENSGALG00010009672	DDX47	case	control	-0.197929771	0.140450989	-1.409244408	0.158762911	0.304313778	down_in_case
Yang2025_S	ENSGALG00010009676	ACO1	case	control	-0.196362398	0.115465607	-1.700613736	0.089015543	0.202420299	down_in_case
Yang2025_S	ENSGALG00010009677	SLC26A5	case	control	-0.432994487	0.189182535	-2.288765651	0.022092972	0.072798668	down_in_case
Yang2025_S	ENSGALG00010009678	JARID2	case	control	0.742843484	0.218391526	3.401429989	0.000670343	0.005065743	up_in_case
Yang2025_S	ENSGALG00010009679	ANK3	case	control	0.397176286	0.130570502	3.041853105	0.002351266	0.013539212	up_in_case
Yang2025_S	ENSGALG00010009683	ADAMTS19	case	control	0.190867223	0.164314663	1.161595803	0.245399688	0.410965587	up_in_case
Yang2025_S	ENSGALG00010009686	MAT2B	case	control	-0.189874751	0.126938721	-1.495798516	0.134706168	0.271026632	down_in_case
Yang2025_S	ENSGALG00010009689	ENSGALG00010009689	case	control	-0.701225969	0.683472552	-1.025975318	0.304903218	0.474542948	down_in_case
Yang2025_S	ENSGALG00010009690	RBM43	case	control	-0.613654308	0.188858969	-3.249272778	0.001157005	0.007857361	down_in_case
Yang2025_S	ENSGALG00010009695	ATAD2B	case	control	0.420325277	0.189579461	2.21714565	0.02661314	0.083808838	up_in_case
Yang2025_S	ENSGALG00010009697	ENSGALG00010009697	case	control	-0.624529999	0.373531486	-1.671960792	0.094532054	0.210701243	down_in_case
Yang2025_S	ENSGALG00010009699	POLR1C	case	control	0.216354954	0.123533955	1.751380454	0.079880398	0.187207087	up_in_case
Yang2025_S	ENSGALG00010009700	SH3TC1	case	control	0.05493067	0.13522455	0.406218174	0.684582302	0.802663389	up_in_case
Yang2025_S	ENSGALG00010009702	ENSGALG00010009702	case	control	0.335077987	0.230899476	1.451185568	0.146728194	0.288508168	up_in_case
Yang2025_S	ENSGALG00010009704	RGP1	case	control	0.004812888	0.195476272	0.024621342	0.980356996	0.988085834	up_in_case
Yang2025_S	ENSGALG00010009705	DTNBP1	case	control	0.112370145	0.081434053	1.379891335	0.167620105	0.315732112	up_in_case
Yang2025_S	ENSGALG00010009710	ENSGALG00010009710	case	control	-0.037388654	0.158323473	-0.236153574	0.813313503	0.887604217	down_in_case
Yang2025_S	ENSGALG00010009712	UBXN2A	case	control	-0.015117893	0.152318569	-0.099251808	0.920938341	0.953225565	down_in_case
Yang2025_S	ENSGALG00010009715	NUDCD2	case	control	0.009466991	0.094750155	0.099915304	0.920411566	0.952963458	up_in_case
Yang2025_S	ENSGALG00010009717	EDN1	case	control	-0.446790337	0.176684505	-2.52874657	0.011447065	0.044769815	down_in_case
Yang2025_S	ENSGALG00010009718	ENSGALG00010009718	case	control	0.259977329	0.335683074	0.774472558	0.43865139	0.602524113	up_in_case
Yang2025_S	ENSGALG00010009719	YIPF3	case	control	-0.270072537	0.147642753	-1.829229891	0.067365176	0.165328238	down_in_case
Yang2025_S	ENSGALG00010009720	RELN	case	control	-0.176372069	0.158671075	-1.111557791	0.266328329	0.433602847	down_in_case
Yang2025_S	ENSGALG00010009721	ZHX1	case	control	0.200430205	0.153405188	1.306541245	0.191368563	0.346511183	up_in_case
Yang2025_S	ENSGALG00010009722	GBA2	case	control	0.012379395	0.131928378	0.093834206	0.925240859	0.955206005	up_in_case
Yang2025_S	ENSGALG00010009724	ISOC1	case	control	-0.260961258	0.150399757	-1.73511755	0.082719986	0.191797506	down_in_case
Yang2025_S	ENSGALG00010009726	PRR16	case	control	-0.350105456	0.146773683	-2.385342172	0.017063242	0.060364631	down_in_case
Yang2025_S	ENSGALG00010009727	CDH20	case	control	-0.610637253	0.142345943	-4.289811423	1.78825E-05	0.000265098	down_in_case
Yang2025_S	ENSGALG00010009728	CCDC149	case	control	-0.043784684	0.133806049	-0.327224996	0.743497717	0.843144448	down_in_case
Yang2025_S	ENSGALG00010009730	C8orf76	case	control	-0.067896171	0.144560843	-0.469671937	0.63858942	0.768870559	down_in_case
Yang2025_S	ENSGALG00010009731	NMI	case	control	-0.106347717	0.120661185	-0.881374714	0.378115039	0.546430012	down_in_case
Yang2025_S	ENSGALG00010009732	HMMR	case	control	0.417028963	0.158189675	2.636259059	0.008382571	0.035537305	up_in_case
Yang2025_S	ENSGALG00010009736	WISP1	case	control	0.359204535	0.240494307	1.493609303	0.135277766	0.271784267	up_in_case
Yang2025_S	ENSGALG00010009738	ENSGALG00010009738	case	control	-0.127615555	0.235133317	-0.542737014	0.587310885	0.728714773	down_in_case
Yang2025_S	ENSGALG00010009740	LRRC73	case	control	0.059432511	0.281434749	0.211176877	0.832749254	0.900679979	up_in_case
Yang2025_S	ENSGALG00010009741	C2H6orf52	case	control	0.089907148	0.104574636	0.859741444	0.389931584	0.557187923	up_in_case
Yang2025_S	ENSGALG00010009743	CCNG1	case	control	0.117014585	0.13500642	0.866733484	0.386088055	0.553586638	up_in_case
Yang2025_S	ENSGALG00010009744	DAPK1	case	control	0.062912051	0.118750998	0.529781238	0.596263615	0.735694482	up_in_case
Yang2025_S	ENSGALG00010009749	NDRG1	case	control	-0.145566838	0.119587084	-1.217245489	0.223510827	0.384921442	down_in_case
Yang2025_S	ENSGALG00010009751	ENSGALG00010009751	case	control	1.271638109	0.34699294	3.664737698	0.000247592	0.002227371	up_in_case
Yang2025_S	ENSGALG00010009761	PDP2	case	control	-0.23981798	0.09630699	-2.490140966	0.012769244	0.04856425	down_in_case
Yang2025_S	ENSGALG00010009762	MAN2A1	case	control	-0.360876275	0.107191225	-3.366658761	0.000760848	0.005615685	down_in_case
Yang2025_S	ENSGALG00010009765	STX10	case	control	-0.121279256	0.114222925	-1.061776839	0.288337007	0.457263707	down_in_case
Yang2025_S	ENSGALG00010009769	TMCC3	case	control	-0.09203353	0.25765682	-0.357194233	0.720946403	0.828226556	down_in_case
Yang2025_S	ENSGALG00010009774	PIAS2	case	control	-0.009514576	0.126034239	-0.075491992	0.939823269	0.96346547	down_in_case
Yang2025_S	ENSGALG00010009775	KIAA1614	case	control	0.273644575	0.23175199	1.180764724	0.237696201	0.401719796	up_in_case
Yang2025_S	ENSGALG00010009777	ST3GAL1	case	control	-0.034333876	0.112927375	-0.304035011	0.761101218	0.854874912	down_in_case
Yang2025_S	ENSGALG00010009778	ENSGALG00010009778	case	control	-0.402269272	0.123451278	-3.258526585	0.001119924	0.007672577	down_in_case
Yang2025_S	ENSGALG00010009779	PPP1R3E	case	control	-0.045946401	0.281378781	-0.163290213	0.87028994	0.923654015	down_in_case
Yang2025_S	ENSGALG00010009780	MFSD2B	case	control	-0.443326569	0.300416767	-1.475705145	0.140023064	0.278865428	down_in_case
Yang2025_S	ENSGALG00010009784	ENSGALG00010009784	case	control	-0.024932653	0.298593991	-0.083500185	0.93345383	0.959480647	down_in_case
Yang2025_S	ENSGALG00010009786	DIPK2B	case	control	0.071512002	0.115038782	0.62163386	0.534182651	0.685688582	up_in_case
Yang2025_S	ENSGALG00010009788	PAK1IP1	case	control	-0.098845958	0.112681178	-0.877218005	0.380368238	0.548301583	down_in_case
Yang2025_S	ENSGALG00010009790	MC1R	case	control	7.18458187	0.735106981	9.773518766	1.46281E-22	1.07286E-19	up_in_case
Yang2025_S	ENSGALG00010009791	GABRG2	case	control	-0.219626966	0.362500049	-0.605867411	0.544602802	0.694323007	down_in_case
Yang2025_S	ENSGALG00010009793	TNFAIP6	case	control	-0.399624495	0.20014966	-1.996628395	0.045865566	0.125345491	down_in_case
Yang2025_S	ENSGALG00010009795	NDUFA6	case	control	-0.518294719	0.25518049	-2.031090694	0.042245795	0.117762233	down_in_case
Yang2025_S	ENSGALG00010009796	ENSGALG00010009796	case	control	-2.284072413	0.570403131	-4.004312542	6.21981E-05	0.000727733	down_in_case
Yang2025_S	ENSGALG00010009798	YIPF4	case	control	-0.518246979	0.075168151	-6.894502182	5.40538E-12	6.1239E-10	down_in_case
Yang2025_S	ENSGALG00010009801	ENSGALG00010009801	case	control	0.879879546	0.562663877	1.563774721	0.117870483	0.246366459	up_in_case
Yang2025_S	ENSGALG00010009804	XPR1	case	control	-0.379477278	0.046145511	-8.223492725	1.97661E-16	5.50881E-14	down_in_case
Yang2025_S	ENSGALG00010009806	WDCP	case	control	-0.116935298	0.11117876	-1.05177732	0.292901727	0.461561186	down_in_case
Yang2025_S	ENSGALG00010009809	SMDT1	case	control	-0.084920036	0.26200273	-0.324118899	0.745848019	0.845197377	down_in_case
Yang2025_S	ENSGALG00010009811	ENSGALG00010009811	case	control	0.311838403	0.1530355	2.037686702	0.041581275	0.116415465	up_in_case
Yang2025_S	ENSGALG00010009822	ENSGALG00010009822	case	control	0.13621523	0.236852772	0.57510507	0.565220239	0.71088981	up_in_case
Yang2025_S	ENSGALG00010009830	RNASEL	case	control	-0.289031675	0.175233706	-1.649406852	0.099064312	0.21760107	down_in_case
Yang2025_S	ENSGALG00010009831	N6AMT1	case	control	0.172041664	0.140818014	1.221730508	0.221809555	0.383345672	up_in_case
Yang2025_S	ENSGALG00010009833	SOD3	case	control	-0.449920181	0.196836301	-2.285758155	0.022268411	0.073151888	down_in_case
Yang2025_S	ENSGALG00010009840	ENSGALG00010009840	case	control	0.00611985	0.210657169	0.029051232	0.976823731	0.986451097	up_in_case
Yang2025_S	ENSGALG00010009841	GABRA1	case	control	0.229559521	0.308118665	0.745036078	0.45624991	0.617866132	up_in_case
Yang2025_S	ENSGALG00010009842	PHETA2	case	control	-0.453257663	0.11643837	-3.892682987	9.91417E-05	0.001058651	down_in_case
Yang2025_S	ENSGALG00010009847	ENSGALG00010009847	case	control	-0.784197323	0.217789498	-3.600712296	0.000317347	0.002731454	down_in_case
Yang2025_S	ENSGALG00010009850	RIF1	case	control	0.127039193	0.111720437	1.137116866	0.255489434	0.421783559	up_in_case
Yang2025_S	ENSGALG00010009852	ENSGALG00010009852	case	control	-0.341208269	0.264709251	-1.288992611	0.197400655	0.35379783	down_in_case
Yang2025_S	ENSGALG00010009857	ORC5	case	control	-0.243914766	0.218776595	-1.114903385	0.264891826	0.432030384	down_in_case
Yang2025_S	ENSGALG00010009858	ST8SIA5	case	control	0.07149623	0.293819342	0.243333982	0.807746682	0.88400859	up_in_case
Yang2025_S	ENSGALG00010009866	NAGA	case	control	-0.576027334	0.205355882	-2.805019888	0.005031348	0.02408408	down_in_case
Yang2025_S	ENSGALG00010009868	LTN1	case	control	0.257465435	0.092176092	2.793191048	0.005219086	0.024737401	up_in_case
Yang2025_S	ENSGALG00010009876	SELENOI	case	control	-0.29284838	0.102327934	-2.861861563	0.004211608	0.020908002	down_in_case
Yang2025_S	ENSGALG00010009883	ENSGALG00010009883	case	control	0.359596728	0.191430818	1.878468325	0.060317126	0.153100028	up_in_case
Yang2025_S	ENSGALG00010009884	GFOD1	case	control	-0.041951884	0.142921498	-0.293530959	0.76911634	0.859404714	down_in_case
Yang2025_S	ENSGALG00010009910	ENSGALG00010009910	case	control	0.173804799	0.169122702	1.027684612	0.304098209	0.473687518	up_in_case
Yang2025_S	ENSGALG00010009914	RWDD2B	case	control	-0.15593871	0.163058641	-0.956335153	0.338902935	0.507044493	down_in_case
Yang2025_S	ENSGALG00010009941	CACNA1E	case	control	0.423806078	0.614240357	0.689967816			up_in_case
Yang2025_S	ENSGALG00010009943	DHX15	case	control	-0.227965767	0.053780513	-4.238817253	2.247E-05	0.000320164	down_in_case
Yang2025_S	ENSGALG00010009948	WBP2NL	case	control	0.070972832	0.069306702	1.024039955	0.305816404	0.475460402	up_in_case
Yang2025_S	ENSGALG00010009972	PTK2B	case	control	-0.160925301	0.383301671	-0.419839811	0.67460248	0.795379098	down_in_case
Yang2025_S	ENSGALG00010009985	USP16	case	control	0.486079445	0.13690915	3.550379533	0.000384676	0.003200836	up_in_case
Yang2025_S	ENSGALG00010009989	TBC1D7	case	control	-0.437857688	0.151987379	-2.880881895	0.003965642	0.020051242	down_in_case
Yang2025_S	ENSGALG00010009991	LHFPL3	case	control	0.049882392	0.453264556	0.110051385	0.912368623	0.948087752	up_in_case
Yang2025_S	ENSGALG00010009993	ENSGALG00010009993	case	control	1.36908742	0.405316539	3.377822731	0.000730622	0.005444499	up_in_case
Yang2025_S	ENSGALG00010009995	SEPTIN3	case	control	0.602093187	0.352460038	1.708259437	0.087588214	0.199990458	up_in_case
Yang2025_S	ENSGALG00010009999	GABRB2	case	control	-0.490576519	0.252771685	-1.940789049	0.05228387	0.137779071	down_in_case
Yang2025_S	ENSGALG00010010006	PPARGC1A	case	control	0.694282106	0.199188506	3.485553054	0.000491121	0.003897363	up_in_case

Yang2025_S	ENSGALG00010010007	CTIF	case	control	0.098682605	0.11036299	0.894163939	0.371234183	0.53982556	up_in_case
Yang2025_S	ENSGALG00010010009	ELF2	case	control	-0.244236231	0.064714072	-3.774082248	0.000160598	0.001566081	down_in_case
Yang2025_S	ENSGALG00010010011	EPB41L4A	case	control	0.539775518	0.239654567	2.252306415	0.024302915	0.07850281	up_in_case
Yang2025_S	ENSGALG00010010014	GLUL	case	control	-0.218120698	0.179499513	-1.215160391	0.224304924	0.385744677	down_in_case
Yang2025_S	ENSGALG00010010017	CCDC59	case	control	0.119880929	0.123230242	0.972820687	0.330642429	0.500000244	up_in_case
Yang2025_S	ENSGALG00010010022	KMT2E	case	control	0.551768085	0.256496095	2.151175387	0.031462359	0.094897829	up_in_case
Yang2025_S	ENSGALG00010010035	ATP10B	case	control	0.206838042	0.511080112	0.404707672	0.685692402	0.803694476	up_in_case
Yang2025_S	ENSGALG00010010037	NAA15	case	control	0.223115135	0.116699392	1.911879161	0.055891695	0.144848572	up_in_case
Yang2025_S	ENSGALG00010010042	METTL25	case	control	-0.377619303	0.128286968	-2.94355154	0.003244698	0.017264172	down_in_case
Yang2025_S	ENSGALG00010010049	ZBTB7C	case	control	-0.134314139	0.311070861	-0.43177988	0.665901404	0.789166564	down_in_case
Yang2025_S	ENSGALG00010010052	ZNF346	case	control	-0.064320515	0.080546439	-0.798551937	0.424550266	0.589541065	down_in_case
Yang2025_S	ENSGALG00010010059	ENSGALG00010010059	case	control	-0.109678499	0.221580409	-0.494982832	0.620612239	0.755748583	down_in_case
Yang2025_S	ENSGALG00010010061	CYP2D6	case	control	-0.516326452	0.210613681	-2.451533299	0.014224902	0.052663128	down_in_case
Yang2025_S	ENSGALG00010010062	RGS7BP	case	control	0.121881976	0.214574326	0.568017519	0.570023072	0.714451476	up_in_case
Yang2025_S	ENSGALG00010010064	ENSGALG00010010064	case	control	0.447831708	0.18544497	2.414903506	0.015739379	0.0570128	up_in_case
Yang2025_S	ENSGALG00010010071	GLT8D2	case	control	-0.446064483	0.15807724	-2.821813452	0.004775295	0.023169822	down_in_case
Yang2025_S	ENSGALG00010010077	CCT8	case	control	-0.041772478	0.141963979	-0.294247023	0.768569151	0.858999929	down_in_case
Yang2025_S	ENSGALG00010010080	CDC42EP3	case	control	-0.740265182	0.138278237	-5.353446756	8.62944E-08	3.01051E-06	down_in_case
Yang2025_S	ENSGALG00010010084	HK3	case	control	0.85032897	0.19032413	4.4677938	7.90304E-06	0.000134904	up_in_case
Yang2025_S	ENSGALG00010010085	TDG	case	control	0.150578138	0.09296658	1.619701797	0.10529635	0.227594949	up_in_case
Yang2025_S	ENSGALG00010010094	SLC2A9	case	control	-0.18018287	0.278983446	-0.645855058	0.518373227	0.672582022	down_in_case
Yang2025_S	ENSGALG00010010095	C12orf73	case	control	-0.266705193	0.224283542	-1.189142955	0.234383418	0.39758161	down_in_case
Yang2025_S	ENSGALG00010010099	TNR	case	control	0.715974022	0.792665558	0.903248558			up_in_case
Yang2025_S	ENSGALG00010010100	IRF2BP2	case	control	0.313960357	0.121504709	2.583935714	0.009768	0.039823604	up_in_case
Yang2025_S	ENSGALG00010010106	C7orf73	case	control	-0.9583746	0.290254962	-3.301837092	0.000960538	0.006788332	down_in_case
Yang2025_S	ENSGALG00010010107	MGST2	case	control	-0.135690676	0.414947205	-0.327007085	0.743662527	0.843200758	down_in_case
Yang2025_S	ENSGALG00010010111	CNOT4	case	control	0.727069777	0.306090858	2.375339731	0.017532805	0.061619075	up_in_case
Yang2025_S	ENSGALG00010010115	NEB	case	control	0.838904159	0.191922027	4.371067622	1.2364E-05	0.000196457	up_in_case
Yang2025_S	ENSGALG00010010116	DRD5	case	control	-0.149769516	0.51095148	-0.293118861	0.769431301	0.859542486	down_in_case
Yang2025_S	ENSGALG00010010119	SRPK2	case	control	0.07390683	0.110709547	0.667574131	0.504405475	0.660157664	up_in_case
Yang2025_S	ENSGALG00010010122	THBS4	case	control	0.391808583	0.216412859	1.81046812	0.070223224	0.170332572	up_in_case
Yang2025_S	ENSGALG00010010124	ENSGALG00010010124	case	control	1.22500193	0.30229411	4.052351303	5.07055E-05	0.000614418	up_in_case
Yang2025_S	ENSGALG00010010125	AHCTF1	case	control	0.07736145	0.070831777	1.092185654	0.274751507	0.442100637	up_in_case
Yang2025_S	ENSGALG00010010127	FAM13C	case	control	0.85724778	0.30662942	2.795712753	0.00517854	0.024612197	up_in_case
Yang2025_S	ENSGALG00010010136	EPHX2	case	control	0.336353253	0.144694472	2.32457569	0.02009466	0.067949305	up_in_case
Yang2025_S	ENSGALG00010010142	ENSGALG00010010142	case	control	0.342776676	0.265267402	1.292192984	0.196290321	0.35235162	up_in_case
Yang2025_S	ENSGALG00010010146	PHACTR1	case	control	1.047856649	0.33463213	3.13136891	0.001739934	0.010790381	up_in_case
Yang2025_S	ENSGALG00010010150	NTRK2	case	control	-0.092774184	0.319225739	-0.290622505	0.771340046	0.860647253	down_in_case
Yang2025_S	ENSGALG00010010153	ENSGALG00010010153	case	control	0.347005733	0.555519561	0.624650791	0.532200277	0.684607387	up_in_case
Yang2025_S	ENSGALG00010010154	IQSEC3	case	control	-0.279183737	0.320776513	-0.870337216	0.384116146	0.551877359	down_in_case
Yang2025_S	ENSGALG00010010155	BRINP3	case	control	0.094524103	0.367483517	0.257219981	0.79700896	0.877137882	up_in_case
Yang2025_S	ENSGALG00010010156	ENSGALG00010010156	case	control	-0.200593484	0.074988172	-2.675001645	0.007472888	0.032479725	down_in_case
Yang2025_S	ENSGALG00010010161	SEPSECS	case	control	-0.630483427	0.107293219	-5.876265347	4.19626E-09	2.10341E-07	down_in_case
Yang2025_S	ENSGALG00010010164	ENSGALG00010010164	case	control	1.196465903	0.624421676	1.916118465	0.055350019	0.143846048	up_in_case
Yang2025_S	ENSGALG00010010165	ENSGALG00010010165	case	control	3.642641132	0.764304592	4.765954788	1.87961E-06	4.12479E-05	up_in_case
Yang2025_S	ENSGALG00010010167	PUS7	case	control	-0.133608468	0.101892496	-1.311268974	0.189766907	0.344547472	down_in_case
Yang2025_S	ENSGALG00010010168	SNCB	case	control	-0.014463682	0.392963392	-0.03680669	0.97063914	0.982321994	down_in_case
Yang2025_S	ENSGALG00010010171	ENSGALG00010010171	case	control	-0.170169825	0.612156448	-0.277984207	0.781024482	0.867424576	down_in_case
Yang2025_S	ENSGALG00010010178	LGI2	case	control	0.368092318	0.182996936	2.011467107	0.044276141	0.122272697	up_in_case
Yang2025_S	ENSGALG00010010183	NKX3-2	case	control	2.201780659	0.728358486	3.022935411	0.002503357	0.014215271	up_in_case
Yang2025_S	ENSGALG00010010185	PHYHIPL	case	control	0.77590041	0.404197215	1.919608503	0.054907369	0.142855525	up_in_case
Yang2025_S	ENSGALG00010010186	TNPO1	case	control	-0.054113887	0.119614612	-0.452401978	0.650979423	0.778783574	down_in_case
Yang2025_S	ENSGALG00010010188	DSP	case	control	-0.810187901	0.744550248	-1.088157452	0.276525604	0.443819143	down_in_case
Yang2025_S	ENSGALG00010010190	RNF44	case	control	1.185846075	0.309674362	3.829332424	0.000128491	0.00130031	up_in_case
Yang2025_S	ENSGALG00010010191	RINT1	case	control	0.019669956	0.10480457	0.187682237	0.851125756	0.911640077	up_in_case
Yang2025_S	ENSGALG00010010195	ENSGALG00010010195	case	control	1.452279656	0.226460913	6.412937394	1.42742E-10	1.09896E-08	up_in_case
Yang2025_S	ENSGALG00010010196	EFCAB10	case	control	1.123178546	0.355493825	3.159488201	0.001580465	0.010015361	up_in_case
Yang2025_S	ENSGALG00010010197	SCCPDH	case	control	-0.301684089	0.102399253	-2.946155173	0.003217509	0.017152255	down_in_case
Yang2025_S	ENSGALG00010010201	RIOK1	case	control	0.571824009	0.135409583	4.222921277	2.41156E-05	0.000339789	up_in_case
Yang2025_S	ENSGALG00010010205	ARL5A	case	control	-0.317612091	0.127974454	-2.481839775	0.013070603	0.049413689	down_in_case
Yang2025_S	ENSGALG00010010206	SCARA5	case	control	0.121279198	0.122759416	0.98794212	0.323180996	0.492457865	up_in_case
Yang2025_S	ENSGALG00010010214	FAF2	case	control	0.100798361	0.12047969	0.836641933	0.402793867	0.569247641	up_in_case
Yang2025_S	ENSGALG00010010215	ENSGALG00010010215	case	control	-0.599407423	0.14193819	-4.223017222	2.41053E-05	0.000339789	down_in_case
Yang2025_S	ENSGALG00010010216	ATXN7L1	case	control	0.760147729	0.249616934	3.04525706	0.002324815	0.013425735	up_in_case
Yang2025_S	ENSGALG00010010218	PBK	case	control	-0.331133161	0.285632567	-1.159297641	0.246334883	0.412036562	down_in_case
Yang2025_S	ENSGALG00010010222	PAH	case	control	-2.688333549	1.097448488	-2.449621626	0.01430064	0.05288732	down_in_case
Yang2025_S	ENSGALG00010010223	ENSGALG00010010223	case	control	-0.025591432	0.250338708	-0.102227228	0.918576316	0.951982818	down_in_case
Yang2025_S	ENSGALG00010010226	BICC1	case	control	-0.262186995	0.126259481	-2.076572731	0.037841012	0.108618402	down_in_case
Yang2025_S	ENSGALG00010010231	C1orf53	case	control	-0.454319589	0.279828674	-1.623563386	0.104469025	0.226333312	down_in_case
Yang2025_S	ENSGALG00010010234	ENSGALG00010010234	case	control	-0.639787253	0.175012178	-3.655672764	0.000256508	0.002291311	down_in_case
Yang2025_S	ENSGALG00010010243	ESCO2	case	control	-0.222244372	0.186446196	-1.192002718	0.23326018	0.396422324	down_in_case
Yang2025_S	ENSGALG00010010246	CLTB	case	control	-0.188521868	0.124713851	-1.511635364	0.13062665	0.264922481	down_in_case
Yang2025_S	ENSGALG00010010252	WDR7	case	control	0.209796602	0.133393375	1.572766278	0.115772947	0.243332732	up_in_case
Yang2025_S	ENSGALG00010010253	IL10RB	case	control	-0.222346095	0.10895076	-2.040794343	0.041271273	0.11581055	down_in_case
Yang2025_S	ENSGALG00010010254	WDR41	case	control	-0.054982301	0.17889588	-0.307342468	0.758582714	0.85303826	down_in_case
Yang2025_S	ENSGALG00010010255	CACNB4	case	control	0.125309834	0.201796189	0.620972251	0.534617881	0.686057664	up_in_case
Yang2025_S	ENSGALG00010010257	ENSGALG00010010257	case	control	-0.069552891	0.182293541	-0.381543366	0.702800099	0.81634023	down_in_case
Yang2025_S	ENSGALG00010010259	IGF1	case	control	-0.346621869	0.230371655	-1.504620301	0.132421724	0.267511847	down_in_case
Yang2025_S	ENSGALG00010010262	HSPA9	case	control	0.090325442	0.069522541	1.299225279	0.193866629	0.349377348	up_in_case
Yang2025_S	ENSGALG00010010263	RAB4A	case	control	0.218410403	0.089625472	2.436923326	0.01481282	0.054406747	up_in_case
Yang2025_S	ENSGALG00010010266	EFNA5	case	control	0.534849856	0.295086156	1.812521005	0.069905731	0.169858128	up_in_case
Yang2025_S	ENSGALG00010010267	CCSER2	case	control	-0.016178903	0.173506178	-0.093246844	0.925707461	0.95539335	down_in_case
Yang2025_S	ENSGALG00010010270	SSR1	case	control	-0.351524734	0.110742483	-3.174253671	0.001502223	0.00962459	down_in_case
Yang2025_S	ENSGALG00010010271	HUNK	case	control	0.359764495	0.137187435	2.622430358	0.008730511	0.036710824	up_in_case
Yang2025_S	ENSGALG00010010274	MIS18A	case	control	-0.229262298	0.095325476	-2.405047507	0.016170364	0.058075777	down_in_case
Yang2025_S	ENSGALG00010010275	PDE8B	case	control	-0.435727217	0.56193152	-0.775409817	0.438097536	0.602000707	down_in_case
Yang2025_S	ENSGALG00010010279	CCSAP	case	control	-0.208377043	0.119714707	-1.740613568	0.081751338	0.190156353	down_in_case
Yang2025_S	ENSGALG00010010280	HSP90B1	case	control	-0.061163642	0.053884298	-1.135092112	0.256336726	0.422589461	down_in_case
Yang2025_S	ENSGALG00010010284	CCDC25	case	control	0.314436132	0.115391216	2.724957268	0.006430981	0.028983092	up_in_case
Yang2025_S	ENSGALG00010010285	ENSGALG00010010285	case	control	3.668435675	0.409614962	8.955814647	3.37236E-19	1.51593E-16	up_in_case
Yang2025_S	ENSGALG00010010287	STAM2	case	control	-0.019848318	0.070986294	-0.279607751	0.77977846	0.866455054	down_in_case
Yang2025_S	ENSGALG00010010288	ENSGALG00010010288	case	control	0.516921933	0.192174147	2.68986198	0.007148158	0.03143408	up_in_case
Yang2025_S	ENSGALG00010010290	ENSGALG00010010290	case	control	0.490685445	0.146178882	3.356746463	0.000788654	0.005784154	up_in_case
Yang2025_S	ENSGALG00010010291	TCF20	case	control	-0.005187034	0.100644848	-0.051537998	0.958896824	0.975131522	down_in_case
Yang2025_S	ENSGALG00010010295	ENSGALG00010010295	case	control	-0.250721283	0.137621856	-1.821812976	0.068483368	0.167266615	down_in_case
Yang2025_S	ENSGALG00010010298	KCNIP4	case	control	-0.855624422	0.304981271	-2.805498248	0.005023885	0.024065948	down_in_case
Yang2025_S	ENSGALG00010010300	DENND1B	case	control	0.340093815	0.20292454	1.675961986	0.093745639	0.209383792	up_in_case
Yang2025_S	ENSGALG00010010301	ENSGALG00010010301	case	control	1.520869427	0.388560945	3.9141078	9.07391E-05	0.00098708	up_in_case
Yang2025_S	ENSGALG00010010302	WDR36	case	control	0.360368498	0.166929971	2.15880046	0.030865651	0.09358417	up_in_case
Yang2025_S	ENSGALG00010010303	ETF1	case	control	-0.16355226	0.075913338	-2.154460131	0.031204105	0.094282135	down_in_case
Yang2025_S	ENSGALG00010010305	URB1	case	control	0.115986583	0.100925232	1.149232765	0.250460014	0.416209439	up_in_case
Yang2025_S	ENSGALG00010010308	PACRGL	case	control	-0.46491218	0.247540305	-1.878127199	0.060363766	0.153190509	down_in_case
Yang2025_S	ENSGALG00010010310	NT5DC3	case	control	-0.503373335	0.155698064	-3.233009598	0.001224934	0.008202529	down_in_case
Yang2025_S	ENSGALG00010010311	CTSV	case	control	0.053905109	0.144441083	0.373197903	0.709001157	0.820319754	up_in_case
Yang2025_S	ENSGALG00010010313	GRID1	case	control	0.227803808	0.263048008	0.86601609	0.386481338	0.553961885	up_in_case
Yang2025_S	ENSGALG00010010314	CRB1	case	control	0.036401	0.261678424	0.139105852	0.889366504	0.935415671	up_in_case
Yang2025_S	ENSGALG00010010317	ENSGALG00010010317	case	control	0.363583111	0.16724215	2.173992083	0.02970573	0.090838127	up_in_case
Yang2025_S	ENSGALG00010010321	NUP133	case	control	-0.179297884	0.120263851	-1.49087096	0.135995377	0.272597178	down_in_case
Yang2025_S	ENSGALG00010010322	ENSGALG00010010322	case	control	0.151606241	0.182322967	0.831525747	0.405676689	0.571829321	up_in_case
Yang2025_S	ENSGALG00010010323	FBP2	case	control	0.040270402	0.235660156	0.170883371	0.864315472	0.919898886	up_in_case
Yang2025_S	ENSGALG00010010327	FBP1	case	control	-1.844169185	0.49998121	-3.688476985	0.0002256	0.00205186	down_in_case
Yang2025_S	ENSGALG00010010330	ALPK2	case	control	-0.232187201	0.159638258	-1.454458373	0.145819266	0.287247876	down_in_case
Yang2025_S	ENSGALG00010010333	WAPL	case	control	0.188949334	0.134911571	1.400542092	0.161351048	0.307162138	up_in_case
Yang2025_S	ENSGALG00010010334	ZBTB41	case	control	0.066386408	0.188595783	0.352003672	0.724835509	0.830640034	up_in_case
Yang2025_S	ENSGALG00010010336	FMNL2	case	control	0.036861177	0.101636893	0.362675164	0.716847554	0.825653282	up_in_case
Yang2025_S	ENSGALG00010010337	SCARA3	case	control	0.313003754	0.223089873	1.403038828	0.160605261	0.306244432	up_in_case
Yang2025_S	ENSGALG00010010338	CNST	case	control	-0.110560192	0.118058977	-0.936482725	0.349024703	0.517355519	down_in_case
Yang2025_S	ENSGALG00010010345	ENSGALG00010010345	case	control	0.391847251	0.227470667	1.722627606	0.08495589	0.195582409	up_in_case
Yang2025_S	ENSGALG00010010346	TFB2M	case	control	-0.39865719	0.178577299	-2.232406879	0.025588084	0.081297297	down_in_case
Yang2025_S	ENSGALG00010010347	FBXW11	case	control	-0.310524184	0.060063292	-5.169949443	2.34157E-07	7.01717E-06	down_in_case
Yang2025_S	ENSGALG00010010349	RREB1	case	control	0.27587843	0.17111044	1.612282867	0.1069004	0.229708107	up_in_case
Yang2025_S	ENSGALG00010010351	NAMPTP1	case	control	-0.110810485	0.155190357	-0.714029447	0.475208971	0.634781155	down_in_case
Yang2025_S	ENSGALG00010010352	MALT1	case	control	0.30400269	0.092085244	3.301318196	0.000962317	0.006793254	up_in_case
Yang2025_S	ENSGALG00010010356	STAB2	case	control	-0.570842406	0.25235887	-2.262026322	0.023695777	0.077041683	down_in_case
Yang2025_S	ENSGALG00010010358	ASPM	case	control	-0.050479775	0.123584648	-0.408463158	0.682933673	0.801622642	down_in_case
Yang2025_S	ENSGALG00010010361	PIGM	case	control	0.014192775	0.215592481	0.065831495	0.947511981	0.967543554	up_in_case

Yang2025_S	ENSGALG00010010366	CLU	case	control	0.221266699	0.200847437	1.101665533	0.270607107	0.438019518	up_in_case
Yang2025_S	ENSGALG00010010368	ENSGALG00010010368	case	control	0.582418938	0.194792261	2.989949059	0.00279024	0.015392712	up_in_case
Yang2025_S	ENSGALG00010010369	ENSGALG00010010369	case	control	0.550480074	0.323967241	1.699184375	0.089284449	0.202667992	up_in_case
Yang2025_S	ENSGALG00010010371	SOCS1L	case	control	-0.099750956	0.260760018	-0.382539303	0.702061382	0.816183066	down_in_case
Yang2025_S	ENSGALG00010010374	CMTM4	case	control	-0.136587089	0.177902106	-0.767765439	0.442626551	0.605892454	down_in_case
Yang2025_S	ENSGALG00010010379	SLC35F6	case	control	-0.561211177	0.276848286	-2.027143407	0.042647749	0.11859836	down_in_case
Yang2025_S	ENSGALG00010010381	MAST4	case	control	0.234836777	0.207798686	1.130116757	0.25842703	0.424782128	up_in_case
Yang2025_S	ENSGALG00010010382	SEC11C	case	control	0.540297061	0.160292089	3.370703213	0.000749766	0.005554485	up_in_case
Yang2025_S	ENSGALG00010010383	SLIT2	case	control	0.093108545	0.202071172	0.460771047	0.644962876	0.774450219	up_in_case
Yang2025_S	ENSGALG00010010386	PRPF40A	case	control	0.238080042	0.11414166	2.085829513	0.036994069	0.106980761	up_in_case
Yang2025_S	ENSGALG00010010390	KCNK3	case	control	0.249192812	0.229352462	1.086505938	0.277255215	0.444628464	up_in_case
Yang2025_S	ENSGALG00010010393	FANCC	case	control	-0.145613465	0.199345981	-0.730455986	0.465111507	0.626094364	down_in_case
Yang2025_S	ENSGALG00010010394	MRPL42	case	control	-0.385179292	0.174306363	-2.209783319	0.027120204	0.084944941	down_in_case
Yang2025_S	ENSGALG00010010397	PICK1	case	control	0.145091042	0.069081905	2.100275655	0.0357046	0.104350586	up_in_case
Yang2025_S	ENSGALG00010010399	FGF18	case	control	1.012798779	0.18800886	5.386973664	7.16539E-08	2.56683E-06	up_in_case
Yang2025_S	ENSGALG00010010403	ENSGALG00010010403	case	control	0.686242315	0.640470466	1.071465978	0.28395996	0.452071524	up_in_case
Yang2025_S	ENSGALG00010010404	COP1	case	control	0.142241576	0.107389056	1.324544428	0.185322298	0.33886248	up_in_case
Yang2025_S	ENSGALG00010010405	EVA1C	case	control	-0.235919729	0.244119316	-0.96641156	0.333838282	0.502651157	down_in_case
Yang2025_S	ENSGALG00010010406	PTCH1	case	control	0.26956488	0.147712409	1.824930495	0.068011519	0.166445471	up_in_case
Yang2025_S	ENSGALG00010010407	SOX10	case	control	0.715074318	0.149651061	4.778277652	1.76803E-06	3.91072E-05	up_in_case
Yang2025_S	ENSGALG00010010408	GAS8	case	control	0.384759532	0.14862447	2.588803391	0.009631007	0.039461357	up_in_case
Yang2025_S	ENSGALG00010010410	CACNA2D1	case	control	-0.05379191	0.131794558	-0.4081497	0.683163774	0.801622642	down_in_case
Yang2025_S	ENSGALG00010010411	CD180	case	control	-0.44675223	0.476113402	-0.938331558	0.348074047	0.516513541	down_in_case
Yang2025_S	ENSGALG00010010416	SLC35B3	case	control	-0.669550698	0.146701036	-4.564048865	5.01764E-06	9.24878E-05	down_in_case
Yang2025_S	ENSGALG00010010417	OTOF	case	control	1.1593576	0.636335993	1.821926803	0.068466093	0.167264201	up_in_case
Yang2025_S	ENSGALG00010010418	BRINP2	case	control	0.252663672	0.284502245	0.888090259	0.374492202	0.54252509	up_in_case
Yang2025_S	ENSGALG00010010420	NPM1	case	control	-0.068917733	0.122114801	-0.56436838	0.572503453	0.715833643	down_in_case
Yang2025_S	ENSGALG00010010421	POLR2F	case	control	-0.130269597	0.224943082	-0.579122488	0.562506527	0.708791794	down_in_case
Yang2025_S	ENSGALG00010010422	CDH1	case	control	0.238288765	0.751296473	0.317170084	0.751114549	0.848540188	up_in_case
Yang2025_S	ENSGALG00010010423	C21orf59	case	control	0.647238922	0.200580558	3.2268278	0.001251708	0.008340391	up_in_case
Yang2025_S	ENSGALG00010010425	BLOC1S5	case	control	-0.600129561	0.172637311	-3.476244847	0.000508488	0.004010061	down_in_case
Yang2025_S	ENSGALG00010010430	ASTN1	case	control	1.329642006	0.773310687	1.719415013	0.08553883	0.196502407	up_in_case
Yang2025_S	ENSGALG00010010431	SOCS2	case	control	0.031164502	0.222541611	0.140038986	0.888629187	0.935063635	up_in_case
Yang2025_S	ENSGALG00010010434	PROM1	case	control	0.550203986	0.17864649	3.079847734	0.002071064	0.012280972	up_in_case
Yang2025_S	ENSGALG00010010435	GAS1	case	control	0.16941511	0.125700134	1.347771915	0.177731754	0.329084771	up_in_case
Yang2025_S	ENSGALG00010010438	LDB3	case	control	0.545827528	0.220031069	2.480683895	0.01311306	0.04952046	up_in_case
Yang2025_S	ENSGALG00010010439	HGF	case	control	-0.156912249	0.194674894	-0.806022004	0.420230162	0.585916689	down_in_case
Yang2025_S	ENSGALG00010010444	TXNDC5	case	control	-0.403497906	0.158066612	-2.552708006	0.010688906	0.042557117	down_in_case
Yang2025_S	ENSGALG00010010445	LMAN1L	case	control	0.059875956	0.081662089	0.733216072	0.463426654	0.624393172	up_in_case
Yang2025_S	ENSGALG00010010446	C22orf23	case	control	0.14867233	0.540835465	0.274893826	0.78339781	0.868952359	up_in_case
Yang2025_S	ENSGALG00010010447	BTF3	case	control	0.67640495	0.251496551	2.689519789	0.00715549	0.031434981	up_in_case
Yang2025_S	ENSGALG00010010448	GALNT13	case	control	0.069995465	0.306037238	0.228715517	0.819090034	0.891999032	up_in_case
Yang2025_S	ENSGALG00010010449	PAPPA2	case	control	-0.93089876	0.479836268	-1.94003418	0.052375536	0.137942373	down_in_case
Yang2025_S	ENSGALG00010010451	RANBP17	case	control	0.374479728	0.494472572	0.757331647	0.448851196	0.611232426	up_in_case
Yang2025_S	ENSGALG00010010453	DRC1	case	control	0.567158141	0.475012395	1.193985983	0.232483451	0.395562502	up_in_case
Yang2025_S	ENSGALG00010010456	BMP6	case	control	0.20425326	0.161580397	1.264096785	0.20619529	0.364674332	up_in_case
Yang2025_S	ENSGALG00010010457	AGGF1	case	control	0.51110347	0.181544682	2.815303998	0.004873111	0.023521581	up_in_case
Yang2025_S	ENSGALG00010010460	SEMA3C	case	control	-0.397956447	0.135175725	-2.943993426	0.003240069	0.017248432	down_in_case
Yang2025_S	ENSGALG00010010461	SLC7A5	case	control	0.055276673	0.185636125	0.297768943	0.76587952	0.857438026	up_in_case
Yang2025_S	ENSGALG00010010463	ACAA2	case	control	0.442191164	0.199954078	2.211463592	0.02700375	0.084675351	up_in_case
Yang2025_S	ENSGALG00010010465	BMPR1A	case	control	-0.329254721	0.092167278	-3.572360247	0.000353778	0.002993261	down_in_case
Yang2025_S	ENSGALG00010010466	CRADD	case	control	-0.029481521	0.19015945	-0.155035792	0.8767931	0.927511717	down_in_case
Yang2025_S	ENSGALG00010010468	ENSGALG00010010468	case	control	-1.680602612	0.828149582	-2.029346688	0.042422991	0.118161979	down_in_case
Yang2025_S	ENSGALG00010010471	KCNJ3	case	control	-0.285128172	0.141183229	-2.019561209	0.043428922	0.120410273	down_in_case
Yang2025_S	ENSGALG00010010472	MMRN2	case	control	0.035276511	0.130819149	0.269658622	0.787422897	0.871820917	up_in_case
Yang2025_S	ENSGALG00010010473	TRIM35	case	control	-0.118776214	0.210899768	-0.563187977	0.573306889	0.716341214	down_in_case
Yang2025_S	ENSGALG00010010475	CCDC71L	case	control	-0.208706756	0.154712053	-1.349001273	0.177336557	0.328527642	down_in_case
Yang2025_S	ENSGALG00010010478	ENSGALG00010010478	case	control	0.592454009	0.530981583	1.115771297	0.264520043	0.431575553	up_in_case
Yang2025_S	ENSGALG00010010486	ENSGALG00010010486	case	control	-0.322856062	0.279373222	-1.155644268	0.247826693	0.413638156	down_in_case
Yang2025_S	ENSGALG00010010494	ENSGALG00010010494	case	control	-0.836034716	0.237415291	-3.521402158	0.000429271	0.003496138	down_in_case
Yang2025_S	ENSGALG00010010496	MRPL17	case	control	0.572717278	0.322975721	1.773251799	0.076186992	0.180739826	up_in_case
Yang2025_S	ENSGALG00010010499	NR4A2	case	control	0.010564775	0.216591105	0.048777511	0.961096604	0.976727041	up_in_case
Yang2025_S	ENSGALG00010010505	CD38	case	control	0.844311498	0.271582025	3.108863689	0.001878083	0.011438415	up_in_case
Yang2025_S	ENSGALG00010010511	GABRP	case	control	0.141384033	0.328226069	0.430751993	0.666648709	0.789228274	up_in_case
Yang2025_S	ENSGALG00010010512	PLXNC1	case	control	-0.596629537	0.123928803	-4.814292756	1.47722E-06	3.36357E-05	down_in_case
Yang2025_S	ENSGALG00010010515	RXRG	case	control	0.132132335	0.205148	0.644082982	0.519521625	0.673507661	up_in_case
Yang2025_S	ENSGALG00010010517	STMN4	case	control	0.806616659	0.663864567	1.215031948			up_in_case
Yang2025_S	ENSGALG00010010521	SNCG	case	control	0.320062192	0.289648093	1.105003622	0.26915802	0.436320695	up_in_case
Yang2025_S	ENSGALG00010010526	ENSGALG00010010526	case	control	1.135559353	0.360306179	3.15165107	0.001623502	0.010222998	up_in_case
Yang2025_S	ENSGALG00010010528	GPD2	case	control	0.209887696	0.087191417	2.407205933	0.016075102	0.057879651	up_in_case
Yang2025_S	ENSGALG00010010534	SYNJ1	case	control	0.616452385	0.224541743	2.745379885	0.006044089	0.027560333	up_in_case
Yang2025_S	ENSGALG00010010535	LMX1A	case	control	-1.059164408	0.388751954	-2.724524972	0.006439406	0.029011679	down_in_case
Yang2025_S	ENSGALG00010010537	ENSGALG00010010537	case	control	0.884758332	0.350844	2.521799813	0.011675613	0.045434144	up_in_case
Yang2025_S	ENSGALG00010010544	ENSGALG00010010544	case	control	0.24311207	0.191291476	1.270898605	0.203764723	0.361895548	up_in_case
Yang2025_S	ENSGALG00010010546	MICALL1	case	control	0.257425476	0.145987025	1.763344902	0.077842337	0.183883482	up_in_case
Yang2025_S	ENSGALG00010010547	BST1	case	control	-0.200420893	0.167585871	-1.195929531	0.23172406	0.394800681	down_in_case
Yang2025_S	ENSGALG00010010567	ERMN	case	control	1.79145103	0.935863396	1.914222778			up_in_case
Yang2025_S	ENSGALG00010010572	AQP3	case	control	-0.73290006	0.364681445	-2.009699342	0.04446302	0.122700173	down_in_case
Yang2025_S	ENSGALG00010010573	CEP83	case	control	0.565254174	0.170414777	3.316931692	0.000910119	0.006487214	up_in_case
Yang2025_S	ENSGALG00010010575	PAXBP1	case	control	0.212310783	0.082334403	2.578639977	0.00991901	0.040286042	up_in_case
Yang2025_S	ENSGALG00010010577	PBX1	case	control	0.275380477	0.153111162	1.798565656	0.072087414	0.173465397	up_in_case
Yang2025_S	ENSGALG00010010578	GLUD2	case	control	-0.113727185	0.096647685	-1.176719179	0.23930761	0.403576371	down_in_case
Yang2025_S	ENSGALG00010010585	FBXL5	case	control	0.247511345	0.073830278	3.352436845	0.000801035	0.005856466	up_in_case
Yang2025_S	ENSGALG00010010586	CYTIP	case	control	0.210283157	0.53903733	0.390108709	0.696456163	0.811871895	up_in_case
Yang2025_S	ENSGALG00010010588	SLC25A3	case	control	-0.270811431	0.161498447	-1.676867085	0.093568476	0.209222836	down_in_case
Yang2025_S	ENSGALG00010010593	EIF3L	case	control	0.18396963	0.134882226	1.363927891	0.172590262	0.322262535	up_in_case
Yang2025_S	ENSGALG00010010596	SHLD2	case	control	-0.696223844	0.135477942	-5.139019927	2.76175E-07	7.91872E-06	down_in_case
Yang2025_S	ENSGALG00010010599	F2RL1	case	control	-0.50751406	0.145364048	-3.491331354	0.00048062	0.003829295	down_in_case
Yang2025_S	ENSGALG00010010604	HIVEP1	case	control	0.496514059	0.221821676	2.23834779	0.025198384	0.080371427	up_in_case
Yang2025_S	ENSGALG00010010605	NUF2	case	control	-0.026322459	0.151381766	-0.173881304	0.861958746	0.918779821	down_in_case
Yang2025_S	ENSGALG00010010614	IKBIP	case	control	-0.215970447	0.132016693	-1.635932871	0.101853637	0.222013208	down_in_case
Yang2025_S	ENSGALG00010010618	APAF1	case	control	-0.089975586	0.104287194	-0.862767352	0.388265393	0.555254111	down_in_case
Yang2025_S	ENSGALG00010010619	LRRC52	case	control	-0.268317952	0.298184196	-0.899839615	0.368205609	0.536765219	down_in_case
Yang2025_S	ENSGALG00010010620	ANKRD54	case	control	-0.362807021	0.149276042	-2.430443741	0.015080347	0.055286671	down_in_case
Yang2025_S	ENSGALG00010010623	SMAD2	case	control	0.204395932	0.181558446	1.125785862	0.26025617	0.426667027	up_in_case
Yang2025_S	ENSGALG00010010625	FAM169A	case	control	0.085522962	0.390035782	0.219269528	0.826440097	0.896360737	up_in_case
Yang2025_S	ENSGALG00010010626	ADTRP	case	control	-0.645267327	0.532730992	-1.211244204	0.225801826	0.387744725	down_in_case
Yang2025_S	ENSGALG00010010633	EPCIP	case	control	0.849532916	0.351342603	2.417961579	0.015607725	0.056683253	up_in_case
Yang2025_S	ENSGALG00010010635	ALDH9A1	case	control	-0.36802338	0.148426628	-2.479497009	0.013156783	0.049658661	down_in_case
Yang2025_S	ENSGALG00010010638	ACVR1C	case	control	-0.339396774	0.372796121	-0.910408544	0.362607094	0.531272195	down_in_case
Yang2025_S	ENSGALG00010010642	CC2D2A	case	control	-0.163238621	0.118274719	-1.380164946	0.167535864	0.315616097	down_in_case
Yang2025_S	ENSGALG00010010645	FAM219A	case	control	-0.421619137	0.143484691	-2.938425938	0.003298835	0.017498948	down_in_case
Yang2025_S	ENSGALG00010010646	MCM3AP	case	control	-0.206429924	0.122402237	-1.686488165	0.091701813	0.206540288	down_in_case
Yang2025_S	ENSGALG00010010648	CENPM	case	control	-0.02213312	0.370103271	-0.059802552	0.952312893	0.97070296	down_in_case
Yang2025_S	ENSGALG00010010651	NUAK1	case	control	0.003632985	0.091916571	0.039524806	0.968471977	0.981359584	up_in_case
Yang2025_S	ENSGALG00010010655	C1QTNF7	case	control	-0.434123013	0.211743434	-2.050231286	0.040341866	0.113911305	down_in_case
Yang2025_S	ENSGALG00010010657	TMEM170B	case	control	-0.623335033	0.147519707	-4.225435687	2.38479E-05	0.000337039	down_in_case
Yang2025_S	ENSGALG00010010659	ADAMTS1	case	control	-0.030932012	0.130496436	-0.237033384	0.812630892	0.887252024	down_in_case
Yang2025_S	ENSGALG00010010661	CPEB2	case	control	0.790055453	0.283254194	2.789210081	0.005283678	0.024967125	up_in_case
Yang2025_S	ENSGALG00010010662	MGST3	case	control	-0.24401288	0.24136377	-1.01097559	0.312028113	0.481678493	down_in_case
Yang2025_S	ENSGALG00010010663	EFR3B	case	control	0.183349507	0.132663087	1.382068757	0.16695059	0.314768838	up_in_case
Yang2025_S	ENSGALG00010010666	C12orf75	case	control	0.218227262	0.174526702	1.25039469	0.211155403	0.371099829	up_in_case
Yang2025_S	ENSGALG00010010671	SIGMAR1	case	control	0.117592706	0.156298527	0.752359659	0.451834799	0.613616804	up_in_case
Yang2025_S	ENSGALG00010010678	KCNIP1	case	control	-0.136587543	0.298535479	-0.457525327	0.647293502	0.775872645	down_in_case
Yang2025_S	ENSGALG00010010680	ENSGALG00010010680	case	control	0.673977113	0.527755238	1.277063806	0.201579722	0.359484697	up_in_case
Yang2025_S	ENSGALG00010010682	SMIM13	case	control	-0.637464114	0.101545987	-6.277590433	3.4386E-10	2.36044E-08	down_in_case
Yang2025_S	ENSGALG00010010685	STIM2	case	control	0.066668877	0.116632172	0.571616526	0.567581797	0.712288575	up_in_case
Yang2025_S	ENSGALG00010010688	IQGAP2	case	control	0.106237443	0.152241737	0.697820749	0.485289302	0.642701722	up_in_case
Yang2025_S	ENSGALG00010010689	POFUT2	case	control	-0.303867482	0.091697694	-3.313796327	0.000920385	0.006550341	down_in_case
Yang2025_S	ENSGALG00010010690	MTPN	case	control	-0.316566728	0.084290507	-3.755662868	0.000172883	0.001661858	down_in_case
Yang2025_S	ENSGALG00010010693	KCNMB1	case	control	0.225125	0.294080633	0.765521339	0.443961165	0.607160485	up_in_case
Yang2025_S	ENSGALG00010010697	NEDD9	case	control	0.206760234	0.174044	1.187976798	0.234842551	0.398028421	up_in_case
Yang2025_S	ENSGALG00010010698	GPRIN2	case	control	-0.586512445	0.23147199	-2.533837656	0.011282099	0.044273738	down_in_case
Yang2025_S	ENSGALG00010010699	DNAJC27	case	control	0.7599676	0.150385406	5.053466433	4.33863E-07	1.18779E-05	up_in_case
Yang2025_S	ENSGALG00010010706	SREBF2	case	control	0.102025226	0.101035712	1.009793711	0.312594134	0.482229123	up_in_case
Yang2025_S	ENSGALG00010010710	UCK2	case	control	-0.167412324	0.133293073	-1.255971669	0.209126242	0.368369888	down_in_case

Yang2025_S	ENSGALG00010010711	ENSGALG00010010711	case	control	0.424442479	0.380924742	1.114242349	0.265175232	0.432416631	up_in_case
Yang2025_S	ENSGALG00010010712	ADAMTS5	case	control	-0.023201355	0.186669157	-0.124291316	0.901084623	0.941063795	down_in_case
Yang2025_S	ENSGALG00010010713	NSA2	case	control	0.221048216	0.222289416	0.994416288	0.320020241	0.489300205	up_in_case
Yang2025_S	ENSGALG00010010719	SH3BP4	case	control	-0.448604949	0.129753234	-3.457370089	0.000545475	0.004255989	down_in_case
Yang2025_S	ENSGALG00010010722	TBC1D19	case	control	-0.657894306	0.142108638	-4.629516661	3.6652E-06	7.20375E-05	down_in_case
Yang2025_S	ENSGALG00010010724	ADCY3	case	control	0.051011659	0.111664151	0.456831116	0.647792439	0.776181224	up_in_case
Yang2025_S	ENSGALG00010010733	GFM2	case	control	0.339917527	0.157915984	2.152521348	0.031356316	0.094623856	up_in_case
Yang2025_S	ENSGALG00010010734	AMN1	case	control	-0.348294688	0.207535256	-1.678243469	0.093299579	0.208755561	down_in_case
Yang2025_S	ENSGALG00010010740	CCDC134	case	control	-0.54658528	0.215116301	-2.540882661	0.011057302	0.043625002	down_in_case
Yang2025_S	ENSGALG00010010742	LCP2	case	control	-0.071873639	0.227489975	-0.315942004	0.752046529	0.84889315	down_in_case
Yang2025_S	ENSGALG00010010743	KLHL20	case	control	-0.034629424	0.124800456	-0.277478347	0.78141283	0.867786721	down_in_case
Yang2025_S	ENSGALG00010010745	ATVR1	case	control	-0.136793508	0.050639904	-2.701298727	0.006906927	0.030681552	down_in_case
Yang2025_S	ENSGALG00010010748	SF3B1	case	control	0.214293459	0.078188304	2.740735484	0.006130184	0.027898142	up_in_case
Yang2025_S	ENSGALG00010010749	NCOA4	case	control	-0.332574786	0.089344975	-3.722366997	0.000197364	0.00184954	down_in_case
Yang2025_S	ENSGALG00010010751	SLC30A5	case	control	0.062922426	0.1428396	0.440511079	0.65956699	0.785024428	up_in_case
Yang2025_S	ENSGALG00010010752	ENSGALG00010010752	case	control	-0.437904509	0.222100378	-1.971651342	0.048649422	0.130748253	down_in_case
Yang2025_S	ENSGALG00010010753	ENSGALG00010010753	case	control	-0.011685156	0.233398556	-0.050065247	0.960070394	0.975838719	down_in_case
Yang2025_S	ENSGALG00010010754	DNAI1	case	control	0.564196835	0.316670959	1.781650067	0.074806314	0.178497601	up_in_case
Yang2025_S	ENSGALG00010010756	CENPL	case	control	-0.148534582	0.16655281	-0.891816729	0.372491174	0.540806887	down_in_case
Yang2025_S	ENSGALG00010010759	ENSGALG00010010759	case	control	1.545746439	0.348495308	4.435487084	9.18643E-06	0.000152034	up_in_case
Yang2025_S	ENSGALG00010010761	MEI1	case	control	0.666259844	0.324491855	2.053240576	0.040049251	0.113478307	up_in_case
Yang2025_S	ENSGALG00010010762	MRPL39	case	control	-0.084451441	0.137794824	-0.61287818	0.539956892	0.690770674	down_in_case
Yang2025_S	ENSGALG00010010766	CENPO	case	control	0.139655477	0.18440444	0.757332507	0.448850681	0.611232426	up_in_case
Yang2025_S	ENSGALG00010010768	ENSGALG00010010768	case	control	0.095370927	0.249712286	0.381923248	0.702518295	0.81634023	up_in_case
Yang2025_S	ENSGALG00010010769	HEXB	case	control	-0.092431051	0.136773887	-0.675794574	0.49917108	0.655992704	down_in_case
Yang2025_S	ENSGALG00010010771	ENSGALG00010010771	case	control	-0.464923523	0.202045048	-2.301088427	0.021386633	0.071105219	down_in_case
Yang2025_S	ENSGALG00010010774	KPTN	case	control	0.37786673	0.191502582	1.973167805	0.048476447	0.13040913	up_in_case
Yang2025_S	ENSGALG00010010776	GCAT	case	control	-0.380330178	0.174583687	-2.178497802	0.029368996	0.090072231	down_in_case
Yang2025_S	ENSGALG00010010778	UPP2	case	control	0.020038031	0.336937999	0.059470973	0.952576985	0.970844746	up_in_case
Yang2025_S	ENSGALG00010010779	DOCK2	case	control	-0.310255291	0.205421244	-1.510336935	0.130957472	0.265284543	down_in_case
Yang2025_S	ENSGALG00010010780	PARG	case	control	-0.365381577	0.066855577	-5.46523707	4.62289E-08	1.75054E-06	down_in_case
Yang2025_S	ENSGALG00010010781	NAPA	case	control	0.60036343	0.126157298	4.758848203	1.94701E-06	4.23931E-05	up_in_case
Yang2025_S	ENSGALG00010010783	F2RL2	case	control	0.098881753	0.259720983	0.380723003	0.703408798	0.816901542	up_in_case
Yang2025_S	ENSGALG00010010784	CCDC148	case	control	0.411681701	0.240405443	1.712447505	0.086814231	0.19858114	up_in_case
Yang2025_S	ENSGALG00010010786	NABP1	case	control	0.49260565	0.138159156	3.565494067	0.000363171	0.003056035	up_in_case
Yang2025_S	ENSGALG00010010789	ENSGALG00010010789	case	control	0.405536507	0.201558336	2.01200563	0.044219344	0.122164265	up_in_case
Yang2025_S	ENSGALG00010010791	H-10	case	control	-0.643170448	0.258048464	-2.492440519	0.012686858	0.048303651	down_in_case
Yang2025_S	ENSGALG00010010792	XRCC6	case	control	0.025941356	0.128116828	0.202482035	0.839539902	0.904934138	up_in_case
Yang2025_S	ENSGALG00010010796	CAVIN2	case	control	0.034164587	0.129812799	0.263183499	0.792409148	0.874700688	up_in_case
Yang2025_S	ENSGALG00010010799	DARS2	case	control	0.059014415	0.104388374	0.565335137	0.571845833	0.715513306	up_in_case
Yang2025_S	ENSGALG00010010800	BICRA	case	control	0.119539001	0.122006404	0.979776446	0.327196481	0.496892215	up_in_case
Yang2025_S	ENSGALG00010010805	PTRHD1	case	control	0.133017197	0.297830975	0.446619754	0.65514963	0.781986044	up_in_case
Yang2025_S	ENSGALG00010010809	CENPH	case	control	0.028412085	0.172203492	0.164991338	0.8689508	0.92285873	up_in_case
Yang2025_S	ENSGALG00010010810	GALT	case	control	0.311319444	0.206391332	1.508393985	0.131453722	0.266019113	up_in_case
Yang2025_S	ENSGALG00010010813	EHD2	case	control	0.662724693	0.394596207	1.679500922	0.093054459	0.208394777	up_in_case
Yang2025_S	ENSGALG00010010816	AP2S1	case	control	-0.155047757	0.278039879	-0.55764575	0.577086309	0.719937128	down_in_case
Yang2025_S	ENSGALG00010010821	RGS8	case	control	-0.505301831	0.591850577	-0.853765884			down_in_case
Yang2025_S	ENSGALG00010010823	TMEFF2	case	control	-0.174974487	0.1619566	-1.080378865	0.279973503	0.447821077	down_in_case
Yang2025_S	ENSGALG00010010824	PKP4	case	control	0.233442555	0.148119387	1.576043213	0.115015845	0.242252992	up_in_case
Yang2025_S	ENSGALG00010010825	MAK	case	control	0.306558549	0.349459115	0.877237238	0.380357794	0.548301583	up_in_case
Yang2025_S	ENSGALG00010010826	NCOA1	case	control	0.194277595	0.209728753	0.926327901	0.354275601	0.523412902	up_in_case
Yang2025_S	ENSGALG00010010828	SPDL1	case	control	0.214112531	0.107028459	2.000519597	0.045444186	0.124781228	up_in_case
Yang2025_S	ENSGALG00010010829	LAMB4	case	control	-0.368233398	0.123457197	-2.98268068	0.002857359	0.015688457	down_in_case
Yang2025_S	ENSGALG00010010831	DESI1	case	control	-0.745368372	0.129333366	-5.763156048	8.25554E-09	3.84752E-07	down_in_case
Yang2025_S	ENSGALG00010010834	KGD4	case	control	-0.177387894	0.246473428	-0.719703926	0.471707309	0.631861675	down_in_case
Yang2025_S	ENSGALG00010010836	DIP2A	case	control	0.501518481	0.112245507	4.468049519	7.8936E-06	0.000134904	up_in_case
Yang2025_S	ENSGALG00010010837	RBPJ	case	control	-0.199061013	0.133476893	-1.491351855	0.135869141	0.272422515	down_in_case
Yang2025_S	ENSGALG00010010838	JAM2	case	control	-0.382484159	0.146250849	-2.615261117	0.008915926	0.037355212	down_in_case
Yang2025_S	ENSGALG00010010840	RGS16	case	control	1.183694731	0.698273927	1.69517246	0.09004271	0.20375188	up_in_case
Yang2025_S	ENSGALG00010010841	PMM1	case	control	-0.093980933	0.213160632	-0.440892544	0.659290792	0.784762722	down_in_case
Yang2025_S	ENSGALG00010010843	ZNF395	case	control	0.118846157	0.139406729	0.852513777	0.393928996	0.56100159	up_in_case
Yang2025_S	ENSGALG00010010844	CDC14B	case	control	0.04515614	0.13343491	0.338413241	0.735051797	0.838348894	up_in_case
Yang2025_S	ENSGALG00010010845	FKRP	case	control	0.021642673	0.157597491	0.137328789	0.890770916	0.936115589	up_in_case
Yang2025_S	ENSGALG00010010848	RGR	case	control	-0.320798376	0.538132075	-0.596133162	0.551086272	0.699206701	down_in_case
Yang2025_S	ENSGALG00010010849	ACBD6	case	control	0.205089654	0.139810972	1.466906721	0.142401432	0.282110315	up_in_case
Yang2025_S	ENSGALG00010010850	ENSGALG00010010850	case	control	0.117276004	0.209125395	0.560792741	0.574938836	0.717707845	up_in_case
Yang2025_S	ENSGALG00010010851	PNOC	case	control	-0.900224131	0.347930597	-2.58736696	0.009671253	0.039591339	down_in_case
Yang2025_S	ENSGALG00010010853	SMIM20	case	control	-0.620369149	0.289897936	-2.139957111	0.032358233	0.096959632	down_in_case
Yang2025_S	ENSGALG00010010854	LRIT1	case	control	-0.444797866	0.380633261	-1.168573302	0.24257559	0.407558578	down_in_case
Yang2025_S	ENSGALG00010010855	ATP5PF	case	control	-0.795859491	0.296800811	-2.681581253	0.007327512	0.031968966	down_in_case
Yang2025_S	ENSGALG00010010857	SEL1L3	case	control	0.07096215	0.1476165	0.480719639	0.630715771	0.763258635	up_in_case
Yang2025_S	ENSGALG00010010859	ENSGALG00010010859	case	control	-0.593172331	0.238535025	-2.486730534	0.012892301	0.048952102	down_in_case
Yang2025_S	ENSGALG00010010861	ENSGALG00010010861	case	control	0.451684075	0.22594405	1.999097011	0.045597859	0.124996358	up_in_case
Yang2025_S	ENSGALG00010010862	STRN4	case	control	0.331319352	0.110070151	3.010074486	0.002611836	0.014652988	up_in_case
Yang2025_S	ENSGALG00010010865	ENSGALG00010010865	case	control	0.034031716	0.077328799	0.440091094	0.659871132	0.785118189	up_in_case
Yang2025_S	ENSGALG00010010867	QSOX1	case	control	-0.010921263	0.17850536	-0.06118171	0.951214496	0.969867125	down_in_case
Yang2025_S	ENSGALG00010010868	SLC39A10	case	control	-0.035009798	0.160644782	-0.21793299	0.827481323	0.896722314	down_in_case
Yang2025_S	ENSGALG00010010869	ENSGALG00010010869	case	control	-0.361355708	0.258680271	-1.396920246	0.162437555	0.308682304	down_in_case
Yang2025_S	ENSGALG00010010870	NOL6	case	control	-0.043776833	0.162970184	-0.268618662	0.78822315	0.872340982	down_in_case
Yang2025_S	ENSGALG00010010874	ENSGALG00010010874	case	control	-0.310614883	0.275693871	-1.1266659	0.259883765	0.426332672	down_in_case
Yang2025_S	ENSGALG00010010876	ENSGALG00010010876	case	control	-0.036033224	0.189282915	-0.19036702	0.84902154	0.910351313	down_in_case
Yang2025_S	ENSGALG00010010880	ELP3	case	control	-0.194009908	0.082433979	-2.353518661	0.018596674	0.064271989	down_in_case
Yang2025_S	ENSGALG00010010882	CEP350	case	control	0.134079478	0.196201134	0.683377693	0.494368212	0.65187557	up_in_case
Yang2025_S	ENSGALG00010010884	DNAH7	case	control	0.212986743	0.268502565	0.793239135	0.427638511	0.592065837	up_in_case
Yang2025_S	ENSGALG00010010885	SNRPD2	case	control	0.350783643	0.186934302	1.876507624	0.060585609	0.153557741	up_in_case
Yang2025_S	ENSGALG00010010887	GABPA	case	control	-0.2865105	0.046946561	-6.102907066	1.04156E-09	6.31052E-08	down_in_case
Yang2025_S	ENSGALG00010010888	GNPAT	case	control	-0.255335498	0.101887926	-2.506042743	0.012209086	0.047024216	down_in_case
Yang2025_S	ENSGALG00010010889	CCL19	case	control	-0.262793411	0.559650265	-0.469567205	0.63866426	0.76888004	down_in_case
Yang2025_S	ENSGALG00010010890	NOL12	case	control	-0.301658776	0.169912195	-1.77538037	0.075835103	0.180211828	down_in_case
Yang2025_S	ENSGALG00010010891	POLR3H	case	control	0.178896933	0.162684069	1.099658588	0.271480904	0.438681581	up_in_case
Yang2025_S	ENSGALG00010010893	CCL21	case	control	-1.17701233	0.37092574	-3.17317512	0.001507815	0.009647111	down_in_case
Yang2025_S	ENSGALG00010010894	SPAG1	case	control	0.176138613	0.213911814	0.823416948	0.410270929	0.575626801	up_in_case
Yang2025_S	ENSGALG00010010896	LY86	case	control	-0.468499365	0.204336643	-2.292781937	0.021860562	0.072237832	down_in_case
Yang2025_S	ENSGALG00010010897	CEP170	case	control	-0.09025384	0.172670309	-0.522694614	0.601186787	0.739907168	down_in_case
Yang2025_S	ENSGALG00010010904	TOR1AIP1	case	control	-0.296599122	0.109349535	-2.712394915	0.006679896	0.029844292	down_in_case
Yang2025_S	ENSGALG00010010905	ENC1	case	control	-0.171137466	0.137577469	-1.243935273	0.213523386	0.373875393	down_in_case
Yang2025_S	ENSGALG00010010906	ATP6V1C1	case	control	-0.231115759	0.107013275	-2.159692415	0.030796489	0.093476165	down_in_case
Yang2025_S	ENSGALG00010010908	STK17B	case	control	-0.253429576	0.10803849	-2.345734156	0.018989646	0.065244208	down_in_case
Yang2025_S	ENSGALG00010010909	SLIT3	case	control	-0.235314718	0.255418922	-0.921289291	0.356899411	0.526117982	down_in_case
Yang2025_S	ENSGALG00010010912	BAALC	case	control	1.412388079	0.321155738	4.397829191	1.09339E-05	0.000177787	up_in_case
Yang2025_S	ENSGALG00010010913	IFT56	case	control	-0.068556888	0.110967684	-0.617809485	0.53670093	0.687907236	down_in_case
Yang2025_S	ENSGALG00010010915	APP	case	control	-0.107030804	0.087327119	-1.22563077	0.220337653	0.381653847	down_in_case
Yang2025_S	ENSGALG00010010917	TANC1	case	control	0.41860116	0.122907552	3.405821313	0.000659653	0.005009787	up_in_case
Yang2025_S	ENSGALG00010010918	TOR1AIP2	case	control	-0.189665917	0.114434379	-1.657420778	0.097434433	0.214935702	down_in_case
Yang2025_S	ENSGALG00010010919	CDHR1	case	control	-0.100673914	0.655664844	-0.153544779	0.87796868	0.928121192	down_in_case
Yang2025_S	ENSGALG00010010920	UBN2	case	control	0.444473099	0.191993007	2.315048379	0.020610285	0.069189187	up_in_case
Yang2025_S	ENSGALG00010010921	ENSGALG00010010921	case	control	0.270610753	0.215556757	1.255403713	0.209332242	0.368499848	up_in_case
Yang2025_S	ENSGALG00010010922	DYM	case	control	-0.076214562	0.08509285	-0.895663528	0.370432495	0.538997267	down_in_case
Yang2025_S	ENSGALG00010010923	F13A1	case	control	-0.557868876	0.193306108	-2.885935065	0.003902527	0.019840098	down_in_case
Yang2025_S	ENSGALG00010010924	ENSGALG00010010924	case	control	0.190433796	0.113362216	1.679870095	0.092982593	0.208355043	up_in_case
Yang2025_S	ENSGALG00010010925	FBXO16	case	control	-0.163056995	0.525338681	-0.310384521	0.756268563	0.852017336	down_in_case
Yang2025_S	ENSGALG00010010928	HECW2	case	control	0.231559962	0.168130029	1.377267127	0.168429675	0.316743255	up_in_case
Yang2025_S	ENSGALG00010010929	FMC1	case	control	-0.374395297	0.407528105	-0.918698103	0.358253502	0.527388818	down_in_case
Yang2025_S	ENSGALG00010010930	FAM163A	case	control	-0.324870554	0.336792911	-0.964600334	0.334745032	0.503363766	down_in_case
Yang2025_S	ENSGALG00010010931	CYYR1	case	control	-0.005413766	0.179109878	-0.030225949	0.975886854	0.985647844	down_in_case
Yang2025_S	ENSGALG00010010932	GHITM	case	control	-0.129568447	0.154215035	-0.840180381	0.400807257	0.567491275	down_in_case
Yang2025_S	ENSGALG00010010933	ANAPC4	case	control	-0.362812574	0.092384766	-3.927190506	8.59439E-05	0.000943932	down_in_case
Yang2025_S	ENSGALG00010010934	ENSGALG00010010934	case	control	0.769303025	0.111183136	6.919241989	4.54067E-12	5.36222E-10	up_in_case
Yang2025_S	ENSGALG00010010935	LGALS1A	case	control	0.663513603	0.224496245	2.955566598	0.003120953	0.016819447	up_in_case
Yang2025_S	ENSGALG00010010937	CDK7	case	control	-0.191229935	0.190264283	-1.005075321	0.314860583	0.484066882	down_in_case
Yang2025_S	ENSGALG00010010938	FZD6	case	control	-0.278028384	0.096034121	-2.895099991	0.003790378	0.019425477	down_in_case
Yang2025_S	ENSGALG00010010939	ZCCHC4	case	control	-0.137615272	0.166898571	-0.824544341	0.409630332	0.575105568	down_in_case
Yang2025_S	ENSGALG00010010940	NRG3	case	control	-0.147255772	0.30687063	-0.479862709	0.631325019	0.763452777	down_in_case
Yang2025_S	ENSGALG00010010942	NRN1	case	control	-0.222770782	0.238211715	-0.935179793	0.349695651	0.518184697	down_in_case
Yang2025_S	ENSGALG00010010943	WDSUB1	case	control	0.197830686	0.092638055	2.135522874	0.032718324	0.097755112	up_in_case
Yang2025_S	ENSGALG00010010945	PI4K2B	case	control	-0.357182834	0.121113947	-2.949146993	0.003186524	0.017078542	down_in_case

Yang2025_S	ENSGALG00010010946	SH2D4B	case	control	0.265283194	0.402146958	0.65966729	0.509467362	0.664864927	up_in_case
Yang2025_S	ENSGALG00010010947	LUC7L2	case	control	-0.030234352	0.06611118	-0.457325852	0.647436851	0.775888589	down_in_case
Yang2025_S	ENSGALG00010010948	PRDM15	case	control	0.450766673	0.138384941	3.257339062	0.00112462	0.007685915	up_in_case
Yang2025_S	ENSGALG00010010949	FZD3	case	control	-0.18410679	0.125074426	-1.471977895	0.141026847	0.280263707	down_in_case
Yang2025_S	ENSGALG00010010951	ENSGALG00010010951	case	control	0.434439683	0.198010139	2.194027466	0.028233435	0.08744897	up_in_case
Yang2025_S	ENSGALG00010010952	ENSGALG00010010952	case	control	-0.1291764	0.499154365	-0.258790485	0.7957969	0.876582922	down_in_case
Yang2025_S	ENSGALG00010010953	EXTL3	case	control	-0.402168952	0.077914114	-5.161695725	2.44723E-07	7.22503E-06	down_in_case
Yang2025_S	ENSGALG00010010954	MTERF2	case	control	-0.382195376	0.236301149	-1.61740803	0.105790234	0.228166987	down_in_case
Yang2025_S	ENSGALG00010010955	GTF3C3	case	control	0.073942686	0.071861498	1.028961099	0.303497955	0.473069799	up_in_case
Yang2025_S	ENSGALG00010010956	AXDND1	case	control	-0.094127132	0.390868465	-0.240815365	0.80969822	0.88536917	down_in_case
Yang2025_S	ENSGALG00010010957	ZMAT2	case	control	0.164340395	0.09545681	1.721620434	0.085138299	0.195872907	up_in_case
Yang2025_S	ENSGALG00010010958	LCORL	case	control	0.378852583	0.181994122	2.081674824	0.037372182	0.107661757	up_in_case
Yang2025_S	ENSGALG00010010959	TMEM263	case	control	-0.21337428	0.094887089	-2.248717739	0.02453046	0.078956682	down_in_case
Yang2025_S	ENSGALG00010010960	POLDIP3	case	control	-0.344520617	0.077831775	-4.426477719	9.57843E-06	0.000157959	down_in_case
Yang2025_S	ENSGALG00010010961	C2orf66	case	control	1.539177096	0.428080394	3.595532795	0.000323728	0.002777843	up_in_case
Yang2025_S	ENSGALG00010010962	ANKDD1B	case	control	1.635144962	0.343636491	4.758356596	1.95175E-06	4.24301E-05	up_in_case
Yang2025_S	ENSGALG00010010963	TSPAN14	case	control	-0.712774348	0.113650587	-6.271629289	3.57289E-10	2.4406E-08	down_in_case
Yang2025_S	ENSGALG00010010964	INTS9	case	control	-0.280083559	0.103845303	-2.697123024	0.006994143	0.030947968	down_in_case
Yang2025_S	ENSGALG00010010965	BAZ2B	case	control	0.919370393	0.205427959	4.47539077	7.62717E-06	0.000131703	up_in_case
Yang2025_S	ENSGALG00010010966	ENSGALG00010010966	case	control	0.233846885	0.497897771	0.469668471	0.638591897	0.768870559	up_in_case
Yang2025_S	ENSGALG00010010967	PRXL2A	case	control	-0.255412996	0.250247953	-1.0206397	0.307425173	0.477109899	down_in_case
Yang2025_S	ENSGALG00010010968	RRP7A	case	control	0.163121181	0.153984219	1.059337004	0.289446328	0.458240693	up_in_case
Yang2025_S	ENSGALG00010010970	TPM2	case	control	-0.163807823	0.139935681	-1.170593675	0.241762141	0.406858182	down_in_case
Yang2025_S	ENSGALG00010010971	LCORL	case	control	0.03989589	0.131738493	0.302841557	0.762010612	0.855286959	up_in_case
Yang2025_S	ENSGALG00010010972	EXOSC3	case	control	0.382505932	0.144552169	2.646144533	0.008141502	0.034665518	up_in_case
Yang2025_S	ENSGALG00010010973	PGAP1	case	control	-0.556295877	0.148977361	-3.734096722	0.00018839	0.001777397	down_in_case
Yang2025_S	ENSGALG00010010974	NCAPG	case	control	-0.276931617	0.145825285	-1.899064472	0.057556	0.148087676	down_in_case
Yang2025_S	ENSGALG00010010976	DYDC2	case	control	0.548146299	0.476704913	1.14986501	0.250199474	0.415924106	up_in_case
Yang2025_S	ENSGALG00010010977	FARS2	case	control	-0.223141923	0.112436419	-1.984605394	0.047188392	0.128029857	down_in_case
Yang2025_S	ENSGALG00010010978	SERHL	case	control	0.00950542	0.175254021	0.054237957	0.956745579	0.973512205	up_in_case
Yang2025_S	ENSGALG00010010979	ENSGALG00010010979	case	control	1.507554641	0.398009073	3.787739386	0.000152024	0.001497143	up_in_case
Yang2025_S	ENSGALG00010010980	POLK	case	control	-0.061949676	0.173875826	-0.356286881	0.721625733	0.828660518	down_in_case
Yang2025_S	ENSGALG00010010981	RIC8B	case	control	0.17036029	0.150831245	1.129476124	0.258697036	0.425010987	up_in_case
Yang2025_S	ENSGALG00010010982	ZBTB21	case	control	-0.029826981	0.148342316	-0.201068594	0.840644939	0.905354913	down_in_case
Yang2025_S	ENSGALG00010010983	MED28	case	control	-0.441201748	0.172950512	-2.551028871	0.010740543	0.042713888	down_in_case
Yang2025_S	ENSGALG00010010984	MAT1A	case	control	-0.256292228	0.1872268	-1.368886445	0.171034773	0.320001285	down_in_case
Yang2025_S	ENSGALG00010010985	CERT1	case	control	0.191666965	0.1518141	1.262510963	0.206764984	0.365504256	up_in_case
Yang2025_S	ENSGALG00010010986	NARS	case	control	-0.091495891	0.085202656	-1.073861952	0.282884558	0.451080938	down_in_case
Yang2025_S	ENSGALG00010010987	MARCHF7	case	control	0.028702555	0.058075745	0.494226206	0.621146434	0.7558516	up_in_case
Yang2025_S	ENSGALG00010010988	ANKRD44	case	control	0.213884304	0.111664819	1.915413525	0.055439788	0.144025623	up_in_case
Yang2025_S	ENSGALG00010010989	TRIQK	case	control	-0.316604555	0.12792906	-2.474844698	0.013329412	0.050133701	down_in_case
Yang2025_S	ENSGALG00010010990	MED18	case	control	0.220460277	0.172616309	1.277169458	0.201542427	0.359484697	up_in_case
Yang2025_S	ENSGALG00010010991	C2CD2	case	control	-0.274098694	0.137648453	-1.991295132	0.04644845	0.126466307	down_in_case
Yang2025_S	ENSGALG00010010992	PCDHA6	case	control	0.379607177	0.209849771	1.808947304	0.07045919	0.170756316	up_in_case
Yang2025_S	ENSGALG00010010993	ENSGALG00010010993	case	control	0.110172023	0.243561789	0.452337058	0.651026183	0.778783574	up_in_case
Yang2025_S	ENSGALG00010010994	NAA20	case	control	-0.201129207	0.161231099	-1.247459133	0.212229196	0.372462529	down_in_case
Yang2025_S	ENSGALG00010010995	HEBP1	case	control	0.094904297	0.131233873	0.723169221	0.469575926	0.630049827	up_in_case
Yang2025_S	ENSGALG00010010996	RFX4	case	control	1.570069888	0.435893024	3.601961496	0.000315825	0.002721721	up_in_case
Yang2025_S	ENSGALG00010010997	LAP3	case	control	0.025704299	0.140159802	0.183392806	0.85448982	0.913978788	up_in_case
Yang2025_S	ENSGALG00010010998	SOAT1	case	control	-0.648039886	0.104816656	-6.182604065	6.30527E-10	4.01844E-08	down_in_case
Yang2025_S	ENSGALG00010011001	ANGPT1	case	control	0.681999674	0.232778994	2.929816224	0.003391625	0.017855043	up_in_case
Yang2025_S	ENSGALG00010011003	FECH	case	control	-0.255247394	0.12388281	-2.060393967	0.039360893	0.111983268	down_in_case
Yang2025_S	ENSGALG00010011004	UMODL1	case	control	0.206586737	0.227890021	0.906519449	0.364660975	0.533552152	up_in_case
Yang2025_S	ENSGALG00010011006	TMEM64	case	control	-0.544536817	0.095272164	-5.715592004	1.09323E-08	5.01123E-07	down_in_case
Yang2025_S	ENSGALG00010011007	ENSGALG00010011007	case	control	0.808881006	0.179805325	4.49864879	6.83867E-06	0.000120905	up_in_case
Yang2025_S	ENSGALG00010011013	ENSGALG00010011013	case	control	0.369414489	0.360564974	1.024543469	0.30557865	0.475143772	up_in_case
Yang2025_S	ENSGALG00010011015	LYRM4	case	control	-0.077993987	0.244204811	-0.319379403	0.749438828	0.847783798	down_in_case
Yang2025_S	ENSGALG00010011018	QDPR	case	control	-0.229488787	0.150515169	-1.524688763	0.127336746	0.260183851	down_in_case
Yang2025_S	ENSGALG00010011019	NECAB1	case	control	-0.075518426	0.205496517	-0.367492488	0.713251694	0.822778341	down_in_case
Yang2025_S	ENSGALG00010011020	SFTPA2	case	control	1.397296965	0.458557857	3.047155214	0.002310184	0.013349802	up_in_case
Yang2025_S	ENSGALG00010011021	PPP1R3G	case	control	0.308951736	0.253447943	1.218994848	0.222846148	0.384326015	up_in_case
Yang2025_S	ENSGALG00010011022	C5orf22	case	control	0.256642826	0.117561858	2.18304499	0.029032497	0.089288865	up_in_case
Yang2025_S	ENSGALG00010011025	POLR3B	case	control	-0.001414836	0.074282255	-0.01904675	0.984803811	0.99091928	down_in_case
Yang2025_S	ENSGALG00010011027	FAM234B	case	control	0.215705181	0.109924672	1.962299971	0.049727571	0.132775186	up_in_case
Yang2025_S	ENSGALG00010011028	HMBOX1	case	control	0.169475758	0.194366236	0.871940318	0.383240937	0.550935517	up_in_case
Yang2025_S	ENSGALG00010011029	HMGCR	case	control	-0.124456394	0.128640932	-0.967471181	0.333308541	0.502478777	down_in_case
Yang2025_S	ENSGALG00010011033	DROSHA	case	control	0.254308335	0.191231418	1.329845996	0.183569018	0.336893752	up_in_case
Yang2025_S	ENSGALG00010011034	FAM180A	case	control	1.520246152	0.482171882	3.152913324	0.001616498	0.010183499	up_in_case
Yang2025_S	ENSGALG00010011038	GSG1	case	control	0.286994266	0.605517945	0.473964924	0.63552492	0.766393522	up_in_case
Yang2025_S	ENSGALG00010011040	CDH6	case	control	0.036200268	0.275716605	0.131295206	0.895541784	0.938334765	up_in_case
Yang2025_S	ENSGALG00010011042	SLC13A4	case	control	0.804685099	0.448418176	1.794497063	0.072733867	0.174719262	up_in_case
Yang2025_S	ENSGALG00010011044	MBL2	case	control	1.134013767	0.343363005	3.3026673	0.000957699	0.006777827	up_in_case
Yang2025_S	ENSGALG00010011045	ENSGALG00010011045	case	control	0.081457734	0.35008873	0.232677396	0.816011923	0.889689863	up_in_case
Yang2025_S	ENSGALG00010011049	ABL2	case	control	-0.145604236	0.095184991	-1.529697436	0.126091638	0.258509193	down_in_case
Yang2025_S	ENSGALG00010011050	SYT10	case	control	-0.03433493	0.355697386	-0.096528486	0.923100851	0.953733233	down_in_case
Yang2025_S	ENSGALG00010011055	TOR3A	case	control	-0.095291518	0.169366134	-0.562636199	0.573682637	0.716589059	down_in_case
Yang2025_S	ENSGALG00010011056	ENSGALG00010011056	case	control	-0.063684768	0.621363717	-0.102491933	0.918366216	0.951977476	down_in_case
Yang2025_S	ENSGALG00010011059	ENSGALG00010011059	case	control	0.919066363	0.251472104	3.654744803	0.000257438	0.0022952	up_in_case
Yang2025_S	ENSGALG00010011061	KIF13B	case	control	-0.073091984	0.141320917	-0.517205703	0.605012572	0.743002572	down_in_case
Yang2025_S	ENSGALG00010011062	TMEM55A	case	control	-0.516832054	0.12783791	-4.04287001	5.28009E-05	0.000635938	down_in_case
Yang2025_S	ENSGALG00010011064	MYLIP	case	control	-0.240096363	0.113185548	-2.12126343	0.03389964	0.10024567	down_in_case
Yang2025_S	ENSGALG00010011067	TMTC2	case	control	-0.654155198	0.136469327	-4.793422907	1.63959E-06	3.68512E-05	down_in_case
Yang2025_S	ENSGALG00010011069	ENSGALG00010011069	case	control	-0.097158786	0.58330305	-0.166566566	0.867711102	0.922385705	down_in_case
Yang2025_S	ENSGALG00010011076	PKP2	case	control	0.175698905	0.239453593	0.733749293	0.46310155	0.624112195	up_in_case
Yang2025_S	ENSGALG00010011077	RASGEF1A	case	control	0.599026364	0.157433834	3.804940466	0.000141838	0.001413817	up_in_case
Yang2025_S	ENSGALG00010011080	EMP1	case	control	0.428990647	0.161000897	2.664523328	0.007709752	0.03328234	up_in_case
Yang2025_S	ENSGALG00010011086	OTUD6A	case	control	0.085199973	0.118115817	0.721325687	0.470709156	0.631008378	up_in_case
Yang2025_S	ENSGALG00010011090	GRIN2B	case	control	0.695723689	0.320264466	2.172341179	0.029829938	0.091150657	up_in_case
Yang2025_S	ENSGALG00010011091	YARS2	case	control	-0.057882751	0.114983096	-0.503402271	0.614681475	0.750840319	down_in_case
Yang2025_S	ENSGALG00010011094	CSGALNACT2	case	control	-0.1446312	0.099650428	-1.451385642	0.146672506	0.288439368	down_in_case
Yang2025_S	ENSGALG00010011095	TNN	case	control	0.356302858	0.794377539	0.448530882	0.653770104	0.78083339	up_in_case
Yang2025_S	ENSGALG00010011096	LDB2	case	control	-0.031395758	0.118183575	-0.265652462	0.790506878	0.873638936	down_in_case
Yang2025_S	ENSGALG00010011099	CCBE1	case	control	0.453898352	0.206176161	2.201507439	0.027700121	0.086315112	up_in_case
Yang2025_S	ENSGALG00010011101	ENSGALG00010011101	case	control	0.763799546	0.444564201	1.718086037	0.085780923	0.196863828	up_in_case
Yang2025_S	ENSGALG00010011104	ABCG1	case	control	0.026713343	0.247823695	0.107791722	0.91416091	0.949666936	up_in_case
Yang2025_S	ENSGALG00010011106	USP40	case	control	-0.025449207	0.138333248	-0.183970283	0.854036768	0.913827938	down_in_case
Yang2025_S	ENSGALG00010011107	ENSGALG00010011107	case	control	0.088466219	0.199902798	0.442546175	0.658094024	0.78434316	up_in_case
Yang2025_S	ENSGALG00010011109	ENSGALG00010011109	case	control	0.523087096	0.583684546	0.896181164	0.370156014	0.538707473	up_in_case
Yang2025_S	ENSGALG00010011111	MRPS14	case	control	0.010228898	0.195351532	0.052361493	0.958240656	0.974606492	up_in_case
Yang2025_S	ENSGALG00010011116	TAPT1	case	control	-0.304333262	0.089583706	-3.397194367	0.000680806	0.005136454	down_in_case
Yang2025_S	ENSGALG00010011117	SLC6A15	case	control	4.15083069	0.670910299	6.18686387	6.13729E-10	3.97782E-08	up_in_case
Yang2025_S	ENSGALG00010011118	GMPR2	case	control	-0.080954668	0.110978107	-0.729465211	0.465717141	0.626727992	down_in_case
Yang2025_S	ENSGALG00010011119	ENSGALG00010011119	case	control	-0.490924109	0.339206614	-1.447271632	0.147820861	0.289923879	down_in_case
Yang2025_S	ENSGALG00010011121	NUP35	case	control	-0.47991441	0.126958831	-3.780078984	0.000156779	0.00153313	down_in_case
Yang2025_S	ENSGALG00010011124	CACYBP	case	control	-0.156875666	0.126961466	-1.235616374	0.216601195	0.377374404	down_in_case
Yang2025_S	ENSGALG00010011126	ATXN1	case	control	0.607561692	0.287341673	2.114422473	0.0344792	0.101514399	up_in_case
Yang2025_S	ENSGALG00010011128	GCNT4	case	control	-0.09002384	0.30209073	-0.298002658	0.765701135	0.857307193	down_in_case
Yang2025_S	ENSGALG00010011129	ENSGALG00010011129	case	control	-0.548351322	0.184930645	-2.965172818	0.003025129	0.016447592	down_in_case
Yang2025_S	ENSGALG00010011130	DNM1L	case	control	0.074589829	0.060220185	1.238618414	0.215486846	0.376339039	up_in_case
Yang2025_S	ENSGALG00010011131	RET	case	control	-0.158705566	0.280572569	-0.565648904	0.571632477	0.715439066	down_in_case
Yang2025_S	ENSGALG00010011133	CDC37L1	case	control	-0.156307604	0.087043812	-1.795734823	0.072536701	0.174275677	down_in_case
Yang2025_S	ENSGALG00010011134	ENSGALG00010011134	case	control	0.846575778	0.52409447	1.615311412	0.106243274	0.228896106	up_in_case
Yang2025_S	ENSGALG00010011136	ENSGALG00010011136	case	control	-0.666862987	0.39311026	-1.696376448	0.089814611	0.203506765	down_in_case
Yang2025_S	ENSGALG00010011141	MSX1	case	control	1.228062708	0.721140516	1.7029451	0.088578343	0.201788329	up_in_case
Yang2025_S	ENSGALG00010011142	DUSP19	case	control	-0.850890014	0.192499782	-4.420212875	9.86037E-06	0.000162033	down_in_case
Yang2025_S	ENSGALG00010011143	AK3	case	control	-0.389377181	0.277348083	-1.403929594	0.160339816	0.306031412	down_in_case
Yang2025_S	ENSGALG00010011144	ATF7IP	case	control	0.502633523	0.251428854	1.999108355	0.045596631	0.124996358	up_in_case
Yang2025_S	ENSGALG00010011146	BMS1	case	control	0.6058995	0.150497394	4.025980008	5.67385E-05	0.000676348	up_in_case
Yang2025_S	ENSGALG00010011148	ENSGALG00010011148	case	control	-0.342598088	0.15234575	-2.24881947	0.024523984	0.078956682	down_in_case
Yang2025_S	ENSGALG00010011150	FGD4	case	control	0.196294266	0.219383395	0.894754436	0.370918372	0.539422617	up_in_case
Yang2025_S	ENSGALG00010011152	SLC18A3	case	control	-0.284798402	0.716569221	-0.39744716	0.691037745	0.80731145	down_in_case
Yang2025_S	ENSGALG00010011154	SLC26A7	case	control	1.086630477	0.638209485	1.702623516	0.088638546	0.201826494	up_in_case
Yang2025_S	ENSGALG00010011161	ENSGALG00010011161	case	control	-0.03614756	0.11894472	-0.303902181	0.761202416	0.854874912	down_in_case
Yang2025_S	ENSGALG00010011166	SOX7	case	control	-0.210889593	0.105699182	-1.995186606	0.046022531	0.125602032	down_in_case
Yang2025_S	ENSGALG00010011170	RBM24	case	control	0.211797187	0.109498576	1.934246036	0.053082885	0.139363095	up_in_case
Yang2025_S	ENSGALG00010011175	GPR52	case	control	-0.082421457	0.496946383	-0.165855834	0.868270405	0.922673138	down_in_case

Yang2025_S	ENSGALG00010011178	RSPO2	case	control	-0.297482107	0.296890508	-1.00199265	0.316347143	0.485548787	down_in_case
Yang2025_S	ENSGALG00010011181	ENSGALG00010011181	case	control	-0.448818727	0.244730499	-1.833930499	0.066664312	0.164186494	down_in_case
Yang2025_S	ENSGALG00010011184	FAM8A1	case	control	-0.411311251	0.093116489	-4.417168799	1.00002E-05	0.000163945	down_in_case
Yang2025_S	ENSGALG00010011187	STK32B	case	control	1.839201595	1.084037716	1.696621408	0.08976826	0.203434819	up_in_case
Yang2025_S	ENSGALG00010011190	ENSGALG00010011190	case	control	-0.349726508	0.254722057	-1.372973004	0.169760739	0.318601468	down_in_case
Yang2025_S	ENSGALG00010011192	RPL17	case	control	0.48509969	0.264694067	1.832680636	0.066850079	0.164469605	up_in_case
Yang2025_S	ENSGALG00010011193	FAM20B	case	control	-0.300714371	0.109654477	-2.742381146	0.006099552	0.027776881	down_in_case
Yang2025_S	ENSGALG00010011198	PINX1	case	control	0.181294952	0.11944687	1.517787381	0.129068007	0.262602231	up_in_case
Yang2025_S	ENSGALG00010011200	PCDHB4	case	control	0.981942624	0.777877797	1.262335329	0.206828149	0.365569541	up_in_case
Yang2025_S	ENSGALG00010011203	ENSGALG00010011203	case	control	-0.441241918	0.207330956	-2.128200856	0.033320434	0.098938898	down_in_case
Yang2025_S	ENSGALG00010011205	CYTL1	case	control	2.818973253	0.894324198	3.152070871			up_in_case
Yang2025_S	ENSGALG00010011210	NUP153	case	control	-0.128835795	0.099613985	-1.293350471	0.195889872	0.352086336	down_in_case
Yang2025_S	ENSGALG00010011211	SLC37A1	case	control	-0.403433132	0.155272224	-2.598231175	0.009370538	0.038704444	down_in_case
Yang2025_S	ENSGALG00010011212	NCKAP1	case	control	-0.133411276	0.134319349	-0.993239445	0.32059328	0.490070402	down_in_case
Yang2025_S	ENSGALG00010011213	XKR6	case	control	1.027050833	0.220810888	4.651268976	3.29899E-06	6.57674E-05	up_in_case
Yang2025_S	ENSGALG00010011218	ENSGALG00010011218	case	control	0.240714214	0.517814778	0.464865478	0.642027794	0.771596145	up_in_case
Yang2025_S	ENSGALG00010011222	RCL1	case	control	-0.269101781	0.104611574	-2.572390133	0.010099901	0.040806839	down_in_case
Yang2025_S	ENSGALG00010011231	PLBD1	case	control	0.053119868	0.14104913	0.376605426	0.706466855	0.819145477	up_in_case
Yang2025_S	ENSGALG00010011235	ENSGALG00010011235	case	control	0.299805423	0.133582131	2.244352749	0.024809714	0.079531485	up_in_case
Yang2025_S	ENSGALG00010011237	ENSGALG00010011237	case	control	-0.519041495	0.286923716	-1.808987773	0.070452903	0.170756316	down_in_case
Yang2025_S	ENSGALG00010011239	RALGPS2	case	control	0.161551784	0.127125	1.270810489	0.203796076	0.361895548	up_in_case
Yang2025_S	ENSGALG00010011243	ALX1	case	control	1.150149615	0.552850642	2.080398444	0.037489002	0.107846664	up_in_case
Yang2025_S	ENSGALG00010011244	PDE9A	case	control	0.02348279	0.142991424	0.164225167	0.869553889	0.9231632	up_in_case
Yang2025_S	ENSGALG00010011246	DNAJB5	case	control	-0.26176246	0.159552248	-1.640606529	0.100879119	0.220440728	down_in_case
Yang2025_S	ENSGALG00010011247	ENSGALG00010011247	case	control	0.255414745	0.151151113	1.689797314	0.091066738	0.205508501	up_in_case
Yang2025_S	ENSGALG00010011250	ENSGALG00010011250	case	control	0.517319341	0.199901443	2.587871968	0.009657087	0.039544961	up_in_case
Yang2025_S	ENSGALG00010011251	FRZB	case	control	-0.330856202	0.092230523	-3.587274498	0.000334153	0.00285845	down_in_case
Yang2025_S	ENSGALG00010011253	KDM4C	case	control	0.21287572	0.174072877	1.222911482	0.221363131	0.382773511	up_in_case
Yang2025_S	ENSGALG00010011262	ENSGALG00010011262	case	control	-0.377867461	0.274432262	-1.376906118	0.168541275	0.316867603	down_in_case
Yang2025_S	ENSGALG00010011267	EVC2	case	control	0.077664964	0.170539464	0.455407578	0.648816044	0.776913715	up_in_case
Yang2025_S	ENSGALG00010011271	ANGPTL1	case	control	0.154530199	0.179330216	0.861707536	0.388848477	0.555811215	up_in_case
Yang2025_S	ENSGALG00010011273	RUNX1T1	case	control	0.196219734	0.212015375	0.925497664	0.354707098	0.523883775	up_in_case
Yang2025_S	ENSGALG00010011274	ENSGALG00010011274	case	control	-1.707371791	0.614703098	-2.777555209	0.005476953	0.025636999	down_in_case
Yang2025_S	ENSGALG00010011275	WDR4	case	control	-0.088764471	0.143831612	-0.617141597	0.537141334	0.688345088	down_in_case
Yang2025_S	ENSGALG00010011278	ENSGALG00010011278	case	control	-0.675186886	0.258428256	-2.612666644	0.008983888	0.037583453	down_in_case
Yang2025_S	ENSGALG00010011280	FAM199X	case	control	-0.456797895	0.095545951	-4.780923651	1.74492E-06	3.86767E-05	down_in_case
Yang2025_S	ENSGALG00010011282	CRY1	case	control	0.143650261	0.118044663	1.216914485	0.223636754	0.385023247	up_in_case
Yang2025_S	ENSGALG00010011283	LONRF3	case	control	-0.179101291	0.171033302	-1.047172037	0.29502024	0.463570012	down_in_case
Yang2025_S	ENSGALG00010011285	ENSGALG00010011285	case	control	-0.026044671	0.458988935	-0.05674357	0.954749466	0.972132325	down_in_case
Yang2025_S	ENSGALG00010011287	ENSGALG00010011287	case	control	0.363119459	0.162205692	2.238635744	0.025179627	0.080366032	up_in_case
Yang2025_S	ENSGALG00010011288	ENSGALG00010011288	case	control	-0.248471097	0.327141024	-0.759522895	0.447539824	0.610326525	down_in_case
Yang2025_S	ENSGALG00010011293	NDUFV3	case	control	-0.014428886	0.148769354	-0.096988292	0.922735692	0.953669203	down_in_case
Yang2025_S	ENSGALG00010011295	FAM167A	case	control	0.599457578	0.524508641	1.142893616	0.253082763	0.419297147	up_in_case
Yang2025_S	ENSGALG00010011297	OSR2	case	control	0.115788818	0.109267318	1.059683907	0.289288426	0.458047758	up_in_case
Yang2025_S	ENSGALG00010011300	EVC	case	control	-0.111298123	0.116971072	-0.951501269	0.341349975	0.509556711	down_in_case
Yang2025_S	ENSGALG00010011304	COMMD5	case	control	-0.444142638	0.170807227	-2.600256711	0.009315405	0.038560076	down_in_case
Yang2025_S	ENSGALG00010011306	ECI2	case	control	-0.393632335	0.225230922	-1.747683366	0.080518871	0.18812063	down_in_case
Yang2025_S	ENSGALG00010011307	ENSGALG00010011307	case	control	-1.012986636	0.42003027	-2.411699127	0.015878379	0.057411833	down_in_case
Yang2025_S	ENSGALG00010011311	DOCK8	case	control	-0.182828042	0.178953286	-1.021652331	0.30694548	0.476577746	down_in_case
Yang2025_S	ENSGALG00010011312	VPS13B	case	control	-0.026367233	0.168003163	-0.156944861	0.875288304	0.926623301	down_in_case
Yang2025_S	ENSGALG00010011313	RASSF9	case	control	-0.102558857	0.274626629	-0.373448334	0.708814792	0.820223694	down_in_case
Yang2025_S	ENSGALG00010011316	PARPBP	case	control	-0.211632814	0.183232486	-1.154996135	0.248092009	0.413833152	down_in_case
Yang2025_S	ENSGALG00010011318	PRP4K	case	control	0.7712	0.209099264	3.688200447	0.000225846	0.00205186	up_in_case
Yang2025_S	ENSGALG00010011321	MAP2	case	control	0.122267021	0.201446604	0.606945058	0.543887375	0.693926988	up_in_case
Yang2025_S	ENSGALG00010011326	COX6C	case	control	-0.375117573	0.25990069	-1.443311186	0.14893283	0.291322149	down_in_case
Yang2025_S	ENSGALG00010011329	PCDHGC5	case	control	-0.057789114	0.096693364	-0.597653365	0.550071249	0.698554894	down_in_case
Yang2025_S	ENSGALG00010011331	PMCH	case	control	-0.482689018	0.53582916	-0.900826335	0.367680663	0.536337281	down_in_case
Yang2025_S	ENSGALG00010011333	ENSGALG00010011333	case	control	0.069114171	0.204078576	0.338664508	0.734862481	0.838270192	up_in_case
Yang2025_S	ENSGALG00010011335	VCP	case	control	0.220712938	0.152470781	1.447575303	0.147735862	0.289842325	up_in_case
Yang2025_S	ENSGALG00010011339	ENSGALG00010011339	case	control	-1.560970233	0.529227132	-2.949527977	0.003182598	0.017077203	down_in_case
Yang2025_S	ENSGALG00010011340	PKNOX1	case	control	0.044726218	0.177592221	0.251847843	0.801158675	0.879898025	up_in_case
Yang2025_S	ENSGALG00010011342	RASAL2	case	control	0.313203601	0.165843007	1.888554768	0.058951513	0.150765933	up_in_case
Yang2025_S	ENSGALG00010011343	PXDC1	case	control	-0.616737542	0.112459062	-5.484107131	4.15563E-08	1.59969E-06	down_in_case
Yang2025_S	ENSGALG00010011345	CRMP1	case	control	0.748659022	0.243427836	3.07548649	0.002101594	0.012419728	up_in_case
Yang2025_S	ENSGALG00010011347	RGS22	case	control	1.173450044	0.493230424	2.379111237	0.017354438	0.061130964	up_in_case
Yang2025_S	ENSGALG00010011348	SLC22A23	case	control	-0.324486635	0.168603477	-1.92455483	0.054285075	0.141725599	down_in_case
Yang2025_S	ENSGALG00010011349	ENSGALG00010011349	case	control	0.476745401	0.131369955	3.629029178	0.000284489	0.002496445	up_in_case
Yang2025_S	ENSGALG00010011351	KANK1	case	control	-0.131414949	0.107473171	-1.222769808	0.221416652	0.382809063	down_in_case
Yang2025_S	ENSGALG00010011353	ENSGALG00010011353	case	control	-0.019128853	0.288679551	-0.066263277	0.947168219	0.967511299	down_in_case
Yang2025_S	ENSGALG00010011357	UNC80	case	control	1.082088675	0.433607453	2.495549067	0.012576236	0.048013657	up_in_case
Yang2025_S	ENSGALG00010011359	RNF19A	case	control	0.20334991	0.093315484	2.179165784	0.029319355	0.089974191	up_in_case
Yang2025_S	ENSGALG00010011360	TCF4	case	control	0.55723727	0.243552148	2.287958757	0.022139923	0.072884438	up_in_case
Yang2025_S	ENSGALG00010011361	NUP37	case	control	-0.382162138	0.18020216	-2.120741162	0.033943591	0.100313257	down_in_case
Yang2025_S	ENSGALG00010011363	FDFT1	case	control	-0.137577233	0.123074725	-1.117834987	0.263637476	0.43063981	down_in_case
Yang2025_S	ENSGALG00010011365	PSMG4	case	control	-0.862991067	0.308550112	-2.796923526	0.005159174	0.024548498	down_in_case
Yang2025_S	ENSGALG00010011367	IER3IP1	case	control	-0.090540779	0.291906827	-0.310170131	0.756431583	0.852063222	down_in_case
Yang2025_S	ENSGALG00010011368	ENSGALG00010011368	case	control	1.461753608	0.40678759	3.593407579	0.000326381	0.002797125	up_in_case
Yang2025_S	ENSGALG00010011371	ENSGALG00010011371	case	control	-0.017891071	0.27797148	-0.064362973	0.948681212	0.967992436	down_in_case
Yang2025_S	ENSGALG00010011372	VMA21	case	control	-0.292971831	0.112726399	-2.598963794	0.009350563	0.038664719	down_in_case
Yang2025_S	ENSGALG00010011374	ENSGALG00010011374	case	control	0.453877575	0.231462866	1.960908815	0.049889661	0.13309234	up_in_case
Yang2025_S	ENSGALG00010011375	C12orf50	case	control	-0.38085345	0.157991865	-2.410588994	0.015926785	0.057512245	down_in_case
Yang2025_S	ENSGALG00010011377	SEC16B	case	control	0.776775606	0.274141697	2.833482151	0.004604388	0.022513034	up_in_case
Yang2025_S	ENSGALG00010011378	TUBB2B	case	control	0.061469292	0.212023663	0.289917133	0.771879634	0.861102952	up_in_case
Yang2025_S	ENSGALG00010011379	CELF4	case	control	1.807786594	0.790273135	2.287546564	0.022163941	0.072934492	up_in_case
Yang2025_S	ENSGALG00010011381	RLIG1	case	control	0.038774584	0.158771017	0.244217018	0.807062748	0.88380506	up_in_case
Yang2025_S	ENSGALG00010011382	ENSGALG00010011382	case	control	-0.129284813	0.21103785	-0.61261434	0.540131374	0.690770674	down_in_case
Yang2025_S	ENSGALG00010011384	PUM3	case	control	0.467700552	0.155632109	3.005167471	0.002654347	0.014819038	up_in_case
Yang2025_S	ENSGALG00010011386	CTSB	case	control	-0.167016538	0.204426984	-0.816998495	0.413929288	0.57930146	down_in_case
Yang2025_S	ENSGALG00010011388	GPR50	case	control	0.110979276	0.202730858	0.547421727	0.584089032	0.726137983	up_in_case
Yang2025_S	ENSGALG00010011389	RC3H1	case	control	-0.048885587	0.157784421	-0.309825185	0.756693898	0.852119509	down_in_case
Yang2025_S	ENSGALG00010011394	SERPINC1	case	control	-1.370606632	0.594749826	-2.304509512	0.021194059	0.070740266	down_in_case
Yang2025_S	ENSGALG00010011398	ENSGALG00010011398	case	control	0.212596924	0.213632911	0.995150624	0.319663012	0.488914946	up_in_case
Yang2025_S	ENSGALG00010011399	JAKMIP1	case	control	0.909901658	0.265257305	3.430260513	0.000603002	0.004632898	up_in_case
Yang2025_S	ENSGALG00010011400	BPHL	case	control	0.354902805	0.248091149	1.430533922	0.152563837	0.29613833	up_in_case
Yang2025_S	ENSGALG00010011401	ANKRD46	case	control	-0.273710595	0.064743142	-4.227638452	2.36157E-05	0.000334096	down_in_case
Yang2025_S	ENSGALG00010011403	SV2C	case	control	1.684679143	0.734729765	2.292923498	0.02185241	0.072228019	up_in_case
Yang2025_S	ENSGALG00010011406	UBE2R2	case	control	0.252180077	0.132056666	1.909635344	0.056180181	0.145272003	up_in_case
Yang2025_S	ENSGALG00010011407	CEP290	case	control	0.971992653	0.200588115	4.845714083	1.26157E-06	2.95462E-05	up_in_case
Yang2025_S	ENSGALG00010011411	ENSGALG00010011411	case	control	0.285223641	0.265592527	1.073914405	0.282861046	0.451080938	up_in_case
Yang2025_S	ENSGALG00010011412	KANSL1L	case	control	-0.190704089	0.085710651	-2.224975387	0.02608289	0.082493209	down_in_case
Yang2025_S	ENSGALG00010011413	ZBTB37	case	control	-0.116554487	0.167140443	-0.697344611	0.485587156	0.642912781	down_in_case
Yang2025_S	ENSGALG00010011414	PABPC1	case	control	0.267881636	0.187227017	1.430785157	0.152491798	0.296039734	up_in_case
Yang2025_S	ENSGALG00010011416	ENSGALG00010011416	case	control	-0.686733497	0.613216395	-1.119887699	0.262761621	0.429591972	down_in_case
Yang2025_S	ENSGALG00010011417	WFS1	case	control	-0.220950626	0.194547736	-1.135714195	0.256076198	0.422348422	down_in_case
Yang2025_S	ENSGALG00010011418	RIPK1	case	control	0.305672932	0.163238235	1.872557199	0.061129556	0.154458816	up_in_case
Yang2025_S	ENSGALG00010011419	RNF180	case	control	0.184817233	0.145849255	1.267179818	0.205091	0.363328641	up_in_case
Yang2025_S	ENSGALG00010011420	HMGB3	case	control	0.176265874	0.104834839	1.681367336	0.092691586	0.207962848	up_in_case
Yang2025_S	ENSGALG00010011421	ENSGALG00010011421	case	control	0.604616717	0.205211388	2.946311712	0.003215881	0.017150137	up_in_case
Yang2025_S	ENSGALG00010011422	KATNAL2	case	control	0.264317767	0.294805312	0.896584137	0.369940866	0.538520117	up_in_case
Yang2025_S	ENSGALG00010011423	GDF6	case	control	0.865383529	0.591547787	1.462913982	0.143490917	0.283824831	up_in_case
Yang2025_S	ENSGALG00010011424	ACADL	case	control	-0.03066057	0.159705989	-0.191981342	0.847756821	0.909738128	down_in_case
Yang2025_S	ENSGALG00010011425	WASHC3	case	control	0.085681358	0.131377061	0.652178988	0.514285713	0.669022724	up_in_case
Yang2025_S	ENSGALG00010011426	NQO2	case	control	0.363663897	0.191689612	1.897149742	0.057808178	0.148489762	up_in_case
Yang2025_S	ENSGALG00010011427	TMTC3	case	control	-0.084126792	0.100377409	-0.838104837	0.401971827	0.56850476	down_in_case
Yang2025_S	ENSGALG00010011428	UQCRB	case	control	-0.091623666	0.229431535	-0.399350796	0.689634744	0.8062131	down_in_case
Yang2025_S	ENSGALG00010011429	UBAP2	case	control	0.485794989	0.242268159	2.005195361	0.044942166	0.123695256	up_in_case
Yang2025_S	ENSGALG00010011430	ENSGALG00010011430	case	control	0.552455492	0.328347777	1.682531545	0.092465813	0.207685843	up_in_case
Yang2025_S	ENSGALG00010011431	DRAM1	case	control	-0.348167865	0.399082444	-0.8724209	0.382978803	0.550697515	down_in_case
Yang2025_S	ENSGALG00010011433	RNASE6	case	control	-0.380413157	0.310564499	-1.224908699	0.220609622	0.381982491	down_in_case
Yang2025_S	ENSGALG00010011434	NPL	case	control	-0.002526362	0.161277713	-0.015664672	0.987501911	0.992344352	down_in_case
Yang2025_S	ENSGALG00010011436	UBAP2L2	case	control	-8.591524212	2.036847572	-4.218049662			down_in_case
Yang2025_S	ENSGALG00010011437	DMRT1	case	control	-0.539887404	0.529659803	-1.019309755	0.308055934	0.477663229	down_in_case
Yang2025_S	ENSGALG00010011438	ENSGALG00010011438	case	control	-0.058272511	0.253221767	-0.230124414	0.817995094	0.891343405	down_in_case
Yang2025_S	ENSGALG00010011439	ENSGALG00010011439	case	control	1.504036015	0.320237179	4.696631481	2.64487E-06	5.48047E-05	up_in_case
Yang2025_S	ENSGALG00010011440	FUNDC1	case	control	-0.067607602	0.154701173	-0.437020618	0.662096395	0.786355857	down_in_case
Yang2025_S	ENSGALG00010011441	CD99L2	case	control	-0.176194261	0.089158682	-1.976187367	0.04813356	0.129847868	down_in_case

Yang2025_S	ENSGALG00010011442	ENSGALG00010011442	case	control	-9.98425255	2.561673124	-3.897551352			down_in_case
Yang2025_S	ENSGALG00010011443	ENSGALG00010011443	case	control	-0.13479796	0.360999631	-0.373401932	0.708849322	0.820223694	down_in_case
Yang2025_S	ENSGALG00010011445	MTERF3	case	control	-0.352149813	0.131942466	-2.668964911	0.00760854	0.032947484	down_in_case
Yang2025_S	ENSGALG00010011446	ENSGALG00010011446	case	control	0.794691346	0.230209125	3.45204103	0.000556363	0.004319176	up_in_case
Yang2025_S	ENSGALG00010011447	LAMC1	case	control	-0.116787503	0.108398375	-1.077391646	0.281305335	0.449231015	down_in_case
Yang2025_S	ENSGALG00010011449	DUSP22	case	control	-0.117046695	0.112473036	-1.040664499	0.298031285	0.46721408	down_in_case
Yang2025_S	ENSGALG00010011450	PTDSS1	case	control	-0.325244915	0.097248448	-3.344474097	0.000824387	0.005983248	down_in_case
Yang2025_S	ENSGALG00010011451	CRHBP	case	control	-0.007563595	0.746889563	-0.010126792	0.991920127	0.994918806	down_in_case
Yang2025_S	ENSGALG00010011452	KITLG	case	control	-0.232993994	0.146149461	-1.594217258	0.110887382	0.23576681	down_in_case
Yang2025_S	ENSGALG00010011453	GNPTAB	case	control	-0.077594339	0.070204495	-1.105261686	0.269046215	0.436202327	down_in_case
Yang2025_S	ENSGALG00010011456	KIAA1328	case	control	0.520385267	0.163849283	3.175999652	0.001493211	0.009585992	up_in_case
Yang2025_S	ENSGALG00010011457	ENSGALG00010011457	case	control	0.625665269	0.377152189	1.658919894	0.097131935	0.214676212	up_in_case
Yang2025_S	ENSGALG00010011458	TPGS2	case	control	0.542278419	0.228613996	2.372026331	0.01769083	0.062086233	up_in_case
Yang2025_S	ENSGALG00010011459	ENSGALG00010011459	case	control	0.541332126	0.408660076	1.324651361	0.185286812	0.33886248	up_in_case
Yang2025_S	ENSGALG00010011460	LAMC2	case	control	-1.052174824	0.54172214	-1.942277686	0.052103493	0.137459707	down_in_case
Yang2025_S	ENSGALG00010011461	IRF4	case	control	-0.229555827	0.266088247	-0.862705622	0.388299341	0.555254111	down_in_case
Yang2025_S	ENSGALG00010011463	IMP3	case	control	0.203954176	0.403571039	0.505373669	0.613296409	0.749608408	up_in_case
Yang2025_S	ENSGALG00010011464	FANCG	case	control	-0.054220998	0.19828774	-0.273446042	0.784510364	0.869701824	down_in_case
Yang2025_S	ENSGALG00010011465	PPP2R2C	case	control	1.302543096	0.230917194	5.640736718	1.69324E-08	7.37354E-07	up_in_case
Yang2025_S	ENSGALG00010011466	MTMR1	case	control	-0.283757166	0.068246889	-4.157803655	3.21322E-05	0.000430955	down_in_case
Yang2025_S	ENSGALG00010011467	ENSGALG00010011467	case	control	-12.07316951	2.741120104	-4.404465713			down_in_case
Yang2025_S	ENSGALG00010011468	EFHC2	case	control	-0.200147471	0.280931134	-0.71244318	0.476190385	0.63550578	down_in_case
Yang2025_S	ENSGALG00010011469	CPS1	case	control	0.444832054	0.193337724	2.300803193	0.021402758	0.071127196	up_in_case
Yang2025_S	ENSGALG00010011471	NMNAT2	case	control	0.211079176	0.444590085	0.474772568			up_in_case
Yang2025_S	ENSGALG00010011475	NDP	case	control	0.221809973	0.313237022	0.708121831	0.478869604	0.637653887	up_in_case
Yang2025_S	ENSGALG00010011477	SDC2	case	control	-0.376716216	0.124253935	-3.031825249	0.002430799	0.013899541	down_in_case
Yang2025_S	ENSGALG00010011478	HDAC3	case	control	-0.430253409	0.122760668	-3.504814831	0.000456925	0.003676241	down_in_case
Yang2025_S	ENSGALG00010011480	BLTP3B	case	control	-0.245532838	0.117349003	-2.092329995	0.036409006	0.10567788	down_in_case
Yang2025_S	ENSGALG00010011482	DMRT2	case	control	-0.226822335	0.14082556	-1.610661693	0.107253479	0.230076545	down_in_case
Yang2025_S	ENSGALG00010011484	CHPT1	case	control	-0.454090538	0.099792962	-4.550326276	5.35628E-06	9.82102E-05	down_in_case
Yang2025_S	ENSGALG00010011485	MTM1	case	control	0.282291847	0.128659816	2.194094912	0.028228587	0.08744897	up_in_case
Yang2025_S	ENSGALG00010011487	MAN2B2	case	control	-0.267488728	0.107164649	-2.496053786	0.012558356	0.04797168	down_in_case
Yang2025_S	ENSGALG00010011493	ENSGALG00010011493	case	control	-0.30795021	0.422077047	-0.729606626	0.465630671	0.626672146	down_in_case
Yang2025_S	ENSGALG00010011496	ACTR6	case	control	-0.067202533	0.119310805	-0.563256052	0.573260539	0.716341214	down_in_case
Yang2025_S	ENSGALG00010011497	CPQ	case	control	-1.069322724	0.216922166	-4.929522624	8.24308E-07	2.06426E-05	down_in_case
Yang2025_S	ENSGALG00010011499	SMG7	case	control	0.121018703	0.12157352	0.995436366	0.319524079	0.488856637	up_in_case
Yang2025_S	ENSGALG00010011503	NCF2	case	control	-0.409859417	0.452420526	-0.905925778	0.364975139	0.533730925	down_in_case
Yang2025_S	ENSGALG00010011505	MYL1	case	control	-0.228020192	0.141943928	-1.606410333	0.108183782	0.231501997	down_in_case
Yang2025_S	ENSGALG00010011509	EXOC2	case	control	-0.458964665	0.073293886	-6.26197748	3.80126E-10	2.54666E-08	down_in_case
Yang2025_S	ENSGALG00010011510	ENSGALG00010011510	case	control	-0.783330176	0.376866342	-2.078535778	0.037660039	0.108249308	down_in_case
Yang2025_S	ENSGALG00010011515	SERINC5	case	control	-0.038030359	0.155883198	-0.243967018	0.807256364	0.88385352	down_in_case
Yang2025_S	ENSGALG00010011518	ENSGALG00010011518	case	control	1.0117416	0.614410465	1.646686795	0.09962244	0.218327344	up_in_case
Yang2025_S	ENSGALG00010011523	PIGO	case	control	0.007446388	0.075952446	0.098040137	0.921900422	0.953371606	up_in_case
Yang2025_S	ENSGALG00010011525	ENSGALG00010011525	case	control	0.428339867	0.310240511	1.380670324	0.16738035	0.315408408	up_in_case
Yang2025_S	ENSGALG00010011529	SCYL2	case	control	-0.146388102	0.099788875	-1.466978175	0.142381993	0.282110315	down_in_case
Yang2025_S	ENSGALG00010011530	ARPC5	case	control	-0.237960634	0.120855118	-1.9689744	0.048956031	0.131206208	down_in_case
Yang2025_S	ENSGALG00010011531	MTDH	case	control	-0.100218607	0.043550899	-2.301183402	0.021381267	0.071105219	down_in_case
Yang2025_S	ENSGALG00010011533	ENSGALG00010011533	case	control	-0.647508191	0.246202718	-2.629979862	0.008538993	0.036046914	down_in_case
Yang2025_S	ENSGALG00010011536	FCHSD1	case	control	0.212832409	0.226301523	0.940481559	0.346970605	0.515375905	up_in_case
Yang2025_S	ENSGALG00010011538	ENSGALG00010011538	case	control	-0.879830094	0.304484604	-2.889571697	0.00385767	0.019662911	down_in_case
Yang2025_S	ENSGALG00010011539	LANCL1	case	control	-0.401664924	0.117271383	-3.425089004	0.000614598	0.004700562	down_in_case
Yang2025_S	ENSGALG00010011543	ENSGALG00010011543	case	control	4.827313963	0.963445522	5.010469041	5.42975E-07	1.43031E-05	up_in_case
Yang2025_S	ENSGALG00010011544	STOML2	case	control	0.09479971	0.174244836	0.544060372	0.586399929	0.728231264	up_in_case
Yang2025_S	ENSGALG00010011545	USP12-like	case	control	-0.30000771	0.075043265	-3.997796628	6.39348E-05	0.000744926	down_in_case
Yang2025_S	ENSGALG00010011546	MAOA	case	control	-0.38666776	0.102591054	-3.769020253	0.00016389	0.001589284	down_in_case
Yang2025_S	ENSGALG00010011549	KIAA0232	case	control	0.298937062	0.127900912	2.337255117	0.019425923	0.066364362	up_in_case
Yang2025_S	ENSGALG00010011551	GMDS	case	control	0.218884351	0.126180107	1.73469778	0.08279435	0.191887953	up_in_case
Yang2025_S	ENSGALG00010011555	ENSGALG00010011555	case	control	-6.737402087	2.951334948	-2.282832076			down_in_case
Yang2025_S	ENSGALG00010011556	LAPTM4B	case	control	-0.425672952	0.115269236	-3.692858288	0.000221748	0.00202361	down_in_case
Yang2025_S	ENSGALG00010011558	MYBPC1	case	control	0.249793585	0.131155163	1.904565393	0.056836589	0.1467787	up_in_case
Yang2025_S	ENSGALG00010011559	MTX3	case	control	-0.151281644	0.103229658	-1.465486256	0.142788299	0.282715964	down_in_case
Yang2025_S	ENSGALG00010011562	ENSGALG00010011562	case	control	0.564877225	0.632535854	0.893035899	0.371837948	0.540223975	up_in_case
Yang2025_S	ENSGALG00010011564	ARAP3	case	control	-0.092515651	0.106072802	-0.872190134	0.383104661	0.550821652	down_in_case
Yang2025_S	ENSGALG00010011570	RGL1	case	control	-0.076943931	0.153983463	-0.499689571	0.617293677	0.753177219	down_in_case
Yang2025_S	ENSGALG00010011571	ATOSB	case	control	0.19149517	0.144135624	1.328576273	0.183987804	0.337427658	up_in_case
Yang2025_S	ENSGALG00010011572	PTCD2	case	control	-0.139181885	0.17104011	-0.813738282	0.415794898	0.581328575	down_in_case
Yang2025_S	ENSGALG00010011573	CMAS	case	control	-0.148832409	0.11203462	-1.328450164	0.184029437	0.337427658	down_in_case
Yang2025_S	ENSGALG00010011574	GAS2L3	case	control	0.344380495	0.144297555	2.386599661	0.017004998	0.060204431	up_in_case
Yang2025_S	ENSGALG00010011575	ENSGALG00010011575	case	control	0.786619413	0.482429543	1.630537399	0.102987968	0.224100145	up_in_case
Yang2025_S	ENSGALG00010011577	ENSGALG00010011577	case	control	0.200480098	0.212926711	0.941545087	0.346425595	0.514872085	up_in_case
Yang2025_S	ENSGALG00010011578	MYLK4	case	control	0.603557725	0.263278978	2.292464557	0.021878851	0.072269491	up_in_case
Yang2025_S	ENSGALG00010011586	ENSGALG00010011586	case	control	-0.258498687	0.251734656	-1.026869685	0.30448183	0.474171856	down_in_case
Yang2025_S	ENSGALG00010011588	COLGALT2	case	control	-0.125144125	0.127065822	-0.98487636	0.324684807	0.494316921	down_in_case
Yang2025_S	ENSGALG00010011589	RERG	case	control	-0.258510367	0.149756541	-1.726204181	0.084310686	0.194547013	down_in_case
Yang2025_S	ENSGALG00010011593	WRNIP1	case	control	-0.343981591	0.168517997	-2.041215759	0.041229386	0.115739624	down_in_case
Yang2025_S	ENSGALG00010011595	ENSGALG00010011595	case	control	0.743117911	0.233744235	3.179192473	0.00147686	0.009511943	up_in_case
Yang2025_S	ENSGALG00010011597	TBC1D14	case	control	-0.092846227	0.059953073	-1.548648339	0.121466279	0.251917338	down_in_case
Yang2025_S	ENSGALG00010011603	ENSGALG00010011603	case	control	-0.430551156	0.391529342	-1.099665108	0.271478062	0.438681581	down_in_case
Yang2025_S	ENSGALG00010011605	APPL2	case	control	0.465140954	0.086821686	5.35742825	8.44149E-08	2.95558E-06	up_in_case
Yang2025_S	ENSGALG00010011607	TSEN15	case	control	-0.332272188	0.18773516	-1.769898547	0.076744043	0.181782805	down_in_case
Yang2025_S	ENSGALG00010011609	ENSGALG00010011609	case	control	0.872248111	0.688102965	1.267612777	0.204936267	0.363100684	up_in_case
Yang2025_S	ENSGALG00010011612	ENSGALG00010011612	case	control	0.356060739	0.306113963	1.163163994	0.244762972	0.410145745	up_in_case
Yang2025_S	ENSGALG00010011617	MAMLD1	case	control	0.287410799	0.20328649	1.413821445	0.157414335	0.302728231	up_in_case
Yang2025_S	ENSGALG00010011619	PCDH1	case	control	0.285788987	0.132325251	2.159746415	0.030792306	0.093476165	up_in_case
Yang2025_S	ENSGALG00010011621	WASHC4	case	control	-0.053876678	0.088962026	-0.605614335	0.544770881	0.694354909	down_in_case
Yang2025_S	ENSGALG00010011622	BACH1	case	control	-0.400463662	0.099977794	-4.005526076	6.18796E-05	0.000724961	down_in_case
Yang2025_S	ENSGALG00010011625	ERBB4	case	control	-0.263840777	0.240768027	-1.095829789	0.273153269	0.440499739	down_in_case
Yang2025_S	ENSGALG00010011628	MATN2	case	control	-0.177373039	0.137306736	-1.291801444	0.196425915	0.352504202	down_in_case
Yang2025_S	ENSGALG00010011630	DELE1	case	control	-0.093827025	0.12240512	-0.766528598	0.443361842	0.606662144	down_in_case
Yang2025_S	ENSGALG00010011633	TADA2B	case	control	-0.457787853	0.169416984	-2.702136715	0.006889543	0.030623853	down_in_case
Yang2025_S	ENSGALG00010011634	AVD	case	control	0.71403528	0.445552471	1.602584042	0.109026519	0.232876233	up_in_case
Yang2025_S	ENSGALG00010011635	PCDH12	case	control	0.14247585	0.125273275	1.137320387	0.255404375	0.421739539	up_in_case
Yang2025_S	ENSGALG00010011636	GRIK1	case	control	-0.238045955	0.462697631	-0.514474117	0.606920552	0.744282561	down_in_case
Yang2025_S	ENSGALG00010011637	CECR2	case	control	0.183641801	0.17892408	1.02636717	0.304718547	0.474388666	up_in_case
Yang2025_S	ENSGALG00010011638	GRPEL1	case	control	0.150504848	0.109157441	1.378786876	0.167960474	0.316176124	up_in_case
Yang2025_S	ENSGALG00010011639	ENSGALG00010011639	case	control	0.083500483	0.263334303	0.317089273	0.751175864	0.848540188	up_in_case
Yang2025_S	ENSGALG00010011641	ENSGALG00010011641	case	control	0.800931547	0.372556395	2.149826328	0.031568955	0.095157557	up_in_case
Yang2025_S	ENSGALG00010011643	C1orf21	case	control	-0.166322979	0.159252206	-1.044399838	0.296300438	0.464971465	down_in_case
Yang2025_S	ENSGALG00010011647	FBXO38	case	control	0.247109492	0.086247942	2.865105967	0.004168697	0.020761542	up_in_case
Yang2025_S	ENSGALG00010011648	ENSGALG00010011648	case	control	-0.172067256	0.291655045	-0.589968386	0.555211845	0.70275463	down_in_case
Yang2025_S	ENSGALG00010011649	MRPS27	case	control	0.235732752	0.16731775	1.408892674	0.158866906	0.304385172	up_in_case
Yang2025_S	ENSGALG00010011650	RPL30	case	control	-0.541306195	0.202705371	-2.670408745	0.007575896	0.032826963	down_in_case
Yang2025_S	ENSGALG00010011651	SERPINB6	case	control	-0.275658023	0.10367796	-2.65879097	0.00784216	0.033770241	down_in_case
Yang2025_S	ENSGALG00010011653	NR2C1	case	control	0.043566505	0.08783125	0.496025104	0.619876699	0.755266399	up_in_case
Yang2025_S	ENSGALG00010011655	SORCS2	case	control	-0.311394255	0.143076148	-2.17642325	0.029523627	0.09044004	down_in_case
Yang2025_S	ENSGALG00010011657	ALDH1L2	case	control	0.207398677	0.128978755	1.608006504	0.107833754	0.231001286	up_in_case
Yang2025_S	ENSGALG00010011658	TENM1	case	control	0.964971481	0.51220464	1.883956927	0.059570802	0.151784248	up_in_case
Yang2025_S	ENSGALG00010011659	CD14	case	control	0.366711152	0.194601416	1.884421811	0.059507942	0.151681575	up_in_case
Yang2025_S	ENSGALG00010011660	ENSGALG00010011660	case	control	0.095118327	0.13587323	0.700052006	0.483894827	0.641978329	up_in_case
Yang2025_S	ENSGALG00010011662	RIDA	case	control	-0.984801986	0.204791576	-4.808801253	1.51838E-06	3.45165E-05	down_in_case
Yang2025_S	ENSGALG00010011663	UTP20	case	control	0.113243544	0.117147682	0.966673361	0.333707348	0.502562617	up_in_case
Yang2025_S	ENSGALG00010011664	SERPINB10B	case	control	-0.217110391	0.45804802	-0.47399046	0.635506711	0.766393522	down_in_case
Yang2025_S	ENSGALG00010011667	ENSGALG00010011667	case	control	-0.872166385	0.349303699	-2.496871307	0.012529442	0.047874355	down_in_case
Yang2025_S	ENSGALG00010011669	ENSGALG00010011669	case	control	-0.664534532	0.562229624	-1.181962857	0.237220439	0.401075809	down_in_case
Yang2025_S	ENSGALG00010011670	TMCO6	case	control	-0.131953036	0.227745965	-0.579386934	0.562328118	0.708639198	down_in_case
Yang2025_S	ENSGALG00010011671	SLC41A2	case	control	-0.717183002	0.148279263	-4.836704642	1.32009E-06	3.07104E-05	down_in_case
Yang2025_S	ENSGALG00010011673	POP1	case	control	0.223589453	0.145587452	1.535774204	0.124593769	0.256154347	up_in_case
Yang2025_S	ENSGALG00010011674	ENSGALG00010011674	case	control	0.947010505	0.704852012	1.343559341	0.179090925	0.330720464	up_in_case
Yang2025_S	ENSGALG00010011677	EDEM3	case	control	0.137922941	0.114794889	1.201472836	0.229567838	0.391941658	up_in_case
Yang2025_S	ENSGALG00010011681	MAP1B	case	control	0.209531051	0.296459138	0.706778857	0.479703915	0.638459796	up_in_case
Yang2025_S	ENSGALG00010011682	USP15	case	control	-0.151281864	0.093667658	-1.61509177	0.106290823	0.228927762	down_in_case
Yang2025_S	ENSGALG00010011683	NIPAL2	case	control	-1.058515229	0.409022968	-2.587911464	0.00965598	0.039544961	down_in_case
Yang2025_S	ENSGALG00010011685	NDUFA2	case	control	-0.096087093	0.308131435	-0.311838007	0.755163638	0.851213493	down_in_case
Yang2025_S	ENSGALG00010011688	PDE3A	case	control	0.320330024	0.203068752	1.577446168	0.114692899	0.241755491	up_in_case
Yang2025_S	ENSGALG00010011692	ARL1	case	control	0.410766583	0.097413966	4.216711419	2.47891E-05	0.000347172	up_in_case
Yang2025_S	ENSGALG00010011696	ZFC3H1	case	control	0.807555957	0.174876747	4.617857843	3.87722E-06	7.51447E-05	up_in_case

Yang2025_S	ENSGALG00010011699	TIAM1	case	control	0.199599532	0.15841955	1.259942551	0.207690087	0.366449615	up_in_case
Yang2025_S	ENSGALG00010011702	NUP205	case	control	-0.273241196	0.059733989	-4.574300175	4.77814E-06	8.90153E-05	down_in_case
Yang2025_S	ENSGALG00010011703	YWHAZ	case	control	-0.561592306	0.100312721	-5.598415656	2.1632E-08	9.10699E-07	down_in_case
Yang2025_S	ENSGALG00010011708	IK	case	control	0.165876509	0.081058902	2.046370064	0.040719975	0.114609746	up_in_case
Yang2025_S	ENSGALG00010011712	DCAF12L2	case	control	-0.601848734	0.153176115	-3.92912911	8.52541E-05	0.000939886	down_in_case
Yang2025_S	ENSGALG00010011713	AVPR1A	case	control	0.255486552	0.615748488	0.414920308	0.678200243	0.797865799	up_in_case
Yang2025_S	ENSGALG00010011720	IKZF2	case	control	-0.05906125	0.292219735	-0.202112461	0.839828807	0.905035528	down_in_case
Yang2025_S	ENSGALG00010011723	SPRY3	case	control	-0.524049764	0.166026906	-3.156414691	0.001597216	0.010103134	down_in_case
Yang2025_S	ENSGALG00010011724	ARHGEF28	case	control	0.893032241	0.226478408	3.943123091	8.04273E-05	0.000898761	up_in_case
Yang2025_S	ENSGALG00010011726	WDR55	case	control	0.326164112	0.169335701	1.926139085	0.054087009	0.141381069	up_in_case
Yang2025_S	ENSGALG00010011729	FAM129A	case	control	0.002101985	0.152948118	0.013743126	0.989034917	0.993402007	up_in_case
Yang2025_S	ENSGALG00010011734	ENSGALG00010011734	case	control	0.079118429	0.321319481	0.246229791	0.805504356	0.883340611	up_in_case
Yang2025_S	ENSGALG00010011738	FGD6	case	control	0.094833895	0.126594367	0.749116229	0.453787154	0.615725243	up_in_case
Yang2025_S	ENSGALG00010011745	AFAP1L1	case	control	0.244925903	0.076995284	3.181050725	0.001467419	0.009466893	up_in_case
Yang2025_S	ENSGALG00010011746	SOD1	case	control	-0.088998702	0.132835728	-0.669990696	0.502863722	0.659189921	down_in_case
Yang2025_S	ENSGALG00010011747	UTP15	case	control	0.130676848	0.228713026	0.571357261	0.567757494	0.712380757	up_in_case
Yang2025_S	ENSGALG00010011751	ZNF706	case	control	-0.746741689	0.137195161	-5.442915648	5.24155E-08	1.94776E-06	down_in_case
Yang2025_S	ENSGALG00010011752	RNF2	case	control	0.124492575	0.181048594	0.687619669	0.491692319	0.649174578	up_in_case
Yang2025_S	ENSGALG00010011758	SERPINB11	case	control	-0.397046703	0.536366862	-0.740252113	0.459147032	0.62028249	down_in_case
Yang2025_S	ENSGALG00010011759	CREB3	case	control	-0.130062856	0.130948418	-0.993237319	0.320594315	0.490070402	down_in_case
Yang2025_S	ENSGALG00010011764	PCYOX1L	case	control	0.289795601	0.191708447	1.511647535	0.130623552	0.264922481	up_in_case
Yang2025_S	ENSGALG00010011770	CHST11	case	control	0.174623326	0.321850518	0.542560338	0.587432553	0.728753962	up_in_case
Yang2025_S	ENSGALG00010011772	GRHL2	case	control	0.404203651	0.497502764	0.812465135	0.416524784	0.581743963	up_in_case
Yang2025_S	ENSGALG00010011774	ANKRA2	case	control	-0.191819091	0.180050503	-1.065362702	0.286711828	0.455308185	down_in_case
Yang2025_S	ENSGALG00010011775	VEZT	case	control	0.02067711	0.107621483	0.192128089	0.847641873	0.909738128	up_in_case
Yang2025_S	ENSGALG00010011776	IL17RA	case	control	-0.194323825	0.099476037	-1.953473743	0.050763486	0.134740795	down_in_case
Yang2025_S	ENSGALG00010011780	SERPINB5	case	control	-0.636507504	0.77900865	-0.817073731	0.413886294	0.57930146	down_in_case
Yang2025_S	ENSGALG00010011784	GRPEL2	case	control	-0.280839246	0.119214571	-2.355745977	0.018485554	0.063919651	down_in_case
Yang2025_S	ENSGALG00010011789	TXNRD1	case	control	-0.316054783	0.186450799	-1.695110908	0.090054383	0.20375188	down_in_case
Yang2025_S	ENSGALG00010011790	AMY2A	case	control	0.323232955	0.44868083	0.720407321	0.471274244	0.631462172	up_in_case
Yang2025_S	ENSGALG00010011792	HDHD5	case	control	-0.122746347	0.095522642	-1.284997409	0.19879319	0.355614291	down_in_case
Yang2025_S	ENSGALG00010011793	TLN1	case	control	0.203655081	0.079062109	2.575887297	0.009998323	0.040507454	up_in_case
Yang2025_S	ENSGALG00010011796	METAP2	case	control	0.094389104	0.089585165	1.053624264	0.292054976	0.460695731	up_in_case
Yang2025_S	ENSGALG00010011797	SCAF4	case	control	0.292016734	0.206360947	1.415077505	0.157045773	0.302258038	up_in_case
Yang2025_S	ENSGALG00010011802	VPS4B	case	control	-0.164897268	0.08352156	-1.974307795	0.048346755	0.130211062	down_in_case
Yang2025_S	ENSGALG00010011808	SMARCA2	case	control	0.244329274	0.163627704	1.493202358	0.135384225	0.271919743	up_in_case
Yang2025_S	ENSGALG00010011810	FGFR4	case	control	-0.136940345	0.157558794	-0.869138064	0.384771619	0.552306604	down_in_case
Yang2025_S	ENSGALG00010011814	ENSGALG00010011814	case	control	-0.740974588	0.449440298	-1.648660771	0.099217151	0.217823349	down_in_case
Yang2025_S	ENSGALG00010011817	KDSR	case	control	-0.694631722	0.112025036	-6.200682882	5.62187E-10	3.69532E-08	down_in_case
Yang2025_S	ENSGALG00010011819	USP44	case	control	0.516793844	0.167607311	3.083360991	0.002046767	0.01218876	up_in_case
Yang2025_S	ENSGALG00010011823	ENSGALG00010011823	case	control	0.660943439	0.468527459	1.410682396	0.158338284	0.303708739	up_in_case
Yang2025_S	ENSGALG00010011825	TAFA2	case	control	1.600854722	0.673133193	2.37821391	0.017396731	0.061233504	up_in_case
Yang2025_S	ENSGALG00010011837	VLDLR	case	control	-0.098321085	0.131726487	-0.746403299	0.455423827	0.61692729	down_in_case
Yang2025_S	ENSGALG00010011839	CBS	case	control	-0.756222801	0.183973758	-4.110492771	3.94816E-05	0.000509422	down_in_case
Yang2025_S	ENSGALG00010011841	KCNMB4	case	control	-0.630099827	0.287501029	-2.191643729	0.028405242	0.087844439	down_in_case
Yang2025_S	ENSGALG00010011843	BCL2	case	control	-0.392960658	0.126215277	-3.113415964	0.001849352	0.011293039	down_in_case
Yang2025_S	ENSGALG00010011846	UBAP1	case	control	0.089668048	0.145555925	0.616038463	0.537869135	0.689087652	up_in_case
Yang2025_S	ENSGALG00010011850	SPAG16	case	control	0.720186324	0.320349114	2.248129598	0.024567927	0.079010862	up_in_case
Yang2025_S	ENSGALG00010011856	NFYB	case	control	0.06030286	0.099421611	0.606536739	0.544158393	0.694082125	up_in_case
Yang2025_S	ENSGALG00010011857	ENSGALG00010011857	case	control	-0.245552228	0.416794642	-0.589144397	0.555764413	0.703011282	down_in_case
Yang2025_S	ENSGALG00010011859	NTN4	case	control	0.419841805	0.136381207	3.078443248	0.002080852	0.012322165	up_in_case
Yang2025_S	ENSGALG00010011860	CECR1	case	control	-0.279930533	0.133673454	-2.094137048	0.036247774	0.105391644	down_in_case
Yang2025_S	ENSGALG00010011861	ENSGALG00010011861	case	control	-0.570826424	0.420600414	-1.357170381	0.17472708	0.325206605	down_in_case
Yang2025_S	ENSGALG00010011864	ENSGALG00010011864	case	control	1.091204886	0.27093764	4.027513062	5.63699E-05	0.000673684	up_in_case
Yang2025_S	ENSGALG00010011866	KLF8	case	control	0.151014698	0.130100984	1.16074985	0.245743643	0.41139328	up_in_case
Yang2025_S	ENSGALG00010011867	ENSGALG00010011867	case	control	-0.547521566	0.453867699	-1.206346184	0.227684037	0.389885959	down_in_case
Yang2025_S	ENSGALG00010011868	SNRPF	case	control	0.012891394	0.217712	0.059213061	0.952782407	0.970968469	up_in_case
Yang2025_S	ENSGALG00010011871	CCT2	case	control	0.287346158	0.123381883	2.328916939	0.019863466	0.067478646	up_in_case
Yang2025_S	ENSGALG00010011873	RFX3	case	control	0.618558842	0.203223205	3.043741202	0.00233656	0.013471233	up_in_case
Yang2025_S	ENSGALG00010011874	KCNS2	case	control	0.743232912	0.428996427	1.732492081	0.083185986	0.192621588	up_in_case
Yang2025_S	ENSGALG00010011875	PSD2	case	control	-0.11414245	0.242244644	-0.471186683	0.637507428	0.767885384	down_in_case
Yang2025_S	ENSGALG00010011876	AMDHD1	case	control	0.241701624	0.37872692	0.638194994	0.523346755	0.676703817	up_in_case
Yang2025_S	ENSGALG00010011880	U2AF1	case	control	0.60181037	0.172739876	3.483911101	0.000494144	0.003912438	up_in_case
Yang2025_S	ENSGALG00010011881	RIOK2	case	control	0.172350602	0.190787537	0.903364049	0.366332714	0.535122787	up_in_case
Yang2025_S	ENSGALG00010011882	ENSGALG00010011882	case	control	-0.426184111	0.407707596	-1.045318052	0.295875997	0.46446232	down_in_case
Yang2025_S	ENSGALG00010011883	HCFC2	case	control	0.27134271	0.155986881	1.73952263	0.081942875	0.190471053	up_in_case
Yang2025_S	ENSGALG00010011885	NCALD	case	control	-0.230204733	0.235275636	-0.978446969	0.327853308	0.497564348	down_in_case
Yang2025_S	ENSGALG00010011886	NTNG1	case	control	-0.393887479	0.231178645	-1.703822944	0.08841417	0.20151316	down_in_case
Yang2025_S	ENSGALG00010011887	PGM5	case	control	-0.036093102	0.112864875	-0.319790389	0.749127234	0.847674443	down_in_case
Yang2025_S	ENSGALG00010011888	CAND1	case	control	-0.246090816	0.064501309	-3.815284086	0.000136026	0.001368846	down_in_case
Yang2025_S	ENSGALG00010011891	HAL	case	control	1.329721514	0.513423868	2.589909811	0.009600108	0.039415882	up_in_case
Yang2025_S	ENSGALG00010011892	ENSGALG00010011892	case	control	0.612789369	0.766662188	0.799295151	0.42411929	0.589066312	up_in_case
Yang2025_S	ENSGALG00010011894	VWC2L	case	control	1.73864512	0.290182663	5.99155409	2.07845E-09	1.16787E-07	up_in_case
Yang2025_S	ENSGALG00010011895	PHF21B	case	control	1.596292859	0.669072786	2.385828408	0.0170407	0.060300192	up_in_case
Yang2025_S	ENSGALG00010011896	SIK1	case	control	-0.087539707	0.128174089	-0.682975073	0.494622594	0.651987937	down_in_case
Yang2025_S	ENSGALG00010011898	ENSGALG00010011898	case	control	-0.292785169	0.10821218	-2.705658168	0.006816918	0.03034944	down_in_case
Yang2025_S	ENSGALG00010011899	PHLPP1	case	control	-0.102373673	0.092964294	-1.101214975	0.270803106	0.438097295	down_in_case
Yang2025_S	ENSGALG00010011902	TP53I3	case	control	-0.604996896	0.278104725	-2.175428324	0.029598034	0.090568424	down_in_case
Yang2025_S	ENSGALG00010011903	LTA4H	case	control	-0.327914475	0.177128833	-1.851276662	0.064129762	0.15955155	down_in_case
Yang2025_S	ENSGALG00010011904	KIF24	case	control	0.2088037	0.224703245	0.929242034	0.352763668	0.521621574	up_in_case
Yang2025_S	ENSGALG00010011906	ENSGALG00010011906	case	control	0.633742161	2.824039865	0.224409779	0.822438462	0.893829354	up_in_case
Yang2025_S	ENSGALG00010011907	BIK	case	control	-0.113722074	0.333914053	-0.34057289	0.733425141	0.837110275	down_in_case
Yang2025_S	ENSGALG00010011908	ENSGALG00010011908	case	control	0.257496583	0.270594445	0.951595969	0.341301927	0.50953957	up_in_case
Yang2025_S	ENSGALG00010011909	C8orf48	case	control	0.072193034	0.294173855	0.245409414	0.80613944	0.88355774	up_in_case
Yang2025_S	ENSGALG00010011911	NUDT2	case	control	0.05891691	0.224507041	0.262427895	0.792991569	0.875273221	up_in_case
Yang2025_S	ENSGALG00010011912	TCP11X2	case	control	0.162939436	0.175665949	0.927552763	0.35363961	0.52258409	up_in_case
Yang2025_S	ENSGALG00010011913	CXXC5	case	control	-0.025875507	0.094879901	-0.272718528	0.785069591	0.87009674	down_in_case
Yang2025_S	ENSGALG00010011915	RRM2B	case	control	-0.522954611	0.189608584	-2.758074559	0.005814293	0.026810781	down_in_case
Yang2025_S	ENSGALG00010011916	MCAT	case	control	0.198136919	0.135094172	1.466657785	0.142469172	0.282204394	up_in_case
Yang2025_S	ENSGALG00010011917	HSF2BP	case	control	-0.228563247	0.283386952	-0.806541183	0.419930872	0.585642184	down_in_case
Yang2025_S	ENSGALG00010011921	MEGF10	case	control	-0.237990307	0.145952834	-1.630597371	0.102975304	0.224100145	down_in_case
Yang2025_S	ENSGALG00010011922	PIK3R1	case	control	0.288794865	0.189671803	1.522603051	0.127858046	0.260787744	up_in_case
Yang2025_S	ENSGALG00010011923	BARD1	case	control	-0.521993039	0.174871903	-2.98500233	0.002835762	0.015600607	down_in_case
Yang2025_S	ENSGALG00010011925	RRP1B	case	control	-0.285706014	0.083373802	-3.426808022	0.000610721	0.004673472	down_in_case
Yang2025_S	ENSGALG00010011926	DYRK2	case	control	-0.053074717	0.097840539	-0.542461413	0.587500683	0.728753962	down_in_case
Yang2025_S	ENSGALG00010011927	ENSGALG00010011927	case	control	1.345463527	0.563062111	2.389547264	0.016869153	0.059875611	up_in_case
Yang2025_S	ENSGALG00010011929	ELK3	case	control	0.050631123	0.148291122	0.341430576	0.732779461	0.836784543	up_in_case
Yang2025_S	ENSGALG00010011930	TSPO	case	control	-0.344906455	0.170513223	-2.022754886	0.043098425	0.119636763	down_in_case
Yang2025_S	ENSGALG00010011931	ENSGALG00010011931	case	control	-5.255466509	1.183761229	-4.439633923	9.0112E-06	0.000149846	down_in_case
Yang2025_S	ENSGALG00010011932	VAV3	case	control	-0.17745341	0.155921321	-1.138095864	0.255080455	0.421454368	down_in_case
Yang2025_S	ENSGALG00010011935	ST8SIA4	case	control	-0.051787195	0.170392265	-0.303929258	0.761181787	0.854874912	down_in_case
Yang2025_S	ENSGALG00010011936	CKAP4	case	control	-0.310845851	0.078911918	-3.939149589	8.17709E-05	0.000906506	down_in_case
Yang2025_S	ENSGALG00010011937	SLC1A1	case	control	-1.687445202	0.546809081	-3.085986063	0.002028783	0.012107536	down_in_case
Yang2025_S	ENSGALG00010011941	ENSGALG00010011941	case	control	0.136794989	0.257379373	0.531491653	0.595078124	0.735096401	up_in_case
Yang2025_S	ENSGALG00010011943	TTLL12	case	control	-0.053401222	0.083209696	-0.641766815	0.521024599	0.674640196	down_in_case
Yang2025_S	ENSGALG00010011947	THAP1	case	control	0.312172108	0.21482157	1.453169291	0.146176756	0.287626814	up_in_case
Yang2025_S	ENSGALG00010011949	ABCA12	case	control	-0.181323548	0.484230686	-0.374456954	0.708064377	0.81991666	down_in_case
Yang2025_S	ENSGALG00010011950	PDXK	case	control	-0.200580074	0.168459448	-1.190672748	0.23378208	0.396948128	down_in_case
Yang2025_S	ENSGALG00010011952	UBE2D2	case	control	-0.284854958	0.092423408	-3.082065067	0.002055699	0.012210642	down_in_case
Yang2025_S	ENSGALG00010011953	CDK17	case	control	-0.07031642	0.116696301	-0.602559122	0.546802021	0.695855204	down_in_case
Yang2025_S	ENSGALG00010011954	ENSGALG00010011954	case	control	1.253072117	0.325734303	3.846914812	0.000119615	0.001226511	up_in_case
Yang2025_S	ENSGALG00010011956	ENSGALG00010011956	case	control	0.13585226	0.557651966	0.243614779	0.807529181	0.883899076	up_in_case
Yang2025_S	ENSGALG00010011957	ENSGALG00010011957	case	control	-0.548145459	0.14996481	-3.655160568	0.000257021	0.00229295	down_in_case
Yang2025_S	ENSGALG00010011960	CFAP54	case	control	0.737317455	0.277663469	2.65543558	0.007920606	0.034006208	up_in_case
Yang2025_S	ENSGALG00010011961	AGPAT3	case	control	-0.295470568	0.094739709	-3.118761625	0.001816128	0.011133037	down_in_case
Yang2025_S	ENSGALG00010011962	CPSF6	case	control	0.286989558	0.187147297	1.533495607	0.125153788	0.257107512	up_in_case
Yang2025_S	ENSGALG00010011963	ZCCHC2	case	control	0.452887624	0.18117155	2.499772312	0.012427315	0.047614691	up_in_case
Yang2025_S	ENSGALG00010011964	ENSGALG00010011964	case	control	-11.18745064	2.949624627	-3.79283877	0.000148935	0.001472412	down_in_case
Yang2025_S	ENSGALG00010011965	ATIC	case	control	-0.357901903	0.208296281	-1.71823472	0.085753811	0.19684952	down_in_case
Yang2025_S	ENSGALG00010011966	SLC25A24	case	control	-0.20176544	0.090840847	-2.221087167	0.026345058	0.083190207	down_in_case
Yang2025_S	ENSGALG00010011967	MTMR9	case	control	-0.624069285	0.102882056	-6.065871054	1.3124E-09	7.68418E-08	down_in_case
Yang2025_S	ENSGALG00010011968	ENSGALG00010011968	case	control	0.35819454	0.298127998	1.201479039	0.229565433	0.391941658	up_in_case
Yang2025_S	ENSGALG00010011969	TMEM173	case	control	0.702557778	0.300141043	2.340758769	0.019244597	0.065906478	up_in_case
Yang2025_S	ENSGALG00010011971	RNF170	case	control	-0.08176169	0.134563803	-0.607605375	0.543449235	0.693431471	down_in_case
Yang2025_S	ENSGALG00010011972	IPO11	case	control	0.082020972	0.142799443	0.57437879	0.565711502	0.711094153	up_in_case
Yang2025_S	ENSGALG00010011973	TRAPPC10	case	control	-0.039394933	0.081267388	-0.484756971	0.627848749	0.760921231	down_in_case

Yang2025_S	ENSGALG00010011976	TNFRSF11A	case	control	-0.331166541	0.16287454	-2.033261555	0.042026105	0.117247155	down_in_case
Yang2025_S	ENSGALG00010011977	GATD3A	case	control	-0.097232383	0.180369459	-0.539073429	0.589836189	0.730805307	down_in_case
Yang2025_S	ENSGALG00010011978	ENSGALG00010011978	case	control	-6.718930941	2.951423644	-2.276505087	0.022815796	0.074633362	down_in_case
Yang2025_S	ENSGALG00010011979	NEDD1	case	control	-0.244324648	0.064773967	-3.77195748	0.000161972	0.001575072	down_in_case
Yang2025_S	ENSGALG00010011981	SCUBE1	case	control	0.842959479	0.278446072	3.0273707	0.002466912	0.014048395	up_in_case
Yang2025_S	ENSGALG00010011982	STK3	case	control	-0.486326941	0.108474791	-4.483317631	7.34914E-06	0.000128173	down_in_case
Yang2025_S	ENSGALG00010011983	LYZ	case	control	0.08488886	0.498194974	0.170392847	0.864701197	0.920045088	up_in_case
Yang2025_S	ENSGALG00010011984	FN1	case	control	-0.081334795	0.220884436	-0.368223293	0.712706743	0.822694538	down_in_case
Yang2025_S	ENSGALG00010011988	HINTW	case	control	-11.43117077	2.778123257	-4.11470972			down_in_case
Yang2025_S	ENSGALG00010011989	H1-010	case	control	-0.220847606	0.277634298	-0.79546226	0.426344656	0.590920308	down_in_case
Yang2025_S	ENSGALG00010011991	CSNK1A1	case	control	-0.461761783	0.060002326	-7.695731435	1.40687E-14	2.84127E-12	down_in_case
Yang2025_S	ENSGALG00010011992	YEATS4	case	control	-0.22376497	0.141927852	-1.576610699	0.114885129	0.242087446	down_in_case
Yang2025_S	ENSGALG00010011993	RELCH	case	control	-0.31773136	0.086022785	-3.693572122	0.000221126	0.002020267	down_in_case
Yang2025_S	ENSGALG00010011996	LRRC70	case	control	0.074306361	0.18580739	0.399910689	0.689222299	0.805798535	up_in_case
Yang2025_S	ENSGALG00010011997	DENND5B	case	control	-0.083740008	0.200517387	-0.417619683	0.676225204	0.796416347	down_in_case
Yang2025_S	ENSGALG00010011998	MPPED1	case	control	-0.052005042	0.107516182	-0.483695018	0.628602329	0.761322631	down_in_case
Yang2025_S	ENSGALG00010012001	EEIG2	case	control	0.248103095	0.203150907	1.221274857	0.22198197	0.383500961	up_in_case
Yang2025_S	ENSGALG00010012004	LGR5	case	control	-0.732779839	0.272435208	-2.68973986	0.007150774	0.03143408	down_in_case
Yang2025_S	ENSGALG00010012007	MREG	case	control	-0.262958189	0.214861114	-1.22385193	0.22100809	0.38238968	down_in_case
Yang2025_S	ENSGALG00010012008	ZNF366	case	control	-0.303911711	0.193401642	-1.57140192	0.11608932	0.243793873	down_in_case
Yang2025_S	ENSGALG00010012010	HENMT1	case	control	0.248139045	0.272073245	0.912030305	0.361752765	0.530312397	up_in_case
Yang2025_S	ENSGALG00010012011	HOOK3	case	control	0.305607465	0.136157881	2.244508087	0.024799729	0.079527187	up_in_case
Yang2025_S	ENSGALG00010012015	ENSGALG00010012015	case	control	-0.5200435	0.277669377	-1.872887479	0.061083925	0.154371507	down_in_case
Yang2025_S	ENSGALG00010012016	WDR91	case	control	-0.163673191	0.106021832	-1.54376876	0.122644362	0.253632828	down_in_case
Yang2025_S	ENSGALG00010012017	N4BP3	case	control	0.040610072	0.147707096	0.2749365	0.783365024	0.868952359	up_in_case
Yang2025_S	ENSGALG00010012019	ENSGALG00010012019	case	control	-0.129832631	0.338794373	-0.38321956	0.701556976	0.815838811	down_in_case
Yang2025_S	ENSGALG00010012021	ENSGALG00010012021	case	control	0.650957219	0.214955806	3.028330479	0.00245909	0.01402512	up_in_case
Yang2025_S	ENSGALG00010012024	UBR5	case	control	0.039740667	0.12506747	0.317753828	0.750671677	0.848540188	up_in_case
Yang2025_S	ENSGALG00010012026	SYT1	case	control	0.945961482	0.345147394	2.740746416	0.00612998	0.027898142	up_in_case
Yang2025_S	ENSGALG00010012028	TMEM140	case	control	0.200678933	0.187251736	1.071706664	0.283851807	0.452051587	up_in_case
Yang2025_S	ENSGALG00010012031	GRK6	case	control	-0.061908128	0.160064502	-0.386769877	0.69892658	0.813564352	down_in_case
Yang2025_S	ENSGALG00010012034	ARHGEF9	case	control	-0.031646914	0.140451488	-0.225322736	0.821728213	0.893494394	down_in_case
Yang2025_S	ENSGALG00010012040	B4GALT7	case	control	0.307351462	0.189511287	1.621810852	0.104843854	0.226933692	up_in_case
Yang2025_S	ENSGALG00010012041	ICOSLG	case	control	-0.162204478	0.125913008	-1.288226539	0.197667116	0.354138759	down_in_case
Yang2025_S	ENSGALG00010012042	FNDC7	case	control	0.716755128	0.350774416	2.043350641	0.04101774	0.115331357	up_in_case
Yang2025_S	ENSGALG00010012043	NUP50	case	control	0.105878355	0.099758514	1.06134655	0.288532438	0.45746951	up_in_case
Yang2025_S	ENSGALG00010012044	ENSGALG00010012044	case	control	2.766779849	0.670784602	4.124691953	3.71231E-05	0.000482566	up_in_case
Yang2025_S	ENSGALG00010012045	ENSGALG00010012045	case	control	-0.055396621	0.466055189	-0.118862793	0.90538406	0.943381503	down_in_case
Yang2025_S	ENSGALG00010012046	LOXHD1	case	control	0.236240403	0.262289914	0.90068428	0.367756209	0.536343719	up_in_case
Yang2025_S	ENSGALG00010012047	LMAN2	case	control	-0.458413687	0.179993854	-2.546829658	0.010870648	0.043071505	down_in_case
Yang2025_S	ENSGALG00010012048	PDE6H	case	control	-0.008941137	0.320335111	-0.027911823	0.977732479	0.986796704	down_in_case
Yang2025_S	ENSGALG00010012049	IFNAR1	case	control	-0.35328151	0.127877911	-2.762646859	0.005733476	0.026528111	down_in_case
Yang2025_S	ENSGALG00010012051	ARK2C	case	control	0.485353635	0.233162938	2.081607132	0.037378369	0.107661757	up_in_case
Yang2025_S	ENSGALG00010012052	KLF10	case	control	0.105217212	0.131121226	0.802442251	0.422297181	0.588177033	up_in_case
Yang2025_S	ENSGALG00010012054	FAM60A	case	control	-0.377155021	0.107981148	-3.492785809	0.00047801	0.003810678	down_in_case
Yang2025_S	ENSGALG00010012055	ENSGALG00010012055	case	control	-0.16369418	0.132468613	-1.235720493	0.216562478	0.377374404	down_in_case
Yang2025_S	ENSGALG00010012058	ENSGALG00010012058	case	control	1.141849237	0.347323958	3.287562548	0.001010587	0.007074163	up_in_case
Yang2025_S	ENSGALG00010012059	ARHGDIB	case	control	0.470614763	0.229074005	2.054422386	0.039934828	0.113246201	up_in_case
Yang2025_S	ENSGALG00010012060	ENSGALG00010012060	case	control	0.13713295	0.177243962	0.773696032	0.439110567	0.602798321	up_in_case
Yang2025_S	ENSGALG00010012061	AZIN1	case	control	-0.522854889	0.105121049	-4.973836287	6.56407E-07	1.68144E-05	down_in_case
Yang2025_S	ENSGALG00010012062	HMGA2	case	control	0.329055918	0.192033234	1.713536306	0.08661392	0.198283072	up_in_case
Yang2025_S	ENSGALG00010012063	PTPRD	case	control	0.747732447	0.269555515	2.773946017	0.005538087	0.02583637	up_in_case
Yang2025_S	ENSGALG00010012064	RGS14	case	control	0.4805776	0.187480087	2.563352767	0.010366666	0.041605579	up_in_case
Yang2025_S	ENSGALG00010012066	STXBP3	case	control	0.500474378	0.099409574	5.034468586	4.79176E-07	1.2926E-05	up_in_case
Yang2025_S	ENSGALG00010012067	ENSGALG00010012067	case	control	0.198126978	0.108268271	1.829963443	0.067255406	0.165175198	up_in_case
Yang2025_S	ENSGALG00010012070	DNAJC28	case	control	0.359795626	0.160882406	2.236388892	0.025326308	0.080660274	up_in_case
Yang2025_S	ENSGALG00010012071	ENSGALG00010012071	case	control	-0.334254111	0.142458353	-2.346328629	0.018959383	0.065191228	down_in_case
Yang2025_S	ENSGALG00010012072	CAPRIN2	case	control	0.713653127	0.161168889	4.427983173	9.51183E-06	0.000157233	up_in_case
Yang2025_S	ENSGALG00010012074	KIAA0930	case	control	-0.262562378	0.070698633	-3.713825412	0.00020415	0.001904167	down_in_case
Yang2025_S	ENSGALG00010012075	PIGN	case	control	-0.348365304	0.109405975	-3.184152456	0.001451785	0.009383408	down_in_case
Yang2025_S	ENSGALG00010012077	IFNGR2	case	control	-0.418078095	0.067663838	-6.178752342	6.46102E-10	4.07395E-08	down_in_case
Yang2025_S	ENSGALG00010012078	TMBIM4	case	control	-0.538044963	0.218769977	-2.459409522	0.013916577	0.051852273	down_in_case
Yang2025_S	ENSGALG00010012079	ENSGALG00010012079	case	control	-1.151933675	0.360008076	-3.199743979	0.001375497	0.009011543	down_in_case
Yang2025_S	ENSGALG00010012081	CHIC1	case	control	-0.133762874	0.098555996	-1.357227152	0.174709046	0.325206605	down_in_case
Yang2025_S	ENSGALG00010012082	HAUS1	case	control	-0.105458505	0.249428539	-0.422800475	0.672440841	0.793789768	down_in_case
Yang2025_S	ENSGALG00010012083	MGP	case	control	-0.280740775	0.395101885	-0.710552862	0.477361362	0.636346544	down_in_case
Yang2025_S	ENSGALG00010012084	TMEM50B	case	control	-0.485212827	0.124215995	-3.906202471	9.3758E-05	0.001014377	down_in_case
Yang2025_S	ENSGALG00010012085	IPO8	case	control	0.307514784	0.124251816	2.474931919	0.013326158	0.050133701	up_in_case
Yang2025_S	ENSGALG00010012086	CIBAR1	case	control	0.255925235	0.161947176	1.580300699	0.11403802	0.240812215	up_in_case
Yang2025_S	ENSGALG00010012087	TMEM171	case	control	-0.712915206	0.248845567	-2.864890122	0.00417154	0.020768276	down_in_case
Yang2025_S	ENSGALG00010012088	ATP5F1AZ	case	control	-0.085126298	0.238021554	-0.357641132	0.720611893	0.827910523	down_in_case
Yang2025_S	ENSGALG00010012089	ENSGALG00010012089	case	control	0.708972762	0.286969022	2.47055503	0.013490356	0.050575225	up_in_case
Yang2025_S	ENSGALG00010012090	HELB	case	control	0.487346852	0.131507691	3.705842964	0.000210689	0.001954676	up_in_case
Yang2025_S	ENSGALG00010012091	ENSGALG00010012091	case	control	-0.232508508	0.619727247	-0.375178772	0.707527516	0.819805489	down_in_case
Yang2025_S	ENSGALG00010012092	OC3	case	control	0.156523892	0.568097126	0.275523119	0.782914367	0.868623543	up_in_case
Yang2025_S	ENSGALG00010012093	ENSGALG00010012093	case	control	0.153431621	0.131016047	1.171090301	0.241562482	0.406690007	up_in_case
Yang2025_S	ENSGALG00010012094	ENSGALG00010012094	case	control	0.696074084	0.369708605	1.882764087	0.059732346	0.152058868	up_in_case
Yang2025_S	ENSGALG00010012095	RNF38	case	control	0.419617603	0.238834151	1.75694138	0.078927801	0.185536254	up_in_case
Yang2025_S	ENSGALG00010012096	ENSGALG00010012096	case	control	-0.174687472	0.140302967	-1.245073258	0.213104823	0.373430215	down_in_case
Yang2025_S	ENSGALG00010012097	GART	case	control	-0.060143985	0.096443117	-0.623621331	0.532876299	0.684831801	down_in_case
Yang2025_S	ENSGALG00010012098	ART4	case	control	-0.543147163	0.517280329	-1.050005446	0.293715609	0.46232091	down_in_case
Yang2025_S	ENSGALG00010012099	UPK3A	case	control	-1.582948267	0.443594605	-3.568456986	0.00035909	0.003025342	down_in_case
Yang2025_S	ENSGALG00010012100	UBE2F	case	control	-0.056836847	0.116547348	-0.487671732	0.625782378	0.759473736	down_in_case
Yang2025_S	ENSGALG00010012101	ENSGALG00010012101	case	control	-0.047392995	0.116145614	-0.408048083	0.683238374	0.801627242	down_in_case
Yang2025_S	ENSGALG00010012102	SON	case	control	0.153325467	0.133041198	1.152466069	0.249129605	0.414727702	up_in_case
Yang2025_S	ENSGALG00010012103	ENSGALG00010012103	case	control	0.026929388	0.104099701	0.258688432	0.795875646	0.876582922	up_in_case
Yang2025_S	ENSGALG00010012105	DONSON	case	control	0.0797519	0.144362111	0.552443434	0.580644579	0.723340087	up_in_case
Yang2025_S	ENSGALG00010012106	TMTC1	case	control	-0.302673835	0.116311707	-2.602264573	0.009261038	0.038382711	down_in_case
Yang2025_S	ENSGALG00010012107	CLCC1	case	control	-0.147301942	0.108689525	-1.355254258	0.175336563	0.325802166	down_in_case
Yang2025_S	ENSGALG00010012108	PSTPIP2	case	control	0.033489229	0.360147983	0.092987413	0.925913561	0.955465452	up_in_case
Yang2025_S	ENSGALG00010012110	ENSGALG00010012110	case	control	0.351686439	0.174779702	2.012169805	0.04420204	0.122140677	up_in_case
Yang2025_S	ENSGALG00010012111	DRD1	case	control	-0.767429849	0.389552849	-1.97002756	0.048835212	0.131070624	down_in_case
Yang2025_S	ENSGALG00010012112	MBD2	case	control	-0.555679022	0.287553302	-1.932438326	0.053305427	0.139836433	down_in_case
Yang2025_S	ENSGALG00010012113	WDR47	case	control	0.071029718	0.07127479	0.996561591	0.318977357	0.48837294	up_in_case
Yang2025_S	ENSGALG00010012114	TAL2	case	control	-0.195966665	0.35828498	-0.546957524	0.584407914	0.726469606	down_in_case
Yang2025_S	ENSGALG00010012116	RBM12B	case	control	-0.144157392	0.091869691	-1.569150721	0.116612824	0.244471145	down_in_case
Yang2025_S	ENSGALG00010012118	ENSGALG00010012118	case	control	-0.893150605	0.866668211	-1.030556554	0.302748819	0.472483457	down_in_case
Yang2025_S	ENSGALG00010012119	EPG5	case	control	-0.103651278	0.110948895	-0.934225418	0.350187627	0.518582846	down_in_case
Yang2025_S	ENSGALG00010012120	ENSGALG00010012120	case	control	0.268225062	0.193606956	1.385410255	0.165927057	0.313560285	up_in_case
Yang2025_S	ENSGALG00010012121	SREK1	case	control	1.142496564	0.242092315	4.719259935	2.36704E-06	5.02051E-05	up_in_case
Yang2025_S	ENSGALG00010012122	GRIP1	case	control	0.788644529	0.263394964	2.994151893	0.002752089	0.015248652	up_in_case
Yang2025_S	ENSGALG00010012123	ENSGALG00010012123	case	control	0.547690804	0.169215284	3.236650913	0.001209413	0.008118095	up_in_case
Yang2025_S	ENSGALG00010012124	ERAP1	case	control	-0.096722208	0.199659959	-0.48443468	0.628077411	0.761060746	down_in_case
Yang2025_S	ENSGALG00010012125	RNF152	case	control	-0.448976984	0.124239897	-3.613790687	0.000301753	0.002620207	down_in_case
Yang2025_S	ENSGALG00010012127	ENSGALG00010012127	case	control	-0.126674493	0.052859296	-2.396446862	0.016554894	0.059092061	down_in_case
Yang2025_S	ENSGALG00010012129	CRYZL1	case	control	-0.225886638	0.181411319	-1.245162869	0.213071888	0.373430215	down_in_case
Yang2025_S	ENSGALG00010012130	TMEM67	case	control	-0.191429816	0.154859831	-1.236148946	0.216403204	0.377317191	down_in_case
Yang2025_S	ENSGALG00010012131	SLC14A1	case	control	-0.69990781	0.378224642	-1.850508223	0.064240334	0.159699414	down_in_case
Yang2025_S	ENSGALG00010012133	RAB3IP	case	control	0.142223163	0.181689987	0.782779316	0.433756716	0.597669682	up_in_case
Yang2025_S	ENSGALG00010012135	CAMSAP2	case	control	0.428693905	0.185336815	2.313053153	0.020719717	0.069472488	up_in_case
Yang2025_S	ENSGALG00010012136	ENSGALG00010012136	case	control	0.58885047	0.233384447	2.523092165	0.011632791	0.0453308	up_in_case
Yang2025_S	ENSGALG00010012138	DDX11	case	control	0.426093423	0.17673568	2.410907758	0.015912872	0.05749287	up_in_case
Yang2025_S	ENSGALG00010012140	GLOD5	case	control	-0.988265417	0.459527268	-2.150613219	0.031506741	0.095011131	down_in_case
Yang2025_S	ENSGALG00010012141	ENSGALG00010012141	case	control	0.281498216	0.163329038	1.723503787	0.084797461	0.195379071	up_in_case
Yang2025_S	ENSGALG00010012144	H1-01	case	control	-0.328678619	0.145118358	-2.264900349	0.023518795	0.076645092	down_in_case
Yang2025_S	ENSGALG00010012147	ARFGAP3	case	control	-0.082187674	0.097614557	-0.84196124	0.39980965	0.566799497	down_in_case
Yang2025_S	ENSGALG00010012148	SETBP1	case	control	0.933773187	0.245821801	3.7985776	0.000145529	0.001445435	up_in_case
Yang2025_S	ENSGALG00010012149	SFXN1	case	control	-0.435455946	0.217747707	-1.999818745	0.04551984	0.124915117	down_in_case
Yang2025_S	ENSGALG00010012154	CWC27	case	control	0.778589466	0.240468498	3.237802346	0.001204542	0.0080893	up_in_case
Yang2025_S	ENSGALG00010012156	ENSGALG00010012156	case	control	0.996725372	0.632400847	1.576097464	0.115003343	0.242252992	up_in_case
Yang2025_S	ENSGALG00010012157	H2BC32	case	control	0.636937206	0.459988124	1.38468185	0.166149771	0.313896023	up_in_case
Yang2025_S	ENSGALG00010012159	WASHC1	case	control	-0.185713884	0.145750332	-1.274191841	0.202595436	0.360465705	down_in_case
Yang2025_S	ENSGALG00010012163	PDP1	case	control	-0.3979608	0.086613768	-4.594659837	4.33456E-06	8.25561E-05	down_in_case
Yang2025_S	ENSGALG00010012168	ENSGALG00010012168	case	control	-1.321010793	0.829773093	-1.592014498	0.111381447	0.236564619	down_in_case
Yang2025_S	ENSGALG00010012171	CDH17	case	control	-0.578867112	0.321003207	-1.80330632	0.071340121	0.172291955	down_in_case
Yang2025_S	ENSGALG00010012172	CNOT2	case	control	0.005326982	0.097149344	0.054832921	0.956271573	0.973310221	up_in_case

Yang2025_S	ENSGALG00010012175	PACSIN2	case	control	0.056550616	0.102186161	0.55340777	0.57998422	0.722711267	up_in_case
Yang2025_S	ENSGALG00010012179	ENSGALG00010012179	case	control	-0.776084392	0.36522238	-2.124963953	0.033589621	0.099610846	down_in_case
Yang2025_S	ENSGALG00010012180	DDX59	case	control	-0.150066624	0.119216276	-1.258776314	0.208111138	0.366952892	down_in_case
Yang2025_S	ENSGALG00010012186	KIF20A	case	control	-0.336110474	0.131895974	-2.548299719	0.010824942	0.042976682	down_in_case
Yang2025_S	ENSGALG00010012187	ENSGALG00010012187	case	control	-1.876115193	0.736199035	-2.548380403	0.010822439	0.042976682	down_in_case
Yang2025_S	ENSGALG00010012188	ADAMTS6	case	control	0.532532102	0.287318291	1.853457018	0.063816881	0.158943386	up_in_case
Yang2025_S	ENSGALG00010012190	CAST	case	control	-0.004378472	0.108284748	-0.040434795	0.96774649	0.980909757	down_in_case
Yang2025_S	ENSGALG00010012191	BEST3	case	control	0.237102684	0.202843983	1.168891875	0.242447197	0.407391981	up_in_case
Yang2025_S	ENSGALG00010012193	TTLL1	case	control	-0.108645624	0.36285788	-0.299416465	0.764622301	0.856649925	down_in_case
Yang2025_S	ENSGALG00010012194	LRRFIP1	case	control	0.778074661	0.169879949	4.580144191	4.64655E-06	8.67959E-05	up_in_case
Yang2025_S	ENSGALG00010012195	ITSN1	case	control	0.348669813	0.188931963	1.845478171	0.064968011	0.161135348	up_in_case
Yang2025_S	ENSGALG00010012196	CENPK	case	control	-0.110800057	0.193501345	-0.572606131	0.566911408	0.711703646	down_in_case
Yang2025_S	ENSGALG00010012198	TMEM5	case	control	-0.098533743	0.143057011	-0.688772553	0.490966411	0.648433033	down_in_case
Yang2025_S	ENSGALG00010012199	ENSGALG00010012199	case	control	-1.028625114	0.440420026	-2.335554816	0.019514456	0.066585196	down_in_case
Yang2025_S	ENSGALG00010012200	GEM	case	control	-0.576116657	0.131480145	-4.381776871	1.17715E-05	0.000188764	down_in_case
Yang2025_S	ENSGALG00010012205	SLC6A13	case	control	-0.268548851	0.416457392	-0.644841121	0.519030151	0.673246301	down_in_case
Yang2025_S	ENSGALG00010012207	GRIA3	case	control	-0.14838951	0.244575593	-0.60672248	0.544035101	0.694051921	down_in_case
Yang2025_S	ENSGALG00010012209	KIF14	case	control	0.231115668	0.180945272	1.277268342	0.201507526	0.359484697	up_in_case
Yang2025_S	ENSGALG00010012210	PPWD1	case	control	-0.162449345	0.157092008	-1.03410318	0.30108793	0.470734915	down_in_case
Yang2025_S	ENSGALG00010012211	ENSGALG00010012211	case	control	-12.04678065	2.755048542	-4.372620105			down_in_case
Yang2025_S	ENSGALG00010012212	RAD54B	case	control	0.129673483	0.153989926	0.842090691	0.399737192	0.566784389	up_in_case
Yang2025_S	ENSGALG00010012215	RAB17	case	control	-1.067523771	0.610906517	-1.747442107	0.080560679	0.188168465	down_in_case
Yang2025_S	ENSGALG00010012216	BRD8	case	control	-0.023476382	0.090039094	-0.260735428	0.794296543	0.875942305	down_in_case
Yang2025_S	ENSGALG00010012219	KDM5A	case	control	-0.223653797	0.098864111	-2.262234446	0.023682922	0.077022565	down_in_case
Yang2025_S	ENSGALG00010012220	ENSGALG00010012220	case	control	1.246862123	0.541466309	2.302751071	0.021292853	0.070946213	up_in_case
Yang2025_S	ENSGALG00010012228	NHP2	case	control	0.368100818	0.24120996	1.52605978	0.126994977	0.259673516	up_in_case
Yang2025_S	ENSGALG00010012229	FSBP	case	control	-0.259218372	0.116995127	-2.215633929	0.026716583	0.083953402	down_in_case
Yang2025_S	ENSGALG00010012230	PTPRQ	case	control	-0.45352653	0.223577997	-2.028493571	0.042509899	0.118356731	down_in_case
Yang2025_S	ENSGALG00010012232	PCSK1	case	control	0.388348628	0.329764244	1.177655355	0.238934032	0.403141511	up_in_case
Yang2025_S	ENSGALG00010012234	C1GALT1C1	case	control	-0.353628349	0.080938062	-4.369122997	1.24746E-05	0.000197989	down_in_case
Yang2025_S	ENSGALG00010012240	TRIM14	case	control	0.086274769	0.303273751	0.284478193	0.776043944	0.863751785	up_in_case
Yang2025_S	ENSGALG00010012242	CCDC77	case	control	0.087779521	0.155526056	0.564403953	0.572479249	0.715833643	up_in_case
Yang2025_S	ENSGALG00010012243	ENSGALG00010012243	case	control	-4.580229007	1.766185315	-2.593289033	0.009506284	0.03912288	down_in_case
Yang2025_S	ENSGALG00010012244	ENSGALG00010012244	case	control	-0.205765095	0.065197161	-3.156043809	0.001599248	0.0101114	down_in_case
Yang2025_S	ENSGALG00010012245	ENSGALG00010012245	case	control	-0.05115756	0.090559996	-0.564902406	0.572140147	0.715753025	down_in_case
Yang2025_S	ENSGALG00010012247	TRIM23	case	control	-0.254115384	0.169388128	-1.500195956	0.13356365	0.26935014	down_in_case
Yang2025_S	ENSGALG00010012248	H2AC39	case	control	0.225799782	0.312696516	0.722105205	0.470229799	0.630487082	up_in_case
Yang2025_S	ENSGALG00010012250	NANS	case	control	-0.306152122	0.154661717	-1.979495171	0.047760283	0.129230979	down_in_case
Yang2025_S	ENSGALG00010012253	MYF6	case	control	0.154769872	0.147220796	1.051277244	0.293131275	0.461705274	up_in_case
Yang2025_S	ENSGALG00010012259	IL1RAPL2	case	control	0.030721619	0.240446495	0.127769046	0.898331747	0.939723381	up_in_case
Yang2025_S	ENSGALG00010012260	ENSGALG00010012260	case	control	-0.273459363	0.767608942	-0.356248277	0.72165464	0.828660518	down_in_case
Yang2025_S	ENSGALG00010012263	SHLD3	case	control	-0.05288447	0.204041945	-0.259184307	0.795493039	0.876525276	down_in_case
Yang2025_S	ENSGALG00010012264	MYF5	case	control	-0.037230415	0.20378567	-0.182693978	0.855038141	0.91435473	down_in_case
Yang2025_S	ENSGALG00010012268	ENSGALG00010012268	case	control	-0.064729649	0.209368187	-0.309166594	0.757194804	0.852371725	down_in_case
Yang2025_S	ENSGALG00010012271	ENSGALG00010012271	case	control	0.48167901	0.235282052	2.047240777	0.04063445	0.114468744	up_in_case
Yang2025_S	ENSGALG00010012274	B4GALNT3	case	control	-0.129170195	0.131554465	-0.981876176	0.326160854	0.496081187	down_in_case
Yang2025_S	ENSGALG00010012275	VIRMA	case	control	-0.008044167	0.10459109	-0.076910637	0.938694635	0.962878892	down_in_case
Yang2025_S	ENSGALG00010012277	H3C33	case	control	0.660708186	0.237835386	2.778006238	0.005469357	0.025624213	up_in_case
Yang2025_S	ENSGALG00010012282	ENSGALG00010012282	case	control	-2.383039238	0.935902907	-2.546246218	0.010888836	0.043131303	down_in_case
Yang2025_S	ENSGALG00010012283	CUPIN1A	case	control	0.774667238	0.632723567	1.224337576	0.220824907	0.382260259	up_in_case
Yang2025_S	ENSGALG00010012285	FBLN1	case	control	-0.425350999	0.151536335	-2.806924153	0.005001702	0.023976166	down_in_case
Yang2025_S	ENSGALG00010012286	ENSGALG00010012286	case	control	-13.8591303	2.948783576	-4.699948283			down_in_case
Yang2025_S	ENSGALG00010012288	MCTS1	case	control	-0.54926736	0.159076702	-3.452846033	0.000554706	0.004308708	down_in_case
Yang2025_S	ENSGALG00010012290	ENSGALG00010012290	case	control	-0.042548415	0.212208598	-0.200502784	0.841087381	0.905691419	down_in_case
Yang2025_S	ENSGALG00010012291	NR5A2	case	control	0.307504554	0.193957117	1.585425477	0.112869697	0.238924384	up_in_case
Yang2025_S	ENSGALG00010012293	SRGAP1	case	control	0.158403399	0.184441584	0.858826925	0.39043601	0.557794321	up_in_case
Yang2025_S	ENSGALG00010012294	ENSGALG00010012294	case	control	1.021465941	0.581766121	1.755801693	0.079122275	0.185867988	up_in_case
Yang2025_S	ENSGALG00010012295	CUL4B	case	control	-0.055212621	0.120478298	-0.458278562	0.646752324	0.775661305	down_in_case
Yang2025_S	ENSGALG00010012297	ABLIM3	case	control	0.486079251	0.166155971	2.925439558	0.0034397	0.018019631	up_in_case
Yang2025_S	ENSGALG00010012300	NINJ2	case	control	0.710604546	0.299031534	2.376353212	0.017484717	0.061481082	up_in_case
Yang2025_S	ENSGALG00010012303	CA9	case	control	0.442299674	0.143317091	3.086161397	0.002027587	0.012105582	up_in_case
Yang2025_S	ENSGALG00010012307	ENSGALG00010012307	case	control	0.2948609	0.239265589	1.232358156	0.217815325	0.378744266	up_in_case
Yang2025_S	ENSGALG00010012308	TRABD2A	case	control	-0.345821882	0.317853948	-1.087989892	0.27659957	0.443852488	down_in_case
Yang2025_S	ENSGALG00010012309	TRAPPC13	case	control	0.103735845	0.171924561	0.603380019	0.546255912	0.695420805	up_in_case
Yang2025_S	ENSGALG00010012311	ENSGALG00010012311	case	control	4.895891593	0.287396119	17.03534344	4.49097E-65	2.08606E-61	up_in_case
Yang2025_S	ENSGALG00010012312	SH3TC2	case	control	-0.043221829	0.176647938	-0.244677799	0.806705919	0.883622621	down_in_case
Yang2025_S	ENSGALG00010012314	ADRB2	case	control	-0.226783466	0.176487017	-1.284986683	0.198796938	0.355614291	down_in_case
Yang2025_S	ENSGALG00010012315	ATP6V1E1	case	control	-0.468418661	0.120007973	-3.903229493	9.49176E-05	0.001024537	down_in_case
Yang2025_S	ENSGALG00010012316	ENSGALG00010012316	case	control	1.683615097	0.834288654	2.018024683	0.043588691	0.120757138	up_in_case
Yang2025_S	ENSGALG00010012318	POLR1D	case	control	-0.17470271	0.381063335	-0.458461087	0.646621213	0.775661305	down_in_case
Yang2025_S	ENSGALG00010012320	DPY19L4	case	control	-0.37403523	0.097106133	-3.851818824	0.000117244	0.001210216	down_in_case
Yang2025_S	ENSGALG00010012322	ENSGALG00010012322	case	control	0.413882827	0.228777753	1.809104344	0.070434794	0.170756316	up_in_case
Yang2025_S	ENSGALG00010012323	SMAD7B	case	control	-6.341383038	2.951791153	-2.148316974	0.031688583	0.095476845	down_in_case
Yang2025_S	ENSGALG00010012324	ENSGALG00010012324	case	control	0.529597891	0.425127855	1.245737922	0.212860627	0.373249381	up_in_case
Yang2025_S	ENSGALG00010012325	MLPH	case	control	5.771916207	0.632241397	9.129291811	6.89488E-20	3.69539E-17	up_in_case
Yang2025_S	ENSGALG00010012326	SGTB	case	control	-0.263413019	0.097924819	-2.689951538	0.00714624	0.03143408	down_in_case
Yang2025_S	ENSGALG00010012327	UBTD2	case	control	-0.088058037	0.074642351	-1.179732895	0.238106466	0.401988564	down_in_case
Yang2025_S	ENSGALG00010012329	ATXN10	case	control	-0.413083756	0.13168955	-3.136799812	0.001708027	0.010630352	down_in_case
Yang2025_S	ENSGALG00010012331	ENSGALG00010012331	case	control	-0.273733618	0.503760359	-0.543380624	0.586867764	0.728487644	down_in_case
Yang2025_S	ENSGALG00010012332	ENSGALG00010012332	case	control	0.116800426	0.63110364	0.185073289	0.853171552	0.913330907	up_in_case
Yang2025_S	ENSGALG00010012334	ARHGEF39	case	control	-0.435169154	0.258168221	-1.685603102	0.091872271	0.206890773	down_in_case
Yang2025_S	ENSGALG00010012336	ENSGALG00010012336	case	control	-11.61016863	2.9494519	-3.936381748			down_in_case
Yang2025_S	ENSGALG00010012337	ACSS3	case	control	-0.08540371	0.16351978	-0.522283662	0.601472843	0.740025081	down_in_case
Yang2025_S	ENSGALG00010012339	WNK1	case	control	0.150558835	0.202764917	0.74252902	0.457766876	0.619138253	up_in_case
Yang2025_S	ENSGALG00010012340	ENHO	case	control	0.668914901	0.278785887	2.399385806	0.016422601	0.058784729	up_in_case
Yang2025_S	ENSGALG00010012341	GOPC	case	control	-0.1954175	0.09368914	-2.08580739	0.036996074	0.106980761	down_in_case
Yang2025_S	ENSGALG00010012342	ENSGALG00010012342	case	control	-3.596230127	2.032221559	-1.769605342			down_in_case
Yang2025_S	ENSGALG00010012343	BCL2L13	case	control	-0.594884306	0.104184166	-5.709930109	1.13023E-08	5.16383E-07	down_in_case
Yang2025_S	ENSGALG00010012346	NLN	case	control	-0.12013574	0.119657055	-1.004000481	0.315378382	0.484623994	down_in_case
Yang2025_S	ENSGALG00010012347	ENSGALG00010012347	case	control	1.460939712	0.374048326	3.905751234	9.39331E-05	0.001014696	up_in_case
Yang2025_S	ENSGALG00010012348	ENSGALG00010012348	case	control	0.356253087	0.874364674	0.407442224	0.68368322	0.801946606	up_in_case
Yang2025_S	ENSGALG00010012350	H2AC32	case	control	0.076182408	0.268374157	0.28386641	0.776512759	0.863997549	up_in_case
Yang2025_S	ENSGALG00010012352	PTPRC	case	control	-0.240877445	0.160506702	-1.500731385	0.133425052	0.269125824	down_in_case
Yang2025_S	ENSGALG00010012353	PIGG	case	control	-0.109192204	0.174779378	-0.62474306	0.532139707	0.684607387	down_in_case
Yang2025_S	ENSGALG00010012354	DCBLD1	case	control	0.038503862	0.124921984	0.308223266	0.75791245	0.85295517	up_in_case
Yang2025_S	ENSGALG00010012358	STK10	case	control	-0.003452055	0.122874178	-0.028094225	0.977587	0.986721342	down_in_case
Yang2025_S	ENSGALG00010012360	ROS1	case	control	0.151066628	0.260668718	0.579534932	0.562228284	0.708639198	up_in_case
Yang2025_S	ENSGALG00010012361	WIF1	case	control	-0.020543461	0.489390981	-0.041977604	0.966516551	0.980027659	down_in_case
Yang2025_S	ENSGALG00010012362	COL6A3	case	control	0.251834932	0.226316449	1.112755756	0.265813342	0.433026528	up_in_case
Yang2025_S	ENSGALG00010012366	INTS8	case	control	-0.096519684	0.092214219	-1.046689827	0.295242657	0.463781583	down_in_case
Yang2025_S	ENSGALG00010012367	RAD52	case	control	0.031552098	0.14434596	0.218586639	0.826972061	0.896430655	up_in_case
Yang2025_S	ENSGALG00010012372	LEMD3	case	control	0.116278214	0.081902332	1.419717996	0.155689797	0.300489933	up_in_case
Yang2025_S	ENSGALG00010012376	VGLL2	case	control	0.363956135	0.223321327	1.629741954	0.103156046	0.224430836	up_in_case
Yang2025_S	ENSGALG00010012378	RPS19BP1	case	control	-0.179100614	0.203512037	-0.880049242	0.378832634	0.546880012	down_in_case
Yang2025_S	ENSGALG00010012381	IFNA3	case	control	3.846694316	0.787015332	4.887699338	1.02021E-06	2.45963E-05	up_in_case
Yang2025_S	ENSGALG00010012382	SH3PXD2B	case	control	-0.044499011	0.11469584	-0.387974063	0.698035228	0.813236427	down_in_case
Yang2025_S	ENSGALG00010012384	NRK	case	control	-0.011161467	0.151736572	-0.073558186	0.941361944	0.963855699	down_in_case
Yang2025_S	ENSGALG00010012387	ERBIN	case	control	0.176480341	0.155274538	1.136569742	0.255718195	0.422007703	up_in_case
Yang2025_S	ENSGALG00010012390	MMP16	case	control	-0.236346607	0.169134792	-1.397386101	0.162297496	0.308500288	down_in_case
Yang2025_S	ENSGALG00010012391	NEURL1B	case	control	-0.078835745	0.111990971	-0.703947333	0.481465573	0.639826699	down_in_case
Yang2025_S	ENSGALG00010012393	ENSGALG00010012393	case	control	-7.282957582	1.699039352	-4.286514949			down_in_case
Yang2025_S	ENSGALG00010012398	ENSGALG00010012398	case	control	1.860000539	0.578408224	3.215722843	0.001301164	0.008614782	up_in_case
Yang2025_S	ENSGALG00010012399	ERC1	case	control	0.317496292	0.205277698	1.546667246	0.121943505	0.252681449	up_in_case
Yang2025_S	ENSGALG00010012401	RIPK2	case	control	-0.177551711	0.102128881	-1.738506374	0.082121628	0.190740921	down_in_case
Yang2025_S	ENSGALG00010012404	VAMP7	case	control	-0.208648221	0.065286341	-3.19589392	0.001393984	0.009115514	down_in_case
Yang2025_S	ENSGALG00010012405	MSRB3	case	control	0.038372312	0.093861493	0.408818469	0.682672885	0.801436112	up_in_case
Yang2025_S	ENSGALG00010012406	ENSGALG00010012406	case	control	0.562162497	0.230804387	2.435666431	0.014864385	0.054581081	up_in_case
Yang2025_S	ENSGALG00010012407	ATF4	case	control	-0.388770967	0.114182798	-3.404812066	0.000662096	0.005022487	down_in_case
Yang2025_S	ENSGALG00010012410	CACNA1I	case	control	0.270500456	0.399587546	0.676949167	0.498438208	0.655443656	up_in_case
Yang2025_S	ENSGALG00010012412	PURA	case	control	-0.041172513	0.178865567	-0.230186917	0.817946527	0.891343405	down_in_case
Yang2025_S	ENSGALG00010012413	NEK7	case	control	0.238089471	0.146748831	1.622428397	0.104711653	0.226785444	up_in_case
Yang2025_S	ENSGALG00010012414	ENSGALG00010012414	case	control	0.999535235	0.245433344	4.072532357	4.65047E-05	0.000574507	up_in_case
Yang2025_S	ENSGALG00010012415	BID	case	control	-0.468315064	0.090933738	-5.150069407	2.6039E-07	7.54373E-06	down_in_case
Yang2025_S	ENSGALG00010012416	ENSGALG00010012416	case	control	1.891312853	0.705106681	2.682307377	0.007311624	0.031929642	up_in_case
Yang2025_S	ENSGALG00010012418	ENSGALG00010012418	case	control	1.788131138	0.436185853	4.099470726	4.14096E-05	0.000528912	up_in_case
Yang2025_S	ENSGALG00010012419	HACE1	case	control	0.225346769	0.091047727	2.475040019	0.013322125	0.050133353	up_in_case

Yang2025_S	ENSGALG00010012422	ENSGALG00010012422	case	control	0.989454019	0.734664668	1.346810405	0.178041303	0.329404418	up_in_case
Yang2025_S	ENSGALG00010012426	MIEF1	case	control	-0.478058065	0.100342619	-4.764257388	1.8955E-06	4.15312E-05	down_in_case
Yang2025_S	ENSGALG00010012427	C7	case	control	-0.092340454	0.248928252	-0.370952084	0.710673221	0.821299662	down_in_case
Yang2025_S	ENSGALG00010012428	CCNE2	case	control	-0.191686145	0.166290825	-1.152716306	0.249026846	0.414727702	down_in_case
Yang2025_S	ENSGALG00010012429	CXCR7	case	control	-0.180559688	0.162553435	-1.110771281	0.266666811	0.433808313	down_in_case
Yang2025_S	ENSGALG00010012431	LAMP2	case	control	-0.14812037	0.134566431	-1.10072303	0.27101722	0.438276077	down_in_case
Yang2025_S	ENSGALG00010012433	ENSGALG00010012433	case	control	-0.071517037	0.865752742	-0.082606769	0.934164219	0.959922758	down_in_case
Yang2025_S	ENSGALG00010012436	ENSGALG00010012436	case	control	-0.506874089	0.225313299	-2.249641236	0.024471729	0.078828834	down_in_case
Yang2025_S	ENSGALG00010012437	ENSGALG00010012437	case	control	-12.05578815	2.949136356	-4.087904623			down_in_case
Yang2025_S	ENSGALG00010012439	TENT2	case	control	0.596057133	0.158345435	3.764283653	0.000167027	0.001612976	up_in_case
Yang2025_S	ENSGALG00010012441	FST	case	control	-0.747330041	0.117852549	-6.341229316	2.27939E-10	1.65434E-08	down_in_case
Yang2025_S	ENSGALG00010012442	CPLX1	case	control	1.269770378	0.828967448	1.531749385			up_in_case
Yang2025_S	ENSGALG00010012445	SOBP	case	control	0.473011641	0.271156468	1.744423224	0.081085318	0.189117286	up_in_case
Yang2025_S	ENSGALG00010012447	COPS8	case	control	-0.551900051	0.169868865	-3.248977089	0.001158208	0.007857658	down_in_case
Yang2025_S	ENSGALG00010012449	SUB1	case	control	-0.303436754	0.083454635	-3.635948479	0.00027696	0.002435028	down_in_case
Yang2025_S	ENSGALG00010012450	PPFIA2	case	control	0.688934012	0.256087826	2.690225552	0.007140374	0.03142802	up_in_case
Yang2025_S	ENSGALG00010012454	IQCA1	case	control	0.470159018	0.283502915	1.65839218	0.097238334	0.214715409	up_in_case
Yang2025_S	ENSGALG00010012456	ENSGALG00010012456	case	control	-0.097520861	0.091404774	-1.066912122	0.286011521	0.454533956	down_in_case
Yang2025_S	ENSGALG00010012458	MRPS30	case	control	0.119513027	0.217307066	0.549973035	0.582337869	0.724801554	up_in_case
Yang2025_S	ENSGALG00010012463	ENSGALG00010012463	case	control	0.651581337	0.766668929	0.849886193	0.395388362	0.562619915	up_in_case
Yang2025_S	ENSGALG00010012464	ENSGALG00010012464	case	control	-0.566900103	0.554642285	-1.022100402	0.306733383	0.476368145	down_in_case
Yang2025_S	ENSGALG00010012466	PDSS2	case	control	-0.143529889	0.08035708	-1.786151117	0.074074794	0.177116034	down_in_case
Yang2025_S	ENSGALG00010012469	CETN2	case	control	-0.276265973	0.182836419	-1.511000787	0.13078825	0.264980266	down_in_case
Yang2025_S	ENSGALG00010012470	CHD1	case	control	0.666715607	0.230387055	2.893893522	0.003804972	0.019472009	up_in_case
Yang2025_S	ENSGALG00010012474	ASB18	case	control	0.249922793	0.599908597	0.416601452	0.676969947	0.796968504	up_in_case
Yang2025_S	ENSGALG00010012477	ENSGALG00010012477	case	control	-0.674239407	0.219967408	-3.065178665	0.0021754	0.012747771	down_in_case
Yang2025_S	ENSGALG00010012483	ENSGALG00010012483	case	control	0.168805688	0.269310667	0.626806543	0.530786054	0.68321667	up_in_case
Yang2025_S	ENSGALG00010012486	RADX	case	control	0.03416509	0.090012244	0.379560472	0.704271707	0.81756109	up_in_case
Yang2025_S	ENSGALG00010012497	PLCXD3	case	control	0.049845717	0.440254114	0.113220333	0.909855876	0.946252827	up_in_case
Yang2025_S	ENSGALG00010012498	KCNT2	case	control	-0.037232775	0.275633393	-0.135080786	0.892548002	0.93692327	down_in_case
Yang2025_S	ENSGALG00010012506	TP53INP1	case	control	-0.696462303	0.092012644	-7.569202133	3.75523E-14	6.796E-12	down_in_case
Yang2025_S	ENSGALG00010012508	MDM1	case	control	-0.230476602	0.107570306	-2.142567128	0.032147873	0.096446951	down_in_case
Yang2025_S	ENSGALG00010012509	MICAL3	case	control	0.25252558	0.190549324	1.325250462	0.185088093	0.338655801	up_in_case
Yang2025_S	ENSGALG00010012511	OXCT1	case	control	-0.502182269	0.138029787	-3.638216642	0.000274532	0.002419741	down_in_case
Yang2025_S	ENSGALG00010012517	MGAT3	case	control	-0.766687619	0.169822447	-4.514642389	6.34237E-06	0.000113893	down_in_case
Yang2025_S	ENSGALG00010012519	ENSGALG00010012519	case	control	-10.56892918	2.113873103	-4.999793586			down_in_case
Yang2025_S	ENSGALG00010012521	HOMER1	case	control	0.460718093	0.217076069	2.122380855	0.033805769	0.10008044	up_in_case
Yang2025_S	ENSGALG00010012529	BEND3	case	control	-0.071337474	0.089234599	-0.799437381	0.424036842	0.589058204	down_in_case
Yang2025_S	ENSGALG00010012532	FAM174A	case	control	0.785799927	0.230376667	3.410935401	0.000647404	0.00493252	up_in_case
Yang2025_S	ENSGALG00010012535	JMY	case	control	0.233988697	0.1477667	1.583500865	0.11330735	0.239705164	up_in_case
Yang2025_S	ENSGALG00010012536	FBXO4	case	control	0.15663232	0.165073874	0.948861959	0.342690825	0.511120266	up_in_case
Yang2025_S	ENSGALG00010012538	RAP1B	case	control	-0.442882878	0.099588839	-4.447113573	8.70318E-06	0.000145768	down_in_case
Yang2025_S	ENSGALG00010012547	ENSGALG00010012547	case	control	0.742419025	0.375974245	1.974653942	0.048307433	0.130130307	up_in_case
Yang2025_S	ENSGALG00010012550	TAB1	case	control	-0.191042592	0.106921608	-1.786753833	0.073977284	0.176973984	down_in_case
Yang2025_S	ENSGALG00010012551	PCGF3	case	control	0.588458312	0.189608735	3.103540102	0.001912203	0.011585457	up_in_case
Yang2025_S	ENSGALG00010012553	ENSGALG00010012553	case	control	-0.640419951	0.358875967	-1.784516127	0.074339837	0.17762785	down_in_case
Yang2025_S	ENSGALG00010012555	ENSGALG00010012555	case	control	0.216012553	0.16172301	1.335694613	0.181649111	0.334206543	up_in_case
Yang2025_S	ENSGALG00010012561	OSGIN2	case	control	0.023275184	0.115362615	0.201756732	0.840106909	0.905085473	up_in_case
Yang2025_S	ENSGALG00010012565	SYNGR1	case	control	-0.059551151	0.053853521	-1.10579866	0.268813675	0.436028234	down_in_case
Yang2025_S	ENSGALG00010012569	C6orf203	case	control	0.10832625	0.123889462	0.874378244	0.381912302	0.549788009	up_in_case
Yang2025_S	ENSGALG00010012571	AGAP1	case	control	0.018353099	0.1829135	0.100337585	0.92007632	0.952943376	up_in_case
Yang2025_S	ENSGALG00010012574	PHAX	case	control	0.05592354	0.176192917	0.317399479	0.750940502	0.848540188	up_in_case
Yang2025_S	ENSGALG00010012578	NDUFS4	case	control	0.186886035	0.274716301	0.680287388	0.496322509	0.653401432	up_in_case
Yang2025_S	ENSGALG00010012582	CD24	case	control	0.018694114	0.252089836	0.074156555	0.940885815	0.963707742	up_in_case
Yang2025_S	ENSGALG00010012583	NDUFAF6	case	control	0.233496119	0.166772132	1.400090747	0.161486146	0.307377331	up_in_case
Yang2025_S	ENSGALG00010012586	CDC73	case	control	-0.067699189	0.104591705	-0.647271113	0.517456494	0.671957021	down_in_case
Yang2025_S	ENSGALG00010012587	MBLAC2	case	control	-0.560179438	0.198441951	-2.822888187	0.004759317	0.023116444	down_in_case
Yang2025_S	ENSGALG00010012588	PEX26	case	control	-0.381279193	0.117436793	-3.24667578	0.001167613	0.007902229	down_in_case
Yang2025_S	ENSGALG00010012599	DMGDH	case	control	0.08949402	0.185012657	0.483718365	0.628585758	0.761322631	up_in_case
Yang2025_S	ENSGALG00010012600	NUP107	case	control	0.275526407	0.101535011	2.713609855	0.00665545	0.029744609	up_in_case
Yang2025_S	ENSGALG00010012601	TUBA8A	case	control	0.201307061	0.235323035	0.855449873	0.392302144	0.55948525	up_in_case
Yang2025_S	ENSGALG00010012604	ENSGALG00010012604	case	control	-10.1163589	2.4665408	-4.101435864	4.10594E-05	0.000525402	down_in_case
Yang2025_S	ENSGALG00010012606	ATP5PO	case	control	-0.27234135	0.225842287	-1.205891744	0.227859234	0.390075974	down_in_case
Yang2025_S	ENSGALG00010012607	LMNB1	case	control	-0.000557931	0.141978686	-0.003929679	0.996864578	0.997723759	down_in_case
Yang2025_S	ENSGALG00010012610	RPL3	case	control	-0.598458421	0.243463417	-2.458104083	0.013967269	0.051957792	down_in_case
Yang2025_S	ENSGALG00010012614	LHFPL2	case	control	0.038633953	0.11627438	0.332265395	0.739688868	0.840645875	up_in_case
Yang2025_S	ENSGALG00010012618	B3GALT2	case	control	-0.641500798	0.288938879	-2.220195498	0.0264055	0.083309136	down_in_case
Yang2025_S	ENSGALG00010012624	PDGFB	case	control	0.171315542	0.115466616	1.483680284	0.137893759	0.275649051	up_in_case
Yang2025_S	ENSGALG00010012629	NUS1	case	control	0.05635688	0.21116317	0.266887828	0.789555529	0.87314152	up_in_case
Yang2025_S	ENSGALG00010012631	GLRX2	case	control	-0.67653326	0.173783027	-3.892976616	9.90217E-05	0.001058558	down_in_case
Yang2025_S	ENSGALG00010012633	USP18	case	control	-0.006556807	0.200604829	-0.032685188	0.973925636	0.984417469	down_in_case
Yang2025_S	ENSGALG00010012640	PRRC1	case	control	-0.038898722	0.080356038	-0.484079643	0.628329348	0.761238868	down_in_case
Yang2025_S	ENSGALG00010012648	MRPS6	case	control	-0.682032568	0.106222869	-6.420769582	1.35587E-10	1.06745E-08	down_in_case
Yang2025_S	ENSGALG00010012650	DSE	case	control	-0.33749316	0.116265192	-2.902787611	0.003698574	0.019063006	down_in_case
Yang2025_S	ENSGALG00010012653	CBX7	case	control	-0.108213269	0.221300016	-0.488988982	0.624849497	0.75873804	down_in_case
Yang2025_S	ENSGALG00010012654	TENT5B	case	control	0.30423072	0.140239285	2.169368733	0.030054701	0.091744196	up_in_case
Yang2025_S	ENSGALG00010012662	SLC35E3	case	control	-0.150793018	0.115602735	-1.304407011	0.192094846	0.347306047	down_in_case
Yang2025_S	ENSGALG00010012667	SCAMP1	case	control	-0.566326473	0.080214237	-7.060173994	1.66294E-12	2.16571E-10	down_in_case
Yang2025_S	ENSGALG00010012669	ERCC8	case	control	0.322807851	0.189817077	1.700625969	0.089013245	0.202420299	up_in_case
Yang2025_S	ENSGALG00010012670	ENSGALG00010012670	case	control	1.522923599	0.401636264	3.791798044	0.000149561	0.001476795	up_in_case
Yang2025_S	ENSGALG00010012671	NBN	case	control	0.600216597	0.205070882	2.926873828	0.003423878	0.017963755	up_in_case
Yang2025_S	ENSGALG00010012672	RO60	case	control	-0.299960156	0.128683961	-2.330983244	0.019754243	0.067222315	down_in_case
Yang2025_S	ENSGALG00010012673	ENSGALG00010012673	case	control	0.308094199	0.333944122	0.92259207	0.35621983	0.525672279	up_in_case
Yang2025_S	ENSGALG00010012674	ENSGALG00010012674	case	control	0.775694053	0.176975253	4.383065097	1.17021E-05	0.000188301	up_in_case
Yang2025_S	ENSGALG00010012676	ENSGALG00010012676	case	control	0.338283087	0.242527607	1.394823008	0.163069219	0.309629318	up_in_case
Yang2025_S	ENSGALG00010012680	TRPM8	case	control	-0.036226075	0.258466639	-0.140157642	0.888535438	0.93504549	down_in_case
Yang2025_S	ENSGALG00010012682	ENSGALG00010012682	case	control	-0.214725786	0.189417158	-1.133613176	0.256956844	0.423356841	down_in_case
Yang2025_S	ENSGALG00010012683	MDM2	case	control	-0.246142309	0.1061609	-2.318577837	0.020417938	0.068792061	down_in_case
Yang2025_S	ENSGALG00010012685	NPTXR	case	control	0.248835642	0.20305736	1.225445077	0.220407572	0.381727506	up_in_case
Yang2025_S	ENSGALG00010012688	NDUFAF2	case	control	0.183287189	0.189914046	0.965106017	0.334491713	0.503200046	up_in_case
Yang2025_S	ENSGALG00010012690	NT5DC1	case	control	-0.532625393	0.202719478	-2.627401166	0.008603983	0.036288289	down_in_case
Yang2025_S	ENSGALG00010012693	UCHL5	case	control	-0.188613793	0.117348916	-1.607290462	0.107990666	0.231214263	down_in_case
Yang2025_S	ENSGALG00010012696	DNAL4	case	control	-0.072346796	0.146273842	-0.494598321	0.620883688	0.755766438	down_in_case
Yang2025_S	ENSGALG00010012699	RNF128	case	control	-0.17285577	0.149483596	-1.156352773	0.247536891	0.413203949	down_in_case
Yang2025_S	ENSGALG00010012704	SMIM15	case	control	-0.285375126	0.131287114	-2.173672023	0.029729775	0.090881504	down_in_case
Yang2025_S	ENSGALG00010012705	CPM	case	control	0.101220065	0.111450118	0.908209585	0.363767501	0.532636348	up_in_case
Yang2025_S	ENSGALG00010012706	TBC1D8B	case	control	-0.286091192	0.128070732	-2.233853024	0.025492745	0.08104983	down_in_case
Yang2025_S	ENSGALG00010012708	SPP2	case	control	-0.906093414	0.566394874	-1.599755673	0.109652796	0.233676665	down_in_case
Yang2025_S	ENSGALG00010012709	PPP1R8	case	control	0.11397097	0.143076421	0.796574093	0.425698428	0.590379098	up_in_case
Yang2025_S	ENSGALG00010012711	ELL2	case	control	0.634106789	0.286829944	2.210741254	0.02705376	0.084794005	up_in_case
Yang2025_S	ENSGALG00010012712	EEF1E1	case	control	-0.43775178	0.21487669	-2.037221315	0.041627869	0.116506196	down_in_case
Yang2025_S	ENSGALG00010012714	RGS2	case	control	-0.100062146	0.129539327	-0.772446087	0.439850267	0.603302507	down_in_case
Yang2025_S	ENSGALG00010012719	COL10A1	case	control	0.785268482	0.938960036	0.836317257	0.402976447	0.569303519	up_in_case
Yang2025_S	ENSGALG00010012720	ACO2	case	control	-0.304903541	0.147312354	-2.069775777	0.038473347	0.110028015	down_in_case
Yang2025_S	ENSGALG00010012722	ENSGALG00010012722	case	control	0.51331697	0.148726541	3.451414688	0.000557656	0.004326802	up_in_case
Yang2025_S	ENSGALG00010012725	ENSGALG00010012725	case	control	1.061779316	0.278625715	3.810772871	0.000138533	0.001388818	up_in_case
Yang2025_S	ENSGALG00010012726	IL7R	case	control	-0.536837177	0.609672199	-0.880534127	0.378570026	0.546557533	down_in_case
Yang2025_S	ENSGALG00010012730	CAP2	case	control	0.202882356	0.216126109	0.938722106	0.34787344	0.516385585	up_in_case
Yang2025_S	ENSGALG00010012732	MGST1	case	control	0.046418133	0.178984705	0.25934134	0.795371885	0.876525276	up_in_case
Yang2025_S	ENSGALG00010012734	TMEM19	case	control	-0.415635248	0.172914809	-2.403699546	0.016230106	0.058221897	down_in_case
Yang2025_S	ENSGALG00010012738	RAB21	case	control	0.118098916	0.144973647	0.814623336	0.415287949	0.580852912	up_in_case
Yang2025_S	ENSGALG00010012741	ENSGALG00010012741	case	control	0.203306223	0.288513818	0.704667198	0.481017369	0.639475009	up_in_case
Yang2025_S	ENSGALG00010012742	ENSGALG00010012742	case	control	-0.196143181	0.150859147	-1.30017427	0.193541247	0.349035496	down_in_case
Yang2025_S	ENSGALG00010012743	ENSGALG00010012743	case	control	1.113103021	0.318322505	3.496777651	0.000470914	0.003764169	up_in_case
Yang2025_S	ENSGALG00010012744	ARL4C	case	control	-0.392710007	0.147060132	-2.670404287	0.007575997	0.032826963	down_in_case
Yang2025_S	ENSGALG00010012745	MARCHF3	case	control	-0.081001232	0.450163244	-0.179937463	0.857201664	0.915649212	down_in_case
Yang2025_S	ENSGALG00010012747	TOB2	case	control	-0.094273195	0.107310784	-0.878506261	0.379669042	0.547738649	down_in_case
Yang2025_S	ENSGALG00010012748	ENSGALG00010012748	case	control	0.874242598	0.350736659	2.492589745	0.012681528	0.04830163	up_in_case
Yang2025_S	ENSGALG00010012749	RGS4	case	control	-0.394123412	0.172992579	-2.278267743	0.02271063	0.074341704	down_in_case
Yang2025_S	ENSGALG00010012751	AP3B1	case	control	0.138754462	0.135756689	1.022081951	0.306742115	0.476368145	up_in_case
Yang2025_S	ENSGALG00010012753	GTPBP1	case	control	-0.225208322	0.066870875	-3.367808824	0.000757681	0.005600398	down_in_case
Yang2025_S	ENSGALG00010012755	CLVS2	case	control	-0.019569834	0.500770357	-0.039079459	0.968827038	0.981620757	down_in_case
Yang2025_S	ENSGALG00010012756	RPA2	case	control	-0.301529532	0.224084538	-1.345606147	0.178429568	0.329938434	down_in_case
Yang2025_S	ENSGALG00010012758	NAB1	case	control	-0.225233894	0.072764333	-3.09538868	0.001965551	0.011826037	down_in_case
Yang2025_S	ENSGALG00010012759	ATP1B4	case	control	0.241201052	0.108832107	2.216267412	0.026673193	0.083853276	up_in_case
Yang2025_S	ENSGALG00010012760	PLEKHF2	case	control	-0.353505656	0.085770282	-4.121540096	3.76348E-05	0.000488306	down_in_case

Yang2025_S	ENSGALG00010012761	SMPDL3A	case	control	-0.415647567	0.188999557	-2.199198626	0.027863802	0.086592198	down_in_case
Yang2025_S	ENSGALG00010012763	SMPDL3B	case	control	-0.383731935	0.155751344	-2.463747179	0.013749306	0.051352606	down_in_case
Yang2025_S	ENSGALG00010012765	PHF5A	case	control	-0.123604362	0.161730366	-0.764261934	0.444711165	0.608030816	down_in_case
Yang2025_S	ENSGALG00010012767	JOSD1	case	control	-0.219809428	0.166109097	-1.323283504	0.185741115	0.339271522	down_in_case
Yang2025_S	ENSGALG00010012768	EPS8	case	control	-0.290148578	0.121109755	-2.395749021	0.016586443	0.059188754	down_in_case
Yang2025_S	ENSGALG00010012769	NEMP2	case	control	-0.203359303	0.154156612	-1.319173408	0.187111146	0.341147955	down_in_case
Yang2025_S	ENSGALG00010012771	ITGA2	case	control	0.407634892	0.226110488	1.802812841	0.071417613	0.172394495	up_in_case
Yang2025_S	ENSGALG00010012772	TOMM22	case	control	-0.052767938	0.215587765	-0.244763137	0.806639838	0.883622621	down_in_case
Yang2025_S	ENSGALG00010012773	XKR8	case	control	-0.22403416	0.287342506	-0.779676364	0.435581395	0.599429857	down_in_case
Yang2025_S	ENSGALG00010012775	KCNE2	case	control	0.199142874	0.136123915	1.462952885	0.143480271	0.283824831	up_in_case
Yang2025_S	ENSGALG00010012778	TBC1D15	case	control	-0.063020422	0.092137194	-0.683984597	0.493984892	0.651431766	down_in_case
Yang2025_S	ENSGALG00010012780	FABP7	case	control	1.523102888	0.327821605	4.646133344	3.38214E-06	6.73288E-05	up_in_case
Yang2025_S	ENSGALG00010012781	SPIN1L	case	control	-12.97387336	2.948983729	-4.399438774			down_in_case
Yang2025_S	ENSGALG00010012786	TEF	case	control	0.014263432	0.11878711	0.120075585	0.904423273	0.942925206	up_in_case
Yang2025_S	ENSGALG00010012790	PITX1	case	control	1.582203933	0.458216455	3.452961836	0.000554467	0.004308708	up_in_case
Yang2025_S	ENSGALG00010012791	CFAP418	case	control	-0.00504776	0.185298177	-0.027241282	0.97826729	0.9869076	down_in_case
Yang2025_S	ENSGALG00010012792	CBY1	case	control	0.011755195	0.251612536	0.046719432	0.962736843	0.977894738	up_in_case
Yang2025_S	ENSGALG00010012795	TBCA	case	control	0.028519979	0.213563814	0.133543124	0.893763861	0.937584125	up_in_case
Yang2025_S	ENSGALG00010012796	MOCS2	case	control	0.288098591	0.173726263	1.658347944	0.097247257	0.214715409	up_in_case
Yang2025_S	ENSGALG00010012797	ZC3H7B	case	control	-0.366525358	0.126996938	-2.886096035	0.003900531	0.019840098	down_in_case
Yang2025_S	ENSGALG00010012798	MFSD6	case	control	-0.03325458	0.149608935	-0.222276698	0.824098491	0.894517251	down_in_case
Yang2025_S	ENSGALG00010012801	DMC1	case	control	-0.07327794	0.328423223	-0.223120458	0.823441757	0.894361721	down_in_case
Yang2025_S	ENSGALG00010012803	NUP155	case	control	-0.107075405	0.133324318	-0.803119842	0.42190547	0.587690196	down_in_case
Yang2025_S	ENSGALG00010012804	ENSGALG00010012804	case	control	0.681057397	0.190676263	3.571799592	0.000354537	0.002997857	up_in_case
Yang2025_S	ENSGALG00010012808	EYA3	case	control	0.218902942	0.20304941	1.078077215	0.280999299	0.448948089	up_in_case
Yang2025_S	ENSGALG00010012809	RHOBTB3	case	control	0.045470418	0.214258988	0.212221755	0.831934035	0.900007824	up_in_case
Yang2025_S	ENSGALG00010012812	DECR1	case	control	0.008000171	0.139176621	0.057482149	0.954161126	0.971804947	up_in_case
Yang2025_S	ENSGALG00010012818	RGS18	case	control	-0.548762328	0.149385583	-3.67346244	0.000239286	0.002163821	down_in_case
Yang2025_S	ENSGALG00010012819	RANGAP1	case	control	0.058936333	0.104362712	0.564725963	0.572260172	0.715833643	up_in_case
Yang2025_S	ENSGALG00010012821	ZBTB33	case	control	-0.122023693	0.11387417	-1.071566031	0.283914997	0.452051587	down_in_case
Yang2025_S	ENSGALG00010012824	HIBCH	case	control	-0.067106407	0.121786713	-0.551015831	0.581622822	0.724170302	down_in_case
Yang2025_S	ENSGALG00010012827	ENSGALG00010012827	case	control	-0.65584774	0.569002146	-1.152627885	0.249063153	0.414727702	down_in_case
Yang2025_S	ENSGALG00010012829	RGS5	case	control	-0.203598456	0.184317879	-1.104605031	0.26933077	0.4364098	down_in_case
Yang2025_S	ENSGALG00010012830	ARRDC3	case	control	0.446695871	0.148493911	3.008176347	0.002628206	0.014702548	up_in_case
Yang2025_S	ENSGALG00010012831	ENSGALG00010012831	case	control	0.028030826	0.299042259	0.093735334	0.925319401	0.955206005	up_in_case
Yang2025_S	ENSGALG00010012832	CHADL	case	control	2.285393911	0.549232953	4.161064804	3.16767E-05	0.000426076	up_in_case
Yang2025_S	ENSGALG00010012833	JAK2	case	control	-0.184549471	0.151556056	-1.217697765	0.223338847	0.384728306	down_in_case
Yang2025_S	ENSGALG00010012836	ENSGALG00010012836	case	control	-0.851887524	0.50889722	-1.673987382	0.094133079	0.210013522	down_in_case
Yang2025_S	ENSGALG00010012837	PKIB	case	control	-0.821305845	0.332410599	-2.470757094	0.013482737	0.050569864	down_in_case
Yang2025_S	ENSGALG00010012838	C2orf88	case	control	1.06392653	0.17255098	6.165867789	7.00976E-10	4.37814E-08	up_in_case
Yang2025_S	ENSGALG00010012839	TGFBI	case	control	0.015372005	0.188038159	0.081749393	0.934846	0.960081549	up_in_case
Yang2025_S	ENSGALG00010012841	L3MBTL2	case	control	-0.137844064	0.153529665	-0.89783342	0.369274364	0.537929988	down_in_case
Yang2025_S	ENSGALG00010012842	DDX17	case	control	0.164843948	0.091963641	1.792490438	0.073054439	0.17530801	up_in_case
Yang2025_S	ENSGALG00010012843	ENSGALG00010012843	case	control	0.766969935	0.219285213	3.497590755	0.000469481	0.003761077	up_in_case
Yang2025_S	ENSGALG00010012845	HDHD2	case	control	0.023618533	0.183029821	0.129041994	0.89732442	0.93902049	up_in_case
Yang2025_S	ENSGALG00010012848	SERINC1	case	control	-0.089532569	0.069633624	-1.285766316	0.198524629	0.355400913	down_in_case
Yang2025_S	ENSGALG00010012849	TRHDE	case	control	0.213210815	0.342755315	0.622049625	0.533909238	0.685508673	up_in_case
Yang2025_S	ENSGALG00010012850	CALB1	case	control	-0.088405322	0.63359781	-0.139529085	0.889032075	0.935275703	down_in_case
Yang2025_S	ENSGALG00010012851	ENSGALG00010012851	case	control	0.313392553	0.29850493	1.04987396	0.293776065	0.462363843	up_in_case
Yang2025_S	ENSGALG00010012852	MSTN	case	control	-0.380268454	0.195102425	-1.949070873	0.051286962	0.135690869	down_in_case
Yang2025_S	ENSGALG00010012854	LECT2	case	control	0.269224925	0.383157846	0.702647558	0.482275416	0.640547216	up_in_case
Yang2025_S	ENSGALG00010012857	NSDHL	case	control	0.173687099	0.260559586	0.666592627	0.50503238	0.6607165	up_in_case
Yang2025_S	ENSGALG00010012859	FBXL21	case	control	-0.356961034	0.113142857	-3.154958635	0.001605209	0.010135289	down_in_case
Yang2025_S	ENSGALG00010012860	ENSGALG00010012860	case	control	0.09479136	0.493219199	0.192189111	0.847594075	0.909738128	up_in_case
Yang2025_S	ENSGALG00010012861	WDR70	case	control	-0.132846044	0.17965067	-0.73946868	0.459622451	0.620804387	down_in_case
Yang2025_S	ENSGALG00010012863	KCNE1	case	control	-0.796257924	0.389801205	-2.042728225	0.041079349	0.115411438	down_in_case
Yang2025_S	ENSGALG00010012865	EP300	case	control	0.580688754	0.240311029	2.416404929	0.015674619	0.056866914	up_in_case
Yang2025_S	ENSGALG00010012867	A4GALT	case	control	-0.073588896	0.087647823	-0.83959753	0.401134085	0.56776986	down_in_case
Yang2025_S	ENSGALG00010012868	CTHRC1	case	control	1.044374636	0.251918232	4.145688963	3.38793E-05	0.000447073	up_in_case
Yang2025_S	ENSGALG00010012869	ENSGALG00010012869	case	control	0.359518483	0.313875941	1.145415866	0.252036929	0.417812825	up_in_case
Yang2025_S	ENSGALG00010012870	KCNC2	case	control	0.309251364	0.165287354	1.870992289	0.06134615	0.154893748	up_in_case
Yang2025_S	ENSGALG00010012871	ENSGALG00010012871	case	control	-0.184226697	0.214778844	-0.857750672	0.391030153	0.558397065	down_in_case
Yang2025_S	ENSGALG00010012873	PTAFR	case	control	-0.928151451	0.158629389	-5.851068682	4.88424E-09	2.40639E-07	down_in_case
Yang2025_S	ENSGALG00010012875	HSF2	case	control	-0.215499697	0.078087731	-2.759712605	0.005785223	0.026694398	down_in_case
Yang2025_S	ENSGALG00010012876	KDELR3	case	control	-0.407324422	0.1332658	-3.056481281	0.002239515	0.013035771	down_in_case
Yang2025_S	ENSGALG00010012877	SLC25A32	case	control	-0.020833977	0.072943331	-0.285618669	0.775170203	0.863270828	down_in_case
Yang2025_S	ENSGALG00010012878	GDNF	case	control	0.462494697	0.214168737	2.159487441	0.030812371	0.093483646	up_in_case
Yang2025_S	ENSGALG00010012879	RCAN1	case	control	-0.30144402	0.139421208	-2.162110225	0.030609681	0.093051027	down_in_case
Yang2025_S	ENSGALG00010012880	CLK4	case	control	0.452307856	0.140077309	3.228987344	0.001242294	0.008290884	up_in_case
Yang2025_S	ENSGALG00010012882	ENSGALG00010012882	case	control	0.761031785	0.397858039	1.912822437	0.055770788	0.144669756	up_in_case
Yang2025_S	ENSGALG00010012883	DCAF13	case	control	-0.101249208	0.095531572	-1.059850748	0.289212505	0.458026624	down_in_case
Yang2025_S	ENSGALG00010012884	ENSGALG00010012884	case	control	-0.374217506	0.329679804	-1.135093811	0.256336014	0.422589461	down_in_case
Yang2025_S	ENSGALG00010012885	PMS1	case	control	-0.085259199	0.081221957	-1.049706285	0.293853173	0.462432972	down_in_case
Yang2025_S	ENSGALG00010012887	KCNJ4	case	control	0.316308555	0.390663292	0.809670532	0.418129559	0.583714225	up_in_case
Yang2025_S	ENSGALG00010012888	ENSGALG00010012888	case	control	-9.223443086	2.949219691	-3.127418115			down_in_case
Yang2025_S	ENSGALG00010012889	RIC1	case	control	-0.022058259	0.08804097	-0.250545383	0.802165613	0.880663886	down_in_case
Yang2025_S	ENSGALG00010012890	MFAP3	case	control	-0.370048234	0.067649634	-5.470069974	4.49858E-08	1.70811E-06	down_in_case
Yang2025_S	ENSGALG00010012891	CAPS2	case	control	0.014439312	0.474480823	0.030431814	0.975722673	0.985553454	up_in_case
Yang2025_S	ENSGALG00010012892	ORMDL1	case	control	-0.51270336	0.185104268	-2.769808416	0.005608927	0.026088252	down_in_case
Yang2025_S	ENSGALG00010012893	ENSGALG00010012893	case	control	-0.142076712	0.26322945	-0.539744744	0.589373078	0.730296447	down_in_case
Yang2025_S	ENSGALG00010012894	CLIC6	case	control	0.90014255	0.48240333	1.865954262	0.062047765	0.156184177	up_in_case
Yang2025_S	ENSGALG00010012896	STK38L	case	control	-0.208096987	0.079824974	-2.606915828	0.009136182	0.037981113	down_in_case
Yang2025_S	ENSGALG00010012897	ENSGALG00010012897	case	control	-0.515727887	0.535513653	-0.963052734	0.33552106	0.503994298	down_in_case
Yang2025_S	ENSGALG00010012898	FAM114A2	case	control	0.018734455	0.100170143	0.187026334	0.851639986	0.912050661	up_in_case
Yang2025_S	ENSGALG00010012899	ENSGALG00010012899	case	control	0.333580904	0.13796908	2.417794652	0.015614886	0.056694486	up_in_case
Yang2025_S	ENSGALG00010012900	RBX1	case	control	-0.630720187	0.258218615	-2.442582179	0.014582609	0.053730476	down_in_case
Yang2025_S	ENSGALG00010012903	RGMB	case	control	-0.605936513	0.134699476	-4.4984326	6.84563E-06	0.000120905	down_in_case
Yang2025_S	ENSGALG00010012904	ERMP1	case	control	-0.413136993	0.183962966	-2.24576176	0.024719272	0.079369367	down_in_case
Yang2025_S	ENSGALG00010012905	NKAIN1	case	control	-0.203027387	0.225887431	-0.898798952	0.368759758	0.537461273	down_in_case
Yang2025_S	ENSGALG00010012908	STRAP	case	control	0.027955574	0.088273645	0.316692188	0.751477177	0.848540188	up_in_case
Yang2025_S	ENSGALG00010012910	XPNPEP3	case	control	-0.352512393	0.088788466	-3.970249821	7.17973E-05	0.000816974	down_in_case
Yang2025_S	ENSGALG00010012911	CSNK1E	case	control	-0.086746559	0.121776581	-0.712341884	0.476253094	0.63550578	down_in_case
Yang2025_S	ENSGALG00010012912	NKAPL	case	control	1.065296106	0.211397227	5.039309748	4.67214E-07	1.26913E-05	up_in_case
Yang2025_S	ENSGALG00010012913	EGFLAM	case	control	-0.170147582	0.075295564	-2.259729162	0.023838065	0.077341895	down_in_case
Yang2025_S	ENSGALG00010012915	ENSGALG00010012915	case	control	-0.731243423	0.292430853	-2.500568651	0.01239941	0.047546994	down_in_case
Yang2025_S	ENSGALG00010012917	RUNX1	case	control	0.726314468	0.32404976	2.241367094	0.025002306	0.079918856	up_in_case
Yang2025_S	ENSGALG00010012918	GLRX	case	control	-0.566835625	0.444707845	-1.274624749	0.202442093	0.360376925	down_in_case
Yang2025_S	ENSGALG00010012921	EBF1	case	control	0.732975677	0.217420849	3.37122995	0.000748334	0.005546929	up_in_case
Yang2025_S	ENSGALG00010012922	SNRNP40	case	control	0.115009525	0.139321184	0.825499194	0.40908824	0.574651492	up_in_case
Yang2025_S	ENSGALG00010012923	MLANA	case	control	7.434355313	0.888718749	8.365250901	5.99871E-17	1.89982E-14	up_in_case
Yang2025_S	ENSGALG00010012924	GLIPR1	case	control	0.567939648	0.243954247	2.328058043	0.019909022	0.067552628	up_in_case
Yang2025_S	ENSGALG00010012925	SETD4	case	control	-0.177540718	0.175518274	-1.011522699	0.311766323	0.481428323	down_in_case
Yang2025_S	ENSGALG00010012926	OSGEPL1	case	control	0.645837749	0.21978674	2.938474581	0.003298317	0.017498948	up_in_case
Yang2025_S	ENSGALG00010012927	ENSGALG00010012927	case	control	-10.49862172	2.531554937	-4.147104043	3.36707E-05	0.000445585	down_in_case
Yang2025_S	ENSGALG00010012930	IL6ST	case	control	-0.360476822	0.096295322	-3.743451037	0.00018151	0.001730056	down_in_case
Yang2025_S	ENSGALG00010012932	ATF6	case	control	0.196148155	0.138744375	1.413737709	0.157438929	0.302733749	up_in_case
Yang2025_S	ENSGALG00010012933	ZCCHC17	case	control	0.540889192	0.151301993	3.574897988	0.000350364	0.002969786	up_in_case
Yang2025_S	ENSGALG00010012934	NDUFA1	case	control	-0.646107603	0.232151633	-2.783127536	0.005383764	0.02536266	down_in_case
Yang2025_S	ENSGALG00010012938	UPF3B	case	control	0.948819326	0.235618065	4.026937939	5.65079E-05	0.000674754	up_in_case
Yang2025_S	ENSGALG00010012939	GJA1	case	control	-0.256023226	0.124850819	-2.050633133	0.040302687	0.113911305	down_in_case
Yang2025_S	ENSGALG00010012940	FBN2	case	control	0.2540984	0.18826083	1.349714648	0.177107532	0.328190619	up_in_case
Yang2025_S	ENSGALG00010012943	ASNSD1	case	control	-0.465548111	0.13260154	-3.510880132	0.000446626	0.003612147	down_in_case
Yang2025_S	ENSGALG00010012944	NCK1	case	control	-0.233838673	0.09031168	-2.589240659	0.009618785	0.039457687	down_in_case
Yang2025_S	ENSGALG00010012945	CLINT1	case	control	0.026999935	0.085475562	0.315878997	0.752094354	0.84889315	up_in_case
Yang2025_S	ENSGALG00010012948	ST13P5	case	control	-0.310482409	0.07151602	-4.341438589	1.41553E-05	0.000220395	down_in_case
Yang2025_S	ENSGALG00010012949	ENSGALG00010012949	case	control	-0.787618498	0.194036445	-4.059126612	4.92566E-05	0.000602097	down_in_case
Yang2025_S	ENSGALG00010012952	BRD10	case	control	0.769232508	0.205510475	3.743033088	0.000181812	0.001731753	up_in_case
Yang2025_S	ENSGALG00010012954	RIMS2	case	control	0.542710511	0.484579874	1.119960898	0.262730425	0.429591972	up_in_case
Yang2025_S	ENSGALG00010012955	RPL39L	case	control	-0.769821385	0.234855299	-3.277854018	0.001045995	0.00726255	down_in_case
Yang2025_S	ENSGALG00010012956	FABP3	case	control	1.161666415	0.275485957	4.216789942	2.47805E-05	0.000347172	up_in_case
Yang2025_S	ENSGALG00010012957	DERA	case	control	-0.160052419	0.208054628	-0.769280744	0.441726666	0.605034384	down_in_case
Yang2025_S	ENSGALG00010012958	OLFML2B	case	control	-0.067511572	0.128905198	-0.523730406	0.600466064	0.739243272	down_in_case
Yang2025_S	ENSGALG00010012959	TBC1D32	case	control	-0.197882787	0.157777232	-1.254191017	0.209772584	0.369041909	down_in_case
Yang2025_S	ENSGALG00010012961	ANKRD55	case	control	1.35024392	0.462058256	2.922237407	0.003475265	0.018164975	up_in_case
Yang2025_S	ENSGALG00010012962	SLC25A17	case	control	-0.379639461	0.129151051	-2.939499573	0.003287427	0.017461665	down_in_case
Yang2025_S	ENSGALG00010012963	UIMC1	case	control	0.320281513	0.126460354	2.532663416	0.011319959	0.04434738	up_in_case

Yang2025_S	ENSGALG00010012964	CBR3	case	control	-0.458622493	0.159903495	-2.868120501	0.004129182	0.020638506	down_in_case
Yang2025_S	ENSGALG00010012965	ENSGALG00010012965	case	control	0.192717985	0.114757822	1.679345101	0.093084806	0.208408864	up_in_case
Yang2025_S	ENSGALG00010012966	GLIPR1L	case	control	-0.362111496	0.321475828	-1.126403494	0.259994769	0.426388973	down_in_case
Yang2025_S	ENSGALG00010012967	ECRG4	case	control	0.089337447	0.244746126	0.365020883	0.715095818	0.824104714	up_in_case
Yang2025_S	ENSGALG00010012968	LSM11	case	control	-0.127204615	0.142731147	-0.89121833	0.372812053	0.541159996	down_in_case
Yang2025_S	ENSGALG00010012969	SLC12A2	case	control	0.329551563	0.171305512	1.923765079	0.054384036	0.141865662	up_in_case
Yang2025_S	ENSGALG00010012970	UNC5A	case	control	0.381374186	0.162594014	2.345561051	0.018998467	0.065244208	up_in_case
Yang2025_S	ENSGALG00010012971	ENSGALG00010012971	case	control	0.911830435	0.456185205	1.998816324	0.045628231	0.125040196	up_in_case
Yang2025_S	ENSGALG00010012973	GLDC	case	control	0.642978884	0.271375487	2.369332953	0.017820203	0.062455868	up_in_case
Yang2025_S	ENSGALG00010012974	SLC49A3	case	control	0.074591401	0.207394765	0.359659035	0.71910213	0.826584854	up_in_case
Yang2025_S	ENSGALG00010012975	KRR1	case	control	-0.04236998	0.105532779	-0.40148644	0.68806202	0.804847162	down_in_case
Yang2025_S	ENSGALG00010012976	SLC9A2	case	control	0.248460856	0.238942038	1.039837351	0.298415473	0.467711125	up_in_case
Yang2025_S	ENSGALG00010012977	NKRF	case	control	0.065273759	0.072440391	0.901068564	0.367551866	0.536205533	up_in_case
Yang2025_S	ENSGALG00010012978	HSD17B7	case	control	-0.484427497	0.238837515	-2.02827222	0.042532473	0.118395926	down_in_case
Yang2025_S	ENSGALG00010012979	HTR1A	case	control	-0.642352316	0.279033317	-2.302063148	0.021331611	0.071011945	down_in_case
Yang2025_S	ENSGALG00010012980	KICS2	case	control	-0.230601319	0.09873287	-2.335608375	0.019511662	0.066585196	down_in_case
Yang2025_S	ENSGALG00010012982	DDR2	case	control	0.253696792	0.112981159	2.245478744	0.024737415	0.079391037	up_in_case
Yang2025_S	ENSGALG00010012985	ARNTL2	case	control	0.087003661	0.111680938	0.779037694	0.435957511	0.599828981	up_in_case
Yang2025_S	ENSGALG00010012986	TMEM184B	case	control	-0.325286354	0.104188405	-3.122097442	0.001795675	0.011061982	down_in_case
Yang2025_S	ENSGALG00010012988	RPL18A	case	control	-0.296162826	0.242181343	-1.222896951	0.22136862	0.382773511	down_in_case
Yang2025_S	ENSGALG00010012991	MFSD9	case	control	-0.142244858	0.102614877	-1.386201129	0.165685497	0.313188741	down_in_case
Yang2025_S	ENSGALG00010012992	HARS1	case	control	-0.04324659	0.11490132	-0.376380267	0.706634214	0.819145477	down_in_case
Yang2025_S	ENSGALG00010012993	DOP1B	case	control	-0.076046736	0.125475378	-0.606068994	0.544468939	0.694251587	down_in_case
Yang2025_S	ENSGALG00010012995	SERINC2	case	control	0.282416237	0.148426163	1.902738913	0.057074622	0.147120764	up_in_case
Yang2025_S	ENSGALG00010012997	UBE2A	case	control	-0.510973292	0.120106048	-4.254351054	2.09656E-05	0.000302126	down_in_case
Yang2025_S	ENSGALG00010012998	MAFF	case	control	0.0444898	0.234503601	0.189719047	0.849529295	0.910630056	up_in_case
Yang2025_S	ENSGALG00010013001	ADGRV1	case	control	0.399149465	0.434781408	0.918046304	0.358594625	0.527612204	up_in_case
Yang2025_S	ENSGALG00010013004	UAP1	case	control	0.147093949	0.127346845	1.155065514	0.248063599	0.413833152	up_in_case
Yang2025_S	ENSGALG00010013005	SLC40A1	case	control	-0.648278614	0.10958463	-5.915780484	3.30306E-09	1.71747E-07	down_in_case
Yang2025_S	ENSGALG00010013008	STEEP1	case	control	0.062740423	0.150654834	0.416451441	0.677079693	0.796968504	up_in_case
Yang2025_S	ENSGALG00010013012	TMEM182	case	control	-0.289696161	0.106616036	-2.717191262	0.006583855	0.029538319	down_in_case
Yang2025_S	ENSGALG00010013015	PLA2G6	case	control	0.12083672	0.084848976	1.424138808	0.154406294	0.29871605	up_in_case
Yang2025_S	ENSGALG00010013016	QRSL1	case	control	0.114640407	0.163869238	0.69958467	0.484186719	0.642111652	up_in_case
Yang2025_S	ENSGALG00010013017	SCMH1	case	control	-0.126486003	0.105184128	-1.202519863	0.229162175	0.391392929	down_in_case
Yang2025_S	ENSGALG00010013018	ID1	case	control	0.512962063	0.272125355	1.885021199	0.059426976	0.151609297	up_in_case
Yang2025_S	ENSGALG00010013021	SLC25A5	case	control	-0.367057746	0.185740139	-1.976189684	0.048133298	0.129847868	down_in_case
Yang2025_S	ENSGALG00010013027	UHMK1	case	control	-0.308678038	0.133172919	-2.317873943	0.020456173	0.068870928	down_in_case
Yang2025_S	ENSGALG00010013029	MYLK2	case	control	0.34956343	0.143856597	2.429943688	0.015101169	0.055319345	up_in_case
Yang2025_S	ENSGALG00010013032	BAIAP2L2	case	control	0.602558701	0.439870296	1.369855401	0.170732044	0.319726284	up_in_case
Yang2025_S	ENSGALG00010013033	MAP3K1	case	control	0.182184069	0.151029972	1.206277576	0.227710481	0.389885959	up_in_case
Yang2025_S	ENSGALG00010013036	ENSGALG00010013036	case	control	0.128924294	0.623820706	0.206668827	0.836268504	0.90301446	up_in_case
Yang2025_S	ENSGALG00010013037	MORC4	case	control	0.127537661	0.117871643	1.082004608	0.27925048	0.447076682	up_in_case
Yang2025_S	ENSGALG00010013040	SLC25A43	case	control	-0.303690492	0.101888075	-2.980628416	0.002876576	0.01577532	down_in_case
Yang2025_S	ENSGALG00010013041	LRP12	case	control	-0.543529159	0.085755561	-6.338121432	2.32583E-10	1.6793E-08	down_in_case
Yang2025_S	ENSGALG00010013042	SLC25A48	case	control	-0.077763058	0.207969684	-0.373915356	0.708467291	0.820178758	down_in_case
Yang2025_S	ENSGALG00010013044	ENSGALG00010013044	case	control	0.415565966	0.107083416	3.880768663	0.000104127	0.001102175	up_in_case
Yang2025_S	ENSGALG00010013045	FSD1L	case	control	0.497006283	0.164650854	3.018546657	0.002539903	0.014368617	up_in_case
Yang2025_S	ENSGALG00010013046	SLC12A5	case	control	0.367651748	0.334257068	1.099907177	0.271372567	0.438664312	up_in_case
Yang2025_S	ENSGALG00010013047	CTPS1	case	control	-0.169772482	0.133731491	-1.269502654	0.204261845	0.362415745	down_in_case
Yang2025_S	ENSGALG00010013048	WDR75	case	control	0.298803821	0.173382713	1.723377241	0.084820328	0.195399449	up_in_case
Yang2025_S	ENSGALG00010013051	XPOT	case	control	-0.584178822	0.118142702	-4.944688184	7.62658E-07	1.91489E-05	down_in_case
Yang2025_S	ENSGALG00010013053	MRTFA	case	control	0.43212863	0.298607467	1.447146094	0.14785601	0.289923879	up_in_case
Yang2025_S	ENSGALG00010013056	ENSGALG00010013056	case	control	-0.662405806	0.252805263	-2.620221576	0.008787265	0.036874807	down_in_case
Yang2025_S	ENSGALG00010013058	ENSGALG00010013058	case	control	-0.735087263	0.60186317	-1.221352793	0.221952473	0.383500961	down_in_case
Yang2025_S	ENSGALG00010013062	ENSGALG00010013062	case	control	0.579818166	0.258474339	2.243232992	0.024881794	0.079670909	up_in_case
Yang2025_S	ENSGALG00010013063	PARP12	case	control	-0.249301504	0.111301434	-2.239876846	0.025098919	0.080168349	down_in_case
Yang2025_S	ENSGALG00010013065	FKTN	case	control	0.018448703	0.13470017	0.136961247	0.891061427	0.936209077	up_in_case
Yang2025_S	ENSGALG00010013069	ENSGALG00010013069	case	control	-2.073017585	2.979640625	-0.695727386			down_in_case
Yang2025_S	ENSGALG00010013071	ENSGALG00010013071	case	control	-0.143564669	0.169640012	-0.846290142	0.397390906	0.564259453	down_in_case
Yang2025_S	ENSGALG00010013074	ENSGALG00010013074	case	control	0.61408327	0.699153161	0.878324385	0.379767706	0.547738649	up_in_case
Yang2025_S	ENSGALG00010013076	RTN4IP1	case	control	0.244143783	0.177104558	1.378529083	0.168039995	0.316223812	up_in_case
Yang2025_S	ENSGALG00010013078	COL5A2	case	control	-0.500261184	0.141763335	-3.528847456	0.000417374	0.003425266	down_in_case
Yang2025_S	ENSGALG00010013079	ENSGALG00010013079	case	control	-0.409103431	0.34751555	-1.177223382	0.239106359	0.403334597	down_in_case
Yang2025_S	ENSGALG00010013080	ARHGEF7	case	control	0.222289188	0.143218498	1.552098302	0.120638704	0.250536564	up_in_case
Yang2025_S	ENSGALG00010013081	NCOA5	case	control	0.224873506	0.101290235	2.220090677	0.026412613	0.083309136	up_in_case
Yang2025_S	ENSGALG00010013082	CHAF1B	case	control	0.009319985	0.135768527	0.06864614	0.945271291	0.966281942	up_in_case
Yang2025_S	ENSGALG00010013084	SGSM3	case	control	0.006702398	0.108090658	0.062007194	0.950557103	0.969409634	up_in_case
Yang2025_S	ENSGALG00010013085	LYSMD3	case	control	0.178102511	0.151820945	1.173108966	0.240752112	0.405570681	up_in_case
Yang2025_S	ENSGALG00010013086	CXCL14	case	control	0.685074802	0.326249715	2.099847971	0.035742217	0.104394842	up_in_case
Yang2025_S	ENSGALG00010013087	GPN2	case	control	0.144487271	0.215942195	0.669101615	0.50343066	0.659581257	up_in_case
Yang2025_S	ENSGALG00010013089	TBK1	case	control	0.073853734	0.077969637	0.94721146	0.343531037	0.512044604	up_in_case
Yang2025_S	ENSGALG00010013093	GPATCH3	case	control	-0.025645117	0.123467332	-0.207707708	0.835457199	0.902558033	down_in_case
Yang2025_S	ENSGALG00010013095	ADSL	case	control	0.004674223	0.222962293	0.020964188	0.983274223	0.989952048	up_in_case
Yang2025_S	ENSGALG00010013097	ENSGALG00010013097	case	control	0.789258447	0.423589478	1.863262636	0.062425324	0.156753593	up_in_case
Yang2025_S	ENSGALG00010013099	SIM2	case	control	-0.133795305	0.19431944	-0.688532782	0.491117334	0.648570892	down_in_case
Yang2025_S	ENSGALG00010013100	ZFPM2	case	control	0.174240607	0.171590175	1.015446296	0.309893134	0.479711267	up_in_case
Yang2025_S	ENSGALG00010013103	AIRIM	case	control	0.836079041	0.182238883	4.587819163	4.479E-06	8.43445E-05	up_in_case
Yang2025_S	ENSGALG00010013104	HLCS	case	control	-0.232737051	0.130680614	-1.780960805	0.074918854	0.178674351	down_in_case
Yang2025_S	ENSGALG00010013106	SETD9	case	control	0.049199939	0.243782282	0.201819174	0.840058091	0.905085473	up_in_case
Yang2025_S	ENSGALG00010013110	ANKRD10	case	control	-0.223586585	0.145982309	-1.531600549	0.125621037	0.257770684	down_in_case
Yang2025_S	ENSGALG00010013111	RFESD	case	control	-0.578418245	0.272683569	-2.121206815	0.033904402	0.10024567	down_in_case
Yang2025_S	ENSGALG00010013112	CD40	case	control	0.104611152	0.22928973	0.456240025	0.648217386	0.77651223	up_in_case
Yang2025_S	ENSGALG00010013114	PIGP	case	control	-0.508811512	0.219593958	-2.317056061	0.020500678	0.068970775	down_in_case
Yang2025_S	ENSGALG00010013115	ENSGALG00010013115	case	control	1.332188214	0.262251767	5.079806438	3.7782E-07	1.0467E-05	up_in_case
Yang2025_S	ENSGALG00010013116	TARS	case	control	-0.189727697	0.183866945	-1.031874963	0.302130696	0.471677263	down_in_case
Yang2025_S	ENSGALG00010013117	ARSK	case	control	-0.025816603	0.205995303	-0.125326172	0.900265333	0.940592827	down_in_case
Yang2025_S	ENSGALG00010013119	RNF114	case	control	-0.057337206	0.115312464	-0.497233377	0.619024487	0.754737155	down_in_case
Yang2025_S	ENSGALG00010013121	PCBD2	case	control	-0.024839443	0.252637946	-0.098320315	0.921677947	0.953337584	down_in_case
Yang2025_S	ENSGALG00010013123	PPFIBP1	case	control	0.098723943	0.159293348	0.61976187	0.535414575	0.686763817	up_in_case
Yang2025_S	ENSGALG00010013125	POU3F3	case	control	2.610485718	0.704827985	3.703720303	0.000212461	0.001965895	up_in_case
Yang2025_S	ENSGALG00010013129	RASSF3	case	control	0.15230712	0.161207816	0.944787438	0.344767407	0.513393226	up_in_case
Yang2025_S	ENSGALG00010013131	SPATA2	case	control	-0.240457826	0.074992357	-3.206431091	0.001343925	0.008826552	down_in_case
Yang2025_S	ENSGALG00010013132	SKIC3	case	control	-0.018876455	0.120993011	-0.156012772	0.876022954	0.927040476	down_in_case
Yang2025_S	ENSGALG00010013133	TXNDC15	case	control	-0.032941518	0.218700809	-0.150623666	0.880272592	0.929358176	down_in_case
Yang2025_S	ENSGALG00010013134	ENSGALG00010013134	case	control	-9.557469491	2.951136968	-3.238571979	0.001201297	0.008075289	down_in_case
Yang2025_S	ENSGALG00010013135	TTC3	case	control	0.495430001	0.163659439	3.027200897	0.002468299	0.014050547	up_in_case
Yang2025_S	ENSGALG00010013137	TMEM121	case	control	-0.239753647	0.199308129	-1.202929592	0.229003568	0.391225863	down_in_case
Yang2025_S	ENSGALG00010013139	TMEM185A	case	control	-0.428919686	0.13713539	-3.127709685	0.001761741	0.010891686	down_in_case
Yang2025_S	ENSGALG00010013143	ENSGALG00010013143	case	control	-0.432457897	0.647298061	-0.668097007	0.504071672	0.659862729	down_in_case
Yang2025_S	ENSGALG00010013144	TEDC1	case	control	-0.089001651	0.251919407	-0.353294142	0.723867937	0.830470694	down_in_case
Yang2025_S	ENSGALG00010013145	ENSGALG00010013145	case	control	0.540819993	0.233016014	2.320956333	0.020289201	0.068441058	up_in_case
Yang2025_S	ENSGALG00010013146	TNRC6B	case	control	0.384878855	0.193848827	1.98545878	0.047093452	0.127823773	up_in_case
Yang2025_S	ENSGALG00010013149	MCTP1	case	control	0.060801881	0.19957251	0.3046606	0.76062466	0.854644786	up_in_case
Yang2025_S	ENSGALG00010013150	GNS	case	control	-0.356344485	0.11567414	-3.080589	0.002065916	0.012260877	down_in_case
Yang2025_S	ENSGALG00010013151	ENSGALG00010013151	case	control	1.116266071	1.037620051	1.075794623	0.282019118	0.450009894	up_in_case
Yang2025_S	ENSGALG00010013152	TBXAS1	case	control	-0.348861101	0.238212553	-1.464495037	0.143058738	0.283090531	down_in_case
Yang2025_S	ENSGALG00010013154	ENSGALG00010013154	case	control	-0.751005491	0.641847678	-1.170068096	0.241973566	0.407104558	down_in_case
Yang2025_S	ENSGALG00010013156	ABCC9	case	control	0.045883946	0.115520364	0.397193575	0.69122472	0.80746219	up_in_case
Yang2025_S	ENSGALG00010013158	CRIP1	case	control	0.11966775	0.197560521	0.60572704	0.544696025	0.694323007	up_in_case
Yang2025_S	ENSGALG00010013159	ENSGALG00010013159	case	control	-0.982124696	2.067013788	-0.475141821	0.634685892	0.765810711	down_in_case
Yang2025_S	ENSGALG00010013160	CRYBG1	case	control	0.032482372	0.652958317	0.049746472			up_in_case
Yang2025_S	ENSGALG00010013162	IDS	case	control	-0.259141594	0.167055945	-1.551226407	0.120847436	0.250895265	down_in_case
Yang2025_S	ENSGALG00010013163	ENSGALG00010013163	case	control	1.213077981	0.289616826	4.188561817	2.80728E-05	0.000385413	up_in_case
Yang2025_S	ENSGALG00010013164	FAM83F	case	control	0.545502893	0.660913013	0.825377746	0.409157165	0.574651492	up_in_case
Yang2025_S	ENSGALG00010013165	NUDC	case	control	0.1643707	0.093486673	1.758226015	0.07870906	0.185178247	up_in_case
Yang2025_S	ENSGALG00010013168	VPS26C	case	control	-0.04130136	0.114729145	-0.359990135	0.71885451	0.826504753	down_in_case
Yang2025_S	ENSGALG00010013170	COL3A1	case	control	-0.807010036	0.167077643	-4.830149745	1.3643E-06	3.14761E-05	down_in_case
Yang2025_S	ENSGALG00010013171	SLC9A8	case	control	0.586829478	0.097787942	6.00104131	1.96056E-09	1.11059E-07	up_in_case
Yang2025_S	ENSGALG00010013173	KCNJ8	case	control	-0.304874152	0.09321781	-3.270556893	0.001073359	0.007408254	down_in_case
Yang2025_S	ENSGALG00010013174	ADAMTS12	case	control	0.254123132	0.208547667	1.218537399	0.223019822	0.384474516	up_in_case
Yang2025_S	ENSGALG00010013175	HIPK2	case	control	0.483609817	0.216242731	2.236421153	0.025324197	0.080660274	up_in_case
Yang2025_S	ENSGALG00010013176	ENSGALG00010013176	case	control	-0.263449304	0.611040767	-0.431148491	0.666360404	0.789228274	down_in_case
Yang2025_S	ENSGALG00010013177	OXR1	case	control	-0.125462058	0.086773691	-1.445853642	0.148218257	0.290298738	down_in_case
Yang2025_S	ENSGALG00010013179	TBC1D30	case	control	-0.004916185	0.27636335	-0.017788844	0.985807304	0.991427886	down_in_case
Yang2025_S	ENSGALG00010013180	C5orf24	case	control	-0.212607707	0.082078994	-2.590281581	0.009589746	0.039396552	down_in_case
Yang2025_S	ENSGALG00010013181	DYRK1A	case	control	0.164200332	0.194136484	0.845798423	0.397665205	0.564488859	up_in_case

Yang2025_S	ENSGALG00010013183	CLTA	case	control	0.579998025	0.200076537	2.89888076	0.003744973	0.019242698	up_in_case
Yang2025_S	ENSGALG00010013184	LDHB	case	control	-0.230781717	0.254763093	-0.905867934	0.365005758	0.533730925	down_in_case
Yang2025_S	ENSGALG00010013185	ENSGALG00010013185	case	control	-0.321169986	0.34401552	-0.933591559	0.350514621	0.518901651	down_in_case
Yang2025_S	ENSGALG00010013186	DDX46	case	control	0.467029068	0.176319132	2.64877136	0.008078497	0.034468417	up_in_case
Yang2025_S	ENSGALG00010013187	KDF1	case	control	-0.553784705	0.281736297	-1.965613628	0.049343259	0.132001979	down_in_case
Yang2025_S	ENSGALG00010013188	ENSGALG00010013188	case	control	-0.635932364	0.295134383	-2.154721372	0.031183644	0.094273917	down_in_case
Yang2025_S	ENSGALG00010013190	SLC45A2	case	control	4.580049531	0.686696105	6.669689104	2.56346E-11	2.49803E-09	up_in_case
Yang2025_S	ENSGALG00010013191	ABRA	case	control	0.04454377	0.21605468	0.206168968	0.836658927	0.903156104	up_in_case
Yang2025_S	ENSGALG00010013192	B4GALT5	case	control	-0.250080043	0.080625277	-3.101757299	0.001923756	0.01164027	down_in_case
Yang2025_S	ENSGALG00010013196	MRPS9	case	control	0.315011366	0.131060131	2.403563649	0.01623614	0.05822198	up_in_case
Yang2025_S	ENSGALG00010013198	CAMLG	case	control	-0.219377049	0.095549361	-2.295955163	0.021678445	0.071772187	down_in_case
Yang2025_S	ENSGALG00010013202	ENSGALG00010013202	case	control	-0.013656203	0.547136003	-0.02495943	0.980087324	0.987885487	down_in_case
Yang2025_S	ENSGALG00010013203	AMACR	case	control	-0.445959986	0.231064718	-1.930021985	0.053604114	0.140382132	down_in_case
Yang2025_S	ENSGALG00010013204	ENSGALG00010013204	case	control	0.316942749	0.385402127	0.822368968	0.410866935	0.576288952	up_in_case
Yang2025_S	ENSGALG00010013205	KCNJ6	case	control	-0.225389482	0.274764376	-0.820300962	0.412044558	0.57730152	down_in_case
Yang2025_S	ENSGALG00010013206	AFF2	case	control	0.179630009	0.20063827	0.895292849	0.370630562	0.539185371	up_in_case
Yang2025_S	ENSGALG00010013207	C1QTNF3	case	control	0.180937222	0.162836348	1.111159909	0.266499524	0.433641132	up_in_case
Yang2025_S	ENSGALG00010013208	ENSGALG00010013208	case	control	0.224545097	0.47810603	0.469655439	0.638601209	0.768870559	up_in_case
Yang2025_S	ENSGALG00010013209	KIAA1549	case	control	0.221845191	0.242775153	0.9137887	0.360827885	0.529388985	up_in_case
Yang2025_S	ENSGALG00010013212	ENSGALG00010013212	case	control	0.019693493	0.139653337	0.141016992	0.887856519	0.934532864	up_in_case
Yang2025_S	ENSGALG00010013213	SH2D1A	case	control	0.717302872	0.488804323	1.467464255	0.142249806	0.281890081	up_in_case
Yang2025_S	ENSGALG00010013214	RAI14	case	control	0.280280014	0.17323464	1.6179213	0.105679559	0.228069484	up_in_case
Yang2025_S	ENSGALG00010013215	SF3A3	case	control	-0.260159797	0.109378527	-2.378527153	0.017381957	0.061206526	down_in_case
Yang2025_S	ENSGALG00010013217	TGFBRAP1	case	control	0.066646325	0.153035748	0.435495139	0.663203067	0.786972416	up_in_case
Yang2025_S	ENSGALG00010013220	CSTF1	case	control	-0.141905008	0.1020325	-1.390782423	0.164291415	0.311356027	down_in_case
Yang2025_S	ENSGALG00010013221	C2orf49	case	control	0.214539607	0.151972112	1.411703792	0.158037196	0.303298214	up_in_case
Yang2025_S	ENSGALG00010013225	GNE	case	control	0.383420918	0.126163336	3.039083544	0.00237299	0.013641758	up_in_case
Yang2025_S	ENSGALG00010013226	DUSP6	case	control	-0.505928056	0.090519743	-5.589145944	2.28189E-08	9.54899E-07	down_in_case
Yang2025_S	ENSGALG00010013227	CITED4	case	control	-0.169102064	0.174267355	-0.970359962	0.331867099	0.501199525	down_in_case
Yang2025_S	ENSGALG00010013228	RTFDC1	case	control	-0.176823806	0.098160974	-1.801365636	0.071645269	0.172729554	down_in_case
Yang2025_S	ENSGALG00010013230	ENSGALG00010013230	case	control	0.389586826	0.153395766	2.539749545	0.011093188	0.043750129	up_in_case
Yang2025_S	ENSGALG00010013232	FHL2	case	control	-0.435978531	0.173895837	-2.5071246	0.01217178	0.046911148	down_in_case
Yang2025_S	ENSGALG00010013233	LRRCC1	case	control	0.952451373	0.214464493	4.441067895	8.95135E-06	0.000149207	up_in_case
Yang2025_S	ENSGALG00010013235	GCNT7	case	control	-0.37196585	0.18254841	-2.037628534	0.041587096	0.116415465	down_in_case
Yang2025_S	ENSGALG00010013236	MON2	case	control	-0.103158161	0.097511442	-1.057908265	0.290097267	0.458768735	down_in_case
Yang2025_S	ENSGALG00010013238	RAD1	case	control	0.422029908	0.211363846	1.996698657	0.045857928	0.125345491	up_in_case
Yang2025_S	ENSGALG00010013241	SEC24A	case	control	-0.321189735	0.084828334	-3.786349701	0.000152876	0.001501292	down_in_case
Yang2025_S	ENSGALG00010013242	SULT4A1	case	control	0.543766103	0.24071213	2.258989204	0.023884056	0.077431301	up_in_case
Yang2025_S	ENSGALG00010013244	TRIM24	case	control	0.049991984	0.156444443	0.319551037	0.749308696	0.847740252	up_in_case
Yang2025_S	ENSGALG00010013246	LNPEP	case	control	-0.270961891	0.132162897	-2.050211501	0.040343796	0.113911305	down_in_case
Yang2025_S	ENSGALG00010013247	ATG5	case	control	-0.248399411	0.063413671	-3.917127132	8.96105E-05	0.000978622	down_in_case
Yang2025_S	ENSGALG00010013248	ENSGALG00010013248	case	control	-3.335654057	0.930484451	-3.584857387	0.000337262	0.00287975	down_in_case
Yang2025_S	ENSGALG00010013249	AURKA	case	control	-0.514997186	0.146467781	-3.516112439	0.000437915	0.003552804	down_in_case
Yang2025_S	ENSGALG00010013250	E2F5	case	control	0.352291389	0.118843228	2.964337101	0.003033358	0.016473049	up_in_case
Yang2025_S	ENSGALG00010013251	MTA1	case	control	0.044033053	0.131973346	0.333651103	0.738642851	0.840267568	up_in_case
Yang2025_S	ENSGALG00010013252	PPM1H	case	control	0.227325205	0.203111664	1.119212949	0.263049303	0.429931038	up_in_case
Yang2025_S	ENSGALG00010013253	SHB	case	control	-0.224852581	0.105600728	-2.12927113	0.033231835	0.098759996	down_in_case
Yang2025_S	ENSGALG00010013254	SAR1B	case	control	-0.499755536	0.108288058	-4.615056789	3.92988E-06	7.5954E-05	down_in_case
Yang2025_S	ENSGALG00010013256	BRIX1	case	control	0.363914909	0.192191141	1.893505119	0.058290728	0.149508797	up_in_case
Yang2025_S	ENSGALG00010013257	STX12	case	control	-0.062938638	0.077414894	-0.813004251	0.416215621	0.581527689	down_in_case
Yang2025_S	ENSGALG00010013258	RBIS	case	control	-0.226817433	0.234983104	-0.965249965	0.334419626	0.503200046	down_in_case
Yang2025_S	ENSGALG00010013259	PTPRB	case	control	-0.073755035	0.144602053	-0.510055243	0.61001276	0.747299148	down_in_case
Yang2025_S	ENSGALG00010013260	FAM210B	case	control	-0.420754395	0.117457456	-3.582185491	0.000340732	0.002902925	down_in_case
Yang2025_S	ENSGALG00010013261	JADE2	case	control	-0.218368412	0.108494041	-2.012722633	0.044143817	0.122097595	down_in_case
Yang2025_S	ENSGALG00010013262	CA13	case	control	-0.69143413	0.230335076	-3.001862071	0.002683337	0.014950941	down_in_case
Yang2025_S	ENSGALG00010013263	ATP11A	case	control	-0.177465315	0.159638492	-1.111669955	0.266280083	0.433602847	down_in_case
Yang2025_S	ENSGALG00010013264	DNAJC21	case	control	0.763487657	0.188303699	4.054554754	5.02299E-05	0.000610693	up_in_case
Yang2025_S	ENSGALG00010013265	PACS2	case	control	-0.076805515	0.107068801	-0.717347293	0.473159833	0.632832543	down_in_case
Yang2025_S	ENSGALG00010013266	KIAA1755	case	control	0.505605662	0.126992712	3.981375421	6.85176E-05	0.000786485	up_in_case
Yang2025_S	ENSGALG00010013267	PNPLA3	case	control	-0.185948858	0.178259218	-1.043137407	0.296884658	0.465835796	down_in_case
Yang2025_S	ENSGALG00010013268	PTPRR	case	control	-0.189172532	0.284470409	-0.664998979	0.506051146	0.66157179	down_in_case
Yang2025_S	ENSGALG00010013269	INPP5B	case	control	-0.014601203	0.082988036	-0.175943472	0.860338352	0.917628392	down_in_case
Yang2025_S	ENSGALG00010013271	GULP1	case	control	-0.141154198	0.118488069	-1.191294607	0.23353795	0.39672697	down_in_case
Yang2025_S	ENSGALG00010013273	STAG2	case	control	0.195704168	0.138786866	1.410105827	0.158508437	0.303951434	up_in_case
Yang2025_S	ENSGALG00010013274	MTF1	case	control	-0.202011868	0.10898241	-1.853619026	0.063793684	0.158923125	down_in_case
Yang2025_S	ENSGALG00010013275	MAPRE1	case	control	0.018014588	0.091514824	0.196848847	0.843945825	0.907425895	up_in_case
Yang2025_S	ENSGALG00010013276	POC1B	case	control	-0.28333151	0.103183758	-2.74589254	0.006034653	0.027544345	down_in_case
Yang2025_S	ENSGALG00010013277	YRDC	case	control	0.035182338	0.31118921	0.113057707	0.909984805	0.946316288	up_in_case
Yang2025_S	ENSGALG00010013278	SAMM50	case	control	-0.523969605	0.183195569	-2.860165282	0.004234203	0.020997727	down_in_case
Yang2025_S	ENSGALG00010013280	FRS2	case	control	0.395490528	0.202090448	1.956997636	0.050347746	0.133915706	up_in_case
Yang2025_S	ENSGALG00010013281	BIRC8	case	control	0.064616632	0.13245598	0.487834768	0.625666883	0.759465854	up_in_case
Yang2025_S	ENSGALG00010013282	SEC22B	case	control	-0.348998115	0.10246306	-3.406087194	0.000659011	0.005009787	down_in_case
Yang2025_S	ENSGALG00010013283	PAWR	case	control	-0.413306049	0.213130532	-1.939215577	0.052475094	0.138126262	down_in_case
Yang2025_S	ENSGALG00010013284	MANEAL	case	control	0.545028399	0.251897021	2.163695292	0.030487743	0.092801812	up_in_case
Yang2025_S	ENSGALG00010013285	ATP2B1	case	control	0.182699397	0.100139619	1.824446698	0.068084568	0.166589041	up_in_case
Yang2025_S	ENSGALG00010013286	RNF14	case	control	-0.752212509	0.189256658	-3.974562996	7.05086E-05	0.000806019	down_in_case
Yang2025_S	ENSGALG00010013288	CA3A	case	control	0.765792116	0.473822418	1.616200684	0.106050931	0.228587737	up_in_case
Yang2025_S	ENSGALG00010013289	TM9SF4	case	control	-0.499624974	0.140157618	-3.564736466	0.000364222	0.003062069	down_in_case
Yang2025_S	ENSGALG00010013290	AKR1D1	case	control	-0.049067487	0.157446959	-0.311644551	0.755310672	0.851230337	down_in_case
Yang2025_S	ENSGALG00010013292	PRDM1	case	control	0.184901886	0.213983529	0.864094011	0.387536249	0.554846155	up_in_case
Yang2025_S	ENSGALG00010013293	TUBGCP3	case	control	-0.138467089	0.088435176	-1.56574675	0.117407917	0.245731349	down_in_case
Yang2025_S	ENSGALG00010013295	CA3B	case	control	0.132233566	0.37362909	0.353916676	0.723401329	0.830088729	up_in_case
Yang2025_S	ENSGALG00010013296	AGXT2	case	control	-0.172159675	0.569045545	-0.302541117	0.762239594	0.855286959	down_in_case
Yang2025_S	ENSGALG00010013297	FCHO2	case	control	0.791258978	0.269575746	2.935200918	0.003333318	0.017628001	up_in_case
Yang2025_S	ENSGALG00010013298	EPYC	case	control	1.454741482	0.67809875	2.145323939	0.031926956	0.095981439	up_in_case
Yang2025_S	ENSGALG00010013299	PLAGL2	case	control	0.006067347	0.160864303	0.037717174	0.969913183	0.982096151	up_in_case
Yang2025_S	ENSGALG00010013300	GNPDA1	case	control	0.212437897	0.140953743	1.507146191	0.131773189	0.266472122	up_in_case
Yang2025_S	ENSGALG00010013301	PARVB	case	control	-0.353452643	0.119306569	-2.962558095	0.003050942	0.016529891	down_in_case
Yang2025_S	ENSGALG00010013303	TFPI	case	control	0.485481538	0.160672745	3.021555016	0.002514799	0.014257009	up_in_case
Yang2025_S	ENSGALG00010013304	KCNJ15	case	control	-0.368995267	0.18324496	-2.013672118	0.044043969	0.121848861	down_in_case
Yang2025_S	ENSGALG00010013305	THOC2	case	control	0.665581861	0.174910616	3.805268518	0.00014165	0.001412955	up_in_case
Yang2025_S	ENSGALG00010013306	ARSB	case	control	-0.265632811	0.153369964	-1.73197414	0.083278167	0.192770973	down_in_case
Yang2025_S	ENSGALG00010013307	POFUT1	case	control	-0.522792319	0.202192108	-2.585621779	0.009720353	0.039722322	down_in_case
Yang2025_S	ENSGALG00010013309	ENSGALG00010013309	case	control	0.238243282	0.19259158	1.237038929	0.216072631	0.377031318	up_in_case
Yang2025_S	ENSGALG00010013310	MYO16	case	control	0.1994958	0.334212079	0.596913792	0.550564938	0.698863401	up_in_case
Yang2025_S	ENSGALG00010013311	RSPO1	case	control	0.194426228	0.343889698	0.565373807	0.571819537	0.715513306	up_in_case
Yang2025_S	ENSGALG00010013312	ENSGALG00010013312	case	control	0.61931184	0.350070409	1.76910651	0.076876102	0.181981041	up_in_case
Yang2025_S	ENSGALG00010013313	GAK	case	control	0.03765339	0.175442968	0.214618978	0.830064397	0.898682881	up_in_case
Yang2025_S	ENSGALG00010013314	KIT	case	control	0.645595264	0.162666719	3.968822072	7.22288E-05	0.0008203	up_in_case
Yang2025_S	ENSGALG00010013316	KDM7A	case	control	-0.116742834	0.14623986	-0.79829695	0.424698188	0.589576534	down_in_case
Yang2025_S	ENSGALG00010013317	PREP	case	control	-0.441749568	0.06975998	-6.332421104	2.41344E-10	1.73357E-08	down_in_case
Yang2025_S	ENSGALG00010013318	KERA	case	control	-0.304720658	0.139430211	-2.185470828	0.028854343	0.088917573	down_in_case
Yang2025_S	ENSGALG00010013319	ENSGALG00010013319	case	control	-7.686529081	1.477070942	-5.203899734			down_in_case
Yang2025_S	ENSGALG00010013320	CALCRL	case	control	0.117781142	0.175037375	0.672891386	0.501016392	0.65780053	up_in_case
Yang2025_S	ENSGALG00010013322	IRS2	case	control	-0.147706717	0.14850532	-0.994622392	0.319919953	0.489200542	down_in_case
Yang2025_S	ENSGALG00010013323	NOTCH2	case	control	0.016066315	0.143458421	0.111992834	0.910829088	0.946982269	up_in_case
Yang2025_S	ENSGALG00010013324	PARVG	case	control	-0.18845725	0.120541663	-1.563420028	0.117953833	0.246483768	down_in_case
Yang2025_S	ENSGALG00010013325	CA2	case	control	-0.212746433	0.170386315	-1.248612206	0.211806946	0.372007629	down_in_case
Yang2025_S	ENSGALG00010013327	SLF1	case	control	0.174149536	0.193064232	0.902028999	0.367041463	0.535797485	up_in_case
Yang2025_S	ENSGALG00010013328	SLC37A3	case	control	-0.531151607	0.171464619	-3.097732983	0.00195007	0.011763737	down_in_case
Yang2025_S	ENSGALG00010013329	LIFR	case	control	-0.105395428	0.132610362	-0.794775205	0.426744277	0.591149772	down_in_case
Yang2025_S	ENSGALG00010013330	ERG	case	control	0.481973428	0.218440785	2.206426005	0.027354186	0.085409048	up_in_case
Yang2025_S	ENSGALG00010013331	KIF3B	case	control	0.051198867	0.092734558	0.552101271	0.580878971	0.723567396	up_in_case
Yang2025_S	ENSGALG00010013332	LUM	case	control	-0.81668347	0.126524347	-6.454753475	1.08395E-10	9.21029E-09	down_in_case
Yang2025_S	ENSGALG00010013333	PPP1R12A	case	control	0.403662075	0.146428242	2.756722809	0.005838382	0.026877388	up_in_case
Yang2025_S	ENSGALG00010013334	SH3BGRL3	case	control	-0.321722708	0.129958654	-2.475577404	0.013302092	0.050080702	down_in_case
Yang2025_S	ENSGALG00010013335	POPDC3	case	control	-0.993520622	0.137281291	-7.237115972	4.58326E-13	6.39517E-11	down_in_case
Yang2025_S	ENSGALG00010013337	RAB19	case	control	-0.066314574	0.30667249	-0.216239069	0.828801401	0.897524675	down_in_case
Yang2025_S	ENSGALG00010013339	ATP6V0D2	case	control	0.925744902	0.698487307	1.3253568	0.185052838	0.338635758	up_in_case
Yang2025_S	ENSGALG00010013340	LIN28B	case	control	0.842119802	0.495268658	1.700329281	0.089069006	0.202508826	up_in_case
Yang2025_S	ENSGALG00010013341	ENSGALG00010013341	case	control	-0.869128902	1.424850454	-0.609979033	0.541875697	0.692235723	down_in_case
Yang2025_S	ENSGALG00010013343	SHISAL1	case	control	0.715563359	0.398337444	1.796374829	0.072434924	0.17406116	up_in_case
Yang2025_S	ENSGALG00010013344	OSMR	case	control	0.087312974	0.115964634	0.752927605	0.451493419	0.613451715	up_in_case
Yang2025_S	ENSGALG00010013345	DCN	case	control	-0.540111048	0.11979613	-4.50858509	6.52614E-06	0.000116592	down_in_case
Yang2025_S	ENSGALG00010013346	HMGCS2	case	control	-1.182109725	0.842274666	-1.403472967	0.160475848	0.306207167	down_in_case
Yang2025_S	ENSGALG00010013347	ENSGALG00010013347	case	control	-0.972500669	0.607661282	-1.600399265	0.109510038	0.233479556	down_in_case

Yang2025_S	ENSGALG00010013348	MKRN4P	case	control	0.153298093	0.07351752	2.085191292	0.03705194	0.107009075	up_in_case
Yang2025_S	ENSGALG00010013349	ETS2	case	control	-0.185752127	0.104824882	-1.772023237	0.0763907	0.181099763	down_in_case
Yang2025_S	ENSGALG00010013351	EDAR	case	control	0.012087642	0.429580317	0.028138259	0.97755188	0.986721342	up_in_case
Yang2025_S	ENSGALG00010013352	ASXL1	case	control	-0.145941212	0.102400707	-1.425197314	0.154100172	0.298289471	down_in_case
Yang2025_S	ENSGALG00010013353	NDFIP1	case	control	-0.50153137	0.17387821	-2.884383094	0.003921813	0.019901846	down_in_case
Yang2025_S	ENSGALG00010013354	WWP1	case	control	-0.100732847	0.09864101	-1.021206569	0.307156581	0.476754313	down_in_case
Yang2025_S	ENSGALG00010013355	FAM171B	case	control	-0.357834152	0.244737109	-1.462116442	0.143709304	0.284135804	down_in_case
Yang2025_S	ENSGALG00010013356	AMER1	case	control	-0.361286864	0.116404562	-3.103717398	0.001911058	0.011583554	down_in_case
Yang2025_S	ENSGALG00010013357	RICTOR	case	control	0.378539908	0.180680619	2.095077549	0.036164099	0.105230053	up_in_case
Yang2025_S	ENSGALG00010013358	RPS16	case	control	-0.626490027	0.248151185	-2.524630406	0.011582003	0.045170782	down_in_case
Yang2025_S	ENSGALG00010013359	BTG1	case	control	-0.463910222	0.119381637	-3.885942875	0.000101933	0.001083481	down_in_case
Yang2025_S	ENSGALG00010013360	PRR5	case	control	-0.062702819	0.160043949	-0.391785	0.695217079	0.810834449	down_in_case
Yang2025_S	ENSGALG00010013361	ENSGALG00010013361	case	control	-0.941231423	0.323736673	-2.90739821	0.003644489	0.018844512	down_in_case
Yang2025_S	ENSGALG00010013362	ENSGALG00010013362	case	control	-0.049248179	0.261866433	-0.188066023	0.850824896	0.911457943	down_in_case
Yang2025_S	ENSGALG00010013363	HS3ST5	case	control	-0.204839575	0.183952195	-1.113547875	0.265473199	0.432573553	down_in_case
Yang2025_S	ENSGALG00010013364	OTOGL	case	control	0.623912852	0.37904767	1.646001023	0.099763548	0.218516983	up_in_case
Yang2025_S	ENSGALG00010013365	CEP85	case	control	0.077819171	0.133346468	0.583586294	0.559498684	0.706203234	up_in_case
Yang2025_S	ENSGALG00010013367	NOL4L	case	control	0.036860147	0.168662792	0.218543442	0.827005715	0.896430655	up_in_case
Yang2025_S	ENSGALG00010013369	MTMR8	case	control	-0.393714122	0.106737485	-3.688620933	0.000225473	0.00205186	down_in_case
Yang2025_S	ENSGALG00010013370	CNKSR1	case	control	0.282199773	0.206062335	1.369487405	0.170846969	0.319821671	up_in_case
Yang2025_S	ENSGALG00010013371	FAT1	case	control	0.385346296	0.240258542	1.60388177	0.108740116	0.232406981	up_in_case
Yang2025_S	ENSGALG00010013372	SPRY4	case	control	0.353996816	0.151018701	2.344059469	0.01907513	0.065454797	up_in_case
Yang2025_S	ENSGALG00010013373	ENSGALG00010013373	case	control	-1.012613584	1.013450658	-0.999174036	0.317710391	0.487211874	down_in_case
Yang2025_S	ENSGALG00010013374	RMDN1	case	control	0.014591259	0.226660752	0.064374883	0.948671729	0.967992436	up_in_case
Yang2025_S	ENSGALG00010013375	PSMG1	case	control	-0.98757314	0.213818966	-4.618734985	3.86087E-06	7.50365E-05	down_in_case
Yang2025_S	ENSGALG00010013376	ARHGAP8	case	control	-1.088821323	0.59906795	-1.81752558	0.069136675	0.168518378	down_in_case
Yang2025_S	ENSGALG00010013378	SH3RF3	case	control	-0.146858082	0.142654238	-1.029468764	0.30325945	0.472856713	down_in_case
Yang2025_S	ENSGALG00010013379	ZNF593	case	control	-0.31353306	0.309608591	-1.012675582	0.311215145	0.481009654	down_in_case
Yang2025_S	ENSGALG00010013380	PHGDH	case	control	-0.021761735	0.15619136	-0.139327395	0.889191443	0.935302141	down_in_case
Yang2025_S	ENSGALG00010013381	DENND2A	case	control	-0.059428789	0.09843813	-0.603717169	0.546031698	0.695242148	down_in_case
Yang2025_S	ENSGALG00010013382	ASB12	case	control	-0.300160084	0.203260309	-1.476727484	0.139748702	0.278518044	down_in_case
Yang2025_S	ENSGALG00010013383	SEPTIN10	case	control	-0.1685666	0.088610062	-1.902341529	0.05712652	0.147227308	down_in_case
Yang2025_S	ENSGALG00010013385	PGRMC1	case	control	-0.758942596	0.1862782	-4.074242689	4.61643E-05	0.000571822	down_in_case
Yang2025_S	ENSGALG00010013386	ITGAV	case	control	-0.471877591	0.121904182	-3.870889279	0.000108439	0.001133607	down_in_case
Yang2025_S	ENSGALG00010013387	NDST1	case	control	-0.074710261	0.084837578	-0.880626989	0.378519746	0.546541566	down_in_case
Yang2025_S	ENSGALG00010013388	SOWAHC	case	control	0.69740936	0.288957611	2.413535185	0.015798602	0.057185798	up_in_case
Yang2025_S	ENSGALG00010013389	NAP1L1	case	control	-0.314638257	0.077599994	-4.054617023	5.02165E-05	0.000610693	down_in_case
Yang2025_S	ENSGALG00010013390	SLC16A2	case	control	-0.060499445	0.150317033	-0.402478975	0.687331559	0.804465418	down_in_case
Yang2025_S	ENSGALG00010013391	HMGN1	case	control	0.11744974	0.109532032	1.072286694	0.283591281	0.451836558	up_in_case
Yang2025_S	ENSGALG00010013392	HDAC2	case	control	0.167564838	0.127860951	1.310523952	0.190018647	0.344869737	up_in_case
Yang2025_S	ENSGALG00010013393	ADCK2	case	control	-0.049330294	0.216379944	-0.227979974	0.819661809	0.89227305	down_in_case
Yang2025_S	ENSGALG00010013394	DNMT3B	case	control	0.28998352	0.189949574	1.526634218	0.126851993	0.25949538	up_in_case
Yang2025_S	ENSGALG00010013396	FYB1	case	control	-0.339743145	0.181669207	-1.870119604	0.061467211	0.1551151	down_in_case
Yang2025_S	ENSGALG00010013397	MATN1	case	control	1.570109458	0.895571489	1.753192769			up_in_case
Yang2025_S	ENSGALG00010013398	CHAMP1	case	control	-0.158000885	0.234818005	-0.672865288	0.501032998	0.65780053	down_in_case
Yang2025_S	ENSGALG00010013399	FGF1	case	control	-0.294592272	0.162983117	-1.807501766	0.07068408	0.171122768	down_in_case
Yang2025_S	ENSGALG00010013400	BBS10	case	control	0.11019205	0.227699035	0.483937272	0.628430388	0.761295093	up_in_case
Yang2025_S	ENSGALG00010013402	MARCKS	case	control	0.084814855	0.099370031	0.853525502	0.393367952	0.560317122	up_in_case
Yang2025_S	ENSGALG00010013403	CPNE3	case	control	-0.428177382	0.110159994	-3.886868235	0.000101546	0.001081009	down_in_case
Yang2025_S	ENSGALG00010013404	ENSGALG00010013404	case	control	-0.259988478	0.246140271	-1.056261446	0.290848785	0.459417118	down_in_case
Yang2025_S	ENSGALG00010013405	RPS14	case	control	-0.450405038	0.342721395	-1.314201695	0.188778338	0.343508512	down_in_case
Yang2025_S	ENSGALG00010013406	ENSGALG00010013406	case	control	-0.645433399	0.616269522	-1.047323251	0.294950516	0.463570012	down_in_case
Yang2025_S	ENSGALG00010013407	ZC3H15	case	control	0.364995312	0.111753597	3.266072168	0.001090505	0.007503886	up_in_case
Yang2025_S	ENSGALG00010013408	RLIM	case	control	0.015212515	0.087279564	0.174296408	0.861632522	0.918583934	up_in_case
Yang2025_S	ENSGALG00010013409	DAB2	case	control	0.468799546	0.195529311	2.39759217	0.016503228	0.05900338	up_in_case
Yang2025_S	ENSGALG00010013410	KIAA0825	case	control	0.316974648	0.194695638	1.628052128	0.103513833	0.224893244	up_in_case
Yang2025_S	ENSGALG00010013411	ENSGALG00010013411	case	control	0.928703833	0.694731011	1.336781889	0.181293843	0.333852062	up_in_case
Yang2025_S	ENSGALG00010013412	COMMD7	case	control	-0.246845493	0.171018076	-1.443388319	0.148911113	0.291320561	down_in_case
Yang2025_S	ENSGALG00010013413	WRB	case	control	-0.309082901	0.112910103	-2.737424669	0.00619223	0.028107077	down_in_case
Yang2025_S	ENSGALG00010013414	LAPTM5	case	control	-0.528904636	0.127942145	-4.133935984	3.56603E-05	0.000467036	down_in_case
Yang2025_S	ENSGALG00010013416	NDUFB2	case	control	0.51145891	0.324289695	1.577166705	0.114757172	0.241854384	up_in_case
Yang2025_S	ENSGALG00010013417	AKR1B1	case	control	-0.035978048	0.122715639	-0.293182259	0.769382844	0.859542486	down_in_case
Yang2025_S	ENSGALG00010013418	HCN1	case	control	0.613281528	0.491197595	1.248543425	0.211832116	0.372007629	up_in_case
Yang2025_S	ENSGALG00010013419	ENSGALG00010013419	case	control	0.226243743	0.323932294	0.698429107	0.484908879	0.642503112	up_in_case
Yang2025_S	ENSGALG00010013420	ENSGALG00010013420	case	control	-12.16169236	2.949227202	-4.123687845	3.72854E-05	0.000484224	down_in_case
Yang2025_S	ENSGALG00010013423	ZNF804A	case	control	0.5247502	0.288045401	1.821762121	0.068491088	0.167266615	up_in_case
Yang2025_S	ENSGALG00010013425	ENSGALG00010013425	case	control	-0.607347155	0.343319235	-1.769044938	0.076886376	0.181981041	down_in_case
Yang2025_S	ENSGALG00010013427	NEXMIF	case	control	-0.04334571	0.251735309	-0.172187643	0.863290012	0.919290108	down_in_case
Yang2025_S	ENSGALG00010013428	BPGM	case	control	-0.45809184	0.141217238	-3.243880473	0.001179132	0.007949302	down_in_case
Yang2025_S	ENSGALG00010013429	PDE4DIP	case	control	0.645332748	0.105692564	6.105753563	1.02317E-09	6.22613E-08	up_in_case
Yang2025_S	ENSGALG00010013431	TMEM175	case	control	-0.253012916	0.168044942	-1.505626488	0.132163085	0.267105524	down_in_case
Yang2025_S	ENSGALG00010013434	CORIN	case	control	0.697672321	0.370915902	1.88094476	0.059979434	0.152492868	up_in_case
Yang2025_S	ENSGALG00010013435	SDC3	case	control	0.546346477	0.236321582	2.311877198	0.020784452	0.069622917	up_in_case
Yang2025_S	ENSGALG00010013436	NPAS2	case	control	0.468495723	0.291564758	1.60683248	0.108091121	0.231339237	up_in_case
Yang2025_S	ENSGALG00010013437	ENSGALG00010013437	case	control	0.508032468	0.200037744	2.539683056	0.011095297	0.043750129	up_in_case
Yang2025_S	ENSGALG00010013438	RPL37	case	control	-0.432188703	0.253642495	-1.703928607	0.088394426	0.201507483	down_in_case
Yang2025_S	ENSGALG00010013440	EEA1	case	control	0.859912477	0.222052813	3.872558356	0.000107699	0.001128409	up_in_case
Yang2025_S	ENSGALG00010013442	ENSGALG00010013442	case	control	-0.104550557	0.140677172	-0.743194902	0.45736369	0.618928382	down_in_case
Yang2025_S	ENSGALG00010013444	OSBPL8	case	control	0.009296518	0.139065705	0.066849825	0.946701258	0.967176102	up_in_case
Yang2025_S	ENSGALG00010013446	ATP5I	case	control	-0.357902875	0.284479487	-1.258097302	0.208356569	0.367292699	down_in_case
Yang2025_S	ENSGALG00010013449	LCA5L	case	control	1.01702831	0.327657556	3.103936692	0.001909642	0.011583554	up_in_case
Yang2025_S	ENSGALG00010013450	CD74	case	control	0.042384957	0.178663915	0.237232891	0.812476122	0.887252024	up_in_case
Yang2025_S	ENSGALG00010013451	PRKAB2	case	control	0.025847143	0.116910185	0.22108547	0.825025884	0.895244953	up_in_case
Yang2025_S	ENSGALG00010013452	LSS	case	control	0.014238297	0.146594581	0.097127034	0.922625512	0.953661386	up_in_case
Yang2025_S	ENSGALG00010013453	PUM1	case	control	0.152691258	0.119763019	1.274944963	0.202328723	0.3602824	up_in_case
Yang2025_S	ENSGALG00010013455	PDE6B	case	control	1.67967341	0.640229678	2.623548183	0.008701914	0.036623694	up_in_case
Yang2025_S	ENSGALG00010013457	SH3BGR	case	control	0.000743987	0.134335926	0.00553826	0.995581131	0.996868798	up_in_case
Yang2025_S	ENSGALG00010013458	LAMA4	case	control	-0.061894884	0.133747526	-0.462774049	0.643526334	0.772918926	down_in_case
Yang2025_S	ENSGALG00010013459	ATP10D	case	control	-0.481203376	0.183837585	-2.617546228	0.008856449	0.037150698	down_in_case
Yang2025_S	ENSGALG00010013461	S100B	case	control	0.137827425	0.498485274	0.27649247	0.782169843	0.868350602	up_in_case
Yang2025_S	ENSGALG00010013463	ENSGALG00010013463	case	control	0.134709758	0.151193366	0.890976643	0.372941701	0.541179069	up_in_case
Yang2025_S	ENSGALG00010013464	ABCB7	case	control	0.173667773	0.092186106	1.883882287	0.0595809	0.151784248	up_in_case
Yang2025_S	ENSGALG00010013465	CALD1	case	control	0.302331335	0.201938976	1.497142063	0.134356297	0.27047092	up_in_case
Yang2025_S	ENSGALG00010013466	BRAF	case	control	0.08644993	0.149561706	0.578021827	0.563249383	0.709342987	up_in_case
Yang2025_S	ENSGALG00010013468	DEPDC1B	case	control	-0.125101958	0.179040832	-0.69873423	0.484718138	0.642460458	down_in_case
Yang2025_S	ENSGALG00010013470	RPL31	case	control	-0.659310596	0.283203014	-2.328049359	0.019909483	0.067552628	down_in_case
Yang2025_S	ENSGALG00010013472	ENSGALG00010013472	case	control	-0.193568066	0.189772488	-1.020000674	0.307728141	0.477367432	down_in_case
Yang2025_S	ENSGALG00010013476	ZDHHC17	case	control	0.008337812	0.104331694	0.079916383	0.93630376	0.961133915	up_in_case
Yang2025_S	ENSGALG00010013477	RFC1	case	control	0.373499489	0.130030168	2.87240641	0.004073587	0.02042657	up_in_case
Yang2025_S	ENSGALG00010013480	TBC1D8	case	control	0.003192915	0.100730062	0.031697734	0.974713102	0.984858808	up_in_case
Yang2025_S	ENSGALG00010013482	ENSGALG00010013482	case	control	0.223747214	0.254916722	0.877726704	0.380092049	0.548016834	up_in_case
Yang2025_S	ENSGALG00010013483	ENSGALG00010013483	case	control	-0.184089725	0.358742097	-0.513153395	0.607844024	0.7451664	down_in_case
Yang2025_S	ENSGALG00010013484	TCOF1	case	control	0.381986701	0.124274429	3.073735307	0.002113969	0.012476984	up_in_case
Yang2025_S	ENSGALG00010013486	PLPP6	case	control	0.011657544	0.292510091	0.039853476	0.968209942	0.981213144	up_in_case
Yang2025_S	ENSGALG00010013487	ENSGALG00010013487	case	control	0.32854015	0.182606686	1.799168239	0.071992072	0.173265893	up_in_case
Yang2025_S	ENSGALG00010013488	UPRT	case	control	0.208408163	0.12378128	1.683680779	0.092243378	0.207391331	up_in_case
Yang2025_S	ENSGALG00010013491	CNOT11	case	control	-0.248520064	0.106383158	-2.336084661	0.01948683	0.066539811	down_in_case
Yang2025_S	ENSGALG00010013493	ENSGALG00010013493	case	control	-0.502192797	0.101023464	-4.971051043	6.65909E-07	1.69953E-05	down_in_case
Yang2025_S	ENSGALG00010013494	ASB1	case	control	-0.31774808	0.121942987	-2.60571015	0.009168401	0.038069627	down_in_case
Yang2025_S	ENSGALG00010013497	FAM172A	case	control	0.046286842	0.205061963	0.225721247	0.821418232	0.893392686	up_in_case
Yang2025_S	ENSGALG00010013498	KDR	case	control	-0.038760221	0.080660639	-0.480534516	0.630847366	0.763258635	down_in_case
Yang2025_S	ENSGALG00010013501	AKR1B10	case	control	0.097571463	0.255049247	0.382559307	0.702046547	0.816183066	up_in_case
Yang2025_S	ENSGALG00010013502	PAFAH2	case	control	-0.173478003	0.29435185	-0.589355913	0.555622544	0.702978042	down_in_case
Yang2025_S	ENSGALG00010013506	RNF149	case	control	-0.154464389	0.096979905	-1.592746335	0.111217108	0.236323636	down_in_case
Yang2025_S	ENSGALG00010013508	TUBE1	case	control	0.37365013	0.181162636	2.062512107	0.039159006	0.111489649	up_in_case
Yang2025_S	ENSGALG00010013509	ENSGALG00010013509	case	control	-0.178099965	0.149548833	-1.190915117	0.23368691	0.396883252	down_in_case
Yang2025_S	ENSGALG00010013510	MRPS33	case	control	-0.046641005	0.172832294	-0.269862792	0.787265813	0.871716258	down_in_case
Yang2025_S	ENSGALG00010013511	FAM78B	case	control	0.496251668	0.161535924	3.072082399	0.00212571	0.012519769	up_in_case
Yang2025_S	ENSGALG00010013512	ENSGALG00010013512	case	control	-8.854938372	1.851066265	-4.783696046			down_in_case
Yang2025_S	ENSGALG00010013514	LMBRD2	case	control	0.019976508	0.160405613	0.124537461	0.900889742	0.940944226	up_in_case
Yang2025_S	ENSGALG00010013515	RBP4L	case	control	0.152664981	0.406954987	0.375139722	0.707556556	0.819805489	up_in_case
Yang2025_S	ENSGALG00010013516	CSRP2	case	control	-0.188832815	0.246505833	-0.766037917	0.443653741	0.606823212	down_in_case
Yang2025_S	ENSGALG00010013517	STMN1	case	control	-0.054057433	0.2034709	-0.265676485	0.790488375	0.873638936	down_in_case
Yang2025_S	ENSGALG00010013520	CREG2	case	control	-0.581072213	0.176753494	-3.28747228	0.001010911	0.007074163	down_in_case
Yang2025_S	ENSGALG00010013523	TMEM178B	case	control	0.014222482	0.292988024	0.048542879	0.961283592	0.97684583	up_in_case
Yang2025_S	ENSGALG00010013524	ZDHHC15	case	control	-0.424614618	0.154132472	-2.754868012	0.005871582	0.026974651	down_in_case

Yang2025_S	ENSGALG00010013526	SIRPA	case	control	-0.586652318	0.388447552	-1.510248463	0.130980037	0.265291688	down_in_case
Yang2025_S	ENSGALG00010013527	ADARB1	case	control	0.219806073	0.16094248	1.36574305	0.172019627	0.321326207	up_in_case
Yang2025_S	ENSGALG00010013528	PRDX6	case	control	0.038548384	0.121590934	0.317033374	0.751218279	0.848540188	up_in_case
Yang2025_S	ENSGALG00010013531	ARSI	case	control	-0.006367589	0.155442001	-0.040964403	0.967324275	0.9806958	down_in_case
Yang2025_S	ENSGALG00010013532	WDR19	case	control	0.744706131	0.1356631	5.489378708	4.0335E-08	1.56565E-06	up_in_case
Yang2025_S	ENSGALG00010013533	SKP2	case	control	-0.412347146	0.130697281	-3.154978762	0.001605098	0.010135289	down_in_case
Yang2025_S	ENSGALG00010013537	LMO3	case	control	0.151020519	0.348827583	0.432937436	0.665060222	0.78846471	up_in_case
Yang2025_S	ENSGALG00010013538	E2F7	case	control	-0.190346416	0.161292139	-1.180134487	0.23794673	0.401816249	down_in_case
Yang2025_S	ENSGALG00010013539	NSFL1C	case	control	0.047574689	0.126169	0.377071145	0.706120734	0.818822704	up_in_case
Yang2025_S	ENSGALG00010013544	ENSGALG00010013544	case	control	0.231923679	0.223833151	1.036145353	0.300134337	0.469771086	up_in_case
Yang2025_S	ENSGALG00010013546	IDUA	case	control	0.045963343	0.169245953	0.271577207	0.785947127	0.870744983	up_in_case
Yang2025_S	ENSGALG00010013549	PCP4	case	control	-0.172738979	0.345104335	-0.50054132	0.616693969	0.752836658	down_in_case
Yang2025_S	ENSGALG00010013550	KLHL5	case	control	-0.084988072	0.091893691	-0.924852089	0.355042851	0.524212983	down_in_case
Yang2025_S	ENSGALG00010013551	UBE2N	case	control	-0.527190147	0.099700092	-5.28775988	1.23823E-07	4.08881E-06	down_in_case
Yang2025_S	ENSGALG00010013552	FGF16	case	control	-0.543504509	0.176968807	-3.071188182	0.002132087	0.012546721	down_in_case
Yang2025_S	ENSGALG00010013553	TADA1	case	control	-0.198262504	0.184043307	-1.077260059	0.281364101	0.449273375	down_in_case
Yang2025_S	ENSGALG00010013554	ENSGALG00010013554	case	control	-11.62735444	2.949388127	-3.94229377	8.0706E-05	0.000899722	down_in_case
Yang2025_S	ENSGALG00010013556	RASL11B	case	control	0.072407781	0.148475543	0.4876748	0.625780204	0.759473736	up_in_case
Yang2025_S	ENSGALG00010013558	SNAPC3	case	control	-0.0934925	0.119058138	-0.785267614	0.43229668	0.596288661	down_in_case
Yang2025_S	ENSGALG00010013561	AGK	case	control	-0.002603316	0.14831498	-0.017552617	0.985995757	0.991474302	down_in_case
Yang2025_S	ENSGALG00010013562	RERGL	case	control	0.225635649	0.10589249	2.130799362	0.033105675	0.098448054	up_in_case
Yang2025_S	ENSGALG00010013563	FKBP1A	case	control	-0.725074816	0.168033072	-4.315072064	1.59551E-05	0.000242208	down_in_case
Yang2025_S	ENSGALG00010013565	ENSGALG00010013565	case	control	-5.662853258	2.954707952	-1.916552617			down_in_case
Yang2025_S	ENSGALG00010013569	ENSGALG00010013569	case	control	0.394690184	0.390970246	1.00951463	0.312727889	0.482229123	up_in_case
Yang2025_S	ENSGALG00010013572	DENND11	case	control	-0.189342644	0.122346908	-1.547588309	0.121721448	0.252333885	down_in_case
Yang2025_S	ENSGALG00010013573	ENSGALG00010013573	case	control	-0.590860317	0.339005722	-1.742921369	0.081347351	0.189487516	down_in_case
Yang2025_S	ENSGALG00010013574	NR2F1	case	control	1.015711012	0.193462001	5.250183534	1.51948E-07	4.90137E-06	up_in_case
Yang2025_S	ENSGALG00010013576	CAMK2A	case	control	-0.2375571	0.150553689	-1.577889596	0.114590976	0.241577193	down_in_case
Yang2025_S	ENSGALG00010013579	H2AC32	case	control	2.214993237	1.468586712	1.508248181	0.13149102	0.266039612	up_in_case
Yang2025_S	ENSGALG00010013580	RANBP3L	case	control	0.448002909	0.291128026	1.538851879	0.123840461	0.255057172	up_in_case
Yang2025_S	ENSGALG00010013582	MAP4K4	case	control	0.348031731	0.170575671	2.040336285	0.041316843	0.115891749	up_in_case
Yang2025_S	ENSGALG00010013583	FASLG	case	control	-0.030148084	0.651516388	-0.046273715	0.963092089	0.977979365	down_in_case
Yang2025_S	ENSGALG00010013588	ENSGALG00010013588	case	control	0.535514974	0.40156387	1.333573596	0.182343641	0.335218818	up_in_case
Yang2025_S	ENSGALG00010013589	DSCAM	case	control	1.073463128	0.755203196	1.421422914			up_in_case
Yang2025_S	ENSGALG00010013592	H3C33	case	control	1.351645409	0.437299959	3.090888484	0.001995586	0.011945226	up_in_case
Yang2025_S	ENSGALG00010013595	WEE2	case	control	2.582012027	0.910307537	2.836417279	0.00456228	0.022369813	up_in_case
Yang2025_S	ENSGALG00010013596	SDCBP2	case	control	-0.641790599	0.181990039	-3.526514988	0.000421067	0.003447458	down_in_case
Yang2025_S	ENSGALG00010013598	PAQR7	case	control	0.269716846	0.23470632	1.149167376	0.25048697	0.416209439	up_in_case
Yang2025_S	ENSGALG00010013604	FYN	case	control	-0.138328826	0.07982581	-1.732883468	0.083116383	0.192492405	down_in_case
Yang2025_S	ENSGALG00010013609	PIGC	case	control	-0.068845869	0.267108106	-0.257745338	0.796603453	0.87676085	down_in_case
Yang2025_S	ENSGALG00010013612	TWIST2	case	control	0.257267788	0.186937366	1.376224523	0.168752132	0.317135665	up_in_case
Yang2025_S	ENSGALG00010013614	ATRX	case	control	0.509375776	0.193669218	2.630132868	0.00853515	0.036041612	up_in_case
Yang2025_S	ENSGALG00010013617	PIK3C2G	case	control	0.104527234	0.240690799	0.43428014	0.664085024	0.78757658	up_in_case
Yang2025_S	ENSGALG00010013619	IL1R2	case	control	0.95786613	0.246311506	3.888840373	0.000100724	0.001073736	up_in_case
Yang2025_S	ENSGALG00010013621	PDGFRA	case	control	-0.199947188	0.167979538	-1.190306812	0.233925824	0.397095427	down_in_case
Yang2025_S	ENSGALG00010013625	SSBP1	case	control	0.364380116	0.173632027	2.098576644	0.035854237	0.104634302	up_in_case
Yang2025_S	ENSGALG00010013627	MYCBP	case	control	-0.212612314	0.292558377	-0.726734666	0.467388513	0.628370374	down_in_case
Yang2025_S	ENSGALG00010013632	SLC1A3	case	control	0.234438163	0.646863578	0.362422883	0.717036042	0.825708392	up_in_case
Yang2025_S	ENSGALG00010013635	BCL6	case	control	0.719712987	0.192735172	3.73420678	0.000188308	0.001777397	up_in_case
Yang2025_S	ENSGALG00010013638	COMMD8	case	control	0.167520398	0.134934172	1.241497214	0.214422127	0.374996528	up_in_case
Yang2025_S	ENSGALG00010013639	ENSGALG00010013639	case	control	0.447092474	0.226917959	1.970282455	0.048806008	0.131042721	up_in_case
Yang2025_S	ENSGALG00010013645	RRAGC	case	control	-0.354254141	0.087014982	-4.071185597	4.67745E-05	0.000577327	down_in_case
Yang2025_S	ENSGALG00010013646	CLDN1	case	control	-0.348996081	0.253388436	-1.377316529	0.168414407	0.316743255	down_in_case
Yang2025_S	ENSGALG00010013648	NAV3	case	control	0.258081452	0.174528228	1.478737589	0.139210462	0.277624717	up_in_case
Yang2025_S	ENSGALG00010013650	BACE2	case	control	-0.114316065	0.130792345	-0.874027185	0.382103449	0.550006359	down_in_case
Yang2025_S	ENSGALG00010013651	ENSGALG00010013651	case	control	0.218953684	0.132728853	1.649631395	0.09901835	0.217534401	up_in_case
Yang2025_S	ENSGALG00010013654	SLC6A7	case	control	1.247192265	0.383675235	3.25064573	0.001151432	0.007828722	up_in_case
Yang2025_S	ENSGALG00010013657	MAGT1	case	control	-0.396038904	0.157578651	-2.513277662	0.011961515	0.046236813	down_in_case
Yang2025_S	ENSGALG00010013661	GABRA4	case	control	-0.01137189	0.653691146	-0.017396427	0.986120359	0.991528047	down_in_case
Yang2025_S	ENSGALG00010013663	OPA1	case	control	-0.023833851	0.090474957	-0.263430368	0.792218887	0.874629234	down_in_case
Yang2025_S	ENSGALG00010013664	REV3L	case	control	0.071634331	0.160416363	0.446552522	0.655198182	0.781986044	up_in_case
Yang2025_S	ENSGALG00010013666	COX7B	case	control	-0.827988745	0.299856131	-2.7612867	0.005757411	0.026583672	down_in_case
Yang2025_S	ENSGALG00010013669	NPR3	case	control	-0.040130313	0.235457334	-0.170435606	0.864667572	0.920045088	down_in_case
Yang2025_S	ENSGALG00010013672	ENSGALG00010013672	case	control	0.532785238	0.369518211	1.441837564	0.149348203	0.291684262	up_in_case
Yang2025_S	ENSGALG00010013675	CHRNA6	case	control	1.473053555	0.452186439	3.25762435	0.00112349	0.007681961	up_in_case
Yang2025_S	ENSGALG00010013678	ATP7A	case	control	-0.249078034	0.09711626	-2.564740811	0.01032529	0.041502985	down_in_case
Yang2025_S	ENSGALG00010013679	MFSD4B	case	control	-0.591323569	0.122470747	-4.828284164	1.37714E-06	3.17199E-05	down_in_case
Yang2025_S	ENSGALG00010013686	UQCRQ	case	control	-0.468783514	0.240202677	-1.951616522	0.05098375	0.135170959	down_in_case
Yang2025_S	ENSGALG00010013690	CREB3L2	case	control	-0.374224998	0.142778358	-2.621020472	0.0087667	0.03680746	down_in_case
Yang2025_S	ENSGALG00010013696	CETN3	case	control	0.496811936	0.231861346	2.142711343	0.032136284	0.096446951	up_in_case
Yang2025_S	ENSGALG00010013697	HDAC4	case	control	0.168711309	0.170746194	0.988082399	0.323112295	0.492457317	up_in_case
Yang2025_S	ENSGALG00010013709	PSMF1	case	control	-0.373594458	0.163356736	-2.286985328	0.022196679	0.072950644	down_in_case
Yang2025_S	ENSGALG00010013715	IL1R1	case	control	0.307878427	0.146324687	2.104077134	0.035371719	0.103636732	up_in_case
Yang2025_S	ENSGALG00010013719	NCDN	case	control	0.170132207	0.146855148	1.158503525	0.246658614	0.412380058	up_in_case
Yang2025_S	ENSGALG00010013720	SLC16A10	case	control	-0.644973368	0.148526253	-4.342487297	1.40879E-05	0.000219702	down_in_case
Yang2025_S	ENSGALG00010013732	FNTA	case	control	0.400462023	0.152730314	2.622020556	0.008741016	0.036722945	up_in_case
Yang2025_S	ENSGALG00010013737	AFF4	case	control	-0.121559545	0.126269224	-0.962701291	0.335697448	0.504042334	down_in_case
Yang2025_S	ENSGALG00010013740	GNPDA2	case	control	0.236599613	0.112070382	2.111169855	0.034757715	0.102183282	up_in_case
Yang2025_S	ENSGALG00010013744	ENSGALG00010013744	case	control	0.445420014	0.32107862	1.387261519	0.165362033	0.312959381	up_in_case
Yang2025_S	ENSGALG00010013746	ENSGALG00010013746	case	control	0.497148008	0.218467595	2.275614416	0.022869098	0.074772613	up_in_case
Yang2025_S	ENSGALG00010013749	NELL2	case	control	-0.222466948	0.268962166	-0.82713101	0.40816281	0.573940338	down_in_case
Yang2025_S	ENSGALG00010013751	FUT10	case	control	-0.117814672	0.183259914	-0.642882935	0.520300063	0.674015188	down_in_case
Yang2025_S	ENSGALG00010013753	PLEKHA5	case	control	0.424044619	0.212075036	1.999502758	0.045553984	0.124959599	up_in_case
Yang2025_S	ENSGALG00010013755	ANGPT4	case	control	0.232075114	0.132758156	1.748104386	0.080445954	0.18799503	up_in_case
Yang2025_S	ENSGALG00010013757	DGKI	case	control	0.770306745	0.289834558	2.657746373	0.007866507	0.033833263	up_in_case
Yang2025_S	ENSGALG00010013760	RPF2	case	control	-0.276319169	0.139890411	-1.975254537	0.04823927	0.129996949	down_in_case
Yang2025_S	ENSGALG00010013766	DNM3	case	control	-0.175604803	0.149834907	-1.171988602	0.241201632	0.406278828	down_in_case
Yang2025_S	ENSGALG00010013767	PGK2	case	control	-0.239233066	0.161602548	-1.480379295	0.138772052	0.27696771	down_in_case
Yang2025_S	ENSGALG00010013768	FAM110A	case	control	0.443968848	0.180721147	2.456651339	0.014023872	0.052098815	up_in_case
Yang2025_S	ENSGALG00010013771	PELO	case	control	0.155281545	0.200075851	0.776113381	0.437682044	0.601548405	up_in_case
Yang2025_S	ENSGALG00010013772	KIAA0319L	case	control	-0.145340319	0.075550039	-1.923762323	0.054384382	0.141865662	down_in_case
Yang2025_S	ENSGALG00010013775	DBX2	case	control	0.319365535	0.339887861	0.939620302	0.347412359	0.515898468	up_in_case
Yang2025_S	ENSGALG00010013777	CPLANE1	case	control	0.3255575	0.226401194	1.437967244	0.150443348	0.293247735	up_in_case
Yang2025_S	ENSGALG00010013778	ENSGALG00010013778	case	control	0.359064056	0.813884105	0.441173447	0.659087435	0.784658933	up_in_case
Yang2025_S	ENSGALG00010013779	SLC52A3	case	control	0.447383185	0.26280005	1.702371005	0.088685842	0.201889204	up_in_case
Yang2025_S	ENSGALG00010013783	NDUFA10	case	control	-0.023779733	0.143890114	-0.165263145	0.868736866	0.922842524	down_in_case
Yang2025_S	ENSGALG00010013784	ANO6	case	control	0.163356477	0.13284021	1.229721609	0.218801372	0.379842671	up_in_case
Yang2025_S	ENSGALG00010013787	ZCCHC10	case	control	-0.349158587	0.126840277	-2.752742221	0.005909841	0.027090011	down_in_case
Yang2025_S	ENSGALG00010013791	AEBP2	case	control	0.018536081	0.090866048	0.203993478	0.838358596	0.904449915	up_in_case
Yang2025_S	ENSGALG00010013792	FGF10	case	control	-0.101675282	0.247795633	-0.410319104	0.68157188	0.800413294	down_in_case
Yang2025_S	ENSGALG00010013793	METTL13	case	control	0.014264792	0.164244813	0.086850792	0.930790114	0.958156182	up_in_case
Yang2025_S	ENSGALG00010013794	GTF3C6	case	control	0.14889583	0.137995208	1.078992757	0.280590956	0.448449934	up_in_case
Yang2025_S	ENSGALG00010013797	IL1RL1	case	control	-0.684035698	0.243076591	-2.814074754	0.004891785	0.023603541	down_in_case
Yang2025_S	ENSGALG00010013799	SCRT2	case	control	0.334534792	0.38066548	0.878815678	0.379501224	0.547561561	up_in_case
Yang2025_S	ENSGALG00010013802	PYROXD1	case	control	0.061080178	0.16083435	0.379770728	0.704115613	0.817518003	up_in_case
Yang2025_S	ENSGALG00010013805	HSPA4	case	control	-0.001645488	0.07264314	-0.022651661	0.981928135	0.98883219	down_in_case
Yang2025_S	ENSGALG00010013808	PLAAT1	case	control	0.062990951	0.143382936	0.43931972	0.660429887	0.785514721	up_in_case
Yang2025_S	ENSGALG00010013814	AMD1	case	control	-0.227182521	0.12310183	-1.845484522	0.064967088	0.161135348	down_in_case
Yang2025_S	ENSGALG00010013815	SRXN1	case	control	-0.036678257	0.290336162	-0.126330309	0.899470466	0.940222773	down_in_case
Yang2025_S	ENSGALG00010013820	MASP1	case	control	0.401260971	0.612102534	0.655545352	0.512116697	0.66732244	up_in_case
Yang2025_S	ENSGALG00010013823	GUF1	case	control	0.359690106	0.116283921	3.093205857	0.001980067	0.011882962	up_in_case
Yang2025_S	ENSGALG00010013828	PTN	case	control	0.749675087	0.281484656	2.663289353	0.007738084	0.033342673	up_in_case
Yang2025_S	ENSGALG00010013831	TCERG1	case	control	0.510293097	0.166861	3.058192728	0.002226763	0.012973435	up_in_case
Yang2025_S	ENSGALG00010013836	IL18R1	case	control	-0.493210944	0.293432118	-1.680834895	0.092794989	0.20809433	down_in_case
Yang2025_S	ENSGALG00010013837	IFI27L2	case	control	0.667399311	0.276231805	2.416084244	0.015688431	0.056887126	up_in_case
Yang2025_S	ENSGALG00010013842	CDK19	case	control	0.455164048	0.179062719	2.541925254	0.011024375	0.043519734	up_in_case
Yang2025_S	ENSGALG00010013846	YIPF7	case	control	-0.330320892	0.242999293	-1.359349191	0.174035963	0.324266767	down_in_case
Yang2025_S	ENSGALG00010013848	ITPA	case	control	-0.308118637	0.254884	-1.208858292	0.226717292	0.388789744	down_in_case
Yang2025_S	ENSGALG00010013859	MYOC	case	control	0.247114207	0.370342232	0.667259052	0.504606677	0.660366361	up_in_case
Yang2025_S	ENSGALG00010013861	TCF15	case	control	0.529647358	0.25594702	2.069363254	0.038512012	0.110107691	up_in_case
Yang2025_S	ENSGALG00010013866	KCTD8	case	control	0.43786577	0.546645252	0.801005346	0.423128551	0.588714652	up_in_case
Yang2025_S	ENSGALG00010013867	RECQL	case	control	0.20795044	0.155009943	1.341529688	0.179748537	0.331761042	up_in_case
Yang2025_S	ENSGALG00010013871	ENSGALG00010013871	case	control	0.779828781	0.295502203	2.638994816	0.008315225	0.035284002	up_in_case
Yang2025_S	ENSGALG00010013872	GLS2	case	control	0.08427377	0.127698941	0.659941026	0.509291675	0.664697902	up_in_case
Yang2025_S	ENSGALG00010013878	ARID2	case	control	0.389805286	0.185435111	2.10211154	0.035543506	0.104010659	up_in_case
Yang2025_S	ENSGALG00010013880	ZMYM4	case	control	0.09058567	0.139133348	0.651070874	0.515000737	0.669640317	up_in_case

Yang2025_S	ENSGALG00010013889	ENSGALG00010013889	case	control	0.639681871	0.143225792	4.466247732	7.96034E-06	0.000135442	up_in_case
Yang2025_S	ENSGALG00010013890	SFPQ	case	control	0.221869731	0.086707257	2.558836925	0.010502299	0.041993511	up_in_case
Yang2025_S	ENSGALG00010013893	CSNK2A1	case	control	-0.21705685	0.089056269	-2.437300072	0.014797394	0.054378083	down_in_case
Yang2025_S	ENSGALG00010013897	AMOT	case	control	0.01809836	0.132531312	0.136559124	0.891379288	0.936336879	up_in_case
Yang2025_S	ENSGALG00010013898	EMB	case	control	-0.243133774	0.311055288	-0.781641669	0.434425191	0.598370568	down_in_case
Yang2025_S	ENSGALG00010013899	WNT5B	case	control	-0.031653089	0.092493637	-0.3422191	0.732186012	0.836362093	down_in_case
Yang2025_S	ENSGALG00010013901	NFXL1	case	control	0.336549057	0.118657498	2.836306717	0.004563859	0.022369813	up_in_case
Yang2025_S	ENSGALG00010013908	TBC1D20	case	control	-0.29460003	0.115846461	-2.543021407	0.01098985	0.043432659	down_in_case
Yang2025_S	ENSGALG00010013910	ADIPOR2	case	control	0.005399799	0.070471954	0.076623379	0.93892316	0.962932392	up_in_case
Yang2025_S	ENSGALG00010013911	TNIK	case	control	-0.05128998	0.120454603	-0.425803402	0.670251107	0.792192467	down_in_case
Yang2025_S	ENSGALG00010013914	NRG1	case	control	0.552552736	0.229356805	2.409140356	0.015990147	0.05765141	up_in_case
Yang2025_S	ENSGALG00010013915	ENSGALG00010013915	case	control	1.026342125	0.34406232	2.983012283	0.002854265	0.015683828	up_in_case
Yang2025_S	ENSGALG00010013917	TRPC5	case	control	-0.133335391	0.722741955	-0.184485472	0.853632623	0.913552769	down_in_case
Yang2025_S	ENSGALG00010013921	PPP2R2B	case	control	0.050709054	0.101451503	0.499835415	0.617190972	0.753177219	up_in_case
Yang2025_S	ENSGALG00010013928	CCNO	case	control	-0.729795274	0.37657653	-1.937973336	0.052626476	0.138446279	down_in_case
Yang2025_S	ENSGALG00010013930	SLC22A16	case	control	0.0563807	0.144223701	0.390925345	0.695852419	0.811317405	up_in_case
Yang2025_S	ENSGALG00010013934	STAT1	case	control	0.012361862	0.078309674	0.157858688	0.874568152	0.92642396	up_in_case
Yang2025_S	ENSGALG00010013936	DLGAP3	case	control	0.480163666	0.266002889	1.805106958	0.071057946	0.171729532	up_in_case
Yang2025_S	ENSGALG00010013938	ENSGALG00010013938	case	control	0.330621335	0.381884311	0.865763075	0.386620102	0.554088715	up_in_case
Yang2025_S	ENSGALG00010013940	UPF3A	case	control	-0.263562417	0.318081209	-0.828601029	0.407330206	0.573290215	down_in_case
Yang2025_S	ENSGALG00010013942	CACNA2D4	case	control	0.56978456	0.531605337	1.071818737	0.283801456	0.45202575	up_in_case
Yang2025_S	ENSGALG00010013944	METTL24	case	control	-0.298120926	0.186189999	-1.601165087	0.109340359	0.233260549	down_in_case
Yang2025_S	ENSGALG00010013945	SCAF11	case	control	0.686416143	0.182053756	3.770403635	0.000162984	0.001582703	up_in_case
Yang2025_S	ENSGALG00010013947	ENSGALG00010013947	case	control	-0.981277614	0.328046095	-2.991279663	0.002778109	0.015342695	down_in_case
Yang2025_S	ENSGALG00010013949	PRRC2C	case	control	0.574105153	0.225880066	2.541637094	0.011033467	0.04354329	up_in_case
Yang2025_S	ENSGALG00010013953	DHX29	case	control	0.066176877	0.152478644	0.434007513	0.664282986	0.787744312	up_in_case
Yang2025_S	ENSGALG00010013954	LPL	case	control	-0.460718713	0.152854013	-3.014109363	0.002577349	0.014522991	down_in_case
Yang2025_S	ENSGALG00010013958	TUSC3	case	control	-0.358655322	0.180858706	-1.983069161	0.047359705	0.128396398	down_in_case
Yang2025_S	ENSGALG00010013959	STK32A	case	control	-0.414198268	0.483798559	-0.85613787	0.391921524	0.559056857	down_in_case
Yang2025_S	ENSGALG00010013964	SLC38A1	case	control	0.08907299	0.139088691	0.640404253	0.521909819	0.675243084	up_in_case
Yang2025_S	ENSGALG00010013967	MLLT3	case	control	-0.171624921	0.196233159	-0.874596945	0.381793251	0.549730208	down_in_case
Yang2025_S	ENSGALG00010013971	ENSGALG00010013971	case	control	-0.025675017	0.292297069	-0.087838777	0.930004817	0.957822654	down_in_case
Yang2025_S	ENSGALG00010013973	ENSGALG00010013973	case	control	-0.019381642	0.406293242	-0.04770358	0.961952481	0.977454267	down_in_case
Yang2025_S	ENSGALG00010013976	CDC40	case	control	-0.23555056	0.060910954	-3.867129691	0.000110124	0.001146918	down_in_case
Yang2025_S	ENSGALG00010013977	SLC38A2	case	control	-0.147519032	0.105807744	-1.394217724	0.163251867	0.309933892	down_in_case
Yang2025_S	ENSGALG00010013981	RLF	case	control	-0.41903811	0.097110616	-4.315059742	1.5956E-05	0.000242208	down_in_case
Yang2025_S	ENSGALG00010013989	ENSGALG00010013989	case	control	0.414503525	0.186009582	2.228398775	0.025853935	0.081936452	up_in_case
Yang2025_S	ENSGALG00010013994	CHRM2	case	control	0.093063726	0.694070067	0.134084051	0.893336111	0.937397493	up_in_case
Yang2025_S	ENSGALG00010013995	REM1	case	control	-0.377488855	0.338376931	-1.115586849	0.264599024	0.431653875	down_in_case
Yang2025_S	ENSGALG00010014001	CDC16	case	control	-0.212912678	0.084332678	-2.524675874	0.011580504	0.045170782	down_in_case
Yang2025_S	ENSGALG00010014002	ZDHHC18	case	control	0.083918448	0.238184472	0.352325435	0.724594215	0.830611257	up_in_case
Yang2025_S	ENSGALG00010014010	EIF5A2	case	control	0.372972215	0.167294538	2.229434496	0.025785009	0.081773806	up_in_case
Yang2025_S	ENSGALG00010014011	SFN	case	control	0.5366236	0.762225118	0.704022457	0.481418788	0.639825543	up_in_case
Yang2025_S	ENSGALG00010014014	PARP8	case	control	0.553168951	0.189204744	2.923652645	0.003459506	0.01810297	up_in_case
Yang2025_S	ENSGALG00010014018	DPYSL3	case	control	-0.183861568	0.129700741	-1.417583025	0.156312539	0.301399644	down_in_case
Yang2025_S	ENSGALG00010014019	IRAG2	case	control	-0.214723044	0.258897981	-0.829373188	0.406893268	0.572964601	down_in_case
Yang2025_S	ENSGALG00010014020	PDXP	case	control	-0.620463108	0.262924357	-2.359854045	0.018282126	0.063420819	down_in_case
Yang2025_S	ENSGALG00010014026	SH3BP1	case	control	0.320049825	0.427068608	0.749410796	0.453609646	0.61560678	up_in_case
Yang2025_S	ENSGALG00010014030	WASF1	case	control	0.181927529	0.326490258	0.557221921	0.577375814	0.720233817	up_in_case
Yang2025_S	ENSGALG00010014031	RPL22L1	case	control	-1.467181917	0.19863044	-7.386490805	1.50755E-13	2.36041E-11	down_in_case
Yang2025_S	ENSGALG00010014032	PSD3	case	control	-0.002754485	0.183817457	-0.014984893	0.988044233	0.992746152	down_in_case
Yang2025_S	ENSGALG00010014033	SLC38A4	case	control	0.437726831	0.276877794	1.580938738	0.113892046	0.240576877	up_in_case
Yang2025_S	ENSGALG00010014035	PSIP1	case	control	0.721424186	0.190077185	3.795427555	0.000147389	0.00145975	up_in_case
Yang2025_S	ENSGALG00010014041	SKIV2L2	case	control	0.04783412	0.134077601	0.356764434	0.721268164	0.828487557	up_in_case
Yang2025_S	ENSGALG00010014042	SGCZ	case	control	1.339877034	0.353835946	3.786718253	0.00015265	0.001501184	up_in_case
Yang2025_S	ENSGALG00010014045	GOLT1B	case	control	-0.59736394	0.087547652	-6.823300525	8.89723E-12	9.61107E-10	down_in_case
Yang2025_S	ENSGALG00010014047	HM13	case	control	0.247187881	0.114758479	2.153983585	0.031241459	0.094344438	up_in_case
Yang2025_S	ENSGALG00010014056	FMO3	case	control	0.156112531	0.199796493	0.781357715	0.434592133	0.598435775	up_in_case
Yang2025_S	ENSGALG00010014062	GGA1	case	control	0.090156566	0.081593445	1.104948638	0.269181846	0.436320695	up_in_case
Yang2025_S	ENSGALG00010014065	CHUNK-1	case	control	1.040955486	0.509738137	2.042137738	0.04113787	0.115543605	up_in_case
Yang2025_S	ENSGALG00010014066	BCL2L1	case	control	0.135304203	0.12571535	1.076274314	0.281804593	0.449770587	up_in_case
Yang2025_S	ENSGALG00010014076	PRRX1	case	control	0.734991035	0.253451989	2.899922145	0.003732554	0.019185959	up_in_case
Yang2025_S	ENSGALG00010014077	SLITRK2	case	control	-0.205572316	0.154275861	-1.332498257	0.182696514	0.335646133	down_in_case
Yang2025_S	ENSGALG00010014080	MYO1B	case	control	0.327335359	0.128313411	2.551061165	0.010739548	0.042713888	up_in_case
Yang2025_S	ENSGALG00010014082	HNRNPR	case	control	0.368519416	0.163910762	2.248292981	0.024557514	0.078995604	up_in_case
Yang2025_S	ENSGALG00010014083	ENSGALG00010014083	case	control	0.024360435	0.209797333	0.116114133	0.907562088	0.944642792	up_in_case
Yang2025_S	ENSGALG00010014089	FIG4	case	control	-0.126855065	0.120239763	-1.055017587	0.291417281	0.460050187	down_in_case
Yang2025_S	ENSGALG00010014091	CHIC2	case	control	-0.405826001	0.095834937	-4.234635246	2.28923E-05	0.000325514	down_in_case
Yang2025_S	ENSGALG00010014092	TYRP1	case	control	8.734131882	0.476450176	18.33167939	4.62405E-75	6.44362E-71	up_in_case
Yang2025_S	ENSGALG00010014093	PLD1	case	control	-0.190954398	0.125324455	-1.523680257	0.127588604	0.260505083	down_in_case
Yang2025_S	ENSGALG00010014096	JAKMIP2	case	control	0.598165943	0.197015022	3.036143818	0.00239625	0.01374146	up_in_case
Yang2025_S	ENSGALG00010014099	TPX2	case	control	-0.115525699	0.106940108	-1.080284106	0.280015685	0.447821077	down_in_case
Yang2025_S	ENSGALG00010014102	TRIM2	case	control	0.074171758	0.167253311	0.443469596	0.657426106	0.783900793	up_in_case
Yang2025_S	ENSGALG00010014104	ALDH1A1	case	control	-0.345376523	0.711543542	-0.485390567	0.627399322	0.760683246	down_in_case
Yang2025_S	ENSGALG00010014109	AMIGO2	case	control	0.708787015	0.210106311	3.373468466	0.000742275	0.005516591	up_in_case
Yang2025_S	ENSGALG00010014111	GORAB	case	control	0.266780958	0.081798245	3.261450857	0.001108436	0.007608897	up_in_case
Yang2025_S	ENSGALG00010014114	ANXA1	case	control	-0.425434747	0.203263616	-2.093019673	0.036347398	0.105609048	down_in_case
Yang2025_S	ENSGALG00010014120	SOX5	case	control	0.795814773	0.225980686	3.521605268	0.000428942	0.003495504	up_in_case
Yang2025_S	ENSGALG00010014122	ABCA1	case	control	-0.197495058	0.151807783	-1.30095476	0.19327394	0.348643671	down_in_case
Yang2025_S	ENSGALG00010014125	PLPP1	case	control	-0.486887359	0.110597007	-4.402355648	1.07082E-05	0.000174729	down_in_case
Yang2025_S	ENSGALG00010014128	COL4A1	case	control	-0.444654448	0.112474596	-3.9533767	7.7056E-05	0.00086525	down_in_case
Yang2025_S	ENSGALG00010014131	CDC23	case	control	0.443271519	0.079713106	5.560836095	2.68485E-08	1.09396E-06	up_in_case
Yang2025_S	ENSGALG00010014134	RPAP3	case	control	0.004307689	0.075864098	0.05678165	0.954719131	0.972132325	up_in_case
Yang2025_S	ENSGALG00010014135	ZBTB24	case	control	0.057268045	0.153316763	0.373527617	0.708755795	0.820223694	up_in_case
Yang2025_S	ENSGALG00010014142	PPIL6	case	control	0.466360748	0.402536157	1.158556169	0.246637144	0.412380058	up_in_case
Yang2025_S	ENSGALG00010014143	NFATC2	case	control	0.53862444	0.292149498	1.843660333	0.065232657	0.161520655	up_in_case
Yang2025_S	ENSGALG00010014145	NTMT2	case	control	-0.945118584	0.277078258	-3.411016767	0.000647211	0.00493252	down_in_case
Yang2025_S	ENSGALG00010014147	ENSGALG00010014147	case	control	-0.165512616	0.289237281	-0.57223818	0.567160624	0.711952374	down_in_case
Yang2025_S	ENSGALG00010014148	KCNG1	case	control	0.453202105	0.246309858	1.839967382	0.065773026	0.162505152	up_in_case
Yang2025_S	ENSGALG00010014151	ATP23	case	control	-0.346565984	0.149015726	-2.325700735	0.020034521	0.067795302	down_in_case
Yang2025_S	ENSGALG00010014153	FOCAD	case	control	0.094076433	0.139608742	0.673857752	0.500401754	0.657219458	up_in_case
Yang2025_S	ENSGALG00010014154	DNAI7	case	control	0.333309676	0.342691621	0.972622777	0.330740818	0.500094759	up_in_case
Yang2025_S	ENSGALG00010014156	CD164	case	control	-0.664871462	0.085509232	-7.775434858	7.51887E-15	1.58751E-12	down_in_case
Yang2025_S	ENSGALG00010014159	CDC42EP1	case	control	-0.008126731	0.081999811	-0.099106709	0.921053545	0.953250803	down_in_case
Yang2025_S	ENSGALG00010014161	GFRA3	case	control	0.95564569	0.222138541	4.302025602	1.69244E-05	0.00025332	up_in_case
Yang2025_S	ENSGALG00010014162	COL18A1	case	control	-0.289653785	0.168305168	-1.721003513	0.085250186	0.196097945	down_in_case
Yang2025_S	ENSGALG00010014165	LUZP1	case	control	0.283747213	0.199252303	1.424059896	0.154429134	0.298718766	up_in_case
Yang2025_S	ENSGALG00010014166	FGF12	case	control	0.946985542	0.242151337	3.910717791	9.20222E-05	0.000998699	up_in_case
Yang2025_S	ENSGALG00010014167	SLC38A9	case	control	0.423681058	0.223266052	1.897651048	0.057742065	0.148347285	up_in_case
Yang2025_S	ENSGALG00010014170	CEMIP2	case	control	-0.13584209	0.103953027	-1.306764157	0.191292822	0.34646029	down_in_case
Yang2025_S	ENSGALG00010014171	DLC1	case	control	-0.076578617	0.12307133	-0.622229537	0.533790947	0.685501506	down_in_case
Yang2025_S	ENSGALG00010014173	MND1	case	control	-0.135673444	0.16384032	-0.828083371	0.407623287	0.573644769	down_in_case
Yang2025_S	ENSGALG00010014176	ABHD17B	case	control	-0.17997092	0.138492459	-1.29949978	0.193772469	0.349316863	down_in_case
Yang2025_S	ENSGALG00010014178	LRTM2	case	control	0.088797157	0.261819593	0.339153982	0.734493734	0.837986751	up_in_case
Yang2025_S	ENSGALG00010014179	CTBPL	case	control	-0.35918158	0.071832529	-5.000263606	5.7252E-07	1.49682E-05	down_in_case
Yang2025_S	ENSGALG00010014180	SLC19A1	case	control	0.043945051	0.173423372	0.253397511	0.799961051	0.879137007	up_in_case
Yang2025_S	ENSGALG00010014184	SLC44A1	case	control	-0.348565265	0.140462505	-2.481553807	0.013081096	0.049426538	down_in_case
Yang2025_S	ENSGALG00010014186	MOCS3	case	control	-0.407140246	0.162574444	-2.50433116	0.012268314	0.047174107	down_in_case
Yang2025_S	ENSGALG00010014189	DDX4	case	control	0.922986043	0.500976773	1.842372926	0.065420619	0.161819739	up_in_case
Yang2025_S	ENSGALG00010014190	HACD4	case	control	-1.052262438	0.229303799	-4.588944627	4.45493E-06	8.42326E-05	down_in_case
Yang2025_S	ENSGALG00010014192	KDM1A	case	control	-0.250312376	0.070373566	-3.556909098	0.000375244	0.003142442	down_in_case
Yang2025_S	ENSGALG00010014193	CARD10	case	control	0.182733503	0.433043886	0.421974559	0.673043587	0.794212046	up_in_case
Yang2025_S	ENSGALG00010014194	LRIG3	case	control	0.081789117	0.096699964	0.845802976	0.397662665	0.564488859	up_in_case
Yang2025_S	ENSGALG00010014197	SH3GL2	case	control	-0.297392051	0.363841794	-0.817366383	0.413719081	0.579185557	down_in_case
Yang2025_S	ENSGALG00010014198	KDM3B	case	control	-0.160693929	0.091155815	-1.762848917	0.077925975	0.18392589	down_in_case
Yang2025_S	ENSGALG00010014199	LNX1	case	control	0.595122559	0.23034051	2.583664333	0.009775688	0.039843293	up_in_case
Yang2025_S	ENSGALG00010014200	DCP1B	case	control	-0.402562567	0.12670912	-3.177060706	0.001487759	0.009562694	down_in_case
Yang2025_S	ENSGALG00010014203	TMEM38B	case	control	-0.5074614	0.139568041	-3.635942707	0.000276966	0.002435028	down_in_case
Yang2025_S	ENSGALG00010014204	KIFAP3	case	control	-0.113919156	0.100093797	-1.13812403	0.255068695	0.421454368	down_in_case
Yang2025_S	ENSGALG00010014205	DPM1	case	control	-0.11587736	0.161420932	-0.717858325	0.472844647	0.632501065	down_in_case
Yang2025_S	ENSGALG00010014209	CCDC50	case	control	-0.492172075	0.0794633	-6.193702899	5.8767E-10	3.82672E-08	down_in_case
Yang2025_S	ENSGALG00010014210	ENSGALG00010014210	case	control	0.277289789	0.222799662	1.244570059	0.213289832	0.37353196	up_in_case
Yang2025_S	ENSGALG00010014213	ETFRF1	case	control	0.182883057	0.156119334	1.171431187	0.241425503	0.406606766	up_in_case
Yang2025_S	ENSGALG00010014214	REEP2	case	control	0.427108194	0.158945543	2.6871354	0.007206773	0.031595501	up_in_case
Yang2025_S	ENSGALG00010014217	RASA3	case	control	-0.106050233	0.086753686	-1.222429135	0.221545387	0.382984117	down_in_case
Yang2025_S	ENSGALG00010014218	IL31RA	case	control	0.133488532	0.148036646	0.90172627	0.367202295	0.535863858	up_in_case

Yang2025_S	ENSGALG00010014220	C9orf85	case	control	-0.286028074	0.223676749	-1.278756398	0.200982853	0.358833575	down_in_case
Yang2025_S	ENSGALG00010014221	SCYL3	case	control	-0.180011431	0.105883869	-1.700083616	0.089115199	0.202547756	down_in_case
Yang2025_S	ENSGALG00010014222	TBX22	case	control	-0.839871974	0.338288403	-2.482709921	0.013038722	0.049338987	down_in_case
Yang2025_S	ENSGALG00010014224	NMUR2	case	control	-1.389438324	0.67702113	-2.052282068	0.040142258	0.113603241	down_in_case
Yang2025_S	ENSGALG00010014225	ZMPSTE24	case	control	-0.02109644	0.093216356	-0.226316936	0.820954927	0.893065296	down_in_case
Yang2025_S	ENSGALG00010014226	SLC16A7	case	control	-0.194606171	0.216712253	-0.897993393	0.369189072	0.537861967	down_in_case
Yang2025_S	ENSGALG00010014234	GLRA1	case	control	1.385554846	0.690641065	2.006186593	0.044836344	0.123469567	up_in_case
Yang2025_S	ENSGALG00010014236	WDR44	case	control	0.131385386	0.136379251	0.963382519	0.335355596	0.503972114	up_in_case
Yang2025_S	ENSGALG00010014238	PHLDA1	case	control	0.254419712	0.138314282	1.83943196	0.065851677	0.162645005	up_in_case
Yang2025_S	ENSGALG00010014240	PIGV	case	control	0.404125399	0.230670865	1.751956842	0.079781229	0.187006128	up_in_case
Yang2025_S	ENSGALG00010014241	ENSGALG00010014241	case	control	-0.381219817	1.022512015	-0.372826736	0.709277403	0.820435014	down_in_case
Yang2025_S	ENSGALG00010014243	ENSGALG00010014243	case	control	-0.29863518	0.563206551	-0.530240957	0.595944878	0.735496579	down_in_case
Yang2025_S	ENSGALG00010014244	SESN1	case	control	-0.364695834	0.137861683	-2.645374884	0.008160046	0.034720683	down_in_case
Yang2025_S	ENSGALG00010014246	KRAS	case	control	0.353361399	0.153270723	2.305472243	0.02114014	0.07062275	up_in_case
Yang2025_S	ENSGALG00010014249	ENSGALG00010014249	case	control	0.645310128	0.233506366	2.763565459	0.005717362	0.026485874	up_in_case
Yang2025_S	ENSGALG00010014250	ENSGALG00010014250	case	control	-0.209679345	0.206462722	-1.015579679	0.309829586	0.479666179	down_in_case
Yang2025_S	ENSGALG00010014257	NOP16	case	control	0.081801381	0.27950778	0.29266227	0.769780312	0.8596641	up_in_case
Yang2025_S	ENSGALG00010014258	ENSGALG00010014258	case	control	0.5718187	0.286879661	1.993235413	0.046235675	0.126035628	up_in_case
Yang2025_S	ENSGALG00010014260	ADNP	case	control	0.131892851	0.141112839	0.934662307	0.349962358	0.518359423	up_in_case
Yang2025_S	ENSGALG00010014263	ZNF131	case	control	0.485138927	0.191385767	2.534874639	0.011248757	0.044180224	up_in_case
Yang2025_S	ENSGALG00010014265	ZNF462	case	control	1.069998319	0.219233483	4.880633674	1.05746E-06	2.52755E-05	up_in_case
Yang2025_S	ENSGALG00010014267	SCFD2	case	control	-0.133661652	0.104427383	-1.279948313	0.200563314	0.358223625	down_in_case
Yang2025_S	ENSGALG00010014272	PARD6B	case	control	-0.479249181	0.181930413	-2.634244458	0.008432475	0.035716274	down_in_case
Yang2025_S	ENSGALG00010014274	FIRRM	case	control	-0.261437578	0.149810982	-1.745116242	0.080964637	0.188953645	down_in_case
Yang2025_S	ENSGALG00010014278	G3BP1	case	control	-0.290782231	0.122923678	-2.365551009	0.018003263	0.062866639	down_in_case
Yang2025_S	ENSGALG00010014284	TMEM131L	case	control	0.112978096	0.124722366	0.905836693	0.365022297	0.533730925	up_in_case
Yang2025_S	ENSGALG00010014288	ARMC2	case	control	0.032178533	0.302639833	0.106326166	0.915323572	0.950120376	up_in_case
Yang2025_S	ENSGALG00010014291	OSTN	case	control	-0.357772089	0.585786928	-0.610754647	0.541362022	0.6917183	down_in_case
Yang2025_S	ENSGALG00010014292	NIM1K	case	control	0.233100414	0.301856809	0.772221819	0.439983061	0.603342251	up_in_case
Yang2025_S	ENSGALG00010014294	TMPO	case	control	-0.255433771	0.081898954	-3.1188893	0.001815342	0.011133037	down_in_case
Yang2025_S	ENSGALG00010014295	CACNA1C	case	control	0.137654393	0.20489431	0.671831213	0.501691155	0.658354482	up_in_case
Yang2025_S	ENSGALG00010014298	METTL18	case	control	0.082800458	0.154159277	0.537109796	0.591191775	0.731899198	up_in_case
Yang2025_S	ENSGALG00010014300	ELFN2	case	control	1.062822113	0.738093272	1.439956377	0.149879741	0.292394558	up_in_case
Yang2025_S	ENSGALG00010014301	ARID1A	case	control	0.304208414	0.16371983	1.858103655	0.063154296	0.157800809	up_in_case
Yang2025_S	ENSGALG00010014302	FOXO3	case	control	0.00282964	0.147386892	0.019198725	0.984682575	0.990868839	up_in_case
Yang2025_S	ENSGALG00010014303	GABRA2	case	control	0.433526634	0.465779423	0.930755229	0.351980194	0.520794648	up_in_case
Yang2025_S	ENSGALG00010014305	ARL10	case	control	0.168039957	0.346285173	0.485264661	0.62748862	0.760683246	up_in_case
Yang2025_S	ENSGALG00010014306	ENSGALG00010014306	case	control	0.691800241	0.363569232	1.902801942	0.057066394	0.147120764	up_in_case
Yang2025_S	ENSGALG00010014308	ENSGALG00010014308	case	control	0.330665716	0.209428343	1.578896683	0.114359757	0.241308784	up_in_case
Yang2025_S	ENSGALG00010014310	COL6A1	case	control	-0.157783579	0.144276365	-1.093620421	0.274121489	0.441502884	down_in_case
Yang2025_S	ENSGALG00010014312	KIAA1191	case	control	-0.214919934	0.147896564	-1.453177324	0.146174526	0.287626814	down_in_case
Yang2025_S	ENSGALG00010014314	ENSGALG00010014314	case	control	-0.046020099	0.239857563	-0.191864283	0.847848516	0.909738128	down_in_case
Yang2025_S	ENSGALG00010014317	RAD23B	case	control	-0.052499325	0.102836964	-0.51051026	0.609694029	0.747105724	down_in_case
Yang2025_S	ENSGALG00010014318	ENSGALG00010014318	case	control	-0.133926063	0.345439842	-0.38769721	0.69824012	0.813407129	down_in_case
Yang2025_S	ENSGALG00010014319	ENSGALG00010014319	case	control	0.435654663	0.296859972	1.467542623	0.142228503	0.281890081	up_in_case
Yang2025_S	ENSGALG00010014323	KLF4	case	control	-0.065654853	0.177102031	-0.370717674	0.710847825	0.821433323	down_in_case
Yang2025_S	ENSGALG00010014324	COL4A2	case	control	-0.309175515	0.109315223	-2.828293318	0.004679691	0.022807149	down_in_case
Yang2025_S	ENSGALG00010014328	RASSF8	case	control	0.223992584	0.103457969	2.165058789	0.030383183	0.092604913	up_in_case
Yang2025_S	ENSGALG00010014329	IER5	case	control	0.382343757	0.215512639	1.774112922	0.076044474	0.180555417	up_in_case
Yang2025_S	ENSGALG00010014334	HMGCS1	case	control	0.084889812	0.144561981	0.587220868	0.557055379	0.704215432	up_in_case
Yang2025_S	ENSGALG00010014338	BHLHE41	case	control	0.384071656	0.171718282	2.236638127	0.025310001	0.080658664	up_in_case
Yang2025_S	ENSGALG00010014340	EFNB1	case	control	0.136785698	0.073541373	1.859982928	0.062887941	0.157332758	up_in_case
Yang2025_S	ENSGALG00010014344	LSG1	case	control	0.3625622	0.10919019	3.320464964	0.000898676	0.0064155	up_in_case
Yang2025_S	ENSGALG00010014346	AFG1L	case	control	-0.064878458	0.128276372	-0.505770913	0.61301748	0.749464694	down_in_case
Yang2025_S	ENSGALG00010014347	KIAA0040	case	control	0.561212296	0.337444216	1.663126139	0.096287189	0.213622349	up_in_case
Yang2025_S	ENSGALG00010014350	PTGER4	case	control	0.362095301	0.211801369	1.709598489	0.087340145	0.199587557	up_in_case
Yang2025_S	ENSGALG00010014355	RAB20	case	control	0.266746659	0.16115263	1.655242349	0.097875348	0.21570346	up_in_case
Yang2025_S	ENSGALG00010014361	SSPN	case	control	0.008000676	0.0793708	0.100801253	0.919708232	0.952659943	up_in_case
Yang2025_S	ENSGALG00010014362	NAXD	case	control	0.07542649	0.078316573	0.963097427	0.335498633	0.503994298	up_in_case
Yang2025_S	ENSGALG00010014367	OPHN1	case	control	0.154574446	0.106311555	1.453975969	0.145952969	0.287408606	up_in_case
Yang2025_S	ENSGALG00010014371	TTC33	case	control	0.033655464	0.147383625	0.228352806	0.819371975	0.89209692	up_in_case
Yang2025_S	ENSGALG00010014372	CCDC171	case	control	0.387859734	0.214181059	1.810896514	0.070156872	0.170276639	up_in_case
Yang2025_S	ENSGALG00010014375	H2BC35	case	control	1.034969625	0.416607564	2.484279484	0.012981388	0.049199796	up_in_case
Yang2025_S	ENSGALG00010014379	CARS2	case	control	-0.030220079	0.086703519	-0.348545008	0.727430918	0.832465302	down_in_case
Yang2025_S	ENSGALG00010014380	SNX3	case	control	-0.151568206	0.156749279	-0.966946748	0.333570655	0.502561032	down_in_case
Yang2025_S	ENSGALG00010014388	MFNG	case	control	-0.533483686	0.229236492	-2.327219723	0.019953574	0.067636354	down_in_case
Yang2025_S	ENSGALG00010014389	COL6A2	case	control	-0.163246702	0.156519269	-1.042981501	0.29695686	0.465896628	down_in_case
Yang2025_S	ENSGALG00010014390	SPARC	case	control	-0.001157553	0.18910753	-0.006121136	0.99511607	0.996546349	down_in_case
Yang2025_S	ENSGALG00010014393	ATP8A1	case	control	0.0297565	0.169956309	0.175083234	0.861014232	0.918138454	up_in_case
Yang2025_S	ENSGALG00010014396	ING1	case	control	-0.058649772	0.099403853	-0.590015079	0.55518054	0.70275463	down_in_case
Yang2025_S	ENSGALG00010014397	PRKAA1	case	control	-0.188021669	0.116464994	-1.614405004	0.106439607	0.229052593	down_in_case
Yang2025_S	ENSGALG00010014399	GAS6	case	control	0.024119321	0.100132491	0.240874076	0.809652715	0.88536917	up_in_case
Yang2025_S	ENSGALG00010014400	RIPOR3	case	control	0.322505008	0.177623817	1.815663083	0.06942207	0.169066157	up_in_case
Yang2025_S	ENSGALG00010014403	H2BC37	case	control	2.984461193	0.728822818	4.094906362	4.22339E-05	0.000535027	up_in_case
Yang2025_S	ENSGALG00010014407	YIPF6	case	control	-0.126694716	0.09912624	-1.278114808	0.201208948	0.359053233	down_in_case
Yang2025_S	ENSGALG00010014408	ENSGALG00010014408	case	control	0.154147306	0.325357823	0.473777776	0.635658384	0.766393522	up_in_case
Yang2025_S	ENSGALG00010014411	ATP13A3	case	control	-0.091729214	0.105958392	-0.865709757	0.386649348	0.554088715	down_in_case
Yang2025_S	ENSGALG00010014414	TMEM255B	case	control	1.09281276	0.399578503	2.734913791	0.006239662	0.028293141	up_in_case
Yang2025_S	ENSGALG00010014416	H2BC32	case	control	1.325750453	0.442211829	2.997998619	0.002717589	0.015096657	up_in_case
Yang2025_S	ENSGALG00010014418	RAC2	case	control	-0.847313089	0.210479898	-4.025624763	5.68242E-05	0.000676791	down_in_case
Yang2025_S	ENSGALG00010014427	TMEM267	case	control	-0.158284951	0.181274479	-0.873178351	0.382565873	0.550444547	down_in_case
Yang2025_S	ENSGALG00010014431	ENSGALG00010014431	case	control	-0.277035697	0.51133652	-0.541787426	0.587964954	0.729264943	down_in_case
Yang2025_S	ENSGALG00010014439	ENSGALG00010014439	case	control	0.908922338	0.279596821	3.250832156	0.001150678	0.007828722	up_in_case
Yang2025_S	ENSGALG00010014449	ENSGALG00010014449	case	control	-0.127763143	0.650144386	-0.196515029	0.844207071	0.907437946	down_in_case
Yang2025_S	ENSGALG00010014453	NR2E1	case	control	-0.614678305	0.214444668	-2.866372531	0.004152053	0.020708254	down_in_case
Yang2025_S	ENSGALG00010014455	C4orf19	case	control	0.868053793	0.337275903	2.573720163	0.010061161	0.040697324	up_in_case
Yang2025_S	ENSGALG00010014465	SIMC1	case	control	0.463606684	0.177628077	2.609985372	0.009054609	0.03777724	up_in_case
Yang2025_S	ENSGALG00010014468	ENSGALG00010014468	case	control	0.424605001	0.548576395	0.774012526	0.438923384	0.602719487	up_in_case
Yang2025_S	ENSGALG00010014469	TMCO3	case	control	0.252842698	0.116833224	2.164133524	0.030454103	0.092776908	up_in_case
Yang2025_S	ENSGALG00010014470	RPS6KA1	case	control	-0.303309754	0.150090491	-2.020845912	0.043295718	0.120064843	down_in_case
Yang2025_S	ENSGALG00010014474	PTPN1	case	control	-0.228090284	0.075816565	-3.008449204	0.002625847	0.014695252	down_in_case
Yang2025_S	ENSGALG00010014476	C5orf34	case	control	0.37625499	0.202042051	1.862260798	0.062566338	0.156950841	up_in_case
Yang2025_S	ENSGALG00010014477	ITPR2	case	control	-0.290147334	0.150281159	-1.930696676	0.053520574	0.140242422	down_in_case
Yang2025_S	ENSGALG00010014482	THOC3	case	control	-0.038858578	0.174734498	-0.222386413	0.824013088	0.894494226	down_in_case
Yang2025_S	ENSGALG00010014487	OSTM1	case	control	-0.571074202	0.102274202	-5.583756139	2.35379E-08	9.82037E-07	down_in_case
Yang2025_S	ENSGALG00010014488	ENSGALG00010014488	case	control	0.201855348	0.143551838	1.406149515	0.159679732	0.305181248	up_in_case
Yang2025_S	ENSGALG00010014492	CPLX2	case	control	0.821506583	0.326482243	2.516236647	0.011861551	0.045926844	up_in_case
Yang2025_S	ENSGALG00010014494	KLHL9	case	control	-0.259419988	0.07306919	-3.550333446	0.000384743	0.003200836	down_in_case
Yang2025_S	ENSGALG00010014504	SEC63	case	control	0.222169078	0.125757754	1.766643176	0.077288005	0.182729616	up_in_case
Yang2025_S	ENSGALG00010014505	CYTH4	case	control	-0.411435365	0.201409875	-2.04277653	0.041074565	0.115411438	down_in_case
Yang2025_S	ENSGALG00010014512	HMGN4	case	control	-0.564302796	0.122531044	-4.605386328	4.11701E-06	7.90227E-05	down_in_case
Yang2025_S	ENSGALG00010014515	ETNK1	case	control	-0.211671205	0.154472445	-1.370284549	0.170598094	0.319613396	down_in_case
Yang2025_S	ENSGALG00010014517	DCUN1D2	case	control	0.26460233	0.120121999	2.202779939	0.027610263	0.086092865	up_in_case
Yang2025_S	ENSGALG00010014522	FAT2	case	control	-0.041202402	0.398773014	-0.103322944	0.917706666	0.951576932	down_in_case
Yang2025_S	ENSGALG00010014537	GKAP1	case	control	0.798151799	0.186884073	4.270839063	1.94739E-05	0.000284454	up_in_case
Yang2025_S	ENSGALG00010014539	PAIP1	case	control	-0.279148605	0.092502454	-3.017742699	0.00254665	0.014390743	down_in_case
Yang2025_S	ENSGALG00010014547	DHDDS	case	control	0.089733528	0.154858287	0.579455768	0.562281684	0.708639198	up_in_case
Yang2025_S	ENSGALG00010014550	SCML4	case	control	0.782972967	0.920037748	0.851022656	0.394756768	0.561835927	up_in_case
Yang2025_S	ENSGALG00010014567	UTP11	case	control	0.098385108	0.19422904	0.506541699	0.612476425	0.749197593	up_in_case
Yang2025_S	ENSGALG00010014570	KIF27	case	control	0.358163808	0.206235975	1.736669887	0.082445456	0.191224605	up_in_case
Yang2025_S	ENSGALG00010014572	IL1RAP	case	control	-0.07966668	0.111108423	-0.717017467	0.473363319	0.632929363	down_in_case
Yang2025_S	ENSGALG00010014573	CEBPB	case	control	-0.175558288	0.224650446	-0.781473135	0.434524271	0.598435775	down_in_case
Yang2025_S	ENSGALG00010014576	HRH2	case	control	-0.051755063	0.298460553	-0.17340671	0.862331751	0.918905584	down_in_case
Yang2025_S	ENSGALG00010014578	ENSGALG00010014578	case	control	0.557297732	0.172818052	3.224765725	0.001260758	0.008390001	up_in_case
Yang2025_S	ENSGALG00010014579	NNT	case	control	-0.112376329	0.122241695	-0.919296229	0.35794065	0.527051558	down_in_case
Yang2025_S	ENSGALG00010014592	QNG1	case	control	0.199264055	0.168954774	1.179392866	0.238241775	0.402168277	up_in_case
Yang2025_S	ENSGALG00010014599	SSTR3	case	control	0.680022719	0.492326573	1.381243174	0.167204204	0.315132795	up_in_case
Yang2025_S	ENSGALG00010014603	ENSGALG00010014603	case	control	-0.535620412	0.289924902	-1.847445354	0.064682621	0.160525793	down_in_case
Yang2025_S	ENSGALG00010014611	TMEM189	case	control	-0.194396206	0.105888396	-1.835859383	0.066378456	0.163598123	down_in_case
Yang2025_S	ENSGALG00010014614	GHR	case	control	-0.226764662	0.131797005	-1.720560057	0.085330687	0.196250721	down_in_case
Yang2025_S	ENSGALG00010014617	GRTP1	case	control	-0.398951202	0.121358455	-3.287378703	0.001011247	0.007074163	down_in_case
Yang2025_S	ENSGALG00010014626	C2CD5	case	control	0.412785361	0.110299068	3.742419284	0.000182257	0.001733619	up_in_case
Yang2025_S	ENSGALG00010014632	NWD2	case	control	0.571052678	0.264855676	2.156090011	0.031076637	0.094039727	up_in_case
Yang2025_S	ENSGALG00010014634	CCDC152	case	control	-1.238321082	0.390310663	-3.172655009	0.001510519	0.009659973	down_in_case
Yang2025_S	ENSGALG00010014641	DOCK11	case	control	-0.016736558	0.106799188	-0.156710533	0.875472986	0.926735549	down_in_case
Yang2025_S	ENSGALG00010014645	SLC36A1	case	control	0.110969364	0.146548056	0.757221674	0.448917068	0.611262394	up_in_case

Yang2025_S	ENSGALG00010014648	C1QTNF6	case	control	-0.161375859	0.147707921	-1.092533549	0.274598653	0.442015967	down_in_case
Yang2025_S	ENSGALG00010014653	FAM162B	case	control	0.56488232	0.232795431	2.426518071	0.015244491	0.055697951	up_in_case
Yang2025_S	ENSGALG00010014654	LAMP1	case	control	-0.282781747	0.079454806	-3.55902635	0.000372232	0.003119095	down_in_case
Yang2025_S	ENSGALG00010014655	FHL3	case	control	-0.411779925	0.175894675	-2.341059648	0.019229095	0.065869576	down_in_case
Yang2025_S	ENSGALG00010014658	SELENOP1	case	control	-0.21591152	0.118992365	-1.814498936	0.069600946	0.169294674	down_in_case
Yang2025_S	ENSGALG00010014668	GP5	case	control	0.337368048	0.181860801	1.855089415	0.063583459	0.158633675	up_in_case
Yang2025_S	ENSGALG00010014676	UBE2V1	case	control	-0.171922764	0.095818947	-1.794246014	0.072773911	0.174785323	down_in_case
Yang2025_S	ENSGALG00010014677	ENSGALG00010014677	case	control	0.442469864	0.159277376	2.777983134	0.005469746	0.025624213	up_in_case
Yang2025_S	ENSGALG00010014682	IL13RA1	case	control	-0.637014348	0.141481428	-4.50245914	6.71717E-06	0.000119393	down_in_case
Yang2025_S	ENSGALG00010014687	CUL4A	case	control	0.272741727	0.140252546	1.944647251	0.051817448	0.136782751	up_in_case
Yang2025_S	ENSGALG00010014690	CSF3R	case	control	0.055924342	0.245121211	0.228149748	0.819529827	0.892199073	up_in_case
Yang2025_S	ENSGALG00010014691	PABIR2	case	control	-0.034992477	0.06835289	-0.511938512	0.608694044	0.746077183	down_in_case
Yang2025_S	ENSGALG00010014696	SPINK5	case	control	-0.17968744	0.293347492	-0.612541253	0.540179713	0.690770674	down_in_case
Yang2025_S	ENSGALG00010014697	ENSGALG00010014697	case	control	0.523708111	0.430954528	1.215228236	0.224279054	0.385744677	up_in_case
Yang2025_S	ENSGALG00010014702	ENSGALG00010014702	case	control	0.364134776	0.366906845	0.992444761	0.320980613	0.490388723	up_in_case
Yang2025_S	ENSGALG00010014703	PCID2	case	control	-0.532673613	0.140324095	-3.796023869	0.000147035	0.001457838	down_in_case
Yang2025_S	ENSGALG00010014705	ARAP2	case	control	-0.006677088	0.285926254	-0.023352483	0.981369108	0.98867905	down_in_case
Yang2025_S	ENSGALG00010014707	KPNA5	case	control	-0.469536019	0.080249206	-5.850973993	4.88703E-09	2.40639E-07	down_in_case
Yang2025_S	ENSGALG00010014710	NPEPL1	case	control	-0.374281512	0.102558769	-3.649434519	0.000262818	0.002337187	down_in_case
Yang2025_S	ENSGALG00010014713	ST8SIA1	case	control	-0.389572487	0.190421373	-2.045844333	0.040771689	0.114691473	down_in_case
Yang2025_S	ENSGALG00010014716	TMEM35A	case	control	-0.64982993	0.315721232	-2.058239559	0.039567143	0.112409406	down_in_case
Yang2025_S	ENSGALG00010014717	ELOVL7	case	control	-0.508914392	0.18748986	-2.714356879	0.006640459	0.029705595	down_in_case
Yang2025_S	ENSGALG00010014719	ENSGALG00010014719	case	control	-0.183280101	0.47482803	-0.385992589	0.699502157	0.814060678	down_in_case
Yang2025_S	ENSGALG00010014722	KCTD17	case	control	0.115422375	0.332731987	0.346892934	0.728671755	0.833097929	up_in_case
Yang2025_S	ENSGALG00010014726	PWP2	case	control	-0.115009214	0.106959893	-1.075255509	0.28226035	0.450336504	down_in_case
Yang2025_S	ENSGALG00010014728	INTS13	case	control	-0.072982931	0.09436202	-0.77343545	0.439264717	0.602950535	down_in_case
Yang2025_S	ENSGALG00010014729	MRPS15	case	control	0.462019247	0.164980919	2.800440492	0.005103292	0.024354236	up_in_case
Yang2025_S	ENSGALG00010014734	ZUP1	case	control	-0.098583381	0.10365388	-0.951082399	0.341562549	0.509710229	down_in_case
Yang2025_S	ENSGALG00010014735	STX16	case	control	-0.060657924	0.09554401	-0.634868934	0.5255139	0.678750226	down_in_case
Yang2025_S	ENSGALG00010014736	KIF2A	case	control	0.023962199	0.099979437	0.239671274	0.810585106	0.885991329	up_in_case
Yang2025_S	ENSGALG00010014737	RMI1	case	control	-0.124642459	0.181773128	-0.685703437	0.492900139	0.650417153	down_in_case
Yang2025_S	ENSGALG00010014738	BRWD1	case	control	0.354154422	0.15199587	2.33002661	0.019804745	0.067319665	up_in_case
Yang2025_S	ENSGALG00010014739	TRMT2B	case	control	0.343240534	0.127223802	2.697927023	0.006977273	0.030895553	up_in_case
Yang2025_S	ENSGALG00010014740	TRMT9B	case	control	0.138742541	0.555854139	0.249602425	0.802894823	0.881249162	up_in_case
Yang2025_S	ENSGALG00010014744	IL2RB	case	control	1.42277474	0.437468976	3.252287177	0.001144803	0.007797081	up_in_case
Yang2025_S	ENSGALG00010014745	LSM10	case	control	-0.260111062	0.371330798	-0.700483406	0.483625465	0.641840081	down_in_case
Yang2025_S	ENSGALG00010014748	FGFR1OP2	case	control	0.360734807	0.14374058	2.509623983	0.012085978	0.046640295	up_in_case
Yang2025_S	ENSGALG00010014750	LONRF1	case	control	0.004721483	0.126097849	0.037443008	0.970131781	0.982113067	up_in_case
Yang2025_S	ENSGALG00010014753	ENSGALG00010014753	case	control	0.289345694	0.409318323	0.706896511	0.479630792	0.638423449	up_in_case
Yang2025_S	ENSGALG00010014754	DIMT1	case	control	-0.06760226	0.176169465	-0.383734261	0.701175417	0.815599285	down_in_case
Yang2025_S	ENSGALG00010014758	FXN	case	control	0.13411994	0.149566658	0.896723514	0.36986647	0.53851105	up_in_case
Yang2025_S	ENSGALG00010014762	STK40	case	control	0.054031527	0.075271407	0.717822722	0.472866602	0.632501065	up_in_case
Yang2025_S	ENSGALG00010014766	F10	case	control	-1.180112448	0.20295763	-5.81457544	6.0788E-09	2.92097E-07	down_in_case
Yang2025_S	ENSGALG00010014767	LRRC15	case	control	0.270255865	0.184385276	1.465712827	0.142726538	0.282633837	up_in_case
Yang2025_S	ENSGALG00010014769	CCDC69	case	control	0.191103541	0.203106414	0.940903527	0.3467543	0.515250711	up_in_case
Yang2025_S	ENSGALG00010014777	APCDD1L	case	control	0.580678372	0.387905904	1.496956779			up_in_case
Yang2025_S	ENSGALG00010014780	EVA1B	case	control	0.075776139	0.207605682	0.365000312	0.715111173	0.824104714	up_in_case
Yang2025_S	ENSGALG00010014783	ENSGALG00010014783	case	control	0.143856325	0.085704676	1.678511967	0.093247196	0.208705378	up_in_case
Yang2025_S	ENSGALG00010014792	ENSGALG00010014792	case	control	0.033715176	0.221665336	0.152099453	0.879108496	0.92862622	up_in_case
Yang2025_S	ENSGALG00010014793	TLR2A	case	control	-0.383008187	0.172514974	-2.220144598	0.026408954	0.083309136	down_in_case
Yang2025_S	ENSGALG00010014795	THRAP3	case	control	0.293765677	0.084642834	3.470650291	0.0005192	0.004076083	up_in_case
Yang2025_S	ENSGALG00010014796	PRLR	case	control	0.534023477	0.338160215	1.579202559	0.114289603	0.241197277	up_in_case
Yang2025_S	ENSGALG00010014797	ZDHHC9	case	control	-0.255691251	0.114695894	-2.229297335	0.025794128	0.081784112	down_in_case
Yang2025_S	ENSGALG00010014801	MPST	case	control	-0.170971294	0.179761993	-0.951098122	0.341554568	0.509710229	down_in_case
Yang2025_S	ENSGALG00010014802	ENSGALG00010014802	case	control	0.492502328	0.499811038	0.985377054	0.324438896	0.494050488	up_in_case
Yang2025_S	ENSGALG00010014805	SPEF2	case	control	0.620628852	0.33202108	1.869245329	0.06158869	0.155337267	up_in_case
Yang2025_S	ENSGALG00010014806	MAP7D1	case	control	0.499433088	0.182994188	2.729229232	0.006348256	0.028665893	up_in_case
Yang2025_S	ENSGALG00010014807	HES1	case	control	0.514757715	0.16038084	3.209596073	0.001329216	0.008753605	up_in_case
Yang2025_S	ENSGALG00010014810	ENTREP1	case	control	0.126259236	0.127238368	0.992304737	0.321048893	0.490388723	up_in_case
Yang2025_S	ENSGALG00010014812	RWDD1	case	control	0.263430371	0.129527569	2.03377839	0.041973944	0.117215814	up_in_case
Yang2025_S	ENSGALG00010014813	SASH3	case	control	0.498207302	0.387457117	1.285838563	0.198499409	0.355400913	up_in_case
Yang2025_S	ENSGALG00010014816	TST	case	control	-0.190013675	0.155219962	-1.224157465	0.220892831	0.382303216	down_in_case
Yang2025_S	ENSGALG00010014818	ENSGALG00010014818	case	control	0.054495281	0.380365299	0.143270906	0.886076233	0.933293447	up_in_case
Yang2025_S	ENSGALG00010014821	C3AR1L	case	control	-0.363819948	0.137305322	-2.649714838	0.008055973	0.034414467	down_in_case
Yang2025_S	ENSGALG00010014824	ANXA6	case	control	0.263068808	0.074496654	3.53128356	0.000413548	0.003395871	up_in_case
Yang2025_S	ENSGALG00010014829	ENSGALG00010014829	case	control	0.659909218	0.467042882	1.412952092	0.157669811	0.302926901	up_in_case
Yang2025_S	ENSGALG00010014831	VAPB	case	control	-0.33610237	0.096283273	-3.490765927	0.000481638	0.003833024	down_in_case
Yang2025_S	ENSGALG00010014837	APBA1	case	control	0.240073218	0.16375456	1.466055161	0.14263326	0.282489266	up_in_case
Yang2025_S	ENSGALG00010014839	TM7SF3	case	control	-0.326971654	0.15162124	-2.156502963	0.031044413	0.093962617	down_in_case
Yang2025_S	ENSGALG00010014840	NCF4	case	control	-0.55050525	0.239588641	-2.297710135	0.021578292	0.071525572	down_in_case
Yang2025_S	ENSGALG00010014847	PAICS	case	control	-0.440299513	0.091225248	-4.826509371	1.38947E-06	3.19509E-05	down_in_case
Yang2025_S	ENSGALG00010014851	CALHM4	case	control	-0.570303867	0.451079248	-1.264309696	0.206118891	0.364639678	down_in_case
Yang2025_S	ENSGALG00010014856	MMGT1	case	control	-0.162169588	0.069504467	-2.333225392	0.019636319	0.066919076	down_in_case
Yang2025_S	ENSGALG00010014859	TRAPPC3	case	control	-0.282685815	0.134356443	-2.103998951	0.035378539	0.103636732	down_in_case
Yang2025_S	ENSGALG00010014861	MED21	case	control	-0.399568715	0.193556726	-2.064349425	0.038984598	0.111094146	down_in_case
Yang2025_S	ENSGALG00010014862	ENSGALG00010014862	case	control	1.113692	0.375884797	2.962854598	0.003048005	0.016520401	up_in_case
Yang2025_S	ENSGALG00010014865	TLR2	case	control	-0.984076435	0.336206006	-2.92700433	0.003422441	0.017962982	down_in_case
Yang2025_S	ENSGALG00010014866	FAM26E	case	control	-0.559627141	0.10760622	-5.200695094	1.98545E-07	6.15092E-06	down_in_case
Yang2025_S	ENSGALG00010014867	TP63	case	control	0.599953005	0.202486148	2.962933571	0.003047223	0.016520401	up_in_case
Yang2025_S	ENSGALG00010014876	PCNT	case	control	0.782821439	0.201732582	3.880490855	0.000104246	0.001102175	up_in_case
Yang2025_S	ENSGALG00010014877	ENSGALG00010014877	case	control	-0.391369877	0.124073367	-3.154342346	0.001608603	0.010147523	down_in_case
Yang2025_S	ENSGALG00010014878	TMCO1	case	control	-0.317586806	0.151632314	-2.094453339	0.036219615	0.105347598	down_in_case
Yang2025_S	ENSGALG00010014880	SFRP2	case	control	-0.628238275	0.12752387	-4.926436697	8.37427E-07	2.07643E-05	down_in_case
Yang2025_S	ENSGALG00010014881	ENSGALG00010014881	case	control	1.086605498	0.339922615	3.196626085	0.00139045	0.009096679	up_in_case
Yang2025_S	ENSGALG00010014882	XPNPEP2	case	control	-0.447936536	0.363776774	-1.231350015	0.218191983	0.379209938	down_in_case
Yang2025_S	ENSGALG00010014883	PPAT	case	control	-0.459411419	0.096717866	-4.750016063	2.034E-06	4.40122E-05	down_in_case
Yang2025_S	ENSGALG00010014886	RABGAP1L	case	control	-0.306553797	0.09644029	-3.178690107	0.001479422	0.009522282	down_in_case
Yang2025_S	ENSGALG00010014887	MTMR12	case	control	0.454921427	0.114903796	3.959150557	7.52168E-05	0.000848701	up_in_case
Yang2025_S	ENSGALG00010014889	PVALB	case	control	0.82092703	0.397888862	2.063206862	0.039092978	0.111357452	up_in_case
Yang2025_S	ENSGALG00010014893	SLC9A6	case	control	-0.125143543	0.110955163	-1.127874895	0.259372758	0.425769746	down_in_case
Yang2025_S	ENSGALG00010014894	RAB22A	case	control	-0.163683298	0.146968132	-1.113733268	0.265393633	0.432573553	down_in_case
Yang2025_S	ENSGALG00010014897	COL8A2	case	control	0.384909056	0.331108049	1.162487767			up_in_case
Yang2025_S	ENSGALG00010014898	ENSGALG00010014898	case	control	0.032760437	0.399541452	0.081995088	0.934650619	0.960002682	up_in_case
Yang2025_S	ENSGALG00010014900	UBLCP1	case	control	-0.194066198	0.086248589	-2.250079675	0.024443888	0.078775574	down_in_case
Yang2025_S	ENSGALG00010014902	AASDH	case	control	0.460896247	0.140889655	3.271327802	0.001070438	0.007395413	up_in_case
Yang2025_S	ENSGALG00010014903	MCF2L	case	control	-0.124194601	0.116898397	-1.062414922	0.288047363	0.456960382	down_in_case
Yang2025_S	ENSGALG00010014905	TEKT2	case	control	1.426597821	0.52081537	2.739162286	0.006159596	0.027995424	up_in_case
Yang2025_S	ENSGALG00010014906	IFT27	case	control	-0.578459423	0.129066011	-4.481888118	7.39855E-06	0.000128874	down_in_case
Yang2025_S	ENSGALG00010014907	ENSGALG00010014907	case	control	0.511353462	0.205152541	2.49255242	0.012682861	0.04830163	up_in_case
Yang2025_S	ENSGALG00010014908	ENSGALG00010014908	case	control	-0.322925129	0.093238793	-3.463420317	0.000533355	0.004173102	down_in_case
Yang2025_S	ENSGALG00010014909	FBXL14	case	control	0.564120917	0.144723039	3.897934437	9.70167E-05	0.001041546	up_in_case
Yang2025_S	ENSGALG00010014910	CACNG2	case	control	2.306556119	0.618433784	3.72967354	0.000191728	0.001800592	up_in_case
Yang2025_S	ENSGALG00010014912	ENSGALG00010014912	case	control	-1.128527128	0.362790473	-3.110685679	0.001866535	0.011378849	down_in_case
Yang2025_S	ENSGALG00010014914	PTAR1	case	control	0.270830086	0.140732316	1.924434228	0.054300178	0.141725599	up_in_case
Yang2025_S	ENSGALG00010014916	DYNLT2B	case	control	-0.622505901	0.258353597	-2.409511257	0.015973903	0.057622662	down_in_case
Yang2025_S	ENSGALG00010014917	EIF3D	case	control	-0.142239759	0.135665044	-1.048462853	0.294425408	0.462981655	down_in_case
Yang2025_S	ENSGALG00010014918	PLRG1	case	control	-0.108106017	0.097951602	-1.103667678	0.269737321	0.436966935	down_in_case
Yang2025_S	ENSGALG00010014922	MAN1A1	case	control	-0.271030456	0.137020945	-1.978022089	0.047926215	0.129523933	down_in_case
Yang2025_S	ENSGALG00010014923	CIMIP1	case	control	0.161351966	0.398633838	0.404762343	0.685652211	0.803694476	up_in_case
Yang2025_S	ENSGALG00010014928	SUCO	case	control	0.200005308	0.089449725	2.235952185	0.025354903	0.08068522	up_in_case
Yang2025_S	ENSGALG00010014929	FHL1	case	control	-0.209903139	0.252556894	-0.831112292	0.405910196	0.571984891	down_in_case
Yang2025_S	ENSGALG00010014931	RNF145	case	control	-0.1563117	0.103595896	-1.508859975	0.13133457	0.265932467	down_in_case
Yang2025_S	ENSGALG00010014934	MAMDC2	case	control	-0.389244431	0.160665782	-2.422696513	0.015405791	0.056110741	down_in_case
Yang2025_S	ENSGALG00010014935	PCYT1A	case	control	-0.089753533	0.137039145	-0.654948137	0.512501146	0.667572691	down_in_case
Yang2025_S	ENSGALG00010014936	FOXRED2	case	control	-0.112958036	0.162452603	-0.695329184	0.486849029	0.644040101	down_in_case
Yang2025_S	ENSGALG00010014940	VAMP4	case	control	0.154017426	0.142407426	1.081526649	0.279462913	0.447262627	up_in_case
Yang2025_S	ENSGALG00010014942	PMEPA1	case	control	0.443516535	0.092997948	4.769100239	1.85051E-06	4.06732E-05	up_in_case
Yang2025_S	ENSGALG00010014943	APLN	case	control	0.398431207	0.231041467	1.724500851	0.084617466	0.195061105	up_in_case
Yang2025_S	ENSGALG00010014944	FGB	case	control	-1.969957896	0.701910878	-2.806564135			down_in_case
Yang2025_S	ENSGALG00010014945	TXN2	case	control	0.155534132	0.223064897	0.697259562	0.48564037	0.642922151	up_in_case
Yang2025_S	ENSGALG00010014946	PCK1	case	control	-1.624826577	0.618329358	-2.627768772	0.008594692	0.036260075	down_in_case
Yang2025_S	ENSGALG00010014948	RBM38	case	control	0.516855447	0.235864658	2.191322141	0.028428489	0.087896826	up_in_case
Yang2025_S	ENSGALG00010014949	AGO3	case	control	-0.113169554	0.118164216	-0.957731184	0.338198328	0.50635796	down_in_case
Yang2025_S	ENSGALG00010014952	C21orf58	case	control	0.468006767	0.547236934	0.8552178	0.392430584	0.559491893	up_in_case
Yang2025_S	ENSGALG00010014956	FGG	case	control	-4.403616518	1.833805444	-2.401354262			down_in_case
Yang2025_S	ENSGALG00010014965	ABHD13	case	control	-0.77346432	0.113009071	-6.844267546	7.68681E-12	8.43431E-10	down_in_case

Yang2025_S	ENSGALG00010014967	HNRNPH1	case	control	0.043228212	0.091482592	0.472529372	0.636548982	0.767065829	up_in_case
Yang2025_S	ENSGALG00010014971	SMC5	case	control	0.975536654	0.238405653	4.091919138	4.27818E-05	0.000539515	up_in_case
Yang2025_S	ENSGALG00010014972	FGA	case	control	-3.4681953	1.744564602	-1.988000499			down_in_case
Yang2025_S	ENSGALG00010014974	LIG4	case	control	0.099677501	0.196891212	0.506256732	0.612676433	0.749275006	up_in_case
Yang2025_S	ENSGALG00010014976	ITGB2	case	control	-0.428457104	0.185200359	-2.313478805	0.020696329	0.069411153	down_in_case
Yang2025_S	ENSGALG00010014980	ENSGALG00010014980	case	control	-0.541209517	0.347149391	-1.559010415	0.118993915	0.248111607	down_in_case
Yang2025_S	ENSGALG00010014984	LPP	case	control	0.064458304	0.270093629	0.238651702	0.811375671	0.886368766	up_in_case
Yang2025_S	ENSGALG00010014985	ENSGALG00010014985	case	control	0.198262885	0.098755104	2.007621657	0.044683511	0.123153626	up_in_case
Yang2025_S	ENSGALG00010014987	KLF9	case	control	-0.19802874	0.15294606	-1.294761962	0.195402358	0.351436739	down_in_case
Yang2025_S	ENSGALG00010014990	AGO1	case	control	-0.284448338	0.087297225	-3.258389235	0.001120466	0.007672577	down_in_case
Yang2025_S	ENSGALG00010014992	ARL15	case	control	-0.548582391	0.100425452	-5.462583231	4.69255E-08	1.76732E-06	down_in_case
Yang2025_S	ENSGALG00010014994	ENSGALG00010014994	case	control	-0.479773318	0.13477868	-3.559712261	0.000371261	0.003112832	down_in_case
Yang2025_S	ENSGALG00010014995	LRAT	case	control	1.418126567	0.434215972	3.265947497	0.001090985	0.007503886	up_in_case
Yang2025_S	ENSGALG00010014997	RAE1	case	control	-0.066381827	0.109678561	-0.605239772	0.545019693	0.694544986	down_in_case
Yang2025_S	ENSGALG00010015002	ADAM19	case	control	-0.296138259	0.152207945	-1.945616307	0.051700847	0.136526682	down_in_case
Yang2025_S	ENSGALG00010015006	HSPB3	case	control	0.29660533	0.233258437	1.271573855	0.203524571	0.361610978	up_in_case
Yang2025_S	ENSGALG00010015009	AGO4	case	control	-0.161142227	0.085886961	-1.876212931	0.060626047	0.153606533	down_in_case
Yang2025_S	ENSGALG00010015013	SNX18	case	control	-0.30873818	0.094458114	-3.268519412	0.001081118	0.007454416	down_in_case
Yang2025_S	ENSGALG00010015016	ARGLU1	case	control	0.780216093	0.129190121	6.039286021	1.54798E-09	8.95532E-08	up_in_case
Yang2025_S	ENSGALG00010015017	FAM184A	case	control	0.472086414	0.25056969	1.884052349	0.059557895	0.151781138	up_in_case
Yang2025_S	ENSGALG00010015018	MAP7D3	case	control	0.437138844	0.170868028	2.558341949	0.010517262	0.042017787	up_in_case
Yang2025_S	ENSGALG00010015020	NIPAL4	case	control	-0.95900652	1.076866679	-0.890552693	0.373169188	0.541255415	down_in_case
Yang2025_S	ENSGALG00010015024	CRACD	case	control	0.450160901	0.243420954	1.849310391	0.064413006	0.159970636	up_in_case
Yang2025_S	ENSGALG00010015025	TRAF3IP1	case	control	1.0925272	0.32704365	3.340615842	0.000835928	0.00605125	up_in_case
Yang2025_S	ENSGALG00010015027	CLSPN	case	control	0.561392664	0.192809071	2.911650683	0.003595245	0.018652171	up_in_case
Yang2025_S	ENSGALG00010015028	MCM9	case	control	-0.633213581	0.298531512	-2.121094607	0.033913842	0.100252309	down_in_case
Yang2025_S	ENSGALG00010015029	TFRC	case	control	-0.095257867	0.155228909	-0.613660607	0.539439625	0.690457572	down_in_case
Yang2025_S	ENSGALG00010015031	MYH9	case	control	0.590984068	0.155094039	3.810488614	0.000138692	0.001389417	up_in_case
Yang2025_S	ENSGALG00010015032	C1orf216	case	control	1.854819851	0.286937391	6.464197102	1.01838E-10	8.70622E-09	up_in_case
Yang2025_S	ENSGALG00010015033	BMP7	case	control	0.017874864	0.218718991	0.081725251	0.934865198	0.960081549	up_in_case
Yang2025_S	ENSGALG00010015035	SLC35F1	case	control	-0.314710587	0.116273208	-2.706647499	0.006796638	0.030268825	down_in_case
Yang2025_S	ENSGALG00010015037	CYFIP2	case	control	0.206120906	0.223628082	0.921712978	0.356678309	0.525982811	up_in_case
Yang2025_S	ENSGALG00010015038	PCSK5	case	control	0.020008118	0.159525906	0.125422374	0.900189176	0.940592827	up_in_case
Yang2025_S	ENSGALG00010015039	FAM114A1	case	control	-0.279361387	0.083574802	-3.342650903	0.000829822	0.006013298	down_in_case
Yang2025_S	ENSGALG00010015040	ZC4H2	case	control	-0.010408879	0.149143352	-0.069791105	0.944359927	0.965988078	down_in_case
Yang2025_S	ENSGALG00010015041	TFAP2C	case	control	0.169843534	0.587347457	0.28917046	0.772450936	0.861541844	up_in_case
Yang2025_S	ENSGALG00010015042	EFNB2	case	control	0.134865633	0.121476553	1.110219457	0.26690447	0.433992275	up_in_case
Yang2025_S	ENSGALG00010015043	COQ10B	case	control	-0.042703551	0.170501826	-0.250458025	0.802233162	0.880663886	down_in_case
Yang2025_S	ENSGALG00010015044	ZC3H12B	case	control	0.235308577	0.183745607	1.280621512	0.20032664	0.357937137	up_in_case
Yang2025_S	ENSGALG00010015046	PSMB2	case	control	-0.287954505	0.172806277	-1.666342851	0.095645146	0.212583268	down_in_case
Yang2025_S	ENSGALG00010015047	NPY2R	case	control	0.091522665	0.285553516	0.320509676	0.748581997	0.847468529	up_in_case
Yang2025_S	ENSGALG00010015049	DCK2	case	control	-0.583918407	0.252129402	-2.315947298	0.020561146	0.069090807	down_in_case
Yang2025_S	ENSGALG00010015050	HIVEP3	case	control	0.596168651	0.2587841	2.303729827	0.021237814	0.070852033	up_in_case
Yang2025_S	ENSGALG00010015052	ENSGALG00010015052	case	control	-0.343564064	0.1970555	-1.74348884	0.081248262	0.189330949	down_in_case
Yang2025_S	ENSGALG00010015054	ENSGALG00010015054	case	control	-0.224742832	0.109534859	-2.051792764	0.040189807	0.113711243	down_in_case
Yang2025_S	ENSGALG00010015055	ZNF335	case	control	-0.112046681	0.054116647	-2.070466043	0.038408723	0.109925149	down_in_case
Yang2025_S	ENSGALG00010015056	ENSGALG00010015056	case	control	0.873169756	0.453493172	1.925430877	0.054175476	0.141538687	up_in_case
Yang2025_S	ENSGALG00010015057	LAS1L	case	control	0.411853021	0.100545874	4.096170275	4.20041E-05	0.0005326	up_in_case
Yang2025_S	ENSGALG00010015059	ENSGALG00010015059	case	control	0.448090134	0.120945124	3.704904501	0.00021147	0.001959335	up_in_case
Yang2025_S	ENSGALG00010015061	MMP9	case	control	0.460649894	0.640959673	0.718687795	0.472333305	0.632182768	up_in_case
Yang2025_S	ENSGALG00010015062	ENSGALG00010015062	case	control	-0.477593322	0.264610933	-1.804888843	0.071092078	0.171782228	down_in_case
Yang2025_S	ENSGALG00010015063	EEF1AKMT4	case	control	0.142288444	0.259111532	0.549139758	0.582909542	0.725318731	up_in_case
Yang2025_S	ENSGALG00010015064	RFK	case	control	-0.361401701	0.139687537	-2.587215066	0.009675518	0.039597164	down_in_case
Yang2025_S	ENSGALG00010015066	RUFY1	case	control	0.315905274	0.088468005	3.570842057	0.000355835	0.003005192	up_in_case
Yang2025_S	ENSGALG00010015068	ESM1	case	control	0.180712588	0.171928567	1.051091106	0.293216749	0.461705274	up_in_case
Yang2025_S	ENSGALG00010015069	HSPD1	case	control	0.399369137	0.137039594	2.914260935	0.003565318	0.018517593	up_in_case
Yang2025_S	ENSGALG00010015070	EDN2	case	control	0.003235045	0.4152494	0.007790607	0.993784058	0.995970515	up_in_case
Yang2025_S	ENSGALG00010015071	SNAI1	case	control	0.058689378	0.172489766	0.340248462	0.733669425	0.837320511	up_in_case
Yang2025_S	ENSGALG00010015072	MAP9	case	control	0.739624668	0.224157812	3.299571233	0.000968326	0.00682876	up_in_case
Yang2025_S	ENSGALG00010015075	ENSGALG00010015075	case	control	-0.40085409	0.222985759	-1.797666774	0.07222983	0.173718101	down_in_case
Yang2025_S	ENSGALG00010015080	ASF1A	case	control	-0.635620916	0.149725561	-4.24523983	2.1836E-05	0.000312086	down_in_case
Yang2025_S	ENSGALG00010015083	CEP135	case	control	0.340623811	0.13167084	2.586934288	0.009683406	0.039606936	up_in_case
Yang2025_S	ENSGALG00010015084	FOXO6	case	control	-0.208890961	0.132889864	-1.571910405	0.115971331	0.243602728	down_in_case
Yang2025_S	ENSGALG00010015085	PSMD2	case	control	-0.160905417	0.112776205	-1.426767439	0.153646939	0.297618863	down_in_case
Yang2025_S	ENSGALG00010015086	FOXN3	case	control	0.169904971	0.187561419	0.905863115	0.365008309	0.533730925	up_in_case
Yang2025_S	ENSGALG00010015087	GABRA3	case	control	1.097522128	0.778481028	1.409825144			up_in_case
Yang2025_S	ENSGALG00010015088	ENSGALG00010015088	case	control	-0.064189937	0.323342846	-0.198519737	0.842638444	0.906661008	down_in_case
Yang2025_S	ENSGALG00010015091	MSN	case	control	-0.087641261	0.065614007	-1.335709627	0.181644202	0.334206543	down_in_case
Yang2025_S	ENSGALG00010015092	HSPE1	case	control	-0.731466759	0.252078762	-2.901738932	0.003710977	0.019096181	down_in_case
Yang2025_S	ENSGALG00010015094	CDK5RAP1	case	control	-0.343272176	0.139212802	-2.465808965	0.013670422	0.051126498	down_in_case
Yang2025_S	ENSGALG00010015095	RBFOX2	case	control	0.285326811	0.105202259	2.712173795	0.006684354	0.029853607	up_in_case
Yang2025_S	ENSGALG00010015097	MFSD2A	case	control	-0.066439322	0.178815532	-0.371552297	0.710226212	0.821055439	down_in_case
Yang2025_S	ENSGALG00010015098	HAVCR1	case	control	-0.707410438	0.168791205	-4.191038482	2.77681E-05	0.000381982	down_in_case
Yang2025_S	ENSGALG00010015100	MOB4	case	control	-0.24775698	0.080372254	-3.082618284	0.002051882	0.012203573	down_in_case
Yang2025_S	ENSGALG00010015101	CAP1	case	control	-0.060785	0.052705793	-1.153288782	0.248791873	0.4145378	down_in_case
Yang2025_S	ENSGALG00010015103	ITPK1	case	control	-0.363401967	0.231077747	-1.572639387	0.115802342	0.24335781	down_in_case
Yang2025_S	ENSGALG00010015104	CNGA2	case	control	0.540239073	0.398337766	1.356233627	0.175024846	0.325456529	up_in_case
Yang2025_S	ENSGALG00010015107	MB	case	control	1.164370725	0.500439194	2.326697707	0.01998136	0.067665852	up_in_case
Yang2025_S	ENSGALG00010015108	ENSGALG00010015108	case	control	0.175461013	0.181474616	0.966862563	0.333612744	0.502561032	up_in_case
Yang2025_S	ENSGALG00010015109	PPT1	case	control	-0.050201235	0.174446683	-0.28777409	0.773519672	0.862181781	down_in_case
Yang2025_S	ENSGALG00010015110	ENSGALG00010015110	case	control	0.143356994	0.613958416	0.233496259	0.815376074	0.889255026	up_in_case
Yang2025_S	ENSGALG00010015111	ADAMTS2	case	control	0.125183842	0.272509001	0.459375072	0.645964846	0.775210701	up_in_case
Yang2025_S	ENSGALG00010015115	CLDN2	case	control	0.214426479	0.20379217	1.052182127	0.292715998	0.461417951	up_in_case
Yang2025_S	ENSGALG00010015117	RASD2	case	control	-0.036028908	0.136337035	-0.264263547	0.791576847	0.874058904	down_in_case
Yang2025_S	ENSGALG00010015118	GPX8	case	control	-0.563378068	0.185970155	-3.029400428	0.002450397	0.013994376	down_in_case
Yang2025_S	ENSGALG00010015119	TLR1A	case	control	0.189035116	0.263072679	0.718566127	0.472408291	0.632182768	up_in_case
Yang2025_S	ENSGALG00010015121	UNC79	case	control	0.409790399	0.385187807	1.06387168	0.287386834	0.456172176	up_in_case
Yang2025_S	ENSGALG00010015122	ENSGALG00010015122	case	control	0.270401868	0.219727974	1.230621038	0.218464633	0.379541786	up_in_case
Yang2025_S	ENSGALG00010015123	CEP85L	case	control	0.352407521	0.262103192	1.344537307	0.178774701	0.330401254	up_in_case
Yang2025_S	ENSGALG00010015124	UXS1	case	control	0.095758984	0.129334504	0.740397815	0.459058644	0.620223212	up_in_case
Yang2025_S	ENSGALG00010015126	MCM5	case	control	-0.026918561	0.154869531	-0.173814443	0.862011294	0.918779821	down_in_case
Yang2025_S	ENSGALG00010015127	RPL11	case	control	-0.481027422	0.433560522	-1.1094816	0.267222478	0.434306651	down_in_case
Yang2025_S	ENSGALG00010015128	OCRL	case	control	-0.129099684	0.064693906	-1.995546296	0.04598333	0.125519629	down_in_case
Yang2025_S	ENSGALG00010015129	PRRG3	case	control	0.254612759	0.142870853	1.782118284	0.074729945	0.178345912	up_in_case
Yang2025_S	ENSGALG00010015131	DOK5	case	control	0.364918307	0.184922885	1.973353963	0.048455249	0.13040913	up_in_case
Yang2025_S	ENSGALG00010015132	GCNT1	case	control	-0.312960821	0.349065719	-0.896567048	0.369949988	0.538520117	down_in_case
Yang2025_S	ENSGALG00010015133	RFTN2	case	control	-0.324618107	0.122248129	-2.655403479	0.00792136	0.034006208	down_in_case
Yang2025_S	ENSGALG00010015134	PLN	case	control	0.328489353	0.565416852	0.580968451	0.561261717	0.707863338	up_in_case
Yang2025_S	ENSGALG00010015136	TCEB3	case	control	-0.053766874	0.087579854	-0.613918292	0.539269322	0.690384243	down_in_case
Yang2025_S	ENSGALG00010015138	ENSGALG00010015138	case	control	-1.003164559	0.568444589	-1.764753467	0.077605208	0.18341733	down_in_case
Yang2025_S	ENSGALG00010015140	ASCC3	case	control	-0.187431395	0.101079317	-1.854300171	0.063696228	0.158756382	down_in_case
Yang2025_S	ENSGALG00010015141	PFDN4	case	control	-1.063396353	0.181681691	-5.853073831	4.8257E-09	2.40165E-07	down_in_case
Yang2025_S	ENSGALG00010015142	HNRNPAB	case	control	0.206620183	0.091842019	2.249734774	0.024465787	0.078827916	up_in_case
Yang2025_S	ENSGALG00010015143	EXOC1	case	control	-0.015561877	0.076509357	-0.203398355	0.838823686	0.90451196	down_in_case
Yang2025_S	ENSGALG00010015145	CLIC2	case	control	-0.173344251	0.102238476	-1.695489383	0.089982623	0.203688734	down_in_case
Yang2025_S	ENSGALG00010015146	ENSGALG00010015146	case	control	-0.178875746	0.119683559	-1.494572416	0.13502607	0.271435124	down_in_case
Yang2025_S	ENSGALG00010015148	ENSGALG00010015148	case	control	0.160424669	0.137324338	1.168217314	0.242719118	0.407701412	up_in_case
Yang2025_S	ENSGALG00010015149	ENSGALG00010015149	case	control	-0.086925578	0.108387039	-0.801992368	0.422557373	0.588421804	down_in_case
Yang2025_S	ENSGALG00010015151	ENSGALG00010015151	case	control	0.458540858	0.301800202	1.519352389	0.128673819	0.262061812	up_in_case
Yang2025_S	ENSGALG00010015152	BOLL	case	control	0.305573546	0.248506831	1.229638415	0.218832538	0.379849454	up_in_case
Yang2025_S	ENSGALG00010015153	ERCC6L	case	control	-0.230019567	0.173322373	-1.327119882	0.18446903	0.337789215	down_in_case
Yang2025_S	ENSGALG00010015155	HMOX1	case	control	-0.155968712	0.18772003	-0.830858127	0.406053781	0.572031601	down_in_case
Yang2025_S	ENSGALG00010015156	CNOT6	case	control	-0.397618424	0.095279861	-4.173163364	3.00399E-05	0.000406414	down_in_case
Yang2025_S	ENSGALG00010015157	CDC42BPB	case	control	0.152403962	0.129592871	1.176021189	0.239586409	0.403948773	up_in_case
Yang2025_S	ENSGALG00010015158	PIN4	case	control	0.212881627	0.295270396	0.720971793	0.470926869	0.63116087	up_in_case
Yang2025_S	ENSGALG00010015159	ENSGALG00010015159	case	control	0.724501452	0.545999192	1.326927698	0.184532601	0.337861226	up_in_case
Yang2025_S	ENSGALG00010015160	SIM1	case	control	0.751848947	0.403048461	1.865405823	0.062124542	0.156292741	up_in_case
Yang2025_S	ENSGALG00010015161	GFPT2	case	control	-0.075383314	0.137497578	-0.548251945	0.583518919	0.725623429	down_in_case
Yang2025_S	ENSGALG00010015163	PLK2	case	control	-0.090721295	0.143100163	-0.633970587	0.526100017	0.679376847	down_in_case
Yang2025_S	ENSGALG00010015164	ENSGALG00010015164	case	control	-0.904372736	0.905506655	-0.998747751	0.317916902	0.487365231	down_in_case
Yang2025_S	ENSGALG00010015165	KLF3	case	control	0.008015355	0.228327989	0.035104564	0.971996362	0.983072239	up_in_case
Yang2025_S	ENSGALG00010015166	YTHDF2	case	control	-0.199684898	0.095009336	-2.101739745	0.03557608	0.104040435	down_in_case
Yang2025_S	ENSGALG00010015167	RAB3C	case	control	0.742821991	0.578138459	1.284851369	0.198844228	0.35565323	up_in_case
Yang2025_S	ENSGALG00010015168	GPR6	case	control	2.457073116	1.19328396	2.059085012	0.039486095	0.112247805	up_in_case
Yang2025_S	ENSGALG00010015171	NHSL2	case	control	0.044718196	0.15675502	0.2852735	0.775434614	0.863270828	up_in_case
Yang2025_S	ENSGALG00010015172	PPP4R3A	case	control	0.037832153	0.169226624	0.223559105	0.823100388	0.894251148	up_in_case

Yang2025_S	ENSGALG00010015173	ENS-1	case	control	-0.137650804	0.629240043	-0.218757222	0.826839172	0.896430655	down_in_case
Yang2025_S	ENSGALG00010015174	ST6GAL2	case	control	0.033381241	0.22519126	0.148235064	0.88215726	0.930642851	up_in_case
Yang2025_S	ENSGALG00010015175	MAPK9	case	control	-0.149067997	0.120758664	-1.234429003	0.217043087	0.377920207	down_in_case
Yang2025_S	ENSGALG00010015177	TC2N	case	control	-0.33347076	0.167000732	-1.99682214	0.045844508	0.125337103	down_in_case
Yang2025_S	ENSGALG00010015180	PITHD1	case	control	0.282351486	0.176052222	1.603793936	0.108759482	0.232412725	up_in_case
Yang2025_S	ENSGALG00010015181	VPS13A	case	control	0.20038377	0.140135937	1.429924215	0.152738771	0.296354048	up_in_case
Yang2025_S	ENSGALG00010015182	PHYKPL	case	control	0.2976796	0.150658876	1.97585172	0.048171574	0.129889876	up_in_case
Yang2025_S	ENSGALG00010015186	ENSGALG00010015186	case	control	0.152363809	0.256884169	0.59312261	0.5530991	0.701249746	up_in_case
Yang2025_S	ENSGALG00010015190	FBLN5	case	control	0.483487317	0.200816838	2.407603479	0.016057611	0.057864703	up_in_case
Yang2025_S	ENSGALG00010015191	TOM1	case	control	-0.007282874	0.171581306	-0.042445614	0.966143467	0.979997759	down_in_case
Yang2025_S	ENSGALG00010015192	ENSGALG00010015192	case	control	0.265326119	0.210632697	1.259662543	0.207791123	0.366574161	up_in_case
Yang2025_S	ENSGALG00010015193	LYPLA2	case	control	-0.148583648	0.114295957	-1.29999041	0.193604256	0.349058779	down_in_case
Yang2025_S	ENSGALG00010015194	TMLHE	case	control	-0.442287341	0.090848234	-4.868419813	1.12494E-06	2.6751E-05	down_in_case
Yang2025_S	ENSGALG00010015196	PDE4D	case	control	0.409572195	0.248442297	1.64856065	0.099237676	0.217823349	up_in_case
Yang2025_S	ENSGALG00010015198	NME5	case	control	-0.342966631	0.189270725	-1.812042675	0.069979603	0.169974707	down_in_case
Yang2025_S	ENSGALG00010015199	ENSGALG00010015199	case	control	-0.088879734	0.221496469	-0.401269303	0.688221861	0.804966563	down_in_case
Yang2025_S	ENSGALG00010015200	TBC1D1	case	control	-0.069818238	0.111008028	-0.628947646	0.529383332	0.681915024	down_in_case
Yang2025_S	ENSGALG00010015201	GCC2	case	control	0.575375725	0.176862795	3.253232127	0.001141002	0.007781465	up_in_case
Yang2025_S	ENSGALG00010015203	HMGXB4	case	control	0.137699978	0.071389814	1.928846293	0.053749947	0.140711161	up_in_case
Yang2025_S	ENSGALG00010015204	ENSGALG00010015204	case	control	0.464592876	0.168649375	2.754785635	0.00587306	0.026974651	up_in_case
Yang2025_S	ENSGALG00010015205	BPIFB3	case	control	1.042712278	0.727278262	1.433718472	0.151652619	0.294903606	up_in_case
Yang2025_S	ENSGALG00010015207	AKR1E2	case	control	-0.071277377	0.14947065	-0.476865367	0.633457995	0.764858952	down_in_case
Yang2025_S	ENSGALG00010015212	GNAQ	case	control	-0.093269077	0.164508622	-0.566955555	0.570744378	0.715003907	down_in_case
Yang2025_S	ENSGALG00010015213	RUBCN	case	control	0.185216074	0.110871427	1.670548307	0.09481093	0.211153957	up_in_case
Yang2025_S	ENSGALG00010015218	PLCL1	case	control	0.238233253	0.159672696	1.492009963	0.135696534	0.272233112	up_in_case
Yang2025_S	ENSGALG00010015227	FYTTD1	case	control	-0.282028946	0.054138794	-5.209368812	1.89484E-07	5.92918E-06	down_in_case
Yang2025_S	ENSGALG00010015233	ENSGALG00010015233	case	control	-0.0324078	0.468310744	-0.069201488	0.944829239	0.966042662	down_in_case
Yang2025_S	ENSGALG00010015234	TRIP11	case	control	0.515837844	0.193535577	2.665338592	0.007691084	0.033212042	up_in_case
Yang2025_S	ENSGALG00010015241	ENSGALG00010015241	case	control	0.262059112	0.212587257	1.232713175	0.217682795	0.378608305	up_in_case
Yang2025_S	ENSGALG00010015248	ENSGALG00010015248	case	control	0.185114	0.120836783	1.531934191	0.125538675	0.25764086	up_in_case
Yang2025_S	ENSGALG00010015250	RORB	case	control	0.573075177	0.589941984	0.971409381	0.331344459	0.500681527	up_in_case
Yang2025_S	ENSGALG00010015253	ENSGALG00010015253	case	control	-0.122954473	0.118580311	-1.03688776	0.299788169	0.469440177	down_in_case
Yang2025_S	ENSGALG00010015260	RASGEF1C	case	control	-0.119256707	0.198055078	-0.602139102	0.547081548	0.695956285	down_in_case
Yang2025_S	ENSGALG00010015261	IQCG	case	control	-0.436374337	0.347835647	-1.25454174	0.209645164	0.368898925	down_in_case
Yang2025_S	ENSGALG00010015285	ALG13	case	control	1.002951441	0.337941716	2.967823721	0.002999163	0.016331901	up_in_case
Yang2025_S	ENSGALG00010015291	ZFP64	case	control	0.263615586	0.0948417	2.77953248	0.005443721	0.025575944	up_in_case
Yang2025_S	ENSGALG00010015296	RPL35A	case	control	-0.384872011	0.330438694	-1.164730459	0.244128116	0.409377291	down_in_case
Yang2025_S	ENSGALG00010015298	ENSGALG00010015298	case	control	0.075893026	0.314046534	0.241661721	0.809042293	0.884860242	up_in_case
Yang2025_S	ENSGALG00010015299	GALE	case	control	-0.004326013	0.187411714	-0.023082938	0.981584116	0.98867905	down_in_case
Yang2025_S	ENSGALG00010015300	ATXN3	case	control	-0.391509609	0.156727054	-2.498034641	0.012488399	0.047743714	down_in_case
Yang2025_S	ENSGALG00010015306	BCAT1	case	control	-0.601546195	0.125599892	-4.789384631	1.67294E-06	3.74195E-05	down_in_case
Yang2025_S	ENSGALG00010015310	PGM2	case	control	-0.292635852	0.088016399	-3.324787814	0.000884858	0.006342851	down_in_case
Yang2025_S	ENSGALG00010015314	GMEB1	case	control	0.234451571	0.096881873	2.419973562	0.015521636	0.056458886	up_in_case
Yang2025_S	ENSGALG00010015318	SALL4	case	control	-0.139783086	0.169940926	-0.822539271	0.410770045	0.576269061	down_in_case
Yang2025_S	ENSGALG00010015320	TRPM6	case	control	1.106442107	0.406802983	2.719847574	0.006531201	0.02933042	up_in_case
Yang2025_S	ENSGALG00010015327	LARGE1	case	control	-0.153818037	0.117685951	-1.307021241	0.191205498	0.346347148	down_in_case
Yang2025_S	ENSGALG00010015333	MED7	case	control	0.190504507	0.115442309	1.650213937	0.098899187	0.217434006	up_in_case
Yang2025_S	ENSGALG00010015339	ENSGALG00010015339	case	control	-3.350351818	0.92831169	-3.609080715	0.000307284	0.002661282	down_in_case
Yang2025_S	ENSGALG00010015348	CPSF2	case	control	-0.013455571	0.103664817	-0.129798821	0.896725596	0.938786882	down_in_case
Yang2025_S	ENSGALG00010015353	RELL1	case	control	-0.613207141	0.084981615	-7.215762391	5.36326E-13	7.39971E-11	down_in_case
Yang2025_S	ENSGALG00010015356	ENSGALG00010015356	case	control	-0.589418963	0.203481131	-2.896676271	0.003771387	0.019349882	down_in_case
Yang2025_S	ENSGALG00010015362	ATP9A	case	control	0.302126418	0.234192667	1.290076338	0.197024155	0.353213894	up_in_case
Yang2025_S	ENSGALG00010015364	CLOCK	case	control	0.469463941	0.183189861	2.562717926	0.010385639	0.041659147	up_in_case
Yang2025_S	ENSGALG00010015370	ENSGALG00010015370	case	control	-0.120554932	0.115596208	-1.042896945	0.296996025	0.465905618	down_in_case
Yang2025_S	ENSGALG00010015374	LIMS1	case	control	-0.058792617	0.110936185	-0.529967903	0.596134185	0.735599917	down_in_case
Yang2025_S	ENSGALG00010015384	TAF12	case	control	-0.045200764	0.128827749	-0.350862017	0.725691868	0.831416277	down_in_case
Yang2025_S	ENSGALG00010015386	SLC24A4	case	control	0.965459836	0.343858486	2.807724328	0.004989292	0.023941384	up_in_case
Yang2025_S	ENSGALG00010015388	PSMD1	case	control	-0.097132047	0.106543393	-0.911666546	0.36194428	0.530524197	down_in_case
Yang2025_S	ENSGALG00010015390	CCNB3	case	control	-0.17290152	0.19812048	-0.872708972	0.382821726	0.550528458	down_in_case
Yang2025_S	ENSGALG00010015391	SYN3	case	control	0.716715639	0.482468688	1.485517417	0.137406813	0.27507024	up_in_case
Yang2025_S	ENSGALG00010015392	TMEM165	case	control	-0.19948558	0.123986834	-1.60892551	0.107632631	0.230676824	down_in_case
Yang2025_S	ENSGALG00010015393	DCX	case	control	-0.350583597	0.292253628	-1.199586807	0.230299853	0.392710285	down_in_case
Yang2025_S	ENSGALG00010015397	SRD5A3	case	control	-0.278530973	0.221150666	-1.259462514	0.207863322	0.366655113	down_in_case
Yang2025_S	ENSGALG00010015404	BEND4	case	control	-0.101941211	0.248017453	-0.411024346	0.681054683	0.80021054	down_in_case
Yang2025_S	ENSGALG00010015407	TIMP3	case	control	-0.18778527	0.098215212	-1.911977452	0.055879086	0.144842832	down_in_case
Yang2025_S	ENSGALG00010015409	FAM13B	case	control	0.20324955	0.11116262	1.828398341	0.06748979	0.165519018	up_in_case
Yang2025_S	ENSGALG00010015410	HTR2B	case	control	-0.397918016	0.242189779	-1.64300086	0.100382755	0.219597126	down_in_case
Yang2025_S	ENSGALG00010015413	CARNMT1	case	control	0.517475332	0.077043586	6.716656919	1.85941E-11	1.85078E-09	up_in_case
Yang2025_S	ENSGALG00010015416	CAPN6	case	control	-0.856856462	0.161747847	-5.29748296	1.1741E-07	3.93295E-06	down_in_case
Yang2025_S	ENSGALG00010015422	ENSGALG00010015422	case	control	0.412870251	0.614142329	0.672271283	0.501411008	0.658073309	up_in_case
Yang2025_S	ENSGALG00010015430	HMGCL	case	control	0.169867854	0.187358818	0.906644567	0.364594786	0.533511325	up_in_case
Yang2025_S	ENSGALG00010015431	RIN3	case	control	-0.272145695	0.152607585	-1.783303863	0.074536853	0.178007034	down_in_case
Yang2025_S	ENSGALG00010015436	SLC30A9	case	control	0.081281591	0.097363216	0.834828535	0.403814257	0.570126816	up_in_case
Yang2025_S	ENSGALG00010015440	ENSGALG00010015440	case	control	0.149376791	0.456596476	0.327152747	0.743552359	0.843144448	up_in_case
Yang2025_S	ENSGALG00010015443	PAK3	case	control	-0.017193752	0.127858336	-0.13447502	0.893026963	0.937284833	down_in_case
Yang2025_S	ENSGALG00010015449	HDX	case	control	-0.153866714	0.097210394	-1.582821631	0.113462125	0.239869088	down_in_case
Yang2025_S	ENSGALG00010015466	NMRK1	case	control	0.427798722	0.174009307	2.458481845	0.013952583	0.051925851	up_in_case
Yang2025_S	ENSGALG00010015469	TMEM33	case	control	-0.259006035	0.08172023	-3.1694237	0.001527416	0.009723406	down_in_case
Yang2025_S	ENSGALG00010015472	LGMN	case	control	-0.221270694	0.102042122	-2.168425048	0.030126362	0.091922673	down_in_case
Yang2025_S	ENSGALG00010015477	ARMC9	case	control	-0.047757952	0.10411577	-0.458700461	0.646449283	0.77557217	down_in_case
Yang2025_S	ENSGALG00010015478	FBXO7	case	control	-0.100817335	0.107026881	-0.941981437	0.346202144	0.514727257	down_in_case
Yang2025_S	ENSGALG00010015481	GOLGA5	case	control	0.264965221	0.113893963	2.326420229	0.019996143	0.067698314	up_in_case
Yang2025_S	ENSGALG00010015483	SGCD	case	control	-0.46057339	0.115629552	-3.983180619	6.7999E-05	0.000781176	down_in_case
Yang2025_S	ENSGALG00010015485	BCAS1	case	control	0.716032375	0.193865713	3.69344514	0.000221236	0.002020267	up_in_case
Yang2025_S	ENSGALG00010015486	MTFR1L	case	control	-0.548261251	0.169419935	-3.236108263	0.001211714	0.008125716	down_in_case
Yang2025_S	ENSGALG00010015487	RPS6KA6	case	control	0.270897601	0.131050456	2.067124442	0.03872243	0.110573167	up_in_case
Yang2025_S	ENSGALG00010015490	RANBP2	case	control	0.006413112	0.101309367	0.063302263	0.949525813	0.968633161	up_in_case
Yang2025_S	ENSGALG00010015493	OSTF1	case	control	0.334992649	0.106715706	3.139112887	0.001694602	0.010579871	up_in_case
Yang2025_S	ENSGALG00010015494	CHRDL1	case	control	-0.441348351	0.264325798	-1.66971349	0.094976063	0.211454137	down_in_case
Yang2025_S	ENSGALG00010015498	MID2	case	control	-0.169457903	0.089011876	-1.903767352	0.05694049	0.146929537	down_in_case
Yang2025_S	ENSGALG00010015505	CHGA	case	control	1.031915667	0.635323892	1.624235575	0.104325541	0.226127923	up_in_case
Yang2025_S	ENSGALG00010015507	AMMECR1	case	control	-0.090726035	0.120139171	-0.755174475	0.450144302	0.612282169	down_in_case
Yang2025_S	ENSGALG00010015508	FUCA1	case	control	-0.063174781	0.122144158	-0.51721492	0.605006138	0.743002572	down_in_case
Yang2025_S	ENSGALG00010015511	BPIFB4	case	control	0.079620056	0.461705374	0.172447756	0.863085531	0.919290108	up_in_case
Yang2025_S	ENSGALG00010015516	SELENON	case	control	-0.573310016	0.108354024	-5.291081903	1.21595E-07	4.04677E-06	down_in_case
Yang2025_S	ENSGALG00010015520	UBE3A	case	control	-0.396783573	0.05392289	-7.358351353	1.86195E-13	2.88293E-11	down_in_case
Yang2025_S	ENSGALG00010015521	DKC1	case	control	0.196516086	0.092774982	2.118201275	0.034158025	0.100803067	up_in_case
Yang2025_S	ENSGALG00010015523	MRPL22	case	control	-0.237983342	0.137591966	-1.729631085	0.083696209	0.193513633	down_in_case
Yang2025_S	ENSGALG00010015532	NXT2	case	control	-0.331369647	0.110322999	-3.003631615	0.002667781	0.014882119	down_in_case
Yang2025_S	ENSGALG00010015533	PSAT1	case	control	-0.257053843	0.132894277	-1.93427324	0.053079542	0.139363095	down_in_case
Yang2025_S	ENSGALG00010015540	ACSL4	case	control	0.034654627	0.092806326	0.373408028	0.708844785	0.820223694	up_in_case
Yang2025_S	ENSGALG00010015541	RASEF	case	control	0.224892465	0.454395787	0.494926387	0.620652083	0.755748583	up_in_case
Yang2025_S	ENSGALG00010015542	CNGA3	case	control	0.280289997	0.355514218	0.788407277	0.430458516	0.5944935	up_in_case
Yang2025_S	ENSGALG00010015544	RTCB	case	control	0.088101974	0.129446357	0.680606053	0.496120796	0.653321044	up_in_case
Yang2025_S	ENSGALG00010015555	PNRC2	case	control	-0.70000853	0.156429429	-4.474915826	7.64414E-06	0.000131783	down_in_case
Yang2025_S	ENSGALG00010015557	IRS4	case	control	-0.230399788	0.131282779	-1.754988661	0.079261247	0.186124426	down_in_case
Yang2025_S	ENSGALG00010015558	MPP1	case	control	-0.043751385	0.163698272	-0.267268457	0.789262471	0.872955991	down_in_case
Yang2025_S	ENSGALG00010015562	IRF9	case	control	0.424502593	0.395773938	1.072588545	0.283455767	0.451733315	up_in_case
Yang2025_S	ENSGALG00010015563	B3GNT7	case	control	1.060555912	0.334082683	3.17453123	0.001500787	0.009619813	up_in_case
Yang2025_S	ENSGALG00010015564	PKD2L2	case	control	0.178683292	0.250520678	0.713247681	0.475692506	0.635122648	up_in_case
Yang2025_S	ENSGALG00010015565	ENSGALG00010015565	case	control	4.01608143	1.685982735	2.382041848	0.017216941	0.060769521	up_in_case
Yang2025_S	ENSGALG00010015567	ENSGALG00010015567	case	control	-0.262692164	0.326261569	-0.805158159	0.420728418	0.586402331	down_in_case
Yang2025_S	ENSGALG00010015570	LIMCH1	case	control	0.244596542	0.202552111	1.207573401	0.227211396	0.389397466	up_in_case
Yang2025_S	ENSGALG00010015571	DUSP15	case	control	0.393215354	0.252352656	1.558197802	0.119186366	0.248371767	up_in_case
Yang2025_S	ENSGALG00010015573	MAN1C1	case	control	0.021599551	0.081604838	0.264684687	0.791252375	0.873985268	up_in_case
Yang2025_S	ENSGALG00010015574	C14orf132	case	control	-0.58294041	0.317315621	-1.837099633	0.066195188	0.163261938	down_in_case
Yang2025_S	ENSGALG00010015580	GEMIN5	case	control	-0.220699265	0.067359251	-3.276450721	0.001051207	0.00729147	down_in_case
Yang2025_S	ENSGALG00010015583	PRDM4	case	control	0.271672129	0.183542759	1.480157157	0.138831311	0.277042018	up_in_case
Yang2025_S	ENSGALG00010015584	TTLL9	case	control	0.266539301	0.348840299	0.764072562	0.444824002	0.608113428	up_in_case
Yang2025_S	ENSGALG00010015585	BDKRB1	case	control	-1.521796674	0.246136668	-6.182730447	6.30022E-10	4.01844E-08	down_in_case
Yang2025_S	ENSGALG00010015588	BDKRB2	case	control	1.295071095	0.599413532	2.160563661	0.030729061	0.093352837	up_in_case
Yang2025_S	ENSGALG00010015589	FRMD3	case	control	-0.551978463	0.191397706	-2.883934583	0.003927403	0.019907242	down_in_case
Yang2025_S	ENSGALG00010015596	UCHL1	case	control	-0.693725898	0.286662009	-2.420013384	0.015519936	0.056458886	down_in_case
Yang2025_S	ENSGALG00010015605	LDLRAP1	case	control	-0.131202158	0.090604777	-1.448071078	0.147597173	0.289767062	down_in_case
Yang2025_S	ENSGALG00010015606	TMEM131	case	control	0.425702208	0.252598329	1.685293046	0.091932047	0.206925064	up_in_case
Yang2025_S	ENSGALG00010015610	ENSGALG00010015610	case	control	0.60995768	0.162188964	3.760784109	0.000169382	0.001633448	up_in_case

Yang2025_S	ENSGALG00010015613	PWP1	case	control	0.0103514	0.129745741	0.079782197	0.936410484	0.961149333	up_in_case
Yang2025_S	ENSGALG00010015614	ATG2B	case	control	0.250087089	0.132569145	1.886465282	0.059232283	0.151227896	up_in_case
Yang2025_S	ENSGALG00010015616	NADK2	case	control	0.429868251	0.127818183	3.363122843	0.000770661	0.005673088	up_in_case
Yang2025_S	ENSGALG00010015617	SRSF10	case	control	-0.05581493	0.0755472	-0.738808715	0.460023159	0.621056625	down_in_case
Yang2025_S	ENSGALG00010015618	NCL	case	control	0.309737501	0.155137942	1.996529651	0.045876302	0.125346234	up_in_case
Yang2025_S	ENSGALG00010015620	PDRG1	case	control	-0.482657768	0.359256845	-1.343489413	0.179113552	0.330720464	down_in_case
Yang2025_S	ENSGALG00010015621	IDNK	case	control	-0.592857465	0.22666135	-2.615608993	0.008906849	0.037328403	down_in_case
Yang2025_S	ENSGALG00010015624	ITGA1	case	control	-0.030171328	0.1553848	-0.194171684	0.846041454	0.908638741	down_in_case
Yang2025_S	ENSGALG00010015626	XKR7	case	control	0.205992838	0.417685198	0.493177251	0.62188735	0.756525554	up_in_case
Yang2025_S	ENSGALG00010015628	UBQLN1	case	control	-0.000179728	0.122949763	-0.001461798	0.998833654	0.998977031	down_in_case
Yang2025_S	ENSGALG00010015631	GSKIP	case	control	-0.777403089	0.113256578	-6.864087774	6.69175E-12	7.45996E-10	down_in_case
Yang2025_S	ENSGALG00010015632	COL4A5	case	control	-0.403540786	0.14016139	-2.879115178	0.003987927	0.020134695	down_in_case
Yang2025_S	ENSGALG00010015633	CCM2L	case	control	0.340027587	0.241528885	1.407813344	0.159186348	0.304622598	up_in_case
Yang2025_S	ENSGALG00010015635	ABTB3	case	control	0.079750666	0.187060716	0.426335724	0.66986323	0.792069929	up_in_case
Yang2025_S	ENSGALG00010015636	APBB2	case	control	-0.122436657	0.095728973	-1.278992697	0.200899628	0.358730947	down_in_case
Yang2025_S	ENSGALG00010015643	ENSGALG00010015643	case	control	0.217754307	0.226286417	0.962295089	0.335901395	0.504123419	up_in_case
Yang2025_S	ENSGALG00010015645	MACO1	case	control	0.273604955	0.123130599	2.222071176	0.026278496	0.083017647	up_in_case
Yang2025_S	ENSGALG00010015647	PDZD2	case	control	-0.077699959	0.178093169	-0.436288262	0.662627597	0.78678558	down_in_case
Yang2025_S	ENSGALG00010015653	HCK	case	control	1.097612748	0.476644877	2.30278935	0.021290698	0.070946213	up_in_case
Yang2025_S	ENSGALG00010015655	ENSGALG00010015655	case	control	-0.186905642	0.289301187	-0.646059023	0.518241132	0.67253587	down_in_case
Yang2025_S	ENSGALG00010015660	KLHL3	case	control	0.415885212	0.150813513	2.757612395	0.005822519	0.026833867	up_in_case
Yang2025_S	ENSGALG00010015668	ZNF217	case	control	-0.005731217	0.152518102	-0.037577293	0.970024713	0.982096151	down_in_case
Yang2025_S	ENSGALG00010015669	INPP4A	case	control	-0.203592585	0.111644077	-1.82358608	0.068214673	0.166825458	down_in_case
Yang2025_S	ENSGALG00010015670	ENSGALG00010015670	case	control	1.058150265	0.256828773	4.120061215	3.78772E-05	0.000490538	up_in_case
Yang2025_S	ENSGALG00010015674	RFKL	case	control	0.055396722	0.122870827	0.450853334	0.652095259	0.779725962	up_in_case
Yang2025_S	ENSGALG00010015676	TGM2	case	control	0.149926179	0.123827181	1.210769539	0.225983743	0.387961495	up_in_case
Yang2025_S	ENSGALG00010015677	GRHL3	case	control	-0.029968292	0.225784363	-0.132729707	0.894407148	0.937885741	down_in_case
Yang2025_S	ENSGALG00010015680	ENSGALG00010015680	case	control	0.565735607	0.116988435	4.835825072	1.32594E-06	3.07951E-05	up_in_case
Yang2025_S	ENSGALG00010015684	PDE6D	case	control	-0.562892395	0.123170219	-4.570036474	4.87639E-06	9.02974E-05	down_in_case
Yang2025_S	ENSGALG00010015686	CNOT8	case	control	-0.352448506	0.083582016	-4.216798319	2.47795E-05	0.000347172	down_in_case
Yang2025_S	ENSGALG00010015693	COA5	case	control	0.167856703	0.168441375	0.996528929	0.318993218	0.48837294	up_in_case
Yang2025_S	ENSGALG00010015695	ASAH1	case	control	-0.166607612	0.088827306	-1.875635087	0.060705405	0.153749513	down_in_case
Yang2025_S	ENSGALG00010015697	FAXDC2	case	control	-0.216637715	0.152329562	-1.422164624	0.154978466	0.299490351	down_in_case
Yang2025_S	ENSGALG00010015698	TNFAIP2	case	control	-0.018866555	0.149999005	-0.12577787	0.899907759	0.940539611	down_in_case
Yang2025_S	ENSGALG00010015701	UNC50	case	control	-0.12383976	0.225716769	-0.548651128	0.583244892	0.725536713	down_in_case
Yang2025_S	ENSGALG00010015703	GK5	case	control	-0.389223035	0.15274375	-2.54820924	0.010827751	0.042976682	down_in_case
Yang2025_S	ENSGALG00010015706	ENSGALG00010015706	case	control	-0.258148631	0.23466784	-1.100059689	0.271306116	0.438641458	down_in_case
Yang2025_S	ENSGALG00010015709	HNRNPA0	case	control	0.070712531	0.089678613	0.788510529	0.430398142	0.594469037	up_in_case
Yang2025_S	ENSGALG00010015711	LBFABP	case	control	0.291061768	0.304775444	0.955003999	0.339575675	0.507560552	up_in_case
Yang2025_S	ENSGALG00010015716	MGAT4A	case	control	-0.279952306	0.164299584	-1.703913659	0.088397219	0.201507483	down_in_case
Yang2025_S	ENSGALG00010015719	XRN1	case	control	-0.114460978	0.122584116	-0.933734177	0.350441031	0.518847829	down_in_case
Yang2025_S	ENSGALG00010015729	RHCE	case	control	0.27432388	0.154621105	1.774168408	0.076035298	0.180555417	up_in_case
Yang2025_S	ENSGALG00010015731	PHF6	case	control	0.058121569	0.13816076	0.420680728	0.673988236	0.794944163	up_in_case
Yang2025_S	ENSGALG00010015733	COL4A6	case	control	-0.277679349	0.130449806	-2.128629838	0.033284898	0.098854444	down_in_case
Yang2025_S	ENSGALG00010015734	TSHZ2	case	control	0.384443757	0.203984693	1.884669632	0.059474455	0.151651698	up_in_case
Yang2025_S	ENSGALG00010015742	HPRT1	case	control	-0.042107806	0.132439389	-0.317940197	0.750530301	0.848502332	down_in_case
Yang2025_S	ENSGALG00010015746	PAPOLB	case	control	-0.108221886	0.121057475	-0.893971113	0.371337347	0.539862903	down_in_case
Yang2025_S	ENSGALG00010015749	ATR	case	control	0.019707195	0.103558023	0.190301	0.84907327	0.910351313	up_in_case
Yang2025_S	ENSGALG00010015751	MYOM3	case	control	0.755571732	0.203656329	3.710033146	0.000207232	0.001926471	up_in_case
Yang2025_S	ENSGALG00010015753	RPRD1B	case	control	-0.350876002	0.056730006	-6.18501613	6.20961E-10	4.00606E-08	down_in_case
Yang2025_S	ENSGALG00010015756	PCM1	case	control	0.34477374	0.129442436	2.663529454	0.007732564	0.033342673	up_in_case
Yang2025_S	ENSGALG00010015758	PCDH19	case	control	-0.191944766	0.110202096	-1.741752414	0.081551777	0.189846977	down_in_case
Yang2025_S	ENSGALG00010015759	EIF5	case	control	-0.376022636	0.125442391	-2.997572294	0.002721393	0.015107015	down_in_case
Yang2025_S	ENSGALG00010015764	TTI1	case	control	0.022576288	0.093841918	0.240577857	0.809882314	0.885439022	up_in_case
Yang2025_S	ENSGALG00010015765	ENSGALG00010015765	case	control	1.204378543	1.41303016	0.852337463	0.394026819	0.56108356	up_in_case
Yang2025_S	ENSGALG00010015766	TLE1	case	control	0.23733199	0.127308692	1.864224565	0.062290175	0.156526714	up_in_case
Yang2025_S	ENSGALG00010015769	TMEM50A	case	control	-0.469996737	0.198658634	-2.36585105	0.01798868	0.062856634	down_in_case
Yang2025_S	ENSGALG00010015774	PLS1	case	control	0.280510186	0.264388989	1.060975294	0.288701129	0.457632833	up_in_case
Yang2025_S	ENSGALG00010015776	SPOCK1	case	control	0.211218495	0.405559375	0.520807822	0.602500653	0.740872548	up_in_case
Yang2025_S	ENSGALG00010015778	PABIR3	case	control	-0.470110942	0.106266478	-4.423887498	9.69405E-06	0.000159488	down_in_case
Yang2025_S	ENSGALG00010015782	ATG4A	case	control	-0.090878983	0.120380452	-0.754931402	0.450290143	0.612312663	down_in_case
Yang2025_S	ENSGALG00010015785	FGL1	case	control	0.179304867	0.153088751	1.171247823	0.241499178	0.406651348	up_in_case
Yang2025_S	ENSGALG00010015788	TRPC1	case	control	0.048783604	0.119826114	0.407119969	0.683919877	0.802021669	up_in_case
Yang2025_S	ENSGALG00010015792	MARK3	case	control	-0.16129788	0.081624626	-1.976093341	0.048144206	0.129847868	down_in_case
Yang2025_S	ENSGALG00010015793	RSRP1	case	control	0.61656192	0.200693306	3.072159874	0.002125159	0.012519769	up_in_case
Yang2025_S	ENSGALG00010015795	PSMD10	case	control	-0.09501931	0.18771198	-0.50619737	0.612718101	0.749275006	down_in_case
Yang2025_S	ENSGALG00010015796	LARP1	case	control	0.12167435	0.185248229	0.656817885	0.511298023	0.666606142	up_in_case
Yang2025_S	ENSGALG00010015797	PIP5K1B	case	control	0.03040799	0.123531069	0.246156618	0.805560996	0.883340611	up_in_case
Yang2025_S	ENSGALG00010015798	IL22RA1	case	control	0.348497692	0.187901966	1.854678262	0.063642185	0.158664256	up_in_case
Yang2025_S	ENSGALG00010015800	BVES	case	control	-0.622221144	0.177311988	-3.509188233	0.000449477	0.003626785	down_in_case
Yang2025_S	ENSGALG00010015801	VSTM2L	case	control	0.141410477	0.236203245	0.598681348	0.549385398	0.697938328	up_in_case
Yang2025_S	ENSGALG00010015803	SYF2	case	control	0.307028683	0.120705653	2.543614775	0.010971201	0.043383564	up_in_case
Yang2025_S	ENSGALG00010015804	CKB	case	control	-0.299591645	0.229602494	-1.304827487	0.191951597	0.347247242	down_in_case
Yang2025_S	ENSGALG00010015807	ENSGALG00010015807	case	control	0.17390707	0.624414284	0.278512318	0.780619111	0.867112579	up_in_case
Yang2025_S	ENSGALG00010015809	PCOLCE2	case	control	-0.031765275	0.337411932	-0.094143899	0.924994849	0.955083226	down_in_case
Yang2025_S	ENSGALG00010015810	LIPT1	case	control	-0.177218233	0.14457053	-1.225825436	0.220264373	0.381644323	down_in_case
Yang2025_S	ENSGALG00010015811	CTNNBL1	case	control	-0.094726077	0.135935801	-0.696844221	0.485900288	0.643082963	down_in_case
Yang2025_S	ENSGALG00010015813	ENSGALG00010015813	case	control	0.0528429	0.17619022	0.299919601	0.764238483	0.856605457	up_in_case
Yang2025_S	ENSGALG00010015814	PAQR9	case	control	0.27592188	0.401291362	0.687584898	0.491714221	0.649174578	up_in_case
Yang2025_S	ENSGALG00010015816	MITD1	case	control	-0.11863558	0.142819032	-0.830670659	0.406159706	0.572105075	down_in_case
Yang2025_S	ENSGALG00010015820	ENSGALG00010015820	case	control	0.03163698	0.306806619	0.103117008	0.917870107	0.951604797	up_in_case
Yang2025_S	ENSGALG00010015825	RUNX3	case	control	-0.436828776	0.922026988	-0.473770054	0.635663892	0.766393522	down_in_case
Yang2025_S	ENSGALG00010015827	ENSGALG00010015827	case	control	0.24022448	0.143037232	1.679454193	0.093063559	0.208394777	up_in_case
Yang2025_S	ENSGALG00010015829	SMAD5	case	control	-0.371059213	0.121617176	-3.051042841	0.00228048	0.013219007	down_in_case
Yang2025_S	ENSGALG00010015831	LYG2	case	control	0.088346308	0.355084053	0.248803932	0.80351245	0.881718717	up_in_case
Yang2025_S	ENSGALG00010015832	RAB39B	case	control	0.171991854	0.078848944	2.181282913	0.029162497	0.089589815	up_in_case
Yang2025_S	ENSGALG00010015834	STARD8	case	control	-0.038986095	0.10751504	-0.362610614	0.716895781	0.825653282	down_in_case
Yang2025_S	ENSGALG00010015837	GDA	case	control	-0.184794412	0.23196656	-0.796642467	0.425658707	0.590379098	down_in_case
Yang2025_S	ENSGALG00010015838	IFNLR1	case	control	0.301869039	1.595304628	0.189223446	0.849917693	0.910869597	up_in_case
Yang2025_S	ENSGALG00010015839	U2SURP	case	control	0.747626899	0.197602307	3.783492774	0.000154643	0.001514369	up_in_case
Yang2025_S	ENSGALG00010015840	MTUS1	case	control	0.113921546	0.10768868	1.057878567	0.290110808	0.458768735	up_in_case
Yang2025_S	ENSGALG00010015843	ENSGALG00010015843	case	control	0.104912768	0.322997999	0.324809345	0.745325372	0.844742501	up_in_case
Yang2025_S	ENSGALG00010015845	SAP30L	case	control	-0.262219377	0.10290602	-2.548144188	0.01082977	0.042976682	down_in_case
Yang2025_S	ENSGALG00010015848	UBE2B	case	control	-0.491118359	0.150175156	-3.270303626	0.001074321	0.00741122	down_in_case
Yang2025_S	ENSGALG00010015852	ZFAND5	case	control	-0.511765796	0.18432081	-2.776494933	0.005494849	0.025694874	down_in_case
Yang2025_S	ENSGALG00010015855	NIPAL3	case	control	0.253033696	0.101488831	2.493217171	0.012659139	0.048242337	up_in_case
Yang2025_S	ENSGALG00010015856	TXNDC9	case	control	-0.282323507	0.082179447	-3.435451523	0.000591567	0.004564933	down_in_case
Yang2025_S	ENSGALG00010015858	CHST2	case	control	0.025676413	0.141247286	0.181783408	0.855752706	0.914658189	up_in_case
Yang2025_S	ENSGALG00010015861	PPP2CA	case	control	-0.524370389	0.098893661	-5.302366037	1.14311E-07	3.84765E-06	down_in_case
Yang2025_S	ENSGALG00010015862	PDGFRL	case	control	-0.020055656	0.134914727	-0.148654316	0.881826411	0.930434697	down_in_case
Yang2025_S	ENSGALG00010015863	ENSGALG00010015863	case	control	0.144230929	0.313388794	0.460230013	0.645351131	0.774738131	up_in_case
Yang2025_S	ENSGALG00010015865	VRK1	case	control	-0.057168906	0.093347885	-0.612428503	0.540254288	0.690770674	down_in_case
Yang2025_S	ENSGALG00010015866	ARHGEF6	case	control	-0.18479377	0.080099663	-2.307048049	0.021052142	0.070434956	down_in_case
Yang2025_S	ENSGALG00010015867	PICALML	case	control	-0.133910261	0.057058515	-2.346893542	0.018930663	0.065168987	down_in_case
Yang2025_S	ENSGALG00010015869	SLC7A2	case	control	0.131903689	0.132046642	0.99891741	0.317834702	0.487348874	up_in_case
Yang2025_S	ENSGALG00010015870	EIF5B	case	control	0.55168591	0.202372662	2.726089105	0.006408969	0.028902585	up_in_case
Yang2025_S	ENSGALG00010015872	TMC1	case	control	1.594290778	0.304768314	5.231156611	1.68453E-07	5.34713E-06	up_in_case
Yang2025_S	ENSGALG00010015874	CLIC4	case	control	-0.237221309	0.091368703	-2.59630816	0.009423151	0.038861086	down_in_case
Yang2025_S	ENSGALG00010015875	DNTTIP1	case	control	0.170790915	0.197914944	0.86295108	0.388164365	0.555232028	up_in_case
Yang2025_S	ENSGALG00010015876	SKP1	case	control	-0.82227212	0.11873235	-6.925426126	4.34665E-12	5.2224E-10	down_in_case
Yang2025_S	ENSGALG00010015877	REV1	case	control	0.010258747	0.09221147	0.111252394	0.911416205	0.94745131	up_in_case
Yang2025_S	ENSGALG00010015879	TRMT61A	case	control	-0.247425987	0.187150414	-1.322070211	0.186144772	0.339741637	down_in_case
Yang2025_S	ENSGALG00010015881	GAB3	case	control	-0.37289357	0.178538132	-2.088593428	0.036744337	0.106430554	down_in_case
Yang2025_S	ENSGALG00010015886	AFF3	case	control	0.375724495	0.258004957	1.456268517	0.145318405	0.286544782	up_in_case
Yang2025_S	ENSGALG00010015887	SLC9A9	case	control	-0.363503425	0.201119224	-1.807402679	0.070699517	0.171130409	down_in_case
Yang2025_S	ENSGALG00010015889	SH3BP5	case	control	-0.31385208	0.081004761	-3.874489311	0.000106849	0.001122877	down_in_case
Yang2025_S	ENSGALG00010015892	UBE2C	case	control	0.206911925	0.222173187	0.931309166	0.351693662	0.520481221	up_in_case
Yang2025_S	ENSGALG00010015893	TCF7	case	control	-0.549338325	0.333230573	-1.648523185	0.099245357	0.217823349	down_in_case
Yang2025_S	ENSGALG00010015894	CEP78	case	control	0.635914063	0.190459173	3.338847135	0.000841268	0.006080434	up_in_case
Yang2025_S	ENSGALG00010015895	RBMXL1	case	control	0.446045855	0.147924695	3.015357615	0.002566764	0.01448678	up_in_case
Yang2025_S	ENSGALG00010015896	LONRF2	case	control	-0.212337398	0.099602915	-2.131839195	0.033020068	0.098310239	down_in_case
Yang2025_S	ENSGALG00010015898	ENSGALG00010015898	case	control	1.095118745	0.239383332	4.57474937	4.7679E-06	8.89434E-05	up_in_case
Yang2025_S	ENSGALG00010015899	SMARCA1	case	control	-0.269135115	0.081431655	-3.305042943	0.000949618	0.006730892	down_in_case
Yang2025_S	ENSGALG00010015900	ENSGALG00010015900	case	control	0.212476783	0.244942577	0.86745549	0.385692489	0.553182886	up_in_case
Yang2025_S	ENSGALG00010015904	MTMR7	case	control	0.763750989	0.231339127	3.301434555	0.000961918	0.006793254	up_in_case
Yang2025_S	ENSGALG00010015905	ANPEP	case	control	0.283812125	0.179854345	1.578010949	0.114563095	0.241554959	up_in_case

Yang2025_S	ENSGALG00010015906	BAG5	case	control	0.261405187	0.16192406	1.61436903	0.106447405	0.229052593	up_in_case
Yang2025_S	ENSGALG00010015908	CHRD	case	control	-0.002797022	0.216199038	-0.012937256	0.989677851	0.993814287	down_in_case
Yang2025_S	ENSGALG00010015909	TNNC2	case	control	0.300225637	0.174877742	1.716774438	0.086020393	0.197250976	up_in_case
Yang2025_S	ENSGALG00010015910	C5orf15	case	control	-0.763634955	0.071939545	-10.61495398	2.5392E-26	2.94864E-23	down_in_case
Yang2025_S	ENSGALG00010015911	TM9SF2L	case	control	-0.585169395	0.079886717	-7.324989902	2.38917E-13	3.54181E-11	down_in_case
Yang2025_S	ENSGALG00010015912	ENSGALG00010015912	case	control	0.303183188	0.353184495	0.858427232	0.390656596	0.557995045	up_in_case
Yang2025_S	ENSGALG00010015913	CHST10	case	control	-0.244950412	0.114863259	-2.132539277	0.032962539	0.09816905	down_in_case
Yang2025_S	ENSGALG00010015914	SRRM1	case	control	0.642072908	0.22939805	2.798946672	0.00512696	0.024442078	up_in_case
Yang2025_S	ENSGALG00010015916	COA8	case	control	-0.495810997	0.192526648	-2.57528504	0.010015751	0.040548951	down_in_case
Yang2025_S	ENSGALG00010015917	TMEM63A	case	control	-0.216874631	0.138029576	-1.571218555	0.116131892	0.243793873	down_in_case
Yang2025_S	ENSGALG00010015918	BICD1	case	control	0.953212947	0.183631783	5.19089306	2.09288E-07	6.38168E-06	up_in_case
Yang2025_S	ENSGALG00010015919	ENSGALG00010015919	case	control	-0.07904444	0.264967872	-0.298317072	0.765461176	0.857247499	down_in_case
Yang2025_S	ENSGALG00010015921	SNX21	case	control	0.948934874	0.152590604	6.218829019	5.00879E-10	3.30794E-08	up_in_case
Yang2025_S	ENSGALG00010015923	RCAN3	case	control	-0.336582168	0.105450317	-3.191855431	0.001413621	0.009207045	down_in_case
Yang2025_S	ENSGALG00010015927	GLA	case	control	-0.110838412	0.118460523	-0.935656954	0.34944984	0.517930602	down_in_case
Yang2025_S	ENSGALG00010015928	CAPN7	case	control	-0.077837239	0.097583066	-0.797651092	0.425072993	0.589920541	down_in_case
Yang2025_S	ENSGALG00010015929	SLC6A12	case	control	-0.183828519	0.622587765	-0.295265229	0.767791276	0.858750416	down_in_case
Yang2025_S	ENSGALG00010015931	RBM47	case	control	-0.052433055	0.207932354	-0.252164004	0.8009143	0.879768309	down_in_case
Yang2025_S	ENSGALG00010015932	ACOT8	case	control	-0.03656042	0.205880571	-0.177580718	0.859052269	0.916888279	down_in_case
Yang2025_S	ENSGALG00010015933	ENSGALG00010015933	case	control	-0.240894204	0.360827545	-0.667615895	0.504378809	0.660175664	down_in_case
Yang2025_S	ENSGALG00010015934	TMC2	case	control	-0.047416798	0.684440178	-0.069278222	0.944768161	0.966042662	down_in_case
Yang2025_S	ENSGALG00010015935	EPHX1	case	control	-0.232007426	0.144822155	-1.602016108	0.109152048	0.233051555	down_in_case
Yang2025_S	ENSGALG00010015936	RPL36AL	case	control	0.271152856	0.277221703	0.978108326	0.32802075	0.497738067	up_in_case
Yang2025_S	ENSGALG00010015937	ENSGALG00010015937	case	control	0.388771651	0.307793309	1.263093247	0.206555671	0.365226909	up_in_case
Yang2025_S	ENSGALG00010015939	SUN2	case	control	-0.105605017	0.148022819	-0.713437411	0.47557513	0.635087632	down_in_case
Yang2025_S	ENSGALG00010015942	AP3S2	case	control	0.111779415	0.230733657	0.484452143	0.628065021	0.761060746	up_in_case
Yang2025_S	ENSGALG00010015946	TLE4	case	control	0.494486269	0.186432701	2.652358017	0.007993174	0.034219624	up_in_case
Yang2025_S	ENSGALG00010015954	CTSA	case	control	0.000108855	0.125046681	0.000870511	0.999305433	0.999305433	up_in_case
Yang2025_S	ENSGALG00010015957	ENSGALG00010015957	case	control	-0.375068013	0.18925909	-1.981770134	0.047504974	0.128740141	down_in_case
Yang2025_S	ENSGALG00010015959	TRNAU1AP	case	control	-0.100503932	0.127940257	-0.78555362	0.432129045	0.596150703	down_in_case
Yang2025_S	ENSGALG00010015963	DIPK2A	case	control	-0.663199716	0.110779572	-5.986660732	2.14193E-09	1.19391E-07	down_in_case
Yang2025_S	ENSGALG00010015964	KLC1	case	control	0.438332782	0.175107885	2.503215558	0.012307056	0.047296974	up_in_case
Yang2025_S	ENSGALG00010015967	ZSWIM1	case	control	-0.003926578	0.319982459	-0.012271228	0.990209223	0.993918139	down_in_case
Yang2025_S	ENSGALG00010015970	THPO	case	control	-0.063468628	0.372527868	-0.170372831	0.864716937	0.920045088	down_in_case
Yang2025_S	ENSGALG00010015971	ENSGALG00010015971	case	control	-0.323059754	0.112039671	-2.883440757	0.003933566	0.019924158	down_in_case
Yang2025_S	ENSGALG00010015972	FSTL4	case	control	0.151769558	0.257454463	0.589500588	0.555525518	0.702919104	up_in_case
Yang2025_S	ENSGALG00010015974	VPS37A	case	control	0.185259778	0.149782494	1.236858683	0.216139551	0.377053661	up_in_case
Yang2025_S	ENSGALG00010015975	BTK	case	control	-0.432968288	0.188743207	-2.293954286	0.021793125	0.072066254	down_in_case
Yang2025_S	ENSGALG00010015979	ENSGALG00010015979	case	control	-0.576464019	0.117400418	-4.910238236	9.09658E-07	2.22387E-05	down_in_case
Yang2025_S	ENSGALG00010015980	EXOSC7	case	control	0.434997933	0.172375715	2.523545342	0.011617808	0.045285077	up_in_case
Yang2025_S	ENSGALG00010015983	CNOT7	case	control	-0.528910055	0.075791958	-6.97844558	2.98464E-12	3.78099E-10	down_in_case
Yang2025_S	ENSGALG00010015984	PDCL3	case	control	0.051877445	0.112605727	0.460699883	0.645013939	0.774450219	up_in_case
Yang2025_S	ENSGALG00010015985	TIMM8A	case	control	0.008649592	0.237884895	0.03636041	0.970994982	0.98240189	up_in_case
Yang2025_S	ENSGALG00010015986	NEURL2	case	control	-0.138200903	0.253582799	-0.544993208	0.585758189	0.727693711	down_in_case
Yang2025_S	ENSGALG00010015987	POLR2H	case	control	-0.591481505	0.293906684	-2.012480617	0.044169298	0.122122851	down_in_case
Yang2025_S	ENSGALG00010015989	ENAH	case	control	0.270391024	0.196057663	1.379140297	0.167851502	0.316092369	up_in_case
Yang2025_S	ENSGALG00010015992	ATP10A	case	control	0.029105286	0.172654481	0.16857533	0.866130687	0.921322324	up_in_case
Yang2025_S	ENSGALG00010015994	ENSGALG00010015994	case	control	-0.378711796	0.223273645	-1.696177782	0.089852217	0.203558875	down_in_case
Yang2025_S	ENSGALG00010016004	XRCC3	case	control	0.031869012	0.10734189	0.296892591	0.766548513	0.858049123	up_in_case
Yang2025_S	ENSGALG00010016006	PLTP	case	control	0.681721533	0.188742398	3.611915185	0.000303944	0.002635631	up_in_case
Yang2025_S	ENSGALG00010016008	TAF7	case	control	0.361467354	0.132736799	2.723188728	0.006465511	0.029101065	up_in_case
Yang2025_S	ENSGALG00010016011	ADD1	case	control	-0.235138326	0.08808863	-2.669337997	0.007600093	0.032921135	down_in_case
Yang2025_S	ENSGALG00010016015	ZDHHC3	case	control	-0.294479058	0.069206651	-4.255068743	2.08985E-05	0.000301783	down_in_case
Yang2025_S	ENSGALG00010016016	ZDHHC2	case	control	-0.553901883	0.16638029	-3.329131611	0.000871172	0.00627055	down_in_case
Yang2025_S	ENSGALG00010016017	RAPGEF6	case	control	-0.000278876	0.127194472	-0.002192514	0.998250628	0.998537256	down_in_case
Yang2025_S	ENSGALG00010016021	ENSGALG00010016021	case	control	1.44447455	0.390776673	3.696419592	0.000218661	0.002007278	up_in_case
Yang2025_S	ENSGALG00010016022	EPHB3	case	control	0.112045623	0.089975844	1.245285608	0.213026784	0.373430215	up_in_case
Yang2025_S	ENSGALG00010016025	PCIF1	case	control	-0.189666339	0.119176704	-1.591471604	0.111503481	0.236751639	down_in_case
Yang2025_S	ENSGALG00010016027	COPS2	case	control	-0.012716006	0.119212256	-0.106666932	0.915053218	0.950120376	down_in_case
Yang2025_S	ENSGALG00010016040	RPSA2	case	control	0.179806928	0.160304298	1.121660052	0.262007008	0.428864024	up_in_case
Yang2025_S	ENSGALG00010016041	ENSGALG00010016041	case	control	-0.174056656	0.14091213	-1.235214143	0.216750817	0.377505641	down_in_case
Yang2025_S	ENSGALG00010016043	ADAMTSL1	case	control	-0.044820448	0.176129819	-0.254473935	0.799129439	0.878430916	down_in_case
Yang2025_S	ENSGALG00010016047	SRP9	case	control	-0.401997044	0.101252157	-3.97025659	7.17953E-05	0.000816974	down_in_case
Yang2025_S	ENSGALG00010016049	ZFYVE21	case	control	-0.661399599	0.167820839	-3.941105298	8.1107E-05	0.000901297	down_in_case
Yang2025_S	ENSGALG00010016053	DRP2	case	control	0.264079449	0.205966208	1.282149394	0.199790245	0.357161906	up_in_case
Yang2025_S	ENSGALG00010016057	SOHO1	case	control	0.40606182	0.424301617	0.95701219	0.338561102	0.506696269	up_in_case
Yang2025_S	ENSGALG00010016059	EPDR1	case	control	-0.157268822	0.132349564	-1.188283644	0.234721679	0.398010051	down_in_case
Yang2025_S	ENSGALG00010016060	ENSGALG00010016060	case	control	1.336557689	0.489194763	2.73215862	0.006292084	0.028467594	up_in_case
Yang2025_S	ENSGALG00010016066	C14orf2	case	control	-0.388522005	0.184225703	-2.108945709	0.034949268	0.102594913	down_in_case
Yang2025_S	ENSGALG00010016067	AHSG	case	control	-0.114015311	0.197967554	-0.575929283	0.564662982	0.710666841	down_in_case
Yang2025_S	ENSGALG00010016069	GALK2	case	control	-0.152664114	0.136687388	-1.116885152	0.264043434	0.431074903	down_in_case
Yang2025_S	ENSGALG00010016076	RAB3GAP2	case	control	-0.120006657	0.081491672	-1.472624795	0.140852235	0.280079349	down_in_case
Yang2025_S	ENSGALG00010016083	CDH22	case	control	0.598996501	0.452537367	1.323639869	0.185622678	0.339173522	up_in_case
Yang2025_S	ENSGALG00010016086	TDRD9	case	control	0.435182894	0.276988221	1.571124193	0.116153804	0.243802268	up_in_case
Yang2025_S	ENSGALG00010016087	IARS2	case	control	0.270438648	0.073044718	3.70237101	0.000213594	0.001969531	up_in_case
Yang2025_S	ENSGALG00010016094	FGF7	case	control	0.179501185	0.213486613	0.840807685	0.400455681	0.567352044	up_in_case
Yang2025_S	ENSGALG00010016097	FNIP1	case	control	-0.069953654	0.127991287	-0.546550126	0.584687842	0.726623122	down_in_case
Yang2025_S	ENSGALG00010016100	SFRP4	case	control	-0.437931757	0.435103228	-1.006500821	0.314174717	0.483549513	down_in_case
Yang2025_S	ENSGALG00010016103	BPNT1	case	control	-0.04488653	0.124570618	-0.360329992	0.718600373	0.826485326	down_in_case
Yang2025_S	ENSGALG00010016107	UVSSA	case	control	0.083797031	0.10664233	0.78577645	0.431998466	0.596029566	up_in_case
Yang2025_S	ENSGALG00010016109	MLH1	case	control	-0.199977403	0.122889251	-1.627297757	0.103673874	0.225065499	down_in_case
Yang2025_S	ENSGALG00010016111	EPB41	case	control	-0.078227179	0.124578707	-0.627933784	0.530047318	0.6824549	down_in_case
Yang2025_S	ENSGALG00010016113	DTWD1	case	control	-0.033660374	0.112322057	-0.299677333	0.76442329	0.856649925	down_in_case
Yang2025_S	ENSGALG00010016114	HAUS6	case	control	-0.108437843	0.161751991	-0.670395724	0.50260556	0.658995905	down_in_case
Yang2025_S	ENSGALG00010016115	RD3L	case	control	-0.134465266	0.266305461	-0.504928684	0.613608926	0.749924609	down_in_case
Yang2025_S	ENSGALG00010016117	ENSGALG00010016117	case	control	-0.204257299	0.223285725	-0.914779922	0.360307177	0.528847747	down_in_case
Yang2025_S	ENSGALG00010016118	EPRS	case	control	0.240056744	0.072320855	3.319329454	0.000902339	0.006438348	up_in_case
Yang2025_S	ENSGALG00010016119	FETUB	case	control	-3.063359509	1.289349597	-2.375895192			down_in_case
Yang2025_S	ENSGALG00010016121	SLC35C2	case	control	0.232966172	0.101646842	2.291917473	0.021910406	0.072282553	up_in_case
Yang2025_S	ENSGALG00010016122	AFG2B	case	control	-0.356873105	0.138211826	-2.582073582	0.009820864	0.039980644	down_in_case
Yang2025_S	ENSGALG00010016127	ENSGALG00010016127	case	control	0.174368751	0.294846644	0.591387943	0.554260517	0.702191868	up_in_case
Yang2025_S	ENSGALG00010016129	CENPI	case	control	-0.598505795	0.154577138	-3.871890769	0.000107994	0.001130655	down_in_case
Yang2025_S	ENSGALG00010016131	SMYD1	case	control	0.37285218	0.102080317	3.652537434	0.000259662	0.002313546	up_in_case
Yang2025_S	ENSGALG00010016137	STARD3NL	case	control	0.202331323	0.102147231	1.980781284	0.047615807	0.128940199	up_in_case
Yang2025_S	ENSGALG00010016139	LURAP1L	case	control	0.031094842	0.260170315	0.119517256	0.90486557	0.943174637	up_in_case
Yang2025_S	ENSGALG00010016141	RRP15	case	control	0.658179865	0.201002291	3.274489372	0.001058531	0.007327686	up_in_case
Yang2025_S	ENSGALG00010016147	FABP1	case	control	0.155676819	0.35914652	0.433463254	0.664678256	0.788145975	up_in_case
Yang2025_S	ENSGALG00010016150	POLG	case	control	0.595867689	0.095131486	6.2636222	3.76136E-10	2.5321E-08	up_in_case
Yang2025_S	ENSGALG00010016159	ASPG	case	control	-0.074990267	0.227123361	-0.330174168	0.741268364	0.841650343	down_in_case
Yang2025_S	ENSGALG00010016168	PLIN2	case	control	-0.016033407	0.192923594	-0.083107549	0.933766024	0.959611277	down_in_case
Yang2025_S	ENSGALG00010016169	RHOH	case	control	-0.100997612	0.126134638	-0.800712742	0.423297964	0.588714652	down_in_case
Yang2025_S	ENSGALG00010016173	ENSGALG00010016173	case	control	0.039707767	0.302130653	0.131425815	0.895438468	0.938334765	up_in_case
Yang2025_S	ENSGALG00010016177	ENSGALG00010016177	case	control	-3.921663678	2.381283951	-1.646869403			down_in_case
Yang2025_S	ENSGALG00010016181	FANCI	case	control	-0.138265151	0.121700399	-1.136110904	0.255910152	0.422124523	down_in_case
Yang2025_S	ENSGALG00010016182	ELMO2	case	control	0.042031591	0.082920558	0.506889874	0.612232091	0.749030218	up_in_case
Yang2025_S	ENSGALG00010016190	THNSL2	case	control	-0.001047275	0.163116907	-0.006420395	0.994877301	0.996498552	down_in_case
Yang2025_S	ENSGALG00010016193	TCAIM	case	control	-0.21142256	0.135626154	-1.558862749	0.119028869	0.248117769	down_in_case
Yang2025_S	ENSGALG00010016197	SNRPN	case	control	0.328589843	0.222639505	1.47588292	0.139975325	0.278840188	up_in_case
Yang2025_S	ENSGALG00010016199	RLBP1	case	control	1.357675201	0.499757345	2.716668829	0.006594255	0.029565942	up_in_case
Yang2025_S	ENSGALG00010016200	MICU3	case	control	-0.238896445	0.097800051	-2.442702661	0.014577742	0.053726749	down_in_case
Yang2025_S	ENSGALG00010016206	KNG1	case	control	-2.178403271	0.926590597	-2.350987889			down_in_case
Yang2025_S	ENSGALG00010016209	ADAMTS7	case	control	-0.228553276	0.120197174	-1.901486281	0.057238348	0.147406464	down_in_case
Yang2025_S	ENSGALG00010016211	TACC3	case	control	-0.198185541	0.083592776	-2.370845304	0.017747457	0.062232214	down_in_case
Yang2025_S	ENSGALG00010016215	SPATA17	case	control	1.068211116	0.399248424	2.675554997	0.007460563	0.032457992	up_in_case
Yang2025_S	ENSGALG00010016218	N4BP2	case	control	0.375574066	0.191932653	1.9568013	0.050370834	0.133928177	up_in_case
Yang2025_S	ENSGALG00010016221	TMEM200B	case	control	-0.053667002	0.201316026	-0.266580872	0.789791885	0.873209532	down_in_case
Yang2025_S	ENSGALG00010016224	MOSPD1	case	control	-0.205300148	0.159054446	-1.290753906	0.196789026	0.352919281	down_in_case
Yang2025_S	ENSGALG00010016225	P4HA2	case	control	-0.393201349	0.11619336	-3.384025974	0.000714312	0.005345835	down_in_case
Yang2025_S	ENSGALG00010016229	ENSGALG00010016229	case	control	0.824237722	0.296255679	2.782183702	0.005399447	0.02541077	up_in_case
Yang2025_S	ENSGALG00010016235	DDX27	case	control	0.368895727	0.113984026	3.23638091	0.001210557	0.008121866	up_in_case
Yang2025_S	ENSGALG00010016236	GPATCH2	case	control	-0.316354253	0.098788129	-3.202350895	0.001363108	0.008938784	down_in_case
Yang2025_S	ENSGALG00010016240	EIF4A2	case	control	-0.34027651	0.126831912	-2.682893479	0.007298823	0.031890641	down_in_case
Yang2025_S	ENSGALG00010016241	TOPAZ1	case	control	2.377569116	0.820338979	2.898276417	0.003752197	0.019272713	up_in_case
Yang2025_S	ENSGALG00010016242	PDS5A	case	control	-0.118639434	0.1147159	-1.034202174	0.301041658	0.470715384	down_in_case
Yang2025_S	ENSGALG00010016245	FAM96A	case	control	-0.106024556	0.221190005	-0.479337012	0.631698896	0.76360027	down_in_case
Yang2025_S	ENSGALG00010016247	KIF26A	case	control	-0.189745371	0.124177172	-1.52802136	0.126507235	0.259094403	down_in_case

Yang2025_S	ENSGALG00010016251	MPDZ	case	control	0.389506627	0.1335299	2.916999324	0.003534165	0.018390065	up_in_case
Yang2025_S	ENSGALG00010016253	EDN3	case	control	2.087775189	0.130206401	16.03435139	7.35475E-58	2.56221E-54	up_in_case
Yang2025_S	ENSGALG00010016257	ENSGALG00010016257	case	control	0.079779203	0.125910362	0.633619043	0.52632947	0.679475002	up_in_case
Yang2025_S	ENSGALG00010016258	CTNNB1	case	control	-0.328402371	0.084244228	-3.898218064	9.69031E-05	0.00104113	down_in_case
Yang2025_S	ENSGALG00010016260	INTS6L	case	control	-0.012815584	0.08073462	-0.158737154	0.873875965	0.926324476	down_in_case
Yang2025_S	ENSGALG00010016262	SPG11	case	control	-0.136643497	0.080514085	-1.697137794	0.089670612	0.203337778	down_in_case
Yang2025_S	ENSGALG00010016266	PDLIM4	case	control	-0.208379152	0.096908442	-2.150268315	0.031533997	0.09507275	down_in_case
Yang2025_S	ENSGALG00010016270	RFC4	case	control	-0.08580028	0.158939328	-0.53983039	0.589314007	0.730288189	down_in_case
Yang2025_S	ENSGALG00010016274	ZNF831	case	control	2.424602015	0.513637485	4.720453791	2.35319E-06	4.99874E-05	up_in_case
Yang2025_S	ENSGALG00010016276	ACTR2	case	control	-0.591316095	0.113766533	-5.197627802	2.01848E-07	6.19548E-06	down_in_case
Yang2025_S	ENSGALG00010016279	PRELID3B	case	control	1.206922072	0.084881682	14.21887561	6.99681E-46	1.95001E-42	up_in_case
Yang2025_S	ENSGALG00010016281	SLC22A4	case	control	-0.567719783	0.177876142	-3.191657834	0.001414588	0.009207045	down_in_case
Yang2025_S	ENSGALG00010016286	ULK4	case	control	-0.207010737	0.136484249	-1.516737196	0.129333049	0.262949525	down_in_case
Yang2025_S	ENSGALG00010016287	BCL11B	case	control	1.243364014	0.576558726	2.156526228	0.031042598	0.093962617	up_in_case
Yang2025_S	ENSGALG00010016289	IRF1	case	control	-0.411878451	0.197690944	-2.083446221	0.037210569	0.107333736	down_in_case
Yang2025_S	ENSGALG00010016292	PHKA1	case	control	0.095671419	0.113821738	0.84053732	0.400607186	0.567380947	up_in_case
Yang2025_S	ENSGALG00010016295	UBE2K	case	control	-0.233661269	0.088048293	-2.653785329	0.007959445	0.034118974	down_in_case
Yang2025_S	ENSGALG00010016299	SRSF4	case	control	0.675909737	0.157272599	4.297695483	1.72583E-05	0.000256939	up_in_case
Yang2025_S	ENSGALG00010016303	ENSGALG00010016303	case	control	0.0393424	0.26560674	0.148122748	0.882245897	0.930665903	up_in_case
Yang2025_S	ENSGALG00010016304	EIF3J	case	control	0.492185069	0.107933705	4.560068297	5.1137E-06	9.41339E-05	up_in_case
Yang2025_S	ENSGALG00010016305	DENND4C	case	control	0.537270196	0.207364144	2.590950322	0.009571131	0.039343276	up_in_case
Yang2025_S	ENSGALG00010016307	RAD50	case	control	0.613870443	0.168671756	3.639438264	0.000273233	0.002409815	up_in_case
Yang2025_S	ENSGALG00010016308	RPS6	case	control	0.126217157	0.239606406	0.526768711	0.598354214	0.737425132	up_in_case
Yang2025_S	ENSGALG00010016310	HDAC8	case	control	-0.125505539	0.138756481	-0.904502173	0.365729183	0.534505864	down_in_case
Yang2025_S	ENSGALG00010016312	MECR	case	control	-0.051496154	0.196826705	-0.261631948	0.793605211	0.875535478	down_in_case
Yang2025_S	ENSGALG00010016314	ACER2	case	control	0.1639825	0.128471201	1.276414477	0.201809041	0.359761566	up_in_case
Yang2025_S	ENSGALG00010016315	RPS4Y1	case	control	-0.656999422	0.252016742	-2.606967368	0.009134807	0.037981113	down_in_case
Yang2025_S	ENSGALG00010016317	CTDSPL2	case	control	-0.282392846	0.101771621	-2.774770053	0.005524075	0.02580556	down_in_case
Yang2025_S	ENSGALG00010016319	RBM41	case	control	-0.093635705	0.143363153	-0.653136483	0.513668294	0.668518435	down_in_case
Yang2025_S	ENSGALG00010016322	EBF4	case	control	0.344050164	0.119679321	2.874767016	0.004043257	0.020311028	up_in_case
Yang2025_S	ENSGALG00010016324	TUBB1	case	control	-0.173425242	0.174766649	-0.99232458	0.321039217	0.490388723	down_in_case
Yang2025_S	ENSGALG00010016328	USH2A	case	control	-0.234316579	0.2660031	-0.880879128	0.378383246	0.546524196	down_in_case
Yang2025_S	ENSGALG00010016332	ENSGALG00010016332	case	control	0.145168301	0.155288314	0.934830815	0.349875496	0.518359423	up_in_case
Yang2025_S	ENSGALG00010016335	ABHD4	case	control	-0.646597766	0.102667397	-6.297985409	3.01539E-10	2.10097E-08	down_in_case
Yang2025_S	ENSGALG00010016336	SLC24A2	case	control	-0.936233296	0.577507004	-1.621163536	0.104982571	0.227022366	down_in_case
Yang2025_S	ENSGALG00010016338	PTPRU	case	control	0.072102446	0.144451935	0.499144895	0.617677312	0.75346911	up_in_case
Yang2025_S	ENSGALG00010016339	SETD3	case	control	-0.06289371	0.10709667	-0.587261115	0.557028353	0.704215432	down_in_case
Yang2025_S	ENSGALG00010016340	RGS12	case	control	0.255479861	0.194613425	1.312755587	0.18926532	0.344174897	up_in_case
Yang2025_S	ENSGALG00010016341	SLX4	case	control	0.120427446	0.085891485	1.402088296	0.160888882	0.306617419	up_in_case
Yang2025_S	ENSGALG00010016344	SMIM14	case	control	-0.470043509	0.084626188	-5.554350483	2.78646E-08	1.12223E-06	down_in_case
Yang2025_S	ENSGALG00010016345	CTSZ	case	control	-0.124155416	0.134213475	-0.925059249	0.354935089	0.524109406	down_in_case
Yang2025_S	ENSGALG00010016346	TRAP1	case	control	0.178650529	0.081110881	2.202547022	0.027626691	0.08612482	up_in_case
Yang2025_S	ENSGALG00010016347	ANO10	case	control	-0.356713956	0.068594545	-5.200325421	1.9894E-07	6.15092E-06	down_in_case
Yang2025_S	ENSGALG00010016350	CASC4	case	control	0.361192688	0.146700994	2.462101163	0.01381257	0.051561255	up_in_case
Yang2025_S	ENSGALG00010016353	C1orf232	case	control	-0.135051846	0.322628881	-0.418598128	0.675509858	0.796044678	down_in_case
Yang2025_S	ENSGALG00010016355	MCF2L2	case	control	0.146781708	0.254382619	0.577011544	0.563931657	0.710009726	up_in_case
Yang2025_S	ENSGALG00010016361	NELFCD	case	control	-0.424161207	0.115332852	-3.677713686	0.000235334	0.002132235	down_in_case
Yang2025_S	ENSGALG00010016362	NUP62	case	control	0.442214153	0.151465694	2.919566403	0.003505187	0.018296325	up_in_case
Yang2025_S	ENSGALG00010016366	KIF3A	case	control	0.169581821	0.130152807	1.302944016	0.192593875	0.347867227	up_in_case
Yang2025_S	ENSGALG00010016368	CCNK	case	control	0.674740126	0.345857929	1.950917036	0.051066916	0.135284782	up_in_case
Yang2025_S	ENSGALG00010016369	MSANTD1	case	control	-0.065206487	0.32634013	-0.199811426	0.841628065	0.906085528	down_in_case
Yang2025_S	ENSGALG00010016370	TMEM255A	case	control	0.449781566	0.10512456	4.278558376	1.88108E-05	0.000275921	up_in_case
Yang2025_S	ENSGALG00010016372	DNAAF6	case	control	-0.075112271	0.157047053	-0.478278766	0.632451808	0.764107503	down_in_case
Yang2025_S	ENSGALG00010016373	SRL	case	control	-0.342719468	0.117522918	-2.916192633	0.003543317	0.018423924	down_in_case
Yang2025_S	ENSGALG00010016374	FRMD5	case	control	0.071599836	0.249368426	0.287124706	0.774016836	0.862393767	up_in_case
Yang2025_S	ENSGALG00010016375	CCNI2	case	control	-0.090485482	0.104930388	-0.862338203	0.388501436	0.555486098	down_in_case
Yang2025_S	ENSGALG00010016377	FRMPD3	case	control	-0.785616295	0.1893279	-4.149500912	3.33201E-05	0.000442246	down_in_case
Yang2025_S	ENSGALG00010016378	WDR76	case	control	0.183438835	0.14879367	1.232840318	0.217635346	0.378575459	up_in_case
Yang2025_S	ENSGALG00010016379	TFAP4	case	control	0.509059609	0.098131411	5.18752969	2.13102E-07	6.48379E-06	up_in_case
Yang2025_S	ENSGALG00010016382	UGDH	case	control	-0.63817083	0.091403129	-6.981936378	2.91139E-12	3.72204E-10	down_in_case
Yang2025_S	ENSGALG00010016383	FAM110D	case	control	-0.118805267	0.259239999	-0.458282934	0.646749183	0.775661305	down_in_case
Yang2025_S	ENSGALG00010016384	GNAS	case	control	0.001389425	0.119450261	0.011631826	0.990719355	0.994000591	up_in_case
Yang2025_S	ENSGALG00010016385	HTT	case	control	-0.041052473	0.101581668	-0.404132698	0.686115141	0.803987092	down_in_case
Yang2025_S	ENSGALG00010016387	CCDC85C	case	control	0.263070769	0.26785837	0.982126371	0.326037595	0.49600446	up_in_case
Yang2025_S	ENSGALG00010016390	ENSGALG00010016390	case	control	0.007878448	0.463419883	0.01700067	0.986436082	0.991702388	up_in_case
Yang2025_S	ENSGALG00010016392	STMN3	case	control	0.265137941	0.37114162	0.714384825	0.474989253	0.634609323	up_in_case
Yang2025_S	ENSGALG00010016393	BLM	case	control	-0.279324903	0.142321662	-1.962630974	0.049689069	0.132723246	down_in_case
Yang2025_S	ENSGALG00010016394	PDIK1L	case	control	-0.044674263	0.117943107	-0.378778074	0.704852671	0.817692472	down_in_case
Yang2025_S	ENSGALG00010016395	ENSGALG00010016395	case	control	0.015528529	0.161850232	0.095943818	0.923565194	0.95391835	up_in_case
Yang2025_S	ENSGALG00010016397	LIAS	case	control	0.087442549	0.089750827	0.97428126	0.329916907	0.499445094	up_in_case
Yang2025_S	ENSGALG00010016398	SEPTIN6	case	control	0.121581433	0.099343543	1.223848365	0.221009435	0.38238968	up_in_case
Yang2025_S	ENSGALG00010016399	ACSM5	case	control	-0.409097625	0.335618383	-1.21893688	0.22286815	0.384326015	down_in_case
Yang2025_S	ENSGALG00010016400	ENSGALG00010016400	case	control	-0.367020917	0.137272768	-2.67366151	0.007502814	0.032590932	down_in_case
Yang2025_S	ENSGALG00010016402	SEPTIN8	case	control	0.03749208	0.096833965	0.387179027	0.698623676	0.81355977	up_in_case
Yang2025_S	ENSGALG00010016403	GRK4	case	control	0.263415056	0.14962759	1.760471158	0.078327954	0.184576558	up_in_case
Yang2025_S	ENSGALG00010016404	ESRRG	case	control	0.006130786	0.10207788	0.060059887	0.952107938	0.970565041	up_in_case
Yang2025_S	ENSGALG00010016407	RPL9	case	control	-0.568276139	0.217543038	-2.612246959	0.008994925	0.037618332	down_in_case
Yang2025_S	ENSGALG00010016409	GMEB2	case	control	0.413272738	0.119652544	3.453940263	0.00055246	0.004298451	up_in_case
Yang2025_S	ENSGALG00010016410	TRAK1	case	control	-0.123983121	0.108880846	-1.1387046	0.254826384	0.421184398	down_in_case
Yang2025_S	ENSGALG00010016411	CTSH	case	control	0.130588519	0.150402173	0.868262184	0.385250819	0.552709788	up_in_case
Yang2025_S	ENSGALG00010016412	TMSB15B	case	control	-0.20124968	0.270179107	-0.744875064	0.456347251	0.617937902	down_in_case
Yang2025_S	ENSGALG00010016414	KLB	case	control	1.325623888	0.486863903	2.722781212	0.006473491	0.029127576	up_in_case
Yang2025_S	ENSGALG00010016415	RASGRF1	case	control	1.152290567	0.376458698	3.060868487	0.00220696	0.012894754	up_in_case
Yang2025_S	ENSGALG00010016416	CYP46A1	case	control	0.49309659	0.098304128	5.016031372	5.27497E-07	1.40589E-05	up_in_case
Yang2025_S	ENSGALG00010016417	NOP14	case	control	0.437383421	0.180555821	2.422427701	0.015417193	0.056137598	up_in_case
Yang2025_S	ENSGALG00010016418	NFIB	case	control	0.363700568	0.243889501	1.491251433	0.135895495	0.272436156	up_in_case
Yang2025_S	ENSGALG00010016421	ENSGALG00010016421	case	control	0.064408506	0.419131646	0.153671303	0.877868913	0.92809932	up_in_case
Yang2025_S	ENSGALG00010016422	KCTD3	case	control	-0.165187147	0.085365091	-1.935066737	0.052982107	0.139197899	down_in_case
Yang2025_S	ENSGALG00010016423	TRMT44	case	control	0.014811632	0.116105558	0.127570397	0.898488959	0.939723381	up_in_case
Yang2025_S	ENSGALG00010016425	TRIM63	case	control	0.140391118	0.156286217	0.898294942	0.369028329	0.537683998	up_in_case
Yang2025_S	ENSGALG00010016426	HELZ2	case	control	-0.092457471	0.169593104	-0.545172347	0.585634988	0.727605524	down_in_case
Yang2025_S	ENSGALG00010016428	ADRA1D	case	control	-0.058791291	0.21900904	-0.268442302	0.788358882	0.872372034	down_in_case
Yang2025_S	ENSGALG00010016429	ENSGALG00010016429	case	control	0.782822099	0.502487289	1.557894329	0.1192583	0.24848451	up_in_case
Yang2025_S	ENSGALG00010016431	B3GNT5	case	control	-0.537395285	0.12727019	-4.222475703	2.41633E-05	0.000340117	down_in_case
Yang2025_S	ENSGALG00010016433	TMED3	case	control	0.168567638	0.12185705	1.383322813	0.166565906	0.31447643	up_in_case
Yang2025_S	ENSGALG00010016434	GPR139	case	control	0.226873995	0.544728914	0.416489723	0.677051686	0.796968504	up_in_case
Yang2025_S	ENSGALG00010016436	SLC30A2	case	control	-0.62996977	0.556049455	-1.132938381	0.257240131	0.423566256	down_in_case
Yang2025_S	ENSGALG00010016441	ENSGALG00010016441	case	control	0.113859124	0.426585444	0.266908133	0.789539894	0.87314152	up_in_case
Yang2025_S	ENSGALG00010016445	ZDHHC21	case	control	-0.400561144	0.183107723	-2.187570998	0.028700866	0.088490253	down_in_case
Yang2025_S	ENSGALG00010016447	ID3	case	control	0.403796981	0.133078014	3.034287714	0.002411044	0.013820606	up_in_case
Yang2025_S	ENSGALG00010016449	FMR1	case	control	0.554895085	0.193577542	2.866526145	0.004150039	0.020705618	up_in_case
Yang2025_S	ENSGALG00010016450	ENSGALG00010016450	case	control	0.716613298	0.201652411	3.553705581	0.000379844	0.003170742	up_in_case
Yang2025_S	ENSGALG00010016451	USP46	case	control	0.167148339	0.158879067	1.052047582	0.29277772	0.461417951	up_in_case
Yang2025_S	ENSGALG00010016452	GLCE	case	control	-0.412694877	0.138176527	-2.986722019	0.00281986	0.015531521	down_in_case
Yang2025_S	ENSGALG00010016453	MYCL	case	control	-0.577150774	0.16938484	-3.407334288	0.000656007	0.004992606	down_in_case
Yang2025_S	ENSGALG00010016454	ANKS3	case	control	-0.111802919	0.135471316	-0.825288499	0.40920782	0.574655947	down_in_case
Yang2025_S	ENSGALG00010016455	RBM27	case	control	0.275514774	0.149806583	1.83913663	0.065895092	0.162694563	up_in_case
Yang2025_S	ENSGALG00010016456	LAMP3	case	control	1.186271205	0.51814034	2.28947857	0.022051562	0.072679402	up_in_case
Yang2025_S	ENSGALG00010016457	SMOX	case	control	-0.153242806	0.136787969	-1.12029448	0.262588294	0.429530212	down_in_case
Yang2025_S	ENSGALG00010016458	ZC3H12A	case	control	-0.83402664	0.183662681	-4.541078431	5.59672E-06	0.000102349	down_in_case
Yang2025_S	ENSGALG00010016459	PAQR5	case	control	0.110706766	0.287295988	0.385340452	0.699985195	0.814486781	up_in_case
Yang2025_S	ENSGALG00010016460	LBR	case	control	-0.303883643	0.041990639	-7.236937805	4.58928E-13	6.39517E-11	down_in_case
Yang2025_S	ENSGALG00010016461	HPCAL4	case	control	0.093447533	0.311983409	0.29952725	0.764537783	0.856649925	up_in_case
Yang2025_S	ENSGALG00010016462	RNF24	case	control	-0.025847333	0.172525298	-0.149817641	0.880908491	0.929747771	down_in_case
Yang2025_S	ENSGALG00010016463	FREM1	case	control	-0.104978731	0.189184646	-0.554900901	0.578962446	0.721760751	down_in_case
Yang2025_S	ENSGALG00010016464	PANK2	case	control	-0.022052707	0.136397147	-0.16168012	0.87155776	0.924500068	down_in_case
Yang2025_S	ENSGALG00010016465	STK35	case	control	-0.478316744	0.217486475	-2.199294201	0.02785701	0.086590995	down_in_case
Yang2025_S	ENSGALG00010016466	TMEM164	case	control	-0.142728713	0.161942102	-0.881356431	0.378124931	0.546430012	down_in_case
Yang2025_S	ENSGALG00010016468	TTC39B	case	control	0.277288002	0.171493078	1.61690493	0.105898804	0.228295148	up_in_case
Yang2025_S	ENSGALG00010016469	MAVS	case	control	0.015949592	0.151519398	0.105264358	0.916166044	0.950686859	up_in_case
Yang2025_S	ENSGALG00010016470	TNIP1	case	control	0.41277858	0.079664408	5.181468006	2.20146E-07	6.669E-06	up_in_case
Yang2025_S	ENSGALG00010016471	POMGNT2	case	control	-0.294901842	0.108590185	-2.715732025	0.006612942	0.029611615	down_in_case
Yang2025_S	ENSGALG00010016472	TFDP1	case	control	-0.397080497	0.073453759	-5.405856759	6.44993E-08	2.33454E-06	down_in_case
Yang2025_S	ENSGALG00010016473	RAB9B	case	control	-0.42982751	0.220470697	-1.949590198	0.051224983	0.135578373	down_in_case
Yang2025_S	ENSGALG00010016474	MAPK8IP3	case	control	0.590994819	0.147120718	4.01707405	5.89252E-05	0.000696457	up_in_case
Yang2025_S	ENSGALG00010016475	MCCC1	case	control	0.204985366	0.069760339	2.938422738	0.003298869	0.017498948	up_in_case

Yang2025_S	ENSGALG00010016477	CNIH3	case	control	-0.179946687	0.326812612	-0.550611207	0.581900224	0.724386245	down_in_case
Yang2025_S	ENSGALG00010016479	ENSGALG00010016479	case	control	0.860059224	0.308300962	2.789674149	0.005276111	0.024939827	up_in_case
Yang2025_S	ENSGALG00010016480	GPX3	case	control	-0.472256849	0.171426	-2.754872948	0.005871493	0.026974651	down_in_case
Yang2025_S	ENSGALG00010016482	EML1	case	control	-0.162818248	0.108108676	-1.506060884	0.132051545	0.266957533	down_in_case
Yang2025_S	ENSGALG00010016483	WDR26	case	control	-0.267922054	0.085785921	-3.123147138	0.001789283	0.011027711	down_in_case
Yang2025_S	ENSGALG00010016491	DNAJC8	case	control	0.456523055	0.218828979	2.086209321	0.036959666	0.106919857	up_in_case
Yang2025_S	ENSGALG00010016496	PLP1	case	control	0.856306973	0.517231938	1.655557034			up_in_case
Yang2025_S	ENSGALG00010016498	KIF23	case	control	0.114275029	0.114703981	0.996260361	0.319123658	0.488518969	up_in_case
Yang2025_S	ENSGALG00010016500	ENSGALG00010016500	case	control	-0.292953785	0.27465436	-1.066627106	0.286140255	0.454659573	down_in_case
Yang2025_S	ENSGALG00010016502	YIPF5	case	control	-0.240976165	0.154728342	-1.557414511	0.119372103	0.248614807	down_in_case
Yang2025_S	ENSGALG00010016504	SNRK	case	control	0.115156052	0.140232647	0.821178627	0.411544529	0.576948995	up_in_case
Yang2025_S	ENSGALG00010016505	KCTD16	case	control	0.25237908	0.433474388	0.58222374	0.560415984	0.70698866	up_in_case
Yang2025_S	ENSGALG00010016507	SPATA18	case	control	-0.401719466	0.131700542	-3.050249147	0.002286516	0.013242976	down_in_case
Yang2025_S	ENSGALG00010016508	CREBBP	case	control	0.686168491	0.211002883	3.251938939	0.001146206	0.007802827	up_in_case
Yang2025_S	ENSGALG00010016510	THG1L	case	control	0.069194381	0.136207289	0.508007921	0.611447788	0.748728025	up_in_case
Yang2025_S	ENSGALG00010016514	GASK1A	case	control	-0.078645852	0.206108229	-0.381575509	0.702776253	0.81634023	down_in_case
Yang2025_S	ENSGALG00010016518	TMEM42	case	control	-0.362980727	0.165840513	-2.188733747	0.028616198	0.08833999	down_in_case
Yang2025_S	ENSGALG00010016519	MEX3B	case	control	0.330069321	0.116012772	2.845111924	0.004439582	0.021828555	up_in_case
Yang2025_S	ENSGALG00010016520	CHMP1B	case	control	0.229608487	0.131625913	1.74440186	0.081089041	0.189117286	up_in_case
Yang2025_S	ENSGALG00010016526	EIF1B	case	control	0.046965157	0.130124423	0.360925	0.718155514	0.826385646	up_in_case
Yang2025_S	ENSGALG00010016527	MRPS34	case	control	0.572870671	0.121978722	4.696480339	2.64683E-06	5.48047E-05	up_in_case
Yang2025_S	ENSGALG00010016529	DCUN1D1	case	control	-0.178242947	0.083085486	-2.145295832	0.031929202	0.095981439	down_in_case
Yang2025_S	ENSGALG00010016533	CNIH4	case	control	-0.069490192	0.259002973	-0.268298821	0.788469314	0.872424955	down_in_case
Yang2025_S	ENSGALG00010016534	TRIT1	case	control	0.044287155	0.180944553	0.244755394	0.806645834	0.883622621	up_in_case
Yang2025_S	ENSGALG00010016535	BNC2	case	control	0.339897813	0.278739336	1.219411001	0.222688236	0.384197174	up_in_case
Yang2025_S	ENSGALG00010016541	UBE2J2	case	control	-0.222627068	0.063950296	-3.481251589	0.000499076	0.003949251	down_in_case
Yang2025_S	ENSGALG00010016542	ENOX2	case	control	0.057541501	0.087876379	0.654800541	0.512596182	0.667572691	up_in_case
Yang2025_S	ENSGALG00010016543	TCEA3	case	control	0.04014241	0.151560647	0.264860377	0.791117023	0.873985268	up_in_case
Yang2025_S	ENSGALG00010016544	ADCY9	case	control	-0.132526346	0.055863444	-2.372326808	0.017676448	0.062061301	down_in_case
Yang2025_S	ENSGALG00010016546	C1QTNF12	case	control	0.535593549	0.355024016	1.508612162	0.131397924	0.265983451	up_in_case
Yang2025_S	ENSGALG00010016547	SGCB	case	control	-0.360622789	0.116971048	-3.083008961	0.00204919	0.012192767	down_in_case
Yang2025_S	ENSGALG00010016548	EVL	case	control	-0.022438434	0.147530064	-0.152093975	0.879112817	0.92862622	down_in_case
Yang2025_S	ENSGALG00010016549	PTPRA	case	control	0.072597993	0.102154852	0.710666129	0.477291153	0.63634254	up_in_case
Yang2025_S	ENSGALG00010016550	FBLIM1	case	control	-0.123593878	0.317951469	-0.388719317	0.697483791	0.81272988	down_in_case
Yang2025_S	ENSGALG00010016552	ENSGALG00010016552	case	control	1.434230719	0.387555584	3.700709731	0.000214997	0.001979697	up_in_case
Yang2025_S	ENSGALG00010016553	SLC25A14	case	control	0.032150793	0.173866186	0.184916883	0.853294228	0.913330907	up_in_case
Yang2025_S	ENSGALG00010016556	ASAP3	case	control	-0.30453855	0.146139591	-2.083888069	0.03717035	0.107323239	down_in_case
Yang2025_S	ENSGALG00010016558	NR2F2	case	control	0.199111743	0.139115017	1.431274253	0.15235163	0.295881053	up_in_case
Yang2025_S	ENSGALG00010016559	ROGDI	case	control	0.033113061	0.150285204	0.220334805	0.825610416	0.895600276	up_in_case
Yang2025_S	ENSGALG00010016560	DCUN1D4	case	control	-0.120755319	0.109642917	-1.101350842	0.270743991	0.438097295	down_in_case
Yang2025_S	ENSGALG00010016562	GLIS2	case	control	0.52532602	0.226044925	2.323989448	0.02012606	0.06802845	up_in_case
Yang2025_S	ENSGALG00010016564	SPEN	case	control	0.131314717	0.231917834	0.566212246	0.571249506	0.715397298	up_in_case
Yang2025_S	ENSGALG00010016565	NVL	case	control	-0.174502662	0.079652999	-2.190785828	0.028467295	0.087963567	down_in_case
Yang2025_S	ENSGALG00010016566	CNTLN	case	control	0.781545193	0.276128085	2.830371971	0.004649391	0.022701216	up_in_case
Yang2025_S	ENSGALG00010016567	E2F2	case	control	-0.086028353	0.203849277	-0.422019415	0.673010847	0.794212046	down_in_case
Yang2025_S	ENSGALG00010016568	PAM16	case	control	-0.405922676	0.370447541	-1.095762911	0.273182543	0.440499739	down_in_case
Yang2025_S	ENSGALG00010016569	YY1	case	control	-0.234452349	0.1186135	-1.976607628	0.048085999	0.129790124	down_in_case
Yang2025_S	ENSGALG00010016570	ENSGALG00010016570	case	control	0.639431923	0.320214125	1.99688856	0.04583729	0.125337103	up_in_case
Yang2025_S	ENSGALG00010016572	RAB33A	case	control	0.602840664	0.123725234	4.87241484	1.10242E-06	2.63053E-05	up_in_case
Yang2025_S	ENSGALG00010016573	DEGS1	case	control	-0.220264483	0.076544857	-2.87758698	0.004007294	0.020210512	down_in_case
Yang2025_S	ENSGALG00010016575	CWH43	case	control	-2.139331135	0.53015127	-4.035322097	5.45274E-05	0.000653907	down_in_case
Yang2025_S	ENSGALG00010016576	MRPS26	case	control	-0.17062946	0.140593383	-1.213637914	0.224886022	0.386505516	down_in_case
Yang2025_S	ENSGALG00010016577	ZBTB17	case	control	0.282916522	0.090232761	3.135408007	0.001716152	0.01067139	up_in_case
Yang2025_S	ENSGALG00010016578	GRIK3	case	control	1.170282283	0.625963702	1.869568922	0.061543704	0.155251904	up_in_case
Yang2025_S	ENSGALG00010016580	MARS2	case	control	-0.230287601	0.206320719	-1.116163234	0.264352269	0.431442065	down_in_case
Yang2025_S	ENSGALG00010016581	H2AFYMACROH2A1	case	control	0.200032075	0.117794453	1.698145122	0.089480375	0.203046577	up_in_case
Yang2025_S	ENSGALG00010016583	VPS41	case	control	0.049158261	0.112446261	0.437171146	0.661987233	0.786355857	up_in_case
Yang2025_S	ENSGALG00010016584	EHBP1	case	control	0.260779145	0.185428012	1.406363268	0.159616282	0.305110135	up_in_case
Yang2025_S	ENSGALG00010016588	ATP11B	case	control	-0.03738315	0.142830984	-0.261729976	0.793529628	0.875535478	down_in_case
Yang2025_S	ENSGALG00010016589	FBXO28	case	control	-0.667374069	0.141023063	-4.73237536	2.21908E-06	4.74276E-05	down_in_case
Yang2025_S	ENSGALG00010016591	HOMER2	case	control	1.216498483	0.258217535	4.711138153	2.46337E-06	5.18344E-05	up_in_case
Yang2025_S	ENSGALG00010016592	TMEM129	case	control	-0.493387133	0.127140552	-3.880643321	0.000104181	0.001102175	down_in_case
Yang2025_S	ENSGALG00010016597	FRYL	case	control	-0.082565986	0.12891276	-0.640479547	0.521860882	0.675243084	down_in_case
Yang2025_S	ENSGALG00010016598	AIFM1	case	control	-0.071235257	0.190498453	-0.373941393	0.70844792	0.820178758	down_in_case
Yang2025_S	ENSGALG00010016599	ENSGALG00010016599	case	control	-0.439432441	0.336464511	-1.306029095	0.191542663	0.346698115	down_in_case
Yang2025_S	ENSGALG00010016600	GNL2	case	control	0.214351151	0.121146359	1.769356939	0.076834327	0.181934808	up_in_case
Yang2025_S	ENSGALG00010016602	HSPB7	case	control	0.385339601	0.484163416	0.79588748	0.426097439	0.590812718	up_in_case
Yang2025_S	ENSGALG00010016603	ENSGALG00010016603	case	control	0.065367122	0.130110397	0.502397375	0.615388025	0.751505751	up_in_case
Yang2025_S	ENSGALG00010016604	TP53BP2	case	control	0.014850228	0.088495346	0.167808016	0.866734316	0.921769266	up_in_case
Yang2025_S	ENSGALG00010016605	WDPCP	case	control	-0.014627286	0.159227957	-0.091863806	0.926806249	0.955608219	down_in_case
Yang2025_S	ENSGALG00010016606	VASN	case	control	0.25998606	0.127375873	2.041093448	0.04124154	0.115750424	up_in_case
Yang2025_S	ENSGALG00010016607	FAM103A1	case	control	-0.754408512	0.150412175	-5.015608017	5.2866E-07	1.40589E-05	down_in_case
Yang2025_S	ENSGALG00010016609	SOX2	case	control	1.049292881	0.475370204	2.207317312	0.027291899	0.085237232	up_in_case
Yang2025_S	ENSGALG00010016611	DNALI1	case	control	0.037015377	0.201128588	0.184038367	0.853983356	0.913827938	up_in_case
Yang2025_S	ENSGALG00010016612	GRIA1	case	control	0.977525991	0.297989946	3.280399231	0.001036603	0.007220353	up_in_case
Yang2025_S	ENSGALG00010016613	SNIP1	case	control	0.295385315	0.115395654	2.559761174	0.010474412	0.041906669	up_in_case
Yang2025_S	ENSGALG00010016614	DNAJC19	case	control	0.857937511	0.166520391	5.152146875	2.57521E-07	7.47616E-06	up_in_case
Yang2025_S	ENSGALG00010016615	BCORL1	case	control	0.311344088	0.151167417	2.059597855	0.039437002	0.112154003	up_in_case
Yang2025_S	ENSGALG00010016616	SLBP	case	control	-0.399088729	0.189676854	-2.104045491	0.035374479	0.103636732	down_in_case
Yang2025_S	ENSGALG00010016619	SLC25A47	case	control	0.244632058	0.191855343	1.275085979	0.202278812	0.3602824	up_in_case
Yang2025_S	ENSGALG00010016620	ENSGALG00010016620	case	control	-0.005123585	0.165801189	-0.030901977	0.975347713	0.985317557	down_in_case
Yang2025_S	ENSGALG00010016622	KIAA1143	case	control	-0.689945856	0.128682256	-5.361623881	8.24771E-08	2.89501E-06	down_in_case
Yang2025_S	ENSGALG00010016623	DNAJA3	case	control	0.329636405	0.108256456	3.044958383	0.002327125	0.013433508	up_in_case
Yang2025_S	ENSGALG00010016624	MDH1	case	control	-0.379737565	0.202557879	-1.874711405	0.060832437	0.153954023	down_in_case
Yang2025_S	ENSGALG00010016626	CAPN2	case	control	-0.556687267	0.284651742	-1.955678413	0.050503049	0.13417731	down_in_case
Yang2025_S	ENSGALG00010016629	LARS1	case	control	0.111209814	0.0741786	1.499216937	0.133817363	0.269731323	up_in_case
Yang2025_S	ENSGALG00010016630	ENSGALG00010016630	case	control	0.090768297	0.608344899	0.149205324	0.881391619	0.930187232	up_in_case
Yang2025_S	ENSGALG00010016631	FAM53A	case	control	-0.203392887	0.091518685	-2.222419251	0.026254986	0.082962182	down_in_case
Yang2025_S	ENSGALG00010016637	POU6F2	case	control	2.2798451	0.641027648	3.556547218	0.000375761	0.003144882	up_in_case
Yang2025_S	ENSGALG00010016640	OCIAD1	case	control	-0.435686006	0.116157675	-3.750815496	0.00017626	0.001690961	down_in_case
Yang2025_S	ENSGALG00010016643	BTBD1	case	control	-0.224924244	0.127482345	-1.764356031	0.077672056	0.183530065	down_in_case
Yang2025_S	ENSGALG00010016644	NMRAL1	case	control	-0.000584752	0.210966287	-0.002771782	0.997788441	0.998289914	down_in_case
Yang2025_S	ENSGALG00010016647	EGR1	case	control	-0.260648717	0.23979416	-1.086968579	0.277050696	0.444371714	down_in_case
Yang2025_S	ENSGALG00010016648	ADRA2C	case	control	0.227599883	0.207754488	1.095523305	0.273287441	0.440617898	up_in_case
Yang2025_S	ENSGALG00010016651	ENSGALG00010016651	case	control	0.047907172	0.136046656	0.352137815	0.72473491	0.830611257	up_in_case
Yang2025_S	ENSGALG00010016652	FXR1	case	control	0.079447972	0.08388967	0.947053106	0.343611719	0.512110086	up_in_case
Yang2025_S	ENSGALG00010016653	EPHA2	case	control	0.195271648	0.160668606	1.215369027	0.224225376	0.385703075	up_in_case
Yang2025_S	ENSGALG00010016654	SMIM3	case	control	0.034161763	0.334683177	0.102071946	0.918699569	0.952039748	up_in_case
Yang2025_S	ENSGALG00010016655	TRANK1	case	control	-0.12272752	0.164967662	-0.743948954	0.456907357	0.618522331	down_in_case
Yang2025_S	ENSGALG00010016657	SLC25A29	case	control	0.270699283	0.124904779	2.167245198	0.030216163	0.092136155	up_in_case
Yang2025_S	ENSGALG00010016658	ZAR1	case	control	0.168381964	0.656076652	0.256649834	0.797449101	0.87739029	up_in_case
Yang2025_S	ENSGALG00010016661	UBE4B	case	control	-0.009899978	0.08132995	-0.121726102	0.903115941	0.94209185	down_in_case
Yang2025_S	ENSGALG00010016664	TM6SF1	case	control	0.035735065	0.240803411	0.148399331	0.882027628	0.930576544	up_in_case
Yang2025_S	ENSGALG00010016667	WARS1	case	control	-0.092447617	0.080951543	-1.142011802	0.253449114	0.41980428	down_in_case
Yang2025_S	ENSGALG00010016671	CASS4	case	control	-0.127567265	0.265109506	-0.481187063	0.630383554	0.763047479	down_in_case
Yang2025_S	ENSGALG00010016672	DCTN4	case	control	-0.232345557	0.059327873	-3.916296763	8.99196E-05	0.000981229	down_in_case
Yang2025_S	ENSGALG00010016673	LRPAP1	case	control	0.231543895	0.148374627	1.560535645	0.118633353	0.24747841	up_in_case
Yang2025_S	ENSGALG00010016674	SLC10A4	case	control	-0.092490991	0.469370039	-0.197053462	0.843785701	0.907404981	down_in_case
Yang2025_S	ENSGALG00010016675	SKIL	case	control	0.170134474	0.122514617	1.388687147	0.164927904	0.312392326	up_in_case
Yang2025_S	ENSGALG00010016677	MEAF6	case	control	-0.064353779	0.13002406	-0.494937466	0.620644263	0.755748583	down_in_case
Yang2025_S	ENSGALG00010016678	NOX1	case	control	0.252861676	0.192982649	1.310281925	0.19010048	0.344973328	up_in_case
Yang2025_S	ENSGALG00010016680	BPIFB3	case	control	-0.543462861	0.545224657	-0.996768677	0.318876805	0.488345495	down_in_case
Yang2025_S	ENSGALG00010016681	SUSD4	case	control	-0.120585467	0.20772193	-0.580513896	0.561568118	0.708132954	down_in_case
Yang2025_S	ENSGALG00010016682	GPR141	case	control	-0.992924888	0.749718539	-1.324396873	0.185371273	0.33886248	down_in_case
Yang2025_S	ENSGALG00010016683	RBM22	case	control	-0.360913014	0.115624374	-3.121426751	0.00179977	0.011065404	down_in_case
Yang2025_S	ENSGALG00010016688	ENSGALG00010016688	case	control	1.005026827	0.718148003	1.399470336	0.161671988	0.30768904	up_in_case
Yang2025_S	ENSGALG00010016691	ENSGALG00010016691	case	control	-0.037136437	0.37493078	-0.099048782	0.921099538	0.953250803	down_in_case
Yang2025_S	ENSGALG00010016692	PRKCI	case	control	-0.468350806	0.079551061	-5.887423771	3.92262E-09	1.9877E-07	down_in_case
Yang2025_S	ENSGALG00010016696	DOK7	case	control	-0.300668307	0.108765743	-2.764365866	0.005703355	0.026439206	down_in_case
Yang2025_S	ENSGALG00010016699	SYNPO	case	control	0.737708489	0.195750242	3.768621079	0.000164152	0.001590721	up_in_case
Yang2025_S	ENSGALG00010016700	CLUAP1	case	control	0.388381033	0.128587733	3.020358343	0.002524758	0.014296018	up_in_case
Yang2025_S	ENSGALG00010016702	WDR25	case	control	-0.542906374	0.153000811	-3.548388862	0.000387596	0.003220718	down_in_case
Yang2025_S	ENSGALG00010016704	CSTF2	case	control	0.436352979	0.107015258	4.07748377	4.55257E-05	0.000566935	up_in_case
Yang2025_S	ENSGALG00010016706	RTN4	case	control	0.038495952	0.114447712	0.336362796	0.736597301	0.8392761	up_in_case
Yang2025_S	ENSGALG00010016707	KIF1B	case	control	0.016715854	0.131994638	0.1266404	0.89922502	0.940208647	up_in_case
Yang2025_S	ENSGALG00010016712	ENSGALG00010016712	case	control	0.137267441	0.105242781	1.30429317	0.192133643	0.347306047	up_in_case

Yang2025_S	ENSGALG00010016720	INTS15	case	control	0.106926562	0.089587555	1.193542586	0.232656944	0.395740151	up_in_case
Yang2025_S	ENSGALG00010016724	PGD	case	control	-0.07993034	0.118071481	-0.676965675	0.498427733	0.655443656	down_in_case
Yang2025_S	ENSGALG00010016729	ELMO1	case	control	0.083589099	0.158910324	0.526014273	0.59887829	0.737810006	up_in_case
Yang2025_S	ENSGALG00010016731	C4orf48	case	control	-0.325060042	0.283925321	-1.144878666	0.252259423	0.418131921	down_in_case
Yang2025_S	ENSGALG00010016732	ARHGAP26	case	control	0.337940492	0.168199756	2.009161608	0.044519998	0.12277581	up_in_case
Yang2025_S	ENSGALG00010016733	PHC3	case	control	1.002809011	0.336972149	2.975940337	0.002920917	0.015968213	up_in_case
Yang2025_S	ENSGALG00010016735	ENSGALG00010016735	case	control	-0.336698802	0.380043207	-0.885948744	0.375645148	0.543743133	down_in_case
Yang2025_S	ENSGALG00010016736	CENPS	case	control	-0.723544813	0.192459058	-3.759473937	0.000170271	0.00164089	down_in_case
Yang2025_S	ENSGALG00010016738	NAA60	case	control	-0.171427665	0.095209232	-1.800536157	0.07177602	0.172955012	down_in_case
Yang2025_S	ENSGALG00010016741	NELFA	case	control	-0.181037493	0.094267222	-1.920471287	0.054798397	0.142651907	down_in_case
Yang2025_S	ENSGALG00010016742	TRMT12	case	control	0.034038189	0.249593087	0.136374728	0.891525052	0.936343201	up_in_case
Yang2025_S	ENSGALG00010016745	BEGAIN	case	control	-0.008260475	0.444726008	-0.018574301	0.985180704	0.991089563	down_in_case
Yang2025_S	ENSGALG00010016746	HDGFL3	case	control	0.163834442	0.101224932	1.618518666	0.105550867	0.227897635	up_in_case
Yang2025_S	ENSGALG00010016750	SYTL4	case	control	0.234856773	0.144434183	1.626047023	0.103939652	0.225537069	up_in_case
Yang2025_S	ENSGALG00010016751	DFFA	case	control	-0.080544153	0.151571473	-0.531393879	0.595145862	0.735096401	down_in_case
Yang2025_S	ENSGALG00010016752	ENSGALG00010016752	case	control	-0.721033268	0.154528499	-4.666021303	3.07088E-06	6.18534E-05	down_in_case
Yang2025_S	ENSGALG00010016754	ENSGALG00010016754	case	control	0.041181744	0.231066006	0.17822502	0.858546261	0.916699268	up_in_case
Yang2025_S	ENSGALG00010016756	NUDT16L1	case	control	0.031012784	0.185445494	0.167233958	0.867185966	0.922021773	up_in_case
Yang2025_S	ENSGALG00010016757	SRPX2	case	control	-0.338367371	0.1703111	-1.986760528	0.046948941	0.127506039	down_in_case
Yang2025_S	ENSGALG00010016758	NR3C1	case	control	-0.120019976	0.122419288	-0.980400863	0.326888284	0.496633126	down_in_case
Yang2025_S	ENSGALG00010016760	DLK1	case	control	-0.481241855	0.130044211	-3.700601903	0.000215089	0.001979697	down_in_case
Yang2025_S	ENSGALG00010016761	GPR160	case	control	-0.253168768	0.165211164	-1.532395039	0.125424981	0.2574544	down_in_case
Yang2025_S	ENSGALG00010016762	EML6	case	control	0.693631561	0.220581648	3.144556988	0.001663385	0.01040961	up_in_case
Yang2025_S	ENSGALG00010016763	UBALD1	case	control	0.198175217	0.160050643	1.238203193	0.215640728	0.376513412	up_in_case
Yang2025_S	ENSGALG00010016766	AOAH	case	control	0.243122424	0.327219332	0.742995293	0.45748453	0.618996692	up_in_case
Yang2025_S	ENSGALG00010016767	SLAIN2	case	control	-0.075978342	0.106144258	-0.715802662	0.474113208	0.633560372	down_in_case
Yang2025_S	ENSGALG00010016768	GALNT10	case	control	-0.275066139	0.17822417	-1.543371693	0.122740618	0.253654235	down_in_case
Yang2025_S	ENSGALG00010016769	BNC1	case	control	0.226680029	0.426755571	0.531170639	0.595300537	0.735157124	up_in_case
Yang2025_S	ENSGALG00010016772	TSPAN6	case	control	-0.530072453	0.126149494	-4.201938793	2.64639E-05	0.000367305	down_in_case
Yang2025_S	ENSGALG00010016773	RPS27A	case	control	-0.735386421	0.292227494	-2.516486082	0.011853158	0.045926844	down_in_case
Yang2025_S	ENSGALG00010016774	PEX14	case	control	0.212897963	0.149106634	1.427823545	0.153342654	0.29727739	up_in_case
Yang2025_S	ENSGALG00010016777	TNMD	case	control	1.412787336	0.298492106	4.73308106	2.21137E-06	4.74084E-05	up_in_case
Yang2025_S	ENSGALG00010016778	MTIF2	case	control	0.241186073	0.082108738	2.937398355	0.003309787	0.017528156	up_in_case
Yang2025_S	ENSGALG00010016779	CTNNA2	case	control	-0.153049675	0.213011867	-0.718503043	0.472447172	0.632182768	down_in_case
Yang2025_S	ENSGALG00010016780	PRORSD1P	case	control	-0.08924389	0.123017912	-0.725454437	0.468173288	0.628857566	down_in_case
Yang2025_S	ENSGALG00010016781	CDIP1	case	control	0.267348234	0.127524065	2.096453195	0.036042008	0.104991463	up_in_case
Yang2025_S	ENSGALG00010016783	CLBA1	case	control	-0.11806832	0.40811947	-0.289298425	0.772353017	0.861501584	down_in_case
Yang2025_S	ENSGALG00010016785	PDGFRB	case	control	-0.079956498	0.12757898	-0.62672157	0.530841762	0.683225266	down_in_case
Yang2025_S	ENSGALG00010016786	DISP1	case	control	-0.324056716	0.104719834	-3.094511353	0.001971374	0.011851206	down_in_case
Yang2025_S	ENSGALG00010016787	PPP2R5C	case	control	-0.016582672	0.101310696	-0.163681355	0.869981996	0.923531585	down_in_case
Yang2025_S	ENSGALG00010016788	ANLN	case	control	-0.36788285	0.119002349	-3.091391504	0.001992208	0.011930131	down_in_case
Yang2025_S	ENSGALG00010016789	DISP3	case	control	-0.434704133	0.157002921	-2.768764627	0.005626927	0.026145791	down_in_case
Yang2025_S	ENSGALG00010016791	LRRTM1	case	control	0.610785302	0.575330701	1.061624732	0.288406081	0.457321204	up_in_case
Yang2025_S	ENSGALG00010016792	HMOX2	case	control	0.043980904	0.168383523	0.261194822	0.793942271	0.875638151	up_in_case
Yang2025_S	ENSGALG00010016793	SLC9A1	case	control	0.1155035	0.09751489	1.184470394	0.236226909	0.399832622	up_in_case
Yang2025_S	ENSGALG00010016796	BROX	case	control	-0.141381895	0.099014144	-1.427895938	0.153321813	0.29727739	down_in_case
Yang2025_S	ENSGALG00010016797	SEC62	case	control	0.420493063	0.134528307	3.125684656	0.001773917	0.010952383	up_in_case
Yang2025_S	ENSGALG00010016799	WDTC1	case	control	-0.569900411	0.10967871	-5.196089687	2.03524E-07	6.23321E-06	down_in_case
Yang2025_S	ENSGALG00010016800	RPL14	case	control	0.077977248	0.173971386	0.448218815	0.653995285	0.780927532	up_in_case
Yang2025_S	ENSGALG00010016802	TMEM222	case	control	-0.053205795	0.139165434	-0.382320473	0.702223671	0.816213761	down_in_case
Yang2025_S	ENSGALG00010016804	CSF1R	case	control	-0.534554039	0.114528388	-4.667437039	3.0498E-06	6.15927E-05	down_in_case
Yang2025_S	ENSGALG00010016807	VSIG4	case	control	-0.07697599	0.271054795	-0.283986822	0.776420479	0.863982227	down_in_case
Yang2025_S	ENSGALG00010016808	ENTPD3	case	control	-0.808968568	0.551257363	-1.467497075	0.142240884	0.281890081	down_in_case
Yang2025_S	ENSGALG00010016810	SH3GL3	case	control	-0.245824034	0.129783749	-1.894104885	0.05821109	0.14933202	down_in_case
Yang2025_S	ENSGALG00010016812	LRRC31	case	control	0.067255706	0.223213348	0.301306826	0.763180536	0.855638848	up_in_case
Yang2025_S	ENSGALG00010016813	ENSGALG00010016813	case	control	-0.951759077	0.62233061	-1.5293464	0.126178593	0.25864941	down_in_case
Yang2025_S	ENSGALG00010016814	HMGXB3	case	control	0.145279801	0.091235694	1.592357061	0.111304498	0.236461294	up_in_case
Yang2025_S	ENSGALG00010016816	MAP3K6	case	control	0.885736991	0.271659011	3.260473449	0.001112264	0.007627986	up_in_case
Yang2025_S	ENSGALG00010016817	GGCT	case	control	-0.124441482	0.203595403	-0.611219508	0.54105427	0.691515294	down_in_case
Yang2025_S	ENSGALG00010016820	EIF2AK3	case	control	-0.072367675	0.131542125	-0.550148284	0.582217671	0.724716682	down_in_case
Yang2025_S	ENSGALG00010016823	HSF3	case	control	-0.059885575	0.065501277	-0.914265773	0.360577211	0.529132628	down_in_case
Yang2025_S	ENSGALG00010016825	ENSGALG00010016825	case	control	0.44035439	0.251739813	1.749244129	0.080248829	0.187754774	up_in_case
Yang2025_S	ENSGALG00010016828	LACTBL1	case	control	-0.024621553	0.288533197	-0.08533352	0.931996243	0.95863854	down_in_case
Yang2025_S	ENSGALG00010016831	AIDA	case	control	-0.067449862	0.115750695	-0.582716693	0.560084034	0.706633863	down_in_case
Yang2025_S	ENSGALG00010016832	DYNC1H1	case	control	-0.17161083	0.113136159	-1.516852178	0.12930401	0.262928845	down_in_case
Yang2025_S	ENSGALG00010016834	ENSGALG00010016834	case	control	-0.337186207	0.110743235	-3.044756706	0.002328686	0.013436952	down_in_case
Yang2025_S	ENSGALG00010016835	CCDC88A	case	control	0.428815659	0.162860297	2.633027617	0.008462747	0.035804457	up_in_case
Yang2025_S	ENSGALG00010016836	HEPH	case	control	-0.006908853	0.129169911	-0.053486553	0.957344245	0.973836926	down_in_case
Yang2025_S	ENSGALG00010016840	LRRC34	case	control	0.15907696	0.160608099	0.990466614	0.3219461	0.491274518	up_in_case
Yang2025_S	ENSGALG00010016842	NPY7R	case	control	-0.022300835	0.261676682	-0.085222859	0.932084218	0.95863854	down_in_case
Yang2025_S	ENSGALG00010016845	MIA3	case	control	0.291319223	0.171148696	1.702141059	0.088728929	0.201933304	up_in_case
Yang2025_S	ENSGALG00010016847	EPHB2	case	control	-0.283849181	0.118327522	-2.398843285	0.016446952	0.058856773	down_in_case
Yang2025_S	ENSGALG00010016848	ENSGALG00010016848	case	control	0.018163801	0.196700521	0.092342413	0.926425991	0.955608219	up_in_case
Yang2025_S	ENSGALG00010016849	WASF2	case	control	-0.063960124	0.122675668	-0.521375796	0.602105009	0.7406067	down_in_case
Yang2025_S	ENSGALG00010016851	TEC	case	control	-0.128241784	0.140214974	-0.914608337	0.36039728	0.528924287	down_in_case
Yang2025_S	ENSGALG00010016852	AHDC1	case	control	0.288284276	0.119642209	2.409553276	0.015972064	0.057622662	up_in_case
Yang2025_S	ENSGALG00010016854	TAF1A	case	control	0.381483577	0.148950522	2.56114293	0.010432842	0.041788347	up_in_case
Yang2025_S	ENSGALG00010016856	NOD1	case	control	0.202045415	0.149045905	1.355591854	0.175229065	0.325792798	up_in_case
Yang2025_S	ENSGALG00010016858	ENSGALG00010016858	case	control	0.710039163	0.226389586	3.13635965	0.001710593	0.010641568	up_in_case
Yang2025_S	ENSGALG00010016859	RPIA	case	control	-0.601224982	0.129743299	-4.633957865	3.5874E-06	7.0808E-05	down_in_case
Yang2025_S	ENSGALG00010016860	VSX1	case	control	-1.481611113	0.479446024	-3.090256336	0.001999838	0.011965541	down_in_case
Yang2025_S	ENSGALG00010016861	FGR	case	control	-0.104785898	0.102220506	-1.025096654	0.305317584	0.474807136	down_in_case
Yang2025_S	ENSGALG00010016863	MYNN	case	control	-0.114588063	0.107040117	-1.070515119	0.284387507	0.452648762	down_in_case
Yang2025_S	ENSGALG00010016864	ENSGALG00010016864	case	control	1.006907819	0.325305333	3.095269945	0.001966338	0.011826037	up_in_case
Yang2025_S	ENSGALG00010016865	CFAP36	case	control	0.380399601	0.173096535	2.197615338	0.027976529	0.086846277	up_in_case
Yang2025_S	ENSGALG00010016866	ZNRF2	case	control	0.308223657	0.157574919	1.956045149	0.050459836	0.134088065	up_in_case
Yang2025_S	ENSGALG00010016867	FLT4	case	control	-0.183122631	0.082753979	-2.212855906	0.026907582	0.084411785	down_in_case
Yang2025_S	ENSGALG00010016869	HSP90AA1	case	control	-0.035879318	0.17439126	-0.205740346	0.836993741	0.903377568	down_in_case
Yang2025_S	ENSGALG00010016872	EDA2R	case	control	0.04916107	0.215866993	0.227737781	0.819850099	0.892338603	up_in_case
Yang2025_S	ENSGALG00010016873	ENTPD6	case	control	0.249298884	0.159555792	1.56245587	0.118180634	0.246762396	up_in_case
Yang2025_S	ENSGALG00010016874	ADAMTSL3	case	control	0.16438321	0.121283496	1.355363389	0.175301808	0.325797639	up_in_case
Yang2025_S	ENSGALG00010016875	SESN2	case	control	0.176908001	0.18119897	0.97631902	0.328906397	0.498511056	up_in_case
Yang2025_S	ENSGALG00010016876	MTURN	case	control	0.426015658	0.169751465	2.509643484	0.012085311	0.046640295	up_in_case
Yang2025_S	ENSGALG00010016877	UBE2D3	case	control	-0.090419814	0.111938789	-0.807761234	0.419228046	0.584954723	down_in_case
Yang2025_S	ENSGALG00010016878	SUCLG1	case	control	-0.580267662	0.179132497	-3.239321008	0.001198146	0.008061888	down_in_case
Yang2025_S	ENSGALG00010016879	RNF130	case	control	-0.404989993	0.157210706	-2.576096784	0.009992267	0.040500944	down_in_case
Yang2025_S	ENSGALG00010016880	AR	case	control	-0.115366969	0.104761676	-1.101232554	0.270795457	0.438097295	down_in_case
Yang2025_S	ENSGALG00010016881	C1QB	case	control	-0.263266197	0.417732665	-0.630226504	0.528546401	0.68121477	down_in_case
Yang2025_S	ENSGALG00010016882	ENSGALG00010016882	case	control	-0.163314618	0.052178706	-3.129909295	0.001748603	0.01081526	down_in_case
Yang2025_S	ENSGALG00010016883	TMEM251	case	control	-0.382330711	0.133508724	-2.863713315	0.004187068	0.0208307	down_in_case
Yang2025_S	ENSGALG00010016884	SBK1	case	control	0.219163942	0.179986137	1.217671235	0.223348932	0.384728306	up_in_case
Yang2025_S	ENSGALG00010016885	CDCA4	case	control	-0.153290092	0.079205505	-1.935346433	0.052947798	0.139133993	down_in_case
Yang2025_S	ENSGALG00010016887	TJP2	case	control	0.259679268	0.106140461	2.446562473	0.01442258	0.05329585	up_in_case
Yang2025_S	ENSGALG00010016888	ENSGALG00010016888	case	control	-0.071357642	0.137102727	-0.520468438	0.60273712	0.740991775	down_in_case
Yang2025_S	ENSGALG00010016889	FUNDC2	case	control	-0.564903326	0.146668283	-3.85157113	0.000117362	0.001210544	down_in_case
Yang2025_S	ENSGALG00010016890	UBR7	case	control	-0.415030299	0.095692786	-4.337111664	1.44367E-05	0.000223777	down_in_case
Yang2025_S	ENSGALG00010016891	ENSGALG00010016891	case	control	-0.595298143	0.339220311	-1.754901233	0.079276203	0.186124426	down_in_case
Yang2025_S	ENSGALG00010016892	MAL	case	control	0.13053579	0.325929757	0.40050283	0.688786199	0.805423822	up_in_case
Yang2025_S	ENSGALG00010016893	TBC1D9B	case	control	-0.278427094	0.089621379	-3.106703967	0.001891857	0.011507217	down_in_case
Yang2025_S	ENSGALG00010016894	BTBD7	case	control	-0.138036093	0.15703594	-0.879009559	0.379396093	0.547523256	down_in_case
Yang2025_S	ENSGALG00010016895	ENSGALG00010016895	case	control	0.894676269	0.227966245	3.924599749	8.68741E-05	0.00095247	up_in_case
Yang2025_S	ENSGALG00010016896	SPTBN1	case	control	0.38127184	0.175227677	2.175865396	0.029565327	0.090547875	up_in_case
Yang2025_S	ENSGALG00010016899	SAXO2	case	control	-0.558986384	0.206927304	-2.701366002	0.00690553	0.030681552	down_in_case
Yang2025_S	ENSGALG00010016900	CISD2	case	control	0.64224573	0.132881254	4.833230505	1.34335E-06	3.10957E-05	up_in_case
Yang2025_S	ENSGALG00010016901	MECOM	case	control	0.270286834	0.14417141	1.874760286	0.060825709	0.153954023	up_in_case
Yang2025_S	ENSGALG00010016902	MRNIP	case	control	0.095639166	0.19977067	0.478744783	0.632120203	0.763944976	up_in_case
Yang2025_S	ENSGALG00010016903	PPM1A	case	control	-0.366611675	0.148966731	-2.461030537	0.013853857	0.051660022	down_in_case
Yang2025_S	ENSGALG00010016904	EAF1	case	control	-0.069803408	0.072691864	-0.960264384	0.33692217	0.505058347	down_in_case
Yang2025_S	ENSGALG00010016905	C1QC	case	control	0.165809907	0.449845871	0.368592707	0.712431333	0.822496891	up_in_case
Yang2025_S	ENSGALG00010016907	SQSTM1	case	control	-0.10582151	0.184285413	-0.574226188	0.56581475	0.711159786	down_in_case
Yang2025_S	ENSGALG00010016908	CMC4	case	control	-0.383860893	0.082148301	-4.672779476	2.97151E-06	6.02329E-05	down_in_case
Yang2025_S	ENSGALG00010016909	MRPS5	case	control	0.164694698	0.095302315	1.728129033	0.083965093	0.193910106	up_in_case
Yang2025_S	ENSGALG00010016910	NSD2	case	control	0.091171693	0.118277147	0.770831016	0.440807101	0.604115554	up_in_case
Yang2025_S	ENSGALG00010016911	AXIN1	case	control	0.096286647	0.116566403	0.826024004	0.408790475	0.574475118	up_in_case
Yang2025_S	ENSGALG00010016912	CCDC88C	case	control	0.453192242	0.105158159	4.309625118	1.63532E-05	0.000246358	up_in_case
Yang2025_S	ENSGALG00010016913	BRCC3	case	control	-0.123929748	0.1634489	-0.758217082	0.448321036	0.610868646	down_in_case

Yang2025_S	ENSGALG00010016915	PLOD2	case	control	-0.360530438	0.175845865	-2.050263958	0.04033868	0.113911305	down_in_case
Yang2025_S	ENSGALG00010016916	ACYP2	case	control	-0.717238333	0.24233927	-2.959645513	0.003079932	0.01664812	down_in_case
Yang2025_S	ENSGALG00010016917	METTL6	case	control	0.149464246	0.099704697	1.49906925	0.133855668	0.269743851	up_in_case
Yang2025_S	ENSGALG00010016918	RCOR1	case	control	-0.113008223	0.107121533	-1.054953372	0.291446651	0.460050187	down_in_case
Yang2025_S	ENSGALG00010016919	ARHGDIG	case	control	0.264696268	0.252920929	1.046557392	0.295303762	0.463820338	up_in_case
Yang2025_S	ENSGALG00010016920	C1QA	case	control	-0.126087834	0.425159586	-0.296565897	0.766797951	0.858190462	down_in_case
Yang2025_S	ENSGALG00010016921	EFL1	case	control	-0.080795092	0.113446219	-0.712188499	0.476348058	0.635571638	down_in_case
Yang2025_S	ENSGALG00010016922	ENSGALG00010016922	case	control	-0.076540671	0.127815596	-0.598836713	0.549281778	0.697870311	down_in_case
Yang2025_S	ENSGALG00010016923	ENSGALG00010016923	case	control	-0.480595664	0.162769903	-2.952607663	0.003151022	0.016946927	down_in_case
Yang2025_S	ENSGALG00010016924	LETM1	case	control	0.180345508	0.11795262	1.528965677	0.126272953	0.258728657	up_in_case
Yang2025_S	ENSGALG00010016925	PHACTR4	case	control	0.224154741	0.167567958	1.337694531	0.180996034	0.333445233	up_in_case
Yang2025_S	ENSGALG00010016926	ENSGALG00010016926	case	control	0.409235173	0.204501166	2.001138578	0.045377458	0.124696288	up_in_case
Yang2025_S	ENSGALG00010016927	MGAT4B	case	control	-0.196615038	0.130827433	-1.502857877	0.132875697	0.268100715	down_in_case
Yang2025_S	ENSGALG00010016928	RGS11	case	control	1.295981629	0.300601302	4.311297453	1.62299E-05	0.000245032	up_in_case
Yang2025_S	ENSGALG00010016929	EPHA8	case	control	0.14442584	0.565997348	0.255170525	0.798591396	0.878185851	up_in_case
Yang2025_S	ENSGALG00010016930	C10H15ORF48	case	control	-0.420107627	0.627488761	-0.669506217	0.503172616	0.659353397	down_in_case
Yang2025_S	ENSGALG00010016931	SLC30A4	case	control	0.095692195	0.134329644	0.712368406	0.476236674	0.63550578	up_in_case
Yang2025_S	ENSGALG00010016932	OGT	case	control	0.93895646	0.122804333	7.645955467	2.07399E-14	3.95906E-12	up_in_case
Yang2025_S	ENSGALG00010016934	SLC25A38	case	control	-0.293741911	0.104022689	-2.823825398	0.004745423	0.023056997	down_in_case
Yang2025_S	ENSGALG00010016935	PRIMA1	case	control	0.250299122	0.440760351	0.567880303	0.570116247	0.714503994	up_in_case
Yang2025_S	ENSGALG00010016936	RCC1	case	control	0.132084111	0.191936192	0.688166778	0.491347762	0.648813709	up_in_case
Yang2025_S	ENSGALG00010016937	DHRS7	case	control	-0.134367484	0.134389366	-0.999837174	0.317389313	0.48693384	down_in_case
Yang2025_S	ENSGALG00010016938	PSME4	case	control	-0.110788255	0.076515314	-1.447922627	0.147638691	0.289807741	down_in_case
Yang2025_S	ENSGALG00010016939	FAM234A	case	control	-0.444819365	0.199789709	-2.226437827	0.025984869	0.082276562	down_in_case
Yang2025_S	ENSGALG00010016940	FGFR3	case	control	0.277163054	0.283408855	0.977961871	0.328093183	0.497765759	up_in_case
Yang2025_S	ENSGALG00010016941	SLC8A1	case	control	-0.18249463	0.173462421	-1.052070125	0.292767378	0.461417951	down_in_case
Yang2025_S	ENSGALG00010016942	ZBTB40	case	control	0.68158923	0.202760776	3.361543802	0.000775081	0.005699603	up_in_case
Yang2025_S	ENSGALG00010016943	PCNX4	case	control	0.105098699	0.091331273	1.150741634	0.249838543	0.415552649	up_in_case
Yang2025_S	ENSGALG00010016944	OPRD1	case	control	-0.47383328	0.483229251	-0.980555874	0.326811803	0.496633126	down_in_case
Yang2025_S	ENSGALG00010016945	BDH2	case	control	-0.093782582	0.124242359	-0.754835809	0.450347505	0.612312663	down_in_case
Yang2025_S	ENSGALG00010016946	TAF1	case	control	0.241861818	0.123825082	1.953253849	0.050789523	0.134784232	up_in_case
Yang2025_S	ENSGALG00010016947	CANX	case	control	-0.207227676	0.056009317	-3.699878682	0.000215703	0.001984036	down_in_case
Yang2025_S	ENSGALG00010016949	LUC7L	case	control	0.603633848	0.108597216	5.558465221	2.72157E-08	1.10247E-06	up_in_case
Yang2025_S	ENSGALG00010016950	ENSGALG00010016950	case	control	0.260414819	0.845555924	0.307980598	0.758097096	0.85295517	up_in_case
Yang2025_S	ENSGALG00010016952	GINS1	case	control	0.61338567	0.253562906	2.419067045	0.015560372	0.056570254	up_in_case
Yang2025_S	ENSGALG00010016953	GJA4	case	control	0.012139359	0.119772385	0.101353569	0.919269793	0.952418183	up_in_case
Yang2025_S	ENSGALG00010016955	ENSGALG00010016955	case	control	0.315288499	0.266603181	1.182613416	0.236962393	0.400736765	up_in_case
Yang2025_S	ENSGALG00010016956	CENPE	case	control	0.182886664	0.100806659	1.81423198	0.069642019	0.169365014	up_in_case
Yang2025_S	ENSGALG00010016957	WNT4	case	control	-0.077029872	0.207213841	-0.371740958	0.710085727	0.820961139	down_in_case
Yang2025_S	ENSGALG00010016958	TLR15	case	control	1.384548805	0.62468549	2.216393413	0.02666457	0.083853276	up_in_case
Yang2025_S	ENSGALG00010016959	PERCC1	case	control	0.653116408	0.732641872	0.891453837	0.372685747	0.541033012	up_in_case
Yang2025_S	ENSGALG00010016961	EIF4A3	case	control	-0.041520356	0.104303223	-0.398073563	0.690575961	0.807042605	down_in_case
Yang2025_S	ENSGALG00010016962	SQOR	case	control	-0.275477931	0.122837162	-2.242626956	0.024920881	0.079740931	down_in_case
Yang2025_S	ENSGALG00010016964	MAML1	case	control	-0.074758083	0.110226515	-0.678222319	0.497630743	0.654505371	down_in_case
Yang2025_S	ENSGALG00010016965	SLC25A33	case	control	-0.177231929	0.260960129	-0.679153284	0.497040744	0.654038032	down_in_case
Yang2025_S	ENSGALG00010016966	KDM3A	case	control	-0.014351322	0.097907124	-0.146580974	0.883462772	0.931385514	down_in_case
Yang2025_S	ENSGALG00010016967	ERLEC1	case	control	-0.082041708	0.095003715	-0.863563156	0.387827911	0.554921649	down_in_case
Yang2025_S	ENSGALG00010016968	C15orf61	case	control	-0.424698586	0.251432352	-1.689116708	0.091197067	0.205730086	down_in_case
Yang2025_S	ENSGALG00010016969	CHMP3	case	control	0.326569607	0.139403163	2.34262695	0.019148518	0.065658121	up_in_case
Yang2025_S	ENSGALG00010016970	SYNE3	case	control	0.18325785	0.171199261	1.070435988	0.284423108	0.452653724	up_in_case
Yang2025_S	ENSGALG00010016972	ASB3	case	control	0.229860533	0.138543053	1.659127091	0.097090185	0.214652028	up_in_case
Yang2025_S	ENSGALG00010016973	PLSCR1	case	control	0.196254685	0.12270952	1.599343597	0.109744277	0.233728642	up_in_case
Yang2025_S	ENSGALG00010016975	MALL	case	control	0.118313613	0.279766353	0.422901508	0.672367123	0.793789768	up_in_case
Yang2025_S	ENSGALG00010016977	CLCN7	case	control	-0.197949281	0.209088864	-0.946723214	0.343779838	0.512251074	down_in_case
Yang2025_S	ENSGALG00010016978	FAM181A	case	control	-0.758037576	0.223207062	-3.396118251	0.000683488	0.005145548	down_in_case
Yang2025_S	ENSGALG00010016979	PLSCR5	case	control	-0.926124892	0.282964059	-3.272941787	0.001064344	0.007360612	down_in_case
Yang2025_S	ENSGALG00010016980	NPHP1	case	control	0.314420063	0.096611679	3.254472627	0.001136031	0.007752494	up_in_case
Yang2025_S	ENSGALG00010016981	ENSGALG00010016981	case	control	1.092247831	0.635488647	1.718752705	0.085659411	0.196746973	up_in_case
Yang2025_S	ENSGALG00010016982	GLRX5	case	control	-0.350815505	0.197132855	-1.779589223	0.075143211	0.179086822	down_in_case
Yang2025_S	ENSGALG00010016983	ZFAND2A	case	control	0.405609279	0.240467532	1.686752782	0.091650898	0.206458983	up_in_case
Yang2025_S	ENSGALG00010016984	RNF103	case	control	-0.335818412	0.103356525	-3.249126366	0.0011576	0.007857361	down_in_case
Yang2025_S	ENSGALG00010016985	ENSGALG00010016985	case	control	-0.262676349	0.209199426	-1.255626531	0.209251407	0.368410542	down_in_case
Yang2025_S	ENSGALG00010016986	GFER	case	control	-0.296674125	0.112411971	-2.639168424	0.008310968	0.035276679	down_in_case
Yang2025_S	ENSGALG00010016987	ASB2	case	control	-0.362518656	0.228443759	-1.586905494	0.11253405	0.238358716	down_in_case
Yang2025_S	ENSGALG00010016988	ECI1	case	control	-0.11826865	0.17501259	-0.67577224	0.499185262	0.655992704	down_in_case
Yang2025_S	ENSGALG00010016989	RHOG2	case	control	0.094623583	0.083343318	1.135346962	0.256229973	0.422552032	up_in_case
Yang2025_S	ENSGALG00010016991	PDLIM3	case	control	0.551535233	0.201383687	2.738728455	0.006167729	0.028004988	up_in_case
Yang2025_S	ENSGALG00010016993	ENSGALG00010016993	case	control	-0.444140893	0.127001671	-3.497126386	0.000470299	0.003762122	down_in_case
Yang2025_S	ENSGALG00010016994	ATL2	case	control	0.239008829	0.074506874	3.20787621	0.00133719	0.008797804	up_in_case
Yang2025_S	ENSGALG00010016996	ZIC4	case	control	0.585539117	0.730808278	0.801221244	0.423003575	0.588714652	up_in_case
Yang2025_S	ENSGALG00010016999	ITGB1BP2	case	control	0.120245751	0.141288706	0.851064142	0.394733723	0.561835927	up_in_case
Yang2025_S	ENSGALG00010017001	TPCN3	case	control	-0.361630026	0.168058541	-2.151809866	0.031412332	0.09476745	down_in_case
Yang2025_S	ENSGALG00010017003	CPZ	case	control	-0.589193794	0.22675981	-2.598316665	0.009368206	0.038704444	down_in_case
Yang2025_S	ENSGALG00010017004	CX3CR1	case	control	-0.95093201	0.737371002	-1.289624907	0.197180924	0.353449469	down_in_case
Yang2025_S	ENSGALG00010017006	DNAH3	case	control	0.838084139	0.200060732	4.189148623	2.80003E-05	0.000384797	up_in_case
Yang2025_S	ENSGALG00010017008	RTN1	case	control	0.474540502	0.414521003	1.14479242	0.252295156	0.418141413	up_in_case
Yang2025_S	ENSGALG00010017010	NONO	case	control	-0.008276226	0.06169984	-0.134136906	0.893294316	0.937397493	down_in_case
Yang2025_S	ENSGALG00010017012	SYNGR3	case	control	0.160011815	0.174897309	0.914890094	0.360249331	0.528847747	up_in_case
Yang2025_S	ENSGALG00010017016	ZIC1	case	control	2.449067816	0.915569414	2.674912222	0.007474882	0.032479725	up_in_case
Yang2025_S	ENSGALG00010017017	ENSGALG00010017017	case	control	1.041080282	0.283507857	3.672139087	0.000240529	0.002173649	up_in_case
Yang2025_S	ENSGALG00010017018	HP1BP3	case	control	-0.209581318	0.089631635	-2.338251655	0.019374197	0.066241564	down_in_case
Yang2025_S	ENSGALG00010017020	GJB5	case	control	0.452135937	0.422485681	1.070180499	0.28453807	0.452733274	up_in_case
Yang2025_S	ENSGALG00010017022	CCR4	case	control	0.807789868	0.318125753	2.539215575	0.011110134	0.043783857	up_in_case
Yang2025_S	ENSGALG00010017024	BUB1	case	control	-0.219483192	0.120061101	-1.828095783	0.067535178	0.165541372	down_in_case
Yang2025_S	ENSGALG00010017033	LDAF1	case	control	-0.298543015	0.180068583	-1.657940602	0.097329456	0.21479319	down_in_case
Yang2025_S	ENSGALG00010017034	ZMYM3	case	control	0.486647867	0.122395263	3.976035147	7.00738E-05	0.000802365	up_in_case
Yang2025_S	ENSGALG00010017035	PIK3CD	case	control	0.092117916	0.147655321	0.623871292	0.532712115	0.68481027	up_in_case
Yang2025_S	ENSGALG00010017038	ZNF598	case	control	0.156611874	0.135894158	1.152454791	0.249134237	0.414727702	up_in_case
Yang2025_S	ENSGALG00010017039	MAP2K5	case	control	-0.506897265	0.127142417	-3.98684624	6.69574E-05	0.000772835	down_in_case
Yang2025_S	ENSGALG00010017041	JKAMP	case	control	-0.21504599	0.132036969	-1.628680146	0.103380748	0.224709206	down_in_case
Yang2025_S	ENSGALG00010017042	HNRNPLL	case	control	-0.557774851	0.143234293	-3.894143204	9.85464E-05	0.001054719	down_in_case
Yang2025_S	ENSGALG00010017045	GLB1	case	control	0.418132021	0.111993321	3.733544265	0.000188804	0.001780097	up_in_case
Yang2025_S	ENSGALG00010017049	TRAF3	case	control	0.516911077	0.165747844	3.118659424	0.001816758	0.011133037	up_in_case
Yang2025_S	ENSGALG00010017050	L3HYPDH	case	control	-0.338655133	0.33356406	-1.015262656	0.309980641	0.479793427	down_in_case
Yang2025_S	ENSGALG00010017054	GALM	case	control	-0.113098651	0.128362951	-0.881084847	0.378271898	0.546523474	down_in_case
Yang2025_S	ENSGALG00010017055	GPR135	case	control	-0.111212008	0.304996158	-0.364634129	0.715384535	0.824215254	down_in_case
Yang2025_S	ENSGALG00010017056	AGTR1	case	control	-0.060023551	0.205926574	-0.291480354	0.770683967	0.860110387	down_in_case
Yang2025_S	ENSGALG00010017057	NDUFAB1	case	control	-0.049135612	0.267921077	-0.183395845	0.854487436	0.913978788	down_in_case
Yang2025_S	ENSGALG00010017060	CLSTN1	case	control	-0.279616618	0.114493236	-2.442210799	0.01459762	0.053771567	down_in_case
Yang2025_S	ENSGALG00010017063	TMPPE	case	control	-0.744181291	0.214779925	-3.464854979	0.000530517	0.004153237	down_in_case
Yang2025_S	ENSGALG00010017065	VRK2	case	control	0.575525825	0.093406106	6.161543905	7.20391E-10	4.44188E-08	up_in_case
Yang2025_S	ENSGALG00010017070	BLOC1S6	case	control	0.94142818	0.192187268	4.898494007	9.6574E-07	2.34453E-05	up_in_case
Yang2025_S	ENSGALG00010017077	CTNNBIP1	case	control	-0.33122031	0.095054491	-3.484530877	0.000493001	0.003905607	down_in_case
Yang2025_S	ENSGALG00010017079	ENSGALG00010017079	case	control	0.242655503	0.871574253	0.278410591	0.78069719	0.867130188	up_in_case
Yang2025_S	ENSGALG00010017080	PALB2	case	control	-0.146010826	0.127443057	-1.145694628	0.251921527	0.417720905	down_in_case
Yang2025_S	ENSGALG00010017083	ENSGALG00010017083	case	control	0.120489178	0.701949714	0.171649301	0.863713246	0.919409694	up_in_case
Yang2025_S	ENSGALG00010017085	ENSGALG00010017085	case	control	-0.264232324	0.133493243	-1.979368534	0.047774529	0.129244429	down_in_case
Yang2025_S	ENSGALG00010017089	CRTAP	case	control	-0.012451146	0.098626005	-0.126246079	0.899537138	0.940222773	down_in_case
Yang2025_S	ENSGALG00010017092	ANKS4B	case	control	0.495253367	0.517786628	0.956481572	0.33882899	0.506988294	up_in_case
Yang2025_S	ENSGALG00010017094	SORBS2	case	control	1.129404325	0.2447551	4.614426113	3.94183E-06	7.60795E-05	up_in_case
Yang2025_S	ENSGALG00010017096	DCTN5	case	control	-0.357710904	0.118145277	-3.027720718	0.002464057	0.014037872	down_in_case
Yang2025_S	ENSGALG00010017098	DAAM1	case	control	0.11173335	0.127873947	0.873777281	0.382239555	0.550145446	up_in_case
Yang2025_S	ENSGALG00010017100	SUSD5	case	control	1.631756539	0.641084095	2.545308096			up_in_case
Yang2025_S	ENSGALG00010017101	CSMD2	case	control	-0.275310238	0.217596091	-1.265235219	0.205787021	0.364237538	down_in_case
Yang2025_S	ENSGALG00010017104	FANCL	case	control	0.018853984	0.142006558	0.1327684	0.894376546	0.937885741	up_in_case
Yang2025_S	ENSGALG00010017105	PLK1	case	control	-0.265123894	0.196325416	-1.350430824	0.176877829	0.327852161	down_in_case
Yang2025_S	ENSGALG00010017106	CRYM	case	control	-0.453962788	0.246181742	-1.844014846	0.065180977	0.161446305	down_in_case
Yang2025_S	ENSGALG00010017109	ITGB1BP3	case	control	-0.116950376	0.16178886	-0.722858026	0.469767113	0.63023055	down_in_case
Yang2025_S	ENSGALG00010017110	BRF1	case	control	0.080338076	0.124379882	0.645908923	0.51833834	0.672582022	up_in_case
Yang2025_S	ENSGALG00010017111	GJB1	case	control	0.074467607	0.241852546	0.307904995	0.758154625	0.85295517	up_in_case
Yang2025_S	ENSGALG00010017112	LZIC	case	control	-0.343886614	0.073247307	-4.694870399	2.66776E-06	5.51561E-05	down_in_case
Yang2025_S	ENSGALG00010017115	LGALSL	case	control	-0.359091612	0.133848517	-2.682821009	0.007300405	0.031890641	down_in_case
Yang2025_S	ENSGALG00010017116	ENSGALG00010017116	case	control	0.25787547	0.315412341	0.817582055	0.413595878	0.579125659	up_in_case
Yang2025_S	ENSGALG00010017119	BTBD6	case	control	0.339211516	0.116272497	2.917383936	0.00352981	0.018386369	up_in_case
Yang2025_S	ENSGALG00010017120	ERN2	case	control	-0.044674248	0.121529579	-0.367599792	0.71317167	0.822771185	down_in_case
Yang2025_S	ENSGALG00010017122	NCBP2	case	control	1.215936467	0.141566566	8.589149962	8.76151E-18	3.21294E-15	up_in_case

Yang2025_S	ENSGALG00010017127	SOX8	case	control	0.355329666	0.184950697	1.921212902	0.054704872	0.142488298	up_in_case
Yang2025_S	ENSGALG00010017131	TLR3	case	control	0.283568518	0.434641747	0.652418964	0.514130934	0.6689035	up_in_case
Yang2025_S	ENSGALG00010017132	TMEM108	case	control	0.923679507	0.315338441	2.929168747	0.003398699	0.017885523	up_in_case
Yang2025_S	ENSGALG00010017133	WDR20	case	control	0.355278744	0.124082013	2.86325743	0.004193097	0.020845812	up_in_case
Yang2025_S	ENSGALG00010017135	ENSGALG00010017135	case	control	-0.337375866	0.178006322	-1.895302719	0.058052308	0.148952111	down_in_case
Yang2025_S	ENSGALG00010017136	ENSGALG00010017136	case	control	0.016190728	0.484383303	0.033425445	0.973335319	0.983997944	up_in_case
Yang2025_S	ENSGALG00010017139	COL9A2	case	control	2.642365764	0.890000757	2.968947772	0.002988214	0.016285006	up_in_case
Yang2025_S	ENSGALG00010017140	UQCRC2	case	control	-0.139900578	0.166816721	-0.83864841	0.401666636	0.56824615	down_in_case
Yang2025_S	ENSGALG00010017141	AFTPH	case	control	-0.103055851	0.091854269	-1.121949494	0.261883915	0.428730305	down_in_case
Yang2025_S	ENSGALG00010017142	RBP7	case	control	-1.368221277	0.48021373	-2.849192332	0.004383038	0.02161033	down_in_case
Yang2025_S	ENSGALG00010017143	FAM149A	case	control	-0.333282979	0.229227346	-1.453940747	0.145962735	0.287408606	down_in_case
Yang2025_S	ENSGALG00010017144	CPB1	case	control	-0.216527012	0.446096249	-0.485381827	0.627405521	0.760683246	down_in_case
Yang2025_S	ENSGALG00010017147	SRSF7L	case	control	-0.124865439	0.181992338	-0.686102724	0.492648333	0.650161429	down_in_case
Yang2025_S	ENSGALG00010017148	MOK	case	control	1.183359546	0.378449766	3.126860292	0.001766839	0.01091564	up_in_case
Yang2025_S	ENSGALG00010017152	CYP4V2	case	control	-0.667279693	0.229953189	-2.901806655	0.003710175	0.019096181	down_in_case
Yang2025_S	ENSGALG00010017153	SLC35F4	case	control	-0.053286994	0.544495057	-0.097864973	0.922039514	0.953373943	down_in_case
Yang2025_S	ENSGALG00010017154	PIGZ	case	control	-0.303816229	0.257439874	-1.18014441	0.237942784	0.401816249	down_in_case
Yang2025_S	ENSGALG00010017162	BCL11A	case	control	-0.016671354	0.138236762	-0.120600004	0.904007865	0.942633164	down_in_case
Yang2025_S	ENSGALG00010017163	CDV3	case	control	-0.112457257	0.07811364	-1.439662226	0.149962985	0.292434117	down_in_case
Yang2025_S	ENSGALG00010017167	SMAP2	case	control	-0.44340611	0.137687948	-3.220369801	0.001280253	0.008491352	down_in_case
Yang2025_S	ENSGALG00010017169	CCDC198	case	control	1.109008754	0.312079854	3.553605719	0.000379988	0.003170742	up_in_case
Yang2025_S	ENSGALG00010017174	DHX57	case	control	-0.231827647	0.100134627	-2.315159632	0.020604198	0.06918542	down_in_case
Yang2025_S	ENSGALG00010017183	NFYC	case	control	0.234101608	0.107235074	2.183069395	0.0290307	0.089288865	up_in_case
Yang2025_S	ENSGALG00010017184	CARD11	case	control	0.204312392	0.205966159	0.991970685	0.321211826	0.490476309	up_in_case
Yang2025_S	ENSGALG00010017185	NAA30	case	control	-0.471377573	0.114767835	-4.107227197	4.00437E-05	0.000514294	down_in_case
Yang2025_S	ENSGALG00010017187	ZNF839	case	control	0.548572932	0.13130153	4.177962986	2.94132E-05	0.000400657	up_in_case
Yang2025_S	ENSGALG00010017188	ENSGALG00010017188	case	control	0.273118798	0.127166699	2.147722631	0.031735796	0.095577766	up_in_case
Yang2025_S	ENSGALG00010017190	STK26	case	control	-0.312973893	0.061112243	-5.121296075	3.03443E-07	8.64719E-06	down_in_case
Yang2025_S	ENSGALG00010017198	TOPBP1	case	control	-0.282090516	0.084763105	-3.327987043	0.000874759	0.006289872	down_in_case
Yang2025_S	ENSGALG00010017200	KCNQ4	case	control	-0.164939439	0.244534142	-0.674504747	0.499990469	0.65674118	down_in_case
Yang2025_S	ENSGALG00010017201	AP5M1	case	control	-0.650348806	0.211265835	-3.078343481	0.002081548	0.012322165	down_in_case
Yang2025_S	ENSGALG00010017207	ENSGALG00010017207	case	control	0.368256309	0.222533488	1.654835473	0.097957876	0.215851203	up_in_case
Yang2025_S	ENSGALG00010017208	CINP	case	control	-0.095740119	0.175071606	-0.546862629	0.584473112	0.726485846	down_in_case
Yang2025_S	ENSGALG00010017211	UBFD1	case	control	0.311768042	0.112839486	2.762933909	0.005728436	0.026520187	up_in_case
Yang2025_S	ENSGALG00010017212	TINAGL1	case	control	0.287415186	0.133967646	2.145407447	0.031920284	0.095981439	up_in_case
Yang2025_S	ENSGALG00010017219	EARS2	case	control	0.123963262	0.124127253	0.998678846	0.317950292	0.487365231	up_in_case
Yang2025_S	ENSGALG00010017221	SPPL2A	case	control	-0.394228701	0.084214788	-4.681228892	2.8516E-06	5.81802E-05	down_in_case
Yang2025_S	ENSGALG00010017223	NOC2L	case	control	0.263552047	0.167477802	1.57365361	0.115567552	0.242974327	up_in_case
Yang2025_S	ENSGALG00010017226	PEF1	case	control	0.147388179	0.193821897	0.760431	0.446996999	0.609660682	up_in_case
Yang2025_S	ENSGALG00010017228	RAP2C	case	control	-0.372596459	0.098479466	-3.783493913	0.000154642	0.001514369	down_in_case
Yang2025_S	ENSGALG00010017240	MELTF	case	control	0.774613337	0.190607839	4.063911225	4.82572E-05	0.000592479	up_in_case
Yang2025_S	ENSGALG00010017246	GGA2	case	control	0.384703211	0.12073398	3.186370648	0.001440699	0.009316074	up_in_case
Yang2025_S	ENSGALG00010017248	TECPR2	case	control	-0.357484561	0.08893975	-4.019401465	5.83462E-05	0.000690785	down_in_case
Yang2025_S	ENSGALG00010017250	COL16A1	case	control	0.51473326	0.212221112	2.425457368	0.015289111	0.055781136	up_in_case
Yang2025_S	ENSGALG00010017252	EXOC5	case	control	-0.298259766	0.076418659	-3.902970408	9.50193E-05	0.001024841	down_in_case
Yang2025_S	ENSGALG00010017256	CARHSP1	case	control	-0.356950698	0.152120842	-2.346494368	0.018950953	0.065189219	down_in_case
Yang2025_S	ENSGALG00010017263	COG7	case	control	-0.28320801	0.126114582	-2.245640482	0.024727045	0.079376037	down_in_case
Yang2025_S	ENSGALG00010017264	PIAS1	case	control	0.428567332	0.210741298	2.033618167	0.041990109	0.11723746	up_in_case
Yang2025_S	ENSGALG00010017266	PMM2	case	control	-0.610262266	0.13841541	-4.408918527	1.03888E-05	0.000169716	down_in_case
Yang2025_S	ENSGALG00010017272	NRXN1	case	control	-0.037498564	0.263106001	-0.142522646	0.886667193	0.933580779	down_in_case
Yang2025_S	ENSGALG00010017274	ENSGALG00010017274	case	control	0.713560242	1.030416515	0.692496899	0.488625332	0.645953326	up_in_case
Yang2025_S	ENSGALG00010017278	CCR5	case	control	-0.678684999	0.17659254	-3.843225769	0.000121428	0.001242862	down_in_case
Yang2025_S	ENSGALG00010017280	SOS1	case	control	0.310684143	0.251395343	1.235838895	0.216518454	0.377374404	up_in_case
Yang2025_S	ENSGALG00010017283	INF2	case	control	0.160622032	0.203576172	0.789002126	0.430110764	0.59424881	up_in_case
Yang2025_S	ENSGALG00010017284	ENSGALG00010017284	case	control	-0.411044136	0.233927817	-1.757140908	0.078893795	0.185487604	down_in_case
Yang2025_S	ENSGALG00010017289	ADGRB2	case	control	0.302616443	0.288554457	1.04873252	0.294301242	0.462876728	up_in_case
Yang2025_S	ENSGALG00010017290	DACT1	case	control	-0.108423206	0.096910099	-1.118801927	0.263224649	0.430167174	down_in_case
Yang2025_S	ENSGALG00010017296	SCNN1G	case	control	-0.016050483	0.128704222	-0.124708287	0.900754496	0.940930578	down_in_case
Yang2025_S	ENSGALG00010017299	USP31	case	control	-0.179347436	0.097588941	-1.837784414	0.066094179	0.163070536	down_in_case
Yang2025_S	ENSGALG00010017300	TRPM7	case	control	0.273565532	0.118983071	2.299197099	0.02149375	0.071313191	up_in_case
Yang2025_S	ENSGALG00010017301	LHCGR	case	control	0.208719694	0.385610424	0.541270881	0.588320888	0.729576539	up_in_case
Yang2025_S	ENSGALG00010017304	ANKRD9	case	control	0.042804546	0.304635142	0.14051086	0.888256373	0.934883124	up_in_case
Yang2025_S	ENSGALG00010017305	HS3ST2	case	control	0.512523584	0.365720215	1.401408952	0.161091817	0.30687826	up_in_case
Yang2025_S	ENSGALG00010017307	TMEM186	case	control	0.348384739	0.133379832	2.611974651	0.009002093	0.037637013	up_in_case
Yang2025_S	ENSGALG00010017310	SAMD11	case	control	0.55295746	0.142846362	3.870994351	0.000108392	0.001133607	up_in_case
Yang2025_S	ENSGALG00010017312	PTP4A2	case	control	0.36787723	0.163364866	2.251874822	0.024330184	0.078572679	up_in_case
Yang2025_S	ENSGALG00010017313	MAP4K3	case	control	0.021538191	0.11079563	0.194395674	0.845866077	0.908520409	up_in_case
Yang2025_S	ENSGALG00010017314	CALML4	case	control	0.185165753	0.141543858	1.308186419	0.190810087	0.345801508	up_in_case
Yang2025_S	ENSGALG00010017318	ADSS1	case	control	-0.120151599	0.125447298	-0.95778547	0.338170948	0.50635796	down_in_case
Yang2025_S	ENSGALG00010017322	KHDRBS1	case	control	0.156136346	0.066871	2.334888756	0.019549233	0.066671209	up_in_case
Yang2025_S	ENSGALG00010017323	ENSGALG00010017323	case	control	0.319006314	0.134722883	2.367870303	0.017890806	0.062610244	up_in_case
Yang2025_S	ENSGALG00010017326	STON1	case	control	-0.07111116	0.132845533	-0.535292065	0.592447913	0.732738232	down_in_case
Yang2025_S	ENSGALG00010017327	CLN6	case	control	0.075432765	0.187003085	0.403377113	0.686670821	0.804164762	up_in_case
Yang2025_S	ENSGALG00010017332	FGFR1	case	control	-0.1948104	0.092260039	-2.111536065	0.034726261	0.102112355	down_in_case
Yang2025_S	ENSGALG00010017334	KDM8	case	control	0.013150694	0.221194941	0.059452959	0.952591332	0.970844746	up_in_case
Yang2025_S	ENSGALG00010017335	THUMPD2	case	control	0.016953039	0.115771445	0.146435412	0.883577674	0.931389877	up_in_case
Yang2025_S	ENSGALG00010017337	ABAT	case	control	0.197319126	0.191967292	1.027878886	0.304006803	0.473598076	up_in_case
Yang2025_S	ENSGALG00010017338	MBNL3	case	control	0.374709021	0.205708122	1.82155677	0.068522265	0.167313434	up_in_case
Yang2025_S	ENSGALG00010017340	SIVA1	case	control	-0.20885577	0.369063555	-0.565907327	0.571456781	0.715439066	down_in_case
Yang2025_S	ENSGALG00010017341	TA3	case	control	0.01307272	0.121461719	0.107628316	0.914290534	0.949730794	up_in_case
Yang2025_S	ENSGALG00010017343	DLG1	case	control	0.184643736	0.102342136	1.804180997	0.071202937	0.171990455	up_in_case
Yang2025_S	ENSGALG00010017344	RERE	case	control	0.530839954	0.314611402	1.687287716	0.091548042	0.206327343	up_in_case
Yang2025_S	ENSGALG00010017346	GM2A	case	control	0.341445356	0.230527685	1.481146857	0.138567444	0.276678225	up_in_case
Yang2025_S	ENSGALG00010017347	TMEM39B	case	control	-0.462676302	0.11727094	-3.945361928	7.96795E-05	0.000891835	down_in_case
Yang2025_S	ENSGALG00010017349	ENSGALG00010017349	case	control	-0.176040635	0.108706212	-1.619416508	0.105357677	0.227679965	down_in_case
Yang2025_S	ENSGALG00010017351	CCR2	case	control	-0.743862088	0.179275769	-4.149261746	3.33549E-05	0.000442246	down_in_case
Yang2025_S	ENSGALG00010017352	GPCPD1	case	control	0.184459512	0.105705209	1.745037099	0.080978411	0.188954129	up_in_case
Yang2025_S	ENSGALG00010017354	FEM1B	case	control	-0.397327422	0.080781097	-4.918569291	8.71791E-07	2.15016E-05	down_in_case
Yang2025_S	ENSGALG00010017356	NSMCE1	case	control	0.011953698	0.183976451	0.064974067	0.948194648	0.967921209	up_in_case
Yang2025_S	ENSGALG00010017359	EHD3	case	control	-0.133660651	0.107692911	-1.24112766	0.214558593	0.375049246	down_in_case
Yang2025_S	ENSGALG00010017361	METTL22	case	control	-0.28585763	0.114392298	-2.498923741	0.012457111	0.047689519	down_in_case
Yang2025_S	ENSGALG00010017362	ENO1	case	control	-0.601990244	0.217204449	-2.77153735	0.005579227	0.02599349	down_in_case
Yang2025_S	ENSGALG00010017363	TIMM9	case	control	0.008185666	0.123877143	0.066078908	0.947315003	0.967543554	up_in_case
Yang2025_S	ENSGALG00010017364	AKT1	case	control	-0.011840664	0.126686935	-0.093463968	0.925534973	0.955297892	down_in_case
Yang2025_S	ENSGALG00010017365	CD8BP	case	control	0.574881668	0.786189114	0.731225678	0.464641318	0.625582297	up_in_case
Yang2025_S	ENSGALG00010017367	TIMD4	case	control	-0.401853801	0.25423994	-1.580608464	0.113967589	0.240699963	down_in_case
Yang2025_S	ENSGALG00010017368	ERLIN2	case	control	-0.275264984	0.069625783	-3.953492106	7.70188E-05	0.00086525	down_in_case
Yang2025_S	ENSGALG00010017372	ENSGALG00010017372	case	control	-0.561372938	0.092110372	-6.094568103	1.09733E-09	6.61963E-08	down_in_case
Yang2025_S	ENSGALG00010017373	RP1-27O5.3	case	control	0.407289674	0.105069331	3.87638972	0.000106018	0.001116673	up_in_case
Yang2025_S	ENSGALG00010017375	BDH1A	case	control	-0.143950355	0.180304652	-0.798372937	0.424654103	0.589574069	down_in_case
Yang2025_S	ENSGALG00010017376	GALNT14	case	control	0.005177039	0.356353062	0.014527838	0.98840887	0.992897751	up_in_case
Yang2025_S	ENSGALG00010017377	USP8	case	control	0.475142433	0.1699829	2.795236661	0.005186173	0.024631672	up_in_case
Yang2025_S	ENSGALG00010017378	ENSGALG00010017378	case	control	0.490996295	0.511737917	0.959468272	0.337322896	0.50545355	up_in_case
Yang2025_S	ENSGALG00010017380	MARCKSL1	case	control	-0.103440677	0.174726241	-0.592015696	0.553840078	0.70174227	down_in_case
Yang2025_S	ENSGALG00010017381	CA6	case	control	0.639679532	0.31208115	2.049721783	0.040391588	0.113961688	up_in_case
Yang2025_S	ENSGALG00010017383	ENSGALG00010017383	case	control	0.02673958	0.128583313	0.207955287	0.83526388	0.902514414	up_in_case
Yang2025_S	ENSGALG00010017384	ARID4A	case	control	0.53060297	0.175572536	3.022129671	0.00251003	0.014237733	up_in_case
Yang2025_S	ENSGALG00010017386	ENSGALG00010017386	case	control	0.335126304	0.911633662	0.367610717	0.713163523	0.822771185	up_in_case
Yang2025_S	ENSGALG00010017387	HS6ST2	case	control	-0.215863789	0.093709309	-2.303546908	0.021248091	0.070852393	down_in_case
Yang2025_S	ENSGALG00010017388	PLPBP	case	control	-0.085120096	0.169797827	-0.501302623	0.616158159	0.752314374	down_in_case
Yang2025_S	ENSGALG00010017390	DDX24	case	control	0.200926329	0.107560599	1.868029097	0.061758015	0.155623498	up_in_case
Yang2025_S	ENSGALG00010017391	CRAMP1	case	control	-0.045821272	0.120691633	-0.379655743	0.704200976	0.817548997	down_in_case
Yang2025_S	ENSGALG00010017393	VDAC1	case	control	-0.712876542	0.117299221	-6.07741922	1.22132E-09	7.27313E-08	down_in_case
Yang2025_S	ENSGALG00010017395	BRF2	case	control	-0.569775173	0.192531412	-2.959388108	0.003082506	0.016653006	down_in_case
Yang2025_S	ENSGALG00010017396	CCR8L	case	control	0.327913647	0.245836456	1.33386908	0.182246767	0.335129149	up_in_case
Yang2025_S	ENSGALG00010017398	SLC22A5	case	control	-0.442951402	0.111200551	-3.983356167	6.79488E-05	0.000781176	down_in_case
Yang2025_S	ENSGALG00010017399	CPXM1	case	control	-0.110218218	0.181056029	-0.608751989	0.542688842	0.692728656	down_in_case
Yang2025_S	ENSGALG00010017400	GPC4	case	control	0.070755694	0.084540061	0.836948697	0.402621406	0.569134641	up_in_case
Yang2025_S	ENSGALG00010017402	SLC2A5	case	control	-0.124377617	0.254345429	-0.489010625	0.624834175	0.75873804	down_in_case
Yang2025_S	ENSGALG00010017403	ITGA11	case	control	0.574329625	0.240919245	2.383909282	0.017129825	0.060538703	up_in_case
Yang2025_S	ENSGALG00010017406	IFT140	case	control	0.173214086	0.140069274	1.236631562	0.216223897	0.377153586	up_in_case
Yang2025_S	ENSGALG00010017407	EPN2	case	control	0.052594972	0.114847034	0.457956729	0.646983529	0.775812364	up_in_case
Yang2025_S	ENSGALG00010017409	GPC3	case	control	0.265276048	0.471706513	0.562375207	0.573860408	0.716746866	up_in_case
Yang2025_S	ENSGALG00010017411	RAB11FIP1	case	control	-0.345179328	0.130267828	-2.649766515	0.008054741	0.034414467	down_in_case
Yang2025_S	ENSGALG00010017414	ENSGALG00010017414	case	control	0.030993177	0.243946329	0.127049165	0.898901486	0.940010804	up_in_case
Yang2025_S	ENSGALG00010017415	HDAC1	case	control	-0.003958215	0.08920107	-0.044374074	0.964606227	0.978937279	down_in_case

Yang2025_S	ENSGALG00010017418	CHGB	case	control	1.452670257	0.339480427	4.279098716	1.87652E-05	0.000275545	up_in_case
Yang2025_S	ENSGALG00010017419	B9D1	case	control	-0.145494508	0.109763809	-1.325523497	0.184997581	0.338579104	down_in_case
Yang2025_S	ENSGALG00010017421	GPR157	case	control	-0.568949488	0.302791332	-1.879015108	0.06024243	0.152938289	down_in_case
Yang2025_S	ENSGALG00010017422	ENSGALG00010017422	case	control	0.115166891	0.301514697	0.38196112	0.702490204	0.81634023	up_in_case
Yang2025_S	ENSGALG00010017423	TMEM204	case	control	-0.187940961	0.061400074	-3.060924006	0.002206551	0.012894754	down_in_case
Yang2025_S	ENSGALG00010017424	APOD	case	control	0.12733313	0.495469916	0.256994674	0.797182884	0.877249614	up_in_case
Yang2025_S	ENSGALG00010017425	H6PD	case	control	-0.157059655	0.114025585	-1.377407136	0.168386408	0.316743255	down_in_case
Yang2025_S	ENSGALG00010017428	PSMA3	case	control	-0.071927601	0.193711837	-0.371312368	0.710404887	0.821193869	down_in_case
Yang2025_S	ENSGALG00010017429	TELO2	case	control	-0.245597368	0.156038208	-1.573956599	0.115497484	0.242900307	down_in_case
Yang2025_S	ENSGALG00010017430	CACNA1H	case	control	0.203573047	0.202784209	1.003890036	0.31543162	0.484623994	up_in_case
Yang2025_S	ENSGALG00010017431	FYCO1	case	control	0.3255093	0.118779736	2.740444724	0.00613561	0.027913721	up_in_case
Yang2025_S	ENSGALG00010017433	RNPS1	case	control	-0.476990864	0.123087334	-3.87522296	0.000106527	0.001120344	down_in_case
Yang2025_S	ENSGALG00010017434	FGF13	case	control	-0.420171651	0.103165624	-4.072787391	4.64538E-05	0.000574507	down_in_case
Yang2025_S	ENSGALG00010017435	ACTR10	case	control	-0.352236007	0.198111412	-1.777969293	0.075408898	0.179566473	down_in_case
Yang2025_S	ENSGALG00010017437	DRG2	case	control	-0.085854529	0.102624559	-0.836588529	0.402823896	0.569247641	down_in_case
Yang2025_S	ENSGALG00010017439	PPP4R4	case	control	0.469450726	0.377081187	1.244959289	0.213146715	0.373430215	up_in_case
Yang2025_S	ENSGALG00010017441	SPSB1	case	control	-0.216301639	0.145301815	-1.488636865	0.136583019	0.273696342	down_in_case
Yang2025_S	ENSGALG00010017442	LCK	case	control	0.791143904	0.246739379	3.206394974	0.001344094	0.008826552	up_in_case
Yang2025_S	ENSGALG00010017447	CAPN13	case	control	-0.590658377	0.58729615	-1.005724925	0.31454791	0.483757728	down_in_case
Yang2025_S	ENSGALG00010017449	TRMT6	case	control	0.418548581	0.149721042	2.795522772	0.005181585	0.024618271	up_in_case
Yang2025_S	ENSGALG00010017450	ENSGALG00010017450	case	control	-0.25280454	0.17546033	-1.440807391	0.149639105	0.292088658	down_in_case
Yang2025_S	ENSGALG00010017451	GABPB1	case	control	0.098346936	0.096803594	1.015943027	0.309656518	0.479558077	up_in_case
Yang2025_S	ENSGALG00010017452	ARMH4	case	control	-1.15113273	0.632394802	-1.820275445	0.068717069	0.167642219	down_in_case
Yang2025_S	ENSGALG00010017453	PPP1R2	case	control	1.261885	0.19612066	6.434227785	1.24103E-10	1.00545E-08	up_in_case
Yang2025_S	ENSGALG00010017454	ENSGALG00010017454	case	control	0.265664642	0.184593531	1.43918717	0.150097499	0.292614529	up_in_case
Yang2025_S	ENSGALG00010017455	CCR9	case	control	0.200303029	0.153073858	1.30853845	0.190690742	0.345729311	up_in_case
Yang2025_S	ENSGALG00010017457	ZMAT4	case	control	-0.030333834	0.236838469	-0.128078156	0.898087123	0.939625215	down_in_case
Yang2025_S	ENSGALG00010017458	ENSGALG00010017458	case	control	0.190826395	0.097444384	1.958310858	0.050193547	0.133558733	up_in_case
Yang2025_S	ENSGALG00010017459	FAM167B	case	control	1.01014743	0.255200608	3.958248518	7.55014E-05	0.000850535	up_in_case
Yang2025_S	ENSGALG00010017462	MCM8	case	control	0.330624853	0.150684667	2.194150599	0.028224585	0.08744897	up_in_case
Yang2025_S	ENSGALG00010017463	PPP1R36	case	control	-0.50561169	0.241595445	-2.092803074	0.036366737	0.105622779	down_in_case
Yang2025_S	ENSGALG00010017464	SH3BP2	case	control	-0.015968991	0.176743603	-0.09035117	0.928008158	0.956493616	down_in_case
Yang2025_S	ENSGALG00010017465	TMEM234	case	control	0.106939355	0.16606584	0.643957567	0.51960295	0.673525808	up_in_case
Yang2025_S	ENSGALG00010017467	SFRP1	case	control	-0.196074262	0.124215831	-1.578496567	0.114451576	0.24137236	down_in_case
Yang2025_S	ENSGALG00010017468	AMDHD2	case	control	-0.347567508	0.202259646	-1.718422403	0.085719597	0.19684952	down_in_case
Yang2025_S	ENSGALG00010017470	HSPA2	case	control	0.024712111	0.114280549	0.216240743	0.828800096	0.897524675	up_in_case
Yang2025_S	ENSGALG00010017471	CRLS1	case	control	-0.023417019	0.148758116	-0.157416748	0.874916415	0.926528291	down_in_case
Yang2025_S	ENSGALG00010017472	LZTFL1	case	control	-0.407466354	0.121744163	-3.346906684	0.000817187	0.005940296	down_in_case
Yang2025_S	ENSGALG00010017473	EIF3I	case	control	0.032056452	0.164046436	0.195410839	0.845071332	0.907946724	up_in_case
Yang2025_S	ENSGALG00010017475	STUM	case	control	-0.010636134	0.112512315	-0.094533067	0.924685717	0.954905548	down_in_case
Yang2025_S	ENSGALG00010017476	ATP6V0C	case	control	-0.721703946	0.186004711	-3.880030462	0.000104443	0.001103426	down_in_case
Yang2025_S	ENSGALG00010017477	MCF2	case	control	0.408681593	0.148840642	2.74576613	0.006036978	0.027545937	up_in_case
Yang2025_S	ENSGALG00010017479	SSTR5	case	control	-0.552687683	0.420485085	-1.314404964	0.188709961	0.343433891	down_in_case
Yang2025_S	ENSGALG00010017480	DCDC2B	case	control	0.628753203	0.213800763	2.940837041	0.003273267	0.01740289	up_in_case
Yang2025_S	ENSGALG00010017482	CORO2B	case	control	-0.415816209	0.141841878	-2.931547538	0.003372777	0.017789422	down_in_case
Yang2025_S	ENSGALG00010017484	IQCC	case	control	0.189668036	0.17942014	1.057116749	0.290458308	0.459060511	up_in_case
Yang2025_S	ENSGALG00010017485	LBH	case	control	-0.417699737	0.105110864	-3.973896903	7.07062E-05	0.000807615	down_in_case
Yang2025_S	ENSGALG00010017487	C1QTNF8	case	control	0.738844379	0.44719725	1.65216664	0.098500585	0.216807084	up_in_case
Yang2025_S	ENSGALG00010017489	TNFRSF1B	case	control	-0.256448607	0.180658563	-1.419520901	0.155747208	0.300541039	down_in_case
Yang2025_S	ENSGALG00010017491	CCDC28B	case	control	1.015600406	0.395594268	2.567277859	0.010250044	0.041281608	up_in_case
Yang2025_S	ENSGALG00010017493	YPEL5	case	control	-0.725736133	0.097714922	-7.427075794	1.11025E-13	1.77831E-11	down_in_case
Yang2025_S	ENSGALG00010017494	TBC1D24	case	control	-0.248692502	0.10567662	-2.353335118	0.018605857	0.064273971	down_in_case
Yang2025_S	ENSGALG00010017495	NT5M	case	control	-0.327181972	0.111983069	-2.921709294	0.003481163	0.018188979	down_in_case
Yang2025_S	ENSGALG00010017496	PARP1	case	control	0.119051394	0.088596638	1.343746177	0.17903048	0.330719549	up_in_case
Yang2025_S	ENSGALG00010017499	SNRPD1	case	control	-0.23765298	0.222368397	-1.068735409	0.285188911	0.453458179	down_in_case
Yang2025_S	ENSGALG00010017500	ACAP2	case	control	0.030253343	0.094601763	0.319796826	0.749122354	0.847674443	up_in_case
Yang2025_S	ENSGALG00010017502	NAT8B	case	control	0.121075757	0.350596422	0.345342249	0.729837088	0.83410808	up_in_case
Yang2025_S	ENSGALG00010017503	MED9	case	control	-0.315883734	0.194302586	-1.625730984	0.104006896	0.225612717	down_in_case
Yang2025_S	ENSGALG00010017505	TNFRSF8	case	control	-0.045655952	0.368152742	-0.124013615	0.901304495	0.941137577	down_in_case
Yang2025_S	ENSGALG00010017509	RASD1	case	control	0.69057492	0.354016795	1.950684065	0.05109464	0.135284782	up_in_case
Yang2025_S	ENSGALG00010017510	HDC	case	control	-1.801650679	0.432971689	-4.161128139	3.16679E-05	0.000426076	down_in_case
Yang2025_S	ENSGALG00010017513	TNIP2	case	control	0.147686299	0.184479026	0.800558755	0.423387136	0.588714652	up_in_case
Yang2025_S	ENSGALG00010017514	TXLNA	case	control	0.137617541	0.073503439	1.872259908	0.061170655	0.154534639	up_in_case
Yang2025_S	ENSGALG00010017516	AAK1	case	control	0.261731672	0.1589639	1.646484963	0.099663953	0.218367483	up_in_case
Yang2025_S	ENSGALG00010017517	LIN9	case	control	-0.1345006	0.122871287	-1.094646301	0.273671623	0.4409822	down_in_case
Yang2025_S	ENSGALG00010017518	PEMT	case	control	0.107322618	0.092661269	1.158225213	0.246772142	0.412520369	up_in_case
Yang2025_S	ENSGALG00010017519	NTN3	case	control	0.093481761	0.14055007	0.665113582	0.505977848	0.66157179	up_in_case
Yang2025_S	ENSGALG00010017520	ZBTB1	case	control	-0.089660239	0.061635209	-1.454691899	0.145754576	0.287161038	down_in_case
Yang2025_S	ENSGALG00010017521	MIIP	case	control	0.094790877	0.127418456	0.743933647	0.456916618	0.618522331	up_in_case
Yang2025_S	ENSGALG00010017522	XXYLT1	case	control	-0.237956938	0.186724648	-1.274373472	0.202531089	0.360448412	down_in_case
Yang2025_S	ENSGALG00010017524	ACBD3	case	control	0.112701171	0.115525035	0.975556257	0.32928441	0.498812725	up_in_case
Yang2025_S	ENSGALG00010017526	PLEKHA2	case	control	0.023338983	0.148653834	0.157002226	0.875243094	0.926623301	up_in_case
Yang2025_S	ENSGALG00010017528	RAI1	case	control	0.055776408	0.104360226	0.534460396	0.593023043	0.733319381	up_in_case
Yang2025_S	ENSGALG00010017529	MFN2	case	control	-0.058375796	0.076406705	-0.764014058	0.444858865	0.608113428	down_in_case
Yang2025_S	ENSGALG00010017530	TLR1B	case	control	-0.101242317	0.151137999	-0.669866728	0.502942751	0.659189921	down_in_case
Yang2025_S	ENSGALG00010017531	SACM1L	case	control	-0.041868046	0.076176282	-0.549620501	0.582579695	0.724973035	down_in_case
Yang2025_S	ENSGALG00010017532	MIXL1	case	control	-0.325741858	0.416631122	-0.781847158	0.434304403	0.598263331	down_in_case
Yang2025_S	ENSGALG00010017533	FAM193A	case	control	0.284211031	0.175312373	1.621169269	0.104981342	0.227022366	up_in_case
Yang2025_S	ENSGALG00010017534	ATP11C	case	control	-0.218013097	0.100361139	-2.172285999	0.029834097	0.091150657	down_in_case
Yang2025_S	ENSGALG00010017535	LIMD1	case	control	-0.500679111	0.187921115	-2.664304708	0.007714765	0.033293665	down_in_case
Yang2025_S	ENSGALG00010017536	GSC	case	control	0.300556684	0.62985114	0.477186854	0.633229071	0.764715063	up_in_case
Yang2025_S	ENSGALG00010017537	MANBA	case	control	-0.173624169	0.169190062	-1.026207842	0.304793626	0.474452544	down_in_case
Yang2025_S	ENSGALG00010017538	H3-3A	case	control	-0.035039939	0.093522568	-0.374668273	0.707907191	0.819872311	down_in_case
Yang2025_S	ENSGALG00010017539	KPNA6	case	control	-0.172698047	0.102805586	-1.679850814	0.092986346	0.208355043	down_in_case
Yang2025_S	ENSGALG00010017540	EIF4G1	case	control	0.207890045	0.106091483	1.959535665	0.050050087	0.133304275	up_in_case
Yang2025_S	ENSGALG00010017541	SREBF1	case	control	0.393529524	0.110936159	3.547351277	0.000389125	0.003229578	up_in_case
Yang2025_S	ENSGALG00010017542	PLOD1	case	control	-0.322536071	0.088235007	-3.65542068	0.000256761	0.002292094	down_in_case
Yang2025_S	ENSGALG00010017543	ZBTB25	case	control	0.193875207	0.234332107	0.8273523	0.408037408	0.573879823	up_in_case
Yang2025_S	ENSGALG00010017544	SDE2	case	control	-0.023659054	0.062524546	-0.378396263	0.705136244	0.817817192	down_in_case
Yang2025_S	ENSGALG00010017545	DICER1	case	control	0.183946706	0.171650149	1.071637323	0.283882963	0.452051587	up_in_case
Yang2025_S	ENSGALG00010017546	NFU1	case	control	0.031561638	0.21825252	0.144610648	0.88501829	0.932602077	up_in_case
Yang2025_S	ENSGALG00010017547	RNF4	case	control	-0.215042758	0.05457985	-3.939966099	8.14931E-05	0.000904866	down_in_case
Yang2025_S	ENSGALG00010017550	TM2D2	case	control	-0.141876414	0.171701564	-0.826296573	0.408635877	0.574475118	down_in_case
Yang2025_S	ENSGALG00010017551	NALF2	case	control	0.368277947	0.204786084	1.798354363	0.07212087	0.17351594	up_in_case
Yang2025_S	ENSGALG00010017552	LARS2	case	control	-0.136495121	0.099003865	-1.37868478	0.167991964	0.316176124	down_in_case
Yang2025_S	ENSGALG00010017553	EDA	case	control	-0.439894162	0.163265766	-2.694344156	0.00705273	0.031143073	down_in_case
Yang2025_S	ENSGALG00010017554	FAM131A	case	control	0.474122981	0.344146412	1.377678117	0.168302691	0.316632644	up_in_case
Yang2025_S	ENSGALG00010017556	TOM1L2	case	control	-0.058008193	0.116089634	-0.499684514	0.617297239	0.753177219	down_in_case
Yang2025_S	ENSGALG00010017558	ENSGALG00010017558	case	control	0.206829249	0.087748279	2.35707471	0.018419541	0.063754669	up_in_case
Yang2025_S	ENSGALG00010017559	DUSP10	case	control	-0.0499343	0.143231176	-0.348627313	0.72736912	0.832465302	down_in_case
Yang2025_S	ENSGALG00010017561	CFAP99	case	control	1.228631684	0.497488724	2.469667402	0.013523873	0.050632769	up_in_case
Yang2025_S	ENSGALG00010017562	SLC30A10	case	control	-0.598166808	0.480084532	-1.245961426	0.212778557	0.373152428	down_in_case
Yang2025_S	ENSGALG00010017563	ALK	case	control	0.334483081	0.12486415	2.678775955	0.007389182	0.03221785	up_in_case
Yang2025_S	ENSGALG00010017565	ENSGALG00010017565	case	control	-0.128621035	0.094372792	-1.362903785	0.172912836	0.322691893	down_in_case
Yang2025_S	ENSGALG00010017568	CDCP1	case	control	0.607979795	0.235758737	2.578821909	0.009913788	0.040279066	up_in_case
Yang2025_S	ENSGALG00010017569	CLMN	case	control	0.581490562	0.195234364	2.978423219	0.002897356	0.015876782	up_in_case
Yang2025_S	ENSGALG00010017571	ADAM9	case	control	-0.048422441	0.078826506	-0.614291357	0.539022813	0.690248383	down_in_case
Yang2025_S	ENSGALG00010017573	ENSGALG00010017573	case	control	0.899287374	0.452065315	1.98928638	0.046669602	0.126895787	up_in_case
Yang2025_S	ENSGALG00010017574	DYNC1LI1	case	control	-0.034971437	0.078970489	-0.442841851	0.65788013	0.784222379	down_in_case
Yang2025_S	ENSGALG00010017575	CLCN2	case	control	0.660003072	0.516193705	1.27859574	0.201039451	0.358888643	up_in_case
Yang2025_S	ENSGALG00010017576	ENSGALG00010017576	case	control	-0.191718309	0.242278526	-0.791313671	0.428760973	0.593030686	down_in_case
Yang2025_S	ENSGALG00010017577	CLPX	case	control	-0.143285693	0.101389075	-1.413226154	0.157589239	0.302855612	down_in_case
Yang2025_S	ENSGALG00010017578	BSDC1	case	control	-0.325616696	0.162305641	-2.006194569	0.044835493	0.123469567	down_in_case
Yang2025_S	ENSGALG00010017579	P3H2	case	control	0.002946629	0.080271333	0.036708362	0.970717541	0.982321994	up_in_case
Yang2025_S	ENSGALG00010017580	LYPLAL1	case	control	-0.369403413	0.11757327	-3.141899627	0.001678556	0.010489091	down_in_case
Yang2025_S	ENSGALG00010017581	EFCAB11	case	control	-0.297463247	0.448845874	-0.662729155	0.507504034	0.663109886	down_in_case
Yang2025_S	ENSGALG00010017582	CLCN6	case	control	-0.067393351	0.080964989	-0.832376454	0.405196488	0.571557148	down_in_case
Yang2025_S	ENSGALG00010017583	MTHFD1	case	control	-0.024073074	0.108969178	-0.220916355	0.825157563	0.895248415	down_in_case
Yang2025_S	ENSGALG00010017585	ENSGALG00010017585	case	control	0.209622432	0.207058212	1.012384057	0.311354458	0.481091883	up_in_case
Yang2025_S	ENSGALG00010017586	ZFYVE28	case	control	0.414132046	0.207152598	1.999164147	0.045590596	0.124996358	up_in_case
Yang2025_S	ENSGALG00010017587	MB21D2	case	control	-0.045022174	0.141786138	-0.317535793	0.750837085	0.848540188	down_in_case
Yang2025_S	ENSGALG00010017589	ATPAF2	case	control	0.280600519	0.136909933	2.049526382	0.040410671	0.113992449	up_in_case
Yang2025_S	ENSGALG00010017590	ENSGALG00010017590	case	control	0.352007553	0.214551588	1.640666271	0.10086671	0.220440728	up_in_case
Yang2025_S	ENSGALG00010017591	ESR2	case	control	-0.374522407	0.380381099	-0.984597836	0.324821653	0.494471241	down_in_case
Yang2025_S	ENSGALG00010017592	GID4	case	control	0.020148062	0.176508134	0.114148063	0.909120423	0.945629103	up_in_case
Yang2025_S	ENSGALG00010017593	MTHFR	case	control	-0.396828195	0.117480195	-3.377830582	0.000730601	0.005444499	down_in_case

Yang2025_S	ENSGALG00010017594	CEP68	case	control	-0.137625936	0.1630549	-0.844046613	0.398643359	0.565576787	down_in_case
Yang2025_S	ENSGALG00010017595	CCNF	case	control	-0.278092604	0.155584695	-1.787403341	0.073872322	0.176844323	down_in_case
Yang2025_S	ENSGALG00010017596	TDP1	case	control	-0.145993898	0.132735874	-1.099882753	0.27138321	0.438664312	down_in_case
Yang2025_S	ENSGALG00010017597	CNOT10	case	control	0.146378009	0.082181203	1.781161663	0.074886045	0.178626674	up_in_case
Yang2025_S	ENSGALG00010017598	ENSGALG00010017598	case	control	0.771617208	0.158610206	4.86486481	1.14535E-06	2.71899E-05	up_in_case
Yang2025_S	ENSGALG00010017599	KCNK13	case	control	-0.208045084	0.226268224	-0.919462222	0.357853857	0.527023197	down_in_case
Yang2025_S	ENSGALG00010017600	MXD4	case	control	-0.15167687	0.076153641	-1.991721851	0.046401585	0.126438421	down_in_case
Yang2025_S	ENSGALG00010017601	ENSGALG00010017601	case	control	0.581719294	0.193247012	3.010236936	0.00261044	0.014652988	up_in_case
Yang2025_S	ENSGALG00010017604	GATM	case	control	-0.040509164	0.205188308	-0.197424328	0.843495493	0.9071629	down_in_case
Yang2025_S	ENSGALG00010017605	GFPT1	case	control	0.120916504	0.085346352	1.416774121	0.156548978	0.301605145	up_in_case
Yang2025_S	ENSGALG00010017606	ENSGALG00010017606	case	control	-0.007984098	0.301751183	-0.026459212	0.978891066	0.987322453	down_in_case
Yang2025_S	ENSGALG00010017608	RBBP4	case	control	-0.386375068	0.090809466	-4.254788461	2.09247E-05	0.000301848	down_in_case
Yang2025_S	ENSGALG00010017609	TRIM71	case	control	0.008394172	0.229566606	0.036565301	0.970831611	0.982321994	up_in_case
Yang2025_S	ENSGALG00010017610	PSMC1	case	control	0.0105316	0.122738017	0.085805526	0.931621013	0.958515861	up_in_case
Yang2025_S	ENSGALG00010017612	CLDN11	case	control	0.190978322	0.478561488	0.399067469	0.689843492	0.806389485	up_in_case
Yang2025_S	ENSGALG00010017613	HAUS3	case	control	-0.675279243	0.132698107	-5.088838562	3.60263E-07	1.00205E-05	down_in_case
Yang2025_S	ENSGALG00010017615	AGTRAP	case	control	-0.193694583	0.110657367	-1.750399345	0.080049429	0.187445604	down_in_case
Yang2025_S	ENSGALG00010017616	PDCD7	case	control	0.150397109	0.124681313	1.206252206	0.22772026	0.389885959	up_in_case
Yang2025_S	ENSGALG00010017619	POLN	case	control	0.165670873	0.456563877	0.362864609	0.716706025	0.825601262	up_in_case
Yang2025_S	ENSGALG00010017620	UBAP1L	case	control	-0.308011337	0.389231541	-0.791331904	0.428750336	0.593030686	down_in_case
Yang2025_S	ENSGALG00010017621	SYNE2	case	control	0.435478303	0.172074814	2.530749815	0.0113819	0.044577509	up_in_case
Yang2025_S	ENSGALG00010017622	RAB1B	case	control	-0.258901009	0.064867587	-3.99122305	6.57334E-05	0.000762694	down_in_case
Yang2025_S	ENSGALG00010017625	YBX1	case	control	0.170965422	0.126233515	1.354358396	0.175622064	0.326175325	up_in_case
Yang2025_S	ENSGALG00010017626	COPS7B	case	control	-0.058069158	0.090298683	-0.643078678	0.520173049	0.673937343	down_in_case
Yang2025_S	ENSGALG00010017628	NRDE2	case	control	0.093072796	0.1014261	0.917641477	0.358806596	0.527673053	up_in_case
Yang2025_S	ENSGALG00010017629	ENSGALG00010017629	case	control	0.283971028	0.114832276	2.472919957	0.013401415	0.050323019	up_in_case
Yang2025_S	ENSGALG00010017631	NAT8L	case	control	0.316429481	0.251551039	1.257913635	0.208422992	0.367316858	up_in_case
Yang2025_S	ENSGALG00010017632	AWAT1	case	control	0.062052622	0.318713694	0.194697069	0.845630107	0.908336972	up_in_case
Yang2025_S	ENSGALG00010017633	HLX	case	control	0.055179595	0.144258733	0.382504366	0.702087291	0.816183066	up_in_case
Yang2025_S	ENSGALG00010017634	PPIH	case	control	0.031049277	0.233688849	0.132865891	0.894299442	0.937885741	up_in_case
Yang2025_S	ENSGALG00010017636	ZBTB8OS	case	control	0.647847414	0.14490497	4.470843297	7.79118E-06	0.000133707	up_in_case
Yang2025_S	ENSGALG00010017638	GFRA2	case	control	0.252900894	0.193128039	1.309498587	0.190365516	0.345212552	up_in_case
Yang2025_S	ENSGALG00010017639	ENSGALG00010017639	case	control	0.858423333	0.153161362	5.604698988	2.08617E-08	8.85868E-07	up_in_case
Yang2025_S	ENSGALG00010017640	P2RY4	case	control	0.116595272	0.516771766	0.225622372	0.821495139	0.893392686	up_in_case
Yang2025_S	ENSGALG00010017641	GPRC5B	case	control	-0.403980612	0.121644953	-3.320981285	0.000897016	0.006406925	down_in_case
Yang2025_S	ENSGALG00010017642	MTARC1	case	control	-0.144384566	0.128278409	-1.125556257	0.260353392	0.426776205	down_in_case
Yang2025_S	ENSGALG00010017643	CALM1	case	control	-0.30055394	0.071361206	-4.21172733	2.53425E-05	0.000354566	down_in_case
Yang2025_S	ENSGALG00010017644	RBBP6	case	control	0.591390655	0.186094262	3.177909133	0.001483412	0.009539156	up_in_case
Yang2025_S	ENSGALG00010017645	SYNC	case	control	0.631872748	0.155323814	4.068099603	4.73981E-05	0.000582445	up_in_case
Yang2025_S	ENSGALG00010017646	MAEA	case	control	-0.274656338	0.08627564	-3.183474949	0.001455187	0.009401035	down_in_case
Yang2025_S	ENSGALG00010017647	ITGA9	case	control	-0.312295299	0.138653787	-2.252338757	0.024300873	0.07850281	down_in_case
Yang2025_S	ENSGALG00010017648	ENSGALG00010017648	case	control	1.062654574	0.189366647	5.611624808	2.00436E-08	8.59406E-07	up_in_case
Yang2025_S	ENSGALG00010017649	ENSGALG00010017649	case	control	-0.132672859	0.138923176	-0.955008822	0.339573236	0.507560552	down_in_case
Yang2025_S	ENSGALG00010017651	PPCS	case	control	-0.02235382	0.224874935	-0.099405566	0.920816264	0.953182308	down_in_case
Yang2025_S	ENSGALG00010017652	C1orf115	case	control	-0.477165483	0.18832614	-2.533718807	0.011285926	0.044276287	down_in_case
Yang2025_S	ENSGALG00010017653	ENSGALG00010017653	case	control	-0.56886502	0.286155884	-1.987954996	0.046816667	0.12723489	down_in_case
Yang2025_S	ENSGALG00010017654	KBTBD13	case	control	0.343224904	0.202237667	1.697136389	0.089670878	0.203337778	up_in_case
Yang2025_S	ENSGALG00010017655	ENSGALG00010017655	case	control	0.469045573	0.150340096	3.119896719	0.001809145	0.011101026	up_in_case
Yang2025_S	ENSGALG00010017656	FSD2	case	control	0.298557239	0.15535801	1.921737019	0.054638857	0.142350043	up_in_case
Yang2025_S	ENSGALG00010017657	DIS3L2	case	control	0.055666289	0.095283535	0.584217294	0.559074128	0.705950663	up_in_case
Yang2025_S	ENSGALG00010017658	WHAMM	case	control	0.660118993	0.166146116	3.973123241	7.09363E-05	0.00080958	up_in_case
Yang2025_S	ENSGALG00010017659	TNRC6A	case	control	0.471818238	0.185580283	2.542394211	0.011009592	0.043476488	up_in_case
Yang2025_S	ENSGALG00010017660	CLIP4	case	control	0.353156427	0.151845479	2.32576188	0.020031257	0.067795302	up_in_case
Yang2025_S	ENSGALG00010017661	NHSL3	case	control	0.364683092	0.222845014	1.636487556	0.101737588	0.221812888	up_in_case
Yang2025_S	ENSGALG00010017662	GOLGA4	case	control	1.250504386	0.270219226	4.627740241	3.69677E-06	7.25557E-05	up_in_case
Yang2025_S	ENSGALG00010017663	ZMYND12	case	control	-0.282601762	0.227698241	-1.241124047	0.214559927	0.375049246	down_in_case
Yang2025_S	ENSGALG00010017664	ENSGALG00010017664	case	control	0.27119872	0.148578072	1.825294379	0.067956618	0.166369549	up_in_case
Yang2025_S	ENSGALG00010017665	CTBP1	case	control	-0.06622135	0.080633291	-0.821265629	0.411494981	0.576937575	down_in_case
Yang2025_S	ENSGALG00010017666	PCARE	case	control	1.837648256	0.636478604	2.887211362	0.00388673	0.019788669	up_in_case
Yang2025_S	ENSGALG00010017667	SLC2A1	case	control	-0.163981066	0.095727161	-1.713004582	0.086711697	0.198379165	down_in_case
Yang2025_S	ENSGALG00010017669	ENSGALG00010017669	case	control	0.379770579	0.204262399	1.859229014	0.062994684	0.15748671	up_in_case
Yang2025_S	ENSGALG00010017670	FAM179A	case	control	0.095491633	0.165084787	0.578439935	0.562967139	0.709231449	up_in_case
Yang2025_S	ENSGALG00010017671	ANTXR1	case	control	0.063218479	0.202058887	0.312871559	0.754378246	0.850506542	up_in_case
Yang2025_S	ENSGALG00010017672	SPRED2	case	control	-0.413744325	0.090851795	-4.554057793	5.26209E-06	9.67377E-05	down_in_case
Yang2025_S	ENSGALG00010017673	WDR89	case	control	-0.122769097	0.113941036	-1.077479204	0.281266236	0.449231015	down_in_case
Yang2025_S	ENSGALG00010017676	SPON2	case	control	-1.040542292	0.19266725	-5.4007222	6.63731E-08	2.39614E-06	down_in_case
Yang2025_S	ENSGALG00010017677	WDR43	case	control	-0.263632971	0.106704224	-2.47068918	0.013485297	0.050569864	down_in_case
Yang2025_S	ENSGALG00010017678	YARS	case	control	0.056313208	0.168126994	0.33494448	0.737666968	0.839956627	up_in_case
Yang2025_S	ENSGALG00010017679	RASL12	case	control	0.201633885	0.199734723	1.009508425	0.312730863	0.482229123	up_in_case
Yang2025_S	ENSGALG00010017684	TRMT61B	case	control	0.424350784	0.169414999	2.504800551	0.012252046	0.047133539	up_in_case
Yang2025_S	ENSGALG00010017685	PHC2	case	control	0.524324439	0.1072653	4.888108615	1.01809E-06	2.45878E-05	up_in_case
Yang2025_S	ENSGALG00010017687	TTC7B	case	control	-0.191246579	0.110724822	-1.72722408	0.084127428	0.194162473	down_in_case
Yang2025_S	ENSGALG00010017688	SPDYA	case	control	0.693145457	0.144491076	4.797150635	1.60939E-06	3.62893E-05	up_in_case
Yang2025_S	ENSGALG00010017691	LZTS1	case	control	0.396176639	0.256842482	1.54248875	0.12295487	0.253871108	up_in_case
Yang2025_S	ENSGALG00010017692	EPCAM	case	control	-1.18094303	0.978579343	-1.206793335	0.227511743	0.389782235	down_in_case
Yang2025_S	ENSGALG00010017693	MTFMT	case	control	0.783809821	0.195672124	4.005730636	6.18261E-05	0.000724961	up_in_case
Yang2025_S	ENSGALG00010017694	ATP6V1B2	case	control	-0.419161031	0.096669197	-4.336035103	1.45076E-05	0.000224128	down_in_case
Yang2025_S	ENSGALG00010017695	FNDC5	case	control	0.12660328	0.152287503	0.831343859	0.405779405	0.571858415	up_in_case
Yang2025_S	ENSGALG00010017696	SLC18A1	case	control	0.515043358	0.217084767	2.372544908	0.017666015	0.062040304	up_in_case
Yang2025_S	ENSGALG00010017698	ENSGALG00010017698	case	control	0.534943388	0.734893162	0.727919942	0.466662595	0.627757821	up_in_case
Yang2025_S	ENSGALG00010017699	RNF19B	case	control	0.673829997	0.186912139	3.605062793	0.000312077	0.002697766	up_in_case
Yang2025_S	ENSGALG00010017700	VPS35L	case	control	-0.113374233	0.082981426	-1.366260358	0.171857259	0.321152058	down_in_case
Yang2025_S	ENSGALG00010017701	PPP2R5E	case	control	-0.51651726	0.061959176	-8.336412629	7.65784E-17	2.37138E-14	down_in_case
Yang2025_S	ENSGALG00010017702	ECEL1	case	control	1.653476102	1.085732524	1.522912932			up_in_case
Yang2025_S	ENSGALG00010017703	RPS6KA5	case	control	-0.065833889	0.144610743	-0.455248946	0.648930151	0.776943178	down_in_case
Yang2025_S	ENSGALG00010017704	ZNF362	case	control	0.88590389	0.300923342	2.943952056	0.003240502	0.017248432	up_in_case
Yang2025_S	ENSGALG00010017705	MAK16	case	control	-0.126947899	0.102863144	-1.234143679	0.21714937	0.377963586	down_in_case
Yang2025_S	ENSGALG00010017706	FGFRL1	case	control	0.342702408	0.145655526	2.352828058	0.018631247	0.064311723	up_in_case
Yang2025_S	ENSGALG00010017707	AK2	case	control	0.135712926	0.125412619	1.082131345	0.27919417	0.44703789	up_in_case
Yang2025_S	ENSGALG00010017708	MEIS1	case	control	0.384296573	0.164803851	2.331842192	0.019708995	0.067150327	up_in_case
Yang2025_S	ENSGALG00010017710	RAB41	case	control	0.390945286	0.109811613	3.560145189	0.00037065	0.003109576	up_in_case
Yang2025_S	ENSGALG00010017711	LRRFIP2	case	control	0.973026731	0.16025381	6.07178532	1.26496E-09	7.43899E-08	up_in_case
Yang2025_S	ENSGALG00010017712	MSH2	case	control	-0.386134147	0.096598241	-3.997320681	6.40635E-05	0.000745802	down_in_case
Yang2025_S	ENSGALG00010017713	ALKBH5	case	control	-0.175958929	0.092519282	-1.901862235	0.057189167	0.147307033	down_in_case
Yang2025_S	ENSGALG00010017714	ATAD3A	case	control	0.159924324	0.082509712	1.938248494	0.052592913	0.138384109	up_in_case
Yang2025_S	ENSGALG00010017715	CILP	case	control	-0.091329003	0.161787768	-0.564498813	0.572414708	0.715833643	down_in_case
Yang2025_S	ENSGALG00010017717	LLGL1	case	control	-0.109492912	0.086825155	-1.261073617	0.207282325	0.366000912	down_in_case
Yang2025_S	ENSGALG00010017718	ATOH8	case	control	0.746564482	0.186312002	4.007065963	6.14777E-05	0.000722337	up_in_case
Yang2025_S	ENSGALG00010017720	ENSGALG00010017720	case	control	0.213943267	0.354406653	0.603666058	0.546065684	0.695242148	up_in_case
Yang2025_S	ENSGALG00010017721	VWA1	case	control	-0.114950207	0.10448571	-1.100152427	0.271265714	0.438627028	down_in_case
Yang2025_S	ENSGALG00010017723	KCNK12	case	control	-0.834499194	0.324925587	-2.568277865	0.010220519	0.04119842	down_in_case
Yang2025_S	ENSGALG00010017724	FLII	case	control	0.235042605	0.061776517	3.804724115	0.000141962	0.001414042	up_in_case
Yang2025_S	ENSGALG00010017725	ENSGALG00010017725	case	control	-0.074535942	0.435685062	-0.171077572	0.864162771	0.919806616	down_in_case
Yang2025_S	ENSGALG00010017726	TTI2	case	control	0.277757078	0.247712687	1.121287252	0.26216561	0.428989875	up_in_case
Yang2025_S	ENSGALG00010017727	AZIN2	case	control	0.172792429	0.182694035	0.94580223	0.344249458	0.51273153	up_in_case
Yang2025_S	ENSGALG00010017729	MIEF2	case	control	-0.278386486	0.170895218	-1.628989327	0.103315278	0.22463698	down_in_case
Yang2025_S	ENSGALG00010017730	TARP	case	control	-0.988991044	0.696315261	-1.420320795	0.15551431	0.300229259	down_in_case
Yang2025_S	ENSGALG00010017732	RHOJ	case	control	0.231529106	0.174839216	1.324240129	0.185423307	0.338913141	up_in_case
Yang2025_S	ENSGALG00010017733	TRIM62	case	control	0.261275715	0.232171024	1.12535884	0.260437005	0.426863052	up_in_case
Yang2025_S	ENSGALG00010017734	TOP3A	case	control	-0.225784928	0.163021899	-1.384997534	0.166053221	0.313756155	down_in_case
Yang2025_S	ENSGALG00010017737	ENSGALG00010017737	case	control	1.110976396	0.28301206	3.925544362	8.65338E-05	0.000949487	up_in_case
Yang2025_S	ENSGALG00010017738	NDUFS5	case	control	0.418344837	0.196159718	2.132674549	0.032951433	0.098156952	up_in_case
Yang2025_S	ENSGALG00010017739	MRPL20	case	control	0.631757799	0.162107799	3.897146247	9.73328E-05	0.001044136	up_in_case
Yang2025_S	ENSGALG00010017741	SMCR8	case	control	-0.288246841	0.093303286	-3.089353571	0.002005926	0.011992619	down_in_case
Yang2025_S	ENSGALG00010017742	DGLUCY	case	control	0.130522355	0.210729208	0.61938426	0.535663248	0.686899591	up_in_case
Yang2025_S	ENSGALG00010017743	KCNH5	case	control	0.379396229	0.401755383	0.944346347	0.344992689	0.513673803	up_in_case
Yang2025_S	ENSGALG00010017744	ETAA1	case	control	-0.223720313	0.068404033	-3.27057195	0.001073302	0.007408254	down_in_case
Yang2025_S	ENSGALG00010017745	SHMT1	case	control	0.039651618	0.132918918	0.298314331	0.765463267	0.857247499	up_in_case
Yang2025_S	ENSGALG00010017746	MSH6	case	control	0.190308425	0.108177166	1.759229169	0.078538592	0.184870824	up_in_case
Yang2025_S	ENSGALG00010017747	DUSP26	case	control	-0.095296622	0.191574164	-0.497439842	0.618878916	0.754737155	down_in_case
Yang2025_S	ENSGALG00010017748	ENSGALG00010017748	case	control	-1.011949941	0.38178477	-2.650577028	0.00803544	0.03434781	down_in_case
Yang2025_S	ENSGALG00010017749	TMEM88B	case	control	0.401584838	0.354255219	1.133603165	0.256961045	0.423356841	up_in_case
Yang2025_S	ENSGALG00010017751	ST3GAL5	case	control	-0.015235455	0.180100077	-0.084594384	0.932583864	0.958940093	down_in_case
Yang2025_S	ENSGALG00010017753	GNRHR	case	control	0.513449073	0.356550035	1.440047742	0.149853892	0.292394558	up_in_case
Yang2025_S	ENSGALG00010017754	PDZD11	case	control	0.012023961	0.139839305	0.085984132	0.931479031	0.958515861	up_in_case
Yang2025_S	ENSGALG00010017755	C1D	case	control	-0.698584814	0.141770497	-4.927575392	8.32563E-07	2.07334E-05	down_in_case

Yang2025_S	ENSGALG00010017756	EIF2B5	case	control	-0.181061749	0.102309896	-1.769738368	0.076770735	0.181815125	down_in_case
Yang2025_S	ENSGALG00010017757	ENSGALG00010017757	case	control	-0.873757394	0.196218618	-4.452979052	8.4687E-06	0.000142354	down_in_case
Yang2025_S	ENSGALG00010017759	ENSGALG00010017759	case	control	0.216719239	0.503209075	0.430674345	0.666705175	0.789228274	up_in_case
Yang2025_S	ENSGALG00010017761	PARP16	case	control	-0.312474278	0.150446036	-2.076985789	0.037802871	0.108570281	down_in_case
Yang2025_S	ENSGALG00010017762	FBXO11	case	control	0.060925974	0.138768668	0.439047046	0.660627447	0.785682639	up_in_case
Yang2025_S	ENSGALG00010017763	POLR1A	case	control	-0.070994296	0.094115461	-0.75433192	0.45064994	0.612664089	down_in_case
Yang2025_S	ENSGALG00010017764	IGDCC3	case	control	-0.411489496	0.218206422	-1.885780872	0.05932449	0.151380106	down_in_case
Yang2025_S	ENSGALG00010017765	SYT16	case	control	-0.575642267	0.231366646	-2.488008873	0.012846053	0.048789792	down_in_case
Yang2025_S	ENSGALG00010017766	DNAAF10	case	control	-0.201292068	0.123525985	-1.629552418	0.103196128	0.224447954	down_in_case
Yang2025_S	ENSGALG00010017767	KIF4B	case	control	0.1527783	0.133004739	1.148668098	0.250692865	0.416426878	up_in_case
Yang2025_S	ENSGALG00010017768	FOXN2	case	control	-0.163177031	0.132756622	-1.229144194	0.219017748	0.380028927	down_in_case
Yang2025_S	ENSGALG00010017770	PTCD3	case	control	-0.225002127	0.118204853	-1.903493153	0.056976226	0.146975882	down_in_case
Yang2025_S	ENSGALG00010017771	PNO1	case	control	-0.175632262	0.159503075	-1.101121482	0.270843789	0.438097295	down_in_case
Yang2025_S	ENSGALG00010017772	ENSGALG00010017772	case	control	0.209335924	0.294757499	0.710197111	0.477581912	0.636547484	up_in_case
Yang2025_S	ENSGALG00010017773	DVL3	case	control	0.080410551	0.117539996	0.68411225	0.493904287	0.651387113	up_in_case
Yang2025_S	ENSGALG00010017774	ASH2L	case	control	-0.232715237	0.098894535	-2.353165801	0.018614332	0.064285184	down_in_case
Yang2025_S	ENSGALG00010017775	PRPSAP2	case	control	0.202617736	0.087977633	2.30305964	0.021275488	0.070926775	up_in_case
Yang2025_S	ENSGALG00010017776	GPR68	case	control	0.677802416	0.217624563	3.114549235	0.001842262	0.011259615	up_in_case
Yang2025_S	ENSGALG00010017778	PPP3R1	case	control	-0.266218178	0.073182016	-3.637754093	0.000275026	0.002422557	down_in_case
Yang2025_S	ENSGALG00010017779	SNAPC1	case	control	0.014137039	0.133961422	0.105530669	0.915954735	0.95053837	up_in_case
Yang2025_S	ENSGALG00010017780	TAF9	case	control	0.017735531	0.148754844	0.119226578	0.905095851	0.943344105	up_in_case
Yang2025_S	ENSGALG00010017781	CYP26C1	case	control	-0.622256279	0.409202538	-1.52065596	0.128346196	0.261630228	down_in_case
Yang2025_S	ENSGALG00010017782	IGDCC4	case	control	-0.32613478	0.128140559	-2.545133113	0.010923609	0.043244457	down_in_case
Yang2025_S	ENSGALG00010017783	STAR	case	control	0.507331935	0.412730175	1.229209702	0.218993192	0.380028927	up_in_case
Yang2025_S	ENSGALG00010017786	KAZALD1	case	control	-0.071673473	0.152801789	-0.469061741	0.639025505	0.769182035	down_in_case
Yang2025_S	ENSGALG00010017787	CNRIP1	case	control	0.232936948	0.169748617	1.372246515	0.16998671	0.318896715	up_in_case
Yang2025_S	ENSGALG00010017789	IMMT	case	control	0.233576139	0.075331863	3.100628718	0.001931103	0.011678209	up_in_case
Yang2025_S	ENSGALG00010017790	R3HCC1L	case	control	0.813593304	0.105280021	7.727898383	1.09337E-14	2.27404E-12	up_in_case
Yang2025_S	ENSGALG00010017791	CCNL2	case	control	0.614279235	0.097466309	6.302477624	2.92925E-10	2.05121E-08	up_in_case
Yang2025_S	ENSGALG00010017792	LSM1	case	control	0.747917491	0.25270452	2.959652208	0.003079865	0.01664812	up_in_case
Yang2025_S	ENSGALG00010017793	FAM83G	case	control	-0.867840851	0.737590263	-1.176589353	0.239359449	0.403614948	down_in_case
Yang2025_S	ENSGALG00010017795	MACF1	case	control	0.259981946	0.168004019	1.547474567	0.121748853	0.252353155	up_in_case
Yang2025_S	ENSGALG00010017796	MXI1	case	control	-0.167835153	0.078601694	-2.135261274	0.032739674	0.097776974	down_in_case
Yang2025_S	ENSGALG00010017797	PPP1R21	case	control	0.292886961	0.096434806	3.037149913	0.002388266	0.013712605	up_in_case
Yang2025_S	ENSGALG00010017798	BAG4	case	control	0.399970423	0.135072329	2.961157377	0.003064853	0.016592356	up_in_case
Yang2025_S	ENSGALG00010017799	GRAPL	case	control	0.70398779	0.353641965	1.990679443	0.04651614	0.126577312	up_in_case
Yang2025_S	ENSGALG00010017801	SMNDC1	case	control	-0.592346462	0.089098884	-6.648191693	2.96716E-11	2.81274E-09	down_in_case
Yang2025_S	ENSGALG00010017803	ENSGALG00010017803	case	control	0.149697916	0.13001262	1.151410652	0.249563334	0.415194013	up_in_case
Yang2025_S	ENSGALG00010017804	DUSP5	case	control	-0.219527349	0.144867179	-1.515369804	0.12967878	0.263498658	down_in_case
Yang2025_S	ENSGALG00010017805	DPP8	case	control	-0.245950357	0.096939845	-2.537144105	0.011176094	0.043981607	down_in_case
Yang2025_S	ENSGALG00010017806	ENSGALG00010017806	case	control	-0.314560426	0.328607529	-0.957252644	0.338439751	0.506623476	down_in_case
Yang2025_S	ENSGALG00010017807	MRPL35	case	control	-0.137636873	0.130116638	-1.057796101	0.290148411	0.458768735	down_in_case
Yang2025_S	ENSGALG00010017808	SFRP5	case	control	0.442840878	0.185140211	2.391921647	0.01676042	0.05962636	up_in_case
Yang2025_S	ENSGALG00010017809	MOSMO	case	control	-0.321166681	0.121273674	-2.64828029	0.008090242	0.034507964	down_in_case
Yang2025_S	ENSGALG00010017810	HIF1A	case	control	-0.08771185	0.111357158	-0.787662429	0.430894187	0.594800446	down_in_case
Yang2025_S	ENSGALG00010017811	HACD3	case	control	-0.207515446	0.073833301	-2.810594199	0.004945011	0.023769828	down_in_case
Yang2025_S	ENSGALG00010017813	DDHD2	case	control	0.088145574	0.14531083	0.606600165	0.544116291	0.694082125	up_in_case
Yang2025_S	ENSGALG00010017814	ENSGALG00010017814	case	control	-0.67143968	0.233476851	-2.875829775	0.00402967	0.020278497	down_in_case
Yang2025_S	ENSGALG00010017815	VWA9	case	control	-0.20824238	0.108897751	-1.912274382	0.05584101	0.144786171	down_in_case
Yang2025_S	ENSGALG00010017816	PRKCH	case	control	0.438191661	0.449055742	0.975806831	0.329160199	0.498695269	up_in_case
Yang2025_S	ENSGALG00010017817	REEP1	case	control	-0.089178099	0.090594341	-0.984367215	0.324934992	0.494589745	down_in_case
Yang2025_S	ENSGALG00010017818	SLC38A6	case	control	0.57711639	0.140825313	4.098101231	4.16553E-05	0.000529885	up_in_case
Yang2025_S	ENSGALG00010017820	FLRT2	case	control	-0.437224879	0.168150507	-2.600199583	0.009316956	0.038560076	down_in_case
Yang2025_S	ENSGALG00010017821	ENSGALG00010017821	case	control	-0.400846406	0.532656376	-0.752542209	0.451725057	0.613616804	down_in_case
Yang2025_S	ENSGALG00010017822	VWA3A	case	control	0.308394002	0.267050678	1.154814527	0.248166387	0.413858138	up_in_case
Yang2025_S	ENSGALG00010017823	PPIE	case	control	0.454565488	0.148278172	3.065626454	0.002172145	0.012734942	up_in_case
Yang2025_S	ENSGALG00010017824	YAE1	case	control	0.158177545	0.186079271	0.850054624	0.395294717	0.562544106	up_in_case
Yang2025_S	ENSGALG00010017825	PLPP5	case	control	0.039908436	0.240698973	0.165802269	0.868312561	0.922673138	up_in_case
Yang2025_S	ENSGALG00010017826	PABPC4	case	control	-0.100311792	0.140891342	-0.711979815	0.476477275	0.635622327	down_in_case
Yang2025_S	ENSGALG00010017828	ENSGALG00010017828	case	control	0.035102295	0.172286063	0.20374425	0.838553361	0.904482259	up_in_case
Yang2025_S	ENSGALG00010017830	DENND4A	case	control	0.078228631	0.114920938	0.680716956	0.496050605	0.653290349	up_in_case
Yang2025_S	ENSGALG00010017831	PLEK	case	control	-0.098432948	0.125354445	-0.785236996	0.432314628	0.596288661	down_in_case
Yang2025_S	ENSGALG00010017832	POU3F1	case	control	0.5262338	0.356681118	1.475362093	0.140115221	0.279009089	up_in_case
Yang2025_S	ENSGALG00010017833	ENSGALG00010017833	case	control	-0.14059667	0.18203071	-0.77237885	0.439890077	0.603302507	down_in_case
Yang2025_S	ENSGALG00010017834	ENSGALG00010017834	case	control	0.60608555	0.317148883	1.911044255	0.055998894	0.145018507	up_in_case
Yang2025_S	ENSGALG00010017838	B3GNT2	case	control	0.052038681	0.136493432	0.381254105	0.703014705	0.816511911	up_in_case
Yang2025_S	ENSGALG00010017841	GALC	case	control	-0.351269555	0.136872496	-2.566399864	0.010276029	0.041362353	down_in_case
Yang2025_S	ENSGALG00010017843	NSD3	case	control	0.001307599	0.122961183	0.010634239	0.991515265	0.994655908	up_in_case
Yang2025_S	ENSGALG00010017848	CYP3A4	case	control	0.510779582	0.202802251	2.518609041	0.01178194	0.045752858	up_in_case
Yang2025_S	ENSGALG00010017849	FBXO48	case	control	-0.085832808	0.282188116	-0.304168755	0.760999327	0.854858978	down_in_case
Yang2025_S	ENSGALG00010017850	TRMT5	case	control	-0.079759424	0.133079544	-0.599336473	0.548948531	0.697637736	down_in_case
Yang2025_S	ENSGALG00010017852	LETM2	case	control	0.246047434	0.120268141	2.045823864	0.040773703	0.114691473	up_in_case
Yang2025_S	ENSGALG00010017855	SDR42E2	case	control	0.813357036	0.306074141	2.657385666	0.00787493	0.033859039	up_in_case
Yang2025_S	ENSGALG00010017856	FASTKD5	case	control	0.001097737	0.122170513	0.008985287	0.992830875	0.995259207	up_in_case
Yang2025_S	ENSGALG00010017858	GPR65	case	control	-0.203239323	0.247722647	-0.820430936	0.411970486	0.577291884	down_in_case
Yang2025_S	ENSGALG00010017859	EXOC6	case	control	0.236232127	0.128072178	1.844523386	0.065106901	0.161348864	up_in_case
Yang2025_S	ENSGALG00010017860	CYSLTR1	case	control	-0.778713443	0.336209679	-2.31615415	0.020549854	0.069090807	down_in_case
Yang2025_S	ENSGALG00010017861	PRSS56	case	control	0.043141622	0.473652303	0.091082892	0.927426726	0.956035762	up_in_case
Yang2025_S	ENSGALG00010017865	UBOX5	case	control	-0.195374526	0.141291503	-1.382776194	0.1667335	0.314529759	down_in_case
Yang2025_S	ENSGALG00010017866	RALA	case	control	-0.50655309	0.091003024	-5.566332497	2.60157E-08	1.06573E-06	down_in_case
Yang2025_S	ENSGALG00010017867	APLF	case	control	-0.10893796	0.102964099	-1.058018868	0.29004684	0.458768735	down_in_case
Yang2025_S	ENSGALG00010017868	CHRND	case	control	0.530319976	0.206659983	2.566147395	0.010283512	0.04138052	up_in_case
Yang2025_S	ENSGALG00010017870	RAB11A	case	control	-0.253966955	0.085599235	-2.966930172	0.003007893	0.016373041	down_in_case
Yang2025_S	ENSGALG00010017873	HSPG2	case	control	-0.055073548	0.149726418	-0.367827862	0.713001592	0.822771185	down_in_case
Yang2025_S	ENSGALG00010017876	COMMD1	case	control	-0.312142603	0.28477302	-1.096110167	0.273030566	0.4403603	down_in_case
Yang2025_S	ENSGALG00010017878	CHRNG	case	control	0.140530006	0.171868427	0.817660396	0.413551131	0.579121194	up_in_case
Yang2025_S	ENSGALG00010017880	CDK13	case	control	0.355945383	0.182502964	1.950353986	0.051133942	0.135363124	up_in_case
Yang2025_S	ENSGALG00010017881	KCNK10	case	control	0.167703182	0.520661235	0.32209654	0.74737956	0.846657521	up_in_case
Yang2025_S	ENSGALG00010017882	LZTS3	case	control	0.256787273	0.201392474	1.275058931	0.202288384	0.3602824	up_in_case
Yang2025_S	ENSGALG00010017886	DDRGK1	case	control	0.510255598	0.164024714	3.110845834	0.001865523	0.011378849	up_in_case
Yang2025_S	ENSGALG00010017887	PROKR1	case	control	-0.647839266	0.302093989	-2.144495723	0.031993188	0.096124423	down_in_case
Yang2025_S	ENSGALG00010017888	MPLKIP	case	control	-0.048808919	0.091081546	-0.535881543	0.592040421	0.732433649	down_in_case
Yang2025_S	ENSGALG00010017890	ANAPC1	case	control	-0.12513685	0.068249798	-1.833512376	0.06672641	0.164281364	down_in_case
Yang2025_S	ENSGALG00010017891	GOLGA7B	case	control	0.487379928	0.216282009	2.25344646	0.024231013	0.078397763	up_in_case
Yang2025_S	ENSGALG00010017894	KAT6A	case	control	0.220162007	0.172478466	1.276460839	0.201792662	0.359761566	up_in_case
Yang2025_S	ENSGALG00010017895	MNAT1	case	control	0.330826968	0.102721914	3.22060751	0.001279192	0.008488353	up_in_case
Yang2025_S	ENSGALG00010017898	NHERF2	case	control	-0.216792296	0.096561851	-2.245113301	0.02476086	0.079447981	down_in_case
Yang2025_S	ENSGALG00010017903	ENSGALG00010017903	case	control	0.455330205	0.531279574	0.85704444	0.391420325	0.558607918	up_in_case
Yang2025_S	ENSGALG00010017904	MEGF11	case	control	-0.279207414	0.277769571	-1.005176384	0.314811924	0.484045478	down_in_case
Yang2025_S	ENSGALG00010017905	EIF4E2	case	control	0.598140347	0.11445275	5.226089782	1.73132E-07	5.48318E-06	up_in_case
Yang2025_S	ENSGALG00010017909	NTHL1	case	control	0.022739946	0.228793727	0.099390599	0.920828147	0.953182308	up_in_case
Yang2025_S	ENSGALG00010017915	BFSP1	case	control	-1.292358061	0.607454227	-2.127498673	0.033378671	0.099069603	down_in_case
Yang2025_S	ENSGALG00010017918	SIX4	case	control	-0.088717177	0.174892563	-0.507266718	0.611967688	0.748892787	down_in_case
Yang2025_S	ENSGALG00010017920	AP3M2	case	control	-0.015942747	0.129051902	-0.123537479	0.901681498	0.94126389	down_in_case
Yang2025_S	ENSGALG00010017921	SPATA7	case	control	0.23928855	0.1249788	1.914633131	0.055539307	0.144203511	up_in_case
Yang2025_S	ENSGALG00010017922	MERTK	case	control	-0.043306008	0.128617786	-0.336703108	0.73634072	0.839269421	down_in_case
Yang2025_S	ENSGALG00010017927	PCSK2	case	control	0.129733798	0.139750356	0.928325348	0.353238829	0.522102576	up_in_case
Yang2025_S	ENSGALG00010017930	TSC2	case	control	0.157611383	0.094056076	1.675717181	0.093793603	0.209423787	up_in_case
Yang2025_S	ENSGALG00010017933	SIX1	case	control	-0.003035147	0.130493517	-0.02325899	0.981443684	0.98867905	down_in_case
Yang2025_S	ENSGALG00010017937	PTPN21	case	control	0.778439578	0.26096907	2.982880608	0.002855494	0.015684392	up_in_case
Yang2025_S	ENSGALG00010017941	DIS3L	case	control	0.275838501	0.0934597	2.951416505	0.003163201	0.016999306	up_in_case
Yang2025_S	ENSGALG00010017946	PTGDR	case	control	0.266790491	0.232467814	1.147644855	0.251115205	0.416804479	up_in_case
Yang2025_S	ENSGALG00010017948	PLAT	case	control	0.081585092	0.137586907	0.592971333	0.553200337	0.701250496	up_in_case
Yang2025_S	ENSGALG00010017949	SFXN3	case	control	-0.017107842	0.100272538	-0.170613439	0.86452773	0.920045088	down_in_case
Yang2025_S	ENSGALG00010017950	FBLN7	case	control	0.734309303	0.392412847	1.871267233	0.06130805	0.154825603	up_in_case
Yang2025_S	ENSGALG00010017954	PTGER2	case	control	-0.122952435	0.296313357	-0.414940578	0.678185404	0.797865799	down_in_case
Yang2025_S	ENSGALG00010017955	IKBKB	case	control	0.292676328	0.076514702	3.825099223	0.000130719	0.001320939	up_in_case
Yang2025_S	ENSGALG00010017956	TIPIN	case	control	0.056369711	0.139641231	0.403675267	0.68645153	0.804089064	up_in_case
Yang2025_S	ENSGALG00010017957	THUMPD1	case	control	-0.153852169	0.080034282	-1.922328345	0.054564456	0.142202298	down_in_case
Yang2025_S	ENSGALG00010017958	CAPN10	case	control	-0.333859573	0.16473738	-2.026616992	0.042701597	0.118724414	down_in_case
Yang2025_S	ENSGALG00010017959	PDZD7	case	control	0.05405806	0.187809221	0.287834961	0.773473074	0.862181781	up_in_case
Yang2025_S	ENSGALG00010017962	TXNDC16	case	control	-0.198051751	0.109666076	-1.805952743	0.070925721	0.171528971	down_in_case
Yang2025_S	ENSGALG00010017963	LPAR4	case	control	-0.329309607	0.107467025	-3.064285134	0.002181908	0.012780536	down_in_case
Yang2025_S	ENSGALG00010017967	ACSM3	case	control	0.591898926	0.954287065	0.620252488	0.53509157	0.686479207	up_in_case
Yang2025_S	ENSGALG00010017972	RNPEPL1	case	control	-0.309231242	0.082805613	-3.734423668	0.000188146	0.001777397	down_in_case
Yang2025_S	ENSGALG00010017973	PKD1	case	control	-0.105646403	0.186353044	-0.566915361	0.570771687	0.715003907	down_in_case

Yang2025_S	ENSGALG00010017974	MXRA8	case	control	-0.116690449	0.124957202	-0.933843319	0.35038472	0.518819581	down_in_case
Yang2025_S	ENSGALG00010017976	GPR137C	case	control	-0.309586782	0.155024076	-1.997023885	0.045822588	0.125326353	down_in_case
Yang2025_S	ENSGALG00010017979	ENSGALG00010017979	case	control	0.256358667	0.183371205	1.398031212	0.162103695	0.308258049	up_in_case
Yang2025_S	ENSGALG00010017983	SLC4A11	case	control	-0.394428432	0.149895241	-2.631360598	0.008504375	0.035922541	down_in_case
Yang2025_S	ENSGALG00010017985	ABCC5	case	control	1.18306464	0.17684936	6.689674431	2.23668E-11	2.2105E-09	up_in_case
Yang2025_S	ENSGALG00010017987	ERI2	case	control	0.631350077	0.221895394	2.845259946	0.004437519	0.021827332	up_in_case
Yang2025_S	ENSGALG00010017991	RAB26	case	control	-0.381692783	0.136482603	-2.796640567	0.005163694	0.02455001	down_in_case
Yang2025_S	ENSGALG00010017993	REXO5	case	control	0.279912227	0.109565626	2.55474493	0.010626564	0.042393692	up_in_case
Yang2025_S	ENSGALG00010017996	ZC3H14	case	control	0.395264804	0.145248763	2.721295491	0.006502661	0.029239941	up_in_case
Yang2025_S	ENSGALG00010017997	ENSGALG00010017997	case	control	3.654143192	0.539447522	6.773862223	1.25389E-11	1.31376E-09	up_in_case
Yang2025_S	ENSGALG00010018000	MAP2K1	case	control	-0.378220417	0.147116502	-2.570890502	0.010143739	0.040948148	down_in_case
Yang2025_S	ENSGALG00010018001	TRAF7	case	control	-0.35126566	0.11718687	-2.997483073	0.00272219	0.015107015	down_in_case
Yang2025_S	ENSGALG00010018002	ZC3H8	case	control	0.101779222	0.163633086	0.621996595	0.533944107	0.685508673	up_in_case
Yang2025_S	ENSGALG00010018004	CDC42BPA	case	control	0.007737676	0.190721141	0.040570626	0.967638202	0.980871342	up_in_case
Yang2025_S	ENSGALG00010018006	DCUN1D3	case	control	-0.291625419	0.100991227	-2.887631203	0.003881547	0.019769501	down_in_case
Yang2025_S	ENSGALG00010018007	DNAAF9	case	control	0.737747426	0.132438152	5.570505278	2.54002E-08	1.04587E-06	up_in_case
Yang2025_S	ENSGALG00010018008	ABCF3	case	control	0.0859924	0.135802219	0.63321793	0.526591339	0.679616917	up_in_case
Yang2025_S	ENSGALG00010018010	ENSGALG00010018010	case	control	-0.225238859	0.172687307	-1.30431624	0.19212578	0.347306047	down_in_case
Yang2025_S	ENSGALG00010018011	LIPA	case	control	-0.675997949	0.112977903	-5.983452771	2.18456E-09	1.21282E-07	down_in_case
Yang2025_S	ENSGALG00010018012	DVL1	case	control	-0.284898459	0.084645677	-3.365776849	0.000763284	0.005628979	down_in_case
Yang2025_S	ENSGALG00010018014	LYRM1	case	control	0.077330828	0.072956947	1.059951526	0.289166653	0.458006059	up_in_case
Yang2025_S	ENSGALG00010018016	IFIT5	case	control	-0.647912056	0.384926318	-1.683210594	0.092334331	0.207461931	down_in_case
Yang2025_S	ENSGALG00010018019	NAPB	case	control	1.358664686	0.287312727	4.728870523	2.25772E-06	4.81797E-05	up_in_case
Yang2025_S	ENSGALG00010018020	SNAPC5	case	control	-0.517440832	0.167873258	-3.082330307	0.002053868	0.012207967	down_in_case
Yang2025_S	ENSGALG00010018021	ATRN	case	control	-0.059258799	0.082742152	-0.716186348	0.473876292	0.63330451	down_in_case
Yang2025_S	ENSGALG00010018022	XPO6	case	control	0.067048044	0.09642756	0.695320341	0.48685457	0.644040101	up_in_case
Yang2025_S	ENSGALG00010018025	CPTP	case	control	-0.2941032	0.119593982	-2.459180598	0.013925455	0.051871481	down_in_case
Yang2025_S	ENSGALG00010018026	CCDC39	case	control	0.99904228	0.508834015	1.963395235	0.049600267	0.132536859	up_in_case
Yang2025_S	ENSGALG00010018027	EML5	case	control	1.284719528	0.435797793	2.947971621	0.003198664	0.017097578	up_in_case
Yang2025_S	ENSGALG00010018030	POLB	case	control	0.0298052	0.090980126	0.327601214	0.743213204	0.842897046	up_in_case
Yang2025_S	ENSGALG00010018032	ERO1A	case	control	0.16077254	0.130694724	1.230137952	0.21864545	0.37976123	up_in_case
Yang2025_S	ENSGALG00010018033	COQ8A	case	control	0.076191329	0.163407299	0.466266376	0.641024843	0.770789645	up_in_case
Yang2025_S	ENSGALG00010018035	ITM2A	case	control	-0.631543484	0.117602046	-5.370174295	7.86606E-08	2.77503E-06	down_in_case
Yang2025_S	ENSGALG00010018036	RPL4	case	control	-0.319623084	0.135098024	-2.365860536	0.017988219	0.062856634	down_in_case
Yang2025_S	ENSGALG00010018037	GZF1	case	control	0.472009821	0.138360557	3.411447834	0.000646189	0.004928647	up_in_case
Yang2025_S	ENSGALG00010018039	TTC14	case	control	0.866281783	0.177857338	4.870655287	1.11229E-06	2.64953E-05	up_in_case
Yang2025_S	ENSGALG00010018042	ZWILCH	case	control	-0.174790273	0.129636985	-1.348305598	0.177560113	0.328854355	down_in_case
Yang2025_S	ENSGALG00010018043	PSMC6	case	control	0.056902243	0.100584461	0.565716037	0.571586832	0.715439066	up_in_case
Yang2025_S	ENSGALG00010018044	INTS11	case	control	-0.024424408	0.113535478	-0.215125782	0.829669254	0.898324868	down_in_case
Yang2025_S	ENSGALG00010018045	PSEN2	case	control	0.422617203	0.094400596	4.476848909	7.57528E-06	0.000131132	up_in_case
Yang2025_S	ENSGALG00010018046	CAPN15	case	control	0.037826311	0.156264006	0.242066692	0.808728487	0.884725346	up_in_case
Yang2025_S	ENSGALG00010018048	GFRA4	case	control	0.23843712	0.161973196	1.472077636	0.140999914	0.28025015	up_in_case
Yang2025_S	ENSGALG00010018049	SUGCT	case	control	-0.48128338	0.229454304	-2.097512978	0.03594819	0.104864564	down_in_case
Yang2025_S	ENSGALG00010018051	GNPNAT1	case	control	-0.045945181	0.104927588	-0.437875128	0.661476807	0.786223072	down_in_case
Yang2025_S	ENSGALG00010018052	PPP1RL	case	control	-0.228347089	0.149678305	-1.525585752	0.127113063	0.259869671	down_in_case
Yang2025_S	ENSGALG00010018053	DIAPH2	case	control	-0.263975128	0.129214307	-2.042924928	0.04105987	0.115403245	down_in_case
Yang2025_S	ENSGALG00010018054	SMAD6	case	control	0.055498684	0.168736301	0.328907793	0.742225384	0.842187992	up_in_case
Yang2025_S	ENSGALG00010018055	PEX5L	case	control	1.124072381	0.548022443	2.051142968	0.040253026	0.11380116	up_in_case
Yang2025_S	ENSGALG00010018057	SSTR4	case	control	0.251240732	0.119171329	2.108231359	0.035010982	0.10275443	up_in_case
Yang2025_S	ENSGALG00010018058	ITPKB	case	control	0.087967499	0.076575246	1.148772018	0.25065	0.416405312	up_in_case
Yang2025_S	ENSGALG00010018060	CASKIN1	case	control	0.425235722	0.285117573	1.491439892	0.135846041	0.272415395	up_in_case
Yang2025_S	ENSGALG00010018061	NDUFAF5	case	control	0.128626012	0.132329556	0.972012726	0.331044218	0.500336353	up_in_case
Yang2025_S	ENSGALG00010018062	GOLGA7	case	control	-0.227056563	0.107175011	-2.1185588	0.034127771	0.10077781	down_in_case
Yang2025_S	ENSGALG00010018063	DECR2	case	control	-0.524833258	0.251652291	-2.085549294	0.037019469	0.106996968	down_in_case
Yang2025_S	ENSGALG00010018064	TTC8	case	control	-0.051999707	0.158024458	-0.329061131	0.742109482	0.842167591	down_in_case
Yang2025_S	ENSGALG00010018067	ENSGALG00010018067	case	control	-0.504651741	0.210503783	-2.397352359	0.016514035	0.05902105	down_in_case
Yang2025_S	ENSGALG00010018068	ADAM33	case	control	-0.232096199	0.088357184	-2.626794877	0.008619327	0.036331013	down_in_case
Yang2025_S	ENSGALG00010018069	ENSGALG00010018069	case	control	0.05469553	0.44323575	0.123400538	0.901789931	0.94127694	up_in_case
Yang2025_S	ENSGALG00010018071	GPAT4	case	control	-0.271899703	0.153035725	-1.776707389	0.075616396	0.179814757	down_in_case
Yang2025_S	ENSGALG00010018075	MLST8	case	control	0.287483499	0.164381438	1.748880549	0.080311669	0.187870256	up_in_case
Yang2025_S	ENSGALG00010018078	NKX6-3	case	control	0.292847082	0.539814068	0.542496202	0.587476723	0.728753962	up_in_case
Yang2025_S	ENSGALG00010018080	BRICD5	case	control	-0.732291437	0.414637184	-1.766101704	0.077378788	0.182913216	down_in_case
Yang2025_S	ENSGALG00010018081	DIO3	case	control	-0.146061553	0.179120624	-0.815436823	0.414822314	0.580318135	down_in_case
Yang2025_S	ENSGALG00010018082	RAB11FIP3	case	control	0.982920339	0.15548845	6.321500648	2.59035E-10	1.82306E-08	up_in_case
Yang2025_S	ENSGALG00010018085	PGP	case	control	-0.114924578	0.157243824	-0.730868628	0.4648594	0.625815451	down_in_case
Yang2025_S	ENSGALG00010018087	ENSGALG00010018087	case	control	0.016222769	0.107005048	0.151607507	0.879496512	0.928681389	up_in_case
Yang2025_S	ENSGALG00010018089	TASP1	case	control	-0.299383798	0.14062783	-2.128908612	0.033261822	0.098806969	down_in_case
Yang2025_S	ENSGALG00010018092	ENSGALG00010018092	case	control	0.095375541	0.315105164	0.302678445	0.762134926	0.855286959	up_in_case
Yang2025_S	ENSGALG00010018095	E4F1	case	control	0.277026787	0.077583809	3.570677846	0.000356059	0.003005255	up_in_case
Yang2025_S	ENSGALG00010018099	SMAD3	case	control	0.091561121	0.136549022	0.670536629	0.502515764	0.658949865	up_in_case
Yang2025_S	ENSGALG00010018102	ZDHHC4	case	control	-0.542196607	0.202586818	-2.676366665	0.007442517	0.032389593	down_in_case
Yang2025_S	ENSGALG00010018103	BTRC	case	control	0.21247263	0.12570625	1.690231234	0.090983725	0.205354423	up_in_case
Yang2025_S	ENSGALG00010018110	CFAP65	case	control	3.872460045	2.999640642	1.290974656	0.196712466	0.352836686	up_in_case
Yang2025_S	ENSGALG00010018111	NKX2-2	case	control	0.138786195	0.441975821	0.314013093	0.753511094	0.849941485	up_in_case
Yang2025_S	ENSGALG00010018115	KLHL4	case	control	-0.204660807	0.160546663	-1.274774597	0.202389035	0.360328503	down_in_case
Yang2025_S	ENSGALG00010018119	GRID2IP	case	control	0.445811019	0.456160301	0.977312183	0.328414623	0.498036541	up_in_case
Yang2025_S	ENSGALG00010018121	FERMT2	case	control	0.146090841	0.109302954	1.336568094	0.18136366	0.333857674	up_in_case
Yang2025_S	ENSGALG00010018123	USP13	case	control	0.124057441	0.127415749	0.97364291	0.330233873	0.499707788	up_in_case
Yang2025_S	ENSGALG00010018128	SEPTIN12	case	control	0.494108124	0.138023841	3.579875194	0.000343758	0.002926251	up_in_case
Yang2025_S	ENSGALG00010018137	NDUFB5	case	control	-0.08141677	0.225123509	-0.361653788	0.717610764	0.82609715	down_in_case
Yang2025_S	ENSGALG00010018140	CHPF	case	control	0.289443124	0.099878858	2.897941856	0.003756202	0.019286174	up_in_case
Yang2025_S	ENSGALG00010018142	DDHD1	case	control	0.289337762	0.097952311	2.953863546	0.003138227	0.016884632	up_in_case
Yang2025_S	ENSGALG00010018143	MRPL47	case	control	-0.288250143	0.16954109	-1.700178665	0.089097324	0.202540165	down_in_case
Yang2025_S	ENSGALG00010018145	ENSGALG00010018145	case	control	0.222218995	0.288433298	0.770434611	0.441042129	0.604378215	up_in_case
Yang2025_S	ENSGALG00010018147	ISM1	case	control	0.087614571	0.263721364	0.332224018	0.739720109	0.840645875	up_in_case
Yang2025_S	ENSGALG00010018148	XRN2	case	control	-0.062459962	0.073605027	-0.848582818	0.396113471	0.563249104	down_in_case
Yang2025_S	ENSGALG00010018149	ENSGALG00010018149	case	control	0.235981108	0.179117598	1.317464674	0.187682915	0.341968824	up_in_case
Yang2025_S	ENSGALG00010018150	AAGAB	case	control	0.06205662	0.072220048	0.859271379	0.390190811	0.557501174	up_in_case
Yang2025_S	ENSGALG00010018154	SLC16A12	case	control	-0.440764262	0.252806169	-1.743486972	0.081248588	0.189330949	down_in_case
Yang2025_S	ENSGALG00010018157	FBXL2	case	control	0.054625753	0.129318702	0.422411854	0.672724427	0.79397094	up_in_case
Yang2025_S	ENSGALG00010018160	ENSGALG00010018160	case	control	0.766139221	0.172567051	4.439661082	9.01007E-06	0.000149846	up_in_case
Yang2025_S	ENSGALG00010018162	ENSGALG00010018162	case	control	0.548667348	0.433234104	1.266445423	0.205353657	0.363701476	up_in_case
Yang2025_S	ENSGALG00010018163	CCT7	case	control	-0.059598544	0.119107762	-0.500374979	0.616811068	0.752847704	down_in_case
Yang2025_S	ENSGALG00010018165	DACH2	case	control	0.311923722	0.196495811	1.587431914	0.112414856	0.238230813	up_in_case
Yang2025_S	ENSGALG00010018166	MSS51	case	control	-0.532690131	0.265861575	-2.003637162	0.045108942	0.124056267	down_in_case
Yang2025_S	ENSGALG00010018168	ENSGALG00010018168	case	control	0.444073277	0.313661608	1.415771858	0.156842312	0.302003263	up_in_case
Yang2025_S	ENSGALG00010018170	SLC5A11	case	control	1.740808484	0.666754563	2.610868498	0.009031261	0.037725008	up_in_case
Yang2025_S	ENSGALG00010018172	ACTL6A	case	control	-0.475459133	0.103789438	-4.580997285	4.62764E-06	8.65586E-05	down_in_case
Yang2025_S	ENSGALG00010018174	BMP4	case	control	0.267044102	0.189086021	1.412288969	0.157864893	0.303127597	up_in_case
Yang2025_S	ENSGALG00010018177	KIF20B	case	control	0.198615549	0.118591702	1.674784547	0.093976512	0.209764968	up_in_case
Yang2025_S	ENSGALG00010018179	PRADC1	case	control	-0.000701334	0.221478633	-0.003166599	0.997473424	0.998118064	down_in_case
Yang2025_S	ENSGALG00010018183	CYP17A1	case	control	-0.214099036	0.273099563	-0.783959643	0.433063791	0.5971006	down_in_case
Yang2025_S	ENSGALG00010018185	GNB4	case	control	-0.717159752	0.096141872	-7.459390371	8.69237E-14	1.40847E-11	down_in_case
Yang2025_S	ENSGALG00010018186	SPTLC3	case	control	0.486939214	0.568637515	0.85632622	0.391817362	0.558965493	up_in_case
Yang2025_S	ENSGALG00010018188	UBP1	case	control	-0.066980902	0.046473065	-1.441284342	0.149504369	0.291948344	down_in_case
Yang2025_S	ENSGALG00010018189	MIS18BP1	case	control	0.656952692	0.14770341	4.447782827	8.67612E-06	0.000145489	up_in_case
Yang2025_S	ENSGALG00010018192	IQCH	case	control	0.746260864	0.201967196	3.694960762	0.000219921	0.002013532	up_in_case
Yang2025_S	ENSGALG00010018193	ANK1	case	control	0.354587187	0.169074782	2.097221027	0.035974014	0.104896557	up_in_case
Yang2025_S	ENSGALG00010018198	BTBD3	case	control	-0.212699219	0.076148396	-2.793219955	0.005218619	0.024737401	down_in_case
Yang2025_S	ENSGALG00010018199	BORCS7	case	control	-0.079697605	0.09012071	-0.884342834	0.376511172	0.544713786	down_in_case
Yang2025_S	ENSGALG00010018206	KIZ	case	control	0.359230397	0.177002617	2.029520263	0.042405327	0.118136392	up_in_case
Yang2025_S	ENSGALG00010018209	GMPPA	case	control	-0.152234939	0.129481855	-1.175724112	0.23970514	0.404051183	down_in_case
Yang2025_S	ENSGALG00010018215	CFAP70	case	control	0.070728663	0.175283341	0.403510469	0.686572735	0.804117588	up_in_case
Yang2025_S	ENSGALG00010018216	FANCM	case	control	0.229851439	0.07036108	3.266741212	0.001087931	0.007491868	up_in_case
Yang2025_S	ENSGALG00010018217	ENSGALG00010018217	case	control	-0.70266662	0.498721187	-1.408936774	0.158853864	0.304385172	down_in_case
Yang2025_S	ENSGALG00010018219	SLC24A5	case	control	5.370697049	0.70525523	7.615253055	2.63175E-14	4.8898E-12	up_in_case
Yang2025_S	ENSGALG00010018226	JAG1	case	control	0.01836024	0.143923138	0.12756976	0.898489463	0.939723381	up_in_case
Yang2025_S	ENSGALG00010018229	AS3MT	case	control	-0.297030959	0.234266579	-1.267918624	0.204827013	0.363045589	down_in_case
Yang2025_S	ENSGALG00010018231	SMYD5	case	control	-0.029161725	0.160146756	-0.182093762	0.855509144	0.914560196	down_in_case
Yang2025_S	ENSGALG00010018233	RASSF2	case	control	0.436442018	0.140595118	3.104247326	0.001907638	0.011577933	up_in_case
Yang2025_S	ENSGALG00010018240	SOUL	case	control	0.019091134	0.419749555	0.045482202	0.963722961	0.978325888	up_in_case
Yang2025_S	ENSGALG00010018241	RALGAPA2	case	control	-0.112532124	0.113943114	-0.987616719	0.323340395	0.492593025	down_in_case
Yang2025_S	ENSGALG00010018244	CHM	case	control	-0.247709	0.090614746	-2.733650004	0.006263659	0.028375839	down_in_case
Yang2025_S	ENSGALG00010018251	USP48	case	control	0.096987751	0.070116541	1.383236394	0.166592394	0.31447643	up_in_case
Yang2025_S	ENSGALG00010018255	MFN1	case	control	-0.145383977	0.063309724	-2.296392536	0.021653447	0.071723506	down_in_case
Yang2025_S	ENSGALG00010018258	OSCP1	case	control	0.054907843	0.194705537	0.282004526	0.77794004	0.865102103	up_in_case

Yang2025_S	ENSGALG00010018259	WDR90	case	control	-0.17604427	0.143461172	-1.227121364	0.219776979	0.381023021	down_in_case
Yang2025_S	ENSGALG00010018261	PRNP	case	control	-0.288540623	0.110078974	-2.621214669	0.008761707	0.036797586	down_in_case
Yang2025_S	ENSGALG00010018263	SLX4IP	case	control	0.367703032	0.175441794	2.095869083	0.036093806	0.105091347	up_in_case
Yang2025_S	ENSGALG00010018264	CFAP61	case	control	0.197152317	0.589805298	0.334266779	0.738178256	0.84012692	up_in_case
Yang2025_S	ENSGALG00010018265	PDCD4	case	control	0.134119648	0.093733677	1.430858702	0.152470715	0.296039734	up_in_case
Yang2025_S	ENSGALG00010018267	ADPRHL2	case	control	-0.28397172	0.180933141	-1.569484283	0.116535139	0.244345043	down_in_case
Yang2025_S	ENSGALG00010018274	ZNF711	case	control	0.550266278	0.1156957	4.756151518	1.97318E-06	4.27625E-05	up_in_case
Yang2025_S	ENSGALG00010018275	ZNF639	case	control	-0.230584935	0.121839346	-1.892532596	0.058420055	0.149702733	down_in_case
Yang2025_S	ENSGALG00010018276	ARHGAP17	case	control	0.838827736	0.26520779	3.16290761	0.001562019	0.009916508	up_in_case
Yang2025_S	ENSGALG00010018278	PUSL1	case	control	0.979388418	0.178903359	5.474399274	4.38998E-08	1.67601E-06	up_in_case
Yang2025_S	ENSGALG00010018283	POF1B	case	control	-0.038132372	0.202387696	-0.188412499	0.850553302	0.911353565	down_in_case
Yang2025_S	ENSGALG00010018284	ENSGALG00010018284	case	control	0.454623287	0.12218873	3.720664644	0.000198699	0.001860802	up_in_case
Yang2025_S	ENSGALG00010018285	BBIP1	case	control	0.360830588	0.16630463	2.169696586	0.030029839	0.091688388	up_in_case
Yang2025_S	ENSGALG00010018287	MKKS	case	control	-0.343372466	0.083684923	-4.10315806	4.07549E-05	0.000521984	down_in_case
Yang2025_S	ENSGALG00010018288	CRNKL1	case	control	-0.006755021	0.063695806	-0.106051269	0.915541675	0.950251246	down_in_case
Yang2025_S	ENSGALG00010018289	HEYL	case	control	0.413147101	0.126062332	3.277323955	0.001047961	0.007272575	up_in_case
Yang2025_S	ENSGALG00010018290	RHOT2	case	control	0.17565791	0.138175298	1.271268543	0.203633129	0.36171162	up_in_case
Yang2025_S	ENSGALG00010018294	FKBP3	case	control	0.487233637	0.156946937	3.10444821	0.001906343	0.0115779	up_in_case
Yang2025_S	ENSGALG00010018296	PIK3CA	case	control	-0.182619265	0.130022134	-1.404524438	0.16016274	0.305861008	down_in_case
Yang2025_S	ENSGALG00010018298	ENSGALG00010018298	case	control	0.776480936	0.169171177	4.589912707	4.43431E-06	8.39568E-05	up_in_case
Yang2025_S	ENSGALG00010018299	SHOC2	case	control	-0.011330432	0.086325864	-0.131251878	0.895576058	0.938334765	down_in_case
Yang2025_S	ENSGALG00010018301	ENSGALG00010018301	case	control	-0.73329272	0.445965895	-1.644279816	0.100118417	0.219184973	down_in_case
Yang2025_S	ENSGALG00010018302	RAB11FIP5	case	control	-0.23834803	0.128033108	-1.861612477	0.062657733	0.157059394	down_in_case
Yang2025_S	ENSGALG00010018306	DES	case	control	0.491689116	0.179524304	2.738844294	0.006165557	0.028004247	up_in_case
Yang2025_S	ENSGALG00010018308	ACAP3	case	control	0.249024982	0.095682383	2.602621033	0.009251415	0.038357177	up_in_case
Yang2025_S	ENSGALG00010018309	DPYSL2	case	control	-0.081945206	0.089349683	-0.917129227	0.359074927	0.5279845	down_in_case
Yang2025_S	ENSGALG00010018310	APOOL	case	control	-0.098946157	0.090807944	-1.089620051	0.275880547	0.443004773	down_in_case
Yang2025_S	ENSGALG00010018311	NKX2-3	case	control	2.387019059	0.942364454	2.533010503	0.011308756	0.044328417	up_in_case
Yang2025_S	ENSGALG00010018312	PRPF39	case	control	0.474546154	0.07047104	6.733917262	1.65155E-11	1.65571E-09	up_in_case
Yang2025_S	ENSGALG00010018313	MYEF2	case	control	0.420603314	0.123630733	3.402093496	0.000668718	0.005058946	up_in_case
Yang2025_S	ENSGALG00010018314	SLC24A3	case	control	-0.244220686	0.167332235	-1.459495747	0.14442871	0.285274852	down_in_case
Yang2025_S	ENSGALG00010018315	CLASP2	case	control	0.140813745	0.099716422	1.412141975	0.157908162	0.303134073	up_in_case
Yang2025_S	ENSGALG00010018317	ZMAT3	case	control	-0.142589796	0.151711313	-0.939875827	0.347281258	0.515758748	down_in_case
Yang2025_S	ENSGALG00010018319	STUB1	case	control	0.254729737	0.077757274	3.275960234	0.001053034	0.007295887	up_in_case
Yang2025_S	ENSGALG00010018322	NT5C1A	case	control	-0.154984615	0.145490276	-1.065257552	0.286759396	0.455331835	down_in_case
Yang2025_S	ENSGALG00010018323	GOT1	case	control	-0.459504759	0.241288239	-1.904381089	0.05686057	0.146813424	down_in_case
Yang2025_S	ENSGALG00010018325	ENSGALG00010018325	case	control	0.16902633	0.256892081	0.65796629	0.510559792	0.665854067	up_in_case
Yang2025_S	ENSGALG00010018326	LCMT1	case	control	-0.172668416	0.148899808	-1.159628197	0.246200216	0.411910194	down_in_case
Yang2025_S	ENSGALG00010018327	SNAP25	case	control	1.826694697	0.636288752	2.870858066			up_in_case
Yang2025_S	ENSGALG00010018329	ENSGALG00010018329	case	control	-0.698535798	0.252168022	-2.770120463	0.005603556	0.026077107	down_in_case
Yang2025_S	ENSGALG00010018333	ENSGALG00010018333	case	control	0.721134015	0.337248187	2.138288784	0.032493312	0.09720788	up_in_case
Yang2025_S	ENSGALG00010018336	JMJD8	case	control	-0.039379943	0.180246055	-0.218478807	0.827056069	0.896430655	down_in_case
Yang2025_S	ENSGALG00010018337	TMEM151B	case	control	-0.027584191	0.191462833	-0.144070735	0.885444614	0.932769179	down_in_case
Yang2025_S	ENSGALG00010018338	PDCD6IP	case	control	0.258243178	0.169439172	1.524105518	0.127482355	0.260330572	up_in_case
Yang2025_S	ENSGALG00010018339	FAM76A	case	control	-0.288364321	0.092469812	-3.11846986	0.001817927	0.011135304	down_in_case
Yang2025_S	ENSGALG00010018340	DNPEP	case	control	0.228244566	0.117693785	1.939308577	0.052463775	0.138122559	up_in_case
Yang2025_S	ENSGALG00010018342	BNIP3L	case	control	-0.291046672	0.096831671	-3.005697099	0.002649728	0.014799183	down_in_case
Yang2025_S	ENSGALG00010018344	SFXN5	case	control	0.156401738	0.188632447	0.829134859	0.4070281	0.573038652	up_in_case
Yang2025_S	ENSGALG00010018345	WDR24	case	control	-0.303754308	0.104973781	-2.893620726	0.003808279	0.019478922	down_in_case
Yang2025_S	ENSGALG00010018348	TOGARAM1	case	control	0.153927824	0.088077627	1.747638181	0.0805267	0.18812063	up_in_case
Yang2025_S	ENSGALG00010018349	KCNMB2	case	control	0.153158203	0.203385498	0.753043874	0.45142355	0.613451715	up_in_case
Yang2025_S	ENSGALG00010018350	CNNM1	case	control	0.430991693	0.463523019	0.929817238	0.35246572	0.521291637	up_in_case
Yang2025_S	ENSGALG00010018351	RIN2	case	control	-0.26644694	0.078654518	-3.387560544	0.000705172	0.005288786	down_in_case
Yang2025_S	ENSGALG00010018354	AARS2	case	control	-0.287959994	0.213002112	-1.351911446	0.176403645	0.327234398	down_in_case
Yang2025_S	ENSGALG00010018355	PRKCB	case	control	-0.120700319	0.172029861	-0.701624229	0.482913537	0.641139495	down_in_case
Yang2025_S	ENSGALG00010018358	FBXL16	case	control	0.535582527	0.462782439	1.157309529	0.247145922	0.41283637	up_in_case
Yang2025_S	ENSGALG00010018359	CACNG3	case	control	0.648450809	0.600489298	1.079870718	0.280199754	0.448030244	up_in_case
Yang2025_S	ENSGALG00010018361	ASIC4	case	control	0.542440202	0.228111028	2.377965706	0.017408445	0.061259262	up_in_case
Yang2025_S	ENSGALG00010018363	TCTE1	case	control	-0.13506388	0.202652011	-0.666481814	0.505103184	0.6607165	down_in_case
Yang2025_S	ENSGALG00010018365	HPSE2	case	control	-0.065590124	0.222007586	-0.295440912	0.767657084	0.858737931	down_in_case
Yang2025_S	ENSGALG00010018367	PAK5	case	control	-0.100572251	0.228513764	-0.44011463	0.659854087	0.785118189	down_in_case
Yang2025_S	ENSGALG00010018370	ZBTB8A	case	control	-0.611393464	0.128968267	-4.740650399	2.13033E-06	4.58828E-05	down_in_case
Yang2025_S	ENSGALG00010018371	ENSGALG00010018371	case	control	-0.142825183	0.158730191	-0.899798475	0.368227506	0.536765219	down_in_case
Yang2025_S	ENSGALG00010018372	NLGN3	case	control	0.313452024	0.22151707	1.415024241	0.157061389	0.302258038	up_in_case
Yang2025_S	ENSGALG00010018374	HPS1	case	control	0.09789916	0.265437473	0.368821924	0.712260462	0.822451901	up_in_case
Yang2025_S	ENSGALG00010018376	PYGB	case	control	0.203540736	0.137182811	1.483718942	0.137883499	0.275649051	up_in_case
Yang2025_S	ENSGALG00010018377	RAP1GAP	case	control	0.410001067	0.194981824	2.10276558	0.035486266	0.10388679	up_in_case
Yang2025_S	ENSGALG00010018378	ARPP21	case	control	1.009111282	0.267889637	3.766891822	0.000165293	0.001600547	up_in_case
Yang2025_S	ENSGALG00010018379	SRBD1	case	control	-0.560158724	0.104745425	-5.347810899	8.90244E-08	3.09365E-06	down_in_case
Yang2025_S	ENSGALG00010018380	SLC20A1	case	control	0.030815666	0.148795994	0.207100104	0.835931681	0.902860639	up_in_case
Yang2025_S	ENSGALG00010018381	HAPSTR1	case	control	-0.122303151	0.085864159	-1.424379539	0.154336634	0.298664213	down_in_case
Yang2025_S	ENSGALG00010018383	METRN	case	control	0.020196045	0.204883905	0.098573117	0.921477215	0.953337584	up_in_case
Yang2025_S	ENSGALG00010018384	LAMP5	case	control	0.058217019	0.225498537	0.258170275	0.796275498	0.876731298	up_in_case
Yang2025_S	ENSGALG00010018385	SPR	case	control	0.208651483	0.252596677	0.826026237	0.408789209	0.574475118	up_in_case
Yang2025_S	ENSGALG00010018387	NT5DC4	case	control	-0.374453235	0.211097115	-1.773843446	0.076089049	0.1806169	down_in_case
Yang2025_S	ENSGALG00010018388	SMC3	case	control	0.625341786	0.174707556	3.579363134	0.000344433	0.0029302	up_in_case
Yang2025_S	ENSGALG00010018391	STAC	case	control	0.451416912	0.190847476	2.365328173	0.0180141	0.062866639	up_in_case
Yang2025_S	ENSGALG00010018393	PRKCE	case	control	0.24239736	0.175264745	1.383035471	0.16665399	0.314529759	up_in_case
Yang2025_S	ENSGALG00010018394	KLHL28	case	control	-0.16638331	0.116386282	-1.429578363	0.152838069	0.296505429	down_in_case
Yang2025_S	ENSGALG00010018395	MED12	case	control	0.174572023	0.099197504	1.759842893	0.078434448	0.184690477	up_in_case
Yang2025_S	ENSGALG00010018396	TBL1XR1	case	control	0.216397157	0.158042653	1.369232628	0.17092657	0.319897285	up_in_case
Yang2025_S	ENSGALG00010018397	FAM173A	case	control	-0.499569602	0.295919033	-1.688196926	0.091373433	0.206000452	down_in_case
Yang2025_S	ENSGALG00010018398	LRFN5	case	control	-0.470170191	0.235341038	-1.997824927	0.045735644	0.125211434	down_in_case
Yang2025_S	ENSGALG00010018399	ABHD12	case	control	-0.635050208	0.126583459	-5.016849843	5.25256E-07	1.40488E-05	down_in_case
Yang2025_S	ENSGALG00010018401	ENSGALG00010018401	case	control	-0.27159065	0.16124857	-1.684298035	0.092124085	0.207230189	down_in_case
Yang2025_S	ENSGALG00010018404	IL2RG	case	control	0.051395435	0.162214825	0.316835624	0.751368332	0.848540188	up_in_case
Yang2025_S	ENSGALG00010018405	DCLK3	case	control	0.721891692	0.256834839	2.810723394	0.004943026	0.023769828	up_in_case
Yang2025_S	ENSGALG00010018406	TMEM260	case	control	0.214385703	0.209370507	1.023953685	0.305857151	0.475470705	up_in_case
Yang2025_S	ENSGALG00010018410	EXOC6B	case	control	0.808919294	0.197895375	4.087610917	4.35838E-05	0.000548608	up_in_case
Yang2025_S	ENSGALG00010018413	SLC15A2	case	control	0.225293976	0.276927198	0.813549472	0.415903094	0.581363191	up_in_case
Yang2025_S	ENSGALG00010018414	EPAS1	case	control	-0.232258832	0.139378847	-1.666385088	0.095636739	0.212583268	down_in_case
Yang2025_S	ENSGALG00010018415	PELI2	case	control	0.269448529	0.112852071	2.387625915	0.016957593	0.060082394	up_in_case
Yang2025_S	ENSGALG00010018416	DUT	case	control	-0.260222022	0.268466474	-0.969290571	0.332400232	0.501678462	down_in_case
Yang2025_S	ENSGALG00010018418	PLCB4	case	control	0.504556042	0.187274047	2.694212304	0.007055521	0.031143073	up_in_case
Yang2025_S	ENSGALG00010018419	USP7	case	control	-0.244680834	0.084441604	-2.897633665	0.003759895	0.019298024	down_in_case
Yang2025_S	ENSGALG00010018420	RHOQ	case	control	-0.159192645	0.140522682	-1.132860844	0.257272695	0.423569826	down_in_case
Yang2025_S	ENSGALG00010018422	ENSGALG00010018422	case	control	0.089296632	0.19903663	0.448644212	0.653688334	0.780828642	up_in_case
Yang2025_S	ENSGALG00010018423	IGFALS	case	control	-1.630786797	0.776973926	-2.098895138	0.035826146	0.104574222	down_in_case
Yang2025_S	ENSGALG00010018425	CYP26B1	case	control	0.483857037	0.160553723	3.013676843	0.002581026	0.014526088	up_in_case
Yang2025_S	ENSGALG00010018426	ENSGALG00010018426	case	control	0.310212072	0.243318705	1.274920776	0.202337284	0.3602824	up_in_case
Yang2025_S	ENSGALG00010018427	PIGF	case	control	0.370982866	0.153537876	2.416230293	0.015682139	0.056879389	up_in_case
Yang2025_S	ENSGALG00010018428	PPP2R5D	case	control	-0.031185538	0.092334294	-0.337745998	0.735554608	0.838716428	down_in_case
Yang2025_S	ENSGALG00010018430	LOXL4	case	control	0.071484597	0.324412657	0.220350827	0.825597939	0.895600276	up_in_case
Yang2025_S	ENSGALG00010018434	HSPBAP1	case	control	0.088480156	0.15813714	0.559515344	0.575810062	0.718602294	up_in_case
Yang2025_S	ENSGALG00010018436	FOXO4	case	control	-0.616298449	0.108771363	-5.665999149	1.4617E-08	6.46629E-07	down_in_case
Yang2025_S	ENSGALG00010018438	PPP2R2A	case	control	-0.010304755	0.081393522	-0.126604116	0.899253739	0.940208647	down_in_case
Yang2025_S	ENSGALG00010018440	MEA1	case	control	0.459191619	0.209450076	2.192367878	0.028352954	0.087741154	up_in_case
Yang2025_S	ENSGALG00010018441	CRIPT	case	control	-0.711043903	0.137798958	-5.160009277	2.46938E-07	7.23621E-06	down_in_case
Yang2025_S	ENSGALG00010018443	PLCB1	case	control	0.310893664	0.180266517	1.724633445	0.084593552	0.195038245	up_in_case
Yang2025_S	ENSGALG00010018444	NUBP2	case	control	-0.023563855	0.240036682	-0.098167725	0.92179911	0.953337584	down_in_case
Yang2025_S	ENSGALG00010018446	FBN1	case	control	-0.476715408	0.227106759	-2.099080677	0.03580979	0.10454838	down_in_case
Yang2025_S	ENSGALG00010018448	KLHDC3	case	control	0.145031705	0.117009843	1.239482949	0.215166699	0.375968396	up_in_case
Yang2025_S	ENSGALG00010018451	PIK3R4	case	control	-0.006061909	0.089140513	-0.068003969	0.945782475	0.96645001	down_in_case
Yang2025_S	ENSGALG00010018452	SPSB3	case	control	-0.258694343	0.120758868	-2.142238884	0.032174264	0.096502017	down_in_case
Yang2025_S	ENSGALG00010018453	TMX4	case	control	-0.166400494	0.086952482	-1.913694591	0.055659191	0.144460946	down_in_case
Yang2025_S	ENSGALG00010018455	RBM20	case	control	0.064422312	0.150668181	0.427577423	0.668958806	0.791176862	up_in_case
Yang2025_S	ENSGALG00010018458	SEMA5B	case	control	-0.243593829	0.149728075	-1.62690818	0.1037566	0.225210004	down_in_case
Yang2025_S	ENSGALG00010018459	SNX12	case	control	-0.496145678	0.175303555	-2.830208876	0.004651762	0.022704837	down_in_case
Yang2025_S	ENSGALG00010018462	KTN1	case	control	0.498573605	0.151849707	3.28333596	0.001025863	0.007154857	up_in_case
Yang2025_S	ENSGALG00010018465	NAALADL2	case	control	0.250032327	0.234311496	1.067093722	0.285929517	0.454480189	up_in_case
Yang2025_S	ENSGALG00010018466	DYSF	case	control	0.220567342	0.152495781	1.446383242	0.14806974	0.29017144	up_in_case
Yang2025_S	ENSGALG00010018467	TLX1	case	control	1.263857965	0.416983745	3.030952599	0.002437835	0.013934058	up_in_case
Yang2025_S	ENSGALG00010018469	KLHDC7A	case	control	0.399698459	0.16765624	2.384035695	0.017123941	0.060533263	up_in_case
Yang2025_S	ENSGALG00010018470	IQCB1	case	control	0.16062626	0.147256153	1.090794897	0.275363142	0.442336123	up_in_case
Yang2025_S	ENSGALG00010018471	HHEX	case	control	-0.258543787	0.119253801	-2.168012966	0.0301577	0.09199815	down_in_case
Yang2025_S	ENSGALG00010018472	LMF1	case	control	-0.411438533	0.143929946	-2.858602698	0.004255113	0.021086418	down_in_case

Yang2025_S	ENSGALG00010018476	ENSGALG00010018476	case	control	0.909459908	0.180551135	5.037132056	4.72559E-07	1.28115E-05	up_in_case
Yang2025_S	ENSGALG00010018480	EME2	case	control	0.235119358	0.298915541	0.786574552	0.431530961	0.595561448	up_in_case
Yang2025_S	ENSGALG00010018482	TWNK	case	control	0.080099993	0.191388626	0.41852013	0.675566872	0.796044678	up_in_case
Yang2025_S	ENSGALG00010018484	NLGN1	case	control	0.235817751	0.266055665	0.886347413	0.375430347	0.543601609	up_in_case
Yang2025_S	ENSGALG00010018485	ATP2C1	case	control	-0.100681108	0.064666363	-1.556931666	0.11948671	0.248774437	down_in_case
Yang2025_S	ENSGALG00010018486	ENSGALG00010018486	case	control	0.222015888	0.18231165	1.21778223	0.223306739	0.384728306	up_in_case
Yang2025_S	ENSGALG00010018487	ENSGALG00010018487	case	control	-0.209250697	0.149717006	-1.39764147	0.162220759	0.30839649	down_in_case
Yang2025_S	ENSGALG00010018488	ENSGALG00010018488	case	control	0.202195672	0.198934593	1.016392719	0.309442411	0.47927976	up_in_case
Yang2025_S	ENSGALG00010018489	SLC7A3	case	control	-0.07176296	0.136820727	-0.524503569	0.599928338	0.738711796	down_in_case
Yang2025_S	ENSGALG00010018490	NME3	case	control	-0.461645227	0.198275158	-2.328305937	0.019895864	0.067545131	down_in_case
Yang2025_S	ENSGALG00010018491	TIA1	case	control	0.528785767	0.102408517	5.163494019	2.42382E-07	7.2017E-06	up_in_case
Yang2025_S	ENSGALG00010018492	ENSGALG00010018492	case	control	-0.210788793	0.144495218	-1.458794257	0.144621744	0.285546114	down_in_case
Yang2025_S	ENSGALG00010018493	ENSGALG00010018493	case	control	0.940672753	0.504746735	1.86365297	0.062370454	0.156656863	up_in_case
Yang2025_S	ENSGALG00010018494	HAO1	case	control	-0.461877015	0.582495156	-0.792928508	0.427819477	0.592197465	down_in_case
Yang2025_S	ENSGALG00010018495	ENSGALG00010018495	case	control	0.221257769	0.362850577	0.609776538	0.542009845	0.692292135	up_in_case
Yang2025_S	ENSGALG00010018496	GNG13	case	control	0.140474323	0.543783915	0.25832747	0.796154188	0.876681834	up_in_case
Yang2025_S	ENSGALG00010018497	ASTE1	case	control	-0.291509286	0.220241146	-1.323591398	0.185638784	0.339173522	down_in_case
Yang2025_S	ENSGALG00010018498	ENSGALG00010018498	case	control	-0.655997966	0.166928092	-3.929823668	8.50082E-05	0.000937917	down_in_case
Yang2025_S	ENSGALG00010018501	ATG14	case	control	0.214137216	0.117948405	1.815516004	0.069444649	0.169079849	up_in_case
Yang2025_S	ENSGALG00010018502	ARHGEF10L	case	control	0.287862624	0.121696558	2.365413031	0.018009973	0.062866639	up_in_case
Yang2025_S	ENSGALG00010018503	BMP2	case	control	0.388988912	0.252794523	1.538755299	0.123864047	0.255068049	up_in_case
Yang2025_S	ENSGALG00010018504	SLCO3A1	case	control	-0.010814634	0.091002031	-0.118839482	0.905402528	0.943381503	down_in_case
Yang2025_S	ENSGALG00010018505	CHTF18	case	control	-0.021059177	0.101000537	-0.208505591	0.834834218	0.902234747	down_in_case
Yang2025_S	ENSGALG00010018506	ENSGALG00010018506	case	control	0.856039511	0.236818176	3.614754261	0.000300633	0.002613422	up_in_case
Yang2025_S	ENSGALG00010018508	ENSGALG00010018508	case	control	0.374120593	0.134108719	2.78968135	0.005275994	0.024939827	up_in_case
Yang2025_S	ENSGALG00010018509	ENSGALG00010018509	case	control	0.172564029	0.099030175	1.742539873	0.081414021	0.189557958	up_in_case
Yang2025_S	ENSGALG00010018510	ENSGALG00010018510	case	control	-0.221576769	0.16525006	-1.340857417	0.17996675	0.332009299	down_in_case
Yang2025_S	ENSGALG00010018511	SOCS5	case	control	-0.429537596	0.093365614	-4.60059736	4.21281E-06	8.04185E-05	down_in_case
Yang2025_S	ENSGALG00010018512	FERMT1	case	control	0.065587002	0.119058197	0.55088187	0.581714656	0.724219935	up_in_case
Yang2025_S	ENSGALG00010018513	RPUSD1	case	control	-0.08981072	0.13743534	-0.653476177	0.513449343	0.668371471	down_in_case
Yang2025_S	ENSGALG00010018514	EAF2	case	control	0.335489626	0.200824431	1.670561813	0.09480826	0.211153957	up_in_case
Yang2025_S	ENSGALG00010018515	SEMA4G	case	control	0.296150149	0.234772829	1.26143281	0.207152953	0.365818832	up_in_case
Yang2025_S	ENSGALG00010018516	LRRN4	case	control	-0.042096806	0.228952691	-0.183866832	0.854117925	0.913827938	down_in_case
Yang2025_S	ENSGALG00010018517	ECT2	case	control	-0.585179964	0.133643666	-4.378658438	1.19412E-05	0.000190608	down_in_case
Yang2025_S	ENSGALG00010018519	OGDH	case	control	-0.023630402	0.089130287	-0.265122025	0.790915461	0.873951863	down_in_case
Yang2025_S	ENSGALG00010018520	ENSGALG00010018520	case	control	0.435868765	0.208644092	2.089053954	0.036702867	0.106353597	up_in_case
Yang2025_S	ENSGALG00010018521	ENSGALG00010018521	case	control	0.736625823	0.104218259	7.068107144	1.57061E-12	2.06476E-10	up_in_case
Yang2025_S	ENSGALG00010018522	NEK11	case	control	0.020227422	0.29047633	0.069635355	0.944483896	0.965993889	up_in_case
Yang2025_S	ENSGALG00010018523	TEX11	case	control	0.177285141	0.167527268	1.058246479	0.289943086	0.458766539	up_in_case
Yang2025_S	ENSGALG00010018524	SEC22A	case	control	0.061512641	0.102651902	0.599235277	0.549016001	0.697656503	up_in_case
Yang2025_S	ENSGALG00010018525	ST8SIA2	case	control	-0.062675646	0.153900676	-0.40724737	0.683826313	0.801979438	down_in_case
Yang2025_S	ENSGALG00010018526	ENSGALG00010018526	case	control	0.107626924	0.12456753	0.864004645	0.387585339	0.55485943	up_in_case
Yang2025_S	ENSGALG00010018527	CCT4	case	control	-0.348336246	0.155529336	-2.239681939	0.025111579	0.080185576	down_in_case
Yang2025_S	ENSGALG00010018528	ENSGALG00010018528	case	control	-0.224119671	0.609230613	-0.367873292	0.712967716	0.822771185	down_in_case
Yang2025_S	ENSGALG00010018529	UBR2	case	control	0.034921053	0.083421896	0.418607766	0.675502813	0.796044678	up_in_case
Yang2025_S	ENSGALG00010018530	FAM161A	case	control	0.45271603	0.228789732	1.978742779	0.047844974	0.12934291	up_in_case
Yang2025_S	ENSGALG00010018531	ADCY5	case	control	-0.075169717	0.129524847	-0.580349783	0.561678762	0.708132954	down_in_case
Yang2025_S	ENSGALG00010018532	MFSD13A	case	control	-0.036299666	0.119495874	-0.303773388	0.761300542	0.854874912	down_in_case
Yang2025_S	ENSGALG00010018533	PCYOX1	case	control	-0.042163154	0.132105874	-0.319161843	0.749603789	0.847783798	down_in_case
Yang2025_S	ENSGALG00010018534	NCEH1	case	control	-0.0807631	0.130510954	-0.618822387	0.536033373	0.687178017	down_in_case
Yang2025_S	ENSGALG00010018535	FAM174B	case	control	-0.146336108	0.067621032	-2.16406205	0.030459588	0.092776908	down_in_case
Yang2025_S	ENSGALG00010018536	ENSGALG00010018536	case	control	1.487903917	0.46677678	3.187613398	0.001434522	0.009293381	up_in_case
Yang2025_S	ENSGALG00010018539	CHD2	case	control	0.010013062	0.121419915	0.082466392	0.934275842	0.959922758	up_in_case
Yang2025_S	ENSGALG00010018540	TNFSF10	case	control	-0.087002956	0.134795953	-0.645441899	0.518640859	0.672803981	down_in_case
Yang2025_S	ENSGALG00010018542	ZMIZ2	case	control	0.172282865	0.121814877	1.414300697	0.157273633	0.302541147	up_in_case
Yang2025_S	ENSGALG00010018543	MRPL3	case	control	-0.218616632	0.113986443	-1.917917848	0.055121429	0.14330692	down_in_case
Yang2025_S	ENSGALG00010018544	HACD2	case	control	-0.226918592	0.106301243	-2.134674867	0.032787577	0.097878083	down_in_case
Yang2025_S	ENSGALG00010018545	ENSGALG00010018545	case	control	3.312023997	0.684966851	4.83530552	1.32941E-06	3.08242E-05	up_in_case
Yang2025_S	ENSGALG00010018546	SNRPGP15	case	control	-0.600872577	0.285649338	-2.103532184	0.035419275	0.103734257	down_in_case
Yang2025_S	ENSGALG00010018548	RGMA	case	control	-0.097198239	0.073963578	-1.314136524	0.188800265	0.343508512	down_in_case
Yang2025_S	ENSGALG00010018549	GHSR	case	control	-0.05078296	0.35983041	-0.141130262	0.887767038	0.934509266	down_in_case
Yang2025_S	ENSGALG00010018551	FBXO34	case	control	0.037272587	0.103418965	0.360403796	0.718545188	0.826485326	up_in_case
Yang2025_S	ENSGALG00010018552	DTD1	case	control	-0.034112984	0.132179949	-0.258079873	0.796345264	0.876731298	down_in_case
Yang2025_S	ENSGALG00010018554	PPIA	case	control	-0.174288072	0.153015763	-1.13902038	0.254694654	0.421184398	down_in_case
Yang2025_S	ENSGALG00010018555	UBN1	case	control	0.13932565	0.125106304	1.113658109	0.265425887	0.432573553	up_in_case
Yang2025_S	ENSGALG00010018556	MRPL2	case	control	0.13261545	0.25347861	0.523182016	0.600847595	0.739647636	up_in_case
Yang2025_S	ENSGALG00010018557	TBCC	case	control	0.10020637	0.180529113	0.555070418	0.578846496	0.721680766	up_in_case
Yang2025_S	ENSGALG00010018558	ENSGALG00010018558	case	control	0.132179705	0.259083263	0.510182339	0.609923724	0.747255767	up_in_case
Yang2025_S	ENSGALG00010018559	DLG3	case	control	0.165960089	0.12033738	1.379123337	0.16785673	0.316092369	up_in_case
Yang2025_S	ENSGALG00010018560	ENSGALG00010018560	case	control	0.097649906	0.089185806	1.094904123	0.273558643	0.440851126	up_in_case
Yang2025_S	ENSGALG00010018561	ACTR1A	case	control	-0.299919081	0.11934987	-2.512940152	0.011972964	0.046242589	down_in_case
Yang2025_S	ENSGALG00010018562	ENSGALG00010018562	case	control	0.389915235	0.358221313	1.088475812	0.276385109	0.443661617	up_in_case
Yang2025_S	ENSGALG00010018564	DLGAP5	case	control	-0.40407478	0.098430221	-4.105190196	4.03982E-05	0.00051837	down_in_case
Yang2025_S	ENSGALG00010018566	ENSGALG00010018566	case	control	0.319968728	0.15495227	2.064950252	0.038927708	0.111000124	up_in_case
Yang2025_S	ENSGALG00010018568	FAM136A	case	control	1.575984448	0.202187831	7.794655288	6.45845E-15	1.40015E-12	up_in_case
Yang2025_S	ENSGALG00010018573	SEC23B	case	control	0.278878979	0.08918981	3.126803137	0.001767182	0.01091564	up_in_case
Yang2025_S	ENSGALG00010018574	ENSGALG00010018574	case	control	0.371554897	0.307208941	1.209453396	0.226488705	0.388474461	up_in_case
Yang2025_S	ENSGALG00010018576	PTK7	case	control	0.166692707	0.082379236	2.023479637	0.043023721	0.119500808	up_in_case
Yang2025_S	ENSGALG00010018577	NARFL	case	control	-0.233461706	0.15517871	-1.504469952	0.132460404	0.267551201	down_in_case
Yang2025_S	ENSGALG00010018579	FNDC3B	case	control	0.014635513	0.170848958	0.085663459	0.931733951	0.958561285	up_in_case
Yang2025_S	ENSGALG00010018581	SUFU	case	control	-0.161481602	0.070211606	-2.299927466	0.02145233	0.071204252	down_in_case
Yang2025_S	ENSGALG00010018582	RCC2	case	control	-0.074791402	0.08823236	-0.847664075	0.396625079	0.563803986	down_in_case
Yang2025_S	ENSGALG00010018583	PPL	case	control	0.691524463	0.268066184	2.57967809	0.009889245	0.040212032	up_in_case
Yang2025_S	ENSGALG00010018585	GDPD2	case	control	0.045138511	0.541864398	0.083302227	0.933611229	0.959571654	up_in_case
Yang2025_S	ENSGALG00010018587	SRF	case	control	0.144668485	0.120441609	1.201150382	0.229692873	0.39210709	up_in_case
Yang2025_S	ENSGALG00010018588	POLR3F	case	control	0.225840079	0.131782806	1.713729479	0.08657842	0.198236984	up_in_case
Yang2025_S	ENSGALG00010018590	TGFA	case	control	0.981145751	0.336835835	2.912830667	0.003581688	0.018588759	up_in_case
Yang2025_S	ENSGALG00010018593	LGALS3	case	control	0.51325643	0.459020759	1.118155161	0.263500731	0.430466903	up_in_case
Yang2025_S	ENSGALG00010018594	BICRAL	case	control	0.384697165	0.200078999	1.922726358	0.054514425	0.142125072	up_in_case
Yang2025_S	ENSGALG00010018598	ENSGALG00010018598	case	control	0.146824177	0.122815215	1.195488497	0.231896228	0.395045714	up_in_case
Yang2025_S	ENSGALG00010018601	CPNE4	case	control	0.152032785	0.376313477	0.404005687	0.686208537	0.804028922	up_in_case
Yang2025_S	ENSGALG00010018602	MCTP2	case	control	0.02257098	0.176231195	0.128075963	0.898088858	0.939625215	up_in_case
Yang2025_S	ENSGALG00010018604	FAM43A	case	control	0.354742097	0.17957334	1.975471957	0.048214614	0.129955637	up_in_case
Yang2025_S	ENSGALG00010018606	DZANK1	case	control	0.59790643	0.260412078	2.296001149	0.021675816	0.071772187	up_in_case
Yang2025_S	ENSGALG00010018607	HAGHL	case	control	-0.217835713	0.203712506	-1.069329109	0.284921399	0.453136235	down_in_case
Yang2025_S	ENSGALG00010018608	TRIM8	case	control	0.631463999	0.188605579	3.348066392	0.000813775	0.005928557	up_in_case
Yang2025_S	ENSGALG00010018609	ENSGALG00010018609	case	control	-0.153728355	0.151944919	-1.011737381	0.311663637	0.481329135	down_in_case
Yang2025_S	ENSGALG00010018611	NUDCD3	case	control	0.129843378	0.107793801	1.204553296	0.228375796	0.390624366	up_in_case
Yang2025_S	ENSGALG00010018613	PADI3	case	control	0.312822352	0.275131167	1.136993512	0.255540998	0.421815187	up_in_case
Yang2025_S	ENSGALG00010018615	HTR2C	case	control	0.132456347	0.30249401	0.437880892	0.661472628	0.786223072	up_in_case
Yang2025_S	ENSGALG00010018616	ENSGALG00010018616	case	control	0.690618347	0.293819821	2.350482496	0.01874909	0.064638437	up_in_case
Yang2025_S	ENSGALG00010018618	FAHD1	case	control	0.09777358	0.237063497	0.41243625	0.680019698	0.799187023	up_in_case
Yang2025_S	ENSGALG00010018619	MAPK1IP1L	case	control	-0.234106269	0.091503521	-2.55844001	0.010514296	0.042017787	down_in_case
Yang2025_S	ENSGALG00010018620	ENSGALG00010018620	case	control	2.185241356	0.869559402	2.513044365	0.011969428	0.046242589	up_in_case
Yang2025_S	ENSGALG00010018623	ARRDC4	case	control	0.194607999	0.162928461	1.194438327	0.232306552	0.395454654	up_in_case
Yang2025_S	ENSGALG00010018624	ENSGALG00010018624	case	control	0.739604408	0.439399955	1.683214572	0.092333561	0.207461931	up_in_case
Yang2025_S	ENSGALG00010018627	MYLK	case	control	0.38197925	0.188875232	2.022389315	0.043136148	0.119717631	up_in_case
Yang2025_S	ENSGALG00010018628	XDH	case	control	1.256544984	0.717084669	1.752296539	0.079722831	0.18693213	up_in_case
Yang2025_S	ENSGALG00010018631	KAT14	case	control	-0.197443814	0.104514849	-1.889146044	0.058872262	0.150639914	down_in_case
Yang2025_S	ENSGALG00010018633	ENSGALG00010018633	case	control	0.529889733	0.267354369	1.981975219	0.047482014	0.128702951	up_in_case
Yang2025_S	ENSGALG00010018638	SEC14L5	case	control	-0.429623024	0.187814709	-2.287483368	0.022167625	0.072934492	down_in_case
Yang2025_S	ENSGALG00010018644	ESF1	case	control	0.606879498	0.136723069	4.438749834	9.04829E-06	0.000150284	up_in_case
Yang2025_S	ENSGALG00010018649	HAGH	case	control	-0.029824167	0.188567064	-0.158162121	0.874329051	0.926381944	down_in_case
Yang2025_S	ENSGALG00010018654	ENSGALG00010018654	case	control	-0.367585535	0.313848396	-1.171220051	0.241510338	0.406651348	down_in_case
Yang2025_S	ENSGALG00010018655	BIRC5	case	control	-1.260278425	0.268457039	-4.694525543	2.67226E-06	5.51674E-05	down_in_case
Yang2025_S	ENSGALG00010018665	IL13RA2	case	control	-0.028466245	0.273954851	-0.103908527	0.91724194	0.951236618	down_in_case
Yang2025_S	ENSGALG00010018670	MRAS	case	control	0.02804287	0.157707969	0.177815175	0.858868129	0.916888279	up_in_case
Yang2025_S	ENSGALG00010018672	PKDCC	case	control	-0.231787266	0.11882014	-1.95074056	0.051087916	0.135284782	down_in_case
Yang2025_S	ENSGALG00010018673	NAGPA	case	control	-0.098740019	0.110891912	-0.890416777	0.373242137	0.541255415	down_in_case
Yang2025_S	ENSGALG00010018684	PADI2	case	control	0.579731686	0.283506274	2.044863693	0.040868298	0.114934356	up_in_case
Yang2025_S	ENSGALG00010018685	ARL3	case	control	-0.235927083	0.109859116	-2.147542155	0.031750145	0.095579665	down_in_case
Yang2025_S	ENSGALG00010018693	ENSGALG00010018693	case	control	1.04712907	0.160130597	6.539219193	6.18408E-11	5.48885E-09	up_in_case
Yang2025_S	ENSGALG00010018694	MGME1	case	control	-0.106675532	0.145819909	-0.7315567	0.464439185	0.625431005	down_in_case
Yang2025_S	ENSGALG00010018698	MRPS16	case	control	0.511089512	0.321938822	1.587536131	0.112391271	0.238230813	up_in_case
Yang2025_S	ENSGALG00010018701	SFXN2	case	control	-0.072003866	0.130000925	-0.553871948	0.579666484	0.722444545	down_in_case
Yang2025_S	ENSGALG00010018704	PGPEP1L	case	control	0.307421981	0.222703668	1.380408252	0.167460981	0.315517681	up_in_case

Yang2025_S	ENSGALG00010018707	SLC45A1	case	control	0.22021743	0.152632247	1.442797536	0.149077513	0.291497965	up_in_case
Yang2025_S	ENSGALG00010018709	SNX5	case	control	-0.311132422	0.112274617	-2.771173308	0.005585469	0.026005182	down_in_case
Yang2025_S	ENSGALG00010018713	ENSGALG00010018713	case	control	0.156696027	0.159438207	0.98280099	0.325705395	0.495654109	up_in_case
Yang2025_S	ENSGALG00010018715	DNAJC13	case	control	0.085988561	0.090020722	0.955208525	0.339472256	0.507560552	up_in_case
Yang2025_S	ENSGALG00010018719	ERRFI1	case	control	-0.119803275	0.152821786	-0.783941075	0.433074687	0.5971006	down_in_case
Yang2025_S	ENSGALG00010018721	LRCH2	case	control	-0.059610984	0.142357671	-0.41874093	0.675405479	0.796044678	down_in_case
Yang2025_S	ENSGALG00010018724	RRBP1	case	control	0.508818176	0.145269579	3.502578983	0.000460777	0.003698692	up_in_case
Yang2025_S	ENSGALG00010018726	EBF2	case	control	-0.003243289	0.221296287	-0.014655868	0.988306728	0.992866719	down_in_case
Yang2025_S	ENSGALG00010018727	PARK7	case	control	0.416735298	0.146301257	2.848473797	0.004392947	0.021639531	up_in_case
Yang2025_S	ENSGALG00010018729	HS3ST6	case	control	1.529428718	0.681656397	2.243694513	0.024852063	0.0796123	up_in_case
Yang2025_S	ENSGALG00010018730	IGF1R	case	control	-0.141294689	0.149762341	-0.943459404	0.345445971	0.514128976	down_in_case
Yang2025_S	ENSGALG00010018732	ENSGALG00010018732	case	control	0.291354861	0.108626996	2.682158876	0.007314871	0.031933812	up_in_case
Yang2025_S	ENSGALG00010018736	EEF2KMT	case	control	0.586844193	0.136044091	4.313632353	1.60594E-05	0.000243157	up_in_case
Yang2025_S	ENSGALG00010018747	FLRT3	case	control	0.109609789	0.186612255	0.58736651	0.556957581	0.704215432	up_in_case
Yang2025_S	ENSGALG00010018750	SOCS4	case	control	-0.703985824	0.110114609	-6.39321005	1.62439E-10	1.23021E-08	down_in_case
Yang2025_S	ENSGALG00010018752	PLS3	case	control	-0.125621985	0.081499699	-1.541379742	0.123224392	0.254314558	down_in_case
Yang2025_S	ENSGALG00010018753	SYNM	case	control	0.291740606	0.212303113	1.374170175	0.169388858	0.318117754	up_in_case
Yang2025_S	ENSGALG00010018756	EML4	case	control	0.259294216	0.149603183	1.733213228	0.083057777	0.19238865	up_in_case
Yang2025_S	ENSGALG00010018758	ENSGALG00010018758	case	control	0.210067619	0.204583299	1.026807268	0.304511226	0.474171856	up_in_case
Yang2025_S	ENSGALG00010018764	TMEM177	case	control	-0.432915754	0.203713764	-2.125117843	0.033576781	0.099593964	down_in_case
Yang2025_S	ENSGALG00010018767	NDUFB6	case	control	0.022290135	0.289065347	0.077111057	0.938535197	0.962857098	up_in_case
Yang2025_S	ENSGALG00010018774	MSRB1	case	control	-0.187297626	0.250482591	-0.747747081	0.454612727	0.61636781	down_in_case
Yang2025_S	ENSGALG00010018780	WDHD1	case	control	-0.227623549	0.177632837	-1.2814272	0.200043655	0.357523193	down_in_case
Yang2025_S	ENSGALG00010018783	ENSGALG00010018783	case	control	0.263438263	0.099455755	2.648798585	0.008077846	0.034468417	up_in_case
Yang2025_S	ENSGALG00010018786	DSTN	case	control	0.142301546	0.10150633	1.401898245	0.160945635	0.306663926	up_in_case
Yang2025_S	ENSGALG00010018796	PER3	case	control	0.605158991	0.212451232	2.848460721	0.004393128	0.021639531	up_in_case
Yang2025_S	ENSGALG00010018799	ENSGALG00010018799	case	control	1.046440927	0.414474976	2.524738496	0.011578441	0.045170782	up_in_case
Yang2025_S	ENSGALG00010018806	GCH1	case	control	-0.504893029	0.241772364	-2.088299179	0.036770855	0.106484179	down_in_case
Yang2025_S	ENSGALG00010018807	RD3	case	control	-0.207729875	0.2870626	-0.723639635	0.469287002	0.629889652	down_in_case
Yang2025_S	ENSGALG00010018808	MATCAP2	case	control	-0.008476287	0.091827551	-0.092306574	0.926454465	0.955608219	down_in_case
Yang2025_S	ENSGALG00010018811	VAMP3	case	control	-0.067266164	0.125170772	-0.537395134	0.590994704	0.731720231	down_in_case
Yang2025_S	ENSGALG00010018815	ENSGALG00010018815	case	control	-0.900250187	0.260069258	-3.461578633	0.000537017	0.004194694	down_in_case
Yang2025_S	ENSGALG00010018819	LRRC28	case	control	-0.061499917	0.091995687	-0.668508703	0.503808929	0.659828705	down_in_case
Yang2025_S	ENSGALG00010018822	TRAF5	case	control	-0.290875767	0.195611507	-1.487007441	0.137012846	0.274518189	down_in_case
Yang2025_S	ENSGALG00010018823	RAB40C	case	control	0.032985325	0.079821308	0.413239599	0.679431078	0.798841721	up_in_case
Yang2025_S	ENSGALG00010018829	PTPN4	case	control	0.094274609	0.121559978	0.775539862	0.43802072	0.60195451	up_in_case
Yang2025_S	ENSGALG00010018831	WBP1L	case	control	-0.552314645	0.094445093	-5.847997241	4.97527E-09	2.43264E-07	down_in_case
Yang2025_S	ENSGALG00010018832	EEPD1	case	control	-0.306101641	0.123232264	-2.48394074	0.012993743	0.049216583	down_in_case
Yang2025_S	ENSGALG00010018834	SAMD4A	case	control	0.13997301	0.138634674	1.009653691	0.312661236	0.482229123	up_in_case
Yang2025_S	ENSGALG00010018835	CAMK2B	case	control	0.93417467	0.20040484	4.66143767	3.14008E-06	6.29598E-05	up_in_case
Yang2025_S	ENSGALG00010018837	RCOR3	case	control	0.119479198	0.152930037	0.781267045	0.434645448	0.598435775	up_in_case
Yang2025_S	ENSGALG00010018838	WFIKKN1	case	control	0.483578042	0.315534308	1.532568821	0.125382129	0.257433323	up_in_case
Yang2025_S	ENSGALG00010018839	RPL3L	case	control	-0.128393554	0.400336142	-0.32071437	0.748426857	0.847361736	down_in_case
Yang2025_S	ENSGALG00010018843	MCRIP2	case	control	0.318433925	0.287702152	1.106818013	0.268372619	0.435464886	up_in_case
Yang2025_S	ENSGALG00010018847	CGRRF1	case	control	-0.626308713	0.208280651	-3.00704223	0.002638031	0.014739761	down_in_case
Yang2025_S	ENSGALG00010018848	MEF2A	case	control	0.250240709	0.193481673	1.293356138	0.195887913	0.352086336	up_in_case
Yang2025_S	ENSGALG00010018849	KCNH1	case	control	1.141259195	0.510345391	2.236248655	0.025335488	0.080660274	up_in_case
Yang2025_S	ENSGALG00010018852	NDUFB10	case	control	0.455130462	0.202174328	2.251178304	0.024374246	0.078660288	up_in_case
Yang2025_S	ENSGALG00010018854	LYSMD4	case	control	0.562076097	0.089332137	6.291980888	3.1344E-10	2.17303E-08	up_in_case
Yang2025_S	ENSGALG00010018855	SYNDIG1	case	control	-0.04813557	0.530078464	-0.090808387	0.927644846	0.956189876	down_in_case
Yang2025_S	ENSGALG00010018857	ENSGALG00010018857	case	control	0.182310765	0.170542863	1.069002607	0.285068495	0.453318438	up_in_case
Yang2025_S	ENSGALG00010018858	EPB41L5	case	control	0.180926469	0.144856196	1.249007457	0.211662346	0.371850075	up_in_case
Yang2025_S	ENSGALG00010018860	AGTR2	case	control	-0.253674717	0.093426223	-2.715241075	0.006622754	0.029638414	down_in_case
Yang2025_S	ENSGALG00010018864	ABCA3	case	control	0.002794808	0.08604015	0.032482606	0.974087187	0.984421577	up_in_case
Yang2025_S	ENSGALG00010018865	ENSGALG00010018865	case	control	-0.186452462	0.128990461	-1.445474811	0.148324563	0.290376902	down_in_case
Yang2025_S	ENSGALG00010018867	RPS2	case	control	-0.779272437	0.20057832	-3.885127946	0.000102276	0.001086293	down_in_case
Yang2025_S	ENSGALG00010018872	KCTD9	case	control	-0.258076371	0.115853428	-2.227611002	0.025906466	0.082084268	down_in_case
Yang2025_S	ENSGALG00010018874	ENSGALG00010018874	case	control	0.051014041	0.174737895	0.291946067	0.770327861	0.859931005	up_in_case
Yang2025_S	ENSGALG00010018876	GMFB	case	control	-0.765172015	0.066645507	-11.48122424	1.63943E-30	2.85567E-27	down_in_case
Yang2025_S	ENSGALG00010018880	TMEM17	case	control	-0.423948974	0.169988234	-2.493990104	0.012631607	0.048162579	down_in_case
Yang2025_S	ENSGALG00010018881	ENSGALG00010018881	case	control	-0.522462032	0.886722766	-0.589205614	0.555723352	0.703011282	down_in_case
Yang2025_S	ENSGALG00010018882	HHAT	case	control	-0.125530662	0.149453422	-0.839931673	0.400946698	0.567573368	down_in_case
Yang2025_S	ENSGALG00010018883	TBL3	case	control	-0.245496361	0.194560522	-1.261799457	0.207020958	0.365783019	down_in_case
Yang2025_S	ENSGALG00010018885	EFEMP1	case	control	-0.461858126	0.143184332	-3.225619168	0.001257005	0.008369022	down_in_case
Yang2025_S	ENSGALG00010018887	ADAMTS17	case	control	-0.166932459	0.137328055	-1.215574334	0.224147116	0.385616057	down_in_case
Yang2025_S	ENSGALG00010018891	GNRH1	case	control	-0.759792589	0.45246277	-1.679237805	0.093105707	0.208422173	down_in_case
Yang2025_S	ENSGALG00010018892	APMAP	case	control	-0.4628958	0.122755743	-3.770868798	0.00016268	0.001580857	down_in_case
Yang2025_S	ENSGALG00010018894	SERTAD4	case	control	-0.172945197	0.118986155	-1.453490087	0.14608773	0.287573459	down_in_case
Yang2025_S	ENSGALG00010018895	SEPTIN7	case	control	0.229606948	0.168809218	1.360156456	0.173780419	0.323955714	up_in_case
Yang2025_S	ENSGALG00010018897	CCDC85A	case	control	-0.284850062	0.097145072	-2.932213191	0.003365556	0.017764783	down_in_case
Yang2025_S	ENSGALG00010018899	ACSS1	case	control	-0.458382554	0.122727138	-3.73497307	0.000187735	0.001774824	down_in_case
Yang2025_S	ENSGALG00010018901	TCF7L2	case	control	0.860751607	0.324677134	2.651100179	0.008023004	0.034305174	up_in_case
Yang2025_S	ENSGALG00010018903	CAMTA1	case	control	0.16506607	0.16401249	1.006423784	0.314211757	0.483549513	up_in_case
Yang2025_S	ENSGALG00010018904	UGP2	case	control	0.053239835	0.11663677	0.456458416	0.648060367	0.776435493	up_in_case
Yang2025_S	ENSGALG00010018908	ENSGALG00010018908	case	control	-0.087051989	0.241054113	-0.361130485	0.718001905	0.826342629	down_in_case
Yang2025_S	ENSGALG00010018909	TTBK1	case	control	0.039649188	0.130139187	0.304667557	0.760619361	0.854644786	up_in_case
Yang2025_S	ENSGALG00010018911	CNIH1	case	control	-0.050708203	0.112740184	-0.449779322	0.65286957	0.780176355	down_in_case
Yang2025_S	ENSGALG00010018913	ENSGALG00010018913	case	control	0.412159147	0.235774147	1.748110017	0.080444979	0.18799503	up_in_case
Yang2025_S	ENSGALG00010018915	DOCK5	case	control	0.01854609	0.208517889	0.088942439	0.929127656	0.95718603	up_in_case
Yang2025_S	ENSGALG00010018916	CERS3	case	control	-0.071119897	0.314269557	-0.226302216	0.820966375	0.893065296	down_in_case
Yang2025_S	ENSGALG00010018917	DNAJC11	case	control	0.045598116	0.115910422	0.393390997	0.694030718	0.809993137	up_in_case
Yang2025_S	ENSGALG00010018919	RALB	case	control	0.162804413	0.108251821	1.503941569	0.132596412	0.267704246	up_in_case
Yang2025_S	ENSGALG00010018920	ACAD11	case	control	0.271393287	0.128122189	2.118237977	0.034154918	0.100803067	up_in_case
Yang2025_S	ENSGALG00010018922	EEF2K	case	control	0.041066075	0.145827276	0.28160764	0.778244381	0.865371485	up_in_case
Yang2025_S	ENSGALG00010018923	VPS54	case	control	0.225287709	0.106869789	2.108057954	0.035025977	0.102776792	up_in_case
Yang2025_S	ENSGALG00010018925	LINS1	case	control	-0.392512225	0.232063174	-1.691402471	0.090759961	0.205015408	down_in_case
Yang2025_S	ENSGALG00010018926	SLC22A7	case	control	-0.631745315	0.837790895	-0.754060851	0.450812684	0.612765777	down_in_case
Yang2025_S	ENSGALG00010018932	CDKN3	case	control	-0.452137839	0.211836845	-2.134368262	0.032812647	0.097895772	down_in_case
Yang2025_S	ENSGALG00010018933	PHF13	case	control	-0.013904856	0.097111942	-0.143183795	0.886145028	0.933295364	down_in_case
Yang2025_S	ENSGALG00010018936	SYT14	case	control	0.317391611	0.220802591	1.437445139	0.150591551	0.293413487	up_in_case
Yang2025_S	ENSGALG00010018937	INHBB	case	control	0.874329345	0.311339006	2.808287198	0.004980579	0.023916912	up_in_case
Yang2025_S	ENSGALG00010018941	KLHL21	case	control	-0.374229182	0.107931868	-3.467272355	0.000525769	0.00412301	down_in_case
Yang2025_S	ENSGALG00010018943	ACKR4	case	control	0.216190014	0.332685339	0.649833306	0.515799902	0.67029544	up_in_case
Yang2025_S	ENSGALG00010018944	NEFL	case	control	0.906064199	0.585027302	1.548755411			up_in_case
Yang2025_S	ENSGALG00010018945	HABP2	case	control	0.636981505	0.335595919	1.898060941	0.057688055	0.148290978	up_in_case
Yang2025_S	ENSGALG00010018947	ENSGALG00010018947	case	control	-0.692383281	0.295191846	-2.345536609	0.018999713	0.065244208	down_in_case
Yang2025_S	ENSGALG00010018948	POLR3E	case	control	-0.140523091	0.107722357	-1.304493275	0.192065451	0.347306047	down_in_case
Yang2025_S	ENSGALG00010018950	TTL	case	control	-0.173687602	0.055220114	-3.145368413	0.001658778	0.010388796	down_in_case
Yang2025_S	ENSGALG00010018951	YKT6	case	control	0.092349316	0.186575606	0.494969938	0.620621341	0.755748583	up_in_case
Yang2025_S	ENSGALG00010018955	ZBTB48	case	control	0.043098573	0.097226736	0.443279026	0.657563924	0.783979576	up_in_case
Yang2025_S	ENSGALG00010018958	PELI3	case	control	-0.321007018	0.070927974	-4.525816836	6.01627E-06	0.000108738	down_in_case
Yang2025_S	ENSGALG00010018959	UBA5	case	control	-0.129688928	0.069230499	-1.873291823	0.061028098	0.154286384	down_in_case
Yang2025_S	ENSGALG00010018960	RBFOX1	case	control	0.544540677	0.257055083	2.118381282	0.03414279	0.1008008	up_in_case
Yang2025_S	ENSGALG00010018961	ASB7	case	control	-0.150212557	0.092299861	-1.627440772	0.103643518	0.225041102	down_in_case
Yang2025_S	ENSGALG00010018968	GLI2	case	control	0.326922024	0.222180783	1.471423492	0.141176624	0.280475777	up_in_case
Yang2025_S	ENSGALG00010018969	DIEXF	case	control	0.110656057	0.110263298	1.003562002	0.315589778	0.484813532	up_in_case
Yang2025_S	ENSGALG00010018971	POLR1B	case	control	-0.195239653	0.068106181	-2.866695057	0.004147825	0.020701983	down_in_case
Yang2025_S	ENSGALG00010018973	NEFM	case	control	1.656032653	1.250233136	1.324579077			up_in_case
Yang2025_S	ENSGALG00010018974	TAS1R1	case	control	1.077994886	0.233852015	4.609731018	4.0319E-06	7.77103E-05	up_in_case
Yang2025_S	ENSGALG00010018977	NPHP3	case	control	0.479562269	0.117022696	4.098027868	4.16685E-05	0.000529885	up_in_case
Yang2025_S	ENSGALG00010018983	MDGA2	case	control	0.728876738	0.251098986	2.902746639	0.003699058	0.019063006	up_in_case
Yang2025_S	ENSGALG00010018985	NOL9	case	control	0.056155293	0.149902435	0.374612281	0.707948838	0.819872311	up_in_case
Yang2025_S	ENSGALG00010018986	CTDSP1	case	control	-0.382887032	0.147860552	-2.589514426	0.00961114	0.039449552	down_in_case
Yang2025_S	ENSGALG00010018987	MRPS10	case	control	-0.408461254	0.215191388	-1.898130115	0.057678944	0.148290978	down_in_case
Yang2025_S	ENSGALG00010018990	GCK	case	control	1.006205699	0.357926908	2.811204403	0.004935642	0.023757572	up_in_case
Yang2025_S	ENSGALG00010018991	RYBP	case	control	-0.443498552	0.108255547	-4.096774404	4.18947E-05	0.000532181	down_in_case
Yang2025_S	ENSGALG00010018998	RPS29	case	control	0.185589842	0.232985813	0.796571428	0.425699977	0.590379098	up_in_case
Yang2025_S	ENSGALG00010018999	THUMPD3	case	control	0.381239109	0.084061475	4.535241745	5.75375E-06	0.000104809	up_in_case
Yang2025_S	ENSGALG00010019000	TRERF1	case	control	0.301639969	0.219870552	1.371897994	0.170095195	0.319057281	up_in_case
Yang2025_S	ENSGALG00010019001	ALDH1A3	case	control	-0.573273177	0.118366539	-4.843202999	1.27763E-06	2.9822E-05	down_in_case
Yang2025_S	ENSGALG00010019004	ECE2	case	control	0.271682131	0.214932218	1.264036327	0.206216989	0.364674332	up_in_case
Yang2025_S	ENSGALG00010019005	ENSGALG00010019005	case	control	-0.310347909	0.111056713	-2.794499329	0.005198015	0.024679502	down_in_case
Yang2025_S	ENSGALG00010019006	ENSGALG00010019006	case	control	-0.131547803	0.088520184	-1.486076932	0.137258773	0.274951188	down_in_case
Yang2025_S	ENSGALG00010019008	HERPUD2	case	control	-0.24381112	0.081018249	-3.009335842	0.002618195	0.014670105	down_in_case
Yang2025_S	ENSGALG00010019009	NRAP	case	control	0.520651573	0.159879505	3.256524787	0.001127851	0.007700442	up_in_case
Yang2025_S	ENSGALG00010019011	VIL1	case	control	0.333193237	0.474824991	0.701717987	0.482855053	0.641122932	up_in_case

Yang2025_S	ENSGALG00010019012	ADAM28	case	control	-0.522678478	0.266605037	-1.96049739	0.049937683	0.133157598	down_in_case
Yang2025_S	ENSGALG00010019013	LRRK1	case	control	0.010477736	0.113560656	0.092265546	0.926487062	0.955608219	up_in_case
Yang2025_S	ENSGALG00010019016	PLEKHG5	case	control	0.335086975	0.122772373	2.729335316	0.006346214	0.028665893	up_in_case
Yang2025_S	ENSGALG00010019017	IRAK2	case	control	-0.193969476	0.085123544	-2.278681858	0.022685984	0.074278474	down_in_case
Yang2025_S	ENSGALG00010019018	SH3BGRL	case	control	-0.574159361	0.105552479	-5.439563024	5.34114E-08	1.97424E-06	down_in_case
Yang2025_S	ENSGALG00010019019	TBX20	case	control	-0.663437294	0.193409527	-3.430220338	0.000603091	0.004632898	down_in_case
Yang2025_S	ENSGALG00010019020	KCNK2	case	control	-0.018505676	0.188425762	-0.098212025	0.921763934	0.953337584	down_in_case
Yang2025_S	ENSGALG00010019021	LRR1	case	control	-0.138981882	0.135573585	-1.025139835	0.305297212	0.474807136	down_in_case
Yang2025_S	ENSGALG00010019023	AP2M1	case	control	-0.439678877	0.14351009	-3.063748881	0.002185823	0.012792711	down_in_case
Yang2025_S	ENSGALG00010019024	CNPY3	case	control	0.693582511	0.155119965	4.471265277	7.77582E-06	0.000133707	up_in_case
Yang2025_S	ENSGALG00010019025	DNAAF2	case	control	0.209036051	0.254802156	0.820385721	0.411996253	0.577291884	up_in_case
Yang2025_S	ENSGALG00010019026	CHSY1	case	control	-0.173223641	0.063475645	-2.728978012	0.006353094	0.028678447	down_in_case
Yang2025_S	ENSGALG00010019027	GNLY	case	control	0.451652228	0.453049557	0.996915727	0.318805417	0.488345495	up_in_case
Yang2025_S	ENSGALG00010019028	TNFRSF25	case	control	1.17837779	0.362907461	3.247047569	0.001166089	0.007897142	up_in_case
Yang2025_S	ENSGALG00010019029	CENPF	case	control	0.258041121	0.176131862	1.465045102	0.142908613	0.282880913	up_in_case
Yang2025_S	ENSGALG00010019030	CDR2	case	control	-0.04655602	0.14509525	-0.320865222	0.74831253	0.847301138	down_in_case
Yang2025_S	ENSGALG00010019032	GNMT	case	control	0.044708625	0.224026743	0.199568248	0.841818262	0.906153624	up_in_case
Yang2025_S	ENSGALG00010019034	CASP7	case	control	-0.731778038	0.202071913	-3.621374337	0.000293042	0.002557008	down_in_case
Yang2025_S	ENSGALG00010019035	ENSGALG00010019035	case	control	-0.087971257	0.387708122	-0.226900734	0.820500931	0.89283777	down_in_case
Yang2025_S	ENSGALG00010019036	REL	case	control	0.336616949	0.168388708	1.999047046	0.045603264	0.124996358	up_in_case
Yang2025_S	ENSGALG00010019040	HMGN5	case	control	-0.463374701	0.120074024	-3.859075299	0.000113817	0.001180967	down_in_case
Yang2025_S	ENSGALG00010019041	KATNIP	case	control	0.242226389	0.090691029	2.670896894	0.007564888	0.032811822	up_in_case
Yang2025_S	ENSGALG00010019042	PEX6	case	control	0.137028525	0.161256249	0.849756367	0.395460553	0.562665183	up_in_case
Yang2025_S	ENSGALG00010019043	METTL9	case	control	-0.493397513	0.100140013	-4.927076571	8.34691E-07	2.07334E-05	down_in_case
Yang2025_S	ENSGALG00010019045	LBX1	case	control	-0.356502452	0.106657218	-3.342506562	0.000830254	0.0060133	down_in_case
Yang2025_S	ENSGALG00010019046	SELENOS	case	control	-0.03161198	0.122605843	-0.257834206	0.796534864	0.876754607	down_in_case
Yang2025_S	ENSGALG00010019047	GHRL	case	control	0.553161826	0.550537635	1.004766596	0.315009252	0.484188609	up_in_case
Yang2025_S	ENSGALG00010019049	POLD2	case	control	0.408670977	0.167107459	2.445557963	0.01446282	0.053402065	up_in_case
Yang2025_S	ENSGALG00010019050	TNS1	case	control	0.293882475	0.154773621	1.898789166	0.057592202	0.148153468	up_in_case
Yang2025_S	ENSGALG00010019051	IGSF6	case	control	-0.444025116	0.265329524	-1.673485518	0.094231755	0.210166375	down_in_case
Yang2025_S	ENSGALG00010019059	BRWD3	case	control	0.233736594	0.179320347	1.303458296	0.192418345	0.347640301	up_in_case
Yang2025_S	ENSGALG00010019062	PUS10	case	control	0.335488062	0.124239604	2.700331058	0.006927051	0.030731758	up_in_case
Yang2025_S	ENSGALG00010019064	ENSGALG00010019064	case	control	-0.505544388	0.113423571	-4.457136932	8.30615E-06	0.000139959	down_in_case
Yang2025_S	ENSGALG00010019065	CAND2	case	control	-0.35984207	0.089021681	-4.042184601	5.29555E-05	0.00063725	down_in_case
Yang2025_S	ENSGALG00010019067	GTF3C1	case	control	0.206984364	0.066907387	3.093595078	0.001977472	0.011872499	up_in_case
Yang2025_S	ENSGALG00010019068	SNRPA1	case	control	-0.061940824	0.165281156	-0.374760353	0.707838702	0.819872311	down_in_case
Yang2025_S	ENSGALG00010019072	AEBP1	case	control	0.017404783	0.18793781	0.092609269	0.92621398	0.955608219	up_in_case
Yang2025_S	ENSGALG00010019076	ESPN	case	control	0.431504964	0.37038985	1.165002128	0.244018131	0.409242106	up_in_case
Yang2025_S	ENSGALG00010019079	CAPG	case	control	0.460031077	0.346027579	1.329463617	0.183695061	0.337036298	up_in_case
Yang2025_S	ENSGALG00010019081	PCSK6	case	control	-0.248930225	0.191990182	-1.296577888	0.194776465	0.350627831	down_in_case
Yang2025_S	ENSGALG00010019082	POLR3K	case	control	0.569189079	0.222205302	2.561545891	0.010420747	0.041751902	up_in_case
Yang2025_S	ENSGALG00010019083	OTOA	case	control	1.229415835	0.223189605	5.508392004	3.62126E-08	1.41748E-06	up_in_case
Yang2025_S	ENSGALG00010019087	PEX13	case	control	-0.180218329	0.087847286	-2.051495691	0.040218699	0.113750268	down_in_case
Yang2025_S	ENSGALG00010019093	RPL32	case	control	-0.237994335	0.247495511	-0.961610715	0.336245187	0.504367781	down_in_case
Yang2025_S	ENSGALG00010019094	SNRNP25	case	control	0.778662775	0.209303298	3.720260425	0.000199017	0.001862531	up_in_case
Yang2025_S	ENSGALG00010019097	STC1	case	control	0.420039128	0.251938778	1.66722698	0.09546928	0.212382569	up_in_case
Yang2025_S	ENSGALG00010019101	POLE2	case	control	-0.433383817	0.15582103	-2.781292217	0.005414298	0.025463462	down_in_case
Yang2025_S	ENSGALG00010019104	POLL	case	control	-0.312949658	0.145740645	-2.147305286	0.031768985	0.095615726	down_in_case
Yang2025_S	ENSGALG00010019108	KDELR2	case	control	-0.379731694	0.106336769	-3.571029092	0.000355581	0.003004868	down_in_case
Yang2025_S	ENSGALG00010019110	MBD4	case	control	0.737024814	0.142812271	5.160794735	2.45904E-07	7.23621E-06	up_in_case
Yang2025_S	ENSGALG00010019113	KLHDC1	case	control	0.362062583	0.137763797	2.628140274	0.00858531	0.036231466	up_in_case
Yang2025_S	ENSGALG00010019114	TENT5D	case	control	0.836435687	0.417321054	2.004297842	0.045038165	0.123910529	up_in_case
Yang2025_S	ENSGALG00010019115	DBNL	case	control	0.746124396	0.085935657	8.682360954	3.87637E-18	1.54335E-15	up_in_case
Yang2025_S	ENSGALG00010019120	DAGLB	case	control	-0.039477327	0.12080773	-0.326778156	0.743835683	0.843328471	down_in_case
Yang2025_S	ENSGALG00010019123	PLEKHO2	case	control	-0.140913346	0.124440586	-1.132374496	0.257477023	0.423769667	down_in_case
Yang2025_S	ENSGALG00010019124	BMPER	case	control	-0.197469257	0.147479174	-1.338963677	0.180582498	0.33286941	down_in_case
Yang2025_S	ENSGALG00010019125	ENSGALG00010019125	case	control	0.045568305	0.130594292	0.348930297	0.727141639	0.832465302	up_in_case
Yang2025_S	ENSGALG00010019127	TMBIM1	case	control	-0.101418291	0.147771093	-0.686320236	0.492511191	0.650042001	down_in_case
Yang2025_S	ENSGALG00010019130	RPS6KC1	case	control	-0.064033503	0.074008989	-0.86521251	0.386922161	0.554308657	down_in_case
Yang2025_S	ENSGALG00010019132	ENSGALG00010019132	case	control	-0.67920947	0.26544661	-2.558742301	0.010505158	0.041993511	down_in_case
Yang2025_S	ENSGALG00010019135	KIAA1841	case	control	0.327132844	0.263269969	1.242575615	0.21402426	0.374498378	up_in_case
Yang2025_S	ENSGALG00010019139	DPCD	case	control	0.088898763	0.154637145	0.574886216	0.565368253	0.710983359	up_in_case
Yang2025_S	ENSGALG00010019141	ACOT7	case	control	0.400026294	0.169244953	2.363593641	0.018098652	0.063035421	up_in_case
Yang2025_S	ENSGALG00010019144	IFT122	case	control	0.037256551	0.075099725	0.496094374	0.619827828	0.755266399	up_in_case
Yang2025_S	ENSGALG00010019145	RAC1	case	control	0.274117687	0.248569266	1.102781897	0.270121891	0.43748821	up_in_case
Yang2025_S	ENSGALG00010019146	KLHDC2	case	control	-0.302525791	0.139272686	-2.172183222	0.029841846	0.091154345	down_in_case
Yang2025_S	ENSGALG00010019148	ENSGALG00010019148	case	control	0.897851326	0.332872723	2.697281167	0.006990822	0.030945711	up_in_case
Yang2025_S	ENSGALG00010019149	COX5B	case	control	0.63516092	0.159319545	3.986710603	6.69957E-05	0.000772835	up_in_case
Yang2025_S	ENSGALG00010019157	ENSGALG00010019157	case	control	0.691741631	0.450460379	1.535632576	0.12462852	0.256187996	up_in_case
Yang2025_S	ENSGALG00010019158	ENSGALG00010019158	case	control	0.811719636	0.305948719	2.653123175	0.007975076	0.034173643	up_in_case
Yang2025_S	ENSGALG00010019166	PNPT1	case	control	0.046086784	0.081513908	0.565385527	0.571811567	0.715513306	up_in_case
Yang2025_S	ENSGALG00010019169	GPR153	case	control	0.900107646	0.386721547	2.327534252	0.019936848	0.067612553	up_in_case
Yang2025_S	ENSGALG00010019172	NEMF	case	control	0.568850133	0.122978475	4.625607301	3.73502E-06	7.2998E-05	up_in_case
Yang2025_S	ENSGALG00010019173	RHO	case	control	0.085803617	0.401902423	0.213493654	0.830941938	0.899143959	up_in_case
Yang2025_S	ENSGALG00010019174	ENSGALG00010019174	case	control	1.01551263	0.405437458	2.504733124	0.012254382	0.047133539	up_in_case
Yang2025_S	ENSGALG00010019183	ANGEL2	case	control	-0.042631643	0.078728453	-0.541502362	0.58816137	0.729443636	down_in_case
Yang2025_S	ENSGALG00010019184	PNKD	case	control	0.100185708	0.181632086	0.551585956	0.581232061	0.723877805	up_in_case
Yang2025_S	ENSGALG00010019185	ANKDD1A	case	control	0.272625899	0.193273435	1.410570982	0.158371153	0.303729978	up_in_case
Yang2025_S	ENSGALG00010019189	ICMT	case	control	-0.529510066	0.219216645	-2.415464699	0.015715146	0.056954631	down_in_case
Yang2025_S	ENSGALG00010019194	ENSGALG00010019194	case	control	0.466771596	0.381310002	1.224126283	0.220904592	0.382303216	up_in_case
Yang2025_S	ENSGALG00010019204	ARF6	case	control	0.392908507	0.153964262	2.551946152	0.010712307	0.042638104	up_in_case
Yang2025_S	ENSGALG00010019205	ENSGALG00010019205	case	control	0.318628173	0.560203068	0.568772631	0.56951045	0.713937399	up_in_case
Yang2025_S	ENSGALG00010019206	RNF207	case	control	0.630873641	0.156849387	4.022161986	5.76664E-05	0.000685649	up_in_case
Yang2025_S	ENSGALG00010019208	PLXND1	case	control	-0.10082844	0.127766913	-0.789159239	0.430018942	0.594239782	down_in_case
Yang2025_S	ENSGALG00010019209	VCPKMT	case	control	0.732722834	0.098109979	7.468382315	8.11867E-14	1.33098E-11	up_in_case
Yang2025_S	ENSGALG00010019212	RPL22	case	control	0.355885699	0.178852675	1.989825982	0.046610108	0.126758755	up_in_case
Yang2025_S	ENSGALG00010019213	RHBDF1	case	control	0.191895316	0.077293961	2.48266894	0.013040222	0.049338987	up_in_case
Yang2025_S	ENSGALG00010019215	ENSGALG00010019215	case	control	-0.25296367	0.175742241	-1.439401646	0.150036758	0.29253704	down_in_case
Yang2025_S	ENSGALG00010019218	RMND5A	case	control	0.006038382	0.085957266	0.070248651	0.943995752	0.965757345	up_in_case
Yang2025_S	ENSGALG00010019221	VASH2	case	control	-0.199881314	0.164293308	-1.216612631	0.223751635	0.385077218	down_in_case
Yang2025_S	ENSGALG00010019223	AAMP	case	control	0.268562849	0.156144064	1.71996836	0.085438193	0.19638851	up_in_case
Yang2025_S	ENSGALG00010019227	CLCN5	case	control	0.211375664	0.137473517	1.537573696	0.124152883	0.255511804	up_in_case
Yang2025_S	ENSGALG00010019230	SPG21	case	control	-0.699056386	0.174527899	-4.005413396	6.19091E-05	0.000724961	down_in_case
Yang2025_S	ENSGALG00010019231	ENSGALG00010019231	case	control	-0.013965541	0.246558494	-0.0566419	0.954830457	0.972132325	down_in_case
Yang2025_S	ENSGALG00010019232	SOS2	case	control	-0.195227427	0.109814672	-1.777790018	0.075438348	0.179566843	down_in_case
Yang2025_S	ENSGALG00010019233	ARPC2	case	control	0.008645735	0.101430489	0.085238032	0.932072155	0.95863854	up_in_case
Yang2025_S	ENSGALG00010019236	MPG	case	control	0.157516494	0.149836209	1.051257872	0.29314017	0.461705274	up_in_case
Yang2025_S	ENSGALG00010019237	SORCS3	case	control	-0.414151293	0.337889798	-1.225699311	0.22031185	0.381653847	down_in_case
Yang2025_S	ENSGALG00010019239	CYTH3	case	control	0.193261138	0.133654826	1.445972009	0.148185053	0.290298738	up_in_case
Yang2025_S	ENSGALG00010019240	ENSGALG00010019240	case	control	-0.158190607	0.370035517	-0.427501144	0.669014352	0.791176862	down_in_case
Yang2025_S	ENSGALG00010019243	SERTAD2	case	control	-0.108121577	0.160218484	-0.674838345	0.499778475	0.656527654	down_in_case
Yang2025_S	ENSGALG00010019246	IL17RC	case	control	0.079547577	0.276847277	0.28733379	0.773856752	0.862350567	up_in_case
Yang2025_S	ENSGALG00010019247	HBZ	case	control	-1.271088096	0.48990452	-2.59456291	0.009471128	0.039024295	down_in_case
Yang2025_S	ENSGALG00010019248	RMI2	case	control	0.619858254	0.269359994	2.301226122	0.021378853	0.071105219	up_in_case
Yang2025_S	ENSGALG00010019250	CXCR1	case	control	-0.329519881	0.378026957	-0.871683553	0.383381035	0.55104845	down_in_case
Yang2025_S	ENSGALG00010019252	TATDN3	case	control	-0.248466287	0.166760818	-1.489956037	0.136235796	0.273039812	down_in_case
Yang2025_S	ENSGALG00010019253	HBAD	case	control	-0.670231572	0.276637482	-2.422779326	0.01540228	0.056110741	down_in_case
Yang2025_S	ENSGALG00010019255	ENSGALG00010019255	case	control	12.12997363	2.586425542	4.689859976	2.73392E-06	5.62735E-05	up_in_case
Yang2025_S	ENSGALG00010019256	BBS9	case	control	-0.203063842	0.179189158	-1.133237327	0.257114603	0.423409595	down_in_case
Yang2025_S	ENSGALG00010019257	SLC1A4	case	control	-0.092537992	0.11714223	-0.789962697	0.429549554	0.593708891	down_in_case
Yang2025_S	ENSGALG00010019259	L2HGDH	case	control	-0.526101668	0.149901423	-3.509650921	0.000448695	0.003624678	down_in_case
Yang2025_S	ENSGALG00010019260	TRAIL-LIKE	case	control	-0.180157292	0.122337984	-1.472619427	0.140853684	0.280079349	down_in_case
Yang2025_S	ENSGALG00010019262	TMC3	case	control	0.802637514	0.388735126	2.064741415	0.038947474	0.111033766	up_in_case
Yang2025_S	ENSGALG00010019263	SNRPB2	case	control	-0.148100632	0.116029508	-1.276404896	0.201812426	0.359761566	down_in_case
Yang2025_S	ENSGALG00010019264	HK2	case	control	0.551788352	0.084373279	6.539847228	6.15817E-11	5.48885E-09	up_in_case
Yang2025_S	ENSGALG00010019266	ATP5S	case	control	-0.143926029	0.199211105	-0.72247995	0.469999448	0.630360183	down_in_case
Yang2025_S	ENSGALG00010019267	STARD5	case	control	0.235858006	0.253887925	0.928984731	0.352897	0.521708009	up_in_case
Yang2025_S	ENSGALG00010019268	NSL1	case	control	-0.153696948	0.127047726	-1.209757564	0.226371934	0.388368501	down_in_case
Yang2025_S	ENSGALG00010019269	SOCS1	case	control	0.037490135	0.288895915	0.129770387	0.896748092	0.938786882	up_in_case
Yang2025_S	ENSGALG00010019270	ENSGALG00010019270	case	control	1.524897367	0.364751748	4.180644439	2.90684E-05	0.000396786	up_in_case
Yang2025_S	ENSGALG00010019271	CDKL1	case	control	0.623219159	0.249767904	2.495193136	0.012588859	0.048048685	up_in_case
Yang2025_S	ENSGALG00010019275	CTAGE1	case	control	0.001656971	0.505454102	0.003278183	0.997384393	0.998100647	up_in_case
Yang2025_S	ENSGALG00010019277	IGFBP5	case	control	0.067847717	0.155138557	0.437336263	0.6618675	0.786315287	up_in_case
Yang2025_S	ENSGALG00010019278	JPT2	case	control	-0.456674581	0.07040823	-6.486096562	8.80887E-11	7.62433E-09	down_in_case
Yang2025_S	ENSGALG00010019280	TLNRD1	case	control	0.144399888	0.119178638	1.2116256	0.225655731	0.387589376	up_in_case
Yang2025_S	ENSGALG00010019282	IGFBP2	case	control	-0.258480477	0.234519908	-1.102168595	0.270388382	0.437767179	down_in_case

Yang2025_S	ENSGALG00010019283	NT5C3A	case	control	-0.34127642	0.161215196	-2.116899819	0.034268351	0.101000311	down_in_case
Yang2025_S	ENSGALG00010019284	CHD5	case	control	0.451915499	0.147486561	3.064113069	0.002183164	0.012782516	up_in_case
Yang2025_S	ENSGALG00010019285	BATF3	case	control	-0.973390651	0.321835849	-3.024494179	0.002490493	0.014159533	down_in_case
Yang2025_S	ENSGALG00010019287	AHSA2	case	control	0.48808872	0.099992527	4.881251971	1.05414E-06	2.52397E-05	up_in_case
Yang2025_S	ENSGALG00010019288	RPL37A	case	control	-1.192725231	0.319740511	-3.730291253	0.000191259	0.001799587	down_in_case
Yang2025_S	ENSGALG00010019289	FKBP9	case	control	-0.223143183	0.105351996	-2.118072677	0.034168913	0.100813848	down_in_case
Yang2025_S	ENSGALG00010019291	ADD2	case	control	0.154749612	0.295442297	0.523789633	0.600424865	0.739243272	up_in_case
Yang2025_S	ENSGALG00010019292	ENSGALG00010019292	case	control	-0.157979062	0.130591903	-1.209715604	0.22638804	0.388368501	down_in_case
Yang2025_S	ENSGALG00010019294	SMARCAL1	case	control	0.126041431	0.092134909	1.368009494	0.171309104	0.320385501	up_in_case
Yang2025_S	ENSGALG00010019295	MAP4K5	case	control	-0.06116335	0.136289876	-0.44877398	0.653594711	0.780783737	down_in_case
Yang2025_S	ENSGALG00010019296	ENSGALG00010019296	case	control	0.20332941	0.155096088	1.31098993	0.189861166	0.344628806	up_in_case
Yang2025_S	ENSGALG00010019297	SLC25A26	case	control	-0.340826085	0.181037379	-1.882628255	0.059750765	0.152076444	down_in_case
Yang2025_S	ENSGALG00010019298	PGAP6	case	control	-0.376578319	0.143721662	-2.620191796	0.008788033	0.036874807	down_in_case
Yang2025_S	ENSGALG00010019300	MESDC2	case	control	-0.150171175	0.082792734	-1.813820701	0.069705334	0.169459845	down_in_case
Yang2025_S	ENSGALG00010019302	USP34	case	control	0.293881564	0.157167805	1.869858551	0.061503463	0.155178482	up_in_case
Yang2025_S	ENSGALG00010019305	SNRNP27	case	control	-0.044103412	0.148913071	-0.296168841	0.767101143	0.858460844	down_in_case
Yang2025_S	ENSGALG00010019307	ATF3	case	control	0.122918866	0.233675879	0.526022911	0.598872288	0.737810006	up_in_case
Yang2025_S	ENSGALG00010019310	CRELD1	case	control	0.626545007	0.135441721	4.625938005	3.72907E-06	7.29839E-05	up_in_case
Yang2025_S	ENSGALG00010019312	KBTBD2	case	control	-0.269866887	0.063998276	-4.216783721	2.47811E-05	0.000347172	down_in_case
Yang2025_S	ENSGALG00010019313	MRPL28	case	control	0.319576214	0.146642371	2.179289739	0.029310151	0.089974191	up_in_case
Yang2025_S	ENSGALG00010019314	GKN2	case	control	-0.92979696	0.90584997	-1.026435933	0.304686148	0.474388666	down_in_case
Yang2025_S	ENSGALG00010019316	CLEC16A	case	control	0.150643068	0.157726843	0.955088339	0.339533026	0.507560552	up_in_case
Yang2025_S	ENSGALG00010019318	NOG2	case	control	0.183264469	0.268721991	0.681985379	0.495248196	0.652541946	up_in_case
Yang2025_S	ENSGALG00010019319	ACTR3	case	control	0.190446618	0.077620009	2.453576338	0.014144351	0.052476448	up_in_case
Yang2025_S	ENSGALG00010019322	EGR3	case	control	1.605044891	0.320696043	5.004878998	5.58971E-07	1.4669E-05	up_in_case
Yang2025_S	ENSGALG00010019323	LRIG1	case	control	-0.079143868	0.125535379	-0.630450704	0.528399746	0.681088748	down_in_case
Yang2025_S	ENSGALG00010019325	SLITRK4	case	control	-0.177005588	0.262276306	-0.674882116	0.499750664	0.656527654	down_in_case
Yang2025_S	ENSGALG00010019328	ENSGALG00010019328	case	control	1.408769849	0.751737933	1.874017244	0.060928048	0.154121954	up_in_case
Yang2025_S	ENSGALG00010019331	CEMIP	case	control	0.40276211	0.287083263	1.402945288	0.160633155	0.306255714	up_in_case
Yang2025_S	ENSGALG00010019334	ATL1	case	control	-0.256020954	0.096707049	-2.647386687	0.008111654	0.034578127	down_in_case
Yang2025_S	ENSGALG00010019335	HIPK3	case	control	0.474144047	0.236041128	2.008734876	0.044565258	0.12287631	up_in_case
Yang2025_S	ENSGALG00010019336	DEXI	case	control	0.348329983	0.230887438	1.508657147	0.131386422	0.265983451	up_in_case
Yang2025_S	ENSGALG00010019337	XPO1	case	control	-0.161906311	0.09656538	-1.676649662	0.09361101	0.209271764	down_in_case
Yang2025_S	ENSGALG00010019344	DNAJC24	case	control	0.168703304	0.102452424	1.646650197	0.099629966	0.218327344	up_in_case
Yang2025_S	ENSGALG00010019345	ENSGALG00010019345	case	control	0.418618221	0.351572537	1.190702279	0.233770483	0.396948128	up_in_case
Yang2025_S	ENSGALG00010019346	POU3F4	case	control	0.968034012	0.357642057	2.706711905	0.00679532	0.030268825	up_in_case
Yang2025_S	ENSGALG00010019350	SAV1	case	control	0.001815912	0.083137148	0.021842368	0.982573697	0.989318242	up_in_case
Yang2025_S	ENSGALG00010019355	ENTPD4	case	control	-0.078303599	0.101021045	-0.775121654	0.438267777	0.602175259	down_in_case
Yang2025_S	ENSGALG00010019356	AVL9	case	control	-0.286213579	0.126011465	-2.271329663	0.023127029	0.075491953	down_in_case
Yang2025_S	ENSGALG00010019362	ENSGALG00010019362	case	control	0.045237907	0.309921945	0.145965486	0.883948635	0.931615809	up_in_case
Yang2025_S	ENSGALG00010019365	ENSGALG00010019365	case	control	1.163211911	0.267874535	4.342375856	1.4095E-05	0.000219702	up_in_case
Yang2025_S	ENSGALG00010019371	TMEM87A	case	control	-0.298257033	0.094862955	-3.144083286	0.00166608	0.010420479	down_in_case
Yang2025_S	ENSGALG00010019373	DENND5A	case	control	0.218709314	0.188779182	1.158545724	0.246641404	0.412380058	up_in_case
Yang2025_S	ENSGALG00010019376	ENSGALG00010019376	case	control	0.209369303	0.548759046	0.381532304	0.702808306	0.81634023	up_in_case
Yang2025_S	ENSGALG00010019388	GPR75	case	control	-0.421142131	0.125502831	-3.355638488	0.00079182	0.00580432	down_in_case
Yang2025_S	ENSGALG00010019390	TVP23A	case	control	0.999481251	0.429571915	2.326691332	0.0199817	0.067665852	up_in_case
Yang2025_S	ENSGALG00010019391	GANC	case	control	0.793041675	0.123644713	6.413874522	1.41867E-10	1.09829E-08	up_in_case
Yang2025_S	ENSGALG00010019392	NIN	case	control	0.844619621	0.18716679	4.512657515	6.40204E-06	0.000114816	up_in_case
Yang2025_S	ENSGALG00010019398	SLC35F5	case	control	-0.579913221	0.11944963	-4.85487667	1.20462E-06	2.83075E-05	down_in_case
Yang2025_S	ENSGALG00010019399	CD93	case	control	0.177465254	0.107345852	1.653210166	0.098288098	0.216441948	up_in_case
Yang2025_S	ENSGALG00010019403	PACC1	case	control	0.09905346	0.124932296	0.792857116	0.427861075	0.592197465	up_in_case
Yang2025_S	ENSGALG00010019411	LSM5	case	control	-0.251450247	0.296756336	-0.847328992	0.396811771	0.563896801	down_in_case
Yang2025_S	ENSGALG00010019418	ZNF703	case	control	0.49376616	0.219690931	2.247549125	0.024604955	0.079093436	up_in_case
Yang2025_S	ENSGALG00010019422	IMMP1L	case	control	-0.135288013	0.223876473	-0.604297591	0.545645808	0.695063205	down_in_case
Yang2025_S	ENSGALG00010019423	LYPD1	case	control	0.057849976	0.166501028	0.347445162	0.72825691	0.83298531	up_in_case
Yang2025_S	ENSGALG00010019426	GRM7	case	control	-0.070967621	0.207983953	-0.341216811	0.732940369	0.836899708	down_in_case
Yang2025_S	ENSGALG00010019430	ENSGALG00010019430	case	control	-0.253644527	0.254855749	-0.995247424	0.319615942	0.488896614	down_in_case
Yang2025_S	ENSGALG00010019441	LMCD1	case	control	0.062801743	0.16508773	0.380414357	0.703637859	0.817099464	up_in_case
Yang2025_S	ENSGALG00010019443	NCKAP5	case	control	0.027301782	0.207773802	0.131401466	0.895457729	0.938334765	up_in_case
Yang2025_S	ENSGALG00010019451	CAV3	case	control	0.176076209	0.171049577	1.029386992	0.303297859	0.472863691	up_in_case
Yang2025_S	ENSGALG00010019458	ELP4	case	control	-0.303310876	0.108704738	-2.79022683	0.005267113	0.024922653	down_in_case
Yang2025_S	ENSGALG00010019461	MGAT5	case	control	-0.429689752	0.121383918	-3.539923241	0.000400243	0.00329828	down_in_case
Yang2025_S	ENSGALG00010019470	TGFB2	case	control	0.208781239	0.131907673	1.582783131	0.113470903	0.239869088	up_in_case
Yang2025_S	ENSGALG00010019471	NUBP1	case	control	-0.423301713	0.191886316	-2.20600261	0.027383817	0.085463268	down_in_case
Yang2025_S	ENSGALG00010019472	CAPN3	case	control	0.431848018	0.141196304	3.058493777	0.002224527	0.012970202	up_in_case
Yang2025_S	ENSGALG00010019473	OXTR	case	control	0.731605612	0.481282648	1.520116328	0.128481742	0.261745372	up_in_case
Yang2025_S	ENSGALG00010019474	TMEM163	case	control	2.259543275	0.572211647	3.948789381	7.85474E-05	0.000881287	up_in_case
Yang2025_S	ENSGALG00010019477	NCAPH	case	control	-0.167964194	0.153995331	-1.090709656	0.27540066	0.442336123	down_in_case
Yang2025_S	ENSGALG00010019481	PYGL	case	control	-0.071520581	0.115840077	-0.61740792	0.536965699	0.6881833	down_in_case
Yang2025_S	ENSGALG00010019483	SCUBE2	case	control	0.320342489	0.26773777	1.196478512	0.231509879	0.394532245	up_in_case
Yang2025_S	ENSGALG00010019495	EMP2	case	control	-0.317453074	0.1117624	-2.840428226	0.004505301	0.022113903	down_in_case
Yang2025_S	ENSGALG00010019498	ENSGALG00010019498	case	control	1.153348669	0.245330768	4.701198614	2.58639E-06	5.40648E-05	up_in_case
Yang2025_S	ENSGALG00010019509	ZNF106	case	control	0.084667854	0.162284246	0.521725652	0.601861361	0.740372358	up_in_case
Yang2025_S	ENSGALG00010019515	ENSGALG00010019515	case	control	-0.777357823	0.130595027	-5.952430523	2.64189E-09	1.41595E-07	down_in_case
Yang2025_S	ENSGALG00010019524	HHIPL2	case	control	-0.249359381	0.39477354	-0.631651708	0.527614491	0.680517209	down_in_case
Yang2025_S	ENSGALG00010019529	SNAP23	case	control	-0.120255375	0.074050039	-1.623974508	0.104381249	0.226213485	down_in_case
Yang2025_S	ENSGALG00010019534	PPP2R5A	case	control	-0.19614466	0.068514921	-2.862802096	0.004199128	0.020863669	down_in_case
Yang2025_S	ENSGALG00010019540	UNC5D	case	control	-0.178080441	0.321942573	-0.553143498	0.580165153	0.722872086	down_in_case
Yang2025_S	ENSGALG00010019546	HSD17B12	case	control	-0.381308804	0.085933147	-4.43727268	9.11059E-06	0.000151138	down_in_case
Yang2025_S	ENSGALG00010019550	ACMSD	case	control	1.045772629	0.376134793	2.780313467	0.005430645	0.025523115	up_in_case
Yang2025_S	ENSGALG00010019557	TRIM9	case	control	0.272661667	0.173578963	1.570822074	0.116223983	0.243848448	up_in_case
Yang2025_S	ENSGALG00010019572	NFKBIE	case	control	-0.214902223	0.238601751	-0.900673288	0.367762055	0.536343719	down_in_case
Yang2025_S	ENSGALG00010019577	MXD1	case	control	-0.116921446	0.17896027	-0.653337446	0.513538757	0.668425422	down_in_case
Yang2025_S	ENSGALG00010019580	CD5	case	control	0.537398805	0.57033314	0.942254215	0.346062503	0.514727257	up_in_case
Yang2025_S	ENSGALG00010019581	SLC35B2	case	control	-0.134008704	0.093565582	-1.432243579	0.152074124	0.295599514	down_in_case
Yang2025_S	ENSGALG00010019584	RAD18	case	control	0.395442982	0.1424089	2.77681369	0.005489464	0.025678306	up_in_case
Yang2025_S	ENSGALG00010019585	PDE1C	case	control	-0.399620025	0.215214096	-1.856848749	0.063332676	0.15818979	down_in_case
Yang2025_S	ENSGALG00010019586	GLYR1	case	control	-0.227800488	0.100674457	-2.26274366	0.023651496	0.076972615	down_in_case
Yang2025_S	ENSGALG00010019587	CCNT2	case	control	0.444673868	0.162684082	2.733358191	0.006269211	0.028391765	up_in_case
Yang2025_S	ENSGALG00010019588	CHMP7	case	control	-0.234182394	0.149471776	-1.5667332	0.117177067	0.245432501	down_in_case
Yang2025_S	ENSGALG00010019591	ENSGALG00010019591	case	control	0.10193886	0.122995282	0.828803012	0.407215884	0.573235943	up_in_case
Yang2025_S	ENSGALG00010019592	ALKBH3	case	control	-0.135303948	0.139015762	-0.973299334	0.330404554	0.499777142	down_in_case
Yang2025_S	ENSGALG00010019594	TMX1	case	control	-0.441435512	0.3479953	-1.268509981	0.204615889	0.362809824	down_in_case
Yang2025_S	ENSGALG00010019595	GRIN2A	case	control	0.308829339	0.222556146	1.387646869	0.165244602	0.312837821	up_in_case
Yang2025_S	ENSGALG00010019598	KCNAB2	case	control	0.247702105	0.14039576	1.764313287	0.077679248	0.183530065	up_in_case
Yang2025_S	ENSGALG00010019600	MAP3K19	case	control	1.007500045	0.457547533	2.201957112	0.027668338	0.086235359	up_in_case
Yang2025_S	ENSGALG00010019602	SLC29A1	case	control	-0.082503186	0.124062671	-0.665012168	0.50604271	0.66157179	down_in_case
Yang2025_S	ENSGALG00010019610	CORO7	case	control	0.138696354	0.127902268	1.084393231	0.278190484	0.445789373	up_in_case
Yang2025_S	ENSGALG00010019613	FRMD6	case	control	0.197730559	0.092772381	2.13135155	0.033060191	0.098375777	up_in_case
Yang2025_S	ENSGALG00010019614	MICALL2	case	control	-0.293332067	0.148209401	-1.979173155	0.047796514	0.12927881	down_in_case
Yang2025_S	ENSGALG00010019617	ANO3	case	control	0.585197704	0.161525037	3.622953528	0.000291258	0.002543034	up_in_case
Yang2025_S	ENSGALG00010019618	NPHP4	case	control	0.044852587	0.103050587	0.43524824	0.663382251	0.787011039	up_in_case
Yang2025_S	ENSGALG00010019621	ENSGALG00010019621	case	control	0.256034406	0.10024797	2.554010891	0.010648993	0.042458859	up_in_case
Yang2025_S	ENSGALG00010019622	C2orf42	case	control	0.258645147	0.098937711	2.614222056	0.008943089	0.037444206	up_in_case
Yang2025_S	ENSGALG00010019624	SRGAP3	case	control	-0.028676942	0.15158254	-0.189183678	0.84994886	0.910869597	down_in_case
Yang2025_S	ENSGALG00010019625	INTS1	case	control	-0.09340967	0.071220024	-1.311564704	0.189667049	0.344497335	down_in_case
Yang2025_S	ENSGALG00010019630	AJAP1	case	control	0.144657	0.465146999	0.310992011	0.755806695	0.851700195	up_in_case
Yang2025_S	ENSGALG00010019634	GNG2	case	control	0.273972416	0.165282819	1.657597668	0.097398701	0.214890895	up_in_case
Yang2025_S	ENSGALG00010019635	LRRC57	case	control	-0.373955979	0.123114345	-3.037468779	0.002385741	0.013703752	down_in_case
Yang2025_S	ENSGALG00010019640	MGRN1	case	control	-0.208125285	0.13664139	-1.523149655	0.127721268	0.260704093	down_in_case
Yang2025_S	ENSGALG00010019641	CAPN11	case	control	0.320321181	0.066838351	4.792475807	1.64736E-06	3.6966E-05	up_in_case
Yang2025_S	ENSGALG00010019643	RAB3GAP1	case	control	0.035838596	0.070060557	0.511537411	0.608974799	0.746355657	up_in_case
Yang2025_S	ENSGALG00010019645	ENSGALG00010019645	case	control	0.175406681	0.123571169	1.419479015	0.155759411	0.300541039	up_in_case
Yang2025_S	ENSGALG00010019646	VPS37C	case	control	0.308646837	0.177646344	1.737422963	0.082312541	0.1910117	up_in_case
Yang2025_S	ENSGALG00010019648	PRDM16	case	control	0.545940191	0.229166569	2.382285485	0.017205553	0.060744713	up_in_case
Yang2025_S	ENSGALG00010019649	TMEM63B	case	control	0.078878963	0.10262993	0.768576603	0.442144699	0.605493563	up_in_case
Yang2025_S	ENSGALG00010019650	KBTBD8	case	control	-0.46504967	0.105733928	-4.398301273	1.09101E-05	0.000177609	down_in_case
Yang2025_S	ENSGALG00010019652	TNFRSF10B	case	control	-0.690762933	0.385613066	-1.791336949	0.07323924	0.175660725	down_in_case
Yang2025_S	ENSGALG00010019655	RTRAF	case	control	-0.261330099	0.152301501	-1.715873428	0.086185211	0.197563895	down_in_case
Yang2025_S	ENSGALG00010019657	PPP1R17	case	control	0.80342396	0.319531735	2.514379234	0.011924213	0.046143825	up_in_case
Yang2025_S	ENSGALG00010019659	MRPL14	case	control	-0.556170633	0.187291065	-2.969552408	0.002982339	0.016265714	down_in_case
Yang2025_S	ENSGALG00010019660	ENSGALG00010019660	case	control	-0.833035534	0.566810052	-1.469690828	0.141645508	0.281045237	down_in_case
Yang2025_S	ENSGALG00010019666	TTC34	case	control	0.314588023	0.420259534	0.74855654	0.454124535	0.615885683	up_in_case
Yang2025_S	ENSGALG00010019667	ITPRID1	case	control	0.455898767	0.50815257	0.897169067	0.369628709	0.538362799	up_in_case
Yang2025_S	ENSGALG00010019670	ENSGALG00010019670	case	control	0.095681185	0.137009639	0.698353677	0.484956039	0.642504506	up_in_case
Yang2025_S	ENSGALG00010019673	NID2	case	control	0.067290119	0.086010099	0.782351368	0.434008106	0.597914281	up_in_case

Yang2025_S	ENSGALG00010019675	MAFK	case	control	0.107350295	0.080354951	1.335951231	0.181565214	0.334158823	up_in_case
Yang2025_S	ENSGALG00010019679	RHOBTB2	case	control	-0.016602	0.113396694	-0.146406386	0.883600586	0.931389877	down_in_case
Yang2025_S	ENSGALG00010019680	CCDC34	case	control	-0.175026569	0.162175784	-1.079239848	0.28048082	0.448376762	down_in_case
Yang2025_S	ENSGALG00010019682	TMEM158	case	control	-0.012764906	0.298922648	-0.042703041	0.965938255	0.979939965	down_in_case
Yang2025_S	ENSGALG00010019683	TMEM184A	case	control	-0.462274845	0.209405611	-2.207557108	0.027275162	0.085235024	down_in_case
Yang2025_S	ENSGALG00010019684	PIGQ	case	control	-0.232207068	0.157503597	-1.474296926	0.140401662	0.279443115	down_in_case
Yang2025_S	ENSGALG00010019690	ENSGALG00010019690	case	control	-0.486204677	0.224821998	-2.162620572	0.030570375	0.092951816	down_in_case
Yang2025_S	ENSGALG00010019692	MMEL1	case	control	1.290458664	0.394147545	3.274049736	0.001060179	0.007335452	up_in_case
Yang2025_S	ENSGALG00010019693	HAUS2	case	control	-0.101449365	0.175994125	-0.57643609	0.564320457	0.710370873	down_in_case
Yang2025_S	ENSGALG00010019696	ENSGALG00010019696	case	control	0.092199913	0.276108587	0.333926278	0.73843519	0.84021347	up_in_case
Yang2025_S	ENSGALG00010019697	EIF4EBP1	case	control	0.230100027	0.202967601	1.133678606	0.256929387	0.423356841	up_in_case
Yang2025_S	ENSGALG00010019700	HES5B	case	control	-0.465488718	0.208122418	-2.23661018	0.025311829	0.080658664	down_in_case
Yang2025_S	ENSGALG00010019706	NPRL3	case	control	-0.036414697	0.090897725	-0.400611751	0.688705993	0.805397618	down_in_case
Yang2025_S	ENSGALG00010019713	NUDT14	case	control	-0.284635552	0.127593503	-2.230799729	0.025694398	0.081579274	down_in_case
Yang2025_S	ENSGALG00010019716	VEGFA	case	control	0.585551071	0.078053009	7.501966625	6.28674E-14	1.04292E-11	up_in_case
Yang2025_S	ENSGALG00010019717	ZRANB3	case	control	0.377173535	0.173137084	2.178467637	0.029371239	0.090072231	up_in_case
Yang2025_S	ENSGALG00010019724	HES5A	case	control	0.395164482	0.380422689	1.038751089	0.298920514	0.468292003	up_in_case
Yang2025_S	ENSGALG00010019726	PEBP4	case	control	0.035223801	0.403671702	0.087258535	0.930466013	0.958016143	up_in_case
Yang2025_S	ENSGALG00010019731	PANK4	case	control	0.413355245	0.101811866	4.059990858	4.90746E-05	0.000600927	up_in_case
Yang2025_S	ENSGALG00010019732	HBA1	case	control	-0.164858854	0.235975127	-0.698628098	0.484784479	0.642460458	down_in_case
Yang2025_S	ENSGALG00010019735	ENSGALG00010019735	case	control	0.409089967	0.255576235	1.6006573	0.109452843	0.233422196	up_in_case
Yang2025_S	ENSGALG00010019738	ADRA2A	case	control	-0.582671394	0.218756883	-2.663556847	0.007731935	0.033342673	down_in_case
Yang2025_S	ENSGALG00010019744	KBP	case	control	1.218831677	0.476034644	2.560384404	0.010455644	0.041855616	up_in_case
Yang2025_S	ENSGALG00010019752	GPAM	case	control	0.170310547	0.162766203	1.046350804	0.295399098	0.463870456	up_in_case
Yang2025_S	ENSGALG00010019761	MRPS18A	case	control	-0.359871009	0.166505438	-2.161316848	0.030670872	0.093216708	down_in_case
Yang2025_S	ENSGALG00010019767	SUCLG2	case	control	-0.110565845	0.101016507	-1.094532449	0.273721524	0.441011613	down_in_case
Yang2025_S	ENSGALG00010019771	VWCE	case	control	0.37946168	0.295806355	1.282804356	0.199560628	0.356888776	up_in_case
Yang2025_S	ENSGALG00010019772	ENSGALG00010019772	case	control	0.034674023	0.200047771	0.173328713	0.862393055	0.918905584	up_in_case
Yang2025_S	ENSGALG00010019778	ABHD17C	case	control	-0.199303079	0.129741449	-1.536155797	0.124500174	0.256037474	down_in_case
Yang2025_S	ENSGALG00010019785	ACSL5	case	control	-0.431044329	0.305062415	-1.412970943	0.157664268	0.302926901	down_in_case
Yang2025_S	ENSGALG00010019786	R3HDM1	case	control	0.458970139	0.208986353	2.196172779	0.028079579	0.087107955	up_in_case
Yang2025_S	ENSGALG00010019789	ARNT2	case	control	0.200159465	0.150745074	1.327801029	0.184243847	0.3375543	up_in_case
Yang2025_S	ENSGALG00010019794	RSPH9	case	control	-0.021870434	0.228033189	-0.095908994	0.923592851	0.95391835	down_in_case
Yang2025_S	ENSGALG00010019799	DOK2	case	control	-0.412395769	0.290315606	-1.42050844	0.155459713	0.300229259	down_in_case
Yang2025_S	ENSGALG00010019800	EXT2	case	control	-0.607149134	0.070671171	-8.5911854	8.60766E-18	3.21294E-15	down_in_case
Yang2025_S	ENSGALG00010019801	FIBIN	case	control	0.033538025	0.212446691	0.157865603	0.874562703	0.92642396	up_in_case
Yang2025_S	ENSGALG00010019803	FAH	case	control	-1.017447308	0.523190277	-1.944698426	0.051811285	0.136782751	down_in_case
Yang2025_S	ENSGALG00010019804	ELFN1	case	control	0.131947306	0.245151402	0.538227827	0.590419771	0.731268288	up_in_case
Yang2025_S	ENSGALG00010019807	ZDHHC6	case	control	-0.352275668	0.117020515	-3.010375296	0.002609251	0.014652988	down_in_case
Yang2025_S	ENSGALG00010019810	ZFAND6	case	control	-0.603859272	0.128102609	-4.713871755	2.43054E-06	5.13953E-05	down_in_case
Yang2025_S	ENSGALG00010019811	UBXN4	case	control	0.190827384	0.10706325	1.782379886	0.074687303	0.178274678	up_in_case
Yang2025_S	ENSGALG00010019813	ENSGALG00010019813	case	control	-0.918655935	0.276880662	-3.317876832	0.000907045	0.006468612	down_in_case
Yang2025_S	ENSGALG00010019820	BBOX1	case	control	0.058626571	0.16221143	0.361420717	0.717784964	0.826161185	up_in_case
Yang2025_S	ENSGALG00010019823	LCT	case	control	0.110538164	0.298746617	0.370006413	0.711377712	0.821704942	up_in_case
Yang2025_S	ENSGALG00010019825	BCL2A1	case	control	-0.098280671	0.317339098	-0.309702369	0.756787301	0.852119509	down_in_case
Yang2025_S	ENSGALG00010019830	JAG2	case	control	-0.092937211	0.083321804	-1.115400854	0.264678684	0.431733286	down_in_case
Yang2025_S	ENSGALG00010019832	MAD2L1BP	case	control	-0.447299718	0.126443839	-3.537536687	0.000403878	0.003322338	down_in_case
Yang2025_S	ENSGALG00010019833	MTHFS	case	control	-0.213080555	0.153257903	-1.39033976	0.16442573	0.311545093	down_in_case
Yang2025_S	ENSGALG00010019834	PLCH2	case	control	0.650429372	0.259281483	2.508583966	0.012121616	0.046751927	up_in_case
Yang2025_S	ENSGALG00010019836	MINAR1	case	control	0.136928443	0.29917089	0.457693068	0.647172969	0.77583924	up_in_case
Yang2025_S	ENSGALG00010019838	ALX4	case	control	-0.486667539	0.206020022	-2.36223419	0.018165163	0.063203881	down_in_case
Yang2025_S	ENSGALG00010019843	MCM6	case	control	-0.253899707	0.128163997	-1.981053282	0.047585299	0.128882631	down_in_case
Yang2025_S	ENSGALG00010019846	ENSGALG00010019846	case	control	0.047074977	0.108020853	0.435795274	0.662985273	0.786952362	up_in_case
Yang2025_S	ENSGALG00010019848	MAD1L1	case	control	0.179526243	0.127248719	1.41082947	0.158294903	0.303699891	up_in_case
Yang2025_S	ENSGALG00010019854	CCDC14	case	control	0.412378211	0.142924001	2.885297147	0.003910444	0.019865853	up_in_case
Yang2025_S	ENSGALG00010019855	TTC7A	case	control	-0.041307322	0.136773109	-0.302013478	0.762641788	0.855408866	down_in_case
Yang2025_S	ENSGALG00010019859	BIN3	case	control	0.185990053	0.130649395	1.423581433	0.154567674	0.298882672	up_in_case
Yang2025_S	ENSGALG00010019865	ENSGALG00010019865	case	control	-0.312932411	0.159674916	-1.959809466	0.050018064	0.133287571	down_in_case
Yang2025_S	ENSGALG00010019867	PEX11A	case	control	-0.783440803	0.425749711	-1.840144061	0.06574709	0.16247308	down_in_case
Yang2025_S	ENSGALG00010019871	TAFA1	case	control	0.42580822	0.654943401	0.650145065	0.515598522	0.670229982	up_in_case
Yang2025_S	ENSGALG00010019872	VTI1A	case	control	0.489249914	0.111390709	4.392196781	1.12211E-05	0.000182033	up_in_case
Yang2025_S	ENSGALG00010019873	LGR4	case	control	-0.263752555	0.119419732	-2.208617876	0.027201231	0.085064891	down_in_case
Yang2025_S	ENSGALG00010019875	PLEKHH1	case	control	0.596709518	0.312463648	1.909692606	0.056172804	0.145272003	up_in_case
Yang2025_S	ENSGALG00010019876	MRM2	case	control	-0.451332972	0.191923369	-2.351631138	0.0186913	0.064471105	down_in_case
Yang2025_S	ENSGALG00010019878	MCFD2	case	control	-0.622655921	0.13217328	-4.710906173	2.46618E-06	5.18344E-05	down_in_case
Yang2025_S	ENSGALG00010019891	PIGH	case	control	0.133152064	0.170523828	0.780841396	0.434895783	0.598663711	up_in_case
Yang2025_S	ENSGALG00010019896	DDB1	case	control	-0.104436066	0.079632976	-1.311467585	0.189699838	0.344497335	down_in_case
Yang2025_S	ENSGALG00010019898	NUDT1	case	control	0.225115143	0.170267414	1.322126991	0.186125867	0.339741637	up_in_case
Yang2025_S	ENSGALG00010019899	ENSGALG00010019899	case	control	0.464612442	0.233719121	1.987909417	0.046821709	0.12723489	up_in_case
Yang2025_S	ENSGALG00010019905	ENSGALG00010019905	case	control	-0.323686859	0.062697397	-5.16268418	2.43434E-07	7.20944E-06	down_in_case
Yang2025_S	ENSGALG00010019906	ENSGALG00010019906	case	control	-0.261140463	0.111675706	-2.338382017	0.01936744	0.066241564	down_in_case
Yang2025_S	ENSGALG00010019910	ENSGALG00010019910	case	control	1.682077713	0.557548959	3.016914813	0.002553616	0.014424254	up_in_case
Yang2025_S	ENSGALG00010019912	ARG2	case	control	0.217941805	0.36318721	0.600081168	0.548452143	0.697241385	up_in_case
Yang2025_S	ENSGALG00010019916	NOC3L	case	control	0.024466505	0.076235199	0.320934499	0.74826003	0.847301138	up_in_case
Yang2025_S	ENSGALG00010019919	LIN7C	case	control	-0.549549316	0.125221014	-4.388634934	1.14064E-05	0.000184824	down_in_case
Yang2025_S	ENSGALG00010019921	CALM2	case	control	0.0919587	0.100282825	0.916993506	0.359146043	0.528033352	up_in_case
Yang2025_S	ENSGALG00010019925	RDH11	case	control	-0.409777288	0.152359082	-2.689549466	0.007154854	0.031434981	down_in_case
Yang2025_S	ENSGALG00010019926	KIF7	case	control	0.131922128	0.081405884	1.620547827	0.105114649	0.227272713	up_in_case
Yang2025_S	ENSGALG00010019931	ZFYVE26	case	control	-0.130962455	0.135988192	-0.963042848	0.335526021	0.503994298	down_in_case
Yang2025_S	ENSGALG00010019932	KALRN	case	control	-0.045455038	0.105606248	-0.430419972	0.66689017	0.789291194	down_in_case
Yang2025_S	ENSGALG00010019934	TKFC	case	control	0.582181137	0.214328116	2.716307817	0.006601451	0.02957917	up_in_case
Yang2025_S	ENSGALG00010019935	ENSGALG00010019935	case	control	1.325320473	0.702243833	1.887265377	0.059124641	0.151008407	up_in_case
Yang2025_S	ENSGALG00010019938	TAFA4	case	control	-0.410755953	0.509634826	-0.805980933	0.420253843	0.585916689	down_in_case
Yang2025_S	ENSGALG00010019947	ENSGALG00010019947	case	control	-0.978614237	0.421612346	-2.321123293	0.020280191	0.068427229	down_in_case
Yang2025_S	ENSGALG00010019948	TICRR	case	control	-0.204281986	0.112577401	-1.814591418	0.069586722	0.169289626	down_in_case
Yang2025_S	ENSGALG00010019954	CYB561A3	case	control	0.296640328	0.219564189	1.351041481	0.176682145	0.327663787	up_in_case
Yang2025_S	ENSGALG00010019956	SLC29A3	case	control	-0.054187596	0.144659366	-0.374587538	0.707967242	0.819872311	down_in_case
Yang2025_S	ENSGALG00010019961	HTATSF1	case	control	-0.161603932	0.075342138	-2.144934263	0.031958103	0.096039717	down_in_case
Yang2025_S	ENSGALG00010019962	SNX8	case	control	-0.367867522	0.118738275	-3.098137663	0.001947409	0.011752772	down_in_case
Yang2025_S	ENSGALG00010019966	ENSGALG00010019966	case	control	-1.770937293	0.702848363	-2.519657705	0.011746901	0.045660546	down_in_case
Yang2025_S	ENSGALG00010019967	CEP170B	case	control	0.380361062	0.238302856	1.596124643	0.110460968	0.235003601	up_in_case
Yang2025_S	ENSGALG00010019968	RHCG	case	control	0.378288885	0.657106684	0.575688688	0.564825623	0.710723581	up_in_case
Yang2025_S	ENSGALG00010019972	PPP1CB	case	control	-0.174094509	0.105971086	-1.642849154	0.100414147	0.21963132	down_in_case
Yang2025_S	ENSGALG00010019975	ENSGALG00010019975	case	control	0.725844559	0.127971917	5.671905035	1.41218E-08	6.32758E-07	up_in_case
Yang2025_S	ENSGALG00010019977	ZMIZ1	case	control	0.074840387	0.210983713	0.354721157	0.722798499	0.829601934	up_in_case
Yang2025_S	ENSGALG00010019979	ENSGALG00010019979	case	control	-0.088976549	0.430161916	-0.206844321	0.836131442	0.902989756	down_in_case
Yang2025_S	ENSGALG00010019982	PLCE1	case	control	0.506864186	0.212966213	2.380021596	0.017311623	0.061046432	up_in_case
Yang2025_S	ENSGALG00010019985	UMPS	case	control	-0.409532192	0.132411045	-3.092885431	0.001982206	0.011890679	down_in_case
Yang2025_S	ENSGALG00010019989	ENSGALG00010019989	case	control	0.228030428	0.214734734	1.061916827	0.288273445	0.45721494	up_in_case
Yang2025_S	ENSGALG00010019990	MARCHF8	case	control	-0.090943244	0.119570471	-0.760582805	0.446906293	0.609596633	down_in_case
Yang2025_S	ENSGALG00010019991	EIF3B	case	control	-0.338486142	0.095331866	-3.550608579	0.000384342	0.003200836	down_in_case
Yang2025_S	ENSGALG00010019992	CHAC2	case	control	-0.362504949	0.169843679	-2.134344653	0.032814579	0.097895772	down_in_case
Yang2025_S	ENSGALG00010019996	ITGB5	case	control	-0.23480444	0.107529582	-2.183626464	0.028989707	0.089196637	down_in_case
Yang2025_S	ENSGALG00010019997	FSHR	case	control	0.128882871	0.3201134	0.402616293	0.687230522	0.804465418	up_in_case
Yang2025_S	ENSGALG00010019999	ERLIN1	case	control	-0.182427585	0.079227751	-2.302571792	0.021302948	0.070950425	down_in_case
Yang2025_S	ENSGALG00010020003	ENSGALG00010020003	case	control	0.033726086	0.233644055	0.144348146	0.885225561	0.932729113	up_in_case
Yang2025_S	ENSGALG00010020005	EOGT	case	control	-0.010381175	0.083717446	-0.124002529	0.901313273	0.941137577	down_in_case
Yang2025_S	ENSGALG00010020006	PLB1	case	control	0.063148032	0.424364064	0.148806266	0.881706506	0.930378628	up_in_case
Yang2025_S	ENSGALG00010020008	EIF2S1	case	control	0.052489177	0.103703812	0.506145106	0.612754788	0.749275006	up_in_case
Yang2025_S	ENSGALG00010020009	MACROD2	case	control	0.423723021	0.241392315	1.755329372	0.079202985	0.186026226	up_in_case
Yang2025_S	ENSGALG00010020010	SORBS3	case	control	1.337817856	0.331238295	4.038838127	5.37166E-05	0.000645294	up_in_case
Yang2025_S	ENSGALG00010020011	TLE3	case	control	0.671737394	0.146320536	4.590862047	4.41419E-06	8.36894E-05	up_in_case
Yang2025_S	ENSGALG00010020013	ADAM8	case	control	0.987210383	0.831008884	1.187966101	0.234846766	0.398028421	up_in_case
Yang2025_S	ENSGALG00010020015	ATP6V1D	case	control	0.262716815	0.117080586	2.243897338	0.024839007	0.079588772	up_in_case
Yang2025_S	ENSGALG00010020017	ENSGALG00010020017	case	control	1.16072343	0.402393328	2.884549394	0.003919743	0.019898584	up_in_case
Yang2025_S	ENSGALG00010020018	ENSGALG00010020018	case	control	0.515860877	0.278592854	1.851665863	0.064073819	0.15946931	up_in_case
Yang2025_S	ENSGALG00010020019	PEX10	case	control	0.310069923	0.149780763	2.07015852	0.038437502	0.109962347	up_in_case
Yang2025_S	ENSGALG00010020023	FOSL2	case	control	-0.252155211	0.234038592	-1.077408681	0.281297728	0.449231015	down_in_case
Yang2025_S	ENSGALG00010020024	ENSGALG00010020024	case	control	5.062292587	1.65900951	3.051394556	0.00227781	0.013209025	up_in_case
Yang2025_S	ENSGALG00010020025	LOXL2	case	control	-0.065613384	0.152727236	-0.429611548	0.667478235	0.789853024	down_in_case
Yang2025_S	ENSGALG00010020027	RER1	case	control	-0.409481049	0.125681849	-3.258076271	0.001121703	0.007673503	down_in_case
Yang2025_S	ENSGALG00010020030	MRPL33	case	control	0.219519282	0.143726826	1.527336885	0.126677262	0.259290195	up_in_case
Yang2025_S	ENSGALG00010020032	CHST12	case	control	-0.102104221	0.134531343	-0.758962327	0.447875093	0.610420332	down_in_case
Yang2025_S	ENSGALG00010020036	TMF1	case	control	0.376915672	0.148773225	2.533491297	0.011293254	0.044280107	up_in_case
Yang2025_S	ENSGALG00010020037	MPP5	case	control	0.038751777	0.13670229	0.283475696	0.776812209	0.86419273	up_in_case
Yang2025_S	ENSGALG00010020038	SKI	case	control	0.207704786	0.210112029	0.988543051	0.322886761	0.49219489	up_in_case

Yang2025_S	ENSGALG00010020041	CDH23	case	control	-0.049248419	0.162222035	-0.303586494	0.761442943	0.854874912	down_in_case
Yang2025_S	ENSGALG00010020046	BDNF	case	control	0.036830072	0.285105727	0.1291804	0.897214905	0.93902049	up_in_case
Yang2025_S	ENSGALG00010020048	ENSGALG00010020048	case	control	-0.349739635	0.247323281	-1.414099123	0.157332801	0.302613192	down_in_case
Yang2025_S	ENSGALG00010020053	LFNG	case	control	-0.069074137	0.112467171	-0.614171551	0.539101971	0.690286314	down_in_case
Yang2025_S	ENSGALG00010020055	R3HCC1	case	control	-0.084855496	0.070316075	-1.206772367	0.227519821	0.389782235	down_in_case
Yang2025_S	ENSGALG00010020056	UACA	case	control	1.015628067	0.216152219	4.698670544	2.6186E-06	5.43819E-05	up_in_case
Yang2025_S	ENSGALG00010020057	RBKS	case	control	-0.126640103	0.154937661	-0.817361655	0.413721782	0.579185557	down_in_case
Yang2025_S	ENSGALG00010020059	PRKCZ	case	control	0.248711579	0.168799819	1.473411406	0.140640137	0.279734556	up_in_case
Yang2025_S	ENSGALG00010020061	UBA3	case	control	-0.074133482	0.097169523	-0.762929357	0.445505527	0.608519851	down_in_case
Yang2025_S	ENSGALG00010020063	ENSGALG00010020063	case	control	-0.017344934	0.093618994	-0.185271531	0.853016067	0.913297079	down_in_case
Yang2025_S	ENSGALG00010020064	TMEM138	case	control	-0.380995851	0.237721637	-1.602697408	0.109001476	0.232858435	down_in_case
Yang2025_S	ENSGALG00010020065	TTYH3	case	control	0.082294314	0.172635991	0.476692687	0.633580971	0.764886312	up_in_case
Yang2025_S	ENSGALG00010020066	ENSGALG00010020066	case	control	-0.127395652	0.497667872	-0.255985284			down_in_case
Yang2025_S	ENSGALG00010020069	TMEM216	case	control	0.290388454	0.316392887	0.91780968	0.358718513	0.527627452	up_in_case
Yang2025_S	ENSGALG00010020070	ARL6IP5	case	control	-0.352370593	0.118907189	-2.963408648	0.003042524	0.016516387	down_in_case
Yang2025_S	ENSGALG00010020071	ENSGALG00010020071	case	control	0.476194427	0.233819715	2.036588006	0.041691346	0.116637001	up_in_case
Yang2025_S	ENSGALG00010020074	ENSGALG00010020074	case	control	-0.389455666	0.122771143	-3.172208509	0.001512843	0.009670401	down_in_case
Yang2025_S	ENSGALG00010020075	CPSF7	case	control	-0.003534711	0.074514962	-0.047436253	0.962165536	0.977528196	down_in_case
Yang2025_S	ENSGALG00010020080	SLC4A1AP	case	control	0.170512504	0.083862031	2.033250351	0.042027236	0.117247155	up_in_case
Yang2025_S	ENSGALG00010020083	CFAP74	case	control	0.156890746	0.359983179	0.435827991	0.662961533	0.786952362	up_in_case
Yang2025_S	ENSGALG00010020084	ENSGALG00010020084	case	control	0.017286184	0.113917646	0.151742816	0.879389786	0.928681389	up_in_case
Yang2025_S	ENSGALG00010020087	ENSGALG00010020087	case	control	0.257380139	0.53652009	0.479721345	0.631425548	0.763463342	up_in_case
Yang2025_S	ENSGALG00010020088	LMOD3	case	control	0.217991331	0.10173101	2.142820876	0.032127484	0.096444742	up_in_case
Yang2025_S	ENSGALG00010020090	MORF4L2	case	control	-0.04023238	0.078095004	-0.515172261	0.606432651	0.743766699	down_in_case
Yang2025_S	ENSGALG00010020091	SUPT7L	case	control	-0.232277298	0.095564222	-2.430588476	0.015074325	0.055279137	down_in_case
Yang2025_S	ENSGALG00010020092	SDHAF2	case	control	-0.008906712	0.254562575	-0.034988303	0.972089068	0.983073064	down_in_case
Yang2025_S	ENSGALG00010020094	CLEC3B	case	control	-0.145852497	0.134977673	-1.08056757	0.279889515	0.44780305	down_in_case
Yang2025_S	ENSGALG00010020095	LRFN2	case	control	-0.117724962	0.257165596	-0.457778816	0.647111356	0.77583924	down_in_case
Yang2025_S	ENSGALG00010020096	GPHN	case	control	0.27380019	0.119510167	2.291020062	0.021962253	0.072419309	up_in_case
Yang2025_S	ENSGALG00010020097	RETSAT	case	control	0.193673273	0.203356717	0.952381984	0.340903293	0.509108069	up_in_case
Yang2025_S	ENSGALG00010020098	GABRD	case	control	0.137286136	0.341450224	0.402067787	0.687634139	0.804616821	up_in_case
Yang2025_S	ENSGALG00010020100	MOCS1	case	control	-0.410309663	0.150057771	-2.734344643	0.006250458	0.028325248	down_in_case
Yang2025_S	ENSGALG00010020101	KIF15	case	control	0.406089549	0.17304472	2.346731807	0.018938881	0.065168987	up_in_case
Yang2025_S	ENSGALG00010020102	TMEM127	case	control	0.423436036	0.178572872	2.371222631	0.017729348	0.062200017	up_in_case
Yang2025_S	ENSGALG00010020103	PSAP	case	control	-0.65430352	0.126807268	-5.15982663	2.47179E-07	7.23621E-06	down_in_case
Yang2025_S	ENSGALG00010020104	BRAT1	case	control	-0.335227964	0.181402316	-1.847980613	0.064605147	0.160362082	down_in_case
Yang2025_S	ENSGALG00010020106	ENSGALG00010020106	case	control	0.06494528	0.103506973	0.627448352	0.530365381	0.682775331	up_in_case
Yang2025_S	ENSGALG00010020108	RPAP1	case	control	0.10379179	0.095036547	1.092125012	0.274778157	0.442100637	up_in_case
Yang2025_S	ENSGALG00010020109	WDFY4	case	control	0.106800456	0.220072677	0.485296302	0.627466178	0.760683246	up_in_case
Yang2025_S	ENSGALG00010020111	ADRA2B	case	control	0.545233287	0.12744973	4.278026228	1.88558E-05	0.000276003	up_in_case
Yang2025_S	ENSGALG00010020113	CIAO1	case	control	0.314603008	0.167534898	1.877835667	0.060403649	0.153208018	up_in_case
Yang2025_S	ENSGALG00010020116	GNA12	case	control	-0.35610001	0.10698932	-3.328369686	0.000873559	0.006284481	down_in_case
Yang2025_S	ENSGALG00010020117	AKAP13	case	control	-0.108388371	0.146897686	-0.737849406	0.460605966	0.621607424	down_in_case
Yang2025_S	ENSGALG00010020118	GNB1	case	control	-0.451621375	0.063482188	-7.114143224	1.1261E-12	1.50887E-10	down_in_case
Yang2025_S	ENSGALG00010020120	DAAM2	case	control	0.263189119	0.19823191	1.327682913	0.184282881	0.33758143	up_in_case
Yang2025_S	ENSGALG00010020121	ENSGALG00010020121	case	control	0.273980339	0.309560001	0.885063762	0.376122243	0.544320641	up_in_case
Yang2025_S	ENSGALG00010020123	ENSGALG00010020123	case	control	-0.221941626	0.182344676	-1.217154403	0.223545475	0.384921442	down_in_case
Yang2025_S	ENSGALG00010020125	NAV2	case	control	0.389487132	0.192941208	2.018682974	0.043520181	0.120615298	up_in_case
Yang2025_S	ENSGALG00010020126	NADK	case	control	0.018865784	0.119878466	0.157374257	0.874949901	0.926528291	up_in_case
Yang2025_S	ENSGALG00010020127	FRMD4B	case	control	-0.02129269	0.163685276	-0.130083113	0.89650067	0.938786882	down_in_case
Yang2025_S	ENSGALG00010020128	KIF6	case	control	0.259721771	0.667729429	0.388962594	0.697303817	0.812588116	up_in_case
Yang2025_S	ENSGALG00010020129	MGAT2	case	control	-0.394251901	0.126773351	-3.10989571	0.001871534	0.01140351	down_in_case
Yang2025_S	ENSGALG00010020130	PDXDC1	case	control	0.340286838	0.107998886	3.150836548	0.001628036	0.010242292	up_in_case
Yang2025_S	ENSGALG00010020131	SNRNP200	case	control	0.119438403	0.096404944	1.238924047	0.215373627	0.376235615	up_in_case
Yang2025_S	ENSGALG00010020132	SHC4	case	control	0.097937811	0.171036688	0.572612882	0.566906835	0.711703646	up_in_case
Yang2025_S	ENSGALG00010020136	KCNK17	case	control	1.109915433	0.340766	3.257119055	0.001125492	0.007688105	up_in_case
Yang2025_S	ENSGALG00010020137	NTAN1	case	control	0.299501238	0.11837336	2.530140547	0.011401684	0.044615885	up_in_case
Yang2025_S	ENSGALG00010020138	ENSGALG00010020138	case	control	-0.318658829	0.111844831	-2.849115381	0.004384098	0.02161033	down_in_case
Yang2025_S	ENSGALG00010020140	SECISBP2L	case	control	-0.012568724	0.148190729	-0.084814511	0.932408857	0.958830892	down_in_case
Yang2025_S	ENSGALG00010020141	THOC7	case	control	-0.052218806	0.15667106	-0.333302181	0.738906193	0.840291756	down_in_case
Yang2025_S	ENSGALG00010020142	SLC35E2B	case	control	-0.470755856	0.104940868	-4.485915409	7.26015E-06	0.000126939	down_in_case
Yang2025_S	ENSGALG00010020143	KCNK5	case	control	-0.469661505	0.223516225	-2.101241216	0.035619797	0.104146427	down_in_case
Yang2025_S	ENSGALG00010020147	ABHD2	case	control	-0.448097415	0.156571526	-2.861934269	0.004210642	0.020908002	down_in_case
Yang2025_S	ENSGALG00010020149	FAHD2A	case	control	0.084421601	0.297492488	0.283777254	0.776581087	0.864004587	up_in_case
Yang2025_S	ENSGALG00010020152	ATXN7	case	control	-0.033145414	0.158307896	-0.209373094	0.834156995	0.90182508	down_in_case
Yang2025_S	ENSGALG00010020153	AXL	case	control	-0.416305235	0.128426709	-3.24157832	0.001188698	0.008006043	down_in_case
Yang2025_S	ENSGALG00010020155	MFGE8	case	control	-0.004071091	0.160482683	-0.025367792	0.979761601	0.987771517	down_in_case
Yang2025_S	ENSGALG00010020160	SYT7	case	control	0.734584845	0.457964098	1.604022777	0.108709032	0.232402761	up_in_case
Yang2025_S	ENSGALG00010020163	CDC2L1	case	control	0.950906471	0.329045094	2.889897125	0.003853679	0.019656303	up_in_case
Yang2025_S	ENSGALG00010020164	C10orf71	case	control	0.207233508	0.199949478	1.03642935	0.300001884	0.469616519	up_in_case
Yang2025_S	ENSGALG00010020166	HAPLN3	case	control	-0.26059303	0.204671517	-1.273225675	0.202937972	0.360844793	down_in_case
Yang2025_S	ENSGALG00010020168	COX7A2L	case	control	-0.812050371	0.200598004	-4.048147819	5.16245E-05	0.000623386	down_in_case
Yang2025_S	ENSGALG00010020170	SORCS1	case	control	-0.15566002	0.230079728	-0.676548175	0.49869267	0.65565453	down_in_case
Yang2025_S	ENSGALG00010020171	RRN3	case	control	-0.143193223	0.137003473	-1.045179509	0.295940012	0.464510483	down_in_case
Yang2025_S	ENSGALG00010020175	ACAN	case	control	1.994355009	0.854991084	2.332603284			up_in_case
Yang2025_S	ENSGALG00010020176	ENSGALG00010020176	case	control	-0.164365642	0.269894064	-0.609000584	0.542524052	0.69269601	down_in_case
Yang2025_S	ENSGALG00010020177	KCNG3	case	control	-0.414313036	0.176627078	-2.345693773	0.018991704	0.065244208	down_in_case
Yang2025_S	ENSGALG00010020179	PSMD6	case	control	-0.126018346	0.081505162	-1.54608423	0.122084227	0.25289783	down_in_case
Yang2025_S	ENSGALG00010020180	TCF7L1	case	control	0.632145873	0.223142595	2.832923377	0.004612444	0.022536609	up_in_case
Yang2025_S	ENSGALG00010020181	LEUTX	case	control	0.102286563	0.210960163	0.48486198	0.627774253	0.760897123	up_in_case
Yang2025_S	ENSGALG00010020183	XPNPEP1	case	control	0.122634178	0.09836328	1.246747547	0.212490079	0.372787265	up_in_case
Yang2025_S	ENSGALG00010020184	RSL1D1	case	control	-0.009761181	0.092166977	-0.105907577	0.915655683	0.950298796	down_in_case
Yang2025_S	ENSGALG00010020186	MGA	case	control	0.243136236	0.192821522	1.260939306	0.207330715	0.366039974	up_in_case
Yang2025_S	ENSGALG00010020187	MMP23B	case	control	-0.05474016	0.1475202	-0.371068911	0.710586206	0.821267212	down_in_case
Yang2025_S	ENSGALG00010020193	VSTM4	case	control	0.078992838	0.13373663	0.590659706	0.554748451	0.702573813	up_in_case
Yang2025_S	ENSGALG00010020194	PRICKLE2	case	control	-0.051307195	0.154222347	-0.332683274	0.739373376	0.84045746	down_in_case
Yang2025_S	ENSGALG00010020199	DET1	case	control	-0.690816084	0.166230754	-4.155765806	3.242E-05	0.000433563	down_in_case
Yang2025_S	ENSGALG00010020202	MAPKBP1	case	control	0.137013212	0.155049345	0.883674881	0.376871744	0.545122249	up_in_case
Yang2025_S	ENSGALG00010020204	GSPT2	case	control	-0.101391622	0.109211665	-0.928395537	0.353202433	0.522102576	down_in_case
Yang2025_S	ENSGALG00010020206	MRPS11	case	control	0.187920661	0.204794531	0.917605859	0.358825249	0.527673053	up_in_case
Yang2025_S	ENSGALG00010020211	ADD3	case	control	0.149377819	0.148346597	1.006951436	0.313958112	0.48342611	up_in_case
Yang2025_S	ENSGALG00010020212	JMJD7	case	control	-0.244162754	0.255158854	-0.956904887	0.338615264	0.506722907	down_in_case
Yang2025_S	ENSGALG00010020215	PRMT3	case	control	-0.083348634	0.116742442	-0.713953151	0.475256149	0.634783326	down_in_case
Yang2025_S	ENSGALG00010020224	MRPL46	case	control	-0.062567353	0.263535165	-0.237415576	0.812334407	0.887252024	down_in_case
Yang2025_S	ENSGALG00010020226	TCIM	case	control	0.060991155	0.183210375	0.332902299	0.739208034	0.840406621	up_in_case
Yang2025_S	ENSGALG00010020228	TM9SF3	case	control	-0.206604409	0.073570319	-2.808257614	0.004981036	0.023916912	down_in_case
Yang2025_S	ENSGALG00010020230	ENSGALG00010020230	case	control	0.228394605	0.241671441	0.945062452	0.344626993	0.513238981	up_in_case
Yang2025_S	ENSGALG00010020236	SLC35G1	case	control	0.187692172	0.255452833	0.734742967	0.462496047	0.623416755	up_in_case
Yang2025_S	ENSGALG00010020237	MIB2	case	control	0.148515899	0.096216189	1.543564557	0.122693857	0.253632828	up_in_case
Yang2025_S	ENSGALG00010020240	SV2B	case	control	-0.317019143	0.389674083	-0.813549468	0.415903096	0.581363191	down_in_case
Yang2025_S	ENSGALG00010020244	SLC25A37	case	control	-0.559007492	0.216762052	-2.578899247	0.009911569	0.040279066	down_in_case
Yang2025_S	ENSGALG00010020246	ADAMTS9	case	control	0.31926174	0.133296214	2.395129838	0.016614481	0.059258456	up_in_case
Yang2025_S	ENSGALG00010020248	NOLC1	case	control	-0.142616352	0.088878745	-1.60461708	0.1085781	0.232238807	down_in_case
Yang2025_S	ENSGALG00010020250	ENSGALG00010020250	case	control	0.270011579	0.203854717	1.324529464	0.185327264	0.33886248	up_in_case
Yang2025_S	ENSGALG00010020252	DMTN	case	control	0.580449265	0.238860675	2.430074621	0.015095714	0.055313905	up_in_case
Yang2025_S	ENSGALG00010020254	ENSGALG00010020254	case	control	0.430262323	0.144042728	2.987046468	0.002816869	0.015521182	up_in_case
Yang2025_S	ENSGALG00010020257	ZNF710	case	control	0.254260609	0.101094373	2.515081711	0.01190048	0.046064774	up_in_case
Yang2025_S	ENSGALG00010020259	ENSGALG00010020259	case	control	-0.131814251	0.228985611	-0.575644253	0.564855663	0.710723581	down_in_case
Yang2025_S	ENSGALG00010020261	SSU72	case	control	0.214654929	0.137254697	1.563916822	0.117837103	0.246333639	up_in_case
Yang2025_S	ENSGALG00010020265	ENSGALG00010020265	case	control	0.52785176	0.221808832	2.379759881	0.017323923	0.061069785	up_in_case
Yang2025_S	ENSGALG00010020268	CPLANE2	case	control	0.352454631	0.307008419	1.148029202	0.250956509	0.416666144	up_in_case
Yang2025_S	ENSGALG00010020269	ENSGALG00010020269	case	control	-0.460155131	0.779198693	-0.590549157	0.55482254	0.702586501	down_in_case
Yang2025_S	ENSGALG00010020272	ENSGALG00010020272	case	control	-0.044113881	0.091764488	-0.48072933	0.630708883	0.763258635	down_in_case
Yang2025_S	ENSGALG00010020277	IDH2	case	control	-0.193891432	0.177382261	-1.093071151	0.274362561	0.441737989	down_in_case
Yang2025_S	ENSGALG00010020282	MAGI1	case	control	0.679600584	0.181423485	3.745935014	0.000179723	0.00171518	up_in_case
Yang2025_S	ENSGALG00010020283	FBXO42	case	control	-0.128367685	0.085963377	-1.493283411	0.135363016	0.271916337	down_in_case
Yang2025_S	ENSGALG00010020284	HEG1	case	control	0.042744632	0.244593853	0.174757588	0.861270116	0.918270777	up_in_case
Yang2025_S	ENSGALG00010020285	ADAMTS14	case	control	-0.160090263	0.187186356	-0.855245363	0.392415328	0.559491893	down_in_case
Yang2025_S	ENSGALG00010020287	GALNT16	case	control	-0.252796971	0.135249153	-1.869120549	0.061606045	0.155352919	down_in_case
Yang2025_S	ENSGALG00010020290	NELL1	case	control	-0.008675989	0.564060909	-0.015381297	0.987727984	0.992499961	down_in_case
Yang2025_S	ENSGALG00010020291	SNX29	case	control	0.041344354	0.168855018	0.2448512	0.806571648	0.883622621	up_in_case
Yang2025_S	ENSGALG00010020292	NPM2	case	control	0.211659972	0.441554626	0.479351725	0.63168843	0.76360027	up_in_case
Yang2025_S	ENSGALG00010020293	SEMA4B	case	control	0.089669219	0.171616338	0.522498149	0.601323535	0.73996394	up_in_case
Yang2025_S	ENSGALG00010020295	LGI1	case	control	0.311527104	0.161752525	1.925948944	0.054110749	0.141416596	up_in_case
Yang2025_S	ENSGALG00010020298	ENSGALG00010020298	case	control	0.432095805	0.3113338	1.387885945	0.165171778	0.312769226	up_in_case
Yang2025_S	ENSGALG00010020300	EHD4	case	control	-0.208539097	0.092082504	-2.264698358	0.023531196	0.076667576	down_in_case

Yang2025_S	ENSGALG00010020303	EXD2	case	control	-0.152947543	0.09219035	-1.659040701	0.097107591	0.214656453	down_in_case
Yang2025_S	ENSGALG00010020304	ZFP36L2	case	control	0.12772311	0.207686748	0.614979585	0.538568203	0.689856412	up_in_case
Yang2025_S	ENSGALG00010020305	XPO7	case	control	-0.115907234	0.066609298	-1.740105926	0.08184042	0.190328146	down_in_case
Yang2025_S	ENSGALG00010020307	SHQ1	case	control	0.823876767	0.278539815	2.957842015	0.003098009	0.016713414	up_in_case
Yang2025_S	ENSGALG00010020309	GXYLT2	case	control	-0.564369958	0.069159246	-8.160441132	3.33803E-16	9.12068E-14	down_in_case
Yang2025_S	ENSGALG00010020311	SGPL1	case	control	-0.265857242	0.113424348	-2.343916867	0.019082424	0.065463708	down_in_case
Yang2025_S	ENSGALG00010020313	FRA10AC1	case	control	0.385591604	0.149276013	2.58307813	0.009792314	0.039878987	up_in_case
Yang2025_S	ENSGALG00010020314	THADA	case	control	-0.069364292	0.132654163	-0.522895708	0.601046831	0.739827541	down_in_case
Yang2025_S	ENSGALG00010020315	ANO5	case	control	-0.115458708	0.108640494	-1.06275942	0.287891067	0.456764433	down_in_case
Yang2025_S	ENSGALG00010020316	PDPK1	case	control	-0.092408856	0.106620146	-0.866711028	0.386100362	0.553586638	down_in_case
Yang2025_S	ENSGALG00010020319	NTRK3	case	control	0.129240047	0.17297356	0.747166484	0.454963073	0.616542878	up_in_case
Yang2025_S	ENSGALG00010020320	PDE6C	case	control	0.406644683	0.325140924	1.250672102	0.211054133	0.370968635	up_in_case
Yang2025_S	ENSGALG00010020321	SZRD1	case	control	-0.293336284	0.136864531	-2.143260068	0.032092221	0.096359643	down_in_case
Yang2025_S	ENSGALG00010020323	ARHGAP25	case	control	-0.047785826	0.123482036	-0.386986057	0.69876653	0.813564352	down_in_case
Yang2025_S	ENSGALG00010020324	PLEKHH2	case	control	0.442722776	0.187500926	2.361176477	0.018217059	0.063284276	up_in_case
Yang2025_S	ENSGALG00010020328	SLC17A6	case	control	1.605527052	0.872024543	1.841148928			up_in_case
Yang2025_S	ENSGALG00010020329	PPP4R2	case	control	-0.139667169	0.096470141	-1.447776142	0.147679667	0.289842325	down_in_case
Yang2025_S	ENSGALG00010020336	OSBP	case	control	0.250474933	0.081675636	3.066703204	0.002164336	0.012698959	up_in_case
Yang2025_S	ENSGALG00010020338	NECAP2	case	control	0.250836723	0.107505382	2.33324805	0.01963513	0.066919076	up_in_case
Yang2025_S	ENSGALG00010020340	DCAF5	case	control	0.027838538	0.099804565	0.27893051	0.780298153	0.866825157	up_in_case
Yang2025_S	ENSGALG00010020341	DYNC2LI1	case	control	-0.571948633	0.135140821	-4.232241795	2.31373E-05	0.000328329	down_in_case
Yang2025_S	ENSGALG00010020342	CPPED1	case	control	-0.205609497	0.199099447	-1.032697477	0.301745495	0.471339925	down_in_case
Yang2025_S	ENSGALG00010020343	PATL1	case	control	0.603777336	0.190472317	3.169895481	0.001524938	0.00971651	up_in_case
Yang2025_S	ENSGALG00010020345	AGBL1	case	control	0.008822733	0.190709517	0.04626268	0.963100884	0.977979365	up_in_case
Yang2025_S	ENSGALG00010020346	PCBD1	case	control	0.482681896	0.242402191	1.991243947	0.046454075	0.126466307	up_in_case
Yang2025_S	ENSGALG00010020350	PDZRN3	case	control	0.240392468	0.106698332	2.253010555	0.024258483	0.078446829	up_in_case
Yang2025_S	ENSGALG00010020351	CROCC	case	control	1.923255593	0.209369178	9.185953766	4.07896E-20	2.36835E-17	up_in_case
Yang2025_S	ENSGALG00010020356	STX3	case	control	0.812015472	0.328305706	2.47335169	0.013385235	0.050285074	up_in_case
Yang2025_S	ENSGALG00010020357	RBP4	case	control	-1.05538576	0.512345426	-2.059910571	0.039407091	0.112091817	down_in_case
Yang2025_S	ENSGALG00010020362	CDS2	case	control	-0.503687484	0.078989674	-6.376624377	1.81034E-10	1.34904E-08	down_in_case
Yang2025_S	ENSGALG00010020364	MRPL16	case	control	-0.138754903	0.267615307	-0.518486421	0.604118932	0.742376155	down_in_case
Yang2025_S	ENSGALG00010020365	UNC5B	case	control	0.064168381	0.155593285	0.412410994	0.680038206	0.799187023	up_in_case
Yang2025_S	ENSGALG00010020369	CNTN3	case	control	0.965907311	0.336432402	2.871029382	0.004091375	0.02050101	up_in_case
Yang2025_S	ENSGALG00010020370	CEP55	case	control	-0.186414747	0.180433503	-1.033149296	0.301534037	0.471168065	down_in_case
Yang2025_S	ENSGALG00010020373	MS4A4A	case	control	0.143048366	0.170424205	0.839366483	0.401263686	0.567822846	up_in_case
Yang2025_S	ENSGALG00010020374	PCNA	case	control	0.074253556	0.15330121	0.484363793	0.62812771	0.761060746	up_in_case
Yang2025_S	ENSGALG00010020375	LRPPRC	case	control	-0.115692187	0.062777298	-1.842898474	0.065343835	0.161705975	down_in_case
Yang2025_S	ENSGALG00010020377	TMEM230	case	control	-0.150580163	0.233045517	-0.646140568	0.518188325	0.672529972	down_in_case
Yang2025_S	ENSGALG00010020379	COL13A1	case	control	0.714776	0.205847403	3.472358608	0.000515907	0.004058764	up_in_case
Yang2025_S	ENSGALG00010020382	MFAP2	case	control	-0.045656509	0.171674262	-0.265948479	0.790278888	0.873525526	down_in_case
Yang2025_S	ENSGALG00010020383	BAAT	case	control	0.713955135	0.171704786	4.158038634	3.20992E-05	0.000430927	up_in_case
Yang2025_S	ENSGALG00010020385	SLC12A8	case	control	0.022029204	0.308353845	0.071441315	0.943046529	0.965069647	up_in_case
Yang2025_S	ENSGALG00010020386	PPM1B	case	control	-0.211787395	0.071848056	-2.947712265	0.003201349	0.017102867	down_in_case
Yang2025_S	ENSGALG00010020388	MYOF	case	control	0.110647751	0.205664449	0.538001351	0.590576116	0.731331927	up_in_case
Yang2025_S	ENSGALG00010020389	ACTN1	case	control	0.408179491	0.162406014	2.513327445	0.011959827	0.046236813	up_in_case
Yang2025_S	ENSGALG00010020390	KLHL25	case	control	-0.291792316	0.18895923	-1.544207793	0.122538001	0.253611622	down_in_case
Yang2025_S	ENSGALG00010020392	SLC23A2	case	control	0.218642884	0.130559807	1.674656922	0.094001564	0.209787283	up_in_case
Yang2025_S	ENSGALG00010020393	SHISA9	case	control	0.139521012	0.294725317	0.473393372	0.635932558	0.766651111	up_in_case
Yang2025_S	ENSGALG00010020396	CHL1	case	control	0.580289502	0.313416383	1.851497029	0.064098082	0.159501209	up_in_case
Yang2025_S	ENSGALG00010020399	ATP13A2	case	control	0.895768951	0.099348772	9.016406854	1.9436E-19	9.35776E-17	up_in_case
Yang2025_S	ENSGALG00010020402	SLC3A1	case	control	0.65240193	0.209311633	3.116892846	0.00182768	0.011180298	up_in_case
Yang2025_S	ENSGALG00010020405	UBE2I	case	control	-0.360121058	0.105024232	-3.428933038	0.000605959	0.004642132	down_in_case
Yang2025_S	ENSGALG00010020413	CYP26A1	case	control	0.338808905	0.342041003	0.990550555	0.321905092	0.491265738	up_in_case
Yang2025_S	ENSGALG00010020415	TSR3	case	control	0.373136314	0.111511977	3.346154585	0.000819407	0.005950201	up_in_case
Yang2025_S	ENSGALG00010020416	ENSGALG00010020416	case	control	-2.566119796	1.073797543	-2.389761284	0.016859327	0.059860169	down_in_case
Yang2025_S	ENSGALG00010020422	ERCC4	case	control	-0.243834087	0.080022031	-3.047086961	0.002310708	0.013349802	down_in_case
Yang2025_S	ENSGALG00010020428	SEMA4C	case	control	0.141150293	0.101850901	1.385852177	0.165792046	0.313347642	up_in_case
Yang2025_S	ENSGALG00010020429	ZNF148	case	control	0.285924116	0.151886631	1.882483761	0.059770363	0.152076444	up_in_case
Yang2025_S	ENSGALG00010020433	GNPTG	case	control	0.193823401	0.155820215	1.243891238	0.213539594	0.373875393	up_in_case
Yang2025_S	ENSGALG00010020436	CNTN4	case	control	-0.070305063	0.203056194	-0.346234517	0.729166477	0.833478373	down_in_case
Yang2025_S	ENSGALG00010020437	SNX4	case	control	-0.137267151	0.060771305	-2.258749428	0.023898976	0.077449356	down_in_case
Yang2025_S	ENSGALG00010020438	HTR7	case	control	-0.368675105	0.207844837	-1.773799678	0.076096291	0.1806169	down_in_case
Yang2025_S	ENSGALG00010020440	PAX7	case	control	0.31650455	0.202071243	1.566301789	0.117277982	0.245545061	up_in_case
Yang2025_S	ENSGALG00010020443	PREPL	case	control	0.316437724	0.25554388	1.238291147	0.215608125	0.376503662	up_in_case
Yang2025_S	ENSGALG00010020444	ENSGALG00010020444	case	control	-0.842353813	1.145478498	-0.735372872	0.462112439	0.623020205	down_in_case
Yang2025_S	ENSGALG00010020449	PRPF19	case	control	-0.191099947	0.101388237	-1.884833515	0.059452319	0.151622998	down_in_case
Yang2025_S	ENSGALG00010020450	ANKRD39	case	control	0.412577684	0.300337219	1.373714805	0.169530239	0.318297478	up_in_case
Yang2025_S	ENSGALG00010020453	ENSGALG00010020453	case	control	0.106674995	0.126896395	0.840646385	0.400546065	0.567352044	up_in_case
Yang2025_S	ENSGALG00010020460	MAPK8	case	control	0.287228239	0.09398652	3.056057811	0.00224268	0.013048746	up_in_case
Yang2025_S	ENSGALG00010020462	CNNM4	case	control	-0.157761808	0.143079828	-1.102613904	0.27019487	0.43755555	down_in_case
Yang2025_S	ENSGALG00010020467	ALDH4A1	case	control	-0.04954484	0.115907763	-0.427450577	0.669051176	0.791176862	down_in_case
Yang2025_S	ENSGALG00010020468	ENSGALG00010020468	case	control	0.759064775	0.426144998	1.781235915	0.074873919	0.178626674	up_in_case
Yang2025_S	ENSGALG00010020473	TMEM109	case	control	0.358496462	0.290042217	1.236014762	0.216453077	0.377317191	up_in_case
Yang2025_S	ENSGALG00010020476	TMEM132A	case	control	0.143533626	0.156337632	0.918100298	0.358566359	0.527612204	up_in_case
Yang2025_S	ENSGALG00010020481	RPP30	case	control	-0.767950411	0.240459338	-3.19368097	0.001404713	0.009177061	down_in_case
Yang2025_S	ENSGALG00010020484	TRNT1	case	control	0.078356735	0.126474738	0.619544555	0.53555768	0.686884148	up_in_case
Yang2025_S	ENSGALG00010020485	LMAN2L	case	control	0.160266365	0.077230079	2.075180637	0.037969796	0.108847791	up_in_case
Yang2025_S	ENSGALG00010020486	MRTFB	case	control	0.82501558	0.286482067	2.879815784	0.003979076	0.020104577	up_in_case
Yang2025_S	ENSGALG00010020489	IFFO2	case	control	0.445586643	0.140747746	3.165852775	0.00154629	0.00983266	up_in_case
Yang2025_S	ENSGALG00010020493	DEPDC7	case	control	-0.352359434	0.17564143	-2.006129386	0.044842445	0.123469567	down_in_case
Yang2025_S	ENSGALG00010020497	OSBPL11	case	control	-0.263432062	0.076669379	-3.435948837	0.000590482	0.004561183	down_in_case
Yang2025_S	ENSGALG00010020507	ANKRD1	case	control	0.034133751	0.445700569	0.076584491	0.938954097	0.962932392	up_in_case
Yang2025_S	ENSGALG00010020509	CRBN	case	control	-0.289691894	0.139655981	-2.074325014	0.038049136	0.1090528	down_in_case
Yang2025_S	ENSGALG00010020514	CAMKMT	case	control	-0.509033464	0.149881249	-3.396245152	0.000683171	0.005145548	down_in_case
Yang2025_S	ENSGALG00010020517	UBR4	case	control	0.154212628	0.135169806	1.140880732	0.253919559	0.420423574	up_in_case
Yang2025_S	ENSGALG00010020520	ARHGAP22	case	control	-0.195350215	0.134487129	-1.452556963	0.146346803	0.287920753	down_in_case
Yang2025_S	ENSGALG00010020521	ENSGALG00010020521	case	control	0.070893254	0.708337658	0.100083983	0.920277651	0.952963458	up_in_case
Yang2025_S	ENSGALG00010020524	ENSGALG00010020524	case	control	-0.649070376	0.308811532	-2.10183335	0.035567877	0.104040435	down_in_case
Yang2025_S	ENSGALG00010020525	ENSGALG00010020525	case	control	-1.371383087	0.598267915	-2.292255782	0.021890888	0.072269491	down_in_case
Yang2025_S	ENSGALG00010020530	SRSF5A	case	control	0.151181512	0.057186554	2.643654869	0.008201624	0.034876297	up_in_case
Yang2025_S	ENSGALG00010020533	ENSGALG00010020533	case	control	0.714192721	0.235524694	3.032347517	0.002426597	0.013881208	up_in_case
Yang2025_S	ENSGALG00010020535	LMLN	case	control	0.25094913	0.167606489	1.497251879	0.134327731	0.270460472	up_in_case
Yang2025_S	ENSGALG00010020537	CCDC167	case	control	-0.506735893	0.176109899	-2.8773845	0.004009867	0.020213904	down_in_case
Yang2025_S	ENSGALG00010020540	PCGF5	case	control	0.316862199	0.176377133	1.796503851	0.072414421	0.174041903	up_in_case
Yang2025_S	ENSGALG00010020544	BMERB1	case	control	-0.026341834	0.155020382	-0.169924972	0.865069142	0.920279296	down_in_case
Yang2025_S	ENSGALG00010020545	STYX	case	control	0.287804744	0.090893061	3.166410512	0.001543328	0.009820219	up_in_case
Yang2025_S	ENSGALG00010020546	LRRC20	case	control	-0.32341829	0.180977626	-1.787062291	0.073927421	0.176905528	down_in_case
Yang2025_S	ENSGALG00010020548	CMTR1	case	control	-0.265510626	0.138102779	-1.922558168	0.054535562	0.142153583	down_in_case
Yang2025_S	ENSGALG00010020550	HECTD2	case	control	0.058988802	0.177309129	0.33268903	0.739369031	0.84045746	up_in_case
Yang2025_S	ENSGALG00010020554	ZFP36L1	case	control	0.405299541	0.228001488	1.777617968	0.075466621	0.17957154	up_in_case
Yang2025_S	ENSGALG00010020556	EMC1	case	control	-0.043827251	0.104310932	-0.420159709	0.674368786	0.795238135	down_in_case
Yang2025_S	ENSGALG00010020557	ENSGALG00010020557	case	control	-0.194290953	0.200896713	-0.967118626	0.333484735	0.502561032	down_in_case
Yang2025_S	ENSGALG00010020558	EIF4EBP2	case	control	-0.206481081	0.119907845	-1.721998104	0.085069862	0.195780103	down_in_case
Yang2025_S	ENSGALG00010020562	LRRN1	case	control	-0.107672684	0.286053397	-0.376407639	0.706613868	0.819145477	down_in_case
Yang2025_S	ENSGALG00010020568	PLA2G4F	case	control	0.793618019	0.585501536	1.355449936	0.175274249	0.325797639	up_in_case
Yang2025_S	ENSGALG00010020569	DTX3L	case	control	-0.374774691	0.139922463	-2.678445493	0.007396477	0.03223957	down_in_case
Yang2025_S	ENSGALG00010020571	MRTO4	case	control	0.496601185	0.168450689	2.948050777	0.003197845	0.017097578	up_in_case
Yang2025_S	ENSGALG00010020572	PPP1R3C	case	control	-0.281626278	0.18351794	-1.534598074	0.124882586	0.256672395	down_in_case
Yang2025_S	ENSGALG00010020574	DTL	case	control	-0.069818979	0.164167013	-0.425292375	0.670623552	0.792363845	down_in_case
Yang2025_S	ENSGALG00010020576	AKR7L	case	control	-0.062245166	0.181708418	-0.342555217	0.731933097	0.836160658	down_in_case
Yang2025_S	ENSGALG00010020578	TNKS2	case	control	-0.41291981	0.099324468	-4.157281887	3.22057E-05	0.000431525	down_in_case
Yang2025_S	ENSGALG00010020580	ENSGALG00010020580	case	control	-0.140951737	0.152161781	-0.926328124	0.354275485	0.523412902	down_in_case
Yang2025_S	ENSGALG00010020582	KANSL3	case	control	-0.118427044	0.14669233	-0.807315859	0.419484529	0.585253996	down_in_case
Yang2025_S	ENSGALG00010020584	PARP9	case	control	-0.467337252	0.125015283	-3.738240957	0.000185312	0.001755491	down_in_case
Yang2025_S	ENSGALG00010020586	C11orf16	case	control	-0.108132918	0.091605215	-1.180423156	0.237831957	0.401719796	down_in_case
Yang2025_S	ENSGALG00010020588	PALD1	case	control	-0.048530511	0.111383751	-0.435705485	0.663050425	0.786952362	down_in_case
Yang2025_S	ENSGALG00010020590	INTS7	case	control	-0.19708974	0.102205784	-1.928361904	0.053810127	0.140815797	down_in_case
Yang2025_S	ENSGALG00010020594	PQLC2	case	control	-0.056204393	0.21094161	-0.266445264	0.789896309	0.87324118	down_in_case
Yang2025_S	ENSGALG00010020595	ZFYVE27	case	control	0.08967199	0.127041324	0.705848989	0.480282053	0.638755271	up_in_case
Yang2025_S	ENSGALG00010020596	SUMF1	case	control	-0.256682982	0.161491726	-1.58944974	0.111958893	0.237404326	down_in_case
Yang2025_S	ENSGALG00010020597	ARID5A	case	control	0.428818348	0.145949673	2.938124749	0.003302041	0.017509111	up_in_case
Yang2025_S	ENSGALG00010020599	PARN	case	control	-0.155655194	0.08605975	-1.808687506	0.070499565	0.170798036	down_in_case
Yang2025_S	ENSGALG00010020601	AKIP1	case	control	-0.501708424	0.217170224	-2.310208158	0.020876633	0.069898096	down_in_case
Yang2025_S	ENSGALG00010020602	DENND2B	case	control	0.161699421	0.067291006	2.402987143	0.016261759	0.058298846	up_in_case
Yang2025_S	ENSGALG00010020603	VPS39	case	control	0.112050864	0.131585146	0.851546452	0.394465871	0.561570075	up_in_case
Yang2025_S	ENSGALG00010020605	STAMBP	case	control	0.111719614	0.084351561	1.324452242	0.185352894	0.33886248	up_in_case

Yang2025_S	ENSGALG00010020607	ENSGALG00010020607	case	control	-0.306044115	0.20718302	-1.477167944	0.139630624	0.278362339	down_in_case
Yang2025_S	ENSGALG00010020608	CAPZB	case	control	0.271746575	0.137398983	1.977791749	0.047952205	0.129523933	up_in_case
Yang2025_S	ENSGALG00010020609	AVPI1	case	control	0.645532067	0.275080541	2.346702038	0.018940394	0.065168987	up_in_case
Yang2025_S	ENSGALG00010020611	LPGAT1	case	control	-0.166413229	0.101600253	-1.637921401	0.101438091	0.221384463	down_in_case
Yang2025_S	ENSGALG00010020612	RPL27A	case	control	-0.188467348	0.281438036	-0.669658409	0.503075571	0.659301992	down_in_case
Yang2025_S	ENSGALG00010020613	ACTG2	case	control	-0.086205044	0.27663086	-0.311624828	0.755325663	0.851230337	down_in_case
Yang2025_S	ENSGALG00010020614	FAIM	case	control	-0.027229241	0.110804458	-0.245741385	0.805882434	0.883489148	down_in_case
Yang2025_S	ENSGALG00010020616	PI4K2A	case	control	-0.308650243	0.229173862	-1.34679514	0.17804622	0.329404418	down_in_case
Yang2025_S	ENSGALG00010020617	DGUOK	case	control	0.373695389	0.254357966	1.469171163	0.14178637	0.281211651	up_in_case
Yang2025_S	ENSGALG00010020618	ENSGALG00010020618	case	control	2.248369156	0.542246346	4.146397984	3.37747E-05	0.000446537	up_in_case
Yang2025_S	ENSGALG00010020619	QARS	case	control	0.273566566	0.136910156	1.998146622	0.045700766	0.12516513	up_in_case
Yang2025_S	ENSGALG00010020620	MARF1	case	control	-0.594411577	0.101225431	-5.872156534	4.30162E-09	2.1485E-07	down_in_case
Yang2025_S	ENSGALG00010020622	ABCG2	case	control	-0.231402272	0.103311339	-2.239853565	0.025100431	0.080168349	down_in_case
Yang2025_S	ENSGALG00010020623	NEK2	case	control	-0.044643259	0.178096338	-0.250669155	0.80206991	0.880663886	down_in_case
Yang2025_S	ENSGALG00010020624	MINOS1	case	control	-0.806856013	0.257175627	-3.137373563	0.001704688	0.010614309	down_in_case
Yang2025_S	ENSGALG00010020625	SLC30A1	case	control	-0.279001927	0.062339529	-4.475521863	7.62249E-06	0.000131703	down_in_case
Yang2025_S	ENSGALG00010020626	MORN4	case	control	-0.159527559	0.281914988	-0.565871152	0.571481373	0.715439066	down_in_case
Yang2025_S	ENSGALG00010020629	TRIM66	case	control	0.44586816	0.405598555	1.09928439	0.271644039	0.438869152	up_in_case
Yang2025_S	ENSGALG00010020630	QRICH1	case	control	0.017557692	0.068666777	0.255694132	0.798187026	0.877949026	up_in_case
Yang2025_S	ENSGALG00010020632	BLOC1S2	case	control	-0.218094738	0.154684168	-1.409935747	0.158558656	0.304005899	down_in_case
Yang2025_S	ENSGALG00010020633	HOGA1	case	control	-0.597371162	0.414057183	-1.442726239	0.149097605	0.291497965	down_in_case
Yang2025_S	ENSGALG00010020635	NDE1	case	control	-0.035015366	0.149848999	-0.233671003	0.815240401	0.889255026	down_in_case
Yang2025_S	ENSGALG00010020636	NBL1	case	control	-0.032323554	0.16480489	-0.196132252	0.844506653	0.907479967	down_in_case
Yang2025_S	ENSGALG00010020638	IMPDH2	case	control	-0.013045942	0.133223032	-0.097925574	0.921991393	0.953373943	down_in_case
Yang2025_S	ENSGALG00010020640	PTPN14	case	control	-0.005459905	0.192656644	-0.028340082	0.977390912	0.986666355	down_in_case
Yang2025_S	ENSGALG00010020642	ANKRD2	case	control	0.650114154	0.547184848	1.188107012	0.234791252	0.398028421	up_in_case
Yang2025_S	ENSGALG00010020646	CEP70	case	control	0.028545705	0.140693459	0.202892905	0.83921874	0.904799934	up_in_case
Yang2025_S	ENSGALG00010020648	TET3	case	control	-0.194296116	0.132304619	-1.468551273	0.14195454	0.281483435	down_in_case
Yang2025_S	ENSGALG00010020649	MYH11	case	control	0.706988106	0.23267703	3.038495494	0.002377627	0.013662774	up_in_case
Yang2025_S	ENSGALG00010020651	LMO1	case	control	-0.037672941	0.288063873	-0.130779818	0.895949489	0.938655449	down_in_case
Yang2025_S	ENSGALG00010020653	NDUFAF3	case	control	0.120168756	0.292856254	0.410333582	0.681561261	0.800413294	up_in_case
Yang2025_S	ENSGALG00010020654	SAYSD1	case	control	-0.266234754	0.262297102	-1.015012182	0.310100021	0.47981832	down_in_case
Yang2025_S	ENSGALG00010020655	RIC3	case	control	-0.371305267	0.174099148	-2.132723061	0.032947451	0.098156952	down_in_case
Yang2025_S	ENSGALG00010020657	DALRD3	case	control	0.261920026	0.162195255	1.614843944	0.106344494	0.228983577	up_in_case
Yang2025_S	ENSGALG00010020659	RAD51B	case	control	-0.459739189	0.191106002	-2.40567635	0.016142559	0.058005817	down_in_case
Yang2025_S	ENSGALG00010020662	BFAR	case	control	-0.45206198	0.109296776	-4.136096216	3.53264E-05	0.000463098	down_in_case
Yang2025_S	ENSGALG00010020664	ESYT3	case	control	-0.007548518	0.550186312	-0.01371993	0.989053423	0.993402007	down_in_case
Yang2025_S	ENSGALG00010020666	TMCO4	case	control	-0.053343228	0.225440038	-0.236618252	0.812952959	0.887326661	down_in_case
Yang2025_S	ENSGALG00010020667	UBTD1	case	control	0.254151807	0.263264987	0.965384004	0.33435251	0.503153589	up_in_case
Yang2025_S	ENSGALG00010020670	ENSGALG00010020670	case	control	0.387051262	0.18766431	2.062466019	0.03916339	0.111489649	up_in_case
Yang2025_S	ENSGALG00010020673	MMS19	case	control	-0.056194727	0.120650647	-0.465763997	0.641384439	0.771022442	down_in_case
Yang2025_S	ENSGALG00010020674	TRPV4	case	control	2.269877374	0.989800017	2.293268677	0.021832541	0.07217947	up_in_case
Yang2025_S	ENSGALG00010020675	SMYD2	case	control	-0.342253276	0.126504576	-2.705461629	0.006820953	0.030357706	down_in_case
Yang2025_S	ENSGALG00010020677	CYR61	case	control	0.149891828	0.24318446	0.616370916	0.537649744	0.688933258	up_in_case
Yang2025_S	ENSGALG00010020679	OTUD3	case	control	0.143693155	0.140455381	1.023051976	0.306283272	0.475973836	up_in_case
Yang2025_S	ENSGALG00010020681	BCL10	case	control	-0.676513066	0.10493132	-6.447198673	1.13936E-10	9.48003E-09	down_in_case
Yang2025_S	ENSGALG00010020682	ZDHHC16	case	control	0.454180818	0.179094962	2.535977631	0.01121339	0.044091023	up_in_case
Yang2025_S	ENSGALG00010020684	P4HTM	case	control	-0.010566129	0.208329123	-0.050718446	0.959549878	0.975582048	down_in_case
Yang2025_S	ENSGALG00010020685	RPL6	case	control	-0.840513922	0.172939374	-4.860165156	1.17288E-06	2.77018E-05	down_in_case
Yang2025_S	ENSGALG00010020686	C1orf52	case	control	-0.219807449	0.129094831	-1.702682037	0.088627588	0.201826494	down_in_case
Yang2025_S	ENSGALG00010020691	ENSGALG00010020691	case	control	0.238943716	0.357194319	0.668946014	0.503529916	0.659649279	up_in_case
Yang2025_S	ENSGALG00010020692	TUB	case	control	0.104761347	0.163600531	0.64034845	0.521946089	0.675243084	up_in_case
Yang2025_S	ENSGALG00010020693	PTGFR	case	control	-0.776418076	0.177377361	-4.377210657	1.20208E-05	0.000191659	down_in_case
Yang2025_S	ENSGALG00010020696	MOB1A	case	control	-0.115086975	0.147842849	-0.778441271	0.436308916	0.600193954	down_in_case
Yang2025_S	ENSGALG00010020699	PLA2G10	case	control	0.197507137	0.230543093	0.856703769	0.39160862	0.558759275	up_in_case
Yang2025_S	ENSGALG00010020700	MTHFD2	case	control	-0.028206772	0.122754248	-0.229782454	0.81826082	0.89137465	down_in_case
Yang2025_S	ENSGALG00010020701	PTPN11	case	control	-0.243531858	0.087222699	-2.792069753	0.005237206	0.024806415	down_in_case
Yang2025_S	ENSGALG00010020704	PROX1	case	control	0.402484788	0.327251846	1.229893102	0.218737138	0.379825796	up_in_case
Yang2025_S	ENSGALG00010020705	EXOSC1	case	control	0.612529835	0.277744911	2.205368341	0.027428258	0.085563638	up_in_case
Yang2025_S	ENSGALG00010020707	SLC4A5	case	control	0.518514578	0.392491604	1.32108451	0.186473187	0.340099334	up_in_case
Yang2025_S	ENSGALG00010020708	FAM214A	case	control	-0.053406391	0.134322331	-0.397598753	0.69092598	0.80731145	down_in_case
Yang2025_S	ENSGALG00010020711	PLA2G2E	case	control	0.044102644	0.221626943	0.198994958	0.842266689	0.906369665	up_in_case
Yang2025_S	ENSGALG00010020712	ZC3H7A	case	control	0.03185871	0.075986067	0.41927041	0.675018519	0.79580229	up_in_case
Yang2025_S	ENSGALG00010020714	PGAM1	case	control	0.103582468	0.127353515	0.813345971	0.416019727	0.581409577	up_in_case
Yang2025_S	ENSGALG00010020715	ENSGALG00010020715	case	control	-0.481094553	1.046923837	-0.459531569	0.645852488	0.775189872	down_in_case
Yang2025_S	ENSGALG00010020716	RRAS	case	control	-0.295497686	0.112903041	-2.617269496	0.008863633	0.037169644	down_in_case
Yang2025_S	ENSGALG00010020719	RPH3A	case	control	0.027895914	0.402622771	0.069285486	0.944762379	0.966042662	up_in_case
Yang2025_S	ENSGALG00010020720	ENSGALG00010020720	case	control	0.373349243	0.39308097	0.949802386	0.342212673	0.51051639	up_in_case
Yang2025_S	ENSGALG00010020721	PTDSS2	case	control	-0.299268605	0.147934523	-2.022980167	0.043075192	0.119619929	down_in_case
Yang2025_S	ENSGALG00010020723	RPS24	case	control	0.096086561	0.210617151	0.456214326	0.648235865	0.77651223	up_in_case
Yang2025_S	ENSGALG00010020724	TACR1	case	control	-0.058906308	0.184270523	-0.319672984	0.749216241	0.847704476	down_in_case
Yang2025_S	ENSGALG00010020725	FOPNL	case	control	-0.442499807	0.245899074	-1.799517985	0.071936782	0.173192649	down_in_case
Yang2025_S	ENSGALG00010020728	TMEM37	case	control	-0.281876942	0.259052092	-1.088109114	0.276546941	0.443819143	down_in_case
Yang2025_S	ENSGALG00010020730	RRP12	case	control	0.150272932	0.134331043	1.118676138	0.263278329	0.430204445	up_in_case
Yang2025_S	ENSGALG00010020732	ARIH2	case	control	-0.010992021	0.080125789	-0.137184555	0.890884919	0.936155459	down_in_case
Yang2025_S	ENSGALG00010020733	TMEM229B	case	control	-0.110040592	0.133885276	-0.821902119	0.411132607	0.576579292	down_in_case
Yang2025_S	ENSGALG00010020736	LRRTM4	case	control	0.322398466	0.196145745	1.64366791	0.100244818	0.219398702	up_in_case
Yang2025_S	ENSGALG00010020737	POLR3A	case	control	-0.203020692	0.093317485	-2.175591132	0.029585847	0.090568424	down_in_case
Yang2025_S	ENSGALG00010020742	THAP10	case	control	-0.558306646	0.239583248	-2.330324222	0.019789021	0.067303873	down_in_case
Yang2025_S	ENSGALG00010020743	PURB	case	control	0.331950285	0.176509142	1.880640743	0.060020805	0.152570216	up_in_case
Yang2025_S	ENSGALG00010020744	CHST3	case	control	-0.093691398	0.134307833	-0.697586996	0.485435516	0.642773082	down_in_case
Yang2025_S	ENSGALG00010020745	ABCC1	case	control	-0.008141477	0.077531114	-0.105009155	0.916368545	0.950826185	down_in_case
Yang2025_S	ENSGALG00010020747	DBI	case	control	0.15427901	0.227139882	0.679224663	0.496995523	0.654038032	up_in_case
Yang2025_S	ENSGALG00010020750	AIFM2	case	control	0.686226081	0.305704201	2.244738799	0.024784906	0.079506828	up_in_case
Yang2025_S	ENSGALG00010020753	SYDE2	case	control	0.328079396	0.105715895	3.10340651	0.001913067	0.011585652	up_in_case
Yang2025_S	ENSGALG00010020754	SLC25A20	case	control	-0.683663792	0.215046844	-3.179138926	0.001477133	0.009511943	down_in_case
Yang2025_S	ENSGALG00010020756	MACROH2A2	case	control	-0.08444049	0.135341429	-0.623907184	0.532688542	0.68481027	down_in_case
Yang2025_S	ENSGALG00010020758	DNAI3	case	control	2.492351726	0.861668303	2.892472333	0.003822228	0.019531629	up_in_case
Yang2025_S	ENSGALG00010020759	ARHGAP19	case	control	-0.009744537	0.151268555	-0.064418789	0.94863677	0.967992436	down_in_case
Yang2025_S	ENSGALG00010020760	TXNDC11	case	control	0.17112971	0.075757036	2.258928257	0.023887848	0.077431301	up_in_case
Yang2025_S	ENSGALG00010020762	TTBK2	case	control	0.065237193	0.221131439	0.295015457	0.767982072	0.858788123	up_in_case
Yang2025_S	ENSGALG00010020764	PRKAR2A	case	control	-0.100005127	0.095504382	-1.047126068	0.295041438	0.463570012	down_in_case
Yang2025_S	ENSGALG00010020765	GLT1D1	case	control	0.301373455	0.307352096	0.980547911	0.326815732	0.496633126	up_in_case
Yang2025_S	ENSGALG00010020766	C2orf76	case	control	-0.384519252	0.154782468	-2.484255852	0.01298225	0.049199796	down_in_case
Yang2025_S	ENSGALG00010020767	UBXN10	case	control	0.00850462	0.104981875	0.081010369	0.935433706	0.960523815	up_in_case
Yang2025_S	ENSGALG00010020770	B4GALNT4	case	control	-0.128602679	0.142182512	-0.90449013	0.365735566	0.534505864	down_in_case
Yang2025_S	ENSGALG00010020771	LINGO1	case	control	0.164724794	0.219425725	0.750708668	0.452828005	0.614665717	up_in_case
Yang2025_S	ENSGALG00010020773	ZCCHC24	case	control	-0.484432081	0.133613438	-3.625623962	0.000288265	0.002524806	down_in_case
Yang2025_S	ENSGALG00010020775	UBR1	case	control	0.064670609	0.112541678	0.57463697	0.565536844	0.711072339	up_in_case
Yang2025_S	ENSGALG00010020776	MCOLN3	case	control	0.859841834	0.624409707	1.377047513	0.168497559	0.316828158	up_in_case
Yang2025_S	ENSGALG00010020778	SLIT1	case	control	0.037433129	0.199015106	0.188091899	0.850804612	0.911457943	up_in_case
Yang2025_S	ENSGALG00010020779	SLC15A4	case	control	-0.70546813	0.129462134	-5.449223698	5.05902E-08	1.88496E-06	down_in_case
Yang2025_S	ENSGALG00010020781	SNN	case	control	-0.214958593	0.107424919	-2.00101238	0.045391056	0.124709062	down_in_case
Yang2025_S	ENSGALG00010020782	PPIF	case	control	0.047164552	0.199184338	0.236788457	0.812820907	0.887252024	up_in_case
Yang2025_S	ENSGALG00010020786	DDX19	case	control	0.136159482	0.09631818	1.413642605	0.157466865	0.302745691	up_in_case
Yang2025_S	ENSGALG00010020787	TMEM132C	case	control	0.765051678	0.432234667	1.769991482	0.07672856	0.181777028	up_in_case
Yang2025_S	ENSGALG00010020790	PRPH2L	case	control	0.202326673	0.374425763	0.540365254	0.588945165	0.730011217	up_in_case
Yang2025_S	ENSGALG00010020794	STEAP3	case	control	-0.288447497	0.156008412	-1.848922719	0.064468973	0.16005258	down_in_case
Yang2025_S	ENSGALG00010020797	DGCR8	case	control	0.477425914	0.16156566	2.954996206	0.003126729	0.016842276	up_in_case
Yang2025_S	ENSGALG00010020798	C1QL2	case	control	-0.117405358	0.481849683	-0.243655568	0.807497588	0.883899076	down_in_case
Yang2025_S	ENSGALG00010020803	ABCC6	case	control	0.753141289	0.221162882	3.405369296	0.000660746	0.005014978	up_in_case
Yang2025_S	ENSGALG00010020806	HTD2	case	control	-0.216776586	0.269456512	-0.804495628	0.421110793	0.586788292	down_in_case
Yang2025_S	ENSGALG00010020809	ENSGALG00010020809	case	control	0.180918407	0.141667187	1.277066419	0.201578799	0.359484697	up_in_case
Yang2025_S	ENSGALG00010020810	IFITM5	case	control	0.76729999	0.219052061	3.502820221	0.00046036	0.003697474	up_in_case
Yang2025_S	ENSGALG00010020811	MCOLN2	case	control	-1.019012176	0.893622744	-1.140315847	0.254154738	0.420623073	down_in_case
Yang2025_S	ENSGALG00010020813	NPY4R	case	control	0.181638101	0.35985128	0.504758802	0.613728255	0.750004669	up_in_case
Yang2025_S	ENSGALG00010020816	TRMT2A	case	control	-0.101987625	0.161627633	-0.631003642	0.528038143	0.680874574	down_in_case
Yang2025_S	ENSGALG00010020818	TMEM62	case	control	0.295780949	0.138446766	2.136423674	0.032644896	0.097619448	up_in_case
Yang2025_S	ENSGALG00010020820	VWA5B1	case	control	0.99441917	0.464679327	2.140011645	0.032353826	0.096959632	up_in_case
Yang2025_S	ENSGALG00010020827	ENSGALG00010020827	case	control	0.047806238	0.104332695	0.45820956	0.646801892	0.775661305	up_in_case
Yang2025_S	ENSGALG00010020828	CAMK2N1	case	control	-0.342265549	0.286623771	-1.194128273	0.232427795	0.395516098	down_in_case
Yang2025_S	ENSGALG00010020831	MUL1	case	control	-0.157629406	0.193974892	-0.812627886	0.416431438	0.581743963	down_in_case
Yang2025_S	ENSGALG00010020833	RANBP1	case	control	-0.348134022	0.10443911	-3.333368315	0.000858013	0.006185415	down_in_case
Yang2025_S	ENSGALG00010020835	PTGR2	case	control	0.160526496	0.111281019	1.442532587	0.149152185	0.291546599	up_in_case
Yang2025_S	ENSGALG00010020839	PGGHG	case	control	0.032563123	0.139051454	0.234180387	0.814844941	0.888973949	up_in_case
Yang2025_S	ENSGALG00010020840	LPAR3	case	control	-0.013787017	0.264225792	-0.052178924	0.958386126	0.974683307	down_in_case

Yang2025_S	ENSGALG00010020843	HMG20A	case	control	0.103083727	0.106047647	0.972051054	0.33102515	0.500336353	up_in_case
Yang2025_S	ENSGALG00010020845	RAB35	case	control	-0.684604401	0.103558053	-6.610827265	3.82178E-11	3.58856E-09	down_in_case
Yang2025_S	ENSGALG00010020847	MIDEAS	case	control	0.193927331	0.23218667	0.835221637	0.403592929	0.569987582	up_in_case
Yang2025_S	ENSGALG00010020854	ENSGALG00010020854	case	control	-0.820908603	0.326183037	-2.516711511	0.011845578	0.045916029	down_in_case
Yang2025_S	ENSGALG00010020855	DNAL1	case	control	-0.02735698	0.13334575	-0.205158243	0.837448493	0.903728394	down_in_case
Yang2025_S	ENSGALG00010020856	VTG2	case	control	0.916584084	0.461726704	1.985122531	0.047130841	0.127900344	up_in_case
Yang2025_S	ENSGALG00010020861	CDA	case	control	0.013728569	0.279200148	0.049171068	0.960782968	0.976479517	up_in_case
Yang2025_S	ENSGALG00010020866	LCOR	case	control	0.224340767	0.126716064	1.770420901	0.07665705	0.181669387	up_in_case
Yang2025_S	ENSGALG00010020870	RIOX1	case	control	-0.142974674	0.118787159	-1.203620622	0.228736245	0.39109688	down_in_case
Yang2025_S	ENSGALG00010020873	PINK1	case	control	-0.378547752	0.24306076	-1.557420261	0.119370739	0.248614807	down_in_case
Yang2025_S	ENSGALG00010020875	ENSGALG00010020875	case	control	-0.154551525	0.159395428	-0.969610777	0.332240539	0.501593688	down_in_case
Yang2025_S	ENSGALG00010020878	XYLT1	case	control	0.540691666	0.211551981	2.555833617	0.010593376	0.042273394	up_in_case
Yang2025_S	ENSGALG00010020880	PXK	case	control	0.141378414	0.202611718	0.697780046	0.48531476	0.642701722	up_in_case
Yang2025_S	ENSGALG00010020886	GBF1	case	control	0.215330283	0.104068567	2.069119326	0.038534891	0.110150503	up_in_case
Yang2025_S	ENSGALG00010020887	ARPP19	case	control	-0.439259182	0.0851231	-5.160281776	2.46578E-07	7.23621E-06	down_in_case
Yang2025_S	ENSGALG00010020889	PPRC1	case	control	0.225216502	0.079995127	2.815377766	0.004871993	0.023521581	up_in_case
Yang2025_S	ENSGALG00010020890	RPS15A	case	control	-0.059940774	0.279670831	-0.214326156	0.830292722	0.898799025	down_in_case
Yang2025_S	ENSGALG00010020894	PSMD13	case	control	0.21013017	0.127343094	1.650110443	0.098920349	0.217434006	up_in_case
Yang2025_S	ENSGALG00010020896	MARCO	case	control	0.508044319	0.471527877	1.077442807	0.281282489	0.449231015	up_in_case
Yang2025_S	ENSGALG00010020898	ENSGALG00010020898	case	control	-0.207842664	0.236723079	-0.877999154	0.379944178	0.547860318	down_in_case
Yang2025_S	ENSGALG00010020900	PDHB	case	control	-0.548942939	0.195481042	-2.808164591	0.004982475	0.023916912	down_in_case
Yang2025_S	ENSGALG00010020903	AGMAT	case	control	0.31547863	0.481419411	0.655309327	0.512268617	0.667457988	up_in_case
Yang2025_S	ENSGALG00010020905	KCTD6	case	control	-0.244789448	0.125339218	-1.953015604	0.050817746	0.134807785	down_in_case
Yang2025_S	ENSGALG00010020906	SIRT3	case	control	0.073293855	0.094723159	0.773769116	0.439067339	0.602798321	up_in_case
Yang2025_S	ENSGALG00010020912	PDE8A	case	control	0.587939474	0.162533315	3.61734745	0.000297638	0.002589002	up_in_case
Yang2025_S	ENSGALG00010020913	TMEM132B	case	control	0.015380483	0.265699207	0.057886822	0.95383878	0.97161867	up_in_case
Yang2025_S	ENSGALG00010020915	EN1	case	control	0.513241487	0.302025794	1.699329983	0.089257026	0.20264139	up_in_case
Yang2025_S	ENSGALG00010020916	ARL6IP1	case	control	-0.950967433	0.23303809	-4.080738191	4.48929E-05	0.000561564	down_in_case
Yang2025_S	ENSGALG00010020918	ENSGALG00010020918	case	control	-0.510565806	0.387944372	-1.316079941	0.188147213	0.342633483	down_in_case
Yang2025_S	ENSGALG00010020920	ACOX2	case	control	0.044101009	0.178461212	0.247118174	0.804816769	0.882663441	up_in_case
Yang2025_S	ENSGALG00010020921	LDB1	case	control	-0.052902428	0.079917395	-0.661963868	0.507994379	0.663564086	down_in_case
Yang2025_S	ENSGALG00010020922	INSIG2	case	control	-0.679912127	0.150105401	-4.529564702	5.91053E-06	0.000107104	down_in_case
Yang2025_S	ENSGALG00010020924	DNAJC16	case	control	-0.188050599	0.102913113	-1.827275392	0.067658373	0.165785024	down_in_case
Yang2025_S	ENSGALG00010020925	RIC8A	case	control	-0.505950815	0.211073221	-2.397039348	0.016528149	0.059041208	down_in_case
Yang2025_S	ENSGALG00010020927	ENSGALG00010020927	case	control	0.163938238	0.148377043	1.104876022	0.269213314	0.43632095	up_in_case
Yang2025_S	ENSGALG00010020928	AACS	case	control	0.531887161	0.222053968	2.395305817	0.016606508	0.059245184	up_in_case
Yang2025_S	ENSGALG00010020932	CASP9	case	control	-0.407583712	0.200680444	-2.031008619	0.04225412	0.117762233	down_in_case
Yang2025_S	ENSGALG00010020934	ABCD3	case	control	-0.224304399	0.080083887	-2.800868052	0.005096535	0.024330325	down_in_case
Yang2025_S	ENSGALG00010020935	BRI3BP	case	control	0.077657785	0.076124199	1.020145839	0.3076593	0.477313777	up_in_case
Yang2025_S	ENSGALG00010020936	BET1L	case	control	-0.170075301	0.106165306	-1.601985696	0.109158773	0.233051555	down_in_case
Yang2025_S	ENSGALG00010020938	SMG1	case	control	-0.102925721	0.123250397	-0.835094438	0.403664538	0.570030942	down_in_case
Yang2025_S	ENSGALG00010020940	ENSGALG00010020940	case	control	0.907221328	0.152940352	5.93186372	2.99515E-09	1.575E-07	up_in_case
Yang2025_S	ENSGALG00010020941	ENSGALG00010020941	case	control	0.561820017	0.11987557	4.686693189	2.77655E-06	5.68988E-05	up_in_case
Yang2025_S	ENSGALG00010020943	FAM107A	case	control	-0.147711813	0.370875219	-0.398279004	0.690424535	0.806933313	down_in_case
Yang2025_S	ENSGALG00010020944	MYO5A	case	control	0.424875829	0.150151996	2.829638238	0.004660066	0.022729445	up_in_case
Yang2025_S	ENSGALG00010020945	NUMB	case	control	0.64070415	0.214967493	2.980469937	0.002878065	0.015777275	up_in_case
Yang2025_S	ENSGALG00010020946	CTBS	case	control	0.169431102	0.151368032	1.119332133	0.262998472	0.429898382	up_in_case
Yang2025_S	ENSGALG00010020949	HPS6	case	control	-0.137076376	0.174078704	-0.787439089	0.431024872	0.594921908	down_in_case
Yang2025_S	ENSGALG00010020950	EFHD2	case	control	0.40370585	0.335605571	1.202917604	0.229008207	0.391225863	up_in_case
Yang2025_S	ENSGALG00010020951	ENSGALG00010020951	case	control	2.322300502	0.728750684	3.186687236	0.001439123	0.009314528	up_in_case
Yang2025_S	ENSGALG00010020952	GNG5	case	control	-0.183685097	0.15559718	-1.180516873	0.237794704	0.401719796	down_in_case
Yang2025_S	ENSGALG00010020954	PLIN1	case	control	-1.325718492	0.81417724	-1.628292253	0.103462931	0.224841171	down_in_case
Yang2025_S	ENSGALG00010020957	RPF1	case	control	-0.208324583	0.105610658	-1.972571576	0.048544394	0.130541515	down_in_case
Yang2025_S	ENSGALG00010020959	ENSGALG00010020959	case	control	0.064649317	0.233790197	0.276527066	0.782143274	0.868350602	up_in_case
Yang2025_S	ENSGALG00010020960	CLEC19A	case	control	0.22621388	0.177324253	1.275707503	0.202058935	0.360067002	up_in_case
Yang2025_S	ENSGALG00010020962	MYO5C	case	control	0.487750378	0.430874796	1.132000252	0.257634328	0.423914791	up_in_case
Yang2025_S	ENSGALG00010020963	CCDC93	case	control	0.632237487	0.184143703	3.433391836	0.00059608	0.004591694	up_in_case
Yang2025_S	ENSGALG00010020965	DHX37	case	control	0.261781987	0.094776748	2.762090842	0.00574325	0.026549948	up_in_case
Yang2025_S	ENSGALG00010020966	PAPLN	case	control	-0.177958168	0.230227168	-0.772967717	0.439541486	0.603152202	down_in_case
Yang2025_S	ENSGALG00010020967	VPS51	case	control	-0.06818462	0.179852672	-0.379113745	0.7046034	0.81762269	down_in_case
Yang2025_S	ENSGALG00010020969	RBP3	case	control	-0.793325681	0.548042522	-1.447562279	0.147739507	0.289842325	down_in_case
Yang2025_S	ENSGALG00010020970	ARMH3	case	control	-0.330898203	0.065985881	-5.014681903	5.31213E-07	1.40999E-05	down_in_case
Yang2025_S	ENSGALG00010020971	TMEM51	case	control	0.08028414	0.350130573	0.229297715	0.818637529	0.891611492	up_in_case
Yang2025_S	ENSGALG00010020973	GNB5	case	control	-0.092357484	0.176709944	-0.522650178	0.601217715	0.739907168	down_in_case
Yang2025_S	ENSGALG00010020975	HCCS	case	control	-0.095955698	0.115391828	-0.831564069	0.40565505	0.571829321	down_in_case
Yang2025_S	ENSGALG00010020976	GDF10	case	control	-0.156100377	0.373069272	-0.418421963	0.675638632	0.79606192	down_in_case
Yang2025_S	ENSGALG00010020978	BCL2L10	case	control	-0.323599555	0.286529566	-1.129375788	0.258739342	0.425030383	down_in_case
Yang2025_S	ENSGALG00010020979	ENSGALG00010020979	case	control	-0.014908702	0.272304439	-0.054750126	0.956337534	0.973310221	down_in_case
Yang2025_S	ENSGALG00010020981	CIT	case	control	0.484351113	0.150323022	3.222068762	0.001272686	0.008453231	up_in_case
Yang2025_S	ENSGALG00010020982	TLCD4	case	control	-0.10852974	0.173230534	-0.626504677	0.53098397	0.683345182	down_in_case
Yang2025_S	ENSGALG00010020984	PSEN1	case	control	-0.253589461	0.110258163	-2.299960872	0.021450437	0.071204252	down_in_case
Yang2025_S	ENSGALG00010020986	CFAP20DC	case	control	0.16763875	0.303981258	0.551477256	0.581306554	0.723905883	up_in_case
Yang2025_S	ENSGALG00010020987	NADSYN1	case	control	0.063026593	0.125188655	0.503452915	0.614645876	0.750840319	up_in_case
Yang2025_S	ENSGALG00010020989	MAPK4	case	control	-0.001169007	0.118691048	-0.009849157	0.992141637	0.994997748	down_in_case
Yang2025_S	ENSGALG00010020990	UBB	case	control	-0.21656992	0.197543486	-1.096315166	0.272940875	0.4403603	down_in_case
Yang2025_S	ENSGALG00010020991	ALG14	case	control	0.075897277	0.177228254	0.428245919	0.668472088	0.790827621	up_in_case
Yang2025_S	ENSGALG00010020992	KCNH7	case	control	0.599379339	0.415258144	1.443389727	0.148910716	0.291320561	up_in_case
Yang2025_S	ENSGALG00010020994	DHCR7	case	control	-0.127449921	0.150340869	-0.847739684	0.396582961	0.563801628	down_in_case
Yang2025_S	ENSGALG00010020998	SYT17	case	control	0.189275128	0.206081165	0.918449432	0.358383622	0.52752464	up_in_case
Yang2025_S	ENSGALG00010020999	IFIH1	case	control	0.089573818	0.255161476	0.351047576	0.725552656	0.831325133	up_in_case
Yang2025_S	ENSGALG00010021000	NFKB2	case	control	0.125816477	0.109762078	1.146265448	0.251685335	0.417428605	up_in_case
Yang2025_S	ENSGALG00010021002	SLC44A3	case	control	-0.660637209	0.163757167	-4.034249132	5.47772E-05	0.000656337	down_in_case
Yang2025_S	ENSGALG00010021003	KAZN	case	control	0.105940528	0.150512179	0.703866815	0.481515719	0.639832321	up_in_case
Yang2025_S	ENSGALG00010021004	PSD	case	control	0.602053777	0.19061172	3.15853493	0.001585643	0.010043607	up_in_case
Yang2025_S	ENSGALG00010021010	LEO1	case	control	0.608507949	0.180003876	3.380526928	0.00072347	0.005402761	up_in_case
Yang2025_S	ENSGALG00010021011	SCARB1	case	control	-0.304198857	0.149128404	-2.039845192	0.041365747	0.115982231	down_in_case
Yang2025_S	ENSGALG00010021013	FAP	case	control	-0.460645817	0.177848474	-2.590102718	0.00959473	0.039405411	down_in_case
Yang2025_S	ENSGALG00010021014	FBXL15	case	control	-0.188273889	0.162188724	-1.16083217	0.245710158	0.411386645	down_in_case
Yang2025_S	ENSGALG00010021015	RBM25	case	control	0.833182494	0.213831307	3.896447653	9.76139E-05	0.001046346	up_in_case
Yang2025_S	ENSGALG00010021016	FHIT	case	control	-0.086910018	0.198479538	-0.437878983	0.661474012	0.786223072	down_in_case
Yang2025_S	ENSGALG00010021017	COQ7	case	control	0.415311975	0.122059309	3.402542409	0.00066762	0.005053386	up_in_case
Yang2025_S	ENSGALG00010021018	F3	case	control	-0.581972944	0.178121942	-3.267272621	0.001085891	0.007483623	down_in_case
Yang2025_S	ENSGALG00010021019	VSIG10	case	control	-0.103569917	0.182922627	-0.56619522	0.571261079	0.715397298	down_in_case
Yang2025_S	ENSGALG00010021020	ZDHHC13	case	control	-0.17616991	0.122633871	-1.436551812	0.150845384	0.293743771	down_in_case
Yang2025_S	ENSGALG00010021021	GCG	case	control	-0.503026387	0.505364543	-0.995373327	0.319554726	0.488856637	down_in_case
Yang2025_S	ENSGALG00010021022	ZFYVE1	case	control	-0.315876756	0.118840694	-2.657984788	0.007860944	0.033830221	down_in_case
Yang2025_S	ENSGALG00010021023	MGEA5	case	control	0.235743209	0.114601434	2.057070319	0.039679462	0.112659598	up_in_case
Yang2025_S	ENSGALG00010021024	ENSGALG00010021024	case	control	0.244236918	0.128693378	1.897820396	0.057719746	0.148317288	up_in_case
Yang2025_S	ENSGALG00010021025	CUEDC2	case	control	0.217466378	0.139467647	1.559260385	0.118934764	0.248070039	up_in_case
Yang2025_S	ENSGALG00010021026	ADGRA2	case	control	-0.085391986	0.09573507	-0.891961385	0.37241363	0.540750645	down_in_case
Yang2025_S	ENSGALG00010021027	PLA2G4A	case	control	0.441221109	0.086133606	5.122519857	3.01479E-07	8.60884E-06	up_in_case
Yang2025_S	ENSGALG00010021028	ENSGALG00010021028	case	control	1.0158135	0.344619379	2.947638934	0.003202108	0.017102867	up_in_case
Yang2025_S	ENSGALG00010021029	PRDM2	case	control	0.518090689	0.222934768	2.323956431	0.020127829	0.06802845	up_in_case
Yang2025_S	ENSGALG00010021030	CSRP3	case	control	1.124920049	0.506901177	2.219209778	0.026472456	0.083460108	up_in_case
Yang2025_S	ENSGALG00010021031	DDX54	case	control	0.104892104	0.116765325	0.898315525	0.369017358	0.537683998	up_in_case
Yang2025_S	ENSGALG00010021032	TMC7	case	control	-0.437314491	0.117975838	-3.706814029	0.000209883	0.001949813	down_in_case
Yang2025_S	ENSGALG00010021033	RNF122	case	control	-0.522231731	0.280673917	-1.860635067	0.062795729	0.157186723	down_in_case
Yang2025_S	ENSGALG00010021034	NPM3	case	control	0.767372271	0.253798078	3.023554309	0.002498242	0.014196859	up_in_case
Yang2025_S	ENSGALG00010021036	E2F8	case	control	0.066616416	0.173878089	0.383121395	0.701629757	0.815855363	up_in_case
Yang2025_S	ENSGALG00010021038	PTGS2	case	control	1.197131653	0.183096093	6.538269806	6.22346E-11	5.48885E-09	up_in_case
Yang2025_S	ENSGALG00010021040	FGF8	case	control	1.322428205	0.649596121	2.035769862	0.04177347	0.116749133	up_in_case
Yang2025_S	ENSGALG00010021041	GLTP	case	control	-0.084233827	0.29308286	-0.287406186	0.773801325	0.862350567	down_in_case
Yang2025_S	ENSGALG00010021042	LRRC38	case	control	-0.380887052	0.098836177	-3.853721012	0.000116336	0.001202629	down_in_case
Yang2025_S	ENSGALG00010021043	PTPRG	case	control	0.095131821	0.150493597	0.632132013	0.527300618	0.680175332	up_in_case
Yang2025_S	ENSGALG00010021044	EIF4G2	case	control	-0.232812354	0.066565251	-3.497505822	0.00046963	0.003761077	down_in_case
Yang2025_S	ENSGALG00010021045	DPP4	case	control	0.399055449	0.147960227	2.697045402	0.006995773	0.030947968	up_in_case
Yang2025_S	ENSGALG00010021046	ENSGALG00010021046	case	control	0.062033778	0.185276116	0.334817998	0.737762383	0.839996634	up_in_case
Yang2025_S	ENSGALG00010021047	TMC5	case	control	0.545779689	0.470553141	1.159868337	0.246102416	0.411858315	up_in_case
Yang2025_S	ENSGALG00010021048	API5	case	control	-0.54509465	0.091219891	-5.975611699	2.29228E-09	1.26257E-07	down_in_case
Yang2025_S	ENSGALG00010021050	CTR9	case	control	0.280067242	0.092673431	3.022087761	0.002510378	0.014237733	up_in_case
Yang2025_S	ENSGALG00010021051	GDE1	case	control	0.500927949	0.12207194	4.103547047	4.06864E-05	0.000521586	up_in_case
Yang2025_S	ENSGALG00010021052	CEP15	case	control	-0.552579402	0.175538682	-3.147906755	0.001644441	0.010326855	down_in_case
Yang2025_S	ENSGALG00010021053	ENSGALG00010021053	case	control	-1.482580685	0.534556158	-2.773479759	0.005546029	0.025864764	down_in_case
Yang2025_S	ENSGALG00010021055	IRAG1	case	control	0.665214436	0.169553906	3.923321205	8.73366E-05	0.000956789	up_in_case
Yang2025_S	ENSGALG00010021057	ZDHHC8	case	control	0.336753355	0.139679463	2.410900975	0.015913168	0.05749287	up_in_case
Yang2025_S	ENSGALG00010021058	CCP110	case	control	0.21316363	0.132068084	1.614043478	0.106517996	0.22909836	up_in_case

Yang2025_S	ENSGALG00010021062	MYO9A	case	control	0.137807061	0.163843268	0.841090774	0.400297083	0.56728769	up_in_case
Yang2025_S	ENSGALG00010021064	LYVE1	case	control	0.051020893	0.326240913	0.156390236	0.875725433	0.926804429	up_in_case
Yang2025_S	ENSGALG00010021065	CNNM2	case	control	0.029521577	0.150392949	0.196296284	0.84437827	0.907479967	up_in_case
Yang2025_S	ENSGALG00010021066	ENSGALG00010021066	case	control	0.220032879	0.437389178	0.503059723	0.614922283	0.751068631	up_in_case
Yang2025_S	ENSGALG00010021067	SLC4A10	case	control	0.527774709	0.318184405	1.658707028	0.097174843	0.214715409	up_in_case
Yang2025_S	ENSGALG00010021069	PGAM5	case	control	-0.148886102	0.123212807	-1.208365475	0.226906714	0.388923131	down_in_case
Yang2025_S	ENSGALG00010021070	RNF141	case	control	-0.238287174	0.076715295	-3.106123426	0.001895576	0.011524803	down_in_case
Yang2025_S	ENSGALG00010021071	HIF1AN	case	control	-0.410915891	0.100830731	-4.075304108	4.59543E-05	0.000570234	down_in_case
Yang2025_S	ENSGALG00010021072	ENSGALG00010021072	case	control	-0.703865067	0.087309308	-8.061741403	7.52152E-16	1.89645E-13	down_in_case
Yang2025_S	ENSGALG00010021073	NDUFB8	case	control	0.311496634	0.181969993	1.7118022	0.086933127	0.198820469	up_in_case
Yang2025_S	ENSGALG00010021074	TTC17	case	control	0.058791513	0.09722623	0.604687776	0.545386472	0.694850275	up_in_case
Yang2025_S	ENSGALG00010021075	DHRS3	case	control	-0.291167523	0.140444528	-2.073185245	0.038155043	0.109311374	down_in_case
Yang2025_S	ENSGALG00010021077	SEC31B	case	control	0.397848266	0.18285548	2.175752495	0.029573773	0.090553839	up_in_case
Yang2025_S	ENSGALG00010021078	ENSGALG00010021078	case	control	-0.089748929	0.300185325	-0.298978401	0.764956526	0.856955477	down_in_case
Yang2025_S	ENSGALG00010021080	TTLL7	case	control	0.673682339	0.196808453	3.423035588	0.00061926	0.004733617	up_in_case
Yang2025_S	ENSGALG00010021081	KCNH6	case	control	0.46856799	0.235438889	1.990189437	0.046570071	0.126674594	up_in_case
Yang2025_S	ENSGALG00010021082	SENP8	case	control	0.087460226	0.124068409	0.704935501	0.480850376	0.639313996	up_in_case
Yang2025_S	ENSGALG00010021083	SCD	case	control	0.733801964	0.305798455	2.399626134	0.016411824	0.058776349	up_in_case
Yang2025_S	ENSGALG00010021085	AMPD3	case	control	-0.134870258	0.146909825	-0.918047912	0.358593783	0.527612204	down_in_case
Yang2025_S	ENSGALG00010021086	PSMD14	case	control	-0.046868016	0.110241288	-0.425140317	0.670734389	0.792364624	down_in_case
Yang2025_S	ENSGALG00010021087	THBS1	case	control	0.483701826	0.396291121	1.220571948	0.222248133	0.383789756	up_in_case
Yang2025_S	ENSGALG00010021088	ANKLE2	case	control	0.138983902	0.119398768	1.164031293	0.244411331	0.409753596	up_in_case
Yang2025_S	ENSGALG00010021090	ADM	case	control	0.233249976	0.244541125	0.953827195	0.340171121	0.508287001	up_in_case
Yang2025_S	ENSGALG00010021091	TANK	case	control	-0.200668584	0.097070215	-2.06725187	0.038710427	0.110561549	down_in_case
Yang2025_S	ENSGALG00010021092	VPS13D	case	control	-0.225884361	0.120921164	-1.868029995	0.06175789	0.155623498	down_in_case
Yang2025_S	ENSGALG00010021093	GRAMD2	case	control	0.059161794	0.261316223	0.22639924	0.820890919	0.893065296	up_in_case
Yang2025_S	ENSGALG00010021094	ENSGALG00010021094	case	control	0.261542815	0.233088533	1.122074993	0.261830555	0.428730305	up_in_case
Yang2025_S	ENSGALG00010021095	NT5C2	case	control	0.15427499	0.133927483	1.151929291	0.249350131	0.415037515	up_in_case
Yang2025_S	ENSGALG00010021096	ZFAND4	case	control	0.000807727	0.10686405	0.007558455	0.993969283	0.995970515	up_in_case
Yang2025_S	ENSGALG00010021097	KATNBL1	case	control	-0.148097158	0.087080295	-1.700696571	0.088999979	0.202420299	down_in_case
Yang2025_S	ENSGALG00010021098	SDK1	case	control	-0.41694295	0.110126123	-3.786049471	0.000153061	0.001501729	down_in_case
Yang2025_S	ENSGALG00010021099	HMCN1	case	control	0.305273351	0.172152138	1.773276562	0.076182891	0.180739826	up_in_case
Yang2025_S	ENSGALG00010021100	EMC7	case	control	-0.473426727	0.124403275	-3.805580884	0.000141472	0.001412184	down_in_case
Yang2025_S	ENSGALG00010021101	TMEM240	case	control	-0.176729012	0.41044462	-0.430579433	0.666774199	0.789228274	down_in_case
Yang2025_S	ENSGALG00010021102	GOLGA3	case	control	0.049033758	0.065224347	0.751770776	0.452188919	0.614037477	up_in_case
Yang2025_S	ENSGALG00010021103	RBMS1	case	control	-0.030529238	0.119947994	-0.254520618	0.799093378	0.878430916	down_in_case
Yang2025_S	ENSGALG00010021104	INA	case	control	0.093295735	0.273809049	0.340732841	0.733304713	0.837109952	up_in_case
Yang2025_S	ENSGALG00010021105	ENSGALG00010021105	case	control	2.3930192	0.52158601	4.587966618	4.47584E-06	8.43445E-05	up_in_case
Yang2025_S	ENSGALG00010021106	ITGB6	case	control	-0.234417204	0.199514506	-1.174938147	0.240019463	0.404483155	down_in_case
Yang2025_S	ENSGALG00010021108	PCGF6	case	control	-0.120087234	0.126384801	-0.950171482	0.342025127	0.510291235	down_in_case
Yang2025_S	ENSGALG00010021110	PKLR	case	control	-0.214743284	0.141273159	-1.520057209	0.128496598	0.261745372	down_in_case
Yang2025_S	ENSGALG00010021111	AVEN	case	control	-0.056417872	0.108135793	-0.521731708	0.601857145	0.740372358	down_in_case
Yang2025_S	ENSGALG00010021112	TAF5	case	control	-0.092304652	0.093008783	-0.992429419	0.320988094	0.490388723	down_in_case
Yang2025_S	ENSGALG00010021113	SYP	case	control	0.436729294	0.20703966	2.109399199	0.034910139	0.102523241	up_in_case
Yang2025_S	ENSGALG00010021114	SBF2	case	control	-0.034744954	0.135643502	-0.256149048	0.79783575	0.877701206	down_in_case
Yang2025_S	ENSGALG00010021115	ALPL	case	control	-0.064552518	0.310077246	-0.208182054	0.83508682	0.902437754	down_in_case
Yang2025_S	ENSGALG00010021116	FAM21C	case	control	0.465288839	0.094400676	4.928871892	8.27058E-07	2.06542E-05	up_in_case
Yang2025_S	ENSGALG00010021118	USMG5	case	control	-0.447623271	0.211647909	-2.114943033	0.034434803	0.101426544	down_in_case
Yang2025_S	ENSGALG00010021120	PDCD11	case	control	0.165187775	0.085502273	1.931969396	0.053363283	0.139909192	up_in_case
Yang2025_S	ENSGALG00010021121	PLA2R1	case	control	-0.17827472	0.107938646	-1.651630137	0.098609973	0.216910808	down_in_case
Yang2025_S	ENSGALG00010021123	RYR3	case	control	0.452724033	0.194187452	2.331376354	0.019733523	0.067201038	up_in_case
Yang2025_S	ENSGALG00010021124	CALHM2	case	control	-0.467188865	0.135465139	-3.448775591	0.000563134	0.004364449	down_in_case
Yang2025_S	ENSGALG00010021125	LY75	case	control	0.516361366	0.114124917	4.524527843	6.05305E-06	0.000109261	up_in_case
Yang2025_S	ENSGALG00010021126	ECE1	case	control	-0.172165449	0.07840693	-2.195793766	0.028106708	0.08715331	down_in_case
Yang2025_S	ENSGALG00010021127	SWAP70	case	control	-0.17506769	0.060533682	-2.892070736	0.003827118	0.019549445	down_in_case
Yang2025_S	ENSGALG00010021129	TXNRD3	case	control	-0.12789038	0.121348007	-1.053914132	0.291922232	0.46053847	down_in_case
Yang2025_S	ENSGALG00010021130	ENSGALG00010021130	case	control	-3.827706611	1.033467883	-3.703749942	0.000212436	0.001965895	down_in_case
Yang2025_S	ENSGALG00010021131	EDC3	case	control	-0.307944519	0.069810115	-4.411173372	1.02812E-05	0.000168208	down_in_case
Yang2025_S	ENSGALG00010021133	ENSGALG00010021133	case	control	0.102883261	0.079355455	1.296486319	0.19480799	0.350639285	up_in_case
Yang2025_S	ENSGALG00010021134	CHFR	case	control	-0.126405032	0.073029965	-1.730865294	0.083475793	0.193132189	down_in_case
Yang2025_S	ENSGALG00010021135	CLK3	case	control	0.4499027	0.104051077	4.323863938	1.5332E-05	0.000233754	up_in_case
Yang2025_S	ENSGALG00010021136	WEE1	case	control	-0.548244175	0.149849982	-3.658620222	0.000253577	0.002269488	down_in_case
Yang2025_S	ENSGALG00010021137	PLAU	case	control	0.155532527	0.215199301	0.722737135	0.469841396	0.630269528	up_in_case
Yang2025_S	ENSGALG00010021138	DDX18	case	control	-0.062985962	0.124279732	-0.506807995	0.612289546	0.749034749	down_in_case
Yang2025_S	ENSGALG00010021139	ARHGAP29	case	control	0.248335306	0.098517915	2.520712166	0.011711761	0.045538905	up_in_case
Yang2025_S	ENSGALG00010021140	SLC11A1	case	control	-0.161861743	0.296789997	-0.545374658	0.585495866	0.727562412	down_in_case
Yang2025_S	ENSGALG00010021141	SGSM1	case	control	-0.138435895	0.20039741	-0.690806809	0.489686956	0.647111213	down_in_case
Yang2025_S	ENSGALG00010021142	WNT6	case	control	0.401377124	0.760742962	0.527612012	0.597768654	0.737029393	up_in_case
Yang2025_S	ENSGALG00010021143	ABCA4	case	control	0.941407416	0.425582471	2.212044621	0.026963582	0.084568426	up_in_case
Yang2025_S	ENSGALG00010021144	FOXK1	case	control	-0.21227382	0.132336223	-1.604049248	0.108703198	0.232402761	down_in_case
Yang2025_S	ENSGALG00010021145	EIF4G3	case	control	0.396999201	0.170897103	2.323030603	0.020177508	0.068146772	up_in_case
Yang2025_S	ENSGALG00010021146	AP5Z1	case	control	-0.27866349	0.123447333	-2.257347185	0.023986389	0.077678441	down_in_case
Yang2025_S	ENSGALG00010021147	ARID3B	case	control	0.241457366	0.176100935	1.371130517	0.170334274	0.319247895	up_in_case
Yang2025_S	ENSGALG00010021148	GPATCH8	case	control	0.695162747	0.153392336	4.531926207	5.84483E-06	0.00010619	up_in_case
Yang2025_S	ENSGALG00010021149	ENSGALG00010021149	case	control	2.143352818	1.83322648	1.169169681	0.242335274	0.407289535	up_in_case
Yang2025_S	ENSGALG00010021150	ENSGALG00010021150	case	control	0.764944776	0.231632616	3.302405292	0.000958595	0.006780718	up_in_case
Yang2025_S	ENSGALG00010021152	STK11IP	case	control	0.077842469	0.092275004	0.843592147	0.398897354	0.565821929	up_in_case
Yang2025_S	ENSGALG00010021153	PTPRF	case	control	0.313314878	0.156590542	2.000854423	0.04540808	0.124731244	up_in_case
Yang2025_S	ENSGALG00010021154	CHST13	case	control	-0.255596535	0.153160463	-1.668815372	0.095153975	0.211782566	down_in_case
Yang2025_S	ENSGALG00010021155	RADIL	case	control	0.451991066	0.245334598	1.842345395	0.065424644	0.161819739	up_in_case
Yang2025_S	ENSGALG00010021156	DDOST	case	control	-0.353282237	0.19059316	-1.853593472	0.063797342	0.158923125	down_in_case
Yang2025_S	ENSGALG00010021158	ACTG1L	case	control	-0.227539193	0.15739144	-1.445689762	0.148264237	0.290319492	down_in_case
Yang2025_S	ENSGALG00010021159	MMD2	case	control	-0.018106848	0.294441012	-0.061495674	0.95096446	0.969683137	down_in_case
Yang2025_S	ENSGALG00010021160	ZNF143	case	control	0.023657348	0.118911614	0.19894901	0.842302631	0.906369665	up_in_case
Yang2025_S	ENSGALG00010021161	ENSGALG00010021161	case	control	0.290950592	0.116883579	2.48923412	0.012801863	0.048635214	up_in_case
Yang2025_S	ENSGALG00010021162	WIPI2	case	control	0.153538138	0.13548544	1.133244556	0.257111568	0.423409595	up_in_case
Yang2025_S	ENSGALG00010021163	INHA	case	control	0.553417883	0.260807198	2.121942519	0.033842565	0.100128975	up_in_case
Yang2025_S	ENSGALG00010021164	SLC41A3	case	control	-0.17762777	0.143510284	-1.237735482	0.215814158	0.376769017	down_in_case
Yang2025_S	ENSGALG00010021165	PTRF	case	control	0.09593364	0.111090252	0.863564875	0.387826967	0.554921649	up_in_case
Yang2025_S	ENSGALG00010021166	NEURL1	case	control	0.770810048	0.262401754	2.937518653	0.003308503	0.017528156	up_in_case
Yang2025_S	ENSGALG00010021167	KIAA1671	case	control	-0.263698088	0.121260105	-2.174648353	0.029656478	0.090707423	down_in_case
Yang2025_S	ENSGALG00010021168	IVNS1ABP	case	control	0.358414031	0.090276541	3.970179061	7.18186E-05	0.000816974	up_in_case
Yang2025_S	ENSGALG00010021169	CAMK2G	case	control	-0.047762016	0.09623004	-0.496331663	0.61966043	0.755266399	down_in_case
Yang2025_S	ENSGALG00010021170	UBL7	case	control	-0.340173339	0.129228342	-2.632343132	0.008479817	0.035862292	down_in_case
Yang2025_S	ENSGALG00010021171	ENSGALG00010021171	case	control	0.52229774	0.116935423	4.466548509	7.94916E-06	0.000135418	up_in_case
Yang2025_S	ENSGALG00010021172	SLC29A4	case	control	0.083267648	0.358118648	0.232514136	0.816138709	0.889758482	up_in_case
Yang2025_S	ENSGALG00010021173	KLF15	case	control	-0.584138498	0.13339902	-4.37888149	1.1929E-05	0.000190608	down_in_case
Yang2025_S	ENSGALG00010021175	IPO7	case	control	-0.419894941	0.089990847	-4.665973891	3.07159E-06	6.18534E-05	down_in_case
Yang2025_S	ENSGALG00010021176	ENSGALG00010021176	case	control	-0.339908858	0.11347856	-2.995357524	0.002741233	0.015200591	down_in_case
Yang2025_S	ENSGALG00010021177	OBSL1	case	control	0.517165163	0.26226109	1.971947738	0.048615573	0.1307077	up_in_case
Yang2025_S	ENSGALG00010021178	NDST2	case	control	-0.17302746	0.095426521	-1.813200974	0.06980083	0.169632815	down_in_case
Yang2025_S	ENSGALG00010021180	TMEM198	case	control	0.196668134	0.125373822	1.568653892	0.116728608	0.244640269	up_in_case
Yang2025_S	ENSGALG00010021181	DAGLA	case	control	-0.017250862	0.120719486	-0.142900396	0.886368846	0.933444955	down_in_case
Yang2025_S	ENSGALG00010021182	ZSWIM8	case	control	-0.161319382	0.114026744	-1.41475041	0.157141688	0.302329066	down_in_case
Yang2025_S	ENSGALG00010021183	CFAP100	case	control	1.015314988	0.566641784	1.791811011	0.073163244	0.175508661	up_in_case
Yang2025_S	ENSGALG00010021184	DRAXIN	case	control	-0.185106308	0.394959133	-0.468672054	0.639304065	0.769251546	down_in_case
Yang2025_S	ENSGALG00010021186	SWT1	case	control	0.445113335	0.137033389	3.248210801	0.001161332	0.007875018	up_in_case
Yang2025_S	ENSGALG00010021187	SEMA7A	case	control	0.057386699	0.081673615	0.702634497	0.482283559	0.640547216	up_in_case
Yang2025_S	ENSGALG00010021188	TNRC18	case	control	-0.156681482	0.10145144	-1.544398798	0.122491751	0.253553558	down_in_case
Yang2025_S	ENSGALG00010021189	ENSGALG00010021189	case	control	0.067207925	0.500581027	0.134259832	0.893197115	0.937392815	up_in_case
Yang2025_S	ENSGALG00010021190	ENSGALG00010021190	case	control	-0.220286981	0.062206954	-3.541195402	0.000398318	0.0032902	down_in_case
Yang2025_S	ENSGALG00010021191	MYRF	case	control	0.481886889	0.294023019	1.638942729	0.101225188	0.221023659	up_in_case
Yang2025_S	ENSGALG00010021192	ENSGALG00010021192	case	control	0.102422233	0.096713003	1.059032701	0.289584887	0.458355907	up_in_case
Yang2025_S	ENSGALG00010021193	USP37	case	control	-0.029965077	0.090406163	-0.331449495	0.740304984	0.841043905	down_in_case
Yang2025_S	ENSGALG00010021194	CYP11A1	case	control	0.273824255	0.393052787	0.696660257	0.486015436	0.643174274	up_in_case
Yang2025_S	ENSGALG00010021195	GCLM	case	control	-0.38906808	0.135980846	-2.861197677	0.004220438	0.020944375	down_in_case
Yang2025_S	ENSGALG00010021196	ATP6V0A1	case	control	0.33455145	0.108347043	3.087776469	0.002016601	0.01204515	up_in_case
Yang2025_S	ENSGALG00010021197	CHCHD1	case	control	0.53078903	0.276368086	1.92058728	0.05478376	0.142651907	up_in_case
Yang2025_S	ENSGALG00010021199	TMEM258	case	control	-0.411214085	0.401038777	-1.02537238	0.305187516	0.474807136	down_in_case
Yang2025_S	ENSGALG00010021200	FTO	case	control	0.030419226	0.206131328	0.147572065	0.882680502	0.930983409	up_in_case
Yang2025_S	ENSGALG00010021201	TRMT1L	case	control	0.245229473	0.141625119	1.731539402	0.083355604	0.19288614	up_in_case
Yang2025_S	ENSGALG00010021202	UROC1	case	control	0.751700921	0.314481135	2.390289395	0.016835101	0.059821971	up_in_case
Yang2025_S	ENSGALG00010021203	FUT11	case	control	-0.132429698	0.113518545	-1.166590874	0.243375631	0.408410334	down_in_case
Yang2025_S	ENSGALG00010021204	NAGLU	case	control	0.120105363	0.196457886	0.611354248	0.540965085	0.691464727	up_in_case
Yang2025_S	ENSGALG00010021206	HSD17B1	case	control	0.277718672	0.592475815	0.468742631	0.639253611	0.769251546	up_in_case
Yang2025_S	ENSGALG00010021207	MAD2L2	case	control	0.033074025	0.13121391	0.252061885	0.80099323	0.87978566	up_in_case

Yang2025_S	ENSGALG00010021210	ENSGALG00010021210	case	control	-0.136112341	0.105257617	-1.293135317	0.195964262	0.352137344	down_in_case
Yang2025_S	ENSGALG00010021211	FEN1	case	control	0.026339329	0.129482743	0.203419613	0.838807071	0.90451196	up_in_case
Yang2025_S	ENSGALG00010021212	MYO18B	case	control	0.399815975	0.086525136	4.620807236	3.8225E-06	7.4603E-05	up_in_case
Yang2025_S	ENSGALG00010021213	FBXO44	case	control	-0.125342338	0.288431802	-0.434564903	0.663878275	0.787398396	down_in_case
Yang2025_S	ENSGALG00010021214	FBXL18	case	control	-0.283448285	0.120304737	-2.356085827	0.01846865	0.063877051	down_in_case
Yang2025_S	ENSGALG00010021215	SH3PXD2A	case	control	0.314617862	0.168798853	1.863862556	0.062341008	0.156611132	up_in_case
Yang2025_S	ENSGALG00010021217	FBXO2	case	control	0.122898373	0.305304206	0.40254399	0.687283721	0.804465418	up_in_case
Yang2025_S	ENSGALG00010021218	DPF3	case	control	0.076622366	0.260204802	0.294469456	0.7683992	0.858994096	up_in_case
Yang2025_S	ENSGALG00010021219	STRA6	case	control	0.071325678	0.255394947	0.279275994	0.780033027	0.866599716	up_in_case
Yang2025_S	ENSGALG00010021220	SYNPO2L	case	control	0.112892237	0.191659702	0.589024377	0.555844921	0.703011282	up_in_case
Yang2025_S	ENSGALG00010021222	ENSGALG00010021222	case	control	-0.577159915	0.337357932	-1.710823608	0.08711368	0.199168028	down_in_case
Yang2025_S	ENSGALG00010021223	DFFB	case	control	0.247008466	0.115472286	2.139114714	0.032426379	0.097091017	up_in_case
Yang2025_S	ENSGALG00010021224	MYOZ1	case	control	-0.061879921	0.154443127	-0.400664778	0.688666946	0.805397618	down_in_case
Yang2025_S	ENSGALG00010021227	CHCHD4	case	control	-0.424426425	0.185192754	-2.291809022	0.021916666	0.072286092	down_in_case
Yang2025_S	ENSGALG00010021228	ESRP2	case	control	-1.53502665	1.015785132	-1.511172591	0.130744484	0.264980266	down_in_case
Yang2025_S	ENSGALG00010021229	GLO1	case	control	-0.551103284	0.172883981	-3.187705892	0.001434063	0.009293381	down_in_case
Yang2025_S	ENSGALG00010021230	C1orf174	case	control	-0.46687772	0.093459745	-4.995495339	5.86849E-07	1.5257E-05	down_in_case
Yang2025_S	ENSGALG00010021231	STN1	case	control	-0.432099913	0.17708642	-2.440051093	0.014685185	0.054022716	down_in_case
Yang2025_S	ENSGALG00010021232	ACTB	case	control	0.137407524	0.151180416	0.908897644	0.363404158	0.53216025	up_in_case
Yang2025_S	ENSGALG00010021233	COASY	case	control	-0.080982199	0.169225935	-0.478544848	0.632262462	0.763944976	down_in_case
Yang2025_S	ENSGALG00010021234	ISLR2	case	control	0.625692529	0.261424184	2.393399572	0.016693051	0.059447396	up_in_case
Yang2025_S	ENSGALG00010021235	XRCC5	case	control	-0.291235663	0.091239495	-3.19199117	0.001412957	0.009207045	down_in_case
Yang2025_S	ENSGALG00010021236	BTBD9	case	control	-0.42355276	0.103857048	-4.078228366	4.53802E-05	0.000565994	down_in_case
Yang2025_S	ENSGALG00010021237	FSCN1	case	control	-0.229340854	0.166341616	-1.378734069	0.167976761	0.316176124	down_in_case
Yang2025_S	ENSGALG00010021238	NDUFS8	case	control	0.187256546	0.226859473	0.825429699	0.40912768	0.574651492	up_in_case
Yang2025_S	ENSGALG00010021239	USP54	case	control	0.181764757	0.136953083	1.327204566	0.184441022	0.337782317	up_in_case
Yang2025_S	ENSGALG00010021240	SLK	case	control	0.332775774	0.189312974	1.757807545	0.078780261	0.185283196	up_in_case
Yang2025_S	ENSGALG00010021241	TMEM43	case	control	-0.261033366	0.108571697	-2.404248743	0.016205742	0.0581631	down_in_case
Yang2025_S	ENSGALG00010021242	BCAR3	case	control	0.247594756	0.224706278	1.101859541	0.27052274	0.437933827	up_in_case
Yang2025_S	ENSGALG00010021243	DYL1	case	control	-0.499749232	0.220861345	-2.262728369	0.023652439	0.076972615	down_in_case
Yang2025_S	ENSGALG00010021244	PPP3CB	case	control	0.103982331	0.099177828	1.048443318	0.294434404	0.462981655	up_in_case
Yang2025_S	ENSGALG00010021245	ZFAND3	case	control	-0.274409046	0.067147949	-4.086633346	4.37678E-05	0.000549959	down_in_case
Yang2025_S	ENSGALG00010021246	PECR	case	control	0.450321311	0.120200378	3.746421743	0.000179375	0.001714396	up_in_case
Yang2025_S	ENSGALG00010021247	GNAO1	case	control	0.551007306	0.270968531	2.033473423	0.042004716	0.117247155	up_in_case
Yang2025_S	ENSGALG00010021248	ANXA7	case	control	-0.055903205	0.145511834	-0.384183222	0.700842653	0.815280271	down_in_case
Yang2025_S	ENSGALG00010021250	ISLR	case	control	-0.71052349	0.173453188	-4.096341491	4.19731E-05	0.0005326	down_in_case
Yang2025_S	ENSGALG00010021251	RNF216	case	control	0.114975083	0.13275041	0.866099642	0.386435522	0.553953193	up_in_case
Yang2025_S	ENSGALG00010021252	XPC	case	control	0.428931884	0.112495735	3.812872393	0.000137361	0.001380048	up_in_case
Yang2025_S	ENSGALG00010021253	TMEM169	case	control	0.081448736	0.107895379	0.754886231	0.450317248	0.612312663	up_in_case
Yang2025_S	ENSGALG00010021254	FNBP1L	case	control	0.14352444	0.16150049	0.888693527	0.374167811	0.542240147	up_in_case
Yang2025_S	ENSGALG00010021255	EIF3F	case	control	-0.619893485	0.136801487	-4.531335866	5.86119E-06	0.000106348	down_in_case
Yang2025_S	ENSGALG00010021256	ENSGALG00010021256	case	control	0.156803345	0.161837627	0.968893006	0.332598575	0.501814762	up_in_case
Yang2025_S	ENSGALG00010021257	RGS6	case	control	0.538814828	0.254472903	2.117376046	0.034227945	0.100923915	up_in_case
Yang2025_S	ENSGALG00010021258	LSM3	case	control	-0.934892641	0.196401793	-4.760102364	1.93495E-06	4.22625E-05	down_in_case
Yang2025_S	ENSGALG00010021259	CEP104	case	control	-0.036904687	0.101887167	-0.362211339	0.717194107	0.825804198	down_in_case
Yang2025_S	ENSGALG00010021260	KDM4A	case	control	-0.071509672	0.073442951	-0.973676442	0.330217218	0.499707788	down_in_case
Yang2025_S	ENSGALG00010021261	JUP	case	control	0.303811024	0.109795364	2.767066045	0.005656329	0.026256146	up_in_case
Yang2025_S	ENSGALG00010021262	MDGA1	case	control	-0.006376348	0.2690787	-0.023696962	0.981094329	0.988594337	down_in_case
Yang2025_S	ENSGALG00010021263	STOML1	case	control	0.384504321	0.175730914	2.188028909	0.028667497	0.08845916	up_in_case
Yang2025_S	ENSGALG00010021264	CBFB	case	control	0.053581879	0.156607597	0.342140994	0.732244789	0.836362093	up_in_case
Yang2025_S	ENSGALG00010021265	SIPA1L1	case	control	0.163339572	0.156227717	1.045522363	0.29578161	0.464418787	up_in_case
Yang2025_S	ENSGALG00010021266	COQ5	case	control	-0.076000285	0.160639385	-0.473111155	0.636133879	0.766761145	down_in_case
Yang2025_S	ENSGALG00010021267	LRRC47	case	control	0.103947223	0.108167827	0.960980965	0.336561739	0.504733946	up_in_case
Yang2025_S	ENSGALG00010021269	NUDT13	case	control	0.571060791	0.13947884	4.094246785	4.23543E-05	0.000535578	up_in_case
Yang2025_S	ENSGALG00010021271	ENSGALG00010021271	case	control	-0.312028659	0.113416223	-2.751181895	0.005938066	0.027192557	down_in_case
Yang2025_S	ENSGALG00010021272	ENSGALG00010021272	case	control	-0.111397086	0.171002822	-0.651434195	0.514766243	0.669522829	down_in_case
Yang2025_S	ENSGALG00010021275	MAP3K2	case	control	0.145536748	0.124472007	1.169232752	0.242309869	0.407289535	up_in_case
Yang2025_S	ENSGALG00010021277	ENSGALG00010021277	case	control	0.306888681	0.255058933	1.203206952	0.228896245	0.391167275	up_in_case
Yang2025_S	ENSGALG00010021287	COL17A1	case	control	-1.750948754	0.629642458	-2.780861951	0.005421479	0.025488632	down_in_case
Yang2025_S	ENSGALG00010021290	ERCC3	case	control	0.131374534	0.095476965	1.375981462	0.168827373	0.317234282	up_in_case
Yang2025_S	ENSGALG00010021293	PCNX1	case	control	-0.243972806	0.090788758	-2.687257902	0.00720413	0.031595501	down_in_case
Yang2025_S	ENSGALG00010021296	GATC	case	control	-0.093650183	0.14775366	-0.633826486	0.526194066	0.679376847	down_in_case
Yang2025_S	ENSGALG00010021298	RPGRIP1L	case	control	0.120906298	0.15551245	0.777470217	0.4368814	0.600744257	up_in_case
Yang2025_S	ENSGALG00010021299	CYP27C1	case	control	-0.58032242	0.187411162	-3.096520041	0.001958066	0.011793634	down_in_case
Yang2025_S	ENSGALG00010021301	SMURF1	case	control	-0.154635798	0.122580877	-1.261500171	0.207128698	0.365818832	down_in_case
Yang2025_S	ENSGALG00010021302	P4HA1	case	control	-0.043267459	0.090893255	-0.476024963	0.634056594	0.765245369	down_in_case
Yang2025_S	ENSGALG00010021307	MAP3K9	case	control	0.209632384	0.265358902	0.789995672	0.429530296	0.593708891	up_in_case
Yang2025_S	ENSGALG00010021309	ST3GAL3	case	control	-0.514643289	0.168352448	-3.056939747	0.002236092	0.013021289	down_in_case
Yang2025_S	ENSGALG00010021311	TRIAP1	case	control	-0.132280193	0.160601605	-0.823654239	0.410136048	0.575553457	down_in_case
Yang2025_S	ENSGALG00010021314	ZP3	case	control	0.479589983	0.233471423	2.054169959	0.039959245	0.11326934	up_in_case
Yang2025_S	ENSGALG00010021315	COX6A1	case	control	-0.183140355	0.269712265	-0.679021237	0.497124406	0.654053936	down_in_case
Yang2025_S	ENSGALG00010021316	PLA2G12B	case	control	0.843463451	0.485325682	1.737932862	0.082222644	0.190908805	up_in_case
Yang2025_S	ENSGALG00010021318	TP73	case	control	0.195469686	0.166396751	1.174720565	0.240106529	0.404580953	up_in_case
Yang2025_S	ENSGALG00010021320	SLC6A6	case	control	-0.330848505	0.159347382	-2.076271981	0.037868803	0.108670052	down_in_case
Yang2025_S	ENSGALG00010021325	ARPC1A	case	control	-0.313632993	0.136557355	-2.296712563	0.021635172	0.071680011	down_in_case
Yang2025_S	ENSGALG00010021329	PSMC3IP	case	control	0.445990213	0.225695379	1.97607153	0.048146676	0.129847868	up_in_case
Yang2025_S	ENSGALG00010021330	COMMD4	case	control	0.604066546	0.254065743	2.377599358	0.017425748	0.061289196	up_in_case
Yang2025_S	ENSGALG00010021332	OIT3	case	control	1.20697887	0.160337265	7.527750152	5.1622E-14	8.99192E-12	up_in_case
Yang2025_S	ENSGALG00010021335	NFATC3	case	control	-0.037328833	0.216705643	-0.172255934	0.863236326	0.919290108	down_in_case
Yang2025_S	ENSGALG00010021338	PDIA5	case	control	-0.129543974	0.090956072	-1.424247671	0.15437479	0.29869657	down_in_case
Yang2025_S	ENSGALG00010021341	L3MBTL1	case	control	0.455519544	0.170833821	2.666448254	0.007665741	0.033143685	up_in_case
Yang2025_S	ENSGALG00010021342	SFR1	case	control	-0.955041381	0.221712647	-4.307563835	1.65062E-05	0.000247892	down_in_case
Yang2025_S	ENSGALG00010021345	NEIL1	case	control	0.135474498	0.198005344	0.68419617	0.493851301	0.651378881	up_in_case
Yang2025_S	ENSGALG00010021346	RPS21	case	control	-1.413760481	0.305123267	-4.633407661	3.59695E-06	7.08961E-05	down_in_case
Yang2025_S	ENSGALG00010021348	SLC4A3	case	control	-0.230277452	0.105520283	-2.182305107	0.029087022	0.089417087	down_in_case
Yang2025_S	ENSGALG00010021350	CABLES2	case	control	-0.306704929	0.111130309	-2.759867516	0.005782481	0.026690582	down_in_case
Yang2025_S	ENSGALG00010021352	CFAP43	case	control	0.351792936	0.237756469	1.479635602	0.13897052	0.277244695	up_in_case
Yang2025_S	ENSGALG00010021353	IPO13	case	control	-0.214592165	0.109441751	-1.960788859	0.049903658	0.13309234	down_in_case
Yang2025_S	ENSGALG00010021354	ENSGALG00010021354	case	control	-0.258101715	0.088847352	-2.905001792	0.00367251	0.018968283	down_in_case
Yang2025_S	ENSGALG00010021358	MMP15	case	control	-0.022796391	0.106735566	-0.213578212	0.830875992	0.89914242	down_in_case
Yang2025_S	ENSGALG00010021360	PTPRN	case	control	0.676322197	0.260367672	2.59756594	0.009388709	0.038741979	up_in_case
Yang2025_S	ENSGALG00010021361	WRAP73	case	control	-0.026123695	0.169636952	-0.153997665	0.877611577	0.928025294	down_in_case
Yang2025_S	ENSGALG00010021362	ARPC1B	case	control	-0.174413286	0.236217829	-0.738357841	0.460297027	0.621298433	down_in_case
Yang2025_S	ENSGALG00010021363	ENSGALG00010021363	case	control	-0.435897212	0.168752726	-2.58305287	0.009793031	0.039878987	down_in_case
Yang2025_S	ENSGALG00010021367	GRIP2	case	control	0.152972722	0.146084538	1.047152039	0.295029462	0.463570012	up_in_case
Yang2025_S	ENSGALG00010021373	CEP250	case	control	0.943871835	0.211269344	4.467623257	7.90934E-06	0.000134904	up_in_case
Yang2025_S	ENSGALG00010021375	TUBG1	case	control	0.06546472	0.124259174	0.526840134	0.59830461	0.737425132	up_in_case
Yang2025_S	ENSGALG00010021378	KIF11	case	control	-0.085259618	0.124931018	-0.682453566	0.494952194	0.652275282	down_in_case
Yang2025_S	ENSGALG00010021381	DNAJB2	case	control	0.156024504	0.185282847	0.842088228	0.39973857	0.566784389	up_in_case
Yang2025_S	ENSGALG00010021382	TPRG1L	case	control	-0.324840245	0.108326036	-2.998727328	0.002711098	0.01507548	down_in_case
Yang2025_S	ENSGALG00010021383	MSI1	case	control	-0.015233103	0.092314178	-0.165013691	0.868933206	0.92285873	down_in_case
Yang2025_S	ENSGALG00010021384	ARTN	case	control	0.269993472	0.339578243	0.795084719	0.426564223	0.59104827	up_in_case
Yang2025_S	ENSGALG00010021385	GDF5	case	control	0.766503149	0.658782359	1.163514988	0.244620621	0.409956506	up_in_case
Yang2025_S	ENSGALG00010021387	SLC2A14	case	control	-0.121701941	0.095219832	-1.278115476	0.201208713	0.359053233	down_in_case
Yang2025_S	ENSGALG00010021388	DPH2	case	control	-0.176091041	0.316783033	-0.555872704	0.57829788	0.721061288	down_in_case
Yang2025_S	ENSGALG00010021390	GSTO2	case	control	0.459990017	0.372155366	1.236040266	0.216443597	0.377317191	up_in_case
Yang2025_S	ENSGALG00010021391	ENSGALG00010021391	case	control	0.584216965	0.404512285	1.444250241	0.148668597	0.290927805	up_in_case
Yang2025_S	ENSGALG00010021393	CCDC174	case	control	0.362267575	0.122707057	2.952296181	0.003154202	0.016957488	up_in_case
Yang2025_S	ENSGALG00010021394	MAN2C1	case	control	0.162701517	0.164592522	0.988510993	0.322902453	0.49219489	up_in_case
Yang2025_S	ENSGALG00010021396	MED6	case	control	-0.258448284	0.126765621	-2.038788451	0.041471145	0.11618424	down_in_case
Yang2025_S	ENSGALG00010021400	B4GALT2	case	control	-0.145657504	0.098411202	-1.48009069	0.138849046	0.277042018	down_in_case
Yang2025_S	ENSGALG00010021401	PDAP1	case	control	0.262656012	0.126358631	2.078655086	0.037649064	0.108240088	up_in_case
Yang2025_S	ENSGALG00010021402	ENSGALG00010021402	case	control	-0.139054081	0.104814949	-1.32666268	0.184620291	0.337977373	down_in_case
Yang2025_S	ENSGALG00010021404	UQCC1	case	control	-0.064081504	0.101598	-0.630735877	0.528213236	0.680974322	down_in_case
Yang2025_S	ENSGALG00010021405	IDE	case	control	-0.21842795	0.099973958	-2.184848486	0.028899958	0.089018769	down_in_case
Yang2025_S	ENSGALG00010021407	SIN3A	case	control	0.086332309	0.097441389	0.885992179	0.375621742	0.543743133	up_in_case
Yang2025_S	ENSGALG00010021408	TTC9	case	control	0.144920905	0.169482524	0.855078751	0.392507552	0.559491893	up_in_case
Yang2025_S	ENSGALG00010021409	MEGF6	case	control	0.961500057	0.266567518	3.606966303	0.000309798	0.002679723	up_in_case
Yang2025_S	ENSGALG00010021411	MCU	case	control	-0.136145487	0.085851422	-1.585826817	0.112778601	0.238840395	down_in_case
Yang2025_S	ENSGALG00010021412	ZNF319	case	control	-0.072997321	0.092572856	-0.788539145	0.430381411	0.594469037	down_in_case
Yang2025_S	ENSGALG00010021413	SYNJ2BP	case	control	0.409798514	0.222518603	1.841637095	0.065528251	0.162021761	up_in_case
Yang2025_S	ENSGALG00010021414	BUD31	case	control	0.118529237	0.156349219	0.758105716	0.448387698	0.610899743	up_in_case
Yang2025_S	ENSGALG00010021415	SIRT4	case	control	0.293593542	0.172240005	1.704560694	0.088276387	0.201330844	up_in_case
Yang2025_S	ENSGALG00010021416	PLEKHH3	case	control	0.381559462	0.151835955	2.512971721	0.011971893	0.046242589	up_in_case
Yang2025_S	ENSGALG00010021417	STK16	case	control	0.008857206	0.199023725	0.044503269	0.964503246	0.978920581	up_in_case
Yang2025_S	ENSGALG00010021419	MANSC1	case	control	-0.016559006	0.121655307	-0.136114132	0.891731056	0.936424676	down_in_case

Yang2025_S	ENSGALG00010021420	PLXNB3	case	control	-0.193386754	0.167108289	-1.157254109	0.247168557	0.41283637	down_in_case
Yang2025_S	ENSGALG00010021421	GLB1L	case	control	0.630706043	0.186873843	3.375036514	0.000738059	0.005491111	up_in_case
Yang2025_S	ENSGALG00010021424	PTCD1	case	control	-0.003022705	0.090365155	-0.0334499	0.973315818	0.983997944	down_in_case
Yang2025_S	ENSGALG00010021425	PTPN9	case	control	0.021768483	0.112779638	0.193017849	0.84694499	0.909398862	up_in_case
Yang2025_S	ENSGALG00010021426	SLC38A7	case	control	-0.061460262	0.10984285	-0.559529015	0.575800735	0.718602294	down_in_case
Yang2025_S	ENSGALG00010021427	RPLP0	case	control	-0.310758858	0.177107572	-1.754633382	0.079322038	0.186180326	down_in_case
Yang2025_S	ENSGALG00010021429	MED8	case	control	-0.062406756	0.132354891	-0.471510766	0.637276033	0.767673023	down_in_case
Yang2025_S	ENSGALG00010021431	CPSF4	case	control	-0.065821849	0.105215536	-0.625590593	0.531583511	0.683990417	down_in_case
Yang2025_S	ENSGALG00010021432	SYT9	case	control	-0.032755232	0.23991067	-0.136530951	0.891401558	0.936336879	down_in_case
Yang2025_S	ENSGALG00010021433	ENSGALG00010021433	case	control	0.039546275	0.147252979	0.268560101	0.78826822	0.872340982	up_in_case
Yang2025_S	ENSGALG00010021434	ANKZF1	case	control	0.423241402	0.162282663	2.60805063	0.009105948	0.03791901	up_in_case
Yang2025_S	ENSGALG00010021435	GCN1	case	control	-0.084077826	0.080711201	-1.041711987	0.29754523	0.466609586	down_in_case
Yang2025_S	ENSGALG00010021436	ENSGALG00010021436	case	control	0.027379586	0.58036991	0.047176095	0.96237288	0.977667571	up_in_case
Yang2025_S	ENSGALG00010021438	GOT2	case	control	-0.278718141	0.206146597	-1.352038523	0.176362992	0.327202542	down_in_case
Yang2025_S	ENSGALG00010021439	ITPRIP	case	control	-0.547577255	0.131194485	-4.173782584	2.99584E-05	0.000405704	down_in_case
Yang2025_S	ENSGALG00010021441	ENSGALG00010021441	case	control	0.497694625	0.253059553	1.966709495	0.049216712	0.131789946	up_in_case
Yang2025_S	ENSGALG00010021443	SNUPN	case	control	-0.229710121	0.240602676	-0.954728038	0.339715247	0.507714712	down_in_case
Yang2025_S	ENSGALG00010021444	ATG9A	case	control	0.043622463	0.068895546	0.633168125	0.526623859	0.679616917	up_in_case
Yang2025_S	ENSGALG00010021446	HOXB8	case	control	1.178950525	0.5675204	2.077371185	0.037767314	0.108506737	up_in_case
Yang2025_S	ENSGALG00010021447	OCSTAMP	case	control	1.251949549	0.605983037	2.065981179	0.038830257	0.11079026	up_in_case
Yang2025_S	ENSGALG00010021448	SZT2	case	control	0.076551435	0.117216422	0.653077736	0.513706165	0.668518435	up_in_case
Yang2025_S	ENSGALG00010021450	OLFML1	case	control	-0.499615248	0.230203136	-2.170323375	0.029982357	0.091563477	down_in_case
Yang2025_S	ENSGALG00010021452	SLC25A1	case	control	0.008810246	0.216433833	0.04070642	0.967529944	0.980832953	up_in_case
Yang2025_S	ENSGALG00010021454	ATP5J2	case	control	-0.366851735	0.347647519	-1.055240478	0.291315356	0.460049805	down_in_case
Yang2025_S	ENSGALG00010021455	CFAP58	case	control	0.691229609	0.406675982	1.699706005	0.089186239	0.202610082	up_in_case
Yang2025_S	ENSGALG00010021457	USB1	case	control	0.03286564	0.23202331	0.14164801	0.887358043	0.934149303	up_in_case
Yang2025_S	ENSGALG00010021458	USP30	case	control	0.231153478	0.111070255	2.081146555	0.037420494	0.107716294	up_in_case
Yang2025_S	ENSGALG00010021459	ARHGEF16	case	control	0.393029339	0.110230221	3.565531635	0.000363119	0.003056035	up_in_case
Yang2025_S	ENSGALG00010021461	SNX33	case	control	0.156995049	0.120209514	1.306011839	0.191548531	0.346698115	up_in_case
Yang2025_S	ENSGALG00010021462	SETD5	case	control	-0.101067703	0.087287678	-1.157869077	0.246917468	0.412664299	down_in_case
Yang2025_S	ENSGALG00010021465	ABCB6	case	control	0.363224641	0.207724216	1.748590742	0.080361788	0.187892871	up_in_case
Yang2025_S	ENSGALG00010021466	HES4	case	control	0.166393642	0.149733081	1.111268401	0.266452837	0.433641132	up_in_case
Yang2025_S	ENSGALG00010021467	LHFPL4	case	control	0.807180101	0.222404215	3.629338136	0.000284149	0.002495031	up_in_case
Yang2025_S	ENSGALG00010021469	CSPG4	case	control	0.20404668	0.137464561	1.484358434	0.137713856	0.275447478	up_in_case
Yang2025_S	ENSGALG00010021473	SLC8A3	case	control	0.216353083	0.114900689	1.88295722	0.059706166	0.152019993	up_in_case
Yang2025_S	ENSGALG00010021474	PPFIBP2	case	control	0.601614517	0.225884898	2.663367596	0.007736285	0.033342673	up_in_case
Yang2025_S	ENSGALG00010021476	ALKBH2	case	control	-0.074505664	0.21955077	-0.339355057	0.734342271	0.837882547	down_in_case
Yang2025_S	ENSGALG00010021477	ZFAND2B	case	control	0.61393589	0.22227738	2.762025945	0.005744391	0.026549948	up_in_case
Yang2025_S	ENSGALG00010021478	SLC13A3	case	control	0.394307874	0.238733224	1.651667361	0.09860238	0.216910808	up_in_case
Yang2025_S	ENSGALG00010021479	SKAP1	case	control	-0.986593233	0.586221732	-1.682969393	0.092381017	0.207533366	down_in_case
Yang2025_S	ENSGALG00010021480	ENSGALG00010021480	case	control	-0.136632624	0.157540668	-0.867284778	0.385785995	0.553249752	down_in_case
Yang2025_S	ENSGALG00010021481	MICU1	case	control	0.800693245	0.137302128	5.83161569	5.48932E-09	2.65603E-07	up_in_case
Yang2025_S	ENSGALG00010021483	UNG	case	control	-0.494877174	0.293969471	-1.683430501	0.092291783	0.207461931	down_in_case
Yang2025_S	ENSGALG00010021484	MARCHF5	case	control	-0.37539142	0.093308063	-4.0231402	5.74273E-05	0.00068339	down_in_case
Yang2025_S	ENSGALG00010021485	SMOC1	case	control	0.18532819	0.290165012	0.638699298	0.523018569	0.676467771	up_in_case
Yang2025_S	ENSGALG00010021487	KLHL17	case	control	-0.10539619	0.125742818	-0.838188548	0.401924818	0.568495974	down_in_case
Yang2025_S	ENSGALG00010021488	HYI	case	control	0.3662649	0.204945715	1.787131288	0.073916272	0.176905528	up_in_case
Yang2025_S	ENSGALG00010021489	CNPPD1	case	control	-0.053126562	0.089220857	-0.595450036	0.551542688	0.699594698	down_in_case
Yang2025_S	ENSGALG00010021490	ACACB	case	control	0.107709993	0.17297213	0.622701432	0.533480742	0.685355779	up_in_case
Yang2025_S	ENSGALG00010021492	PARP6	case	control	0.210845557	0.10137695	2.079817523	0.037542273	0.107977622	up_in_case
Yang2025_S	ENSGALG00010021493	UNC93B1	case	control	0.154188061	0.148559286	1.037889084	0.299321696	0.468867787	up_in_case
Yang2025_S	ENSGALG00010021494	SLC2A10	case	control	-0.406209705	0.264017439	-1.538571489	0.123908943	0.255086243	down_in_case
Yang2025_S	ENSGALG00010021496	MTMR14	case	control	0.12556427	0.14053247	0.893489384	0.371595155	0.540012356	up_in_case
Yang2025_S	ENSGALG00010021498	B3GALT6	case	control	-0.305810382	0.110585547	-2.765373866	0.005685759	0.026366406	down_in_case
Yang2025_S	ENSGALG00010021502	DNAJB12	case	control	0.026486591	0.086039643	0.307841717	0.758202776	0.85295517	up_in_case
Yang2025_S	ENSGALG00010021503	KCNS1	case	control	0.752389171	0.43861648	1.715369134	0.086277571	0.197743083	up_in_case
Yang2025_S	ENSGALG00010021504	ODR4	case	control	-0.023234235	0.104125628	-0.223136566	0.82342922	0.894361721	down_in_case
Yang2025_S	ENSGALG00010021505	IHH	case	control	1.226224804	0.766852928	1.599035173	0.109812788	0.233771861	up_in_case
Yang2025_S	ENSGALG00010021506	MAX	case	control	0.950900533	0.2277128	4.175876518	2.96841E-05	0.000402773	up_in_case
Yang2025_S	ENSGALG00010021509	DDIT4	case	control	0.459153482	0.241188535	1.903711893	0.056947717	0.146929537	up_in_case
Yang2025_S	ENSGALG00010021510	NTSR1	case	control	-0.464254142	0.41557392	-1.117139742	0.26393458	0.431024068	down_in_case
Yang2025_S	ENSGALG00010021512	CPNE9	case	control	0.406183292	0.399570109	1.016550744	0.309367196	0.479216527	up_in_case
Yang2025_S	ENSGALG00010021513	SDF4	case	control	-0.315671862	0.045388848	-6.954833043	3.5298E-12	4.39176E-10	down_in_case
Yang2025_S	ENSGALG00010021514	TPR	case	control	0.537268905	0.181505526	2.960069137	0.0030757	0.016638153	up_in_case
Yang2025_S	ENSGALG00010021516	SRSF5	case	control	0.97517438	0.108158137	9.016190588	1.94743E-19	9.35776E-17	up_in_case
Yang2025_S	ENSGALG00010021518	CPEB3	case	control	-0.072893912	0.180812088	-0.403147338	0.686639838	0.80422764	down_in_case
Yang2025_S	ENSGALG00010021519	FNTB	case	control	0.303172221	0.185212307	1.63689026	0.101653401	0.22175018	up_in_case
Yang2025_S	ENSGALG00010021521	ENSGALG00010021521	case	control	0.535859486	0.268419758	1.996348885	0.04589596	0.125354804	up_in_case
Yang2025_S	ENSGALG00010021523	CELF6	case	control	0.574456213	0.196763552	2.919525532	0.003505647	0.018296325	up_in_case
Yang2025_S	ENSGALG00010021525	ENSGALG00010021525	case	control	-0.154837627	0.139905443	-1.106730542	0.268410446	0.435475558	down_in_case
Yang2025_S	ENSGALG00010021526	KCNJ12	case	control	-0.328762134	0.171184038	-1.920518627	0.054792423	0.142651907	down_in_case
Yang2025_S	ENSGALG00010021528	BRPF1	case	control	-0.160556893	0.095503791	-1.681157269	0.092732371	0.208007484	down_in_case
Yang2025_S	ENSGALG00010021529	BTAF1	case	control	0.089746896	0.139257236	0.644468454	0.519271707	0.673246301	up_in_case
Yang2025_S	ENSGALG00010021530	MLF2	case	control	0.011689764	0.099768703	0.117168646	0.906726413	0.944055033	up_in_case
Yang2025_S	ENSGALG00010021531	RAB15	case	control	1.057119873	0.286354479	3.691647771	0.000222806	0.002030608	up_in_case
Yang2025_S	ENSGALG00010021532	DPP10	case	control	0.132621012	0.158580615	0.836300273	0.402986	0.569303519	up_in_case
Yang2025_S	ENSGALG00010021533	SSX2IP	case	control	0.62444879	0.221415815	2.820253789	0.004798568	0.023266544	up_in_case
Yang2025_S	ENSGALG00010021534	KIFC3	case	control	0.540065171	0.101126616	5.340484948	9.26983E-08	3.21331E-06	up_in_case
Yang2025_S	ENSGALG00010021535	SUSD6	case	control	-0.132540666	0.063495369	-2.087406796	0.036851378	0.106695191	down_in_case
Yang2025_S	ENSGALG00010021538	TNFRSF18	case	control	0.272625891	0.497720699	0.54774875	0.583864433	0.725923526	up_in_case
Yang2025_S	ENSGALG00010021539	HEXA	case	control	-0.213219162	0.130029647	-1.639773438	0.101052283	0.220715292	down_in_case
Yang2025_S	ENSGALG00010021540	STK4	case	control	-0.077272262	0.07897421	-0.97844932	0.327852145	0.497564348	down_in_case
Yang2025_S	ENSGALG00010021541	CYP27A1	case	control	-0.236909475	0.168932128	-1.402394432	0.160797495	0.306485172	down_in_case
Yang2025_S	ENSGALG00010021543	PLEKHD1	case	control	1.134081214	0.227744508	4.97962046	6.37091E-07	1.63798E-05	up_in_case
Yang2025_S	ENSGALG00010021545	CYB5R2	case	control	0.603178426	0.202927126	2.97238934	0.002954917	0.016128778	up_in_case
Yang2025_S	ENSGALG00010021546	OGG1	case	control	0.029495711	0.336029018	0.087777273	0.930053701	0.957822654	up_in_case
Yang2025_S	ENSGALG00010021549	ANAPC16	case	control	0.33123047	0.168636562	1.96416759	0.04951066	0.132322794	up_in_case
Yang2025_S	ENSGALG00010021550	MAP2K3	case	control	0.17140397	0.129998387	1.318508439	0.187333502	0.341426182	up_in_case
Yang2025_S	ENSGALG00010021552	ENSGALG00010021552	case	control	0.247477891	0.180403119	1.371804948	0.170124167	0.319068676	up_in_case
Yang2025_S	ENSGALG00010021553	SLC39A9	case	control	-0.621761921	0.110747159	-5.61424715	1.9742E-08	8.51717E-07	down_in_case
Yang2025_S	ENSGALG00010021554	SNX11	case	control	-0.263036684	0.257045403	-1.023308262	0.306162119	0.475891704	down_in_case
Yang2025_S	ENSGALG00010021556	CAMK1	case	control	0.00322613	0.20211651	0.015961734	0.987264919	0.992277608	up_in_case
Yang2025_S	ENSGALG00010021558	CHURC1	case	control	0.424517931	0.260842693	1.627486381	0.103633839	0.225041102	up_in_case
Yang2025_S	ENSGALG00010021561	TGIF2	case	control	-0.587030014	0.153242679	-3.830721419	0.000127768	0.001296232	down_in_case
Yang2025_S	ENSGALG00010021562	LRP6	case	control	0.184208555	0.199859684	0.921689414	0.356690604	0.525982811	up_in_case
Yang2025_S	ENSGALG00010021564	PRKAG3	case	control	1.298631682	0.25083655	5.177202767	2.25237E-07	6.79369E-06	up_in_case
Yang2025_S	ENSGALG00010021565	CBX1	case	control	0.012491736	0.096815839	0.129025753	0.897337272	0.93902049	up_in_case
Yang2025_S	ENSGALG00010021566	ENSGALG00010021566	case	control	0.257843217	0.116319356	2.21668368	0.026644715	0.083851423	up_in_case
Yang2025_S	ENSGALG00010021568	TADA3	case	control	0.065821357	0.218012582	0.301915404	0.762716553	0.855408866	up_in_case
Yang2025_S	ENSGALG00010021570	KATNB1	case	control	0.136060001	0.095580282	1.423515382	0.154586806	0.298882672	up_in_case
Yang2025_S	ENSGALG00010021572	MYL9	case	control	-0.291588007	0.124828423	-2.335910354	0.019495914	0.066554523	down_in_case
Yang2025_S	ENSGALG00010021573	NATD1	case	control	-0.223698102	0.193786421	-1.154353854	0.248355126	0.414119	down_in_case
Yang2025_S	ENSGALG00010021574	TTLL4	case	control	-0.150185362	0.130983671	-1.146596061	0.251548605	0.417277905	down_in_case
Yang2025_S	ENSGALG00010021575	ADGRL2	case	control	0.076520331	0.150719473	0.507700363	0.611663494	0.74886055	up_in_case
Yang2025_S	ENSGALG00010021576	ERH	case	control	-0.015017262	0.148353373	-0.101226295	0.919370823	0.952452042	down_in_case
Yang2025_S	ENSGALG00010021577	ARIH1	case	control	0.154721549	0.115142571	1.343738873	0.179032842	0.330719549	up_in_case
Yang2025_S	ENSGALG00010021579	C20H20ORF24	case	control	-0.301285928	0.101174514	-2.977883627	0.002902462	0.015895503	down_in_case
Yang2025_S	ENSGALG00010021580	KCTD10	case	control	-0.078029191	0.072469907	-1.076711614	0.281609122	0.449510094	down_in_case
Yang2025_S	ENSGALG00010021582	STK36	case	control	0.175990496	0.11727225	1.500700264	0.133433105	0.269125824	up_in_case
Yang2025_S	ENSGALG00010021583	NFE2L1	case	control	0.014932795	0.078981625	0.189066696	0.850040544	0.910897799	up_in_case
Yang2025_S	ENSGALG00010021584	OVCH2	case	control	0.352505703	0.41118099	0.857300583	0.391278787	0.55852861	up_in_case
Yang2025_S	ENSGALG00010021586	BBS4	case	control	-0.265608038	0.182036787	-1.459089898	0.144540366	0.285454932	down_in_case
Yang2025_S	ENSGALG00010021588	RNF25	case	control	0.327394419	0.124401745	2.631751027	0.008494609	0.035892169	up_in_case
Yang2025_S	ENSGALG00010021593	DCAF7	case	control	-0.64827618	0.131973738	-4.912160478	9.00783E-07	2.20993E-05	down_in_case
Yang2025_S	ENSGALG00010021594	ADPGK	case	control	-0.432095257	0.125006497	-3.456582398	0.000547072	0.004263758	down_in_case
Yang2025_S	ENSGALG00010021595	UBE3B	case	control	-0.082577569	0.0926938	-0.890863992	0.37300214	0.541210414	down_in_case
Yang2025_S	ENSGALG00010021597	GGT7	case	control	-0.304803124	0.124913339	-2.440116692	0.014682518	0.054022716	down_in_case
Yang2025_S	ENSGALG00010021599	ARPC4	case	control	0.126343728	0.219514284	0.575560393	0.564912359	0.710730744	up_in_case
Yang2025_S	ENSGALG00010021600	FMN1	case	control	0.21792485	0.271029254	0.80406394	0.421360049	0.586989132	up_in_case
Yang2025_S	ENSGALG00010021602	PLEKHG4	case	control	-0.242586888	0.124564468	-1.947480625	0.05147714	0.136090676	down_in_case
Yang2025_S	ENSGALG00010021604	SPTB	case	control	0.25710562	0.100025669	2.57039641	0.01015822	0.04098286	up_in_case
Yang2025_S	ENSGALG00010021605	MAP3K3	case	control	0.012996153	0.109959965	0.118189862	0.905917222	0.943647271	up_in_case
Yang2025_S	ENSGALG00010021606	RNF8	case	control	-0.071302852	0.099494279	-0.716652787	0.473588365	0.63298042	down_in_case
Yang2025_S	ENSGALG00010021607	SLC9A5	case	control	0.463492791	0.218558714	2.120678621	0.033948857	0.100313257	up_in_case
Yang2025_S	ENSGALG00010021609	ENSGALG00010021609	case	control	0.330456462	0.221355461	1.492876933	0.135469404	0.272051614	up_in_case
Yang2025_S	ENSGALG00010021610	TMEM11	case	control	0.068200161	0.149755383	0.455410411	0.648814007	0.776913715	up_in_case

Yang2025_S	ENSGALG00010021611	GREM1	case	control	0.119424785	0.426285358	0.280152209	0.779360731	0.866220799	up_in_case
Yang2025_S	ENSGALG00010021612	BCS1L	case	control	0.21287956	0.190590227	1.116948979	0.264016141	0.431074903	up_in_case
Yang2025_S	ENSGALG00010021613	ADGRL4	case	control	-0.501020542	0.07818462	-6.408172614	1.47274E-10	1.12146E-08	down_in_case
Yang2025_S	ENSGALG00010021615	FLVCR1	case	control	-0.054575573	0.245131453	-0.222637988	0.823817268	0.894465038	down_in_case
Yang2025_S	ENSGALG00010021616	ENSGALG00010021616	case	control	2.169497192	0.776391991	2.794332266	0.005200701	0.024683846	up_in_case
Yang2025_S	ENSGALG00010021617	SORBS1	case	control	0.458108622	0.178704804	2.563493605	0.010362461	0.041605579	up_in_case
Yang2025_S	ENSGALG00010021618	NEO1	case	control	0.071884241	0.203964642	0.352434813	0.724512198	0.830611257	up_in_case
Yang2025_S	ENSGALG00010021619	FHOD1	case	control	0.186977667	0.153395738	1.218923485	0.222873235	0.384326015	up_in_case
Yang2025_S	ENSGALG00010021621	ZNF518A	case	control	-0.069365512	0.168964395	-0.410533305	0.681414777	0.800413294	down_in_case
Yang2025_S	ENSGALG00010021622	PTHLH	case	control	-0.249429474	0.217439333	-1.147122148	0.251331141	0.417042981	down_in_case
Yang2025_S	ENSGALG00010021623	FAR1	case	control	-0.021010603	0.102194693	-0.205593874	0.837108163	0.903431092	down_in_case
Yang2025_S	ENSGALG00010021624	HSD11B2	case	control	-0.271331027	0.390045973	-0.695638582	0.486655198	0.643918982	down_in_case
Yang2025_S	ENSGALG00010021626	ZNF142	case	control	-0.058681154	0.098626064	-0.594986272	0.551852647	0.699924151	down_in_case
Yang2025_S	ENSGALG00010021627	NCOA6	case	control	0.48548306	0.203228885	2.388848712	0.016901261	0.059928515	up_in_case
Yang2025_S	ENSGALG00010021628	REC114	case	control	-0.211295056	0.300924791	-0.702152372	0.482584144	0.640824285	down_in_case
Yang2025_S	ENSGALG00010021629	DHRS7B	case	control	-0.170436304	0.130275657	-1.308274373	0.190780264	0.345801508	down_in_case
Yang2025_S	ENSGALG00010021630	VTG1	case	control	0.446219911	0.400945438	1.112919287	0.265743096	0.432962708	up_in_case
Yang2025_S	ENSGALG00010021632	LIMD2	case	control	0.059832186	0.204590868	0.292447981	0.769944128	0.859723027	up_in_case
Yang2025_S	ENSGALG00010021633	CCNJ	case	control	0.025622897	0.123913154	0.206781089	0.836180826	0.902989756	up_in_case
Yang2025_S	ENSGALG00010021634	PLCD4	case	control	0.83521646	0.15839213	5.273093162	1.34143E-07	4.39833E-06	up_in_case
Yang2025_S	ENSGALG00010021635	FOXJ2	case	control	-0.019457602	0.095394293	-0.203970288	0.838376717	0.904449915	down_in_case
Yang2025_S	ENSGALG00010021636	C1orf159	case	control	-0.23130787	0.13936998	-1.659667817	0.096981297	0.214581514	down_in_case
Yang2025_S	ENSGALG00010021637	ATP6V0D1	case	control	-0.056480886	0.136906838	-0.412549777	0.679936504	0.799187023	down_in_case
Yang2025_S	ENSGALG00010021638	SRC	case	control	0.123055894	0.092445419	1.331119429	0.183149717	0.336345717	up_in_case
Yang2025_S	ENSGALG00010021639	SCG5	case	control	1.031750775	0.428928739	2.405413025	0.016154197	0.058032671	up_in_case
Yang2025_S	ENSGALG00010021640	PLEKHG3	case	control	0.511365022	0.112446033	4.547648407	5.42487E-06	9.93371E-05	up_in_case
Yang2025_S	ENSGALG00010021642	NPTN	case	control	0.049067377	0.108724454	0.451300284	0.65177314	0.779409484	up_in_case
Yang2025_S	ENSGALG00010021643	ENSGALG00010021643	case	control	-0.186868091	0.133754732	-1.397095173	0.162384953	0.30862443	down_in_case
Yang2025_S	ENSGALG00010021644	TARDBP	case	control	0.127698604	0.087588651	1.457935514	0.144858318	0.285867306	up_in_case
Yang2025_S	ENSGALG00010021645	ASCC1	case	control	0.267436185	0.153645566	1.740604642	0.081752903	0.190156353	up_in_case
Yang2025_S	ENSGALG00010021646	RPUSD3	case	control	0.39906907	0.206652683	1.931110033	0.053469446	0.140161162	up_in_case
Yang2025_S	ENSGALG00010021647	ARHGAP11B	case	control	-0.443803039	0.103204953	-4.300210667	1.70636E-05	0.000254856	down_in_case
Yang2025_S	ENSGALG00010021648	BRE	case	control	-0.259205661	0.071102502	-3.645520965	0.000266851	0.002368512	down_in_case
Yang2025_S	ENSGALG00010021649	PXN	case	control	-0.013944314	0.069230519	-0.201418603	0.84037127	0.905270072	down_in_case
Yang2025_S	ENSGALG00010021650	CD276	case	control	-0.036917056	0.083298539	-0.443189721	0.657628513	0.783989505	down_in_case
Yang2025_S	ENSGALG00010021651	RNF113B	case	control	0.350921608	0.099669916	3.52083779	0.000430186	0.003501541	up_in_case
Yang2025_S	ENSGALG00010021652	BORCS5	case	control	-0.145853928	0.123553237	-1.180494598	0.237803558	0.401719796	down_in_case
Yang2025_S	ENSGALG00010021655	USP22	case	control	0.306469037	0.14727206	2.080972027	0.037436467	0.107740017	up_in_case
Yang2025_S	ENSGALG00010021656	SETD6	case	control	0.383357024	0.0961436	3.987337946	6.68188E-05	0.000772073	up_in_case
Yang2025_S	ENSGALG00010021657	JAGN1	case	control	0.278823225	0.205777184	1.354976386	0.175425081	0.325896347	up_in_case
Yang2025_S	ENSGALG00010021658	STRADA	case	control	-0.065380649	0.090509637	-0.722361187	0.470072443	0.630397411	down_in_case
Yang2025_S	ENSGALG00010021659	SPOCK2	case	control	0.382043721	0.172305376	2.217247827	0.026606161	0.083805799	up_in_case
Yang2025_S	ENSGALG00010021660	RNF223	case	control	0.573500357	0.257726831	2.22522566	0.026066093	0.082458798	up_in_case
Yang2025_S	ENSGALG00010021661	DUSP16	case	control	-0.274807679	0.113148729	-2.428729708	0.015151823	0.055475738	down_in_case
Yang2025_S	ENSGALG00010021662	RASGRP1	case	control	-0.203728388	0.193226442	-1.054350461	0.291722494	0.46027547	down_in_case
Yang2025_S	ENSGALG00010021663	CNOT9	case	control	-0.117694205	0.120912619	-0.973382318	0.330363324	0.499777142	down_in_case
Yang2025_S	ENSGALG00010021664	TYSND1	case	control	0.820468809	0.192022342	4.272777844	1.93053E-05	0.000282287	up_in_case
Yang2025_S	ENSGALG00010021665	CNOT1	case	control	-0.075884226	0.11288231	-0.67224197	0.501429666	0.658073309	down_in_case
Yang2025_S	ENSGALG00010021666	CCDC47	case	control	-0.111947664	0.114641133	-0.976505216	0.328814164	0.498425474	down_in_case
Yang2025_S	ENSGALG00010021667	TBX15	case	control	-0.123200412	0.101010151	-1.219683478	0.222584887	0.384122383	down_in_case
Yang2025_S	ENSGALG00010021668	PYGO1	case	control	-0.315431185	0.188203482	-1.676011418	0.093735956	0.209383792	down_in_case
Yang2025_S	ENSGALG00010021669	ENSGALG00010021669	case	control	-0.168521773	0.150346045	-1.120892627	0.26233357	0.429214312	down_in_case
Yang2025_S	ENSGALG00010021670	BLCAP	case	control	-0.60169373	0.164350422	-3.661041585	0.000251192	0.002252484	down_in_case
Yang2025_S	ENSGALG00010021671	TNFRSF13B	case	control	-0.945831538	0.563562278	-1.678308814	0.093286828	0.208755561	down_in_case
Yang2025_S	ENSGALG00010021674	ING4	case	control	0.397690868	0.119682955	3.322869718	0.000890965	0.006373511	up_in_case
Yang2025_S	ENSGALG00010021675	DDX42	case	control	0.181361404	0.084728406	2.140502947	0.032314143	0.096879858	up_in_case
Yang2025_S	ENSGALG00010021676	TP53INP2	case	control	0.639699678	0.191067326	3.348032825	0.000813874	0.005928557	up_in_case
Yang2025_S	ENSGALG00010021677	ENSGALG00010021677	case	control	0.287210484	0.158099263	1.816646576	0.069271247	0.168757837	up_in_case
Yang2025_S	ENSGALG00010021678	DNAAF4	case	control	0.649634576	0.24361423	2.666652829	0.007661077	0.033133799	up_in_case
Yang2025_S	ENSGALG00010021679	MASP2	case	control	0.193603162	0.449444238	0.430761251	0.666641977	0.789228274	up_in_case
Yang2025_S	ENSGALG00010021680	PIGT	case	control	0.327147092	0.147355004	2.220128835	0.026410023	0.083309136	up_in_case
Yang2025_S	ENSGALG00010021681	BTBD10	case	control	-0.317041744	0.133056283	-2.38276418	0.017183198	0.060681161	down_in_case
Yang2025_S	ENSGALG00010021682	MYL4	case	control	0.475192316	0.245760143	1.933561359	0.053167082	0.139552324	up_in_case
Yang2025_S	ENSGALG00010021683	HFM1	case	control	0.640676735	0.340602525	1.88100994	0.059970567	0.152492868	up_in_case
Yang2025_S	ENSGALG00010021684	BIN1	case	control	-0.079286518	0.138032146	-0.574406183	0.56569297	0.711094153	down_in_case
Yang2025_S	ENSGALG00010021685	DBNDD2	case	control	0.159949144	0.152275233	1.050395007	0.29353654	0.462091244	up_in_case
Yang2025_S	ENSGALG00010021686	CCPG1	case	control	0.186435558	0.183898146	1.013797918	0.310679189	0.480394418	up_in_case
Yang2025_S	ENSGALG00010021687	ZNF384	case	control	0.028402631	0.093794866	0.302816481	0.762029723	0.855286959	up_in_case
Yang2025_S	ENSGALG00010021688	ITPR1	case	control	0.150793542	0.159130654	0.947608382	0.343328857	0.511907514	up_in_case
Yang2025_S	ENSGALG00010021689	MPRIP	case	control	0.216590697	0.140900569	1.537188227	0.124247222	0.255592714	up_in_case
Yang2025_S	ENSGALG00010021691	PIGB	case	control	0.130319082	0.179021228	0.727953238	0.466642212	0.627757821	up_in_case
Yang2025_S	ENSGALG00010021693	PPA1	case	control	0.181134984	0.175230345	1.03369644	0.301278098	0.470873743	up_in_case
Yang2025_S	ENSGALG00010021695	FNBP4	case	control	0.966955022	0.205779487	4.698986462	2.61456E-06	5.43789E-05	up_in_case
Yang2025_S	ENSGALG00010021697	PIANP	case	control	0.24453666	0.24133377	1.013271619	0.31093044	0.480673174	up_in_case
Yang2025_S	ENSGALG00010021698	GYPC	case	control	-0.230389397	0.138258236	-1.666370146	0.095639713	0.212583268	down_in_case
Yang2025_S	ENSGALG00010021699	SYS1	case	control	-0.07831363	0.271520547	-0.288426165	0.773020542	0.86190515	down_in_case
Yang2025_S	ENSGALG00010021700	FLCN	case	control	-0.102676082	0.091420681	-1.123116572	0.261387989	0.428169933	down_in_case
Yang2025_S	ENSGALG00010021701	DR1	case	control	-0.086418852	0.100055509	-0.86370908	0.387747724	0.554921649	down_in_case
Yang2025_S	ENSGALG00010021702	PLD6	case	control	0.568146443	0.322730742	1.760434843	0.078334106	0.184576558	up_in_case
Yang2025_S	ENSGALG00010021703	CD82	case	control	-0.180622983	0.139075754	-1.298738122	0.194033817	0.349517996	down_in_case
Yang2025_S	ENSGALG00010021704	COPS7A	case	control	-0.297466979	0.103047078	-2.886709503	0.003892935	0.019813019	down_in_case
Yang2025_S	ENSGALG00010021705	CCDC18	case	control	1.040479023	0.153716197	6.768831417	1.29827E-11	1.3501E-09	up_in_case
Yang2025_S	ENSGALG00010021706	ENSGALG00010021706	case	control	0.208617506	0.18292744	1.14043856	0.254103636	0.42058845	up_in_case
Yang2025_S	ENSGALG00010021707	DLX2	case	control	2.639412837	0.683080937	3.863982575	0.000111553	0.00116007	up_in_case
Yang2025_S	ENSGALG00010021708	CNTNAP5	case	control	0.730372862	0.51579792	1.41600583	0.156773797	0.301913055	up_in_case
Yang2025_S	ENSGALG00010021710	ENSGALG00010021710	case	control	0.339664792	0.282239257	1.203464025	0.228796805	0.391152432	up_in_case
Yang2025_S	ENSGALG00010021711	ENSGALG00010021711	case	control	2.4218971	0.3773804	6.417654708	1.3839E-10	1.07735E-08	up_in_case
Yang2025_S	ENSGALG00010021713	RAB27A	case	control	-0.142475828	0.101531684	-1.403264704	0.160537919	0.306244432	down_in_case
Yang2025_S	ENSGALG00010021714	TSPAN18	case	control	-0.593187305	0.091211768	-6.503407566	7.85207E-11	6.85909E-09	down_in_case
Yang2025_S	ENSGALG00010021715	MMAB	case	control	0.391320765	0.14784501	2.646831064	0.008124993	0.034613812	up_in_case
Yang2025_S	ENSGALG00010021716	TFCP2L1	case	control	-0.301512254	0.290893739	-1.036503074	0.299967506	0.469615458	down_in_case
Yang2025_S	ENSGALG00010021717	SDC4	case	control	-0.48318649	0.155926579	-3.098807747	0.001943011	0.011731305	down_in_case
Yang2025_S	ENSGALG00010021718	SST7	case	control	0.604081263	0.656284942	0.920455771	0.357334636	0.526536761	up_in_case
Yang2025_S	ENSGALG00010021719	BHLHE40	case	control	0.201666778	0.149154755	1.352064019	0.176354837	0.327202542	up_in_case
Yang2025_S	ENSGALG00010021720	CACNB1	case	control	0.021919921	0.099002177	0.221408474	0.824774395	0.895111463	up_in_case
Yang2025_S	ENSGALG00010021721	TP53I11	case	control	-0.212757532	0.19160862	-1.110375574	0.266837219	0.433984203	down_in_case
Yang2025_S	ENSGALG00010021722	AARS1	case	control	-0.04032601	0.107911619	-0.373694788	0.708631403	0.820223694	down_in_case
Yang2025_S	ENSGALG00010021723	COPS3	case	control	0.133195436	0.104958392	1.269030829	0.204430069	0.362664928	up_in_case
Yang2025_S	ENSGALG00010021725	SH2D6	case	control	0.467170831	0.303190843	1.540847427	0.123353924	0.254468827	up_in_case
Yang2025_S	ENSGALG00010021726	ARNTL	case	control	0.262518698	0.157158493	1.670407326	0.094838801	0.211182278	up_in_case
Yang2025_S	ENSGALG00010021727	MVK	case	control	-0.431362912	0.175563114	-2.457024729	0.014009304	0.052072461	down_in_case
Yang2025_S	ENSGALG00010021728	TMED5	case	control	0.176419932	0.091564575	1.926726932	0.05401367	0.141242351	up_in_case
Yang2025_S	ENSGALG00010021729	RBPJL	case	control	1.473850703	0.618180397	2.384175735	0.017117426	0.060525585	up_in_case
Yang2025_S	ENSGALG00010021730	ARL8B	case	control	-0.18222038	0.070040554	-2.601641066	0.00927789	0.038432639	down_in_case
Yang2025_S	ENSGALG00010021731	SRM	case	control	0.475593185	0.117262322	4.055805641	4.99618E-05	0.000609114	up_in_case
Yang2025_S	ENSGALG00010021732	CASR	case	control	0.405013485	0.278852795	1.452427562	0.146382757	0.287950836	up_in_case
Yang2025_S	ENSGALG00010021733	ENSGALG00010021733	case	control	-0.185675105	0.121042222	-1.53396974	0.125037097	0.256952065	down_in_case
Yang2025_S	ENSGALG00010021734	PARD3B	case	control	-0.161152327	0.188043011	-0.856997162	0.391446453	0.558607918	down_in_case
Yang2025_S	ENSGALG00010021735	PIGBOS1	case	control	0.943090721	0.407648849	2.313488004	0.020695824	0.069411153	up_in_case
Yang2025_S	ENSGALG00010021736	EDEM1	case	control	-0.250093231	0.108387835	-2.307392077	0.021032973	0.070387721	down_in_case
Yang2025_S	ENSGALG00010021737	AGBL2	case	control	1.603317444	0.577102642	2.77821886	0.005465779	0.025624213	up_in_case
Yang2025_S	ENSGALG00010021738	PRDM11	case	control	-0.373189452	0.110883587	-3.365596848	0.000763782	0.005628979	down_in_case
Yang2025_S	ENSGALG00010021739	EXOSC10	case	control	0.176114817	0.076964617	2.288256904	0.022122565	0.072844503	up_in_case
Yang2025_S	ENSGALG00010021740	PDLIM1	case	control	0.196663758	0.164136645	1.198170939	0.230850476	0.393601051	up_in_case
Yang2025_S	ENSGALG00010021741	RPL19	case	control	0.063994494	0.219077115	0.292109443	0.770202947	0.859929338	up_in_case
Yang2025_S	ENSGALG00010021742	MATN4	case	control	1.404947734	0.521594054	2.69356547	0.007069226	0.031174484	up_in_case
Yang2025_S	ENSGALG00010021743	SYT13	case	control	-0.631634242	0.629963584	-1.002651992	0.316028801	0.485273977	down_in_case
Yang2025_S	ENSGALG00010021744	LITAF	case	control	-0.278668711	0.315914497	-0.882101688	0.37772182	0.546068426	down_in_case
Yang2025_S	ENSGALG00010021745	RSL24D1	case	control	-0.044541718	0.123610813	-0.360338362	0.718594115	0.826485326	down_in_case
Yang2025_S	ENSGALG00010021746	MTOR	case	control	-0.052278688	0.112156592	-0.466122298	0.641127964	0.770838258	down_in_case
Yang2025_S	ENSGALG00010021747	AKTIP	case	control	-0.002330642	0.129564229	-0.017988312	0.985648177	0.991410954	down_in_case
Yang2025_S	ENSGALG00010021748	CDCA7	case	control	-0.383598312	0.176988611	-2.167361555	0.030207296	0.092129279	down_in_case
Yang2025_S	ENSGALG00010021749	CLASP1	case	control	0.179301047	0.144455933	1.241216219	0.214525885	0.375049246	up_in_case
Yang2025_S	ENSGALG00010021750	HELLS	case	control	0.012054706	0.10422511	0.115660282	0.907921785	0.944876033	up_in_case
Yang2025_S	ENSGALG00010021751	MTCH2	case	control	0.0682178	0.186189735	0.366388617	0.714075112	0.823558969	up_in_case
Yang2025_S	ENSGALG00010021752	CSTB	case	control	-0.390505407	0.169705534	-2.301076444	0.021387311	0.071105219	down_in_case

Yang2025_S	ENSGALG00010021753	MTF2	case	control	0.136666209	0.070980225	1.92541245	0.054177779	0.141538687	up_in_case
Yang2025_S	ENSGALG00010021754	SLC17A9	case	control	0.322344218	0.416303848	0.774300358	0.438753192	0.602595511	up_in_case
Yang2025_S	ENSGALG00010021755	ENSGALG00010021755	case	control	-0.032511901	0.132978189	-0.244490475	0.806850978	0.883642595	down_in_case
Yang2025_S	ENSGALG00010021756	USP42	case	control	1.098922129	0.313186673	3.508840652	0.000450064	0.003627327	up_in_case
Yang2025_S	ENSGALG00010021757	ZP4	case	control	0.580797042	0.376155433	1.544034704	0.122579925	0.253623053	up_in_case
Yang2025_S	ENSGALG00010021758	RASSF10	case	control	1.303424	0.671637823	1.940664978	0.052298927	0.137792692	up_in_case
Yang2025_S	ENSGALG00010021759	NIFK	case	control	-0.099477611	0.138291267	-0.719334003	0.47193515	0.631861675	down_in_case
Yang2025_S	ENSGALG00010021760	CSTA	case	control	-0.258518485	0.292144947	-0.884898019	0.376211637	0.544393474	down_in_case
Yang2025_S	ENSGALG00010021761	MAFB	case	control	0.833732689	0.361556593	2.305953492	0.021113231	0.070588502	up_in_case
Yang2025_S	ENSGALG00010021762	LAMB2	case	control	0.19362193	0.140534642	1.377752329	0.168279769	0.316632268	up_in_case
Yang2025_S	ENSGALG00010021763	ENSGALG00010021763	case	control	0.562948441	0.83420198	0.674834698	0.499780793	0.656527654	up_in_case
Yang2025_S	ENSGALG00010021765	EIF2AK1	case	control	0.296609051	0.076934243	3.855358038	0.00011556	0.001197274	up_in_case
Yang2025_S	ENSGALG00010021766	AGRN	case	control	-0.115352677	0.116603465	-0.98927315	0.322529517	0.491841631	down_in_case
Yang2025_S	ENSGALG00010021767	CCDC58	case	control	0.20415135	0.197456172	1.033907161	0.301179567	0.470772548	up_in_case
Yang2025_S	ENSGALG00010021768	DIPK1A	case	control	-0.417403783	0.131873905	-3.165173458	0.001549905	0.009848577	down_in_case
Yang2025_S	ENSGALG00010021769	ENSGALG00010021769	case	control	0.795279284	0.195434015	4.069298197	4.7155E-05	0.000580481	up_in_case
Yang2025_S	ENSGALG00010021770	RALGAPB	case	control	-0.090031917	0.117256865	-0.767817873	0.442595395	0.605892454	down_in_case
Yang2025_S	ENSGALG00010021771	ANKRD61	case	control	0.20342667	0.123458644	1.647731285	0.099407825	0.218011968	up_in_case
Yang2025_S	ENSGALG00010021772	TSN	case	control	-0.298925497	0.163240629	-1.831195451	0.067071375	0.164839438	down_in_case
Yang2025_S	ENSGALG00010021774	FBXL20	case	control	-0.202325927	0.078792253	-2.567840354	0.010233427	0.0412266	down_in_case
Yang2025_S	ENSGALG00010021775	CCDC36	case	control	-0.059515319	0.282391695	-0.210754497	0.833078847	0.900896612	down_in_case
Yang2025_S	ENSGALG00010021776	PMS2	case	control	-0.241826296	0.111760564	-2.163789152	0.030480535	0.092800143	down_in_case
Yang2025_S	ENSGALG00010021778	RBL2	case	control	-0.214198738	0.082084433	-2.609492809	0.009067655	0.037820346	down_in_case
Yang2025_S	ENSGALG00010021779	UNC13C	case	control	0.650161791	0.232378122	2.797861455	0.005144217	0.024507579	up_in_case
Yang2025_S	ENSGALG00010021781	RAPGEF4	case	control	0.30512383	0.257042533	1.187055803	0.23520561	0.398394333	up_in_case
Yang2025_S	ENSGALG00010021782	ADAM11	case	control	0.616906931	0.311771431	1.978715394	0.047848059	0.12934291	up_in_case
Yang2025_S	ENSGALG00010021783	GIT2	case	control	-0.149599834	0.099734596	-1.499979341	0.133619754	0.26942429	down_in_case
Yang2025_S	ENSGALG00010021784	ANGPTL7	case	control	1.571029548	0.557101939	2.820003732	0.004802309	0.023276583	up_in_case
Yang2025_S	ENSGALG00010021785	CCDC71	case	control	-0.10277042	0.088495148	-1.161311352	0.245515305	0.411109802	down_in_case
Yang2025_S	ENSGALG00010021786	FAM162A	case	control	0.583826134	0.255849906	2.281908732	0.022494729	0.073790973	up_in_case
Yang2025_S	ENSGALG00010021788	RPL5	case	control	-0.334568	0.143503265	-2.33143127	0.01973063	0.067201038	down_in_case
Yang2025_S	ENSGALG00010021789	CHD9	case	control	0.49678209	0.184813337	2.688020783	0.007187692	0.031536679	up_in_case
Yang2025_S	ENSGALG00010021790	PHF21A	case	control	0.279101126	0.151121108	1.846870568	0.064765901	0.160703852	up_in_case
Yang2025_S	ENSGALG00010021791	PLEKHM2	case	control	0.201029012	0.121903067	1.649089034	0.099129396	0.217709714	up_in_case
Yang2025_S	ENSGALG00010021792	KLHDC8B	case	control	0.450912629	0.290537245	1.551995952	0.120663192	0.25055008	up_in_case
Yang2025_S	ENSGALG00010021793	ENSGALG00010021793	case	control	-0.258777181	0.159823683	-1.619141646	0.10541679	0.227713993	down_in_case
Yang2025_S	ENSGALG00010021794	ENSGALG00010021794	case	control	0.578369403	0.172670733	3.34955087	0.000809427	0.005905426	up_in_case
Yang2025_S	ENSGALG00010021795	NDRG3	case	control	-0.130939048	0.096607258	-1.355374852	0.175298158	0.325797639	down_in_case
Yang2025_S	ENSGALG00010021797	SLC25A34	case	control	1.28970251	0.315530223	4.087413553	4.36209E-05	0.000548608	up_in_case
Yang2025_S	ENSGALG00010021798	RANBP10	case	control	0.048626157	0.152762937	0.318311223	0.750248872	0.848252984	up_in_case
Yang2025_S	ENSGALG00010021799	ENSGALG00010021799	case	control	0.441121196	0.26187822	1.684451635	0.092094419	0.207223596	up_in_case
Yang2025_S	ENSGALG00010021800	TMEM82	case	control	0.780155324	0.247380627	3.153663783	0.001612347	0.010166543	up_in_case
Yang2025_S	ENSGALG00010021801	VAMP1	case	control	0.410237452	0.243568083	1.68428247	0.092127092	0.207230189	up_in_case
Yang2025_S	ENSGALG00010021803	TLL2	case	control	-0.218383286	0.141887129	-1.539133872	0.123771617	0.255028462	down_in_case
Yang2025_S	ENSGALG00010021805	FUT8	case	control	-0.345802598	0.103698319	-3.334698213	0.00085392	0.006159098	down_in_case
Yang2025_S	ENSGALG00010021807	CDC42	case	control	-0.693368343	0.067890275	-10.21307311	1.73288E-24	1.50923E-21	down_in_case
Yang2025_S	ENSGALG00010021810	ANKRD13A	case	control	-0.270453321	0.07872483	-3.435425896	0.000591623	0.004564933	down_in_case
Yang2025_S	ENSGALG00010021812	TEAD1	case	control	-0.133473933	0.1383418	-0.964812755	0.334638606	0.503363766	down_in_case
Yang2025_S	ENSGALG00010021817	EVI5	case	control	0.183546806	0.114030061	1.609635249	0.107477508	0.230450697	up_in_case
Yang2025_S	ENSGALG00010021821	PPP1R19L	case	control	0.341943886	0.172877965	1.97794951	0.047934403	0.129523933	up_in_case
Yang2025_S	ENSGALG00010021823	ENSGALG00010021823	case	control	0.911315634	0.239087227	3.811645003	0.000138045	0.001384923	up_in_case
Yang2025_S	ENSGALG00010021826	FBXO40	case	control	-0.436766617	0.238694085	-1.829817511	0.067277231	0.165199686	down_in_case
Yang2025_S	ENSGALG00010021827	CCZ1	case	control	-0.199783782	0.087946488	-2.271651624	0.02310756	0.075463757	down_in_case
Yang2025_S	ENSGALG00010021829	ERCC6	case	control	0.311581675	0.133657211	2.331199893	0.019742822	0.067216277	up_in_case
Yang2025_S	ENSGALG00010021831	ENSGALG00010021831	case	control	0.288991116	0.274684944	1.052082114	0.292761877	0.461417951	up_in_case
Yang2025_S	ENSGALG00010021835	NUDT8	case	control	-0.14551148	0.208793587	-0.696915467	0.485855698	0.643082963	down_in_case
Yang2025_S	ENSGALG00010021837	ENSGALG00010021837	case	control	0.897107438	0.36279476	2.472768455	0.013407098	0.050330794	up_in_case
Yang2025_S	ENSGALG00010021839	C1orf50	case	control	-0.154753169	0.173835582	-0.890227231	0.373343886	0.541255415	down_in_case
Yang2025_S	ENSGALG00010021840	ENSGALG00010021840	case	control	1.273843099	0.330453219	3.854836403	0.000115807	0.001198048	up_in_case
Yang2025_S	ENSGALG00010021841	CENPT	case	control	0.186282173	0.122799353	1.516963801	0.129275824	0.262909895	up_in_case
Yang2025_S	ENSGALG00010021842	ENSGALG00010021842	case	control	0.164628766	0.168515859	0.976933369	0.328602141	0.498180008	up_in_case
Yang2025_S	ENSGALG00010021843	ENSGALG00010021843	case	control	-0.089566476	0.351286368	-0.254967128	0.798748488	0.878289291	down_in_case
Yang2025_S	ENSGALG00010021847	P3H1	case	control	0.106546317	0.144194323	0.738907848	0.459962956	0.621056625	up_in_case
Yang2025_S	ENSGALG00010021848	LARGE2	case	control	0.015006122	0.097938587	0.15321971	0.878225015	0.928321746	up_in_case
Yang2025_S	ENSGALG00010021851	ENSGALG00010021851	case	control	1.988449082	0.430379819	4.620219155			up_in_case
Yang2025_S	ENSGALG00010021853	ENSGALG00010021853	case	control	0.786151067	0.44665927	1.760068848	0.078396134	0.184660265	up_in_case
Yang2025_S	ENSGALG00010021854	TAPBPL	case	control	0.927356981	0.213026162	4.353253957	1.34132E-05	0.000211919	up_in_case
Yang2025_S	ENSGALG00010021860	ENSGALG00010021860	case	control	-0.106145252	0.234041391	-0.453531966	0.650165731	0.778021422	down_in_case
Yang2025_S	ENSGALG00010021862	PDK1	case	control	-0.319686117	0.094827886	-3.371224767	0.000748348	0.005546929	down_in_case
Yang2025_S	ENSGALG00010021863	CLDN19	case	control	0.171273404	0.192452543	0.889951368	0.373492	0.541413817	up_in_case
Yang2025_S	ENSGALG00010021866	SCNN1A	case	control	0.363194569	0.206793052	1.756319012	0.079033953	0.185723125	up_in_case
Yang2025_S	ENSGALG00010021867	CXCL12	case	control	-0.472775121	0.085144367	-5.552629481	2.81404E-08	1.13008E-06	down_in_case
Yang2025_S	ENSGALG00010021869	GJC3	case	control	-0.051316103	0.09584882	-0.535385865	0.592383062	0.732723058	down_in_case
Yang2025_S	ENSGALG00010021870	ENSGALG00010021870	case	control	0.121169136	0.178598537	0.678444171	0.497490111	0.654382168	up_in_case
Yang2025_S	ENSGALG00010021871	ENSGALG00010021871	case	control	0.513225278	0.161796235	3.172047106	0.001513685	0.009671341	up_in_case
Yang2025_S	ENSGALG00010021874	TMCC1	case	control	-0.119729816	0.108957802	-1.098864084	0.271827355	0.438985389	down_in_case
Yang2025_S	ENSGALG00010021877	PLEKHN1	case	control	-0.054054056	0.183660056	-0.294315798	0.768516602	0.858999929	down_in_case
Yang2025_S	ENSGALG00010021879	CREBL2	case	control	-0.325711252	0.120856478	-2.695025177	0.007038331	0.031106612	down_in_case
Yang2025_S	ENSGALG00010021881	PHF20	case	control	0.176389173	0.134397373	1.312445092	0.189370001	0.344271928	up_in_case
Yang2025_S	ENSGALG00010021887	NUTF2	case	control	-0.300133297	0.17076245	-1.757607117	0.078814382	0.185332165	down_in_case
Yang2025_S	ENSGALG00010021892	NDUFV1	case	control	-0.13748821	0.240745734	-0.571093025	0.567936587	0.712477164	down_in_case
Yang2025_S	ENSGALG00010021893	FAM43B	case	control	0.260498611	0.253270531	1.028538971	0.303696369	0.473273194	up_in_case
Yang2025_S	ENSGALG00010021897	ENSGALG00010021897	case	control	-0.085343115	0.224939564	-0.379404644	0.704387402	0.81756109	down_in_case
Yang2025_S	ENSGALG00010021902	LMTK2	case	control	-0.205120557	0.113409057	-1.808678798	0.070500919	0.170798036	down_in_case
Yang2025_S	ENSGALG00010021903	MORC2	case	control	0.305939827	0.149495652	2.046479767	0.040709191	0.114602541	up_in_case
Yang2025_S	ENSGALG00010021905	RBSN	case	control	0.156552101	0.096284442	1.625933518	0.103963799	0.225554342	up_in_case
Yang2025_S	ENSGALG00010021908	EDC4	case	control	-0.078970539	0.061986207	-1.274001788	0.202662782	0.360493474	down_in_case
Yang2025_S	ENSGALG00010021913	ENSGALG00010021913	case	control	-0.142305148	0.163558275	-0.870057773	0.384268832	0.551947636	down_in_case
Yang2025_S	ENSGALG00010021916	MRPS25	case	control	-0.396261676	0.170070569	-2.329983831	0.019807006	0.067319665	down_in_case
Yang2025_S	ENSGALG00010021919	PARVA	case	control	-0.540402524	0.114830625	-4.706083614	2.52521E-06	5.29952E-05	down_in_case
Yang2025_S	ENSGALG00010021922	MRPL51	case	control	-0.699240577	0.247546249	-2.824686618	0.004732688	0.023011168	down_in_case
Yang2025_S	ENSGALG00010021924	ENSGALG00010021924	case	control	1.162164999	0.666651305	1.743287669	0.081283378	0.189380351	up_in_case
Yang2025_S	ENSGALG00010021927	ENSGALG00010021927	case	control	0.831309799	0.544118422	1.527810428	0.126559613	0.259131839	up_in_case
Yang2025_S	ENSGALG00010021931	ITGA6	case	control	-0.198625902	0.079512736	-2.498038848	0.012488251	0.047743714	down_in_case
Yang2025_S	ENSGALG00010021932	RPAP2	case	control	0.115006024	0.105924549	1.08573532	0.27759611	0.444990428	up_in_case
Yang2025_S	ENSGALG00010021933	PRR14L	case	control	0.322962825	0.151324021	2.134246922	0.032822574	0.097898665	up_in_case
Yang2025_S	ENSGALG00010021934	EFTUD2	case	control	0.080523499	0.083272663	0.966985997	0.333551034	0.502561032	up_in_case
Yang2025_S	ENSGALG00010021935	CETP	case	control	0.088895639	0.31041584	0.286375974	0.774590174	0.862822868	up_in_case
Yang2025_S	ENSGALG00010021938	CABP2	case	control	0.986056678	0.166395703	5.925974404	3.10451E-09	1.62027E-07	up_in_case
Yang2025_S	ENSGALG00010021943	OCM3	case	control	-0.013610339	0.26637843	-0.051094	0.959250617	0.975386303	down_in_case
Yang2025_S	ENSGALG00010021945	C1QTNF4	case	control	-0.111232363	0.179124424	-0.620978202	0.534613966	0.686057664	down_in_case
Yang2025_S	ENSGALG00010021947	CDK2AP2	case	control	0.381565083	0.292912061	1.302660878	0.192690564	0.347996761	up_in_case
Yang2025_S	ENSGALG00010021949	ENSGALG00010021949	case	control	-0.3250529	0.225309495	-1.442695079	0.149106386	0.291497965	down_in_case
Yang2025_S	ENSGALG00010021951	PSKH1	case	control	-0.492585664	0.113588187	-4.336592352	1.44709E-05	0.000223809	down_in_case
Yang2025_S	ENSGALG00010021953	PITPNM1	case	control	0.326170492	0.108979704	2.992947118	0.002762976	0.015290736	up_in_case
Yang2025_S	ENSGALG00010021958	BHLHA15	case	control	0.564568687	0.535234199	1.054806827	0.291513682	0.460050187	up_in_case
Yang2025_S	ENSGALG00010021960	CDKN1B	case	control	0.018661613	0.093233985	0.20015891	0.841356302	0.905910993	up_in_case
Yang2025_S	ENSGALG00010021965	PEX16	case	control	-0.029519021	0.186594743	-0.158198569	0.874300331	0.926381944	down_in_case
Yang2025_S	ENSGALG00010021966	ADGRA1	case	control	-0.157592076	0.144119264	-1.093483771	0.274181451	0.441548424	down_in_case
Yang2025_S	ENSGALG00010021969	CCDC103	case	control	0.032266718	0.438241583	0.073627697	0.941306632	0.963855699	up_in_case
Yang2025_S	ENSGALG00010021970	ERGIC3	case	control	-0.032647807	0.101631774	-0.321236218	0.748031387	0.847252165	down_in_case
Yang2025_S	ENSGALG00010021971	ICOS	case	control	-0.902107305	0.657225393	-1.372599591	0.169876859	0.318733544	down_in_case
Yang2025_S	ENSGALG00010021972	PISD	case	control	-0.379592487	0.113029227	-3.358356914	0.000784073	0.005759651	down_in_case
Yang2025_S	ENSGALG00010021974	GFAP	case	control	1.001922434	0.629227079	1.592306605	0.11131583	0.236461294	up_in_case
Yang2025_S	ENSGALG00010021978	NR2C2	case	control	-0.149679034	0.120220435	-1.245038197	0.21311771	0.373430215	down_in_case
Yang2025_S	ENSGALG00010021980	GLMN	case	control	-0.533522456	0.133927476	-3.983666909	6.786E-05	0.000780866	down_in_case
Yang2025_S	ENSGALG00010021988	FAM180B	case	control	-0.975047998	0.205907863	-4.735360681	2.18666E-06	4.69508E-05	down_in_case
Yang2025_S	ENSGALG00010021989	NCAPD2	case	control	-0.048158818	0.127245116	-0.378472823	0.705079379	0.817817192	down_in_case
Yang2025_S	ENSGALG00010021994	ENSGALG00010021994	case	control	0.207811356	0.238867857	0.869984597	0.384308822	0.551947636	up_in_case
Yang2025_S	ENSGALG00010021999	ANKRD22	case	control	-1.187989742	0.638816736	-1.859672228	0.062931914	0.157414506	down_in_case
Yang2025_S	ENSGALG00010022002	KIF18B	case	control	0.152187319	0.156155988	0.974585222	0.329766047	0.499325205	up_in_case
Yang2025_S	ENSGALG00010022005	NLRC5	case	control	0.300048102	0.193314246	1.552126181	0.120632035	0.250536564	up_in_case
Yang2025_S	ENSGALG00010022006	SFI1	case	control	0.779361952	0.146898665	5.305439298	1.12402E-07	3.79255E-06	up_in_case
Yang2025_S	ENSGALG00010022019	BTBD8	case	control	0.747371868	0.199767212	3.741213888	0.000183134	0.001738397	up_in_case
Yang2025_S	ENSGALG00010022021	TECPR1	case	control	-0.031922005	0.078077893	-0.408848192	0.682651071	0.801436112	down_in_case
Yang2025_S	ENSGALG00010022022	STAMBPL1	case	control	-0.51777223	0.281218979	-1.841171004	0.065596503	0.162129704	down_in_case

Yang2025_S	ENSGALG00010022024	MICAL2	case	control	0.44778655	0.144734754	3.093842608	0.001975823	0.011872499	up_in_case
Yang2025_S	ENSGALG00010022027	EIF4ENIF1	case	control	-0.191879955	0.071421901	-2.686570254	0.007218977	0.031624155	down_in_case
Yang2025_S	ENSGALG00010022028	FGD5	case	control	0.042792934	0.135092337	0.316768033	0.751419622	0.848540188	up_in_case
Yang2025_S	ENSGALG00010022029	ENSGALG00010022029	case	control	1.248605688	0.557035489	2.241519099	0.02499247	0.079915114	up_in_case
Yang2025_S	ENSGALG00010022030	ZNF341	case	control	0.108824204	0.112232752	0.969629657	0.332231125	0.501593688	up_in_case
Yang2025_S	ENSGALG00010022032	KBTBD4	case	control	-0.111742795	0.116379394	-0.96015962	0.336974886	0.505081751	down_in_case
Yang2025_S	ENSGALG00010022033	RAPH1	case	control	0.624851061	0.254523217	2.454986494	0.014088988	0.05231283	up_in_case
Yang2025_S	ENSGALG00010022034	ENSGALG00010022034	case	control	0.100830267	0.199818403	0.504609514	0.613833126	0.750067048	up_in_case
Yang2025_S	ENSGALG00010022037	DRG1	case	control	-0.382208142	0.205925922	-1.856046772	0.063446892	0.158389902	down_in_case
Yang2025_S	ENSGALG00010022038	GAPDH	case	control	-0.746202718	0.212236701	-3.51589859	0.000438268	0.003552804	down_in_case
Yang2025_S	ENSGALG00010022041	MAPK8IP1	case	control	0.056279118	0.188800012	0.298088531	0.765635595	0.857302693	up_in_case
Yang2025_S	ENSGALG00010022042	AIP	case	control	0.32335532	0.131061222	2.467208193	0.013617116	0.050954487	up_in_case
Yang2025_S	ENSGALG00010022044	PXMP4	case	control	0.022850797	0.109252764	0.209155316	0.834326993	0.901896412	up_in_case
Yang2025_S	ENSGALG00010022046	CRY2	case	control	-0.196064962	0.131350946	-1.492680241	0.135520908	0.272115829	down_in_case
Yang2025_S	ENSGALG00010022049	EPHX4	case	control	1.585466645	0.481802535	3.290698015	0.000999391	0.007015878	up_in_case
Yang2025_S	ENSGALG00010022050	ENSGALG00010022050	case	control	1.175782588	0.368257553	3.192826806	0.001408874	0.009189101	up_in_case
Yang2025_S	ENSGALG00010022051	BAIAP2L1	case	control	0.16989191	0.114359299	1.485597695	0.137385565	0.27506722	up_in_case
Yang2025_S	ENSGALG00010022052	IFFO1	case	control	-0.154417386	0.103306295	-1.494752918	0.134978938	0.271413707	down_in_case
Yang2025_S	ENSGALG00010022054	ENSGALG00010022054	case	control	1.329649948	0.294028784	4.522176123	6.12071E-06	0.000110054	up_in_case
Yang2025_S	ENSGALG00010022055	E2F1	case	control	-0.166417036	0.233700049	-0.712096709	0.476404892	0.635586613	down_in_case
Yang2025_S	ENSGALG00010022058	NLRP3	case	control	0.296204785	0.143111742	2.06974481	0.038476248	0.110028015	up_in_case
Yang2025_S	ENSGALG00010022059	NPTX2	case	control	-0.301156101	0.211749881	-1.422225595	0.154960771	0.299490351	down_in_case
Yang2025_S	ENSGALG00010022062	MITF	case	control	0.216247228	0.128045257	1.688834344	0.09125118	0.205791422	up_in_case
Yang2025_S	ENSGALG00010022065	PABPC1L	case	control	0.352868734	0.160619244	2.196926871	0.028025669	0.086960076	up_in_case
Yang2025_S	ENSGALG00010022066	DAD1	case	control	-0.442284217	0.238458955	-1.854760357	0.063630455	0.158664256	down_in_case
Yang2025_S	ENSGALG00010022067	TMEM130	case	control	0.044956682	0.225891405	0.199019002	0.842247881	0.906369665	up_in_case
Yang2025_S	ENSGALG00010022069	NOP2	case	control	0.157866452	0.107134644	1.473533169	0.140607327	0.279709222	up_in_case
Yang2025_S	ENSGALG00010022071	ACTA2	case	control	-0.194851701	0.159711003	-1.220026784	0.222454722	0.38403203	down_in_case
Yang2025_S	ENSGALG00010022072	SMTN	case	control	0.088666943	0.115369055	0.768550487	0.442160208	0.605493563	up_in_case
Yang2025_S	ENSGALG00010022073	PTPMT1	case	control	-0.281529145	0.256845073	-1.09610491	0.273032866	0.4403603	down_in_case
Yang2025_S	ENSGALG00010022076	YWHAB	case	control	-0.382411069	0.074220089	-5.152393008	2.57183E-07	7.47616E-06	down_in_case
Yang2025_S	ENSGALG00010022079	ABI2	case	control	-0.119334689	0.161526317	-0.738794091	0.460032041	0.621056625	down_in_case
Yang2025_S	ENSGALG00010022081	FAS	case	control	0.303812376	0.186528242	1.628774138	0.103360841	0.224700987	up_in_case
Yang2025_S	ENSGALG00010022082	TGFBR3	case	control	-0.66129945	0.126210606	-5.239650379	1.60881E-07	5.11844E-06	down_in_case
Yang2025_S	ENSGALG00010022084	SELENOM	case	control	-0.25895715	0.212052415	-1.221194061	0.222012552	0.38350625	down_in_case
Yang2025_S	ENSGALG00010022085	MLLT6	case	control	0.382957184	0.159751406	2.397206971	0.016520589	0.059029335	up_in_case
Yang2025_S	ENSGALG00010022087	SLC35C1	case	control	-0.378272894	0.099323745	-3.808484008	0.000139821	0.001398716	down_in_case
Yang2025_S	ENSGALG00010022088	DKK3	case	control	-0.163198796	0.125406009	-1.301363441	0.193134081	0.348522136	down_in_case
Yang2025_S	ENSGALG00010022090	TRRAP	case	control	-0.05684655	0.126340695	-0.449946474	0.652749036	0.780167124	down_in_case
Yang2025_S	ENSGALG00010022093	CHD4	case	control	0.238899485	0.125462631	1.904148543	0.056890841	0.14686437	up_in_case
Yang2025_S	ENSGALG00010022097	INPP5J	case	control	-0.765677596	0.199943292	-3.829473793	0.000128418	0.00130031	down_in_case
Yang2025_S	ENSGALG00010022098	CDC7	case	control	0.089030022	0.133171534	0.668536432	0.503791235	0.659828705	up_in_case
Yang2025_S	ENSGALG00010022100	ENTPD1	case	control	-0.598651721	0.125219404	-4.780822326	1.7458E-06	3.86767E-05	down_in_case
Yang2025_S	ENSGALG00010022101	ENSGALG00010022101	case	control	0.736923918	0.293199416	2.513388082	0.011957771	0.046236813	up_in_case
Yang2025_S	ENSGALG00010022104	EXOC3L1	case	control	-0.079094673	0.20357779	-0.388523094	0.697628967	0.812831075	down_in_case
Yang2025_S	ENSGALG00010022105	DLG5	case	control	0.583597633	0.196724869	2.966567648	0.003011441	0.016380068	up_in_case
Yang2025_S	ENSGALG00010022108	USP47	case	control	0.112696039	0.070260849	1.603966378	0.108721464	0.232402761	up_in_case
Yang2025_S	ENSGALG00010022111	PCGF2	case	control	0.591255218	0.307220378	1.924531248			up_in_case
Yang2025_S	ENSGALG00010022115	E2F4	case	control	0.105632283	0.138403585	0.763219271	0.445332638	0.608402971	up_in_case
Yang2025_S	ENSGALG00010022116	CYP20A1	case	control	-0.177960998	0.118787941	-1.498140264	0.134096812	0.270073576	down_in_case
Yang2025_S	ENSGALG00010022117	FAM20C	case	control	0.002501749	0.14471902	0.017286939	0.986207705	0.991544327	up_in_case
Yang2025_S	ENSGALG00010022120	FOXL3	case	control	-0.124306638	0.438708827	-0.283346561	0.776911189	0.864233848	down_in_case
Yang2025_S	ENSGALG00010022122	PSMB3	case	control	-0.040390718	0.173561021	-0.232717682	0.815980638	0.889689863	down_in_case
Yang2025_S	ENSGALG00010022124	ENSGALG00010022124	case	control	0.068408013	0.200536087	0.341125697	0.733008956	0.836909448	up_in_case
Yang2025_S	ENSGALG00010022125	CBFA2T2	case	control	0.082842895	0.141749801	0.584430416	0.558930768	0.705950663	up_in_case
Yang2025_S	ENSGALG00010022129	ELMO3	case	control	0.658732539	0.152319357	4.324680394	1.52753E-05	0.000233145	up_in_case
Yang2025_S	ENSGALG00010022131	ENSGALG00010022131	case	control	-1.773994095	2.231659479	-0.794921498	0.426659168	0.591121049	down_in_case
Yang2025_S	ENSGALG00010022134	CH25H	case	control	-0.740868856	0.242589456	-3.054002712	0.0022581	0.013116561	down_in_case
Yang2025_S	ENSGALG00010022135	CHST1	case	control	0.018020925	0.274968263	0.065538199	0.947745494	0.96767537	up_in_case
Yang2025_S	ENSGALG00010022137	PDDC1	case	control	-0.330495425	0.175594243	-1.882154103	0.059815095	0.152130563	down_in_case
Yang2025_S	ENSGALG00010022138	SPAG17	case	control	0.426038041	0.249302599	1.70891937	0.087465886	0.199809364	up_in_case
Yang2025_S	ENSGALG00010022142	TMEM208	case	control	-0.651243923	0.319727141	-2.036874068	0.041662663	0.116580164	down_in_case
Yang2025_S	ENSGALG00010022143	MATCAP1	case	control	0.046927813	0.198159235	0.236818705	0.81279744	0.887252024	up_in_case
Yang2025_S	ENSGALG00010022144	SNTA1	case	control	0.390685938	0.173956324	2.245885231	0.02471136	0.07936225	up_in_case
Yang2025_S	ENSGALG00010022148	TALDO1	case	control	-0.474762266	0.185488589	-2.559522761	0.010481599	0.041923388	down_in_case
Yang2025_S	ENSGALG00010022149	NBEAL1	case	control	0.243129028	0.121661645	1.998403263	0.045672958	0.125138158	up_in_case
Yang2025_S	ENSGALG00010022150	CPNE2	case	control	-0.098400897	0.166508931	-0.590964675	0.55454409	0.70237883	down_in_case
Yang2025_S	ENSGALG00010022151	PIP4K2B	case	control	-0.185386106	0.114328112	-1.621526865	0.104904693	0.227022366	down_in_case
Yang2025_S	ENSGALG00010022152	ZNF644	case	control	0.09710635	0.098228175	0.988579404	0.322868967	0.49219489	up_in_case
Yang2025_S	ENSGALG00010022154	CPN1	case	control	0.09936794	0.156055648	0.636746834	0.524289762	0.677608777	up_in_case
Yang2025_S	ENSGALG00010022157	EPS8L2	case	control	-0.06028872	0.292335977	-0.206230927	0.836610531	0.903156104	down_in_case
Yang2025_S	ENSGALG00010022158	CWC25	case	control	0.232142896	0.10281198	2.257936243	0.023949635	0.077577443	up_in_case
Yang2025_S	ENSGALG00010022159	KIAA2013	case	control	-0.232812779	0.075654911	-3.077298955	0.002088857	0.012354933	down_in_case
Yang2025_S	ENSGALG00010022161	CELF1	case	control	-0.170133282	0.116135775	-1.464951542	0.142934139	0.28288414	down_in_case
Yang2025_S	ENSGALG00010022162	POLE	case	control	0.013348037	0.128384555	0.103969184	0.917193804	0.951236618	up_in_case
Yang2025_S	ENSGALG00010022164	PDGFA	case	control	-0.044318089	0.092507099	-0.479077706	0.631883349	0.763751797	down_in_case
Yang2025_S	ENSGALG00010022165	FAM192A	case	control	0.142217504	0.109911079	1.293932383	0.195688778	0.351815652	up_in_case
Yang2025_S	ENSGALG00010022166	SDHB	case	control	-0.423833053	0.15582578	-2.719916136	0.006529847	0.02933042	down_in_case
Yang2025_S	ENSGALG00010022167	WDR3	case	control	-0.099615376	0.12886502	-0.773021075	0.439509908	0.603152202	down_in_case
Yang2025_S	ENSGALG00010022169	TOX2	case	control	0.609699178	0.354531942	1.719729888	0.085481552	0.196435592	up_in_case
Yang2025_S	ENSGALG00010022170	RSPRY1	case	control	-0.037640802	0.10511213	-0.358101412	0.720267422	0.82765814	down_in_case
Yang2025_S	ENSGALG00010022171	FOXP1	case	control	0.424821416	0.2303352	1.844361684	0.065130447	0.161378518	up_in_case
Yang2025_S	ENSGALG00010022172	GIPC2	case	control	-0.110061582	0.179434409	-0.613380579	0.539624725	0.690511528	down_in_case
Yang2025_S	ENSGALG00010022173	RPL23	case	control	-0.107817176	0.184768767	-0.583524899	0.559540001	0.706203234	down_in_case
Yang2025_S	ENSGALG00010022174	KCNMA1	case	control	0.790508403	0.178601819	4.426093798	9.59548E-06	0.000158053	up_in_case
Yang2025_S	ENSGALG00010022176	ARL2BPL	case	control	0.387747973	0.265291298	1.461593263	0.143852702	0.284338638	up_in_case
Yang2025_S	ENSGALG00010022178	ENSGALG00010022178	case	control	-0.130399217	0.338719096	-0.384977459	0.700254116	0.814731661	down_in_case
Yang2025_S	ENSGALG00010022180	PLLP	case	control	0.049027371	0.276285531	0.177451822	0.859153505	0.916926101	up_in_case
Yang2025_S	ENSGALG00010022181	ENSGALG00010022181	case	control	-0.019724254	0.154662009	-0.127531345	0.898519866	0.939723381	down_in_case
Yang2025_S	ENSGALG00010022182	CNTNAP1	case	control	-0.09102792	0.204257947	-0.445651789	0.655848795	0.782537285	down_in_case
Yang2025_S	ENSGALG00010022184	PRKAR1B	case	control	-0.066133718	0.080755479	-0.81893785	0.412821874	0.578248698	down_in_case
Yang2025_S	ENSGALG00010022186	DNAJB4	case	control	0.257670122	0.10215373	2.522376053	0.011656502	0.04538034	up_in_case
Yang2025_S	ENSGALG00010022190	ACVR1B	case	control	-0.518486288	0.151109443	-3.431197137	0.000600924	0.004623894	down_in_case
Yang2025_S	ENSGALG00010022193	GDAP2	case	control	-0.458181514	0.133613542	-3.429154774	0.000605464	0.004640893	down_in_case
Yang2025_S	ENSGALG00010022194	ENSGALG00010022194	case	control	-0.641803427	0.13732929	-4.673463527	2.96162E-06	6.01607E-05	down_in_case
Yang2025_S	ENSGALG00010022196	PLXNA1	case	control	-0.065639565	0.156040504	-0.420657223	0.674005402	0.794944163	down_in_case
Yang2025_S	ENSGALG00010022199	RBM39	case	control	0.423596115	0.100855365	4.200035526	2.66873E-05	0.00036967	up_in_case
Yang2025_S	ENSGALG00010022200	DNMBP	case	control	-0.207303219	0.073251041	-2.830037848	0.00465425	0.022709023	down_in_case
Yang2025_S	ENSGALG00010022202	CX3CL1	case	control	0.115880771	0.160218391	0.723267599	0.469515495	0.630049827	up_in_case
Yang2025_S	ENSGALG00010022203	DNAAF5	case	control	0.098379436	0.136760996	0.719353022	0.471923434	0.631861675	up_in_case
Yang2025_S	ENSGALG00010022204	ENSGALG00010022204	case	control	0.998245843	0.418840214	2.383357209	0.017155539	0.060610029	up_in_case
Yang2025_S	ENSGALG00010022205	GALNT18	case	control	-0.21152409	0.106635265	-1.983622294	0.047297962	0.12825396	down_in_case
Yang2025_S	ENSGALG00010022207	ENSGALG00010022207	case	control	-0.008159884	0.13929035	-0.058581832	0.953285181	0.971338769	down_in_case
Yang2025_S	ENSGALG00010022209	ZFYVE19	case	control	-0.111034089	0.092969278	-1.194309467	0.232356936	0.39549211	down_in_case
Yang2025_S	ENSGALG00010022210	EZH1	case	control	0.164886584	0.08794586	1.874864648	0.060811347	0.153954023	up_in_case
Yang2025_S	ENSGALG00010022211	ENSGALG00010022211	case	control	0.893090194	0.316246664	2.824030401	0.004742389	0.023050291	up_in_case
Yang2025_S	ENSGALG00010022213	IDH1	case	control	-0.689579218	0.170082371	-4.054383839	5.02666E-05	0.000610693	down_in_case
Yang2025_S	ENSGALG00010022214	ENSGALG00010022214	case	control	-0.624061737	0.297298231	-2.099110159	0.035807191	0.10454838	down_in_case
Yang2025_S	ENSGALG00010022215	ENSGALG00010022215	case	control	0.439611863	0.265318316	1.656922405			up_in_case
Yang2025_S	ENSGALG00010022216	ROMO1	case	control	-0.203796928	0.345685642	-0.589544092	0.555496343	0.702919104	down_in_case
Yang2025_S	ENSGALG00010022218	ENSGALG00010022218	case	control	0.720905347	0.233684589	3.084950315	0.002035862	0.012129	up_in_case
Yang2025_S	ENSGALG00010022219	EIF4E3	case	control	-0.222365465	0.150840225	-1.474178821	0.140433451	0.279443115	down_in_case
Yang2025_S	ENSGALG00010022220	PPP1R14D	case	control	0.079018977	0.304102795	0.25984298	0.794984896	0.876474756	up_in_case
Yang2025_S	ENSGALG00010022221	CUTC	case	control	0.07105842	0.271539144	0.261687573	0.793562322	0.875535478	up_in_case
Yang2025_S	ENSGALG00010022222	ENSGALG00010022222	case	control	0.34099614	0.264257113	1.290395315	0.196913438	0.353060829	up_in_case
Yang2025_S	ENSGALG00010022223	DOK4	case	control	0.617091852	0.165137866	3.736828305	0.000186356	0.001764179	up_in_case
Yang2025_S	ENSGALG00010022224	NHEJ1	case	control	-0.065715453	0.25694408	-0.255757803	0.798137859	0.877949026	down_in_case
Yang2025_S	ENSGALG00010022226	FBRSL1	case	control	0.833699172	0.282080733	2.955533913	0.003121284	0.016819447	up_in_case
Yang2025_S	ENSGALG00010022227	TMEM80	case	control	-0.797412549	0.152626184	-5.224611705	1.74521E-07	5.51462E-06	down_in_case
Yang2025_S	ENSGALG00010022228	FUBP1	case	control	0.636097486	0.141713545	4.488614602	7.16879E-06	0.000125815	up_in_case
Yang2025_S	ENSGALG00010022229	RAPSN	case	control	0.491388742	0.123908771	3.965730084	7.31716E-05	0.000828981	up_in_case
Yang2025_S	ENSGALG00010022230	NFS1	case	control	0.05511632	0.068891853	0.800041192	0.423686932	0.588783805	up_in_case
Yang2025_S	ENSGALG00010022231	ZNF503	case	control	0.361252161	0.204414108	1.767256505	0.077185281	0.18252846	up_in_case
Yang2025_S	ENSGALG00010022232	GPR27	case	control	-0.57818354	0.108007241	-5.353192381	8.64159E-08	3.01051E-06	down_in_case
Yang2025_S	ENSGALG00010022233	FAM46C	case	control	-0.115889953	0.154726406	-0.748999188	0.453857695	0.615725243	down_in_case
Yang2025_S	ENSGALG00010022234	COX15	case	control	-0.238612565	0.200005953	-1.193027317	0.232858675	0.395997136	down_in_case

Yang2025_S	ENSGALG00010022235	RAMP2	case	control	0.097165355	0.161846775	0.600353979	0.548270352	0.697154505	up_in_case
Yang2025_S	ENSGALG00010022236	SHF	case	control	-0.568506497	0.126371484	-4.498692903	6.83725E-06	0.000120905	down_in_case
Yang2025_S	ENSGALG00010022237	SUN1	case	control	-0.060085783	0.076281511	-0.787684753	0.430881126	0.594800446	down_in_case
Yang2025_S	ENSGALG00010022238	COMTD1	case	control	0.102345601	0.173168601	0.591017082	0.554508975	0.70237883	up_in_case
Yang2025_S	ENSGALG00010022239	DEAF1	case	control	-0.032454803	0.117932921	-0.275197146	0.78316478	0.868832195	down_in_case
Yang2025_S	ENSGALG00010022240	ENSGALG00010022240	case	control	0.105303257	0.114980294	0.915837424	0.359752172	0.528458584	up_in_case
Yang2025_S	ENSGALG00010022241	POLR2C	case	control	0.092493636	0.116253987	0.79561689	0.426254746	0.590854461	up_in_case
Yang2025_S	ENSGALG00010022242	USP19	case	control	-0.113670928	0.058041152	-1.958454028	0.05017676	0.133539564	down_in_case
Yang2025_S	ENSGALG00010022243	ENSGALG00010022243	case	control	-0.262637644	0.55581695	-0.472525431	0.636551794	0.767065829	down_in_case
Yang2025_S	ENSGALG00010022244	ENTPD7	case	control	0.115675029	0.173872543	0.665286347	0.505867362	0.661529813	up_in_case
Yang2025_S	ENSGALG00010022245	PIKFYVE	case	control	0.088885885	0.091936359	0.966819717	0.333634166	0.502561032	up_in_case
Yang2025_S	ENSGALG00010022246	RHOV	case	control	-0.071604673	0.528128754	-0.135581849	0.892151855	0.936719116	down_in_case
Yang2025_S	ENSGALG00010022247	GALNT9	case	control	0.288787082	0.317037508	0.91089248	0.362352028	0.531065998	up_in_case
Yang2025_S	ENSGALG00010022248	FIGNL2	case	control	-0.234588685	0.229371801	-1.022744224	0.306428796	0.476093797	down_in_case
Yang2025_S	ENSGALG00010022250	SLC25A28	case	control	0.065411556	0.1482737	0.441154134	0.659101416	0.784658933	up_in_case
Yang2025_S	ENSGALG00010022251	WNK4	case	control	0.493006679	0.177689262	2.774544023	0.005527915	0.025806197	up_in_case
Yang2025_S	ENSGALG00010022252	COQ9	case	control	-0.249045489	0.208386338	-1.195114282	0.232042383	0.395198069	down_in_case
Yang2025_S	ENSGALG00010022253	ALPK3	case	control	-0.041189025	0.116851022	-0.352491784	0.724469479	0.830611257	down_in_case
Yang2025_S	ENSGALG00010022255	NOC4L	case	control	-0.319936977	0.113513462	-2.818493686	0.004824956	0.02335386	down_in_case
Yang2025_S	ENSGALG00010022256	PSMC3	case	control	-0.036333232	0.134160138	-0.270819876	0.786529572	0.871165336	down_in_case
Yang2025_S	ENSGALG00010022257	KCNK18	case	control	-0.802591047	0.543818335	-1.475844036	0.139985766	0.278840188	down_in_case
Yang2025_S	ENSGALG00010022258	ENSGALG00010022258	case	control	0.737585843	0.761246944	0.968917969	0.332586119	0.501814762	up_in_case
Yang2025_S	ENSGALG00010022259	GET4	case	control	-0.008175089	0.093562704	-0.087375507	0.930373037	0.958009922	down_in_case
Yang2025_S	ENSGALG00010022264	SCN8A	case	control	1.008641131	0.222782976	4.52746054	5.96967E-06	0.000108036	up_in_case
Yang2025_S	ENSGALG00010022266	COA3	case	control	0.536709051	0.343528118	1.562343874	0.118207002	0.246773718	up_in_case
Yang2025_S	ENSGALG00010022269	CNTD1	case	control	-0.765148214	0.486677667	-1.572186821	0.115907231	0.243504789	down_in_case
Yang2025_S	ENSGALG00010022271	SLC18A2	case	control	-0.342171311	0.281767879	-1.214373023	0.224605312	0.386166511	down_in_case
Yang2025_S	ENSGALG00010022274	CCDC91	case	control	0.859483196	0.205753056	4.177256039	2.95047E-05	0.000401366	up_in_case
Yang2025_S	ENSGALG00010022275	VDAC2	case	control	-0.858138483	0.204742562	-4.191304796	2.77355E-05	0.000381952	down_in_case
Yang2025_S	ENSGALG00010022276	BECN1	case	control	0.24247955	0.072957049	3.323593161	0.000888657	0.006363536	up_in_case
Yang2025_S	ENSGALG00010022279	EP400	case	control	0.371444053	0.204994206	1.811973426	0.069990303	0.169974707	up_in_case
Yang2025_S	ENSGALG00010022280	PDZD8	case	control	-0.342694177	0.105606881	-3.244998566	0.001174512	0.007929424	down_in_case
Yang2025_S	ENSGALG00010022282	TMT1A	case	control	-0.467606	0.258550643	-1.808566376	0.070518396	0.170810681	down_in_case
Yang2025_S	ENSGALG00010022290	NEXN	case	control	1.236262641	0.270572931	4.569055133	4.89928E-06	9.04258E-05	up_in_case
Yang2025_S	ENSGALG00010022293	DNAJC10	case	control	-0.330894041	0.088845782	-3.72436411	0.000195808	0.001836196	down_in_case
Yang2025_S	ENSGALG00010022296	CIAPIN1	case	control	0.189007878	0.163086709	1.15894103	0.246480223	0.412230186	up_in_case
Yang2025_S	ENSGALG00010022297	VPS18	case	control	-0.423584266	0.08194684	-5.169012809	2.35334E-07	7.02222E-06	down_in_case
Yang2025_S	ENSGALG00010022299	MRGBP	case	control	-0.123776952	0.097295732	-1.272172473	0.203311846	0.361371246	down_in_case
Yang2025_S	ENSGALG00010022302	ENSGALG00010022302	case	control	-0.324263962	0.210127459	-1.543177474	0.122787721	0.253709134	down_in_case
Yang2025_S	ENSGALG00010022303	DUSP13A	case	control	0.158045688	0.18943397	0.834304894	0.404109195	0.570373994	up_in_case
Yang2025_S	ENSGALG00010022304	ADAP1	case	control	0.830560456	0.201337942	4.125205842	3.70403E-05	0.000481939	up_in_case
Yang2025_S	ENSGALG00010022307	ENSGALG00010022307	case	control	0.85304819	0.299379475	2.84938769	0.004380347	0.02161033	up_in_case
Yang2025_S	ENSGALG00010022308	OGFR	case	control	0.357082485	0.17974064	1.986654131	0.046960738	0.127513228	up_in_case
Yang2025_S	ENSGALG00010022309	WSCD2	case	control	-0.132559253	0.410352111	-0.323037823	0.746666598	0.846019462	down_in_case
Yang2025_S	ENSGALG00010022310	DCLRE1A	case	control	0.173405252	0.120763568	1.435906999	0.151028807	0.293977709	up_in_case
Yang2025_S	ENSGALG00010022311	DLL4	case	control	-0.270586664	0.103651445	-2.61054406	0.009039833	0.037749496	down_in_case
Yang2025_S	ENSGALG00010022312	ZNF592	case	control	0.217897076	0.139124487	1.566202193	0.117301289	0.245545061	up_in_case
Yang2025_S	ENSGALG00010022313	ENSGALG00010022313	case	control	0.502574329	0.30880577	1.627477133	0.103635801	0.225041102	up_in_case
Yang2025_S	ENSGALG00010022314	DUSP13B	case	control	0.073697863	0.317918892	0.231813413	0.816682939	0.890142883	up_in_case
Yang2025_S	ENSGALG00010022316	INO80	case	control	0.120929964	0.139797655	0.865035708	0.38701919	0.554390668	up_in_case
Yang2025_S	ENSGALG00010022317	PSME3	case	control	-0.251723282	0.100964635	-2.493182714	0.012660368	0.048242337	down_in_case
Yang2025_S	ENSGALG00010022320	COL9A3	case	control	1.673386984	0.788145944	2.123194311	0.033737572	0.099964506	up_in_case
Yang2025_S	ENSGALG00010022322	ENSGALG00010022322	case	control	0.529623617	0.333189854	1.589555054	0.111935135	0.237404326	up_in_case
Yang2025_S	ENSGALG00010022323	DUSP29	case	control	0.845740442	0.325534051	2.59800915	0.0093766	0.038714938	up_in_case
Yang2025_S	ENSGALG00010022325	MIGA1	case	control	-0.038525334	0.157813849	-0.244118839	0.807138783	0.88381887	down_in_case
Yang2025_S	ENSGALG00010022326	COX19	case	control	-0.461742736	0.235049043	-1.964452738	0.049477612	0.132285212	down_in_case
Yang2025_S	ENSGALG00010022328	ENSGALG00010022328	case	control	0.285919966	0.226929736	1.259949313	0.207687648	0.366449615	up_in_case
Yang2025_S	ENSGALG00010022329	CYP2AC7	case	control	-0.413126865	0.516777747	-0.799428512	0.424041983	0.589058204	down_in_case
Yang2025_S	ENSGALG00010022332	KAT6B	case	control	0.51713305	0.231356174	2.235224763	0.025402597	0.080800087	up_in_case
Yang2025_S	ENSGALG00010022333	ENSGALG00010022333	case	control	-0.174157023	0.131140056	-1.328023094	0.184170478	0.337527816	down_in_case
Yang2025_S	ENSGALG00010022334	PDE1A	case	control	0.13343586	0.250457373	0.532768742	0.594193674	0.734509126	up_in_case
Yang2025_S	ENSGALG00010022340	GFOD2	case	control	-0.280019491	0.14219723	-1.969233086	0.048926331	0.131188844	down_in_case
Yang2025_S	ENSGALG00010022343	GATA5	case	control	0.414147005	0.442151642	0.936662822	0.348932026	0.517328203	up_in_case
Yang2025_S	ENSGALG00010022345	USP33	case	control	0.215595261	0.071368498	3.020874299	0.00252046	0.014277482	up_in_case
Yang2025_S	ENSGALG00010022347	SEC11A	case	control	0.017688264	0.10913851	0.162071704	0.871249388	0.924369153	up_in_case
Yang2025_S	ENSGALG00010022348	G6PC1	case	control	-0.216830493	0.370656936	-0.58498971	0.558554636	0.705598663	down_in_case
Yang2025_S	ENSGALG00010022353	SLC39A13	case	control	-0.453010987	0.104434539	-4.337750628	1.43948E-05	0.000223625	down_in_case
Yang2025_S	ENSGALG00010022354	FAR2	case	control	-0.128502209	0.167872037	-0.765477153	0.443987466	0.607160485	down_in_case
Yang2025_S	ENSGALG00010022356	TCFL5	case	control	0.647069849	0.231922174	2.790030111	0.005270314	0.024929337	up_in_case
Yang2025_S	ENSGALG00010022357	ENSGALG00010022357	case	control	0.594605529	0.530520317	1.120796903	0.262374323	0.429230593	up_in_case
Yang2025_S	ENSGALG00010022359	ZZZ3	case	control	0.006820652	0.079008241	0.086328367	0.931205389	0.958442063	up_in_case
Yang2025_S	ENSGALG00010022363	KIF18A	case	control	-0.222881077	0.13645344	-1.633385544	0.102387935	0.223073152	down_in_case
Yang2025_S	ENSGALG00010022365	PLCG1	case	control	-0.037242637	0.068892842	-0.540587908	0.588791655	0.730011217	down_in_case
Yang2025_S	ENSGALG00010022366	AARSD1	case	control	0.377438476	0.128570755	2.935647963	0.003328518	0.017609302	up_in_case
Yang2025_S	ENSGALG00010022373	AK5	case	control	1.035521882	0.37621656	2.752462259	0.005914896	0.027104269	up_in_case
Yang2025_S	ENSGALG00010022375	NMB	case	control	-0.018001508	0.463680072	-0.038823122	0.96903141	0.981620757	down_in_case
Yang2025_S	ENSGALG00010022377	ZHX3	case	control	0.079922498	0.154583633	0.517017852	0.605143697	0.743098116	up_in_case
Yang2025_S	ENSGALG00010022379	METTL15	case	control	0.51138731	0.179330438	2.851648143	0.004349321	0.021484507	up_in_case
Yang2025_S	ENSGALG00010022385	ENSGALG00010022385	case	control	-0.120695767	0.592787935	-0.203606989	0.838660631	0.904482259	down_in_case
Yang2025_S	ENSGALG00010022386	SPI1	case	control	-0.283231209	0.153664128	-1.843183651	0.065302201	0.161631647	down_in_case
Yang2025_S	ENSGALG00010022388	CYP2W1	case	control	-0.276088435	0.462914393	-0.596413591	0.550898963	0.699095753	down_in_case
Yang2025_S	ENSGALG00010022391	ENSGALG00010022391	case	control	0.253647312	0.198091596	1.280454681	0.200385274	0.357995998	up_in_case
Yang2025_S	ENSGALG00010022392	EMILIN3	case	control	0.459690272	0.168478641	2.728478039	0.006362733	0.028712655	up_in_case
Yang2025_S	ENSGALG00010022395	PTGES3L	case	control	0.444114451	0.159805711	2.779089982	0.005451142	0.025593554	up_in_case
Yang2025_S	ENSGALG00010022396	PPP1R1C	case	control	0.137499711	0.346407601	0.39693041	0.691418779	0.80762118	up_in_case
Yang2025_S	ENSGALG00010022397	ATF1	case	control	-0.105324697	0.103404776	-1.018567041	0.308408558	0.478021501	down_in_case
Yang2025_S	ENSGALG00010022400	NHLRC2	case	control	0.270120347	0.123865029	2.180763605	0.029200906	0.089688037	up_in_case
Yang2025_S	ENSGALG00010022401	LCAT	case	control	-0.343524467	0.246613188	-1.392968761	0.163629238	0.310312116	down_in_case
Yang2025_S	ENSGALG00010022406	ITPRID2	case	control	-0.158208812	0.092528445	-1.709839744	0.087295511	0.19951828	down_in_case
Yang2025_S	ENSGALG00010022407	RUNDC1	case	control	0.052121311	0.085080123	0.612614428	0.540131316	0.690770674	up_in_case
Yang2025_S	ENSGALG00010022408	RAD51AP1	case	control	0.163494224	0.099180265	1.648455212	0.099259294	0.217823349	up_in_case
Yang2025_S	ENSGALG00010022409	CARNS1	case	control	0.617549364	0.288230571	2.142553309	0.032148984	0.096446951	up_in_case
Yang2025_S	ENSGALG00010022411	ADRB1	case	control	-0.51461691	0.265139815	-1.940926563	0.052267186	0.137761156	down_in_case
Yang2025_S	ENSGALG00010022412	CHAC1	case	control	0.456480253	0.423057584	1.079002645	0.280586549	0.448449934	up_in_case
Yang2025_S	ENSGALG00010022415	DIP2B	case	control	-0.032144365	0.101233492	-0.317526981	0.75084377	0.848540188	down_in_case
Yang2025_S	ENSGALG00010022416	CCDC186	case	control	-0.162568483	0.120375414	-1.350512356	0.176851693	0.327847325	down_in_case
Yang2025_S	ENSGALG00010022419	DUOX2	case	control	-0.094454925	0.52053761	-0.181456485	0.856009286	0.914761457	down_in_case
Yang2025_S	ENSGALG00010022421	CMKLR1	case	control	-0.197966893	0.175667028	-1.126943942	0.259766183	0.426264927	down_in_case
Yang2025_S	ENSGALG00010022422	RPS6KB2	case	control	0.233557286	0.113455748	2.058576051	0.039534869	0.11234062	up_in_case
Yang2025_S	ENSGALG00010022425	VWA2	case	control	-0.207750713	0.463300243	-0.44841486	0.653853819	0.78083339	down_in_case
Yang2025_S	ENSGALG00010022426	EXD1	case	control	-1.469015821	0.462878855	-3.173650743	0.001505347	0.009640171	down_in_case
Yang2025_S	ENSGALG00010022427	RPL27	case	control	-0.448911727	0.277299244	-1.618871081	0.105475004	0.227769128	down_in_case
Yang2025_S	ENSGALG00010022429	ADK	case	control	0.014881785	0.139614959	0.10659162	0.915112967	0.950120376	up_in_case
Yang2025_S	ENSGALG00010022431	LARP4	case	control	-0.073025748	0.100595459	-0.725934838	0.467878718	0.628604892	down_in_case
Yang2025_S	ENSGALG00010022433	LNPK	case	control	-0.083134238	0.120685918	-0.688847875	0.490919004	0.648431879	down_in_case
Yang2025_S	ENSGALG00010022435	PIGK	case	control	-0.523993954	0.137238381	-3.818129808	0.000134467	0.001356843	down_in_case
Yang2025_S	ENSGALG00010022436	AFAP1L2	case	control	-0.02613643	0.09474488	-0.275861137	0.782654725	0.868542931	down_in_case
Yang2025_S	ENSGALG00010022438	RPN2	case	control	-0.135580305	0.088016346	-1.540399156	0.123463087	0.254539484	down_in_case
Yang2025_S	ENSGALG00010022439	IFI35	case	control	0.30330387	0.154641489	1.961335679	0.049839878	0.132998602	up_in_case
Yang2025_S	ENSGALG00010022440	SORD	case	control	0.003869091	0.235355331	0.016439359	0.98688388	0.99200944	up_in_case
Yang2025_S	ENSGALG00010022441	FICD	case	control	-0.307515836	0.164906499	-1.864789066	0.062210977	0.156437055	down_in_case
Yang2025_S	ENSGALG00010022448	FERRY3	case	control	1.049415042	0.279302097	3.75727592	0.000171773	0.001653078	up_in_case
Yang2025_S	ENSGALG00010022449	ENSGALG00010022449	case	control	0.504788458	0.161621376	3.123277825	0.001788488	0.011027693	up_in_case
Yang2025_S	ENSGALG00010022451	CHP1	case	control	-0.326639845	0.129095086	-2.530226783	0.011398882	0.044615885	down_in_case
Yang2025_S	ENSGALG00010022452	AP3M1	case	control	-0.339097603	0.132411531	-2.560937103	0.010439025	0.041801097	down_in_case
Yang2025_S	ENSGALG00010022453	ENSGALG00010022453	case	control	-0.157878644	0.119517269	-1.32096931	0.186511597	0.340099334	down_in_case
Yang2025_S	ENSGALG00010022454	ABLIM1	case	control	0.131866522	0.221117057	0.596365217	0.550931272	0.699095753	up_in_case
Yang2025_S	ENSGALG00010022455	C7orf50	case	control	-0.072178066	0.215684683	-0.334646231	0.737891966	0.840006907	down_in_case
Yang2025_S	ENSGALG00010022456	ATP5G3	case	control	-0.841789387	0.290755107	-2.89518349	0.00378937	0.019425477	down_in_case
Yang2025_S	ENSGALG00010022459	FGF6	case	control	1.24929876	0.573965147	2.176610838	0.029509616	0.090436881	up_in_case
Yang2025_S	ENSGALG00010022460	VAT1	case	control	-0.162631112	0.158355141	-1.027002414	0.304419327	0.474134719	down_in_case
Yang2025_S	ENSGALG00010022462	ITGA5	case	control	0.081678398	0.151694216	0.538441082	0.590272571	0.731150958	up_in_case
Yang2025_S	ENSGALG00010022463	DUSP12	case	control	-0.129494803	0.2060397	-0.628494427	0.529680097	0.682108137	down_in_case
Yang2025_S	ENSGALG00010022466	NEDD4	case	control	0.339798157	0.157120235	2.16266324	0.03056709	0.092951816	up_in_case
Yang2025_S	ENSGALG00010022467	SART3	case	control	0.178043296	0.067201456	2.64939641	0.008063569	0.034436357	up_in_case
Yang2025_S	ENSGALG00010022468	MYBPC3	case	control	0.326434924	0.225605017	1.446931139	0.147916211	0.289984862	up_in_case

Yang2025_S	ENSGALG00010022470	ENSGALG00010022470	case	control	0.63067412	0.286671404	2.199989646	0.02780763	0.086532428	up_in_case
Yang2025_S	ENSGALG00010022471	ST6GALNAC5	case	control	-0.349783071	0.160800563	-2.175260243	0.02961062	0.090587045	down_in_case
Yang2025_S	ENSGALG00010022472	QSER1	case	control	0.237986656	0.221764041	1.073152596	0.283202656	0.451484842	up_in_case
Yang2025_S	ENSGALG00010022473	ZNF385A	case	control	0.276321257	0.163316535	1.69193681	0.090658022	0.204818344	up_in_case
Yang2025_S	ENSGALG00010022474	VCL	case	control	-0.055009319	0.100045263	-0.549844313	0.582426162	0.724846706	down_in_case
Yang2025_S	ENSGALG00010022476	OIP5	case	control	-0.904499241	0.173649896	-5.208751979	1.90115E-07	5.92918E-06	down_in_case
Yang2025_S	ENSGALG00010022478	ATP6V1A	case	control	-0.379706313	0.124424787	-3.051693494	0.002275543	0.013201372	down_in_case
Yang2025_S	ENSGALG00010022479	ENSGALG00010022479	case	control	-1.302344749	0.405572223	-3.211129055	0.001322145	0.008719402	down_in_case
Yang2025_S	ENSGALG00010022480	PIF1	case	control	-0.632155132	0.226826411	-2.786955577	0.005320577	0.025107431	down_in_case
Yang2025_S	ENSGALG00010022481	CORO1B	case	control	0.137632638	0.097706938	1.408627071	0.15894547	0.304454312	up_in_case
Yang2025_S	ENSGALG00010022482	PRTG	case	control	0.895251654	0.466784207	1.917913332	0.055122001	0.14330692	up_in_case
Yang2025_S	ENSGALG00010022483	TOP2A	case	control	-0.301997256	0.129871787	-2.325349189	0.020053296	0.06784236	down_in_case
Yang2025_S	ENSGALG00010022485	ATF2	case	control	-0.147678357	0.135277982	-1.091665881	0.274979987	0.442234588	down_in_case
Yang2025_S	ENSGALG00010022489	NUSAP1	case	control	-0.118768099	0.141881535	-0.837093417	0.40254006	0.569077381	down_in_case
Yang2025_S	ENSGALG00010022493	ENSGALG00010022493	case	control	-0.257610931	0.206472539	-1.247676483	0.212149557	0.372424297	down_in_case
Yang2025_S	ENSGALG00010022494	FHIP2A	case	control	-0.425921941	0.08110123	-5.251732222	1.50675E-07	4.88293E-06	down_in_case
Yang2025_S	ENSGALG00010022496	TUBGCP2	case	control	0.029484304	0.087793503	0.335836973	0.736993807	0.839601758	up_in_case
Yang2025_S	ENSGALG00010022497	CORO1C	case	control	-0.080801916	0.096112148	-0.840704506	0.400513496	0.567352044	down_in_case
Yang2025_S	ENSGALG00010022498	NAA50	case	control	-0.429330265	0.105954446	-4.052026875	5.07758E-05	0.000614736	down_in_case
Yang2025_S	ENSGALG00010022500	ENSGALG00010022500	case	control	-0.277980219	0.390188557	-0.712425351	0.476201422	0.63550578	down_in_case
Yang2025_S	ENSGALG00010022502	ENSGALG00010022502	case	control	0.638069133	0.546397042	1.167775598	0.242897294	0.407804071	up_in_case
Yang2025_S	ENSGALG00010022504	RAD9A	case	control	0.088257105	0.152833572	0.577471979	0.563620663	0.709746425	up_in_case
Yang2025_S	ENSGALG00010022505	ZNF511	case	control	0.543230458	0.135519999	4.008489252	6.11084E-05	0.000718604	up_in_case
Yang2025_S	ENSGALG00010022506	EBNA1BP2	case	control	0.398045899	0.132041384	3.014554124	0.002573573	0.014517279	up_in_case
Yang2025_S	ENSGALG00010022507	ENSGALG00010022507	case	control	0.873921295	0.362629348	2.409957438	0.015954382	0.057590643	up_in_case
Yang2025_S	ENSGALG00010022508	USF3	case	control	0.519612768	0.275287647	1.887526642	0.059089526	0.151004404	up_in_case
Yang2025_S	ENSGALG00010022511	IGFBP4	case	control	0.633191719	0.145583545	4.349335756	1.36551E-05	0.000214042	up_in_case
Yang2025_S	ENSGALG00010022513	NDUFAF1	case	control	-0.146310043	0.201505451	-0.726084792	0.467786792	0.628604892	down_in_case
Yang2025_S	ENSGALG00010022515	CHRNA1	case	control	0.108238252	0.203376312	0.53220678	0.594582787	0.734726536	up_in_case
Yang2025_S	ENSGALG00010022516	TRUB1	case	control	-0.554606222	0.197808458	-2.803753829	0.005051146	0.024155015	down_in_case
Yang2025_S	ENSGALG00010022517	HNRNPA1	case	control	-0.141495591	0.145639947	-0.971543826	0.33127754	0.500634695	down_in_case
Yang2025_S	ENSGALG00010022518	ECHS1	case	control	0.123054037	0.187906125	0.654869749	0.512551619	0.667572691	up_in_case
Yang2025_S	ENSGALG00010022520	TMOD2	case	control	-0.324680421	0.234906959	-1.382166037	0.166920726	0.314755117	down_in_case
Yang2025_S	ENSGALG00010022521	RTF1	case	control	-0.087266422	0.067372508	-1.295282371	0.195222838	0.351204525	down_in_case
Yang2025_S	ENSGALG00010022522	TNS4	case	control	-0.121052024	0.338045969	-0.358093382	0.720273431	0.82765814	down_in_case
Yang2025_S	ENSGALG00010022524	SOX18	case	control	0.225701446	0.162733593	1.38693826	0.16546059	0.313072856	up_in_case
Yang2025_S	ENSGALG00010022525	ISCU	case	control	-0.32267874	0.213063529	-1.514471954	0.129906182	0.263768417	down_in_case
Yang2025_S	ENSGALG00010022526	PPP1CA	case	control	-0.107279907	0.189032922	-0.567519698	0.570361148	0.714697493	down_in_case
Yang2025_S	ENSGALG00010022528	SIDT1	case	control	0.023234028	0.28322891	0.082032685	0.934620722	0.960002682	up_in_case
Yang2025_S	ENSGALG00010022529	CCR7	case	control	-0.179218045	0.362648644	-0.494191962	0.621170616	0.7558516	down_in_case
Yang2025_S	ENSGALG00010022531	ENSGALG00010022531	case	control	0.482143645	0.414574943	1.162983081	0.244836367	0.410170086	up_in_case
Yang2025_S	ENSGALG00010022532	CBX5	case	control	0.124723851	0.135582882	0.919908539	0.357620556	0.526771874	up_in_case
Yang2025_S	ENSGALG00010022533	TMEM119	case	control	-0.267459533	0.158689433	-1.685427491	0.091906123	0.206900134	down_in_case
Yang2025_S	ENSGALG00010022534	CFAP57	case	control	0.178828663	0.525070621	0.34058021	0.733419629	0.837110275	up_in_case
Yang2025_S	ENSGALG00010022536	SMUG1	case	control	-0.552833637	0.306877952	-1.801477211	0.071627696	0.172727596	down_in_case
Yang2025_S	ENSGALG00010022538	WIPF1	case	control	0.372192544	0.231884597	1.605076615	0.108476945	0.232093694	up_in_case
Yang2025_S	ENSGALG00010022539	LYSMD2	case	control	-0.320211322	0.166207673	-1.926573647	0.054032786	0.141265829	down_in_case
Yang2025_S	ENSGALG00010022540	GPR146	case	control	-0.110955467	0.167168593	-0.66373393	0.506860619	0.662393578	down_in_case
Yang2025_S	ENSGALG00010022541	ITPKA	case	control	-0.362534234	0.149897325	-2.418550387	0.015582487	0.056624139	down_in_case
Yang2025_S	ENSGALG00010022542	ENSGALG00010022542	case	control	2.044533499	0.757074445	2.700571274	0.00692205	0.030724674	up_in_case
Yang2025_S	ENSGALG00010022544	SPICE1	case	control	0.034352819	0.102636042	0.334705218	0.737847465	0.840006907	up_in_case
Yang2025_S	ENSGALG00010022545	CSTF3	case	control	0.268892741	0.104899941	2.56332596	0.010367466	0.041605579	up_in_case
Yang2025_S	ENSGALG00010022546	MTG1	case	control	0.443108215	0.25206749	1.757895135	0.078765354	0.185279406	up_in_case
Yang2025_S	ENSGALG00010022547	SMARCE1	case	control	0.001869606	0.117926437	0.015854002	0.987350867	0.992277608	up_in_case
Yang2025_S	ENSGALG00010022548	ENSGALG00010022548	case	control	0.42767889	0.142653315	2.998029812	0.002717311	0.015096657	up_in_case
Yang2025_S	ENSGALG00010022550	TRIM45	case	control	0.381713726	0.242596932	1.573448284	0.115615054	0.243037529	up_in_case
Yang2025_S	ENSGALG00010022551	GPER1	case	control	0.730018628	0.605498708	1.205648531	0.227953038	0.390140701	up_in_case
Yang2025_S	ENSGALG00010022552	GPR155	case	control	-0.107639594	0.082731534	-1.301070934	0.193234176	0.348617069	down_in_case
Yang2025_S	ENSGALG00010022553	PRPF6	case	control	-0.299356409	0.07738482	-3.868412535	0.000109546	0.00114261	down_in_case
Yang2025_S	ENSGALG00010022554	SCG3	case	control	-0.296541272	0.2476519	-1.197411658	0.231146142	0.394008745	down_in_case
Yang2025_S	ENSGALG00010022557	WNT1	case	control	0.263139688	0.351189592	0.749280997	0.453687859	0.615652967	up_in_case
Yang2025_S	ENSGALG00010022558	NYAP2	case	control	1.229601445	0.437830533	2.808395833	0.004978899	0.023916912	up_in_case
Yang2025_S	ENSGALG00010022559	SSH1	case	control	0.009318418	0.090036233	0.103496307	0.91756908	0.95150507	up_in_case
Yang2025_S	ENSGALG00010022564	VTCN1	case	control	-0.770502627	0.363553692	-2.119364055	0.034059712	0.100598153	down_in_case
Yang2025_S	ENSGALG00010022565	ARF3	case	control	-0.11486061	0.14918529	-0.769919141	0.441347859	0.6047377	down_in_case
Yang2025_S	ENSGALG00010022566	ENSGALG00010022566	case	control	-0.172733186	0.714260176	-0.241835108	0.808907934	0.884782737	down_in_case
Yang2025_S	ENSGALG00010022567	PAOX	case	control	-0.078275374	0.233139474	-0.335744834	0.737063294	0.839612278	down_in_case
Yang2025_S	ENSGALG00010022568	SCRN3	case	control	0.329140205	0.121161847	2.716533397	0.006596954	0.029568528	up_in_case
Yang2025_S	ENSGALG00010022569	KIAA1549L	case	control	0.219654378	0.381989981	0.575026543			up_in_case
Yang2025_S	ENSGALG00010022571	RIPOR1	case	control	0.059752139	0.113348915	0.527152279	0.598087845	0.737227255	up_in_case
Yang2025_S	ENSGALG00010022572	ENSGALG00010022572	case	control	0.825935034	0.429356997	1.923655697	0.054397755	0.141873987	up_in_case
Yang2025_S	ENSGALG00010022573	AP4E1	case	control	-0.147585253	0.113599467	-1.299172053	0.19388489	0.349377348	down_in_case
Yang2025_S	ENSGALG00010022574	ITPRIPL2	case	control	-0.111571763	0.192532024	-0.579497168	0.562253757	0.708639198	down_in_case
Yang2025_S	ENSGALG00010022575	TMEM41B	case	control	-0.272504952	0.082532093	-3.301805911	0.000960645	0.006788332	down_in_case
Yang2025_S	ENSGALG00010022578	ZNF512B	case	control	0.155582422	0.067265372	2.312964555	0.020724588	0.069472488	up_in_case
Yang2025_S	ENSGALG00010022579	TNFAIP8L3	case	control	-0.137202743	0.126359815	-1.08580994	0.277563088	0.444988683	down_in_case
Yang2025_S	ENSGALG00010022581	TMEM125	case	control	1.735493461	0.791417888	2.192891376	0.028315206	0.087663275	up_in_case
Yang2025_S	ENSGALG00010022582	ENSGALG00010022582	case	control	-1.737150986	0.294284799	-5.902958596	3.5704E-09	1.83592E-07	down_in_case
Yang2025_S	ENSGALG00010022583	FKBP11	case	control	-0.198650786	0.230196161	-0.862963071	0.388157771	0.555232028	down_in_case
Yang2025_S	ENSGALG00010022585	CIR1	case	control	0.828629268	0.178723703	4.636370287	3.54581E-06	7.01858E-05	up_in_case
Yang2025_S	ENSGALG00010022588	TCP11L1	case	control	-0.122554253	0.109273008	-1.121541869	0.26205728	0.428864024	down_in_case
Yang2025_S	ENSGALG00010022590	ENSGALG00010022590	case	control	-0.076859694	0.169445911	-0.453594268	0.65012088	0.778021422	down_in_case
Yang2025_S	ENSGALG00010022593	MANBAL	case	control	-0.001805841	0.153230693	-0.011785114	0.990597057	0.994000591	down_in_case
Yang2025_S	ENSGALG00010022597	SLCO4A1	case	control	0.69812545	0.243730844	2.864329516	0.00417893	0.020797641	up_in_case
Yang2025_S	ENSGALG00010022599	RNLS	case	control	-0.033633644	0.241986363	-0.138989831	0.889458185	0.935441494	down_in_case
Yang2025_S	ENSGALG00010022603	ATRNL1	case	control	0.131450212	0.22004842	0.597369491	0.550260718	0.698668165	up_in_case
Yang2025_S	ENSGALG00010022608	P3H4	case	control	0.234007777	0.147834953	1.582898846	0.113444522	0.239869088	up_in_case
Yang2025_S	ENSGALG00010022614	DAO	case	control	-0.607754218	0.174147221	-3.489887544	0.000483224	0.00384345	down_in_case
Yang2025_S	ENSGALG00010022616	GLDN	case	control	0.204791698	0.16634909	1.231095991	0.218286965	0.379327703	up_in_case
Yang2025_S	ENSGALG00010022619	MADD	case	control	0.342462011	0.127574073	2.68441702	0.007265642	0.031782669	up_in_case
Yang2025_S	ENSGALG00010022622	MPPED2	case	control	-0.237450352	0.145586137	-1.630995624	0.102891243	0.224029605	down_in_case
Yang2025_S	ENSGALG00010022623	TOP1	case	control	0.350873354	0.171316245	2.048103227	0.040549886	0.114325721	up_in_case
Yang2025_S	ENSGALG00010022625	ENSGALG00010022625	case	control	-0.389856176	0.107885209	-3.613620236	0.000301951	0.002620207	down_in_case
Yang2025_S	ENSGALG00010022627	CTCF	case	control	-0.17583725	0.082190308	-2.139391547	0.032403971	0.09705953	down_in_case
Yang2025_S	ENSGALG00010022630	ENSGALG00010022630	case	control	0.959079462	0.338736423	2.831344369	0.004635279	0.022640241	up_in_case
Yang2025_S	ENSGALG00010022637	DMXL2	case	control	0.299369767	0.166182715	1.801449492	0.071632061	0.172727596	up_in_case
Yang2025_S	ENSGALG00010022642	SEC13	case	control	0.021034776	0.086686859	0.242652424	0.808274666	0.884506634	up_in_case
Yang2025_S	ENSGALG00010022643	SP3	case	control	0.350775752	0.18358151	1.910735742	0.056038549	0.145055048	up_in_case
Yang2025_S	ENSGALG00010022644	ARMC8	case	control	-0.103142432	0.076577043	-1.346910621	0.17800902	0.329404418	down_in_case
Yang2025_S	ENSGALG00010022647	SVIP	case	control	-0.615667374	0.101945157	-6.039201753	1.54878E-09	8.95532E-08	down_in_case
Yang2025_S	ENSGALG00010022649	PATZ1	case	control	-0.104894107	0.109130203	-0.961183099	0.336460113	0.504635849	down_in_case
Yang2025_S	ENSGALG00010022650	NR1H3	case	control	-0.368763391	0.057513993	-6.411716018	1.43891E-10	1.10171E-08	down_in_case
Yang2025_S	ENSGALG00010022652	CARMIL2	case	control	0.935218285	0.263777892	3.545476378	0.000391904	0.003246839	up_in_case
Yang2025_S	ENSGALG00010022655	NRIP3	case	control	2.455621972	1.113015193	2.206278932			up_in_case
Yang2025_S	ENSGALG00010022656	FKBP10	case	control	-0.102394504	0.128761136	-0.79522834	0.426480689	0.59104827	down_in_case
Yang2025_S	ENSGALG00010022657	MRPS35	case	control	-0.137212569	0.154557766	-0.887775316	0.374661623	0.542601301	down_in_case
Yang2025_S	ENSGALG00010022659	TIE1	case	control	-0.405359328	0.106449433	-3.807998924	0.000140096	0.001400456	down_in_case
Yang2025_S	ENSGALG00010022660	METAP1D	case	control	0.277160196	0.294710007	0.940450575	0.34698649	0.515375905	up_in_case
Yang2025_S	ENSGALG00010022661	ENSGALG00010022661	case	control	0.227554537	0.399504109	0.569592482	0.568954129	0.713496741	up_in_case
Yang2025_S	ENSGALG00010022662	GFRA1	case	control	0.032573622	0.224749177	0.14493322	0.884763597	0.932404199	up_in_case
Yang2025_S	ENSGALG00010022664	TRPC4AP	case	control	-0.062869452	0.086667791	-0.725407341	0.468202171	0.628857566	down_in_case
Yang2025_S	ENSGALG00010022666	ENSGALG00010022666	case	control	0.220197724	0.087032825	2.530053751	0.011404505	0.044615885	up_in_case
Yang2025_S	ENSGALG00010022667	PTEN	case	control	-0.186294262	0.136059554	-1.369211172	0.170933275	0.319897285	down_in_case
Yang2025_S	ENSGALG00010022671	DBR1	case	control	-0.201907385	0.086533461	-2.333286819	0.019633097	0.066919076	down_in_case
Yang2025_S	ENSGALG00010022673	KLHL42	case	control	-0.258451835	0.207866715	-1.243353635	0.213737548	0.374127965	down_in_case
Yang2025_S	ENSGALG00010022674	ENSGALG00010022674	case	control	0.737985664	0.456109499	1.618001085	0.105662364	0.228069484	up_in_case
Yang2025_S	ENSGALG00010022681	PPP2R3A	case	control	-0.306289851	0.123826867	-2.473533078	0.013378442	0.050277397	down_in_case
Yang2025_S	ENSGALG00010022682	EIF1	case	control	-0.889980222	0.210712651	-4.223667719	2.40358E-05	0.000339351	down_in_case
Yang2025_S	ENSGALG00010022684	ENSGALG00010022684	case	control	1.096031183	0.296045153	3.702243297	0.000213702	0.001969531	up_in_case
Yang2025_S	ENSGALG00010022685	ATAD1	case	control	0.161409901	0.115789611	1.39399295	0.163319734	0.309978274	up_in_case
Yang2025_S	ENSGALG00010022689	MPL	case	control	0.519998323	0.298369828	1.742797947	0.081368915	0.189487516	up_in_case
Yang2025_S	ENSGALG00010022691	ASIC2	case	control	0.663632301	0.865858142	0.766444604			up_in_case
Yang2025_S	ENSGALG00010022695	PIK3IP1	case	control	-0.214621974	0.172434529	-1.244657757	0.21325758	0.37352242	down_in_case
Yang2025_S	ENSGALG00010022698	SEMA6D	case	control	0.324160068	0.160728926	2.016812257	0.043715109	0.121059231	up_in_case
Yang2025_S	ENSGALG00010022702	CDC20	case	control	-0.533029957	0.177383942	-3.004950454	0.002656241	0.014823677	down_in_case
Yang2025_S	ENSGALG00010022707	SALL1	case	control	2.177803606	0.486172905	4.479483701	7.48238E-06	0.000129847	up_in_case

Yang2025_S	ENSGALG00010022708	SPRED1	case	control	-0.50106231	0.088458196	-5.664396658	1.47543E-08	6.50636E-07	down_in_case
Yang2025_S	ENSGALG00010022709	RNF5	case	control	0.441804267	0.151364112	2.918817821	0.003513615	0.018326254	up_in_case
Yang2025_S	ENSGALG00010022711	LRRC49	case	control	0.103704337	0.153265772	0.676630765	0.498640254	0.655647475	up_in_case
Yang2025_S	ENSGALG00010022712	DDX23	case	control	0.172182475	0.066030304	2.607628065	0.009117196	0.037937722	up_in_case
Yang2025_S	ENSGALG00010022717	ELOVL1	case	control	-0.194758754	0.218751587	-0.890319274	0.373294475	0.541255415	down_in_case
Yang2025_S	ENSGALG00010022718	C12orf43	case	control	0.299365628	0.20457227	1.463373452	0.143365219	0.283656727	up_in_case
Yang2025_S	ENSGALG00010022719	MSL2	case	control	-0.166339482	0.108610141	-1.53152809	0.12563893	0.257770684	down_in_case
Yang2025_S	ENSGALG00010022724	LUZP2	case	control	-0.141307833	0.344003067	-0.410774921	0.681237584	0.800321665	down_in_case
Yang2025_S	ENSGALG00010022727	ACP2	case	control	0.111343375	0.124148207	0.896858503	0.369794425	0.538462416	up_in_case
Yang2025_S	ENSGALG00010022729	ENSGALG00010022729	case	control	-0.255722868	0.138291842	-1.849153671	0.064435626	0.159998299	down_in_case
Yang2025_S	ENSGALG00010022731	LRRC8B	case	control	-0.168987159	0.15827528	-1.06767879	0.285665429	0.454164034	down_in_case
Yang2025_S	ENSGALG00010022733	PCCB	case	control	-0.086935761	0.162230989	-0.535876419	0.592043963	0.732433649	down_in_case
Yang2025_S	ENSGALG00010022734	ENSGALG00010022734	case	control	-0.586603679	0.331600905	-1.769005063	0.07689303	0.181981041	down_in_case
Yang2025_S	ENSGALG00010022737	CYLD	case	control	-0.06068664	0.074712078	-0.812273496	0.416634715	0.581743963	down_in_case
Yang2025_S	ENSGALG00010022741	HNF1A	case	control	1.679293448	0.523545888	3.207538222	0.001338763	0.008803992	up_in_case
Yang2025_S	ENSGALG00010022742	IFT46	case	control	0.164779482	0.149904353	1.099230803	0.271667406	0.438869152	up_in_case
Yang2025_S	ENSGALG00010022743	ZNF326	case	control	0.749951977	0.187541328	3.998862468	6.36476E-05	0.000742201	up_in_case
Yang2025_S	ENSGALG00010022745	PAPSS2	case	control	0.012405623	0.243019275	0.051047897	0.959287354	0.975386303	up_in_case
Yang2025_S	ENSGALG00010022747	NT5C3B	case	control	-0.243738554	0.147887511	-1.648134803	0.099325012	0.217864637	down_in_case
Yang2025_S	ENSGALG00010022748	ENSGALG00010022748	case	control	0.67251496	0.376678251	1.785383039	0.074199208	0.177322237	up_in_case
Yang2025_S	ENSGALG00010022749	HSPA12A	case	control	-0.469735163	0.10061011	-4.668866388	3.02866E-06	6.12546E-05	down_in_case
Yang2025_S	ENSGALG00010022751	SLC25A12	case	control	0.021074468	0.136703287	0.154162119	0.87748191	0.928025294	up_in_case
Yang2025_S	ENSGALG00010022754	SNX20	case	control	-0.641963615	0.181791637	-3.53131545	0.000413498	0.003395871	down_in_case
Yang2025_S	ENSGALG00010022755	MINPP1	case	control	0.035630018	0.129065011	0.276062561	0.782500016	0.868509576	up_in_case
Yang2025_S	ENSGALG00010022759	THSD4	case	control	-0.244533013	0.202115274	-1.209869042	0.226329148	0.388368501	down_in_case
Yang2025_S	ENSGALG00010022760	ELAVL4	case	control	0.270049746	0.384097274	0.703076446	0.48200811	0.640303433	up_in_case
Yang2025_S	ENSGALG00010022764	MFAP5	case	control	-0.807022401	0.161441019	-4.998868341	5.76678E-07	1.50206E-05	down_in_case
Yang2025_S	ENSGALG00010022765	KRT12	case	control	0.239908391	0.503982213	0.476025511	0.634056203	0.765245369	up_in_case
Yang2025_S	ENSGALG00010022767	CACNB3	case	control	0.904510505	0.31097599	2.908618459	0.003630296	0.018799025	up_in_case
Yang2025_S	ENSGALG00010022769	FXYD6	case	control	0.035673157	0.18605705	0.191732358	0.847951858	0.909778961	up_in_case
Yang2025_S	ENSGALG00010022770	HAT1	case	control	-0.16253183	0.123033928	-1.321032604	0.186490493	0.340099334	down_in_case
Yang2025_S	ENSGALG00010022771	SGMS1	case	control	-0.13081986	0.099748622	-1.311495422	0.189690439	0.344497335	down_in_case
Yang2025_S	ENSGALG00010022776	CDH4	case	control	0.118465792	0.217571759	0.544490669	0.586103867	0.72799335	up_in_case
Yang2025_S	ENSGALG00010022778	GDF11	case	control	0.225226651	0.250717474	0.898328495	0.369010446	0.537683998	up_in_case
Yang2025_S	ENSGALG00010022780	DYNC1I2	case	control	0.011154103	0.089991498	0.123946186	0.901357884	0.941137577	up_in_case
Yang2025_S	ENSGALG00010022782	TMPRSS13	case	control	-0.251266591	0.339054331	-0.741080612	0.458644565	0.619844051	down_in_case
Yang2025_S	ENSGALG00010022783	STAG1	case	control	-0.064703735	0.113460954	-0.570273145	0.568492453	0.712981939	down_in_case
Yang2025_S	ENSGALG00010022786	KRT40	case	control	0.01115373	0.406921457	0.027410031	0.978132698	0.9869076	up_in_case
Yang2025_S	ENSGALG00010022787	ENO4	case	control	0.737779349	0.39176964	1.883196842	0.059673697	0.151965089	up_in_case
Yang2025_S	ENSGALG00010022788	CYBRD1	case	control	-0.405322436	0.13938894	-2.907852202	0.003639203	0.018838147	down_in_case
Yang2025_S	ENSGALG00010022789	ENSGALG00010022789	case	control	-0.59752863	0.400349061	-1.492519126	0.135563107	0.27212214	down_in_case
Yang2025_S	ENSGALG00010022792	DDB2	case	control	-0.001242814	0.131721127	-0.009435188	0.992471921	0.995042536	down_in_case
Yang2025_S	ENSGALG00010022795	TAF4	case	control	-0.042651248	0.134466712	-0.317188155	0.751100837	0.848540188	down_in_case
Yang2025_S	ENSGALG00010022796	RIMKLB	case	control	0.083242118	0.098597227	0.844264285	0.398521739	0.56546181	up_in_case
Yang2025_S	ENSGALG00010022797	EFHD1	case	control	-0.359378083	0.092880259	-3.8692623	0.000109165	0.001140342	down_in_case
Yang2025_S	ENSGALG00010022799	DCAF17	case	control	0.108294922	0.144757703	0.74811164	0.454392822	0.616189565	up_in_case
Yang2025_S	ENSGALG00010022800	KLHL10	case	control	-0.417354983	0.374590366	-1.114163687	0.265208971	0.432416631	down_in_case
Yang2025_S	ENSGALG00010022801	LSM14B	case	control	0.187050539	0.073701742	2.537939192	0.011150736	0.043906613	up_in_case
Yang2025_S	ENSGALG00010022803	ENSGALG00010022803	case	control	-0.47115062	0.313031997	-1.505119683	0.132293309	0.26729118	down_in_case
Yang2025_S	ENSGALG00010022804	SNX19	case	control	-0.100638458	0.141258868	-0.712439927	0.476192398	0.63550578	down_in_case
Yang2025_S	ENSGALG00010022805	ENSGALG00010022805	case	control	1.325738096	0.203866896	6.502959131	7.87552E-11	6.85909E-09	up_in_case
Yang2025_S	ENSGALG00010022806	SPPL3	case	control	-0.191044901	0.056115934	-3.404467971	0.000662931	0.005026083	down_in_case
Yang2025_S	ENSGALG00010022807	ADCY6	case	control	0.546012102	0.105942297	5.153863168	2.55174E-07	7.43902E-06	up_in_case
Yang2025_S	ENSGALG00010022808	PSMA7	case	control	-0.273951826	0.194668158	-1.407275999	0.159345563	0.304718049	down_in_case
Yang2025_S	ENSGALG00010022809	MYO1E	case	control	0.628133678	0.180706564	3.475987063	0.000508977	0.004011647	up_in_case
Yang2025_S	ENSGALG00010022811	GIGYF2	case	control	0.387414261	0.169378752	2.287266002	0.022180302	0.072934492	up_in_case
Yang2025_S	ENSGALG00010022813	USP24	case	control	0.04439327	0.105003654	0.422778337	0.672456994	0.793789768	up_in_case
Yang2025_S	ENSGALG00010022815	LRRC8D	case	control	0.114395615	0.151140501	0.756882593	0.449120205	0.611419505	up_in_case
Yang2025_S	ENSGALG00010022816	METTL8	case	control	0.329067208	0.241641012	1.361801975	0.173260388	0.323158836	up_in_case
Yang2025_S	ENSGALG00010022817	KRT23	case	control	0.200909143	0.912777283	0.220107518	0.82578742	0.895722557	up_in_case
Yang2025_S	ENSGALG00010022819	PACSIN3	case	control	0.167364205	0.120732072	1.386244781	0.165672172	0.313188741	up_in_case
Yang2025_S	ENSGALG00010022820	SHTN1	case	control	0.074297989	0.217507915	0.34158752	0.73266133	0.836718213	up_in_case
Yang2025_S	ENSGALG00010022821	ENSGALG00010022821	case	control	-0.982046529	0.775346583	-1.266590388			down_in_case
Yang2025_S	ENSGALG00010022822	KCNJ13	case	control	5.369124695	0.585586547	9.168797889	4.78323E-20	2.66617E-17	up_in_case
Yang2025_S	ENSGALG00010022823	IGHMBP2	case	control	-0.039357201	0.110988186	-0.354607118	0.722883943	0.829631671	down_in_case
Yang2025_S	ENSGALG00010022824	PUS1	case	control	-0.039068122	0.089837017	-0.434877771	0.663651151	0.787263027	down_in_case
Yang2025_S	ENSGALG00010022825	SS18L1	case	control	0.93606114	0.297583447	3.1455417	0.001657796	0.010387312	up_in_case
Yang2025_S	ENSGALG00010022826	SNORC	case	control	3.054829673	0.849115283	3.597661865	0.000321091	0.002760271	up_in_case
Yang2025_S	ENSGALG00010022827	CMTR2	case	control	-0.604834349	0.11773565	-5.137223521	2.78827E-07	7.97835E-06	down_in_case
Yang2025_S	ENSGALG00010022828	PRKG1	case	control	-0.320416943	0.153338758	-2.089601786	0.036653586	0.106254987	down_in_case
Yang2025_S	ENSGALG00010022830	MTG2	case	control	0.2739598	0.168451632	1.626341031	0.103877128	0.225436501	up_in_case
Yang2025_S	ENSGALG00010022831	LRRC8C	case	control	0.116435056	0.178801573	0.651197049	0.514919295	0.6695969	up_in_case
Yang2025_S	ENSGALG00010022833	HRH3	case	control	-0.085308321	0.452907176	-0.188357185	0.85059666	0.911353565	down_in_case
Yang2025_S	ENSGALG00010022834	CHRNA7	case	control	-0.191764591	0.248237317	-0.772505091	0.439815333	0.603302507	down_in_case
Yang2025_S	ENSGALG00010022837	ITGB3	case	control	-0.26992491	0.153887823	-1.75403683	0.079424197	0.186388713	down_in_case
Yang2025_S	ENSGALG00010022838	ARFGAP2	case	control	0.055618416	0.097081183	0.572906239	0.56670818	0.711703646	up_in_case
Yang2025_S	ENSGALG00010022839	SYT12	case	control	0.755777415	0.236292278	3.198485459	0.001381515	0.009046716	up_in_case
Yang2025_S	ENSGALG00010022840	MARVELD1	case	control	0.359042648	0.245616946	1.461799173	0.143796251	0.284267379	up_in_case
Yang2025_S	ENSGALG00010022841	ENSGALG00010022841	case	control	0.442151198	0.394832805	1.119844126	0.262780192	0.429591972	up_in_case
Yang2025_S	ENSGALG00010022842	PHC1	case	control	0.430599623	0.18580499	2.317481477	0.020477519	0.068909495	up_in_case
Yang2025_S	ENSGALG00010022845	HYDIN	case	control	0.518392439	0.307124061	1.687892631	0.091431841	0.206098788	up_in_case
Yang2025_S	ENSGALG00010022848	SGIP1	case	control	0.662890998	0.611419716	1.084183221	0.27828357	0.445836002	up_in_case
Yang2025_S	ENSGALG00010022850	ULK1	case	control	0.299663476	0.158848456	1.886473965	0.059231114	0.151227896	up_in_case
Yang2025_S	ENSGALG00010022858	HERC4	case	control	-0.00956387	0.077692246	-0.123099409	0.902028379	0.941343928	down_in_case
Yang2025_S	ENSGALG00010022860	MOB3C	case	control	-0.007398971	0.119934821	-0.061691601	0.950808429	0.969594984	down_in_case
Yang2025_S	ENSGALG00010022862	ACADS	case	control	0.162593473	0.117717329	1.381219523	0.167211474	0.315132795	up_in_case
Yang2025_S	ENSGALG00010022863	FAM81A	case	control	0.378446686	0.235153365	1.609361132	0.107537399	0.230508177	up_in_case
Yang2025_S	ENSGALG00010022864	CSTPP1	case	control	0.044528901	0.135207947	0.329336415	0.741901423	0.842162493	up_in_case
Yang2025_S	ENSGALG00010022866	KLHL11	case	control	-0.40965705	0.12120862	-3.379768277	0.00072547	0.0054133	down_in_case
Yang2025_S	ENSGALG00010022867	ATPAF1	case	control	0.163191817	0.15441399	1.05684606	0.290581849	0.459190859	up_in_case
Yang2025_S	ENSGALG00010022868	VAC14	case	control	0.270415896	0.081269451	3.327399063	0.000876607	0.006296662	up_in_case
Yang2025_S	ENSGALG00010022872	KRT13	case	control	-0.258738867	0.422324117	-0.612654728	0.540104663	0.690770674	down_in_case
Yang2025_S	ENSGALG00010022873	NGEF	case	control	0.203884718	0.335145639	0.608346624	0.542957605	0.692931058	up_in_case
Yang2025_S	ENSGALG00010022874	EFCAB14	case	control	-0.268391438	0.057543881	-4.664117763	3.09944E-06	6.22344E-05	down_in_case
Yang2025_S	ENSGALG00010022875	SPON1	case	control	-0.117774317	0.356041234	-0.33078842	0.740804308	0.841450817	down_in_case
Yang2025_S	ENSGALG00010022877	KYAT3	case	control	-0.410970211	0.171968584	-2.389798197	0.016857633	0.059860169	down_in_case
Yang2025_S	ENSGALG00010022879	ENSGALG00010022879	case	control	-0.336160925	0.278961913	-1.20504237	0.228186946	0.390349262	down_in_case
Yang2025_S	ENSGALG00010022881	CCNB1	case	control	-0.508507276	0.195214786	-2.604860454	0.009191169	0.038135652	down_in_case
Yang2025_S	ENSGALG00010022885	M6PR	case	control	-0.322558596	0.117938728	-2.734967565	0.006238642	0.028293141	down_in_case
Yang2025_S	ENSGALG00010022886	PLA2G15	case	control	-0.719022975	0.155160855	-4.634048812	3.58582E-06	7.0808E-05	down_in_case
Yang2025_S	ENSGALG00010022888	OSBPL2	case	control	-0.098376299	0.092412256	-1.064537369	0.287085335	0.455797441	down_in_case
Yang2025_S	ENSGALG00010022890	MYPN	case	control	0.45913961	0.2200282	2.086730744	0.036912481	0.106827709	up_in_case
Yang2025_S	ENSGALG00010022891	NTM	case	control	-0.153943036	0.170543303	-0.902662452	0.36670507	0.53547471	down_in_case
Yang2025_S	ENSGALG00010022900	MMP17	case	control	-0.551723413	0.136560253	-4.040146394	5.34178E-05	0.000642259	down_in_case
Yang2025_S	ENSGALG00010022911	SMPD3	case	control	-0.602068647	0.597670613	-1.007358624	0.313762467	0.483178249	down_in_case
Yang2025_S	ENSGALG00010022917	CYP4B7	case	control	-0.364629226	0.39852289	-0.914951775	0.360216948	0.528884747	down_in_case
Yang2025_S	ENSGALG00010022919	ACLY	case	control	-0.04009026	0.094133515	-0.425887206	0.670190038	0.792192467	down_in_case
Yang2025_S	ENSGALG00010022921	ENSGALG00010022921	case	control	0.555068591	0.2407648	2.305439129	0.021141992	0.07062275	up_in_case
Yang2025_S	ENSGALG00010022923	CD63	case	control	0.37389496	0.252246951	1.482257599	0.138271766	0.276162187	up_in_case
Yang2025_S	ENSGALG00010022928	RNF111	case	control	0.616355698	0.253689696	2.429565361	0.015116939	0.055362561	up_in_case
Yang2025_S	ENSGALG00010022929	LRP4	case	control	-0.360262926	0.182746323	-1.971382629	0.048680126	0.130780328	down_in_case
Yang2025_S	ENSGALG00010022930	GTF2B	case	control	-0.143241203	0.112324508	-1.275244425	0.202222742	0.3602824	down_in_case
Yang2025_S	ENSGALG00010022931	RDH5	case	control	0.046228387	0.220847405	0.209322756	0.834196289	0.90182508	up_in_case
Yang2025_S	ENSGALG00010022932	OPCML	case	control	-0.037827936	0.187398621	-0.201858132	0.840027634	0.905085473	down_in_case
Yang2025_S	ENSGALG00010022933	UNC119B	case	control	-0.294396661	0.101254599	-2.907489292	0.003643428	0.018844512	down_in_case
Yang2025_S	ENSGALG00010022937	BLOC1S1	case	control	0.438916664	0.175423286	2.502043331	0.012347881	0.047414639	up_in_case
Yang2025_S	ENSGALG00010022942	NEU2	case	control	-0.339767162	0.369367941	-0.919860996	0.357645404	0.526771874	down_in_case
Yang2025_S	ENSGALG00010022945	KCNA4	case	control	0.406698151	0.459841352	0.884431445	0.376463354	0.544701157	up_in_case
Yang2025_S	ENSGALG00010022946	KRT17	case	control	0.341625763	0.416171767	0.820876836	0.411716427	0.577073869	up_in_case
Yang2025_S	ENSGALG00010022949	INSC	case	control	0.090985976	0.174980283	0.519978447	0.603078598	0.741346176	up_in_case
Yang2025_S	ENSGALG00010022950	ITGA7	case	control	0.436400734	0.147970978	2.949231931	0.003185648	0.017078542	up_in_case
Yang2025_S	ENSGALG00010022953	ENSGALG00010022953	case	control	-0.738858746	2.234380272	-0.33067726	0.740888281	0.841450817	down_in_case
Yang2025_S	ENSGALG00010022954	ADRM1	case	control	0.046485537	0.131973006	0.352235193	0.724661886	0.830611257	up_in_case
Yang2025_S	ENSGALG00010022956	PBLD	case	control	-0.263881151	0.22366401	-1.179810518	0.238075585	0.40198513	down_in_case
Yang2025_S	ENSGALG00010022957	SLTM	case	control	0.938156976	0.248695393	3.77231345	0.000161741	0.001574164	up_in_case
Yang2025_S	ENSGALG00010022958	SFSWAP	case	control	0.966895861	0.172332013	5.610657266	2.0156E-08	8.61575E-07	up_in_case

Yang2025_S	ENSGALG00010022959	ADAMTS8	case	control	-0.30193803	0.171526702	-1.760297532	0.078357372	0.184600166	down_in_case
Yang2025_S	ENSGALG00010022967	ADAMTS15	case	control	-0.406881064	0.082871421	-4.909787448	9.11752E-07	2.22398E-05	down_in_case
Yang2025_S	ENSGALG00010022969	TTN	case	control	1.570556044	0.198583857	7.908780024	2.59923E-15	6.13904E-13	up_in_case
Yang2025_S	ENSGALG00010022971	CKAP5	case	control	-0.180930651	0.06937218	-2.608115384	0.009104226	0.03791901	down_in_case
Yang2025_S	ENSGALG00010022972	ENSGALG00010022972	case	control	-2.968021744	0.810636129	-3.661348957			down_in_case
Yang2025_S	ENSGALG00010022974	INPP5D	case	control	0.209666673	0.133489327	1.570662447	0.116261076	0.243880491	up_in_case
Yang2025_S	ENSGALG00010022976	MINDY2	case	control	-0.445209581	0.058960248	-7.551012752	4.31887E-14	7.71582E-12	down_in_case
Yang2025_S	ENSGALG00010022978	ARL14EP	case	control	-0.374837431	0.157541053	-2.379300022	0.017345552	0.061115111	down_in_case
Yang2025_S	ENSGALG00010022979	LAMA5	case	control	-0.03513259	0.114120395	-0.307855485	0.7581923	0.85295517	down_in_case
Yang2025_S	ENSGALG00010022981	HNRNPH3	case	control	0.484203037	0.174889001	2.768630581	0.005629242	0.02614783	up_in_case
Yang2025_S	ENSGALG00010022983	PKN2	case	control	-0.113442056	0.104065454	-1.090102924	0.275667808	0.442714176	down_in_case
Yang2025_S	ENSGALG00010022985	ENSGALG00010022985	case	control	0.942836041	0.471895743	1.997975303	0.045719338	0.125191388	up_in_case
Yang2025_S	ENSGALG00010022986	ALG9	case	control	-0.858780431	0.123532569	-6.951854366	3.60515E-12	4.44583E-10	down_in_case
Yang2025_S	ENSGALG00010022987	F2	case	control	-1.230965013	0.545095824	-2.258254344			down_in_case
Yang2025_S	ENSGALG00010022990	SLC25A22	case	control	-0.124408289	0.164362685	-0.756913222	0.449101853	0.611419505	down_in_case
Yang2025_S	ENSGALG00010022991	TAL1	case	control	-0.132797537	0.186172441	-0.713303948	0.475657695	0.635122648	down_in_case
Yang2025_S	ENSGALG00010022992	SLC7A6	case	control	0.027454241	0.150615463	0.182280363	0.855362709	0.91449128	up_in_case
Yang2025_S	ENSGALG00010022994	LIPC	case	control	-2.117430829	0.842471195	-2.513356946			down_in_case
Yang2025_S	ENSGALG00010022997	ARHGAP1	case	control	-0.271506771	0.094163886	-2.883342878	0.003934789	0.019924158	down_in_case
Yang2025_S	ENSGALG00010022999	ENSGALG00010022999	case	control	0.993489718	0.460279872	2.158447021	0.030893093	0.093646999	up_in_case
Yang2025_S	ENSGALG00010023002	ATG16L1	case	control	0.012128179	0.135476778	0.089522201	0.928666911	0.956889486	up_in_case
Yang2025_S	ENSGALG00010023003	ENSGALG00010023003	case	control	-0.198515091	0.687424509	-0.288780934	0.772749024	0.861736367	down_in_case
Yang2025_S	ENSGALG00010023005	IRF7	case	control	-0.017258981	0.173052909	-0.099732393	0.920556783	0.953042999	down_in_case
Yang2025_S	ENSGALG00010023007	LMO4	case	control	0.164981592	0.117687706	1.401859192	0.160957299	0.306663926	up_in_case
Yang2025_S	ENSGALG00010023015	METTL2B	case	control	-0.408361667	0.13350206	-3.058841688	0.002221945	0.012970202	down_in_case
Yang2025_S	ENSGALG00010023017	BUB1B	case	control	-0.127181884	0.110243371	-1.153646544	0.248645108	0.414408513	down_in_case
Yang2025_S	ENSGALG00010023019	CABP1	case	control	0.109998588	0.353789277	0.31091555	0.755864823	0.851700195	up_in_case
Yang2025_S	ENSGALG00010023024	PRMT7	case	control	-0.143053013	0.156696167	-0.912932429	0.361278081	0.529882123	down_in_case
Yang2025_S	ENSGALG00010023026	PHRF1	case	control	0.183789422	0.118354224	1.552875901	0.120452785	0.250262347	up_in_case
Yang2025_S	ENSGALG00010023028	TMEM61	case	control	0.325219335	0.417442196	0.779076331	0.435934752	0.599828981	up_in_case
Yang2025_S	ENSGALG00010023030	HS2ST1	case	control	-0.263119631	0.073854597	-3.562670988	0.000367101	0.003081655	down_in_case
Yang2025_S	ENSGALG00010023041	CD151	case	control	-0.199194323	0.074732018	-2.665448218	0.007688577	0.033211507	down_in_case
Yang2025_S	ENSGALG00010023042	ENSGALG00010023042	case	control	-0.940479242	0.865634627	-1.086462132	0.277274586	0.444628464	down_in_case
Yang2025_S	ENSGALG00010023043	CNP	case	control	0.422652582	0.170488707	2.479064971	0.013172731	0.049691934	up_in_case
Yang2025_S	ENSGALG00010023046	SLC7A6OS	case	control	-0.099722358	0.101563738	-0.981869719	0.326164035	0.496081187	down_in_case
Yang2025_S	ENSGALG00010023048	ARFGAP1	case	control	-0.231316092	0.093441728	-2.475511715	0.01330454	0.050080702	down_in_case
Yang2025_S	ENSGALG00010023050	RUFY2	case	control	0.416320568	0.153506061	2.712079015	0.006686265	0.029853607	up_in_case
Yang2025_S	ENSGALG00010023052	RAD54L	case	control	-0.227899945	0.193285169	-1.179086564	0.238363708	0.402325372	down_in_case
Yang2025_S	ENSGALG00010023053	MAN1A2	case	control	-0.355858732	0.10512782	-3.385010082	0.000711756	0.005329568	down_in_case
Yang2025_S	ENSGALG00010023054	AQP9	case	control	0.241134508	0.201879595	1.194447153	0.232303101	0.395454654	up_in_case
Yang2025_S	ENSGALG00010023056	SAG	case	control	0.603747359	0.440985543	1.369086513	0.170972234	0.319927229	up_in_case
Yang2025_S	ENSGALG00010023057	PAFAH1B2	case	control	-0.646042593	0.118811313	-5.437551147	5.40178E-08	1.99137E-06	down_in_case
Yang2025_S	ENSGALG00010023058	PPARA	case	control	0.091745634	0.15904786	0.576842932	0.564045565	0.710088982	up_in_case
Yang2025_S	ENSGALG00010023059	SELENOF	case	control	-0.460551862	0.179561812	-2.564865303	0.010321587	0.041502985	down_in_case
Yang2025_S	ENSGALG00010023060	ALDH1A2	case	control	-0.319565077	0.119462672	-2.675020334	0.007472472	0.032479725	down_in_case
Yang2025_S	ENSGALG00010023062	STX2	case	control	0.32167392	0.17966575	1.79040201	0.073389307	0.175960082	up_in_case
Yang2025_S	ENSGALG00010023063	ENSGALG00010023063	case	control	-0.052958397	0.06868173	-0.771069638	0.440665656	0.604091536	down_in_case
Yang2025_S	ENSGALG00010023064	ATG13	case	control	-0.559818822	0.08295861	-6.74817026	1.49721E-11	1.51794E-09	down_in_case
Yang2025_S	ENSGALG00010023065	DNA2	case	control	-0.146736729	0.155775058	-0.941978331	0.346203734	0.514727257	down_in_case
Yang2025_S	ENSGALG00010023066	TLR21	case	control	-0.032339252	0.204109724	-0.158440525	0.874109683	0.926381944	down_in_case
Yang2025_S	ENSGALG00010023067	C15orf62	case	control	-0.08853003	0.614107158	-0.144160557	0.885373687	0.932764975	down_in_case
Yang2025_S	ENSGALG00010023068	CDPF1	case	control	-0.127870212	0.099832286	-1.280850278	0.20024626	0.357839399	down_in_case
Yang2025_S	ENSGALG00010023069	ENSGALG00010023069	case	control	0.180177746	0.264784905	0.680468344	0.49620796	0.653374083	up_in_case
Yang2025_S	ENSGALG00010023070	SIDT2	case	control	0.325025368	0.099325891	3.272312615	0.001066716	0.007373354	up_in_case
Yang2025_S	ENSGALG00010023071	POLR2M	case	control	0.161210696	0.103178667	1.562442123	0.11818387	0.246762396	up_in_case
Yang2025_S	ENSGALG00010023073	POMGNT1	case	control	-0.621676876	0.172914904	-3.595276417	0.000324047	0.00277883	down_in_case
Yang2025_S	ENSGALG00010023074	CCNT1	case	control	0.455868602	0.152508462	2.989136435	0.002797672	0.015427604	up_in_case
Yang2025_S	ENSGALG00010023075	PKDREJ	case	control	0.21464612	0.229625729	0.934765111	0.349909363	0.518359423	up_in_case
Yang2025_S	ENSGALG00010023076	DNAJC7	case	control	0.389369626	0.125426009	3.10437706	0.001906802	0.0115779	up_in_case
Yang2025_S	ENSGALG00010023077	KANSL2	case	control	0.547092878	0.098217626	5.570210781	2.54431E-08	1.04587E-06	up_in_case
Yang2025_S	ENSGALG00010023078	TAGLN	case	control	0.114797329	0.243249109	0.471933193	0.636974475	0.767508803	up_in_case
Yang2025_S	ENSGALG00010023079	SLC25A16	case	control	0.064606975	0.090756507	0.711871547	0.476544323	0.635650918	up_in_case
Yang2025_S	ENSGALG00010023080	MCRS1	case	control	-0.070481552	0.136737236	-0.515452509	0.606236847	0.743766699	down_in_case
Yang2025_S	ENSGALG00010023081	PRLHR2	case	control	0.042245966	0.355189855	0.118939112	0.905323595	0.943381503	up_in_case
Yang2025_S	ENSGALG00010023083	HARBI1	case	control	0.033465972	0.183457393	0.182418225	0.855254524	0.914445776	up_in_case
Yang2025_S	ENSGALG00010023084	ENSGALG00010023084	case	control	0.978037092	0.246371659	3.969762988	7.19442E-05	0.000817734	up_in_case
Yang2025_S	ENSGALG00010023086	PCSK7	case	control	0.099582177	0.136860807	0.727616466	0.466848398	0.627886539	up_in_case
Yang2025_S	ENSGALG00010023087	SH3GLB1	case	control	0.227844632	0.122391346	1.861607369	0.062658454	0.157059394	up_in_case
Yang2025_S	ENSGALG00010023089	ENSGALG00010023089	case	control	1.313241496	0.447930425	2.931797938	0.003370059	0.017781816	up_in_case
Yang2025_S	ENSGALG00010023090	LRRC41	case	control	-0.18912284	0.100356374	-1.884512496	0.059495686	0.15167808	down_in_case
Yang2025_S	ENSGALG00010023091	ENSGALG00010023091	case	control	0.202082518	0.100933243	2.00214035	0.045269639	0.124449086	up_in_case
Yang2025_S	ENSGALG00010023093	POP5	case	control	0.469785586	0.23277594	2.018187898	0.043571696	0.120734059	up_in_case
Yang2025_S	ENSGALG00010023094	CCDC32	case	control	-0.616248789	0.097439866	-6.324401018	2.54217E-10	1.80515E-08	down_in_case
Yang2025_S	ENSGALG00010023096	SPATS2	case	control	0.358194419	0.120744111	2.966558092	0.003011535	0.016380068	up_in_case
Yang2025_S	ENSGALG00010023097	LURAP1	case	control	-0.132939348	0.249897332	-0.531975859	0.594742716	0.734858995	down_in_case
Yang2025_S	ENSGALG00010023102	RNF10	case	control	-0.365406435	0.072498804	-5.040171893	4.65114E-07	1.26589E-05	down_in_case
Yang2025_S	ENSGALG00010023103	DGKD	case	control	-0.004927999	0.103507403	-0.047610113	0.962026972	0.977458685	down_in_case
Yang2025_S	ENSGALG00010023104	ENSGALG00010023104	case	control	0.225529961	0.135426886	1.665326339	0.095847666	0.212918417	up_in_case
Yang2025_S	ENSGALG00010023107	ZC3HAV1	case	control	-0.248817875	0.110498722	-2.25177151	0.024336715	0.078575561	down_in_case
Yang2025_S	ENSGALG00010023108	TET1	case	control	0.552110971	0.240207689	2.298473344	0.021534863	0.071415593	up_in_case
Yang2025_S	ENSGALG00010023110	LIMA1	case	control	-0.03940344	0.0543575	-0.724894267	0.468516898	0.629159003	down_in_case
Yang2025_S	ENSGALG00010023112	PCMT1L	case	control	-0.31624247	0.216412575	-1.461294334	0.143934684	0.284419998	down_in_case
Yang2025_S	ENSGALG00010023113	MYZAP	case	control	0.69313792	0.227763335	3.04323749	0.002340475	0.013488224	up_in_case
Yang2025_S	ENSGALG00010023114	UQCRHL	case	control	-0.438478688	0.304005616	-1.442337459	0.149207198	0.291592092	down_in_case
Yang2025_S	ENSGALG00010023115	RPLP2	case	control	-0.549466141	0.301675057	-1.821384064	0.068548496	0.167348159	down_in_case
Yang2025_S	ENSGALG00010023116	PROC	case	control	-1.143471619	0.848844173	-1.347092499	0.177950444	0.329404418	down_in_case
Yang2025_S	ENSGALG00010023117	RPUSD2	case	control	-0.030225871	0.100796111	-0.299871397	0.764275253	0.856605457	down_in_case
Yang2025_S	ENSGALG00010023118	PIDD1	case	control	-0.121162487	0.168389265	-0.719538071	0.471809454	0.631861675	down_in_case
Yang2025_S	ENSGALG00010023119	NDRG4	case	control	0.596351184	0.216838208	2.750212654	0.00595566	0.027255213	up_in_case
Yang2025_S	ENSGALG00010023120	CHRM4	case	control	-0.263462607	0.255715501	-1.030295803	0.302871168	0.47251443	down_in_case
Yang2025_S	ENSGALG00010023122	ENSGALG00010023122	case	control	-0.279715336	0.193338717	-1.446763175	0.147963264	0.290036303	down_in_case
Yang2025_S	ENSGALG00010023123	RNF214	case	control	0.352830191	0.118277257	2.983077228	0.00285366	0.015683828	up_in_case
Yang2025_S	ENSGALG00010023124	NSUN4	case	control	-0.205644677	0.233457245	-0.880866545	0.378390057	0.546524196	down_in_case
Yang2025_S	ENSGALG00010023125	COL20A1	case	control	0.024268461	0.123449907	0.196585498	0.84415192	0.907437946	up_in_case
Yang2025_S	ENSGALG00010023126	CGNL1	case	control	0.168316241	0.17012053	0.989394059	0.32247038	0.491805269	up_in_case
Yang2025_S	ENSGALG00010023127	CERS5	case	control	-0.262643069	0.132793807	-1.97782619	0.047948318	0.129523933	down_in_case
Yang2025_S	ENSGALG00010023128	IWS1	case	control	0.799675398	0.182459393	4.38275819	1.17186E-05	0.000188349	up_in_case
Yang2025_S	ENSGALG00010023130	DYNLT5	case	control	0.098751533	0.488153781	0.202295951	0.839685365	0.904950933	up_in_case
Yang2025_S	ENSGALG00010023131	SOX6	case	control	0.203579095	0.21249145	0.958057816	0.338033605	0.506287435	up_in_case
Yang2025_S	ENSGALG00010023134	KNL1	case	control	-0.033311503	0.098811387	-0.33712211	0.736024851	0.839001402	down_in_case
Yang2025_S	ENSGALG00010023135	GINS3	case	control	-0.32703281	0.19512405	-1.676025126	0.093733271	0.209383792	down_in_case
Yang2025_S	ENSGALG00010023136	MDK	case	control	0.072402407	0.155806945	0.464693064	0.642151276	0.771677995	up_in_case
Yang2025_S	ENSGALG00010023137	ETV6	case	control	0.296753	0.188267438	1.576231145	0.114972543	0.242235015	up_in_case
Yang2025_S	ENSGALG00010023140	CCAR1	case	control	0.557066969	0.161943098	3.439893242	0.000581944	0.004500214	up_in_case
Yang2025_S	ENSGALG00010023142	TLK2	case	control	0.021575107	0.092484435	0.233283654	0.815541151	0.889315747	up_in_case
Yang2025_S	ENSGALG00010023144	C11orf58	case	control	-0.092515468	0.138457162	-0.668188393	0.504013344	0.659851437	down_in_case
Yang2025_S	ENSGALG00010023147	RAD51	case	control	-0.305316231	0.240262991	-1.270758472	0.203814587	0.361895548	down_in_case
Yang2025_S	ENSGALG00010023149	ADGRD1	case	control	-0.635558527	0.15873882	-4.003800246	6.2333E-05	0.0007287	down_in_case
Yang2025_S	ENSGALG00010023155	CFAP263	case	control	1.034329468	0.24062436	4.298523509	1.7194E-05	0.000256529	up_in_case
Yang2025_S	ENSGALG00010023157	STOX1	case	control	-1.286749088	0.345383563	-3.725565505	0.000194878	0.001828702	down_in_case
Yang2025_S	ENSGALG00010023159	DAW1	case	control	0.091740266	0.459194119	0.199785369	0.841648445	0.906085528	up_in_case
Yang2025_S	ENSGALG00010023160	ENSGALG00010023160	case	control	0.44636341	0.100131025	4.457793297	8.28077E-06	0.0001397	up_in_case
Yang2025_S	ENSGALG00010023161	ODF2L	case	control	0.15240233	0.499744898	0.304960253	0.760396425	0.854595062	up_in_case
Yang2025_S	ENSGALG00010023162	CHRNA4	case	control	-0.248880389	0.255397803	-0.974481322	0.329817609	0.49934902	down_in_case
Yang2025_S	ENSGALG00010023163	CRTAC1	case	control	0.436868392	0.162189979	2.693559704	0.007069348	0.031174484	up_in_case
Yang2025_S	ENSGALG00010023164	NKIRAS2	case	control	-0.156105141	0.167012335	-0.934692285	0.349946904	0.518359423	down_in_case
Yang2025_S	ENSGALG00010023165	CSNK2A2	case	control	-0.217706765	0.121350173	-1.794037539	0.072807177	0.174835088	down_in_case
Yang2025_S	ENSGALG00010023166	KDM2A	case	control	0.091465501	0.105545072	0.866601344	0.386160477	0.553615869	up_in_case
Yang2025_S	ENSGALG00010023167	GPD1	case	control	-0.863209767	0.251493408	-3.432335555	0.000598407	0.00460707	down_in_case
Yang2025_S	ENSGALG00010023168	SPHKAP	case	control	1.65406081	0.50958227	3.245915147	0.001170737	0.007913093	up_in_case
Yang2025_S	ENSGALG00010023169	RAN	case	control	-0.425156629	0.218928534	-1.9419882	0.052138529	0.137526102	down_in_case
Yang2025_S	ENSGALG00010023170	TCF12	case	control	-0.462401368	0.096685353	-4.78253792	1.73096E-06	3.85318E-05	down_in_case
Yang2025_S	ENSGALG00010023171	ENSGALG00010023171	case	control	-0.096644645	0.179599842	-0.538110967	0.590500441	0.731303204	down_in_case
Yang2025_S	ENSGALG00010023172	DHX58	case	control	-0.516857494	0.294530767	-1.754850605	0.079284865	0.186124426	down_in_case
Yang2025_S	ENSGALG00010023174	KCNQ2	case	control	0.753660591	0.306232305	2.461074739	0.013852151	0.051660022	up_in_case
Yang2025_S	ENSGALG00010023175	GRK2	case	control	0.297953887	0.077491351	3.844995398	0.000120555	0.001235242	up_in_case

Yang2025_S	ENSGALG00010023177	LZTS2	case	control	0.227001528	0.153104939	1.482653205	0.138166573	0.275996445	up_in_case
Yang2025_S	ENSGALG00010023178	CFAP20	case	control	0.108481217	0.119352716	0.908912852	0.363396129	0.53216025	up_in_case
Yang2025_S	ENSGALG00010023179	KAT2A	case	control	0.080010671	0.105350975	0.759467773	0.447572785	0.610326525	up_in_case
Yang2025_S	ENSGALG00010023180	EEF1A2	case	control	-0.283849567	0.148907029	-1.906220069	0.05662166	0.146304993	down_in_case
Yang2025_S	ENSGALG00010023181	PLEKHA7	case	control	0.088371935	0.153789087	0.57463073	0.565541064	0.711072339	up_in_case
Yang2025_S	ENSGALG00010023182	EPHB6	case	control	-0.231571143	0.153582558	-1.507795843	0.131606786	0.266212886	down_in_case
Yang2025_S	ENSGALG00010023183	ENSGALG00010023183	case	control	0.068751077	0.799712332	0.085969759	0.931490457	0.958515861	up_in_case
Yang2025_S	ENSGALG00010023184	SMARCD1	case	control	0.006952688	0.072100511	0.096430495	0.923178673	0.953733233	up_in_case
Yang2025_S	ENSGALG00010023185	FBXW4	case	control	-0.55078958	0.230627628	-2.388220283	0.016930191	0.060015825	down_in_case
Yang2025_S	ENSGALG00010023186	MIER1	case	control	0.162355087	0.099481966	1.63200521	0.102678389	0.223601085	up_in_case
Yang2025_S	ENSGALG00010023187	ENSGALG00010023187	case	control	1.349425345	0.274665337	4.912980142	8.97024E-07	2.20848E-05	up_in_case
Yang2025_S	ENSGALG00010023188	KIF1BP	case	control	-0.107216227	0.058218177	-1.841628042	0.065529576	0.162021761	down_in_case
Yang2025_S	ENSGALG00010023189	RMDN3	case	control	-0.166269809	0.072999481	-2.277684813	0.022745363	0.074420436	down_in_case
Yang2025_S	ENSGALG00010023190	SF3B3	case	control	-0.397676433	0.123048056	-3.231879042	0.001229791	0.008227142	down_in_case
Yang2025_S	ENSGALG00010023193	PPDPF	case	control	-0.027414315	0.204889214	-0.133800672	0.893560195	0.93756203	down_in_case
Yang2025_S	ENSGALG00010023194	MRC2	case	control	-0.095885282	0.154002549	-0.622621392	0.53353335	0.685360181	down_in_case
Yang2025_S	ENSGALG00010023195	ENSGALG00010023195	case	control	1.193418006	0.242213915	4.927124044	8.34488E-07	2.07334E-05	up_in_case
Yang2025_S	ENSGALG00010023196	DGKZ	case	control	-0.20777795	0.071542103	-2.904275127	0.003681045	0.018998284	down_in_case
Yang2025_S	ENSGALG00010023198	PTK6	case	control	0.026715443	0.368663531	0.072465651	0.94223134	0.964518749	up_in_case
Yang2025_S	ENSGALG00010023199	RPS13	case	control	-0.190348808	0.274231295	-0.694117743	0.487608372	0.644792434	down_in_case
Yang2025_S	ENSGALG00010023200	VPS26A	case	control	-0.376271693	0.092686448	-4.059619275	4.91528E-05	0.000601356	down_in_case
Yang2025_S	ENSGALG00010023201	ZNF280D	case	control	0.231790299	0.127196614	1.822299291	0.068409586	0.167180783	up_in_case
Yang2025_S	ENSGALG00010023202	PID1	case	control	-0.326026465	0.092415924	-3.52781695	0.000419002	0.003434582	down_in_case
Yang2025_S	ENSGALG00010023203	COG4	case	control	-0.003530613	0.09369009	-0.037683957	0.969939667	0.982096151	down_in_case
Yang2025_S	ENSGALG00010023204	SRGN	case	control	-0.455009383	0.677902108	-0.671202196	0.50209173	0.658677885	down_in_case
Yang2025_S	ENSGALG00010023205	DNAJC17	case	control	0.744443228	0.152494565	4.881768909	1.05138E-06	2.52169E-05	up_in_case
Yang2025_S	ENSGALG00010023206	HSPB9	case	control	0.305545611	0.338586876	0.902414217	0.366836872	0.535611044	up_in_case
Yang2025_S	ENSGALG00010023207	MNS1	case	control	0.769550714	0.230902229	3.332798976	0.00085977	0.006194881	up_in_case
Yang2025_S	ENSGALG00010023208	BACE1	case	control	-0.17268545	0.057027301	-3.028118917	0.002460812	0.01402512	down_in_case
Yang2025_S	ENSGALG00010023209	PIK3C2A	case	control	0.251035722	0.125536368	1.999705156	0.045532111	0.124924192	up_in_case
Yang2025_S	ENSGALG00010023210	ZNHIT6	case	control	-0.499358347	0.205626185	-2.428476446	0.01516241	0.055499917	down_in_case
Yang2025_S	ENSGALG00010023211	TLK1	case	control	0.081115141	0.129886656	0.624507117	0.532294598	0.684607387	up_in_case
Yang2025_S	ENSGALG00010023212	RIMBP2	case	control	0.852673684	0.314704795	2.709439757	0.006739694	0.030044031	up_in_case
Yang2025_S	ENSGALG00010023213	FCSK	case	control	0.538122297	0.227498694	2.365386313	0.018011272	0.062866639	up_in_case
Yang2025_S	ENSGALG00010023214	SUPV3L1	case	control	0.053882933	0.093083321	0.578867753	0.56267841	0.708944272	up_in_case
Yang2025_S	ENSGALG00010023215	TRIP12	case	control	-0.007529072	0.102895131	-0.073172282	0.941669023	0.964072894	down_in_case
Yang2025_S	ENSGALG00010023216	SLC35D1	case	control	0.036841903	0.165404524	0.22273818	0.823739285	0.894465038	up_in_case
Yang2025_S	ENSGALG00010023217	CREB3L1	case	control	0.123172424	0.270049692	0.45611022	0.648310721	0.776535147	up_in_case
Yang2025_S	ENSGALG00010023218	SSH3	case	control	0.639250978	0.200951101	3.181127018	0.001467033	0.009466893	up_in_case
Yang2025_S	ENSGALG00010023219	TEX9	case	control	0.689174924	0.181264406	3.802042216	0.000143508	0.0014274	up_in_case
Yang2025_S	ENSGALG00010023220	ST3GAL2	case	control	0.032226456	0.100732429	0.319921364	0.749027942	0.847674443	up_in_case
Yang2025_S	ENSGALG00010023221	AMY1A	case	control	0.095008186	0.168150946	0.565017258	0.572062026	0.715719548	up_in_case
Yang2025_S	ENSGALG00010023222	ENSGALG00010023222	case	control	-1.021668114	0.651626692	-1.567873334	0.116910696	0.244911388	down_in_case
Yang2025_S	ENSGALG00010023223	CEP164	case	control	0.647751729	0.238429077	2.716748043	0.006592677	0.029565942	up_in_case
Yang2025_S	ENSGALG00010023225	PDPR	case	control	-0.027554179	0.127157793	-0.216692809	0.828447753	0.897350908	down_in_case
Yang2025_S	ENSGALG00010023226	ENSGALG00010023226	case	control	0.423994863	0.295061894	1.43696923	0.150726737	0.29355375	up_in_case
Yang2025_S	ENSGALG00010023227	RNPC3	case	control	0.93212034	0.177218728	5.259716905	1.44277E-07	4.68649E-06	up_in_case
Yang2025_S	ENSGALG00010023228	GORASP2	case	control	0.114559171	0.098499075	1.163048188	0.244809952	0.410170086	up_in_case
Yang2025_S	ENSGALG00010023230	ENSGALG00010023230	case	control	-0.433697878	0.111974676	-3.87317823	0.000107425	0.001127237	down_in_case
Yang2025_S	ENSGALG00010023231	ASIC1	case	control	-0.06899047	0.209077025	-0.329976332	0.741417846	0.841751481	down_in_case
Yang2025_S	ENSGALG00010023233	SEZ6L	case	control	0.778710283	0.82196558	0.94737578	0.343447328	0.51202937	up_in_case
Yang2025_S	ENSGALG00010023234	ENSGALG00010023234	case	control	0.386902298	0.270541673	1.430102407	0.152687629	0.296305324	up_in_case
Yang2025_S	ENSGALG00010023235	ENSGALG00010023235	case	control	0.443180254	0.353971589	1.252022105	0.210561809	0.370286793	up_in_case
Yang2025_S	ENSGALG00010023238	NUCB2	case	control	0.040951732	0.156097204	0.262347636	0.79305344	0.875273221	up_in_case
Yang2025_S	ENSGALG00010023239	RBPMS2	case	control	0.654453149	0.321664782	2.034581293	0.041893021	0.117013279	up_in_case
Yang2025_S	ENSGALG00010023240	PIWIL1	case	control	0.982002526	0.395280373	2.484318961	0.012979949	0.049199796	up_in_case
Yang2025_S	ENSGALG00010023243	HK1	case	control	0.197791906	0.122705901	1.611918453	0.106979686	0.229772184	up_in_case
Yang2025_S	ENSGALG00010023244	PPIB	case	control	0.119853906	0.170146296	0.704416778	0.48117326	0.639560224	up_in_case
Yang2025_S	ENSGALG00010023245	TANC2	case	control	0.225492745	0.206661521	1.091121097	0.275219601	0.442336123	up_in_case
Yang2025_S	ENSGALG00010023248	PRDM10	case	control	0.30836304	0.164554744	1.873923735	0.060940937	0.154121954	up_in_case
Yang2025_S	ENSGALG00010023249	IL12RB2	case	control	-0.326195435	0.321645145	-1.014146926	0.310512649	0.480190186	down_in_case
Yang2025_S	ENSGALG00010023250	FZD10	case	control	-0.419450397	0.651531608	-0.643791325	0.51971076	0.673564866	down_in_case
Yang2025_S	ENSGALG00010023252	GLG1	case	control	0.050138448	0.119163114	0.420754766	0.673934166	0.794944163	up_in_case
Yang2025_S	ENSGALG00010023254	MYO3B	case	control	-0.032209442	0.292948468	-0.109949173	0.912449684	0.948101286	down_in_case
Yang2025_S	ENSGALG00010023255	SNX22	case	control	0.589546507	0.365962519	1.610947777	0.107191105	0.230035295	up_in_case
Yang2025_S	ENSGALG00010023257	PAK6	case	control	0.415469748	0.306572472	1.355208918	0.175351004	0.325802166	up_in_case
Yang2025_S	ENSGALG00010023258	ENSGALG00010023258	case	control	-0.06494844	0.472672542	-0.137406839	0.890709226	0.936115589	down_in_case
Yang2025_S	ENSGALG00010023259	TSPAN15	case	control	-0.373259946	0.220368841	-1.693796387	0.090303981	0.204183997	down_in_case
Yang2025_S	ENSGALG00010023260	ENSGALG00010023260	case	control	-0.68681419	0.394916035	-1.739139789	0.082010177	0.190563919	down_in_case
Yang2025_S	ENSGALG00010023261	SNX1	case	control	0.088218578	0.096683954	0.912442803	0.361535666	0.530148322	up_in_case
Yang2025_S	ENSGALG00010023262	POLD4	case	control	-0.080768402	0.506049961	-0.159605588	0.873191777	0.9258105	down_in_case
Yang2025_S	ENSGALG00010023263	SRP14	case	control	-0.343856379	0.118182334	-2.909541268	0.003619596	0.018757557	down_in_case
Yang2025_S	ENSGALG00010023264	RACGAP1	case	control	0.304320511	0.173032795	1.758744702	0.078620881	0.185002022	up_in_case
Yang2025_S	ENSGALG00010023265	RAB5C	case	control	-0.250708269	0.095078205	-2.636863711	0.008367644	0.035484821	down_in_case
Yang2025_S	ENSGALG00010023266	RFWD3	case	control	0.364625799	0.170504089	2.138516449	0.032474851	0.097194382	up_in_case
Yang2025_S	ENSGALG00010023267	DSCAML1	case	control	-0.37287356	0.615561304	-0.605745614	0.544683689	0.694323007	down_in_case
Yang2025_S	ENSGALG00010023268	KCNJ11	case	control	-0.035099821	0.148191359	-0.236854708	0.812769508	0.887252024	down_in_case
Yang2025_S	ENSGALG00010023269	OPRL1	case	control	-0.430013129	0.447797915	-0.960283903	0.336912349	0.505058347	down_in_case
Yang2025_S	ENSGALG00010023270	ENSGALG00010023270	case	control	-0.640763319	0.593148202	-1.080275245	0.28001963	0.447821077	down_in_case
Yang2025_S	ENSGALG00010023271	PATL2	case	control	-0.037882625	0.242104035	-0.156472505	0.87566059	0.926804429	down_in_case
Yang2025_S	ENSGALG00010023273	HDAC7	case	control	-0.236087946	0.117204646	-2.01432242	0.043975694	0.121684132	down_in_case
Yang2025_S	ENSGALG00010023274	ZBTB38	case	control	0.204301072	0.187174165	1.091502519	0.275051824	0.442234588	up_in_case
Yang2025_S	ENSGALG00010023275	TWIST3	case	control	-1.181592707	0.660630074	-1.788584496	0.073681757	0.17644875	down_in_case
Yang2025_S	ENSGALG00010023276	ABCC8	case	control	0.137241433	0.107700933	1.274282682	0.202563252	0.360454465	up_in_case
Yang2025_S	ENSGALG00010023277	KCNH4	case	control	-0.051580808	0.546480105	-0.094387349	0.924801465	0.95495431	down_in_case
Yang2025_S	ENSGALG00010023278	ENSGALG00010023278	case	control	1.000743489	0.271543255	3.685392545	0.00022835	0.002071656	up_in_case
Yang2025_S	ENSGALG00010023279	FA2H	case	control	0.293593759	0.521408188	0.563078536	0.573381406	0.716341214	up_in_case
Yang2025_S	ENSGALG00010023281	B2M	case	control	0.282649189	0.227733206	1.241141744	0.214553391	0.375049246	up_in_case
Yang2025_S	ENSGALG00010023282	SERBP1	case	control	-0.128102072	0.068018433	-1.883343471	0.059653836	0.151942278	down_in_case
Yang2025_S	ENSGALG00010023285	SLC48A1	case	control	-0.193914936	0.152866471	-1.268524968	0.20461054	0.362809824	down_in_case
Yang2025_S	ENSGALG00010023287	RGS19	case	control	0.106990608	0.157141792	0.680853937	0.495963917	0.653237919	up_in_case
Yang2025_S	ENSGALG00010023289	NFRKB	case	control	-0.168413163	0.095743388	-1.759005686	0.078576543	0.184928918	down_in_case
Yang2025_S	ENSGALG00010023290	SLC16A14	case	control	0.135730957	0.268480269	0.505552819	0.61317061	0.749520391	up_in_case
Yang2025_S	ENSGALG00010023293	COL11A1	case	control	1.141116138	0.397591633	2.870070805	0.004103799	0.020548487	up_in_case
Yang2025_S	ENSGALG00010023295	ENSGALG00010023295	case	control	0.682660901	0.49810595	1.370513443	0.170526682	0.319522565	up_in_case
Yang2025_S	ENSGALG00010023299	PASK	case	control	-0.301732439	0.167634814	-1.799938995	0.071870271	0.173062421	down_in_case
Yang2025_S	ENSGALG00010023304	ENSGALG00010023304	case	control	0.037247657	0.166664274	0.223489147	0.823154829	0.894251148	up_in_case
Yang2025_S	ENSGALG00010023305	PLCB2	case	control	0.199225955	0.182114188	1.093961744	0.273971757	0.441312731	up_in_case
Yang2025_S	ENSGALG00010023309	PIK3R3	case	control	-0.401736046	0.106665435	-3.76631891	0.000165672	0.001602111	down_in_case
Yang2025_S	ENSGALG00010023311	ETS1	case	control	-0.17616536	0.070932087	-2.483577863	0.01300699	0.049239989	down_in_case
Yang2025_S	ENSGALG00010023312	UBR3	case	control	-0.000359782	0.09557306	-0.003764472	0.996996393	0.997784023	down_in_case
Yang2025_S	ENSGALG00010023317	RAPGEF3	case	control	0.169338249	0.154066134	1.099127007	0.271712671	0.438891396	up_in_case
Yang2025_S	ENSGALG00010023319	EIF2AK4	case	control	0.269224363	0.126346724	2.13083769	0.033102516	0.098448054	up_in_case
Yang2025_S	ENSGALG00010023320	ENSGALG00010023320	case	control	-0.468645854	0.106828154	-4.386913345	1.14971E-05	0.000186076	down_in_case
Yang2025_S	ENSGALG00010023322	PRKAB1	case	control	-0.085040141	0.109502766	-0.776602678	0.437393221	0.601329374	down_in_case
Yang2025_S	ENSGALG00010023324	LAG3	case	control	0.178488211	0.906745075	0.196844975	0.843948855	0.907425895	up_in_case
Yang2025_S	ENSGALG00010023328	TCEA2	case	control	0.107311791	0.126878683	0.845782669	0.397673995	0.564488859	up_in_case
Yang2025_S	ENSGALG00010023331	CD4	case	control	-0.542355563	0.409998816	-1.322822268	0.18589449	0.339462681	down_in_case
Yang2025_S	ENSGALG00010023332	PPP1R7	case	control	-0.002495885	0.110918609	-0.02250195	0.982047556	0.988859939	down_in_case
Yang2025_S	ENSGALG00010023333	ENDOU	case	control	-0.291057395	0.520900665	-0.558757964	0.576326914	0.719118513	down_in_case
Yang2025_S	ENSGALG00010023334	RPS17	case	control	0.728056786	0.169184765	4.303323576	1.68255E-05	0.000252111	up_in_case
Yang2025_S	ENSGALG00010023335	WDR59	case	control	-0.013299202	0.092170951	-0.144288433	0.885272712	0.932729113	down_in_case
Yang2025_S	ENSGALG00010023336	GPR162	case	control	0.018452238	0.149591217	0.12335108	0.901829094	0.94127694	up_in_case
Yang2025_S	ENSGALG00010023337	FLI1	case	control	0.119974594	0.134654069	0.890983799	0.372937862	0.541179069	up_in_case
Yang2025_S	ENSGALG00010023338	HDLBP	case	control	-0.292415245	0.060170739	-4.859758217	1.17529E-06	2.77118E-05	down_in_case
Yang2025_S	ENSGALG00010023340	P3H3	case	control	0.703872676	0.109368963	6.435762518	1.22855E-10	1.00116E-08	up_in_case
Yang2025_S	ENSGALG00010023342	FITM2	case	control	-0.233189553	0.141095219	-1.652710518	0.098389793	0.216597435	down_in_case
Yang2025_S	ENSGALG00010023343	TMEM233	case	control	0.035917951	0.142112518	0.252743048	0.80046678	0.879415372	up_in_case
Yang2025_S	ENSGALG00010023347	STAT5A	case	control	-0.111425023	0.150101537	-0.742330997	0.457886816	0.619194565	down_in_case
Yang2025_S	ENSGALG00010023349	ENSGALG00010023349	case	control	1.721523014	0.695863633	2.473937323	0.013363314	0.050247647	up_in_case
Yang2025_S	ENSGALG00010023350	ZNRF1	case	control	0.233775473	0.130402429	1.792723306	0.073017178	0.175248773	up_in_case
Yang2025_S	ENSGALG00010023351	GNB3	case	control	0.85262419	0.366904543	2.323831104	0.020134548	0.068031313	up_in_case
Yang2025_S	ENSGALG00010023354	SEPTIN2	case	control	-0.589457189	0.124825309	-4.722257012	2.33242E-06	4.96217E-05	down_in_case
Yang2025_S	ENSGALG00010023356	ENSGALG00010023356	case	control	0.121336338	0.190111291	0.638238464	0.523318462	0.676703817	up_in_case
Yang2025_S	ENSGALG00010023359	CDCA3	case	control	-0.1120347	0.22760204	-0.492239435	0.62255009	0.756869264	down_in_case
Yang2025_S	ENSGALG00010023362	GADD45A	case	control	-0.0508654	0.152520102	-0.333499646	0.738757157	0.840267568	down_in_case

Yang2025_S	ENSGALG00010023363	CPEB1	case	control	0.383789882	0.271192475	1.415193701	0.157011711	0.302245917	up_in_case
Yang2025_S	ENSGALG00010023366	GDAP1L1	case	control	1.234565431	0.207059979	5.962356586	2.48626E-09	1.36216E-07	up_in_case
Yang2025_S	ENSGALG00010023368	ENSGALG00010023368	case	control	1.834991697	0.383739233	4.781871484	1.73671E-06	3.85981E-05	up_in_case
Yang2025_S	ENSGALG00010023369	ACE	case	control	-0.436483379	0.163064698	-2.676749686	0.007434015	0.0323627	down_in_case
Yang2025_S	ENSGALG00010023374	ENSGALG00010023374	case	control	0.471404719	0.314561297	1.498610043	0.133974826	0.269905913	up_in_case
Yang2025_S	ENSGALG00010023375	ST14	case	control	0.029632184	0.114531453	0.258725298	0.795847199	0.876582922	up_in_case
Yang2025_S	ENSGALG00010023377	OSER1	case	control	0.067005747	0.134249233	0.499114561	0.61769868	0.75346911	up_in_case
Yang2025_S	ENSGALG00010023379	CCDC60	case	control	0.983893342	0.508520093	1.934817041	0.053012751	0.139252156	up_in_case
Yang2025_S	ENSGALG00010023380	USP5	case	control	0.011285824	0.13484501	0.083694783	0.933299105	0.959399477	up_in_case
Yang2025_S	ENSGALG00010023381	HOXB7	case	control	0.146033225	0.139154485	1.049432404	0.293979151	0.462526755	up_in_case
Yang2025_S	ENSGALG00010023383	HOXB6	case	control	0.374282139	0.126832196	2.951002604	0.003167442	0.017008983	up_in_case
Yang2025_S	ENSGALG00010023384	AP3B2	case	control	0.63532647	0.265673068	2.391384546	0.016784962	0.059683197	up_in_case
Yang2025_S	ENSGALG00010023387	ENSGALG00010023387	case	control	-0.220419428	0.112792223	-1.954207676	0.050676662	0.134587247	down_in_case
Yang2025_S	ENSGALG00010023388	FARP2	case	control	-0.224983956	0.122529851	-1.836156283	0.066334546	0.163518822	down_in_case
Yang2025_S	ENSGALG00010023389	GNG12	case	control	-0.13498953	0.072523783	-1.861313963	0.062699852	0.157087818	down_in_case
Yang2025_S	ENSGALG00010023390	HSPB8	case	control	0.041756432	0.151530952	0.275563716	0.782883181	0.868623543	up_in_case
Yang2025_S	ENSGALG00010023392	TPI1	case	control	-0.572830845	0.205468067	-2.787931246	0.00530458	0.025048906	down_in_case
Yang2025_S	ENSGALG00010023394	HOXB5	case	control	0.766789103	0.170122037	4.507288511	6.56613E-06	0.000117006	up_in_case
Yang2025_S	ENSGALG00010023396	ENSGALG00010023396	case	control	0.476204752	0.232288944	2.050053452	0.040359215	0.113914916	up_in_case
Yang2025_S	ENSGALG00010023397	ENSGALG00010023397	case	control	0.98428479	0.238571701	4.125739912	3.69545E-05	0.000481722	up_in_case
Yang2025_S	ENSGALG00010023399	SRRM4	case	control	0.766022625	0.599752097	1.277232092	0.20152032	0.359484697	up_in_case
Yang2025_S	ENSGALG00010023401	LRRC23	case	control	0.31446928	0.336589613	0.934280999	0.350158963	0.518582846	up_in_case
Yang2025_S	ENSGALG00010023405	DNAJA2	case	control	-0.052260411	0.092703948	-0.563734467	0.572934857	0.716104335	down_in_case
Yang2025_S	ENSGALG00010023408	ZBTB44	case	control	-0.012529412	0.143800185	-0.087130711	0.930567614	0.958016143	down_in_case
Yang2025_S	ENSGALG00010023411	ENSGALG00010023411	case	control	0.767901526	0.272068567	2.822455877	0.004765738	0.023139567	up_in_case
Yang2025_S	ENSGALG00010023412	GSS	case	control	0.081782722	0.191183986	0.42776973	0.668818777	0.791103443	up_in_case
Yang2025_S	ENSGALG00010023414	SUDS3	case	control	-0.35668467	0.059976124	-5.947111042	2.72916E-09	1.44604E-07	down_in_case
Yang2025_S	ENSGALG00010023415	HOXB4	case	control	-0.068184923	0.090603207	-0.752566327	0.451710559	0.613616804	down_in_case
Yang2025_S	ENSGALG00010023421	OLFM3	case	control	0.039810123	0.12400758	0.321029756	0.748187842	0.847297649	up_in_case
Yang2025_S	ENSGALG00010023422	WLS	case	control	-0.353023547	0.145898769	-2.419647197	0.015535572	0.056494831	down_in_case
Yang2025_S	ENSGALG00010023428	HOXB3	case	control	0.772215526	0.268737415	2.873494659	0.004059579	0.020363657	up_in_case
Yang2025_S	ENSGALG00010023429	BMF	case	control	-0.340182795	0.187279205	-1.816447239	0.069301794	0.168802745	down_in_case
Yang2025_S	ENSGALG00010023431	BCAR1	case	control	0.244861925	0.114550979	2.137580381	0.032550815	0.097359007	up_in_case
Yang2025_S	ENSGALG00010023432	TMOD3	case	control	-0.411021596	0.096320797	-4.267215502	1.97928E-05	0.000287844	down_in_case
Yang2025_S	ENSGALG00010023434	ATN1	case	control	0.613863033	0.299727996	2.048067055	0.04055343	0.114325721	up_in_case
Yang2025_S	ENSGALG00010023437	KCNJ5	case	control	0.160862318	0.226828627	0.709179966	0.478212804	0.637205529	up_in_case
Yang2025_S	ENSGALG00010023438	HOXB2	case	control	0.471622244	0.187240498	2.518804686	0.011775396	0.045752858	up_in_case
Yang2025_S	ENSGALG00010023439	ACSS2	case	control	0.180790607	0.148123159	1.220542473	0.222259299	0.383789756	up_in_case
Yang2025_S	ENSGALG00010023441	ENSGALG00010023441	case	control	0.212874451	0.231022549	0.921444475	0.356818418	0.526054237	up_in_case
Yang2025_S	ENSGALG00010023442	STK25	case	control	-0.269289264	0.078933815	-3.411583017	0.000645868	0.004928647	down_in_case
Yang2025_S	ENSGALG00010023443	ENSGALG00010023443	case	control	0.512518848	0.265123798	1.9331303	0.053220148	0.139665305	up_in_case
Yang2025_S	ENSGALG00010023447	C12orf57	case	control	-0.617302669	0.280764763	-2.198647235	0.027903016	0.086675997	down_in_case
Yang2025_S	ENSGALG00010023448	FAM219B	case	control	0.21282393	0.112645676	1.889321792	0.058848723	0.150607339	up_in_case
Yang2025_S	ENSGALG00010023453	ARHGAP32	case	control	0.448073255	0.261501243	1.713465109	0.086627007	0.198283072	up_in_case
Yang2025_S	ENSGALG00010023454	HOXB1	case	control	-0.662270287	0.370223603	-1.788838645	0.073640805	0.176441647	down_in_case
Yang2025_S	ENSGALG00010023455	CFDP1	case	control	0.499605233	0.120902127	4.13231138	3.59133E-05	0.000469468	up_in_case
Yang2025_S	ENSGALG00010023456	MPI	case	control	-0.014486872	0.126389616	-0.114620744	0.908745737	0.945380504	down_in_case
Yang2025_S	ENSGALG00010023466	CLCF1	case	control	0.7216379	0.436542366	1.653076439	0.098315308	0.216467659	up_in_case
Yang2025_S	ENSGALG00010023469	ENSGALG00010023469	case	control	0.374819161	0.266171005	1.408189297	0.159075025	0.304493196	up_in_case
Yang2025_S	ENSGALG00010023472	PTPN6	case	control	-0.181378677	0.119610559	-1.516410248	0.129415649	0.263040705	down_in_case
Yang2025_S	ENSGALG00010023473	BOK	case	control	0.845154529	0.208360119	4.056220231	4.98732E-05	0.000608567	up_in_case
Yang2025_S	ENSGALG00010023475	ENSGALG00010023475	case	control	1.35492108	0.566975313	2.38973558	0.016860507	0.059860169	up_in_case
Yang2025_S	ENSGALG00010023476	SCAMP2	case	control	-0.185719562	0.120568899	-1.540360443	0.123472518	0.254539484	down_in_case
Yang2025_S	ENSGALG00010023478	TAOK3	case	control	0.606798105	0.21911823	2.769272578	0.005618161	0.026113768	up_in_case
Yang2025_S	ENSGALG00010023479	MCCC2L	case	control	-0.165159903	0.179092784	-0.922203006	0.356422696	0.525804601	down_in_case
Yang2025_S	ENSGALG00010023482	MTCL2	case	control	0.119135323	0.110035291	1.082701029	0.278941147	0.44673542	up_in_case
Yang2025_S	ENSGALG00010023485	BARX2	case	control	-0.00099619	0.139676378	-0.007132132	0.99430943	0.996239712	down_in_case
Yang2025_S	ENSGALG00010023487	GRN	case	control	0.238486116	0.285811151	0.834418514	0.404045188	0.570373994	up_in_case
Yang2025_S	ENSGALG00010023489	ETV5	case	control	0.084943611	0.101552374	0.836451258	0.402901086	0.56929899	up_in_case
Yang2025_S	ENSGALG00010023490	PHB2	case	control	-0.295828531	0.161882575	-1.827426651	0.067635645	0.16575848	down_in_case
Yang2025_S	ENSGALG00010023496	PLEKHA3	case	control	-0.25469576	0.206704033	-1.232176059	0.217883324	0.378815237	down_in_case
Yang2025_S	ENSGALG00010023498	DISP2	case	control	-0.20222556	0.303172369	-0.667031632	0.504751928	0.660443016	down_in_case
Yang2025_S	ENSGALG00010023499	PEBP1	case	control	0.028440972	0.194590545	0.146158039	0.88379663	0.93152606	up_in_case
Yang2025_S	ENSGALG00010023506	RPE65	case	control	0.259387025	0.382021142	0.678986048	0.497146702	0.654053936	up_in_case
Yang2025_S	ENSGALG00010023510	ASPHD2	case	control	-0.150821453	0.204712181	-0.736748798	0.461275125	0.622307717	down_in_case
Yang2025_S	ENSGALG00010023513	CDCA9	case	control	0.00492781	0.185543197	0.026558826	0.978811613	0.987313777	up_in_case
Yang2025_S	ENSGALG00010023514	ULK3	case	control	0.135262107	0.131895228	1.025526925	0.305114628	0.474793114	up_in_case
Yang2025_S	ENSGALG00010023518	METTL5	case	control	-0.160959029	0.126173018	-1.275700869	0.202061281	0.360067002	down_in_case
Yang2025_S	ENSGALG00010023519	DGKG	case	control	0.465390072	0.189280643	2.458730401	0.013942928	0.051922688	up_in_case
Yang2025_S	ENSGALG00010023522	EMG1	case	control	-0.417721855	0.228096333	-1.831339631	0.067049866	0.164816045	down_in_case
Yang2025_S	ENSGALG00010023526	KNSTRN	case	control	0.225508952	0.207889057	1.084756239	0.278029632	0.445582855	up_in_case
Yang2025_S	ENSGALG00010023527	TMEM98	case	control	0.153732032	0.140794293	1.09189108	0.274880979	0.442215013	up_in_case
Yang2025_S	ENSGALG00010023531	CPLX3	case	control	1.025893624	0.626451996	1.637625279	0.101499886	0.221484641	up_in_case
Yang2025_S	ENSGALG00010023533	FKBP7	case	control	0.023924308	0.11506203	0.207925305	0.835287291	0.902514414	up_in_case
Yang2025_S	ENSGALG00010023536	ENSGALG00010023536	case	control	0.385977276	0.337246625	1.144495591	0.252418165	0.418295533	up_in_case
Yang2025_S	ENSGALG00010023543	ENSGALG00010023543	case	control	-0.224319983	0.897321592	-0.249988393	0.802596325	0.880990924	down_in_case
Yang2025_S	ENSGALG00010023544	SSB	case	control	0.58940438	0.171813077	3.430497781	0.000602475	0.004632898	up_in_case
Yang2025_S	ENSGALG00010023545	PTPRT	case	control	0.374812148	0.182627272	2.05233394	0.04013722	0.113603241	up_in_case
Yang2025_S	ENSGALG00010023546	SLC11A2	case	control	-0.315953772	0.132135226	-2.391139609	0.016796165	0.059707795	down_in_case
Yang2025_S	ENSGALG00010023551	ENSGALG00010023551	case	control	0.38504864	0.114019721	3.37703544	0.000732716	0.005457188	up_in_case
Yang2025_S	ENSGALG00010023552	SGPP2	case	control	-0.764580233	0.42785463	-1.787009373	0.073935974	0.176905528	down_in_case
Yang2025_S	ENSGALG00010023555	HPS4	case	control	-0.172200951	0.100873501	-1.707097989	0.087803841	0.200384298	down_in_case
Yang2025_S	ENSGALG00010023556	IVD	case	control	0.217001466	0.119045931	1.822838161	0.068327906	0.167043749	up_in_case
Yang2025_S	ENSGALG00010023569	LPCAT3	case	control	-0.084015801	0.124878485	-0.672780433	0.501086988	0.657809437	down_in_case
Yang2025_S	ENSGALG00010023571	APLP2	case	control	-0.115842364	0.076901961	-1.50636424	0.131973695	0.266838863	down_in_case
Yang2025_S	ENSGALG00010023572	MYBL2	case	control	-0.066789619	0.159754242	-0.418077281	0.675890615	0.796197456	down_in_case
Yang2025_S	ENSGALG00010023574	DEPDC1	case	control	-0.149218061	0.144927998	-1.029601342	0.303197185	0.47281253	down_in_case
Yang2025_S	ENSGALG00010023578	SRRD	case	control	0.213019207	0.232081295	0.917864606	0.358689754	0.527627452	up_in_case
Yang2025_S	ENSGALG00010023580	RDM1	case	control	0.055205209	0.196859131	0.280430013	0.779147614	0.866148845	up_in_case
Yang2025_S	ENSGALG00010023585	TFIP11	case	control	-0.424891641	0.133184582	-3.190246463	0.001421515	0.009243496	down_in_case
Yang2025_S	ENSGALG00010023587	SLC12A3	case	control	1.289405149	0.531816002	2.424532442	0.015328114	0.055886255	up_in_case
Yang2025_S	ENSGALG00010023588	FARSB	case	control	0.161443867	0.109460237	1.474908799	0.140237064	0.279204908	up_in_case
Yang2025_S	ENSGALG00010023590	S1PR1	case	control	0.016175547	0.102694089	0.157511958	0.874841385	0.926528291	up_in_case
Yang2025_S	ENSGALG00010023592	BAHD1	case	control	-0.37314882	0.091055052	-4.098057279	4.16632E-05	0.000529885	down_in_case
Yang2025_S	ENSGALG00010023593	CSK	case	control	0.107812906	0.113721677	0.94804182	0.343108165	0.511687958	up_in_case
Yang2025_S	ENSGALG00010023597	MOGAT1	case	control	-0.877494368	0.234261427	-3.745791103	0.000179826	0.00171518	down_in_case
Yang2025_S	ENSGALG00010023600	CHST14	case	control	-0.388384763	0.106617157	-3.642797963	0.000269691	0.002386119	down_in_case
Yang2025_S	ENSGALG00010023601	TPST2	case	control	-0.375206675	0.155693682	-2.409903017	0.015956762	0.057590643	down_in_case
Yang2025_S	ENSGALG00010023602	GH	case	control	1.427177319	0.518592956	2.752018326	0.005922921	0.027132117	up_in_case
Yang2025_S	ENSGALG00010023603	C1S	case	control	-0.660580056	0.256188692	-2.5784903	0.009923309	0.040291756	down_in_case
Yang2025_S	ENSGALG00010023604	ENSGALG00010023604	case	control	0.927172016	0.684704013	1.354120902	0.175697809	0.326232465	up_in_case
Yang2025_S	ENSGALG00010023605	INSYN1	case	control	-0.527753691	0.407784204	-1.294198466	0.195596876	0.351695803	down_in_case
Yang2025_S	ENSGALG00010023606	LRRC7	case	control	0.613777365	0.318351765	1.927984802	0.053857017	0.14091204	up_in_case
Yang2025_S	ENSGALG00010023612	GPT2	case	control	-0.155677591	0.24439788	-0.63698421	0.524135129	0.677471758	down_in_case
Yang2025_S	ENSGALG00010023613	COPB1	case	control	-0.506644699	0.076962495	-6.583007691	4.61025E-11	4.19895E-09	down_in_case
Yang2025_S	ENSGALG00010023617	ENSGALG00010023617	case	control	0.41441351	0.331013274	1.251954354	0.210586497	0.370286793	up_in_case
Yang2025_S	ENSGALG00010023620	IRS1	case	control	-0.02696302	0.096253327	-0.280125588	0.779381154	0.866220799	down_in_case
Yang2025_S	ENSGALG00010023621	CD79B	case	control	1.042027138	0.544394168	1.914104154	0.055606849	0.144351982	up_in_case
Yang2025_S	ENSGALG00010023623	OSBPL6	case	control	0.001011126	0.218060338	0.004636908	0.996300296	0.997408509	up_in_case
Yang2025_S	ENSGALG00010023624	PSMA1	case	control	-0.273838915	0.178831356	-1.531269018	0.125702919	0.257864004	down_in_case
Yang2025_S	ENSGALG00010023626	LOXL1	case	control	-0.009848532	0.122449607	-0.080429263	0.935895854	0.960856754	down_in_case
Yang2025_S	ENSGALG00010023627	IFT52	case	control	0.007783837	0.145486238	0.05350222	0.957331762	0.973836926	up_in_case
Yang2025_S	ENSGALG00010023631	ENSGALG00010023631	case	control	-0.693960256	0.25024084	-2.773169461	0.00555132	0.02588078	down_in_case
Yang2025_S	ENSGALG00010023635	SCN4A	case	control	-0.404067164	0.128769818	-3.137902728	0.001701613	0.010607942	down_in_case
Yang2025_S	ENSGALG00010023636	PDE3B	case	control	-0.254437756	0.084424779	-3.013780549	0.002580144	0.014526088	down_in_case
Yang2025_S	ENSGALG00010023637	RHBDD1	case	control	0.10857887	0.122220497	0.888385107	0.374333634	0.542351755	up_in_case
Yang2025_S	ENSGALG00010023638	DCTN2	case	control	0.246308974	0.120006574	2.052462347	0.040124751	0.113599838	up_in_case
Yang2025_S	ENSGALG00010023639	KLHL23	case	control	0.407005504	0.206409526	1.971834887	0.048628458	0.130717123	up_in_case
Yang2025_S	ENSGALG00010023641	LRRC40	case	control	-0.30989927	0.093786395	-3.304309425	0.000952107	0.006745098	down_in_case
Yang2025_S	ENSGALG00010023642	PAX6	case	control	0.45154852	0.343526377	1.314450797	0.188694546	0.343433891	up_in_case
Yang2025_S	ENSGALG00010023643	SCN4B	case	control	-0.163564547	0.216894298	-0.754121011	0.450776562	0.612765777	down_in_case
Yang2025_S	ENSGALG00010023646	DPH5	case	control	0.071945021	0.134040327	0.536741612	0.591446108	0.732083986	up_in_case
Yang2025_S	ENSGALG00010023648	SCN2B	case	control	0.665593077	0.11885017	5.600270293	2.14018E-08	9.03739E-07	up_in_case
Yang2025_S	ENSGALG00010023652	SLC30A7	case	control	-0.633653082	0.123795728	-5.118537531	3.07914E-07	8.7567E-06	down_in_case
Yang2025_S	ENSGALG00010023655	C1R	case	control	-0.321007596	0.12661867	-2.535231139	0.011237315	0.044147727	down_in_case
Yang2025_S	ENSGALG00010023658	ENSGALG00010023658	case	control	-0.17308572	0.270266745	-0.640425517	0.521895998	0.675243084	down_in_case

Yang2025_S	ENSGALG00010023660	CRYBB1	case	control	0.015768328	0.37324353	0.042246756	0.96630199	0.980015883	up_in_case
Yang2025_S	ENSGALG00010023663	PHOSPHO2	case	control	-0.229397263	0.175859717	-1.304433255	0.192085903	0.347306047	down_in_case
Yang2025_S	ENSGALG00010023664	SLC6A9	case	control	-0.053807375	0.122658664	-0.438675697	0.660896539	0.785935592	down_in_case
Yang2025_S	ENSGALG00010023665	C16orf87	case	control	-0.145057843	0.137102391	-1.058025631	0.290043757	0.458768735	down_in_case
Yang2025_S	ENSGALG00010023666	ENSGALG00010023666	case	control	-0.090083023	0.234890489	-0.38351073	0.701341116	0.815723934	down_in_case
Yang2025_S	ENSGALG00010023668	RCN1	case	control	-0.203028417	0.117398033	-1.729402203	0.083737136	0.193576143	down_in_case
Yang2025_S	ENSGALG00010023670	EXTL2	case	control	-0.29356628	0.114789069	-2.557441075	0.010544542	0.042114702	down_in_case
Yang2025_S	ENSGALG00010023671	ENSGALG00010023671	case	control	0.204986842	0.370782063	0.552849941	0.580366168	0.723057894	up_in_case
Yang2025_S	ENSGALG00010023676	ENSGALG00010023676	case	control	1.436755543	0.305643318	4.700758892	2.59196E-06	5.40704E-05	up_in_case
Yang2025_S	ENSGALG00010023677	MN1	case	control	0.054546284	0.125185461	0.435723796	0.663307139	0.786952362	up_in_case
Yang2025_S	ENSGALG00010023686	MYLK3	case	control	-0.529744928	0.237845271	-2.227267015	0.025929434	0.082138363	down_in_case
Yang2025_S	ENSGALG00010023689	WT1	case	control	-0.222140341	0.227914154	-0.974666721	0.329725606	0.499318226	down_in_case
Yang2025_S	ENSGALG00010023693	SRSF11	case	control	0.776509591	0.199578865	3.890740593	9.99387E-05	0.001066345	up_in_case
Yang2025_S	ENSGALG00010023694	PPCDC	case	control	-0.432735793	0.204909056	-2.111843184	0.034699902	0.102056381	down_in_case
Yang2025_S	ENSGALG00010023695	CLSTN3	case	control	-0.025086047	0.114727244	-0.218658153	0.826916349	0.896430655	down_in_case
Yang2025_S	ENSGALG00010023696	LRRC4C	case	control	0.461277682	0.225164308	2.048627008	0.040498602	0.114217368	up_in_case
Yang2025_S	ENSGALG00010023698	ENSGALG00010023698	case	control	0.260211438	0.227972739	1.141414709	0.253697386	0.420165567	up_in_case
Yang2025_S	ENSGALG00010023699	PPIG	case	control	1.061977718	0.357662797	2.969214937	0.002985617	0.016277218	up_in_case
Yang2025_S	ENSGALG00010023702	COL4A4	case	control	-0.01379687	0.162061565	-0.085133512	0.932155249	0.95864084	down_in_case
Yang2025_S	ENSGALG00010023703	PITPNB	case	control	0.628695168	0.090360455	6.957636168	3.46029E-12	4.34407E-10	up_in_case
Yang2025_S	ENSGALG00010023704	ANKRD13C	case	control	-0.172005567	0.054158712	-3.175953805	0.001493447	0.009585992	down_in_case
Yang2025_S	ENSGALG00010023706	CDK5RAP3	case	control	0.497224667	0.13426321	3.703357517	0.000212765	0.001967279	up_in_case
Yang2025_S	ENSGALG00010023707	MPZL2	case	control	-0.572353416	0.449623237	-1.27296227	0.203031431	0.360964913	down_in_case
Yang2025_S	ENSGALG00010023710	CDC14A	case	control	-0.025171018	0.113339502	-0.222085134	0.82424761	0.894609428	down_in_case
Yang2025_S	ENSGALG00010023711	ENSGALG00010023711	case	control	-0.096435438	0.313682785	-0.307429806	0.758516244	0.85303826	down_in_case
Yang2025_S	ENSGALG00010023712	ARHGAP40	case	control	-0.265578055	0.129700089	-2.047632015	0.04059607	0.114422782	down_in_case
Yang2025_S	ENSGALG00010023714	EIF3M	case	control	-0.274805585	0.14063231	-1.954071472	0.050692765	0.134604361	down_in_case
Yang2025_S	ENSGALG00010023715	CD3E	case	control	-0.744526885	0.648721956	-1.147682575	0.251099627	0.416804479	down_in_case
Yang2025_S	ENSGALG00010023716	FASTKD1	case	control	-0.241849921	0.122999811	-1.966262537	0.049268292	0.131838918	down_in_case
Yang2025_S	ENSGALG00010023720	SCAMP5	case	control	0.107854267	0.131302045	0.821421074	0.411406464	0.57687151	up_in_case
Yang2025_S	ENSGALG00010023724	CYP2R1	case	control	-0.587313302	0.141561716	-4.148814517	3.34201E-05	0.00044269	down_in_case
Yang2025_S	ENSGALG00010023726	PNPO	case	control	0.639713383	0.141937254	4.507015352	6.57459E-06	0.000117008	up_in_case
Yang2025_S	ENSGALG00010023730	SP2	case	control	0.174412955	0.184805865	0.943763095	0.345290723	0.514062631	up_in_case
Yang2025_S	ENSGALG00010023731	ACTR5	case	control	-0.236026937	0.133867859	-1.76313372	0.077877939	0.183905962	down_in_case
Yang2025_S	ENSGALG00010023732	TM2D1	case	control	0.086513253	0.152445744	0.567501923	0.57037322	0.714697493	up_in_case
Yang2025_S	ENSGALG00010023733	ORC6	case	control	0.021029359	0.205677029	0.102244569	0.918562552	0.951982818	up_in_case
Yang2025_S	ENSGALG00010023738	ENSGALG00010023738	case	control	-0.034328073	0.205360961	-0.167159683	0.867244406	0.922021773	down_in_case
Yang2025_S	ENSGALG00010023740	PPP1R16B	case	control	-0.377843014	0.145934695	-2.58912395	0.009622045	0.039459447	down_in_case
Yang2025_S	ENSGALG00010023743	CD3D	case	control	0.851620958	0.230284396	3.698127064	0.000217196	0.001995141	up_in_case
Yang2025_S	ENSGALG00010023744	MT4	case	control	-0.28619092	0.277730663	-1.030462091	0.302793139	0.472491253	down_in_case
Yang2025_S	ENSGALG00010023746	TTC28	case	control	-0.055293707	0.139975423	-0.395024396	0.692824884	0.80892712	down_in_case
Yang2025_S	ENSGALG00010023748	ENSGALG00010023748	case	control	-0.956734684	0.208834322	-4.581309593	4.62073E-06	8.65456E-05	down_in_case
Yang2025_S	ENSGALG00010023749	RPP25	case	control	0.25897993	0.162745098	1.591322464	0.111537024	0.23678678	up_in_case
Yang2025_S	ENSGALG00010023750	ENSGALG00010023750	case	control	-0.265256156	0.142585389	-1.860331961	0.062838574	0.157241711	down_in_case
Yang2025_S	ENSGALG00010023751	PRRG4	case	control	0.188998119	0.266690634	0.708679252	0.478523543	0.637375795	up_in_case
Yang2025_S	ENSGALG00010023752	C1QL1	case	control	0.282112195	0.288644515	0.977368979	0.328386515	0.498036541	up_in_case
Yang2025_S	ENSGALG00010023759	CALCA	case	control	0.844087469	0.371534237	2.271896866	0.02309274	0.075433037	up_in_case
Yang2025_S	ENSGALG00010023760	ZW10	case	control	0.229399143	0.081869878	2.80199689	0.005078736	0.024253664	up_in_case
Yang2025_S	ENSGALG00010023762	BBS5	case	control	0.011978481	0.133705767	0.089588368	0.928614328	0.956889486	up_in_case
Yang2025_S	ENSGALG00010023764	COX5A	case	control	-1.114180664	0.205207117	-5.429542019	5.64989E-08	2.06102E-06	down_in_case
Yang2025_S	ENSGALG00010023766	ENSGALG00010023766	case	control	0.133204041	0.337406357	0.394788178	0.69299922	0.809060333	up_in_case
Yang2025_S	ENSGALG00010023767	ENSGALG00010023767	case	control	-0.052908356	0.19645928	-0.269309528	0.7876915	0.872049023	down_in_case
Yang2025_S	ENSGALG00010023769	ENSGALG00010023769	case	control	0.379617021	0.62127562	0.611028356	0.541180808	0.691613587	up_in_case
Yang2025_S	ENSGALG00010023770	SCRN2	case	control	0.267049632	0.230675911	1.157683224	0.246993333	0.412678801	up_in_case
Yang2025_S	ENSGALG00010023773	OVOA	case	control	0.281581276	0.360952174	0.780106886	0.435327964	0.599199444	up_in_case
Yang2025_S	ENSGALG00010023775	ENSGALG00010023775	case	control	-0.490793016	0.197696143	-2.482562428	0.013044121	0.049340343	down_in_case
Yang2025_S	ENSGALG00010023777	OAZ2	case	control	-0.784247456	0.223782093	-3.504513897	0.000457442	0.003678275	down_in_case
Yang2025_S	ENSGALG00010023778	FANCF	case	control	-0.069599051	0.435555381	-0.159793804	0.873043505	0.925723729	down_in_case
Yang2025_S	ENSGALG00010023781	COL4A3	case	control	0.157918864	0.186044394	0.848823556	0.39597948	0.56321143	up_in_case
Yang2025_S	ENSGALG00010023782	TMPRSS5	case	control	1.792608353	0.543670486	3.297233156	0.000976424	0.006875427	up_in_case
Yang2025_S	ENSGALG00010023783	VPS35	case	control	-0.22704015	0.043919747	-5.169432113	2.34806E-07	7.02152E-06	down_in_case
Yang2025_S	ENSGALG00010023786	ENSGALG00010023786	case	control	-0.439566134	0.251426482	-1.748288927	0.08041401	0.18798343	down_in_case
Yang2025_S	ENSGALG00010023788	RTCA	case	control	0.513150817	0.093144408	5.509196172	3.60476E-08	1.415E-06	up_in_case
Yang2025_S	ENSGALG00010023791	VTI1B	case	control	0.125734446	0.105349222	1.193501417	0.232673058	0.395740151	up_in_case
Yang2025_S	ENSGALG00010023792	ZNF609	case	control	0.172802123	0.121874093	1.417874124	0.156227518	0.301277397	up_in_case
Yang2025_S	ENSGALG00010023793	RBM45	case	control	-0.054062824	0.090111545	-0.599954462	0.548536584	0.697241385	down_in_case
Yang2025_S	ENSGALG00010023798	ARCN1	case	control	-0.064402731	0.068256241	-0.943543473	0.34540299	0.514128976	down_in_case
Yang2025_S	ENSGALG00010023799	NMT1	case	control	0.08999464	0.118563908	0.759039079	0.44782918	0.610420332	up_in_case
Yang2025_S	ENSGALG00010023800	PEX5	case	control	-0.192721351	0.134648254	-1.431294845	0.152345731	0.295881053	down_in_case
Yang2025_S	ENSGALG00010023802	ENSGALG00010023802	case	control	0.012962415	0.258988655	0.050050127	0.960082443	0.975838719	up_in_case
Yang2025_S	ENSGALG00010023805	GALNT6	case	control	0.642940525	0.401518519	1.601272406	0.109316598	0.233245566	up_in_case
Yang2025_S	ENSGALG00010023806	PATJ	case	control	0.286800822	0.187568091	1.529049107	0.12625227	0.258724322	up_in_case
Yang2025_S	ENSGALG00010023807	MRPL10	case	control	0.184020724	0.274245983	0.671006088	0.502216651	0.658733908	up_in_case
Yang2025_S	ENSGALG00010023808	DHX35	case	control	-0.00362516	0.13225114	-0.027411184	0.978131778	0.9869076	down_in_case
Yang2025_S	ENSGALG00010023809	CDON	case	control	-0.10221813	0.096516932	-1.059069403	0.289568173	0.458355907	down_in_case
Yang2025_S	ENSGALG00010023811	MRPL44	case	control	-0.022438455	0.240110108	-0.09345069	0.925545522	0.955297892	down_in_case
Yang2025_S	ENSGALG00010023815	PLCD3	case	control	0.321622854	0.157693242	2.039547486	0.041395417	0.116018732	up_in_case
Yang2025_S	ENSGALG00010023817	BBS2	case	control	0.123798012	0.128566581	0.96290973	0.335592826	0.504040314	up_in_case
Yang2025_S	ENSGALG00010023818	ALOX5	case	control	0.003195459	0.272426804	0.011729606	0.990641343	0.994000591	up_in_case
Yang2025_S	ENSGALG00010023819	BIN2	case	control	0.131702542	0.350832708	0.375399839	0.707363125	0.819785857	up_in_case
Yang2025_S	ENSGALG00010023821	KANK4	case	control	0.239005257	0.082748182	2.888344502	0.003872755	0.019731932	up_in_case
Yang2025_S	ENSGALG00010023822	CHEK2	case	control	0.364081984	0.14173599	2.568733492	0.010207092	0.041156201	up_in_case
Yang2025_S	ENSGALG00010023823	MUC2	case	control	0.502089094	0.532998123	0.942009122	0.346187969	0.514727257	up_in_case
Yang2025_S	ENSGALG00010023826	DBT	case	control	-0.283044396	0.100476257	-2.817027669	0.004847035	0.023436306	down_in_case
Yang2025_S	ENSGALG00010023828	CHEK1	case	control	0.374011081	0.172916447	2.162958395	0.03054438	0.09293361	up_in_case
Yang2025_S	ENSGALG00010023829	USP1	case	control	0.196453366	0.089929572	2.184524635	0.028923719	0.089061042	up_in_case
Yang2025_S	ENSGALG00010023830	AGFG1	case	control	0.313500741	0.147654459	2.123205376	0.033736646	0.099964506	up_in_case
Yang2025_S	ENSGALG00010023831	SATB2	case	control	-1.1221652	0.658469951	-1.704201077	0.088343528	0.201451001	down_in_case
Yang2025_S	ENSGALG00010023832	ENSGALG00010023832	case	control	-0.446323383	0.176169125	-2.533493786	0.011293174	0.044280107	down_in_case
Yang2025_S	ENSGALG00010023833	PIP4K2C	case	control	0.426615693	0.128818607	3.31175521	0.000927126	0.006591584	up_in_case
Yang2025_S	ENSGALG00010023835	INAFM2	case	control	-0.107082509	0.409953421	-0.261206527	0.793933245	0.875638151	down_in_case
Yang2025_S	ENSGALG00010023836	NDUFS3	case	control	-0.259550491	0.25585355	-1.01444944	0.310368343	0.480100209	down_in_case
Yang2025_S	ENSGALG00010023837	STT3A	case	control	0.151041025	0.130722599	1.155431626	0.247913717	0.41373385	up_in_case
Yang2025_S	ENSGALG00010023840	SMAGP	case	control	-0.962150435	0.233487986	-4.120770628	3.77607E-05	0.000489484	down_in_case
Yang2025_S	ENSGALG00010023841	OSBPL7	case	control	0.318856267	0.142185377	2.242539096	0.024926551	0.079740931	up_in_case
Yang2025_S	ENSGALG00010023847	PIK3AP1	case	control	-0.508554597	0.195822186	-2.597023169	0.009403558	0.038791765	down_in_case
Yang2025_S	ENSGALG00010023850	DAZAP2	case	control	0.389243997	0.13462307	2.891361754	0.003835763	0.019586427	up_in_case
Yang2025_S	ENSGALG00010023851	HSCB	case	control	0.516240765	0.251357207	2.053813261	0.039993769	0.113344147	up_in_case
Yang2025_S	ENSGALG00010023852	LRRC39	case	control	0.031454525	0.130750538	0.240568994	0.809889184	0.885439022	up_in_case
Yang2025_S	ENSGALG00010023854	SHCBP1	case	control	-0.513565017	0.195049789	-2.632994475	0.008463572	0.035804457	down_in_case
Yang2025_S	ENSGALG00010023857	ACBD4	case	control	0.901972393	0.216207236	4.171795582	3.02209E-05	0.00040807	up_in_case
Yang2025_S	ENSGALG00010023859	ENSGALG00010023859	case	control	0.995723676	0.427147723	2.331099106	0.019748134	0.067217942	up_in_case
Yang2025_S	ENSGALG00010023860	TRIP4	case	control	0.275047409	0.089524816	3.072303538	0.002124136	0.012519769	up_in_case
Yang2025_S	ENSGALG00010023861	ENSGALG00010023861	case	control	0.549599669	0.157956142	3.479444748	0.000502454	0.003971467	up_in_case
Yang2025_S	ENSGALG00010023862	TRMT13	case	control	0.067059019	0.151174388	0.443587171	0.657341082	0.783900793	up_in_case
Yang2025_S	ENSGALG00010023863	EYA2	case	control	0.237691161	0.163006089	1.458173508	0.144792724	0.285791304	up_in_case
Yang2025_S	ENSGALG00010023866	AMBRA1	case	control	-0.143310425	0.089535652	-1.600596205	0.109466383	0.233422196	down_in_case
Yang2025_S	ENSGALG00010023867	ENSGALG00010023867	case	control	0.713602683	0.406224484	1.756670786	0.07897394	0.185613401	up_in_case
Yang2025_S	ENSGALG00010023869	DOCK7	case	control	0.214114536	0.108685288	1.970041578	0.048833606	0.131070624	up_in_case
Yang2025_S	ENSGALG00010023871	UQCRFS1	case	control	-0.521755236	0.212747434	-2.452463126	0.014188192	0.052560292	down_in_case
Yang2025_S	ENSGALG00010023874	HEXIM1	case	control	0.519251532	0.11271557	4.60674184	4.09027E-06	7.86179E-05	up_in_case
Yang2025_S	ENSGALG00010023876	POU6F1	case	control	1.156836824	0.421256493	2.746157847	0.006029774	0.027531097	up_in_case
Yang2025_S	ENSGALG00010023879	EI24	case	control	-0.216397371	0.106288792	-2.035937813	0.0417566	0.116741493	down_in_case
Yang2025_S	ENSGALG00010023880	ENSGALG00010023880	case	control	0.362501908	0.425070998	0.852803201	0.393768449	0.560830267	up_in_case
Yang2025_S	ENSGALG00010023881	VSTM2B	case	control	-0.153976542	0.142948176	-1.077149401	0.281413526	0.449300812	down_in_case
Yang2025_S	ENSGALG00010023883	TBX21	case	control	0.123832843	0.197020255	0.628528485	0.529657792	0.682108137	up_in_case
Yang2025_S	ENSGALG00010023884	C2orf69	case	control	-0.538565737	0.135885644	-3.963374801	7.38976E-05	0.000835847	down_in_case
Yang2025_S	ENSGALG00010023886	CYP2C18	case	control	1.002256747	0.594165784	1.686830131	0.09163602	0.206458842	up_in_case
Yang2025_S	ENSGALG00010023887	RASA2	case	control	-0.13211767	0.121542997	-1.08700356	0.277035236	0.444371714	down_in_case
Yang2025_S	ENSGALG00010023888	CTH	case	control	-0.209621943	0.121274639	-1.728489528	0.083900497	0.193857308	down_in_case
Yang2025_S	ENSGALG00010023889	SAMD8	case	control	-0.295010478	0.120462855	-2.448974657	0.014326353	0.052968354	down_in_case
Yang2025_S	ENSGALG00010023890	SASS6	case	control	0.200574966	0.106004281	1.892140248	0.058472297	0.14975399	up_in_case
Yang2025_S	ENSGALG00010023891	ENSGALG00010023891	case	control	-0.933756098	0.409503036	-2.280217765	0.022594776	0.074049437	down_in_case
Yang2025_S	ENSGALG00010023896	POP4	case	control	0.230166164	0.173779178	1.32447493	0.185345364	0.33886248	up_in_case
Yang2025_S	ENSGALG00010023902	PTGER3	case	control	-0.627402706	0.234872813	-2.671244484	0.007557058	0.032795891	down_in_case
Yang2025_S	ENSGALG00010023903	TBKBP1	case	control	0.703142917	0.378663724	1.856905938	0.063324538	0.15818979	up_in_case

Yang2025_S	ENSGALG00010023909	FOXRED1	case	control	-0.229586668	0.222866581	-1.030152961	0.302938207	0.47251443	down_in_case
Yang2025_S	ENSGALG00010023910	RNF7	case	control	-0.403935739	0.096328092	-4.193332707	2.74886E-05	0.000379261	down_in_case
Yang2025_S	ENSGALG00010023911	TFCP2	case	control	0.239695507	0.105301886	2.276269842	0.022829864	0.074661853	up_in_case
Yang2025_S	ENSGALG00010023913	ZMYND8	case	control	0.338233325	0.185976615	1.818687389	0.068959137	0.168203321	up_in_case
Yang2025_S	ENSGALG00010023914	CCDC117	case	control	-0.650178108	0.117296859	-5.543013797	2.97309E-08	1.18372E-06	down_in_case
Yang2025_S	ENSGALG00010023917	ZRANB2	case	control	0.023656101	0.089460224	0.264431501	0.791447441	0.873985268	up_in_case
Yang2025_S	ENSGALG00010023918	MFSD14A	case	control	-0.538157627	0.145050777	-3.71013266	0.000207151	0.001926471	down_in_case
Yang2025_S	ENSGALG00010023920	ENSGALG00010023920	case	control	0.359647002	0.411985265	0.872960837	0.382684424	0.550444617	up_in_case
Yang2025_S	ENSGALG00010023921	GRK7	case	control	0.477175185	0.194879078	2.448570623	0.014342431	0.053013734	up_in_case
Yang2025_S	ENSGALG00010023923	CSNK1G1	case	control	-0.083171873	0.122119972	-0.681066919	0.495829148	0.653122146	down_in_case
Yang2025_S	ENSGALG00010023925	NCOA3	case	control	0.379741939	0.198135643	1.916575597	0.055291871	0.143721734	up_in_case
Yang2025_S	ENSGALG00010023926	ZYX	case	control	0.309976268	0.202615664	1.529873168	0.126048126	0.258496047	up_in_case
Yang2025_S	ENSGALG00010023927	KPNB1	case	control	0.061023362	0.076338542	0.799378142	0.424071181	0.589058204	up_in_case
Yang2025_S	ENSGALG00010023930	SRPRA	case	control	-0.318968281	0.104286289	-3.058583112	0.002223864	0.012970202	down_in_case
Yang2025_S	ENSGALG00010023931	CSRNP2	case	control	0.552896663	0.190443474	2.90320614	0.003693634	0.019049146	up_in_case
Yang2025_S	ENSGALG00010023937	XBP1	case	control	-0.158232322	0.215690007	-0.733609887	0.463186533	0.624166361	down_in_case
Yang2025_S	ENSGALG00010023940	ATP1B3	case	control	-0.238212303	0.097058574	-2.454314885	0.014115332	0.052396684	down_in_case
Yang2025_S	ENSGALG00010023942	NEGR1	case	control	-0.374396731	0.125365688	-2.986437005	0.00282249	0.015539864	down_in_case
Yang2025_S	ENSGALG00010023945	LETMD1	case	control	0.617620907	0.209576454	2.946995694	0.003208777	0.017131917	up_in_case
Yang2025_S	ENSGALG00010023946	ZNRF3	case	control	0.086359002	0.16283992	0.530330659	0.595882693	0.735484972	up_in_case
Yang2025_S	ENSGALG00010023947	ENSGALG00010023947	case	control	-0.767610838	0.775791956	-0.989454495	0.322440824	0.491805269	down_in_case
Yang2025_S	ENSGALG00010023948	SULF2	case	control	0.044260696	0.179061596	0.247181403	0.804767837	0.882663441	up_in_case
Yang2025_S	ENSGALG00010023953	SLC35A3	case	control	-0.107663018	0.110716609	-0.972419756	0.330841767	0.500138862	down_in_case
Yang2025_S	ENSGALG00010023954	PLEKHF1	case	control	0.251623716	0.09816533	2.563264615	0.010369299	0.041605579	up_in_case
Yang2025_S	ENSGALG00010023955	C1QL4	case	control	1.301360724	0.951500592	1.367693026	0.171408184	0.320527783	up_in_case
Yang2025_S	ENSGALG00010023956	KREMEN1	case	control	0.000279584	0.151636777	0.001843773	0.998528883	0.998743898	up_in_case
Yang2025_S	ENSGALG00010023957	YBX3	case	control	-0.032217657	0.148391646	-0.217112336	0.828120801	0.897066492	down_in_case
Yang2025_S	ENSGALG00010023962	FPGT	case	control	-0.459253736	0.137217415	-3.346905599	0.00081719	0.005940296	down_in_case
Yang2025_S	ENSGALG00010023966	PEAK1	case	control	0.153254394	0.202241088	0.757780703	0.448582276	0.611045359	up_in_case
Yang2025_S	ENSGALG00010023967	PRPH	case	control	1.778536557	0.422946532	4.205109686	2.60956E-05	0.000363278	up_in_case
Yang2025_S	ENSGALG00010023968	TYW5	case	control	-0.058197154	0.220031865	-0.264494207	0.791399128	0.873985268	down_in_case
Yang2025_S	ENSGALG00010023972	ENSGALG00010023972	case	control	0.505095586	0.425422839	1.18727896	0.235117604	0.398342105	up_in_case
Yang2025_S	ENSGALG00010023974	PREX1	case	control	0.251389415	0.16537186	1.520146261	0.12847422	0.261745372	up_in_case
Yang2025_S	ENSGALG00010023978	TFDP2	case	control	0.183603562	0.175406024	1.046734643	0.295221981	0.463781583	up_in_case
Yang2025_S	ENSGALG00010023979	FAM118B	case	control	-0.022149632	0.167761631	-0.132030381	0.894960261	0.938174682	down_in_case
Yang2025_S	ENSGALG00010023981	ENSGALG00010023981	case	control	0.495281268	0.327404704	1.512749398	0.130343327	0.264540381	up_in_case
Yang2025_S	ENSGALG00010023984	FMNL1	case	control	0.145304386	0.177937067	0.816605495	0.414153914	0.579557621	up_in_case
Yang2025_S	ENSGALG00010023986	SYCE3	case	control	0.95842111	0.309114576	3.100536771	0.001931702	0.011678209	up_in_case
Yang2025_S	ENSGALG00010023987	TNNI3K	case	control	0.547491851	0.273502443	2.001780481	0.045308346	0.124530928	up_in_case
Yang2025_S	ENSGALG00010023989	ENSGALG00010023989	case	control	0.249759131	0.148306104	1.684078565	0.092166486	0.207285343	up_in_case
Yang2025_S	ENSGALG00010023991	TSPAN3	case	control	-0.359060463	0.109192389	-3.288328666	0.001007841	0.007068075	down_in_case
Yang2025_S	ENSGALG00010023992	FAM131B	case	control	0.381213827	0.1550571	2.458538361	0.013950387	0.051925851	up_in_case
Yang2025_S	ENSGALG00010023993	C19orf12	case	control	-0.330712296	0.15651852	-2.112927573	0.034606967	0.101869052	down_in_case
Yang2025_S	ENSGALG00010023996	MYO7B	case	control	0.396597804	0.465608023	0.851784731	0.394333582	0.561463009	up_in_case
Yang2025_S	ENSGALG00010023997	ARFGEF2	case	control	0.252314756	0.126151719	2.000089715	0.045490577	0.124859404	up_in_case
Yang2025_S	ENSGALG00010023999	DGCR14	case	control	-0.041636847	0.106359404	-0.391473112	0.69544756	0.811035379	down_in_case
Yang2025_S	ENSGALG00010024001	ENSGALG00010024001	case	control	0.026703604	0.502766393	0.053113342	0.957641601	0.9740683	up_in_case
Yang2025_S	ENSGALG00010024002	DGCR2	case	control	-0.091021933	0.097085913	-0.93754006	0.348480828	0.516824216	down_in_case
Yang2025_S	ENSGALG00010024003	ERICH3	case	control	1.401107792	0.323494167	4.331168642	1.4832E-05	0.000228128	up_in_case
Yang2025_S	ENSGALG00010024004	EIF4B	case	control	0.124463747	0.089976739	1.383288039	0.166576564	0.31447643	up_in_case
Yang2025_S	ENSGALG00010024005	STYK1	case	control	-0.127049321	0.267925446	-0.474196547	0.635359755	0.766393522	down_in_case
Yang2025_S	ENSGALG00010024008	CCNE1	case	control	0.051294111	0.125613601	0.408348388	0.683017919	0.801622642	up_in_case
Yang2025_S	ENSGALG00010024009	CSE1L	case	control	-0.36712109	0.085110299	-4.313474339	1.60709E-05	0.000243157	down_in_case
Yang2025_S	ENSGALG00010024010	ENSGALG00010024010	case	control	-0.173849427	0.093704688	-1.855290598	0.06355474	0.158616537	down_in_case
Yang2025_S	ENSGALG00010024011	AGPS	case	control	-0.377453094	0.078522759	-4.806926043	1.53269E-06	3.4785E-05	down_in_case
Yang2025_S	ENSGALG00010024013	PTPRJ	case	control	0.196849047	0.178237927	1.104417281	0.269412168	0.436490938	up_in_case
Yang2025_S	ENSGALG00010024016	CRYZ	case	control	0.141985788	0.13714919	1.035265227	0.300545064	0.470149918	up_in_case
Yang2025_S	ENSGALG00010024017	TYW3	case	control	0.359422371	0.181761341	1.977441234	0.047991777	0.1296057	up_in_case
Yang2025_S	ENSGALG00010024018	KRT18	case	control	-1.176318576	1.074851748	-1.094400766	0.273779248	0.441053621	down_in_case
Yang2025_S	ENSGALG00010024019	MAP3K14	case	control	-0.067863583	0.078020906	-0.869812804	0.384402713	0.551947636	down_in_case
Yang2025_S	ENSGALG00010024020	MAIP1	case	control	-0.545302579	0.143774764	-3.79275587	0.000148985	0.001472412	down_in_case
Yang2025_S	ENSGALG00010024023	LHX8	case	control	-0.688225954	0.465493947	-1.478485293	0.139277931	0.277698951	down_in_case
Yang2025_S	ENSGALG00010024024	AGL	case	control	-0.14046922	0.135610204	-1.035830752	0.300281108	0.469895255	down_in_case
Yang2025_S	ENSGALG00010024025	CLCN1	case	control	0.326536699	0.213548785	1.529096497	0.126240524	0.258724322	up_in_case
Yang2025_S	ENSGALG00010024026	TIRAP	case	control	0.027899405	0.190278992	0.146623673	0.883429067	0.931385514	up_in_case
Yang2025_S	ENSGALG00010024029	SCAPER	case	control	0.187432415	0.149409542	1.254487584	0.209664836	0.368898925	up_in_case
Yang2025_S	ENSGALG00010024030	STAU1	case	control	0.156489061	0.092673248	1.688610939	0.091294013	0.205854704	up_in_case
Yang2025_S	ENSGALG00010024031	LIMS2	case	control	-0.489680653	0.129888262	-3.770014656	0.000163238	0.001584068	down_in_case
Yang2025_S	ENSGALG00010024034	LRP2	case	control	-0.127047242	0.273224944	-0.464991374	0.641937634	0.77155813	down_in_case
Yang2025_S	ENSGALG00010024035	OSBPL5	case	control	0.089752135	0.158652811	0.565714119	0.571588135	0.715439066	up_in_case
Yang2025_S	ENSGALG00010024038	NKD1	case	control	0.376805234	0.227425	1.65683295	0.097553253	0.215129701	up_in_case
Yang2025_S	ENSGALG00010024039	MRPL45	case	control	-0.041561685	0.132574661	-0.313496446	0.75390352	0.850181881	down_in_case
Yang2025_S	ENSGALG00010024042	DCPS	case	control	-0.448338917	0.134845119	-3.324843498	0.000884682	0.006342851	down_in_case
Yang2025_S	ENSGALG00010024045	PSTPIP1	case	control	-0.412223548	0.185795667	-2.218693016	0.026507616	0.08353316	down_in_case
Yang2025_S	ENSGALG00010024046	ENSGALG00010024046	case	control	1.048552927	0.737620171	1.421535051	0.155161271	0.299719944	up_in_case
Yang2025_S	ENSGALG00010024049	RCN2	case	control	-0.388589252	0.073221606	-5.307029889	1.11426E-07	3.76874E-06	down_in_case
Yang2025_S	ENSGALG00010024051	ENSGALG00010024051	case	control	0.004063561	0.63164133	0.006433335	0.994866976	0.996498552	up_in_case
Yang2025_S	ENSGALG00010024052	ENSGALG00010024052	case	control	0.911347487	0.153207713	5.948443902	2.70704E-09	1.43979E-07	up_in_case
Yang2025_S	ENSGALG00010024053	ISL2	case	control	0.759474716	0.436799377	1.738726648	0.082082856	0.190701001	up_in_case
Yang2025_S	ENSGALG00010024058	SLC44A5	case	control	-0.241057673	0.116293075	-2.072846325	0.038186584	0.109379248	down_in_case
Yang2025_S	ENSGALG00010024060	FRRS1	case	control	-0.538565709	0.149450501	-3.603639375	0.000313792	0.00270923	down_in_case
Yang2025_S	ENSGALG00010024061	ENSGALG00010024061	case	control	-1.819429085	1.468767152	-1.238745762	0.215439666	0.376303803	down_in_case
Yang2025_S	ENSGALG00010024062	ST3GAL4	case	control	0.14887743	0.131860092	1.129056018	0.258874204	0.425201795	up_in_case
Yang2025_S	ENSGALG00010024063	BRD7	case	control	0.297377262	0.144572014	2.056949014	0.039691131	0.112669771	up_in_case
Yang2025_S	ENSGALG00010024064	GPR17	case	control	0.593119802	0.227367221	2.608642531	0.009090215	0.037891756	up_in_case
Yang2025_S	ENSGALG00010024068	CASP2	case	control	-0.144485167	0.129292321	-1.117507715	0.263777303	0.430817711	down_in_case
Yang2025_S	ENSGALG00010024073	RABGGTB	case	control	-0.257034529	0.117638942	-2.18494424	0.028892935	0.089016815	down_in_case
Yang2025_S	ENSGALG00010024074	ADCY7	case	control	-0.015329707	0.209708943	-0.073099921	0.941726604	0.964072894	down_in_case
Yang2025_S	ENSGALG00010024075	SPATS2L	case	control	0.258497316	0.16350115	1.581012218	0.113875244	0.240576877	up_in_case
Yang2025_S	ENSGALG00010024077	SFT2D3	case	control	0.392075039	0.11132309	3.521956129	0.000428375	0.003492923	up_in_case
Yang2025_S	ENSGALG00010024078	KIRREL3	case	control	0.300598864	0.271825864	1.105850857	0.268791078	0.436028234	up_in_case
Yang2025_S	ENSGALG00010024080	TENT4B	case	control	-0.018823727	0.140923462	-0.133574117	0.893739353	0.937584125	down_in_case
Yang2025_S	ENSGALG00010024081	CARS	case	control	0.214572767	0.1238795	1.732108763	0.083254199	0.19274751	up_in_case
Yang2025_S	ENSGALG00010024083	PALMD	case	control	0.2782239	0.171505536	1.62224443	0.104751022	0.226803215	up_in_case
Yang2025_S	ENSGALG00010024086	GABARAPL1	case	control	0.063585887	0.066331457	0.958608318	0.337756101	0.505926181	up_in_case
Yang2025_S	ENSGALG00010024091	ETFA	case	control	0.203446013	0.204854367	0.993125093	0.320648997	0.490100227	up_in_case
Yang2025_S	ENSGALG00010024095	RBM12	case	control	-0.154337158	0.102033633	-1.512610638	0.13037859	0.264573417	down_in_case
Yang2025_S	ENSGALG00010024096	MYO1D	case	control	0.473082686	0.154450595	3.06300332	0.002191276	0.012819242	up_in_case
Yang2025_S	ENSGALG00010024101	CNEP1R1	case	control	-0.1519578	0.095841134	-1.585517551	0.112848792	0.238916427	down_in_case
Yang2025_S	ENSGALG00010024102	SGF29	case	control	0.278129216	0.074923237	3.712188972	0.000205474	0.001914671	up_in_case
Yang2025_S	ENSGALG00010024104	PLPPR5	case	control	0.077335203	0.130629204	0.592020783	0.553836672	0.70174227	up_in_case
Yang2025_S	ENSGALG00010024106	WDR33	case	control	-0.119810389	0.067590121	-1.772602063	0.07629467	0.180933666	down_in_case
Yang2025_S	ENSGALG00010024107	NFE2L2	case	control	-0.212284155	0.065521334	-3.239924177	0.001195615	0.008048741	down_in_case
Yang2025_S	ENSGALG00010024110	CPNE1	case	control	-0.056217643	0.071530288	-0.785927817	0.431909777	0.596025227	down_in_case
Yang2025_S	ENSGALG00010024112	NAP1L4	case	control	0.165581236	0.072623497	2.279995374	0.022607963	0.074075232	up_in_case
Yang2025_S	ENSGALG00010024113	ENSGALG00010024113	case	control	0.713044961	0.219601338	3.246997337	0.001166295	0.007897142	up_in_case
Yang2025_S	ENSGALG00010024115	POLR2D	case	control	-0.295384602	0.251212992	-1.175833302	0.239661496	0.404026487	down_in_case
Yang2025_S	ENSGALG00010024116	MCAM	case	control	0.324531067	0.167179794	1.941209878	0.052232825	0.137704076	up_in_case
Yang2025_S	ENSGALG00010024118	TMEM266	case	control	0.271463304	0.146700013	1.850465435	0.064246495	0.159699414	up_in_case
Yang2025_S	ENSGALG00010024119	AMMECR1L	case	control	-0.336575477	0.11427845	-2.945222635	0.003227224	0.017190887	down_in_case
Yang2025_S	ENSGALG00010024121	NRG4	case	control	0.037146968	0.153370639	0.242203908	0.808622167	0.884678488	up_in_case
Yang2025_S	ENSGALG00010024122	SOCS7	case	control	0.204776777	0.08882063	2.305509178	0.021138073	0.07062275	up_in_case
Yang2025_S	ENSGALG00010024123	PLPPR4	case	control	0.113358081	0.461559909	0.245597763	0.805993621	0.883489148	up_in_case
Yang2025_S	ENSGALG00010024124	DIDO1	case	control	0.737611059	0.170366535	4.329553677	1.49412E-05	0.000228797	up_in_case
Yang2025_S	ENSGALG00010024126	CDK5R1	case	control	0.085110978	0.181939314	0.467798722	0.639928535	0.769816044	up_in_case
Yang2025_S	ENSGALG00010024128	PHLDA2	case	control	1.614159805	0.200107398	8.066467412	7.23614E-16	1.86733E-13	up_in_case
Yang2025_S	ENSGALG00010024129	GID8	case	control	-0.800755722	0.083035815	-9.64349805	5.23722E-22	3.47527E-19	down_in_case
Yang2025_S	ENSGALG00010024131	ENSGALG00010024131	case	control	-0.413048856	1.886719558	-0.218924352	0.826708977	0.896430655	down_in_case
Yang2025_S	ENSGALG00010024132	ENSGALG00010024132	case	control	-0.159702104	0.130033153	-1.228164514	0.219385217	0.380477037	down_in_case
Yang2025_S	ENSGALG00010024133	PSMD11	case	control	0.11729997	0.070601592	1.661435199	0.096626073	0.214135549	up_in_case
Yang2025_S	ENSGALG00010024135	FBXO22	case	control	-0.06896032	0.095285764	-0.723721127	0.46923696	0.629883156	down_in_case
Yang2025_S	ENSGALG00010024136	SAP130	case	control	0.555322084	0.238668377	2.32675183	0.019978478	0.067665852	up_in_case
Yang2025_S	ENSGALG00010024137	ENSGALG00010024137	case	control	0.397047794	0.498578729	0.796359272	0.425823244	0.590491283	up_in_case
Yang2025_S	ENSGALG00010024138	CBL	case	control	-0.182872792	0.180677586	-1.012149854	0.311466409	0.481091883	down_in_case
Yang2025_S	ENSGALG00010024140	KRT6A	case	control	-0.764636933	1.609768978	-0.474997931	0.634788448	0.765868141	down_in_case
Yang2025_S	ENSGALG00010024141	YTHDF1	case	control	-0.24615381	0.100723947	-2.443845962	0.01453163	0.05357673	down_in_case

Yang2025_S	ENSGALG00010024142	DNAJC6	case	control	0.341221908	0.394890904	0.864091586			up_in_case
Yang2025_S	ENSGALG00010024143	UBE2Q2	case	control	-0.246612297	0.088637687	-2.782251052	0.005398326	0.02541077	down_in_case
Yang2025_S	ENSGALG00010024144	HNRNPA3	case	control	0.352586619	0.105439743	3.343963172	0.000825907	0.005991158	up_in_case
Yang2025_S	ENSGALG00010024145	RAP1GAP3	case	control	0.223114781	0.134770298	1.655518944	0.097819277	0.215648097	up_in_case
Yang2025_S	ENSGALG00010024146	CDKN1C	case	control	0.352835649	0.180301252	1.956922899	0.050356534	0.133915706	up_in_case
Yang2025_S	ENSGALG00010024147	ENSGALG00010024147	case	control	0.330898064	0.275578294	1.200740663	0.229851816	0.39223427	up_in_case
Yang2025_S	ENSGALG00010024148	UGGT1	case	control	-0.224717129	0.080651141	-2.786285787	0.005331584	0.025150856	down_in_case
Yang2025_S	ENSGALG00010024149	TCIRG1	case	control	0.186071768	0.099965458	1.861360624	0.062693267	0.157087818	up_in_case
Yang2025_S	ENSGALG00010024150	NHERF4	case	control	-0.092894236	0.426110275	-0.218005153	0.827425096	0.896722314	down_in_case
Yang2025_S	ENSGALG00010024151	LEPROT	case	control	-0.336574567	0.092058172	-3.656107439	0.000256074	0.002290367	down_in_case
Yang2025_S	ENSGALG00010024152	ZNF423	case	control	-0.241746375	0.104106927	-2.322096938	0.020227717	0.068283244	down_in_case
Yang2025_S	ENSGALG00010024153	KCNQ1	case	control	-0.319412216	0.238372842	-1.339968987	0.180255428	0.332388433	down_in_case
Yang2025_S	ENSGALG00010024155	SNX7	case	control	0.025616157	0.064832552	0.395112589	0.692759799	0.80892712	up_in_case
Yang2025_S	ENSGALG00010024157	CHRNB4	case	control	-0.323321459	0.241720697	-1.337582846	0.181032459	0.333468251	down_in_case
Yang2025_S	ENSGALG00010024158	ENSGALG00010024158	case	control	-0.290794212	0.183229623	-1.587048032	0.112501767	0.238326561	down_in_case
Yang2025_S	ENSGALG00010024159	NLRX1	case	control	0.173741621	0.171630629	1.012299623	0.311394815	0.481091883	up_in_case
Yang2025_S	ENSGALG00010024162	SRCIN1	case	control	0.9487025	0.223195371	4.250547387	2.13249E-05	0.000306353	up_in_case
Yang2025_S	ENSGALG00010024163	HS6ST1	case	control	0.097422193	0.093015711	1.047373517	0.294927341	0.463570012	up_in_case
Yang2025_S	ENSGALG00010024164	SNAP29	case	control	-0.644420625	0.101903893	-6.323807704	2.55195E-10	1.80515E-08	down_in_case
Yang2025_S	ENSGALG00010024165	LEPR	case	control	0.535164805	0.308079275	1.737100961	0.082369352	0.191111708	up_in_case
Yang2025_S	ENSGALG00010024167	CHRNA3	case	control	-0.116725115	0.304520766	-0.383307569	0.701491727	0.815831015	down_in_case
Yang2025_S	ENSGALG00010024168	BIRC7	case	control	1.122637261	0.470095077	2.388106824	0.016935418	0.060019088	up_in_case
Yang2025_S	ENSGALG00010024169	FAM168B	case	control	-0.13496643	0.11243343	-1.200411919	0.229979402	0.392403939	down_in_case
Yang2025_S	ENSGALG00010024170	NUP160	case	control	-0.271444799	0.076055706	-3.569026095	0.000358311	0.003020605	down_in_case
Yang2025_S	ENSGALG00010024172	ABCG4	case	control	0.105685412	0.309947139	0.340978827	0.733119521	0.836967108	up_in_case
Yang2025_S	ENSGALG00010024173	CBLN1	case	control	1.45214723	0.469078249	3.095746249	0.001963183	0.011817258	up_in_case
Yang2025_S	ENSGALG00010024174	PLEKHB2	case	control	0.252912908	0.085569292	2.955650337	0.003120106	0.016819447	up_in_case
Yang2025_S	ENSGALG00010024175	DHRS9	case	control	-0.032368565	0.434707857	-0.074460501	0.94064397	0.963601685	down_in_case
Yang2025_S	ENSGALG00010024178	N4BP1	case	control	-0.170222078	0.10038484	-1.695695074	0.089943642	0.203683654	down_in_case
Yang2025_S	ENSGALG00010024179	ATG4C	case	control	-0.029167371	0.106974316	-0.272657698	0.785116355	0.87009674	down_in_case
Yang2025_S	ENSGALG00010024180	HINFP	case	control	0.141189999	0.139959145	1.008794384	0.313073255	0.482490136	up_in_case
Yang2025_S	ENSGALG00010024181	FOXD3	case	control	0.196603852	0.179354122	1.096176939	0.273001349	0.4403603	up_in_case
Yang2025_S	ENSGALG00010024183	MTX2	case	control	-0.708010832	0.242703979	-2.917178513	0.003532136	0.018386369	down_in_case
Yang2025_S	ENSGALG00010024184	ENSGALG00010024184	case	control	-0.218308357	0.143052384	-1.52607283	0.126991728	0.259673516	down_in_case
Yang2025_S	ENSGALG00010024185	CHRNA5	case	control	0.165198724	0.155298829	1.063747391	0.28744315	0.4562096	up_in_case
Yang2025_S	ENSGALG00010024186	HAO2	case	control	-0.642020412	0.598787683	-1.072200432	0.283630016	0.451836558	down_in_case
Yang2025_S	ENSGALG00010024188	TSSC4	case	control	0.22249922	0.081472303	2.730979884	0.006314633	0.028551072	up_in_case
Yang2025_S	ENSGALG00010024189	KAT7	case	control	-0.312725676	0.072652299	-4.304415424	1.67427E-05	0.000251141	down_in_case
Yang2025_S	ENSGALG00010024190	WARS2	case	control	0.054779495	0.14945031	0.366539856	0.713962278	0.823529869	up_in_case
Yang2025_S	ENSGALG00010024191	DPYD	case	control	-0.161063918	0.10021087	-1.607249974	0.107999543	0.231214263	down_in_case
Yang2025_S	ENSGALG00010024192	ALG6	case	control	0.617797372	0.181390283	3.405901137	0.000659461	0.005009787	up_in_case
Yang2025_S	ENSGALG00010024193	GSTK1	case	control	-0.053075272	0.187578642	-0.282949444	0.777215592	0.864503454	down_in_case
Yang2025_S	ENSGALG00010024194	NKAIN4	case	control	-0.029693846	0.150899484	-0.196778976	0.844000504	0.907425895	down_in_case
Yang2025_S	ENSGALG00010024195	PSMA4	case	control	0.097688115	0.110848385	0.881276842	0.378167997	0.546430012	up_in_case
Yang2025_S	ENSGALG00010024196	MRPL21	case	control	-0.373479618	0.225093727	-1.65921824	0.097071823	0.214645487	down_in_case
Yang2025_S	ENSGALG00010024197	KRT7	case	control	-0.288683931	0.228848072	-1.26146543	0.207141207	0.365818832	down_in_case
Yang2025_S	ENSGALG00010024198	PI4KA	case	control	0.044785156	0.076974192	0.581820407	0.560687657	0.707226757	up_in_case
Yang2025_S	ENSGALG00010024199	SLC35B1	case	control	-0.153065811	0.150890968	-1.014413342	0.310385561	0.480100209	down_in_case
Yang2025_S	ENSGALG00010024201	IREB2	case	control	-0.192862185	0.088513652	-2.178897614	0.029339275	0.090013826	down_in_case
Yang2025_S	ENSGALG00010024203	SPOP	case	control	0.065699874	0.106099838	0.6192269	0.535766893	0.686899591	up_in_case
Yang2025_S	ENSGALG00010024204	PTBP2	case	control	-0.345280119	0.152689363	-2.261323991	0.023739202	0.077128329	down_in_case
Yang2025_S	ENSGALG00010024205	ANKRD13D	case	control	0.666543115	0.127866361	5.212810538	1.86001E-07	5.83766E-06	up_in_case
Yang2025_S	ENSGALG00010024206	KRT80	case	control	-0.156479324	0.322133619	-0.485759061	0.627138004	0.760589041	down_in_case
Yang2025_S	ENSGALG00010024207	KCTD18	case	control	-0.227530022	0.090963936	-2.50132121	0.01237309	0.047485268	down_in_case
Yang2025_S	ENSGALG00010024208	SERPIND1	case	control	-1.13305852	0.502963628	-2.252764331	0.024274012	0.078464013	down_in_case
Yang2025_S	ENSGALG00010024209	RWDD3	case	control	-0.207113526	0.147607612	-1.403135808	0.160576345	0.306244432	down_in_case
Yang2025_S	ENSGALG00010024211	TRPM5	case	control	0.738016129	0.436393191	1.69117242	0.090803877	0.205060935	up_in_case
Yang2025_S	ENSGALG00010024212	RIMS4	case	control	-0.706536852	0.266006578	-2.656087894	0.007905301	0.033958189	down_in_case
Yang2025_S	ENSGALG00010024213	ENSGALG00010024213	case	control	1.059110042	0.201803357	5.248228056	1.53569E-07	4.94223E-06	up_in_case
Yang2025_S	ENSGALG00010024214	CRABP1	case	control	1.39587928	0.50503827	2.763907932	0.005711365	0.026467534	up_in_case
Yang2025_S	ENSGALG00010024215	ENSGALG00010024215	case	control	1.048242482	0.31880956	3.287989492	0.001009056	0.007073036	up_in_case
Yang2025_S	ENSGALG00010024218	NGFR	case	control	-0.234686565	0.092657694	-2.53283407	0.01131445	0.044338262	down_in_case
Yang2025_S	ENSGALG00010024220	SIAH1	case	control	-0.29596004	0.135111014	-2.190495295	0.028488336	0.088003759	down_in_case
Yang2025_S	ENSGALG00010024222	ENSGALG00010024222	case	control	-0.112818374	0.29668043	-0.380269014	0.703745734	0.817156637	down_in_case
Yang2025_S	ENSGALG00010024223	ENSGALG00010024223	case	control	-0.018333065	0.328358104	-0.055832533	0.955475218	0.972716771	down_in_case
Yang2025_S	ENSGALG00010024225	BCDIN3D	case	control	-0.205290342	0.210930221	-0.973261875	0.330423166	0.499777142	down_in_case
Yang2025_S	ENSGALG00010024227	MUC6	case	control	1.355262247	0.474698236	2.854997435	0.004303718	0.021296983	up_in_case
Yang2025_S	ENSGALG00010024228	ENSGALG00010024228	case	control	0.419788241	0.332950396	1.260813161	0.207376171	0.366073846	up_in_case
Yang2025_S	ENSGALG00010024230	LONP2	case	control	0.123691083	0.106275814	1.1638686	0.244477266	0.40976554	up_in_case
Yang2025_S	ENSGALG00010024233	FAIM2	case	control	-0.107316676	0.356245309	-0.301243759	0.763228624	0.855638848	down_in_case
Yang2025_S	ENSGALG00010024234	ENSGALG00010024234	case	control	0.129501971	0.202912968	0.638214365	0.523334148	0.676703817	up_in_case
Yang2025_S	ENSGALG00010024238	ATF7	case	control	0.035676901	0.130408235	0.27357859	0.784408489	0.869658071	up_in_case
Yang2025_S	ENSGALG00010024240	WDR61	case	control	-0.488546121	0.190330056	-2.566836425	0.010263101	0.041322253	down_in_case
Yang2025_S	ENSGALG00010024242	KMT2A	case	control	0.197493881	0.196216987	1.006507564	0.314171475	0.483549513	up_in_case
Yang2025_S	ENSGALG00010024243	MPP3	case	control	0.794033482	0.167671116	4.735660509	2.18343E-06	4.69508E-05	up_in_case
Yang2025_S	ENSGALG00010024245	TARBP2	case	control	0.40839552	0.24262527	1.683235718	0.092329469	0.207461931	up_in_case
Yang2025_S	ENSGALG00010024250	AP2A2	case	control	-0.160998595	0.056925967	-2.828210096	0.004680908	0.022807149	down_in_case
Yang2025_S	ENSGALG00010024251	ENSGALG00010024251	case	control	0.032047609	0.186622163	0.171724561	0.863654076	0.919409694	up_in_case
Yang2025_S	ENSGALG00010024254	ENSGALG00010024254	case	control	-0.287492377	0.290573547	-0.989396247	0.32246931	0.491805269	down_in_case
Yang2025_S	ENSGALG00010024255	CFAP97D1	case	control	1.324867454	0.363515786	3.644594002	0.000267814	0.002375552	up_in_case
Yang2025_S	ENSGALG00010024258	DNAJA4	case	control	0.271039692	0.14743459	1.838372472	0.066007539	0.162914462	up_in_case
Yang2025_S	ENSGALG00010024259	ENSGALG00010024259	case	control	-0.73524752	0.415325745	-1.770291219	0.07667864	0.181689653	down_in_case
Yang2025_S	ENSGALG00010024263	PHKB	case	control	0.035677786	0.172128121	0.207274594	0.835795415	0.902783436	up_in_case
Yang2025_S	ENSGALG00010024264	AMER3	case	control	-0.382222596	0.204336517	-1.87055452	0.061406853	0.155006599	down_in_case
Yang2025_S	ENSGALG00010024266	ENSGALG00010024266	case	control	-0.222745893	0.315388026	-0.706259827	0.480026571	0.638593662	down_in_case
Yang2025_S	ENSGALG00010024267	MAP3K12	case	control	0.22498123	0.225723819	0.996710188	0.318905203	0.488345495	up_in_case
Yang2025_S	ENSGALG00010024270	RAB18L	case	control	-0.541896557	0.351205393	-1.542961947	0.122840009	0.253709134	down_in_case
Yang2025_S	ENSGALG00010024274	ACSBG1	case	control	-0.200580707	0.152088083	-1.318845652	0.187220719	0.341303076	down_in_case
Yang2025_S	ENSGALG00010024277	ITGB3BP	case	control	-0.42624065	0.147239724	-2.894875362	0.003793091	0.019425477	down_in_case
Yang2025_S	ENSGALG00010024280	ENSGALG00010024280	case	control	0.105234327	0.116633588	0.902264342	0.366916463	0.535671127	up_in_case
Yang2025_S	ENSGALG00010024282	HIC2	case	control	-0.297293512	0.151979765	-1.956138782	0.050448808	0.134084329	down_in_case
Yang2025_S	ENSGALG00010024283	UBE4A	case	control	-0.14897952	0.075603066	-1.970548639	0.048775526	0.130986117	down_in_case
Yang2025_S	ENSGALG00010024285	CCND2	case	control	-0.259367186	0.117427236	-2.208748117	0.027192166	0.085055629	down_in_case
Yang2025_S	ENSGALG00010024286	ADA	case	control	-0.455950481	0.130603097	-3.491115385	0.000481008	0.003830202	down_in_case
Yang2025_S	ENSGALG00010024288	CHID1	case	control	0.260496194	0.114535674	2.274367324	0.02294391	0.074999623	up_in_case
Yang2025_S	ENSGALG00010024290	SOST	case	control	-0.175892047	0.482019955	-0.364906151	0.715181462	0.824117562	down_in_case
Yang2025_S	ENSGALG00010024291	ENSGALG00010024291	case	control	0.279669328	0.219888096	1.271871161	0.2034189	0.361515415	up_in_case
Yang2025_S	ENSGALG00010024300	MEOX1	case	control	0.242721731	0.21033708	1.15396549	0.248514318	0.414250874	up_in_case
Yang2025_S	ENSGALG00010024304	PKIG	case	control	-0.039419431	0.124314062	-0.317095511	0.751171131	0.848540188	down_in_case
Yang2025_S	ENSGALG00010024305	ENSGALG00010024305	case	control	-0.710960857	0.410588711	-1.731564551	0.083351122	0.19288614	down_in_case
Yang2025_S	ENSGALG00010024309	ABCB11	case	control	0.51426012	0.235483343	2.183849243	0.028973328	0.089165928	up_in_case
Yang2025_S	ENSGALG00010024311	KLHL6	case	control	-0.290933563	0.147732213	-1.96933057	0.048915143	0.131184087	down_in_case
Yang2025_S	ENSGALG00010024313	SERINC3	case	control	-0.411931408	0.153169711	-2.689379029	0.007158509	0.031438329	down_in_case
Yang2025_S	ENSGALG00010024314	ITFG1	case	control	0.112286908	0.122589805	0.91595633	0.3596898	0.528442526	up_in_case
Yang2025_S	ENSGALG00010024321	UBE2L3	case	control	-0.674104185	0.106552757	-6.326482777	2.50812E-10	1.79234E-08	down_in_case
Yang2025_S	ENSGALG00010024322	IDH3A	case	control	0.077370405	0.12420714	0.622914308	0.533340835	0.685302401	up_in_case
Yang2025_S	ENSGALG00010024323	TTPAL	case	control	-0.269728516	0.13575779	-1.986836386	0.046940531	0.127506039	down_in_case
Yang2025_S	ENSGALG00010024325	PCBP2	case	control	-0.00385109	0.090986394	-0.042326001	0.966238818	0.980015883	down_in_case
Yang2025_S	ENSGALG00010024326	KLHL24	case	control	-0.06645273	0.093506513	-0.71067488	0.47728573	0.63634254	down_in_case
Yang2025_S	ENSGALG00010024327	PPP1R1B	case	control	0.634946664	0.287247839	2.210448882	0.027074024	0.084838437	up_in_case
Yang2025_S	ENSGALG00010024328	YDJC	case	control	-0.1794735	0.138810172	-1.292941982	0.196031126	0.352203938	down_in_case
Yang2025_S	ENSGALG00010024330	SDF2L1	case	control	-0.504125986	0.142385641	-3.540567595	0.000399267	0.003292184	down_in_case
Yang2025_S	ENSGALG00010024332	EFCAB7	case	control	-0.194403259	0.181178326	-1.072994011	0.283273803	0.451546608	down_in_case
Yang2025_S	ENSGALG00010024333	ETV4	case	control	0.242096343	0.158959562	1.523005846	0.127757243	0.260734795	up_in_case
Yang2025_S	ENSGALG00010024334	USP28	case	control	0.025878421	0.115090111	0.224853558	0.8220932	0.893732933	up_in_case
Yang2025_S	ENSGALG00010024335	AMHR2	case	control	-0.115316629	0.399819752	-0.288421541	0.773024081	0.86190515	down_in_case
Yang2025_S	ENSGALG00010024336	TSPAN4	case	control	0.352822003	0.198773135	1.774998427	0.075898147	0.18030015	up_in_case
Yang2025_S	ENSGALG00010024338	NDUFA9	case	control	-0.048923352	0.139792695	-0.349970731	0.726360663	0.831977301	down_in_case
Yang2025_S	ENSGALG00010024339	TOP3B	case	control	0.289466375	0.073584369	3.933802489	8.36125E-05	0.000923981	up_in_case
Yang2025_S	ENSGALG00010024340	NETO2	case	control	-0.154362795	0.328351606	-0.470114328	0.638273338	0.768675046	down_in_case
Yang2025_S	ENSGALG00010024343	POLR2L	case	control	-0.075589444	0.337868583	-0.223724393	0.822971765	0.894251148	down_in_case
Yang2025_S	ENSGALG00010024344	YEATS2	case	control	0.134238176	0.116558045	1.151685206	0.249450454	0.415154911	up_in_case
Yang2025_S	ENSGALG00010024345	STARD3	case	control	-0.271328371	0.19367551	-1.400943106	0.161231087	0.307101585	down_in_case
Yang2025_S	ENSGALG00010024346	PPM1F	case	control	0.035410793	0.154459819	0.229255696	0.818670186	0.891611492	up_in_case
Yang2025_S	ENSGALG00010024347	OTOG	case	control	1.360597713	0.338791645	4.016030893	5.91865E-05	0.000698952	up_in_case
Yang2025_S	ENSGALG00010024349	R3HDML	case	control	-0.403105737	0.552255501	-0.729926159	0.465435321	0.626469738	down_in_case

Yang2025_S	ENSGALG00010024350	PGM1	case	control	-0.303184618	0.169440005	-1.789333151	0.073561178	0.176281173	down_in_case
Yang2025_S	ENSGALG00010024351	SP1	case	control	0.372811594	0.181754479	2.051182434	0.040249184	0.11380116	up_in_case
Yang2025_S	ENSGALG00010024352	MAP6D1	case	control	-0.047155291	0.470620386	-0.100198149	0.920187016	0.952943376	down_in_case
Yang2025_S	ENSGALG00010024354	C2CD2L	case	control	-0.14791523	0.126466979	-1.169595655	0.242163727	0.407289535	down_in_case
Yang2025_S	ENSGALG00010024356	PARL	case	control	-0.314039893	0.186170605	-1.686839302	0.091634256	0.206458842	down_in_case
Yang2025_S	ENSGALG00010024358	MAPK3	case	control	-0.552499737	0.068596837	-8.054303342	7.99328E-16	1.95415E-13	down_in_case
Yang2025_S	ENSGALG00010024360	FTL	case	control	0.04918116	0.194891933	0.252350926	0.800769828	0.879678956	up_in_case
Yang2025_S	ENSGALG00010024361	H2AX	case	control	0.306084244	0.214209273	1.428902869	0.153032153	0.296840625	up_in_case
Yang2025_S	ENSGALG00010024362	DHX8	case	control	-0.01022038	0.082981359	-0.123164772	0.901976621	0.941343928	down_in_case
Yang2025_S	ENSGALG00010024363	EDEM2	case	control	-0.139286937	0.142578713	-0.976912567	0.32861244	0.498180008	down_in_case
Yang2025_S	ENSGALG00010024365	ENSGALG00010024365	case	control	0.656431788	0.187912431	3.493285601	0.000477116	0.00380573	up_in_case
Yang2025_S	ENSGALG00010024366	PARD6A	case	control	0.331423369	0.410227036	0.807902309	0.419146822	0.584899957	up_in_case
Yang2025_S	ENSGALG00010024367	ENSGALG00010024367	case	control	0.562709636	0.267293332	2.105213889	0.035272694	0.103435393	up_in_case
Yang2025_S	ENSGALG00010024368	DPAGT1	case	control	0.00649241	0.095109167	0.068262719	0.945576501	0.966310395	up_in_case
Yang2025_S	ENSGALG00010024369	MYOD1	case	control	-0.104522991	0.155662788	-0.671470637	0.501920759	0.658593764	down_in_case
Yang2025_S	ENSGALG00010024370	HERPUD1	case	control	-0.155404695	0.123827634	-1.2550082	0.209475783	0.368659389	down_in_case
Yang2025_S	ENSGALG00010024371	YPEL1	case	control	-0.167180808	0.128448245	-1.301542172	0.193072939	0.348492568	down_in_case
Yang2025_S	ENSGALG00010024373	TCAP	case	control	1.271518445	0.624659434	2.035538688	0.0417967	0.116767645	up_in_case
Yang2025_S	ENSGALG00010024374	ENSGALG00010024374	case	control	-0.653909768	0.58041254	-1.12662929	0.25989925	0.426332672	down_in_case
Yang2025_S	ENSGALG00010024377	CIB2	case	control	-0.38179627	0.245681869	-1.554027049	0.120177965	0.249840361	down_in_case
Yang2025_S	ENSGALG00010024378	ZDHHC1	case	control	-0.011642192	0.111618498	-0.104303426	0.916928559	0.951194779	down_in_case
Yang2025_S	ENSGALG00010024383	DUSP28	case	control	0.275977422	0.196094566	1.407369041	0.159317986	0.304707129	up_in_case
Yang2025_S	ENSGALG00010024386	TPPP3	case	control	0.507979463	0.262835732	1.932687993	0.053274645	0.139781996	up_in_case
Yang2025_S	ENSGALG00010024387	PPIL2	case	control	-0.106395755	0.071952273	-1.478699012	0.139220776	0.277624717	down_in_case
Yang2025_S	ENSGALG00010024389	AAAS	case	control	0.004907012	0.236076359	0.0207857	0.983416605	0.990023869	up_in_case
Yang2025_S	ENSGALG00010024391	TBC1D2B	case	control	-0.284716378	0.136259819	-2.089510911	0.036661757	0.106256568	down_in_case
Yang2025_S	ENSGALG00010024392	CHMP4B	case	control	0.548643415	0.107264292	5.114874708	3.13949E-07	8.91015E-06	up_in_case
Yang2025_S	ENSGALG00010024393	OGFOD1	case	control	0.13639627	0.11508566	1.185171726	0.235949557	0.399411695	up_in_case
Yang2025_S	ENSGALG00010024396	ENSGALG00010024396	case	control	0.848375243	0.271785091	3.121492938	0.001799366	0.011065404	up_in_case
Yang2025_S	ENSGALG00010024398	PHB	case	control	-0.086181806	0.260718671	-0.33055479	0.740980801	0.841460961	down_in_case
Yang2025_S	ENSGALG00010024399	ROR1	case	control	0.170280246	0.214150612	0.795142468	0.426530634	0.59104827	up_in_case
Yang2025_S	ENSGALG00010024400	KCNA1	case	control	1.167664946	0.444963061	2.624183999	0.008685685	0.036577521	up_in_case
Yang2025_S	ENSGALG00010024401	KCNC1	case	control	-0.099201827	0.160178597	-0.619320111	0.535705498	0.686899591	down_in_case
Yang2025_S	ENSGALG00010024402	GPC1	case	control	-0.013487867	0.122557011	-0.110053819	0.912366693	0.948087752	down_in_case
Yang2025_S	ENSGALG00010024403	HMBS	case	control	-0.075242661	0.164574755	-0.457194431	0.647531301	0.775935049	down_in_case
Yang2025_S	ENSGALG00010024406	NUDT21	case	control	-0.061146818	0.213488794	-0.286416991	0.774558762	0.862822868	down_in_case
Yang2025_S	ENSGALG00010024407	HIRA	case	control	0.228058861	0.111923515	2.037631333	0.041586816	0.116415465	up_in_case
Yang2025_S	ENSGALG00010024410	MYG1	case	control	-0.028816408	0.292891962	-0.098385792	0.921625956	0.953337584	down_in_case
Yang2025_S	ENSGALG00010024412	PIGU	case	control	0.329436217	0.190890013	1.725790745	0.084385065	0.194621961	up_in_case
Yang2025_S	ENSGALG00010024413	AMFR	case	control	-0.490151544	0.136239799	-3.59771189	0.000321029	0.002760271	down_in_case
Yang2025_S	ENSGALG00010024415	SIK2	case	control	-0.049555775	0.127010338	-0.390171188	0.696409965	0.811871895	down_in_case
Yang2025_S	ENSGALG00010024416	VDR	case	control	0.285144739	0.38153343	0.747365019	0.454843255	0.616500414	up_in_case
Yang2025_S	ENSGALG00010024419	PGAP3	case	control	0.202358897	0.148120031	1.366181845	0.171881894	0.321155028	up_in_case
Yang2025_S	ENSGALG00010024420	TMEM106C	case	control	-0.336300253	0.220211424	-1.527169877	0.126718775	0.259337072	down_in_case
Yang2025_S	ENSGALG00010024421	DAPK2	case	control	0.292623592	0.118002154	2.479815692	0.013145031	0.049627745	up_in_case
Yang2025_S	ENSGALG00010024423	SERGEF	case	control	-0.193144066	0.119279082	-1.619261838	0.105390938	0.227693445	down_in_case
Yang2025_S	ENSGALG00010024424	SAMHD1	case	control	-0.11176876	0.074777328	-1.494687806	0.134995938	0.271413707	down_in_case
Yang2025_S	ENSGALG00010024425	MRPL40	case	control	0.068936398	0.157932393	0.436493087	0.662479014	0.786676187	up_in_case
Yang2025_S	ENSGALG00010024426	PHAF1	case	control	-0.19331054	0.096190203	-2.009669733	0.044466155	0.122700173	down_in_case
Yang2025_S	ENSGALG00010024427	C22orf39	case	control	0.039787848	0.355645214	0.111875112	0.910922431	0.947008659	up_in_case
Yang2025_S	ENSGALG00010024428	SAAL1	case	control	-0.346325532	0.093458112	-3.705676526	0.000210827	0.001954676	down_in_case
Yang2025_S	ENSGALG00010024430	ZNF652	case	control	0.237480832	0.269889623	0.87991835	0.378903543	0.546925717	up_in_case
Yang2025_S	ENSGALG00010024431	FEZ1	case	control	0.206920378	0.151998085	1.361335422	0.173407714	0.323355612	up_in_case
Yang2025_S	ENSGALG00010024433	ENSGALG00010024433	case	control	-0.128129367	0.129721353	-0.987727647	0.323286051	0.492564085	down_in_case
Yang2025_S	ENSGALG00010024434	B3GNT9	case	control	-0.074801918	0.103059461	-0.725813209	0.467953288	0.628644468	down_in_case
Yang2025_S	ENSGALG00010024437	HERC1	case	control	-0.058737761	0.122541133	-0.479330979	0.631703187	0.76360027	down_in_case
Yang2025_S	ENSGALG00010024438	RBL1	case	control	0.042031724	0.092304248	0.455360665	0.648849789	0.776913715	up_in_case
Yang2025_S	ENSGALG00010024439	UFD1	case	control	0.134273673	0.146916383	0.913946215	0.360745108	0.529323268	up_in_case
Yang2025_S	ENSGALG00010024440	PKNOX2	case	control	-0.035644704	0.163781182	-0.21763614	0.827712624	0.896833482	down_in_case
Yang2025_S	ENSGALG00010024441	SAA	case	control	0.178352456	0.501010621	0.35598538	0.721851514	0.828788074	up_in_case
Yang2025_S	ENSGALG00010024446	GRM4	case	control	1.292619694	0.46672836	2.769533211	0.005613668	0.02610159	up_in_case
Yang2025_S	ENSGALG00010024449	CACHD1	case	control	0.117756007	0.148675093	0.792035875	0.428339758	0.592683401	up_in_case
Yang2025_S	ENSGALG00010024451	PHOSPHO1	case	control	-0.116938137	0.238256889	-0.490806951	0.623562994	0.75777015	down_in_case
Yang2025_S	ENSGALG00010024452	SLC16A1	case	control	-0.293453068	0.19408028	-1.512018984	0.130529033	0.264840139	down_in_case
Yang2025_S	ENSGALG00010024453	HTR3A	case	control	1.243621981	0.345377131	3.600765281	0.000317282	0.002731454	up_in_case
Yang2025_S	ENSGALG00010024454	HPS5	case	control	-0.234626841	0.118902326	-1.973273778	0.048464379	0.13040913	down_in_case
Yang2025_S	ENSGALG00010024457	CHD6	case	control	-0.124769454	0.125172384	-0.996780994	0.318870826	0.488345495	down_in_case
Yang2025_S	ENSGALG00010024459	AHCYL1	case	control	0.00577403	0.127808193	0.045177305	0.963965983	0.97850131	up_in_case
Yang2025_S	ENSGALG00010024460	IMP4	case	control	-0.144212628	0.261707909	-0.551044212	0.581603367	0.724170302	down_in_case
Yang2025_S	ENSGALG00010024462	ZBTB32	case	control	-0.207734039	0.235824035	-0.880885781	0.378379644	0.546524196	down_in_case
Yang2025_S	ENSGALG00010024463	FBXL22	case	control	0.065658143	0.201497895	0.32585027	0.744537643	0.84405565	up_in_case
Yang2025_S	ENSGALG00010024465	SENP1	case	control	0.400626217	0.168016581	2.384444528	0.017104927	0.060496739	up_in_case
Yang2025_S	ENSGALG00010024466	ADGRG3	case	control	1.493633001	0.637113884	2.344373651	0.019059067	0.065415787	up_in_case
Yang2025_S	ENSGALG00010024467	ENSGALG00010024467	case	control	0.123581101	0.266824693	0.463154664	0.64325351	0.772775763	up_in_case
Yang2025_S	ENSGALG00010024469	GTF2H1	case	control	0.600875169	0.146210415	4.109660522	3.96241E-05	0.000509955	up_in_case
Yang2025_S	ENSGALG00010024471	CCDC61	case	control	0.334022983	0.150668857	2.216934472	0.02662757	0.083835333	up_in_case
Yang2025_S	ENSGALG00010024472	LDHA	case	control	-0.399945653	0.247246366	-1.617599723	0.105748889	0.228148439	down_in_case
Yang2025_S	ENSGALG00010024473	RAVER2	case	control	-0.030583096	0.063729879	-0.479886302	0.631308242	0.763452777	down_in_case
Yang2025_S	ENSGALG00010024474	STRIP1	case	control	0.164473747	0.114557135	1.435735511	0.151077617	0.294031646	up_in_case
Yang2025_S	ENSGALG00010024475	ADGRG1	case	control	-0.180814413	0.15168765	-1.192018028	0.233254176	0.396422324	down_in_case
Yang2025_S	ENSGALG00010024476	KCNB1	case	control	0.019621361	0.222085868	0.088350335	0.929598234	0.957495113	up_in_case
Yang2025_S	ENSGALG00010024479	ERBB2	case	control	0.08038639	0.076075424	1.056666998	0.290663592	0.459190859	up_in_case
Yang2025_S	ENSGALG00010024480	CDC45	case	control	-0.352058616	0.189246267	-1.860320007	0.062840265	0.157241711	down_in_case
Yang2025_S	ENSGALG00010024482	USP3	case	control	-0.335480411	0.058099774	-5.774211958	7.73141E-09	3.62751E-07	down_in_case
Yang2025_S	ENSGALG00010024486	GPR37L1	case	control	0.230094416	0.236194729	0.97417253	0.329970881	0.499472543	up_in_case
Yang2025_S	ENSGALG00010024488	ADGRG5	case	control	0.218100635	0.596237834	0.365794692	0.714518281	0.823693931	up_in_case
Yang2025_S	ENSGALG00010024490	TGM3	case	control	-0.491481425	0.604363037	-0.813222178	0.416090686	0.581450432	down_in_case
Yang2025_S	ENSGALG00010024492	NTF3	case	control	-0.007156302	0.188460499	-0.037972424	0.969709668	0.982096151	down_in_case
Yang2025_S	ENSGALG00010024493	JAK1	case	control	0.064354259	0.089997603	0.715066365	0.474568036	0.634107353	up_in_case
Yang2025_S	ENSGALG00010024494	DDAH1	case	control	0.914839079	0.297617516	3.073875123	0.002112979	0.012476422	up_in_case
Yang2025_S	ENSGALG00010024495	CLDN5	case	control	0.542440244	0.180839467	2.999567806	0.002703629	0.015045957	up_in_case
Yang2025_S	ENSGALG00010024496	UEVLD	case	control	-0.380483407	0.123980813	-3.068889425	0.002148561	0.012627667	down_in_case
Yang2025_S	ENSGALG00010024497	ABI3	case	control	0.112161654	0.23495548	0.477374071	0.633095775	0.764620352	up_in_case
Yang2025_S	ENSGALG00010024498	CCDC102A	case	control	0.541577692	0.087892302	6.161833048	7.19076E-10	4.44188E-08	up_in_case
Yang2025_S	ENSGALG00010024499	CA12	case	control	0.345676584	1.09095455	0.316856999	0.751352112	0.848540188	up_in_case
Yang2025_S	ENSGALG00010024500	GNGT2	case	control	-0.169101461	0.332652992	-0.508341921	0.611213576	0.748594951	down_in_case
Yang2025_S	ENSGALG00010024501	ARL8A	case	control	-0.008947317	0.153816226	-0.058168875	0.953614113	0.971460828	down_in_case
Yang2025_S	ENSGALG00010024502	VPS8	case	control	-0.148452221	0.053315826	-2.784393146	0.005362799	0.025272439	down_in_case
Yang2025_S	ENSGALG00010024504	CASC3	case	control	0.282674032	0.084645609	3.339500241	0.000839293	0.006072453	up_in_case
Yang2025_S	ENSGALG00010024505	DNASE2B	case	control	-0.801718339	0.14773525	-5.42672341	5.7398E-08	2.08836E-06	down_in_case
Yang2025_S	ENSGALG00010024506	RAB8B	case	control	-0.333674464	0.079287914	-4.208389973	2.57197E-05	0.000359009	down_in_case
Yang2025_S	ENSGALG00010024508	RPS27L	case	control	-0.414237032	0.257522389	-1.608547641	0.107715291	0.230782992	down_in_case
Yang2025_S	ENSGALG00010024509	SEPTIN5	case	control	-0.132593573	0.099758875	-1.329140627	0.183801579	0.337187335	down_in_case
Yang2025_S	ENSGALG00010024510	CNN3	case	control	-0.409096804	0.059650762	-6.85819918	6.9734E-12	7.71225E-10	down_in_case
Yang2025_S	ENSGALG00010024511	ENSGALG00010024511	case	control	0.131373924	0.149180804	0.880635584	0.378515092	0.546541566	up_in_case
Yang2025_S	ENSGALG00010024512	CCNDBP1	case	control	-0.321214158	0.175487142	-1.830414207	0.067188025	0.165038802	down_in_case
Yang2025_S	ENSGALG00010024513	SPTY2D1	case	control	0.379396819	0.151750334	2.500138276	0.012414484	0.047578612	up_in_case
Yang2025_S	ENSGALG00010024514	FAF1	case	control	-0.347289947	0.081611759	-4.255391022	2.08684E-05	0.000301661	down_in_case
Yang2025_S	ENSGALG00010024515	C3orf70	case	control	-0.14253743	0.090573553	-1.573720195	0.115552151	0.242974327	down_in_case
Yang2025_S	ENSGALG00010024516	EHHADH	case	control	-0.444349996	0.180198615	-2.465890184	0.013667323	0.051126498	down_in_case
Yang2025_S	ENSGALG00010024517	LACTB	case	control	0.071320694	0.121742065	0.585834436	0.55798678	0.705009138	up_in_case
Yang2025_S	ENSGALG00010024519	MIEN1	case	control	0.305909054	0.172976935	1.768496212	0.076977985	0.182089326	up_in_case
Yang2025_S	ENSGALG00010024520	MAP3K13	case	control	-0.029580031	0.200967171	-0.147188374	0.882983337	0.931020944	down_in_case
Yang2025_S	ENSGALG00010024521	HEPACAM	case	control	1.531825501	0.5326512	2.87585103	0.004029398	0.020278497	up_in_case
Yang2025_S	ENSGALG00010024522	DNTTIP2	case	control	-0.055476292	0.104170719	-0.532551679	0.594343959	0.734561691	down_in_case
Yang2025_S	ENSGALG00010024523	TOX3	case	control	0.857365652	0.366226429	2.341080772	0.019228007	0.065869576	up_in_case
Yang2025_S	ENSGALG00010024524	TMEM41A	case	control	0.132805369	0.185265658	0.716837487	0.473474378	0.632930794	up_in_case
Yang2025_S	ENSGALG00010024525	GP1BB	case	control	0.960093137	0.241671041	3.972727272	7.10544E-05	0.000810264	up_in_case
Yang2025_S	ENSGALG00010024526	TGM6	case	control	0.151264637	0.479595412	0.31540051	0.75245758	0.849234338	up_in_case
Yang2025_S	ENSGALG00010024527	ATP6V0B	case	control	-0.697266101	0.27046386	-2.578037977	0.009936308	0.040332784	down_in_case
Yang2025_S	ENSGALG00010024528	TBX1	case	control	1.209648319	0.252211524	4.796165932	1.61731E-06	3.64091E-05	up_in_case
Yang2025_S	ENSGALG00010024530	ENSGALG00010024530	case	control	1.020836697	0.61797032	1.651918649	0.098551136	0.216884093	up_in_case
Yang2025_S	ENSGALG00010024531	CEP63	case	control	0.304051016	0.143203466	2.1232099	0.033736267	0.099964506	up_in_case
Yang2025_S	ENSGALG00010024532	TMEM86A	case	control	-0.183602228	0.101409475	-1.810503678	0.070217714	0.170332572	down_in_case
Yang2025_S	ENSGALG00010024533	CDKN2C	case	control	-0.198423302	0.166797629	-1.189605054	0.234201658	0.397418426	down_in_case
Yang2025_S	ENSGALG00010024534	VWF	case	control	-0.121144436	0.150546985	-0.804695198	0.420995591	0.586716028	down_in_case

Yang2025_S	ENSGALG00010024535	ENSGALG00010024535	case	control	0.179869111	0.145417561	1.236914647	0.216118772	0.377053661	up_in_case
Yang2025_S	ENSGALG00010024536	URI1	case	control	-0.266559576	0.050143649	-5.315918936	1.0612E-07	3.62448E-06	down_in_case
Yang2025_S	ENSGALG00010024537	EPB42	case	control	-0.172520192	0.199463021	-0.864923185	0.387080951	0.554422146	down_in_case
Yang2025_S	ENSGALG00010024539	RAPGEFL1	case	control	0.401724952	0.568815318	0.706248477	0.480033628	0.638593662	up_in_case
Yang2025_S	ENSGALG00010024540	PTPN5	case	control	0.737209049	0.357084626	2.06452195	0.038968255	0.111070287	up_in_case
Yang2025_S	ENSGALG00010024541	WIPF2	case	control	0.060013879	0.141301368	0.424722564	0.671038933	0.792653008	up_in_case
Yang2025_S	ENSGALG00010024542	ZNFX1	case	control	0.241151063	0.200499552	1.202751135	0.229072639	0.391287966	up_in_case
Yang2025_S	ENSGALG00010024544	ZNF536	case	control	0.027696908	0.222427234	0.124521208	0.900902609	0.940944226	up_in_case
Yang2025_S	ENSGALG00010024545	PHACTR3	case	control	0.410774558	0.306353118	1.3408532	0.179968119	0.332009299	up_in_case
Yang2025_S	ENSGALG00010024546	KY	case	control	-0.43934396	0.179706402	-2.444787473	0.014493752	0.053502102	down_in_case
Yang2025_S	ENSGALG00010024547	PFKM	case	control	-0.115357447	0.151944053	-0.759210017	0.447726935	0.610420332	down_in_case
Yang2025_S	ENSGALG00010024548	CDC6	case	control	0.308183288	0.274650606	1.122092147	0.261823263	0.428730305	up_in_case
Yang2025_S	ENSGALG00010024549	GNB1L	case	control	-0.24473288	0.145644641	-1.680342499	0.092890697	0.20827544	down_in_case
Yang2025_S	ENSGALG00010024551	RNF11	case	control	-0.66119779	0.127169232	-5.199353502	1.99983E-07	6.1654E-06	down_in_case
Yang2025_S	ENSGALG00010024552	CD81	case	control	-0.245568854	0.10654852	-2.304760818	0.021179973	0.070726796	down_in_case
Yang2025_S	ENSGALG00010024553	ASB8	case	control	0.34755364	0.177907547	1.953563221	0.050752894	0.134738345	up_in_case
Yang2025_S	ENSGALG00010024554	ST6GALNAC3	case	control	-0.839128795	0.17422444	-4.816366732	1.46196E-06	3.33973E-05	down_in_case
Yang2025_S	ENSGALG00010024555	EPHB1	case	control	-0.144689012	0.063975319	-2.261637986	0.023719779	0.077101731	down_in_case
Yang2025_S	ENSGALG00010024556	ENSGALG00010024556	case	control	0.372376232	0.314579519	1.183726876	0.236521196	0.400184904	up_in_case
Yang2025_S	ENSGALG00010024557	TSPAN32	case	control	-0.423544992	0.348373165	-1.215779614	0.224068887	0.38552907	down_in_case
Yang2025_S	ENSGALG00010024558	PFDN5	case	control	-0.151696111	0.236034248	-0.642686865	0.520427306	0.67408974	down_in_case
Yang2025_S	ENSGALG00010024559	ENSGALG00010024559	case	control	0.085228586	0.329017213	0.259039899	0.795604456	0.876561361	up_in_case
Yang2025_S	ENSGALG00010024560	A4GNT	case	control	0.295427792	0.489236347	0.603854954	0.54594008	0.695242148	up_in_case
Yang2025_S	ENSGALG00010024561	ROBO3	case	control	1.052838152	0.355502086	2.961552672	0.003060921	0.016577512	up_in_case
Yang2025_S	ENSGALG00010024563	ESPL1	case	control	-0.039328256	0.214411466	-0.183424221	0.854465172	0.913978788	down_in_case
Yang2025_S	ENSGALG00010024564	PPP1R3D	case	control	-0.05445505	0.17464444	-0.311805232	0.755188548	0.851213493	down_in_case
Yang2025_S	ENSGALG00010024565	FAM217B	case	control	0.083247237	0.151918658	0.547972433	0.583710832	0.725797309	up_in_case
Yang2025_S	ENSGALG00010024566	MTSS2	case	control	0.663746653	0.273474206	2.427090524	0.015220458	0.055645469	up_in_case
Yang2025_S	ENSGALG00010024568	DZIP1L	case	control	0.60747811	0.279476361	2.17362967	0.029732958	0.090881504	up_in_case
Yang2025_S	ENSGALG00010024569	ASCL2	case	control	-0.318315446	0.282117445	-1.128308268	0.259189753	0.425611561	down_in_case
Yang2025_S	ENSGALG00010024570	TPM1	case	control	0.285540795	0.132315328	2.158032629	0.030925295	0.093703846	up_in_case
Yang2025_S	ENSGALG00010024571	KCNA3	case	control	1.112507278	0.690701691	1.610691405	0.107247	0.230076545	up_in_case
Yang2025_S	ENSGALG00010024573	ENSGALG00010024573	case	control	1.375412064	0.266823916	5.15475555	2.53962E-07	7.4192E-06	up_in_case
Yang2025_S	ENSGALG00010024575	TXNRD2	case	control	0.332485361	0.146719924	2.266122769	0.023443868	0.076418782	up_in_case
Yang2025_S	ENSGALG00010024576	CSAD	case	control	-0.011783503	0.21784817	-0.054090436	0.956863112	0.973560709	down_in_case
Yang2025_S	ENSGALG00010024579	ZNF740	case	control	0.208530964	0.145259373	1.435576651	0.151122843	0.294078594	up_in_case
Yang2025_S	ENSGALG00010024580	IGF2	case	control	0.146297618	0.182926351	0.799762401	0.423848471	0.588924962	up_in_case
Yang2025_S	ENSGALG00010024581	RARA	case	control	0.251487657	0.102813861	2.44604817	0.01444317	0.053357788	up_in_case
Yang2025_S	ENSGALG00010024582	TLN2	case	control	-0.117537828	0.10660858	-1.102517523	0.270236745	0.437572512	down_in_case
Yang2025_S	ENSGALG00010024585	TBCCD1	case	control	-0.019527411	0.120535623	-0.162005308	0.871301674	0.924369153	down_in_case
Yang2025_S	ENSGALG00010024586	DLGAP4	case	control	0.094428941	0.124802949	0.756624278	0.449274992	0.611510746	up_in_case
Yang2025_S	ENSGALG00010024587	ENSGALG00010024587	case	control	-0.467462407	0.202767776	-2.305407769	0.021143747	0.07062275	down_in_case
Yang2025_S	ENSGALG00010024588	TTC39A	case	control	1.030424695	0.462765895	2.226665157	0.025969661	0.082247096	up_in_case
Yang2025_S	ENSGALG00010024589	ADORA1	case	control	-0.01080041	0.253006284	-0.042688308	0.96595	0.979939965	down_in_case
Yang2025_S	ENSGALG00010024590	MRPL23	case	control	-0.637021576	0.304110362	-2.094705262	0.036197201	0.105304384	down_in_case
Yang2025_S	ENSGALG00010024592	COMT	case	control	-0.294140737	0.175107148	-1.679775729	0.093000959	0.208355043	down_in_case
Yang2025_S	ENSGALG00010024593	SRSF6	case	control	0.274078125	0.070964724	3.862174172	0.000112382	0.001167822	up_in_case
Yang2025_S	ENSGALG00010024594	TNNI2	case	control	-0.104212198	0.192969763	-0.540044186	0.589166561	0.730170404	down_in_case
Yang2025_S	ENSGALG00010024595	MYBPHL	case	control	0.621834394	0.239737818	2.593810188	0.009491887	0.039084592	up_in_case
Yang2025_S	ENSGALG00010024596	EPS15	case	control	-0.046669618	0.126177812	-0.369871828	0.711477993	0.82171568	down_in_case
Yang2025_S	ENSGALG00010024597	DNAJB11	case	control	-0.115564846	0.163065265	-0.70870302	0.478508791	0.637375795	down_in_case
Yang2025_S	ENSGALG00010024598	LRRC3C	case	control	0.311730121	0.13862923	2.248660845	0.024534082	0.078956682	up_in_case
Yang2025_S	ENSGALG00010024599	ENSGALG00010024599	case	control	0.502359634	0.310930237	1.615666712	0.106166392	0.228765837	up_in_case
Yang2025_S	ENSGALG00010024600	CD9	case	control	-0.376407746	0.207969196	-1.809920666	0.070308091	0.170508744	down_in_case
Yang2025_S	ENSGALG00010024601	ENSGALG00010024601	case	control	-0.337136972	0.337341118	-0.999394838	0.317603459	0.487155113	down_in_case
Yang2025_S	ENSGALG00010024602	PROCR	case	control	-0.032166401	0.215903054	-0.148985392	0.88156516	0.930299924	down_in_case
Yang2025_S	ENSGALG00010024604	MFSD5	case	control	0.237634813	0.206795347	1.149130365	0.250502229	0.416209439	up_in_case
Yang2025_S	ENSGALG00010024605	LSP1P1	case	control	-0.354796535	0.127501592	-2.782683169	0.005391142	0.025388837	down_in_case
Yang2025_S	ENSGALG00010024607	RARG	case	control	-0.216660053	0.183485139	-1.180804367	0.237680449	0.401719796	down_in_case
Yang2025_S	ENSGALG00010024608	DENND2D	case	control	0.208255751	0.371139314	0.561125547	0.574711954	0.717496947	up_in_case
Yang2025_S	ENSGALG00010024609	PRR33	case	control	0.317257272	0.342779005	0.925544642	0.354682674	0.523883775	up_in_case
Yang2025_S	ENSGALG00010024610	PFKL	case	control	0.195520896	0.139863463	1.397941195	0.162130727	0.308267387	up_in_case
Yang2025_S	ENSGALG00010024611	ENSGALG00010024611	case	control	0.64658599	0.530681869	1.218406032	0.223069714	0.384474516	up_in_case
Yang2025_S	ENSGALG00010024613	ENSGALG00010024613	case	control	-0.255843838	0.094087883	-2.719200715	0.006543989	0.029378376	down_in_case
Yang2025_S	ENSGALG00010024615	SYT8	case	control	-0.180604209	0.749381886	-0.241004236	0.809551833	0.885348046	down_in_case
Yang2025_S	ENSGALG00010024616	ARVCF	case	control	-0.192342938	0.161321306	-1.192297179	0.23314474	0.396348903	down_in_case
Yang2025_S	ENSGALG00010024618	VPS13C	case	control	-0.042608712	0.182487439	-0.233488466	0.815382125	0.889552026	down_in_case
Yang2025_S	ENSGALG00010024619	ENSGALG00010024619	case	control	-0.400401418	0.372424667	-1.075120564	0.282320754	0.450336504	down_in_case
Yang2025_S	ENSGALG00010024620	MSANTD2	case	control	0.096277403	0.092552263	1.040249041	0.298224213	0.467463937	up_in_case
Yang2025_S	ENSGALG00010024621	THRA	case	control	0.046800055	0.140345244	0.333463774	0.73878423	0.840267568	up_in_case
Yang2025_S	ENSGALG00010024622	PRMT8	case	control	0.512098695	0.130811091	3.914795679	9.04808E-05	0.000985039	up_in_case
Yang2025_S	ENSGALG00010024623	NRDC	case	control	0.050801854	0.068537707	0.741224896	0.458557091	0.619785942	up_in_case
Yang2025_S	ENSGALG00010024624	CTSD	case	control	-0.309862605	0.155152911	-1.997143358	0.045809612	0.125315458	down_in_case
Yang2025_S	ENSGALG00010024625	SPRYD3	case	control	0.774474926	0.157394953	4.920582983	8.62868E-07	2.13193E-05	up_in_case
Yang2025_S	ENSGALG00010024626	TSHZ3	case	control	-0.073794879	0.16726744	-0.441178981	0.659083428	0.784658933	down_in_case
Yang2025_S	ENSGALG00010024627	CEPT1	case	control	-0.277209951	0.111149953	-2.494017709	0.012630624	0.048162579	down_in_case
Yang2025_S	ENSGALG00010024628	TANGO2	case	control	-0.118422354	0.104452412	-1.133744558	0.256901713	0.423356841	down_in_case
Yang2025_S	ENSGALG00010024629	ENSGALG00010024629	case	control	-0.20455568	0.13831528	-1.478908757	0.139164703	0.277592346	down_in_case
Yang2025_S	ENSGALG00010024630	CFAP410	case	control	-0.577666686	0.240048286	-2.406460359	0.016107952	0.057911329	down_in_case
Yang2025_S	ENSGALG00010024631	EPB41L1	case	control	0.136643049	0.164227112	0.83203709	0.405388009	0.571694968	up_in_case
Yang2025_S	ENSGALG00010024632	IFITM10	case	control	0.09663517	0.306882229	0.314893341	0.752842637	0.84953131	up_in_case
Yang2025_S	ENSGALG00010024633	NRGN	case	control	-1.708501101	0.408672062	-4.180616344	2.9072E-05	0.000396786	down_in_case
Yang2025_S	ENSGALG00010024634	ENSGALG00010024634	case	control	1.441247609	0.30932772	4.65929019	3.17302E-06	6.35287E-05	up_in_case
Yang2025_S	ENSGALG00010024635	ENSGALG00010024635	case	control	0.067691718	0.318548138	0.21250075	0.831716393	0.899842231	up_in_case
Yang2025_S	ENSGALG00010024636	PANX3	case	control	0.161693805	0.184729168	0.875301973	0.381409621	0.549404907	up_in_case
Yang2025_S	ENSGALG00010024637	DUSP8	case	control	0.246866517	0.182879446	1.349886617	0.177052355	0.328132008	up_in_case
Yang2025_S	ENSGALG00010024638	ZNF507	case	control	0.046669073	0.17221846	0.270987632	0.786400545	0.87110426	up_in_case
Yang2025_S	ENSGALG00010024639	AAR2	case	control	-0.640234805	0.169340422	-3.780755932	0.000156353	0.00153004	down_in_case
Yang2025_S	ENSGALG00010024640	GSDMA	case	control	-0.133825154	0.226853028	-0.589920068	0.555244239	0.70275463	down_in_case
Yang2025_S	ENSGALG00010024641	TRA2B	case	control	0.083461835	0.08087284	1.032013161	0.302065952	0.471677263	up_in_case
Yang2025_S	ENSGALG00010024642	RAB3B	case	control	-0.364894224	0.2298693	-1.587398685	0.112422377	0.238230813	down_in_case
Yang2025_S	ENSGALG00010024643	ETV7	case	control	0.754757333	0.525020222	1.437577642	0.150553928	0.293386068	up_in_case
Yang2025_S	ENSGALG00010024644	EIF6	case	control	-0.091043949	0.111015241	-0.820103155	0.412157304	0.57740143	down_in_case
Yang2025_S	ENSGALG00010024645	PARP11	case	control	-0.2118224	0.078683102	-2.69209517	0.007100468	0.031291911	down_in_case
Yang2025_S	ENSGALG00010024646	RORA	case	control	0.670937265	0.274741654	2.44206605	0.014603475	0.053778916	up_in_case
Yang2025_S	ENSGALG00010024647	RPUSD4	case	control	0.444975341	0.196794645	2.261115091	0.023752131	0.077134921	up_in_case
Yang2025_S	ENSGALG00010024648	MMP24	case	control	0.007627221	0.094983927	0.080300124	0.935998559	0.960891405	up_in_case
Yang2025_S	ENSGALG00010024649	NCOR2	case	control	0.414838099	0.19348301	2.144054394	0.032028529	0.096189127	up_in_case
Yang2025_S	ENSGALG00010024650	PRKAG1	case	control	0.269659831	0.148913099	1.810853666	0.070163507	0.170276639	up_in_case
Yang2025_S	ENSGALG00010024651	PSMD3	case	control	-0.39058067	0.233600779	-1.672000719	0.09452418	0.210701243	down_in_case
Yang2025_S	ENSGALG00010024652	PUS3	case	control	-0.13831414	0.186697892	-0.74084468	0.458787621	0.619977259	down_in_case
Yang2025_S	ENSGALG00010024653	RFLNA	case	control	0.146592984	0.162314205	0.903143285	0.366449854	0.535158113	up_in_case
Yang2025_S	ENSGALG00010024654	CCDC92	case	control	-0.134496941	0.212694759	-0.632347228	0.527160009	0.680119871	down_in_case
Yang2025_S	ENSGALG00010024655	IGF2BP2	case	control	0.17505809	0.084840932	2.063368301	0.039077649	0.111336545	up_in_case
Yang2025_S	ENSGALG00010024656	HYLS1	case	control	-0.440451855	0.203957836	-2.159524068	0.030809532	0.093483646	down_in_case
Yang2025_S	ENSGALG00010024657	DPY19L3	case	control	0.066643533	0.186816847	0.356731924	0.721292504	0.828487557	up_in_case
Yang2025_S	ENSGALG00010024659	BEND5	case	control	0.421807382	0.117732541	3.582759505	0.000339984	0.002899432	up_in_case
Yang2025_S	ENSGALG00010024660	KMT2D	case	control	0.157398192	0.163911599	0.96026268	0.336923028	0.505058347	up_in_case
Yang2025_S	ENSGALG00010024662	MOB2	case	control	-0.002269093	0.11133813	-0.020380193	0.983740084	0.990134928	down_in_case
Yang2025_S	ENSGALG00010024663	ICE2	case	control	0.135870795	0.123808937	1.097423163	0.272456452	0.43983789	up_in_case
Yang2025_S	ENSGALG00010024664	DNAH10	case	control	0.293492671	0.264674107	1.1088832	0.267480572	0.434500554	up_in_case
Yang2025_S	ENSGALG00010024665	MAP1LC3A	case	control	-0.478068519	0.140485831	-3.402966099	0.000666586	0.005051044	down_in_case
Yang2025_S	ENSGALG00010024666	MED24	case	control	0.087781711	0.085111265	1.031375941	0.302364559	0.471989484	up_in_case
Yang2025_S	ENSGALG00010024667	RBM7	case	control	0.630737595	0.111696303	5.646897689	1.63369E-08	7.13651E-07	up_in_case
Yang2025_S	ENSGALG00010024668	ENSGALG00010024668	case	control	1.03613409	0.202639847	5.113180369	3.1678E-07	8.9722E-06	up_in_case
Yang2025_S	ENSGALG00010024669	STIL	case	control	0.068071339	0.118663949	0.573648018	0.566206011	0.711223655	up_in_case
Yang2025_S	ENSGALG00010024670	SENP2	case	control	0.040378965	0.116401405	0.346894137	0.728670852	0.833097929	up_in_case
Yang2025_S	ENSGALG00010024671	KCTD20	case	control	-0.346560375	0.089683237	-3.864271474	0.000111421	0.001159563	down_in_case
Yang2025_S	ENSGALG00010024672	DYNLRB1	case	control	-0.481289008	0.086946948	-5.535433045	3.1046E-08	1.23192E-06	down_in_case
Yang2025_S	ENSGALG00010024673	REXO2	case	control	-0.205591816	0.193766935	-1.06102631	0.288677945	0.457632833	down_in_case
Yang2025_S	ENSGALG00010024674	DHH	case	control	0.031081424	0.337784296	0.092015598	0.926685647	0.955608219	up_in_case
Yang2025_S	ENSGALG00010024675	NR1D1	case	control	0.692166787	0.402315844	1.720456197	0.085349549	0.196261711	up_in_case
Yang2025_S	ENSGALG00010024676	ENSGALG00010024676	case	control	0.201215853	0.105280646	1.911233084	0.055974634	0.145009579	up_in_case
Yang2025_S	ENSGALG00010024677	LMBR1L	case	control	0.336147524	0.252036498	1.33372558	0.182293808	0.335171424	up_in_case
Yang2025_S	ENSGALG00010024678	AMOTL2	case	control	-0.081103032	0.094607716	-0.857256002	0.391303418	0.55852861	down_in_case
Yang2025_S	ENSGALG00010024679	MSL1	case	control	0.223362842	0.119019382	1.876693013	0.060560181	0.153521215	up_in_case

Yang2025_S	ENSGALG00010024680	BRSK1	case	control	0.011240169	0.310493105	0.036201025	0.971122069	0.98240189	up_in_case
Yang2025_S	ENSGALG00010024681	ATP6V0A2	case	control	-0.169971495	0.111933586	-1.518503086	0.128887622	0.262388461	down_in_case
Yang2025_S	ENSGALG00010024682	ENSGALG00010024682	case	control	-0.693995462	0.287582525	-2.413204563	0.015812942	0.057219772	down_in_case
Yang2025_S	ENSGALG00010024683	SHISA4	case	control	0.974336202	0.30959555	3.147125995	0.001648839	0.010341155	up_in_case
Yang2025_S	ENSGALG00010024684	ENSGALG00010024684	case	control	-0.512556159	0.385402463	-1.32992445	0.183543165	0.336893752	down_in_case
Yang2025_S	ENSGALG00010024685	ENSGALG00010024685	case	control	0.266445496	0.306236898	0.870063333	0.384265795	0.551947636	up_in_case
Yang2025_S	ENSGALG00010024686	CMPK1	case	control	-0.667049346	0.137376174	-4.855640745	1.19998E-06	2.82462E-05	down_in_case
Yang2025_S	ENSGALG00010024687	RYK	case	control	-0.178051804	0.079585623	-2.237235789	0.025270933	0.080583629	down_in_case
Yang2025_S	ENSGALG00010024688	CADM1	case	control	0.048164729	0.108005675	0.445946281	0.65563605	0.782417432	up_in_case
Yang2025_S	ENSGALG00010024689	FOXD2	case	control	0.323680969	0.155817366	2.077309978	0.037772959	0.108506737	up_in_case
Yang2025_S	ENSGALG00010024690	TUBA1C	case	control	1.037926139	0.326388145	3.18003627	0.001472566	0.009495701	up_in_case
Yang2025_S	ENSGALG00010024691	TCTN2	case	control	-0.45772709	0.163248881	-2.803860502	0.005049475	0.024155015	down_in_case
Yang2025_S	ENSGALG00010024692	DRAM2	case	control	-0.014776097	0.121590065	-0.121523886	0.903276097	0.942152126	down_in_case
Yang2025_S	ENSGALG00010024693	ANXA2	case	control	-0.309712505	0.128414911	-2.411811078	0.015873504	0.057409105	down_in_case
Yang2025_S	ENSGALG00010024694	GTF2H3	case	control	-0.317074734	0.138202527	-2.294275959	0.021774653	0.07203936	down_in_case
Yang2025_S	ENSGALG00010024695	TRABD2B	case	control	0.487188714	0.255075797	1.909976245	0.056136272	0.145212354	up_in_case
Yang2025_S	ENSGALG00010024696	TRADD	case	control	0.090338244	0.098138694	0.920516054	0.357303147	0.526536761	up_in_case
Yang2025_S	ENSGALG00010024697	IGF2BP1	case	control	-0.002704973	0.094545385	-0.028610316	0.977175385	0.986591725	down_in_case
Yang2025_S	ENSGALG00010024698	ENSGALG00010024698	case	control	0.038262683	0.196241877	0.194977156	0.845410831	0.908215124	up_in_case
Yang2025_S	ENSGALG00010024699	FBXL8	case	control	0.012593636	0.099668776	0.126354876	0.899451019	0.940222773	up_in_case
Yang2025_S	ENSGALG00010024700	TOLLIP	case	control	-0.455699853	0.070974774	-6.420588948	1.35748E-10	1.06745E-08	down_in_case
Yang2025_S	ENSGALG00010024701	EIF2B1	case	control	-0.479880517	0.211979997	-2.263800936	0.023586361	0.07681139	down_in_case
Yang2025_S	ENSGALG00010024702	INAVA	case	control	1.082317019	0.315587883	3.429526531	0.000604635	0.004640893	up_in_case
Yang2025_S	ENSGALG00010024705	SLCO2A1	case	control	-0.037998112	0.281723463	-0.134877342	0.892708854	0.937021534	down_in_case
Yang2025_S	ENSGALG00010024706	ENSGALG00010024706	case	control	1.085115667	0.443707504	2.445565284	0.014462526	0.053402065	up_in_case
Yang2025_S	ENSGALG00010024707	ENSGALG00010024707	case	control	1.2993329	0.29037106	4.474732784	7.65069E-06	0.000131783	up_in_case
Yang2025_S	ENSGALG00010024708	BNIP2	case	control	0.536833903	0.157821108	3.401534251	0.000670087	0.005065743	up_in_case
Yang2025_S	ENSGALG00010024709	TEAD4	case	control	0.051766162	0.098526002	0.525406099	0.599300914	0.738096744	up_in_case
Yang2025_S	ENSGALG00010024710	HSDL1	case	control	-0.10157003	0.228648617	-0.444218871	0.65688435	0.783571599	down_in_case
Yang2025_S	ENSGALG00010024711	DDX55	case	control	0.298381437	0.130923822	2.279046182	0.02266432	0.074224983	up_in_case
Yang2025_S	ENSGALG00010024712	LRIF1	case	control	0.151490291	0.12602232	1.20209095	0.229328292	0.391628647	up_in_case
Yang2025_S	ENSGALG00010024713	RND2	case	control	0.589717032	0.43604357	1.352426849	0.176238805	0.327059229	up_in_case
Yang2025_S	ENSGALG00010024714	SRPRB	case	control	-0.486798828	0.190567349	-2.554471329	0.010634919	0.042414881	down_in_case
Yang2025_S	ENSGALG00010024715	DNAAF1	case	control	0.392842419	0.134160661	2.928149094	0.003409865	0.017923979	up_in_case
Yang2025_S	ENSGALG00010024716	GTF2A2	case	control	-0.192264799	0.189759303	-1.013203547	0.310962947	0.480673174	down_in_case
Yang2025_S	ENSGALG00010024717	TF	case	control	-1.712408806	0.594063124	-2.882536782			down_in_case
Yang2025_S	ENSGALG00010024718	ENSGALG00010024718	case	control	0.34726356	0.396042755	0.876833514	0.380577073	0.54844561	up_in_case
Yang2025_S	ENSGALG00010024719	PYM1	case	control	0.763879525	0.150363363	5.080223718	3.76991E-07	1.04649E-05	up_in_case
Yang2025_S	ENSGALG00010024720	BUD13	case	control	-0.107822525	0.129358945	-0.833514259	0.404554759	0.570814573	down_in_case
Yang2025_S	ENSGALG00010024721	ENSGALG00010024721	case	control	1.170959007	0.49783659	2.352095105	0.018668001	0.064406684	up_in_case
Yang2025_S	ENSGALG00010024722	BRCA1	case	control	0.207280885	0.094243943	2.199408031	0.027848922	0.086585169	up_in_case
Yang2025_S	ENSGALG00010024723	ITCH	case	control	-0.009896519	0.085360947	-0.115937309	0.907702227	0.944718092	down_in_case
Yang2025_S	ENSGALG00010024724	HOXD3	case	control	0.139615249	0.163476999	0.854036045	0.393085018	0.560013816	up_in_case
Yang2025_S	ENSGALG00010024726	TMED2	case	control	-0.399154429	0.074303029	-5.371980602	7.78765E-08	2.75434E-06	down_in_case
Yang2025_S	ENSGALG00010024728	SLC5A9	case	control	0.830918695	0.429159946	1.936151551	0.052849142	0.138900943	up_in_case
Yang2025_S	ENSGALG00010024729	FOXM1	case	control	-0.030113315	0.121018861	-0.248831586	0.803491058	0.881718717	down_in_case
Yang2025_S	ENSGALG00010024731	AOX1	case	control	0.450198613	0.260601356	1.727537493	0.084071178	0.194122927	up_in_case
Yang2025_S	ENSGALG00010024732	AHCY	case	control	-0.392004864	0.16663062	-2.352537988	0.018645785	0.064345967	down_in_case
Yang2025_S	ENSGALG00010024733	MBTPS1	case	control	-0.389153623	0.092570959	-4.20384134	2.62423E-05	0.000364593	down_in_case
Yang2025_S	ENSGALG00010024734	ZPR1	case	control	0.069388135	0.124721171	0.556346085	0.577974288	0.720851312	up_in_case
Yang2025_S	ENSGALG00010024735	OTUD7A	case	control	0.178569726	0.144673025	1.234298696	0.217091622	0.37795749	up_in_case
Yang2025_S	ENSGALG00010024736	DGKA	case	control	-0.062050383	0.176109261	-0.352340264	0.724583096	0.830611257	down_in_case
Yang2025_S	ENSGALG00010024737	STK38	case	control	-0.127944512	0.080217733	-1.594965443	0.110719964	0.235482707	down_in_case
Yang2025_S	ENSGALG00010024738	NBR1	case	control	0.07429195	0.071731382	1.03569662	0.3003437	0.469940429	up_in_case
Yang2025_S	ENSGALG00010024739	ASIP	case	control	-0.837455393	0.348241511	-2.404812086	0.016180784	0.058098227	down_in_case
Yang2025_S	ENSGALG00010024741	RILPL2	case	control	-0.028976636	0.175840698	-0.164789131	0.869109959	0.922939315	down_in_case
Yang2025_S	ENSGALG00010024742	PAK2	case	control	-0.063106471	0.086662614	-0.728185634	0.466499959	0.627660222	down_in_case
Yang2025_S	ENSGALG00010024744	KLF13	case	control	0.395035182	0.233371686	1.69272969	0.090506931	0.204510148	up_in_case
Yang2025_S	ENSGALG00010024745	PMEL	case	control	8.97454135	0.504960183	17.77277031	1.14875E-70	8.00391E-67	up_in_case
Yang2025_S	ENSGALG00010024746	HOXD4	case	control	0.437838596	0.27006307	1.621245722	0.104964951	0.227022366	up_in_case
Yang2025_S	ENSGALG00010024747	TULP3	case	control	-0.407011045	0.089983867	-4.523155764	6.09244E-06	0.000109829	down_in_case
Yang2025_S	ENSGALG00010024748	TRPM1	case	control	2.380362025	0.324769043	7.329399388	2.31186E-13	3.46407E-11	up_in_case
Yang2025_S	ENSGALG00010024749	APOA5	case	control	-0.65573067	0.454673136	-1.442202359	0.149245295	0.291605888	down_in_case
Yang2025_S	ENSGALG00010024751	MTMR10	case	control	-0.100838767	0.088586984	-1.138302295	0.254994276	0.421411911	down_in_case
Yang2025_S	ENSGALG00010024752	MPP2	case	control	0.110687244	0.169129761	0.654451606	0.512820896	0.667802933	up_in_case
Yang2025_S	ENSGALG00010024753	HOXD8	case	control	0.039936524	0.12633039	0.316127609	0.751905651	0.84889315	up_in_case
Yang2025_S	ENSGALG00010024754	EIF2S2	case	control	0.646619209	0.109081266	5.927866737	3.06895E-09	1.60774E-07	up_in_case
Yang2025_S	ENSGALG00010024755	PIGX	case	control	-0.169991655	0.241265921	-0.704582125	0.481070326	0.639484402	down_in_case
Yang2025_S	ENSGALG00010024756	CDK2	case	control	0.212441784	0.191699785	1.108200431	0.267775265	0.434850054	up_in_case
Yang2025_S	ENSGALG00010024757	PPFIA1	case	control	0.249440323	0.148303527	1.681958134	0.092576958	0.207806043	up_in_case
Yang2025_S	ENSGALG00010024758	SPATA6	case	control	-0.257906983	0.146406787	-1.761578057	0.078140614	0.184276435	down_in_case
Yang2025_S	ENSGALG00010024759	APOC3	case	control	-0.805873809	0.636500602	-1.266100623	0.20547706	0.363873786	down_in_case
Yang2025_S	ENSGALG00010024760	ENSGALG00010024760	case	control	-0.084673481	0.186229958	-0.454671641	0.649345486	0.777306876	down_in_case
Yang2025_S	ENSGALG00010024761	NECAB2	case	control	-0.254226278	0.23840839	-1.066347868	0.286266418	0.454808179	down_in_case
Yang2025_S	ENSGALG00010024762	FAN1	case	control	0.210636366	0.133695997	1.57548746	0.115143971	0.242376319	up_in_case
Yang2025_S	ENSGALG00010024763	HOXD9	case	control	0.420717768	0.310991138	1.3528288	0.17611033	0.326864337	up_in_case
Yang2025_S	ENSGALG00010024764	ENSGALG00010024764	case	control	0.064726459	0.257549149	0.251316922	0.801569093	0.880210032	up_in_case
Yang2025_S	ENSGALG00010024765	SNRNP35	case	control	0.198384436	0.124461501	1.593942176	0.110948987	0.235825828	up_in_case
Yang2025_S	ENSGALG00010024767	CTTN	case	control	0.516038325	0.145871376	3.537625666	0.000403742	0.003322338	up_in_case
Yang2025_S	ENSGALG00010024768	ENSGALG00010024768	case	control	0.436938739	0.529940116	0.824505876	0.409652178	0.575105568	up_in_case
Yang2025_S	ENSGALG00010024769	RALY	case	control	-0.034045831	0.142291633	-0.239267973	0.810897798	0.886194088	down_in_case
Yang2025_S	ENSGALG00010024770	CDC27	case	control	0.282180989	0.136147172	2.072617332	0.038207907	0.109417834	up_in_case
Yang2025_S	ENSGALG00010024771	AOX2	case	control	0.245820977	0.254539886	0.965746394	0.334171098	0.502989226	up_in_case
Yang2025_S	ENSGALG00010024772	ELAPOR1	case	control	0.634113893	0.151500919	4.185544867	2.84483E-05	0.000389417	up_in_case
Yang2025_S	ENSGALG00010024773	APOA1	case	control	0.019305534	0.285078663	0.067720024	0.946008509	0.96654279	up_in_case
Yang2025_S	ENSGALG00010024775	ITFG2	case	control	0.136424541	0.075448476	1.808181539	0.070578251	0.170925952	up_in_case
Yang2025_S	ENSGALG00010024776	MPHOSPH10	case	control	0.825092862	0.209292749	3.942290721	8.07071E-05	0.000899722	up_in_case
Yang2025_S	ENSGALG00010024778	RAB5B	case	control	0.227882311	0.182572294	1.248175751	0.211966702	0.372150182	up_in_case
Yang2025_S	ENSGALG00010024779	ENSGALG00010024779	case	control	2.354533762	0.883114532	2.666170329	0.007672081	0.03316081	up_in_case
Yang2025_S	ENSGALG00010024781	CEP19	case	control	-0.126963843	0.236876408	-0.535991932	0.591964126	0.732433649	down_in_case
Yang2025_S	ENSGALG00010024783	UBE2T	case	control	-0.012198212	0.208757842	-0.058432354	0.953404244	0.971389057	down_in_case
Yang2025_S	ENSGALG00010024784	ENSGALG00010024784	case	control	0.167608974	0.162002056	1.034610166	0.300851004	0.470470064	up_in_case
Yang2025_S	ENSGALG00010024785	MCEE	case	control	-0.35653955	0.142955866	-2.494053313	0.012629357	0.048162579	down_in_case
Yang2025_S	ENSGALG00010024786	PDCD5	case	control	-0.213590909	0.102858489	-2.076551104	0.03784301	0.108618402	down_in_case
Yang2025_S	ENSGALG00010024787	SARS1	case	control	-0.02346939	0.146115673	-0.160621991	0.872391133	0.925114134	down_in_case
Yang2025_S	ENSGALG00010024788	SIK3	case	control	0.762983764	0.216579645	3.522878444	0.000426887	0.003482829	up_in_case
Yang2025_S	ENSGALG00010024789	SUOX	case	control	-0.18965223	0.18210377	-1.041451421	0.297666088	0.466746589	down_in_case
Yang2025_S	ENSGALG00010024791	SHANK2	case	control	2.038074283	0.473813342	4.301428651	1.697E-05	0.000253731	up_in_case
Yang2025_S	ENSGALG00010024792	BZW1	case	control	-0.321759328	0.107256101	-2.999916319	0.002700538	0.015034756	down_in_case
Yang2025_S	ENSGALG00010024793	FKBP4	case	control	0.094947933	0.119941782	0.791616825	0.428584134	0.592962659	up_in_case
Yang2025_S	ENSGALG00010024794	DDX6	case	control	-0.366003489	0.089961354	-4.068452447	4.73264E-05	0.000582077	down_in_case
Yang2025_S	ENSGALG00010024795	NRROS	case	control	0.022425911	0.203210365	0.110358106	0.912125377	0.947976367	up_in_case
Yang2025_S	ENSGALG00010024797	IKZF4	case	control	0.144001761	0.163152353	0.882621414	0.377440855	0.545888771	up_in_case
Yang2025_S	ENSGALG00010024798	FBXO45	case	control	-0.217966399	0.060848341	-3.582125566	0.00034081	0.002902925	down_in_case
Yang2025_S	ENSGALG00010024799	ENSGALG00010024799	case	control	1.030728393	0.203829362	5.056820004	4.26305E-07	1.17171E-05	up_in_case
Yang2025_S	ENSGALG00010024800	ENSGALG00010024800	case	control	0.362701841	0.141551249	2.562335858	0.010397072	0.041692771	up_in_case
Yang2025_S	ENSGALG00010024802	CD59	case	control	-0.300116856	0.128791446	-2.330254579	0.0197927	0.067303873	down_in_case
Yang2025_S	ENSGALG00010024803	CLK1	case	control	0.52618391	0.194962342	2.698900234	0.006956903	0.030824942	up_in_case
Yang2025_S	ENSGALG00010024804	WDR53	case	control	0.103292824	0.143293825	0.720846305	0.471004082	0.63116087	up_in_case
Yang2025_S	ENSGALG00010024805	ENSGALG00010024805	case	control	1.023323764	0.263709317	3.880499086	0.000104242	0.001102175	up_in_case
Yang2025_S	ENSGALG00010024806	ANKRD27	case	control	0.222544732	0.157620013	1.411906569	0.157977473	0.303225357	up_in_case
Yang2025_S	ENSGALG00010024807	PPIL3	case	control	-0.106933335	0.17443372	-0.613031328	0.539855625	0.690743563	down_in_case
Yang2025_S	ENSGALG00010024808	CELSR2	case	control	0.398422873	0.298891177	1.333003127	0.182530778	0.335385863	up_in_case
Yang2025_S	ENSGALG00010024809	KANSL1	case	control	0.258803265	0.155455435	1.6648068	0.095951307	0.213046759	up_in_case
Yang2025_S	ENSGALG00010024810	ADIPOR1	case	control	-0.238988913	0.168657052	-1.417011091	0.156479685	0.301513331	down_in_case
Yang2025_S	ENSGALG00010024811	NIF3L1	case	control	-0.169239131	0.160432016	-1.054896242	0.291472781	0.460050187	down_in_case
Yang2025_S	ENSGALG00010024812	SBNO1	case	control	0.193568252	0.090273576	2.144240441	0.032013626	0.096165096	up_in_case
Yang2025_S	ENSGALG00010024813	RNF168	case	control	0.294383353	0.184090047	1.599126935	0.109792401	0.233771861	up_in_case
Yang2025_S	ENSGALG00010024814	APBA2	case	control	0.427518781	0.217119431	1.969048922	0.048947474	0.131206208	up_in_case
Yang2025_S	ENSGALG00010024816	NFIA	case	control	0.458492535	0.239460369	1.914690674	0.055531964	0.144203511	up_in_case
Yang2025_S	ENSGALG00010024817	UCKL1	case	control	0.262282422	0.108243279	2.423082753	0.015389421	0.056095102	up_in_case
Yang2025_S	ENSGALG00010024818	FBXO3	case	control	-0.326119172	0.073665286	-4.427040052	9.5535E-06	0.000157735	down_in_case
Yang2025_S	ENSGALG00010024820	NUDT19	case	control	-0.110644914	0.109741565	-1.008231599	0.313343292	0.482746133	down_in_case
Yang2025_S	ENSGALG00010024821	NECAP1	case	control	-0.345353103	0.089764209	-3.847336337	0.000119409	0.001225305	down_in_case
Yang2025_S	ENSGALG00010024822	RPS26	case	control	-0.03049304	0.176722435	-0.172547646	0.863007007	0.919290108	down_in_case
Yang2025_S	ENSGALG00010024823	ASB10	case	control	-0.117606829	0.31246207	-0.376387536	0.706628811	0.819145477	down_in_case

Yang2025_S	ENSGALG00010024824	DNAJC5	case	control	-0.100960934	0.131511918	-0.767694177	0.442668896	0.605892454	down_in_case
Yang2025_S	ENSGALG00010024825	UBXN7	case	control	-0.296454556	0.133810583	-2.215479155	0.026727194	0.083953402	down_in_case
Yang2025_S	ENSGALG00010024826	RBM33	case	control	1.073432881	0.306697642	3.499971087	0.000465309	0.003732916	up_in_case
Yang2025_S	ENSGALG00010024827	ENSGALG00010024827	case	control	1.150349828	0.390031053	2.949380106	0.003184121	0.017078542	up_in_case
Yang2025_S	ENSGALG00010024828	TIMM17B	case	control	0.296929622	0.264751616	1.121540355	0.262057924	0.428864024	up_in_case
Yang2025_S	ENSGALG00010024829	ERBB3	case	control	0.294582517	0.129589906	2.273190301	0.023014715	0.075213426	up_in_case
Yang2025_S	ENSGALG00010024830	ORC2	case	control	0.131734621	0.120080256	1.097054793	0.27261744	0.439910158	up_in_case
Yang2025_S	ENSGALG00010024831	SLC7A9	case	control	0.881818994	0.461614818	1.910291782	0.056095655	0.145134229	up_in_case
Yang2025_S	ENSGALG00010024833	MAPT	case	control	0.451701855	0.219553488	2.057365873	0.039651045	0.112601858	up_in_case
Yang2025_S	ENSGALG00010024834	INSIG1	case	control	0.330969435	0.201508423	1.642459556	0.100494801	0.219773235	up_in_case
Yang2025_S	ENSGALG00010024835	ENTREP2	case	control	0.268243077	0.146719614	1.828270055	0.067509032	0.165535519	up_in_case
Yang2025_S	ENSGALG00010024836	TXNDC12	case	control	-0.36958503	0.118021763	-3.131498982	0.001739164	0.010790381	down_in_case
Yang2025_S	ENSGALG00010024837	TNFRSF1A	case	control	-0.412778136	0.147047648	-2.807104652	0.0049989	0.023970981	down_in_case
Yang2025_S	ENSGALG00010024838	EN2	case	control	2.180388754	1.053182853	2.070285087	0.038425655	0.109951028	up_in_case
Yang2025_S	ENSGALG00010024839	PA2G4	case	control	0.332298984	0.179222313	1.854116141	0.063722546	0.158793576	up_in_case
Yang2025_S	ENSGALG00010024840	BTF3L4	case	control	-0.367689301	0.083846283	-4.385278475	1.15837E-05	0.000186828	down_in_case
Yang2025_S	ENSGALG00010024841	ENSGALG00010024841	case	control	-0.063684833	0.215996919	-0.294841397	0.768115041	0.858788123	down_in_case
Yang2025_S	ENSGALG00010024842	ZFYVE9	case	control	-0.080510338	0.111982058	-0.718957482	0.472167118	0.632111517	down_in_case
Yang2025_S	ENSGALG00010024843	BCL9L	case	control	0.209106749	0.159311267	1.312567231	0.189328818	0.344245443	up_in_case
Yang2025_S	ENSGALG00010024844	TM4SF19	case	control	-0.206311901	0.550234449	-0.374952716	0.707695633	0.819872311	down_in_case
Yang2025_S	ENSGALG00010024845	HYCC2	case	control	0.079314143	0.157457477	0.50371786	0.614459656	0.750766796	up_in_case
Yang2025_S	ENSGALG00010024846	LMO2	case	control	0.248822119	0.098537681	2.525146901	0.011564994	0.045155269	up_in_case
Yang2025_S	ENSGALG00010024847	PPIL1	case	control	-0.058093992	0.367193323	-0.158210917	0.874290602	0.926381944	down_in_case
Yang2025_S	ENSGALG00010024848	ESYT1	case	control	0.133120532	0.106017251	1.255649726	0.209242994	0.368410542	up_in_case
Yang2025_S	ENSGALG00010024849	CENATAC	case	control	0.613568998	0.173276381	3.540984616	0.000398637	0.003290879	up_in_case
Yang2025_S	ENSGALG00010024850	CDK2AP1	case	control	-0.06632605	0.104470884	-0.634875937	0.525509332	0.678750226	down_in_case
Yang2025_S	ENSGALG00010024851	CPNE5	case	control	-0.615431705	0.688489601	-0.89388671	0.371382509	0.539872237	down_in_case
Yang2025_S	ENSGALG00010024852	CEP89	case	control	0.633422056	0.164889635	3.841491055	0.000122289	0.00124934	up_in_case
Yang2025_S	ENSGALG00010024853	TJP1	case	control	0.010276102	0.155900518	0.065914485	0.947445908	0.967543554	up_in_case
Yang2025_S	ENSGALG00010024854	C12orf65	case	control	0.434802222	0.176950987	2.45719015	0.014002855	0.052062375	up_in_case
Yang2025_S	ENSGALG00010024855	CAPRIN1	case	control	0.017904079	0.116520924	0.153655482	0.877881388	0.92809932	up_in_case
Yang2025_S	ENSGALG00010024856	SORT1	case	control	0.11951816	0.092251184	1.295573178	0.195122575	0.351084161	up_in_case
Yang2025_S	ENSGALG00010024857	SLC37A4	case	control	-0.343766296	0.254736475	-1.349497735	0.177177148	0.328275967	down_in_case
Yang2025_S	ENSGALG00010024858	SLC25A39	case	control	-0.302198819	0.243229486	-1.242443188	0.214073089	0.374527119	down_in_case
Yang2025_S	ENSGALG00010024859	TARSL2	case	control	-0.07080752	0.110229657	-0.642363602	0.520637127	0.674229718	down_in_case
Yang2025_S	ENSGALG00010024860	NEU4	case	control	1.097634952	0.396836265	2.765964325	0.005675474	0.026327475	up_in_case
Yang2025_S	ENSGALG00010024861	ORC1	case	control	0.139350928	0.075057927	1.856578435	0.063371154	0.158257533	up_in_case
Yang2025_S	ENSGALG00010024862	METTL16	case	control	-0.032038077	0.113301276	-0.282768897	0.777353998	0.864588392	down_in_case
Yang2025_S	ENSGALG00010024863	LMOD1	case	control	0.428905252	0.227381664	1.886278973	0.059257371	0.151264237	up_in_case
Yang2025_S	ENSGALG00010024864	MPHOSPH9	case	control	0.07509148	0.141488433	0.530725225	0.595609203	0.735407961	up_in_case
Yang2025_S	ENSGALG00010024865	SNAP47	case	control	-0.192849601	0.122430899	-1.575171001	0.115216979	0.24245675	down_in_case
Yang2025_S	ENSGALG00010024866	TRAPPC4	case	control	-0.297853284	0.206848829	-1.439956343	0.149879751	0.292394558	down_in_case
Yang2025_S	ENSGALG00010024867	TM2D3	case	control	-0.244691705	0.173240493	-1.412439436	0.157820612	0.303091266	down_in_case
Yang2025_S	ENSGALG00010024868	FAAP24	case	control	0.321010913	0.233714542	1.373517073	0.169591657	0.318369897	up_in_case
Yang2025_S	ENSGALG00010024869	NDUFB3	case	control	-0.425529254	0.27893955	-1.525524989	0.127128206	0.259869671	down_in_case
Yang2025_S	ENSGALG00010024870	TPD52L2	case	control	-0.176270151	0.142156493	-1.23997256	0.214985544	0.375698965	down_in_case
Yang2025_S	ENSGALG00010024871	POLQ	case	control	0.029257039	0.109886559	0.266247654	0.790048485	0.873340127	up_in_case
Yang2025_S	ENSGALG00010024872	RUNDC3A	case	control	0.55069899	0.314503042	1.751013239	0.07994363	0.18731336	up_in_case
Yang2025_S	ENSGALG00010024873	HYOU1	case	control	-0.018083394	0.08012165	-0.225699226	0.82143536	0.893392686	down_in_case
Yang2025_S	ENSGALG00010024874	JMJD4	case	control	-0.236742599	0.086860662	-2.725544504	0.006419552	0.028940944	down_in_case
Yang2025_S	ENSGALG00010024875	GAL3ST2	case	control	0.223514697	0.913428294	0.244698679	0.806689751	0.883622621	up_in_case
Yang2025_S	ENSGALG00010024876	CFLAR	case	control	-0.118139079	0.097021779	-1.21765526	0.223355005	0.384728306	down_in_case
Yang2025_S	ENSGALG00010024877	PRPF38A	case	control	0.048191677	0.114225979	0.421897691	0.673099696	0.794212046	up_in_case
Yang2025_S	ENSGALG00010024878	RHPN2	case	control	-0.283377616	0.197430369	-1.435329417	0.151193249	0.294174521	down_in_case
Yang2025_S	ENSGALG00010024879	D2HGDH	case	control	-0.449880283	0.140219025	-3.208411141	0.001334705	0.008785603	down_in_case
Yang2025_S	ENSGALG00010024880	NAT10	case	control	-0.074626374	0.08208592	-0.909125146	0.36328407	0.532096229	down_in_case
Yang2025_S	ENSGALG00010024881	CASP10	case	control	0.154584798	0.186741933	0.827799067	0.407784303	0.573744868	up_in_case
Yang2025_S	ENSGALG00010024882	MYL6	case	control	1.381067502	0.128190308	10.77357187	4.58847E-27	5.81276E-24	up_in_case
Yang2025_S	ENSGALG00010024883	ING5	case	control	-0.339610001	0.106283883	-3.195310455	0.001396805	0.00912968	down_in_case
Yang2025_S	ENSGALG00010024884	ZBTB46	case	control	-0.245113279	0.1137046	-2.155702396	0.031106911	0.094109503	down_in_case
Yang2025_S	ENSGALG00010024885	SLC4A1	case	control	-0.221623411	0.186834982	-1.186198687	0.235543845	0.398821807	down_in_case
Yang2025_S	ENSGALG00010024886	ABTB2	case	control	0.590232542	0.19723873	2.992477916	0.002767227	0.01530818	up_in_case
Yang2025_S	ENSGALG00010024887	CASP18	case	control	-0.174593097	0.261269764	-0.668248381	0.503975057	0.659851437	down_in_case
Yang2025_S	ENSGALG00010024888	PITPNM2	case	control	0.454348589	0.201007807	2.260352948	0.023799354	0.077234281	up_in_case
Yang2025_S	ENSGALG00010024889	SLC2A4RG	case	control	0.143138402	0.353106283	0.405369174	0.685206166	0.803259689	up_in_case
Yang2025_S	ENSGALG00010024890	SMARCC2	case	control	0.318089416	0.104766466	3.036175867	0.002395996	0.01374146	up_in_case
Yang2025_S	ENSGALG00010024891	PKP1	case	control	0.189057568	0.495332769	0.381677892	0.7027003	0.81634023	up_in_case
Yang2025_S	ENSGALG00010024893	ARL6IP4	case	control	0.503767399	0.127402248	3.954148447	7.68077E-05	0.000863854	up_in_case
Yang2025_S	ENSGALG00010024894	TUT4	case	control	0.307504595	0.147127549	2.090054492	0.036612905	0.106181234	up_in_case
Yang2025_S	ENSGALG00010024896	RNF41	case	control	0.250895915	0.132939299	1.887296801	0.059120417	0.151008407	up_in_case
Yang2025_S	ENSGALG00010024897	ADAL	case	control	-0.567590158	0.090327502	-6.283691485	3.30627E-10	2.28083E-08	down_in_case
Yang2025_S	ENSGALG00010024898	NABP2	case	control	0.394124424	0.134711268	2.925697528	0.003436849	0.018011468	up_in_case
Yang2025_S	ENSGALG00010024899	OGFOD2	case	control	0.393367259	0.128780218	3.054562756	0.002253889	0.013097555	up_in_case
Yang2025_S	ENSGALG00010024900	DTYMK	case	control	-0.322717331	0.191812206	-1.682465042	0.092478698	0.207685843	down_in_case
Yang2025_S	ENSGALG00010024901	ZGPAT	case	control	-0.282033176	0.182713731	-1.543579537	0.122690225	0.253632828	down_in_case
Yang2025_S	ENSGALG00010024902	LARP6	case	control	0.017683604	0.218968913	0.080758515	0.935634001	0.960658694	up_in_case
Yang2025_S	ENSGALG00010024903	CASP8	case	control	-0.366866615	0.1755015	-2.090390191	0.036582763	0.106115905	down_in_case
Yang2025_S	ENSGALG00010024904	ALS2CL	case	control	0.239325518	0.143638972	1.666160055	0.09568154	0.212583268	up_in_case
Yang2025_S	ENSGALG00010024905	GPX7	case	control	-0.593819603	0.088857941	-6.682797226	2.34424E-11	2.30049E-09	down_in_case
Yang2025_S	ENSGALG00010024907	PHLDB1	case	control	0.231414489	0.10740148	2.1546676	0.031187855	0.094273917	up_in_case
Yang2025_S	ENSGALG00010024908	ENSGALG00010024908	case	control	-0.497754196	0.170295728	-2.922881279	0.003468087	0.018141064	down_in_case
Yang2025_S	ENSGALG00010024910	UBTF	case	control	0.093823662	0.122359818	0.766784905	0.443209412	0.606513126	up_in_case
Yang2025_S	ENSGALG00010024911	CAT	case	control	-0.458373677	0.102134978	-4.487920652	7.19217E-06	0.000126067	down_in_case
Yang2025_S	ENSGALG00010024915	STXBP5L	case	control	-0.352308902	0.27028164	-1.3034881	0.192408177	0.347640301	down_in_case
Yang2025_S	ENSGALG00010024917	TRAK2	case	control	-0.189053648	0.116392104	-1.624282425	0.104315546	0.226127923	down_in_case
Yang2025_S	ENSGALG00010024918	ATG4B	case	control	-0.280303169	0.120258151	-2.330845497	0.019761508	0.067230619	down_in_case
Yang2025_S	ENSGALG00010024919	ANKRD52	case	control	-0.200598411	0.118358048	-1.694843855	0.090105046	0.203833411	down_in_case
Yang2025_S	ENSGALG00010024920	VIPR2	case	control	-0.327396143	0.183546326	-1.783724853	0.074468386	0.177904501	down_in_case
Yang2025_S	ENSGALG00010024921	STRADB	case	control	0.057861738	0.163946193	0.352931269	0.724139968	0.830489928	up_in_case
Yang2025_S	ENSGALG00010024924	MNX1	case	control	2.321895555	0.546034228	4.252289392	2.11596E-05	0.000304292	up_in_case
Yang2025_S	ENSGALG00010024925	GTF2E1	case	control	0.067960614	0.096011183	0.707840605	0.479044247	0.637733449	up_in_case
Yang2025_S	ENSGALG00010024927	NOM1	case	control	-0.389255161	0.136500887	-2.851667622	0.004349055	0.021484507	down_in_case
Yang2025_S	ENSGALG00010024928	THAP4	case	control	-0.039465858	0.125760989	-0.313816381	0.753660502	0.850041205	down_in_case
Yang2025_S	ENSGALG00010024930	TMEM237	case	control	-0.103781666	0.100737834	-1.030215376	0.302908913	0.47251443	down_in_case
Yang2025_S	ENSGALG00010024931	UBE3C	case	control	-0.40120734	0.103573797	-3.873637464	0.000107223	0.001125961	down_in_case
Yang2025_S	ENSGALG00010024932	ANKK1	case	control	1.427832099	1.076947935	1.325813488	0.184901484	0.338447679	up_in_case
Yang2025_S	ENSGALG00010024933	CS	case	control	-0.12295595	0.180474054	-0.681294334	0.495685268	0.653056085	down_in_case
Yang2025_S	ENSGALG00010024934	G6PC3	case	control	0.316967627	0.186919754	1.695741731	0.089934802	0.203683654	up_in_case
Yang2025_S	ENSGALG00010024935	KLHL30	case	control	-0.008545296	0.147969091	-0.057750548	0.953947329	0.971658215	down_in_case
Yang2025_S	ENSGALG00010024937	ILKAP	case	control	0.638642385	0.099933457	6.390676421	1.65154E-10	1.24401E-08	up_in_case
Yang2025_S	ENSGALG00010024938	ENSGALG00010024938	case	control	0.62888558	0.206053697	3.052047064	0.002272865	0.013191324	up_in_case
Yang2025_S	ENSGALG00010024939	COQ10A	case	control	0.243226712	0.172084807	1.413411888	0.157534652	0.302792466	up_in_case
Yang2025_S	ENSGALG00010024940	RABL3	case	control	-0.17973191	0.114053683	-1.57585363	0.11505954	0.24227274	down_in_case
Yang2025_S	ENSGALG00010024941	PDHX	case	control	0.186010996	0.077011414	2.415369169	0.015719269	0.05695476	up_in_case
Yang2025_S	ENSGALG00010024942	DNAJB6	case	control	0.163535419	0.10003	1.634863727	0.102077618	0.222466626	up_in_case
Yang2025_S	ENSGALG00010024943	ABCB9	case	control	-0.039116395	0.191557478	-0.20420187	0.83819575	0.904449915	down_in_case
Yang2025_S	ENSGALG00010024944	ENSGALG00010024944	case	control	0.045451386	0.224154142	0.202768444	0.839316024	0.904799934	up_in_case
Yang2025_S	ENSGALG00010024945	APIP	case	control	-0.067964309	0.131291915	-0.517657992	0.604696912	0.742811308	down_in_case
Yang2025_S	ENSGALG00010024946	KIF1A	case	control	0.625081032	0.509804004	1.226120287			up_in_case
Yang2025_S	ENSGALG00010024947	MPP4	case	control	1.515636231	0.207615383	7.300211628	2.87315E-13	4.21446E-11	up_in_case
Yang2025_S	ENSGALG00010024949	PAN2	case	control	0.145786718	0.120905423	1.205791386	0.227897938	0.390094308	up_in_case
Yang2025_S	ENSGALG00010024950	NCAM1	case	control	-0.133412926	0.101862543	-1.309734893	0.190285535	0.345174295	down_in_case
Yang2025_S	ENSGALG00010024953	NCAPG2	case	control	-0.272875063	0.178542201	-1.528350501	0.126425538	0.259003215	down_in_case
Yang2025_S	ENSGALG00010024956	VPS37B	case	control	-0.509703188	0.099810351	-5.106716699	3.27805E-07	9.2282E-06	down_in_case
Yang2025_S	ENSGALG00010024960	ARFRP1	case	control	-0.469997588	0.146228376	-3.214133939	0.001308386	0.008652403	down_in_case
Yang2025_S	ENSGALG00010024961	LSM12	case	control	0.329803374	0.161762405	2.038813496	0.041468645	0.11618424	up_in_case
Yang2025_S	ENSGALG00010024963	TNFRSF6B	case	control	-0.954617823	0.328685102	-2.904353798	0.00368012	0.018998284	down_in_case
Yang2025_S	ENSGALG00010024964	ITM2C	case	control	-0.262678232	0.217135224	-1.209744912	0.22637679	0.388368501	down_in_case
Yang2025_S	ENSGALG00010024965	STAT2	case	control	0.464141338	0.104103165	4.458474823	8.25449E-06	0.000139653	up_in_case
Yang2025_S	ENSGALG00010024966	AGAP3	case	control	0.26342205	0.075390542	3.494099443	0.000475664	0.00379632	up_in_case
Yang2025_S	ENSGALG00010024968	TMEM101	case	control	-0.119661716	0.243020214	-0.492394088	0.622440778	0.756868433	down_in_case
Yang2025_S	ENSGALG00010024970	CAB39	case	control	-0.196414617	0.102794524	-1.910749812	0.05603674	0.145055048	down_in_case
Yang2025_S	ENSGALG00010024973	RTEL1	case	control	0.024900547	0.140507985	0.177218023	0.85933714	0.916995034	up_in_case
Yang2025_S	ENSGALG00010024974	CD44	case	control	0.095930249	0.149742953	0.640632813	0.521761276	0.675243084	up_in_case
Yang2025_S	ENSGALG00010024977	LRP3	case	control	-0.115086435	0.128563712	-0.895170443	0.370695982	0.539211745	down_in_case
Yang2025_S	ENSGALG00010024983	APOF	case	control	0.531858139	0.695130801	0.765119512	0.444200382	0.607392044	up_in_case

Yang2025_S	ENSGALG00010024984	SHISAL2A	case	control	0.727751511	0.379111815	1.919622344	0.05490562	0.142855525	up_in_case
Yang2025_S	ENSGALG00010024986	PTS	case	control	0.078553473	0.144730309	0.542757581	0.587296723	0.728714773	up_in_case
Yang2025_S	ENSGALG00010024989	TIMELESS	case	control	0.52211603	0.185013073	2.822049386	0.004771783	0.023160848	up_in_case
Yang2025_S	ENSGALG00010024994	NDUFB4	case	control	0.280071644	0.209083612	1.339519826	0.180401505	0.332613782	up_in_case
Yang2025_S	ENSGALG00010024996	COA7	case	control	0.063141934	0.212844112	0.296658119	0.766727535	0.858180578	up_in_case
Yang2025_S	ENSGALG00010024997	HES6	case	control	-0.523501626	0.388181721	-1.348599375	0.177465682	0.328723152	down_in_case
Yang2025_S	ENSGALG00010025003	ENSGALG00010025003	case	control	0.461920984	0.24156155	1.912228931	0.055846837	0.144786171	up_in_case
Yang2025_S	ENSGALG00010025005	HIP1R	case	control	0.609485275	0.104359518	5.840246147	5.21238E-09	2.53082E-07	up_in_case
Yang2025_S	ENSGALG00010025006	ENSGALG00010025006	case	control	1.33949585	0.297693473	4.499580848	6.80876E-06	0.000120866	up_in_case
Yang2025_S	ENSGALG00010025007	ZYG11B	case	control	-0.04715134	0.098001363	-0.481129425	0.630424516	0.763047479	down_in_case
Yang2025_S	ENSGALG00010025008	ENSGALG00010025008	case	control	-1.714080192	0.685850959	-2.499202151	0.012447328	0.047678263	down_in_case
Yang2025_S	ENSGALG00010025026	DENR	case	control	-0.218521695	0.148660969	-1.469933214	0.141579843	0.281045237	down_in_case
Yang2025_S	ENSGALG00010025028	HES6	case	control	0.485659888	0.172694893	2.812242326	0.004919742	0.023705605	up_in_case
Yang2025_S	ENSGALG00010025031	ECHDC2	case	control	-0.204685942	0.154834741	-1.321963926	0.186180164	0.339761731	down_in_case
Yang2025_S	ENSGALG00010025032	FSTL1	case	control	-0.581146109	0.110388785	-5.264539407	1.40541E-07	4.58652E-06	down_in_case
Yang2025_S	ENSGALG00010025039	CEBPA	case	control	-0.034538345	0.218195773	-0.158290624	0.874227796	0.926381944	down_in_case
Yang2025_S	ENSGALG00010025044	TMEM9	case	control	0.750684605	0.228411763	3.286540924	0.00101426	0.007088123	up_in_case
Yang2025_S	ENSGALG00010025047	PER2	case	control	0.723829484	0.189614918	3.817365706	0.000134884	0.001360066	up_in_case
Yang2025_S	ENSGALG00010025048	ESYT2	case	control	-0.064183156	0.148965642	-0.430858787	0.666571051	0.789228274	down_in_case
Yang2025_S	ENSGALG00010025055	ALS2	case	control	0.1226584	0.130722925	0.938308256	0.348086018	0.516513541	up_in_case
Yang2025_S	ENSGALG00010025056	RBMS2	case	control	0.20107554	0.116781468	1.721810346	0.08510388	0.195826051	up_in_case
Yang2025_S	ENSGALG00010025057	SCP2	case	control	-0.0757501	0.090098495	-0.840747675	0.400489306	0.567352044	down_in_case
Yang2025_S	ENSGALG00010025060	GPR156	case	control	-0.110280976	0.20782826	-0.530635132	0.595671645	0.735419897	down_in_case
Yang2025_S	ENSGALG00010025064	GPN3	case	control	-0.215751476	0.129482465	-1.666260186	0.095661603	0.212583268	down_in_case
Yang2025_S	ENSGALG00010025067	SLC1A2	case	control	0.261988005	0.26318039	0.995469325	0.319508056	0.488856637	up_in_case
Yang2025_S	ENSGALG00010025069	PODN	case	control	-0.586406708	0.202586753	-2.89459553	0.003796474	0.019435658	down_in_case
Yang2025_S	ENSGALG00010025071	CDK15	case	control	0.805650212	0.338726313	2.378469526	0.017384674	0.061206526	up_in_case
Yang2025_S	ENSGALG00010025073	IL18	case	control	0.666029883	0.334592479	1.990570396	0.046528138	0.12658524	up_in_case
Yang2025_S	ENSGALG00010025080	BAZ2A	case	control	0.304331972	0.179485645	1.695578344	0.089965762	0.203683654	up_in_case
Yang2025_S	ENSGALG00010025092	COL1A1	case	control	-0.283102436	0.216804584	-1.305795433	0.191622133	0.34678629	down_in_case
Yang2025_S	ENSGALG00010025097	LMBR1	case	control	-0.361876978	0.087719005	-4.125411324	3.70073E-05	0.000481939	down_in_case
Yang2025_S	ENSGALG00010025098	ATP5B	case	control	-0.39850618	0.178735313	-2.229588403	0.02577478	0.081759974	down_in_case
Yang2025_S	ENSGALG00010025102	ARPC3	case	control	-0.194603851	0.134925907	-1.442301598	0.14921731	0.291592092	down_in_case
Yang2025_S	ENSGALG00010025103	GSK3B	case	control	0.17897539	0.171024122	1.046492085	0.295333897	0.463820338	up_in_case
Yang2025_S	ENSGALG00010025113	SDHD	case	control	-0.365653084	0.153031685	-2.38939461	0.016876165	0.059885245	down_in_case
Yang2025_S	ENSGALG00010025114	CEBPG	case	control	-0.390308158	0.068367169	-5.708999865	1.13642E-08	5.17517E-07	down_in_case
Yang2025_S	ENSGALG00010025115	MGAT4F	case	control	-0.00692266	0.207664507	-0.033335789	0.973406814	0.983998836	down_in_case
Yang2025_S	ENSGALG00010025116	ANAPC7	case	control	-0.029561769	0.083247095	-0.355108714	0.722508149	0.82933699	down_in_case
Yang2025_S	ENSGALG00010025121	DYNC2I1	case	control	0.880449234	0.296593771	2.968535818	0.002992222	0.016300476	up_in_case
Yang2025_S	ENSGALG00010025125	PIK3CB	case	control	0.01726854	0.141033859	0.122442511	0.902548573	0.941675229	up_in_case
Yang2025_S	ENSGALG00010025126	ENSGALG00010025126	case	control	0.873974177	0.203614816	4.292291671	1.76838E-05	0.000262993	up_in_case
Yang2025_S	ENSGALG00010025127	PAMR1	case	control	-0.069072851	0.222256778	-0.310779503	0.755968253	0.851747866	down_in_case
Yang2025_S	ENSGALG00010025130	ATP2A2	case	control	0.149114659	0.145022055	1.028220561	0.303846089	0.473453566	up_in_case
Yang2025_S	ENSGALG00010025131	SUMO1	case	control	-0.641882338	0.168793961	-3.802756542	0.000143095	0.001424306	down_in_case
Yang2025_S	ENSGALG00010025138	CFAP91	case	control	0.853913821	0.310646733	2.748826018	0.005980912	0.027334868	up_in_case
Yang2025_S	ENSGALG00010025139	COPB2	case	control	-0.046150363	0.06757536	-0.682946611	0.494640579	0.651987937	down_in_case
Yang2025_S	ENSGALG00010025144	PIH1D2	case	control	0.445379003	0.15640466	2.847606981	0.004404929	0.021679022	up_in_case
Yang2025_S	ENSGALG00010025145	CPT2	case	control	-0.064034461	0.110728064	-0.578303806	0.563059025	0.709231449	down_in_case
Yang2025_S	ENSGALG00010025147	COX17	case	control	-0.331255166	0.286034417	-1.158095481	0.246825074	0.412559363	down_in_case
Yang2025_S	ENSGALG00010025149	IFT81	case	control	-0.12007862	0.096041173	-1.250282727	0.211196286	0.371124873	down_in_case
Yang2025_S	ENSGALG00010025154	TMEM183A	case	control	0.022909722	0.120926794	0.189451166	0.849739226	0.910785025	up_in_case
Yang2025_S	ENSGALG00010025155	CZIB	case	control	-0.835430215	0.239489963	-3.488372555	0.00048597	0.003860888	down_in_case
Yang2025_S	ENSGALG00010025156	DLAT	case	control	-0.217330419	0.121407504	-1.790090506	0.073439363	0.176019526	down_in_case
Yang2025_S	ENSGALG00010025157	NOP58	case	control	-0.021203761	0.129859529	-0.163282292	0.870296176	0.923654015	down_in_case
Yang2025_S	ENSGALG00010025160	MRPS22	case	control	0.012184082	0.188517911	0.064630897	0.948467884	0.967992436	up_in_case
Yang2025_S	ENSGALG00010025161	ENSGALG00010025161	case	control	-0.001549784	0.151066021	-0.010258981	0.991814661	0.994884631	down_in_case
Yang2025_S	ENSGALG00010025163	LDLRAD3	case	control	0.168741253	0.160377334	1.052151505	0.292730045	0.461417951	up_in_case
Yang2025_S	ENSGALG00010025164	C11orf52	case	control	-0.731030317	0.154070179	-4.744787865	2.08725E-06	4.50943E-05	down_in_case
Yang2025_S	ENSGALG00010025167	PTGES3	case	control	-0.205231386	0.10650028	-1.927050189	0.053973376	0.141189973	down_in_case
Yang2025_S	ENSGALG00010025168	POPDC2	case	control	0.079224591	0.146629	0.540306426	0.588985728	0.730011217	up_in_case
Yang2025_S	ENSGALG00010025170	COMMD9	case	control	-0.419809949	0.159059011	-2.63933458	0.008306895	0.035270136	down_in_case
Yang2025_S	ENSGALG00010025172	RBP1	case	control	1.888500136	0.756729374	2.49560834	0.012574135	0.048013657	up_in_case
Yang2025_S	ENSGALG00010025182	PLA1A	case	control	0.296828538	0.125709937	2.361217773	0.018215031	0.063284276	up_in_case
Yang2025_S	ENSGALG00010025186	TMEM120B	case	control	-0.152172124	0.191474091	-0.794740027	0.426764744	0.591149772	down_in_case
Yang2025_S	ENSGALG00010025188	PPFIA4	case	control	0.721058018	0.495531053	1.45512176	0.145635556	0.287007704	up_in_case
Yang2025_S	ENSGALG00010025190	PRR5L	case	control	-0.493026598	0.191469002	-2.574968239	0.010024929	0.040574321	down_in_case
Yang2025_S	ENSGALG00010025192	CRYAB	case	control	0.182864376	0.242081471	0.755383611	0.450018844	0.612226971	up_in_case
Yang2025_S	ENSGALG00010025195	P2RX7	case	control	0.43008052	0.255761245	1.681570325	0.092652189	0.207907931	up_in_case
Yang2025_S	ENSGALG00010025200	PEPD	case	control	-0.245086754	0.147703742	-1.659313104	0.097052716	0.214637295	down_in_case
Yang2025_S	ENSGALG00010025204	TRAF6	case	control	-0.405359272	0.085958896	-4.715733802	2.40841E-06	5.10049E-05	down_in_case
Yang2025_S	ENSGALG00010025207	BMPR2	case	control	0.337773037	0.197800694	1.707643339	0.087702542	0.200218696	up_in_case
Yang2025_S	ENSGALG00010025210	HSPB2	case	control	0.464318598	0.252779401	1.836852989	0.066231601	0.163322838	up_in_case
Yang2025_S	ENSGALG00010025212	P2RX4	case	control	0.389533592	0.169530678	2.297717421	0.021577877	0.071525572	up_in_case
Yang2025_S	ENSGALG00010025214	LRP8	case	control	1.238636219	0.345133194	3.58886436	0.000332122	0.002844569	up_in_case
Yang2025_S	ENSGALG00010025215	MYOG	case	control	0.024965488	0.191037191	0.130683915	0.896025357	0.938664363	up_in_case
Yang2025_S	ENSGALG00010025216	DPP6	case	control	-0.221525618	0.300026945	-0.738352412	0.460300325	0.621298433	down_in_case
Yang2025_S	ENSGALG00010025219	NACA	case	control	0.457295528	0.129601979	3.528460989	0.000417984	0.003428252	up_in_case
Yang2025_S	ENSGALG00010025222	FAM117B	case	control	0.083340573	0.229202741	0.363610715	0.716148725	0.825027487	up_in_case
Yang2025_S	ENSGALG00010025228	FDXACB1	case	control	-0.06307615	0.131250959	-0.480576678	0.630817394	0.763258635	down_in_case
Yang2025_S	ENSGALG00010025229	ENSGALG00010025229	case	control	-0.332913632	0.118689032	-2.804923295	0.005032856	0.02408408	down_in_case
Yang2025_S	ENSGALG00010025230	CHST8	case	control	0.719304073	0.329553091	2.182665227	0.029060472	0.089355182	up_in_case
Yang2025_S	ENSGALG00010025232	PRIM1	case	control	0.228075272	0.19721112	1.1565031	0.247475432	0.41315085	up_in_case
Yang2025_S	ENSGALG00010025233	WDR12	case	control	0.000707825	0.126216975	0.005608001	0.995525486	0.996868798	up_in_case
Yang2025_S	ENSGALG00010025236	ENSGALG00010025236	case	control	0.062264093	0.328189579	0.189719898	0.849528628	0.910630056	up_in_case
Yang2025_S	ENSGALG00010025238	IGSF9B	case	control	0.248439503	0.279464717	0.888983433	0.374011984	0.542111191	up_in_case
Yang2025_S	ENSGALG00010025242	CLSTN2	case	control	0.455561134	0.301457695	1.511194249	0.130738967	0.264980266	up_in_case
Yang2025_S	ENSGALG00010025243	PAXIP1	case	control	0.465914531	0.223750758	2.082292525	0.037315758	0.107525867	up_in_case
Yang2025_S	ENSGALG00010025245	ENSGALG00010025245	case	control	0.506132538	0.139486737	3.628535216	0.000285034	0.00249808	up_in_case
Yang2025_S	ENSGALG00010025247	SGCA	case	control	-0.031109226	0.151094064	-0.205893107	0.83687441	0.903318738	down_in_case
Yang2025_S	ENSGALG00010025248	C11orf74	case	control	-0.305416034	0.21062934	-1.450016573	0.147053898	0.288895163	down_in_case
Yang2025_S	ENSGALG00010025250	GLIS1	case	control	0.478227111	0.484028184	0.988015011	0.323145296	0.492457317	up_in_case
Yang2025_S	ENSGALG00010025252	PPP1R9B	case	control	0.570878433	0.077860592	7.332058734	2.26644E-13	3.43291E-11	up_in_case
Yang2025_S	ENSGALG00010025256	ANAPC5	case	control	-0.475144145	0.158325833	-3.001052545	0.002690481	0.014984755	down_in_case
Yang2025_S	ENSGALG00010025257	LPCAT2	case	control	-0.07841719	0.131100631	-0.598145021	0.549743172	0.69832918	down_in_case
Yang2025_S	ENSGALG00010025262	CARF	case	control	0.049783131	0.126831137	0.392515054	0.694677691	0.810341003	up_in_case
Yang2025_S	ENSGALG00010025263	SAMD14	case	control	0.161614823	0.497077608	0.32512996	0.745082715	0.844536167	up_in_case
Yang2025_S	ENSGALG00010025268	ZBTB39	case	control	-0.151343641	0.14312973	-1.057387875	0.290334603	0.458917048	down_in_case
Yang2025_S	ENSGALG00010025270	NDC1	case	control	-0.218477524	0.14627193	-1.493639445	0.135269884	0.271784267	down_in_case
Yang2025_S	ENSGALG00010025271	RNF34	case	control	-0.199720429	0.084153042	-2.373300158	0.01762993	0.061944801	down_in_case
Yang2025_S	ENSGALG00010025272	NCAPD3	case	control	-0.395010563	0.155605576	-2.538537332	0.011131693	0.043856414	down_in_case
Yang2025_S	ENSGALG00010025274	PDK2	case	control	0.170551063	0.209909368	0.812498579	0.416505602	0.581743963	up_in_case
Yang2025_S	ENSGALG00010025280	HSPB11	case	control	0.048064351	0.153520462	0.313081074	0.754219068	0.850395882	up_in_case
Yang2025_S	ENSGALG00010025286	ITGA3	case	control	0.628084616	0.101996122	6.157926433	7.37036E-10	4.52449E-08	up_in_case
Yang2025_S	ENSGALG00010025289	KDM2B	case	control	-0.115856675	0.073808209	-1.56969903	0.116485147	0.244276979	down_in_case
Yang2025_S	ENSGALG00010025291	DUS2	case	control	0.023294564	0.12860397	0.1811341	0.85626232	0.914961692	up_in_case
Yang2025_S	ENSGALG00010025292	ATP1A1	case	control	-0.249527364	0.126241346	-1.976589858	0.048088009	0.129790124	down_in_case
Yang2025_S	ENSGALG00010025297	SPSB4	case	control	-0.387884559	0.103667834	-3.741609557	0.000182845	0.001737204	down_in_case
Yang2025_S	ENSGALG00010025298	ENSGALG00010025298	case	control	0.214418847	0.496688706	0.431696643	0.665961907	0.789166564	up_in_case
Yang2025_S	ENSGALG00010025301	MORN3	case	control	-0.094592577	0.247423237	-0.382310804	0.702230842	0.816213761	down_in_case
Yang2025_S	ENSGALG00010025303	TMCC2	case	control	0.009802279	0.107012346	0.09159952	0.927016233	0.95575401	up_in_case
Yang2025_S	ENSGALG00010025304	DDX28	case	control	-0.092030479	0.176550574	-0.521269779	0.60217885	0.740632152	down_in_case
Yang2025_S	ENSGALG00010025305	GPR182	case	control	-0.210291778	0.306705406	-0.685647447	0.492935454	0.650417153	down_in_case
Yang2025_S	ENSGALG00010025312	PLEKHM3	case	control	-0.177525124	0.120795836	-1.469629496	0.141662127	0.281045237	down_in_case
Yang2025_S	ENSGALG00010025313	DIO1	case	control	-0.296186113	0.597076668	-0.496060438	0.619851771	0.755266399	down_in_case
Yang2025_S	ENSGALG00010025315	LRRC42	case	control	0.397807971	0.140755991	2.826224085	0.00471003	0.022914314	up_in_case
Yang2025_S	ENSGALG00010025316	SLC25A36	case	control	-0.18531972	0.14227678	-1.302529621	0.192735399	0.348032627	down_in_case
Yang2025_S	ENSGALG00010025318	NUAK2	case	control	-0.241032026	0.553747569	-0.435274193	0.663363415	0.787011039	down_in_case
Yang2025_S	ENSGALG00010025319	LDLRAD1	case	control	0.752940601	0.310750262	2.42297656	0.01539392	0.056096829	up_in_case
Yang2025_S	ENSGALG00010025320	JAM3	case	control	-0.313898215	0.070066681	-4.47999266	7.46456E-06	0.000129699	down_in_case
Yang2025_S	ENSGALG00010025323	KLHDC8A	case	control	-0.002454211	0.194284896	-0.01263202	0.989921374	0.993843973	down_in_case
Yang2025_S	ENSGALG00010025325	DPEP2	case	control	-0.085527575	0.182916268	-0.467577741	0.640086586	0.769927197	down_in_case
Yang2025_S	ENSGALG00010025327	TMEM59	case	control	-0.507072919	0.131666677	-3.851186408	0.000117547	0.001211551	down_in_case
Yang2025_S	ENSGALG00010025329	MYO1A	case	control	0.075918722	0.149748521	0.506974774	0.612172519	0.749023097	up_in_case
Yang2025_S	ENSGALG00010025330	USH1C	case	control	0.715408272	0.340679316	2.099946306	0.035733565	0.104391452	up_in_case
Yang2025_S	ENSGALG00010025331	ENSGALG00010025331	case	control	0.325741897	0.444282229	0.73318687	0.463444462	0.624393172	up_in_case
Yang2025_S	ENSGALG00010025333	FZD5	case	control	-0.601391915	0.100557333	-5.980587342	2.22334E-09	1.22946E-07	down_in_case

Yang2025_S	ENSGALG00010025334	RHOF	case	control	0.540401111	0.288198625	1.875099545	0.06077903	0.153908011	up_in_case
Yang2025_S	ENSGALG00010025336	ADAM10	case	control	0.327856932	0.171590717	1.910691541	0.056044233	0.145055048	up_in_case
Yang2025_S	ENSGALG00010025337	NEMP1	case	control	0.120776585	0.2105504	0.573623159	0.566222837	0.711223655	up_in_case
Yang2025_S	ENSGALG00010025338	FADD	case	control	0.082826989	0.096980182	0.854060977	0.393071204	0.560013816	up_in_case
Yang2025_S	ENSGALG00010025340	METTL21A	case	control	-0.404624104	0.197212006	-2.051721453	0.040196741	0.113711243	down_in_case
Yang2025_S	ENSGALG00010025341	VPS26B	case	control	-0.277866649	0.112932917	-2.460457562	0.013875998	0.051728741	down_in_case
Yang2025_S	ENSGALG00010025342	TCEANC2	case	control	-0.177300839	0.1514678	-1.170551354	0.241779161	0.406858182	down_in_case
Yang2025_S	ENSGALG00010025343	NAB2	case	control	0.343557643	0.215669321	1.592983378	0.11116392	0.236246641	up_in_case
Yang2025_S	ENSGALG00010025344	ENSGALG00010025344	case	control	0.790383549	0.242539989	3.258776217	0.001118939	0.007669658	up_in_case
Yang2025_S	ENSGALG00010025345	CCM2	case	control	0.033757873	0.138033178	0.244563472	0.806794451	0.883642595	up_in_case
Yang2025_S	ENSGALG00010025346	PXYLP1	case	control	0.196256103	0.157197482	1.24846849	0.211859541	0.372008909	up_in_case
Yang2025_S	ENSGALG00010025348	HIPK1	case	control	-0.06744205	0.17439224	-0.386726209	0.698958911	0.813564352	down_in_case
Yang2025_S	ENSGALG00010025349	SETD1A	case	control	0.342115052	0.225849626	1.514791311	0.129825262	0.263680074	up_in_case
Yang2025_S	ENSGALG00010025351	CREB1	case	control	-0.384767315	0.10866613	-3.540820993	0.000398884	0.003290971	down_in_case
Yang2025_S	ENSGALG00010025353	SLC12A4	case	control	0.098721857	0.072487064	1.361923788	0.173221938	0.323138917	up_in_case
Yang2025_S	ENSGALG00010025355	STAT6	case	control	0.314192997	0.072556306	4.330333421	1.48884E-05	0.000228491	up_in_case
Yang2025_S	ENSGALG00010025357	OLFML3	case	control	-0.36529668	0.193545305	-1.88739623	0.059107052	0.151008407	down_in_case
Yang2025_S	ENSGALG00010025358	INCENP	case	control	0.069874047	0.187902988	0.371862352	0.709995337	0.820924745	up_in_case
Yang2025_S	ENSGALG00010025359	CDCP2	case	control	-0.46293719	0.316614996	-1.462145496	0.143701344	0.284135804	down_in_case
Yang2025_S	ENSGALG00010025360	THYN1	case	control	0.25796113	0.133508098	1.932175907	0.053337798	0.139895015	up_in_case
Yang2025_S	ENSGALG00010025362	G6PC2	case	control	0.0568551	0.157527466	0.360921824	0.718157888	0.826385646	up_in_case
Yang2025_S	ENSGALG00010025364	EPHA4	case	control	-0.121377967	0.132683529	-0.914793028	0.360300295	0.528847747	down_in_case
Yang2025_S	ENSGALG00010025365	SIKE1	case	control	-0.289694502	0.151391049	-1.913551062	0.055677543	0.144481669	down_in_case
Yang2025_S	ENSGALG00010025368	LRP1	case	control	0.117984187	0.196763102	0.599625569	0.548755803	0.697456413	up_in_case
Yang2025_S	ENSGALG00010025369	ENSGALG00010025369	case	control	-0.693081016	0.639253136	-1.084204327	0.278274214	0.445836002	down_in_case
Yang2025_S	ENSGALG00010025370	HPD	case	control	0.132808542	0.368917028	0.359995696	0.718850352	0.826504753	up_in_case
Yang2025_S	ENSGALG00010025373	CYB5RL	case	control	-0.633938709	0.220160876	-2.87943398	0.003983897	0.02012164	down_in_case
Yang2025_S	ENSGALG00010025377	MYO1G	case	control	0.196346228	0.217743999	0.901729688	0.367200479	0.535863858	up_in_case
Yang2025_S	ENSGALG00010025378	PAX3	case	control	0.31350978	0.209836243	1.494068781	0.135157643	0.271621251	up_in_case
Yang2025_S	ENSGALG00010025379	ACAD8	case	control	-0.437625082	0.154329351	-2.835656854	0.004573155	0.022391749	down_in_case
Yang2025_S	ENSGALG00010025380	SPC25	case	control	-0.039461092	0.162825723	-0.242351709	0.80850765	0.884622652	down_in_case
Yang2025_S	ENSGALG00010025381	MRPL37	case	control	-0.166487309	0.1800788	-0.924524757	0.355213168	0.524408888	down_in_case
Yang2025_S	ENSGALG00010025382	MAB21L3	case	control	0.865889232	0.437023481	1.981333427	0.047553894	0.128822612	up_in_case
Yang2025_S	ENSGALG00010025386	TPCN2	case	control	-0.301007731	0.131602523	-2.287248937	0.022181297	0.072934492	down_in_case
Yang2025_S	ENSGALG00010025387	PSMD9	case	control	0.016018215	0.121950626	0.131350002	0.895498439	0.938334765	up_in_case
Yang2025_S	ENSGALG00010025389	SNED1	case	control	0.279103837	0.110920118	2.516259827	0.011860771	0.045926844	up_in_case
Yang2025_S	ENSGALG00010025390	BCL7A	case	control	-0.432003998	0.090323309	-4.782862853	1.72816E-06	3.85311E-05	down_in_case
Yang2025_S	ENSGALG00010025392	SLC6A2	case	control	-0.560809527	0.239619186	-2.340419967	0.019262066	0.065950097	down_in_case
Yang2025_S	ENSGALG00010025398	TSPAN2	case	control	0.535138963	0.292960347	1.826660054	0.067750898	0.165928979	up_in_case
Yang2025_S	ENSGALG00010025400	SLC22A15	case	control	0.359257348	0.292632715	1.227673223	0.219569662	0.380749532	up_in_case
Yang2025_S	ENSGALG00010025403	ENSGALG00010025403	case	control	0.683733001	0.212351781	3.219812889	0.001282743	0.008499773	up_in_case
Yang2025_S	ENSGALG00010025404	CERS6	case	control	-0.316067428	0.12424828	-2.543837458	0.010964209	0.043368225	down_in_case
Yang2025_S	ENSGALG00010025405	TAMALIN	case	control	0.089868429	0.307008523	0.292722912	0.769733955	0.8596641	up_in_case
Yang2025_S	ENSGALG00010025406	ENSGALG00010025406	case	control	-0.065934	0.098663041	-0.668274558	0.50395835	0.659851437	down_in_case
Yang2025_S	ENSGALG00010025407	MLXIP	case	control	-0.08215271	0.167959958	-0.489120805	0.624756173	0.75873804	down_in_case
Yang2025_S	ENSGALG00010025413	NECAB3	case	control	0.270117779	0.103605326	2.607180446	0.009129124	0.037974429	up_in_case
Yang2025_S	ENSGALG00010025414	MMP2	case	control	-0.425190077	0.128617967	-3.305837327	0.00094693	0.006718674	down_in_case
Yang2025_S	ENSGALG00010025415	ENSGALG00010025415	case	control	0.760590996	0.243625062	3.12197353	0.001796431	0.011061982	up_in_case
Yang2025_S	ENSGALG00010025417	B3GAT1	case	control	-0.135472897	0.134033497	-1.010739103	0.312141316	0.481746511	down_in_case
Yang2025_S	ENSGALG00010025418	CASQ2	case	control	-0.095662875	0.098710579	-0.969124848	0.332482901	0.501748888	down_in_case
Yang2025_S	ENSGALG00010025419	CCND1	case	control	0.434254084	0.116421865	3.73000452	0.000191476	0.00180042	up_in_case
Yang2025_S	ENSGALG00010025420	STK39	case	control	-0.177650529	0.170909285	-1.039443402	0.298598568	0.467945461	down_in_case
Yang2025_S	ENSGALG00010025421	NR4A1	case	control	0.08410749	0.194685638	0.432016919	0.665729117	0.789123447	up_in_case
Yang2025_S	ENSGALG00010025422	SSBP3	case	control	-0.093877109	0.09770719	-0.960800418	0.336652528	0.504815774	down_in_case
Yang2025_S	ENSGALG00010025423	NGF	case	control	0.267735737	0.275770067	0.97086584	0.331615091	0.500873216	up_in_case
Yang2025_S	ENSGALG00010025424	IRX6	case	control	0.404806868	0.339420702	1.192640478	0.233010206	0.396168524	up_in_case
Yang2025_S	ENSGALG00010025425	ENSGALG00010025425	case	control	0.371528043	0.100753769	3.687485301	0.000226481	0.002056036	up_in_case
Yang2025_S	ENSGALG00010025426	ENSGALG00010025426	case	control	0.125442811	0.0939211	1.335619062	0.181673816	0.334207872	up_in_case
Yang2025_S	ENSGALG00010025427	SLC35G2	case	control	-0.134223399	0.143134993	-0.937739938	0.348378074	0.516794462	down_in_case
Yang2025_S	ENSGALG00010025428	VANGL1	case	control	-0.021538332	0.141595435	-0.152111767	0.879098783	0.92862622	down_in_case
Yang2025_S	ENSGALG00010025429	SCN3B	case	control	-0.294175838	0.30663163	-0.959378648	0.337368028	0.50545355	down_in_case
Yang2025_S	ENSGALG00010025432	IRX3	case	control	1.437372874	0.566596758	2.536853331	0.011185381	0.044005725	up_in_case
Yang2025_S	ENSGALG00010025433	CHPF2	case	control	-0.017232979	0.113817188	-0.151409286	0.879652865	0.928774259	down_in_case
Yang2025_S	ENSGALG00010025434	SYT6	case	control	-0.625041788	0.144920273	-4.313004483	1.61051E-05	0.00024341	down_in_case
Yang2025_S	ENSGALG00010025435	SCLY	case	control	-0.160364569	0.249538275	-0.642645179	0.52045436	0.67408974	down_in_case
Yang2025_S	ENSGALG00010025438	EXOSC6	case	control	1.107792073	0.354913888	3.121298184	0.001800556	0.011065404	up_in_case
Yang2025_S	ENSGALG00010025442	ABCF2	case	control	-0.072368517	0.114169145	-0.633871059	0.526164975	0.679376847	down_in_case
Yang2025_S	ENSGALG00010025445	B3GNT4	case	control	0.88629949	0.171478399	5.168578039	2.35882E-07	7.02353E-06	up_in_case
Yang2025_S	ENSGALG00010025448	CHD1L	case	control	0.416583055	0.120323886	3.462180847	0.000535817	0.004190017	up_in_case
Yang2025_S	ENSGALG00010025449	ENSGALG00010025449	case	control	0.301994262	0.090879457	3.323020096	0.000890485	0.006373348	up_in_case
Yang2025_S	ENSGALG00010025451	LRRC43	case	control	1.643697214	0.327859286	5.013422785	5.34702E-07	1.41542E-05	up_in_case
Yang2025_S	ENSGALG00010025452	TRIM33	case	control	0.009792646	0.113644175	0.086169361	0.931331787	0.958501363	up_in_case
Yang2025_S	ENSGALG00010025458	BCAS2	case	control	0.14424744	0.203795585	0.707804537	0.479066648	0.637733449	up_in_case
Yang2025_S	ENSGALG00010025459	DIABLO	case	control	-0.13426735	0.090410974	-1.485078019	0.137523158	0.27518455	down_in_case
Yang2025_S	ENSGALG00010025464	VPS33A	case	control	-0.068318911	0.108259832	-0.631064263	0.527998506	0.680874574	down_in_case
Yang2025_S	ENSGALG00010025465	ENSGALG00010025465	case	control	-0.271844336	0.153074019	-1.775901214	0.075749201	0.180069108	down_in_case
Yang2025_S	ENSGALG00010025470	ORAOV1	case	control	0.36531614	0.101259373	3.607726676	0.000308892	0.002673543	up_in_case
Yang2025_S	ENSGALG00010025472	ZCCHC8	case	control	0.628200632	0.169124674	3.714423317	0.000203668	0.001900943	up_in_case
Yang2025_S	ENSGALG00010025473	PARS2	case	control	0.113856296	0.157429034	0.723222985	0.469542899	0.630049827	up_in_case
Yang2025_S	ENSGALG00010025477	ENSGALG00010025477	case	control	0.337370635	0.160515387	2.101796228	0.03557113	0.104040435	up_in_case
Yang2025_S	ENSGALG00010025480	FGF19	case	control	-0.215679146	0.374820506	-0.575419815	0.565007406	0.710786152	down_in_case
Yang2025_S	ENSGALG00010025481	TMEM39A	case	control	0.14659105	0.088140581	1.663150479	0.096282318	0.213622349	up_in_case
Yang2025_S	ENSGALG00010025483	FGF4	case	control	0.760426361	0.212473339	3.578926022	0.000345009	0.002933314	up_in_case
Yang2025_S	ENSGALG00010025485	DENND2C	case	control	0.408686828	0.102980932	3.968567976	7.23058E-05	0.000820506	up_in_case
Yang2025_S	ENSGALG00010025486	B3GALT1	case	control	0.077126077	0.181183422	0.425679547	0.670341368	0.792224172	up_in_case
Yang2025_S	ENSGALG00010025487	FGF3	case	control	-0.158515297	0.601242197	-0.263646328	0.792052457	0.874514776	down_in_case
Yang2025_S	ENSGALG00010025488	SLC4A8	case	control	-0.185774081	0.212808419	-0.872963962	0.382682721	0.550444617	down_in_case
Yang2025_S	ENSGALG00010025490	ENSGALG00010025490	case	control	2.240614819	0.507948574	4.411105641	1.02844E-05	0.000168208	up_in_case
Yang2025_S	ENSGALG00010025491	ZMYND11	case	control	0.161445564	0.144922732	1.114011321	0.26527433	0.432451777	up_in_case
Yang2025_S	ENSGALG00010025496	ATG101	case	control	-0.070023632	0.194614615	-0.359806645	0.718991734	0.826543483	down_in_case
Yang2025_S	ENSGALG00010025499	STAC3	case	control	0.998012007	0.123820793	8.0601326	7.62117E-16	1.89645E-13	up_in_case
Yang2025_S	ENSGALG00010025504	AMPD1	case	control	-0.124110758	0.152518068	-0.813744619	0.415791267	0.581328575	down_in_case
Yang2025_S	ENSGALG00010025505	ENSGALG00010025505	case	control	-0.001951088	0.278729944	-0.006999924	0.994414914	0.996273767	down_in_case
Yang2025_S	ENSGALG00010025506	R3HDM2	case	control	0.626126933	0.188342649	3.324403354	0.000886079	0.006348337	up_in_case
Yang2025_S	ENSGALG00010025508	ENSGALG00010025508	case	control	-0.124592618	0.121832819	-1.022652343	0.306472252	0.47610823	down_in_case
Yang2025_S	ENSGALG00010025512	NRAS	case	control	0.00596579	0.072441834	0.082352824	0.93436615	0.959922758	up_in_case
Yang2025_S	ENSGALG00010025513	RSRC2	case	control	0.568314843	0.13134438	4.326906448	1.51218E-05	0.000231055	up_in_case
Yang2025_S	ENSGALG00010025520	ACOT11	case	control	0.522548459	0.233714847	2.235837676	0.025362406	0.080690669	up_in_case
Yang2025_S	ENSGALG00010025522	SUMO3	case	control	-0.165602069	0.113724044	-1.456174634	0.14534435	0.286555393	down_in_case
Yang2025_S	ENSGALG00010025523	B4GALT4	case	control	0.158298826	0.165823862	0.95462031	0.339769742	0.507741701	up_in_case
Yang2025_S	ENSGALG00010025527	KNTC1	case	control	-0.045442764	0.144570671	-0.314329064	0.753271126	0.849770657	down_in_case
Yang2025_S	ENSGALG00010025531	ENSGALG00010025531	case	control	-0.087532711	0.132485225	-0.660697911	0.508806064	0.664188525	down_in_case
Yang2025_S	ENSGALG00010025533	INHBC	case	control	-0.013932472	0.440223417	-0.031648638	0.974752255	0.984858808	down_in_case
Yang2025_S	ENSGALG00010025538	ANO1	case	control	0.251146532	0.170286477	1.474847188	0.140253632	0.279204908	up_in_case
Yang2025_S	ENSGALG00010025541	TTC21B	case	control	0.292603078	0.113219398	2.584389975	0.009755142	0.039792471	up_in_case
Yang2025_S	ENSGALG00010025544	CSDE1	case	control	0.191426002	0.104137243	1.838208861	0.066031635	0.162945073	up_in_case
Yang2025_S	ENSGALG00010025548	ENSGALG00010025548	case	control	-0.044569411	0.171786044	-0.259447218	0.795290202	0.876525276	down_in_case
Yang2025_S	ENSGALG00010025549	DHCR24	case	control	0.166862162	0.295949444	0.563819821	0.572876761	0.716095952	up_in_case
Yang2025_S	ENSGALG00010025553	UBE2G2	case	control	-0.390347186	0.201086894	-1.941186605	0.052235647	0.137704076	down_in_case
Yang2025_S	ENSGALG00010025554	DIP2C	case	control	-0.154021356	0.124653259	-1.235598316	0.216607911	0.377374404	down_in_case
Yang2025_S	ENSGALG00010025557	VPS29	case	control	-1.073399355	0.163503464	-6.564994576	5.20348E-11	4.70847E-09	down_in_case
Yang2025_S	ENSGALG00010025558	GALNT3	case	control	1.773557564	0.307831201	5.76146135	8.33888E-09	3.87341E-07	up_in_case
Yang2025_S	ENSGALG00010025562	GRAMD1B	case	control	-0.153084855	0.158907115	-0.963360606	0.335366589	0.503972114	down_in_case
Yang2025_S	ENSGALG00010025563	TSPEAR	case	control	0.29423036	0.305869322	0.961947927	0.336075762	0.504276488	up_in_case
Yang2025_S	ENSGALG00010025565	ENSGALG00010025565	case	control	-0.347978638	0.158714367	-2.19248355	0.02834461	0.087734815	down_in_case
Yang2025_S	ENSGALG00010025568	MYL3	case	control	0.682432301	0.311192463	2.192958965	0.028310336	0.087663275	up_in_case
Yang2025_S	ENSGALG00010025569	PPTC7	case	control	0.157593832	0.063233537	2.492250789	0.012693637	0.048316263	up_in_case
Yang2025_S	ENSGALG00010025571	SRSF3	case	control	0.321588616	0.109889292	2.926478187	0.003428235	0.01797985	up_in_case
Yang2025_S	ENSGALG00010025572	ENSGALG00010025572	case	control	0.215261282	0.141838956	1.5176457	0.129103739	0.262636585	up_in_case
Yang2025_S	ENSGALG00010025573	CLMP	case	control	-0.47061318	0.122707423	-3.835246225	0.000125439	0.001277768	down_in_case
Yang2025_S	ENSGALG00010025574	ARHGAP31	case	control	-0.34949635	0.122371676	-2.856023238	0.004289837	0.021250936	down_in_case
Yang2025_S	ENSGALG00010025575	TRPM2	case	control	0.802477166	0.551381146	1.455394643			up_in_case
Yang2025_S	ENSGALG00010025577	PTH2R	case	control	0.077845316	0.283802155	0.274294309	0.783858457	0.869324919	up_in_case
Yang2025_S	ENSGALG00010025578	ORMDL2	case	control	0.787791666	0.158633062	4.96612534	6.83038E-07	1.73057E-05	up_in_case
Yang2025_S	ENSGALG00010025579	CSRNP3	case	control	1.437586849	0.236766232	6.071756254	1.26519E-09	7.43899E-08	up_in_case
Yang2025_S	ENSGALG00010025580	RASSF7	case	control	-0.251459445	0.177531187	-1.416424065	0.156651382	0.301719006	down_in_case

Yang2025_S	ENSGALG00010025582	DMAP1	case	control	-0.069652106	0.070188165	-0.99236254	0.321020706	0.490388723	down_in_case
Yang2025_S	ENSGALG00010025585	SARNP	case	control	0.088452433	0.092182791	0.959533036	0.337290285	0.50545355	up_in_case
Yang2025_S	ENSGALG00010025586	TCTN1	case	control	-0.20876492	0.096689771	-2.159121054	0.030840777	0.093529102	down_in_case
Yang2025_S	ENSGALG00010025587	LRRC56	case	control	0.658194926	0.246692953	2.668073478	0.007628757	0.033014513	up_in_case
Yang2025_S	ENSGALG00010025588	ENSGALG00010025588	case	control	-0.183247948	0.289391517	-0.633218106	0.526591224	0.679616917	down_in_case
Yang2025_S	ENSGALG00010025590	HRAS	case	control	0.180316864	0.116817503	1.54357746	0.122690729	0.253632828	up_in_case
Yang2025_S	ENSGALG00010025591	OTOS	case	control	0.346501814	0.296362658	1.169181759	0.242330409	0.407289535	up_in_case
Yang2025_S	ENSGALG00010025592	HSPA8	case	control	0.136061745	0.188322781	0.722492226	0.469991903	0.630360183	up_in_case
Yang2025_S	ENSGALG00010025594	UPK1B	case	control	0.201463449	0.339042547	0.594212884	0.552369737	0.700452478	up_in_case
Yang2025_S	ENSGALG00010025595	ERI3	case	control	0.036801064	0.089845624	0.409603302	0.682096975	0.800894956	up_in_case
Yang2025_S	ENSGALG00010025597	SCN2A	case	control	2.53663704	0.923118781	2.747898854	0.005997851	0.027403296	up_in_case
Yang2025_S	ENSGALG00010025598	ENSGALG00010025598	case	control	1.108497784	0.455684916	2.432597053	0.014990973	0.055002426	up_in_case
Yang2025_S	ENSGALG00010025599	COPS9	case	control	0.411034348	0.197276157	2.083548026	0.037201299	0.107333736	up_in_case
Yang2025_S	ENSGALG00010025600	ENSGALG00010025600	case	control	0.007537268	0.07064309	0.106695055	0.915030906	0.950120376	up_in_case
Yang2025_S	ENSGALG00010025604	HVCN1	case	control	-0.436992403	0.204613773	-2.135693985	0.032704365	0.097734361	down_in_case
Yang2025_S	ENSGALG00010025606	CCDC12	case	control	-0.072823528	0.141006295	-0.516455861	0.60553606	0.743383402	down_in_case
Yang2025_S	ENSGALG00010025608	ENSGALG00010025608	case	control	-0.983671084	0.759661842	-1.294880208	0.195361557	0.351408713	down_in_case
Yang2025_S	ENSGALG00010025610	RNH1	case	control	-0.445765241	0.1997503	-2.231612371	0.025640593	0.081426998	down_in_case
Yang2025_S	ENSGALG00010025612	TMEM53	case	control	-0.246009454	0.203320868	-1.209956736	0.226295495	0.388368501	down_in_case
Yang2025_S	ENSGALG00010025613	CDKN1A	case	control	0.080594194	0.272867186	0.29536052	0.767718489	0.858737931	up_in_case
Yang2025_S	ENSGALG00010025618	ADIPOQ	case	control	-0.114456953	0.193556733	-0.591335426	0.554295698	0.702191868	down_in_case
Yang2025_S	ENSGALG00010025621	PPP1CC	case	control	-0.359838661	0.077918739	-4.61812734	3.87219E-06	7.51447E-05	down_in_case
Yang2025_S	ENSGALG00010025623	ENSGALG00010025623	case	control	0.535970612	0.433081395	1.23757478	0.215873771	0.376778682	up_in_case
Yang2025_S	ENSGALG00010025624	ARMH1	case	control	0.653058036	0.297041793	2.198539235	0.027910702	0.08668055	up_in_case
Yang2025_S	ENSGALG00010025628	ENSGALG00010025628	case	control	0.898581497	0.234378603	3.833888781	0.000126133	0.001282968	up_in_case
Yang2025_S	ENSGALG00010025629	ST6GAL1	case	control	-0.063066995	0.102795451	-0.613519322	0.539533011	0.690457572	down_in_case
Yang2025_S	ENSGALG00010025635	CHKA	case	control	0.072074862	0.147649753	0.48814753	0.625445347	0.75932923	up_in_case
Yang2025_S	ENSGALG00010025637	C6orf89	case	control	-0.382939532	0.082589769	-4.636646105	3.54108E-06	7.01858E-05	down_in_case
Yang2025_S	ENSGALG00010025639	MYL2	case	control	0.464372854	0.304569886	1.524684072	0.127337916	0.260183851	up_in_case
Yang2025_S	ENSGALG00010025640	SLC38A11	case	control	5.129717676	0.580674165	8.834072509	1.00932E-18	4.26209E-16	up_in_case
Yang2025_S	ENSGALG00010025642	RPS8	case	control	-0.353496273	0.178409504	-1.981375795	0.047549146	0.128822612	down_in_case
Yang2025_S	ENSGALG00010025644	ENSGALG00010025644	case	control	0.373818288	0.520039109	0.718827259	0.47224736	0.632158209	up_in_case
Yang2025_S	ENSGALG00010025645	NBEAL2	case	control	0.06610067	0.128308876	0.515168333	0.606435396	0.743766699	up_in_case
Yang2025_S	ENSGALG00010025647	JHY	case	control	0.241786665	0.513622157	0.470748121	0.637820616	0.768196222	up_in_case
Yang2025_S	ENSGALG00010025650	PPP1R1A	case	control	0.242300462	0.316270799	0.766117083	0.443606639	0.606818349	up_in_case
Yang2025_S	ENSGALG00010025652	KMT5B	case	control	0.197420521	0.137045666	1.440545521	0.149713121	0.292192205	up_in_case
Yang2025_S	ENSGALG00010025653	IGSF11	case	control	-0.112072392	0.127005103	-0.882424324	0.377547387	0.545929525	down_in_case
Yang2025_S	ENSGALG00010025658	PDE1B	case	control	0.902146139	0.373315249	2.41657993	0.015667086	0.056854386	up_in_case
Yang2025_S	ENSGALG00010025659	COBLL1	case	control	0.507613296	0.191973334	2.644186488	0.008188753	0.034832194	up_in_case
Yang2025_S	ENSGALG00010025660	KIF2C	case	control	0.020400607	0.135902453	0.150112133	0.880676148	0.929643369	up_in_case
Yang2025_S	ENSGALG00010025661	SNRPE	case	control	0.097797901	0.164086237	0.596015261	0.551165032	0.699242964	up_in_case
Yang2025_S	ENSGALG00010025666	CUX2	case	control	0.797208695	0.307237705	2.594761914	0.009465646	0.039013245	up_in_case
Yang2025_S	ENSGALG00010025674	GRB14	case	control	-0.86135116	0.404182807	-2.131093023	0.033081479	0.098418107	down_in_case
Yang2025_S	ENSGALG00010025679	ENSGALG00010025679	case	control	-5.359548723	2.233704115	-2.399399584	0.016421983	0.058784729	down_in_case
Yang2025_S	ENSGALG00010025685	NCKAP1L	case	control	-0.066504136	0.11786217	-0.564253447	0.572581659	0.715855501	down_in_case
Yang2025_S	ENSGALG00010025688	BEST4	case	control	0.062717386	0.481599582	0.130227243	0.896386641	0.938786882	up_in_case
Yang2025_S	ENSGALG00010025691	ENSGALG00010025691	case	control	0.091224695	0.288792904	0.315882743	0.752091511	0.84889315	up_in_case
Yang2025_S	ENSGALG00010025695	ENSGALG00010025695	case	control	-3.162775337	2.558292982	-1.236283475	0.216353212	0.377284696	down_in_case
Yang2025_S	ENSGALG00010025696	MTCH1	case	control	0.233800292	0.093900021	2.48988541	0.012778429	0.048585922	up_in_case
Yang2025_S	ENSGALG00010025701	ENSGALG00010025701	case	control	-0.913783514	2.565271759	-0.356213142	0.721680951	0.828660518	down_in_case
Yang2025_S	ENSGALG00010025702	GTSF2	case	control	1.408262468	0.26836344	5.247594341	1.54098E-07	4.94231E-06	up_in_case
Yang2025_S	ENSGALG00010025703	UBASH3B	case	control	-0.564342711	0.084783276	-6.656297563	2.80811E-11	2.7152E-09	down_in_case
Yang2025_S	ENSGALG00010025705	ACSL3	case	control	-0.085883419	0.087596719	-0.980441046	0.326868457	0.496633126	down_in_case
Yang2025_S	ENSGALG00010025706	ENSGALG00010025706	case	control	-1.673814041	2.617954054	-0.639359594			down_in_case
Yang2025_S	ENSGALG00010025708	HOXC8	case	control	1.140560239	0.277103821	4.116003286	3.85499E-05	0.000497862	up_in_case
Yang2025_S	ENSGALG00010025710	ENSGALG00010025710	case	control	-1.739891235	2.954272436	-0.588940686	0.555901064	0.703011282	down_in_case
Yang2025_S	ENSGALG00010025720	PHETA1	case	control	0.009451355	0.327274866	0.028878951	0.976961133	0.986518362	up_in_case
Yang2025_S	ENSGALG00010025723	SETD2	case	control	-0.020742306	0.136191468	-0.152302537	0.878948324	0.92862622	down_in_case
Yang2025_S	ENSGALG00010025727	GLI1	case	control	0.354428304	0.126731178	2.796693845	0.005162843	0.02455001	up_in_case
Yang2025_S	ENSGALG00010025734	PLK3	case	control	0.670053059	0.223803973	2.99392834	0.002754106	0.015253764	up_in_case
Yang2025_S	ENSGALG00010025736	KIF9	case	control	0.046874455	0.243944921	0.192151796	0.847623304	0.909738128	up_in_case
Yang2025_S	ENSGALG00010025737	ENSGALG00010025737	case	control	0.153498386	0.095375326	1.609414008	0.107525844	0.230508177	up_in_case
Yang2025_S	ENSGALG00010025738	SOX13	case	control	0.192474397	0.115769348	1.662567856	0.09639897	0.213802267	up_in_case
Yang2025_S	ENSGALG00010025743	SH2B3	case	control	0.058241338	0.093559839	0.622503617	0.533610766	0.685396444	up_in_case
Yang2025_S	ENSGALG00010025745	COX14	case	control	-0.246341548	0.358810289	-0.68655096	0.492365741	0.649911585	down_in_case
Yang2025_S	ENSGALG00010025748	ETNK2	case	control	-0.315766842	0.114349874	-2.761409618	0.005755244	0.026582476	down_in_case
Yang2025_S	ENSGALG00010025753	SORL1	case	control	-0.021822573	0.187057774	-0.116662207	0.90712774	0.944261228	down_in_case
Yang2025_S	ENSGALG00010025755	ENSGALG00010025755	case	control	1.046459436	0.509085416	2.055567498	0.039824223	0.113001538	up_in_case
Yang2025_S	ENSGALG00010025762	ENSGALG00010025762	case	control	-0.497946484	2.454380643	-0.202880709	0.839228273	0.904799934	down_in_case
Yang2025_S	ENSGALG00010025782	ENSGALG00010025782	case	control	-1.427300365	2.611373568	-0.546570733	0.584673681	0.726623122	down_in_case
Yang2025_S	ENSGALG00010025784	ENSGALG00010025784	case	control	-2.163007567	2.974797162	-0.727110942	0.467157996	0.628242297	down_in_case
Yang2025_S	ENSGALG00010025787	KCNE4	case	control	-0.280538821	0.107655706	-2.605889004	0.009163615	0.038061096	down_in_case
Yang2025_S	ENSGALG00010025790	LRP5	case	control	0.232910836	0.212620854	1.095427996	0.273329175	0.440634203	up_in_case
Yang2025_S	ENSGALG00010025791	FIGN	case	control	0.175322719	0.24139495	0.726289922	0.467661057	0.628604892	up_in_case
Yang2025_S	ENSGALG00010025799	SC5D	case	control	1.076772099	0.376023563	2.863576129	0.004188881	0.020832285	up_in_case
Yang2025_S	ENSGALG00010025800	ARL2BP	case	control	-0.187649247	0.242238162	-0.774647748	0.438547835	0.602441254	down_in_case
Yang2025_S	ENSGALG00010025801	PTPN7	case	control	0.42268664	0.51065165	0.827739693	0.407817935	0.573744868	up_in_case
Yang2025_S	ENSGALG00010025804	ENSGALG00010025804	case	control	-4.433631189	2.557557184	-1.733541372	0.082999491	0.192308052	down_in_case
Yang2025_S	ENSGALG00010025805	ATXN2	case	control	0.431408558	0.251079407	1.718215617	0.085757294	0.19684952	up_in_case
Yang2025_S	ENSGALG00010025806	TECTA	case	control	0.379661089	0.584085793	0.650009113	0.515686335	0.670281604	up_in_case
Yang2025_S	ENSGALG00010025809	ENSGALG00010025809	case	control	0.364345758	0.184953897	1.969927446	0.048846686	0.131076175	up_in_case
Yang2025_S	ENSGALG00010025822	KLF7	case	control	0.390826152	0.184562882	2.117577209	0.03421089	0.100894974	up_in_case
Yang2025_S	ENSGALG00010025829	ENSGALG00010025829	case	control	-0.141511596	0.123091565	-1.149644949	0.250290137	0.416005851	down_in_case
Yang2025_S	ENSGALG00010025830	TBCEL	case	control	0.305024919	0.214767576	1.420255912	0.155533191	0.300229259	up_in_case
Yang2025_S	ENSGALG00010025831	BRAP	case	control	-0.20810942	0.084244409	-2.470305433	0.013499773	0.050596918	down_in_case
Yang2025_S	ENSGALG00010025832	ENKD1	case	control	-0.577014259	0.265241555	-2.175429333	0.029597958	0.090568424	down_in_case
Yang2025_S	ENSGALG00010025835	PPP6R3	case	control	0.133731742	0.111757124	1.196628336	0.231451451	0.394480916	up_in_case
Yang2025_S	ENSGALG00010025836	ENSGALG00010025836	case	control	0.371666459	0.102730435	3.617880713	0.000297025	0.002585917	up_in_case
Yang2025_S	ENSGALG00010025837	ENSGALG00010025837	case	control	-0.231729591	0.255350201	-0.907497194	0.364143933	0.532963523	down_in_case
Yang2025_S	ENSGALG00010025846	GRIK4	case	control	-0.09116268	0.258227473	-0.353032462	0.724064104	0.830489928	down_in_case
Yang2025_S	ENSGALG00010025847	DEF6	case	control	0.662547408	0.168651705	3.928495156	8.54791E-05	0.000941621	up_in_case
Yang2025_S	ENSGALG00010025849	OBSCN	case	control	0.761750687	0.216202147	3.523326194	0.000426167	0.003478987	up_in_case
Yang2025_S	ENSGALG00010025852	BTBD19	case	control	-0.076840084	0.183386987	-0.419005106	0.675212401	0.795963523	down_in_case
Yang2025_S	ENSGALG00010025858	ENSGALG00010025858	case	control	-1.407650741	1.605112001	-0.876979762	0.38049763	0.548431369	down_in_case
Yang2025_S	ENSGALG00010025862	PTCH2	case	control	0.623408668	0.226378288	2.753835957	0.005890128	0.027008535	up_in_case
Yang2025_S	ENSGALG00010025865	LGR6	case	control	0.418061597	0.521895282	0.801044982	0.423105605	0.588714652	up_in_case
Yang2025_S	ENSGALG00010025869	FASTKD2	case	control	0.012502342	0.135370457	0.092356505	0.926414796	0.955608219	up_in_case
Yang2025_S	ENSGALG00010025870	ENSGALG00010025870	case	control	0.076743278	0.240601678	0.318964018	0.749753798	0.847830819	up_in_case
Yang2025_S	ENSGALG00010025874	MDH1B	case	control	-0.107995381	0.288124577	-0.374821829	0.707792979	0.819872311	down_in_case
Yang2025_S	ENSGALG00010025876	ENSGALG00010025876	case	control	-1.774531165	1.517625953	-1.169280982	0.242290443	0.407289535	down_in_case
Yang2025_S	ENSGALG00010025877	ENSGALG00010025877	case	control	-4.544623555	1.415234863	-3.211215094	0.00132175	0.008719402	down_in_case
Yang2025_S	ENSGALG00010025881	GAL	case	control	-0.455613729	0.76280345	-0.5972885	0.550314781	0.698673148	down_in_case
Yang2025_S	ENSGALG00010025882	LDHD	case	control	0.447861025	0.237133973	1.88864134	0.058939904	0.150765933	up_in_case
Yang2025_S	ENSGALG00010025886	ALDH2	case	control	-0.14022733	0.126837409	-1.105567602	0.268913719	0.436139743	down_in_case
Yang2025_S	ENSGALG00010025887	ZNF76	case	control	0.361630527	0.112073143	3.226736734	0.001252106	0.008340391	up_in_case
Yang2025_S	ENSGALG00010025890	SCG2	case	control	0.510684974	0.460212216	1.10967279	0.267140052	0.434223332	up_in_case
Yang2025_S	ENSGALG00010025891	NUP93	case	control	-0.33312531	0.116026798	-2.871106641	0.004090375	0.02050101	down_in_case
Yang2025_S	ENSGALG00010025892	ZC3H11A	case	control	0.476316363	0.078964058	6.032065402	1.61877E-09	9.28296E-08	up_in_case
Yang2025_S	ENSGALG00010025897	MAPKAPK5	case	control	0.055481521	0.092381402	0.600570245	0.548126261	0.69703773	up_in_case
Yang2025_S	ENSGALG00010025898	ENSGALG00010025898	case	control	-2.157593088	1.577798497	-1.367470619	0.17147784	0.320615015	down_in_case
Yang2025_S	ENSGALG00010025899	ENSGALG00010025899	case	control	0.147843601	0.193635816	0.763513713	0.445157087	0.60828241	up_in_case
Yang2025_S	ENSGALG00010025902	ENSGALG00010025902	case	control	-2.318257506	1.471842705	-1.575071506	0.11523994	0.242468453	down_in_case
Yang2025_S	ENSGALG00010025903	ENSGALG00010025903	case	control	0.832331856	0.856901575	0.971327256	0.33138534	0.500689007	up_in_case
Yang2025_S	ENSGALG00010025904	RPL10A	case	control	-0.257975519	0.25413587	-1.015108647	0.31005404	0.47981832	down_in_case
Yang2025_S	ENSGALG00010025905	CPT1A	case	control	-0.060930301	0.109886564	-0.55448363	0.579247906	0.722052024	down_in_case
Yang2025_S	ENSGALG00010025907	ARHGEF12	case	control	0.167537916	0.115273437	1.453395679	0.146113926	0.2875844	up_in_case
Yang2025_S	ENSGALG00010025910	HEATR3	case	control	-0.191886369	0.110102929	-1.742790774	0.081370169	0.189487516	down_in_case
Yang2025_S	ENSGALG00010025913	EIF2B3	case	control	-0.034879729	0.114426429	-0.304822315	0.760501485	0.854644209	down_in_case
Yang2025_S	ENSGALG00010025917	SCUBE3	case	control	0.461992475	0.466250202	0.990868149	0.321749967	0.491136574	up_in_case
Yang2025_S	ENSGALG00010025919	RGS9BP	case	control	0.737838132	0.387191894	1.90561358	0.056700359	0.146481183	up_in_case
Yang2025_S	ENSGALG00010025920	GTSF1	case	control	-0.153760425	0.468049539	-0.328513142	0.742523709	0.842447418	down_in_case
Yang2025_S	ENSGALG00010025924	TMEM116	case	control	0.061703796	0.105609185	0.58426543	0.559041747	0.705950663	up_in_case
Yang2025_S	ENSGALG00010025925	KLHL18	case	control	0.063326505	0.088329432	0.716935487	0.473413904	0.632929363	up_in_case
Yang2025_S	ENSGALG00010025926	ENSGALG00010025926	case	control	0.389430269	0.402122529	0.968436836	0.33282625	0.501995216	up_in_case
Yang2025_S	ENSGALG00010025927	ENSGALG00010025927	case	control	-0.727799191	2.098392105	-0.346836604	0.728714076	0.833097929	down_in_case
Yang2025_S	ENSGALG00010025929	NELFE	case	control	0.191124955	0.174486647	1.095355769	0.273360804	0.440634217	up_in_case

Yang2025_S	ENSGALG00010025931	TMEM136	case	control	0.046326812	0.169020298	0.274090227	0.784015284	0.86942965	up_in_case
Yang2025_S	ENSGALG00010025932	AP1S3	case	control	-0.009256052	0.398570212	-0.023223139	0.981472281	0.98867905	down_in_case
Yang2025_S	ENSGALG00010025933	SKIC2	case	control	0.547605383	0.158191549	3.461660151	0.000536855	0.004194694	up_in_case
Yang2025_S	ENSGALG00010025935	CATH3	case	control	0.227556249	0.325688195	0.698693574	0.484743551	0.642460458	up_in_case
Yang2025_S	ENSGALG00010025936	GOSR2	case	control	-0.274272405	0.067386296	-4.070151056	4.69827E-05	0.000579384	down_in_case
Yang2025_S	ENSGALG00010025938	ERP29	case	control	-0.567335563	0.140127971	-4.048696041	5.15038E-05	0.000622468	down_in_case
Yang2025_S	ENSGALG00010025940	HECTD3	case	control	-0.089713591	0.145928444	-0.614777961	0.538701366	0.68996356	down_in_case
Yang2025_S	ENSGALG00010025941	ENSGALG00010025941	case	control	1.641146827	0.727435948	2.256070561	0.024066212	0.077900737	up_in_case
Yang2025_S	ENSGALG00010025942	ENSGALG00010025942	case	control	2.165592763	1.916514512	1.129964187	0.258491315	0.424823267	up_in_case
Yang2025_S	ENSGALG00010025943	CATH1	case	control	0.589977175	0.440011651	1.340821711	0.179978345	0.332009299	up_in_case
Yang2025_S	ENSGALG00010025946	KRT5	case	control	-0.766602801	1.31032196	-0.58504919	0.558514642	0.705598663	down_in_case
Yang2025_S	ENSGALG00010025949	GPATCH1	case	control	0.324919416	0.097033132	3.348540949	0.000812383	0.00592389	up_in_case
Yang2025_S	ENSGALG00010025951	TBRG4	case	control	0.127125123	0.138699112	0.916553261	0.359376786	0.528174132	up_in_case
Yang2025_S	ENSGALG00010025954	TUBA1A	case	control	0.244382746	0.171622003	1.423959293	0.154458256	0.298733629	up_in_case
Yang2025_S	ENSGALG00010025957	ENSGALG00010025957	case	control	-0.207761577	0.096325631	-2.156867017	0.031016028	0.093937914	down_in_case
Yang2025_S	ENSGALG00010025958	HOXC9	case	control	0.020536299	0.442115332	0.046450095	0.96295151	0.977970213	up_in_case
Yang2025_S	ENSGALG00010025959	UROD	case	control	-0.435484819	0.226356345	-1.923890485	0.054368312	0.141865662	down_in_case
Yang2025_S	ENSGALG00010025960	CMKLR2	case	control	-0.432989553	0.103411559	-4.187051775	2.82601E-05	0.000387603	down_in_case
Yang2025_S	ENSGALG00010025962	ENSGALG00010025962	case	control	-0.134052611	0.201609106	-0.664913475	0.506105837	0.66157179	down_in_case
Yang2025_S	ENSGALG00010025963	OAF	case	control	-0.309507208	0.179015611	-1.728939763	0.083819877	0.19373277	down_in_case
Yang2025_S	ENSGALG00010025968	FADS2	case	control	-0.008054065	0.207794535	-0.038759752	0.969081934	0.981620757	down_in_case
Yang2025_S	ENSGALG00010025970	KLHL12	case	control	0.072334426	0.123226406	0.587004262	0.557200844	0.704335429	up_in_case
Yang2025_S	ENSGALG00010025971	TRAFD1	case	control	0.184571931	0.099770362	1.849967533	0.064318229	0.159820707	up_in_case
Yang2025_S	ENSGALG00010025974	EEF1B2	case	control	0.468243025	0.146819528	3.189242127	0.001426463	0.00925899	up_in_case
Yang2025_S	ENSGALG00010025976	NAA25	case	control	0.062921496	0.103375292	0.608670556	0.542742827	0.692728656	up_in_case
Yang2025_S	ENSGALG00010025980	HECTD4	case	control	0.045449494	0.14255564	0.318819329	0.74986352	0.847886088	up_in_case
Yang2025_S	ENSGALG00010025982	SERPINE2	case	control	-0.332878561	0.091942388	-3.620512455	0.00029402	0.002563936	down_in_case
Yang2025_S	ENSGALG00010025983	RNF26	case	control	-0.185168343	0.162576104	-1.138964084	0.254718135	0.421184398	down_in_case
Yang2025_S	ENSGALG00010025984	NDUFS1	case	control	0.183857505	0.083997841	2.188836063	0.028608758	0.088336591	up_in_case
Yang2025_S	ENSGALG00010025985	CHCHD10	case	control	0.522586469	0.319043684	1.637977791	0.101426327	0.221384463	up_in_case
Yang2025_S	ENSGALG00010025986	RAB3IL1	case	control	-0.415748342	0.14298855	-2.907563876	0.003642559	0.018844512	down_in_case
Yang2025_S	ENSGALG00010025988	NSF	case	control	-0.081171024	0.115953565	-0.700030427	0.483908302	0.641978329	down_in_case
Yang2025_S	ENSGALG00010025990	FAM124B	case	control	-0.011961175	0.25560266	-0.046795973	0.962675839	0.977894738	down_in_case
Yang2025_S	ENSGALG00010025992	MMP11	case	control	0.485732356	0.229127079	2.119925579	0.034012322	0.10047948	up_in_case
Yang2025_S	ENSGALG00010025994	INO80D	case	control	0.284930895	0.23077669	1.234660638	0.216956831	0.377817226	up_in_case
Yang2025_S	ENSGALG00010025996	CUL3	case	control	-0.165728349	0.087578094	-1.892349353	0.058444449	0.149737709	down_in_case
Yang2025_S	ENSGALG00010025997	CCDC43	case	control	0.48936421	0.115223285	4.247094773	2.1656E-05	0.00031047	up_in_case
Yang2025_S	ENSGALG00010025998	SMARCB1	case	control	-0.131194369	0.115455013	-1.136324577	0.255820748	0.422123196	down_in_case
Yang2025_S	ENSGALG00010025999	DBF4B	case	control	0.115582243	0.17600539	0.65669718	0.511375648	0.666606142	up_in_case
Yang2025_S	ENSGALG00010026001	ANKS1A	case	control	0.211309158	0.156151157	1.353234655	0.175980677	0.326667209	up_in_case
Yang2025_S	ENSGALG00010026002	FTH1	case	control	-0.464976326	0.253899711	-1.831338535	0.067050029	0.164816045	down_in_case
Yang2025_S	ENSGALG00010026003	NECTIN1	case	control	0.042019497	0.117014889	0.359095303	0.719523795	0.827001327	up_in_case
Yang2025_S	ENSGALG00010026005	DERL3	case	control	-0.080948883	0.180140667	-0.449364843	0.653168488	0.780408376	down_in_case
Yang2025_S	ENSGALG00010026006	ITGA2B	case	control	0.686023486	0.197305603	3.476958964	0.000507135	0.004002416	up_in_case
Yang2025_S	ENSGALG00010026008	NRP2	case	control	-0.002407705	0.132200081	-0.018212586	0.985469262	0.991302546	down_in_case
Yang2025_S	ENSGALG00010026009	ENSGALG00010026009	case	control	0.580589309	0.185179837	3.135272817	0.001716944	0.010671547	up_in_case
Yang2025_S	ENSGALG00010026010	ZSWIM5	case	control	0.286594836	0.089284618	3.209901562	0.001327804	0.008748607	up_in_case
Yang2025_S	ENSGALG00010026011	GJC2	case	control	-0.084957857	0.108735998	-0.781322274	0.434612973	0.598435775	down_in_case
Yang2025_S	ENSGALG00010026013	RABIF	case	control	0.605156999	0.151320393	3.999176758	6.35632E-05	0.000741837	up_in_case
Yang2025_S	ENSGALG00010026016	SLC2A11	case	control	0.561094784	0.399131835	1.405788099	0.159787057	0.305269076	up_in_case
Yang2025_S	ENSGALG00010026017	PLEKHM1	case	control	0.003099825	0.112511788	0.027551114	0.978020172	0.9869076	up_in_case
Yang2025_S	ENSGALG00010026023	THY1	case	control	0.45246947	0.304554423	1.485676893	0.137364605	0.275064776	up_in_case
Yang2025_S	ENSGALG00010026030	IBA57	case	control	-0.291305857	0.135241733	-2.153964243	0.031242976	0.094344438	down_in_case
Yang2025_S	ENSGALG00010026036	TUB5A	case	control	0.137890996	0.159368053	0.865236124	0.386909202	0.554308657	up_in_case
Yang2025_S	ENSGALG00010026040	GUK1	case	control	-0.232137756	0.149871185	-1.5489152	0.121402107	0.251824079	down_in_case
Yang2025_S	ENSGALG00010026044	CERKL	case	control	-0.643599006	0.272630302	-2.360702394	0.018240362	0.063307458	down_in_case
Yang2025_S	ENSGALG00010026051	TAF11	case	control	-0.3760323	0.21547386	-1.745141148	0.080960303	0.188953645	down_in_case
Yang2025_S	ENSGALG00010026059	ENSGALG00010026059	case	control	0.556700756	0.750193586	0.742076134	0.458041209	0.619208794	up_in_case
Yang2025_S	ENSGALG00010026061	MPEG1	case	control	-0.31260464	0.178559666	-1.750701303	0.079997375	0.187355197	down_in_case
Yang2025_S	ENSGALG00010026062	ITGA4	case	control	0.18429749	0.154881081	1.189928998	0.2340743	0.397299071	up_in_case
Yang2025_S	ENSGALG00010026064	ENSGALG00010026064	case	control	-0.295194292	0.257116237	-1.148096653	0.250928666	0.416666144	down_in_case
Yang2025_S	ENSGALG00010026066	SELENOP2	case	control	0.51988803	0.361643917	1.437568851	0.150556424	0.293386068	up_in_case
Yang2025_S	ENSGALG00010026068	BLTP3A	case	control	-0.109928847	0.141983212	-0.774238344	0.438789857	0.602595511	down_in_case
Yang2025_S	ENSGALG00010026073	HPDL	case	control	0.25874295	0.405696414	0.637774801	0.523620286	0.676931875	up_in_case
Yang2025_S	ENSGALG00010026076	C1orf35	case	control	0.464193405	0.169244703	2.742735199	0.00609298	0.027759119	up_in_case
Yang2025_S	ENSGALG00010026078	UBE2E3	case	control	-0.772454055	0.081394473	-9.490251919	2.30477E-21	1.45986E-18	down_in_case
Yang2025_S	ENSGALG00010026081	KPNA1	case	control	-0.188834908	0.153397902	-1.231013622	0.218317769	0.37933393	down_in_case
Yang2025_S	ENSGALG00010026083	MUTYH	case	control	0.166794984	0.320338261	0.520683928	0.602586972	0.740872548	up_in_case
Yang2025_S	ENSGALG00010026088	SNRPC	case	control	-0.47411022	0.191093092	-2.481043215	0.013099849	0.049483978	down_in_case
Yang2025_S	ENSGALG00010026090	USP2	case	control	-0.106493443	0.08630995	-1.233848972	0.217259187	0.378107502	down_in_case
Yang2025_S	ENSGALG00010026091	DTX4	case	control	-0.208093486	0.122014048	-1.705487938	0.08810346	0.200969343	down_in_case
Yang2025_S	ENSGALG00010026093	TOE1	case	control	-0.078136672	0.096602202	-0.808849807	0.418601543	0.584264877	down_in_case
Yang2025_S	ENSGALG00010026094	C6orf106	case	control	-0.467073666	0.108092267	-4.32106457	1.55278E-05	0.000236481	down_in_case
Yang2025_S	ENSGALG00010026095	MIF	case	control	0.339881821	0.236067119	1.439767737	0.149933122	0.292416802	up_in_case
Yang2025_S	ENSGALG00010026097	ARF1	case	control	-0.541059266	0.108584299	-4.982849903	6.26546E-07	1.61385E-05	down_in_case
Yang2025_S	ENSGALG00010026098	STAT3	case	control	-0.227611145	0.13273846	-1.714733953	0.086394015	0.197944855	down_in_case
Yang2025_S	ENSGALG00010026102	IPO9	case	control	-0.105386486	0.042764981	-2.46431738	0.01372745	0.051284722	down_in_case
Yang2025_S	ENSGALG00010026104	DDX51	case	control	0.325361856	0.105690273	3.078446542	0.002080829	0.012322165	up_in_case
Yang2025_S	ENSGALG00010026105	CNTF	case	control	0.30662267	0.394383392	0.777473584	0.436879413	0.600744257	up_in_case
Yang2025_S	ENSGALG00010026106	TESK2	case	control	-0.177144786	0.113935561	-1.554780472	0.119998361	0.249674543	down_in_case
Yang2025_S	ENSGALG00010026111	ENSGALG00010026111	case	control	-0.527313142	0.131158421	-4.020429169	5.80922E-05	0.000689536	down_in_case
Yang2025_S	ENSGALG00010026112	ZFP91	case	control	0.019934342	0.056693871	0.351613697	0.725127991	0.830906879	up_in_case
Yang2025_S	ENSGALG00010026113	MFRP	case	control	0.159439841	0.340334501	0.468479805	0.63944151	0.769350496	up_in_case
Yang2025_S	ENSGALG00010026114	CWC22	case	control	0.078609897	0.136980129	0.573878104	0.566050291	0.711190394	up_in_case
Yang2025_S	ENSGALG00010026115	MMACHC	case	control	-0.282682173	0.234525011	-1.20533913	0.22807241	0.390244348	down_in_case
Yang2025_S	ENSGALG00010026116	DLX3	case	control	0.549544146	0.55204107	0.995476924	0.319504362	0.488856637	up_in_case
Yang2025_S	ENSGALG00010026117	LZTR1	case	control	-0.146891218	0.08604056	-1.707232231	0.087778897	0.200360184	down_in_case
Yang2025_S	ENSGALG00010026120	C1QTNF5	case	control	0.04904722	0.269444387	0.18203096	0.855558429	0.914560196	up_in_case
Yang2025_S	ENSGALG00010026121	ENSGALG00010026121	case	control	0.147951845	0.265830114	0.556565404	0.577824397	0.720728873	up_in_case
Yang2025_S	ENSGALG00010026124	PACSIN1	case	control	0.646496252	0.387745449	1.667321316	0.09545053	0.212374763	up_in_case
Yang2025_S	ENSGALG00010026127	ENSGALG00010026127	case	control	-2.62603215	0.574635573	-4.569908778	4.87937E-06	9.02974E-05	down_in_case
Yang2025_S	ENSGALG00010026128	PRDX1	case	control	-0.722440372	0.239371584	-3.018070732	0.002543895	0.014381006	down_in_case
Yang2025_S	ENSGALG00010026132	DDTL	case	control	-0.840104647	0.228971646	-3.66903353	0.000243469	0.002194529	down_in_case
Yang2025_S	ENSGALG00010026137	LPXN	case	control	0.733729658	0.318979964	2.300237448	0.021434771	0.071185305	up_in_case
Yang2025_S	ENSGALG00010026143	RNPEP	case	control	-0.270124539	0.229451874	-1.177260112	0.239091702	0.403334597	down_in_case
Yang2025_S	ENSGALG00010026148	RPS10	case	control	0.270798125	0.1923	1.408206576	0.15906991	0.304493196	up_in_case
Yang2025_S	ENSGALG00010026149	LASP1	case	control	0.197512493	0.084590769	2.334917802	0.019547715	0.066671209	up_in_case
Yang2025_S	ENSGALG00010026154	ZNF385B	case	control	0.288854529	0.202730919	1.424817341	0.154210008	0.29846062	up_in_case
Yang2025_S	ENSGALG00010026162	MED1	case	control	-0.045804592	0.151849788	-0.301644095	0.76292339	0.855571981	down_in_case
Yang2025_S	ENSGALG00010026163	CTNND1	case	control	-0.144731211	0.062162458	-2.328273626	0.019897579	0.067545131	down_in_case
Yang2025_S	ENSGALG00010026166	WNT9A	case	control	-0.358484158	0.233757167	-1.533575047	0.125134231	0.257107512	down_in_case
Yang2025_S	ENSGALG00010026167	CABIN1	case	control	0.248609443	0.165891491	1.498626849	0.133970464	0.269905913	up_in_case
Yang2025_S	ENSGALG00010026173	CDK12	case	control	0.045253492	0.136535792	0.331440508	0.740311772	0.841043905	up_in_case
Yang2025_S	ENSGALG00010026183	SESTD1	case	control	0.318430219	0.113631653	2.802302086	0.005073934	0.024239036	up_in_case
Yang2025_S	ENSGALG00010026184	NUDT3	case	control	-0.400609624	0.135198734	-2.963116682	0.003045411	0.016520401	down_in_case
Yang2025_S	ENSGALG00010026192	SMIM29	case	control	0.10940849	0.248796052	0.439751714	0.660116944	0.785343602	up_in_case
Yang2025_S	ENSGALG00010026199	TBX6	case	control	0.590367462	0.486491627	1.213520294	0.22493096	0.386535076	up_in_case
Yang2025_S	ENSGALG00010026203	TMX2	case	control	0.062337081	0.18683882	0.333640949	0.738650514	0.840267568	up_in_case
Yang2025_S	ENSGALG00010026205	ENSGALG00010026205	case	control	-0.668863006	0.442938971	-1.510056803	0.131028931	0.26535215	down_in_case
Yang2025_S	ENSGALG00010026209	MED19	case	control	0.054657594	0.154297923	0.354234152	0.723163411	0.829884059	up_in_case
Yang2025_S	ENSGALG00010026213	AKR1A1	case	control	-0.23777552	0.111556601	-2.131433896	0.033053413	0.098375777	down_in_case
Yang2025_S	ENSGALG00010026218	FTSJ3	case	control	0.472975676	0.098881589	4.783253185	1.72481E-06	3.85179E-05	up_in_case
Yang2025_S	ENSGALG00010026220	ENSGALG00010026220	case	control	-0.328012195	0.140217237	-2.339314347	0.019319171	0.066096893	down_in_case
Yang2025_S	ENSGALG00010026221	CRKL	case	control	-0.259048336	0.124317984	-2.083755919	0.037182375	0.107323239	down_in_case
Yang2025_S	ENSGALG00010026229	CRHR2	case	control	-0.413561128	0.148146973	-2.791559755	0.005245467	0.0248371	down_in_case
Yang2025_S	ENSGALG00010026232	PSMC5	case	control	-0.103002235	0.233272459	-0.441553347	0.65881245	0.784658933	down_in_case
Yang2025_S	ENSGALG00010026234	ZDHHC5	case	control	0.083577367	0.106771625	0.78276758	0.433763609	0.597669682	up_in_case
Yang2025_S	ENSGALG00010026237	KLHL22	case	control	-0.164785257	0.092785428	-1.775982071	0.075735872	0.180068142	down_in_case
Yang2025_S	ENSGALG00010026242	CCDC141	case	control	0.655855239	0.254617993	2.575840115	0.009999687	0.040507454	up_in_case
Yang2025_S	ENSGALG00010026244	CLP1	case	control	-0.102708224	0.202269722	-0.507778539	0.611608662	0.748859213	down_in_case
Yang2025_S	ENSGALG00010026252	ENO2	case	control	1.07954975	0.480282742	2.247737961			up_in_case
Yang2025_S	ENSGALG00010026259	MED15	case	control	0.967430545	0.299769928	3.227243482	0.001249891	0.0083336	up_in_case
Yang2025_S	ENSGALG00010026261	IFT70A	case	control	-0.139679535	0.113073683	-1.235296592	0.216720142	0.377499397	down_in_case
Yang2025_S	ENSGALG00010026262	NAV1	case	control	0.077825414	0.139405198	0.558267661	0.576661625	0.719471729	up_in_case
Yang2025_S	ENSGALG00010026264	FZD7	case	control	-0.180860445	0.187474821	-0.964718588	0.334685782	0.503363766	down_in_case
Yang2025_S	ENSGALG00010026266	ENSGALG00010026266	case	control	1.77188327	0.230036444	7.702619794	1.33305E-14	2.73176E-12	up_in_case

Yang2025_S	ENSGALG00010026268	GARS1	case	control	-0.605696855	0.141336403	-4.285497879	1.8233E-05	0.000269721	down_in_case
Yang2025_S	ENSGALG00010026272	LRRC58	case	control	-0.21302499	0.097267186	-2.190101292	0.028516892	0.088072448	down_in_case
Yang2025_S	ENSGALG00010026277	SMARCD2	case	control	-0.103190327	0.081209763	-1.27066405	0.20384819	0.3619091	down_in_case
Yang2025_S	ENSGALG00010026287	NASP	case	control	0.546041999	0.140609898	3.883382385	0.000103013	0.001093291	up_in_case
Yang2025_S	ENSGALG00010026291	SERPING1	case	control	0.17026772	0.118943493	1.431500916	0.152286705	0.295847655	up_in_case
Yang2025_S	ENSGALG00010026295	SMPD4	case	control	0.00243861	0.103186365	0.023633068	0.981145295	0.988594337	up_in_case
Yang2025_S	ENSGALG00010026298	SNF8	case	control	0.277008892	0.155733558	1.77873604	0.075283048	0.17932808	up_in_case
Yang2025_S	ENSGALG00010026300	HMGA1	case	control	0.091547727	0.109538762	0.835756451	0.40329193	0.569677957	up_in_case
Yang2025_S	ENSGALG00010026305	ATP5MC1	case	control	-0.484057394	0.416491385	-1.162226666	0.245143406	0.41058574	down_in_case
Yang2025_S	ENSGALG00010026315	SYT2	case	control	0.348119389	0.399868397	0.870584902	0.383980843	0.551739848	up_in_case
Yang2025_S	ENSGALG00010026316	ENSGALG00010026316	case	control	-0.320633204	0.242510168	-1.32214334	0.186120424	0.339741637	down_in_case
Yang2025_S	ENSGALG00010026320	ENSGALG00010026320	case	control	-0.049336654	0.161464285	-0.305557693	0.759941437	0.854396292	down_in_case
Yang2025_S	ENSGALG00010026321	GPBP1L1	case	control	-0.214438175	0.065419601	-3.277888763	0.001045866	0.00726255	down_in_case
Yang2025_S	ENSGALG00010026325	TMEM69	case	control	-0.18034043	0.121428112	-1.485162104	0.137500888	0.275179502	down_in_case
Yang2025_S	ENSGALG00010026329	ENSGALG00010026329	case	control	-0.091832297	0.249692922	-0.367780935	0.713036586	0.822771185	down_in_case
Yang2025_S	ENSGALG00010026334	ENSGALG00010026334	case	control	-0.191268578	0.114771334	-1.666518731	0.095610141	0.212583268	down_in_case
Yang2025_S	ENSGALG00010026335	IPP	case	control	0.053639966	0.099263747	0.540378217	0.588936227	0.730011217	up_in_case
Yang2025_S	ENSGALG00010026344	TIMM10	case	control	-0.035680271	0.290141969	-0.122975216	0.902126724	0.941376059	down_in_case
Yang2025_S	ENSGALG00010026346	FZD2	case	control	-0.114090371	0.157151338	-0.725990456	0.467844621	0.628604892	down_in_case
Yang2025_S	ENSGALG00010026354	ENSGALG00010026354	case	control	-0.165775442	0.178218135	-0.930182787	0.352276452	0.521067017	down_in_case
Yang2025_S	ENSGALG00010026355	PPP1R12B	case	control	0.029023489	0.071889521	0.403723505	0.686416053	0.804089064	up_in_case
Yang2025_S	ENSGALG00010026360	CTDSPL	case	control	0.040252562	0.104660283	0.384602079	0.700532254	0.814987223	up_in_case
Yang2025_S	ENSGALG00010026362	ENSGALG00010026362	case	control	-0.213235896	0.320891881	-0.664510101	0.506363886	0.661806486	down_in_case
Yang2025_S	ENSGALG00010026367	RBP	case	control	-0.433495696	0.602591594	-0.719385568	0.471903386	0.631861675	down_in_case
Yang2025_S	ENSGALG00010026377	ENSGALG00010026377	case	control	0.142776148	0.24343544	0.586505184	0.557536079	0.704567449	up_in_case
Yang2025_S	ENSGALG00010026388	SNRPD3	case	control	0.067731952	0.11605628	0.583612989	0.559480719	0.706203234	up_in_case
Yang2025_S	ENSGALG00010026393	SLC43A3	case	control	0.182827058	0.173936318	1.051114916	0.293205814	0.461705274	up_in_case
Yang2025_S	ENSGALG00010026402	TFEB	case	control	0.173408507	0.112904842	1.53588194	0.124567338	0.256137798	up_in_case
Yang2025_S	ENSGALG00010026403	GUCD1	case	control	-0.319486404	0.096888342	-3.297470029	0.000975601	0.006873103	down_in_case
Yang2025_S	ENSGALG00010026409	VILL	case	control	-0.309776474	0.181128091	-1.710261901	0.087217454	0.199372575	down_in_case
Yang2025_S	ENSGALG00010026410	ENSGALG00010026410	case	control	2.338471918	0.817195019	2.861583665			up_in_case
Yang2025_S	ENSGALG00010026422	P2RX3	case	control	0.82624092	0.446692067	1.849687918	0.064358543	0.159892368	up_in_case
Yang2025_S	ENSGALG00010026427	PGC	case	control	0.145522155	0.428804776	0.339366917	0.734333338	0.837882547	up_in_case
Yang2025_S	ENSGALG00010026433	VPS11	case	control	0.179762316	0.078662412	2.285237796	0.022298888	0.073234741	up_in_case
Yang2025_S	ENSGALG00010026434	ENSGALG00010026434	case	control	0.128516407	0.203990945	0.630010348	0.528687814	0.681334013	up_in_case
Yang2025_S	ENSGALG00010026436	PLCD1	case	control	-0.476472431	0.074843273	-6.366269306	1.93681E-10	1.43561E-08	down_in_case
Yang2025_S	ENSGALG00010026438	TOMM6	case	control	-0.727455513	0.316144183	-2.301024508	0.021390246	0.071105219	down_in_case
Yang2025_S	ENSGALG00010026443	UPB1	case	control	0.462123154	0.35088135	1.317035385	0.187826764	0.34218407	up_in_case
Yang2025_S	ENSGALG00010026448	ADORA2A	case	control	-0.182575596	0.116558667	-1.566383692	0.117258819	0.245545061	down_in_case
Yang2025_S	ENSGALG00010026457	SSRP1	case	control	0.187044925	0.055894225	3.346408751	0.000818656	0.005947849	up_in_case
Yang2025_S	ENSGALG00010026458	DLEC1	case	control	-0.065992592	0.187004176	-0.352893684	0.724168146	0.830489928	down_in_case
Yang2025_S	ENSGALG00010026480	USP49	case	control	0.413651283	0.197954073	2.089632594	0.036650816	0.106254987	up_in_case
Yang2025_S	ENSGALG00010026481	DIXDC1	case	control	-0.278993185	0.093690378	-2.977821104	0.002903054	0.015895503	down_in_case
Yang2025_S	ENSGALG00010026484	TNKS1BP1	case	control	0.183024717	0.125625276	1.456909968	0.145141234	0.286308881	up_in_case
Yang2025_S	ENSGALG00010026485	ACAA1	case	control	-0.436689059	0.108906766	-4.00975142	6.07827E-05	0.00071644	down_in_case
Yang2025_S	ENSGALG00010026492	SPECC1L	case	control	0.125773474	0.19892301	0.632272126	0.527209074	0.680120204	up_in_case
Yang2025_S	ENSGALG00010026498	ENSGALG00010026498	case	control	-0.159021568	0.184650753	-0.861201839	0.389126887	0.556095085	down_in_case
Yang2025_S	ENSGALG00010026507	APLNR	case	control	0.201263231	0.158052125	1.273397817	0.202876912	0.360782257	up_in_case
Yang2025_S	ENSGALG00010026508	CSRP1	case	control	0.316780292	0.203746163	1.554779176	0.11999867	0.249674543	up_in_case
Yang2025_S	ENSGALG00010026516	ENSGALG00010026516	case	control	-0.133445625	0.096516914	-1.382613885	0.166783289	0.314542326	down_in_case
Yang2025_S	ENSGALG00010026522	MED20	case	control	-0.382470095	0.23690708	-1.614430836	0.106434008	0.229052593	down_in_case
Yang2025_S	ENSGALG00010026523	LRRC55	case	control	0.254234964	0.448449873	0.566919469	0.570768896	0.715003907	up_in_case
Yang2025_S	ENSGALG00010026536	BCR	case	control	-0.122519455	0.158908389	-0.771006845	0.440702874	0.604091536	down_in_case
Yang2025_S	ENSGALG00010026537	FJX1	case	control	0.443207359	0.353839494	1.252566111	0.210363655	0.369988329	up_in_case
Yang2025_S	ENSGALG00010026550	MYD88	case	control	-0.728202308	0.173413953	-4.199214051	2.67843E-05	0.000370277	down_in_case
Yang2025_S	ENSGALG00010026553	PLPP3	case	control	-0.146313759	0.103282897	-1.416631051	0.156590825	0.301644062	down_in_case
Yang2025_S	ENSGALG00010026559	RAB36	case	control	0.293954758	0.429531396	0.684361519	0.493746909	0.651302838	up_in_case
Yang2025_S	ENSGALG00010026560	TNNI1	case	control	0.011763499	0.268532929	0.043806542	0.965058612	0.979253751	up_in_case
Yang2025_S	ENSGALG00010026565	RSPH14	case	control	-0.113697789	0.391765962	-0.290218652	0.771648968	0.860923008	down_in_case
Yang2025_S	ENSGALG00010026566	ENSGALG00010026566	case	control	0.25226671	0.182959005	1.378815491	0.167951649	0.316176124	up_in_case
Yang2025_S	ENSGALG00010026568	BYSL	case	control	0.104828705	0.230873384	0.454052792	0.64979083	0.777706366	up_in_case
Yang2025_S	ENSGALG00010026569	GNAZ	case	control	-0.107223358	0.174541626	-0.614313962	0.539007878	0.690248383	down_in_case
Yang2025_S	ENSGALG00010026581	DNER	case	control	0.155930062	0.277374904	0.562163555	0.57400459	0.716798455	up_in_case
Yang2025_S	ENSGALG00010026584	ENSGALG00010026584	case	control	-0.155188297	0.099358684	-1.56189968	0.118311624	0.246955135	down_in_case
Yang2025_S	ENSGALG00010026588	CCND3	case	control	-0.089235625	0.142597745	-0.625785664	0.531455536	0.6838889	down_in_case
Yang2025_S	ENSGALG00010026595	PRKAA2	case	control	-0.322944697	0.142274069	-2.26987742	0.023215021	0.075725965	down_in_case
Yang2025_S	ENSGALG00010026612	MFF	case	control	-0.134626859	0.100838932	-1.335068276	0.181854001	0.334495183	down_in_case
Yang2025_S	ENSGALG00010026625	ENSGALG00010026625	case	control	0.348973363	0.732092394	0.4766794	0.633590434	0.764886312	up_in_case
Yang2025_S	ENSGALG00010026630	XIRP1	case	control	0.350562561	0.197125689	1.778370754	0.075342983	0.179440175	up_in_case
Yang2025_S	ENSGALG00010026633	SLC5A1	case	control	0.176042981	0.359828558	0.489241269	0.624670895	0.75873804	up_in_case
Yang2025_S	ENSGALG00010026638	LAD1	case	control	0.554480952	0.330543911	1.677480462	0.093448567	0.209021795	up_in_case
Yang2025_S	ENSGALG00010026643	YWHAH	case	control	-0.061530589	0.114671056	-0.536583436	0.591555388	0.732154218	down_in_case
Yang2025_S	ENSGALG00010026659	LIPH	case	control	1.023856787	0.225712045	4.536119409	5.72987E-06	0.00010451	up_in_case
Yang2025_S	ENSGALG00010026666	ENSGALG00010026666	case	control	0.780344077	0.311786977	2.502811644	0.01232111	0.047337928	up_in_case
Yang2025_S	ENSGALG00010026671	TAF8	case	control	0.015317101	0.109846757	0.13944063	0.889101968	0.935278623	up_in_case
Yang2025_S	ENSGALG00010026673	DEPDC5	case	control	0.096909604	0.076296687	1.27016793	0.204024819	0.362134041	up_in_case
Yang2025_S	ENSGALG00010026681	PLXDC1	case	control	-0.207102058	0.148460948	-1.394993507	0.163017797	0.309573863	down_in_case
Yang2025_S	ENSGALG00010026686	ENSGALG00010026686	case	control	1.631366081	0.366703514	4.448733157	8.63782E-06	0.000145022	up_in_case
Yang2025_S	ENSGALG00010026690	KRT14	case	control	0.123424875	0.366869228	0.336427439	0.736548561	0.8392761	up_in_case
Yang2025_S	ENSGALG00010026692	NMNAT3	case	control	0.682268115	0.447653962	1.524097124	0.127484451	0.260330572	up_in_case
Yang2025_S	ENSGALG00010026696	CSRNP1	case	control	-0.284669202	0.092271336	-3.085131463	0.002034622	0.0121268	down_in_case
Yang2025_S	ENSGALG00010026697	CHN1	case	control	-0.379651829	0.089769763	-4.229172664	2.34552E-05	0.000332163	down_in_case
Yang2025_S	ENSGALG00010026700	C8B	case	control	-1.0974124	0.665099001	-1.649998568	0.098943229	0.217434006	down_in_case
Yang2025_S	ENSGALG00010026704	WDFY1	case	control	-0.211204887	0.081971229	-2.576573382	0.009978502	0.040466052	down_in_case
Yang2025_S	ENSGALG00010026706	KRT24	case	control	-0.45484432	0.492287165	-0.923941049	0.355517009	0.524801856	down_in_case
Yang2025_S	ENSGALG00010026707	GORASP1	case	control	-0.269263597	0.082835536	-3.250580723	0.001151696	0.007828722	down_in_case
Yang2025_S	ENSGALG00010026715	WDR48	case	control	-0.304929657	0.116159778	-2.625088164	0.008662653	0.036502593	down_in_case
Yang2025_S	ENSGALG00010026724	OLA1	case	control	0.160116913	0.138307116	1.157691068	0.24699013	0.412678801	up_in_case
Yang2025_S	ENSGALG00010026731	ENSGALG00010026731	case	control	0.667021215	0.898570704	0.742313556	0.45789738	0.619194565	up_in_case
Yang2025_S	ENSGALG00010026740	DAB1	case	control	0.266469626	0.194384478	1.370837983	0.170425468	0.319375859	up_in_case
Yang2025_S	ENSGALG00010026743	TNNT2	case	control	1.390531299	0.244058398	5.697535132	1.21552E-08	5.49944E-07	up_in_case
Yang2025_S	ENSGALG00010026746	HOXD10	case	control	0.94534564	0.45192021	2.091841921	0.036452659	0.105760525	up_in_case
Yang2025_S	ENSGALG00010026755	ENSGALG00010026755	case	control	-0.818574815	0.340100261	-2.406863237	0.016090194	0.057907246	down_in_case
Yang2025_S	ENSGALG00010026759	OMA1	case	control	0.035713327	0.098794527	0.361490947	0.717732472	0.826161185	up_in_case
Yang2025_S	ENSGALG00010026770	ENSGALG00010026770	case	control	1.078008686	0.682943014	1.578475312	0.114456455	0.24137236	up_in_case
Yang2025_S	ENSGALG00010026775	BTG2	case	control	-0.497497579	0.148334033	-3.353900443	0.00079681	0.005831697	down_in_case
Yang2025_S	ENSGALG00010026780	MYSM1	case	control	0.172368061	0.100519508	1.714772219	0.086386997	0.197944855	up_in_case
Yang2025_S	ENSGALG00010026782	HOXB9	case	control	2.027577981	0.585040602	3.465704732	0.000528844	0.004143257	up_in_case
Yang2025_S	ENSGALG00010026786	ENSGALG00010026786	case	control	0.8390543	0.566585011	1.480897452	0.138633902	0.276736157	up_in_case
Yang2025_S	ENSGALG00010026796	DCAKD	case	control	0.084533912	0.253645509	0.333275809	0.738926098	0.840291756	up_in_case
Yang2025_S	ENSGALG00010026801	FMOD	case	control	1.564260869	0.348791782	4.484798517	7.29829E-06	0.000127446	up_in_case
Yang2025_S	ENSGALG00010026822	PRELP	case	control	-0.307188639	0.258566098	-1.188046851	0.234814952	0.398028421	down_in_case
Yang2025_S	ENSGALG00010026825	ADAM23	case	control	0.268705478	0.139322803	1.928653971	0.053773834	0.140747253	up_in_case
Yang2025_S	ENSGALG00010026827	FAM117A	case	control	-0.249005115	0.092760944	-2.68437454	0.007266565	0.031782669	down_in_case
Yang2025_S	ENSGALG00010026834	ENSGALG00010026834	case	control	-3.550267793	1.892397334	-1.876068904	0.060645819	0.15362652	down_in_case
Yang2025_S	ENSGALG00010026839	FGGY	case	control	-0.340980497	0.168617872	-2.022208514	0.043154815	0.11972175	down_in_case
Yang2025_S	ENSGALG00010026845	ENSGALG00010026845	case	control	-2.038904702	2.655350076	-0.767847796	0.442577615	0.605892454	down_in_case
Yang2025_S	ENSGALG00010026846	MICAL1	case	control	0.052825649	0.221272401	0.238735823	0.811310437	0.886368766	up_in_case
Yang2025_S	ENSGALG00010026851	ENSGALG00010026851	case	control	-4.035832948	2.326264638	-1.734898464	0.082758791	0.191855558	down_in_case
Yang2025_S	ENSGALG00010026854	SMPD2	case	control	0.450427211	0.145464849	3.09646772	0.001958412	0.011793634	up_in_case
Yang2025_S	ENSGALG00010026856	UBE2Z	case	control	-0.56445374	0.155008354	-3.641440771	0.000271117	0.002394175	down_in_case
Yang2025_S	ENSGALG00010026874	TEAD3	case	control	0.096893031	0.097576672	0.992993807	0.320712972	0.49014425	up_in_case
Yang2025_S	ENSGALG00010026885	TULP1	case	control	0.79119778	0.471905627	1.676601707	0.093620393	0.209271764	up_in_case
Yang2025_S	ENSGALG00010026886	JUN	case	control	-0.465236651	0.106932605	-4.350746423	1.35675E-05	0.000212909	down_in_case
Yang2025_S	ENSGALG00010026889	ENSGALG00010026889	case	control	-0.162145619	0.27179854	-0.596565451	0.550797543	0.699094978	down_in_case
Yang2025_S	ENSGALG00010026893	FKBP5	case	control	0.793079551	0.182285497	4.350755065	1.3567E-05	0.000212909	up_in_case
Yang2025_S	ENSGALG00010026894	IP6K1	case	control	-0.141985023	0.157179425	-0.903330842	0.366350332	0.535122787	down_in_case
Yang2025_S	ENSGALG00010026897	ENSGALG00010026897	case	control	0.769446544	0.247574774	3.107935964	0.001883989	0.011466028	up_in_case
Yang2025_S	ENSGALG00010026898	PNPLA2	case	control	-0.411575747	0.131421504	-3.131723016	0.001737837	0.010786978	down_in_case
Yang2025_S	ENSGALG00010026899	HOOK1	case	control	0.548200318	0.190758484	2.873792594	0.004055752	0.020351784	up_in_case
Yang2025_S	ENSGALG00010026901	FAM212A	case	control	0.110023218	0.199363778	0.551871655	0.58103629	0.723698668	up_in_case
Yang2025_S	ENSGALG00010026907	ENSGALG00010026907	case	control	0.513371207	0.23273493	2.205819331	0.027396652	0.08548418	up_in_case
Yang2025_S	ENSGALG00010026909	ENSGALG00010026909	case	control	-0.325691376	0.110797357	-2.939522971	0.003287179	0.017461665	down_in_case
Yang2025_S	ENSGALG00010026916	ENSGALG00010026916	case	control	0.912968725	0.323794161	2.819596014	0.004808414	0.023298072	up_in_case
Yang2025_S	ENSGALG00010026917	AMT	case	control	0.222107211	0.190332975	1.166940256	0.243234497	0.408271828	up_in_case
Yang2025_S	ENSGALG00010026919	NICN1	case	control	0.398888599	0.23820264	1.674576737	0.094017307	0.209788818	up_in_case

Yang2025_S	ENSGALG00010026920	GATA2	case	control	0.552524552	0.248738834	2.221303936	0.026330382	0.083162711	up_in_case
Yang2025_S	ENSGALG00010026924	TPH1	case	control	-0.330099349	0.251964288	-1.310103709	0.190160754	0.345037774	down_in_case
Yang2025_S	ENSGALG00010026942	CDK18	case	control	0.197860953	0.183900129	1.07591525	0.281965161	0.449975323	up_in_case
Yang2025_S	ENSGALG00010026943	EEFSEC	case	control	-0.0870329	0.095840891	-0.908097775	0.363826566	0.532666862	down_in_case
Yang2025_S	ENSGALG00010026950	LHFPL5	case	control	0.588697608	0.3101875	1.897876635	0.057712335	0.148317288	up_in_case
Yang2025_S	ENSGALG00010026951	RUVBL1	case	control	-0.044325213	0.073621082	-0.602072287	0.547126021	0.695956285	down_in_case
Yang2025_S	ENSGALG00010026954	MFSD4A	case	control	-0.446084023	0.089221889	-4.999715091	5.74151E-07	1.49828E-05	down_in_case
Yang2025_S	ENSGALG00010026958	CLTCL1	case	control	0.108187651	0.115253551	0.938692556	0.347888616	0.516385585	up_in_case
Yang2025_S	ENSGALG00010026960	SEC61A1	case	control	-0.512924156	0.094562024	-5.424208752	5.82118E-08	2.11245E-06	down_in_case
Yang2025_S	ENSGALG00010026964	SRPK1	case	control	-0.022811171	0.06329302	-0.360405795	0.718543693	0.826485326	down_in_case
Yang2025_S	ENSGALG00010026972	TNNT3	case	control	0.533027259	0.146851281	3.629707923	0.000283742	0.00249303	up_in_case
Yang2025_S	ENSGALG00010026973	MGLL	case	control	-0.282318911	0.14842196	-1.902137062	0.057153238	0.14726893	down_in_case
Yang2025_S	ENSGALG00010026977	ELK4	case	control	-0.092944805	0.220655011	-0.421222269	0.673592784	0.794726564	down_in_case
Yang2025_S	ENSGALG00010026980	LIMK2	case	control	0.038506613	0.086209609	0.446662655	0.655118649	0.781986044	up_in_case
Yang2025_S	ENSGALG00010026982	ABTB1	case	control	-0.579915016	0.13196655	-4.394409169	1.11074E-05	0.000180399	down_in_case
Yang2025_S	ENSGALG00010026999	MLEC	case	control	-0.224274667	0.141059627	-1.58992811	0.111851011	0.237308744	down_in_case
Yang2025_S	ENSGALG00010027001	SLC26A8	case	control	0.255030024	0.200785402	1.270162185	0.204026865	0.362134041	up_in_case
Yang2025_S	ENSGALG00010027003	SEPTIN2L	case	control	0.074195494	0.0827775	0.896324412	0.370079525	0.538652411	up_in_case
Yang2025_S	ENSGALG00010027012	RILPL1	case	control	0.412318426	0.183472727	2.247300904	0.024620803	0.079126128	up_in_case
Yang2025_S	ENSGALG00010027015	ENSGALG00010027015	case	control	0.090391807	0.392894584	0.230066309	0.818040244	0.891343405	up_in_case
Yang2025_S	ENSGALG00010027025	ORAI1	case	control	0.199427698	0.21779992	0.915646332	0.359852422	0.528458584	up_in_case
Yang2025_S	ENSGALG00010027026	SLC45A3	case	control	-0.514023465	0.294448548	-1.745715738	0.080860359	0.188805144	down_in_case
Yang2025_S	ENSGALG00010027027	ARHGEF3	case	control	-0.06920816	0.111753652	-0.619292151	0.535723914	0.686899591	down_in_case
Yang2025_S	ENSGALG00010027036	NUCKS1	case	control	0.046979744	0.142063564	0.330695235	0.740874702	0.841450817	up_in_case
Yang2025_S	ENSGALG00010027040	CLIP1	case	control	0.765774909	0.163366272	4.687472508	2.766E-06	5.67661E-05	up_in_case
Yang2025_S	ENSGALG00010027041	MAPK14	case	control	-0.38431907	0.094303087	-4.075360455	4.59431E-05	0.000570234	down_in_case
Yang2025_S	ENSGALG00010027043	IL17RD	case	control	-0.314717759	0.102586915	-3.067815798	0.002156295	0.01266244	down_in_case
Yang2025_S	ENSGALG00010027045	MAPK13	case	control	0.29356011	0.190177297	1.5436128	0.122682162	0.253632828	up_in_case
Yang2025_S	ENSGALG00010027046	HESX1	case	control	0.539259212	0.29285151	1.841408333	0.065561743	0.162072535	up_in_case
Yang2025_S	ENSGALG00010027050	GSTT1	case	control	-0.229897618	0.123198273	-1.86607825	0.062030418	0.156182419	down_in_case
Yang2025_S	ENSGALG00010027052	APPL1	case	control	0.090554907	0.106964139	0.846591277	0.397222978	0.564193476	up_in_case
Yang2025_S	ENSGALG00010027059	ASB14	case	control	-0.184047351	0.140669644	-1.308365793	0.190749269	0.345790434	down_in_case
Yang2025_S	ENSGALG00010027063	RAB7L1	case	control	-0.324537438	0.108253661	-2.997934974	0.002718157	0.015096657	down_in_case
Yang2025_S	ENSGALG00010027073	DNAH12	case	control	0.084646214	0.341407027	0.247933426	0.804185917	0.882249311	up_in_case
Yang2025_S	ENSGALG00010027074	BRPF3	case	control	0.055208525	0.064337556	0.858107271	0.390833233	0.55819013	up_in_case
Yang2025_S	ENSGALG00010027075	SLC41A1	case	control	-0.066349243	0.065915096	-1.006586458	0.314133545	0.483549513	down_in_case
Yang2025_S	ENSGALG00010027076	VDHAP	case	control	0.073495869	0.243202339	0.302200503	0.762499221	0.855408866	up_in_case
Yang2025_S	ENSGALG00010027077	ARF4	case	control	-0.237405096	0.074672282	-3.179293417	0.001476346	0.009511943	down_in_case
Yang2025_S	ENSGALG00010027078	PDE12	case	control	-0.309446835	0.191970074	-1.611953513	0.106972056	0.229772184	down_in_case
Yang2025_S	ENSGALG00010027079	PM20D1	case	control	-0.23304025	0.144056103	-1.617704798	0.105726232	0.228134877	down_in_case
Yang2025_S	ENSGALG00010027080	TBC1D22B	case	control	0.054383377	0.100644211	0.540352759	0.58895378	0.730011217	up_in_case
Yang2025_S	ENSGALG00010027081	PDE4B	case	control	0.380349815	0.194903934	1.951473257	0.051000774	0.135180018	up_in_case
Yang2025_S	ENSGALG00010027082	SLC26A9	case	control	0.219789786	0.468211701	0.46942395	0.638766632	0.768936854	up_in_case
Yang2025_S	ENSGALG00010027083	DENND6A	case	control	-0.087242789	0.076860774	-1.135075594	0.256343646	0.422589461	down_in_case
Yang2025_S	ENSGALG00010027084	OSBPL9	case	control	-0.110314273	0.069205664	-1.594006441	0.110934592	0.235825828	down_in_case
Yang2025_S	ENSGALG00010027085	PIM1	case	control	-0.260080872	0.207091141	-1.255876379	0.209160794	0.368384184	down_in_case
Yang2025_S	ENSGALG00010027086	CACNA1S	case	control	-0.170445558	0.138695335	-1.228920627	0.219101567	0.380127034	down_in_case
Yang2025_S	ENSGALG00010027087	RAB7B	case	control	0.102602756	0.296497018	0.346049875	0.729305232	0.833568603	up_in_case
Yang2025_S	ENSGALG00010027088	AGBL4	case	control	0.335207423	0.302303627	1.108843535	0.267497686	0.434500554	up_in_case
Yang2025_S	ENSGALG00010027092	ENSGALG00010027092	case	control	-0.311535399	0.117447029	-2.652560927	0.007988371	0.034219624	down_in_case
Yang2025_S	ENSGALG00010027093	SLMAP	case	control	0.447075933	0.128296859	3.484698972	0.000492691	0.003905375	up_in_case
Yang2025_S	ENSGALG00010027095	SCN5A	case	control	0.412173066	0.15544004	2.651653115	0.008009879	0.034259737	up_in_case
Yang2025_S	ENSGALG00010027096	YIPF1	case	control	-0.042365977	0.155357863	-0.272699278	0.78508439	0.87009674	down_in_case
Yang2025_S	ENSGALG00010027097	ENSGALG00010027097	case	control	0.047663627	0.107101058	0.445034134	0.656295086	0.78293571	up_in_case
Yang2025_S	ENSGALG00010027098	EXOG	case	control	-0.861478054	0.197721471	-4.357028341	1.3184E-05	0.000208772	down_in_case
Yang2025_S	ENSGALG00010027099	GNA11	case	control	-0.156630965	0.123557781	-1.267673828	0.204914455	0.363100684	down_in_case
Yang2025_S	ENSGALG00010027100	MIER2	case	control	-0.054113928	0.148358813	-0.364750344	0.715297774	0.824183437	down_in_case
Yang2025_S	ENSGALG00010027101	RNF220	case	control	0.015884386	0.061236589	0.259393702	0.795331489	0.876525276	up_in_case
Yang2025_S	ENSGALG00010027102	DUS3L	case	control	0.223806177	0.106341088	2.104606811	0.035325548	0.103568591	up_in_case
Yang2025_S	ENSGALG00010027103	ACVR2B	case	control	-0.077698993	0.185962588	-0.417820564	0.676078315	0.796310651	down_in_case
Yang2025_S	ENSGALG00010027105	CHERP	case	control	-0.040854169	0.098571739	-0.414461275	0.678536322	0.798126416	down_in_case
Yang2025_S	ENSGALG00010027106	CYP2J23	case	control	0.344485141	0.139947059	2.46153898	0.013834236	0.05161448	up_in_case
Yang2025_S	ENSGALG00010027107	KIF21B	case	control	0.239090798	0.141004058	1.695630615	0.089955856	0.203683654	up_in_case
Yang2025_S	ENSGALG00010027108	ENSGALG00010027108	case	control	-0.09310829	0.126497476	-0.736048602	0.46170112	0.622646386	down_in_case
Yang2025_S	ENSGALG00010027109	C19orf44	case	control	0.129107669	0.149866027	0.861487231	0.388969751	0.555927536	up_in_case
Yang2025_S	ENSGALG00010027110	IKBKE	case	control	0.573453828	0.293899239	1.951191947	0.051034217	0.135227573	up_in_case
Yang2025_S	ENSGALG00010027112	XYLB	case	control	-0.118560674	0.186654113	-0.63518919	0.525305033	0.678606251	down_in_case
Yang2025_S	ENSGALG00010027113	PIAS4	case	control	0.242723237	0.116765481	2.078724249	0.037642703	0.108240088	up_in_case
Yang2025_S	ENSGALG00010027114	FLNB	case	control	-0.122992047	0.121473896	-1.012497755	0.311300119	0.48108763	down_in_case
Yang2025_S	ENSGALG00010027116	CCDC94	case	control	0.394275681	0.118071005	3.339309937	0.000839868	0.006073462	up_in_case
Yang2025_S	ENSGALG00010027117	RASSF5	case	control	-0.147560392	0.316615616	-0.466055317	0.641175906	0.770838258	down_in_case
Yang2025_S	ENSGALG00010027118	ABHD6	case	control	0.120060293	0.191004159	0.628574237	0.529627831	0.682108137	up_in_case
Yang2025_S	ENSGALG00010027119	NUB1	case	control	0.017380615	0.097342004	0.178552056	0.858289443	0.91663576	up_in_case
Yang2025_S	ENSGALG00010027120	ENSGALG00010027120	case	control	1.773549559	0.504302039	3.516839956	0.000436717	0.003546417	up_in_case
Yang2025_S	ENSGALG00010027122	DNASE1L3	case	control	-0.091979655	0.261219282	-0.352116638	0.724750791	0.830611257	down_in_case
Yang2025_S	ENSGALG00010027123	WDR86	case	control	-0.320937764	0.152263216	-2.107782643	0.035049795	0.10282503	down_in_case
Yang2025_S	ENSGALG00010027124	IP6K2	case	control	-0.416665784	0.129396599	-3.220067516	0.001281604	0.008496267	down_in_case
Yang2025_S	ENSGALG00010027125	CRYGN	case	control	1.466390839	0.468337127	3.131058283	0.001741776	0.010796996	up_in_case
Yang2025_S	ENSGALG00010027126	ENSGALG00010027126	case	control	-0.753389709	0.364298384	-2.068056685	0.038634693	0.110390496	down_in_case
Yang2025_S	ENSGALG00010027127	S1PR4	case	control	0.667763533	0.77212811	0.864835154	0.387129273	0.55443437	up_in_case
Yang2025_S	ENSGALG00010027128	EIF2D	case	control	0.300911168	0.115527109	2.60468016	0.009196006	0.038138793	up_in_case
Yang2025_S	ENSGALG00010027129	NCLN	case	control	-0.043895283	0.102413837	-0.428606958	0.668209281	0.790583829	down_in_case
Yang2025_S	ENSGALG00010027130	NCKIPSD	case	control	0.151454623	0.125881609	1.203151316	0.22891777	0.391167275	up_in_case
Yang2025_S	ENSGALG00010027131	UBA52	case	control	0.103187397	0.233447533	0.442015367	0.658478086	0.784599583	up_in_case
Yang2025_S	ENSGALG00010027132	ENSGALG00010027132	case	control	3.156381538	0.772440998	4.086242892	4.38415E-05	0.000550388	up_in_case
Yang2025_S	ENSGALG00010027133	CELF5	case	control	0.542189496	0.249114258	2.176469146	0.029520198	0.09044004	up_in_case
Yang2025_S	ENSGALG00010027134	DYRK3	case	control	-0.25121157	0.087751526	-2.862760125	0.004199684	0.020863669	down_in_case
Yang2025_S	ENSGALG00010027135	PRKAG2	case	control	-0.221163838	0.090023361	-2.456738289	0.014020478	0.052098815	down_in_case
Yang2025_S	ENSGALG00010027136	MAPKAPK2	case	control	-0.115949318	0.097741916	-1.186280383	0.235511591	0.398816255	down_in_case
Yang2025_S	ENSGALG00010027137	FKBP8	case	control	0.045474595	0.154223054	0.294862501	0.768098919	0.858788123	up_in_case
Yang2025_S	ENSGALG00010027139	HSD11B1L	case	control	-0.073120938	0.169427207	-0.431577311	0.666048652	0.789166564	down_in_case
Yang2025_S	ENSGALG00010027143	GALNT11	case	control	-0.198684156	0.117298843	-1.693828773	0.090297825	0.204183997	down_in_case
Yang2025_S	ENSGALG00010027145	CELSR3	case	control	0.337089397	0.180212604	1.870509552	0.061413092	0.155006599	up_in_case
Yang2025_S	ENSGALG00010027146	KXD1	case	control	-0.355934383	0.176116275	-2.021019256	0.043277772	0.120038963	down_in_case
Yang2025_S	ENSGALG00010027147	KMT2C	case	control	0.424594084	0.242497525	1.75092131	0.079959465	0.18731336	up_in_case
Yang2025_S	ENSGALG00010027148	ELL	case	control	0.279294172	0.162668392	1.716954154	0.085987549	0.197208113	up_in_case
Yang2025_S	ENSGALG00010027149	C1orf116	case	control	0.325065076	0.216145976	1.503914542	0.132603372	0.267704246	up_in_case
Yang2025_S	ENSGALG00010027150	YOD1	case	control	-0.251232789	0.084463305	-2.974460781	0.00293504	0.016026559	down_in_case
Yang2025_S	ENSGALG00010027151	MICOS13	case	control	0.154176257	0.31362505	0.491594202	0.623006243	0.757291695	up_in_case
Yang2025_S	ENSGALG00010027152	C4BPS	case	control	0.677511495	0.316752824	2.13892803	0.032441498	0.097115418	up_in_case
Yang2025_S	ENSGALG00010027153	ENSGALG00010027153	case	control	-0.493924629	0.105706149	-4.672619655	2.97382E-06	6.02329E-05	down_in_case
Yang2025_S	ENSGALG00010027154	PLPP2	case	control	0.41356667	0.148378716	2.787237155	0.005315956	0.025094121	up_in_case
Yang2025_S	ENSGALG00010027155	ENSGALG00010027155	case	control	-0.373778046	0.209219567	-1.786534836	0.074012702	0.176997941	down_in_case
Yang2025_S	ENSGALG00010027156	ISYNA1	case	control	0.036854548	0.122229698	0.301518772	0.763018938	0.855610276	up_in_case
Yang2025_S	ENSGALG00010027157	ENSGALG00010027157	case	control	-0.724578398	0.240677995	-3.010571854	0.002607562	0.014651767	down_in_case
Yang2025_S	ENSGALG00010027158	XRCC2	case	control	-0.145883756	0.13278244	-1.098667531	0.27191311	0.439062478	down_in_case
Yang2025_S	ENSGALG00010027159	TMUB1	case	control	0.832765306	0.171226206	4.863538857	1.15305E-06	2.73262E-05	up_in_case
Yang2025_S	ENSGALG00010027160	FASTK	case	control	0.000137381	0.106606327	0.001288676	0.998971786	0.999043479	up_in_case
Yang2025_S	ENSGALG00010027161	C4BPG	case	control	-0.098996972	0.120180668	-0.823734574	0.41009039	0.575547344	down_in_case
Yang2025_S	ENSGALG00010027162	SLC4A2	case	control	0.145141596	0.125146135	1.159776898	0.246139652	0.411858315	up_in_case
Yang2025_S	ENSGALG00010027163	ENSGALG00010027163	case	control	1.082595897	0.204395441	5.296575564	1.17995E-07	3.94305E-06	up_in_case
Yang2025_S	ENSGALG00010027164	ENSGALG00010027164	case	control	-0.558170256	0.461788157	-1.208714965	0.22677237	0.388836346	down_in_case
Yang2025_S	ENSGALG00010027165	ENSGALG00010027165	case	control	0.917292631	0.150548722	6.092995152	1.10817E-09	6.65621E-08	up_in_case
Yang2025_S	ENSGALG00010027166	CDK5	case	control	0.242676804	0.146680558	1.65445788	0.098034515	0.215985923	up_in_case
Yang2025_S	ENSGALG00010027167	ENSGALG00010027167	case	control	0.926433982	0.444246173	2.085406784	0.037032392	0.106996968	up_in_case
Yang2025_S	ENSGALG00010027168	SSBP4	case	control	0.158574127	0.22624052	0.700909486	0.483359504	0.641609325	up_in_case
Yang2025_S	ENSGALG00010027169	ABCB8	case	control	0.075572111	0.220607023	0.342564392	0.731926194	0.836160658	up_in_case
Yang2025_S	ENSGALG00010027170	TLE2	case	control	0.759789843	0.144998842	5.239971804	1.60601E-07	5.11844E-06	up_in_case
Yang2025_S	ENSGALG00010027171	ATG9B	case	control	0.032776973	0.168151534	0.194925213	0.845451496	0.908215124	up_in_case
Yang2025_S	ENSGALG00010027172	ENSGALG00010027172	case	control	0.209870715	0.348429923	0.602332638	0.54695274	0.695862907	up_in_case
Yang2025_S	ENSGALG00010027173	ENSGALG00010027173	case	control	0.270081747	0.127217423	2.122993386	0.033754406	0.09997187	up_in_case
Yang2025_S	ENSGALG00010027174	SHISA5	case	control	-0.093162415	0.07645814	-1.218476087	0.223043107	0.384474516	down_in_case
Yang2025_S	ENSGALG00010027175	NOS3	case	control	0.596124011	0.12258064	4.863117123	1.15551E-06	2.7338E-05	up_in_case
Yang2025_S	ENSGALG00010027176	ENSGALG00010027176	case	control	0.951305577	0.171506431	5.546763292	2.91006E-08	1.16194E-06	up_in_case
Yang2025_S	ENSGALG00010027178	C4BPM	case	control	-0.011512153	0.096969831	-0.11871891	0.905498055	0.943410496	down_in_case
Yang2025_S	ENSGALG00010027183	CD34	case	control	0.700631714	0.234174967	2.991915498	0.00277233	0.015320323	up_in_case

Yang2025_S	ENSGALG00010027184	MPND	case	control	1.2031992	0.214693787	5.604257186	2.0915E-08	8.85868E-07	up_in_case
Yang2025_S	ENSGALG00010027185	ATRIP	case	control	0.238431752	0.063617499	3.747895705	0.000178324	0.001706696	up_in_case
Yang2025_S	ENSGALG00010027186	PLXNA2	case	control	-0.16425363	0.140689105	-1.1674936	0.243011091	0.407945977	down_in_case
Yang2025_S	ENSGALG00010027187	ENSGALG00010027187	case	control	-0.265614483	0.227192632	-1.169115742	0.242357002	0.407289535	down_in_case
Yang2025_S	ENSGALG00010027188	KCNH2	case	control	0.494384176	0.14862253	3.326441657	0.000879624	0.006315077	up_in_case
Yang2025_S	ENSGALG00010027189	ENSGALG00010027189	case	control	1.503236129	0.580623463	2.589003416	0.009625414	0.039461357	up_in_case
Yang2025_S	ENSGALG00010027190	IPPK	case	control	-0.020418845	0.0869829	-0.234745508	0.814406264	0.888605848	down_in_case
Yang2025_S	ENSGALG00010027191	AOC1	case	control	-1.086953434	1.029579981	-1.055725106	0.291093824	0.459752061	down_in_case
Yang2025_S	ENSGALG00010027192	SH3GL1	case	control	-0.187046275	0.06697458	-2.792795034	0.005225479	0.024759283	down_in_case
Yang2025_S	ENSGALG00010027193	CAMK1G	case	control	0.1280014	0.422993146	0.302608685	0.762188095	0.855286959	up_in_case
Yang2025_S	ENSGALG00010027194	ENSGALG00010027194	case	control	-0.143299586	0.516697125	-0.277337688	0.781520823	0.867837491	down_in_case
Yang2025_S	ENSGALG00010027195	CENPP	case	control	-0.015702552	0.220579777	-0.07118763	0.943248426	0.965205377	down_in_case
Yang2025_S	ENSGALG00010027196	ENSGALG00010027196	case	control	-1.034691457	0.392667809	-2.635030001	0.008412985	0.035644555	down_in_case
Yang2025_S	ENSGALG00010027197	ENSGALG00010027197	case	control	0.45439828	0.488967926	0.929300789	0.352733226	0.521621574	up_in_case
Yang2025_S	ENSGALG00010027198	ECM2	case	control	-0.145959963	0.238524852	-0.611927697	0.540585596	0.691106448	down_in_case
Yang2025_S	ENSGALG00010027199	LAMB3	case	control	-0.189874913	0.327143874	-0.580401862	0.56164365	0.708132954	down_in_case
Yang2025_S	ENSGALG00010027200	G0S2	case	control	-0.115019312	0.225709695	-0.509589594	0.610339016	0.747633104	down_in_case
Yang2025_S	ENSGALG00010027201	ASPN	case	control	0.344102481	0.323943602	1.062229596	0.288131467	0.457041775	up_in_case
Yang2025_S	ENSGALG00010027202	CHAF1A	case	control	0.020113233	0.069392758	0.289846279	0.771933841	0.861102952	up_in_case
Yang2025_S	ENSGALG00010027203	ENSGALG00010027203	case	control	-0.100689955	0.294344837	-0.342081606	0.732289479	0.836362093	down_in_case
Yang2025_S	ENSGALG00010027204	OMD	case	control	-1.258393769	1.18710387	-1.060053632	0.289120201	0.45798454	down_in_case
Yang2025_S	ENSGALG00010027205	OGN	case	control	0.279515499	0.305251908	0.915687966	0.359830578	0.528458584	up_in_case
Yang2025_S	ENSGALG00010027206	ENSGALG00010027206	case	control	-0.893694771	0.607705289	-1.47060555	0.141397822	0.28080072	down_in_case
Yang2025_S	ENSGALG00010027207	NOL8	case	control	0.403562921	0.137459731	2.935862867	0.003326213	0.017603791	up_in_case
Yang2025_S	ENSGALG00010027208	UBXN6	case	control	-0.276376568	0.124199047	-2.225271239	0.026063035	0.082458798	down_in_case
Yang2025_S	ENSGALG00010027209	TRAF3IP3	case	control	0.406723423	0.262330228	1.550425303	0.121039469	0.251256518	up_in_case
Yang2025_S	ENSGALG00010027210	ENSGALG00010027210	case	control	0.207833393	0.234062837	0.88793845	0.37457386	0.54258698	up_in_case
Yang2025_S	ENSGALG00010027211	HDGFRP2	case	control	0.059233414	0.066403931	0.89201667	0.372383998	0.540750645	up_in_case
Yang2025_S	ENSGALG00010027212	IRF6	case	control	-0.367377806	0.540607663	-0.679564555	0.496780219	0.653818696	down_in_case
Yang2025_S	ENSGALG00010027213	KCNC4	case	control	-0.401363722	0.287402641	-1.396520646	0.162557768	0.308826516	down_in_case
Yang2025_S	ENSGALG00010027214	PLIN4	case	control	0.377705619	0.17094773	2.209480169	0.02714126	0.08496623	up_in_case
Yang2025_S	ENSGALG00010027215	ENSGALG00010027215	case	control	0.532344519	0.204640801	2.601360613	0.009285479	0.038452645	up_in_case
Yang2025_S	ENSGALG00010027216	ENSGALG00010027216	case	control	0.975962741	0.44137895	2.21116739	0.027024248	0.08472056	up_in_case
Yang2025_S	ENSGALG00010027217	SEMA6B	case	control	0.354238494	0.117729879	3.008909025	0.002621876	0.014684825	up_in_case
Yang2025_S	ENSGALG00010027218	IARS1	case	control	-0.111717954	0.094079496	-1.18748461	0.235036523	0.398301588	down_in_case
Yang2025_S	ENSGALG00010027219	ENSGALG00010027219	case	control	0.016930699	0.130467265	0.129769706	0.896748632	0.938786882	up_in_case
Yang2025_S	ENSGALG00010027221	LRRC61	case	control	-0.618814875	0.267018942	-2.317494296	0.020476821	0.068909495	down_in_case
Yang2025_S	ENSGALG00010027222	GUCA1B	case	control	0.552178435	0.319928793	1.725941671	0.084357907	0.19459153	up_in_case
Yang2025_S	ENSGALG00010027223	SIRT6	case	control	-0.061974422	0.133822687	-0.463108488	0.643286606	0.772775763	down_in_case
Yang2025_S	ENSGALG00010027224	RARRES2	case	control	0.494540911	0.276530235	1.788379169	0.073714855	0.176497681	up_in_case
Yang2025_S	ENSGALG00010027225	CREB3L3	case	control	-0.711345668	0.533541472	-1.333252813	0.182448853	0.335323762	down_in_case
Yang2025_S	ENSGALG00010027227	ENSGALG00010027227	case	control	0.688923891	0.138384401	4.978334898	6.41336E-07	1.64586E-05	up_in_case
Yang2025_S	ENSGALG00010027229	MDM4	case	control	0.108041059	0.129510377	0.834227043	0.404153055	0.570373994	up_in_case
Yang2025_S	ENSGALG00010027230	MAP2K2	case	control	0.181681259	0.13041025	1.393151687	0.163573926	0.310249443	up_in_case
Yang2025_S	ENSGALG00010027231	ENSGALG00010027231	case	control	0.886063154	0.188017405	4.712665597	2.44497E-06	5.15442E-05	up_in_case
Yang2025_S	ENSGALG00010027232	ARRDC2	case	control	-0.254238842	0.223468742	-1.137693082	0.255248663	0.421600115	down_in_case
Yang2025_S	ENSGALG00010027233	ENSGALG00010027233	case	control	0.677907479	0.209908589	3.229536647	0.00123991	0.008278938	up_in_case
Yang2025_S	ENSGALG00010027236	PEX11G	case	control	-0.119462722	0.165197505	-0.723150884	0.46958719	0.630049827	down_in_case
Yang2025_S	ENSGALG00010027237	CTC-575C13.4	case	control	0.268424935	0.204990124	1.309453011	0.190380945	0.345212552	up_in_case
Yang2025_S	ENSGALG00010027239	ENSGALG00010027239	case	control	0.061797273	0.083450849	0.740522992	0.458982715	0.620180756	up_in_case
Yang2025_S	ENSGALG00010027240	SLC6A1	case	control	0.589181528	0.465497925	1.265701727	0.20561989	0.364034197	up_in_case
Yang2025_S	ENSGALG00010027241	LRRN2	case	control	0.028259825	0.108414486	0.260664657	0.794351124	0.875942305	up_in_case
Yang2025_S	ENSGALG00010027242	ENSGALG00010027242	case	control	0.461603986	0.127329468	3.625272236	0.000288657	0.002526657	up_in_case
Yang2025_S	ENSGALG00010027243	HRH1	case	control	0.188301552	0.205431611	0.916614297	0.35934479	0.528174132	up_in_case
Yang2025_S	ENSGALG00010027244	ARHGEF18	case	control	0.083946636	0.110288751	0.761153199	0.446565568	0.609310799	up_in_case
Yang2025_S	ENSGALG00010027245	LRIG2	case	control	-0.25572405	0.096265683	-2.656440408	0.007897041	0.033943634	down_in_case
Yang2025_S	ENSGALG00010027246	ZNF767	case	control	0.003594739	0.116163164	0.030945599	0.975312925	0.985317557	up_in_case
Yang2025_S	ENSGALG00010027247	ATG7	case	control	-0.034725204	0.086033964	-0.403622038	0.686490678	0.804089064	down_in_case
Yang2025_S	ENSGALG00010027248	ENSGALG00010027248	case	control	-0.546221356	0.382684555	-1.427341001	0.153481628	0.297505423	down_in_case
Yang2025_S	ENSGALG00010027249	C2CD4C	case	control	-0.92284814	0.376691838	-2.449875594	0.014290558	0.052864063	down_in_case
Yang2025_S	ENSGALG00010027250	ENSGALG00010027250	case	control	0.32992676	0.173927617	1.896919912	0.057838509	0.148527461	up_in_case
Yang2025_S	ENSGALG00010027251	ENSGALG00010027251	case	control	-0.273594157	0.163732578	-1.670981794	0.094725274	0.211064389	down_in_case
Yang2025_S	ENSGALG00010027252	MAGI3	case	control	-0.037538687	0.13158281	-0.285285644	0.775425311	0.863270828	down_in_case
Yang2025_S	ENSGALG00010027253	VGLL4	case	control	-0.113221098	0.103723842	-1.0915629	0.275025271	0.442234588	down_in_case
Yang2025_S	ENSGALG00010027254	INSR	case	control	-0.582072659	0.11198165	-5.197928931	2.01521E-07	6.19548E-06	down_in_case
Yang2025_S	ENSGALG00010027255	ENSGALG00010027255	case	control	0.878205498	0.287850076	3.050912856	0.002281468	0.013219232	up_in_case
Yang2025_S	ENSGALG00010027256	PHTF1	case	control	0.333466003	0.113094315	2.948565557	0.003192524	0.017090981	up_in_case
Yang2025_S	ENSGALG00010027257	TAFA3	case	control	-0.26032833	0.325118846	-0.800717441	0.423295243	0.588714652	down_in_case
Yang2025_S	ENSGALG00010027258	ENSGALG00010027258	case	control	-0.051249737	0.208529188	-0.245767691	0.805862069	0.883489148	down_in_case
Yang2025_S	ENSGALG00010027259	ENSGALG00010027259	case	control	-0.001110556	0.084286374	-0.01317598	0.989487393	0.99369464	down_in_case
Yang2025_S	ENSGALG00010027260	LEMD2	case	control	0.036273945	0.060652969	0.59805721	0.54980176	0.698339945	up_in_case
Yang2025_S	ENSGALG00010027261	ENSGALG00010027261	case	control	0.404954043	0.150886308	2.68383558	0.007278289	0.031823959	up_in_case
Yang2025_S	ENSGALG00010027262	USE1	case	control	0.596412469	0.118576719	5.029760252	4.91094E-07	1.32112E-05	up_in_case
Yang2025_S	ENSGALG00010027263	TAMM41	case	control	-0.02594694	0.15025774	-0.172682887	0.862900696	0.919290108	down_in_case
Yang2025_S	ENSGALG00010027264	ENSGALG00010027264	case	control	1.002282755	0.189055172	5.301535764	1.14833E-07	3.85588E-06	up_in_case
Yang2025_S	ENSGALG00010027265	IP6K3	case	control	0.282261059	0.135476714	2.083465491	0.037208814	0.107333736	up_in_case
Yang2025_S	ENSGALG00010027267	MYO9B	case	control	0.181863795	0.120929959	1.503877085	0.132613018	0.267704246	up_in_case
Yang2025_S	ENSGALG00010027268	UQCRC1	case	control	0.032132372	0.168309765	0.190912105	0.848594456	0.910118044	up_in_case
Yang2025_S	ENSGALG00010027269	CDC25A	case	control	-0.140586624	0.123574461	-1.137667304	0.255259431	0.421600115	down_in_case
Yang2025_S	ENSGALG00010027270	ENSGALG00010027270	case	control	0.362680168	0.262178108	1.38333506	0.166562152	0.31447643	up_in_case
Yang2025_S	ENSGALG00010027271	INKA2	case	control	-0.330338133	0.112460975	-2.937357892	0.003310218	0.017528156	down_in_case
Yang2025_S	ENSGALG00010027272	DDX20	case	control	-0.099211242	0.100325206	-0.988896467	0.322713799	0.492068803	down_in_case
Yang2025_S	ENSGALG00010027273	HAUS8	case	control	0.037890213	0.10343339	0.366324767	0.714122751	0.823558969	up_in_case
Yang2025_S	ENSGALG00010027274	COPG1	case	control	-0.270799852	0.109227614	-2.479225185	0.013166815	0.049683067	down_in_case
Yang2025_S	ENSGALG00010027275	CPAMD8	case	control	-0.455798645	0.196923164	-2.314601469	0.020634752	0.069237966	down_in_case
Yang2025_S	ENSGALG00010027276	ENSGALG00010027276	case	control	0.394931859	0.250017672	1.579615774	0.114194885	0.241033886	up_in_case
Yang2025_S	ENSGALG00010027277	KCND3	case	control	0.830895416	0.414296126	2.005559222	0.044903296	0.123612689	up_in_case
Yang2025_S	ENSGALG00010027278	H1-10	case	control	-0.498802973	0.255416065	-1.952903679	0.05083101	0.134817305	down_in_case
Yang2025_S	ENSGALG00010027279	F2RL3	case	control	-0.415791845	0.234704753	-1.771552722	0.076468834	0.181254158	down_in_case
Yang2025_S	ENSGALG00010027280	HMCES	case	control	0.073644516	0.165997737	0.443647709	0.657297307	0.783900793	up_in_case
Yang2025_S	ENSGALG00010027281	ENSGALG00010027281	case	control	1.217008668	0.263416599	4.620091035	3.83572E-06	7.47562E-05	up_in_case
Yang2025_S	ENSGALG00010027282	ENSGALG00010027282	case	control	-0.182468992	0.302216019	-0.603770087	0.54599651	0.695242148	down_in_case
Yang2025_S	ENSGALG00010027283	RAB7A	case	control	-0.092557889	0.097749932	-0.94688443	0.343697673	0.512183411	down_in_case
Yang2025_S	ENSGALG00010027284	CTTNBP2NL	case	control	0.170397134	0.102634938	1.660225426	0.096869111	0.214430327	up_in_case
Yang2025_S	ENSGALG00010027285	PTPRS	case	control	0.008027136	0.121056899	0.066308789	0.947131986	0.967511299	up_in_case
Yang2025_S	ENSGALG00010027286	RPN1	case	control	-0.185982465	0.102309429	-1.817842863	0.069088153	0.168429569	down_in_case
Yang2025_S	ENSGALG00010027287	DHX30	case	control	-0.290925291	0.1611303	-1.805528136	0.070992077	0.171607266	down_in_case
Yang2025_S	ENSGALG00010027288	DAG1	case	control	0.164539222	0.175918545	0.93531482	0.34962608	0.518136703	up_in_case
Yang2025_S	ENSGALG00010027289	SMARCC1	case	control	0.165770892	0.132913461	1.247209204	0.212320799	0.372537186	up_in_case
Yang2025_S	ENSGALG00010027290	CSPG5	case	control	0.624236892	0.283143584	2.204665505	0.027477575	0.085698302	up_in_case
Yang2025_S	ENSGALG00010027291	MST1	case	control	0.193235411	0.164174505	1.177012296	0.239190599	0.403427862	up_in_case
Yang2025_S	ENSGALG00010027292	KDM4B	case	control	-0.166997099	0.073985965	-2.257145636	0.023998976	0.077701146	down_in_case
Yang2025_S	ENSGALG00010027293	ELP6	case	control	0.203823753	0.22509868	0.905486223	0.365207855	0.533867458	up_in_case
Yang2025_S	ENSGALG00010027295	ENSGALG00010027295	case	control	0.952790564	0.254695401	3.740902109	0.000183361	0.00173937	up_in_case
Yang2025_S	ENSGALG00010027299	PGPEP1	case	control	-0.299567571	0.170842186	-1.753475402	0.079520438	0.186583146	down_in_case
Yang2025_S	ENSGALG00010027301	SCAP	case	control	-0.025177651	0.066941429	-0.376114631	0.706831678	0.819306224	down_in_case
Yang2025_S	ENSGALG00010027303	APEH	case	control	-0.017910394	0.11015769	-0.162588681	0.870842299	0.924115691	down_in_case
Yang2025_S	ENSGALG00010027304	PTPN23	case	control	0.094888078	0.087613232	1.083033645	0.27879349	0.446550262	up_in_case
Yang2025_S	ENSGALG00010027305	LSM4	case	control	-0.024061156	0.116696388	-0.206185957	0.836645657	0.903156104	down_in_case
Yang2025_S	ENSGALG00010027306	ENSGALG00010027306	case	control	-0.739963421	0.306591514	-2.413515659	0.015799449	0.057185798	down_in_case
Yang2025_S	ENSGALG00010027307	JUND	case	control	-0.370559333	0.240470442	-1.540976641	0.123322471	0.25444161	down_in_case
Yang2025_S	ENSGALG00010027309	ENSGALG00010027309	case	control	1.379893716	0.604753879	2.2817443	0.022504441	0.073805457	up_in_case
Yang2025_S	ENSGALG00010027310	ARHGEF2	case	control	0.086859204	0.143225738	0.606449688	0.544216182	0.694092303	up_in_case
Yang2025_S	ENSGALG00010027311	NFASC	case	control	0.808765292	0.337698736	2.394931358	0.016623477	0.059275372	up_in_case
Yang2025_S	ENSGALG00010027313	BSN	case	control	0.390044393	0.245399502	1.589426182	0.111964207	0.237404326	up_in_case
Yang2025_S	ENSGALG00010027314	ENSGALG00010027314	case	control	0.878365866	0.270642638	3.245482207	0.001172519	0.007920044	up_in_case
Yang2025_S	ENSGALG00010027315	RAB3A	case	control	0.722599861	0.166823556	4.331521748	1.48082E-05	0.000228014	up_in_case
Yang2025_S	ENSGALG00010027316	TRAIP	case	control	-0.017930429	0.285925651	-0.062710109	0.949997347	0.968980606	down_in_case
Yang2025_S	ENSGALG00010027317	MPV17L2	case	control	-0.193110381	0.189516054	-1.018965814	0.308219196	0.477863207	down_in_case
Yang2025_S	ENSGALG00010027319	MST1R	case	control	0.400307729	0.345240234	1.15950486	0.246250457	0.411944791	up_in_case
Yang2025_S	ENSGALG00010027320	MON1A	case	control	0.164163339	0.147056184	1.116330743	0.264280587	0.431386901	up_in_case
Yang2025_S	ENSGALG00010027321	IFI30	case	control	0.734059776	0.232457527	3.157823211	0.001589519	0.010063585	up_in_case
Yang2025_S	ENSGALG00010027322	CRABP2	case	control	0.306196175	0.201734786	1.517815451	0.129060929	0.262602231	up_in_case
Yang2025_S	ENSGALG00010027323	ISG20L2	case	control	0.068960592	0.15627125	0.441287771	0.659004678	0.784658933	up_in_case
Yang2025_S	ENSGALG00010027324	PIK3R2	case	control	-0.170806612	0.06616469	-2.581537253	0.009836137	0.040019438	down_in_case
Yang2025_S	ENSGALG00010027325	METTL25B	case	control	0.369913218	0.269242895	1.373901503	0.169472263	0.318231503	up_in_case

Yang2025_S	ENSGALG00010027326	MRPL24	case	control	0.120754222	0.243047655	0.496833521	0.619306455	0.754901631	up_in_case
Yang2025_S	ENSGALG00010027327	RBM6	case	control	0.448310996	0.158181921	2.834148125	0.004594803	0.022474053	up_in_case
Yang2025_S	ENSGALG00010027328	RBM15	case	control	-0.201655839	0.135822061	-1.484706078	0.137621701	0.275342197	down_in_case
Yang2025_S	ENSGALG00010027329	MINDY4	case	control	-0.030738613	0.155341947	-0.197877094	0.843141226	0.907018562	down_in_case
Yang2025_S	ENSGALG00010027330	ENSGALG00010027330	case	control	0.139025363	0.115756812	1.201012365	0.229746405	0.392150435	up_in_case
Yang2025_S	ENSGALG00010027331	SLC16A4	case	control	0.118562096	0.155863917	0.760676998	0.446850017	0.609579539	up_in_case
Yang2025_S	ENSGALG00010027332	HDGF	case	control	-0.137058872	0.084875615	-1.61482036	0.106349603	0.228983577	down_in_case
Yang2025_S	ENSGALG00010027333	PROK1	case	control	0.203834333	0.584693821	0.348617218	0.7273767	0.832465302	up_in_case
Yang2025_S	ENSGALG00010027334	PRCC	case	control	-0.380626877	0.07395706	-5.146592883	2.6526E-07	7.653E-06	down_in_case
Yang2025_S	ENSGALG00010027335	MAST3	case	control	0.502205858	0.189802285	2.645942109	0.008146376	0.034673105	up_in_case
Yang2025_S	ENSGALG00010027336	RAD54L2	case	control	-0.181811928	0.07679825	-2.367396765	0.017913717	0.062657541	down_in_case
Yang2025_S	ENSGALG00010027337	AQP1	case	control	0.001575417	0.13096609	0.012029201	0.990402317	0.994000591	up_in_case
Yang2025_S	ENSGALG00010027338	ENSGALG00010027338	case	control	0.856925531	0.451209142	1.899175904	0.057541352	0.148077329	up_in_case
Yang2025_S	ENSGALG00010027339	LAMTOR5	case	control	-0.003175316	0.180529203	-0.017588935	0.985966784	0.991474302	down_in_case
Yang2025_S	ENSGALG00010027346	SIN3B	case	control	-0.403397371	0.062593485	-6.444718151	1.15815E-10	9.54963E-09	down_in_case
Yang2025_S	ENSGALG00010027348	ENSGALG00010027348	case	control	0.071439943	0.239106198	0.298779133	0.765108574	0.857056911	up_in_case
Yang2025_S	ENSGALG00010027350	INSRR	case	control	0.581899659	0.269710874	2.157494243	0.030967175	0.093810345	up_in_case
Yang2025_S	ENSGALG00010027351	TMEM38A	case	control	-0.381243013	0.126778846	-3.007150055	0.002637096	0.014739761	down_in_case
Yang2025_S	ENSGALG00010027353	ENSGALG00010027353	case	control	0.002036793	0.456240829	0.004464294	0.996438021	0.997440113	up_in_case
Yang2025_S	ENSGALG00010027355	ADCYAP1R1	case	control	0.118535179	0.223584704	0.530157821	0.596002513	0.735502569	up_in_case
Yang2025_S	ENSGALG00010027356	ENSGALG00010027356	case	control	0.883033367	0.108994631	8.101622601	5.4231E-16	1.42586E-13	up_in_case
Yang2025_S	ENSGALG00010027358	RNF126	case	control	0.129683584	0.110768149	1.170766008	0.241692845	0.406839703	up_in_case
Yang2025_S	ENSGALG00010027359	ENSGALG00010027359	case	control	0.253349595	0.332823391	0.76121331	0.446529669	0.609310799	up_in_case
Yang2025_S	ENSGALG00010027360	SMIM7	case	control	-0.14186489	0.178989848	-0.792586238	0.428018929	0.592357114	down_in_case
Yang2025_S	ENSGALG00010027361	FANCD2	case	control	-0.198509426	0.28370986	-0.699691671	0.484119879	0.642111652	down_in_case
Yang2025_S	ENSGALG00010027362	ENSGALG00010027362	case	control	-0.697788525	0.255149233	-2.734825091	0.006241343	0.028293141	down_in_case
Yang2025_S	ENSGALG00010027363	VIPR1	case	control	0.043272471	0.194480028	0.22250342	0.823922011	0.894465038	up_in_case
Yang2025_S	ENSGALG00010027364	FSTL3	case	control	-0.212284175	0.123426768	-1.719920076	0.085446971	0.19638851	down_in_case
Yang2025_S	ENSGALG00010027365	PLEKHA6	case	control	0.594741804	0.230217332	2.583392821	0.009783386	0.039863007	up_in_case
Yang2025_S	ENSGALG00010027366	USP4	case	control	-0.098904296	0.099179002	-0.997230201	0.318652785	0.488175677	down_in_case
Yang2025_S	ENSGALG00010027367	SEC22C	case	control	-0.531146402	0.156472933	-3.394493806	0.000687556	0.005167795	down_in_case
Yang2025_S	ENSGALG00010027368	EMC3	case	control	0.007689184	0.199358933	0.038569547	0.969233582	0.981703006	up_in_case
Yang2025_S	ENSGALG00010027369	ENSGALG00010027369	case	control	-0.216738741	0.10059536	-2.154560032	0.031196279	0.094278932	down_in_case
Yang2025_S	ENSGALG00010027370	NUP210L	case	control	0.8065859	0.336395755	2.397729125	0.01649706	0.05900338	up_in_case
Yang2025_S	ENSGALG00010027371	PRSS57	case	control	0.967548051	0.608604268	1.589781902	0.111883975	0.237342547	up_in_case
Yang2025_S	ENSGALG00010027372	PIK3C2B	case	control	0.227977576	0.072556361	3.142075674	0.001677547	0.010487489	up_in_case
Yang2025_S	ENSGALG00010027373	MLN	case	control	-0.730806677	0.692676534	-1.055047545	0.291403581	0.460050187	down_in_case
Yang2025_S	ENSGALG00010027374	NKTR	case	control	0.420241594	0.16670234	2.520909982	0.011705179	0.04553648	up_in_case
Yang2025_S	ENSGALG00010027375	FUT7	case	control	0.013501979	0.392042048	0.03444013	0.972526183	0.983322621	up_in_case
Yang2025_S	ENSGALG00010027376	DSTYK	case	control	-0.228145901	0.091084853	-2.504762247	0.012253373	0.047133539	down_in_case
Yang2025_S	ENSGALG00010027378	ZBTB47	case	control	0.058257065	0.150384833	0.387386571	0.698470044	0.813470962	up_in_case
Yang2025_S	ENSGALG00010027379	OPTC	case	control	0.210131688	0.430223402	0.488424588	0.625249129	0.75915715	up_in_case
Yang2025_S	ENSGALG00010027380	GPX1	case	control	-0.446904869	0.245931645	-1.817191394	0.069187812	0.16861353	down_in_case
Yang2025_S	ENSGALG00010027381	KLHL40	case	control	-0.036664772	0.101907034	-0.359786466	0.719006825	0.826543483	down_in_case
Yang2025_S	ENSGALG00010027382	PALM	case	control	0.579191559	0.141781014	4.085113684	4.40552E-05	0.000552574	up_in_case
Yang2025_S	ENSGALG00010027383	ASS1	case	control	0.116851601	0.217922809	0.536206382	0.591815923	0.732346585	up_in_case
Yang2025_S	ENSGALG00010027384	HHATL	case	control	-0.078982327	0.12056548	-0.655099015	0.512404006	0.667571973	down_in_case
Yang2025_S	ENSGALG00010027385	CNTN2	case	control	0.388008123	0.642668559	0.603745301	0.546012991	0.695242148	up_in_case
Yang2025_S	ENSGALG00010027386	RHOA	case	control	-0.149105747	0.085373098	-1.746519103	0.080720791	0.188510846	down_in_case
Yang2025_S	ENSGALG00010027387	ENSGALG00010027387	case	control	0.329927214	0.270521291	1.219597959	0.222617321	0.384122383	up_in_case
Yang2025_S	ENSGALG00010027388	URM1	case	control	-0.706000013	0.174259862	-4.051420713	5.09076E-05	0.000615796	down_in_case
Yang2025_S	ENSGALG00010027391	TPM3	case	control	0.504569419	0.090915472	5.549874033	2.85876E-08	1.14473E-06	up_in_case
Yang2025_S	ENSGALG00010027392	RBBP5	case	control	-0.105557083	0.097671458	-1.080736224	0.279814465	0.447773837	down_in_case
Yang2025_S	ENSGALG00010027393	TEX264	case	control	0.265244022	0.150486886	1.762572343	0.077972645	0.183973731	up_in_case
Yang2025_S	ENSGALG00010027394	SLC27A4	case	control	-0.299470285	0.13554774	-2.209334398	0.02715139	0.08496623	down_in_case
Yang2025_S	ENSGALG00010027395	BCL2L15	case	control	0.544514637	0.421360641	1.292276933	0.196261257	0.352344836	up_in_case
Yang2025_S	ENSGALG00010027397	RFX5	case	control	0.316526601	0.154180815	2.052957113	0.040076737	0.113510027	up_in_case
Yang2025_S	ENSGALG00010027399	MAPKAP1	case	control	-0.059824675	0.106797227	-0.560170673	0.575363029	0.718173039	down_in_case
Yang2025_S	ENSGALG00010027401	ENSGALG00010027401	case	control	0.272582745	0.210880385	1.292594118	0.196151475	0.352329312	up_in_case
Yang2025_S	ENSGALG00010027402	AP4B1	case	control	-0.009876281	0.159009896	-0.062111107	0.950474352	0.969396186	down_in_case
Yang2025_S	ENSGALG00010027403	ACKR2	case	control	-0.324473538	0.32866118	-0.987258481	0.323515939	0.492806582	down_in_case
Yang2025_S	ENSGALG00010027405	DCLRE1B	case	control	-0.086854084	0.158252741	-0.54883147	0.583121111	0.725517204	down_in_case
Yang2025_S	ENSGALG00010027407	ENSGALG00010027407	case	control	-0.503141297	1.500674533	-0.335276761	0.737416322	0.839871887	down_in_case
Yang2025_S	ENSGALG00010027410	ALAS1	case	control	0.055155028	0.152297623	0.362152917	0.717237762	0.825804198	up_in_case
Yang2025_S	ENSGALG00010027412	ENSGALG00010027412	case	control	0.856343371	0.273581766	3.13011859	0.001747357	0.010812356	up_in_case
Yang2025_S	ENSGALG00010027414	PBX3	case	control	0.063076633	0.109808964	0.57442153	0.565682587	0.711094153	up_in_case
Yang2025_S	ENSGALG00010027417	WNT2B	case	control	-0.56211801	0.385887291	-1.456689618	0.145202077	0.286355922	down_in_case
Yang2025_S	ENSGALG00010027418	ENSGALG00010027418	case	control	0.04167373	0.96228224	0.04330718	0.965456668	0.979586331	up_in_case
Yang2025_S	ENSGALG00010027419	PTBP1	case	control	-0.271790107	0.058342275	-4.658544891	3.18452E-06	6.36676E-05	down_in_case
Yang2025_S	ENSGALG00010027421	PLPPR3	case	control	0.48556589	0.152299845	3.188223141	0.0014315	0.009282436	up_in_case
Yang2025_S	ENSGALG00010027423	PEX11B	case	control	0.489318184	0.188317249	2.598371557	0.009366708	0.038704444	up_in_case
Yang2025_S	ENSGALG00010027425	CFD	case	control	0.385327623	0.190179877	2.026121947	0.04275229	0.118841643	up_in_case
Yang2025_S	ENSGALG00010027427	UBAC1	case	control	-0.124079342	0.080538787	-1.540615977	0.123410277	0.254539484	down_in_case
Yang2025_S	ENSGALG00010027438	ST7L	case	control	-0.211228616	0.131085814	-1.611376625	0.107097659	0.229940435	down_in_case
Yang2025_S	ENSGALG00010027440	TWF2	case	control	0.173227153	0.100092143	1.730676826	0.083509421	0.193177918	up_in_case
Yang2025_S	ENSGALG00010027443	MED16	case	control	-0.181724298	0.1502587	-1.209409499	0.226505561	0.388474461	down_in_case
Yang2025_S	ENSGALG00010027444	SELENBP1	case	control	-0.299426892	0.130434044	-2.295619174	0.021697665	0.071818757	down_in_case
Yang2025_S	ENSGALG00010027445	PPM1M	case	control	-0.109233867	0.176236054	-0.619815663	0.535379155	0.686763817	down_in_case
Yang2025_S	ENSGALG00010027448	ADAMTSL5	case	control	0.27333776	0.202330336	1.350947984	0.176712096	0.327675723	up_in_case
Yang2025_S	ENSGALG00010027450	CAMSAP1	case	control	0.021963161	0.117961874	0.186188641	0.852296831	0.912683958	up_in_case
Yang2025_S	ENSGALG00010027455	R3HDM4	case	control	-0.146692346	0.262298925	-0.559256375	0.575986764	0.718758445	down_in_case
Yang2025_S	ENSGALG00010027457	CAPZA1	case	control	-0.256648866	0.099611616	-2.576495339	0.009980755	0.040466052	down_in_case
Yang2025_S	ENSGALG00010027460	WDR82	case	control	-0.4391145	0.110897917	-3.95962802	7.50666E-05	0.000847693	down_in_case
Yang2025_S	ENSGALG00010027467	GLYCTK	case	control	0.005038053	0.144246901	0.034926597	0.972138272	0.983073064	up_in_case
Yang2025_S	ENSGALG00010027468	BSG	case	control	0.003033854	0.105691034	0.028704928	0.977099926	0.98658702	up_in_case
Yang2025_S	ENSGALG00010027471	ENSGALG00010027471	case	control	0.023845237	0.156685226	0.152185613	0.879040541	0.92862622	up_in_case
Yang2025_S	ENSGALG00010027473	DNAH1	case	control	0.336056166	0.498931309	0.673551971	0.500596197	0.657350924	up_in_case
Yang2025_S	ENSGALG00010027475	ARID3A	case	control	0.704048696	0.134189626	5.246670091	1.54873E-07	4.94989E-06	up_in_case
Yang2025_S	ENSGALG00010027479	HCN2	case	control	-0.198572006	0.251935486	-0.788185934	0.430587956	0.594613335	down_in_case
Yang2025_S	ENSGALG00010027487	MPZ	case	control	0.380671649	0.181581261	2.096425842	0.036044432	0.104991463	up_in_case
Yang2025_S	ENSGALG00010027490	ATP2B4	case	control	-0.258693541	0.120922654	-2.139330662	0.032408898	0.09705953	down_in_case
Yang2025_S	ENSGALG00010027492	PCP4L1	case	control	-0.454051842	0.536242623	-0.846728369	0.397146543	0.56417904	down_in_case
Yang2025_S	ENSGALG00010027497	KCNT1	case	control	0.208909736	0.183176475	1.140483438	0.254084949	0.42058845	up_in_case
Yang2025_S	ENSGALG00010027499	MOV10	case	control	-0.098210528	0.200067494	-0.490886979	0.623506387	0.75777015	down_in_case
Yang2025_S	ENSGALG00010027501	BAP1	case	control	0.23778187	0.157281306	1.511825376	0.130578292	0.264901514	up_in_case
Yang2025_S	ENSGALG00010027502	THOP1	case	control	-0.170171796	0.146726181	-1.159791623	0.246133656	0.411858315	down_in_case
Yang2025_S	ENSGALG00010027510	RHOC	case	control	0.106655353	0.256840978	0.415258321	0.677952809	0.79777676	up_in_case
Yang2025_S	ENSGALG00010027511	POLRMT	case	control	0.04948787	0.083807532	0.590494298	0.554859307	0.702586501	up_in_case
Yang2025_S	ENSGALG00010027513	SEMA3G	case	control	0.277633975	0.152486946	1.820706507	0.068651483	0.167540877	up_in_case
Yang2025_S	ENSGALG00010027516	PPM1J	case	control	-0.256976037	0.279026135	-0.920974793	0.357063587	0.526248661	down_in_case
Yang2025_S	ENSGALG00010027520	FANCE	case	control	-0.11688694	0.316118669	-0.369756524	0.711563912	0.82171568	down_in_case
Yang2025_S	ENSGALG00010027522	PSMD5	case	control	-0.243073652	0.102779788	-2.364994685	0.01803033	0.062876037	down_in_case
Yang2025_S	ENSGALG00010027524	ENSGALG00010027524	case	control	-0.234499388	0.211307153	-1.109756035	0.267104169	0.434215655	down_in_case
Yang2025_S	ENSGALG00010027525	ENSGALG00010027525	case	control	0.132247009	0.288230663	0.458823525	0.6463609	0.77557217	up_in_case
Yang2025_S	ENSGALG00010027527	ENSGALG00010027527	case	control	0.288691357	0.570900302	0.505677359	0.613083165	0.749479244	up_in_case
Yang2025_S	ENSGALG00010027529	ACY1	case	control	0.021225264	0.216091251	0.098223616	0.92175473	0.953337584	up_in_case
Yang2025_S	ENSGALG00010027530	PHF19	case	control	0.01620064	0.172701367	0.093807246	0.925262275	0.955206005	up_in_case
Yang2025_S	ENSGALG00010027531	UFC1	case	control	0.171718043	0.213246316	0.805256785	0.420671514	0.586381669	up_in_case
Yang2025_S	ENSGALG00010027533	WDR18	case	control	-0.20422237	0.18646298	-1.095243516	0.273409966	0.440662489	down_in_case
Yang2025_S	ENSGALG00010027534	KCNN3	case	control	0.128940286	0.173616927	0.742671167	0.45768079	0.619081907	up_in_case
Yang2025_S	ENSGALG00010027536	USP21	case	control	-0.13459744	0.180140276	-0.747181269	0.454954149	0.616542878	down_in_case
Yang2025_S	ENSGALG00010027537	SGTA	case	control	0.082094131	0.121843775	0.673765495	0.500460415	0.657234557	up_in_case
Yang2025_S	ENSGALG00010027538	ABHD14A	case	control	0.340918716	0.214561787	1.588906958	0.1120814	0.237616661	up_in_case
Yang2025_S	ENSGALG00010027539	DDR2L	case	control	-0.454329633	0.116140434	-3.911898857	9.15733E-05	0.000995357	down_in_case
Yang2025_S	ENSGALG00010027540	PPOX	case	control	0.239667191	0.275633712	0.869513344	0.384566412	0.552068915	up_in_case
Yang2025_S	ENSGALG00010027543	TUBB4B	case	control	-0.0062985	0.167088992	-0.037695483	0.969930478	0.982096151	down_in_case
Yang2025_S	ENSGALG00010027545	PPARD	case	control	-0.197667676	0.091589588	-2.158189389	0.03091311	0.093687296	down_in_case
Yang2025_S	ENSGALG00010027547	B4GALT3	case	control	0.255806949	0.130028352	1.967316697	0.049146711	0.131627796	up_in_case
Yang2025_S	ENSGALG00010027548	PCBP4	case	control	-0.057872948	0.125988117	-0.459352431	0.645981102	0.775210701	down_in_case
Yang2025_S	ENSGALG00010027553	FGF22	case	control	-0.279291761	0.329931271	-0.846514972	0.397265525	0.564196401	down_in_case
Yang2025_S	ENSGALG00010027554	ENSGALG00010027554	case	control	0.108802071	0.162263998	0.670525026	0.502523159	0.658949865	up_in_case
Yang2025_S	ENSGALG00010027555	ENSGALG00010027555	case	control	0.846216724	0.369208882	2.291972825	0.021907211	0.072282553	up_in_case
Yang2025_S	ENSGALG00010027557	C6orf132	case	control	-0.016734591	1.248079997	-0.013408268	0.989302071	0.993580134	down_in_case
Yang2025_S	ENSGALG00010027559	ENSGALG00010027559	case	control	-0.31244484	0.235892445	-1.324522454	0.185329591	0.33886248	down_in_case
Yang2025_S	ENSGALG00010027561	ADAMTS4	case	control	-0.35350906	0.296811477	-1.191022208	0.233644867	0.396860216	down_in_case
Yang2025_S	ENSGALG00010027562	SLC39A3	case	control	-0.337059416	0.290504541	-1.160255239	0.245944903	0.411680747	down_in_case

Yang2025_S	ENSGALG00010027563	WDR77	case	control	-0.27779473	0.27551993	-1.008256388	0.313331394	0.482746133	down_in_case
Yang2025_S	ENSGALG00010027567	PARP3	case	control	0.093046866	0.210828103	0.441339959	0.658966902	0.784658933	up_in_case
Yang2025_S	ENSGALG00010027568	NELFB	case	control	-0.323371982	0.114421391	-2.826149724	0.004711124	0.022914314	down_in_case
Yang2025_S	ENSGALG00010027569	ATP5PB	case	control	-0.089572772	0.26488369	-0.33815888	0.735243462	0.838430249	down_in_case
Yang2025_S	ENSGALG00010027570	NDUFS2	case	control	-0.090075084	0.192556926	-0.467784181	0.639938934	0.769816044	down_in_case
Yang2025_S	ENSGALG00010027571	RBM10	case	control	0.482660496	0.171395434	2.816063905	0.0048616	0.023490426	up_in_case
Yang2025_S	ENSGALG00010027574	GNG7	case	control	-0.453081549	0.243667557	-1.859425012	0.062966919	0.157459692	down_in_case
Yang2025_S	ENSGALG00010027575	FCER1G	case	control	-0.229774728	0.391210321	-0.587343217	0.556973222	0.704215432	down_in_case
Yang2025_S	ENSGALG00010027576	GADD45B	case	control	0.16095676	0.229931184	0.700021444	0.483913913	0.641978329	up_in_case
Yang2025_S	ENSGALG00010027578	ENSGALG00010027578	case	control	-0.006500914	0.237647796	-0.027355245	0.978176394	0.9869076	down_in_case
Yang2025_S	ENSGALG00010027579	C26H6ORF125	case	control	-0.160422975	0.240790927	-0.666233472	0.505261881	0.66080003	down_in_case
Yang2025_S	ENSGALG00010027583	GLE1	case	control	0.071469428	0.096221555	0.742759023	0.457627588	0.619070036	up_in_case
Yang2025_S	ENSGALG00010027586	HJV	case	control	0.022639068	0.091308346	0.247940843	0.804180178	0.882249311	up_in_case
Yang2025_S	ENSGALG00010027590	SEMA3F	case	control	0.24253704	0.102428706	2.367861985	0.017891209	0.062610244	up_in_case
Yang2025_S	ENSGALG00010027592	MAP1S	case	control	0.016225509	0.079518351	0.204047354	0.838316494	0.904449915	up_in_case
Yang2025_S	ENSGALG00010027593	ENSGALG00010027593	case	control	1.456323797	0.298250054	4.882895337	1.04539E-06	2.51165E-05	up_in_case
Yang2025_S	ENSGALG00010027594	ITPR3	case	control	0.017580141	0.128823064	0.136467344	0.891451839	0.936336879	up_in_case
Yang2025_S	ENSGALG00010027595	GNAT1	case	control	-0.830889162	0.288359693	-2.881433091	0.003958713	0.020023471	down_in_case
Yang2025_S	ENSGALG00010027597	EXOSC2	case	control	-0.297748369	0.155191131	-1.918591393	0.055036066	0.143163632	down_in_case
Yang2025_S	ENSGALG00010027600	KIRREL1	case	control	-0.14810694	0.105158854	-1.408411517	0.159009251	0.304492774	down_in_case
Yang2025_S	ENSGALG00010027605	FCHO1	case	control	0.614389321	0.329488483	1.864676164	0.06222681	0.156437055	up_in_case
Yang2025_S	ENSGALG00010027607	ABL1	case	control	-0.52250289	0.129087605	-4.047661204	5.1732E-05	0.000624143	down_in_case
Yang2025_S	ENSGALG00010027608	GNAI2	case	control	0.607958645	0.14921119	4.074484252	4.61164E-05	0.000571737	up_in_case
Yang2025_S	ENSGALG00010027609	RPL36	case	control	1.135592117	0.202302656	5.613332716	1.98466E-08	8.5359E-07	up_in_case
Yang2025_S	ENSGALG00010027612	BAK1	case	control	0.064824271	0.136288143	0.475641311	0.634329939	0.765447497	up_in_case
Yang2025_S	ENSGALG00010027614	LONP1	case	control	0.002427317	0.096334318	0.025196808	0.979897983	0.987837546	up_in_case
Yang2025_S	ENSGALG00010027616	SLC38A3	case	control	0.335577424	0.195873109	1.713238868	0.086668604	0.198313135	up_in_case
Yang2025_S	ENSGALG00010027618	PPP1R26	case	control	-0.194333985	0.108369505	-1.793253424	0.072932411	0.175105642	down_in_case
Yang2025_S	ENSGALG00010027619	JAK3	case	control	-0.025164701	0.336109455	-0.074870553	0.940317706	0.963543774	down_in_case
Yang2025_S	ENSGALG00010027621	TSPO2	case	control	0.024868029	0.164031411	0.151605287	0.879498263	0.928681389	up_in_case
Yang2025_S	ENSGALG00010027623	APOBEC2	case	control	0.100827381	0.156393036	0.644705052	0.519118342	0.673246301	up_in_case
Yang2025_S	ENSGALG00010027626	SEMA3B	case	control	0.377491338	0.112186653	3.364850695	0.000765851	0.005640662	up_in_case
Yang2025_S	ENSGALG00010027627	OARD1	case	control	-0.380478623	0.083915834	-4.534050423	5.78632E-06	0.000105264	down_in_case
Yang2025_S	ENSGALG00010027628	PIERCE1	case	control	0.254482298	0.249860313	1.018498274	0.30844122	0.478021501	up_in_case
Yang2025_S	ENSGALG00010027632	LAMC3	case	control	0.918836246	0.435516117	2.109764047	0.034878685	0.10245246	up_in_case
Yang2025_S	ENSGALG00010027634	OASL	case	control	0.371301007	0.198242875	1.872960161	0.061073886	0.154371507	up_in_case
Yang2025_S	ENSGALG00010027635	CD48	case	control	0.151218089	0.166560801	0.907885216	0.363938869	0.532719343	up_in_case
Yang2025_S	ENSGALG00010027636	SLC1A6	case	control	-1.017857768	0.491998469	-2.068823041	0.038562695	0.110207375	down_in_case
Yang2025_S	ENSGALG00010027638	MRPS2	case	control	-0.104601487	0.162640882	-0.643143871	0.52013075	0.673937343	down_in_case
Yang2025_S	ENSGALG00010027641	HYAL3	case	control	0.40418818	0.221095125	1.828118914	0.067531707	0.165541372	up_in_case
Yang2025_S	ENSGALG00010027642	DIPK1B	case	control	-0.144110297	0.190148902	-0.7578813	0.448522046	0.611023043	down_in_case
Yang2025_S	ENSGALG00010027644	NAA80	case	control	0.281125925	0.306983103	0.915770029	0.359787526	0.528458584	up_in_case
Yang2025_S	ENSGALG00010027648	NFYA	case	control	-0.324760977	0.088384862	-3.674395929	0.000238413	0.002158728	down_in_case
Yang2025_S	ENSGALG00010027649	FBLN2	case	control	-0.2850854	0.099495293	-2.865315445	0.00416594	0.020761542	down_in_case
Yang2025_S	ENSGALG00010027650	CCDC124	case	control	0.202717128	0.096530568	2.100030412	0.035726166	0.104391452	up_in_case
Yang2025_S	ENSGALG00010027654	AIF1L	case	control	-0.608468642	0.152924484	-3.978883067	6.92398E-05	0.00079412	down_in_case
Yang2025_S	ENSGALG00010027663	COPA	case	control	-0.010447097	0.058062467	-0.17992857	0.857208645	0.915649212	down_in_case
Yang2025_S	ENSGALG00010027664	TREMB1	case	control	-0.493888916	0.409049435	-1.207406423	0.227275663	0.38945971	down_in_case
Yang2025_S	ENSGALG00010027665	TRAF1	case	control	0.350562886	0.17497215	2.003535334	0.045119859	0.124061806	up_in_case
Yang2025_S	ENSGALG00010027667	PTPDC1	case	control	-0.025508134	0.147718209	-0.172681039	0.862902148	0.919290108	down_in_case
Yang2025_S	ENSGALG00010027668	RANBP3	case	control	-0.16681101	0.089712999	-1.859385064	0.062972577	0.157459692	down_in_case
Yang2025_S	ENSGALG00010027672	CNTRL	case	control	0.939284266	0.17292113	5.431865176	5.57681E-08	2.03971E-06	up_in_case
Yang2025_S	ENSGALG00010027674	TREM2	case	control	0.027930467	0.173863311	0.160646121	0.872372127	0.925114134	up_in_case
Yang2025_S	ENSGALG00010027685	ENSGALG00010027685	case	control	-0.326723995	0.378929428	-0.862229142	0.388561436	0.555514888	down_in_case
Yang2025_S	ENSGALG00010027692	PHF2	case	control	0.179804992	0.191780963	0.937553911	0.348473706	0.516824216	up_in_case
Yang2025_S	ENSGALG00010027693	RAB14	case	control	-0.197004223	0.069016515	-2.854450454	0.004311136	0.021326118	down_in_case
Yang2025_S	ENSGALG00010027695	TREMB2	case	control	-0.257383793	0.286055695	-0.899768114	0.368243666	0.536765219	down_in_case
Yang2025_S	ENSGALG00010027697	CATH2	case	control	-0.775812182	0.546912137	-1.418531661	0.156035602	0.300948943	down_in_case
Yang2025_S	ENSGALG00010027698	FAM120A	case	control	-0.186627889	0.096669516	-1.930576424	0.053535456	0.140255043	down_in_case
Yang2025_S	ENSGALG00010027699	VANGL2	case	control	-0.256624441	0.109529347	-2.342974259	0.019130703	0.065613179	down_in_case
Yang2025_S	ENSGALG00010027700	TCF3	case	control	0.008174024	0.088976026	0.091867712	0.926803145	0.955608219	up_in_case
Yang2025_S	ENSGALG00010027701	NHLH1	case	control	0.999751377	0.531119984	1.882345624	0.059789104	0.152092216	up_in_case
Yang2025_S	ENSGALG00010027702	FOXP4	case	control	0.531096001	0.204878998	2.592242277	0.009535259	0.039218961	up_in_case
Yang2025_S	ENSGALG00010027703	NCSTN	case	control	-0.359305513	0.1391656	-2.581855815	0.009827063	0.039994196	down_in_case
Yang2025_S	ENSGALG00010027704	MDFI	case	control	0.336177058	0.111141045	3.024778642	0.002488152	0.014151997	up_in_case
Yang2025_S	ENSGALG00010027705	S100A11	case	control	0.223620294	0.196016682	1.14082277	0.253943683	0.420423574	up_in_case
Yang2025_S	ENSGALG00010027706	GSN	case	control	-0.490488345	0.165803527	-2.958250375	0.003093907	0.016697752	down_in_case
Yang2025_S	ENSGALG00010027707	HIGD1C	case	control	0.092387993	0.191043215	0.483597351	0.628671655	0.761322631	up_in_case
Yang2025_S	ENSGALG00010027708	ENSGALG00010027708	case	control	-0.1323855	0.367689578	-0.360046919	0.718812046	0.826504753	down_in_case
Yang2025_S	ENSGALG00010027709	PSMA5	case	control	0.060376703	0.205384376	0.293969308	0.768781357	0.859099295	up_in_case
Yang2025_S	ENSGALG00010027710	ENSGALG00010027710	case	control	0.078454024	0.233972907	0.335312428	0.73738942	0.839871887	up_in_case
Yang2025_S	ENSGALG00010027711	WNK2	case	control	0.212051435	0.183984259	1.152552047	0.249094295	0.414727702	up_in_case
Yang2025_S	ENSGALG00010027712	STOM	case	control	-0.606309532	0.157279434	-3.854982913	0.000115738	0.001198048	down_in_case
Yang2025_S	ENSGALG00010027713	ENSGALG00010027713	case	control	0.112473204	0.355087827	0.316747564	0.751435155	0.848540188	up_in_case
Yang2025_S	ENSGALG00010027714	SYPL2	case	control	-0.15098556	0.244292673	-0.61805194	0.536541101	0.687765637	down_in_case
Yang2025_S	ENSGALG00010027715	NINJ1	case	control	-0.267265123	0.109978655	-2.4301545	0.015092388	0.055313905	down_in_case
Yang2025_S	ENSGALG00010027717	ATXN7L2	case	control	0.307008939	0.133932898	2.292259366	0.021890681	0.072269491	up_in_case
Yang2025_S	ENSGALG00010027718	ENSGALG00010027718	case	control	-0.429956424	0.373374268	-1.15154273	0.249509027	0.415194013	down_in_case
Yang2025_S	ENSGALG00010027720	DAB2IP	case	control	0.121475517	0.149180013	0.814288147	0.415479898	0.581063065	up_in_case
Yang2025_S	ENSGALG00010027721	SUSD3	case	control	0.036765265	0.277587806	0.132445532	0.894631902	0.937909687	up_in_case
Yang2025_S	ENSGALG00010027722	GNAI3	case	control	-0.073430019	0.086863421	-0.845350303	0.397915283	0.564658806	down_in_case
Yang2025_S	ENSGALG00010027723	ENSGALG00010027723	case	control	-0.106977006	0.724685099	-0.147618608	0.882643769	0.930983409	down_in_case
Yang2025_S	ENSGALG00010027724	TTLL11	case	control	-0.419128252	0.184417661	-2.272712112	0.023043534	0.075289953	down_in_case
Yang2025_S	ENSGALG00010027725	BTN1A1	case	control	-0.832617714	0.542987509	-1.533401231	0.125177026	0.257107512	down_in_case
Yang2025_S	ENSGALG00010027727	GSTM2	case	control	-0.330351321	0.386460838	-0.854811894	0.392655293	0.559588004	down_in_case
Yang2025_S	ENSGALG00010027728	CRNN	case	control	-1.593209619	2.033708339	-0.783401232	0.433391533	0.597478335	down_in_case
Yang2025_S	ENSGALG00010027729	EPS8L3	case	control	0.122602398	0.349856158	0.350436587	0.726011073	0.831645236	up_in_case
Yang2025_S	ENSGALG00010027730	ENSGALG00010027730	case	control	0.097679306	0.140995248	0.692784382	0.488444875	0.645776027	up_in_case
Yang2025_S	ENSGALG00010027731	OLFML2A	case	control	0.195527633	0.110010537	1.777353673	0.075510068	0.179602633	up_in_case
Yang2025_S	ENSGALG00010027732	EEF2	case	control	-0.624223892	0.122178529	-5.109112835	3.23675E-07	9.13039E-06	down_in_case
Yang2025_S	ENSGALG00010027733	RPL35	case	control	-0.476780554	0.336352059	-1.417504487	0.156335483	0.301402181	down_in_case
Yang2025_S	ENSGALG00010027734	CSF1	case	control	0.25718979	0.126816448	2.028047565	0.042555394	0.118412422	up_in_case
Yang2025_S	ENSGALG00010027736	ENSGALG00010027736	case	control	-1.898855293	1.496717187	-1.268680088	0.204555187	0.362809824	down_in_case
Yang2025_S	ENSGALG00010027738	ENSGALG00010027738	case	control	0.730510778	0.249471413	2.928234421	0.003408929	0.017923979	up_in_case
Yang2025_S	ENSGALG00010027739	FGD3	case	control	-0.0193439	0.091289992	-0.211895076	0.832188892	0.900143771	down_in_case
Yang2025_S	ENSGALG00010027740	MARCHF2	case	control	-0.490794182	0.117502171	-4.176894588	2.95516E-05	0.000401366	down_in_case
Yang2025_S	ENSGALG00010027741	RAB11B	case	control	-0.283326359	0.105440116	-2.687083142	0.007207901	0.031595501	down_in_case
Yang2025_S	ENSGALG00010027743	C1orf74	case	control	0.155615721	0.372012546	0.418307723	0.675722144	0.796093006	up_in_case
Yang2025_S	ENSGALG00010027744	ENSGALG00010027744	case	control	-1.450954913	1.192401277	-1.216834417	0.223667222	0.385028134	down_in_case
Yang2025_S	ENSGALG00010027745	BICD2	case	control	0.244920288	0.134922067	1.815272288	0.069482075	0.169096632	up_in_case
Yang2025_S	ENSGALG00010027746	UQCR11	case	control	0.0089215	0.351159268	0.025405851	0.979731245	0.987771517	up_in_case
Yang2025_S	ENSGALG00010027751	ANGPTL4	case	control	0.413235873	0.23179287	1.782780779	0.074621996	0.178149308	up_in_case
Yang2025_S	ENSGALG00010027752	SCAI	case	control	0.068612713	0.12638305	0.542894898	0.587202169	0.728714773	up_in_case
Yang2025_S	ENSGALG00010027759	ENSGALG00010027759	case	control	-1.388936178	1.05903778	-1.311507676	0.189686302	0.344497335	down_in_case
Yang2025_S	ENSGALG00010027767	ENSGALG00010027767	case	control	-0.213686871	0.790640805	-0.270270482	0.786952173	0.871438218	down_in_case
Yang2025_S	ENSGALG00010027772	ENSGALG00010027772	case	control	-0.609772157	0.877948568	-0.694542004	0.487342369	0.644563013	down_in_case
Yang2025_S	ENSGALG00010027775	MVB12B	case	control	-0.118577718	0.100620486	-1.178464968	0.23861129	0.402694481	down_in_case
Yang2025_S	ENSGALG00010027776	TKT	case	control	-0.20445725	0.10324025	-1.980402498	0.04765832	0.128980129	down_in_case
Yang2025_S	ENSGALG00010027784	PEAR1	case	control	0.152459897	0.092946651	1.640294677	0.100943912	0.220547729	up_in_case
Yang2025_S	ENSGALG00010027787	DCP1A	case	control	-0.060254592	0.173597491	-0.347093677	0.728520943	0.833097929	down_in_case
Yang2025_S	ENSGALG00010027789	UAP1L1	case	control	-0.517717092	0.08256861	-6.27014421	3.60714E-10	2.45197E-08	down_in_case
Yang2025_S	ENSGALG00010027801	MBD3	case	control	0.036870495	0.08542763	0.431599179	0.666032756	0.789166564	up_in_case
Yang2025_S	ENSGALG00010027803	SAPCD2	case	control	-0.312747542	0.267140315	-1.170723867	0.241709788	0.406839703	down_in_case
Yang2025_S	ENSGALG00010027811	MEX3D	case	control	0.418424778	0.122965352	3.402785992	0.000667025	0.005051627	up_in_case
Yang2025_S	ENSGALG00010027813	LRRC71	case	control	1.070721782	0.343085564	3.120859329	0.001803242	0.011069679	up_in_case
Yang2025_S	ENSGALG00010027814	ENSGALG00010027814	case	control	0.492386501	0.071210281	6.914542324	4.69376E-12	5.49644E-10	up_in_case
Yang2025_S	ENSGALG00010027816	ZMYND19	case	control	-0.090455959	0.200781044	-0.450520415	0.652335237	0.779880879	down_in_case
Yang2025_S	ENSGALG00010027821	NMRK2	case	control	0.002429399	0.195595714	0.012420512	0.99009012	0.993885564	up_in_case
Yang2025_S	ENSGALG00010027822	DPH7	case	control	0.225845299	0.125248949	1.803171215	0.07136133	0.172313313	up_in_case
Yang2025_S	ENSGALG00010027826	ARHGEF11	case	control	0.100504095	0.084722105	1.186279484	0.235511946	0.398816255	up_in_case
Yang2025_S	ENSGALG00010027830	CACNA1D	case	control	0.422396996	0.239931942	1.760486715	0.078325318	0.184576558	up_in_case
Yang2025_S	ENSGALG00010027831	ATCAY	case	control	0.056573174	0.172292925	0.328354599	0.742643566	0.842447418	up_in_case
Yang2025_S	ENSGALG00010027838	ENSGALG00010027838	case	control	-0.294268956	0.374918751	-0.784887274	0.432519664	0.596512422	down_in_case
Yang2025_S	ENSGALG00010027842	SLAMF8	case	control	0.094264634	0.28644194	0.3290881	0.742089098	0.842167591	up_in_case
Yang2025_S	ENSGALG00010027847	NRTN	case	control	1.457740201	1.167308737	1.248804326	0.211736651	0.371933724	up_in_case
Yang2025_S	ENSGALG00010027860	ENSGALG00010027860	case	control	0.527522559	0.347908586	1.516267723	0.12945167	0.263075546	up_in_case

Yang2025_S	ENSGALG00010027861	PNPLA7	case	control	-0.155877733	0.080895674	-1.926898265	0.05399231	0.141212995	down_in_case
Yang2025_S	ENSGALG00010027863	CHDH	case	control	-0.270076075	0.116846847	-2.311368103	0.020812531	0.069700222	down_in_case
Yang2025_S	ENSGALG00010027864	ACTR8	case	control	0.010856215	0.145151593	0.074792258	0.940380001	0.963543774	up_in_case
Yang2025_S	ENSGALG00010027865	SELENOK	case	control	0.40076232	0.172894443	2.317959517	0.020451521	0.068870928	up_in_case
Yang2025_S	ENSGALG00010027866	KCNA2	case	control	-0.119998298	0.138335757	-0.867442377	0.385699671	0.553182886	down_in_case
Yang2025_S	ENSGALG00010027867	UBAP2L	case	control	0.702062215	0.299883407	2.341117245	0.019226128	0.065869576	up_in_case
Yang2025_S	ENSGALG00010027868	CACNA2D3	case	control	0.397375623	0.529152168	0.750966635	0.452672736	0.614514815	up_in_case
Yang2025_S	ENSGALG00010027869	NSMF	case	control	-0.180763049	0.137279254	-1.316754301	0.187920995	0.34231099	down_in_case
Yang2025_S	ENSGALG00010027870	HAX1	case	control	-0.307477984	0.252060522	-1.219857761	0.2225188	0.384095068	down_in_case
Yang2025_S	ENSGALG00010027872	KDM5B	case	control	-0.189704192	0.102510964	-1.850574656	0.064230769	0.159699414	down_in_case
Yang2025_S	ENSGALG00010027873	RFX2	case	control	-0.082842403	0.159388113	-0.519752704	0.603235948	0.741408796	down_in_case
Yang2025_S	ENSGALG00010027874	FRS3	case	control	0.020469636	0.104216068	0.196415352	0.844285081	0.907451801	up_in_case
Yang2025_S	ENSGALG00010027875	WNT5A	case	control	-0.552100921	0.134724916	-4.097986744	4.16759E-05	0.000529885	down_in_case
Yang2025_S	ENSGALG00010027876	ANAPC2	case	control	0.16544588	0.12606577	1.312377499	0.189392795	0.344271928	up_in_case
Yang2025_S	ENSGALG00010027877	OAZ1	case	control	-0.629463135	0.185311995	-3.39677491	0.00068185	0.00514101	down_in_case
Yang2025_S	ENSGALG00010027879	ENSGALG00010027879	case	control	1.187599248	0.187278743	6.341345673	2.27767E-10	1.65434E-08	up_in_case
Yang2025_S	ENSGALG00010027880	AMH	case	control	-0.422305783	0.191863573	-2.201073281	0.027730836	0.086372196	down_in_case
Yang2025_S	ENSGALG00010027881	ENSGALG00010027881	case	control	0.19961289	0.301085036	0.662978446	0.507344358	0.662963582	up_in_case
Yang2025_S	ENSGALG00010027883	ATP8B2	case	control	-0.048600196	0.07818075	-0.621638904	0.534179334	0.685688582	down_in_case
Yang2025_S	ENSGALG00010027884	SF3A2	case	control	0.268910164	0.199713272	1.346481192	0.178147382	0.329504084	up_in_case
Yang2025_S	ENSGALG00010027885	PLEKHJ1	case	control	-0.002943825	0.11561864	-0.025461507	0.979686852	0.987771517	down_in_case
Yang2025_S	ENSGALG00010027886	IL6R	case	control	-0.199841081	0.256999146	-0.777594337	0.4368082	0.600744257	down_in_case
Yang2025_S	ENSGALG00010027887	ERC2	case	control	0.896530886	0.377965346	2.371992287	0.01769246	0.062086233	up_in_case
Yang2025_S	ENSGALG00010027888	PFKFB2	case	control	0.114336866	0.144033589	0.793820851	0.427299732	0.591655581	up_in_case
Yang2025_S	ENSGALG00010027889	RBM8A	case	control	0.555221547	0.167990073	3.305085445	0.000949474	0.006730892	up_in_case
Yang2025_S	ENSGALG00010027890	ENSGALG00010027890	case	control	-0.700318515	0.362672705	-1.930993166	0.053483898	0.140172675	down_in_case
Yang2025_S	ENSGALG00010027891	CCDC66	case	control	0.838450415	0.174079904	4.816468725	1.46121E-06	3.33973E-05	up_in_case
Yang2025_S	ENSGALG00010027892	SHE	case	control	-0.021562002	0.239802974	-0.089915489	0.928354373	0.956760728	down_in_case
Yang2025_S	ENSGALG00010027893	DOT1L	case	control	0.380420629	0.203865276	1.866039362	0.062035859	0.156182419	up_in_case
Yang2025_S	ENSGALG00010027894	RSBN1	case	control	-0.414337098	0.095527693	-4.337350603	1.4421E-05	0.000223777	down_in_case
Yang2025_S	ENSGALG00010027895	LIX1L	case	control	0.406204649	0.144431419	2.812439638	0.004916725	0.023699261	up_in_case
Yang2025_S	ENSGALG00010027896	TOR4A	case	control	-0.548019828	0.238283361	-2.299866122	0.021455806	0.071204252	down_in_case
Yang2025_S	ENSGALG00010027897	AP3D1	case	control	0.028677673	0.114502114	0.250455402	0.80223519	0.880663886	up_in_case
Yang2025_S	ENSGALG00010027898	TASOR	case	control	0.035668802	0.10253158	0.347881129	0.727929458	0.832815845	up_in_case
Yang2025_S	ENSGALG00010027899	PMVK	case	control	1.87554807	0.184389376	10.17167099	2.65317E-24	2.17482E-21	up_in_case
Yang2025_S	ENSGALG00010027901	MOB3A	case	control	-0.269473878	0.149137915	-1.806877065	0.070781449	0.17126923	down_in_case
Yang2025_S	ENSGALG00010027902	UBE2Q1	case	control	-0.122918783	0.114326801	-1.075152824	0.282306313	0.450336504	down_in_case
Yang2025_S	ENSGALG00010027903	NDUFA8	case	control	-0.054209226	0.273660554	-0.198089295	0.8429752	0.906953321	down_in_case
Yang2025_S	ENSGALG00010027905	SYN2	case	control	0.163262105	0.403693544	0.404420897	0.685903236	0.803806374	up_in_case
Yang2025_S	ENSGALG00010027906	LHX6	case	control	-0.226126592	0.128414758	-1.760908131	0.078253954	0.184481279	down_in_case
Yang2025_S	ENSGALG00010027907	MKNK2	case	control	-0.223170267	0.110084141	-2.027269903	0.042634818	0.118586065	down_in_case
Yang2025_S	ENSGALG00010027908	PBXIP1	case	control	0.870714454	0.157687755	5.521763273	3.35614E-08	1.32487E-06	up_in_case
Yang2025_S	ENSGALG00010027909	TIMP4	case	control	-0.263788845	0.506236779	-0.521077993	0.602312441	0.740731079	down_in_case
Yang2025_S	ENSGALG00010027910	CHRNB2	case	control	0.167185566	0.208930836	0.800195744	0.423597395	0.588783805	up_in_case
Yang2025_S	ENSGALG00010027911	PTPN22	case	control	0.658118304	0.489841392	1.343533467	0.179099297	0.330720464	up_in_case
Yang2025_S	ENSGALG00010027912	RGS3	case	control	0.21923977	0.169542961	1.293122221	0.195968791	0.352137344	up_in_case
Yang2025_S	ENSGALG00010027913	PYGO2	case	control	-0.163412748	0.11784136	-1.386718114	0.165527735	0.313096942	down_in_case
Yang2025_S	ENSGALG00010027914	BTBD2	case	control	-0.396379405	0.169562761	-2.337656003	0.0194051	0.066325747	down_in_case
Yang2025_S	ENSGALG00010027915	GSTM3	case	control	-0.227444626	0.182544351	-1.245969124	0.212775731	0.373152428	down_in_case
Yang2025_S	ENSGALG00010027916	ADAR	case	control	0.177631124	0.07108896	2.498716034	0.012464414	0.047704371	up_in_case
Yang2025_S	ENSGALG00010027917	PPARG	case	control	-0.762943613	0.229137224	-3.329636274	0.000869595	0.006262432	down_in_case
Yang2025_S	ENSGALG00010027918	CSNK1G2	case	control	0.233457606	0.090266799	2.586306483	0.009701063	0.039655122	up_in_case
Yang2025_S	ENSGALG00010027919	TSEN2	case	control	-0.092958799	0.212559711	-0.437330287	0.661871834	0.786315287	down_in_case
Yang2025_S	ENSGALG00010027920	SCAMP4	case	control	-0.358106668	0.118242054	-3.028589716	0.002456981	0.014020839	down_in_case
Yang2025_S	ENSGALG00010027921	MKRN2OS	case	control	-0.997196961	0.26393695	-3.77816354	0.000157989	0.001543884	down_in_case
Yang2025_S	ENSGALG00010027922	SHC1	case	control	-0.20401571	0.157474269	-1.295549499	0.195130737	0.351084161	down_in_case
Yang2025_S	ENSGALG00010027923	AMPD2	case	control	0.571127014	0.169954788	3.360464394	0.000778116	0.005718903	up_in_case
Yang2025_S	ENSGALG00010027924	ENSGALG00010027924	case	control	1.41139051	0.495523002	2.848284546	0.00439556	0.021643864	up_in_case
Yang2025_S	ENSGALG00010027925	MKRN2	case	control	0.158401525	0.077924312	2.032761279	0.042076647	0.117338018	up_in_case
Yang2025_S	ENSGALG00010027926	CKS1B	case	control	-0.349535879	0.361627404	-0.966563582	0.333762248	0.502590979	down_in_case
Yang2025_S	ENSGALG00010027927	ENSGALG00010027927	case	control	-0.292403821	0.135386075	-2.159777665	0.030789885	0.093476165	down_in_case
Yang2025_S	ENSGALG00010027928	FLAD1	case	control	0.250957444	0.193041734	1.300016523	0.193595306	0.349058779	up_in_case
Yang2025_S	ENSGALG00010027929	CDK5RAP2	case	control	0.526874674	0.151912135	3.468285628	0.00052379	0.004109807	up_in_case
Yang2025_S	ENSGALG00010027930	RAF1	case	control	-0.063173783	0.068534775	-0.921777054	0.356644879	0.525982811	down_in_case
Yang2025_S	ENSGALG00010027931	ABHD17A	case	control	0.049758929	0.144934194	0.343320839	0.731357105	0.835708181	up_in_case
Yang2025_S	ENSGALG00010027932	POLE3	case	control	-0.032875006	0.079722272	-0.41236915	0.680068871	0.799187023	down_in_case
Yang2025_S	ENSGALG00010027933	ENSGALG00010027933	case	control	1.142209379	0.191810538	5.954883337	2.60257E-09	1.40569E-07	up_in_case
Yang2025_S	ENSGALG00010027934	CNBP	case	control	0.009285655	0.123001183	0.075492404	0.939822941	0.96346547	up_in_case
Yang2025_S	ENSGALG00010027935	ISY1	case	control	0.655065621	0.101521073	6.452508821	1.10013E-10	9.29114E-09	up_in_case
Yang2025_S	ENSGALG00010027936	ENSGALG00010027936	case	control	0.136316391	0.155015155	0.879374603	0.379198198	0.547294344	up_in_case
Yang2025_S	ENSGALG00010027937	HDHD3	case	control	-0.042164704	0.208364862	-0.202359953	0.839635334	0.904950933	down_in_case
Yang2025_S	ENSGALG00010027938	ENSGALG00010027938	case	control	0.360220246	0.155295011	2.319586727	0.020363245	0.068654521	up_in_case
Yang2025_S	ENSGALG00010027939	BSPRY	case	control	0.258953955	0.754737115	0.343104837	0.731519592	0.835825313	up_in_case
Yang2025_S	ENSGALG00010027940	ZBTB7B	case	control	0.832672954	0.399323726	2.085207816	0.037050441	0.107009075	up_in_case
Yang2025_S	ENSGALG00010027941	KLF2	case	control	-0.236024395	0.139038385	-1.697548454	0.089593019	0.203269084	down_in_case
Yang2025_S	ENSGALG00010027942	KHDC4	case	control	0.363833989	0.090831931	4.005573625	6.18672E-05	0.000724961	up_in_case
Yang2025_S	ENSGALG00010027943	RAB43	case	control	-0.292903094	0.130600314	-2.242744176	0.024913316	0.079739976	down_in_case
Yang2025_S	ENSGALG00010027944	WDR31	case	control	0.206330696	0.31620673	0.652518356	0.514066835	0.6689035	up_in_case
Yang2025_S	ENSGALG00010027946	AP1M1	case	control	-0.127113617	0.088148305	-1.442042668	0.149290337	0.291653	down_in_case
Yang2025_S	ENSGALG00010027947	GP9	case	control	-0.915863681	0.458350655	-1.998172513	0.04569796	0.12516513	down_in_case
Yang2025_S	ENSGALG00010027948	PRPF4	case	control	-0.123407837	0.149812802	-0.823746942	0.410083361	0.575547344	down_in_case
Yang2025_S	ENSGALG00010027950	CDC26	case	control	-0.260283714	0.208792276	-1.246615628	0.212538469	0.372825222	down_in_case
Yang2025_S	ENSGALG00010027951	FAM32A	case	control	-0.140781985	0.189686159	-0.742183749	0.457976014	0.619195168	down_in_case
Yang2025_S	ENSGALG00010027952	CIB3	case	control	-0.623267402	0.162862993	-3.826943075	0.000129745	0.001312039	down_in_case
Yang2025_S	ENSGALG00010027953	SLC31A1	case	control	-0.151356612	0.18329886	-0.825736785	0.408953421	0.574646155	down_in_case
Yang2025_S	ENSGALG00010027954	CFAP92	case	control	0.418997055	0.377707151	1.109317243	0.267293349	0.434371174	up_in_case
Yang2025_S	ENSGALG00010027955	ENSGALG00010027955	case	control	0.613677118	0.227830413	2.693569786	0.007069134	0.031174484	up_in_case
Yang2025_S	ENSGALG00010027956	RAB8A	case	control	0.270812737	0.095538733	2.83458581	0.004588513	0.02245117	up_in_case
Yang2025_S	ENSGALG00010027957	ENTREP3	case	control	0.764213687	0.370060557	2.065104408	0.038913122	0.110981245	up_in_case
Yang2025_S	ENSGALG00010027958	ENSGALG00010027958	case	control	-0.081469761	0.197873287	-0.41172693	0.680539584	0.799672747	down_in_case
Yang2025_S	ENSGALG00010027959	TPM4	case	control	0.569384799	0.162383182	3.506427165	0.000454166	0.003656824	up_in_case
Yang2025_S	ENSGALG00010027960	PPP6C	case	control	0.191596096	0.101274973	1.891840515	0.058512233	0.149828734	up_in_case
Yang2025_S	ENSGALG00010027962	SCAMP3	case	control	-0.079040259	0.160563798	-0.492267002	0.622530604	0.756869264	down_in_case
Yang2025_S	ENSGALG00010027964	RABEPK	case	control	-0.028914789	0.159234219	-0.181586531	0.85590722	0.914722533	down_in_case
Yang2025_S	ENSGALG00010027965	ACAD9	case	control	0.283419477	0.122390546	2.315697476	0.020574792	0.069119993	up_in_case
Yang2025_S	ENSGALG00010027966	CLK2	case	control	0.191025845	0.073154443	2.611267837	0.009020721	0.03769228	up_in_case
Yang2025_S	ENSGALG00010027968	FKBP15	case	control	0.281100256	0.088936151	3.160697327	0.00157392	0.009978421	up_in_case
Yang2025_S	ENSGALG00010027969	HSPA5	case	control	0.080177704	0.105648038	0.758913327	0.447904406	0.610420332	up_in_case
Yang2025_S	ENSGALG00010027970	GRIN3B	case	control	0.81242385	0.150871043	5.384889211	7.24893E-08	2.5901E-06	up_in_case
Yang2025_S	ENSGALG00010027971	ENSGALG00010027971	case	control	-0.009254867	0.259220185	-0.035702725	0.971519398	0.982732492	down_in_case
Yang2025_S	ENSGALG00010027972	TMEM259	case	control	-0.297206634	0.093190909	-3.189223466	0.001426556	0.00925899	down_in_case
Yang2025_S	ENSGALG00010027973	GAPVD1	case	control	0.123588232	0.10243633	1.206488287	0.227629273	0.389873884	up_in_case
Yang2025_S	ENSGALG00010027974	ENSGALG00010027974	case	control	0.659176303	0.509942839	1.292647437	0.196133025	0.352329312	up_in_case
Yang2025_S	ENSGALG00010027975	IQSEC1	case	control	0.9174674	0.329428338	2.785028774	0.005352297	0.025240021	up_in_case
Yang2025_S	ENSGALG00010027976	LRSAM1	case	control	0.063522498	0.110279736	0.57601242	0.564606787	0.710666841	up_in_case
Yang2025_S	ENSGALG00010027977	BOLA1	case	control	0.016810619	0.309191351	0.05436963	0.956640675	0.973476545	up_in_case
Yang2025_S	ENSGALG00010027979	RPL12	case	control	0.107743112	0.230519261	0.467393098	0.64021866	0.770019595	up_in_case
Yang2025_S	ENSGALG00010027980	NUP210	case	control	0.524934705	0.192465208	2.727426478	0.006383048	0.028795006	up_in_case
Yang2025_S	ENSGALG00010027981	CNN2	case	control	-0.225772675	0.249345587	-0.90546088	0.365221276	0.533867458	down_in_case
Yang2025_S	ENSGALG00010027982	ENTPD2	case	control	-0.473979292	0.180717434	-2.622764617	0.008721951	0.036685899	down_in_case
Yang2025_S	ENSGALG00010027983	POGZ	case	control	0.001923781	0.150313619	0.012798448	0.989789595	0.993843973	up_in_case
Yang2025_S	ENSGALG00010027984	ARHGAP45	case	control	0.155814929	0.161593812	0.964238212	0.334926511	0.503527989	up_in_case
Yang2025_S	ENSGALG00010027985	PSMB4	case	control	0.126984689	0.196358196	0.646699207	0.517826636	0.672248386	up_in_case
Yang2025_S	ENSGALG00010027986	CGN	case	control	1.293816539	0.227460016	5.688105361	1.28457E-08	5.77433E-07	up_in_case
Yang2025_S	ENSGALG00010027987	TMEM141	case	control	0.610338168	0.227759396	2.679749676	0.007367723	0.032134343	up_in_case
Yang2025_S	ENSGALG00010027988	HDAC11	case	control	0.122272915	0.126219307	0.968733848	0.332678	0.501880257	up_in_case
Yang2025_S	ENSGALG00010027989	ENSGALG00010027989	case	control	-0.386627204	0.311691983	-1.240414334	0.214822182	0.375460567	down_in_case
Yang2025_S	ENSGALG00010027990	ENSGALG00010027990	case	control	0.612260357	0.322155008	1.900514785	0.057365597	0.147693554	up_in_case
Yang2025_S	ENSGALG00010027991	ENSGALG00010027991	case	control	-0.290341491	0.204058983	-1.422831219	0.154785089	0.299199641	down_in_case
Yang2025_S	ENSGALG00010027993	CLIC3	case	control	0.00470602	0.188074701	0.025022076	0.980037355	0.987885487	up_in_case
Yang2025_S	ENSGALG00010027994	TUFT1	case	control	0.46049405	0.107882982	4.268458669	1.96828E-05	0.000286604	up_in_case
Yang2025_S	ENSGALG00010027995	POLR2E	case	control	-0.217693226	0.188650536	-1.153949686	0.248520797	0.414250874	down_in_case
Yang2025_S	ENSGALG00010027996	GPX4	case	control	0.375410644	0.219008156	1.714140016	0.086503014	0.198096878	up_in_case
Yang2025_S	ENSGALG00010027998	SNX27	case	control	-0.035236639	0.100548222	-0.350445167	0.726004634	0.831645236	down_in_case
Yang2025_S	ENSGALG00010027999	ENSGALG00010027999	case	control	0.087720082	0.457014922	0.191941396	0.847788111	0.909738128	up_in_case
Yang2025_S	ENSGALG00010028000	MRPL9	case	control	0.815883833	0.212509329	3.839284785	0.000123393	0.001259696	up_in_case

Yang2025_S	ENSGALG00010028001	SBNO2	case	control	0.278672059	0.131259282	2.123065541	0.03374836	0.09997187	up_in_case
Yang2025_S	ENSGALG00010028002	ABCA2	case	control	0.313064011	0.132171499	2.368619651	0.017854604	0.062529256	up_in_case
Yang2025_S	ENSGALG00010028004	ENSGALG00010028004	case	control	0.396488052	0.343760198	1.15338557	0.248752162	0.414537356	up_in_case
Yang2025_S	ENSGALG00010028005	IER5L	case	control	0.333128152	0.132499473	2.514184739	0.011930792	0.046156464	up_in_case
Yang2025_S	ENSGALG00010028006	ENSGALG00010028006	case	control	-0.161494592	0.531690362	-0.303738046	0.76132747	0.854874912	down_in_case
Yang2025_S	ENSGALG00010028007	STK11	case	control	-0.041745218	0.086498324	-0.482613026	0.629370528	0.761970314	down_in_case
Yang2025_S	ENSGALG00010028008	NTMT1	case	control	-0.476179979	0.197820498	-2.40713164	0.016078373	0.057879651	down_in_case
Yang2025_S	ENSGALG00010028009	RRP9	case	control	0.457101962	0.119333342	3.830463096	0.000127902	0.001296232	up_in_case
Yang2025_S	ENSGALG00010028010	ASB6	case	control	-0.119866477	0.175973007	-0.681163998	0.495767726	0.653102974	down_in_case
Yang2025_S	ENSGALG00010028011	ENSGALG00010028011	case	control	-0.491828681	0.151371717	-3.249145157	0.001157524	0.007857361	down_in_case
Yang2025_S	ENSGALG00010028012	PRRX2	case	control	0.195591051	0.231767372	0.843911072	0.398719101	0.565626659	up_in_case
Yang2025_S	ENSGALG00010028013	CBARP	case	control	0.447872035	0.153696732	2.913998431	0.003568317	0.018526266	up_in_case
Yang2025_S	ENSGALG00010028014	ENSGALG00010028014	case	control	0.202710454	0.695257215	0.291561813	0.770621676	0.860110387	up_in_case
Yang2025_S	ENSGALG00010028015	DUSP7	case	control	-0.120500748	0.124081299	-0.971143504	0.331476823	0.500772932	down_in_case
Yang2025_S	ENSGALG00010028019	RPL29	case	control	-0.611334845	0.263883982	-2.316680386	0.020521149	0.06902298	down_in_case
Yang2025_S	ENSGALG00010028020	ENSGALG00010028020	case	control	-0.021953216	0.228218164	-0.09619399	0.923366504	0.953825963	down_in_case
Yang2025_S	ENSGALG00010028021	ATP5F1D	case	control	0.001906283	0.301353045	0.006325747	0.994952818	0.996498552	up_in_case
Yang2025_S	ENSGALG00010028022	ENSGALG00010028022	case	control	-0.150678283	0.150322201	-1.002368791	0.316165509	0.485376928	down_in_case
Yang2025_S	ENSGALG00010028024	RBM3	case	control	-0.079267271	0.13598892	-0.582895069	0.55996394	0.706610296	down_in_case
Yang2025_S	ENSGALG00010028025	TNNC1	case	control	0.92163651	0.290671885	3.170710887	0.001520664	0.009707033	up_in_case
Yang2025_S	ENSGALG00010028026	ANKMY1	case	control	0.509840187	0.140685891	3.623961025	0.000290125	0.002536322	up_in_case
Yang2025_S	ENSGALG00010028027	ENSGALG00010028027	case	control	0.166856565	0.363748196	0.458714482	0.646439213	0.77557217	up_in_case
Yang2025_S	ENSGALG00010028028	FAM174C	case	control	0.228009102	0.275585004	0.827363965	0.408030799	0.573879823	up_in_case
Yang2025_S	ENSGALG00010028029	EFNA2	case	control	-0.108000545	0.173845921	-0.621242907	0.534439812	0.685955493	down_in_case
Yang2025_S	ENSGALG00010028030	PFDN2	case	control	0.259541178	0.341979053	0.758938817	0.447889157	0.610420332	up_in_case
Yang2025_S	ENSGALG00010028031	NISCH	case	control	-0.246309129	0.091208251	-2.700513669	0.006923249	0.030724674	down_in_case
Yang2025_S	ENSGALG00010028032	ENSGALG00010028032	case	control	-0.093180618	0.230316431	-0.404576511	0.685788827	0.803739891	down_in_case
Yang2025_S	ENSGALG00010028033	ENSGALG00010028033	case	control	-0.211805977	0.245190329	-0.863843113	0.387674081	0.554921649	down_in_case
Yang2025_S	ENSGALG00010028034	MUM1L1	case	control	0.094064151	0.07909332	1.189280604	0.234329265	0.397538144	up_in_case
Yang2025_S	ENSGALG00010028035	ENSGALG00010028035	case	control	0.94008464	1.276107181	0.736681568	0.461316018	0.622307717	up_in_case
Yang2025_S	ENSGALG00010028036	NDUFS7	case	control	0.333963232	0.200958046	1.661855494	0.096541752	0.214016754	up_in_case
Yang2025_S	ENSGALG00010028037	ENSGALG00010028037	case	control	-0.188011239	0.226095546	-0.83155658	0.405659279	0.571829321	down_in_case
Yang2025_S	ENSGALG00010028039	STAB1	case	control	-0.200018098	0.129556517	-1.54386751	0.122620432	0.253632828	down_in_case
Yang2025_S	ENSGALG00010028042	DPP7	case	control	0.213901872	0.155512663	1.375462735	0.168988031	0.317493355	up_in_case
Yang2025_S	ENSGALG00010028043	NT5DC2	case	control	0.286494821	0.174053102	1.646019619	0.09975972	0.218516983	up_in_case
Yang2025_S	ENSGALG00010028044	GAMT	case	control	-0.256989683	0.203261392	-1.264331024	0.206111239	0.364639678	down_in_case
Yang2025_S	ENSGALG00010028045	ENSGALG00010028045	case	control	-0.217090732	0.421122196	-0.515505319	0.606199954	0.743766699	down_in_case
Yang2025_S	ENSGALG00010028046	SMIM4	case	control	-0.453687674	0.243815866	-1.860779943	0.062775259	0.157183298	down_in_case
Yang2025_S	ENSGALG00010028047	ENSGALG00010028047	case	control	-0.370706424	0.425170799	-0.871900011	0.383262928	0.550935517	down_in_case
Yang2025_S	ENSGALG00010028048	S100A10	case	control	0.012045938	0.320777144	0.037552357	0.970044595	0.982096151	up_in_case
Yang2025_S	ENSGALG00010028049	MAN1B1	case	control	-0.438670546	0.10863628	-4.037974652	5.39147E-05	0.000647115	down_in_case
Yang2025_S	ENSGALG00010028050	PBRM1	case	control	-0.126886437	0.127457193	-0.995521983	0.319482458	0.488856637	down_in_case
Yang2025_S	ENSGALG00010028051	GNL3	case	control	0.360324448	0.083261146	4.327642193	1.50714E-05	0.000230538	up_in_case
Yang2025_S	ENSGALG00010028052	ENSGALG00010028052	case	control	0.85252191	0.221454497	3.849648215	0.000118288	0.001216715	up_in_case
Yang2025_S	ENSGALG00010028054	RPS15	case	control	-0.557440345	0.346208617	-1.610128453	0.107369817	0.230290656	down_in_case
Yang2025_S	ENSGALG00010028055	ENSGALG00010028055	case	control	0.458912861	0.148160963	3.09739388	0.001952303	0.011772107	up_in_case
Yang2025_S	ENSGALG00010028056	SLC25A25	case	control	0.253266485	0.145564254	1.739894772	0.081877497	0.190350837	up_in_case
Yang2025_S	ENSGALG00010028057	APC2	case	control	0.432640511	0.561087871	0.771074432			up_in_case
Yang2025_S	ENSGALG00010028058	GLT8D1	case	control	-0.309001602	0.125196289	-2.468137071	0.01358183	0.0508361	down_in_case
Yang2025_S	ENSGALG00010028060	ENSGALG00010028060	case	control	-0.050236685	0.31577754	-0.159088848	0.873598874	0.926171661	down_in_case
Yang2025_S	ENSGALG00010028061	NAIF1	case	control	-0.327135518	0.072815453	-4.492666099	7.0337E-06	0.000123756	down_in_case
Yang2025_S	ENSGALG00010028062	ENSGALG00010028062	case	control	0.679613323	0.851126514	0.798486843	0.424588025	0.589541065	up_in_case
Yang2025_S	ENSGALG00010028064	SPCS1	case	control	0.053778463	0.17758886	0.302825658	0.762022729	0.855286959	up_in_case
Yang2025_S	ENSGALG00010028065	REEP6	case	control	-0.120244704	0.438302215	-0.274341994	0.783821815	0.869324919	down_in_case
Yang2025_S	ENSGALG00010028066	ENSGALG00010028066	case	control	0.73523776	0.160841844	4.571184588	4.84975E-06	9.01083E-05	up_in_case
Yang2025_S	ENSGALG00010028067	FAM102A	case	control	0.011421694	0.155025245	0.073676349	0.941267919	0.963855699	up_in_case
Yang2025_S	ENSGALG00010028068	POLE4	case	control	0.230087621	0.309739601	0.742842118	0.457577273	0.619062067	up_in_case
Yang2025_S	ENSGALG00010028069	NEK4	case	control	-0.257071181	0.09993482	-2.572388481	0.010099949	0.040806839	down_in_case
Yang2025_S	ENSGALG00010028070	MEX3A	case	control	0.244343994	0.167532538	1.458486794	0.144706413	0.285661405	up_in_case
Yang2025_S	ENSGALG00010028071	DPM2	case	control	0.036140387	0.265905331	0.13591449	0.891888881	0.936513567	up_in_case
Yang2025_S	ENSGALG00010028072	ENSGALG00010028072	case	control	0.275400009	0.327711061	0.840374471	0.400698458	0.56739488	up_in_case
Yang2025_S	ENSGALG00010028073	ST6GALNAC4	case	control	-0.011705308	0.170807443	-0.068529262	0.945364327	0.966306161	down_in_case
Yang2025_S	ENSGALG00010028074	ENSGALG00010028074	case	control	-0.640399279	0.431844982	-1.482937873	0.138090918	0.275884866	down_in_case
Yang2025_S	ENSGALG00010028075	RAB25	case	control	-0.442502259	0.480428865	-0.921056771	0.357020788	0.52624124	down_in_case
Yang2025_S	ENSGALG00010028076	ST6GALNAC6	case	control	0.047854348	0.126314706	0.378850167	0.704799131	0.817692472	up_in_case
Yang2025_S	ENSGALG00010028077	ENSGALG00010028077	case	control	0.642702558	0.707830578	0.907989254	0.363883899	0.532694835	up_in_case
Yang2025_S	ENSGALG00010028078	LAMTOR2	case	control	0.689696563	0.184122996	3.745846957	0.000179786	0.00171518	up_in_case
Yang2025_S	ENSGALG00010028080	MUSTN1	case	control	0.686543365	0.427175771	1.607168319	0.10801745	0.231217075	up_in_case
Yang2025_S	ENSGALG00010028081	UBQLN4	case	control	-0.141646776	0.084660163	-1.673121937	0.094303294	0.210292271	down_in_case
Yang2025_S	ENSGALG00010028082	AK1	case	control	-0.075688893	0.206708945	-0.366161671	0.714244441	0.823558969	down_in_case
Yang2025_S	ENSGALG00010028083	ELAVL1	case	control	-0.309388752	0.072452085	-4.270253237	1.95251E-05	0.000284903	down_in_case
Yang2025_S	ENSGALG00010028084	ENG	case	control	0.191150514	0.110821294	1.724853656	0.084553849	0.194978965	up_in_case
Yang2025_S	ENSGALG00010028085	ENSGALG00010028085	case	control	1.286844836	0.149073271	8.632297551	6.01317E-18	2.3276E-15	up_in_case
Yang2025_S	ENSGALG00010028086	MATK	case	control	0.810426396	0.359719816	2.252937873	0.024263066	0.078446829	up_in_case
Yang2025_S	ENSGALG00010028087	STIMATE	case	control	-0.121866426	0.093834638	-1.298736035	0.194034533	0.349517996	down_in_case
Yang2025_S	ENSGALG00010028088	NAXE	case	control	-0.010919521	0.24547711	-0.044482847	0.964519525	0.978920581	down_in_case
Yang2025_S	ENSGALG00010028090	ENSGALG00010028090	case	control	1.285224625	0.510584199	2.517164903	0.011830344	0.045882508	up_in_case
Yang2025_S	ENSGALG00010028091	FPGS	case	control	-0.00588146	0.215395781	-0.027305361	0.978216181	0.9869076	down_in_case
Yang2025_S	ENSGALG00010028093	SFMBT1	case	control	-0.270236544	0.184020013	-1.468517147	0.141963802	0.281483435	down_in_case
Yang2025_S	ENSGALG00010028095	CDK9	case	control	0.023937087	0.221057131	0.108284616	0.913769925	0.94940232	up_in_case
Yang2025_S	ENSGALG00010028096	TOR2A	case	control	0.041996448	0.171568034	0.244780145	0.806626669	0.883622621	up_in_case
Yang2025_S	ENSGALG00010028097	TTC24	case	control	0.738670946	0.264377502	2.794000778	0.005206035	0.02470075	up_in_case
Yang2025_S	ENSGALG00010028098	RFT1	case	control	-0.216271252	0.154623111	-1.398699396	0.161903145	0.308002776	down_in_case
Yang2025_S	ENSGALG00010028099	PCASP2	case	control	0.31732603	0.213956379	1.483134236	0.13803875	0.275859741	up_in_case
Yang2025_S	ENSGALG00010028101	MRPL54	case	control	0.376972796	0.294855733	1.278499125	0.201073493	0.358903436	up_in_case
Yang2025_S	ENSGALG00010028102	CERCAM	case	control	-0.107177572	0.095138401	-1.126543764	0.259935427	0.426341829	down_in_case
Yang2025_S	ENSGALG00010028103	APBA3	case	control	-0.145399848	0.139479969	-1.042442499	0.297206571	0.466183428	down_in_case
Yang2025_S	ENSGALG00010028104	GOLGA1	case	control	0.677720674	0.168594771	4.019820252	5.82426E-05	0.000690145	up_in_case
Yang2025_S	ENSGALG00010028105	PRKCD	case	control	0.179029659	0.116975078	1.530494038	0.125894488	0.258218971	up_in_case
Yang2025_S	ENSGALG00010028106	ARPC5L	case	control	0.388030501	0.20441923	1.898209384	0.057668505	0.148290978	up_in_case
Yang2025_S	ENSGALG00010028107	NOTCH1	case	control	0.207914882	0.150235073	1.383930382	0.166379773	0.314245342	up_in_case
Yang2025_S	ENSGALG00010028108	IQGAP3	case	control	0.598926825	0.169865361	3.525891451	0.00042206	0.003453555	up_in_case
Yang2025_S	ENSGALG00010028109	TJP3	case	control	-0.244685865	0.176457667	-1.386654769	0.165547059	0.313096942	down_in_case
Yang2025_S	ENSGALG00010028110	COQ4	case	control	0.418926665	0.2053215	2.040344856	0.04131599	0.115891749	up_in_case
Yang2025_S	ENSGALG00010028111	TRUB2	case	control	-0.245877901	0.190774042	-1.288843588	0.197452469	0.353845185	down_in_case
Yang2025_S	ENSGALG00010028112	SWI5	case	control	0.055980619	0.229639199	0.243776407	0.807403994	0.883899076	up_in_case
Yang2025_S	ENSGALG00010028113	PIP5K1C	case	control	0.201007928	0.065911537	3.04966229	0.002290988	0.013257857	up_in_case
Yang2025_S	ENSGALG00010028114	PFKFB4	case	control	0.339095796	0.105262402	3.221433197	0.001275512	0.008467965	up_in_case
Yang2025_S	ENSGALG00010028115	CACTIN	case	control	0.353284921	0.081882194	4.314551226	1.59927E-05	0.000242502	up_in_case
Yang2025_S	ENSGALG00010028116	HYAL1	case	control	0.222088017	0.138679865	1.60144385	0.109278647	0.233236016	up_in_case
Yang2025_S	ENSGALG00010028117	ENSGALG00010028117	case	control	1.072534732	0.546406998	1.962886154	0.049659404	0.132669439	up_in_case
Yang2025_S	ENSGALG00010028118	ENSGALG00010028118	case	control	0.392524886	0.086218973	4.552650917	5.29741E-06	9.72588E-05	up_in_case
Yang2025_S	ENSGALG00010028119	HYAL2	case	control	0.36615098	0.096678278	3.78731384	0.000152285	0.001498649	up_in_case
Yang2025_S	ENSGALG00010028120	TBXA2R	case	control	0.655579817	0.165204462	3.968293651	7.23891E-05	0.000820783	up_in_case
Yang2025_S	ENSGALG00010028121	TUSC2	case	control	-0.280452945	0.151167145	-1.855250658	0.063560441	0.158616537	down_in_case
Yang2025_S	ENSGALG00010028122	SEC16A	case	control	-0.089102285	0.112219265	-0.794001682	0.427194452	0.591568587	down_in_case
Yang2025_S	ENSGALG00010028123	RASSF1	case	control	0.356496718	0.248473201	1.434749164	0.151358589	0.294372915	up_in_case
Yang2025_S	ENSGALG00010028124	SUGP2	case	control	0.237555955	0.123399425	1.925097742	0.054217131	0.141614944	up_in_case
Yang2025_S	ENSGALG00010028125	HMG20B	case	control	0.207931305	0.122315393	1.699960243	0.089138404	0.202567459	up_in_case
Yang2025_S	ENSGALG00010028126	ZMYND10	case	control	-0.095237814	0.142170702	-0.669883547	0.502932029	0.659189921	down_in_case
Yang2025_S	ENSGALG00010028127	MFSD12	case	control	0.468392023	0.153120132	3.058983926	0.002220891	0.012970202	up_in_case
Yang2025_S	ENSGALG00010028128	DNM1	case	control	0.250247594	0.17958195	1.393500812	0.1634684	0.310159741	up_in_case
Yang2025_S	ENSGALG00010028129	CYB561D2	case	control	0.736150894	0.21831307	3.371996435	0.000746254	0.005539158	up_in_case
Yang2025_S	ENSGALG00010028130	TMEM115	case	control	-0.173072651	0.204412786	-0.84668212	0.397172328	0.56417904	down_in_case
Yang2025_S	ENSGALG00010028131	TMEM161A	case	control	-0.097539097	0.16898465	-0.577206844	0.563799734	0.709907771	down_in_case
Yang2025_S	ENSGALG00010028132	CACNA2D2	case	control	0.288613363	0.124805474	2.312505644	0.020749835	0.069540391	up_in_case
Yang2025_S	ENSGALG00010028133	ENSGALG00010028133	case	control	0.747333931	0.32219439	2.319512549	0.020367261	0.068654521	up_in_case
Yang2025_S	ENSGALG00010028134	INPP5E	case	control	-0.260072254	0.084377208	-3.082257179	0.002054373	0.012207967	down_in_case
Yang2025_S	ENSGALG00010028136	C3orf18	case	control	0.157827282	0.129408755	1.21960281	0.222615481	0.384122383	up_in_case
Yang2025_S	ENSGALG00010028137	NUP214	case	control	0.048614564	0.145401363	0.334347374	0.738117446	0.840126326	up_in_case
Yang2025_S	ENSGALG00010028138	ENSGALG00010028138	case	control	0.374493986	0.288756608	1.296919191	0.194658993	0.350475305	up_in_case
Yang2025_S	ENSGALG00010028139	FZR1	case	control	-0.209722764	0.094062176	-2.229618476	0.025772782	0.081759974	down_in_case
Yang2025_S	ENSGALG00010028140	HEMK1	case	control	-0.158019929	0.182981034	-0.86358638	0.387815149	0.554921649	down_in_case
Yang2025_S	ENSGALG00010028141	CISH	case	control	-0.050068542	0.094843604	-0.527906367	0.597564325	0.736842657	down_in_case
Yang2025_S	ENSGALG00010028142	PMPCA	case	control	0.145154674	0.086551936	1.677081763	0.093526494	0.209162526	up_in_case
Yang2025_S	ENSGALG00010028144	FAM78A	case	control	0.211902435	0.140969267	1.503181792	0.132792172	0.267988256	up_in_case

Yang2025_S	ENSGALG00010028145	ENSGALG00010028145	case	control	0.08346254	0.276947921	0.301365469	0.763135823	0.855638848	up_in_case
Yang2025_S	ENSGALG00010028146	MAPKAPK3	case	control	-0.195497404	0.122110903	-1.600982377	0.109380823	0.233311153	down_in_case
Yang2025_S	ENSGALG00010028147	SLC25A42	case	control	-0.24870726	0.170182803	-1.461412408	0.143902298	0.284396329	down_in_case
Yang2025_S	ENSGALG00010028148	ENSGALG00010028148	case	control	0.038892286	0.212775025	0.182785954	0.85496597	0.91434772	up_in_case
Yang2025_S	ENSGALG00010028149	SNAPC4	case	control	0.590413855	0.126557841	4.665170113	3.08362E-06	6.20061E-05	up_in_case
Yang2025_S	ENSGALG00010028150	ARMC6	case	control	-0.158582013	0.127388022	-1.244873813	0.213178138	0.373430215	down_in_case
Yang2025_S	ENSGALG00010028151	DOCK3	case	control	1.156360839	0.276631968	4.180141759	2.91327E-05	0.000397226	up_in_case
Yang2025_S	ENSGALG00010028152	ENSGALG00010028152	case	control	0.09639271	0.361612133	0.266563815	0.789805019	0.873209532	up_in_case
Yang2025_S	ENSGALG00010028153	MANF	case	control	0.02040329	0.210161219	0.097083994	0.922659692	0.953661386	up_in_case
Yang2025_S	ENSGALG00010028154	ENSGALG00010028154	case	control	0.0765689	0.195873675	0.390909601	0.695864057	0.811317405	up_in_case
Yang2025_S	ENSGALG00010028155	CIZ1	case	control	0.271469721	0.074512857	3.643260114	0.000269207	0.002384865	up_in_case
Yang2025_S	ENSGALG00010028156	DOHH	case	control	-0.132007101	0.312096605	-0.422968717	0.672318085	0.793789768	down_in_case
Yang2025_S	ENSGALG00010028157	PRRC2B	case	control	-0.022015865	0.183621513	-0.119898072	0.904563891	0.943001259	down_in_case
Yang2025_S	ENSGALG00010028158	DDX49	case	control	0.018620327	0.096927533	0.192105658	0.847659443	0.909738128	up_in_case
Yang2025_S	ENSGALG00010028159	DCAF1	case	control	0.128467317	0.106293396	1.208610523	0.226812512	0.388857327	up_in_case
Yang2025_S	ENSGALG00010028160	ENSGALG00010028160	case	control	1.02535787	0.23803815	4.307535868	1.65083E-05	0.000247892	up_in_case
Yang2025_S	ENSGALG00010028161	ENSGALG00010028161	case	control	2.358416972	0.452786295	5.208675702	1.90193E-07	5.92918E-06	up_in_case
Yang2025_S	ENSGALG00010028162	CARD9	case	control	0.493063815	0.154527398	3.190785717	0.001418865	0.009230569	up_in_case
Yang2025_S	ENSGALG00010028163	GBP	case	control	0.986942331	0.307242816	3.212255192	0.001316973	0.008697642	up_in_case
Yang2025_S	ENSGALG00010028164	BBLN	case	control	-0.302711581	0.240969631	-1.256222951	0.209035148	0.368255978	down_in_case
Yang2025_S	ENSGALG00010028165	NFIC	case	control	0.915292158	0.310152258	2.9511059	0.003166383	0.017008983	up_in_case
Yang2025_S	ENSGALG00010028167	ENSGALG00010028167	case	control	-0.360642225	0.246552184	-1.462741956	0.143538001	0.283877667	down_in_case
Yang2025_S	ENSGALG00010028168	PTGES2	case	control	0.018180216	0.140957379	0.128976689	0.897376094	0.93902049	up_in_case
Yang2025_S	ENSGALG00010028169	DNLZ	case	control	0.351137275	0.268776449	1.306428732	0.1914068	0.346531605	up_in_case
Yang2025_S	ENSGALG00010028170	COPE	case	control	-0.249492834	0.196528775	-1.26949773	0.2042636	0.362415745	down_in_case
Yang2025_S	ENSGALG00010028171	RNF123	case	control	-0.245356778	0.091649665	-2.677115912	0.007425894	0.032337448	down_in_case
Yang2025_S	ENSGALG00010028172	CERS1	case	control	-0.409974705	0.209158314	-1.960116705	0.049982151	0.133235534	down_in_case
Yang2025_S	ENSGALG00010028173	GDF3	case	control	0.020155163	0.189305649	0.106468893	0.915210335	0.950120376	up_in_case
Yang2025_S	ENSGALG00010028174	GMPPB	case	control	0.586379247	0.243578662	2.407350635	0.016068734	0.057876915	up_in_case
Yang2025_S	ENSGALG00010028175	POMT1	case	control	-0.316448902	0.15842631	-1.997451701	0.045776136	0.12524847	down_in_case
Yang2025_S	ENSGALG00010028176	UPF1	case	control	-0.142879215	0.088334512	-1.617478966	0.105774932	0.228166987	down_in_case
Yang2025_S	ENSGALG00010028177	ENSGALG00010028177	case	control	1.183968306	0.321017942	3.688168642	0.000225874	0.00205186	up_in_case
Yang2025_S	ENSGALG00010028178	ENSGALG00010028178	case	control	-0.128648867	0.14414953	-0.892468167	0.372142048	0.540468936	down_in_case
Yang2025_S	ENSGALG00010028179	MCM2	case	control	-0.145500052	0.192291534	-0.756663849	0.449251279	0.611510746	down_in_case
Yang2025_S	ENSGALG00010028180	ODF2	case	control	0.576883431	0.151331583	3.812049131	0.000137819	0.001383656	up_in_case
Yang2025_S	ENSGALG00010028181	BGLAP	case	control	0.058169157	0.37845197	0.153702877	0.877844017	0.92809932	up_in_case
Yang2025_S	ENSGALG00010028182	SMG5	case	control	0.179370003	0.119956044	1.495297761	0.134836749	0.271240562	up_in_case
Yang2025_S	ENSGALG00010028183	CRTC1	case	control	0.420111606	0.210474555	1.996020881	0.04593165	0.125403123	up_in_case
Yang2025_S	ENSGALG00010028184	TMEM79	case	control	-0.047467548	0.164895678	-0.28786411	0.773450761	0.862181781	down_in_case
Yang2025_S	ENSGALG00010028185	GLMP	case	control	0.25698076	0.176162234	1.458773282	0.144627518	0.285546114	up_in_case
Yang2025_S	ENSGALG00010028186	CCT3	case	control	0.303208764	0.110298849	2.74897486	0.005978197	0.027331423	up_in_case
Yang2025_S	ENSGALG00010028187	TPRA1	case	control	-0.418891671	0.163486642	-2.562237903	0.010400005	0.041692771	down_in_case
Yang2025_S	ENSGALG00010028188	ENSGALG00010028188	case	control	0.644737472	0.16340169	3.945720958	7.95602E-05	0.000891215	up_in_case
Yang2025_S	ENSGALG00010028189	GPSM1	case	control	-0.205648403	0.100656341	-2.043074505	0.041045063	0.115384902	down_in_case
Yang2025_S	ENSGALG00010028190	ILF2	case	control	0.073508867	0.128038984	0.574113172	0.56589122	0.711190394	up_in_case
Yang2025_S	ENSGALG00010028191	KLHL26	case	control	-0.140525875	0.07945825	-1.768549837	0.076969028	0.182089326	down_in_case
Yang2025_S	ENSGALG00010028192	ENSGALG00010028192	case	control	1.634342977	0.780527206	2.093896234	0.036269225	0.105425876	up_in_case
Yang2025_S	ENSGALG00010028193	BRINP1	case	control	-0.060434371	0.326797763	-0.184928963	0.853284753	0.913330907	down_in_case
Yang2025_S	ENSGALG00010028194	ENSGALG00010028194	case	control	0.127531316	0.103928369	1.227107837	0.219782063	0.381023021	up_in_case
Yang2025_S	ENSGALG00010028195	ENSGALG00010028195	case	control	0.017265927	0.241281741	0.071559196	0.942952714	0.965065006	up_in_case
Yang2025_S	ENSGALG00010028196	ENSGALG00010028196	case	control	0.370170096	0.167536366	2.209491013	0.027140507	0.08496623	up_in_case
Yang2025_S	ENSGALG00010028197	INTS3	case	control	0.003778119	0.07040529	0.053662436	0.957204111	0.973836554	up_in_case
Yang2025_S	ENSGALG00010028198	UCK1	case	control	-0.268600815	0.120073358	-2.236972618	0.02528813	0.080620016	down_in_case
Yang2025_S	ENSGALG00010028199	DPP9	case	control	-0.045007634	0.060545643	-0.743367004	0.457259515	0.618872508	down_in_case
Yang2025_S	ENSGALG00010028201	ENSGALG00010028201	case	control	0.356169161	0.258743572	1.376533368	0.168656564	0.316998816	up_in_case
Yang2025_S	ENSGALG00010028202	QSOX2	case	control	-0.48167828	0.110136791	-4.37345484	1.22296E-05	0.000194764	down_in_case
Yang2025_S	ENSGALG00010028205	PRRT1B	case	control	0.033952833	0.454988398	0.074623513	0.940514265	0.963601685	up_in_case
Yang2025_S	ENSGALG00010028206	RBM15B	case	control	-0.164026567	0.082224008	-1.994874382	0.046056582	0.125645747	down_in_case
Yang2025_S	ENSGALG00010028208	AMIGO3	case	control	0.137930923	0.287057855	0.480498689	0.630872835	0.763258635	up_in_case
Yang2025_S	ENSGALG00010028211	GATAD2B	case	control	0.326048714	0.209713861	1.554731345	0.120010066	0.249674543	up_in_case
Yang2025_S	ENSGALG00010028216	NKX2-3	case	control	-1.625864146	1.489551078	-1.091512853	0.27504728	0.442234588	down_in_case
Yang2025_S	ENSGALG00010028219	ENSGALG00010028219	case	control	-1.763608844	1.853811626	-0.951341991	0.341430797	0.509622768	down_in_case
Yang2025_S	ENSGALG00010028221	TNFAIP8L1	case	control	-0.416563292	0.17501842	-2.38011114	0.017307417	0.061046432	down_in_case
Yang2025_S	ENSGALG00010028232	C9orf69	case	control	-0.69173424	0.193562519	-3.573699317	0.000351973	0.002979796	down_in_case
Yang2025_S	ENSGALG00010028234	EDCH3	case	control	-0.829612274	2.389821083	-0.347144094	0.728483069	0.833097929	down_in_case
Yang2025_S	ENSGALG00010028239	EDMPN2	case	control	-1.282192788	2.438961606	-0.525712576	0.599087925	0.738003026	down_in_case
Yang2025_S	ENSGALG00010028242	ENSGALG00010028242	case	control	0.481606877	0.180480992	2.668463158	0.007619914	0.032986485	up_in_case
Yang2025_S	ENSGALG00010028245	JAC1	case	control	-1.191209074	1.154000668	-1.032242967	0.301958311	0.471619487	down_in_case
Yang2025_S	ENSGALG00010028247	MVP	case	control	0.197493093	0.161608107	1.222049414	0.221688941	0.383184743	up_in_case
Yang2025_S	ENSGALG00010028262	C1orf43	case	control	-0.020807836	0.176383657	-0.117969184	0.906092075	0.943676612	down_in_case
Yang2025_S	ENSGALG00010028270	RAPGEF1	case	control	0.070219941	0.160776918	0.436753869	0.662289857	0.786518592	up_in_case
Yang2025_S	ENSGALG00010028283	ENSGALG00010028283	case	control	-0.310988929	0.078899539	-3.941581065	8.09463E-05	0.000900228	down_in_case
Yang2025_S	ENSGALG00010028286	KCTD12A	case	control	-3.960702601	1.788840003	-2.214117861	0.026820672	0.084177042	down_in_case
Yang2025_S	ENSGALG00010028292	ENSGALG00010028292	case	control	-0.721131504	0.44432557	-1.622979977	0.104593685	0.226568165	down_in_case
Yang2025_S	ENSGALG00010028297	ENSGALG00010028297	case	control	0.147382788	0.662224941	0.222556988	0.823880315	0.894465038	up_in_case
Yang2025_S	ENSGALG00010028301	FBN3	case	control	-0.177754248	0.162969664	-1.090719854	0.275396172	0.442336123	down_in_case
Yang2025_S	ENSGALG00010028311	ENSGALG00010028311	case	control	-0.281147186	0.383858362	-0.732424284	0.463909641	0.624899067	down_in_case
Yang2025_S	ENSGALG00010028314	MEF2D	case	control	0.667538296	0.198948244	3.355336454	0.000792685	0.005807606	up_in_case
Yang2025_S	ENSGALG00010028316	TIMM44	case	control	0.437025697	0.144045783	3.033936072	0.002413856	0.013831036	up_in_case
Yang2025_S	ENSGALG00010028318	NACC2	case	control	0.027442758	0.176079829	0.155854071	0.876148049	0.927040476	up_in_case
Yang2025_S	ENSGALG00010028321	RHBG	case	control	-0.024914585	0.23401601	-0.106465302	0.915213184	0.950120376	down_in_case
Yang2025_S	ENSGALG00010028322	STRBP	case	control	0.188176863	0.111852426	1.68236729	0.09249764	0.207694911	up_in_case
Yang2025_S	ENSGALG00010028326	CRB2	case	control	0.824361158	0.289253244	2.849963396	0.004372426	0.021590984	up_in_case
Yang2025_S	ENSGALG00010028328	HNRNPM	case	control	0.472701347	0.109414782	4.320269518	1.55839E-05	0.000237076	up_in_case
Yang2025_S	ENSGALG00010028329	HAPLN2	case	control	0.32190867	0.634517803	0.507328035	0.611924671	0.748892787	up_in_case
Yang2025_S	ENSGALG00010028333	ENSGALG00010028333	case	control	-0.134905571	0.430888351	-0.313087069	0.754214513	0.850395882	down_in_case
Yang2025_S	ENSGALG00010028337	ENSGALG00010028337	case	control	-0.074802695	0.282791944	-0.264514944	0.791383151	0.873985268	down_in_case
Yang2025_S	ENSGALG00010028339	BCAN	case	control	0.163111679	0.334293121	0.487930108	0.625599347	0.759450031	up_in_case
Yang2025_S	ENSGALG00010028344	LSM7	case	control	-0.533682377	0.167711418	-3.182146949	0.001461876	0.009439873	down_in_case
Yang2025_S	ENSGALG00010028346	DENND1A	case	control	0.317814789	0.157062774	2.023488962	0.04302276	0.119500808	up_in_case
Yang2025_S	ENSGALG00010028348	NES	case	control	1.015820285	0.20177935	5.034312416	4.79567E-07	1.2926E-05	up_in_case
Yang2025_S	ENSGALG00010028355	RBM18	case	control	-0.526863024	0.155219544	-3.394308535	0.000688021	0.005168505	down_in_case
Yang2025_S	ENSGALG00010028357	SPPL2C	case	control	-0.462227188	0.105547984	-4.379308539	1.19056E-05	0.000190477	down_in_case
Yang2025_S	ENSGALG00010028360	ENSGALG00010028360	case	control	0.171998699	0.863022472	0.199298054	0.842029601	0.906285819	up_in_case
Yang2025_S	ENSGALG00010028362	TMPRSS9	case	control	-0.540059472	0.37862165	-1.426382966	0.153757828	0.29775088	down_in_case
Yang2025_S	ENSGALG00010028364	MHM2	case	control	-0.321212579	0.498304512	-0.64461102	0.519179292	0.673246301	down_in_case
Yang2025_S	ENSGALG00010028366	MRRF	case	control	0.39559981	0.121101451	3.266680997	0.001088162	0.007491868	up_in_case
Yang2025_S	ENSGALG00010028368	TIMM13	case	control	-0.101923141	0.378988694	-0.268934517	0.787980074	0.87222991	down_in_case
Yang2025_S	ENSGALG00010028370	S100A12	case	control	0.430696587	0.385891817	1.116107076	0.264376304	0.431442065	up_in_case
Yang2025_S	ENSGALG00010028372	PTGS1	case	control	0.176651002	0.151487849	1.166106739	0.243571292	0.408640255	up_in_case
Yang2025_S	ENSGALG00010028374	FDPS	case	control	0.122244242	0.216541591	0.564530082	0.572393433	0.715833643	up_in_case
Yang2025_S	ENSGALG00010028375	LMNB2	case	control	-0.33066317	0.11994199	-2.756859139	0.005835949	0.026875064	down_in_case
Yang2025_S	ENSGALG00010028377	PDCL	case	control	-0.113582923	0.087614941	-1.296387606	0.19484198	0.350655171	down_in_case
Yang2025_S	ENSGALG00010028380	RUSC1	case	control	-0.265671693	0.091911022	-2.890531366	0.003845911	0.019631054	down_in_case
Yang2025_S	ENSGALG00010028384	ENSGALG00010028384	case	control	-0.823815011	1.178884803	-0.698808746	0.484671562	0.642460458	down_in_case
Yang2025_S	ENSGALG00010028386	NCAN	case	control	1.22074275	0.665924764	1.833154157			up_in_case
Yang2025_S	ENSGALG00010028391	MED26	case	control	-0.072230397	0.139482805	-0.517844453	0.6045668	0.742782433	down_in_case
Yang2025_S	ENSGALG00010028392	ASH1L	case	control	0.163886485	0.142334326	1.151419267	0.249559791	0.415194013	up_in_case
Yang2025_S	ENSGALG00010028394	ZBTB26	case	control	-0.176516374	0.090553308	-1.949308948	0.051258541	0.135641429	down_in_case
Yang2025_S	ENSGALG00010028399	SLC35E1	case	control	-0.362264482	0.065205428	-5.555741225	2.76436E-08	1.11656E-06	down_in_case
Yang2025_S	ENSGALG00010028401	ENSGALG00010028401	case	control	-1.480710549	1.509337225	-0.981033612	0.326576167	0.496491259	down_in_case
Yang2025_S	ENSGALG00010028405	S100A6	case	control	-0.641731809	0.416029761	-1.542514188	0.122948693	0.253871108	down_in_case
Yang2025_S	ENSGALG00010028412	ENSGALG00010028412	case	control	0.740846988	0.457891801	1.617952071	0.105672927	0.228069484	up_in_case
Yang2025_S	ENSGALG00010028414	KBTBD12	case	control	0.037192559	0.192884707	0.192822747	0.847097788	0.909492848	up_in_case
Yang2025_S	ENSGALG00010028415	FSD1	case	control	0.190574345	0.140154188	1.359747772	0.173909755	0.324105452	up_in_case
Yang2025_S	ENSGALG00010028418	ENSGALG00010028418	case	control	-0.201255425	0.121392265	-1.657893312	0.097339002	0.21479319	down_in_case
Yang2025_S	ENSGALG00010028419	ENSGALG00010028419	case	control	0.687738905	0.234800088	2.929040233	0.003400104	0.017886165	up_in_case
Yang2025_S	ENSGALG00010028420	ENSGALG00010028420	case	control	1.591728393	0.46417153	3.429181437	0.000605405	0.004640893	up_in_case
Yang2025_S	ENSGALG00010028421	ZBTB7A	case	control	0.281533196	0.11903837	2.365062587	0.018027024	0.062876037	up_in_case
Yang2025_S	ENSGALG00010028422	RXRA	case	control	-0.260548079	0.136651719	-1.90665789	0.056564903	0.146188744	down_in_case
Yang2025_S	ENSGALG00010028425	DAP3	case	control	0.193981651	0.14944752	1.297991766	0.194290164	0.349933235	up_in_case
Yang2025_S	ENSGALG00010028426	GPR21	case	control	0.103493558	0.17249567	0.599977716	0.548521087	0.697241385	up_in_case
Yang2025_S	ENSGALG00010028427	ENSGALG00010028427	case	control	0.605975985	0.293109727	2.067403192	0.038696178	0.110543509	up_in_case
Yang2025_S	ENSGALG00010028428	RABGAP1	case	control	0.094488703	0.133364245	0.70850102	0.478634179	0.637401307	up_in_case
Yang2025_S	ENSGALG00010028429	REXO1	case	control	0.416317867	0.140169542	2.97010221	0.002977007	0.016242987	up_in_case
Yang2025_S	ENSGALG00010028430	NR2C2AP	case	control	-0.167061686	0.240441102	-0.694813342	0.487172286	0.644399222	down_in_case

Yang2025_S	ENSGALG00010028431	ENSGALG00010028431	case	control	0.682937686	0.512153522	1.333462831	0.182379965	0.335241369	up_in_case
Yang2025_S	ENSGALG00010028432	RFXANK	case	control	-0.332582278	0.254552886	-1.30653509	0.191370654	0.346511183	down_in_case
Yang2025_S	ENSGALG00010028433	MSTO1	case	control	0.613098783	0.121291788	5.054742718	4.30971E-07	1.1822E-05	up_in_case
Yang2025_S	ENSGALG00010028434	COL5A1	case	control	-0.019622294	0.198745928	-0.098730546	0.921352215	0.953337584	down_in_case
Yang2025_S	ENSGALG00010028435	ATP8B3	case	control	1.039725023	0.337004375	3.085197405	0.002034171	0.0121268	up_in_case
Yang2025_S	ENSGALG00010028436	S100A1	case	control	0.821922887	0.198368278	4.143418986	3.42166E-05	0.000449819	up_in_case
Yang2025_S	ENSGALG00010028437	ENSGALG00010028437	case	control	-0.008111841	0.09693177	-0.083686094	0.933306014	0.959399477	down_in_case
Yang2025_S	ENSGALG00010028438	MED27	case	control	-0.404989009	0.132850862	-3.048448484	0.002300263	0.013300486	down_in_case
Yang2025_S	ENSGALG00010028439	BORCS8	case	control	0.070197238	0.189487771	0.370457881	0.711041354	0.821520709	up_in_case
Yang2025_S	ENSGALG00010028440	PSMB7	case	control	0.125949438	0.208228746	0.604860955	0.545271388	0.694802194	up_in_case
Yang2025_S	ENSGALG00010028442	CHTOP	case	control	0.725889533	0.086322121	8.409078948	4.13243E-17	1.3392E-14	up_in_case
Yang2025_S	ENSGALG00010028443	ENSGALG00010028443	case	control	0.226203555	0.232068643	0.974726927	0.329695732	0.499318226	up_in_case
Yang2025_S	ENSGALG00010028444	MEF2B	case	control	0.795161752	0.199196003	3.991855955	6.55582E-05	0.000761295	up_in_case
Yang2025_S	ENSGALG00010028445	SYT11	case	control	-0.343551896	0.14153172	-2.427384444	0.015208131	0.055638042	down_in_case
Yang2025_S	ENSGALG00010028446	ENSGALG00010028446	case	control	0.077572779	0.222572722	0.348527792	0.727443846	0.832465302	up_in_case
Yang2025_S	ENSGALG00010028447	OLFM1	case	control	0.059014937	0.136957367	0.430900057	0.666541042	0.789228274	up_in_case
Yang2025_S	ENSGALG00010028448	RIT1	case	control	-0.221937648	0.124871526	-1.777327911	0.075514304	0.179602633	down_in_case
Yang2025_S	ENSGALG00010028449	EFCAB12	case	control	0.300819198	0.330591266	0.909942969	0.362852587	0.531575988	up_in_case
Yang2025_S	ENSGALG00010028450	PTGDS	case	control	0.40286907	0.27979952	1.439849042	0.149910113	0.292412853	up_in_case
Yang2025_S	ENSGALG00010028451	TMEM59L	case	control	0.390063589	0.234241108	1.665222611	0.095868352	0.212927986	up_in_case
Yang2025_S	ENSGALG00010028453	ENSGALG00010028453	case	control	-0.246181337	0.115396664	-2.133348815	0.032896123	0.098055073	down_in_case
Yang2025_S	ENSGALG00010028454	C8G	case	control	0.483459354	0.183110681	2.640257531	0.008284305	0.03518494	up_in_case
Yang2025_S	ENSGALG00010028455	ENSA	case	control	-0.392061459	0.178227823	-2.199776968	0.027822723	0.086532428	down_in_case
Yang2025_S	ENSGALG00010028456	FBXW5	case	control	-0.397319759	0.124691242	-3.186428755	0.00144041	0.009316074	down_in_case
Yang2025_S	ENSGALG00010028457	KANK3	case	control	0.067353067	0.147854542	0.455536001	0.648723674	0.776913715	up_in_case
Yang2025_S	ENSGALG00010028458	TRAF2	case	control	-0.480890137	0.179058754	-2.685655556	0.007238767	0.031700886	down_in_case
Yang2025_S	ENSGALG00010028459	MCL1	case	control	-0.239704721	0.189967984	-1.26181642	0.207014852	0.365783019	down_in_case
Yang2025_S	ENSGALG00010028460	CRLF1	case	control	0.070831298	0.133443821	0.530794892	0.59556092	0.735407961	up_in_case
Yang2025_S	ENSGALG00010028461	ENSGALG00010028461	case	control	0.37418911	0.18106986	2.066545532	0.038776998	0.110660959	up_in_case
Yang2025_S	ENSGALG00010028462	EDF1	case	control	-0.09872421	0.130116323	-0.758738089	0.448009248	0.610503507	down_in_case
Yang2025_S	ENSGALG00010028463	RPS28	case	control	-0.886394509	0.411522764	-2.153937975	0.031245036	0.094344438	down_in_case
Yang2025_S	ENSGALG00010028464	ENSGALG00010028464	case	control	0.429214545	0.122488615	3.504117866	0.000458123	0.003681625	up_in_case
Yang2025_S	ENSGALG00010028465	ENSGALG00010028465	case	control	0.076194874	0.268141939	0.284158733	0.776288739	0.863955241	up_in_case
Yang2025_S	ENSGALG00010028467	ENSGALG00010028467	case	control	0.497316766	0.402502378	1.235562307	0.216621303	0.377374404	up_in_case
Yang2025_S	ENSGALG00010028468	PHPT1	case	control	-0.288883086	0.270642469	-1.067397468	0.285792391	0.454314051	down_in_case
Yang2025_S	ENSGALG00010028470	AJM1	case	control	-0.00092089	0.400559018	-0.002299012	0.998165655	0.998523934	down_in_case
Yang2025_S	ENSGALG00010028471	TPGS1	case	control	-0.34460382	0.272342079	-1.265334469	0.205751455	0.36422085	down_in_case
Yang2025_S	ENSGALG00010028472	RPRD2	case	control	-0.071248191	0.164971424	-0.431882017	0.665827166	0.789166564	down_in_case
Yang2025_S	ENSGALG00010028473	CDC34	case	control	-0.176641231	0.165124258	-1.069747312	0.284733064	0.452940096	down_in_case
Yang2025_S	ENSGALG00010028474	PEX19	case	control	0.239117111	0.225390939	1.060899397	0.288735623	0.457635454	up_in_case
Yang2025_S	ENSGALG00010028475	AMBP	case	control	-2.452202358	1.40697115	-1.742894556			down_in_case
Yang2025_S	ENSGALG00010028476	ENSGALG00010028476	case	control	0.309729648	0.058292858	5.313337851	1.07635E-07	3.65828E-06	up_in_case
Yang2025_S	ENSGALG00010028477	NRARP	case	control	-0.239101509	0.155530437	-1.537329372	0.124212672	0.255592714	down_in_case
Yang2025_S	ENSGALG00010028478	PEA15	case	control	0.378014004	0.117300392	3.222615016	0.001270262	0.008441152	up_in_case
Yang2025_S	ENSGALG00010028479	CASQ1	case	control	-0.104721269	0.246324479	-0.425135454	0.670737934	0.792364624	down_in_case
Yang2025_S	ENSGALG00010028480	F11R	case	control	-0.39815682	0.18201829	-2.18745501	0.028709324	0.088490253	down_in_case
Yang2025_S	ENSGALG00010028481	ENSGALG00010028481	case	control	-0.023191733	0.167654937	-0.138330155	0.889979495	0.935608284	down_in_case
Yang2025_S	ENSGALG00010028482	CD320	case	control	0.090199752	0.223333661	0.403878895	0.686301777	0.804070562	up_in_case
Yang2025_S	ENSGALG00010028483	EXD3	case	control	0.420426341	0.111451657	3.772275381	0.000161766	0.001574164	up_in_case
Yang2025_S	ENSGALG00010028485	USF1	case	control	0.40915733	0.173653446	2.356171669	0.018464382	0.063877051	up_in_case
Yang2025_S	ENSGALG00010028486	ARHGAP30	case	control	0.39114337	0.190510639	2.05313137	0.040059838	0.11348523	up_in_case
Yang2025_S	ENSGALG00010028488	PTGES	case	control	-0.031372164	0.245664178	-0.127703454	0.898383657	0.939723381	down_in_case
Yang2025_S	ENSGALG00010028490	NECTIN4	case	control	1.22014549	0.349722642	3.488894747	0.000485022	0.003855552	up_in_case
Yang2025_S	ENSGALG00010028491	TOR1B	case	control	0.010752496	0.116737852	0.092108048	0.926612194	0.955608219	up_in_case
Yang2025_S	ENSGALG00010028492	ENSGALG00010028492	case	control	0.525135789	0.180699448	2.906128354	0.003659313	0.018907129	up_in_case
Yang2025_S	ENSGALG00010028493	TOR1A	case	control	0.089658304	0.113435769	0.790388288	0.42930104	0.593600912	up_in_case
Yang2025_S	ENSGALG00010028494	ENSGALG00010028494	case	control	0.234012787	0.282016058	0.829785326	0.406660169	0.572752118	up_in_case
Yang2025_S	ENSGALG00010028495	LINGO3	case	control	0.244048545	0.289449029	0.843148605	0.399145338	0.566086872	up_in_case
Yang2025_S	ENSGALG00010028496	ENSGALG00010028496	case	control	-0.348419351	0.156312582	-2.228991081	0.025814498	0.08183008	down_in_case
Yang2025_S	ENSGALG00010028497	ADMP	case	control	1.45909173	0.827819495	1.762572323	0.077972648	0.183973731	up_in_case
Yang2025_S	ENSGALG00010028498	C9orf78	case	control	0.093116024	0.13238952	0.703348906	0.481838339	0.640138932	up_in_case
Yang2025_S	ENSGALG00010028500	VPS45	case	control	-0.118340287	0.101578371	-1.165014614	0.244013077	0.409242106	down_in_case
Yang2025_S	ENSGALG00010028501	USP20	case	control	0.130167684	0.088560263	1.469820432	0.141610394	0.281045237	up_in_case
Yang2025_S	ENSGALG00010028502	PLEKHO1	case	control	-0.087594612	0.0920337	-0.951766709	0.341215309	0.509464838	down_in_case
Yang2025_S	ENSGALG00010028503	NDOR1	case	control	0.356181208	0.166845163	2.134800927	0.032777275	0.097868293	up_in_case
Yang2025_S	ENSGALG00010028506	SHD	case	control	0.987688686	0.712463386	1.386300973	0.16565502	0.313188741	up_in_case
Yang2025_S	ENSGALG00010028507	ANP32E	case	control	-0.004102699	0.089284436	-0.045950881	0.963349399	0.978089172	down_in_case
Yang2025_S	ENSGALG00010028510	CILP2	case	control	1.758804134	0.536131761	3.280544561	0.001036069	0.007220353	up_in_case
Yang2025_S	ENSGALG00010028511	YJEFN3	case	control	0.590051012	0.537855427	1.097043895	0.272622204	0.439910158	up_in_case
Yang2025_S	ENSGALG00010028512	NDUFA13	case	control	0.253980442	0.341279211	0.744201327	0.456754685	0.618429512	up_in_case
Yang2025_S	ENSGALG00010028513	ENSGALG00010028513	case	control	-0.992548724	2.416496563	-0.410738728	0.681264126	0.800321665	down_in_case
Yang2025_S	ENSGALG00010028514	FNBP1	case	control	0.316279806	0.132123915	2.393811947	0.016674295	0.059410971	up_in_case
Yang2025_S	ENSGALG00010028516	GATAD2A	case	control	-0.037695506	0.091130443	-0.413643393	0.679135289	0.798628713	down_in_case
Yang2025_S	ENSGALG00010028517	RNF208	case	control	0.306216947	0.202603361	1.511410994	0.13068377	0.264980266	up_in_case
Yang2025_S	ENSGALG00010028518	MAU2	case	control	0.257335593	0.104628984	2.459505798	0.013912845	0.051852232	up_in_case
Yang2025_S	ENSGALG00010028519	ENSGALG00010028519	case	control	-1.130555491	1.738627953	-0.650257284	0.515526044	0.670198286	down_in_case
Yang2025_S	ENSGALG00010028523	SUGP1	case	control	0.516387076	0.146182148	3.532490687	0.000411665	0.003384394	up_in_case
Yang2025_S	ENSGALG00010028528	UHRF1	case	control	0.022064543	0.142265614	0.155094002	0.876747211	0.927511717	up_in_case
Yang2025_S	ENSGALG00010028535	TICAM1	case	control	-0.238596064	0.111097877	-2.147620369	0.031743926	0.095579665	down_in_case
Yang2025_S	ENSGALG00010028536	GPR107	case	control	-0.345349895	0.082679362	-4.17697823	2.95407E-05	0.000401366	down_in_case
Yang2025_S	ENSGALG00010028537	ENSGALG00010028537	case	control	-2.260988683	1.812563667	-1.247398215	0.212251521	0.372462529	down_in_case
Yang2025_S	ENSGALG00010028538	FEM1A	case	control	-0.812270891	0.132676266	-6.122201934	9.2291E-10	5.64068E-08	down_in_case
Yang2025_S	ENSGALG00010028539	ENSGALG00010028539	case	control	-1.181442845	1.7769665	-0.664865007	0.506136839	0.66157179	down_in_case
Yang2025_S	ENSGALG00010028541	ENSGALG00010028541	case	control	-0.316832339	0.531276209	-0.596360863			down_in_case
Yang2025_S	ENSGALG00010028542	ENSGALG00010028542	case	control	-3.613157625	2.124553052	-1.700667169	0.089005503	0.202420299	down_in_case
Yang2025_S	ENSGALG00010028543	ANP32B	case	control	0.184702805	0.104766098	1.763001662	0.077900209	0.18392589	up_in_case
Yang2025_S	ENSGALG00010028544	ZNF414	case	control	0.574973663	0.233977007	2.457393872	0.013994915	0.052046743	up_in_case
Yang2025_S	ENSGALG00010028545	ENSGALG00010028545	case	control	-2.754581744	1.689243813	-1.630659661	0.102962153	0.224100145	down_in_case
Yang2025_S	ENSGALG00010028546	ENSGALG00010028546	case	control	-3.598680211	1.929846433	-1.864749521	0.062216522	0.156437055	down_in_case
Yang2025_S	ENSGALG00010028547	ZNF618	case	control	-0.361214614	0.131826138	-2.740083405	0.00614236	0.027935307	down_in_case
Yang2025_S	ENSGALG00010028548	MYO1F	case	control	-0.084849145	0.171031941	-0.496101162	0.619823039	0.755266399	down_in_case
Yang2025_S	ENSGALG00010028549	ADAMTS10	case	control	-0.16824627	0.101601253	-1.655946801	0.097732593	0.215491088	down_in_case
Yang2025_S	ENSGALG00010028551	NCS1	case	control	0.044720715	0.233972828	0.191136361	0.848418761	0.910118044	up_in_case
Yang2025_S	ENSGALG00010028552	ENSGALG00010028552	case	control	-1.143675655	1.518888299	-0.752968902	0.451468601	0.613451715	down_in_case
Yang2025_S	ENSGALG00010028554	ENSGALG00010028554	case	control	-0.152280445	1.49656664	-0.101753201	0.918952573	0.952231121	down_in_case
Yang2025_S	ENSGALG00010028555	ENSGALG00010028555	case	control	-2.768581133	1.799987474	-1.538111333	0.124021395	0.255278899	down_in_case
Yang2025_S	ENSGALG00010028556	SPTAN1	case	control	0.237117845	0.124121876	1.910363044	0.056086486	0.145134229	up_in_case
Yang2025_S	ENSGALG00010028558	ENSGALG00010028558	case	control	-1.429342295	1.853755828	-0.771052084	0.44067606	0.604091536	down_in_case
Yang2025_S	ENSGALG00010028559	DYNC2I2	case	control	0.007696055	0.166849779	0.046125653	0.963210099	0.978018999	up_in_case
Yang2025_S	ENSGALG00010028561	SET	case	control	0.556963995	0.236714941	2.352889055	0.018628191	0.064311723	up_in_case
Yang2025_S	ENSGALG00010028562	ACSBG2	case	control	0.377556597	0.136669315	2.76255572	0.005735077	0.026528111	up_in_case
Yang2025_S	ENSGALG00010028563	ENSGALG00010028563	case	control	-2.002983665	2.984417199	-0.67114734	0.502126672	0.658677885	down_in_case
Yang2025_S	ENSGALG00010028565	PKN1	case	control	0.229811895	0.126853625	1.811630496	0.07004331	0.170073797	up_in_case
Yang2025_S	ENSGALG00010028566	ZDHHC12	case	control	-0.020235987	0.11433204	-0.17699314	0.859513778	0.916995034	down_in_case
Yang2025_S	ENSGALG00010028567	ENSGALG00010028567	case	control	-0.321361558	1.809317614	-0.177614784	0.859025513	0.916888279	down_in_case
Yang2025_S	ENSGALG00010028568	MLLT1	case	control	-0.638156636	0.09344763	-6.829029678	8.54909E-12	9.30715E-10	down_in_case
Yang2025_S	ENSGALG00010028569	ZER1	case	control	-0.038869734	0.067591117	-0.575071637	0.565242849	0.71088981	down_in_case
Yang2025_S	ENSGALG00010028572	TBC1D13	case	control	-0.2736798	0.128213008	-2.134571245	0.032796048	0.097882402	down_in_case
Yang2025_S	ENSGALG00010028573	KCNN1	case	control	0.531650066	0.100554922	5.287161057	1.24229E-07	4.09252E-06	up_in_case
Yang2025_S	ENSGALG00010028574	ENDOG	case	control	0.029315971	0.17540488	0.167133158	0.867265277	0.922021773	up_in_case
Yang2025_S	ENSGALG00010028575	SETDB1	case	control	0.182465931	0.070602474	2.5844127	0.0097545	0.039792471	up_in_case
Yang2025_S	ENSGALG00010028577	CERS2	case	control	0.136560642	0.120975182	1.128831877	0.258968763	0.425306978	up_in_case
Yang2025_S	ENSGALG00010028578	SPOUT1	case	control	-0.012551818	0.141807175	-0.088513279	0.929468731	0.957432493	down_in_case
Yang2025_S	ENSGALG00010028579	OCLN	case	control	-0.031127349	0.168478024	-0.184756139	0.853420311	0.913395702	down_in_case
Yang2025_S	ENSGALG00010028580	PRUNE1	case	control	-0.152373271	0.174529875	-0.873049791	0.382635939	0.550444617	down_in_case
Yang2025_S	ENSGALG00010028581	NR2F6	case	control	-0.029165887	0.115342787	-0.252862687	0.800374324	0.879383127	down_in_case
Yang2025_S	ENSGALG00010028582	KYAT1	case	control	-0.182695012	0.179167599	-1.019687783	0.307876558	0.477544505	down_in_case
Yang2025_S	ENSGALG00010028583	USHBP1	case	control	1.011470185	0.173062224	5.844546322	5.07951E-09	2.47493E-07	up_in_case
Yang2025_S	ENSGALG00010028584	LRRC8A	case	control	-0.386408919	0.083230245	-4.642650278	3.43968E-06	6.82792E-05	down_in_case
Yang2025_S	ENSGALG00010028585	MINDY1	case	control	0.080570245	0.180438592	0.446524462	0.655218447	0.781986044	up_in_case
Yang2025_S	ENSGALG00010028586	BABAM1	case	control	-0.206323953	0.133445956	-1.546123686	0.122074699	0.25289783	down_in_case
Yang2025_S	ENSGALG00010028587	ANKLE1	case	control	0.742257685	0.161904283	4.584546318	4.54973E-06	8.53304E-05	up_in_case
Yang2025_S	ENSGALG00010028588	ABHD8	case	control	1.049232007	0.500471896	2.096485367	0.036039157	0.104991463	up_in_case
Yang2025_S	ENSGALG00010028589	PHYHD1	case	control	-0.06884301	0.254306454	-0.27070886	0.786614962	0.871165336	down_in_case
Yang2025_S	ENSGALG00010028590	ENSGALG00010028590	case	control	0.277507787	0.219992558	1.261441706	0.20714975	0.365818832	up_in_case
Yang2025_S	ENSGALG00010028591	GABPB2	case	control	0.117901166	0.125166883	0.941951765	0.346217336	0.514727257	up_in_case

Yang2025_S	ENSGALG00010028592	SEMA6C	case	control	0.354476616	0.180262916	1.966442263	0.049247546	0.13182233	up_in_case
Yang2025_S	ENSGALG00010028593	DOLK	case	control	0.061257838	0.161589204	0.379096107	0.704616497	0.81762269	up_in_case
Yang2025_S	ENSGALG00010028594	ENSGALG00010028594	case	control	-0.395939032	0.257014606	-1.540531247	0.123430912	0.254539484	down_in_case
Yang2025_S	ENSGALG00010028595	SCNM1	case	control	-0.061010684	0.123382059	-0.494485864	0.620963087	0.755797067	down_in_case
Yang2025_S	ENSGALG00010028596	DDA1	case	control	-0.045347469	0.117210479	-0.386889203	0.698838234	0.813564352	down_in_case
Yang2025_S	ENSGALG00010028597	NUP188	case	control	-0.327572848	0.118330175	-2.768295131	0.00563504	0.026166039	down_in_case
Yang2025_S	ENSGALG00010028598	TMOD4	case	control	-0.244317817	0.140322647	-1.741114654	0.081663483	0.190011795	down_in_case
Yang2025_S	ENSGALG00010028599	VPS72	case	control	-0.169775746	0.13816679	-1.228773911	0.219156586	0.380127835	down_in_case
Yang2025_S	ENSGALG00010028600	PSMD4	case	control	0.129140891	0.198701267	0.649924852	0.515740764	0.670289829	up_in_case
Yang2025_S	ENSGALG00010028601	ANO8	case	control	0.364884383	0.099069099	3.683130122	0.000230387	0.002088777	up_in_case
Yang2025_S	ENSGALG00010028602	ZNF687	case	control	0.507823288	0.1790699	2.835894191	0.004569758	0.022382981	up_in_case
Yang2025_S	ENSGALG00010028603	SH3GLB2	case	control	-0.190072758	0.190432788	-0.99810941	0.318226308	0.487734668	down_in_case
Yang2025_S	ENSGALG00010028604	PI4KB	case	control	-0.303996221	0.07427784	-4.092690634	4.26396E-05	0.00053821	down_in_case
Yang2025_S	ENSGALG00010028605	GTPBP3	case	control	0.645464409	0.285681779	2.259382493	0.023859602	0.077375741	up_in_case
Yang2025_S	ENSGALG00010028606	PRPF3	case	control	0.243013665	0.124571083	1.950803172	0.051080464	0.135284782	up_in_case
Yang2025_S	ENSGALG00010028607	ENSGALG00010028607	case	control	0.520490065	0.109789244	4.740811079	2.12864E-06	4.58828E-05	up_in_case
Yang2025_S	ENSGALG00010028608	MIGA2	case	control	-0.349524244	0.196290525	-1.780647552	0.074970046	0.17873526	down_in_case
Yang2025_S	ENSGALG00010028609	MRPS21	case	control	0.068027986	0.371434758	0.18314922	0.854680937	0.914113045	up_in_case
Yang2025_S	ENSGALG00010028610	ENSGALG00010028610	case	control	0.571782321	0.246647556	2.318216044	0.020437583	0.068841603	up_in_case
Yang2025_S	ENSGALG00010028612	DIRAS1	case	control	0.844737325	0.497238173	1.698858556	0.089345837	0.202774307	up_in_case
Yang2025_S	ENSGALG00010028613	ENSGALG00010028613	case	control	0.261436095	0.353714508	0.73911612	0.459836489	0.620973105	up_in_case
Yang2025_S	ENSGALG00010028618	DOLPP1	case	control	-0.013481802	0.150044136	-0.089852241	0.928404634	0.956760728	down_in_case
Yang2025_S	ENSGALG00010028626	CRAT	case	control	-0.010967298	0.12293436	-0.089212634	0.928912925	0.957072208	down_in_case
Yang2025_S	ENSGALG00010028642	APH1A	case	control	0.065215493	0.135410358	0.481613767	0.630080344	0.762763409	up_in_case
Yang2025_S	ENSGALG00010028649	ENSGALG00010028649	case	control	0.168033292	0.212144568	0.792069736	0.428320015	0.592683401	up_in_case
Yang2025_S	ENSGALG00010028658	PTPA	case	control	-0.035504113	0.168809063	-0.210321134	0.833417042	0.901192401	down_in_case
Yang2025_S	ENSGALG00010028662	FCRL4	case	control	0.865101612	0.157660991	5.487099916	4.08586E-08	1.58157E-06	up_in_case
Yang2025_S	ENSGALG00010028669	ZBTB34	case	control	-0.22802106	0.108546504	-2.100676214	0.035669399	0.104269576	down_in_case
Yang2025_S	ENSGALG00010028671	CADM3	case	control	0.415863213	0.144608666	2.875783488	0.004030261	0.020278497	up_in_case
Yang2025_S	ENSGALG00010028675	ZBTB43	case	control	-0.312327896	0.096554848	-3.234719968	0.001217621	0.008161398	down_in_case
Yang2025_S	ENSGALG00010028678	ENSGALG00010028678	case	control	0.824844849	0.238005604	3.465653055	0.000528945	0.004143257	up_in_case
Yang2025_S	ENSGALG00010028683	CRPL2	case	control	2.27839821	0.330449472	6.894845966	5.39232E-12	6.1239E-10	up_in_case
Yang2025_S	ENSGALG00010028686	TAGLN2	case	control	0.285555134	0.229373174	1.244936922	0.213154938	0.373430215	up_in_case
Yang2025_S	ENSGALG00010028689	CFAP45	case	control	0.101242049	0.942664323	0.107399894	0.914471738	0.949848217	up_in_case
Yang2025_S	ENSGALG00010028692	RALGPS1	case	control	0.38284211	0.160256192	2.388938021	0.016897153	0.059928515	up_in_case
Yang2025_S	ENSGALG00010028695	VSIG8	case	control	-0.359608874	1.008408429	-0.356610341	0.721383535	0.828523824	down_in_case
Yang2025_S	ENSGALG00010028700	ANGPTL2	case	control	0.214755422	0.243863521	0.880637748	0.37851392	0.546541566	up_in_case
Yang2025_S	ENSGALG00010028712	FBXW2	case	control	-0.126349486	0.099665172	-1.26773961	0.204890954	0.363100684	down_in_case
Yang2025_S	ENSGALG00010028726	SLC2A8	case	control	0.025325536	0.146970453	0.17231719	0.863188171	0.919290108	up_in_case
Yang2025_S	ENSGALG00010028727	ENSGALG00010028727	case	control	-0.143157045	0.301947535	-0.474112315	0.635419817	0.766393522	down_in_case
Yang2025_S	ENSGALG00010028734	ENSGALG00010028734	case	control	0.95389646	0.175508331	5.435049475	5.47812E-08	2.01419E-06	up_in_case
Yang2025_S	ENSGALG00010028735	ENSGALG00010028735	case	control	0.981399736	0.34209823	2.868765902	0.004120767	0.020611229	up_in_case
Yang2025_S	ENSGALG00010028738	MIDN	case	control	0.655001287	0.174761132	3.747980337	0.000178264	0.001706696	up_in_case
Yang2025_S	ENSGALG00010028739	GARNL3	case	control	-0.157056674	0.046267797	-3.394513789	0.000687506	0.005167795	down_in_case
Yang2025_S	ENSGALG00010028740	DAZAP1	case	control	0.048460136	0.073906902	0.655691613	0.512022566	0.667262177	up_in_case
Yang2025_S	ENSGALG00010028742	ENSGALG00010028742	case	control	0.399594807	0.267869077	1.491754153	0.135763607	0.272289272	up_in_case
Yang2025_S	ENSGALG00010028743	ARRDC1	case	control	0.137495256	0.105324146	1.305448574	0.191740147	0.346954805	up_in_case
Yang2025_S	ENSGALG00010028744	HOMER3	case	control	-0.050712188	0.102944385	-0.49261733	0.622283001	0.756808659	down_in_case
Yang2025_S	ENSGALG00010028745	ENSGALG00010028745	case	control	0.099105703	0.285006164	0.347731789	0.72804162	0.832875789	up_in_case
Yang2025_S	ENSGALG00010028746	MYDGF	case	control	-0.492638871	0.297074099	-1.658303004	0.097256323	0.214715409	down_in_case
Yang2025_S	ENSGALG00010028747	ENSGALG00010028747	case	control	-0.512345092	0.190163499	-2.694234675	0.007055047	0.031143073	down_in_case
Yang2025_S	ENSGALG00010028748	PIAS3	case	control	0.690479568	0.129349411	5.338095938	9.39278E-08	3.24785E-06	up_in_case
Yang2025_S	ENSGALG00010028750	OTUD7B	case	control	0.210541623	0.095684804	2.200366359	0.027780913	0.086470185	up_in_case
Yang2025_S	ENSGALG00010028751	EHMT1	case	control	-0.114233306	0.084546344	-1.351132416	0.176653019	0.327653377	down_in_case
Yang2025_S	ENSGALG00010028752	MTMR11	case	control	0.012679636	0.195768146	0.064768639	0.948358212	0.967992436	up_in_case
Yang2025_S	ENSGALG00010028753	SV2A	case	control	1.135843025	0.620137324	1.831599197	0.067011157	0.164778625	up_in_case
Yang2025_S	ENSGALG00010028756	ENSGALG00010028756	case	control	-0.633935484	0.976669727	-0.649078666	0.51628753	0.670687679	down_in_case
Yang2025_S	ENSGALG00010028760	SSR2	case	control	-0.351676835	0.197642765	-1.779355973	0.07518142	0.179116616	down_in_case
Yang2025_S	ENSGALG00010028761	CTSS	case	control	-0.571558393	0.218965812	-2.610263165	0.009047259	0.037764947	down_in_case
Yang2025_S	ENSGALG00010028762	CACNA1B	case	control	1.379748852	0.583096514	2.366244385	0.017969578	0.062821393	up_in_case
Yang2025_S	ENSGALG00010028763	CTSK	case	control	0.622506306	0.198831712	3.130820026	0.00174319	0.010800954	up_in_case
Yang2025_S	ENSGALG00010028764	ARNT	case	control	-0.042555869	0.071415557	-0.595890739	0.55124822	0.699284838	down_in_case
Yang2025_S	ENSGALG00010028768	NTNG2	case	control	0.615604751	0.20681588	2.976583566	0.002914796	0.015947266	up_in_case
Yang2025_S	ENSGALG00010028771	SETX	case	control	0.330409308	0.144386235	2.288371242	0.022115911	0.072839806	up_in_case
Yang2025_S	ENSGALG00010028772	TTF1	case	control	-0.106060289	0.109708873	-0.966743043	0.333672504	0.502562617	down_in_case
Yang2025_S	ENSGALG00010028777	CFAP77	case	control	0.661992316	0.457919067	1.44565353	0.148274404	0.290319492	up_in_case
Yang2025_S	ENSGALG00010028782	MLLT11	case	control	-1.111246609	0.293477606	-3.786478376	0.000152797	0.001501292	down_in_case
Yang2025_S	ENSGALG00010028783	DDX31	case	control	-0.466182308	0.090543293	-5.148722672	2.62266E-07	7.58233E-06	down_in_case
Yang2025_S	ENSGALG00010028786	GTF3C4	case	control	-0.221073354	0.111027595	-1.991156824	0.046463649	0.126466307	down_in_case
Yang2025_S	ENSGALG00010028800	ENSGALG00010028800	case	control	-0.135175401	0.25089147	-0.538780377	0.590038405	0.730925875	down_in_case
Yang2025_S	ENSGALG00010028806	SPACA9	case	control	0.594460525	0.272983138	2.177645585	0.029432433	0.090240034	up_in_case
Yang2025_S	ENSGALG00010028818	TSC1	case	control	0.120949827	0.108384676	1.115931064	0.264451644	0.43151448	up_in_case
Yang2025_S	ENSGALG00010028823	GFI1B	case	control	-0.238491724	0.242118071	-0.985022403	0.324613067	0.494261701	down_in_case
Yang2025_S	ENSGALG00010028829	GTF3C5	case	control	-0.076336701	0.115489969	-0.660981223	0.508624356	0.664188525	down_in_case
Yang2025_S	ENSGALG00010028833	GBGT1	case	control	-0.216760035	0.118140234	-1.834768961	0.066539929	0.163967094	down_in_case
Yang2025_S	ENSGALG00010028849	ENSGALG00010028849	case	control	0.159246722	0.313800333	0.507477862	0.611819565	0.748892787	up_in_case
Yang2025_S	ENSGALG00010028853	ENSGALG00010028853	case	control	-1.048545774	2.273048915	-0.461294857	0.644587074	0.774071086	down_in_case
Yang2025_S	ENSGALG00010028854	RPL7A	case	control	-0.187913052	0.207788664	-0.904346984	0.365811441	0.534505864	down_in_case
Yang2025_S	ENSGALG00010028855	SURF6	case	control	0.837881494	0.216172581	3.875984125	0.000106195	0.00111769	up_in_case
Yang2025_S	ENSGALG00010028856	ETV3	case	control	0.11048951	0.092700569	1.191896781	0.233301721	0.396422324	up_in_case
Yang2025_S	ENSGALG00010028857	MED22	case	control	-0.714365039	0.152628725	-4.680410188	2.86302E-06	5.83277E-05	down_in_case
Yang2025_S	ENSGALG00010028858	ENSGALG00010028858	case	control	0.469086526	0.560163598	0.83740987	0.40236222	0.568941404	up_in_case
Yang2025_S	ENSGALG00010028859	SURF1	case	control	-0.288985716	0.165686539	-1.744171359	0.081129214	0.189158584	down_in_case
Yang2025_S	ENSGALG00010028860	SURF2	case	control	0.00675864	0.168568358	0.040094357	0.968017901	0.981113495	up_in_case
Yang2025_S	ENSGALG00010028861	SURF4	case	control	-0.411938253	0.085061869	-4.842807434	1.28017E-06	2.98315E-05	down_in_case
Yang2025_S	ENSGALG00010028862	ENSGALG00010028862	case	control	-3.742092328	1.744351288	-2.145263029	0.031931823	0.095981439	down_in_case
Yang2025_S	ENSGALG00010028865	STKLD1	case	control	-0.003977137	0.16655129	-0.023879355	0.980948842	0.988594337	down_in_case
Yang2025_S	ENSGALG00010028866	HORMAD1	case	control	0.597306593	0.443709604	1.346165571	0.178249126	0.329648516	up_in_case
Yang2025_S	ENSGALG00010028867	REXO4	case	control	0.268713223	0.129764166	2.070781407	0.038379228	0.109863301	up_in_case
Yang2025_S	ENSGALG00010028868	ENSGALG00010028868	case	control	-0.027106558	2.80414214	-0.009666614	0.992287278	0.995042536	down_in_case
Yang2025_S	ENSGALG00010028869	ADAMTS13	case	control	-0.162283939	0.199318995	-0.814192042	0.415534944	0.58108173	down_in_case
Yang2025_S	ENSGALG00010028870	CACFD1	case	control	-0.346176109	0.209120253	-1.655392548	0.097844897	0.215670458	down_in_case
Yang2025_S	ENSGALG00010028871	PIP5K1A	case	control	-0.123594955	0.128370458	-0.96279905	0.335648378	0.504042334	down_in_case
Yang2025_S	ENSGALG00010028872	SLC2A6	case	control	-0.619272705	0.398588584	-1.553663929	0.120264601	0.249983177	down_in_case
Yang2025_S	ENSGALG00010028873	TMEM8C	case	control	-0.302303675	0.253671099	-1.191715086	0.233372981	0.39649506	down_in_case
Yang2025_S	ENSGALG00010028874	CRPL1	case	control	6.858666231	0.520874334	13.1676026	1.34811E-39	3.13098E-36	up_in_case
Yang2025_S	ENSGALG00010028875	DUSP23	case	control	0.592242454	0.244145981	2.425771875	0.015275869	0.055758469	up_in_case
Yang2025_S	ENSGALG00010028876	ADAMTSL2	case	control	0.698449324	0.174165395	4.010264629	6.06507E-05	0.000715638	up_in_case
Yang2025_S	ENSGALG00010028877	FAM163B	case	control	1.010070948	1.08852771	0.927923965	0.353447012	0.522354875	up_in_case
Yang2025_S	ENSGALG00010028878	IGSF9	case	control	0.303411139	0.117286284	2.586927719	0.00968359	0.039606936	up_in_case
Yang2025_S	ENSGALG00010028881	SARDH	case	control	-0.376264855	0.138166883	-2.723263677	0.006464044	0.029101065	down_in_case
Yang2025_S	ENSGALG00010028884	VAV2	case	control	-0.206380885	0.080904625	-2.550915795	0.010744028	0.042715558	down_in_case
Yang2025_S	ENSGALG00010028885	BRD3	case	control	-0.174487674	0.161654401	-1.079387091	0.280415203	0.448323297	down_in_case
Yang2025_S	ENSGALG00010028887	SF3B4	case	control	0.041609627	0.169999204	0.244763661	0.806639433	0.883622621	up_in_case
Yang2025_S	ENSGALG00010028888	WDR5B	case	control	-0.203364449	0.089532408	-2.271405993	0.023122412	0.075491953	down_in_case
Yang2025_S	ENSGALG00010028889	FUBP3	case	control	0.354202315	0.098511874	3.595529139	0.000323733	0.002777843	up_in_case
Yang2025_S	ENSGALG00010028890	ENSGALG00010028890	case	control	1.020972491	0.22267946	4.584942369	4.54112E-06	8.52836E-05	up_in_case
Yang2025_S	ENSGALG00010028891	SLC31A2	case	control	1.175524736	0.500263385	2.349811662	0.018782912	0.064739026	up_in_case
Yang2025_S	ENSGALG00010028892	NIBAN2	case	control	0.07795553	0.157570971	0.494732812	0.620788737	0.755766438	up_in_case
Yang2025_S	ENSGALG00010028893	STXBP1	case	control	0.148270973	0.196361241	0.755092869	0.450193262	0.612282169	up_in_case
Yang2025_S	ENSGALG00010028894	AKNA	case	control	0.435038446	0.121632601	3.576659895	0.000348012	0.002951646	up_in_case
Yang2025_S	ENSGALG00010028896	ATP6V1G1	case	control	-0.253003009	0.216234096	-1.170042165	0.241984001	0.407104558	down_in_case
Yang2025_S	ENSGALG00010028897	TMEM268	case	control	-0.144538535	0.296622205	-0.487281576	0.626058802	0.759743047	down_in_case
Yang2025_S	ENSGALG00010028898	TNFSF15	case	control	-0.661971773	0.312840039	-2.116007195	0.034344196	0.101202446	down_in_case
Yang2025_S	ENSGALG00010028900	TNC	case	control	1.456218243	0.477047012	3.052567578	0.002268926	0.013173954	up_in_case
Yang2025_S	ENSGALG00010028901	PAPPA	case	control	-0.058212596	0.184148729	-0.316117281	0.75191349	0.84889315	down_in_case
Yang2025_S	ENSGALG00010028903	ASTN2	case	control	0.954210668	0.219278591	4.351590659	1.35153E-05	0.000212569	up_in_case
Yang2025_S	ENSGALG00010028904	TRIM32	case	control	-0.113955657	0.088860631	-1.282408812	0.199699275	0.357045085	down_in_case
Yang2025_S	ENSGALG00010028905	COLGALT1	case	control	0.294824877	0.093962849	3.137674958	0.001702936	0.010608142	up_in_case
Yang2025_S	ENSGALG00010028909	ENSGALG00010028909	case	control	-0.170909735	0.150054029	-1.138987976	0.254708169	0.421184398	down_in_case
Yang2025_S	ENSGALG00010028911	LMX1B	case	control	1.112915298	1.038935873	1.071206921	0.2840764	0.452205236	up_in_case
Yang2025_S	ENSGALG00010028912	EXFABP	case	control	0.422676257	0.331842235	1.273726526	0.202760352	0.360620997	up_in_case
Yang2025_S	ENSGALG00010028914	TLR4	case	control	-0.390385174	0.142193489	-2.745450432	0.00604279	0.027560333	down_in_case
Yang2025_S	ENSGALG00010028917	NR6A1	case	control	0.234474812	0.180286492	1.300567833	0.193406423	0.348837498	up_in_case
Yang2025_S	ENSGALG00010028918	AGPAT2	case	control	-0.419195571	0.16540794	-2.534313474	0.011266789	0.044238576	down_in_case
Yang2025_S	ENSGALG00010028919	EGFL7	case	control	-0.202957623	0.139974794	-1.449958359	0.147070132	0.288895163	down_in_case

Yang2025_S	ENSGALG00010028921	MRPL41	case	control	-0.282610021	0.276529488	-1.021988733	0.306786233	0.47638357	down_in_case
Yang2025_S	ENSGALG00010028923	ENSGALG00010028923	case	control	0.984207203	0.373909514	2.6322069	0.008483218	0.035865792	up_in_case
Yang2025_S	ENSGALG00010028924	TMEM203	case	control	0.080161629	0.248178065	0.323000458	0.746694895	0.846019462	up_in_case
Yang2025_S	ENSGALG00010028925	ENSGALG00010028925	case	control	0.124035555	0.423269796	0.293041356	0.769490541	0.859542486	up_in_case
Yang2025_S	ENSGALG00010028926	ENSGALG00010028926	case	control	-1.054582375	0.690904467	-1.526379442	0.126915394	0.259586969	down_in_case
Yang2025_S	ENSGALG00010029140	NPDC1	case	control	0.179509788	0.101871097	1.762126771	0.078047881	0.184120065	up_in_case
Yang2025_S	ENSGALG00010029141	C5	case	control	-1.247301871	0.398456561	-3.130333373	0.00174608	0.010809253	down_in_case
Yang2025_S	ENSGALG00010029142	ENSGALG00010029142	case	control	-0.169161022	0.221992119	-0.762013638	0.446051865	0.608967643	down_in_case
Yang2025_S	ENSGALG00010029143	SH2D3C	case	control	0.217888185	0.097214061	2.24132377	0.02500511	0.079918856	up_in_case
Yang2025_S	ENSGALG00010029144	ENSGALG00010029144	case	control	-0.319468567	0.20369989	-1.568329599	0.116804232	0.244725149	down_in_case
Yang2025_S	ENSGALG00010029145	RC3H2	case	control	0.149149182	0.172312705	0.865572751	0.386724504	0.554139431	up_in_case
Yang2025_S	ENSGALG00010029146	NEK6	case	control	-0.384277209	0.088531916	-4.340549995	1.42127E-05	0.000221042	down_in_case
Yang2025_S	ENSGALG00010029147	RABL6	case	control	0.15094881	0.141879619	1.063921729	0.287364159	0.456172176	up_in_case
Yang2025_S	ENSGALG00010029148	RALGDS	case	control	-0.306792514	0.087766779	-3.495542597	0.000473099	0.003778014	down_in_case
Yang2025_S	ENSGALG00010029149	ENSGALG00010029149	case	control	-0.064954531	0.294004826	-0.220930151	0.82514682	0.895248415	down_in_case
Yang2025_S	ENSGALG00010029150	NARF	case	control	-0.453349489	0.12147876	-3.731923921	0.000190023	0.001790378	down_in_case
Yang2025_S	ENSGALG00010029151	CYBC1	case	control	-0.430001721	0.128941687	-3.334854166	0.000853441	0.006158833	down_in_case
Yang2025_S	ENSGALG00010029152	HEXD	case	control	0.355635553	0.135370854	2.627120559	0.008611082	0.036307239	up_in_case
Yang2025_S	ENSGALG00010029153	OGFOD3	case	control	0.007529152	0.125067794	0.060200563	0.951995898	0.970549759	up_in_case
Yang2025_S	ENSGALG00010029154	UTS2R	case	control	0.585330301	0.203569172	2.875338608	0.004035944	0.020283804	up_in_case
Yang2025_S	ENSGALG00010029155	ENSGALG00010029155	case	control	0.355030642	0.174369315	2.036084396	0.041741881	0.11673151	up_in_case
Yang2025_S	ENSGALG00010029156	ENSGALG00010029156	case	control	0.483157968	0.774172133	0.624096305	0.532564341	0.684787177	up_in_case
Yang2025_S	ENSGALG00010029158	CD7	case	control	-0.335704953	0.721966379	-0.464986962	0.641940794	0.77155813	down_in_case
Yang2025_S	ENSGALG00010029160	DUS1L	case	control	0.349136968	0.108901169	3.205998354	0.001345948	0.008834564	up_in_case
Yang2025_S	ENSGALG00010029162	ELN	case	control	0.618411221	0.407606699	1.517176295	0.12922218	0.262839158	up_in_case
Yang2025_S	ENSGALG00010029163	ANKFY1	case	control	-0.406639965	0.120744741	-3.367765426	0.0007578	0.005600398	down_in_case
Yang2025_S	ENSGALG00010029164	CYB5D2	case	control	-0.358124314	0.234405543	-1.527797975	0.126562705	0.259131839	down_in_case
Yang2025_S	ENSGALG00010029165	RAC3	case	control	-0.164835684	0.164970763	-0.999181193	0.317706925	0.487211874	down_in_case
Yang2025_S	ENSGALG00010029166	ZZEF1	case	control	-0.186671301	0.108771502	-1.716178389	0.086129397	0.197468436	down_in_case
Yang2025_S	ENSGALG00010029167	ENSGALG00010029167	case	control	0.151210412	0.181425991	0.833455072	0.404588126	0.570814573	up_in_case
Yang2025_S	ENSGALG00010029168	RFNG	case	control	-0.365637967	0.149758661	-2.441514668	0.014625794	0.05384688	down_in_case
Yang2025_S	ENSGALG00010029169	SBDS	case	control	-0.201788724	0.078498264	-2.570613852	0.010151845	0.040969002	down_in_case
Yang2025_S	ENSGALG00010029170	DCXR	case	control	0.242851059	0.203041176	1.196068027	0.231670013	0.394756864	up_in_case
Yang2025_S	ENSGALG00010029171	ENSGALG00010029171	case	control	0.047575745	0.096351251	0.493774032	0.621465775	0.756078712	up_in_case
Yang2025_S	ENSGALG00010029172	LRRC45	case	control	0.233376386	0.097513945	2.393261648	0.016699328	0.059454555	up_in_case
Yang2025_S	ENSGALG00010029173	ZNHIT3	case	control	0.491371764	0.263391153	1.86555911	0.062103075	0.156266947	up_in_case
Yang2025_S	ENSGALG00010029174	CALN1	case	control	-0.433856298	0.427652698	-1.014506165	0.310341289	0.480100209	down_in_case
Yang2025_S	ENSGALG00010029175	CENPX	case	control	-0.592726003	0.361207356	-1.640957729	0.10080619	0.220350472	down_in_case
Yang2025_S	ENSGALG00010029177	ASPSCR1	case	control	-0.40467304	0.103609024	-3.905770222	9.39257E-05	0.001014696	down_in_case
Yang2025_S	ENSGALG00010029178	NOTUM	case	control	0.076263838	0.121442422	0.627983506	0.530014745	0.6824549	up_in_case
Yang2025_S	ENSGALG00010029179	PIGW	case	control	-0.299277168	0.171054804	-1.749598146	0.08018768	0.187643211	down_in_case
Yang2025_S	ENSGALG00010029180	CA4	case	control	-0.181969975	0.27537053	-0.660818626	0.508728636	0.664188525	down_in_case
Yang2025_S	ENSGALG00010029181	MYO19	case	control	0.454757541	0.104860446	4.336788176	1.4458E-05	0.000223809	up_in_case
Yang2025_S	ENSGALG00010029182	HEATR6	case	control	-0.194485398	0.118048517	-1.647503955	0.099454504	0.218080018	down_in_case
Yang2025_S	ENSGALG00010029183	ENSGALG00010029183	case	control	0.226386618	0.191274237	1.183570881	0.236582973	0.400192246	up_in_case
Yang2025_S	ENSGALG00010029184	DYNLL2	case	control	-0.611653853	0.196631264	-3.110664298	0.00186667	0.011378849	down_in_case
Yang2025_S	ENSGALG00010029186	ZNF302	case	control	0.407296537	0.1446679	2.815389841	0.00487181	0.023521581	up_in_case
Yang2025_S	ENSGALG00010029187	SRSF1	case	control	0.068799506	0.110292881	0.623789184	0.532766044	0.684816421	up_in_case
Yang2025_S	ENSGALG00010029188	ENSGALG00010029188	case	control	1.787496179	0.861228187	2.075519829	0.037938383	0.108802504	up_in_case
Yang2025_S	ENSGALG00010029189	ENSGALG00010029189	case	control	1.306793807	0.515353816	2.535721611	0.01122159	0.044098381	up_in_case
Yang2025_S	ENSGALG00010029190	DHRS7C	case	control	-0.619714196	0.155419403	-3.987366988	6.68106E-05	0.000772073	down_in_case
Yang2025_S	ENSGALG00010029191	GSG1L2	case	control	0.700897272	0.494058464	1.418652494	0.156000354	0.300922609	up_in_case
Yang2025_S	ENSGALG00010029192	DTX2	case	control	-0.261431097	0.16701209	-1.565342346	0.117502659	0.245855789	down_in_case
Yang2025_S	ENSGALG00010029194	DHRS11	case	control	-0.185731559	0.161914627	-1.147095617	0.251342105	0.417042981	down_in_case
Yang2025_S	ENSGALG00010029196	MRM1	case	control	0.198413058	0.264145892	0.751149512	0.452562681	0.614425269	up_in_case
Yang2025_S	ENSGALG00010029197	LHX1	case	control	0.345019994	0.33386858	1.033400609	0.301416462	0.471037164	up_in_case
Yang2025_S	ENSGALG00010029198	PPM1D	case	control	-0.230333654	0.082712265	-2.784758159	0.005356766	0.025252549	down_in_case
Yang2025_S	ENSGALG00010029199	APPBP2	case	control	-0.168395083	0.107873979	-1.561035246	0.118515435	0.247343506	down_in_case
Yang2025_S	ENSGALG00010029200	GAS7	case	control	-0.059629733	0.212680754	-0.280372021	0.7791921	0.866148845	down_in_case
Yang2025_S	ENSGALG00010029201	PIGS	case	control	-0.130649879	0.106324883	-1.228779904	0.219154338	0.380127835	down_in_case
Yang2025_S	ENSGALG00010029202	PSMD12	case	control	0.064377561	0.097475514	0.660448542	0.50896603	0.66433511	up_in_case
Yang2025_S	ENSGALG00010029203	ALDOC	case	control	0.37995415	0.156655898	2.425405968	0.015291276	0.055781136	up_in_case
Yang2025_S	ENSGALG00010029204	HELZ	case	control	0.518403016	0.281098757	1.844202447	0.065153642	0.161407289	up_in_case
Yang2025_S	ENSGALG00010029205	CACNG1	case	control	-0.15520542	0.16935517	-0.91644926	0.359431309	0.528174132	down_in_case
Yang2025_S	ENSGALG00010029206	CACNG4	case	control	0.565870546	0.239953567	2.358250195	0.018361313	0.063600522	up_in_case
Yang2025_S	ENSGALG00010029207	SPAG5	case	control	0.559780669	0.135017042	4.146000098	3.38334E-05	0.000446889	up_in_case
Yang2025_S	ENSGALG00010029208	CACNG5	case	control	-0.702124969	0.376981553	-1.862491583	0.062533831	0.156925794	down_in_case
Yang2025_S	ENSGALG00010029209	PRKCA	case	control	0.239913417	0.089172847	2.690431282	0.007135973	0.031418573	up_in_case
Yang2025_S	ENSGALG00010029210	RSKR	case	control	0.295025403	0.187438902	1.573981707	0.115491679	0.242900307	up_in_case
Yang2025_S	ENSGALG00010029211	SDF2	case	control	0.316403424	0.208103107	1.520416624	0.128406299	0.261676189	up_in_case
Yang2025_S	ENSGALG00010029212	SUPT6H	case	control	0.06911702	0.150772227	0.458420104	0.646650651	0.775661305	up_in_case
Yang2025_S	ENSGALG00010029213	APOH	case	control	1.384028142	1.071092667	1.292164707			up_in_case
Yang2025_S	ENSGALG00010029214	ENSGALG00010029214	case	control	-0.231202465	0.29314487	-0.78869695	0.430289151	0.594436336	down_in_case
Yang2025_S	ENSGALG00010029215	ENSGALG00010029215	case	control	-0.15987193	0.310221099	-0.515348346	0.606309621	0.743766699	down_in_case
Yang2025_S	ENSGALG00010029216	RAB34	case	control	0.132283876	0.183490106	0.720931932	0.470951395	0.63116087	up_in_case
Yang2025_S	ENSGALG00010029217	CEP112	case	control	0.514904877	0.190965982	2.696317274	0.007011085	0.031005862	up_in_case
Yang2025_S	ENSGALG00010029218	RPL23A	case	control	-0.641888438	0.242291297	-2.649242654	0.008067239	0.034441474	down_in_case
Yang2025_S	ENSGALG00010029219	TLCD1	case	control	0.030463017	0.213940445	0.142390171	0.886771826	0.933602704	up_in_case
Yang2025_S	ENSGALG00010029220	NEK8	case	control	0.122701456	0.130698594	0.938812364	0.34782709	0.516385585	up_in_case
Yang2025_S	ENSGALG00010029221	AXIN2	case	control	0.318116234	0.174197923	1.82617696	0.067823611	0.166073101	up_in_case
Yang2025_S	ENSGALG00010029222	TRAF4	case	control	0.400477893	0.15873629	2.522913276	0.01163871	0.045341186	up_in_case
Yang2025_S	ENSGALG00010029223	NME2	case	control	0.607667979	0.19289338	3.15027908	0.001631146	0.0102526	up_in_case
Yang2025_S	ENSGALG00010029224	FAM222B	case	control	0.350239856	0.196135409	1.785704367	0.074147138	0.177257763	up_in_case
Yang2025_S	ENSGALG00010029225	ENSGALG00010029225	case	control	4.650783196	1.200929572	3.872652739	0.000107657	0.001128409	up_in_case
Yang2025_S	ENSGALG00010029226	MBTD1	case	control	-0.164686674	0.105683037	-1.558307547	0.119160361	0.248354716	down_in_case
Yang2025_S	ENSGALG00010029227	UTP18	case	control	0.153119544	0.094256017	1.624506837	0.104267682	0.226072842	up_in_case
Yang2025_S	ENSGALG00010029228	ERAL1	case	control	0.1991532	0.118884064	1.675188363	0.09389728	0.209621691	up_in_case
Yang2025_S	ENSGALG00010029229	CBX4	case	control	0.575667781	0.11544709	4.986420866	6.15081E-07	1.5902E-05	up_in_case
Yang2025_S	ENSGALG00010029230	PPM1E	case	control	0.046182552	0.174477204	0.264691037	0.791247483	0.873985268	up_in_case
Yang2025_S	ENSGALG00010029231	CBX8	case	control	-0.183872643	0.232696721	-0.790181498	0.42942178	0.59365005	down_in_case
Yang2025_S	ENSGALG00010029232	CA10	case	control	0.062480599	0.34239537	0.182480853	0.855205379	0.914445776	up_in_case
Yang2025_S	ENSGALG00010029233	CBX2	case	control	-0.50582844	0.172978623	-2.924225146	0.003453149	0.018076495	down_in_case
Yang2025_S	ENSGALG00010029234	TOM1L1	case	control	-0.529208785	0.25608141	-2.066564632	0.038775196	0.110660959	down_in_case
Yang2025_S	ENSGALG00010029235	TRIM37	case	control	-0.204698588	0.084575794	-2.420297557	0.015507811	0.056438066	down_in_case
Yang2025_S	ENSGALG00010029237	COX11	case	control	0.152778625	0.134783029	1.133515294	0.256997922	0.423367543	up_in_case
Yang2025_S	ENSGALG00010029238	CAMKK1	case	control	0.144643908	0.227395247	0.636090286	0.524717576	0.678098806	up_in_case
Yang2025_S	ENSGALG00010029239	RBFOX3	case	control	0.785994151	0.369463631	2.127392481	0.033387486	0.099074664	up_in_case
Yang2025_S	ENSGALG00010029240	MIS12	case	control	-0.344246911	0.229000943	-1.503255432	0.132773188	0.267988256	down_in_case
Yang2025_S	ENSGALG00010029241	STXBP4	case	control	-0.325501809	0.138797465	-2.345156719	0.019019083	0.065294636	down_in_case
Yang2025_S	ENSGALG00010029242	DERL2	case	control	-0.324105248	0.14446501	-2.243486136	0.024865483	0.079636981	down_in_case
Yang2025_S	ENSGALG00010029243	NME1	case	control	0.681269087	0.266867367	2.552837746	0.010684926	0.042553427	up_in_case
Yang2025_S	ENSGALG00010029244	HLF	case	control	0.100527222	0.158373794	0.634746565	0.525593719	0.678790405	up_in_case
Yang2025_S	ENSGALG00010029245	SKA2	case	control	0.147665505	0.11989016	1.231673272	0.218071158	0.379047223	up_in_case
Yang2025_S	ENSGALG00010029246	DHX33	case	control	-0.284224968	0.139621086	-2.035687994	0.041781695	0.116749133	down_in_case
Yang2025_S	ENSGALG00010029247	CCDC92B	case	control	0.418463186	0.264327757	1.583122374	0.113393575	0.239814762	up_in_case
Yang2025_S	ENSGALG00010029248	C1QBP	case	control	0.040366264	0.180149231	0.224071255	0.822701859	0.894045886	up_in_case
Yang2025_S	ENSGALG00010029249	PRR11	case	control	0.0944843	0.239633978	0.394285905	0.693369969	0.809425359	up_in_case
Yang2025_S	ENSGALG00010029250	SPAG9	case	control	0.321701867	0.108143455	2.974769634	0.002932086	0.016016709	up_in_case
Yang2025_S	ENSGALG00010029251	ENGASE	case	control	0.259710069	0.294275207	0.882541454	0.377484073	0.54589462	up_in_case
Yang2025_S	ENSGALG00010029252	RPAIN	case	control	-0.414130746	0.321381039	-1.28859732	0.197538115	0.353953149	down_in_case
Yang2025_S	ENSGALG00010029253	NUP88	case	control	-0.023313961	0.103517293	-0.225218032	0.821809663	0.893494394	down_in_case
Yang2025_S	ENSGALG00010029254	MMD	case	control	-0.549197975	0.097900875	-5.609735099	2.02637E-08	8.63529E-07	down_in_case
Yang2025_S	ENSGALG00010029255	RAP1GAP2	case	control	-0.046070897	0.181629496	-0.253653169	0.799763516	0.878989242	down_in_case
Yang2025_S	ENSGALG00010029256	RABEP1	case	control	0.261981658	0.08984535	2.915917835	0.003546439	0.018433281	up_in_case
Yang2025_S	ENSGALG00010029257	YPEL2	case	control	0.078483746	0.112178382	0.699633424	0.484156264	0.642111652	up_in_case
Yang2025_S	ENSGALG00010029258	TMEM100	case	control	-0.443753343	0.140215748	-3.164789616	0.001551951	0.009857083	down_in_case
Yang2025_S	ENSGALG00010029259	PTRH2	case	control	-0.43502597	0.261081454	-1.66624616	0.095664396	0.212583268	down_in_case
Yang2025_S	ENSGALG00010029260	DHX40	case	control	0.356795888	0.104047468	3.429164552	0.000605442	0.004640893	up_in_case
Yang2025_S	ENSGALG00010029261	IFT22	case	control	-0.198275719	0.127981627	-1.549251436	0.121321289	0.251766517	down_in_case
Yang2025_S	ENSGALG00010029262	CLTC	case	control	-0.362843276	0.083366212	-4.35240212	1.34654E-05	0.000212503	down_in_case
Yang2025_S	ENSGALG00010029263	TOB1	case	control	0.142088031	0.094189287	1.508537069	0.131417127	0.265983684	up_in_case
Yang2025_S	ENSGALG00010029264	COL26A1	case	control	-0.460175632	0.201482254	-2.283951183	0.0223744	0.073448119	down_in_case
Yang2025_S	ENSGALG00010029266	CANT1	case	control	0.150004417	0.14906274	1.006317325	0.314262949	0.483574889	up_in_case
Yang2025_S	ENSGALG00010029267	ENSGALG00010029267	case	control	0.385138395	0.162904835	2.364192531	0.018069419	0.062965081	up_in_case
Yang2025_S	ENSGALG00010029268	PCTP	case	control	-1.029016367	0.219468393	-4.688676815	2.74977E-06	5.65163E-05	down_in_case
Yang2025_S	ENSGALG00010029269	WSCD1	case	control	-0.200484664	0.116288141	-1.724033617	0.084701775	0.195223161	down_in_case

Yang2025_S	ENSGALG00010029270	TIMP2	case	control	0.294196894	0.09941381	2.959316159	0.003083226	0.016653006	up_in_case
Yang2025_S	ENSGALG00010029271	MYL10	case	control	-0.680357047	0.186477819	-3.648460994	0.000263816	0.002343069	down_in_case
Yang2025_S	ENSGALG00010029273	SLC13A5	case	control	0.092867509	0.321540478	0.288820585	0.772718678	0.861736367	up_in_case
Yang2025_S	ENSGALG00010029274	VMP1	case	control	-0.213158497	0.096061161	-2.218987314	0.026487588	0.083488924	down_in_case
Yang2025_S	ENSGALG00010029275	PCYT2	case	control	0.283463029	0.091867255	3.085571997	0.00203161	0.012119216	up_in_case
Yang2025_S	ENSGALG00010029276	TUBD1	case	control	-0.053218926	0.115699647	-0.459974833	0.645534285	0.774874688	down_in_case
Yang2025_S	ENSGALG00010029277	WFIKKN2	case	control	-0.589760536	0.368168285	-1.601877619	0.109182675	0.233066876	down_in_case
Yang2025_S	ENSGALG00010029278	MED31	case	control	0.214324262	0.107632171	1.991265813	0.046451672	0.126466307	up_in_case
Yang2025_S	ENSGALG00010029279	ENSGALG00010029279	case	control	-0.449261767	0.172465402	-2.604938514	0.009189075	0.038135652	down_in_case
Yang2025_S	ENSGALG00010029280	USP36	case	control	0.295868148	0.102595225	2.883839356	0.003928591	0.019907242	up_in_case
Yang2025_S	ENSGALG00010029281	KIAA0753	case	control	0.492477125	0.165154761	2.981912974	0.002864534	0.015721655	up_in_case
Yang2025_S	ENSGALG00010029282	LUC7L3	case	control	1.728369783	0.320387313	5.394626176	6.86664E-08	2.47252E-06	up_in_case
Yang2025_S	ENSGALG00010029283	RPS6KB1	case	control	-0.213445947	0.101483646	-2.103254615	0.035443518	0.103783448	down_in_case
Yang2025_S	ENSGALG00010029284	CYTH1	case	control	0.14280828	0.147615536	0.967433944	0.333327148	0.502478777	up_in_case
Yang2025_S	ENSGALG00010029285	CUX1	case	control	0.366436306	0.101097109	3.62459729	0.000289412	0.002531674	up_in_case
Yang2025_S	ENSGALG00010029286	MAFG	case	control	-0.166252762	0.149563773	-1.111584431	0.26631687	0.433602847	down_in_case
Yang2025_S	ENSGALG00010029287	ANKRD40	case	control	0.141915477	0.147427592	0.962611374	0.335742588	0.504047938	up_in_case
Yang2025_S	ENSGALG00010029288	PYCR1	case	control	-0.158371704	0.133947544	-1.182341229	0.237070333	0.400870657	down_in_case
Yang2025_S	ENSGALG00010029289	PITPNM3	case	control	0.416021752	0.230127138	1.807790924	0.070639047	0.171043461	up_in_case
Yang2025_S	ENSGALG00010029290	ENSGALG00010029290	case	control	0.670700902	0.170373936	3.936640293	8.26303E-05	0.000915305	up_in_case
Yang2025_S	ENSGALG00010029291	RNFT1	case	control	-0.037137168	0.120081546	-0.309266235	0.757119013	0.852355263	down_in_case
Yang2025_S	ENSGALG00010029292	ENSGALG00010029292	case	control	0.791318715	0.227908058	3.472096253	0.000516411	0.004058764	up_in_case
Yang2025_S	ENSGALG00010029293	DNAH17	case	control	0.248169314	0.172115598	1.441875795	0.149337416	0.291684262	up_in_case
Yang2025_S	ENSGALG00010029294	PGS1	case	control	-0.271581846	0.117257611	-2.316112743	0.020552114	0.069090807	down_in_case
Yang2025_S	ENSGALG00010029295	SOCS3	case	control	0.101980581	0.22676878	0.449711733	0.65291831	0.780176355	up_in_case
Yang2025_S	ENSGALG00010029296	FBXO39	case	control	0.461819815	0.142576651	3.239098485	0.001199082	0.008064287	up_in_case
Yang2025_S	ENSGALG00010029297	SH2B2	case	control	0.471075256	0.208055868	2.264176739	0.023563247	0.076754054	up_in_case
Yang2025_S	ENSGALG00010029298	ENDOV	case	control	0.112518011	0.170939506	0.658232927	0.51038847	0.665692936	up_in_case
Yang2025_S	ENSGALG00010029299	PRKRIP1	case	control	-0.193840591	0.108168371	-1.792026527	0.073128717	0.175456038	down_in_case
Yang2025_S	ENSGALG00010029300	TEKT1	case	control	1.085205791	0.212629414	5.103742569	3.33001E-07	9.35559E-06	up_in_case
Yang2025_S	ENSGALG00010029301	RNF213	case	control	0.345918404	0.182248436	1.898059662	0.057688223	0.148290978	up_in_case
Yang2025_S	ENSGALG00010029303	SLC26A11	case	control	0.243621583	0.204098855	1.193645026	0.232616853	0.395740151	up_in_case
Yang2025_S	ENSGALG00010029304	ORAI3	case	control	0.027906672	0.110288256	0.253033943	0.800241984	0.879307053	up_in_case
Yang2025_S	ENSGALG00010029305	SMTNL2	case	control	0.038104023	0.113241722	0.336483961	0.736505945	0.8392761	up_in_case
Yang2025_S	ENSGALG00010029306	CEP95	case	control	-0.288616426	0.119890298	-2.407337626	0.016069306	0.057876915	down_in_case
Yang2025_S	ENSGALG00010029307	SGSH	case	control	0.410141896	0.148686035	2.758442621	0.00580775	0.026789473	up_in_case
Yang2025_S	ENSGALG00010029308	GAA	case	control	0.195302579	0.21251885	0.91898944	0.358101096	0.527220155	up_in_case
Yang2025_S	ENSGALG00010029309	ALKBH4	case	control	-0.303265347	0.314049358	-0.965661412	0.334213634	0.502998919	down_in_case
Yang2025_S	ENSGALG00010029310	LRWD1	case	control	-0.225751636	0.077980704	-2.894967932	0.003791973	0.019425477	down_in_case
Yang2025_S	ENSGALG00010029311	ENSGALG00010029311	case	control	0.399357468	0.284480708	1.403812124	0.160374803	0.306056269	up_in_case
Yang2025_S	ENSGALG00010029312	SMURF2	case	control	-0.065126445	0.139675249	-0.466270479	0.641021907	0.770789645	down_in_case
Yang2025_S	ENSGALG00010029313	CCDC40	case	control	0.766416574	0.293181114	2.614140334	0.008945229	0.037444206	up_in_case
Yang2025_S	ENSGALG00010029314	MED13	case	control	0.199579758	0.16308739	1.22375959	0.221042933	0.382400157	up_in_case
Yang2025_S	ENSGALG00010029315	TBC1D16	case	control	-0.063823569	0.106733955	-0.59796874	0.549860791	0.698351269	down_in_case
Yang2025_S	ENSGALG00010029316	INTS2	case	control	-0.051105194	0.07956226	-0.642329593	0.520659203	0.674229718	down_in_case
Yang2025_S	ENSGALG00010029317	KPNA2	case	control	-0.374773991	0.175513814	-2.135296264	0.032736818	0.097776974	down_in_case
Yang2025_S	ENSGALG00010029318	C17orf58	case	control	-0.169981269	0.155828834	-1.090820387	0.275351924	0.442336123	down_in_case
Yang2025_S	ENSGALG00010029319	BRIP1	case	control	-0.161473922	0.120358552	-1.341607386	0.17972333	0.331758459	down_in_case
Yang2025_S	ENSGALG00010029320	TBX4	case	control	1.571089999	0.694918582	2.260826002	0.023770034	0.077163208	up_in_case
Yang2025_S	ENSGALG00010029321	TBX2	case	control	0.490456275	0.132152095	3.711301547	0.000206196	0.001919402	up_in_case
Yang2025_S	ENSGALG00010029322	BPTF	case	control	0.425943518	0.234279629	1.818098824	0.06904903	0.168363645	up_in_case
Yang2025_S	ENSGALG00010029323	NOL11	case	control	-0.451499125	0.134671798	-3.352588537	0.000800596	0.00585633	down_in_case
Yang2025_S	ENSGALG00010029324	BCAS3	case	control	-0.233714674	0.098659701	-2.368897034	0.01784122	0.062513803	down_in_case
Yang2025_S	ENSGALG00010029325	LRRC75A	case	control	0.397306186	0.232500915	1.708837085	0.087481131	0.199811435	up_in_case
Yang2025_S	ENSGALG00010029326	NCBP3	case	control	-0.089497219	0.089123612	-1.004192004	0.315286075	0.484560655	down_in_case
Yang2025_S	ENSGALG00010029327	CRK	case	control	-0.492708215	0.070217143	-7.016921964	2.26809E-12	2.92647E-10	down_in_case
Yang2025_S	ENSGALG00010029328	ENSGALG00010029328	case	control	-0.371154626	0.134594478	-2.757576913	0.005823151	0.026833867	down_in_case
Yang2025_S	ENSGALG00010029329	ENSGALG00010029329	case	control	0.031485862	0.198271795	0.158801518	0.873825253	0.926324476	up_in_case
Yang2025_S	ENSGALG00010029330	AFMID	case	control	0.632168695	0.23496224	2.690511864	0.00713425	0.031418573	up_in_case
Yang2025_S	ENSGALG00010029331	TK1	case	control	-0.414574302	0.262390572	-1.579989321	0.114109312	0.240889753	down_in_case
Yang2025_S	ENSGALG00010029332	YWHAE	case	control	0.578451346	0.176399456	3.279212749	0.001040971	0.00723849	up_in_case
Yang2025_S	ENSGALG00010029333	ENSGALG00010029333	case	control	-0.870104372	0.131629844	-6.61023627	3.83707E-11	3.58856E-09	down_in_case
Yang2025_S	ENSGALG00010029336	SYNGR2	case	control	0.045574808	0.131094936	0.347647359	0.728105035	0.832879959	up_in_case
Yang2025_S	ENSGALG00010029337	MYO1C	case	control	0.278792279	0.149495401	1.864888669	0.062197011	0.156437055	up_in_case
Yang2025_S	ENSGALG00010029338	INPP5K	case	control	0.075130753	0.098701026	0.761195262	0.446540448	0.609310799	up_in_case
Yang2025_S	ENSGALG00010029339	PITPNA	case	control	-0.139553667	0.083870138	-1.663925569	0.096127305	0.213369543	down_in_case
Yang2025_S	ENSGALG00010029340	NDEL1	case	control	0.0102004	0.098920646	0.103116996	0.917870116	0.951604797	up_in_case
Yang2025_S	ENSGALG00010029341	SLC43A2	case	control	-0.74921169	0.142692828	-5.250521008	1.5167E-07	4.90137E-06	down_in_case
Yang2025_S	ENSGALG00010029342	TMC6	case	control	1.193693459	0.155502207	7.676376322	1.63652E-14	3.21196E-12	up_in_case
Yang2025_S	ENSGALG00010029343	SCARF1	case	control	0.078512033	0.121799613	0.644600023	0.51918642	0.673246301	up_in_case
Yang2025_S	ENSGALG00010029344	ENSGALG00010029344	case	control	0.352304122	0.243077897	1.449346597	0.147240814	0.289148922	up_in_case
Yang2025_S	ENSGALG00010029345	RILP	case	control	0.580285641	0.147212809	3.94181488	8.08674E-05	0.000900069	up_in_case
Yang2025_S	ENSGALG00010029346	ARL16	case	control	-0.204799973	0.214992257	-0.95259232	0.34079667	0.509057948	down_in_case
Yang2025_S	ENSGALG00010029347	PRPF8	case	control	-0.337252329	0.077630919	-4.344304219	1.39718E-05	0.00021827	down_in_case
Yang2025_S	ENSGALG00010029348	HGS	case	control	0.124954546	0.088694287	1.408822939	0.158887531	0.304385172	up_in_case
Yang2025_S	ENSGALG00010029349	MYH10	case	control	0.558769421	0.207778239	2.689258621	0.007161092	0.031439764	up_in_case
Yang2025_S	ENSGALG00010029350	MRPL12	case	control	0.690513947	0.24180086	2.855713363	0.004294026	0.021256912	up_in_case
Yang2025_S	ENSGALG00010029351	ENSGALG00010029351	case	control	0.065074978	0.174778673	0.372327911	0.70964872	0.820660159	up_in_case
Yang2025_S	ENSGALG00010029352	AATF	case	control	0.422093021	0.146670696	2.877827904	0.004004235	0.020202396	up_in_case
Yang2025_S	ENSGALG00010029353	GCGR	case	control	-0.678290094	0.481556313	-1.408537434	0.158971991	0.304463261	down_in_case
Yang2025_S	ENSGALG00010029354	TLCD2	case	control	0.486273993	0.37368387	1.301297784	0.193156545	0.348522136	up_in_case
Yang2025_S	ENSGALG00010029355	MCRIP1	case	control	0.299857924	0.192106901	1.560890951	0.118549483	0.247372638	up_in_case
Yang2025_S	ENSGALG00010029357	ARHGDIA	case	control	0.02438446	0.102893084	0.236988327	0.812665846	0.887252024	up_in_case
Yang2025_S	ENSGALG00010029359	ENSGALG00010029359	case	control	-0.668499081	0.341285042	-1.958770528	0.050139667	0.133491833	down_in_case
Yang2025_S	ENSGALG00010029360	ACACA	case	control	-0.101566173	0.100427198	-1.011341296	0.311853108	0.481461672	down_in_case
Yang2025_S	ENSGALG00010029361	ALYREF	case	control	0.447852462	0.102981698	4.348854879	1.3685E-05	0.000214271	up_in_case
Yang2025_S	ENSGALG00010029362	TADA2A	case	control	0.068119969	0.138177242	0.492989788	0.622019803	0.756554591	up_in_case
Yang2025_S	ENSGALG00010029363	PIK3R6	case	control	0.292737919	0.113074323	2.588898272	0.009628353	0.039461357	up_in_case
Yang2025_S	ENSGALG00010029364	ANAPC11	case	control	-0.327744685	0.386790613	-0.847343947	0.396803438	0.563896801	down_in_case
Yang2025_S	ENSGALG00010029365	PIK3R5	case	control	-0.242689079	0.119689995	-2.027647167	0.042596271	0.118502504	down_in_case
Yang2025_S	ENSGALG00010029366	MGAT5B	case	control	-0.740952174	0.404721135	-1.830772128	0.067134562	0.164963573	down_in_case
Yang2025_S	ENSGALG00010029367	DUSP14	case	control	-0.654266846	0.099387137	-6.58301329	4.61008E-11	4.19895E-09	down_in_case
Yang2025_S	ENSGALG00010029368	DDX52	case	control	-0.046500483	0.092598085	-0.502175426	0.615544127	0.751630513	down_in_case
Yang2025_S	ENSGALG00010029369	MFSD11	case	control	-0.633469679	0.119103052	-5.318668729	1.04529E-07	3.57891E-06	down_in_case
Yang2025_S	ENSGALG00010029370	SRSF2	case	control	0.530048016	0.103717724	5.110486366	3.21331E-07	9.08264E-06	up_in_case
Yang2025_S	ENSGALG00010029371	AUTS2	case	control	0.917201361	0.350887613	2.613946251	0.008950312	0.037454232	up_in_case
Yang2025_S	ENSGALG00010029372	METTL23	case	control	-0.187881446	0.221770187	-0.847189827	0.396889323	0.563924581	down_in_case
Yang2025_S	ENSGALG00010029373	GATSL2	case	control	-0.151714848	0.1720294	-0.881912325	0.377824222	0.546159806	down_in_case
Yang2025_S	ENSGALG00010029374	RCC1L	case	control	-0.337802982	0.136008403	-2.483691996	0.013002822	0.049237588	down_in_case
Yang2025_S	ENSGALG00010029375	JMJD6	case	control	-0.04137925	0.193633099	-0.213699258	0.83078159	0.899110085	down_in_case
Yang2025_S	ENSGALG00010029376	ENSGALG00010029376	case	control	0.398005786	0.147484752	2.698623293	0.006962694	0.030840796	up_in_case
Yang2025_S	ENSGALG00010029377	NCF1C	case	control	0.154031663	0.269504415	0.571536695	0.567635894	0.712292317	up_in_case
Yang2025_S	ENSGALG00010029378	ST6GALNAC1	case	control	0.312928826	0.372738941	0.83953886	0.401166992	0.56776986	up_in_case
Yang2025_S	ENSGALG00010029379	GTF2I	case	control	0.218139836	0.130340576	1.673614181	0.09420645	0.210143569	up_in_case
Yang2025_S	ENSGALG00010029380	RGS9	case	control	0.309954072	0.186919679	1.658220657	0.097272937	0.214715409	up_in_case
Yang2025_S	ENSGALG00010029382	GTF2IRD1	case	control	-0.111301657	0.07771554	-1.432167325	0.15209594	0.295600688	down_in_case
Yang2025_S	ENSGALG00010029383	GNA13	case	control	-0.48657522	0.108888552	-4.468561771	7.87473E-06	0.00013488	down_in_case
Yang2025_S	ENSGALG00010029384	WDR81	case	control	-0.224100105	0.099923308	-2.242721047	0.024914809	0.079739976	down_in_case
Yang2025_S	ENSGALG00010029385	CLIP2	case	control	-0.046621817	0.107229311	-0.43478613	0.663717673	0.787274921	down_in_case
Yang2025_S	ENSGALG00010029386	SLC16A6	case	control	-0.313884547	0.16674064	-1.882471763	0.05977199	0.152076444	down_in_case
Yang2025_S	ENSGALG00010029387	SERPINF1	case	control	-0.498810795	0.154052259	-3.237932353	0.001203994	0.0080893	down_in_case
Yang2025_S	ENSGALG00010029388	ST6GALNAC2	case	control	1.915701522	0.18246848	10.49880789	8.74779E-26	8.12669E-23	up_in_case
Yang2025_S	ENSGALG00010029389	SMYD4	case	control	0.211337061	0.156070232	1.354115123	0.175699652	0.326232465	up_in_case
Yang2025_S	ENSGALG00010029390	RPA1	case	control	0.062011217	0.080611356	0.769261552	0.441738057	0.605034384	up_in_case
Yang2025_S	ENSGALG00010029391	ARSG	case	control	-0.086761844	0.121939437	-0.711515866	0.476764623	0.635883903	down_in_case
Yang2025_S	ENSGALG00010029392	RTN4RL1	case	control	-0.246243929	0.360778278	-0.682535352	0.494900497	0.652268838	down_in_case
Yang2025_S	ENSGALG00010029394	CYGB	case	control	-0.153320024	0.093633631	-1.637446101	0.101537292	0.221531575	down_in_case
Yang2025_S	ENSGALG00010029395	DPH1	case	control	0.644070823	0.131106971	4.912559707	8.9895E-07	2.20932E-05	up_in_case
Yang2025_S	ENSGALG00010029396	WIPI1	case	control	0.154389204	0.126008713	1.225226421	0.220489922	0.381822675	up_in_case
Yang2025_S	ENSGALG00010029397	OVCA2	case	control	0.451422713	0.25202718	1.791166782	0.073266535	0.17569595	up_in_case
Yang2025_S	ENSGALG00010029398	RHBDF2	case	control	0.198205643	0.098557562	2.011064793	0.044318613	0.122341497	up_in_case
Yang2025_S	ENSGALG00010029399	PRKAR1A	case	control	-0.431577512	0.063887398	-6.755283968	1.42556E-11	1.47149E-09	down_in_case
Yang2025_S	ENSGALG00010029400	AANAT	case	control	1.481878534	0.709952817	2.087291574	0.036861786	0.106703155	up_in_case
Yang2025_S	ENSGALG00010029401	HIC1	case	control	0.125074413	0.121433932	1.029979108	0.303019813	0.472588818	up_in_case
Yang2025_S	ENSGALG00010029402	SMG6	case	control	0.114611996	0.069389619	1.651716762	0.098592304	0.216910808	up_in_case
Yang2025_S	ENSGALG00010029403	FAM20A	case	control	-0.346213858	0.162851451	-2.125948872	0.033507517	0.099409676	down_in_case

Yang2025_S	ENSGALG00010029404	SRR	case	control	0.066933828	0.166537618	0.40191417	0.687747194	0.804674261	up_in_case
Yang2025_S	ENSGALG00010029405	POLDIP2	case	control	-0.114415856	0.13054109	-0.876473886	0.380772467	0.548657257	down_in_case
Yang2025_S	ENSGALG00010029406	TMEM199	case	control	0.171489305	0.274561412	0.624593618	0.53223781	0.684607387	up_in_case
Yang2025_S	ENSGALG00010029407	UBE2O	case	control	0.130486916	0.094431251	1.381819196	0.167027224	0.31487072	up_in_case
Yang2025_S	ENSGALG00010029408	KIF12	case	control	0.439685833	0.303992939	1.446368575	0.148073851	0.29017144	up_in_case
Yang2025_S	ENSGALG00010029409	ENSGALG00010029409	case	control	-0.101246802	0.169438236	-0.597544004	0.550144237	0.698583921	down_in_case
Yang2025_S	ENSGALG00010029410	VTN	case	control	-1.052353746	0.510443761	-2.061644841	0.039241562	0.111689371	down_in_case
Yang2025_S	ENSGALG00010029411	SARM1	case	control	0.261016785	0.169296421	1.541773797	0.123128572	0.254154443	up_in_case
Yang2025_S	ENSGALG00010029412	SLC46A1	case	control	-0.322664135	0.238863625	-1.350829938	0.176749915	0.327702245	down_in_case
Yang2025_S	ENSGALG00010029413	B3GNTL1	case	control	-0.0119146	0.218909162	-0.054427142	0.956594854	0.973476545	down_in_case
Yang2025_S	ENSGALG00010029416	UNC119	case	control	-0.204565928	0.337845064	-0.605502195	0.544845367	0.694386335	down_in_case
Yang2025_S	ENSGALG00010029417	ENSGALG00010029417	case	control	0.016044887	0.16592264	0.096701013	0.922963835	0.953733233	up_in_case
Yang2025_S	ENSGALG00010029418	ABCA5	case	control	-0.028494755	0.126498553	-0.225257557	0.821778915	0.893494394	down_in_case
Yang2025_S	ENSGALG00010029419	RFC2	case	control	-0.096515342	0.163712228	-0.589542659	0.555497304	0.702919104	down_in_case
Yang2025_S	ENSGALG00010029420	LAT2	case	control	0.093800682	0.477265944	0.196537556	0.84418944	0.907437946	up_in_case
Yang2025_S	ENSGALG00010029421	TBCD	case	control	-0.119245606	0.078312526	-1.522688796	0.127836582	0.260782772	down_in_case
Yang2025_S	ENSGALG00010029422	EIF4H	case	control	-0.167362647	0.066278436	-2.525144788	0.011565063	0.045155269	down_in_case
Yang2025_S	ENSGALG00010029423	MYBBP1A	case	control	-0.025355213	0.072608296	-0.349205453	0.726935073	0.832429965	down_in_case
Yang2025_S	ENSGALG00010029424	MAP2K6	case	control	-0.222302229	0.147811253	-1.503960114	0.132591637	0.267704246	down_in_case
Yang2025_S	ENSGALG00010029425	SPNS2	case	control	0.070557471	0.186062838	0.379213127	0.704529605	0.81762269	up_in_case
Yang2025_S	ENSGALG00010029426	ZNF750	case	control	-0.210354657	2.178123647	-0.096576086	0.923063048	0.953733233	down_in_case
Yang2025_S	ENSGALG00010029427	SPNS3	case	control	-0.451240028	0.392415473	-1.14990376	0.250183512	0.415924106	down_in_case
Yang2025_S	ENSGALG00010029428	FN3K	case	control	0.195978446	0.25876161	0.75737064	0.44882784	0.611232426	up_in_case
Yang2025_S	ENSGALG00010029429	UBE2G1	case	control	-0.149496787	0.090606014	-1.649965378	0.098950017	0.217434006	down_in_case
Yang2025_S	ENSGALG00010029430	FN3KRP	case	control	-0.741842533	0.165684	-4.47745428	7.55384E-06	0.000130924	down_in_case
Yang2025_S	ENSGALG00010029431	ENSGALG00010029431	case	control	5.451744988	1.285770151	4.240061868	2.23458E-05	0.00031872	up_in_case
Yang2025_S	ENSGALG00010029432	ENSGALG00010029432	case	control	0.051007997	2.908004243	0.017540551			up_in_case
Yang2025_S	ENSGALG00010029436	WDR45B	case	control	-0.078814557	0.079033691	-0.997227332	0.318654177	0.488175677	down_in_case
Yang2025_S	ENSGALG00010029437	ENSGALG00010029437	case	control	1.709449547	1.152417078	1.483360131	0.137978754	0.275779395	up_in_case
Yang2025_S	ENSGALG00010029438	ENSGALG00010029438	case	control	0.200430832	0.414402987	0.483661648	0.628626015	0.761322631	up_in_case
Yang2025_S	ENSGALG00010029439	ENSGALG00010029439	case	control	1.030540833	0.405827461	2.539357073	0.011105641	0.043778532	up_in_case
Yang2025_S	ENSGALG00010029440	FOXK2	case	control	0.243831546	0.21205753	1.149836771	0.250211107	0.415924106	up_in_case
Yang2025_S	ENSGALG00010029441	ENSGALG00010029441	case	control	-9.215926009	2.969807123	-3.103206918			down_in_case
Yang2025_S	ENSGALG00010029442	ENSGALG00010029442	case	control	1.076277455	0.445012897	2.418530929	0.015583321	0.056624139	up_in_case
Yang2025_S	ENSGALG00010029443	ENSGALG00010029443	case	control	0.266398183	0.370208604	0.719589389	0.471777847	0.631861675	up_in_case
Yang2025_S	ENSGALG00010029445	SPHK1	case	control	0.4583733	0.220058133	2.082964599	0.037254451	0.107415843	up_in_case
Yang2025_S	ENSGALG00010029446	PRPSAP1	case	control	-0.130557175	0.124158083	-1.051539879	0.293010704	0.461680705	down_in_case
Yang2025_S	ENSGALG00010029449	UBALD2	case	control	0.836951054	0.310876701	2.692228304	0.007097634	0.031289317	up_in_case
Yang2025_S	ENSGALG00010029451	ENSGALG00010029451	case	control	0.218196629	0.223596267	0.975850944	0.329138335	0.498695269	up_in_case
Yang2025_S	ENSGALG00010029452	ENSGALG00010029452	case	control	-5.419088196	2.295311549	-2.360937972	0.018228779	0.063284276	down_in_case
Yang2025_S	ENSGALG00010029454	ENSGALG00010029454	case	control	-1.071058701	0.650440344	-1.646667079	0.099626495	0.218327344	down_in_case
Yang2025_S	ENSGALG00010029455	ENSGALG00010029455	case	control	0.831165212	0.292594771	2.840670079	0.004501886	0.022104927	up_in_case
Yang2025_S	ENSGALG00010029456	RNF157	case	control	1.112412406	0.499780947	2.225799947	0.026027584	0.082377616	up_in_case
Yang2025_S	ENSGALG00010029457	ENSGALG00010029457	case	control	0.194024843	0.344309703	0.563518372	0.573081953	0.716223948	up_in_case
Yang2025_S	ENSGALG00010029458	ENSGALG00010029458	case	control	-0.5009392	1.903003912	-0.263236033	0.79236866	0.874700688	down_in_case
Yang2025_S	ENSGALG00010029459	FOXJ1	case	control	0.586024388	0.471473172	1.242964441	0.213880937	0.374331934	up_in_case
Yang2025_S	ENSGALG00010029460	ENSGALG00010029460	case	control	0.227679073	0.340742715	0.668184711	0.504015694	0.659851437	up_in_case
Yang2025_S	ENSGALG00010029461	ENSGALG00010029461	case	control	0.909169354	0.261872104	3.471806813	0.000516968	0.004060852	up_in_case
Yang2025_S	ENSGALG00010029462	ENSGALG00010029462	case	control	-6.841902924	1.489131368	-4.594559669	4.33664E-06	8.25561E-05	down_in_case
Yang2025_S	ENSGALG00010029463	ENSGALG00010029463	case	control	1.873740504	0.704834717	2.658411199	0.007851004	0.03379788	up_in_case
Yang2025_S	ENSGALG00010029464	ENSGALG00010029464	case	control	1.65811473	0.668888199	2.478911621	0.013178396	0.049699849	up_in_case
Yang2025_S	ENSGALG00010029465	ENSGALG00010029465	case	control	4.679617083	2.924841568	1.59995575	0.1096084	0.233617782	up_in_case
Yang2025_S	ENSGALG00010029466	SLC39A11	case	control	-0.378333558	0.223799387	-1.690503097	0.090931746	0.205270352	down_in_case
Yang2025_S	ENSGALG00010029468	ENSGALG00010029468	case	control	0.14760766	0.290399251	0.50829215	0.611248474	0.748594951	up_in_case
Yang2025_S	ENSGALG00010029469	ENSGALG00010029469	case	control	1.059277601	0.302938523	3.49667514	0.000471095	0.003764169	up_in_case
Yang2025_S	ENSGALG00010029470	SSTR2	case	control	-1.136395492	0.394039145	-2.883965989	0.003927012	0.019907242	down_in_case
Yang2025_S	ENSGALG00010029472	ENSGALG00010029472	case	control	0.420307704	0.648236998	0.648385861	0.516735413	0.671206933	up_in_case
Yang2025_S	ENSGALG00010029473	COG1	case	control	0.01423682	0.106627838	0.133518794	0.893783102	0.937584125	up_in_case
Yang2025_S	ENSGALG00010029474	ENSGALG00010029474	case	control	2.115546646	1.352332346	1.564368886	0.117730963	0.24618562	up_in_case
Yang2025_S	ENSGALG00010029477	FAM104A	case	control	-0.321117227	0.116501977	-2.756324259	0.005845502	0.026892395	down_in_case
Yang2025_S	ENSGALG00010029479	ENSGALG00010029479	case	control	0.760225869	0.488959401	1.554783213	0.119997708	0.249674543	up_in_case
Yang2025_S	ENSGALG00010029480	ENSGALG00010029480	case	control	-0.039112592	0.175066063	-0.223416188	0.823211606	0.894251148	down_in_case
Yang2025_S	ENSGALG00010029481	ENSGALG00010029481	case	control	0.455806876	0.707229573	0.644496347	0.519253626	0.673246301	up_in_case
Yang2025_S	ENSGALG00010029482	DDX25	case	control	-0.384678958	0.159843198	-2.406601988	0.016101707	0.057911329	down_in_case
Yang2025_S	ENSGALG00010029484	ENSGALG00010029484	case	control	1.674456999	0.511126223	3.276014656	0.001052831	0.007295887	up_in_case
Yang2025_S	ENSGALG00010029487	CDC42EP4	case	control	-0.352953342	0.156068984	-2.261521363	0.023726991	0.077107189	down_in_case
Yang2025_S	ENSGALG00010029489	ENSGALG00010029489	case	control	1.899793833	0.754788266	2.516989094	0.011836249	0.045892636	up_in_case
Yang2025_S	ENSGALG00010029491	ENSGALG00010029491	case	control	0.212136613	0.300769739	0.705312355	0.480615873	0.639124171	up_in_case
Yang2025_S	ENSGALG00010029492	SDK2	case	control	0.242733924	0.170073305	1.427231187	0.153513268	0.297525367	up_in_case
Yang2025_S	ENSGALG00010029494	MYH7	case	control	1.194042763	0.210554474	5.670944617	1.42012E-08	6.34276E-07	up_in_case
Yang2025_S	ENSGALG00010029495	RPL38	case	control	-1.510234651	0.277115222	-5.449843723	5.04141E-08	1.88343E-06	down_in_case
Yang2025_S	ENSGALG00010029496	TTYH2	case	control	-0.165635294	0.093936515	-1.763268451	0.077855224	0.183883482	down_in_case
Yang2025_S	ENSGALG00010029497	ENSGALG00010029497	case	control	0.52714778	0.626176906	0.8418512	0.399871249	0.566799497	up_in_case
Yang2025_S	ENSGALG00010029498	ENSGALG00010029498	case	control	-0.718445834	0.16741929	-4.29129663	1.77633E-05	0.000263893	down_in_case
Yang2025_S	ENSGALG00010029501	ENSGALG00010029501	case	control	-0.085243655	0.418328079	-0.203772252	0.838531478	0.904482259	down_in_case
Yang2025_S	ENSGALG00010029502	BTBD17	case	control	1.73176804	0.397923875	4.352008385	1.34896E-05	0.000212569	up_in_case
Yang2025_S	ENSGALG00010029504	GPR142	case	control	0.806059624	0.374475905	2.152500637	0.031357945	0.094623856	up_in_case
Yang2025_S	ENSGALG00010029505	VEZF1	case	control	0.368357371	0.256289908	1.437268342	0.150641761	0.293470283	up_in_case
Yang2025_S	ENSGALG00010029506	OXLD1	case	control	0.068413473	0.263586531	0.25954844	0.795212112	0.876525276	up_in_case
Yang2025_S	ENSGALG00010029507	VPS37D	case	control	0.006482161	0.282131085	0.022975707	0.981669651	0.98869374	up_in_case
Yang2025_S	ENSGALG00010029508	ENSGALG00010029508	case	control	0.114258393	0.216289661	0.528265626	0.597314986	0.736600383	up_in_case
Yang2025_S	ENSGALG00010029509	DNAJC30	case	control	0.270248967	0.241282695	1.120051178	0.262691954	0.429591972	up_in_case
Yang2025_S	ENSGALG00010029510	PDE6G	case	control	-0.005269878	0.358703534	-0.014691459	0.988278334	0.992866719	down_in_case
Yang2025_S	ENSGALG00010029511	CUEDC1	case	control	-0.100206588	0.098445521	-1.017888745	0.308730831	0.478390318	down_in_case
Yang2025_S	ENSGALG00010029512	TSPAN10	case	control	4.15800911	0.979317509	4.245823312	2.17792E-05	0.000311595	up_in_case
Yang2025_S	ENSGALG00010029513	WBSCR22	case	control	-0.148266929	0.209418081	-0.70799488	0.478948437	0.637697924	down_in_case
Yang2025_S	ENSGALG00010029514	MRPS23	case	control	0.286879392	0.129968409	2.207300946	0.027293041	0.085237232	up_in_case
Yang2025_S	ENSGALG00010029515	NPLOC4	case	control	-0.044809172	0.119435344	-0.375175145	0.707530214	0.819805489	down_in_case
Yang2025_S	ENSGALG00010029516	ENSGALG00010029516	case	control	0.698282596	0.212008851	3.293648314	0.000988962	0.006946159	up_in_case
Yang2025_S	ENSGALG00010029517	MSI2	case	control	0.535767632	0.158865934	3.372451349	0.000745022	0.005534056	up_in_case
Yang2025_S	ENSGALG00010029518	FAAP100	case	control	0.719958672	0.111997491	6.428346463	1.28999E-10	1.03311E-08	up_in_case
Yang2025_S	ENSGALG00010029519	STX1A	case	control	0.116023425	0.157315588	0.737520206	0.46080606	0.621800372	up_in_case
Yang2025_S	ENSGALG00010029520	MKS1	case	control	0.485074847	0.140462836	3.453403487	0.00055356	0.004304611	up_in_case
Yang2025_S	ENSGALG00010029521	ACTG1	case	control	0.087259652	0.116091478	0.751645634	0.452264193	0.614079853	up_in_case
Yang2025_S	ENSGALG00010029522	AKAP1	case	control	0.194226785	0.067197548	2.890384987	0.003847703	0.019633007	up_in_case
Yang2025_S	ENSGALG00010029523	FSCN2	case	control	1.83559739	0.50466466	3.637261601	0.000275552	0.002425659	up_in_case
Yang2025_S	ENSGALG00010029524	ENSGALG00010029524	case	control	0.382239975	0.106947464	3.574091048	0.000351447	0.002977148	up_in_case
Yang2025_S	ENSGALG00010029525	SLC38A10	case	control	0.246218367	0.113106455	2.176872815	0.029490058	0.090396824	up_in_case
Yang2025_S	ENSGALG00010029526	BCL7B	case	control	-0.210705017	0.16234752	-1.297864097	0.194334039	0.349967025	down_in_case
Yang2025_S	ENSGALG00010029527	NDUFAF8	case	control	0.072127553	0.252841679	0.285267657	0.77543909	0.863270828	up_in_case
Yang2025_S	ENSGALG00010029528	TBL2	case	control	-0.384711395	0.282452577	-1.362038895	0.173185611	0.323114404	down_in_case
Yang2025_S	ENSGALG00010029529	TEPSIN	case	control	0.591405967	0.186360385	3.173453236	0.001506372	0.009642301	up_in_case
Yang2025_S	ENSGALG00010029530	ENSGALG00010029530	case	control	0.182572314	0.20503029	0.890465079	0.373216211	0.541255415	up_in_case
Yang2025_S	ENSGALG00010029531	CEP131	case	control	0.93941546	0.138675707	6.77418909	1.25106E-11	1.31376E-09	up_in_case
Yang2025_S	ENSGALG00010029532	EVI2A	case	control	0.27517218	0.311255122	0.884072774	0.376656929	0.54486809	up_in_case
Yang2025_S	ENSGALG00010029533	MLXIPL	case	control	-0.271357638	0.33918303	-0.800033061	0.423691642	0.588783805	down_in_case
Yang2025_S	ENSGALG00010029534	NF1	case	control	0.437776459	0.191178041	2.289888825	0.022027762	0.072618137	up_in_case
Yang2025_S	ENSGALG00010029536	BAZ1B	case	control	0.103834907	0.123917293	0.837937181	0.402065986	0.568580222	up_in_case
Yang2025_S	ENSGALG00010029537	MYH1B	case	control	0.602413982	0.210092727	2.867371897	0.004138963	0.020679976	up_in_case
Yang2025_S	ENSGALG00010029538	FZD9	case	control	0.260245976	0.131903817	1.972998072	0.048495782	0.130435963	up_in_case
Yang2025_S	ENSGALG00010029539	FKBP6	case	control	0.240967148	0.692221099	0.348107199	0.727759678	0.832758344	up_in_case
Yang2025_S	ENSGALG00010029541	ENSGALG00010029541	case	control	-0.434261806	0.276618203	-1.569895984	0.116439312	0.244217612	down_in_case
Yang2025_S	ENSGALG00010029542	AATK	case	control	0.15673566	0.109512039	1.431218527	0.152367595	0.295881053	up_in_case
Yang2025_S	ENSGALG00010029543	NSUN5	case	control	-0.071082724	0.246559448	-0.288298519	0.773118241	0.86194117	down_in_case
Yang2025_S	ENSGALG00010029544	ENSGALG00010029544	case	control	0.377804442	0.204769223	1.845025513	0.065033827	0.161223277	up_in_case
Yang2025_S	ENSGALG00010029545	OMG	case	control	0.934282919	0.401799964	2.325243906	0.020058922	0.067844921	up_in_case
Yang2025_S	ENSGALG00010029546	WSB1	case	control	0.383430228	0.104514945	3.668664121	0.000243821	0.002196282	up_in_case
Yang2025_S	ENSGALG00010029547	HIP1	case	control	0.559654465	0.080847351	6.922359935	4.44181E-12	5.29031E-10	up_in_case
Yang2025_S	ENSGALG00010029548	BAIAP2	case	control	0.16859514	0.169472155	0.994825022	0.319821374	0.489103473	up_in_case
Yang2025_S	ENSGALG00010029549	KSR1	case	control	0.14435962	0.148893803	0.96954754	0.332272073	0.501593688	up_in_case
Yang2025_S	ENSGALG00010029550	CHMP6	case	control	0.12639369	0.169556901	0.745435243	0.456008645	0.617659454	up_in_case
Yang2025_S	ENSGALG00010029552	NOS2	case	control	0.716124024	0.15660625	4.572767846	4.81323E-06	8.95493E-05	up_in_case
Yang2025_S	ENSGALG00010029553	MYH1C	case	control	0.862744378	0.166392032	5.18501016	2.16003E-07	6.55774E-06	up_in_case
Yang2025_S	ENSGALG00010029554	ENSGALG00010029554	case	control	0.117102852	0.965238995	0.121320059	0.903437532	0.942249982	up_in_case
Yang2025_S	ENSGALG00010029555	LYRM9	case	control	0.258243991	0.274216597	0.94175186	0.346319697	0.514824513	up_in_case
Yang2025_S	ENSGALG00010029556	NPTX1	case	control	0.299921922	0.31592757	0.949337604	0.342448934	0.510814161	up_in_case

Yang2025_S	ENSGALG00010029558	ENSGALG00010029558	case	control	1.212773519	0.476608272	2.544591838	0.010940554	0.043286944	up_in_case
Yang2025_S	ENSGALG00010029560	ENSGALG00010029560	case	control	-0.112700014	0.229251779	-0.491599302	0.623002637	0.757291695	down_in_case
Yang2025_S	ENSGALG00010029561	SCO1	case	control	0.170927212	0.103006828	1.659377496	0.097039748	0.214637295	up_in_case
Yang2025_S	ENSGALG00010029563	ADPRM	case	control	-0.068842577	0.165315539	-0.416431373	0.677094375	0.796968504	down_in_case
Yang2025_S	ENSGALG00010029564	NLK	case	control	0.146597653	0.128734613	1.138758641	0.254803837	0.421184398	up_in_case
Yang2025_S	ENSGALG00010029566	TMEM97	case	control	-0.079605534	0.120790499	-0.659038042	0.50987134	0.665142963	down_in_case
Yang2025_S	ENSGALG00010029567	IFT20	case	control	0.012110138	0.1443267	0.083907818	0.933129724	0.959399477	up_in_case
Yang2025_S	ENSGALG00010029569	TMEM220	case	control	0.515902435	0.184247164	2.800056309	0.005109369	0.024374893	up_in_case
Yang2025_S	ENSGALG00010029570	TNFAIP1	case	control	-0.39646744	0.105566887	-3.755604151	0.000172924	0.001661858	down_in_case
Yang2025_S	ENSGALG00010029571	EPX	case	control	-1.394233765	0.490917297	-2.840058341	0.004510528	0.022131765	down_in_case
Yang2025_S	ENSGALG00010029572	TSPOAP1	case	control	0.614987362	0.147025371	4.18286556	2.87858E-05	0.00039365	up_in_case
Yang2025_S	ENSGALG00010029573	MAP2K4	case	control	-0.237168132	0.080865908	-2.932856854	0.003358587	0.017734714	down_in_case
Yang2025_S	ENSGALG00010029574	SGSM2	case	control	-0.092885376	0.155024923	-0.599164148	0.549063428	0.697656503	down_in_case
Yang2025_S	ENSGALG00010029576	ATP2A3	case	control	-0.627020404	0.125352429	-5.00206028	5.67208E-07	1.48572E-05	down_in_case
Yang2025_S	ENSGALG00010029577	NOG	case	control	0.534744745	0.78679241	0.679651632	0.496725069	0.653807862	up_in_case
Yang2025_S	ENSGALG00010029578	C17orf67	case	control	0.525931286	0.671893198	0.782760248	0.433767916	0.597669682	up_in_case
Yang2025_S	ENSGALG00010029579	GALNT17	case	control	-0.029133855	0.185032042	-0.157453025	0.874887826	0.926528291	down_in_case
Yang2025_S	ENSGALG00010029580	P2RX1	case	control	0.203372251	0.276433006	0.735701766	0.461912215	0.622866791	up_in_case
Yang2025_S	ENSGALG00010029581	DGKE	case	control	-0.160833911	0.145597773	-1.104645407	0.269313268	0.4364098	down_in_case
Yang2025_S	ENSGALG00010029582	SCPEP1	case	control	-0.52497065	0.104641081	-5.016869538	5.25202E-07	1.40488E-05	down_in_case
Yang2025_S	ENSGALG00010029583	LIMK1	case	control	0.451856116	0.144643587	3.123927757	0.001784543	0.011008235	up_in_case
Yang2025_S	ENSGALG00010029584	TRIM25	case	control	-0.374041813	0.088087573	-4.246249481	2.17379E-05	0.000311323	down_in_case
Yang2025_S	ENSGALG00010029585	ENSGALG00010029585	case	control	0.741483386	0.315703111	2.348673046	0.018840442	0.064921257	up_in_case
Yang2025_S	ENSGALG00010029586	COIL	case	control	0.253389984	0.099904384	2.536324976	0.011202272	0.044059742	up_in_case
Yang2025_S	ENSGALG00010029588	RAB11FIP4	case	control	0.051532999	0.229526291	0.224518936	0.822353534	0.893806762	up_in_case
Yang2025_S	ENSGALG00010029590	UTP6	case	control	0.172220898	0.091614229	1.879848793	0.060128688	0.152733175	up_in_case
Yang2025_S	ENSGALG00010029591	SUZ12	case	control	-0.214367632	0.077572048	-2.763464906	0.005719124	0.026485874	down_in_case
Yang2025_S	ENSGALG00010029592	MNT	case	control	0.27202897	0.185078584	1.469802523	0.141615246	0.281045237	up_in_case
Yang2025_S	ENSGALG00010029593	PAFAH1B1	case	control	-0.225855811	0.075066228	-3.00875396	0.002623215	0.014686418	down_in_case
Yang2025_S	ENSGALG00010029594	CRLF3	case	control	-0.242543629	0.076309269	-3.178429472	0.001480752	0.009526447	down_in_case
Yang2025_S	ENSGALG00010029595	CLUH	case	control	-0.055968101	0.077194648	-0.725025665	0.468436285	0.629111376	down_in_case
Yang2025_S	ENSGALG00010029596	ATAD5	case	control	-0.035644186	0.120449435	-0.295926555	0.767286171	0.858585699	down_in_case
Yang2025_S	ENSGALG00010029597	ADAP2	case	control	0.023757856	0.199718687	0.1189566	0.90530974	0.943381503	up_in_case
Yang2025_S	ENSGALG00010029598	SUPT4H1	case	control	-0.058707969	0.329787166	-0.17801775	0.858709036	0.916802821	down_in_case
Yang2025_S	ENSGALG00010029599	RNF43	case	control	0.595784909	0.380880929	1.564228775	0.117763852	0.246217446	up_in_case
Yang2025_S	ENSGALG00010029600	RHOT1	case	control	-0.114184281	0.074466589	-1.533362584	0.125186542	0.257107512	down_in_case
Yang2025_S	ENSGALG00010029601	HSF5	case	control	-0.047525803	0.294394931	-0.161435536	0.87175038	0.924634004	down_in_case
Yang2025_S	ENSGALG00010029602	MTMR4	case	control	-0.001817766	0.078745187	-0.023084151	0.981583148	0.98867905	down_in_case
Yang2025_S	ENSGALG00010029603	RHBDL3	case	control	-0.174586637	0.499559918	-0.349480875	0.726728326	0.832329449	down_in_case
Yang2025_S	ENSGALG00010029604	ENSGALG00010029604	case	control	-0.130346504	0.114722936	-1.136185219	0.255879055	0.422123196	down_in_case
Yang2025_S	ENSGALG00010029605	C17orf75	case	control	-0.331646505	0.100904898	-3.286723549	0.001013603	0.007087083	down_in_case
Yang2025_S	ENSGALG00010029606	ABHD11	case	control	0.100652956	0.194761047	0.516802294	0.605294179	0.743151928	up_in_case
Yang2025_S	ENSGALG00010029607	ZNF207	case	control	0.217587433	0.163574516	1.33020374	0.183451152	0.336810514	up_in_case
Yang2025_S	ENSGALG00010029608	ENSGALG00010029608	case	control	0.450784781	0.194638556	2.316009687	0.02055774	0.069090807	up_in_case
Yang2025_S	ENSGALG00010029609	METTL27	case	control	0.161149496	0.140491003	1.147044954	0.251363042	0.417042981	up_in_case
Yang2025_S	ENSGALG00010029610	ERN1	case	control	0.431199337	0.083269504	5.178358406	2.23847E-07	6.76639E-06	up_in_case
Yang2025_S	ENSGALG00010029611	YWHAG	case	control	0.121967128	0.130067281	0.937723365	0.348386593	0.516794462	up_in_case
Yang2025_S	ENSGALG00010029612	HSPB1	case	control	0.23656498	0.147473327	1.604120455	0.108687504	0.232402761	up_in_case
Yang2025_S	ENSGALG00010029614	MDH2	case	control	0.061756573	0.154263528	0.400331651	0.688912259	0.805503635	up_in_case
Yang2025_S	ENSGALG00010029615	ENSGALG00010029615	case	control	-0.144175033	0.35325572	-0.408132198	0.683176623	0.801622642	down_in_case
Yang2025_S	ENSGALG00010029616	TMEM120A	case	control	0.192180464	0.214756938	0.894874298	0.370854288	0.539385711	up_in_case
Yang2025_S	ENSGALG00010029617	POR	case	control	-0.109523214	0.107498979	-1.018830271	0.308283552	0.477909811	down_in_case
Yang2025_S	ENSGALG00010029618	TEX2	case	control	0.176847683	0.102389268	1.727209167	0.084130105	0.194162473	up_in_case
Yang2025_S	ENSGALG00010029619	ENSGALG00010029619	case	control	-0.330781154	0.190271036	-1.738473503	0.082127415	0.190740921	down_in_case
Yang2025_S	ENSGALG00010029620	ENSGALG00010029620	case	control	0.41910731	0.449560147	0.932260818	0.351201752	0.519863651	up_in_case
Yang2025_S	ENSGALG00010029621	TAF15	case	control	0.413633979	0.092567698	4.468448383	7.8789E-06	0.00013488	up_in_case
Yang2025_S	ENSGALG00010029622	MMP28	case	control	0.06304154	0.143802223	0.438390581	0.661103173	0.786114235	up_in_case
Yang2025_S	ENSGALG00010029623	ENSGALG00010029623	case	control	0.187459213	0.464814713	0.403298793	0.686728429	0.804164762	up_in_case
Yang2025_S	ENSGALG00010029624	RASL10B	case	control	0.247755304	0.216086234	1.146557557	0.251564527	0.417277905	up_in_case
Yang2025_S	ENSGALG00010029625	PECAM1	case	control	0.339742261	0.10141715	3.34994882	0.000808265	0.00590313	up_in_case
Yang2025_S	ENSGALG00010029626	AP2B1	case	control	-0.308915916	0.074802374	-4.129760864	3.63141E-05	0.000473817	down_in_case
Yang2025_S	ENSGALG00010029627	PEX12	case	control	-0.334691082	0.184074921	-1.818232923	0.06902854	0.16834314	down_in_case
Yang2025_S	ENSGALG00010029628	UNC45B	case	control	0.039657586	0.127029121	0.312192875	0.754893947	0.851019104	up_in_case
Yang2025_S	ENSGALG00010029629	ENSGALG00010029629	case	control	0.867074403	0.225010724	3.853480352	0.000116451	0.00120292	up_in_case
Yang2025_S	ENSGALG00010029630	RAD51D	case	control	0.287697391	0.270573028	1.063289247	0.2876508	0.456487177	up_in_case
Yang2025_S	ENSGALG00010029631	LIG3	case	control	0.060765866	0.067968538	0.894029323	0.371306202	0.539862903	up_in_case
Yang2025_S	ENSGALG00010029632	TMEM132E	case	control	-0.072262114	0.239308318	-0.301962401	0.762680726	0.855408866	down_in_case
Yang2025_S	ENSGALG00010029633	POLG2	case	control	1.105617082	0.148984679	7.421011954	1.16229E-13	1.84051E-11	up_in_case
Yang2025_S	ENSGALG00010029634	DDX5	case	control	-0.064166922	0.065006695	-0.987081755	0.323602561	0.492884653	down_in_case
Yang2025_S	ENSGALG00010029635	ENSGALG00010029635	case	control	0.043465777	0.372323785	0.116741874	0.907064606	0.944261228	up_in_case
Yang2025_S	ENSGALG00010029636	SPATA20	case	control	-0.016124855	0.13485666	-0.119570324	0.904823529	0.943174637	down_in_case
Yang2025_S	ENSGALG00010029638	EPN3	case	control	0.438574616	0.133790121	3.278079227	0.001045161	0.00726255	up_in_case
Yang2025_S	ENSGALG00010029641	ENSGALG00010029641	case	control	0.166055431	0.4534261	0.366223803	0.714198082	0.823558969	up_in_case
Yang2025_S	ENSGALG00010029642	NAT9	case	control	0.009201109	0.197832727	0.046509541	0.96290413	0.977970213	up_in_case
Yang2025_S	ENSGALG00010029644	MYCBPAP	case	control	0.484637995	0.129528405	3.741557652	0.000182883	0.001737204	up_in_case
Yang2025_S	ENSGALG00010029645	ENSGALG00010029645	case	control	1.439628021	0.377193246	3.816685577	0.000135256	0.001362832	up_in_case
Yang2025_S	ENSGALG00010029646	MRPS17	case	control	-0.576995603	0.164556007	-3.506378246	0.000454249	0.003656824	down_in_case
Yang2025_S	ENSGALG00010029647	RSAD1	case	control	1.206342097	0.296212212	4.072560306	4.64992E-05	0.000574507	up_in_case
Yang2025_S	ENSGALG00010029648	NIPSNAP2	case	control	0.177472023	0.126371923	1.404362761	0.160210854	0.305868236	up_in_case
Yang2025_S	ENSGALG00010029649	PSPH	case	control	-0.048602502	0.18157025	-0.267678775	0.788946589	0.872675878	down_in_case
Yang2025_S	ENSGALG00010029650	ACSF2	case	control	0.069676323	0.11670697	0.597019382	0.55049444	0.698837572	up_in_case
Yang2025_S	ENSGALG00010029651	ENSGALG00010029651	case	control	1.032388167	0.527360541	1.957651525	0.050270917	0.133739066	up_in_case
Yang2025_S	ENSGALG00010029652	LRRC59	case	control	-0.060812808	0.123074315	-0.494114536	0.621225293	0.755852131	down_in_case
Yang2025_S	ENSGALG00010029653	CCT6A	case	control	0.16119962	0.113244049	1.423471002	0.154599662	0.298882672	up_in_case
Yang2025_S	ENSGALG00010029654	EME1	case	control	-0.058184781	0.14256371	-0.408131781	0.683176928	0.801622642	down_in_case
Yang2025_S	ENSGALG00010029655	PHKG1	case	control	0.057049707	0.187025316	0.305037354	0.760337703	0.854595062	up_in_case
Yang2025_S	ENSGALG00010029656	MRPL27	case	control	-0.038118964	0.243351524	-0.156641567	0.875527341	0.926735549	down_in_case
Yang2025_S	ENSGALG00010029657	CHCHD2	case	control	-0.074737863	0.191188227	-0.390912474	0.695861934	0.811317405	down_in_case
Yang2025_S	ENSGALG00010029658	XYLT2	case	control	-0.021815775	0.114933755	-0.189811727	0.849456666	0.910630056	down_in_case
Yang2025_S	ENSGALG00010029659	VKORC1L1	case	control	-0.57687654	0.093310189	-6.182353123	6.31531E-10	4.01844E-08	down_in_case
Yang2025_S	ENSGALG00010029660	CD300LG	case	control	-0.580609671	0.36664641	-1.583568403	0.113291969	0.239705164	down_in_case
Yang2025_S	ENSGALG00010029663	GUSB	case	control	0.028781417	0.098114013	0.293346645	0.769257203	0.859493195	up_in_case
Yang2025_S	ENSGALG00010029665	ASL1	case	control	0.138238456	0.219018913	0.631171318	0.527928513	0.680859216	up_in_case
Yang2025_S	ENSGALG00010029666	SLC9A3R1	case	control	-0.177421785	0.17639277	-1.005833658	0.314495594	0.483757728	down_in_case
Yang2025_S	ENSGALG00010029667	TMEM104	case	control	-0.198195032	0.116603738	-1.699731366	0.089181467	0.202610082	down_in_case
Yang2025_S	ENSGALG00010029668	CRCP	case	control	0.166138646	0.1323164	1.255616427	0.209255073	0.368410542	up_in_case
Yang2025_S	ENSGALG00010029669	HID1	case	control	0.102780759	0.081456906	1.26178079	0.207027676	0.365783019	up_in_case
Yang2025_S	ENSGALG00010029670	TPST1	case	control	0.376584296	0.148823787	2.530403929	0.011393128	0.044608946	up_in_case
Yang2025_S	ENSGALG00010029671	GRIN2C	case	control	0.019295832	0.135092407	0.142834319	0.886421032	0.933444955	up_in_case
Yang2025_S	ENSGALG00010029672	KCTD7	case	control	-0.065554624	0.240094245	-0.273037048	0.784824737	0.869981124	down_in_case
Yang2025_S	ENSGALG00010029673	TMEM248	case	control	-0.566532758	0.077208316	-7.337716762	2.17268E-13	3.32707E-11	down_in_case
Yang2025_S	ENSGALG00010029674	FDXR	case	control	-0.912628862	0.194881433	-4.682995432	2.82713E-06	5.78503E-05	down_in_case
Yang2025_S	ENSGALG00010029675	DHRS13	case	control	0.452902036	0.284734289	1.590612908	0.111696718	0.237053573	up_in_case
Yang2025_S	ENSGALG00010029676	FADS6	case	control	0.9421181	1.181846443	0.797157791	0.425359396	0.590200457	up_in_case
Yang2025_S	ENSGALG00010029677	PHF12	case	control	0.002592593	0.091040397	0.028477389	0.977281402	0.986627279	up_in_case
Yang2025_S	ENSGALG00010029682	CDR2L	case	control	-0.215601668	0.112032288	-1.924460101	0.054296937	0.141725599	down_in_case
Yang2025_S	ENSGALG00010029683	MRPL58	case	control	0.226616325	0.203500774	1.113589494	0.265455335	0.432573553	up_in_case
Yang2025_S	ENSGALG00010029684	ENSGALG00010029684	case	control	0.828151815	0.513120954	1.613950492	0.106538165	0.229106379	up_in_case
Yang2025_S	ENSGALG00010029685	ATP5PD	case	control	-0.512844713	0.246404288	-2.081314073	0.037405168	0.107694425	down_in_case
Yang2025_S	ENSGALG00010029686	KCTD2	case	control	-0.326253801	0.077597091	-4.204459171	2.61707E-05	0.000363961	down_in_case
Yang2025_S	ENSGALG00010029687	SLC16A5	case	control	-0.343611424	0.244001596	-1.408234329	0.159061694	0.304493196	down_in_case
Yang2025_S	ENSGALG00010029689	ENSGALG00010029689	case	control	0.852021783	0.152639314	5.581928789	2.37866E-08	9.89451E-07	up_in_case
Yang2025_S	ENSGALG00010029690	ENSGALG00010029690	case	control	-0.161642754	0.147231237	-1.09788356	0.272255338	0.439564144	down_in_case
Yang2025_S	ENSGALG00010029691	NUFIP2	case	control	-0.249438162	0.113310284	-2.201372674	0.027709652	0.086325508	down_in_case
Yang2025_S	ENSGALG00010029692	CRYBA1	case	control	-0.215474347	0.456942848	-0.47155645	0.637243418	0.767673023	down_in_case
Yang2025_S	ENSGALG00010029693	HN1	case	control	-0.1378322	0.121721745	-1.13235478	0.257485308	0.423769667	down_in_case
Yang2025_S	ENSGALG00010029694	ARMC7	case	control	-0.431318215	0.205966987	-2.094113338	0.036249885	0.105391644	down_in_case
Yang2025_S	ENSGALG00010029695	TAOK1	case	control	-0.164060921	0.117744363	-1.39336539	0.163509326	0.310169134	down_in_case
Yang2025_S	ENSGALG00010029696	SUMO2	case	control	-0.505306948	0.091932358	-5.496508049	3.87385E-08	1.50788E-06	down_in_case
Yang2025_S	ENSGALG00010029697	GIT1	case	control	0.559553246	0.121147396	4.618780617	3.86002E-06	7.50365E-05	up_in_case
Yang2025_S	ENSGALG00010029698	NUP85	case	control	-0.007091633	0.127785831	-0.055496238	0.955743127	0.972847368	down_in_case
Yang2025_S	ENSGALG00010029699	ANKRD13B	case	control	0.757261111	0.152387245	4.969320837	6.71878E-07	1.71163E-05	up_in_case
Yang2025_S	ENSGALG00010029700	GGA3	case	control	0.175077157	0.09592213	1.825200892	0.067970719	0.166374842	up_in_case
Yang2025_S	ENSGALG00010029701	MRPS7	case	control	-0.106566902	0.083644954	-1.274038629	0.202649727	0.360493474	down_in_case
Yang2025_S	ENSGALG00010029702	CORO6	case	control	0.197072448	0.233098528	0.845446986	0.39786132	0.564639729	up_in_case
Yang2025_S	ENSGALG00010029703	MIF4GD	case	control	-0.09360934	0.101481056	-0.922431672	0.356303455	0.52574001	down_in_case

Yang2025_S	ENSGALG00010029704	SLC25A19	case	control	-0.316205065	0.164416949	-1.923190206	0.054456167	0.141999754	down_in_case
Yang2025_S	ENSGALG00010029705	GRB2	case	control	-0.440770769	0.063827036	-6.905706334	4.99542E-12	5.80094E-10	down_in_case
Yang2025_S	ENSGALG00010029706	SSH2	case	control	0.227561498	0.142517876	1.596722494	0.110327579	0.23475566	up_in_case
Yang2025_S	ENSGALG00010029707	NSRP1	case	control	0.917071311	0.253487428	3.617817725	0.000297098	0.002585917	up_in_case
Yang2025_S	ENSGALG00010029708	TMEM94	case	control	-0.128018676	0.098525308	-1.29934814	0.19382448	0.349365429	down_in_case
Yang2025_S	ENSGALG00010029709	SLC6A4	case	control	0.070025828	0.213242545	0.328385822	0.742619962	0.842447418	up_in_case
Yang2025_S	ENSGALG00010029710	BLMH	case	control	-0.075353233	0.10379882	-0.725954621	0.46786659	0.628604892	down_in_case
Yang2025_S	ENSGALG00010029711	CASKIN2	case	control	0.063676114	0.096955683	0.65675484	0.511338566	0.666606142	up_in_case
Yang2025_S	ENSGALG00010029713	CPD	case	control	-0.202619857	0.093579361	-2.1652195	0.03037088	0.092587663	down_in_case
Yang2025_S	ENSGALG00010029716	GOSR1	case	control	-0.024409766	0.094632345	-0.257943164	0.796450772	0.876731298	down_in_case
Yang2025_S	ENSGALG00010029717	TRARG1	case	control	0.323634564	0.353210778	0.91626469	0.359528083	0.528260632	up_in_case
Yang2025_S	ENSGALG00010029718	ABR	case	control	0.096381062	0.078627655	1.22579088	0.22027738	0.381644323	up_in_case
Yang2025_S	ENSGALG00010029720	TIMM22	case	control	0.087042292	0.234549431	0.371104257	0.71055988	0.821267212	up_in_case
Yang2025_S	ENSGALG00010029721	EXOC7	case	control	0.069914689	0.086617353	0.807167225	0.419570146	0.585277587	up_in_case
Yang2025_S	ENSGALG00010029722	SPECC1	case	control	-0.004404621	0.126582841	-0.034796351	0.972242131	0.983106748	down_in_case
Yang2025_S	ENSGALG00010029723	ADORA2B	case	control	-0.297267862	0.180363002	-1.64816431	0.099318958	0.217864637	down_in_case
Yang2025_S	ENSGALG00010029724	CACNA1G	case	control	0.31256316	0.20588899	1.518114981	0.128985415	0.262549191	up_in_case
Yang2025_S	ENSGALG00010029725	TSEN54	case	control	0.344888212	0.106745361	3.230943339	0.001233824	0.008242252	up_in_case
Yang2025_S	ENSGALG00010029726	SEPTIN9	case	control	-0.334919967	0.071243108	-4.701085835	2.58782E-06	5.40648E-05	down_in_case
Yang2025_S	ENSGALG00010029727	ZSWIM7	case	control	-0.234760096	0.232630097	-1.009156164	0.312899746	0.482294504	down_in_case
Yang2025_S	ENSGALG00010029728	TNRC6C	case	control	0.382881441	0.202588733	1.889944399	0.058765397	0.150421712	up_in_case
Yang2025_S	ENSGALG00010029729	RASL10A	case	control	0.063396811	0.540740003	0.117240837	0.906669206	0.944055033	up_in_case
Yang2025_S	ENSGALG00010029730	TTC19	case	control	0.515810935	0.1319518	3.909086	9.2646E-05	0.001003754	up_in_case
Yang2025_S	ENSGALG00010029731	NCOR1	case	control	0.386325407	0.208753733	1.850627543	0.064223155	0.159699414	up_in_case
Yang2025_S	ENSGALG00010029732	PIGL	case	control	-0.171526828	0.129813554	-1.321332194	0.186390624	0.340056735	down_in_case
Yang2025_S	ENSGALG00010029733	ENSGALG00010029733	case	control	0.081672669	0.307767745	0.265371111	0.790723588	0.873809136	up_in_case
Yang2025_S	ENSGALG00010029734	SEC14L1	case	control	-0.151393856	0.111068555	-1.363066758	0.172861472	0.322639246	down_in_case
Yang2025_S	ENSGALG00010029735	SRP68	case	control	0.346624896	0.070437392	4.921035342	8.60876E-07	2.13078E-05	up_in_case
Yang2025_S	ENSGALG00010029736	ENSGALG00010029736	case	control	-0.973651726	0.150527091	-6.468282371	9.91231E-11	8.52643E-09	down_in_case
Yang2025_S	ENSGALG00010029737	ENSGALG00010029737	case	control	0.120930962	0.874540816	0.138279381	0.890019621	0.935608284	up_in_case
Yang2025_S	ENSGALG00010029738	TRPV2	case	control	-0.291909399	0.090338501	-3.231284512	0.001232352	0.008236367	down_in_case
Yang2025_S	ENSGALG00010029739	P2RX5	case	control	0.153664335	0.119378342	1.287204473	0.198023029	0.354594051	up_in_case
Yang2025_S	ENSGALG00010029740	HASPIN	case	control	0.016715816	0.180890209	0.092408628	0.926373385	0.955608219	up_in_case
Yang2025_S	ENSGALG00010029741	EMC6	case	control	-0.704996286	0.16073475	-4.386085067	1.15409E-05	0.000186386	down_in_case
Yang2025_S	ENSGALG00010029742	TAX1BP3	case	control	-0.164002005	0.074825915	-2.191780803	0.028395338	0.087833304	down_in_case
Yang2025_S	ENSGALG00010029743	MYOCD	case	control	-0.216159687	0.158536255	-1.363471633	0.172733919	0.322444362	down_in_case
Yang2025_S	ENSGALG00010029744	CTNS	case	control	-0.463682855	0.145225541	-3.192846462	0.001408778	0.009189101	down_in_case
Yang2025_S	ENSGALG00010029745	SHPK	case	control	0.363597812	0.233918322	1.554379365	0.120093952	0.249707977	up_in_case
Yang2025_S	ENSGALG00010029746	TRPV1	case	control	0.582495123	0.29695816	1.961539375	0.049816136	0.132960709	up_in_case
Yang2025_S	ENSGALG00010029747	TRPV3	case	control	0.981044948	0.230618049	4.253981653	2.10003E-05	0.000302313	up_in_case
Yang2025_S	ENSGALG00010029749	ASPA	case	control	0.773228065	0.294702922	2.623754318	0.008696649	0.03661263	up_in_case
Yang2025_S	ENSGALG00010029750	TEN1	case	control	0.18808771	0.254287509	0.739665549	0.459502957	0.620703151	up_in_case
Yang2025_S	ENSGALG00010029752	ENSGALG00010029752	case	control	0.116914201	0.137945671	0.847538019	0.396695305	0.563846295	up_in_case
Yang2025_S	ENSGALG00010029753	AKAP10	case	control	-0.0015852	0.077334536	-0.020497958	0.983646141	0.990111888	down_in_case
Yang2025_S	ENSGALG00010029754	NLE1	case	control	-0.081755411	0.21924257	-0.372899348	0.709223358	0.820435014	down_in_case
Yang2025_S	ENSGALG00010029755	ACOX1	case	control	-0.256104729	0.104500054	-2.450761699	0.014255429	0.052748117	down_in_case
Yang2025_S	ENSGALG00010029756	ELAC2	case	control	-0.075454986	0.085900641	-0.878398404	0.37972755	0.547738649	down_in_case
Yang2025_S	ENSGALG00010029757	ULK2	case	control	-0.161826185	0.116260103	-1.391932231	0.163942921	0.310864689	down_in_case
Yang2025_S	ENSGALG00010029758	DNAH9	case	control	-0.084733045	0.325412812	-0.260386322	0.794565793	0.876109695	down_in_case
Yang2025_S	ENSGALG00010029759	ALDH3A2	case	control	-0.227237825	0.162771265	-1.396056146	0.162697588	0.309050013	down_in_case
Yang2025_S	ENSGALG00010029760	SHISA6	case	control	0.387376099	0.479959531	0.807101588	0.419607957	0.585277587	up_in_case
Yang2025_S	ENSGALG00010029761	MRPL38	case	control	-0.153396453	0.154824367	-0.990777203	0.321794383	0.491150573	down_in_case
Yang2025_S	ENSGALG00010029762	SLC47A2	case	control	0.559284551	0.377336069	1.482192125	0.138289181	0.276162187	up_in_case
Yang2025_S	ENSGALG00010029763	GEMIN4	case	control	-0.304628915	0.214271592	-1.421695297	0.155114726	0.299712106	down_in_case
Yang2025_S	ENSGALG00010029764	NXN	case	control	-0.25410321	0.18159925	-1.399252525	0.161737271	0.30777125	down_in_case
Yang2025_S	ENSGALG00010029765	KCNJ16	case	control	0.366429781	0.335510346	1.092156426	0.274764351	0.442100637	up_in_case
Yang2025_S	ENSGALG00010029766	ENSGALG00010029766	case	control	0.07168307	0.214578793	0.334064091	0.738331196	0.84021347	up_in_case
Yang2025_S	ENSGALG00010029767	GLOD4	case	control	0.163168153	0.106993159	1.525033503	0.127250741	0.260082001	up_in_case
Yang2025_S	ENSGALG00010029768	KCNJ2	case	control	0.018806256	0.111602992	0.168510318	0.866181827	0.921322324	up_in_case
Yang2025_S	ENSGALG00010029770	TLCD3A	case	control	-0.278208917	0.218209804	-1.274960668	0.202323164	0.3602824	down_in_case
Yang2025_S	ENSGALG00010029771	SOX9	case	control	0.75246151	0.326775752	2.302684656	0.021296592	0.070946213	up_in_case
Yang2025_S	ENSGALG00010029772	VPS53	case	control	0.033591166	0.066093182	0.5082395	0.611285393	0.748594951	up_in_case
Yang2025_S	ENSGALG00010029773	TRIM65	case	control	0.820755089	0.256285558	3.202502303	0.001362392	0.008938293	up_in_case
Yang2025_S	ENSGALG00010029774	RFLNB	case	control	-0.028709281	0.093294232	-0.307728358	0.758289039	0.852983352	down_in_case
Yang2025_S	ENSGALG00010029775	ENSGALG00010029775	case	control	0.077791397	0.179736236	0.432808645	0.665153792	0.788508558	up_in_case
Yang2025_S	ENSGALG00010029776	WBP2	case	control	-0.151980453	0.13376152	-1.136204592	0.255870949	0.422123196	down_in_case
Yang2025_S	ENSGALG00010029777	ENSGALG00010029777	case	control	-0.039193042	0.138020542	-0.283965281	0.776436988	0.863982227	down_in_case
Yang2025_S	ENSGALG00010029778	DOC2B	case	control	-0.563234592	0.277044717	-2.033009679	0.042051545	0.117291489	down_in_case
Yang2025_S	ENSGALG00010029780	TEX14	case	control	0.650506769	0.266124751	2.444367788	0.014510625	0.053550203	up_in_case
Yang2025_S	ENSGALG00010029781	UNC13D	case	control	0.278258355	0.289199215	0.962168431	0.335965004	0.504164585	up_in_case
Yang2025_S	ENSGALG00010029782	UNK	case	control	0.343661426	0.147544249	2.329209229	0.019847984	0.067442492	up_in_case
Yang2025_S	ENSGALG00010029783	RAD51C	case	control	0.061792849	0.141263026	0.437431158	0.661798691	0.786315287	up_in_case
Yang2025_S	ENSGALG00010029785	ENSGALG00010029785	case	control	-2.768143066	1.164153959	-2.377815276	0.017415548	0.061268786	down_in_case
Yang2025_S	ENSGALG00010029787	H3-3B	case	control	0.045564804	0.134719795	0.338219071	0.735198105	0.838430249	up_in_case
Yang2025_S	ENSGALG00010029791	GALK1	case	control	0.547729784	0.174038641	3.147173409	0.001648572	0.010341155	up_in_case
Yang2025_S	ENSGALG00010029804	ITGB4	case	control	-0.081824474	0.182478656	-0.448405725	0.653860411	0.78083339	down_in_case
Yang2025_S	ENSGALG00010029812	SAP30BP	case	control	0.718633803	0.141615803	5.074531142	3.88453E-07	1.07403E-05	up_in_case
Yang2025_S	ENSGALG00010029835	RECQL5	case	control	-0.225192574	0.11038888	-2.039993275	0.041350996	0.115964203	down_in_case
Yang2025_S	ENSGALG00010029841	SMIM5	case	control	-0.171348483	0.112184033	-1.527387443	0.126664697	0.259290195	down_in_case
Yang2025_S	ENSGALG00010029859	CSNK1D	case	control	-0.037132676	0.092187155	-0.402796641	0.687097833	0.804394548	down_in_case
Yang2025_S	ENSGALG00010029864	SLC16A3	case	control	0.129989727	0.19786515	0.656961205	0.511205862	0.666571881	up_in_case
Yang2025_S	ENSGALG00010029879	ENSGALG00010029879	case	control	1.102695355	0.29082081	3.791665926	0.00014964	0.001476795	up_in_case
Yang2025_S	ENSGALG00010029895	FASN	case	control	-0.182701955	0.149840167	-1.21931227	0.222725693	0.384214228	down_in_case
Yang2025_S	ENSGALG00010029907	NTN1	case	control	0.423333558	0.27368547	1.546788577	0.121914236	0.252658369	up_in_case
Yang2025_S	ENSGALG00010029921	STX8	case	control	0.190134039	0.161050932	1.180583289	0.237768305	0.401719796	up_in_case
Yang2025_S	ENSGALG00010029925	GGNBP2	case	control	0.463713709	0.11504529	4.030705738	5.56096E-05	0.000665739	up_in_case
Yang2025_S	ENSGALG00010029926	BLTP2	case	control	0.233319213	0.048873312	4.773959555	1.80639E-06	3.98922E-05	up_in_case
Yang2025_S	ENSGALG00010029927	ENSGALG00010029927	case	control	0.883308621	0.710288427	1.243591458	0.213649962	0.374021635	up_in_case
Yang2025_S	ENSGALG00010029928	USP43	case	control	0.648808021	0.21482382	3.020186594	0.00252619	0.014298318	up_in_case
Yang2025_S	ENSGALG00010029929	GDPD1	case	control	0.034576433	0.060784209	0.568839077	0.569465353	0.713937399	up_in_case
Yang2025_S	ENSGALG00010029930	ARHGAP44	case	control	0.726282172	0.188755453	3.847741413	0.000119212	0.001225086	up_in_case
Yang2025_S	ENSGALG00010029932	RASA4B	case	control	0.220961319	0.333552382	0.662448631	0.507683746	0.663220493	up_in_case
Yang2025_S	ENSGALG00010029933	TVP23B	case	control	-0.482639185	0.156276094	-3.08837503	0.002012543	0.012026067	down_in_case
Yang2025_S	ENSGALG00010029934	TEKT3	case	control	0.511332819	0.39265033	1.30226	0.192827521	0.348108757	up_in_case
Yang2025_S	ENSGALG00010029935	SYNRG	case	control	0.250237249	0.148797492	1.681730294	0.092621151	0.207871757	up_in_case
Yang2025_S	ENSGALG00010029936	SERPINF2	case	control	-1.119410963	0.439470441	-2.547181467	0.010859695	0.043052587	down_in_case
Yang2025_S	ENSGALG00010029937	PMP22	case	control	-0.207046285	0.08552429	-2.42090622	0.015481871	0.056358377	down_in_case
Yang2025_S	ENSGALG00010029938	ENSGALG00010029938	case	control	0.050049549	0.192650898	0.259794011	0.795022671	0.876474756	up_in_case
Yang2025_S	ENSGALG00010029939	ENSGALG00010029939	case	control	3.156818374	1.313339093	2.403658271	0.016231939	0.058221897	up_in_case
Yang2025_S	ENSGALG00010029941	COX10	case	control	-0.049267737	0.111537438	-0.441714797	0.658695601	0.784658933	down_in_case
Yang2025_S	ENSGALG00010029943	HS3ST3A1	case	control	0.366016258	0.28280475	1.294236601	0.195583708	0.351695803	up_in_case
Yang2025_S	ENSGALG00010029946	METRNL	case	control	0.017796958	0.09792167	0.181746878	0.855781376	0.914658189	up_in_case
Yang2025_S	ENSGALG00010029948	MYADML2	case	control	-0.197700414	0.150734144	-1.311583482	0.189660709	0.344497335	down_in_case
Yang2025_S	ENSGALG00010029950	RFFL	case	control	-0.326560646	0.127902196	-2.553205937	0.010673637	0.042520619	down_in_case
Yang2025_S	ENSGALG00010029956	CCLI5	case	control	0.590443965	0.193251393	3.055315432	0.002248239	0.013075632	up_in_case
Yang2025_S	ENSGALG00010029962	ENSGALG00010029962	case	control	1.128789909	0.626318804	1.802260927	0.071504363	0.172509227	up_in_case
Yang2025_S	ENSGALG00010029966	RABGEF1	case	control	0.006376027	0.069741327	0.091423944	0.927155737	0.955827121	up_in_case
Yang2025_S	ENSGALG00010030004	FLOT2	case	control	-0.026570305	0.118860491	-0.223541942	0.823113744	0.894251148	down_in_case
Yang2025_S	ENSGALG00010030014	ENSGALG00010030014	case	control	0.339929918	0.236865557	1.435117549	0.151253604	0.294250868	up_in_case
Yang2025_S	ENSGALG00010030102	GPS1	case	control	0.098411758	0.097779165	1.006469616	0.31418972	0.483549513	up_in_case
Yang2025_S	ENSGALG00010030103	C1QTNF1	case	control	-0.005543213	0.205798895	-0.026935096	0.978511501	0.987082508	down_in_case
Yang2025_S	ENSGALG00010030104	P4HB	case	control	-0.269742923	0.092466131	-2.917207858	0.003531803	0.018386369	down_in_case
Yang2025_S	ENSGALG00010030105	RAB40B	case	control	0.350757776	0.184620162	1.899888786	0.057447716	0.147863672	up_in_case
Yang2025_S	ENSGALG00010030106	GPRC5C	case	control	0.393664665	0.250610289	1.570824034	0.116223528	0.243848448	up_in_case
Yang2025_S	ENSGALG00010030107	RPTOR	case	control	-0.289176962	0.07454594	-3.879177886	0.00010481	0.001105623	down_in_case
Yang2025_S	ENSGALG00010030108	LLGL2	case	control	0.205180031	0.129845947	1.580180478	0.114065541	0.240833836	up_in_case
Yang2025_S	ENSGALG00010030109	FBF1	case	control	0.442646777	0.09162266	4.831193267	1.35717E-06	3.13635E-05	up_in_case

Note, Blank pvalue and padj cells indicate NA values returned by DESeq2, mainly for genes for which reliable cohort-level significance testing was not available; these entries were retained for completeness but were not interpreted as significant DEGs.
