# Supplementary material for: Cross-cohort transcriptomic meta-analysis identifies conserved melanogenesis signatures and developmental background in black-boned chicken breast muscle
Source: Anim Biosci. 2026 Jun 24;39(10):260430. doi: 10.5713/ab.260430 (PMC13662945; doi:10.5713/ab.260430)
Supplement: Supplementary file 13 [file ab-260430-Supplementary-5.pdf]

Supplement 5. Random-effects meta-analysis results across the three pigmentation-related cohorts.

gene_id	symbol	k	meta_log2FC	meta_se	meta_z	meta_pvalue	tau2	Q	I2	direction_consistency	cohorts	meta_fdr
ENSGALG00010024745	PMEL	3	8.91582778	0.24761795	36.00638715	6.6456E-284	0	1.13763347	0	1	CRA004775;PRJNA827465;Yang2025_Silkie_Wencha	9.3962E-280
ENSGALG00010014092	TYRP1	3	8.832436078	0.301617845	29.28353288	1.681E-188	0	0.262061567	0	1	CRA004775;PRJNA827465;Yang2025_Silkie_Wencha	1.1884E-184
ENSGALG00010016279	PRELID3B	3	1.286821488	0.049797398	25.84113883	3.0604E-147	0	1.664249548	0	1	CRA004775;PRJNA827465;Yang2025_Silkie_Wencha	1.4423E-143
ENSGALG00010005140	DCT	3	7.779409913	0.397246028	19.58335482	2.14422E-85	0	0.096906619	0	1	CRA004775;PRJNA827465;Yang2025_Silkie_Wencha	7.5793E-82
ENSGALG00010001409	GPNMB	3	6.353508602	0.41142663	15.44262849	8.45818E-54	0	0.710119296	0	1	CRA004775;PRJNA827465;Yang2025_Silkie_Wencha	2.3918E-50
ENSGALG00010007864	TYR	3	6.704858539	0.443324799	15.1240322	1.12442E-51	0	0.160698885	0	1	CRA004775;PRJNA827465;Yang2025_Silkie_Wencha	2.64969E-48
ENSGALG00010012325	MLPH	3	5.41105141	0.372955822	14.50855862	1.06944E-47	0	0.588744115	0	1	CRA004775;PRJNA827465;Yang2025_Silkie_Wencha	2.16012E-44
ENSGALG00010025640	SLC38A11	2	5.043745532	0.364194906	13.84902824	1.28945E-43	0	0.036134961	0	1	PRJNA827465;Yang2025_Silkie_Wenchang	2.27894E-40
ENSGALG00010002896	OCA2	3	3.38211729	0.246098993	13.74291398	5.61688E-43	0.024083512	2.263293063	11.63318475	1	CRA004775;PRJNA827465;Yang2025_Silkie_Wencha	8.82411E-40
ENSGALG00010012923	MLANA	3	7.072751818	0.572625063	12.35145347	4.78392E-35	0.432319745	3.564422794	43.8899335	1	CRA004775;PRJNA827465;Yang2025_Silkie_Wencha	6.76399E-32
ENSGALG00010018219	SLC24A5	2	4.898060665	0.429264939	11.41034412	3.71258E-30	0	0.713424919	0	1	PRJNA827465;Yang2025_Silkie_Wenchang	4.77201E-27
ENSGALG00010016253	EDN3	3	1.944732901	0.171915022	11.31217547	1.14222E-29	0.055159161	5.887088154	66.02734752	1	CRA004775;PRJNA827465;Yang2025_Silkie_Wencha	1.34582E-26
ENSGALG00010013190	SLC45A2	2	4.671732192	0.429058084	10.8883444	1.31E-27	0	0.02924125	0	1	PRJNA827465;Yang2025_Silkie_Wenchang	1.32301E-24
ENSGALG00010024748	TRPM1	2	2.328204354	0.213787395	10.89027887	1.28247E-27	0	0.045514941	0	1	PRJNA827465;Yang2025_Silkie_Wenchang	1.32301E-24
ENSGALG00010012311	ENSGALG00010012311	3	4.445685227	0.422866676	10.51320777	7.5095E-26	0.36982257	6.977429586	71.33614929	1	CRA004775;PRJNA827465;Yang2025_Silkie_Wencha	7.07845E-23
ENSGALG00010028874	CRPL1	3	5.822667805	0.574299019	10.13873889	3.71869E-24	0.686611098	6.602827939	69.70994824	1	CRA004775;PRJNA827465;Yang2025_Silkie_Wencha	3.28616E-21
ENSGALG00010008126	CDH3	3	2.121539826	0.221715526	9.568747252	1.08209E-21	0	0.871998106	0	1	CRA004775;PRJNA827465;Yang2025_Silkie_Wencha	8.99979E-19
ENSGALG00010013314	KIT	3	0.691366909	0.087606392	7.891740446	2.98001E-15	0	0.38694861	0	1	CRA004775;PRJNA827465;Yang2025_Silkie_Wencha	2.3408E-12
ENSGALG00010011117	SLC6A15	2	3.617522337	0.512888258	7.053236801	1.74803E-12	0.097351535	1.226631069	18.47589505	1	PRJNA827465;Yang2025_Silkie_Wenchang	1.30081E-09
ENSGALG00010002210	GPR143	2	3.394091803	0.48208661	7.040419155	1.91662E-12	0.004348218	1.008646706	0.857258125	1	PRJNA827465;Yang2025_Silkie_Wenchang	1.35496E-09
ENSGALG00010001365	ENSGALG0001000136	3	-0.603539101	0.088941869	-6.785770428	1.15469E-11	0	1.850504586	0	1	CRA004775;PRJNA827465;Yang2025_Silkie_Wencha	7.77434E-09
ENSGALG00010016006	PLTP	3	0.574339269	0.094590134	6.071872851	1.26427E-09	0	0.634977066	0	1	CRA004775;PRJNA827465;Yang2025_Silkie_Wencha	8.12523E-07
ENSGALG00010007845	TFAP2B	2	2.732606089	0.471233898	5.798831752	6.67785E-09	0	0.154425362	0	1	PRJNA827465;Yang2025_Silkie_Wenchang	4.10513E-06
ENSGALG00010006123	MLH3	3	0.435845446	0.076881879	5.669026979	1.43611E-08	0	0.562081826	0	1	CRA004775;PRJNA827465;Yang2025_Silkie_Wencha	8.46047E-06
ENSGALG00010015386	SLC24A4	3	1.135225986	0.20153518	5.632892397	1.77212E-08	0	0.542036133	0	1	CRA004775;PRJNA827465;Yang2025_Silkie_Wencha	1.00224E-05
ENSGALG00010021707	DLX2	2	2.563205596	0.480699171	5.332244682	9.70061E-08	0	0.024657564	0	1	PRJNA827465;Yang2025_Silkie_Wenchang	5.27527E-05
ENSGALG00010006096	EDIL3	3	-0.45002363	0.084853312	-5.303548184	1.13573E-07	0	1.725504521	0	0.666666667	CRA004775;PRJNA827465;Yang2025_Silkie_Wencha	5.94745E-05
ENSGALG00010029213	APOH	3	2.516450083	0.482748483	5.212756064	1.86055E-07	0.040497599	2.106629637	5.061622352	1	CRA004775;PRJNA827465;Yang2025_Silkie_Wencha	9.39514E-05
ENSGALG00010001351	CAPN1	3	-0.321388065	0.061785593	-5.201666765	1.97509E-07	0	0.033746742	0	1	CRA004775;PRJNA827465;Yang2025_Silkie_Wencha	9.62959E-05
ENSGALG00010002352	ENSGALG0001000235	3	-0.379516046	0.073840595	-5.139666656	2.75226E-07	0	0.336861992	0	1	CRA004775;PRJNA827465;Yang2025_Silkie_Wencha	0.000129714
ENSGALG00010022482	PRTG	3	1.133459945	0.221539072	5.116298144	3.1159E-07	0	1.437558804	0	1	CRA004775;PRJNA827465;Yang2025_Silkie_Wencha	0.000142115
ENSGALG00010022822	KCNJ13	2	4.483795155	0.879368507	5.098880754	3.41668E-07	1.214185762	4.652095426	78.50431024	1	PRJNA827465;Yang2025_Silkie_Wenchang	0.000146389
ENSGALG00010018941	KLHL21	3	-0.340254554	0.066661624	-5.10420441	3.32189E-07	0	0.460414303	0	1	CRA004775;PRJNA827465;Yang2025_Silkie_Wencha	0.000146389
ENSGALG00010003176	PRAG1	3	-0.411448902	0.081911606	-5.023084321	5.08482E-07	0	1.842130408	0	0.666666667	CRA004775;PRJNA827465;Yang2025_Silkie_Wencha	0.000211454
ENSGALG00010029190	DHRS7C	3	-0.574179555	0.114771916	-5.002787911	5.65071E-07	0	1.011963887	0	0.666666667	CRA004775;PRJNA827465;Yang2025_Silkie_Wencha	0.000228273
ENSGALG00010005145	AKAP7	3	-0.415642232	0.083215808	-4.994750928	5.89117E-07	0	1.224613885	0	1	CRA004775;PRJNA827465;Yang2025_Silkie_Wencha	0.000231376
ENSGALG00010007014	ENSGALG0001000701	3	1.429199532	0.287911255	4.964028011	6.9046E-07	0	0.718825319	0	1	CRA004775;PRJNA827465;Yang2025_Silkie_Wencha	0.000263849
ENSGALG00010014161	GFRA3	3	0.840720004	0.17030169	4.936650963	7.94755E-07	0	1.089075363	0	1	CRA004775;PRJNA827465;Yang2025_Silkie_Wencha	0.000295712
ENSGALG00010010880	ELP3	3	-0.229417252	0.046551685	-4.928226606	8.29793E-07	0	0.726052401	0	1	CRA004775;PRJNA827465;Yang2025_Silkie_Wencha	0.000300832
ENSGALG00010018229	AS3MT	3	-0.510545421	0.104763543	-4.873311883	1.09743E-06	0	1.047640621	0	1	CRA004775;PRJNA827465;Yang2025_Silkie_Wencha	0.000387913
ENSGALG00010024665	MAP1LC3A	3	-0.360680639	0.074524621	-4.839751396	1.30002E-06	0	1.19149914	0	1	CRA004775;PRJNA827465;Yang2025_Silkie_Wencha	0.000448315
ENSGALG00010022049	EPHX4	3	1.462157012	0.302763688	4.829367161	1.36968E-06	0	0.167303764	0	1	CRA004775;PRJNA827465;Yang2025_Silkie_Wencha	0.000461092
ENSGALG00010002465	ENSGALG0001000246	2	3.315485858	0.687294216	4.823968804	1.40729E-06	0.292750606	1.447883474	30.9336685	1	CRA004775;Yang2025_Silkie_Wenchang	0.000462738
ENSGALG00010017434	FGF13	3	-0.411204567	0.0859635	-4.783478665	1.72287E-06	0	1.5878493	0	0.666666667	CRA004775;PRJNA827465;Yang2025_Silkie_Wencha	0.000553629
ENSGALG00010024886	ABTB2	3	0.427751468	0.089961837	4.75481028	1.98633E-06	0	0.988188618	0	1	CRA004775;PRJNA827465;Yang2025_Silkie_Wencha	0.000624104
ENSGALG00010004929	ENSGALG0001000492	3	0.520621034	0.11043395	4.714320502	2.42519E-06	0	0.76676332	0	1	CRA004775;PRJNA827465;Yang2025_Silkie_Wencha	0.000729568
ENSGALG00010019999	ERLIN1	3	-0.180880719	0.03833969	-4.717845151	2.38356E-06	0	1.667011421	0	1	CRA004775;PRJNA827465;Yang2025_Silkie_Wencha	0.000729568
ENSGALG00010027425	CFD	3	0.467184556	0.099893674	4.676818252	2.9136E-06	0.00099183	2.04869819	2.377030924	0.666666667	CRA004775;PRJNA827465;Yang2025_Silkie_Wencha	0.000855937
ENSGALG00010010585	FBXL5	3	0.207617783	0.04442792	4.673137569	2.96633E-06	0	0.844994204	0	0.666666667	CRA004775;PRJNA827465;Yang2025_Silkie_Wencha	0.000855937
ENSGALG00010010407	SOX10	3	0.663891197	0.142367456	4.663251313	3.11252E-06	0.026858235	3.696987561	45.90190075	1	CRA004775;PRJNA827465;Yang2025_Silkie_Wencha	0.000870057
ENSGALG00010002350	BEND6	3	0.540765299	0.116005418	4.661552106	3.13834E-06	0.009575203	2.545095864	21.41749833	1	CRA004775;PRJNA827465;Yang2025_Silkie_Wencha	0.000870057
ENSGALG00010022741	HNF1A	3	1.359969153	0.292412892	4.65085223	3.30566E-06	0	1.093573999	0	1	CRA004775;PRJNA827465;Yang2025_Silkie_Wencha	0.000878972
ENSGALG00010013520	CREG2	3	-0.450935734	0.097055257	-4.646175253	3.38146E-06	0	1.120133998	0	1	CRA004775;PRJNA827465;Yang2025_Silkie_Wencha	0.000878972
ENSGALG00010020546	LRRC20	3	-0.390044479	0.083990962	-4.643886304	3.41916E-06	0	0.465754213	0	1	CRA004775;PRJNA827465;Yang2025_Silkie_Wencha	0.000878972
ENSGALG00010028478	PEA15	3	0.326434999	0.070159166	4.65277762	3.27493E-06	0	0.662938226	0	1	CRA004775;PRJNA827465;Yang2025_Silkie_Wencha	0.000878972
ENSGALG00010008171	KIF21A	3	0.601635339	0.130151186	4.622588215	3.78982E-06	0.008023044	2.338230312	14.46522656	1	CRA004775;PRJNA827465;Yang2025_Silkie_Wencha	0.000956861
ENSGALG00010021083	SCD	3	0.92776779	0.201302885	4.60881517	4.0497E-06	0	1.150684558	0	1	CRA004775;PRJNA827465;Yang2025_Silkie_Wencha	0.001004539
ENSGALG00010021117	CYP1A2	2	2.031815692	0.441531225	4.601748593	4.18959E-06	0	0.497012559	0	1	CRA004775;PRJNA827465	0.001012003
ENSGALG00010007954	PSCA	3	1.724027682	0.374780716	4.600097087	4.22294E-06	0	1.29719256	0	1	CRA004775;PRJNA827465;Yang2025_Silkie_Wencha	0.001012003
ENSGALG00010021042	LRRC38	3	-0.29500299	0.064208416	-4.594459887	4.33872E-06	0	1.368181737	0	1	CRA004775;PRJNA827465;Yang2025_Silkie_Wencha	0.001022418
ENSGALG00010022625	ENSGALG0001002262	3	-0.355437669	0.077450938	-4.589197733	4.44953E-06	0	1.053768477	0	1	CRA004775;PRJNA827465;Yang2025_Silkie_Wencha	0.001031342
ENSGALG00010014995	LRAT	3	1.000239239	0.219709186	4.552559943	5.29918E-06	0	1.273797523	0	1	CRA004775;PRJNA827465;Yang2025_Silkie_Wencha	0.001208589
ENSGALG00010012618	B3GALT2	3	-0.750117929	0.165718724	-4.526452475	5.99821E-06	0	0.522500691	0	1	CRA004775;PRJNA827465;Yang2025_Silkie_Wencha	0.001346169
ENSGALG00010019534	PPP2R5A	3	-0.173583216	0.038871714	-4.465540626	7.98668E-06	0	1.539449984	0	0.666666667	CRA004775;PRJNA827465;Yang2025_Silkie_Wencha	0.001764433
ENSGALG00010028763	CTSK	3	0.411918772	0.092484359	4.453929017	8.4313E-06	0	1.836563676	0	1	CRA004775;PRJNA827465;Yang2025_Silkie_Wencha	0.001834002
ENSGALG00010022948	PDZK1IP1	2	1.876258093	0.423808395	4.42717633	9.54918E-06	0	0.212326572	0	1	CRA004775;PRJNA827465	0.002029388
ENSGALG00010009259	MGAT4D	2	1.746174025	0.394560361	4.425619493	9.61659E-06	0	0.04612006	0	1	CRA004775;Yang2025_Silkie_Wenchang	0.002029388
ENSGALG00010025485	DENND2C	3	0.305817818	0.069178542	4.420703455	9.83801E-06	0.001329369	2.176312741	8.101443227	1	CRA004775;PRJNA827465;Yang2025_Silkie_Wencha	0.002045583
ENSGALG00010022211	ENSGALG0001002221	3	1.009373226	0.230415847	4.380658887	1.18321E-05	0.010093534	2.127532467	5.994384067	1	CRA004775;PRJNA827465;Yang2025_Silkie_Wencha	0.002424551
ENSGALG00010004835	HHLA2	3	0.973684219	0.224557601	4.336010962	1.45092E-05	0.065870122	3.525360541	43.26821393	1	CRA004775;PRJNA827465;Yang2025_Silkie_Wencha	0.002930645
ENSGALG00010020024	ENSGALG0001002002	2	4.690361764	1.087112901	4.314512097	1.59956E-05	0	0.088081952	0	1	PRJNA827465;Yang2025_Silkie_Wenchang	0.003185373
ENSGALG00010017240	MELTF	3	0.627407471	0.146456455	4.283918177	1.8363E-05	0	1.576297331	0	1	CRA004775;PRJNA827465;Yang2025_Silkie_Wencha	0.003606043
ENSGALG00010023635	SCN4A	3	-0.44374128	0.103858045	-4.272602338	1.93205E-05	0	1.099224069	0	1	CRA004775;PRJNA827465;Yang2025_Silkie_Wencha	0.003696156
ENSGALG00010023821	KANK4	3	0.235429532	0.05510575	4.272322431	1.93448E-05	0	0.533427243	0	1	CRA004775;PRJNA827465;Yang2025_Silkie_Wencha	0.003696156
ENSGALG00010002890	VXN	3	0.652817342	0.153441474	4.254503847	2.09513E-05	0.016343521	2.524005939	20.76088376	1	CRA004775;PRJNA827465;Yang2025_Silkie_Wencha	0.00394974
ENSGALG00010005519	PTBP3	3	0.578672898	0.13623206	4.247699814	2.15977E-05	0	0.027638173	0	1	CRA004775;PRJNA827465;Yang2025_Silkie_Wencha	0.004018016
ENSGALG00010003512	ENSGALG0001000351	3	-1.054320794	0.24866748	-4.239882086	2.23637E-05	0	0.372604548	0	1	CRA004775;PRJNA827465;Yang2025_Silkie_Wencha	0.004042433
ENSGALG00010008752	GOLM1	3	-0.6379402	0.150540843	-4.237655303	2.25866E-05	0.020491189	2.850144973	29.82813088	1	CRA004775;PRJNA827465;Yang2025_Silkie_Wencha	0.004042433
ENSGALG00010023196	DGKZ	3	-0.176463182	0.041588647	-4.243061365	2.20491E-05	0	1.535849035	0	1	CRA004775;PRJNA827465;Yang2025_Silkie_Wencha	0.004042433
ENSGALG00010016414	KLB	3	1.449674242	0.343268478	4.223149909	2.40911E-05	0	0.83509063	0	1	CRA004775;PRJNA827465;Yang2025_Silkie_Wencha	0.00415396
ENSGALG00010014244	SESN1	3	-0.332791781	0.078878896	-4.223736364	2.40283E-05	0	0.487945559	0	0.666666667	CRA004775;PRJNA827465;Yang2025_Silkie_Wencha	0.00415396
ENSGALG00010021146	AP5Z1	3	-0.221275486	0.052379349	-4.224479464	2.39494E-05	0	1.050542526	0	1	CRA004775;PRJNA827465;Yang2025_Silkie_Wencha	0.00415396
ENSGALG00010007970	SPRTN	3	0.229762692	0.054518486	4.21439971	2.50443E-05	0	0.469932722	0	1	CRA004775;PRJNA827465;Yang2025_Silkie_Wencha	0.004266288
ENSGALG00010006944	MAB21L2	3	1.29237895	0.309084595	4.181311429	2.89833E-05	0.020334261	2.133516214	6.25803606	1	CRA004775;PRJNA827465;Yang2025_Silkie_Wencha	0.004860124
ENSGALG00010001267	BCHE	3	-0.282622486	0.067621707	-4.179478747	2.92178E-05	0	1.27371309	0	1	CRA004775;PRJNA827465;Yang2025_Silkie_Wencha	0.004860124
ENSGALG00010011891	HAL	2	1.508276129	0.362227467	4.163892218	3.12868E-05	0	0.240807212	0	1	PRJNA827465;Yang2025_Silkie_Wenchang	0.005143766
ENSGALG00010015009	AGO4	3	-0.155771766	0.037707797	-4.131022663	3.61153E-05	0	0.032028877	0	1	CRA004775;PRJNA827465;Yang2025_Silkie_Wencha	0.005869358
ENSGALG00010001699	ASB15	3	-0.477175068	0.115722493	-4.123442704	3.73251E-05	0	1.646445047	0	0.666666667	CRA004775;PRJNA827465;Yang2025_Silkie_Wencha	0.005997046
ENSGALG00010013928	CCNO	3	-0.920899596	0.223516213	-4.120057266	3.78778E-05	0	0.979947055	0	1	CRA004775;PRJNA827465;Yang2025_Silkie_Wencha	0.006017467
ENSGALG00010004339	B4GALT6	3	0.474388627	0.115290939	4.11470869	3.87669E-05	0	0.98436257	0	1	CRA004775;PRJNA827465;Yang2025_Silkie_Wencha	0.006090275
ENSGALG00010007320	ENSGALG0001000732	3	-0.428058105	0.104290583	-4.104475116	4.05234E-05	0	0.579750338	0	1	CRA004775;PRJNA827465;Yang2025_Silkie_Wencha	0.006271184
ENSGALG00010008284	BCKDHA	3	-0.269878465	0.065777961	-4.102870908	4.08055E-05	0	0.973933978	0	1	CRA004775;PRJNA827465;Yang2025_Silkie_Wencha	0.006271184
ENSGALG00010014797	ZDHHC9	3	-0.202478413	0.049431276	-4.096159914	4.2006E-05	0	1.062929476	0	1	CRA004775;PRJNA827465;Yang2025_Silkie_Wencha	0.006386264
ENSGALG00010004973	SLN	3	-0.642222	0.157373335	-4.080881935	4.48651E-05	0	0.692066081	0	1	CRA004775;PRJNA827465;Yang2025_Silkie_Wencha	0.006748385
ENSGALG00010011365	PSMG4	3	-0.526377672	0.129371811	-4.068719971	4.72721E-05	0.006916652	2.23250654	10.41459616	1	CRA004775;PRJNA827465;Yang2025_Silkie_Wencha	0.007035584
ENSGALG00010000440	ENSGALG0001000044	3	1.062678213	0.261485174	4.064009424	4.82369E-05	0	0.457194346	0	1	CRA004775;PRJNA827465;Yang2025_Silkie_Wencha	0.007104388
ENSGALG00010028380	RUSC1	3	-0.246122002	0.060610161	-4.060738274	4.89178E-05	0	1.824001129	0	1	CRA004775;PRJNA827465;Yang2025_Silkie_Wencha	0.007130398
ENSGALG00010014238	PHLDA1	3	0.305564252	0.075329472	4.056370564	4.98412E-05	0	0.528967188	0	1	CRA004775;PRJNA827465;Yang2025_Silkie_Wencha	0.007190859
ENSGALG00010000224	MYBPC2	3	-0.67421461	0.166450562	-4.050539711	5.10996E-05	0	0.949756917	0	1	CRA004775;PRJNA827465;Yang2025_Silkie_Wencha	0.007297957
ENSGALG00010000085	TDG	3	0.175712661	0.043490091	4.040291875	5.33847E-05	0	0.149376513	0	1	CRA004775;PRJNA827465;Yang2025_Silkie_Wencha	0.007548066
ENSGALG00010001487	CDH19	3	0.446511728	0.110674721	4.034451352	5.473E-05	0.000253989	2.010995721	0.54677995	1	CRA004775;PRJNA827465;Yang2025_Silkie_Wencha	0.007661662
ENSGALG00010021197	CHCHD1	3	0.302663454	0.075405847	4.013792885	5.97508E-05	0	1.041139969	0	1	CRA004775;PRJNA827465;Yang2025_Silkie_Wencha	0.008282513
ENSGALG00010024909	SLC39A5	2	2.527801801	0.633736666	3.988725816	6.64291E-05	0	0.543661011	0	1	CRA004775;PRJNA827465	0.009118852
ENSGALG00010016229	ENSGALG0001001622	3	0.866153627	0.217482938	3.98262795	6.81574E-05	0	0.08631852	0	1	CRA004775;PRJNA827465;Yang2025_Silkie_Wencha	0.009177882
ENSGALG00010021752	CSTB	3	-0.283335556	0.071115535	-3.984397453	6.76516E-05	0	0.997803151	0	1	CRA004775;PRJNA827465;Yang2025_Silkie_Wencha	0.009177882
ENSGALG00010005068	PLCXD2	3	-0.463926877	0.116681446	-3.976012405	7.00805E-05	0	1.03840816	0	1	CRA004775;PRJNA827465;Yang2025_Silkie_Wencha	0.009347809
ENSGALG00010007767	VAT1L	3	0.814559553	0.205392724	3.965863729	7.31306E-05	0.066557812	4.319999369	53.70369694	1	CRA004775;PRJNA827465;Yang2025_Silkie_Wencha	0.009581207
ENSGALG00010010352	MALT1	3	0.264265207	0.066637977	3.965684738	7.31855E-05	0	0.392366784	0	1	CRA004775;PRJNA827465;Yang2025_Silkie_Wencha	0.009581207
ENSGALG00010011044	MBL2	2	1.137839997	0.287199997	3.961817444	7.43814E-05	0	0.000412089	0	1	PRJNA827465;Yang2025_Silkie_Wenchang	0.009648428
ENSGALG00010021533	SSX2IP	2	0.512281065	0.129524444	3.955091787	7.65053E-05	0	0.390146667	0	1	PRJNA827465;Yang2025_Silkie_Wenchang	0.00983371
ENSGALG00010023167	GPD1	3	-0.721409624	0.182880265	-3.944710083	7.99866E-05	0	0.896247978	0	1	CRA004775;PRJNA827465;Yang2025_Silkie_Wencha	0.010177097
ENSGALG00010012026	SYT1	2	0.723460756	0.183609067	3.940223479	8.14057E-05	0	0.579604299	0	1	PRJNA827465;Yang2025_Silkie_Wenchang	0.010276748
ENSGALG00010014887	MTMR12	3	0.324692103	0.082584452	3.931780028	8.43192E-05	0.003198041	2.322538584	13.88732943	1	CRA004775;PRJNA827465;Yang2025_Silkie_Wencha	0.010550347
ENSGALG00010009214	PCDH10	3	0.710296327	0.180782847	3.929002875	8.52988E-05	0.011996228	2.230521644	10.33487592	1	CRA004775;PRJNA827465;Yang2025_Silkie_Wencha	0.010579298
ENSGALG00010007896	TIMMDC1	3	-0.241091737	0.061398028	-3.926701638	8.61187E-05	0	0.523084078	0	1	CRA004775;PRJNA827465;Yang2025_Silkie_Wencha	0.010588107
ENSGALG00010013709	PSMF1	3	-0.230405749	0.058782196	-3.919651932	8.86769E-05	0	1.837587001	0	1	CRA004775;PRJNA827465;Yang2025_Silkie_Wencha	0.010808649
ENSGALG00010009021	HNF4beta	3	1.113103944	0.284695496	3.909805252	9.23706E-05	0	1.314941526	0	1	CRA004775;PRJNA827465;Yang2025_Silkie_Wencha	0.01097502
ENSGALG00010009993	ENSGALG0001000999	3	1.05424689	0.269615892	3.91018082	9.22271E-05	0	1.09773953	0	1	CRA004775;PRJNA827465;Yang2025_Silkie_Wencha	0.01097502
ENSGALG00010026909	ENSGALG0001002690	3	-0.265574837	0.067901553	-3.911174714	9.18483E-05	0	0.759344736	0	1	CRA004775;PRJNA827465;Yang2025_Silkie_Wencha	0.01097502
ENSGALG00010003632	TECRL	2	-1.084467047	0.278838353	-3.889232004	0.000100562	0	0.820977549	0	1	PRJNA827465;Yang2025_Silkie_Wenchang	0.011755221
ENSGALG00010018415	PELI2	3	0.222762512	0.057278083	3.889140461	0.0001006	0	0.415657028	0	1	CRA004775;PRJNA827465;Yang2025_Silkie_Wencha	0.011755221
ENSGALG00010009790	MC1R	2	5.730142408	1.478682318	3.875168004	0.000106551	3.761513276	7.139395547	85.99321198	1	PRJNA827465;Yang2025_Silkie_Wenchang	0.012333708
ENSGALG00010016241	TOPAZ1	2	1.974169494	0.509782669	3.872570829	0.000107693	0	0.393948323	0	1	PRJNA827465;Yang2025_Silkie_Wenchang	0.012333708
ENSGALG00010024042	DCPS	3	-0.276897576	0.071554937	-3.869720077	0.00010896	0.002348638	2.279575282	12.26435838	1	CRA004775;PRJNA827465;Yang2025_Silkie_Wencha	0.012333708
ENSGALG00010019458	ELP4	3	-0.216226033	0.055878966	-3.869542474	0.00010904	0	1.559827689	0	1	CRA004775;PRJNA827465;Yang2025_Silkie_Wencha	0.012333708
ENSGALG00010024837	TNFRSF1A	3	-0.268375186	0.069407194	-3.866676819	0.000110328	0	1.280370976	0	1	CRA004775;PRJNA827465;Yang2025_Silkie_Wencha	0.012370065
ENSGALG00010020497	OSBPL11	3	-0.179597329	0.04646819	-3.864952138	0.000111111	0	1.926147152	0	1	CRA004775;PRJNA827465;Yang2025_Silkie_Wencha	0.012370065
ENSGALG00010020941	ENSGALG00010020941	3	0.429347596	0.111306108	3.857358804	0.000114619	0.011420499	2.846579561	29.74023886	1	CRA004775;PRJNA827465;Yang2025_Silkie_Wencha	0.012660906
ENSGALG00010012079	ENSGALG0001001207	3	-0.855622589	0.223531479	-3.827749865	0.00012932	0.030144187	2.463757897	18.82319271	1	CRA004775;PRJNA827465;Yang2025_Silkie_Wencha	0.014107786
ENSGALG00010023670	EXTL2	3	-0.208343819	0.054440466	-3.82700286	0.000129713	0	1.292403231	0	0.666666667	CRA004775;PRJNA827465;Yang2025_Silkie_Wencha	0.014107786
ENSGALG00010005345	NOX3	2	2.916781507	0.763443655	3.820558975	0.00013315	0.848950553	3.645345059	72.56775466	1	PRJNA827465;Yang2025_Silkie_Wenchang	0.014371005
ENSGALG00010005190	ENSGALG0001000519	3	-0.598529168	0.157031579	-3.811521054	0.000138114	0	1.860264835	0	1	CRA004775;PRJNA827465;Yang2025_Silkie_Wencha	0.014586384
ENSGALG00010002404	OSBPL3	3	0.46173134	0.121125941	3.811993826	0.00013785	0	1.657074135	0	0.666666667	CRA004775;PRJNA827465;Yang2025_Silkie_Wencha	0.014586384
ENSGALG00010020375	LRPPRC	3	-0.150625705	0.039520861	-3.811296192	0.00013824	0	1.724738888	0	0.666666667	CRA004775;PRJNA827465;Yang2025_Silkie_Wencha	0.014586384
ENSGALG00010009040	CRELD2	3	0.263186278	0.069136812	3.806745907	0.000140807	0.002633257	2.275700449	12.11497099	0.666666667	CRA004775;PRJNA827465;Yang2025_Silkie_Wencha	0.014747216
ENSGALG00010022412	CHAC1	3	0.862111026	0.227535419	3.788909135	0.00015131	0	1.675692657	0	1	CRA004775;PRJNA827465;Yang2025_Silkie_Wencha	0.015730702
ENSGALG00010000142	ENSGALG0001000014	3	-1.071050826	0.283278774	-3.780907438	0.000156258	0	0.659280577	0	1	CRA004775;PRJNA827465;Yang2025_Silkie_Wencha	0.016126486
ENSGALG00010012722	ENSGALG0001001272	3	0.393922743	0.104720337	3.761664216	0.000168787	0	1.803604219	0	1	CRA004775;PRJNA827465;Yang2025_Silkie_Wencha	0.017046236
ENSGALG00010021774	FBXL20	3	-0.178568424	0.047434174	-3.764552231	0.000166848	0	0.466769677	0	1	CRA004775;PRJNA827465;Yang2025_Silkie_Wencha	0.017046236
ENSGALG00010024798	FBXO45	3	-0.151291482	0.040211685	-3.762376046	0.000168307	0.000256418	2.090063509	4.309127865	1	CRA004775;PRJNA827465;Yang2025_Silkie_Wencha	0.017046236
ENSGALG00010004386	CFAP44	3	0.290726334	0.077399674	3.756170021	0.000172534	0	0.336877841	0	1	CRA004775;PRJNA827465;Yang2025_Silkie_Wencha	0.017301072
ENSGALG00010024791	SHANK2	2	1.584352784	0.422564159	3.749378057	0.000177274	0.159924585	1.803613376	44.55574498	1	PRJNA827465;Yang2025_Silkie_Wenchang	0.017651214
ENSGALG00010024277	ITGB3BP	3	-0.399739763	0.106676676	-3.747208658	0.000178813	0	0.074549881	0	1	CRA004775;PRJNA827465;Yang2025_Silkie_Wencha	0.017680012
ENSGALG00010019935	ENSGALG0001001993	3	1.541443444	0.412228072	3.73929761	0.000184535	0	0.155834171	0	1	CRA004775;PRJNA827465;Yang2025_Silkie_Wencha	0.018119045
ENSGALG00010006531	FAM161B	3	0.296396833	0.079328497	3.736322291	0.000186731	0	0.206119423	0	1	CRA004775;PRJNA827465;Yang2025_Silkie_Wencha	0.018208234
ENSGALG00010017848	CYP3A4	3	0.440708616	0.118359687	3.723468918	0.000196504	0	0.825405403	0	1	CRA004775;PRJNA827465;Yang2025_Silkie_Wencha	0.018633292
ENSGALG00010027712	STOM	3	-0.42260031	0.113469244	-3.724359968	0.000195811	0.007432249	2.470426867	19.04233124	1	CRA004775;PRJNA827465;Yang2025_Silkie_Wencha	0.018633292
ENSGALG00010016983	ZFAND2A	3	0.385192378	0.103467492	3.722834797	0.000196998	0	1.232556366	0	0.666666667	CRA004775;PRJNA827465;Yang2025_Silkie_Wencha	0.018633292
ENSGALG00010028127	MFSD12	3	0.336929077	0.090485639	3.723564116	0.00019643	0	1.292231291	0	1	CRA004775;PRJNA827465;Yang2025_Silkie_Wencha	0.018633292
ENSGALG00010002100	BTD	3	-0.288308411	0.077496311	-3.720285613	0.000198998	0	0.427790305	0	1	CRA004775;PRJNA827465;Yang2025_Silkie_Wencha	0.018633292
ENSGALG00010019896	DDB1	3	-0.172434474	0.046329784	-3.721892487	0.000197735	0	1.110947916	0	1	CRA004775;PRJNA827465;Yang2025_Silkie_Wencha	0.018633292
ENSGALG00010002689	COG6	3	-0.179644912	0.048338072	-3.716426898	0.00020206	0	0.489737223	0	1	CRA004775;PRJNA827465;Yang2025_Silkie_Wencha	0.018795574
ENSGALG00010024860	NEU4	2	0.96613973	0.260121774	3.714182458	0.000203862	0	0.192516414	0	1	PRJNA827465;Yang2025_Silkie_Wenchang	0.018839217
ENSGALG00010008832	MTSS1	3	0.220587574	0.059472066	3.70909552	0.000208001	0	1.35900931	0	0.666666667	CRA004775;PRJNA827465;Yang2025_Silkie_Wencha	0.019096919
ENSGALG00010029688	PIPOX	2	1.821276663	0.491975748	3.701964319	0.000213937	0	0.000923197	0	1	CRA004775;PRJNA827465	0.019515169
ENSGALG00010017452	ARMH4	3	-1.311385718	0.354618391	-3.698019478	0.000217288	0	0.243874666	0	1	CRA004775;PRJNA827465;Yang2025_Silkie_Wencha	0.019568396
ENSGALG00010022929	LRP4	3	-0.338364291	0.091479853	-3.698784803	0.000216634	0	0.10144386	0	1	CRA004775;PRJNA827465;Yang2025_Silkie_Wencha	0.019568396
ENSGALG00010010271	HUNK	3	0.280613097	0.075937995	3.695292408	0.000219634	0	0.860122701	0	1	CRA004775;PRJNA827465;Yang2025_Silkie_Wencha	0.019654451
ENSGALG00010000137	ENSGALG0001000013	3	-0.963238764	0.261548088	-3.682836188	0.000230653	0.03724498	2.392153328	16.3933191	1	CRA004775;PRJNA827465;Yang2025_Silkie_Wencha	0.020510744
ENSGALG00010024342	HNF4A	2	2.077862829	0.567425071	3.661915792	0.000250336	0	0.921709634	0	1	CRA004775;PRJNA827465	0.021848783
ENSGALG00010002399	CSMD1	2	-0.663895219	0.181241316	-3.663045665	0.000249234	0	0.010823109	0	1	PRJNA827465;Yang2025_Silkie_Wenchang	0.021848783
ENSGALG00010016547	SGCB	3	-0.298133472	0.08139655	-3.662728627	0.000249543	0	0.742465318	0	1	CRA004775;PRJNA827465;Yang2025_Silkie_Wencha	0.021848783
ENSGALG00010021166	NEURL1	3	0.544812103	0.148864308	3.659789992	0.000252422	0.007287909	2.182474255	8.36088923	0.666666667	CRA004775;PRJNA827465;Yang2025_Silkie_Wencha	0.021895676
ENSGALG00010012961	ANKRD55	2	1.283843962	0.352338127	3.643783807	0.000268659	0	0.049341626	0	1	PRJNA827465;Yang2025_Silkie_Wenchang	0.023162019
ENSGALG00010015054	ENSGALG0001001505	3	-0.292238223	0.080291791	-3.639702384	0.000272953	0	0.885074767	0	1	CRA004775;PRJNA827465;Yang2025_Silkie_Wencha	0.02338962
ENSGALG00010026628	FYB2	2	1.964711858	0.540526098	3.634814057	0.000278181	0	0.184716376	0	1	CRA004775;PRJNA827465	0.023424158
ENSGALG00010017421	GPR157	3	-0.380508934	0.104630779	-3.636682599	0.000276172	0	0.570620847	0	1	CRA004775;PRJNA827465;Yang2025_Silkie_Wencha	0.023424158
ENSGALG00010028042	DPP7	3	0.230853491	0.063514127	3.634679411	0.000278327	0	0.042144145	0	1	CRA004775;PRJNA827465;Yang2025_Silkie_Wencha	0.023424158
ENSGALG00010000571	ENSGALG0001000057	2	2.493056502	0.688053382	3.623347499	0.000290815	0	0.210419762	0	1	CRA004775;PRJNA827465	0.023991454
ENSGALG00010021784	ANGPTL7	2	1.316540471	0.363035331	3.626480287	0.000287311	0	0.362689394	0	1	PRJNA827465;Yang2025_Silkie_Wenchang	0.023991454
ENSGALG00010011894	VWC2L	3	1.310216769	0.361456177	3.624828824	0.000289153	0.237605741	5.647422489	64.58561399	0.666666667	CRA004775;PRJNA827465;Yang2025_Silkie_Wencha	0.023991454
ENSGALG00010015320	TRPM6	2	0.982045579	0.271213985	3.620925292	0.000293551	0	0.168326116	0	1	PRJNA827465;Yang2025_Silkie_Wenchang	0.023991454
ENSGALG00010009307	DIRAS2	3	0.933246304	0.257730692	3.621013453	0.000293451	0.110124701	4.520651687	55.75859105	1	CRA004775;PRJNA827465;Yang2025_Silkie_Wencha	0.023991454
ENSGALG00010002953	WDFY2	3	-0.287494879	0.079790527	-3.603120466	0.00031442	0	0.022927653	0	1	CRA004775;PRJNA827465;Yang2025_Silkie_Wencha	0.025549313
ENSGALG00010027947	GP9	3	-0.882279806	0.245153182	-3.598891919	0.000319576	0	1.614840934	0	1	CRA004775;PRJNA827465;Yang2025_Silkie_Wencha	0.025673208
ENSGALG00010025230	CHST8	2	0.558203985	0.155097746	3.599046404	0.000319386	0	0.30695754	0	1	PRJNA827465;Yang2025_Silkie_Wenchang	0.025673208
ENSGALG00010005881	CORO2A	3	1.027727294	0.285926192	3.594379675	0.000325165	0.066035436	2.716379117	26.37257489	1	CRA004775;PRJNA827465;Yang2025_Silkie_Wencha	0.025974651
ENSGALG00010008392	CD36	3	-0.689932039	0.19206253	-3.592226129	0.000327865	0	0.973592429	0	1	CRA004775;PRJNA827465;Yang2025_Silkie_Wencha	0.026043176
ENSGALG00010020396	CHL1	3	0.60261296	0.167962662	3.587779291	0.000333506	0	1.859353505	0	1	CRA004775;PRJNA827465;Yang2025_Silkie_Wencha	0.02634328

ENSGALG00010028134	INPP5E	3	-0.177878863	0.049638179	-3.58350902	0.000339009	0	1.461102348	0	1	CRA004775;PRJNA827465;Yang2025_Silkie_Wencha	0.026629161
ENSGALG00010007214	MTHFSD	3	0.3092519	0.086390234	3.579709014	0.000343977	0.004820954	2.417013305	17.25324822	1	CRA004775;PRJNA827465;Yang2025_Silkie_Wencha	0.026870117
ENSGALG00010021812	TEAD1	3	-0.192678557	0.053857955	-3.577531963	0.000346854	0	0.843570788	0	0.666666667	CRA004775;PRJNA827465;Yang2025_Silkie_Wencha	0.026945968
ENSGALG00010008298	ENSGALG00010008298	3	0.200023252	0.055995856	3.572108149	0.000354119	0	0.270105115	0	1	CRA004775;PRJNA827465;Yang2025_Silkie_Wencha	0.027360055
ENSGALG00010020944	MYO5A	3	0.262236265	0.073475864	3.569012355	0.00035833	0	1.947724642	0	0.666666667	CRA004775;PRJNA827465;Yang2025_Silkie_Wencha	0.027534898
ENSGALG00010011119	ENSGALG00010011119	3	-0.511668285	0.143885716	-3.556074216	0.000376438	0.004039103	2.084944776	4.074197884	1	CRA004775;PRJNA827465;Yang2025_Silkie_Wencha	0.028615343
ENSGALG00010018832	EEPD1	3	-0.277544757	0.07802748	-3.557012946	0.000375096	0	0.285056496	0	1	CRA004775;PRJNA827465;Yang2025_Silkie_Wencha	0.028615343
ENSGALG00010013833	TMPRSS2	2	2.169328817	0.609563237	3.551770655	0.000382648	0	0.189895216	0	1	CRA004775;PRJNA827465	0.028931889
ENSGALG00010018259	WDR90	3	-0.23816415	0.067095425	-3.54963322	0.000385768	0	1.865363839	0	1	CRA004775;PRJNA827465;Yang2025_Silkie_Wencha	0.029012643
ENSGALG00010016373	SRL	3	-0.270360868	0.076206555	-3.547737715	0.000388555	0	0.675333545	0	1	CRA004775;PRJNA827465;Yang2025_Silkie_Wencha	0.02906761
ENSGALG00010021332	OIT3	3	0.805086348	0.227394627	3.540480964	0.000399398	0.115638508	8.871446557	77.45576229	1	CRA004775;PRJNA827465;Yang2025_Silkie_Wencha	0.029565941
ENSGALG00010027914	BTBD2	3	-0.274277905	0.077452112	-3.5412579	0.000398224	0	0.661589732	0	1	CRA004775;PRJNA827465;Yang2025_Silkie_Wencha	0.029565941
ENSGALG00010012419	HACE1	3	0.186299092	0.052673666	3.536854498	0.000404923	0	1.825388955	0	1	CRA004775;PRJNA827465;Yang2025_Silkie_Wencha	0.029818759
ENSGALG00010009116	S1PR3	3	0.306692425	0.086815164	3.532705689	0.00041133	0	1.509250918	0	0.666666667	CRA004775;PRJNA827465;Yang2025_Silkie_Wencha	0.03013366
ENSGALG00010003803	LACTB2	3	-0.216520643	0.061331364	-3.53034124	0.000415024	0	1.128351585	0	1	CRA004775;PRJNA827465;Yang2025_Silkie_Wencha	0.030247549
ENSGALG00010003339	ENSGALG00010003339	2	-1.850875603	0.52496152	-3.525735761	0.000422308	0	0.004966449	0	1	PRJNA827465;Yang2025_Silkie_Wenchang	0.030313479
ENSGALG00010020766	C2orf76	3	-0.322477585	0.091464765	-3.525702885	0.000422361	0	0.579429386	0	1	CRA004775;PRJNA827465;Yang2025_Silkie_Wencha	0.030313479
ENSGALG00010015469	TMEM33	3	-0.17956713	0.050900607	-3.527799411	0.00041903	0	1.735808854	0	1	CRA004775;PRJNA827465;Yang2025_Silkie_Wencha	0.030313479
ENSGALG00010021316	PLA2G12B	3	1.287896651	0.365962413	3.519204718	0.000432843	0.052059144	2.285454238	12.49004391	1	CRA004775;PRJNA827465;Yang2025_Silkie_Wencha	0.030753573
ENSGALG00010007695	RASGRP3	3	0.223579115	0.063515525	3.520070337	0.000431432	0	0.4101443	0	1	CRA004775;PRJNA827465;Yang2025_Silkie_Wencha	0.030753573
ENSGALG00010002142	CLDN34	3	1.412284611	0.402745756	3.506640581	0.000453802	0.156374119	2.922031512	31.55446846	1	CRA004775;PRJNA827465;Yang2025_Silkie_Wencha	0.031762888
ENSGALG00010000730	INTS5	3	-0.298044345	0.085016895	-3.505707248	0.000455396	0	0.144528658	0	1	CRA004775;PRJNA827465;Yang2025_Silkie_Wencha	0.031762888
ENSGALG00010000434	TP53	3	-0.271228085	0.077391982	-3.504608854	0.000457292	0.002566407	2.216837143	9.781374496	0.666666667	CRA004775;PRJNA827465;Yang2025_Silkie_Wencha	0.031762888
ENSGALG00010005978	VPS16	3	-0.210346455	0.060029935	-3.504026013	0.000458281	0	0.913074734	0	1	CRA004775;PRJNA827465;Yang2025_Silkie_Wencha	0.031762888
ENSGALG00010019588	CHMP7	3	-0.173254414	0.049382817	-3.508394734	0.00045082	0	0.282765895	0	1	CRA004775;PRJNA827465;Yang2025_Silkie_Wencha	0.031762888
ENSGALG00010016924	LETM1	3	0.187253334	0.053553466	3.496567972	0.000471284	0	0.316483444	0	1	CRA004775;PRJNA827465;Yang2025_Silkie_Wencha	0.032504831
ENSGALG00010001817	SLC25A6	3	0.155438973	0.044567694	3.487705081	0.000487185	0	1.20667394	0	0.666666667	CRA004775;PRJNA827465;Yang2025_Silkie_Wencha	0.033438401
ENSGALG00010000614	KAT8	3	-0.364509786	0.104643888	-3.483335647	0.000495207	0	0.188305147	0	1	CRA004775;PRJNA827465;Yang2025_Silkie_Wencha	0.033824796
ENSGALG00010016856	NOD1	3	0.30552595	0.087840013	3.478209286	0.000504776	0	1.001592706	0	1	CRA004775;PRJNA827465;Yang2025_Silkie_Wencha	0.03431261
ENSGALG00010011980	AvBD9	2	2.333742657	0.672399246	3.470769294	0.00051897	0	0.137299444	0	1	CRA004775;PRJNA827465	0.034941487
ENSGALG00010021666	CCDC47	3	-0.204210833	0.058822328	-3.471655067	0.00051726	0	1.016228544	0	1	CRA004775;PRJNA827465;Yang2025_Silkie_Wencha	0.034941487
ENSGALG00010008072	NRSN1	3	-0.454346232	0.131022181	-3.467704683	0.000524924	0	0.889198043	0	1	CRA004775;PRJNA827465;Yang2025_Silkie_Wencha	0.035174871
ENSGALG00010028466	MAMDC4	2	1.982211231	0.572254402	3.463863668	0.000532476	0	0.011788799	0	1	CRA004775;PRJNA827465	0.035345927
ENSGALG00010028171	RNF123	3	-0.196836873	0.056820622	-3.464180204	0.00053185	0	0.525833308	0	1	CRA004775;PRJNA827465;Yang2025_Silkie_Wencha	0.035345927
ENSGALG00010001268	CBLN2	3	-0.536435077	0.1550929	-3.458798412	0.000542591	0	1.403721354	0	0.666666667	CRA004775;PRJNA827465;Yang2025_Silkie_Wencha	0.035841831
ENSGALG00010016760	DLK1	3	-0.4152001	0.120126889	-3.456346068	0.000547552	0.013191143	2.837658198	29.51934799	0.666666667	CRA004775;PRJNA827465;Yang2025_Silkie_Wencha	0.035841831
ENSGALG00010020691	ENSGALG00010020691	3	0.334412806	0.096731269	3.457132415	0.000545957	0	0.157109817	0	1	CRA004775;PRJNA827465;Yang2025_Silkie_Wencha	0.035841831
ENSGALG00010001976	THRSP	3	-1.684290035	0.488207472	-3.449947266	0.000560696	0	0.427651217	0	1	CRA004775;PRJNA827465;Yang2025_Silkie_Wencha	0.036365512
ENSGALG00010003589	SLC25A30	3	0.344562448	0.099853866	3.450667079	0.000559203	0	0.644083984	0	1	CRA004775;PRJNA827465;Yang2025_Silkie_Wencha	0.036365512
ENSGALG00010005350	ARHGAP20	3	-0.522296899	0.151792348	-3.440864492	0.000579859	0	0.992311561	0	1	CRA004775;PRJNA827465;Yang2025_Silkie_Wencha	0.037273845
ENSGALG00010010988	ANKRD44	3	0.207150348	0.060203931	3.44081098	0.000579974	0	1.139931553	0	0.666666667	CRA004775;PRJNA827465;Yang2025_Silkie_Wencha	0.037273845
ENSGALG00010007506	RAB38	3	3.332327553	0.969644868	3.436647439	0.000588962	2.588030389	26.4763383	92.44608534	1	CRA004775;PRJNA827465;Yang2025_Silkie_Wencha	0.037655794
ENSGALG00010007857	ENSGALG00010007857	3	0.650050992	0.189210322	3.435600005	0.000591243	0	0.251119789	0	1	CRA004775;PRJNA827465;Yang2025_Silkie_Wencha	0.037655794
ENSGALG00010001780	ENSGALG00010001780	3	0.353744145	0.103031816	3.43334864	0.000596175	0	0.574314086	0	1	CRA004775;PRJNA827465;Yang2025_Silkie_Wencha	0.037799632
ENSGALG00010016199	RLBP1	2	0.948156819	0.276388743	3.43051894	0.000602428	0	0.967344581	0	1	PRJNA827465;Yang2025_Silkie_Wenchang	0.038025573
ENSGALG00010024174	PLEKHB2	3	0.164139152	0.047867216	3.429051584	0.000605694	0	1.566852946	0	1	CRA004775;PRJNA827465;Yang2025_Silkie_Wencha	0.038061836
ENSGALG00010024687	RYK	3	-0.16419862	0.048053423	-3.417001514	0.000633149	0.000631103	2.154835056	7.185471353	0.666666667	CRA004775;PRJNA827465;Yang2025_Silkie_Wencha	0.039611051
ENSGALG00010012066	STXBP3	3	0.366619128	0.10734746	3.415256671	0.000637219	0.013228933	3.275386663	38.93850694	1	CRA004775;PRJNA827465;Yang2025_Silkie_Wencha	0.039690063
ENSGALG00010008139	ENSGALG00010008139	3	0.927746733	0.272005291	3.410767237	0.000647804	0.05406674	2.617229873	23.58332675	1	CRA004775;PRJNA827465;Yang2025_Silkie_Wencha	0.040086841
ENSGALG00010022572	ENSGALG00010022572	2	0.771030642	0.226098423	3.410154884	0.00064926	0	0.022626748	0	1	PRJNA827465;Yang2025_Silkie_Wenchang	0.040086841
ENSGALG00010008425	MAGI2	3	0.260917561	0.076603082	3.406097434	0.000658987	0	1.537542569	0	1	CRA004775;PRJNA827465;Yang2025_Silkie_Wencha	0.040510483
ENSGALG00010023546	SLC11A2	3	-0.249124701	0.073265147	-3.400316699	0.000673078	0	1.288997569	0	1	CRA004775;PRJNA827465;Yang2025_Silkie_Wencha	0.041175892
ENSGALG00010004899	GATAD1	3	-0.248322477	0.073076785	-3.398103485	0.000678547	0	0.118640387	0	1	CRA004775;PRJNA827465;Yang2025_Silkie_Wencha	0.041175892
ENSGALG00010024558	PFDN5	3	-0.151019036	0.044429732	-3.399053494	0.000676195	0	0.333048456	0	1	CRA004775;PRJNA827465;Yang2025_Silkie_Wencha	0.041175892
ENSGALG00010014865	TLR2	3	-0.648545092	0.191052318	-3.394594203	0.000687304	0	1.494906816	0	1	CRA004775;PRJNA827465;Yang2025_Silkie_Wencha	0.041352297
ENSGALG00010016980	NPHP1	3	0.221772188	0.065314116	3.395471006	0.000685106	0.001390957	2.216744151	9.777589834	1	CRA004775;PRJNA827465;Yang2025_Silkie_Wencha	0.041352297
ENSGALG00010013965	SLC2A2	2	2.147457363	0.633429994	3.390204729	0.000698404	0	0.027965137	0	1	CRA004775;PRJNA827465	0.041665572
ENSGALG00010000789	ROPN1L	3	1.467270698	0.432715136	3.390846715	0.000696771	0.296312456	4.425503859	54.80740581	0.666666667	CRA004775;PRJNA827465;Yang2025_Silkie_Wencha	0.041665572
ENSGALG00010008132	TDO2	3	1.023978635	0.302399848	3.382867556	0.000717332	0	1.903939992	0	1	CRA004775;PRJNA827465;Yang2025_Silkie_Wencha	0.042614956
ENSGALG00010016958	TLR15	3	0.899282718	0.266604628	3.373094931	0.000743283	0	0.749466995	0	1	CRA004775;PRJNA827465;Yang2025_Silkie_Wencha	0.043606977
ENSGALG00010003830	DMB2	3	-0.691791087	0.204995716	-3.37466119	0.000739066	0	1.099253627	0	1	CRA004775;PRJNA827465;Yang2025_Silkie_Wencha	0.043606977
ENSGALG00010018573	SEC23B	3	0.18895662	0.056008957	3.37368577	0.00074169	0.00107279	2.20953998	9.483421055	0.666666667	CRA004775;PRJNA827465;Yang2025_Silkie_Wencha	0.043606977
ENSGALG00010002435	ATP7B	2	1.790864958	0.531626777	3.368650784	0.000755371	0.262241956	1.821514256	45.10062182	1	PRJNA827465;Yang2025_Silkie_Wenchang	0.044068854
ENSGALG00010015028	MCM9	3	-0.600910151	0.178421347	-3.367927452	0.000757355	0	0.020847317	0	1	CRA004775;PRJNA827465;Yang2025_Silkie_Wencha	0.044068854
ENSGALG00010006603	MEST	3	-0.324156241	0.09635619	-3.364145473	0.000767811	0	1.806969975	0	1	CRA004775;PRJNA827465;Yang2025_Silkie_Wencha	0.044492107
ENSGALG00010029441	ENSGALG00010029441	2	-7.067735365	2.102087207	-3.362246504	0.000773111	0.213739327	1.024772863	2.417400379	1	PRJNA827465;Yang2025_Silkie_Wenchang	0.044616379
ENSGALG00010025891	NUP93	3	-0.219474176	0.065421913	-3.354750023	0.000794367	0	1.778015013	0	1	CRA004775;PRJNA827465;Yang2025_Silkie_Wencha	0.04554129
ENSGALG00010010311	CTSV	3	0.18623485	0.05552076	3.354328162	0.000795579	0	1.232357347	0	1	CRA004775;PRJNA827465;Yang2025_Silkie_Wencha	0.04554129
ENSGALG00010011020	SFTPA2	2	1.12092437	0.334598622	3.350056739	0.00080795	0	0.776876869	0	1	PRJNA827465;Yang2025_Silkie_Wenchang	0.045877937
ENSGALG00010024502	VPS8	3	-0.119211011	0.03558141	-3.350373477	0.000807027	0	0.553197557	0	1	CRA004775;PRJNA827465;Yang2025_Silkie_Wencha	0.045877937
ENSGALG00010007604	ENSGALG00010007604	3	-0.968890615	0.289367439	-3.34830559	0.000813073	0	0.359264549	0	1	CRA004775;PRJNA827465;Yang2025_Silkie_Wencha	0.045984159
ENSGALG00010002377	ART1A	3	0.570242817	0.170492341	3.344682887	0.000823767	0	1.272335154	0	1	CRA004775;PRJNA827465;Yang2025_Silkie_Wencha	0.046403352
ENSGALG00010021121	PLA2R1	3	-0.236546544	0.070906646	-3.336027838	0.000849847	0	1.258595267	0	0.666666667	CRA004775;PRJNA827465;Yang2025_Silkie_Wencha	0.047682461
ENSGALG00010010828	SPDL1	3	0.195201184	0.058577997	3.332329439	0.000861222	0	0.061195696	0	1	CRA004775;PRJNA827465;Yang2025_Silkie_Wencha	0.048129738
ENSGALG00010028422	RXRA	3	-0.264561293	0.079547019	-3.325847968	0.0008815	0	0.066904146	0	1	CRA004775;PRJNA827465;Yang2025_Silkie_Wencha	0.049069007
ENSGALG00010028888	WDR5B	3	-0.144146793	0.043371858	-3.323509731	0.000888923	0	0.672753253	0	1	CRA004775;PRJNA827465;Yang2025_Silkie_Wencha	0.049288178
ENSGALG00010015300	ATXN3	3	-0.226477333	0.06816937	-3.322274108	0.000892869	0	1.647609976	0	0.666666667	CRA004775;PRJNA827465;Yang2025_Silkie_Wencha	0.049313592
ENSGALG00010001420	FAM221A	2	0.905447121	0.272772193	3.319426044	0.000902027	0	0.354957424	0	1	PRJNA827465;Yang2025_Silkie_Wenchang	0.049625521
ENSGALG00010028599	VPS72	3	-0.205305618	0.061938178	-3.314686126	0.000917461	0	0.143386367	0	1	CRA004775;PRJNA827465;Yang2025_Silkie_Wencha	0.050278985
ENSGALG00010013672	ENSGALG00010013672	3	1.070180468	0.323179307	3.311413957	0.000928258	0.125905077	3.33594985	40.04706036	1	CRA004775;PRJNA827465;Yang2025_Silkie_Wencha	0.050479374
ENSGALG00010029347	PRPF8	3	-0.237233892	0.071619371	-3.312426367	0.000924905	0.006669375	3.608260994	44.57163704	1	CRA004775;PRJNA827465;Yang2025_Silkie_Wencha	0.050479374
ENSGALG00010013510	MRPS33	3	-0.226048542	0.068370489	-3.306229715	0.000945605	0	1.471959171	0	1	CRA004775;PRJNA827465;Yang2025_Silkie_Wencha	0.051225722
ENSGALG00010022345	USP33	3	0.148883974	0.045114111	3.300164186	0.000966283	0	1.465556761	0	1	CRA004775;PRJNA827465;Yang2025_Silkie_Wencha	0.052146078
ENSGALG00010009615	ENSGALG00010009615	3	-0.767496121	0.233329925	-3.289317145	0.001004308	0	1.347059173	0	1	CRA004775;PRJNA827465;Yang2025_Silkie_Wencha	0.053873475
ENSGALG00010011459	ENSGALG00010011459	3	0.568022436	0.172710631	3.288867814	0.001005913	0	0.56358036	0	1	CRA004775;PRJNA827465;Yang2025_Silkie_Wencha	0.053873475
ENSGALG00010023149	ADGRD1	3	-0.441806298	0.134505313	-3.28467544	0.001020999	0.017609061	2.918791494	31.47849019	1	CRA004775;PRJNA827465;Yang2025_Silkie_Wencha	0.054270325
ENSGALG00010008190	TSNARE1	3	-0.233463534	0.07107083	-3.284941727	0.001020035	0	1.807831592	0	1	CRA004775;PRJNA827465;Yang2025_Silkie_Wencha	0.054270325
ENSGALG00010022564	VTCN1	3	-0.720210797	0.220200746	-3.270700984	0.001072813	0	0.514148422	0	1	CRA004775;PRJNA827465;Yang2025_Silkie_Wencha	0.056810862
ENSGALG00010023731	ACTR5	3	-0.209326581	0.064051611	-3.268092384	0.00108275	0	0.052141014	0	1	CRA004775;PRJNA827465;Yang2025_Silkie_Wencha	0.057123152
ENSGALG00010017115	LGALSL	3	-0.276032292	0.084514732	-3.266084926	0.001090455	0	1.037551286	0	0.666666667	CRA004775;PRJNA827465;Yang2025_Silkie_Wencha	0.057315796
ENSGALG00010013303	TFPI	3	0.391937612	0.120150897	3.262044826	0.001106117	0	0.805475304	0	1	CRA004775;PRJNA827465;Yang2025_Silkie_Wencha	0.057923635
ENSGALG00010026162	MED1	3	-0.121461859	0.037258242	-3.259999723	0.001114123	0	0.366932653	0	1	CRA004775;PRJNA827465;Yang2025_Silkie_Wencha	0.058127632
ENSGALG00010010829	LAMB4	3	-0.349424278	0.10727269	-3.257346099	0.001124592	0	0.10837075	0	1	CRA004775;PRJNA827465;Yang2025_Silkie_Wencha	0.058458123
ENSGALG00010001706	IQUB	3	0.510209223	0.15675376	3.254845199	0.001134542	0	0.80657195	0	1	CRA004775;PRJNA827465;Yang2025_Silkie_Wencha	0.058673641
ENSGALG00010002864	RNASEH2B	3	-0.278954676	0.085720876	-3.254221012	0.001137038	0.004975366	2.501998645	20.06390555	1	CRA004775;PRJNA827465;Yang2025_Silkie_Wencha	0.058673641
ENSGALG00010007654	HTR1F	2	1.385719799	0.42625948	3.250883237	0.001150471	0	0.019458552	0	1	PRJNA827465;Yang2025_Silkie_Wenchang	0.059150942
ENSGALG00010005194	ENSGALG00010005194	2	3.483278863	1.076444302	3.23591184	0.001212548	1.552143772	3.01840567	66.86992706	1	CRA004775;PRJNA827465	0.059727703
ENSGALG00010024305	ENSGALG00010024305	3	-0.813332073	0.25124974	-3.237145932	0.001207317	0	0.110965984	0	1	CRA004775;PRJNA827465;Yang2025_Silkie_Wencha	0.059727703
ENSGALG00010012979	HTR1A	3	-0.57927861	0.178908104	-3.237855624	0.001204317	0	0.955306494	0	1	CRA004775;PRJNA827465;Yang2025_Silkie_Wencha	0.059727703
ENSGALG00010029144	ENSGALG00010029144	3	-0.336444362	0.103838732	-3.240066173	0.00119502	0	0.237223248	0	1	CRA004775;PRJNA827465;Yang2025_Silkie_Wencha	0.059727703
ENSGALG00010029386	SLC16A6	3	-0.303256916	0.093800997	-3.232981801	0.001225054	0	1.678841142	0	0.666666667	CRA004775;PRJNA827465;Yang2025_Silkie_Wencha	0.059727703
ENSGALG00010025144	PIH1D2	3	0.293565096	0.090581992	3.240877011	0.001191626	0	1.490622311	0	1	CRA004775;PRJNA827465;Yang2025_Silkie_Wencha	0.059727703
ENSGALG00010027041	MAPK14	3	-0.283352987	0.087351039	-3.243842196	0.001179291	0.0110093	3.982445995	49.77960774	1	CRA004775;PRJNA827465;Yang2025_Silkie_Wencha	0.059727703
ENSGALG00010016507	SPATA18	3	-0.278329036	0.086022564	-3.235535225	0.001214149	0	1.658894028	0	1	CRA004775;PRJNA827465;Yang2025_Silkie_Wencha	0.059727703
ENSGALG00010019065	CAND2	3	-0.258401524	0.079700782	-3.242145394	0.001186335	0.005681895	2.805387045	28.7085893	0.666666667	CRA004775;PRJNA827465;Yang2025_Silkie_Wencha	0.059727703
ENSGALG00010020873	PINK1	3	-0.257670924	0.079678911	-3.233866022	0.001221267	0	0.27925386	0	1	CRA004775;PRJNA827465;Yang2025_Silkie_Wencha	0.059727703
ENSGALG00010019713	NUDT14	3	-0.235616415	0.072631617	-3.243992404	0.001178669	0	0.26463307	0	1	CRA004775;PRJNA827465;Yang2025_Silkie_Wencha	0.059727703
ENSGALG00010019344	DNAJC24	3	0.190026183	0.058566695	3.244611703	0.001176109	0	1.916854003	0	0.666666667	CRA004775;PRJNA827465;Yang2025_Silkie_Wencha	0.059727703
ENSGALG00010019690	ENSGALG00010019690	3	-0.189771124	0.058697387	-3.233042113	0.001224795	0	1.874384422	0	1	CRA004775;PRJNA827465;Yang2025_Silkie_Wencha	0.059727703
ENSGALG00010006548	TMEM209	3	-0.177390592	0.054726109	-3.241425284	0.001189336	0	1.197569248	0	1	CRA004775;PRJNA827465;Yang2025_Silkie_Wencha	0.059727703
ENSGALG00010012968	LSM11	3	-0.169687912	0.052425302	-3.236756003	0.001208967	0	0.603606659	0	1	CRA004775;PRJNA827465;Yang2025_Silkie_Wencha	0.059727703
ENSGALG00010027269	CDC25A	3	-0.222482028	0.068841598	-3.231796401	0.001230147	0	1.878811265	0	1	CRA004775;PRJNA827465;Yang2025_Silkie_Wencha	0.059769912
ENSGALG00010001599	TBLX1	3	-0.120270246	0.037251586	-3.228594003	0.001244004	0	1.854086751	0	0.666666667	CRA004775;PRJNA827465;Yang2025_Silkie_Wencha	0.060236191
ENSGALG00010022707	SALL1	2	1.599877834	0.495710234	3.227445644	0.001249008	0.343362739	3.149260617	68.24651492	1	PRJNA827465;Yang2025_Silkie_Wenchang	0.060272081
ENSGALG00010015616	NADK2	3	0.289136159	0.089615798	3.226397201	0.001253593	0.001940089	2.168784234	7.782435478	1	CRA004775;PRJNA827465;Yang2025_Silkie_Wencha	0.060287568
ENSGALG00010010122	THBS4	3	0.435585056	0.135116762	3.223767719	0.00126516	0	1.589627334	0	0.666666667	CRA004775;PRJNA827465;Yang2025_Silkie_Wencha	0.060637606
ENSGALG00010017361	METTL22	3	-0.173738446	0.053956739	-3.219958225	0.001282093	0	1.393141753	0	1	CRA004775;PRJNA827465;Yang2025_Silkie_Wencha	0.061241587
ENSGALG00010001358	TCERG1L	2	0.997113347	0.310055678	3.215917071	0.001300284	0.053899914	1.378713022	27.46858963	1	PRJNA827465;Yang2025_Silkie_Wenchang	0.061694345
ENSGALG00010004739	ENSGALG00010004739	2	0.849201293	0.264141171	3.214952404	0.001304662	0	0.275455508	0	1	PRJNA827465;Yang2025_Silkie_Wenchang	0.061694345
ENSGALG00010008002	GJA8	3	0.623301532	0.193854315	3.215309048	0.001303042	0.061572181	4.880212323	59.01817652	1	CRA004775;PRJNA827465;Yang2025_Silkie_Wencha	0.061694345
ENSGALG00010002440	LRRC51	3	-0.516031032	0.160635135	-3.212441866	0.001316118	0	0.059327453	0	1	CRA004775;PRJNA827465;Yang2025_Silkie_Wencha	0.062028633
ENSGALG00010002455	NYX	3	0.37027764	0.115533112	3.204948209	0.001350868	0	0.645057045	0	1	CRA004775;PRJNA827465;Yang2025_Silkie_Wencha	0.06262912
ENSGALG00010027880	AMH	3	-0.304634759	0.094959429	-3.208051708	0.001336375	0	0.889041327	0	0.666666667	CRA004775;PRJNA827465;Yang2025_Silkie_Wencha	0.06262912
ENSGALG00010024170	NUP160	3	-0.225043608	0.070203108	-3.205607458	0.001347777	0.006379897	3.575825921	44.06886565	1	CRA004775;PRJNA827465;Yang2025_Silkie_Wencha	0.06262912
ENSGALG00010000350	ENSGALG00010000350	3	-0.173529617	0.054102756	-3.207408076	0.001339369	0	0.023214441	0	1	CRA004775;PRJNA827465;Yang2025_Silkie_Wencha	0.06262912
ENSGALG00010027872	KDM5B	3	-0.133075337	0.04152222	-3.204918658	0.001351007	0	0.429332909	0	1	CRA004775;PRJNA827465;Yang2025_Silkie_Wencha	0.06262912
ENSGALG00010026686	ENSGALG00010026686	3	1.050291733	0.328523011	3.197011159	0.001388596	0.179072426	4.488749678	55.44416277	1	CRA004775;PRJNA827465;Yang2025_Silkie_Wencha	0.063799486
ENSGALG00010012140	GLOD5	2	-0.926166867	0.289720205	-3.196763122	0.00138979	0	0.030309717	0	1	PRJNA827465;Yang2025_Silkie_Wenchang	0.063799486
ENSGALG00010004329	COQ3	3	-0.265327862	0.082996196	-3.196867743	0.001389286	0	0.852503912	0	1	CRA004775;PRJNA827465;Yang2025_Silkie_Wencha	0.063799486
ENSGALG00010028044	GAMT	3	-0.425700568	0.133280885	-3.19401067	0.001403109	0	1.963464541	0	1	CRA004775;PRJNA827465;Yang2025_Silkie_Wencha	0.064202468
ENSGALG00010027249	C2CD4C	2	-0.738614189	0.231617424	-3.188940514	0.001427953	0	0.384613108	0	1	PRJNA827465;Yang2025_Silkie_Wenchang	0.065045886
ENSGALG00010003634	CYP4F11	3	0.597741007	0.187475057	3.188376188	0.001430743	0	1.211824448	0	1	CRA004775;PRJNA827465;Yang2025_Silkie_Wencha	0.065045886
ENSGALG00010017997	ENSGALG00010017997	3	2.349084849	0.737537367	3.185038417	0.001447348	1.191502856	7.871698951	74.59252427	1	CRA004775;PRJNA827465;Yang2025_Silkie_Wencha	0.065589911
ENSGALG00010016122	AFG2B	3	-0.234859047	0.073881627	-3.178855897	0.001478576	0	1.520080902	0	1	CRA004775;PRJNA827465;Yang2025_Silkie_Wencha	0.066791
ENSGALG00010011683	NIPAL2	2	-0.779898194	0.245597529	-3.175513201	0.001495717	0	0.725614696	0	1	PRJNA827465;Yang2025_Silkie_Wenchang	0.067350143
ENSGALG00010026481	DIXDC1	3	-0.206695454	0.06525098	-3.167698828	0.001536506	0	1.163104302	0	1	CRA004775;PRJNA827465;Yang2025_Silkie_Wencha	0.068967171
ENSGALG00010002586	GPR63	3	0.243236182	0.076932481	3.161683855	0.001568598	0	0.827594464	0	1	CRA004775;PRJNA827465;Yang2025_Silkie_Wencha	0.070184815
ENSGALG00010021743	SYT13	2	-0.679246514	0.214909449	-3.160617265	0.001574352	0	0.006464599	0	1	PRJNA827465;Yang2025_Silkie_Wenchang	0.070220081
ENSGALG00010009493	ENSGALG00010009493	3	0.869464677	0.27519462	3.159453762	0.001580652	0	0.067100104	0	1	CRA004775;PRJNA827465;Yang2025_Silkie_Wencha	0.070279358
ENSGALG00010029594	CRLF3	3	-0.191866847	0.060886795	-3.151206206	0.001625977	0.003097751	2.693356544	25.74321418	1	CRA004775;PRJNA827465;Yang2025_Silkie_Wencha	0.072067969
ENSGALG00010009193	EDARADD	3	-0.478873426	0.152202276	-3.146296088	0.001653525	0	0.950395539	0	1	CRA004775;PRJNA827465;Yang2025_Silkie_Wencha	0.073059977
ENSGALG00010020402	SLC3A1	3	0.562258662	0.178819155	3.14428654	0.001664923	0.033528015	3.055546798	34.54526694	1	CRA004775;PRJNA827465;Yang2025_Silkie_Wencha	0.073334424
ENSGALG00010016030	TGM4	2	1.164933503	0.370970571	3.140231584	0.001688143	0	0.757270463	0	1	CRA004775;PRJNA827465	0.073442024
ENSGALG00010027636	SLC1A6	3	-0.905264802	0.288119955	-3.141971895	0.001678141	0	1.93813908	0	1	CRA004775;PRJNA827465;Yang2025_Silkie_Wencha	0.073442024
ENSGALG00010016480	GPX3	3	-0.296576568	0.094402248	-3.141626127	0.001680124	0	1.519985021	0	1	CRA004775;PRJNA827465;Yang2025_Silkie_Wencha	0.073442024
ENSGALG00010007789	WWOX	3	-0.194203544	0.061838263	-3.140507728	0.001686553	0	1.903489311	0	1	CRA004775;PRJNA827465;Yang2025_Silkie_Wencha	0.073442024
ENSGALG00010027376	DSTYK	3	-0.127898773	0.040827927	-3.132629583	0.001732479	0	1.54028707	0	1	CRA004775;PRJNA827465;Yang2025_Silkie_Wencha	0.075139648
ENSGALG00010027528	ENSGALG00010027528	2	1.574773711	0.503525811	3.127493522	0.001763037	0	0.962071173	0	1	CRA004775;PRJNA827465	0.076231134
ENSGALG00010000645	ENSGALG00010000645	2	0.659554466	0.211224682	3.122525543	0.001793065	0	0.54105183	0	1	PRJNA827465;Yang2025_Silkie_Wenchang	0.077058214
ENSGALG00010009200	ADGRA3	3	0.177040143	0.056682806	3.123348228	0.001788061	0	1.289689175	0	1	CRA004775;PRJNA827465;Yang2025_Silkie_Wencha	0.077058214
ENSGALG00010004903	ENSGALG00010004903	2	1.060343518	0.339809224	3.120408286	0.001806005	0	0.335929799	0	1	PRJNA827465;Yang2025_Silkie_Wenchang	0.077379117
ENSGALG00010018308	ACAP3	3	0.204545484	0.065676029	3.114461825	0.001842808	0	0.796720441	0	0.666666667	CRA004775;PRJNA827465;Yang2025_Silkie_Wencha	0.078498204
ENSGALG00010010575	PAXBP1	3	0.138612304	0.044506982	3.114394564	0.001843228	0	1.586579142	0	1	CRA004775;PRJNA827465;Yang2025_Silkie_Wencha	0.078498204
ENSGALG00010003193	ACOT9	3	-0.342424527	0.110028623	-3.11214043	0.001857361	0.017576457	3.76065134	46.81772334	1	CRA004775;PRJNA827465;Yang2025_Silkie_Wencha	0.078862554
ENSGALG00010003948	SCD5	3	0.261677914	0.084132621	3.110302636	0.001868957	0	0.96349391	0	1	CRA004775;PRJNA827465;Yang2025_Silkie_Wencha	0.079117335
ENSGALG00010002701	TMEM196	2	0.938748853	0.302397944	3.104349328	0.00190698	0	0.008740577	0	1	PRJNA827465;Yang2025_Silkie_Wenchang	0.080485953
ENSGALG00010001214	TES	3	-0.187711146	0.060502645	-3.102527932	0.001918754	0	0.232082901	0	1	CRA004775;PRJNA827465;Yang2025_Silkie_Wencha	0.08074187
ENSGALG00010013411	ENSGALG00010013411	3	1.590613947	0.514149808	3.093677994	0.001976919	0.325091665	3.371608695	40.6811353	1	CRA004775;PRJNA827465;Yang2025_Silkie_Wencha	0.082569084
ENSGALG00010012852	MSTN	3	-0.334827205	0.108234646	-3.093530735	0.001977901	0	1.620671151	0	0.666666667	CRA004775;PRJNA827465;Yang2025_Silkie_Wencha	0.082569084
ENSGALG00010009376	CDO1	3	-0.279957059	0.09050546	-3.093261537	0.001979696	0	0.83917523	0	1	CRA004775;PRJNA827465;Yang2025_Silkie_Wencha	0.082569084
ENSGALG00010011505	MYL1	3	-0.283931922	0.091858938	-3.090955843	0.001995133	0	0.298812872	0	1	CRA004775;PRJNA827465;Yang2025_Silkie_Wencha	0.082968193
ENSGALG00010005326	MASTL	3	-0.246326225	0.079852995	-3.084746216	0.002037259	0.000874795	2.068494672	3.311329403	1	CRA004775;PRJNA827465;Yang2025_Silkie_Wencha	0.083979026
ENSGALG00010022433	LNPK	3	-0.210683695	0.068283455	-3.085428148	0.002032593	0.002031868	2.265246337	11.70938155	1	CRA004775;PRJNA827465;Yang2025_Silkie_Wencha	0.083979026
ENSGALG00010021133	ENSGALG00010021133	3	0.117165665	0.037981187	3.084834248	0.002036656	0	0.556275906	0	0.666666667	CRA004775;PRJNA827465;Yang2025_Silkie_Wencha	0.083979026
ENSGALG00010018416	DUT	3	-0.187108783	0.060708327	-3.082094199	0.002055498	0	0.307298766	0	1	CRA004775;PRJNA827465;Yang2025_Silkie_Wencha	0.084484544
ENSGALG00010011784	GRPEL2	3	-0.181675061	0.05902248	-3.078065545	0.002083491	0	0.938835209	0	1	CRA004775;PRJNA827465;Yang2025_Silkie_Wencha	0.085203465
ENSGALG00010019213	RHBDF1	3	0.149143863	0.048457254	3.077843862	0.002085041	0	1.162966802	0	0.666666667	CRA004775;PRJNA827465;Yang2025_Silkie_Wencha	0.085203465
ENSGALG00010011531	MTDH	3	-0.09727646	0.031674234	-3.071154277	0.002132329	0	1.043555955	0	0.666666667	CRA004775;PRJNA827465;Yang2025_Silkie_Wencha	0.086884743
ENSGALG00010010894	SPAG1	3	0.289545859	0.094356328	3.068642739	0.002150336	0	0.814363329	0	1	CRA004775;PRJNA827465;Yang2025_Silkie_Wencha	0.087366659
ENSGALG00010024936	EHF	2	1.247597861	0.407231129	3.06361123	0.002186829	0	0.902815174	0	1	CRA004775;PRJNA827465	0.088594769
ENSGALG00010005696	NUBPL	3	-0.228451329	0.074677051	-3.059190535	0.002219359	0	1.270640097	0	1	CRA004775;PRJNA827465;Yang2025_Silkie_Wencha	0.089655782
ENSGALG00010011243	ALX1	2	1.003197666	0.328232856	3.05635968	0.002240423	0	0.109116265	0	1	PRJNA827465;Yang2025_Silkie_Wenchang	0.090248842
ENSGALG00010008092	SPIRE2	3	0.877490232	0.287681018	3.050219439	0.002286742	0	0.72365872	0	1	CRA004775;PRJNA827465;Yang2025_Silkie_Wencha	0.09159277
ENSGALG00010023641	LRRC40	3	-0.226281443	0.074164822	-3.051061631	0.002280338	0.00580144	3.008723384	33.52662426	1	CRA004775;PRJNA827465;Yang2025_Silkie_Wencha	0.09159277
ENSGALG00010010754	DNAI1	3	0.529441267	0.173680278	3.04836723	0.002300886	0	1.932354137	0	0.666666667	CRA004775;PRJNA827465;Yang2025_Silkie_Wencha	0.091898931
ENSGALG00010023854	SHCBP1	3	-0.476051622	0.156324887	-3.045270845	0.002324709	0.02079934	2.744589222	27.1293502	0.666666667	CRA004775;PRJNA827465;Yang2025_Silkie_Wencha	0.092167207
ENSGALG00010029956	CCLI5	3	0.410657388	0.134864887	3.044954076	0.002327158	0.008091791	2.287511058	12.56872866	0.666666667	CRA004775;PRJNA827465;Yang2025_Silkie_Wencha	0.092167207
ENSGALG00010019585	PDE1C	3	-0.370838315	0.121787714	-3.044956691	0.002327138	0	0.033820733	0	1	CRA004775;PRJNA827465;Yang2025_Silkie_Wencha	0.092167207
ENSGALG00010015483	SGCD	3	-0.33809867	0.111149445	-3.041838574	0.00235138	0.013617756	3.177005094	37.04763004	0.666666667	CRA004775;PRJNA827465;Yang2025_Silkie_Wencha	0.092866357
ENSGALG00010003522	ENSGALG00010003522	3	-0.914173292	0.300946708	-3.037658387	0.002384241	0	0.42083375	0	1	CRA004775;PRJNA827465;Yang2025_Silkie_Wencha	0.093641054
ENSGALG00010002159	GALNT15	3	-0.28906858	0.095157104	-3.037803458	0.002383093	0	0.776519108	0	1	CRA004775;PRJNA827465;Yang2025_Silkie_Wencha	0.093641054

ENSGALG00010016518	TMEM42	3	-0.268399982	0.088411935	-3.035789019	0.002399072	0.00692568	2.671656204	25.14006867	1	CRA004775;PRJNA827465;Yang2025_Silkie_Wencha	0.093962535
ENSGALG00010021710	ENSGALG0001002171	3	0.51415609	0.169520703	3.0329988	0.002421366	0	0.92116692	0	1	CRA004775;PRJNA827465;Yang2025_Silkie_Wencha	0.094573726
ENSGALG00010012111	DRD1	3	-0.671308599	0.221519561	-3.030470972	0.002441727	0	1.214119862	0	1	CRA004775;PRJNA827465;Yang2025_Silkie_Wencha	0.095106259
ENSGALG00010018333	ENSGALG0001001833	3	0.503122833	0.166081787	3.02936789	0.002450661	0	1.079041394	0	1	CRA004775;PRJNA827465;Yang2025_Silkie_Wencha	0.095192006
ENSGALG00010005634	HADH	3	-0.233071754	0.077012015	-3.02643364	0.002474571	0.002799577	2.189701245	8.663339135	0.666666667	CRA004775;PRJNA827465;Yang2025_Silkie_Wencha	0.095857435
ENSGALG00010012948	ST13P5	3	-0.209435046	0.069255812	-3.024078981	0.002493913	0.005841949	3.381012498	40.84612225	1	CRA004775;PRJNA827465;Yang2025_Silkie_Wencha	0.096342729
ENSGALG00010003222	ITGA8	3	0.240961803	0.079820713	3.018787919	0.002537881	0.004767616	2.448904591	18.33083218	1	CRA004775;PRJNA827465;Yang2025_Silkie_Wencha	0.097774122
ENSGALG00010011136	ENSGALG0001001113	3	-0.651303773	0.215860557	-3.017243085	0.002550852	0	0.79307552	0	1	CRA004775;PRJNA827465;Yang2025_Silkie_Wencha	0.098006771
ENSGALG00010018945	HABP2	3	0.974048842	0.322936373	3.016225249	0.002559431	0.1412375	3.631968971	44.93345026	1	CRA004775;PRJNA827465;Yang2025_Silkie_Wencha	0.098069886
ENSGALG00010020136	KCNK17	3	1.113133687	0.369380187	3.013517578	0.002582381	0.292889561	7.217804415	72.29074265	1	CRA004775;PRJNA827465;Yang2025_Silkie_Wencha	0.098681844
ENSGALG00010015596	UCHL1	3	-0.497862232	0.165881247	-3.00131715	0.002688144	0.00384119	2.074155345	3.575206884	1	CRA004775;PRJNA827465;Yang2025_Silkie_Wencha	0.102446555
ENSGALG00010017509	RASD1	3	0.587320585	0.195773453	3.000001152	0.002699786	0	1.732333157	0	1	CRA004775;PRJNA827465;Yang2025_Silkie_Wencha	0.102613635
ENSGALG00010022542	ENSGALG0001002254	2	1.489327137	0.496902509	2.99722201	0.002724522	0	0.944841291	0	1	PRJNA827465;Yang2025_Silkie_Wenchang	0.103000051
ENSGALG00010016987	ASB2	3	-0.396083532	0.13212487	-2.997796937	0.002719388	0.010514073	2.339154872	14.49903449	0.666666667	CRA004775;PRJNA827465;Yang2025_Silkie_Wencha	0.103000051
ENSGALG00010016086	TDRD9	2	0.553374283	0.184718285	2.995774255	0.00273749	0	0.327903054	0	1	PRJNA827465;Yang2025_Silkie_Wenchang	0.103214326
ENSGALG00010003511	UBXN2B	3	-0.200817691	0.067125406	-2.991679338	0.002774475	0	0.71258602	0	1	CRA004775;PRJNA827465;Yang2025_Silkie_Wencha	0.104330599
ENSGALG00010025674	GRB14	2	-0.759572225	0.254095101	-2.989322591	0.002795968	0	0.104848302	0	1	PRJNA827465;Yang2025_Silkie_Wenchang	0.104859911
ENSGALG00010028133	ENSGALG0001002813	3	0.541106778	0.181369023	2.983457534	0.002850116	0	0.600351512	0	1	CRA004775;PRJNA827465;Yang2025_Silkie_Wencha	0.106607904
ENSGALG00010020277	IDH2	3	-0.208830399	0.07002661	-2.982441081	0.002859597	0	0.025519917	0	1	CRA004775;PRJNA827465;Yang2025_Silkie_Wencha	0.106680316
ENSGALG00010027822	DPH7	3	0.221057424	0.074222362	2.97831298	0.002898399	0	0.677679382	0	1	CRA004775;PRJNA827465;Yang2025_Silkie_Wencha	0.107843307
ENSGALG00010018323	GOT1	3	-0.265274386	0.089132857	-2.976168332	0.002918746	0	0.792554771	0	1	CRA004775;PRJNA827465;Yang2025_Silkie_Wencha	0.108315355
ENSGALG00010002483	COQ2	3	-0.240013706	0.080731701	-2.972979671	0.00294924	0	1.221590999	0	1	CRA004775;PRJNA827465;Yang2025_Silkie_Wencha	0.109160477
ENSGALG00010011637	CECR2	3	0.192235138	0.064734491	2.969593735	0.002981938	0	1.49893279	0	0.666666667	CRA004775;PRJNA827465;Yang2025_Silkie_Wencha	0.110082571
ENSGALG00010026068	BLTP3A	3	-0.139152683	0.046892013	-2.967513509	0.003002191	0	0.06679564	0	1	CRA004775;PRJNA827465;Yang2025_Silkie_Wencha	0.110541607
ENSGALG00010024588	TTC39A	3	0.811010746	0.273459943	2.96573874	0.003019569	0	0.505354171	0	1	CRA004775;PRJNA827465;Yang2025_Silkie_Wencha	0.110815531
ENSGALG00010021723	COPS3	3	0.13307224	0.044878681	2.965154904	0.003025306	0	0.058786562	0	1	CRA004775;PRJNA827465;Yang2025_Silkie_Wencha	0.110815531
ENSGALG00010008030	GJA5	3	0.371497764	0.125328021	2.964203559	0.003034675	0.017761179	3.208716151	37.6697749	1	CRA004775;PRJNA827465;Yang2025_Silkie_Wencha	0.110871488
ENSGALG00010004385	ADAMTS3	3	0.535439473	0.180776392	2.961888264	0.003057587	0.03137546	2.89360191	30.88199198	1	CRA004775;PRJNA827465;Yang2025_Silkie_Wencha	0.111420684
ENSGALG00010023387	ENSGALG0001002338	3	-0.212677243	0.071919353	-2.957162902	0.00310484	0	0.101171001	0	1	CRA004775;PRJNA827465;Yang2025_Silkie_Wencha	0.112851769
ENSGALG00010021241	TMEM43	3	-0.198193553	0.067119752	-2.952835026	0.003148702	0	1.285750452	0	1	CRA004775;PRJNA827465;Yang2025_Silkie_Wencha	0.114152554
ENSGALG00010025389	SNED1	3	0.166628701	0.056505945	2.948870291	0.003189378	0	1.954250667	0	0.666666667	CRA004775;PRJNA827465;Yang2025_Silkie_Wencha	0.115331499
ENSGALG00010005553	PPP1R14C	3	-0.353733702	0.120040666	-2.946782246	0.003210992	0.017773429	3.426046179	41.62367068	1	CRA004775;PRJNA827465;Yang2025_Silkie_Wencha	0.115816889
ENSGALG00010021202	UROC1	2	0.734533027	0.249389408	2.945325676	0.003226149	0	0.008030246	0	1	PRJNA827465;Yang2025_Silkie_Wenchang	0.116067478
ENSGALG00010028053	ORM1	2	1.7521319	0.59573345	2.941133993	0.00327013	0	0.07128269	0	1	CRA004775;PRJNA827465	0.116539638
ENSGALG00010008466	C11orf97	2	-1.064796649	0.361964282	-2.9417147	0.003264005	0.135577495	2.065875374	51.59436951	1	PRJNA827465;Yang2025_Silkie_Wenchang	0.116539638
ENSGALG00010004146	NAAA	3	-0.28959014	0.098393025	-2.943197855	0.003248407	0	0.850984002	0	1	CRA004775;PRJNA827465;Yang2025_Silkie_Wencha	0.116539638
ENSGALG00010016968	C15orf61	3	-0.236779027	0.080511506	-2.940934001	0.003272242	0	0.817693593	0	1	CRA004775;PRJNA827465;Yang2025_Silkie_Wencha	0.116539638
ENSGALG00010001476	IGF2BP3	3	-0.179218495	0.060961128	-2.93988152	0.003283378	0	0.842724344	0	0.666666667	CRA004775;PRJNA827465;Yang2025_Silkie_Wencha	0.116642405
ENSGALG00010018142	DDHD1	3	0.194305689	0.066125342	2.938445139	0.00329863	0.002128257	2.310889264	13.45323069	0.666666667	CRA004775;PRJNA827465;Yang2025_Silkie_Wencha	0.116890562
ENSGALG00010004131	NUDT6	3	0.474833162	0.161709187	2.936340055	0.0033211	0.045099633	4.814011966	58.45461095	1	CRA004775;PRJNA827465;Yang2025_Silkie_Wencha	0.117392595
ENSGALG00010024908	ENSGALG0001002490	3	-0.274687205	0.0936378	-2.933507663	0.003351554	0.009450551	2.778362863	28.01516222	1	CRA004775;PRJNA827465;Yang2025_Silkie_Wencha	0.117926472
ENSGALG00010009558	TMEM26	3	-0.263112617	0.089706878	-2.93302614	0.003356756	0	0.91003461	0	0.666666667	CRA004775;PRJNA827465;Yang2025_Silkie_Wencha	0.117926472
ENSGALG00010008026	PURG	3	0.230098279	0.078461862	2.93261301	0.003361226	0	0.276233269	0	1	CRA004775;PRJNA827465;Yang2025_Silkie_Wencha	0.117926472
ENSGALG00010009668	TFAP2A	2	2.400526163	0.819369862	2.929722309	0.00339265	0.870660532	2.795179692	64.22412474	1	PRJNA827465;Yang2025_Silkie_Wenchang	0.118734366
ENSGALG00010011649	MRPS27	3	0.307470188	0.104991289	2.928530457	0.003405685	0	0.359958143	0	1	CRA004775;PRJNA827465;Yang2025_Silkie_Wencha	0.118896235
ENSGALG00010023328	TCEA2	3	0.178736862	0.061170436	2.921948461	0.003478491	0.001124987	2.136684414	6.397033303	0.666666667	CRA004775;PRJNA827465;Yang2025_Silkie_Wencha	0.121138876
ENSGALG00010013225	GNE	3	0.230903426	0.07910542	2.918983615	0.003511747	0.003190568	2.323959485	13.9399799	1	CRA004775;PRJNA827465;Yang2025_Silkie_Wencha	0.121996531
ENSGALG00010001701	NDUFA5	3	-0.25631692	0.087889881	-2.916341645	0.003541625	0	1.339818402	0	1	CRA004775;PRJNA827465;Yang2025_Silkie_Wencha	0.122272589
ENSGALG00010028164	BBLN	3	-0.223787756	0.076756428	-2.915557193	0.00355054	0	1.50477599	0	1	CRA004775;PRJNA827465;Yang2025_Silkie_Wencha	0.122272589
ENSGALG00010020070	ARL6IP5	3	-0.208967828	0.071681464	-2.915228252	0.003554285	0.004820708	2.725460643	26.61790935	1	CRA004775;PRJNA827465;Yang2025_Silkie_Wencha	0.122272589
ENSGALG00010004917	ENSGALG0001000491	3	-0.205412533	0.070487559	-2.915412284	0.003552189	0.001795288	2.222958176	10.02979626	1	CRA004775;PRJNA827465;Yang2025_Silkie_Wencha	0.122272589
ENSGALG00010014077	SLITRK2	3	-0.200658178	0.068874748	-2.91337801	0.003575415	0	1.379901702	0	0.666666667	CRA004775;PRJNA827465;Yang2025_Silkie_Wencha	0.122403858
ENSGALG00010028455	ENSA	3	-0.200441547	0.068799491	-2.913416125	0.003574979	0.002809251	2.253436015	11.24664793	1	CRA004775;PRJNA827465;Yang2025_Silkie_Wencha	0.122403858
ENSGALG00010003768	ARSJ	3	-0.162751195	0.055899627	-2.911489833	0.003597096	0	0.314202305	0	1	CRA004775;PRJNA827465;Yang2025_Silkie_Wencha	0.122848653
ENSGALG00010019041	KATNIP	3	0.160718634	0.05523152	2.909697133	0.003617792	0	1.66590827	0	1	CRA004775;PRJNA827465;Yang2025_Silkie_Wencha	0.122976339
ENSGALG00010021730	ARL8B	3	-0.10537273	0.036214801	-2.909659232	0.00361823	0	1.904755116	0	1	CRA004775;PRJNA827465;Yang2025_Silkie_Wencha	0.122976339
ENSGALG00010021660	RNF223	3	0.663057528	0.228272037	2.904681343	0.003676272	0.052550877	2.98834348	33.07328916	1	CRA004775;PRJNA827465;Yang2025_Silkie_Wencha	0.124649414
ENSGALG00010029412	SLC46A1	3	-0.214205402	0.073811582	-2.902056787	0.003707213	0	1.380538067	0	1	CRA004775;PRJNA827465;Yang2025_Silkie_Wencha	0.125397821
ENSGALG00010005518	HPGDS	3	2.567087562	0.88557832	2.898769656	0.0037463	2.023526149	14.49891662	86.20586591	1	CRA004775;PRJNA827465;Yang2025_Silkie_Wencha	0.12641751
ENSGALG00010001129	TM4SF4	2	2.015639818	0.695945734	2.896260039	0.003776393	0	0.360387919	0	1	CRA004775;PRJNA827465	0.126721512
ENSGALG00010025748	ETNK2	3	-0.241325909	0.083304847	-2.896901171	0.003768685	0	1.441014326	0	0.666666667	CRA004775;PRJNA827465;Yang2025_Silkie_Wencha	0.126721512
ENSGALG00010017774	ASH2L	3	-0.115765191	0.039977231	-2.895778165	0.003782197	0	1.725086226	0	1	CRA004775;PRJNA827465;Yang2025_Silkie_Wencha	0.126721512
ENSGALG00010021353	IPO13	3	-0.181798216	0.062822062	-2.89385436	0.003805385	0	0.675994954	0	1	CRA004775;PRJNA827465;Yang2025_Silkie_Wencha	0.127197009
ENSGALG00010005892	ENSGALG0001000589	3	-1.526428434	0.52794481	-2.891265158	0.003836942	0.288633703	3.051616922	34.46097426	1	CRA004775;PRJNA827465;Yang2025_Silkie_Wencha	0.127949359
ENSGALG00010012773	XKR8	3	-0.341051303	0.118122486	-2.887268245	0.003886028	0	1.848833033	0	1	CRA004775;PRJNA827465;Yang2025_Silkie_Wencha	0.129281284
ENSGALG00010008999	TRAPPC9	3	-0.156982434	0.054397402	-2.885844332	0.003903652	0	1.707626122	0	1	CRA004775;PRJNA827465;Yang2025_Silkie_Wencha	0.129562755
ENSGALG00010002590	ATP6V1C2	3	0.368133963	0.127725862	2.882219432	0.003948846	0	1.099920401	0	1	CRA004775;PRJNA827465;Yang2025_Silkie_Wencha	0.130146242
ENSGALG00010028046	SMIM4	3	-0.279427744	0.096945297	-2.882323869	0.003947538	0	0.698986996	0	1	CRA004775;PRJNA827465;Yang2025_Silkie_Wencha	0.130146242
ENSGALG00010027134	DYRK3	3	-0.175040283	0.060722248	-2.882638382	0.003943599	0	1.643845742	0	0.666666667	CRA004775;PRJNA827465;Yang2025_Silkie_Wencha	0.130146242
ENSGALG00010017458	ENSGALG0001001745	3	0.19325278	0.067165947	2.877243446	0.00401166	0	0.67968732	0	0.666666667	CRA004775;PRJNA827465;Yang2025_Silkie_Wencha	0.131908969
ENSGALG00010007889	DCAF6	3	-0.133861623	0.046552117	-2.875521682	0.004033604	0	0.928348264	0	0.666666667	CRA004775;PRJNA827465;Yang2025_Silkie_Wencha	0.132322814
ENSGALG00010011574	GAS2L3	3	0.224025075	0.077997462	2.872209792	0.004076123	0	1.020706499	0	1	CRA004775;PRJNA827465;Yang2025_Silkie_Wencha	0.133099997
ENSGALG00010020558	EIF4EBP2	3	-0.159098258	0.055380415	-2.872825328	0.00406819	0	0.403450752	0	1	CRA004775;PRJNA827465;Yang2025_Silkie_Wencha	0.133099997
ENSGALG00010023864	EPHA1	2	1.154959323	0.402493242	2.869512334	0.004111053	0	0.474971428	0	1	CRA004775;PRJNA827465	0.133623398
ENSGALG00010029625	PECAM1	3	0.285006049	0.099300324	2.870142201	0.004102872	0.011908675	3.401613378	41.20437046	1	CRA004775;PRJNA827465;Yang2025_Silkie_Wencha	0.133623398
ENSGALG00010023317	RAPGEF3	3	0.199974339	0.069731652	2.867769974	0.00413376	0	0.691414974	0	0.666666667	CRA004775;PRJNA827465;Yang2025_Silkie_Wencha	0.133780369
ENSGALG00010021348	SLC4A3	3	-0.190939705	0.066583108	-2.867689891	0.004134806	0	0.769009611	0	1	CRA004775;PRJNA827465;Yang2025_Silkie_Wencha	0.133780369
ENSGALG00010002788	ENSGALG0001000278	3	-0.897587278	0.314198595	-2.856751403	0.004280009	0.097999042	2.982307435	32.93783275	1	CRA004775;PRJNA827465;Yang2025_Silkie_Wencha	0.138162211
ENSGALG00010005967	GJD2	3	0.469986031	0.164581309	2.855646452	0.004294931	0.021504646	2.633545664	24.05675635	0.666666667	CRA004775;PRJNA827465;Yang2025_Silkie_Wencha	0.138328085
ENSGALG00010021616	ENSGALG0001002161	3	1.781834215	0.624528137	2.853088772	0.004329652	0.709090087	5.09274243	60.7284282	1	CRA004775;PRJNA827465;Yang2025_Silkie_Wencha	0.139129446
ENSGALG00010018397	FAM173A	3	-0.28987908	0.101685865	-2.850731308	0.004361881	0	0.971939714	0	1	CRA004775;PRJNA827465;Yang2025_Silkie_Wencha	0.139847251
ENSGALG00010004237	RUFY3	3	0.165704221	0.058146642	2.849764223	0.004375165	0	0.258088429	0	1	CRA004775;PRJNA827465;Yang2025_Silkie_Wencha	0.139955783
ENSGALG00010021505	IHH	3	0.964256486	0.33917341	2.842960139	0.004469666	0	0.700965387	0	1	CRA004775;PRJNA827465;Yang2025_Silkie_Wencha	0.14169643
ENSGALG00010016944	OPRD1	2	-0.753067595	0.264846238	-2.843414357	0.0044633	0	0.477280063	0	1	PRJNA827465;Yang2025_Silkie_Wenchang	0.14169643
ENSGALG00010025315	LRRC42	3	0.22406829	0.078780075	2.844225407	0.004451954	0.002087087	2.201576891	9.156023197	1	CRA004775;PRJNA827465;Yang2025_Silkie_Wencha	0.14169643
ENSGALG00010001547	PWWP2B	3	-0.168027617	0.059099881	-2.843112617	0.004467528	0	1.401166958	0	0.666666667	CRA004775;PRJNA827465;Yang2025_Silkie_Wencha	0.14169643
ENSGALG00010020676	CYP2C23a	2	1.759029756	0.619598047	2.838985315	0.004525724	0.117017297	1.169157509	14.46832506	1	CRA004775;PRJNA827465	0.143152598
ENSGALG00010022645	ENSGALG0001002264	2	1.259014494	0.443686177	2.837623891	0.00454507	0.017432901	1.045247297	4.328860488	1	CRA004775;PRJNA827465	0.14344364
ENSGALG00010027927	ENSGALG0001002792	3	-0.252172637	0.088945402	-2.835139664	0.004580566	0	0.400058913	0	1	CRA004775;PRJNA827465;Yang2025_Silkie_Wencha	0.144241908
ENSGALG00010011927	ENSGALG0001001192	2	1.138397988	0.401655189	2.834266853	0.004593096	0	0.275355294	0	1	PRJNA827465;Yang2025_Silkie_Wenchang	0.144315075
ENSGALG00010017445	F9	2	1.390429221	0.490976918	2.831964537	0.004626298	0	0.040460615	0	1	CRA004775;PRJNA827465	0.144395643
ENSGALG00010023089	ENSGALG0001002308	2	1.086962555	0.383704824	2.8328092	0.004614092	0	0.958621598	0	1	PRJNA827465;Yang2025_Silkie_Wenchang	0.144395643
ENSGALG00010001844	HOXA10	3	0.265884075	0.093864612	2.832633822	0.004616624	0	1.278268855	0	1	CRA004775;PRJNA827465;Yang2025_Silkie_Wencha	0.144395643
ENSGALG00010004334	ENSGALG0001000433	3	0.327069952	0.11552262	2.831219992	0.004637082	0.001444103	2.062142878	3.013509831	1	CRA004775;PRJNA827465;Yang2025_Silkie_Wencha	0.144413426
ENSGALG00010027278	H1-10	3	-0.186644736	0.065947609	-2.830197169	0.004651933	0	1.769550901	0	1	CRA004775;PRJNA827465;Yang2025_Silkie_Wencha	0.144557528
ENSGALG00010028683	CRPL2	3	1.366243512	0.484169995	2.821826068	0.004775107	0.58125249	11.62097211	82.78973583	1	CRA004775;PRJNA827465;Yang2025_Silkie_Wencha	0.147413175
ENSGALG00010005364	MAP7	3	1.239395274	0.439157009	2.822214487	0.004769327	0.314844606	4.545780537	56.00315537	1	CRA004775;PRJNA827465;Yang2025_Silkie_Wencha	0.147413175
ENSGALG00010018166	MSS51	3	-0.388277124	0.137580248	-2.82218654	0.004769742	0	0.450880656	0	1	CRA004775;PRJNA827465;Yang2025_Silkie_Wencha	0.147413175
ENSGALG00010025382	MAB21L3	3	0.72793958	0.258057994	2.820837163	0.004789851	0.055487067	2.710582203	26.21511358	1	CRA004775;PRJNA827465;Yang2025_Silkie_Wencha	0.147546193
ENSGALG00010025980	HECTD4	3	-0.128133949	0.045452949	-2.8190459	0.004816663	0	1.707761791	0	0.666666667	CRA004775;PRJNA827465;Yang2025_Silkie_Wencha	0.148049561
ENSGALG00010028176	UPF1	3	-0.108164324	0.038397995	-2.816926315	0.004848565	0	0.461904158	0	1	CRA004775;PRJNA827465;Yang2025_Silkie_Wencha	0.14870685
ENSGALG00010017764	IGDCC3	3	-0.384735395	0.136802539	-2.812341048	0.004918233	0	0.79807371	0	1	CRA004775;PRJNA827465;Yang2025_Silkie_Wencha	0.150517077
ENSGALG00010022029	ENSGALG0001002202	3	1.153794719	0.410828805	2.808456235	0.004977965	0.12278067	2.595021236	22.92933975	0.666666667	CRA004775;PRJNA827465;Yang2025_Silkie_Wencha	0.151787954
ENSGALG00010000901	PCDH9	2	-0.56279602	0.200493388	-2.807055261	0.004999666	0	0.594921973	0	1	PRJNA827465;Yang2025_Silkie_Wenchang	0.151787954
ENSGALG00010015711	LBFABP	3	0.529447736	0.188626355	2.806859811	0.005002701	0.006403918	2.119149108	5.622497623	1	CRA004775;PRJNA827465;Yang2025_Silkie_Wencha	0.151787954
ENSGALG00010019104	POLL	3	-0.260588792	0.092836635	-2.806960766	0.005001133	0	0.242903877	0	1	CRA004775;PRJNA827465;Yang2025_Silkie_Wencha	0.151787954
ENSGALG00010027196	ENSGALG0001002719	3	-0.642394631	0.229140643	-2.803494928	0.005055203	0	1.516061526	0	1	CRA004775;PRJNA827465;Yang2025_Silkie_Wencha	0.152894511
ENSGALG00010012138	DDX11	3	0.270815167	0.096611424	2.803138152	0.005060799	0	1.157787254	0	1	CRA004775;PRJNA827465;Yang2025_Silkie_Wencha	0.152894511
ENSGALG00010024765	SNRNP35	3	0.148729366	0.05313781	2.798936705	0.005127119	0	0.416455354	0	0.666666667	CRA004775;PRJNA827465;Yang2025_Silkie_Wencha	0.154567869
ENSGALG00010007353	ING2	3	-0.243636195	0.087082045	-2.797777589	0.005145553	0	0.930963434	0	1	CRA004775;PRJNA827465;Yang2025_Silkie_Wencha	0.154793565
ENSGALG00010010449	PAPPA2	3	-0.925514047	0.33141188	-2.792639928	0.005227985	0	0.059449861	0	1	CRA004775;PRJNA827465;Yang2025_Silkie_Wencha	0.156640176
ENSGALG00010029186	ZNF302	3	0.234053649	0.083812954	2.7925713	0.005229094	0.003175122	2.259769062	11.49538093	1	CRA004775;PRJNA827465;Yang2025_Silkie_Wencha	0.156640176
ENSGALG00010006580	ALDH6A1	3	-0.290265619	0.104028858	-2.790241329	0.005266877	0.017332079	4.926306578	59.40163349	0.666666667	CRA004775;PRJNA827465;Yang2025_Silkie_Wencha	0.157438415
ENSGALG00010027351	TMEM38A	3	-0.289075871	0.103662303	-2.788630601	0.00529314	0.00689478	2.483997117	19.48460865	0.666666667	CRA004775;PRJNA827465;Yang2025_Silkie_Wencha	0.157557279
ENSGALG00010027084	OSBPL9	3	-0.088549951	0.031752143	-2.788786649	0.005290591	0	0.128560227	0	1	CRA004775;PRJNA827465;Yang2025_Silkie_Wencha	0.157557279
ENSGALG00010026066	SELENOP2	3	0.866033582	0.310871188	2.785827753	0.005339123	0.074743626	2.66379848	24.91924538	1	CRA004775;PRJNA827465;Yang2025_Silkie_Wencha	0.158592155
ENSGALG00010002669	IL4I1	3	-0.840923824	0.302529301	-2.779644227	0.005441848	0	0.944507241	0	1	CRA004775;PRJNA827465;Yang2025_Silkie_Wencha	0.160631089
ENSGALG00010022304	ADAP1	3	0.743501101	0.267469764	2.779757573	0.005439949	0.141798896	6.370556404	68.60556797	0.666666667	CRA004775;PRJNA827465;Yang2025_Silkie_Wencha	0.160631089
ENSGALG00010022323	DUSP29	3	0.631603103	0.227180609	2.780180519	0.005432869	0	0.982985876	0	1	CRA004775;PRJNA827465;Yang2025_Silkie_Wencha	0.160631089
ENSGALG00010022234	COX15	3	-0.213656632	0.076896944	-2.778480157	0.005461385	0	0.207830667	0	1	CRA004775;PRJNA827465;Yang2025_Silkie_Wencha	0.160871919
ENSGALG00010011375	C12orf50	3	-0.25255976	0.090927661	-2.777589964	0.005476368	0	1.279511725	0	1	CRA004775;PRJNA827465;Yang2025_Silkie_Wencha	0.160977887
ENSGALG00010012696	DNAL4	3	-0.161663762	0.058280178	-2.773906444	0.00553876	0	0.545121127	0	1	CRA004775;PRJNA827465;Yang2025_Silkie_Wencha	0.162474136
ENSGALG00010005990	SLC2A12	3	-0.335296286	0.120955392	-2.772065631	0.00557018	0.019407625	3.628906199	44.88697447	1	CRA004775;PRJNA827465;Yang2025_Silkie_Wencha	0.163057519
ENSGALG00010004395	BOC	3	0.208749706	0.075326566	2.771262732	0.005583935	0	0.997777614	0	1	CRA004775;PRJNA827465;Yang2025_Silkie_Wencha	0.163122435
ENSGALG00010017387	HS6ST2	3	-0.134254399	0.048467566	-2.769984349	0.005605899	0	1.767951148	0	1	CRA004775;PRJNA827465;Yang2025_Silkie_Wencha	0.163426393
ENSGALG00010005531	SUSD1	3	0.291615329	0.105405623	2.766601271	0.005664399	0	1.798378397	0	1	CRA004775;PRJNA827465;Yang2025_Silkie_Wencha	0.164505976
ENSGALG00010029199	APPBP2	3	-0.130412299	0.047139857	-2.766497576	0.005666201	0	1.911815331	0	0.666666667	CRA004775;PRJNA827465;Yang2025_Silkie_Wencha	0.164505976
ENSGALG00010003764	HDAC9	3	-0.322300827	0.116601873	-2.764113644	0.005707766	0.01720735	3.457042511	42.14708111	0.666666667	CRA004775;PRJNA827465;Yang2025_Silkie_Wencha	0.165373152
ENSGALG00010024254	ENSGALG0001002425	3	-0.41653365	0.150741941	-2.763223345	0.005723359	0	1.061570717	0	1	CRA004775;PRJNA827465;Yang2025_Silkie_Wencha	0.165477424
ENSGALG00010019934	TKFC	3	0.334150769	0.120985503	2.761907516	0.005746475	0.006617145	2.325710436	14.00477164	1	CRA004775;PRJNA827465;Yang2025_Silkie_Wencha	0.165477424
ENSGALG00010014840	NCF4	3	-0.329936828	0.119436131	-2.762454085	0.005736863	0	1.485631115	0	1	CRA004775;PRJNA827465;Yang2025_Silkie_Wencha	0.165477424
ENSGALG00010016588	ATP11B	3	-0.114817793	0.041597322	-2.760220821	0.00577623	0	0.901527968	0	0.666666667	CRA004775;PRJNA827465;Yang2025_Silkie_Wencha	0.165996181
ENSGALG00010027309	ENSGALG0001002730	2	1.16516128	0.422376783	2.758582683	0.005805262	0	0.246149701	0	1	CRA004775;Yang2025_Silkie_Wenchang	0.166348009
ENSGALG00010017729	MIEF2	3	-0.233325785	0.084613675	-2.757542253	0.005823769	0	0.350097995	0	1	CRA004775;PRJNA827465;Yang2025_Silkie_Wencha	0.166348009
ENSGALG00010009554	ABCB10	3	-0.188355289	0.068304528	-2.757581321	0.005823073	0	1.22978712	0	1	CRA004775;PRJNA827465;Yang2025_Silkie_Wencha	0.166348009
ENSGALG00010018049	SUGCT	3	-0.399491906	0.145000466	-2.755107745	0.005867281	0	0.260753614	0	1	CRA004775;PRJNA827465;Yang2025_Silkie_Wencha	0.166816164
ENSGALG00010005530	IL17D	3	-0.27717899	0.100622336	-2.75464675	0.005875553	0	0.686591853	0	1	CRA004775;PRJNA827465;Yang2025_Silkie_Wencha	0.166816164
ENSGALG00010027198	ECM2	3	-0.249931928	0.090714376	-2.755152376	0.005866481	0	1.906976942	0	0.666666667	CRA004775;PRJNA827465;Yang2025_Silkie_Wencha	0.166816164
ENSGALG00010006472	DCLK1	3	-0.26446434	0.096081242	-2.752507507	0.005914079	0	1.880220216	0	1	CRA004775;PRJNA827465;Yang2025_Silkie_Wencha	0.167238327
ENSGALG00010011670	TMCO6	3	-0.240970911	0.08754177	-2.752639245	0.0059117	0	0.935437638	0	1	CRA004775;PRJNA827465;Yang2025_Silkie_Wencha	0.167238327
ENSGALG00010015188	SULT1C3	2	1.789543305	0.650415093	2.751386497	0.005934358	0	0.312970213	0	1	CRA004775;PRJNA827465	0.167476825
ENSGALG00010007579	GATB	3	-0.185131334	0.06730256	-2.750732432	0.005946219	0	0.605270704	0	1	CRA004775;PRJNA827465;Yang2025_Silkie_Wencha	0.167477273
ENSGALG00010009096	ENSGALG0001000909	2	2.102189075	0.764708623	2.74900663	0.005977618	0	0.473584727	0	1	CRA004775;Yang2025_Silkie_Wenchang	0.168018839
ENSGALG00010027524	ENSGALG0001002752	3	-0.188657046	0.068643216	-2.748371321	0.005989214	0	0.053649665	0	1	CRA004775;PRJNA827465;Yang2025_Silkie_Wencha	0.168018839
ENSGALG00010009002	ENSGALG0001000900	3	-0.204191832	0.074317262	-2.74756934	0.006003881	0	0.690138272	0	1	CRA004775;PRJNA827465;Yang2025_Silkie_Wencha	0.168096785
ENSGALG00010027125	CRYGN	2	1.0791787	0.392926167	2.746517774	0.006023162	0.084984549	1.379626365	27.51660701	1	CRA004775;Yang2025_Silkie_Wenchang	0.168303341
ENSGALG00010011917	HSF2BP	3	-0.442069892	0.161232508	-2.741816146	0.006110053	0	1.365853455	0	1	CRA004775;PRJNA827465;Yang2025_Silkie_Wencha	0.170394557
ENSGALG00010004216	SNX14	3	0.120061188	0.043808922	2.740564769	0.006133369	0	0.886981964	0	1	CRA004775;PRJNA827465;Yang2025_Silkie_Wencha	0.170708085
ENSGALG00010002907	GABRG3	2	-0.606360482	0.22137299	-2.739089728	0.006160956	0	0.060347904	0	1	PRJNA827465;Yang2025_Silkie_Wenchang	0.171139002
ENSGALG00010010310	NT5DC3	3	-0.350695947	0.128165009	-2.736284649	0.006213725	0.021983034	3.601959715	44.4746705	0.666666667	CRA004775;PRJNA827465;Yang2025_Silkie_Wencha	0.17226639
ENSGALG00010010369	ENSGALG0001001036	2	0.492112131	0.180121453	2.732112816	0.006292959	0	0.046983425	0	1	PRJNA827465;Yang2025_Silkie_Wenchang	0.173105341
ENSGALG00010023060	ALDH1A2	3	-0.218494277	0.079958718	-2.732588539	0.006283878	0	1.554034881	0	1	CRA004775;PRJNA827465;Yang2025_Silkie_Wencha	0.173105341
ENSGALG00010012720	ACO2	3	-0.188563292	0.068985619	-2.733371034	0.006268967	0	1.981403515	0	0.666666667	CRA004775;PRJNA827465;Yang2025_Silkie_Wencha	0.173105341
ENSGALG00010012127	ENSGALG0001001212	3	-0.101998704	0.03731625	-2.733358875	0.006269198	0	1.235575088	0	1	CRA004775;PRJNA827465;Yang2025_Silkie_Wencha	0.173105341
ENSGALG00010025092	COL1A1	3	-0.339489818	0.124335853	-2.730425785	0.006325257	0	0.345782724	0	1	CRA004775;PRJNA827465;Yang2025_Silkie_Wencha	0.173655947
ENSGALG00010007999	SDC1	3	0.241310886	0.088451065	2.728185175	0.006368385	0	0.504822271	0	0.666666667	CRA004775;PRJNA827465;Yang2025_Silkie_Wencha	0.17416362
ENSGALG00010016712	ENSGALG0001001671	3	0.190315337	0.069744465	2.728751821	0.006357453	0	0.460248399	0	1	CRA004775;PRJNA827465;Yang2025_Silkie_Wencha	0.17416362
ENSGALG00010015867	PICALML	3	-0.077669703	0.028478342	-2.727325319	0.006385005	0	1.924236995	0	0.666666667	CRA004775;PRJNA827465;Yang2025_Silkie_Wencha	0.174281065
ENSGALG00010003962	UMAD1	3	-0.414942627	0.152185366	-2.72656062	0.00639982	0.040781457	5.267185813	62.02905933	1	CRA004775;PRJNA827465;Yang2025_Silkie_Wencha	0.174348842
ENSGALG00010021793	ENSGALG0001002179	3	-0.272147603	0.099896089	-2.724306879	0.00644366	0	0.973819148	0	0.666666667	CRA004775;PRJNA827465;Yang2025_Silkie_Wencha	0.174401721
ENSGALG00010000606	UBE2QL1	3	-0.211922441	0.07778236	-2.72455657	0.00643879	0	0.996601023	0	1	CRA004775;PRJNA827465;Yang2025_Silkie_Wencha	0.174401721
ENSGALG00010008110	DEF8	3	0.187925851	0.068994077	2.723796884	0.006453618	0	1.54071134	0	0.666666667	CRA004775;PRJNA827465;Yang2025_Silkie_Wencha	0.174401721
ENSGALG00010013760	RPF2	3	-0.165137022	0.060638686	-2.723294842	0.006463435	0	1.753943659	0	1	CRA004775;PRJNA827465;Yang2025_Silkie_Wencha	0.174401721
ENSGALG00010020437	SNX4	3	-0.091673398	0.033661774	-2.723367977	0.006462004	0	1.581247099	0	0.666666667	CRA004775;PRJNA827465;Yang2025_Silkie_Wencha	0.174401721
ENSGALG00010022999	ENSGALG0001002299	2	0.73304679	0.269380378	2.721233061	0.006503889	0	0.48696716	0	1	PRJNA827465;Yang2025_Silkie_Wenchang	0.17449428
ENSGALG00010014222	TBX22	3	-0.561086341	0.206125912	-2.722056314	0.006487709	0	1.262623066	0	1	CRA004775;PRJNA827465;Yang2025_Silkie_Wencha	0.17449428
ENSGALG00010024640	GSDMA	3	-0.323381201	0.118836068	-2.721237805	0.006503796	0	1.465695711	0	1	CRA004775;PRJNA827465;Yang2025_Silkie_Wencha	0.17449428
ENSGALG00010028473	CDC34	3	-0.163942116	0.060259451	-2.720604213	0.006516273	0	1.062306082	0	1	CRA004775;PRJNA827465;Yang2025_Silkie_Wencha	0.17449542
ENSGALG00010022774	A1CF	2	2.220858898	0.816756554	2.719119774	0.00654559	0.424546804	1.426456452	29.8962125	1	CRA004775;PRJNA827465	0.174949147
ENSGALG00010024540	PTPN5	2	0.545220734	0.200676887	2.716908464	0.006589483	0	0.422515663	0	1	PRJNA827465;Yang2025_Silkie_Wenchang	0.17522954
ENSGALG00010010752	ENSGALG0001001075	3	-0.376733613	0.138672291	-2.716718753	0.006593261	0	1.34265681	0	1	CRA004775;PRJNA827465;Yang2025_Silkie_Wencha	0.17522954
ENSGALG00010009491	RBM34	3	0.219184076	0.08064661	2.71783374	0.006571085	0	1.884991325	0	1	CRA004775;PRJNA827465;Yang2025_Silkie_Wencha	0.17522954
ENSGALG00010005263	ENSGALG0001000526	3	-0.684462081	0.25215711	-2.714427049	0.006639052	0.015933006	2.17187101	7.913499886	0.666666667	CRA004775;PRJNA827465;Yang2025_Silkie_Wencha	0.176026335
ENSGALG00010003431	PM20D2	3	-0.366511992	0.135046249	-2.713973879	0.006648141	0	1.094325702	0	0.666666667	CRA004775;PRJNA827465;Yang2025_Silkie_Wencha	0.176026335
ENSGALG00010002956	SCRG1	3	0.43428387	0.160084981	2.712833322	0.006671065	0	0.93145661	0	1	CRA004775;PRJNA827465;Yang2025_Silkie_Wencha	0.176303161
ENSGALG00010018860	AGTR2	3	-0.196414291	0.072431541	-2.711723196	0.006693446	0	1.974787822	0	0.666666667	CRA004775;PRJNA827465;Yang2025_Silkie_Wencha	0.176564618
ENSGALG00010002991	BLB1	3	-0.556943458	0.205506984	-2.710095037	0.006726393	0.044078137	3.032611194	34.05023354	1	CRA004775;PRJNA827465;Yang2025_Silkie_Wencha	0.177103302
ENSGALG00010020760	TXNDC11	3	0.142564577	0.052617004	2.709477277	0.006738932	0	0.71876632	0	0.666666667	CRA004775;PRJNA827465;Yang2025_Silkie_Wencha	0.177103645
ENSGALG00010017300	TRPM7	3	0.145818903	0.053839595	2.708395245	0.006760945	0	1.701890492	0	1	CRA004775;PRJNA827465;Yang2025_Silkie_Wencha	0.177282732
ENSGALG00010008059	ENSGALG0001000805	3	-0.142568202	0.052648783	-2.707910708	0.006770824	0	0.956072538	0	1	CRA004775;PRJNA827465;Yang2025_Silkie_Wencha	0.177282732
ENSGALG00010027907	MKNK2	3	-0.179079823	0.066171385	-2.706303084	0.006803692	0	0.261630568	0	1	CRA004775;PRJNA827465;Yang2025_Silkie_Wencha	0.177814049

ENSGALG00010024879	D2HGDH	3	-0.324712424	0.120053758	-2.704725202	0.006836091	0.024763679	4.765626605	58.03280102	1	CRA004775;PRJNA827465;Yang2025_Silkie_Wencha	0.178331176
ENSGALG00010017796	MXI1	3	-0.122723636	0.045444642	-2.700508351	0.00692336	0	0.509355062	0	1	CRA004775;PRJNA827465;Yang2025_Silkie_Wencha	0.180275112
ENSGALG00010001173	IFT80	3	-0.183247462	0.067886817	-2.6993085	0.006948373	0.000627003	2.085167303	4.084435007	1	CRA004775;PRJNA827465;Yang2025_Silkie_Wencha	0.18059384
ENSGALG00010000307	ZMAT5	3	0.194117688	0.072062884	2.693726316	0.007065816	0	0.587648017	0	0.666666667	CRA004775;PRJNA827465;Yang2025_Silkie_Wencha	0.183309301
ENSGALG00010000813	ENSGALG0001000081	3	-0.445265305	0.165412507	-2.691847875	0.007105735	0	0.700365846	0	1	CRA004775;PRJNA827465;Yang2025_Silkie_Wencha	0.183670906
ENSGALG00010028751	EHMT1	3	-0.105034864	0.039018564	-2.691920267	0.007104193	0	1.565189729	0	1	CRA004775;PRJNA827465;Yang2025_Silkie_Wencha	0.183670906
ENSGALG00010017808	SFRP5	3	0.361846243	0.134695571	2.686400465	0.007222646	0.000327353	2.00990866	0.492990576	1	CRA004775;PRJNA827465;Yang2025_Silkie_Wencha	0.186012746
ENSGALG00010001840	HOXA9	3	0.24018898	0.089398452	2.686724144	0.007215652	0	1.732604899	0	1	CRA004775;PRJNA827465;Yang2025_Silkie_Wencha	0.186012746
ENSGALG00010028074	ENSGALG0001002807	3	-0.589423881	0.21950802	-2.685204302	0.007248548	0	0.165954963	0	1	CRA004775;PRJNA827465;Yang2025_Silkie_Wencha	0.186244407
ENSGALG00010029206	CACNG4	3	0.382059317	0.142324429	2.684425429	0.007265459	0	1.252381176	0	1	CRA004775;PRJNA827465;Yang2025_Silkie_Wencha	0.186244407
ENSGALG00010021131	EDC3	3	-0.225851107	0.08414209	-2.684163275	0.007271159	0.013219902	6.061801576	67.0065083	1	CRA004775;PRJNA827465;Yang2025_Silkie_Wencha	0.186244407
ENSGALG00010015588	BDKRB2	3	0.865197776	0.322670258	2.681368222	0.007332179	0	1.102217487	0	1	CRA004775;PRJNA827465;Yang2025_Silkie_Wencha	0.186498173
ENSGALG00010029543	NSUN5	3	-0.206364252	0.076947126	-2.681896828	0.007320603	0	0.538597268	0	1	CRA004775;PRJNA827465;Yang2025_Silkie_Wencha	0.186498173
ENSGALG00010010981	RIC8B	3	0.149206444	0.055614715	2.682859104	0.007299574	0	0.448968242	0	1	CRA004775;PRJNA827465;Yang2025_Silkie_Wencha	0.186498173
ENSGALG00010005247	THAP7	3	-0.145544467	0.054318744	-2.681292994	0.007333827	0	1.265472906	0	0.666666667	CRA004775;PRJNA827465;Yang2025_Silkie_Wencha	0.186498173
ENSGALG00010019649	TMEM63B	3	0.12573414	0.046927943	2.679302165	0.007377578	0	0.26640295	0	1	CRA004775;PRJNA827465;Yang2025_Silkie_Wencha	0.187273935
ENSGALG00010024622	PRMT8	3	0.350636286	0.131031226	2.675975008	0.00745122	0.018632745	3.14940493	36.49593989	1	CRA004775;PRJNA827465;Yang2025_Silkie_Wencha	0.188130002
ENSGALG00010009106	THSD7B	3	0.308025944	0.115067442	2.676916586	0.007430313	0.01325273	2.958176254	32.39077632	1	CRA004775;PRJNA827465;Yang2025_Silkie_Wencha	0.188130002
ENSGALG00010006128	ACYP1	3	-0.208748469	0.077993717	-2.676478029	0.007440044	0	1.639949684	0	0.666666667	CRA004775;PRJNA827465;Yang2025_Silkie_Wencha	0.188130002
ENSGALG00010009999	GABRB2	2	-0.380349923	0.142232181	-2.674136874	0.007492187	0	0.278263103	0	1	PRJNA827465;Yang2025_Silkie_Wenchang	0.188827141
ENSGALG00010020954	PLIN1	3	-1.515385347	0.567517557	-2.670199943	0.007580609	0.332476619	3.0390564	34.19009927	1	CRA004775;PRJNA827465;Yang2025_Silkie_Wencha	0.189066285
ENSGALG00010008824	GREM2	3	-1.354230267	0.507502798	-2.668419314	0.007620908	0.633815479	13.3694882	85.04056423	1	CRA004775;PRJNA827465;Yang2025_Silkie_Wencha	0.189066285
ENSGALG00010002270	IMMP2L	3	-0.455257858	0.170419286	-2.671521697	0.007550819	0.012135456	2.290075894	12.70095834	1	CRA004775;PRJNA827465;Yang2025_Silkie_Wencha	0.189066285
ENSGALG00010020071	ENSGALG0001002007	3	0.312611219	0.11703957	2.67098742	0.007562848	0.016284319	2.893490956	30.87934158	0.666666667	CRA004775;PRJNA827465;Yang2025_Silkie_Wencha	0.189066285
ENSGALG00010016314	ACER2	3	0.182504135	0.068395012	2.668383688	0.007621716	0	0.359959509	0	1	CRA004775;PRJNA827465;Yang2025_Silkie_Wencha	0.189066285
ENSGALG00010025156	DLAT	3	-0.176563424	0.066124909	-2.670149963	0.007581738	0	0.258044094	0	1	CRA004775;PRJNA827465;Yang2025_Silkie_Wencha	0.189066285
ENSGALG00010028771	SETX	3	0.151268663	0.0565989	2.672646802	0.007525547	0.000789135	2.085225935	4.087131934	1	CRA004775;PRJNA827465;Yang2025_Silkie_Wencha	0.189066285
ENSGALG00010028153	MANF	3	0.143615352	0.053821375	2.668370172	0.007622023	0	1.859062972	0	0.666666667	CRA004775;PRJNA827465;Yang2025_Silkie_Wencha	0.189066285
ENSGALG00010025835	PPP6R3	3	0.09692043	0.036310216	2.66923308	0.007602467	0	0.135200591	0	1	CRA004775;PRJNA827465;Yang2025_Silkie_Wencha	0.189066285
ENSGALG00010021729	RBPJL	3	1.13147084	0.424437529	2.665812428	0.007680253	0.218203876	3.336293833	40.05324171	1	CRA004775;PRJNA827465;Yang2025_Silkie_Wencha	0.18984578
ENSGALG00010022400	NHLRC2	3	0.168421604	0.063178176	2.665819363	0.007680095	0	1.053357554	0	1	CRA004775;PRJNA827465;Yang2025_Silkie_Wencha	0.18984578
ENSGALG00010000435	P2RX6	3	0.478082568	0.179380797	2.665182531	0.007694655	0	1.066244121	0	1	CRA004775;PRJNA827465;Yang2025_Silkie_Wencha	0.189868622
ENSGALG00010003995	MLNR	3	-0.385056775	0.14481438	-2.658967818	0.007838045	0	0.487029922	0	1	CRA004775;PRJNA827465;Yang2025_Silkie_Wencha	0.193069891
ENSGALG00010001972	FAM204A	3	-0.190060738	0.071508202	-2.65788724	0.00786322	0	1.849681627	0	1	CRA004775;PRJNA827465;Yang2025_Silkie_Wencha	0.193353154
ENSGALG00010005429	ZDHHC20	3	-0.139240275	0.05240734	-2.656884976	0.007886635	0	0.989500403	0	1	CRA004775;PRJNA827465;Yang2025_Silkie_Wencha	0.193592235
ENSGALG00010004576	STEAP2	3	-0.221500317	0.083463371	-2.653862588	0.007957623	0	1.919458722	0	0.666666667	CRA004775;PRJNA827465;Yang2025_Silkie_Wencha	0.19499623
ENSGALG00010018764	TMEM177	3	-0.216606139	0.081671714	-2.652156168	0.007997954	0	1.766936175	0	1	CRA004775;PRJNA827465;Yang2025_Silkie_Wencha	0.195645453
ENSGALG00010015498	MID2	3	-0.11947953	0.045067378	-2.651131161	0.008022268	0	0.423943469	0	1	CRA004775;PRJNA827465;Yang2025_Silkie_Wencha	0.195901293
ENSGALG00010009801	ENSGALG0001000980	3	0.935232058	0.353434724	2.646123865	0.008142	0.061685762	2.383148994	16.07742507	1	CRA004775;PRJNA827465;Yang2025_Silkie_Wencha	0.196607443
ENSGALG00010021994	ENSGALG0001002199	2	0.374868648	0.14150295	2.649193632	0.008068409	0	0.753565773	0	1	PRJNA827465;Yang2025_Silkie_Wenchang	0.196607443
ENSGALG00010007811	PRCP	3	-0.292496107	0.110434346	-2.64859728	0.008082658	0.0122233	2.970536209	32.67208817	1	CRA004775;PRJNA827465;Yang2025_Silkie_Wencha	0.196607443
ENSGALG00010010740	CCDC134	3	-0.28799998	0.108849579	-2.645852967	0.008148523	0.011055679	2.66869759	25.05707626	1	CRA004775;PRJNA827465;Yang2025_Silkie_Wencha	0.196607443
ENSGALG00010028175	POMT1	3	-0.19021179	0.07188156	-2.64618339	0.008140567	0	1.902568932	0	1	CRA004775;PRJNA827465;Yang2025_Silkie_Wencha	0.196607443
ENSGALG00010005265	DYNC2H1	3	-0.164710565	0.062207685	-2.647752693	0.008102878	0	1.429218404	0	0.666666667	CRA004775;PRJNA827465;Yang2025_Silkie_Wencha	0.196607443
ENSGALG00010015128	OCRL	3	-0.107449665	0.040607316	-2.646066692	0.008143376	0	0.22916524	0	1	CRA004775;PRJNA827465;Yang2025_Silkie_Wencha	0.196607443
ENSGALG00010029452	ENSGALG0001002945	3	-3.577523608	1.352779676	-2.644572263	0.008179424	2.888687321	4.237117129	52.79809504	1	CRA004775;PRJNA827465;Yang2025_Silkie_Wencha	0.196681761
ENSGALG00010017356	NSMCE1	3	-0.199003392	0.075249618	-2.644576764	0.008179315	0.001549727	2.12200265	5.749410842	0.666666667	CRA004775;PRJNA827465;Yang2025_Silkie_Wencha	0.196681761
ENSGALG00010021795	NDRG3	3	-0.135718432	0.051343393	-2.64334758	0.008209072	0	1.635919988	0	1	CRA004775;PRJNA827465;Yang2025_Silkie_Wencha	0.197059531
ENSGALG00010012631	GLRX2	3	-0.404630851	0.153300858	-2.639455872	0.008303923	0.036824466	4.281533269	53.28776225	1	CRA004775;PRJNA827465;Yang2025_Silkie_Wencha	0.198998596
ENSGALG00010019865	ENSGALG0001001986	3	-0.173145943	0.065620831	-2.638582004	0.008325356	0	1.546672745	0	0.666666667	CRA004775;PRJNA827465;Yang2025_Silkie_Wencha	0.199174639
ENSGALG00010018593	LGALS3	3	0.732900391	0.278026306	2.636097288	0.008386568	0.032997553	2.302495758	13.13773356	1	CRA004775;PRJNA827465;Yang2025_Silkie_Wencha	0.200300149
ENSGALG00010029324	BCAS3	3	-0.143366678	0.054401624	-2.635338172	0.00840535	0	1.296942438	0	1	CRA004775;PRJNA827465;Yang2025_Silkie_Wencha	0.20041018
ENSGALG00010009069	GRID2	2	0.630729913	0.239415849	2.63445346	0.008427286	0	0.853418044	0	1	PRJNA827465;Yang2025_Silkie_Wenchang	0.200584443
ENSGALG00010016940	FGFR3	3	0.36508267	0.138800464	2.630269815	0.008531712	0	0.129709357	0	1	CRA004775;PRJNA827465;Yang2025_Silkie_Wencha	0.200584443
ENSGALG00010020123	ENSGALG0001002012	3	-0.246057049	0.093458426	-2.632796846	0.008468498	0	0.411608621	0	1	CRA004775;PRJNA827465;Yang2025_Silkie_Wencha	0.200584443
ENSGALG00010016873	ENTPD6	3	0.217935274	0.082867445	2.629926315	0.008540338	0	1.755417521	0	0.666666667	CRA004775;PRJNA827465;Yang2025_Silkie_Wencha	0.200584443
ENSGALG00010018042	ZWILCH	3	-0.213051369	0.0809257	-2.632678739	0.008471444	0	0.17756474	0	1	CRA004775;PRJNA827465;Yang2025_Silkie_Wencha	0.200584443
ENSGALG00010024421	DAPK2	3	0.194734059	0.073940785	2.633648791	0.008447281	0	1.253168756	0	1	CRA004775;PRJNA827465;Yang2025_Silkie_Wencha	0.200584443
ENSGALG00010022997	ARHGAP1	3	-0.186199158	0.070780705	-2.630648552	0.008522212	0.005160413	2.95999749	32.43237515	1	CRA004775;PRJNA827465;Yang2025_Silkie_Wencha	0.200584443
ENSGALG00010024426	PHAF1	3	-0.160524347	0.061034046	-2.630078728	0.00853651	0	0.254847663	0	1	CRA004775;PRJNA827465;Yang2025_Silkie_Wencha	0.200584443
ENSGALG00010024152	ZNF423	3	-0.124812737	0.047432966	-2.631350064	0.008504638	0	1.918452948	0	1	CRA004775;PRJNA827465;Yang2025_Silkie_Wencha	0.200584443
ENSGALG00010023330	TSPAN1	2	1.698503397	0.648402014	2.619522088	0.008805307	0.007502593	1.008999245	0.891898043	1	CRA004775;PRJNA827465	0.201779961
ENSGALG00010029676	FADS6	2	1.6583989	0.632751977	2.620930411	0.008769016	0	0.514919128	0	1	PRJNA827465;Yang2025_Silkie_Wenchang	0.201779961
ENSGALG00010005015	NRXN3	2	0.649791921	0.248055779	2.619539537	0.008804857	0	0.92603813	0	1	PRJNA827465;Yang2025_Silkie_Wenchang	0.201779961
ENSGALG00010009073	PRKAR2B	3	-0.592620808	0.226022133	-2.621959185	0.00874259	0	0.384412743	0	1	CRA004775;PRJNA827465;Yang2025_Silkie_Wencha	0.201779961
ENSGALG00010004368	GJD4	2	0.453195293	0.17294181	2.620507402	0.008779902	0	0.027629059	0	1	PRJNA827465;Yang2025_Silkie_Wenchang	0.201779961
ENSGALG00010011578	MYLK4	3	0.391919646	0.149404851	2.623205621	0.008710668	0	1.326953297	0	1	CRA004775;PRJNA827465;Yang2025_Silkie_Wencha	0.201779961
ENSGALG00010027215	ENSGALG0001002721	3	0.322734761	0.122919574	2.625576634	0.008650233	0	1.649140948	0	1	CRA004775;PRJNA827465;Yang2025_Silkie_Wencha	0.201779961
ENSGALG00010025098	ATP5B	3	-0.200374017	0.076426774	-2.621777768	0.008747245	0	1.682222988	0	0.666666667	CRA004775;PRJNA827465;Yang2025_Silkie_Wencha	0.201779961
ENSGALG00010007699	SLC39A6	3	-0.200128494	0.076368509	-2.620563074	0.008778469	0.005602039	2.848614268	29.79042398	0.666666667	CRA004775;PRJNA827465;Yang2025_Silkie_Wencha	0.201779961
ENSGALG00010029636	SPATA20	3	-0.187868315	0.071701314	-2.620151642	0.008789068	0.003859702	2.495141153	19.84421413	1	CRA004775;PRJNA827465;Yang2025_Silkie_Wencha	0.201779961
ENSGALG00010019996	ITGB5	3	-0.170367953	0.064936052	-2.623626606	0.00869991	0	1.573668119	0	0.666666667	CRA004775;PRJNA827465;Yang2025_Silkie_Wencha	0.201779961
ENSGALG00010003025	MFHAS1	3	-0.163625274	0.062406487	-2.621927345	0.008743407	0	0.691301613	0	1	CRA004775;PRJNA827465;Yang2025_Silkie_Wencha	0.201779961
ENSGALG00010029382	GTF2IRD1	3	-0.144771316	0.05513049	-2.625975487	0.008640104	0	0.414446058	0	1	CRA004775;PRJNA827465;Yang2025_Silkie_Wencha	0.201779961
ENSGALG00010023282	SERBP1	3	-0.100833397	0.038492199	-2.619580034	0.008803811	0	0.856766944	0	1	CRA004775;PRJNA827465;Yang2025_Silkie_Wencha	0.201779961
ENSGALG00010005649	TOMM70	3	-0.100365194	0.03823238	-2.625135904	0.008661439	0	1.757727019	0	0.666666667	CRA004775;PRJNA827465;Yang2025_Silkie_Wencha	0.201779961
ENSGALG00010024309	ABCB11	3	0.447572469	0.170908724	2.6187807	0.008824466	0	1.02664217	0	1	CRA004775;PRJNA827465;Yang2025_Silkie_Wencha	0.201891781
ENSGALG00010000247	ENSGALG0001000024	3	0.470701268	0.180165862	2.612599646	0.008985649	0	0.741778241	0	1	CRA004775;PRJNA827465;Yang2025_Silkie_Wencha	0.205247328
ENSGALG00010022051	BAIAP2L1	3	0.327816312	0.125542934	2.611188873	0.009022805	0.024243372	4.196917183	52.34597414	1	CRA004775;PRJNA827465;Yang2025_Silkie_Wencha	0.205415894
ENSGALG00010005630	NIT2	3	-0.198635842	0.076086276	-2.610665858	0.009036614	0	0.389224634	0	1	CRA004775;PRJNA827465;Yang2025_Silkie_Wencha	0.205415894
ENSGALG00010003695	NOA1	3	-0.118085347	0.045215544	-2.611609541	0.009011711	0	0.194432648	0	1	CRA004775;PRJNA827465;Yang2025_Silkie_Wencha	0.205415894
ENSGALG00010011154	SLC26A7	2	1.017879466	0.390483515	2.606715592	0.009141526	0	0.018548161	0	1	PRJNA827465;Yang2025_Silkie_Wenchang	0.206544958
ENSGALG00010012955	RPL39L	3	-0.399670299	0.153413412	-2.60518486	0.00918247	0.037171333	3.982355616	49.778468	1	CRA004775;PRJNA827465;Yang2025_Silkie_Wencha	0.206544958
ENSGALG00010010295	ENSGALG0001001029	3	-0.264583546	0.101569201	-2.604958427	0.009188541	0	0.023487599	0	1	CRA004775;PRJNA827465;Yang2025_Silkie_Wencha	0.206544958
ENSGALG00010000279	ENSGALG0001000027	3	-0.245832249	0.094286126	-2.607300351	0.009125927	0	1.253420255	0	1	CRA004775;PRJNA827465;Yang2025_Silkie_Wencha	0.206544958
ENSGALG00010013372	SPRY4	3	0.236945263	0.090864743	2.607669995	0.009116079	0.004174854	2.307533959	13.32738606	0.666666667	CRA004775;PRJNA827465;Yang2025_Silkie_Wencha	0.206544958
ENSGALG00010002438	RARB	3	0.157862204	0.0605699	2.606281419	0.009153122	0	0.753645358	0	1	CRA004775;PRJNA827465;Yang2025_Silkie_Wencha	0.206544958
ENSGALG00010017033	LDAF1	3	-0.147927105	0.056774274	-2.605530535	0.00917321	0	1.390541486	0	1	CRA004775;PRJNA827465;Yang2025_Silkie_Wencha	0.206544958
ENSGALG00010015356	ENSGALG0001001535	3	-0.320997419	0.123387686	-2.601535295	0.009280751	0.018788305	3.265908581	38.76129871	1	CRA004775;PRJNA827465;Yang2025_Silkie_Wencha	0.208167985
ENSGALG00010024874	JMJD4	3	-0.151157358	0.058110924	-2.601186618	0.00929019	0	1.819971228	0	1	CRA004775;PRJNA827465;Yang2025_Silkie_Wencha	0.208167985
ENSGALG00010022062	MITF	3	0.183462892	0.070563885	2.59995451	0.009323612	0	1.999803562	0	1	CRA004775;PRJNA827465;Yang2025_Silkie_Wencha	0.208586311
ENSGALG00010021110	PKLR	3	-0.253644145	0.097692904	-2.596341538	0.009422236	0	0.437438058	0	0.666666667	CRA004775;PRJNA827465;Yang2025_Silkie_Wencha	0.210127742
ENSGALG00010018786	DSTN	3	0.107074696	0.041234424	2.596730713	0.009411568	0	1.37508803	0	0.666666667	CRA004775;PRJNA827465;Yang2025_Silkie_Wencha	0.210127742
ENSGALG00010012973	GLDC	3	0.669157226	0.258021944	2.59341208	0.009502883	0.114026449	4.745008885	57.85044773	1	CRA004775;PRJNA827465;Yang2025_Silkie_Wencha	0.211259853
ENSGALG00010017018	HP1BP3	3	-0.101597102	0.039168499	-2.593847216	0.009490865	4.39567E-05	2.010859776	0.540056369	1	CRA004775;PRJNA827465;Yang2025_Silkie_Wencha	0.211259853
ENSGALG00010016544	ADCY9	3	-0.102087373	0.039414372	-2.590105266	0.009594659	0	0.956430533	0	1	CRA004775;PRJNA827465;Yang2025_Silkie_Wencha	0.212965275
ENSGALG00010029189	ENSGALG0001002918	3	0.852009688	0.32922224	2.587946941	0.009654985	0	1.516523946	0	1	CRA004775;PRJNA827465;Yang2025_Silkie_Wencha	0.213968393
ENSGALG00010026443	UPB1	3	0.569952123	0.220523559	2.584540741	0.009750878	0	0.268301446	0	1	CRA004775;PRJNA827465;Yang2025_Silkie_Wencha	0.215491536
ENSGALG00010025613	CDKN1A	3	0.341648306	0.132195175	2.584423417	0.009754196	0	1.44151046	0	1	CRA004775;PRJNA827465;Yang2025_Silkie_Wencha	0.215491536
ENSGALG00010027365	PLEKHA6	3	0.301836948	0.116856885	2.582962456	0.009795598	0.007953171	2.296761894	12.92088198	1	CRA004775;PRJNA827465;Yang2025_Silkie_Wencha	0.215984806
ENSGALG00010023180	EEF1A2	3	-0.247917693	0.096013921	-2.582101541	0.009820069	0	0.104983265	0	1	CRA004775;PRJNA827465;Yang2025_Silkie_Wencha	0.215984806
ENSGALG00010017705	MAK16	3	-0.125677946	0.048674248	-2.582021321	0.009822352	0	1.722798231	0	0.666666667	CRA004775;PRJNA827465;Yang2025_Silkie_Wencha	0.215984806
ENSGALG00010027081	PDE4B	3	0.306664161	0.118876308	2.579691158	0.009888871	0	0.826951491	0	0.666666667	CRA004775;PRJNA827465;Yang2025_Silkie_Wencha	0.217109858
ENSGALG00010021122	CYP1A1	2	1.834049334	0.711563995	2.577490356	0.009952066	0.457913195	1.822643859	45.13464631	1	CRA004775;PRJNA827465	0.217148548
ENSGALG00010000476	ENSGALG0001000047	3	-0.498528099	0.193391229	-2.577821662	0.00994253	0.031865726	2.7039435	26.03395745	1	CRA004775;PRJNA827465;Yang2025_Silkie_Wencha	0.217148548
ENSGALG00010012112	MBD2	3	-0.409621471	0.158905398	-2.577769391	0.009944034	0	0.488626626	0	1	CRA004775;PRJNA827465;Yang2025_Silkie_Wencha	0.217148548
ENSGALG00010014928	SUCO	3	0.126788121	0.049180668	2.578007294	0.00993719	0.000696129	2.143630979	6.70035935	0.666666667	CRA004775;PRJNA827465;Yang2025_Silkie_Wencha	0.217148548
ENSGALG00010017581	EFCAB11	2	-0.57249002	0.222469582	-2.573340651	0.010072202	0	0.497728327	0	1	PRJNA827465;Yang2025_Silkie_Wenchang	0.218210914
ENSGALG00010018007	DNAAF9	3	0.441140164	0.171345933	2.574558708	0.010036805	0.061247041	7.867985025	74.58053118	1	CRA004775;PRJNA827465;Yang2025_Silkie_Wencha	0.218210914
ENSGALG00010000200	GAL3ST1	3	0.387112185	0.150443254	2.573144192	0.010077921	0	1.48261876	0	1	CRA004775;PRJNA827465;Yang2025_Silkie_Wencha	0.218210914
ENSGALG00010023800	PEX5	3	-0.114061067	0.044294483	-2.575062587	0.010022195	0	0.387667862	0	1	CRA004775;PRJNA827465;Yang2025_Silkie_Wencha	0.218210914
ENSGALG00010009034	SNX17	3	-0.104845783	0.04074532	-2.573198145	0.01007635	0	1.189020149	0	1	CRA004775;PRJNA827465;Yang2025_Silkie_Wencha	0.218210914
ENSGALG00010024362	DHX8	3	-0.089723809	0.034897752	-2.571048349	0.010139117	0	1.647622518	0	1	CRA004775;PRJNA827465;Yang2025_Silkie_Wencha	0.219200269
ENSGALG00010015190	FBLN5	3	0.397246909	0.154725115	2.567436516	0.010245355	0	1.569810825	0	1	CRA004775;PRJNA827465;Yang2025_Silkie_Wencha	0.221158887
ENSGALG00010013526	SIRPA	3	-0.586732328	0.228782986	-2.564580254	0.010330069	0	1.843934577	0	1	CRA004775;PRJNA827465;Yang2025_Silkie_Wencha	0.221298244
ENSGALG00010002644	KCNF1	2	-0.478909535	0.186675801	-2.565461254	0.010303873	0	0.002460476	0	1	PRJNA827465;Yang2025_Silkie_Wenchang	0.221298244
ENSGALG00010021188	TNRC18	3	-0.14546364	0.056712379	-2.564936316	0.010319474	0	0.202826552	0	1	CRA004775;PRJNA827465;Yang2025_Silkie_Wencha	0.221298244
ENSGALG00010019972	PPP1CB	3	-0.119213323	0.046482789	-2.564676634	0.0103272	0	0.363216361	0	1	CRA004775;PRJNA827465;Yang2025_Silkie_Wencha	0.221298244
ENSGALG00010000323	AP1B1	3	-0.114124339	0.044487104	-2.5653353	0.010307614	0	1.987945515	0	1	CRA004775;PRJNA827465;Yang2025_Silkie_Wencha	0.221298244
ENSGALG00010025138	CFAP91	3	0.713463583	0.278316868	2.56349386	0.010362453	0.116568407	4.103376245	51.25964863	0.666666667	CRA004775;PRJNA827465;Yang2025_Silkie_Wencha	0.22165617
ENSGALG00010005258	ITIH5	3	-0.345531281	0.134847266	-2.562389961	0.010395452	0.014614521	2.618612474	23.623674	0.666666667	CRA004775;PRJNA827465;Yang2025_Silkie_Wencha	0.222026134
ENSGALG00010016565	NVL	3	-0.123315894	0.048145691	-2.561306944	0.010427918	0	1.419947093	0	1	CRA004775;PRJNA827465;Yang2025_Silkie_Wencha	0.222383608
ENSGALG00010008850	CYSTM1	3	-0.199073756	0.077758936	-2.560139928	0.010463003	0	0.320110902	0	1	CRA004775;PRJNA827465;Yang2025_Silkie_Wencha	0.222795773
ENSGALG00010011080	EMP1	3	0.266281062	0.104037229	2.559478609	0.010482931	0	1.773502756	0	1	CRA004775;PRJNA827465;Yang2025_Silkie_Wencha	0.222884447
ENSGALG00010021032	TMC7	3	-0.272701171	0.106649271	-2.556990478	0.010558211	0.014979786	3.652377321	45.24114503	1	CRA004775;PRJNA827465;Yang2025_Silkie_Wencha	0.223999332
ENSGALG00010009811	ENSGALG0001000981	3	0.213041637	0.083343817	2.556178055	0.010582895	0	0.780563008	0	1	CRA004775;PRJNA827465;Yang2025_Silkie_Wencha	0.223999332
ENSGALG00010028123	RASSF1	3	0.212761784	0.08323371	2.556197297	0.01058231	0	0.725146429	0	1	CRA004775;PRJNA827465;Yang2025_Silkie_Wencha	0.223999332
ENSGALG00010006244	EPHA3	3	-0.392852381	0.153784842	-2.554558532	0.010632255	0.035711654	4.263113886	53.08593546	1	CRA004775;PRJNA827465;Yang2025_Silkie_Wencha	0.224707707
ENSGALG00010029522	AKAP1	3	0.119608791	0.046848067	2.553121153	0.010676235	0.000640405	2.178678147	8.201218106	1	CRA004775;PRJNA827465;Yang2025_Silkie_Wencha	0.225080317
ENSGALG00010024737	STK38	3	-0.108545921	0.042517971	-2.552942143	0.010681724	0	0.234283566	0	1	CRA004775;PRJNA827465;Yang2025_Silkie_Wencha	0.225080317
ENSGALG00010021243	DYL1	3	-0.220513347	0.086416674	-2.551745359	0.010718482	0.005989829	2.410779513	17.03928173	1	CRA004775;PRJNA827465;Yang2025_Silkie_Wencha	0.225518785
ENSGALG00010023921	GRK7	3	0.325710338	0.12775252	2.549541402	0.01078647	0	1.620597307	0	1	CRA004775;PRJNA827465;Yang2025_Silkie_Wencha	0.225606366
ENSGALG00010014658	SELENOP1	3	-0.213260068	0.083643801	-2.549621902	0.01078398	0	0.068662898	0	1	CRA004775;PRJNA827465;Yang2025_Silkie_Wencha	0.225606366
ENSGALG00010020080	SLC4A1AP	3	0.114471221	0.044893131	2.549860487	0.010776603	0.000669889	2.173257977	7.972269245	0.666666667	CRA004775;PRJNA827465;Yang2025_Silkie_Wencha	0.225606366
ENSGALG00010027775	MVB12B	3	-0.101910255	0.03995695	-2.550501327	0.010756811	0	1.169405836	0	0.666666667	CRA004775;PRJNA827465;Yang2025_Silkie_Wencha	0.225606366
ENSGALG00010020346	PCBD1	3	0.415751265	0.163150691	2.548265426	0.010826007	0	1.307726555	0	1	CRA004775;PRJNA827465;Yang2025_Silkie_Wencha	0.226025242
ENSGALG00010019738	ADRA2A	3	-0.370690104	0.14551069	-2.547511133	0.010849439	0	1.771240833	0	1	CRA004775;PRJNA827465;Yang2025_Silkie_Wencha	0.226025242
ENSGALG00010019297	SLC25A26	3	-0.254405386	0.099878859	-2.547139488	0.010861001	0	1.854466861	0	1	CRA004775;PRJNA827465;Yang2025_Silkie_Wencha	0.226025242
ENSGALG00010002387	MCMBP	3	0.114707129	0.045039066	2.546836312	0.010870441	0	0.611573762	0	1	CRA004775;PRJNA827465;Yang2025_Silkie_Wencha	0.226025242
ENSGALG00010014199	LNX1	3	0.385176413	0.151328377	2.545301954	0.010918328	0	1.661923221	0	1	CRA004775;PRJNA827465;Yang2025_Silkie_Wencha	0.226687574
ENSGALG00010027938	ENSGALG0001002793	3	0.217996396	0.085717799	2.543187041	0.010984641	0	1.261507928	0	1	CRA004775;PRJNA827465;Yang2025_Silkie_Wencha	0.22772997
ENSGALG00010005509	COCH	3	0.370123863	0.145727233	2.539840051	0.011090318	0	1.050879153	0	1	CRA004775;PRJNA827465;Yang2025_Silkie_Wencha	0.229584187
ENSGALG00010021617	SORBS1	3	0.236641335	0.093205809	2.53891188	0.011119783	0.007546757	2.556800042	21.77722281	1	CRA004775;PRJNA827465;Yang2025_Silkie_Wencha	0.229857614
ENSGALG00010019471	NUBP1	3	-0.268863311	0.105970575	-2.537150636	0.011175885	0.01244739	2.969286823	32.64375862	1	CRA004775;PRJNA827465;Yang2025_Silkie_Wencha	0.230008506
ENSGALG00010008007	CBR4	3	-0.158654095	0.062521035	-2.537611462	0.011161182	0	1.039416032	0	1	CRA004775;PRJNA827465;Yang2025_Silkie_Wencha	0.230008506
ENSGALG00010024437	HERC1	3	-0.09368494	0.036918539	-2.537612342	0.011161154	0	0.306472731	0	0.666666667	CRA004775;PRJNA827465;Yang2025_Silkie_Wencha	0.230008506
ENSGALG00010024950	NCAM1	3	-0.165440718	0.065244379	-2.535708382	0.011222014	0.000721159	2.092245086	4.408904428	1	CRA004775;PRJNA827465;Yang2025_Silkie_Wencha	0.230622175
ENSGALG00010028242	ENSGALG0001002824	3	0.337392986	0.133123265	2.53444044	0.011262707	0	1.464065716	0	1	CRA004775;PRJNA827465;Yang2025_Silkie_Wencha	0.23112252
ENSGALG00010015894	CEP78	3	0.392315163	0.154846652	2.533572142	0.01129065	0.021560863	2.811300956	28.85855939	1	CRA004775;PRJNA827465;Yang2025_Silkie_Wencha	0.231360139
ENSGALG00010007010	ENSGALG0001000701	2	1.679429017	0.664830873	2.526099621	0.011533677	0	0.388445323	0	1	PRJNA827465;Yang2025_Silkie_Wenchang	0.232637699
ENSGALG00010025330	USH1C	3	0.964744179	0.381911221	2.526095401	0.011533816	0.276575141	6.075649441	67.08170839	1	CRA004775;PRJNA827465;Yang2025_Silkie_Wencha	0.232637699
ENSGALG00010012892	ORMDL1	3	-0.449242288	0.177493971	-2.531028439	0.011372863	0.058665894	5.373090003	62.77747072	1	CRA004775;PRJNA827465;Yang2025_Silkie_Wencha	0.232637699
ENSGALG00010005354	M1AP	3	0.379005035	0.149969229	2.527218669	0.011496989	0.000420221	2.011785532	0.585824498	1	CRA004775;PRJNA827465;Yang2025_Silkie_Wencha	0.232637699
ENSGALG00010022392	EMILIN3	3	0.278697825	0.11032774	2.526090213	0.011533986	0.005716832	2.283898539	12.4304357	0.666666667	CRA004775;PRJNA827465;Yang2025_Silkie_Wencha	0.232637699
ENSGALG00010027086	CACNA1S	3	-0.244058227	0.096606651	-2.52630875	0.011526813	0	0.608116994	0	1	CRA004775;PRJNA827465;Yang2025_Silkie_Wencha	0.232637699
ENSGALG00010013576	CAMK2A	3	-0.225718265	0.089290053	-2.527921723	0.011473993	0	0.009595152	0	1	CRA004775;PRJNA827465;Yang2025_Silkie_Wencha	0.232637699
ENSGALG00010011702	NUP205	3	-0.179104179	0.070843171	-2.528178474	0.011465605	0.007433863	4.389954448	54.44144071	0.666666667	CRA004775;PRJNA827465;Yang2025_Silkie_Wencha	0.232637699
ENSGALG00010025642	RPS8	3	-0.145584037	0.057629444	-2.526209304	0.011530077	0	1.921279677	0	1	CRA004775;PRJNA827465;Yang2025_Silkie_Wencha	0.232637699
ENSGALG00010011312	VPS13B	3	-0.122344598	0.048425332	-2.526458617	0.011521896	0	0.703257407	0	0.666666667	CRA004775;PRJNA827465;Yang2025_Silkie_Wencha	0.232637699
ENSGALG00010024106	WDR33	3	-0.081831338	0.032375951	-2.527534612	0.01148665	0	0.786463351	0	0.666666667	CRA004775;PRJNA827465;Yang2025_Silkie_Wencha	0.232637699
ENSGALG00010017608	RBBP4	3	-0.299680688	0.118754758	-2.523525734	0.011618456	0.03317443	10.74828172	81.39237459	1	CRA004775;PRJNA827465;Yang2025_Silkie_Wencha	0.232681791
ENSGALG00010013487	ENSGALG0001001348	3	0.274988551	0.108909723	2.524921966	0.011572398	0	0.394759535	0	1	CRA004775;PRJNA827465;Yang2025_Silkie_Wencha	0.232681791
ENSGALG00010001668	UBE2E1	3	-0.249725844	0.098951801	-2.523711958	0.011612303	0.016361272	4.681284281	57.27668136	1	CRA004775;PRJNA827465;Yang2025_Silkie_Wencha	0.232681791
ENSGALG00010009237	DENND3	3	0.23912749	0.094750141	2.523769215	0.011610412	0	0.511924604	0	1	CRA004775;PRJNA827465;Yang2025_Silkie_Wencha	0.232681791
ENSGALG00010028131	TMEM161A	3	-0.231115818	0.091517866	-2.525362825	0.011557889	0	1.566644226	0	1	CRA004775;PRJNA827465;Yang2025_Silkie_Wencha	0.232681791
ENSGALG00010016550	FBLIM1	3	-0.277956082	0.110195113	-2.522399349	0.01165573	0	0.577284013	0	1	CRA004775;PRJNA827465;Yang2025_Silkie_Wencha	0.232768881
ENSGALG00010000714	FBXL7	3	-0.133810435	0.053043921	-2.522634692	0.011647933	0	1.144519468	0	0.666666667	CRA004775;PRJNA827465;Yang2025_Silkie_Wencha	0.232768881
ENSGALG00010011895	PHF21B	2	1.023084279	0.405962561	2.520144409	0.01173067	0.045715285	1.140789533	12.3414117	1	PRJNA827465;Yang2025_Silkie_Wenchang	0.233605547
ENSGALG00010011422	KATNAL2	3	0.460296636	0.182640697	2.520230396	0.011727804	0	0.725432969	0	1	CRA004775;PRJNA827465;Yang2025_Silkie_Wencha	0.233605547
ENSGALG00010003525	NOL10	3	-0.12499854	0.049630353	-2.518590587	0.011782557	0	0.972849273	0	1	CRA004775;PRJNA827465;Yang2025_Silkie_Wencha	0.234308825
ENSGALG00010002705	JADE3	3	0.098926093	0.039288481	2.517941377	0.011804297	0	0.01298049	0	1	CRA004775;PRJNA827465;Yang2025_Silkie_Wencha	0.234411446
ENSGALG00010003126	ENSGALG0001000312	3	1.058055769	0.420724152	2.514844378	0.011908494	0.304503081	5.129417874	61.00922075	1	CRA004775;PRJNA827465;Yang2025_Silkie_Wencha	0.234463182
ENSGALG00010012283	CUPIN1A	3	0.835941552	0.332350216	2.515242992	0.011895037	0	0.047189565	0	1	CRA004775;PRJNA827465;Yang2025_Silkie_Wencha	0.234463182
ENSGALG00010011841	KCNMB4	3	-0.302162772	0.120172163	-2.514415681	0.011922981	0	1.580987643	0	1	CRA004775;PRJNA827465;Yang2025_Silkie_Wencha	0.234463182
ENSGALG00010011478	HDAC3	3	-0.300875812	0.11959	-2.515894398	0.011873075	0.031551837	7.957742633	74.86724449	1	CRA004775;PRJNA827465;Yang2025_Silkie_Wencha	0.234463182
ENSGALG00010002819	BMP3	3	-0.274855772	0.10919204	-2.517177736	0.011829914	0	0.39210574	0	1	CRA004775;PRJNA827465;Yang2025_Silkie_Wencha	0.234463182
ENSGALG00010004236	DBF4	3	0.198988152	0.079109708	2.515344272	0.01189162	0	1.329124669	0	1	CRA004775;PRJNA827465;Yang2025_Silkie_Wencha	0.234463182
ENSGALG00010010922	DYM	3	-0.121057082	0.048132876	-2.515060238	0.011901205	0	0.45646361	0	1	CRA004775;PRJNA827465;Yang2025_Silkie_Wencha	0.234463182
ENSGALG00010011839	CBS	3	-0.508768245	0.202405786	-2.513605243	0.011950412	0.061461605	4.433753697	54.89149518	1	CRA004775;PRJNA827465;Yang2025_Silkie_Wencha	0.234676207
ENSGALG00010024702	INAVA	3	0.643250453	0.256041471	2.512290102	0.011995044	0.084947968	3.519059292	43.16662966	1	CRA004775;PRJNA827465;Yang2025_Silkie_Wencha	0.235225969
ENSGALG00010006598	ENSGALG0001000659	3	0.6616178	0.263473345	2.51113751	0.012034281	0.163925801	10.13540132	80.26718492	1	CRA004775;PRJNA827465;Yang2025_Silkie_Wencha	0.235668556

ENSGALG00010011326	COX6C	3	-0.154934182	0.061733083	-2.509743165	0.0120819	0	0.770363386	0	1	CRA004775;PRJNA827465;Yang2025_Silkie_Wencha	0.236273837
ENSGALG00010000431	AIFM3	3	1.419089913	0.566151649	2.506554411	0.012191429	0.280132491	1.709640939	41.5081859	1	PRJNA827465;Yang2025_Silkie_Wenchang	0.237758094
ENSGALG00010000346	ENSGALG0001000034(	3	-0.139919058	0.055818896	-2.50666113	0.012187749	0	0.198409081	0	1	CRA004775;PRJNA827465;Yang2025_Silkie_Wencha	0.237758094
ENSGALG00010012085	IPO8	3	0.144009707	0.057479371	2.505415512	0.012230762	0.002249662	2.331330056	14.21206129	1	CRA004775;PRJNA827465;Yang2025_Silkie_Wencha	0.238196607
ENSGALG00010023342	FITM2	3	-0.181921663	0.072647746	-2.50416114	0.012274212	0	0.192512322	0	1	CRA004775;PRJNA827465;Yang2025_Silkie_Wencha	0.238714004
ENSGALG00010016303	ENSGALG0001001630:	3	0.817397575	0.326640465	2.502438193	0.012334116	0.270643824	14.50016635	86.20705479	1	CRA004775;PRJNA827465;Yang2025_Silkie_Wencha	0.238808216
ENSGALG00010011667	ENSGALG0001001166	3	-0.698816107	0.27937191	-2.501382856	0.012370936	0.073575554	2.895415399	30.92528275	0.666666667	CRA004775;PRJNA827465;Yang2025_Silkie_Wencha	0.238808216
ENSGALG00010016979	PLSCR5	2	-0.668767079	0.267460741	-2.500430816	0.012404236	0.057699633	1.674194291	40.269776	1	PRJNA827465;Yang2025_Silkie_Wenchang	0.238808216
ENSGALG00010025373	CYB5RL	3	-0.331441906	0.132460221	-2.502199554	0.012342433	0.026788631	3.860589067	48.19443444	1	CRA004775;PRJNA827465;Yang2025_Silkie_Wencha	0.238808216
ENSGALG00010012230	PTPRQ	2	-0.293482915	0.117307246	-2.501831095	0.012355285	0	0.707057535	0	1	PRJNA827465;Yang2025_Silkie_Wenchang	0.238808216
ENSGALG00010012144	HI-01	3	-0.205772678	0.08232009	-2.499665376	0.012431066	0	1.073001162	0	1	CRA004775;PRJNA827465;Yang2025_Silkie_Wencha	0.238808216
ENSGALG00010014474	PTPN1	3	-0.135607076	0.054241658	-2.500054044	0.012417436	0.002396585	2.617716345	23.59752791	1	CRA004775;PRJNA827465;Yang2025_Silkie_Wencha	0.238808216
ENSGALG00010007798	HGSNAT	3	-0.129964701	0.051953372	-2.501564302	0.012364599	0	0.403256651	0	1	CRA004775;PRJNA827465;Yang2025_Silkie_Wencha	0.238808216
ENSGALG00010016832	DYNC1H1	3	-0.12131301	0.048511004	-2.500731791	0.0123937	0.000400408	2.062439972	3.029986972	0.666666667	CRA004775;PRJNA827465;Yang2025_Silkie_Wencha	0.238808216
ENSGALG00010013449	LCA5L	3	0.595292334	0.238491745	2.496071026	0.012557745	0.062888089	3.13142964	36.13140865	1	CRA004775;PRJNA827465;Yang2025_Silkie_Wencha	0.240588026
ENSGALG00010006220	SGCG	3	-0.179044421	0.071725966	-2.496228781	0.012552162	0	1.144162068	0	0.666666667	CRA004775;PRJNA827465;Yang2025_Silkie_Wencha	0.240588026
ENSGALG00010007304	F5	3	0.62033804	0.248733765	2.493984044	0.012631822	0.086563126	3.723876523	46.29252641	1	CRA004775;PRJNA827465;Yang2025_Silkie_Wencha	0.240877762
ENSGALG00010008877	FNDC4	3	-0.298777309	0.119774136	-2.494506069	0.012613257	0.005771806	2.285002735	12.47275248	1	CRA004775;PRJNA827465;Yang2025_Silkie_Wencha	0.240877762
ENSGALG00010001440	CDCA7L	3	-0.129502158	0.051915381	-2.494485278	0.012613996	0	0.831613454	0	0.666666667	CRA004775;PRJNA827465;Yang2025_Silkie_Wencha	0.240877762
ENSGALG00010023289	NFRKB	3	-0.119276268	0.047830546	-2.493725835	0.012641014	0	0.789202318	0	1	CRA004775;PRJNA827465;Yang2025_Silkie_Wencha	0.240877762
ENSGALG00010004577	ZPLD1	3	-0.813899506	0.327208372	-2.487404281	0.012867908	0	0.287569133	0	1	CRA004775;PRJNA827465;Yang2025_Silkie_Wencha	0.242622177
ENSGALG00010026124	PACSIN1	3	0.331513047	0.133279564	2.487350935	0.012869838	0	0.867688634	0	1	CRA004775;PRJNA827465;Yang2025_Silkie_Wencha	0.242622177
ENSGALG00010017638	GFRA2	3	0.309108471	0.124189458	2.489007346	0.012810032	0	0.144695644	0	1	CRA004775;PRJNA827465;Yang2025_Silkie_Wencha	0.242622177
ENSGALG00010022471	ST6GALNAC5	3	-0.253979197	0.101992402	-2.490177628	0.012767927	0	1.317430544	0	0.666666667	CRA004775;PRJNA827465;Yang2025_Silkie_Wencha	0.242622177
ENSGALG00010000050	CIB1	3	-0.18697104	0.07511981	-2.488971138	0.012811337	0	0.201304442	0	1	CRA004775;PRJNA827465;Yang2025_Silkie_Wencha	0.242622177
ENSGALG00010008067	MPC2	3	0.170157571	0.068393394	2.487924086	0.012849116	0	0.431195007	0	0.666666667	CRA004775;PRJNA827465;Yang2025_Silkie_Wencha	0.242622177
ENSGALG00010025569	PPTC7	3	0.149686016	0.060156768	2.488265598	0.012836783	0.003737998	3.056962543	34.57558044	1	CRA004775;PRJNA827465;Yang2025_Silkie_Wencha	0.242622177
ENSGALG00010009197	FH	3	-0.1315261	0.052858331	-2.488275678	0.012836419	0	0.879630581	0	0.666666667	CRA004775;PRJNA827465;Yang2025_Silkie_Wencha	0.242622177
ENSGALG00010007106	SLC7A1	3	-0.403613927	0.162486518	-2.483984096	0.012992161	0.049549922	7.217957397	72.29132994	1	CRA004775;PRJNA827465;Yang2025_Silkie_Wencha	0.244602084
ENSGALG00010021105	ENSGALG0001002110:	2	1.740918095	0.701083814	2.483181126	0.013021486	0.644702401	2.876860389	65.23988429	1	CRA004775;Yang2025_Silkie_Wenchang	0.244828176
ENSGALG00010002458	ENSGALG0001000245:	3	0.177306475	0.07145564	2.481311984	0.013089975	0	0.679692236	0	1	CRA004775;PRJNA827465;Yang2025_Silkie_Wencha	0.245463066
ENSGALG00010025582	DMAP1	3	-0.106568847	0.042944707	-2.481536244	0.013081741	0	1.761372631	0	1	CRA004775;PRJNA827465;Yang2025_Silkie_Wencha	0.245463066
ENSGALG00010021143	ABCA4	2	0.600778383	0.24233006	2.479174	0.013168705	0	0.947968777	0	1	PRJNA827465;Yang2025_Silkie_Wenchang	0.245976529
ENSGALG00010023094	CCDC32	3	-0.368903928	0.148787289	-2.479404862	0.013160183	0.050820483	9.78145421	79.55314254	1	CRA004775;PRJNA827465;Yang2025_Silkie_Wencha	0.245976529
ENSGALG00010017778	PPP3R1	3	-0.179946126	0.072587362	-2.479151176	0.013169548	0.007239697	3.871285969	48.33758043	0.666666667	CRA004775;PRJNA827465;Yang2025_Silkie_Wencha	0.245976529
ENSGALG00010028497	ADMP	2	1.23892015	0.500180303	2.476947097	0.013251154	0	0.111411152	0	1	CRA004775;Yang2025_Silkie_Wenchang	0.247174233
ENSGALG00010016916	ACYP2	3	-0.423883653	0.17135875	-2.473662152	0.01337361	0.030328538	2.941189162	32.00029342	0.666666667	CRA004775;PRJNA827465;Yang2025_Silkie_Wencha	0.248474994
ENSGALG00010029943	HS3ST3A1	3	0.316992624	0.128101553	2.474541607	0.013340728	0	0.727947243	0	0.666666667	CRA004775;PRJNA827465;Yang2025_Silkie_Wencha	0.248474994
ENSGALG00010014389	COL6A2	3	-0.167823633	0.067839762	-2.473824093	0.01336755	0	0.145958779	0	0.666666667	CRA004775;PRJNA827465;Yang2025_Silkie_Wencha	0.248474994
ENSGALG00010028806	SPACA9	3	0.445853962	0.180323146	2.472527636	0.013416134	0	0.974960849	0	1	CRA004775;PRJNA827465;Yang2025_Silkie_Wencha	0.248937951
ENSGALG00010020776	MCOLN3	3	0.738228808	0.29876071	2.470970189	0.013474705	0	0.4174098	0	1	CRA004775;PRJNA827465;Yang2025_Silkie_Wencha	0.249697058
ENSGALG00010029320	TBX4	2	1.035148958	0.419180234	2.469460328	0.013531702	0	0.935003312	0	1	PRJNA827465;Yang2025_Silkie_Wenchang	0.250097696
ENSGALG00010004139	PPEF2	2	0.904104189	0.366049419	2.469889074	0.013515155	0	0.148852611	0	1	CRA004775;Yang2025_Silkie_Wenchang	0.250097696
ENSGALG00010016978	FAM181A	3	-0.529431988	0.214542251	-2.467728323	0.013597347	0.064282537	3.965205443	49.56125152	0.666666667	CRA004775;PRJNA827465;Yang2025_Silkie_Wencha	0.250982893
ENSGALG00010012107	CLCC1	3	-0.155828364	0.063163151	-2.467077104	0.013622102	0	1.652862623	0	0.666666667	CRA004775;PRJNA827465;Yang2025_Silkie_Wencha	0.251111998
ENSGALG00010003205	ENSGALG0001000320:	3	-0.345852865	0.140214693	-2.466595037	0.013640453	0	0.787297455	0	0.666666667	CRA004775;PRJNA827465;Yang2025_Silkie_Wencha	0.251122863
ENSGALG00010024834	INSIG1	3	0.330761647	0.134219529	2.464333241	0.013726842	0	0.45690585	0	1	CRA004775;PRJNA827465;Yang2025_Silkie_Wencha	0.252384684
ENSGALG00010002122	ENSGALG0001000212:	3	-1.047061578	0.425407412	-2.461314843	0.013842883	0	0.81467262	0	1	CRA004775;PRJNA827465;Yang2025_Silkie_Wencha	0.253663346
ENSGALG00010030107	RPTOR	3	-0.204932108	0.083256283	-2.461461166	0.013837238	0.007843726	3.212670071	37.74648638	1	CRA004775;PRJNA827465;Yang2025_Silkie_Wencha	0.253663346
ENSGALG00010023879	EI24	3	-0.118473834	0.04813808	-2.461125033	0.013850209	0	1.448282746	0	1	CRA004775;PRJNA827465;Yang2025_Silkie_Wencha	0.253663346
ENSGALG00010023716	FASTKD1	3	-0.172915614	0.070306047	-2.459465892	0.013906911	0	0.558006495	0	1	CRA004775;PRJNA827465;Yang2025_Silkie_Wencha	0.254372338
ENSGALG00010002423	ENSGALG0001000242:	3	-0.73752235	0.30018313	-2.456908056	0.014013855	0	1.275029848	0	1	CRA004775;PRJNA827465;Yang2025_Silkie_Wencha	0.25441759
ENSGALG00010025919	RGS9BP	3	0.551213579	0.224473158	2.455587942	0.014065433	0	0.593413808	0	1	CRA004775;PRJNA827465;Yang2025_Silkie_Wencha	0.25441759
ENSGALG00010012915	ENSGALG0001001291:	3	-0.413368275	0.168241516	-2.45699329	0.01401053	0	1.766288887	0	1	CRA004775;PRJNA827465;Yang2025_Silkie_Wencha	0.25441759
ENSGALG00010020607	ENSGALG0001002060:	3	-0.233233133	0.094994703	-2.455437256	0.014071331	0	0.286276776	0	1	CRA004775;PRJNA827465;Yang2025_Silkie_Wencha	0.25441759
ENSGALG00010017734	TOP3A	3	-0.219459202	0.089299748	-2.457556798	0.013988569	0	0.003034299	0	1	CRA004775;PRJNA827465;Yang2025_Silkie_Wencha	0.25441759
ENSGALG00010029241	STXBP4	3	-0.171155324	0.069669073	-2.456690127	0.014022358	0	1.690531361	0	1	CRA004775;PRJNA827465;Yang2025_Silkie_Wencha	0.25441759
ENSGALG00010011485	MTM1	3	0.13685462	0.055724274	2.455924695	0.01405226	0	1.604193149	0	1	CRA004775;PRJNA827465;Yang2025_Silkie_Wencha	0.25441759
ENSGALG00010024250	AP2A2	3	-0.096609882	0.039300017	-2.458259373	0.01396123	0.00123586	2.591035529	22.81078442	1	CRA004775;PRJNA827465;Yang2025_Silkie_Wencha	0.25441759
ENSGALG00010015055	ZNF335	3	-0.077224226	0.031445971	-2.455774899	0.014058119	0	1.472792411	0	1	CRA004775;PRJNA827465;Yang2025_Silkie_Wencha	0.25441759
ENSGALG00010020816	TRMT2A	3	-0.120135991	0.048961952	-2.453660149	0.014141056	0	0.926937608	0	1	CRA004775;PRJNA827465;Yang2025_Silkie_Wencha	0.25528843
ENSGALG00010029340	NDEL1	3	0.105528183	0.043014961	2.45329024	0.014155607	0	1.71737391	0	0.666666667	CRA004775;PRJNA827465;Yang2025_Silkie_Wencha	0.25528843
ENSGALG00010029747	TRPV3	3	0.668424537	0.272554527	2.452442678	0.014188998	0.130125469	5.165716734	61.28320419	1	CRA004775;PRJNA827465;Yang2025_Silkie_Wencha	0.2554927
ENSGALG00010016877	UBE2D3	3	-0.101485867	0.041387565	-2.452085931	0.014203074	0	0.078030767	0	1	CRA004775;PRJNA827465;Yang2025_Silkie_Wencha	0.2554927
ENSGALG00010014274	FIRRM	3	-0.200367895	0.081829507	-2.448601996	0.014341182	0	1.431047289	0	1	CRA004775;PRJNA827465;Yang2025_Silkie_Wencha	0.257649265
ENSGALG00010020100	MOCS1	3	-0.257645205	0.105314404	-2.446438432	0.014427544	0.011490861	2.920681125	31.52282243	0.666666667	CRA004775;PRJNA827465;Yang2025_Silkie_Wencha	0.258871877
ENSGALG00010020392	SLC23A2	3	0.20758134	0.084892813	2.445216888	0.014476506	0	0.091363275	0	1	CRA004775;PRJNA827465;Yang2025_Silkie_Wencha	0.259131384
ENSGALG00010024904	ALS2CL	3	0.190029517	0.077716497	2.445163187	0.014478661	0	1.814034595	0	0.666666667	CRA004775;PRJNA827465;Yang2025_Silkie_Wencha	0.259131384
ENSGALG00010024350	PGM1	3	-0.264637472	0.108271286	-2.444207347	0.014517081	0	0.382998624	0	0.666666667	CRA004775;PRJNA827465;Yang2025_Silkie_Wencha	0.259162881
ENSGALG00010027136	MAPKAPK2	3	-0.144235572	0.059002186	-2.444580153	0.014502085	0	0.67553	0	0.666666667	CRA004775;PRJNA827465;Yang2025_Silkie_Wencha	0.259162881
ENSGALG00010003869	LY96	3	-0.408723461	0.167265372	-2.443562922	0.014543033	0	0.868369369	0	1	CRA004775;PRJNA827465;Yang2025_Silkie_Wencha	0.2592988
ENSGALG00010002345	SCML2	3	-0.218492574	0.089438945	-2.442924328	0.014568792	0	1.771412344	0	0.666666667	CRA004775;PRJNA827465;Yang2025_Silkie_Wencha	0.259322464
ENSGALG00010010964	INTS9	3	-0.177561023	0.072692827	-2.44262096	0.014581042	0.004261536	2.673197763	25.18323831	1	CRA004775;PRJNA827465;Yang2025_Silkie_Wencha	0.259322464
ENSGALG00010014224	NMUR2	2	-0.905368795	0.371011871	-2.440269073	0.014676326	0	0.730644243	0	1	PRJNA827465;Yang2025_Silkie_Wenchang	0.260035804
ENSGALG00010008225	CELSR1	3	-0.216661582	0.088770035	-2.440706282	0.014658572	0	0.172549444	0	1	CRA004775;PRJNA827465;Yang2025_Silkie_Wencha	0.260035804
ENSGALG00010010456	BMP6	3	0.214616376	0.087938238	2.440535335	0.014665511	0	0.243470342	0	1	CRA004775;PRJNA827465;Yang2025_Silkie_Wencha	0.260035804
ENSGALG00010006053	TMEM243	3	-0.378228062	0.155096807	-2.438657948	0.014741915	0	0.52606676	0	1	CRA004775;PRJNA827465;Yang2025_Silkie_Wencha	0.260871005
ENSGALG00010015466	NMRK1	3	0.276740025	0.113568437	2.436768813	0.01481915	0	1.552771257	0	1	CRA004775;PRJNA827465;Yang2025_Silkie_Wencha	0.261909958
ENSGALG00010027751	ANGPTL4	3	0.280518238	0.115164928	2.43579572	0.014859073	0	0.461599499	0	1	CRA004775;PRJNA827465;Yang2025_Silkie_Wencha	0.262287688
ENSGALG00010002197	GSTA2	3	0.469686455	0.192966776	2.43402758	0.014931857	0.031926626	2.764182617	27.64588027	0.666666667	CRA004775;PRJNA827465;Yang2025_Silkie_Wencha	0.263243804
ENSGALG00010021040	FGF8	2	0.937830114	0.385603198	2.432111867	0.01501107	0	0.541250097	0	1	PRJNA827465;Yang2025_Silkie_Wenchang	0.263948695
ENSGALG00010011984	FN1	3	-0.276697006	0.113749059	-2.432521267	0.014994111	0	1.064581976	0	1	CRA004775;PRJNA827465;Yang2025_Silkie_Wencha	0.263948695
ENSGALG00010018427	PIGF	3	0.215383961	0.088573143	2.431707327	0.015027845	0	1.539751104	0	1	CRA004775;PRJNA827465;Yang2025_Silkie_Wencha	0.263948695
ENSGALG00010027076	VDHAP	3	0.208232162	0.08566207	2.430856061	0.015063197	0	0.362639343	0	1	CRA004775;PRJNA827465;Yang2025_Silkie_Wencha	0.264241374
ENSGALG00010011339	ENSGALG0001001133!	3	-1.099313963	0.452496924	-2.429439638	0.015122183	0.396059274	5.681305874	64.79682586	1	CRA004775;PRJNA827465;Yang2025_Silkie_Wencha	0.264885614
ENSGALG00010021800	TMEM82	3	0.568669893	0.234152856	2.428626765	0.015156126	0.067859788	3.390487699	41.01143619	1	CRA004775;PRJNA827465;Yang2025_Silkie_Wencha	0.264885614
ENSGALG00010018510	ENSGALG0001001851(	3	-0.15875742	0.065364261	-2.428810764	0.015148437	0	0.209785653	0	1	CRA004775;PRJNA827465;Yang2025_Silkie_Wencha	0.264885614
ENSGALG00010020449	PRPF19	3	-0.158939097	0.065481998	-2.427218194	0.015215102	0.004902639	3.01941168	33.76193073	1	CRA004775;PRJNA827465;Yang2025_Silkie_Wencha	0.26558806
ENSGALG00010016069	GALK2	3	-0.194870739	0.080305757	-2.426609815	0.015240637	0	1.972357339	0	0.666666667	CRA004775;PRJNA827465;Yang2025_Silkie_Wencha	0.265705756
ENSGALG00010009129	ENSGALG0001000912!	3	0.974401578	0.402011101	2.423817588	0.015358318	0.343520056	7.0837558	71.7663898	1	CRA004775;PRJNA827465;Yang2025_Silkie_Wencha	0.266019863
ENSGALG00010022134	CH25H	3	-0.40128167	0.16560942	-2.423060665	0.015390356	0.028230801	2.993233386	33.18262421	1	CRA004775;PRJNA827465;Yang2025_Silkie_Wencha	0.266019863
ENSGALG00010015793	RSRP1	3	0.338229624	0.139475095	2.425018065	0.015307625	0.023029877	3.179992876	37.10677734	1	CRA004775;PRJNA827465;Yang2025_Silkie_Wencha	0.266019863
ENSGALG00010000788	GATD3AL1	3	-0.160044563	0.06602338	-2.42405891	0.015348116	0	1.205690968	0	1	CRA004775;PRJNA827465;Yang2025_Silkie_Wencha	0.266019863
ENSGALG00010021648	BRE	3	-0.159484542	0.065775558	-2.424677915	0.015321974	0.005281689	3.405846578	41.27744882	1	CRA004775;PRJNA827465;Yang2025_Silkie_Wencha	0.266019863
ENSGALG00010009094	DARS	3	-0.123441191	0.050941828	-2.423179441	0.015385325	0	0.625035089	0	1	CRA004775;PRJNA827465;Yang2025_Silkie_Wencha	0.266019863
ENSGALG00010006592	STARD13	3	0.108646481	0.044809902	2.424608761	0.015324892	0	1.33923798	0	1	CRA004775;PRJNA827465;Yang2025_Silkie_Wencha	0.266019863
ENSGALG00010012830	ARRDC3	3	0.27037395	0.11165639	2.4214821	0.015457362	0.008736207	2.54876631	21.53066398	1	CRA004775;PRJNA827465;Yang2025_Silkie_Wencha	0.266851826
ENSGALG00010012316	ENSGALG0001001231(	2	1.31394365	0.543054298	2.419543781	0.01553999	0	0.340680376	0	1	PRJNA827465;Yang2025_Silkie_Wenchang	0.267053839
ENSGALG00010026839	FGGY	3	-0.26988455	0.111539912	-2.419623117	0.0155366	0.008543913	2.51931765	20.61342483	0.666666667	CRA004775;PRJNA827465;Yang2025_Silkie_Wencha	0.267053839
ENSGALG00010016483	WDR26	3	-0.168930699	0.069822359	-2.419435555	0.015544615	0.0053823	3.105787468	35.60409331	1	CRA004775;PRJNA827465;Yang2025_Silkie_Wencha	0.267053839
ENSGALG00010026091	DTX4	3	-0.132679316	0.054818305	-2.420346949	0.015505705	0	1.134718806	0	1	CRA004775;PRJNA827465;Yang2025_Silkie_Wencha	0.267053839
ENSGALG00010011142	DUSP19	3	-0.485254723	0.200654284	-2.418362144	0.015590552	0.086600149	7.492115565	73.30527028	0.666666667	CRA004775;PRJNA827465;Yang2025_Silkie_Wencha	0.267282588
ENSGALG00010021438	GOT2	3	-0.163891655	0.067773082	-2.418241141	0.015595738	0	0.348296972	0	1	CRA004775;PRJNA827465;Yang2025_Silkie_Wencha	0.267282588
ENSGALG00010003820	SH3YL1	3	-0.425977521	0.176260295	-2.416752567	0.015659658	0	0.03601124	0	1	CRA004775;PRJNA827465;Yang2025_Silkie_Wencha	0.268053153
ENSGALG00010019250	CXCR1	3	-0.463024731	0.191727517	-2.415014487	0.015734584	0	0.375524841	0	1	CRA004775;PRJNA827465;Yang2025_Silkie_Wencha	0.269010013
ENSGALG00010000590	DNAH5	2	-0.762731934	0.316445592	-2.410309872	0.015938977	0	0.041113185	0	1	PRJNA827465;Yang2025_Silkie_Wenchang	0.271192767
ENSGALG00010004338	ENSGALG0001000433!	3	0.262507829	0.108879623	2.410991357	0.015909225	0	1.237893719	0	1	CRA004775;PRJNA827465;Yang2025_Silkie_Wencha	0.271192767
ENSGALG00010022219	EIF4E3	3	-0.181952941	0.07548572	-2.410428641	0.015933788	0	0.654406015	0	1	CRA004775;PRJNA827465;Yang2025_Silkie_Wencha	0.271192767
ENSGALG00010023347	STAT5A	3	-0.158846388	0.065888551	-2.410834451	0.015916071	0	0.444196199	0	1	CRA004775;PRJNA827465;Yang2025_Silkie_Wencha	0.271192767
ENSGALG00010001753	PNPLA8	3	-0.129925097	0.053948586	-2.408313297	0.016026421	0	1.091748445	0	0.666666667	CRA004775;PRJNA827465;Yang2025_Silkie_Wencha	0.272352848
ENSGALG00010006163	ENSGALG0001000616:	3	0.7040264	0.292626926	2.405883866	0.016133393	0	1.158561228	0	1	CRA004775;PRJNA827465;Yang2025_Silkie_Wencha	0.273185673
ENSGALG00010013242	SULT4A1	3	0.341363901	0.141841181	2.406662855	0.016099024	0.004375246	2.144177074	6.724121608	1	CRA004775;PRJNA827465;Yang2025_Silkie_Wencha	0.273185673
ENSGALG00010027192	SH3GL1	3	-0.115432828	0.047974517	-2.406127978	0.016122616	0.001169331	2.349648798	14.88089618	1	CRA004775;PRJNA827465;Yang2025_Silkie_Wencha	0.273185673
ENSGALG00010023748	ENSGALG0001002374!	3	-0.600843823	0.250011768	-2.403262163	0.016249533	0.14736211	10.05121198	80.10190209	1	CRA004775;PRJNA827465;Yang2025_Silkie_Wencha	0.274823148
ENSGALG00010013119	RNF114	3	-0.112566911	0.046887031	-2.400811246	0.016358772	0	0.30344117	0	1	CRA004775;PRJNA827465;Yang2025_Silkie_Wencha	0.276340121
ENSGALG00010027059	ASB14	3	-0.196330466	0.081878874	-2.397815898	0.016493152	0	0.316115695	0	0.666666667	CRA004775;PRJNA827465;Yang2025_Silkie_Wencha	0.278277664
ENSGALG00010013938	ENSGALG0001001393!	3	0.558281778	0.232898629	2.397102042	0.016525321	0	1.628748789	0	1	CRA004775;PRJNA827465;Yang2025_Silkie_Wencha	0.278453059
ENSGALG00010017941	DIS3L	3	0.15778302	0.065833125	2.396711662	0.016542936	0.003670642	2.690356813	25.66041833	1	CRA004775;PRJNA827465;Yang2025_Silkie_Wencha	0.278453059
ENSGALG00010012015	ENSGALG0001001201:	3	-0.397864826	0.166092233	-2.395445104	0.0166002	0	0.375218715	0	1	CRA004775;PRJNA827465;Yang2025_Silkie_Wencha	0.278540968
ENSGALG00010020295	LGI1	3	0.204039809	0.085160194	2.395952846	0.016577223	0	0.80047627	0	1	CRA004775;PRJNA827465;Yang2025_Silkie_Wencha	0.278540968
ENSGALG00010013976	CDC40	3	-0.156243331	0.065241261	-2.394854557	0.016626959	0.006303954	4.298123992	53.4680711	1	CRA004775;PRJNA827465;Yang2025_Silkie_Wencha	0.278540968
ENSGALG00010016542	ENOX2	3	0.095999414	0.040084088	2.394950688	0.016622601	0	0.477117166	0	1	CRA004775;PRJNA827465;Yang2025_Silkie_Wencha	0.278540968
ENSGALG00010003379	ENSGALG0001000337!	2	0.953442985	0.398311939	2.393709281	0.016678963	0	0.002551962	0	1	CRA004775;PRJNA827465	0.278713118
ENSGALG00010025727	GLI1	3	0.213586281	0.08921329	2.394108326	0.016660827	0.00238649	2.187782414	8.583230817	1	CRA004775;PRJNA827465;Yang2025_Silkie_Wencha	0.278713118
ENSGALG00010024433	ENSGALG0001002443:	3	-0.165908364	0.069321239	-2.393326574	0.016696373	0	0.251137287	0	1	CRA004775;PRJNA827465;Yang2025_Silkie_Wencha	0.278713118
ENSGALG00010012992	HARS1	3	-0.08537605	0.035697872	-2.391628576	0.016773808	0	1.412752314	0	1	CRA004775;PRJNA827465;Yang2025_Silkie_Wencha	0.279675549
ENSGALG00010018162	ENSGALG0001001816:	3	0.497306065	0.207983801	2.391080761	0.016798857	0	0.13278374	0	1	CRA004775;PRJNA827465;Yang2025_Silkie_Wencha	0.2797633
ENSGALG00010027254	INSR	3	-0.360328317	0.150786441	-2.389659947	0.016863979	0.041665164	6.816320437	70.65865641	1	CRA004775;PRJNA827465;Yang2025_Silkie_Wencha	0.28051741
ENSGALG00010021629	DHRS7B	3	-0.164298461	0.06878504	-2.388578396	0.016913699	0.000550992	2.063245461	3.065338681	1	CRA004775;PRJNA827465;Yang2025_Silkie_Wencha	0.281013861
ENSGALG00010027199	LAMB3	3	-0.65038971	0.272408869	-2.387549686	0.01696111	0.083843245	3.19458982	37.39415346	1	CRA004775;PRJNA827465;Yang2025_Silkie_Wencha	0.281333871
ENSGALG00010020236	SLC35G1	3	0.362802038	0.151971866	2.387297384	0.016972756	0	0.894072348	0	1	CRA004775;PRJNA827465;Yang2025_Silkie_Wencha	0.281333871
ENSGALG00010013396	FYB1	3	-0.241317162	0.101141253	-2.385941987	0.017035438	0	0.822577541	0	1	CRA004775;PRJNA827465;Yang2025_Silkie_Wencha	0.282042224
ENSGALG00010021469	CSPG4	3	0.15440091	0.064725715	2.38546473	0.017057558	0	1.667217056	0	1	CRA004775;PRJNA827465;Yang2025_Silkie_Wencha	0.282078143
ENSGALG00010003110	BF1	3	-0.319266384	0.133912533	-2.384141184	0.017119033	0.018136579	3.016901619	33.70682068	1	CRA004775;PRJNA827465;Yang2025_Silkie_Wencha	0.282764032
ENSGALG00010017766	DNAAF10	3	-0.158369516	0.066458645	-2.382978395	0.017173202	0	0.97972844	0	0.666666667	CRA004775;PRJNA827465;Yang2025_Silkie_Wencha	0.283327778
ENSGALG00010015118	GPX8	3	-0.332934013	0.139793763	-2.381608495	0.017237212	0.015155404	2.634517649	24.08477505	1	CRA004775;PRJNA827465;Yang2025_Silkie_Wencha	0.284052383
ENSGALG00010001521	TBC1D5	3	-0.15144983	0.063636143	-2.379934158	0.017315732	0	0.976753612	0	0.666666667	CRA004775;PRJNA827465;Yang2025_Silkie_Wencha	0.285014122
ENSGALG00010014096	JAKMIP2	3	0.345339943	0.145205368	2.378286341	0.017393314	0.027804545	3.423366928	41.57798325	1	CRA004775;PRJNA827465;Yang2025_Silkie_Wencha	0.285958209
ENSGALG00010027218	IARS1	3	-0.104405386	0.043940931	-2.376039473	0.017499591	0	0.096868721	0	1	CRA004775;PRJNA827465;Yang2025_Silkie_Wencha	0.287371327
ENSGALG00010015913	CHST10	3	-0.114916397	0.04839645	-2.374479896	0.017573693	0	1.947442059	0	1	CRA004775;PRJNA827465;Yang2025_Silkie_Wencha	0.28825342
ENSGALG00010026166	WNT9A	3	-0.268840125	0.113270551	-2.373433545	0.017623564	0	0.192503027	0	1	CRA004775;PRJNA827465;Yang2025_Silkie_Wencha	0.288448355
ENSGALG00010009170	RPL26L1	3	-0.154059768	0.064911696	-2.373374553	0.017626379	0	1.394336538	0	1	CRA004775;PRJNA827465;Yang2025_Silkie_Wencha	0.288448355
ENSGALG00010009035	ENSGALG0001000903:	3	-0.140789002	0.059354255	-2.372011937	0.017691519	0	0.104078409	0	1	CRA004775;PRJNA827465;Yang2025_Silkie_Wencha	0.289179641
ENSGALG00010019169	GPR153	3	0.479675157	0.202498119	2.36878821	0.01784647	0.027584748	2.479688294	19.34470131	0.666666667	CRA004775;PRJNA827465;Yang2025_Silkie_Wencha	0.290524057
ENSGALG00010004006	NKAIN3	3	-0.42528154	0.179614882	-2.367741108	0.017897054	0	0.195097752	0	1	CRA004775;PRJNA827465;Yang2025_Silkie_Wencha	0.290524057
ENSGALG00010004949	RAB39A	3	-0.35751393	0.150870165	-2.369679458	0.017803513	0.018979116	2.713266924	26.28812218	0.666666667	CRA004775;PRJNA827465;Yang2025_Silkie_Wencha	0.290524057
ENSGALG00010002177	FBXO9	3	-0.257932447	0.108864041	-2.369308043	0.017821403	0.014432394	3.200797932	37.51558073	1	CRA004775;PRJNA827465;Yang2025_Silkie_Wencha	0.290524057
ENSGALG00010023991	TSPAN3	3	-0.20522932	0.086671123	-2.367908865	0.017888942	0.011846397	4.195681721	52.33194192	1	CRA004775;PRJNA827465;Yang2025_Silkie_Wencha	0.290524057
ENSGALG00010010932	GHITM	3	-0.1572731	0.066422226	-2.367778218	0.01789526	0	0.333026078	0	1	CRA004775;PRJNA827465;Yang2025_Silkie_Wencha	0.290524057
ENSGALG00010029472	ENSGALG0001002947:	3	0.755943337	0.319694038	2.364583776	0.018050344	0	0.574351211	0	1	CRA004775;PRJNA827465;Yang2025_Silkie_Wencha	0.290689571
ENSGALG00010010893	CCL21	3	-0.704737391	0.297785195	-2.366596464	0.017952495	0.086266566	2.922589911	31.56754588	0.666666667	CRA004775;PRJNA827465;Yang2025_Silkie_Wencha	0.290689571
ENSGALG00010004963	ENSGALG0001000496:	3	0.529909712	0.223910892	2.366609805	0.017951848	0.052475422	3.049453137	34.41447008	0.666666667	CRA004775;PRJNA827465;Yang2025_Silkie_Wencha	0.290689571
ENSGALG00010029451	ENSGALG00010029451	3	0.32470786	0.137231624	2.366129975	0.017975133	0.012971188	2.477605297	19.27689197	0.666666667	CRA004775;PRJNA827465;Yang2025_Silkie_Wencha	0.290689571
ENSGALG00010020861	CDA	3	0.283743724	0.120007351	2.364386183	0.018059976	0	1.288092149	0	1	CRA004775;PRJNA827465;Yang2025_Silkie_Wencha	0.290689571
ENSGALG00010004457	AGA	3	0.166615623	0.070427812	2.365764571	0.017992882	0	1.907413844	0	1	CRA004775;PRJNA827465;Yang2025_Silkie_Wencha	0.290689571
ENSGALG00010019706	NPRL3	3	-0.135048919	0.057118123	-2.364379497	0.018060302	0	1.947475448	0	1	CRA004775;PRJNA827465;Yang2025_Silkie_Wencha	0.290689571
ENSGALG00010017957	THUMPD1	3	-0.113380249	0.047958242	-2.364145245	0.018071726	0	0.468521713	0	1	CRA004775;PRJNA827465;Yang2025_Silkie_Wencha	0.290689571
ENSGALG00010010263	RAB4A	3	0.152396744	0.064479065	2.363507339	0.018102868	0	1.177598135	0	1	CRA004775;PRJNA827465;Yang2025_Silkie_Wencha	0.290859605
ENSGALG00010022470	ENSGALG0001002247(	3	0.529057457	0.224048195	2.361355585	0.018208262	0.06556108	3.504360234	42.92824178	0.666666667	CRA004775;PRJNA827465;Yang2025_Silkie_Wencha	0.291219217
ENSGALG00010017101	CSMD2	3	-0.418233626	0.177190192	-2.360365562	0.018256934	0.040959295	3.523508379	43.23839239	1	CRA004775;PRJNA827465;Yang2025_Silkie_Wencha	0.291219217
ENSGALG00010008376	MMRN1	3	-0.366057951	0.155020098	-2.361358017	0.018208143	0	0.776903004	0	1	CRA004775;PRJNA827465;Yang2025_Silkie_Wencha	0.291219217
ENSGALG00010019483	SCUBE2	3	0.33781191	0.143133859	2.3601118	0.018269428	0	0.198602817	0	1	CRA004775;PRJNA827465;Yang2025_Silkie_Wencha	0.291219217
ENSGALG00010029154	UTS2R	3	0.322457214	0.13661436	2.360346406	0.018257877	0.016367426	2.708261876	26.15189773	1	CRA004775;PRJNA827465;Yang2025_Silkie_Wencha	0.291219217
ENSGALG00010016993	ENSGALG0001001699:	3	-0.297000752	0.125832023	-2.360295459	0.018260385	0.023383153	4.092491371	51.13001303	0.666666667	CRA004775;PRJNA827465;Yang2025_Silkie_Wencha	0.291219217
ENSGALG00010018619	MAPK1IP1L	3	-0.154290748	0.065341229	-2.361307697	0.018210614	0.006997702	4.313829424	53.63748068	1	CRA004775;PRJNA827465;Yang2025_Silkie_Wencha	0.291219217
ENSGALG00010018566	ENSGALG0001001856(	3	0.191152698	0.081086582	2.357390023	0.018403906	0	1.716460307	0	0.666666667	CRA004775;PRJNA827465;Yang2025_Silkie_Wencha	0.292702839
ENSGALG00010005557	CMSS1	3	-0.175757063	0.074547875	-2.357640144	0.018391512	0	1.739579741	0	0.666666667	CRA004775;PRJNA827465;Yang2025_Silkie_Wencha	0.292702839
ENSGALG00010022233	FAM46C	3	-0.203247054	0.086304337	-2.355003947	0.018522509	0	1.866999119	0	0.666666667	CRA004775;PRJNA827465;Yang2025_Silkie_Wencha	0.293598379
ENSGALG00010008240	RCAN2	3	0.188119631	0.079873836	2.355209677	0.018512257	0	0.98174494	0	1	CRA004775;PRJNA827465;Yang2025_Silkie_Wencha	0.293598379
ENSGALG00010024188	TSSC4	3	0.137265563	0.058268136	2.355756908	0.01848501	0.003200536	2.778887608	28.02875531	0.666666667	CRA004775;PRJNA827465;Yang2025_Silkie_Wencha	0.293598379
ENSGALG00010028438	MED27	3	-0.298402165	0.126746587	-2.354321105	0.018556573	0.031999257	6.320868735	68.35877972	1	CRA004775;PRJNA827465;Yang2025_Silkie_Wencha	0.293808949
ENSGALG00010018067	ENSGALG0001001806:	3	-0.26726854	0.113607205	-2.352566818	0.01864434	0.000924071	2.033929478	1.66817378	1	CRA004775;PRJNA827465;Yang2025_Silkie_Wencha	0.294538904
ENSGALG00010023636	PDE3B	3	-0.152939568	0.065009038	-2.352589296	0.018643213	0.003389942	2.672221462	25.15590388	1	CRA004775;PRJNA827465;Yang2025_Silkie_Wencha	0.294538904
ENSGALG00010025708	HOXC8	3	0.670864747	0.285534047	2.349508765	0.018798202	0.132423328	4.56453337	56.18391108	1	CRA004775;PRJNA827465;Yang2025_Silkie_Wencha	0.294664935
ENSGALG00010012818	RGS18	3	-0.41754155	0.177574192	-2.351363929	0.01870473	0.055196924	5.501367312	63.64540147	1	CRA004775;PRJNA827465;Yang2025_Silkie_Wencha	0.294664935
ENSGALG00010019051	IGSF6	3	-0.358648808	0.152643588	-2.349583182	0.018794444	0	1.618686058	0	1	CRA004775;PRJNA827465;Yang2025_Silkie_Wencha	0.294664935
ENSGALG00010007436	PNAT3	3	-0.229629473	0.097678066	-2.350880627	0.018729041	0.001036443	2.05279887	2.572043027	1	CRA004775;PRJNA827465;Yang2025_Silkie_Wencha	0.294664935
ENSGALG00010007925	STK17A	3	-0.219518914	0.093373328	-2.350980937	0.018723993	0	0.223679818	0	1	CRA004775;PRJNA827465;Yang2025_Silkie_Wencha	0.294664935
ENSGALG00010021411	MCU	3	-0.139336936	0.059291722	-2.35002345	0.018772228	0	1.751823487	0	0.666666667	CRA004775;PRJNA827465;Yang2025_Silkie_Wencha	0.294664935
ENSGALG00010003889	ITM2B	3	-0.110001666	0.046796505	-2.350638513	0.018741231	0.000336979	2.0727092	3.507930608	1	CRA004775;PRJNA827465;Yang2025_Silkie_Wencha	0.294664935
ENSGALG00010023859	ENSGALG0001002385!	3	0.636014931	0.270832551	2.348369607	0.0188558	0	1.822526122	0	0.666666667	CRA004775;PRJNA827465;Yang2025_Silkie_Wencha	0.295044638

ENSGALG00010012436	ENSGALG0001001243	3	-0.371499155	0.158316514	-2.346559723	0.018947629	0	0.84670725	0	1	CRA004775;PRJNA827465;Yang2025_Silkie_Wencha	0.295044638
ENSGALG00010029690	ENSGALG0001002969	3	-0.173473267	0.073910094	-2.347084911	0.018920942	0	0.690458665	0	0.666666667	CRA004775;PRJNA827465;Yang2025_Silkie_Wencha	0.295044638
ENSGALG00010025340	METTL21A	3	-0.172361761	0.073421131	-2.34757704	0.018895965	0	1.814835556	0	1	CRA004775;PRJNA827465;Yang2025_Silkie_Wencha	0.295044638
ENSGALG00010007457	SHANK3	3	-0.167534652	0.071380485	-2.347065203	0.018921943	0	0.546073476	0	1	CRA004775;PRJNA827465;Yang2025_Silkie_Wencha	0.295044638
ENSGALG00010013438	RPL37	3	-0.147062368	0.062667025	-2.346726497	0.018939151	0	1.518817043	0	1	CRA004775;PRJNA827465;Yang2025_Silkie_Wencha	0.295044638
ENSGALG00010024045	PSTPIP1	3	-0.368838705	0.157305782	-2.344724391	0.019041149	0.025194886	2.991632794	33.14687538	1	CRA004775;PRJNA827465;Yang2025_Silkie_Wencha	0.295685188
ENSGALG00010003506	DYNC1I1	3	0.334728465	0.142770472	2.34452167	0.019051503	0	1.673074705	0	1	CRA004775;PRJNA827465;Yang2025_Silkie_Wencha	0.295685188
ENSGALG00010003307	COL1A2	3	-0.240162289	0.102433598	-2.344565593	0.01904926	0	1.759185867	0	1	CRA004775;PRJNA827465;Yang2025_Silkie_Wencha	0.295685188
ENSGALG00010005540	PCMT1	3	-0.115639088	0.049345835	-2.343441722	0.019106747	0	1.22589819	0	0.666666667	CRA004775;PRJNA827465;Yang2025_Silkie_Wencha	0.296217429
ENSGALG00010012550	TAB1	3	-0.230826587	0.09855513	-2.342106261	0.019175255	0.016884142	5.017806982	60.14195032	1	CRA004775;PRJNA827465;Yang2025_Silkie_Wencha	0.296953916
ENSGALG00010012711	ELL2	3	0.272562118	0.116518384	2.339219868	0.019324057	0	1.927798701	0	1	CRA004775;PRJNA827465;Yang2025_Silkie_Wencha	0.298278217
ENSGALG00010001805	ENSGALG0001000180	3	-0.19489667	0.083291475	-2.339935379	0.019287077	0	1.630620454	0	1	CRA004775;PRJNA827465;Yang2025_Silkie_Wencha	0.298278217
ENSGALG00010016738	NAA60	3	-0.127230294	0.054388116	-2.339303197	0.019319747	0	0.322310662	0	1	CRA004775;PRJNA827465;Yang2025_Silkie_Wencha	0.298278217
ENSGALG00010001382	BNIP3	3	-0.210760763	0.090161032	-2.337603709	0.019407815	0	0.482661826	0	1	CRA004775;PRJNA827465;Yang2025_Silkie_Wencha	0.299244386
ENSGALG00010004495	MRPL15	3	-0.299506649	0.128176062	-2.336681623	0.019455745	0	0.156228895	0	1	CRA004775;PRJNA827465;Yang2025_Silkie_Wencha	0.299656618
ENSGALG00010009730	C8orf76	3	-0.164551241	0.070447062	-2.335814085	0.019500933	0	0.590978463	0	1	CRA004775;PRJNA827465;Yang2025_Silkie_Wencha	0.300025783
ENSGALG00010022236	SHF	3	-0.349363567	0.149594608	-2.33540214	0.019522423	0.040938365	6.043736285	66.90788768	1	CRA004775;PRJNA827465;Yang2025_Silkie_Wencha	0.30002993
ENSGALG00010026746	HOXD10	2	0.61905182	0.265186676	2.334400167	0.019574778	0	0.795081205	0	1	PRJNA827465;Yang2025_Silkie_Wenchang	0.300507912
ENSGALG00010015620	PDRG1	3	-0.195599662	0.08382545	-2.333416205	0.019626312	0	0.690524507	0	1	CRA004775;PRJNA827465;Yang2025_Silkie_Wencha	0.300972256
ENSGALG00010017352	GPCPD1	3	0.141966918	0.060855877	2.332838262	0.019656636	0	0.385487473	0	1	CRA004775;PRJNA827465;Yang2025_Silkie_Wencha	0.301110695
ENSGALG00010025164	C11orf52	3	-0.494591202	0.212136518	-2.331476006	0.019728274	0.093047386	9.326612398	78.55598673	1	CRA004775;PRJNA827465;Yang2025_Silkie_Wencha	0.301508218
ENSGALG00010026522	MED20	3	-0.190160223	0.081574304	-2.331128976	0.01974656	0	1.526155609	0	1	CRA004775;PRJNA827465;Yang2025_Silkie_Wencha	0.301508218
ENSGALG00010028892	NIBAN2	3	0.150319494	0.064480426	2.331242269	0.019740588	0	0.890413648	0	1	CRA004775;PRJNA827465;Yang2025_Silkie_Wencha	0.301508218
ENSGALG00010029346	ARL16	3	-0.229890947	0.098665631	-2.330000284	0.019806136	0	0.047754365	0	1	CRA004775;PRJNA827465;Yang2025_Silkie_Wencha	0.302091649
ENSGALG00010003189	ANTXR2	3	-0.136050503	0.058475141	-2.326638299	0.019984524	0	0.187934263	0	1	CRA004775;PRJNA827465;Yang2025_Silkie_Wencha	0.30448404
ENSGALG00010023621	CD79B	3	0.673673304	0.289709094	2.325344005	0.020053573	0	1.60810938	0	1	CRA004775;PRJNA827465;Yang2025_Silkie_Wencha	0.305207179
ENSGALG00010005003	EXPH5	3	0.782278416	0.336503829	2.32472367	0.020086741	0	1.7338157	0	0.666666667	CRA004775;PRJNA827465;Yang2025_Silkie_Wencha	0.305383256
ENSGALG00010001273	ENSGALG0001000127	3	0.955809799	0.411234214	2.324246783	0.020112271	0.216674813	3.49633328	42.79721525	1	CRA004775;PRJNA827465;Yang2025_Silkie_Wencha	0.305442967
ENSGALG00010009153	SECISBP2	3	0.154183761	0.066365925	2.323236831	0.020166433	0	0.685843781	0	1	CRA004775;PRJNA827465;Yang2025_Silkie_Wencha	0.305936908
ENSGALG00010027208	UBXN6	3	-0.150543464	0.06484617	-2.321547486	0.020257314	0	1.592890177	0	1	CRA004775;PRJNA827465;Yang2025_Silkie_Wencha	0.306986246
ENSGALG00010015774	PLS1	3	0.904287578	0.389843449	2.319617222	0.020361593	0.323091053	7.410439122	73.01104608	1	CRA004775;PRJNA827465;Yang2025_Silkie_Wencha	0.307577531
ENSGALG00010001604	ENSGALG0001000160	3	-0.377250889	0.162581606	-2.320378666	0.020320402	0	0.0862997	0	1	CRA004775;PRJNA827465;Yang2025_Silkie_Wencha	0.307577531
ENSGALG00010000186	THOC5	3	-0.089530272	0.038592605	-2.319881527	0.020347287	0	1.244668122	0	1	CRA004775;PRJNA827465;Yang2025_Silkie_Wencha	0.307577531
ENSGALG00010028081	UBQLN4	3	-0.108223886	0.046675233	-2.318657635	0.020413607	0	0.736027484	0	1	CRA004775;PRJNA827465;Yang2025_Silkie_Wencha	0.308034145
ENSGALG00010011883	HCFC2	3	0.197283655	0.085215226	2.315122125	0.020606249	0	0.582358833	0	1	CRA004775;PRJNA827465;Yang2025_Silkie_Wencha	0.310278766
ENSGALG00010024711	DDX55	3	0.174092858	0.075186575	2.315477964	0.020586789	0	1.406620965	0	1	CRA004775;PRJNA827465;Yang2025_Silkie_Wencha	0.310278766
ENSGALG00010015563	B3GNT7	3	0.707437788	0.305949009	2.312273505	0.020762616	0.131496492	3.813837685	47.55938335	1	CRA004775;PRJNA827465;Yang2025_Silkie_Wencha	0.311637606
ENSGALG00010005176	BAMBI	3	0.516282067	0.223226324	2.312818925	0.020732597	0	1.703065709	0	1	CRA004775;PRJNA827465;Yang2025_Silkie_Wencha	0.311637606
ENSGALG00010013276	POC1B	3	-0.164429597	0.071101165	-2.312614659	0.020743835	0.00209167	2.262701632	11.61008717	1	CRA004775;PRJNA827465;Yang2025_Silkie_Wencha	0.311637606
ENSGALG00010018006	DCUN1D3	3	-0.175726524	0.076076713	-2.309859574	0.02089593	0.004656089	2.670267729	25.1011433	0.666666667	CRA004775;PRJNA827465;Yang2025_Silkie_Wencha	0.313305993
ENSGALG00010002638	FABP9	3	0.750139005	0.324948312	2.308487158	0.020972056	0.09454611	2.79757035	28.50939386	1	CRA004775;PRJNA827465;Yang2025_Silkie_Wencha	0.314114305
ENSGALG00010027522	PSMD5	3	-0.121120153	0.052507234	-2.306732686	0.021069727	0	1.908522877	0	1	CRA004775;PRJNA827465;Yang2025_Silkie_Wencha	0.315243247
ENSGALG00010000605	MARCHF11	2	0.894744388	0.388302155	2.304247801	0.021208737	0	0.123479921	0	1	PRJNA827465;Yang2025_Silkie_Wenchang	0.316987671
ENSGALG00010006913	HSPH1	3	0.225815741	0.098025314	2.303647211	0.021242455	0.002621176	2.155152435	7.199139719	1	CRA004775;PRJNA827465;Yang2025_Silkie_Wencha	0.317156364
ENSGALG00010017712	MSH2	3	-0.236791956	0.102822042	-2.302929903	0.021282787	0.018881528	5.558827492	64.02118967	1	CRA004775;PRJNA827465;Yang2025_Silkie_Wencha	0.317215943
ENSGALG00010029835	RECQL5	3	-0.17050934	0.074048981	-2.302656146	0.021298197	0	0.801886774	0	1	CRA004775;PRJNA827465;Yang2025_Silkie_Wencha	0.317215943
ENSGALG00010006132	MIPEP	3	-0.157347325	0.068341162	-2.302379989	0.021313752	0.00317951	2.519482749	20.61862693	1	CRA004775;PRJNA827465;Yang2025_Silkie_Wencha	0.317215943
ENSGALG00010029212	SUPT6H	3	-0.118565165	0.051510303	-2.301775744	0.021347822	0.001120197	2.125004181	5.882538119	0.666666667	CRA004775;PRJNA827465;Yang2025_Silkie_Wencha	0.317388914
ENSGALG00010008096	CPSF1	3	-0.162591592	0.070650539	-2.301349628	0.021371877	0	0.75919127	0	1	CRA004775;PRJNA827465;Yang2025_Silkie_Wencha	0.317412778
ENSGALG00010029660	CD300LG	3	-0.350584257	0.152461526	-2.299493295	0.021476944	0	0.515239312	0	1	CRA004775;PRJNA827465;Yang2025_Silkie_Wencha	0.318638517
ENSGALG00010001852	CPXM2	3	0.33116274	0.144046679	2.298996003	0.021505166	0	1.640098139	0	1	CRA004775;PRJNA827465;Yang2025_Silkie_Wencha	0.318722794
ENSGALG00010029701	MRPS7	3	-0.101301245	0.04407716	-2.298270698	0.021546387	0	0.084175192	0	1	CRA004775;PRJNA827465;Yang2025_Silkie_Wencha	0.318999337
ENSGALG00010008444	SLC7A11	3	0.642534465	0.279669564	2.297477267	0.021591558	0	0.346410798	0	1	CRA004775;PRJNA827465;Yang2025_Silkie_Wencha	0.319333729
ENSGALG00010004350	MOXD1	3	-0.245029088	0.106710598	-2.296202008	0.021664334	0	1.813625878	0	0.666666667	CRA004775;PRJNA827465;Yang2025_Silkie_Wencha	0.320075251
ENSGALG00010007679	TMEM135	3	-0.354896902	0.154604919	-2.295508477	0.021704001	0.043655665	5.961069343	66.44897275	0.666666667	CRA004775;PRJNA827465;Yang2025_Silkie_Wencha	0.320326589
ENSGALG00010024933	CS	3	-0.161969642	0.070619551	-2.29355243	0.021816221	0	0.056522446	0	1	CRA004775;PRJNA827465;Yang2025_Silkie_Wencha	0.321647072
ENSGALG00010011253	KDM4C	3	0.143547617	0.062603653	2.292959125	0.021850358	0	0.185290684	0	1	CRA004775;PRJNA827465;Yang2025_Silkie_Wencha	0.321814809
ENSGALG00010005421	ENSGALG0001000542	3	-0.612757992	0.267286855	-2.292510764	0.021876187	0.117738181	4.62105427	56.71983312	1	CRA004775;PRJNA827465;Yang2025_Silkie_Wencha	0.321859948
ENSGALG00010006608	KL	3	0.446551069	0.194933705	2.290784285	0.021975893	0	0.603335407	0	1	CRA004775;PRJNA827465;Yang2025_Silkie_Wencha	0.321986685
ENSGALG00010002247	RFTN1	3	-0.337737775	0.147412688	-2.291103827	0.021957409	0.035311413	4.853607532	58.79353683	1	CRA004775;PRJNA827465;Yang2025_Silkie_Wencha	0.321986685
ENSGALG00010016889	FUNDC2	3	-0.331648877	0.14470075	-2.291963773	0.021907734	0.036410612	5.267198334	62.02914959	1	CRA004775;PRJNA827465;Yang2025_Silkie_Wencha	0.321986685
ENSGALG00010009135	TENM4	3	-0.221975937	0.096874705	-2.291371482	0.021941937	0	0.84857441	0	1	CRA004775;PRJNA827465;Yang2025_Silkie_Wencha	0.321986685
ENSGALG00010018497	ASTE1	3	-0.200922291	0.087727022	-2.29031246	0.02200321	0	0.387483501	0	1	CRA004775;PRJNA827465;Yang2025_Silkie_Wencha	0.322053195
ENSGALG00010002090	AQP11	3	0.313855744	0.137164774	2.288165795	0.022127868	0	0.277050717	0	1	CRA004775;PRJNA827465;Yang2025_Silkie_Wencha	0.323542837
ENSGALG00010021754	SLC17A9	3	0.838571623	0.366638818	2.287187233	0.022184897	0.195820594	3.886746228	48.54307736	1	CRA004775;PRJNA827465;Yang2025_Silkie_Wencha	0.324041588
ENSGALG00010021113	SYP	3	1.048074447	0.458410595	2.286322474	0.0222354	0.499205477	10.6716055	81.25867753	1	CRA004775;PRJNA827465;Yang2025_Silkie_Wencha	0.324444088
ENSGALG00010014517	DCUN1D2	3	0.169515866	0.074196148	2.284699017	0.022330482	0.000574973	2.059138186	2.871987237	0.666666667	CRA004775;PRJNA827465;Yang2025_Silkie_Wencha	0.325495556
ENSGALG00010017418	CHGB	3	0.772738638	0.338392869	2.283554732	0.022397713	0.241091191	7.049597197	71.62958472	1	CRA004775;PRJNA827465;Yang2025_Silkie_Wencha	0.325844641
ENSGALG00010013434	CORIN	2	0.522774905	0.229092009	2.281942996	0.022492706	0	0.359468146	0	1	PRJNA827465;Yang2025_Silkie_Wenchang	0.325844641
ENSGALG00010004871	C11orf87	3	-0.311787659	0.136632522	-2.281943237	0.022492692	0	1.682682436	0	1	CRA004775;PRJNA827465;Yang2025_Silkie_Wencha	0.325844641
ENSGALG00010005905	CITED2	3	0.288782695	0.126494471	2.282966935	0.022432316	0.015005197	2.813183815	28.90617423	1	CRA004775;PRJNA827465;Yang2025_Silkie_Wencha	0.325844641
ENSGALG00010008693	C4orf46	3	-0.150593942	0.065976185	-2.282550006	0.022456889	0	0.430568788	0	1	CRA004775;PRJNA827465;Yang2025_Silkie_Wencha	0.325844641
ENSGALG00010023188	KIF1BP	3	-0.081032389	0.035490191	-2.283233392	0.022416624	0	0.880825996	0	1	CRA004775;PRJNA827465;Yang2025_Silkie_Wencha	0.325844641
ENSGALG00010008674	RXFP1	3	-0.28362732	0.12433424	-2.281168235	0.022538494	0	0.142140682	0	1	CRA004775;PRJNA827465;Yang2025_Silkie_Wencha	0.326173758
ENSGALG00010005010	NKAIN2	3	-0.580648747	0.254722261	-2.279536718	0.02263518	0.061538357	2.825400479	29.21357468	0.666666667	CRA004775;PRJNA827465;Yang2025_Silkie_Wencha	0.327088578
ENSGALG00010014120	SOX5	3	0.441869859	0.19386028	2.279321263	0.022647975	0.061057045	4.438969286	54.94449564	1	CRA004775;PRJNA827465;Yang2025_Silkie_Wencha	0.327088578
ENSGALG00010013125	POU3F3	2	1.779668849	0.781516354	2.277199754	0.0227743	0.804241992	2.906371562	65.59283702	1	PRJNA827465;Yang2025_Silkie_Wenchang	0.327605279
ENSGALG00010023099	DNAJC22	2	1.698490752	0.745924822	2.277026721	0.02278463	0.165205761	1.155300562	13.44243805	1	CRA004775;PRJNA827465	0.327605279
ENSGALG00010027388	URM1	3	-0.389031801	0.170765839	-2.278159402	0.022717082	0.056878138	6.100188229	67.21412644	1	CRA004775;PRJNA827465;Yang2025_Silkie_Wencha	0.327605279
ENSGALG00010010786	NABP1	3	0.308802851	0.135660425	2.276292817	0.02282849	0.025196378	3.863551462	48.23415659	0.666666667	CRA004775;PRJNA827465;Yang2025_Silkie_Wencha	0.327605279
ENSGALG00010018048	GFRA4	3	0.2848984	0.125174984	2.276001092	0.022845944	0.009909093	2.479783541	19.34779924	1	CRA004775;PRJNA827465;Yang2025_Silkie_Wencha	0.327605279
ENSGALG00010016664	TM6SF1	3	-0.268287244	0.117855539	-2.276407591	0.022821626	0.002359174	2.097845947	4.664114997	0.666666667	CRA004775;PRJNA827465;Yang2025_Silkie_Wencha	0.327605279
ENSGALG00010027280	HMCES	3	0.142842585	0.062740319	2.276727098	0.022802527	0	1.219729867	0	0.666666667	CRA004775;PRJNA827465;Yang2025_Silkie_Wencha	0.327605279
ENSGALG00010006988	USPL1	3	0.231319367	0.101675141	2.275082825	0.022900962	0.015729323	4.079304889	50.97203925	1	CRA004775;PRJNA827465;Yang2025_Silkie_Wencha	0.328061498
ENSGALG00010024728	SLC5A9	3	0.987600973	0.434801749	2.271382246	0.023123848	0.373222544	6.058778096	66.99004373	1	CRA004775;PRJNA827465;Yang2025_Silkie_Wencha	0.329251783
ENSGALG00010001111	SUCNR1	3	0.563967296	0.248346274	2.270890908	0.023153583	0	0.506941676	0	1	CRA004775;PRJNA827465;Yang2025_Silkie_Wencha	0.329251783
ENSGALG00010016799	WDTC1	3	-0.3795889	0.167119891	-2.271356796	0.023125388	0.059254841	10.54993204	81.04253191	1	CRA004775;PRJNA827465;Yang2025_Silkie_Wencha	0.329251783
ENSGALG00010005497	MKX	3	0.318212384	0.139995664	2.273015994	0.023025216	0	0.499114653	0	1	CRA004775;PRJNA827465;Yang2025_Silkie_Wencha	0.329251783
ENSGALG00010005498	SMO	3	-0.303431099	0.133633933	-2.27061415	0.023170346	0.032603862	7.083334054	71.76470876	1	CRA004775;PRJNA827465;Yang2025_Silkie_Wencha	0.329251783
ENSGALG00010001532	ENSGALG0001000153	3	0.238975123	0.10520555	2.271506823	0.023116314	0	0.400590756	0	1	CRA004775;PRJNA827465;Yang2025_Silkie_Wencha	0.329251783
ENSGALG00010015508	FUCA1	3	-0.131651116	0.057972601	-2.270919592	0.023151846	0	0.642299481	0	1	CRA004775;PRJNA827465;Yang2025_Silkie_Wencha	0.329251783
ENSGALG00010014878	TMCO1	3	-0.128032951	0.056375971	-2.271055346	0.023143628	0.001450895	2.175737169	8.077132254	1	CRA004775;PRJNA827465;Yang2025_Silkie_Wencha	0.329251783
ENSGALG00010003266	ENSGALG0001000326	2	1.301862564	0.573983434	2.268118706	0.023321973	0.300400253	1.77778428	43.75020574	1	PRJNA827465;Yang2025_Silkie_Wenchang	0.329731876
ENSGALG00010011377	SEC16B	3	0.682933497	0.300902603	2.269616449	0.023230865	0.171571358	5.546209959	63.93933849	1	CRA004775;PRJNA827465;Yang2025_Silkie_Wencha	0.329731876
ENSGALG00010029341	SLC43A2	3	-0.455800509	0.201059577	-2.266992278	0.023390697	0.074155024	7.165636413	72.08901088	0.666666667	CRA004775;PRJNA827465;Yang2025_Silkie_Wencha	0.329731876
ENSGALG00010028598	TMOD4	3	-0.249293189	0.109860786	-2.269173546	0.023257774	0	0.084231963	0	1	CRA004775;PRJNA827465;Yang2025_Silkie_Wencha	0.329731876
ENSGALG00010020550	HECTD2	3	0.238222748	0.105057975	2.267536076	0.023357498	0	1.615566296	0	1	CRA004775;PRJNA827465;Yang2025_Silkie_Wencha	0.329731876
ENSGALG00010017098	DAAM1	3	0.124662713	0.054945988	2.268822839	0.023279102	0	1.069291067	0	1	CRA004775;PRJNA827465;Yang2025_Silkie_Wencha	0.329731876
ENSGALG00010014611	TMEM189	3	-0.103802588	0.045768056	-2.268013932	0.023328358	0	1.029621642	0	0.666666667	CRA004775;PRJNA827465;Yang2025_Silkie_Wencha	0.329731876
ENSGALG00010021435	GCN1	3	-0.089107266	0.039304716	-2.26708839	0.023384827	0	0.896100057	0	0.666666667	CRA004775;PRJNA827465;Yang2025_Silkie_Wencha	0.329731876
ENSGALG00010023396	ENSGALG0001002339	3	0.306218043	0.135155306	2.265675336	0.023471269	0	0.87500654	0	1	CRA004775;PRJNA827465;Yang2025_Silkie_Wencha	0.330486684
ENSGALG00010017972	RNPEPL1	3	-0.182861078	0.080720767	-2.265353577	0.023490991	0.010260902	4.479148141	55.34865254	1	CRA004775;PRJNA827465;Yang2025_Silkie_Wencha	0.330486684
ENSGALG00010020048	ENSGALG0001002004	3	-0.399471733	0.176437426	-2.264098624	0.02356805	0.030227422	2.886760795	30.71819447	0.666666667	CRA004775;PRJNA827465;Yang2025_Silkie_Wencha	0.331241206
ENSGALG00010019120	DAGLB	3	-0.140282206	0.061989955	-2.262982843	0.023636747	0	1.411987121	0	1	CRA004775;PRJNA827465;Yang2025_Silkie_Wencha	0.331876827
ENSGALG00010003816	KLHL8	3	-0.174015239	0.076923263	-2.262192638	0.023685504	0	0.416058372	0	1	CRA004775;PRJNA827465;Yang2025_Silkie_Wencha	0.332231489
ENSGALG00010016941	SLC8A1	3	-0.273974421	0.121160586	-2.261250386	0.023743757	0	0.54442179	0	1	CRA004775;PRJNA827465;Yang2025_Silkie_Wencha	0.332389084
ENSGALG00010004226	RUNDC3B	3	-0.258265588	0.11419944	-2.261531127	0.023726387	0	1.919328578	0	0.666666667	CRA004775;PRJNA827465;Yang2025_Silkie_Wencha	0.332389084
ENSGALG00010003256	ENSGALG0001000325	2	1.124957416	0.497839286	2.259679878	0.023841126	0.261463057	1.634362252	38.81405429	1	CRA004775;Yang2025_Silkie_Wenchang	0.332643509
ENSGALG00010026638	LAD1	3	0.908944511	0.402167155	2.260116223	0.023814039	0.283166368	5.175333341	61.35514627	1	CRA004775;PRJNA827465;Yang2025_Silkie_Wencha	0.332643509
ENSGALG00010023222	ENSGALG0001002322	2	-0.798859929	0.353617384	-2.25910819	0.023876656	0	0.16571466	0	1	PRJNA827465;Yang2025_Silkie_Wenchang	0.332643509
ENSGALG00010005240	VIP	3	0.305373377	0.135138482	2.259707019	0.02383944	0	0.688346689	0	1	CRA004775;PRJNA827465;Yang2025_Silkie_Wencha	0.332643509
ENSGALG00010001798	PSTK	3	0.194142142	0.08595368	2.258683314	0.023903091	0	0.115177866	0	1	CRA004775;PRJNA827465;Yang2025_Silkie_Wencha	0.332643509
ENSGALG00010027570	NDUFS2	3	-0.158686651	0.070253448	-2.258773843	0.023897456	0	0.336385581	0	1	CRA004775;PRJNA827465;Yang2025_Silkie_Wencha	0.332643509
ENSGALG00010016289	IRF1	3	-0.330404369	0.146321866	-2.258065568	0.023941572	0.016379307	2.627921114	23.89421473	1	CRA004775;PRJNA827465;Yang2025_Silkie_Wencha	0.332851411
ENSGALG00010027123	WDR86	3	-0.341526664	0.151301108	-2.257264787	0.023991534	0.041945097	5.604029979	64.3113972	1	CRA004775;PRJNA827465;Yang2025_Silkie_Wencha	0.333205812
ENSGALG00010001466	PRKX	3	-0.138244071	0.061253908	-2.256902043	0.024014196	0	0.832352536	0	0.666666667	CRA004775;PRJNA827465;Yang2025_Silkie_Wencha	0.333205812
ENSGALG00010006279	HACD1	3	-0.14704374	0.065169187	-2.256338403	0.024049446	0.000208418	2.025939228	1.280355698	0.666666667	CRA004775;PRJNA827465;Yang2025_Silkie_Wencha	0.333367763
ENSGALG00010023487	GRN	3	0.153742005	0.068186539	2.254726619	0.024150494	0	0.269616789	0	1	CRA004775;PRJNA827465;Yang2025_Silkie_Wencha	0.334440579
ENSGALG00010029580	P2RX1	3	0.409733835	0.181853925	2.253093163	0.024253275	0.01981414	2.477796503	19.28312121	1	CRA004775;PRJNA827465;Yang2025_Silkie_Wencha	0.334871665
ENSGALG00010019141	ACOT7	3	0.251943607	0.111857451	2.252363217	0.024299328	0.008763366	2.563393195	21.97841501	0.666666667	CRA004775;PRJNA827465;Yang2025_Silkie_Wencha	0.334871665
ENSGALG00010000324	ENSGALG0001000032	3	-0.232052772	0.103009262	-2.252736958	0.024275739	0	0.007864924	0	1	CRA004775;PRJNA827465;Yang2025_Silkie_Wencha	0.334871665
ENSGALG00010007393	PLCG2	3	0.218446824	0.0969861	2.252351874	0.024300044	0	0.138110531	0	1	CRA004775;PRJNA827465;Yang2025_Silkie_Wencha	0.334871665
ENSGALG00010024508	RPS27L	3	-0.204238382	0.090631874	-2.253493967	0.024228021	0	1.898032713	0	0.666666667	CRA004775;PRJNA827465;Yang2025_Silkie_Wencha	0.334871665
ENSGALG00010002346	FBXO15	3	-0.958063702	0.425516272	-2.25153247	0.024351833	0.356326089	6.226835451	67.88095629	1	CRA004775;PRJNA827465;Yang2025_Silkie_Wencha	0.334932455
ENSGALG00010009065	EIF2B4	3	-0.127616849	0.056673715	-2.251781966	0.024336054	0	1.563283129	0	1	CRA004775;PRJNA827465;Yang2025_Silkie_Wencha	0.334932455
ENSGALG00010003756	MHCY7	3	-0.703195225	0.312595473	-2.249537457	0.024478323	0	0.193893153	0	1	CRA004775;PRJNA827465;Yang2025_Silkie_Wencha	0.335692533
ENSGALG00010008162	HSBP1L1	3	-0.365662633	0.162520258	-2.249951097	0.02445205	0	0.503161757	0	1	CRA004775;PRJNA827465;Yang2025_Silkie_Wencha	0.335692533
ENSGALG00010008354	TMEM71	3	-0.307849949	0.136831616	-2.249845157	0.024458776	0	0.425868007	0	1	CRA004775;PRJNA827465;Yang2025_Silkie_Wencha	0.335692533
ENSGALG00010015946	TLE4	3	0.247692327	0.110190767	2.247850111	0.024585749	0.008972254	2.582887288	22.56727543	1	CRA004775;PRJNA827465;Yang2025_Silkie_Wencha	0.336839059
ENSGALG00010011445	MTERF3	3	-0.209656998	0.093355119	-2.245800765	0.024716772	0.005920537	2.533014811	21.04270407	0.666666667	CRA004775;PRJNA827465;Yang2025_Silkie_Wencha	0.338306335
ENSGALG00010013358	RPS16	3	-0.274908473	0.12243717	-2.245302415	0.024748725	0.017735646	2.915688187	31.40555946	1	CRA004775;PRJNA827465;Yang2025_Silkie_Wencha	0.338416078
ENSGALG00010028108	IQGAP3	3	0.378031525	0.168456866	2.244084992	0.024826933	0.047670607	4.984034148	59.87186402	1	CRA004775;PRJNA827465;Yang2025_Silkie_Wencha	0.339157499
ENSGALG00010004127	EPHA7	3	-0.296397254	0.132348894	-2.239514408	0.025122465	0	1.023386581	0	0.666666667	CRA004775;PRJNA827465;Yang2025_Silkie_Wencha	0.342863447
ENSGALG00010002918	LRATD1	3	0.37124863	0.166006712	2.236347113	0.025329043	0	1.53963331	0	1	CRA004775;PRJNA827465;Yang2025_Silkie_Wencha	0.342973112
ENSGALG00010027994	TUFT1	3	0.273598166	0.122351788	2.236159929	0.025341297	0.024681467	4.874641921	58.97134533	1	CRA004775;PRJNA827465;Yang2025_Silkie_Wencha	0.342973112
ENSGALG00010023749	RPP25	3	0.242356413	0.108294537	2.237937564	0.025225127	0	0.431527752	0	1	CRA004775;PRJNA827465;Yang2025_Silkie_Wencha	0.342973112
ENSGALG00010021224	MYOZ1	3	-0.215235299	0.096257136	-2.236045124	0.025348815	0	1.61498002	0	1	CRA004775;PRJNA827465;Yang2025_Silkie_Wencha	0.342973112
ENSGALG00010014914	PTAR1	3	0.187915161	0.084036328	2.236118169	0.025344032	0	0.642727048	0	1	CRA004775;PRJNA827465;Yang2025_Silkie_Wencha	0.342973112
ENSGALG00010029270	TIMP2	3	0.178143054	0.079651151	2.236540864	0.025316364	0.006764328	3.039673483	34.20345931	1	CRA004775;PRJNA827465;Yang2025_Silkie_Wencha	0.342973112
ENSGALG00010018345	WDR24	3	-0.177298583	0.079270642	-2.236623532	0.025310956	0.004852479	2.619946077	23.66255102	1	CRA004775;PRJNA827465;Yang2025_Silkie_Wencha	0.342973112
ENSGALG00010007784	INTS10	3	-0.120916386	0.054048354	-2.237189058	0.025273986	0	1.818195657	0	1	CRA004775;PRJNA827465;Yang2025_Silkie_Wencha	0.342973112
ENSGALG00010020429	ZNF148	3	0.117978647	0.052744067	2.236813615	0.025298524	0	1.390320908	0	1	CRA004775;PRJNA827465;Yang2025_Silkie_Wencha	0.342973112
ENSGALG00010000285	MYLPF	3	-0.255652324	0.11435352	-2.235631439	0.025375923	0	0.057437998	0	1	CRA004775;PRJNA827465;Yang2025_Silkie_Wencha	0.342995536
ENSGALG00010025592	HSPA8	3	0.244277077	0.109300626	2.234910131	0.025423249	0	0.911494837	0	1	CRA004775;PRJNA827465;Yang2025_Silkie_Wencha	0.342995536
ENSGALG00010020989	MAPK4	3	0.07911984	0.035398718	2.235104657	0.025410479	0	0.946403326	0	0.666666667	CRA004775;PRJNA827465;Yang2025_Silkie_Wencha	0.342995536
ENSGALG00010025116	ANAPC7	3	-0.09718389	0.043498148	-2.23420756	0.025469419	0	0.930764426	0	1	CRA004775;PRJNA827465;Yang2025_Silkie_Wencha	0.343290866
ENSGALG00010027986	CGN	3	0.854600711	0.382659519	2.233318833	0.025527926	0.335673452	10.26418717	80.51477465	1	CRA004775;PRJNA827465;Yang2025_Silkie_Wencha	0.343530702
ENSGALG00010018298	ENSGALG0001001829	3	0.768063035	0.343929467	2.23319927	0.025535806	0.287875867	15.94509817	87.45696026	1	CRA004775;PRJNA827465;Yang2025_Silkie_Wencha	0.343530702
ENSGALG00010004332	ENSGALG0001000433	3	-0.864632536	0.387467866	-2.231494822	0.02564837	0	1.151589238	0	1	CRA004775;PRJNA827465;Yang2025_Silkie_Wencha	0.344389408
ENSGALG00010014912	ENSGALG0001001491	3	-0.696113737	0.312052352	-2.23075946	0.025697067	0.156111022	4.324374567	53.75053735	1	CRA004775;PRJNA827465;Yang2025_Silkie_Wencha	0.344389408
ENSGALG00010014197	SH3GL2	3	-0.316496635	0.141830614	-2.231511418	0.025647272	0	0.062676686	0	1	CRA004775;PRJNA827465;Yang2025_Silkie_Wencha	0.344389408
ENSGALG00010006446	LSAMP	3	-0.196226407	0.087952903	-2.231039574	0.025678508	0	0.574279578	0	0.666666667	CRA004775;PRJNA827465;Yang2025_Silkie_Wencha	0.344389408
ENSGALG00010018554	PPIA	3	-0.113787749	0.051019719	-2.230270013	0.025729523	0	0.209309063	0	1	CRA004775;PRJNA827465;Yang2025_Silkie_Wencha	0.344497842
ENSGALG00010001142	PPM1L	3	-0.321322568	0.144120223	-2.229545325	0.025777643	0.024129583	3.26234824	38.69446629	0.666666667	CRA004775;PRJNA827465;Yang2025_Silkie_Wencha	0.344815606
ENSGALG00010017741	SMCR8	3	-0.167920693	0.075366043	-2.228068333	0.025875959	0.006420812	3.148880043	36.48535438	1	CRA004775;PRJNA827465;Yang2025_Silkie_Wencha	0.345634299
ENSGALG00010029418	ABCA5	3	-0.153147256	0.068740889	-2.227891702	0.025887738	0	1.449113034	0	1	CRA004775;PRJNA827465;Yang2025_Silkie_Wencha	0.345634299
ENSGALG00010013382	ASB12	3	-0.211880264	0.095188031	-2.225912879	0.026020018	0	0.477228323	0	1	CRA004775;PRJNA827465;Yang2025_Silkie_Wencha	0.347072668
ENSGALG00010011274	ENSGALG0001001127	3	-1.249442688	0.561989908	-2.223247553	0.026199112	0.561336019	4.909934415	59.26625835	1	CRA004775;PRJNA827465;Yang2025_Silkie_Wencha	0.34791097
ENSGALG00010027373	MLN	2	-1.081947132	0.486399322	-2.224400987	0.026121477	0	0.506954213	0	1	PRJNA827465;Yang2025_Silkie_Wenchang	0.34791097
ENSGALG00010004780	ENSGALG0001000478	3	0.354279737	0.159311445	2.223818489	0.026160659	0.000506725	2.011949772	0.593939891	1	CRA004775;PRJNA827465;Yang2025_Silkie_Wencha	0.34791097
ENSGALG00010014310	COL6A1	3	-0.151856481	0.068306994	-2.22314688	0.026205897	0	0.182557155	0	0.666666667	CRA004775;PRJNA827465;Yang2025_Silkie_Wencha	0.34791097
ENSGALG00010006359	SGK1	3	0.138665018	0.062370674	2.223240639	0.026199578	0	1.31454615	0	1	CRA004775;PRJNA827465;Yang2025_Silkie_Wencha	0.34791097
ENSGALG00010016336	SLC24A2	2	-0.631081587	0.284152054	-2.220929176	0.026355759	0	0.368385676	0	1	PRJNA827465;Yang2025_Silkie_Wenchang	0.349572301
ENSGALG00010009348	ERGIC2	3	-0.137892078	0.062106646	-2.220246736	0.026402023	0.005486575	3.811456004	47.52661456	1	CRA004775;PRJNA827465;Yang2025_Silkie_Wencha	0.349641467
ENSGALG00010020204	GSPT2	3	-0.087069928	0.039218518	-2.220122825	0.026410431	0	0.164797574	0	1	CRA004775;PRJNA827465;Yang2025_Silkie_Wencha	0.349641467
ENSGALG00010000577	ENSGALG0001000057	3	0.590819856	0.266192504	2.219521016	0.026451299	0.115924277	4.313665917	53.63572334	1	CRA004775;PRJNA827465;Yang2025_Silkie_Wencha	0.349854927
ENSGALG00010021924	ENSGALG0001002192	3	0.858358114	0.386986774	2.218055425	0.026551054	0	0.444779756	0	1	CRA004775;PRJNA827465;Yang2025_Silkie_Wencha	0.349865188
ENSGALG00010007985	APELA	2	0.32562548	0.146806526	2.218058618	0.026550836	0	0.20783377	0	1	PRJNA827465;Yang2025_Silkie_Wenchang	0.349865188
ENSGALG00010010776	GCAT	3	-0.189581602	0.085471166	-2.218076718	0.026549602	0	1.62310544	0	1	CRA004775;PRJNA827465;Yang2025_Silkie_Wencha	0.349865188
ENSGALG00010018911	CNIH1	3	-0.081252364	0.036629105	-2.218245993	0.026538064	0	0.309768675	0	1	CRA004775;PRJNA827465;Yang2025_Silkie_Wencha	0.349865188
ENSGALG00010013298	EPYC	2	1.11960049	0.505478193	2.214933317	0.026764642	0	0.549751079	0	1	PRJNA827465;Yang2025_Silkie_Wenchang	0.350025678
ENSGALG00010015653	HCK	3	0.571205197	0.257834512	2.215394646	0.026732989	0.033167965	2.390882839	16.34889142	1	CRA004775;PRJNA827465;Yang2025_Silkie_Wencha	0.350025678
ENSGALG00010008947	SPMIP2	2	0.520543558	0.23491948	2.215838203	0.026702585	0	0.52486835	0	1	PRJNA827465;Yang2025_Silkie_Wenchang	0.350025678
ENSGALG00010025587	LRRC56	2	0.420818063	0.189846217	2.216626015	0.026648658	0.031531948	1.713229181	41.63069301	1	PRJNA827465;Yang2025_Silkie_Wenchang	0.350025678
ENSGALG00010016340	RGS12	3	0.342485072	0.154631834	2.214841947	0.026770915	0.040804346	4.632791865	56.82948731	1	CRA004775;PRJNA827465;Yang2025_Silkie_Wencha	0.350025678
ENSGALG00010013322	IRS2	3	-0.272693367	0.12311418	-2.21496312	0.026762596	0.011465321	2.610433359	23.3843686	0.666666667	CRA004775;PRJNA827465;Yang2025_Silkie_Wencha	0.350025678
ENSGALG00010002277	RGS10	3	-0.257078839	0.11597068	-2.216757186	0.026639689	0.020703694	4.543052767	55.97673849	1	CRA004775;PRJNA827465;Yang2025_Silkie_Wencha	0.350025678
ENSGALG00010017629	ENSGALG0001001762	3	0.180305644	0.081399236	2.215077835	0.026754723	0.008456044	3.40469184	41.25753243	1	CRA004775;PRJNA827465;Yang2025_Silkie_Wencha	0.350025678
ENSGALG00010028871	PIP5K1A	3	-0.116048682	0.052404036	-2.214498955	0.026794475	0	0.488797904	0	1	CRA004775;PRJNA827465;Yang2025_Silkie_Wencha	0.350025678
ENSGALG00010028535	TICAM1	3	-0.100808	0.045526691	-2.214261528	0.026810793	0.0002104	2.03266236	1.60687582	0.666666667	CRA004775;PRJNA827465;Yang2025_Silkie_Wencha	0.350025678
ENSGALG00010018027	EML5	2	0.838037771	0.378632834	2.213325672	0.026875201	0.152967697	2.079806809	51.9186111	1	PRJNA827465;Yang2025_Silkie_Wenchang	0.350205925

ENSGALG00010005273	ENSGALG0001000527	3	-0.651225196	0.294576684	-2.210715345	0.027055555	0.059951528	2.587088178	22.69300996	1	CRA004775;PRJNA827465;Yang2025_Silkie_Wencha	0.350205925
ENSGALG00010016450	ENSGALG0001001645	3	0.456087702	0.206223017	2.211623648	0.02699268	0.046477427	3.121572289	35.9297234	0.666666667	CRA004775;PRJNA827465;Yang2025_Silkie_Wencha	0.350205925
ENSGALG00010002523	DNAJB13	3	0.397112394	0.179679369	2.210116809	0.027097057	0	1.424656517	0	1	CRA004775;PRJNA827465;Yang2025_Silkie_Wencha	0.350205925
ENSGALG00010014994	ENSGALG0001001499	3	-0.257338653	0.116419896	-2.210435355	0.027074962	0.024860313	5.225879652	61.72893114	1	CRA004775;PRJNA827465;Yang2025_Silkie_Wencha	0.350205925
ENSGALG00010021017	COQ7	3	0.248684903	0.112423554	2.212035599	0.026964205	0.018188397	3.936921768	49.19888894	1	CRA004775;PRJNA827465;Yang2025_Silkie_Wencha	0.350205925
ENSGALG00010003136	NUP98	3	-0.167938099	0.07598566	-2.210128839	0.027096222	0.009432717	4.782666426	58.18232296	1	CRA004775;PRJNA827465;Yang2025_Silkie_Wencha	0.350205925
ENSGALG00010014400	RIPOR3	3	0.163002137	0.073727675	2.210867728	0.027044998	0	1.227382136	0	1	CRA004775;PRJNA827465;Yang2025_Silkie_Wencha	0.350205925
ENSGALG00010016943	PCNX4	3	0.119864777	0.054170814	2.212718783	0.02691704	0	1.645149541	0	0.666666667	CRA004775;PRJNA827465;Yang2025_Silkie_Wencha	0.350205925
ENSGALG00010006351	IGF2R	3	-0.095655911	0.043246211	-2.211891131	0.026974188	0	1.202063748	0	0.666666667	CRA004775;PRJNA827465;Yang2025_Silkie_Wencha	0.350205925
ENSGALG00010027036	NUCKS1	3	-0.085381301	0.038609339	-2.211415792	0.027007057	0	1.155451647	0	0.666666667	CRA004775;PRJNA827465;Yang2025_Silkie_Wencha	0.350205925
ENSGALG00010008123	ENSGALG0001000812	3	-0.223422161	0.101143649	-2.208958885	0.027177501	0.013695858	3.673446951	45.55522302	1	CRA004775;PRJNA827465;Yang2025_Silkie_Wencha	0.350924825
ENSGALG00010027267	MYO9B	3	0.106808638	0.048414398	2.206133774	0.027374635	0	0.871162262	0	0.666666667	CRA004775;PRJNA827465;Yang2025_Silkie_Wencha	0.353147772
ENSGALG00010019692	MMEL1	3	0.812043015	0.368208937	2.205386491	0.027426985	0.274064556	6.383378967	68.6686313	1	CRA004775;PRJNA827465;Yang2025_Silkie_Wencha	0.353226434
ENSGALG00010023383	HOXB6	3	0.219246912	0.099416667	2.205333553	0.027430697	0.008360147	2.721316361	26.50615605	1	CRA004775;PRJNA827465;Yang2025_Silkie_Wencha	0.353226434
ENSGALG00010002012	MID1	3	-0.310775588	0.140954145	-2.204799205	0.027468188	0.021868184	3.171615559	36.94065492	1	CRA004775;PRJNA827465;Yang2025_Silkie_Wencha	0.353387359
ENSGALG00010014011	SFN	2	1.03555205	0.460868992	2.203559074	0.027555368	0	0.622302253	0	1	PRJNA827465;Yang2025_Silkie_Wenchang	0.354186683
ENSGALG00010028451	TMEM59L	3	0.287160313	0.130378126	2.202519113	0.027628661	0	0.425667359	0	1	CRA004775;PRJNA827465;Yang2025_Silkie_Wencha	0.354484239
ENSGALG00010017817	REEP1	3	-0.146710278	0.06660886	-2.202564	0.027625494	0	1.079475954	0	1	CRA004775;PRJNA827465;Yang2025_Silkie_Wencha	0.354484239
ENSGALG00010011148	ENSGALG0001001114	3	-0.240194467	0.109081904	-2.201964366	0.027667826	0.013045615	3.055004192	34.53364138	1	CRA004775;PRJNA827465;Yang2025_Silkie_Wencha	0.354664902
ENSGALG00010019298	PGAP6	3	-0.241350957	0.109661171	-2.2008789	0.027744598	0.017525451	3.812798212	47.5450866	1	CRA004775;PRJNA827465;Yang2025_Silkie_Wencha	0.355326876
ENSGALG00010029161	HNF1B	2	1.33182476	0.605253799	2.200440148	0.027775682	0	0.988340844	0	1	CRA004775;PRJNA827465	0.355403048
ENSGALG00010012347	ENSGALG0001001234	3	0.823048053	0.374196865	2.199505472	0.02784200	0.238431445	4.907819232	59.24870282	0.666666667	CRA004775;PRJNA827465;Yang2025_Silkie_Wencha	0.35560799
ENSGALG00010027245	LRIG2	3	-0.139593072	0.060903392	-2.199796547	0.027821333	0.003012795	2.576919859	22.38796278	0.666666667	CRA004775;PRJNA827465;Yang2025_Silkie_Wencha	0.35560799
ENSGALG00010029724	CACNA1G	3	0.203279383	0.092453722	2.19871498	0.027898195	0	0.952338686	0	0.666666667	CRA004775;PRJNA827465;Yang2025_Silkie_Wencha	0.356004135
ENSGALG00010017568	CDCP1	3	0.528822715	0.240709465	2.196919839	0.028025188	0.085990662	3.955648	49.43938389	1	CRA004775;PRJNA827465;Yang2025_Silkie_Wencha	0.356980295
ENSGALG00010012986	TMEM184B	3	-0.178216312	0.081117122	-2.197024594	0.02801869	0.009230862	3.702591142	45.98377397	1	CRA004775;PRJNA827465;Yang2025_Silkie_Wencha	0.356980295
ENSGALG00010020753	SYDE2	3	0.221164747	0.100719849	2.196039298	0.028089131	0.015442686	4.143154223	51.7275995	1	CRA004775;PRJNA827465;Yang2025_Silkie_Wencha	0.357472744
ENSGALG00010021612	BCS1L	3	0.167258971	0.076229248	2.194157419	0.028224095	0	1.841641964	0	0.666666667	CRA004775;PRJNA827465;Yang2025_Silkie_Wencha	0.358867332
ENSGALG00010002817	SHFM1	3	-0.17172891	0.078287564	-2.193565626	0.028266652	0.003036178	2.203826755	9.248764893	0.666666667	CRA004775;PRJNA827465;Yang2025_Silkie_Wencha	0.359085526
ENSGALG00010020938	SMG1	3	-0.1019818	0.046502667	-2.193031214	0.02830513	0	1.854670111	0	0.666666667	CRA004775;PRJNA827465;Yang2025_Silkie_Wencha	0.359251558
ENSGALG00010024750	APOA4	2	1.82072119	0.832084546	2.188239135	0.028652188	0.101559148	1.078386944	7.268907027	1	CRA004775;PRJNA827465	0.362276731
ENSGALG00010006035	ADH1C	3	1.105384475	0.505055655	2.188638943	0.028623093	0.465385193	5.116283901	60.90912782	1	CRA004775;PRJNA827465;Yang2025_Silkie_Wencha	0.362276731
ENSGALG00010003323	STMN2	3	1.004452468	0.459079076	2.187972664	0.028671594	0.227310105	3.098326047	35.44901441	1	CRA004775;PRJNA827465;Yang2025_Silkie_Wencha	0.362276731
ENSGALG00010003035	DGAT2	3	-0.540235109	0.246842539	-2.188581882	0.028627244	0.001693735	2.017920938	0.888089197	1	CRA004775;PRJNA827465;Yang2025_Silkie_Wencha	0.362276731
ENSGALG00010016482	EML1	3	-0.138433305	0.063245842	-2.188812759	0.028610452	0	0.153965434	0	1	CRA004775;PRJNA827465;Yang2025_Silkie_Wencha	0.362276731
ENSGALG00010023736	KIF5A	2	-0.883318453	0.404154769	-2.185594533	0.028845283	0	0.088572216	0	1	CRA004775;PRJNA827465	0.363202693
ENSGALG00010029585	ENSGALG0001002958	3	0.360216205	0.164835024	2.185313509	0.028865868	0.000326371	2.007879623	0.392435017	1	CRA004775;PRJNA827465;Yang2025_Silkie_Wencha	0.363202693
ENSGALG00010010215	ENSGALG0001001021	3	-0.341404643	0.156234116	-2.185211853	0.028873317	0.051538378	6.998461362	71.42228989	0.666666667	CRA004775;PRJNA827465;Yang2025_Silkie_Wencha	0.363202693
ENSGALG00010005333	PLXNA4	3	-0.252576766	0.115546888	-2.185924441	0.028821134	0	1.037428849	0	1	CRA004775;PRJNA827465;Yang2025_Silkie_Wencha	0.363202693
ENSGALG00010014250	ENSGALG0001001425	3	-0.250669286	0.114699616	-2.185441376	0.0288565	0	0.124159957	0	1	CRA004775;PRJNA827465;Yang2025_Silkie_Wencha	0.363202693
ENSGALG00010026090	USP2	3	-0.144708435	0.066252484	-2.184196374	0.028947821	0	0.478272669	0	1	CRA004775;PRJNA827465;Yang2025_Silkie_Wencha	0.363816218
ENSGALG00010010422	CDH1	3	1.454231874	0.665981407	2.183592301	0.02899222	0.829247736	5.3120753	62.3499313	1	CRA004775;PRJNA827465;Yang2025_Silkie_Wencha	0.364050618
ENSGALG00010021447	OCSTAMP	2	0.930348377	0.426451272	2.181605348	0.029138672	0	0.557996556	0	1	CRA004775;Yang2025_Silkie_Wenchang	0.364917344
ENSGALG00010003017	PGM3	3	0.286736847	0.131402053	2.182133691	0.029099667	0.021880062	3.418703605	41.498292	0.666666667	CRA004775;PRJNA827465;Yang2025_Silkie_Wencha	0.364917344
ENSGALG00010008088	SLC35A4	3	-0.137350714	0.062957403	-2.181645177	0.02913573	0	1.582374515	0	1	CRA004775;PRJNA827465;Yang2025_Silkie_Wencha	0.364917344
ENSGALG00010024521	HEPACAM	3	0.882091126	0.404515427	2.180611829	0.029212139	0.32172008	5.902075654	66.11361634	1	CRA004775;PRJNA827465;Yang2025_Silkie_Wencha	0.365513663
ENSGALG00010024211	TRPM5	3	1.149583333	0.527324481	2.180030275	0.029255217	0.590011361	7.301172587	72.60921547	1	CRA004775;PRJNA827465;Yang2025_Silkie_Wencha	0.365729018
ENSGALG00010016935	PRIMA1	3	0.315235173	0.144702911	2.178499177	0.029368893	0	0.534816232	0	1	CRA004775;PRJNA827465;Yang2025_Silkie_Wencha	0.366758613
ENSGALG00010000263	MTMR3	3	-0.112001432	0.051426949	-2.177874321	0.029415395	0	0.719504061	0	1	CRA004775;PRJNA827465;Yang2025_Silkie_Wencha	0.366758613
ENSGALG00010013392	HDAC2	3	0.091892489	0.042188939	2.178118048	0.029397249	0	0.415572047	0	1	CRA004775;PRJNA827465;Yang2025_Silkie_Wencha	0.366758613
ENSGALG00010010851	PNOC	3	-0.747407143	0.34339388	-2.17653018	0.02951564	0.178757345	4.339998859	53.9170491	0.666666667	CRA004775;PRJNA827465;Yang2025_Silkie_Wencha	0.367684253
ENSGALG00010011538	ENSGALG0001001153	3	-0.490551282	0.225527764	-2.175125909	0.029620682	0.061423388	3.272411469	38.88299136	1	CRA004775;PRJNA827465;Yang2025_Silkie_Wencha	0.368667983
ENSGALG00010019594	TMX1	3	-0.459427802	0.21187277	-2.16841363	0.03012723	0	0.949119776	0	1	CRA004775;PRJNA827465;Yang2025_Silkie_Wencha	0.373329447
ENSGALG00010015431	RIN3	3	-0.171648349	0.079144422	-2.16879908	0.030097941	0	1.076616137	0	1	CRA004775;PRJNA827465;Yang2025_Silkie_Wencha	0.373329447
ENSGALG00010024204	PTBP2	3	-0.169718533	0.077791357	-2.169718077	0.03002821	0.00213497	2.184606969	8.450351556	0.666666667	CRA004775;PRJNA827465;Yang2025_Silkie_Wencha	0.373329447
ENSGALG00010028189	GPSM1	3	-0.144255723	0.066518638	-2.168651176	0.030109177	0	1.263072636	0	1	CRA004775;PRJNA827465;Yang2025_Silkie_Wencha	0.373329447
ENSGALG00010022151	PIP4K2B	3	-0.133283986	0.061456789	-2.168743098	0.030102193	0	0.564580774	0	1	CRA004775;PRJNA827465;Yang2025_Silkie_Wencha	0.373329447
ENSGALG00010007892	ENSGALG0001000789	3	1.22527864	0.56612028	2.164343308	0.030438011	0.652153938	6.266579963	68.08466481	1	CRA004775;PRJNA827465;Yang2025_Silkie_Wencha	0.373371282
ENSGALG00010027046	HESX1	2	0.399217329	0.184417481	2.164747759	0.030407007	0	0.378954498	0	1	PRJNA827465;Yang2025_Silkie_Wenchang	0.373371282
ENSGALG00010007165	ENSGALG0001000716	3	-0.37678479	0.173898676	-2.166691543	0.030258382	0	0.030746717	0	1	CRA004775;PRJNA827465;Yang2025_Silkie_Wencha	0.373371282
ENSGALG00010029354	TLCD2	3	0.328111421	0.151487849	2.165925668	0.030316867	0	1.856631889	0	1	CRA004775;PRJNA827465;Yang2025_Silkie_Wencha	0.373371282
ENSGALG00010030106	GPRC5C	3	0.243918405	0.11264373	2.165397085	0.030357289	0	0.452151007	0	1	CRA004775;PRJNA827465;Yang2025_Silkie_Wencha	0.373371282
ENSGALG00010007994	FAM65B	3	-0.175858281	0.081225515	-2.165062066	0.030382933	0	1.215577627	0	1	CRA004775;PRJNA827465;Yang2025_Silkie_Wencha	0.373371282
ENSGALG00010018057	SSTR4	3	0.17254807	0.079659555	2.166068716	0.030305936	0	1.842934796	0	0.666666667	CRA004775;PRJNA827465;Yang2025_Silkie_Wencha	0.373371282
ENSGALG00010009543	MAF1	3	-0.147119165	0.067944897	-2.165271725	0.030366882	0.000312812	2.036929692	1.813007685	1	CRA004775;PRJNA827465;Yang2025_Silkie_Wencha	0.373371282
ENSGALG00010020611	LPGAT1	3	-0.131921139	0.06087421	-2.167110499	0.03022643	0	0.186551763	0	1	CRA004775;PRJNA827465;Yang2025_Silkie_Wencha	0.373371282
ENSGALG00010017503	MED9	3	-0.122904984	0.05678822	-2.164269005	0.03044371	0	1.354643067	0	1	CRA004775;PRJNA827465;Yang2025_Silkie_Wencha	0.373371282
ENSGALG00010001702	PLEKHA1	3	0.105909045	0.048881841	2.166633712	0.030262795	0	1.712501702	0	1	CRA004775;PRJNA827465;Yang2025_Silkie_Wencha	0.373371282
ENSGALG00010014676	UBE2V1	3	-0.07403046	0.034206536	-2.164219702	0.030447492	0	1.510376173	0	0.666666667	CRA004775;PRJNA827465;Yang2025_Silkie_Wencha	0.373371282
ENSGALG00010029321	TBX2	3	0.284627863	0.131566356	2.163378776	0.030512059	0.028339221	4.686104544	57.3206278	1	CRA004775;PRJNA827465;Yang2025_Silkie_Wencha	0.373838819
ENSGALG00010024214	CRABP1	2	0.858993616	0.397252896	2.162334434	0.030592407	0.190992426	2.239086982	55.33893912	1	PRJNA827465;Yang2025_Silkie_Wenchang	0.37426944
ENSGALG00010025365	SIKE1	3	-0.100295933	0.046385328	-2.162233967	0.030600147	0	1.755348437	0	1	CRA004775;PRJNA827465;Yang2025_Silkie_Wencha	0.37426944
ENSGALG00010023807	MRPL10	3	0.228569573	0.105770265	2.161000287	0.030695317	0.007695431	2.283950442	12.4324257	0.666666667	CRA004775;PRJNA827465;Yang2025_Silkie_Wencha	0.374290312
ENSGALG00010017495	NT5M	3	-0.218446993	0.101082607	-2.161074002	0.030689623	0.015778221	4.288111756	53.35942452	1	CRA004775;PRJNA827465;Yang2025_Silkie_Wencha	0.374290312
ENSGALG00010000799	ENSGALG0001000079	3	-0.201470161	0.093200927	-2.161675506	0.030643197	0	0.948868343	0	0.666666667	CRA004775;PRJNA827465;Yang2025_Silkie_Wencha	0.374290312
ENSGALG00010023252	GLG1	3	-0.094752422	0.04384982	-2.160839472	0.030707742	0	1.710107916	0	0.666666667	CRA004775;PRJNA827465;Yang2025_Silkie_Wencha	0.374290312
ENSGALG00010027224	RARRES2	3	0.172116195	0.079696178	2.159654313	0.03079944	0	1.567149308	0	1	CRA004775;PRJNA827465;Yang2025_Silkie_Wencha	0.375084659
ENSGALG00010007514	VIT	3	0.887406388	0.411308372	2.157520852	0.030965104	0	1.801357891	0	1	CRA004775;PRJNA827465;Yang2025_Silkie_Wencha	0.375324038
ENSGALG00010017748	ENSGALG0001001774	2	-0.677567138	0.314212386	-2.156398565	0.031052557	0.064781672	1.485464982	32.68101153	1	PRJNA827465;Yang2025_Silkie_Wenchang	0.375324038
ENSGALG00010024137	ENSGALG0001002413	3	0.477632101	0.221368158	2.157636878	0.030956075	0	0.623215706	0	1	CRA004775;PRJNA827465;Yang2025_Silkie_Wencha	0.375324038
ENSGALG00010014793	TLR2A	3	-0.321408085	0.148981834	-2.157364268	0.030977293	0.021573392	2.920196081	31.5114484	1	CRA004775;PRJNA827465;Yang2025_Silkie_Wencha	0.375324038
ENSGALG00010018514	EAF2	3	0.260481842	0.120798762	2.156328743	0.031058004	0	0.467754108	0	0.666666667	CRA004775;PRJNA827465;Yang2025_Silkie_Wencha	0.375324038
ENSGALG00010013958	TUSC3	3	-0.245455115	0.113760498	-2.157648028	0.030955208	0	0.816651716	0	1	CRA004775;PRJNA827465;Yang2025_Silkie_Wencha	0.375324038
ENSGALG00010001182	ZCCHC7	3	0.208220902	0.096551267	2.156583847	0.031038104	0	1.211610717	0	1	CRA004775;PRJNA827465;Yang2025_Silkie_Wencha	0.375324038
ENSGALG00010011306	ECI2	3	-0.203810839	0.094426808	-2.158400171	0.030896732	0	0.925154683	0	1	CRA004775;PRJNA827465;Yang2025_Silkie_Wencha	0.375324038
ENSGALG00010017550	TM2D2	3	-0.131047167	0.060769416	-2.156465784	0.031047313	0	1.227021914	0	1	CRA004775;PRJNA827465;Yang2025_Silkie_Wencha	0.375324038
ENSGALG00010028362	TMPRSS9	3	-0.497562427	0.230931279	-2.154590877	0.031193864	0	0.881020168	0	0.666666667	CRA004775;PRJNA827465;Yang2025_Silkie_Wencha	0.376643926
ENSGALG00010027200	G0S2	3	-0.292132705	0.135680615	-2.15309096	0.031311531	0	0.983698849	0	1	CRA004775;PRJNA827465;Yang2025_Silkie_Wencha	0.376928949
ENSGALG00010003703	TDRP	3	-0.26729012	0.124151738	-2.152930963	0.031324105	0.00398637	2.169022229	7.792554012	1	CRA004775;PRJNA827465;Yang2025_Silkie_Wencha	0.376928949
ENSGALG00010007883	DHTKD1	3	-0.179701338	0.083445226	-2.153524485	0.031277482	0.007051105	2.971047156	32.68366691	0.666666667	CRA004775;PRJNA827465;Yang2025_Silkie_Wencha	0.376928949
ENSGALG00010022200	DNMBP	3	-0.166754864	0.077441213	-2.153309049	0.031294398	0.010320008	5.048510073	60.3843516	1	CRA004775;PRJNA827465;Yang2025_Silkie_Wencha	0.376928949
ENSGALG00010016598	AIFM1	3	-0.109538221	0.050917428	-2.151291341	0.031453211	0	0.078748882	0	1	CRA004775;PRJNA827465;Yang2025_Silkie_Wencha	0.378160676
ENSGALG00010010924	ENSGALG0001001092	3	0.159954214	0.074372077	2.150729427	0.031497562	0	1.016107491	0	0.666666667	CRA004775;PRJNA827465;Yang2025_Silkie_Wencha	0.378372161
ENSGALG00010022816	METTL8	3	0.275108391	0.127961994	2.149922662	0.031561333	0	0.134565109	0	1	CRA004775;PRJNA827465;Yang2025_Silkie_Wencha	0.378816369
ENSGALG00010021783	GIT2	3	-0.08074853	0.037567057	-2.149450478	0.031598708	0	0.67879463	0	1	CRA004775;PRJNA827465;Yang2025_Silkie_Wencha	0.378943281
ENSGALG00010003305	CALR	3	0.102821846	0.047844742	2.149073059	0.031628609	0.001900972	2.670819146	25.11660689	0.666666667	CRA004775;PRJNA827465;Yang2025_Silkie_Wencha	0.378980426
ENSGALG00010028086	MATK	3	0.478182126	0.22272698	2.146942976	0.031797822	0	1.420241823	0	1	CRA004775;PRJNA827465;Yang2025_Silkie_Wencha	0.379543257
ENSGALG00010011475	NDP	3	0.409879828	0.190963229	2.146380902	0.031842602	0	0.596442421	0	1	CRA004775;PRJNA827465;Yang2025_Silkie_Wencha	0.379543257
ENSGALG00010005109	ABHD3	3	0.390422704	0.181916422	2.146165254	0.031859797	0	1.718083818	0	1	CRA004775;PRJNA827465;Yang2025_Silkie_Wencha	0.379543257
ENSGALG00010001670	SHOX	3	0.343974523	0.160133323	2.148050867	0.031709715	0	0.229189704	0	1	CRA004775;PRJNA827465;Yang2025_Silkie_Wencha	0.379543257
ENSGALG00010021476	ALKBH2	3	-0.202982002	0.09460112	-2.145661715	0.031899978	0	1.176037232	0	1	CRA004775;PRJNA827465;Yang2025_Silkie_Wencha	0.379543257
ENSGALG00010007997	TMEM192	3	-0.17676687	0.082319175	-2.147335297	0.031766598	0.00584202	2.718961211	26.44249605	1	CRA004775;PRJNA827465;Yang2025_Silkie_Wencha	0.379543257
ENSGALG00010021465	ABCB6	3	0.172515638	0.080410137	2.145446375	0.031917175	0	1.952494956	0	0.666666667	CRA004775;PRJNA827465;Yang2025_Silkie_Wencha	0.379543257
ENSGALG00010011992	YEATS4	3	-0.131557099	0.061281191	-2.146777792	0.031810976	0	1.199570836	0	0.666666667	CRA004775;PRJNA827465;Yang2025_Silkie_Wencha	0.379543257
ENSGALG00010014099	TPX2	3	-0.128384715	0.059838203	-2.145530922	0.031910422	0	0.109897449	0	1	CRA004775;PRJNA827465;Yang2025_Silkie_Wencha	0.379543257
ENSGALG00010027575	FCER1G	3	-0.415238894	0.193588039	-2.144961516	0.031955924	0	1.051783087	0	1	CRA004775;PRJNA827465;Yang2025_Silkie_Wencha	0.379601191
ENSGALG00010016117	ENSGALG0001001611	3	-0.308995999	0.144073306	-2.144713733	0.031975742	0	0.985536907	0	1	CRA004775;PRJNA827465;Yang2025_Silkie_Wencha	0.379601191
ENSGALG00010001159	RTTN	3	0.174535286	0.081400236	2.144161926	0.032019915	0	0.16966915	0	1	CRA004775;PRJNA827465;Yang2025_Silkie_Wencha	0.379806688
ENSGALG00010010938	FZD6	3	-0.164346691	0.076666604	-2.143654248	0.032060601	0.003791973	2.502380764	20.07611195	1	CRA004775;PRJNA827465;Yang2025_Silkie_Wencha	0.379970522
ENSGALG00010003243	VPS50	3	-0.116626464	0.054414566	-2.143294943	0.032089423	0	1.350530474	0	0.666666667	CRA004775;PRJNA827465;Yang2025_Silkie_Wencha	0.37999359
ENSGALG00010020951	ENSGALG0001002095	2	1.497052348	0.699681859	2.13961864	0.032385599	0.63951166	2.744839677	63.56799967	1	PRJNA827465;Yang2025_Silkie_Wenchang	0.3822245
ENSGALG00010028762	CACNA1B	2	0.861075189	0.402444118	2.139614296	0.03238595	0.090982092	1.357853939	26.35437645	1	PRJNA827465;Yang2025_Silkie_Wenchang	0.3822245
ENSGALG00010021137	PLAU	3	0.261574698	0.122242014	2.139810108	0.032370116	0	0.856575232	0	1	CRA004775;PRJNA827465;Yang2025_Silkie_Wencha	0.3822245
ENSGALG00010011607	TSEN15	3	-0.19872287	0.092850705	-2.140240824	0.03233531	0	0.827482678	0	1	CRA004775;PRJNA827465;Yang2025_Silkie_Wencha	0.3822245
ENSGALG00010000817	ENSGALG0001000081	3	-0.159656155	0.074639592	-2.139027709	0.032433424	0	0.065174014	0	1	CRA004775;PRJNA827465;Yang2025_Silkie_Wencha	0.382465544
ENSGALG00010017463	PPP1R36	3	-0.354628936	0.165881408	-2.137846191	0.032529229	0	0.787978116	0	1	CRA004775;PRJNA827465;Yang2025_Silkie_Wencha	0.382828766
ENSGALG00010023120	CHRM4	3	-0.279049147	0.130540368	-2.137646384	0.032545454	0	0.129388879	0	1	CRA004775;PRJNA827465;Yang2025_Silkie_Wencha	0.382828766
ENSGALG00010004736	CD47	3	0.109255039	0.051096468	2.138211166	0.032499609	0	1.355780546	0	0.666666667	CRA004775;PRJNA827465;Yang2025_Silkie_Wencha	0.382828766
ENSGALG00010020673	MMS19	3	-0.151478517	0.070897297	-2.13659086	0.032631284	0.004080923	2.63817564	24.19003613	1	CRA004775;PRJNA827465;Yang2025_Silkie_Wencha	0.383519302
ENSGALG00010014748	FGFR1OP2	3	0.184258044	0.086284195	2.135478511	0.032721944	0.00458996	2.434651564	17.85272154	0.666666667	CRA004775;PRJNA827465;Yang2025_Silkie_Wencha	0.384265416
ENSGALG00010005455	ENSGALG0001000545	3	-0.634367051	0.297270215	-2.133974475	0.03284487	0	0.730404847	0	1	CRA004775;PRJNA827465;Yang2025_Silkie_Wencha	0.385182938
ENSGALG00010014653	FAM162B	3	0.387023658	0.181401166	2.133523542	0.032881803	0.038193297	3.211858626	37.73075864	1	CRA004775;PRJNA827465;Yang2025_Silkie_Wencha	0.385182938
ENSGALG00010008335	HNRNPUL1	3	-0.145944755	0.068404245	-2.133562818	0.032878584	0.006034927	3.433955708	41.75813057	1	CRA004775;PRJNA827465;Yang2025_Silkie_Wencha	0.385182938
ENSGALG00010021055	IRAG1	3	0.433317559	0.203137577	2.133123602	0.032914588	0.080650975	6.676388056	70.04368255	1	CRA004775;PRJNA827465;Yang2025_Silkie_Wencha	0.385247815
ENSGALG00010018986	CTDSP1	3	-0.193973338	0.090953824	-2.132657328	0.032952847	0.007859143	2.735716777	26.89301696	1	CRA004775;PRJNA827465;Yang2025_Silkie_Wencha	0.385376593
ENSGALG00010020094	CLEC3B	3	-0.166805299	0.078247132	-2.131775252	0.033025327	0	0.048299555	0	1	CRA004775;PRJNA827465;Yang2025_Silkie_Wencha	0.385905041
ENSGALG00010017887	PROKR1	3	-0.432657739	0.203164823	-2.129589825	0.033205492	0	1.082492131	0	0.666666667	CRA004775;PRJNA827465;Yang2025_Silkie_Wencha	0.387689883
ENSGALG00010024505	DNASE2B	3	-0.56696568	0.266332958	-2.128785278	0.033272029	0.14838262	11.29558494	82.29396697	1	CRA004775;PRJNA827465;Yang2025_Silkie_Wencha	0.388146225
ENSGALG00010008592	KIAA0513	3	-0.145138599	0.068197392	-2.12821334	0.033319399	0	0.239824183	0	1	CRA004775;PRJNA827465;Yang2025_Silkie_Wencha	0.388378389
ENSGALG00010025941	ENSGALG0001002594	2	0.972147169	0.457026755	2.127112163	0.033410765	0.127270939	1.359397859	26.4380186	1	PRJNA827465;Yang2025_Silkie_Wenchang	0.388905456
ENSGALG00010012133	RAB3IP	3	0.238271725	0.112022149	2.127005471	0.033419629	0	0.480524742	0	1	CRA004775;PRJNA827465;Yang2025_Silkie_Wencha	0.388905456
ENSGALG00010014777	APCDD1L	3	0.421405588	0.198206897	2.126089424	0.033495814	0	1.575103129	0	1	CRA004775;PRJNA827465;Yang2025_Silkie_Wencha	0.38947148
ENSGALG00010009842	PHETA2	3	-0.253570701	0.11930333	-2.125428531	0.033550872	0.024754837	5.185770663	61.43292618	1	CRA004775;PRJNA827465;Yang2025_Silkie_Wencha	0.389791105
ENSGALG00010021459	ARHGEF16	3	0.255756195	0.12037784	2.124611925	0.033619008	0.026615632	5.600937751	64.29169384	1	CRA004775;PRJNA827465;Yang2025_Silkie_Wencha	0.389941882
ENSGALG00010017687	TTC7B	3	-0.129814166	0.06109365	-2.124838945	0.033600054	0	0.660819876	0	1	CRA004775;PRJNA827465;Yang2025_Silkie_Wencha	0.389941882
ENSGALG00010010642	CC2D2A	3	-0.221534355	0.104289735	-2.12422013	0.033651741	0.015960029	4.004760536	50.05943597	0.666666667	CRA004775;PRJNA827465;Yang2025_Silkie_Wencha	0.390001608
ENSGALG00010027886	IL6R	3	-0.216958553	0.102184679	-2.12320041	0.033737062	0	0.116882986	0	1	CRA004775;PRJNA827465;Yang2025_Silkie_Wencha	0.390670201
ENSGALG00010012123	ENSGALG0001001212	3	0.328875274	0.154932742	2.122697055	0.033779246	0.028623411	3.327734126	39.89904469	1	CRA004775;PRJNA827465;Yang2025_Silkie_Wencha	0.390838591
ENSGALG00010010904	TOR1AIP1	3	-0.173172068	0.081641751	-2.121121424	0.033911586	0.007541097	3.084551505	35.16075201	1	CRA004775;PRJNA827465;Yang2025_Silkie_Wencha	0.391728683
ENSGALG00010014726	PWP2	3	-0.122820246	0.057896201	-2.121386966	0.033889251	0	1.247187821	0	1	CRA004775;PRJNA827465;Yang2025_Silkie_Wencha	0.391728683
ENSGALG00010011633	TADA2B	3	-0.312173466	0.147232123	-2.120280946	0.03398236	0.036374614	4.61185823	56.63353251	1	CRA004775;PRJNA827465;Yang2025_Silkie_Wencha	0.392196231
ENSGALG00010017577	CLPX	3	-0.130958954	0.061773607	-2.119982301	0.034007538	0.00203944	2.333422655	14.28899537	0.666666667	CRA004775;PRJNA827465;Yang2025_Silkie_Wencha	0.392196231
ENSGALG00010014980	ENSGALG0001001498	3	-0.450684753	0.212642646	-2.119446694	0.034052734	0	0.284875518	0	1	CRA004775;PRJNA827465;Yang2025_Silkie_Wencha	0.392397399
ENSGALG00010025862	PTCH2	3	0.43189929	0.203856045	2.118648433	0.034120189	0.043591457	3.054724376	34.52764461	1	CRA004775;PRJNA827465;Yang2025_Silkie_Wencha	0.392854525
ENSGALG00010029313	CCDC40	3	0.416789791	0.196980934	2.115888996	0.03435425	0.025841517	2.483491683	19.46822234	1	CRA004775;PRJNA827465;Yang2025_Silkie_Wencha	0.394906288
ENSGALG00010000643	VKORC1	3	-0.312635771	0.147747286	-2.116017015	0.03434336	0.004736343	2.11394213	5.390030703	1	CRA004775;PRJNA827465;Yang2025_Silkie_Wencha	0.394906288
ENSGALG00010003356	ENSGALG0001000335	3	0.666732957	0.315442575	2.113642895	0.034545779	0.215560286	7.782102676	74.30000498	1	CRA004775;PRJNA827465;Yang2025_Silkie_Wencha	0.396655455
ENSGALG00010004924	ENSGALG0001000492	3	0.638101507	0.301971391	2.113119073	0.034590578	0.15400271	5.054273225	60.42952347	1	CRA004775;PRJNA827465;Yang2025_Silkie_Wencha	0.396655455
ENSGALG00010020601	AKIP1	3	-0.228579339	0.108158366	-2.113376407	0.034568564	0.003667032	2.178236855	8.182620486	1	CRA004775;PRJNA827465;Yang2025_Silkie_Wencha	0.396655455
ENSGALG00010004251	CCN2	3	0.284685128	0.134788498	2.112087699	0.034678928	0	0.643602609	0	1	CRA004775;PRJNA827465;Yang2025_Silkie_Wencha	0.397346322
ENSGALG00010022038	GAPDH	3	-0.435451966	0.206315301	-2.110614012	0.034805502	0.067740881	4.627270139	56.7779719	1	CRA004775;PRJNA827465;Yang2025_Silkie_Wencha	0.398151292
ENSGALG00010018014	LYRM1	3	0.086306439	0.040890025	2.110696681	0.034798391	0	0.067962132	0	1	CRA004775;PRJNA827465;Yang2025_Silkie_Wencha	0.398151292
ENSGALG00010027668	RANBP3	3	-0.077877112	0.036909482	-2.10994866	0.034862778	0	1.661730165	0	1	CRA004775;PRJNA827465;Yang2025_Silkie_Wencha	0.398484093
ENSGALG00010029474	ENSGALG0001002947	2	1.550666085	0.735198585	2.109179911	0.034929055	0	0.247685757	0	1	PRJNA827465;Yang2025_Silkie_Wenchang	0.398598214
ENSGALG00010017544	SDE2	3	-0.075243149	0.035674143	-2.109178866	0.034929145	0	1.53928701	0	0.666666667	CRA004775;PRJNA827465;Yang2025_Silkie_Wencha	0.398598214
ENSGALG00010000616	PSENEN	3	-0.430352225	0.204093239	-2.10860598	0.034978606	0	1.466947978	0	0.666666667	CRA004775;PRJNA827465;Yang2025_Silkie_Wencha	0.398840737
ENSGALG00010019319	ACTR3	3	0.101456484	0.048135746	2.107716027	0.03505556	0.000566825	2.12965201	6.087943443	0.666666667	CRA004775;PRJNA827465;Yang2025_Silkie_Wencha	0.399396104
ENSGALG00010001140	NMD3	3	-0.230833615	0.109553145	-2.107046903	0.035113514	0.019505718	4.734738609	57.75901976	0.666666667	CRA004775;PRJNA827465;Yang2025_Silkie_Wencha	0.39973428
ENSGALG00010018983	MDGA2	3	0.577007509	0.274184526	2.10444957	0.035339249	0.138732524	5.945458145	66.36087664	1	CRA004775;PRJNA827465;Yang2025_Silkie_Wencha	0.399762875
ENSGALG00010019657	PPP1R17	3	0.422844912	0.201050124	2.103181546	0.035449903	0.011267847	2.201074058	9.135270009	1	CRA004775;PRJNA827465;Yang2025_Silkie_Wencha	0.399762875
ENSGALG00010007388	CYP1B1	3	0.383104592	0.181885844	2.106291417	0.035179046	0.054413552	4.950802796	59.6025113	1	CRA004775;PRJNA827465;Yang2025_Silkie_Wencha	0.399762875
ENSGALG00010022879	ENSGALG0001002287	3	-0.356634714	0.169548283	-2.103440436	0.035427287	0	0.264938899	0	1	CRA004775;PRJNA827465;Yang2025_Silkie_Wencha	0.399762875
ENSGALG00010008360	ZC3H3	3	-0.297283035	0.141169176	-2.105863641	0.035216199	0.044500631	8.481837735	76.42020441	1	CRA004775;PRJNA827465;Yang2025_Silkie_Wencha	0.399762875
ENSGALG00010020252	DMTN	3	0.278255739	0.132224154	2.104424433	0.03534144	0.011952022	2.4955935	19.85874302	1	CRA004775;PRJNA827465;Yang2025_Silkie_Wencha	0.399762875
ENSGALG00010029626	AP2B1	3	-0.222206764	0.105618486	-2.103862426	0.035390449	0.021603832	7.569159987	73.57698868	1	CRA004775;PRJNA827465;Yang2025_Silkie_Wencha	0.399762875
ENSGALG00010023422	WLS	3	-0.195805416	0.093102366	-2.103119656	0.035455311	0.008934824	2.978331581	32.84830967	1	CRA004775;PRJNA827465;Yang2025_Silkie_Wencha	0.399762875
ENSGALG00010016672	DCTN4	3	-0.192682519	0.091494495	-2.105946589	0.035208992	0.017273586	9.974913832	79.94970149	1	CRA004775;PRJNA827465;Yang2025_Silkie_Wencha	0.399762875
ENSGALG00010007085	PRIMPOL	3	-0.182230292	0.086592761	-2.104451792	0.035339056	0	0.480368951	0	1	CRA004775;PRJNA827465;Yang2025_Silkie_Wencha	0.399762875
ENSGALG00010021190	ENSGALG0001002119	3	-0.13355334	0.063437515	-2.105273827	0.035267479	0.004849865	3.405704546	41.27499984	0.666666667	CRA004775;PRJNA827465;Yang2025_Silkie_Wencha	0.399762875
ENSGALG00010021753	MTF2	3	0.092353974	0.043910509	2.103231688	0.035445522	0	1.152926494	0	0.666666667	CRA004775;PRJNA827465;Yang2025_Silkie_Wencha	0.399762875
ENSGALG00010026354	ENSGALG0001002635	3	-0.304825391	0.145033748	-2.101754899	0.035574752	0.014013026	2.520895089	20.66310062	0.666666667	CRA004775;PRJNA827465;Yang2025_Silkie_Wencha	0.40001726
ENSGALG00010022240	ENSGALG0001002224	3	0.174780496	0.083142022	2.102192017	0.035536459	0	0.769792358	0	1	CRA004775;PRJNA827465;Yang2025_Silkie_Wencha	0.40001726
ENSGALG00010006047	ZBTB11	3	0.161637679	0.076905182	2.101778782	0.035572659	0	0.451583657	0	1	CRA004775;PRJNA827465;Yang2025_Silkie_Wencha	0.40001726
ENSGALG00010006270	STAM	3	-0.118163321	0.056226236	-2.10156909	0.03559104	0.000236954	2.024457264	1.208089932	0.666666667	CRA004775;PRJNA827465;Yang2025_Silkie_Wencha	0.40001726
ENSGALG00010010941	RIPK4	2	1.561758726	0.743695989	2.099996166	0.035729178	0.595878702	2.166085152	53.83376322	1	CRA004775;PRJNA827465	0.400932423
ENSGALG00010010337	SCARA3	3	0.167624045	0.079813646	2.10019281	0.035711884	0	1.55913098	0	0.666666667	CRA004775;PRJNA827465;Yang2025_Silkie_Wencha	0.400932423
ENSGALG00010012880	CLK4	3	0.249668805	0.118911473	2.099619135	0.035762358	0.02251386	4.294827271	53.43235306	1	CRA004775;PRJNA827465;Yang2025_Silkie_Wencha	0.400986504
ENSGALG00010029437	ENSGALG0001002943	3	1.343414038	0.639998589	2.099089062	0.03580905	0	0.7632348	0	1	CRA004775;PRJNA827465;Yang2025_Silkie_Wencha	0.401085844
ENSGALG00010020413	CYP26A1	3	0.419998403	0.200106473	2.098874644	0.035827953	0	1.179332783	0	1	CRA004775;PRJNA827465;Yang2025_Silkie_Wencha	0.401085844
ENSGALG00010016808	ENTPD3	2	-0.567860488	0.270648986	-2.098143783	0.035892446	0	0.252058044	0	1	PRJNA827465;Yang2025_Silkie_Wenchang	0.401489947
ENSGALG00010018730	IGF1R	3	-0.138757417	0.066163671	-2.097184371	0.035977257	0	0.843844135	0	0.666666667	CRA004775;PRJNA827465;Yang2025_Silkie_Wencha	0.402120508

ENSGALG00010020928	AACS	3	0.278074017	0.137521446	2.022041107	0.043172105	0.015338312	2.653961246	24.64094933	0.666666667	CRA004775;PRJNA827465;Yang2025_Silkie_Wencha	0.437793079
ENSGALG00010028098	RFT1	3	-0.205511626	0.101622254	-2.022309261	0.043144413	0	1.138405744	0	1	CRA004775;PRJNA827465;Yang2025_Silkie_Wencha	0.437793079
ENSGALG00010016655	TRANK1	3	-0.133337412	0.065948806	-2.021832094	0.043193701	0	0.076608771	0	1	CRA004775;PRJNA827465;Yang2025_Silkie_Wencha	0.437793079
ENSGALG00010021025	CUEDC2	3	0.132145651	0.065369036	2.021532801	0.04322464	0	0.511643284	0	1	CRA004775;PRJNA827465;Yang2025_Silkie_Wencha	0.437793079
ENSGALG00010016341	SLX4	3	0.086260283	0.042662478	2.021923854	0.043184219	0	0.218207785	0	1	CRA004775;PRJNA827465;Yang2025_Silkie_Wencha	0.437793079
ENSGALG00010028872	SLC2A6	3	-0.36643734	0.181329965	-2.020831692	0.043297191	0	0.688591329	0	1	CRA004775;PRJNA827465;Yang2025_Silkie_Wencha	0.437896268
ENSGALG00010005534	ENSGALG0001000553	3	0.20737885	0.102714715	2.018978985	0.043489404	0	0.719247901	0	1	CRA004775;PRJNA827465;Yang2025_Silkie_Wencha	0.439525861
ENSGALG00010008459	EPB41L4B	3	0.653874658	0.323968027	2.018330833	0.043556818	0.211008826	6.381454695	68.65918359	1	CRA004775;PRJNA827465;Yang2025_Silkie_Wencha	0.439892746
ENSGALG00010006585	TTC29	3	0.281652333	0.139597747	2.017599415	0.043632998	0	0.931064519	0	0.666666667	CRA004775;PRJNA827465;Yang2025_Silkie_Wencha	0.440347578
ENSGALG00010028048	S100A10	3	0.185643997	0.092026298	2.017292907	0.043664955	0	1.583882061	0	0.666666667	CRA004775;PRJNA827465;Yang2025_Silkie_Wencha	0.440355781
ENSGALG00010024831	SLC7A9	3	0.949531619	0.471696449	2.013014133	0.044113143	0.360970365	4.468339715	55.2406458	1	CRA004775;PRJNA827465;Yang2025_Silkie_Wencha	0.440635492
ENSGALG00010001588	ENSGALG0001000158	3	0.597131159	0.296295087	2.015325884	0.043870515	0	1.870340776	0	1	CRA004775;PRJNA827465;Yang2025_Silkie_Wencha	0.440635492
ENSGALG00010011957	ENSGALG0001001195	3	-0.306665066	0.152196143	-2.014933228	0.043911646	0.036722233	4.507686857	55.63134566	0.666666667	CRA004775;PRJNA827465;Yang2025_Silkie_Wencha	0.440635492
ENSGALG00010025378	PAX3	3	0.289747693	0.143857869	2.014124747	0.043996438	0	0.980242441	0	1	CRA004775;PRJNA827465;Yang2025_Silkie_Wencha	0.440635492
ENSGALG00010012191	BEST3	3	0.277935802	0.137954817	2.01468719	0.043937435	0	0.088149424	0	1	CRA004775;PRJNA827465;Yang2025_Silkie_Wencha	0.440635492
ENSGALG00010018398	LRFN5	3	-0.267380332	0.132775995	-2.013770129	0.044033673	0	1.149496135	0	1	CRA004775;PRJNA827465;Yang2025_Silkie_Wencha	0.440635492
ENSGALG00010004630	ENSGALG0001000463	3	0.264630554	0.131436884	2.013366004	0.044076139	0	1.62512722	0	0.666666667	CRA004775;PRJNA827465;Yang2025_Silkie_Wencha	0.440635492
ENSGALG00010011304	COMMD5	3	-0.201354677	0.099870023	-2.016167324	0.043782482	0.01178355	3.0356264	34.11573968	1	CRA004775;PRJNA827465;Yang2025_Silkie_Wencha	0.440635492
ENSGALG00010028147	SLC25A42	3	-0.184076183	0.091331484	-2.015473464	0.043855064	0	0.828436761	0	0.666666667	CRA004775;PRJNA827465;Yang2025_Silkie_Wencha	0.440635492
ENSGALG00010023104	ENSGALG0001002310	3	0.181192339	0.089925732	2.014910913	0.043913984	0	1.484538514	0	0.666666667	CRA004775;PRJNA827465;Yang2025_Silkie_Wencha	0.440635492
ENSGALG00010002342	BAG2	3	-0.144918683	0.07199628	-2.012863468	0.044128995	0	1.511679657	0	0.666666667	CRA004775;PRJNA827465;Yang2025_Silkie_Wencha	0.440635492
ENSGALG00010029764	NXN	3	-0.129599147	0.064372081	-2.013281908	0.044084981	0	0.767167005	0	1	CRA004775;PRJNA827465;Yang2025_Silkie_Wencha	0.440635492
ENSGALG00010000444	RBM19	3	0.097792435	0.04856051	2.013826372	0.044027766	0	1.711837754	0	0.666666667	CRA004775;PRJNA827465;Yang2025_Silkie_Wencha	0.440635492
ENSGALG00010018188	UBP1	3	-0.053656294	0.026639445	-2.014167087	0.043991994	0	0.133404708	0	1	CRA004775;PRJNA827465;Yang2025_Silkie_Wencha	0.440635492
ENSGALG00010009437	DPYSL5	3	0.384621467	0.191158255	2.012057844	0.04421384	0	1.679538838	0	0.666666667	CRA004775;PRJNA827465;Yang2025_Silkie_Wencha	0.44088761
ENSGALG00010001235	CCDC102B	2	-0.380841461	0.18928205	-2.012031574	0.044216609	0	0.130543331	0	1	PRJNA827465;Yang2025_Silkie_Wenchang	0.44088761
ENSGALG00010016751	DFFA	3	-0.132738399	0.065982412	-2.011723965	0.044249043	0	0.867500821	0	1	CRA004775;PRJNA827465;Yang2025_Silkie_Wencha	0.440900084
ENSGALG00010014114	ANXA1	3	-0.289132192	0.143754892	-2.011285922	0.044295265	0	1.624004082	0	1	CRA004775;PRJNA827465;Yang2025_Silkie_Wencha	0.441049821
ENSGALG00010000734	SLAIN1	2	-0.441079583	0.219358909	-2.010766673	0.044350108	0	0.343229593	0	1	PRJNA827465;Yang2025_Silkie_Wenchang	0.441285133
ENSGALG00010023522	EMG1	3	-0.268231656	0.133480722	-2.009516066	0.044482433	0.022793599	3.265291577	38.74972716	1	CRA004775;PRJNA827465;Yang2025_Silkie_Wencha	0.442284591
ENSGALG00010022867	ATPAF1	3	0.145685397	0.072509163	2.00919982	0.044515947	0	0.368545497	0	0.666666667	CRA004775;PRJNA827465;Yang2025_Silkie_Wencha	0.442284591
ENSGALG00010017140	UQCRC2	3	-0.131722075	0.065568226	-2.008931498	0.044544399	0	0.065469908	0	1	CRA004775;PRJNA827465;Yang2025_Silkie_Wencha	0.442284591
ENSGALG00010027050	GSTT1	3	-0.260942961	0.129979033	-2.007577331	0.044688225	0.019006668	3.189845231	37.30103328	1	CRA004775;PRJNA827465;Yang2025_Silkie_Wencha	0.443392947
ENSGALG00010020055	R3HCC1	3	-0.082603998	0.041151991	-2.007290449	0.044718745	0	1.124735156	0	1	CRA004775;PRJNA827465;Yang2025_Silkie_Wencha	0.443392947
ENSGALG00010019392	NIN	3	0.456896197	0.227850566	2.005244944	0.044936867	0.106872576	8.1728502	75.52873293	1	CRA004775;PRJNA827465;Yang2025_Silkie_Wencha	0.444620272
ENSGALG00010016447	ID3	3	0.226204614	0.112777211	2.005765278	0.044881297	0.017658753	3.744315909	46.58570354	1	CRA004775;PRJNA827465;Yang2025_Silkie_Wencha	0.444620272
ENSGALG00010010406	PTCH1	3	0.14996455	0.074784992	2.005276015	0.044933547	0	1.1462635	0	0.666666667	CRA004775;PRJNA827465;Yang2025_Silkie_Wencha	0.444620272
ENSGALG00010010961	C2orf66	2	1.006805847	0.502810233	2.00235751	0.045246295	0.352984453	3.287593784	69.58261678	1	PRJNA827465;Yang2025_Silkie_Wenchang	0.446330405
ENSGALG00010014203	TMEM38B	3	-0.360431106	0.180021145	-2.002159837	0.045267544	0.062520933	6.923073819	71.11109816	1	CRA004775;PRJNA827465;Yang2025_Silkie_Wencha	0.446330405
ENSGALG00010001156	ALDH7A1	3	-0.238143295	0.118885355	-2.003133982	0.045162909	0	1.855639375	0	1	CRA004775;PRJNA827465;Yang2025_Silkie_Wencha	0.446330405
ENSGALG00010020632	BLOC1S2	3	-0.130540265	0.065185407	-2.002599531	0.04522029	0	0.411764481	0	1	CRA004775;PRJNA827465;Yang2025_Silkie_Wencha	0.446330405
ENSGALG00010022149	NBEAL1	3	0.125089488	0.06245399	2.002906276	0.045187349	0	1.359737456	0	1	CRA004775;PRJNA827465;Yang2025_Silkie_Wencha	0.446330405
ENSGALG00010018684	PADI2	2	0.430757869	0.21519671	2.001693563	0.045317699	0	0.651472984	0	1	PRJNA827465;Yang2025_Silkie_Wenchang	0.446513553
ENSGALG00010015186	ENSGALG0001001518	3	0.359000478	0.179433956	2.000738795	0.045420546	0	1.277105845	0	1	CRA004775;PRJNA827465;Yang2025_Silkie_Wencha	0.446956912
ENSGALG00010029560	ENSGALG0001002956	3	-0.240192088	0.120054687	-2.000688959	0.04542592	0	1.396758486	0	1	CRA004775;PRJNA827465;Yang2025_Silkie_Wencha	0.446956912
ENSGALG00010005118	PRTFDC1	3	-0.47137797	0.23575698	-1.999423177	0.045562586	0.130840018	13.64743525	85.34523181	1	CRA004775;PRJNA827465;Yang2025_Silkie_Wencha	0.447399495
ENSGALG00010013301	PARVB	3	-0.221532984	0.110800111	-1.999393172	0.04556583	0.015150302	3.452364834	42.06869504	1	CRA004775;PRJNA827465;Yang2025_Silkie_Wencha	0.447399495
ENSGALG00010006070	TMEM242	3	-0.151251458	0.075628132	-1.999936442	0.045507127	0	1.073538691	0	0.666666667	CRA004775;PRJNA827465;Yang2025_Silkie_Wencha	0.447399495
ENSGALG00010012094	ENSGALG0001001209	3	0.44768824	0.224032059	1.998322219	0.045681738	0	1.431026738	0	1	CRA004775;PRJNA827465;Yang2025_Silkie_Wencha	0.448226298
ENSGALG00010013271	GULP1	3	-0.120722382	0.060448078	-1.997125227	0.045811581	0	1.725203061	0	0.666666667	CRA004775;PRJNA827465;Yang2025_Silkie_Wencha	0.449188589
ENSGALG00010027725	BTN1A1	3	-0.508350844	0.254586321	-1.996772023	0.045849954	0.012483236	2.128224209	6.024938943	1	CRA004775;PRJNA827465;Yang2025_Silkie_Wencha	0.449253293
ENSGALG00010015478	FBXO7	3	-0.080987356	0.040565511	-1.996458399	0.04588405	0	1.565281237	0	1	CRA004775;PRJNA827465;Yang2025_Silkie_Wencha	0.449276024
ENSGALG00010025870	ENSGALG0001002587	3	-0.223444824	0.111938759	-1.996134542	0.04591928	0	1.99584667	0	0.666666667	CRA004775;PRJNA827465;Yang2025_Silkie_Wencha	0.449309827
ENSGALG00010011664	SERPINB10B	3	-0.714336798	0.357980216	-1.995464455	0.045992247	0.140940894	3.155457301	36.61774477	1	CRA004775;PRJNA827465;Yang2025_Silkie_Wencha	0.449712574

ENSGALG00010013563	FKBP1A	3	-0.363843947	0.182412146	-1.99462566	0.046083723	0.07180766	8.065745926	75.20378129	1	CRA004775;PRJNA827465;Yang2025_Silkie_Wencha	0.449882461
ENSGALG00010023479	MCCC2L	3	-0.325543278	0.163247585	-1.994169031	0.046133585	0.039109965	4.036186012	50.44826988	1	CRA004775;PRJNA827465;Yang2025_Silkie_Wencha	0.449882461
ENSGALG00010016465	STK35	3	-0.296340575	0.14854516	-1.994952747	0.046048034	0.017305596	2.595510679	22.94387321	1	CRA004775;PRJNA827465;Yang2025_Silkie_Wencha	0.449882461
ENSGALG00010024547	PFKM	3	-0.218548626	0.109595497	-1.99413873	0.046136896	0	1.282244833	0	0.666666667	CRA004775;PRJNA827465;Yang2025_Silkie_Wencha	0.449882461
ENSGALG00010020170	SORCS1	3	-0.256905649	0.12888564	-1.993283724	0.046230388	0	1.509583425	0	0.666666667	CRA004775;PRJNA827465;Yang2025_Silkie_Wencha	0.449897326
ENSGALG00010017354	FEM1B	3	-0.249043328	0.124943233	-1.993251823	0.046233879	0.039273475	14.85981694	86.5755319	1	CRA004775;PRJNA827465;Yang2025_Silkie_Wencha	0.449897326
ENSGALG00010024693	ANXA2	3	-0.194490859	0.097567731	-1.993393276	0.0462184	0.004270273	2.306714593	13.29659915	1	CRA004775;PRJNA827465;Yang2025_Silkie_Wencha	0.449897326
ENSGALG00010000105	CACNA1F	3	-0.456002636	0.228902255	-1.992128194	0.046356994	0.052285977	2.998924599	33.04149691	1	CRA004775;PRJNA827465;Yang2025_Silkie_Wencha	0.450785104
ENSGALG00010020704	PROX1	3	0.400143695	0.200902588	1.991729919	0.046400699	0	0.424785422	0	1	CRA004775;PRJNA827465;Yang2025_Silkie_Wencha	0.4508999
ENSGALG00010029749	ASPA	3	0.425406319	0.213761094	1.99010171	0.046579733	0.024507264	2.406242797	16.88286807	1	CRA004775;PRJNA827465;Yang2025_Silkie_Wencha	0.451646616
ENSGALG00010009025	UTP23	3	0.337643382	0.169612121	1.99067956	0.046516127	0.060349939	7.678269786	73.95246513	1	CRA004775;PRJNA827465;Yang2025_Silkie_Wencha	0.451646616
ENSGALG00010021622	PTHLH	3	-0.219850361	0.11047854	-1.989982501	0.046592863	0	1.216535219	0	0.666666667	CRA004775;PRJNA827465;Yang2025_Silkie_Wencha	0.451646616
ENSGALG00010021668	PYGO1	3	-0.181194844	0.091058654	-1.989869569	0.046605305	0	0.70706988	0	1	CRA004775;PRJNA827465;Yang2025_Silkie_Wencha	0.451646616
ENSGALG00010019208	PLXND1	3	-0.126229858	0.063486646	-1.988289904	0.046779637	0	0.263167921	0	1	CRA004775;PRJNA827465;Yang2025_Silkie_Wencha	0.453025537
ENSGALG00010029374	RCC1L	3	-0.187108856	0.094189896	-1.98736662	0.046881784	0.01150199	3.412947682	41.39962911	1	CRA004775;PRJNA827465;Yang2025_Silkie_Wencha	0.453483009
ENSGALG00010015669	INPP4A	3	-0.096237376	0.048426604	-1.987283203	0.046891022	0	1.589161744	0	0.666666667	CRA004775;PRJNA827465;Yang2025_Silkie_Wencha	0.453483009
ENSGALG00010001542	MTO1	3	-0.087785168	0.044213572	-1.985480116	0.04709108	0	0.671717071	0	0.666666667	CRA004775;PRJNA827465;Yang2025_Silkie_Wencha	0.455106482
ENSGALG00010013654	SLC6A7	2	0.788970525	0.398024415	1.982216405	0.047455025	0.221183912	3.168691765	68.44123461	1	PRJNA827465;Yang2025_Silkie_Wenchang	0.455593089
ENSGALG00010002289	ENSGALG0001000228	3	0.726235211	0.366232579	1.982989093	0.047368648	0.319539108	10.84213654	81.55379155	1	CRA004775;PRJNA827465;Yang2025_Silkie_Wencha	0.455593089
ENSGALG00010019834	PLCH2	3	0.585530007	0.295488873	1.98156364	0.0475281	0.182615575	7.276159181	72.51297078	1	CRA004775;PRJNA827465;Yang2025_Silkie_Wencha	0.455593089
ENSGALG00010012547	ENSGALG0001001254	2	0.527284053	0.266020707	1.982116575	0.047466195	0	0.655663799	0	1	PRJNA827465;Yang2025_Silkie_Wenchang	0.455593089
ENSGALG00010016016	ZDHHC2	3	-0.428110057	0.215886868	-1.983029637	0.047364119	0.092058692	7.250915218	72.41727506	1	CRA004775;PRJNA827465;Yang2025_Silkie_Wencha	0.455593089
ENSGALG00010002225	PRDX3	3	-0.341636781	0.172367603	-1.982024327	0.047476518	0.054055309	5.342145861	62.56186087	0.666666667	CRA004775;PRJNA827465;Yang2025_Silkie_Wencha	0.455593089
ENSGALG00010006249	C9orf72	3	-0.221965467	0.111920712	-1.983238521	0.047340793	0	0.66813999	0	1	CRA004775;PRJNA827465;Yang2025_Silkie_Wencha	0.455593089
ENSGALG00010019967	CEP170B	3	0.202285633	0.102022135	1.982762207	0.047393997	0	1.038025801	0	1	CRA004775;PRJNA827465;Yang2025_Silkie_Wencha	0.455593089
ENSGALG00010020655	RIC3	3	-0.195762	0.098786357	-1.981670406	0.047516142	0	1.52411268	0	1	CRA004775;PRJNA827465;Yang2025_Silkie_Wencha	0.455593089
ENSGALG00010023299	PASK	3	-0.16516206	0.083282182	-1.983157248	0.047349868	0	1.187509404	0	0.666666667	CRA004775;PRJNA827465;Yang2025_Silkie_Wencha	0.455593089
ENSGALG00010009623	XPO5	3	-0.097362645	0.049102527	-1.98284388	0.047384871	0.001576847	2.48353192	19.46952708	1	CRA004775;PRJNA827465;Yang2025_Silkie_Wencha	0.455593089
ENSGALG00010002805	FAM171A1	3	-0.068686088	0.03462327	-1.983812885	0.047276703	0	0.342960317	0	1	CRA004775;PRJNA827465;Yang2025_Silkie_Wencha	0.455593089
ENSGALG00010014789	F7	2	1.377845604	0.69568068	1.980571898	0.047639303	0	0.099587758	0	1	CRA004775;PRJNA827465	0.456349665
ENSGALG00010002022	ERNI	2	-1.252710015	0.632945134	-1.97917631	0.04779615	0	0.791321167	0	1	PRJNA827465;Yang2025_Silkie_Wenchang	0.457232676
ENSGALG00010009092	EED	3	-0.347092064	0.175368096	-1.979220124	0.047791228	0.057843444	5.512539983	63.71908402	1	CRA004775;PRJNA827465;Yang2025_Silkie_Wencha	0.457232676
ENSGALG00010016683	RBM22	3	-0.242046629	0.122330665	-1.978625952	0.047858136	0.033665441	8.9060131	77.54326231	1	CRA004775;PRJNA827465;Yang2025_Silkie_Wencha	0.45751601
ENSGALG00010022567	PAOX	3	-0.317599972	0.160572088	-1.977927651	0.047936869	0.036026882	3.693908945	45.85681376	1	CRA004775;PRJNA827465;Yang2025_Silkie_Wencha	0.457649825
ENSGALG00010013561	AGK	3	-0.160929028	0.0813525	-1.978169429	0.047909596	0.001106455	2.099426149	4.531240823	1	CRA004775;PRJNA827465;Yang2025_Silkie_Wencha	0.457649825
ENSGALG00010024472	LDHA	3	-0.344824485	0.174371652	-1.977526057	0.047982198	0	0.770232509	0	0.666666667	CRA004775;PRJNA827465;Yang2025_Silkie_Wencha	0.457773481
ENSGALG00010025047	PER2	3	0.407290755	0.206020601	1.97694188	0.0480482	0.092009753	8.09982496	75.30810839	1	CRA004775;PRJNA827465;Yang2025_Silkie_Wencha	0.457885658
ENSGALG00010001997	INTS4	3	-0.211174816	0.10682396	-1.976848786	0.048058725	0.023657793	8.926251524	77.59417831	1	CRA004775;PRJNA827465;Yang2025_Silkie_Wencha	0.457885658
ENSGALG00010003495	ENSGALG0001000349	3	-0.330346201	0.167167683	-1.976136744	0.048139292	0	1.082319216	0	0.666666667	CRA004775;PRJNA827465;Yang2025_Silkie_Wencha	0.458344409
ENSGALG00010002955	BLEC1	3	-0.480157012	0.243228047	-1.974102154	0.048370128	0	1.216580618	0	1	CRA004775;PRJNA827465;Yang2025_Silkie_Wencha	0.460232333
ENSGALG00010025445	B3GNT4	3	0.508899241	0.257998967	1.972485572	0.048554202	0.138447962	7.865658999	74.57301416	1	CRA004775;PRJNA827465;Yang2025_Silkie_Wencha	0.460434513
ENSGALG00010026184	NUDT3	3	-0.211316502	0.107109524	-1.972901138	0.048506827	0.015718711	3.630862777	44.91667345	1	CRA004775;PRJNA827465;Yang2025_Silkie_Wencha	0.460434513
ENSGALG00010025504	AMPD1	3	-0.193328334	0.097980581	-1.973129288	0.048480834	0	0.352856974	0	1	CRA004775;PRJNA827465;Yang2025_Silkie_Wencha	0.460434513
ENSGALG00010028439	BORCS8	3	-0.180629028	0.091571684	-1.972542386	0.048547723	0.003877616	2.296303774	12.90350941	0.666666667	CRA004775;PRJNA827465;Yang2025_Silkie_Wencha	0.460434513
ENSGALG00010004233	NHSL1	3	0.108108631	0.054798086	1.972854135	0.048512183	0	0.032325588	0	1	CRA004775;PRJNA827465;Yang2025_Silkie_Wencha	0.460434513
ENSGALG00010024953	NCAPG2	3	-0.222915903	0.11314242	-1.970223937	0.048812711	0	0.20401188	0	0.666666667	CRA004775;PRJNA827465;Yang2025_Silkie_Wencha	0.462015333
ENSGALG00010021011	SCARB1	3	-0.148106796	0.075205068	-1.970248962	0.048809845	0.004097436	2.402900228	16.76724748	0.666666667	CRA004775;PRJNA827465;Yang2025_Silkie_Wencha	0.462015333
ENSGALG00010019068	SNRPA1	3	-0.142581106	0.07238044	-1.969884488	0.048851611	0	0.376699186	0	1	CRA004775;PRJNA827465;Yang2025_Silkie_Wencha	0.462015333
ENSGALG00010008104	TCF25	3	-0.095497052	0.048474221	-1.970058528	0.048831663	0	0.021245699	0	1	CRA004775;PRJNA827465;Yang2025_Silkie_Wencha	0.462015333
ENSGALG00010029459	FOXJ1	2	0.559697105	0.28418568	1.969476808	0.048898363	0	0.004897535	0	1	PRJNA827465;Yang2025_Silkie_Wenchang	0.462148368
ENSGALG00010015889	SH3BP5	3	-0.183561574	0.093225289	-1.969010503	0.048951885	0.013373441	4.639740398	56.89414001	0.666666667	CRA004775;PRJNA827465;Yang2025_Silkie_Wencha	0.462345158
ENSGALG00010020377	TMEM230	3	-0.187853862	0.09542441	-1.968614333	0.048997395	0.002813444	2.138899452	6.493968299	1	CRA004775;PRJNA827465;Yang2025_Silkie_Wencha	0.46246607
ENSGALG00010018311	NKX2-3	2	1.429811546	0.726660911	1.967646154	0.049108765	0.556231537	1.955589288	48.86451843	1	CRA004775;Yang2025_Silkie_Wenchang	0.462918764
ENSGALG00010011398	ENSGALG0001001139	3	0.226993332	0.115406011	1.966910816	0.049193493	0	1.412506503	0	0.666666667	CRA004775;PRJNA827465;Yang2025_Silkie_Wencha	0.462918764
ENSGALG00010023147	RAD51	3	-0.156468547	0.079551914	-1.966873443	0.049197803	0	1.269384267	0	1	CRA004775;PRJNA827465;Yang2025_Silkie_Wencha	0.462918764
ENSGALG00010022799	DCAF17	3	0.139719054	0.071034737	1.966911863	0.049193393	0	1.130052468	0	0.666666667	CRA004775;PRJNA827465;Yang2025_Silkie_Wencha	0.462918764
ENSGALG00010013265	PACS2	3	-0.118252001	0.060124799	-1.96677583	0.04920906	0	0.393221292	0	1	CRA004775;PRJNA827465;Yang2025_Silkie_Wencha	0.462918764
ENSGALG00010012334	ARHGEF39	3	-0.332985975	0.169557414	-1.963853819	0.049547047	0	0.275513582	0	1	CRA004775;PRJNA827465;Yang2025_Silkie_Wencha	0.46578836
ENSGALG00010004063	ENSGALG0001000406	3	0.922036143	0.469619791	1.963367306	0.04960351	0.503054615	8.431946857	76.28068542	1	CRA004775;PRJNA827465;Yang2025_Silkie_Wencha	0.466009319
ENSGALG00010027189	ENSGALG0001002718	3	0.858398107	0.437562202	1.961773897	0.049788814	0.273808817	3.816014953	47.5893039	1	CRA004775;PRJNA827465;Yang2025_Silkie_Wencha	0.467439602
ENSGALG00010001612	HPX	3	0.662396707	0.337754389	1.961178881	0.049858183	0	1.412900597	0	1	CRA004775;PRJNA827465;Yang2025_Silkie_Wencha	0.467780257
ENSGALG00010029400	AANAT	3	0.772647917	0.39428421	1.959621757	0.050040016	0.049568323	2.236274409	10.56553739	1	CRA004775;PRJNA827465;Yang2025_Silkie_Wencha	0.467865406
ENSGALG00010021172	SLC29A4	3	-0.4241091	0.216352641	-1.960267725	0.049964506	0.062825071	3.50419082	42.92920016	0.666666667	CRA004775;PRJNA827465;Yang2025_Silkie_Wencha	0.467865406
ENSGALG00010012926	OSGEPL1	3	0.352582516	0.179943998	1.959401366	0.050065801	0.037129137	3.209628949	37.68750121	0.666666667	CRA004775;PRJNA827465;Yang2025_Silkie_Wencha	0.467865406
ENSGALG00010014655	FHL3	3	-0.183116138	0.09343682	-1.959785627	0.050020852	0.005589135	2.39665645	16.55040921	1	CRA004775;PRJNA827465;Yang2025_Silkie_Wencha	0.467865406
ENSGALG00010011246	DNAJB5	3	-0.181344407	0.092512608	-1.960212891	0.049970912	0	0.978311857	0	1	CRA004775;PRJNA827465;Yang2025_Silkie_Wencha	0.467865406
ENSGALG00010008289	MTMR2	3	-0.11992514	0.06120085	-1.959533464	0.050050345	0.00181382	2.319548731	13.77633187	1	CRA004775;PRJNA827465;Yang2025_Silkie_Wencha	0.467865406
ENSGALG00010005381	FGF9	3	0.698098344	0.356664979	1.95729434	0.050312873	0.231582522	6.348177821	68.49489639	0.666666667	CRA004775;PRJNA827465;Yang2025_Silkie_Wencha	0.469716942
ENSGALG00010011399	JAKMIP1	3	0.530400655	0.27100728	1.957145412	0.050330375	0.090402321	3.441324125	41.88283557	1	CRA004775;PRJNA827465;Yang2025_Silkie_Wencha	0.469716942
ENSGALG00010005478	ENSGALG0001000547	2	1.116981852	0.571751669	1.953613627	0.050746928	0	0.579794269	0	1	CRA004775;PRJNA827465	0.470424615
ENSGALG00010026801	FMOD	3	0.922407049	0.471750995	1.955283738	0.05054959	0.505688925	8.75489456	78.15563576	1	CRA004775;PRJNA827465;Yang2025_Silkie_Wencha	0.470424615
ENSGALG00010019648	PRDM16	3	0.336552331	0.172131187	1.955208328	0.050558486	0.027284426	2.85071752	29.84222443	1	CRA004775;PRJNA827465;Yang2025_Silkie_Wencha	0.470424615
ENSGALG00010013837	IFI27L2	3	0.335755347	0.171809857	1.954226338	0.050674456	0.036811586	3.397959932	41.14108481	0.666666667	CRA004775;PRJNA827465;Yang2025_Silkie_Wencha	0.470424615
ENSGALG00010029330	AFMID	3	0.312561878	0.159956483	1.954043197	0.050696109	0.028345149	3.02327507	33.8465752	1	CRA004775;PRJNA827465;Yang2025_Silkie_Wencha	0.470424615
ENSGALG00010008149	DNAJA1	3	0.211144495	0.108067625	1.953818218	0.050722719	0.005239883	2.307292367	13.31834829	1	CRA004775;PRJNA827465;Yang2025_Silkie_Wencha	0.470424615
ENSGALG00010008019	PPP2CB	3	-0.1638036	0.08381335	-1.954385562	0.050655637	0.01334684	6.019013261	66.77196223	1	CRA004775;PRJNA827465;Yang2025_Silkie_Wencha	0.470424615
ENSGALG00010016653	EPHA2	3	0.161361445	0.082521454	1.955387811	0.050537314	0	0.797810358	0	0.666666667	CRA004775;PRJNA827465;Yang2025_Silkie_Wencha	0.470424615
ENSGALG00010006682	ARHGAP10	3	0.147840061	0.075683446	1.953400241	0.050772188	0	0.877492646	0	1	CRA004775;PRJNA827465;Yang2025_Silkie_Wencha	0.470424615
ENSGALG00010020686	C1orf52	3	-0.123049825	0.062944886	-1.954881988	0.050597001	0	1.397159334	0	0.666666667	CRA004775;PRJNA827465;Yang2025_Silkie_Wencha	0.470424615
ENSGALG00010020428	SEMA4C	3	0.110452401	0.056505415	1.954722406	0.050615844	0	0.164659702	0	1	CRA004775;PRJNA827465;Yang2025_Silkie_Wencha	0.470424615
ENSGALG00010001819	HOXA6	3	0.137229411	0.070287441	1.952403001	0.050890377	0.003688372	2.579113168	22.45396501	1	CRA004775;PRJNA827465;Yang2025_Silkie_Wencha	0.471210897
ENSGALG00010029470	SSTR2	3	-1.36296068	0.698396367	-1.951557516	0.050990761	1.188294616	11.97895683	83.30405536	1	CRA004775;PRJNA827465;Yang2025_Silkie_Wencha	0.471259193
ENSGALG00010000511	FERMT3	3	0.405731063	0.207875735	1.95179617	0.050962409	0.069224235	4.466315785	55.2203629	0.666666667	CRA004775;PRJNA827465;Yang2025_Silkie_Wencha	0.471259193
ENSGALG00010026264	FZD7	3	-0.132477852	0.067884552	-1.951516931	0.050995584	0	0.080795254	0	1	CRA004775;PRJNA827465;Yang2025_Silkie_Wencha	0.471259193
ENSGALG00010013256	BRIX1	3	0.225638008	0.115662864	1.950825008	0.051077866	0	0.891516572	0	1	CRA004775;PRJNA827465;Yang2025_Silkie_Wencha	0.471711265
ENSGALG00010017094	SORBS2	3	0.642134237	0.329651702	1.947917251	0.051424865	0.282124773	15.96972539	87.47630313	1	CRA004775;PRJNA827465;Yang2025_Silkie_Wencha	0.472124676
ENSGALG00010013170	COL3A1	3	-0.457625387	0.234849384	-1.948590958	0.051344293	0.10300346	7.039398355	71.58848101	0.666666667	CRA004775;PRJNA827465;Yang2025_Silkie_Wencha	0.472124676
ENSGALG00010022886	PLA2G15	3	-0.412600005	0.211748323	-1.948539659	0.051350424	0.08011071	6.247443604	67.98690589	0.666666667	CRA004775;PRJNA827465;Yang2025_Silkie_Wencha	0.472124676
ENSGALG00010010678	KCNIP1	3	-0.382663179	0.196315578	-1.949224728	0.051268593	0	1.201343805	0	1	CRA004775;PRJNA827465;Yang2025_Silkie_Wencha	0.472124676
ENSGALG00010014929	FHL1	3	-0.259601352	0.133289353	-1.947652582	0.051456548	0	0.182236049	0	1	CRA004775;PRJNA827465;Yang2025_Silkie_Wencha	0.472124676
ENSGALG00010029289	PITPNM3	3	0.237679308	0.122021162	1.947853187	0.051432533	0	1.872344358	0	0.666666667	CRA004775;PRJNA827465;Yang2025_Silkie_Wencha	0.472124676
ENSGALG00010025836	ENSGALG0001002583	3	0.225907388	0.11597959	1.947820207	0.05143648	0.023263956	5.378169586	62.81262671	1	CRA004775;PRJNA827465;Yang2025_Silkie_Wencha	0.472124676
ENSGALG00010026117	LZTR1	3	-0.162809999	0.083547958	-1.948701113	0.051331129	0.013250063	5.800128864	65.5180075	1	CRA004775;PRJNA827465;Yang2025_Silkie_Wencha	0.472124676
ENSGALG00010021771	ANKRD61	3	0.117905837	0.06046147	1.950098735	0.051164352	0	1.062563063	0	0.666666667	CRA004775;PRJNA827465;Yang2025_Silkie_Wencha	0.472124676
ENSGALG00010012875	HSF2	3	-0.11578326	0.059403455	-1.949099744	0.051283514	0.003864826	3.047686242	34.37644687	1	CRA004775;PRJNA827465;Yang2025_Silkie_Wencha	0.472124676
ENSGALG00010008005	ANKRD42	2	0.472177043	0.24249126	1.947192005	0.05151172	0	0.020048099	0	1	PRJNA827465;Yang2025_Silkie_Wenchang	0.472155789
ENSGALG00010019724	HES5A	2	0.331890725	0.170480421	1.946796728	0.051559109	0	0.034615622	0	1	PRJNA827465;Yang2025_Silkie_Wenchang	0.472155789
ENSGALG00010018452	SPSB3	3	-0.121915448	0.062623886	-1.946788301	0.05156012	0	1.804036678	0	1	CRA004775;PRJNA827465;Yang2025_Silkie_Wencha	0.472155789
ENSGALG00010004177	CSPP1	3	0.437136589	0.224639993	1.945942853	0.051661605	0.124759968	12.34165503	83.79471801	1	CRA004775;PRJNA827465;Yang2025_Silkie_Wencha	0.47224413
ENSGALG00010019287	AHSA2	3	0.308809545	0.158699723	1.945873246	0.051669967	0.06396379	14.30886523	86.02265122	1	CRA004775;PRJNA827465;Yang2025_Silkie_Wencha	0.47224413
ENSGALG00010024062	ST3GAL4	3	0.139795216	0.071824014	1.946357617	0.051611797	0	0.870776062	0	0.666666667	CRA004775;PRJNA827465;Yang2025_Silkie_Wencha	0.47224413
ENSGALG00010011898	ENSGALG0001001189	3	-0.157029101	0.080774925	-1.944032775	0.051891499	0.006918108	2.999723937	33.32719804	1	CRA004775;PRJNA827465;Yang2025_Silkie_Wencha	0.47396247
ENSGALG00010006086	ENSGALG0001000608	3	0.504356143	0.259573421	1.943019212	0.052013837	0	0.047181077	0	1	CRA004775;PRJNA827465;Yang2025_Silkie_Wencha	0.474682345
ENSGALG00010008314	INVS	3	0.241693821	0.12440337	1.942823735	0.052037459	0.021856529	3.687683219	45.76540659	1	CRA004775;PRJNA827465;Yang2025_Silkie_Wencha	0.474682345
ENSGALG00010012863	KCNE1	2	-0.526895024	0.27141503	-1.941289044	0.052223228	0	0.926896109	0	1	PRJNA827465;Yang2025_Silkie_Wenchang	0.475774649
ENSGALG00010001877	OAT	3	-0.256710504	0.132237853	-1.94127852	0.052224503	0.03999124	10.04864077	80.09681065	1	CRA004775;PRJNA827465;Yang2025_Silkie_Wencha	0.475774649
ENSGALG00010022343	GATA5	3	0.83766473	0.43186255	1.93965587	0.052421526	0.351789644	5.395382192	62.93126364	1	CRA004775;PRJNA827465;Yang2025_Silkie_Wencha	0.47586049
ENSGALG00010027095	SCN5A	3	0.243781485	0.125690491	1.939538006	0.052435861	0.008749862	2.387430479	16.22792717	0.666666667	CRA004775;PRJNA827465;Yang2025_Silkie_Wencha	0.47586049
ENSGALG00010004087	NCOA7	3	0.166641487	0.085870574	1.940612243	0.052305328	0.001576538	2.10434011	4.958329196	1	CRA004775;PRJNA827465;Yang2025_Silkie_Wencha	0.47586049
ENSGALG00010013872	GLS2	3	0.127195467	0.065565989	1.939961103	0.052384417	0	0.938383354	0	1	CRA004775;PRJNA827465;Yang2025_Silkie_Wencha	0.47586049
ENSGALG00010013491	CNOT11	3	-0.118356286	0.060990725	-1.940562037	0.052311423	0.003061343	2.529057773	20.91916516	0.666666667	CRA004775;PRJNA827465;Yang2025_Silkie_Wencha	0.47586049
ENSGALG00010007381	EGFR	3	-0.114803717	0.059177007	-1.940005459	0.052379026	0	1.528052705	0	0.666666667	CRA004775;PRJNA827465;Yang2025_Silkie_Wencha	0.47586049
ENSGALG00010020764	PRKAR2A	3	-0.10142692	0.052302304	-1.939243807	0.052471658	0	0.090119166	0	1	CRA004775;PRJNA827465;Yang2025_Silkie_Wencha	0.475879903
ENSGALG00010017307	TMEM186	3	0.208805798	0.107720388	1.938405552	0.052573764	0.009425687	2.689213951	25.6288255	0.666666667	CRA004775;PRJNA827465;Yang2025_Silkie_Wencha	0.476500284
ENSGALG00010002600	TRPA1	2	-0.978849312	0.505421749	-1.936698044	0.052782265	0.126755648	1.311304417	23.74005708	1	PRJNA827465;Yang2025_Silkie_Wenchang	0.477166525
ENSGALG00010016456	LAMP3	3	0.693923794	0.358164557	1.937444064	0.052691085	0.140861825	3.085710731	35.18511051	1	CRA004775;PRJNA827465;Yang2025_Silkie_Wencha	0.477166525
ENSGALG00010022787	ENO4	3	0.438092183	0.226145889	1.937210462	0.052719622	0	1.170214544	0	1	CRA004775;PRJNA827465;Yang2025_Silkie_Wencha	0.477166525
ENSGALG00010002601	PLCXD1	3	-0.366711446	0.189328908	-1.936901502	0.052757385	0.07379355	6.894885456	70.99299165	1	CRA004775;PRJNA827465;Yang2025_Silkie_Wencha	0.477166525
ENSGALG00010014263	ZNF131	3	0.310247329	0.160369494	1.93457822	0.053042075	0.040705431	4.24337102	52.86766133	1	CRA004775;PRJNA827465;Yang2025_Silkie_Wencha	0.47920888
ENSGALG00010028899	TNFSF8	2	0.806443397	0.417440471	1.931876408	0.053374762	0	0.804790807	0	1	CRA004775;PRJNA827465	0.479323642
ENSGALG00010016609	SOX2	2	0.663879758	0.343701895	1.931556874	0.053414223	0.040697437	1.204841386	17.00152306	1	PRJNA827465;Yang2025_Silkie_Wenchang	0.479323642
ENSGALG00010018016	IFIT5	3	-0.426059892	0.22061265	-1.93125776	0.053451184	0	1.890595378	0	0.666666667	CRA004775;PRJNA827465;Yang2025_Silkie_Wencha	0.479323642
ENSGALG00010019771	VWCE	3	0.372311699	0.192721603	1.931862816	0.05337644	0	0.109069478	0	1	CRA004775;PRJNA827465;Yang2025_Silkie_Wencha	0.479323642
ENSGALG00010020742	THAP10	3	-0.307906566	0.159313753	-1.932705493	0.053272488	0.014860846	2.429219107	17.66901578	0.666666667	CRA004775;PRJNA827465;Yang2025_Silkie_Wencha	0.479323642
ENSGALG00010023992	FAM131B	3	0.212863838	0.110220698	1.931251042	0.053452015	0.009783456	2.643812575	24.35167232	0.666666667	CRA004775;PRJNA827465;Yang2025_Silkie_Wencha	0.479323642
ENSGALG00010029937	PMP22	3	-0.145975297	0.075511597	-1.933150713	0.053217634	0.00522437	2.836898524	29.50047444	0.666666667	CRA004775;PRJNA827465;Yang2025_Silkie_Wencha	0.479323642
ENSGALG00010021458	USP30	3	0.134735329	0.069710266	1.932790351	0.05326203	0.003437204	2.534821143	21.09896961	1	CRA004775;PRJNA827465;Yang2025_Silkie_Wencha	0.479323642
ENSGALG00010005629	NRP1	3	-0.134595141	0.069696034	-1.931173602	0.053461587	0	1.234008387	0	0.666666667	CRA004775;PRJNA827465;Yang2025_Silkie_Wencha	0.479323642
ENSGALG00010018501	ATG14	3	0.107460675	0.055599943	1.932747942	0.053267256	0	1.290978192	0	1	CRA004775;PRJNA827465;Yang2025_Silkie_Wencha	0.479323642
ENSGALG00010018539	CHD2	3	-0.089944708	0.046561846	-1.931725565	0.053393388	0.000140265	2.023291006	1.151144649	0.666666667	CRA004775;PRJNA827465;Yang2025_Silkie_Wencha	0.479323642
ENSGALG00010028764	ARNT	3	-0.076458842	0.039574572	-1.932019437	0.053357107	0	0.547311086	0	1	CRA004775;PRJNA827465;Yang2025_Silkie_Wencha	0.479323642
ENSGALG00010007956	HS1BP3	3	-0.182386756	0.094466755	-1.930697794	0.053520436	0	0.429706508	0	1	CRA004775;PRJNA827465;Yang2025_Silkie_Wencha	0.479547175
ENSGALG00010021757	ZP4	2	0.478612611	0.248085358	1.929225548	0.053702868	0	0.130607963	0	1	PRJNA827465;Yang2025_Silkie_Wenchang	0.480354509
ENSGALG00010029310	LRWD1	3	-0.22013185	0.114077513	-1.929669081	0.053647853	0.031680002	12.89039084	84.48456664	1	CRA004775;PRJNA827465;Yang2025_Silkie_Wencha	0.480354509
ENSGALG00010019008	HERPUD2	3	-0.133440171	0.069180326	-1.92887458	0.053746434	0.007835019	4.438402335	54.93874036	1	CRA004775;PRJNA827465;Yang2025_Silkie_Wencha	0.480354509
ENSGALG00010023215	TRIP12	3	-0.064868468	0.033628186	-1.928990966	0.053731984	0	1.839058203	0	0.666666667	CRA004775;PRJNA827465;Yang2025_Silkie_Wencha	0.480354509
ENSGALG00010013194	GRAP2	2	-0.800692566	0.415625771	-1.926474783	0.054045118	0	0.683781469	0	1	CRA004775;PRJNA827465	0.481614646
ENSGALG00010008416	MSX2	3	0.78612586	0.408692004	1.923516616	0.054415202	0	0.496715968	0	1	CRA004775;PRJNA827465;Yang2025_Silkie_Wencha	0.481614646
ENSGALG00010010638	ACVR1C	3	-0.572461911	0.297503762	-1.924217387	0.054327341	0.099964116	3.203280384	37.56400439	1	CRA004775;PRJNA827465;Yang2025_Silkie_Wencha	0.481614646
ENSGALG00010002215	YLEC18	3	-0.502358322	0.26115919	-1.923571297	0.054408342	0	1.166069957	0	1	CRA004775;PRJNA827465;Yang2025_Silkie_Wencha	0.481614646
ENSGALG00010029430	FN3KRP	3	-0.353245718	0.183614091	-1.92384864	0.054373559	0.076527502	8.877644718	77.47150214	0.666666667	CRA004775;PRJNA827465;Yang2025_Silkie_Wencha	0.481614646
ENSGALG00010021100	EMC7	3	-0.27797347	0.144342373	-1.925792569	0.05413028	0.045862692	8.539072566	76.57825268	1	CRA004775;PRJNA827465;Yang2025_Silkie_Wencha	0.481614646
ENSGALG00010023259	TSPAN15	3	-0.266558814	0.138494663	-1.924686549	0.054268584	0	0.401851969	0	1	CRA004775;PRJNA827465;Yang2025_Silkie_Wencha	0.481614646
ENSGALG00010012910	XPNPEP3	3	-0.210158566	0.109253034	-1.923594778	0.054405396	0.021424535	6.091866707	67.16934076	1	CRA004775;PRJNA827465;Yang2025_Silkie_Wencha	0.481614646
ENSGALG00010008336	PAIP2B	3	-0.20539269	0.106693688	-1.925068796	0.054220752	0.020251785	5.492028507	63.58358305	1	CRA004775;PRJNA827465;Yang2025_Silkie_Wencha	0.481614646
ENSGALG00010013715	IL1R1	3	0.204114372	0.106122789	1.923379273	0.054432435	0	1.137149632	0	1	CRA004775;PRJNA827465;Yang2025_Silkie_Wencha	0.481614646
ENSGALG00010024313	SERINC3	3	-0.191136699	0.099204635	-1.926691215	0.054018123	0.01511726	3.972926993	49.65928134	1	CRA004775;PRJNA827465;Yang2025_Silkie_Wencha	0.481614646
ENSGALG00010005307	TIAM2	3	0.130944312	0.067955987	1.92689883	0.053992239	0	0.913004916	0	1	CRA004775;PRJNA827465;Yang2025_Silkie_Wencha	0.481614646
ENSGALG00010019030	CDR2	3	0.128698273	0.06682974	1.925763476	0.054133914	0	1.853706312	0	0.666666667	CRA004775;PRJNA827465;Yang2025_Silkie_Wencha	0.481614646
ENSGALG00010016603	ENSGALG0001001660	3	0.122692559	0.063689918	1.92640474	0.054053856	0	0.262115123	0	1	CRA004775;PRJNA827465;Yang2025_Silkie_Wencha	0.481614646
ENSGALG00010009506	HAS2	3	0.120738177	0.062711821	1.925285759	0.054193618	0	1.693391295	0	1	CRA004775;PRJNA827465;Yang2025_Silkie_Wencha	0.481614646
ENSGALG00010022983	PKN2	3	-0.077145938	0.040094625	-1.924096755	0.054342457	0	0.348871288	0	0.666666667	CRA004775;PRJNA827465;Yang2025_Silkie_Wencha	0.481614646
ENSGALG00010000367	HSD17B10	3	-0.312899821	0.162712095	-1.92302742	0.054476606	0.041743701	3.884854533	48.51802087	1	CRA004775;PRJNA827465;Yang2025_Silkie_Wencha	0.481620299
ENSGALG00010009149	FUT4	3	0.24909607	0.129564747	1.922560537	0.054535264	0	1.163401186	0	0.666666667	CRA004775;PRJNA827465;Yang2025_Silkie_Wencha	0.481620299
ENSGALG00010009322	ENSGALG0001000932	3	0.183723027	0.095553198	1.92273029	0.054513931	0	0.843542123	0	0.666666667	CRA004775;PRJNA827465;Yang2025_Silkie_Wencha	0.481620299
ENSGALG00010004716	ADH6	3	1.325288316	0.689452822	1.922232058	0.054576565	0.978194961	6.394789427	68.72453702	1	CRA004775;PRJNA827465;Yang2025_Silkie_Wencha	0.481684175
ENSGALG00010011407	CEP290	3	0.575452508	0.299652661	1.920398458	0.054807588	0.220429608	13.64956365	85.34751695	1	CRA004775;PRJNA827465;Yang2025_Silkie_Wencha	0.482485307
ENSGALG00010003681	NT5C1B	3	-0.252844026	0.131607972	-1.921190808	0.054707657	0.031697537	5.206999986	61.59016698	1	CRA004775;PRJNA827465;Yang2025_Silkie_Wencha	0.482485307
ENSGALG00010022088	DKK3	3	-0.168657164	0.087822483	-1.920432651	0.054803273	0	0.454806847	0	1	CRA004775;PRJNA827465;Yang2025_Silkie_Wencha	0.482485307
ENSGALG00010012571	AGAP1	3	-0.136095298	0.070877139	-1.920157897	0.054837958	0	1.145206269	0	0.666666667	CRA004775;PRJNA827465;Yang2025_Silkie_Wencha	0.482485307
ENSGALG00010028854	RPL7A	3	-0.121267518	0.06315401	-1.920187137	0.054834266	0	0.363439401	0	1	CRA004775;PRJNA827465;Yang2025_Silkie_Wencha	0.482485307
ENSGALG00010003389	ENSGALG0001000338	2	-1.257828918	0.655708589	-1.918274276	0.055076242	0.708283889	5.669145984	82.36065886	1	PRJNA827465;Yang2025_Silkie_Wenchang	0.482705868
ENSGALG00010018426	ENSGALG0001001842	3	0.663954122	0.346194707	1.917863294	0.055128348	0.243302728	7.093305996	71.80440261	1	CRA004775;PRJNA827465;Yang2025_Silkie_Wencha	0.482705868
ENSGALG00010023986	SYCE3	3	0.535861323	0.27943046	1.917691159	0.055150183	0.084924894	3.120419143	35.90604633	1	CRA004775;PRJNA827465;Yang2025_Silkie_Wencha	0.482705868
ENSGALG00010029454	ENSGALG0001002945	3	-0.375597138	0.195804538	-1.918224883	0.055082502	0	1.977410071	0	1	CRA004775;PRJNA827465;Yang2025_Silkie_Wencha	0.482705868
ENSGALG00010008580	PANX1	3	-0.304005252	0.158466411	-1.918420765	0.05505768	0	1.946563599	0	0.666666667	CRA004775;PRJNA827465;Yang2025_Silkie_Wencha	0.482705868
ENSGALG00010015647	PDZD2	3	-0.220675115	0.115082841	-1.917532733	0.055170287	0.008996329	2.476105809	19.22800744	1	CRA004775;PRJNA827465;Yang2025_Silkie_Wencha	0.482705868
ENSGALG00010013012	TMEM182	3	-0.162686022	0.084779338	-1.918934805	0.054992585	0.005593424	2.62288804	23.74817494	0.666666667	CRA004775;PRJNA827465;Yang2025_Silkie_Wencha	0.482705868
ENSGALG00010006084	TMEM68	3	-0.137629524	0.071703034	-1.919437949	0.054928932	0	1.05137219	0	1	CRA004775;PRJNA827465;Yang2025_Silkie_Wencha	0.482705868
ENSGALG00010023518	METTL5	3	-0.091731849	0.047822819	-1.918160629	0.055090647	0	0.540637509	0	0.666666667	CRA004775;PRJNA827465;Yang2025_Silkie_Wencha	0.482705868
ENSGALG00010027126	ENSGALG0001002712	3	-0.67151385	0.350692099	-1.914824575	0.055514879	0.210808179	4.686918042	57.32803556	1	CRA004775;PRJNA827465;Yang2025_Silkie_Wencha	0.483625926
ENSGALG00010006260	MAP3K4	3	0.449735449	0.234720904	1.916043447	0.055359566	0.128917081	22.18590374	90.98526694	1	CRA004775;PRJNA827465;Yang2025_Silkie_Wencha	0.483625926
ENSGALG00010000610	ENSGALG0001000061	3	-0.302100942	0.157694389	-1.915736784	0.055398608	0.045573282	5.115993231	60.90690684	1	CRA004775;PRJNA827465;Yang2025_Silkie_Wencha	0.483625926
ENSGALG00010022749	HSPA12A	3	-0.279005986	0.145697405	-1.914968811	0.055496481	0.035491953	5.536723104	63.87755063	0.666666667	CRA004775;PRJNA827465;Yang2025_Silkie_Wencha	0.483625926
ENSGALG00010003006	CYP7B1	3	-0.242574095	0.126663383	-1.915108291	0.055478695	0	1.262029156	0	1	CRA004775;PRJNA827465;Yang2025_Silkie_Wencha	0.483625926
ENSGALG00010022252	COQ9	3	-0.152057735	0.079363473	-1.915966243	0.055369393	0	0.258535335	0	1	CRA004775;PRJNA827465;Yang2025_Silkie_Wencha	0.483625926
ENSGALG00010014334	HMGCS1	3	0.139103901	0.072642045	1.914922687	0.055502364	0	0.27702418	0	1	CRA004775;PRJNA827465;Yang2025_Silkie_Wencha	0.483625926
ENSGALG00010004446	ACAT2	3	0.159324605	0.083239297	1.914055133	0.055613112	0	0.035515895	0	1	CRA004775;PRJNA827465;Yang2025_Silkie_Wencha	0.484183365
ENSGALG00010024475	ADGRG1	3	-0.176627602	0.092308041	-1.913458464	0.055689386	0	1.454908474	0	0.666666667	CRA004775;PRJNA827465;Yang2025_Silkie_Wencha	0.484549064
ENSGALG00010005505	FABP2	2	1.674897406	0.875966015	1.912057521	0.055868817	0.768137617	2.001977367	50.04938534	1	CRA004775;PRJNA827465	0.485811315
ENSGALG00010028918	AGPAT2	3	-0.209138137	0.109443072	-1.91093081	0.056013473	0.01213355	2.822374635	29.13768514	0.666666667	CRA004775;PRJNA827465;Yang2025_Silkie_Wencha	0.486769819

ENSGALG00010010953	EXTL3	3	-0.227823938	0.119259673	-1.910318324	0.05609224	0.031542451	11.00909819	81.83320772	1	CRA004775;PRJNA827465;Yang2025_Silkie_Wencha	0.48685585
ENSGALG00010005899	ENSGALG0001000589	3	0.200956377	0.105183282	1.910535339	0.056064321	0	0.607503032	0	1	CRA004775;PRJNA827465;Yang2025_Silkie_Wencha	0.48685585
ENSGALG00010025125	PIK3CB	3	0.122685419	0.064284037	1.908489639	0.056327961	0	0.80888509	0	1	CRA004775;PRJNA827465;Yang2025_Silkie_Wencha	0.488601866
ENSGALG00010024288	CHID1	3	0.133594861	0.070021546	1.907910767	0.05640275	0.000248672	2.026516623	1.308482857	1	CRA004775;PRJNA827465;Yang2025_Silkie_Wencha	0.488950637
ENSGALG00010008155	MAP10	3	-0.247848809	0.129962765	-1.907075529	0.056510808	0	1.324247064	0	0.666666667	CRA004775;PRJNA827465;Yang2025_Silkie_Wencha	0.48928739
ENSGALG00010012229	FSBP	3	-0.141137106	0.073998687	-1.907292039	0.056482781	0	1.732240323	0	0.666666667	CRA004775;PRJNA827465;Yang2025_Silkie_Wencha	0.48928739
ENSGALG00010025755	ENSGALG0001002575	3	0.610411593	0.32037946	1.90527693	0.056744083	0.159768798	4.13835237	51.67158759	1	CRA004775;PRJNA827465;Yang2025_Silkie_Wencha	0.49024163
ENSGALG00010002467	NEK3	3	0.455631648	0.239108794	1.905541155	0.056709763	0.107205431	6.003313486	66.68506476	1	CRA004775;PRJNA827465;Yang2025_Silkie_Wencha	0.49024163
ENSGALG00010024386	TPPP3	3	0.325491158	0.170864108	1.904970923	0.056783851	0	1.641712948	0	0.666666667	CRA004775;PRJNA827465;Yang2025_Silkie_Wencha	0.49024163
ENSGALG00010007651	CTNNAL1	3	0.322921697	0.169522499	1.904889906	0.056794384	0.059754974	8.357201818	76.06854491	1	CRA004775;PRJNA827465;Yang2025_Silkie_Wencha	0.49024163
ENSGALG00010019316	CLEC16A	3	0.128306621	0.067322892	1.905839409	0.056671044	0	0.227268669	0	0.666666667	CRA004775;PRJNA827465;Yang2025_Silkie_Wencha	0.49024163
ENSGALG00010006113	EIF2B2	3	-0.207565169	0.109066877	-1.903099953	0.057027504	0.021824079	5.492437065	63.5862919	1	CRA004775;PRJNA827465;Yang2025_Silkie_Wencha	0.491653585
ENSGALG00010027906	LHX6	3	-0.169885328	0.089265197	-1.903153001	0.057020584	0	0.435961626	0	0.666666667	CRA004775;PRJNA827465;Yang2025_Silkie_Wencha	0.491653585
ENSGALG00010001672	ENSGALG0001000167	2	1.372826184	0.722462022	1.900205328	0.057406179	0.417527487	1.652012127	39.46775671	1	CRA004775;Yang2025_Silkie_Wenchang	0.492209614
ENSGALG00010013704	LEAP2	2	1.274965123	0.670576315	1.901297577	0.057263046	0.301716796	1.488761737	32.83008455	1	CRA004775;PRJNA827465	0.492209614
ENSGALG00010021390	GSTO2	3	0.793023599	0.417110553	1.901231206	0.057271735	0.350692453	6.805599441	70.6124344	1	CRA004775;PRJNA827465;Yang2025_Silkie_Wencha	0.492209614
ENSGALG00010013619	IL1R2	3	0.513304053	0.270215157	1.899612364	0.057484009	0.150490238	7.180458146	72.14662408	1	CRA004775;PRJNA827465;Yang2025_Silkie_Wencha	0.492209614
ENSGALG00010005561	CLYBL	3	-0.512561497	0.269774414	-1.899963341	0.057437931	0.136439412	5.92728057	66.25771336	0.666666667	CRA004775;PRJNA827465;Yang2025_Silkie_Wencha	0.492209614
ENSGALG00010020912	PDE8A	3	0.342258237	0.18006506	1.90074763	0.057335077	0.051586279	4.594082211	56.46573334	1	CRA004775;PRJNA827465;Yang2025_Silkie_Wencha	0.492209614
ENSGALG00010017679	RASL12	3	0.251927171	0.132614786	1.899691417	0.057473627	0	0.116170244	0	1	CRA004775;PRJNA827465;Yang2025_Silkie_Wencha	0.492209614
ENSGALG00010002930	SERAF	3	0.25105709	0.132164235	1.899584174	0.057487711	0	1.285341682	0	1	CRA004775;PRJNA827465;Yang2025_Silkie_Wencha	0.492209614
ENSGALG00010028061	NAIF1	3	-0.166873694	0.087804441	-1.900515421	0.057365513	0.016155498	7.189505301	72.18167431	1	CRA004775;PRJNA827465;Yang2025_Silkie_Wencha	0.492209614
ENSGALG00010002774	TBX18	3	0.166833085	0.08775773	1.901064281	0.057293593	0.001468015	2.101550503	4.832170481	1	CRA004775;PRJNA827465;Yang2025_Silkie_Wencha	0.492209614
ENSGALG00010026030	IBA57	3	-0.13380334	0.070444447	-1.89941643	0.057509745	0	1.913233916	0	1	CRA004775;PRJNA827465;Yang2025_Silkie_Wencha	0.492209614
ENSGALG00010005367	H2AZ1	3	-0.093593854	0.049240747	-1.900739927	0.057336086	0	1.623946674	0	0.666666667	CRA004775;PRJNA827465;Yang2025_Silkie_Wencha	0.492209614
ENSGALG00010017826	PABPC4	3	-0.235509435	0.124055433	-1.898420962	0.05764065	0.013441477	2.777519643	27.99330853	0.666666667	CRA004775;PRJNA827465;Yang2025_Silkie_Wencha	0.492882785
ENSGALG00010005213	ENSGALG0001000521	3	-0.221369519	0.116615333	-1.898288275	0.057658118	0	0.356852043	0	1	CRA004775;PRJNA827465;Yang2025_Silkie_Wencha	0.492882785
ENSGALG00010017050	L3HYPDH	3	-0.512131465	0.2698477	-1.897853737	0.057715352	0.139983499	5.622834765	64.43075275	1	CRA004775;PRJNA827465;Yang2025_Silkie_Wencha	0.493073936
ENSGALG00010029627	PEX12	3	-0.163154505	0.086034202	-1.89639121	0.057908333	0	1.235673218	0	1	CRA004775;PRJNA827465;Yang2025_Silkie_Wencha	0.494423868
ENSGALG00010015487	RPS6KA6	3	0.158143838	0.08342201	1.895708798	0.057998561	0.004072593	2.406819189	16.90277321	1	CRA004775;PRJNA827465;Yang2025_Silkie_Wencha	0.494895388
ENSGALG00010000189	ENSGALG0001000018	3	0.654305586	0.345392929	1.894380375	0.058174539	0	1.088434377	0	0.666666667	CRA004775;PRJNA827465;Yang2025_Silkie_Wencha	0.495847685
ENSGALG00010017347	TMEM39B	3	-0.330610175	0.17452554	-1.89433692	0.058180303	0.071845299	12.41807573	83.89444514	1	CRA004775;PRJNA827465;Yang2025_Silkie_Wencha	0.495847685
ENSGALG00010013598	PAQR7	3	0.27878628	0.147203305	1.893886006	0.058240142	0	0.880472357	0	0.666666667	CRA004775;PRJNA827465;Yang2025_Silkie_Wencha	0.496058659
ENSGALG00010010453	DRC1	2	0.480416542	0.253723508	1.893464843	0.058296079	0	0.046657888	0	1	PRJNA827465;Yang2025_Silkie_Wenchang	0.496091978
ENSGALG00010019246	IL17RC	3	0.141066286	0.07450704	1.893328271	0.058314228	0	0.692161095	0	0.666666667	CRA004775;PRJNA827465;Yang2025_Silkie_Wencha	0.496091978
ENSGALG00010028833	GBGT1	3	-0.141765219	0.074893101	-1.892900916	0.058371047	0.000166876	2.01581332	0.784463536	1	CRA004775;PRJNA827465;Yang2025_Silkie_Wencha	0.496276753
ENSGALG00010013017	SCMH1	3	-0.106608758	0.056337012	-1.892339579	0.058445751	0	0.100793071	0	1	CRA004775;PRJNA827465;Yang2025_Silkie_Wencha	0.496613263
ENSGALG00010029771	SOX9	3	0.478390224	0.25292609	1.891422998	0.058567901	0.092566221	3.852491403	48.08554282	1	CRA004775;PRJNA827465;Yang2025_Silkie_Wencha	0.497143276
ENSGALG00010017049	TRAF3	3	0.427718996	0.226271546	1.890290684	0.058719094	0.109251757	8.942007739	77.63365836	1	CRA004775;PRJNA827465;Yang2025_Silkie_Wencha	0.497143276
ENSGALG00010011915	RRM2B	3	-0.253843324	0.134275163	-1.890471159	0.058694974	0.023081559	3.389423655	40.99291787	0.666666667	CRA004775;PRJNA827465;Yang2025_Silkie_Wencha	0.497143276
ENSGALG00010027928	FLAD1	3	0.138768131	0.073408785	1.890347752	0.058711466	0	0.82545333	0	0.666666667	CRA004775;PRJNA827465;Yang2025_Silkie_Wencha	0.497143276
ENSGALG00010001958	RAB11FIP2	3	-0.110687904	0.058541276	-1.890766856	0.058655473	0	1.708767066	0	0.666666667	CRA004775;PRJNA827465;Yang2025_Silkie_Wencha	0.497143276
ENSGALG00010026102	IPO9	3	-0.064234705	0.033981353	-1.890292737	0.05871882	0.00062991	2.384777039	16.13471752	1	CRA004775;PRJNA827465;Yang2025_Silkie_Wencha	0.497143276
ENSGALG00010011539	LANCL1	3	-0.2370301	0.125417668	-1.889925902	0.058767871	0.030371847	6.357435102	68.54077206	1	CRA004775;PRJNA827465;Yang2025_Silkie_Wencha	0.497258483
ENSGALG00010027184	MPND	3	0.708665136	0.375167533	1.88892981	0.058901234	0.344473955	13.4512813	85.13152796	1	CRA004775;PRJNA827465;Yang2025_Silkie_Wencha	0.498088848
ENSGALG00010021731	SRM	3	0.241922621	0.128093231	1.888644847	0.058939433	0.031974453	6.390885251	68.70543091	0.666666667	CRA004775;PRJNA827465;Yang2025_Silkie_Wencha	0.498113957
ENSGALG00010027877	OAZ1	3	-0.326856543	0.17314544	-1.887757153	0.05905856	0.065643565	7.951158455	74.84643261	1	CRA004775;PRJNA827465;Yang2025_Silkie_Wencha	0.498524762
ENSGALG00010022588	TCP11L1	3	-0.112031268	0.05934201	-1.88789136	0.059040537	0	0.091675898	0	1	CRA004775;PRJNA827465;Yang2025_Silkie_Wencha	0.498524762
ENSGALG00010024534	VWF	3	-0.153190338	0.081169464	-1.887290251	0.059121297	0.000814221	2.057775806	2.807682247	0.666666667	CRA004775;PRJNA827465;Yang2025_Silkie_Wencha	0.498756575
ENSGALG00010015485	BCAS1	3	0.55130308	0.292593795	1.884192657	0.059538921	0.189146868	8.277059608	75.83682981	1	CRA004775;PRJNA827465;Yang2025_Silkie_Wencha	0.499130355
ENSGALG00010027395	BCL2L15	3	0.353134103	0.187456318	1.883820763	0.059589224	0	0.263161549	0	1	CRA004775;PRJNA827465;Yang2025_Silkie_Wencha	0.499130355
ENSGALG00010018796	PER3	3	0.300617171	0.159529191	1.884402272	0.059510583	0.035120209	3.619248397	44.73990784	0.666666667	CRA004775;PRJNA827465;Yang2025_Silkie_Wencha	0.499130355
ENSGALG00010024377	CIB2	3	-0.250331845	0.132785586	-1.885233574	0.05939831	0.001974252	2.061141779	2.966403338	1	CRA004775;PRJNA827465;Yang2025_Silkie_Wencha	0.499130355
ENSGALG00010000684	COBL	3	-0.200947649	0.106563866	-1.88570157	0.059335181	0	0.312404872	0	1	CRA004775;PRJNA827465;Yang2025_Silkie_Wencha	0.499130355
ENSGALG00010023694	PPCDC	3	-0.176765065	0.093813304	-1.884221716	0.059534991	0.001916282	2.135926442	6.363816612	1	CRA004775;PRJNA827465;Yang2025_Silkie_Wencha	0.499130355
ENSGALG00010006135	ZC2HC1C	3	0.175635851	0.093133649	1.885847425	0.059315518	0	0.802418289	0	1	CRA004775;PRJNA827465;Yang2025_Silkie_Wencha	0.499130355
ENSGALG00010020614	FAIM	3	-0.131935664	0.069938555	-1.886451101	0.059234192	0	1.553037197	0	1	CRA004775;PRJNA827465;Yang2025_Silkie_Wencha	0.499130355
ENSGALG00010018540	TNFSF10	3	-0.106831793	0.056709639	-1.883838364	0.059586843	0	0.105392511	0	1	CRA004775;PRJNA827465;Yang2025_Silkie_Wencha	0.499130355
ENSGALG00010021400	B4GALT2	3	-0.102356723	0.054322541	-1.884240338	0.059532474	0	0.820714308	0	1	CRA004775;PRJNA827465;Yang2025_Silkie_Wencha	0.499130355
ENSGALG00010026498	ENSGALG0001002649	3	-0.095031261	0.05044223	-1.883962343	0.059570069	0	0.438399524	0	1	CRA004775;PRJNA827465;Yang2025_Silkie_Wencha	0.499130355
ENSGALG00010022090	TRRAP	3	-0.085127009	0.045182267	-1.884080073	0.059554145	0	0.250820148	0	0.666666667	CRA004775;PRJNA827465;Yang2025_Silkie_Wencha	0.499130355
ENSGALG00010018154	SLC16A12	2	-0.32806624	0.174192334	-1.88335636	0.05965209	0	0.378361773	0	1	PRJNA827465;Yang2025_Silkie_Wenchang	0.499361104
ENSGALG00010008106	ENSGALG0001000810	3	-0.491189223	0.260958896	-1.882247474	0.059802423	0.09626318	3.842292314	47.94773962	1	CRA004775;PRJNA827465;Yang2025_Silkie_Wencha	0.499436771
ENSGALG00010022986	ALG9	3	-0.423045972	0.224708215	-1.882645778	0.059748388	0.138823932	25.63072953	92.19686666	1	CRA004775;PRJNA827465;Yang2025_Silkie_Wencha	0.499436771
ENSGALG00010022483	TOP2A	3	-0.19614859	0.104203172	-1.88236678	0.059786233	0.008009362	2.582984653	22.57019422	0.666666667	CRA004775;PRJNA827465;Yang2025_Silkie_Wencha	0.499436771
ENSGALG00010018780	WDHD1	3	-0.161508984	0.085799575	-1.882398417	0.059781941	0	0.193970357	0	1	CRA004775;PRJNA827465;Yang2025_Silkie_Wencha	0.499436771
ENSGALG00010013318	KERA	2	-0.191246859	0.10166926	-1.881068663	0.059962579	0.003772158	1.219930694	18.02813022	1	PRJNA827465;Yang2025_Silkie_Wenchang	0.499593935
ENSGALG00010024473	RAVER2	3	-0.075949258	0.040374426	-1.881122912	0.059955201	0	0.998655924	0	1	CRA004775;PRJNA827465;Yang2025_Silkie_Wencha	0.499593935
ENSGALG00010005991	FARP1	3	-0.075477161	0.040122233	-1.881180469	0.059947374	0	0.032389224	0	1	CRA004775;PRJNA827465;Yang2025_Silkie_Wencha	0.499593935
ENSGALG00010012565	SYNGR1	3	-0.070616914	0.037528738	-1.881675676	0.059880064	0	0.141160209	0	1	CRA004775;PRJNA827465;Yang2025_Silkie_Wencha	0.499593935
ENSGALG00010023711	ENSGALG0001002371	3	-0.517274954	0.275081581	-1.880441982	0.060047867	0.079077045	3.04873646	34.39905264	1	CRA004775;PRJNA827465;Yang2025_Silkie_Wencha	0.499957806
ENSGALG00010012131	SLC14A1	3	-0.492633264	0.262007155	-1.880228285	0.060076972	0	0.708505561	0	1	CRA004775;PRJNA827465;Yang2025_Silkie_Wencha	0.499957806
ENSGALG00010008418	TMEM245	3	-0.108019058	0.057477355	-1.879332457	0.060199113	0	1.358440812	0	0.666666667	CRA004775;PRJNA827465;Yang2025_Silkie_Wencha	0.50067956
ENSGALG00010018887	ADAMTS17	3	-0.183114122	0.097500141	-1.878090839	0.060368739	0	0.255733601	0	1	CRA004775;PRJNA827465;Yang2025_Silkie_Wencha	0.501795183
ENSGALG00010000233	HORMAD2	2	0.52504142	0.279682393	1.877277345	0.060480092	0.049691654	1.426953823	29.92064747	1	PRJNA827465;Yang2025_Silkie_Wenchang	0.501835689
ENSGALG00010002576	HELQ	3	0.138517166	0.073784791	1.877313259	0.060475172	0	1.743615095	0	0.666666667	CRA004775;PRJNA827465;Yang2025_Silkie_Wencha	0.501835689
ENSGALG00010014407	YIPF6	3	-0.073902119	0.039360011	-1.877593973	0.060436731	0	1.425193878	0	1	CRA004775;PRJNA827465;Yang2025_Silkie_Wencha	0.501835689
ENSGALG00010029178	NOTUM	3	0.141241515	0.075265023	1.876588997	0.060574447	0	0.562556564	0	1	CRA004775;PRJNA827465;Yang2025_Silkie_Wencha	0.502232506
ENSGALG00010021127	SWAP70	3	-0.129105654	0.068804596	-1.876410331	0.060598957	0.006009632	3.609370861	44.58868105	0.666666667	CRA004775;PRJNA827465;Yang2025_Silkie_Wencha	0.502232506
ENSGALG00010023277	KCNH4	3	-0.363647513	0.193885412	-1.875579543	0.060713038	0	1.151419774	0	1	CRA004775;PRJNA827465;Yang2025_Silkie_Wencha	0.502588781
ENSGALG00010019284	CHD5	3	0.231154199	0.123233106	1.875747567	0.060689951	0.02328604	4.098177897	51.19782376	1	CRA004775;PRJNA827465;Yang2025_Silkie_Wencha	0.502588781
ENSGALG00010029604	ENSGALG0001002960	3	-0.120013847	0.06399693	-1.875306304	0.060750597	0	0.011794994	0	1	CRA004775;PRJNA827465;Yang2025_Silkie_Wencha	0.502605433
ENSGALG00010008119	CCDC83	3	0.549771732	0.2933257	1.874270587	0.060893139	0.0721473	2.674074132	25.20775786	0.666666667	CRA004775;PRJNA827465;Yang2025_Silkie_Wencha	0.502608347
ENSGALG00010001385	MME	3	0.286478253	0.152820784	1.874602686	0.060847403	0.014949721	2.46222341	18.77260235	0.666666667	CRA004775;PRJNA827465;Yang2025_Silkie_Wencha	0.502608347
ENSGALG00010024769	RALY	3	-0.19575584	0.104431966	-1.874482003	0.06086402	0.006716082	2.441793742	18.093	1	CRA004775;PRJNA827465;Yang2025_Silkie_Wencha	0.502608347
ENSGALG00010002627	MRPS31	3	-0.107313009	0.05725171	-1.874407047	0.060874343	0	0.283151475	0	0.666666667	CRA004775;PRJNA827465;Yang2025_Silkie_Wencha	0.502608347
ENSGALG00010016338	PTPRU	3	0.142213089	0.075921422	1.873161553	0.06104608	0	1.148696223	0	0.666666667	CRA004775;PRJNA827465;Yang2025_Silkie_Wencha	0.503283102
ENSGALG00010021215	SH3PXD2A	3	0.139692116	0.07456709	1.873374915	0.061016631	0	1.456093467	0	1	CRA004775;PRJNA827465;Yang2025_Silkie_Wencha	0.503283102
ENSGALG00010004397	ENSGALG0001000439	3	0.15127624	0.080772847	1.872860067	0.061087711	0	1.411781088	0	0.666666667	CRA004775;PRJNA827465;Yang2025_Silkie_Wencha	0.503332834
ENSGALG00010013207	C1QTNF3	3	0.173306893	0.092565119	1.872269969	0.061169263	0	1.188952703	0	1	CRA004775;PRJNA827465;Yang2025_Silkie_Wencha	0.503711248
ENSGALG00010025242	CLSTN2	3	0.25768804	0.137660694	1.871907165	0.061219448	0	0.56187626	0	1	CRA004775;PRJNA827465;Yang2025_Silkie_Wencha	0.503831069
ENSGALG00010011886	NTNG1	3	-0.271563817	0.145231924	-1.869863108	0.06150283	0.017265427	2.605770327	23.24726476	0.666666667	CRA004775;PRJNA827465;Yang2025_Silkie_Wencha	0.505868827
ENSGALG00010016182	ELMO2	3	0.06883696	0.036823249	1.869388535	0.061568778	0	1.160508634	0	0.666666667	CRA004775;PRJNA827465;Yang2025_Silkie_Wencha	0.506116836
ENSGALG00010011381	RLIG1	3	0.138980007	0.074362444	1.868954249	0.06162918	0	0.731194985	0	1	CRA004775;PRJNA827465;Yang2025_Silkie_Wencha	0.506318985
ENSGALG00010003252	PHEX	3	-0.245889483	0.131614456	-1.868255899	0.061726411	0	0.73577739	0	1	CRA004775;PRJNA827465;Yang2025_Silkie_Wencha	0.506529146
ENSGALG00010026951	RUVBL1	3	-0.081569457	0.043660534	-1.868265221	0.061725112	0	0.589844792	0	1	CRA004775;PRJNA827465;Yang2025_Silkie_Wencha	0.506529146
ENSGALG00010029263	TOB1	3	0.100687244	0.053901578	1.867983238	0.061764407	0	1.461732721	0	1	CRA004775;PRJNA827465;Yang2025_Silkie_Wencha	0.506546958
ENSGALG00010024529	GRB7	2	1.600959889	0.857560559	1.866876773	0.061918798	0.601199864	1.681889697	40.54306878	1	CRA004775;PRJNA827465	0.507173571
ENSGALG00010017649	ENSGALG0001001764	3	-0.147771575	0.079149452	-1.866994278	0.061902387	0	0.396545407	0	1	CRA004775;PRJNA827465;Yang2025_Silkie_Wencha	0.507173571
ENSGALG00010019146	KLHDC2	3	-0.128490133	0.068834072	-1.866664721	0.061948423	0.003045945	2.315465986	13.624298	0.666666667	CRA004775;PRJNA827465;Yang2025_Silkie_Wencha	0.507173571
ENSGALG00010020750	AIFM2	3	0.523505068	0.280672414	1.865181763	0.062155931	0.125055917	4.291028735	53.39113011	0.666666667	CRA004775;PRJNA827465;Yang2025_Silkie_Wencha	0.508519576
ENSGALG00010010943	WDSUB1	3	0.161168591	0.086418585	1.864976047	0.062184762	0.008897792	3.330240726	39.94428138	0.666666667	CRA004775;PRJNA827465;Yang2025_Silkie_Wencha	0.508519576
ENSGALG00010011287	ENSGALG0001001128	3	0.185602091	0.099566928	1.864093676	0.06230855	0	1.941535806	0	1	CRA004775;PRJNA827465;Yang2025_Silkie_Wencha	0.509237338
ENSGALG00010023066	TLR21	3	-0.226778844	0.12172325	-1.863069254	0.062452523	0	1.548800934	0	1	CRA004775;PRJNA827465;Yang2025_Silkie_Wencha	0.510119137
ENSGALG00010021525	ENSGALG0001002152	3	-0.156149192	0.083892906	-1.861291965	0.062702957	0	0.865225355	0	0.666666667	CRA004775;PRJNA827465;Yang2025_Silkie_Wencha	0.511847924
ENSGALG00010024811	NIF3L1	3	-0.125454416	0.067410418	-1.861053823	0.062736576	0	0.16054487	0	1	CRA004775;PRJNA827465;Yang2025_Silkie_Wencha	0.511847924
ENSGALG00010015234	TRIP11	3	0.418618878	0.225030713	1.860274414	0.062846712	0.114071982	8.957385743	77.67205681	1	CRA004775;PRJNA827465;Yang2025_Silkie_Wencha	0.5118604
ENSGALG00010003861	NUDT15	3	-0.185803626	0.099875978	-1.860343505	0.062836942	0.014817764	3.917210019	48.94325322	1	CRA004775;PRJNA827465;Yang2025_Silkie_Wencha	0.5118604
ENSGALG00010010857	SEL1L3	3	0.107861747	0.057966093	1.8607731	0.062776226	0	0.407624954	0	1	CRA004775;PRJNA827465;Yang2025_Silkie_Wencha	0.5118604
ENSGALG00010005567	NDST3	3	-0.23846054	0.128326726	-1.858229746	0.063136396	0.012023762	2.51868032	20.59333675	0.666666667	CRA004775;PRJNA827465;Yang2025_Silkie_Wencha	0.513923717
ENSGALG00010021342	SFR1	3	-0.616212481	0.331767808	-1.85742096	0.063251287	0.220754332	9.240094807	78.35520044	1	CRA004775;PRJNA827465;Yang2025_Silkie_Wencha	0.514148149
ENSGALG00010022911	SMPD3	3	-0.565314253	0.304505189	-1.856501213	0.06338215	0	1.662076066	0	1	CRA004775;PRJNA827465;Yang2025_Silkie_Wencha	0.514148149
ENSGALG00010006546	SMOC2	3	-0.199266068	0.10731267	-1.85687364	0.063329134	0	1.272954879	0	0.666666667	CRA004775;PRJNA827465;Yang2025_Silkie_Wencha	0.514148149
ENSGALG00010013379	ZNF593	3	-0.189335573	0.101939337	-1.857335725	0.063263405	0.008217032	2.426055546	17.5616567	1	CRA004775;PRJNA827465;Yang2025_Silkie_Wencha	0.514148149
ENSGALG00010024431	FEZ1	3	0.12318203	0.066348495	1.856591183	0.063369339	0	1.068152426	0	0.666666667	CRA004775;PRJNA827465;Yang2025_Silkie_Wencha	0.514148149
ENSGALG00010000449	PLBD2	3	-0.102310985	0.055108532	-1.856536197	0.063377169	0.000174674	2.027959132	1.378683219	1	CRA004775;PRJNA827465;Yang2025_Silkie_Wencha	0.514148149
ENSGALG00010025186	TMEM120B	3	-0.146819254	0.079110158	-1.855883717	0.063470134	0	0.49752999	0	1	CRA004775;PRJNA827465;Yang2025_Silkie_Wencha	0.514301805
ENSGALG00010003412	ASB5	3	-0.122146957	0.065816984	-1.855857712	0.063473842	0	0.288087121	0	0.666666667	CRA004775;PRJNA827465;Yang2025_Silkie_Wencha	0.514301805
ENSGALG00010012861	WDR70	3	-0.202202276	0.109084852	-1.853698176	0.06359486	0	0.446858765	0	1	CRA004775;PRJNA827465;Yang2025_Silkie_Wencha	0.515016608
ENSGALG00010029629	ENSGALG0001002962	3	0.494986678	0.266886441	1.854671507	0.06364315	0.142678793	6.679984898	70.05981255	1	CRA004775;PRJNA827465;Yang2025_Silkie_Wencha	0.515083284
ENSGALG00010021087	THBS1	3	0.41503694	0.224018271	1.852692363	0.063926465	0	0.076104859	0	1	CRA004775;PRJNA827465;Yang2025_Silkie_Wencha	0.516006876
ENSGALG00010017991	RAB26	3	-0.24504194	0.132225196	-1.853216688	0.063851307	0.023674646	3.784749553	47.15634491	1	CRA004775;PRJNA827465;Yang2025_Silkie_Wencha	0.516006876
ENSGALG00010008094	PIM3	3	0.188424489	0.101708146	1.852599777	0.063939744	0.014265356	3.850208942	48.05476716	0.666666667	CRA004775;PRJNA827465;Yang2025_Silkie_Wencha	0.516006876
ENSGALG00010018458	SEMA5B	3	-0.157157825	0.084786763	-1.853565573	0.063801337	0	1.673514432	0	0.666666667	CRA004775;PRJNA827465;Yang2025_Silkie_Wencha	0.516006876
ENSGALG00010023593	CSK	3	0.080752219	0.043585864	1.852715813	0.063923102	0	0.310081599	0	0.666666667	CRA004775;PRJNA827465;Yang2025_Silkie_Wencha	0.516006876
ENSGALG00010007870	AvBD3	2	0.504472775	0.272716394	1.849807296	0.064341329	0	0.067914177	0	1	PRJNA827465;Yang2025_Silkie_Wenchang	0.516231528
ENSGALG00010006197	ENSGALG0001000619	3	-0.499231815	0.269937586	-1.849372485	0.064404046	0	1.429967749	0	1	CRA004775;PRJNA827465;Yang2025_Silkie_Wencha	0.516231528
ENSGALG00010016546	C1QTNF12	3	0.427880707	0.231075059	1.851695762	0.064069523	0	0.858687531	0	1	CRA004775;PRJNA827465;Yang2025_Silkie_Wencha	0.516231528
ENSGALG00010013596	SDCBP2	3	-0.304524827	0.16472713	-1.848662253	0.064506597	0.054318204	6.181793432	67.64692929	0.666666667	CRA004775;PRJNA827465;Yang2025_Silkie_Wencha	0.516231528
ENSGALG00010007900	FHOD3	3	-0.211258389	0.114190879	-1.85004608	0.064306908	0	1.373639219	0	0.666666667	CRA004775;PRJNA827465;Yang2025_Silkie_Wencha	0.516231528
ENSGALG00010022729	ENSGALG0001002272	3	-0.189376797	0.102430351	-1.848834793	0.064481672	0.012083236	3.156947413	36.64766187	1	CRA004775;PRJNA827465;Yang2025_Silkie_Wencha	0.516231528
ENSGALG00010025940	HECTD3	3	-0.15905474	0.086004862	-1.849369158	0.064404526	0.010513059	3.524516212	43.25462333	1	CRA004775;PRJNA827465;Yang2025_Silkie_Wencha	0.516231528
ENSGALG00010023107	ZC3HAV1	3	-0.154038372	0.083326936	-1.848602377	0.064515249	0	1.780246359	0	1	CRA004775;PRJNA827465;Yang2025_Silkie_Wencha	0.516231528
ENSGALG00010012742	ENSGALG0001001274	3	-0.150828563	0.081506424	-1.850511338	0.064239885	0	1.211601996	0	0.666666667	CRA004775;PRJNA827465;Yang2025_Silkie_Wencha	0.516231528
ENSGALG00010029424	MAP2K6	3	-0.135878945	0.073468947	-1.849474503	0.064389326	0	0.705695395	0	0.666666667	CRA004775;PRJNA827465;Yang2025_Silkie_Wencha	0.516231528
ENSGALG00010002281	TGFBR2	3	-0.117240317	0.063410478	-1.848910787	0.064470696	0	0.418080445	0	1	CRA004775;PRJNA827465;Yang2025_Silkie_Wencha	0.516231528
ENSGALG00010002809	SH3KBP1	3	-0.105501177	0.057000729	-1.850874175	0.064187657	0	1.762103966	0	0.666666667	CRA004775;PRJNA827465;Yang2025_Silkie_Wencha	0.516231528
ENSGALG00010022209	ZFYVE19	3	-0.100557384	0.054391962	-1.84875451	0.064493269	0	0.667643088	0	0.666666667	CRA004775;PRJNA827465;Yang2025_Silkie_Wencha	0.516231528
ENSGALG00010009268	PWWP2A	3	-0.092506955	0.049958614	-1.851671778	0.064072969	0	0.530372516	0	1	CRA004775;PRJNA827465;Yang2025_Silkie_Wencha	0.516231528
ENSGALG00010029429	UBE2G1	3	-0.080840998	0.043716151	-1.849224972	0.064425334	0.000240716	2.049746752	2.42697066	0.666666667	CRA004775;PRJNA827465;Yang2025_Silkie_Wencha	0.516231528
ENSGALG00010005254	ENSGALG0001000525	3	0.612573141	0.331618941	1.847220005	0.064715261	0	1.742045255	0	0.666666667	CRA004775;PRJNA827465;Yang2025_Silkie_Wencha	0.516391366
ENSGALG00010005295	ENSGALG0001000529	3	0.495986413	0.268525695	1.847072452	0.06473664	0	0.290927065	0	1	CRA004775;PRJNA827465;Yang2025_Silkie_Wencha	0.516391366
ENSGALG00010027370	NUP210L	3	0.487221177	0.263794296	1.84697389	0.064750925	0.092672635	5.940716939	44.35280649	0.666666667	CRA004775;PRJNA827465;Yang2025_Silkie_Wencha	0.516391366
ENSGALG00010007399	WWC2	3	0.350073926	0.189428945	1.848048757	0.06459529	0.069452394	6.581335802	69.61103247	1	CRA004775;PRJNA827465;Yang2025_Silkie_Wencha	0.516391366
ENSGALG00010021242	BCAR3	3	0.24484463	0.132566991	1.84695019	0.06475436	0	1.241027559	0	1	CRA004775;PRJNA827465;Yang2025_Silkie_Wencha	0.516391366
ENSGALG00010012772	TOMM22	3	-0.110002733	0.059536399	-1.847655131	0.064652249	0	0.783158629	0	1	CRA004775;PRJNA827465;Yang2025_Silkie_Wencha	0.516391366
ENSGALG00010010196	EFCAB10	3	0.584825127	0.31695389	1.84514261	0.065016796	0.145487373	3.937986792	49.21262804	1	CRA004775;PRJNA827465;Yang2025_Silkie_Wencha	0.517026143
ENSGALG00010025568	MYL3	2	0.440847578	0.238813847	1.845988342	0.064893898	0.018554483	1.194237333	16.26455042	1	PRJNA827465;Yang2025_Silkie_Wenchang	0.517026143
ENSGALG00010002832	LPIN1	3	-0.238692627	0.129356025	-1.845237795	0.065002955	0	0.825843634	0	0.666666667	CRA004775;PRJNA827465;Yang2025_Silkie_Wencha	0.517026143
ENSGALG00010024800	ENSGALG0001002480	3	0.19496044	0.105635858	1.845589594	0.064951819	0.016368098	3.840217218	47.91961271	1	CRA004775;PRJNA827465;Yang2025_Silkie_Wencha	0.517026143
ENSGALG00010027364	FSTL3	3	-0.130234502	0.070579133	-1.845226725	0.065004564	0	0.951563034	0	0.666666667	CRA004775;PRJNA827465;Yang2025_Silkie_Wencha	0.517026143
ENSGALG00010009868	LTN1	3	0.149262825	0.080909217	1.844818568	0.065063935	0.009447707	3.911802311	48.87267196	1	CRA004775;PRJNA827465;Yang2025_Silkie_Wencha	0.517110164
ENSGALG00010004705	ENSGALG0001000470	3	0.49240411	0.26701232	1.844125057	0.065164917	0.138229425	5.725438394	65.0681771	1	CRA004775;PRJNA827465;Yang2025_Silkie_Wencha	0.517183837
ENSGALG00010002603	INSYN2A	3	0.16553917	0.089771717	1.84400137	0.065182941	0	1.832883743	0	1	CRA004775;PRJNA827465;Yang2025_Silkie_Wencha	0.517183837
ENSGALG00010029235	TRIM37	3	-0.115872562	0.062835302	-1.844067886	0.065173247	0.004436368	3.09987813	35.48133455	0.666666667	CRA004775;PRJNA827465;Yang2025_Silkie_Wencha	0.517183837
ENSGALG00010019076	ESPN	3	0.438126745	0.237833381	1.842158333	0.065451993	0.042710853	2.667902018	25.03472817	1	CRA004775;PRJNA827465;Yang2025_Silkie_Wencha	0.518736397
ENSGALG00010000621	UPP1	3	-0.231868268	0.125863486	-1.842220296	0.065442933	0	1.050993294	0	1	CRA004775;PRJNA827465;Yang2025_Silkie_Wencha	0.518736397
ENSGALG00010029541	ENSGALG0001002954	3	-0.23725311	0.128826405	-1.841649697	0.065526407	0	0.734243532	0	1	CRA004775;PRJNA827465;Yang2025_Silkie_Wencha	0.519035217
ENSGALG00010012426	MIEF1	3	-0.321702662	0.174756706	-1.840860185	0.06564205	0.070755984	10.88114737	81.61958539	1	CRA004775;PRJNA827465;Yang2025_Silkie_Wencha	0.519039076
ENSGALG00010024849	CENATAC	3	0.293849765	0.15973047	1.83966005	0.065818162	0.051080284	6.07969243	67.10359902	1	CRA004775;PRJNA827465;Yang2025_Silkie_Wencha	0.519039076
ENSGALG00010029294	PGS1	3	-0.168686929	0.091688223	-1.839788391	0.06579931	0.014257347	4.582467042	56.35538713	1	CRA004775;PRJNA827465;Yang2025_Silkie_Wencha	0.519039076
ENSGALG00010019243	SERTAD2	3	-0.135741291	0.07378478	-1.839692293	0.065813426	0.001833789	2.145421758	6.778236393	0.666666667	CRA004775;PRJNA827465;Yang2025_Silkie_Wencha	0.519039076
ENSGALG00010015927	GLA	3	-0.132605188	0.072051564	-1.840420678	0.0657065	0	0.823938227	0	1	CRA004775;PRJNA827465;Yang2025_Silkie_Wencha	0.519039076
ENSGALG00010020648	TET3	3	-0.126764919	0.068844613	-1.841319359	0.065574772	0	0.39361374	0	1	CRA004775;PRJNA827465;Yang2025_Silkie_Wencha	0.519039076
ENSGALG00010024645	PARP11	3	-0.121490483	0.066040226	-1.839643649	0.065820572	0.002843256	2.49843299	19.94982424	1	CRA004775;PRJNA827465;Yang2025_Silkie_Wencha	0.519039076
ENSGALG00010021112	TAF5	3	-0.080396014	0.043692477	-1.840042478	0.065762001	0	0.021038351	0	1	CRA004775;PRJNA827465;Yang2025_Silkie_Wencha	0.519039076
ENSGALG00010011141	MSX1	3	0.705900027	0.384393022	1.836401773	0.066298258	0	1.215003268	0	1	CRA004775;PRJNA827465;Yang2025_Silkie_Wencha	0.519328799
ENSGALG00010015115	CLDN2	3	0.676997692	0.368388851	1.837725788	0.066102822	0.360520944	19.54346827	89.76640189	1	CRA004775;PRJNA827465;Yang2025_Silkie_Wencha	0.519328799
ENSGALG00010007404	FOLH1	3	-0.363125363	0.197816129	-1.835671156	0.066406306	0.062829667	4.464251057	55.1996522	0.666666667	CRA004775;PRJNA827465;Yang2025_Silkie_Wencha	0.519328799
ENSGALG00010003081	DOP1A	3	0.296832196	0.161627375	1.836521797	0.066280521	0.052219913	6.804678563	70.60845738	0.666666667	CRA004775;PRJNA827465;Yang2025_Silkie_Wencha	0.519328799
ENSGALG00010000652	SRD5A1	3	0.22453211	0.122272087	1.836331713	0.066308612	0.008396428	2.396329414	16.53902054	0.666666667	CRA004775;PRJNA827465;Yang2025_Silkie_Wencha	0.519328799
ENSGALG00010022949	INSC	2	0.223206148	0.121590238	1.83572425	0.06639845	0.00164826	1.058597932	5.535428505	1	PRJNA827465;Yang2025_Silkie_Wenchang	0.519328799
ENSGALG00010014976	ITGB2	3	-0.218973637	0.119118933	-1.838277356	0.066021546	0.006259668	2.276574948	12.14873019	1	CRA004775;PRJNA827465;Yang2025_Silkie_Wencha	0.519328799
ENSGALG00010011950	PDXK	3	-0.180613016	0.098375568	-1.835953986	0.066364462	0	0.195244106	0	1	CRA004775;PRJNA827465;Yang2025_Silkie_Wencha	0.519328799
ENSGALG00010011466	MTMR1	3	-0.142973285	0.077816176	-1.837320873	0.06616254	0.012044559	6.687948499	70.0954635	0.666666667	CRA004775;PRJNA827465;Yang2025_Silkie_Wencha	0.519328799
ENSGALG00010022215	ENSGALG0001002221	3	0.137268997	0.074701531	1.837566031	0.066126378	0	1.71978027	0	1	CRA004775;PRJNA827465;Yang2025_Silkie_Wencha	0.519328799
ENSGALG00010017406	IFT140	3	0.116954768	0.063688344	1.836360628	0.066304339	0	0.207004645	0	1	CRA004775;PRJNA827465;Yang2025_Silkie_Wencha	0.519328799
ENSGALG00010009508	TOMM20	3	-0.114363879	0.062268982	-1.836610714	0.066267384	0	0.832057048	0	0.666666667	CRA004775;PRJNA827465;Yang2025_Silkie_Wencha	0.519328799
ENSGALG00010027583	GLE1	3	0.08492889	0.046186167	1.83883827	0.065938978	0	0.337194488	0	0.666666667	CRA004775;PRJNA827465;Yang2025_Silkie_Wencha	0.519328799
ENSGALG00010016885	CDCA4	3	-0.077986325	0.042405016	-1.839082551	0.065903045	0	1.392726444	0	1	CRA004775;PRJNA827465;Yang2025_Silkie_Wencha	0.519328799
ENSGALG00010024169	FAM168B	3	-0.073862521	0.040237628	-1.835657916	0.066408266	0	0.383590975	0	1	CRA004775;PRJNA827465;Yang2025_Silkie_Wencha	0.519328799

ENSGALG00010021538	TNFRSF18	3	0.453410372	0.247698235	1.830494963	0.067175959	0	0.533938823	0	1	CRA004775;PRJNA827465;Yang2025_Silkie_Wencha	0.520153828
ENSGALG00010005395	ENSGALG0001000539	2	0.439978687	0.239913223	1.833907618	0.066667709	0	0.006402278	0	1	PRJNA827465;Yang2025_Silkie_Wenchang	0.520153828
ENSGALG00010009286	ENSGALG0001000928	3	0.432318329	0.235975679	1.832046128	0.066944549	0.100623041	5.007380438	60.05895648	0.666666667	CRA004775;PRJNA827465;Yang2025_Silkie_Wencha	0.520153828
ENSGALG00010012343	BCL2L13	3	-0.339730056	0.185363001	-1.832782455	0.066834929	0.076749057	12.49357606	83.99177313	1	CRA004775;PRJNA827465;Yang2025_Silkie_Wencha	0.520153828
ENSGALG00010014014	PARP8	3	0.319764917	0.17458141	1.831609202	0.067009665	0.03787472	3.445229	41.94870646	0.666666667	CRA004775;PRJNA827465;Yang2025_Silkie_Wencha	0.520153828
ENSGALG00010008400	MAP1LC3C	3	-0.295682851	0.161417683	-1.831787239	0.066983125	0	0.557353952	0	0.666666667	CRA004775;PRJNA827465;Yang2025_Silkie_Wencha	0.520153828
ENSGALG00010005402	ABCC4	3	0.192119627	0.104827425	1.832722946	0.066843783	0	0.025934117	0	1	CRA004775;PRJNA827465;Yang2025_Silkie_Wencha	0.520153828
ENSGALG00010014886	RABGAP1L	3	-0.169814869	0.092758272	-1.830724799	0.06714163	0.013314141	4.296548429	53.45100764	1	CRA004775;PRJNA827465;Yang2025_Silkie_Wencha	0.520153828
ENSGALG00010005588	PLAGL1	3	-0.166773938	0.091087983	-1.830910429	0.067113914	0.007501839	2.611709036	23.42179116	0.666666667	CRA004775;PRJNA827465;Yang2025_Silkie_Wencha	0.520153828
ENSGALG00010021684	BIN1	3	-0.154119087	0.084064264	-1.833348443	0.06675077	0.002272833	2.18393707	8.42226971	0.666666667	CRA004775;PRJNA827465;Yang2025_Silkie_Wencha	0.520153828
ENSGALG00010029329	ENSGALG0001002932	3	-0.153100829	0.083454349	-1.834545847	0.066573009	0	1.891122269	0	0.666666667	CRA004775;PRJNA827465;Yang2025_Silkie_Wencha	0.520153828
ENSGALG00010009524	FAM185A	3	-0.151541359	0.082623178	-1.834126498	0.066635219	0	1.269975887	0	0.666666667	CRA004775;PRJNA827465;Yang2025_Silkie_Wencha	0.520153828
ENSGALG00010011022	C5orf22	3	0.148296627	0.080898662	1.833115948	0.06678533	0.000308534	2.027762319	1.369111088	0.666666667	CRA004775;PRJNA827465;Yang2025_Silkie_Wencha	0.520153828
ENSGALG00010016675	SKIL	3	0.125643514	0.068609231	1.831291679	0.067057019	0	1.638057388	0	1	CRA004775;PRJNA827465;Yang2025_Silkie_Wencha	0.520153828
ENSGALG00010020723	RPS24	3	-0.117227343	0.06400301	-1.831591086	0.067012366	0	1.310347907	0	0.666666667	CRA004775;PRJNA827465;Yang2025_Silkie_Wencha	0.520153828
ENSGALG00010025160	MRPS22	3	-0.113143528	0.061762436	-1.831914907	0.066964099	0	0.672827912	0	0.666666667	CRA004775;PRJNA827465;Yang2025_Silkie_Wencha	0.520153828
ENSGALG00010023208	BACE1	3	-0.107657453	0.058736491	-1.832888755	0.066819116	0.004610224	3.764576957	46.87318063	1	CRA004775;PRJNA827465;Yang2025_Silkie_Wencha	0.520153828
ENSGALG00010022526	PPP1CA	3	-0.100575635	0.054937072	-1.830742532	0.067138982	0	0.024295462	0	1	CRA004775;PRJNA827465;Yang2025_Silkie_Wencha	0.520153828
ENSGALG00010019391	GANC	3	0.49040442	0.26799114	1.829927732	0.067260746	0.183109759	18.80448467	89.36423925	1	CRA004775;PRJNA827465;Yang2025_Silkie_Wencha	0.520240529
ENSGALG00010010951	ENSGALG0001001095	3	0.245470538	0.134134651	1.83003077	0.067245338	0	1.755573648	0	1	CRA004775;PRJNA827465;Yang2025_Silkie_Wencha	0.520240529
ENSGALG00010000632	ENSGALG0001000063	2	0.667352478	0.365190477	1.827409308	0.067638251	0	0.184036809	0	1	PRJNA827465;Yang2025_Silkie_Wenchang	0.520676908
ENSGALG00010010434	PROM1	3	0.321323017	0.175716409	1.828645484	0.067452734	0.046867303	4.42848241	54.83780187	0.666666667	CRA004775;PRJNA827465;Yang2025_Silkie_Wencha	0.520676908
ENSGALG00010010231	C1orf53	3	-0.242147959	0.132512623	-1.827357681	0.067646008	0	1.873600604	0	1	CRA004775;PRJNA827465;Yang2025_Silkie_Wencha	0.520676908
ENSGALG00010020489	IFFO2	3	0.235548038	0.128791544	1.828909184	0.067413214	0.022577466	3.717092891	46.19451118	0.666666667	CRA004775;PRJNA827465;Yang2025_Silkie_Wencha	0.520676908
ENSGALG00010009883	ENSGALG0001000988	3	0.235497114	0.128849441	1.827692163	0.067595765	0	0.889087711	0	1	CRA004775;PRJNA827465;Yang2025_Silkie_Wencha	0.520676908
ENSGALG00010020596	SUMF1	3	-0.224384597	0.122731811	-1.828251334	0.06751184	0.025508378	4.55862191	56.12709192	1	CRA004775;PRJNA827465;Yang2025_Silkie_Wencha	0.520676908
ENSGALG00010029209	PRKCA	3	0.217744377	0.119122847	1.827897694	0.067564907	0.025970283	6.392195682	68.71184645	1	CRA004775;PRJNA827465;Yang2025_Silkie_Wencha	0.520676908
ENSGALG00010020364	MRPL16	3	0.136150522	0.074507474	1.827340465	0.067648595	0	1.997067707	0	0.666666667	CRA004775;PRJNA827465;Yang2025_Silkie_Wencha	0.520676908
ENSGALG00010024356	PARL	3	-0.105798553	0.057857423	-1.828608111	0.067458337	0	1.39870094	0	1	CRA004775;PRJNA827465;Yang2025_Silkie_Wencha	0.520676908
ENSGALG00010024684	ENSGALG0001002468	3	-0.452459408	0.247663944	-1.826908673	0.067713502	0	0.331935871	0	1	CRA004775;PRJNA827465;Yang2025_Silkie_Wencha	0.520892929
ENSGALG00010024792	BZW1	3	-0.225637956	0.123525535	-1.82665031	0.067752364	0.034482323	9.244204458	78.36482297	1	CRA004775;PRJNA827465;Yang2025_Silkie_Wencha	0.520908468
ENSGALG00010012418	ENSGALG0001001241	3	0.942366913	0.516733325	1.823700676	0.068197337	0.487719935	5.500653116	63.64068124	1	CRA004775;PRJNA827465;Yang2025_Silkie_Wencha	0.521211969
ENSGALG00010009500	CCDC146	3	0.60648715	0.332525607	1.823881042	0.068170058	0.153288259	3.730321738	46.38532168	0.666666667	CRA004775;PRJNA827465;Yang2025_Silkie_Wencha	0.521211969
ENSGALG00010019148	ENSGALG0001001914	2	0.580597839	0.318247075	1.824361902	0.068097378	0.091126726	1.817737291	44.98654976	1	PRJNA827465;Yang2025_Silkie_Wenchang	0.521211969
ENSGALG00010027891	CCDC66	3	0.45130746	0.247605452	1.824306519	0.068105745	0.147277509	11.93342597	83.24035357	1	CRA004775;PRJNA827465;Yang2025_Silkie_Wencha	0.521211969
ENSGALG00010019832	MAD2L1BP	3	-0.290567922	0.159228848	-1.82484472	0.068024465	0.045050981	6.223087471	67.86161195	1	CRA004775;PRJNA827465;Yang2025_Silkie_Wencha	0.521211969
ENSGALG00010029238	CAMKK1	3	0.251181381	0.137627219	1.825085061	0.067988194	0	1.603357049	0	0.666666667	CRA004775;PRJNA827465;Yang2025_Silkie_Wencha	0.521211969
ENSGALG00010021098	SDK1	3	-0.248015964	0.135957977	-1.824210463	0.06812026	0.029521466	5.142751531	61.11031249	0.666666667	CRA004775;PRJNA827465;Yang2025_Silkie_Wencha	0.521211969
ENSGALG00010028056	SLC25A25	3	0.227438385	0.124711459	1.823716822	0.068194894	0.017317936	3.159948339	36.70782604	0.666666667	CRA004775;PRJNA827465;Yang2025_Silkie_Wencha	0.521211969
ENSGALG00010019622	C2orf42	3	0.127557285	0.069879408	1.825391617	0.067941953	0.004899103	2.896167655	30.94322435	0.666666667	CRA004775;PRJNA827465;Yang2025_Silkie_Wencha	0.521211969
ENSGALG00010021429	MED8	3	-0.117239463	0.064241164	-1.824989713	0.068002582	0	0.517963872	0	1	CRA004775;PRJNA827465;Yang2025_Silkie_Wencha	0.521211969
ENSGALG00010013104	HLCS	3	-0.106361657	0.058299658	-1.8243959	0.068092241	0	1.167574443	0	1	CRA004775;PRJNA827465;Yang2025_Silkie_Wencha	0.521211969
ENSGALG00010013037	MORC4	3	0.073189961	0.040139772	1.823377595	0.068246221	0.000139888	2.024128874	1.19206215	1	CRA004775;PRJNA827465;Yang2025_Silkie_Wencha	0.521303794
ENSGALG00010018472	LMF1	3	-0.201791649	0.110817652	-1.820934174	0.068616864	0.016017441	3.505367307	42.94463819	0.666666667	CRA004775;PRJNA827465;Yang2025_Silkie_Wencha	0.523851963
ENSGALG00010011129	ENSGALG0001001112	3	-0.276102758	0.151830425	-1.818494271	0.068988622	0.04334082	5.393398626	62.91763063	0.666666667	CRA004775;PRJNA827465;Yang2025_Silkie_Wencha	0.526405894
ENSGALG00010015207	AKR1E2	3	-0.157780067	0.086795635	-1.817834118	0.06908949	0	0.919053649	0	1	CRA004775;PRJNA827465;Yang2025_Silkie_Wencha	0.526693155
ENSGALG00010021443	SNUPN	3	-0.132511458	0.07289832	-1.817760338	0.069100771	0.002015952	2.133660671	6.264382754	1	CRA004775;PRJNA827465;Yang2025_Silkie_Wencha	0.526693155
ENSGALG00010020945	NUMB	3	0.395838715	0.217828995	1.817199385	0.069186589	0.118512607	13.71936907	85.42207014	1	CRA004775;PRJNA827465;Yang2025_Silkie_Wencha	0.526860875
ENSGALG00010016654	SMIM3	3	-0.198563303	0.109273067	-1.817129404	0.069197301	0	1.762751627	0	0.666666667	CRA004775;PRJNA827465;Yang2025_Silkie_Wencha	0.526860875
ENSGALG00010016060	ENSGALG0001001606	3	1.038232003	0.571554453	1.816505843	0.069292812	0.33603313	2.930118768	31.74338113	1	CRA004775;PRJNA827465;Yang2025_Silkie_Wencha	0.527129604
ENSGALG00010006619	LIN52	3	-0.351308757	0.193408055	-1.816412234	0.06930716	0.084142182	16.32360123	87.74780165	1	CRA004775;PRJNA827465;Yang2025_Silkie_Wencha	0.527129604
ENSGALG00010023585	TFIP11	3	-0.188543768	0.103822105	-1.816027204	0.069366199	0.0201069	5.300565784	62.26817888	1	CRA004775;PRJNA827465;Yang2025_Silkie_Wencha	0.527294995
ENSGALG00010017776	GPR68	3	0.380582789	0.209632921	1.815472431	0.069451339	0.073155688	4.560569977	56.14583243	1	CRA004775;PRJNA827465;Yang2025_Silkie_Wencha	0.527375124
ENSGALG00010008290	FAM13A	3	-0.233294137	0.128502076	-1.815489253	0.069448756	0.026674981	5.207370953	61.59290325	0.666666667	CRA004775;PRJNA827465;Yang2025_Silkie_Wencha	0.527375124
ENSGALG00010001248	PTPRZ1	3	0.759810599	0.418826206	1.814142926	0.069655724	0.408596209	10.28256281	80.54921775	1	CRA004775;PRJNA827465;Yang2025_Silkie_Wencha	0.527510598
ENSGALG00010021481	MICU1	3	0.41808143	0.230402775	1.814567683	0.069590373	0.12792941	13.05125983	84.67580888	1	CRA004775;PRJNA827465;Yang2025_Silkie_Wencha	0.527510598
ENSGALG00010019752	GPAM	3	0.156933805	0.086483552	1.814608692	0.069584066	0	1.568284251	0	0.666666667	CRA004775;PRJNA827465;Yang2025_Silkie_Wencha	0.527510598
ENSGALG00010015052	ENSGALG0001001505	3	-0.13600191	0.074928354	-1.815092724	0.069509661	0	1.356216178	0	1	CRA004775;PRJNA827465;Yang2025_Silkie_Wencha	0.527510598
ENSGALG00010021425	PTPN9	3	0.113369764	0.062490917	1.814179865	0.069650039	0	1.095156269	0	1	CRA004775;PRJNA827465;Yang2025_Silkie_Wencha	0.527510598
ENSGALG00010014306	ENSGALG0001001430	3	0.387892797	0.213849597	1.813857979	0.069699594	0	1.511495902	0	0.666666667	CRA004775;PRJNA827465;Yang2025_Silkie_Wencha	0.527560253
ENSGALG00010017575	CLCN2	2	1.361128423	0.750782383	1.812946672	0.069840047	0.788795003	3.294072546	69.64244151	1	CRA004775;Yang2025_Silkie_Wenchang	0.528177812
ENSGALG00010009617	SLC25A46	3	-0.111965167	0.061762165	-1.812843934	0.069855896	0	0.728849909	0	0.666666667	CRA004775;PRJNA827465;Yang2025_Silkie_Wencha	0.528177812
ENSGALG00010021004	PSD	3	0.305119249	0.168401768	1.811852995	0.070008914	0.042196205	4.029047612	50.56047616	1	CRA004775;PRJNA827465;Yang2025_Silkie_Wencha	0.528335453
ENSGALG00010003019	TAPBP	3	-0.210346502	0.116109433	-1.811622851	0.070044492	0.001961498	2.092536696	4.422225728	1	CRA004775;PRJNA827465;Yang2025_Silkie_Wencha	0.528335453
ENSGALG00010015674	RFKL	3	0.139964614	0.077244265	1.811974195	0.069990184	0	0.783336833	0	1	CRA004775;PRJNA827465;Yang2025_Silkie_Wencha	0.528335453
ENSGALG00010027113	PIAS4	3	0.130532914	0.07205794	1.811499404	0.070063581	0.004351399	2.615899455	23.54446208	0.666666667	CRA004775;PRJNA827465;Yang2025_Silkie_Wencha	0.528335453
ENSGALG00010007581	LPCAT1	3	-0.117751408	0.064978577	-1.812151996	0.069962714	0	1.677495426	0	1	CRA004775;PRJNA827465;Yang2025_Silkie_Wencha	0.528335453
ENSGALG00010020924	DNAJC16	3	-0.094737607	0.052322154	-1.810659517	0.070193573	0	1.456451609	0	1	CRA004775;PRJNA827465;Yang2025_Silkie_Wencha	0.528335453
ENSGALG00010002137	PDK3	3	-0.256494142	0.141704	-1.810069882	0.070284951	0.036953626	6.083854879	67.12610607	0.666666667	CRA004775;PRJNA827465;Yang2025_Silkie_Wencha	0.529440021
ENSGALG00010022554	SCG3	3	-0.209520195	0.115810993	-1.80915636	0.070426715	0	1.980183579	0	0.666666667	CRA004775;PRJNA827465;Yang2025_Silkie_Wencha	0.530225414
ENSGALG00010016804	CSF1R	3	-0.392050101	0.216781954	-1.808501045	0.070528554	0.1010499	13.37427955	85.04592346	1	CRA004775;PRJNA827465;Yang2025_Silkie_Wencha	0.530709542
ENSGALG00010024218	NGFR	3	-0.155369113	0.085943322	-1.807809019	0.07063623	0.003023403	2.237432934	10.61184585	0.666666667	CRA004775;PRJNA827465;Yang2025_Silkie_Wencha	0.531039026
ENSGALG00010005288	ENSGALG0001000528	3	0.106216495	0.058756611	1.807736905	0.070647458	0.002819212	2.69958024	25.91440808	1	CRA004775;PRJNA827465;Yang2025_Silkie_Wencha	0.531039026
ENSGALG00010009030	ZDHHC7	3	0.074434805	0.041182039	1.80745798	0.070690901	0	0.498902238	0	0.666666667	CRA004775;PRJNA827465;Yang2025_Silkie_Wencha	0.531083234
ENSGALG00010023955	C1QL4	3	0.620839737	0.343699311	1.8063456	0.070864373	0	0.8654385	0	1	CRA004775;PRJNA827465;Yang2025_Silkie_Wencha	0.532103756
ENSGALG00010014284	TMEM131L	3	0.088736307	0.049140252	1.8057764	0.070953273	0	1.47554292	0	0.666666667	CRA004775;PRJNA827465;Yang2025_Silkie_Wencha	0.532488498
ENSGALG00010002855	RACK1	3	-0.259442804	0.143726772	-1.805111189	0.071057284	0.037686265	5.11173384	60.87433222	1	CRA004775;PRJNA827465;Yang2025_Silkie_Wencha	0.532703577
ENSGALG00010011015	LYRM4	3	-0.19821885	0.109806426	-1.805166213	0.071048676	0	0.850755101	0	1	CRA004775;PRJNA827465;Yang2025_Silkie_Wencha	0.532703577
ENSGALG00010029359	ENSGALG0001002935	3	-0.423674901	0.234790776	-1.804478472	0.071156331	0.033169855	2.468184459	18.96877915	1	CRA004775;PRJNA827465;Yang2025_Silkie_Wencha	0.533061159
ENSGALG00010017663	ZMYND12	3	-0.249129538	0.138073545	-1.80432492	0.071180385	0	1.789316176	0	1	CRA004775;PRJNA827465;Yang2025_Silkie_Wencha	0.533061159
ENSGALG00010026692	NMNAT3	2	0.613166406	0.340157024	1.802598102	0.071451355	0	0.056384463	0	1	CRA004775;Yang2025_Silkie_Wenchang	0.533418574
ENSGALG00010007853	C1orf131	3	0.569107684	0.315682453	1.802785294	0.071421941	0.246320522	12.89890509	84.494808	1	CRA004775;PRJNA827465;Yang2025_Silkie_Wencha	0.533418574
ENSGALG00010005537	GRIN3A	3	-0.342007133	0.189732196	-1.802578272	0.071454472	0	0.425045841	0	1	CRA004775;PRJNA827465;Yang2025_Silkie_Wencha	0.533418574
ENSGALG00010000599	RETREG1	3	-0.324403954	0.179951842	-1.802726494	0.071431179	0.046042748	4.160380115	51.92746949	0.666666667	CRA004775;PRJNA827465;Yang2025_Silkie_Wencha	0.533418574
ENSGALG00010001929	POLR2I	3	0.14963263	0.08297066	1.803440279	0.071319097	0.003430471	2.248286531	11.0433669	0.666666667	CRA004775;PRJNA827465;Yang2025_Silkie_Wencha	0.533418574
ENSGALG00010011389	RC3H1	3	-0.111551791	0.061874168	-1.802881464	0.071406833	0	1.317524682	0	0.666666667	CRA004775;PRJNA827465;Yang2025_Silkie_Wencha	0.533418574
ENSGALG00010027257	TAFA3	3	-0.328881032	0.182576511	-1.801332658	0.071650463	0	0.187545227	0	1	CRA004775;PRJNA827465;Yang2025_Silkie_Wencha	0.534468122
ENSGALG00010008936	IFT172	3	0.16687923	0.09264869	1.801204414	0.071670667	0.01286529	4.14248524	51.71980384	0.666666667	CRA004775;PRJNA827465;Yang2025_Silkie_Wencha	0.534468122
ENSGALG00010024034	LRP2	3	-0.263179127	0.146172277	-1.800472233	0.071786104	0	0.391958387	0	1	CRA004775;PRJNA827465;Yang2025_Silkie_Wencha	0.535046772
ENSGALG00010023073	POMGNT1	3	-0.33465248	0.186073689	-1.798494359	0.072098702	0.076127107	8.382398354	76.14048014	1	CRA004775;PRJNA827465;Yang2025_Silkie_Wencha	0.535776387
ENSGALG00010018465	NAALADL2	3	0.291110097	0.161858752	1.798544061	0.072090833	0.029487035	2.992642163	33.16942385	1	CRA004775;PRJNA827465;Yang2025_Silkie_Wencha	0.535776387
ENSGALG00010009609	MMADHC	3	-0.257018876	0.142862163	-1.799068906	0.072007781	0.049640015	20.81006807	90.38926738	1	CRA004775;PRJNA827465;Yang2025_Silkie_Wencha	0.535776387
ENSGALG00010027185	ATRIP	3	0.14142008	0.078594154	1.799371484	0.071959937	0.009234192	4.423869383	54.79070861	0.666666667	CRA004775;PRJNA827465;Yang2025_Silkie_Wencha	0.535776387
ENSGALG00010027784	PEAR1	3	0.119922701	0.066650707	1.799271252	0.071975783	0	0.784631048	0	0.666666667	CRA004775;PRJNA827465;Yang2025_Silkie_Wencha	0.535776387
ENSGALG00010013082	CHAF1B	3	-0.106961194	0.059478646	-1.798312513	0.072127498	0	1.214460988	0	0.666666667	CRA004775;PRJNA827465;Yang2025_Silkie_Wencha	0.535776387
ENSGALG00010016270	RFC4	3	-0.093743091	0.052132346	-1.798175191	0.07214925	0	1.205308248	0	1	CRA004775;PRJNA827465;Yang2025_Silkie_Wencha	0.535776387
ENSGALG00010029215	ENSGALG0001002921	3	-0.240610007	0.133913778	-1.796753178	0.072374812	0	0.484898975	0	0.666666667	CRA004775;PRJNA827465;Yang2025_Silkie_Wencha	0.536111765
ENSGALG00010027638	MRPS2	3	-0.122658559	0.068268564	-1.796706292	0.07238226	0	0.478682421	0	1	CRA004775;PRJNA827465;Yang2025_Silkie_Wencha	0.536111765
ENSGALG00010008130	RCSD1	3	-0.111675233	0.06214862	-1.796906074	0.072350532	0	1.480453115	0	1	CRA004775;PRJNA827465;Yang2025_Silkie_Wencha	0.536111765
ENSGALG00010027533	WDR18	3	-0.103868557	0.05781089	-1.796695342	0.072383999	0.000329093	2.035019031	1.720820832	1	CRA004775;PRJNA827465;Yang2025_Silkie_Wencha	0.536111765
ENSGALG00010028199	DPP9	3	-0.075869431	0.042207174	-1.797548242	0.072248627	0	1.57172969	0	1	CRA004775;PRJNA827465;Yang2025_Silkie_Wencha	0.536111765
ENSGALG00010024722	BRCA1	3	0.124243526	0.069163158	1.796383078	0.072433613	0	1.699672076	0	0.666666667	CRA004775;PRJNA827465;Yang2025_Silkie_Wencha	0.536198355
ENSGALG00010009582	TIGD5	3	-0.170038739	0.094669288	-1.796134134	0.072473187	0	1.201137392	0	1	CRA004775;PRJNA827465;Yang2025_Silkie_Wencha	0.536210565
ENSGALG00010025323	KLHDC8A	3	0.20810248	0.115927482	1.79510912	0.072636316	0	1.893627964	0	0.666666667	CRA004775;PRJNA827465;Yang2025_Silkie_Wencha	0.536855657
ENSGALG00010008380	SIL1	3	0.107242923	0.059738871	1.795194994	0.072622638	0	1.296466886	0	0.666666667	CRA004775;PRJNA827465;Yang2025_Silkie_Wencha	0.536855657
ENSGALG00010015327	LARGE1	3	-0.138321199	0.077077013	-1.794584316	0.072719954	0	1.935476544	0	0.666666667	CRA004775;PRJNA827465;Yang2025_Silkie_Wencha	0.537193013
ENSGALG00010000857	ENSGALG0001000085	3	-0.669689466	0.373340847	-1.79377497	0.072849094	0.160192544	3.229023788	38.06177559	1	CRA004775;PRJNA827465;Yang2025_Silkie_Wencha	0.537304817
ENSGALG00010020481	RPP30	3	-0.317371819	0.176902703	-1.794047314	0.072805617	0.06154028	5.920193917	66.21732281	1	CRA004775;PRJNA827465;Yang2025_Silkie_Wencha	0.537304817
ENSGALG00010014329	IER5	3	0.16502304	0.091985562	1.794010243	0.072811534	0	1.684072837	0	0.666666667	CRA004775;PRJNA827465;Yang2025_Silkie_Wencha	0.537304817
ENSGALG00010002655	FABP4	3	-0.720879837	0.401940307	-1.793499741	0.072893052	0	1.965052174	0	1	CRA004775;PRJNA827465;Yang2025_Silkie_Wencha	0.537348729
ENSGALG00010021478	SLC13A3	3	0.265311914	0.147971119	1.792997965	0.07297325	0	1.896777255	0	0.666666667	CRA004775;PRJNA827465;Yang2025_Silkie_Wencha	0.537659601
ENSGALG00010005851	SLC15A1	2	1.667912561	0.930534815	1.792423597	0.073065138	1.036931192	2.459941545	59.34862754	1	CRA004775;PRJNA827465	0.53805624
ENSGALG00010015413	CARNMT1	3	0.298516241	0.166682859	1.790923456	0.073305579	0.067252311	15.52933817	87.12115109	1	CRA004775;PRJNA827465;Yang2025_Silkie_Wencha	0.539545852
ENSGALG00010022453	ENSGALG0001002245	3	-0.219549071	0.122614544	-1.790563048	0.073363441	0.020320493	3.696353516	45.89262117	1	CRA004775;PRJNA827465;Yang2025_Silkie_Wencha	0.539690789
ENSGALG00010021235	XRCC5	3	-0.167924763	0.093820584	-1.789849902	0.073478044	0.010770974	3.474517431	42.43804962	1	CRA004775;PRJNA827465;Yang2025_Silkie_Wencha	0.540252766
ENSGALG00010014388	MFNG	3	-0.458812228	0.256519323	-1.788606893	0.073678147	0.123215003	5.634958882	64.50728316	1	CRA004775;PRJNA827465;Yang2025_Silkie_Wencha	0.540656488
ENSGALG00010007439	HSD17B2	2	0.376880828	0.210711272	1.788612562	0.073677233	0	0.020169522	0	1	PRJNA827465;Yang2025_Silkie_Wenchang	0.540656488
ENSGALG00010024676	ENSGALG0001002467	3	0.239884578	0.134111067	1.788700837	0.073663008	0.030836346	5.678847603	64.78158704	1	CRA004775;PRJNA827465;Yang2025_Silkie_Wencha	0.540656488
ENSGALG00010007801	TTC13	3	0.21488489	0.120153871	1.788414209	0.073709206	0.027715908	5.88233434	65.99989248	1	CRA004775;PRJNA827465;Yang2025_Silkie_Wencha	0.540656488
ENSGALG00010022182	CNTNAP1	3	-0.211237693	0.118136307	-1.788084442	0.073762385	0	0.681804452	0	1	CRA004775;PRJNA827465;Yang2025_Silkie_Wencha	0.540656488
ENSGALG00010013663	OPA1	3	-0.069857846	0.039066431	-1.788180913	0.073746825	0	1.006503367	0	0.666666667	CRA004775;PRJNA827465;Yang2025_Silkie_Wencha	0.540656488
ENSGALG00010011368	ENSGALG0001001136	2	0.907136059	0.507462158	1.787593509	0.073841613	0.399642324	4.345111968	76.98563334	1	PRJNA827465;Yang2025_Silkie_Wenchang	0.540956774
ENSGALG00010012463	ENSGALG0001001246	2	0.803992877	0.450158141	1.786023185	0.074095504	0	0.060314026	0	1	PRJNA827465;Yang2025_Silkie_Wenchang	0.542101243
ENSGALG00010012671	NBN	3	0.27581358	0.154456558	1.785703269	0.074147316	0.034480041	3.832205688	47.81073453	1	CRA004775;PRJNA827465;Yang2025_Silkie_Wencha	0.542101243
ENSGALG00010018314	SLC24A3	3	-0.227772203	0.127545739	-1.78580802	0.074130348	0.024655488	3.936938917	49.19911021	1	CRA004775;PRJNA827465;Yang2025_Silkie_Wencha	0.542101243
ENSGALG00010028054	RPS15	3	-0.112916217	0.063239734	-1.785526425	0.074175969	0	1.92047167	0	1	CRA004775;PRJNA827465;Yang2025_Silkie_Wencha	0.542101243
ENSGALG00010019830	JAG2	3	-0.096712806	0.05416741	-1.785442686	0.07418954	0	1.813605714	0	0.666666667	CRA004775;PRJNA827465;Yang2025_Silkie_Wencha	0.542101243
ENSGALG00010001441	HMGCLL1	3	0.246975434	0.138389449	1.784640643	0.074319625	0	0.903039058	0	0.666666667	CRA004775;PRJNA827465;Yang2025_Silkie_Wencha	0.542771266
ENSGALG00010022271	SLC18A2	3	-0.323827033	0.181593072	-1.783256532	0.074544554	0	0.351189371	0	1	CRA004775;PRJNA827465;Yang2025_Silkie_Wencha	0.543852139
ENSGALG00010029642	NAT9	3	-0.158029404	0.088613901	-1.783347781	0.074529708	0	0.965533419	0	0.666666667	CRA004775;PRJNA827465;Yang2025_Silkie_Wencha	0.543852139
ENSGALG00010027632	LAMC3	3	0.498523492	0.2796777	1.78249282	0.074668901	0	1.880204471	0	0.666666667	CRA004775;PRJNA827465;Yang2025_Silkie_Wencha	0.543976721
ENSGALG00010009717	EDN1	3	-0.268573739	0.150677339	-1.782442805	0.07467705	0.027256776	3.327465336	39.89418978	0.666666667	CRA004775;PRJNA827465;Yang2025_Silkie_Wencha	0.543976721
ENSGALG00010010663	EFR3B	3	0.1313324	0.073680793	1.782450955	0.074675722	0	1.146908015	0	0.666666667	CRA004775;PRJNA827465;Yang2025_Silkie_Wencha	0.543976721
ENSGALG00010023473	BOK	3	0.388506539	0.218162373	1.780813686	0.074942893	0.1079089	8.734075319	77.10118213	0.666666667	CRA004775;PRJNA827465;Yang2025_Silkie_Wencha	0.544510567
ENSGALG00010023987	TNNI3K	3	0.344100531	0.193182913	1.781216182	0.074877141	0	1.761795268	0	1	CRA004775;PRJNA827465;Yang2025_Silkie_Wencha	0.544510567
ENSGALG00010013609	PIGC	3	-0.182448929	0.102427758	-1.781244976	0.074872439	0	0.273213663	0	1	CRA004775;PRJNA827465;Yang2025_Silkie_Wencha	0.544510567
ENSGALG00010024024	AGL	3	-0.157149896	0.088201892	-1.781706635	0.074797084	0.002949092	2.228659715	10.25996538	0.666666667	CRA004775;PRJNA827465;Yang2025_Silkie_Wencha	0.544510567
ENSGALG00010029677	PHF12	3	-0.068796569	0.038630279	-1.780897546	0.07492919	0	1.860209606	0	0.666666667	CRA004775;PRJNA827465;Yang2025_Silkie_Wencha	0.544510567
ENSGALG00010006641	ENSGALG0001000664	2	0.867309384	0.487275526	1.779915751	0.07508975	0.283907698	2.458283887	59.32121569	1	PRJNA827465;Yang2025_Silkie_Wenchang	0.544527604
ENSGALG00010009744	DAPK1	3	0.134563707	0.075584241	1.780314335	0.075024532	0.001757718	2.176852831	8.124243786	0.666666667	CRA004775;PRJNA827465;Yang2025_Silkie_Wencha	0.544527604
ENSGALG00010003894	COPS4	3	-0.098085647	0.055108708	-1.779857481	0.075099288	0	0.887456925	0	1	CRA004775;PRJNA827465;Yang2025_Silkie_Wencha	0.544527604
ENSGALG00010016076	RAB3GAP2	3	-0.069306728	0.038935781	-1.780026658	0.075071598	0	1.04775584	0	0.666666667	CRA004775;PRJNA827465;Yang2025_Silkie_Wencha	0.544527604
ENSGALG00010029619	ENSGALG0001002961	3	-0.129917068	0.073057062	-1.778295829	0.075355281	0	1.332114379	0	1	CRA004775;PRJNA827465;Yang2025_Silkie_Wencha	0.5461037
ENSGALG00010026061	MPEG1	3	-0.263956921	0.148471915	-1.777823913	0.07543278	0.028008369	3.46103367	42.21379534	1	CRA004775;PRJNA827465;Yang2025_Silkie_Wencha	0.546385284
ENSGALG00010027219	ENSGALG0001002721	3	-0.093208841	0.052436161	-1.777567979	0.075474837	0	1.573942899	0	0.666666667	CRA004775;PRJNA827465;Yang2025_Silkie_Wencha	0.546409993
ENSGALG00010021916	MRPS25	3	-0.192617372	0.108397388	-1.776955845	0.075575505	0.015490826	3.304482306	39.4761474	0.666666667	CRA004775;PRJNA827465;Yang2025_Silkie_Wencha	0.546858785
ENSGALG00010012486	RADX	3	0.079303223	0.044655321	1.775896377	0.075749998	0	1.802019231	0	1	CRA004775;PRJNA827465;Yang2025_Silkie_Wencha	0.547841036
ENSGALG00010027933	ENSGALG0001002793	3	0.579212789	0.326220932	1.775523064	0.075811561	0.272869049	14.8779926	86.55732629	1	CRA004775;PRJNA827465;Yang2025_Silkie_Wencha	0.547922396
ENSGALG00010029552	NOS2	3	0.364713142	0.20543073	1.77535825	0.075838753	0.08909329	7.997301273	74.99156363	1	CRA004775;PRJNA827465;Yang2025_Silkie_Wencha	0.547922396
ENSGALG00010015121	UNC79	2	0.364925372	0.205580132	1.775100385	0.075881313	0	0.018970294	0	1	PRJNA827465;Yang2025_Silkie_Wenchang	0.547949893
ENSGALG00010022105	DLG5	3	0.353572847	0.199250558	1.774513707	0.075978217	0.085999823	8.084426013	75.26107609	1	CRA004775;PRJNA827465;Yang2025_Silkie_Wencha	0.548369581
ENSGALG00010015151	ENSGALG0001001515	2	0.360028759	0.203048625	1.773115968	0.076209493	0	0.194658668	0	1	PRJNA827465;Yang2025_Silkie_Wenchang	0.549197766
ENSGALG00010003432	LACC1	3	-0.330673586	0.186477832	-1.773259497	0.076185717	0	1.354997351	0	1	CRA004775;PRJNA827465;Yang2025_Silkie_Wencha	0.549197766
ENSGALG00010001237	MELK	3	0.199252372	0.112371808	1.77315267	0.076203412	0.004753242	2.221839672	9.984504036	1	CRA004775;PRJNA827465;Yang2025_Silkie_Wencha	0.549197766
ENSGALG00010009371	PHTF2	3	0.169227398	0.095472935	1.772516972	0.076308781	0.007534213	2.711940733	26.25207566	1	CRA004775;PRJNA827465;Yang2025_Silkie_Wencha	0.549633137
ENSGALG00010013385	PGRMC1	3	-0.349315425	0.197130397	-1.772001836	0.076394252	0.085423137	7.814464937	74.40643709	1	CRA004775;PRJNA827465;Yang2025_Silkie_Wencha	0.549968603
ENSGALG00010012099	UPK3A	2	-0.98948544	0.558777874	-1.770802828	0.076593495	0.467654666	3.939776948	74.6178524	1	PRJNA827465;Yang2025_Silkie_Wenchang	0.550542847
ENSGALG00010004040	LGALS1B	3	0.220304897	0.124459321	1.770095604	0.076711216	0	0.334311175	0	1	CRA004775;PRJNA827465;Yang2025_Silkie_Wencha	0.550542847
ENSGALG00010011447	LAMC1	3	-0.151841621	0.085778506	-1.770159307	0.076700606	0.003101523	2.278605345	12.22701183	0.666666667	CRA004775;PRJNA827465;Yang2025_Silkie_Wencha	0.550542847
ENSGALG00010014935	PCYT1A	3	-0.130496243	0.073697675	-1.77069687	0.076611123	0	1.04206478	0	1	CRA004775;PRJNA827465;Yang2025_Silkie_Wencha	0.550542847
ENSGALG00010016422	KCTD3	3	-0.100910781	0.056989259	-1.77069825	0.076610894	0	1.815436641	0	1	CRA004775;PRJNA827465;Yang2025_Silkie_Wencha	0.550542847
ENSGALG00010021556	CAMK1	3	-0.094132199	0.05318554	-1.769883296	0.076746584	0	1.447383547	0	0.666666667	CRA004775;PRJNA827465;Yang2025_Silkie_Wencha	0.550542847
ENSGALG00010010588	SLC25A3	3	-0.092316763	0.052144565	-1.770400481	0.07666045	0	1.389222074	0	1	CRA004775;PRJNA827465;Yang2025_Silkie_Wencha	0.550542847
ENSGALG00010002039	AAMDC	3	0.145255756	0.082096341	1.769332898	0.076838336	0	0.230554105	0	1	CRA004775;PRJNA827465;Yang2025_Silkie_Wencha	0.550921521
ENSGALG00010026127	ENSGALG0001002612	3	-1.207226748	0.682618205	-1.768524101	0.076973327	1.09575099	9.629920295	79.23139612	1	CRA004775;PRJNA827465;Yang2025_Silkie_Wencha	0.551185652
ENSGALG00010013783	NDUFA10	3	-0.110105738	0.062270723	-1.768178251	0.077031109	0.001918164	2.210826103	9.536078048	0.666666667	CRA004775;PRJNA827465;Yang2025_Silkie_Wencha	0.551185652
ENSGALG00010024522	DNTTIP2	3	-0.094997189	0.053721878	-1.768314767	0.077008297	0	1.144688626	0	0.666666667	CRA004775;PRJNA827465;Yang2025_Silkie_Wencha	0.551185652
ENSGALG00010022578	ZNF512B	3	0.079909802	0.045182773	1.768590027	0.076962316	0.000643351	2.20710245	9.383454297	1	CRA004775;PRJNA827465;Yang2025_Silkie_Wencha	0.551185652
ENSGALG00010022452	AP3M1	3	-0.170185562	0.096272535	-1.767747805	0.077103074	0.010919174	3.2495709	38.45341243	1	CRA004775;PRJNA827465;Yang2025_Silkie_Wencha	0.551421531
ENSGALG00010002812	TRIM13	3	-0.281335004	0.159190211	-1.767288346	0.077179951	0.056120437	10.80238928	81.48557742	1	CRA004775;PRJNA827465;Yang2025_Silkie_Wencha	0.551692275
ENSGALG00010002621	SLC25A15	3	0.243239646	0.13774675	1.765846721	0.077421568	0.014120749	2.610290561	23.3801773	0.666666667	CRA004775;PRJNA827465;Yang2025_Silkie_Wencha	0.552744979
ENSGALG00010027988	HDAC11	3	0.172104137	0.09746182	1.765862123	0.077418983	0.009683712	2.995652066	33.23657234	1	CRA004775;PRJNA827465;Yang2025_Silkie_Wencha	0.552744979
ENSGALG00010007984	EIF3H	3	-0.097388877	0.055155644	-1.765710077	0.077444501	0.001074326	2.157251301	7.289428973	0.666666667	CRA004775;PRJNA827465;Yang2025_Silkie_Wencha	0.552744979
ENSGALG00010008243	YTHDC2	3	0.205541064	0.11646496	1.764831786	0.077592041	0	1.821098029	0	0.666666667	CRA004775;PRJNA827465;Yang2025_Silkie_Wencha	0.5535186
ENSGALG00010013114	PIGP	3	-0.30802801	0.174688255	-1.763301202	0.077849703	0.053780547	4.85423846	58.79889263	1	CRA004775;PRJNA827465;Yang2025_Silkie_Wencha	0.554796848
ENSGALG00010003882	ENSGALG0001000388	3	-0.098163768	0.055667103	-1.76340715	0.077831845	0	1.263332094	0	1	CRA004775;PRJNA827465;Yang2025_Silkie_Wencha	0.554796848
ENSGALG00010012092	OC3	3	0.57397724	0.325651088	1.762552808	0.077975942	0	1.590993469	0	1	CRA004775;PRJNA827465;Yang2025_Silkie_Wencha	0.554857499
ENSGALG00010024157	CHRNB4	2	-0.297812282	0.168947492	-1.762750539	0.077942573	0	0.021773603	0	1	PRJNA827465;Yang2025_Silkie_Wenchang	0.554857499
ENSGALG00010013410	KIAA0825	3	0.230960111	0.13103245	1.762617662	0.077964996	0	0.543685793	0	1	CRA004775;PRJNA827465;Yang2025_Silkie_Wencha	0.554857499
ENSGALG00010014918	PLRG1	3	-0.071791575	0.040740928	-1.762148738	0.0780417	0	0.166915196	0	1	CRA004775;PRJNA827465;Yang2025_Silkie_Wencha	0.555063645
ENSGALG00010010106	C7orf73	3	-0.535486547	0.304116175	-1.76079601	0.078272935	0.219172773	10.56303239	81.0660431	1	CRA004775;PRJNA827465;Yang2025_Silkie_Wencha	0.555454199

ENSGALG00010028748	PIAS3	3	0.351420165	0.199560462	1.760970892	0.07824333	0.09174057	10.46793347	80.89403219	1	CRA004775;PRJNA827465;Yang2025_Silkie_Wencha	0.555454199
ENSGALG00010028011	ENSGALG00010028011	3	-0.265146652	0.150592141	-1.760693816	0.07829024	0.038306634	4.99360165	59.84874761	1	CRA004775;PRJNA827465;Yang2025_Silkie_Wencha	0.555454199
ENSGALG00010000466	FBXO21	3	-0.125011919	0.070982666	-1.761161222	0.078211119	0.006238885	3.298645781	39.36905831	1	CRA004775;PRJNA827465;Yang2025_Silkie_Wencha	0.555454199
ENSGALG00010018519	OGDH	3	-0.118222136	0.067146385	-1.760662696	0.07829551	0.001437475	2.196207984	8.933943645	1	CRA004775;PRJNA827465;Yang2025_Silkie_Wencha	0.555454199
ENSGALG00010017466	SPIA4	2	1.397935675	0.794462944	1.759598337	0.078475934	0	0.022281183	0	1	CRA004775;PRJNA827465	0.555477525
ENSGALG00010023531	CPLX3	2	0.835764875	0.474806356	1.760222592	0.078370073	0	0.216459395	0	1	CRA004775;Yang2025_Silkie_Wenchang	0.555477525
ENSGALG00010004326	ENSGALG00010004326	3	-0.810382458	0.460579453	-1.759484606	0.078495233	0.215226812	2.999319105	33.3181989	1	CRA004775;PRJNA827465;Yang2025_Silkie_Wencha	0.555477525
ENSGALG00010002334	OSBPL10	3	-0.120005401	0.068185658	-1.759980092	0.078411182	0	0.315827514	0	0.666666667	CRA004775;PRJNA827465;Yang2025_Silkie_Wencha	0.555477525
ENSGALG00010021420	PLXNB3	3	-0.109953941	0.062484183	-1.759708398	0.078457261	0	0.671076525	0	0.666666667	CRA004775;PRJNA827465;Yang2025_Silkie_Wencha	0.555477525
ENSGALG00010023079	SLC25A16	3	0.107624732	0.061182085	1.759088992	0.078562394	0	0.417243618	0	1	CRA004775;PRJNA827465;Yang2025_Silkie_Wencha	0.555674684
ENSGALG00010011042	SLC13A4	2	0.62265564	0.354227497	1.757784931	0.078784166	0	0.438279907	0	1	PRJNA827465;Yang2025_Silkie_Wenchang	0.556240571
ENSGALG00010002912	RPS3	3	-0.450675688	0.256435533	-1.757461933	0.078839105	0.166694167	14.85853709	86.53972469	1	CRA004775;PRJNA827465;Yang2025_Silkie_Wencha	0.556240571
ENSGALG00010018574	ENSGALG00010018574	3	0.28623289	0.16284902	1.757658049	0.07880571	0	0.225477027	0	1	CRA004775;PRJNA827465;Yang2025_Silkie_Wencha	0.556240571
ENSGALG00010002763	SEPTIN11	3	0.088252408	0.050202706	1.757921326	0.078760897	0	1.395002507	0	1	CRA004775;PRJNA827465;Yang2025_Silkie_Wencha	0.556240571
ENSGALG00010018348	TOGARAM1	3	0.082138324	0.046735887	1.757498139	0.078832939	0	0.93001583	0	1	CRA004775;PRJNA827465;Yang2025_Silkie_Wencha	0.556240571
ENSGALG00010020970	ARMH3	3	-0.203375959	0.115788521	-1.756443181	0.079012765	0.029337033	12.2455661	83.66755785	1	CRA004775;PRJNA827465;Yang2025_Silkie_Wencha	0.557111902
ENSGALG00010009400	CNTFR	3	-0.149310362	0.085035798	-1.755853005	0.079113511	0	0.572538982	0	1	CRA004775;PRJNA827465;Yang2025_Silkie_Wencha	0.557111902
ENSGALG00010028194	ENSGALG00010028194	3	0.126740757	0.07220245	1.755352576	0.079199018	0	0.114448001	0	1	CRA004775;PRJNA827465;Yang2025_Silkie_Wencha	0.557111902
ENSGALG00010025815	CASQ2	3	-0.125942043	0.071695754	-1.75619944	0.079054362	0	0.342407598	0	0.666666667	CRA004775;PRJNA827465;Yang2025_Silkie_Wencha	0.557111902
ENSGALG00010023866	AMBRA1	3	-0.078165796	0.044527801	-1.755438026	0.079184413	0	1.935132752	0	1	CRA004775;PRJNA827465;Yang2025_Silkie_Wencha	0.557111902
ENSGALG00010006157	SNX9	3	0.072304223	0.041181287	1.755754315	0.079130368	0	1.097063828	0	1	CRA004775;PRJNA827465;Yang2025_Silkie_Wencha	0.557111902
ENSGALG00010015814	PAQR9	3	0.436261801	0.248726041	1.753985223	0.079433039	0	0.261547867	0	1	CRA004775;PRJNA827465;Yang2025_Silkie_Wencha	0.557371584
ENSGALG00010014214	REEP2	3	0.204336357	0.116459348	1.754572398	0.079332476	0.017090495	3.392132615	41.04004098	1	CRA004775;PRJNA827465;Yang2025_Silkie_Wencha	0.557371584
ENSGALG00010015046	PSMB2	3	-0.190880108	0.108808229	-1.754280078	0.079382528	0.017416549	3.604643782	44.51601542	1	CRA004775;PRJNA827465;Yang2025_Silkie_Wencha	0.557371584
ENSGALG00010020130	PDXDC1	3	0.17153121	0.097791472	1.754050807	0.079421802	0.01574932	4.541555616	55.96222596	0.666666667	CRA004775;PRJNA827465;Yang2025_Silkie_Wencha	0.557371584
ENSGALG00010028426	GPR21	3	0.157959443	0.090020115	1.754712754	0.079308453	0	1.795001038	0	1	CRA004775;PRJNA827465;Yang2025_Silkie_Wencha	0.557371584
ENSGALG00010003690	ENSGALG00010003690	2	0.563834184	0.321538103	1.753553248	0.079507088	0.023766212	1.12619532	11.20545591	1	PRJNA827465;Yang2025_Silkie_Wenchang	0.557614442
ENSGALG00010010648	CENPM	3	-0.287212321	0.163863179	-1.752756921	0.079643741	0.016772865	2.370843972	15.64185481	0.666666667	CRA004775;PRJNA827465;Yang2025_Silkie_Wencha	0.558019251
ENSGALG00010016908	CMC4	3	-0.20849758	0.118949341	-1.752826688	0.079631761	0.027395727	7.216525875	72.28583345	1	CRA004775;PRJNA827465;Yang2025_Silkie_Wencha	0.558019251
ENSGALG00010027732	EEF2	3	-0.331108573	0.189042273	-1.751505462	0.079858881	0.086079513	13.4008897	85.07561778	1	CRA004775;PRJNA827465;Yang2025_Silkie_Wencha	0.558858638
ENSGALG00010028043	NT5DC2	3	0.105494613	0.060235465	1.751370443	0.079882121	0	1.263255175	0	1	CRA004775;PRJNA827465;Yang2025_Silkie_Wencha	0.558858638
ENSGALG00010027119	NUB1	3	-0.097178983	0.055486379	-1.751402517	0.0798766	0.000440861	2.071082863	3.43215928	0.666666667	CRA004775;PRJNA827465;Yang2025_Silkie_Wencha	0.558858638
ENSGALG00010005934	SLITRK6	2	-0.353562224	0.201926732	-1.750943126	0.079955707	0	0.179754885	0	1	PRJNA827465;Yang2025_Silkie_Wenchang	0.559028357
ENSGALG00010021745	RSL24D1	3	-0.099533806	0.056851431	-1.750770463	0.079985456	0	0.652549589	0	0.666666667	CRA004775;PRJNA827465;Yang2025_Silkie_Wencha	0.559028357
ENSGALG00010016471	POMGNT2	3	-0.16083009	0.091903992	-1.749979369	0.080121874	0.008341831	2.943455401	32.05264808	1	CRA004775;PRJNA827465;Yang2025_Silkie_Wencha	0.559465751
ENSGALG00010004135	NUP54	3	-0.083859382	0.047921053	-1.749948641	0.080127176	0	1.128809473	0	1	CRA004775;PRJNA827465;Yang2025_Silkie_Wencha	0.559465751
ENSGALG00010004352	CHMP2A	3	-0.213027065	0.121827028	-1.748602656	0.080359727	0.025921138	4.701250951	57.45813144	1	CRA004775;PRJNA827465;Yang2025_Silkie_Wencha	0.560812526
ENSGALG00010028604	PI4KB	3	-0.18131301	0.10372304	-1.748049525	0.080455452	0.022558095	8.316900499	75.95257993	1	CRA004775;PRJNA827465;Yang2025_Silkie_Wencha	0.56120357
ENSGALG00010014943	APLN	3	0.206344478	0.118089787	1.747352448	0.080576221	0	1.337721329	0	1	CRA004775;PRJNA827465;Yang2025_Silkie_Wencha	0.561768828
ENSGALG00010008197	STARD4	3	0.242853652	0.139036001	1.746696178	0.080690054	0	1.494654413	0	1	CRA004775;PRJNA827465;Yang2025_Silkie_Wencha	0.562008211
ENSGALG00010004378	TRIM28	3	-0.085972639	0.049217422	-1.746792831	0.080673281	0	0.994736481	0	1	CRA004775;PRJNA827465;Yang2025_Silkie_Wencha	0.562008211
ENSGALG00010020101	KIF15	3	0.194882964	0.111591104	1.746402329	0.080741066	0.008259104	2.431060567	17.73137916	0.666666667	CRA004775;PRJNA827465;Yang2025_Silkie_Wencha	0.562086621
ENSGALG00010012016	WDR91	3	-0.175266408	0.100374603	-1.746123052	0.080789572	0.021605361	7.280167912	72.52810616	1	CRA004775;PRJNA827465;Yang2025_Silkie_Wencha	0.562147519
ENSGALG00010024300	MEOX1	3	0.208096901	0.119231973	1.745311221	0.08093071	0	0.046798522	0	1	CRA004775;PRJNA827465;Yang2025_Silkie_Wencha	0.562852585
ENSGALG00010003724	ENSGALG00010003724	3	0.189753938	0.108818089	1.743772009	0.081198853	0	1.970338625	0	0.666666667	CRA004775;PRJNA827465;Yang2025_Silkie_Wencha	0.563418588
ENSGALG00010008450	GIN1	3	-0.180221279	0.103338688	-1.743986527	0.081161439	0	0.274321007	0	1	CRA004775;PRJNA827465;Yang2025_Silkie_Wencha	0.563418588
ENSGALG00010022276	BECN1	3	0.149851907	0.085912811	1.744232375	0.081118578	0.0128502	5.610483687	64.3524496	1	CRA004775;PRJNA827465;Yang2025_Silkie_Wencha	0.563418588
ENSGALG00010001469	ARHGEF26	3	0.133927652	0.076783081	1.744233885	0.081118315	0	0.054645015	0	1	CRA004775;PRJNA827465;Yang2025_Silkie_Wencha	0.563418588
ENSGALG00010020141	THOC7	3	-0.112552187	0.064547891	-1.743700454	0.081211336	0	0.521468926	0	0.666666667	CRA004775;PRJNA827465;Yang2025_Silkie_Wencha	0.563418588
ENSGALG00010002253	ENSGALG00010002253	3	0.271186913	0.155605628	1.742783448	0.081371449	0	0.025401445	0	1	CRA004775;PRJNA827465;Yang2025_Silkie_Wencha	0.564252532
ENSGALG00010002814	TERF1	3	-0.149095833	0.085589645	-1.741984485	0.081511159	0	0.42817742	0	1	CRA004775;PRJNA827465;Yang2025_Silkie_Wencha	0.564944255
ENSGALG00010019262	TMC3	2	0.445975893	0.256164525	1.740974448	0.081688058	0.041712778	1.399408353	28.54122976	1	PRJNA827465;Yang2025_Silkie_Wenchang	0.565426071
ENSGALG00010013340	LIN28B	2	0.406521223	0.233511752	1.740902628	0.081700648	0	0.994667821	0	1	PRJNA827465;Yang2025_Silkie_Wenchang	0.565426071
ENSGALG00010029759	ALDH3A2	3	-0.126443908	0.072617841	-1.74122373	0.081644369	0	1.539532038	0	1	CRA004775;PRJNA827465;Yang2025_Silkie_Wencha	0.565426071
ENSGALG00010012179	ENSGALG00010012179	3	-0.35114686	0.201813154	-1.739960223	0.081866003	0.049358349	3.333875074	40.00974976	1	CRA004775;PRJNA827465;Yang2025_Silkie_Wencha	0.566016339
ENSGALG00010017918	SIX4	3	-0.114304388	0.065688629	-1.740093975	0.081842518	0	1.639115606	0	0.666666667	CRA004775;PRJNA827465;Yang2025_Silkie_Wencha	0.566016339
ENSGALG00010014710	NPEPL1	3	-0.208511802	0.119863154	-1.739582142	0.081932417	0.024320171	5.488100536	63.55751891	1	CRA004775;PRJNA827465;Yang2025_Silkie_Wencha	0.566198655
ENSGALG00010011734	ENSGALG00010011734	3	0.732473841	0.421259115	1.73877268	0.082074756	0.439585627	11.70462645	82.9127396	1	CRA004775;PRJNA827465;Yang2025_Silkie_Wencha	0.566905215
ENSGALG00010014151	ATP23	3	-0.148930522	0.085678109	-1.738256415	0.082165642	0.007387357	2.789124958	28.29292232	1	CRA004775;PRJNA827465;Yang2025_Silkie_Wencha	0.567255869
ENSGALG00010013115	ENSGALG00010013115	3	0.649853988	0.374435986	1.735554307	0.082642671	0.345278514	11.73916772	82.96301707	0.666666667	CRA004775;PRJNA827465;Yang2025_Silkie_Wencha	0.567776836
ENSGALG00010012441	FST	3	-0.39752544	0.229047369	-1.735559947	0.082641673	0.118730712	13.29444204	84.9561193	1	CRA004775;PRJNA827465;Yang2025_Silkie_Wencha	0.567776836
ENSGALG00010004996	GPR158	3	0.330927241	0.190651121	1.73577391	0.082603819	0	0.326337324	0	1	CRA004775;PRJNA827465;Yang2025_Silkie_Wencha	0.567776836
ENSGALG00010023631	ENSGALG00010023631	3	-0.31393522	0.180860144	-1.735789951	0.082600982	0.046801156	3.721012865	46.25119363	0.666666667	CRA004775;PRJNA827465;Yang2025_Silkie_Wencha	0.567776836
ENSGALG00010017782	IGDCC4	3	-0.277958768	0.160032859	-1.736885604	0.082407365	0.046254922	5.920960594	66.22169717	1	CRA004775;PRJNA827465;Yang2025_Silkie_Wencha	0.567776836
ENSGALG00010023050	RUFY2	3	0.25182181	0.144945627	1.737353616	0.082324773	0.041879214	6.274536909	68.1251377	1	CRA004775;PRJNA827465;Yang2025_Silkie_Wencha	0.567776836
ENSGALG00010017096	DCTN5	3	-0.246865076	0.142184103	-1.736235422	0.082522216	0.049055111	11.30720972	82.31217029	1	CRA004775;PRJNA827465;Yang2025_Silkie_Wencha	0.567776836
ENSGALG00010001796	SLCO2B1	3	-0.230558064	0.13277164	-1.736500839	0.082475316	0.030171703	5.490510527	63.57351489	1	CRA004775;PRJNA827465;Yang2025_Silkie_Wencha	0.567776836
ENSGALG00010024077	SFT2D3	3	0.208206855	0.119906515	1.736409855	0.082491391	0.023217491	4.877123814	58.99222418	0.666666667	CRA004775;PRJNA827465;Yang2025_Silkie_Wencha	0.567776836
ENSGALG00010017380	MARCKSL1	3	0.098001969	0.056431526	1.736652848	0.082448465	0	1.534529259	0	0.666666667	CRA004775;PRJNA827465;Yang2025_Silkie_Wencha	0.567776836
ENSGALG00010024500	GNGT2	3	-0.302177495	0.174222344	-1.73443594	0.082840763	0	1.534409703	0	1	CRA004775;PRJNA827465;Yang2025_Silkie_Wencha	0.568861363
ENSGALG00010021951	PSKH1	3	-0.343954221	0.198357729	-1.734009669	0.082916368	0.092507811	14.65511354	86.35288635	1	CRA004775;PRJNA827465;Yang2025_Silkie_Wencha	0.569104137
ENSGALG00010011175	GPR52	3	-0.44816192	0.258561728	-1.733287918	0.083044507	0	0.769503475	0	1	CRA004775;PRJNA827465;Yang2025_Silkie_Wencha	0.569707077
ENSGALG00010006537	DRD3	2	0.238175401	0.137434049	1.733015974	0.08309283	0	0.327499572	0	1	PRJNA827465;Yang2025_Silkie_Wenchang	0.569762133
ENSGALG00010012962	SLC25A17	3	-0.184819053	0.106664084	-1.732720571	0.083145346	0.01757513	4.147257589	51.77536102	1	CRA004775;PRJNA827465;Yang2025_Silkie_Wencha	0.569845881
ENSGALG00010020212	JMJD7	3	-0.354972953	0.204958939	-1.731922283	0.083287401	0.081235815	5.694686237	64.87954004	1	CRA004775;PRJNA827465;Yang2025_Silkie_Wencha	0.570542907
ENSGALG00010001244	ENSGALG00010001244	3	-0.187312866	0.10821805	-1.730883773	0.083472496	0	0.410011452	0	1	CRA004775;PRJNA827465;Yang2025_Silkie_Wencha	0.57137787
ENSGALG00010024857	SLC37A4	3	-0.104515084	0.060385941	-1.73078504	0.083490111	0	0.974977644	0	1	CRA004775;PRJNA827465;Yang2025_Silkie_Wencha	0.57137787
ENSGALG00010023392	TPI1	3	-0.288635788	0.166798318	-1.730447833	0.083550294	0.035536216	3.441834992	41.89146183	0.666666667	CRA004775;PRJNA827465;Yang2025_Silkie_Wencha	0.571513114
ENSGALG00010017798	BAG4	3	0.177882159	0.102887217	1.728904365	0.083826213	0.017351439	4.350910126	54.03260601	0.666666667	CRA004775;PRJNA827465;Yang2025_Silkie_Wencha	0.572731502
ENSGALG00010026827	FAM117A	3	-0.151292857	0.087514645	-1.728771865	0.083849933	0.008726259	3.246299949	38.39139847	1	CRA004775;PRJNA827465;Yang2025_Silkie_Wencha	0.572731502
ENSGALG00010025737	ENSGALG00010025737	3	0.121673594	0.070374809	1.728936751	0.083820416	0	1.61879377	0	0.666666667	CRA004775;PRJNA827465;Yang2025_Silkie_Wencha	0.572731502
ENSGALG00010013247	ATG5	3	-0.141952495	0.08212729	-1.728444899	0.083908492	0.011907836	5.51021802	63.70379552	1	CRA004775;PRJNA827465;Yang2025_Silkie_Wencha	0.572854739
ENSGALG00010013394	DNMT3B	3	0.384978442	0.222802641	1.727889944	0.084007958	0.088582304	6.119307692	67.31656421	1	CRA004775;PRJNA827465;Yang2025_Silkie_Wencha	0.573036373
ENSGALG00010021676	TP53INP2	3	0.33930994	0.19637762	1.727844237	0.084016154	0.058541491	4.265795863	53.11543111	1	CRA004775;PRJNA827465;Yang2025_Silkie_Wencha	0.573036373
ENSGALG00010018418	PLCB4	3	0.356104255	0.206268039	1.726415091	0.084272763	0.088887762	7.35122312	72.79364308	1	CRA004775;PRJNA827465;Yang2025_Silkie_Wencha	0.573817736
ENSGALG00010017815	VWA9	3	-0.126579296	0.073307601	-1.726687181	0.084223859	0.005696064	2.983300572	32.96015765	1	CRA004775;PRJNA827465;Yang2025_Silkie_Wencha	0.573817736
ENSGALG00010005302	PSPC1	3	-0.10351713	0.059964661	-1.726302253	0.08429305	0.003194459	2.651375145	24.56744554	1	CRA004775;PRJNA827465;Yang2025_Silkie_Wencha	0.573817736
ENSGALG00010012988	RPL18A	3	-0.102250406	0.059225127	-1.726470012	0.08426289	0	1.923352958	0	1	CRA004775;PRJNA827465;Yang2025_Silkie_Wencha	0.573817736
ENSGALG00010016458	ZC3H12A	3	-0.436326999	0.252930082	-1.725089383	0.084511366	0.158955875	14.09688086	85.81246433	1	CRA004775;PRJNA827465;Yang2025_Silkie_Wencha	0.574482306
ENSGALG00010011967	MTMR9	3	-0.312473688	0.181156648	-1.724881155	0.084548892	0.077933505	15.2769307	86.90836505	1	CRA004775;PRJNA827465;Yang2025_Silkie_Wencha	0.574482306
ENSGALG00010013317	PREP	3	-0.234363052	0.135842607	-1.725254376	0.08448164	0.047935995	19.86393297	89.93150046	1	CRA004775;PRJNA827465;Yang2025_Silkie_Wencha	0.574482306
ENSGALG00010004265	PIP4K2A	3	-0.184355422	0.106881552	-1.724857264	0.084553199	0.023101864	6.336832252	68.43848913	0.666666667	CRA004775;PRJNA827465;Yang2025_Silkie_Wencha	0.574482306
ENSGALG00010020199	DET1	3	-0.436353698	0.253129635	-1.723834899	0.084737653	0.173059734	23.35509215	91.43655702	1	CRA004775;PRJNA827465;Yang2025_Silkie_Wencha	0.575242561
ENSGALG00010012244	ENSGALG00010012244	3	-0.114511755	0.066430374	-1.723786105	0.084746464	0.006118973	4.033934774	50.42061629	1	CRA004775;PRJNA827465;Yang2025_Silkie_Wencha	0.575242561
ENSGALG00010024373	TCAP	3	0.515445015	0.299156883	1.722992331	0.084889912	0	1.979422947	0	1	CRA004775;PRJNA827465;Yang2025_Silkie_Wencha	0.575903082
ENSGALG00010022945	KCNA4	2	0.455869103	0.264644455	1.722571909	0.084965969	0	0.017096785	0	1	PRJNA827465;Yang2025_Silkie_Wenchang	0.575903082
ENSGALG00010015881	GAB3	3	-0.183247954	0.106375375	-1.722653883	0.084951135	0	1.968070821	0	1	CRA004775;PRJNA827465;Yang2025_Silkie_Wencha	0.575903082
ENSGALG00010028911	LMX1B	2	1.046853023	0.607876513	1.722147509	0.085042801	0	0.006147887	0	1	PRJNA827465;Yang2025_Silkie_Wenchang	0.576049146
ENSGALG00010021705	CCDC18	3	0.546711946	0.317502995	1.721911149	0.085085615	0.232898483	14.03671788	85.75165493	1	CRA004775;PRJNA827465;Yang2025_Silkie_Wencha	0.576049146
ENSGALG00010025904	RPL10A	3	-0.107095383	0.06220046	-1.721777987	0.085109744	0	1.243557923	0	1	CRA004775;PRJNA827465;Yang2025_Silkie_Wencha	0.576049146
ENSGALG00010010308	PACRGL	3	-0.229927422	0.133567314	-1.721434803	0.085171953	0	1.379376441	0	0.666666667	CRA004775;PRJNA827465;Yang2025_Silkie_Wencha	0.576194378
ENSGALG00010017351	CCR2	3	-0.499944295	0.290551416	-1.720674098	0.085309979	0.193642741	12.27900465	83.71203483	1	CRA004775;PRJNA827465;Yang2025_Silkie_Wencha	0.576484755
ENSGALG00010025658	PDE1B	3	0.429675683	0.24971087	1.720692749	0.085306593	0.051277319	2.749237867	27.25256611	1	CRA004775;PRJNA827465;Yang2025_Silkie_Wencha	0.576484755
ENSGALG00010029296	FBXO39	3	0.238579871	0.138703148	1.720075383	0.08541874	0.029637762	4.241842897	52.85068192	0.666666667	CRA004775;PRJNA827465;Yang2025_Silkie_Wencha	0.576484755
ENSGALG00010020927	ENSGALG00010020927	3	0.13674824	0.079497318	1.720161682	0.085403056	0	0.541208756	0	0.666666667	CRA004775;PRJNA827465;Yang2025_Silkie_Wencha	0.576484755
ENSGALG00010003676	POLR2B	3	-0.11842118	0.068832568	-1.720423695	0.085355453	0.007440182	4.237135857	52.79830368	1	CRA004775;PRJNA827465;Yang2025_Silkie_Wencha	0.576484755
ENSGALG00010021756	USP42	3	0.463468049	0.269601846	1.719083364	0.085599193	0.161263668	8.155588311	75.47693773	1	CRA004775;PRJNA827465;Yang2025_Silkie_Wencha	0.577302777
ENSGALG00010009387	DEPTOR	3	-0.210090581	0.122219569	-1.718960259	0.085621608	0.020102877	3.756412177	46.7577064	0.666666667	CRA004775;PRJNA827465;Yang2025_Silkie_Wencha	0.577302777
ENSGALG00010019322	EGR3	3	0.783975233	0.456432238	1.717615821	0.085866713	0.52950543	14.91122851	86.58728891	1	CRA004775;PRJNA827465;Yang2025_Silkie_Wencha	0.57812831
ENSGALG00010008194	PDF	3	0.355300677	0.20684374	1.717725063	0.085846776	0.074856909	4.932057134	59.44896935	0.666666667	CRA004775;PRJNA827465;Yang2025_Silkie_Wencha	0.57812831
ENSGALG00010016519	MEX3B	3	0.204260321	0.118906785	1.717818882	0.085829656	0.021038902	4.215885985	52.5603869	1	CRA004775;PRJNA827465;Yang2025_Silkie_Wencha	0.57812831
ENSGALG00010024423	SERGEF	3	-0.131548797	0.076609074	-1.717143801	0.085952901	0	0.590466811	0	1	CRA004775;PRJNA827465;Yang2025_Silkie_Wencha	0.578157976
ENSGALG00010012180	DDX59	3	-0.11536171	0.06717954	-1.717214954	0.085939904	0	0.162410785	0	1	CRA004775;PRJNA827465;Yang2025_Silkie_Wencha	0.578157976
ENSGALG00010027875	WNT5A	3	-0.303768903	0.177025639	-1.715959929	0.086169377	0.057506355	5.556123658	64.00368093	0.666666667	CRA004775;PRJNA827465;Yang2025_Silkie_Wencha	0.579086354
ENSGALG00010020184	RSL1D1	3	-0.084137211	0.049032693	-1.715941046	0.086172833	0	0.910247684	0	1	CRA004775;PRJNA827465;Yang2025_Silkie_Wencha	0.579086354
ENSGALG00010013293	TUBGCP3	3	-0.078103539	0.045529755	-1.71543949	0.08626468	0	0.702912835	0	1	CRA004775;PRJNA827465;Yang2025_Silkie_Wencha	0.579428179
ENSGALG00010014802	ENSGALG00010014802	3	0.279666691	0.16306734	1.715038035	0.086338254	0	1.291801082	0	0.666666667	CRA004775;PRJNA827465;Yang2025_Silkie_Wencha	0.579646994
ENSGALG00010001124	GPR171	2	0.648993767	0.378705415	1.713716627	0.086580782	0	0.344889364	0	1	CRA004775;Yang2025_Silkie_Wenchang	0.579690909
ENSGALG00010003408	ENSGALG00010003408	3	0.584593712	0.341137085	1.713662154	0.086590791	0.237646079	6.574474046	69.57931561	1	CRA004775;PRJNA827465;Yang2025_Silkie_Wencha	0.579690909
ENSGALG00010005457	SEMA3D	3	-0.26436038	0.154259889	-1.713733761	0.086577633	0.035918798	4.237743157	52.80506803	0.666666667	CRA004775;PRJNA827465;Yang2025_Silkie_Wencha	0.579690909
ENSGALG00010028037	ENSGALG00010028037	3	-0.236755754	0.138115166	-1.714190846	0.086493681	0	1.357606273	0	1	CRA004775;PRJNA827465;Yang2025_Silkie_Wencha	0.579690909
ENSGALG00010015481	GOLGA5	3	0.133621045	0.077970154	1.71374607	0.086575372	0.007121367	3.126161394	36.02377651	0.666666667	CRA004775;PRJNA827465;Yang2025_Silkie_Wencha	0.579690909
ENSGALG00010028137	NUP214	3	-0.088274097	0.051507093	-1.713824088	0.086561038	0	1.211520297	0	0.666666667	CRA004775;PRJNA827465;Yang2025_Silkie_Wencha	0.579690909
ENSGALG00010023782	TMPRSS5	3	0.996960344	0.581938325	1.713171828	0.086680933	0.738189221	7.360379035	72.82748632	0.666666667	CRA004775;PRJNA827465;Yang2025_Silkie_Wencha	0.580019739
ENSGALG00010010345	ENSGALG00010010345	3	0.256020156	0.149536786	1.712088131	0.086880428	0	1.891294436	0	0.666666667	CRA004775;PRJNA827465;Yang2025_Silkie_Wencha	0.580804906
ENSGALG00010020759	ARHGAP19	3	-0.117868321	0.068839546	-1.712218172	0.08685647	0	1.584283986	0	0.666666667	CRA004775;PRJNA827465;Yang2025_Silkie_Wencha	0.580804906
ENSGALG00010027983	POGZ	3	-0.085588916	0.050024175	-1.710951081	0.087090144	0	0.442972932	0	0.666666667	CRA004775;PRJNA827465;Yang2025_Silkie_Wencha	0.581931731
ENSGALG00010025236	ENSGALG00010025236	3	0.301519203	0.176263715	1.71061414	0.087152367	0	0.995606077	0	1	CRA004775;PRJNA827465;Yang2025_Silkie_Wencha	0.58206997
ENSGALG00010021717	SDC4	3	-0.255536288	0.149440977	-1.709947926	0.087275503	0.036801987	4.659160679	57.07381355	0.666666667	CRA004775;PRJNA827465;Yang2025_Silkie_Wencha	0.58206997
ENSGALG00010001548	AKAP17A	3	0.204916666	0.119817788	1.710235768	0.087222284	0.028285166	6.248885371	67.99429208	1	CRA004775;PRJNA827465;Yang2025_Silkie_Wencha	0.58206997
ENSGALG00010021496	MTMR14	3	0.129160987	0.075526043	1.710151655	0.087237833	0.002388444	2.22161073	9.975227768	0.666666667	CRA004775;PRJNA827465;Yang2025_Silkie_Wencha	0.58206997
ENSGALG00010008024	MRS2	3	-0.12781777	0.074763009	-1.709639182	0.087332616	0	0.221384214	0	1	CRA004775;PRJNA827465;Yang2025_Silkie_Wencha	0.582176262
ENSGALG00010002293	GYG2	3	-0.299589988	0.17537784	-1.70825452	0.087589126	0.023398033	2.590308983	22.78913393	0.666666667	CRA004775;PRJNA827465;Yang2025_Silkie_Wencha	0.583269421
ENSGALG00010000089	ENSGALG00010000089	3	0.228806993	0.133971121	1.707882946	0.087658064	0	0.196600434	0	1	CRA004775;PRJNA827465;Yang2025_Silkie_Wencha	0.583269421
ENSGALG00010018001	TRAF7	3	-0.177384658	0.10385625	-1.707982512	0.087639587	0.022201909	6.556903068	69.49779523	1	CRA004775;PRJNA827465;Yang2025_Silkie_Wencha	0.583269421
ENSGALG00010009383	ASXL2	3	-0.098050771	0.057411351	-1.707863835	0.087661611	0	0.596213695	0	1	CRA004775;PRJNA827465;Yang2025_Silkie_Wencha	0.583269421
ENSGALG00010017967	ACSM3	2	0.946725064	0.554612939	1.707001403	0.087821792	0	0.208768377	0	1	PRJNA827465;Yang2025_Silkie_Wenchang	0.58378576
ENSGALG00010028025	TNNC1	3	0.504063772	0.295267408	1.707143279	0.087795425	0.146571854	5.027652616	60.22000419	1	CRA004775;PRJNA827465;Yang2025_Silkie_Wencha	0.58378576
ENSGALG00010000107	ENSGALG00010000107	3	-0.464134509	0.272135717	-1.705525881	0.08809639	0.013133522	2.106958276	5.076430685	1	CRA004775;PRJNA827465;Yang2025_Silkie_Wencha	0.585089713
ENSGALG00010002995	RWDD2A	3	-0.323889078	0.189908279	-1.705502673	0.088100714	0.07969461	8.038436755	75.11954051	1	CRA004775;PRJNA827465;Yang2025_Silkie_Wencha	0.585089713
ENSGALG00010007549	SH3D19	3	-0.121556259	0.071285454	-1.705204253	0.088156337	0	1.860471701	0	0.666666667	CRA004775;PRJNA827465;Yang2025_Silkie_Wencha	0.585184249
ENSGALG00010009741	C2H6orf52	3	0.074881855	0.043920312	1.704948169	0.088204091	0	0.028952304	0	1	CRA004775;PRJNA827465;Yang2025_Silkie_Wencha	0.58522649
ENSGALG00010022373	AK5	3	1.087660583	0.63806123	1.704633555	0.088262789	0.961271208	14.38708665	86.09864493	1	CRA004775;PRJNA827465;Yang2025_Silkie_Wencha	0.585341265
ENSGALG00010022426	EXD1	3	-0.656486719	0.385298508	-1.703839245	0.088411124	0.28845869	5.712503199	64.98907869	1	CRA004775;PRJNA827465;Yang2025_Silkie_Wencha	0.585460093
ENSGALG00010005841	ENSGALG00010005841	3	-0.391641794	0.230045611	-1.702452793	0.088670521	0.035423659	2.497824159	19.93031243	0.666666667	CRA004775;PRJNA827465;Yang2025_Silkie_Wencha	0.585460093
ENSGALG00010016399	ACSM5	3	-0.355890221	0.209078154	-1.702187501	0.088720225	0	0.064839891	0	1	CRA004775;PRJNA827465;Yang2025_Silkie_Wencha	0.585460093
ENSGALG00010023271	PATL2	3	-0.343328474	0.201674057	-1.70239286	0.088681748	0.048891557	3.321059318	39.77825119	1	CRA004775;PRJNA827465;Yang2025_Silkie_Wencha	0.585460093
ENSGALG00010025639	MYL2	2	0.330674399	0.194039902	1.704156696	0.088351817	0	0.324348485	0	1	PRJNA827465;Yang2025_Silkie_Wenchang	0.585460093
ENSGALG00010011178	RSPO2	3	-0.262732897	0.154203221	-1.703809398	0.088416702	0	1.11800102	0	0.666666667	CRA004775;PRJNA827465;Yang2025_Silkie_Wencha	0.585460093
ENSGALG00010011458	TPGS2	3	0.256911468	0.15094591	1.702010135	0.088753468	0.013685989	2.481147278	19.39212889	1	CRA004775;PRJNA827465;Yang2025_Silkie_Wencha	0.585460093
ENSGALG00010027479	HCN2	3	-0.227972529	0.133909736	-1.702434309	0.088673983	0	0.019453869	0	1	CRA004775;PRJNA827465;Yang2025_Silkie_Wencha	0.585460093
ENSGALG00010026780	MYSM1	3	0.104217249	0.061177711	1.703516637	0.088471427	0	0.833437059	0	1	CRA004775;PRJNA827465;Yang2025_Silkie_Wencha	0.585460093
ENSGALG00010002609	PDIA6	3	0.087690531	0.051525641	1.701881435	0.088777597	0.00201118	2.52446046	20.7751505	1	CRA004775;PRJNA827465;Yang2025_Silkie_Wencha	0.585460093
ENSGALG00010017571	ADAM9	3	-0.06664037	0.039127535	-1.703157917	0.08853852	0	0.181097641	0	1	CRA004775;PRJNA827465;Yang2025_Silkie_Wencha	0.585460093
ENSGALG00010024738	NBR1	3	0.066564332	0.039083538	1.703129616	0.088543815	0	0.153247003	0	1	CRA004775;PRJNA827465;Yang2025_Silkie_Wencha	0.585460093
ENSGALG00010013264	DNAJC21	3	0.405320003	0.238282722	1.701004588	0.088942126	0.126045291	8.983201311	77.73622197	1	CRA004775;PRJNA827465;Yang2025_Silkie_Wencha	0.586271662
ENSGALG00010025346	PXYLP1	3	0.1959088	0.115238236	1.700032971	0.089124724	0	1.991832934	0	0.666666667	CRA004775;PRJNA827465;Yang2025_Silkie_Wencha	0.586985024
ENSGALG00010017594	CEP68	3	-0.137848487	0.081097033	-1.699796923	0.089169131	0	0.208126651	0	1	CRA004775;PRJNA827465;Yang2025_Silkie_Wencha	0.586985024
ENSGALG00010024862	METTL16	3	-0.130222827	0.076612196	-1.699766292	0.089174894	0.005503641	2.773555729	27.8903979	0.666666667	CRA004775;PRJNA827465;Yang2025_Silkie_Wencha	0.586985024
ENSGALG00010009672	DDX47	3	-0.096817269	0.056982006	-1.69908495	0.089303179	0	1.29851913	0	1	CRA004775;PRJNA827465;Yang2025_Silkie_Wencha	0.587555906
ENSGALG00010008864	ADAT1	3	0.25900716	0.152472333	1.698715795	0.089372746	0.042891339	5.384008665	62.85295726	0.666666667	CRA004775;PRJNA827465;Yang2025_Silkie_Wencha	0.587591282
ENSGALG00010013416	NDUFB2	3	0.11783856	0.069382304	1.698395028	0.08943323	0	1.652547449	0	0.666666667	CRA004775;PRJNA827465;Yang2025_Silkie_Wencha	0.587591282
ENSGALG00010021111	AVEN	3	-0.10674162	0.062841454	-1.698586106	0.089397196	0.000557575	2.071415152	3.447650375	0.666666667	CRA004775;PRJNA827465;Yang2025_Silkie_Wencha	0.587591282
ENSGALG00010010771	ENSGALG00010010771	3	-0.216570233	0.127576814	-1.697567337	0.089589452	0.027514164	4.385518443	54.39535768	1	CRA004775;PRJNA827465;Yang2025_Silkie_Wencha	0.588344292
ENSGALG00010005431	GRM3	2	-0.922263535	0.543393625	-1.697229214	0.089653334	0	0.42305616	0	1	PRJNA827465;Yang2025_Silkie_Wenchang	0.588490478
ENSGALG00010001508	CD109	3	0.349505209	0.206021881	1.696447033	0.089801253	0.017884412	2.270256681	11.90423457	1	CRA004775;PRJNA827465;Yang2025_Silkie_Wencha	0.588940789
ENSGALG00010008056	GTF2E2	3	-0.16568386	0.097666426	-1.696425945	0.089805244	0.014409629	4.06341075	50.78026508	1	CRA004775;PRJNA827465;Yang2025_Silkie_Wencha	0.588940789
ENSGALG00010029225	ENSGALG00010029225	3	2.219371756	1.308925489	1.695567681	0.089967783	3.941115825	9.009164088	77.80038214	1	CRA004775;PRJNA827465;Yang2025_Silkie_Wencha	0.589283661
ENSGALG00010022534	CFAP57	2	0.516709909	0.304950364	1.694406595	0.090188047	0	0.624854773	0	1	PRJNA827465;Yang2025_Silkie_Wenchang	0.589283661
ENSGALG00010027516	PPM1J	3	-0.272382087	0.160755125	-1.694391308	0.09019095	0	0.379038755	0	1	CRA004775;PRJNA827465;Yang2025_Silkie_Wencha	0.589283661
ENSGALG00010005829	NPAS3	3	-0.268818596	0.158582631	-1.695132653	0.090050259	0.032032471	3.19247995	37.35277806	0.666666667	CRA004775;PRJNA827465;Yang2025_Silkie_Wencha	0.589283661
ENSGALG00010024803	CLK1	3	0.253406473	0.149477143	1.695285769	0.090021223	0.039379056	4.837283766	58.65448262	1	CRA004775;PRJNA827465;Yang2025_Silkie_Wencha	0.589283661
ENSGALG00010010064	ENSGALG00010010064	3	0.242533151	0.14313791	1.694401925	0.090188934	0.022768851	3.168264644	36.8739602	1	CRA004775;PRJNA827465;Yang2025_Silkie_Wencha	0.589283661
ENSGALG00010003103	YTHDF3	3	-0.150214449	0.088629017	-1.694867593	0.090100541	0.010938864	3.809393136	47.4981991	0.666666667	CRA004775;PRJNA827465;Yang2025_Silkie_Wencha	0.589283661
ENSGALG00010027600	KIRREL1	3	-0.102620289	0.060545639	-1.694924537	0.090089737	0	0.288454046	0	1	CRA004775;PRJNA827465;Yang2025_Silkie_Wencha	0.589283661
ENSGALG00010024427	C22orf39	3	-0.148144043	0.087444719	-1.694145115	0.090237711	0	0.678461427	0	0.666666667	CRA004775;PRJNA827465;Yang2025_Silkie_Wencha	0.589316858
ENSGALG00010009163	SAMD12	3	-0.284502538	0.167964758	-1.693822803	0.09029896	0.052011588	5.308262514	62.32288824	0.666666667	CRA004775;PRJNA827465;Yang2025_Silkie_Wencha	0.589444594
ENSGALG00010024924	MNX1	3	1.07594831	0.635367222	1.693427473	0.090374129	0.897109829	7.727132609	74.11717773	1	CRA004775;PRJNA827465;Yang2025_Silkie_Wencha	0.589663042
ENSGALG00010026059	ENSGALG00010026059	2	0.850864571	0.502545791	1.693108541	0.090434809	0	0.278922342	0	1	PRJNA827465;Yang2025_Silkie_Wenchang	0.589786791
ENSGALG00010013964	SLC38A1	3	0.115824119	0.068420246	1.692834	0.090487069	0	1.65068505	0	0.666666667	CRA004775;PRJNA827465;Yang2025_Silkie_Wencha	0.589855541
ENSGALG00010028322	STRBP	3	0.116270203	0.068702902	1.692362341	0.090576908	0	0.746862983	0	1	CRA004775;PRJNA827465;Yang2025_Silkie_Wencha	0.59016908

ENSGALG00010003186	CYP21A1	3	-0.322166259	0.190480995	-1.691330199	0.090773755	0	1.561923995	0	0.666666667	CRA004775;PRJNA827465;Yang2025_Silkie_Wencha	0.591179239
ENSGALG00010009070	ENSGALG0001000907	3	2.232833841	1.324950225	1.685220922	0.091945956	4.009496217	9.076015424	77.96389818	1	CRA004775;PRJNA827465;Yang2025_Silkie_Wencha	0.591870389
ENSGALG00010026633	SLC5A1	3	0.695804407	0.412423811	1.687110171	0.09158217	0.279856487	4.740359509	57.80910717	1	CRA004775;PRJNA827465;Yang2025_Silkie_Wencha	0.591870389
ENSGALG00010013376	ARHGAP8	3	-0.472899056	0.28057784	-1.685446921	0.091902377	0	1.796376238	0	1	CRA004775;PRJNA827465;Yang2025_Silkie_Wencha	0.591870389
ENSGALG00010028450	PTGDS	3	0.309737019	0.183569298	1.687302957	0.091545113	0	0.196178476	0	1	CRA004775;PRJNA827465;Yang2025_Silkie_Wencha	0.591870389
ENSGALG00010013679	MFSD4B	3	-0.294384501	0.174307732	-1.688878041	0.091242804	0.065595715	9.789186627	79.56929338	0.666666667	CRA004775;PRJNA827465;Yang2025_Silkie_Wencha	0.591870389
ENSGALG00010004542	ERMARD	3	-0.285333611	0.168847517	-1.689889299	0.091049135	0.054246041	5.560934741	64.0348234	1	CRA004775;PRJNA827465;Yang2025_Silkie_Wencha	0.591870389
ENSGALG00010024821	NECAP1	3	-0.270244917	0.160288235	-1.685993473	0.091797056	0.061682687	16.10864294	87.58430485	1	CRA004775;PRJNA827465;Yang2025_Silkie_Wencha	0.591870389
ENSGALG00010014125	PLPP1	3	-0.255532962	0.151341478	-1.688452929	0.091324317	0.042442907	6.007849832	66.71021986	0.666666667	CRA004775;PRJNA827465;Yang2025_Silkie_Wencha	0.591870389
ENSGALG00010010784	CCDC148	3	0.24859721	0.14743045	1.686199904	0.091757302	0	1.491348781	0	1	CRA004775;PRJNA827465;Yang2025_Silkie_Wencha	0.591870389
ENSGALG00010027539	DDR2L	3	-0.236024521	0.140072748	-1.685013854	0.091985899	0.034825145	5.802222741	65.53045119	0.666666667	CRA004775;PRJNA827465;Yang2025_Silkie_Wencha	0.591870389
ENSGALG00010020699	PLA2G10	3	0.217013307	0.128813292	1.684712063	0.092044138	0	0.139921594	0	1	CRA004775;PRJNA827465;Yang2025_Silkie_Wencha	0.591870389
ENSGALG00010024840	BTF3L4	3	-0.208795751	0.123585797	-1.689480158	0.091127451	0.036331078	12.05970503	83.41584645	1	CRA004775;PRJNA827465;Yang2025_Silkie_Wencha	0.591870389
ENSGALG00010014738	BRWD1	3	0.200901869	0.11925285	1.684671424	0.092051983	0.024275387	4.610607379	56.62176725	1	CRA004775;PRJNA827465;Yang2025_Silkie_Wencha	0.591870389
ENSGALG00010029487	CDC42EP4	3	-0.194380758	0.115076358	-1.689145898	0.091191474	0.015206714	3.112088834	35.73448232	1	CRA004775;PRJNA827465;Yang2025_Silkie_Wencha	0.591870389
ENSGALG00010024794	DDX6	3	-0.187135985	0.110695808	-1.690542651	0.090924186	0.024005693	7.548215967	73.5036728	0.666666667	CRA004775;PRJNA827465;Yang2025_Silkie_Wencha	0.591870389
ENSGALG00010022506	EBNA1BP2	3	0.179988463	0.106800404	1.685278861	0.091934782	0.018759131	4.43193172	54.87295098	1	CRA004775;PRJNA827465;Yang2025_Silkie_Wencha	0.591870389
ENSGALG00010007780	SIRT5	3	-0.165441559	0.09811546	-1.686192564	0.091758716	0.004782509	2.178271551	8.18408295	1	CRA004775;PRJNA827465;Yang2025_Silkie_Wencha	0.591870389
ENSGALG00010007542	MRPL36	3	-0.157358922	0.09312578	-1.689746088	0.091076542	0	0.970671666	0	1	CRA004775;PRJNA827465;Yang2025_Silkie_Wencha	0.591870389
ENSGALG00010024940	RABL3	3	-0.147124152	0.087325281	-1.684783043	0.092030438	0.010699808	3.725057993	46.30956072	1	CRA004775;PRJNA827465;Yang2025_Silkie_Wencha	0.591870389
ENSGALG00010018607	HAGHL	3	-0.142691996	0.084430225	-1.690058233	0.091016815	0	1.512845663	0	1	CRA004775;PRJNA827465;Yang2025_Silkie_Wencha	0.591870389
ENSGALG00010008995	ECD	3	0.140677031	0.08333199	1.688151574	0.091382136	0.010321741	4.060321319	50.7428146	1	CRA004775;PRJNA827465;Yang2025_Silkie_Wencha	0.591870389
ENSGALG00010025562	GRAMD1B	3	-0.138822262	0.082254387	-1.687718651	0.09146525	0	0.12970426	0	1	CRA004775;PRJNA827465;Yang2025_Silkie_Wencha	0.591870389
ENSGALG00010026999	MLEC	3	-0.135603551	0.080382733	-1.686973639	0.091608421	0.006357433	2.70589218	26.08722496	1	CRA004775;PRJNA827465;Yang2025_Silkie_Wencha	0.591870389
ENSGALG00010021550	MAP2K3	3	0.113028696	0.067084142	1.684879497	0.092011823	0	0.373807262	0	0.666666667	CRA004775;PRJNA827465;Yang2025_Silkie_Wencha	0.591870389
ENSGALG00010002628	TRIM7.2	3	-0.107454978	0.06369593	-1.68699913	0.091603519	0.003108241	2.544629735	21.40310345	1	CRA004775;PRJNA827465;Yang2025_Silkie_Wencha	0.591870389
ENSGALG00010022354	FAR2	3	-0.094764339	0.056179412	-1.686816149	0.091638709	0	0.160692557	0	1	CRA004775;PRJNA827465;Yang2025_Silkie_Wencha	0.591870389
ENSGALG00010025976	NAA25	3	0.079829738	0.047353253	1.685834303	0.091827719	0	0.101216717	0	1	CRA004775;PRJNA827465;Yang2025_Silkie_Wencha	0.591870389
ENSGALG00010027332	HDGF	3	-0.071618717	0.04246479	-1.686543524	0.091691159	0	1.3534396	0	0.666666667	CRA004775;PRJNA827465;Yang2025_Silkie_Wencha	0.591870389
ENSGALG00010004472	NEIL3	3	-0.226693842	0.134621891	-1.683930008	0.092195196	0	0.299895376	0	1	CRA004775;PRJNA827465;Yang2025_Silkie_Wencha	0.592521763
ENSGALG00010013097	ENSGALG0001001309	2	0.437697814	0.260023513	1.683300903	0.092316856	0.01177906	1.086531749	7.964033147	1	PRJNA827465;Yang2025_Silkie_Wenchang	0.593034086
ENSGALG00010022778	GDF11	3	0.248661108	0.147744989	1.683042577	0.092366849	0	0.013407839	0	1	CRA004775;PRJNA827465;Yang2025_Silkie_Wencha	0.593085778
ENSGALG00010004952	PEX1	3	0.112881155	0.067080921	1.682760943	0.092421379	0.003331012	2.595022906	22.92938935	1	CRA004775;PRJNA827465;Yang2025_Silkie_Wencha	0.593166534
ENSGALG00010027149	C1orf116	3	0.246001476	0.146305649	1.681421583	0.092681056	0	1.166339873	0	0.666666667	CRA004775;PRJNA827465;Yang2025_Silkie_Wencha	0.593217498
ENSGALG00010005293	BLZF1	3	-0.143530148	0.085335804	-1.681945226	0.092579462	0.007353546	2.952175317	32.25334592	1	CRA004775;PRJNA827465;Yang2025_Silkie_Wencha	0.593217498
ENSGALG00010010095	C12orf73	3	-0.143470422	0.085287855	-1.682190524	0.092531901	0.002406269	2.121494358	5.726829174	1	CRA004775;PRJNA827465;Yang2025_Silkie_Wencha	0.593217498
ENSGALG00010012219	KDM5A	3	-0.094776607	0.056366605	-1.6814319	0.092679054	0.003387647	2.695289549	25.7964696	1	CRA004775;PRJNA827465;Yang2025_Silkie_Wencha	0.593217498
ENSGALG00010017717	LLGL1	3	-0.088557187	0.052647163	-1.682088475	0.092551685	0	0.22512876	0	1	CRA004775;PRJNA827465;Yang2025_Silkie_Wencha	0.593217498
ENSGALG00010006532	KLHDC10	3	-0.087691564	0.052145428	-1.681673114	0.092632244	0.0021067	2.638257053	24.19237549	1	CRA004775;PRJNA827465;Yang2025_Silkie_Wencha	0.593217498
ENSGALG00010018713	ENSGALG0001001871	3	0.133888586	0.079647674	1.681010621	0.092760852	0	1.800912231	0	0.666666667	CRA004775;PRJNA827465;Yang2025_Silkie_Wencha	0.593459584
ENSGALG00010012959	TBC1D32	3	-0.133784774	0.079612275	-1.680454102	0.092868997	0	1.462531021	0	0.666666667	CRA004775;PRJNA827465;Yang2025_Silkie_Wencha	0.593882746
ENSGALG00010025381	MRPL37	3	-0.116731591	0.069499963	-1.679592136	0.093036699	0	0.520368428	0	0.666666667	CRA004775;PRJNA827465;Yang2025_Silkie_Wencha	0.594686204
ENSGALG00010022502	ENSGALG0001002250	3	0.95022865	0.566122335	1.678486419	0.09325218	0.664421792	6.663126469	69.98406064	1	CRA004775;PRJNA827465;Yang2025_Silkie_Wencha	0.595256238
ENSGALG00010005705	ECHDC3	3	-0.132950597	0.079192412	-1.678829995	0.093185181	0	1.762918379	0	1	CRA004775;PRJNA827465;Yang2025_Silkie_Wencha	0.595256238
ENSGALG00010028150	ARMC6	3	-0.093271203	0.055563777	-1.67863324	0.093223544	0	1.235827673	0	1	CRA004775;PRJNA827465;Yang2025_Silkie_Wencha	0.595256238
ENSGALG00010010356	STAB2	3	-0.315497999	0.188012508	-1.678069207	0.093333589	0.011847928	2.228286825	10.24494795	1	CRA004775;PRJNA827465;Yang2025_Silkie_Wencha	0.595384772
ENSGALG00010024762	FAN1	3	0.108451622	0.064633341	1.67795167	0.093356534	0	1.014836264	0	0.666666667	CRA004775;PRJNA827465;Yang2025_Silkie_Wencha	0.595384772
ENSGALG00010002883	MAP3K15	3	0.37162699	0.221671838	1.676473628	0.093645458	0.055036073	3.177979218	37.0669264	1	CRA004775;PRJNA827465;Yang2025_Silkie_Wencha	0.596689106
ENSGALG00010029675	DHRS13	3	0.246310833	0.146915578	1.67654674	0.093631149	0	1.33616084	0	1	CRA004775;PRJNA827465;Yang2025_Silkie_Wencha	0.596689106
ENSGALG00010019114	TENT5D	3	0.548754412	0.327390853	1.676144605	0.093709872	0.155851912	3.871975419	48.34677952	0.666666667	CRA004775;PRJNA827465;Yang2025_Silkie_Wencha	0.596830574
ENSGALG00010028139	FZR1	3	-0.148641944	0.088694797	-1.675881214	0.093761462	0.012091616	4.209719379	52.490895	1	CRA004775;PRJNA827465;Yang2025_Silkie_Wencha	0.596890281
ENSGALG00010024462	ZBTB32	3	-0.534835886	0.319562528	-1.673650192	0.094199368	0.204509647	7.370242899	72.86385229	1	CRA004775;PRJNA827465;Yang2025_Silkie_Wencha	0.598266301
ENSGALG00010019430	ENSGALG0001001943	3	-0.307259661	0.183626161	-1.673289137	0.09427039	0.034988092	3.054669236	34.52646276	1	CRA004775;PRJNA827465;Yang2025_Silkie_Wencha	0.598266301
ENSGALG00010026438	TOMM6	3	-0.279444793	0.166988902	-1.673433323	0.094242022	0.041463412	3.497417366	42.81494626	1	CRA004775;PRJNA827465;Yang2025_Silkie_Wencha	0.598266301
ENSGALG00010016702	WDR25	3	-0.272632741	0.162876137	-1.673865473	0.094157041	0.050983324	5.929775997	66.27191312	1	CRA004775;PRJNA827465;Yang2025_Silkie_Wencha	0.598266301
ENSGALG00010029445	SPHK1	3	0.234333793	0.140045267	1.673271785	0.094273804	0.024119034	3.25191743	38.49782343	0.666666667	CRA004775;PRJNA827465;Yang2025_Silkie_Wencha	0.598266301
ENSGALG00010024921	STRADB	3	-0.108639793	0.064893604	-1.674121741	0.094106676	0	1.440187438	0	0.666666667	CRA004775;PRJNA827465;Yang2025_Silkie_Wencha	0.598266301
ENSGALG00010014066	BCL2L1	3	0.108306957	0.064688056	1.674296053	0.09407243	0	0.963795057	0	0.666666667	CRA004775;PRJNA827465;Yang2025_Silkie_Wencha	0.598266301
ENSGALG00010022745	PAPSS2	3	0.395129671	0.236443985	1.671134371	0.09469514	0.102347975	5.240110419	61.83286534	1	CRA004775;PRJNA827465;Yang2025_Silkie_Wencha	0.598461398
ENSGALG00010020677	CYR61	3	0.245634316	0.147009082	1.670878511	0.094745677	0	0.250248142	0	1	CRA004775;PRJNA827465;Yang2025_Silkie_Wencha	0.598461398
ENSGALG00010029754	NLE1	3	-0.219633143	0.131400702	-1.671476172	0.094627661	0.009382921	2.348319399	14.83270969	1	CRA004775;PRJNA827465;Yang2025_Silkie_Wencha	0.598461398
ENSGALG00010015887	SLC9A9	3	-0.170754347	0.10210267	-1.672378872	0.094449636	0	1.855193994	0	1	CRA004775;PRJNA827465;Yang2025_Silkie_Wencha	0.598461398
ENSGALG00010017383	ENSGALG0001001738	3	0.146592104	0.087673157	1.672029502	0.094518505	0.003644226	2.317627704	13.70486311	0.666666667	CRA004775;PRJNA827465;Yang2025_Silkie_Wencha	0.598461398
ENSGALG00010013378	SH3RF3	3	-0.132497046	0.079282467	-1.671202362	0.094681714	0	1.441934928	0	0.666666667	CRA004775;PRJNA827465;Yang2025_Silkie_Wencha	0.598461398
ENSGALG00010012624	PDGFB	3	0.124988448	0.074771308	1.671609756	0.0946013	0	0.515438415	0	1	CRA004775;PRJNA827465;Yang2025_Silkie_Wencha	0.598461398
ENSGALG00010029266	CANT1	3	0.106274261	0.063555042	1.672160972	0.094492584	0	0.613282621	0	0.666666667	CRA004775;PRJNA827465;Yang2025_Silkie_Wencha	0.598461398
ENSGALG00010004568	RSU1	3	-0.091885488	0.054996397	-1.670754676	0.094770144	0	1.340461513	0	0.666666667	CRA004775;PRJNA827465;Yang2025_Silkie_Wencha	0.598461398
ENSGALG00010027366	USP4	3	-0.068580068	0.041010842	-1.672242364	0.09447654	0	0.939723332	0	1	CRA004775;PRJNA827465;Yang2025_Silkie_Wencha	0.598461398
ENSGALG00010018529	UBR2	3	0.061428978	0.036738484	1.672060765	0.09451234	0	1.193005063	0	1	CRA004775;PRJNA827465;Yang2025_Silkie_Wencha	0.598461398
ENSGALG00010002366	ENSGALG0001000236	3	0.977045965	0.585129513	1.669794368	0.094960055	0.966593208	36.96489573	94.58946127	1	CRA004775;PRJNA827465;Yang2025_Silkie_Wencha	0.599193562
ENSGALG00010019093	RPL32	3	-0.134114731	0.080320734	-1.669739855	0.094970845	0	0.215864836	0	1	CRA004775;PRJNA827465;Yang2025_Silkie_Wencha	0.599193562
ENSGALG00010011525	ENSGALG0001001152	3	0.274372476	0.164369347	1.66924357	0.095069119	0	0.649560191	0	1	CRA004775;PRJNA827465;Yang2025_Silkie_Wencha	0.599278764
ENSGALG00010012555	ENSGALG0001001255	3	0.183376313	0.109847154	1.669377005	0.095042688	0	0.153896293	0	1	CRA004775;PRJNA827465;Yang2025_Silkie_Wencha	0.599278764
ENSGALG00010012964	CBR3	3	-0.221594384	0.13281327	-1.66846569	0.095223317	0.030972376	4.876865015	58.99004804	1	CRA004775;PRJNA827465;Yang2025_Silkie_Wencha	0.599493285
ENSGALG00010004424	MRPL18	3	0.124039481	0.074330781	1.668749876	0.09516696	0	0.942391294	0	0.666666667	CRA004775;PRJNA827465;Yang2025_Silkie_Wencha	0.599493285
ENSGALG00010025635	CHKA	3	0.117684584	0.070536113	1.668430237	0.09523035	0	0.742182748	0	1	CRA004775;PRJNA827465;Yang2025_Silkie_Wencha	0.599493285
ENSGALG00010020179	PSMD6	3	-0.067947267	0.040757259	-1.667120605	0.095490426	0	0.804449664	0	1	CRA004775;PRJNA827465;Yang2025_Silkie_Wencha	0.600862986
ENSGALG00010001609	ENSGALG0001000160	3	-0.579434884	0.347692814	-1.66651383	0.095611116	0	0.343730408	0	1	CRA004775;PRJNA827465;Yang2025_Silkie_Wencha	0.601115063
ENSGALG00010017576	ENSGALG0001001757	3	-0.294275274	0.1765837	-1.666491722	0.095615516	0.03784996	3.28158235	39.05379214	1	CRA004775;PRJNA827465;Yang2025_Silkie_Wencha	0.601115063
ENSGALG00010021667	TBX15	3	-0.124755264	0.074908134	-1.66544349	0.095824309	0	0.672444885	0	0.666666667	CRA004775;PRJNA827465;Yang2025_Silkie_Wencha	0.602159957
ENSGALG00010000808	ADCY2	3	0.170497701	0.102399246	1.665028874	0.095906995	0.002123872	2.119755008	5.649474004	1	CRA004775;PRJNA827465;Yang2025_Silkie_Wencha	0.60241182
ENSGALG00010015112	CBLN4	2	0.638142479	0.383519498	1.663911434	0.09613013	0.092653019	1.400434794	28.59360504	1	CRA004775;PRJNA827465	0.603009718
ENSGALG00010017613	HAUS3	3	-0.423587882	0.254541103	-1.664123701	0.096087712	0.140027856	14.11040517	85.82606257	1	CRA004775;PRJNA827465;Yang2025_Silkie_Wencha	0.603009718
ENSGALG00010023335	WDR59	3	-0.19933425	0.119771533	-1.664287364	0.096055016	0.033587819	10.22625963	80.4425071	1	CRA004775;PRJNA827465;Yang2025_Silkie_Wencha	0.603009718
ENSGALG00010021036	E2F8	3	-0.13892362	0.083513662	-1.663483757	0.09621564	0	1.872132287	0	0.666666667	CRA004775;PRJNA827465;Yang2025_Silkie_Wencha	0.603100167
ENSGALG00010028121	TUSC2	3	-0.107313209	0.064513882	-1.663412668	0.096229859	0.000683585	2.059163372	2.873175243	1	CRA004775;PRJNA827465;Yang2025_Silkie_Wencha	0.603100167
ENSGALG00010001281	LRRN3	3	0.296111842	0.178062326	1.662967395	0.096318962	0.056742232	5.262366076	61.99428221	1	CRA004775;PRJNA827465;Yang2025_Silkie_Wencha	0.603265909
ENSGALG00010013085	LYSMD3	3	0.160340943	0.09642514	1.66285413	0.096341638	0	0.124200559	0	1	CRA004775;PRJNA827465;Yang2025_Silkie_Wencha	0.603265909
ENSGALG00010012891	CAPS2	2	0.363864333	0.218856187	1.662572756	0.096397988	0	0.688907495	0	1	PRJNA827465;Yang2025_Silkie_Wenchang	0.603349392
ENSGALG00010023054	AQP9	3	0.263799627	0.158689688	1.662361488	0.096440316	0	1.45935887	0	0.666666667	CRA004775;PRJNA827465;Yang2025_Silkie_Wencha	0.603349392
ENSGALG00010000655	ENSGALG0001000065	2	0.758509368	0.456967515	1.659875909	0.096939419	0	0.27294384	0	1	PRJNA827465;Yang2025_Silkie_Wenchang	0.603786949
ENSGALG00010010107	MGST2	3	0.492418568	0.296370954	1.661494018	0.096614269	0.142813819	4.379491301	54.33259567	0.666666667	CRA004775;PRJNA827465;Yang2025_Silkie_Wencha	0.603786949
ENSGALG00010012809	RHOBTB3	3	0.422828705	0.254848001	1.659140756	0.097087432	0.122755987	5.814161239	65.60122917	1	CRA004775;PRJNA827465;Yang2025_Silkie_Wencha	0.603786949
ENSGALG00010027917	PPARG	3	-0.398841712	0.240223655	-1.660293242	0.096855474	0.08238991	3.905122192	48.78521333	0.666666667	CRA004775;PRJNA827465;Yang2025_Silkie_Wencha	0.603786949
ENSGALG00010011262	ENSGALG0001001126	3	-0.278164051	0.16766098	-1.659086396	0.097098384	0	0.463509878	0	1	CRA004775;PRJNA827465;Yang2025_Silkie_Wencha	0.603786949
ENSGALG00010011360	TCF4	3	0.263901732	0.158810403	1.66174084	0.096564749	0.01389608	2.433317005	17.80766764	1	CRA004775;PRJNA827465;Yang2025_Silkie_Wencha	0.603786949
ENSGALG00010021029	PRDM2	3	0.232867372	0.140211094	1.660834145	0.096746761	0.035716695	4.950832942	59.60275729	1	CRA004775;PRJNA827465;Yang2025_Silkie_Wencha	0.603786949
ENSGALG00010017580	LYPLAL1	3	-0.206046662	0.124177992	-1.659284862	0.097058404	0.017884574	3.260347943	38.65685395	1	CRA004775;PRJNA827465;Yang2025_Silkie_Wencha	0.603786949
ENSGALG00010016258	CTNNB1	3	-0.176267606	0.106203969	-1.65970828	0.096973153	0.020410636	5.955086607	66.41526594	1	CRA004775;PRJNA827465;Yang2025_Silkie_Wencha	0.603786949
ENSGALG00010015260	RASGEF1C	3	-0.155460105	0.093671304	-1.659634268	0.09698805	0	0.903994778	0	0.666666667	CRA004775;PRJNA827465;Yang2025_Silkie_Wencha	0.603786949
ENSGALG00010016732	ARHGAP26	3	0.142217644	0.085673005	1.660005319	0.096913383	0.007569564	2.678115286	25.32061593	0.666666667	CRA004775;PRJNA827465;Yang2025_Silkie_Wencha	0.603786949
ENSGALG00010009715	NUDCD2	3	-0.121320404	0.073126953	-1.659038141	0.097108107	0.006561768	3.304517178	39.4767861	0.666666667	CRA004775;PRJNA827465;Yang2025_Silkie_Wencha	0.603786949
ENSGALG00010019977	ZMIZ1	3	-0.113899526	0.06863831	-1.659416241	0.097031946	0	1.09561142	0	0.666666667	CRA004775;PRJNA827465;Yang2025_Silkie_Wencha	0.603786949
ENSGALG00010024850	CDK2AP1	3	-0.106821947	0.064348346	-1.660057383	0.096902909	0	0.3090308	0	1	CRA004775;PRJNA827465;Yang2025_Silkie_Wencha	0.603786949
ENSGALG00010002765	MAP7D2	3	0.567231335	0.34197001	1.65871661	0.097172911	0.02643871	2.146317637	6.817147414	1	CRA004775;PRJNA827465;Yang2025_Silkie_Wencha	0.603924301
ENSGALG00010028881	SARDH	3	-0.198874856	0.119950531	-1.657973955	0.097322724	0.015327514	3.078521305	35.03374504	1	CRA004775;PRJNA827465;Yang2025_Silkie_Wencha	0.604324105
ENSGALG00010029148	RALGDS	3	-0.18462627	0.111354296	-1.65800761	0.09731593	0.019588903	4.867827722	58.91391162	1	CRA004775;PRJNA827465;Yang2025_Silkie_Wencha	0.604324105
ENSGALG00010016436	SLC30A2	2	-0.748131234	0.452369911	-1.653804146	0.098167313	0	0.133541874	0	1	CRA004775;Yang2025_Silkie_Wenchang	0.604390365
ENSGALG00010005256	OPRM1	2	0.599491343	0.362198612	1.655145335	0.097895021	0	0.020792714	0	1	PRJNA827465;Yang2025_Silkie_Wenchang	0.604390365
ENSGALG00010017111	GJB1	3	0.437036531	0.264228209	1.654011634	0.098125149	0.094986991	3.623026555	44.79753406	1	CRA004775;PRJNA827465;Yang2025_Silkie_Wencha	0.604390365
ENSGALG00010025126	ENSGALG0001002512	3	0.381195385	0.230445777	1.654165205	0.098093951	0.128282893	11.22286772	82.17924287	1	CRA004775;PRJNA827465;Yang2025_Silkie_Wencha	0.604390365
ENSGALG00010019237	SORCS3	3	-0.291166866	0.175845361	-1.655812038	0.097759889	0	1.472312395	0	1	CRA004775;PRJNA827465;Yang2025_Silkie_Wencha	0.604390365
ENSGALG00010024183	MTX2	3	-0.288752811	0.174434593	-1.655364377	0.097850608	0.054447044	4.856586614	58.81881332	1	CRA004775;PRJNA827465;Yang2025_Silkie_Wencha	0.604390365
ENSGALG00010017904	MEGF11	2	-0.258003864	0.155964725	-1.654244985	0.098077746	0	0.008509985	0	1	PRJNA827465;Yang2025_Silkie_Wenchang	0.604390365
ENSGALG00010015204	ENSGALG0001001520	3	0.229404778	0.13851187	1.656210242	0.09767925	0.027689057	3.853294923	48.09636844	0.666666667	CRA004775;PRJNA827465;Yang2025_Silkie_Wencha	0.604390365
ENSGALG00010025398	TSPAN2	3	0.181199504	0.10946365	1.655339499	0.097855651	0	1.700090289	0	1	CRA004775;PRJNA827465;Yang2025_Silkie_Wencha	0.604390365
ENSGALG00010015227	FYTTD1	3	-0.168852412	0.102088661	-1.65397812	0.098131958	0.022909658	14.31844113	86.031999	1	CRA004775;PRJNA827465;Yang2025_Silkie_Wencha	0.604390365
ENSGALG00010003560	ADGRL3	3	-0.127582952	0.076992808	-1.657076232	0.097504064	0	0.890904181	0	0.666666667	CRA004775;PRJNA827465;Yang2025_Silkie_Wencha	0.604390365
ENSGALG00010013277	YRDC	3	-0.126494706	0.076418489	-1.655289274	0.097865834	0	0.456681956	0	0.666666667	CRA004775;PRJNA827465;Yang2025_Silkie_Wencha	0.604390365
ENSGALG00010021778	RBL2	3	-0.118350011	0.071536421	-1.654402176	0.098045825	0.007646736	4.089446917	51.09363098	0.666666667	CRA004775;PRJNA827465;Yang2025_Silkie_Wencha	0.604390365
ENSGALG00010015071	SNAI1	3	0.110828504	0.066964569	1.655031998	0.097918007	0	1.407659249	0	0.666666667	CRA004775;PRJNA827465;Yang2025_Silkie_Wencha	0.604390365
ENSGALG00010028262	C1orf43	3	-0.10463914	0.063251207	-1.654342179	0.098058008	0	0.440883259	0	1	CRA004775;PRJNA827465;Yang2025_Silkie_Wencha	0.604390365
ENSGALG00010029595	CLUH	3	-0.089785425	0.054229224	-1.655664937	0.097789692	0	0.383033115	0	1	CRA004775;PRJNA827465;Yang2025_Silkie_Wencha	0.604390365
ENSGALG00010000717	NDFIP2	3	-0.08439562	0.050953836	-1.656315329	0.097657978	0	1.35086383	0	0.666666667	CRA004775;PRJNA827465;Yang2025_Silkie_Wencha	0.604390365
ENSGALG00010021722	AARS1	3	-0.081285316	0.049038955	-1.657566233	0.09740505	0	0.182441874	0	1	CRA004775;PRJNA827465;Yang2025_Silkie_Wencha	0.604390365
ENSGALG00010012848	SERINC1	3	-0.068528304	0.041439361	-1.653700796	0.098188321	0	0.494574171	0	1	CRA004775;PRJNA827465;Yang2025_Silkie_Wencha	0.604390365
ENSGALG00010028050	PBRM1	3	-0.066118285	0.039947028	-1.655149032	0.097894271	0	0.402370964	0	0.666666667	CRA004775;PRJNA827465;Yang2025_Silkie_Wencha	0.604390365
ENSGALG00010001471	BMP5	3	0.369062782	0.223316473	1.652644684	0.098403198	0.057399621	3.147854188	36.46465558	0.666666667	CRA004775;PRJNA827465;Yang2025_Silkie_Wencha	0.605254051
ENSGALG00010014194	LRIG3	3	0.101114848	0.061185668	1.652590421	0.098414249	0	1.465051109	0	1	CRA004775;PRJNA827465;Yang2025_Silkie_Wencha	0.605254051
ENSGALG00010019001	ALDH1A3	3	-0.314578322	0.190388866	-1.652293689	0.098474695	0.073433908	7.43793684	73.11082303	1	CRA004775;PRJNA827465;Yang2025_Silkie_Wencha	0.605280145
ENSGALG00010027274	COPG1	3	-0.124743279	0.075503634	-1.652149343	0.09850411	0.010083958	4.783703397	58.19138784	1	CRA004775;PRJNA827465;Yang2025_Silkie_Wencha	0.605280145
ENSGALG00010030109	FBF1	3	0.218387605	0.132252761	1.65128957	0.098679461	0.036941312	8.79796564	77.26747203	0.666666667	CRA004775;PRJNA827465;Yang2025_Silkie_Wencha	0.605831046
ENSGALG00010005394	PHYH	3	-0.127569497	0.077248243	-1.651422626	0.098652308	0	0.838307789	0	1	CRA004775;PRJNA827465;Yang2025_Silkie_Wencha	0.605831046
ENSGALG00010001552	TUBGCP6	3	-0.124262228	0.075313827	-1.649925823	0.098958108	0.006471031	3.175963281	37.02697975	0.666666667	CRA004775;PRJNA827465;Yang2025_Silkie_Wencha	0.60727808
ENSGALG00010022754	SNX20	3	-0.355259853	0.215369094	-1.649539618	0.099037134	0.070012626	4.2526989	52.97104152	1	CRA004775;PRJNA827465;Yang2025_Silkie_Wencha	0.607499364
ENSGALG00010015905	ANPEP	3	0.156712986	0.095038867	1.648935744	0.099160799	0	0.987072851	0	1	CRA004775;PRJNA827465;Yang2025_Silkie_Wencha	0.607994163
ENSGALG00010023202	PID1	3	-0.169683057	0.10292738	-1.648570655	0.099235625	0.017836109	5.199027868	61.53126986	1	CRA004775;PRJNA827465;Yang2025_Silkie_Wencha	0.608189206
ENSGALG00010029419	RFC2	3	-0.101795542	0.061757296	-1.648316064	0.09928783	0	1.030085984	0	1	CRA004775;PRJNA827465;Yang2025_Silkie_Wencha	0.608245506
ENSGALG00010022476	OIP5	3	-0.426683727	0.258971255	-1.647610374	0.09943265	0.172109093	15.32231054	86.94713832	1	CRA004775;PRJNA827465;Yang2025_Silkie_Wencha	0.608341948
ENSGALG00010003543	SOX11	3	0.300482132	0.182350578	1.64782659	0.099388261	0.071975613	11.6396423	82.81734139	1	CRA004775;PRJNA827465;Yang2025_Silkie_Wencha	0.608341948
ENSGALG00010012535	JMY	3	0.13209962	0.080172862	1.647684962	0.099417335	0	0.67967796	0	1	CRA004775;PRJNA827465;Yang2025_Silkie_Wencha	0.608341948
ENSGALG00010028073	ST6GALNAC4	3	-0.166361342	0.101012156	-1.64694378	0.099569602	0	1.434414893	0	1	CRA004775;PRJNA827465;Yang2025_Silkie_Wencha	0.608916351
ENSGALG00010021258	LSM3	3	-0.477070748	0.289839927	-1.645980087	0.099767859	0.211143529	15.3568954	86.97653434	1	CRA004775;PRJNA827465;Yang2025_Silkie_Wencha	0.609075025
ENSGALG00010007624	TPPP	3	0.320604574	0.194779576	1.64598661	0.099766516	0.053521815	3.649337692	45.19553494	1	CRA004775;PRJNA827465;Yang2025_Silkie_Wencha	0.609075025
ENSGALG00010023172	DHX58	3	-0.296133925	0.179849055	-1.646569259	0.099646613	0.033762429	3.011077271	33.57858933	1	CRA004775;PRJNA827465;Yang2025_Silkie_Wencha	0.609075025
ENSGALG00010009198	KCNN2	3	0.137360982	0.083439041	1.646243536	0.099713629	0	1.491259074	0	0.666666667	CRA004775;PRJNA827465;Yang2025_Silkie_Wencha	0.609075025
ENSGALG00010006239	EYA4	3	0.332222772	0.201890682	1.645557726	0.099854849	0.078791865	6.215159672	67.82061756	1	CRA004775;PRJNA827465;Yang2025_Silkie_Wencha	0.60934236
ENSGALG00010021746	MTOR	3	-0.095003073	0.057747712	-1.645140023	0.099940939	0	0.465588135	0	0.666666667	CRA004775;PRJNA827465;Yang2025_Silkie_Wencha	0.60934236
ENSGALG00010027946	AP1M1	3	-0.079650068	0.048410126	-1.645318338	0.09990418	0	1.332512854	0	1	CRA004775;PRJNA827465;Yang2025_Silkie_Wencha	0.60934236
ENSGALG00010001146	DIPK1C	3	-0.247231776	0.15031485	-1.64475949	0.100019419	0.03227202	4.063331779	50.77930849	1	CRA004775;PRJNA827465;Yang2025_Silkie_Wencha	0.609558003
ENSGALG00010011625	ERBB4	3	-0.18652286	0.113421696	-1.644507764	0.100071362	0	1.369181792	0	0.666666667	CRA004775;PRJNA827465;Yang2025_Silkie_Wencha	0.609611799
ENSGALG00010027847	NRTN	3	0.742469768	0.451962857	1.642767222	0.100431104	0.020761469	2.059201754	2.874985599	0.666666667	CRA004775;PRJNA827465;Yang2025_Silkie_Wencha	0.611045074
ENSGALG00010007863	AvBD1	3	0.497853889	0.30306271	1.642742153	0.100436293	0	1.411501108	0	1	CRA004775;PRJNA827465;Yang2025_Silkie_Wencha	0.611045074
ENSGALG00010005219	NALCN	3	-0.223829935	0.13623171	-1.643009071	0.100381057	0	1.625855216	0	0.666666667	CRA004775;PRJNA827465;Yang2025_Silkie_Wencha	0.611045074
ENSGALG00010013565	ENSGALG0001001356	2	-3.272102192	1.992857156	-1.641915068	0.100607607	0.956871226	1.13194181	11.65623614	1	PRJNA827465;Yang2025_Silkie_Wenchang	0.611088208
ENSGALG00010026220	ENSGALG0001002622	3	-0.269720581	0.164215397	-1.64248046	0.100490472	0.055937967	7.506141606	73.35515229	1	CRA004775;PRJNA827465;Yang2025_Silkie_Wencha	0.611088208
ENSGALG00010028088	NAXE	3	-0.153384448	0.093420392	-1.641873304	0.100616263	0	0.624160756	0	0.666666667	CRA004775;PRJNA827465;Yang2025_Silkie_Wencha	0.611088208
ENSGALG00010027324	PIK3R2	3	-0.092006923	0.056032507	-1.642027579	0.100584289	0.003157356	2.968521859	32.62640147	1	CRA004775;PRJNA827465;Yang2025_Silkie_Wencha	0.611088208
ENSGALG00010021153	PTPRF	3	0.279144797	0.170094954	1.641111573	0.100774257	0.051782756	5.406156415	63.00513995	1	CRA004775;PRJNA827465;Yang2025_Silkie_Wencha	0.611784979
ENSGALG00010001134	ENSGALG0001000113	3	0.512494897	0.312607057	1.639422033	0.101125396	0.068055701	2.54387934	21.37991891	1	CRA004775;PRJNA827465;Yang2025_Silkie_Wencha	0.611813162
ENSGALG00010015072	MAP9	3	0.351857107	0.214562264	1.639883451	0.101029402	0.087008948	5.836875675	65.73509337	1	CRA004775;PRJNA827465;Yang2025_Silkie_Wencha	0.611813162
ENSGALG00010018724	RRBP1	3	0.253859328	0.154903515	1.638822263	0.101250281	0.042557489	5.44598039	63.27566652	0.666666667	CRA004775;PRJNA827465;Yang2025_Silkie_Wencha	0.611813162
ENSGALG00010012415	BID	3	-0.241565994	0.14739005	-1.638957273	0.101222158	0.050007556	10.56346063	81.06681067	1	CRA004775;PRJNA827465;Yang2025_Silkie_Wencha	0.611813162
ENSGALG00010001461	ASB9	3	0.239014208	0.145865557	1.638592502	0.101298155	0	1.977743292	0	1	CRA004775;PRJNA827465;Yang2025_Silkie_Wencha	0.611813162
ENSGALG00010011211	SLC37A1	3	-0.18781022	0.114579635	-1.639123921	0.101187454	0.014246824	3.07627364	34.98627776	1	CRA004775;PRJNA827465;Yang2025_Silkie_Wencha	0.611813162
ENSGALG00010027972	TMEM259	3	-0.175649186	0.107186025	-1.638732153	0.101269055	0.021579562	6.291978574	68.21349634	1	CRA004775;PRJNA827465;Yang2025_Silkie_Wencha	0.611813162
ENSGALG00010009253	SLC45A4	3	-0.15022161	0.091582694	-1.640283817	0.100946169	0	1.804069052	0	1	CRA004775;PRJNA827465;Yang2025_Silkie_Wencha	0.611813162
ENSGALG00010023783	VPS35	3	-0.130639964	0.079667131	-1.639822623	0.101042053	0.013556823	11.16219717	82.08238066	1	CRA004775;PRJNA827465;Yang2025_Silkie_Wencha	0.611813162
ENSGALG00010027513	SEMA3G	3	0.130198865	0.079400502	1.639773838	0.1010522	0.001538871	2.118176268	5.579151748	1	CRA004775;PRJNA827465;Yang2025_Silkie_Wencha	0.611813162
ENSGALG00010015472	LGMN	3	-0.103805107	0.063325215	-1.639238125	0.101163677	0.001545281	2.22379638	10.06370828	0.666666667	CRA004775;PRJNA827465;Yang2025_Silkie_Wencha	0.611813162
ENSGALG00010011025	POLR3B	3	-0.067512061	0.041163405	-1.640099056	0.100984573	0	1.356686421	0	1	CRA004775;PRJNA827465;Yang2025_Silkie_Wencha	0.611813162
ENSGALG00010008211	ADGRB1	3	-0.172748409	0.105461441	-1.638024362	0.101416612	0	0.943099867	0	0.666666667	CRA004775;PRJNA827465;Yang2025_Silkie_Wencha	0.612228078
ENSGALG00010011340	PKNOX1	3	0.109560255	0.066892819	1.637847768	0.101453454	0.000863491	2.067679411	3.273206231	1	CRA004775;PRJNA827465;Yang2025_Silkie_Wencha	0.612228078
ENSGALG00010007548	HSBP1	3	-0.196630085	0.120072415	-1.63759583	0.101506034	0.029729599	7.788760311	74.32197269	1	CRA004775;PRJNA827465;Yang2025_Silkie_Wencha	0.612284048
ENSGALG00010009654	NDUFA12	3	-0.105623227	0.064507786	-1.637371768	0.101552813	0	0.463221533	0	0.666666667	CRA004775;PRJNA827465;Yang2025_Silkie_Wencha	0.612305002
ENSGALG00010004534	CUBN	3	-0.222796725	0.136120422	-1.636761936	0.101680222	0	1.652064815	0	0.666666667	CRA004775;PRJNA827465;Yang2025_Silkie_Wencha	0.612811875
ENSGALG00010019399	CD93	3	0.113018733	0.069083298	1.635977671	0.101844261	0	1.352895225	0	0.666666667	CRA004775;PRJNA827465;Yang2025_Silkie_Wencha	0.613538987
ENSGALG00010029512	TSPAN10	3	2.279017218	1.395791512	1.632777673	0.102515764	5.166005526	18.02595417	88.90488691	0.666666667	CRA004775;PRJNA827465;Yang2025_Silkie_Wencha	0.613680652
ENSGALG00010014764	ENSGALG0001001476	2	1.922944746	1.17636102	1.634655274	0.102121333	1.185398105	1.609415623	37.86564605	1	CRA004775;PRJNA827465	0.613680652
ENSGALG00010004375	ENSGALG0001000437	3	1.041554806	0.638766099	1.630573081	0.102980433	0.96882454	13.56267926	85.25365113	1	CRA004775;PRJNA827465;Yang2025_Silkie_Wencha	0.613680652
ENSGALG00010010195	ENSGALG0001001019	3	1.039178341	0.636008231	1.633907064	0.102278366	1.024005118	27.22867051	92.65480112	0.666666667	CRA004775;PRJNA827465;Yang2025_Silkie_Wencha	0.613680652

ENSGALG00010005520	ZIC2	2	-0.958834982	0.587719879	-1.631448953	0.102795623	0	0.273101211	0	1	CRA004775;PRJNA827465	0.613680652
ENSGALG00010016981	ENSGALG00010016981	3	0.875225268	0.53683344	1.630347892	0.103027991	0.394681152	3.686296805	45.74500899	1	CRA004775;PRJNA827465;Yang2025_Silkie_Wencha	0.613680652
ENSGALG00010012430	F13B	2	0.848400047	0.520131347	1.631126544	0.102863621	0	0.766198089	0	1	CRA004775;PRJNA827465	0.613680652
ENSGALG00010019390	TVP23A	3	0.424722014	0.259787355	1.634883324	0.102073509	0.047957679	2.606771431	23.27674087	1	CRA004775;PRJNA827465;Yang2025_Silkie_Wencha	0.613680652
ENSGALG00010022100	ENTPD1	3	-0.393562024	0.241164694	-1.631922225	0.102695871	0.133029177	11.81418303	83.07119507	1	CRA004775;PRJNA827465;Yang2025_Silkie_Wencha	0.613680652
ENSGALG00010024495	CLDN5	3	0.349902743	0.214390662	1.632080145	0.102662604	0.090311565	6.793222261	70.55889056	1	CRA004775;PRJNA827465;Yang2025_Silkie_Wencha	0.613680652
ENSGALG00010006715	IRF8	3	-0.32824869	0.201138627	-1.631952524	0.102689488	0.088024369	8.420284219	76.24783264	1	CRA004775;PRJNA827465;Yang2025_Silkie_Wencha	0.613680652
ENSGALG00010027654	AIF1L	3	-0.295188089	0.180920304	-1.631591822	0.102765502	0.076806305	10.36769213	80.70930372	1	CRA004775;PRJNA827465;Yang2025_Silkie_Wencha	0.613680652
ENSGALG00010014512	HMGN4	3	-0.281260762	0.172294369	-1.632443143	0.102586167	0.069351924	12.0163969	83.35607573	1	CRA004775;PRJNA827465;Yang2025_Silkie_Wencha	0.613680652
ENSGALG00010020324	PLEKHH2	3	0.259355178	0.159105096	1.630087186	0.103083072	0.038896535	4.142989199	51.7256767	1	CRA004775;PRJNA827465;Yang2025_Silkie_Wencha	0.613680652
ENSGALG00010028337	ENSGALG00010028337	3	-0.255286181	0.156335027	-1.632952952	0.102480995	0	0.588077324	0	1	CRA004775;PRJNA827465;Yang2025_Silkie_Wencha	0.613680652
ENSGALG00010010457	AGGF1	3	0.24386007	0.149546585	1.630662918	0.102961465	0.040494707	5.062190294	60.49141016	1	CRA004775;PRJNA827465;Yang2025_Silkie_Wencha	0.613680652
ENSGALG00010011463	IMP3	3	0.16142081	0.098732707	1.634927426	0.102064262	0	1.695654327	0	0.666666667	CRA004775;PRJNA827465;Yang2025_Silkie_Wencha	0.613680652
ENSGALG00010005167	GUCY1A2	3	-0.159034525	0.097518799	-1.630808909	0.102930647	0	0.801382308	0	0.666666667	CRA004775;PRJNA827465;Yang2025_Silkie_Wencha	0.613680652
ENSGALG00010027146	KXD1	3	-0.157282932	0.096466423	-1.630442256	0.10300806	0.007447674	2.533230378	21.04942299	1	CRA004775;PRJNA827465;Yang2025_Silkie_Wencha	0.613680652
ENSGALG00010025130	ATP2A2	3	0.145780768	0.089348864	1.631590606	0.102765759	0	1.178429873	0	0.666666667	CRA004775;PRJNA827465;Yang2025_Silkie_Wencha	0.613680652
ENSGALG00010023665	C16orf87	3	-0.140983978	0.086401029	-1.631739564	0.102734362	0	0.001641374	0	1	CRA004775;PRJNA827465;Yang2025_Silkie_Wencha	0.613680652
ENSGALG00010004928	HDDC2	3	0.123951081	0.076034532	1.630194567	0.103060382	0	0.529328615	0	1	CRA004775;PRJNA827465;Yang2025_Silkie_Wencha	0.613680652
ENSGALG00010023836	NDUFS3	3	-0.113453273	0.069568003	-1.630825501	0.102927145	0	0.99741176	0	1	CRA004775;PRJNA827465;Yang2025_Silkie_Wencha	0.613680652
ENSGALG00010011133	CDC37L1	3	-0.103806882	0.063488134	-1.635059604	0.102036553	0	1.959562466	0	0.666666667	CRA004775;PRJNA827465;Yang2025_Silkie_Wencha	0.613680652
ENSGALG00010015146	ENSGALG00010015146	3	-0.100403957	0.061494605	-1.632727896	0.102526238	0	0.67652083	0	0.666666667	CRA004775;PRJNA827465;Yang2025_Silkie_Wencha	0.613680652
ENSGALG00010024616	ARVCF	3	-0.099137917	0.060739801	-1.632173886	0.10264286	0	1.121047654	0	0.666666667	CRA004775;PRJNA827465;Yang2025_Silkie_Wencha	0.613680652
ENSGALG00010024657	DPY19L3	3	0.089804596	0.054930033	1.634890621	0.102071979	0	0.347331115	0	1	CRA004775;PRJNA827465;Yang2025_Silkie_Wencha	0.613680652
ENSGALG00010019087	PEX13	3	-0.078749882	0.048291094	-1.630733028	0.102946664	0.000432165	2.096543546	4.604891042	0.666666667	CRA004775;PRJNA827465;Yang2025_Silkie_Wencha	0.613680652
ENSGALG00010013574	NR2F1	3	0.516970792	0.317194475	1.629822813	0.103138951	0.2499624	15.35205257	86.97242606	1	CRA004775;PRJNA827465;Yang2025_Silkie_Wencha	0.613754892
ENSGALG00010007698	ADAMTS18	3	-0.469234616	0.287970869	-1.62945168	0.103217436	0.136513265	5.085443554	60.67206373	1	CRA004775;PRJNA827465;Yang2025_Silkie_Wencha	0.61385001
ENSGALG00010005436	AQR	3	-0.180501773	0.11079675	-1.629125163	0.103286525	0.022770995	6.034238589	66.85580176	1	CRA004775;PRJNA827465;Yang2025_Silkie_Wencha	0.61385001
ENSGALG00010004717	ALKBH8	3	-0.164066071	0.100698284	-1.629283682	0.103252979	0	0.162249462	0	1	CRA004775;PRJNA827465;Yang2025_Silkie_Wencha	0.61385001
ENSGALG00010021470	ENSGALG00010021470	2	0.542111499	0.333457449	1.625729162	0.104007284	0.010129615	1.04330928	4.151144883	1	CRA004775;PRJNA827465	0.61562049
ENSGALG00010023162	CHRNA4	3	-0.513442524	0.315751251	-1.626098145	0.103928779	0.183780409	6.073751426	67.0714216	1	CRA004775;PRJNA827465;Yang2025_Silkie_Wencha	0.61562049
ENSGALG00010000811	ENSGALG00010000811	3	-0.477177099	0.293439559	-1.626151203	0.103917494	0	0.753164211	0	1	CRA004775;PRJNA827465;Yang2025_Silkie_Wencha	0.61562049
ENSGALG00010024788	SIK3	3	0.357219155	0.219846274	1.624858809	0.104192647	0.104309142	7.781839895	74.29913713	1	CRA004775;PRJNA827465;Yang2025_Silkie_Wencha	0.61562049
ENSGALG00010021647	ARHGAP11B	3	-0.241482105	0.148579118	-1.625276203	0.10410372	0.03798878	5.640364425	64.54129823	0.666666667	CRA004775;PRJNA827465;Yang2025_Silkie_Wencha	0.61562049
ENSGALG00010023438	HOXB2	3	0.225655833	0.138692252	1.627025518	0.103731678	0.01768376	2.82715057	29.2573936	1	CRA004775;PRJNA827465;Yang2025_Silkie_Wencha	0.61562049
ENSGALG00010029276	TUBD1	3	-0.174735631	0.107498603	-1.625468843	0.104062697	0.014019628	3.396831714	41.01481151	0.666666667	CRA004775;PRJNA827465;Yang2025_Silkie_Wencha	0.61562049
ENSGALG00010017119	BTBD6	3	0.170047046	0.104624974	1.625300723	0.104098497	0.015590396	3.885187278	48.52243002	1	CRA004775;PRJNA827465;Yang2025_Silkie_Wencha	0.61562049
ENSGALG00010017001	TPCN3	3	-0.168587375	0.10362166	-1.626951115	0.103747481	0.012131682	3.039194685	34.19309366	1	CRA004775;PRJNA827465;Yang2025_Silkie_Wencha	0.61562049
ENSGALG00010027265	IP6K3	3	0.133216285	0.0818747	1.627075105	0.103721148	0	1.925029491	0	0.666666667	CRA004775;PRJNA827465;Yang2025_Silkie_Wencha	0.61562049
ENSGALG00010007867	ZNF516	3	-0.112065965	0.068914352	-1.62616295	0.103914995	0	1.367659397	0	1	CRA004775;PRJNA827465;Yang2025_Silkie_Wencha	0.61562049
ENSGALG00010015523	MRPL22	3	-0.102974795	0.063367233	-1.625048004	0.104152331	0	1.286538316	0	1	CRA004775;PRJNA827465;Yang2025_Silkie_Wencha	0.61562049
ENSGALG00010019843	MCM6	3	-0.088761504	0.054618692	-1.625112242	0.104138645	0.000608708	2.07558938	3.641827248	1	CRA004775;PRJNA827465;Yang2025_Silkie_Wencha	0.61562049
ENSGALG00010029617	POR	3	-0.07183939	0.044177737	-1.626144655	0.103918887	0	0.26815505	0	1	CRA004775;PRJNA827465;Yang2025_Silkie_Wencha	0.61562049
ENSGALG00010019716	VEGFA	3	0.323618392	0.199249517	1.624186587	0.104335993	0.100672975	23.06574119	91.32913188	1	CRA004775;PRJNA827465;Yang2025_Silkie_Wencha	0.615779275
ENSGALG00010023624	PSMA1	3	-0.120186373	0.074003014	-1.624074018	0.104360012	0.005187373	2.465845397	18.89191422	1	CRA004775;PRJNA827465;Yang2025_Silkie_Wencha	0.615779275
ENSGALG00010006227	AGPAT4	3	0.106957973	0.065864226	1.623916043	0.104393728	0	1.696843338	0	0.666666667	CRA004775;PRJNA827465;Yang2025_Silkie_Wencha	0.615779275
ENSGALG00010018021	ATRN	3	-0.09296797	0.057229684	-1.624471147	0.104275293	0	1.517316978	0	1	CRA004775;PRJNA827465;Yang2025_Silkie_Wencha	0.615779275
ENSGALG00010007781	MBP	3	0.400043193	0.246337099	1.62396937	0.10444196	0.10546403	4.766593274	57.98842949	1	CRA004775;PRJNA827465;Yang2025_Silkie_Wencha	0.615802366
ENSGALG00010023279	FA2H	3	0.936456349	0.577026651	1.622899649	0.104610859	0.837104959	13.16747072	84.81105413	1	CRA004775;PRJNA827465;Yang2025_Silkie_Wencha	0.61638448
ENSGALG00010003678	ZNFY1	3	0.343766867	0.21183255	1.622823629	0.104627113	0	1.359787306	0	0.666666667	CRA004775;PRJNA827465;Yang2025_Silkie_Wencha	0.61638448
ENSGALG00010012878	GDNF	3	0.276098267	0.170187047	1.622322454	0.104734323	0.017404302	2.457210769	18.60690075	1	CRA004775;PRJNA827465;Yang2025_Silkie_Wencha	0.616394264
ENSGALG00010019992	CHAC2	3	-0.150991612	0.093099536	-1.621829898	0.104839775	0.003373585	2.228774029	10.26456815	1	CRA004775;PRJNA827465;Yang2025_Silkie_Wencha	0.616394264
ENSGALG00010016580	MARS2	3	-0.103820956	0.064003469	-1.622114504	0.104778833	0	0.912349832	0	1	CRA004775;PRJNA827465;Yang2025_Silkie_Wencha	0.616394264
ENSGALG00010025435	SCLY	3	-0.101204488	0.062402673	-1.621797329	0.104846751	0	1.66999088	0	1	CRA004775;PRJNA827465;Yang2025_Silkie_Wencha	0.616394264
ENSGALG00010016410	TRAK1	3	-0.092296785	0.056901647	-1.622040657	0.104794643	0	0.875745718	0	0.666666667	CRA004775;PRJNA827465;Yang2025_Silkie_Wencha	0.616394264
ENSGALG00010010136	EPHX2	3	0.15625341	0.096381707	1.621193635	0.104976117	0.010757829	3.124530636	35.99038599	1	CRA004775;PRJNA827465;Yang2025_Silkie_Wencha	0.616898306
ENSGALG00010022166	SDHB	3	-0.199770142	0.123256587	-1.620766455	0.105067735	0.021342093	3.701611824	45.96948316	1	CRA004775;PRJNA827465;Yang2025_Silkie_Wencha	0.617180186
ENSGALG00010009444	NFIL3	3	-0.196557829	0.12130989	-1.620295164	0.105168887	0.012453624	2.738442694	26.96578956	0.666666667	CRA004775;PRJNA827465;Yang2025_Silkie_Wencha	0.617517814
ENSGALG00010007855	RNF182	2	0.527738851	0.325909605	1.619279833	0.105387068	0.178541074	6.206818748	83.88868693	1	PRJNA827465;Yang2025_Silkie_Wenchang	0.6177727
ENSGALG00010024523	TOX3	3	0.509048619	0.31432796	1.619482462	0.105343497	0.166220545	4.675071211	57.21990298	1	CRA004775;PRJNA827465;Yang2025_Silkie_Wencha	0.6177727
ENSGALG00010011001	ANGPT1	3	0.329650839	0.203508431	1.619838734	0.105266923	0.06023155	3.929947398	49.10873359	1	CRA004775;PRJNA827465;Yang2025_Silkie_Wencha	0.6177727
ENSGALG00010012232	PCSK1	3	0.310757086	0.191891692	1.61944002	0.105352622	0	0.188410801	0	1	CRA004775;PRJNA827465;Yang2025_Silkie_Wencha	0.6177727
ENSGALG00010015087	GABRA3	2	0.538891428	0.333123187	1.617694138	0.10572853	0	0.630362414	0	1	PRJNA827465;Yang2025_Silkie_Wenchang	0.619260847
ENSGALG00010010985	CERT1	3	0.154289145	0.095364233	1.617893208	0.105685614	0	0.82186249	0	1	CRA004775;PRJNA827465;Yang2025_Silkie_Wencha	0.619260847
ENSGALG00010000728	OTULIN	2	0.328129358	0.202941418	1.616867373	0.105906913	0.02564486	1.438313637	30.47413482	1	PRJNA827465;Yang2025_Silkie_Wenchang	0.620048794
ENSGALG00010001118	P2RY12	3	-0.284118861	0.175774151	-1.616385909	0.106010903	0	0.636897766	0	0.666666667	CRA004775;PRJNA827465;Yang2025_Silkie_Wencha	0.620061489
ENSGALG00010017437	DRG2	3	-0.080789734	0.049985968	-1.616248275	0.106040645	0	0.210129656	0	1	CRA004775;PRJNA827465;Yang2025_Silkie_Wencha	0.620061489
ENSGALG00010009130	HEATR1	3	-0.066134679	0.040917851	-1.616279392	0.10603392	0	0.055332776	0	1	CRA004775;PRJNA827465;Yang2025_Silkie_Wencha	0.620061489
ENSGALG00010020569	DTX3L	3	-0.205809186	0.127360466	-1.615958171	0.106103357	0.018612809	3.240298132	38.27728441	1	CRA004775;PRJNA827465;Yang2025_Silkie_Wencha	0.620118052
ENSGALG00010016681	SUSD4	3	-0.18989738	0.117525459	-1.615797808	0.106138036	0	1.470979489	0	1	CRA004775;PRJNA827465;Yang2025_Silkie_Wencha	0.620118052
ENSGALG00010015839	U2SURP	3	0.357668033	0.221504161	1.614723764	0.106370529	0.123056388	13.7146166	85.41701851	1	CRA004775;PRJNA827465;Yang2025_Silkie_Wencha	0.620195014
ENSGALG00010015893	TCF7	3	-0.324159718	0.200718899	-1.614993505	0.106312101	0	0.719015011	0	1	CRA004775;PRJNA827465;Yang2025_Silkie_Wencha	0.620195014
ENSGALG00010006311	ABCD4	3	0.2494421	0.154479295	1.614728373	0.10636953	0.038160782	4.535432913	55.90277624	0.666666667	CRA004775;PRJNA827465;Yang2025_Silkie_Wencha	0.620195014
ENSGALG00010028443	ENSGALG00010028443	3	0.220336318	0.136446757	1.614815349	0.106350688	0	1.710255159	0	0.666666667	CRA004775;PRJNA827465;Yang2025_Silkie_Wencha	0.620195014
ENSGALG00010022565	ARF3	3	-0.121064161	0.074954756	-1.615163164	0.106275365	0	0.906938437	0	1	CRA004775;PRJNA827465;Yang2025_Silkie_Wencha	0.620195014
ENSGALG00010024898	NABP2	3	0.181831388	0.112623315	1.614509291	0.106417003	0.018953081	4.00035474	50.00443386	1	CRA004775;PRJNA827465;Yang2025_Silkie_Wencha	0.620210226
ENSGALG00010025621	PPP1CC	3	-0.196051644	0.121480641	-1.61385092	0.106559767	0.029729461	10.98662496	81.7960474	1	CRA004775;PRJNA827465;Yang2025_Silkie_Wencha	0.620786379
ENSGALG00010017723	KCNK12	3	-0.414270034	0.2568033	-1.613180336	0.106705334	0.076004305	3.190124367	37.30651943	1	CRA004775;PRJNA827465;Yang2025_Silkie_Wencha	0.621378386
ENSGALG00010004367	TSPAN13	3	-0.654044581	0.405502923	-1.612921987	0.106761458	0.449736104	24.25035458	91.75269791	1	CRA004775;PRJNA827465;Yang2025_Silkie_Wencha	0.621449259
ENSGALG00010028205	PRRT1B	3	0.344480482	0.213619937	1.612585824	0.10683452	0	0.82963668	0	1	CRA004775;PRJNA827465;Yang2025_Silkie_Wencha	0.621618636
ENSGALG00010009373	ARHGAP15	3	-0.200376638	0.124307681	-1.611940917	0.106974797	0	0.784151359	0	1	CRA004775;PRJNA827465;Yang2025_Silkie_Wencha	0.622178798
ENSGALG00010017505	TNFRSF8	3	-0.338928904	0.210293504	-1.611694596	0.107028414	0	1.003717663	0	1	CRA004775;PRJNA827465;Yang2025_Silkie_Wencha	0.622234683
ENSGALG00010024476	KCNB1	3	-0.203516877	0.126314294	-1.611194356	0.107137368	0	1.503557252	0	0.666666667	CRA004775;PRJNA827465;Yang2025_Silkie_Wencha	0.622612101
ENSGALG00010005267	CYP51A1	3	0.155745769	0.096712086	1.610406469	0.107309149	0	1.552113	0	0.666666667	CRA004775;PRJNA827465;Yang2025_Silkie_Wencha	0.623354174
ENSGALG00010029778	DOC2B	3	-0.266072125	0.165262935	-1.609992738	0.107399442	0	1.844392079	0	1	CRA004775;PRJNA827465;Yang2025_Silkie_Wencha	0.623622467
ENSGALG00010010958	LCORL	3	0.263147146	0.163477773	1.609681491	0.107467408	0.0441087	4.614986076	56.6629245	1	CRA004775;PRJNA827465;Yang2025_Silkie_Wencha	0.623706433
ENSGALG00010014906	IFT27	3	-0.257461923	0.159961675	-1.609522555	0.107502127	0.059531717	9.417052534	78.76193222	0.666666667	CRA004775;PRJNA827465;Yang2025_Silkie_Wencha	0.623706433
ENSGALG00010028597	NUP188	3	-0.253822856	0.157742278	-1.60909846	0.107594814	0.052996626	9.773912284	79.53736496	1	CRA004775;PRJNA827465;Yang2025_Silkie_Wencha	0.623988136
ENSGALG00010002599	KIFC1	3	-0.323371185	0.200999483	-1.608816006	0.10765658	0	1.083015589	0	1	CRA004775;PRJNA827465;Yang2025_Silkie_Wencha	0.624090358
ENSGALG00010005143	GTF3A	3	0.104178917	0.064767514	1.608505724	0.107724464	0	1.12809602	0	1	CRA004775;PRJNA827465;Yang2025_Silkie_Wencha	0.624227947
ENSGALG00010004082	FAM241A	3	-0.206899246	0.128672976	-1.607946376	0.107846924	0.026138427	4.267105348	53.129819	0.666666667	CRA004775;PRJNA827465;Yang2025_Silkie_Wencha	0.624592159
ENSGALG00010028198	UCK1	3	-0.126121354	0.078442695	-1.607815162	0.107875667	0.007024877	3.124264924	35.98494209	1	CRA004775;PRJNA827465;Yang2025_Silkie_Wencha	0.624592159
ENSGALG00010019260	TRAIL-LIKE	3	-0.099964128	0.062188665	-1.607433245	0.107959362	0	0.762414453	0	0.666666667	CRA004775;PRJNA827465;Yang2025_Silkie_Wencha	0.624820884
ENSGALG00010004201	CPA6	2	0.552683081	0.344159147	1.60589392	0.10829722	0	0.391360192	0	1	PRJNA827465;Yang2025_Silkie_Wenchang	0.625751691
ENSGALG00010022827	CMTR2	3	-0.361770281	0.225273711	-1.605914331	0.108292734	0.126976437	19.18895152	89.5773357	1	CRA004775;PRJNA827465;Yang2025_Silkie_Wencha	0.625751691
ENSGALG00010023190	SF3B3	3	-0.23093376	0.143800824	-1.605927932	0.108289746	0.047909138	9.901453702	79.8009458	1	CRA004775;PRJNA827465;Yang2025_Silkie_Wencha	0.625751691
ENSGALG00010024424	SAMHD1	3	-0.084849922	0.052823503	-1.606291073	0.108209971	0	0.579262177	0	0.666666667	CRA004775;PRJNA827465;Yang2025_Silkie_Wencha	0.625751691
ENSGALG00010001745	CADPS2	3	-0.195906323	0.122008846	-1.605673107	0.108345753	0.011221648	2.622166667	23.72719762	0.666666667	CRA004775;PRJNA827465;Yang2025_Silkie_Wencha	0.62577639
ENSGALG00010011873	RFX3	3	0.386201359	0.240645261	1.604857531	0.108525162	0.116103998	7.222586617	72.30908944	1	CRA004775;PRJNA827465;Yang2025_Silkie_Wencha	0.625790073
ENSGALG00010020356	STX3	3	0.352425683	0.219597041	1.604874461	0.108521435	0.049539436	3.039446706	34.19855014	1	CRA004775;PRJNA827465;Yang2025_Silkie_Wencha	0.625790073
ENSGALG00010027374	NKTR	3	0.236510917	0.147362122	1.604964107	0.108501704	0.038033091	4.952031601	59.6125356	1	CRA004775;PRJNA827465;Yang2025_Silkie_Wencha	0.625790073
ENSGALG00010028448	RIT1	3	-0.080835284	0.050352881	-1.605375555	0.108411181	0.000729541	2.119203638	5.624926084	1	CRA004775;PRJNA827465;Yang2025_Silkie_Wencha	0.625790073
ENSGALG00010010298	KCNIP4	2	-0.502897635	0.313434052	-1.604476703	0.108609016	0.129643516	2.86291539	65.07057095	1	PRJNA827465;Yang2025_Silkie_Wenchang	0.626018293
ENSGALG00010024824	DNAJC5	3	-0.112711741	0.070269295	-1.603997038	0.108714706	0	0.953879812	0	0.666666667	CRA004775;PRJNA827465;Yang2025_Silkie_Wencha	0.626372136
ENSGALG00010006031	ERCC5	3	0.377996065	0.235725418	1.60354394	0.108814616	0.140328535	17.52130748	88.58532674	1	CRA004775;PRJNA827465;Yang2025_Silkie_Wencha	0.626674466
ENSGALG00010015201	GCC2	3	0.334317478	0.208537042	1.603156322	0.108900146	0.093799799	8.249451602	75.7559642	1	CRA004775;PRJNA827465;Yang2025_Silkie_Wencha	0.626674466
ENSGALG00010008234	COMMD10	3	-0.217589974	0.135724775	-1.60317064	0.108896986	0	0.869645637	0	1	CRA004775;PRJNA827465;Yang2025_Silkie_Wencha	0.626674466
ENSGALG00010013901	NFXL1	3	0.167445722	0.10451824	1.60207177	0.10913974	0.014622864	3.666567663	45.45307264	0.666666667	CRA004775;PRJNA827465;Yang2025_Silkie_Wencha	0.627724048
ENSGALG00010019016	PLEKHG5	3	0.166565633	0.103978168	1.601928913	0.10917133	0.014061245	3.538177133	43.47371754	1	CRA004775;PRJNA827465;Yang2025_Silkie_Wencha	0.627724048
ENSGALG00010003701	SARAF	3	-0.190925548	0.119206061	-1.601642954	0.109234587	0.026244068	5.293184826	62.21556462	1	CRA004775;PRJNA827465;Yang2025_Silkie_Wencha	0.627832447
ENSGALG00010024162	SRCIN1	3	0.618208131	0.386042812	1.601397854	0.109288828	0.336272217	12.04352807	83.39357049	1	CRA004775;PRJNA827465;Yang2025_Silkie_Wencha	0.627888962
ENSGALG00010024692	DRAM2	3	-0.089796022	0.056080705	-1.601192819	0.109334219	0	0.522516692	0	1	CRA004775;PRJNA827465;Yang2025_Silkie_Wencha	0.627894606
ENSGALG00010005558	TAGAP	3	-0.430102947	0.26871866	-1.600569708	0.109472256	0	0.285344713	0	1	CRA004775;PRJNA827465;Yang2025_Silkie_Wencha	0.627924574
ENSGALG00010020675	SMYD2	3	-0.237491336	0.148382831	-1.600531097	0.109480814	0.038558853	4.961525479	59.68981701	0.666666667	CRA004775;PRJNA827465;Yang2025_Silkie_Wencha	0.627924574
ENSGALG00010014567	UTP11	3	-0.145476831	0.09090214	-1.600367501	0.10951708	0.000308385	2.019525835	0.966852463	0.666666667	CRA004775;PRJNA827465;Yang2025_Silkie_Wencha	0.627924574
ENSGALG00010012198	TMEM5	3	-0.122644189	0.076633422	-1.600400785	0.109509701	0	1.299137851	0	0.666666667	CRA004775;PRJNA827465;Yang2025_Silkie_Wencha	0.627924574
ENSGALG00010001827	PDZRN4	3	0.244799409	0.152994135	1.600057473	0.109585834	0.001819812	2.037606306	1.845611991	1	CRA004775;PRJNA827465;Yang2025_Silkie_Wencha	0.628064089
ENSGALG00010001192	ENSGALG00010001192	3	0.128635912	0.080436311	1.599226882	0.109770199	0	1.87633732	0	1	CRA004775;PRJNA827465;Yang2025_Silkie_Wencha	0.628865822
ENSGALG00010028686	TAGLN2	3	0.117774697	0.073662667	1.598838342	0.109856527	0	0.812821026	0	0.666666667	CRA004775;PRJNA827465;Yang2025_Silkie_Wencha	0.629105483
ENSGALG00010004081	RPS20	3	-0.348341213	0.21796296	-1.59816701	0.110005814	0.096678228	6.373144226	68.61831571	1	CRA004775;PRJNA827465;Yang2025_Silkie_Wencha	0.629288039
ENSGALG00010005695	KIF5B	3	0.153605542	0.096117929	1.59809459	0.110021927	0.014958283	4.282330805	53.29646188	1	CRA004775;PRJNA827465;Yang2025_Silkie_Wencha	0.629288039
ENSGALG00010013625	SSBP1	3	0.148780744	0.093087795	1.598284111	0.109979762	0.007385689	2.552820807	21.65529227	0.666666667	CRA004775;PRJNA827465;Yang2025_Silkie_Wencha	0.629288039
ENSGALG00010010976	DYDC2	2	0.493414181	0.309031251	1.596648167	0.110344156	0	0.022737511	0	1	PRJNA827465;Yang2025_Silkie_Wenchang	0.629791195
ENSGALG00010021682	MYL4	3	0.433733873	0.271543845	1.597288544	0.110201403	0.130370807	6.036702398	66.86932918	1	CRA004775;PRJNA827465;Yang2025_Silkie_Wencha	0.629791195
ENSGALG00010020925	RIC8A	3	-0.344985674	0.216063616	-1.596685645	0.110335797	0.103863182	8.682362029	76.96479376	1	CRA004775;PRJNA827465;Yang2025_Silkie_Wencha	0.629791195
ENSGALG00010014632	NWD2	3	0.231793334	0.145112342	1.597337144	0.110190575	0.01228483	2.351994087	14.96577263	1	CRA004775;PRJNA827465;Yang2025_Silkie_Wencha	0.629791195
ENSGALG00010022660	METAP1D	3	0.21642827	0.135523328	1.596981664	0.110269794	0	1.207017497	0	0.666666667	CRA004775;PRJNA827465;Yang2025_Silkie_Wencha	0.629791195
ENSGALG00010010167	PUS7	3	-0.093344391	0.058468135	-1.596500232	0.110377154	0	0.23284807	0	1	CRA004775;PRJNA827465;Yang2025_Silkie_Wencha	0.629791195
ENSGALG00010011823	ENSGALG00010011823	3	0.804392959	0.504043313	1.59588063	0.110515447	0.505198236	6.117969112	67.30941325	1	CRA004775;PRJNA827465;Yang2025_Silkie_Wencha	0.629952166
ENSGALG00010028333	ENSGALG00010028333	3	-0.409009357	0.256307655	-1.595775037	0.110539029	0.052734705	2.645402655	24.39714248	0.666666667	CRA004775;PRJNA827465;Yang2025_Silkie_Wencha	0.629952166
ENSGALG00010021908	EDC4	3	-0.066477246	0.041648207	-1.596161076	0.110452835	0	0.181890916	0	1	CRA004775;PRJNA827465;Yang2025_Silkie_Wencha	0.629952166
ENSGALG00010025336	ADAM10	3	0.117180843	0.073448278	1.595419884	0.110618373	0.004044432	2.320655188	13.81744215	1	CRA004775;PRJNA827465;Yang2025_Silkie_Wencha	0.63015035
ENSGALG00010029420	LAT2	3	0.33142526	0.2078543	1.594507598	0.11082239	0	0.318003292	0	1	CRA004775;PRJNA827465;Yang2025_Silkie_Wencha	0.630380959
ENSGALG00010005238	TMEM63C	3	-0.238053275	0.149301985	-1.59444146	0.110837192	0.040878929	6.490779644	69.18706057	1	CRA004775;PRJNA827465;Yang2025_Silkie_Wencha	0.630380959
ENSGALG00010020003	ENSGALG00010020003	3	-0.165730266	0.103908809	-1.594958778	0.110721454	0	1.026138379	0	0.666666667	CRA004775;PRJNA827465;Yang2025_Silkie_Wencha	0.630380959
ENSGALG00010005512	SMARCAD1	3	-0.109688665	0.068780341	-1.594767687	0.110764195	0	0.360405709	0	1	CRA004775;PRJNA827465;Yang2025_Silkie_Wencha	0.630380959
ENSGALG00010005757	ARHGAP12	3	0.301565803	0.189195241	1.593939689	0.110949544	0.085059179	11.12026255	82.01481313	1	CRA004775;PRJNA827465;Yang2025_Silkie_Wencha	0.630766225
ENSGALG00010001230	PTX3	3	-0.576852272	0.36206876	-1.593211939	0.111112654	0.267125331	7.605009558	73.70154521	1	CRA004775;PRJNA827465;Yang2025_Silkie_Wencha	0.631185943
ENSGALG00010002330	KHDRBS2	3	0.279767329	0.175579707	1.593392159	0.111072244	0	1.109293355	0	0.666666667	CRA004775;PRJNA827465;Yang2025_Silkie_Wencha	0.631185943
ENSGALG00010013364	OTOGL	2	0.396721325	0.249267323	1.591549669	0.111485927	0	0.632991544	0	1	PRJNA827465;Yang2025_Silkie_Wenchang	0.631406421
ENSGALG00010005023	POGLUT3	3	-0.343956113	0.216018238	-1.592254969	0.111327427	0.104290461	10.47373942	80.90462328	1	CRA004775;PRJNA827465;Yang2025_Silkie_Wencha	0.631406421
ENSGALG00010029582	SCPEP1	3	-0.260548625	0.163649563	-1.592113174	0.111359278	0.056822437	8.82675261	77.34161035	0.666666667	CRA004775;PRJNA827465;Yang2025_Silkie_Wencha	0.631406421
ENSGALG00010003260	ACP5	3	0.23733989	0.149134053	1.591453363	0.111507583	0	1.061085454	0	1	CRA004775;PRJNA827465;Yang2025_Silkie_Wencha	0.631406421
ENSGALG00010019189	ICMT	3	-0.230322697	0.144725215	-1.591448295	0.111508723	0.038967828	5.188361847	61.45218743	1	CRA004775;PRJNA827465;Yang2025_Silkie_Wencha	0.631406421
ENSGALG00010018444	NUBP2	3	-0.150182512	0.094297709	-1.59264222	0.111240477	0.005187212	2.35731301	15.1576396	1	CRA004775;PRJNA827465;Yang2025_Silkie_Wencha	0.631406421
ENSGALG00010017337	ABAT	3	0.111500622	0.07003769	1.592008842	0.111382718	0	0.880381043	0	1	CRA004775;PRJNA827465;Yang2025_Silkie_Wencha	0.631406421
ENSGALG00010024830	ORC2	3	0.109187787	0.068585252	1.592000963	0.111384488	0	1.612261648	0	0.666666667	CRA004775;PRJNA827465;Yang2025_Silkie_Wencha	0.631406421
ENSGALG00010014033	SLC38A4	3	0.432628159	0.271995364	1.590571813	0.111705972	0.046266227	2.431798904	17.75635738	0.666666667	CRA004775;PRJNA827465;Yang2025_Silkie_Wencha	0.631782707
ENSGALG00010006361	SERTM1	3	-0.415596722	0.261289994	-1.590557358	0.111709228	0.142269568	8.702507581	77.01811827	0.666666667	CRA004775;PRJNA827465;Yang2025_Silkie_Wencha	0.631782707
ENSGALG00010029338	INPP5K	3	0.110018805	0.069157638	1.590840994	0.111645365	0	0.441493793	0	1	CRA004775;PRJNA827465;Yang2025_Silkie_Wencha	0.631782707
ENSGALG00010010845	FKRP	3	-0.17777944	0.111812192	-1.589982599	0.111838727	0.01003925	2.656188536	24.70414005	0.666666667	CRA004775;PRJNA827465;Yang2025_Silkie_Wencha	0.632114254
ENSGALG00010004896	UBA6	3	-0.115780119	0.072822248	-1.589900369	0.111857265	0.004382654	2.689854257	25.64652919	1	CRA004775;PRJNA827465;Yang2025_Silkie_Wencha	0.632114254
ENSGALG00010025031	ECHDC2	3	-0.13648142	0.085864113	-1.589504807	0.11194647	0	0.560746451	0	0.666666667	CRA004775;PRJNA827465;Yang2025_Silkie_Wencha	0.632365615
ENSGALG00010007991	PCF11	3	0.422501012	0.265910283	1.588885569	0.11208623	0.186551759	20.95056417	90.4537177	1	CRA004775;PRJNA827465;Yang2025_Silkie_Wencha	0.632491381
ENSGALG00010001763	ENSGALG00010001763	3	0.354683968	0.22332202	1.588217626	0.112237137	0	1.111828336	0	1	CRA004775;PRJNA827465;Yang2025_Silkie_Wencha	0.632491381
ENSGALG00010018233	RASSF2	3	0.257449787	0.162098464	1.588230887	0.112234139	0.048186088	6.008560338	66.71415635	0.666666667	CRA004775;PRJNA827465;Yang2025_Silkie_Wencha	0.632491381
ENSGALG00010010861	ENSGALG00010010861	3	0.235069616	0.148001792	1.588288989	0.112221006	0	1.633618047	0	1	CRA004775;PRJNA827465;Yang2025_Silkie_Wencha	0.632491381
ENSGALG00010023264	RACGAP1	3	0.118140639	0.074381423	1.588308398	0.11221662	0	1.649759523	0	1	CRA004775;PRJNA827465;Yang2025_Silkie_Wencha	0.632491381
ENSGALG00010007680	CHMP2B	3	-0.075008148	0.047199448	-1.58917427	0.112021054	0	0.3188831	0	1	CRA004775;PRJNA827465;Yang2025_Silkie_Wencha	0.632491381
ENSGALG00010003034	ERI1	3	-0.162890509	0.102576266	-1.587994133	0.112287666	0.022024635	8.892348122	77.50875278	1	CRA004775;PRJNA827465;Yang2025_Silkie_Wencha	0.632524026
ENSGALG00010011876	AMDHD1	3	0.418344699	0.263611898	1.586971991	0.112518988	0	1.809238427	0	0.666666667	CRA004775;PRJNA827465;Yang2025_Silkie_Wencha	0.633574662
ENSGALG00010022117	FAM20C	3	-0.10673147	0.067267335	-1.586676049	0.112586034	0	1.157902546	0	0.666666667	CRA004775;PRJNA827465;Yang2025_Silkie_Wencha	0.633699813
ENSGALG00010005651	USP6NL	3	0.289038124	0.182286864	1.585622342	0.112825005	0.065284314	5.875040812	65.95768329	1	CRA004775;PRJNA827465;Yang2025_Silkie_Wencha	0.633889872
ENSGALG00010028861	SURF4	3	-0.287009589	0.181144649	-1.584422123	0.113097691	0.07737418	21.91748974	90.87486741	1	CRA004775;PRJNA827465;Yang2025_Silkie_Wencha	0.633889872
ENSGALG00010007998	RPL13	3	-0.268471179	0.169451406	-1.584354982	0.11311296	0.049448274	4.50952664	55.64944705	1	CRA004775;PRJNA827465;Yang2025_Silkie_Wencha	0.633889872
ENSGALG00010029218	RPL23A	3	-0.261809136	0.165219679	-1.584612301	0.113054448	0.048212674	4.710909778	57.54535547	1	CRA004775;PRJNA827465;Yang2025_Silkie_Wencha	0.633889872
ENSGALG00010019023	AP2M1	3	-0.211934053	0.133661285	-1.585605386	0.112828854	0.041608078	9.753833579	79.4952417	1	CRA004775;PRJNA827465;Yang2025_Silkie_Wencha	0.633889872
ENSGALG00010012291	NR5A2	3	0.142205002	0.089653006	1.586171043	0.112700515	0	1.00358112	0	0.666666667	CRA004775;PRJNA827465;Yang2025_Silkie_Wencha	0.633889872
ENSGALG00010027268	UQCRC1	3	-0.111779406	0.070513383	-1.585222563	0.112915776	0	1.006176927	0	0.666666667	CRA004775;PRJNA827465;Yang2025_Silkie_Wencha	0.633889872
ENSGALG00010022489	NUSAP1	3	-0.104142752	0.065693984	-1.585270757	0.11290483	0	1.140392909	0	0.666666667	CRA004775;PRJNA827465;Yang2025_Silkie_Wencha	0.633889872
ENSGALG00010018808	MATCAP2	3	0.090216729	0.056879435	1.586104523	0.112715601	0.000920736	2.169773528	7.82448147	0.666666667	CRA004775;PRJNA827465;Yang2025_Silkie_Wencha	0.633889872
ENSGALG00010001686	WASL	3	-0.061033172	0.038507079	-1.584985758	0.11296957	0	1.201251893	0	0.666666667	CRA004775;PRJNA827465;Yang2025_Silkie_Wencha	0.633889872
ENSGALG00010027074	BRPF3	3	0.054070465	0.034118523	1.58478329	0.11301558	0	0.890709806	0	0.666666667	CRA004775;PRJNA827465;Yang2025_Silkie_Wencha	0.633889872
ENSGALG00010012121	SREK1	3	0.47098774	0.297680084	1.582194323	0.113605216	0.224079585	13.6995316	85.40096072	1	CRA004775;PRJNA827465;Yang2025_Silkie_Wencha	0.634336062
ENSGALG00010001897	TLR7	3	0.341277904	0.215507419	1.583601648	0.113284399	0.051927205	3.14983651	36.50464099	0.666666667	CRA004775;PRJNA827465;Yang2025_Silkie_Wencha	0.634336062
ENSGALG00010028492	ENSGALG00010028492	3	0.251100727	0.158685864	1.582376148	0.113563726	0.036474888	3.93029639	49.1132525	0.666666667	CRA004775;PRJNA827465;Yang2025_Silkie_Wencha	0.634336062
ENSGALG00010023931	CSRNP2	3	0.241188982	0.152352018	1.583103298	0.113397922	0.038243066	4.435142463	54.90561991	1	CRA004775;PRJNA827465;Yang2025_Silkie_Wencha	0.634336062
ENSGALG00010026088	SNRPC	3	-0.207379819	0.131084087	-1.582036569	0.113641223	0.027083004	4.004299228	50.05368265	1	CRA004775;PRJNA827465;Yang2025_Silkie_Wencha	0.634336062
ENSGALG00010004247	MEOX2	3	0.192291559	0.121446359	1.583345601	0.113342715	0.015652638	2.762373451	27.59849326	1	CRA004775;PRJNA827465;Yang2025_Silkie_Wencha	0.634336062
ENSGALG00010024613	ENSGALG00010024613	3	-0.154854051	0.097804786	-1.583297269	0.113353725	0.016906225	5.302679217	62.28321725	1	CRA004775;PRJNA827465;Yang2025_Silkie_Wencha	0.634336062
ENSGALG00010001835	MECP2	3	-0.121864574	0.077018136	-1.582284124	0.113584723	0	1.45470748	0	1	CRA004775;PRJNA827465;Yang2025_Silkie_Wencha	0.634336062
ENSGALG00010019767	SUCLG2	3	-0.097394085	0.061529394	-1.582887112	0.113447197	0	0.122228716	0	1	CRA004775;PRJNA827465;Yang2025_Silkie_Wencha	0.634336062

ENSGALG00010003617	COG3	3	0.077188462	0.048770413	1.58269036	0.113492056	0	0.580190635	0	0.666666667	CRA004775;PRJNA827465;Yang2025_Silkie_Wencha	0.634336062
ENSGALG00010022581	TMEM125	3	0.827287298	0.523288605	1.580938874	0.113892015	0.389768747	3.790174885	47.23198637	1	CRA004775;PRJNA827465;Yang2025_Silkie_Wencha	0.634609848
ENSGALG00010003571	RPL7	3	-0.345241731	0.218354796	-1.581104414	0.113854166	0.108054932	8.984434212	77.73927715	1	CRA004775;PRJNA827465;Yang2025_Silkie_Wencha	0.634609848
ENSGALG00010019581	SLC35B2	3	-0.233254655	0.147642682	-1.579859231	0.114139108	0.052481169	11.85624411	83.1312515	1	CRA004775;PRJNA827465;Yang2025_Silkie_Wencha	0.634609848
ENSGALG00010025520	ACOT11	3	0.218891201	0.138548579	1.579887735	0.114132579	0.016848551	2.599642218	23.06633635	1	CRA004775;PRJNA827465;Yang2025_Silkie_Wencha	0.634609848
ENSGALG00010005372	CDIN1	3	-0.217848787	0.137781784	-1.581114578	0.113851842	0.034962004	5.588081398	64.20954067	0.666666667	CRA004775;PRJNA827465;Yang2025_Silkie_Wencha	0.634609848
ENSGALG00010027394	SLC27A4	3	-0.208750062	0.132107824	-1.580149123	0.11407272	0.035871906	6.48452067	69.15731938	0.666666667	CRA004775;PRJNA827465;Yang2025_Silkie_Wencha	0.634609848
ENSGALG00010017198	TOPBP1	3	-0.172800516	0.109375448	-1.579883965	0.114133442	0.021593135	6.520347823	69.3267897	1	CRA004775;PRJNA827465;Yang2025_Silkie_Wencha	0.634609848
ENSGALG00010010279	CCSAP	3	-0.134527702	0.085147223	-1.579942319	0.114120077	0	0.773501612	0	1	CRA004775;PRJNA827465;Yang2025_Silkie_Wencha	0.634609848
ENSGALG00010005430	MCM4	3	-0.119151501	0.075402725	-1.580201514	0.114060725	0.004754734	2.651087945	24.55927373	1	CRA004775;PRJNA827465;Yang2025_Silkie_Wencha	0.634609848
ENSGALG00010021965	PEX16	3	-0.093994036	0.059460496	-1.579987427	0.114109746	0	1.269710425	0	1	CRA004775;PRJNA827465;Yang2025_Silkie_Wencha	0.634609848
ENSGALG00010003787	SMIM8	3	-0.227336549	0.143978207	-1.578964991	0.114344087	0	0.937244758	0	0.666666667	CRA004775;PRJNA827465;Yang2025_Silkie_Wencha	0.635499627
ENSGALG00010014020	PDXP	3	-0.322334773	0.204191476	-1.578590738	0.11442996	0.095189786	9.555534677	79.06972171	1	CRA004775;PRJNA827465;Yang2025_Silkie_Wencha	0.635652261
ENSGALG00010020116	GNA12	3	-0.180140806	0.114134008	-1.578327167	0.114490468	0.021473001	5.139498478	61.0856972	0.666666667	CRA004775;PRJNA827465;Yang2025_Silkie_Wencha	0.635652261
ENSGALG00010014974	LIG4	3	0.170017145	0.107724579	1.578257686	0.114506423	0	0.300389905	0	1	CRA004775;PRJNA827465;Yang2025_Silkie_Wencha	0.635652261
ENSGALG00010025917	SCUBE3	3	0.769020295	0.487371656	1.577892941	0.114590207	0.566190724	10.22711025	80.44413376	1	CRA004775;PRJNA827465;Yang2025_Silkie_Wencha	0.63584464
ENSGALG00010003836	CD1F	3	0.30715842	0.194685576	1.577715343	0.11463102	0.034145516	2.835708097	29.47087882	1	CRA004775;PRJNA827465;Yang2025_Silkie_Wencha	0.63584464
ENSGALG00010016783	CLBA1	3	0.273250896	0.173236481	1.577328825	0.114719883	0	1.607909974	0	0.666666667	CRA004775;PRJNA827465;Yang2025_Silkie_Wencha	0.63608801
ENSGALG00010024052	ENSGALG0001002405	3	0.425529018	0.269820401	1.577082443	0.114776556	0.179599264	13.54555809	85.2350122	1	CRA004775;PRJNA827465;Yang2025_Silkie_Wencha	0.636152776
ENSGALG00010022193	GDAP2	3	-0.274767969	0.174285522	-1.576539266	0.11489577	0.076921709	14.09969573	85.81529674	1	CRA004775;PRJNA827465;Yang2025_Silkie_Wencha	0.636596158
ENSGALG00010024002	DGCR2	3	-0.057029141	0.036179607	-1.576278639	0.114961602	0	0.151858788	0	1	CRA004775;PRJNA827465;Yang2025_Silkie_Wencha	0.636679239
ENSGALG00010008415	ENSGALG0001000841	2	0.576678772	0.365977665	1.57572122	0.115090065	0	0.839736614	0	1	CRA004775;Yang2025_Silkie_Wenchang	0.636891753
ENSGALG00010029248	C1QBP	3	-0.090941559	0.057710742	-1.575816839	0.11506802	0	0.727552339	0	0.666666667	CRA004775;PRJNA827465;Yang2025_Silkie_Wencha	0.636891753
ENSGALG00010006177	NEK9	3	-0.193885105	0.123086634	-1.575192196	0.115212088	0.023937132	4.331575743	53.82742636	1	CRA004775;PRJNA827465;Yang2025_Silkie_Wencha	0.637317572
ENSGALG00010019081	PCSK6	3	-0.127269735	0.080817838	-1.574772832	0.11530889	0	1.497803622	0	0.666666667	CRA004775;PRJNA827465;Yang2025_Silkie_Wencha	0.637603594
ENSGALG00010012114	TAL2	3	-0.396292537	0.252062852	-1.572197308	0.1159048	0	1.356698894	0	1	CRA004775;PRJNA827465;Yang2025_Silkie_Wencha	0.638973582
ENSGALG00010000202	ENSGALG0001000020	3	0.379091173	0.241048069	1.572681221	0.11579265	0.059635406	3.035865595	34.12093067	1	CRA004775;PRJNA827465;Yang2025_Silkie_Wencha	0.638973582
ENSGALG00010023777	OAZ2	3	-0.354208854	0.225326757	-1.571978662	0.1159555	0.112986016	8.572723162	76.6701903	1	CRA004775;PRJNA827465;Yang2025_Silkie_Wencha	0.638973582
ENSGALG00010008152	ENSGALG0001000815	3	-0.302180678	0.192252221	-1.571792911	0.115998586	0.056013151	4.117034528	51.42134499	0.666666667	CRA004775;PRJNA827465;Yang2025_Silkie_Wencha	0.638973582
ENSGALG00010011819	USP44	3	0.267008699	0.169947469	1.571124894	0.116153641	0.062730155	7.682167941	73.96568241	1	CRA004775;PRJNA827465;Yang2025_Silkie_Wencha	0.638973582
ENSGALG00010026898	PNPLA2	3	-0.23177849	0.141105112	-1.571718323	0.116015891	0.025618684	3.635463908	44.98638823	0.666666667	CRA004775;PRJNA827465;Yang2025_Silkie_Wencha	0.638973582
ENSGALG00010025924	TMEM116	3	0.210992298	0.134174486	1.572521757	0.115829597	0.02747815	4.399652665	54.54186609	1	CRA004775;PRJNA827465;Yang2025_Silkie_Wencha	0.638973582
ENSGALG00010022650	NR1H3	3	-0.207922721	0.132268164	-1.571978587	0.115955517	0.039113877	18.28272916	89.06071417	1	CRA004775;PRJNA827465;Yang2025_Silkie_Wencha	0.638973582
ENSGALG00010017263	COG7	3	-0.207394927	0.131872383	-1.572694162	0.115789652	0.037811548	7.964875418	74.88975163	1	CRA004775;PRJNA827465;Yang2025_Silkie_Wencha	0.638973582
ENSGALG00010008196	PDCD6	3	-0.157324944	0.100048955	-1.572479632	0.115839359	0.017374225	4.758219689	57.96747248	1	CRA004775;PRJNA827465;Yang2025_Silkie_Wencha	0.638973582
ENSGALG00010015834	STARD8	3	-0.100822925	0.064154494	-1.571564486	0.116051588	0	0.665504868	0	1	CRA004775;PRJNA827465;Yang2025_Silkie_Wencha	0.638973582
ENSGALG00010010445	LMAN1L	3	0.087303334	0.055559616	1.571345172	0.116102494	0	0.789882833	0	0.666666667	CRA004775;PRJNA827465;Yang2025_Silkie_Wencha	0.638973582
ENSGALG00010001185	PTPN2	3	-0.062153341	0.039568401	-1.570782222	0.116233243	0	0.190275776	0	0.666666667	CRA004775;PRJNA827465;Yang2025_Silkie_Wencha	0.638973582
ENSGALG00010028596	DDA1	3	0.052249103	0.033263228	1.570776673	0.116234532	0	1.360399429	0	0.666666667	CRA004775;PRJNA827465;Yang2025_Silkie_Wencha	0.638973582
ENSGALG00010017539	KPNA6	3	-0.048069261	0.030589615	-1.571424187	0.116084151	0	1.666444723	0	1	CRA004775;PRJNA827465;Yang2025_Silkie_Wencha	0.638973582
ENSGALG00010003264	ENSGALG0001000326	3	-0.554262962	0.352929641	-1.570463056	0.116307423	0.141026705	3.057403838	34.58502357	1	CRA004775;PRJNA827465;Yang2025_Silkie_Wencha	0.639125787
ENSGALG00010004056	SHROOM3	3	0.145965342	0.092963986	1.570127834	0.116385374	0	1.405668543	0	1	CRA004775;PRJNA827465;Yang2025_Silkie_Wencha	0.639305675
ENSGALG00010021346	RPS21	3	-0.545745615	0.347644768	-1.569836987	0.116453304	0.306278754	14.53048209	86.23583176	1	CRA004775;PRJNA827465;Yang2025_Silkie_Wencha	0.639428946
ENSGALG00010006280	VRTN	2	1.218352566	0.777018704	1.567983576	0.116884965	1.026593409	6.673980692	85.01643852	1	PRJNA827465;Yang2025_Silkie_Wenchang	0.639565219
ENSGALG00010012470	CHD1	3	0.407342579	0.259749731	1.568211748	0.116831724	0.149641558	7.858870645	74.55105077	0.666666667	CRA004775;PRJNA827465;Yang2025_Silkie_Wencha	0.639565219
ENSGALG00010007872	KLHL2	3	0.342218402	0.218048636	1.569459033	0.116541018	0.125761768	18.18607989	89.00257773	1	CRA004775;PRJNA827465;Yang2025_Silkie_Wencha	0.639565219
ENSGALG00010006121	ENSGALG0001000612	3	0.258261696	0.164691461	1.568154744	0.116845023	0	0.296464329	0	1	CRA004775;PRJNA827465;Yang2025_Silkie_Wencha	0.639565219
ENSGALG00010022224	NHEJ1	3	-0.205836612	0.131259833	-1.568161458	0.116843457	0	1.251689241	0	1	CRA004775;PRJNA827465;Yang2025_Silkie_Wencha	0.639565219
ENSGALG00010025837	ENSGALG0001002583	3	-0.205663984	0.131150241	-1.568155592	0.116844825	0	0.597045933	0	1	CRA004775;PRJNA827465;Yang2025_Silkie_Wencha	0.639565219
ENSGALG00010012390	MMP16	3	-0.147553228	0.094033579	-1.569154661	0.116611906	0	0.399070203	0	1	CRA004775;PRJNA827465;Yang2025_Silkie_Wencha	0.639565219
ENSGALG00010027360	SMIM7	3	-0.11209546	0.071482153	-1.568160089	0.116843776	0	0.480351355	0	0.666666667	CRA004775;PRJNA827465;Yang2025_Silkie_Wencha	0.639565219
ENSGALG00010012606	ATP5PO	3	-0.109604468	0.06986104	-1.568892604	0.116672965	0	0.678156199	0	0.666666667	CRA004775;PRJNA827465;Yang2025_Silkie_Wencha	0.639565219
ENSGALG00010002926	GALNTL6	2	0.507027303	0.323615956	1.566756193	0.117171691	0.038929059	1.22704861	18.50363612	1	PRJNA827465;Yang2025_Silkie_Wenchang	0.639895918
ENSGALG00010016100	SFRP4	3	-0.358260117	0.2286571	-1.566800758	0.11716127	0	0.775906433	0	0.666666667	CRA004775;PRJNA827465;Yang2025_Silkie_Wencha	0.639895918
ENSGALG00010015957	ENSGALG0001001595	3	-0.329996536	0.210580274	-1.567091901	0.117095549	0.067946851	4.449316495	55.04927549	1	CRA004775;PRJNA827465;Yang2025_Silkie_Wencha	0.639895918
ENSGALG00010013359	BTG1	3	-0.20738786	0.132337512	-1.567113191	0.117088236	0.037895909	7.770567912	74.26185547	1	CRA004775;PRJNA827465;Yang2025_Silkie_Wencha	0.639895918
ENSGALG00010009155	PAK4	3	-0.101582076	0.064832571	-1.566837068	0.117152781	0	0.291238003	0	1	CRA004775;PRJNA827465;Yang2025_Silkie_Wencha	0.639895918
ENSGALG00010008245	FBXO32	3	-0.214664983	0.137035075	-1.566496634	0.117232396	0	0.79011797	0	1	CRA004775;PRJNA827465;Yang2025_Silkie_Wencha	0.63998025
ENSGALG00010004487	ABHD10	3	-0.176423171	0.112696449	-1.565472311	0.117472205	0.017575009	3.709674411	46.0869128	1	CRA004775;PRJNA827465;Yang2025_Silkie_Wencha	0.640794561
ENSGALG00010026612	MFF	3	-0.084083568	0.053708712	-1.565548042	0.117454462	0	0.41250709	0	1	CRA004775;PRJNA827465;Yang2025_Silkie_Wencha	0.640794561
ENSGALG00010000254	ENSGALG0001000025	2	0.453856506	0.290086895	1.564553639	0.117687607	0	0.111351452	0	1	PRJNA827465;Yang2025_Silkie_Wenchang	0.640816016
ENSGALG00010018082	RAB11FIP3	3	0.433679305	0.277201871	1.5644891	0.117702751	0.197747903	19.69632483	89.8458214	0.666666667	CRA004775;PRJNA827465;Yang2025_Silkie_Wencha	0.640816016
ENSGALG00010015751	MYOM3	3	0.398739704	0.254867889	1.56449565	0.117701214	0.109074635	5.657097316	64.64618004	0.666666667	CRA004775;PRJNA827465;Yang2025_Silkie_Wencha	0.640816016
ENSGALG00010026272	LRRC58	3	-0.095794263	0.061201833	-1.565218839	0.117531606	0.001628141	2.274939671	12.08558076	1	CRA004775;PRJNA827465;Yang2025_Silkie_Wencha	0.640816016
ENSGALG00010007130	IRF2	3	-0.063852416	0.040808722	-1.564675717	0.117658965	0	0.606178189	0	0.666666667	CRA004775;PRJNA827465;Yang2025_Silkie_Wencha	0.640816016
ENSGALG00010003257	ENSGALG0001000325	2	2.325069295	1.487104114	1.563487905	0.117937878	3.827602075	6.791932163	85.27664918	1	CRA004775;Yang2025_Silkie_Wenchang	0.641253343
ENSGALG00010024270	RAB18L	3	-0.594016664	0.380145692	-1.562602643	0.118146086	0.237393295	4.441970469	54.97493705	0.666666667	CRA004775;PRJNA827465;Yang2025_Silkie_Wencha	0.641253343
ENSGALG00010019823	LCT	3	0.273413622	0.174931432	1.56297596	0.118058249	0.010778503	2.25271859	11.21838259	1	CRA004775;PRJNA827465;Yang2025_Silkie_Wencha	0.641253343
ENSGALG00010008136	ELP1	3	0.174543772	0.111675021	1.56296162	0.118061623	0	1.826039708	0	0.666666667	CRA004775;PRJNA827465;Yang2025_Silkie_Wencha	0.641253343
ENSGALG00010002361	ENSGALG0001000236	3	0.17392201	0.11133986	1.562082169	0.118268633	0.023688076	6.353252215	68.52005977	1	CRA004775;PRJNA827465;Yang2025_Silkie_Wencha	0.641253343
ENSGALG00010010710	UCK2	3	-0.119116551	0.07625755	-1.562029606	0.118281015	0.003297003	2.373922553	15.75125325	1	CRA004775;PRJNA827465;Yang2025_Silkie_Wencha	0.641253343
ENSGALG00010008561	EEF1D	3	-0.108584318	0.069507645	-1.562192448	0.118242659	0	1.63075099	0	1	CRA004775;PRJNA827465;Yang2025_Silkie_Wencha	0.641253343
ENSGALG00010005610	MTHFD1L	3	-0.100812592	0.064539656	-1.562025566	0.118281966	0	0.624421799	0	1	CRA004775;PRJNA827465;Yang2025_Silkie_Wencha	0.641253343
ENSGALG00010018631	KAT14	3	-0.097333415	0.062267431	-1.563151301	0.118017012	0.003198448	2.635870572	24.12374031	1	CRA004775;PRJNA827465;Yang2025_Silkie_Wencha	0.641253343
ENSGALG00010024406	NUDT21	3	-0.089410683	0.057229297	-1.562323616	0.118211771	0	1.625765138	0	1	CRA004775;PRJNA827465;Yang2025_Silkie_Wencha	0.641253343
ENSGALG00010016989	RHOG2	3	0.074416608	0.047601472	1.563325773	0.117975989	0	0.365188892	0	1	CRA004775;PRJNA827465;Yang2025_Silkie_Wencha	0.641253343
ENSGALG00010007126	ENSGALG0001000712	3	0.954166106	0.611022275	1.561589724	0.118384672	0.213593073	2.468949776	18.99389695	0.666666667	CRA004775;PRJNA827465;Yang2025_Silkie_Wencha	0.641318343
ENSGALG00010024694	GTF2H3	3	-0.174514107	0.1117482	-1.561672641	0.118365127	0.020836708	4.513473751	55.68823238	1	CRA004775;PRJNA827465;Yang2025_Silkie_Wencha	0.641318343
ENSGALG00010022681	PPP2R3A	3	-0.170829475	0.109429561	-1.561090749	0.118502341	0.013645055	3.223707631	37.9596344	0.666666667	CRA004775;PRJNA827465;Yang2025_Silkie_Wencha	0.641464239
ENSGALG00010006344	HINT3	3	-0.145486326	0.093193189	-1.561126167	0.118493985	0.007220125	2.709914645	26.19693748	0.666666667	CRA004775;PRJNA827465;Yang2025_Silkie_Wencha	0.641464239
ENSGALG00010018467	TLX1	3	1.00249277	0.642570525	1.560128782	0.118729451	0.883854854	8.025501116	75.07943777	0.666666667	CRA004775;PRJNA827465;Yang2025_Silkie_Wencha	0.641892976
ENSGALG00010017169	CCDC198	2	0.668370332	0.428527648	1.559690105	0.118833132	0.280404329	4.21738397	76.28861856	1	PRJNA827465;Yang2025_Silkie_Wenchang	0.641892976
ENSGALG00010010478	ENSGALG0001001047	2	0.476300833	0.305326074	1.559974315	0.118765951	0	0.071490802	0	1	PRJNA827465;Yang2025_Silkie_Wenchang	0.641892976
ENSGALG00010013188	ENSGALG0001001318	3	-0.275373178	0.176493668	-1.560243948	0.118702244	0.014156661	2.26934201	11.86872711	1	CRA004775;PRJNA827465;Yang2025_Silkie_Wencha	0.641892976
ENSGALG00010014080	MYO1B	3	0.15517176	0.099433479	1.560558488	0.11862796	0.011184954	3.13138766	36.13055242	1	CRA004775;PRJNA827465;Yang2025_Silkie_Wencha	0.641892976
ENSGALG00010005915	PAPSS1	3	0.109034749	0.06991714	1.559485259	0.118881571	0	0.150089111	0	1	CRA004775;PRJNA827465;Yang2025_Silkie_Wencha	0.641892976
ENSGALG00010019646	VPS37C	3	0.097651513	0.062620802	1.55941015	0.118899335	0	1.681738327	0	0.666666667	CRA004775;PRJNA827465;Yang2025_Silkie_Wencha	0.641892976
ENSGALG00010005297	IL22RA2	2	-0.902255616	0.578730813	-1.559024673	0.118990541	0.331517193	1.733405544	42.31009566	1	CRA004775;PRJNA827465	0.642140174
ENSGALG00010018255	MFN1	3	-0.095235644	0.061105015	-1.558556929	0.119101284	0.003958292	3.095919255	35.39883197	0.666666667	CRA004775;PRJNA827465;Yang2025_Silkie_Wencha	0.642492581
ENSGALG00010022302	ENSGALG0001002230	3	-0.246331245	0.158103764	-1.558035301	0.11922488	0.032027992	3.42822967	41.66085145	1	CRA004775;PRJNA827465;Yang2025_Silkie_Wencha	0.642668922
ENSGALG00010007375	CHORDC1	3	0.110592783	0.070975101	1.558191275	0.119187913	0.004637915	2.595061434	22.93053361	0.666666667	CRA004775;PRJNA827465;Yang2025_Silkie_Wencha	0.642668922
ENSGALG00010029762	SLC47A2	3	0.342826116	0.22007317	1.557782424	0.119284834	0	0.499953613	0	1	CRA004775;PRJNA827465;Yang2025_Silkie_Wencha	0.642700661
ENSGALG00010004003	HNRNPDL	3	0.185615144	0.119179992	1.557435446	0.119367136	0.027313384	6.824888959	70.69549392	1	CRA004775;PRJNA827465;Yang2025_Silkie_Wencha	0.642700661
ENSGALG00010029686	KCTD2	3	-0.176858154	0.113544382	-1.557612549	0.119325122	0.032469255	14.17829368	85.89393022	1	CRA004775;PRJNA827465;Yang2025_Silkie_Wencha	0.642700661
ENSGALG00010028760	SSR2	3	-0.213295861	0.137023379	-1.556638459	0.119556347	0.036672039	5.758722506	65.27007513	1	CRA004775;PRJNA827465;Yang2025_Silkie_Wencha	0.643474379
ENSGALG00010014403	H2BC37	2	1.766149744	1.136437435	1.554110846	0.120157979	2.244590131	7.396468611	86.48003456	1	CRA004775;Yang2025_Silkie_Wenchang	0.644488811
ENSGALG00010021314	ZP3	3	1.008344663	0.648805792	1.554154842	0.120147486	1.108596847	27.59906092	92.75337662	0.666666667	CRA004775;PRJNA827465;Yang2025_Silkie_Wencha	0.644488811
ENSGALG00010016959	PERCC1	2	0.799915603	0.515104267	1.552919775	0.120442302	0	0.079393833	0	1	PRJNA827465;Yang2025_Silkie_Wenchang	0.644488811
ENSGALG00010002768	TRIM39.2	3	0.484829924	0.312085095	1.553518356	0.120299347	0	1.456682291	0	0.666666667	CRA004775;PRJNA827465;Yang2025_Silkie_Wencha	0.644488811
ENSGALG00010003597	RNF144A	3	-0.40635341	0.261627696	-1.553174284	0.120381503	0.137612554	10.48997115	80.93417064	1	CRA004775;PRJNA827465;Yang2025_Silkie_Wencha	0.644488811
ENSGALG00010021893	FAM43B	3	0.264908959	0.170468746	1.554003095	0.120183678	0	0.672274201	0	0.666666667	CRA004775;PRJNA827465;Yang2025_Silkie_Wencha	0.644488811
ENSGALG00010017696	SLC18A1	3	0.255730321	0.16469123	1.552786519	0.120474144	0.025044859	2.863356616	30.15190673	0.666666667	CRA004775;PRJNA827465;Yang2025_Silkie_Wencha	0.644488811
ENSGALG00010021325	ARPC1A	3	-0.116218643	0.074809296	-1.553532114	0.120296063	0.00701977	3.066189499	34.77245941	1	CRA004775;PRJNA827465;Yang2025_Silkie_Wencha	0.644488811
ENSGALG00010021070	RNF141	3	-0.115422019	0.074318382	-1.553074977	0.120405223	0.008603837	4.35430356	54.06842972	0.666666667	CRA004775;PRJNA827465;Yang2025_Silkie_Wencha	0.644488811
ENSGALG00010001198	IFRD1	3	0.103320411	0.066502931	1.553621919	0.120274628	0	0.956379304	0	1	CRA004775;PRJNA827465;Yang2025_Silkie_Wencha	0.644488811
ENSGALG00010013893	CSNK2A1	3	-0.096947613	0.062326967	-1.555468173	0.119834609	0.005152433	3.403298449	41.23348187	1	CRA004775;PRJNA827465;Yang2025_Silkie_Wencha	0.644488811
ENSGALG00010009411	SMARCA5	3	0.096445875	0.062052359	1.554266057	0.120120966	0	1.812389155	0	0.666666667	CRA004775;PRJNA827465;Yang2025_Silkie_Wencha	0.644488811
ENSGALG00010022245	PIKFYVE	3	0.079682338	0.051257379	1.554553508	0.120052444	0	0.119216984	0	1	CRA004775;PRJNA827465;Yang2025_Silkie_Wencha	0.644488811
ENSGALG00010012911	CSNK1E	3	-0.078192508	0.050277048	-1.555232691	0.119890661	0	0.597270778	0	0.666666667	CRA004775;PRJNA827465;Yang2025_Silkie_Wencha	0.644488811
ENSGALG00010027861	PNPLA7	3	-0.07633841	0.04910675	-1.554540064	0.120055648	0	1.94999444	0	1	CRA004775;PRJNA827465;Yang2025_Silkie_Wencha	0.644488811
ENSGALG00010023332	PPP1R7	3	-0.07473081	0.048084489	-1.554156275	0.120147145	0	1.155856786	0	0.666666667	CRA004775;PRJNA827465;Yang2025_Silkie_Wencha	0.644488811
ENSGALG00010029737	ENSGALG0001002973	3	0.694944654	0.447620925	1.552529418	0.120535599	0	1.69319447	0	1	CRA004775;PRJNA827465;Yang2025_Silkie_Wencha	0.644573691
ENSGALG00010002356	WNT11	3	-0.513453519	0.331253615	-1.550031443	0.121133969	0.223661757	7.610840878	73.72169472	1	CRA004775;PRJNA827465;Yang2025_Silkie_Wencha	0.645188996
ENSGALG00010004369	UBE2M	3	-0.332726312	0.214410598	-1.551818407	0.12070568	0.102631712	12.38791159	83.85522866	1	CRA004775;PRJNA827465;Yang2025_Silkie_Wencha	0.645188996
ENSGALG00010022614	DAO	3	-0.32135256	0.207306205	-1.550134792	0.121109167	0.08310688	6.709814077	70.19291478	0.666666667	CRA004775;PRJNA827465;Yang2025_Silkie_Wencha	0.645188996
ENSGALG00010018071	GPAT4	3	-0.312356982	0.201389843	-1.551006633	0.120900094	0.096414769	12.09999906	83.47107309	1	CRA004775;PRJNA827465;Yang2025_Silkie_Wencha	0.645188996
ENSGALG00010011018	QDPR	3	-0.277300105	0.178761177	-1.551232264	0.120846033	0.071107777	8.181394121	75.55428854	1	CRA004775;PRJNA827465;Yang2025_Silkie_Wencha	0.645188996
ENSGALG00010005159	ARHGAP21	3	0.228925679	0.147656979	1.550388476	0.121048303	0.042238041	5.910541669	66.16215379	1	CRA004775;PRJNA827465;Yang2025_Silkie_Wencha	0.645188996
ENSGALG00010021071	HIF1AN	3	-0.217142032	0.140095812	-1.549953771	0.121152612	0.03916242	8.411731584	76.2236826	1	CRA004775;PRJNA827465;Yang2025_Silkie_Wencha	0.645188996
ENSGALG00010009173	LYAR	3	0.182390577	0.117639931	1.550413835	0.12104222	0.018191658	3.352144041	40.33669271	1	CRA004775;PRJNA827465;Yang2025_Silkie_Wencha	0.645188996
ENSGALG00010005029	FAT4	3	-0.154470475	0.099572203	-1.551341332	0.120819907	0	0.651760756	0	0.666666667	CRA004775;PRJNA827465;Yang2025_Silkie_Wencha	0.645188996
ENSGALG00010029538	FZD9	3	0.152979853	0.098646106	1.550794645	0.120950904	0.008054248	2.663729376	24.91729761	0.666666667	CRA004775;PRJNA827465;Yang2025_Silkie_Wencha	0.645188996
ENSGALG00010014812	RWDD1	3	0.113921806	0.073421062	1.551622971	0.120752463	0.004502609	2.599048981	23.04877611	1	CRA004775;PRJNA827465;Yang2025_Silkie_Wencha	0.645188996
ENSGALG00010015758	PCDH19	3	-0.103669282	0.066906982	-1.549453878	0.121272651	0	1.035136499	0	0.666666667	CRA004775;PRJNA827465;Yang2025_Silkie_Wencha	0.645585093
ENSGALG00010013047	CTPS1	3	-0.070488596	0.045498655	-1.549245721	0.121322662	0	1.916606835	0	1	CRA004775;PRJNA827465;Yang2025_Silkie_Wencha	0.645608251
ENSGALG00010001885	SMPX	3	-0.215828627	0.139350337	-1.548820268	0.121424932	0	0.683635381	0	1	CRA004775;PRJNA827465;Yang2025_Silkie_Wencha	0.645909372
ENSGALG00010011648	ENSGALG0001001164	3	-0.200864249	0.129737457	-1.548236365	0.121565399	0	0.108033093	0	1	CRA004775;PRJNA827465;Yang2025_Silkie_Wencha	0.646413379
ENSGALG00010028364	MHM2	3	-0.637721977	0.41220089	-1.547114507	0.121835636	0.171114456	2.985316236	33.00542247	1	CRA004775;PRJNA827465;Yang2025_Silkie_Wencha	0.646673329
ENSGALG00010007992	LAPTM4A	3	-0.418650978	0.270653452	-1.546815583	0.121907722	0.199493021	27.27068342	92.66611706	1	CRA004775;PRJNA827465;Yang2025_Silkie_Wencha	0.646673329
ENSGALG00010020320	PDE6C	2	0.340862705	0.220279304	1.547411397	0.121764075	0	0.07565995	0	1	PRJNA827465;Yang2025_Silkie_Wenchang	0.646673329
ENSGALG00010017693	MTFMT	3	0.337401711	0.218142279	1.546704807	0.121934443	0.107295495	8.174693482	75.53425086	1	CRA004775;PRJNA827465;Yang2025_Silkie_Wencha	0.646673329
ENSGALG00010020584	PARP9	3	-0.281446111	0.181947752	-1.546851274	0.121899113	0.066453602	8.590778453	76.7192227	1	CRA004775;PRJNA827465;Yang2025_Silkie_Wencha	0.646673329
ENSGALG00010002134	ANKRD28	3	0.266204219	0.172044608	1.547297657	0.121791486	0.072603242	14.05993035	85.77517846	1	CRA004775;PRJNA827465;Yang2025_Silkie_Wencha	0.646673329
ENSGALG00010015994	ENSGALG0001001599	3	-0.214715663	0.138791846	-1.547033703	0.121855119	0.027692012	3.715328096	46.16895336	1	CRA004775;PRJNA827465;Yang2025_Silkie_Wencha	0.646673329
ENSGALG00010026899	HOOK1	3	0.252604915	0.163348861	1.54641369	0.12200469	0.039165366	3.943150733	49.27913906	1	CRA004775;PRJNA827465;Yang2025_Silkie_Wencha	0.646803267
ENSGALG00010004191	CABLES1	3	-0.355123923	0.229718105	-1.545911772	0.122125877	0.131711545	17.48084358	88.55890455	1	CRA004775;PRJNA827465;Yang2025_Silkie_Wencha	0.647002808
ENSGALG00010025360	THYN1	3	0.133633934	0.086445287	1.545878767	0.122133849	0.009309141	3.269976313	38.83747745	0.666666667	CRA004775;PRJNA827465;Yang2025_Silkie_Wencha	0.647002808
ENSGALG00010029456	RNF157	3	0.724033965	0.468509614	1.545398309	0.122249949	0.435464209	6.05489573	66.96887793	1	CRA004775;PRJNA827465;Yang2025_Silkie_Wencha	0.647070266
ENSGALG00010024911	CAT	3	-0.230240439	0.1490141	-1.545091637	0.1223241	0.042413986	7.512854199	73.37895896	0.666666667	CRA004775;PRJNA827465;Yang2025_Silkie_Wencha	0.647070266
ENSGALG00010014218	IL31RA	3	0.158294801	0.102431608	1.545370654	0.122256634	0	1.488941813	0	0.666666667	CRA004775;PRJNA827465;Yang2025_Silkie_Wencha	0.647070266
ENSGALG00010020104	BRAT1	3	-0.145606508	0.094239503	-1.545068717	0.122329643	0.006797756	2.568040833	22.1196184	1	CRA004775;PRJNA827465;Yang2025_Silkie_Wencha	0.647070266
ENSGALG00010000042	CKMT1B	3	0.597041312	0.386616357	1.544273284	0.122522142	0.232963424	4.235515068	52.78024118	1	CRA004775;PRJNA827465;Yang2025_Silkie_Wencha	0.647846134
ENSGALG00010023996	MYO7B	3	0.797739715	0.51677384	1.54369214	0.122662931	0.624610028	9.141002494	78.12056171	1	CRA004775;PRJNA827465;Yang2025_Silkie_Wencha	0.647858689
ENSGALG00010027380	GPX1	3	-0.26385008	0.170919835	-1.543706613	0.122659424	0.046692813	4.033492258	50.41517692	1	CRA004775;PRJNA827465;Yang2025_Silkie_Wencha	0.647858689
ENSGALG00010028008	NTMT1	3	-0.205042997	0.132802714	-1.543966918	0.122596347	0.025394909	3.576455342	44.07870898	1	CRA004775;PRJNA827465;Yang2025_Silkie_Wencha	0.647858689
ENSGALG00010023476	SCAMP2	3	-0.193093189	0.125100296	-1.543507047	0.122707799	0.033313155	7.401905182	72.97992948	1	CRA004775;PRJNA827465;Yang2025_Silkie_Wencha	0.647858689
ENSGALG00010003619	HNF4G	3	0.799246251	0.518628862	1.541075536	0.123298403	0.506905483	5.740374138	65.15906539	1	CRA004775;PRJNA827465;Yang2025_Silkie_Wencha	0.648071434
ENSGALG00010013164	FAM83F	3	0.701508617	0.454881332	1.542179396	0.123030006	0.22468195	3.108069208	35.65136855	1	CRA004775;PRJNA827465;Yang2025_Silkie_Wencha	0.648071434
ENSGALG00010005369	DPT	3	-0.615555188	0.399643566	-1.540260472	0.123496875	0.383188529	19.36642709	89.67284987	0.666666667	CRA004775;PRJNA827465;Yang2025_Silkie_Wencha	0.648071434
ENSGALG00010011438	ENSGALG0001001143	3	0.461966038	0.29994487	1.540169828	0.123518962	0.2132568	10.34699039	80.67070786	0.666666667	CRA004775;PRJNA827465;Yang2025_Silkie_Wencha	0.648071434
ENSGALG00010001195	ZNF608	3	0.444077823	0.288282184	1.540427568	0.123456166	0.187597599	8.883218563	77.48563783	1	CRA004775;PRJNA827465;Yang2025_Silkie_Wencha	0.648071434
ENSGALG00010008231	ZCCHC6	3	0.358234488	0.232362372	1.54170611	0.123145027	0.133464553	12.29723111	83.7361762	1	CRA004775;PRJNA827465;Yang2025_Silkie_Wencha	0.648071434
ENSGALG00010010661	CPEB2	3	0.341133813	0.221304531	1.541467822	0.123202969	0.076453116	4.144091665	51.73851928	1	CRA004775;PRJNA827465;Yang2025_Silkie_Wencha	0.648071434
ENSGALG00010020803	ABCC6	3	0.34017633	0.220901243	1.539947557	0.123573137	0.091673722	5.487899049	63.55618093	0.666666667	CRA004775;PRJNA827465;Yang2025_Silkie_Wencha	0.648071434
ENSGALG00010006237	ENSGALG0001000623	3	0.320835517	0.208250318	1.54062438	0.123408231	0	1.981757722	0	0.666666667	CRA004775;PRJNA827465;Yang2025_Silkie_Wencha	0.648071434
ENSGALG00010012690	NT5DC1	3	-0.245375192	0.159331072	-1.540033522	0.123552183	0.036411945	3.750006456	46.66675849	0.666666667	CRA004775;PRJNA827465;Yang2025_Silkie_Wencha	0.648071434
ENSGALG00010017535	LIMD1	3	-0.228403789	0.148039609	-1.542855935	0.122865734	0.037234964	4.59013326	56.42828026	1	CRA004775;PRJNA827465;Yang2025_Silkie_Wencha	0.648071434
ENSGALG00010022792	DDB2	3	-0.196440711	0.127504186	-1.540660881	0.123399342	0.020954449	3.489692995	42.68836821	1	CRA004775;PRJNA827465;Yang2025_Silkie_Wencha	0.648071434
ENSGALG00010019592	ALKBH3	3	-0.125888166	0.081724539	-1.540396156	0.123463818	0	0.009143556	0	1	CRA004775;PRJNA827465;Yang2025_Silkie_Wencha	0.648071434
ENSGALG00010003113	WBP4	3	0.123510522	0.080099256	1.541968401	0.123081273	0	1.750334884	0	1	CRA004775;PRJNA827465;Yang2025_Silkie_Wencha	0.648071434
ENSGALG00010000288	VARS2	3	-0.119605044	0.077658054	-1.540149899	0.123523819	0.005841012	2.817707847	29.02032047	0.666666667	CRA004775;PRJNA827465;Yang2025_Silkie_Wencha	0.648071434
ENSGALG00010006352	GEMIN2	3	-0.103295287	0.067054217	-1.540474128	0.123444824	0	0.259508003	0	1	CRA004775;PRJNA827465;Yang2025_Silkie_Wencha	0.648071434
ENSGALG00010007835	GUCY1A1	3	-0.098479694	0.063876657	-1.54171647	0.123142508	0.000232187	2.03049468	1.501835	0.666666667	CRA004775;PRJNA827465;Yang2025_Silkie_Wencha	0.648071434
ENSGALG00010017520	ZBTB1	3	-0.051657279	0.033507993	-1.541640487	0.123160981	0	0.721367477	0	0.666666667	CRA004775;PRJNA827465;Yang2025_Silkie_Wencha	0.648071434
ENSGALG00010018882	HHAT	3	-0.175623162	0.114069627	-1.539613711	0.123654541	0.01459691	3.125686477	36.01405596	1	CRA004775;PRJNA827465;Yang2025_Silkie_Wencha	0.648090143
ENSGALG00010012337	ACSS3	3	-0.131112548	0.085172912	-1.53936909	0.123714216	0	0.14703123	0	1	CRA004775;PRJNA827465;Yang2025_Silkie_Wencha	0.648090143
ENSGALG00010029153	OGFOD3	3	0.11145798	0.072403231	1.53940617	0.123705169	0	1.051812684	0	1	CRA004775;PRJNA827465;Yang2025_Silkie_Wencha	0.648090143
ENSGALG00010021763	ENSGALG0001002176	2	1.521073349	0.988764614	1.538357389	0.123961255	1.199765858	2.583884508	61.29857983	1	CRA004775;Yang2025_Silkie_Wenchang	0.648475836
ENSGALG00010010368	ENSGALG0001001036	3	0.265563568	0.172642272	1.538230263	0.123992324	0.047528476	4.35126828	54.03638959	1	CRA004775;PRJNA827465;Yang2025_Silkie_Wencha	0.648475836
ENSGALG00010024209	RWDD3	3	-0.149132073	0.096950265	-1.538232745	0.123991717	0	0.352685838	0	0.666666667	CRA004775;PRJNA827465;Yang2025_Silkie_Wencha	0.648475836
ENSGALG00010023415	HOXB4	3	-0.104578874	0.067993162	-1.538079283	0.124029231	0	0.606003174	0	0.666666667	CRA004775;PRJNA827465;Yang2025_Silkie_Wencha	0.648475836
ENSGALG00010028102	CERCAM	3	-0.083398876	0.054227615	-1.537941059	0.124063027	0	0.403720135	0	1	CRA004775;PRJNA827465;Yang2025_Silkie_Wencha	0.648475836
ENSGALG00010024855	CAPRIN1	3	-0.071060489	0.04618075	-1.538746971	0.12386608	0.00116845	2.217135803	9.793527441	0.666666667	CRA004775;PRJNA827465;Yang2025_Silkie_Wencha	0.648475836
ENSGALG00010029317	KPNA2	3	-0.160298946	0.104246289	-1.537694507	0.124123327	0.007060187	2.395913845	16.52454433	0.666666667	CRA004775;PRJNA827465;Yang2025_Silkie_Wencha	0.648551266
ENSGALG00010029933	TVP23B	3	-0.21791353	0.141818225	-1.536569297	0.124398816	0.041438952	6.76784225	70.44848378	1	CRA004775;PRJNA827465;Yang2025_Silkie_Wencha	0.648834387
ENSGALG00010028456	FBXW5	3	-0.209678633	0.136461902	-1.536536058	0.124406961	0.036341322	6.745988923	70.35275298	1	CRA004775;PRJNA827465;Yang2025_Silkie_Wencha	0.648834387
ENSGALG00010020975	HCCS	3	-0.155193633	0.100979203	-1.536887089	0.124320962	0.014868845	3.951920836	49.39169879	0.666666667	CRA004775;PRJNA827465;Yang2025_Silkie_Wencha	0.648834387
ENSGALG00010009407	PTPN12	3	0.104215324	0.067806139	1.536960012	0.124303102	0.003952818	2.412195629	17.08798509	1	CRA004775;PRJNA827465;Yang2025_Silkie_Wencha	0.648834387
ENSGALG00010000039	PDIA3	3	0.062980713	0.040981457	1.536810019	0.124339839	0	1.84924939	0	0.666666667	CRA004775;PRJNA827465;Yang2025_Silkie_Wencha	0.648834387
ENSGALG00010019270	ENSGALG0001001927	3	0.698531847	0.454690553	1.536279659	0.124469806	0.488254222	9.651000134	79.27675917	0.666666667	CRA004775;PRJNA827465;Yang2025_Silkie_Wencha	0.648922783
ENSGALG00010029758	DNAH9	3	-0.301594555	0.196362775	-1.535904938	0.124561697	0	1.267975853	0	1	CRA004775;PRJNA827465;Yang2025_Silkie_Wencha	0.649026259

ENSGALG00010016962	SQOR	3	-0.121117629	0.07886164	-1.535824373	0.12458146	0.004405454	2.558074591	21.81619696	0.666666667	CRA004775;PRJNA827465;Yang2025_Silkie_Wencha	0.649026259
ENSGALG00010025943	CATH1	3	0.708059328	0.461126912	1.535497734	0.124661613	0.385808308	5.186892592	61.44126826	1	CRA004775;PRJNA827465;Yang2025_Silkie_Wencha	0.649204622
ENSGALG00010014880	SFRP2	3	-0.347615589	0.226474707	-1.534898061	0.12480887	0.10605425	11.6203993	82.78888746	1	CRA004775;PRJNA827465;Yang2025_Silkie_Wencha	0.64925408
ENSGALG00010011881	RIOK2	3	0.1488646	0.096980565	1.534994156	0.124785264	0	1.301821303	0	0.666666667	CRA004775;PRJNA827465;Yang2025_Silkie_Wencha	0.649254088
ENSGALG00010027951	FAM32A	3	-0.098112074	0.063919323	-1.534936055	0.124799536	0	0.193541457	0	1	CRA004775;PRJNA827465;Yang2025_Silkie_Wencha	0.649254088
ENSGALG00010029495	RPL38	3	-0.543416672	0.354130635	-1.534509073	0.124904463	0.335262348	21.95776799	90.8916061	1	CRA004775;PRJNA827465;Yang2025_Silkie_Wencha	0.649297726
ENSGALG00010012188	ADAMTS6	3	0.242821119	0.158279977	1.534124049	0.124999138	0	1.890688759	0	1	CRA004775;PRJNA827465;Yang2025_Silkie_Wencha	0.649297726
ENSGALG00010018503	BMP2	3	0.191003428	0.124476057	1.534459185	0.124916727	0	1.044776266	0	0.666666667	CRA004775;PRJNA827465;Yang2025_Silkie_Wencha	0.649297726
ENSGALG00010028488	PTGES	3	-0.113303568	0.073855993	-1.534114749	0.125001426	0	1.138411134	0	1	CRA004775;PRJNA827465;Yang2025_Silkie_Wencha	0.649297726
ENSGALG00010016548	EVL	3	-0.106149934	0.069201289	-1.533930015	0.125046871	0.003936554	2.44383577	18.16144013	0.666666667	CRA004775;PRJNA827465;Yang2025_Silkie_Wencha	0.649297726
ENSGALG00010008424	PPIP5K2	3	0.164819939	0.107462682	1.533741162	0.125093343	0	1.907078589	0	1	CRA004775;PRJNA827465;Yang2025_Silkie_Wencha	0.649300579
ENSGALG00010004380	ISCA2	3	-0.352765816	0.230130044	-1.532897703	0.125301062	0.119561193	8.794339751	77.25809945	1	CRA004775;PRJNA827465;Yang2025_Silkie_Wencha	0.64962421
ENSGALG00010014697	ENSGALG0001001469	2	0.35066913	0.228762732	1.532894485	0.125301855	0	0.224474246	0	1	PRJNA827465;Yang2025_Silkie_Wenchang	0.649624214
ENSGALG00010022608	P3H4	3	0.165557563	0.10802519	1.532582944	0.125378647	0.013640162	3.129586267	36.0937891	0.666666667	CRA004775;PRJNA827465;Yang2025_Silkie_Wencha	0.649624214
ENSGALG00010022579	TNFAIP8L3	3	-0.127378537	0.083115124	-1.532555464	0.125385422	0	0.13455266	0	0.666666667	CRA004775;PRJNA827465;Yang2025_Silkie_Wencha	0.649624214
ENSGALG00010028921	MRPL41	3	-0.105879205	0.069069037	-1.532947472	0.125288798	0	0.854501074	0	1	CRA004775;PRJNA827465;Yang2025_Silkie_Wencha	0.649624214
ENSGALG00010004224	NT5E	3	-0.198212653	0.12935695	-1.532292261	0.12545033	0.002875424	2.109914822	5.209443582	0.666666667	CRA004775;PRJNA827465;Yang2025_Silkie_Wencha	0.649722424
ENSGALG00010009571	DNAJC2	3	0.580275694	0.378871093	1.531591361	0.125623306	0.37296025	17.66627663	88.67899534	1	CRA004775;PRJNA827465;Yang2025_Silkie_Wencha	0.650008427
ENSGALG00010024001	ENSGALG0001002400	3	0.575652108	0.376365865	1.529501373	0.126140199	0.167066098	3.294187467	39.28700112	1	CRA004775;PRJNA827465;Yang2025_Silkie_Wencha	0.650008427
ENSGALG00010004429	ENSGALG0001000442	3	0.561292264	0.366544579	1.531306957	0.125693547	0.250244827	5.384728768	62.85792496	0.666666667	CRA004775;PRJNA827465;Yang2025_Silkie_Wencha	0.650008427
ENSGALG00010029516	ENSGALG0001002951	3	0.391043972	0.255632367	1.529712287	0.126087961	0.122387087	6.225205596	67.87254703	0.666666667	CRA004775;PRJNA827465;Yang2025_Silkie_Wencha	0.650008427
ENSGALG00010010831	DESI1	3	-0.372440291	0.243628326	-1.528723268	0.126333062	0.162412002	26.09246537	92.33495198	1	CRA004775;PRJNA827465;Yang2025_Silkie_Wencha	0.650008427
ENSGALG00010013305	THOC2	3	0.34709155	0.226889453	1.529781795	0.126070749	0.128742305	13.52061526	85.20777375	1	CRA004775;PRJNA827465;Yang2025_Silkie_Wencha	0.650008427
ENSGALG00010022800	KLHL10	3	-0.328487333	0.214484468	-1.531520376	0.125640835	0	1.988839686	0	1	CRA004775;PRJNA827465;Yang2025_Silkie_Wencha	0.650008427
ENSGALG00010021779	UNC13C	3	0.327035698	0.213623117	1.530900322	0.12579403	0.056103654	3.415290591	41.43982929	1	CRA004775;PRJNA827465;Yang2025_Silkie_Wencha	0.650008427
ENSGALG00010008127	TBC1D2	3	0.318875768	0.208333938	1.53059924	0.12586847	0.085358378	6.569588491	69.55669289	0.666666667	CRA004775;PRJNA827465;Yang2025_Silkie_Wencha	0.650008427
ENSGALG00010028427	ENSGALG0001002842	3	0.26888474	0.175737169	1.53009601	0.125992966	0.018074339	2.48253628	19.47722972	0.666666667	CRA004775;PRJNA827465;Yang2025_Silkie_Wencha	0.650008427
ENSGALG00010002243	ARSH	3	0.265245417	0.173420677	1.529491299	0.126142694	0.041788659	3.809761991	47.50328224	0.666666667	CRA004775;PRJNA827465;Yang2025_Silkie_Wencha	0.650008427
ENSGALG00010021277	ENSGALG0001002127	3	0.206460188	0.135038425	1.528899558	0.126289346	0.006904402	2.246031431	10.95405111	1	CRA004775;PRJNA827465;Yang2025_Silkie_Wencha	0.650008427
ENSGALG00010028496	ENSGALG0001002849	3	-0.191997759	0.125585705	-1.528818576	0.126309426	0.019253884	3.366066941	40.58347517	0.666666667	CRA004775;PRJNA827465;Yang2025_Silkie_Wencha	0.650008427
ENSGALG00010029240	MIS12	3	-0.18584348	0.121526188	-1.529246353	0.126203383	0.011250063	2.47233897	19.10494378	0.666666667	CRA004775;PRJNA827465;Yang2025_Silkie_Wencha	0.650008427
ENSGALG00010012732	MGST1	3	0.136406635	0.089134928	1.530338766	0.125932897	0	0.336633012	0	1	CRA004775;PRJNA827465;Yang2025_Silkie_Wencha	0.650008427
ENSGALG00010020654	SAYSD1	3	-0.126175356	0.082521326	-1.529003014	0.126263697	0	1.698860898	0	1	CRA004775;PRJNA827465;Yang2025_Silkie_Wencha	0.650008427
ENSGALG00010015043	COQ10B	3	0.122769504	0.080239946	1.530029739	0.126009368	0	1.259416848	0	0.666666667	CRA004775;PRJNA827465;Yang2025_Silkie_Wencha	0.650008427
ENSGALG00010013412	COMMD7	3	-0.094110728	0.061483066	-1.530677208	0.125849189	0	1.739270346	0	1	CRA004775;PRJNA827465;Yang2025_Silkie_Wencha	0.650008427
ENSGALG00010013328	SLC37A3	3	-0.302362907	0.197886645	-1.527960147	0.126522433	0.094088273	11.30424797	82.30753602	1	CRA004775;PRJNA827465;Yang2025_Silkie_Wencha	0.650745974
ENSGALG00010002860	NIPA2	3	-0.374893176	0.24547286	-1.527228617	0.126704173	0.161023216	21.6082746	90.74428645	1	CRA004775;PRJNA827465;Yang2025_Silkie_Wencha	0.650970311
ENSGALG00010023112	PCMT1L	3	-0.126754674	0.08299628	-1.527233199	0.126703034	0	0.915336038	0	1	CRA004775;PRJNA827465;Yang2025_Silkie_Wencha	0.650970311
ENSGALG00010007891	GDI1	3	-0.058728363	0.038449428	-1.527418372	0.12665701	0	1.86401247	0	1	CRA004775;PRJNA827465;Yang2025_Silkie_Wencha	0.650970311
ENSGALG00010027105	CHERP	3	-0.057456934	0.037642032	-1.526403611	0.126909379	0	0.204719034	0	1	CRA004775;PRJNA827465;Yang2025_Silkie_Wencha	0.651787762
ENSGALG00010023680	MPZL3	2	0.93529391	0.613226829	1.525200571	0.127209078	0.356970853	1.903358529	47.46129094	1	CRA004775;PRJNA827465	0.652379092
ENSGALG00010017127	SOX8	3	0.224766474	0.147353566	1.525354827	0.127170619	0.030783649	3.753710842	46.71939091	1	CRA004775;PRJNA827465;Yang2025_Silkie_Wencha	0.652379092
ENSGALG00010015296	RPL35A	3	-0.108687219	0.07125	-1.525441663	0.127148974	0	1.037408991	0	1	CRA004775;PRJNA827465;Yang2025_Silkie_Wencha	0.652379092
ENSGALG00010027137	FKBP8	3	-0.077334627	0.050688044	-1.525697601	0.127085192	0	1.674459112	0	0.666666667	CRA004775;PRJNA827465;Yang2025_Silkie_Wencha	0.652379092
ENSGALG00010005417	LOXL3	3	0.30184444	0.198002725	1.524445884	0.127397365	0.086929538	7.74301779	74.17027761	1	CRA004775;PRJNA827465;Yang2025_Silkie_Wencha	0.652642701
ENSGALG00010003474	RPS7	3	-0.27348214	0.179382823	-1.52457262	0.127365731	0.065172378	6.428600279	68.88902851	1	CRA004775;PRJNA827465;Yang2025_Silkie_Wencha	0.652642701
ENSGALG00010025470	ORAOV1	3	0.198262443	0.130077134	1.524254629	0.127445116	0.026269787	4.208830755	52.48086425	1	CRA004775;PRJNA827465;Yang2025_Silkie_Wencha	0.652642701
ENSGALG00010004042	PLAG1	3	0.104732547	0.068705486	1.524369496	0.127416436	0	0.884599036	0	1	CRA004775;PRJNA827465;Yang2025_Silkie_Wencha	0.652642701
ENSGALG00010029542	AATK	3	0.11500417	0.075467762	1.523884717	0.127537512	0	1.497995631	0	1	CRA004775;PRJNA827465;Yang2025_Silkie_Wencha	0.652642701
ENSGALG00010019232	SOS2	3	-0.094772272	0.062184248	-1.524055939	0.127494738	0.003737003	2.657534606	24.74227821	0.666666667	CRA004775;PRJNA827465;Yang2025_Silkie_Wencha	0.652643098
ENSGALG00010021927	ENSGALG0001002192	2	0.581081473	0.381494557	1.523171071	0.127715912	0	0.415967333	0	1	PRJNA827465;Yang2025_Silkie_Wenchang	0.652879494
ENSGALG00010022946	KRT17	3	0.397335124	0.260864801	1.523145791	0.127722235	0	1.425873652	0	1	CRA004775;PRJNA827465;Yang2025_Silkie_Wencha	0.652879494
ENSGALG00010013908	TBC1D20	3	-0.134057269	0.088004383	-1.523302179	0.127683122	0.0105628	3.58674826	44.23918673	1	CRA004775;PRJNA827465;Yang2025_Silkie_Wencha	0.652879494
ENSGALG00010005468	PPIL4	3	0.257601623	0.169169859	1.522739485	0.127823895	0.066295893	11.61534188	82.7813936	0.666666667	CRA004775;PRJNA827465;Yang2025_Silkie_Wencha	0.652927043
ENSGALG00010017751	ST3GAL5	3	0.204516954	0.134294854	1.522894946	0.127784991	0.013593317	2.636634331	24.14571956	0.666666667	CRA004775;PRJNA827465;Yang2025_Silkie_Wencha	0.652927043
ENSGALG00010022116	CYP20A1	3	-0.084297956	0.055379127	-1.522197272	0.127959658	0	1.598360045	0	1	CRA004775;PRJNA827465;Yang2025_Silkie_Wencha	0.653384475
ENSGALG00010025597	SCN2A	2	1.446713906	0.950785309	1.521598927	0.128109607	1.239776858	3.05894364	67.30897599	1	PRJNA827465;Yang2025_Silkie_Wenchang	0.653913981
ENSGALG00010005527	NUP43	3	-0.115422956	0.075878206	-1.521160847	0.128219478	0.007175144	3.401208501	41.19737148	1	CRA004775;PRJNA827465;Yang2025_Silkie_Wencha	0.654238615
ENSGALG00010027929	CDK5RAP2	3	0.280247872	0.184287691	1.520708572	0.128332987	0.068054564	7.331985486	72.72225915	1	CRA004775;PRJNA827465;Yang2025_Silkie_Wencha	0.654345509
ENSGALG00010020653	NDUFAF3	3	-0.136558747	0.089794406	-1.52079348	0.128311671	0	1.099636997	0	0.666666667	CRA004775;PRJNA827465;Yang2025_Silkie_Wencha	0.654345509
ENSGALG00010025983	RNF26	3	-0.200836401	0.132103108	-1.520300345	0.128435507	0.031208635	4.991798259	59.93427827	1	CRA004775;PRJNA827465;Yang2025_Silkie_Wencha	0.65463217
ENSGALG00010005291	IFNGR1	3	-0.299788869	0.197266245	-1.519717018	0.128582113	0.085649997	9.398353269	78.71967628	1	CRA004775;PRJNA827465;Yang2025_Silkie_Wencha	0.654671404
ENSGALG00010006851	TMEM167A	3	-0.263255528	0.173209491	-1.519867797	0.128544206	0.065972544	7.954074141	74.85565303	1	CRA004775;PRJNA827465;Yang2025_Silkie_Wencha	0.654671404
ENSGALG00010015298	ENSGALG0001001529	3	0.239824155	0.157778421	1.520006057	0.128509453	0	1.943086483	0	1	CRA004775;PRJNA827465;Yang2025_Silkie_Wencha	0.654671404
ENSGALG00010017532	MIXL1	3	-0.504871375	0.332400673	-1.518863878	0.128796763	0.107568003	2.935062572	31.85835222	0.666666667	CRA004775;PRJNA827465;Yang2025_Silkie_Wencha	0.655310338
ENSGALG00010003812	BG8	3	-0.34047739	0.224167908	-1.518849833	0.128800299	0	1.208215829	0	1	CRA004775;PRJNA827465;Yang2025_Silkie_Wencha	0.655310338
ENSGALG00010021938	CABP2	3	0.442901207	0.29164911	1.518609836	0.128860734	0.219802248	15.84317043	87.37626406	0.666666667	CRA004775;PRJNA827465;Yang2025_Silkie_Wencha	0.655381984
ENSGALG00010021078	ENSGALG0001002107	3	-0.721960563	0.475535666	-1.518204867	0.128962761	0.537292674	10.40366364	80.77600286	1	CRA004775;PRJNA827465;Yang2025_Silkie_Wencha	0.655580248
ENSGALG00010020940	ENSGALG0001002094	3	0.462177525	0.3044842	1.517903146	0.129038817	0.223468193	17.43623384	88.52963307	1	CRA004775;PRJNA827465;Yang2025_Silkie_Wencha	0.655580248
ENSGALG00010003292	GADD45GIP1	3	0.170865459	0.112560132	1.517992704	0.129016238	0.017463215	3.5132088	43.07198592	1	CRA004775;PRJNA827465;Yang2025_Silkie_Wencha	0.655580248
ENSGALG00010023260	ENSGALG0001002326	3	-0.338255342	0.222884553	-1.517625774	0.129108766	0	1.974653764	0	0.666666667	CRA004775;PRJNA827465;Yang2025_Silkie_Wencha	0.655700013
ENSGALG00010025563	TSPEAR	3	0.255176622	0.168195425	1.517143656	0.129230418	0	0.033514348	0	1	CRA004775;PRJNA827465;Yang2025_Silkie_Wencha	0.656082184
ENSGALG00010023227	RNPC3	3	0.423218383	0.279059353	1.516589137	0.12937045	0.206157507	18.50441787	89.19177024	1	CRA004775;PRJNA827465;Yang2025_Silkie_Wencha	0.656082898
ENSGALG00010027444	SELENBP1	3	-0.128691757	0.084866171	-1.516408195	0.129416168	0.005725728	2.645224821	24.39205984	1	CRA004775;PRJNA827465;Yang2025_Silkie_Wencha	0.656082898
ENSGALG00010006557	COQ6	3	-0.118211389	0.077953985	-1.516425213	0.129411868	0	1.095874643	0	1	CRA004775;PRJNA827465;Yang2025_Silkie_Wencha	0.656082898
ENSGALG00010029201	PIGS	3	-0.080395168	0.053004767	-1.516753529	0.129328924	0	1.777952509	0	1	CRA004775;PRJNA827465;Yang2025_Silkie_Wencha	0.656082898
ENSGALG00010018558	ENSGALG0001001855	3	0.222188732	0.146547915	1.516150748	0.129481239	0	0.241346935	0	1	CRA004775;PRJNA827465;Yang2025_Silkie_Wencha	0.656177502
ENSGALG00010029316	INTS2	3	-0.140495212	0.092702468	-1.515549859	0.129633214	0.014183882	5.012964344	60.10344653	1	CRA004775;PRJNA827465;Yang2025_Silkie_Wencha	0.656712294
ENSGALG00010024682	ENSGALG0001002468	3	-0.546502255	0.360692648	-1.515146644	0.129735272	0.30152505	9.641034227	79.25533762	0.666666667	CRA004775;PRJNA827465;Yang2025_Silkie_Wencha	0.656993914
ENSGALG00010005843	GPR12	2	-0.658767822	0.435271959	-1.513462581	0.130162199	0.299854357	4.789494288	79.12096894	1	PRJNA827465;Yang2025_Silkie_Wenchang	0.657062637
ENSGALG00010002928	ASB4	3	-0.510325551	0.337235873	-1.513259979	0.130213634	0.168631385	4.014326075	50.17843687	0.666666667	CRA004775;PRJNA827465;Yang2025_Silkie_Wencha	0.657062637
ENSGALG00010012725	ENSGALG0001001272	3	0.47998863	0.316927607	1.514505583	0.129897659	0.205688613	6.445261746	68.96945262	1	CRA004775;PRJNA827465;Yang2025_Silkie_Wencha	0.657062637
ENSGALG00010007416	DCTD	3	-0.466747348	0.308510214	-1.512907281	0.130303212	0.219419605	16.48164918	87.86529201	1	CRA004775;PRJNA827465;Yang2025_Silkie_Wencha	0.657062637
ENSGALG00010004285	SLC18B1	3	-0.421282372	0.278160771	-1.514528344	0.129891891	0.20609024	18.82889396	89.37802718	1	CRA004775;PRJNA827465;Yang2025_Silkie_Wencha	0.657062637
ENSGALG00010004200	ABCB1	3	0.395372005	0.26139598	1.512540494	0.130396419	0.138114897	6.348629264	68.49713668	1	CRA004775;PRJNA827465;Yang2025_Silkie_Wencha	0.657062637
ENSGALG00010027248	ENSGALG0001002724	2	-0.367465952	0.242955179	-1.512484536	0.130410644	0	0.365515939	0	1	PRJNA827465;Yang2025_Silkie_Wenchang	0.657062637
ENSGALG00010004043	CAB39L	3	-0.236739914	0.156498098	-1.512733488	0.13034737	0.041838357	5.466508618	63.41357639	1	CRA004775;PRJNA827465;Yang2025_Silkie_Wencha	0.657062637
ENSGALG00010009340	SYK	3	0.205608548	0.135894929	1.51299647	0.130280556	0.014230486	2.474727132	19.18300913	1	CRA004775;PRJNA827465;Yang2025_Silkie_Wencha	0.657062637
ENSGALG00010003850	PLA2G12A	3	-0.176167899	0.116480062	-1.512429647	0.130424598	0.029221066	7.692000924	73.99896308	1	CRA004775;PRJNA827465;Yang2025_Silkie_Wencha	0.657062637
ENSGALG00010017248	TECPR2	3	-0.170943648	0.113008657	-1.512659767	0.130366104	0.025506227	8.005697389	75.01779167	0.666666667	CRA004775;PRJNA827465;Yang2025_Silkie_Wencha	0.657062637
ENSGALG00010013538	E2F7	3	-0.136382722	0.090179589	-1.512345794	0.130445917	0	0.165384108	0	1	CRA004775;PRJNA827465;Yang2025_Silkie_Wencha	0.657062637
ENSGALG00010021454	ATP5J2	3	-0.129508255	0.085536845	-1.514063972	0.130009615	0	0.785836078	0	0.666666667	CRA004775;PRJNA827465;Yang2025_Silkie_Wencha	0.657062637
ENSGALG00010021138	DDX18	3	0.083139647	0.054915255	1.513962699	0.1300353	0	1.868464757	0	0.666666667	CRA004775;PRJNA827465;Yang2025_Silkie_Wencha	0.657062637
ENSGALG00010018819	LRRC28	3	-0.074396231	0.049144244	-1.513834058	0.130067932	0	0.291753299	0	0.666666667	CRA004775;PRJNA827465;Yang2025_Silkie_Wencha	0.657062637
ENSGALG00010022190	ACVR1B	3	-0.252306168	0.166859462	-1.512087868	0.130511511	0.056513932	7.101939235	71.83867767	1	CRA004775;PRJNA827465;Yang2025_Silkie_Wencha	0.657158921
ENSGALG00010017711	LRRFIP2	3	0.540090452	0.357300941	1.511584189	0.130639676	0.348645439	33.64286186	94.05520253	1	CRA004775;PRJNA827465;Yang2025_Silkie_Wencha	0.657570091
ENSGALG00010026008	NRP2	3	-0.115889092	0.076677777	-1.51137782	0.130692217	0	1.153569045	0	1	CRA004775;PRJNA827465;Yang2025_Silkie_Wencha	0.657600447
ENSGALG00010021738	PRDM11	3	-0.219760426	0.145428747	-1.511120954	0.130757637	0.04587472	9.85829706	79.71252045	1	CRA004775;PRJNA827465;Yang2025_Silkie_Wencha	0.657695563
ENSGALG00010029427	SPNS3	3	-0.544057734	0.360161338	-1.510594495	0.130891797	0.263710848	6.299605328	68.25197936	1	CRA004775;PRJNA827465;Yang2025_Silkie_Wencha	0.658136246
ENSGALG00010020765	GLT1D1	3	0.244659961	0.162006361	1.510187373	0.13099562	0	0.483731151	0	0.666666667	CRA004775;PRJNA827465;Yang2025_Silkie_Wencha	0.658424126
ENSGALG00010005423	ST3GAL6	3	-0.11084495	0.07341908	-1.509756736	0.131105508	0	0.115460816	0	1	CRA004775;PRJNA827465;Yang2025_Silkie_Wencha	0.65850827
ENSGALG00010021402	ENSGALG0001002140	3	-0.101803521	0.067423194	-1.509918395	0.131064248	0	1.061933504	0	0.666666667	CRA004775;PRJNA827465;Yang2025_Silkie_Wencha	0.65850827
ENSGALG00010025877	ENSGALG0001002587	2	-3.053353774	2.02376016	-1.508752783	0.131361971	4.316601025	1.925858223	48.07509775	1	PRJNA827465;Yang2025_Silkie_Wenchang	0.658603888
ENSGALG00010012702	PTPRO	2	-0.676051111	0.448500601	-1.507358318	0.131718837	0.161413462	1.500309423	33.34708265	0.5	CRA004775;PRJNA827465	0.658603888
ENSGALG00010006623	ENSGALG0001000662	3	0.463364283	0.307344432	1.507638449	0.131647087	0.187832455	6.54486597	69.4416966	1	CRA004775;PRJNA827465;Yang2025_Silkie_Wencha	0.658603888
ENSGALG00010010127	FAM13C	3	0.428616689	0.284367505	1.507263247	0.131743194	0.130476359	4.47484186	55.30568314	1	CRA004775;PRJNA827465;Yang2025_Silkie_Wencha	0.658603888
ENSGALG00010015800	BVES	3	-0.314732005	0.208828345	-1.507132591	0.131776674	0.072327221	5.140288397	61.09167725	0.666666667	CRA004775;PRJNA827465;Yang2025_Silkie_Wencha	0.658603888
ENSGALG00010025949	GPATCH1	3	0.176786867	0.117143917	1.509142533	0.131262362	0.025009753	5.78508738	65.42835279	1	CRA004775;PRJNA827465;Yang2025_Silkie_Wencha	0.658603888
ENSGALG00010005310	LAMTOR3	3	-0.154540238	0.102404928	-1.509109388	0.131270831	0.012460695	3.034874366	34.09941372	1	CRA004775;PRJNA827465;Yang2025_Silkie_Wencha	0.658603888
ENSGALG00010020590	INTS7	3	-0.152727256	0.1013009	-1.507659414	0.131641718	0.016228281	4.538293097	55.93056779	1	CRA004775;PRJNA827465;Yang2025_Silkie_Wencha	0.658603888
ENSGALG00010007011	HMGB1	3	-0.151304736	0.100288718	-1.508691504	0.131377638	0.017323806	5.794269422	65.48313766	1	CRA004775;PRJNA827465;Yang2025_Silkie_Wencha	0.658603888
ENSGALG00010011589	RERG	3	-0.144155886	0.095606759	-1.507800153	0.131605683	0	1.496186894	0	1	CRA004775;PRJNA827465;Yang2025_Silkie_Wencha	0.658603888
ENSGALG00010001153	TSHZ1	3	0.134599236	0.089241935	1.508250975	0.131490305	0	0.537943422	0	0.666666667	CRA004775;PRJNA827465;Yang2025_Silkie_Wencha	0.658603888
ENSGALG00010000623	ENSGALG0001000062	3	-0.121362812	0.080479865	-1.507989756	0.131557149	0	0.009381239	0	1	CRA004775;PRJNA827465;Yang2025_Silkie_Wencha	0.658603888
ENSGALG00010027299	PGPEP1	3	-0.110251584	0.073122758	-1.5077602	0.131615912	0	1.950011713	0	1	CRA004775;PRJNA827465;Yang2025_Silkie_Wencha	0.658603888
ENSGALG00010025831	BRAP	3	-0.091884228	0.060954984	-1.507411239	0.13170528	0.004786091	3.38269653	40.87557125	1	CRA004775;PRJNA827465;Yang2025_Silkie_Wencha	0.658603888
ENSGALG00010021484	MARCHF5	3	-0.179395712	0.119062934	-1.506730149	0.13187984	0.027465573	6.791534976	70.55157623	0.666666667	CRA004775;PRJNA827465;Yang2025_Silkie_Wencha	0.658886594
ENSGALG00010003108	PPP1R3B	3	-0.184236028	0.122291229	-1.506535093	0.131929865	0	1.970242546	0	1	CRA004775;PRJNA827465;Yang2025_Silkie_Wencha	0.658903697
ENSGALG00010016949	LUC7L	3	0.284058248	0.188600199	1.5061397	0.132031315	0.091712235	19.36876724	89.67409761	1	CRA004775;PRJNA827465;Yang2025_Silkie_Wencha	0.659149646
ENSGALG00010025343	NAB2	3	0.169213555	0.112361103	1.505979839	0.132072349	0	1.802697341	0	0.666666667	CRA004775;PRJNA827465;Yang2025_Silkie_Wencha	0.659149646
ENSGALG00010027959	TPM4	3	0.262167658	0.174161358	1.505314738	0.132243178	0.059339577	6.498007193	69.22133293	1	CRA004775;PRJNA827465;Yang2025_Silkie_Wencha	0.659769334
ENSGALG00010009024	PMAIP1	3	-0.200466254	0.133189643	-1.505118939	0.132293501	0.006072738	2.208674774	9.447962926	1	CRA004775;PRJNA827465;Yang2025_Silkie_Wencha	0.659787586
ENSGALG00010010526	ENSGALG0001001052	3	0.543698177	0.361320111	1.504754814	0.132387125	0.216806966	4.617934853	56.69059734	0.666666667	CRA004775;PRJNA827465;Yang2025_Silkie_Wencha	0.660021705
ENSGALG00010019546	HSD17B12	3	-0.191059774	0.127041948	-1.503910934	0.132604301	0.036490652	10.41793148	80.80233103	1	CRA004775;PRJNA827465;Yang2025_Silkie_Wencha	0.660638554
ENSGALG00010026346	FZD2	3	-0.116535415	0.077483283	-1.504007198	0.132579513	0	0.508065193	0	0.666666667	CRA004775;PRJNA827465;Yang2025_Silkie_Wencha	0.660638554
ENSGALG00010009057	NXNL2	3	0.518208719	0.344649431	1.503582111	0.132689	0.156502431	3.561835941	43.84918246	0.666666667	CRA004775;PRJNA827465;Yang2025_Silkie_Wencha	0.660827676
ENSGALG00010018693	ENSGALG0001001869	3	0.510875125	0.339894726	1.503039283	0.132828914	0.303339725	18.15842147	88.98582675	1	CRA004775;PRJNA827465;Yang2025_Silkie_Wencha	0.661281447
ENSGALG00010008860	SQLE	3	0.189154483	0.125862524	1.5028658	0.132873654	0	0.465912231	0	1	CRA004775;PRJNA827465;Yang2025_Silkie_Wencha	0.661281447
ENSGALG00010010962	ANKDD1B	3	0.771684229	0.513635514	1.502396561	0.132994724	0.564837759	8.213583129	75.65009121	0.666666667	CRA004775;PRJNA827465;Yang2025_Silkie_Wencha	0.661651092
ENSGALG00010013595	WEE2	3	1.559989776	1.038511271	1.502140439	0.133060843	2.403310443	8.301338376	75.90749938	0.666666667	CRA004775;PRJNA827465;Yang2025_Silkie_Wencha	0.66174719
ENSGALG00010022616	GLDN	3	0.575597812	0.38412957	1.498447027	0.134017146	0.365011003	15.41757847	87.02779426	0.666666667	CRA004775;PRJNA827465;Yang2025_Silkie_Wencha	0.662245476
ENSGALG00010021988	FAM180B	3	-0.484277582	0.323111142	-1.498795671	0.133926648	0.210634723	7.822938734	74.43416	0.666666667	CRA004775;PRJNA827465;Yang2025_Silkie_Wencha	0.662245476
ENSGALG00010009181	ARID3C	2	0.439056028	0.292990096	1.498535391	0.133994205	0	0.813340068	0	1	PRJNA827465;Yang2025_Silkie_Wenchang	0.662245476
ENSGALG00010005756	TET2	3	0.378358845	0.252199303	1.500237477	0.133552898	0.119029899	6.494770595	69.20599472	1	CRA004775;PRJNA827465;Yang2025_Silkie_Wencha	0.662245476
ENSGALG00010008004	ENY2	3	-0.35428141	0.236370755	-1.498837746	0.13391573	0.132166369	11.05685876	81.91167996	1	CRA004775;PRJNA827465;Yang2025_Silkie_Wencha	0.662245476
ENSGALG00010004451	CLDND1	3	-0.337315309	0.224795402	-1.500543629	0.133473641	0.136321841	23.11048798	91.34592051	1	CRA004775;PRJNA827465;Yang2025_Silkie_Wencha	0.662245476
ENSGALG00010021255	EIF3F	3	-0.291267616	0.194329822	-1.498831276	0.133917409	0.093511163	13.81006607	85.51781005	1	CRA004775;PRJNA827465;Yang2025_Silkie_Wencha	0.662245476
ENSGALG00010017860	CYSLTR1	3	-0.289986398	0.193271027	-1.500413195	0.133507404	0.04773572	3.391295744	41.0254914	1	CRA004775;PRJNA827465;Yang2025_Silkie_Wencha	0.662245476
ENSGALG00010007926	EIF3E	3	-0.272305788	0.181699611	-1.498659172	0.133962074	0.0794869	11.37233291	82.41345892	1	CRA004775;PRJNA827465;Yang2025_Silkie_Wencha	0.662245476
ENSGALG00010018892	APMAP	3	-0.243404094	0.162304946	-1.499671453	0.133699529	0.056657763	9.120185086	78.07062048	1	CRA004775;PRJNA827465;Yang2025_Silkie_Wencha	0.662245476
ENSGALG00010015778	PABIR3	3	-0.236933723	0.158050198	-1.49910425	0.133846589	0.058613704	11.48019788	82.5786975	1	CRA004775;PRJNA827465;Yang2025_Silkie_Wencha	0.662245476
ENSGALG00010009695	ATAD2B	3	0.225192416	0.1501544	1.499739038	0.133682014	0.036499509	4.307229727	53.56644232	1	CRA004775;PRJNA827465;Yang2025_Silkie_Wencha	0.662245476
ENSGALG00010021170	UBL7	3	-0.210721502	0.140482633	-1.499982576	0.133618916	0.035827835	5.526598699	63.81137642	1	CRA004775;PRJNA827465;Yang2025_Silkie_Wencha	0.662245476
ENSGALG00010015643	ENSGALG0001001564	3	0.206816761	0.137960914	1.499096775	0.133848528	0	0.030672376	0	1	CRA004775;PRJNA827465;Yang2025_Silkie_Wencha	0.662245476
ENSGALG00010008161	TMEFF1	3	-0.190935523	0.127307094	-1.499802697	0.133665519	0	1.189871834	0	0.666666667	CRA004775;PRJNA827465;Yang2025_Silkie_Wencha	0.662245476
ENSGALG00010017946	PTGDR	3	0.190211506	0.126812876	1.499938423	0.133630354	0	0.620536922	0	1	CRA004775;PRJNA827465;Yang2025_Silkie_Wencha	0.662245476
ENSGALG00010021176	ENSGALG0001002117	3	-0.160825923	0.107258682	-1.499421032	0.133764441	0.018008203	4.428132255	54.83423067	0.666666667	CRA004775;PRJNA827465;Yang2025_Silkie_Wencha	0.662245476
ENSGALG00010027958	ENSGALG0001002795	3	-0.159598176	0.106525101	-1.498221299	0.134075764	0	0.519206348	0	0.666666667	CRA004775;PRJNA827465;Yang2025_Silkie_Wencha	0.662245476
ENSGALG00010024191	DPYD	3	-0.14154901	0.094410251	-1.49929704	0.13379659	0	0.346873366	0	0.666666667	CRA004775;PRJNA827465;Yang2025_Silkie_Wencha	0.662245476
ENSGALG00010023165	CSNK2A2	3	-0.135692468	0.090574171	-1.498136459	0.1340978	0.014411829	4.753670174	57.92724512	1	CRA004775;PRJNA827465;Yang2025_Silkie_Wencha	0.662245476
ENSGALG00010006151	ENSGALG0001000615	3	0.316704576	0.211434387	1.497885845	0.134162912	0.089319032	6.239076114	67.94397178	1	CRA004775;PRJNA827465;Yang2025_Silkie_Wencha	0.662335688
ENSGALG00010012098	ART4	2	-0.480631666	0.321039332	-1.497111472	0.134364255	0	0.023756145	0	1	PRJNA827465;Yang2025_Silkie_Wenchang	0.662635579
ENSGALG00010005891	RNF6	3	0.322675337	0.215485581	1.497433544	0.134280485	0.118250088	16.64825822	87.98673126	1	CRA004775;PRJNA827465;Yang2025_Silkie_Wencha	0.662635579
ENSGALG00010011643	C1orf21	3	-0.195996566	0.130907889	-1.497209735	0.134338693	0.020875044	3.344625211	40.20256759	0.666666667	CRA004775;PRJNA827465;Yang2025_Silkie_Wencha	0.662635579
ENSGALG00010000661	ENSGALG0001000066	2	0.335747486	0.224290503	1.49693135	0.134411122	0	0.148502069	0	1	PRJNA827465;Yang2025_Silkie_Wenchang	0.662635582
ENSGALG00010009567	NSD1	3	-0.070954352	0.047407475	-1.496691217	0.134473623	0	0.076309512	0	1	CRA004775;PRJNA827465;Yang2025_Silkie_Wencha	0.662712636
ENSGALG00010009697	ENSGALG0001000969	3	-0.368808382	0.24655075	-1.495872073	0.134686995	0.079589518	3.444059901	41.92900073	1	CRA004775;PRJNA827465;Yang2025_Silkie_Wencha	0.662824757
ENSGALG00010010461	SLC7A5	3	0.286327924	0.191479557	1.495344612	0.134824528	0.057370302	4.547606618	56.02082221	1	CRA004775;PRJNA827465;Yang2025_Silkie_Wencha	0.662824757
ENSGALG00010013078	COL5A2	3	-0.25792278	0.172470589	-1.495459498	0.134794563	0.051704466	5.900237393	66.10305879	1	CRA004775;PRJNA827465;Yang2025_Silkie_Wencha	0.662824757
ENSGALG00010014246	KRAS	3	0.236388544	0.157994448	1.4961826	0.134606078	0.053124034	7.438950618	73.11448748	1	CRA004775;PRJNA827465;Yang2025_Silkie_Wencha	0.662824757
ENSGALG00010013767	PGK2	3	-0.140904305	0.094220188	-1.495478929	0.134789495	0	1.018735288	0	0.666666667	CRA004775;PRJNA827465;Yang2025_Silkie_Wencha	0.662824757
ENSGALG00010019124	BMPER	3	-0.136264892	0.091082906	-1.496053407	0.134639738	0	0.561651035	0	0.666666667	CRA004775;PRJNA827465;Yang2025_Silkie_Wencha	0.662824757
ENSGALG00010007588	TTC27	3	-0.084663137	0.056594594	-1.495958023	0.134664594	0	0.348997778	0	1	CRA004775;PRJNA827465;Yang2025_Silkie_Wencha	0.662824757
ENSGALG00010012738	RAB21	3	-0.071020033	0.047510456	-1.494829528	0.134958938	0	1.906854075	0	0.666666667	CRA004775;PRJNA827465;Yang2025_Silkie_Wencha	0.663254926
ENSGALG00010012921	EBF1	3	0.316483336	0.211759808	1.49453921	0.135034742	0.089912332	6.488910157	69.17818321	1	CRA004775;PRJNA827465;Yang2025_Silkie_Wencha	0.663396877
ENSGALG00010014065	CHUNK-1	2	0.583269601	0.390498258	1.49365481	0.135265866	0.10213229	1.484965245	32.65835661	1	PRJNA827465;Yang2025_Silkie_Wenchang	0.663691266
ENSGALG00010023119	NDRG4	3	0.362456596	0.24261524	1.493956425	0.135187009	0.127182847	7.812342064	74.39948247	1	CRA004775;PRJNA827465;Yang2025_Silkie_Wencha	0.663691266
ENSGALG00010012610	RPL3	3	-0.251702948	0.168525693	-1.493558299	0.135291106	0.055299416	5.722358792	65.04937784	1	CRA004775;PRJNA827465;Yang2025_Silkie_Wencha	0.663691266
ENSGALG00010028434	COL5A1	3	-0.151279019	0.101300657	-1.493366614	0.135341247	0	1.006871514	0	0.666666667	CRA004775;PRJNA827465;Yang2025_Silkie_Wencha	0.663691266
ENSGALG00010009083	MED30	3	-0.111404209	0.074606065	-1.493232615	0.135376307	0.001838432	2.142405444	6.64698852	1	CRA004775;PRJNA827465;Yang2025_Silkie_Wencha	0.663691266
ENSGALG00010021703	CD82	3	-0.093144073	0.062359695	-1.493658253	0.135264965	0	0.53405014	0	1	CRA004775;PRJNA827465;Yang2025_Silkie_Wencha	0.663691266
ENSGALG00010023168	SPHKAP	2	0.962246547	0.645476946	1.490752774	0.136026415	0.635573372	4.150784541	75.90816892	1	PRJNA827465;Yang2025_Silkie_Wenchang	0.664104722
ENSGALG00010003363	HBE1	3	-0.704235892	0.472513268	-1.490404481	0.136117915	0.375771601	4.930531348	59.43642056	0.666666667	CRA004775;PRJNA827465;Yang2025_Silkie_Wencha	0.664104722
ENSGALG00010013820	MASP1	3	0.556079407	0.37306597	1.490565885	0.136075507	0	0.137933235	0	1	CRA004775;PRJNA827465;Yang2025_Silkie_Wencha	0.664104722
ENSGALG00010022691	ASIC2	3	0.470150111	0.315344529	1.490909363	0.135985293	0	0.060355523	0	1	CRA004775;PRJNA827465;Yang2025_Silkie_Wencha	0.664104722
ENSGALG00010005503	DYNLT1	3	-0.416278047	0.27925049	-1.49069764	0.136040896	0.185443077	10.50161455	80.95530938	1	CRA004775;PRJNA827465;Yang2025_Silkie_Wencha	0.664104722
ENSGALG00010016370	TMEM255A	3	0.263995097	0.176996339	1.491528576	0.135822775	0.059112677	7.636265901	73.8091886	1	CRA004775;PRJNA827465;Yang2025_Silkie_Wencha	0.664104722
ENSGALG00010010689	POFUT2	3	-0.192503207	0.12917731	-1.49022461	0.136165188	0.035999477	9.08067693	77.97521027	1	CRA004775;PRJNA827465;Yang2025_Silkie_Wencha	0.664104722
ENSGALG00010007996	NUDCD1	3	-0.158015821	0.105945874	-1.491476879	0.135836337	0.020435851	6.197284657	67.7278016	0.666666667	CRA004775;PRJNA827465;Yang2025_Silkie_Wencha	0.664104722
ENSGALG00010000653	NSUN2	3	0.15625598	0.10483933	1.490432834	0.136110464	0.021727633	7.724916902	74.10975386	1	CRA004775;PRJNA827465;Yang2025_Silkie_Wencha	0.664104722
ENSGALG00010009646	MVD	3	-0.148217983	0.099302235	-1.49259464	0.135543327	0	1.136100709	0	1	CRA004775;PRJNA827465;Yang2025_Silkie_Wencha	0.664104722

ENSGALG00010021144	FOXK1	3	-0.141926354	0.095198777	-1.490842198	0.13600293	0.012003801	3.486479613	42.63554582	0.666666667	CRA004775;PRJNA827465;Yang2025_Silkie_Wencha	0.664104722
ENSGALG00010029261	IFT22	3	-0.129134668	0.086652924	-1.490251716	0.136158063	0	0.541131405	0	1	CRA004775;PRJNA827465;Yang2025_Silkie_Wencha	0.664104722
ENSGALG00010006022	AFG2A	3	0.109144739	0.073233403	1.490368242	0.136127438	0.007191249	3.715873235	46.17685068	0.666666667	CRA004775;PRJNA827465;Yang2025_Silkie_Wencha	0.664104722
ENSGALG00010024822	RPS26	3	-0.07397729	0.049632416	-1.490503507	0.136091895	0	0.096427376	0	1	CRA004775;PRJNA827465;Yang2025_Silkie_Wencha	0.664104722
ENSGALG00010011124	CACYBP	3	-0.067408507	0.04521863	-1.490724212	0.136033917	0	0.682664458	0	0.666666667	CRA004775;PRJNA827465;Yang2025_Silkie_Wencha	0.664104722
ENSGALG00010003245	ENSGALG0001000324	3	0.864916716	0.58128067	1.487950248	0.136764015	0.856504866	14.73803825	86.42967289	1	CRA004775;PRJNA827465;Yang2025_Silkie_Wencha	0.664420216
ENSGALG00010001911	USP35	3	-0.70595988	0.474546545	-1.487651501	0.136842824	0.343066663	4.090645693	51.10796314	0.666666667	CRA004775;PRJNA827465;Yang2025_Silkie_Wencha	0.664420216
ENSGALG00010013661	GABRA4	2	0.466428476	0.313606858	1.487303178	0.136934756	0	0.69397863	0	0.5	PRJNA827465;Yang2025_Silkie_Wenchang	0.664420216
ENSGALG00010017070	BLOC1S6	3	0.387119852	0.259873294	1.489648458	0.136316693	0.169823243	13.9323621	85.6449329	1	CRA004775;PRJNA827465;Yang2025_Silkie_Wencha	0.664420216
ENSGALG00010017187	ZNF839	3	0.29039871	0.195055634	1.488799397	0.136540201	0.085089204	10.81562573	81.50823587	1	CRA004775;PRJNA827465;Yang2025_Silkie_Wencha	0.664420216
ENSGALG00010012966	GLIPR1L	3	-0.246925595	0.165800647	-1.48928608	0.136412052	0	1.595584062	0	1	CRA004775;PRJNA827465;Yang2025_Silkie_Wencha	0.664420216
ENSGALG00010024546	KY	3	-0.229466128	0.154129399	-1.488788839	0.136542982	0.019315223	2.683119186	25.45988973	0.666666667	CRA004775;PRJNA827465;Yang2025_Silkie_Wencha	0.664420216
ENSGALG00010028172	CERS1	3	-0.215093524	0.144527691	-1.488251292	0.136684635	0.023901036	3.112570872	35.744435	1	CRA004775;PRJNA827465;Yang2025_Silkie_Wencha	0.664420216
ENSGALG00010007917	POGLUT1	3	-0.214716787	0.144319481	-1.487787964	0.136806821	0.045072783	7.725537655	74.11183416	1	CRA004775;PRJNA827465;Yang2025_Silkie_Wencha	0.664420216
ENSGALG00010020126	NADK	3	0.156273336	0.105024586	1.487969071	0.13675905	0.014773575	3.674826188	45.57565724	0.666666667	CRA004775;PRJNA827465;Yang2025_Silkie_Wencha	0.664420216
ENSGALG00010017188	ENSGALG0001001718	3	0.128563697	0.086381666	1.488321568	0.13666611	0.003472537	2.297020068	12.93066927	0.666666667	CRA004775;PRJNA827465;Yang2025_Silkie_Wencha	0.664420216
ENSGALG00010017756	EIF2B5	3	-0.098843554	0.066458203	-1.487304043	0.136934527	0.004319361	2.843479659	29.66364316	1	CRA004775;PRJNA827465;Yang2025_Silkie_Wencha	0.664420216
ENSGALG00010006833	LTV1	3	-0.090943931	0.061140112	-1.48746752	0.136891375	0	0.196466407	0	1	CRA004775;PRJNA827465;Yang2025_Silkie_Wencha	0.664420216
ENSGALG00010024670	SENP2	3	-0.084311823	0.056626287	-1.488916687	0.136509309	0	1.679723054	0	0.666666667	CRA004775;PRJNA827465;Yang2025_Silkie_Wencha	0.664420216
ENSGALG00010021069	PGAM5	3	-0.066631991	0.044793192	-1.487547275	0.136870327	0	1.961361043	0	1	CRA004775;PRJNA827465;Yang2025_Silkie_Wencha	0.664420216
ENSGALG00010017603	CYP3A5	2	1.40562202	0.945533876	1.486590862	0.137122903	1.044928509	2.376081197	57.91389616	1	CRA004775;PRJNA827465	0.664648859
ENSGALG00010012398	ENSGALG0001001239	2	1.085908916	0.730438979	1.486652475	0.137106621	0.798202869	3.929415365	74.55092152	1	PRJNA827465;Yang2025_Silkie_Wenchang	0.664648859
ENSGALG00010007930	CBFA2T3	3	0.100396706	0.067529363	1.48671188	0.137090924	0	1.421094756	0	0.666666667	CRA004775;PRJNA827465;Yang2025_Silkie_Wencha	0.664648859
ENSGALG00010000381	ENSGALG0001000038	3	0.320170671	0.215430631	1.486189168	0.137229092	0.060629505	3.523336252	43.23561939	0.666666667	CRA004775;PRJNA827465;Yang2025_Silkie_Wencha	0.664935617
ENSGALG00010014682	IL13RA1	3	-0.330152077	0.222343907	-1.484871261	0.13757793	0.106846141	10.48841422	80.93134045	1	CRA004775;PRJNA827465;Yang2025_Silkie_Wencha	0.66577468
ENSGALG00010008422	EXO1	3	-0.14874698	0.100168159	-1.484972679	0.137551062	0	0.12365434	0	1	CRA004775;PRJNA827465;Yang2025_Silkie_Wencha	0.66577468
ENSGALG00010007259	ANKRD34B	3	0.134157799	0.090394584	1.48457776	0.13765571	0	1.164344374	0	1	CRA004775;PRJNA827465;Yang2025_Silkie_Wencha	0.66577468
ENSGALG00010005849	SNX25	3	0.080088565	0.053948262	1.48454392	0.13766468	0	1.695596983	0	1	CRA004775;PRJNA827465;Yang2025_Silkie_Wencha	0.66577468
ENSGALG00010012977	NKRF	3	0.069486634	0.046793219	1.484972281	0.137551167	0	1.165982617	0	1	CRA004775;PRJNA827465;Yang2025_Silkie_Wencha	0.66577468
ENSGALG00010020305	XPO7	3	-0.05336294	0.035931681	-1.485122254	0.137511442	0	1.383025905	0	0.666666667	CRA004775;PRJNA827465;Yang2025_Silkie_Wencha	0.66577468
ENSGALG00010013312	ENSGALG0001001331	3	0.440046815	0.296454691	1.484364485	0.137712251	0.100579475	3.216022883	37.81137777	1	CRA004775;PRJNA827465;Yang2025_Silkie_Wencha	0.665679837
ENSGALG00010028360	ENSGALG0001002836	2	0.949959867	0.640181295	1.483891946	0.137837589	0.270247883	1.465177427	31.74888025	1	PRJNA827465;Yang2025_Silkie_Wenchang	0.666057986
ENSGALG00010023207	MNS1	3	0.381097651	0.25692098	1.483326315	0.137987734	0.144125494	8.511532419	76.50246863	1	CRA004775;PRJNA827465;Yang2025_Silkie_Wencha	0.666100571
ENSGALG00010025637	C6orf89	3	-0.239125327	0.161181395	-1.483578967	0.137920653	0.061545628	16.02308093	87.518006	1	CRA004775;PRJNA827465;Yang2025_Silkie_Wencha	0.666100571
ENSGALG00010001194	POT1	3	-0.227556256	0.153396196	-1.483454361	0.137953734	0.047757963	7.407745596	73.00123993	0.666666667	CRA004775;PRJNA827465;Yang2025_Silkie_Wencha	0.666100571
ENSGALG00010017985	ABCC5	3	0.550631955	0.371651569	1.48158114	0.13845178	0.337126161	16.72863399	88.0444512	0.666666667	CRA004775;PRJNA827465;Yang2025_Silkie_Wencha	0.666797829
ENSGALG00010026659	LIPH	3	0.487922018	0.329403326	1.481229787	0.138545351	0.249856596	9.959014851	79.91769236	0.666666667	CRA004775;PRJNA827465;Yang2025_Silkie_Wencha	0.666797829
ENSGALG00010000176	SLA	3	-0.29837162	0.201390625	-1.481556655	0.1384583	0.00475441	2.069768958	3.370857301	1	CRA004775;PRJNA827465;Yang2025_Silkie_Wencha	0.666797829
ENSGALG00010024767	CTTN	3	0.236462074	0.159634424	1.481272449	0.138533987	0.061348842	11.0933863	81.97123993	1	CRA004775;PRJNA827465;Yang2025_Silkie_Wencha	0.666797829
ENSGALG00010022950	ITGA7	3	0.23068048	0.155714496	1.481432282	0.138491418	0.032807141	3.856109029	48.1342466	0.666666667	CRA004775;PRJNA827465;Yang2025_Silkie_Wencha	0.666797829
ENSGALG00010024718	ENSGALG0001002471	3	0.217371104	0.146648371	1.482260603	0.138270967	0	1.004854333	0	0.666666667	CRA004775;PRJNA827465;Yang2025_Silkie_Wencha	0.666797829
ENSGALG00010015899	SMARCA1	3	-0.129024388	0.087101103	-1.481317508	0.138521985	0.012558259	4.841169831	58.68767117	0.666666667	CRA004775;PRJNA827465;Yang2025_Silkie_Wencha	0.666797829
ENSGALG00010000777	MYCBP2	3	-0.088924273	0.060035798	-1.481187493	0.138556618	0	0.552919794	0	0.666666667	CRA004775;PRJNA827465;Yang2025_Silkie_Wencha	0.666797829
ENSGALG00010022517	HNRNPA1	3	-0.081526497	0.055027081	-1.481570433	0.138454631	0	0.932041909	0	1	CRA004775;PRJNA827465;Yang2025_Silkie_Wencha	0.666797829
ENSGALG00010025214	LRP8	3	0.564908179	0.381457349	1.480920948	0.13862764	0.30262527	7.598061132	73.67749528	1	CRA004775;PRJNA827465;Yang2025_Silkie_Wencha	0.666876879
ENSGALG00010029300	TEKT1	3	0.541872115	0.365938959	1.480771867	0.138667376	0.307798594	10.4352279	80.83415122	1	CRA004775;PRJNA827465;Yang2025_Silkie_Wencha	0.666876879
ENSGALG00010001208	MOV10L1	2	0.874189566	0.590765691	1.47975689	0.138938137	0.058785138	1.075897833	7.054317769	1	PRJNA827465;Yang2025_Silkie_Wenchang	0.667688696
ENSGALG00010012294	ENSGALG0001001229	2	0.605590603	0.409345762	1.47941095	0.139030515	0.00206443	1.006196746	0.615858237	1	PRJNA827465;Yang2025_Silkie_Wenchang	0.667688696
ENSGALG00010018867	RPS2	3	-0.376996383	0.254855653	-1.479254542	0.139072297	0.153101955	11.2415445	82.20885039	1	CRA004775;PRJNA827465;Yang2025_Silkie_Wencha	0.667688696
ENSGALG00010021545	CYB5R2	3	0.309620328	0.20922052	1.479875535	0.138906466	0.079144921	5.358756736	62.67791022	1	CRA004775;PRJNA827465;Yang2025_Silkie_Wencha	0.667688696
ENSGALG00010003154	MTFR1	3	0.14143866	0.095604647	1.479411982	0.13903024	0.015334857	4.722565955	57.65014149	1	CRA004775;PRJNA827465;Yang2025_Silkie_Wencha	0.667688696
ENSGALG00010016315	RPS4Y1	3	-0.316556102	0.214183808	-1.477964677	0.139417233	0.101233609	8.030458689	75.09482238	1	CRA004775;PRJNA827465;Yang2025_Silkie_Wencha	0.668891253
ENSGALG00010011776	IL17RA	3	-0.202396099	0.137073497	-1.476551655	0.139795859	0.031661568	5.830788646	65.69932266	1	CRA004775;PRJNA827465;Yang2025_Silkie_Wencha	0.668891253
ENSGALG00010019025	DNAAF2	3	0.18907412	0.127960773	1.477594391	0.139516377	0	1.686848642	0	0.666666667	CRA004775;PRJNA827465;Yang2025_Silkie_Wencha	0.668891253
ENSGALG00010027322	CRABP2	3	0.155381888	0.105201511	1.476992928	0.13967753	0	1.836815251	0	1	CRA004775;PRJNA827465;Yang2025_Silkie_Wencha	0.668891253
ENSGALG00010024118	TMEM266	3	0.114212638	0.077309371	1.477345329	0.139583093	0.001699659	2.157993903	7.321332218	0.666666667	CRA004775;PRJNA827465;Yang2025_Silkie_Wencha	0.668891253
ENSGALG00010023319	EIF2AK4	3	0.106022515	0.071740193	1.47786771	0.139443191	0.003617726	2.485143297	19.52174337	0.666666667	CRA004775;PRJNA827465;Yang2025_Silkie_Wencha	0.668891253
ENSGALG00010024425	MRPL40	3	-0.106019901	0.071776558	-1.477082538	0.139653514	0	1.73440841	0	0.666666667	CRA004775;PRJNA827465;Yang2025_Silkie_Wencha	0.668891253
ENSGALG00010010844	CDC14B	3	0.091391558	0.061880677	1.476685005	0.139760094	0	0.733118085	0	1	CRA004775;PRJNA827465;Yang2025_Silkie_Wencha	0.668891253
ENSGALG00010018326	LCMT1	3	-0.087322558	0.059097815	-1.477593688	0.139516565	0	1.331467682	0	1	CRA004775;PRJNA827465;Yang2025_Silkie_Wencha	0.668891253
ENSGALG00010019640	MGRN1	3	-0.075080324	0.050847815	-1.476569325	0.139791119	0	1.218416665	0	1	CRA004775;PRJNA827465;Yang2025_Silkie_Wencha	0.668891253
ENSGALG00010022257	KCNK18	2	-0.547239942	0.370748962	-1.476039039	0.139933412	0	0.41194534	0	1	PRJNA827465;Yang2025_Silkie_Wenchang	0.669096557
ENSGALG00010025131	SUMO1	3	-0.313371529	0.212288995	-1.476155319	0.139902201	0.113994782	14.59268077	86.29449906	1	CRA004775;PRJNA827465;Yang2025_Silkie_Wencha	0.669096557
ENSGALG00010025513	RSRC2	3	0.269534319	0.182716117	1.475153503	0.140171279	0.08458878	15.40546659	87.01759542	1	CRA004775;PRJNA827465;Yang2025_Silkie_Wencha	0.669980119
ENSGALG00010019278	JPT2	3	-0.26370938	0.178792841	-1.474943733	0.140227671	0.078788169	22.839708	91.24332063	1	CRA004775;PRJNA827465;Yang2025_Silkie_Wencha	0.669980119
ENSGALG00010004974	MTPAP	3	-0.060429527	0.040974111	-1.474822187	0.140260355	0	1.102840048	0	0.666666667	CRA004775;PRJNA827465;Yang2025_Silkie_Wencha	0.669980119
ENSGALG00010024986	PTS	3	0.120663199	0.081868815	1.473860332	0.1405192	0	0.157264894	0	1	CRA004775;PRJNA827465;Yang2025_Silkie_Wencha	0.670989858
ENSGALG00010007642	ZNF318	3	0.385368906	0.261612447	1.47305264	0.140736842	0.176508678	16.8377569	88.12193328	0.666666667	CRA004775;PRJNA827465;Yang2025_Silkie_Wencha	0.671350025
ENSGALG00010024594	TNNI2	3	-0.157714985	0.107051437	-1.473263588	0.140679975	0	0.236633681	0	0.666666667	CRA004775;PRJNA827465;Yang2025_Silkie_Wencha	0.671350025
ENSGALG00010024343	POLR2L	3	0.104358281	0.070844951	1.473051784	0.140737073	0	1.337070025	0	0.666666667	CRA004775;PRJNA827465;Yang2025_Silkie_Wencha	0.671350025
ENSGALG00010001223	GRM8	2	0.588774742	0.400023677	1.471849732	0.141061461	0.126233975	1.591782011	37.17732747	1	PRJNA827465;Yang2025_Silkie_Wenchang	0.671764227
ENSGALG00010020168	COX7A2L	3	-0.36124138	0.245380904	-1.472165817	0.140976106	0.145619278	11.83196754	83.09664058	1	CRA004775;PRJNA827465;Yang2025_Silkie_Wencha	0.671764227
ENSGALG00010017631	NAT8L	3	0.194978852	0.132431574	1.472298832	0.140940199	0	0.779206522	0	0.666666667	CRA004775;PRJNA827465;Yang2025_Silkie_Wencha	0.671764227
ENSGALG00010009020	ARHGAP35	3	-0.084960737	0.057702293	-1.472397953	0.140913446	0	1.444519092	0	1	CRA004775;PRJNA827465;Yang2025_Silkie_Wencha	0.671764227
ENSGALG00010001829	POLD3	3	-0.078319817	0.053211011	-1.471872361	0.141055349	0	0.52979633	0	1	CRA004775;PRJNA827465;Yang2025_Silkie_Wencha	0.671764227
ENSGALG00010023015	METTL2B	3	-0.179459248	0.121952121	-1.471554952	0.141141098	0.028994675	5.927585217	66.25944753	1	CRA004775;PRJNA827465;Yang2025_Silkie_Wencha	0.671917166
ENSGALG00010003713	SPERT	3	0.698394918	0.474676149	1.471308215	0.141207783	0.456307949	6.939311254	71.17869588	1	CRA004775;PRJNA827465;Yang2025_Silkie_Wencha	0.671929952
ENSGALG00010028344	LSM7	3	-0.252941177	0.171929253	-1.471193369	0.14123883	0.05908346	6.791008761	70.54929436	1	CRA004775;PRJNA827465;Yang2025_Silkie_Wencha	0.671929952
ENSGALG00010012517	MGAT3	3	-0.403776226	0.274840695	-1.469128234	0.141798011	0.176684456	14.22414773	85.93940363	1	CRA004775;PRJNA827465;Yang2025_Silkie_Wencha	0.672367651
ENSGALG00010003296	ENSGALG0001000329(	2	-0.383861114	0.261137604	-1.469957247	0.141573333	0	0.298491327	0	1	PRJNA827465;Yang2025_Silkie_Wenchang	0.672367651
ENSGALG00010012340	ENHO	3	0.352233976	0.239753846	1.469148388	0.141792546	0.11873513	6.542351486	69.42995184	0.666666667	CRA004775;PRJNA827465;Yang2025_Silkie_Wencha	0.672367651
ENSGALG00010021299	CYP27C1	2	-0.333458094	0.226808078	-1.47022142	0.141501795	0.077260956	3.930622556	74.55873756	1	PRJNA827465;Yang2025_Silkie_Wenchang	0.672367651
ENSGALG00010001479	TMEM168	3	-0.280158862	0.19064644	-1.469520554	0.141691651	0.088755148	20.80842791	90.38850984	1	CRA004775;PRJNA827465;Yang2025_Silkie_Wencha	0.672367651
ENSGALG00010018822	TRAF5	3	-0.175227882	0.119190458	-1.470150262	0.141521062	0	0.773706674	0	0.666666667	CRA004775;PRJNA827465;Yang2025_Silkie_Wencha	0.672367651
ENSGALG00010023350	ZNRF1	3	0.169771032	0.115474334	1.470205771	0.141506032	0.018683532	3.826370743	47.73114959	1	CRA004775;PRJNA827465;Yang2025_Silkie_Wencha	0.672367651
ENSGALG00010012141	ENSGALG0001001214l	3	0.141359655	0.096222113	1.469097392	0.141806375	0	1.1498263	0	1	CRA004775;PRJNA827465;Yang2025_Silkie_Wencha	0.672367651
ENSGALG00010006616	COPG2	3	-0.108415298	0.073761043	-1.469817856	0.141611092	0.006108769	2.956540809	32.3533775	1	CRA004775;PRJNA827465;Yang2025_Silkie_Wencha	0.672367651
ENSGALG00010017109	ITGB1BP3	3	-0.090647858	0.061700658	-1.469155438	0.141790634	0	0.060493597	0	1	CRA004775;PRJNA827465;Yang2025_Silkie_Wencha	0.672367651
ENSGALG00010016021	ENSGALG0001001602l	3	0.66886615	0.455557712	1.468235819	0.142040178	0.389995912	5.505410514	63.67210047	1	CRA004775;PRJNA827465;Yang2025_Silkie_Wencha	0.67251868
ENSGALG00010023676	ENSGALG0001002367(	3	0.637246959	0.43437193	1.467053726	0.142361441	0.451273159	11.85829044	83.13416246	1	CRA004775;PRJNA827465;Yang2025_Silkie_Wencha	0.67251868
ENSGALG00010003828	ENSGALG0001000382!	2	0.416427334	0.283821089	1.467217731	0.142316835	0	0.015245874	0	1	PRJNA827465;Yang2025_Silkie_Wenchang	0.67251868
ENSGALG00010014703	PCID2	3	-0.268054572	0.182585481	-1.468104531	0.142075831	0.079737277	11.63968492	82.8174043	1	CRA004775;PRJNA827465;Yang2025_Silkie_Wencha	0.67251868
ENSGALG00010025573	CLMP	3	-0.248546209	0.169363963	-1.467527118	0.142232718	0.05590153	8.770095112	77.19523022	1	CRA004775;PRJNA827465;Yang2025_Silkie_Wencha	0.67251868
ENSGALG00010013369	MTMR8	3	-0.198934612	0.135514459	-1.467995475	0.142105452	0.034708253	7.143018092	72.00063091	1	CRA004775;PRJNA827465;Yang2025_Silkie_Wencha	0.67251868
ENSGALG00010022498	NAA50	3	-0.197750223	0.134726081	-1.467794665	0.142160007	0.036273468	8.056187132	75.17436019	0.666666667	CRA004775;PRJNA827465;Yang2025_Silkie_Wencha	0.67251868
ENSGALG00010023909	FOXRED1	3	-0.135981162	0.092648109	-1.46771654	0.142181236	0	0.368048376	0	1	CRA004775;PRJNA827465;Yang2025_Silkie_Wencha	0.67251868
ENSGALG00010013285	ATP2B1	3	0.130430228	0.08885447	1.467908457	0.142129091	0.01116903	3.852038847	48.07944366	1	CRA004775;PRJNA827465;Yang2025_Silkie_Wencha	0.67251868
ENSGALG00010015145	CLIC2	3	-0.106134378	0.072289887	-1.468177404	0.14205604	0.004082608	2.634745712	24.09134625	1	CRA004775;PRJNA827465;Yang2025_Silkie_Wencha	0.67251868
ENSGALG00010021528	BRPF1	3	-0.071895637	0.048998431	-1.467304867	0.14229314	0	1.320669672	0	0.666666667	CRA004775;PRJNA827465;Yang2025_Silkie_Wencha	0.67251868
ENSGALG00010022313	ENSGALG0001002231:	3	0.267923767	0.182738229	1.466161561	0.142604278	0	0.985195201	0	1	CRA004775;PRJNA827465;Yang2025_Silkie_Wencha	0.673231717
ENSGALG00010024568	DZIP1L	3	0.267842392	0.182684251	1.466149328	0.14260761	0.026877626	2.662067065	24.87041269	1	CRA004775;PRJNA827465;Yang2025_Silkie_Wencha	0.673231717
ENSGALG00010012186	KIF20A	3	-0.182419148	0.124481052	-1.465437063	0.142801712	0.019171862	3.488277609	42.66511372	0.666666667	CRA004775;PRJNA827465;Yang2025_Silkie_Wencha	0.673485106
ENSGALG00010001738	PUDP	3	-0.109602766	0.07477985	-1.465672454	0.142737542	0	1.069035471	0	0.666666667	CRA004775;PRJNA827465;Yang2025_Silkie_Wencha	0.673485106
ENSGALG00010009234	PCDH18	3	-0.103729432	0.070784393	-1.465427998	0.142804183	0	0.396857476	0	1	CRA004775;PRJNA827465;Yang2025_Silkie_Wencha	0.673485106
ENSGALG00010001773	NRCAM	3	0.671416865	0.458528932	1.464284625	0.143116196	0.505075871	20.11500055	90.05717154	0.666666667	CRA004775;PRJNA827465;Yang2025_Silkie_Wencha	0.674491635
ENSGALG00010002454	NEK5	2	0.35307173	0.24114918	1.46412163	0.143160718	0	0.002553495	0	1	PRJNA827465;Yang2025_Silkie_Wenchang	0.674491635
ENSGALG00010013746	ENSGALG0001001374(	3	0.276949162	0.189115882	1.464441588	0.143073332	0.058668653	4.423745151	54.789439	0.666666667	CRA004775;PRJNA827465;Yang2025_Silkie_Wencha	0.674491635
ENSGALG00010001559	HDAC10	3	-0.128427814	0.087745704	-1.463636489	0.143293297	0	1.200144792	0	0.666666667	CRA004775;PRJNA827465;Yang2025_Silkie_Wencha	0.674891381
ENSGALG00010020711	PLA2G2E	3	0.507448786	0.346751841	1.463435017	0.143348383	0.276911165	10.22883439	80.44743005	1	CRA004775;PRJNA827465;Yang2025_Silkie_Wencha	0.674926003
ENSGALG00010022682	EIF1	3	-0.403520818	0.275803507	-1.463073559	0.143447252	0.18153991	12.48089804	83.97551207	1	CRA004775;PRJNA827465;Yang2025_Silkie_Wencha	0.674941995
ENSGALG00010025734	PLK3	3	0.282585521	0.193129525	1.463191717	0.143414927	0.065359426	4.907649903	59.24729678	0.666666667	CRA004775;PRJNA827465;Yang2025_Silkie_Wencha	0.674941995
ENSGALG00010010657	TMEM170B	3	-0.387404166	0.264888325	-1.462518842	0.143599085	0.146433016	11.31180617	82.31935758	1	CRA004775;PRJNA827465;Yang2025_Silkie_Wencha	0.675307481
ENSGALG00010012329	ATXN10	3	-0.199194733	0.13620704	-1.462440808	0.143620454	0.031993542	5.097467382	60.76482986	1	CRA004775;PRJNA827465;Yang2025_Silkie_Wencha	0.675307481
ENSGALG00010024656	HYLS1	3	-0.221268457	0.15134862	-1.461978689	0.14374705	0.035116593	4.020638187	50.25665312	1	CRA004775;PRJNA827465;Yang2025_Silkie_Wencha	0.675678037
ENSGALG00010023349	ENSGALG0001002334!	3	1.15976109	0.793820585	1.460986414	0.14401917	1.499817104	10.25717892	80.50146131	0.666666667	CRA004775;PRJNA827465;Yang2025_Silkie_Wencha	0.675720368
ENSGALG00010016496	PLP1	3	0.794698666	0.544108381	1.460552152	0.144138385	0.702222232	10.29063402	80.56485154	1	CRA004775;PRJNA827465;Yang2025_Silkie_Wencha	0.675720368
ENSGALG00010011923	BARD1	3	-0.359420786	0.245958585	-1.461306122	0.143931451	0.114394827	8.070497863	75.21838139	1	CRA004775;PRJNA827465;Yang2025_Silkie_Wencha	0.675720368
ENSGALG00010003519	KCTD4	2	-0.327288317	0.224070835	-1.460646661	0.144112434	0	0.124984145	0	1	PRJNA827465;Yang2025_Silkie_Wenchang	0.675720368
ENSGALG00010008205	ENSGALG0001000820!	3	0.185319755	0.126867875	1.460730352	0.144089456	0	0.48814251	0	1	CRA004775;PRJNA827465;Yang2025_Silkie_Wencha	0.675720368
ENSGALG00010019356	AVL9	3	-0.12638845	0.086521695	-1.460771774	0.144078084	0.008210691	2.967312514	32.59894296	1	CRA004775;PRJNA827465;Yang2025_Silkie_Wencha	0.675720368
ENSGALG00010016500	ENSGALG0001001650(	3	-0.088492175	0.060564143	-1.461131477	0.143979363	0	1.100876491	0	0.666666667	CRA004775;PRJNA827465;Yang2025_Silkie_Wencha	0.675720368
ENSGALG00010017415	HDAC1	3	-0.081739458	0.055949099	-1.460961104	0.144026116	0.00175737	2.351785493	14.95823042	1	CRA004775;PRJNA827465;Yang2025_Silkie_Wencha	0.675720368
ENSGALG00010010965	BAZ2B	3	0.536168573	0.367274474	1.459857982	0.144329109	0.343916149	21.1908621	90.56196963	1	CRA004775;PRJNA827465;Yang2025_Silkie_Wencha	0.67626054
ENSGALG00010002336	PRIM2	3	-0.28499353	0.195346177	-1.45891532	0.144588416	0.080947706	9.066077122	77.93974204	1	CRA004775;PRJNA827465;Yang2025_Silkie_Wencha	0.67626054
ENSGALG00010029783	RAD51C	3	-0.264807076	0.181477578	-1.459172417	0.144517659	0.071794696	8.312806694	75.9407373	0.666666667	CRA004775;PRJNA827465;Yang2025_Silkie_Wencha	0.67626054
ENSGALG00010009385	GPI	3	-0.226145713	0.154930998	-1.459654405	0.144385079	0.02729499	3.196990448	37.44116435	0.666666667	CRA004775;PRJNA827465;Yang2025_Silkie_Wencha	0.67626054
ENSGALG00010017485	LBH	3	-0.215556382	0.147745745	-1.458968467	0.144573787	0.049006868	10.50651694	80.96419572	1	CRA004775;PRJNA827465;Yang2025_Silkie_Wencha	0.67626054
ENSGALG00010020875	ENSGALG0001002087!	3	-0.091424857	0.062654503	-1.459190521	0.144512677	0	0.556161982	0	1	CRA004775;PRJNA827465;Yang2025_Silkie_Wencha	0.67626054
ENSGALG00010026114	CWC22	3	0.068350703	0.046837995	1.459300356	0.144482458	0	1.055835906	0	1	CRA004775;PRJNA827465;Yang2025_Silkie_Wencha	0.67626054
ENSGALG00010001698	RPL15	3	-0.430648534	0.295326175	-1.458213223	0.14478178	0.206017852	10.61194477	81.15331315	1	CRA004775;PRJNA827465;Yang2025_Silkie_Wencha	0.676427194
ENSGALG00010022809	MYO1E	3	0.360267306	0.247110882	1.457917611	0.144863254	0.14937775	13.33375618	85.00047569	1	CRA004775;PRJNA827465;Yang2025_Silkie_Wencha	0.676427194
ENSGALG00010015879	TRMT61A	3	-0.330535613	0.226707177	-1.45798478	0.144844738	0.126730819	12.59843887	84.12501723	1	CRA004775;PRJNA827465;Yang2025_Silkie_Wencha	0.676427194
ENSGALG00010024513	SPTY2D1	3	0.171375189	0.117530161	1.458137959	0.14480252	0.021107673	4.003969007	50.04956341	1	CRA004775;PRJNA827465;Yang2025_Silkie_Wencha	0.676427194
ENSGALG00010025387	PSMD9	3	-0.076515101	0.052474255	-1.458145526	0.144800435	0	1.294477195	0	0.666666667	CRA004775;PRJNA827465;Yang2025_Silkie_Wencha	0.676427194
ENSGALG00010010733	GFM2	3	0.155507607	0.106699467	1.457435652	0.144996162	0.004030708	2.264205436	11.6687926	1	CRA004775;PRJNA827465;Yang2025_Silkie_Wencha	0.676730202
ENSGALG00010028893	STXBP1	3	-0.104087993	0.071423508	-1.457335214	0.145023871	0	1.996321379	0	0.666666667	CRA004775;PRJNA827465;Yang2025_Silkie_Wencha	0.676730202
ENSGALG00010001151	CCDC112	3	0.805714775	0.55355876	1.455518066	0.145525893	0.786776404	16.53988928	87.90802063	1	CRA004775;PRJNA827465;Yang2025_Silkie_Wencha	0.677188568
ENSGALG00010018937	INHBB	3	0.47352646	0.325217874	1.456028397	0.14538477	0.182332308	5.177206477	61.36912814	1	CRA004775;PRJNA827465;Yang2025_Silkie_Wencha	0.677188568
ENSGALG00010029674	FDXR	3	-0.393248792	0.270228315	-1.455246434	0.14560105	0.191353588	16.24031372	87.68496696	0.666666667	CRA004775;PRJNA827465;Yang2025_Silkie_Wencha	0.677188568
ENSGALG00010016281	SLC22A4	3	-0.38642669	0.265442439	-1.455783375	0.145452513	0.127965124	6.457313297	69.02736621	1	CRA004775;PRJNA827465;Yang2025_Silkie_Wencha	0.677188568
ENSGALG00010029934	TEKT3	3	0.33951676	0.233262858	1.455511448	0.145527723	0	0.588729509	0	0.666666667	CRA004775;PRJNA827465;Yang2025_Silkie_Wencha	0.677188568
ENSGALG00010023269	OPRL1	2	-0.328474182	0.225705757	-1.455320355	0.145580594	0	0.068927436	0	1	PRJNA827465;Yang2025_Silkie_Wenchang	0.677188568
ENSGALG00010018068	ADAM33	3	-0.159012486	0.109257382	-1.45539353	0.145560346	0.01785032	4.475737288	55.3146248	1	CRA004775;PRJNA827465;Yang2025_Silkie_Wencha	0.677188568
ENSGALG00010027989	ENSGALG0001002798!	3	-0.13286685	0.091276792	-1.455647667	0.145490044	0	0.836119326	0	1	CRA004775;PRJNA827465;Yang2025_Silkie_Wencha	0.677188568
ENSGALG00010008267	MAML2	3	-0.095218678	0.065383476	-1.456311028	0.145306658	0	1.064031187	0	0.666666667	CRA004775;PRJNA827465;Yang2025_Silkie_Wencha	0.677188568
ENSGALG00010016820	EIF2AK3	3	0.08849538	0.060768623	1.456267653	0.145318644	0	1.968242753	0	0.666666667	CRA004775;PRJNA827465;Yang2025_Silkie_Wencha	0.677188568
ENSGALG00010012074	KIAA0930	3	-0.164888836	0.11332137	-1.455055088	0.145654011	0.023403863	6.450132477	68.99288492	1	CRA004775;PRJNA827465;Yang2025_Silkie_Wencha	0.677212123
ENSGALG00010029966	RABGEF1	3	0.085853831	0.059013134	1.454825833	0.145717485	0.004036006	3.2449929	38.36658317	1	CRA004775;PRJNA827465;Yang2025_Silkie_Wencha	0.677284521
ENSGALG00010024123	PLPPR4	3	-0.549135998	0.377916131	-1.453063137	0.146206225	0.200134982	3.742752703	46.56339441	0.666666667	CRA004775;PRJNA827465;Yang2025_Silkie_Wencha	0.677315181
ENSGALG00010022958	SFSWAP	3	0.374237772	0.257654632	1.45247834	0.146368648	0.180378726	23.56634649	91.51332176	1	CRA004775;PRJNA827465;Yang2025_Silkie_Wencha	0.677315181
ENSGALG00010021877	PLEKHN1	3	-0.348366713	0.23965149	-1.453638837	0.146046464	0.10575042	6.738616497	70.32031722	1	CRA004775;PRJNA827465;Yang2025_Silkie_Wencha	0.677315181
ENSGALG00010015863	ENSGALG0001001586:	3	0.26829609	0.184660965	1.452911773	0.146248252	0	0.272436031	0	1	CRA004775;PRJNA827465;Yang2025_Silkie_Wencha	0.677315181
ENSGALG00010017569	CLMN	3	0.256521109	0.176519168	1.453219568	0.146162801	0.051735064	4.586931966	56.39787085	1	CRA004775;PRJNA827465;Yang2025_Silkie_Wencha	0.677315181
ENSGALG00010024413	AMFR	3	-0.245030731	0.168694998	-1.452507388	0.146360576	0.063271177	9.632708867	79.23740842	1	CRA004775;PRJNA827465;Yang2025_Silkie_Wencha	0.677315181
ENSGALG00010014470	RPS6KA1	3	-0.215501236	0.148236938	-1.45376206	0.146012285	0.038955379	5.503816562	63.6615796	1	CRA004775;PRJNA827465;Yang2025_Silkie_Wencha	0.677315181
ENSGALG00010016400	ENSGALG0001001640(	3	-0.166728564	0.11479644	-1.45238445	0.146394738	0.020203115	4.140801181	51.70016833	1	CRA004775;PRJNA827465;Yang2025_Silkie_Wencha	0.677315181
ENSGALG00010017882	LZTS3	3	0.160737307	0.110519526	1.45437926	0.145841187	0	0.434793387	0	1	CRA004775;PRJNA827465;Yang2025_Silkie_Wencha	0.677315181
ENSGALG00010028021	ATP5F1D	3	-0.149989131	0.103176606	-1.453712581	0.146026009	0	0.289367021	0	0.666666667	CRA004775;PRJNA827465;Yang2025_Silkie_Wencha	0.677315181
ENSGALG00010016706	RTN4	3	-0.116168792	0.07992191	-1.453528724	0.146077011	0.006923811	2.993625733	33.19138134	0.666666667	CRA004775;PRJNA827465;Yang2025_Silkie_Wencha	0.677315181
ENSGALG00010023760	ZW10	3	0.111473667	0.076713594	1.453114902	0.146191854	0.008328795	3.896923679	48.67746549	0.666666667	CRA004775;PRJNA827465;Yang2025_Silkie_Wencha	0.677315181
ENSGALG00010027471	ENSGALG0001002747l	3	-0.103182846	0.070947175	-1.454361594	0.145846082	0	1.191434952	0	0.666666667	CRA004775;PRJNA827465;Yang2025_Silkie_Wencha	0.677315181
ENSGALG00010017565	ENSGALG0001001756!	3	-0.091663777	0.063093327	-1.452828387	0.146271408	0	0.310919908	0	0.666666667	CRA004775;PRJNA827465;Yang2025_Silkie_Wencha	0.677315181
ENSGALG00010008204	ENSGALG0001000820	3	0.262483837	0.180752671	1.452171278	0.146453987	0.05230217	4.268928321	53.14983411	0.666666667	CRA004775;PRJNA827465;Yang2025_Silkie_Wencha	0.677367655
ENSGALG00010006382	PNN	3	0.53532429	0.368737849	1.451774729	0.146564253	0.376245383	33.23492036	93.98223321	1	CRA004775;PRJNA827465;Yang2025_Silkie_Wencha	0.677434446
ENSGALG00010006528	ZC3HC1	3	-0.143177192	0.098617231	-1.451847615	0.146543981	0.010284181	2.855791187	29.9668684	1	CRA004775;PRJNA827465;Yang2025_Silkie_Wencha	0.677434446
ENSGALG00010005985	ENSGALG0001000598!	3	0.612062671	0.421702686	1.451408046	0.146666271	0.414500253	13.11390364	84.74901101	0.666666667	CRA004775;PRJNA827465;Yang2025_Silkie_Wencha	0.677463051
ENSGALG00010015986	NEURL2	3	-0.18217482	0.125506268	-1.451519691	0.146635203	0	0.159697333	0	1	CRA004775;PRJNA827465;Yang2025_Silkie_Wencha	0.677463051
ENSGALG00010013186	DDX46	3	0.310097002	0.21383114	1.45019571	0.147003951	0.110761332	12.33135053	83.78117632	0.666666667	CRA004775;PRJNA827465;Yang2025_Silkie_Wencha	0.678229269
ENSGALG00010016773	RPS27A	3	-0.291087368	0.200774813	-1.449820141	0.147108682	0.080618966	6.124931369	67.34657289	1	CRA004775;PRJNA827465;Yang2025_Silkie_Wencha	0.678229269
ENSGALG00010024836	TXNDC12	3	-0.198307136	0.136784325	-1.449779694	0.147119964	0.042442663	9.714371893	79.41194735	1	CRA004775;PRJNA827465;Yang2025_Silkie_Wencha	0.678229269
ENSGALG00010005014	SULT1B1	3	0.154034509	0.106228386	1.450031525	0.147049728	0.002530966	2.124733249	5.870536859	0.666666667	CRA004775;PRJNA827465;Yang2025_Silkie_Wencha	0.678229269
ENSGALG00010024019	MAP3K14	3	-0.080870441	0.055767887	-1.450125602	0.147023497	0.001426602	2.320714939	13.81966108	1	CRA004775;PRJNA827465;Yang2025_Silkie_Wencha	0.678229269
ENSGALG00010023917	ZRANB2	3	-0.068715637	0.047374055	-1.450490941	0.146921664	0	1.737140383	0	0.666666667	CRA004775;PRJNA827465;Yang2025_Silkie_Wencha	0.678229269
ENSGALG00010005848	WASF3	3	-0.150717932	0.103995757	-1.44927001	0.147262193	0	0.079018271	0	1	CRA004775;PRJNA827465;Yang2025_Silkie_Wencha	0.678333131
ENSGALG00010029360	ACACA	3	-0.071638269	0.049431679	-1.44923801	0.147271126	0	0.117637276	0	1	CRA004775;PRJNA827465;Yang2025_Silkie_Wencha	0.678333131
ENSGALG00010027922	SHC1	3	-0.066016225	0.045554091	-1.449183223	0.147286421	0	1.276409656	0	1	CRA004775;PRJNA827465;Yang2025_Silkie_Wencha	0.678333131
ENSGALG00010020128	KIF6	2	0.521127599	0.359675134	1.448884145	0.14736994	0	0.215904539	0	1	PRJNA827465;Yang2025_Silkie_Wenchang	0.678496769
ENSGALG00010001215	PRDM6	2	1.031006729	0.712113958	1.447811431	0.147669795	0.688893591	3.107747764	67.82235639	1	PRJNA827465;Yang2025_Silkie_Wenchang	0.678571458
ENSGALG00010012952	BRD10	3	0.385087382	0.265847632	1.448526661	0.147469815	0.160694173	10.68179869	81.27656158	1	CRA004775;PRJNA827465;Yang2025_Silkie_Wencha	0.678571458
ENSGALG00010001495	CCNL1	3	0.325560551	0.224831189	1.448022193	0.147610844	0.122528002	11.68924566	82.89025608	1	CRA004775;PRJNA827465;Yang2025_Silkie_Wencha	0.678571458
ENSGALG00010012308	TRABD2A	3	-0.271248519	0.1873411	-1.447885806	0.14764899	0	0.132955537	0	1	CRA004775;PRJNA827465;Yang2025_Silkie_Wencha	0.678571458

ENSGALG00010008451	OPN3	3	-0.23877415	0.164922513	-1.447795972	0.14767412	0.007826017	2.187045161	8.552414188	0.666666667	CRA004775;PRJNA827465;Yang2025_Silkie_Wencha	0.678571458
ENSGALG00010023526	KNSTRN	3	0.123576203	0.085332456	1.448173524	0.147568527	0	0.30598043	0	1	CRA004775;PRJNA827465;Yang2025_Silkie_Wencha	0.678571458
ENSGALG00010019011	VIL1	3	0.789380336	0.54544632	1.447219106	0.147835567	0.648890595	7.615984655	73.73944289	1	CRA004775;PRJNA827465;Yang2025_Silkie_Wencha	0.678661422
ENSGALG00010009612	DERL1	3	-0.329400914	0.227610763	-1.447211503	0.147837696	0.131279662	14.73964095	86.43114844	1	CRA004775;PRJNA827465;Yang2025_Silkie_Wencha	0.678661422
ENSGALG00010023533	FKBP7	3	0.070091356	0.048423908	1.447453522	0.147769945	0	0.481351218	0	0.666666667	CRA004775;PRJNA827465;Yang2025_Silkie_Wencha	0.678661422
ENSGALG00010029282	LUC7L3	3	0.539404644	0.372917661	1.446444348	0.148052611	0.37361167	27.12168805	92.62582773	1	CRA004775;PRJNA827465;Yang2025_Silkie_Wencha	0.679004789
ENSGALG00010005433	GPC5	2	-0.283936543	0.196262553	-1.446717872	0.147975957	0	0.479750944	0	1	PRJNA827465;Yang2025_Silkie_Wenchang	0.679004789
ENSGALG00010010423	C21orf59	3	0.273483234	0.18907461	1.446430243	0.148056564	0.069072824	5.781603657	65.40752153	0.666666667	CRA004775;PRJNA827465;Yang2025_Silkie_Wencha	0.679004789
ENSGALG00010017310	SAMD11	3	0.257180617	0.177848656	1.446064444	0.148159127	0.061324215	7.161247405	72.0719047	0.666666667	CRA004775;PRJNA827465;Yang2025_Silkie_Wencha	0.679217235
ENSGALG00010005392	ENSGALG00010005392	3	-0.119404472	0.082580138	-1.445922411	0.148198965	0	1.339204663	0	0.666666667	CRA004775;PRJNA827465;Yang2025_Silkie_Wencha	0.679217235
ENSGALG00010020636	NBL1	3	-0.133207575	0.092148694	-1.44557203	0.148297277	0	0.545571415	0	1	CRA004775;PRJNA827465;Yang2025_Silkie_Wencha	0.679447567
ENSGALG00010003022	BLB2	3	-0.384091434	0.265772065	-1.445191141	0.148404204	0.113363484	4.300962576	53.49878162	0.666666667	CRA004775;PRJNA827465;Yang2025_Silkie_Wencha	0.679717215
ENSGALG00010015032	C1orf216	3	0.851675137	0.589460408	1.444838577	0.148503233	0.972239317	30.36255004	93.41293799	0.666666667	CRA004775;PRJNA827465;Yang2025_Silkie_Wencha	0.679722095
ENSGALG00010010291	TCF20	3	-0.072357584	0.050084078	-1.444722304	0.148535902	0	1.010368091	0	0.666666667	CRA004775;PRJNA827465;Yang2025_Silkie_Wencha	0.679722095
ENSGALG00010027973	GAPVD1	3	0.05401389	0.037388291	1.444673942	0.148549492	0	0.53739017	0	1	CRA004775;PRJNA827465;Yang2025_Silkie_Wencha	0.679722095
ENSGALG00010022630	ENSGALG00010022630	3	0.4845523	0.335510271	1.444224938	0.148675712	0.18195156	4.732383119	57.7379948	1	CRA004775;PRJNA827465;Yang2025_Silkie_Wencha	0.680079552
ENSGALG00010004762	IFT57	3	0.279574058	0.193632859	1.443835819	0.148785164	0.080279909	7.881199221	74.62315133	1	CRA004775;PRJNA827465;Yang2025_Silkie_Wencha	0.6803601
ENSGALG00010002360	DHRSX	3	-0.177930241	0.123272928	-1.44338456	0.148912171	0.021679076	3.545980256	43.59810672	1	CRA004775;PRJNA827465;Yang2025_Silkie_Wencha	0.680500707
ENSGALG00010005260	ANXA5	3	-0.163914165	0.113557397	-1.443447728	0.148894387	0.022124131	5.269751593	62.04625054	1	CRA004775;PRJNA827465;Yang2025_Silkie_Wencha	0.680500707
ENSGALG00010007851	AvBD7	3	0.438267497	0.303678111	1.44319722	0.148964837	0	0.818124223	0	1	CRA004775;PRJNA827465;Yang2025_Silkie_Wencha	0.680521433
ENSGALG00010018406	TMEM260	3	0.137322582	0.095169834	1.442921308	0.14904264	0	1.441993268	0	0.666666667	CRA004775;PRJNA827465;Yang2025_Silkie_Wencha	0.68065694
ENSGALG00010024851	CPNE5	2	-0.521038474	0.361178122	-1.44260807	0.149130909	0	0.025933947	0	1	PRJNA827465;Yang2025_Silkie_Wenchang	0.680840142
ENSGALG00010010973	PGAP1	3	-0.306163112	0.21230699	-1.442077401	0.14928054	0.099366495	9.866148351	79.72866484	1	CRA004775;PRJNA827465;Yang2025_Silkie_Wencha	0.680863726
ENSGALG00010028494	ENSGALG00010028494	3	0.173952752	0.120614338	1.442222828	0.149239523	0	0.628427769	0	0.666666667	CRA004775;PRJNA827465;Yang2025_Silkie_Wencha	0.680863726
ENSGALG00010022642	SEC13	3	0.05476164	0.037972775	1.442128981	0.149265991	0	0.560005284	0	0.666666667	CRA004775;PRJNA827465;Yang2025_Silkie_Wencha	0.680863726
ENSGALG00010023199	RPS13	3	-0.094372926	0.065460589	-1.44167549	0.149393941	0	0.130113784	0	1	CRA004775;PRJNA827465;Yang2025_Silkie_Wencha	0.681161217
ENSGALG00010014449	ENSGALG00010014449	3	-0.606652952	0.423136813	-1.433704027	0.151656742	0.242266467	3.642406125	45.09124103	1	CRA004775;PRJNA827465;Yang2025_Silkie_Wencha	0.681329478
ENSGALG00010002428	IBSP	3	0.604492768	0.421795867	1.433140566	0.15181767	0	1.302573272	0	0.666666667	CRA004775;PRJNA827465;Yang2025_Silkie_Wencha	0.681329478
ENSGALG00010004957	ENSGALG00010004957	3	0.599491745	0.417881333	1.434598048	0.151401671	0.436392169	14.58410782	86.28644258	0.666666667	CRA004775;PRJNA827465;Yang2025_Silkie_Wencha	0.681329478
ENSGALG00010006814	ENSGALG00010006814	2	0.580682653	0.405197903	1.433084052	0.151833819	0	0.454112011	0	1	CRA004775;PRJNA827465	0.681329478
ENSGALG00010015391	SYN3	3	0.502646966	0.350874254	1.432555852	0.151984806	0.139859877	3.181249756	37.13162582	1	CRA004775;PRJNA827465;Yang2025_Silkie_Wencha	0.681329478
ENSGALG00010003333	ENSGALG00010003333	3	0.483993104	0.336038323	1.440291394	0.149784975	0.082239156	2.539077617	21.23123821	1	CRA004775;PRJNA827465;Yang2025_Silkie_Wencha	0.681329478
ENSGALG00010013362	ENSGALG00010013362	3	-0.447160794	0.311284607	-1.436501465	0.1508597	0.159864242	4.997469979	59.97974959	1	CRA004775;PRJNA827465;Yang2025_Silkie_Wencha	0.681329478
ENSGALG00010000081	ENSGALG00010000081	3	-0.386580133	0.269646693	-1.433654272	0.151670948	0.113860913	4.477782038	55.33503009	0.666666667	CRA004775;PRJNA827465;Yang2025_Silkie_Wencha	0.681329478
ENSGALG00010025415	ENSGALG00010025415	3	0.382716991	0.266087489	1.438312607	0.150345376	0.126347298	5.125262275	60.97760675	0.666666667	CRA004775;PRJNA827465;Yang2025_Silkie_Wencha	0.681329478
ENSGALG00010009989	TBC1D7	3	-0.369235125	0.25645192	-1.439783041	0.149928791	0.171924032	18.41506186	89.13932511	0.666666667	CRA004775;PRJNA827465;Yang2025_Silkie_Wencha	0.681329478
ENSGALG00010022274	CCDC91	3	0.361586732	0.252237385	1.433517605	0.151709971	0.148815054	10.04126148	80.08218385	1	CRA004775;PRJNA827465;Yang2025_Silkie_Wencha	0.681329478
ENSGALG00010014923	CIMIP1	2	0.341421726	0.237064757	1.440204484	0.149809555	0	0.31569817	0	1	PRJNA827465;Yang2025_Silkie_Wenchang	0.681329478
ENSGALG00010017753	GNRHR	3	0.340836497	0.236723769	1.439806824	0.14992206	0	0.511699359	0	1	CRA004775;PRJNA827465;Yang2025_Silkie_Wencha	0.681329478
ENSGALG00010012849	TRHDE	2	0.340065048	0.237221743	1.43353237	0.151705755	0	0.262910831	0	1	PRJNA827465;Yang2025_Silkie_Wenchang	0.681329478
ENSGALG00010013524	ZDHHC15	3	-0.332178488	0.231739907	-1.43341081	0.151740471	0.116525273	9.541413623	79.03874542	0.666666667	CRA004775;PRJNA827465;Yang2025_Silkie_Wencha	0.681329478
ENSGALG00010021521	ENSGALG00010021521	3	0.31712728	0.221270557	1.433210477	0.151797697	0.073407679	4.002211935	50.02763391	0.666666667	CRA004775;PRJNA827465;Yang2025_Silkie_Wencha	0.681329478
ENSGALG00010000151	TBX5	3	0.315388594	0.218490681	1.435249286	0.151216073	0.060074894	3.295469111	39.31061307	1	CRA004775;PRJNA827465;Yang2025_Silkie_Wencha	0.681329478
ENSGALG00010013282	SEC22B	3	-0.293628373	0.204331257	-1.437021326	0.150711934	0.106454588	20.74370015	90.35851856	0.666666667	CRA004775;PRJNA827465;Yang2025_Silkie_Wencha	0.681329478
ENSGALG00010001513	ZRSR2	3	0.274772344	0.191022347	1.438430367	0.150311982	0.09020455	12.87511722	84.46616084	1	CRA004775;PRJNA827465;Yang2025_Silkie_Wencha	0.681329478
ENSGALG00010012551	PCGF3	3	0.271807409	0.189210855	1.43653179	0.150851077	0.067770557	5.663593668	64.68673218	1	CRA004775;PRJNA827465;Yang2025_Silkie_Wencha	0.681329478
ENSGALG00010001788	HOXA1	3	0.271538835	0.188696532	1.439264183	0.150075687	0.007702389	2.146198805	6.811987991	0.666666667	CRA004775;PRJNA827465;Yang2025_Silkie_Wencha	0.681329478
ENSGALG00010004471	C1H3ORF52	3	0.270274063	0.188248294	1.435731807	0.151078671	0	0.359478349	0	1	CRA004775;PRJNA827465;Yang2025_Silkie_Wencha	0.681329478
ENSGALG00010008515	TAFA5	3	0.263448228	0.18301366	1.439500352	0.15000881	0.050247471	4.246982893	52.90774533	0.666666667	CRA004775;PRJNA827465;Yang2025_Silkie_Wencha	0.681329478
ENSGALG00010007932	NPY5R	3	0.262806967	0.182675349	1.438655891	0.150248042	0.042524655	3.458568407	42.17260541	1	CRA004775;PRJNA827465;Yang2025_Silkie_Wencha	0.681329478
ENSGALG00010011237	ENSGALG00010011237	3	-0.261225557	0.182167825	-1.433982961	0.151577125	0	1.396569056	0	1	CRA004775;PRJNA827465;Yang2025_Silkie_Wencha	0.681329478
ENSGALG00010027830	CACNA1D	2	0.217048559	0.151141959	1.436057599	0.150985952	0.007444536	1.17446351	14.85474076	1	PRJNA827465;Yang2025_Silkie_Wenchang	0.681329478
ENSGALG00010027438	ST7L	3	-0.216223624	0.150871937	-1.433159992	0.15181212	0.056396895	12.50210564	84.00269477	1	CRA004775;PRJNA827465;Yang2025_Silkie_Wencha	0.681329478
ENSGALG00010025228	FDXACB1	3	-0.213126667	0.148126303	-1.438817159	0.150202334	0.044877334	6.667746584	70.00485884	1	CRA004775;PRJNA827465;Yang2025_Silkie_Wencha	0.681329478
ENSGALG00010020597	ARID5A	3	0.207198587	0.144478769	1.434083282	0.151548498	0.030902907	4.230452445	52.72373284	0.666666667	CRA004775;PRJNA827465;Yang2025_Silkie_Wencha	0.681329478
ENSGALG00010004000	ENSGALG00010004000	3	0.205268235	0.142624434	1.439222086	0.15008761	0.032504603	4.272856151	53.1929012	0.666666667	CRA004775;PRJNA827465;Yang2025_Silkie_Wencha	0.681329478
ENSGALG00010022152	ZNF644	3	0.201082476	0.139833532	1.438013277	0.150430287	0.04182647	9.175256242	78.20224365	1	CRA004775;PRJNA827465;Yang2025_Silkie_Wencha	0.681329478
ENSGALG00010024083	PALMD	3	0.181654449	0.12651746	1.435805367	0.151057732	0.019131873	3.20319212	37.56228396	1	CRA004775;PRJNA827465;Yang2025_Silkie_Wencha	0.681329478
ENSGALG00010022885	M6PR	3	-0.173345343	0.120542381	-1.438044791	0.150421346	0.032849848	8.872034765	77.45725696	1	CRA004775;PRJNA827465;Yang2025_Silkie_Wencha	0.681329478
ENSGALG00010017211	UBFD1	3	0.172563964	0.119874672	1.439536482	0.149998581	0.025304383	5.726469759	65.07446849	1	CRA004775;PRJNA827465;Yang2025_Silkie_Wencha	0.681329478
ENSGALG00010024189	KAT7	3	-0.163835283	0.114233476	-1.434214286	0.151511122	0.029808064	11.74518989	82.97175254	1	CRA004775;PRJNA827465;Yang2025_Silkie_Wencha	0.681329478
ENSGALG00010005545	RPL34	3	-0.15728208	0.109763199	-1.432921795	0.151880188	0.010207755	2.406746547	16.90026511	1	CRA004775;PRJNA827465;Yang2025_Silkie_Wencha	0.681329478
ENSGALG00010025113	SDHD	3	-0.151799986	0.105387525	-1.440422653	0.149747859	0.014270975	3.275400762	38.93876977	0.666666667	CRA004775;PRJNA827465;Yang2025_Silkie_Wencha	0.681329478
ENSGALG00010022477	ZC3H7B	3	-0.151043281	0.105121391	-1.436846291	0.150761674	0.021661025	5.878406432	65.97717386	1	CRA004775;PRJNA827465;Yang2025_Silkie_Wencha	0.681329478
ENSGALG00010001611	MAPK12	3	-0.133748917	0.09309395	-1.436709011	0.150800693	0	1.141396631	0	1	CRA004775;PRJNA827465;Yang2025_Silkie_Wencha	0.681329478
ENSGALG00010012705	CPM	3	0.126900839	0.088300555	1.43714656	0.150676354	0	0.787877971	0	1	CRA004775;PRJNA827465;Yang2025_Silkie_Wencha	0.681329478
ENSGALG00010016657	SLC25A29	3	0.125216956	0.087315195	1.434079792	0.151549494	0.00373867	2.32614355	14.02078346	0.666666667	CRA004775;PRJNA827465;Yang2025_Silkie_Wencha	0.681329478
ENSGALG00010022238	COMTD1	3	0.121616048	0.084852218	1.433268932	0.151780997	0	0.03684036	0	1	CRA004775;PRJNA827465;Yang2025_Silkie_Wencha	0.681329478
ENSGALG00010001666	PIEZO2	3	-0.121341116	0.084549992	-1.435140487	0.151247068	0	0.101762194	0	1	CRA004775;PRJNA827465;Yang2025_Silkie_Wencha	0.681329478
ENSGALG00010013910	ADIPOR2	3	0.119590642	0.083357309	1.43467493	0.151379751	0.010676657	4.693137484	57.38458532	1	CRA004775;PRJNA827465;Yang2025_Silkie_Wencha	0.681329478
ENSGALG00010014001	CDC16	3	-0.109923545	0.076717203	-1.432840877	0.151903317	0.008233301	3.84319417	47.95995445	1	CRA004775;PRJNA827465;Yang2025_Silkie_Wencha	0.681329478
ENSGALG00010012346	NLN	3	-0.101142292	0.070295475	-1.438816539	0.150202509	0	0.408979233	0	1	CRA004775;PRJNA827465;Yang2025_Silkie_Wencha	0.681329478
ENSGALG00010023966	PEAK1	3	0.094546788	0.065882984	1.435071432	0.151266743	0	0.610195414	0	1	CRA004775;PRJNA827465;Yang2025_Silkie_Wencha	0.681329478
ENSGALG00010029422	EIF4H	3	-0.075690477	0.05265698	-1.437425341	0.150597173	0.00370998	3.557885377	43.78683437	0.666666667	CRA004775;PRJNA827465;Yang2025_Silkie_Wencha	0.681329478
ENSGALG00010005174	TPP2	3	-0.06780737	0.047321543	-1.432906977	0.151884423	0.000800615	2.153345956	7.121287474	0.666666667	CRA004775;PRJNA827465;Yang2025_Silkie_Wencha	0.681329478
ENSGALG00010027930	RAF1	3	-0.063331456	0.044184791	-1.433331598	0.151763096	0	0.014456393	0	1	CRA004775;PRJNA827465;Yang2025_Silkie_Wencha	0.681329478
ENSGALG00010017538	H3-3A	3	-0.062037089	0.043300435	-1.432712829	0.151939922	0	0.154763716	0	1	CRA004775;PRJNA827465;Yang2025_Silkie_Wencha	0.681329478
ENSGALG00010002186	CILK1	3	0.061406286	0.042665077	1.439263457	0.150075893	0	0.486366993	0	0.666666667	CRA004775;PRJNA827465;Yang2025_Silkie_Wencha	0.681329478
ENSGALG00010023186	MIER1	3	0.053490002	0.037221946	1.435994287	0.151003967	0	1.579988869	0	1	CRA004775;PRJNA827465;Yang2025_Silkie_Wencha	0.681329478
ENSGALG00010012243	ENSGALG00010012243	3	-1.691661071	1.181117523	-1.432254656	0.152070955	2.178954727	4.178112091	52.1314901	1	CRA004775;PRJNA827465;Yang2025_Silkie_Wencha	0.681375544
ENSGALG00010028062	ENSGALG00010028062	3	0.962280577	0.671897793	1.432182969	0.152091464	0.810537225	4.995010361	59.96004302	1	CRA004775;PRJNA827465;Yang2025_Silkie_Wencha	0.681375544
ENSGALG00010003344	ZFAND1	3	0.134756618	0.09412922	1.431613024	0.1522546	0	1.809283448	0	0.666666667	CRA004775;PRJNA827465;Yang2025_Silkie_Wencha	0.681890337
ENSGALG00010024633	NRGN	3	-0.745781145	0.521070966	-1.431246784	0.1523595	0.615803598	8.763830559	77.17892893	0.666666667	CRA004775;PRJNA827465;Yang2025_Silkie_Wencha	0.682072194
ENSGALG00010010035	ATP10B	3	0.522711145	0.365457341	1.430293187	0.152632889	0.151046149	3.159284419	36.69452526	1	CRA004775;PRJNA827465;Yang2025_Silkie_Wencha	0.682072194
ENSGALG00010005901	ENSGALG00010005901	2	0.462756979	0.323538067	1.430301489	0.152630508	0.141619223	3.085530497	67.59066225	1	PRJNA827465;Yang2025_Silkie_Wenchang	0.682072194
ENSGALG00010003819	PHIP	3	0.343288172	0.23987764	1.431097006	0.152402415	0.132105915	10.09988237	80.19778917	0.666666667	CRA004775;PRJNA827465;Yang2025_Silkie_Wencha	0.682072194
ENSGALG00010008350	LRRC6	2	0.319981332	0.223647234	1.430741291	0.152504374	0.037786231	1.55067062	35.51177232	1	PRJNA827465;Yang2025_Silkie_Wenchang	0.682072194
ENSGALG00010003874	YES1	3	0.198373267	0.138690997	1.430325484	0.152623624	0.040857235	7.92311786	74.75741198	1	CRA004775;PRJNA827465;Yang2025_Silkie_Wencha	0.682072194
ENSGALG00010012704	SMIM15	3	-0.176641047	0.123444514	-1.430934774	0.15244891	0.024452872	4.519820376	55.75045392	1	CRA004775;PRJNA827465;Yang2025_Silkie_Wencha	0.682072194
ENSGALG00010014744	IL2RB	3	0.73432658	0.513505322	1.430027205	0.152709211	0.611628016	8.870627741	77.45368131	0.666666667	CRA004775;PRJNA827465;Yang2025_Silkie_Wencha	0.682197642
ENSGALG00010024128	PHLDA2	3	0.716579836	0.501163624	1.429832097	0.152765215	0.696996736	34.65800628	94.22932761	1	CRA004775;PRJNA827465;Yang2025_Silkie_Wencha	0.682232271
ENSGALG00010017100	SUSD5	2	0.927812442	0.65068388	1.425903531	0.15389619	0.507937985	2.4761639	59.61495117	1	PRJNA827465;Yang2025_Silkie_Wenchang	0.68233867
ENSGALG00010007933	CEP126	2	0.620416372	0.434987566	1.426285301	0.153786006	0.206520057	2.188032676	54.29684341	1	PRJNA827465;Yang2025_Silkie_Wenchang	0.68233867
ENSGALG00010020010	SORBS3	3	0.528103356	0.369754291	1.428254842	0.153218521	0.336993822	12.45613772	83.94365858	1	CRA004775;PRJNA827465;Yang2025_Silkie_Wencha	0.68233867
ENSGALG00010022985	ENSGALG00010022985	2	0.508373371	0.35651375	1.425957263	0.153880679	0.093174273	1.54456212	35.25673152	1	PRJNA827465;Yang2025_Silkie_Wenchang	0.68233867
ENSGALG00010016415	RASGRF1	3	0.488153593	0.342264246	1.426247697	0.153796856	0.210658945	4.999997779	59.99998223	1	CRA004775;PRJNA827465;Yang2025_Silkie_Wencha	0.68233867
ENSGALG00010006203	MMR1L4	3	-0.432814973	0.303098567	-1.42796773	0.153301147	0.200570053	13.24287269	84.89753661	1	CRA004775;PRJNA827465;Yang2025_Silkie_Wencha	0.68233867
ENSGALG00010013001	ADGRV1	3	0.335710076	0.235288755	1.426800341	0.153637453	0	0.601159115	0	0.666666667	CRA004775;PRJNA827465;Yang2025_Silkie_Wencha	0.68233867
ENSGALG00010015809	PCOLCE2	3	0.298833937	0.209514614	1.426315482	0.153777298	0.064773606	3.823521362	47.69219757	0.666666667	CRA004775;PRJNA827465;Yang2025_Silkie_Wencha	0.68233867
ENSGALG00010016869	HSP90AA1	3	0.240275319	0.168508568	1.425893783	0.153899004	0.056708359	6.284997974	68.17819181	0.666666667	CRA004775;PRJNA827465;Yang2025_Silkie_Wencha	0.68233867
ENSGALG00010007001	SNX30	3	0.231748499	0.16231522	1.427768129	0.153358609	0.057375609	9.646860897	79.26786733	1	CRA004775;PRJNA827465;Yang2025_Silkie_Wencha	0.68233867
ENSGALG00010004406	ENSGALG00010004406	3	0.222295268	0.155859951	1.426250083	0.153796168	0.037827598	4.106010296	51.29091611	0.666666667	CRA004775;PRJNA827465;Yang2025_Silkie_Wencha	0.68233867
ENSGALG00010000760	CDH10	2	0.194131073	0.1358773	1.428723358	0.153083761	0	0.001994483	0	1	PRJNA827465;Yang2025_Silkie_Wenchang	0.68233867
ENSGALG00010024383	DUSP28	3	0.179300277	0.125523416	1.428420944	0.153170734	0	0.421335388	0	1	CRA004775;PRJNA827465;Yang2025_Silkie_Wencha	0.68233867
ENSGALG00010028481	ENSGALG00010028481	3	0.176958078	0.123851554	1.428791746	0.153064099	0.009796672	2.504410935	20.14090132	0.666666667	CRA004775;PRJNA827465;Yang2025_Silkie_Wencha	0.68233867
ENSGALG00010028148	ENSGALG00010028148	3	-0.1706825	0.119554946	-1.427649011	0.153392909	0	1.488795705	0	0.666666667	CRA004775;PRJNA827465;Yang2025_Silkie_Wencha	0.68233867
ENSGALG00010025822	KLF7	3	0.166298526	0.116436617	1.4282322	0.153225035	0.012264665	2.663422858	24.90865677	0.666666667	CRA004775;PRJNA827465;Yang2025_Silkie_Wencha	0.68233867
ENSGALG00010029198	PPM1D	3	-0.119289139	0.083613768	-1.426668631	0.153675432	0.008578158	3.47816692	42.49844686	0.666666667	CRA004775;PRJNA827465;Yang2025_Silkie_Wencha	0.68233867
ENSGALG00010024812	SBNO1	3	0.110658934	0.077567175	1.426620651	0.153689269	0.009944105	4.588585658	56.41358473	1	CRA004775;PRJNA827465;Yang2025_Silkie_Wencha	0.68233867
ENSGALG00010012508	MDM1	3	-0.097233505	0.068079774	-1.428228969	0.153225965	0.003084975	2.469120695	18.99950439	1	CRA004775;PRJNA827465;Yang2025_Silkie_Wencha	0.68233867
ENSGALG00010010305	URB1	3	0.091373361	0.063985858	1.428024322	0.153284858	0	0.435017399	0	1	CRA004775;PRJNA827465;Yang2025_Silkie_Wencha	0.68233867
ENSGALG00010023704	ANKRD13C	3	-0.088430871	0.061924787	-1.428036744	0.153281283	0.005871555	4.760254022	57.98543543	0.666666667	CRA004775;PRJNA827465;Yang2025_Silkie_Wencha	0.68233867
ENSGALG00010028885	BRD3	3	-0.080827088	0.056595251	-1.428160245	0.15324574	0	0.912292113	0	0.666666667	CRA004775;PRJNA827465;Yang2025_Silkie_Wencha	0.68233867
ENSGALG00010024325	PCBP2	3	-0.073453116	0.051495012	-1.426412245	0.153749381	0	0.924342562	0	1	CRA004775;PRJNA827465;Yang2025_Silkie_Wencha	0.68233867
ENSGALG00010027204	OMD	2	-1.053033453	0.739220086	-1.424519535	0.154296134	0	0.048880809	0	1	PRJNA827465;Yang2025_Silkie_Wenchang	0.68245674
ENSGALG00010005859	ENSGALG00010005859	3	-0.554339175	0.3890335	-1.424913728	0.154182141	0	0.136203842	0	1	CRA004775;PRJNA827465;Yang2025_Silkie_Wencha	0.68245674
ENSGALG00010015416	CAPN6	3	-0.402181568	0.282250495	-1.424910052	0.154183203	0.167233521	9.443381303	78.82114535	0.666666667	CRA004775;PRJNA827465;Yang2025_Silkie_Wencha	0.68245674
ENSGALG00010010361	PIGM	3	-0.343889961	0.241395985	-1.424588569	0.154276166	0.114088358	6.160875529	67.53708153	0.666666667	CRA004775;PRJNA827465;Yang2025_Silkie_Wencha	0.68245674
ENSGALG00010022213	IDH1	3	-0.325666889	0.228569541	-1.424804402	0.15421375	0.132237014	13.66120359	85.3600015	0.666666667	CRA004775;PRJNA827465;Yang2025_Silkie_Wencha	0.68245674
ENSGALG00010009511	ZFPM1	3	0.310623725	0.218088902	1.424298632	0.154360043	0.067990304	3.898003266	48.69167972	0.666666667	CRA004775;PRJNA827465;Yang2025_Silkie_Wencha	0.68245674
ENSGALG00010003242	ASAP2	3	0.293216415	0.205740812	1.425173796	0.154106969	0.075688638	5.232720026	61.77896027	1	CRA004775;PRJNA827465;Yang2025_Silkie_Wencha	0.68245674
ENSGALG00010001654	RPS11	3	-0.146432431	0.102788375	-1.424601091	0.154272544	0.01023355	2.53897453	21.22804004	1	CRA004775;PRJNA827465;Yang2025_Silkie_Wencha	0.68245674
ENSGALG00010021734	PARD3B	3	-0.112870642	0.079245877	-1.424309329	0.154356948	0	0.144247474	0	1	CRA004775;PRJNA827465;Yang2025_Silkie_Wencha	0.68245674
ENSGALG00010011322	GATA4	2	1.617821559	1.138400758	1.421135349	0.155277415	1.478030633	2.269950403	55.94617403	1	CRA004775;PRJNA827465	0.682852683
ENSGALG00010000708	ENSGALG00010000708	2	0.521085217	0.366746654	1.420831551	0.155365736	0	0.013318126	0	1	PRJNA827465;Yang2025_Silkie_Wenchang	0.682852683
ENSGALG00010005617	DTD2	3	-0.388540276	0.273362131	-1.421339062	0.155218213	0.145725172	6.072928611	67.06696014	0.666666667	CRA004775;PRJNA827465;Yang2025_Silkie_Wencha	0.682852683
ENSGALG00010010753	ENSGALG00010010753	3	0.218008009	0.153256637	1.422502896	0.154880311	0.006108883	2.178364278	8.187991308	0.666666667	CRA004775;PRJNA827465;Yang2025_Silkie_Wencha	0.682852683
ENSGALG00010021186	SWT1	3	0.199195584	0.140150241	1.421300332	0.155229467	0.042322005	7.415336368	73.02887016	1	CRA004775;PRJNA827465;Yang2025_Silkie_Wencha	0.682852683
ENSGALG00010015064	RFK	3	-0.179353489	0.126183583	-1.421369434	0.155209387	0.018852405	3.299387858	39.38269504	1	CRA004775;PRJNA827465;Yang2025_Silkie_Wencha	0.682852683
ENSGALG00010012930	IL6ST	3	-0.169455982	0.119265785	-1.420826458	0.155367217	0.026745387	6.679783667	70.05891059	0.666666667	CRA004775;PRJNA827465;Yang2025_Silkie_Wencha	0.682852683
ENSGALG00010018263	SLX4IP	3	0.166878207	0.117278907	1.422917481	0.154760078	0.011464318	2.617057812	23.57830266	1	CRA004775;PRJNA827465;Yang2025_Silkie_Wencha	0.682852683
ENSGALG00010019991	EIF3B	3	-0.152147507	0.106997524	-1.421972224	0.155034314	0.02393868	7.369942276	72.86274539	1	CRA004775;PRJNA827465;Yang2025_Silkie_Wencha	0.682852683
ENSGALG00010004588	MINDY3	3	-0.149432822	0.105065865	-1.422277564	0.154945689	0.01659323	4.168809061	52.0246677	1	CRA004775;PRJNA827465;Yang2025_Silkie_Wencha	0.682852683
ENSGALG00010019072	AEBP1	3	-0.147325886	0.103597448	-1.422099558	0.154997351	0	1.384084535	0	0.666666667	CRA004775;PRJNA827465;Yang2025_Silkie_Wencha	0.682852683
ENSGALG00010023954	PLEKHF1	3	0.13985136	0.098252176	1.423391983	0.154622555	0.016946263	5.113666682	60.88912078	1	CRA004775;PRJNA827465;Yang2025_Silkie_Wencha	0.682852683
ENSGALG00010014494	KLHL9	3	-0.135536927	0.095304427	-1.422147226	0.154983515	0.016846418	6.562116814	69.52202991	1	CRA004775;PRJNA827465;Yang2025_Silkie_Wencha	0.682852683
ENSGALG00010016605	WDPCP	3	-0.124805093	0.087816291	-1.421206605	0.155256705	0	1.244461721	0	1	CRA004775;PRJNA827465;Yang2025_Silkie_Wencha	0.682852683
ENSGALG00010005255	BZW2	3	-0.122392922	0.086015998	-1.422908828	0.154762587	0.009094178	3.36865832	40.62918201	0.666666667	CRA004775;PRJNA827465;Yang2025_Silkie_Wencha	0.682852683
ENSGALG00010002413	TSSC1	3	-0.098868165	0.069484893	-1.422872826	0.154773025	0	0.476842491	0	1	CRA004775;PRJNA827465;Yang2025_Silkie_Wencha	0.682852683
ENSGALG00010006803	COX4I1	3	-0.096506596	0.067866168	-1.422013335	0.15502238	0	0.97811278	0	0.666666667	CRA004775;PRJNA827465;Yang2025_Silkie_Wencha	0.682852683
ENSGALG00010017390	DDX24	3	0.082156716	0.057745549	1.422736777	0.154812475	0	1.770958033	0	0.666666667	CRA004775;PRJNA827465;Yang2025_Silkie_Wencha	0.682852683
ENSGALG00010009213	DUS4L	3	0.082078082	0.057720769	1.421985241	0.155030535	0	0.710995721	0	0.666666667	CRA004775;PRJNA827465;Yang2025_Silkie_Wencha	0.682852683
ENSGALG00010012084	TMEM50B	3	-0.211459646	0.14885787	-1.420547302	0.155448408	0.05220988	10.34844535	80.6734255	1	CRA004775;PRJNA827465;Yang2025_Silkie_Wencha	0.682997215
ENSGALG00010007922	IL17A	2	0.257090748	0.181099021	1.419614232	0.15572002	0	0.429053896	0	1	PRJNA827465;Yang2025_Silkie_Wenchang	0.683341205
ENSGALG00010016901	MECOM	3	0.193604922	0.136340734	1.420007917	0.155605376	0.01892703	3.001519375	33.36708014	1	CRA004775;PRJNA827465;Yang2025_Silkie_Wencha	0.683341205
ENSGALG00010024190	WARS2	3	-0.098609264	0.069455923	-1.419738733	0.155683757	0	1.88575411	0	0.666666667	CRA004775;PRJNA827465;Yang2025_Silkie_Wencha	0.683341205
ENSGALG00010009705	DTNBP1	3	0.067244073	0.047364266	1.419721614	0.155688743	0	0.651370723	0	0.666666667	CRA004775;PRJNA827465;Yang2025_Silkie_Wencha	0.683341205
ENSGALG00010001172	TMEM200C	3	-0.173726122	0.122406682	-1.419253589	0.155825098	0	1.441910953	0	1	CRA004775;PRJNA827465;Yang2025_Silkie_Wencha	0.683421021
ENSGALG00010016934	SLC25A38	3	-0.167900867	0.11830503	-1.419220023	0.15583488	0.023781549	5.166875823	61.29188956	1	CRA004775;PRJNA827465;Yang2025_Silkie_Wencha	0.683421021
ENSGALG00010022255	NOC4L	3	-0.181201588	0.12771072	-1.418843994	0.155944503	0.029665274	5.545078022	63.93197729	0.666666667	CRA004775;PRJNA827465;Yang2025_Silkie_Wencha	0.683526718
ENSGALG00010006188	TMED10	3	-0.16509908	0.116364826	-1.418805708	0.155955668	0.032732286	12.0854353	83.45115463	0.666666667	CRA004775;PRJNA827465;Yang2025_Silkie_Wencha	0.683526718
ENSGALG00010019045	LBX1	3	-0.190106465	0.134015745	-1.418538286	0.156033669	0.025552555	4.212779251	52.52540233	0.666666667	CRA004775;PRJNA827465;Yang2025_Silkie_Wencha	0.683656662
ENSGALG00010011441	CD99L2	3	-0.083665489	0.0589877	-1.418354815	0.156087201	0	1.949569264	0	0.666666667	CRA004775;PRJNA827465;Yang2025_Silkie_Wencha	0.683679346
ENSGALG00010017494	TBC1D24	3	-0.140175941	0.098871938	-1.417752542	0.156263024	0.01393052	4.00065533	50.00819028	1	CRA004775;PRJNA827465;Yang2025_Silkie_Wencha	0.684237503
ENSGALG00010025380	SPC25	3	-0.120686406	0.085148497	-1.417363899	0.156376562	0	0.344185255	0	1	CRA004775;PRJNA827465;Yang2025_Silkie_Wencha	0.684522665
ENSGALG00010023331	CD4	3	-0.372791917	0.26306014	-1.417135709	0.156443254	0	1.02941634	0	1	CRA004775;PRJNA827465;Yang2025_Silkie_Wencha	0.684602653
ENSGALG00010004374	ILDR1	2	0.735720037	0.519508147	1.416185753	0.156721126	0	0.012476082	0	1	CRA004775;PRJNA827465	0.684861871
ENSGALG00010026199	TBX6	3	0.417073883	0.294499891	1.416210655	0.156713837	0	0.389599719	0	1	CRA004775;PRJNA827465;Yang2025_Silkie_Wencha	0.684861871
ENSGALG00010017586	ZFYVE28	3	0.1865775	0.131753974	1.416105292	0.156744679	0.015497967	2.747009134	27.19354387	1	CRA004775;PRJNA827465;Yang2025_Silkie_Wencha	0.684861871
ENSGALG00010015291	ZFP64	3	0.109221324	0.077122922	1.416197954	0.156717555	0.0100904	4.622336407	56.7318381	1	CRA004775;PRJNA827465;Yang2025_Silkie_Wencha	0.684861871
ENSGALG00010027934	CNBP	3	-0.058157332	0.04105645	-1.416521205	0.15662296	0	0.353951366	0	0.666666667	CRA004775;PRJNA827465;Yang2025_Silkie_Wencha	0.684861871
ENSGALG00010003767	ENSGALG00010003767	3	-1.491962154	1.053960892	-1.415576389	0.156899568	0	0.186700823	0	1	CRA004775;PRJNA827465;Yang2025_Silkie_Wencha	0.685115194
ENSGALG00010021766	AGRN	3	-0.100067438	0.07068647	-1.415651937	0.156877437	0	0.6928047	0	0.666666667	CRA004775;PRJNA827465;Yang2025_Silkie_Wencha	0.685115194
ENSGALG00010028163	GBP	3	0.494733489	0.349647181	1.414950599	0.157082981	0.184814367	4.046249052	50.57150526	0.666666667	CRA004775;PRJNA827465;Yang2025_Silkie_Wencha	0.685439244
ENSGALG00010013940	UPF3A	3	-0.348220031	0.246087272	-1.415026584	0.157060702	0.057701592	2.878796593	30.52652608	1	CRA004775;PRJNA827465;Yang2025_Silkie_Wencha	0.685439244
ENSGALG00010027568	NELFB	3	-0.22219878	0.157050138	-1.414827034	0.157119216	0.061163314	12.64609482	84.18484102	0.666666667	CRA004775;PRJNA827465;Yang2025_Silkie_Wencha	0.685439244
ENSGALG00010003468	ILVBL	3	-0.189195888	0.133818467	-1.41382495	0.157413306	0.018395725	2.834058558	29.42982797	1	CRA004775;PRJNA827465;Yang2025_Silkie_Wencha	0.686510406
ENSGALG00010012868	CTHRC1	3	0.520969236	0.368638846	1.413223923	0.157589895	0.239475384	5.519710357	63.7662147	0.666666667	CRA004775;PRJNA827465;Yang2025_Silkie_Wencha	0.686883378
ENSGALG00010005317	CHCHD3	3	-0.07794776	0.055156796	-1.413203192	0.157595988	0.000449949	2.043342905	2.121176297	0.666666667	CRA004775;PRJNA827465;Yang2025_Silkie_Wencha	0.686883378
ENSGALG00010017768	FOXN2	3	-0.102427618	0.072509172	-1.412616018	0.157768657	0	0.307594326	0	1	CRA004775;PRJNA827465;Yang2025_Silkie_Wencha	0.687424048
ENSGALG00010023257	PAK6	3	0.259338156	0.183627276	1.412307369	0.157859477	0	0.796314285	0	1	CRA004775;PRJNA827465;Yang2025_Silkie_Wencha	0.687476186
ENSGALG00010009093	SLU7	3	0.190350381	0.134801442	1.412079715	0.157926491	0.036264391	6.687692049	70.09431676	1	CRA004775;PRJNA827465;Yang2025_Silkie_Wencha	0.687476186
ENSGALG00010022806	SPPL3	3	-0.096911006	0.068624746	-1.412187472	0.157894768	0.007999632	5.447934034	63.28883596	1	CRA004775;PRJNA827465;Yang2025_Silkie_Wencha	0.687476186
ENSGALG00010014380	SNX3	3	-0.07136044	0.05054901	-1.41170796	0.158035969	0	0.300033409	0	1	CRA004775;PRJNA827465;Yang2025_Silkie_Wencha	0.687741016
ENSGALG00010018377	RAP1GAP	3	0.387867081	0.274802041	1.411441776	0.158114392	0.159595907	7.083833256	71.76669851	0.666666667	CRA004775;PRJNA827465;Yang2025_Silkie_Wencha	0.687814762
ENSGALG00010029473	COG1	3	-0.124664621	0.088331916	-1.411320243	0.158150208	0.010696978	3.679966823	45.65168394	0.666666667	CRA004775;PRJNA827465;Yang2025_Silkie_Wencha	0.687814762
ENSGALG00010005218	GTPBP8	3	-0.112336644	0.079610803	-1.411072871	0.158223128	0	0.924297771	0	1	CRA004775;PRJNA827465;Yang2025_Silkie_Wencha	0.687920299
ENSGALG00010005494	PPDPFL	3	-1.026926601	0.729866649	-1.407005791	0.159425672	1.289581871	11.09716459	81.97737824	0.666666667	CRA004775;PRJNA827465;Yang2025_Silkie_Wencha	0.68855483
ENSGALG00010001443	ANKRD26	3	0.589693247	0.419211541	1.406672262	0.159524594	0.479875769	29.87025899	93.30437677	1	CRA004775;PRJNA827465;Yang2025_Silkie_Wencha	0.68855483
ENSGALG00010028490	NECTIN4	3	0.533034253	0.379165935	1.405807339	0.159781342	0.304085871	6.833884815	70.73406922	1	CRA004775;PRJNA827465;Yang2025_Silkie_Wencha	0.68855483
ENSGALG00010015738	CRACDL	2	0.505722129	0.359681776	1.406026556	0.159716239	0	0.010447101	0	1	CRA004775;PRJNA827465	0.68855483

ENSGALG00010011960	CFAP54	3	0.385036411	0.273379388	1.408432487	0.159003045	0.098564228	3.702524363	45.98279973	1	CRA004775;PRJNA827465;Yang2025_Silkie_Wencha	0.68855483
ENSGALG00010008412	NEK1	3	0.370850065	0.263088302	1.409603024	0.158656934	0.179418599	16.96867348	88.21357484	1	CRA004775;PRJNA827465;Yang2025_Silkie_Wencha	0.68855483
ENSGALG00010018436	FOXO4	3	-0.331396188	0.235621679	-1.406475795	0.159582887	0.131293809	20.1587446	90.07874726	1	CRA004775;PRJNA827465;Yang2025_Silkie_Wencha	0.68855483
ENSGALG00010008430	ENSGALG0001000843	3	0.327683158	0.23304304	1.406105745	0.159692727	0	0.531517037	0	1	CRA004775;PRJNA827465;Yang2025_Silkie_Wencha	0.68855483
ENSGALG00010001190	LXN	3	0.327138471	0.232019503	1.409961089	0.158551173	0.019055173	2.22882622	10.26666942	0.666666667	CRA004775;PRJNA827465;Yang2025_Silkie_Wencha	0.68855483
ENSGALG00010009333	ADRA1B	3	0.299000615	0.212342505	1.408105343	0.159099879	0.067919689	4.073609635	50.90349397	1	CRA004775;PRJNA827465;Yang2025_Silkie_Wencha	0.68855483
ENSGALG00010021727	MVK	3	-0.288719416	0.205341771	-1.406043276	0.159711275	0.104582395	12.78478193	84.35640114	0.666666667	CRA004775;PRJNA827465;Yang2025_Silkie_Wencha	0.68855483
ENSGALG00010011349	ENSGALG0001001134	3	0.280733553	0.199287641	1.408685214	0.158928269	0.09618365	13.58391364	85.27670263	0.666666667	CRA004775;PRJNA827465;Yang2025_Silkie_Wencha	0.68855483
ENSGALG00010021124	CALHM2	3	-0.241856325	0.171735583	-1.408306425	0.159040354	0.057691916	7.088978834	71.78719183	1	CRA004775;PRJNA827465;Yang2025_Silkie_Wencha	0.68855483
ENSGALG00010027920	SCAMP4	3	-0.229494299	0.162892879	-1.408866364	0.158874687	0.066331748	13.32587873	84.99160888	0.666666667	CRA004775;PRJNA827465;Yang2025_Silkie_Wencha	0.68855483
ENSGALG00010012904	ERMP1	3	-0.226444623	0.160635415	-1.40968057	0.158634025	0.034784364	3.657007939	45.31048242	1	CRA004775;PRJNA827465;Yang2025_Silkie_Wencha	0.68855483
ENSGALG00010029391	ARSG	3	-0.222507447	0.158220715	-1.406310463	0.159631955	0.056482802	8.781203568	77.22407886	1	CRA004775;PRJNA827465;Yang2025_Silkie_Wencha	0.68855483
ENSGALG00010006328	SYNDIG1L	3	0.210312261	0.149578446	1.406033199	0.159714267	0	1.586182546	0	1	CRA004775;PRJNA827465;Yang2025_Silkie_Wencha	0.68855483
ENSGALG00010028103	APBA3	3	-0.199874028	0.141794869	-1.409599869	0.158657866	0.038347605	5.924108223	66.23964444	1	CRA004775;PRJNA827465;Yang2025_Silkie_Wencha	0.68855483
ENSGALG00010008210	CTDP1	3	0.186279792	0.13207985	1.410357388	0.158434181	0.032346534	5.419482054	63.09610439	1	CRA004775;PRJNA827465;Yang2025_Silkie_Wencha	0.68855483
ENSGALG00010005186	ESR1	3	-0.182460254	0.129475784	-1.409223012	0.158769235	0	1.442876329	0	0.666666667	CRA004775;PRJNA827465;Yang2025_Silkie_Wencha	0.68855483
ENSGALG00010021645	ASCC1	3	0.165501953	0.117519871	1.408289098	0.159045482	0.019747902	3.731096466	46.39645429	0.666666667	CRA004775;PRJNA827465;Yang2025_Silkie_Wencha	0.68855483
ENSGALG00010016611	DNALI1	3	0.164054805	0.116553349	1.40755119	0.159264009	0	1.086676153	0	1	CRA004775;PRJNA827465;Yang2025_Silkie_Wencha	0.68855483
ENSGALG00010017578	BSDC1	3	-0.162403251	0.115365179	-1.407731971	0.159210451	0.022573033	4.444019103	54.99569301	1	CRA004775;PRJNA827465;Yang2025_Silkie_Wencha	0.68855483
ENSGALG00010001196	LSMEM1	3	-0.152411426	0.108198466	-1.408628342	0.158945094	0	1.11308155	0	0.666666667	CRA004775;PRJNA827465;Yang2025_Silkie_Wencha	0.68855483
ENSGALG00010003808	TWIST1	3	-0.150471079	0.106911743	-1.407432662	0.159299132	0	1.858607365	0	0.666666667	CRA004775;PRJNA827465;Yang2025_Silkie_Wencha	0.68855483
ENSGALG00010000303	ENSGALG0001000030	3	-0.128117992	0.091044355	-1.407204125	0.159366869	0.013778639	4.580537973	56.33700644	1	CRA004775;PRJNA827465;Yang2025_Silkie_Wencha	0.68855483
ENSGALG00010028460	CRLF1	3	0.126480184	0.089948716	1.406136628	0.159683558	0.001329792	2.092091645	4.401893441	0.666666667	CRA004775;PRJNA827465;Yang2025_Silkie_Wencha	0.68855483
ENSGALG00010029192	DTX2	3	-0.120355317	0.08549367	-1.407768746	0.159199558	0.005277647	2.443345676	18.1450247	1	CRA004775;PRJNA827465;Yang2025_Silkie_Wencha	0.68855483
ENSGALG00010021096	ZFAND4	3	0.085613239	0.060895547	1.405903098	0.159752901	0	0.933111161	0	1	CRA004775;PRJNA827465;Yang2025_Silkie_Wencha	0.68855483
ENSGALG00010002706	ROCK2	3	0.40023545	0.284816297	1.405240693	0.159949717	0.182660521	10.52042156	80.98935495	0.666666667	CRA004775;PRJNA827465;Yang2025_Silkie_Wencha	0.6890704
ENSGALG00010008282	FAM83H	3	0.300091062	0.213660527	1.404930814	0.160041853	0.055464543	3.356801619	40.41947582	1	CRA004775;PRJNA827465;Yang2025_Silkie_Wencha	0.689218184
ENSGALG00010001533	ENSGALG0001000153	3	0.279736644	0.199158775	1.404591105	0.160142903	0.079632701	6.933641706	71.15512908	0.666666667	CRA004775;PRJNA827465;Yang2025_Silkie_Wencha	0.689218184
ENSGALG00010021862	PDK1	3	-0.156497542	0.111427087	-1.40448383	0.160174823	0.022578772	6.331149932	68.41016211	1	CRA004775;PRJNA827465;Yang2025_Silkie_Wencha	0.689218184
ENSGALG00010015605	LDLRAP1	3	-0.062953423	0.044823623	-1.404469778	0.160179005	0	1.260792599	0	1	CRA004775;PRJNA827465;Yang2025_Silkie_Wencha	0.689218184
ENSGALG00010019028	TNFRSF25	3	0.549862162	0.391655679	1.403942777	0.160335589	0.33015459	7.078439715	71.74518566	0.666666667	CRA004775;PRJNA827465;Yang2025_Silkie_Wencha	0.689469358
ENSGALG00010003717	LEPROTL1	3	-0.413099818	0.294276258	-1.403782353	0.16038367	0.238597683	28.42972316	92.96510913	1	CRA004775;PRJNA827465;Yang2025_Silkie_Wencha	0.689469358
ENSGALG00010027698	FAM120A	3	-0.060192787	0.042877649	-1.403826668	0.160370471	0.001131758	2.244825224	10.90620425	0.666666667	CRA004775;PRJNA827465;Yang2025_Silkie_Wencha	0.689469358
ENSGALG00010018528	ENSGALG0001001852	2	-0.577630488	0.411759144	-1.402835846	0.160665795	0	0.619840104	0	1	PRJNA827465;Yang2025_Silkie_Wenchang	0.689557033
ENSGALG00010012371	ATP6V1G3	2	0.505592154	0.360460744	1.402627506	0.160727945	0	0.402657947	0	1	CRA004775;PRJNA827465	0.689557033
ENSGALG00010022652	CARMIL2	3	0.43068723	0.30702948	1.402755298	0.160689821	0.181609267	6.094638067	67.18426955	1	CRA004775;PRJNA827465;Yang2025_Silkie_Wencha	0.689557033
ENSGALG00010006075	SOX21	3	-0.39613847	0.282503641	-1.402241998	0.160842994	0	1.309663493	0	0.666666667	CRA004775;PRJNA827465;Yang2025_Silkie_Wencha	0.689557033
ENSGALG00010022803	ENSGALG0001002280	3	-0.237047949	0.168912164	-1.403379982	0.160503559	0.014062019	2.302406918	13.13438192	1	CRA004775;PRJNA827465;Yang2025_Silkie_Wencha	0.689557033
ENSGALG00010001696	FAM135A	3	-0.141776393	0.101085391	-1.402540895	0.160753787	0.017624069	5.346602704	62.59306871	0.666666667	CRA004775;PRJNA827465;Yang2025_Silkie_Wencha	0.689557033
ENSGALG00010020284	HEG1	3	-0.125467066	0.089475141	-1.402256147	0.160838771	0	0.546621551	0	0.666666667	CRA004775;PRJNA827465;Yang2025_Silkie_Wencha	0.689557033
ENSGALG00010007376	CDKN2AIP	3	-0.089900263	0.064103743	-1.402418317	0.160790367	0.004282483	2.881860685	30.60039264	0.666666667	CRA004775;PRJNA827465;Yang2025_Silkie_Wencha	0.689557033
ENSGALG00010029718	ABR	3	0.074115626	0.052812289	1.4033784	0.16050403	0	0.150707117	0	1	CRA004775;PRJNA827465;Yang2025_Silkie_Wencha	0.689557033
ENSGALG00010000796	ENSGALG0001000079	3	-0.173341798	0.123645582	-1.401924716	0.160937729	0.001620928	2.051357117	2.503567816	0.666666667	CRA004775;PRJNA827465;Yang2025_Silkie_Wencha	0.689754031
ENSGALG00010014154	DNAI7	2	0.332384831	0.237233557	1.401086905	0.161188087	0	1.39858E-05	0	1	PRJNA827465;Yang2025_Silkie_Wenchang	0.690617686
ENSGALG00010008183	ALG10	3	-0.348510896	0.24879101	-1.400817885	0.161268539	0.157673074	28.5413473	92.99262232	1	CRA004775;PRJNA827465;Yang2025_Silkie_Wencha	0.690753067
ENSGALG00010000246	SUPT20H	3	-0.049634796	0.03544112	-1.400486096	0.161367804	0	0.517080118	0	0.666666667	CRA004775;PRJNA827465;Yang2025_Silkie_Wencha	0.690968923
ENSGALG00010019365	ENSGALG0001001936	3	0.562174996	0.401566322	1.399955537	0.161526633	0.362091399	11.00066382	81.81927898	1	CRA004775;PRJNA827465;Yang2025_Silkie_Wencha	0.691230348
ENSGALG00010029262	CLTC	3	-0.193425242	0.138150432	-1.400105953	0.161481593	0.040078934	11.58512361	82.73648113	1	CRA004775;PRJNA827465;Yang2025_Silkie_Wencha	0.691230348
ENSGALG00010010940	NRG3	2	-0.617553794	0.441227544	-1.399626568	0.161625174	0.321692391	5.6448124	82.28461942	1	PRJNA827465;Yang2025_Silkie_Wenchang	0.691233615
ENSGALG00010028399	SLC35E1	3	-0.180789867	0.12915627	-1.399776157	0.16158036	0.040884552	17.91875133	88.83850798	1	CRA004775;PRJNA827465;Yang2025_Silkie_Wencha	0.691233615
ENSGALG00010017092	ANKS4B	3	0.854708438	0.610964389	1.398949715	0.161828063	0.822477986	7.856939439	74.54479552	1	CRA004775;PRJNA827465;Yang2025_Silkie_Wencha	0.691876845
ENSGALG00010029930	ARHGAP44	3	0.473184182	0.338691345	1.397095582	0.16238483	0.248219449	11.06643649	81.92733495	0.666666667	CRA004775;PRJNA827465;Yang2025_Silkie_Wencha	0.691876845
ENSGALG00010002742	PROSER1	3	0.388736126	0.278380626	1.396419469	0.162588216	0.192649774	15.15149345	86.79998109	1	CRA004775;PRJNA827465;Yang2025_Silkie_Wencha	0.691876845
ENSGALG00010017612	CLDN11	3	-0.366765044	0.262252458	-1.398518997	0.161957272	0.008102578	2.073264359	3.533768302	0.666666667	CRA004775;PRJNA827465;Yang2025_Silkie_Wencha	0.691876845
ENSGALG00010019652	TNFRSF10B	3	-0.326500197	0.23347384	-1.398444456	0.161979641	0	1.75290717	0	0.666666667	CRA004775;PRJNA827465;Yang2025_Silkie_Wencha	0.691876845
ENSGALG00010024823	ASB10	3	-0.314115145	0.224947067	-1.396395825	0.162595332	0	0.953514883	0	1	CRA004775;PRJNA827465;Yang2025_Silkie_Wencha	0.691876845
ENSGALG00010019158	ENSGALG0001001915	3	0.311565487	0.222837318	1.398174643	0.162060629	0.081648647	4.416888705	54.71925752	0.666666667	CRA004775;PRJNA827465;Yang2025_Silkie_Wencha	0.691876845
ENSGALG00010006542	RFC3	3	-0.293031193	0.209682664	-1.397498423	0.162263741	0.093365427	10.95311626	81.74035633	1	CRA004775;PRJNA827465;Yang2025_Silkie_Wencha	0.691876845
ENSGALG00010026268	GARS1	3	-0.293141585	0.208901012	-1.397471454	0.162271845	0.109445581	15.96894811	87.47569354	1	CRA004775;PRJNA827465;Yang2025_Silkie_Wencha	0.691876845
ENSGALG00010017167	SMAP2	3	-0.288807113	0.206538438	-1.398321379	0.162016581	0.102592619	13.83127411	85.54001617	0.666666667	CRA004775;PRJNA827465;Yang2025_Silkie_Wencha	0.691876845
ENSGALG00010015542	CNGA3	3	0.27557195	0.197343275	1.396409125	0.162591329	0	1.521666427	0	1	CRA004775;PRJNA827465;Yang2025_Silkie_Wencha	0.691876845
ENSGALG00010024592	COMT	3	-0.17464949	0.125024635	-1.396920611	0.162437446	0.018886231	3.222888189	37.9438602	1	CRA004775;PRJNA827465;Yang2025_Silkie_Wencha	0.691876845
ENSGALG00010014186	MOCS3	3	-0.165274847	0.118358766	-1.396355557	0.162607451	0.021617184	3.995755491	49.94688715	1	CRA004775;PRJNA827465;Yang2025_Silkie_Wencha	0.691876845
ENSGALG00010006998	POLH	3	0.123858011	0.088615829	1.397696228	0.162204307	0.009471708	3.218512515	37.85949283	1	CRA004775;PRJNA827465;Yang2025_Silkie_Wencha	0.691876845
ENSGALG00010017283	INF2	3	0.105522563	0.075490716	1.397821732	0.162166607	0	1.524126748	0	1	CRA004775;PRJNA827465;Yang2025_Silkie_Wencha	0.691876845
ENSGALG00010028586	BABAM1	3	-0.064577034	0.04622919	-1.396888733	0.162447033	0	1.40354155	0	1	CRA004775;PRJNA827465;Yang2025_Silkie_Wencha	0.691876845
ENSGALG00010012327	UBTD2	3	-0.060100066	0.043030555	-1.396683483	0.162508773	0	0.228922295	0	1	CRA004775;PRJNA827465;Yang2025_Silkie_Wencha	0.691876845
ENSGALG00010022243	ENSGALG0001002224	3	0.700860057	0.502019159	1.396082291	0.162689716	0.474816691	5.380601822	62.82943682	0.666666667	CRA004775;PRJNA827465;Yang2025_Silkie_Wencha	0.691979887
ENSGALG00010020064	TMEM138	3	-0.176558598	0.126493895	-1.395787511	0.162778492	0.023157371	3.721075523	46.25209868	1	CRA004775;PRJNA827465;Yang2025_Silkie_Wencha	0.691979887
ENSGALG00010009357	LYST	3	0.123106072	0.088193385	1.395865155	0.162755105	0.006021606	2.501310819	20.04192423	0.666666667	CRA004775;PRJNA827465;Yang2025_Silkie_Wencha	0.691979887
ENSGALG00010017476	ATP6V0C	3	-0.276030418	0.197808832	-1.39544031	0.162883103	0.095115364	11.60207541	82.76170487	1	CRA004775;PRJNA827465;Yang2025_Silkie_Wencha	0.692036983
ENSGALG00010016137	STARD3NL	3	0.098584578	0.070648777	1.395418041	0.162889814	0.002596668	2.353884241	15.03405456	0.666666667	CRA004775;PRJNA827465;Yang2025_Silkie_Wencha	0.692036983
ENSGALG00010007802	ENSGALG0001000780	3	1.371847867	0.985587954	1.391908111	0.163950225	1.564148085	4.319065016	53.69368156	1	CRA004775;PRJNA827465;Yang2025_Silkie_Wencha	0.692450961
ENSGALG00010018628	XDH	3	0.877484501	0.630449697	1.391839039	0.163971145	0.70433663	4.893640791	59.1306333	0.666666667	CRA004775;PRJNA827465;Yang2025_Silkie_Wencha	0.692450961
ENSGALG00010026743	TNNT2	3	0.628572707	0.45159677	1.391889289	0.163955926	0.44660745	12.54258071	84.05431827	0.666666667	CRA004775;PRJNA827465;Yang2025_Silkie_Wencha	0.692450961
ENSGALG00010018329	ENSGALG0001001832	3	-0.442182146	0.31767001	-1.39195433	0.163936228	0.243824063	10.47909717	80.91438635	0.666666667	CRA004775;PRJNA827465;Yang2025_Silkie_Wencha	0.692450961
ENSGALG00010011859	NTN4	3	0.246551011	0.17697572	1.393134671	0.16357907	0.053669828	5.572354799	64.10853091	1	CRA004775;PRJNA827465;Yang2025_Silkie_Wencha	0.692450961
ENSGALG00010013135	TTC3	3	0.242344946	0.173868235	1.393842559	0.163365153	0.067921837	8.689196395	76.98291178	1	CRA004775;PRJNA827465;Yang2025_Silkie_Wencha	0.692450961
ENSGALG00010017041	JKAMP	3	-0.238900542	0.171303238	-1.394606107	0.163134653	0.069598335	11.18128909	82.11297478	0.666666667	CRA004775;PRJNA827465;Yang2025_Silkie_Wencha	0.692450961
ENSGALG00010012890	MFAP3	3	-0.215136745	0.154658305	-1.391045538	0.164211618	0.056550417	19.34284377	89.66025873	1	CRA004775;PRJNA827465;Yang2025_Silkie_Wencha	0.692450961
ENSGALG00010016789	DISP3	3	-0.1892109	0.13592538	-1.392020393	0.163916223	0.029986668	4.471449388	55.27177373	1	CRA004775;PRJNA827465;Yang2025_Silkie_Wencha	0.692450961
ENSGALG00010027079	PM20D1	3	-0.185274401	0.133126611	-1.391715746	0.164008492	0.034258184	5.834465335	65.72093782	1	CRA004775;PRJNA827465;Yang2025_Silkie_Wencha	0.692450961
ENSGALG00010009595	ENSGALG0001000959	3	-0.184100763	0.132339579	-1.391123991	0.164187831	0	0.039943064	0	1	CRA004775;PRJNA827465;Yang2025_Silkie_Wencha	0.692450961
ENSGALG00010008230	TRMU	3	0.182072557	0.130556427	1.394588996	0.163139815	0.035694761	7.00802385	71.46128434	1	CRA004775;PRJNA827465;Yang2025_Silkie_Wencha	0.692450961
ENSGALG00010020999	IFIH1	3	-0.179398258	0.12895775	-1.391139794	0.16418304	0	1.529762271	0	0.666666667	CRA004775;PRJNA827465;Yang2025_Silkie_Wencha	0.692450961
ENSGALG00010023818	ALOX5	3	-0.178366772	0.127994142	-1.393554184	0.163452272	0	0.910768072	0	0.666666667	CRA004775;PRJNA827465;Yang2025_Silkie_Wencha	0.692450961
ENSGALG00010021835	NUDT8	3	-0.139562029	0.100234527	-1.392354842	0.163814972	0	0.016213535	0	1	CRA004775;PRJNA827465;Yang2025_Silkie_Wencha	0.692450961
ENSGALG00010026707	GORASP1	3	-0.132727064	0.095414288	-1.391060666	0.164207031	0.014589052	4.694313256	57.3952591	1	CRA004775;PRJNA827465;Yang2025_Silkie_Wencha	0.692450961
ENSGALG00010009479	MTBP	3	0.131600124	0.094492312	1.392707214	0.163708346	0	1.914150048	0	0.666666667	CRA004775;PRJNA827465;Yang2025_Silkie_Wencha	0.692450961
ENSGALG00010027330	ENSGALG0001002733	3	0.129790797	0.09323703	1.392052033	0.163906642	0	1.266662466	0	1	CRA004775;PRJNA827465;Yang2025_Silkie_Wencha	0.692450961
ENSGALG00010022427	RPL27	3	-0.117656036	0.084419196	-1.393711876	0.163404629	0.004805301	2.267606577	11.80127892	1	CRA004775;PRJNA827465;Yang2025_Silkie_Wencha	0.692450961
ENSGALG00010020855	DNAL1	3	0.101660854	0.07302805	1.392079535	0.163898315	0	1.80075486	0	0.666666667	CRA004775;PRJNA827465;Yang2025_Silkie_Wencha	0.692450961
ENSGALG00010010837	RBPJ	3	-0.100518959	0.072256294	-1.391144674	0.16418156	0	0.806397364	0	1	CRA004775;PRJNA827465;Yang2025_Silkie_Wencha	0.692450961
ENSGALG00010019577	MXD1	3	-0.090102605	0.064734381	-1.391881771	0.163958203	0	0.354372192	0	1	CRA004775;PRJNA827465;Yang2025_Silkie_Wencha	0.692450961
ENSGALG00010014900	UBLCP1	3	-0.089562245	0.064272018	-1.393487357	0.163472466	0.004632017	3.087649591	35.22581041	1	CRA004775;PRJNA827465;Yang2025_Silkie_Wencha	0.692450961
ENSGALG00010016763	UBALD1	3	0.08799859	0.063116384	1.394227361	0.163248958	0	0.890234461	0	1	CRA004775;PRJNA827465;Yang2025_Silkie_Wencha	0.692450961
ENSGALG00010016961	EIF4A3	3	-0.0560724	0.040303445	-1.391255749	0.164147887	0	0.522890359	0	1	CRA004775;PRJNA827465;Yang2025_Silkie_Wencha	0.692450961
ENSGALG00010011963	ZCCHC2	3	0.234635059	0.168709471	1.390764006	0.164297001	0.059512812	7.077673309	71.74212608	1	CRA004775;PRJNA827465;Yang2025_Silkie_Wencha	0.692604443
ENSGALG00010028326	CRB2	3	0.358216423	0.257664108	1.390245718	0.164454275	0.105441908	4.372118428	54.25558495	0.666666667	CRA004775;PRJNA827465;Yang2025_Silkie_Wencha	0.693060803
ENSGALG00010022839	SYT12	3	0.36458435	0.262371352	1.389573775	0.164658344	0.144874385	6.782663775	70.51305996	0.666666667	CRA004775;PRJNA827465;Yang2025_Silkie_Wencha	0.693671355
ENSGALG00010001749	VAPA	3	-0.070953025	0.051065707	-1.389445664	0.164697273	0	1.685353257	0	0.666666667	CRA004775;PRJNA827465;Yang2025_Silkie_Wencha	0.693671355
ENSGALG00010022582	ENSGALG0001002258	3	-0.730629387	0.525967637	-1.38911472	0.164797868	0.729109528	18.5636936	89.226282	1	CRA004775;PRJNA827465;Yang2025_Silkie_Wencha	0.693681769
ENSGALG00010003650	SPACA1	2	0.509061318	0.366422579	1.38927388	0.164749484	0.098417879	1.577299153	36.60048583	1	PRJNA827465;Yang2025_Silkie_Wenchang	0.693681769
ENSGALG00010000283	ENSGALG0001000028	3	-0.386506533	0.278313497	-1.388745202	0.164910244	0	1.87244108	0	0.666666667	CRA004775;PRJNA827465;Yang2025_Silkie_Wencha	0.693741724
ENSGALG00010016211	TACC3	3	-0.101503093	0.073086169	-1.388813982	0.164889322	0.004973701	2.856196741	29.97681247	0.666666667	CRA004775;PRJNA827465;Yang2025_Silkie_Wencha	0.693741724
ENSGALG00010014881	ENSGALG0001001488	2	0.622689661	0.448908786	1.387118452	0.165405646	0.301189618	3.944624762	74.64904623	1	PRJNA827465;Yang2025_Silkie_Wenchang	0.693960232
ENSGALG00010028151	DOCK3	3	0.573548077	0.413590782	1.386752561	0.165517227	0.364936776	8.625156528	76.81201502	1	CRA004775;PRJNA827465;Yang2025_Silkie_Wencha	0.693960232
ENSGALG00010000156	ENSGALG0001000015	3	0.512202136	0.369730402	1.385339514	0.165948677	0.063587423	2.336077826	14.38641395	0.666666667	CRA004775;PRJNA827465;Yang2025_Silkie_Wencha	0.693960232
ENSGALG00010002023	CLCN4	3	-0.374936377	0.270387831	-1.386661432	0.165545026	0.13835135	6.012356783	66.73517437	1	CRA004775;PRJNA827465;Yang2025_Silkie_Wencha	0.693960232
ENSGALG00010001123	CP	3	0.37277642	0.269060268	1.385475539	0.165907107	0.166673196	12.82168693	84.40142853	1	CRA004775;PRJNA827465;Yang2025_Silkie_Wencha	0.693960232
ENSGALG00010022019	BTBD8	3	0.364332869	0.262760113	1.386560783	0.165575734	0.153623055	9.055258703	77.9133864	1	CRA004775;PRJNA827465;Yang2025_Silkie_Wencha	0.693960232
ENSGALG00010017063	TMPPE	3	-0.342831772	0.247497145	-1.385194859	0.165992892	0.126054615	7.516124922	73.39054339	1	CRA004775;PRJNA827465;Yang2025_Silkie_Wencha	0.693960232
ENSGALG00010016416	CYP46A1	3	0.270011927	0.194669405	1.38702806	0.165433207	0.09234863	15.9879783	87.49060098	1	CRA004775;PRJNA827465;Yang2025_Silkie_Wencha	0.693960232
ENSGALG00010013407	ZC3H15	3	0.260712273	0.187881694	1.387640632	0.165246502	0.083444113	14.7142035	86.40769105	0.666666667	CRA004775;PRJNA827465;Yang2025_Silkie_Wencha	0.693960232
ENSGALG00010012943	ASNSD1	3	-0.234384481	0.169048617	-1.386491563	0.165596855	0.062177781	8.435248208	76.28996858	1	CRA004775;PRJNA827465;Yang2025_Silkie_Wencha	0.693960232
ENSGALG00010014372	CCDC171	3	0.188328793	0.135889344	1.385898167	0.165778001	0.016203194	2.810878689	28.84787209	0.666666667	CRA004775;PRJNA827465;Yang2025_Silkie_Wencha	0.693960232
ENSGALG00010025553	UBE2G2	3	-0.187695569	0.135197789	-1.388303535	0.165044636	0.033859173	5.211557698	61.62375789	1	CRA004775;PRJNA827465;Yang2025_Silkie_Wencha	0.693960232
ENSGALG00010007318	RMDN2	3	0.180279733	0.130017358	1.386582038	0.165569248	0.022221512	3.539952355	43.50206446	1	CRA004775;PRJNA827465;Yang2025_Silkie_Wencha	0.693960232
ENSGALG00010026098	STAT3	3	-0.173494601	0.125094448	-1.386908878	0.16546955	0.030614856	6.25840637	68.0429828	1	CRA004775;PRJNA827465;Yang2025_Silkie_Wencha	0.693960232
ENSGALG00010028594	ENSGALG0001002859	3	-0.116457957	0.083991889	-1.386538125	0.165582647	0	1.413821013	0	0.666666667	CRA004775;PRJNA827465;Yang2025_Silkie_Wencha	0.693960232
ENSGALG00010022711	LRRC49	3	0.115503568	0.083350036	1.385765063	0.165818654	0	0.428790781	0	0.666666667	CRA004775;PRJNA827465;Yang2025_Silkie_Wencha	0.693960232
ENSGALG00010028786	GTF3C4	3	-0.109748534	0.079173742	-1.386173374	0.16569397	0.007982968	3.270388957	38.84519467	1	CRA004775;PRJNA827465;Yang2025_Silkie_Wencha	0.693960232
ENSGALG00010000816	TBC1D4	3	-0.100089027	0.072233563	-1.385630497	0.165859761	0	1.31448791	0	0.666666667	CRA004775;PRJNA827465;Yang2025_Silkie_Wencha	0.693960232
ENSGALG00010013911	TNIK	3	-0.095152379	0.068683892	-1.385366735	0.165940357	0	0.503582339	0	1	CRA004775;PRJNA827465;Yang2025_Silkie_Wencha	0.693960232
ENSGALG00010029708	TMEM94	3	-0.094845893	0.06838616	-1.386916494	0.165467228	0.006055802	3.359602525	40.4691482	1	CRA004775;PRJNA827465;Yang2025_Silkie_Wencha	0.693960232
ENSGALG00010029513	WBSCR22	3	-0.071784901	0.051804186	-1.385696926	0.165839467	0	1.812500957	0	1	CRA004775;PRJNA827465;Yang2025_Silkie_Wencha	0.693960232
ENSGALG00010016752	ENSGALG0001001675	3	-0.404290691	0.29190435	-1.385010848	0.16604915	0.203620911	14.95805536	86.62927799	0.666666667	CRA004775;PRJNA827465;Yang2025_Silkie_Wencha	0.693990227
ENSGALG00010011400	BPHL	3	0.230533946	0.166478633	1.384765969	0.16612404	0	0.476133477	0	1	CRA004775;PRJNA827465;Yang2025_Silkie_Wencha	0.694013948
ENSGALG00010027399	MAPKAP1	3	-0.061882398	0.044691038	-1.384671308	0.166152996	0	1.155090423	0	0.666666667	CRA004775;PRJNA827465;Yang2025_Silkie_Wencha	0.694013948
ENSGALG00010029684	ENSGALG0001002968	3	0.3830776	0.276723183	1.384335047	0.166255887	0	1.060942695	0	1	CRA004775;PRJNA827465;Yang2025_Silkie_Wencha	0.694031842
ENSGALG00010009050	PIK3CG	3	0.093477584	0.067529634	1.384245384	0.166283331	0	0.695737736	0	0.666666667	CRA004775;PRJNA827465;Yang2025_Silkie_Wencha	0.694031842
ENSGALG00010030004	FLOT2	3	-0.085164541	0.061527244	-1.384176101	0.166304539	0	1.672772627	0	1	CRA004775;PRJNA827465;Yang2025_Silkie_Wencha	0.694031842
ENSGALG00010029287	ANKRD40	3	0.100991299	0.072979407	1.383832828	0.166409649	0	0.102597931	0	1	CRA004775;PRJNA827465;Yang2025_Silkie_Wencha	0.694265572
ENSGALG00010017435	ACTR10	3	-0.277996779	0.200915579	-1.383649696	0.166465744	0.094443124	9.741344442	79.46895306	0.666666667	CRA004775;PRJNA827465;Yang2025_Silkie_Wencha	0.694294734
ENSGALG00010022329	CYP2AC7	2	-0.379240164	0.274144443	-1.383358934	0.166554836	0	0.005983763	0	1	PRJNA827465;Yang2025_Silkie_Wenchang	0.694443272
ENSGALG00010011657	ALDH1L2	3	0.108914918	0.078740529	1.383212923	0.166599588	0	1.009308886	0	0.666666667	CRA004775;PRJNA827465;Yang2025_Silkie_Wencha	0.694443272
ENSGALG00010029174	CALN1	2	-0.25949078	0.187631076	-1.382984024	0.166669765	0	0.205871098	0	1	PRJNA827465;Yang2025_Silkie_Wenchang	0.694531035
ENSGALG00010006992	SLC35B4	3	-0.24155468	0.174714314	-1.38256949	0.166796909	0.075931386	14.63453944	86.33370044	0.666666667	CRA004775;PRJNA827465;Yang2025_Silkie_Wencha	0.694551493
ENSGALG00010027336	RAD54L2	3	-0.091368755	0.066080629	-1.382685917	0.166761192	0.004599667	3.050830112	34.44407173	0.666666667	CRA004775;PRJNA827465;Yang2025_Silkie_Wencha	0.694551493
ENSGALG00010020602	DENND2B	3	0.081125196	0.058680597	1.382487574	0.166822043	0.003702721	3.098759173	35.45803697	1	CRA004775;PRJNA827465;Yang2025_Silkie_Wencha	0.694551493
ENSGALG00010028182	SMG5	3	0.103326733	0.074750521	1.382287795	0.166883352	0.003746509	2.465530547	18.88155664	0.666666667	CRA004775;PRJNA827465;Yang2025_Silkie_Wencha	0.694602212
ENSGALG00010010900	SLC34A2	2	1.441791663	1.043266056	1.381998058	0.166972297	1.320959193	2.543158793	60.67882184	1	CRA004775;PRJNA827465	0.694767896
ENSGALG00010012873	PTAFR	3	-0.510177507	0.36930874	-1.381439029	0.167144013	0.3525006	24.3052186	91.77131449	0.666666667	CRA004775;PRJNA827465;Yang2025_Silkie_Wencha	0.695096971
ENSGALG00010015895	RBMXL1	3	0.189705021	0.137357858	1.381100604	0.16724803	0.034028275	5.339464547	62.5430606	0.666666667	CRA004775;PRJNA827465;Yang2025_Silkie_Wencha	0.695096971
ENSGALG00010002840	SRPX	3	-0.181974429	0.13174993	-1.381210821	0.167214149	0.025181394	4.045253093	50.55933574	0.666666667	CRA004775;PRJNA827465;Yang2025_Silkie_Wencha	0.695096971
ENSGALG00010014985	ENSGALG0001001498	3	0.093601269	0.067764069	1.381281707	0.16719236	0.004423457	2.824072402	29.18028595	1	CRA004775;PRJNA827465;Yang2025_Silkie_Wencha	0.695096971
ENSGALG00010003928	ENSGALG0001000392	3	-0.316801625	0.229411493	-1.380931795	0.167299933	0.102306613	5.744076788	65.18152396	1	CRA004775;PRJNA827465;Yang2025_Silkie_Wencha	0.69510836
ENSGALG00010010411	CD180	3	-0.646567155	0.468609137	-1.379757891	0.167661202	0.398525978	5.143469676	61.11574237	1	CRA004775;PRJNA827465;Yang2025_Silkie_Wencha	0.695390799
ENSGALG00010022227	TMEM80	3	-0.380034763	0.275459795	-1.379637864	0.167698173	0.196947155	18.65671826	89.28000106	1	CRA004775;PRJNA827465;Yang2025_Silkie_Wencha	0.695390799
ENSGALG00010007854	FAM181B	3	0.267478079	0.193773826	1.380362276	0.167475129	0.076787522	8.229907745	75.69839101	0.666666667	CRA004775;PRJNA827465;Yang2025_Silkie_Wencha	0.695390799
ENSGALG00010005895	MLLT10	3	0.260484451	0.188764961	1.379940692	0.167604906	0.080144335	8.645262414	76.86594224	1	CRA004775;PRJNA827465;Yang2025_Silkie_Wencha	0.695390799
ENSGALG00010005220	ANGEL1	3	-0.224824377	0.162964352	-1.379592371	0.167712188	0.067180574	15.50716626	87.10273711	1	CRA004775;PRJNA827465;Yang2025_Silkie_Wencha	0.695390799
ENSGALG00010003755	SLC35A1	3	0.093142737	0.06747054	1.38049492	0.167434313	0	1.488163151	0	0.666666667	CRA004775;PRJNA827465;Yang2025_Silkie_Wencha	0.695390799
ENSGALG00010014217	RASA3	3	-0.074778178	0.054186746	-1.380008643	0.167583984	0	0.373385234	0	1	CRA004775;PRJNA827465;Yang2025_Silkie_Wencha	0.695390799
ENSGALG00010015613	PWP1	3	-0.084511307	0.061270283	-1.379319669	0.167796215	0	0.691682578	0	0.666666667	CRA004775;PRJNA827465;Yang2025_Silkie_Wencha	0.695535234
ENSGALG00010002476	DDX3X	3	0.159243171	0.115472401	1.379058289	0.167876783	0.024045805	6.695409666	70.12878823	1	CRA004775;PRJNA827465;Yang2025_Silkie_Wencha	0.69566525
ENSGALG00010010635	ALDH9A1	3	-0.195640398	0.141903666	-1.37868459	0.167992023	0.041934412	6.772959998	70.47081334	0.666666667	CRA004775;PRJNA827465;Yang2025_Silkie_Wencha	0.695921937
ENSGALG00010013787	ZCCHC10	3	-0.148625351	0.107818674	-1.378475037	0.16805667	0.018223127	4.238267391	52.8109056	1	CRA004775;PRJNA827465;Yang2025_Silkie_Wencha	0.695921937
ENSGALG00010020700	MTHFD2	3	-0.102299691	0.074225992	-1.378219248	0.168135606	0	0.697422665	0	0.666666667	CRA004775;PRJNA827465;Yang2025_Silkie_Wencha	0.695921937
ENSGALG00010000085	ENSGALG0001000008	3	0.097629282	0.070832326	1.378315346	0.168105947	0	0.694580951	0	0.666666667	CRA004775;PRJNA827465;Yang2025_Silkie_Wencha	0.695921937
ENSGALG00010028452	GOLPH3L	2	0.531787166	0.386042144	1.377536559	0.16834642	0.006094389	1.01766972	1.736292216	0.5	CRA004775;PRJNA827465	0.696051188
ENSGALG00010013436	NPAS2	3	0.420172889	0.305074044	1.377281667	0.168425181	0.207150343	7.97151424	74.9106639	0.666666667	CRA004775;PRJNA827465;Yang2025_Silkie_Wencha	0.696051188
ENSGALG00010005560	RSPH3	3	-0.253535035	0.184056948	-1.377481468	0.16836344	0	0.284622504	0	1	CRA004775;PRJNA827465;Yang2025_Silkie_Wencha	0.696051188
ENSGALG00010017042	HNRNPLL	3	-0.239812982	0.174095553	-1.377479082	0.168364178	0.07147153	11.12642973	82.02478199	1	CRA004775;PRJNA827465;Yang2025_Silkie_Wencha	0.696051188
ENSGALG00010013150	GNS	3	-0.167828708	0.121825431	-1.377616369	0.168321764	0.025080921	5.033043369	60.26261144	0.666666667	CRA004775;PRJNA827465;Yang2025_Silkie_Wencha	0.696051188
ENSGALG00010021091	TANK	3	-0.080402649	0.05838286	-1.377161867	0.168462209	0.00213786	2.433841436	17.82537801	1	CRA004775;PRJNA827465;Yang2025_Silkie_Wencha	0.696051188
ENSGALG00010013248	ENSGALG0001001324	3	-1.417731705	1.030905638	-1.375229365	0.169060347	2.161436028	6.251455735	68.00745163	1	CRA004775;PRJNA827465;Yang2025_Silkie_Wencha	0.696088052
ENSGALG00010017491	CCDC28B	3	0.395671941	0.28784741	1.3745892	0.169258839	0.134288163	4.347399467	53.99548592	0.666666667	CRA004775;PRJNA827465;Yang2025_Silkie_Wencha	0.696088052
ENSGALG00010005866	DNAJC1	3	0.384308558	0.279548161	1.37474901	0.169209271	0.2100218	22.12592891	90.96083148	1	CRA004775;PRJNA827465;Yang2025_Silkie_Wencha	0.696088052
ENSGALG00010018102	ZDHHC4	3	-0.349153913	0.253960353	-1.374836301	0.169182201	0.152832718	10.13980493	80.27575468	0.666666667	CRA004775;PRJNA827465;Yang2025_Silkie_Wencha	0.696088052
ENSGALG00010028470	AJM1	3	-0.324509509	0.235936726	-1.375409053	0.169004664	0	1.214061776	0	1	CRA004775;PRJNA827465;Yang2025_Silkie_Wencha	0.696088052
ENSGALG00010012648	MRPS6	3	-0.311232213	0.226220058	-1.375794059	0.168885401	0.117349388	13.84835307	85.55784945	0.666666667	CRA004775;PRJNA827465;Yang2025_Silkie_Wencha	0.696088052
ENSGALG00010024829	ERBB3	3	0.282403476	0.205099944	1.376906649	0.168541111	0.086001767	8.963456283	77.68717851	0.666666667	CRA004775;PRJNA827465;Yang2025_Silkie_Wencha	0.696088052
ENSGALG00010001505	SSR3	3	-0.238141777	0.173004169	-1.376508898	0.168664135	0.065879853	9.046316792	77.8915547	0.666666667	CRA004775;PRJNA827465;Yang2025_Silkie_Wencha	0.696088052
ENSGALG00010027971	ENSGALG0001002797	3	-0.217854533	0.158475546	-1.37468864	0.169227995	0	1.779601248	0	0.666666667	CRA004775;PRJNA827465;Yang2025_Silkie_Wencha	0.696088052
ENSGALG00010019388	GPR75	3	-0.19527405	0.141995038	-1.375217415	0.169064051	0.03579268	5.484926058	63.53642732	0.666666667	CRA004775;PRJNA827465;Yang2025_Silkie_Wencha	0.696088052
ENSGALG00010004365	TPD52	3	0.178343315	0.129597858	1.376128564	0.168781834	0.017678686	2.952047185	32.2504054	0.666666667	CRA004775;PRJNA827465;Yang2025_Silkie_Wencha	0.696088052
ENSGALG00010020132	SHC4	3	0.129555604	0.094179433	1.375625231	0.168937691	0	0.897446004	0	1	CRA004775;PRJNA827465;Yang2025_Silkie_Wencha	0.696088052
ENSGALG00010004389	ENSGALG0001000438	3	-0.112305954	0.081617774	-1.375998746	0.168822021	0.007206494	3.071576808	34.88686349	1	CRA004775;PRJNA827465;Yang2025_Silkie_Wencha	0.696088052
ENSGALG00010018577	NARFL	3	-0.084448885	0.06141297	-1.375098537	0.169100898	0	1.10575859	0	1	CRA004775;PRJNA827465;Yang2025_Silkie_Wencha	0.696088052
ENSGALG00010015719	XRN1	3	-0.070883727	0.05153723	-1.375388761	0.169010952	0	0.485144316	0	0.666666667	CRA004775;PRJNA827465;Yang2025_Silkie_Wencha	0.696088052

ENSGALG00010015573	MAN1C1	3	-0.067831709	0.049317489	-1.375408806	0.16900474	0	1.902427356	0	0.666666667	CRA004775;PRJNA827465;Yang2025_Silkie_Wencha	0.696088052
ENSGALG00010017433	RNPS1	3	-0.232552334	0.169247711	-1.374035324	0.169430716	0.072513575	15.2950748	86.92389527	1	CRA004775;PRJNA827465;Yang2025_Silkie_Wencha	0.696592293
ENSGALG00010016452	GLCE	3	-0.184072926	0.133984405	-1.373838439	0.169491845	0.03356951	5.814821013	65.6051322	1	CRA004775;PRJNA827465;Yang2025_Silkie_Wencha	0.696632517
ENSGALG00010022242	USP19	3	-0.114713344	0.083507661	-1.373686466	0.16953904	0.013137439	6.825611967	70.69859802	1	CRA004775;PRJNA827465;Yang2025_Silkie_Wencha	0.696632517
ENSGALG00010001343	ENSGALG0001000134:	3	-0.310938623	0.226463228	-1.373020358	0.169746018	0	0.27141805	0	1	CRA004775;PRJNA827465;Yang2025_Silkie_Wencha	0.697280345
ENSGALG00010028112	SWI5	3	0.078081758	0.056886034	1.372599782	0.1698768	0	0.031298867	0	1	CRA004775;PRJNA827465;Yang2025_Silkie_Wencha	0.697614891
ENSGALG00010027306	ENSGALG0001002730(	3	-0.332792521	0.242919367	-1.369971132	0.170695913	0.063450611	3.107851452	35.64685986	0.666666667	CRA004775;PRJNA827465;Yang2025_Silkie_Wencha	0.6977362
ENSGALG00010018901	TCF7L2	3	0.323820241	0.236235336	1.370752769	0.170452039	0.117127508	7.012807761	71.48075253	1	CRA004775;PRJNA827465;Yang2025_Silkie_Wencha	0.6977362
ENSGALG00010015016	ARGLU1	3	0.320868458	0.233941319	1.371576683	0.170195257	0.148340949	24.15621492	91.72055719	1	CRA004775;PRJNA827465;Yang2025_Silkie_Wencha	0.6977362
ENSGALG00010019101	POLE2	3	-0.282750993	0.206078472	-1.372054978	0.170046324	0.09678234	9.484036719	78.91193319	0.666666667	CRA004775;PRJNA827465;Yang2025_Silkie_Wencha	0.6977362
ENSGALG00010008016	GSR	3	-0.267402047	0.195166961	-1.370119435	0.170649622	0.075590962	5.996738628	66.64853808	1	CRA004775;PRJNA827465;Yang2025_Silkie_Wencha	0.6977362
ENSGALG00010010719	SH3BP4	3	-0.255971719	0.186779093	-1.370451661	0.170545955	0.072765181	8.325291928	75.97681838	0.666666667	CRA004775;PRJNA827465;Yang2025_Silkie_Wencha	0.6977362
ENSGALG00010012047	LMAN2	3	-0.219725559	0.160385653	-1.36998263	0.170692324	0.063899455	14.99474272	86.66199189	0.666666667	CRA004775;PRJNA827465;Yang2025_Silkie_Wencha	0.6977362
ENSGALG00010021748	CDCA7	3	-0.209298079	0.152647569	-1.371119632	0.170337666	0.03358594	3.930253358	49.11269535	1	CRA004775;PRJNA827465;Yang2025_Silkie_Wencha	0.6977362
ENSGALG00010027460	WDR82	3	-0.208039262	0.151831834	-1.370195278	0.170625952	0.05772783	14.09541001	85.81098387	1	CRA004775;PRJNA827465;Yang2025_Silkie_Wencha	0.6977362
ENSGALG00010002561	PAAF1	3	-0.154904348	0.112912836	-1.371893165	0.170096698	0.023081367	5.295730192	62.23372552	0.666666667	CRA004775;PRJNA827465;Yang2025_Silkie_Wencha	0.6977362
ENSGALG00010017647	ITGA9	3	-0.151585366	0.110534833	-1.37138097	0.170256227	0.014218718	3.210464408	37.70371679	0.666666667	CRA004775;PRJNA827465;Yang2025_Silkie_Wencha	0.6977362
ENSGALG00010017732	RHOJ	3	0.136425563	0.099475588	1.371447673	0.170235445	0	1.40494771	0	1	CRA004775;PRJNA827465;Yang2025_Silkie_Wencha	0.6977362
ENSGALG00010029373	GATSL2	3	-0.126983925	0.092638161	-1.370751782	0.170452347	0	0.629640611	0	0.666666667	CRA004775;PRJNA827465;Yang2025_Silkie_Wencha	0.6977362
ENSGALG00010014547	DHDDS	3	0.103758579	0.075647218	1.371611304	0.170184473	0.004988962	2.573591562	22.28759103	0.666666667	CRA004775;PRJNA827465;Yang2025_Silkie_Wencha	0.6977362
ENSGALG00010014469	TMCO3	3	0.102145886	0.074482413	1.371409468	0.170247348	0.004427051	2.602218415	23.14250069	1	CRA004775;PRJNA827465;Yang2025_Silkie_Wencha	0.6977362
ENSGALG00010021772	TSN	3	-0.099936989	0.072909789	-1.370693723	0.170470453	0.005000736	2.559585365	21.8623443	1	CRA004775;PRJNA827465;Yang2025_Silkie_Wencha	0.6977362
ENSGALG00010027685	ENSGALG0001002768:	3	-0.309931117	0.226279534	-1.369682496	0.170786035	0.02623628	2.405747709	16.86576309	0.666666667	CRA004775;PRJNA827465;Yang2025_Silkie_Wencha	0.697902818
ENSGALG00010009804	XPR1	3	-0.188314903	0.13755031	-1.369062006	0.170979893	0.049820306	26.88961975	92.56218564	1	CRA004775;PRJNA827465;Yang2025_Silkie_Wencha	0.698291367
ENSGALG00010013323	NOTCH2	3	-0.095723336	0.069917847	-1.369083008	0.170973329	0	1.886038475	0	0.666666667	CRA004775;PRJNA827465;Yang2025_Silkie_Wencha	0.698291367
ENSGALG00010016591	HOMER2	3	0.554424794	0.405109729	1.368579313	0.171130814	0.40651133	12.73407636	84.2941102	1	CRA004775;PRJNA827465;Yang2025_Silkie_Wencha	0.698705915
ENSGALG00010006521	ZNF410	3	-0.332935527	0.243373135	-1.368004429	0.17131069	0.153176855	16.21426896	87.66518549	0.666666667	CRA004775;PRJNA827465;Yang2025_Silkie_Wencha	0.698965588
ENSGALG00010020971	TMEM51	3	0.231435774	0.16916587	1.368099693	0.171280873	0	1.810705687	0	0.666666667	CRA004775;PRJNA827465;Yang2025_Silkie_Wencha	0.698965588
ENSGALG00010013484	TCOF1	3	0.143820575	0.105139523	1.367902106	0.171342721	0.02165687	5.872686649	65.94403687	0.666666667	CRA004775;PRJNA827465;Yang2025_Silkie_Wencha	0.698965588
ENSGALG00010006946	PLPPR1	2	0.332955494	0.243576929	1.366941835	0.171643538	0	0.515136508	0	1	PRJNA827465;Yang2025_Silkie_Wenchang	0.699471865
ENSGALG00010027724	TTLL11	3	-0.158461025	0.11592948	-1.366874288	0.171664712	0.015132084	3.054278651	34.51808992	0.666666667	CRA004775;PRJNA827465;Yang2025_Silkie_Wencha	0.699471865
ENSGALG00010018199	BORCS7	3	-0.150739508	0.110256311	-1.367173519	0.171570924	0.02501727	7.456755381	73.17868298	1	CRA004775;PRJNA827465;Yang2025_Silkie_Wencha	0.699471865
ENSGALG00010027052	APPL1	3	0.08657521	0.063329613	1.367057309	0.171607343	0	0.752992468	0	1	CRA004775;PRJNA827465;Yang2025_Silkie_Wencha	0.699471865
ENSGALG00010028348	NES	3	0.518807246	0.379802361	1.365992685	0.171941259	0.337641269	14.54984904	86.25415291	1	CRA004775;PRJNA827465;Yang2025_Silkie_Wencha	0.699641487
ENSGALG00010027921	MKRN2OS	3	-0.372795656	0.272856183	-1.366271609	0.171853729	0.175798294	9.716035694	79.4154729	0.666666667	CRA004775;PRJNA827465;Yang2025_Silkie_Wencha	0.699641487
ENSGALG00010022194	ENSGALG0001002219	3	-0.36482141	0.267057583	-1.366077707	0.171914575	0.192183603	23.01896171	91.31151081	0.666666667	CRA004775;PRJNA827465;Yang2025_Silkie_Wencha	0.699641487
ENSGALG00010009720	RELN	3	-0.133093836	0.097447753	-1.365796861	0.172002732	0	1.056535002	0	0.666666667	CRA004775;PRJNA827465;Yang2025_Silkie_Wencha	0.699641487
ENSGALG00010017873	HSPG2	3	-0.132168156	0.096719165	-1.36651464	0.171777489	0	1.26998634	0	0.666666667	CRA004775;PRJNA827465;Yang2025_Silkie_Wencha	0.699641487
ENSGALG00010024653	RFLNA	3	0.132059497	0.096702235	1.365630239	0.17205505	0	0.444917158	0	0.666666667	CRA004775;PRJNA827465;Yang2025_Silkie_Wencha	0.699641487
ENSGALG00010013299	PLAGL2	3	-0.073863404	0.054093359	-1.365480092	0.172102206	0	0.404111279	0	0.666666667	CRA004775;PRJNA827465;Yang2025_Silkie_Wencha	0.699641487
ENSGALG00010004211	RNF146	3	0.054859236	0.040173447	1.36555959	0.172077238	0	0.289165084	0	0.666666667	CRA004775;PRJNA827465;Yang2025_Silkie_Wencha	0.699641487
ENSGALG00010008064	DDX60	3	0.517507264	0.379039155	1.365313472	0.172154547	0.305324174	8.615099968	76.78494727	0.666666667	CRA004775;PRJNA827465;Yang2025_Silkie_Wencha	0.699652176
ENSGALG00010017017	ENSGALG0001001701	3	0.44882271	0.328780909	1.365111834	0.172217903	0.22760955	6.828508582	70.71102751	0.666666667	CRA004775;PRJNA827465;Yang2025_Silkie_Wencha	0.699652176
ENSGALG00010029349	MYH10	3	0.280113338	0.20521135	1.364999246	0.172253287	0.092453498	7.983124612	74.94715293	0.666666667	CRA004775;PRJNA827465;Yang2025_Silkie_Wencha	0.699652176
ENSGALG00010005572	ENSGALG0001000557	3	-0.15431873	0.11306722	-1.36484058	0.172303161	0.007979985	2.20285843	9.208872758	1	CRA004775;PRJNA827465;Yang2025_Silkie_Wencha	0.699653762
ENSGALG00010023064	ATG13	3	-0.27639948	0.202562043	-1.364517633	0.172404709	0.110019824	32.17062373	93.7831482	1	CRA004775;PRJNA827465;Yang2025_Silkie_Wencha	0.699831869
ENSGALG00010025195	P2RX7	3	0.224779191	0.164758391	1.364295863	0.172474467	0	1.811573194	0	0.666666667	CRA004775;PRJNA827465;Yang2025_Silkie_Wencha	0.699831869
ENSGALG00010027384	HHATL	3	-0.113644979	0.08330345	-1.364228969	0.172495513	0	0.207775266	0	1	CRA004775;PRJNA827465;Yang2025_Silkie_Wencha	0.699831869
ENSGALG00010019474	TMEM163	3	1.052835762	0.772121586	1.36356214	0.172705415	1.210787895	6.894789536	70.99258811	0.666666667	CRA004775;PRJNA827465;Yang2025_Silkie_Wencha	0.699904999
ENSGALG00010021670	BLCAP	3	-0.286909253	0.210400517	-1.363633782	0.172682854	0.096889402	9.657960482	79.2916941	1	CRA004775;PRJNA827465;Yang2025_Silkie_Wencha	0.699904999
ENSGALG00010009446	ENSGALG0001000944	3	-0.183005065	0.134173753	-1.363940874	0.172586175	0.018319082	2.701167695	25.95794762	0.666666667	CRA004775;PRJNA827465;Yang2025_Silkie_Wencha	0.699904999
ENSGALG00010021755	ENSGALG0001002175	3	-0.1033045	0.075761839	-1.363542672	0.172711545	0	1.223817195	0	0.666666667	CRA004775;PRJNA827465;Yang2025_Silkie_Wencha	0.699904999
ENSGALG00010009344	ENPP2	3	0.364183055	0.267145776	1.363237185	0.172807772	0.135428563	5.486986614	63.55012067	1	CRA004775;PRJNA827465;Yang2025_Silkie_Wencha	0.699936178
ENSGALG00010010888	GNPAT	3	-0.120206905	0.088179693	-1.363203936	0.172818247	0.010563619	3.706094369	46.03483341	1	CRA004775;PRJNA827465;Yang2025_Silkie_Wencha	0.699936178
ENSGALG00010025971	TRAFD1	3	0.123093253	0.090317301	1.362897816	0.172914717	0.009692758	3.334670343	40.02405652	0.666666667	CRA004775;PRJNA827465;Yang2025_Silkie_Wencha	0.700126342
ENSGALG00010021633	CCNJ	3	0.090136697	0.066147201	1.362668346	0.172987058	0	0.753415912	0	0.666666667	CRA004775;PRJNA827465;Yang2025_Silkie_Wencha	0.700218727
ENSGALG00010024596	EPS15	3	-0.059562054	0.04371812	-1.362411154	0.173068166	0	0.78631339	0	1	CRA004775;PRJNA827465;Yang2025_Silkie_Wencha	0.700346535
ENSGALG00010024668	ENSGALG0001002466	3	0.560226893	0.41140284	1.361747753	0.173277505	0.447002626	21.96650822	90.89523023	0.666666667	CRA004775;PRJNA827465;Yang2025_Silkie_Wencha	0.70060954
ENSGALG00010015024	CRACD	3	0.384983655	0.282778033	1.361434093	0.173376548	0.187224354	9.919878402	79.83846254	0.666666667	CRA004775;PRJNA827465;Yang2025_Silkie_Wencha	0.70060954
ENSGALG00010012854	LECT2	3	0.362488402	0.266257534	1.361420262	0.173380917	0	1.171024399	0	1	CRA004775;PRJNA827465;Yang2025_Silkie_Wencha	0.70060954
ENSGALG00010010690	MTPN	3	-0.168541114	0.123786335	-1.361548627	0.173340377	0.028756939	8.142796225	75.43841274	1	CRA004775;PRJNA827465;Yang2025_Silkie_Wencha	0.70060954
ENSGALG00010002424	CASK	3	0.108537928	0.07969653	1.361890267	0.173232518	0.008909355	3.571897494	44.00735174	1	CRA004775;PRJNA827465;Yang2025_Silkie_Wencha	0.70060954
ENSGALG00010016053	DRP2	3	0.410495132	0.301562192	1.361228745	0.173441413	0.172400922	5.995579108	66.62538844	1	CRA004775;PRJNA827465;Yang2025_Silkie_Wencha	0.700653753
ENSGALG00010017799	GRAPL	3	0.489363707	0.35972754	1.360373206	0.173711853	0.27035319	6.615563197	69.76825797	0.666666667	CRA004775;PRJNA827465;Yang2025_Silkie_Wencha	0.7009107
ENSGALG00010002888	ENSGALG0001000288	2	0.393349822	0.289176036	1.360243496	0.173752882	0	0.000444283	0	1	PRJNA827465;Yang2025_Silkie_Wenchang	0.7009107
ENSGALG00010013375	PSMG1	3	-0.365403114	0.268574796	-1.360526452	0.173663388	0.188002841	17.65272787	88.67030629	1	CRA004775;PRJNA827465;Yang2025_Silkie_Wencha	0.7009107
ENSGALG00010007965	EGLN1	3	-0.14472779	0.106350077	-1.360833794	0.17356622	0.014335623	3.566546627	43.92334633	0.666666667	CRA004775;PRJNA827465;Yang2025_Silkie_Wencha	0.7009107
ENSGALG00010026516	ENSGALG0001002651	3	-0.076095712	0.055935755	-1.360412717	0.173699356	0	1.696991295	0	0.666666667	CRA004775;PRJNA827465;Yang2025_Silkie_Wencha	0.7009107
ENSGALG00010017694	ATP6V1B2	3	-0.254674822	0.18727765	-1.359878354	0.173868422	0.083208556	18.5751117	89.23290458	0.666666667	CRA004775;PRJNA827465;Yang2025_Silkie_Wencha	0.700976795
ENSGALG00010027734	CSF1	3	0.110913248	0.081552048	1.360030192	0.17382037	0.002908357	2.282089644	12.3610238	1	CRA004775;PRJNA827465;Yang2025_Silkie_Wencha	0.700976795
ENSGALG00010004859	NPC1	3	-0.172785004	0.127099656	-1.359445075	0.174005596	0.026839473	5.1114178	60.85792375	0.666666667	CRA004775;PRJNA827465;Yang2025_Silkie_Wencha	0.701135839
ENSGALG00010006922	SMYD3	3	-0.169383973	0.1245983	-1.359440488	0.174007049	0.015850915	3.005896555	33.46411084	0.666666667	CRA004775;PRJNA827465;Yang2025_Silkie_Wencha	0.701135839
ENSGALG00010016626	CAPN2	3	-0.192354691	0.141549221	-1.358924409	0.174170543	0.006141214	2.185030433	8.4680941	1	CRA004775;PRJNA827465;Yang2025_Silkie_Wencha	0.701594675
ENSGALG00010025832	ENKD1	3	-0.22454104	0.165263811	-1.358682448	0.174247236	0.029594308	2.923085377	31.57914524	0.666666667	CRA004775;PRJNA827465;Yang2025_Silkie_Wencha	0.701703695
ENSGALG00010003887	GIPC1	3	0.133117331	0.098063463	1.357461045	0.174634763	0.01022923	3.027149744	33.93124988	1	CRA004775;PRJNA827465;Yang2025_Silkie_Wencha	0.703064042
ENSGALG00010007874	ENSGALG0001000787	3	0.961081099	0.708122933	1.357223519	0.1747102	1.201236315	9.946004792	79.89142332	0.666666667	CRA004775;PRJNA827465;Yang2025_Silkie_Wencha	0.703104413
ENSGALG00010002475	ENSGALG0001000247	3	0.205014625	0.151067698	1.357104309	0.17474807	0	0.001315297	0	1	CRA004775;PRJNA827465;Yang2025_Silkie_Wencha	0.703104413
ENSGALG00010027709	PSMA5	3	-0.091286805	0.067280793	-1.356803349	0.174843703	0	1.378400062	0	0.666666667	CRA004775;PRJNA827465;Yang2025_Silkie_Wencha	0.703104413
ENSGALG00010027427	UBAC1	3	-0.088213221	0.065008558	-1.356947824	0.17479779	0.004144008	2.89956918	31.02432582	0.666666667	CRA004775;PRJNA827465;Yang2025_Silkie_Wencha	0.703104413
ENSGALG00010011892	ENSGALG0001001189	2	0.561497434	0.413918465	1.356541157	0.17492705	0	0.00631747	0	1	PRJNA827465;Yang2025_Silkie_Wenchang	0.703228216
ENSGALG00010025935	CATH3	3	0.271101646	0.19986945	1.356393617	0.174973963	0	0.562677333	0	1	CRA004775;PRJNA827465;Yang2025_Silkie_Wencha	0.703228216
ENSGALG00010025429	SCN3B	2	-0.273788179	0.202020824	-1.355247312	0.175338775	0	0.007811547	0	1	PRJNA827465;Yang2025_Silkie_Wenchang	0.704299801
ENSGALG00010024858	SLC25A39	3	-0.164258033	0.121201925	-1.355242792	0.175340215	0.020602949	3.441823801	41.89127289	1	CRA004775;PRJNA827465;Yang2025_Silkie_Wencha	0.704299801
ENSGALG00010017664	ENSGALG0001001766	3	0.205619562	0.151792583	1.354608757	0.175542242	0.046672436	7.015611251	71.49214903	1	CRA004775;PRJNA827465;Yang2025_Silkie_Wencha	0.704911037
ENSGALG00010001756	ENSGALG0001000175	3	0.419650056	0.31002202	1.353613707	0.175859652	0.146163134	4.059793661	50.73641256	0.666666667	CRA004775;PRJNA827465;Yang2025_Silkie_Wencha	0.704956583
ENSGALG00010021671	TNFRSF13B	3	-0.367631403	0.271862124	-1.352271505	0.176288477	0	1.999222507	0	0.666666667	CRA004775;PRJNA827465;Yang2025_Silkie_Wencha	0.704956583
ENSGALG00010028738	MIDN	3	0.352351367	0.260543808	1.35236902	0.176257295	0.138267357	9.758909952	79.50589943	1	CRA004775;PRJNA827465;Yang2025_Silkie_Wencha	0.704956583
ENSGALG00010023857	ACBD4	3	0.3519941	0.260131685	1.353138123	0.176011509	0.163699486	10.65374174	81.22725283	0.666666667	CRA004775;PRJNA827465;Yang2025_Silkie_Wencha	0.704956583
ENSGALG00010012300	NINJ2	3	0.320272813	0.236875037	1.352074985	0.176351329	0.072320478	3.467960306	42.32921303	1	CRA004775;PRJNA827465;Yang2025_Silkie_Wencha	0.704956583
ENSGALG00010016589	FBXO28	3	-0.308674628	0.227995388	-1.353863473	0.175779939	0.13650834	19.89520022	89.84732409	1	CRA004775;PRJNA827465;Yang2025_Silkie_Wencha	0.704956583
ENSGALG00010028747	ENSGALG0001002874	2	-0.290271067	0.214440764	-1.353618882	0.175858	0.059139557	2.795225644	64.22471288	1	PRJNA827465;Yang2025_Silkie_Wenchang	0.704956583
ENSGALG00010010954	MTERF2	3	-0.276432605	0.204400289	-1.352408089	0.176244803	0.056963783	3.685759467	45.73709929	0.666666667	CRA004775;PRJNA827465;Yang2025_Silkie_Wencha	0.704956583
ENSGALG00010003923	ENSGALG0001000392	3	-0.254222403	0.187934266	-1.352719806	0.176145161	0.070620999	6.11610475	67.29944823	1	CRA004775;PRJNA827465;Yang2025_Silkie_Wencha	0.704956583
ENSGALG00010008389	GALNT1	3	-0.227221693	0.167846577	-1.353746364	0.175817311	0.063514199	17.60022001	88.63650569	1	CRA004775;PRJNA827465;Yang2025_Silkie_Wencha	0.704956583
ENSGALG00010026017	PLEKHM1	3	-0.164760779	0.121687239	-1.353969244	0.175746119	0.030328849	6.799946486	70.35800383	0.666666667	CRA004775;PRJNA827465;Yang2025_Silkie_Wencha	0.704956583
ENSGALG00010028482	CD320	3	0.144797205	0.106907655	1.354413819	0.175604391	0.000652838	2.028400809	1.400157639	0.666666667	CRA004775;PRJNA827465;Yang2025_Silkie_Wencha	0.704956583
ENSGALG00010018594	BICRAL	3	0.135298607	0.100063449	1.352128157	0.176334321	0.006841928	2.419878723	17.35123001	1	CRA004775;PRJNA827465;Yang2025_Silkie_Wencha	0.704956583
ENSGALG00010023974	PREX1	3	0.082674948	0.061122301	1.352615116	0.176178621	0	1.441522493	0	0.666666667	CRA004775;PRJNA827465;Yang2025_Silkie_Wencha	0.704956583
ENSGALG00010011091	YARS2	3	0.075156198	0.055563083	1.352628284	0.176174412	0	1.748820271	0	0.666666667	CRA004775;PRJNA827465;Yang2025_Silkie_Wencha	0.704956583
ENSGALG00010025743	SH2B3	3	0.069271858	0.051194049	1.353123259	0.176016256	0	0.267595102	0	0.666666667	CRA004775;PRJNA827465;Yang2025_Silkie_Wencha	0.704956583
ENSGALG00010005831	SEC61A2	3	-0.261470683	0.193425791	-1.351788097	0.176443113	0.088458101	13.8691314	85.57948626	1	CRA004775;PRJNA827465;Yang2025_Silkie_Wencha	0.705124131
ENSGALG00010029879	ENSGALG0001002987	3	0.49265686	0.364645246	1.351057954	0.176676869	0.266404592	6.708242968	70.18593379	1	CRA004775;PRJNA827465;Yang2025_Silkie_Wencha	0.705546542
ENSGALG00010010923	F13A1	3	-0.43331178	0.320683284	-1.35127466	0.176607483	0.217724432	10.30862343	80.95976749	0.666666667	CRA004775;PRJNA827465;Yang2025_Silkie_Wencha	0.705546542
ENSGALG00010023485	BARX2	3	0.144740638	0.107136691	1.350990378	0.176698515	0.003928231	2.21604789	9.749242838	0.666666667	CRA004775;PRJNA827465;Yang2025_Silkie_Wencha	0.705546542
ENSGALG00010008455	FKBP1B	3	-0.100540809	0.074435368	-1.350712867	0.176787429	0	0.09709707	0	1	CRA004775;PRJNA827465;Yang2025_Silkie_Wencha	0.705702275
ENSGALG00010029576	ATP2A3	3	-0.336116119	0.249085114	-1.349402673	0.177207663	0.141450754	15.60716668	87.18537425	1	CRA004775;PRJNA827465;Yang2025_Silkie_Wencha	0.706465666
ENSGALG00010004977	RNF217	3	-0.217792429	0.161081529	-1.349572634	0.17715107	0.041296906	4.261836415	53.03718313	0.666666667	CRA004775;PRJNA827465;Yang2025_Silkie_Wencha	0.706465666
ENSGALG00010027520	FANCE	3	-0.160287978	0.118759073	-1.349690374	0.17711532	0	0.303934269	0	1	CRA004775;PRJNA827465;Yang2025_Silkie_Wencha	0.706465666
ENSGALG00010026316	ENSGALG0001002631	3	-0.15863334	0.117563847	-1.349337777	0.177228497	0	1.853698921	0	1	CRA004775;PRJNA827465;Yang2025_Silkie_Wencha	0.706465666
ENSGALG00010022032	KBTBD4	3	-0.123323813	0.091388579	-1.349444474	0.177194244	0.013438297	4.273727537	53.20244488	1	CRA004775;PRJNA827465;Yang2025_Silkie_Wencha	0.706465666
ENSGALG00010008355	MAFA	3	-0.221099985	0.163971093	-1.348383923	0.177534932	0.042633799	4.96306689	59.70233639	0.666666667	CRA004775;PRJNA827465;Yang2025_Silkie_Wencha	0.707190447
ENSGALG00010028658	PTPA	3	-0.164251478	0.121803007	-1.348501016	0.177497294	0.0268181	4.953275392	59.62267708	1	CRA004775;PRJNA827465;Yang2025_Silkie_Wencha	0.707190447
ENSGALG00010022796	RIMKLB	3	0.10266626	0.076144697	1.348304793	0.177560371	0.006761638	3.170147188	36.91144664	0.666666667	CRA004775;PRJNA827465;Yang2025_Silkie_Wencha	0.707190447
ENSGALG00010018490	NME3	3	-0.337940087	0.250692616	-1.348025694	0.177650119	0.148743628	11.24736791	82.21806189	0.666666667	CRA004775;PRJNA827465;Yang2025_Silkie_Wencha	0.707348642
ENSGALG00010024561	ROBO3	3	0.470766307	0.34930212	1.347733894	0.177743987	0.204456705	4.594122843	56.46611838	0.666666667	CRA004775;PRJNA827465;Yang2025_Silkie_Wencha	0.707393967
ENSGALG00010027860	ENSGALG0001002786(	3	0.293651273	0.217894035	1.347679263	0.177761565	0	0.743880816	0	1	CRA004775;PRJNA827465;Yang2025_Silkie_Wencha	0.707393967
ENSGALG00010021173	KLF15	3	-0.276209897	0.205030072	-1.347167731	0.177926218	0.080382551	7.760871636	74.22969875	0.666666667	CRA004775;PRJNA827465;Yang2025_Silkie_Wencha	0.707849971
ENSGALG00010027334	PRCC	3	-0.179579792	0.133368656	-1.346491723	0.178143988	0.044350171	15.94073073	87.45352372	1	CRA004775;PRJNA827465;Yang2025_Silkie_Wencha	0.708516975
ENSGALG00010011181	ENSGALG0001001118	3	-0.267318918	0.198802738	-1.34464405	0.178740211	0.047377445	3.312347961	39.61987015	0.666666667	CRA004775;PRJNA827465;Yang2025_Silkie_Wencha	0.709949478
ENSGALG00010015975	BTK	3	-0.230971036	0.171833213	-1.344158281	0.178897209	0.043373215	4.020083051	50.24978403	1	CRA004775;PRJNA827465;Yang2025_Silkie_Wencha	0.709949478
ENSGALG00010010909	SLIT3	3	-0.183097972	0.136191208	-1.344418437	0.178813115	0	0.845052643	0	1	CRA004775;PRJNA827465;Yang2025_Silkie_Wencha	0.709949478
ENSGALG00010010960	POLDIP3	3	-0.179803298	0.133764055	-1.344182471	0.178889389	0.044807329	17.20194459	88.3734075	0.666666667	CRA004775;PRJNA827465;Yang2025_Silkie_Wencha	0.709949478
ENSGALG00010027704	MDFI	3	0.165282024	0.12290544	1.344790138	0.178693016	0.023300549	4.762826471	58.0081279	0.666666667	CRA004775;PRJNA827465;Yang2025_Silkie_Wencha	0.709949478
ENSGALG00010028093	SFMBT1	3	-0.146817123	0.109228243	-1.344131516	0.178905862	0	0.963192184	0	1	CRA004775;PRJNA827465;Yang2025_Silkie_Wencha	0.709949478
ENSGALG00010017174	DHX57	3	-0.134093788	0.099718308	-1.344725863	0.17871378	0.01648742	4.885957644	59.06636639	1	CRA004775;PRJNA827465;Yang2025_Silkie_Wencha	0.709949478
ENSGALG00010028569	ZER1	3	-0.046228182	0.034372987	-1.344898614	0.178657978	0	0.796687578	0	1	CRA004775;PRJNA827465;Yang2025_Silkie_Wencha	0.709949478
ENSGALG00010001289	ENSGALG0001000128	3	0.620045779	0.461403983	1.34382407	0.179005284	0.529181212	12.46706787	83.95773552	0.666666667	CRA004775;PRJNA827465;Yang2025_Silkie_Wencha	0.7101447
ENSGALG00010025483	FGF4	3	0.49004876	0.365025521	1.342505474	0.179432157	0.286092667	11.19072453	82.12805618	0.666666667	CRA004775;PRJNA827465;Yang2025_Silkie_Wencha	0.710888159
ENSGALG00010022662	GFRA1	3	0.436262283	0.325120424	1.341848282	0.179645194	0.230092348	8.521509451	76.52997968	1	CRA004775;PRJNA827465;Yang2025_Silkie_Wencha	0.710888159
ENSGALG00010014971	SMC5	3	0.401446252	0.299132497	1.342034902	0.179584679	0.210262198	9.52426677	79.00100818	1	CRA004775;PRJNA827465;Yang2025_Silkie_Wencha	0.710888159
ENSGALG00010027209	TRAF3IP3	3	0.247777425	0.184566041	1.342486533	0.179438294	0.01851544	2.410379447	17.02551222	0.666666667	CRA004775;PRJNA827465;Yang2025_Silkie_Wencha	0.710888159
ENSGALG00010008880	FMN2	2	-0.207151122	0.154358859	-1.342009926	0.179592777	0.002366728	1.034447551	3.33004328	0.5	PRJNA827465;Yang2025_Silkie_Wenchang	0.710888159
ENSGALG00010024401	KCNC1	3	-0.194317316	0.14479996	-1.341970787	0.179605468	0.0252204	3.362217375	40.51544631	0.666666667	CRA004775;PRJNA827465;Yang2025_Silkie_Wencha	0.710888159
ENSGALG00010027106	CYP2J23	3	0.162658499	0.121211318	1.341941507	0.179614962	0.016459987	3.17909967	37.08910674	1	CRA004775;PRJNA827465;Yang2025_Silkie_Wencha	0.710888159
ENSGALG00010001398	WDR11	3	-0.065092908	0.048499456	-1.342136862	0.179551624	0.0013741	2.421541015	17.40796512	0.666666667	CRA004775;PRJNA827465;Yang2025_Silkie_Wencha	0.710888159
ENSGALG00010007989	SPG7	3	-0.051636403	0.038448036	-1.343017967	0.179266156	0	1.575156076	0	0.666666667	CRA004775;PRJNA827465;Yang2025_Silkie_Wencha	0.710888159
ENSGALG00010025902	ENSGALG0001002590	2	-1.658395168	1.236203816	-1.341522446	0.179750887	0	0.682344344	0	1	PRJNA827465;Yang2025_Silkie_Wenchang	0.711107384
ENSGALG00010003791	ITGB8	3	0.370762788	0.276439527	1.341207573	0.179853068	0.162243526	7.534701669	73.45614879	0.666666667	CRA004775;PRJNA827465;Yang2025_Silkie_Wencha	0.711312597
ENSGALG00010007181	GJB2	2	0.657192045	0.490361617	1.340219182	0.180174097	0	0.756049047	0	1	CRA004775;PRJNA827465	0.711730573
ENSGALG00010022348	G6PC1	3	0.654280338	0.488096268	1.340473962	0.180091304	0.523044024	7.787982319	74.31940754	0.666666667	CRA004775;PRJNA827465;Yang2025_Silkie_Wencha	0.711730573
ENSGALG00010020282	MAGI1	3	0.340701351	0.254192112	1.340330144	0.180138035	0.148928825	11.36060769	82.39530794	1	CRA004775;PRJNA827465;Yang2025_Silkie_Wencha	0.711730573
ENSGALG00010020074	ENSGALG0001002007	3	-0.280228439	0.209229042	-1.339338152	0.180460614	0.105607004	11.99413348	83.3251814	0.666666667	CRA004775;PRJNA827465;Yang2025_Silkie_Wencha	0.711730573
ENSGALG00010001302	ENSGALG0001000130	2	0.255197777	0.1903748	1.340501878	0.180082234	0	0.648058654	0	1	PRJNA827465;Yang2025_Silkie_Wenchang	0.711730573
ENSGALG00010027387	ENSGALG0001002738	2	0.226445794	0.16903111	1.339669335	0.180352871	0	0.240043568	0	1	PRJNA827465;Yang2025_Silkie_Wenchang	0.711730573
ENSGALG00010028187	TPRA1	3	-0.198056747	0.14785671	-1.339518156	0.180402048	0.05266096	11.59333042	82.74870182	0.666666667	CRA004775;PRJNA827465;Yang2025_Silkie_Wencha	0.711730573
ENSGALG00010022363	KIF18A	3	-0.122404516	0.091375247	-1.339580689	0.180381705	0	1.129514676	0	1	CRA004775;PRJNA827465;Yang2025_Silkie_Wencha	0.711730573
ENSGALG00010012985	ARNTL2	3	0.106009985	0.0791302	1.339690593	0.180345957	0.002733371	2.307026787	13.30833213	1	CRA004775;PRJNA827465;Yang2025_Silkie_Wencha	0.711730573
ENSGALG00010019042	PEX6	3	0.084069582	0.062769716	1.339333482	0.180462133	0	1.802224519	0	0.666666667	CRA004775;PRJNA827465;Yang2025_Silkie_Wencha	0.711730573
ENSGALG00010029392	RTN4RL1	3	-0.377120023	0.281808944	-1.338211689	0.18082744	0.056369045	2.601366846	23.11734105	0.666666667	CRA004775;PRJNA827465;Yang2025_Silkie_Wencha	0.712332977
ENSGALG00010003833	ACP1	3	-0.241353122	0.180359215	-1.338180148	0.180837719	0.066408372	6.423051475	68.86215208	1	CRA004775;PRJNA827465;Yang2025_Silkie_Wencha	0.712332977
ENSGALG00010018880	TMEM17	3	-0.216346397	0.161649642	-1.338366077	0.180777132	0.028983613	3.178470992	37.07666343	1	CRA004775;PRJNA827465;Yang2025_Silkie_Wencha	0.712332977
ENSGALG00010029528	TBL2	3	-0.18336663	0.137035995	-1.338090986	0.180866779	0.028774961	3.90014142	48.71980821	1	CRA004775;PRJNA827465;Yang2025_Silkie_Wencha	0.712332977
ENSGALG00010027348	ENSGALG0001002734	3	0.12345983	0.092239273	1.338473577	0.180742108	0	0.464137047	0	0.666666667	CRA004775;PRJNA827465;Yang2025_Silkie_Wencha	0.712332977
ENSGALG00010006108	MKLN1	3	-0.169414431	0.126644307	-1.337718493	0.18098822	0.036851195	12.58344755	84.10610453	1	CRA004775;PRJNA827465;Yang2025_Silkie_Wencha	0.712612767
ENSGALG00010007911	IFI6	3	-0.374890123	0.280491668	-1.336546378	0.181370752	0	1.603901655	0	0.666666667	CRA004775;PRJNA827465;Yang2025_Silkie_Wencha	0.712988764
ENSGALG00010006073	CEP97	3	-0.218581217	0.163521187	-1.336714961	0.181315697	0.051173431	5.678532557	64.77963312	1	CRA004775;PRJNA827465;Yang2025_Silkie_Wencha	0.712988764
ENSGALG00010019043	METTL9	3	-0.210859126	0.157747	-1.336691827	0.181323251	0.0574013	10.47058206	80.89886513	0.666666667	CRA004775;PRJNA827465;Yang2025_Silkie_Wencha	0.712988764
ENSGALG00010005412	MTFR2	3	-0.118785853	0.088828134	-1.337254844	0.181139465	0.001609766	2.083473444	4.006455861	0.666666667	CRA004775;PRJNA827465;Yang2025_Silkie_Wencha	0.712988764
ENSGALG00010026901	FAM212A	3	0.115065043	0.086062988	1.336986388	0.18122708	0	0.259272949	0	1	CRA004775;PRJNA827465;Yang2025_Silkie_Wencha	0.712988764
ENSGALG00010013084	SGSM3	3	0.086053725	0.06438743	1.336498844	0.181386278	0	1.916132077	0	0.666666667	CRA004775;PRJNA827465;Yang2025_Silkie_Wencha	0.712988764
ENSGALG00010017658	WHAMM	3	0.356509594	0.266865682	1.335913975	0.181577393	0.16936328	13.10204781	84.73521063	0.666666667	CRA004775;PRJNA827465;Yang2025_Silkie_Wencha	0.71314521
ENSGALG00010024619	ENSGALG0001002461	3	-0.234963714	0.17587445	-1.335974118	0.181557733	0	0.364418776	0	1	CRA004775;PRJNA827465;Yang2025_Silkie_Wencha	0.71314521
ENSGALG00010014200	DCP1B	3	-0.190737542	0.142758772	-1.336082819	0.181522205	0.037078607	6.008814331	66.71556334	1	CRA004775;PRJNA827465;Yang2025_Silkie_Wencha	0.71314521
ENSGALG00010018039	TTC14	3	0.378191354	0.283255807	1.335158341	0.181824528	0.203345343	16.29453833	87.72594866	1	CRA004775;PRJNA827465;Yang2025_Silkie_Wencha	0.713223507
ENSGALG00010010512	PLXNC1	3	-0.281584723	0.210885708	-1.335248012	0.181795188	0.087382813	8.74905034	77.14037613	0.666666667	CRA004775;PRJNA827465;Yang2025_Silkie_Wencha	0.713223507
ENSGALG00010009097	C1QTNF2	3	-0.149699953	0.11212359	-1.335133431	0.18183268	0	0.368738555	0	0.666666667	CRA004775;PRJNA827465;Yang2025_Silkie_Wencha	0.713223507
ENSGALG00010007882	CPE	3	0.120326031	0.090125568	1.33509318	0.181845851	0	0.917457913	0	1	CRA004775;PRJNA827465;Yang2025_Silkie_Wencha	0.713223507
ENSGALG00010029713	CPD	3	-0.092997882	0.069657062	-1.335081887	0.181849547	0.003638164	2.605716552	23.2456808	1	CRA004775;PRJNA827465;Yang2025_Silkie_Wencha	0.713223507
ENSGALG00010020036	TMF1	3	0.200410751	0.150151615	1.334722581	0.18196716	0.045952043	6.939752151	71.18052696	1	CRA004775;PRJNA827465;Yang2025_Silkie_Wencha	0.713486877
ENSGALG00010028914	TLR4	3	-0.263156648	0.197208896	-1.334405561	0.182070978	0.071918507	6.854103275	70.82039882	0.666666667	CRA004775;PRJNA827465;Yang2025_Silkie_Wencha	0.713498216
ENSGALG00010005742	GPR183	3	-0.126345905	0.094677073	-1.334493144	0.182042292	0.010206476	3.276342044	38.95631246	1	CRA004775;PRJNA827465;Yang2025_Silkie_Wencha	0.713498216
ENSGALG00010022297	VPS18	3	-0.218575052	0.163855586	-1.333949343	0.182220459	0.062233108	14.11034552	85.82600265	1	CRA004775;PRJNA827465;Yang2025_Silkie_Wencha	0.713802329
ENSGALG00010015199	ENSGALG0001001519	3	-0.213288613	0.159921692	-1.333706584	0.182300036	0.044951923	4.816096312	58.47259127	1	CRA004775;PRJNA827465;Yang2025_Silkie_Wencha	0.713802329
ENSGALG00010017105	PLK1	3	-0.119983787	0.089961717	-1.33372051	0.18229547	0	1.617369691	0	0.666666667	CRA004775;PRJNA827465;Yang2025_Silkie_Wencha	0.713802329
ENSGALG00010015585	BDKRB1	3	-0.631813185	0.473930138	-1.333135698	0.182487276	0.56803607	16.79952886	88.09490423	0.666666667	CRA004775;PRJNA827465;Yang2025_Silkie_Wencha	0.714149177
ENSGALG00010013289	TM9SF4	3	-0.238912533	0.179211931	-1.333128503	0.182489637	0.079587489	13.10698919	84.74096552	0.666666667	CRA004775;PRJNA827465;Yang2025_Silkie_Wencha	0.714149177
ENSGALG00010001754	APCDD1	3	-0.286105042	0.21464077	-1.332948264	0.182548782	0.062103145	3.720503648	46.24383715	0.666666667	CRA004775;PRJNA827465;Yang2025_Silkie_Wencha	0.714182964
ENSGALG00010027290	CSPG5	3	0.351780022	0.263995271	1.332523953	0.182688076	0.111506318	4.510140171	55.65548023	1	CRA004775;PRJNA827465;Yang2025_Silkie_Wencha	0.714197397
ENSGALG00010001525	EEF1A1	3	-0.313214731	0.235062293	-1.332475433	0.182704009	0.14132422	15.39144856	87.00577147	0.666666667	CRA004775;PRJNA827465;Yang2025_Silkie_Wencha	0.714197397
ENSGALG00010009719	YIPF3	3	-0.167553352	0.125739658	-1.332541811	0.182682212	0.032020389	6.350692476	68.50737132	0.666666667	CRA004775;PRJNA827465;Yang2025_Silkie_Wencha	0.714197397
ENSGALG00010024527	ATP6V0B	3	-0.365619352	0.27448344	-1.332026994	0.182851318	0.183705079	11.96236246	83.28089449	0.666666667	CRA004775;PRJNA827465;Yang2025_Silkie_Wencha	0.714378224

ENSGALG00010024138	CBL	3	-0.086745053	0.065119151	-1.332097432	0.182828174	0.00043166	2.027454571	1.354139876	1	CRA004775;PRJNA827465;Yang2025_Silkie_Wencha	0.714378224
ENSGALG00010010768	ENSGALG00010010768	3	0.081785837	0.061421112	1.331559045	0.18300513	0	0.402824244	0	0.666666667	CRA004775;PRJNA827465;Yang2025_Silkie_Wencha	0.714584241
ENSGALG00010025508	ENSGALG00010025508	3	-0.074800557	0.05616991	-1.331683759	0.182964128	0	0.51682414	0	1	CRA004775;PRJNA827465;Yang2025_Silkie_Wencha	0.714584241
ENSGALG00010001813	HOXA5	3	0.280200854	0.21048017	1.331245857	0.183108127	0.091874766	7.493844332	73.31142854	0.666666667	CRA004775;PRJNA827465;Yang2025_Silkie_Wencha	0.714591722
ENSGALG00010022229	RAPSN	3	0.245094338	0.184097504	1.33132896	0.183080793	0.063765558	8.123297446	75.37945627	0.666666667	CRA004775;PRJNA827465;Yang2025_Silkie_Wencha	0.714591722
ENSGALG00010013160	CRYBG1	3	0.694292411	0.521685155	1.330864801	0.183233501	0.39142033	3.838099899	47.89088214	1	CRA004775;PRJNA827465;Yang2025_Silkie_Wencha	0.714883684
ENSGALG00010012751	AP3B1	3	0.122019155	0.091699272	1.330644753	0.18330593	0	0.163427231	0	1	CRA004775;PRJNA827465;Yang2025_Silkie_Wencha	0.714968976
ENSGALG00010011187	STK32B	2	0.776168399	0.583572432	1.330029242	0.183508637	0.29545859	1.44669379	30.87687199	1	PRJNA827465;Yang2025_Silkie_Wenchang	0.715067429
ENSGALG00010018186	SPTLC3	2	0.486328256	0.365740285	1.329709293	0.183614072	0	1.9689E-06	0	1	PRJNA827465;Yang2025_Silkie_Wenchang	0.715067429
ENSGALG00010026690	KRT14	3	0.40530824	0.304874221	1.329427715	0.183706899	0.092559201	2.978050907	32.84198079	1	CRA004775;PRJNA827465;Yang2025_Silkie_Wencha	0.715067429
ENSGALG00010015971	ENSGALG00010015971	3	-0.189726308	0.142735446	-1.329216491	0.183776556	0.034916195	5.676174714	64.76500283	1	CRA004775;PRJNA827465;Yang2025_Silkie_Wencha	0.715067429
ENSGALG00010027627	OARD1	3	-0.187146266	0.140783525	-1.329319364	0.183742628	0.038770855	8.64544339	76.86642651	0.666666667	CRA004775;PRJNA827465;Yang2025_Silkie_Wencha	0.715067429
ENSGALG00010011549	KIAA0232	3	0.160919393	0.121045837	1.329408731	0.183713159	0.025559063	5.163893542	61.26953463	1	CRA004775;PRJNA827465;Yang2025_Silkie_Wencha	0.715067429
ENSGALG00010013387	NDST1	3	-0.115598469	0.086897836	-1.330280181	0.183425975	0.01121722	4.062142222	50.76489472	1	CRA004775;PRJNA827465;Yang2025_Silkie_Wencha	0.715067429
ENSGALG00010027730	ENSGALG00010027730	3	0.099479256	0.074785978	1.330185945	0.183457014	0	0.903763088	0	0.666666667	CRA004775;PRJNA827465;Yang2025_Silkie_Wencha	0.715067429
ENSGALG00010028191	KLHL26	3	-0.081790485	0.061534228	-1.329186832	0.183786338	0.003166034	2.697920735	25.86883766	1	CRA004775;PRJNA827465;Yang2025_Silkie_Wencha	0.715067429
ENSGALG00010008041	ENSGALG00010008041	3	0.489920291	0.36872283	1.3286953	0.183948516	0.29540537	9.609818733	79.18795291	1	CRA004775;PRJNA827465;Yang2025_Silkie_Wencha	0.715146325
ENSGALG00010005163	GPC6	2	-0.34477552	0.259570316	-1.328254807	0.184093944	0	0.121249695	0	1	PRJNA827465;Yang2025_Silkie_Wenchang	0.715146325
ENSGALG00010015068	ESM1	3	0.211390834	0.159155168	1.328205905	0.184110094	0.02675936	3.078115119	35.02517213	1	CRA004775;PRJNA827465;Yang2025_Silkie_Wencha	0.715146325
ENSGALG00010018385	SPR	3	0.198539278	0.149402967	1.328884445	0.183886096	0.027783959	3.307982781	39.54019315	0.666666667	CRA004775;PRJNA827465;Yang2025_Silkie_Wencha	0.715146325
ENSGALG00010019557	TRIM9	3	0.13784698	0.10375493	1.328582456	0.183985763	0	1.132425027	0	1	CRA004775;PRJNA827465;Yang2025_Silkie_Wencha	0.715146325
ENSGALG00010022256	PSMC3	3	0.058401052	0.043962003	1.32844384	0.184031525	0	1.551149277	0	0.666666667	CRA004775;PRJNA827465;Yang2025_Silkie_Wencha	0.715146325
ENSGALG00010017852	LETM2	3	0.097631725	0.073547684	1.327461578	0.184356042	0.00375195	2.523208075	20.73582755	0.666666667	CRA004775;PRJNA827465;Yang2025_Silkie_Wencha	0.715592083
ENSGALG00010024013	PTPRJ	3	0.095967698	0.072297544	1.327399135	0.184376686	0	0.488571686	0	0.666666667	CRA004775;PRJNA827465;Yang2025_Silkie_Wencha	0.715592083
ENSGALG00010014856	MMGT1	3	-0.074004889	0.055744101	-1.327582448	0.184316086	0.002779193	2.79515939	28.44772976	0.666666667	CRA004775;PRJNA827465;Yang2025_Silkie_Wencha	0.715592083
ENSGALG00010006186	CHIR1AB3	3	-0.31292156	0.235788136	-1.327130217	0.184465611	0	0.926095698	0	1	CRA004775;PRJNA827465;Yang2025_Silkie_Wencha	0.715740745
ENSGALG00010024883	ING5	3	-0.162045339	0.122151389	-1.326594319	0.184642916	0.03183455	8.036733808	75.11426846	1	CRA004775;PRJNA827465;Yang2025_Silkie_Wencha	0.716035707
ENSGALG00010025787	KCNE4	3	-0.143546558	0.108195666	-1.326731134	0.184597638	0.013099452	3.195271224	37.40750441	0.666666667	CRA004775;PRJNA827465;Yang2025_Silkie_Wencha	0.716035707
ENSGALG00010027672	CNTRL	3	0.390664814	0.294862973	1.324902921	0.185203352	0.213909807	15.14877861	86.79761549	0.666666667	CRA004775;PRJNA827465;Yang2025_Silkie_Wencha	0.716100555
ENSGALG00010011712	DCAF12L2	3	-0.286407218	0.216028455	-1.325784692	0.184911025	0.123238072	19.23474398	89.6021491	0.666666667	CRA004775;PRJNA827465;Yang2025_Silkie_Wencha	0.716100555
ENSGALG00010012483	ENSGALG00010012483	3	0.210674839	0.159002857	1.324975177	0.185179384	0	1.586441498	0	0.666666667	CRA004775;PRJNA827465;Yang2025_Silkie_Wencha	0.716100555
ENSGALG00010013823	GUF1	3	0.210332482	0.158722233	1.325160806	0.185117822	0.054617677	8.085861896	75.26546921	0.666666667	CRA004775;PRJNA827465;Yang2025_Silkie_Wencha	0.716100555
ENSGALG00010028055	ENSGALG00010028055	3	0.18127608	0.136775691	1.325353057	0.185054079	0.037049418	6.095688078	67.18992221	0.666666667	CRA004775;PRJNA827465;Yang2025_Silkie_Wencha	0.716100555
ENSGALG00010024009	CSE1L	3	-0.14679369	0.110749515	-1.325456735	0.18501971	0.028940355	10.9269811	81.69668291	0.666666667	CRA004775;PRJNA827465;Yang2025_Silkie_Wencha	0.716100555
ENSGALG00010000215	SF3A1	3	-0.126733998	0.095609608	-1.325536215	0.184993366	0.017443201	5.527381174	63.8164994	0.666666667	CRA004775;PRJNA827465;Yang2025_Silkie_Wencha	0.716100555
ENSGALG00010025329	MYO1A	3	0.112518003	0.084878299	1.325639229	0.184959226	0	0.868157098	0	1	CRA004775;PRJNA827465;Yang2025_Silkie_Wencha	0.716100555
ENSGALG00010023750	ENSGALG00010023750	3	-0.10089309	0.076150148	-1.324923105	0.185196657	0	1.868596611	0	1	CRA004775;PRJNA827465;Yang2025_Silkie_Wencha	0.716100555
ENSGALG00010019294	SMARCAL1	3	0.083530788	0.063055912	1.324709851	0.185267404	0	0.403566726	0	1	CRA004775;PRJNA827465;Yang2025_Silkie_Wencha	0.716100555
ENSGALG00010008343	TNFRSF21	3	0.077032095	0.058143609	1.324859193	0.185217858	0	1.933406375	0	0.666666667	CRA004775;PRJNA827465;Yang2025_Silkie_Wencha	0.716100555
ENSGALG00010004060	ETV1	3	-0.076588185	0.057808107	-1.324869279	0.185214512	0.001229153	2.208667487	9.447664194	0.666666667	CRA004775;PRJNA827465;Yang2025_Silkie_Wencha	0.716100555
ENSGALG00010010762	MRPL39	3	-0.080892757	0.06107251	-1.324536327	0.185324987	0	0.134636991	0	0.666666667	CRA004775;PRJNA827465;Yang2025_Silkie_Wencha	0.716127353
ENSGALG00010013425	ENSGALG00010013425	3	-0.273965411	0.206927718	-1.323966718	0.185514098	0	1.497534766	0	1	CRA004775;PRJNA827465;Yang2025_Silkie_Wencha	0.716170528
ENSGALG00010025985	CHCHD10	3	0.194691107	0.147036153	1.324103651	0.185468623	0	1.677720443	0	1	CRA004775;PRJNA827465;Yang2025_Silkie_Wencha	0.716170528
ENSGALG00010011184	FAM8A1	3	-0.182444185	0.137773086	-1.324236761	0.185424426	0.038686813	9.140201734	78.11864488	0.666666667	CRA004775;PRJNA827465;Yang2025_Silkie_Wencha	0.716170528
ENSGALG00010018267	ADPRHL2	3	-0.088358817	0.066749376	-1.323739966	0.18558942	0	1.392415733	0	1	CRA004775;PRJNA827465;Yang2025_Silkie_Wencha	0.716170528
ENSGALG00010016433	TMED3	3	0.0761624	0.057533593	1.32379009	0.185572768	0	0.782336048	0	1	CRA004775;PRJNA827465;Yang2025_Silkie_Wencha	0.716170528
ENSGALG00010023295	ENSGALG00010023295	3	0.927800004	0.702386366	1.320925419	0.186526233	1.131380561	8.737971112	77.11139148	0.666666667	CRA004775;PRJNA827465;Yang2025_Silkie_Wencha	0.716757878
ENSGALG00010013339	ATP6V0D2	3	0.591126738	0.447113609	1.322095159	0.186136466	0	1.299715329	0	0.666666667	CRA004775;PRJNA827465;Yang2025_Silkie_Wencha	0.716757878
ENSGALG00010020856	VTG2	2	0.495224116	0.375062718	1.320376815	0.186709241	0.105024715	1.582990786	36.82843837	1	PRJNA827465;Yang2025_Silkie_Wenchang	0.716757878
ENSGALG00010002728	RP2	3	-0.374299779	0.283099636	-1.322148572	0.186118682	0.209586691	34.49479897	94.20202448	1	CRA004775;PRJNA827465;Yang2025_Silkie_Wencha	0.716757878
ENSGALG00010007413	GPATCH11	3	0.323194173	0.244453673	1.322108066	0.186132168	0.151511422	23.25294149	91.39893763	0.666666667	CRA004775;PRJNA827465;Yang2025_Silkie_Wencha	0.716757878
ENSGALG00010007485	CTSC	3	-0.301546781	0.228348026	-1.320557863	0.18664883	0.111279191	11.82780185	83.09068731	0.666666667	CRA004775;PRJNA827465;Yang2025_Silkie_Wencha	0.716757878
ENSGALG00010024420	TMEM106C	3	-0.291402129	0.220305188	-1.322720228	0.185928434	0.107297984	7.965785647	74.89262091	0.666666667	CRA004775;PRJNA827465;Yang2025_Silkie_Wencha	0.716757878
ENSGALG00010028232	C9orf69	3	-0.281358981	0.21318584	-1.319782693	0.186907582	0.098162447	8.252405837	75.76464319	1	CRA004775;PRJNA827465;Yang2025_Silkie_Wencha	0.716757878
ENSGALG00010000575	ENSGALG00010000575	3	-0.274462174	0.207857802	-1.320432386	0.186690697	0.070498784	4.536043068	55.90870788	1	CRA004775;PRJNA827465;Yang2025_Silkie_Wencha	0.716757878
ENSGALG00010011650	RPL30	3	-0.218256959	0.165356821	-1.319915065	0.186863377	0.052364593	5.607326482	64.33237825	1	CRA004775;PRJNA827465;Yang2025_Silkie_Wencha	0.716757878
ENSGALG00010013040	SLC25A43	3	-0.156769832	0.118764831	-1.320002145	0.186834302	0.02093173	4.373771901	54.2728783	1	CRA004775;PRJNA827465;Yang2025_Silkie_Wencha	0.716757878
ENSGALG00010005470	SEMA3A	3	-0.154311034	0.116665721	-1.322676721	0.185942908	0.016381195	3.273419829	38.90181815	0.666666667	CRA004775;PRJNA827465;Yang2025_Silkie_Wencha	0.716757878
ENSGALG00010021393	CCDC174	3	0.152489665	0.115358448	1.32187687	0.186209156	0.025102654	5.66444057	64.69201194	1	CRA004775;PRJNA827465;Yang2025_Silkie_Wencha	0.716757878
ENSGALG00010017399	CPXM1	3	-0.134067648	0.101568383	-1.319974225	0.186843624	0	0.88351565	0	1	CRA004775;PRJNA827465;Yang2025_Silkie_Wencha	0.716757878
ENSGALG00010006277	GNG10	3	0.133920453	0.101375684	1.321031306	0.186490926	0	1.131443563	0	0.666666667	CRA004775;PRJNA827465;Yang2025_Silkie_Wencha	0.716757878
ENSGALG00010021905	RBSN	3	0.109279341	0.082682867	1.321668513	0.186278558	0.012964612	5.56297166	64.04799229	0.666666667	CRA004775;PRJNA827465;Yang2025_Silkie_Wencha	0.716757878
ENSGALG00010009335	PTP4A3	3	-0.107434357	0.081387437	-1.320036125	0.186822957	0	0.108603158	0	1	CRA004775;PRJNA827465;Yang2025_Silkie_Wencha	0.716757878
ENSGALG00010008381	C18orf21	3	0.101099529	0.07659146	1.319984368	0.186840237	0	0.177661363	0	1	CRA004775;PRJNA827465;Yang2025_Silkie_Wencha	0.716757878
ENSGALG00010016059	EPDR1	3	-0.096620335	0.073067743	-1.322339118	0.186055252	0	1.908303359	0	0.666666667	CRA004775;PRJNA827465;Yang2025_Silkie_Wencha	0.716757878
ENSGALG00010012216	BRD8	3	-0.08805295	0.066656276	-1.321000139	0.186501318	0.006660667	3.91859056	48.9612408	1	CRA004775;PRJNA827465;Yang2025_Silkie_Wencha	0.716757878
ENSGALG00010019282	IGFBP2	3	-0.087587659	0.066274645	-1.321586234	0.18630597	0	1.484369919	0	1	CRA004775;PRJNA827465;Yang2025_Silkie_Wencha	0.716757878
ENSGALG00010024555	EPHB1	3	-0.082625382	0.062501439	-1.321975685	0.186176248	0.002355657	2.406342309	16.8863053	0.666666667	CRA004775;PRJNA827465;Yang2025_Silkie_Wencha	0.716757878
ENSGALG00010028472	RPRD2	3	-0.073558354	0.055635583	-1.32214581	0.186119602	0	0.652260774	0	0.666666667	CRA004775;PRJNA827465;Yang2025_Silkie_Wencha	0.716757878
ENSGALG00010021807	CDC42	3	-0.302012485	0.228894408	-1.319440207	0.187021988	0.14695923	63.35889967	96.84337952	1	CRA004775;PRJNA827465;Yang2025_Silkie_Wencha	0.716759278
ENSGALG00010026907	ENSGALG00010026907	3	0.200026162	0.151612344	1.319326363	0.187060028	0.038563657	4.466059805	55.21779628	0.666666667	CRA004775;PRJNA827465;Yang2025_Silkie_Wencha	0.716759278
ENSGALG00010027699	VANGL2	3	-0.110199686	0.083527066	-1.319329063	0.187059126	0.007847827	3.103839156	35.56367132	0.666666667	CRA004775;PRJNA827465;Yang2025_Silkie_Wencha	0.716759278
ENSGALG00010002062	GSTA4	3	0.23792493	0.180410539	1.318797287	0.187236892	0	0.645055929	0	1	CRA004775;PRJNA827465;Yang2025_Silkie_Wencha	0.717242593
ENSGALG00010029162	ELN	3	0.34472269	0.26147998	1.318352133	0.187385797	0	1.018261564	0	1	CRA004775;PRJNA827465;Yang2025_Silkie_Wencha	0.717618575
ENSGALG00010020662	BFAR	3	-0.214350632	0.162609806	-1.318190071	0.187440028	0.056749516	10.18016448	80.353952	1	CRA004775;PRJNA827465;Yang2025_Silkie_Wencha	0.717631888
ENSGALG00010000353	ENSGALG00010000353	3	-0.394497461	0.299361946	-1.317794283	0.187572522	0.188982957	7.070868676	71.71493219	0.666666667	CRA004775;PRJNA827465;Yang2025_Silkie_Wencha	0.717660702
ENSGALG00010015837	GDA	3	-0.167144687	0.126844555	-1.317712743	0.187599827	0	0.008358597	0	1	CRA004775;PRJNA827465;Yang2025_Silkie_Wencha	0.717660702
ENSGALG00010028036	NDUFS7	3	0.091741213	0.069616723	1.317804248	0.187569185	0	1.710712626	0	1	CRA004775;PRJNA827465;Yang2025_Silkie_Wencha	0.717660702
ENSGALG00010003774	ENSGALG00010003774	2	0.87830165	0.667079006	1.316638121	0.187959954	0.473927716	2.133821216	53.13571764	1	PRJNA827465;Yang2025_Silkie_Wenchang	0.718249418
ENSGALG00010023155	CFAP263	2	0.580701145	0.441096115	1.316495714	0.188007716	0.342444356	8.287181335	87.93317101	1	PRJNA827465;Yang2025_Silkie_Wenchang	0.718249418
ENSGALG00010019761	MRPS18A	3	-0.187983643	0.142764668	-1.316737858	0.187926509	0.036253759	5.034757729	60.27614222	1	CRA004775;PRJNA827465;Yang2025_Silkie_Wencha	0.718249418
ENSGALG00010028374	FDPS	3	0.13007611	0.098781421	1.316807437	0.187903179	0	0.171681656	0	0.666666667	CRA004775;PRJNA827465;Yang2025_Silkie_Wencha	0.718249418
ENSGALG00010028739	GARNL3	3	-0.07787604	0.059147306	-1.316645587	0.187957451	0.005688944	5.286421378	62.16722321	0.666666667	CRA004775;PRJNA827465;Yang2025_Silkie_Wencha	0.718249418
ENSGALG00010024634	ENSGALG00010024634	3	0.505696499	0.384446906	1.315387093	0.188379841	0.387229296	18.73532041	89.32497573	1	CRA004775;PRJNA827465;Yang2025_Silkie_Wencha	0.718452024
ENSGALG00010027176	ENSGALG00010027176	3	0.405665312	0.308594629	1.314557268	0.188658739	0.258563681	26.61477882	92.48537809	1	CRA004775;PRJNA827465;Yang2025_Silkie_Wencha	0.718452024
ENSGALG00010015364	CLOCK	3	0.381028457	0.28981026	1.314751439	0.188593453	0.215055099	17.56930449	88.61651011	0.666666667	CRA004775;PRJNA827465;Yang2025_Silkie_Wencha	0.718452024
ENSGALG00010008135	WWC1	3	0.338095749	0.257230039	1.314371177	0.188721325	0.171433445	21.21335025	90.57197483	1	CRA004775;PRJNA827465;Yang2025_Silkie_Wencha	0.718452024
ENSGALG00010029741	EMC6	3	-0.310714934	0.236315706	-1.314829807	0.188567107	0.128250784	12.51257799	84.01608365	1	CRA004775;PRJNA827465;Yang2025_Silkie_Wencha	0.718452024
ENSGALG00010029369	MFSD11	3	-0.301953683	0.229712233	-1.314486739	0.188682458	0.136524263	20.07464833	90.03718537	1	CRA004775;PRJNA827465;Yang2025_Silkie_Wencha	0.718452024
ENSGALG00010013045	FSD1L	3	0.255759672	0.19447546	1.315125684	0.188467666	0.069108058	5.848182252	65.8013394	1	CRA004775;PRJNA827465;Yang2025_Silkie_Wencha	0.718452024
ENSGALG00010003851	PHF14	3	0.216590449	0.164588348	1.315952508	0.188189983	0.063157553	9.880125119	79.75734137	1	CRA004775;PRJNA827465;Yang2025_Silkie_Wencha	0.718452024
ENSGALG00010001785	CNTN1	3	-0.195159851	0.148464009	-1.314526343	0.188669139	0	0.172460223	0	1	CRA004775;PRJNA827465;Yang2025_Silkie_Wencha	0.718452024
ENSGALG00010024901	ZGPAT	3	-0.140222733	0.106556071	-1.315952545	0.188189971	0.019865587	4.736375387	57.77361724	1	CRA004775;PRJNA827465;Yang2025_Silkie_Wencha	0.718452024
ENSGALG00010020905	KCTD6	3	-0.085014688	0.064615193	-1.315707409	0.188272266	0.002480932	2.35731963	15.15787786	1	CRA004775;PRJNA827465;Yang2025_Silkie_Wencha	0.718452024
ENSGALG00010029299	PRKRIP1	3	-0.074526169	0.056651792	-1.315512995	0.188337553	0	1.923269838	0	0.666666667	CRA004775;PRJNA827465;Yang2025_Silkie_Wencha	0.718452024
ENSGALG00010012372	LEMD3	3	0.058036272	0.044134304	1.314992353	0.188512472	0	0.72199367	0	1	CRA004775;PRJNA827465;Yang2025_Silkie_Wencha	0.718452024
ENSGALG00010019744	KBP	2	0.672531568	0.511881651	1.313841912	0.18889941	0.333857653	2.735133646	63.43871527	1	PRJNA827465;Yang2025_Silkie_Wenchang	0.718936408
ENSGALG00010008362	RAMP3	3	0.64850211	0.493765055	1.313381949	0.189054277	0.617632279	31.03357655	93.55536737	1	CRA004775;PRJNA827465;Yang2025_Silkie_Wencha	0.71900181
ENSGALG00010024554	ST6GALNAC3	3	-0.438356015	0.333799766	-1.313230443	0.189105309	0.244227553	15.86742313	87.39555891	1	CRA004775;PRJNA827465;Yang2025_Silkie_Wencha	0.71900181
ENSGALG00010011421	ENSGALG00010011421	3	0.308304745	0.234775997	1.313186821	0.189120004	0.132695698	11.45075766	82.53390684	0.666666667	CRA004775;PRJNA827465;Yang2025_Silkie_Wencha	0.71900181
ENSGALG00010018811	VAMP3	3	-0.067194737	0.051159294	-1.313441452	0.189034237	0	0.261436715	0	1	CRA004775;PRJNA827465;Yang2025_Silkie_Wencha	0.71900181
ENSGALG00010029464	ENSGALG00010029464	3	0.697594223	0.532783326	1.309339443	0.190419395	0.513701003	5.057882059	60.45775728	0.666666667	CRA004775;PRJNA827465;Yang2025_Silkie_Wencha	0.719108624
ENSGALG00010017757	ENSGALG00010017757	3	-0.356550279	0.272196806	-1.309898833	0.190230062	0.181822384	13.90978649	85.62163408	1	CRA004775;PRJNA827465;Yang2025_Silkie_Wencha	0.719108624
ENSGALG00010007414	ROBO2	2	0.311297625	0.237666314	1.30980962	0.190260248	0.068420273	2.412836288	58.55499998	1	PRJNA827465;Yang2025_Silkie_Wenchang	0.719108624
ENSGALG00010025073	IL18	3	0.29985536	0.229056166	1.309090976	0.190503536	0.051450857	2.929957018	31.73961298	0.666666667	CRA004775;PRJNA827465;Yang2025_Silkie_Wencha	0.719108624
ENSGALG00010022647	SVIP	3	-0.287754635	0.219262844	-1.312372993	0.189394315	0.114333735	19.45121268	89.71786473	1	CRA004775;PRJNA827465;Yang2025_Silkie_Wencha	0.719108624
ENSGALG00010001202	CFTR	3	-0.279430241	0.213325216	-1.309879099	0.190236739	0.070360401	4.596763203	56.49112404	0.666666667	CRA004775;PRJNA827465;Yang2025_Silkie_Wencha	0.719108624
ENSGALG00010023686	MYLK3	3	-0.248658883	0.189817383	-1.309990053	0.190199201	0.047145137	3.536519041	43.44721528	0.666666667	CRA004775;PRJNA827465;Yang2025_Silkie_Wencha	0.719108624
ENSGALG00010001320	CPED1	3	0.227479813	0.173775462	1.309044502	0.190519277	0.034969614	3.250504527	38.47109016	1	CRA004775;PRJNA827465;Yang2025_Silkie_Wencha	0.719108624
ENSGALG00010024672	DYNLRB1	3	-0.215119509	0.164050543	-1.311300191	0.189756364	0.063747089	16.96070709	88.2080388	0.666666667	CRA004775;PRJNA827465;Yang2025_Silkie_Wencha	0.719108624
ENSGALG00010025549	DHCR24	3	0.213165975	0.162790566	1.309449188	0.190382239	0	0.396648027	0	0.666666667	CRA004775;PRJNA827465;Yang2025_Silkie_Wencha	0.719108624
ENSGALG00010009795	NDUFA6	3	-0.173830517	0.132698446	-1.309966487	0.190207173	0.019266519	2.735074926	26.87586065	1	CRA004775;PRJNA827465;Yang2025_Silkie_Wencha	0.719108624
ENSGALG00010003117	ID2	3	0.173265824	0.132288873	1.309753576	0.190279213	0.028703924	5.113229066	60.88577346	0.666666667	CRA004775;PRJNA827465;Yang2025_Silkie_Wencha	0.719108624
ENSGALG00010010444	TXNDC5	3	-0.171829751	0.131104747	-1.310629513	0.189982963	0.028367128	4.505943325	55.61417763	1	CRA004775;PRJNA827465;Yang2025_Silkie_Wencha	0.719108624
ENSGALG00010004225	CETN4	3	-0.167347625	0.127789026	-1.309583304	0.19033684	0.033470528	7.250619481	72.41615002	0.666666667	CRA004775;PRJNA827465;Yang2025_Silkie_Wencha	0.719108624
ENSGALG00010004489	LIG1	3	-0.159412439	0.121673217	-1.310168688	0.190138776	0.017691023	3.228199031	38.04595129	1	CRA004775;PRJNA827465;Yang2025_Silkie_Wencha	0.719108624
ENSGALG00010026403	GUCD1	3	-0.158791753	0.121026638	-1.312039698	0.189506741	0.027205911	6.227945947	67.8866834	0.666666667	CRA004775;PRJNA827465;Yang2025_Silkie_Wencha	0.719108624
ENSGALG00010004094	DGKB	2	0.158729474	0.121256538	1.309038477	0.190521317	0	0.049422888	0	1	PRJNA827465;Yang2025_Silkie_Wenchang	0.719108624
ENSGALG00010002925	INTS6	3	0.15195575	0.116955359	1.309863666	0.190241961	0.022703827	4.507319138	55.62772596	1	CRA004775;PRJNA827465;Yang2025_Silkie_Wencha	0.719108624
ENSGALG00010007987	ENSGALG00010007987	3	-0.153152651	0.116750516	-1.311794206	0.18958958	0	0.209746507	0	1	CRA004775;PRJNA827465;Yang2025_Silkie_Wencha	0.719108624
ENSGALG00010002581	ATP13A1	3	-0.15223275	0.116253748	-1.309486815	0.190369501	0.026768805	6.277190212	68.13861087	1	CRA004775;PRJNA827465;Yang2025_Silkie_Wencha	0.719108624
ENSGALG00010008121	MPZL1	3	-0.146033994	0.111269235	-1.312438192	0.189372328	0.021149054	5.377809235	62.8101349	1	CRA004775;PRJNA827465;Yang2025_Silkie_Wencha	0.719108624
ENSGALG00010019621	ENSGALG00010019621	3	0.132092211	0.100704162	1.311755231	0.189602735	0.009874583	2.92438135	31.60948509	0.666666667	CRA004775;PRJNA827465;Yang2025_Silkie_Wencha	0.719108624
ENSGALG00010016986	GFER	3	-0.121700018	0.092872027	-1.310405531	0.190058684	0.012181711	3.779263098	47.07963039	0.666666667	CRA004775;PRJNA827465;Yang2025_Silkie_Wencha	0.719108624
ENSGALG00010001570	SMPD1	3	0.085231812	0.065064413	1.309960517	0.190209193	0	0.44497046	0	0.666666667	CRA004775;PRJNA827465;Yang2025_Silkie_Wencha	0.719108624
ENSGALG00010018010	ENSGALG00010018010	3	-0.07227204	0.055196115	-1.309368249	0.190409641	0	0.941636656	0	1	CRA004775;PRJNA827465;Yang2025_Silkie_Wencha	0.719108624
ENSGALG00010001460	MALSU1	3	-0.056122693	0.042862146	-1.30937665	0.190406797	0	1.245910945	0	0.666666667	CRA004775;PRJNA827465;Yang2025_Silkie_Wencha	0.719108624
ENSGALG00010014691	PABIR2	3	-0.044545404	0.033934168	-1.312700653	0.189283838	0	0.043085012	0	1	CRA004775;PRJNA827465;Yang2025_Silkie_Wencha	0.719108624
ENSGALG00010029651	ENSGALG00010029651	3	0.320608594	0.245087244	1.308140677	0.190825599	0.042128928	2.449331937	18.34508137	0.666666667	CRA004775;PRJNA827465;Yang2025_Silkie_Wencha	0.719680752
ENSGALG00010022028	FGD5	3	0.099068001	0.075725419	1.30825293	0.190787534	0	1.259531121	0	1	CRA004775;PRJNA827465;Yang2025_Silkie_Wencha	0.719680752
ENSGALG00010023182	EPHB6	3	0.085926906	0.06567618	1.308342027	0.190757326	0	1.31288033	0	1	CRA004775;PRJNA827465;Yang2025_Silkie_Wencha	0.719680752
ENSGALG00010017666	PCARE	2	1.031414212	0.788822094	1.307537174	0.191030338	0.867148616	3.29449737	69.6463561	1	PRJNA827465;Yang2025_Silkie_Wenchang	0.719724604
ENSGALG00010025588	ENSGALG00010025588	3	-0.23037609	0.176255658	-1.30705642	0.191193551	0	0.067428763	0	0.666666667	CRA004775;PRJNA827465;Yang2025_Silkie_Wencha	0.719724604
ENSGALG00010013399	FGF1	3	-0.162515668	0.124327313	-1.307159822	0.191158438	0	1.615246832	0	0.666666667	CRA004775;PRJNA827465;Yang2025_Silkie_Wencha	0.719724604
ENSGALG00010005212	TAF13	3	-0.128982757	0.098652257	-1.307448961	0.191060278	0.008714267	2.56667826	22.07827405	1	CRA004775;PRJNA827465;Yang2025_Silkie_Wencha	0.719724604
ENSGALG00010011372	VMA21	3	-0.127313569	0.097369237	-1.307533805	0.191031482	0.018780368	6.071805065	67.06086611	1	CRA004775;PRJNA827465;Yang2025_Silkie_Wencha	0.719724604
ENSGALG00010015845	SAP30L	3	-0.120581861	0.092237249	-1.307301135	0.191110459	0.01095298	3.567768053	43.94254418	0.666666667	CRA004775;PRJNA827465;Yang2025_Silkie_Wencha	0.719724604
ENSGALG00010005305	SCAF8	3	-0.092229384	0.070525053	-1.307568074	0.191019851	0.005589102	2.902573343	31.09562571	0.666666667	CRA004775;PRJNA827465;Yang2025_Silkie_Wencha	0.719724604
ENSGALG00010021960	CDKN1B	3	0.066008778	0.050512597	1.306768709	0.19131166	0	0.380994247	0	1	CRA004775;PRJNA827465;Yang2025_Silkie_Wencha	0.719977524
ENSGALG00010022655	NRIP3	3	0.752263653	0.575792443	1.306484068	0.191387993	0.518587032	4.281623244	53.28874387	0.666666667	CRA004775;PRJNA827465;Yang2025_Silkie_Wencha	0.720073134
ENSGALG00010002500	OSR1	3	0.284269045	0.217668711	1.305971097	0.191562387	0.073137709	3.813066651	47.54877941	1	CRA004775;PRJNA827465;Yang2025_Silkie_Wencha	0.72019662
ENSGALG00010026229	CRHR2	3	-0.195157141	0.14948962	-1.30583903	0.191607304	0.030633506	3.864373094	48.2451629	0.666666667	CRA004775;PRJNA827465;Yang2025_Silkie_Wencha	0.72019662
ENSGALG00010017085	ENSGALG00010017085	3	-0.159188488	0.121883938	-1.30606592	0.19150131	0.025737927	4.926527456	59.40345369	0.666666667	CRA004775;PRJNA827465;Yang2025_Silkie_Wencha	0.72019662
ENSGALG00010020059	PRKCZ	3	0.120693983	0.092429978	1.305788294	0.191624562	0.001591237	2.121326106	5.719351929	0.666666667	CRA004775;PRJNA827465;Yang2025_Silkie_Wencha	0.72019662
ENSGALG00010011988	HINTW	3	-3.350794159	2.597866252	-1.289825508	0.197111249	16.84413086	14.49306973	86.20030099	1	CRA004775;PRJNA827465;Yang2025_Silkie_Wencha	0.720732808
ENSGALG00010000078	ENSGALG00010000078	3	0.957356639	0.742466252	1.289427818	0.197249396	1.383176022	12.73015207	84.28926659	0.666666667	CRA004775;PRJNA827465;Yang2025_Silkie_Wencha	0.720732808
ENSGALG00010000598	ENSGALG00010000598	3	0.889836889	0.684668561	1.299660799	0.193717252	0.986234982	7.077167232	71.74010541	0.666666667	CRA004775;PRJNA827465;Yang2025_Silkie_Wencha	0.720732808
ENSGALG00010029645	ENSGALG00010029645	2	0.799710981	0.616352714	1.297489187	0.194462923	0.647109741	6.689295438	85.0507425	1	PRJNA827465;Yang2025_Silkie_Wenchang	0.720732808
ENSGALG00010020087	ENSGALG00010020087	3	0.78014952	0.603284612	1.29316993	0.195952293	0.832023983	8.421370436	76.25089627	1	CRA004775;PRJNA827465;Yang2025_Silkie_Wencha	0.720732808
ENSGALG00010001737	ENSGALG00010001737	3	0.769847096	0.590835847	1.302979516	0.192581755	0	0.732911398	0	1	CRA004775;Yang2025_Silkie_Wenchang	0.720732808
ENSGALG00010024947	MPP4	3	0.756869746	0.587343183	1.288632894	0.197525742	0.914090617	29.5099091	93.22261552	1	CRA004775;PRJNA827465;Yang2025_Silkie_Wencha	0.720732808
ENSGALG00010016552	ENSGALG00010016552	3	0.72261325	0.555221913	1.301485466	0.193092336	0.761495883	13.54108527	85.23013511	0.666666667	CRA004775;PRJNA827465;Yang2025_Silkie_Wencha	0.720732808
ENSGALG00010027593	ENSGALG00010027593	3	0.695441028	0.538015499	1.292604079	0.196148028	0.693349194	12.56923235	84.0881293	1	CRA004775;PRJNA827465;Yang2025_Silkie_Wencha	0.720732808
ENSGALG00010007880	BCO1	3	-0.657207684	0.510355118	-1.287739792	0.197836557	0.667820362	14.13282082	85.85854343	0.666666667	CRA004775;PRJNA827465;Yang2025_Silkie_Wencha	0.720732808
ENSGALG00010024827	ENSGALG00010024827	2	0.659206627	0.504716088	1.287905503	0.19777886	0.352937412	3.25406509	69.26920721	1	PRJNA827465;Yang2025_Silkie_Wenchang	0.720732808
ENSGALG00010004306	PNISR	3	0.531389948	0.409219732	1.298544293	0.194100366	0.468588224	35.96559145	94.4391294	1	CRA004775;PRJNA827465;Yang2025_Silkie_Wencha	0.720732808
ENSGALG00010011109	ENSGALG00010011109	3	0.504789541	0.390403788	1.29299345	0.196013324	0	0.4715116	0	1	CRA004775;PRJNA827465;Yang2025_Silkie_Wencha	0.720732808
ENSGALG00010028014	ENSGALG00010028014	3	-0.503093104	0.386175072	-1.304825296	0.191952343	0.215605357	3.87544432	48.3930142	0.666666667	CRA004775;PRJNA827465;Yang2025_Silkie_Wencha	0.720732808
ENSGALG00010006024	AMER2	3	0.498305976	0.386418142	1.289551193	0.197206531	0.168810104	3.139964274	36.30500778	0.666666667	CRA004775;PRJNA827465;Yang2025_Silkie_Wencha	0.720732808
ENSGALG00010011200	PCDHB4	3	0.496808133	0.381447826	1.302427487	0.192770291	0	1.184330592	0	1	CRA004775;PRJNA827465;Yang2025_Silkie_Wencha	0.720732808
ENSGALG00010015918	BICD1	3	0.477300091	0.369030099	1.293390682	0.195875971	0.326906477	14.41472031	86.12529444	1	CRA004775;PRJNA827465;Yang2025_Silkie_Wencha	0.720732808
ENSGALG00010027231	ENSGALG00010027231	3	0.476712467	0.367312398	1.297837458	0.194331509	0.337942673	17.72593197	88.71709536	0.666666667	CRA004775;PRJNA827465;Yang2025_Silkie_Wencha	0.720732808
ENSGALG00010006942	ALOX5AP	3	-0.449614324	0.347934639	-1.292237888	0.196274775	0.298965012	13.37978597	85.05207778	0.666666667	CRA004775;PRJNA827465;Yang2025_Silkie_Wencha	0.720732808
ENSGALG00010004735	ENSGALG00010004735	3	-0.429123464	0.330174539	-1.299686721	0.193708363	0.285112593	17.88237979	88.81580627	0.666666667	CRA004775;PRJNA827465;Yang2025_Silkie_Wencha	0.720732808
ENSGALG00010013406	ENSGALG00010013406	3	-0.423855613	0.327767781	-1.293158259	0.195956329	0	0.856982123	0	1	CRA004775;PRJNA827465;Yang2025_Silkie_Wencha	0.720732808
ENSGALG00010009144	CREBZF	3	0.416744885	0.322007773	1.294207533	0.195593746	0.247554487	10.53553486	81.01662586	0.666666667	CRA004775;PRJNA827465;Yang2025_Silkie_Wencha	0.720732808
ENSGALG00010011059	ENSGALG00010011059	3	0.4105328	0.315957427	1.299329483	0.19383088	0.2236854	9.331094443	78.56628703	1	CRA004775;PRJNA827465;Yang2025_Silkie_Wencha	0.720732808
ENSGALG00010022848	SGIP1	2	0.4034069	0.310200947	1.300469596	0.19344007	0	0.242542171	0	1	PRJNA827465;Yang2025_Silkie_Wenchang	0.720732808
ENSGALG00010010942	NRN1	3	-0.394011312	0.303938782	-1.296350897	0.194854621	0.162935557	5.238942591	61.82435739	0.666666667	CRA004775;PRJNA827465;Yang2025_Silkie_Wencha	0.720732808
ENSGALG00010015027	CLSPN	3	0.392925472	0.304708683	1.289511897	0.197220183	0.221921003	14.61984398	86.31996345	0.666666667	CRA004775;PRJNA827465;Yang2025_Silkie_Wencha	0.720732808
ENSGALG00010012949	ENSGALG00010012949	3	-0.374607733	0.29072328	-1.288537104	0.197559062	0.202906315	12.17421932	83.57184188	0.666666667	CRA004775;PRJNA827465;Yang2025_Silkie_Wencha	0.720732808
ENSGALG00010014876	PCNT	3	0.364805946	0.283244232	1.28795543	0.19776147	0.210746636	18.34298259	89.09664778	0.666666667	CRA004775;PRJNA827465;Yang2025_Silkie_Wencha	0.720732808
ENSGALG00010001595	AKAIN1	3	0.355340028	0.275189951	1.291253649	0.196615737	0.08941261	3.281686761	39.05573122	0.666666667	CRA004775;PRJNA827465;Yang2025_Silkie_Wencha	0.720732808
ENSGALG00010021584	OVCH2	3	0.341347709	0.265128055	1.28748242	0.19792693	0	0.9501235	0	1	CRA004775;PRJNA827465;Yang2025_Silkie_Wencha	0.720732808
ENSGALG00010007713	SCLT1	3	0.340948923	0.262992365	1.296421372	0.194830353	0.168671557	11.73377342	82.95518476	1	CRA004775;PRJNA827465;Yang2025_Silkie_Wencha	0.720732808
ENSGALG00010011575	ENSGALG00010011575	3	0.336874083	0.261172637	1.289852132	0.197102003	0	1.487003313	0	1	CRA004775;PRJNA827465;Yang2025_Silkie_Wencha	0.720732808
ENSGALG00010023128	IWS1	3	0.336097449	0.260099427	1.292188347	0.196291926	0.177554101	19.10155533	89.52964842	1	CRA004775;PRJNA827465;Yang2025_Silkie_Wencha	0.720732808
ENSGALG00010009360	TNFAIP8	3	-0.318003999	0.244442569	-1.300935431	0.193280557	0.141064603	21.85834323	90.8501757	0.666666667	CRA004775;PRJNA827465;Yang2025_Silkie_Wencha	0.720732808
ENSGALG00010021474	PPFIBP2	3	0.31606332	0.242846522	1.301494115	0.193089378	0.117355681	6.704121413	70.16760472	0.666666667	CRA004775;PRJNA827465;Yang2025_Silkie_Wencha	0.720732808
ENSGALG00010027321	IFI30	3	0.313210951	0.241363505	1.297673193	0.194399659	0.118178735	6.824604188	70.69427113	0.666666667	CRA004775;PRJNA827465;Yang2025_Silkie_Wencha	0.720732808
ENSGALG00010029378	ST6GALNAC1	3	0.311509747	0.238785653	1.304558056	0.192043379	0	0.409842647	0	0.666666667	CRA004775;PRJNA827465;Yang2025_Silkie_Wencha	0.720732808

ENSGALG00010008333	APC	3	0.309263353	0.236980129	1.30501808	0.191886691	0.116701117	6.86373052	70.86132688	1	CRA004775;PRJNA827465;Yang2025_Silkie_Wencha	0.720732808
ENSGALG00010009178	ENSGALG0001000917	3	-0.306971338	0.238432373	-1.287456624	0.197935179	0	1.131904466	0	1	CRA004775;PRJNA827465;Yang2025_Silkie_Wencha	0.720732808
ENSGALG00010013945	SCAF11	3	0.286121446	0.220285516	1.298866357	0.193989797	0.124857963	16.37702274	87.78776807	0.666666667	CRA004775;PRJNA827465;Yang2025_Silkie_Wencha	0.720732808
ENSGALG00010001158	FRMPD1	3	0.279661705	0.216056658	1.294390586	0.195530541	0	0.601908101	0	1	CRA004775;PRJNA827465;Yang2025_Silkie_Wencha	0.720732808
ENSGALG00010002940	BG1	3	-0.279617895	0.216620171	-1.290821136	0.196765707	0	1.348077335	0	0.666666667	CRA004775;PRJNA827465;Yang2025_Silkie_Wencha	0.720732808
ENSGALG00010010998	SOAT1	3	-0.279359385	0.216621964	-1.289617081	0.197183642	0.116900645	18.49956486	89.1889349	0.666666667	CRA004775;PRJNA827465;Yang2025_Silkie_Wencha	0.720732808
ENSGALG00010007033	CEBPZOS	3	-0.27743826	0.212699474	-1.304367403	0.192108344	0.092065103	6.922635616	71.10926949	0.666666667	CRA004775;PRJNA827465;Yang2025_Silkie_Wencha	0.720732808
ENSGALG00010017765	SYT16	3	-0.273642322	0.2110433	-1.296616962	0.194763013	0.065129248	4.018005835	50.22406432	1	CRA004775;PRJNA827465;Yang2025_Silkie_Wencha	0.720732808
ENSGALG00010012062	HMGA2	3	0.27200551	0.209828574	1.29632254	0.194864386	0.083746371	6.386284756	68.68288721	0.666666667	CRA004775;PRJNA827465;Yang2025_Silkie_Wencha	0.720732808
ENSGALG00010024897	ADAL	3	-0.267240014	0.206624352	-1.293361657	0.195886005	0.097324494	18.59930469	89.24690985	0.666666667	CRA004775;PRJNA827465;Yang2025_Silkie_Wencha	0.720732808
ENSGALG00010012884	ENSGALG0001001288	3	-0.265353174	0.205590986	-1.290684866	0.196812975	0	0.183429322	0	1	CRA004775;PRJNA827465;Yang2025_Silkie_Wencha	0.720732808
ENSGALG00010010855	ATP5PF	3	-0.26501532	0.204265832	-1.29740406	0.194492196	0.076845729	5.047075155	60.3730886	0.666666667	CRA004775;PRJNA827465;Yang2025_Silkie_Wencha	0.720732808
ENSGALG00010010150	NTRK2	3	-0.253340034	0.195775064	-1.294036268	0.195652894	0	0.629544073	0	1	CRA004775;PRJNA827465;Yang2025_Silkie_Wencha	0.720732808
ENSGALG00010006341	HHIP	3	0.253122027	0.19438941	1.302138972	0.192868883	0.065498998	5.101884097	60.79879586	1	CRA004775;PRJNA827465;Yang2025_Silkie_Wencha	0.720732808
ENSGALG00010023140	CCAR1	3	0.248303189	0.191315021	1.297876076	0.194329922	0.08895692	11.5113784	82.62588605	0.666666667	CRA004775;PRJNA827465;Yang2025_Silkie_Wencha	0.720732808
ENSGALG00010016878	SUCLG1	3	-0.236425713	0.183056075	-1.291548026	0.196513713	0.064173433	6.078367266	67.09642717	0.666666667	CRA004775;PRJNA827465;Yang2025_Silkie_Wencha	0.720732808
ENSGALG00010003680	TRAPPC5	3	-0.23378259	0.180857049	-1.292637422	0.19613649	0.056136292	4.430069042	54.85397674	1	CRA004775;PRJNA827465;Yang2025_Silkie_Wencha	0.720732808
ENSGALG00010007580	BRD9	3	0.229879152	0.176648028	1.301340039	0.193142088	0.075265501	13.18757929	84.83421441	1	CRA004775;PRJNA827465;Yang2025_Silkie_Wencha	0.720732808
ENSGALG00010014147	ENSGALG0001001414	3	-0.22625877	0.175036498	-1.292637662	0.196136407	0	1.150865979	0	0.666666667	CRA004775;PRJNA827465;Yang2025_Silkie_Wencha	0.720732808
ENSGALG00010017362	ENO1	3	-0.217902663	0.169040462	-1.289056247	0.197378533	0.052802637	5.212634642	61.63168652	1	CRA004775;PRJNA827465;Yang2025_Silkie_Wencha	0.720732808
ENSGALG00010020797	DGCR8	3	0.213487221	0.164955013	1.294214809	0.195591233	0.054446851	6.811811448	70.63923429	1	CRA004775;PRJNA827465;Yang2025_Silkie_Wencha	0.720732808
ENSGALG00010025115	MGAT4F	3	0.210084621	0.163098344	1.288085554	0.197716183	0.032341809	3.312551364	39.62357771	0.666666667	CRA004775;PRJNA827465;Yang2025_Silkie_Wencha	0.720732808
ENSGALG00010016896	SPTBN1	3	0.206246295	0.16017137	1.287660184	0.197864279	0.051027609	6.299874597	68.25333633	0.666666667	CRA004775;PRJNA827465;Yang2025_Silkie_Wencha	0.720732808
ENSGALG00010005253	POMT2	3	-0.205596172	0.159299189	-1.29062912	0.196832313	0.052832276	7.491642633	73.3035851	1	CRA004775;PRJNA827465;Yang2025_Silkie_Wencha	0.720732808
ENSGALG00010021972	PISD	3	-0.205585082	0.159205692	-1.291317408	0.196593637	0.048659343	8.792766814	77.25403116	1	CRA004775;PRJNA827465;Yang2025_Silkie_Wencha	0.720732808
ENSGALG00010010853	SMIM20	3	-0.202998131	0.156712295	-1.295355486	0.195197626	0.031678307	3.083102324	35.13027498	0.666666667	CRA004775;PRJNA827465;Yang2025_Silkie_Wencha	0.720732808
ENSGALG00010017593	MTHFR	3	-0.198994869	0.154559309	-1.287498439	0.197920613	0.060728538	14.59731153	86.29884691	0.666666667	CRA004775;PRJNA827465;Yang2025_Silkie_Wencha	0.720732808
ENSGALG00010012135	CAMSAP2	3	0.191120792	0.148391406	1.287950544	0.197763179	0.037139144	4.566118531	56.19912215	1	CRA004775;PRJNA827465;Yang2025_Silkie_Wencha	0.720732808
ENSGALG00010026094	C6orf106	3	-0.190298081	0.147401476	-1.291018823	0.196697151	0.049463823	10.84557983	81.55930773	0.666666667	CRA004775;PRJNA827465;Yang2025_Silkie_Wencha	0.720732808
ENSGALG00010029177	ASPSCR1	3	-0.180359137	0.139290373	-1.2948428	0.195374464	0.044418836	9.906670239	79.81158198	1	CRA004775;PRJNA827465;Yang2025_Silkie_Wencha	0.720732808
ENSGALG00010024903	CASP8	3	-0.175353574	0.135545828	-1.293684772	0.195774328	0.019386282	3.020071729	33.77640732	1	CRA004775;PRJNA827465;Yang2025_Silkie_Wencha	0.720732808
ENSGALG00010008057	BAG1	3	-0.175138675	0.1344366	-1.302760376	0.192656582	0.039636919	9.678441005	79.33551489	1	CRA004775;PRJNA827465;Yang2025_Silkie_Wencha	0.720732808
ENSGALG00010024639	AAR2	3	-0.174869261	0.13559146	-1.28967755	0.197162637	0.044366759	13.42715715	85.10481423	0.666666667	CRA004775;PRJNA827465;Yang2025_Silkie_Wencha	0.720732808
ENSGALG00010016696	DOK7	3	-0.166204645	0.128996521	-1.288442847	0.197591852	0.025306447	4.645615611	56.94865509	0.666666667	CRA004775;PRJNA827465;Yang2025_Silkie_Wencha	0.720732808
ENSGALG00010027419	PTBP1	3	-0.157569986	0.121140644	-1.300719404	0.193354518	0.028193626	10.30440868	80.59083192	1	CRA004775;PRJNA827465;Yang2025_Silkie_Wencha	0.720732808
ENSGALG00010000821	ENSGALG0001000082	3	-0.153329559	0.118539211	-1.29349232	0.195840839	0	1.174055097	0	1	CRA004775;PRJNA827465;Yang2025_Silkie_Wencha	0.720732808
ENSGALG00010026569	GNAZ	3	-0.151299958	0.116119611	-1.302966455	0.192586214	0.009815552	2.509475259	20.30206342	0.666666667	CRA004775;PRJNA827465;Yang2025_Silkie_Wencha	0.720732808
ENSGALG00010005246	ENSGALG0001000524	3	0.150192503	0.115248328	1.303207657	0.192503877	0	0.014371215	0	1	CRA004775;PRJNA827465;Yang2025_Silkie_Wencha	0.720732808
ENSGALG00010028421	ZBTB7A	3	0.141039324	0.10936135	1.289663343	0.197167572	0.018714362	4.364356143	54.1742256	1	CRA004775;PRJNA827465;Yang2025_Silkie_Wencha	0.720732808
ENSGALG00010028091	FPGS	3	-0.13861216	0.107193835	-1.293098239	0.195977084	0.014158746	3.098684779	35.45648743	1	CRA004775;PRJNA827465;Yang2025_Silkie_Wencha	0.720732808
ENSGALG00010007795	ZNF236	3	0.137879124	0.106022071	1.300475676	0.193437988	0.017730348	4.135022888	51.632674	1	CRA004775;PRJNA827465;Yang2025_Silkie_Wencha	0.720732808
ENSGALG00010005880	SLC25A4	3	-0.1377719	0.105594728	-1.304723278	0.191987092	0	0.88121244	0	0.666666667	CRA004775;PRJNA827465;Yang2025_Silkie_Wencha	0.720732808
ENSGALG00010028179	MCM2	3	-0.135264433	0.10474286	-1.291395259	0.196566654	0.009346112	2.486604244	19.56902651	1	CRA004775;PRJNA827465;Yang2025_Silkie_Wencha	0.720732808
ENSGALG00010003466	TSC22D1	3	-0.13208525	0.102014904	-1.294764247	0.195401569	0.015159815	3.993154664	49.91428662	0.666666667	CRA004775;PRJNA827465;Yang2025_Silkie_Wencha	0.720732808
ENSGALG00010000870	DACH1	3	0.131452295	0.101898885	1.290026819	0.197041346	0	1.131960013	0	1	CRA004775;PRJNA827465;Yang2025_Silkie_Wencha	0.720732808
ENSGALG00010005249	PDGFD	3	-0.12821472	0.098340177	-1.303787763	0.192305956	0	0.365786514	0	1	CRA004775;PRJNA827465;Yang2025_Silkie_Wencha	0.720732808
ENSGALG00010017761	PARP16	3	-0.123751642	0.094945362	-1.303398496	0.19243875	0.006139373	2.478224279	19.29705407	0.666666667	CRA004775;PRJNA827465;Yang2025_Silkie_Wencha	0.720732808
ENSGALG00010009653	ABLIM2	3	-0.123339957	0.094595077	-1.303872901	0.192276921	0	0.863855774	0	1	CRA004775;PRJNA827465;Yang2025_Silkie_Wencha	0.720732808
ENSGALG00010006289	SKIDA1	3	0.121697488	0.09332469	1.304022423	0.192225937	0	0.949741143	0	0.666666667	CRA004775;PRJNA827465;Yang2025_Silkie_Wencha	0.720732808
ENSGALG00010021354	ENSGALG0001002135	3	-0.121099494	0.093822056	-1.290735883	0.196795277	0.014882741	5.189458311	61.46033208	1	CRA004775;PRJNA827465;Yang2025_Silkie_Wencha	0.720732808
ENSGALG00010001520	KCNAB1	3	0.119569395	0.092276825	1.295768407	0.195055285	0	0.739930801	0	0.666666667	CRA004775;PRJNA827465;Yang2025_Silkie_Wencha	0.720732808
ENSGALG00010006761	PPP2R3C	3	-0.119354625	0.091675864	-1.301919821	0.192943796	0.00883556	2.90918664	31.25226231	1	CRA004775;PRJNA827465;Yang2025_Silkie_Wencha	0.720732808
ENSGALG00010021733	ENSGALG0001002173	3	-0.118718867	0.091979853	-1.290705127	0.196805946	0	0.743759087	0	1	CRA004775;PRJNA827465;Yang2025_Silkie_Wencha	0.720732808
ENSGALG00010001957	NDUFC2	3	-0.114046572	0.087641842	-1.301279958	0.193162645	0	0.144746332	0	1	CRA004775;PRJNA827465;Yang2025_Silkie_Wencha	0.720732808
ENSGALG00010021085	AMPD3	3	-0.113429718	0.087423596	-1.297472577	0.194468635	0	0.139345897	0	0.666666667	CRA004775;PRJNA827465;Yang2025_Silkie_Wencha	0.720732808
ENSGALG00010022830	MTG2	3	0.110341039	0.085567077	1.289526809	0.197215002	0	1.986279969	0	0.666666667	CRA004775;PRJNA827465;Yang2025_Silkie_Wencha	0.720732808
ENSGALG00010008146	SOX4	3	-0.109481905	0.084309244	-1.298575339	0.194089706	0.010101525	3.884681854	48.51573243	0.666666667	CRA004775;PRJNA827465;Yang2025_Silkie_Wencha	0.720732808
ENSGALG00010025417	B3GAT1	3	-0.101298128	0.078026393	-1.298254664	0.19419984	0	0.62260295	0	0.666666667	CRA004775;PRJNA827465;Yang2025_Silkie_Wencha	0.720732808
ENSGALG00010013258	RBIS	3	-0.100111147	0.077344071	-1.294360969	0.195540766	0	0.351727981	0	1	CRA004775;PRJNA827465;Yang2025_Silkie_Wencha	0.720732808
ENSGALG00010023268	KCNJ11	3	-0.099022295	0.076821892	-1.288985368	0.197403173	0	0.651655801	0	1	CRA004775;PRJNA827465;Yang2025_Silkie_Wencha	0.720732808
ENSGALG00010016880	AR	3	-0.097690906	0.07552817	-1.293436686	0.195860069	0	0.85060976	0	1	CRA004775;PRJNA827465;Yang2025_Silkie_Wencha	0.720732808
ENSGALG00010025048	ESYT2	3	-0.097390266	0.075555184	-1.288995155	0.197399771	0	0.07385545	0	1	CRA004775;PRJNA827465;Yang2025_Silkie_Wencha	0.720732808
ENSGALG00010030108	LLGL2	3	0.09208394	0.070730157	1.301904934	0.192948886	0	1.905394322	0	0.666666667	CRA004775;PRJNA827465;Yang2025_Silkie_Wencha	0.720732808
ENSGALG00010010125	AHCTF1	3	0.089873817	0.068974528	1.303000099	0.192574728	0.006585942	3.830585675	47.7886629	1	CRA004775;PRJNA827465;Yang2025_Silkie_Wencha	0.720732808
ENSGALG00010001710	LCMT2	3	0.088590571	0.068549025	1.292368068	0.19622971	0	0.952105945	0	0.666666667	CRA004775;PRJNA827465;Yang2025_Silkie_Wencha	0.720732808
ENSGALG00010018649	HAGH	3	0.088052586	0.067786992	1.298959921	0.193957684	0	1.968996893	0	0.666666667	CRA004775;PRJNA827465;Yang2025_Silkie_Wencha	0.720732808
ENSGALG00010020084	ENSGALG0001002008	3	0.084804678	0.065161563	1.301452488	0.193103617	0	0.53983874	0	1	CRA004775;PRJNA827465;Yang2025_Silkie_Wencha	0.720732808
ENSGALG00010014734	ZUP1	3	-0.082582916	0.063840616	-1.293579558	0.195810687	0.000224701	2.03035186	1.494906403	1	CRA004775;PRJNA827465;Yang2025_Silkie_Wencha	0.720732808
ENSGALG00010025026	DENR	3	-0.079098957	0.060750771	-1.302023918	0.192908209	0	1.157523142	0	0.666666667	CRA004775;PRJNA827465;Yang2025_Silkie_Wencha	0.720732808
ENSGALG00010005033	USP14	3	-0.078264004	0.060635931	-1.290719916	0.196800816	0	0.629313183	0	1	CRA004775;PRJNA827465;Yang2025_Silkie_Wencha	0.720732808
ENSGALG00010027114	FLNB	3	-0.077705964	0.059629633	-1.303143424	0.192525801	0	0.188848723	0	1	CRA004775;PRJNA827465;Yang2025_Silkie_Wencha	0.720732808
ENSGALG00010015877	REV1	3	0.076329419	0.058880196	1.296351303	0.194854481	0	0.954860033	0	1	CRA004775;PRJNA827465;Yang2025_Silkie_Wencha	0.720732808
ENSGALG00010019227	CLCN5	3	0.075966824	0.058778591	1.292423355	0.196210573	0	1.743913276	0	0.666666667	CRA004775;PRJNA827465;Yang2025_Silkie_Wencha	0.720732808
ENSGALG00010013558	SNAPC3	3	-0.075189516	0.058147608	-1.293080126	0.195983348	0	1.910014009	0	1	CRA004775;PRJNA827465;Yang2025_Silkie_Wencha	0.720732808
ENSGALG00010017763	POLR1A	3	-0.074889767	0.057683166	-1.298295016	0.194185978	0	0.232431897	0	1	CRA004775;PRJNA827465;Yang2025_Silkie_Wencha	0.720732808
ENSGALG00010024039	MRPL45	3	-0.072310861	0.055631897	-1.299809368	0.193666314	0	0.829626332	0	1	CRA004775;PRJNA827465;Yang2025_Silkie_Wencha	0.720732808
ENSGALG00010025910	HEATR3	3	-0.072077475	0.055883586	-1.289778983	0.197127407	0	1.842053416	0	1	CRA004775;PRJNA827465;Yang2025_Silkie_Wencha	0.720732808
ENSGALG00010029515	NPLOC4	3	-0.071804175	0.055221071	-1.300303921	0.193496825	0	0.119825086	0	1	CRA004775;PRJNA827465;Yang2025_Silkie_Wencha	0.720732808
ENSGALG00010011653	NR2C1	3	0.071153417	0.055156382	1.290030527	0.197040059	0	0.335659463	0	0.666666667	CRA004775;PRJNA827465;Yang2025_Silkie_Wencha	0.720732808
ENSGALG00010006404	SMAD1	3	-0.069952526	0.053762533	-1.301138952	0.193210897	0	0.668524457	0	0.666666667	CRA004775;PRJNA827465;Yang2025_Silkie_Wencha	0.720732808
ENSGALG00010027097	ENSGALG0001002709	3	-0.063678806	0.049229055	-1.293520775	0.195831004	0	1.425011518	0	0.666666667	CRA004775;PRJNA827465;Yang2025_Silkie_Wencha	0.720732808
ENSGALG00010020090	MORF4L2	3	-0.050897524	0.039015137	-1.304558377	0.192043269	0	0.644740831	0	0.666666667	CRA004775;PRJNA827465;Yang2025_Silkie_Wencha	0.720732808
ENSGALG00010002622	AZI2	3	0.050700753	0.039121448	1.295983544	0.194981154	0	0.444584486	0	1	CRA004775;PRJNA827465;Yang2025_Silkie_Wencha	0.720732808
ENSGALG00010008319	ERP44	3	0.040240628	0.03089425	1.302528086	0.192735923	0	0.898593968	0	0.666666667	CRA004775;PRJNA827465;Yang2025_Silkie_Wencha	0.720732808
ENSGALG00010024115	POLR2D	3	-0.175101312	0.136022622	-1.287295525	0.197991303	0.029849477	4.038534212	50.47708166	1	CRA004775;PRJNA827465;Yang2025_Silkie_Wencha	0.720751552
ENSGALG00010029353	GCGR	3	-0.582801916	0.45288718	-1.286858939	0.198143459	0.320457821	4.173807276	52.08211909	0.666666667	CRA004775;PRJNA827465;Yang2025_Silkie_Wencha	0.720753202
ENSGALG00010000407	WSB2	3	-0.160285298	0.12455147	-1.286900095	0.198129112	0.019171012	2.950637955	32.21804808	1	CRA004775;PRJNA827465;Yang2025_Silkie_Wencha	0.720753202
ENSGALG00010027714	SYPL2	3	-0.137197987	0.106614919	-1.286855424	0.198144685	0	0.735367752	0	1	CRA004775;PRJNA827465;Yang2025_Silkie_Wencha	0.720753202
ENSGALG00010018655	BIRC5	3	-0.51752434	0.402771045	-1.284909495	0.198823913	0.407622813	15.14360547	86.79310549	1	CRA004775;PRJNA827465;Yang2025_Silkie_Wencha	0.720956764
ENSGALG00010009151	CCNJL	3	0.320093687	0.249041569	1.285302243	0.198686687	0.096682677	4.330512669	53.8160917	0.666666667	CRA004775;PRJNA827465;Yang2025_Silkie_Wencha	0.720956764
ENSGALG00010013185	ENSGALG0001001318	3	-0.275986577	0.214831219	-1.284666996	0.198908676	0	0.110105875	0	1	CRA004775;PRJNA827465;Yang2025_Silkie_Wencha	0.720956764
ENSGALG00010010604	HIVEP1	3	0.25013497	0.194634573	1.285151793	0.198739246	0.080433216	7.177672288	72.1358134	0.666666667	CRA004775;PRJNA827465;Yang2025_Silkie_Wencha	0.720956764
ENSGALG00010009866	NAGA	3	-0.230567266	0.179502305	-1.284480814	0.198973772	0.059998791	5.509139033	63.69668676	1	CRA004775;PRJNA827465;Yang2025_Silkie_Wencha	0.720956764
ENSGALG00010019925	RDH11	3	-0.185651444	0.144450305	-1.28522708	0.198712943	0.028991161	3.844405384	47.97635004	0.666666667	CRA004775;PRJNA827465;Yang2025_Silkie_Wencha	0.720956764
ENSGALG00010011843	BCL2	3	-0.185029263	0.144063548	-1.284358642	0.199016497	0.036264127	5.425034415	63.13387443	0.666666667	CRA004775;PRJNA827465;Yang2025_Silkie_Wencha	0.720956764
ENSGALG00010022553	PRPF6	3	-0.146870369	0.114301117	-1.284942555	0.198812359	0.028240088	10.29638419	80.57570537	1	CRA004775;PRJNA827465;Yang2025_Silkie_Wencha	0.720956764
ENSGALG00010027538	ABHD14A	3	0.131212175	0.102038755	1.285905289	0.198476118	0	1.43645161	0	0.666666667	CRA004775;PRJNA827465;Yang2025_Silkie_Wencha	0.720956764
ENSGALG00010020646	CEP70	3	-0.102369778	0.07962322	-1.285677453	0.198555653	0	1.573332324	0	0.666666667	CRA004775;PRJNA827465;Yang2025_Silkie_Wencha	0.720956764
ENSGALG00010008708	ZC3H4	3	-0.09908632	0.077134991	-1.284583289	0.198937941	0	1.605395944	0	0.666666667	CRA004775;PRJNA827465;Yang2025_Silkie_Wencha	0.720956764
ENSGALG00010018559	DLG3	3	0.098752737	0.076888407	1.284364456	0.199014463	0.003321309	2.371437332	15.66296218	0.666666667	CRA004775;PRJNA827465;Yang2025_Silkie_Wencha	0.720956764
ENSGALG00010017865	UBOX5	3	-0.088672129	0.06901969	-1.284736699	0.198884309	0.001037617	2.108241318	5.134199652	1	CRA004775;PRJNA827465;Yang2025_Silkie_Wencha	0.720956764
ENSGALG00010024238	ATF7	3	-0.086460446	0.067304789	-1.284610605	0.198928391	0	1.195699823	0	0.666666667	CRA004775;PRJNA827465;Yang2025_Silkie_Wencha	0.720956764
ENSGALG00010009180	NFKBIB	3	-0.075203471	0.058538105	-1.284692608	0.198899722	0	0.783192604	0	1	CRA004775;PRJNA827465;Yang2025_Silkie_Wencha	0.720956764
ENSGALG00010009394	BANP	3	0.067642736	0.052627573	1.285309828	0.198684037	0	0.491045491	0	0.666666667	CRA004775;PRJNA827465;Yang2025_Silkie_Wencha	0.720956764
ENSGALG00010010336	FMNL2	3	0.054743723	0.042628452	1.284206213	0.199069812	0	0.044458183	0	1	CRA004775;PRJNA827465;Yang2025_Silkie_Wencha	0.720965182
ENSGALG00010018317	ZMAT3	3	-0.124945122	0.097319221	-1.283868908	0.199187828	0	0.029056301	0	1	CRA004775;PRJNA827465;Yang2025_Silkie_Wencha	0.721207861
ENSGALG00010025114	CEBPG	3	-0.17099996	0.133222123	-1.283570301	0.199292347	0.038869607	12.08998224	83.45737851	0.666666667	CRA004775;PRJNA827465;Yang2025_Silkie_Wencha	0.721401559
ENSGALG00010008198	GLRB	3	0.840067294	0.655139386	1.282272616	0.199747031	0.933560706	9.2230572	78.31521635	0.666666667	CRA004775;PRJNA827465;Yang2025_Silkie_Wencha	0.721708267
ENSGALG00010009740	LRRC73	3	-0.511658778	0.398947201	-1.282522542	0.199659402	0.383105073	11.40522022	82.46417025	0.666666667	CRA004775;PRJNA827465;Yang2025_Silkie_Wencha	0.721708267
ENSGALG00010027314	ENSGALG0001002731	3	0.373089976	0.2910168	1.282022121	0.199834886	0.159288777	5.860150312	65.87118259	0.666666667	CRA004775;PRJNA827465;Yang2025_Silkie_Wencha	0.721708267
ENSGALG00010017987	ERI2	3	0.276565015	0.21579984	1.281581189	0.199989602	0.072033151	4.343775821	53.95710823	0.666666667	CRA004775;PRJNA827465;Yang2025_Silkie_Wencha	0.721708267
ENSGALG00010015306	BCAT1	3	-0.260818922	0.203259651	-1.283180997	0.199428672	0.092984898	11.01259442	81.83897523	0.666666667	CRA004775;PRJNA827465;Yang2025_Silkie_Wencha	0.721708267
ENSGALG00010024915	STXBP5L	3	-0.244898197	0.191015735	-1.282083895	0.199813218	0.035802308	2.855640446	29.96317157	0.666666667	CRA004775;PRJNA827465;Yang2025_Silkie_Wencha	0.721708267
ENSGALG00010016884	SBK1	3	0.226813916	0.176960758	1.281718715	0.199941337	0.052896533	4.800926775	58.34137671	1	CRA004775;PRJNA827465;Yang2025_Silkie_Wencha	0.721708267
ENSGALG00010024732	AHCY	3	-0.180811369	0.140997058	-1.282376887	0.199710468	0.030918835	4.165199155	51.98308831	0.666666667	CRA004775;PRJNA827465;Yang2025_Silkie_Wencha	0.721708267
ENSGALG00010013144	TEDC1	3	-0.170473264	0.132952184	-1.282214842	0.199767291	0.015263908	2.534152511	21.07815172	1	CRA004775;PRJNA827465;Yang2025_Silkie_Wencha	0.721708267
ENSGALG00010004238	CCNC	3	-0.16952358	0.132273444	-1.281614625	0.199977867	0.033546456	7.06396644	71.68729471	1	CRA004775;PRJNA827465;Yang2025_Silkie_Wencha	0.721708267
ENSGALG00010013405	RPS14	3	-0.130320554	0.101673238	-1.281758661	0.19992732	0.009604573	2.443651688	18.15527516	1	CRA004775;PRJNA827465;Yang2025_Silkie_Wencha	0.721708267
ENSGALG00010022397	ATF1	3	-0.045207494	0.035243365	-1.282723537	0.199588951	0	0.601691722	0	0.666666667	CRA004775;PRJNA827465;Yang2025_Silkie_Wencha	0.721708267
ENSGALG00010008184	SLC66A2	3	-0.288859464	0.225604268	-1.280381204	0.200411101	0.127386096	17.97293304	88.87215573	1	CRA004775;PRJNA827465;Yang2025_Silkie_Wencha	0.722123486
ENSGALG00010004921	RRAGD	3	-0.178448494	0.139371327	-1.28038168	0.200410934	0.036936722	6.220429805	67.84788089	0.666666667	CRA004775;PRJNA827465;Yang2025_Silkie_Wencha	0.722123486
ENSGALG00010018036	RPL4	3	-0.164822378	0.128718396	-1.280488125	0.200373518	0.034765772	7.038004487	71.58285415	0.666666667	CRA004775;PRJNA827465;Yang2025_Silkie_Wencha	0.722123486
ENSGALG00010012007	MREG	3	-0.159287537	0.124376911	-1.280684139	0.200304633	0	0.355943943	0	1	CRA004775;PRJNA827465;Yang2025_Silkie_Wencha	0.722123486
ENSGALG00010016066	C14orf2	3	-0.152122172	0.118759078	-1.280930887	0.200217943	0.017785699	3.202145671	37.54187956	0.666666667	CRA004775;PRJNA827465;Yang2025_Silkie_Wencha	0.722123486
ENSGALG00010013995	REM1	3	-0.129501073	0.101128572	-1.280558698	0.200348715	0	1.123965171	0	0.666666667	CRA004775;PRJNA827465;Yang2025_Silkie_Wencha	0.722123486
ENSGALG00010001779	ENSGALG0001000177	2	1.863768719	1.458342223	1.278005046	0.201247647	3.942310256	13.66724637	92.68323719	1	PRJNA827465;Yang2025_Silkie_Wenchang	0.722423145
ENSGALG00010029558	ENSGALG0001002955	3	0.828782841	0.649528348	1.275976398	0.201963862	1.054052648	12.66472792	84.20810922	0.666666667	CRA004775;PRJNA827465;Yang2025_Silkie_Wencha	0.722423145
ENSGALG00010007574	ENSGALG0001000757	3	0.563357925	0.442186702	1.274027289	0.202653745	0.471945707	12.2287589	83.64511055	1	CRA004775;PRJNA827465;Yang2025_Silkie_Wencha	0.722423145
ENSGALG00010016429	ENSGALG0001001642	3	0.530236941	0.414699278	1.278605895	0.201035873	0.315890479	5.175646459	61.35748421	0.666666667	CRA004775;PRJNA827465;Yang2025_Silkie_Wencha	0.722423145
ENSGALG00010001721	TM4SF1	3	-0.470970386	0.368410501	-1.278384805	0.201113779	0.348343225	17.5272379	88.58918894	1	CRA004775;PRJNA827465;Yang2025_Silkie_Wencha	0.722423145
ENSGALG00010023174	KCNQ2	2	0.396465413	0.310102195	1.278499215	0.201073461	0.129973542	2.96265894	66.24653663	1	PRJNA827465;Yang2025_Silkie_Wenchang	0.722423145
ENSGALG00010012331	ENSGALG0001001233	3	-0.392723457	0.307848538	-1.275703498	0.202060352	0	0.249877623	0	1	CRA004775;PRJNA827465;Yang2025_Silkie_Wencha	0.722423145
ENSGALG00010026329	ENSGALG0001002632	3	-0.354566798	0.277453739	-1.277931232	0.201273675	0.130196209	4.928957805	59.42347086	0.666666667	CRA004775;PRJNA827465;Yang2025_Silkie_Wencha	0.722423145
ENSGALG00010018276	ARHGAP17	3	0.34009525	0.265957555	1.278757619	0.200982422	0.174057356	12.70643698	84.25994633	0.666666667	CRA004775;PRJNA827465;Yang2025_Silkie_Wencha	0.722423145
ENSGALG00010026740	DAB1	3	0.32859332	0.2572383	1.27738879	0.201465021	0.140007879	8.90095016	77.53048872	0.666666667	CRA004775;PRJNA827465;Yang2025_Silkie_Wencha	0.722423145
ENSGALG00010027957	ENTREP3	3	0.315025556	0.246740621	1.276747842	0.201691285	0.057347634	2.879880345	30.55267023	0.666666667	CRA004775;PRJNA827465;Yang2025_Silkie_Wencha	0.722423145
ENSGALG00010009482	ARID4B	3	0.308568683	0.242246998	1.273777117	0.202742417	0.143167089	13.38234988	85.05494164	0.666666667	CRA004775;PRJNA827465;Yang2025_Silkie_Wencha	0.722423145
ENSGALG00010027175	NOS3	3	0.285538631	0.223510495	1.27751778	0.201419507	0.111180911	12.17647072	83.57487941	1	CRA004775;PRJNA827465;Yang2025_Silkie_Wencha	0.722423145
ENSGALG00010007940	GLIPR2	3	-0.283077073	0.221898861	-1.275703138	0.202060479	0.104770749	10.27020533	80.52619266	0.666666667	CRA004775;PRJNA827465;Yang2025_Silkie_Wencha	0.722423145
ENSGALG00010002275	IL1RAPL1	2	0.265265265	0.208009679	1.275254433	0.2022192	0	0.992116144	0	1	PRJNA827465;Yang2025_Silkie_Wenchang	0.722423145
ENSGALG00010028471	TPGS1	3	-0.255201375	0.199537752	-1.278962862	0.200910134	0.07627033	5.533097154	63.85387886	1	CRA004775;PRJNA827465;Yang2025_Silkie_Wencha	0.722423145
ENSGALG00010023918	MFSD14A	3	-0.249186014	0.194700383	-1.279843469	0.200600193	0.088434519	11.66670086	82.8571931	1	CRA004775;PRJNA827465;Yang2025_Silkie_Wencha	0.722423145
ENSGALG00010017395	BRF2	3	-0.244173063	0.191366517	-1.275944543	0.201975124	0.081501189	8.138020327	75.42399847	0.666666667	CRA004775;PRJNA827465;Yang2025_Silkie_Wencha	0.722423145
ENSGALG00010009644	SLC27A6	3	-0.226027538	0.176856703	-1.278026411	0.201240114	0	1.037053497	0	1	CRA004775;PRJNA827465;Yang2025_Silkie_Wencha	0.722423145
ENSGALG00010021786	FAM162A	3	0.224076935	0.17578034	1.274755388	0.202395835	0.040725041	3.411925766	41.38207754	1	CRA004775;PRJNA827465;Yang2025_Silkie_Wencha	0.722423145
ENSGALG00010013203	AMACR	3	-0.218522244	0.171289678	-1.275746712	0.20204507	0.042843296	3.852096691	48.0802233	0.666666667	CRA004775;PRJNA827465;Yang2025_Silkie_Wencha	0.722423145
ENSGALG00010005817	VTA1	3	-0.204367325	0.160356635	-1.274455065	0.202502188	0.064669521	21.64850998	90.76148889	1	CRA004775;PRJNA827465;Yang2025_Silkie_Wencha	0.722423145
ENSGALG00010021839	C1orf50	3	-0.193016158	0.150985165	-1.27837829	0.201116075	0.043807433	5.665524421	64.69876658	1	CRA004775;PRJNA827465;Yang2025_Silkie_Wencha	0.722423145
ENSGALG00010020540	PCGF5	3	0.183586543	0.144071996	1.274269453	0.202567938	0.028216061	3.647072401	45.16149449	0.666666667	CRA004775;PRJNA827465;Yang2025_Silkie_Wencha	0.722423145
ENSGALG00010007963	LDAH	3	-0.178722393	0.140250902	-1.274304765	0.202555429	0.039975576	10.69844804	81.30569974	1	CRA004775;PRJNA827465;Yang2025_Silkie_Wencha	0.722423145
ENSGALG00010020987	NADSYN1	3	-0.176316718	0.137996261	-1.277691994	0.201358049	0.041264362	7.549604397	73.50854568	0.666666667	CRA004775;PRJNA827465;Yang2025_Silkie_Wencha	0.722423145
ENSGALG00010024785	MCEE	3	-0.173085735	0.135511281	-1.277279163	0.201503707	0.029347767	4.578738995	56.31985133	1	CRA004775;PRJNA827465;Yang2025_Silkie_Wencha	0.722423145
ENSGALG00010023225	PDPR	3	-0.152389036	0.119624431	-1.27389559	0.202700421	0.024170292	4.78961965	58.24303085	1	CRA004775;PRJNA827465;Yang2025_Silkie_Wencha	0.722423145
ENSGALG00010006346	NSUN3	3	-0.149486069	0.116957905	-1.278118567	0.201207623	0	0.489856233	0	1	CRA004775;PRJNA827465;Yang2025_Silkie_Wencha	0.722423145
ENSGALG00010017715	CILP	3	-0.147303749	0.115421399	-1.276225642	0.201875767	0	0.515886142	0	1	CRA004775;PRJNA827465;Yang2025_Silkie_Wencha	0.722423145
ENSGALG00010016015	ZDHHC3	3	-0.14229893	0.111301348	-1.278501398	0.201072692	0.027411821	10.45519173	80.87074775	1	CRA004775;PRJNA827465;Yang2025_Silkie_Wencha	0.722423145
ENSGALG00010015992	ATP10A	3	0.142211144	0.111447974	1.27603167	0.201944324	0	1.057872692	0	1	CRA004775;PRJNA827465;Yang2025_Silkie_Wencha	0.722423145
ENSGALG00010016865	CFAP36	3	0.139898635	0.109778066	1.274376933	0.202529863	0.01869589	3.904114186	48.77199014	1	CRA004775;PRJNA827465;Yang2025_Silkie_Wencha	0.722423145
ENSGALG00010000121	ENSGALG0001000012	3	-0.119684554	0.093871455	-1.274983476	0.202315091	0	0.924922155	0	0.666666667	CRA004775;PRJNA827465;Yang2025_Silkie_Wencha	0.722423145
ENSGALG00010028901	PAPPA	3	-0.117747517	0.092367971	-1.27476565	0.202392202	0	0.155377719	0	1	CRA004775;PRJNA827465;Yang2025_Silkie_Wencha	0.722423145
ENSGALG00010014243	ENSGALG0001001424	3	0.112774178	0.088123886	1.279723161	0.200642516	0	0.547033308	0	0.666666667	CRA004775;PRJNA827465;Yang2025_Silkie_Wencha	0.722423145
ENSGALG00010007952	EMC2	3	0.101189807	0.07924806	1.276874254	0.201646645	0.007297848	3.043849169	34.2937219	1	CRA004775;PRJNA827465;Yang2025_Silkie_Wencha	0.722423145
ENSGALG00010015127	RPL11	3	-0.090731887	0.071140253	-1.27539449	0.202169648	0.001498221	2.051181506	2.495220733	1	CRA004775;PRJNA827465;Yang2025_Silkie_Wencha	0.722423145
ENSGALG00010025291	DUS2	3	0.078780488	0.061570882	1.279508835	0.200717931	0	1.859340678	0	0.666666667	CRA004775;PRJNA827465;Yang2025_Silkie_Wencha	0.722423145
ENSGALG00010017556	TOM1L2	3	-0.062598013	0.049094631	-1.275048045	0.202292237	0	1.93193406	0	1	CRA004775;PRJNA827465;Yang2025_Silkie_Wencha	0.722423145
ENSGALG00010019130	RPS6KC1	3	-0.04954093	0.038814538	-1.276349842	0.201831879	0	0.414260734	0	1	CRA004775;PRJNA827465;Yang2025_Silkie_Wencha	0.722423145
ENSGALG00010016667	WARS1	3	-0.049125341	0.038565215	-1.273825153	0.202725389	0	0.932360677	0	1	CRA004775;PRJNA827465;Yang2025_Silkie_Wencha	0.722423145
ENSGALG00010008032	PUM2	3	0.043430645	0.033959947	1.278878472	0.200939855	0	0.236767503	0	0.666666667	CRA004775;PRJNA827465;Yang2025_Silkie_Wencha	0.722423145
ENSGALG00010014198	KDM3B	3	-0.040242893	0.031566401	-1.274864797	0.202357101	0	1.992700503	0	1	CRA004775;PRJNA827465;Yang2025_Silkie_Wencha	0.722423145
ENSGALG00010005379	DZIP1	3	0.191889034	0.150677068	1.273511863	0.202836466	0.039200462	5.194789865	61.49988639	0.666666667	CRA004775;PRJNA827465;Yang2025_Silkie_Wencha	0.722440651
ENSGALG00010024716	GTF2A2	3	-0.067965132	0.053369819	-1.273475047	0.202849522	0	0.661309771	0	1	CRA004775;PRJNA827465;Yang2025_Silkie_Wencha	0.722440651
ENSGALG00010012519	ENSGALG0001001251	3	-3.343665867	2.6309674	-1.270888369	0.203768365	18.46025574	20.93582433	90.44699665	1	CRA004775;PRJNA827465;Yang2025_Silkie_Wencha	0.722505555
ENSGALG00010010996	RFX4	2	0.860201354	0.675887742	1.272698556	0.20312503	0.770734848	6.311244485	84.15526443	1	PRJNA827465;Yang2025_Silkie_Wenchang	0.722505555
ENSGALG00010024213	ENSGALG0001002421	3	0.530271283	0.417051758	1.271475955	0.203559376	0.448672497	19.35878113	89.66877105	0.666666667	CRA004775;PRJNA827465;Yang2025_Silkie_Wencha	0.722505555
ENSGALG00010023876	POU6F1	3	0.450882948	0.354783754	1.270866952	0.203775985	0.201485546	4.298300096	53.46997754	1	CRA004775;PRJNA827465;Yang2025_Silkie_Wencha	0.722505555
ENSGALG00010015025	TRAF3IP1	3	0.353646303	0.278179458	1.271288345	0.203626087	0.17968048	9.829877069	79.6538656	1	CRA004775;PRJNA827465;Yang2025_Silkie_Wencha	0.722505555
ENSGALG00010013032	BAIAP2L2	3	0.344036391	0.270358152	1.272520872	0.203188113	0	1.687763785	0	0.666666667	CRA004775;PRJNA827465;Yang2025_Silkie_Wencha	0.722505555
ENSGALG00010024486	GPR37L1	3	0.343881583	0.270133344	1.273006799	0.203015629	0.136440813	5.72280946	65.05213018	1	CRA004775;PRJNA827465;Yang2025_Silkie_Wencha	0.722505555
ENSGALG00010002375	NPY	3	0.34292108	0.269886484	1.27061228	0.203866616	0	1.258764032	0	0.666666667	CRA004775;PRJNA827465;Yang2025_Silkie_Wencha	0.722505555
ENSGALG00010018627	MYLK	3	0.297627689	0.234000833	1.271908675	0.203405569	0.125114437	8.72516752	77.077804	0.666666667	CRA004775;PRJNA827465;Yang2025_Silkie_Wencha	0.722505555
ENSGALG00010003843	LRCH1	3	0.282245712	0.222203362	1.270213506	0.204008588	0.103295664	7.087828605	71.78261339	1	CRA004775;PRJNA827465;Yang2025_Silkie_Wencha	0.722505555

ENSGALG00010000823	PIBF1	3	-0.272032606	0.213841469	-1.272122788	0.203329496	0.109819597	12.90983858	84.50793953	0.666666667	CRA004775;PRJNA827465;Yang2025_Silkie_Wencha	0.722505555
ENSGALG00010029232	CA10	2	0.268511104	0.211146515	1.271681435	0.203486329	0	0.584274645	0	1	PRJNA827465;Yang2025_Silkie_Wenchang	0.722505555
ENSGALG00010023773	OVOA	3	0.267399706	0.210474348	1.270462215	0.203920034	0	1.143652681	0	1	CRA004775;PRJNA827465;Yang2025_Silkie_Wencha	0.722505555
ENSGALG00010009457	BDP1	3	0.262928799	0.206665278	1.272244675	0.203286199	0.095479311	9.65554501	79.28651363	0.666666667	CRA004775;PRJNA827465;Yang2025_Silkie_Wencha	0.722505555
ENSGALG00010003700	REST	3	0.243117085	0.191456429	1.269829837	0.20414525	0.074699625	7.085491598	71.77330645	1	CRA004775;PRJNA827465;Yang2025_Silkie_Wencha	0.722505555
ENSGALG00010024700	TOLLIP	3	-0.22631199	0.178175368	-1.270164293	0.204026114	0.074984644	20.85755884	90.41115015	1	CRA004775;PRJNA827465;Yang2025_Silkie_Wencha	0.722505555
ENSGALG00010012997	UBE2A	3	-0.206528201	0.162271435	-1.272732946	0.203112822	0.066650644	15.53468068	87.12558023	1	CRA004775;PRJNA827465;Yang2025_Silkie_Wencha	0.722505555
ENSGALG00010003251	ELOF1	3	0.200901707	0.158011229	1.271439431	0.203572362	0.037947118	4.048988368	50.60494578	0.666666667	CRA004775;PRJNA827465;Yang2025_Silkie_Wencha	0.722505555
ENSGALG00010024715	DNAAF1	3	0.185313549	0.145927527	1.269901253	0.204119807	0.031189329	4.249127293	52.93151129	0.666666667	CRA004775;PRJNA827465;Yang2025_Silkie_Wencha	0.722505555
ENSGALG00010007066	INIP	3	-0.153789673	0.121041345	-1.270554886	0.203887045	0	0.700292609	0	1	CRA004775;PRJNA827465;Yang2025_Silkie_Wencha	0.722505555
ENSGALG00010024334	USP28	3	-0.124547089	0.09785539	-1.272766779	0.20310813	0.011605597	3.348598318	40.27351715	0.666666667	CRA004775;PRJNA827465;Yang2025_Silkie_Wencha	0.722505555
ENSGALG00010015409	FAM13B	3	0.107646556	0.08457279	1.272827304	0.20307933	0.007354847	2.969093438	32.63937151	0.666666667	CRA004775;PRJNA827465;Yang2025_Silkie_Wencha	0.722505555
ENSGALG00010010646	MCM3AP	3	-0.097787461	0.07685246	-1.272408624	0.203227971	0.007746136	3.268345122	38.80695198	1	CRA004775;PRJNA827465;Yang2025_Silkie_Wencha	0.722505555
ENSGALG00010026321	GPBP1L1	3	-0.09415021	0.074134073	-1.269999148	0.204084934	0.010042907	6.003720807	66.68732501	0.666666667	CRA004775;PRJNA827465;Yang2025_Silkie_Wencha	0.722505555
ENSGALG00010026003	NECTIN1	3	0.093702151	0.073732839	1.270833343	0.203787944	0.001249337	2.126856613	5.964311782	0.666666667	CRA004775;PRJNA827465;Yang2025_Silkie_Wencha	0.722505555
ENSGALG00010016895	ENSGALG0001001689	3	0.306369381	0.241339561	1.269453626	0.20427932	0.14110917	11.05725836	81.91233365	0.666666667	CRA004775;PRJNA827465;Yang2025_Silkie_Wencha	0.722569455
ENSGALG00010017266	PMM2	3	-0.272008514	0.214280042	-1.269406667	0.20429606	0.108958173	13.71184556	85.41407142	1	CRA004775;PRJNA827465;Yang2025_Silkie_Wencha	0.722569455
ENSGALG00010019587	CCNT2	3	0.18602362	0.146550413	1.269348994	0.204316619	0.034889755	4.493793222	55.49416937	0.666666667	CRA004775;PRJNA827465;Yang2025_Silkie_Wencha	0.722569455
ENSGALG00010004373	MMS22L	3	-0.180810648	0.142490144	-1.268914417	0.204464456	0.026318067	3.673370563	45.55409083	1	CRA004775;PRJNA827465;Yang2025_Silkie_Wencha	0.722761852
ENSGALG00010010970	TPM2	3	-0.123268442	0.097145162	-1.268909738	0.204473259	0	0.618931628	0	1	CRA004775;PRJNA827465;Yang2025_Silkie_Wencha	0.722761852
ENSGALG00010012561	OSGIN2	3	0.091515147	0.072135506	1.268656058	0.204563761	0	1.848046481	0	0.666666667	CRA004775;PRJNA827465;Yang2025_Silkie_Wencha	0.72290103
ENSGALG00010024498	CCDC102A	3	0.227457599	0.179407197	1.267828732	0.204859119	0.078055884	17.62433393	88.65205342	0.666666667	CRA004775;PRJNA827465;Yang2025_Silkie_Wencha	0.723013703
ENSGALG00010007041	NAE1	3	-0.207646977	0.163786233	-1.267792621	0.204872018	0.061035034	10.00906208	80.01810774	1	CRA004775;PRJNA827465;Yang2025_Silkie_Wencha	0.723013703
ENSGALG00010020436	CNTN4	3	-0.13642	0.10759584	-1.267892882	0.204836206	0	1.240682902	0	0.666666667	CRA004775;PRJNA827465;Yang2025_Silkie_Wencha	0.723013703
ENSGALG00010018206	KIZ	3	0.136246433	0.107450797	1.267989041	0.204801864	0.007684994	2.467441548	18.94438181	1	CRA004775;PRJNA827465;Yang2025_Silkie_Wencha	0.723013703
ENSGALG00010011449	DUSP22	3	-0.082044312	0.06469501	-1.268170636	0.204737021	0	1.903556576	0	1	CRA004775;PRJNA827465;Yang2025_Silkie_Wencha	0.723013703
ENSGALG00010011913	CXXC5	3	0.068342796	0.053910544	1.267707398	0.204902462	0	1.894821627	0	0.666666667	CRA004775;PRJNA827465;Yang2025_Silkie_Wencha	0.723013703
ENSGALG00010027999	ENSGALG0001002799	3	-0.425402816	0.335721066	-1.267131733	0.205108191	0.134097836	3.28485808	39.11456899	0.666666667	CRA004775;PRJNA827465;Yang2025_Silkie_Wencha	0.7231575
ENSGALG00010024772	ELAPOR1	3	0.266945736	0.210648477	1.267256901	0.205063446	0.095343801	7.987119017	74.95968201	0.666666667	CRA004775;PRJNA827465;Yang2025_Silkie_Wencha	0.7231575
ENSGALG00010000150	B3GAT3	3	0.128323581	0.101259317	1.26727678	0.20505634	0	0.397009175	0	0.666666667	CRA004775;PRJNA827465;Yang2025_Silkie_Wencha	0.7231575
ENSGALG00010007910	ANKS6	3	0.127942889	0.100976661	1.267054074	0.205135956	0.015679414	4.330258543	53.81338134	0.666666667	CRA004775;PRJNA827465;Yang2025_Silkie_Wencha	0.7231575
ENSGALG00010024051	ENSGALG0001002405	3	0.879314663	0.69616399	1.26308553	0.206558444	1.030160441	6.863623464	70.86087239	1	CRA004775;PRJNA827465;Yang2025_Silkie_Wencha	0.72361988
ENSGALG00010005391	ADGB	2	0.71686096	0.566481844	1.265461493	0.205705944	0.437642157	2.958637159	66.20065435	1	PRJNA827465;Yang2025_Silkie_Wenchang	0.72361988
ENSGALG00010012780	FABP7	3	0.664238573	0.525664658	1.263616572	0.206367683	0.698432046	14.03013053	85.74496512	0.666666667	CRA004775;PRJNA827465;Yang2025_Silkie_Wencha	0.72361988
ENSGALG00010000230	ENSGALG0001000023	2	0.491015682	0.388586833	1.263593206	0.206376073	0	0.38078253	0	1	PRJNA827465;Yang2025_Silkie_Wenchang	0.72361988
ENSGALG00010015123	CEP85L	3	0.489318343	0.387251	1.264401252	0.206086044	0.358954338	13.76882661	85.47443397	0.666666667	CRA004775;PRJNA827465;Yang2025_Silkie_Wencha	0.72361988
ENSGALG00010022957	SLTM	3	0.413935159	0.327435182	1.264174352	0.206167455	0.276980991	16.84417394	88.1264584	0.666666667	CRA004775;PRJNA827465;Yang2025_Silkie_Wencha	0.72361988
ENSGALG00010028090	ENSGALG0001002809	3	0.36926272	0.291632119	1.266193593	0.205443781	0.166974197	6.270174663	68.10296192	1	CRA004775;PRJNA827465;Yang2025_Silkie_Wencha	0.72361988
ENSGALG00010029746	TRPV1	2	0.315917749	0.249471993	1.26634555	0.205389396	0.045115623	1.564614584	36.08649632	1	PRJNA827465;Yang2025_Silkie_Wenchang	0.72361988
ENSGALG00010003746	HOPX	3	-0.303827573	0.24012081	-1.265311297	0.205759759	0.079479047	3.695279409	45.87689377	0.666666667	CRA004775;PRJNA827465;Yang2025_Silkie_Wencha	0.72361988
ENSGALG00010028857	MED22	3	-0.272558738	0.215608512	-1.264137191	0.20618079	0.119839073	15.65381795	87.20885593	1	CRA004775;PRJNA827465;Yang2025_Silkie_Wencha	0.72361988
ENSGALG00010029365	PIK3R5	3	-0.249895581	0.197523437	-1.26514395	0.205819731	0.081511878	8.905814541	77.54276163	0.666666667	CRA004775;PRJNA827465;Yang2025_Silkie_Wencha	0.72361988
ENSGALG00010008022	SLC35F3	3	-0.246503597	0.194980853	-1.264503597	0.206069331	0	1.405609814	0	1	CRA004775;PRJNA827465;Yang2025_Silkie_Wencha	0.72361988
ENSGALG00010009635	TDRD7	3	0.209422576	0.165624735	1.264440217	0.206072065	0.054542364	6.251818622	68.00930864	0.666666667	CRA004775;PRJNA827465;Yang2025_Silkie_Wencha	0.72361988
ENSGALG00010027190	IPPK	3	0.173961322	0.137480105	1.265356342	0.205743618	0.044625326	11.1429647	82.0514553	0.666666667	CRA004775;PRJNA827465;Yang2025_Silkie_Wencha	0.72361988
ENSGALG00010027991	ENSGALG0001002799	3	-0.16677917	0.132018283	-1.263303583	0.206480099	0	0.914350283	0	0.666666667	CRA004775;PRJNA827465;Yang2025_Silkie_Wencha	0.72361988
ENSGALG00010007187	ENPP6	3	-0.152871532	0.121015563	-1.263284886	0.206486816	0.019252148	3.493265863	42.74698582	0.666666667	CRA004775;PRJNA827465;Yang2025_Silkie_Wencha	0.72361988
ENSGALG00010003272	CPSF3	3	0.146302288	0.11574978	1.263953056	0.206246877	0.03089514	10.77941833	81.44612318	0.666666667	CRA004775;PRJNA827465;Yang2025_Silkie_Wencha	0.72361988
ENSGALG00010006288	ARF5	3	0.13837212	0.109380786	1.265049606	0.205853546	0.005152417	2.151058728	7.022529244	0.666666667	CRA004775;PRJNA827465;Yang2025_Silkie_Wencha	0.72361988
ENSGALG00010002400	RBBP7	3	-0.091758068	0.072480302	-1.265972483	0.205522934	0	0.192893374	0	1	CRA004775;PRJNA827465;Yang2025_Silkie_Wencha	0.72361988
ENSGALG00010024515	C3orf70	3	-0.079260985	0.062746318	-1.263193374	0.206518256	0	0.967977494	0	0.666666667	CRA004775;PRJNA827465;Yang2025_Silkie_Wencha	0.72361988
ENSGALG00010021874	TMCC1	3	-0.072087419	0.056979949	-1.265136591	0.205822368	0	0.949314619	0	1	CRA004775;PRJNA827465;Yang2025_Silkie_Wencha	0.72361988
ENSGALG00010019268	NSL1	3	-0.0668249	0.052900632	-1.26321554	0.20651173	0	0.689193478	0	1	CRA004775;PRJNA827465;Yang2025_Silkie_Wencha	0.72361988
ENSGALG00010012586	CDC73	3	-0.062989442	0.049795545	-1.264961393	0.205885168	0	0.264102101	0	0.666666667	CRA004775;PRJNA827465;Yang2025_Silkie_Wencha	0.72361988
ENSGALG00010013089	TBK1	3	0.057163518	0.04517146	1.265487429	0.205696652	0	0.078911728	0	1	CRA004775;PRJNA827465;Yang2025_Silkie_Wencha	0.72361988
ENSGALG00010029227	UTP18	3	0.04947831	0.039156613	1.263600342	0.206373511	0	1.70601935	0	0.666666667	CRA004775;PRJNA827465;Yang2025_Silkie_Wencha	0.72361988
ENSGALG00010002625	FHL5	3	-0.25385298	0.201019085	-1.262830238	0.206650195	0.095338187	15.78620232	87.33070843	1	CRA004775;PRJNA827465;Yang2025_Silkie_Wencha	0.723761979
ENSGALG00010020638	IMPDH2	3	-0.089145232	0.070600785	-1.262666299	0.20670913	0	0.836546955	0	1	CRA004775;PRJNA827465;Yang2025_Silkie_Wencha	0.723789102
ENSGALG00010015670	ENSGALG0001001567	3	0.380445702	0.301411866	1.2623448	0.206824743	0.236898205	18.1446754	88.97748262	0.666666667	CRA004775;PRJNA827465;Yang2025_Silkie_Wencha	0.723991143
ENSGALG00010022186	DNAJB4	3	0.227095375	0.179937562	1.262078759	0.206920448	0.067394556	12.90564702	84.50290793	0.666666667	CRA004775;PRJNA827465;Yang2025_Silkie_Wencha	0.723991143
ENSGALG00010019602	SLC29A1	3	-0.083200337	0.065918315	-1.262173286	0.206886439	0	0.87445638	0	1	CRA004775;PRJNA827465;Yang2025_Silkie_Wencha	0.723991143
ENSGALG00010011964	ENSGALG0001001196	3	-3.329625763	2.643848144	-1.25938616	0.207890886	16.90467882	11.88270589	83.16881678	1	CRA004775;PRJNA827465;Yang2025_Silkie_Wencha	0.724136029
ENSGALG00010019275	CTAGE1	3	0.852162333	0.676485367	1.25969074	0.207780947	1.00105016	8.433463423	76.25829663	1	CRA004775;PRJNA827465;Yang2025_Silkie_Wencha	0.724136029
ENSGALG00010018585	GDPD2	3	0.726697923	0.577785919	1.257728683	0.208489895	0.738880142	7.871609046	74.59223409	1	CRA004775;PRJNA827465;Yang2025_Silkie_Wencha	0.724136029
ENSGALG00010001149	CIDEA	3	-0.566239362	0.450104697	-1.258017002	0.208385607	0.32098356	4.253550621	52.98045849	0.666666667	CRA004775;PRJNA827465;Yang2025_Silkie_Wencha	0.724136029
ENSGALG00010002414	ENSGALG0001000241	3	0.512208679	0.40646581	1.26015194	0.207614556	0.234660439	3.800592501	47.37662616	0.666666667	CRA004775;PRJNA827465;Yang2025_Silkie_Wencha	0.724136029
ENSGALG00010021797	SLC25A34	3	0.508855747	0.405086121	1.256166828	0.209054491	0.401184675	12.35379448	83.81064212	1	CRA004775;PRJNA827465;Yang2025_Silkie_Wencha	0.724136029
ENSGALG00010018590	TGFA	3	0.431627094	0.343013307	1.258339211	0.208269106	0.231068314	5.805963374	65.55265903	0.666666667	CRA004775;PRJNA827465;Yang2025_Silkie_Wencha	0.724136029
ENSGALG00010025557	VPS29	3	-0.429724517	0.341087207	-1.259867002	0.207717344	0.326966808	38.35323402	94.78531589	1	CRA004775;PRJNA827465;Yang2025_Silkie_Wencha	0.724136029
ENSGALG00010015989	ENAH	3	0.415785174	0.330145422	1.259390621	0.207885858	0.285250349	19.78341896	89.89052396	0.666666667	CRA004775;PRJNA827465;Yang2025_Silkie_Wencha	0.724136029
ENSGALG00010017730	TARP	3	-0.380568434	0.302174531	-1.259366868	0.207897851	0	1.402633325	0	0.666666667	CRA004775;PRJNA827465;Yang2025_Silkie_Wencha	0.724136029
ENSGALG00010002434	GPR34	3	-0.2699904	0.21428141	-1.259980509	0.207676394	0.09091671	7.113151037	71.88306575	1	CRA004775;PRJNA827465;Yang2025_Silkie_Wencha	0.724136029
ENSGALG00010029233	CBX2	3	-0.268293483	0.212776608	-1.260916256	0.207339021	0.085210628	6.619255686	69.78512245	0.666666667	CRA004775;PRJNA827465;Yang2025_Silkie_Wencha	0.724136029
ENSGALG00010024551	RNF11	3	-0.257072809	0.20459625	-1.256488373	0.208938959	0.105718635	14.90614513	86.58271483	0.666666667	CRA004775;PRJNA827465;Yang2025_Silkie_Wencha	0.724136029
ENSGALG00010023062	STX2	3	0.251615307	0.199723462	1.259818469	0.207748556	0.095561567	10.81648383	81.50970286	0.666666667	CRA004775;PRJNA827465;Yang2025_Silkie_Wencha	0.724136029
ENSGALG00010022326	COX19	3	-0.240206947	0.191224775	-1.256149719	0.209061693	0.071880779	5.914814828	66.18659995	0.666666667	CRA004775;PRJNA827465;Yang2025_Silkie_Wencha	0.724136029
ENSGALG00010024605	LSP1P1	3	-0.236691094	0.18826972	-1.257191514	0.208684295	0.072982628	8.20167263	75.61473019	0.666666667	CRA004775;PRJNA827465;Yang2025_Silkie_Wencha	0.724136029
ENSGALG00010006103	NXPE3	3	0.215060345	0.170906864	1.2583482	0.208265857	0.0479697	4.962307083	59.69616619	0.666666667	CRA004775;PRJNA827465;Yang2025_Silkie_Wencha	0.724136029
ENSGALG00010027207	NOL8	3	0.19538819	0.154961564	1.260881634	0.207351496	0.051025817	8.029296263	75.09121678	1	CRA004775;PRJNA827465;Yang2025_Silkie_Wencha	0.724136029
ENSGALG00010012650	DSE	3	-0.188447945	0.149619583	-1.259513901	0.207844772	0.038967195	5.878149912	65.97568912	0.666666667	CRA004775;PRJNA827465;Yang2025_Silkie_Wencha	0.724136029
ENSGALG00010021866	SCNN1A	3	0.183997995	0.146257984	1.258037275	0.208378276	0	1.512131271	0	0.666666667	CRA004775;PRJNA827465;Yang2025_Silkie_Wencha	0.724136029
ENSGALG00010010620	ANKRD54	3	-0.183929135	0.146370663	-1.256598362	0.208899108	0.040985603	6.091839738	67.16919542	1	CRA004775;PRJNA827465;Yang2025_Silkie_Wencha	0.724136029
ENSGALG00010013403	CPNE3	3	-0.181488037	0.144417311	-1.256688226	0.208866553	0.042711826	7.332377028	72.72377175	0.666666667	CRA004775;PRJNA827465;Yang2025_Silkie_Wencha	0.724136029
ENSGALG00010019239	CYTH3	3	0.181405974	0.144057128	1.259264128	0.207934945	0.040788966	6.695382444	70.12866678	0.666666667	CRA004775;PRJNA827465;Yang2025_Silkie_Wencha	0.724136029
ENSGALG00010024545	PHACTR3	3	0.171858202	0.136585962	1.258242064	0.208304227	0	0.821929848	0	0.666666667	CRA004775;PRJNA827465;Yang2025_Silkie_Wencha	0.724136029
ENSGALG00010005621	CDH2	3	-0.163761519	0.130311131	-1.256696328	0.208863618	0.026935778	4.638331204	56.8810438	0.666666667	CRA004775;PRJNA827465;Yang2025_Silkie_Wencha	0.724136029
ENSGALG00010016235	DDX27	3	0.150243971	0.119412886	1.258189299	0.208020797	0.030095548	7.353522866	72.80215161	1	CRA004775;PRJNA827465;Yang2025_Silkie_Wencha	0.724136029
ENSGALG00010002536	RGL2	3	-0.144583469	0.11490783	-1.258256021	0.208299181	0	1.969633007	0	0.666666667	CRA004775;PRJNA827465;Yang2025_Silkie_Wencha	0.724136029
ENSGALG00010025341	VPS26B	3	-0.133201076	0.105722607	-1.259911006	0.207701468	0.018163388	4.565984108	56.19783265	1	CRA004775;PRJNA827465;Yang2025_Silkie_Wencha	0.724136029
ENSGALG00010021788	RPL5	3	-0.125031646	0.099519322	-1.256355484	0.208987114	0.013883017	3.579644131	44.12852433	1	CRA004775;PRJNA827465;Yang2025_Silkie_Wencha	0.724136029
ENSGALG00010008163	CHTF8	3	-0.122612977	0.097594926	-1.256345813	0.208990619	0	1.467542175	0	1	CRA004775;PRJNA827465;Yang2025_Silkie_Wencha	0.724136029
ENSGALG00010028070	MEX3A	3	0.119943594	0.09533334	1.258149506	0.208337692	0	0.843018836	0	0.666666667	CRA004775;PRJNA827465;Yang2025_Silkie_Wencha	0.724136029
ENSGALG00010020091	SUPT7L	3	-0.105978061	0.084316501	-1.256907717	0.208787054	0.010377473	4.018303883	50.22775633	1	CRA004775;PRJNA827465;Yang2025_Silkie_Wencha	0.724136029
ENSGALG00010002753	SLC9A7	3	0.102734441	0.081691596	1.257588855	0.208540486	0	0.917189001	0	0.666666667	CRA004775;PRJNA827465;Yang2025_Silkie_Wencha	0.724136029
ENSGALG00010008069	DLG2	3	-0.102633757	0.081492172	-1.259430862	0.207874748	0.002176033	2.175337853	8.060258461	0.666666667	CRA004775;PRJNA827465;Yang2025_Silkie_Wencha	0.724136029
ENSGALG00010001657	SMAP1	3	0.087636905	0.069704196	1.257268723	0.208656346	0.00655156	3.549442669	43.65312567	0.666666667	CRA004775;PRJNA827465;Yang2025_Silkie_Wencha	0.724136029
ENSGALG00010027979	RPL12	3	-0.086267839	0.068631845	-1.256965166	0.20876625	0	0.934290889	0	0.666666667	CRA004775;PRJNA827465;Yang2025_Silkie_Wencha	0.724136029
ENSGALG00010026261	IFT70A	3	-0.08240848	0.065319011	-1.261630862	0.207081646	0	1.406120278	0	1	CRA004775;PRJNA827465;Yang2025_Silkie_Wencha	0.724136029
ENSGALG00010003878	ARHGAP24	3	0.080956951	0.064204829	1.260916844	0.207338809	0	0.070112271	0	1	CRA004775;PRJNA827465;Yang2025_Silkie_Wencha	0.724136029
ENSGALG00010005203	ENSGALG0001000520	3	-0.079260479	0.06305465	-1.257012438	0.208749132	0	0.197301393	0	1	CRA004775;PRJNA827465;Yang2025_Silkie_Wencha	0.724136029
ENSGALG00010005035	NECTIN3	3	-0.073741498	0.058509541	-1.260332878	0.207549304	0	0.706436432	0	0.666666667	CRA004775;PRJNA827465;Yang2025_Silkie_Wencha	0.724136029
ENSGALG00010001174	ENSGALG0001000117	3	-0.061891645	0.049187796	-1.258272381	0.208293266	0	1.270135521	0	1	CRA004775;PRJNA827465;Yang2025_Silkie_Wencha	0.724136029
ENSGALG00010012938	UPF3B	3	0.437226177	0.348215378	1.255619956	0.209253793	0.329079813	24.66110389	91.89006296	0.666666667	CRA004775;PRJNA827465;Yang2025_Silkie_Wencha	0.724446468
ENSGALG00010025986	RAB3IL1	3	-0.259106928	0.206354449	-1.255640134	0.209246473	0.109122858	16.32627923	87.74981138	0.666666667	CRA004775;PRJNA827465;Yang2025_Silkie_Wencha	0.724446468
ENSGALG00010011450	PTDSS1	3	-0.141774773	0.11294261	-1.255281538	0.209376575	0.023320704	6.028684966	66.82526934	0.666666667	CRA004775;PRJNA827465;Yang2025_Silkie_Wencha	0.724694098
ENSGALG00010019966	ENSGALG0001001996	2	-0.91552138	0.729594383	-1.254836114	0.20953826	0.770048526	3.393360481	70.53068763	1	PRJNA827465;Yang2025_Silkie_Wenchang	0.724975059
ENSGALG00010024963	TNFRSF6B	3	-0.418212392	0.333358675	-1.2545418	0.209645142	0.171488047	4.262268281	53.07662802	0.666666667	CRA004775;PRJNA827465;Yang2025_Silkie_Wencha	0.724975059
ENSGALG00010026006	ITGA2B	3	0.323351413	0.257754646	1.254493055	0.209662849	0.120702173	5.84883428	65.80515186	0.666666667	CRA004775;PRJNA827465;Yang2025_Silkie_Wencha	0.724975059
ENSGALG00010022087	SLC35C1	3	-0.200950531	0.160161663	-1.254673107	0.209597452	0.057597743	11.87346962	83.15572394	0.666666667	CRA004775;PRJNA827465;Yang2025_Silkie_Wencha	0.724975059
ENSGALG00010011436	UBAP2L2	3	-2.925899785	2.333097921	-1.254083576	0.209811629	13.72865425	13.35422545	85.02346686	1	CRA004775;PRJNA827465;Yang2025_Silkie_Wencha	0.725312133
ENSGALG00010024697	IGF2BP1	3	-0.108847827	0.086807819	-1.253894272	0.209880437	0.008760457	3.309733822	39.57217989	0.666666667	CRA004775;PRJNA827465;Yang2025_Silkie_Wencha	0.725350708
ENSGALG00010021687	ZNF384	3	-0.101907845	0.08129024	-1.253629532	0.209976692	0.011656036	4.975548521	59.80342687	0.666666667	CRA004775;PRJNA827465;Yang2025_Silkie_Wencha	0.725350708
ENSGALG00010028859	SURF1	3	-0.084130728	0.067108495	-1.253652432	0.209968365	0	1.840476571	0	1	CRA004775;PRJNA827465;Yang2025_Silkie_Wencha	0.725350708
ENSGALG00010020303	EXD2	3	-0.099290334	0.079231586	-1.253166048	0.210145283	0.009781466	4.249214204	52.93247401	1	CRA004775;PRJNA827465;Yang2025_Silkie_Wencha	0.725755779
ENSGALG00010010259	IGF1	3	-0.448748387	0.358139478	-1.252998941	0.210206092	0.247148889	8.025505618	75.07945175	1	CRA004775;PRJNA827465;Yang2025_Silkie_Wencha	0.725788507
ENSGALG00010027962	SCAMP3	3	-0.107250366	0.085605643	-1.252842244	0.210263124	0	1.817965542	0	1	CRA004775;PRJNA827465;Yang2025_Silkie_Wencha	0.725808183
ENSGALG00010015729	RHCE	3	0.140454819	0.112136193	1.252537792	0.210373966	0.004627969	2.231038342	10.35564191	0.666666667	CRA004775;PRJNA827465;Yang2025_Silkie_Wencha	0.726013549
ENSGALG00010024233	FAIM2	3	0.281331888	0.224714558	1.251952211	0.210587278	0.043663598	2.660732711	24.83273529	0.666666667	CRA004775;PRJNA827465;Yang2025_Silkie_Wencha	0.726572358
ENSGALG00010029567	IFT20	3	-0.072470658	0.057900354	-1.251644467	0.210699444	0	0.445424521	0	0.666666667	CRA004775;PRJNA827465;Yang2025_Silkie_Wencha	0.726782004
ENSGALG00010013587	SLC26A1	2	1.047800614	0.838309008	1.249897835	0.21133687	1.017187479	3.522256459	71.60910877	1	CRA004775;PRJNA827465	0.727243903
ENSGALG00010012422	ENSGALG0001001242	2	0.802888523	0.642864595	1.248923223	0.211693157	0	0.275244594	0	1	CRA004775;Yang2025_Silkie_Wenchang	0.727243903
ENSGALG00010007748	CSGALNACT1	3	0.420829889	0.336857678	1.249280978	0.211562323	0.223093091	6.23136358	67.90429616	0.666666667	CRA004775;PRJNA827465;Yang2025_Silkie_Wencha	0.727243903
ENSGALG00010018750	SOCS4	3	-0.296791452	0.237694502	-1.248625648	0.211802027	0.150319626	24.36094924	91.79013929	1	CRA004775;PRJNA827465;Yang2025_Silkie_Wencha	0.727243903
ENSGALG00010021714	TSPAN18	3	-0.289286769	0.231434275	-1.249973751	0.211309136	0.124946136	18.10192628	88.95145208	1	CRA004775;PRJNA827465;Yang2025_Silkie_Wencha	0.727243903
ENSGALG00010013435	SDC3	3	0.281135058	0.224849158	1.250327373	0.211179983	0.103306098	6.518480658	69.31800361	0.666666667	CRA004775;PRJNA827465;Yang2025_Silkie_Wencha	0.727243903
ENSGALG00010009727	CDH20	3	-0.278603169	0.223022255	-1.249216896	0.211585754	0.096214336	6.901169057	71.01940289	0.666666667	CRA004775;PRJNA827465;Yang2025_Silkie_Wencha	0.727243903
ENSGALG00010023498	DISP2	3	-0.256472724	0.205283915	-1.249356159	0.211534836	0	1.15765885	0	0.666666667	CRA004775;PRJNA827465;Yang2025_Silkie_Wencha	0.727243903
ENSGALG00010005399	NDNF	3	0.256141652	0.205146378	1.248579939	0.211818754	0	0.84469609	0	1	CRA004775;PRJNA827465;Yang2025_Silkie_Wencha	0.727243903
ENSGALG00010020163	CDC2L1	3	0.24827495	0.198594353	1.250161176	0.211240676	0.079798338	6.751493381	70.37692423	1	CRA004775;PRJNA827465;Yang2025_Silkie_Wencha	0.727243903
ENSGALG00010028180	ODF2	3	0.239352379	0.191635707	1.248996773	0.211666254	0.076628782	8.803003697	77.28048211	0.666666667	CRA004775;PRJNA827465;Yang2025_Silkie_Wencha	0.727243903
ENSGALG00010013450	CD74	3	-0.226986956	0.18183379	-1.248321091	0.211913493	0.067140726	6.420465799	68.84961212	0.666666667	CRA004775;PRJNA827465;Yang2025_Silkie_Wencha	0.727243903
ENSGALG00010022076	YWHAB	3	-0.169109136	0.135436455	-1.248623461	0.211802827	0.04248016	15.57677476	87.16037158	0.666666667	CRA004775;PRJNA827465;Yang2025_Silkie_Wencha	0.727243903
ENSGALG00010008083	FANCA	3	0.165947831	0.132869499	1.248953545	0.211682066	0	0.343100529	0	1	CRA004775;PRJNA827465;Yang2025_Silkie_Wencha	0.727243903
ENSGALG00010020096	GPHN	3	0.122393591	0.097965221	1.249357579	0.211534317	0.012387346	3.480378533	42.53498633	1	CRA004775;PRJNA827465;Yang2025_Silkie_Wencha	0.727243903
ENSGALG00010024564	PPP1R3D	3	-0.119170501	0.09546255	-1.248348189	0.211903574	0	0.393230149	0	0.666666667	CRA004775;PRJNA827465;Yang2025_Silkie_Wencha	0.727243903
ENSGALG00010015827	ENSGALG0001001582	3	0.116679111	0.093323927	1.250259333	0.211204829	0	1.437051945	0	0.666666667	CRA004775;PRJNA827465;Yang2025_Silkie_Wencha	0.727243903
ENSGALG00010028084	ENG	3	0.094833652	0.075829937	1.250609655	0.211076926	0	1.76740027	0	0.666666667	CRA004775;PRJNA827465;Yang2025_Silkie_Wencha	0.727243903
ENSGALG00010016017	RAPGEF6	3	-0.057813269	0.046275971	-1.249315094	0.211549849	0	0.259397347	0	1	CRA004775;PRJNA827465;Yang2025_Silkie_Wencha	0.727243903
ENSGALG00010008011	ADNP2	3	-0.055984078	0.044805055	-1.24950362	0.21148093	0	0.890887166	0	1	CRA004775;PRJNA827465;Yang2025_Silkie_Wencha	0.727243903
ENSGALG00010019280	TLNRD1	3	0.054970349	0.04394628	1.250853299	0.210988005	0	1.207399744	0	1	CRA004775;PRJNA827465;Yang2025_Silkie_Wencha	0.727243903
ENSGALG00010024151	LEPROT	3	-0.156910142	0.125717882	-1.248113151	0.211989623	0.031008458	8.15562643	75.47705235	1	CRA004775;PRJNA827465;Yang2025_Silkie_Wencha	0.727308175
ENSGALG00010019948	TICRR	3	-0.077135574	0.061807898	-1.247988957	0.212035101	0	1.82827843	0	1	CRA004775;PRJNA827465;Yang2025_Silkie_Wencha	0.727308175
ENSGALG00010006331	SMAD9	3	0.221294302	0.177368734	1.247651135	0.212158843	0.041637035	3.489630152	42.68733612	0.666666667	CRA004775;PRJNA827465;Yang2025_Silkie_Wencha	0.727556121
ENSGALG00010020787	TMEM132C	2	0.352261098	0.282407441	1.247350625	0.212268962	0.063323219	1.526407228	34.48668339	1	PRJNA827465;Yang2025_Silkie_Wenchang	0.727580814
ENSGALG00010015392	TMEM165	3	-0.065251503	0.052310356	-1.247391678	0.212253916	0	1.658622241	0	1	CRA004775;PRJNA827465;Yang2025_Silkie_Wencha	0.727580814
ENSGALG00010022226	FBRSL1	3	0.352795664	0.282994497	1.246652029	0.212525115	0.197284617	12.80374366	84.37956857	0.666666667	CRA004775;PRJNA827465;Yang2025_Silkie_Wencha	0.727707001
ENSGALG00010009518	FGL2	3	-0.309582753	0.248337373	-1.246621678	0.212536249	0.071065942	3.245962851	38.38500034	0.666666667	CRA004775;PRJNA827465;Yang2025_Silkie_Wencha	0.727707001
ENSGALG00010022747	NT5C3B	3	-0.196313839	0.157493188	-1.246490988	0.212584196	0.05461322	7.848786362	74.51835344	0.666666667	CRA004775;PRJNA827465;Yang2025_Silkie_Wencha	0.727707001
ENSGALG00010024049	RCN2	3	-0.182438363	0.146382434	-1.246313222	0.212649426	0.047964741	13.13067859	84.76849474	1	CRA004775;PRJNA827465;Yang2025_Silkie_Wencha	0.727707001
ENSGALG00010013451	PRKAB2	3	-0.119860324	0.096127717	-1.246886195	0.212439229	0.008533098	2.828270284	29.28540065	0.666666667	CRA004775;PRJNA827465;Yang2025_Silkie_Wencha	0.727707001
ENSGALG00010027263	TAMM41	3	0.100952702	0.080970813	1.246778907	0.212478577	0	1.872875439	0	0.666666667	CRA004775;PRJNA827465;Yang2025_Silkie_Wencha	0.727707001
ENSGALG00010020228	TM9SF3	3	-0.090272105	0.072433948	-1.246267914	0.212666053	0.008321463	4.597016574	56.49352209	0.666666667	CRA004775;PRJNA827465;Yang2025_Silkie_Wencha	0.727707001
ENSGALG00010006263	EXOSC8	3	-0.297141699	0.238494539	-1.24590567	0.212799028	0.132725717	10.33167405	80.64205288	1	CRA004775;PRJNA827465;Yang2025_Silkie_Wencha	0.727972342
ENSGALG00010021829	ERCC6	3	0.112970388	0.090682731	1.245776197	0.212846571	0.011580398	3.554873762	43.73921174	0.666666667	CRA004775;PRJNA827465;Yang2025_Silkie_Wencha	0.727972342
ENSGALG00010020537	CCDC167	3	-0.273486237	0.219676606	-1.244949301	0.213150387	0.108426865	9.418415933	78.76500662	0.666666667	CRA004775;PRJNA827465;Yang2025_Silkie_Wencha	0.728275326
ENSGALG00010008223	ENSGALG0001000822	3	-0.209486136	0.168232336	-1.245219206	0.213051185	0.059494092	9.515436363	78.98152093	0.666666667	CRA004775;PRJNA827465;Yang2025_Silkie_Wencha	0.728275326
ENSGALG00010004141	KPNA3	3	-0.134414495	0.107977829	-1.244834207	0.213192699	0.020314354	5.730154103	65.09692473	0.666666667	CRA004775;PRJNA827465;Yang2025_Silkie_Wencha	0.728275326
ENSGALG00010008160	TXNL4A	3	-0.078218468	0.062832562	-1.244871537	0.213178975	0	0.465545666	0	1	CRA004775;PRJNA827465;Yang2025_Silkie_Wencha	0.728275326
ENSGALG00010026325	TMEM69	3	-0.070633387	0.056721171	-1.245273772	0.213031133	0	1.607340263	0	1	CRA004775;PRJNA827465;Yang2025_Silkie_Wencha	0.728275326
ENSGALG00010013470	RPL31	3	-0.23191205	0.186371385	-1.244354389	0.213369162	0.064665972	5.158420246	61.22844001	1	CRA004775;PRJNA827465;Yang2025_Silkie_Wencha	0.728398088
ENSGALG00010025745	COX14	3	-0.203951634	0.163898445	-1.244378089	0.213360443	0	0.234506567	0	1	CRA004775;PRJNA827465;Yang2025_Silkie_Wencha	0.728398088
ENSGALG00010013533	SKP2	3	-0.186332234	0.149797243	-1.243896282	0.213537738	0.051599634	9.438171814	78.80945548	0.666666667	CRA004775;PRJNA827465;Yang2025_Silkie_Wencha	0.728398088
ENSGALG00010026458	DLEC1	3	0.119929748	0.096407211	1.243991473	0.213502701	0	1.449123301	0	0.666666667	CRA004775;PRJNA827465;Yang2025_Silkie_Wencha	0.728398088
ENSGALG00010008073	ZNF276	3	-0.059095214	0.04749539	-1.244230534	0.213414729	0	1.681019048	0	1	CRA004775;PRJNA827465;Yang2025_Silkie_Wencha	0.728398088
ENSGALG00010017407	EPN2	3	0.042743434	0.034357418	1.244081684	0.213469501	0	1.032850046	0	1	CRA004775;PRJNA827465;Yang2025_Silkie_Wencha	0.728398088
ENSGALG00010020854	ENSGALG0001002085	3	-0.331718895	0.266861644	-1.243036991	0.213854203	0.109315714	4.105442117	51.28417494	0.666666667	CRA004775;PRJNA827465;Yang2025_Silkie_Wencha	0.728949993
ENSGALG00010020616	PI4K2A	3	-0.154475743	0.124260386	-1.243161616	0.213808284	0.020322026	3.195460001	37.41120217	1	CRA004775;PRJNA827465;Yang2025_Silkie_Wencha	0.728949993
ENSGALG00010017973	PKD1	3	-0.104029782	0.083673968	-1.243275351	0.213766384	0	0.002397033	0	1	CRA004775;PRJNA827465;Yang2025_Silkie_Wencha	0.728949993
ENSGALG00010018019	NAPB	3	0.568958269	0.457943064	1.242421414	0.214081119	0.480753116	9.534289116	79.0230821	0.666666667	CRA004775;PRJNA827465;Yang2025_Silkie_Wencha	0.729213645
ENSGALG00010028019	RPL29	3	-0.242310764	0.195022982	-1.242472869	0.214062145	0.073503689	5.645680052	64.57468398	1	CRA004775;PRJNA827465;Yang2025_Silkie_Wencha	0.729213645
ENSGALG00010029255	RAP1GAP2	3	-0.101896437	0.082015315	-1.242407432	0.214086275	0	0.413869851	0	1	CRA004775;PRJNA827465;Yang2025_Silkie_Wencha	0.729213645
ENSGALG00010020098	GABRD	3	0.433355817	0.348882363	1.242125892	0.214190117	0.221064592	5.138611312	61.07897877	1	CRA004775;PRJNA827465;Yang2025_Silkie_Wencha	0.729391633
ENSGALG00010000239	ENSGALG0001000023	3	-4.11305614	3.313322895	-1.241368943	0.214469487	28.38171597	16.61895222	87.96554696	1	CRA004775;PRJNA827465;Yang2025_Silkie_Wencha	0.729547928
ENSGALG00010008921	OPA3	3	0.639391223	0.515026341	1.241472857	0.214431119	0.697324266	17.7699349	88.74503474	1	CRA004775;PRJNA827465;Yang2025_Silkie_Wencha	0.729547928
ENSGALG00010009985	USP16	3	0.178596825	0.143885037	1.241246684	0.214514634	0.045627055	8.180121534	75.55048551	0.666666667	CRA004775;PRJNA827465;Yang2025_Silkie_Wencha	0.729547928
ENSGALG00010012749	RGS4	3	-0.155155865	0.124994894	-1.24129763	0.21449582	0.021928881	3.691462173	45.82092661	1	CRA004775;PRJNA827465;Yang2025_Silkie_Wencha	0.729547928
ENSGALG00010009119	CISD1	3	-0.110739779	0.089180572	-1.241747799	0.214329628	0.008225386	2.88266095	30.61965888	0.666666667	CRA004775;PRJNA827465;Yang2025_Silkie_Wencha	0.729547928
ENSGALG00010027169	ABCB8	3	-0.096694119	0.077906071	-1.241162829	0.214545603	0	0.985728026	0	0.666666667	CRA004775;PRJNA827465;Yang2025_Silkie_Wencha	0.729547928
ENSGALG00010020283	FBXO42	3	-0.062687645	0.050516332	-1.240938175	0.214628589	0	0.992197664	0	1	CRA004775;PRJNA827465;Yang2025_Silkie_Wencha	0.729654633
ENSGALG00010010963	TSPAN14	3	-0.327625703	0.26412894	-1.240400629	0.214827249	0.171032563	22.45744447	91.09426719	1	CRA004775;PRJNA827465;Yang2025_Silkie_Wencha	0.73014305
ENSGALG00010005484	GINM1	3	-0.300997784	0.242705153	-1.240178792	0.214909271	0.15478742	20.91806947	90.43888824	1	CRA004775;PRJNA827465;Yang2025_Silkie_Wencha	0.73014305

ENSGALG00010030103	C1QTNF1	3	0.135268477	0.109076013	1.240130368	0.214927178	0	0.688579188	0	0.666666667	CRA004775;PRJNA827465;Yang2025_Silkie_Wencha	0.73014305
ENSGALG00010020313	FRA10AC1	3	0.159510548	0.128705905	1.239341336	0.215219117	0.02761745	4.57022408	56.2384696	1	CRA004775;PRJNA827465;Yang2025_Silkie_Wencha	0.730959185
ENSGALG00010007826	ENSGALG0001000782€	3	-1.354293695	1.094489187	-1.237375125	0.215947849	0	0.942071813	0	0.666666667	CRA004775;PRJNA827465;Yang2025_Silkie_Wencha	0.731012139
ENSGALG00010005268	MTTP	3	0.706242109	0.570384753	1.238185462	0.215647301	0.619624187	5.672748236	64.74372003	1	CRA004775;PRJNA827465;Yang2025_Silkie_Wencha	0.731012139
ENSGALG00010023270	ENSGALG0001002327(	2	-0.517777938	0.418243718	-1.237981388	0.215722962	0	0.085504025	0	1	PRJNA827465;Yang2025_Silkie_Wenchang	0.731012139
ENSGALG00010020056	UACA	3	0.434291025	0.350982317	1.237358704	0.215953943	0.302685568	14.20522138	85.92066997	1	CRA004775;PRJNA827465;Yang2025_Silkie_Wencha	0.731012139
ENSGALG00010022456	ATP5G3	3	-0.356826307	0.288141921	-1.238369988	0.215578904	0.181309334	7.984589866	74.95175039	1	CRA004775;PRJNA827465;Yang2025_Silkie_Wencha	0.731012139
ENSGALG00010014537	GKAP1	3	0.349538738	0.282335004	1.238028348	0.21570555	0.189603186	11.78191173	83.02482614	1	CRA004775;PRJNA827465;Yang2025_Silkie_Wencha	0.731012139
ENSGALG00010010917	TANC1	3	0.189621857	0.153041696	1.239020884	0.215337764	0.053045471	9.901344374	79.80072277	1	CRA004775;PRJNA827465;Yang2025_Silkie_Wencha	0.731012139
ENSGALG00010001896	ARHGEF10	3	-0.185875229	0.150060441	-1.238669087	0.215468071	0.036772074	4.698878434	57.43665157	0.666666667	CRA004775;PRJNA827465;Yang2025_Silkie_Wencha	0.731012139
ENSGALG00010017457	ZMAT4	3	-0.17119134	0.138336341	-1.237500851	0.215901199	0	0.974543512	0	1	CRA004775;PRJNA827465;Yang2025_Silkie_Wencha	0.731012139
ENSGALG00010013981	RLF	3	-0.166884188	0.134882354	-1.237257383	0.215991544	0.040479168	10.21537241	80.42166335	0.666666667	CRA004775;PRJNA827465;Yang2025_Silkie_Wencha	0.731012139
ENSGALG00010021139	ARHGAP29	3	0.157369331	0.127035592	1.238781427	0.215426454	0.036329074	9.331390228	78.56696643	0.666666667	CRA004775;PRJNA827465;Yang2025_Silkie_Wencha	0.731012139
ENSGALG00010007532	DCUN1D5	3	-0.140232256	0.11331966	-1.237492738	0.215904209	0.017088791	3.200914459	37.51785543	1	CRA004775;PRJNA827465;Yang2025_Silkie_Wencha	0.731012139
ENSGALG00010009013	LIPG	3	0.137741552	0.111189879	1.238795771	0.21542114	0	0.168919683	0	1	CRA004775;PRJNA827465;Yang2025_Silkie_Wencha	0.731012139
ENSGALG00010011885	NCALD	3	-0.135233752	0.109260843	-1.237714706	0.215821864	0	0.706635904	0	1	CRA004775;PRJNA827465;Yang2025_Silkie_Wencha	0.731012139
ENSGALG00010022027	EIF4ENIF1	3	-0.083687933	0.067642627	-1.237207022	0.216010235	0.007015618	4.334023528	53.85350386	0.666666667	CRA004775;PRJNA827465;Yang2025_Silkie_Wencha	0.731012139
ENSGALG00010029387	SERPINF1	3	-0.250628005	0.202676946	-1.236588624	0.216239845	0.077780756	7.478971047	73.25835349	1	CRA004775;PRJNA827465;Yang2025_Silkie_Wencha	0.731614064
ENSGALG00010020674	TRPV4	2	1.217911689	0.985861395	1.235378214	0.216689777	1.09885042	2.287925694	56.29228682	1	PRJNA827465;Yang2025_Silkie_Wenchang	0.73198157
ENSGALG00010020720	ENSGALG0001002072(	3	0.28801357	0.233148667	1.23532154	0.21671086	0	0.57421936	0	1	CRA004775;PRJNA827465;Yang2025_Silkie_Wencha	0.73198157
ENSGALG00010019097	STC1	3	0.208941833	0.169104257	1.235579974	0.216614732	0.00968517	1.192290183	16.12780057	1	PRJNA827465;Yang2025_Silkie_Wenchang	0.73198157
ENSGALG00010024685	ENSGALG0001002468:	3	-0.19913563	0.161200726	-1.235327129	0.216708781	0.032807389	3.40913037	41.33401241	0.666666667	CRA004775;PRJNA827465;Yang2025_Silkie_Wencha	0.73198157
ENSGALG00010001808	HOXA4	3	0.184621133	0.149448798	1.235347058	0.216701367	0.034774086	4.217815032	52.58208373	0.666666667	CRA004775;PRJNA827465;Yang2025_Silkie_Wencha	0.73198157
ENSGALG00010010007	CTIF	3	0.080291206	0.064959038	1.236028243	0.216448066	0	0.100083921	0	1	CRA004775;PRJNA827465;Yang2025_Silkie_Wencha	0.73198157
ENSGALG00010016581	H2AFYMACROH2A1	3	0.063005175	0.050996784	1.235473493	0.216654336	0	1.666195808	0	1	CRA004775;PRJNA827465;Yang2025_Silkie_Wencha	0.73198157
ENSGALG00010015904	MTMR7	3	0.498301474	0.403604826	1.234627143	0.216969302	0.38897267	12.94061607	84.54478528	0.666666667	CRA004775;PRJNA827465;Yang2025_Silkie_Wencha	0.732178087
ENSGALG00010010780	PARG	3	-0.16739432	0.135570369	-1.234741198	0.216926838	0.047389526	19.59387459	89.79272838	1	CRA004775;PRJNA827465;Yang2025_Silkie_Wencha	0.732178087
ENSGALG00010012075	PIGN	3	-0.163614048	0.132537931	-1.234469614	0.217027963	0.031571355	5.373206469	62.77827753	0.666666667	CRA004775;PRJNA827465;Yang2025_Silkie_Wencha	0.732178087
ENSGALG00010023209	PIK3C2A	3	0.088906552	0.072016113	1.23453695	0.217002887	0.004737442	2.572800446	22.26369507	1	CRA004775;PRJNA827465;Yang2025_Silkie_Wencha	0.732178087
ENSGALG00010020599	PARN	3	-0.056949027	0.046118449	-1.234842634	0.216889077	0	1.892860753	0	1	CRA004775;PRJNA827465;Yang2025_Silkie_Wencha	0.732178087
ENSGALG00010025710	ENSGALG0001002571(	2	-1.900154607	1.545267255	-1.229660825	0.218824143	0	0.004051239	0	1	PRJNA827465;Yang2025_Silkie_Wenchang	0.732474544
ENSGALG00010006845	CALML3	2	0.893520591	0.729688425	1.224523455	0.220754824	0	0.059087947	0	1	PRJNA827465;Yang2025_Silkie_Wenchang	0.732474544
ENSGALG00010007770	ENSGALG0001000777(	2	0.81439644	0.663992709	1.226514131	0.220005261	0.710546114	5.149423063	80.58034875	1	PRJNA827465;Yang2025_Silkie_Wenchang	0.732474544
ENSGALG00010007223	FOXF1	3	0.680994669	0.553236933	1.230927705	0.218349904	0.720480916	9.428697708	78.78816288	0.666666667	CRA004775;PRJNA827465;Yang2025_Silkie_Wencha	0.732474544
ENSGALG00010021851	ENSGALG0001002185	3	0.680238648	0.553570869	1.228819836	0.219139362	0.805166162	17.22260993	88.3873582	0.666666667	CRA004775;PRJNA827465;Yang2025_Silkie_Wencha	0.732474544
ENSGALG00010024600	CD9	3	0.648672415	0.526296277	1.232523282	0.217753675	0.758664648	33.38642778	94.0095418	0.666666667	CRA004775;PRJNA827465;Yang2025_Silkie_Wencha	0.732474544
ENSGALG00010009136	KHK	3	0.617778696	0.502736801	1.228831259	0.219135079	0.576043207	8.404299042	76.20265545	0.666666667	CRA004775;PRJNA827465;Yang2025_Silkie_Wencha	0.732474544
ENSGALG00010022674	ENSGALG0001002267	3	0.550992638	0.448929444	1.227347961	0.219691836	0.466606639	8.994522414	77.76424464	0.666666667	CRA004775;PRJNA827465;Yang2025_Silkie_Wencha	0.732474544
ENSGALG00010017453	PPP1R2	3	0.520437096	0.425249561	1.22383923	0.221012882	0.504535723	36.72234674	94.55372497	1	CRA004775;PRJNA827465;Yang2025_Silkie_Wencha	0.732474544
ENSGALG00010028177	ENSGALG0001002817	3	0.491707052	0.401042725	1.226071491	0.220171773	0.326830473	6.695001323	70.12696632	0.666666667	CRA004775;PRJNA827465;Yang2025_Silkie_Wencha	0.732474544
ENSGALG00010000589	ENSGALG0001000058	3	0.458064477	0.373995036	1.224787587	0.220655263	0.333690807	10.58463337	81.10468327	1	CRA004775;PRJNA827465;Yang2025_Silkie_Wencha	0.732474544
ENSGALG00010027729	EPS8L3	3	0.429584194	0.349916904	1.227674884	0.219569038	0.261242763	7.175169709	72.12609484	1	CRA004775;PRJNA827465;Yang2025_Silkie_Wencha	0.732474544
ENSGALG00010012838	C2orf88	3	0.417214628	0.339469484	1.229019609	0.219064454	0.309918379	18.06977673	88.93179462	0.666666667	CRA004775;PRJNA827465;Yang2025_Silkie_Wencha	0.732474544
ENSGALG00010005604	ENSGALG0001000560	3	0.390126641	0.316126082	1.234085585	0.217171014	0	1.856232046	0	0.666666667	CRA004775;PRJNA827465;Yang2025_Silkie_Wencha	0.732474544
ENSGALG00010027163	ENSGALG0001002716	3	0.368372135	0.301082502	1.223492341	0.221143795	0.24857841	28.54801129	92.99425806	0.666666667	CRA004775;PRJNA827465;Yang2025_Silkie_Wencha	0.732474544
ENSGALG00010010116	DRD5	2	-0.344035787	0.28030731	-1.227352179	0.219690252	0	0.206792022	0	1	PRJNA827465;Yang2025_Silkie_Wenchang	0.732474544
ENSGALG00010018855	SYNDIG1	2	-0.342817647	0.279055457	-1.228492856	0.21926201	0	0.427536818	0	1	PRJNA827465;Yang2025_Silkie_Wenchang	0.732474544
ENSGALG00010001837	TAB3	3	0.341143894	0.278759683	1.223792084	0.221030671	0.185238238	10.87304659	81.60589138	0.666666667	CRA004775;PRJNA827465;Yang2025_Silkie_Wencha	0.732474544
ENSGALG00010018264	CFAP61	3	0.33804205	0.276355643	1.223213851	0.221248936	0	0.073136628	0	1	CRA004775;PRJNA827465;Yang2025_Silkie_Wencha	0.732474544
ENSGALG00010020103	PSAP	3	-0.324259696	0.263021326	-1.232826632	0.217640453	0.175081542	22.29255529	91.02839502	0.666666667	CRA004775;PRJNA827465;Yang2025_Silkie_Wencha	0.732474544
ENSGALG00010001639	ENSGALG0001000163	3	0.31570723	0.25799169	1.22386512	0.221003114	0.133611064	7.137916839	71.98062061	1	CRA004775;PRJNA827465;Yang2025_Silkie_Wencha	0.732474544
ENSGALG00010019018	SH3BGRL	3	-0.302321605	0.246788683	-1.225022157	0.220566872	0.142514146	20.82451565	90.39593509	0.666666667	CRA004775;PRJNA827465;Yang2025_Silkie_Wencha	0.732474544
ENSGALG00010025424	IRX6	3	0.301231651	0.244746176	1.230792062	0.218400645	0	0.307228703	0	1	CRA004775;PRJNA827465;Yang2025_Silkie_Wencha	0.732474544
ENSGALG00010001776	LMBRD1	3	-0.285885325	0.233569028	-1.223986452	0.220957339	0.119459279	8.608853447	76.7681026	1	CRA004775;PRJNA827465;Yang2025_Silkie_Wencha	0.732474544
ENSGALG00010012072	CAPRIN2	3	0.283719888	0.23059682	1.230362474	0.218561399	0.125220006	11.31776815	82.3286714	0.666666667	CRA004775;PRJNA827465;Yang2025_Silkie_Wencha	0.732474544
ENSGALG00010006071	TEX30	3	-0.278769832	0.227769695	-1.223910984	0.220985809	0.083946199	4.376673029	54.30318905	1	CRA004775;PRJNA827465;Yang2025_Silkie_Wencha	0.732474544
ENSGALG00010007730	COG2	3	-0.25476091	0.208091733	-1.224272136	0.220849585	0.113857298	19.48296088	89.73461984	0.666666667	CRA004775;PRJNA827465;Yang2025_Silkie_Wencha	0.732474544
ENSGALG00010012021	ENSGALG0001001202	3	0.246326053	0.200284978	1.229877823	0.21874286	0.09218679	9.201845335	78.26522912	0.666666667	CRA004775;PRJNA827465;Yang2025_Silkie_Wencha	0.732474544
ENSGALG00010015621	IDNK	3	-0.236961708	0.193247878	-1.226206002	0.220121163	0.057977864	4.186299642	52.22586983	1	CRA004775;PRJNA827465;Yang2025_Silkie_Wencha	0.732474544
ENSGALG00010021922	MRPL51	3	-0.22746324	0.184928706	-1.230005037	0.218695219	0.070806419	6.679473617	70.05752078	1	CRA004775;PRJNA827465;Yang2025_Silkie_Wencha	0.732474544
ENSGALG00010002825	TRIM41	3	-0.226496924	0.184331266	-1.228749349	0.219165797	0.091476386	20.36332143	90.17841953	0.666666667	CRA004775;PRJNA827465;Yang2025_Silkie_Wencha	0.732474544
ENSGALG00010000687	IKZF1	3	-0.222004339	0.180575425	-1.2294272	0.218911677	0	1.368941393	0	1	CRA004775;PRJNA827465;Yang2025_Silkie_Wencha	0.732474544
ENSGALG00010010061	CYP2D6	3	-0.217168855	0.177123312	-1.226088493	0.220165375	0.038665857	3.385625767	40.92652559	0.666666667	CRA004775;PRJNA827465;Yang2025_Silkie_Wencha	0.732474544
ENSGALG00010011250	ENSGALG0001001125(	3	0.216562331	0.177008035	1.223460454	0.221155832	0.066722087	7.183849615	72.15977356	0.666666667	CRA004775;PRJNA827465;Yang2025_Silkie_Wencha	0.732474544
ENSGALG00010013437	ENSGALG0001001343	3	0.215837454	0.176067958	1.22587583	0.220245406	0.053240076	4.746923146	57.86744511	0.666666667	CRA004775;PRJNA827465;Yang2025_Silkie_Wencha	0.732474544
ENSGALG00010015194	TMLHE	3	-0.212896492	0.173937055	-1.223985835	0.220957571	0.075426881	18.72776834	89.32067097	0.666666667	CRA004775;PRJNA827465;Yang2025_Silkie_Wencha	0.732474544
ENSGALG00010005453	UST	3	-0.209321285	0.169766578	-1.232994664	0.217577755	0.069449901	16.32563155	87.74932538	0.666666667	CRA004775;PRJNA827465;Yang2025_Silkie_Wencha	0.732474544
ENSGALG00010029163	ANKFY1	3	-0.205604133	0.167212197	-1.229600095	0.218846895	0.060652161	10.10426658	80.20638129	0.666666667	CRA004775;PRJNA827465;Yang2025_Silkie_Wencha	0.732474544
ENSGALG00010013333	PPP1R12A	3	0.199001458	0.161479649	1.232362459	0.217813718	0.055527829	7.990256592	74.96951472	0.666666667	CRA004775;PRJNA827465;Yang2025_Silkie_Wencha	0.732474544
ENSGALG00010021627	NCOA6	3	0.198591041	0.160984744	1.2336016	0.217351389	0.049658947	5.583037249	64.1772048	1	CRA004775;PRJNA827465;Yang2025_Silkie_Wencha	0.732474544
ENSGALG00010004070	ARL11	3	-0.193581325	0.157421327	-1.229704632	0.218807732	0	1.771729508	0	0.666666667	CRA004775;PRJNA827465;Yang2025_Silkie_Wencha	0.732474544
ENSGALG00010017185	NAA30	3	-0.190247676	0.154798274	-1.229003856	0.21907036	0.054222293	10.38327705	80.73825835	0.666666667	CRA004775;PRJNA827465;Yang2025_Silkie_Wencha	0.732474544
ENSGALG00010029938	ENSGALG0001002993	3	0.190171161	0.155073913	1.226325931	0.220076047	0.004988809	2.127997517	6.014927951	1	CRA004775;PRJNA827465;Yang2025_Silkie_Wencha	0.732474544
ENSGALG00010005322	TFB1M	3	0.186812966	0.151602901	1.23225192	0.217854994	0.04227028	5.650557855	64.60526462	0.666666667	CRA004775;PRJNA827465;Yang2025_Silkie_Wencha	0.732474544
ENSGALG00010010971	LCORL	3	0.183121519	0.149285844	1.226650257	0.219954071	0.038421155	5.225289344	61.72460761	1	CRA004775;PRJNA827465;Yang2025_Silkie_Wencha	0.732474544
ENSGALG00010017154	PIGZ	3	-0.178768828	0.145412341	-1.229392405	0.218924717	0	0.612785922	0	0.666666667	CRA004775;PRJNA827465;Yang2025_Silkie_Wencha	0.732474544
ENSGALG00010025960	CMKLR2	3	-0.176393464	0.14376263	-1.226977163	0.219831174	0.044505369	8.931785257	77.60805995	0.666666667	CRA004775;PRJNA827465;Yang2025_Silkie_Wencha	0.732474544
ENSGALG00010022303	DUSP13A	3	0.157976745	0.129152084	1.223183868	0.221260257	0	0.003107694	0	1	CRA004775;PRJNA827465;Yang2025_Silkie_Wencha	0.732474544
ENSGALG00010013777	CPLANE1	3	0.152819665	0.124790648	1.224608314	0.220722834	0	1.418103676	0	0.666666667	CRA004775;PRJNA827465;Yang2025_Silkie_Wencha	0.732474544
ENSGALG00010024757	PPFIA1	3	0.144711622	0.117979472	1.226583067	0.219979337	0.024963162	4.981178357	59.84885791	1	CRA004775;PRJNA827465;Yang2025_Silkie_Wencha	0.732474544
ENSGALG00010011388	GPR50	3	0.138557368	0.112389202	1.232835229	0.217637245	0	1.348519147	0	1	CRA004775;PRJNA827465;Yang2025_Silkie_Wencha	0.732474544
ENSGALG00010014135	ZBTB24	3	0.137495073	0.112037938	1.227218874	0.219740338	0.016208957	3.426317516	41.62829362	1	CRA004775;PRJNA827465;Yang2025_Silkie_Wencha	0.732474544
ENSGALG00010021698	GYPC	3	-0.129095853	0.105356418	-1.225325002	0.220452792	0.011630624	3.003841472	33.4158902	0.666666667	CRA004775;PRJNA827465;Yang2025_Silkie_Wencha	0.732474544
ENSGALG00010022978	ARL14EP	3	-0.122627001	0.099504533	-1.232376029	0.217808651	0.015486442	4.001172229	50.01464857	0.666666667	CRA004775;PRJNA827465;Yang2025_Silkie_Wencha	0.732474544
ENSGALG00010025368	LRP1	3	-0.121395047	0.098895303	-1.227510742	0.219630687	0.000233586	2.011233445	0.5585351	0.666666667	CRA004775;PRJNA827465;Yang2025_Silkie_Wencha	0.732474544
ENSGALG00010029275	PCYT2	3	0.119166068	0.097287503	1.224885667	0.220618301	0.016785206	5.555067167	63.99683496	0.666666667	CRA004775;PRJNA827465;Yang2025_Silkie_Wencha	0.732474544
ENSGALG00010022058	NLRP3	3	0.115589675	0.094363601	1.22599803	0.220199416	0.007858635	2.739173727	26.96582099	0.666666667	CRA004775;PRJNA827465;Yang2025_Silkie_Wencha	0.732474544
ENSGALG00010019614	MICALL2	3	-0.115035146	0.093269195	-1.233366989	0.217438875	0.006290114	2.502827228	20.0903691	1	CRA004775;PRJNA827465;Yang2025_Silkie_Wencha	0.732474544
ENSGALG00010028301	FBN3	3	-0.113604456	0.092318685	-1.230568389	0.218484334	0	0.932588965	0	0.666666667	CRA004775;PRJNA827465;Yang2025_Silkie_Wencha	0.732474544
ENSGALG00010021817	EVI5	3	0.110134395	0.089489075	1.230702136	0.218434289	0.011727035	3.876452146	48.40643133	0.666666667	CRA004775;PRJNA827465;Yang2025_Silkie_Wencha	0.732474544
ENSGALG00010003977	MYO6	3	0.108516653	0.088697395	1.223448028	0.221160523	0	1.443184021	0	0.666666667	CRA004775;PRJNA827465;Yang2025_Silkie_Wencha	0.732474544
ENSGALG00010024107	NFE2L2	3	-0.104289135	0.085048677	-1.226228783	0.220112593	0.013021302	6.619503871	69.7862553	1	CRA004775;PRJNA827465;Yang2025_Silkie_Wencha	0.732474544
ENSGALG00010026221	CRKL	3	-0.097311843	0.079256994	-1.227801344	0.219521551	0.006218434	2.806538697	28.73784344	1	CRA004775;PRJNA827465;Yang2025_Silkie_Wencha	0.732474544
ENSGALG00010017786	KAZALD1	3	-0.097284323	0.079176744	-1.228698203	0.21918498	0	0.885095379	0	1	CRA004775;PRJNA827465;Yang2025_Silkie_Wencha	0.732474544
ENSGALG00010024283	UBE4A	3	-0.086642389	0.070205487	-1.230206092	0.218619715	0.007545954	4.0261885	50.32522695	0.666666667	CRA004775;PRJNA827465;Yang2025_Silkie_Wencha	0.732474544
ENSGALG00010007879	PLK4	3	-0.088429505	0.071769244	-1.2321365	0.217898099	0	0.528227204	0	0.666666667	CRA004775;PRJNA827465;Yang2025_Silkie_Wencha	0.732474544
ENSGALG00010028060	ENSGALG0001002806(	3	-0.088160284	0.071605865	-1.231188038	0.218252544	0	0.021062218	0	1	CRA004775;PRJNA827465;Yang2025_Silkie_Wencha	0.732474544
ENSGALG00010020603	VPS39	3	0.087106615	0.071001424	1.226830201	0.219886417	0	0.428408398	0	1	CRA004775;PRJNA827465;Yang2025_Silkie_Wencha	0.732474544
ENSGALG00010005956	TXNL4B	3	0.086091959	0.069924223	1.231217948	0.218241361	0	0.776243792	0	0.666666667	CRA004775;PRJNA827465;Yang2025_Silkie_Wencha	0.732474544
ENSGALG00010021244	PPP3CB	3	0.083600776	0.067836575	1.232384974	0.217805311	0.006577974	3.636821534	45.00692483	1	CRA004775;PRJNA827465;Yang2025_Silkie_Wencha	0.732474544
ENSGALG00010003884	TYMS	3	-0.080450967	0.065679682	-1.224898865	0.220613328	0	1.749527334	0	0.666666667	CRA004775;PRJNA827465;Yang2025_Silkie_Wencha	0.732474544
ENSGALG00010018143	MRPL47	3	-0.076886271	0.062467188	-1.23082651	0.218387758	0	1.915629732	0	0.666666667	CRA004775;PRJNA827465;Yang2025_Silkie_Wencha	0.732474544
ENSGALG00010029379	GTF2I	3	0.076711095	0.062579392	1.225825183	0.220264468	0	1.553606991	0	0.666666667	CRA004775;PRJNA827465;Yang2025_Silkie_Wencha	0.732474544
ENSGALG00010011061	KIF13B	3	-0.076637923	0.062645176	-1.223365118	0.221191822	0	1.587168872	0	1	CRA004775;PRJNA827465;Yang2025_Silkie_Wencha	0.732474544
ENSGALG00010008527	CHUK	3	-0.07624708	0.062233111	-1.225185096	0.220505489	0.006322019	4.471911035	55.27639114	0.666666667	CRA004775;PRJNA827465;Yang2025_Silkie_Wencha	0.732474544
ENSGALG00010021747	AKTIP	3	0.070850831	0.057774185	1.22634064	0.220070514	0	1.409757539	0	0.666666667	CRA004775;PRJNA827465;Yang2025_Silkie_Wencha	0.732474544
ENSGALG00010028007	STK11	3	-0.069265734	0.056595512	-1.223907956	0.220986952	0	0.796521216	0	0.666666667	CRA004775;PRJNA827465;Yang2025_Silkie_Wencha	0.732474544
ENSGALG00010027286	RPN1	3	-0.06638161	0.054055376	-1.228029754	0.219435799	0.002433312	2.553608576	21.67946101	1	CRA004775;PRJNA827465;Yang2025_Silkie_Wencha	0.732474544
ENSGALG00010019127	TMBIM1	3	-0.064112969	0.052131504	-1.229831562	0.218760187	0	1.116719168	0	1	CRA004775;PRJNA827465;Yang2025_Silkie_Wencha	0.732474544
ENSGALG00010025988	NSF	3	-0.050971842	0.041589367	-1.225597925	0.220330019	0	1.544544831	0	1	CRA004775;PRJNA827465;Yang2025_Silkie_Wencha	0.732474544
ENSGALG00010011647	FBXO38	3	0.134799286	0.110148001	1.222984392	0.221335591	0.025399562	8.191945487	75.58577504	0.666666667	CRA004775;PRJNA827465;Yang2025_Silkie_Wencha	0.732552417
ENSGALG00010003164	C3	3	-0.467755966	0.382538285	-1.222769024	0.221416948	0.272336244	5.323837759	62.43311516	0.666666667	CRA004775;PRJNA827465;Yang2025_Silkie_Wencha	0.732650182
ENSGALG00010000234	ENSGALG0001000023	3	-3.054602178	2.508960965	-1.217476964	0.223422796	14.82476637	10.53975485	81.02422658	1	CRA004775;PRJNA827465;Yang2025_Silkie_Wencha	0.732804055
ENSGALG00010012832	CHADL	3	1.069165401	0.880793707	1.213865849	0.224798956	2.091178207	20.02389692	90.0119342	0.666666667	CRA004775;PRJNA827465;Yang2025_Silkie_Wencha	0.732804055
ENSGALG00010006062	ENSGALG0001000606	3	-0.868714625	0.71251294	-1.219226453	0.222758254	0.998029752	5.81455677	65.60356912	0.666666667	CRA004775;PRJNA827465;Yang2025_Silkie_Wencha	0.732804055
ENSGALG00010000028	ND4L	3	0.730459481	0.601140094	1.215123543	0.224318976	0.920890187	21.28152206	90.60217594	0.666666667	CRA004775;PRJNA827465;Yang2025_Silkie_Wencha	0.732804055
ENSGALG00010014696	SPINK5	3	0.654096171	0.538707154	1.214196184	0.224672817	0.667522183	8.904331095	77.5390203	0.666666667	CRA004775;PRJNA827465;Yang2025_Silkie_Wencha	0.732804055
ENSGALG00010003639	ENSGALG0001000363	3	-0.589787023	0.483907089	-1.218802198	0.222919277	0.040613097	2.112932631	5.34482875	0.666666667	CRA004775;PRJNA827465;Yang2025_Silkie_Wencha	0.732804055
ENSGALG00010019083	OTOA	3	0.538986937	0.441819959	1.219924375	0.222493545	0.495005252	17.63994163	88.66209401	1	CRA004775;PRJNA827465;Yang2025_Silkie_Wencha	0.732804055
ENSGALG00010000702	DDC	3	0.507797843	0.418117713	1.214485364	0.224562435	0.29856447	4.667390016	57.14949912	0.666666667	CRA004775;PRJNA827465;Yang2025_Silkie_Wencha	0.732804055
ENSGALG00010001711	CREB5	3	0.495557453	0.405577206	1.22185726	0.221761611	0.367717401	8.967503853	77.69724962	0.666666667	CRA004775;PRJNA827465;Yang2025_Silkie_Wencha	0.732804055
ENSGALG00010010142	ENSGALG0001001014	3	0.489304958	0.400646371	1.221288882	0.221976662	0.369495908	10.69951245	81.30755948	0.666666667	CRA004775;PRJNA827465;Yang2025_Silkie_Wencha	0.732804055
ENSGALG00010008905	GABARAPL2	3	-0.451632422	0.369795144	-1.221304363	0.221970802	0.378165853	31.71030225	93.69290149	1	CRA004775;PRJNA827465;Yang2025_Silkie_Wencha	0.732804055
ENSGALG00010021047	TMC5	3	0.435652533	0.358114189	1.216518493	0.223787471	0.106320454	2.750176344	27.27739062	0.666666667	CRA004775;PRJNA827465;Yang2025_Silkie_Wencha	0.732804055
ENSGALG00010000607	ENSGALG0001000060	3	-0.434619752	0.357403625	-1.216047408	0.223966863	0.275203108	8.070644986	75.21883315	1	CRA004775;PRJNA827465;Yang2025_Silkie_Wencha	0.732804055
ENSGALG00010006060	ENSGALG0001000606(	2	-0.424784571	0.347729886	-1.22159351	0.221861384	0	0.015732431	0	1	CRA004775;Yang2025_Silkie_Wenchang	0.732804055
ENSGALG00010025666	CUX2	2	0.41749793	0.343765799	1.214483613	0.224563104	0.167960183	3.374501706	70.36599513	1	PRJNA827465;Yang2025_Silkie_Wenchang	0.732804055
ENSGALG00010024646	RORA	3	0.385178494	0.317178262	1.214391212	0.22459837	0.208391467	7.549172472	73.50702998	0.666666667	CRA004775;PRJNA827465;Yang2025_Silkie_Wencha	0.732804055
ENSGALG00010022516	TRUB1	3	-0.350850578	0.288810744	-1.214811377	0.224438041	0.205266156	13.53756794	85.22629759	0.666666667	CRA004775;PRJNA827465;Yang2025_Silkie_Wencha	0.732804055
ENSGALG00010017790	R3HCC1L	3	0.326126641	0.268079842	1.216528025	0.223783842	0.192630381	26.25756234	92.38314671	0.666666667	CRA004775;PRJNA827465;Yang2025_Silkie_Wencha	0.732804055
ENSGALG00010028484	NOXA1	2	0.326115527	0.266772209	1.222449403	0.221537727	0	0.062702375	0	1	CRA004775;PRJNA827465	0.732804055
ENSGALG00010024905	GPX7	3	-0.320483026	0.262916304	-1.218954556	0.222861441	0.166228753	30.17527977	93.37205814	0.666666667	CRA004775;PRJNA827465;Yang2025_Silkie_Wencha	0.732804055
ENSGALG00010011867	ENSGALG0001001186	3	-0.299902676	0.246923032	-1.214559344	0.224534203	0	1.694338622	0	0.666666667	CRA004775;PRJNA827465;Yang2025_Silkie_Wencha	0.732804055
ENSGALG00010012136	ENSGALG0001001213(	3	0.293703913	0.241888696	1.214210986	0.224667166	0.104172642	5.210401081	61.61523904	0.666666667	CRA004775;PRJNA827465;Yang2025_Silkie_Wencha	0.732804055
ENSGALG00010002626	CHD7	3	0.283915314	0.233936461	1.213642852	0.224884136	0.127547377	10.129355	80.25540618	0.666666667	CRA004775;PRJNA827465;Yang2025_Silkie_Wencha	0.732804055
ENSGALG00010023861	ENSGALG0001002386	3	0.280994501	0.230909652	1.216902362	0.223641367	0.133219487	12.72331663	84.2808282	0.666666667	CRA004775;PRJNA827465;Yang2025_Silkie_Wencha	0.732804055
ENSGALG00010015987	POLR2H	3	-0.280738421	0.230590656	-1.217475271	0.22342344	0.113635072	7.288409277	72.55916999	0.666666667	CRA004775;PRJNA827465;Yang2025_Silkie_Wencha	0.732804055
ENSGALG00010016939	FAM234A	3	-0.275585668	0.226799548	-1.215106782	0.224325367	0.130264434	14.41696168	86.12745151	0.666666667	CRA004775;PRJNA827465;Yang2025_Silkie_Wencha	0.732804055
ENSGALG00010013286	RNF14	3	-0.273341011	0.224726017	-1.216330065	0.223859213	0.118612091	10.79426375	81.47164043	0.666666667	CRA004775;PRJNA827465;Yang2025_Silkie_Wencha	0.732804055
ENSGALG00010008018	ENSGALG0001000801	3	0.269799001	0.221360468	1.218821966	0.222911772	0.058202473	3.330405308	39.9472492	0.666666667	CRA004775;PRJNA827465;Yang2025_Silkie_Wencha	0.732804055
ENSGALG00010006767	ENSGALG0001000676	3	-0.255205158	0.20953012	-1.217987935	0.223228558	0	0.74419101	0	1	CRA004775;PRJNA827465;Yang2025_Silkie_Wencha	0.732804055
ENSGALG00010021789	CHD9	3	0.24987878	0.205071662	1.218494929	0.22303595	0.09351599	9.046797154	77.8927286	0.666666667	CRA004775;PRJNA827465;Yang2025_Silkie_Wencha	0.732804055
ENSGALG00010001801	HOXA3	3	0.245157149	0.201504785	1.216631897	0.223744301	0.078642366	5.649379931	64.59788464	1	CRA004775;PRJNA827465;Yang2025_Silkie_Wencha	0.732804055
ENSGALG00010012200	GEM	3	-0.244390483	0.200783494	-1.217184137	0.223534164	0.08372226	8.438012435	76.29773581	0.666666667	CRA004775;PRJNA827465;Yang2025_Silkie_Wencha	0.732804055
ENSGALG00010024266	ENSGALG0001002426(	3	-0.238249691	0.195051575	-1.221470226	0.221908032	0	0.04994956	0	1	CRA004775;PRJNA827465;Yang2025_Silkie_Wencha	0.732804055
ENSGALG00010019461	MGAT5	3	-0.236328214	0.19466715	-1.214011785	0.224743224	0.075395998	9.762624626	79.51370582	0.666666667	CRA004775;PRJNA827465;Yang2025_Silkie_Wencha	0.732804055
ENSGALG00010028149	SNAPC4	3	0.230587078	0.189341906	1.217834355	0.223286926	0.084956945	12.40151754	83.87294141	0.666666667	CRA004775;PRJNA827465;Yang2025_Silkie_Wencha	0.732804055
ENSGALG00010027203	ENSGALG0001002720	3	-0.224617442	0.184882237	-1.214921701	0.224395956	0	0.382042101	0	1	CRA004775;PRJNA827465;Yang2025_Silkie_Wencha	0.732804055
ENSGALG00010010201	RIOK1	3	0.21837559	0.178861319	1.220921275	0.222115829	0.074570019	10.70347265	81.31447554	0.666666667	CRA004775;PRJNA827465;Yang2025_Silkie_Wencha	0.732804055
ENSGALG00010014942	PMEPA1	3	0.203592354	0.166898409	1.219857966	0.222518723	0.055251402	9.329967936	78.56369911	0.666666667	CRA004775;PRJNA827465;Yang2025_Silkie_Wencha	0.732804055
ENSGALG00010008374	ZNF821	3	0.200799543	0.165438306	1.213742742	0.224845977	0.061030361	10.41484185	80.79663592	0.666666667	CRA004775;PRJNA827465;Yang2025_Silkie_Wencha	0.732804055
ENSGALG00010027616	SLC38A3	3	0.190908247	0.157106534	1.215151536	0.224308301	0	1.954290028	0	0.666666667	CRA004775;PRJNA827465;Yang2025_Silkie_Wencha	0.732804055
ENSGALG00010013556	RASL11B	3	0.185727226	0.152095831	1.221119762	0.222040678	0.032387092	3.829381582	47.7722458	1	CRA004775;PRJNA827465;Yang2025_Silkie_Wencha	0.732804055
ENSGALG00010007943	BLOC1S4	3	-0.175544307	0.144388661	-1.215776259	0.224070165	0.042466167	8.733833615	77.10054842	1	CRA004775;PRJNA827465;Yang2025_Silkie_Wencha	0.732804055
ENSGALG00010001716	TRIL	3	-0.170457081	0.139683079	-1.220313023	0.222346235	0.034835059	7.23253797	72.34718976	0.666666667	CRA004775;PRJNA827465;Yang2025_Silkie_Wencha	0.732804055
ENSGALG00010011076	PKP2	3	0.151016116	0.124275105	1.21517593	0.224298999	0	0.502998483	0	1	CRA004775;PRJNA827465;Yang2025_Silkie_Wencha	0.732804055
ENSGALG00010029160	DUS1L	3	0.149758293	0.123233224	1.215242842	0.224273485	0.031574662	7.441870102	73.1250348	1	CRA004775;PRJNA827465;Yang2025_Silkie_Wencha	0.732804055
ENSGALG00010024506	RAB8B	3	-0.148046623	0.121816877	-1.215321115	0.224243642	0.033705395	10.73715217	81.37308694	1	CRA004775;PRJNA827465;Yang2025_Silkie_Wencha	0.732804055
ENSGALG00010009029	IGSF3	3	-0.147512741	0.121096224	-1.218144844	0.223168935	0.01812735	3.489779293	42.68978545	0.666666667	CRA004775;PRJNA827465;Yang2025_Silkie_Wencha	0.732804055
ENSGALG00010027703	NCSTN	3	-0.144928016	0.118731886	-1.220632644	0.222225141	0.024695572	4.898219948	59.16884049	1	CRA004775;PRJNA827465;Yang2025_Silkie_Wencha	0.732804055
ENSGALG00010016350	CASC4	3	0.140356856	0.114948777	1.221038276	0.222071528	0.026900778	6.361662948	68.56167929	1	CRA004775;PRJNA827465;Yang2025_Silkie_Wencha	0.732804055
ENSGALG00010016917	METTL6	3	0.129913224	0.106953196	1.214673609	0.224490602	0.017211651	4.329262508	53.80275517	1	CRA004775;PRJNA827465;Yang2025_Silkie_Wencha	0.732804055
ENSGALG00010024841	ENSGALG0001002484	3	-0.128879527	0.106131886	-1.214333706	0.224620319	0	0.160607193	0	1	CRA004775;PRJNA827465;Yang2025_Silkie_Wencha	0.732804055
ENSGALG00010020120	DAAM2	3	0.114358804	0.093858133	1.218421886	0.223063692	0	0.777381607	0	0.666666667	CRA004775;PRJNA827465;Yang2025_Silkie_Wencha	0.732804055
ENSGALG00010025477	ENSGALG0001002547	3	0.104800564	0.086223196	1.215456732	0.224191942	0.011883656	3.943253179	49.28045678	1	CRA004775;PRJNA827465;Yang2025_Silkie_Wencha	0.732804055
ENSGALG00010015580	GEMIN5	3	-0.102847651	0.084398942	-1.218589352	0.223000093	0.012850466	6.499104373	69.22652899	1	CRA004775;PRJNA827465;Yang2025_Silkie_Wencha	0.732804055
ENSGALG00010001298	DOCK4	3	0.098769685	0.081307436	1.214768177	0.224454521	0	1.927374427	0	1	CRA004775;PRJNA827465;Yang2025_Silkie_Wencha	0.732804055
ENSGALG00010024902	LARP6	3	-0.095413803	0.078496687	-1.215513764	0.224170202	0	0.829029238	0	0.666666667	CRA004775;PRJNA827465;Yang2025_Silkie_Wencha	0.732804055
ENSGALG00010018872	KCTD9	3	-0.095165493	0.078343581	-1.214719724	0.224473007	0.007446869	3.26228925	38.69335775	1	CRA004775;PRJNA827465;Yang2025_Silkie_Wencha	0.732804055
ENSGALG00010011424	ACADL	3	-0.080785702	0.066452726	-1.215686797	0.224104256	0	0.845057843	0	0.666666667	CRA004775;PRJNA827465;Yang2025_Silkie_Wencha	0.732804055
ENSGALG00010012841	L3MBTL2	3	-0.078202044	0.064279572	-1.216592494	0.2237593	0	1.658022203	0	1	CRA004775;PRJNA827465;Yang2025_Silkie_Wencha	0.732804055
ENSGALG00010004689	BBX	3	0.074514629	0.061305159	1.215470765	0.224186592	0	0.45145117	0	1	CRA004775;PRJNA827465;Yang2025_Silkie_Wencha	0.732804055
ENSGALG00010012824	HIBCH	3	-0.072426601	0.059601913	-1.215172421	0.224300337	0	0.511684296	0	0.666666667	CRA004775;PRJNA827465;Yang2025_Silkie_Wencha	0.732804055
ENSGALG00010006049	AIMP1	3	0.070601615	0.057843451	1.22056366	0.222251273	0	0.136631207	0	1	CRA004775;PRJNA827465;Yang2025_Silkie_Wencha	0.732804055
ENSGALG00010000718	EEF1G	3	-0.066923882	0.055134133	-1.213837569	0.224809757	0	0.192905582	0	1	CRA004775;PRJNA827465;Yang2025_Silkie_Wencha	0.732804055
ENSGALG00010017803	ENSGALG0001001780	3	0.062437106	0.051375545	1.215307913	0.224248675	0	1.483886591	0	1	CRA004775;PRJNA827465;Yang2025_Silkie_Wencha	0.732804055
ENSGALG00010020248	NOLC1	3	-0.056724237	0.046519834	-1.219355959	0.222709118	0	1.709583073	0	0.666666667	CRA004775;PRJNA827465;Yang2025_Silkie_Wencha	0.732804055
ENSGALG00010029695	TAOK1	3	-0.043447539	0.035768726	-1.214679524	0.224488345	0	1.521576765	0	0.666666667	CRA004775;PRJNA827465;Yang2025_Silkie_Wencha	0.732804055
ENSGALG00010012437	ENSGALG0001001243	3	-3.313008513	2.734208035	-1.211688529	0.225631632	18.77510161	15.66587533	87.2333977	0.666666667	CRA004775;PRJNA827465;Yang2025_Silkie_Wencha	0.732807409
ENSGALG00010028903	ASTN2	3	0.390462523	0.321939294	1.212845186	0.225189016	0.235277473	8.538987624	76.57801969	0.666666667	CRA004775;PRJNA827465;Yang2025_Silkie_Wencha	0.732807409
ENSGALG00010015776	SPOCK1	3	0.38032753	0.31407854	1.210931286	0.225921741	0.171654457	4.752450993	57.9164519	1	CRA004775;PRJNA827465;Yang2025_Silkie_Wencha	0.732807409

ENSGALG00010005546	NRF1	3	0.250912143	0.207032101	1.211948533	0.225532082	0.108535114	14.26778981	85.98241195	0.666666667	CRA004775;PRJNA827465;Yang2025_Silkie_Wencha	0.732807409
ENSGALG00010017554	FAM131A	3	0.244146495	0.201479605	1.211767784	0.225601283	0	1.07739078	0	0.666666667	CRA004775;PRJNA827465;Yang2025_Silkie_Wencha	0.732807409
ENSGALG00010021148	GPATCH8	3	0.233670748	0.192954363	1.211015622	0.225589418	0.096394488	16.23015294	87.67725722	0.666666667	CRA004775;PRJNA827465;Yang2025_Silkie_Wencha	0.732807409
ENSGALG00010029179	PIGW	3	-0.23180607	0.191056143	-1.21328771	0.22501984	0.078765006	7.513244245	73.38034097	0.666666667	CRA004775;PRJNA827465;Yang2025_Silkie_Wencha	0.732807409
ENSGALG00010019700	HES5B	3	-0.206790717	0.170721536	-1.211274932	0.225790053	0.028904126	2.928928829	31.71565044	1	CRA004775;PRJNA827465;Yang2025_Silkie_Wencha	0.732807409
ENSGALG00010011982	STK3	3	-0.193065654	0.159424885	-1.21101329	0.225890311	0.057086123	10.86227947	81.58765841	0.666666667	CRA004775;PRJNA827465;Yang2025_Silkie_Wencha	0.732807409
ENSGALG00010003447	ZC2HC1A	3	0.183558242	0.151384603	1.21252913	0.225309897	0.038492967	4.698725695	57.43526799	1	CRA004775;PRJNA827465;Yang2025_Silkie_Wencha	0.732807409
ENSGALG00010001125	CLRN1	3	-0.170236902	0.140344561	-1.212992511	0.225126884	0	0.302679627	0	1	CRA004775;PRJNA827465;Yang2025_Silkie_Wencha	0.732807409
ENSGALG00010023510	ASPHD2	3	-0.164947187	0.135956075	-1.21323881	0.22502853	0	0.75153456	0	1	CRA004775;PRJNA827465;Yang2025_Silkie_Wencha	0.732807409
ENSGALG00010024733	MBTPS1	3	-0.154927741	0.127898754	-1.211331116	0.225768528	0.039844721	12.67561398	84.2216716	1	CRA004775;PRJNA827465;Yang2025_Silkie_Wencha	0.732807409
ENSGALG00010021264	CBFB	3	-0.147961814	0.122161157	-1.211201805	0.225818071	0.019658746	3.505798407	42.95165415	0.666666667	CRA004775;PRJNA827465;Yang2025_Silkie_Wencha	0.732807409
ENSGALG00010009367	DMXL1	3	0.139170497	0.114827293	1.211998419	0.225512986	0.006442048	2.337991383	14.4564854	0.666666667	CRA004775;PRJNA827465;Yang2025_Silkie_Wencha	0.732807409
ENSGALG00010028579	OCLN	3	-0.134744224	0.111231248	-1.211388224	0.22574665	0.006482689	2.353989035	15.03783705	0.666666667	CRA004775;PRJNA827465;Yang2025_Silkie_Wencha	0.732807409
ENSGALG00010007098	NDUFAF7	3	0.079969105	0.065941835	1.212721861	0.225236179	0	1.375459672	0	0.666666667	CRA004775;PRJNA827465;Yang2025_Silkie_Wencha	0.732807409
ENSGALG00010021461	SNX33	3	0.072132485	0.059523452	1.21183302	0.225576306	0.002572658	2.382992325	16.07190762	0.666666667	CRA004775;PRJNA827465;Yang2025_Silkie_Wencha	0.732807409
ENSGALG00010022021	TECPR1	3	-0.052943746	0.043665402	-1.212487321	0.225325893	0	1.141272585	0	0.666666667	CRA004775;PRJNA827465;Yang2025_Silkie_Wencha	0.732807409
ENSGALG00010012908	STRAP	3	-0.045955736	0.037937902	-1.211341021	0.225764733	0	0.901742935	0	0.666666667	CRA004775;PRJNA827465;Yang2025_Silkie_Wencha	0.732807409
ENSGALG00010024525	GP1BB	3	0.393730385	0.325556346	1.209407802	0.226506213	0.227511317	7.684986291	73.97523009	0.666666667	CRA004775;PRJNA827465;Yang2025_Silkie_Wencha	0.73317553
ENSGALG00010017020	GJB5	2	0.351696807	0.290971118	1.208700054	0.226778101	0	0.107513837	0	1	PRJNA827465;Yang2025_Silkie_Wenchang	0.73317553
ENSGALG00010002231	TMEM14A	3	-0.337905424	0.27969918	-1.208103021	0.227007638	0.204317103	26.17047813	92.35780107	1	CRA004775;PRJNA827465;Yang2025_Silkie_Wencha	0.73317553
ENSGALG00010023113	MYZAP	3	0.302195388	0.249879096	1.209366419	0.226522104	0.126576063	7.207119962	72.24966407	1	CRA004775;PRJNA827465;Yang2025_Silkie_Wencha	0.73317553
ENSGALG00010022411	ADRB1	3	-0.255174521	0.210828816	-1.210339869	0.226148505	0.051480804	7.512394959	38.2581696	0.666666667	CRA004775;PRJNA827465;Yang2025_Silkie_Wencha	0.73317553
ENSGALG00010014035	PSIP1	3	0.254312836	0.210456892	1.208384447	0.226899419	0.107043356	11.11981877	82.01409535	1	CRA004775;PRJNA827465;Yang2025_Silkie_Wencha	0.73317553
ENSGALG00010008387	PHF20L1	3	0.252697328	0.208880078	1.209772277	0.226366287	0.102654605	10.85917155	81.58238875	1	CRA004775;PRJNA827465;Yang2025_Silkie_Wencha	0.73317553
ENSGALG00010023447	C12orf57	3	-0.211192056	0.174517343	-1.210149395	0.226221572	0.038508468	3.227947117	38.0411163	1	CRA004775;PRJNA827465;Yang2025_Silkie_Wencha	0.73317553
ENSGALG00010021826	FBXO40	3	-0.206947253	0.1710475	-1.209881775	0.226324262	0.021828807	2.578784527	22.44408251	0.666666667	CRA004775;PRJNA827465;Yang2025_Silkie_Wencha	0.73317553
ENSGALG00010020705	EXOSC1	3	0.204060036	0.168771309	1.209091978	0.226627511	0.034966373	3.211563975	37.72504564	1	CRA004775;PRJNA827465;Yang2025_Silkie_Wencha	0.73317553
ENSGALG00010025892	ZC3H11A	3	0.203725581	0.168474066	1.209240008	0.226570651	0.073958115	20.77594389	90.3734819	1	CRA004775;PRJNA827465;Yang2025_Silkie_Wencha	0.73317553
ENSGALG00010021318	TP73	3	0.169498691	0.140298452	1.208129444	0.226997476	0.010604231	2.39883674	16.62625612	0.666666667	CRA004775;PRJNA827465;Yang2025_Silkie_Wencha	0.73317553
ENSGALG00010000720	RBM26	3	0.167849845	0.138891372	1.208497277	0.226856043	0.042376121	7.963175496	74.88439127	0.666666667	CRA004775;PRJNA827465;Yang2025_Silkie_Wencha	0.73317553
ENSGALG00010005692	SEC24B	3	0.159577303	0.131934159	1.209522269	0.226462261	0.035148695	6.933985875	71.1565608	0.666666667	CRA004775;PRJNA827465;Yang2025_Silkie_Wencha	0.73317553
ENSGALG00010009000	FAM149B1	3	0.13284809	0.109892363	1.208892839	0.226704018	0.020783938	4.720656485	57.63301129	0.666666667	CRA004775;PRJNA827465;Yang2025_Silkie_Wencha	0.73317553
ENSGALG00010018092	ENSGALG00010018092	3	-0.125626754	0.103831064	-1.209914927	0.226311539	0	0.929629338	0	0.666666667	CRA004775;PRJNA827465;Yang2025_Silkie_Wencha	0.73317553
ENSGALG00010010252	WDR7	3	0.102962497	0.085228941	1.208069648	0.227020473	0	1.085227839	0	1	CRA004775;PRJNA827465;Yang2025_Silkie_Wencha	0.73317553
ENSGALG00010016792	HMOX2	3	0.077994247	0.064518684	1.20886296	0.226715499	0	0.063126609	0	1	CRA004775;PRJNA827465;Yang2025_Silkie_Wencha	0.73317553
ENSGALG00010012116	RBM12B	3	-0.051229148	0.042402704	-1.208157576	0.226986656	0	1.629375447	0	1	CRA004775;PRJNA827465;Yang2025_Silkie_Wencha	0.73317553
ENSGALG00010004837	CENPC	3	0.081891143	0.067795327	1.207917228	0.227079102	0	0.036446581	0	1	CRA004775;PRJNA827465;Yang2025_Silkie_Wencha	0.733197402
ENSGALG00010023490	PHB2	3	-0.077802388	0.064433058	-1.20749178	0.227242809	0.003187199	2.36197165	15.32497859	1	CRA004775;PRJNA827465;Yang2025_Silkie_Wencha	0.733558464
ENSGALG00010012286	ENSGALG00010012286	3	-4.090223637	3.393535681	-1.20529855	0.228088069	30.39198941	20.29314897	90.14445711	0.666666667	CRA004775;PRJNA827465;Yang2025_Silkie_Wencha	0.733660518
ENSGALG00010000262	HNRNPKL	3	-3.174337745	2.641798022	-1.2015823	0.229525402	17.20405033	13.74521603	85.44948296	0.666666667	CRA004775;PRJNA827465;Yang2025_Silkie_Wencha	0.733660518
ENSGALG00010029431	ENSGALG00010029431	3	2.290061719	1.909782412	1.199121797	0.23048059	7.975640431	7.508417163	73.36322747	0.666666667	CRA004775;PRJNA827465;Yang2025_Silkie_Wencha	0.733660518
ENSGALG00010016177	ENSGALG00010016177	2	-1.90384678	1.591343834	-1.19637676	0.231549566	0.933446978	1.211080004	17.42907185	1	PRJNA827465;Yang2025_Silkie_Wenchang	0.733660518
ENSGALG00010010975	ATP8B1	2	1.306690597	1.083851168	1.205599658	0.227971891	2.052388295	7.876586467	87.30414496	1	CRA004775;PRJNA827465	0.733660518
ENSGALG00010021711	ENSGALG00010021711	2	1.305885682	1.091370473	1.196555811	0.231479733	2.29415503	26.71586025	96.25690511	1	PRJNA827465;Yang2025_Silkie_Wenchang	0.733660518
ENSGALG00010025898	ENSGALG00010025898	2	-1.241210867	1.034470144	-1.199851802	0.230196902	0	0.591661219	0	1	PRJNA827465;Yang2025_Silkie_Wenchang	0.733660518
ENSGALG00010000646	ENSGALG00010000646	3	-0.918861842	0.761879652	-1.206045915	0.227799787	1.327866229	8.963270276	77.68671547	0.666666667	CRA004775;PRJNA827465;Yang2025_Silkie_Wencha	0.733660518
ENSGALG00010021679	MASP2	2	0.87460406	0.730485756	1.197291053	0.23119313	0.795371541	3.870266782	74.16198789	1	PRJNA827465;Yang2025_Silkie_Wenchang	0.733660518
ENSGALG00010026625	ENSGALG00010026625	2	0.71411547	0.592326543	1.205611125	0.227967468	0	0.720273404	0	1	CRA004775;Yang2025_Silkie_Wenchang	0.733660518
ENSGALG00010002279	ENSGALG00010002279	2	0.676127111	0.562310867	1.202408046	0.229205473	0	0.245868675	0	1	PRJNA827465;Yang2025_Silkie_Wenchang	0.733660518
ENSGALG00010024347	OTOG	3	0.601820959	0.501829919	1.199252847	0.230429644	0.50794264	8.224230371	75.68161506	0.666666667	CRA004775;PRJNA827465;Yang2025_Silkie_Wencha	0.733660518
ENSGALG00010009273	MAL2	3	0.53986709	0.448285258	1.20429365	0.2284761	0.210253855	3.065410274	34.75587862	0.666666667	CRA004775;PRJNA827465;Yang2025_Silkie_Wencha	0.733660518
ENSGALG00010002629	ENSGALG00010002629	2	0.537518099	0.449130752	1.196796471	0.231385894	0	0.02807728	0	1	CRA004775;PRJNA827465	0.733660518
ENSGALG00010029736	ENSGALG00010029736	3	-0.412064864	0.344320169	-1.196749134	0.23140435	0.294948636	24.35000433	91.7864491	0.666666667	CRA004775;PRJNA827465;Yang2025_Silkie_Wencha	0.733660518
ENSGALG00010016791	LRRTM1	2	0.404134417	0.3362321	1.201950726	0.229382619	0	0.195934498	0	1	PRJNA827465;Yang2025_Silkie_Wenchang	0.733660518
ENSGALG00010027098	EXOG	3	-0.330634864	0.276349452	-1.196437561	0.231525851	0.182881246	10.64018103	81.20332733	0.666666667	CRA004775;PRJNA827465;Yang2025_Silkie_Wencha	0.733660518
ENSGALG00010010698	GPRIN2	3	-0.318258532	0.265434017	-1.199011847	0.230523339	0.108672699	4.56867584	56.22363962	0.666666667	CRA004775;PRJNA827465;Yang2025_Silkie_Wencha	0.733660518
ENSGALG00010013501	AKR1B10	3	-0.26998753	0.22400439	-1.205277853	0.228096056	0.078303276	4.265171483	53.10856766	0.666666667	CRA004775;PRJNA827465;Yang2025_Silkie_Wencha	0.733660518
ENSGALG00010009228	ATP6V0E1	3	-0.268938571	0.22362188	-1.202648736	0.22911228	0.109140927	7.759808345	74.22616757	1	CRA004775;PRJNA827465;Yang2025_Silkie_Wencha	0.733660518
ENSGALG00010023185	FBXW4	3	-0.268840556	0.224705277	-1.196414075	0.231535011	0.095153432	5.890874198	66.04918162	0.666666667	CRA004775;PRJNA827465;Yang2025_Silkie_Wencha	0.733660518
ENSGALG00010004981	ENSGALG00010004981	3	0.263023491	0.219008463	1.200974098	0.229761249	0.067948186	3.910123965	48.85072652	0.666666667	CRA004775;PRJNA827465;Yang2025_Silkie_Wencha	0.733660518
ENSGALG00010028202	QSOX2	3	-0.260106524	0.216690315	-1.200360633	0.229999311	0.111691895	19.49258976	89.7396907	0.666666667	CRA004775;PRJNA827465;Yang2025_Silkie_Wencha	0.733660518
ENSGALG00010001180	SPIRE1	3	0.255918841	0.212347501	1.205188851	0.228130406	0.07858809	4.772444032	58.09275108	0.666666667	CRA004775;PRJNA827465;Yang2025_Silkie_Wencha	0.733660518
ENSGALG00010019810	ZFAND6	3	-0.248062195	0.206168101	-1.203203569	0.228897554	0.100758859	13.54108532	85.23013516	0.666666667	CRA004775;PRJNA827465;Yang2025_Silkie_Wencha	0.733660518
ENSGALG00010016599	ENSGALG00010016599	3	-0.247962247	0.206065506	-1.203317581	0.228853448	0	1.411250833	0	1	CRA004775;PRJNA827465;Yang2025_Silkie_Wencha	0.733660518
ENSGALG00010005303	ENSGALG00010005303	3	-0.247022619	0.20544673	-1.202368217	0.229220897	0	1.663914835	0	0.666666667	CRA004775;PRJNA827465;Yang2025_Silkie_Wencha	0.733660518
ENSGALG00010029408	KIF12	3	0.246992837	0.206214591	1.197746658	0.231015659	0	0.826195783	0	1	CRA004775;PRJNA827465;Yang2025_Silkie_Wencha	0.733660518
ENSGALG00010021052	CEP15	3	-0.226574972	0.189383718	-1.196380417	0.23154814	0.07065157	6.29463169	68.22689399	1	CRA004775;PRJNA827465;Yang2025_Silkie_Wencha	0.733660518
ENSGALG00010014839	TM7SF3	3	-0.218581226	0.182515539	-1.197603375	0.231071462	0.06729158	7.339860898	72.7515272	0.666666667	CRA004775;PRJNA827465;Yang2025_Silkie_Wencha	0.733660518
ENSGALG00010012271	ENSGALG00010012271	3	0.21434812	0.177828384	1.205365054	0.228062406	0.032180299	2.958810263	32.40526352	0.666666667	CRA004775;PRJNA827465;Yang2025_Silkie_Wencha	0.733660518
ENSGALG00010029529	TEPSIN	3	0.210756019	0.175634812	1.199967228	0.230152069	0.064056315	6.722777541	70.25039148	0.666666667	CRA004775;PRJNA827465;Yang2025_Silkie_Wencha	0.733660518
ENSGALG00010012538	RAP1B	3	-0.209257292	0.174752911	-1.19744667	0.231132502	0.067575073	14.91235733	86.58830421	1	CRA004775;PRJNA827465;Yang2025_Silkie_Wencha	0.733660518
ENSGALG00010026950	LHFPL5	3	0.204121089	0.170219121	1.199166629	0.23046316	0.018730705	2.45078176	18.39338643	1	CRA004775;PRJNA827465;Yang2025_Silkie_Wencha	0.733660518
ENSGALG00010027710	ENSGALG00010027710	3	0.203583166	0.168675209	1.20695369	0.227449979	0	1.684339217	0	0.666666667	CRA004775;PRJNA827465;Yang2025_Silkie_Wencha	0.733660518
ENSGALG00010026051	TAF11	3	-0.201217353	0.167168545	-1.203679515	0.228713473	0.053732247	5.598744457	64.2777052	0.666666667	CRA004775;PRJNA827465;Yang2025_Silkie_Wencha	0.733660518
ENSGALG00010010997	LAP3	3	-0.195701692	0.163179943	-1.199299915	0.230411348	0.054109679	6.764151063	70.43235757	0.666666667	CRA004775;PRJNA827465;Yang2025_Silkie_Wencha	0.733660518
ENSGALG00010026218	FTSJ3	3	0.195147143	0.163144327	1.196162605	0.231633111	0.05803496	10.36331953	80.70116439	0.666666667	CRA004775;PRJNA827465;Yang2025_Silkie_Wencha	0.733660518
ENSGALG00010014713	ST8SIA1	3	-0.192405237	0.160642911	-1.197720058	0.231026018	0.032994548	3.485132037	42.61336504	1	CRA004775;PRJNA827465;Yang2025_Silkie_Wencha	0.733660518
ENSGALG00010024323	TTPAL	3	-0.190741167	0.158237051	-1.205414069	0.228043493	0.052870489	7.939744273	74.81027183	0.666666667	CRA004775;PRJNA827465;Yang2025_Silkie_Wencha	0.733660518
ENSGALG00010017958	CAPN10	3	-0.187951255	0.155842171	-1.206035906	0.227803646	0.044011675	5.248879781	61.89663159	0.666666667	CRA004775;PRJNA827465;Yang2025_Silkie_Wencha	0.733660518
ENSGALG00010008258	CCDC82	3	0.18464043	0.154355005	1.196206302	0.231616062	0.043109171	5.478804715	63.4956874	0.666666667	CRA004775;PRJNA827465;Yang2025_Silkie_Wencha	0.733660518
ENSGALG00010023169	RAN	3	-0.184440587	0.154094407	-1.19693239	0.231332908	0.046582594	5.798456272	65.50806101	0.666666667	CRA004775;PRJNA827465;Yang2025_Silkie_Wencha	0.733660518
ENSGALG00010022533	TMEM119	3	-0.183438895	0.152383043	-1.203801238	0.228666411	0.037151589	4.518027089	55.73289047	0.666666667	CRA004775;PRJNA827465;Yang2025_Silkie_Wencha	0.733660518
ENSGALG00010020840	LPAR3	2	0.17980558	0.149671321	1.201336229	0.229620802	0	0.790447538	0	0.5	PRJNA827465;Yang2025_Silkie_Wenchang	0.733660518
ENSGALG00010018807	RD3	3	-0.172165825	0.143454712	-1.200140606	0.230084738	0	0.25309423	0	0.666666667	CRA004775;PRJNA827465;Yang2025_Silkie_Wencha	0.733660518
ENSGALG00010022199	RBM39	3	0.169604049	0.141368063	1.199733837	0.230242727	0.048790089	13.0691983	84.6968425	1	CRA004775;PRJNA827465;Yang2025_Silkie_Wencha	0.733660518
ENSGALG00010024918	ATG4B	3	-0.16576715	0.138385899	-1.197861571	0.230970913	0.044882618	9.879296722	79.75564399	0.666666667	CRA004775;PRJNA827465;Yang2025_Silkie_Wencha	0.733660518
ENSGALG00010020246	ADAMTS9	3	0.162997886	0.135398871	1.2038349	0.228653397	0.030308627	4.885102395	59.05920003	0.666666667	CRA004775;PRJNA827465;Yang2025_Silkie_Wencha	0.733660518
ENSGALG00010013413	WRB	3	-0.158045697	0.131501298	-1.201856552	0.229419111	0.038774312	8.645059804	76.86540006	0.666666667	CRA004775;PRJNA827465;Yang2025_Silkie_Wencha	0.733660518
ENSGALG00010001693	NKIRAS1	3	-0.155639383	0.129950442	-1.197682599	0.231040606	0	1.485060888	0	1	CRA004775;PRJNA827465;Yang2025_Silkie_Wencha	0.733660518
ENSGALG00010006300	GCC1	3	0.151259876	0.125771455	1.202656648	0.229109217	0.034034702	7.43731798	73.10858558	0.666666667	CRA004775;PRJNA827465;Yang2025_Silkie_Wencha	0.733660518
ENSGALG00010009606	CHSY3	3	0.149069972	0.123795294	1.204165096	0.228525774	0	0.630263947	0	0.666666667	CRA004775;PRJNA827465;Yang2025_Silkie_Wencha	0.733660518
ENSGALG00010027150	YOD1	3	-0.145132834	0.120950021	-1.199940545	0.230162432	0.029590601	7.586533253	73.63749775	0.666666667	CRA004775;PRJNA827465;Yang2025_Silkie_Wencha	0.733660518
ENSGALG00010011417	WFS1	3	0.144375068	0.119977323	1.203352965	0.228839761	0.026070251	5.168723317	61.30572527	0.666666667	CRA004775;PRJNA827465;Yang2025_Silkie_Wencha	0.733660518
ENSGALG00010029606	ABHD11	3	0.14159571	0.117950251	1.200469756	0.229956952	0.003008251	2.14657528	6.828331704	0.666666667	CRA004775;PRJNA827465;Yang2025_Silkie_Wencha	0.733660518
ENSGALG00010007912	TRAPPC2L	3	-0.133737825	0.111482412	-1.199631598	0.230282449	0.012159432	2.67442761	25.21764312	1	CRA004775;PRJNA827465;Yang2025_Silkie_Wencha	0.733660518
ENSGALG00010008542	MOCOS	3	0.132954933	0.11077777	1.200195061	0.230063593	0	1.004821903	0	1	CRA004775;PRJNA827465;Yang2025_Silkie_Wencha	0.733660518
ENSGALG00010007217	MSH3	3	0.127421565	0.106488077	1.196580581	0.231470073	0	0.738684364	0	0.666666667	CRA004775;PRJNA827465;Yang2025_Silkie_Wencha	0.733660518
ENSGALG00010021765	EIF2AK1	3	0.124504047	0.1033833	1.204295539	0.22847537	0.021379821	7.258801406	72.4472418	0.666666667	CRA004775;PRJNA827465;Yang2025_Silkie_Wencha	0.733660518
ENSGALG00010024644	EIF6	3	-0.112567588	0.0939824	-1.19775179	0.231013661	0.017528186	6.043786049	66.90816015	1	CRA004775;PRJNA827465;Yang2025_Silkie_Wencha	0.733660518
ENSGALG00010028155	CIZ1	3	0.111772285	0.09309794	1.200588168	0.229910993	0.017524588	7.617199065	73.74362961	0.666666667	CRA004775;PRJNA827465;Yang2025_Silkie_Wencha	0.733660518
ENSGALG00010023421	OLFM3	3	-0.110857272	0.092608728	-1.197049928	0.231287095	0.006910235	2.647417177	24.45467161	0.666666667	CRA004775;PRJNA827465;Yang2025_Silkie_Wencha	0.733660518
ENSGALG00010010435	GAS1	3	0.102472394	0.085470834	1.198916515	0.230560409	0	0.551051087	0	0.666666667	CRA004775;PRJNA827465;Yang2025_Silkie_Wencha	0.733660518
ENSGALG00010020257	ZNF710	3	0.101679759	0.084986325	1.196424949	0.23153077	0.010180845	3.786795631	47.18489734	0.666666667	CRA004775;PRJNA827465;Yang2025_Silkie_Wencha	0.733660518
ENSGALG00010022979	LAMA5	3	-0.094996988	0.07932743	-1.197530127	0.231099992	0	1.105670933	0	0.666666667	CRA004775;PRJNA827465;Yang2025_Silkie_Wencha	0.733660518
ENSGALG00010012119	EPG5	3	-0.092591994	0.076834678	-1.205080783	0.228172118	0	0.832884862	0	1	CRA004775;PRJNA827465;Yang2025_Silkie_Wencha	0.733660518
ENSGALG00010013391	HMGN1	3	0.086858056	0.072564955	1.196969748	0.231318346	0.005079015	2.769600804	27.78742709	1	CRA004775;PRJNA827465;Yang2025_Silkie_Wencha	0.733660518
ENSGALG00010007983	RCHY1	3	-0.084986619	0.07069143	-1.202219543	0.229278479	0	0.578517885	0	0.666666667	CRA004775;PRJNA827465;Yang2025_Silkie_Wencha	0.733660518
ENSGALG00010015158	PIN4	3	0.079907797	0.066407978	1.203286098	0.228865627	0	0.347431189	0	0.666666667	CRA004775;PRJNA827465;Yang2025_Silkie_Wencha	0.733660518
ENSGALG00010006063	POGLUT2	3	-0.077725251	0.064415961	-1.20661479	0.227580528	0.004736336	3.226309151	38.00966037	0.666666667	CRA004775;PRJNA827465;Yang2025_Silkie_Wencha	0.733660518
ENSGALG00010017830	DENND4A	3	0.0765173	0.063635919	1.202423112	0.229199639	0	1.913721324	0	1	CRA004775;PRJNA827465;Yang2025_Silkie_Wencha	0.733660518
ENSGALG00010009638	RND3	3	0.075472571	0.06257873	1.206041918	0.227801328	0	0.887600952	0	0.666666667	CRA004775;PRJNA827465;Yang2025_Silkie_Wencha	0.733660518
ENSGALG00010014236	WDR44	3	0.075172125	0.06233435	1.205950242	0.227836677	0.003228965	2.507786012	20.24837883	1	CRA004775;PRJNA827465;Yang2025_Silkie_Wencha	0.733660518
ENSGALG00010001135	GYG1	3	-0.074901216	0.062486852	-1.198671608	0.230655662	0	0.863263736	0	0.666666667	CRA004775;PRJNA827465;Yang2025_Silkie_Wencha	0.733660518
ENSGALG00010013349	ETS2	3	-0.07173749	0.059957858	-1.196465179	0.231515079	0	1.853174063	0	0.666666667	CRA004775;PRJNA827465;Yang2025_Silkie_Wencha	0.733660518
ENSGALG00010018381	HAPSTR1	3	-0.070104825	0.058415009	-1.200116651	0.230094039	0.003280952	2.811745128	28.86979762	0.666666667	CRA004775;PRJNA827465;Yang2025_Silkie_Wencha	0.733660518
ENSGALG00010007002	EXOC4	3	-0.062519261	0.051814182	-1.206605199	0.227584224	0	0.540230964	0	1	CRA004775;PRJNA827465;Yang2025_Silkie_Wencha	0.733660518
ENSGALG00010028712	FBXW2	3	-0.062107382	0.05168143	-1.201734976	0.229466225	0	0.878472443	0	1	CRA004775;PRJNA827465;Yang2025_Silkie_Wencha	0.733660518
ENSGALG00010027301	SCAP	3	-0.057681318	0.047956811	-1.20277635	0.229062879	0.000740864	2.215545203	9.728765735	1	CRA004775;PRJNA827465;Yang2025_Silkie_Wencha	0.733660518
ENSGALG00010009686	MAT2B	3	-0.053568462	0.044432377	-1.205617752	0.227964911	0	1.829678937	0	0.666666667	CRA004775;PRJNA827465;Yang2025_Silkie_Wencha	0.733660518
ENSGALG00010014967	HNRNPH1	3	-0.048130826	0.040056484	-1.201573895	0.229528661	0	1.233859962	0	0.666666667	CRA004775;PRJNA827465;Yang2025_Silkie_Wencha	0.733660518
ENSGALG00010025512	NRAS	3	0.04749582	0.039441399	1.20421233	0.228507521	0.000330662	2.111468652	5.279199944	0.666666667	CRA004775;PRJNA827465;Yang2025_Silkie_Wencha	0.733660518
ENSGALG00010005356	DNAJC3	3	0.041602399	0.034534809	1.204651182	0.228337989	0	0.475706251	0	1	CRA004775;PRJNA827465;Yang2025_Silkie_Wencha	0.733660518
ENSGALG00010003456	CDS1	3	0.293567423	0.245452471	1.196025534	0.231686595	0	1.257844286	0	1	CRA004775;PRJNA827465;Yang2025_Silkie_Wencha	0.733665569
ENSGALG00010007166	RASGRF2	3	0.253771459	0.212221308	1.195786896	0.231779731	0.066377449	3.929800474	49.10683091	0.666666667	CRA004775;PRJNA827465;Yang2025_Silkie_Wencha	0.733796152
ENSGALG00010011467	ENSGALG00010011467	3	-3.448398979	2.887709854	-1.194163941	0.232413845	21.58168336	18.00794217	88.8937893	0.666666667	CRA004775;PRJNA827465;Yang2025_Silkie_Wencha	0.733883805
ENSGALG00010023275	TWIST3	2	-0.632458097	0.529486595	-1.194474238	0.232292512	0.14547278	1.349707601	25.90987859	1	PRJNA827465;Yang2025_Silkie_Wenchang	0.733883805
ENSGALG00010009649	TENM2	3	0.406108058	0.34004912	1.19426293	0.232375133	0.232023291	7.996141665	74.98793689	0.666666667	CRA004775;PRJNA827465;Yang2025_Silkie_Wencha	0.733883805
ENSGALG00010024366	PARD6A	3	0.276786348	0.231790692	1.194121929	0.232430276	0	1.518109262	0	0.666666667	CRA004775;PRJNA827465;Yang2025_Silkie_Wencha	0.733883805
ENSGALG00010012885	PMS1	3	0.19565598	0.163833504	1.194236681	0.232385398	0.060463547	13.12020771	84.75633889	0.666666667	CRA004775;PRJNA827465;Yang2025_Silkie_Wencha	0.733883805
ENSGALG00010000366	EMID1	3	0.190890222	0.159797538	1.194575484	0.232252933	0	0.437839271	0	1	CRA004775;PRJNA827465;Yang2025_Silkie_Wencha	0.733883805
ENSGALG00010013808	PLAAT1	3	-0.182626718	0.152856147	-1.194762019	0.232180024	0.03905384	4.826577416	58.56276968	0.666666667	CRA004775;PRJNA827465;Yang2025_Silkie_Wencha	0.733883805
ENSGALG00010023993	C19orf12	3	-0.174235989	0.145852795	-1.194601647	0.232242705	0.041848812	6.055641613	66.97294642	0.666666667	CRA004775;PRJNA827465;Yang2025_Silkie_Wencha	0.733883805
ENSGALG00010012404	VAMP7	3	-0.155104292	0.129800171	-1.194946749	0.232107836	0.040647885	18.4115833	89.13727316	0.666666667	CRA004775;PRJNA827465;Yang2025_Silkie_Wencha	0.733883805
ENSGALG00010001300	MRPL49	3	0.138562549	0.116011352	1.194387845	0.232326289	0.002135062	2.100811144	4.798677118	1	CRA004775;PRJNA827465;Yang2025_Silkie_Wencha	0.733883805
ENSGALG00010018453	TMX4	3	-0.089444827	0.074887183	-1.19439434	0.23232375	0.006787551	3.35774927	40.43629113	1	CRA004775;PRJNA827465;Yang2025_Silkie_Wencha	0.733883805
ENSGALG00010004505	ENSGALG00010004505	3	-0.089390954	0.074773115	-1.195495916	0.231893331	0	0.113769885	0	0.666666667	CRA004775;PRJNA827465;Yang2025_Silkie_Wencha	0.733883805
ENSGALG00010018423	IGFALS	3	-0.611613964	0.512381449	-1.19366922	0.232607385	0.18350885	2.607092283	23.2861831	1	CRA004775;PRJNA827465;Yang2025_Silkie_Wencha	0.73395131
ENSGALG00010027607	ABL1	3	-0.221163198	0.185265495	-1.193763567	0.232570467	0.07981995	12.33869816	83.79083454	1	CRA004775;PRJNA827465;Yang2025_Silkie_Wencha	0.73395131
ENSGALG00010010288	ENSGALG00010010288	3	0.208760311	0.174881818	1.193722214	0.232586648	0.046844913	4.128771823	51.55944465	0.666666667	CRA004775;PRJNA827465;Yang2025_Silkie_Wencha	0.73395131
ENSGALG00010022290	NEXN	3	0.643622205	0.539491666	1.19301603	0.232863095	0.746646613	22.66042943	91.17404193	0.666666667	CRA004775;PRJNA827465;Yang2025_Silkie_Wencha	0.734102854
ENSGALG00010015898	ENSGALG00010015898	3	0.456654873	0.382757942	1.193064396	0.232844154	0.317722986	10.06266383	80.12454719	0.666666667	CRA004775;PRJNA827465;Yang2025_Silkie_Wencha	0.734102854
ENSGALG00010008179	SEMA6A	3	0.242998943	0.203679805	1.193043871	0.232852192	0.071551578	4.75023846	57.8968505	0.666666667	CRA004775;PRJNA827465;Yang2025_Silkie_Wencha	0.734102854
ENSGALG00010014626	C2CD5	3	0.17198167	0.144156477	1.193020766	0.23286124	0.047935214	9.913121491	79.82472018	1	CRA004775;PRJNA827465;Yang2025_Silkie_Wencha	0.734102854
ENSGALG00010012781	SPIN1L	3	-3.732237184	3.133655492	-1.19101707	0.233646884	25.39237512	17.50980161	88.57782604	0.666666667	CRA004775;PRJNA827465;Yang2025_Silkie_Wencha	0.734277773
ENSGALG00010000249	ENSGALG00010000249	3	-3.682614721	3.113941241	-1.182621776	0.236959078	24.62430973	14.86286489	86.54364408	1	CRA004775;PRJNA827465;Yang2025_Silkie_Wencha	0.734277773
ENSGALG00010015565	ENSGALG00010015565	3	1.803763708	1.520405113	1.186370456	0.235476034	3.17474081	3.684020915	45.7114917	0.666666667	CRA004775;PRJNA827465;Yang2025_Silkie_Wencha	0.734277773
ENSGALG00010024875	GAL3ST2	2	1.61002465	1.363438226	1.180856323	0.237659805	2.947022399	4.81762309	79.24287597	1	CRA004775;Yang2025_Silkie_Wenchang	0.734277773
ENSGALG00010025942	ENSGALG00010025942	2	1.18257865	0.994938646	1.188594547	0.234599255	0	0.360146009	0	1	PRJNA827465;Yang2025_Silkie_Wenchang	0.734277773
ENSGALG00010004504	ZNF804B	2	0.894876724	0.751328211	1.191059661	0.233630164	0.73328852	2.805258968	64.35266721	1	PRJNA827465;Yang2025_Silkie_Wenchang	0.734277773
ENSGALG00010002050	ENSGALG00010002050	3	0.843745206	0.708886888	1.190239544	0.233952255	1.143938091	9.824488819	79.64270674	0.666666667	CRA004775;PRJNA827465;Yang2025_Silkie_Wencha	0.734277773
ENSGALG00010005847	ENSGALG00010005847	3	-0.707151574	0.598216089	-1.182100561	0.237165802	0.679647548	5.512108014	63.71624078	0.666666667	CRA004775;PRJNA827465;Yang2025_Silkie_Wencha	0.734277773
ENSGALG00010025900	FAM237A	2	-0.666747162	0.562146032	-1.186074656	0.235592818	0.171806207	1.288027357	22.36189747	0.5	CRA004775;PRJNA827465	0.734277773
ENSGALG00010001226	VEPH1	2	-0.575027895	0.484055977	-1.187936773	0.234858321	0.265593159	2.290845504	56.34799473	1	PRJNA827465;Yang2025_Silkie_Wenchang	0.734277773
ENSGALG00010027487	MPZ	3	0.50031099	0.421179073	1.187881883	0.234879948	0.407051163	14.60476982	86.30584375	0.666666667	CRA004775;PRJNA827465;Yang2025_Silkie_Wencha	0.734277773
ENSGALG00010007908	IRX1	2	0.473824649	0.401077948	1.181377963	0.237452608	0	0.230800788	0	1	PRJNA827465;Yang2025_Silkie_Wenchang	0.734277773
ENSGALG00010002553	ENSGALG00010002553	3	0.451600977	0.380679779	1.186301457	0.235503271	0.266357029	6.060836535	67.00125488	0.666666667	CRA004775;PRJNA827465;Yang2025_Silkie_Wencha	0.734277773
ENSGALG00010001642	ENSGALG00010001642	3	0.424618987	0.359314908	1.181746088	0.237306465	0.320799353	13.40477049	85.07993851	0.666666667	CRA004775;PRJNA827465;Yang2025_Silkie_Wencha	0.734277773
ENSGALG00010007271	CADM2	2	0.418027179	0.353043412	1.184067357	0.236386401	0.119016906	1.852875619	46.02983657	1	PRJNA827465;Yang2025_Silkie_Wenchang	0.734277773
ENSGALG00010019009	NRAP	3	0.401669435	0.339473786	1.183211932	0.236725165	0.251626545	13.11626124	84.75175232	0.666666667	CRA004775;PRJNA827465;Yang2025_Silkie_Wencha	0.734277773
ENSGALG00010001845	ANKRD12	3	0.400859245	0.338412623	1.184528053	0.236204098	0.292036135	14.619464	86.31960789	0.666666667	CRA004775;PRJNA827465;Yang2025_Silkie_Wencha	0.734277773
ENSGALG00010021479	SKAP1	3	-0.397314399	0.335627208	-1.183796752	0.236493528	0.134757587	3.281824785	39.05829436	0.666666667	CRA004775;PRJNA827465;Yang2025_Silkie_Wencha	0.734277773
ENSGALG00010012506	TP53INP1	3	-0.378095813	0.318138427	-1.188463203	0.234650969	0.258818989	48.65992888	95.88984192	0.666666667	CRA004775;PRJNA827465;Yang2025_Silkie_Wencha	0.734277773
ENSGALG00010018078	NKX6-3	3	0.371466542	0.314216201	1.182200477	0.237126164	0	0.456654891	0	1	CRA004775;PRJNA827465;Yang2025_Silkie_Wencha	0.734277773
ENSGALG00010007430	MEF2C	3	0.371120531	0.31238271	1.1880316	0.23482096	0.218585052	8.775123007	77.2082967	1	CRA004775;PRJNA827465;Yang2025_Silkie_Wencha	0.734277773
ENSGALG00010008623	TRPS1	3	0.328135061	0.277230061	1.183620058	0.236563497	0.167209316	8.883002211	77.48508947	0.666666667	CRA004775;PRJNA827465;Yang2025_Silkie_Wencha	0.734277773
ENSGALG00010025958	HOXC9	2	0.324924249	0.273207233	1.189295926	0.234323238	0	0.766836036	0	1	PRJNA827465;Yang2025_Silkie_Wenchang	0.734277773
ENSGALG00010014359	TMEM271	2	0.315412669	0.265726576	1.186982025	0.235234711	0	0.227994748	0	1	CRA004775;PRJNA827465	0.734277773
ENSGALG00010014045	GOLT1B	3	-0.314632272	0.265544921	-1.184855167	0.236074716	0.188208456	40.04907404	95.00612674	0.666666667	CRA004775;PRJNA827465;Yang2025_Silkie_Wencha	0.734277773
ENSGALG00010012532	FAM174A	3	0.306606149	0.25859948	1.18564101	0.235764099	0.135364824	6.383492526	68.66918866	0.666666667	CRA004775;PRJNA827465;Yang2025_Silkie_Wencha	0.734277773
ENSGALG00010001154	PTGR3	3	-0.283048261	0.238621999	-1.186178399	0.235551855	0.148701189	20.36157621	90.17757771	1	CRA004775;PRJNA827465;Yang2025_Silkie_Wencha	0.734277773
ENSGALG00010000146	ACADM	3	-0.278380781	0.235658823	-1.181287326	0.2374886	0.119719518	10.21730424	80.42536511	0.666666667	CRA004775;PRJNA827465;Yang2025_Silkie_Wencha	0.734277773
ENSGALG00010006345	SMC2	3	0.267707194	0.226454269	1.182168899	0.23713869	0.067172985	3.674538999	45.57140363	0.666666667	CRA004775;PRJNA827465;Yang2025_Silkie_Wencha	0.734277773
ENSGALG00010020383	BAAT	3	0.254857397	0.215704434	1.181512092	0.237399353	0.112812448	11.9534849	83.26847764	0.666666667	CRA004775;PRJNA827465;Yang2025_Silkie_Wencha	0.734277773
ENSGALG00010011077	RASGEF1A	3	0.254103561	0.214138459	1.186632057	0.235372786	0.089026899	7.103303189	71.84408512	0.666666667	CRA004775;PRJNA827465;Yang2025_Silkie_Wencha	0.734277773
ENSGALG00010019024	CNPY3	3	0.247218412	0.20770077	1.190262373	0.233943285	0.107985857	13.91303092	85.62498703	0.666666667	CRA004775;PRJNA827465;Yang2025_Silkie_Wencha	0.734277773
ENSGALG00010010379	SLC35F6	3	-0.237451639	0.200881898	-1.182045974	0.237187459	0.073007981	4.943111571	59.5396549	0.666666667	CRA004775;PRJNA827465;Yang2025_Silkie_Wencha	0.734277773
ENSGALG00010005514	EEF1AKMT1	3	-0.229869436	0.193522755	-1.187816057	0.234905887	0.076770913	8.325858146	75.97845213	0.666666667	CRA004775;PRJNA827465;Yang2025_Silkie_Wencha	0.734277773
ENSGALG00010019264	HK2	3	0.227429925	0.192536137	1.181232409	0.23751041	0.085350846	14.96107496	86.63197661	0.666666667	CRA004775;PRJNA827465;Yang2025_Silkie_Wencha	0.734277773
ENSGALG00010011969	TMEM173	3	0.22360531	0.188408145	1.186813395	0.235301234	0.051018953	3.834486572	47.84177848	1	CRA004775;PRJNA827465;Yang2025_Silkie_Wencha	0.734277773
ENSGALG00010022178	ENSGALG00010022178	3	-0.216869838	0.182442963	-1.188699382	0.234557984	0	0.94513109	0	1	CRA004775;PRJNA827465;Yang2025_Silkie_Wencha	0.734277773
ENSGALG00010008033	ACOT13	3	0.214534718	0.180418315	1.189096119	0.234401845	0.063308173	6.153353294	67.49739688	0.666666667	CRA004775;PRJNA827465;Yang2025_Silkie_Wencha	0.734277773

ENSGALG00010012447	COPS8	3	-0.213798146	0.179922338	-1.188280167	0.234723049	0.067586861	7.529446579	73.43762282	0.666666667	CRA004775;PRJNA827465;Yang2025_Silkie_Wencha	0.734277773
ENSGALG00010027362	ENSGALG0001002736	3	-0.210091899	0.177524698	-1.183451661	0.236630193	0.061985477	5.886889202	66.02619938	1	CRA004775;PRJNA827465;Yang2025_Silkie_Wencha	0.734277773
ENSGALG00010002782	GSTA3	3	0.2066991	0.174771741	1.182680325	0.236935865	0.058657143	5.842984204	65.77091551	0.666666667	CRA004775;PRJNA827465;Yang2025_Silkie_Wencha	0.734277773
ENSGALG00010009279	ELMOD2	3	-0.200800193	0.169907153	-1.181823071	0.237275911	0.068529414	20.1552184	90.07701152	0.666666667	CRA004775;PRJNA827465;Yang2025_Silkie_Wencha	0.734277773
ENSGALG00010010266	EFNA5	3	0.187569103	0.157556822	1.190485438	0.23385565	0.008404365	2.197182252	8.974323894	0.666666667	CRA004775;PRJNA827465;Yang2025_Silkie_Wencha	0.734277773
ENSGALG00010011639	ENSGALG0001001163	3	0.180788834	0.151721317	1.191584918	0.233424042	0	0.876208634	0	1	CRA004775;PRJNA827465;Yang2025_Silkie_Wencha	0.734277773
ENSGALG00010010992	PCDHA6	3	0.175645351	0.148267201	1.184654118	0.23615423	0	1.888662256	0	0.666666667	CRA004775;PRJNA827465;Yang2025_Silkie_Wencha	0.734277773
ENSGALG00010010983	MED28	3	-0.16953796	0.143120408	-1.184582705	0.236182478	0.039059411	5.551640361	63.97461165	0.666666667	CRA004775;PRJNA827465;Yang2025_Silkie_Wencha	0.734277773
ENSGALG00010028119	HYAL2	3	0.168418833	0.14192422	1.186681403	0.235353313	0.040585045	8.267836489	75.80987478	1	CRA004775;PRJNA827465;Yang2025_Silkie_Wencha	0.734277773
ENSGALG00010008008	BTC	3	0.167935512	0.1417117	1.185050439	0.235997505	0	1.960241317	0	0.666666667	CRA004775;PRJNA827465;Yang2025_Silkie_Wencha	0.734277773
ENSGALG00010002285	CAV1	3	-0.167765004	0.14123882	-1.18781086	0.234907935	0.038266061	7.375255457	72.88229524	0.666666667	CRA004775;PRJNA827465;Yang2025_Silkie_Wencha	0.734277773
ENSGALG00010018045	PSEN2	3	0.167487675	0.140779021	1.189720409	0.234156301	0.043971019	10.30555188	80.59298499	0.666666667	CRA004775;PRJNA827465;Yang2025_Silkie_Wencha	0.734277773
ENSGALG00010004440	GTF2H5	3	-0.166144601	0.140139084	-1.18556934	0.235792416	0.039073451	6.416358336	68.82967105	0.666666667	CRA004775;PRJNA827465;Yang2025_Silkie_Wencha	0.734277773
ENSGALG00010028118	ENSGALG0001002811	3	0.163874952	0.13761477	1.190823864	0.233722738	0.047837372	17.59798873	88.63506489	1	CRA004775;PRJNA827465;Yang2025_Silkie_Wencha	0.734277773
ENSGALG00010011989	H1-010	3	-0.16310858	0.137217413	-1.188687185	0.234562785	0	0.05900776	0	1	CRA004775;PRJNA827465;Yang2025_Silkie_Wencha	0.734277773
ENSGALG00010002614	GPAT3	3	-0.162300278	0.136467705	-1.189294403	0.234323837	0.02629619	3.789266416	47.21933534	1	CRA004775;PRJNA827465;Yang2025_Silkie_Wencha	0.734277773
ENSGALG00010012857	NSDHL	3	0.157759747	0.132782665	1.188104994	0.234792047	0	0.015231518	0	1	CRA004775;PRJNA827465;Yang2025_Silkie_Wencha	0.734277773
ENSGALG00010021214	FBXL18	3	-0.156609413	0.131915548	-1.1871945	0.23515091	0.030349109	5.243416905	61.85693344	0.666666667	CRA004775;PRJNA827465;Yang2025_Silkie_Wencha	0.734277773
ENSGALG00010029196	MRM1	3	-0.156317123	0.131233454	-1.191137764	0.233599507	0.013444257	2.477923721	19.28726526	0.666666667	CRA004775;PRJNA827465;Yang2025_Silkie_Wencha	0.734277773
ENSGALG00010013027	UHMK1	3	-0.15609936	0.131157543	-1.190166856	0.233980817	0.034400434	6.584453082	69.62541953	0.666666667	CRA004775;PRJNA827465;Yang2025_Silkie_Wencha	0.734277773
ENSGALG00010021586	BBS4	3	-0.153724114	0.129563784	-1.186474414	0.235435	0.025584129	3.92997448	49.10908429	0.666666667	CRA004775;PRJNA827465;Yang2025_Silkie_Wencha	0.734277773
ENSGALG00010011961	AGPAT3	3	-0.15201849	0.12791112	-1.188469695	0.234648412	0.031389233	6.70576137	70.17490051	0.666666667	CRA004775;PRJNA827465;Yang2025_Silkie_Wencha	0.734277773
ENSGALG00010019717	ZRANB3	3	0.144107513	0.121201936	1.188986884	0.234444828	0.017273881	3.165340952	36.81565335	1	CRA004775;PRJNA827465;Yang2025_Silkie_Wencha	0.734277773
ENSGALG00010018732	ENSGALG0001001873	3	0.14191701	0.119329895	1.18928295	0.234328342	0.016875164	3.376995399	40.77575584	0.666666667	CRA004775;PRJNA827465;Yang2025_Silkie_Wencha	0.734277773
ENSGALG00010013374	RMDN1	3	-0.138131092	0.116022443	-1.190554927	0.233828355	0	1.593430015	0	0.666666667	CRA004775;PRJNA827465;Yang2025_Silkie_Wencha	0.734277773
ENSGALG00010005716	ZBTB2	3	0.136925753	0.11545944	1.185920815	0.235653572	0.021493037	4.151446081	51.824016	0.666666667	CRA004775;PRJNA827465;Yang2025_Silkie_Wencha	0.734277773
ENSGALG00010017643	CALM1	3	-0.133685051	0.112648579	-1.186744088	0.235328579	0.024328169	8.654961638	76.89186754	0.666666667	CRA004775;PRJNA827465;Yang2025_Silkie_Wencha	0.734277773
ENSGALG00010023090	LRRC41	3	-0.131519347	0.110899914	-1.185928302	0.235650615	0.025242745	6.847853614	70.7937682	0.666666667	CRA004775;PRJNA827465;Yang2025_Silkie_Wencha	0.734277773
ENSGALG00010028675	ZBTB43	3	-0.129428937	0.109530454	-1.181670774	0.237336359	0.021872048	5.848365255	65.80240952	0.666666667	CRA004775;PRJNA827465;Yang2025_Silkie_Wencha	0.734277773
ENSGALG00010027237	CTC-575C13.4	3	0.125144643	0.105731656	1.183606186	0.23656899	0.000404457	2.017180646	0.851715767	0.666666667	CRA004775;PRJNA827465;Yang2025_Silkie_Wencha	0.734277773
ENSGALG00010018874	ENSGALG0001001887	3	-0.123799578	0.104287375	-1.187100837	0.235187848	0	1.58769173	0	0.666666667	CRA004775;PRJNA827465;Yang2025_Silkie_Wencha	0.734277773
ENSGALG00010014504	SEC63	3	0.117749568	0.099493773	1.183486809	0.236616271	0.020313923	6.493310294	69.19906936	0.666666667	CRA004775;PRJNA827465;Yang2025_Silkie_Wencha	0.734277773
ENSGALG00010003972	PENK	3	0.116392048	0.098552941	1.181010392	0.237598595	0	0.283013286	0	1	CRA004775;PRJNA827465;Yang2025_Silkie_Wencha	0.734277773
ENSGALG00010023852	LRRC39	3	-0.116237099	0.098000804	-1.186083111	0.235589479	0.005973668	2.447671165	18.28967759	0.666666667	CRA004775;PRJNA827465;Yang2025_Silkie_Wencha	0.734277773
ENSGALG00010000223	CASTOR1	3	0.115662321	0.097745323	1.183506458	0.236068489	0.002524266	2.157380702	7.294989794	1	CRA004775;PRJNA827465;Yang2025_Silkie_Wencha	0.734277773
ENSGALG00010002464	NGLY1	3	0.114764356	0.096742571	1.186285986	0.235509379	0.013708276	3.834747249	47.84532408	1	CRA004775;PRJNA827465;Yang2025_Silkie_Wencha	0.734277773
ENSGALG00010028518	MAU2	3	0.105581861	0.089377301	1.181305091	0.237481546	0.011043228	3.741413372	46.54426546	0.666666667	CRA004775;PRJNA827465;Yang2025_Silkie_Wencha	0.734277773
ENSGALG00010023569	LPCAT3	3	-0.101532234	0.085133261	-1.192627098	0.233015448	0.006800843	2.819866005	29.07464409	1	CRA004775;PRJNA827465;Yang2025_Silkie_Wencha	0.734277773
ENSGALG00010023997	ARFGEF2	3	0.096007052	0.081299165	1.180910678	0.237638209	0.005147187	2.551005464	21.59954071	0.666666667	CRA004775;PRJNA827465;Yang2025_Silkie_Wencha	0.734277773
ENSGALG00010023381	HOXB7	3	0.095858556	0.080577948	1.189637595	0.234188863	0	0.214646002	0	1	CRA004775;PRJNA827465;Yang2025_Silkie_Wencha	0.734277773
ENSGALG00010029766	ENSGALG0001002976	3	0.093390339	0.078464422	1.190225281	0.233957859	0	0.527332498	0	0.666666667	CRA004775;PRJNA827465;Yang2025_Silkie_Wencha	0.734277773
ENSGALG00010010156	ENSGALG0001001015	3	-0.093143476	0.078892791	-1.180633548	0.23774833	0.00969785	4.531015196	55.85978167	1	CRA004775;PRJNA827465;Yang2025_Silkie_Wencha	0.734277773
ENSGALG00010011971	RNF170	3	-0.077649102	0.065375492	-1.187740238	0.234935766	0	1.534792181	0	0.666666667	CRA004775;PRJNA827465;Yang2025_Silkie_Wencha	0.734277773
ENSGALG00010014204	KIFAP3	3	-0.069867545	0.059144729	-1.181297909	0.237484398	0	0.36964611	0	0.666666667	CRA004775;PRJNA827465;Yang2025_Silkie_Wencha	0.734277773
ENSGALG00010002017	ARHGAP28	3	-0.06857874	0.058079004	-1.180783666	0.237688675	0	0.399977782	0	1	CRA004775;PRJNA827465;Yang2025_Silkie_Wencha	0.734277773
ENSGALG00010002623	MID1IP1	3	0.06690705	0.056210204	1.190300792	0.23392819	0	0.150229675	0	1	CRA004775;PRJNA827465;Yang2025_Silkie_Wencha	0.734277773
ENSGALG00010025064	GPN3	3	-0.066682103	0.056333725	-1.18369775	0.23653273	0	1.92988119	0	1	CRA004775;PRJNA827465;Yang2025_Silkie_Wencha	0.734277773
ENSGALG00010019789	ARNT2	3	0.066532701	0.055991051	1.188273828	0.234725545	0	0.970968947	0	1	CRA004775;PRJNA827465;Yang2025_Silkie_Wencha	0.734277773
ENSGALG00010022173	RPL23	3	-0.065862769	0.055623175	-1.184088629	0.236377981	0	0.143334341	0	1	CRA004775;PRJNA827465;Yang2025_Silkie_Wencha	0.734277773
ENSGALG00010024631	EPB41L1	3	-0.061942165	0.05231663	-1.183986142	0.236418548	0	1.634723035	0	0.666666667	CRA004775;PRJNA827465;Yang2025_Silkie_Wencha	0.734277773
ENSGALG00010031165	NUDC	3	0.059584846	0.05027384	1.185205784	0.235936093	0.001652081	2.365400101	15.44770803	0.666666667	CRA004775;PRJNA827465;Yang2025_Silkie_Wencha	0.734277773
ENSGALG00010018548	RGMA	3	-0.059479655	0.05037424	-1.180755378	0.237699915	0	0.912303188	0	0.666666667	CRA004775;PRJNA827465;Yang2025_Silkie_Wencha	0.734277773
ENSGALG00010020827	ENSGALG0001002082	3	-0.055604453	0.046772461	-1.188828877	0.234507012	0	1.79942556	0	0.666666667	CRA004775;PRJNA827465;Yang2025_Silkie_Wencha	0.734277773
ENSGALG00010029711	CASKIN2	3	0.052580184	0.044100367	1.192284487	0.233149715	0	0.016744088	0	1	CRA004775;PRJNA827465;Yang2025_Silkie_Wencha	0.734277773
ENSGALG00010010986	NARS	3	-0.039071925	0.033010855	-1.183608383	0.23656812	0	0.812588458	0	1	CRA004775;PRJNA827465;Yang2025_Silkie_Wencha	0.734277773
ENSGALG00010028160	ENSGALG0001002816	3	0.4164574	0.352821034	1.180364436	0.2378553	0.280481444	9.255806402	78.39194217	0.666666667	CRA004775;PRJNA827465;Yang2025_Silkie_Wencha	0.734447716
ENSGALG00010007433	RNF144B	3	0.263985339	0.223743546	1.179856779	0.238057182	0.077386608	4.144213381	51.73993672	0.666666667	CRA004775;PRJNA827465;Yang2025_Silkie_Wencha	0.73491059
ENSGALG00010017441	SPSB1	3	-0.095082028	0.080606435	-1.179583598	0.238165869	0	1.14205625	0	0.666666667	CRA004775;PRJNA827465;Yang2025_Silkie_Wencha	0.735085621
ENSGALG00010018729	HS3ST6	2	0.805109378	0.68564262	1.174240566	0.240298682	0.530088865	2.283642116	56.21030139	1	PRJNA827465;Yang2025_Silkie_Wenchang	0.735123848
ENSGALG00010010979	ENSGALG0001001097	2	0.795036253	0.678486797	1.171778517	0.24128599	0.809615566	8.147024451	87.72557998	1	PRJNA827465;Yang2025_Silkie_Wenchang	0.735123848
ENSGALG00010022500	ENSGALG0001002250	3	0.646996097	0.54969537	1.177008453	0.239192132	0.723457703	9.978820444	79.95755098	0.666666667	CRA004775;PRJNA827465;Yang2025_Silkie_Wencha	0.735123848
ENSGALG00010018926	SLC22A7	2	-0.597431776	0.510029648	-1.171366761	0.241451387	0	0.002665271	0	1	PRJNA827465;Yang2025_Silkie_Wenchang	0.735123848
ENSGALG00010004841	AKAP9	3	0.518335597	0.442063199	1.172537322	0.240981397	0.527169266	26.52860161	92.46096711	0.666666667	CRA004775;PRJNA827465;Yang2025_Silkie_Wencha	0.735123848
ENSGALG00010024882	MYL6	3	0.514256349	0.436968218	1.176873574	0.239245971	0.545489397	64.02335084	96.87613976	0.666666667	CRA004775;PRJNA827465;Yang2025_Silkie_Wencha	0.735123848
ENSGALG00010011758	SERPINB11	3	-0.475514636	0.405337739	-1.173131909	0.240742912	0.154442484	2.888438289	30.75843068	1	CRA004775;PRJNA827465;Yang2025_Silkie_Wencha	0.735123848
ENSGALG00010000486	ENSGALG0001000048	3	0.426424537	0.362319858	1.176928419	0.239224078	0.247916236	5.5734241	64.11541695	1	CRA004775;PRJNA827465;Yang2025_Silkie_Wencha	0.735123848
ENSGALG00010017648	ENSGALG0001001764	3	0.411365646	0.35091422	1.172268386	0.24108932	0.318190347	18.34743095	89.09929131	0.666666667	CRA004775;PRJNA827465;Yang2025_Silkie_Wencha	0.735123848
ENSGALG00010015061	MMP9	3	0.397883893	0.339626643	1.171533218	0.241384514	0	0.198047558	0	1	CRA004775;PRJNA827465;Yang2025_Silkie_Wencha	0.735123848
ENSGALG00010012680	TRPM8	2	-0.358640402	0.304786589	-1.176693513	0.239317858	0.13004086	3.308274373	69.77276105	1	PRJNA827465;Yang2025_Silkie_Wenchang	0.735123848
ENSGALG00010013828	PTN	3	0.338440238	0.288398274	1.173516865	0.240588598	0.119001975	3.983163345	49.78865221	1	CRA004775;PRJNA827465;Yang2025_Silkie_Wencha	0.735123848
ENSGALG00010008283	NQO1	3	0.309459465	0.263803556	1.173072275	0.240766823	0.134979643	6.003438374	66.65575781	0.666666667	CRA004775;PRJNA827465;Yang2025_Silkie_Wencha	0.735123848
ENSGALG00010021015	RBM25	3	0.304187323	0.259720879	1.171208584	0.241514946	0.170720888	14.73105011	86.42323538	0.666666667	CRA004775;PRJNA827465;Yang2025_Silkie_Wencha	0.735123848
ENSGALG00010017688	SPDYA	3	0.300659242	0.25629903	1.173079907	0.240763763	0.139484012	9.813708313	79.62034395	0.666666667	CRA004775;PRJNA827465;Yang2025_Silkie_Wencha	0.735123848
ENSGALG00010005435	MAD2L1	3	-0.30021562	0.255645421	-1.174343816	0.24025734	0.13115332	7.502946912	73.34380713	0.666666667	CRA004775;PRJNA827465;Yang2025_Silkie_Wencha	0.735123848
ENSGALG00010021250	ISLR	3	-0.280277216	0.237792366	-1.178663643	0.238532138	0.128898483	9.455389643	78.84804249	0.666666667	CRA004775;PRJNA827465;Yang2025_Silkie_Wencha	0.735123848
ENSGALG00010002700	LHFPL6	3	-0.261450105	0.223163302	-1.171564065	0.241372123	0.1131789	12.1665016	83.56142081	1	CRA004775;PRJNA827465;Yang2025_Silkie_Wencha	0.735123848
ENSGALG00010005975	ABRACL	3	-0.260220908	0.22161593	-1.174197669	0.24031586	0.10001273	7.428306815	73.07596401	0.666666667	CRA004775;PRJNA827465;Yang2025_Silkie_Wencha	0.735123848
ENSGALG00010000576	NOS1	3	-0.25815661	0.219253953	-1.177431953	0.239023143	0	0.093021821	0	1	CRA004775;PRJNA827465;Yang2025_Silkie_Wencha	0.735123848
ENSGALG00010021607	SLC9A5	3	0.255142485	0.217772897	1.171598893	0.241358133	0.098578845	6.744121637	70.30454437	0.666666667	CRA004775;PRJNA827465;Yang2025_Silkie_Wencha	0.735123848
ENSGALG00010004939	DDX10	3	0.23909429	0.202951119	1.178088062	0.238761501	0.096495336	11.15961633	82.07823692	0.666666667	CRA004775;PRJNA827465;Yang2025_Silkie_Wencha	0.735123848
ENSGALG00010018354	AARS2	3	-0.230759397	0.196179532	-1.176266426	0.239488427	0.094513775	13.00299544	84.6189287	0.666666667	CRA004775;PRJNA827465;Yang2025_Silkie_Wencha	0.735123848
ENSGALG00010011126	ATXN1	3	0.221218787	0.188114284	1.175980805	0.239602547	0.053600819	3.840018819	47.91692191	1	CRA004775;PRJNA827465;Yang2025_Silkie_Wencha	0.735123848
ENSGALG00010005587	LEF1	3	0.205450519	0.175143662	1.173039987	0.240779771	0	1.823415744	0	0.666666667	CRA004775;PRJNA827465;Yang2025_Silkie_Wencha	0.735123848
ENSGALG00010020473	TMEM109	3	0.179517506	0.153257834	1.171343099	0.241460894	0	1.104796243	0	0.666666667	CRA004775;PRJNA827465;Yang2025_Silkie_Wencha	0.735123848
ENSGALG00010016047	SRP9	3	-0.177182112	0.150827379	-1.174734405	0.24010099	0.048245387	9.161551925	78.16963745	1	CRA004775;PRJNA827465;Yang2025_Silkie_Wencha	0.735123848
ENSGALG00010024900	DTYMK	3	-0.173373989	0.147006918	-1.179359387	0.2382551	0.03434358	4.17007894	52.03927722	1	CRA004775;PRJNA827465;Yang2025_Silkie_Wencha	0.735123848
ENSGALG00010017963	LPAR4	3	-0.171498042	0.145741089	-1.176730897	0.239302932	0.039790512	7.410083131	73.00974949	0.666666667	CRA004775;PRJNA827465;Yang2025_Silkie_Wencha	0.735123848
ENSGALG00010002673	SLC66A3	3	-0.167836597	0.142380007	-1.17879329	0.238480497	0.027087062	3.612186388	44.63187153	0.666666667	CRA004775;PRJNA827465;Yang2025_Silkie_Wencha	0.735123848
ENSGALG00010009080	TLL1	3	0.156872605	0.133669986	1.173581365	0.240562749	0	0.318496245	0	1	CRA004775;PRJNA827465;Yang2025_Silkie_Wencha	0.735123848
ENSGALG00010024011	AGPS	3	-0.153968007	0.131441919	-1.171376736	0.241447379	0.040167152	11.89846681	83.19111166	0.666666667	CRA004775;PRJNA827465;Yang2025_Silkie_Wencha	0.735123848
ENSGALG00010010439	HGF	3	-0.15123759	0.128400581	-1.177857522	0.238853412	0	1.936775591	0	0.666666667	CRA004775;PRJNA827465;Yang2025_Silkie_Wencha	0.735123848
ENSGALG00010027401	ENSGALG0001002740	3	0.149979545	0.127926317	1.172390079	0.241040481	0	1.107507369	0	0.666666667	CRA004775;PRJNA827465;Yang2025_Silkie_Wencha	0.735123848
ENSGALG00010016957	WNT4	3	-0.147654333	0.126017722	-1.171694987	0.241319536	0	0.205466141	0	1	CRA004775;PRJNA827465;Yang2025_Silkie_Wencha	0.735123848
ENSGALG00010029704	SLC25A19	3	-0.144975558	0.123496887	-1.173920746	0.240426772	0.023774584	4.055989238	50.69020447	1	CRA004775;PRJNA827465;Yang2025_Silkie_Wencha	0.735123848
ENSGALG00010011577	ENSGALG0001001157	3	0.144587932	0.122979901	1.175789811	0.239678879	0	0.678396949	0	1	CRA004775;PRJNA827465;Yang2025_Silkie_Wencha	0.735123848
ENSGALG00010008101	CENPQ	3	-0.143855503	0.122562626	-1.173730594	0.240502952	0.012345888	2.672982749	25.17722007	1	CRA004775;PRJNA827465;Yang2025_Silkie_Wencha	0.735123848
ENSGALG00010029249	PRR11	3	-0.142643631	0.121721898	-1.171881421	0.241244667	0.012577852	2.482855649	19.44759249	0.666666667	CRA004775;PRJNA827465;Yang2025_Silkie_Wencha	0.735123848
ENSGALG00010029694	ARMC7	3	-0.140287179	0.119493007	-1.174019985	0.240387021	0.020240988	3.545027277	43.58294468	1	CRA004775;PRJNA827465;Yang2025_Silkie_Wencha	0.735123848
ENSGALG00010015734	TSHZ2	3	0.138577072	0.118248231	1.171916757	0.241230479	0.012583309	2.650448814	24.5410819	1	CRA004775;PRJNA827465;Yang2025_Silkie_Wencha	0.735123848
ENSGALG00010028082	AK1	3	-0.138504362	0.117882188	-1.174938849	0.240019182	0	0.480069528	0	0.666666667	CRA004775;PRJNA827465;Yang2025_Silkie_Wencha	0.735123848
ENSGALG00010017615	AGTRAP	3	-0.13584006	0.115993362	-1.171102009	0.241557776	0.019402791	3.876160496	48.40254932	0.666666667	CRA004775;PRJNA827465;Yang2025_Silkie_Wencha	0.735123848
ENSGALG00010014192	KDM1A	3	-0.134947572	0.115081242	-1.172628736	0.24094472	0.027155593	10.25195764	80.49153078	0.666666667	CRA004775;PRJNA827465;Yang2025_Silkie_Wencha	0.735123848
ENSGALG00010003133	SAT1	3	-0.121784754	0.103313624	-1.178786974	0.238483013	0	0.672859244	0	1	CRA004775;PRJNA827465;Yang2025_Silkie_Wencha	0.735123848
ENSGALG00010000704	ENSGALG0001000070	3	-0.120338111	0.102506274	-1.173958493	0.240411652	0.016192262	4.088663284	51.08425759	1	CRA004775;PRJNA827465;Yang2025_Silkie_Wencha	0.735123848
ENSGALG00010007391	NANP	3	-0.114528446	0.097738129	-1.171788815	0.241281855	0.016921604	5.241197686	61.84078297	1	CRA004775;PRJNA827465;Yang2025_Silkie_Wencha	0.735123848
ENSGALG00010004421	RAP1GDS1	3	0.11414084	0.097145421	1.174948229	0.240015429	0.016059915	4.639138113	56.8885437	1	CRA004775;PRJNA827465;Yang2025_Silkie_Wencha	0.735123848
ENSGALG00010014260	ADNP	3	0.107715666	0.091800936	1.173361308	0.240650946	0.01365848	4.0796724	50.97645585	1	CRA004775;PRJNA827465;Yang2025_Silkie_Wencha	0.735123848
ENSGALG00010024536	URI1	3	-0.106763939	0.090817203	-1.175591581	0.239758121	0.019264367	11.76490643	83.00028979	0.666666667	CRA004775;PRJNA827465;Yang2025_Silkie_Wencha	0.735123848
ENSGALG00010029205	CACNG1	3	-0.106113866	0.090165174	-1.176883071	0.23924218	0	0.493238498	0	1	CRA004775;PRJNA827465;Yang2025_Silkie_Wencha	0.735123848
ENSGALG00010016286	ULK4	3	-0.099009811	0.08414247	-1.176692478	0.239318271	0	1.949398971	0	1	CRA004775;PRJNA827465;Yang2025_Silkie_Wencha	0.735123848
ENSGALG00010011810	FGFR4	3	-0.089382638	0.076316191	-1.171214609	0.241512525	0	0.273864726	0	0.666666667	CRA004775;PRJNA827465;Yang2025_Silkie_Wencha	0.735123848
ENSGALG00010017314	CALML4	3	0.084935663	0.072037268	1.179051687	0.238377595	0	1.449803996	0	0.666666667	CRA004775;PRJNA827465;Yang2025_Silkie_Wencha	0.735123848
ENSGALG00010026388	SNRPD3	3	-0.077859995	0.066147526	-1.177065869	0.239169216	0.00306765	2.451048262	18.40225951	0.666666667	CRA004775;PRJNA827465;Yang2025_Silkie_Wencha	0.735123848
ENSGALG00010024201	IREB2	3	-0.077030211	0.065409327	-1.177664024	0.238930575	0.005344019	3.318582256	39.73330037	0.666666667	CRA004775;PRJNA827465;Yang2025_Silkie_Wencha	0.735123848
ENSGALG00010020261	SSU72	3	0.076739791	0.065132882	1.178203527	0.238715477	0.003100245	2.396981875	16.5617387	0.666666667	CRA004775;PRJNA827465;Yang2025_Silkie_Wencha	0.735123848
ENSGALG00010006323	ANAPC10	3	-0.070940515	0.060509887	-1.172378907	0.241044964	0	1.733712147	0	0.666666667	CRA004775;PRJNA827465;Yang2025_Silkie_Wencha	0.735123848
ENSGALG00010023811	MRPL44	3	-0.068856037	0.05853604	-1.176301588	0.239474382	0	0.224520392	0	0.666666667	CRA004775;PRJNA827465;Yang2025_Silkie_Wencha	0.735123848
ENSGALG00010021638	SRC	3	0.065839117	0.055940764	1.176943483	0.239218065	0	1.699919085	0	0.666666667	CRA004775;PRJNA827465;Yang2025_Silkie_Wencha	0.735123848
ENSGALG00010021665	CNOT1	3	-0.05553401	0.04730653	-1.173918477	0.240427681	0	0.040569172	0	1	CRA004775;PRJNA827465;Yang2025_Silkie_Wencha	0.735123848
ENSGALG00010002894	FCHSD2	3	0.049714277	0.042298825	1.175311054	0.239870294	0	0.52467554	0	1	CRA004775;PRJNA827465;Yang2025_Silkie_Wencha	0.735123848
ENSGALG00010029405	POLDIP2	3	-0.044253074	0.037582445	-1.177493194	0.238998712	0	1.773501875	0	1	CRA004775;PRJNA827465;Yang2025_Silkie_Wencha	0.735123848
ENSGALG00010025289	KDM2B	3	-0.041686755	0.035582892	-1.171539263	0.241382085	0	1.329140423	0	0.666666667	CRA004775;PRJNA827465;Yang2025_Silkie_Wencha	0.735123848
ENSGALG00010009117	ERGIC1	3	-0.041566449	0.035278192	-1.178247709	0.238697868	0	1.063861343	0	1	CRA004775;PRJNA827465;Yang2025_Silkie_Wencha	0.735123848
ENSGALG00010016864	ENSGALG0001001686	3	0.369807706	0.315901714	1.170641655	0.241742846	0.196392424	5.949192696	66.38199328	0.666666667	CRA004775;PRJNA827465;Yang2025_Silkie_Wencha	0.735528751
ENSGALG00010021199	TMEM258	3	-0.096320745	0.082304881	-1.170292015	0.241883474	0	1.232191489	0	1	CRA004775;PRJNA827465;Yang2025_Silkie_Wencha	0.73567528
ENSGALG00010016600	GNL2	3	0.070688651	0.060404063	1.170263194	0.241895069	4.60919E-05	2.005729855	0.28567429	1	CRA004775;PRJNA827465;Yang2025_Silkie_Wencha	0.73567528
ENSGALG00010009133	BOD1L1	3	0.350482691	0.29960176	1.169828546	0.242069974	0.231639245	17.72950657	88.71937021	0.666666667	CRA004775;PRJNA827465;Yang2025_Silkie_Wencha	0.736048894
ENSGALG00010009038	RAD21	3	-0.115076866	0.098383417	-1.169677471	0.242130788	0.019797207	12.24547594	83.66743759	1	CRA004775;PRJNA827465;Yang2025_Silkie_Wencha	0.736075513
ENSGALG00010006079	ENSGALG0001000607	3	0.481169265	0.411579216	1.169080571	0.242371171	0.004360478	2.015116589	0.750159505	0.666666667	CRA004775;PRJNA827465;Yang2025_Silkie_Wencha	0.73664789
ENSGALG00010018035	ITM2A	3	-0.254280946	0.217603892	-1.168549621	0.242585136	0.128529333	26.64003881	92.49250343	0.666666667	CRA004775;PRJNA827465;Yang2025_Silkie_Wencha	0.737139746
ENSGALG00010009243	BCAP29	3	-0.310861151	0.266090803	-1.168252142	0.242705074	0.197124662	30.85742469	93.51857772	0.666666667	CRA004775;PRJNA827465;Yang2025_Silkie_Wencha	0.737203962
ENSGALG00010011049	ABL2	3	-0.060510893	0.051796692	-1.168238566	0.242710548	0	1.192616008	0	1	CRA004775;PRJNA827465;Yang2025_Silkie_Wencha	0.737203962
ENSGALG00010009284	CCN3	3	0.421887057	0.361522875	1.166971958	0.243221694	0.158056839	3.347332853	40.25093745	0.666666667	CRA004775;PRJNA827465;Yang2025_Silkie_Wencha	0.737440145
ENSGALG00010004336	ENSGALG0001000433	2	-0.395129082	0.338719323	-1.166538353	0.243396851	0.092238254	1.665635209	39.96284453	1	PRJNA827465;Yang2025_Silkie_Wenchang	0.737440145
ENSGALG00010029333	ENSGALG0001002933	3	-0.329712108	0.282752178	-1.166081585	0.243581461	0.209602448	26.52664494	92.46041101	0.666666667	CRA004775;PRJNA827465;Yang2025_Silkie_Wencha	0.737440145
ENSGALG00010018215	CFAP70	3	-0.319768802	0.273945619	-1.167271094	0.243100908	0.156883019	7.68993461	73.99197651	0.666666667	CRA004775;PRJNA827465;Yang2025_Silkie_Wencha	0.737440145
ENSGALG00010013614	ATRX	3	0.259576445	0.2226305	1.165951856	0.243633911	0.10740429	8.43222426	76.28146574	0.666666667	CRA004775;PRJNA827465;Yang2025_Silkie_Wencha	0.737440145
ENSGALG00010020963	CCDC93	3	0.255069739	0.21853678	1.167170754	0.243141419	0.115834588	11.43902742	82.5159961	0.666666667	CRA004775;PRJNA827465;Yang2025_Silkie_Wencha	0.737440145
ENSGALG00010014883	PPAT	3	-0.253516781	0.217395273	-1.166155899	0.24355142	0.108508735	13.57077629	85.26244957	0.666666667	CRA004775;PRJNA827465;Yang2025_Silkie_Wencha	0.737440145
ENSGALG00010003990	ICA1	3	0.243605467	0.208974011	1.165721355	0.243727123	0.078747275	5.63489978	64.50691089	1	CRA004775;PRJNA827465;Yang2025_Silkie_Wencha	0.737440145
ENSGALG00010025659	COBLL1	3	0.22839463	0.195832552	1.166275105	0.243503235	0.069748958	5.698127279	64.90074893	1	CRA004775;PRJNA827465;Yang2025_Silkie_Wencha	0.737440145
ENSGALG00010010418	BRINP2	2	0.222613377	0.190744717	1.167074925	0.243180113	0	0.020266185	0	1	PRJNA827465;Yang2025_Silkie_Wenchang	0.737440145
ENSGALG00010028671	CADM3	3	0.209981639	0.180119726	1.165789245	0.243699667	0.04412406	3.746415159	46.61563347	0.666666667	CRA004775;PRJNA827465;Yang2025_Silkie_Wencha	0.737440145
ENSGALG00010013330	ERG	3	0.205990872	0.176420408	1.167613628	0.242962651	0.04930565	4.235254143	52.77733207	0.666666667	CRA004775;PRJNA827465;Yang2025_Silkie_Wencha	0.737440145
ENSGALG00010027803	SAPCD2	3	-0.15008785	0.128606478	-1.167031802	0.243197527	0	0.872792862	0	0.666666667	CRA004775;PRJNA827465;Yang2025_Silkie_Wencha	0.737440145
ENSGALG00010020075	CPSF7	3	-0.112544016	0.096525015	-1.165956989	0.243631836	0.020767234	8.357034438	76.0680656	1	CRA004775;PRJNA827465;Yang2025_Silkie_Wencha	0.737440145
ENSGALG00010029143	SH2D3C	3	0.101079555	0.086602716	1.167163791	0.243144231	0.0087597	3.283598671	39.09121668	0.666666667	CRA004775;PRJNA827465;Yang2025_Silkie_Wencha	0.737440145
ENSGALG00010024678	AMOTL2	3	-0.076172454	0.065302376	-1.166457618	0.243429474	0	0.578719586	0	0.666666667	CRA004775;PRJNA827465;Yang2025_Silkie_Wencha	0.737440145
ENSGALG00010021182	ZSWIM8	3	-0.070791604	0.060693356	-1.166381442	0.243460258	0	0.989317485	0	1	CRA004775;PRJNA827465;Yang2025_Silkie_Wencha	0.737440145
ENSGALG00010018823	RAB40C	3	0.048745338	0.041774993	1.166854498	0.243269134	0	0.143934138	0	1	CRA004775;PRJNA827465;Yang2025_Silkie_Wencha	0.737440145
ENSGALG00010011318	PRP4K	3	0.29995155	0.25740775	1.165277854	0.24390654	0.161094143	11.88039234	83.16553912	0.666666667	CRA004775;PRJNA827465;Yang2025_Silkie_Wencha	0.737509533
ENSGALG00010027964	RABEPK	3	-0.179326162	0.153879015	-1.165371128	0.243868799	0.049240112	6.840311747	70.76156652	1	CRA004775;PRJNA827465;Yang2025_Silkie_Wencha	0.737509533
ENSGALG00010027107	KIF21B	3	0.128680029	0.110410966	1.165464204	0.243831142	0.009923222	2.66614734	24.98539109	0.666666667	CRA004775;PRJNA827465;Yang2025_Silkie_Wencha	0.737509533
ENSGALG00010024258	DNAJA4	3	0.253259843	0.217428217	1.164797494	0.244100973	0.094173251	7.809023302	74.38860248	0.666666667	CRA004775;PRJNA827465;Yang2025_Silkie_Wencha	0.737781886
ENSGALG00010008331	CCDC97	3	-0.101664235	0.087272042	-1.164911842	0.24405468	0.003394673	2.298806339	12.99832588	0.666666667	CRA004775;PRJNA827465;Yang2025_Silkie_Wencha	0.737781886
ENSGALG00010028412	ENSGALG0001002841	3	0.272468601	0.23405631	1.164115597	0.244377169	0.027352767	2.354738581	15.06488166	0.666666667	CRA004775;PRJNA827465;Yang2025_Silkie_Wencha	0.738431302
ENSGALG00010004962	ENSGALG0001000496	3	-0.159672805	0.137205244	-1.16375147	0.244524744	0.038929465	6.724479527	70.2579212	0.666666667	CRA004775;PRJNA827465;Yang2025_Silkie_Wencha	0.738431302
ENSGALG00010019635	LRRC57	3	-0.146681596	0.126014695	-1.164003903	0.24442243	0.031105745	6.296152116	68.23456672	1	CRA004775;PRJNA827465;Yang2025_Silkie_Wencha	0.738431302
ENSGALG00010021842	ENSGALG0001002184	2	0.126841003	0.108983825	1.163851634	0.244484143	0	0.086434904	0	1	PRJNA827465;Yang2025_Silkie_Wenchang	0.738431302
ENSGALG00010013319	ENSGALG0001001331	3	-2.692918959	2.315196534	-1.163149184	0.24476898	14.3389961	18.74609318	89.33111033	1	CRA004775;PRJNA827465;Yang2025_Silkie_Wencha	0.738539945
ENSGALG00010014634	CCDC152	3	-0.645769573	0.555095646	-1.163348293	0.244688219	0.787838169	13.58824649	85.2813974	0.666666667	CRA004775;PRJNA827465;Yang2025_Silkie_Wencha	0.738539945
ENSGALG00010007847	CMC2	3	-0.222430219	0.191189978	-1.163398942	0.244667679	0.060949936	4.526278294	55.81358746	1	CRA004775;PRJNA827465;Yang2025_Silkie_Wencha	0.738539945
ENSGALG00010023201	ZNF280D	3	0.111484834	0.095847545	1.163147514	0.244769657	0.013643776	3.871240661	48.33697578	0.666666667	CRA004775;PRJNA827465;Yang2025_Silkie_Wencha	0.738539945
ENSGALG00010012748	ENSGALG0001001274	2	0.474682229	0.408194256	1.162883167	0.244876908	0.203254836	2.56178566	60.96472802	1	PRJNA827465;Yang2025_Silkie_Wenchang	0.738544734
ENSGALG00010013848	ITPA	3	-0.17956967	0.154451434	-1.162628697	0.244980183	0.035713741	3.863871119	48.23843916	0.666666667	CRA004775;PRJNA827465;Yang2025_Silkie_Wencha	0.738544734
ENSGALG00010023338	HDLBP	3	-0.120794827	0.103885236	-1.162771844	0.244922084	0.02483746	11.79071608	83.03750183	0.666666667	CRA004775;PRJNA827465;Yang2025_Silkie_Wencha	0.738544734
ENSGALG00010011363	FDFT1	3	-0.092939387	0.079931602	-1.16273646	0.244936444	0	1.911001296	0	0.666666667	CRA004775;PRJNA827465;Yang2025_Silkie_Wencha	0.738544734
ENSGALG00010012336	ENSGALG0001001233	3	-3.103174957	2.67027015	-1.162120229	0.245186632	17.72313732	14.62214183	86.32211325	0.666666667	CRA004775;PRJNA827465;Yang2025_Silkie_Wencha	0.738605564
ENSGALG00010009327	NID1	3	-0.250174363	0.215276856	-1.162105243	0.245192718	0.102516248	15.81402956	87.35300201	0.666666667	CRA004775;PRJNA827465;Yang2025_Silkie_Wencha	0.738605564
ENSGALG00010001491	PHF3	3	0.223967461	0.192775069	1.161807188	0.245313794	0.077017613	6.836987381	70.74734984	0.666666667	CRA004775;PRJNA827465;Yang2025_Silkie_Wencha	0.738605564
ENSGALG00010021240	SLK	3	0.17615657	0.151601658	1.161969947	0.245247673	0.039815707	4.740594437	57.81119801	0.666666667	CRA004775;PRJNA827465;Yang2025_Silkie_Wencha	0.738605564
ENSGALG00010024627	CEPT1	3	-0.124005382	0.106703711	-1.162146857	0.245175817	0.019616377	5.090469002	60.71088933	1	CRA004775;PRJNA827465;Yang2025_Silkie_Wencha	0.738605564
ENSGALG00010012883	DCAF13	3	-0.044164608	0.038010955	-1.161891561	0.245279516	0	1.830760335	0	1	CRA004775;PRJNA827465;Yang2025_Silkie_Wencha	0.738605564
ENSGALG00010001286	SRFBP1	3	0.278488229	0.239800207	1.161334398	0.245505936	0.107768774	5.381973075	62.83890737	0.666666667	CRA004775;PRJNA827465;Yang2025_Silkie_Wencha	0.738782325
ENSGALG00010020962	MYO5C	2	0.266258216	0.229280532	1.161277035	0.245529256	0	0.36863148	0	1	PRJNA827465;Yang2025_Silkie_Wenchang	0.738782325
ENSGALG00010025606	CCDC12	3	-0.061903661	0.053298838	-1.161444842	0.245461042	0	0.141916235	0	1	CRA004775;PRJNA827465;Yang2025_Silkie_Wencha	0.738782325
ENSGALG00010029250	SPAG9	3	0.156707641	0.13498241	1.160948603	0.245662802	0.036109404	7.656312321	73.87776365	0.666666667	CRA004775;PRJNA827465;Yang2025_Silkie_Wencha	0.739026884
ENSGALG00010001540	TRABD	3	-0.076456406	0.065882277	-1.160500357	0.245845149	0	1.554882168	0	1	CRA004775;PRJNA827465;Yang2025_Silkie_Wencha	0.739418115
ENSGALG00010011943	TTLL12	3	-0.071055474	0.061237319	-1.160329598	0.245914638	0.003388306	2.760258819	27.54302654	1	CRA004775;PRJNA827465;Yang2025_Silkie_Wencha	0.739469815
ENSGALG00010020685	RPL6	3	-0.319508201	0.275398838	-1.160165391	0.245981475	0.202619964	21.51719593	90.70510857	0.666666667	CRA004775;PRJNA827465;Yang2025_Silkie_Wencha	0.739513517
ENSGALG00010012260	ENSGALG0001001226	3	0.367372815	0.316932226	1.159152605	0.246393986	0	1.710014966	0	0.666666667	CRA004775;PRJNA827465;Yang2025_Silkie_Wencha	0.740005569

ENSGALG00010019307	ATF3	3	0.298348016	0.25740844	1.159045195	0.246437763	0.101954089	4.441511952	54.97028891	1	CRA004775;PRJNA827465;Yang2025_Silkie_Wencha	0.740005569
ENSGALG00010005184	FGF14	2	-0.232587058	0.200676974	-1.159012181	0.24645122	0	0.931289948	0	0.5	PRJNA827465;Yang2025_Silkie_Wenchang	0.740005569
ENSGALG00010002608	RPP38	3	-0.198844939	0.171551056	-1.159100641	0.246415165	0.053439153	5.370583149	62.76009617	0.666666667	CRA004775;PRJNA827465;Yang2025_Silkie_Wencha	0.740005569
ENSGALG00010013253	SHB	3	-0.12690802	0.109439809	-1.159614776	0.246205682	0.020676855	5.240191607	61.83345667	0.666666667	CRA004775;PRJNA827465;Yang2025_Silkie_Wencha	0.740005569
ENSGALG00010006061	RPL24	3	-0.068597452	0.059187132	-1.158992674	0.246459171	0.000502812	2.039021639	1.913743262	0.666666667	CRA004775;PRJNA827465;Yang2025_Silkie_Wencha	0.740005569
ENSGALG00010021080	TTLL7	3	0.317506848	0.274083513	1.158431039	0.246688178	0.169707302	10.49580126	80.94476114	0.666666667	CRA004775;PRJNA827465;Yang2025_Silkie_Wencha	0.740378721
ENSGALG00010003152	ENSGALG0001000315	3	0.129036754	0.111377856	1.158549456	0.246639882	0.000559003	2.028511831	1.405554107	0.666666667	CRA004775;PRJNA827465;Yang2025_Silkie_Wencha	0.740378721
ENSGALG00010019613	FRMD6	3	0.082420579	0.07116718	1.158126236	0.246812525	0.00570245	3.0873656	35.21985216	0.666666667	CRA004775;PRJNA827465;Yang2025_Silkie_Wencha	0.740594713
ENSGALG00010003303	MTHFD2L	3	-0.123344628	0.10651565	-1.157995342	0.246865937	0	0.521586981	0	0.666666667	CRA004775;PRJNA827465;Yang2025_Silkie_Wencha	0.740597812
ENSGALG00010010948	PRDM15	3	0.18632039	0.160933632	1.157746757	0.246967397	0.055374239	8.275203769	75.83141085	1	CRA004775;PRJNA827465;Yang2025_Silkie_Wencha	0.740680574
ENSGALG00010026508	CSRP1	3	0.133238274	0.115091651	1.157671066	0.246998296	0.003416018	2.163235612	7.545900737	0.666666667	CRA004775;PRJNA827465;Yang2025_Silkie_Wencha	0.740680574
ENSGALG00010008699	NAA35	3	0.120610963	0.104220016	1.157272546	0.247161026	0.002712518	2.161949382	7.490896118	0.666666667	CRA004775;PRJNA827465;Yang2025_Silkie_Wencha	0.740854304
ENSGALG00010021798	RANBP10	3	-0.071459546	0.061746762	-1.157300307	0.247149688	0	1.377612514	0	0.666666667	CRA004775;PRJNA827465;Yang2025_Silkie_Wencha	0.740854304
ENSGALG00010016575	CWH43	2	-1.134725719	0.98186374	-1.155685532	0.247809808	1.692228077	8.143343056	87.72003103	1	PRJNA827465;Yang2025_Silkie_Wenchang	0.74091412
ENSGALG00010014258	ENSGALG0001001425	3	0.426144975	0.368405408	1.156728337	0.247383368	0.251356397	5.425696919	63.13837596	0.666666667	CRA004775;PRJNA827465;Yang2025_Silkie_Wencha	0.74091412
ENSGALG00010019288	RPL37A	3	-0.386878457	0.334500377	-1.156586012	0.24744154	0.280025697	13.7917325	85.49855865	0.666666667	CRA004775;PRJNA827465;Yang2025_Silkie_Wencha	0.74091412
ENSGALG00010017755	C1D	3	-0.294035111	0.254264849	-1.156412743	0.247512372	0.157281011	17.07170088	88.2847057	1	CRA004775;PRJNA827465;Yang2025_Silkie_Wencha	0.74091412
ENSGALG00010023081	PRLHR2	3	0.285335769	0.246759501	1.156331438	0.247545614	0.062976618	2.89891218	31.00860337	0.666666667	CRA004775;PRJNA827465;Yang2025_Silkie_Wencha	0.74091412
ENSGALG00010015094	CDK5RAP1	3	-0.283515735	0.245208478	-1.156221328	0.247590638	0.150442457	16.38303432	87.79224922	0.666666667	CRA004775;PRJNA827465;Yang2025_Silkie_Wencha	0.74091412
ENSGALG00010018405	DCLK3	3	0.280821772	0.242925346	1.156000298	0.247681035	0.107960002	5.454448874	63.33268409	0.666666667	CRA004775;PRJNA827465;Yang2025_Silkie_Wencha	0.74091412
ENSGALG00010025190	PRR5L	3	-0.275662961	0.23850141	-1.155812709	0.247757773	0.099318389	5.447878576	63.28846225	0.666666667	CRA004775;PRJNA827465;Yang2025_Silkie_Wencha	0.74091412
ENSGALG00010021171	ENSGALG0001002117	3	0.259216397	0.224033808	1.157041429	0.247255435	0.123551062	14.96619413	86.63654913	0.666666667	CRA004775;PRJNA827465;Yang2025_Silkie_Wencha	0.74091412
ENSGALG00010019617	ANO3	3	0.238646631	0.206454796	1.155926795	0.247711101	0.085568532	7.140422175	71.99045167	0.666666667	CRA004775;PRJNA827465;Yang2025_Silkie_Wencha	0.74091412
ENSGALG00010013971	ENSGALG0001001397	3	-0.188777185	0.16327392	-1.15619926	0.247599662	0	0.923832949	0	1	CRA004775;PRJNA827465;Yang2025_Silkie_Wencha	0.74091412
ENSGALG00010024942	DNAJB6	3	0.054324769	0.04699058	1.156077847	0.247649316	0.001555938	2.391692045	16.37719396	1	CRA004775;PRJNA827465;Yang2025_Silkie_Wencha	0.74091412
ENSGALG00010011991	CSNK1A1	3	-0.186845184	0.161705791	-1.155463776	0.247900558	0.068086748	31.71618583	93.69407151	0.666666667	CRA004775;PRJNA827465;Yang2025_Silkie_Wencha	0.741028751
ENSGALG00010023823	MUC2	3	0.866811793	0.750601085	1.154823528	0.2481627	1.397425063	12.27011222	83.70023057	0.666666667	CRA004775;PRJNA827465;Yang2025_Silkie_Wencha	0.741342154
ENSGALG00010020754	SLC25A20	3	-0.212646204	0.184125812	-1.15489622	0.248132928	0.080082828	10.7252529	81.35242107	0.666666667	CRA004775;PRJNA827465;Yang2025_Silkie_Wencha	0.741342154
ENSGALG00010029259	PTRH2	3	-0.184863128	0.160067739	-1.1549056	0.248129086	0.038348408	3.843030579	47.9577391	0.666666667	CRA004775;PRJNA827465;Yang2025_Silkie_Wencha	0.741342154
ENSGALG00010019660	ENSGALG0001001966	2	-0.439680644	0.38110696	-1.153693558	0.248625826	0	0.878981337	0	1	PRJNA827465;Yang2025_Silkie_Wenchang	0.741912832
ENSGALG00010019813	ENSGALG0001001981	3	-0.383955822	0.333020373	-1.152949936	0.248930934	0.235596098	6.878425822	70.92357973	0.666666667	CRA004775;PRJNA827465;Yang2025_Silkie_Wencha	0.741912832
ENSGALG00010016630	ENSGALG0001001663	3	-0.286288733	0.248193077	-1.153492019	0.248708491	0	1.895933463	0	0.666666667	CRA004775;PRJNA827465;Yang2025_Silkie_Wencha	0.741912832
ENSGALG00010007935	LARP1B	3	0.284863073	0.247046086	1.153076649	0.248878925	0.150772348	12.59243462	84.11744781	0.666666667	CRA004775;PRJNA827465;Yang2025_Silkie_Wencha	0.741912832
ENSGALG00010016251	MPDZ	3	0.25492056	0.221045396	1.153249808	0.248807865	0.119960492	13.69797335	85.39929997	0.666666667	CRA004775;PRJNA827465;Yang2025_Silkie_Wencha	0.741912832
ENSGALG00010024240	WDR61	3	-0.226980798	0.196794495	-1.153282393	0.248794495	0.093009768	10.78128676	81.44933861	0.666666667	CRA004775;PRJNA827465;Yang2025_Silkie_Wencha	0.741912832
ENSGALG00010012905	NKAIN1	3	-0.137207414	0.118879236	-1.154174764	0.248428527	0	0.328691326	0	1	CRA004775;PRJNA827465;Yang2025_Silkie_Wencha	0.741912832
ENSGALG00010011903	LTA4H	3	-0.115961124	0.10056137	-1.153137877	0.248853797	0.01164635	2.859540321	30.05868863	1	CRA004775;PRJNA827465;Yang2025_Silkie_Wencha	0.741912832
ENSGALG00010005278	DAPP1	3	-0.105114005	0.091148443	-1.153217778	0.248821008	0	0.137722348	0	1	CRA004775;PRJNA827465;Yang2025_Silkie_Wencha	0.741912832
ENSGALG00010017744	ETAA1	3	-0.095949545	0.08319477	-1.153292991	0.248790146	0.012068785	5.423254735	63.12177654	0.666666667	CRA004775;PRJNA827465;Yang2025_Silkie_Wencha	0.741912832
ENSGALG00010008988	PPM1G	3	-0.049254663	0.042704541	-1.153382335	0.248753489	0	0.027983153	0	1	CRA004775;PRJNA827465;Yang2025_Silkie_Wencha	0.741912832
ENSGALG00010026550	MYD88	3	-0.334984233	0.290631962	-1.152616309	0.249072012	0.226360855	23.57877522	91.51779521	0.666666667	CRA004775;PRJNA827465;Yang2025_Silkie_Wencha	0.742176856
ENSGALG00010023024	PRMT7	3	-0.102000401	0.088527873	-1.152184026	0.249245461	0.008582691	2.989856254	33.10715198	1	CRA004775;PRJNA827465;Yang2025_Silkie_Wencha	0.742537203
ENSGALG00010016965	SLC25A33	3	-0.10906226	0.095154338	-1.152050469	0.249300335	0	0.642507557	0	1	CRA004775;PRJNA827465;Yang2025_Silkie_Wencha	0.742544225
ENSGALG00010003777	ENSGALG0001000377	3	0.623474003	0.54137942	1.151639645	0.249469183	0.41069558	3.745606786	46.60411211	0.666666667	CRA004775;PRJNA827465;Yang2025_Silkie_Wencha	0.742577848
ENSGALG00010022866	KLHL11	3	-0.215401063	0.18700238	-1.151862686	0.249377504	0.075387537	11.16450298	82.08608118	0.666666667	CRA004775;PRJNA827465;Yang2025_Silkie_Wencha	0.742577848
ENSGALG00010007067	CASP3	3	-0.204991818	0.177981743	-1.15157756	0.249420712	0.067610866	12.17852418	83.5776489	0.666666667	CRA004775;PRJNA827465;Yang2025_Silkie_Wencha	0.742577848
ENSGALG00010028146	MAPKAPK3	3	-0.085909757	0.074608873	-1.151468387	0.249539593	0	1.378940017	0	0.666666667	CRA004775;PRJNA827465;Yang2025_Silkie_Wencha	0.742631091
ENSGALG00010011288	ENSGALG0001001128	3	-0.231092475	0.200719097	-1.151322811	0.249599456	0	0.067236597	0	1	CRA004775;PRJNA827465;Yang2025_Silkie_Wencha	0.742652927
ENSGALG00010026266	ENSGALG0001002626	2	0.947335904	0.823461019	1.150431996	0.249965988	1.305049643	26.5247388	96.2299346	1	PRJNA827465;Yang2025_Silkie_Wenchang	0.742786037
ENSGALG00010001480	JAKMIP3	2	0.378828114	0.329148435	1.150939967	0.249759403	0.151357449	3.147096735	68.22468185	1	PRJNA827465;Yang2025_Silkie_Wenchang	0.742786037
ENSGALG00010013440	EEA1	3	0.362403882	0.315142109	1.149969717	0.250156344	0.26099046	18.54915521	89.21783781	0.666666667	CRA004775;PRJNA827465;Yang2025_Silkie_Wencha	0.742786037
ENSGALG00010014487	OSTM1	3	-0.319059896	0.277501791	-1.14975797	0.25024357	0.193609284	24.81098624	91.93905482	0.666666667	CRA004775;PRJNA827465;Yang2025_Silkie_Wencha	0.742786037
ENSGALG00010022282	TMT1A	3	-0.268613618	0.233547503	-1.150145537	0.250083934	0.080142366	4.025898539	50.32164918	0.666666667	CRA004775;PRJNA827465;Yang2025_Silkie_Wencha	0.742786037
ENSGALG00010022522	HPSE	3	-0.200790793	0.174665604	-1.149572601	0.250319949	0	0.936220264	0	1	CRA004775;PRJNA827465;Yang2025_Silkie_Wencha	0.742786037
ENSGALG00010013878	ARID2	3	0.193084918	0.167832123	1.150464608	0.249952563	0.050008544	5.162422573	61.25849886	0.666666667	CRA004775;PRJNA827465;Yang2025_Silkie_Wencha	0.742786037
ENSGALG00010025661	SNRPE	3	-0.166754203	0.144902256	-1.150804741	0.249812574	0.036263012	4.84699443	58.7373159	0.666666667	CRA004775;PRJNA827465;Yang2025_Silkie_Wencha	0.742786037
ENSGALG00010028551	NCS1	3	-0.148141138	0.128745216	-1.150653539	0.249874798	0	0.989914503	0	0.666666667	CRA004775;PRJNA827465;Yang2025_Silkie_Wencha	0.742786037
ENSGALG00010020462	CNNM4	3	-0.094499309	0.082131433	-1.150586385	0.249902437	0.007063788	2.860980539	30.09389709	1	CRA004775;PRJNA827465;Yang2025_Silkie_Wencha	0.742786037
ENSGALG00010021688	ITPR1	3	0.077462107	0.067362806	1.149923998	0.250175176	0	0.490937409	0	1	CRA004775;PRJNA827465;Yang2025_Silkie_Wencha	0.742786037
ENSGALG00010001843	PRICKLE1	3	0.070409933	0.061237763	1.149779635	0.250234645	0	0.761711395	0	1	CRA004775;PRJNA827465;Yang2025_Silkie_Wencha	0.742786037
ENSGALG00010005428	ZNF770	3	0.05482757	0.047694597	1.149555143	0.250327142	0	0.042522056	0	1	CRA004775;PRJNA827465;Yang2025_Silkie_Wencha	0.742786037
ENSGALG00010014492	CPLX2	3	0.29688108	0.258317652	1.14928685	0.250437719	0.11602102	4.755249198	57.94121577	1	CRA004775;PRJNA827465;Yang2025_Silkie_Wencha	0.742802371
ENSGALG00010022493	ENSGALG0001002249	3	-0.165308168	0.143819483	-1.149414282	0.250385194	0.024646973	3.280861508	39.04040158	0.666666667	CRA004775;PRJNA827465;Yang2025_Silkie_Wencha	0.742802371
ENSGALG00010025157	NOP58	3	0.057139439	0.049726475	1.149074805	0.250525137	0	0.557534438	0	0.666666667	CRA004775;PRJNA827465;Yang2025_Silkie_Wencha	0.742905811
ENSGALG00010000037	ND6	3	0.4386546	0.381836635	1.148801764	0.250637732	0.34799623	11.02218957	81.85478496	0.666666667	CRA004775;PRJNA827465;Yang2025_Silkie_Wencha	0.742928069
ENSGALG00010002343	GPD1L	3	-0.267106127	0.232490937	-1.148888339	0.250602026	0.127757345	13.88682477	85.59785961	0.666666667	CRA004775;PRJNA827465;Yang2025_Silkie_Wencha	0.742928069
ENSGALG00010016892	MAL	3	0.378936099	0.329902459	1.148630722	0.250708284	0.208137932	5.779048339	65.3922258	1	CRA004775;PRJNA827465;Yang2025_Silkie_Wencha	0.742981434
ENSGALG00010016109	MLH1	3	-0.086484198	0.075307147	-1.148419519	0.25079542	0	1.407896665	0	1	CRA004775;PRJNA827465;Yang2025_Silkie_Wencha	0.743083914
ENSGALG00010017619	POLN	3	0.281025331	0.244831063	1.147833642	0.251037246	0	0.097728742	0	1	CRA004775;PRJNA827465;Yang2025_Silkie_Wencha	0.743087395
ENSGALG00010027402	AP4B1	3	-0.176131992	0.15340802	-1.148127669	0.250915863	0.05263589	8.092157098	75.28471116	1	CRA004775;PRJNA827465;Yang2025_Silkie_Wencha	0.743087395
ENSGALG00010027937	HDHD3	3	-0.113893152	0.099212003	-1.14797755	0.250977832	0.011400861	2.782250188	28.11573853	1	CRA004775;PRJNA827465;Yang2025_Silkie_Wencha	0.743087395
ENSGALG00010011947	THAP1	3	0.112798543	0.098250051	1.148076186	0.250937114	0	1.65280722	0	0.666666667	CRA004775;PRJNA827465;Yang2025_Silkie_Wencha	0.743087395
ENSGALG00010009602	TSTD2	3	-0.102141334	0.088990337	-1.147780054	0.251059374	0	1.157358551	0	1	CRA004775;PRJNA827465;Yang2025_Silkie_Wencha	0.743087395
ENSGALG00010007610	TLDC1	3	-0.171403401	0.149411026	-1.147193786	0.251301539	0.050195723	12.52708272	84.03459094	0.666666667	CRA004775;PRJNA827465;Yang2025_Silkie_Wencha	0.743518753
ENSGALG00010029216	RAB34	3	-0.086340001	0.075263303	-1.14717262	0.251310285	0	1.80502856	0	0.666666667	CRA004775;PRJNA827465;Yang2025_Silkie_Wencha	0.743518753
ENSGALG00010012122	GRIP1	3	0.389556675	0.339907258	1.146067539	0.251767208	0.249420433	9.046288205	77.89148483	0.666666667	CRA004775;PRJNA827465;Yang2025_Silkie_Wencha	0.74365156
ENSGALG00010017153	SLC35F4	2	-0.368683771	0.32159175	-1.146434169	0.251615551	0	0.515272026	0	1	PRJNA827465;Yang2025_Silkie_Wenchang	0.74365156
ENSGALG00010012912	NKAPL	3	0.353888007	0.308703136	1.146369975	0.251642101	0.257453848	22.64370787	91.16752428	0.666666667	CRA004775;PRJNA827465;Yang2025_Silkie_Wencha	0.74365156
ENSGALG00010025610	RNH1	3	-0.176652569	0.154095751	-1.14638183	0.251637198	0.048484497	6.425004383	68.87161657	0.666666667	CRA004775;PRJNA827465;Yang2025_Silkie_Wencha	0.74365156
ENSGALG00010008348	ENSGALG0001000834	3	0.164302526	0.1432931	1.146618548	0.251539307	0.042924628	8.904804641	77.54021474	0.666666667	CRA004775;PRJNA827465;Yang2025_Silkie_Wencha	0.74365156
ENSGALG00010000787	SNRNP48	3	-0.106487671	0.092917415	-1.146046433	0.25177594	0	1.226679043	0	0.666666667	CRA004775;PRJNA827465;Yang2025_Silkie_Wencha	0.74365156
ENSGALG00010013180	C5orf24	3	-0.088195215	0.076908195	-1.146759647	0.251480971	0.009277762	4.537237295	55.92031296	0.666666667	CRA004775;PRJNA827465;Yang2025_Silkie_Wencha	0.74365156
ENSGALG00010011792	HDHD5	3	-0.076782566	0.066997114	-1.146057815	0.251771231	0	0.47545494	0	1	CRA004775;PRJNA827465;Yang2025_Silkie_Wencha	0.74365156
ENSGALG00010026982	ABTB1	3	-0.239787943	0.209310393	-1.145609351	0.251956826	0.108851553	15.52832725	87.12031266	1	CRA004775;PRJNA827465;Yang2025_Silkie_Wencha	0.743827862
ENSGALG00010012734	TMEM19	3	-0.19895359	0.173753894	-1.145030973	0.252196327	0.05866	5.925126242	66.24544494	0.666666667	CRA004775;PRJNA827465;Yang2025_Silkie_Wencha	0.743827862
ENSGALG00010007877	MSMO1	3	0.18565897	0.162140967	1.145046644	0.252189836	0.042605627	4.37653973	54.30179723	0.666666667	CRA004775;PRJNA827465;Yang2025_Silkie_Wencha	0.743827862
ENSGALG00010020166	HAPLN3	3	-0.133275894	0.116392049	-1.145060119	0.252184255	0	1.121210775	0	1	CRA004775;PRJNA827465;Yang2025_Silkie_Wencha	0.743827862
ENSGALG00010019021	LRR1	3	-0.124365495	0.108570011	-1.145486626	0.252007632	0.01560077	3.554281255	43.72983293	1	CRA004775;PRJNA827465;Yang2025_Silkie_Wencha	0.743827862
ENSGALG00010002392	DFNA5	3	0.101487738	0.088612502	1.145298182	0.252085659	0	0.261234735	0	1	CRA004775;PRJNA827465;Yang2025_Silkie_Wencha	0.743827862
ENSGALG00010022525	ISCU	3	-0.055425842	0.048406311	-1.145012721	0.252203888	0	1.921411577	0	1	CRA004775;PRJNA827465;Yang2025_Silkie_Wencha	0.743827862
ENSGALG00010021116	FAM21C	3	0.182655692	0.159553575	1.144792224	0.252295237	0.061724949	14.90337176	86.58021801	0.666666667	CRA004775;PRJNA827465;Yang2025_Silkie_Wencha	0.743942097
ENSGALG00010026560	TNNI1	3	0.141487339	0.123614477	1.144585507	0.252380899	0	0.451326665	0	1	CRA004775;PRJNA827465;Yang2025_Silkie_Wencha	0.744039518
ENSGALG00010005313	MPHOSPH8	3	0.329577558	0.288044548	1.144189539	0.252545041	0.209877498	14.68775322	86.38321348	0.666666667	CRA004775;PRJNA827465;Yang2025_Silkie_Wencha	0.744042702
ENSGALG00010002591	ENSGALG0001000259	3	-0.274008059	0.239614892	-1.143535182	0.252816456	0.126915952	7.994414536	74.98253323	0.666666667	CRA004775;PRJNA827465;Yang2025_Silkie_Wencha	0.744042702
ENSGALG00010029307	SGSH	3	0.180406754	0.157671729	1.144192147	0.252543959	0.042517754	4.860212485	58.84953577	0.666666667	CRA004775;PRJNA827465;Yang2025_Silkie_Wencha	0.744042702
ENSGALG00010006028	ARL5B	3	-0.172410833	0.150784246	-1.143427367	0.252861195	0.050689745	15.7980568	87.34021516	0.666666667	CRA004775;PRJNA827465;Yang2025_Silkie_Wencha	0.744042702
ENSGALG00010011759	CREB3	3	-0.131932075	0.115336236	-1.143890936	0.252668871	0.019256413	3.915248411	48.9176729	1	CRA004775;PRJNA827465;Yang2025_Silkie_Wencha	0.744042702
ENSGALG00010021933	PRR14L	3	0.127585611	0.11157185	1.143528689	0.25281915	0.019628034	4.103523966	51.26140321	1	CRA004775;PRJNA827465;Yang2025_Silkie_Wencha	0.744042702
ENSGALG00010027741	RAB11B	3	-0.125511851	0.109725797	-1.143868203	0.252678299	0.028777287	10.56578438	81.07097468	0.666666667	CRA004775;PRJNA827465;Yang2025_Silkie_Wencha	0.744042702
ENSGALG00010015148	ENSGALG0001001514	3	0.09690542	0.084758355	1.143314075	0.252908213	0	0.691670922	0	1	CRA004775;PRJNA827465;Yang2025_Silkie_Wencha	0.744042702
ENSGALG00010001725	MTA2	3	-0.084564179	0.073958829	-1.143395317	0.252874496	0.006248308	3.155852251	36.62567697	0.666666667	CRA004775;PRJNA827465;Yang2025_Silkie_Wencha	0.744042702
ENSGALG00010015101	CAP1	3	-0.038979577	0.034062028	-1.144370402	0.252470058	0	1.583181265	0	1	CRA004775;PRJNA827465;Yang2025_Silkie_Wencha	0.744042702
ENSGALG00010005311	YME1L1	3	-0.148891243	0.130243189	-1.143178725	0.252964394	0.037059003	19.05757564	89.5054857	1	CRA004775;PRJNA827465;Yang2025_Silkie_Wencha	0.744053165
ENSGALG00010020362	CDS2	3	-0.314336691	0.275370604	-1.141504166	0.253660179	0.193098748	38.71211344	94.83365845	0.666666667	CRA004775;PRJNA827465;Yang2025_Silkie_Wencha	0.744241809
ENSGALG00010004892	HEY2	3	0.299213827	0.262044711	1.141842647	0.253519432	0.123608478	5.12512155	60.97653528	0.666666667	CRA004775;PRJNA827465;Yang2025_Silkie_Wencha	0.744241809
ENSGALG00010021843	ENSGALG0001002184	3	-0.298283836	0.261154472	-1.142173953	0.253381721	0.073682218	3.104414071	35.57560447	1	CRA004775;PRJNA827465;Yang2025_Silkie_Wencha	0.744241809
ENSGALG00010010956	AXDND1	2	-0.297057827	0.260150961	-1.141867112	0.253509261	0	0.483913561	0	1	PRJNA827465;Yang2025_Silkie_Wenchang	0.744241809
ENSGALG00010003192	RGCC	3	-0.243205836	0.212861853	-1.14255247	0.253224449	0.072334534	4.604783827	56.56690791	0.666666667	CRA004775;PRJNA827465;Yang2025_Silkie_Wencha	0.744241809
ENSGALG00010024197	KRT7	3	-0.201511567	0.176379321	-1.142489752	0.253250503	0	1.095625836	0	0.666666667	CRA004775;PRJNA827465;Yang2025_Silkie_Wencha	0.744241809
ENSGALG00010028572	TBC1D13	3	-0.14411102	0.12615588	-1.14232504	0.253318937	0.036733254	9.322767048	78.54714175	0.666666667	CRA004775;PRJNA827465;Yang2025_Silkie_Wencha	0.744241809
ENSGALG00010013327	SLF1	3	0.137187228	0.120044329	1.142804734	0.253119673	0	0.445851396	0	0.666666667	CRA004775;PRJNA827465;Yang2025_Silkie_Wencha	0.744241809
ENSGALG00010011244	PDE9A	3	0.102468172	0.08973884	1.141848635	0.253516942	0	0.904430395	0	0.666666667	CRA004775;PRJNA827465;Yang2025_Silkie_Wencha	0.744241809
ENSGALG00010017771	PNO1	3	-0.072935382	0.063862565	-1.142067844	0.25342582	0	0.819490479	0	0.666666667	CRA004775;PRJNA827465;Yang2025_Silkie_Wencha	0.744241809
ENSGALG00010009647	GALNT2	3	0.063450063	0.055580288	1.141592909	0.253623273	0	0.403135656	0	0.666666667	CRA004775;PRJNA827465;Yang2025_Silkie_Wencha	0.744241809
ENSGALG00010003737	SRP72	3	-0.043604491	0.038193494	-1.141673247	0.253589865	0	1.076443885	0	1	CRA004775;PRJNA827465;Yang2025_Silkie_Wencha	0.744241809
ENSGALG00010023791	VTI1B	3	0.055010853	0.048205519	1.141173332	0.253797799	0	0.573784198	0	1	CRA004775;PRJNA827465;Yang2025_Silkie_Wencha	0.744491097
ENSGALG00010011442	ENSGALG0001001144	3	-2.958200818	2.601563968	-1.137085558	0.25550252	16.8500229	13.04848854	84.67255427	0.666666667	CRA004775;PRJNA827465;Yang2025_Silkie_Wencha	0.744535853
ENSGALG00010025615	AQP5	2	1.528144573	1.354927206	1.127842563	0.259386414	3.186693003	7.556453033	86.76627783	1	CRA004775;PRJNA827465	0.744535853
ENSGALG00010016860	VSX1	2	-0.772777967	0.682790242	-1.131794099	0.257721009	0.738778454	4.789896762	79.12272331	1	PRJNA827465;Yang2025_Silkie_Wenchang	0.744535853
ENSGALG00010023187	ENSGALG0001002318	3	0.740987961	0.652794987	1.135100568	0.256333184	1.094532316	22.82799397	91.2388272	0.666666667	CRA004775;PRJNA827465;Yang2025_Silkie_Wencha	0.744535853
ENSGALG00010026782	HOXB9	3	0.740826596	0.654779469	1.131413906	0.257880921	0.987536072	8.712852708	77.0454056	0.666666667	CRA004775;PRJNA827465;Yang2025_Silkie_Wencha	0.744535853
ENSGALG00010002784	TRIM27.2	3	0.664561528	0.586747004	1.132620234	0.257373768	0.756621235	7.994705316	74.98344316	0.666666667	CRA004775;PRJNA827465;Yang2025_Silkie_Wencha	0.744535853
ENSGALG00010008236	ENSGALG0001000823	3	0.575869101	0.508582657	1.132301885	0.257507538	0.625443194	10.32399016	80.62764523	0.666666667	CRA004775;PRJNA827465;Yang2025_Silkie_Wencha	0.744535853
ENSGALG00010003845	CFI	2	0.551517112	0.487197951	1.132018538	0.257626641	0.213265865	1.741492622	42.57799388	1	PRJNA827465;Yang2025_Silkie_Wenchang	0.744535853
ENSGALG00010016067	AHSG	3	0.485308651	0.428670974	1.132123893	0.257582351	0.372354699	7.33450939	72.73164579	0.666666667	CRA004775;PRJNA827465;Yang2025_Silkie_Wencha	0.744535853
ENSGALG00010024799	ENSGALG0001002479	3	0.458337762	0.404450533	1.13323565	0.257115307	0.439474878	24.98012887	91.99363618	0.666666667	CRA004775;PRJNA827465;Yang2025_Silkie_Wencha	0.744535853
ENSGALG00010004769	HTR2A	3	-0.456913404	0.402481001	-1.135242167	0.256273866	0.343988357	8.905600901	77.5422229	0.666666667	CRA004775;PRJNA827465;Yang2025_Silkie_Wencha	0.744535853
ENSGALG00010014291	OSTN	3	-0.449174719	0.398096106	-1.128307241	0.259190186	0.108331087	2.501353011	20.04327293	1	CRA004775;PRJNA827465;Yang2025_Silkie_Wencha	0.744535853
ENSGALG00010001113	IGSF10	3	0.401460748	0.352329899	1.139445584	0.254517352	0.230121473	5.344975197	62.58167856	1	CRA004775;PRJNA827465;Yang2025_Silkie_Wencha	0.744535853
ENSGALG00010004501	TAGLN3	3	0.392864835	0.346117125	1.135063266	0.256348811	0.13972836	3.251000689	38.48048059	0.666666667	CRA004775;PRJNA827465;Yang2025_Silkie_Wencha	0.744535853
ENSGALG00010004729	ENSGALG0001000472	3	0.363671027	0.320708968	1.133959644	0.256811477	0	0.889078344	0	1	CRA004775;PRJNA827465;Yang2025_Silkie_Wencha	0.744535853
ENSGALG00010021183	CFAP100	3	0.352434046	0.310342643	1.135628808	0.256111947	0.080824521	2.603880861	23.19157032	0.666666667	CRA004775;PRJNA827465;Yang2025_Silkie_Wencha	0.744535853
ENSGALG00010003719	RPGR	3	0.324718228	0.287091518	1.131061729	0.258029111	0.212560556	16.16192894	87.62523949	0.666666667	CRA004775;PRJNA827465;Yang2025_Silkie_Wencha	0.744535853
ENSGALG00010025318	NUAK2	3	0.309452377	0.274344534	1.127969901	0.259332631	0	1.763226398	0	0.666666667	CRA004775;PRJNA827465;Yang2025_Silkie_Wencha	0.744535853
ENSGALG00010001938	ALG8	3	-0.299762276	0.264796005	-1.132049843	0.25761348	0.17942143	18.93998699	89.44033066	0.666666667	CRA004775;PRJNA827465;Yang2025_Silkie_Wencha	0.744535853
ENSGALG00010012045	ENSGALG0001001204	3	-0.299450571	0.265376548	-1.128398771	0.259151547	0	0.432118303	0	1	CRA004775;PRJNA827465;Yang2025_Silkie_Wencha	0.744535853
ENSGALG00010011345	CRMP1	3	0.299221313	0.262855122	1.138350703	0.25497407	0.124185056	5.500107883	63.6370769	0.666666667	CRA004775;PRJNA827465;Yang2025_Silkie_Wencha	0.744535853
ENSGALG00010024714	SRPRB	3	-0.283103841	0.251029118	-1.127772917	0.259415834	0.163268308	17.28228642	88.42745716	0.666666667	CRA004775;PRJNA827465;Yang2025_Silkie_Wencha	0.744535853
ENSGALG00010022066	DAD1	3	-0.280869145	0.248636498	-1.129637634	0.258628946	0.154459094	13.76926601	85.47489751	0.666666667	CRA004775;PRJNA827465;Yang2025_Silkie_Wencha	0.744535853
ENSGALG00010007283	STOX2	3	0.272826872	0.241523729	1.129606904	0.258641901	0.131205757	9.406231491	78.73749969	0.666666667	CRA004775;PRJNA827465;Yang2025_Silkie_Wencha	0.744535853
ENSGALG00010028568	MLLT1	3	-0.269735526	0.238046407	-1.133121599	0.257163192	0.152771002	35.06346323	94.29605688	1	CRA004775;PRJNA827465;Yang2025_Silkie_Wencha	0.744535853
ENSGALG00010024228	ENSGALG0001002422	2	0.255436833	0.223896944	1.140867882	0.253924907	0	0.444806673	0	1	PRJNA827465;Yang2025_Silkie_Wenchang	0.744535853
ENSGALG00010022228	FUBP1	3	0.252851662	0.222931512	1.134212297	0.256705507	0.12312116	16.39332471	87.79991224	1	CRA004775;PRJNA827465;Yang2025_Silkie_Wencha	0.744535853
ENSGALG00010012410	CACNA1I	2	0.251461007	0.222986131	1.127697968	0.259447496	0	0.003297037	0	1	PRJNA827465;Yang2025_Silkie_Wenchang	0.744535853
ENSGALG00010028072	ENSGALG0001002807	2	0.249139318	0.220586913	1.129438342	0.258712966	0	0.011741087	0	1	PRJNA827465;Yang2025_Silkie_Wenchang	0.744535853
ENSGALG00010017377	USP8	3	0.237070102	0.20958374	1.131147396	0.257993059	0.111836774	14.90700814	86.58349159	0.666666667	CRA004775;PRJNA827465;Yang2025_Silkie_Wencha	0.744535853
ENSGALG00010029925	GGNBP2	3	0.236423326	0.208881107	1.131855961	0.257694996	0.11195188	19.32452214	89.65045559	0.666666667	CRA004775;PRJNA827465;Yang2025_Silkie_Wencha	0.744535853
ENSGALG00010021363	ENSGALG0001002136	3	-0.235485259	0.207947348	-1.132427325	0.257454822	0.102976351	11.55808126	82.69608981	0.666666667	CRA004775;PRJNA827465;Yang2025_Silkie_Wencha	0.744535853
ENSGALG00010007890	ENSGALG0001000789	3	-0.233192301	0.206617949	-1.128615892	0.259059904	0.100135005	12.12166174	83.50061203	0.666666667	CRA004775;PRJNA827465;Yang2025_Silkie_Wencha	0.744535853
ENSGALG00010025392	SLC6A2	3	-0.232096611	0.205262504	-1.13073068	0.258168464	0.059961587	3.813459242	47.55417921	0.666666667	CRA004775;PRJNA827465;Yang2025_Silkie_Wencha	0.744535853
ENSGALG00010023693	SRSF11	3	0.231683969	0.205036328	1.129965462	0.258490778	0.103884956	12.75600299	84.32110747	0.666666667	CRA004775;PRJNA827465;Yang2025_Silkie_Wencha	0.744535853
ENSGALG00010004065	TIFA	3	-0.230494796	0.203105625	-1.134851859	0.256437394	0	0.854764961	0	0.666666667	CRA004775;PRJNA827465;Yang2025_Silkie_Wencha	0.744535853
ENSGALG00010000256	ENSGALG0001000025	3	-0.227057831	0.199327453	-1.139119712	0.254653227	0	0.411559547	0	1	CRA004775;PRJNA827465;Yang2025_Silkie_Wencha	0.744535853
ENSGALG00010008893	ENSGALG0001000889	3	-0.223592177	0.197657787	-1.131208541	0.257967328	0.068567566	6.199890718	67.74136689	0.666666667	CRA004775;PRJNA827465;Yang2025_Silkie_Wencha	0.744535853
ENSGALG00010024358	MAPK3	3	-0.219206392	0.192429547	-1.139151418	0.254640005	0.098720487	43.08957192	95.35850576	0.666666667	CRA004775;PRJNA827465;Yang2025_Silkie_Wencha	0.744535853
ENSGALG00010020932	CASP9	3	-0.218585079	0.192941584	-1.132908077	0.257252858	0.079134865	7.13287711	71.96082353	0.666666667	CRA004775;PRJNA827465;Yang2025_Silkie_Wencha	0.744535853
ENSGALG00010012439	TENT2	3	0.216882426	0.191672041	1.131528755	0.257832608	0.08414363	8.686491831	76.97574534	0.666666667	CRA004775;PRJNA827465;Yang2025_Silkie_Wencha	0.744535853
ENSGALG00010013551	UBE2N	3	-0.211283924	0.185465683	-1.139207643	0.254616558	0.087293544	18.90489551	89.42072968	1	CRA004775;PRJNA827465;Yang2025_Silkie_Wencha	0.744535853
ENSGALG00010016592	TMEM129	3	-0.209150363	0.185187661	-1.129396862	0.258730456	0.085361362	14.00459162	85.71896951	0.666666667	CRA004775;PRJNA827465;Yang2025_Silkie_Wencha	0.744535853
ENSGALG00010005963	UGCG	3	0.206419895	0.182798015	1.129223944	0.258803375	0.073337928	9.447363266	78.83007201	0.666666667	CRA004775;PRJNA827465;Yang2025_Silkie_Wencha	0.744535853
ENSGALG00010023320	ENSGALG0001002332	3	-0.20480295	0.179891199	-1.138482322	0.254919136	0.074627189	13.49899745	85.18408491	1	CRA004775;PRJNA827465;Yang2025_Silkie_Wencha	0.744535853
ENSGALG00010002589	SLC4A7	3	0.204179166	0.179653057	1.136519298	0.255739294	0.069171985	13.93527924	85.6479374	0.666666667	CRA004775;PRJNA827465;Yang2025_Silkie_Wencha	0.744535853
ENSGALG00010011121	NUP35	3	-0.203484293	0.179278395	-1.135018493	0.25636757	0.077132256	11.78051484	83.02281329	0.666666667	CRA004775;PRJNA827465;Yang2025_Silkie_Wencha	0.744535853
ENSGALG00010024893	ARL6IP4	3	0.202910478	0.178957816	1.133845297	0.256859447	0.079293112	13.63765884	85.33472626	0.666666667	CRA004775;PRJNA827465;Yang2025_Silkie_Wencha	0.744535853
ENSGALG00010018312	PRPF39	3	0.200004076	0.175977355	1.136533026	0.255733552	0.076570357	21.87606173	90.8575866	1	CRA004775;PRJNA827465;Yang2025_Silkie_Wencha	0.744535853
ENSGALG00010017655	ENSGALG0001001765	3	0.198920667	0.175904708	1.130843333	0.258121038	0.061261009	6.872657768	70.8991766	1	CRA004775;PRJNA827465;Yang2025_Silkie_Wencha	0.744535853
ENSGALG00010029428	FN3K	3	0.192390769	0.170578917	1.127869564	0.259375009	0	0.654214524	0	0.666666667	CRA004775;PRJNA827465;Yang2025_Silkie_Wencha	0.744535853
ENSGALG00010028692	RALGPS1	3	0.192324547	0.168894988	1.138722644	0.254818855	0.052844877	5.569736803	64.09166051	0.666666667	CRA004775;PRJNA827465;Yang2025_Silkie_Wencha	0.744535853
ENSGALG00010010655	C1QTNF7	3	-0.191329279	0.167983862	-1.138974163	0.254713931	0.042693187	4.010562715	50.1316862	0.666666667	CRA004775;PRJNA827465;Yang2025_Silkie_Wencha	0.744535853
ENSGALG00010011509	EXOC2	3	-0.191054347	0.167781452	-1.138709585	0.254824304	0.074766615	25.5984547	92.18702838	1	CRA004775;PRJNA827465;Yang2025_Silkie_Wencha	0.744535853
ENSGALG00010026485	ACAA1	3	-0.188694501	0.166981429	-1.130032853	0.258462381	0.071289593	13.66938857	85.36876767	0.666666667	CRA004775;PRJNA827465;Yang2025_Silkie_Wencha	0.744535853
ENSGALG00010027171	ATG9B	3	-0.187100279	0.165253269	-1.132203194	0.257549018	0.028919937	3.088385976	35.24125497	0.666666667	CRA004775;PRJNA827465;Yang2025_Silkie_Wencha	0.744535853
ENSGALG00010011004	UMODL1	2	0.179989119	0.159279155	1.130023068	0.258466505	0	0.026631264	0	1	PRJNA827465;Yang2025_Silkie_Wenchang	0.744535853
ENSGALG00010021252	XPC	3	0.173583274	0.153014931	1.134420495	0.256618206	0.05185659	9.716374113	79.41618986	1	CRA004775;PRJNA827465;Yang2025_Silkie_Wencha	0.744535853
ENSGALG00010013645	RRAGC	3	-0.173173647	0.153316934	-1.129514153	0.258681003	0.055589556	15.49141293	87.08962178	0.666666667	CRA004775;PRJNA827465;Yang2025_Silkie_Wencha	0.744535853
ENSGALG00010017396	CCR8L	3	0.171899767	0.151994828	1.130958002	0.258072769	0	1.11357737	0	1	CRA004775;PRJNA827465;Yang2025_Silkie_Wencha	0.744535853
ENSGALG00010015404	BEND4	2	-0.171082683	0.151426699	-1.129805272	0.258558286	0	0.12390408	0	1	PRJNA827465;Yang2025_Silkie_Wenchang	0.744535853
ENSGALG00010003097	TFPI2	3	-0.170963978	0.15068657	-1.134566787	0.256556876	0.038184256	4.766691654	58.04217799	0.666666667	CRA004775;PRJNA827465;Yang2025_Silkie_Wencha	0.744535853
ENSGALG00010005772	SVEP1	3	-0.163709872	0.144476382	-1.133125498	0.257161555	0.029293058	3.842311591	47.94800077	0.666666667	CRA004775;PRJNA827465;Yang2025_Silkie_Wencha	0.744535853
ENSGALG00010019267	STARD5	3	0.163246058	0.144639398	1.128641714	0.259049006	0	0.287637377	0	1	CRA004775;PRJNA827465;Yang2025_Silkie_Wencha	0.744535853
ENSGALG00010028566	ZDHHC12	3	-0.156300894	0.138572417	-1.127936555	0.259346715	0.035829923	6.071934098	67.06156609	1	CRA004775;PRJNA827465;Yang2025_Silkie_Wencha	0.744535853

ENSGALG00010015393	DCX	3	-0.152178925	0.134098956	-1.134825574	0.256448409	0	1.696402029	0	1	CRA004775;PRJNA827465;Yang2025_Silkie_Wencha	0.744535853
ENSGALG00010021810	ANKRD13A	3	-0.149795769	0.132136046	-1.13364804	0.256942213	0.035593083	10.84817847	81.56372514	0.666666667	CRA004775;PRJNA827465;Yang2025_Silkie_Wencha	0.744535853
ENSGALG00010013334	SH3BGRL3	3	-0.148541927	0.130265587	-1.140300595	0.25416109	0.033639481	6.387813965	68.69038437	0.666666667	CRA004775;PRJNA827465;Yang2025_Silkie_Wencha	0.744535853
ENSGALG00010021320	SLC6A6	3	-0.143552436	0.12611866	-1.138233119	0.255023153	0.016160972	2.946652599	32.12637279	1	CRA004775;PRJNA827465;Yang2025_Silkie_Wencha	0.744535853
ENSGALG00010028405	S100A6	3	-0.143312315	0.126440113	-1.13344026	0.257029415	0	1.937333164	0	1	CRA004775;PRJNA827465;Yang2025_Silkie_Wencha	0.744535853
ENSGALG00010004777	CDK14	3	-0.140098855	0.123506736	-1.134341816	0.256651195	0.02280552	4.060495193	50.74492383	0.666666667	CRA004775;PRJNA827465;Yang2025_Silkie_Wencha	0.744535853
ENSGALG00010024689	FOXD2	3	0.138160148	0.121682002	1.135419742	0.256199492	0.010089788	2.527178728	20.86036585	0.666666667	CRA004775;PRJNA827465;Yang2025_Silkie_Wencha	0.744535853
ENSGALG00010028145	ENSGALG0001002814	3	-0.135031941	0.119353044	-1.131365711	0.257901197	0	1.042238875	0	0.666666667	CRA004775;PRJNA827465;Yang2025_Silkie_Wencha	0.744535853
ENSGALG00010018401	ENSGALG0001001840	3	-0.127474535	0.111764909	-1.140559557	0.254053255	0.023251912	5.243722045	61.85915304	0.666666667	CRA004775;PRJNA827465;Yang2025_Silkie_Wencha	0.744535853
ENSGALG00010028083	ELAVL1	3	-0.125795984	0.110758789	-1.13576525	0.256054824	0.029791477	14.26707593	85.98171055	1	CRA004775;PRJNA827465;Yang2025_Silkie_Wencha	0.744535853
ENSGALG00010028020	ENSGALG0001002802	3	-0.12292332	0.108539177	-1.132524894	0.257413825	0	0.288401083	0	1	CRA004775;PRJNA827465;Yang2025_Silkie_Wencha	0.744535853
ENSGALG00010027733	RPL35	3	-0.119483055	0.105071003	-1.137164881	0.255469365	0.00900196	2.391070148	16.35544436	1	CRA004775;PRJNA827465;Yang2025_Silkie_Wencha	0.744535853
ENSGALG00010002353	CMTM8	3	0.118623512	0.104076362	1.13977381	0.254380548	0	0.634843439	0	1	CRA004775;PRJNA827465;Yang2025_Silkie_Wencha	0.744535853
ENSGALG00010008020	COA6	3	-0.110413028	0.097151444	-1.136504241	0.255745592	0	0.570300219	0	0.666666667	CRA004775;PRJNA827465;Yang2025_Silkie_Wencha	0.744535853
ENSGALG00010027151	MICOS13	3	-0.106429656	0.093993933	-1.132303475	0.25750687	0	1.08444231	0	0.666666667	CRA004775;PRJNA827465;Yang2025_Silkie_Wencha	0.744535853
ENSGALG00010012367	RAD52	3	0.106007406	0.093489638	1.13389471	0.256838717	0.007625083	2.698168369	25.8756413	1	CRA004775;PRJNA827465;Yang2025_Silkie_Wencha	0.744535853
ENSGALG00010022727	ACP2	3	0.103824715	0.091910946	1.129622964	0.258635131	0.00869461	3.016196136	33.69131483	0.666666667	CRA004775;PRJNA827465;Yang2025_Silkie_Wencha	0.744535853
ENSGALG00010013320	CALCRL	3	0.101499761	0.089270655	1.136989087	0.255542848	0.003652448	2.233683565	10.46180258	1	CRA004775;PRJNA827465;Yang2025_Silkie_Wencha	0.744535853
ENSGALG00010029375	JMJD6	3	0.095380707	0.084311444	1.131290158	0.257932986	0	0.632384711	0	0.666666667	CRA004775;PRJNA827465;Yang2025_Silkie_Wencha	0.744535853
ENSGALG00010023806	PATJ	3	0.094434991	0.082917299	1.138905776	0.254742456	0	1.483847803	0	1	CRA004775;PRJNA827465;Yang2025_Silkie_Wencha	0.744535853
ENSGALG00010027883	ATP8B2	3	-0.091168246	0.080683059	-1.129955246	0.258495083	0.011026606	5.04349597	60.34496683	1	CRA004775;PRJNA827465;Yang2025_Silkie_Wencha	0.744535853
ENSGALG00010018002	ZC3H8	3	0.09107955	0.080615585	1.12980076	0.258560188	0.006721578	2.667416587	25.02108558	1	CRA004775;PRJNA827465;Yang2025_Silkie_Wencha	0.744535853
ENSGALG00010017898	NHERF2	3	-0.088908789	0.078181433	-1.13721105	0.255450069	0.008956486	3.888797702	48.57022264	0.666666667	CRA004775;PRJNA827465;Yang2025_Silkie_Wencha	0.744535853
ENSGALG00010021058	CCP110	3	0.088828505	0.078093498	1.137463528	0.255344563	0.006325884	2.740759452	27.02752521	1	CRA004775;PRJNA827465;Yang2025_Silkie_Wencha	0.744535853
ENSGALG00010027337	AQP1	3	0.08730243	0.076596444	1.139771322	0.254381585	0	0.790253104	0	1	CRA004775;PRJNA827465;Yang2025_Silkie_Wencha	0.744535853
ENSGALG00010015816	MITD1	3	-0.083863998	0.074183458	-1.130494593	0.258267875	0	1.495990052	0	1	CRA004775;PRJNA827465;Yang2025_Silkie_Wencha	0.744535853
ENSGALG00010029237	COX11	3	0.080934181	0.071343548	1.13442887	0.256614695	0	0.63703293	0	1	CRA004775;PRJNA827465;Yang2025_Silkie_Wencha	0.744535853
ENSGALG00010008185	PDGFC	3	-0.080363001	0.070681301	-1.136976824	0.255547974	0	0.350510232	0	1	CRA004775;PRJNA827465;Yang2025_Silkie_Wencha	0.744535853
ENSGALG00010026942	CDK18	3	0.077800169	0.068747777	1.131675408	0.257770924	0	1.349930151	0	0.666666667	CRA004775;PRJNA827465;Yang2025_Silkie_Wencha	0.744535853
ENSGALG00010008075	SDHA	3	-0.076487284	0.067484662	-1.133402484	0.257045271	0	1.595467687	0	0.666666667	CRA004775;PRJNA827465;Yang2025_Silkie_Wencha	0.744535853
ENSGALG00010002341	FAM49A	3	0.074085006	0.065426181	1.132344956	0.257489437	0	0.417134559	0	1	CRA004775;PRJNA827465;Yang2025_Silkie_Wencha	0.744535853
ENSGALG00010024326	KLHL24	3	-0.073509212	0.064892983	-1.132775969	0.257308346	0.00057991	2.075657598	3.644994152	0.666666667	CRA004775;PRJNA827465;Yang2025_Silkie_Wencha	0.744535853
ENSGALG00010017363	TIMM9	3	-0.071138335	0.062659777	-1.135311012	0.25624503	0	0.595972483	0	0.666666667	CRA004775;PRJNA827465;Yang2025_Silkie_Wencha	0.744535853
ENSGALG00010024628	TANGO2	3	-0.068230732	0.060020694	-1.136786799	0.255627423	0	0.348789608	0	1	CRA004775;PRJNA827465;Yang2025_Silkie_Wencha	0.744535853
ENSGALG00010021626	ZNF142	3	-0.066368658	0.058331404	-1.137786052	0.255209831	0	0.140100776	0	1	CRA004775;PRJNA827465;Yang2025_Silkie_Wencha	0.744535853
ENSGALG00010010792	XRCC6	3	-0.063938815	0.056637254	-1.128918001	0.258932426	0	1.845182102	0	0.666666667	CRA004775;PRJNA827465;Yang2025_Silkie_Wencha	0.744535853
ENSGALG00010026163	CTNND1	3	-0.061983296	0.054586865	-1.135498372	0.256166564	0.003829856	3.545171041	43.5852325	1	CRA004775;PRJNA827465;Yang2025_Silkie_Wencha	0.744535853
ENSGALG00010003842	ENSGALG0001000384	3	-0.059508373	0.052379451	-1.136101506	0.255914085	0	1.750920115	0	0.666666667	CRA004775;PRJNA827465;Yang2025_Silkie_Wencha	0.744535853
ENSGALG00010015203	HMGXB4	3	0.05852277	0.051860169	1.128472412	0.259120462	0.002440901	2.789860059	28.31181645	0.666666667	CRA004775;PRJNA827465;Yang2025_Silkie_Wencha	0.744535853
ENSGALG00010023117	RPUSD2	3	-0.057326766	0.05070765	-1.130534875	0.258250912	0	0.25452228	0	0.666666667	CRA004775;PRJNA827465;Yang2025_Silkie_Wencha	0.744535853
ENSGALG00010022823	IGHMBP2	3	-0.055427201	0.049127759	-1.128225706	0.25922461	0	0.3863879	0	1	CRA004775;PRJNA827465;Yang2025_Silkie_Wencha	0.744535853
ENSGALG00010018552	DTD1	3	-0.054159269	0.047491939	-1.14038867	0.254124411	0	0.295661533	0	0.666666667	CRA004775;PRJNA827465;Yang2025_Silkie_Wencha	0.744535853
ENSGALG00010017959	PDZD7	3	-0.221487137	0.196470891	-1.127328002	0.259603827	0.050921442	3.698497843	45.92399172	0.666666667	CRA004775;PRJNA827465;Yang2025_Silkie_Wencha	0.744607475
ENSGALG00010018231	SMYD5	3	-0.188578316	0.16730685	-1.12714044	0.259683108	0.068991012	12.18513362	83.58655668	1	CRA004775;PRJNA827465;Yang2025_Silkie_Wencha	0.744607475
ENSGALG00010024701	EIF2B1	3	-0.185170354	0.164254326	-1.127339283	0.25959906	0.05512102	6.428144926	68.88682469	0.666666667	CRA004775;PRJNA827465;Yang2025_Silkie_Wencha	0.744607475
ENSGALG00010025913	EIF2B3	3	-0.05376735	0.04769872	-1.127228378	0.259645935	0	0.511880334	0	1	CRA004775;PRJNA827465;Yang2025_Silkie_Wencha	0.744607475
ENSGALG00010010190	RNF44	3	0.390605362	0.346665678	1.126749449	0.259848429	0.302880005	13.92859341	85.64104831	0.666666667	CRA004775;PRJNA827465;Yang2025_Silkie_Wencha	0.744830842
ENSGALG00010009114	SETD7	3	0.146787161	0.130279794	1.126707039	0.259866366	0.02861115	4.952082677	59.61295215	0.666666667	CRA004775;PRJNA827465;Yang2025_Silkie_Wencha	0.744830842
ENSGALG00010011978	ENSGALG0001001197	3	-1.775777972	1.578575326	-1.124924445	0.260621052	3.578903168	3.838906994	47.9018376	1	CRA004775;PRJNA827465;Yang2025_Silkie_Wencha	0.744969822
ENSGALG00010011085	UGT1A1	2	1.23690836	1.100993581	1.123447386	0.261247534	1.549619253	2.69584366	62.90586079	1	CRA004775;PRJNA827465	0.744969822
ENSGALG00010024046	ENSGALG0001002404	2	0.598545117	0.532353909	1.124336849	0.260870152	0	0.776833013	0	1	PRJNA827465;Yang2025_Silkie_Wenchang	0.744969822
ENSGALG00010011864	ENSGALG0001001186	3	0.450976872	0.401198699	1.124073618	0.260981796	0.352731039	8.456016293	76.34820073	1	CRA004775;PRJNA827465;Yang2025_Silkie_Wencha	0.744969822
ENSGALG00010013388	SOWAHC	3	0.274930655	0.244486813	1.124521406	0.260791894	0.092815194	4.1731626	52.07471667	0.666666667	CRA004775;PRJNA827465;Yang2025_Silkie_Wencha	0.744969822
ENSGALG00010017403	ITGA11	3	0.263368098	0.234467127	1.123262355	0.261326087	0.080879041	4.073899554	50.90698792	0.666666667	CRA004775;PRJNA827465;Yang2025_Silkie_Wencha	0.744969822
ENSGALG00010012322	ENSGALG0001001232	3	0.256102452	0.227530797	1.125572694	0.260346431	0.077841609	4.286908925	53.34633801	0.666666667	CRA004775;PRJNA827465;Yang2025_Silkie_Wencha	0.744969822
ENSGALG00010011770	CHST11	3	0.24023797	0.213844708	1.123422564	0.261258071	0	0.282002071	0	1	CRA004775;PRJNA827465;Yang2025_Silkie_Wencha	0.744969822
ENSGALG00010028573	KCNN1	3	0.235906329	0.21000376	1.123343357	0.261291696	0.091182792	12.95745977	84.56487586	0.666666667	CRA004775;PRJNA827465;Yang2025_Silkie_Wencha	0.744969822
ENSGALG00010007898	GAB1	3	0.234522808	0.208311208	1.125829041	0.260237889	0.097253743	8.453315449	76.34064395	0.666666667	CRA004775;PRJNA827465;Yang2025_Silkie_Wencha	0.744969822
ENSGALG00010013280	FRS2	3	0.21033209	0.186844145	1.12570868	0.260288848	0.063224205	5.380711998	62.83019792	0.666666667	CRA004775;PRJNA827465;Yang2025_Silkie_Wencha	0.744969822
ENSGALG00010016379	TFAP4	3	0.207997214	0.185132542	1.123504339	0.261223359	0.085844195	14.4523017	86.16137387	1	CRA004775;PRJNA827465;Yang2025_Silkie_Wencha	0.744969822
ENSGALG00010018274	ZNF711	3	0.204481296	0.18157601	1.126147094	0.260103263	0.084269448	16.3254783	87.74921038	1	CRA004775;PRJNA827465;Yang2025_Silkie_Wencha	0.744969822
ENSGALG00010020405	UBE2I	3	-0.165633259	0.147105987	-1.125945054	0.260188777	0.048921931	11.031529	81.87014692	0.666666667	CRA004775;PRJNA827465;Yang2025_Silkie_Wencha	0.744969822
ENSGALG00010009166	CLGN	3	-0.156366472	0.138834644	-1.126278479	0.260047664	0	0.572336225	0	1	CRA004775;PRJNA827465;Yang2025_Silkie_Wencha	0.744969822
ENSGALG00010029546	WSB1	3	0.149296462	0.132598727	1.125926817	0.260196497	0.036449816	7.164979717	72.08645273	0.666666667	CRA004775;PRJNA827465;Yang2025_Silkie_Wencha	0.744969822
ENSGALG00010016616	SLBP	3	-0.145646796	0.129453186	-1.125092401	0.260549881	0.020404374	3.100738318	35.49923293	0.666666667	CRA004775;PRJNA827465;Yang2025_Silkie_Wencha	0.744969822
ENSGALG00010021023	MGEA5	3	0.142353826	0.12660004	1.124437448	0.260827493	0.032107813	6.837827796	70.75094519	0.666666667	CRA004775;PRJNA827465;Yang2025_Silkie_Wencha	0.744969822
ENSGALG00010005386	CPOX	3	0.135362461	0.120511195	1.123235574	0.261337458	0.021156889	3.916300816	48.93139997	0.666666667	CRA004775;PRJNA827465;Yang2025_Silkie_Wencha	0.744969822
ENSGALG00010008409	CWC15	3	-0.121808634	0.108344779	-1.124268612	0.26089909	0.02151527	5.815498608	65.60913974	0.666666667	CRA004775;PRJNA827465;Yang2025_Silkie_Wencha	0.744969822
ENSGALG00010009336	LAMB1	3	-0.115215594	0.102396677	-1.125188803	0.260509037	0.01209233	3.291475627	39.23697981	0.666666667	CRA004775;PRJNA827465;Yang2025_Silkie_Wencha	0.744969822
ENSGALG00010013158	CRIP1	3	0.11331551	0.100697671	1.125304179	0.26046016	0	0.776827865	0	0.666666667	CRA004775;PRJNA827465;Yang2025_Silkie_Wencha	0.744969822
ENSGALG00010011333	ENSGALG0001001133	3	-0.109106361	0.096925609	-1.125671134	0.260304747	0	1.613236385	0	0.666666667	CRA004775;PRJNA827465;Yang2025_Silkie_Wencha	0.744969822
ENSGALG00010020226	TCIM	3	0.100320589	0.089279398	1.123670083	0.261153012	0	1.806505422	0	1	CRA004775;PRJNA827465;Yang2025_Silkie_Wencha	0.744969822
ENSGALG00010003531	PPM1K	3	-0.097122406	0.086396144	-1.124152101	0.260948506	0	0.172635111	0	1	CRA004775;PRJNA827465;Yang2025_Silkie_Wencha	0.744969822
ENSGALG00010024440	PKNOX2	3	-0.092227252	0.081994063	-1.124804029	0.260672087	0	0.540356077	0	1	CRA004775;PRJNA827465;Yang2025_Silkie_Wencha	0.744969822
ENSGALG00010027075	SLC41A1	3	-0.038052738	0.033861304	-1.123782412	0.261105344	0	1.89868034	0	1	CRA004775;PRJNA827465;Yang2025_Silkie_Wencha	0.744969822
ENSGALG00010017832	POU3F1	2	0.268355081	0.239071528	1.122488672	0.261654722	0	0.949122552	0	1	PRJNA827465;Yang2025_Silkie_Wenchang	0.745273771
ENSGALG00010015193	LYPLA2	3	-0.132681732	0.118167838	-1.122824395	0.261512083	0.028593011	6.74923407	70.36700788	0.666666667	CRA004775;PRJNA827465;Yang2025_Silkie_Wencha	0.745273771
ENSGALG00010027250	ENSGALG0001002725	3	0.114739316	0.102203133	1.122659475	0.26158214	0.009152543	2.672347812	25.15944253	0.666666667	CRA004775;PRJNA827465;Yang2025_Silkie_Wencha	0.745273771
ENSGALG00010013934	STAT1	3	-0.046234803	0.041189568	-1.12248819	0.261654926	0	1.882836178	0	0.666666667	CRA004775;PRJNA827465;Yang2025_Silkie_Wencha	0.745273771
ENSGALG00010018876	GMFB	3	-0.361339595	0.321982917	-1.122232194	0.261763728	0.264074196	60.01510211	96.66750546	1	CRA004775;PRJNA827465;Yang2025_Silkie_Wencha	0.745382337
ENSGALG00010028221	TNFAIP8L1	3	-0.241642089	0.215338407	-1.122150445	0.261798478	0.090008341	7.833665545	74.46916787	0.666666667	CRA004775;PRJNA827465;Yang2025_Silkie_Wencha	0.745382337
ENSGALG00010013511	FAM78B	3	0.204004245	0.181820636	1.122008207	0.26185895	0.070818063	7.437784603	73.11027266	0.666666667	CRA004775;PRJNA827465;Yang2025_Silkie_Wencha	0.745397503
ENSGALG00010009943	DHX15	3	-0.108382047	0.096606667	-1.121889928	0.261909244	0.020553938	12.47902724	83.97310975	0.666666667	CRA004775;PRJNA827465;Yang2025_Silkie_Wencha	0.745397503
ENSGALG00010020416	ENSGALG0001002041	2	-1.326045907	1.182238695	-1.121639744	0.262015646	1.782430419	2.748633358	63.61828336	1	PRJNA827465;Yang2025_Silkie_Wenchang	0.745550256
ENSGALG00010012211	ENSGALG0001001221	3	-3.366365432	3.002090602	-1.121340385	0.262143001	23.43554924	18.08204512	88.93930423	0.666666667	CRA004775;PRJNA827465;Yang2025_Silkie_Wencha	0.745620137
ENSGALG00010013842	CDK19	3	0.194208886	0.173194489	1.121334101	0.262145675	0.05533375	5.766521242	65.31704443	1	CRA004775;PRJNA827465;Yang2025_Silkie_Wencha	0.745620137
ENSGALG00010023699	PPIG	3	0.383279718	0.341915409	1.121019152	0.26227971	0.28804977	13.05022602	84.67459493	0.666666667	CRA004775;PRJNA827465;Yang2025_Silkie_Wencha	0.745851332
ENSGALG00010000069	ENSGALG0001000006	3	0.499540856	0.445667561	1.120882244	0.26233799	0.50331948	18.10006129	88.95031366	0.666666667	CRA004775;PRJNA827465;Yang2025_Silkie_Wencha	0.74586705
ENSGALG00010017640	P2RY4	3	0.378589086	0.337883122	1.120473506	0.262512037	0	0.630969177	0	1	CRA004775;PRJNA827465;Yang2025_Silkie_Wencha	0.746014105
ENSGALG00010002118	ENSGALG0001000211	3	-0.263784707	0.235429985	-1.120438027	0.262527148	0	0.469444976	0	1	CRA004775;PRJNA827465;Yang2025_Silkie_Wencha	0.746014105
ENSGALG00010017131	TLR3	3	0.258766047	0.230980431	1.120294246	0.262588393	0	0.207746446	0	1	CRA004775;PRJNA827465;Yang2025_Silkie_Wencha	0.746014105
ENSGALG00010011603	ENSGALG0001001160	3	-0.256020221	0.228535367	-1.120265207	0.262600764	0	0.950532506	0	0.666666667	CRA004775;PRJNA827465;Yang2025_Silkie_Wencha	0.746014105
ENSGALG00010005830	USP12	3	-0.168125496	0.150156173	-1.119670889	0.262854035	0	0.198638753	0	1	CRA004775;PRJNA827465;Yang2025_Silkie_Wencha	0.746583607
ENSGALG00010005340	ENSGALG0001000534	2	-0.793263737	0.708739575	-1.119259832	0.263029307	0.508512024	2.024717841	50.61040211	1	CRA004775;PRJNA827465	0.746631474
ENSGALG00010024530	ENSGALG0001002453	2	0.647336369	0.578312326	1.119354266	0.262989034	0.092060325	1.12910038	11.43467274	0.5	CRA004775;Yang2025_Silkie_Wenchang	0.746631474
ENSGALG00010020365	UNC5B	3	-0.093939609	0.083924333	-1.119336968	0.26299641	0	1.887270519	0	0.666666667	CRA004775;PRJNA827465;Yang2025_Silkie_Wencha	0.746631474
ENSGALG00010000318	ENSGALG0001000031	3	-3.52821572	3.17704147	-1.110534991	0.266768559	26.06218769	17.02348543	88.25152459	0.666666667	CRA004775;PRJNA827465;Yang2025_Silkie_Wencha	0.747183902
ENSGALG00010026416	ENSGALG0001002641	2	1.280431963	1.152675053	1.110835148	0.266639314	1.972363801	3.722185726	73.13406494	1	CRA004775;PRJNA827465	0.747183902
ENSGALG00010008265	ENSGALG0001000826	2	1.155239821	1.03717982	1.113827899	0.265353026	1.738873496	5.162393588	80.62914067	1	CRA004775;PRJNA827465	0.747183902
ENSGALG00010029484	ENSGALG0001002948	2	0.860289273	0.770148104	1.117043941	0.263975538	0.994747171	6.09662085	83.59747105	1	PRJNA827465;Yang2025_Silkie_Wenchang	0.747183902
ENSGALG00010008403	GNAT3	2	-0.771343907	0.694292937	-1.110977608	0.266577988	0.760573583	4.707601539	78.75776037	1	PRJNA827465;Yang2025_Silkie_Wenchang	0.747183902
ENSGALG00010020328	SLC17A6	2	0.761309764	0.684016121	1.112999739	0.265708542	0.42015408	1.753433331	42.9690321	1	PRJNA827465;Yang2025_Silkie_Wenchang	0.747183902
ENSGALG00010000386	ENSGALG0001000038	3	-0.659687151	0.5933198	-1.112037649	0.266121963	0.984341897	36.97345371	94.59071361	1	CRA004775;PRJNA827465;Yang2025_Silkie_Wencha	0.747183902
ENSGALG00010021038	PTGS2	3	0.58295497	0.522651083	1.115380773	0.264687285	0.718186051	26.79084472	92.5347632	0.666666667	CRA004775;PRJNA827465;Yang2025_Silkie_Wencha	0.747183902
ENSGALG00010000602	ENSGALG0001000060	2	0.372654744	0.33452452	1.113983346	0.265286331	0	0.16674987	0	1	PRJNA827465;Yang2025_Silkie_Wenchang	0.747183902
ENSGALG00010017639	ENSGALG0001001763	3	0.368835488	0.331192398	1.113659283	0.265425383	0.289170354	22.69249683	91.18651414	0.666666667	CRA004775;PRJNA827465;Yang2025_Silkie_Wencha	0.747183902
ENSGALG00010002632	HPGD	3	0.330500388	0.296449361	1.114861941	0.264909588	0.173725742	6.63051006	69.83640803	0.666666667	CRA004775;PRJNA827465;Yang2025_Silkie_Wencha	0.747183902
ENSGALG00010002298	ENSGALG0001000229	2	0.329840161	0.295907625	1.114672733	0.26499069	0	0.529563826	0	1	PRJNA827465;Yang2025_Silkie_Wenchang	0.747183902
ENSGALG00010024776	MPHOSPH10	3	0.318354208	0.284979829	1.117111371	0.263946709	0.20400816	14.8304397	86.51422317	0.666666667	CRA004775;PRJNA827465;Yang2025_Silkie_Wencha	0.747183902
ENSGALG00010009103	AP3S1	3	-0.304278291	0.273543012	-1.11235995	0.265983417	0.185958607	13.01064065	84.6279668	0.666666667	CRA004775;PRJNA827465;Yang2025_Silkie_Wencha	0.747183902
ENSGALG00010000806	CFAP90	3	0.299782286	0.269433189	1.112640778	0.26586274	0.142903603	5.822485556	65.65040856	1	CRA004775;PRJNA827465;Yang2025_Silkie_Wencha	0.747183902
ENSGALG00010001506	DPYSL4	3	0.29790116	0.267552211	1.113431875	0.265522992	0.128756957	5.312121921	62.35026173	0.666666667	CRA004775;PRJNA827465;Yang2025_Silkie_Wencha	0.747183902
ENSGALG00010009678	JARID2	3	0.292483486	0.261937528	1.11661543	0.264158791	0.152319963	9.370418463	78.65623603	1	CRA004775;PRJNA827465;Yang2025_Silkie_Wencha	0.747183902
ENSGALG00010010216	ATXN7L1	3	0.282111965	0.252704726	1.116369964	0.264263805	0.153881838	11.247422	82.21814742	0.666666667	CRA004775;PRJNA827465;Yang2025_Silkie_Wencha	0.747183902
ENSGALG00010001238	GOLIM4	3	0.281508822	0.253177274	1.112069887	0.266108103	0.131962604	6.645204103	69.90310652	1	CRA004775;PRJNA827465;Yang2025_Silkie_Wencha	0.747183902
ENSGALG00010002381	TDRD3	3	0.269165858	0.241674232	1.113754892	0.265384353	0.137046643	10.28019258	80.54511155	0.666666667	CRA004775;PRJNA827465;Yang2025_Silkie_Wencha	0.747183902
ENSGALG00010021593	DCAF7	3	-0.264325854	0.236513586	-1.117592689	0.263740993	0.15001485	24.31348707	91.77411288	0.666666667	CRA004775;PRJNA827465;Yang2025_Silkie_Wencha	0.747183902
ENSGALG00010021269	NUDT13	3	0.257590024	0.23192992	1.110637319	0.266724493	0.12738918	13.00182202	84.61754056	0.666666667	CRA004775;PRJNA827465;Yang2025_Silkie_Wencha	0.747183902
ENSGALG00010008209	PPARGC1B	3	0.254429456	0.228545827	1.113253561	0.265599545	0.115991349	9.158771524	78.16301024	0.666666667	CRA004775;PRJNA827465;Yang2025_Silkie_Wencha	0.747183902
ENSGALG00010017644	RBBP6	3	0.248270549	0.222179918	1.117430194	0.263810431	0.126402511	15.21493832	86.85502394	0.666666667	CRA004775;PRJNA827465;Yang2025_Silkie_Wencha	0.747183902
ENSGALG00010018831	WBP1L	3	-0.246238313	0.22165755	-1.110895217	0.266613454	0.107374088	19.57841716	89.7846696	0.666666667	CRA004775;PRJNA827465;Yang2025_Silkie_Wencha	0.747183902
ENSGALG00010007777	KIF13A	3	0.243657179	0.218658156	1.114329251	0.265137963	0.117566061	13.34812718	85.01662464	0.666666667	CRA004775;PRJNA827465;Yang2025_Silkie_Wencha	0.747183902
ENSGALG00010016772	TSPAN6	3	-0.242325955	0.218005061	-1.111561145	0.266326887	0.121145679	18.64518111	89.27336781	0.666666667	CRA004775;PRJNA827465;Yang2025_Silkie_Wencha	0.747183902
ENSGALG00010020825	MAK	3	0.2398541	0.214886322	1.116190632	0.264340544	0	1.595468132	0	1	CRA004775;PRJNA827465;Yang2025_Silkie_Wencha	0.747183902
ENSGALG00010022698	SEMA6D	3	0.233378837	0.209667072	1.113092464	0.26566872	0.096914431	9.782645489	79.55563245	0.666666667	CRA004775;PRJNA827465;Yang2025_Silkie_Wencha	0.747183902
ENSGALG00010012969	SLC12A2	3	0.231728856	0.20765155	1.115911998	0.264459805	0.100079948	9.739151577	79.46433029	0.666666667	CRA004775;PRJNA827465;Yang2025_Silkie_Wencha	0.747183902
ENSGALG00010023696	LRRC4C	3	0.228545029	0.205789915	1.110574484	0.266751551	0.05470827	3.57897879	44.11813772	0.666666667	CRA004775;PRJNA827465;Yang2025_Silkie_Wencha	0.747183902
ENSGALG00010004408	SERAC1	3	-0.220138685	0.197920015	-1.112260853	0.26602601	0.087364257	8.046458168	75.14434353	0.666666667	CRA004775;PRJNA827465;Yang2025_Silkie_Wencha	0.747183902
ENSGALG00010021140	SLC11A1	3	-0.218335594	0.195600553	-1.116231989	0.264322845	0	1.890947167	0	1	CRA004775;PRJNA827465;Yang2025_Silkie_Wencha	0.747183902
ENSGALG00010002449	ALG11	3	-0.208751069	0.186810581	-1.117447779	0.263802916	0.073808105	7.748797803	74.18954461	1	CRA004775;PRJNA827465;Yang2025_Silkie_Wencha	0.747183902
ENSGALG00010023309	PIK3R3	3	-0.203214667	0.181722897	-1.11826672	0.263453096	0.067725609	9.221133027	78.31069138	0.666666667	CRA004775;PRJNA827465;Yang2025_Silkie_Wencha	0.747183902
ENSGALG00010010042	METTL25	3	-0.185478951	0.166845905	-1.111678174	0.266276547	0.057785406	7.577340697	73.60551571	0.666666667	CRA004775;PRJNA827465;Yang2025_Silkie_Wencha	0.747183902
ENSGALG00010024391	TBC1D2B	3	-0.177218495	0.159350805	-1.112128021	0.26608311	0.044250293	5.73824173	65.14611803	0.666666667	CRA004775;PRJNA827465;Yang2025_Silkie_Wencha	0.747183902
ENSGALG00010004245	ARFGEF3	3	-0.175461831	0.156991402	-1.117652487	0.263715443	0	0.030136124	0	1	CRA004775;PRJNA827465;Yang2025_Silkie_Wencha	0.747183902
ENSGALG00010021554	SNX11	3	-0.173696051	0.155542622	-1.116710315	0.264118206	0.040560503	4.381210746	54.35051825	0.666666667	CRA004775;PRJNA827465;Yang2025_Silkie_Wencha	0.747183902
ENSGALG00010023363	CPEB1	3	0.167556082	0.150066731	1.116543826	0.264189421	0.016293596	2.489196067	19.65277358	0.666666667	CRA004775;PRJNA827465;Yang2025_Silkie_Wencha	0.747183902
ENSGALG00010018287	MKKS	3	-0.148331111	0.133459241	-1.111433792	0.266381674	0.044857698	14.93591808	86.60946057	0.666666667	CRA004775;PRJNA827465;Yang2025_Silkie_Wencha	0.747183902
ENSGALG00010002239	AGPAT5	3	-0.143214593	0.128526087	-1.114284243	0.265157265	0.031659019	6.333860899	68.42368293	0.666666667	CRA004775;PRJNA827465;Yang2025_Silkie_Wencha	0.747183902
ENSGALG00010010302	WDR36	3	0.141343487	0.126655643	1.115966756	0.264436365	0.013672174	2.785263225	28.19350135	0.666666667	CRA004775;PRJNA827465;Yang2025_Silkie_Wencha	0.747183902
ENSGALG00010028140	HEMK1	3	-0.139899541	0.125340817	-1.116153103	0.264356605	0.009284127	2.455747535	18.55840343	1	CRA004775;PRJNA827465;Yang2025_Silkie_Wencha	0.747183902
ENSGALG00010017106	CRYM	3	-0.129321259	0.116431983	-1.110702195	0.266696557	0.012751977	2.557935761	21.8119536	1	CRA004775;PRJNA827465;Yang2025_Silkie_Wencha	0.747183902
ENSGALG00010018527	CCT4	3	-0.125878502	0.11317564	-1.112240248	0.266034867	0.022706896	4.778087675	58.14224987	1	CRA004775;PRJNA827465;Yang2025_Silkie_Wencha	0.747183902
ENSGALG00010016412	TMSB15B	3	-0.12074605	0.108577345	-1.112074073	0.266106303	0	0.747472437	0	0.666666667	CRA004775;PRJNA827465;Yang2025_Silkie_Wencha	0.747183902
ENSGALG00010017622	RAB1B	3	-0.120252233	0.108294219	-1.110421538	0.266817421	0.023554895	11.26836165	82.25119089	1	CRA004775;PRJNA827465;Yang2025_Silkie_Wencha	0.747183902
ENSGALG00010016893	TBC1D9B	3	-0.118238557	0.106381999	-1.111452675	0.26637355	0.024639794	8.826181452	77.34014408	0.666666667	CRA004775;PRJNA827465;Yang2025_Silkie_Wencha	0.747183902
ENSGALG00010001418	RNMT	3	-0.116010948	0.103846848	-1.117135	0.263936607	0.019940082	6.67112345	70.02004213	0.666666667	CRA004775;PRJNA827465;Yang2025_Silkie_Wencha	0.747183902
ENSGALG00010017797	PPP1R21	3	0.115749622	0.103631129	1.116938737	0.26402052	0.019463572	5.66798653	64.71410104	0.666666667	CRA004775;PRJNA827465;Yang2025_Silkie_Wencha	0.747183902
ENSGALG00010020794	STEAP3	3	-0.110083468	0.098699454	-1.115340185	0.264704671	0.007754245	2.566996831	22.08794432	1	CRA004775;PRJNA827465;Yang2025_Silkie_Wencha	0.747183902
ENSGALG00010005477	TMEM161B	3	0.108879356	0.097724518	1.114145749	0.265216665	0	1.88934465	0	1	CRA004775;PRJNA827465;Yang2025_Silkie_Wencha	0.747183902
ENSGALG00010004002	CDADC1	3	-0.10738356	0.096303435	-1.115054302	0.264827152	0.007684477	2.723018996	26.55210989	1	CRA004775;PRJNA827465;Yang2025_Silkie_Wencha	0.747183902
ENSGALG00010001306	CEP120	3	0.101238986	0.090776473	1.115255771	0.264740833	0	0.607718827	0	1	CRA004775;PRJNA827465;Yang2025_Silkie_Wencha	0.747183902
ENSGALG00010018883	TBL3	3	-0.101062649	0.090866596	-1.112209043	0.266048281	0.007366045	2.571217471	22.21583656	1	CRA004775;PRJNA827465;Yang2025_Silkie_Wencha	0.747183902
ENSGALG00010009126	JPH3	3	0.100335349	0.089923321	1.115787855	0.264512954	0	1.455832624	0	1	CRA004775;PRJNA827465;Yang2025_Silkie_Wencha	0.747183902
ENSGALG00010017534	ATP11C	3	-0.098524412	0.088698436	-1.110779589	0.266663234	0.009427414	3.352207257	40.33781784	1	CRA004775;PRJNA827465;Yang2025_Silkie_Wencha	0.747183902
ENSGALG00010017856	FASTKD5	3	-0.090041774	0.080709144	-1.115632879	0.264579312	0.004093213	2.446126221	18.23807034	0.666666667	CRA004775;PRJNA827465;Yang2025_Silkie_Wencha	0.747183902
ENSGALG00010025262	CARF	3	0.087827836	0.079092837	1.110439817	0.266809548	0	1.82759475	0	0.666666667	CRA004775;PRJNA827465;Yang2025_Silkie_Wencha	0.747183902
ENSGALG00010021203	FUT11	3	-0.086968862	0.07787009	-1.116845535	0.264060375	0.005751185	2.812087234	28.878451	0.666666667	CRA004775;PRJNA827465;Yang2025_Silkie_Wencha	0.747183902
ENSGALG00010023574	DEPDC1	3	-0.084842303	0.076094461	-1.114960296	0.264867436	0	1.178133497	0	0.666666667	CRA004775;PRJNA827465;Yang2025_Silkie_Wencha	0.747183902
ENSGALG00010007382	EIF2AK2	3	-0.081879869	0.073582674	-1.112760171	0.265811446	0	1.368925654	0	1	CRA004775;PRJNA827465;Yang2025_Silkie_Wencha	0.747183902
ENSGALG00010016097	FNIP1	3	-0.079857573	0.071466232	-1.117416863	0.263816128	0	0.111607634	0	1	CRA004775;PRJNA827465;Yang2025_Silkie_Wencha	0.747183902
ENSGALG00010008181	DISC1	3	0.072520564	0.065010945	1.115513163	0.264630581	0	1.284239281	0	0.666666667	CRA004775;PRJNA827465;Yang2025_Silkie_Wencha	0.747183902
ENSGALG00010021381	DNAJB2	3	0.06898395	0.061736644	1.11739067	0.263827323	0	0.261609622	0	1	CRA004775;PRJNA827465;Yang2025_Silkie_Wencha	0.747183902
ENSGALG00010004244	TMEM241	3	-0.0613338	0.055110218	-1.112929724	0.265738613	0	0.991064588	0	1	CRA004775;PRJNA827465;Yang2025_Silkie_Wencha	0.747183902
ENSGALG00010027925	MKRN2	3	0.056645292	0.050774793	1.115618374	0.264585524	0.002544013	2.78222473	28.11508076	1	CRA004775;PRJNA827465;Yang2025_Silkie_Wencha	0.747183902
ENSGALG00010010011	EPB41L4A	3	0.201321988	0.181371153	1.110000044	0.266999007	0.042422625	3.406574611	41.28999865	0.666666667	CRA004775;PRJNA827465;Yang2025_Silkie_Wencha	0.747309962
ENSGALG00010026924	TPH1	3	-0.174693658	0.157406297	-1.109826366	0.267073855	0	0.693401713	0	1	CRA004775;PRJNA827465;Yang2025_Silkie_Wencha	0.747309962
ENSGALG00010018025	CPTP	3	-0.166977794	0.150451861	-1.109842	0.267067117	0.050072065	8.565948501	76.65173915	0.666666667	CRA004775;PRJNA827465;Yang2025_Silkie_Wencha	0.747309962
ENSGALG00010029564	NLK	3	0.046829984	0.04219156	1.109937233	0.267026075	0	0.706724474	0	0.666666667	CRA004775;PRJNA827465;Yang2025_Silkie_Wencha	0.747309962
ENSGALG00010025548	ENSGALG0001002554	3	0.270893091	0.244181562	1.10939208	0.267261078	0.121624616	7.755802803	74.21285648	0.666666667	CRA004775;PRJNA827465;Yang2025_Silkie_Wencha	0.747522472
ENSGALG00010015080	ASF1A	3	-0.249634463	0.225109946	-1.108944615	0.267454076	0.122341169	13.80683767	85.51442373	1	CRA004775;PRJNA827465;Yang2025_Silkie_Wencha	0.747522472
ENSGALG00010021594	ADPGK	3	-0.213476977	0.192474639	-1.109117429	0.267379527	0.08883183	14.23124783	85.94641859	0.666666667	CRA004775;PRJNA827465;Yang2025_Silkie_Wencha	0.747522472
ENSGALG00010013744	ENSGALG0001001374	3	0.205807387	0.185532782	1.109277752	0.26731038	0	0.925727795	0	0.666666667	CRA004775;PRJNA827465;Yang2025_Silkie_Wencha	0.747522472
ENSGALG00010002079	ARX	3	-0.141349732	0.127416044	-1.109355837	0.267276706	0	1.58094016	0	0.666666667	CRA004775;PRJNA827465;Yang2025_Silkie_Wencha	0.747522472
ENSGALG00010028206	RBM15B	3	-0.068093777	0.06140579	-1.108914613	0.267467019	0.004265709	3.115195292	35.79856758	0.666666667	CRA004775;PRJNA827465;Yang2025_Silkie_Wencha	0.747522472
ENSGALG00010028453	ENSGALG0001002845	3	-0.104173174	0.0939675	-1.108608547	0.267599089	0.013919681	4.217979	52.58392704	0.666666667	CRA004775;PRJNA827465;Yang2025_Silkie_Wencha	0.747743779
ENSGALG00010002147	ENSGALG0001000214	2	0.694662992	0.627052803	1.107822163	0.267938627	0.46586711	2.410957626	58.52270529	0.5	PRJNA827465;Yang2025_Silkie_Wenchang	0.748544604
ENSGALG00010025688	BEST4	3	0.306803887	0.277105121	1.107175087	0.268218236	0.010095009	2.086491033	4.145286598	0.666666667	CRA004775;PRJNA827465;Yang2025_Silkie_Wencha	0.749177725
ENSGALG00010000154	ENSGALG0001000015	3	-3.308902512	2.989510166	-1.106837685	0.268364112	22.38973551	13.86316609	85.57328112	0.666666667	CRA004775;PRJNA827465;Yang2025_Silkie_Wencha	0.749192492
ENSGALG00010029526	BCL7B	3	-0.124305507	0.112286374	-1.107039989	0.268276639	0.01866722	3.757305867	46.77037029	1	CRA004775;PRJNA827465;Yang2025_Silkie_Wencha	0.749192492
ENSGALG00010003503	NUFIP1	3	-0.068472689	0.061865727	-1.106795195	0.268382486	0.004105112	3.063963365	34.72506809	0.666666667	CRA004775;PRJNA827465;Yang2025_Silkie_Wencha	0.749192492
ENSGALG00010012078	TMBIM4	3	-0.282846759	0.255644778	-1.106405386	0.268551095	0.153357152	10.63941473	81.2019735	0.666666667	CRA004775;PRJNA827465;Yang2025_Silkie_Wencha	0.749348574

ENSGALG00010015852	ZFAND5	3	-0.214823989	0.194178695	-1.106321106	0.268587559	0.058273976	4.382773978	54.36680034	0.666666667	CRA004775;PRJNA827465;Yang2025_Silkie_Wencha	0.749348574
ENSGALG00010016879	RNF130	3	-0.199877044	0.180671914	-1.106298374	0.268597395	0.073506446	9.70084502	77.95133755	0.666666667	CRA004775;PRJNA827465;Yang2025_Silkie_Wencha	0.749348574
ENSGALG00010015505	CHGA	3	0.930952393	0.842218094	1.105357864	0.269004554	1.836691684	15.12925477	86.78057822	0.666666667	CRA004775;PRJNA827465;Yang2025_Silkie_Wencha	0.749764932
ENSGALG00010012013	FOXD1	2	-0.39416696	0.356584146	-1.105396761	0.268987707	0	0.977402216	0	0.5	CRA004775;PRJNA827465	0.749764932
ENSGALG00010001557	THRB	2	0.299660285	0.27110207	1.105341191	0.269011776	0	0.82509774	0	1	PRJNA827465;Yang2025_Silkie_Wenchang	0.749764932
ENSGALG00010015100	MOB4	3	-0.100637912	0.091029403	-1.105553901	0.268919652	0.014976142	6.037903713	66.87592093	0.666666667	CRA004775;PRJNA827465;Yang2025_Silkie_Wencha	0.749764932
ENSGALG00010011912	TCP11X2	3	0.070800482	0.064039217	1.10558008	0.268908316	0	0.948842069	0	0.666666667	CRA004775;PRJNA827465;Yang2025_Silkie_Wencha	0.749764932
ENSGALG00010013420	ENSGALG0001001342(	3	-3.584453707	3.24487695	-1.104650118	0.269311225	26.95427872	15.50031675	87.09703788	0.666666667	CRA004775;PRJNA827465;Yang2025_Silkie_Wencha	0.749820979
ENSGALG00010024690	TUBA1C	3	0.53636848	0.485917241	1.103826815	0.26966827	0.522981883	8.544649441	76.59353946	0.666666667	CRA004775;PRJNA827465;Yang2025_Silkie_Wencha	0.749820979
ENSGALG00010020265	ENSGALG0001002026:	3	0.419936704	0.380343644	1.104098125	0.269550574	0.354789052	14.14505742	85.86078557	0.666666667	CRA004775;PRJNA827465;Yang2025_Silkie_Wencha	0.749820979
ENSGALG00010022385	ENSGALG0001002238:	3	-0.34412869	0.309128797	-1.104486846	0.269382007	0	0.196156037	0	1	CRA004775;PRJNA827465;Yang2025_Silkie_Wencha	0.749820979
ENSGALG00010026856	UBE2Z	3	-0.216331104	0.195929033	-1.104129904	0.269536791	0.099119768	16.37737156	87.78802818	0.666666667	CRA004775;PRJNA827465;Yang2025_Silkie_Wencha	0.749820979
ENSGALG00010027423	PEX11B	3	0.175972695	0.159350675	1.104310951	0.269458274	0.041238291	4.388941911	54.43093026	0.666666667	CRA004775;PRJNA827465;Yang2025_Silkie_Wencha	0.749820979
ENSGALG00010023115	RPLP2	3	-0.174717628	0.158239388	-1.104134881	0.269534632	0.040622832	4.063897275	50.78615761	1	CRA004775;PRJNA827465;Yang2025_Silkie_Wencha	0.749820979
ENSGALG00010001688	ENSGALG0001000168:	3	0.168711341	0.152817368	1.104006324	0.269590394	0.052208508	9.05885009	77.92214265	1	CRA004775;PRJNA827465;Yang2025_Silkie_Wencha	0.749820979
ENSGALG00010017252	EXOC5	3	-0.147046807	0.133068253	-1.105047996	0.269138793	0.040646306	11.67625893	82.87122603	0.666666667	CRA004775;PRJNA827465;Yang2025_Silkie_Wencha	0.749820979
ENSGALG00010022820	SHTN1	3	0.138863734	0.125759527	1.104200509	0.269506169	0	0.147836525	0	1	CRA004775;PRJNA827465;Yang2025_Silkie_Wencha	0.749820979
ENSGALG00010027739	FGD3	3	0.050132185	0.04541231	1.103933828	0.269621843	0	1.397488334	0	0.666666667	CRA004775;PRJNA827465;Yang2025_Silkie_Wencha	0.749820979
ENSGALG00010020935	BRI3BP	3	0.044196883	0.040019667	1.104379092	0.269428727	0	0.401377947	0	0.666666667	CRA004775;PRJNA827465;Yang2025_Silkie_Wencha	0.749820979
ENSGALG00010024524	TMEM41A	3	0.097668232	0.088506562	1.10351401	0.269804011	0	0.467490877	0	0.666666667	CRA004775;PRJNA827465;Yang2025_Silkie_Wencha	0.750050906
ENSGALG00010027475	ARID3A	3	0.271316702	0.245900552	1.103359467	0.269871092	0.138367111	13.75863387	85.46367308	0.666666667	CRA004775;PRJNA827465;Yang2025_Silkie_Wencha	0.750089908
ENSGALG00010023891	ENSGALG0001002389	3	-0.353877406	0.320940248	-1.102627071	0.270189149	0.174586619	4.602771436	56.5479184	0.666666667	CRA004775;PRJNA827465;Yang2025_Silkie_Wencha	0.750678793
ENSGALG00010003860	TMEM70	3	-0.186318768	0.168973748	-1.102649244	0.270179516	0.067681522	13.70205751	85.40365198	0.666666667	CRA004775;PRJNA827465;Yang2025_Silkie_Wencha	0.750678793
ENSGALG00010021623	FAR1	3	0.122831682	0.11141867	1.102433568	0.270273225	0.022080335	5.671316325	64.73481842	0.666666667	CRA004775;PRJNA827465;Yang2025_Silkie_Wencha	0.750764857
ENSGALG00010006948	ENSGALG0001000694:	2	0.711989106	0.646444482	1.101392504	0.270725867	0.749329717	9.666399327	89.65488631	1	PRJNA827465;Yang2025_Silkie_Wenchang	0.750909341
ENSGALG00010004128	MCMDC2	2	0.708999855	0.643462733	1.101850689	0.270526589	0	0.188368424	0	1	PRJNA827465;Yang2025_Silkie_Wenchang	0.750909341
ENSGALG00010009772	ENSGALG0001000977:	2	-0.667015487	0.606196726	-1.100328421	0.271189053	0	0.981803671	0	0.5	CRA004775;PRJNA827465	0.750909341
ENSGALG00010008268	ENSGALG0001000826:	3	-0.526129029	0.478208287	-1.100208934	0.271241099	0.59186727	15.84517083	87.37785776	0.666666667	CRA004775;PRJNA827465;Yang2025_Silkie_Wencha	0.750909341
ENSGALG00010014166	FGF12	3	0.390321764	0.35480027	1.100116876	0.271281202	0.289385302	10.32785946	80.63490303	0.666666667	CRA004775;PRJNA827465;Yang2025_Silkie_Wencha	0.750909341
ENSGALG00010029928	USP43	3	0.262718347	0.238777764	1.100263034	0.271217533	0.123081598	7.714843656	74.07594905	0.666666667	CRA004775;PRJNA827465;Yang2025_Silkie_Wencha	0.750909341
ENSGALG00010022247	GALNT9	3	0.255245748	0.231796953	1.10116092	0.270826627	0.063346903	3.197810884	37.45721456	0.666666667	CRA004775;PRJNA827465;Yang2025_Silkie_Wencha	0.750909341
ENSGALG00010008588	PYCR3	3	-0.236055439	0.214554554	-1.100211739	0.271239877	0.093928892	6.486893855	69.16860296	0.666666667	CRA004775;PRJNA827465;Yang2025_Silkie_Wencha	0.750909341
ENSGALG00010020845	RAB35	3	-0.235323964	0.213782273	-1.100764624	0.270999112	0.127381082	34.52745862	94.20705881	0.666666667	CRA004775;PRJNA827465;Yang2025_Silkie_Wencha	0.750909341
ENSGALG00010007753	PRSS23	3	-0.189492304	0.172073202	-1.101230765	0.270796235	0.050439187	5.523879842	63.79356436	0.666666667	CRA004775;PRJNA827465;Yang2025_Silkie_Wencha	0.750909341
ENSGALG00010013238	RAD1	3	0.185547357	0.168045237	1.101770932	0.27056127	0.042022954	3.964384903	49.55081182	0.666666667	CRA004775;PRJNA827465;Yang2025_Silkie_Wencha	0.750909341
ENSGALG00010013353	NDFIP1	3	-0.182799925	0.166144145	-1.100248968	0.27122366	0.0589101	7.507720316	73.36075512	1	CRA004775;PRJNA827465;Yang2025_Silkie_Wencha	0.750909341
ENSGALG00010004855	ZEB1	3	0.174266294	0.158364619	1.100411792	0.271152743	0.04343117	4.619673325	56.7068955	0.666666667	CRA004775;PRJNA827465;Yang2025_Silkie_Wencha	0.750909341
ENSGALG00010024802	CD59	3	-0.144192003	0.130948714	-1.101133404	0.270838601	0.026685517	4.440887018	54.96395221	0.666666667	CRA004775;PRJNA827465;Yang2025_Silkie_Wencha	0.750909341
ENSGALG00010005241	C4orf54	3	-0.10658447	0.096858029	-1.100419558	0.27114936	0	1.308079403	0	0.666666667	CRA004775;PRJNA827465;Yang2025_Silkie_Wencha	0.750909341
ENSGALG00010023830	AGFG1	3	0.086533733	0.078609482	1.100805281	0.270981413	0.0097578	3.975492315	49.69176541	1	CRA004775;PRJNA827465;Yang2025_Silkie_Wencha	0.750909341
ENSGALG00010022833	JAK2	3	-0.084236859	0.076510563	-1.100983382	0.27090389	0	1.188312177	0	0.666666667	CRA004775;PRJNA827465;Yang2025_Silkie_Wencha	0.750909341
ENSGALG00010021606	RNF8	3	-0.062610936	0.056817445	-1.101966754	0.270476125	0	1.711649001	0	0.666666667	CRA004775;PRJNA827465;Yang2025_Silkie_Wencha	0.750909341
ENSGALG00010013209	KIAA1549	3	-0.080908496	0.073629028	-1.099905326	0.271373374	0	1.808383682	0	0.666666667	CRA004775;PRJNA827465;Yang2025_Silkie_Wencha	0.751017446
ENSGALG00010017662	GOLGA4	3	0.468408708	0.426031459	1.099469764	0.271563215	0.493796571	24.93340234	91.97863183	0.666666667	CRA004775;PRJNA827465;Yang2025_Silkie_Wencha	0.751201822
ENSGALG00010009469	DNMT3A	3	0.190927513	0.173656356	1.09945594	0.271569242	0.06814123	10.24290542	80.47429007	0.666666667	CRA004775;PRJNA827465;Yang2025_Silkie_Wencha	0.751201822
ENSGALG00010001546	CGAS	3	-0.077121133	0.070149226	-1.0993868	0.271599386	0	1.399978689	0	0.666666667	CRA004775;PRJNA827465;Yang2025_Silkie_Wencha	0.751201822
ENSGALG00010017791	CCNL2	3	0.225758808	0.205411711	1.099055196	0.271743991	0.10928511	13.97852163	89.35468217	0.666666667	CRA004775;PRJNA827465;Yang2025_Silkie_Wencha	0.751442964
ENSGALG00010027618	PPP1R26	3	-0.074324749	0.067632934	-1.098943147	0.271792865	0.003277041	2.507838562	20.25004999	0.666666667	CRA004775;PRJNA827465;Yang2025_Silkie_Wencha	0.751442964
ENSGALG00010009780	MFSD2B	3	-0.186322613	0.169575373	-1.098759862	0.271872824	0	1.265792368	0	1	CRA004775;PRJNA827465;Yang2025_Silkie_Wencha	0.751517079
ENSGALG00010022396	PPP1R1C	3	0.329678496	0.300120349	1.098487648	0.271991608	0.133408618	4.012418317	50.15474854	1	CRA004775;PRJNA827465;Yang2025_Silkie_Wencha	0.751698465
ENSGALG00010006228	ENSGALG0001000622:	2	0.637189713	0.580925137	1.097197713	0.272555228	0.034004492	1.052722345	5.008190908	1	CRA004775;Yang2025_Silkie_Wenchang	0.753094206
ENSGALG00010029404	SRR	3	-0.105530052	0.096191103	-1.097087452	0.272603165	0	1.613219553	0	0.666666667	CRA004775;PRJNA827465;Yang2025_Silkie_Wencha	0.753094206
ENSGALG00010021409	MEGF6	3	0.354709006	0.323535271	1.096353434	0.272924134	0.230509369	7.91876459	74.74353509	0.666666667	CRA004775;PRJNA827465;Yang2025_Silkie_Wencha	0.753098034
ENSGALG00010007500	MPHOSPH6	3	0.16011872	0.146040903	1.096396399	0.272905339	0.036416912	4.650237282	56.99144197	0.666666667	CRA004775;PRJNA827465;Yang2025_Silkie_Wencha	0.753098034
ENSGALG00010023862	TRMT13	3	-0.140408362	0.128035456	-1.096636559	0.272800299	0.018562859	3.194285725	37.38819341	0.666666667	CRA004775;PRJNA827465;Yang2025_Silkie_Wencha	0.753098034
ENSGALG00010005989	FCF1	3	-0.089921629	0.081986021	-1.096792205	0.272732238	0.004996489	2.442274694	18.10912977	1	CRA004775;PRJNA827465;Yang2025_Silkie_Wencha	0.753098034
ENSGALG00010025406	ENSGALG0001002540(	3	-0.062031432	0.056576195	-1.09642284	0.272893773	0	0.993372158	0	0.666666667	CRA004775;PRJNA827465;Yang2025_Silkie_Wencha	0.753098034
ENSGALG00010012378	RPS19BP1	3	-0.06155384	0.056137922	-1.0964752	0.272870871	0	1.410947338	0	1	CRA004775;PRJNA827465;Yang2025_Silkie_Wencha	0.753098034
ENSGALG00010001823	RALBP1	3	0.103278026	0.094236608	1.095944961	0.273102862	0.012718405	3.554731134	43.73695436	0.666666667	CRA004775;PRJNA827465;Yang2025_Silkie_Wencha	0.753444168
ENSGALG00010005446	ENSGALG0001000544(	3	-0.2848341	0.259940336	-1.09576722	0.273180657	0	0.411544863	0	1	CRA004775;PRJNA827465;Yang2025_Silkie_Wencha	0.753511766
ENSGALG00010010952	ENSGALG0001001095:	3	0.291634187	0.266251382	1.095333981	0.273370345	0	1.359711598	0	0.666666667	CRA004775;PRJNA827465;Yang2025_Silkie_Wencha	0.75388791
ENSGALG00010012927	ENSGALG0001001292:	3	-2.909406861	2.657802831	-1.094666175	0.273662912	18.10639535	16.1820518	87.64062787	0.666666667	CRA004775;PRJNA827465;Yang2025_Silkie_Wencha	0.753926646
ENSGALG00010007401	SEC61G	3	-0.547449126	0.500149177	-1.094571684	0.273704327	0.693692239	31.09927778	93.56098249	0.666666667	CRA004775;PRJNA827465;Yang2025_Silkie_Wencha	0.753926646
ENSGALG00010009515	MBD5	3	0.295547467	0.269874507	1.095129254	0.273460014	0.189183886	17.75912652	88.73818486	0.666666667	CRA004775;PRJNA827465;Yang2025_Silkie_Wencha	0.753926646
ENSGALG00010018172	ACTL6A	3	-0.223112927	0.203832479	-1.094589679	0.273696439	0.098983148	15.36076199	86.97981259	0.666666667	CRA004775;PRJNA827465;Yang2025_Silkie_Wencha	0.753926646
ENSGALG00010018285	BBIP1	3	0.157277152	0.143633747	1.094987458	0.273522131	0.035793843	4.847639061	58.74280294	0.666666667	CRA004775;PRJNA827465;Yang2025_Silkie_Wencha	0.753926646
ENSGALG00010028829	GTF3C5	3	-0.060937845	0.05565559	-1.094909702	0.273556198	0	0.043014921	0	1	CRA004775;PRJNA827465;Yang2025_Silkie_Wencha	0.753926646
ENSGALG00010010838	JAM2	3	-0.17049934	0.155806906	-1.09429899	0.273823868	0.042598791	5.393112531	62.91566348	0.666666667	CRA004775;PRJNA827465;Yang2025_Silkie_Wencha	0.754109012
ENSGALG00010013347	ENSGALG0001001334:	3	-0.431257007	0.394318061	-1.093678047	0.274096206	0	1.393078142	0	0.666666667	CRA004775;PRJNA827465;Yang2025_Silkie_Wencha	0.754149077
ENSGALG00010016607	FAM103A1	3	-0.338816425	0.309739966	-1.093873772	0.274010343	0.2550381	25.01325607	92.00423969	0.666666667	CRA004775;PRJNA827465;Yang2025_Silkie_Wencha	0.754149077
ENSGALG00010017006	DNAH3	3	0.333663788	0.30505195	1.093793329	0.27404563	0.213090583	9.921941362	79.84265451	1	CRA004775;PRJNA827465;Yang2025_Silkie_Wencha	0.754149077
ENSGALG00010012934	NDUFA1	3	-0.216716829	0.19810452	-1.093951966	0.273976045	0.078693761	6.222371898	67.85791604	0.666666667	CRA004775;PRJNA827465;Yang2025_Silkie_Wencha	0.754149077
ENSGALG00010010947	LUC7L2	3	-0.036275069	0.033168575	-1.093657759	0.274105107	0	0.153463885	0	0.666666667	CRA004775;PRJNA827465;Yang2025_Silkie_Wencha	0.754149077
ENSGALG00010005398	SEPHS1	3	-0.184596405	0.168818385	-1.093461506	0.274191221	0.063265757	8.642496596	76.85853876	0.666666667	CRA004775;PRJNA827465;Yang2025_Silkie_Wencha	0.754239237
ENSGALG00010005060	POMP	3	-0.191743874	0.175404156	-1.093154679	0.274325892	0.065727376	7.694010525	74.0057543	0.666666667	CRA004775;PRJNA827465;Yang2025_Silkie_Wencha	0.754462903
ENSGALG00010001497	LEKR1	2	0.518696076	0.474579927	1.092958311	0.274412104	0.371234242	5.651677715	82.30613898	1	PRJNA827465;Yang2025_Silkie_Wenchang	0.754516929
ENSGALG00010027863	CHDH	3	-0.118013824	0.107985546	-1.092866851	0.274452264	0.015565877	3.730994947	46.39499575	0.666666667	CRA004775;PRJNA827465;Yang2025_Silkie_Wencha	0.754516929
ENSGALG00010013278	SAMM50	3	-0.175514631	0.160643837	-1.092569966	0.274582655	0.050907911	6.088675862	67.15213545	0.666666667	CRA004775;PRJNA827465;Yang2025_Silkie_Wencha	0.754581956
ENSGALG00010005385	PDSS1	3	-0.070793073	0.064793879	-1.092588901	0.274574338	0	0.75029117	0	1	CRA004775;PRJNA827465;Yang2025_Silkie_Wencha	0.754581956
ENSGALG00010029151	CYBC1	3	-0.175987577	0.161112475	-1.092327442	0.274689203	0.052580826	7.586832025	73.63853591	1	CRA004775;PRJNA827465;Yang2025_Silkie_Wencha	0.754728068
ENSGALG00010017112	LZIC	3	-0.141818527	0.129857902	-1.09210549	0.274786737	0.040816882	13.97309483	85.6867786	1	CRA004775;PRJNA827465;Yang2025_Silkie_Wencha	0.754849363
ENSGALG00010018616	ENSGALG0001001861(	3	1.059394819	0.970227294	1.091903748	0.27487541	2.420038347	25.17578357	92.05585799	0.666666667	CRA004775;PRJNA827465;Yang2025_Silkie_Wencha	0.754946275
ENSGALG00010001463	DSEL	3	-0.12373761	0.113348077	-1.091660429	0.274982384	0.023252693	6.880307261	70.93153076	0.666666667	CRA004775;PRJNA827465;Yang2025_Silkie_Wencha	0.755093403
ENSGALG00010011347	RGS22	2	0.584844029	0.535867912	1.091395874	0.275098727	0.390335209	3.064730274	67.37070115	1	PRJNA827465;Yang2025_Silkie_Wenchang	0.755119569
ENSGALG00010029633	POLG2	3	0.406949173	0.372858749	1.091429862	0.275083779	0.382217932	26.64685628	92.49442419	0.666666667	CRA004775;PRJNA827465;Yang2025_Silkie_Wencha	0.755119569
ENSGALG00010028608	MIGA2	3	-0.256807832	0.235340387	-1.091218703	0.27517666	0.132552148	10.78410779	81.45419131	0.666666667	CRA004775;PRJNA827465;Yang2025_Silkie_Wencha	0.755186878
ENSGALG00010000309	ENSGALG0001000030(	3	-3.631952205	3.341986055	-1.08676462	0.277140847	29.10530714	17.82026292	88.77682103	0.666666667	CRA004775;PRJNA827465;Yang2025_Silkie_Wencha	0.755279869
ENSGALG00010000282	ENSGALG0001000028:	3	-3.602389747	3.327591502	-1.082581725	0.278994123	28.57746099	16.10327647	87.58016728	0.666666667	CRA004775;PRJNA827465;Yang2025_Silkie_Wencha	0.755279869
ENSGALG00010015691	EXOC3L4	2	1.28367493	1.180140424	1.087730667	0.276714025	2.106744866	3.979399539	74.87058059	0.5	CRA004775;PRJNA827465	0.755279869
ENSGALG00010002960	BLEC2	3	-1.269027228	1.164867985	-1.089417209	0.275969946	3.250851844	10.18420439	80.36174527	0.666666667	CRA004775;PRJNA827465;Yang2025_Silkie_Wencha	0.755279869
ENSGALG00010024779	ENSGALG0001002477(	2	1.221632578	1.131372141	1.079779619	0.280240329	1.783576737	3.297139558	69.67068023	1	PRJNA827465;Yang2025_Silkie_Wenchang	0.755279869
ENSGALG00010029502	BTBD17	2	0.893499245	0.825406948	1.082495424	0.279032447	1.225647793	9.92865525	89.92814259	1	PRJNA827465;Yang2025_Silkie_Wenchang	0.755279869
ENSGALG00010011403	SV2C	2	0.807023163	0.744211124	1.084400833	0.278187115	0.784302389	3.216772433	68.91293926	1	PRJNA827465;Yang2025_Silkie_Wenchang	0.755279869
ENSGALG00010011307	ENSGALG0001001130:	3	-0.593362454	0.548321944	-1.082142453	0.279189234	0.578343838	6.113741326	67.28680699	0.666666667	CRA004775;PRJNA827465;Yang2025_Silkie_Wencha	0.755279869
ENSGALG00010002747	FUT9	2	0.563457567	0.520902767	1.081694326	0.279388375	0.375436339	3.174018626	68.49419874	1	PRJNA827465;Yang2025_Silkie_Wenchang	0.755279869
ENSGALG00010016691	ENSGALG0001001669	3	-0.483557716	0.444166026	-1.088686858	0.276291999	0.435700165	8.047024557	75.14609299	1	CRA004775;PRJNA827465;Yang2025_Silkie_Wencha	0.755279869
ENSGALG00010005943	PROX2	2	0.443946238	0.407879326	1.088425447	0.276407332	0	0.7792042	0	1	PRJNA827465;Yang2025_Silkie_Wenchang	0.755279869
ENSGALG00010012148	SETBP1	3	0.43136282	0.397272594	1.085810666	0.277562766	0.391904303	13.91552051	85.62755882	0.666666667	CRA004775;PRJNA827465;Yang2025_Silkie_Wencha	0.755279869
ENSGALG00010017278	CCR5	3	-0.427711584	0.395058865	-1.082652795	0.278962564	0.371011666	17.89492987	88.82364997	0.666666667	CRA004775;PRJNA827465;Yang2025_Silkie_Wencha	0.755279869
ENSGALG00010012360	ROS1	3	0.417506728	0.382780143	1.090722013	0.275395221	0.277993325	6.639822154	69.8787113	1	CRA004775;PRJNA827465;Yang2025_Silkie_Wencha	0.755279869
ENSGALG00010001890	KLHL34	3	0.392909341	0.363164357	1.081905021	0.279294733	0.292612637	12.30259673	83.74326945	0.666666667	CRA004775;PRJNA827465;Yang2025_Silkie_Wencha	0.755279869
ENSGALG00010006448	MAS1	2	-0.372767293	0.34354336	-1.085066212	0.277892331	0	0.462356099	0	1	PRJNA827465;Yang2025_Silkie_Wenchang	0.755279869
ENSGALG00010013361	ENSGALG0001001336	3	-0.345324468	0.316940988	-1.089554463	0.275909452	0.190852283	5.538859087	63.89148075	0.666666667	CRA004775;PRJNA827465;Yang2025_Silkie_Wencha	0.755279869
ENSGALG00010000710	ENSGALG0001000071(	3	-0.329861581	0.302475679	-1.090539189	0.2754757	0.120314535	3.53780002	43.4676921	0.666666667	CRA004775;PRJNA827465;Yang2025_Silkie_Wencha	0.755279869
ENSGALG00010006252	COX7C	3	-0.328994775	0.30398784	-1.082262944	0.279135707	0.186843318	6.282512788	68.165604	1	CRA004775;PRJNA827465;Yang2025_Silkie_Wencha	0.755279869
ENSGALG00010027165	ENSGALG0001002716:	3	0.325035124	0.300795943	1.080583473	0.279882437	0.235520296	19.76562837	89.88142465	0.666666667	CRA004775;PRJNA827465;Yang2025_Silkie_Wencha	0.755279869
ENSGALG00010011696	ZFC3H1	3	0.318552028	0.294288754	1.082447165	0.27905388	0.216911294	16.4551927	87.84578195	1	CRA004775;PRJNA827465;Yang2025_Silkie_Wencha	0.755279869
ENSGALG00010026897	ENSGALG0001002689:	3	0.298922674	0.276297324	1.081887694	0.279302433	0.147083682	5.841615206	65.76289383	0.666666667	CRA004775;PRJNA827465;Yang2025_Silkie_Wencha	0.755279869
ENSGALG00010005318	SLC35D3	2	-0.287595151	0.264405195	-1.087706127	0.276724862	0	0.481367322	0	1	PRJNA827465;Yang2025_Silkie_Wenchang	0.755279869
ENSGALG00010007819	NRIP1	3	0.282827571	0.261407367	1.081941853	0.279278366	0.158237782	13.8768949	85.58755389	0.666666667	CRA004775;PRJNA827465;Yang2025_Silkie_Wencha	0.755279869
ENSGALG00010029290	ENSGALG0001002929(	3	0.273235213	0.250607505	1.090291422	0.275584793	0.124315329	8.597066645	76.73625106	0.666666667	CRA004775;PRJNA827465;Yang2025_Silkie_Wencha	0.755279869
ENSGALG00010012761	SMPDL3A	3	-0.272049954	0.250821934	-1.084633827	0.278083867	0.143229874	9.066009193	77.93957675	0.666666667	CRA004775;PRJNA827465;Yang2025_Silkie_Wencha	0.755279869
ENSGALG00010002682	UBL5	3	0.271199959	0.249852144	1.085441793	0.277726031	0.144987372	12.20517648	83.61351019	0.666666667	CRA004775;PRJNA827465;Yang2025_Silkie_Wencha	0.755279869
ENSGALG00010027012	RILPL1	3	0.270913103	0.248967381	1.088146977	0.276530228	0.148103623	11.68057752	82.87755896	0.666666667	CRA004775;PRJNA827465;Yang2025_Silkie_Wencha	0.755279869
ENSGALG00010029481	ENSGALG0001002948	3	0.269487677	0.247653113	1.088165917	0.276521868	0	0.884157953	0	0.666666667	CRA004775;PRJNA827465;Yang2025_Silkie_Wencha	0.755279869
ENSGALG00010023057	PAFAH1B2	3	-0.26270449	0.24143863	-1.088079774	0.276559892	0.159587222	30.60961137	93.46610457	0.666666667	CRA004775;PRJNA827465;Yang2025_Silkie_Wencha	0.755279869
ENSGALG00010005388	ENSGALG0001000538:	3	-0.255391693	0.235791597	-1.083124659	0.278753095	0	0.768055324	0	1	CRA004775;PRJNA827465;Yang2025_Silkie_Wencha	0.755279869
ENSGALG00010024544	ZNF536	3	0.248263437	0.228509377	1.086447481	0.277281064	0.079377372	4.339037854	53.90683218	1	CRA004775;PRJNA827465;Yang2025_Silkie_Wencha	0.755279869
ENSGALG00010007903	PSMA2	3	-0.235817269	0.217454028	-1.084446545	0.278166856	0.117500661	15.08998357	86.7461751	1	CRA004775;PRJNA827465;Yang2025_Silkie_Wencha	0.755279869
ENSGALG00010006310	ALG5	3	0.235051358	0.216706128	1.084654875	0.278074541	0.109742832	14.44280811	86.15227742	0.666666667	CRA004775;PRJNA827465;Yang2025_Silkie_Wencha	0.755279869
ENSGALG00010012667	SCAMP1	3	-0.231497771	0.213898934	-1.082276414	0.279129723	0.119188652	31.6301522	93.67691945	1	CRA004775;PRJNA827465;Yang2025_Silkie_Wencha	0.755279869
ENSGALG00010018209	GMPPA	3	-0.231406052	0.214035748	-1.081156089	0.279627687	0.118107639	18.45382758	89.16213999	0.666666667	CRA004775;PRJNA827465;Yang2025_Silkie_Wencha	0.755279869
ENSGALG00010005366	AUP1	3	-0.225261067	0.207335475	-1.086456945	0.27727688	0.088167933	6.467574807	69.07650766	0.666666667	CRA004775;PRJNA827465;Yang2025_Silkie_Wencha	0.755279869
ENSGALG00010021222	ENSGALG0001002122:	3	-0.224335392	0.206783408	-1.084881009	0.27797436	0.016455387	2.280991064	12.31881476	0.666666667	CRA004775;PRJNA827465;Yang2025_Silkie_Wencha	0.755279869
ENSGALG00010027242	ENSGALG0001002724:	3	0.217650437	0.201609136	1.079566339	0.280335338	0.091386643	9.740056435	79.46623807	0.666666667	CRA004775;PRJNA827465;Yang2025_Silkie_Wencha	0.755279869
ENSGALG00010013846	YIPF7	3	-0.20905072	0.193642188	-1.079572189	0.280332731	0	0.740041561	0	0.666666667	CRA004775;PRJNA827465;Yang2025_Silkie_Wencha	0.755279869
ENSGALG00010021477	ZFAND2B	3	0.207057935	0.191819964	1.079438919	0.280392109	0.069174638	5.510140681	63.70328607	0.666666667	CRA004775;PRJNA827465;Yang2025_Silkie_Wencha	0.755279869
ENSGALG00010019064	ENSGALG0001001906	3	-0.204995127	0.188935785	-1.084998941	0.277922124	0.07919689	11.73946145	82.96344335	0.666666667	CRA004775;PRJNA827465;Yang2025_Silkie_Wencha	0.755279869
ENSGALG00010010836	DIP2A	3	0.201907071	0.186066716	1.085132663	0.277862903	0.080806407	13.65179088	85.34990744	1	CRA004775;PRJNA827465;Yang2025_Silkie_Wencha	0.755279869
ENSGALG00010029705	GRB2	3	-0.197706745	0.182815756	-1.081453534	0.279495419	0.086704769	38.69200108	94.83097295	0.666666667	CRA004775;PRJNA827465;Yang2025_Silkie_Wencha	0.755279869
ENSGALG00010025577	PTH2R	3	0.189214148	0.175012623	1.081145718	0.2796323	0	0.420494291	0	1	CRA004775;PRJNA827465;Yang2025_Silkie_Wencha	0.755279869
ENSGALG00010008618	EIF4EBP3	3	0.188639097	0.173216665	1.089035495	0.276138233	0.067037981	8.924316024	77.58931895	0.666666667	CRA004775;PRJNA827465;Yang2025_Silkie_Wencha	0.755279869
ENSGALG00010023455	CFDP1	3	0.180952368	0.166741953	1.085223989	0.277822462	0.067117492	12.24744371	83.67006172	0.666666667	CRA004775;PRJNA827465;Yang2025_Silkie_Wencha	0.755279869
ENSGALG00010024428	SAAL1	3	-0.16830908	0.155560342	-1.081953653	0.279273123	0.057988836	15.28405801	86.91446998	0.666666667	CRA004775;PRJNA827465;Yang2025_Silkie_Wencha	0.755279869
ENSGALG00010003835	ENSGALG0001000383:	3	0.16615991	0.153131554	1.085079501	0.277886445	0.02937975	3.051836486	34.46568946	0.666666667	CRA004775;PRJNA827465;Yang2025_Silkie_Wencha	0.755279869
ENSGALG00010013409	DAB2	3	0.164697502	0.151102726	1.089970424	0.275726172	0.035242383	4.08485638	51.0386703	0.666666667	CRA004775;PRJNA827465;Yang2025_Silkie_Wencha	0.755279869
ENSGALG00010007224	DHFR	3	0.16232204	0.150352911	1.079606899	0.280317268	0	1.331462041	0	0.666666667	CRA004775;PRJNA827465;Yang2025_Silkie_Wencha	0.755279869
ENSGALG00010007671	FAM206A	3	-0.156010813	0.144504503	-1.079625957	0.280308778	0.030946581	3.826197852	47.72878776	0.666666667	CRA004775;PRJNA827465;Yang2025_Silkie_Wencha	0.755279869
ENSGALG00010007028	KATNAL1	3	-0.153881054	0.142538017	-1.079579031	0.280329683	0.034260405	4.565055753	56.188925	0.666666667	CRA004775;PRJNA827465;Yang2025_Silkie_Wencha	0.755279869
ENSGALG00010017045	GLB1	3	0.153206897	0.141673258	1.081410134	0.279514716	0.045834141	9.918589688	79.83584297	0.666666667	CRA004775;PRJNA827465;Yang2025_Silkie_Wencha	0.755279869
ENSGALG00010001200	CTTNBP2	3	0.149973228	0.138277378	1.084582526	0.278106598	0.031145778	4.55360618	56.07876656	0.666666667	CRA004775;PRJNA827465;Yang2025_Silkie_Wencha	0.755279869
ENSGALG00010010405	EVA1C	3	-0.149203787	0.13773581	-1.083260679	0.278692733	0	0.957569405	0	0.666666667	CRA004775;PRJNA827465;Yang2025_Silkie_Wencha	0.755279869
ENSGALG00010024755	PIGX	3	-0.147825911	0.136886783	-1.079913696	0.280180614	0.019091704	2.830923975	29.35168807	1	CRA004775;PRJNA827465;Yang2025_Silkie_Wencha	0.755279869
ENSGALG00010019807	ZDHHC6	3	-0.147552065	0.135964927	-1.085221525	0.277823553	0.032472698	5.365834117	62.72713699	1	CRA004775;PRJNA827465;Yang2025_Silkie_Wencha	0.755279869
ENSGALG00010028375	LMNB2	3	-0.146480834	0.134956273	-1.085394783	0.277746842	0.029035334	4.840250686	58.67982611	1	CRA004775;PRJNA827465;Yang2025_Silkie_Wencha	0.755279869
ENSGALG00010028283	ENSGALG0001002828:	3	-0.143354378	0.131602751	-1.089296213	0.276023282	0.03058967	6.394097725	68.72115369	0.666666667	CRA004775;PRJNA827465;Yang2025_Silkie_Wencha	0.755279869
ENSGALG00010010920	UBN2	3	0.141165353	0.130036494	1.085582579	0.277663711	0.0266852	4.072086108	50.88512504	1	CRA004775;PRJNA827465;Yang2025_Silkie_Wencha	0.755279869
ENSGALG00010022619	MADD	3	0.134825569	0.12459181	1.082138293	0.279191083	0.024892936	4.571665919	56.25227137	0.666666667	CRA004775;PRJNA827465;Yang2025_Silkie_Wencha	0.755279869
ENSGALG00010014482	THOC3	3	-0.129258239	0.119051327	-1.085735392	0.277596078	0.022245484	4.043702882	50.54038196	1	CRA004775;PRJNA827465;Yang2025_Silkie_Wencha	0.755279869
ENSGALG00010013215	SF3A3	3	-0.12750749	0.117789715	-1.082501046	0.279029951	0.027408802	6.760375634	70.41584509	0.666666667	CRA004775;PRJNA827465;Yang2025_Silkie_Wencha	0.755279869
ENSGALG00010015917	TMEM63A	3	-0.125156206	0.114734301	-1.090835129	0.275345436	0.019727464	4.019540172	50.24306477	0.666666667	CRA004775;PRJNA827465;Yang2025_Silkie_Wencha	0.755279869
ENSGALG00010009408	ENSGALG0001000940:	3	0.120721464	0.111717679	1.080594093	0.279877711	0.021947089	5.164342326	61.27290032	0.666666667	CRA004775;PRJNA827465;Yang2025_Silkie_Wencha	0.755279869
ENSGALG00010002911	MYBL1	3	-0.120566203	0.111414297	-1.082143018	0.279188984	0.001161896	2.050193344	2.448224912	0.666666667	CRA004775;PRJNA827465;Yang2025_Silkie_Wencha	0.755279869
ENSGALG00010016043	ADAMTSL1	3	0.116469933	0.1073798	1.084654029	0.278074916	0	1.357791145	0	0.666666667	CRA004775;PRJNA827465;Yang2025_Silkie_Wencha	0.755279869
ENSGALG00010012753	GTPBP1	3	-0.115824457	0.106631353	-1.086213887	0.277384375	0.024936936	11.01809145	81.84803595	0.666666667	CRA004775;PRJNA827465;Yang2025_Silkie_Wencha	0.755279869
ENSGALG00010016778	MTIF2	3	0.115788129	0.107137819	1.080740025	0.279812773	0.021853289	7.292368765	72.57406935	0.666666667	CRA004775;PRJNA827465;Yang2025_Silkie_Wencha	0.755279869
ENSGALG00010024607	RARG	3	-0.115367209	0.106068219	-1.087669902	0.276740859	0.015023998	3.343435946	40.18129755	1	CRA004775;PRJNA827465;Yang2025_Silkie_Wencha	0.755279869
ENSGALG00010022310	DCLRE1A	3	0.113652323	0.105135991	1.081003014	0.279695773	0.017495458	4.310712636	53.60395904	0.666666667	CRA004775;PRJNA827465;Yang2025_Silkie_Wencha	0.755279869
ENSGALG00010011930	TSPO	3	-0.113595634	0.104774999	-1.084186451	0.278282138	0.012049925	3.055353656	34.54112928	1	CRA004775;PRJNA827465;Yang2025_Silkie_Wencha	0.755279869
ENSGALG00010017038	ZNF598	3	0.111852419	0.103138338	1.084489253	0.278147929	0.017887234	4.454094131	55.09749141	0.666666667	CRA004775;PRJNA827465;Yang2025_Silkie_Wencha	0.755279869
ENSGALG00010025652	KMT5B	3	0.111205677	0.102903168	1.08068274	0.279838263	0.015595311	3.829756065	47.77735276	0.666666667	CRA004775;PRJNA827465;Yang2025_Silkie_Wencha	0.755279869
ENSGALG00010018095	E4F1	3	0.108412735	0.100062203	1.08345341	0.27860722	0.0207559	7.609999747	73.71879019	0.666666667	CRA004775;PRJNA827465;Yang2025_Silkie_Wencha	0.755279869
ENSGALG00010004430	TCEA1	3	-0.106967137	0.098832948	-1.082302409	0.279118176	0.016792466	5.221332808	61.69560391	0.666666667	CRA004775;PRJNA827465;Yang2025_Silkie_Wencha	0.755279869
ENSGALG00010023193	PPDPF	3	-0.106470209	0.098621219	-1.079587232	0.28032603	0	1.48847809	0	0.666666667	CRA004775;PRJNA827465;Yang2025_Silkie_Wencha	0.755279869
ENSGALG00010006840	ENSGALG0001000684(	3	-0.103054546	0.094688726	-1.08835075	0.276440294	0	0.061496378	0	1	CRA004775;PRJNA827465;Yang2025_Silkie_Wencha	0.755279869
ENSGALG00010024121	NRG4	3	0.102634398	0.095047493	1.079822254	0.280221339	0	1.020984491	0	1	CRA004775;PRJNA827465;Yang2025_Silkie_Wencha	0.755279869
ENSGALG00010021720	CACNB1	3	-0.101362213	0.093455203	-1.084607494	0.278095535	0.009152258	3.074864265	34.95647847	0.666666667	CRA004775;PRJNA827465;Yang2025_Silkie_Wencha	0.755279869
ENSGALG00010025420	STK39	3	-0.100494261	0.092158144	-1.090454469	0.275512999	0	0.947668411	0	1	CRA004775;PRJNA827465;Yang2025_Silkie_Wencha	0.755279869
ENSGALG00010017831	PLEK	3	-0.099749003	0.091926302	-1.085097521	0.277878465	0	0.017863396	0	1	CRA004775;PRJNA827465;Yang2025_Silkie_Wencha	0.755279869
ENSGALG00010025247	SGCA	3	-0.090407516	0.083365875	-1.08446671	0.27815792	0	0.224610395	0	1	CRA004775;PRJNA827465;Yang2025_Silkie_Wencha	0.755279869
ENSGALG00010001906	SKAP2	3	-0.087531112	0.080281929	-1.090296573	0.275582524	0.004209635	2.45819371	18.63944683	0.666666667	CRA004775;PRJNA827465;Yang2025_Silkie_Wencha	0.755279869
ENSGALG00010008218	ARHGAP42	3	0.087041953	0.080123574	1.086346371	0.277325778	0	1.884177475	0	1	CRA004775;PRJNA827465;Yang2025_Silkie_Wencha	0.755279869
ENSGALG00010020453	ENSGALG0001002045:	3	0.084923622	0.078243167	1.085380687	0.277753082	0	1.507036793	0	1	CRA004775;PRJNA827465;Yang2025_Silkie_Wencha	0.755279869
ENSGALG00010023714	EIF3M	3	-0.083704641	0.077455246	-1.080683956	0.279837722	0.005168787	2.724615981	26.59516005	1	CRA004775;PRJNA827465;Yang2025_Silkie_Wencha	0.755279869
ENSGALG00010004901	CUL5	3	-0.08046046	0.073962086	-1.08786088	0.276656528	0.006687441	3.206550631	37.62768064	0.666666667	CRA004775;PRJNA827465;Yang2025_Silkie_Wencha	0.755279869
ENSGALG00010020293	SEMA4B	3	0.078729053	0.072682341	1.083193695	0.278722458	0	0.685435457	0	0.666666667	CRA004775;PRJNA827465;Yang2025_Silkie_Wencha	0.755279869
ENSGALG00010016953	GJA4	3	-0.075850514	0.069764971	-1.087229212	0.276935524	0	1.191202936	0	0.666666667	CRA004775;PRJNA827465;Yang2025_Silkie_Wencha	0.755279869
ENSGALG00010013517	STMN1	3	0.073698025	0.067811873	1.086801203	0.277124676	0	0.62035298	0	0.666666667	CRA004775;PRJNA827465;Yang2025_Silkie_Wencha	0.755279869
ENSGALG00010028197	INTS3	3	-0.069054988	0.063967423	-1.079533687	0.280349885	0.004670647	3.217385769	37.8377309	0.666666667	CRA004775;PRJNA827465;Yang2025_Silkie_Wencha	0.755279869
ENSGALG00010016758	NR3C1	3	-0.065454995	0.060310361	-1.085302661	0.277787628	0.002069495	2.274816427	12.08081777	0.666666667	CRA004775;PRJNA827465;Yang2025_Silkie_Wencha	0.755279869
ENSGALG00010015875	DNTTIP1	3	-0.063882575	0.059000276	-1.082750449	0.278919205	0	1.561286413	0	0.666666667	CRA004775;PRJNA827465;Yang2025_Silkie_Wencha	0.755279869

ENSGALG00010024641	TRA2B	3	0.056948952	0.052369414	1.087446804	0.276839396	0.001786429	2.429916484	17.69264444	0.666666667	CRA004775;PRJNA827465;Yang2025_Silkie_Wencha	0.755279869
ENSGALG00010009159	PTK2	3	-0.03502726	0.032192785	-1.088046888	0.276574409	0	1.343910734	0	0.666666667	CRA004775;PRJNA827465;Yang2025_Silkie_Wencha	0.755279869
ENSGALG00010012976	SLC9A2	3	0.360359478	0.33404947	1.078760812	0.280694369	0.274042127	11.37688867	82.42050127	0.666666667	CRA004775;PRJNA827465;Yang2025_Silkie_Wencha	0.755323286
ENSGALG00010028538	FEM1A	3	-0.306555706	0.284149004	-1.078855467	0.280652164	0.198684422	18.53281955	89.20833392	0.666666667	CRA004775;PRJNA827465;Yang2025_Silkie_Wencha	0.755323286
ENSGALG00010015798	IL22RA1	3	0.254371819	0.235816875	1.078683699	0.280728755	0.09447858	5.010811874	60.08630836	0.666666667	CRA004775;PRJNA827465;Yang2025_Silkie_Wencha	0.755323286
ENSGALG00010022881	CCNB1	3	-0.237261608	0.219954218	-1.078686329	0.280727582	0.091083232	6.833593922	70.73282342	0.666666667	CRA004775;PRJNA827465;Yang2025_Silkie_Wencha	0.755323286
ENSGALG00010013690	CREB3L2	3	-0.175182256	0.16237233	-1.078892293	0.280635745	0.048163091	6.103692028	67.23294703	0.666666667	CRA004775;PRJNA827465;Yang2025_Silkie_Wencha	0.755323286
ENSGALG00010009492	CDC42SE2	3	-0.098177952	0.090999862	-1.078880234	0.280641122	0.002826965	2.217805511	9.820766955	0.666666667	CRA004775;PRJNA827465;Yang2025_Silkie_Wencha	0.755323286
ENSGALG00010019938	TAFA4	2	-0.341925739	0.317044598	-1.078478363	0.280820333	0	0.029756843	0	1	PRJNA827465;Yang2025_Silkie_Wenchang	0.75540434
ENSGALG00010029264	COL26A1	3	-0.2412388	0.223715896	-1.078326593	0.280888034	0.086175135	5.264954905	62.01296999	0.666666667	CRA004775;PRJNA827465;Yang2025_Silkie_Wencha	0.75540434
ENSGALG00010023230	ENSGALG0001002323	3	-0.171811824	0.159359044	-1.078142918	0.280969981	0.053786162	8.615284748	76.78544519	0.666666667	CRA004775;PRJNA827465;Yang2025_Silkie_Wencha	0.75540434
ENSGALG00010017747	DUSP26	3	-0.10635983	0.098651491	-1.078137075	0.280972588	0	0.389240411	0	1	CRA004775;PRJNA827465;Yang2025_Silkie_Wencha	0.75540434
ENSGALG00010022597	SLCO4A1	3	0.294956092	0.273664885	1.077800289	0.28112289	0.115144158	4.230783548	52.7274327	0.666666667	CRA004775;PRJNA827465;Yang2025_Silkie_Wencha	0.755664743
ENSGALG00010003476	ENSGALG0001000347	3	-1.071281745	0.995021104	-1.076642235	0.281640128	2.668301356	20.99218995	90.47264719	0.666666667	CRA004775;PRJNA827465;Yang2025_Silkie_Wencha	0.755905423
ENSGALG00010017003	CPZ	3	-0.293014326	0.272007791	-1.077227698	0.281378554	0.119748813	5.169022754	61.3079668	0.666666667	CRA004775;PRJNA827465;Yang2025_Silkie_Wencha	0.755905423
ENSGALG00010019515	ENSGALG0001001951	3	-0.26792377	0.248841895	-1.076682728	0.281622031	0.169767063	24.87747019	91.96059734	0.666666667	CRA004775;PRJNA827465;Yang2025_Silkie_Wencha	0.755905423
ENSGALG00010012756	RPA2	3	-0.192441329	0.17874006	-1.076654722	0.281634547	0.059736953	5.346076575	62.58938734	0.666666667	CRA004775;PRJNA827465;Yang2025_Silkie_Wencha	0.755905423
ENSGALG00010018564	DLGAP5	3	-0.167874356	0.155834192	-1.07726266	0.281362939	0.04740567	8.81946845	77.32289637	0.666666667	CRA004775;PRJNA827465;Yang2025_Silkie_Wencha	0.755905423
ENSGALG00010021103	RBMS1	3	0.076342057	0.070887476	1.076947036	0.281503928	0.007532532	3.668229769	45.47778831	0.666666667	CRA004775;PRJNA827465;Yang2025_Silkie_Wencha	0.755905423
ENSGALG00010021568	TADA3	3	-0.069850393	0.064861138	-1.076922112	0.281515063	0	1.024993379	0	0.666666667	CRA004775;PRJNA827465;Yang2025_Silkie_Wencha	0.755905423
ENSGALG00010027392	RBBP5	3	-0.041714902	0.038734492	-1.076944598	0.281505017	0	1.250535114	0	1	CRA004775;PRJNA827465;Yang2025_Silkie_Wencha	0.755905423
ENSGALG00010020351	CROCC	3	0.599769181	0.557205279	1.076388188	0.281753683	0.901575425	70.88825661	97.17865822	0.666666667	CRA004775;PRJNA827465;Yang2025_Silkie_Wencha	0.756066677
ENSGALG00010029188	ENSGALG0001002918	3	0.446804559	0.415220262	1.076066366	0.281897577	0.199457809	3.179970686	37.10633848	1	CRA004775;PRJNA827465;Yang2025_Silkie_Wencha	0.756088663
ENSGALG00010000731	ENSGALG0001000073	3	0.380769151	0.353871032	1.076011078	0.281922302	0.114351748	2.745208769	27.14579588	0.666666667	CRA004775;PRJNA827465;Yang2025_Silkie_Wencha	0.756088663
ENSGALG00010018351	RIN2	3	-0.101057934	0.09390398	-1.076183715	0.281845102	0.017525003	6.652261211	69.93503507	0.666666667	CRA004775;PRJNA827465;Yang2025_Silkie_Wencha	0.756088663
ENSGALG00010003184	BET1	3	-0.30727022	0.285621634	-1.075794629	0.282019115	0.218600733	23.84775749	91.61346722	0.666666667	CRA004775;PRJNA827465;Yang2025_Silkie_Wencha	0.7560945
ENSGALG00010015486	MTFR1L	3	-0.212557003	0.197617344	-1.075598923	0.282106669	0.083565365	8.447112319	76.32326972	1	CRA004775;PRJNA827465;Yang2025_Silkie_Wencha	0.7560945
ENSGALG00010006254	PROS1	3	-0.190188815	0.176832864	-1.075528671	0.282138103	0.066747959	9.288887891	78.46889721	0.666666667	CRA004775;PRJNA827465;Yang2025_Silkie_Wencha	0.7560945
ENSGALG00010021582	STK36	3	0.07705063	0.071639815	1.075528048	0.282138382	0	1.355742264	0	0.666666667	CRA004775;PRJNA827465;Yang2025_Silkie_Wencha	0.7560945
ENSGALG00010021231	STN1	3	-0.409017845	0.380671398	-1.074464348	0.282614615	0.372358311	20.88929693	90.42571894	0.666666667	CRA004775;PRJNA827465;Yang2025_Silkie_Wencha	0.756214302
ENSGALG00010012350	H2AC32	3	-0.210537614	0.195950739	-1.074441541	0.282624832	0.043881491	3.204951262	37.59655493	0.666666667	CRA004775;PRJNA827465;Yang2025_Silkie_Wencha	0.756214302
ENSGALG00010007719	LTBP1	3	0.143571025	0.133590078	1.074713239	0.282503135	0.016768729	2.813170965	28.9058495	0.666666667	CRA004775;PRJNA827465;Yang2025_Silkie_Wencha	0.756214302
ENSGALG00010002884	ADHFE1	3	0.136860059	0.127371789	1.074492708	0.282601911	0	1.005992361	0	0.666666667	CRA004775;PRJNA827465;Yang2025_Silkie_Wencha	0.756214302
ENSGALG00010013241	SEC24A	3	-0.129381063	0.120378948	-1.07478147	0.282472578	0.028611165	8.394737733	76.17555112	0.666666667	CRA004775;PRJNA827465;Yang2025_Silkie_Wencha	0.756214302
ENSGALG00010018355	PRKCB	3	-0.109782704	0.102147389	-1.074748028	0.282487555	0	0.600369244	0	1	CRA004775;PRJNA827465;Yang2025_Silkie_Wencha	0.756214302
ENSGALG00010006033	NSUN6	3	0.10616032	0.098744049	1.075106002	0.282327272	0	1.522304199	0	0.666666667	CRA004775;PRJNA827465;Yang2025_Silkie_Wencha	0.756214302
ENSGALG00010010743	KLHL20	3	-0.066128096	0.061551547	-1.074353121	0.282664445	0	0.772078571	0	1	CRA004775;PRJNA827465;Yang2025_Silkie_Wencha	0.756214302
ENSGALG00010024621	THRA	3	-0.065222141	0.060707442	-1.074368126	0.282657722	0	0.802324232	0	0.666666667	CRA004775;PRJNA827465;Yang2025_Silkie_Wencha	0.756214302
ENSGALG00010013356	AMER1	3	-0.166609631	0.155108473	-1.074149131	0.282755847	0.054457957	10.3955038	80.7609132	0.666666667	CRA004775;PRJNA827465;Yang2025_Silkie_Wencha	0.756220945
ENSGALG00010000055	PRC1	3	0.153817312	0.143204585	1.074108852	0.282773898	0.036136833	5.102520106	60.80368213	0.666666667	CRA004775;PRJNA827465;Yang2025_Silkie_Wencha	0.756220945
ENSGALG00010003437	PNRC1	3	0.150173226	0.139840924	1.073886112	0.282873728	0.032498086	4.869965132	58.93194416	0.666666667	CRA004775;PRJNA827465;Yang2025_Silkie_Wencha	0.756344865
ENSGALG00010011361	NUP37	3	-0.160938635	0.149938481	-1.073364453	0.283107627	0.042115074	5.429803041	63.16625143	0.666666667	CRA004775;PRJNA827465;Yang2025_Silkie_Wencha	0.756827139
ENSGALG00010027719	GPR61	2	-0.483414944	0.45092376	-1.072054716	0.283695457	0.111804581	1.283379646	22.08073398	1	CRA004775;PRJNA827465	0.757289974
ENSGALG00010005245	KIAA1217	3	0.313473959	0.292413186	1.072024021	0.283709243	0.192872393	10.79986874	81.48125641	0.666666667	CRA004775;PRJNA827465;Yang2025_Silkie_Wencha	0.757289974
ENSGALG00010023354	SEPTIN2	3	-0.299728195	0.279486789	-1.072423483	0.283529865	0.176558369	16.49658239	87.87627672	0.666666667	CRA004775;PRJNA827465;Yang2025_Silkie_Wencha	0.757289974
ENSGALG00010003382	ASNS	3	-0.253371218	0.23628211	-1.072325016	0.283574074	0.105518121	6.343804743	68.47317846	0.666666667	CRA004775;PRJNA827465;Yang2025_Silkie_Wencha	0.757289974
ENSGALG00010029175	CENPX	3	-0.215608917	0.200999249	-1.072685187	0.283412389	0.049605384	3.180470969	37.11623154	0.666666667	CRA004775;PRJNA827465;Yang2025_Silkie_Wencha	0.757289974
ENSGALG00010002437	TTK	3	-0.182913922	0.170609044	-1.072123242	0.283664681	0.049550695	5.258196683	61.96414626	0.666666667	CRA004775;PRJNA827465;Yang2025_Silkie_Wencha	0.757289974
ENSGALG00010005997	ENSGALG0001000599	3	-0.173445308	0.161788478	-1.072049817	0.283697657	0.005623891	2.113786693	5.383073561	0.666666667	CRA004775;PRJNA827465;Yang2025_Silkie_Wencha	0.757289974
ENSGALG00010029756	ELAC2	3	-0.109842378	0.102403105	-1.07264695	0.283429551	0.020889731	6.621460948	69.79518545	0.666666667	CRA004775;PRJNA827465;Yang2025_Silkie_Wencha	0.757289974
ENSGALG00010009852	ENSGALG0001000985	3	-0.247069553	0.230540462	-1.071697139	0.283856086	0.058476674	3.143996558	36.3866988	0.666666667	CRA004775;PRJNA827465;Yang2025_Silkie_Wencha	0.757395962
ENSGALG00010016761	GPR160	3	-0.138221602	0.128972006	-1.07171786	0.283846776	0.024413832	3.87016851	48.32266361	0.666666667	CRA004775;PRJNA827465;Yang2025_Silkie_Wencha	0.757395962
ENSGALG00010013554	ENSGALG0001001355	3	-3.358996458	3.138462988	-1.070267985	0.284498701	24.86545046	14.07672616	85.79215098	0.666666667	CRA004775;PRJNA827465;Yang2025_Silkie_Wencha	0.757823498
ENSGALG00010024826	RBM33	3	0.34836372	0.325389131	1.070606504	0.284346398	0.261282899	12.69292333	84.24318852	0.666666667	CRA004775;PRJNA827465;Yang2025_Silkie_Wencha	0.757823498
ENSGALG00010001553	CNGA4	3	0.297716335	0.278161424	1.070300582	0.284484032	0.109076114	3.77660151	47.04233437	1	CRA004775;PRJNA827465;Yang2025_Silkie_Wencha	0.757823498
ENSGALG00010029673	TMEM248	3	-0.246758415	0.230515292	-1.070464404	0.284410323	0.138248313	35.92242282	94.4324468	1	CRA004775;PRJNA827465;Yang2025_Silkie_Wencha	0.757823498
ENSGALG00010021309	ST3GAL3	3	-0.222306263	0.207559458	-1.071048582	0.284147586	0.088792484	8.297510538	75.89638494	0.666666667	CRA004775;PRJNA827465;Yang2025_Silkie_Wencha	0.757823498
ENSGALG00010001363	HIBADH	3	0.201056192	0.18769362	1.071193535	0.284082418	0.076383481	11.56338247	82.70402276	0.666666667	CRA004775;PRJNA827465;Yang2025_Silkie_Wencha	0.757823498
ENSGALG00010012876	KDELR3	3	-0.192207139	0.179498353	-1.070801687	0.284258608	0.06538558	8.724607853	77.07633359	0.666666667	CRA004775;PRJNA827465;Yang2025_Silkie_Wencha	0.757823498
ENSGALG00010023200	VPS26A	3	-0.160588311	0.149965007	-1.070838551	0.284242029	0.052596794	14.45151357	86.16061916	0.666666667	CRA004775;PRJNA827465;Yang2025_Silkie_Wencha	0.757823498
ENSGALG00010023017	BUB1B	3	-0.081087897	0.075736203	-1.070662295	0.284321302	0	1.66480691	0	0.666666667	CRA004775;PRJNA827465;Yang2025_Silkie_Wencha	0.757823498
ENSGALG00010029489	ENSGALG0001002948	3	1.224928677	1.145759997	1.069097089	0.285025923	3.324571234	12.91426275	84.51324679	0.666666667	CRA004775;PRJNA827465;Yang2025_Silkie_Wencha	0.75813722
ENSGALG00010009131	CDKL2	3	0.384434716	0.359508079	1.069335401	0.284918564	0.254691436	6.28729955	68.18984074	0.666666667	CRA004775;PRJNA827465;Yang2025_Silkie_Wencha	0.75813722
ENSGALG00010020486	MRTFB	3	0.301786955	0.282234318	1.069278027	0.284944409	0.178716804	8.69880029	77.00832375	0.666666667	CRA004775;PRJNA827465;Yang2025_Silkie_Wencha	0.75813722
ENSGALG00010010161	SEPSECS	3	-0.248031146	0.231848721	-1.069797346	0.284710537	0.143132623	24.55100638	91.85369443	1	CRA004775;PRJNA827465;Yang2025_Silkie_Wencha	0.75813722
ENSGALG00010027948	PRPF4	3	-0.160759627	0.150341603	-1.069295681	0.284936456	0.051741955	9.101698277	78.02607888	0.666666667	CRA004775;PRJNA827465;Yang2025_Silkie_Wencha	0.75813722
ENSGALG00010010321	NUP133	3	-0.130260521	0.121846557	-1.069053775	0.285045439	0.025761629	5.263575153	62.00301236	0.666666667	CRA004775;PRJNA827465;Yang2025_Silkie_Wencha	0.75813722
ENSGALG00010000063	SERF2	3	-0.092738017	0.086706028	-1.069568285	0.284813677	0	0.57372176	0	1	CRA004775;PRJNA827465;Yang2025_Silkie_Wencha	0.75813722
ENSGALG00010004436	NOVA1	3	-0.053661947	0.050187019	-1.069239592	0.284961723	0	0.549660638	0	0.666666667	CRA004775;PRJNA827465;Yang2025_Silkie_Wencha	0.75813722
ENSGALG00010007927	DLL1	3	0.098583311	0.092243575	1.068728209	0.285192157	0	0.799545967	0	1	CRA004775;PRJNA827465;Yang2025_Silkie_Wencha	0.758237372
ENSGALG00010023375	ST14	3	0.083156955	0.077810315	1.068713771	0.285198664	0	1.751135614	0	1	CRA004775;PRJNA827465;Yang2025_Silkie_Wencha	0.758237372
ENSGALG00010018451	PIK3R4	3	0.052685459	0.049302645	1.068613248	0.285243976	0	0.652752002	0	0.666666667	CRA004775;PRJNA827465;Yang2025_Silkie_Wencha	0.758237372
ENSGALG00010022850	ULK1	3	0.169341507	0.15854056	1.068127342	0.285463074	0.043663286	5.302742657	62.28366849	0.666666667	CRA004775;PRJNA827465;Yang2025_Silkie_Wencha	0.758677143
ENSGALG00010012604	ENSGALG0001001260	3	-2.861359135	2.699634565	-1.059906097	0.289187322	18.65484874	15.25952796	86.89343468	0.666666667	CRA004775;PRJNA827465;Yang2025_Silkie_Wencha	0.758891917
ENSGALG00010025804	ENSGALG0001002580	2	-2.230312673	2.105912922	-1.05907165	0.28956715	2.748382971	1.447655715	30.92280231	1	PRJNA827465;Yang2025_Silkie_Wenchang	0.758891917
ENSGALG00010027736	ENSGALG0001002773	2	-1.241412891	1.168529617	-1.062371782	0.288066939	0	0.494146655	0	1	PRJNA827465;Yang2025_Silkie_Wenchang	0.758891917
ENSGALG00010014439	ENSGALG0001001443	3	0.489013195	0.461778324	1.058978237	0.289609691	0.482845823	10.86123707	81.5858913	0.666666667	CRA004775;PRJNA827465;Yang2025_Silkie_Wencha	0.758891917
ENSGALG00010011641	ENSGALG0001001164	3	0.422215271	0.398301817	1.060038528	0.289127072	0.280527953	5.484394624	63.53289402	0.666666667	CRA004775;PRJNA827465;Yang2025_Silkie_Wencha	0.758891917
ENSGALG00010000034	CYTB	3	0.415922289	0.390558048	1.064944867	0.28690088	0.377082519	13.22380443	84.87575939	0.666666667	CRA004775;PRJNA827465;Yang2025_Silkie_Wencha	0.758891917
ENSGALG00010027172	ENSGALG0001002717	3	-0.414457657	0.388998847	-1.065447005	0.286673695	0.254002475	4.712030351	57.55545166	0.666666667	CRA004775;PRJNA827465;Yang2025_Silkie_Wencha	0.758891917
ENSGALG00010019947	ENSGALG0001001994	3	-0.405592928	0.381981977	-1.061811687	0.288321183	0.278312545	5.561284652	64.0370863	0.666666667	CRA004775;PRJNA827465;Yang2025_Silkie_Wencha	0.758891917
ENSGALG00010026132	DDTL	3	-0.387701117	0.365626859	-1.060373791	0.28897458	0.359887495	21.51685381	90.70496078	0.666666667	CRA004775;PRJNA827465;Yang2025_Silkie_Wencha	0.758891917
ENSGALG00010005231	ENSGALG0001000523	3	-0.371360203	0.350836356	-1.058499772	0.289827655	0.213483431	4.752772853	57.91930181	0.666666667	CRA004775;PRJNA827465;Yang2025_Silkie_Wencha	0.758891917
ENSGALG00010009317	LRRTM3	2	0.36818453	0.345815118	1.064686043	0.287018028	0.016931576	1.068678213	6.426463267	1	PRJNA827465;Yang2025_Silkie_Wenchang	0.758891917
ENSGALG00010007762	SH2D4A	3	0.355950174	0.334490735	1.064155554	0.287258238	0.139967575	3.303655539	39.46100081	0.666666667	CRA004775;PRJNA827465;Yang2025_Silkie_Wencha	0.758891917
ENSGALG00010000871	PRR7	3	0.319796844	0.302127038	1.058484691	0.289834527	0.21376775	9.827716002	79.64939158	1	CRA004775;PRJNA827465;Yang2025_Silkie_Wencha	0.758891917
ENSGALG00010009202	FABP6	3	0.316249015	0.297774942	1.062040388	0.28821735	0.199749948	8.211262048	75.64302821	0.666666667	CRA004775;PRJNA827465;Yang2025_Silkie_Wencha	0.758891917
ENSGALG00010007593	SEC61B	3	-0.315760212	0.297268785	-1.062204403	0.288142901	0.196555235	8.232924302	75.70729517	0.666666667	CRA004775;PRJNA827465;Yang2025_Silkie_Wencha	0.758891917
ENSGALG00010017861	PRSS56	3	0.314676568	0.296899061	1.059877277	0.289200434	0	1.372456669	0	1	CRA004775;PRJNA827465;Yang2025_Silkie_Wencha	0.758891917
ENSGALG00010023652	SLC30A7	3	-0.309385267	0.291777848	-1.060345291	0.288987542	0.214351539	22.21278651	90.99617691	0.666666667	CRA004775;PRJNA827465;Yang2025_Silkie_Wencha	0.758891917
ENSGALG00010028329	HAPLN2	3	0.299802441	0.281623382	1.064543398	0.287082606	0.047225585	2.474210401	19.16613077	0.666666667	CRA004775;PRJNA827465;Yang2025_Silkie_Wencha	0.758891917
ENSGALG00010013297	FCHO2	3	0.296039034	0.278958107	1.061231156	0.288584864	0.159632306	6.975545541	71.32840739	1	CRA004775;PRJNA827465;Yang2025_Silkie_Wencha	0.758891917
ENSGALG00010012011	HOOK3	3	0.291446184	0.275143099	1.059253111	0.289484523	0.205017222	25.39358679	92.12399565	0.666666667	CRA004775;PRJNA827465;Yang2025_Silkie_Wencha	0.758891917
ENSGALG00010024212	RIMS4	3	-0.282046342	0.264519287	-1.066260026	0.286306114	0.118488593	4.899873646	59.18262093	1	CRA004775;PRJNA827465;Yang2025_Silkie_Wencha	0.758891917
ENSGALG00010014867	TP63	3	0.280588855	0.265038811	1.058672017	0.289749176	0.12788553	5.822403928	65.64992699	0.666666667	CRA004775;PRJNA827465;Yang2025_Silkie_Wencha	0.758891917
ENSGALG00010002308	GXYLT1	3	-0.26497956	0.248296199	-1.067191369	0.285885429	0.15717028	20.70050646	90.33840064	1	CRA004775;PRJNA827465;Yang2025_Silkie_Wencha	0.758891917
ENSGALG00010027708	ENSGALG0001002770	3	-0.255222035	0.239134678	-1.067273208	0.285848483	0	1.61863363	0	1	CRA004775;PRJNA827465;Yang2025_Silkie_Wencha	0.758891917
ENSGALG00010016453	MYCL	3	-0.247566453	0.232272176	-1.065846359	0.286491101	0.117494138	9.425071391	78.78000158	0.666666667	CRA004775;PRJNA827465;Yang2025_Silkie_Wencha	0.758891917
ENSGALG00010019786	R3HDM1	3	0.245886361	0.23124292	1.063327961	0.287632349	0.131751762	12.66475105	84.18316033	0.666666667	CRA004775;PRJNA827465;Yang2025_Silkie_Wencha	0.758891917
ENSGALG00010004934	CIP2A	3	-0.240126074	0.226556324	-1.059895704	0.28919205	0.099239003	9.365694958	78.64547149	0.666666667	CRA004775;PRJNA827465;Yang2025_Silkie_Wencha	0.758891917
ENSGALG00010025403	ENSGALG0001002540	3	0.230323878	0.216234049	1.065160085	0.286803493	0.099968783	7.776859062	74.28267654	0.666666667	CRA004775;PRJNA827465;Yang2025_Silkie_Wencha	0.758891917
ENSGALG00010006966	PGBD5	2	-0.224147601	0.211147133	-1.061570657	0.288430641	0	0.039395375	0	1	PRJNA827465;Yang2025_Silkie_Wenchang	0.758891917
ENSGALG00010015192	ENSGALG0001001519	3	0.220129828	0.207023038	1.063310781	0.287641038	0.053155508	3.393782066	41.06869677	0.666666667	CRA004775;PRJNA827465;Yang2025_Silkie_Wencha	0.758891917
ENSGALG00010001879	IRAK4	3	0.208979821	0.196912198	1.061284284	0.288560726	0.070937596	5.217532703	61.66770553	0.666666667	CRA004775;PRJNA827465;Yang2025_Silkie_Wencha	0.758891917
ENSGALG00010004044	RNF138	3	-0.203179257	0.190836901	-1.064674888	0.287023078	0.086349002	11.52727236	82.64984172	0.666666667	CRA004775;PRJNA827465;Yang2025_Silkie_Wencha	0.758891917
ENSGALG00010017701	PPP2R5E	3	-0.197346331	0.185567411	-1.063475151	0.287566528	0.09291056	40.90961055	95.11117321	0.666666667	CRA004775;PRJNA827465;Yang2025_Silkie_Wencha	0.758891917
ENSGALG00010028076	ST6GALNAC6	3	-0.185040596	0.173925225	-1.063908904	0.287369969	0.053720904	5.891424538	66.05235309	0.666666667	CRA004775;PRJNA827465;Yang2025_Silkie_Wencha	0.758891917
ENSGALG00010004038	SENP6	3	0.174292701	0.164011427	1.062686324	0.287924225	0.057826363	7.775075162	74.276776	0.666666667	CRA004775;PRJNA827465;Yang2025_Silkie_Wencha	0.758891917
ENSGALG00010006173	TBPL1	3	-0.172421751	0.162638411	-1.060153932	0.289074575	0.061660636	18.7857795	89.35364913	0.666666667	CRA004775;PRJNA827465;Yang2025_Silkie_Wencha	0.758891917
ENSGALG00010003933	TECR	3	-0.170857371	0.160517095	-1.064418531	0.287139143	0.036306566	3.781377296	47.10921859	0.666666667	CRA004775;PRJNA827465;Yang2025_Silkie_Wencha	0.758891917
ENSGALG00010021227	CHCHD4	3	-0.166866908	0.156308891	-1.067545856	0.285725418	0.045656128	5.438771295	63.22698839	0.666666667	CRA004775;PRJNA827465;Yang2025_Silkie_Wencha	0.758891917
ENSGALG00010009778	ENSGALG0001000977	3	-0.16655492	0.157265013	-1.059071674	0.289567139	0.059171555	11.42931522	82.50113885	0.666666667	CRA004775;PRJNA827465;Yang2025_Silkie_Wencha	0.758891917
ENSGALG00010012669	ERCC8	3	0.153637499	0.144052058	1.066541508	0.286178926	0.016606086	2.699595072	25.91481513	0.666666667	CRA004775;PRJNA827465;Yang2025_Silkie_Wencha	0.758891917
ENSGALG00010017710	RAB41	3	0.152908566	0.144167011	1.060634913	0.28885585	0.043253454	6.820433361	70.67635011	0.666666667	CRA004775;PRJNA827465;Yang2025_Silkie_Wencha	0.758891917
ENSGALG00010023555	HPS4	3	-0.152346939	0.143506531	-1.061602823	0.288416032	0.045834153	9.425447633	78.78084864	0.666666667	CRA004775;PRJNA827465;Yang2025_Silkie_Wencha	0.758891917
ENSGALG00010029182	HEATR6	3	-0.150764907	0.141792504	-1.063278398	0.287655718	0.042498369	7.7414752	74.1651307	0.666666667	CRA004775;PRJNA827465;Yang2025_Silkie_Wencha	0.758891917
ENSGALG00010023053	MAN1A2	3	-0.147896637	0.139525126	-1.060000026	0.289144587	0.039372984	8.624646669	76.81064423	1	CRA004775;PRJNA827465;Yang2025_Silkie_Wencha	0.758891917
ENSGALG00010015493	OSTF1	3	0.143699729	0.13572175	1.058781872	0.289699131	0.033095671	6.257076827	68.03619238	0.666666667	CRA004775;PRJNA827465;Yang2025_Silkie_Wencha	0.758891917
ENSGALG00010024894	TUT4	3	0.143479237	0.135051447	1.062404296	0.288052184	0.03456546	5.76401743	65.30197862	0.666666667	CRA004775;PRJNA827465;Yang2025_Silkie_Wencha	0.758891917
ENSGALG00010002448	RAB9A	3	-0.140458898	0.132597162	-1.059290374	0.289467558	0.02732826	4.36143035	54.14348414	0.666666667	CRA004775;PRJNA827465;Yang2025_Silkie_Wencha	0.758891917
ENSGALG00010006516	NBEA	3	0.132493749	0.124399719	1.065064696	0.286846654	0.021812402	3.570183781	43.98047489	0.666666667	CRA004775;PRJNA827465;Yang2025_Silkie_Wencha	0.758891917
ENSGALG00010028010	ASB6	3	-0.127396264	0.120314702	-1.058858655	0.289664156	0.024732183	4.482498757	55.3820289	1	CRA004775;PRJNA827465;Yang2025_Silkie_Wencha	0.758891917
ENSGALG00010021704	COPS7A	3	-0.125525069	0.11840314	-1.060149832	0.28907644	0.026056533	6.331343275	68.41112678	1	CRA004775;PRJNA827465;Yang2025_Silkie_Wencha	0.758891917
ENSGALG00010003914	CAMK2D	3	-0.117263835	0.110182506	-1.064269085	0.287206819	0.016492137	3.40044408	41.18415264	0.666666667	CRA004775;PRJNA827465;Yang2025_Silkie_Wencha	0.758891917
ENSGALG00010011335	VCP	3	0.114173394	0.107065079	1.066392465	0.286246266	0	1.352655125	0	0.666666667	CRA004775;PRJNA827465;Yang2025_Silkie_Wencha	0.758891917
ENSGALG00010019302	USP34	3	0.11267312	0.105679476	1.066177884	0.286343238	0.017312789	3.853885355	48.10432029	1	CRA004775;PRJNA827465;Yang2025_Silkie_Wencha	0.758891917
ENSGALG00010028548	MYO1F	3	-0.108309894	0.102230136	-1.059471286	0.2893852	0	1.765311033	0	1	CRA004775;PRJNA827465;Yang2025_Silkie_Wencha	0.758891917
ENSGALG00010013546	IDUA	3	0.10696092	0.100868258	1.060402173	0.288961674	0	0.201982269	0	1	CRA004775;PRJNA827465;Yang2025_Silkie_Wencha	0.758891917
ENSGALG00010007118	ENSGALG0001000711	3	0.105890154	0.099951874	1.059411398	0.289412461	0	1.637122922	0	0.666666667	CRA004775;PRJNA827465;Yang2025_Silkie_Wencha	0.758891917
ENSGALG00010019610	CORO7	3	0.10390885	0.097456053	1.066212377	0.286327648	0.009316509	2.918795473	31.47858361	0.666666667	CRA004775;PRJNA827465;Yang2025_Silkie_Wencha	0.758891917
ENSGALG00010017313	MAP4K3	3	0.09975953	0.094003929	1.061227234	0.288586645	0.014425483	4.480612767	55.36324819	1	CRA004775;PRJNA827465;Yang2025_Silkie_Wencha	0.758891917
ENSGALG00010001380	SCRN1	3	0.099555728	0.093757977	1.06183742	0.288309499	0	1.15434701	0	0.666666667	CRA004775;PRJNA827465;Yang2025_Silkie_Wencha	0.758891917
ENSGALG00010015614	ATG2B	3	0.096670142	0.091297975	1.05884213	0.289671683	0.01072293	3.258328974	38.61884371	1	CRA004775;PRJNA827465;Yang2025_Silkie_Wencha	0.758891917
ENSGALG00010029527	NDUFAF8	3	-0.09425214	0.088846072	-1.060847573	0.288759178	0	0.628405113	0	0.666666667	CRA004775;PRJNA827465;Yang2025_Silkie_Wencha	0.758891917
ENSGALG00010006568	ENTPD5	3	0.090104644	0.084517983	1.066100269	0.286378318	0	0.935069886	0	0.666666667	CRA004775;PRJNA827465;Yang2025_Silkie_Wencha	0.758891917
ENSGALG00010013281	BIRC8	3	-0.089815089	0.084739157	-1.059900677	0.289189788	0.003766922	2.340290442	14.54052181	0.666666667	CRA004775;PRJNA827465;Yang2025_Silkie_Wencha	0.758891917
ENSGALG00010020806	HTD2	3	-0.08896727	0.083967031	-1.059550027	0.289349359	0	1.230243957	0	1	CRA004775;PRJNA827465;Yang2025_Silkie_Wencha	0.758891917
ENSGALG00010029228	ERAL1	3	0.086590118	0.081285326	1.065261375	0.286757667	0.007921729	3.175592448	37.01962601	0.666666667	CRA004775;PRJNA827465;Yang2025_Silkie_Wencha	0.758891917
ENSGALG00010016541	UBE2J2	3	-0.081949988	0.077422509	-1.058477555	0.289837778	0.012552295	7.773379304	74.27116416	0.666666667	CRA004775;PRJNA827465;Yang2025_Silkie_Wencha	0.758891917
ENSGALG00010009086	UBE2D1	3	-0.07268401	0.068120567	-1.066990677	0.285976046	0.001821683	2.225864749	10.14728093	0.666666667	CRA004775;PRJNA827465;Yang2025_Silkie_Wencha	0.758891917
ENSGALG00010017700	VPS35L	3	-0.07168847	0.067236835	-1.066208264	0.286329507	0.005338677	3.267645796	38.79385574	0.666666667	CRA004775;PRJNA827465;Yang2025_Silkie_Wencha	0.758891917
ENSGALG00010027378	ZBTB47	3	0.070909697	0.066845371	1.060801912	0.288779933	0	0.122059418	0	0.666666667	CRA004775;PRJNA827465;Yang2025_Silkie_Wencha	0.758891917
ENSGALG00010019956	SLC29A3	3	0.069775428	0.065767329	1.060943629	0.28871552	0	1.367086077	0	0.666666667	CRA004775;PRJNA827465;Yang2025_Silkie_Wencha	0.758891917
ENSGALG00010016224	MOSPD1	3	-0.063141518	0.059279155	-1.065155509	0.286805563	0	1.462724853	0	0.666666667	CRA004775;PRJNA827465;Yang2025_Silkie_Wencha	0.758891917
ENSGALG00010017949	SFXN3	3	-0.06178015	0.058051623	-1.064227783	0.287225524	0	0.337654119	0	1	CRA004775;PRJNA827465;Yang2025_Silkie_Wencha	0.758891917
ENSGALG00010028126	ZMYND10	3	-0.058455382	0.054974859	-1.063311179	0.287640857	0	0.588120421	0	1	CRA004775;PRJNA827465;Yang2025_Silkie_Wencha	0.758891917
ENSGALG00010021404	UQCC1	3	-0.058252175	0.054992389	-1.05927704	0.289473628	0	0.301498091	0	1	CRA004775;PRJNA827465;Yang2025_Silkie_Wencha	0.758891917
ENSGALG00010025600	ENSGALG0001002560	3	0.051998647	0.048867015	1.064084788	0.287290291	0.001461663	2.408588348	16.96380986	0.666666667	CRA004775;PRJNA827465;Yang2025_Silkie_Wencha	0.758891917
ENSGALG00010005511	AHCYL2	3	-0.04983212	0.046975808	-1.0608039	0.288779029	0	0.265399748	0	0.666666667	CRA004775;PRJNA827465;Yang2025_Silkie_Wencha	0.758891917
ENSGALG00010022170	RSPRY1	3	0.048965822	0.046103109	1.0620937	0.28819315	0	1.009282723	0	0.666666667	CRA004775;PRJNA827465;Yang2025_Silkie_Wencha	0.758891917
ENSGALG00010008188	VPS4A	3	-0.047918151	0.045114279	-1.06215045	0.28816739	0	0.500282098	0	0.666666667	CRA004775;PRJNA827465;Yang2025_Silkie_Wencha	0.758891917
ENSGALG00010026173	CDK12	3	-0.046565206	0.043838107	-1.062208411	0.288141083	0	0.558229712	0	0.666666667	CRA004775;PRJNA827465;Yang2025_Silkie_Wencha	0.758891917
ENSGALG00010016904	EAF1	3	-0.040469611	0.038188239	-1.059740191	0.289262813	0	0.246126162	0	1	CRA004775;PRJNA827465;Yang2025_Silkie_Wencha	0.758891917
ENSGALG00010011510	ENSGALG0001001151	3	-0.366126862	0.346219357	-1.057499689	0.290283597	0.188459252	4.209292606	52.48607813	0.666666667	CRA004775;PRJNA827465;Yang2025_Silkie_Wencha	0.75935611
ENSGALG00010021790	PHF21A	3	0.191073189	0.18065906	1.057645203	0.290217226	0.08088232	12.93127182	84.53361721	0.666666667	CRA004775;PRJNA827465;Yang2025_Silkie_Wencha	0.75935611
ENSGALG00010004148	ENSGALG0001000414	3	0.158762634	0.150087273	1.05780211	0.29014567	0.035046865	4.293984124	53.42320926	0.666666667	CRA004775;PRJNA827465;Yang2025_Silkie_Wencha	0.75935611
ENSGALG00010015044	ZC3H12B	3	0.085719236	0.081054128	1.057555461	0.290258157	0	0.919869636	0	1	CRA004775;PRJNA827465;Yang2025_Silkie_Wencha	0.75935611
ENSGALG00010017208	CINP	3	-0.083729902	0.079175933	-1.057517092	0.290275658	0	1.608294052	0	1	CRA004775;PRJNA827465;Yang2025_Silkie_Wencha	0.75935611
ENSGALG00010002421	ENSGALG0001000242	3	-0.338176193	0.319853036	-1.057286176	0.290381	0.221788602	8.795975825	77.2623295	0.666666667	CRA004775;PRJNA827465;Yang2025_Silkie_Wencha	0.759391257
ENSGALG00010006819	EMC8	3	-0.179897534	0.170158547	-1.057234779	0.290404451	0.064223625	8.274590119	75.82961849	0.666666667	CRA004775;PRJNA827465;Yang2025_Silkie_Wencha	0.759391257
ENSGALG00010029385	CLIP2	3	-0.064831602	0.061332484	-1.057051622	0.290488028	0	0.97480143	0	0.666666667	CRA004775;PRJNA827465;Yang2025_Silkie_Wencha	0.759469348
ENSGALG00010001786	P2RY8	3	-0.329535074	0.311811365	-1.056841127	0.290584101	0.193229785	7.846062921	74.50950852	0.666666667	CRA004775;PRJNA827465;Yang2025_Silkie_Wencha	0.759580071
ENSGALG00010008339	FAM76B	3	0.367083993	0.348260465	1.054050143	0.291859961	0.317823719	18.93962807	89.44013054	0.666666667	CRA004775;PRJNA827465;Yang2025_Silkie_Wencha	0.759595229
ENSGALG00010011724	ARHGEF28	3	0.359711523	0.341220581	1.054190581	0.291795671	0.281181887	11.32606643	82.34161867	0.666666667	CRA004775;PRJNA827465;Yang2025_Silkie_Wencha	0.759595229
ENSGALG00010024984	SHISAL2A	2	0.359241989	0.340453359	1.055187088	0.291339768	0.10798199	1.861844644	46.28982589	1	PRJNA827465;Yang2025_Silkie_Wenchang	0.759595229
ENSGALG00010011218	ENSGALG0001001121	3	0.329189692	0.312297477	1.054090143	0.291841649	0	0.3324959	0	0.666666667	CRA004775;PRJNA827465;Yang2025_Silkie_Wencha	0.759595229
ENSGALG00010003761	UGT8	3	0.258045942	0.244422654	1.055736602	0.29108857	0.079927196	3.673356935	45.55388885	0.666666667	CRA004775;PRJNA827465;Yang2025_Silkie_Wencha	0.759595229
ENSGALG00010028605	GTPBP3	3	0.230982043	0.218937038	1.05501584	0.29141808	0.071884194	3.942705198	49.27340748	1	CRA004775;PRJNA827465;Yang2025_Silkie_Wencha	0.759595229
ENSGALG00010018391	STAC	3	0.202575134	0.192182139	1.054078882	0.291846804	0.042924094	3.319847172	39.75626297	0.666666667	CRA004775;PRJNA827465;Yang2025_Silkie_Wencha	0.759595229
ENSGALG00010025245	ENSGALG0001002524	3	0.186705146	0.177044856	1.054564081	0.291624739	0.07567814	12.09075555	83.45843655	0.666666667	CRA004775;PRJNA827465;Yang2025_Silkie_Wencha	0.759595229
ENSGALG00010003981	TMEM150C	3	-0.167491408	0.158870605	-1.054263046	0.291762502	0.038943709	4.07943115	50.97355669	0.666666667	CRA004775;PRJNA827465;Yang2025_Silkie_Wencha	0.759595229
ENSGALG00010023414	SUDS3	3	-0.152681827	0.144770384	-1.054648211	0.291586246	0.056091145	28.24316354	92.9186403	0.666666667	CRA004775;PRJNA827465;Yang2025_Silkie_Wencha	0.759595229
ENSGALG00010015753	RPRD1B	3	-0.14248804	0.135040807	-1.055148015	0.291357635	0.044963072	24.29014367	91.76620761	0.666666667	CRA004775;PRJNA827465;Yang2025_Silkie_Wencha	0.759595229
ENSGALG00010002389	COL21A1	3	0.131182747	0.12443937	1.054190057	0.291795911	0	0.441764034	0	1	CRA004775;PRJNA827465;Yang2025_Silkie_Wencha	0.759595229
ENSGALG00010024285	CCND2	3	-0.107815635	0.102158806	-1.055372892	0.291254816	0.017092795	4.516515042	55.71807065	0.666666667	CRA004775;PRJNA827465;Yang2025_Silkie_Wencha	0.759595229
ENSGALG00010011871	CCT2	3	0.102494547	0.09704911	1.056110116	0.290917909	0.014688305	4.125021542	51.51540472	1	CRA004775;PRJNA827465;Yang2025_Silkie_Wencha	0.759595229
ENSGALG00010027502	THOP1	3	-0.088345108	0.083704467	-1.055440785	0.291223778	0.006948306	2.747954315	27.21858624	1	CRA004775;PRJNA827465;Yang2025_Silkie_Wencha	0.759595229
ENSGALG00010026040	GUK1	3	-0.085447847	0.080926813	-1.055865715	0.29102957	0	1.7374975	0	1	CRA004775;PRJNA827465;Yang2025_Silkie_Wencha	0.759595229
ENSGALG00010019680	CCDC34	3	-0.085346327	0.080973132	-1.054007984	0.291879262	0	0.840432162	0	0.666666667	CRA004775;PRJNA827465;Yang2025_Silkie_Wencha	0.759595229
ENSGALG00010016181	FANCI	3	-0.080673454	0.076444404	-1.055321899	0.291278129	0	0.375260936	0	1	CRA004775;PRJNA827465;Yang2025_Silkie_Wencha	0.759595229
ENSGALG00010024652	PUS3	3	-0.071989717	0.068245723	-1.054860485	0.291489137	0	1.250825431	0	1	CRA004775;PRJNA827465;Yang2025_Silkie_Wencha	0.759595229

ENSGALG00010024973	RTEL1	3	0.070469247	0.066772263	1.055367068	0.291257478	0	0.626479345	0	1	CRA004775;PRJNA827465;Yang2025_Silkie_Wencha	0.759595229
ENSGALG00010005358	SHPRH	3	-0.062477817	0.059138711	-1.056462269	0.29075707	0	0.365019478	0	1	CRA004775;PRJNA827465;Yang2025_Silkie_Wencha	0.759595229
ENSGALG00010016796	BROX	3	-0.060418853	0.057227545	-1.055765248	0.291075479	0	1.628990712	0	1	CRA004775;PRJNA827465;Yang2025_Silkie_Wencha	0.759595229
ENSGALG00010011420	HMGB3	3	0.044976763	0.042599206	1.055812249	0.291054001	0.000687238	2.124616701	5.865373305	0.666666667	CRA004775;PRJNA827465;Yang2025_Silkie_Wencha	0.759595229
ENSGALG00010016925	PHACTR4	3	0.039561893	0.037491738	1.055216284	0.291326418	0	1.292136963	0	1	CRA004775;PRJNA827465;Yang2025_Silkie_Wencha	0.759595229
ENSGALG00010011456	KIAA1328	3	0.203115192	0.192767693	1.053678597	0.292030091	0.068435954	5.721384659	65.04342709	0.666666667	CRA004775;PRJNA827465;Yang2025_Silkie_Wencha	0.759662989
ENSGALG00010022759	THSD4	3	-0.097736579	0.09276138	-1.053634385	0.29205034	0	0.766631522	0	0.666666667	CRA004775;PRJNA827465;Yang2025_Silkie_Wencha	0.759662989
ENSGALG00010022971	CKAP5	3	-0.075603105	0.071756992	-1.053599139	0.292066483	0.00799392	4.640008623	56.89663183	0.666666667	CRA004775;PRJNA827465;Yang2025_Silkie_Wencha	0.759662989
ENSGALG00010012442	CPLX1	3	0.554094151	0.52641036	1.052589753	0.292529056	0.392237739	3.62539382	44.83357948	0.666666667	CRA004775;PRJNA827465;Yang2025_Silkie_Wencha	0.759742234
ENSGALG00010018668	OVOL2	2	0.4689737	0.445494293	1.05270417	0.292476597	0.050247905	1.129102199	11.43405787	1	CRA004775;PRJNA827465	0.759742234
ENSGALG00010027940	ZBTB7B	3	0.385318803	0.365913877	1.053031403	0.2923266	0.223478442	4.535629568	55.90468821	0.666666667	CRA004775;PRJNA827465;Yang2025_Silkie_Wencha	0.759742234
ENSGALG00010003248	MRI1	3	0.325680162	0.309299955	1.052972586	0.292353556	0.226050032	10.63487759	81.19395373	0.666666667	CRA004775;PRJNA827465;Yang2025_Silkie_Wencha	0.759742234
ENSGALG00010022006	SF1	3	0.294170467	0.279521745	1.052406377	0.292613144	0.19903127	17.65960419	88.67471785	1	CRA004775;PRJNA827465;Yang2025_Silkie_Wencha	0.759742234
ENSGALG00010006083	CACNB2	3	0.223623816	0.212426846	1.052709769	0.29247403	0.069488051	4.399651041	54.54184931	0.666666667	CRA004775;PRJNA827465;Yang2025_Silkie_Wencha	0.759742234
ENSGALG00010021879	CREBL2	3	-0.12517695	0.118829678	-1.053414865	0.292150894	0.022739301	4.521305098	55.76498474	0.666666667	CRA004775;PRJNA827465;Yang2025_Silkie_Wencha	0.759742234
ENSGALG00010000810	UCHL3	3	-0.065971795	0.062682103	-1.052482156	0.292578393	0	0.748201631	0	1	CRA004775;PRJNA827465;Yang2025_Silkie_Wencha	0.759742234
ENSGALG00010017531	SACM1L	3	0.05108882	0.048512871	1.053098262	0.292295959	0.001434505	2.409524682	16.99607747	0.666666667	CRA004775;PRJNA827465;Yang2025_Silkie_Wencha	0.759742234
ENSGALG00010014362	NAXD	3	0.049500097	0.047037216	1.052360272	0.292634288	0	0.784702362	0	0.666666667	CRA004775;PRJNA827465;Yang2025_Silkie_Wencha	0.759742234
ENSGALG00010022340	GFOD2	3	-0.131991282	0.125454936	-1.052101149	0.292753145	0.029591815	5.564616168	64.05861717	0.666666667	CRA004775;PRJNA827465;Yang2025_Silkie_Wencha	0.759771792
ENSGALG00010007916	MCM3	3	-0.065605539	0.062349765	-1.052217891	0.292699593	0	1.672428987	0	1	CRA004775;PRJNA827465;Yang2025_Silkie_Wencha	0.759771792
ENSGALG00010009078	PTTG2	3	-0.150563272	0.14312569	-1.051965389	0.29281543	0.020763352	2.762033058	27.58957052	1	CRA004775;PRJNA827465;Yang2025_Silkie_Wencha	0.759793973
ENSGALG00010021002	SLC44A3	3	-0.257874472	0.245278265	-1.051354763	0.293095684	0.133651759	9.172724326	78.19622689	0.666666667	CRA004775;PRJNA827465;Yang2025_Silkie_Wencha	0.75982396
ENSGALG00010028026	ANKMY1	3	0.196997843	0.187367663	1.051397984	0.293075842	0.07282323	7.085006181	71.76140834	0.666666667	CRA004775;PRJNA827465;Yang2025_Silkie_Wencha	0.75982396
ENSGALG00010008974	SMCO4	3	-0.121837139	0.115865659	-1.051537955	0.293011587	0.013676575	2.743599819	27.10307144	0.666666667	CRA004775;PRJNA827465;Yang2025_Silkie_Wencha	0.75982396
ENSGALG00010027103	ACVR2B	3	-0.117701936	0.111931957	-1.051548985	0.293006524	0	0.640845364	0	0.666666667	CRA004775;PRJNA827465;Yang2025_Silkie_Wencha	0.75982396
ENSGALG00010027331	SLC16A4	3	0.093106941	0.088537808	1.051606582	0.292980087	0	0.058368139	0	1	CRA004775;PRJNA827465;Yang2025_Silkie_Wencha	0.75982396
ENSGALG00010003806	EGF	3	-0.141446601	0.134553022	-1.051234661	0.293150828	0	1.825523992	0	0.666666667	CRA004775;PRJNA827465;Yang2025_Silkie_Wencha	0.759827599
ENSGALG00010020013	ADAM8	3	0.395551772	0.376383595	1.050927239	0.293292009	0	0.832146686	0	1	CRA004775;PRJNA827465;Yang2025_Silkie_Wencha	0.759956754
ENSGALG00010028029	EFNA2	3	-0.097415751	0.092703665	-1.050829552	0.29333688	0	0.950103423	0	1	CRA004775;PRJNA827465;Yang2025_Silkie_Wencha	0.759956754
ENSGALG00010010246	CLTB	3	-0.083990828	0.079932261	-1.050775079	0.293361904	0.00720434	2.92460825	31.61477267	1	CRA004775;PRJNA827465;Yang2025_Silkie_Wencha	0.759956754
ENSGALG00010025784	ENSGALG0001002578	2	-2.198621534	2.093392856	-1.050267047	0.293595351	0	0.000283931	0	1	PRJNA827465;Yang2025_Silkie_Wenchang	0.760422178
ENSGALG00010018359	CACNG3	3	0.329408273	0.313801154	1.049735696	0.293839647	0	1.511597914	0	1	CRA004775;PRJNA827465;Yang2025_Silkie_Wencha	0.760664209
ENSGALG00010029659	VKORC1L1	3	-0.279312068	0.266113516	-1.049597451	0.29390323	0.195978293	42.36040041	95.27860931	0.666666667	CRA004775;PRJNA827465;Yang2025_Silkie_Wencha	0.760664209
ENSGALG00010023726	PNPO	3	0.250989163	0.239129355	1.049595788	0.293903994	0.12160557	9.045781057	77.89024533	0.666666667	CRA004775;PRJNA827465;Yang2025_Silkie_Wencha	0.760664209
ENSGALG00010022671	DBR1	3	-0.095813151	0.091269919	-1.049786284	0.293816383	0.015218765	5.730688709	65.10127694	0.666666667	CRA004775;PRJNA827465;Yang2025_Silkie_Wencha	0.760664209
ENSGALG00010028912	EXFABP	3	-0.683333377	0.651694234	-1.048549061	0.294385711	1.090408827	17.7929578	88.75959791	0.666666667	CRA004775;PRJNA827465;Yang2025_Silkie_Wencha	0.760935935
ENSGALG00010017142	RBP7	3	-0.52205693	0.497852881	-1.048616872	0.294354487	0.470304587	5.447474825	63.2857413	0.666666667	CRA004775;PRJNA827465;Yang2025_Silkie_Wencha	0.760935935
ENSGALG00010000060	HYPK	3	0.284677821	0.271366155	1.049052461	0.294153145	0.183604209	15.26008732	86.8939151	0.666666667	CRA004775;PRJNA827465;Yang2025_Silkie_Wencha	0.760935935
ENSGALG00010008311	SRP19	3	0.251756117	0.240042517	1.048798023	0.294271087	0.115291799	6.05384387	66.96313874	0.666666667	CRA004775;PRJNA827465;Yang2025_Silkie_Wencha	0.760935935
ENSGALG00010023874	HEXIM1	3	0.242365886	0.231126315	1.048629561	0.294348645	0.129131349	20.29686319	90.14626063	0.666666667	CRA004775;PRJNA827465;Yang2025_Silkie_Wencha	0.760935935
ENSGALG00010018388	SMC3	3	0.216830521	0.206788349	1.048562566	0.294379492	0.110604041	16.60698077	87.9568717	0.666666667	CRA004775;PRJNA827465;Yang2025_Silkie_Wencha	0.760935935
ENSGALG00010010619	LRRC52	3	-0.210542173	0.200736969	-1.048846029	0.294248989	0.038074772	2.83313676	29.40686703	1	CRA004775;PRJNA827465;Yang2025_Silkie_Wencha	0.760935935
ENSGALG00010014221	SCYL3	3	-0.049415898	0.047135144	-1.048387538	0.294460093	0	1.899625397	0	1	CRA004775;PRJNA827465;Yang2025_Silkie_Wencha	0.76098908
ENSGALG00010026259	MED15	3	0.232902578	0.222229902	1.048025381	0.294626913	0.113024034	10.02800958	80.0558627	0.666666667	CRA004775;PRJNA827465;Yang2025_Silkie_Wencha	0.761281054
ENSGALG00010018425	CYP26B1	3	0.216652901	0.206794564	1.047672129	0.294789693	0.085047208	6.651653572	69.93228859	0.666666667	CRA004775;PRJNA827465;Yang2025_Silkie_Wencha	0.761562483
ENSGALG00010008027	C6orf62	3	-0.174722925	0.166829985	-1.047314456	0.294954571	0.055760725	6.481430648	69.10261513	0.666666667	CRA004775;PRJNA827465;Yang2025_Silkie_Wencha	0.761570979
ENSGALG00010009059	MED29	3	-0.412200227	0.39357774	-1.047315905	0.294953903	0.368376418	11.56393938	82.70485571	0.666666667	CRA004775;PRJNA827465;Yang2025_Silkie_Wencha	0.761570979
ENSGALG00010009712	UBXN2A	3	-0.086013384	0.082121173	-1.047395951	0.294916998	0.003701667	2.288085564	12.59068141	0.666666667	CRA004775;PRJNA827465;Yang2025_Silkie_Wencha	0.761570979
ENSGALG00010007000	LRGUK	2	1.031910741	0.985523763	1.047068351	0.295068055	1.673295937	7.184199283	86.08805643	1	PRJNA827465;Yang2025_Silkie_Wenchang	0.76158584
ENSGALG00010028004	ENSGALG0001002800	3	-0.462746102	0.441939188	-1.047080943	0.295062248	0.473530256	10.99641761	81.81225858	0.666666667	CRA004775;PRJNA827465;Yang2025_Silkie_Wencha	0.76158584
ENSGALG00010023752	C1QL1	3	0.394063683	0.376953393	1.045446464	0.295816672	0.309614583	8.214922133	75.65406017	0.666666667	CRA004775;PRJNA827465;Yang2025_Silkie_Wencha	0.762186318
ENSGALG00010027240	SLC6A1	3	0.249894708	0.238965612	1.045735016	0.295683391	0	0.998823762	0	1	CRA004775;PRJNA827465;Yang2025_Silkie_Wencha	0.762186318
ENSGALG00010018709	SNX5	3	-0.181937535	0.173862213	-1.04644668	0.29535485	0.060360613	9.130602884	78.09564138	0.666666667	CRA004775;PRJNA827465;Yang2025_Silkie_Wencha	0.762186318
ENSGALG00010014179	CTBPL	3	-0.165163485	0.157944941	-1.045702916	0.295698216	0.061504236	22.86483124	91.25294222	0.666666667	CRA004775;PRJNA827465;Yang2025_Silkie_Wencha	0.762186318
ENSGALG00010029242	DERL2	3	-0.142967781	0.136759389	-1.045396464	0.295839771	0.035711233	5.741458196	65.16564378	0.666666667	CRA004775;PRJNA827465;Yang2025_Silkie_Wencha	0.762186318
ENSGALG00010014987	KLF9	3	-0.134115439	0.128223864	-1.045947571	0.295585239	0.006299653	2.245576662	10.93601773	0.666666667	CRA004775;PRJNA827465;Yang2025_Silkie_Wencha	0.762186318
ENSGALG00010017979	ENSGALG0001001797	3	0.127627911	0.122076202	1.045477405	0.295802378	0.012994949	2.720485062	26.48369851	0.666666667	CRA004775;PRJNA827465;Yang2025_Silkie_Wencha	0.762186318
ENSGALG00010017604	GATM	3	-0.120760986	0.115425535	-1.046224187	0.295457539	0	0.324833449	0	0.666666667	CRA004775;PRJNA827465;Yang2025_Silkie_Wencha	0.762186318
ENSGALG00010013355	FAM171B	3	-0.089303537	0.08539714	-1.045743876	0.2956793	0	1.582540452	0	0.666666667	CRA004775;PRJNA827465;Yang2025_Silkie_Wencha	0.762186318
ENSGALG00010009143	MCCC2	3	0.082789525	0.079148057	1.046008305	0.295557198	0.004596116	2.517512084	20.55648857	1	CRA004775;PRJNA827465;Yang2025_Silkie_Wencha	0.762186318
ENSGALG00010020476	TMEM132A	3	0.069635146	0.066630869	1.045088379	0.295982125	0	0.396415846	0	0.666666667	CRA004775;PRJNA827465;Yang2025_Silkie_Wencha	0.762414148
ENSGALG00010029962	ENSGALG0001002996	3	0.450822406	0.43153145	1.044703475	0.296160038	0.105298395	2.448564118	18.31947609	0.666666667	CRA004775;PRJNA827465;Yang2025_Silkie_Wencha	0.762420195
ENSGALG00010011536	FCHSD1	3	0.149015876	0.14265125	1.044616685	0.296200165	0.014347619	2.597154755	22.99265203	0.666666667	CRA004775;PRJNA827465;Yang2025_Silkie_Wencha	0.762420195
ENSGALG00010017299	USP31	3	-0.075228801	0.072004005	-1.044786333	0.296121733	0.003658485	2.522528204	20.71446429	0.666666667	CRA004775;PRJNA827465;Yang2025_Silkie_Wencha	0.762420195
ENSGALG00010022547	SMARCE1	3	-0.040282569	0.03855684	-1.044758037	0.296134814	0	0.227348373	0	0.666666667	CRA004775;PRJNA827465;Yang2025_Silkie_Wencha	0.762420195
ENSGALG00010012897	ENSGALG0001001289	3	-0.317969632	0.304467239	-1.044347606	0.296324595	0	0.332050389	0	1	CRA004775;PRJNA827465;Yang2025_Silkie_Wencha	0.762601646
ENSGALG00010002959	DLX5	2	0.357507953	0.342432673	1.044024071	0.296474253	0	0.007324232	0	1	PRJNA827465;Yang2025_Silkie_Wenchang	0.762709146
ENSGALG00010029184	DYNLL2	3	-0.262685962	0.251587256	-1.044114741	0.296432307	0.141923779	10.17231993	80.33880163	0.666666667	CRA004775;PRJNA827465;Yang2025_Silkie_Wencha	0.762709146
ENSGALG00010016393	BLM	3	-0.096698236	0.092637622	-1.043833313	0.296562516	0.007073067	2.630483538	23.96835141	0.666666667	CRA004775;PRJNA827465;Yang2025_Silkie_Wencha	0.762797419
ENSGALG00010007901	FSTL5	3	-0.427682339	0.410032987	-1.043043737	0.296928037	0.297765171	5.380610297	62.82949536	0.666666667	CRA004775;PRJNA827465;Yang2025_Silkie_Wencha	0.763002515
ENSGALG00010015098	HAVCR1	3	-0.327623126	0.314034422	-1.043271382	0.296822621	0.218752366	11.83439141	83.10010265	0.666666667	CRA004775;PRJNA827465;Yang2025_Silkie_Wencha	0.763002515
ENSGALG00010020696	MOB1A	3	-0.158335345	0.151770316	-1.043256344	0.296829584	0.044264949	6.278315238	68.14432019	0.666666667	CRA004775;PRJNA827465;Yang2025_Silkie_Wencha	0.763002515
ENSGALG00010022877	KYAT3	3	-0.145444735	0.139426563	-1.043163738	0.296872465	0.027438158	3.768329623	46.92608662	0.666666667	CRA004775;PRJNA827465;Yang2025_Silkie_Wencha	0.763002515
ENSGALG00010009490	SNTB1	3	0.089876329	0.08613786	1.043400999	0.296762611	0	0.406212049	0	0.666666667	CRA004775;PRJNA827465;Yang2025_Silkie_Wencha	0.763002515
ENSGALG00010016604	TP53BP2	3	0.040377179	0.038713962	1.042961682	0.29696604	0	0.111681068	0	1	CRA004775;PRJNA827465;Yang2025_Silkie_Wencha	0.763002515
ENSGALG00010017393	VDAC1	3	-0.285571229	0.273877213	-1.042698026	0.297088172	0.182349448	20.54710589	90.26626908	0.666666667	CRA004775;PRJNA827465;Yang2025_Silkie_Wencha	0.76304955
ENSGALG00010029171	ENSGALG0001002917	3	-0.047205928	0.045276768	-1.04260815	0.297129813	0	1.850428921	0	0.666666667	CRA004775;PRJNA827465;Yang2025_Silkie_Wencha	0.76304955
ENSGALG00010027998	SNX27	3	-0.045937986	0.044062142	-1.042572677	0.297146249	0	0.623549	0	1	CRA004775;PRJNA827465;Yang2025_Silkie_Wencha	0.76304955
ENSGALG00010021768	DIPK1A	3	-0.164368616	0.157690273	-1.04235101	0.297248971	0.050676361	6.718730361	70.23247113	0.666666667	CRA004775;PRJNA827465;Yang2025_Silkie_Wencha	0.763106073
ENSGALG00010011887	PGM5	3	-0.072698244	0.069748426	-1.04229225	0.297276204	0	0.411693068	0	0.666666667	CRA004775;PRJNA827465;Yang2025_Silkie_Wencha	0.763106073
ENSGALG00010012587	MBLAC2	3	-0.244412872	0.234561497	-1.041999114	0.29741209	0.106143161	5.968094913	66.48846861	0.666666667	CRA004775;PRJNA827465;Yang2025_Silkie_Wencha	0.763316307
ENSGALG00010012888	ENSGALG0001001288	3	-2.356155473	2.262151052	-1.041555325	0.297617891	11.43049965	8.725697144	77.07919531	0.666666667	CRA004775;PRJNA827465;Yang2025_Silkie_Wencha	0.763428765
ENSGALG00010020129	MGAT2	3	-0.158474685	0.152145905	-1.041596782	0.297598661	0.055785964	11.42161049	82.48933456	0.666666667	CRA004775;PRJNA827465;Yang2025_Silkie_Wencha	0.763428765
ENSGALG00010022467	SART3	3	0.071722319	0.068859545	1.041574096	0.297609184	0.007776392	4.848129037	58.7469726	1	CRA004775;PRJNA827465;Yang2025_Silkie_Wencha	0.763428765
ENSGALG00010018644	ESF1	3	0.24346347	0.23396408	1.040601918	0.29806034	0.134346543	16.09968324	87.57739534	0.666666667	CRA004775;PRJNA827465;Yang2025_Silkie_Wencha	0.763455644
ENSGALG00010016421	ENSGALG0001001642	3	0.234799865	0.225580705	1.040868564	0.297936553	0	1.232815308	0	1	CRA004775;PRJNA827465;Yang2025_Silkie_Wencha	0.763455644
ENSGALG00010001407	FOXP2	3	0.22989042	0.220915768	1.040624765	0.298049733	0.104596327	7.866152301	74.57460874	0.666666667	CRA004775;PRJNA827465;Yang2025_Silkie_Wencha	0.763455644
ENSGALG00010022937	BLOC1S1	3	0.22119014	0.21250909	1.040850251	0.297945054	0.09365525	7.386246222	72.9226465	0.666666667	CRA004775;PRJNA827465;Yang2025_Silkie_Wencha	0.763455644
ENSGALG00010029670	TPST1	3	0.137995415	0.132606254	1.040640321	0.29804251	0.028339213	4.48609349	55.41778154	0.666666667	CRA004775;PRJNA827465;Yang2025_Silkie_Wencha	0.763455644
ENSGALG00010028889	FUBP3	3	0.128289494	0.123263025	1.040778395	0.297978409	0.036061608	11.02301978	81.85615159	0.666666667	CRA004775;PRJNA827465;Yang2025_Silkie_Wencha	0.763455644
ENSGALG00010026277	SMARCD2	3	-0.081563697	0.078328149	-1.0413076	0.29773281	0.008698893	3.862392387	48.21862205	0.666666667	CRA004775;PRJNA827465;Yang2025_Silkie_Wencha	0.763455644
ENSGALG00010027246	ZNF767	3	-0.065514157	0.062949203	-1.040746408	0.297993258	0.001046332	2.155813287	7.227587295	0.666666667	CRA004775;PRJNA827465;Yang2025_Silkie_Wencha	0.763455644
ENSGALG00010028463	RPS28	3	-0.264377334	0.254120565	-1.040361823	0.298171832	0.117430285	4.982256881	59.85754955	0.666666667	CRA004775;PRJNA827465;Yang2025_Silkie_Wencha	0.763464601
ENSGALG00010020041	CDH23	3	-0.084692673	0.081405586	-1.040379136	0.298163791	0	0.617572484	0	0.666666667	CRA004775;PRJNA827465;Yang2025_Silkie_Wencha	0.763464601
ENSGALG00010029501	ENSGALG0001002950	3	-0.248627087	0.239098122	-1.039853783	0.298407837	0.097767469	4.809389276	58.41467834	0.666666667	CRA004775;PRJNA827465;Yang2025_Silkie_Wencha	0.763930547
ENSGALG00010022054	ENSGALG0001002205	3	0.505261107	0.486406317	1.038763455	0.298914761	0.566425774	10.17579194	80.34551009	0.666666667	CRA004775;PRJNA827465;Yang2025_Silkie_Wencha	0.763944932
ENSGALG00010003790	ELOVL6	3	0.33352718	0.320841597	1.039538462	0.29855438	0.252706686	18.92469562	89.43179832	0.666666667	CRA004775;PRJNA827465;Yang2025_Silkie_Wencha	0.763944932
ENSGALG00010025248	C11orf74	3	-0.196216434	0.18881996	-1.039172097	0.298724705	0.0615518	4.824083531	58.54134807	0.666666667	CRA004775;PRJNA827465;Yang2025_Silkie_Wencha	0.763944932
ENSGALG00010005157	IMPA1	3	-0.157224762	0.151227198	-1.039659294	0.298498219	0.048248705	8.605659032	76.75947894	1	CRA004775;PRJNA827465;Yang2025_Silkie_Wencha	0.763944932
ENSGALG00010017489	TNFRSF1B	3	-0.120668332	0.116200721	-1.038447363	0.299061829	0.011270902	2.602582619	23.15325609	1	CRA004775;PRJNA827465;Yang2025_Silkie_Wencha	0.763944932
ENSGALG00010008522	NUDT12	3	-0.120228457	0.115678245	-1.039335071	0.29864893	0	1.301436229	0	1	CRA004775;PRJNA827465;Yang2025_Silkie_Wencha	0.763944932
ENSGALG00010027045	MAPK13	3	0.116125717	0.111788418	1.038799181	0.298898143	0	1.374636731	0	1	CRA004775;PRJNA827465;Yang2025_Silkie_Wencha	0.763944932
ENSGALG00010005126	MED23	3	-0.112422529	0.108238952	-1.038651308	0.298966934	0.025248077	11.88938611	83.17827362	0.666666667	CRA004775;PRJNA827465;Yang2025_Silkie_Wencha	0.763944932
ENSGALG00010014990	AGO1	3	-0.107425065	0.103402491	-1.038902102	0.298850269	0.020883939	6.806864177	70.6178947	0.666666667	CRA004775;PRJNA827465;Yang2025_Silkie_Wencha	0.763944932
ENSGALG00010008790	GPN1	3	-0.10367402	0.099824707	-1.038560731	0.299009077	0.017118153	4.925477087	59.39479639	0.666666667	CRA004775;PRJNA827465;Yang2025_Silkie_Wencha	0.763944932
ENSGALG00010000153	NF2	3	0.081473061	0.078442058	1.038640027	0.298972183	0.007718856	3.337391649	40.07296085	0.666666667	CRA004775;PRJNA827465;Yang2025_Silkie_Wencha	0.763944932
ENSGALG00010004187	ARFGEF1	3	-0.050807528	0.048921545	-1.038551191	0.299013516	0	1.792158226	0	0.666666667	CRA004775;PRJNA827465;Yang2025_Silkie_Wencha	0.763944932
ENSGALG00010013163	ENSGALG0001001316	2	0.617931016	0.595771765	1.037194194	0.299645363	0.625266	8.388861996	88.07943198	1	PRJNA827465;Yang2025_Silkie_Wenchang	0.764096402
ENSGALG00010023067	C15orf62	2	0.502429516	0.484861951	1.036232096	0.300093876	0.207196695	1.735683362	42.38580481	0.5	PRJNA827465;Yang2025_Silkie_Wenchang	0.764096402
ENSGALG00010008696	PFN4	3	0.279956715	0.269819588	1.037570019	0.299470282	0.099014855	3.587852035	44.2563411	0.666666667	CRA004775;PRJNA827465;Yang2025_Silkie_Wencha	0.764096402
ENSGALG00010023069	ENSGALG0001002306	3	0.187873736	0.181237359	1.036617047	0.299914365	0	0.928108626	0	0.666666667	CRA004775;PRJNA827465;Yang2025_Silkie_Wencha	0.764096402
ENSGALG00010020485	LMAN2L	3	-0.182516615	0.17586615	-1.037815493	0.299355962	0.077036793	22.31387101	91.03696531	0.666666667	CRA004775;PRJNA827465;Yang2025_Silkie_Wencha	0.764096402
ENSGALG00010016890	UBR7	3	-0.16936528	0.163433404	-1.036295373	0.300064364	0.068989143	17.76052679	88.73907275	0.666666667	CRA004775;PRJNA827465;Yang2025_Silkie_Wencha	0.764096402
ENSGALG00010024674	DHH	3	0.164921174	0.158931059	1.037690024	0.299414391	0	1.575313012	0	1	CRA004775;PRJNA827465;Yang2025_Silkie_Wencha	0.764096402
ENSGALG00010016225	P4HA2	3	-0.150780442	0.145406106	-1.036960868	0.299754095	0.041824448	6.785924228	70.52722764	0.666666667	CRA004775;PRJNA827465;Yang2025_Silkie_Wencha	0.764096402
ENSGALG00010005758	HIVEP2	3	0.137321828	0.132373987	1.037377742	0.299559847	0.025053229	3.655484785	45.28769458	0.666666667	CRA004775;PRJNA827465;Yang2025_Silkie_Wencha	0.764096402
ENSGALG00010019289	FKBP9	3	-0.115729472	0.111648637	-1.036550696	0.299945301	0.020563699	4.715061335	57.5827363	0.666666667	CRA004775;PRJNA827465;Yang2025_Silkie_Wencha	0.764096402
ENSGALG00010029573	MAP2K4	3	-0.100632311	0.097051368	-1.036897395	0.299783678	0.01718377	6.367098893	68.58851993	1	CRA004775;PRJNA827465;Yang2025_Silkie_Wencha	0.764096402
ENSGALG00010029417	ENSGALG0001002941	3	-0.099189728	0.095702858	-1.03643434	0.299999557	0	1.367754436	0	0.666666667	CRA004775;PRJNA827465;Yang2025_Silkie_Wencha	0.764096402
ENSGALG00010011247	ENSGALG0001001124	3	0.094245157	0.090941345	1.03632904	0.300048662	0.008466697	2.797235524	28.50083654	1	CRA004775;PRJNA827465;Yang2025_Silkie_Wencha	0.764096402
ENSGALG00010023189	RMDN3	3	-0.079425626	0.076537985	-1.037728206	0.29939661	0.008219512	3.963402035	49.53830112	0.666666667	CRA004775;PRJNA827465;Yang2025_Silkie_Wencha	0.764096402
ENSGALG00010025428	VANGL1	3	-0.076524926	0.073715855	-1.038106732	0.299220368	0	0.218101833	0	1	CRA004775;PRJNA827465;Yang2025_Silkie_Wencha	0.764096402
ENSGALG00010020193	VSTM4	3	0.074250078	0.071626286	1.036631691	0.299907538	0	0.378002123	0	1	CRA004775;PRJNA827465;Yang2025_Silkie_Wencha	0.764096402
ENSGALG00010000859	BORA	3	0.066726719	0.064295437	1.037814232	0.29935655	0	0.990530901	0	0.666666667	CRA004775;PRJNA827465;Yang2025_Silkie_Wencha	0.764096402
ENSGALG00010018288	CRNKL1	3	0.047391208	0.045718643	1.036583853	0.299929842	0.00198591	2.826818941	29.24909443	0.666666667	CRA004775;PRJNA827465;Yang2025_Silkie_Wencha	0.764096402
ENSGALG00010024549	GNB1L	3	-0.061464777	0.05932924	-1.03599468	0.300204625	0	1.904430892	0	1	CRA004775;PRJNA827465;Yang2025_Silkie_Wencha	0.764240761
ENSGALG00010006015	CDC123	3	-0.250267426	0.241637465	-1.035714499	0.300335356	0.152836195	24.81654599	91.94086074	0.666666667	CRA004775;PRJNA827465;Yang2025_Silkie_Wencha	0.764435932
ENSGALG00010028487	ENTPD8	2	0.926640079	0.894884306	1.035485898	0.300442049	1.098644107	3.183673661	68.58974548	1	CRA004775;PRJNA827465	0.764468139
ENSGALG00010008133	CREG1	3	-0.349916213	0.338060572	-1.035069576	0.30063642	0.296899646	23.69851501	91.56065264	1	CRA004775;PRJNA827465;Yang2025_Silkie_Wencha	0.764468139
ENSGALG00010005949	SLITRK1	3	-0.242846264	0.234635774	-1.03499249	0.300672418	0.008978925	2.098556202	4.696381341	0.666666667	CRA004775;PRJNA827465;Yang2025_Silkie_Wencha	0.764468139
ENSGALG00010018546	SNRPGP15	3	-0.227454882	0.21969994	-1.035297881	0.300529819	0.092366747	5.532846977	63.85224445	0.666666667	CRA004775;PRJNA827465;Yang2025_Silkie_Wencha	0.764468139
ENSGALG00010014992	ARL15	3	-0.18720994	0.180854936	-1.035138683	0.300604149	0.084681845	19.81374947	89.90599935	0.666666667	CRA004775;PRJNA827465;Yang2025_Silkie_Wencha	0.764468139
ENSGALG00010016882	ENSGALG0001001688	3	-0.07637479	0.073775737	-1.035229109	0.300561928	0.009648061	6.584482477	69.62555513	0.666666667	CRA004775;PRJNA827465;Yang2025_Silkie_Wencha	0.764468139
ENSGALG00010010935	LGALS1A	3	0.309959183	0.299610417	1.034540742	0.30088344	0.190263463	8.598285975	76.73955012	0.666666667	CRA004775;PRJNA827465;Yang2025_Silkie_Wencha	0.764592194
ENSGALG00010000053	HDDC3	3	0.256920756	0.248325736	1.034611876	0.300850206	0.143872733	9.216456757	78.29968661	0.666666667	CRA004775;PRJNA827465;Yang2025_Silkie_Wencha	0.764592194
ENSGALG00010018462	KTN1	3	0.218054564	0.210730515	1.03475552	0.300783101	0.11178823	15.00652696	86.67246589	0.666666667	CRA004775;PRJNA827465;Yang2025_Silkie_Wencha	0.764592194
ENSGALG00010017391	CRAMP1	3	-0.056391635	0.05454698	-1.033817726	0.301221383	0	0.010387607	0	1	CRA004775;PRJNA827465;Yang2025_Silkie_Wencha	0.765313412
ENSGALG00010012974	SLC49A3	3	0.245155636	0.23719457	1.033563443	0.301340297	0.107061736	6.129677124	67.37185402	0.666666667	CRA004775;PRJNA827465;Yang2025_Silkie_Wencha	0.765380886
ENSGALG00010027083	DENND6A	3	-0.047641165	0.046095606	-1.033529429	0.301356206	0	1.892323797	0	0.666666667	CRA004775;PRJNA827465;Yang2025_Silkie_Wencha	0.765380886
ENSGALG00010028192	ENSGALG0001002819	2	0.859916218	0.832274056	1.033212813	0.301504318	0.68319419	1.963102781	49.06023212	0.5	CRA004775;Yang2025_Silkie_Wenchang	0.765386666
ENSGALG00010013288	CA3A	3	0.696320721	0.674458918	1.032413838	0.301878292	0.987992843	8.671527483	76.93601267	0.666666667	CRA004775;PRJNA827465;Yang2025_Silkie_Wencha	0.765386666
ENSGALG00010001274	DLGAP1	2	0.560274209	0.542669762	1.032440443	0.301865834	0.435938169	3.758015737	73.39021255	1	PRJNA827465;Yang2025_Silkie_Wenchang	0.765386666
ENSGALG00010021421	GLB1L	3	0.273521819	0.264946075	1.032367882	0.301899812	0.173095375	11.74380397	82.96974298	0.666666667	CRA004775;PRJNA827465;Yang2025_Silkie_Wencha	0.765386666
ENSGALG00010007921	FAM120B	3	-0.244854298	0.237125023	-1.032595781	0.301793103	0.145637578	29.00120046	93.10373375	0.666666667	CRA004775;PRJNA827465;Yang2025_Silkie_Wencha	0.765386666
ENSGALG00010015911	TM9SF2L	3	-0.222869268	0.215790656	-1.032803146	0.301696031	0.129999448	40.22564056	95.02804686	0.666666667	CRA004775;PRJNA827465;Yang2025_Silkie_Wencha	0.765386666
ENSGALG00010012745	MARCHF3	3	0.222331484	0.21515589	1.033350676	0.30143982	0	1.6402717	0	0.666666667	CRA004775;PRJNA827465;Yang2025_Silkie_Wencha	0.765386666
ENSGALG00010012077	IFNGR2	3	-0.159383678	0.15430403	-1.032919739	0.30164146	0.060247445	24.27675953	91.7616682	0.666666667	CRA004775;PRJNA827465;Yang2025_Silkie_Wencha	0.765386666
ENSGALG00010023135	GINS3	3	-0.152798875	0.147986798	-1.032516936	0.301830018	0.039589708	4.999884589	59.99907669	0.666666667	CRA004775;PRJNA827465;Yang2025_Silkie_Wencha	0.765386666
ENSGALG00010023578	SRRD	3	-0.097268979	0.094172697	-1.032878774	0.301660633	0.005182203	2.295653289	12.87883021	0.666666667	CRA004775;PRJNA827465;Yang2025_Silkie_Wencha	0.765386666
ENSGALG00010027015	ENSGALG0001002701	2	0.471012489	0.456413329	1.031986708	0.302078344	0.198819077	1.858096062	46.18146927	1	PRJNA827465;Yang2025_Silkie_Wenchang	0.765701992
ENSGALG00010029462	ENSGALG0001002946	3	-1.663916669	1.616443807	-1.029368705	0.303306449	6.879717824	21.65243997	90.76316571	0.666666667	CRA004775;PRJNA827465;Yang2025_Silkie_Wencha	0.765947595
ENSGALG00010013492	B3GALT5	2	0.514156828	0.498759542	1.03087116	0.302601243	0	0.026032676	0	1	CRA004775;PRJNA827465	0.765947595
ENSGALG00010007948	ENSGALG0001000794	3	-0.473364491	0.460329042	-1.028317678	0.303800418	0.46566223	8.863909444	77.43659259	0.666666667	CRA004775;PRJNA827465;Yang2025_Silkie_Wencha	0.765947595
ENSGALG00010011981	SCUBE1	2	0.438968529	0.42661404	1.028959406	0.303498751	0.267676012	3.748078273	73.31966071	0.5	PRJNA827465;Yang2025_Silkie_Wenchang	0.765947595
ENSGALG00010008006	CPNE7	2	-0.425109794	0.41332035	-1.028523744	0.303703528	0	0.177484641	0	0.5	CRA004775;Yang2025_Silkie_Wenchang	0.765947595
ENSGALG00010023903	TBKBP1	3	0.320293946	0.310474168	1.031628325	0.302246266	0.1894985	5.85733939	65.85480426	0.666666667	CRA004775;PRJNA827465;Yang2025_Silkie_Wencha	0.765947595
ENSGALG00010001558	CCKBR	3	0.25775038	0.250652277	1.028318527	0.303800019	0.035851837	2.460521172	18.71640761	0.666666667	CRA004775;PRJNA827465;Yang2025_Silkie_Wencha	0.765947595
ENSGALG00010007788	PALM2AKAP2	3	0.256581467	0.249301754	1.029200408	0.303385511	0.1089205	5.266345413	62.02299995	0.666666667	CRA004775;PRJNA827465;Yang2025_Silkie_Wencha	0.765947595
ENSGALG00010024667	RBM7	3	0.24763458	0.240390794	1.030133372	0.302947401	0.144553177	20.03005848	90.01500669	1	CRA004775;PRJNA827465;Yang2025_Silkie_Wencha	0.765947595
ENSGALG00010017792	LSM1	3	0.243967494	0.236589498	1.031184798	0.302454168	0.118895451	7.081098043	71.75579284	0.666666667	CRA004775;PRJNA827465;Yang2025_Silkie_Wencha	0.765947595
ENSGALG00010024399	ROR1	3	0.240597686	0.233888252	1.028686492	0.30362702	0.115672526	7.791476656	74.33092482	0.666666667	CRA004775;PRJNA827465;Yang2025_Silkie_Wencha	0.765947595
ENSGALG00010020250	ENSGALG0001002025	3	0.240283376	0.233111426	1.030766189	0.302650477	0.090635695	4.799549691	58.32942403	0.666666667	CRA004775;PRJNA827465;Yang2025_Silkie_Wencha	0.765947595
ENSGALG00010023613	COPB1	3	-0.212579818	0.206298436	-1.030448036	0.302799734	0.115338331	38.16496984	94.75959235	0.666666667	CRA004775;PRJNA827465;Yang2025_Silkie_Wencha	0.765947595
ENSGALG00010027147	KMT2C	3	0.204399892	0.198556953	1.029427019	0.303279058	0.075936383	5.697944309	64.89962184	0.666666667	CRA004775;PRJNA827465;Yang2025_Silkie_Wencha	0.765947595
ENSGALG00010019331	CEMIP	3	0.196589931	0.190874172	1.02994517	0.303035745	0	1.897047475	0	0.666666667	CRA004775;PRJNA827465;Yang2025_Silkie_Wencha	0.765947595
ENSGALG00010005191	MSANTD4	3	0.192838761	0.187114276	1.030593524	0.302731474	0.086138356	16.9090794	88.17203496	0.666666667	CRA004775;PRJNA827465;Yang2025_Silkie_Wencha	0.765947595
ENSGALG00010001991	TOMM7	3	0.1755195	0.17024361	1.030990238	0.302545397	0.038800584	3.262203024	38.6917373	1	CRA004775;PRJNA827465;Yang2025_Silkie_Wencha	0.765947595
ENSGALG00010013686	UQCRQ	3	-0.166300375	0.161476568	-1.029873106	0.303069577	0.033961575	3.372606899	40.69869214	0.666666667	CRA004775;PRJNA827465;Yang2025_Silkie_Wencha	0.765947595
ENSGALG00010024361	H2AX	3	0.154132656	0.149832002	1.028703173	0.303619178	0.004353004	2.116250788	5.493242493	0.666666667	CRA004775;PRJNA827465;Yang2025_Silkie_Wencha	0.765947595
ENSGALG00010025272	NCAPD3	3	-0.152334613	0.148042679	-1.028991193	0.303483814	0.033771574	4.364103402	54.17157166	0.666666667	CRA004775;PRJNA827465;Yang2025_Silkie_Wencha	0.765947595
ENSGALG00010022643	SP3	3	0.135910811	0.132085445	1.028961297	0.303497862	0.03347689	5.498228875	63.62464994	0.666666667	CRA004775;PRJNA827465;Yang2025_Silkie_Wencha	0.765947595
ENSGALG00010009276	ENSGALG0001000927	3	-0.102850953	0.099956296	-1.028959228	0.303498834	0	0.794473378	0	1	CRA004775;PRJNA827465;Yang2025_Silkie_Wencha	0.765947595
ENSGALG00010012965	ENSGALG0001001296	3	0.102360403	0.099322845	1.030582679	0.302736562	0.014169142	3.900889948	48.7296482	0.666666667	CRA004775;PRJNA827465;Yang2025_Silkie_Wencha	0.765947595
ENSGALG00010029761	MRPL38	3	-0.080355632	0.078129035	-1.028498968	0.303715176	0.003455332	2.345873583	14.74391397	1	CRA004775;PRJNA827465;Yang2025_Silkie_Wencha	0.765947595
ENSGALG00010022656	FKBP10	3	-0.073997411	0.07178552	-1.030812496	0.302628757	0	1.324154865	0	1	CRA004775;PRJNA827465;Yang2025_Silkie_Wencha	0.765947595
ENSGALG00010026209	MED19	3	-0.072322776	0.070261605	-1.029335664	0.30332197	0	0.950170654	0	0.666666667	CRA004775;PRJNA827465;Yang2025_Silkie_Wencha	0.765947595
ENSGALG00010027292	KDM4B	3	-0.063764154	0.061917666	-1.029821656	0.303093733	0.004908936	3.481664478	42.5562109	0.666666667	CRA004775;PRJNA827465;Yang2025_Silkie_Wencha	0.765947595
ENSGALG00010024604	MFSD5	3	0.060088362	0.058269939	1.031206891	0.30244381	0	0.832524432	0	0.666666667	CRA004775;PRJNA827465;Yang2025_Silkie_Wencha	0.765947595
ENSGALG00010003165	BMP2K	3	-0.056114639	0.054528727	-1.029083982	0.303440213	0	0.972151687	0	1	CRA004775;PRJNA827465;Yang2025_Silkie_Wencha	0.765947595
ENSGALG00010021414	BUD31	3	-0.054505138	0.052931645	-1.029726897	0.303138226	0	1.476540974	0	0.666666667	CRA004775;PRJNA827465;Yang2025_Silkie_Wencha	0.765947595
ENSGALG00010029443	ENSGALG0001002944	3	-0.51522345	0.501180274	-1.02802021	0.303940321	0.586274929	10.37061278	80.71473651	0.666666667	CRA004775;PRJNA827465;Yang2025_Silkie_Wencha	0.766163701

ENSGALG00010010301	ENSGALG0001001030	2	0.761910469	0.742040631	1.026777291	0.304525345	0.97566339	8.734460899	88.5510965	1	PRJNA827465;Yang2025_Silkie_Wenchang	0.766180997
ENSGALG00010016147	FABP1	3	0.458654105	0.446639519	1.026899963	0.304467572	0.434153266	7.810843774	74.39457173	0.666666667	CRA004775;PRJNA827465;Yang2025_Silkie_Wencha	0.766180997
ENSGALG00010015963	DIPK2A	3	-0.289249493	0.281748172	-1.026624203	0.304597453	0.191676678	28.47819234	92.97708234	1	CRA004775;PRJNA827465;Yang2025_Silkie_Wencha	0.766180997
ENSGALG00010017398	SLC22A5	3	-0.269858173	0.262731459	-1.027125468	0.304361388	0.15687342	15.13495765	86.78555932	0.666666667	CRA004775;PRJNA827465;Yang2025_Silkie_Wencha	0.766180997
ENSGALG00010012967	ECRG4	3	0.15139396	0.147402364	1.027079592	0.304382988	0.012099611	2.367973734	15.53960369	0.666666667	CRA004775;PRJNA827465;Yang2025_Silkie_Wencha	0.766180997
ENSGALG00010017200	KCNQ4	3	-0.141497828	0.137798624	-1.026845002	0.304493455	0	0.868423034	0	1	CRA004775;PRJNA827465;Yang2025_Silkie_Wencha	0.766180997
ENSGALG00010004991	FAM133B	3	0.112156227	0.109154663	1.027498267	0.304185901	0.025344391	7.483461836	73.27440102	0.666666667	CRA004775;PRJNA827465;Yang2025_Silkie_Wencha	0.766180997
ENSGALG00010001535	RAP2B	3	-0.088619148	0.086237957	-1.027611862	0.304132442	0.010251165	3.549929375	43.66085101	1	CRA004775;PRJNA827465;Yang2025_Silkie_Wencha	0.766180997
ENSGALG00010022253	ALPK3	3	-0.088389324	0.086087707	-1.026735724	0.304544923	0	1.041894063	0	0.666666667	CRA004775;PRJNA827465;Yang2025_Silkie_Wencha	0.766180997
ENSGALG00010026062	ITGA4	3	0.085270145	0.083052307	1.026704113	0.304559812	0	0.899765964	0	0.666666667	CRA004775;PRJNA827465;Yang2025_Silkie_Wencha	0.766180997
ENSGALG00010009593	NCBP1	3	-0.079557808	0.077488002	-1.026711312	0.304556421	0.010126467	5.054833764	60.43391151	0.666666667	CRA004775;PRJNA827465;Yang2025_Silkie_Wencha	0.766180997
ENSGALG00010017673	WDR89	3	-0.05301146	0.051609797	-1.027158858	0.304345667	0	0.935168459	0	0.666666667	CRA004775;PRJNA827465;Yang2025_Silkie_Wencha	0.766180997
ENSGALG00010025679	ENSGALG0001002567!	2	-2.610685047	2.54351703	-1.026407536	0.304699527	9.036296318	3.269962179	69.41860654	1	PRJNA827465;Yang2025_Silkie_Wenchang	0.766219691
ENSGALG00010003796	ENSGALG0001000379(	3	-0.653253044	0.636632352	-1.026107207	0.304841054	1.013033277	12.18943448	83.59234792	0.666666667	CRA004775;PRJNA827465;Yang2025_Silkie_Wencha	0.766219691
ENSGALG00010023394	HOXB5	3	0.325182626	0.316835786	1.026344373	0.304729289	0.241047945	13.3178698	84.98258333	0.666666667	CRA004775;PRJNA827465;Yang2025_Silkie_Wencha	0.766219691
ENSGALG00010004522	TSPAN5	3	-0.251262437	0.244946107	-1.02578661	0.304992179	0.164319345	30.03566995	93.34125058	0.666666667	CRA004775;PRJNA827465;Yang2025_Silkie_Wencha	0.766219691
ENSGALG00010021439	ITPRIP	3	-0.235932886	0.229976331	-1.025900731	0.304938378	0.116932505	12.50798064	84.01020871	0.666666667	CRA004775;PRJNA827465;Yang2025_Silkie_Wencha	0.766219691
ENSGALG00010017816	PRKCH	3	0.226472068	0.22073495	1.025990978	0.304895837	0	0.517936459	0	1	CRA004775;PRJNA827465;Yang2025_Silkie_Wencha	0.766219691
ENSGALG00010010486	ENSGALG0001001048(	3	-0.187820643	0.183036629	-1.026136919	0.30482705	0	0.71670387	0	0.666666667	CRA004775;PRJNA827465;Yang2025_Silkie_Wencha	0.766219691
ENSGALG00010001717	STARD10	3	0.721589562	0.704046536	1.024917425	0.305402152	1.249081922	12.90791467	84.50563045	0.666666667	CRA004775;PRJNA827465;Yang2025_Silkie_Wencha	0.766351406
ENSGALG00010015921	SNX21	3	0.366917444	0.358093515	1.024641407	0.305532419	0.320337671	25.32175654	92.10165378	0.666666667	CRA004775;PRJNA827465;Yang2025_Silkie_Wencha	0.766351406
ENSGALG00010026078	UBE2E3	3	-0.279063867	0.272346716	-1.024663968	0.30552177	0.207322591	54.23687749	96.31247208	0.666666667	CRA004775;PRJNA827465;Yang2025_Silkie_Wencha	0.766351406
ENSGALG00010009620	TMOD1	3	-0.275897653	0.269159075	-1.025035672	0.305346356	0.13576306	5.757378738	65.26196919	0.666666667	CRA004775;PRJNA827465;Yang2025_Silkie_Wencha	0.766351406
ENSGALG00010003875	AKAP8	3	0.191869083	0.187241647	1.024713708	0.305498293	0.087961658	17.050823	88.27036091	0.666666667	CRA004775;PRJNA827465;Yang2025_Silkie_Wencha	0.766351406
ENSGALG00010013400	BBS10	3	-0.164679202	0.160618794	-1.025279787	0.30523119	0.031929576	3.342473844	40.16407926	0.666666667	CRA004775;PRJNA827465;Yang2025_Silkie_Wencha	0.766351406
ENSGALG00010008303	TEX10	3	-0.134771201	0.131500645	-1.024871028	0.305424047	0.039780827	11.0849945	81.95759142	0.666666667	CRA004775;PRJNA827465;Yang2025_Silkie_Wencha	0.766351406
ENSGALG00010017346	GM2A	3	0.128584085	0.12544026	1.025062333	0.305333777	0	1.663316289	0	0.666666667	CRA004775;PRJNA827465;Yang2025_Silkie_Wencha	0.766351406
ENSGALG00010007978	JADE1	3	0.04376048	0.042703892	1.024742197	0.305484847	0	0.954931393	0	1	CRA004775;PRJNA827465;Yang2025_Silkie_Wencha	0.766351406
ENSGALG00010007731	ENSGALG0001000773	3	-0.146253821	0.142792542	-1.024239918	0.305721969	0.015040887	2.610147238	23.37597011	0.666666667	CRA004775;PRJNA827465;Yang2025_Silkie_Wencha	0.766554871
ENSGALG00010023930	SRPRA	3	-0.119703629	0.11685798	-1.024351348	0.305669353	0.028329913	7.805072088	74.37563705	1	CRA004775;PRJNA827465;Yang2025_Silkie_Wencha	0.766554871
ENSGALG00010019998	PLEK2	2	0.951501929	0.929471879	1.023701686	0.305976199	1.358955517	4.671698409	78.59450862	1	CRA004775;PRJNA827465	0.766655531
ENSGALG00010022585	CIR1	3	0.278788863	0.272252576	1.024008172	0.305831415	0.197325398	20.88764208	90.42496041	0.666666667	CRA004775;PRJNA827465;Yang2025_Silkie_Wencha	0.766655531
ENSGALG00010016474	MAPK8IP3	3	0.220661135	0.215520776	1.023850875	0.305905716	0.109765558	10.24607303	80.48032651	0.666666667	CRA004775;PRJNA827465;Yang2025_Silkie_Wencha	0.766655531
ENSGALG00010012975	KRR1	3	-0.061424856	0.060003039	-1.023695745	0.305979006	0	0.050627212	0	1	CRA004775;PRJNA827465;Yang2025_Silkie_Wencha	0.766655531
ENSGALG00010002138	MYO7A	3	-0.308432895	0.301363602	-1.023457686	0.306091497	0.181115318	7.560646475	73.54723559	0.666666667	CRA004775;PRJNA827465;Yang2025_Silkie_Wencha	0.766665664
ENSGALG00010028432	RFXANK	3	-0.14184632	0.13858307	-1.023547251	0.306049171	0.024262317	3.194388869	37.39021508	0.666666667	CRA004775;PRJNA827465;Yang2025_Silkie_Wencha	0.766665664
ENSGALG00010005637	TOPORS	3	0.096976645	0.094789935	1.023069006	0.30627522	0	0.491727249	0	1	CRA004775;PRJNA827465;Yang2025_Silkie_Wencha	0.766989963
ENSGALG00010024400	KCNA1	2	0.562223713	0.550137872	1.021968748	0.306795692	0.471218639	4.374146452	77.13839692	1	PRJNA827465;Yang2025_Silkie_Wenchang	0.767017439
ENSGALG00010010759	ENSGALG0001001075!	3	0.553758602	0.541773101	1.022122732	0.306722816	0.689985964	11.91961158	83.22092976	0.666666667	CRA004775;PRJNA827465;Yang2025_Silkie_Wencha	0.767017439
ENSGALG00010008116	URAH	3	0.336262565	0.328921184	1.022319573	0.306629672	0.144711928	3.626141752	44.84495817	0.666666667	CRA004775;PRJNA827465;Yang2025_Silkie_Wencha	0.767017439
ENSGALG00010017136	ENSGALG0001001713(	3	0.277994217	0.271847726	1.022610049	0.306492257	0.068214336	2.75920797	27.51543117	1	CRA004775;PRJNA827465;Yang2025_Silkie_Wencha	0.767017439
ENSGALG00010018704	PGPEP1L	3	0.221405064	0.216611433	1.022130093	0.306719332	0.048267867	3.026693581	33.92129244	0.666666667	CRA004775;PRJNA827465;Yang2025_Silkie_Wencha	0.767017439
ENSGALG00010012108	PSTPIP2	3	0.193924157	0.18974765	1.022010846	0.306775767	0	0.275337043	0	1	CRA004775;PRJNA827465;Yang2025_Silkie_Wencha	0.767017439
ENSGALG00010008636	AGTPBP1	3	0.152598735	0.149178313	1.022928417	0.306341692	0	0.702738874	0	0.666666667	CRA004775;PRJNA827465;Yang2025_Silkie_Wencha	0.767017439
ENSGALG00010010882	CEP350	3	-0.076712622	0.075015993	-1.0226169	0.306489016	0	1.363796739	0	0.666666667	CRA004775;PRJNA827465;Yang2025_Silkie_Wencha	0.767017439
ENSGALG00010024035	OSBPL5	3	0.063141455	0.061762554	1.02232584	0.306626707	0	0.772421445	0	0.666666667	CRA004775;PRJNA827465;Yang2025_Silkie_Wencha	0.767017439
ENSGALG00010021700	FLCN	3	-0.062936163	0.061587455	-1.021899065	0.306828675	0.00287969	2.564429031	22.00992984	1	CRA004775;PRJNA827465;Yang2025_Silkie_Wencha	0.767017439
ENSGALG00010016651	ENSGALG0001001665	3	-0.13594298	0.133058903	-1.02167519	0.306934657	0.025515613	4.021547538	50.26790108	0.666666667	CRA004775;PRJNA827465;Yang2025_Silkie_Wencha	0.767146742
ENSGALG00010015876	SKP1	3	-0.30766429	0.301195005	-1.021478728	0.307027682	0.248732186	39.38450995	94.92186141	1	CRA004775;PRJNA827465;Yang2025_Silkie_Wencha	0.767224474
ENSGALG00010016952	GINS1	3	0.224640623	0.219938272	1.021380324	0.307074284	0.080418553	4.623204704	56.73996442	1	CRA004775;PRJNA827465;Yang2025_Silkie_Wencha	0.767224474
ENSGALG00010016215	SPATA17	2	0.520124058	0.509649594	1.020552287	0.307466605	0.400742827	4.298461195	76.7358607	1	PRJNA827465;Yang2025_Silkie_Wenchang	0.767662074
ENSGALG00010022249	DRD4	2	-0.48096027	0.471236192	-1.020635253	0.307427281	0	0.464823653	0	1	CRA004775;PRJNA827465	0.767662074
ENSGALG00010024225	BCDIN3D	3	-0.092652483	0.090757062	-1.020884556	0.307309137	0	0.460552087	0	0.666666667	CRA004775;PRJNA827465;Yang2025_Silkie_Wencha	0.767662074
ENSGALG00010015985	TIMM8A	3	-0.079517814	0.077911993	-1.020610698	0.307438919	0	1.468248542	0	0.666666667	CRA004775;PRJNA827465;Yang2025_Silkie_Wencha	0.767662074
ENSGALG00010011931	ENSGALG0001001193	3	-1.765096349	1.740771929	-1.013973353	0.310595468	7.761028446	13.69724796	85.39852673	0.666666667	CRA004775;PRJNA827465;Yang2025_Silkie_Wencha	0.767829909
ENSGALG00010023229	HKDC1	2	0.964866351	0.958781464	1.00634648	0.314248929	1.579640141	6.89507047	85.49688499	1	CRA004775;PRJNA827465	0.767829909
ENSGALG00010011050	SYT10	2	-0.843829014	0.828446518	-1.01856788	0.30840816	1.215083675	8.675881984	88.47379434	1	PRJNA827465;Yang2025_Silkie_Wenchang	0.767829909
ENSGALG00010028695	VSIG8	2	-0.790140078	0.77533335	-1.019097241	0.308156804	0	0.445842475	0	1	PRJNA827465;Yang2025_Silkie_Wenchang	0.767829909
ENSGALG00010014882	XPNPEP2	3	0.721116168	0.709876009	1.015833975	0.309708454	1.203814633	10.48595332	80.92686532	0.666666667	CRA004775;PRJNA827465;Yang2025_Silkie_Wencha	0.767829909
ENSGALG00010025558	GALNT3	3	0.653568505	0.644934406	1.013387562	0.310875078	1.055349788	19.12873551	89.54452583	0.666666667	CRA004775;PRJNA827465;Yang2025_Silkie_Wencha	0.767829909
ENSGALG00010002415	PCDH17	2	0.602210421	0.592354628	1.016638333	0.309325511	0.550581725	4.303677047	76.76405573	1	PRJNA827465;Yang2025_Silkie_Wenchang	0.767829909
ENSGALG00010009123	ENSGALG0001000912	3	0.554465488	0.546259733	1.01502171	0.310095479	0.858712258	62.87573976	96.81912291	0.666666667	CRA004775;PRJNA827465;Yang2025_Silkie_Wencha	0.767829909
ENSGALG00010024168	BIRC7	2	0.544411817	0.538493342	1.010990804	0.312020831	0.403330363	3.24616677	69.19443544	1	PRJNA827465;Yang2025_Silkie_Wenchang	0.767829909
ENSGALG00010008091	ENSGALG0001000809	3	0.542648276	0.538303333	1.008071552	0.313420114	0.722905007	14.50248933	86.20926412	0.666666667	CRA004775;PRJNA827465;Yang2025_Silkie_Wencha	0.767829909
ENSGALG00010014031	RPL22L1	3	-0.507662564	0.49855797	-1.018261856	0.308553531	0.692410593	38.41577858	94.79380589	0.666666667	CRA004775;PRJNA827465;Yang2025_Silkie_Wencha	0.767829909
ENSGALG00010000138	ENSGALG0001000013	3	0.483623087	0.477829977	1.01212379	0.311478869	0.438314923	5.915307055	66.18941365	0.666666667	CRA004775;PRJNA827465;Yang2025_Silkie_Wencha	0.767829909
ENSGALG00010021840	ENSGALG0001002184(	3	0.465498248	0.461430487	1.008815545	0.313063105	0.498999901	9.599825762	79.16628854	0.666666667	CRA004775;PRJNA827465;Yang2025_Silkie_Wencha	0.767829909
ENSGALG00010016928	RGS11	3	0.4563508	0.453148171	1.007067509	0.313902333	0.502990128	11.02373758	81.857333	0.666666667	CRA004775;PRJNA827465;Yang2025_Silkie_Wencha	0.767829909
ENSGALG00010002539	SNAP91	3	0.403154739	0.396809394	1.015990913	0.309633714	0.32782913	6.80785273	70.62216121	0.666666667	CRA004775;PRJNA827465;Yang2025_Silkie_Wencha	0.767829909
ENSGALG00010000166	PFDN6	3	0.391554211	0.389003297	1.006557564	0.314147436	0.410486692	27.44936766	92.71385766	1	CRA004775;PRJNA827465;Yang2025_Silkie_Wencha	0.767829909
ENSGALG00010028583	USHBP1	3	0.379099687	0.375168605	1.010478172	0.312266252	0.364902624	20.28679158	90.14136862	1	CRA004775;PRJNA827465;Yang2025_Silkie_Wencha	0.767829909
ENSGALG00010015141	PFDN4	3	-0.350619223	0.346668396	-1.01139656	0.311826667	0.322019806	24.40215251	91.80400172	0.666666667	CRA004775;PRJNA827465;Yang2025_Silkie_Wencha	0.767829909
ENSGALG00010004565	ENSGALG0001000456	3	0.346459837	0.341397513	1.014828238	0.310187711	0.074007527	2.497208597	19.91057524	0.666666667	CRA004775;PRJNA827465;Yang2025_Silkie_Wencha	0.767829909
ENSGALG00010015250	RORB	2	0.342497503	0.338709104	1.011184818	0.311927981	0	0.227879605	0	1	PRJNA827465;Yang2025_Silkie_Wenchang	0.767829909
ENSGALG00010007643	COTL1	3	-0.322123881	0.317757981	-1.013739701	0.310706975	0.26641174	37.50821102	94.6678342	0.666666667	CRA004775;PRJNA827465;Yang2025_Silkie_Wencha	0.767829909
ENSGALG00010022764	MFAP5	3	-0.322055167	0.319725361	-1.007286898	0.313796924	0.229401421	12.74720597	84.31028725	0.666666667	CRA004775;PRJNA827465;Yang2025_Silkie_Wencha	0.767829909
ENSGALG00010002661	ACBD7	3	0.316941304	0.313582879	1.010709846	0.312155323	0.223704168	8.626700911	76.81616622	0.666666667	CRA004775;PRJNA827465;Yang2025_Silkie_Wencha	0.767829909
ENSGALG00010029158	CD7	3	-0.291311913	0.287373972	-1.013703193	0.3107244	0	1.960209756	0	0.666666667	CRA004775;PRJNA827465;Yang2025_Silkie_Wencha	0.767829909
ENSGALG00010028165	NFIC	3	0.273879927	0.269599611	1.015876566	0.309688169	0.165361934	8.85678738	77.41844854	0.666666667	CRA004775;PRJNA827465;Yang2025_Silkie_Wencha	0.767829909
ENSGALG00010005048	ROCK1	3	0.269298682	0.264614094	1.017703472	0.308818897	0.164324051	10.69276668	81.29576695	0.666666667	CRA004775;PRJNA827465;Yang2025_Silkie_Wencha	0.767829909
ENSGALG00010029699	ANKRD13B	3	0.266405259	0.262952583	1.013130414	0.310997873	0.173000384	16.11246501	87.58725001	0.666666667	CRA004775;PRJNA827465;Yang2025_Silkie_Wencha	0.767829909
ENSGALG00010021553	SLC39A9	3	-0.258937271	0.255827479	-1.012155819	0.311463557	0.164431623	28.88734782	93.07655375	0.666666667	CRA004775;PRJNA827465;Yang2025_Silkie_Wencha	0.767829909
ENSGALG00010016441	ENSGALG0001001644	3	-0.258781571	0.254057545	-1.018594315	0.308395604	0	1.81215275	0	0.666666667	CRA004775;PRJNA827465;Yang2025_Silkie_Wencha	0.767829909
ENSGALG00010026044	CERKL	3	-0.258595535	0.255830902	-1.010806488	0.312109057	0.096181149	4.057134641	50.70412553	0.666666667	CRA004775;PRJNA827465;Yang2025_Silkie_Wencha	0.767829909
ENSGALG00010022743	ZNF326	3	0.256148305	0.253634826	1.009909837	0.31253849	0.156087129	13.08309324	84.71309527	0.666666667	CRA004775;PRJNA827465;Yang2025_Silkie_Wencha	0.767829909
ENSGALG00010016304	EIF3J	3	0.239246214	0.237141824	1.008873973	0.313035079	0.138504714	22.58684043	91.14528654	0.666666667	CRA004775;PRJNA827465;Yang2025_Silkie_Wencha	0.767829909
ENSGALG00010024392	CHMP4B	3	0.232902769	0.229402289	1.015259136	0.309982318	0.142328769	29.48997144	93.21803344	0.666666667	CRA004775;PRJNA827465;Yang2025_Silkie_Wencha	0.767829909
ENSGALG00010025334	RHOF	3	0.23249476	0.229037757	1.015093591	0.310061217	0.058296008	3.137196631	36.2488159	0.666666667	CRA004775;PRJNA827465;Yang2025_Silkie_Wencha	0.767829909
ENSGALG00010012303	CA9	3	0.231453641	0.229452977	1.008719277	0.313109284	0.102253541	7.906015248	74.70280619	0.666666667	CRA004775;PRJNA827465;Yang2025_Silkie_Wencha	0.767829909
ENSGALG00010001419	KCND2	2	0.226659528	0.223840402	1.012594359	0.311253955	0	0.515645029	0	1	PRJNA827465;Yang2025_Silkie_Wenchang	0.767829909
ENSGALG00010023612	GPT2	3	0.226360318	0.224012251	1.010481872	0.31226448	0.075683474	4.065939241	50.81087342	0.666666667	CRA004775;PRJNA827465;Yang2025_Silkie_Wencha	0.767829909
ENSGALG00010028768	NTNG2	3	0.225479914	0.221920381	1.016039682	0.30961049	0.091456214	5.785630196	65.43159635	0.666666667	CRA004775;PRJNA827465;Yang2025_Silkie_Wencha	0.767829909
ENSGALG00010020810	IFITM5	3	0.223554004	0.220039711	1.015971176	0.309643113	0.115187764	10.19630084	80.38504324	0.666666667	CRA004775;PRJNA827465;Yang2025_Silkie_Wencha	0.767829909
ENSGALG00010016449	FMR1	3	0.221961581	0.219869774	1.009513845	0.312728265	0.118750902	12.28510133	83.72011801	0.666666667	CRA004775;PRJNA827465;Yang2025_Silkie_Wencha	0.767829909
ENSGALG00010020533	ENSGALG0001002053	3	0.215122301	0.211887108	1.015268476	0.309977867	0.096675113	7.457024516	73.179651	0.666666667	CRA004775;PRJNA827465;Yang2025_Silkie_Wencha	0.767829909
ENSGALG00010003734	ZC3H13	3	0.212235811	0.210949993	1.006095368	0.314369697	0.104511026	10.33380004	80.64603542	0.666666667	CRA004775;PRJNA827465;Yang2025_Silkie_Wencha	0.767829909
ENSGALG00010022875	SPON1	3	-0.211100728	0.209495988	-1.007660006	0.313617711	0	0.551013956	0	1	CRA004775;PRJNA827465;Yang2025_Silkie_Wencha	0.767829909
ENSGALG00010028849	ENSGALG0001002884	3	0.211041659	0.207561955	1.016764653	0.309265401	0	0.092839288	0	1	CRA004775;PRJNA827465;Yang2025_Silkie_Wencha	0.767829909
ENSGALG00010027909	TIMP4	3	-0.203026279	0.199836572	-1.015961579	0.309647683	0	0.05710223	0	1	CRA004775;PRJNA827465;Yang2025_Silkie_Wencha	0.767829909
ENSGALG00010027740	MARCHF2	3	-0.199487552	0.196194851	-1.016782808	0.309256762	0.099079303	16.75119767	88.0605552	0.666666667	CRA004775;PRJNA827465;Yang2025_Silkie_Wencha	0.767829909
ENSGALG00010008274	NFAT5	3	0.198100809	0.196782425	1.006699706	0.314079103	0.068844476	4.967366592	59.73721764	0.666666667	CRA004775;PRJNA827465;Yang2025_Silkie_Wencha	0.767829909
ENSGALG00010019962	SNX8	3	-0.195197366	0.193182111	-1.01043189	0.312288416	0.086253401	10.44930579	80.85997251	0.666666667	CRA004775;PRJNA827465;Yang2025_Silkie_Wencha	0.767829909
ENSGALG00010021013	FAP	3	-0.193681947	0.190909328	-1.014523223	0.310333154	0.056890106	4.571976236	56.2552407	0.666666667	CRA004775;PRJNA827465;Yang2025_Silkie_Wencha	0.767829909
ENSGALG00010027715	NINJ1	3	-0.189431895	0.186562386	-1.015380962	0.309924265	0.067474591	8.731911608	77.09550795	0.666666667	CRA004775;PRJNA827465;Yang2025_Silkie_Wencha	0.767829909
ENSGALG00010009007	TMEM65	3	-0.183541718	0.18207708	-1.00804405	0.313433316	0.086224975	19.58675552	89.78901841	0.666666667	CRA004775;PRJNA827465;Yang2025_Silkie_Wencha	0.767829909
ENSGALG00010008103	RBPMS	3	0.18150434	0.178211073	1.018479582	0.308450099	0.073045008	11.83677778	83.10350979	0.666666667	CRA004775;PRJNA827465;Yang2025_Silkie_Wencha	0.767829909
ENSGALG00010001197	CEP76	3	-0.180175641	0.17837992	-1.010066829	0.312463273	0.068011171	7.696051304	74.01264725	0.666666667	CRA004775;PRJNA827465;Yang2025_Silkie_Wencha	0.767829909
ENSGALG00010028318	NACC2	3	-0.177664509	0.176460591	-1.006822588	0.314020038	0.048617841	4.389553565	54.43728	0.666666667	CRA004775;PRJNA827465;Yang2025_Silkie_Wencha	0.767829909
ENSGALG00010023235	ENSGALG0001002323	3	0.172007108	0.170411546	1.009362994	0.312800579	0	0.775755538	0	1	CRA004775;PRJNA827465;Yang2025_Silkie_Wencha	0.767829909
ENSGALG00010003366	CASD1	3	-0.168107756	0.166935286	-1.007023503	0.31392348	0.065437614	12.46386769	83.95361657	0.666666667	CRA004775;PRJNA827465;Yang2025_Silkie_Wencha	0.767829909
ENSGALG00010016913	BRCC3	3	-0.16738251	0.165719977	-1.010032183	0.312479871	0.061461975	8.262910233	75.79545289	0.666666667	CRA004775;PRJNA827465;Yang2025_Silkie_Wencha	0.767829909
ENSGALG00010026854	SMPD2	3	0.166577894	0.16363124	1.018007893	0.308674205	0.054862279	6.988352309	71.38095059	0.666666667	CRA004775;PRJNA827465;Yang2025_Silkie_Wencha	0.767829909
ENSGALG00010022928	RNF111	3	0.162313735	0.159859043	1.015355353	0.309936467	0.05086258	6.135529177	67.40297467	0.666666667	CRA004775;PRJNA827465;Yang2025_Silkie_Wencha	0.767829909
ENSGALG00010008291	PTGFRN	3	-0.158706053	0.15758695	-1.007101493	0.313886004	0.048589623	9.165333954	78.17864564	0.666666667	CRA004775;PRJNA827465;Yang2025_Silkie_Wencha	0.767829909
ENSGALG00010027346	SIN3B	3	-0.15321623	0.15102774	-1.014490645	0.310348691	0.055941191	21.48849454	90.69269373	0.666666667	CRA004775;PRJNA827465;Yang2025_Silkie_Wencha	0.767829909
ENSGALG00010017590	ENSGALG0001001759(	3	0.150929379	0.149547908	1.00923765	0.312860674	0.019693493	2.725500401	26.6189798	0.666666667	CRA004775;PRJNA827465;Yang2025_Silkie_Wencha	0.767829909
ENSGALG00010013792	FGF10	3	0.146754555	0.145531143	1.008406535	0.313259337	0	1.76388719	0	0.666666667	CRA004775;PRJNA827465;Yang2025_Silkie_Wencha	0.767829909
ENSGALG00010024671	KCTD20	3	-0.146712017	0.144713169	-1.013812476	0.310672241	0.039580005	9.380308888	78.6787405	0.666666667	CRA004775;PRJNA827465;Yang2025_Silkie_Wencha	0.767829909
ENSGALG00010007162	ENSGALG0001000716	3	-0.141570224	0.139430959	-1.015342828	0.309942435	0	0.991963319	0	1	CRA004775;PRJNA827465;Yang2025_Silkie_Wencha	0.767829909
ENSGALG00010008154	KCNQ3	3	-0.139068975	0.138054293	-1.007349873	0.313766671	0	0.222750513	0	0.666666667	CRA004775;PRJNA827465;Yang2025_Silkie_Wencha	0.767829909
ENSGALG00010013465	CALD1	3	0.135826204	0.133487222	1.017522142	0.308905104	0.024725545	3.571955269	44.00825739	0.666666667	CRA004775;PRJNA827465;Yang2025_Silkie_Wencha	0.767829909
ENSGALG00010018339	FAM76A	3	-0.131263245	0.130449138	-1.006240806	0.314299747	0.034877483	8.835839753	77.36491317	0.666666667	CRA004775;PRJNA827465;Yang2025_Silkie_Wencha	0.767829909
ENSGALG00010017895	MNAT1	3	0.130195056	0.128345838	1.014408085	0.310388068	0.033486943	7.798517683	74.35410059	1	CRA004775;PRJNA827465;Yang2025_Silkie_Wencha	0.767829909
ENSGALG00010029950	RFFL	3	-0.129730822	0.127850426	-1.01470778	0.310245144	0.023604614	3.949648721	49.36258535	0.666666667	CRA004775;PRJNA827465;Yang2025_Silkie_Wencha	0.767829909
ENSGALG00010003068	BRD2	3	0.129238129	0.127069658	1.017065213	0.309122408	0.030494155	5.390022948	62.89440659	0.666666667	CRA004775;PRJNA827465;Yang2025_Silkie_Wencha	0.767829909
ENSGALG00010018179	PRADC1	3	-0.129145802	0.12698852	-1.01698801	0.309159133	0.022266995	3.50684626	42.96870031	1	CRA004775;PRJNA827465;Yang2025_Silkie_Wencha	0.767829909
ENSGALG00010013152	TBXAS1	3	-0.127800901	0.127028376	-1.006081516	0.314376359	0	1.20543592	0	1	CRA004775;PRJNA827465;Yang2025_Silkie_Wencha	0.767829909
ENSGALG00010014477	ITPR2	3	-0.12590551	0.124838238	-1.008549238	0.313190862	0.020710688	3.582173049	44.16796808	0.666666667	CRA004775;PRJNA827465;Yang2025_Silkie_Wencha	0.767829909
ENSGALG00010028321	RHBG	3	-0.125783736	0.123750619	-1.016429149	0.309425071	0	0.280224949	0	1	CRA004775;PRJNA827465;Yang2025_Silkie_Wencha	0.767829909
ENSGALG00010017742	DGLUCY	3	-0.124781035	0.123375144	-1.011395253	0.311827292	0.004832516	2.188107947	8.596831218	0.666666667	CRA004775;PRJNA827465;Yang2025_Silkie_Wencha	0.767829909
ENSGALG00010013617	PIK3C2G	3	0.124776451	0.123084281	1.013748056	0.310702987	0	0.642085875	0	1	CRA004775;PRJNA827465;Yang2025_Silkie_Wencha	0.767829909
ENSGALG00010014917	EIF3D	3	-0.124689441	0.123724649	-1.007797893	0.313551498	0.026620893	4.941012626	59.52246733	0.666666667	CRA004775;PRJNA827465;Yang2025_Silkie_Wencha	0.767829909
ENSGALG00010027510	RHOC	3	-0.124245864	0.122633616	-1.013146867	0.310990015	0	1.193495766	0	0.666666667	CRA004775;PRJNA827465;Yang2025_Silkie_Wencha	0.767829909
ENSGALG00010029221	AXIN2	3	0.123514208	0.122550989	1.007859738	0.313521803	0.010813388	2.562733149	21.95832013	0.666666667	CRA004775;PRJNA827465;Yang2025_Silkie_Wencha	0.767829909
ENSGALG00010017625	YBX1	3	0.123131813	0.122019566	1.009115314	0.312919334	0.030518944	6.764491914	70.43384743	0.666666667	CRA004775;PRJNA827465;Yang2025_Silkie_Wencha	0.767829909
ENSGALG00010014861	MED21	3	-0.120692732	0.118791314	-1.016006374	0.309626351	0.01811382	3.269861138	38.83532311	1	CRA004775;PRJNA827465;Yang2025_Silkie_Wencha	0.767829909
ENSGALG00010005362	ATP1B1	3	-0.115599819	0.113759436	-1.016177844	0.309544705	0.023607538	6.50693515	69.26356335	0.666666667	CRA004775;PRJNA827465;Yang2025_Silkie_Wencha	0.767829909
ENSGALG00010025541	TTC21B	3	0.111429386	0.109592922	1.016757145	0.309268974	0.019292503	4.548510149	56.02955837	0.666666667	CRA004775;PRJNA827465;Yang2025_Silkie_Wencha	0.767829909
ENSGALG00010015803	SYF2	3	0.109837669	0.108376552	1.013481853	0.31083006	0.019100989	4.507509697	55.62960183	0.666666667	CRA004775;PRJNA827465;Yang2025_Silkie_Wencha	0.767829909
ENSGALG00010011231	PLBD1	3	-0.104873305	0.104138792	-1.007053213	0.313909203	0.005615965	2.378363941	15.90858045	0.666666667	CRA004775;PRJNA827465;Yang2025_Silkie_Wencha	0.767829909
ENSGALG00010005252	KLHL14	3	-0.104693322	0.103222216	-1.014251836	0.3104626	0	0.349272952	0	0.666666667	CRA004775;PRJNA827465;Yang2025_Silkie_Wencha	0.767829909
ENSGALG00010029244	HLF	3	0.104423566	0.103246266	1.01140283	0.311823667	0.010911616	2.848146989	29.77890509	1	CRA004775;PRJNA827465;Yang2025_Silkie_Wencha	0.767829909
ENSGALG00010023136	MDK	3	-0.10381304	0.102564024	-1.012177916	0.311452993	0.002986744	2.16375332	7.568021667	0.666666667	CRA004775;PRJNA827465;Yang2025_Silkie_Wencha	0.767829909
ENSGALG00010025996	CUL3	3	-0.100939225	0.100270024	-1.006673996	0.314091462	0.016785104	5.14325555	61.11412352	0.666666667	CRA004775;PRJNA827465;Yang2025_Silkie_Wencha	0.767829909
ENSGALG00010016121	SLC35C2	3	0.100512914	0.099333155	1.011876794	0.311596966	0.017912007	5.24189563	61.84586376	0.666666667	CRA004775;PRJNA827465;Yang2025_Silkie_Wencha	0.767829909
ENSGALG00010021293	PCNX1	3	-0.098507088	0.096843321	-1.017179983	0.309067816	0.015952431	5.388099788	62.88116259	0.666666667	CRA004775;PRJNA827465;Yang2025_Silkie_Wencha	0.767829909
ENSGALG00010009164	TMEM128	3	-0.097455674	0.095676818	-1.018592346	0.30839654	0.013551718	3.96608765	49.57247099	0.666666667	CRA004775;PRJNA827465;Yang2025_Silkie_Wencha	0.767829909
ENSGALG00010017522	XXYLT1	3	-0.097372289	0.096227985	-1.011891591	0.31158989	0.011591951	3.065837903	34.76497901	1	CRA004775;PRJNA827465;Yang2025_Silkie_Wencha	0.767829909
ENSGALG00010018486	ENSGALG0001001848(	3	0.096939535	0.096241549	1.007252441	0.313813478	0.01360177	3.593260902	44.34025097	0.666666667	CRA004775;PRJNA827465;Yang2025_Silkie_Wencha	0.767829909
ENSGALG00010002837	DNMT1	3	-0.094326279	0.09309693	-1.013205039	0.310962234	0.017462323	6.787388216	70.5335847	0.666666667	CRA004775;PRJNA827465;Yang2025_Silkie_Wencha	0.767829909
ENSGALG00010023869	DOCK7	3	0.091037741	0.090112289	1.010269988	0.312365956	0.012199956	4.028764447	50.35698844	0.666666667	CRA004775;PRJNA827465;Yang2025_Silkie_Wencha	0.767829909
ENSGALG00010002952	NBAS	3	-0.089056427	0.088314122	-1.008405277	0.313259941	0.012984146	5.023720721	60.18886974	0.666666667	CRA004775;PRJNA827465;Yang2025_Silkie_Wencha	0.767829909
ENSGALG00010014018	DPYSL3	3	-0.08571411	0.084634535	-1.01275572	0.311176856	0	1.321945587	0	0.666666667	CRA004775;PRJNA827465;Yang2025_Silkie_Wencha	0.767829909
ENSGALG00010025280	HSPB11	3	0.085704918	0.08435636	1.01598644	0.309635844	0	0.133434102	0	1	CRA004775;PRJNA827465;Yang2025_Silkie_Wencha	0.767829909
ENSGALG00010024460	IMP4	3	-0.083627761	0.082610542	-1.012313425	0.311388218	0	0.704029889	0	1	CRA004775;PRJNA827465;Yang2025_Silkie_Wencha	0.767829909
ENSGALG00010028368	TIMM13	3	-0.082932274	0.082033471	-1.010956543	0.31203723	0	0.026816752	0	1	CRA004775;PRJNA827465;Yang2025_Silkie_Wencha	0.767829909
ENSGALG00010010799	DARS2	3	-0.082034533	0.080906725	-1.013939598	0.310611575	0.00666828	2.98543197	33.00801961	0.666666667	CRA004775;PRJNA827465;Yang2025_Silkie_Wencha	0.767829909
ENSGALG00010007942	PGRMC2	3	-0.081116985	0.080227353	-1.011088879	0.311973892	0.01141974	5.461755611	63.38173762	0.666666667	CRA004775;PRJNA827465;Yang2025_Silkie_Wencha	0.767829909
ENSGALG00010018923	VPS54	3	0.080686741	0.080152644	1.006663499	0.314096508	0.007243162	3.112862075	35.75044599	0.666666667	CRA004775;PRJNA827465;Yang2025_Silkie_Wencha	0.767829909
ENSGALG00010013458	LAMA4	3	-0.075487624	0.074408795	-1.01449867	0.310344864	5.42835E-07	2.000045976	0.002298747	0.666666667	CRA004775;PRJNA827465;Yang2025_Silkie_Wencha	0.767829909
ENSGALG00010018275	ZNF639	3	-0.073577511	0.073121751	-1.006232886	0.314303556	0.005518082	2.788510886	28.27713136	0.666666667	CRA004775;PRJNA827465;Yang2025_Silkie_Wencha	0.767829909
ENSGALG00010001691	CTPS2	3	-0.070232683	0.069679551	-1.007938225	0.313484119	0	0.269294478	0	0.666666667	CRA004775;PRJNA827465;Yang2025_Silkie_Wencha	0.767829909
ENSGALG00010005377	EMCN	3	-0.069909874	0.068733524	-1.017114646	0.309098894	0.004130397	2.74027932	27.01473951	0.666666667	CRA004775;PRJNA827465;Yang2025_Silkie_Wencha	0.767829909
ENSGALG00010029401	HIC1	3	0.068811998	0.068003166	1.011894029	0.311588725	0	0.440352824	0	1	CRA004775;PRJNA827465;Yang2025_Silkie_Wencha	0.767829909
ENSGALG00010009435	GTDC1	3	0.068573158	0.06760938	1.014255091	0.310461047	0	0.25261957	0	1	CRA004775;PRJNA827465;Yang2025_Silkie_Wencha	0.767829909
ENSGALG00010013444	OSBPL8	3	0.068568781	0.067967314	1.00884936	0.313046884	0	0.843855742	0	0.666666667	CRA004775;PRJNA827465;Yang2025_Silkie_Wencha	0.767829909
ENSGALG00010009100	ENSGALG0001000910(	3	-0.068279823	0.067459936	-1.012153694	0.311464573	0	1.114102052	0	0.666666667	CRA004775;PRJNA827465;Yang2025_Silkie_Wencha	0.767829909
ENSGALG00010012175	PACSIN2	3	0.067441696	0.066507442	1.014047362	0.310560153	0.002590975	2.411696199	17.07081512	1	CRA004775;PRJNA827465;Yang2025_Silkie_Wencha	0.767829909
ENSGALG00010021296	GATC	3	-0.062098029	0.061306292	-1.012914455	0.311101024	0	0.062993933	0	1	CRA004775;PRJNA827465;Yang2025_Silkie_Wencha	0.767829909
ENSGALG00010001395	GNAL	3	-0.060420022	0.059487118	-1.015682455	0.309780626	0	0.456656576	0	0.666666667	CRA004775;PRJNA827465;Yang2025_Silkie_Wencha	0.767829909
ENSGALG00010017600	MXD4	3	-0.058915984	0.058228473	-1.011807128	0.311630281	0.002661963	2.627627807	23.88571947	0.666666667	CRA004775;PRJNA827465;Yang2025_Silkie_Wencha	0.767829909
ENSGALG00010021693	PPA1	3	-0.055658602	0.055217346	-1.007991249	0.313458663	0.000972785	2.090529818	4.330472456	0.666666667	CRA004775;PRJNA827465;Yang2025_Silkie_Wencha	0.767829909
ENSGALG00010024338	NDUFA9	3	-0.055470607	0.054879922	-1.010763237	0.312129763	0	0.026792064	0	1	CRA004775;PRJNA827465;Yang2025_Silkie_Wencha	0.767829909
ENSGALG00010022571	RIPOR1	3	0.050676989	0.049856667	1.016453624	0.309413421	0	0.109880213	0	0.666666667	CRA004775;PRJNA827465;Yang2025_Silkie_Wencha	0.767829909
ENSGALG00010015811	CTNNBL1	3	-0.048919363	0.048572174	-1.007147901	0.313863705	0	1.259248352	0	1	CRA004775;PRJNA827465;Yang2025_Silkie_Wencha	0.767829909
ENSGALG00010028500	VPS45	3	-0.04832721	0.047701756	-1.013111765	0.311006779	0	0.953047218	0	1	CRA004775;PRJNA827465;Yang2025_Silkie_Wencha	0.767829909
ENSGALG00010016366	KIF3A	3	0.048036228	0.04759325	1.009307587	0.312827143	0	1.055091198	0	0.666666667	CRA004775;PRJNA827465;Yang2025_Silkie_Wencha	0.767829909
ENSGALG00010024457	CHD6	3	-0.046099018	0.04519381	-1.020029484	0.307714478	0	0.496996568	0	1	CRA004775;PRJNA827465;Yang2025_Silkie_Wencha	0.767829909
ENSGALG00010004610	TGS1	3	0.045005734	0.044643505	1.008113816	0.313399826	0	0.805355546	0	0.666666667	CRA004775;PRJNA827465;Yang2025_Silkie_Wencha	0.767829909
ENSGALG00010028377	PDCL	3	-0.041206688	0.040715796	-1.012056555	0.311511013	0	1.661018474	0	1	CRA004775;PRJNA827465;Yang2025_Silkie_Wencha	0.767829909
ENSGALG00010010153	ENSGALG0001001015	3	0.28305982	0.281454927	1.005702133	0.314558877	0	1.695733396	0	0.666666667	CRA004775;PRJNA827465;Yang2025_Silkie_Wencha	0.767927291

ENSGALG00010008486	CTU2	3	-0.194122706	0.193030162	-1.005659962	0.314579169	0.075719857	6.381206682	68.6579655	0.666666667	CRA004775;PRJNA827465;Yang2025_Silkie_Wencha	0.767927291
ENSGALG00010026536	BCR	3	-0.049504678	0.049221954	-1.005743875	0.314538792	0	0.684105964	0	1	CRA004775;PRJNA827465;Yang2025_Silkie_Wencha	0.767927291
ENSGALG00010016559	ROGDI	3	-0.166875672	0.165960988	-1.005511443	0.314650642	0.054568079	6.789447415	70.54252169	0.666666667	CRA004775;PRJNA827465;Yang2025_Silkie_Wencha	0.767969175
ENSGALG00010023840	SMAGP	3	-0.319833412	0.318210773	-1.005099259	0.314849057	0.251115803	12.16564792	83.5602673	0.666666667	CRA004775;PRJNA827465;Yang2025_Silkie_Wencha	0.768055697
ENSGALG00010015541	RASEF	2	0.253901941	0.252607547	1.005124127	0.314837084	0	0.005898783	0	1	PRJNA827465;Yang2025_Silkie_Wenchang	0.768055697
ENSGALG00010008226	COX20	3	-0.076929787	0.076538666	-1.00511011	0.314843833	0	0.04256846	0	1	CRA004775;PRJNA827465;Yang2025_Silkie_Wencha	0.768055697
ENSGALG00010018613	PADI3	3	0.306372045	0.305009612	1.004466853	0.315153641	0.184113661	6.016864219	66.76009417	0.666666667	CRA004775;PRJNA827465;Yang2025_Silkie_Wencha	0.768422277
ENSGALG00010012297	ABLIM3	3	0.183034959	0.1822232	1.004454751	0.315159471	0.060213457	5.784353321	65.4239655	0.666666667	CRA004775;PRJNA827465;Yang2025_Silkie_Wencha	0.768422277
ENSGALG00010004240	ENSGALG0001000424	3	-0.164526283	0.163797592	-1.004448729	0.315162373	0.048402057	5.354088155	62.64536664	0.666666667	CRA004775;PRJNA827465;Yang2025_Silkie_Wencha	0.768422277
ENSGALG00010029765	KCNJ16	3	0.45215783	0.450255819	1.004224291	0.315270516	0.430175142	7.672412675	73.93258047	0.666666667	CRA004775;PRJNA827465;Yang2025_Silkie_Wencha	0.768553419
ENSGALG00010013214	RAI14	3	0.181168044	0.180438989	1.004040446	0.315359119	0.059678194	5.795677845	65.49152569	0.666666667	CRA004775;PRJNA827465;Yang2025_Silkie_Wencha	0.768636886
ENSGALG00010012154	CWC27	3	0.274326597	0.27341051	1.003350593	0.315691733	0.156917	6.892268163	70.98197643	0.666666667	CRA004775;PRJNA827465;Yang2025_Silkie_Wencha	0.769107272
ENSGALG00010006273	ARL13B	3	0.219738412	0.219035993	1.003206868	0.31576106	0.116556194	11.56684166	82.70919531	0.666666667	CRA004775;PRJNA827465;Yang2025_Silkie_Wencha	0.769107272
ENSGALG00010011006	TMEM64	3	-0.21010461	0.209436723	-1.003188968	0.315769695	0.110752873	23.20575677	91.38144892	1	CRA004775;PRJNA827465;Yang2025_Silkie_Wencha	0.769107272
ENSGALG00010019291	ADD2	3	0.169116465	0.169166261	1.003252446	0.315739074	0	1.246737272	0	0.666666667	CRA004775;PRJNA827465;Yang2025_Silkie_Wencha	0.769107272
ENSGALG00010007962	ADCY8	3	0.168453739	0.167959226	1.00294424	0.315887765	0	1.844694212	0	0.666666667	CRA004775;PRJNA827465;Yang2025_Silkie_Wencha	0.769262335
ENSGALG00010020659	RAD51B	3	-0.158359723	0.157924295	-1.002757195	0.315978026	0.03726821	3.992379546	49.90456251	0.666666667	CRA004775;PRJNA827465;Yang2025_Silkie_Wencha	0.769349633
ENSGALG00010009833	SOD3	3	-0.169241871	0.168796206	-1.002640255	0.316034466	0.038941299	3.690034587	45.79996602	0.666666667	CRA004775;PRJNA827465;Yang2025_Silkie_Wencha	0.769354565
ENSGALG00010005564	TM9SF2	3	-0.260258673	0.259635318	-1.002504881	0.31609981	0.185522973	38.49614586	94.80467471	1	CRA004775;PRJNA827465;Yang2025_Silkie_Wencha	0.769377654
ENSGALG00010000174	ENSGALG0001000017	3	-0.13046546	0.130153722	-1.002395155	0.316152781	0	1.037937965	0	1	CRA004775;PRJNA827465;Yang2025_Silkie_Wencha	0.769377654
ENSGALG00010007786	SAMSN1	3	-0.149412019	0.149099421	-1.002096578	0.31629695	0	0.316026525	0	1	CRA004775;PRJNA827465;Yang2025_Silkie_Wencha	0.769596039
ENSGALG00010018974	TAS1R1	3	0.365216843	0.364564495	1.00178939	0.316445322	0.346211286	15.69977888	87.26096708	0.666666667	CRA004775;PRJNA827465;Yang2025_Silkie_Wencha	0.769768383
ENSGALG00010000800	LMO7	3	0.176171691	0.175868405	1.001724504	0.316476668	0.076114418	11.69363452	82.8966777	0.666666667	CRA004775;PRJNA827465;Yang2025_Silkie_Wencha	0.769768383
ENSGALG00010027287	DHX30	3	-0.112984548	0.112817961	-1.001476602	0.316596447	0.02523726	6.021481628	66.78558329	0.666666667	CRA004775;PRJNA827465;Yang2025_Silkie_Wencha	0.769794869
ENSGALG00010016568	PAM16	3	-0.084848645	0.084717811	-1.001544352	0.316563709	0	0.947258685	0	0.666666667	CRA004775;PRJNA827465;Yang2025_Silkie_Wencha	0.769794869
ENSGALG00010017703	RPS6KA5	3	-0.062834041	0.062752704	-1.001296146	0.316683656	0	0.253372452	0	0.666666667	CRA004775;PRJNA827465;Yang2025_Silkie_Wencha	0.76987452
ENSGALG00010018189	MIS18BP1	3	0.245347165	0.245347528	1.000988953	0.316832149	0.138632554	12.54040623	84.05155333	0.666666667	CRA004775;PRJNA827465;Yang2025_Silkie_Wencha	0.770103105
ENSGALG00010009112	ENSGALG0001000911	3	0.134080223	0.133990383	1.000670497	0.316986136	0	0.684637513	0	0.666666667	CRA004775;PRJNA827465;Yang2025_Silkie_Wencha	0.770212574
ENSGALG00010002681	PLEKHB1	3	0.101985487	0.101911786	1.000723184	0.316960656	0.010721392	3.029545411	33.98349493	0.666666667	CRA004775;PRJNA827465;Yang2025_Silkie_Wencha	0.770212574
ENSGALG00010019696	ENSGALG0001001969	2	0.443614427	0.443583905	1.000068808	0.31727721	0.24402289	2.424956822	58.76215235	1	PRJNA827465;Yang2025_Silkie_Wenchang	0.770654952
ENSGALG00010011170	RBM24	3	0.134231244	0.134209421	1.000162604	0.317231824	0.030785596	5.443686308	63.26019012	0.666666667	CRA004775;PRJNA827465;Yang2025_Silkie_Wencha	0.770654952
ENSGALG00010006673	BAZ1A	3	0.15685886	0.15701162	0.999901892	0.317357988	0.043326828	5.929212031	66.26870502	0.666666667	CRA004775;PRJNA827465;Yang2025_Silkie_Wencha	0.770718756
ENSGALG00010013635	BCL6	3	0.26206117	0.262138533	0.999704876	0.317453352	0.161398803	11.39314252	82.44558079	1	CRA004775;PRJNA827465;Yang2025_Silkie_Wencha	0.770795112
ENSGALG00010012320	DPY19L4	3	-0.138492035	0.138561441	-0.999499098	0.317552976	0.041815368	9.760938223	79.5101664	0.666666667	CRA004775;PRJNA827465;Yang2025_Silkie_Wencha	0.770795112
ENSGALG00010027282	ENSGALG0001002728	3	0.068861314	0.068892729	0.999544292	0.317531094	0	1.685821531	0	0.666666667	CRA004775;PRJNA827465;Yang2025_Silkie_Wencha	0.770795112
ENSGALG00010009338	KYNU	3	0.569360198	0.569826563	0.999181567	0.317706744	0.584855376	5.320907908	62.41242971	0.666666667	CRA004775;PRJNA827465;Yang2025_Silkie_Wencha	0.770935447
ENSGALG00010013414	LAPTM5	3	-0.270099918	0.270408007	-0.998860649	0.317862201	0.17172485	17.91576533	88.83664771	0.666666667	CRA004775;PRJNA827465;Yang2025_Silkie_Wencha	0.770935447
ENSGALG00010022108	USP47	3	0.100041002	0.100143565	0.998975834	0.317806398	0.02034315	8.702594212	77.01834704	0.666666667	CRA004775;PRJNA827465;Yang2025_Silkie_Wencha	0.770935447
ENSGALG00010027410	ALAS1	3	-0.058931466	0.058986424	-0.999068284	0.317761614	0	1.609577671	0	0.666666667	CRA004775;PRJNA827465;Yang2025_Silkie_Wencha	0.770935447
ENSGALG00010013880	ZMYM4	3	0.041624152	0.041673458	0.998816857	0.317883419	0	0.172935274	0	1	CRA004775;PRJNA827465;Yang2025_Silkie_Wencha	0.770935447
ENSGALG00010029268	PCTP	3	-0.348830617	0.349635988	-0.997696544	0.31842653	0.30681096	13.80318082	85.5105861	0.666666667	CRA004775;PRJNA827465;Yang2025_Silkie_Wencha	0.771095345
ENSGALG00010000651	ENSGALG0001000065	3	0.294557705	0.295145787	0.998007485	0.318275729	0.144880169	4.551877869	56.06209003	0.666666667	CRA004775;PRJNA827465;Yang2025_Silkie_Wencha	0.771095345
ENSGALG00010003691	ERICH1	3	0.25852124	0.259118636	0.997694508	0.318427517	0.14719858	8.903615933	77.53721617	0.666666667	CRA004775;PRJNA827465;Yang2025_Silkie_Wencha	0.771095345
ENSGALG00010025644	ENSGALG0001002564	3	0.220962691	0.221504052	0.997555976	0.318494718	0	0.110128084	0	1	CRA004775;PRJNA827465;Yang2025_Silkie_Wencha	0.771095345
ENSGALG00010024726	TMED2	3	-0.170106849	0.17045298	-0.997969348	0.318294222	0.077784576	30.3122299	93.40200306	0.666666667	CRA004775;PRJNA827465;Yang2025_Silkie_Wencha	0.771095345
ENSGALG00010002146	CACUL1	3	-0.163785976	0.164133356	-0.997883553	0.318335828	0.060623679	11.90726441	83.20353079	0.666666667	CRA004775;PRJNA827465;Yang2025_Silkie_Wencha	0.771095345
ENSGALG00010008293	PLA2G7	3	0.152041236	0.152333683	0.99808022	0.318240461	0.053926656	9.41458682	78.75636989	0.666666667	CRA004775;PRJNA827465;Yang2025_Silkie_Wencha	0.771095345
ENSGALG00010027077	ARF4	3	-0.127469734	0.127665058	-0.998470027	0.318051492	0.038048138	12.95132876	84.55756906	0.666666667	CRA004775;PRJNA827465;Yang2025_Silkie_Wencha	0.771095345
ENSGALG00010001141	MTAP	3	0.114433492	0.114710626	0.997584056	0.318481096	0	0.245641734	0	0.666666667	CRA004775;PRJNA827465;Yang2025_Silkie_Wencha	0.771095345
ENSGALG00010017770	PTCD3	3	-0.087388648	0.087581242	-0.997800968	0.318375881	0.010674904	3.610008468	44.5984679	0.666666667	CRA004775;PRJNA827465;Yang2025_Silkie_Wencha	0.771095345
ENSGALG00010024808	CELSR2	3	0.308038324	0.308851873	0.997365894	0.31858694	0.195447564	6.453041932	69.00686496	0.666666667	CRA004775;PRJNA827465;Yang2025_Silkie_Wencha	0.771186568
ENSGALG00010001741	SATB1	3	0.159936817	0.160382292	0.997222414	0.318656564	0.02173085	2.770547119	27.81209219	0.666666667	CRA004775;PRJNA827465;Yang2025_Silkie_Wencha	0.771198416
ENSGALG00010002946	GDPD5	3	-0.111253777	0.111573881	-0.99713101	0.318700922	0.023073261	8.238098393	75.72255265	0.666666667	CRA004775;PRJNA827465;Yang2025_Silkie_Wencha	0.771198416
ENSGALG00010002927	GABRA5	2	0.564861095	0.566731822	0.996699097	0.318910588	0	0.52310279	0	0.5	CRA004775;PRJNA827465	0.771292319
ENSGALG00010020017	ENSGALG0001002001	3	0.434533252	0.436162479	0.996264632	0.319121583	0.477163632	13.58292656	85.27563267	0.666666667	CRA004775;PRJNA827465;Yang2025_Silkie_Wencha	0.771292319
ENSGALG00010003274	BACH2	3	0.23176908	0.232598955	0.996432166	0.319040211	0.119710682	10.8030445	81.48670035	0.666666667	CRA004775;PRJNA827465;Yang2025_Silkie_Wencha	0.771292319
ENSGALG00010002135	ELOVL5	3	-0.223694841	0.224448346	-0.996642857	0.318937896	0.121516124	20.40462423	90.19830026	0.666666667	CRA004775;PRJNA827465;Yang2025_Silkie_Wencha	0.771292319
ENSGALG00010014465	SIMC1	3	0.171522289	0.172128335	0.996479105	0.319017414	0.055951107	5.834452593	65.72086296	0.666666667	CRA004775;PRJNA827465;Yang2025_Silkie_Wencha	0.771292319
ENSGALG00010024363	EDEM2	3	-0.106054821	0.106397899	-0.996775515	0.318873486	0.016035142	3.750869908	46.67903582	0.666666667	CRA004775;PRJNA827465;Yang2025_Silkie_Wencha	0.771292319
ENSGALG00010001232	TSPAN12	3	0.067275766	0.067527646	0.99626997	0.319118991	0	0.766134283	0	0.666666667	CRA004775;PRJNA827465;Yang2025_Silkie_Wencha	0.771292319
ENSGALG00010000525	ENSGALG0001000052	2	-1.004787787	1.01026328	-0.994580132	0.319940514	1.734644766	6.646856659	84.95529464	1	PRJNA827465;Yang2025_Silkie_Wenchang	0.771329899
ENSGALG00010027379	OPTC	3	-0.686374793	0.690214152	-0.994437437	0.32000995	1.164755573	12.38187307	83.84735501	0.666666667	CRA004775;PRJNA827465;Yang2025_Silkie_Wencha	0.771329899
ENSGALG00010003275	MBTPS2	3	-0.322482042	0.323931504	-0.995525406	0.319480794	0.278087269	23.58334487	91.51943878	0.666666667	CRA004775;PRJNA827465;Yang2025_Silkie_Wencha	0.771329899
ENSGALG00010016508	CREBBP	3	0.30486597	0.306605877	0.994325266	0.320064539	0.247946007	19.31981375	89.64793333	0.666666667	CRA004775;PRJNA827465;Yang2025_Silkie_Wencha	0.771329899
ENSGALG00010027954	CFAP92	3	0.21371553	0.214553283	0.996095359	0.319203815	0	0.494033486	0	1	CRA004775;PRJNA827465;Yang2025_Silkie_Wencha	0.771329899
ENSGALG00010017781	CYP26C1	3	-0.197168151	0.198223356	-0.994676686	0.319893537	0	1.669135329	0	0.666666667	CRA004775;PRJNA827465;Yang2025_Silkie_Wencha	0.771329899
ENSGALG00010017384	ARID4A	3	0.170487392	0.171420671	0.994555626	0.319952438	0.065161053	8.255766227	75.77450784	1	CRA004775;PRJNA827465;Yang2025_Silkie_Wencha	0.771329899
ENSGALG00010021064	LYVE1	3	0.169188789	0.170053928	0.994912563	0.319778792	0	0.370644826	0	1	CRA004775;PRJNA827465;Yang2025_Silkie_Wencha	0.771329899
ENSGALG00010018440	MEA1	3	0.156932844	0.157819088	0.994384433	0.320035744	0.035046061	3.652219005	45.23877135	0.666666667	CRA004775;PRJNA827465;Yang2025_Silkie_Wencha	0.771329899
ENSGALG00010017996	ZC3H14	3	0.137261764	0.138019227	0.994511902	0.319973714	0.039870739	7.186381272	72.16958126	0.666666667	CRA004775;PRJNA827465;Yang2025_Silkie_Wencha	0.771329899
ENSGALG00010021239	USP54	3	0.132229085	0.132888505	0.995037793	0.319717884	0.034511687	6.279670718	68.1511963	0.666666667	CRA004775;PRJNA827465;Yang2025_Silkie_Wencha	0.771329899
ENSGALG00010022037	DRG1	3	-0.131615202	0.132180228	-0.995725342	0.319383613	0.02844385	4.122961902	51.49118407	0.666666667	CRA004775;PRJNA827465;Yang2025_Silkie_Wencha	0.771329899
ENSGALG00010016573	DEGS1	3	-0.124542877	0.125060879	-0.995858003	0.319319143	0.031957717	9.355841077	78.62298019	0.666666667	CRA004775;PRJNA827465;Yang2025_Silkie_Wencha	0.771329899
ENSGALG00010004583	XKR4	3	-0.121541573	0.122155538	-0.994973907	0.319748955	0	1.918444235	0	0.666666667	CRA004775;PRJNA827465;Yang2025_Silkie_Wencha	0.771329899
ENSGALG00010017804	DUSP5	3	-0.119321271	0.11997107	-0.9945837	0.319938778	0.017061701	3.273199179	38.89769945	0.666666667	CRA004775;PRJNA827465;Yang2025_Silkie_Wencha	0.771329899
ENSGALG00010017323	ENSGALG0001001732	3	0.113246015	0.113740696	0.995650798	0.319419844	0.020179441	4.239735463	52.82724554	0.666666667	CRA004775;PRJNA827465;Yang2025_Silkie_Wencha	0.771329899
ENSGALG00010009721	ZHX1	3	0.074079639	0.074489953	0.994491697	0.319983546	0.002713961	2.249765951	11.10186376	1	CRA004775;PRJNA827465;Yang2025_Silkie_Wencha	0.771329899
ENSGALG00010001790	HOXA2	3	0.278055162	0.279735112	0.993994497	0.320225546	0.187708545	10.72671394	81.35496098	0.666666667	CRA004775;PRJNA827465;Yang2025_Silkie_Wencha	0.771410179
ENSGALG00010017641	GPRC5B	3	-0.161091713	0.162070216	-0.993962478	0.320241135	0.04771315	6.623833155	69.80600276	0.666666667	CRA004775;PRJNA827465;Yang2025_Silkie_Wencha	0.771410179
ENSGALG00010021046	ENSGALG0001002104	3	0.152746143	0.153697756	0.99380854	0.320316087	0.024601658	3.022640736	33.83269219	0.666666667	CRA004775;PRJNA827465;Yang2025_Silkie_Wencha	0.771410179
ENSGALG00010014745	LSM10	3	-0.149919118	0.150846806	-0.993850136	0.320295833	0	0.10560884	0	1	CRA004775;PRJNA827465;Yang2025_Silkie_Wencha	0.771410179
ENSGALG00010014690	CSF3R	3	-0.206397672	0.207727183	-0.993599727	0.320417777	0.038778945	2.81656924	28.9916267	0.666666667	CRA004775;PRJNA827465;Yang2025_Silkie_Wencha	0.771416581
ENSGALG00010029657	CHCHD2	3	-0.07778073	0.078283387	-0.993579015	0.320427865	0	0.281476314	0	0.666666667	CRA004775;PRJNA827465;Yang2025_Silkie_Wencha	0.771416581
ENSGALG00010025702	GTSF2	3	0.464586647	0.467809156	0.993111489	0.320655626	0.578342773	18.1805865	88.99925478	0.666666667	CRA004775;PRJNA827465;Yang2025_Silkie_Wencha	0.771463076
ENSGALG00010029292	ENSGALG0001002929	3	0.272150691	0.274224955	0.992435904	0.320984932	0.161326713	8.0919814	75.28417453	0.666666667	CRA004775;PRJNA827465;Yang2025_Silkie_Wencha	0.771463076
ENSGALG00010019230	SPG21	3	-0.27118702	0.273233771	-0.992509158	0.320949215	0.20382764	26.12154212	92.3434842	0.666666667	CRA004775;PRJNA827465;Yang2025_Silkie_Wencha	0.771463076
ENSGALG00010013666	COX7B	3	-0.235675673	0.2375673	-0.992037512	0.321179227	0.114722901	6.342315126	68.46577377	0.666666667	CRA004775;PRJNA827465;Yang2025_Silkie_Wencha	0.771463076
ENSGALG00010029556	NPTX1	3	0.226555626	0.228376503	0.992026864	0.321184421	0	0.136300242	0	1	CRA004775;PRJNA827465;Yang2025_Silkie_Wencha	0.771463076
ENSGALG00010024960	ARFRP1	3	-0.171555253	0.17284604	-0.992532156	0.320938001	0.072587512	11.83893536	83.10658907	0.666666667	CRA004775;PRJNA827465;Yang2025_Silkie_Wencha	0.771463076
ENSGALG00010018991	RYBP	3	-0.171105882	0.172420061	-0.99237804	0.321013147	0.065884838	10.62969237	81.18478004	0.666666667	CRA004775;PRJNA827465;Yang2025_Silkie_Wencha	0.771463076
ENSGALG00010027405	DCLRE1B	3	-0.164640278	0.165980427	-0.991925862	0.321233692	0.044168696	4.91607872	59.31716895	1	CRA004775;PRJNA827465;Yang2025_Silkie_Wencha	0.771463076
ENSGALG00010022734	ENSGALG0001002273	3	-0.130459451	0.131559715	-0.991636772	0.321374745	0.01851066	2.572560259	22.25643723	0.666666667	CRA004775;PRJNA827465;Yang2025_Silkie_Wencha	0.771463076
ENSGALG00010001760	RAB5A	3	-0.104427488	0.105277243	-0.991928408	0.32123245	0.015481434	3.560891789	43.83429437	0.666666667	CRA004775;PRJNA827465;Yang2025_Silkie_Wencha	0.771463076
ENSGALG00010013131	SPATA2	3	-0.090666482	0.091424695	-0.991706692	0.321340626	0.015594903	6.152161277	67.49109931	0.666666667	CRA004775;PRJNA827465;Yang2025_Silkie_Wencha	0.771463076
ENSGALG00010013452	LSS	3	0.089135486	0.089875071	0.991770957	0.321309268	0	1.453190914	0	0.666666667	CRA004775;PRJNA827465;Yang2025_Silkie_Wencha	0.771463076
ENSGALG00010020300	EHD4	3	-0.085466126	0.086125906	-0.992339354	0.321032012	0.009060068	3.446773259	41.9747152	0.666666667	CRA004775;PRJNA827465;Yang2025_Silkie_Wencha	0.771463076
ENSGALG00010013621	PDGFRA	3	-0.075174435	0.075740268	-0.992529289	0.320939399	0	1.333217888	0	0.666666667	CRA004775;PRJNA827465;Yang2025_Silkie_Wencha	0.771463076
ENSGALG00010018137	NDUFB5	3	-0.072438356	0.07303244	-0.991865478	0.321263151	0	0.818260476	0	0.666666667	CRA004775;PRJNA827465;Yang2025_Silkie_Wencha	0.771463076
ENSGALG00010019183	ANGEL2	3	0.068821167	0.069287415	0.99327081	0.320577999	0.006172407	3.534772659	43.41927493	0.666666667	CRA004775;PRJNA827465;Yang2025_Silkie_Wencha	0.771463076
ENSGALG00010017518	PEMT	3	0.051777313	0.052211564	0.991682856	0.321352256	0	0.859163625	0	0.666666667	CRA004775;PRJNA827465;Yang2025_Silkie_Wencha	0.771463076
ENSGALG00010011682	USP15	3	-0.086805177	0.08758827	-0.991059384	0.321656584	0.010346761	3.743055186	46.56771272	0.666666667	CRA004775;PRJNA827465;Yang2025_Silkie_Wencha	0.772008563
ENSGALG00010001426	ATE1	3	-0.104483056	0.105470746	-0.990635413	0.321863639	0.022976469	7.320317482	72.6787806	1	CRA004775;PRJNA827465;Yang2025_Silkie_Wencha	0.772374405
ENSGALG00010022129	ELMO3	3	0.244448635	0.246816259	0.990407345	0.321975057	0.135073013	10.01121889	80.02241263	0.666666667	CRA004775;PRJNA827465;Yang2025_Silkie_Wencha	0.772510661
ENSGALG00010015636	APBB2	3	-0.057082588	0.057649005	-0.990174731	0.322088721	0	1.077054391	0	0.666666667	CRA004775;PRJNA827465;Yang2025_Silkie_Wencha	0.772652261
ENSGALG00010003364	ANKRD6	3	-0.084415375	0.085266662	-0.990016177	0.322166212	0	0.611301077	0	1	CRA004775;PRJNA827465;Yang2025_Silkie_Wencha	0.772707052
ENSGALG00010013134	ENSGALG0001001313	3	-2.325203511	2.35069717	-0.989154852	0.322587384	12.80684365	10.17102694	80.33630221	0.666666667	CRA004775;PRJNA827465;Yang2025_Silkie_Wencha	0.772727123
ENSGALG00010009751	ENSGALG0001000975	3	0.426042473	0.430439653	0.989784444	0.32227949	0.42624309	9.094771613	78.00934333	0.666666667	CRA004775;PRJNA827465;Yang2025_Silkie_Wencha	0.772727123
ENSGALG00010028761	CTSS	3	-0.212433214	0.214745037	-0.989234572	0.322548387	0.082666489	5.315329653	62.37298285	0.666666667	CRA004775;PRJNA827465;Yang2025_Silkie_Wencha	0.772727123
ENSGALG00010022528	SIDT1	3	0.19228523	0.19440615	0.989090263	0.322618981	0	0.789052825	0	1	CRA004775;PRJNA827465;Yang2025_Silkie_Wencha	0.772727123
ENSGALG00010013307	POFUT1	3	-0.188466743	0.190500708	-0.989323059	0.322505106	0.086218537	10.63884111	81.20095996	0.666666667	CRA004775;PRJNA827465;Yang2025_Silkie_Wencha	0.772727123
ENSGALG00010005875	CDK8	3	0.145534136	0.147153823	0.988993238	0.32266645	0.036815576	4.971047496	59.76703096	0.666666667	CRA004775;PRJNA827465;Yang2025_Silkie_Wencha	0.772727123
ENSGALG00010013612	TWIST2	3	0.136076215	0.137531394	0.989419298	0.322458037	0	1.067528422	0	0.666666667	CRA004775;PRJNA827465;Yang2025_Silkie_Wencha	0.772727123
ENSGALG00010008113	DBNDD1	3	0.118789989	0.120018105	0.989767241	0.3222879	0	0.537201095	0	0.666666667	CRA004775;PRJNA827465;Yang2025_Silkie_Wencha	0.772727123
ENSGALG00010027467	GLYCTK	3	0.102743078	0.103853247	0.989310215	0.322511388	0.005427997	2.371204833	15.65469282	0.666666667	CRA004775;PRJNA827465;Yang2025_Silkie_Wencha	0.772727123
ENSGALG00010010168	SNCB	3	-0.182160572	0.184215794	-0.988843398	0.322739768	0	1.135501715	0	1	CRA004775;PRJNA827465;Yang2025_Silkie_Wencha	0.772771816
ENSGALG00010012393	ENSGALG0001001239	3	-2.18827658	2.216717012	-0.987170021	0.323559296	12.77193708	15.51510013	87.1093323	0.666666667	CRA004775;PRJNA827465;Yang2025_Silkie_Wencha	0.77289122
ENSGALG00010023324	LAG3	2	-0.570144078	0.577515503	-0.987235971	0.323526971	0.071787829	1.111958273	10.06856786	0.5	CRA004775;Yang2025_Silkie_Wenchang	0.77289122
ENSGALG00010002140	ENSGALG0001000214	2	-0.315404897	0.319306181	-0.987781995	0.323259428	0	0.094379272	0	1	PRJNA827465;Yang2025_Silkie_Wenchang	0.77289122
ENSGALG00010014807	HES1	3	0.237161805	0.239931289	0.988457176	0.322928798	0.114459976	9.953439304	79.906443	0.666666667	CRA004775;PRJNA827465;Yang2025_Silkie_Wencha	0.77289122
ENSGALG00010009076	TMEM214	3	0.234186552	0.237313933	0.986821757	0.323730027	0.147647976	17.65901417	88.67433946	0.666666667	CRA004775;PRJNA827465;Yang2025_Silkie_Wencha	0.77289122
ENSGALG00010015318	SALL4	3	0.227869152	0.23090016	0.986873079	0.323704863	0.10473078	6.696502043	70.13366102	0.666666667	CRA004775;PRJNA827465;Yang2025_Silkie_Wencha	0.77289122
ENSGALG00010027417	WNT2B	3	-0.221205774	0.223990783	-0.987566414	0.323365042	0	1.463423456	0	0.666666667	CRA004775;PRJNA827465;Yang2025_Silkie_Wencha	0.77289122
ENSGALG00010006549	ZDHHC23	3	0.214753319	0.217553521	0.987128676	0.323579562	0.097044545	6.997336889	71.41769745	0.666666667	CRA004775;PRJNA827465;Yang2025_Silkie_Wencha	0.77289122
ENSGALG00010003826	ENSGALG0001000382	3	0.202610718	0.205393534	0.986451298	0.323911703	0.09172256	8.495128157	76.45709443	0.666666667	CRA004775;PRJNA827465;Yang2025_Silkie_Wencha	0.77289122
ENSGALG00010005926	NUDT5	3	0.195571385	0.198053334	0.987468284	0.323413124	0.043018146	3.022501547	33.82964511	0.666666667	CRA004775;PRJNA827465;Yang2025_Silkie_Wencha	0.77289122
ENSGALG00010024367	ENSGALG0001002436	3	0.186189401	0.188695758	0.986717475	0.323781161	0.046602951	3.355606975	40.39826432	0.666666667	CRA004775;PRJNA827465;Yang2025_Silkie_Wencha	0.77289122
ENSGALG00010015861	PPP2CA	3	-0.177892914	0.180016656	-0.988202526	0.323053471	0.083578589	21.47025194	90.6847856	0.666666667	CRA004775;PRJNA827465;Yang2025_Silkie_Wencha	0.77289122
ENSGALG00010004933	SPTLC2	3	-0.149134568	0.151155241	-0.986631804	0.323823173	0.046727067	8.14817416	75.45462381	0.666666667	CRA004775;PRJNA827465;Yang2025_Silkie_Wencha	0.77289122
ENSGALG00010029685	ATP5PD	3	-0.147533087	0.149546041	-0.986539568	0.323868409	0.033102735	3.593229822	44.33976954	1	CRA004775;PRJNA827465;Yang2025_Silkie_Wencha	0.77289122
ENSGALG00010005779	ARMT1	3	-0.144356623	0.146165991	-0.987621143	0.323338228	0.047648562	10.45711932	80.8742739	0.666666667	CRA004775;PRJNA827465;Yang2025_Silkie_Wencha	0.77289122
ENSGALG00010013080	ARHGEF7	3	0.122219925	0.123655931	0.988387086	0.32296311	0.025770374	4.698657607	57.43465119	0.666666667	CRA004775;PRJNA827465;Yang2025_Silkie_Wencha	0.77289122
ENSGALG00010010977	FARS2	3	-0.122213061	0.123741736	-0.987646246	0.323325929	0.029185212	6.105407054	67.24215138	0.666666667	CRA004775;PRJNA827465;Yang2025_Silkie_Wencha	0.77289122
ENSGALG00010005504	LATS1	3	0.119301476	0.120916409	0.986644217	0.323817086	0.021569862	3.578650442	44.11301041	0.666666667	CRA004775;PRJNA827465;Yang2025_Silkie_Wencha	0.77289122
ENSGALG00010027457	CAPZA1	3	-0.095446812	0.096762927	-0.986398554	0.323937575	0.017014335	5.576358911	64.13430283	1	CRA004775;PRJNA827465;Yang2025_Silkie_Wencha	0.77289122
ENSGALG00010021044	EIF4G2	3	-0.091789536	0.093036028	-0.986602049	0.323837765	0.016566401	8.866385155	77.44289285	0.666666667	CRA004775;PRJNA827465;Yang2025_Silkie_Wencha	0.77289122
ENSGALG00010021178	NDST2	3	-0.065962122	0.066724374	-0.988576113	0.322870578	0.005165804	3.077482403	35.01181361	0.666666667	CRA004775;PRJNA827465;Yang2025_Silkie_Wencha	0.77289122
ENSGALG00010018685	ARL3	3	-0.119785567	0.121474035	-0.986100174	0.324083958	0.02872277	6.165914788	67.56361272	0.666666667	CRA004775;PRJNA827465;Yang2025_Silkie_Wencha	0.772993267
ENSGALG00010027745	BICD2	3	0.108336161	0.109864542	0.986088499	0.324089687	0.018487315	4.087474319	51.07002898	0.666666667	CRA004775;PRJNA827465;Yang2025_Silkie_Wencha	0.772993267
ENSGALG00010029782	UNK	3	0.11311726	0.114741552	0.985843909	0.324209715	0.022796596	4.689042639	57.34737016	0.666666667	CRA004775;PRJNA827465;Yang2025_Silkie_Wencha	0.773021893
ENSGALG00010027969	HSPA5	3	0.112946265	0.114568414	0.985841219	0.324211035	0.020273219	4.458752905	55.14440826	0.666666667	CRA004775;PRJNA827465;Yang2025_Silkie_Wencha	0.773021893
ENSGALG00010018216	FANCM	3	0.093487	0.094864989	0.985474214	0.324391192	0.016064437	6.015366582	66.75181849	0.666666667	CRA004775;PRJNA827465;Yang2025_Silkie_Wencha	0.773321035
ENSGALG00010000442	DGCR6	3	-0.096250135	0.097682481	-0.985336718	0.324458703	0.011710615	3.079997369	35.06487959	0.666666667	CRA004775;PRJNA827465;Yang2025_Silkie_Wencha	0.773351584
ENSGALG00010026731	ENSGALG0001002673	2	0.493250831	0.500773426	0.984978047	0.324634855	0	0.054245683	0	1	PRJNA827465;Yang2025_Silkie_Wenchang	0.773541688
ENSGALG00010020545	STYX	3	0.161227887	0.163697842	0.984911501	0.324667543	0.055235785	8.628302534	76.8204697	0.666666667	CRA004775;PRJNA827465;Yang2025_Silkie_Wencha	0.773541688
ENSGALG00010028740	DAZAP1	3	0.038573324	0.039167091	0.984840161	0.32470259	0	1.894291158	0	1	CRA004775;PRJNA827465;Yang2025_Silkie_Wencha	0.773541688
ENSGALG00010028314	MEF2D	3	0.242295741	0.246130998	0.98441782	0.32491012	0.147870399	12.24251899	83.66349277	0.666666667	CRA004775;PRJNA827465;Yang2025_Silkie_Wencha	0.773905691
ENSGALG00010020624	MINOS1	3	-0.239884675	0.243757686	-0.984111223	0.325060829	0.135717109	8.848622882	77.39761286	0.666666667	CRA004775;PRJNA827465;Yang2025_Silkie_Wencha	0.774021031
ENSGALG00010017373	RP1-27O5.3	3	0.155391588	0.157902783	0.984096576	0.32506803	0.05012095	7.780846515	74.29585591	0.666666667	CRA004775;PRJNA827465;Yang2025_Silkie_Wencha	0.774021031
ENSGALG00010005900	SPTSSA	3	-0.345588822	0.351291174	-0.983767448	0.325229868	0.3421306	31.83649209	93.71790085	0.666666667	CRA004775;PRJNA827465;Yang2025_Silkie_Wencha	0.774145641
ENSGALG00010013947	ENSGALG0001001394	3	-0.31496323	0.320146417	-0.983809947	0.325208968	0.211067902	6.471811075	69.09674932	0.666666667	CRA004775;PRJNA827465;Yang2025_Silkie_Wencha	0.774145641
ENSGALG00010024454	HPS5	3	-0.08250799	0.083889538	-0.983531344	0.325345996	0.007899448	3.032659898	34.05129269	1	CRA004775;PRJNA827465;Yang2025_Silkie_Wencha	0.774291709
ENSGALG00010003982	GLCCI1	3	0.136788394	0.139151619	0.983016911	0.325599116	0.033040119	5.35148641	62.62720585	0.666666667	CRA004775;PRJNA827465;Yang2025_Silkie_Wencha	0.774372733
ENSGALG00010017296	SCNN1G	3	-0.096219849	0.09787123	-0.983127002	0.325544936	0.006459716	2.500377547	20.01207968	1	CRA004775;PRJNA827465;Yang2025_Silkie_Wencha	0.774372733
ENSGALG00010017141	AFTPH	3	-0.064921765	0.066040349	-0.983062558	0.325576657	0.005036424	3.137851523	36.26212122	0.666666667	CRA004775;PRJNA827465;Yang2025_Silkie_Wencha	0.774372733
ENSGALG00010005229	CIPC	3	0.060940172	0.061977888	0.983256682	0.325481124	0	0.101356663	0	1	CRA004775;PRJNA827465;Yang2025_Silkie_Wencha	0.774372733
ENSGALG00010020903	AGMAT	3	0.297409513	0.302679459	0.982589021	0.32580975	0	0.034203771	0	1	CRA004775;PRJNA827465;Yang2025_Silkie_Wencha	0.774446603
ENSGALG00010004222	SPAG6	3	-0.247227985	0.251743276	-0.982063906	0.326068366	0.109367862	4.725296099	57.67461006	0.666666667	CRA004775;PRJNA827465;Yang2025_Silkie_Wencha	0.774446603
ENSGALG00010018977	NPHP3	3	0.16571298	0.168651119	0.982578598	0.325814882	0.066941781	10.37691591	80.72645073	0.666666667	CRA004775;PRJNA827465;Yang2025_Silkie_Wencha	0.774446603
ENSGALG00010018302	RAB11FIP5	3	-0.137776466	0.140284365	-0.982122745	0.326039381	0.037319039	6.505033745	69.25457917	0.666666667	CRA004775;PRJNA827465;Yang2025_Silkie_Wencha	0.774446603
ENSGALG00010005792	ADGRG6	3	0.101447619	0.103255575	0.982490473	0.325858274	0	1.794621569	0	0.666666667	CRA004775;PRJNA827465;Yang2025_Silkie_Wencha	0.774446603
ENSGALG00010017349	ENSGALG0001001734	3	-0.086552413	0.088118132	-0.982231587	0.325985769	0.007549556	2.900947822	31.05701576	0.666666667	CRA004775;PRJNA827465;Yang2025_Silkie_Wencha	0.774446603
ENSGALG00010003470	METTL3	3	-0.07090852	0.072209889	-0.982502649	0.325825278	0	1.245784662	0	0.666666667	CRA004775;PRJNA827465;Yang2025_Silkie_Wencha	0.774446603
ENSGALG00010005789	RNASET2	3	0.068587305	0.069839065	0.982076512	0.326062156	0	0.59155019	0	0.666666667	CRA004775;PRJNA827465;Yang2025_Silkie_Wencha	0.774446603
ENSGALG00010013069	ENSGALG0001001306	3	-1.279178149	1.303315923	-0.981479722	0.32635623	0	0.749057249	0	1	CRA004775;PRJNA827465;Yang2025_Silkie_Wencha	0.774972145
ENSGALG00010012051	ARK2C	3	0.193819456	0.197554725	0.981092488	0.326547134	0.042891432	3.130819405	36.1189599	0.666666667	CRA004775;PRJNA827465;Yang2025_Silkie_Wencha	0.774972145
ENSGALG00010019277	IGFBP5	3	0.190077599	0.193685168	0.981374053	0.326408317	0.081719456	8.521778951	76.53072191	0.666666667	CRA004775;PRJNA827465;Yang2025_Silkie_Wencha	0.774972145
ENSGALG00010020095	LRFN2	2	-0.176587893	0.179990449	-0.981095904	0.32654545	0	0.102699848	0	1	PRJNA827465;Yang2025_Silkie_Wenchang	0.774972145
ENSGALG00010008050	CDK10	3	-0.163923534	0.16708837	-0.98105891	0.326563692	0.058611546	7.035156921	71.57135196	0.666666667	CRA004775;PRJNA827465;Yang2025_Silkie_Wencha	0.774972145
ENSGALG00010004242	ADAM22	3	0.104001015	0.106026535	0.980896103	0.326643979	0	0.168896235	0	1	CRA004775;PRJNA827465;Yang2025_Silkie_Wencha	0.775032594
ENSGALG00010016305	DENND4C	3	0.202035065	0.206073171	0.980404503	0.326886488	0.079492094	5.722896212	65.05265995	0.666666667	CRA004775;PRJNA827465;Yang2025_Silkie_Wencha	0.775477861
ENSGALG00010020620	MARF1	3	-0.196486227	0.200574012	-0.979619564	0.327273944	0.106593511	25.84401062	92.26126305	0.666666667	CRA004775;PRJNA827465;Yang2025_Silkie_Wencha	0.775602586
ENSGALG00010024165	LEPR	3	0.187806345	0.19162535	0.980070457	0.327051341	0.003543151	2.062066427	3.009914048	0.666666667	CRA004775;PRJNA827465;Yang2025_Silkie_Wencha	0.775602586
ENSGALG00010006107	PGF	3	0.171679287	0.175190225	0.979952782	0.327106218	0.044193357	3.875779906	48.3974826	1	CRA004775;PRJNA827465;Yang2025_Silkie_Wencha	0.775602586
ENSGALG00010009410	JMJD1C	3	0.152435401	0.155622533	0.979520121	0.327323003	0.048621367	6.142271488	67.4387561	0.666666667	CRA004775;PRJNA827465;Yang2025_Silkie_Wencha	0.775602586
ENSGALG00010004975	SNW1	3	0.142181655	0.145121863	0.979739731	0.327214609	0.049980926	10.53677083	81.01885731	0.666666667	CRA004775;PRJNA827465;Yang2025_Silkie_Wencha	0.775602586
ENSGALG00010018422	ENSGALG0001001842	3	-0.087219732	0.0890338	-0.979624956	0.327271281	0	1.553764065	0	0.666666667	CRA004775;PRJNA827465;Yang2025_Silkie_Wencha	0.775602586
ENSGALG00010028887	SF3B4	3	-0.057795575	0.058964716	-0.980172198	0.327001126	0	1.415702109	0	0.666666667	CRA004775;PRJNA827465;Yang2025_Silkie_Wencha	0.775602586
ENSGALG00010025608	ENSGALG0001002560	3	-0.48982397	0.500303606	-0.979053445	0.327553573	0.413227022	4.488184044	55.43854752	0.666666667	CRA004775;PRJNA827465;Yang2025_Silkie_Wencha	0.775710797
ENSGALG00010016276	ACTR2	3	-0.257208557	0.262760048	-0.978872391	0.327643035	0.178252496	27.23486079	92.65647063	0.666666667	CRA004775;PRJNA827465;Yang2025_Silkie_Wencha	0.775710797
ENSGALG00010006120	MOGS	3	0.228672393	0.233516814	0.979254506	0.327454243	0.127481648	10.65368568	81.22715406	0.666666667	CRA004775;PRJNA827465;Yang2025_Silkie_Wencha	0.775710797
ENSGALG00010009332	ENSGALG0001000933	3	0.151025654	0.154254763	0.979066391	0.327547177	0.052579066	10.31846337	80.61726898	0.666666667	CRA004775;PRJNA827465;Yang2025_Silkie_Wencha	0.775710797

ENSGALG00010016969	CHMP3	3	0.133616881	0.136487946	0.978964699	0.327597422	0.034206662	5.510969371	63.70874405	0.666666667	CRA004775;PRJNA827465;Yang2025_Silkie_Wencha	0.775710797
ENSGALG00010010416	SLC35B3	3	-0.252096733	0.25765155	-0.978440585	0.327856464	0.170591534	18.5398418	89.21242144	0.666666667	CRA004775;PRJNA827465;Yang2025_Silkie_Wencha	0.775826367
ENSGALG00010016129	CENPI	3	-0.235771658	0.240924582	-0.978611882	0.327771786	0.117114025	9.535234426	79.02516172	0.666666667	CRA004775;PRJNA827465;Yang2025_Silkie_Wencha	0.775826367
ENSGALG00010008151	SIPA1L2	3	0.130332926	0.133197627	0.978492854	0.327830624	0.025525704	3.93203891	49.13580344	0.666666667	CRA004775;PRJNA827465;Yang2025_Silkie_Wencha	0.775826367
ENSGALG00010018495	ENSGALG00010018495	3	0.253134589	0.258858033	0.977889641	0.328128909	0	0.035832473	0	1	CRA004775;PRJNA827465;Yang2025_Silkie_Wencha	0.776081407
ENSGALG00010017372	ENSGALG00010017372	3	-0.234131829	0.239421809	-0.977905188	0.328121219	0.14950353		93.5448526	0.666666667	CRA004775;PRJNA827465;Yang2025_Silkie_Wencha	0.776081407
ENSGALG00010008272	DCP2	3	0.075165664	0.076863965	0.977905108	0.328121259	0	0.71839896	0	0.666666667	CRA004775;PRJNA827465;Yang2025_Silkie_Wencha	0.776081407
ENSGALG00010002533	LPAR2	3	-0.141086632	0.1443463	-0.97741772	0.328362394	0.030096588	3.884306228	48.51075372	0.666666667	CRA004775;PRJNA827465;Yang2025_Silkie_Wencha	0.776503744
ENSGALG00010015684	PDE6D	3	-0.215645039	0.220707135	-0.977064192	0.328537374	0.124591935	18.92246699	89.43055363	0.666666667	CRA004775;PRJNA827465;Yang2025_Silkie_Wencha	0.776527905
ENSGALG00010002024	KLHL31	3	-0.210484515	0.215391446	-0.977218541	0.328460971	0.090218821	9.811614925	79.61599578	0.666666667	CRA004775;PRJNA827465;Yang2025_Silkie_Wencha	0.776527905
ENSGALG00010016828	LACTBL1	3	-0.192400645	0.196896201	-0.977167888	0.328486043	0	0.638052704	0	1	CRA004775;PRJNA827465;Yang2025_Silkie_Wencha	0.776527905
ENSGALG00010024446	GRM4	3	0.532538493	0.545694735	0.975890841	0.329118562	0.593913367	6.401743971	68.75851317	0.666666667	CRA004775;PRJNA827465;Yang2025_Silkie_Wencha	0.776740299
ENSGALG00010025344	ENSGALG00010025344	2	0.382168268	0.391676784	0.975723564	0.329201472	0.261224772	6.663810147	84.89357008	1	PRJNA827465;Yang2025_Silkie_Wenchang	0.776740299
ENSGALG00010028065	REEP6	3	0.364880666	0.373576573	0.976722558	0.328706524	0.215618771	4.133700669	51.61720308	0.666666667	CRA004775;PRJNA827465;Yang2025_Silkie_Wencha	0.776740299
ENSGALG00010011860	CECR1	3	-0.185263056	0.189789182	-0.976151824	0.328989233	0.078394362	8.300213086	75.90423307	0.666666667	CRA004775;PRJNA827465;Yang2025_Silkie_Wencha	0.776740299
ENSGALG00010002546	ATP6AP2	3	-0.177566177	0.181814821	-0.976632027	0.328751357	0.072270453	18.71097962	89.31108878	0.666666667	CRA004775;PRJNA827465;Yang2025_Silkie_Wencha	0.776740299
ENSGALG00010021159	MMD2	3	-0.165685676	0.16983058	-0.975593892	0.329265752	0	0.598170161	0	1	CRA004775;PRJNA827465;Yang2025_Silkie_Wencha	0.776740299
ENSGALG00010024927	NOM1	3	-0.143611214	0.147200877	-0.975613845	0.329255861	0.043731019	6.498868498	69.22541208	0.666666667	CRA004775;PRJNA827465;Yang2025_Silkie_Wencha	0.776740299
ENSGALG00010023123	RNF214	3	0.139902073	0.143408099	0.97555211	0.329286467	0.04561685	8.952215938	77.65916267	0.666666667	CRA004775;PRJNA827465;Yang2025_Silkie_Wencha	0.776740299
ENSGALG00010025999	DBF4B	3	-0.120653384	0.123217575	-0.975632937	0.329246397	0.019256997	3.119527084	35.88771803	0.666666667	CRA004775;PRJNA827465;Yang2025_Silkie_Wencha	0.776740299
ENSGALG00010023058	PPARA	3	0.099519371	0.102005094	0.975631382	0.329247167	0	0.307082109	0	1	CRA004775;PRJNA827465;Yang2025_Silkie_Wencha	0.776740299
ENSGALG00010008469	NOCT	3	-0.060054443	0.061536375	-0.975917795	0.329105203	0	0.161170535	0	0.666666667	CRA004775;PRJNA827465;Yang2025_Silkie_Wencha	0.776740299
ENSGALG00010027259	ENSGALG00010027259	3	-0.054262072	0.05557877	-0.976309328	0.328911199	0.002623977	2.667336303	25.01882878	1	CRA004775;PRJNA827465;Yang2025_Silkie_Wencha	0.776740299
ENSGALG00010016973	PLSCR1	3	0.079111184	0.081117727	0.97526381	0.329429417	0.003526923	2.414049634	17.15166202	0.666666667	CRA004775;PRJNA827465;Yang2025_Silkie_Wencha	0.7768183
ENSGALG00010023482	MTCL2	3	0.065332642	0.066987611	0.97529441	0.329414243	0	1.079475	0	0.666666667	CRA004775;PRJNA827465;Yang2025_Silkie_Wencha	0.7768183
ENSGALG00010004231	ARMC3	2	-0.259876761	0.266630712	-0.974669268	0.329724342	0.04837533	1.490907985	32.92677954	1	PRJNA827465;Yang2025_Silkie_Wenchang	0.777384104
ENSGALG00010016561	DEGS2	2	0.921936294	0.948896886	0.971976039	0.331062469	1.392742564	4.239508551	76.41236035	1	CRA004775;PRJNA827465	0.777514211
ENSGALG00010009458	SCX	3	0.624791766	0.64284495	0.971916736	0.331091973	0.908156415	10.38618497	80.74365124	0.666666667	CRA004775;PRJNA827465;Yang2025_Silkie_Wencha	0.777514211
ENSGALG00010013461	S100B	3	0.564973714	0.580788953	0.972769387	0.330667931	0.704655475	6.831504967	70.72387403	0.666666667	CRA004775;PRJNA827465;Yang2025_Silkie_Wencha	0.777514211
ENSGALG00010004308	DSG2	3	0.531777442	0.547279741	0.971673903	0.331212804	0.622128332	6.568680341	69.55248397	0.666666667	CRA004775;PRJNA827465;Yang2025_Silkie_Wencha	0.777514211
ENSGALG00010013544	ENSGALG00010013544	3	-0.401696736	0.413340021	-0.971830772	0.331134745	0.415662367	11.25360597	82.22791872	0.666666667	CRA004775;PRJNA827465;Yang2025_Silkie_Wencha	0.777514211
ENSGALG00010022097	INPP5J	3	-0.272008002	0.279544631	-0.97303962	0.330533611	0.174283723	8.864468008	77.43801435	0.666666667	CRA004775;PRJNA827465;Yang2025_Silkie_Wencha	0.777514211
ENSGALG00010023159	DAW1	2	-0.266624002	0.27451944	-0.971239057	0.331429249	0	0.947797381	0	0.5	PRJNA827465;Yang2025_Silkie_Wenchang	0.777514211
ENSGALG00010024643	ETV7	3	0.261726137	0.268856795	0.973477859	0.330315859	0.000987232	2.008816672	0.438898766	0.666666667	CRA004775;PRJNA827465;Yang2025_Silkie_Wencha	0.777514211
ENSGALG00010024122	TRIM55	3	0.258405918	0.265768099	0.972298479	0.33090208	0.145538381	10.83181306	81.53587041	0.666666667	CRA004775;PRJNA827465;Yang2025_Silkie_Wencha	0.777514211
ENSGALG00010010572	AQP3	3	-0.24168297	0.248436168	-0.972817171	0.330644177	0.053528582	2.778375998	28.01550252	0.666666667	CRA004775;PRJNA827465;Yang2025_Silkie_Wencha	0.777514211
ENSGALG00010010573	CEP83	3	0.233779674	0.240521361	0.971970525	0.331065212	0.115261193	8.688537851	76.98116721	0.666666667	CRA004775;PRJNA827465;Yang2025_Silkie_Wencha	0.777514211
ENSGALG00010007904	ENSGALG00010007904	3	0.229918789	0.236215347	0.973343995	0.330382364	0.138891755	13.3661871	85.03686964	0.666666667	CRA004775;PRJNA827465;Yang2025_Silkie_Wencha	0.777514211
ENSGALG00010008821	ENSGALG00010008821	3	0.217809301	0.223820469	0.973142901	0.33048285	0.11584853	9.71375023	79.41062975	0.666666667	CRA004775;PRJNA827465;Yang2025_Silkie_Wencha	0.777514211
ENSGALG00010024630	CFAP410	3	-0.201790326	0.207218157	-0.973806197	0.330152775	0.09700527	8.698365843	77.00717542	0.666666667	CRA004775;PRJNA827465;Yang2025_Silkie_Wencha	0.777514211
ENSGALG00010001761	ELOB	3	0.196906301	0.202230269	0.973673735	0.330218562	0.084426636	6.968184972	71.29812127	0.666666667	CRA004775;PRJNA827465;Yang2025_Silkie_Wencha	0.777514211
ENSGALG00010022172	GIPC2	3	0.194922175	0.200682621	0.971295738	0.33140103	0.073985279	5.955230857	66.41607944	0.666666667	CRA004775;PRJNA827465;Yang2025_Silkie_Wencha	0.777514211
ENSGALG00010000741	FAM105A	3	0.18190224	0.187264954	0.971362959	0.331367567	0.054182472	4.530246498	55.8522919	0.666666667	CRA004775;PRJNA827465;Yang2025_Silkie_Wencha	0.777514211
ENSGALG00010029791	GALK1	3	0.181694031	0.187019377	0.971525168	0.331286826	0.083000274	10.02437004	80.04862158	0.666666667	CRA004775;PRJNA827465;Yang2025_Silkie_Wencha	0.777514211
ENSGALG00010012633	USP18	3	0.176525072	0.181389684	0.973181426	0.33046314	0.048688598	4.048927626	50.60420475	0.666666667	CRA004775;PRJNA827465;Yang2025_Silkie_Wencha	0.777514211
ENSGALG00010011593	WRNIP1	3	-0.158983157	0.163624071	-0.971636327	0.331231304	0.052800552	6.199042012	67.73695038	0.666666667	CRA004775;PRJNA827465;Yang2025_Silkie_Wencha	0.777514211
ENSGALG00010010284	CCDC25	3	0.122476455	0.125802968	0.973557786	0.330276155	0.029218127	6.279493741	68.15929869	1	CRA004775;PRJNA827465;Yang2025_Silkie_Wencha	0.777514211
ENSGALG00010029164	CYB5D2	3	-0.120695176	0.123995504	-0.973383489	0.330362742	0.018456391	2.962777765	32.4957807	1	CRA004775;PRJNA827465;Yang2025_Silkie_Wencha	0.777514211
ENSGALG00010007741	ENSGALG00010007741	3	0.104379915	0.107134626	0.974287388	0.329913865	0	1.109415481	0	0.666666667	CRA004775;PRJNA827465;Yang2025_Silkie_Wencha	0.777514211
ENSGALG00010005799	UFSP2	3	-0.095227267	0.09799542	-0.971752229	0.331173827	0.01163467	3.348616181	40.27383575	0.666666667	CRA004775;PRJNA827465;Yang2025_Silkie_Wencha	0.777514211
ENSGALG00010009605	ENSGALG00010009605	3	0.087507003	0.089853284	0.974061096	0.330026205	0	0.27990744	0	0.666666667	CRA004775;PRJNA827465;Yang2025_Silkie_Wencha	0.777514211
ENSGALG00010023388	FARP2	3	-0.078624278	0.080756256	-0.973599835	0.330255268	0.005012205	2.562992843	21.96622767	0.666666667	CRA004775;PRJNA827465;Yang2025_Silkie_Wencha	0.777514211
ENSGALG00010022504	RAD9A	3	-0.077890969	0.080012339	-0.973486954	0.330311341	0	1.730543566	0	0.666666667	CRA004775;PRJNA827465;Yang2025_Silkie_Wencha	0.777514211
ENSGALG00010024758	SPATA6	3	-0.07611926	0.078237791	-0.972921892	0.330592124	0.004147092	2.377429274	15.87552061	1	CRA004775;PRJNA827465;Yang2025_Silkie_Wencha	0.777514211
ENSGALG00010011427	TMTC3	3	-0.060747873	0.062527745	-0.971534682	0.331282091	0.004757663	3.089876376	35.2724913	0.666666667	CRA004775;PRJNA827465;Yang2025_Silkie_Wencha	0.777514211
ENSGALG00010028462	EDF1	3	-0.036993582	0.038082992	-0.971393804	0.331352213	0	0.418693831	0	0.666666667	CRA004775;PRJNA827465;Yang2025_Silkie_Wencha	0.777514211
ENSGALG00010014907	ENSGALG00010014907	3	0.153335165	0.157899471	0.971093595	0.331501673	0.046504669	5.314106778	62.36432418	0.666666667	CRA004775;PRJNA827465;Yang2025_Silkie_Wencha	0.777555102
ENSGALG00010019255	ENSGALG00010019255	2	5.83797982	6.015910752	0.970423276	0.331835551	69.09077122	21.09959403	95.26057232	1	CRA004775;Yang2025_Silkie_Wenchang	0.777711704
ENSGALG00010004887	MYH15	3	0.342337599	0.352697461	0.970626767	0.331734172	0.216110461	5.728909515	65.08934214	0.666666667	CRA004775;PRJNA827465;Yang2025_Silkie_Wencha	0.777711704
ENSGALG00010018361	ASIC4	3	0.230787502	0.237825374	0.970407399	0.331843462	0.09193846	4.747801012	57.8752354	0.666666667	CRA004775;PRJNA827465;Yang2025_Silkie_Wencha	0.777711704
ENSGALG00010029601	HSF5	2	-0.169965119	0.175084909	-0.970758246	0.33166868	0	0.267638086	0	1	PRJNA827465;Yang2025_Silkie_Wenchang	0.777711704
ENSGALG00010018338	PDCD6IP	3	0.052091075	0.053674124	0.970506301	0.331794196	4.22591E-05	2.005121165	0.255404246	1	CRA004775;PRJNA827465;Yang2025_Silkie_Wencha	0.777711704
ENSGALG00010029280	USP36	3	0.11567443	0.119242352	0.970078641	0.332007294	0.024780104	5.632030813	64.48883065	0.666666667	CRA004775;PRJNA827465;Yang2025_Silkie_Wencha	0.777837802
ENSGALG00010025523	B4GALT4	3	0.091072027	0.093875658	0.970134635	0.331979387	0	1.385684033	0	1	CRA004775;PRJNA827465;Yang2025_Silkie_Wencha	0.777837802
ENSGALG00010002575	BGV	3	0.197546288	0.203672427	0.969921606	0.33208557	0	1.078701525	0	0.666666667	CRA004775;PRJNA827465;Yang2025_Silkie_Wencha	0.777892291
ENSGALG00010013205	KCNJ6	2	-0.172263683	0.177652223	-0.96966804	0.332211986	0	0.06423901	0	1	PRJNA827465;Yang2025_Silkie_Wenchang	0.777926806
ENSGALG00010024119	AMMECR1L	3	-0.132735765	0.136894155	-0.969623459	0.332234215	0.037871783	7.704733856	74.04193269	0.666666667	CRA004775;PRJNA827465;Yang2025_Silkie_Wencha	0.777926806
ENSGALG00010027563	WDR77	3	-0.081232634	0.083782903	-0.969560993	0.332265364	0.004112489	2.254926913	11.30532931	1	CRA004775;PRJNA827465;Yang2025_Silkie_Wencha	0.777926806
ENSGALG00010025865	LGR6	2	0.312879093	0.322833847	0.969164467	0.332463137	0	0.065793412	0	1	PRJNA827465;Yang2025_Silkie_Wenchang	0.778260975
ENSGALG00010009067	ENSGALG00010009067	3	3.156395217	3.259069373	0.968495867	0.332796782	30.78468203	61.08658884	96.72595894	0.666666667	CRA004775;PRJNA827465;Yang2025_Silkie_Wencha	0.778328722
ENSGALG00010001534	SLITRK3	3	0.518238979	0.535095972	0.968498881	0.332795277	0.604675442	7.192569234	72.19352453	0.666666667	CRA004775;PRJNA827465;Yang2025_Silkie_Wencha	0.778328722
ENSGALG00010018498	ENSGALG00010018498	3	-0.263483724	0.272051759	-0.968505864	0.332791792	0.146917616	7.931187312	74.78309462	0.666666667	CRA004775;PRJNA827465;Yang2025_Silkie_Wencha	0.778328722
ENSGALG00010021045	DPP4	3	0.22398277	0.231211351	0.968736046	0.332676903	0.108774805	9.540952396	79.03773212	0.666666667	CRA004775;PRJNA827465;Yang2025_Silkie_Wencha	0.778328722
ENSGALG00010029258	TMEM100	3	-0.190215011	0.196412896	-0.968444614	0.332822367	0.075140426	7.549053328	73.50661185	0.666666667	CRA004775;PRJNA827465;Yang2025_Silkie_Wencha	0.778328722
ENSGALG00010022316	INO80	3	0.039350185	0.04062298	0.968668117	0.332710805	0	0.535112996	0	1	CRA004775;PRJNA827465;Yang2025_Silkie_Wencha	0.778328722
ENSGALG00010009732	HMMR	3	0.174306974	0.180066568	0.968014086	0.333037335	0.048330547	4.416687826	54.71719807	0.666666667	CRA004775;PRJNA827465;Yang2025_Silkie_Wencha	0.778521984
ENSGALG00010017880	CDK13	3	0.145668381	0.150465665	0.968117079	0.332985901	0.040247013	4.993244168	59.94588022	0.666666667	CRA004775;PRJNA827465;Yang2025_Silkie_Wencha	0.778521984
ENSGALG00010015746	PAPOLB	3	-0.047700691	0.049280206	-0.967948292	0.333070194	0.001667768	2.299213652	13.01373848	0.666666667	CRA004775;PRJNA827465;Yang2025_Silkie_Wencha	0.778521984
ENSGALG00010018568	FAM136A	3	0.583353135	0.603542216	0.966549017	0.333769532	1.039981268	52.30673866	96.17640088	0.666666667	CRA004775;PRJNA827465;Yang2025_Silkie_Wencha	0.778750948
ENSGALG00010000864	MZT1	3	-0.381479762	0.394573255	-0.966816067	0.333635991	0.436053143	54.59884207	96.33691865	0.666666667	CRA004775;PRJNA827465;Yang2025_Silkie_Wencha	0.778750948
ENSGALG00010017536	GSC	3	0.274025257	0.283554716	0.966392872	0.33384763	0	0.189631623	0	1	CRA004775;PRJNA827465;Yang2025_Silkie_Wencha	0.778750948
ENSGALG00010024754	EIF2S2	3	0.270853672	0.280255637	0.966452181	0.333817964	0.199517236	33.91398877	94.10272848	0.666666667	CRA004775;PRJNA827465;Yang2025_Silkie_Wencha	0.778750948
ENSGALG00010023439	ACSS2	3	-0.203753374	0.21073051	-0.966890715	0.333598669	0.104063504	11.45782167	82.5446751	0.666666667	CRA004775;PRJNA827465;Yang2025_Silkie_Wencha	0.778750948
ENSGALG00010014821	C3AR1L	3	-0.161682041	0.167090829	-0.96762965	0.333229364	0.038548284	3.954188253	49.42071869	0.666666667	CRA004775;PRJNA827465;Yang2025_Silkie_Wencha	0.778750948
ENSGALG00010022804	SNX19	3	-0.154130977	0.159333291	-0.967349483	0.333369355	0.055338878	7.884183283	74.63275614	0.666666667	CRA004775;PRJNA827465;Yang2025_Silkie_Wencha	0.778750948
ENSGALG00010016361	NELFCD	3	-0.14461411	0.149567068	-0.966884704	0.333601674	0.053421426	12.00512795	83.34045245	1	CRA004775;PRJNA827465;Yang2025_Silkie_Wencha	0.778750948
ENSGALG00010027996	GPX4	3	0.111690465	0.115583332	0.966319827	0.333884168	0.013251122	2.640745098	24.26379959	0.666666667	CRA004775;PRJNA827465;Yang2025_Silkie_Wencha	0.778750948
ENSGALG00010011619	PCDH1	3	0.106089281	0.109771548	0.966455181	0.333816464	0.015277426	3.445243239	41.94894637	1	CRA004775;PRJNA827465;Yang2025_Silkie_Wencha	0.778750948
ENSGALG00010005913	NFKBIA	3	-0.096301894	0.099634087	-0.966555697	0.333766191	0.011362492	3.23291742	38.13637219	0.666666667	CRA004775;PRJNA827465;Yang2025_Silkie_Wencha	0.778750948
ENSGALG00010009522	TAF5L	3	-0.090608482	0.093707811	-0.966925606	0.333581225	0.017771271	7.118832841	71.90550692	0.666666667	CRA004775;PRJNA827465;Yang2025_Silkie_Wencha	0.778750948
ENSGALG00010029269	WSCD1	3	-0.068953226	0.071276983	-0.967398208	0.333345006	0.000462033	2.04695355	2.293825872	0.666666667	CRA004775;PRJNA827465;Yang2025_Silkie_Wencha	0.778750948
ENSGALG00010005902	EAPP	3	0.172170174	0.178191644	0.966207902	0.33394016	0.07372763	9.788199682	79.56723335	0.666666667	CRA004775;PRJNA827465;Yang2025_Silkie_Wencha	0.778753079
ENSGALG00010001820	PGM2L1	3	-0.113926547	0.11794259	-0.965949171	0.334069616	0	0.114672737	0	1	CRA004775;PRJNA827465;Yang2025_Silkie_Wencha	0.778926501
ENSGALG00010000024	COX3	3	0.311970371	0.323044583	0.965719248	0.334184685	0.274172752	18.23121786	89.02980582	0.666666667	CRA004775;PRJNA827465;Yang2025_Silkie_Wencha	0.778937895
ENSGALG00010011888	CAND1	3	-0.107001857	0.110797151	-0.965745559	0.334171516	0.02457443	10.78264523	81.45167575	0.666666667	CRA004775;PRJNA827465;Yang2025_Silkie_Wencha	0.778937895
ENSGALG00010027838	ENSGALG00010027838	3	0.576778264	0.598248509	0.964111494	0.334990031	0.882257501	12.0061464	83.34186564	0.666666667	CRA004775;PRJNA827465;Yang2025_Silkie_Wencha	0.778938719
ENSGALG00010010146	PHACTR1	2	0.505658877	0.524097822	0.964817741	0.334636109	0.456678289	5.89408501	83.03383819	0.5	PRJNA827465;Yang2025_Silkie_Wenchang	0.778938719
ENSGALG00010027371	PRSS57	2	0.471030594	0.487832996	0.96555706	0.334265871	0.114111462	1.315226066	23.96744363	0.5	CRA004775;Yang2025_Silkie_Wenchang	0.778938719
ENSGALG00010020916	ARL6IP1	3	-0.340840321	0.353257907	-0.964848384	0.334620757	0.331889434	20.09822256	90.04887127	0.666666667	CRA004775;PRJNA827465;Yang2025_Silkie_Wencha	0.778938719
ENSGALG00010014265	ZNF462	3	0.334856296	0.347455296	0.963739219	0.335176686	0.321452989	20.00528877	90.00264369	0.666666667	CRA004775;PRJNA827465;Yang2025_Silkie_Wencha	0.778938719
ENSGALG00010022275	VDAC2	3	-0.261378404	0.270783221	-0.965268098	0.334410546	0.182436446	13.86313217	85.57324582	0.666666667	CRA004775;PRJNA827465;Yang2025_Silkie_Wencha	0.778938719
ENSGALG00010023340	P3H3	3	0.257173419	0.266514103	0.964952384	0.334568662	0.174235351	22.15956594	90.97455245	0.666666667	CRA004775;PRJNA827465;Yang2025_Silkie_Wencha	0.778938719
ENSGALG00010000636	ENSGALG00010000636	3	0.243880508	0.253052013	0.963756443	0.335168049	0.15317091	12.08968913	83.45697744	0.666666667	CRA004775;PRJNA827465;Yang2025_Silkie_Wencha	0.778938719
ENSGALG00010019259	L2HGDH	3	-0.217571051	0.225720231	-0.963896989	0.335097574	0.125455255	14.23962221	85.95468355	0.666666667	CRA004775;PRJNA827465;Yang2025_Silkie_Wencha	0.778938719
ENSGALG00010023978	TFDP2	3	0.18833483	0.195092269	0.965362857	0.334363099	0.083211469	8.257347069	75.77914573	0.666666667	CRA004775;PRJNA827465;Yang2025_Silkie_Wencha	0.778938719
ENSGALG00010007326	RWDD4	3	0.163148113	0.169266953	0.963850951	0.335120658	0.057270314	6.520757348	69.32871608	0.666666667	CRA004775;PRJNA827465;Yang2025_Silkie_Wencha	0.778938719
ENSGALG00010012210	PPWD1	3	-0.097474986	0.10108355	-0.964301174	0.334894952	0	0.747011995	0	0.666666667	CRA004775;PRJNA827465;Yang2025_Silkie_Wencha	0.778938719
ENSGALG00010023580	RDM1	3	-0.085734058	0.088824578	-0.965206477	0.334441404	0	1.75049424	0	0.666666667	CRA004775;PRJNA827465;Yang2025_Silkie_Wencha	0.778938719
ENSGALG00010020149	FAHD2A	3	0.085207675	0.088408416	0.96379597	0.335148227	0	1.660269951	0	0.666666667	CRA004775;PRJNA827465;Yang2025_Silkie_Wencha	0.778938719
ENSGALG00010008259	MCC	3	0.065733376	0.068106907	0.96514992	0.334469727	0	0.868378629	0	0.666666667	CRA004775;PRJNA827465;Yang2025_Silkie_Wencha	0.778938719
ENSGALG00010022627	CTCF	3	-0.057062034	0.05917096	-0.964358762	0.33486609	0.004632787	3.474777377	42.44235579	1	CRA004775;PRJNA827465;Yang2025_Silkie_Wencha	0.778938719
ENSGALG00010019591	ENSGALG00010019591	3	0.049512613	0.051364007	0.963955408	0.335068283	0	0.552650092	0	0.666666667	CRA004775;PRJNA827465;Yang2025_Silkie_Wencha	0.778938719
ENSGALG00010008385	CTNNA1	3	-0.02783592	0.028857101	-0.964612492	0.33473894	9.80723E-05	2.064672255	3.132325475	0.666666667	CRA004775;PRJNA827465;Yang2025_Silkie_Wencha	0.778938719
ENSGALG00010008213	ISCA1	3	-0.220186798	0.228600137	-0.963196264	0.33544904	0.133933751	18.53438925	89.20924789	0.666666667	CRA004775;PRJNA827465;Yang2025_Silkie_Wencha	0.779315474
ENSGALG00010009378	ENSGALG00010009378	3	-0.17331046	0.179914498	-0.963293463	0.335400273	0.073614314	9.383385972	78.68573236	0.666666667	CRA004775;PRJNA827465;Yang2025_Silkie_Wencha	0.779315474
ENSGALG00010026367	RBP	3	0.418372218	0.434796454	0.962225458	0.335936364	0.211902038	3.184236518	37.19059533	0.666666667	CRA004775;PRJNA827465;Yang2025_Silkie_Wencha	0.779370829
ENSGALG00010007432	NAT	3	-0.304534724	0.316395404	-0.962513109	0.335791922	0.203666801	6.425735444	68.87515807	0.666666667	CRA004775;PRJNA827465;Yang2025_Silkie_Wencha	0.779370829
ENSGALG00010004584	EIF4E	3	-0.171846045	0.178604336	-0.962160544	0.335968965	0.079768812	30.36261085	93.41295118	0.666666667	CRA004775;PRJNA827465;Yang2025_Silkie_Wencha	0.779370829
ENSGALG00010018419	USP7	3	-0.096952696	0.100736591	-0.962437729	0.335829769	0.019852285	7.13837385	71.98241446	0.666666667	CRA004775;PRJNA827465;Yang2025_Silkie_Wencha	0.779370829
ENSGALG00010007188	MAB21L1	3	-0.094941373	0.098646767	-0.96243775	0.335829759	0	0.508890318	0	0.666666667	CRA004775;PRJNA827465;Yang2025_Silkie_Wencha	0.779370829
ENSGALG00010023829	USP1	3	0.090472059	0.09399385	0.962531683	0.335782596	0.015400432	5.468377869	63.42608269	0.666666667	CRA004775;PRJNA827465;Yang2025_Silkie_Wencha	0.779370829
ENSGALG00010013476	ZDHHC17	3	0.050630062	0.052609705	0.962371134	0.335863209	0	0.637983302	0	1	CRA004775;PRJNA827465;Yang2025_Silkie_Wencha	0.779370829
ENSGALG00010021462	SETD5	3	-0.041492419	0.043092811	-0.962861734	0.335616915	0	1.234489119	0	1	CRA004775;PRJNA827465;Yang2025_Silkie_Wencha	0.779370829
ENSGALG00010004639	HBS1L	3	-0.033872028	0.035191631	-0.962502385	0.335797306	0	0.504512031	0	0.666666667	CRA004775;PRJNA827465;Yang2025_Silkie_Wencha	0.779370829
ENSGALG00010001259	ENSGALG00010001259	3	0.264466635	0.274923583	0.961964166	0.336067605	0.100563567	3.594399044	44.35787526	0.666666667	CRA004775;PRJNA827465;Yang2025_Silkie_Wencha	0.779471762
ENSGALG00010021093	GRAMD2	3	0.114371523	0.118916729	0.961778243	0.336161009	0	1.135599629	0	1	CRA004775;PRJNA827465;Yang2025_Silkie_Wencha	0.779560523
ENSGALG00010024717	TF	3	-0.647245325	0.673199554	-0.961446455	0.336327736	0.937641563	6.558943671	69.50728501	0.666666667	CRA004775;PRJNA827465;Yang2025_Silkie_Wencha	0.77964319
ENSGALG00010006080	ENSGALG00010006080	3	0.310509835	0.323054069	0.961169863	0.336466767	0.274898017	20.0367787	90.01835559	0.666666667	CRA004775;PRJNA827465;Yang2025_Silkie_Wencha	0.77964319
ENSGALG00010012063	PTPRD	3	0.310183445	0.322623833	0.961439961	0.336331	0.216732499	8.454963908	76.3452568	0.666666667	CRA004775;PRJNA827465;Yang2025_Silkie_Wencha	0.77964319
ENSGALG00010013459	ATP10D	3	-0.187179978	0.194710331	-0.961325351	0.336388605	0.067504849	5.600164455	64.28676308	0.666666667	CRA004775;PRJNA827465;Yang2025_Silkie_Wencha	0.77964319
ENSGALG00010012366	INTS8	3	-0.046072742	0.047934582	-0.961158731	0.336472363	0	0.500378872	0	1	CRA004775;PRJNA827465;Yang2025_Silkie_Wencha	0.77964319
ENSGALG00010021662	RASGRP1	3	-0.098692439	0.102721376	-0.960778013	0.336663796	0	0.453391427	0	1	CRA004775;PRJNA827465;Yang2025_Silkie_Wencha	0.77995894
ENSGALG00010011359	RNF19A	3	0.114319734	0.119006184	0.960620112	0.336743212	0.027860187	7.286329381	72.55133696	0.666666667	CRA004775;PRJNA827465;Yang2025_Silkie_Wencha	0.780015118
ENSGALG00010000604	ENSGALG00010000604	3	-0.204086318	0.212483332	-0.96048154	0.336812917	0.106673105	9.700412621	79.38232034	0.666666667	CRA004775;PRJNA827465;Yang2025_Silkie_Wencha	0.780048786
ENSGALG00010021740	PDLIM1	3	0.086564704	0.090161004	0.960112468	0.336998614	0	0.975356018	0	1	CRA004775;PRJNA827465;Yang2025_Silkie_Wencha	0.780351032
ENSGALG00010011152	SLC18A3	3	-0.360898163	0.376112404	-0.959548687	0.337282405	0	0.303471331	0	1	CRA004775;PRJNA827465;Yang2025_Silkie_Wencha	0.780397405
ENSGALG00010018289	HEYL	3	0.161962851	0.168773628	0.959645489	0.337233667	0.056362372	7.334853331	72.73292444	0.666666667	CRA004775;PRJNA827465;Yang2025_Silkie_Wencha	0.780397405
ENSGALG00010020027	RER1	3	-0.139977298	0.145855878	-0.95969597	0.337208252	0.048335483	9.414264381	78.7556423	0.666666667	CRA004775;PRJNA827465;Yang2025_Silkie_Wencha	0.780397405
ENSGALG00010020959	ENSGALG00010020959	3	0.129376174	0.13481232	0.959676194	0.337218208	0	0.983271485	0	1	CRA004775;PRJNA827465;Yang2025_Silkie_Wencha	0.780397405
ENSGALG00010004250	MOB1B	3	-0.074131142	0.077258212	-0.95952444	0.337294614	0.008041696	3.627021085	44.85832993	0.666666667	CRA004775;PRJNA827465;Yang2025_Silkie_Wencha	0.780397405
ENSGALG00010022474	VCL	3	0.172091222	0.179414146	0.959184244	0.337465937	0.078986703	16.58068519	87.93777231	0.666666667	CRA004775;PRJNA827465;Yang2025_Silkie_Wencha	0.780666047
ENSGALG00010027183	CD34	3	0.252250136	0.263136705	0.958627706	0.33774633	0.140069262	7.489825291	73.29710744	0.666666667	CRA004775;PRJNA827465;Yang2025_Silkie_Wencha	0.781059104
ENSGALG00010023506	RPE65	2	0.224051359	0.233714248	0.958655113	0.337732518	0	0.01367323	0	1	PRJNA827465;Yang2025_Silkie_Wenchang	0.781059104
ENSGALG00010000033	ND5	3	0.384008494	0.401233004	0.957071054	0.338531393	0.374438511	11.08187635	81.95251475	0.666666667	CRA004775;PRJNA827465;Yang2025_Silkie_Wencha	0.781117856
ENSGALG00010007868	INHBA	3	0.270761306	0.282534614	0.958329677	0.337896544	0.164640507	8.342704577	76.02695886	0.666666667	CRA004775;PRJNA827465;Yang2025_Silkie_Wencha	0.781117856
ENSGALG00010021794	ENSGALG00010021794	3	0.232093598	0.242485729	0.957143328	0.338494917	0.12390863	8.395943042	76.17897132	0.666666667	CRA004775;PRJNA827465;Yang2025_Silkie_Wencha	0.781117856
ENSGALG00010007865	GABRR3	2	0.230811453	0.241150067	0.957127884	0.338502711	0	0.202286945	0	1	PRJNA827465;Yang2025_Silkie_Wenchang	0.781117856
ENSGALG00010013657	MAGT1	3	-0.222375653	0.232127893	-0.957987644	0.338068989	0.135902331	16.4548961	87.84556288	0.666666667	CRA004775;PRJNA827465;Yang2025_Silkie_Wencha	0.781117856
ENSGALG00010018020	SNAPC5	3	-0.214678225	0.224128308	-0.957836281	0.338145321	0.12104865	11.97430443	83.29756845	0.666666667	CRA004775;PRJNA827465;Yang2025_Silkie_Wencha	0.781117856
ENSGALG00010010425	BLOC1S5	3	-0.181914813	0.190079935	-0.957043747	0.338545175	0.084295873	9.942141197	79.88360897	0.666666667	CRA004775;PRJNA827465;Yang2025_Silkie_Wencha	0.781117856
ENSGALG00010005187	KBTBD3	3	-0.149202505	0.155799275	-0.957658528	0.338234975	0	1.523982216	0	1	CRA004775;PRJNA827465;Yang2025_Silkie_Wencha	0.781117856
ENSGALG00010010471	KCNJ3	3	-0.14621605	0.152669875	-0.957726922	0.338200478	0.033430007	4.123777298	51.50077573	0.666666667	CRA004775;PRJNA827465;Yang2025_Silkie_Wencha	0.781117856
ENSGALG00010019215	ENSGALG00010019215	3	-0.131686726	0.137406123	-0.958375971	0.337873207	0.03833823	6.325552093	68.38220647	0.666666667	CRA004775;PRJNA827465;Yang2025_Silkie_Wencha	0.781117856
ENSGALG00010003239	RRM1	3	-0.100153871	0.104549851	-0.957953264	0.338086326	0.019053252	5.620581613	64.41649392	0.666666667	CRA004775;PRJNA827465;Yang2025_Silkie_Wencha	0.781117856
ENSGALG00010012980	KICS2	3	-0.090982758	0.094994278	-0.957770935	0.338178279	0.01645243	5.418801894	63.09147226	0.666666667	CRA004775;PRJNA827465;Yang2025_Silkie_Wencha	0.781117856
ENSGALG00010022838	ARFGAP2	3	-0.088600592	0.092543613	-0.957392836	0.338369013	0.015197227	5.28732025	62.17365498	0.666666667	CRA004775;PRJNA827465;Yang2025_Silkie_Wencha	0.781117856
ENSGALG00010009388	TMED7	3	-0.045202666	0.047212982	-0.95742027	0.338355171	0	0.207273627	0	1	CRA004775;PRJNA827465;Yang2025_Silkie_Wencha	0.781117856
ENSGALG00010012282	ENSGALG00010012282	3	-0.817130226	0.854577505	-0.95618036	0.338981121	1.214927192	4.490296701	55.45951341	0.666666667	CRA004775;PRJNA827465;Yang2025_Silkie_Wencha	0.781358668
ENSGALG00010029927	ENSGALG00010029927	3	0.341106042	0.356655784	0.95640126	0.338869548	0	1.25259547	0	0.666666667	CRA004775;PRJNA827465;Yang2025_Silkie_Wencha	0.781358668
ENSGALG00010024321	UBE2L3	3	-0.236127279	0.246936687	-0.956225993	0.33895807	0.166853409	35.45024785	94.3582905	1	CRA004775;PRJNA827465;Yang2025_Silkie_Wencha	0.781358668
ENSGALG00010017467	SFRP1	3	0.222495433	0.232625393	0.956453766	0.338843032	0.128064894	14.48935445	86.19676254	0.666666667	CRA004775;PRJNA827465;Yang2025_Silkie_Wencha	0.781358668
ENSGALG00010002443	TYK2	3	-0.202381018	0.211581447	-0.956515902	0.338811654	0.120078119	29.28545509	93.17067126	0.666666667	CRA004775;PRJNA827465;Yang2025_Silkie_Wencha	0.781358668
ENSGALG00010017828	ENSGALG00010017828	3	-0.170027009	0.177812027	-0.956217705	0.338962257	0.06899484	8.004035497	75.01260457	0.666666667	CRA004775;PRJNA827465;Yang2025_Silkie_Wencha	0.781358668
ENSGALG00010019524	HHIPL2	3	-0.308613889	0.323285091	-0.95461838	0.339770719	0.121983407	3.266225823	38.76724671	0.666666667	CRA004775;PRJNA827465;Yang2025_Silkie_Wencha	0.781493101
ENSGALG00010005474	COL8A1	3	0.304937496	0.31932321	0.954949363	0.339603305	0.194570891	5.765925763	65.31346253	0.666666667	CRA004775;PRJNA827465;Yang2025_Silkie_Wencha	0.781493101
ENSGALG00010027955	ENSGALG00010027955	3	0.302397736	0.316330861	0.955953947	0.339095502	0.260977475	16.13462874	87.60430108	0.666666667	CRA004775;PRJNA827465;Yang2025_Silkie_Wencha	0.781493101
ENSGALG00010011423	GDF6	3	0.26899383	0.281428029	0.955817481	0.339164455	0	1.365941955	0	1	CRA004775;PRJNA827465;Yang2025_Silkie_Wencha	0.781493101
ENSGALG00010021156	DDOST	3	-0.176158402	0.184518894	-0.95469032	0.339734327	0.07805555	9.213270095	78.29218096	0.666666667	CRA004775;PRJNA827465;Yang2025_Silkie_Wencha	0.781493101
ENSGALG00010003909	DNAJB1	3	-0.147520916	0.154439717	-0.955200635	0.339476245	0.041632175	5.783024074	65.41601809	0.666666667	CRA004775;PRJNA827465;Yang2025_Silkie_Wencha	0.781493101
ENSGALG00010011996	LRRC70	3	0.129723354	0.135867607	0.954777648	0.339690153	0	0.224358168	0	1	CRA004775;PRJNA827465;Yang2025_Silkie_Wencha	0.781493101
ENSGALG00010008918	PLAC9	3	-0.124581361	0.130391321	-0.955442124	0.33935416	0.024008402	3.974805634	49.68307424	0.666666667	CRA004775;PRJNA827465;Yang2025_Silkie_Wencha	0.781493101
ENSGALG00010026553	PLPP3	3	-0.096635853	0.101183651	-0.955054016	0.339550381	0.015608068	4.351060999	54.03419993	0.666666667	CRA004775;PRJNA827465;Yang2025_Silkie_Wencha	0.781493101
ENSGALG00010002571	BCOR	3	0.091436726	0.095711046	0.955341417	0.339405069	0.011599084	3.110312324	35.69777592	0.666666667	CRA004775;PRJNA827465;Yang2025_Silkie_Wencha	0.781493101
ENSGALG00010016011	ADD1	3	-0.079677275	0.08338402	-0.955546088	0.33930161	0.012279666	5.11477409	60.89758874	0.666666667	CRA004775;PRJNA827465;Yang2025_Silkie_Wencha	0.781493101
ENSGALG00010018985	NOL9	3	0.051264679	0.053685179	0.954913068	0.339621661	0	1.509192527	0	0.666666667	CRA004775;PRJNA827465;Yang2025_Silkie_Wencha	0.781493101
ENSGALG00010023144	C11orf58	3	-0.041656404	0.043613795	-0.955119905	0.339517064	0	0.469981384	0	1	CRA004775;PRJNA827465;Yang2025_Silkie_Wencha	0.781493101
ENSGALG00010022259	GET4	3	-0.039149561	0.041014305	-0.954534305	0.339813253	0	1.098492852	0	0.666666667	CRA004775;PRJNA827465;Yang2025_Silkie_Wencha	0.781493101
ENSGALG00010020389	ACTN1	3	0.155025092	0.162452668	0.954278522	0.339942677	0.041607676	4.669780175	57.17143152	0.666666667	CRA004775;PRJNA827465;Yang2025_Silkie_Wencha	0.781634336
ENSGALG00010021677	ENSGALG00010021677	3	0.090731268	0.095098861	0.954073131	0.340046626	0.013066352	3.575615732	44.06557779	0.666666667	CRA004775;PRJNA827465;Yang2025_Silkie_Wencha	0.781634336
ENSGALG00010015191	TOM1	3	-0.087947704	0.092190689	-0.953975994	0.340095794	0	0.390671339	0	0.666666667	CRA004775;PRJNA827465;Yang2025_Silkie_Wencha	0.781634336
ENSGALG00010009104	MTR	3	-0.056898307	0.059637298	-0.954072508	0.340046941	0	1.461656639	0	1	CRA004775;PRJNA827465;Yang2025_Silkie_Wencha	0.781634336

ENSGALG00010016543	TCEA3	3	-0.166509376	0.174563875	-0.953859307	0.340154864	0.040298069	3.683569441	45.70483787	0.666666667	CRA004775;PRJNA827465;Yang2025_Silkie_Wencha	0.78164304
ENSGALG00010027597	EXOSC2	3	-0.137330853	0.144022216	-0.953539371	0.340316858	0.041014931	6.084644344	67.13037136	0.666666667	CRA004775;PRJNA827465;Yang2025_Silkie_Wencha	0.781778875
ENSGALG00010010254	WDR41	3	-0.109179541	0.114501075	-0.953524159	0.340324562	0	0.181729285	0	1	CRA004775;PRJNA827465;Yang2025_Silkie_Wencha	0.781778875
ENSGALG00010022150	CPNE2	3	-0.080536465	0.084479119	-0.953329833	0.340422981	0.003816577	2.285503733	12.49193904	1	CRA004775;PRJNA827465;Yang2025_Silkie_Wencha	0.781877928
ENSGALG00010000326	ENSGALG0001000032(	3	-1.750359578	1.836839656	-0.952919092	0.340631066	8.464217516	12.24050768	83.66080842	0.666666667	CRA004775;PRJNA827465;Yang2025_Silkie_Wencha	0.782216959
ENSGALG00010005017	ENSGALG0001000501	3	-0.102760465	0.107848761	-0.95282008	0.340681239	0	1.266029516	0	1	CRA004775;PRJNA827465;Yang2025_Silkie_Wencha	0.782216959
ENSGALG00010018852	NDUFB10	3	0.183363959	0.192513925	0.952471148	0.340858092	0.069610472	5.772926332	65.35552534	0.666666667	CRA004775;PRJNA827465;Yang2025_Silkie_Wencha	0.782328611
ENSGALG00010029370	SRSF2	3	0.178792684	0.187691374	0.952588709	0.3407985	0.08835283	18.17510859	88.9959392	0.666666667	CRA004775;PRJNA827465;Yang2025_Silkie_Wencha	0.782328611
ENSGALG00010029181	MYO19	3	0.150706081	0.158238778	0.952396645	0.340895861	0.062296513	12.65625279	84.19753435	0.666666667	CRA004775;PRJNA827465;Yang2025_Silkie_Wencha	0.782328611
ENSGALG00010020973	GNB5	3	0.095667566	0.100463622	0.952260771	0.340964748	0	1.723375853	0	0.666666667	CRA004775;PRJNA827465;Yang2025_Silkie_Wencha	0.782359715
ENSGALG00010011861	ENSGALG0001001186	2	-0.24841458	0.260908924	-0.95211224	0.341040062	0	0.955142987	0	1	PRJNA827465;Yang2025_Silkie_Wenchang	0.782405556
ENSGALG00010000029	ND4	3	0.413899561	0.435089801	0.95129686	0.341453701	0.45599958	13.13526952	84.77381833	0.666666667	CRA004775;PRJNA827465;Yang2025_Silkie_Wencha	0.782430115
ENSGALG00010022269	CNTD1	2	-0.34284725	0.36030508	-0.951547088	0.341326727	0.071302704	1.365773613	26.78142333	1	PRJNA827465;Yang2025_Silkie_Wenchang	0.782430115
ENSGALG00010013056	ENSGALG0001001305(	3	-0.205144752	0.215688077	-0.951117718	0.341544621	0.087989555	5.500046957	63.63667409	0.666666667	CRA004775;PRJNA827465;Yang2025_Silkie_Wencha	0.782430115
ENSGALG00010012090	HELB	3	0.178128239	0.187132009	0.951885463	0.341155073	0.071025872	8.529112533	76.55090149	0.666666667	CRA004775;PRJNA827465;Yang2025_Silkie_Wencha	0.782430115
ENSGALG00010014091	CHIC2	3	-0.147197369	0.154694448	-0.951536216	0.341332243	0.058683879	14.2446356	85.9596268	0.666666667	CRA004775;PRJNA827465;Yang2025_Silkie_Wencha	0.782430115
ENSGALG00010009884	GFOD1	3	0.133359017	0.140214163	0.95110946	0.341548813	0.029834947	4.242901642	52.86244724	0.666666667	CRA004775;PRJNA827465;Yang2025_Silkie_Wencha	0.782430115
ENSGALG00010021903	MORC2	3	0.111084002	0.116775352	0.951262405	0.341471187	0.026439146	5.701271705	64.92010724	0.666666667	CRA004775;PRJNA827465;Yang2025_Silkie_Wencha	0.782430115
ENSGALG00010014153	FOCAD	3	0.089603601	0.094144648	0.951765213	0.341216068	0	0.343748436	0	1	CRA004775;PRJNA827465;Yang2025_Silkie_Wencha	0.782430115
ENSGALG00010001778	KAT2B	3	0.051582165	0.054215739	0.95142189	0.34138986	0	0.669478275	0	1	CRA004775;PRJNA827465;Yang2025_Silkie_Wencha	0.782430115
ENSGALG00010002098	ENSGALG0001000209	3	0.527727438	0.555115097	0.950663099	0.341775426	0.730363827	9.575477448	79.11331304	0.666666667	CRA004775;PRJNA827465;Yang2025_Silkie_Wencha	0.782568866
ENSGALG00010029466	SLC39A11	3	-0.219588433	0.230979318	-0.95068439	0.341764614	0.122478577	9.185665464	78.22694493	0.666666667	CRA004775;PRJNA827465;Yang2025_Silkie_Wencha	0.782568866
ENSGALG00010002781	EIF3G	3	-0.051939389	0.054621058	-0.950803418	0.34170417	0	0.385632977	0	0.666666667	CRA004775;PRJNA827465;Yang2025_Silkie_Wencha	0.782568866
ENSGALG00010025579	CSRNP3	2	0.695759442	0.732158	0.950285924	0.341966989	1.030304991	25.53676782	96.08407764	0.5	PRJNA827465;Yang2025_Silkie_Wenchang	0.782753968
ENSGALG00010018925	LINS1	3	-0.137224138	0.144394685	-0.950340645	0.341939193	0.018332338	2.690373282	25.668734	0.666666667	CRA004775;PRJNA827465;Yang2025_Silkie_Wencha	0.782753968
ENSGALG00010022050	ENSGALG0001002205(	3	0.35055578	0.36898418	0.950056394	0.342083599	0.319427176	9.522397137	78.99688522	0.666666667	CRA004775;PRJNA827465;Yang2025_Silkie_Wencha	0.782845771
ENSGALG00010021088	ANKLE2	3	0.05137177	0.05593884	0.949915488	0.342155196	0.002735172	2.472451445	19.10862378	1	CRA004775;PRJNA827465;Yang2025_Silkie_Wencha	0.782845771
ENSGALG00010008441	PHLPP2	3	0.048977005	0.051561252	0.949880061	0.342173199	0	0.577960272	0	0.666666667	CRA004775;PRJNA827465;Yang2025_Silkie_Wencha	0.782845771
ENSGALG00010015422	ENSGALG0001001542	3	-0.229485314	0.242051523	-0.948084572	0.343086402	0.009637111	2.105814871	5.024889526	0.666666667	CRA004775;PRJNA827465;Yang2025_Silkie_Wencha	0.783091987
ENSGALG00010014249	ENSGALG0001001424	3	0.195559728	0.206397402	0.947491231	0.343388523	0.100413792	10.44158718	80.84582386	0.666666667	CRA004775;PRJNA827465;Yang2025_Silkie_Wencha	0.783091987
ENSGALG00010021048	API5	3	-0.189030586	0.199427398	-0.947866684	0.343197327	0.108181504	32.40445659	93.82800944	0.666666667	CRA004775;PRJNA827465;Yang2025_Silkie_Wencha	0.783091987
ENSGALG00010012744	ARL4C	3	-0.157042985	0.165514273	-0.94881838	0.342712992	0.04253222	4.713456922	57.5682979	0.666666667	CRA004775;PRJNA827465;Yang2025_Silkie_Wencha	0.783091987
ENSGALG00010000501	ENSGALG0001000050	3	0.156466401	0.164993623	0.948317872	0.342967655	0.060174401	9.556918746	79.07275291	0.666666667	CRA004775;PRJNA827465;Yang2025_Silkie_Wencha	0.783091987
ENSGALG00010012081	KCNH6	3	0.153991242	0.16224872	0.949100649	0.342566678	0.035537865	3.251826132	38.49609669	1	CRA004775;PRJNA827465;Yang2025_Silkie_Wencha	0.783091987
ENSGALG00010021821	PPP1R19L	3	0.127261275	0.134227665	0.948100192	0.343078451	0.028949306	4.253864724	52.98393039	0.666666667	CRA004775;PRJNA827465;Yang2025_Silkie_Wencha	0.783091987
ENSGALG00010016464	PANK2	3	-0.122920288	0.129574871	-0.948642955	0.342802236	0.034488577	6.610361226	69.74446733	0.666666667	CRA004775;PRJNA827465;Yang2025_Silkie_Wencha	0.783091987
ENSGALG00010017646	MAEA	3	-0.119614698	0.126132075	-0.948328948	0.342962018	0.03584295	11.61851473	82.78609575	0.666666667	CRA004775;PRJNA827465;Yang2025_Silkie_Wencha	0.783091987
ENSGALG00010017077	CTNNBIP1	3	-0.118240014	0.124604446	-0.948922916	0.342659818	0.031708397	7.743599851	74.17221914	0.666666667	CRA004775;PRJNA827465;Yang2025_Silkie_Wencha	0.783091987
ENSGALG00010013100	ZFPM2	3	0.114429915	0.120716606	0.947921909	0.34316921	0	0.275636086	0	0.666666667	CRA004775;PRJNA827465;Yang2025_Silkie_Wencha	0.783091987
ENSGALG00010005466	LATS2	3	0.107863974	0.113761023	0.948162836	0.343046563	0.018789925	3.722544521	46.2733088	0.666666667	CRA004775;PRJNA827465;Yang2025_Silkie_Wencha	0.783091987
ENSGALG00010011311	DOCK8	3	-0.105794985	0.111431011	-0.949421383	0.342406339	0	1.2865354	0	1	CRA004775;PRJNA827465;Yang2025_Silkie_Wencha	0.783091987
ENSGALG00010008221	ENSGALG0001000822	3	-0.095983766	0.101220322	-0.948265766	0.342994174	0	1.097505101	0	0.666666667	CRA004775;PRJNA827465;Yang2025_Silkie_Wencha	0.783091987
ENSGALG00010003300	CASP8AP2	3	0.064647522	0.068167647	0.948360771	0.342945823	0	1.394974661	0	1	CRA004775;PRJNA827465;Yang2025_Silkie_Wencha	0.783091987
ENSGALG00010021126	ECE1	3	-0.063659636	0.067183389	-0.947550231	0.343358473	0.005766349	3.498415927	42.83126874	0.666666667	CRA004775;PRJNA827465;Yang2025_Silkie_Wencha	0.783091987
ENSGALG00010017528	RAI1	3	-0.054861209	0.057806163	-0.949054668	0.342592809	0	1.621968957	0	0.666666667	CRA004775;PRJNA827465;Yang2025_Silkie_Wencha	0.783091987
ENSGALG00010009569	ZC3H18	3	-0.05359098	0.056552785	-0.947627603	0.343319069	0	0.530816904	0	0.666666667	CRA004775;PRJNA827465;Yang2025_Silkie_Wencha	0.783091987
ENSGALG00010013352	ASXL1	3	-0.043448851	0.045844712	-0.947648854	0.343308248	0	1.831190472	0	1	CRA004775;PRJNA827465;Yang2025_Silkie_Wencha	0.783091987
ENSGALG00010016964	MAML1	3	0.043321066	0.045694433	0.948060047	0.343098886	0.000222665	2.033412012	1.643150148	0.666666667	CRA004775;PRJNA827465;Yang2025_Silkie_Wencha	0.783091987
ENSGALG00010001115	PFN2	3	-0.08591778	0.090691574	-0.947362321	0.343454184	0.010203989	3.012313267	33.605843	1	CRA004775;PRJNA827465;Yang2025_Silkie_Wencha	0.783115418
ENSGALG00010018987	MRPS10	3	-0.131357237	0.138675727	-0.947225877	0.343523692	0.022523711	3.095533348	35.39077842	0.666666667	CRA004775;PRJNA827465;Yang2025_Silkie_Wencha	0.78314761
ENSGALG00010029785	ENSGALG0001002978	3	-0.69766902	0.736762459	-0.946938882	0.343669924	0.96826533	5.059146688	60.46764161	0.666666667	CRA004775;PRJNA827465;Yang2025_Silkie_Wencha	0.783228409
ENSGALG00010012900	RBX1	3	-0.213541111	0.225490435	-0.947007402	0.343635007	0.111666231	7.88778115	74.64432694	0.666666667	CRA004775;PRJNA827465;Yang2025_Silkie_Wencha	0.783228409
ENSGALG00010010626	ADTRP	3	-0.347370785	0.366973815	-0.94658194	0.34385185	0.131093995	2.915086199	31.39139416	0.666666667	CRA004775;PRJNA827465;Yang2025_Silkie_Wencha	0.783333451
ENSGALG00010021640	PLEKHG3	3	0.196069844	0.207147608	0.946522363	0.343882221	0.106132288	14.76380271	86.45533546	0.666666667	CRA004775;PRJNA827465;Yang2025_Silkie_Wencha	0.783333451
ENSGALG00010001119	COMMD2	3	-0.126071233	0.133199305	-0.946523825	0.343881476	0.041491463	12.35004341	83.80572494	0.666666667	CRA004775;PRJNA827465;Yang2025_Silkie_Wencha	0.783333451
ENSGALG00010005487	ENSGALG0001000548	3	0.220768548	0.23335331	0.946069921	0.344112924	0.117310518	7.81420879	74.40559814	0.666666667	CRA004775;PRJNA827465;Yang2025_Silkie_Wencha	0.783382652
ENSGALG00010002543	UCP3	3	-0.203635486	0.215212874	-0.946204948	0.344044063	0	0.046313197	0	1	CRA004775;PRJNA827465;Yang2025_Silkie_Wencha	0.783382652
ENSGALG00010026671	TAF8	3	-0.126529557	0.133745759	-0.946045374	0.344125444	0.035461024	7.031412057	71.55621113	0.666666667	CRA004775;PRJNA827465;Yang2025_Silkie_Wencha	0.783382652
ENSGALG00010021532	DPP10	3	0.111749962	0.118121026	0.946063251	0.344116326	0.008568786	2.499458775	19.83835484	0.666666667	CRA004775;PRJNA827465;Yang2025_Silkie_Wencha	0.783382652
ENSGALG00010013357	RICTOR	3	0.233308695	0.246706622	0.945692877	0.344305257	0.128476681	8.425070517	76.26312629	0.666666667	CRA004775;PRJNA827465;Yang2025_Silkie_Wencha	0.783359679
ENSGALG00010005562	HECTD1	3	0.17165101	0.181489223	0.945791774	0.344254802	0.073561036	15.479436	87.07963262	0.666666667	CRA004775;PRJNA827465;Yang2025_Silkie_Wencha	0.783359679
ENSGALG00010029469	ENSGALG0001002946	3	0.390712914	0.413334247	0.94527109	0.344520494	0.33620107	6.441677874	68.95218856	0.666666667	CRA004775;PRJNA827465;Yang2025_Silkie_Wencha	0.783692923
ENSGALG00010005668	LNP1	2	0.165762005	0.175329752	0.945429985	0.3444394	0	0.314998932	0		PRJNA827465;Yang2025_Silkie_Wenchang	0.783692923
ENSGALG00010006627	SF3B5	3	-0.055671913	0.058897427	-0.94523507	0.344538879	0	0.152524569	0	0.666666667	CRA004775;PRJNA827465;Yang2025_Silkie_Wencha	0.783692923
ENSGALG00010002388	CYP2W1	3	0.425140493	0.450170373	0.944399097	0.344965743	0.39660873	5.843938781	65.77650665	0.666666667	CRA004775;PRJNA827465;Yang2025_Silkie_Wencha	0.783926329
ENSGALG00010028436	S100A1	3	0.331497557	0.350934499	0.94461376	0.3448561	0.274131534	10.80181301	81.48458969	0.666666667	CRA004775;PRJNA827465;Yang2025_Silkie_Wencha	0.783926329
ENSGALG00010028855	SURF6	3	0.253268789	0.268076657	0.944762494	0.344780144	0.180433737	14.10671183	85.82235163	0.666666667	CRA004775;PRJNA827465;Yang2025_Silkie_Wencha	0.783926329
ENSGALG00010016369	MSANTD1	2	0.221751712	0.234753606	0.944614722	0.344855608	0.030956383	1.369556692	26.98367247	0.5	PRJNA827465;Yang2025_Silkie_Wenchang	0.783926329
ENSGALG00010018515	SEMA4G	3	0.153580695	0.162625499	0.944382623	0.344974158	0.042851837	4.297172793	53.45777105	0.666666667	CRA004775;PRJNA827465;Yang2025_Silkie_Wencha	0.783926329
ENSGALG00010013221	C2orf49	3	0.0972093	0.102926748	0.944451284	0.344939085	0.014251113	3.488989758	42.67681653	0.666666667	CRA004775;PRJNA827465;Yang2025_Silkie_Wencha	0.783926329
ENSGALG00010017743	KCNH5	2	0.207750312	0.220060492	0.944060014	0.345138981	0	0.260772886	0		PRJNA827465;Yang2025_Silkie_Wenchang	0.783966442
ENSGALG00010026097	ARF1	3	-0.179767919	0.190427579	-0.944022498	0.345158151	0.098084363	25.96063592	92.29602847	0.666666667	CRA004775;PRJNA827465;Yang2025_Silkie_Wencha	0.783966442
ENSGALG00010021576	ERH	3	-0.067095125	0.071068245	-0.944094311	0.345121456	0.004633403	2.483050288	19.45390675	1	CRA004775;PRJNA827465;Yang2025_Silkie_Wencha	0.783966442
ENSGALG00010008799	ENSGALG0001000879	3	0.211542592	0.22414045	0.943794803	0.345274517	0.08551619	4.523773003	55.78911677	0.666666667	CRA004775;PRJNA827465;Yang2025_Silkie_Wencha	0.783978866
ENSGALG00010016292	PHKA1	3	0.070702847	0.074908316	0.94385845	0.345241987	0	0.112404351	0	1	CRA004775;PRJNA827465;Yang2025_Silkie_Wencha	0.783978866
ENSGALG00010005890	SNX32	3	-0.123602566	0.131010381	-0.943456274	0.345447571	0.044114059	16.59830612	87.95057769	0.666666667	CRA004775;PRJNA827465;Yang2025_Silkie_Wencha	0.784113328
ENSGALG00010008313	PSMD7	3	0.10505941	0.111368016	0.943353521	0.345500109	0.021559872	5.034252307	60.27215408	0.666666667	CRA004775;PRJNA827465;Yang2025_Silkie_Wencha	0.784113328
ENSGALG00010027950	CDC26	3	-0.068431094	0.072539644	-0.943361316	0.345496123	0	0.960235907	0	1	CRA004775;PRJNA827465;Yang2025_Silkie_Wencha	0.784113328
ENSGALG00010006266	CENPW	3	-0.211460642	0.224216603	-0.943108761	0.345625275	0.093015221	7.657764386	73.88271695	0.666666667	CRA004775;PRJNA827465;Yang2025_Silkie_Wencha	0.784271507
ENSGALG00010001795	ENSGALG0001000179	3	0.376247249	0.399051978	0.942852735	0.345756234	0.211585009	3.587139627	44.24527038	0.666666667	CRA004775;PRJNA827465;Yang2025_Silkie_Wencha	0.784442777
ENSGALG00010029571	EPX	3	-0.528931486	0.561203146	-0.942495582	0.345938972	0.61908732	6.057976876	66.98567788	0.666666667	CRA004775;PRJNA827465;Yang2025_Silkie_Wencha	0.784523784
ENSGALG00010018319	STUB1	3	0.10181792	0.108013463	0.942641008	0.345864557	0.022178196	7.46093917	73.19372328	0.666666667	CRA004775;PRJNA827465;Yang2025_Silkie_Wencha	0.784523784
ENSGALG00010027543	TUBB4B	3	0.056440829	0.059886861	0.942457621	0.345958398	0	1.432876854	0	0.666666667	CRA004775;PRJNA827465;Yang2025_Silkie_Wencha	0.784523784
ENSGALG00010025155	CZIB	3	-0.244652477	0.259669607	-0.942168318	0.346106472	0.158233542	9.998750659	79.99750101	0.666666667	CRA004775;PRJNA827465;Yang2025_Silkie_Wencha	0.784733708
ENSGALG00010012342	ENSGALG0001001234	2	-1.630529927	1.735233744	-0.939660108	0.347391934	2.745088958	1.814295058	44.88217362	1	PRJNA827465;Yang2025_Silkie_Wenchang	0.784982422
ENSGALG00010011555	ENSGALG0001001155	3	-1.360989688	1.452806105	-0.936800639	0.348861117	3.441417408	4.470708709	55.26436343	1	CRA004775;PRJNA827465;Yang2025_Silkie_Wencha	0.784982422
ENSGALG00010010327	FBP1	3	-0.664864552	0.712268412	-0.933446634	0.350589412	1.173560623	8.97483901	77.71547771	0.666666667	CRA004775;PRJNA827465;Yang2025_Silkie_Wencha	0.784982422
ENSGALG00010023597	MOGAT1	3	-0.493722225	0.524742358	-0.940885022	0.346763784	0.684897352	13.65406602	85.35234855	0.666666667	CRA004775;PRJNA827465;Yang2025_Silkie_Wencha	0.784982422
ENSGALG00010015138	ENSGALG0001001513	3	-0.473220284	0.507791514	-0.931918457	0.351378668	0.428681387	4.500757845	55.56303918	0.666666667	CRA004775;PRJNA827465;Yang2025_Silkie_Wencha	0.784982422
ENSGALG00010012156	ENSGALG0001001215(	2	0.442048694	0.471756044	0.937028151	0.348744077	0.13028915	1.398936637	28.5171341	1	PRJNA827465;Yang2025_Silkie_Wenchang	0.784982422
ENSGALG00010021512	CPNE9	3	-0.418902579	0.445743945	-0.939782993	0.347328884	0.419266113	7.078680072	71.74614505	0.666666667	CRA004775;PRJNA827465;Yang2025_Silkie_Wencha	0.784982422
ENSGALG00010028117	ENSGALG0001002811	3	0.405566289	0.433899971	0.934699966	0.349942944	0.35544038	5.434932765	63.20101671	0.666666667	CRA004775;PRJNA827465;Yang2025_Silkie_Wencha	0.784982422
ENSGALG00010022468	MYBPC3	3	-0.404238018	0.430636303	-0.938699351	0.347885127	0.403799194	13.74775787	85.45217323	0.666666667	CRA004775;PRJNA827465;Yang2025_Silkie_Wencha	0.784982422
ENSGALG00010003315	EPGN	2	0.402295318	0.432033244	0.931167504	0.351766924	0.239064456	2.722022056	63.2626048	1	PRJNA827465;Yang2025_Silkie_Wenchang	0.784982422
ENSGALG00010002766	NANOS1	3	0.366614587	0.391886907	0.93551119	0.349524919	0.283396935	5.574374263	64.12153354	0.666666667	CRA004775;PRJNA827465;Yang2025_Silkie_Wencha	0.784982422
ENSGALG00010029504	GPR142	2	0.358055116	0.381637687	0.938206913	0.348138086	0.20612316	3.22297631	68.97277846	1	PRJNA827465;Yang2025_Silkie_Wenchang	0.784982422
ENSGALG00010009139	CKS2	3	-0.34888785	0.37382214	-0.933299055	0.350665583	0.208159413	4.00669825	50.08358815	0.666666667	CRA004775;PRJNA827465;Yang2025_Silkie_Wencha	0.784982422
ENSGALG00010028735	ENSGALG0001002873	3	0.346692625	0.369743745	0.937656496	0.348420968	0.2756015	6.138858366	67.4206525	0.666666667	CRA004775;PRJNA827465;Yang2025_Silkie_Wencha	0.784982422
ENSGALG00010022309	WSCD2	3	0.332429919	0.3568787	0.931492742	0.351598737	0.250141367	5.839837712	65.75247295	0.666666667	CRA004775;PRJNA827465;Yang2025_Silkie_Wencha	0.784982422
ENSGALG00010023707	MPZL2	3	-0.319110859	0.340884398	-0.936126325	0.349208151	0.138154805	3.295482904	39.31086707	0.666666667	CRA004775;PRJNA827465;Yang2025_Silkie_Wencha	0.784982422
ENSGALG00010021854	TAPBPL	3	0.310863531	0.33320353	0.932953893	0.350843774	0.2716437	14.20893958	85.92435425	0.666666667	CRA004775;PRJNA827465;Yang2025_Silkie_Wencha	0.784982422
ENSGALG00010022473	ZNF385A	3	-0.307453584	0.327634482	-0.93840423	0.348036713	0.265037251	15.42293477	87.03229943	0.666666667	CRA004775;PRJNA827465;Yang2025_Silkie_Wencha	0.784982422
ENSGALG00010013332	LUM	3	-0.296137755	0.316306465	-0.936236808	0.349151276	0.2319399	19.75201673	89.87445167	0.666666667	CRA004775;PRJNA827465;Yang2025_Silkie_Wencha	0.784982422
ENSGALG00010018835	CAMK2B	3	0.295912531	0.317211562	0.932855437	0.350894614	0.267126067	19.22067947	89.59454059	0.666666667	CRA004775;PRJNA827465;Yang2025_Silkie_Wencha	0.784982422
ENSGALG00010008195	ENSGALG0001000819	3	0.292057224	0.311357522	0.938012423	0.348238026	0.132967016	3.710887296	46.10453402	1	CRA004775;PRJNA827465;Yang2025_Silkie_Wencha	0.784982422
ENSGALG00010027412	ENSGALG0001002741	3	0.277038071	0.295563956	0.937320214	0.348593868	0.18345453	6.862421596	70.85576903	0.666666667	CRA004775;PRJNA827465;Yang2025_Silkie_Wencha	0.784982422
ENSGALG00010001114	MED12L	3	0.265636018	0.285180234	0.931467144	0.351611973	0.2215374	27.28657605	92.67038856	0.666666667	CRA004775;PRJNA827465;Yang2025_Silkie_Wencha	0.784982422
ENSGALG00010018185	GNB4	3	-0.257420473	0.27585526	-0.933172248	0.350731041	0.204116606	45.24626711	95.57974585	0.666666667	CRA004775;PRJNA827465;Yang2025_Silkie_Wencha	0.784982422
ENSGALG00010020664	ESYT3	3	0.25666743	0.275678647	0.931038486	0.351833656	0	0.372482338	0	0.666666667	CRA004775;PRJNA827465;Yang2025_Silkie_Wencha	0.784982422
ENSGALG00010013005	SLC40A1	3	-0.255004336	0.27278307	-0.93482464	0.349878679	0.177567387	16.85767722	88.1359693	0.666666667	CRA004775;PRJNA827465;Yang2025_Silkie_Wencha	0.784982422
ENSGALG00010022148	TALDO1	3	-0.252148131	0.2692654	-0.936429749	0.349051967	0.172373399	12.84572041	84.43061241	0.666666667	CRA004775;PRJNA827465;Yang2025_Silkie_Wencha	0.784982422
ENSGALG00010015870	EIF5B	3	0.251793656	0.26801537	0.939474687	0.347487083	0.184204036	16.33571481	87.75688715	0.666666667	CRA004775;PRJNA827465;Yang2025_Silkie_Wencha	0.784982422
ENSGALG00010009690	RBM43	3	-0.244553776	0.261540815	-0.935050142	0.349762459	0.157879212	10.26242753	80.51143363	0.666666667	CRA004775;PRJNA827465;Yang2025_Silkie_Wencha	0.784982422
ENSGALG00010016858	ENSGALG0001001685	3	0.240052131	0.255305387	0.940254861	0.347086847	0.134133743	7.219947057	72.29896585	0.666666667	CRA004775;PRJNA827465;Yang2025_Silkie_Wencha	0.784982422
ENSGALG00010020341	DYNC2LI1	3	-0.236291011	0.252328491	-0.936442053	0.349045635	0.144343389	12.51847343	84.0236111	0.666666667	CRA004775;PRJNA827465;Yang2025_Silkie_Wencha	0.784982422
ENSGALG00010006293	SLC22A3	3	0.229623672	0.245349684	0.935903676	0.349322784	0	0.554004517	0	0.666666667	CRA004775;PRJNA827465;Yang2025_Silkie_Wencha	0.784982422
ENSGALG00010018606	DZANK1	3	0.223361555	0.237423325	0.940773427	0.346820981	0.072513344	3.528851003	43.32432857	0.666666667	CRA004775;PRJNA827465;Yang2025_Silkie_Wencha	0.784982422
ENSGALG00010028136	C3orf18	3	-0.210302638	0.224941133	-0.934922997	0.349827985	0.119009904	15.01929758	86.68379803	0.666666667	CRA004775;PRJNA827465;Yang2025_Silkie_Wencha	0.784982422
ENSGALG00010029517	MSI2	3	0.207457193	0.221494289	0.936625472	0.348951245	0.111524952	11.32768424	82.34414062	0.666666667	CRA004775;PRJNA827465;Yang2025_Silkie_Wencha	0.784982422
ENSGALG00010020779	SLC15A4	3	-0.207186692	0.220114396	-0.941268247	0.346567411	0.134280528	30.08963479	93.35319284	0.666666667	CRA004775;PRJNA827465;Yang2025_Silkie_Wencha	0.784982422
ENSGALG00010005215	VASH1	3	-0.205818866	0.219759005	-0.936566245	0.348981722	0.117040281	16.90397966	88.1684666	0.666666667	CRA004775;PRJNA827465;Yang2025_Silkie_Wencha	0.784982422
ENSGALG00010023805	GALNT6	3	0.201164246	0.215681161	0.932692705	0.350978652	0	1.940905863	0	0.666666667	CRA004775;PRJNA827465;Yang2025_Silkie_Wencha	0.784982422
ENSGALG00010019248	RMI2	3	0.200291138	0.214916646	0.931947997	0.351363401	0.071454556	4.120865417	51.46650527	0.666666667	CRA004775;PRJNA827465;Yang2025_Silkie_Wencha	0.784982422
ENSGALG00010004029	ARL4A	3	0.196704124	0.209541782	0.938734615	0.347867017	0.100596879	13.06619182	84.6933213	0.666666667	CRA004775;PRJNA827465;Yang2025_Silkie_Wencha	0.784982422
ENSGALG00010016572	RAB33A	3	0.19568082	0.210040133	0.931635388	0.351524988	0.115475507	19.89532474	89.947387	0.666666667	CRA004775;PRJNA827465;Yang2025_Silkie_Wencha	0.784982422
ENSGALG00010002741	PDK4	3	-0.193230609	0.207428153	-0.931554404	0.351566857	0.020455679	2.329027608	14.12725236	0.666666667	CRA004775;PRJNA827465;Yang2025_Silkie_Wencha	0.784982422
ENSGALG00010029646	MRPS17	3	-0.188627038	0.202228725	-0.932741073	0.350953673	0.096287775	10.76895967	81.42810391	0.666666667	CRA004775;PRJNA827465;Yang2025_Silkie_Wencha	0.784982422
ENSGALG00010029204	HELZ	3	0.184299231	0.196081072	0.939913423	0.347261971	0.071850786	5.219171781	61.67974376	0.666666667	CRA004775;PRJNA827465;Yang2025_Silkie_Wencha	0.784982422
ENSGALG00010029279	ENSGALG0001002927	3	-0.182785104	0.195837475	-0.933351008	0.350638767	0.081782227	7.984339501	74.95096495	0.666666667	CRA004775;PRJNA827465;Yang2025_Silkie_Wencha	0.784982422
ENSGALG00010024158	ENSGALG0001002415	3	-0.181853337	0.194068023	-0.93705977	0.348727814	0.050840104	3.609173014	44.58564352	0.666666667	CRA004775;PRJNA827465;Yang2025_Silkie_Wencha	0.784982422
ENSGALG00010017250	COL16A1	3	0.172859118	0.184004689	0.939427788	0.347511151	0.055246401	4.481286027	55.36995434	0.666666667	CRA004775;PRJNA827465;Yang2025_Silkie_Wencha	0.784982422
ENSGALG00010010071	GLT8D2	3	-0.171612342	0.182914065	-0.938212937	0.348134991	0.058997862	5.733191308	65.11541491	0.666666667	CRA004775;PRJNA827465;Yang2025_Silkie_Wencha	0.784982422
ENSGALG00010029534	NF1	3	0.157229316	0.168870016	0.931067101	0.351818855	0.052119294	5.369026801	62.74930124	0.666666667	CRA004775;PRJNA827465;Yang2025_Silkie_Wencha	0.784982422
ENSGALG00010023170	TCF12	3	-0.15396398	0.165377172	-0.930986896	0.351860343	0.067452929	15.4884736	87.08717171	0.666666667	CRA004775;PRJNA827465;Yang2025_Silkie_Wencha	0.784982422
ENSGALG00010024126	CDK5R1	3	-0.150943083	0.161840264	-0.932667056	0.350991899	0.033678169	3.512576876	43.0617444	0.666666667	CRA004775;PRJNA827465;Yang2025_Silkie_Wencha	0.784982422
ENSGALG00010008346	NR4A3	3	0.150083633	0.159908567	0.93855905	0.347957186	0	0.79808972	0	1	CRA004775;PRJNA827465;Yang2025_Silkie_Wencha	0.784982422
ENSGALG00010022059	NPTX2	3	-0.147544485	0.157928594	-0.934248076	0.350175941	0.030280131	3.251775448	38.49513805	0.666666667	CRA004775;PRJNA827465;Yang2025_Silkie_Wencha	0.784982422
ENSGALG00010013273	STAG2	3	0.146765465	0.156767779	0.93619662	0.349171963	0.049602357	7.667596593	73.91620731	0.666666667	CRA004775;PRJNA827465;Yang2025_Silkie_Wencha	0.784982422
ENSGALG00010014302	FOXO3	3	-0.146335056	0.15545092	-0.941358569	0.346521138	0.038189068	4.786468232	58.21553799	0.666666667	CRA004775;PRJNA827465;Yang2025_Silkie_Wencha	0.784982422
ENSGALG00010015017	FAM184A	3	0.143593217	0.153410339	0.936007425	0.349269365	0.023900266	2.867218087	30.24597573	0.666666667	CRA004775;PRJNA827465;Yang2025_Silkie_Wencha	0.784982422
ENSGALG00010022137	PDDC1	3	-0.140215574	0.150532611	-0.931463112	0.351614058	0.042990819	5.612550837	64.3655789	0.666666667	CRA004775;PRJNA827465;Yang2025_Silkie_Wencha	0.784982422
ENSGALG00010024351	SP1	3	0.139111832	0.149417692	0.931026511	0.351839851	0.040180976	5.050898444	60.40308428	0.666666667	CRA004775;PRJNA827465;Yang2025_Silkie_Wencha	0.784982422
ENSGALG00010022386	SPI1	3	-0.137487341	0.147619132	-0.931365328	0.35166462	0.029895125	3.865450356	48.25958644	0.666666667	CRA004775;PRJNA827465;Yang2025_Silkie_Wencha	0.784982422
ENSGALG00010011190	ENSGALG0001001119(	3	-0.136512922	0.145146384	-0.940518933	0.346951443	0.011698749	2.411197247	17.05365446	0.666666667	CRA004775;PRJNA827465;Yang2025_Silkie_Wencha	0.784982422
ENSGALG00010018469	KLHDC7A	3	0.13028463	0.138701436	0.939317096	0.347567963	0.029387375	4.079019745	50.96861195	0.666666667	CRA004775;PRJNA827465;Yang2025_Silkie_Wencha	0.784982422
ENSGALG00010023513	CDCA9	3	-0.128171207	0.1375371	-0.931902789	0.351386766	0.017243102	2.800567479	28.58590215	0.666666667	CRA004775;PRJNA827465;Yang2025_Silkie_Wencha	0.784982422
ENSGALG00010024282	HIC2	3	-0.12803642	0.136378032	-0.938834638	0.347815652	0.026033415	3.844213746	47.97375661	0.666666667	CRA004775;PRJNA827465;Yang2025_Silkie_Wencha	0.784982422
ENSGALG00010001567	INPP5A	3	-0.123477525	0.131965008	-0.935683836	0.349435995	0.038774059	12.07068266	83.43092884	0.666666667	CRA004775;PRJNA827465;Yang2025_Silkie_Wencha	0.784982422
ENSGALG00010011316	PARPBP	3	-0.117796484	0.126289147	-0.93275223	0.350947911	0	1.91048698	0	0.666666667	CRA004775;PRJNA827465;Yang2025_Silkie_Wencha	0.784982422
ENSGALG00010014278	G3BP1	3	-0.116334704	0.124559346	-0.933970099	0.350319317	0.028032585	5.732158279	65.10912814	0.666666667	CRA004775;PRJNA827465;Yang2025_Silkie_Wencha	0.784982422
ENSGALG00010006038	ENSGALG0001000603	3	0.114386665	0.122072687	0.93703733	0.348739356	0	0.367085118	0	0.666666667	CRA004775;PRJNA827465;Yang2025_Silkie_Wencha	0.784982422
ENSGALG00010020580	ENSGALG0001002058(	3	-0.111824157	0.119106179	-0.938861089	0.347802069	0.026151965	5.203888288	61.56719958	0.666666667	CRA004775;PRJNA827465;Yang2025_Silkie_Wencha	0.784982422
ENSGALG00010001681	NAPG	3	-0.111069739	0.118163334	-0.939967883	0.347234035	0.031730252	10.91852652	81.68251003	0.666666667	CRA004775;PRJNA827465;Yang2025_Silkie_Wencha	0.784982422
ENSGALG00010007924	ABHD18	3	0.11088309	0.1181893	0.93818214	0.348150815	0.025396355	5.219148852	61.67957541	0.666666667	CRA004775;PRJNA827465;Yang2025_Silkie_Wencha	0.784982422
ENSGALG00010021201	TRMT1L	3	0.109931494	0.116995562	0.939621067	0.347411966	0.025089141	5.226363466	61.73247397	0.666666667	CRA004775;PRJNA827465;Yang2025_Silkie_Wencha	0.784982422
ENSGALG00010020960	CLEC19A	3	0.109088259	0.116461934	0.936685963	0.348920119	0	1.539747987	0	0.666666667	CRA004775;PRJNA827465;Yang2025_Silkie_Wencha	0.784982422
ENSGALG00010017993	REXO5	3	0.105789389	0.113442156	0.932540357	0.35105734	0.020608507	4.642282452	56.91774422	0.666666667	CRA004775;PRJNA827465;Yang2025_Silkie_Wencha	0.784982422
ENSGALG00010024533	CDKN2C	3	-0.102647102	0.109254945	-0.939519048	0.347464317	0.007843183	2.505532412	20.17664628	0.666666667	CRA004775;PRJNA827465;Yang2025_Silkie_Wencha	0.784982422
ENSGALG00010010816	AP2S1	3	-0.092833348	0.098723806	-0.940333963	0.347046284	0.005572514	2.229938576	10.31143094	1	CRA004775;PRJNA827465;Yang2025_Silkie_Wencha	0.784982422
ENSGALG00010006838	ASB13	3	-0.092584661	0.098576502	-0.939216343	0.34761968	0.008000336	2.641893611	24.29672445	1	CRA004775;PRJNA827465;Yang2025_Silkie_Wencha	0.784982422
ENSGALG00010024866	TRAPPC4	3	-0.09255376	0.098329786	-0.94125863	0.346572338	0.010407991	2.656123773	24.70230415	1	CRA004775;PRJNA827465;Yang2025_Silkie_Wencha	0.784982422
ENSGALG00010007885	USP38	3	-0.091759958	0.097644008	-0.939739774	0.347351058	0.016082164	5.527614091	63.81802407	0.666666667	CRA004775;PRJNA827465;Yang2025_Silkie_Wencha	0.784982422
ENSGALG00010002914	SLC27A1	3	-0.091143724	0.097903928	-0.930950636	0.3518791	0	0.642451146	0	0.666666667	CRA004775;PRJNA827465;Yang2025_Silkie_Wencha	0.784982422
ENSGALG00010029926	BLTP2	3	0.090709634	0.096873022	0.936376629	0.349079306	0.018954104	9.963502941	79.9267385	0.666666667	CRA004775;PRJNA827465;Yang2025_Silkie_Wencha	0.784982422
ENSGALG00010001678	SYAP1	3	-0.090197656	0.095923027	-0.940312854	0.347057108	0.018816341	8.197137701	75.60123944	0.666666667	CRA004775;PRJNA827465;Yang2025_Silkie_Wencha	0.784982422
ENSGALG00010014539	PAIP1	3	-0.087648145	0.09390753	-0.933345232	0.350641748	0.018253212	6.875360834	70.91061767	0.666666667	CRA004775;PRJNA827465;Yang2025_Silkie_Wencha	0.784982422
ENSGALG00010003852	TCEB1	3	-0.087035383	0.093202612	-0.933829873	0.350391657	0.013311499	4.257340405	53.02231417	0.666666667	CRA004775;PRJNA827465;Yang2025_Silkie_Wencha	0.784982422
ENSGALG00010029591	SUZ12	3	-0.085359272	0.091560252	-0.932274328	0.351194771	0.013787091	5.463396983	63.39273887	0.666666667	CRA004775;PRJNA827465;Yang2025_Silkie_Wencha	0.784982422
ENSGALG00010020701	PTPN11	3	-0.084526712	0.090299663	-0.936068955	0.349237686	0.015198923	6.115862609	67.29815354	0.666666667	CRA004775;PRJNA827465;Yang2025_Silkie_Wencha	0.784982422
ENSGALG00010001353	GLRX3	3	-0.08178498	0.087533214	-0.934330815	0.350133273	0.007496033	2.627268824	23.87531943	0.666666667	CRA004775;PRJNA827465;Yang2025_Silkie_Wencha	0.784982422
ENSGALG00010029593	PAFAH1B1	3	-0.078713112	0.084084053	-0.936124138	0.349209276	0.013615329	6.935521228	71.16294603	0.666666667	CRA004775;PRJNA827465;Yang2025_Silkie_Wencha	0.784982422
ENSGALG00010016384	GNAS	3	0.077282111	0.082600689	0.935610972	0.349473523	0.010201685	3.861194753	48.20256093	1	CRA004775;PRJNA827465;Yang2025_Silkie_Wencha	0.784982422
ENSGALG00010004624	PHF10	3	-0.075906427	0.081384411	-0.932690003	0.350980048	0.011626621	5.421801555	63.11189224	0.666666667	CRA004775;PRJNA827465;Yang2025_Silkie_Wencha	0.784982422
ENSGALG00010010605	NUF2	3	-0.073202393	0.078160376	-0.936566544	0.348981568	0	1.424647777	0	0.666666667	CRA004775;PRJNA827465;Yang2025_Silkie_Wencha	0.784982422
ENSGALG00010013930	SLC22A16	3	0.070711523	0.075486078	0.9367492	0.348887582	0	1.576163864	0	0.666666667	CRA004775;PRJNA827465;Yang2025_Silkie_Wencha	0.784982422
ENSGALG00010014750	LONRF1	3	0.068419035	0.073305976	0.933335029	0.350647014	0	1.474609933	0	1	CRA004775;PRJNA827465;Yang2025_Silkie_Wencha	0.784982422
ENSGALG00010020268	CPLANE2	3	-0.068411587	0.073128806	-0.935494381	0.349533578	0.001889251	2.076135075	3.667154221	0.666666667	CRA004775;PRJNA827465;Yang2025_Silkie_Wencha	0.784982422
ENSGALG00010017332	FGFR1	3	-0.06835624	0.07340242	-0.931253224	0.351722592	0.006853067	3.416650862	41.46314385	0.666666667	CRA004775;PRJNA827465;Yang2025_Silkie_Wencha	0.784982422
ENSGALG00010002202	SFXN4	3	-0.066340153	0.071224217	-0.931426911	0.351632776	0	1.877984034	0	1	CRA004775;PRJNA827465;Yang2025_Silkie_Wencha	0.784982422
ENSGALG00010007223	SIRT6	3	-0.065446124	0.069762532	-0.938127127	0.348179082	0.000430456	2.665205622	24.958856	1	CRA004775;PRJNA827465;Yang2025_Silkie_Wencha	0.784982422
ENSGALG00010015832	RAB39B	3	0.062455156	0.066759912	0.935518855	0.349520971	0.005903348	3.595774675	44.37916219	0.666666667	CRA004775;PRJNA827465;Yang2025_Silkie_Wencha	0.784982422

ENSGALG00010021741	RPL19	3	-0.059404913	0.063196131	-0.940008693	0.347213102	0	0.683091583	0	0.666666667	CRA004775;PRJNA827465;Yang2025_Silkie_Wencha	0.784982422
ENSGALG00010009311	BNIP1	3	-0.058085535	0.062281867	-0.932623532	0.351014379	0	0.110811472	0	1	CRA004775;PRJNA827465;Yang2025_Silkie_Wencha	0.784982422
ENSGALG00010002054	RSF1	3	-0.052901009	0.056256263	-0.940357684	0.34703412	0	0.97648518	0	0.666666667	CRA004775;PRJNA827465;Yang2025_Silkie_Wencha	0.784982422
ENSGALG00010027722	GNAI3	3	-0.046189866	0.049411615	-0.934797731	0.349892549	0.001633474	2.384331435	16.11904407	1	CRA004775;PRJNA827465;Yang2025_Silkie_Wencha	0.784982422
ENSGALG00010022644	ARMC8	3	-0.034829961	0.037345477	-0.932642014	0.351004833	0	1.408856701	0	1	CRA004775;PRJNA827465;Yang2025_Silkie_Wencha	0.784982422
ENSGALG00010020716	RRAS	3	-0.11075321	0.119016216	-0.93057244	0.352074776	0.023699178	5.266968823	62.02749499	0.666666667	CRA004775;PRJNA827465;Yang2025_Silkie_Wencha	0.785171177
ENSGALG00010002653	EIF1AX	3	-0.08504286	0.091387615	-0.930573144	0.352074412	0.015163266	5.590741545	64.22657024	0.666666667	CRA004775;PRJNA827465;Yang2025_Silkie_Wencha	0.785171177
ENSGALG00010018183	CYP17A1	2	-0.164465102	0.176790111	-0.93028451	0.352223796	0	0.056856805	0	1	PRJNA827465;Yang2025_Silkie_Wenchang	0.785379632
ENSGALG00010010394	MRPL42	3	-0.127219893	0.136785939	-0.930065571	0.352337135	0.028481689	4.024380759	50.30291317	0.666666667	CRA004775;PRJNA827465;Yang2025_Silkie_Wencha	0.785508477
ENSGALG00010007102	CMTM3	3	-0.198775913	0.213760481	-0.929900198	0.35242276	0.114904492	23.71330979	91.56591797	0.666666667	CRA004775;PRJNA827465;Yang2025_Silkie_Wencha	0.785575502
ENSGALG00010022244	ENTPD7	3	0.105622312	0.113598196	0.929788634	0.352480532	0.013752316	3.01744734	33.71881015	0.666666667	CRA004775;PRJNA827465;Yang2025_Silkie_Wencha	0.78558043
ENSGALG00010029243	NME1	3	0.276364918	0.297336944	0.929467135	0.35264705	0.152991613	5.035824759	60.28455922	0.666666667	CRA004775;PRJNA827465;Yang2025_Silkie_Wencha	0.785732329
ENSGALG00010025327	TMEM59	3	-0.164335005	0.176810305	-0.92944246	0.352659832	0.079766053	14.85539175	86.53687473	0.666666667	CRA004775;PRJNA827465;Yang2025_Silkie_Wencha	0.785732329
ENSGALG00010008782	CCDC9	3	0.374996922	0.403568744	0.929202094	0.352784362	0.410644954	12.55344195	84.06811448	0.666666667	CRA004775;PRJNA827465;Yang2025_Silkie_Wencha	0.785789326
ENSGALG00010023592	BAHD1	3	-0.16809144	0.180903274	-0.929178543	0.352796566	0.078591978	18.42186726	89.14333725	0.666666667	CRA004775;PRJNA827465;Yang2025_Silkie_Wencha	0.785789326
ENSGALG00010020651	LMO1	3	0.122050017	0.131391756	0.928901637	0.352940065	0	1.350513977	0	0.666666667	CRA004775;PRJNA827465;Yang2025_Silkie_Wencha	0.785950599
ENSGALG00010019961	HTATSF1	3	-0.057489208	0.061894599	-0.928824303	0.352980147	0.004865019	3.440123983	41.86256048	0.666666667	CRA004775;PRJNA827465;Yang2025_Silkie_Wencha	0.785950599
ENSGALG00010019910	ENSGALG0001001991(	3	0.319003812	0.343530997	0.928602703	0.353095021	0.270137426	13.56243083	85.25338101	0.666666667	CRA004775;PRJNA827465;Yang2025_Silkie_Wencha	0.786027477
ENSGALG00010028049	MAN1B1	3	-0.19938998	0.214734195	-0.928543219	0.35312586	0.111491828	18.75079115	89.33378339	0.666666667	CRA004775;PRJNA827465;Yang2025_Silkie_Wencha	0.786027477
ENSGALG00010021656	SETD6	3	0.144172797	0.15537183	0.92792108	0.353448509	0.052014878	10.62368629	81.1741429	0.666666667	CRA004775;PRJNA827465;Yang2025_Silkie_Wencha	0.786273772
ENSGALG00010021237	FSCN1	3	-0.139979464	0.150844191	-0.927973846	0.353421136	0.03486646	4.288017283	53.35839695	0.666666667	CRA004775;PRJNA827465;Yang2025_Silkie_Wencha	0.786273772
ENSGALG00010019684	PIGQ	3	-0.090103808	0.097077672	-0.928162021	0.353323532	0.005765923	2.463070352	18.8005329	0.666666667	CRA004775;PRJNA827465;Yang2025_Silkie_Wencha	0.786273772
ENSGALG00010003824	SP4	3	-0.061231757	0.06598954	-0.927900953	0.35345895	0	1.620133524	0	0.666666667	CRA004775;PRJNA827465;Yang2025_Silkie_Wencha	0.786273772
ENSGALG00010024167	CHRNA3	2	-0.212918466	0.229504288	-0.927731976	0.353546617	0	0.230978636	0	1	PRJNA827465;Yang2025_Silkie_Wenchang	0.786345072
ENSGALG00010023786	ENSGALG0001002378(	3	-0.174602976	0.188244173	-0.927534558	0.353649058	0.038001376	3.005374175	33.45254589	0.666666667	CRA004775;PRJNA827465;Yang2025_Silkie_Wencha	0.786377777
ENSGALG00010027752	SCAI	3	0.052529059	0.056636611	0.927475329	0.353679795	0	0.572983913	0	1	CRA004775;PRJNA827465;Yang2025_Silkie_Wencha	0.786377777
ENSGALG00010016938	PSME4	3	-0.043012833	0.046380918	-0.927382112	0.353728175	0	1.341813233	0	0.666666667	CRA004775;PRJNA827465;Yang2025_Silkie_Wencha	0.786377777
ENSGALG00010013442	ENSGALG0001001344:	3	-0.063672423	0.068666183	-0.927274818	0.353783866	0	0.2130374	0	0.666666667	CRA004775;PRJNA827465;Yang2025_Silkie_Wencha	0.78637794
ENSGALG00010026917	AMT	3	0.168383622	0.181643849	0.926998754	0.353927181	0.054668291	4.4963813	55.51978654	0.666666667	CRA004775;PRJNA827465;Yang2025_Silkie_Wencha	0.78638225
ENSGALG00010017519	NTN3	3	0.14414884	0.155508808	0.926949685	0.353952659	0.035594309	4.171547804	52.05616491	1	CRA004775;PRJNA827465;Yang2025_Silkie_Wencha	0.78638225
ENSGALG00010008292	REEP5	3	-0.086259556	0.093043888	-0.927084603	0.35388261	0.010798766	3.38012978	40.83067425	0.666666667	CRA004775;PRJNA827465;Yang2025_Silkie_Wencha	0.78638225
ENSGALG00010022780	DYNC1I2	3	-0.032574684	0.035149869	-0.926737019	0.354063092	0	0.510145971	0	0.666666667	CRA004775;PRJNA827465;Yang2025_Silkie_Wencha	0.786504015
ENSGALG00010025876	ENSGALG0001002587(	2	-1.006270602	1.090933807	-0.922393821	0.356323192	0	0.530275594	0	1	PRJNA827465;Yang2025_Silkie_Wenchang	0.786517334
ENSGALG00010022969	TTN	3	0.709696231	0.769627573	0.922129424	0.356461071	1.649224971	71.11188513	97.18753061	0.666666667	CRA004775;PRJNA827465;Yang2025_Silkie_Wencha	0.786517334
ENSGALG00010028900	TNC	3	0.452480163	0.490124946	0.923193497	0.355906377	0.506729344	6.784168685	70.51960096	0.666666667	CRA004775;PRJNA827465;Yang2025_Silkie_Wencha	0.786517334
ENSGALG00010008428	ENSGALG0001000842(	3	0.44044006	0.476412835	0.924492432	0.35522999	0.431204506	5.70428014	64.93860836	0.666666667	CRA004775;PRJNA827465;Yang2025_Silkie_Wencha	0.786517334
ENSGALG00010010399	FGF18	3	0.43112293	0.465938933	0.925277755	0.354821447	0.536896363	19.39930254	89.690351	0.666666667	CRA004775;PRJNA827465;Yang2025_Silkie_Wencha	0.786517334
ENSGALG00010021639	SCG5	3	0.354401864	0.384308451	0.922180772	0.356434291	0.299852955	6.313655849	68.32263196	0.666666667	CRA004775;PRJNA827465;Yang2025_Silkie_Wencha	0.786517334
ENSGALG00010029647	RSAD1	3	0.338617228	0.366545225	0.9238075	0.35558655	0.344938146	14.62009174	86.32019528	0.666666667	CRA004775;PRJNA827465;Yang2025_Silkie_Wencha	0.786517334
ENSGALG00010005353	ENSGALG0001000535:	3	-0.328487348	0.355043162	-0.925203984	0.354859811	0.349314495	45.6198211	95.61594072	0.666666667	CRA004775;PRJNA827465;Yang2025_Silkie_Wencha	0.786517334
ENSGALG00010008177	NREP	3	-0.274087903	0.296801591	-0.923471811	0.355761384	0.201464544	13.29266625	84.95410956	0.666666667	CRA004775;PRJNA827465;Yang2025_Silkie_Wencha	0.786517334
ENSGALG00010025624	ARMH1	3	0.251027448	0.271098283	0.925964726	0.354464312	0.121494144	4.481526007	55.37234422	0.666666667	CRA004775;PRJNA827465;Yang2025_Silkie_Wencha	0.786517334
ENSGALG00010017606	ENSGALG0001001760(	3	-0.248304339	0.268339981	-0.925334859	0.354791752	0.100081859	3.763114311	46.85253131	1	CRA004775;PRJNA827465;Yang2025_Silkie_Wencha	0.786517334
ENSGALG00010021695	FNBP4	3	0.248097607	0.268269991	0.924805663	0.355067004	0.192815072	22.15142145	90.97123404	0.666666667	CRA004775;PRJNA827465;Yang2025_Silkie_Wencha	0.786517334
ENSGALG00010028431	ENSGALG0001002843l	3	0.241634957	0.261296311	0.924754569	0.355093587	0.061315342	2.833269448	29.41017306	0.666666667	CRA004775;PRJNA827465;Yang2025_Silkie_Wencha	0.786517334
ENSGALG00010011965	ATIC	3	-0.209316572	0.22603224	-0.926047416	0.354421339	0.11962997	9.455774477	78.84890334	0.666666667	CRA004775;PRJNA827465;Yang2025_Silkie_Wencha	0.786517334
ENSGALG00010007955	TMA16	3	0.208975447	0.226344633	0.923262216	0.355870573	0.119242123	12.56172291	84.07861713	0.666666667	CRA004775;PRJNA827465;Yang2025_Silkie_Wencha	0.786517334
ENSGALG00010009847	ENSGALG0001000984:	3	-0.207906176	0.224791026	-0.924886459	0.355024971	0.126233214	13.40781774	85.08332945	0.666666667	CRA004775;PRJNA827465;Yang2025_Silkie_Wencha	0.786517334
ENSGALG00010016859	RPIA	3	-0.20007834	0.216041322	-0.926111444	0.354388067	0.109063463	13.16949093	84.81338412	0.666666667	CRA004775;PRJNA827465;Yang2025_Silkie_Wencha	0.786517334
ENSGALG00010023788	RTCA	3	0.188763959	0.204067386	0.925007976	0.354961759	0.105209504	18.88477489	89.40945809	0.666666667	CRA004775;PRJNA827465;Yang2025_Silkie_Wencha	0.786517334
ENSGALG00010016903	PPM1A	3	-0.187657996	0.203216687	-0.923437926	0.355779035	0.086852911	6.973459422	71.31983025	0.666666667	CRA004775;PRJNA827465;Yang2025_Silkie_Wencha	0.786517334
ENSGALG00010006052	RALGAPA1	3	0.185418243	0.200584182	0.924391151	0.3552827	0.095171122	14.18457523	85.900177	0.666666667	CRA004775;PRJNA827465;Yang2025_Silkie_Wencha	0.786517334
ENSGALG00010023639	KLHL23	3	0.17272513	0.186976606	0.923779361	0.355601203	0.037774817	3.11353461	35.76432413	0.666666667	CRA004775;PRJNA827465;Yang2025_Silkie_Wencha	0.786517334
ENSGALG00010003797	ENSGALG0001000379:	3	0.170145387	0.184338419	0.923005569	0.356004303	0.053725867	4.343557227	53.95479108	0.666666667	CRA004775;PRJNA827465;Yang2025_Silkie_Wencha	0.786517334
ENSGALG00010009793	TNFAIP6	3	-0.160577073	0.173688083	-0.924514051	0.355218739	0.035267409	3.272281608	38.88056592	0.666666667	CRA004775;PRJNA827465;Yang2025_Silkie_Wencha	0.786517334
ENSGALG00010027901	MOB3A	3	-0.150569489	0.163233704	-0.922416663	0.356311282	0.058071576	8.232381295	75.70569282	0.666666667	CRA004775;PRJNA827465;Yang2025_Silkie_Wencha	0.786517334
ENSGALG00010004746	LYN	3	-0.137972257	0.149505441	-0.922857762	0.356081335	0.034124202	4.60166171	56.5374396	0.666666667	CRA004775;PRJNA827465;Yang2025_Silkie_Wencha	0.786517334
ENSGALG00010001712	ATP6AP1	3	-0.137296897	0.148753619	-0.922981893	0.356016641	0.039679419	5.697482105	64.89677435	0.666666667	CRA004775;PRJNA827465;Yang2025_Silkie_Wencha	0.786517334
ENSGALG00010008186	NOP53	3	-0.137269528	0.148464482	-0.924595073	0.355176577	0.036999226	4.673487818	57.20540894	0.666666667	CRA004775;PRJNA827465;Yang2025_Silkie_Wencha	0.786517334
ENSGALG00010006524	TAB2	3	0.132041733	0.142680152	0.925438695	0.354737759	0.037471676	5.015087609	60.12033775	0.666666667	CRA004775;PRJNA827465;Yang2025_Silkie_Wencha	0.786517334
ENSGALG00010010270	SSR1	3	-0.12683504	0.137090815	-0.925189921	0.354867125	0.040906265	9.362592054	78.63839428	0.666666667	CRA004775;PRJNA827465;Yang2025_Silkie_Wencha	0.786517334
ENSGALG00010025243	PAXIP1	3	0.119942714	0.129943751	0.92303564	0.355988633	0.024650674	3.591995543	44.3206436	0.666666667	CRA004775;PRJNA827465;Yang2025_Silkie_Wencha	0.786517334
ENSGALG00010008278	CYP39A1	3	0.111417774	0.120780185	0.922483877	0.356276236	0	0.543093592	0	0.666666667	CRA004775;PRJNA827465;Yang2025_Silkie_Wencha	0.786517334
ENSGALG00010003753	SLCO5A1	3	0.104013068	0.112747885	0.922527883	0.356253293	0	0.736023652	0	1	CRA004775;PRJNA827465;Yang2025_Silkie_Wencha	0.786517334
ENSGALG00010018367	PAK5	3	-0.100603805	0.109009536	-0.92288995	0.356064559	0	0.012122624	0	1	CRA004775;PRJNA827465;Yang2025_Silkie_Wencha	0.786517334
ENSGALG00010013516	CSRP2	3	0.097347653	0.105178836	0.925544121	0.354682944	0.006971828	2.343505235	14.65775413	0.666666667	CRA004775;PRJNA827465;Yang2025_Silkie_Wencha	0.786517334
ENSGALG00010018115	KLHL4	3	-0.092679117	0.100370436	-0.923370677	0.355814067	0.000325584	2.016154526	0.801254346	0.666666667	CRA004775;PRJNA827465;Yang2025_Silkie_Wencha	0.786517334
ENSGALG00010012103	ENSGALG0001001210:	3	-0.082681184	0.089304431	-0.925835182	0.354531641	0.003910242	2.337145279	14.42551653	0.666666667	CRA004775;PRJNA827465;Yang2025_Silkie_Wencha	0.786517334
ENSGALG00010022158	CWC25	3	0.079298258	0.085978256	0.922305959	0.356369007	0.010424501	3.78290364	47.13055921	0.666666667	CRA004775;PRJNA827465;Yang2025_Silkie_Wencha	0.786517334
ENSGALG00010013815	SRXN1	3	-0.074670412	0.080791177	-0.924239678	0.355361541	0	0.930126018	0	1	CRA004775;PRJNA827465;Yang2025_Silkie_Wencha	0.786517334
ENSGALG00010007909	GALNS	3	-0.069824099	0.0756444	-0.92305708	0.35597746	0	1.13507446	0	0.666666667	CRA004775;PRJNA827465;Yang2025_Silkie_Wencha	0.786517334
ENSGALG00010008892	ENSGALG0001000889:	3	-0.068340769	0.074104961	-0.922215841	0.356416002	0	1.133476029	0	0.666666667	CRA004775;PRJNA827465;Yang2025_Silkie_Wencha	0.786517334
ENSGALG00010019323	LRIG1	3	-0.062963993	0.068210517	-0.923083356	0.355963768	0.002419335	2.277020589	12.16592377	0.666666667	CRA004775;PRJNA827465;Yang2025_Silkie_Wencha	0.786517334
ENSGALG00010027931	ABHD17A	3	-0.05104104	0.055282451	-0.923277443	0.35586264	0	1.051424906	0	0.666666667	CRA004775;PRJNA827465;Yang2025_Silkie_Wencha	0.786517334
ENSGALG00010029202	PSMD12	3	-0.037860256	0.040982938	-0.923805317	0.355587687	0	1.356029643	0	0.666666667	CRA004775;PRJNA827465;Yang2025_Silkie_Wencha	0.786517334
ENSGALG00010002853	CEP162	3	0.368622997	0.399810313	0.921994719	0.35653133	0.422928589	19.52099146	89.75461874	0.666666667	CRA004775;PRJNA827465;Yang2025_Silkie_Wencha	0.786549614
ENSGALG00010025006	ENSGALG0001002500(	2	0.645253936	0.700093269	0.921668532	0.356701499	0.883481017	10.12223266	90.12075662	0.5	PRJNA827465;Yang2025_Silkie_Wenchang	0.78657771
ENSGALG00010017948	PLAT	3	0.124701959	0.135318539	0.921543784	0.356766593	0.031338127	4.789658569	58.24337014	0.666666667	CRA004775;PRJNA827465;Yang2025_Silkie_Wencha	0.78657771
ENSGALG00010006013	TRMT10C	3	-0.080339829	0.087170291	-0.921642314	0.356715179	0.007179285	2.666741182	25.00209569	0.666666667	CRA004775;PRJNA827465;Yang2025_Silkie_Wencha	0.78657771
ENSGALG00010001803	PPP4R1	3	-0.034353486	0.037273736	-0.92165395	0.356709108	0	1.140554782	0	0.666666667	CRA004775;PRJNA827465;Yang2025_Silkie_Wencha	0.78657771
ENSGALG00010014936	FOXRED2	3	-0.180495196	0.195910062	-0.921316615	0.356885149	0.085054835	8.562703612	76.64289119	0.666666667	CRA004775;PRJNA827465;Yang2025_Silkie_Wencha	0.78671642
ENSGALG00010021674	ING4	3	0.136432813	0.148112839	0.921141034	0.356976799	0.044339028	7.590617499	73.65168249	0.666666667	CRA004775;PRJNA827465;Yang2025_Silkie_Wencha	0.786795786
ENSGALG00010017305	HS3ST2	3	0.28626409	0.310863717	0.920866846	0.35711995	0.096092542	2.975638812	32.78754155	0.666666667	CRA004775;PRJNA827465;Yang2025_Silkie_Wencha	0.786826216
ENSGALG00010028104	GOLGA1	3	0.219439667	0.238370656	0.920581715	0.357268852	0.147861234	16.31492645	87.74128706	0.666666667	CRA004775;PRJNA827465;Yang2025_Silkie_Wencha	0.786826216
ENSGALG00010018747	FLRT3	3	0.134043146	0.145603798	0.920601991	0.357258262	0.03312763	4.119476974	51.45014737	0.666666667	CRA004775;PRJNA827465;Yang2025_Silkie_Wencha	0.786826216
ENSGALG00010024332	EFCAB7	3	-0.091443062	0.099286793	-0.920999247	0.35705082	0	1.17082289	0	0.666666667	CRA004775;PRJNA827465;Yang2025_Silkie_Wencha	0.786826216
ENSGALG00010002569	GTPBP6	3	-0.074051275	0.080428786	-0.920706122	0.357203879	0	1.696344351	0	0.666666667	CRA004775;PRJNA827465;Yang2025_Silkie_Wencha	0.786826216
ENSGALG00010010267	CCSER2	3	0.208070016	0.226054083	0.920443521	0.357341034	0.111855175	8.887793442	77.49722681	0.666666667	CRA004775;PRJNA827465;Yang2025_Silkie_Wencha	0.78686262
ENSGALG00010025200	PEPD	3	-0.127054849	0.138069654	-0.920222841	0.357456321	0.040385583	7.072190975	71.72022069	0.666666667	CRA004775;PRJNA827465;Yang2025_Silkie_Wencha	0.786993914
ENSGALG00010000171	DPF2	3	-0.084278294	0.091595333	-0.920115592	0.357512357	0.014717684	4.933731769	59.46273341	0.666666667	CRA004775;PRJNA827465;Yang2025_Silkie_Wencha	0.786994741
ENSGALG00010029458	ENSGALG0001002945(	3	-0.633245828	0.692971036	-0.916698896	0.359300411	0	0.011909862	0	1	CRA004775;PRJNA827465;Yang2025_Silkie_Wencha	0.787164224
ENSGALG00010000062	ENSGALG0001000006:	2	0.474882804	0.516759628	0.918962662	0.358115102	0.068525878	1.126053701	11.19428861	0.5	CRA004775;Yang2025_Silkie_Wenchang	0.787164224
ENSGALG00010029191	GSG1L2	3	0.465409384	0.506002656	0.919776563	0.357689533	0.553533062	7.26089386	72.45518199	0.666666667	CRA004775;PRJNA827465;Yang2025_Silkie_Wencha	0.787164224
ENSGALG00010004755	PAX9	2	0.443625203	0.482407998	0.919605821	0.357778784	0.323639883	3.27336034	69.45035389	0.5	PRJNA827465;Yang2025_Silkie_Wenchang	0.787164224
ENSGALG00010014190	HACD4	3	-0.35835239	0.390619029	-0.917396142	0.358935094	0.362134468	11.53771986	82.66555243	0.666666667	CRA004775;PRJNA827465;Yang2025_Silkie_Wencha	0.787164224
ENSGALG00010027609	RPL36	3	0.333301948	0.36306763	0.91801615	0.358610411	0.358900827	26.58001585	92.47555001	0.666666667	CRA004775;PRJNA827465;Yang2025_Silkie_Wencha	0.787164224
ENSGALG00010016566	CNTLN	3	0.296619523	0.323279451	0.917532873	0.358863475	0.178753347	5.396922919	62.94184612	0.666666667	CRA004775;PRJNA827465;Yang2025_Silkie_Wencha	0.787164224
ENSGALG00010012091	ENSGALG0001001209:	3	-0.292068416	0.317924881	-0.918671149	0.358267605	0	1.575100492	0	1	CRA004775;PRJNA827465;Yang2025_Silkie_Wencha	0.787164224
ENSGALG00010002174	ENSGALG0001000217:	3	-0.269795884	0.293870018	-0.918078971	0.358577523	0	1.97986657	0	0.666666667	CRA004775;PRJNA827465;Yang2025_Silkie_Wencha	0.787164224
ENSGALG00010013145	ENSGALG0001001314:	3	0.227260472	0.247522413	0.918140984	0.358545061	0.090005396	3.967596229	49.5916448	0.666666667	CRA004775;PRJNA827465;Yang2025_Silkie_Wencha	0.787164224
ENSGALG00010016942	ZBTB40	3	0.218049697	0.237988804	0.916218298	0.35955241	0.126930114	8.527803997	76.54730338	0.666666667	CRA004775;PRJNA827465;Yang2025_Silkie_Wencha	0.787164224
ENSGALG00010003804	PSME1	3	-0.206223576	0.224790739	-0.917402458	0.358931785	0.11156301	8.167258193	75.51197779	0.666666667	CRA004775;PRJNA827465;Yang2025_Silkie_Wencha	0.787164224
ENSGALG00010001880	MTCL1	3	0.189062741	0.205949172	0.918006801	0.358615305	0.076857199	5.560448804	64.03168035	0.666666667	CRA004775;PRJNA827465;Yang2025_Silkie_Wencha	0.787164224
ENSGALG00010023384	AP3B2	3	0.181563268	0.198168661	0.916205754	0.359558988	0.068335981	4.749489164	57.89020817	0.666666667	CRA004775;PRJNA827465;Yang2025_Silkie_Wencha	0.787164224
ENSGALG00010022708	SPRED1	3	-0.174325364	0.19023443	-0.916371257	0.359472206	0.090978403	21.57889606	90.73168528	0.666666667	CRA004775;PRJNA827465;Yang2025_Silkie_Wencha	0.787164224
ENSGALG00010004902	ANKRD29	3	0.171751931	0.187272483	0.91712316	0.359078106	0	0.376259525	0	0.666666667	CRA004775;PRJNA827465;Yang2025_Silkie_Wencha	0.787164224
ENSGALG00010021391	ENSGALG0001002139:	3	0.171148123	0.186516237	0.917604417	0.358826005	0	1.752053898	0	0.666666667	CRA004775;PRJNA827465;Yang2025_Silkie_Wencha	0.787164224
ENSGALG00010004104	SCARB2	3	-0.157076189	0.170985314	-0.918653101	0.358277048	0.066850781	10.0206583	80.04123142	0.666666667	CRA004775;PRJNA827465;Yang2025_Silkie_Wencha	0.787164224
ENSGALG00010024708	BNIP2	3	0.146667169	0.15995367	0.916935319	0.359176535	0.063771654	13.61456199	85.3098469	0.666666667	CRA004775;PRJNA827465;Yang2025_Silkie_Wencha	0.787164224
ENSGALG00010027811	MEX3D	3	0.145940365	0.159248758	0.916430162	0.359441321	0.058029331	9.747585524	79.48209846	0.666666667	CRA004775;PRJNA827465;Yang2025_Silkie_Wencha	0.787164224
ENSGALG00010023074	CCNT1	3	0.132289626	0.145391922	0.916760866	0.359267963	0.047137899	5.763513441	75.9658256	0.666666667	CRA004775;PRJNA827465;Yang2025_Silkie_Wencha	0.787164224
ENSGALG00010012654	TENT5B	3	0.130135824	0.141999073	0.916455443	0.359428067	0.021961149	3.161242171	36.73373022	0.666666667	CRA004775;PRJNA827465;Yang2025_Silkie_Wencha	0.787164224
ENSGALG00010023102	RNF10	3	-0.128827375	0.140289483	-0.91829674	0.358463533	0.046686605	14.28756444	86.00181291	0.666666667	CRA004775;PRJNA827465;Yang2025_Silkie_Wencha	0.787164224
ENSGALG00010009784	ENSGALG0001000978:	3	0.128437537	0.139873167	0.918242862	0.358491734	0	0.410458111	0	0.666666667	CRA004775;PRJNA827465;Yang2025_Silkie_Wencha	0.787164224
ENSGALG00010028429	REXO1	3	0.12555784	0.136613045	0.919076601	0.358055507	0.040567701	7.901871518	74.68954038	0.666666667	CRA004775;PRJNA827465;Yang2025_Silkie_Wencha	0.787164224
ENSGALG00010001647	ARL8BL	3	-0.101751575	0.110754282	-0.91871459	0.358244876	0	1.992842474	0	0.666666667	CRA004775;PRJNA827465;Yang2025_Silkie_Wencha	0.787164224
ENSGALG00010021602	PLEKHG4	3	-0.091382268	0.099666723	-0.916878421	0.359206353	0.012125625	3.32046147	39.7674083	0.666666667	CRA004775;PRJNA827465;Yang2025_Silkie_Wencha	0.787164224
ENSGALG00010020952	GNG5	3	-0.080028383	0.087226769	-0.917475041	0.358893766	0.00833449	2.807094416	28.75195117	1	CRA004775;PRJNA827465;Yang2025_Silkie_Wencha	0.787164224
ENSGALG00010001585	ENSGALG0001000158:	3	0.076499475	0.083312529	0.918223029	0.358502114	0.005053799	2.559405026	21.86294448	0.666666667	CRA004775;PRJNA827465;Yang2025_Silkie_Wencha	0.787164224
ENSGALG00010018165	DACH2	3	0.075156401	0.08201643	0.916357869	0.359479225	0	1.935859099	0	1	CRA004775;PRJNA827465;Yang2025_Silkie_Wencha	0.787164224
ENSGALG00010029614	MDH2	3	-0.067132904	0.073193128	-0.917202183	0.359036651	0	1.090568019	0	0.666666667	CRA004775;PRJNA827465;Yang2025_Silkie_Wencha	0.787164224
ENSGALG00010000043	PPIP5K1	3	-0.062605267	0.068139155	-0.918785491	0.358207783	0	0.98588645	0	1	CRA004775;PRJNA827465;Yang2025_Silkie_Wencha	0.787164224
ENSGALG00010012034	ARHGEF9	3	-0.05771016	0.062943772	-0.916852575	0.359219898	0	1.051443348	0	0.666666667	CRA004775;PRJNA827465;Yang2025_Silkie_Wencha	0.787164224
ENSGALG00010017514	TXLNA	3	0.051100575	0.055723804	0.917033139	0.359125275	0.002494828	2.672286628	25.15772899	0.666666667	CRA004775;PRJNA827465;Yang2025_Silkie_Wencha	0.787164224
ENSGALG00010006081	ZDHHC14	3	-0.049760629	0.054315543	-0.916139778	0.359593587	0	1.537997453	0	0.666666667	CRA004775;PRJNA827465;Yang2025_Silkie_Wencha	0.787164224
ENSGALG00010018837	RCOR3	3	0.047758166	0.052103086	0.916609169	0.359347478	0	0.770889157	0	1	CRA004775;PRJNA827465;Yang2025_Silkie_Wencha	0.787164224
ENSGALG00010024064	GPR17	3	0.191809778	0.209529766	0.915816313	0.359763246	0.08048444	5.422022176	63.11339321	0.666666667	CRA004775;PRJNA827465;Yang2025_Silkie_Wencha	0.787413705
ENSGALG00010027152	C4BPS	3	0.318527488	0.347935793	0.915477783	0.359940861	0.22041905	5.57146921	64.10282594	0.666666667	CRA004775;PRJNA827465;Yang2025_Silkie_Wencha	0.787558624
ENSGALG00010010333	WAPL	3	0.085877553	0.093802388	0.91551564	0.359920996	0.014068123	4.062763356	50.772422	0.666666667	CRA004775;PRJNA827465;Yang2025_Silkie_Wencha	0.787558624
ENSGALG00010007626	DLK2	3	-0.18597869	0.203174821	-0.915362886	0.360001156	0.069255876	5.727765274	65.08236801	0.666666667	CRA004775;PRJNA827465;Yang2025_Silkie_Wencha	0.787568674
ENSGALG00010007950	ARC	3	-0.341202345	0.373097388	-0.914512822	0.360447443	0.13197981	2.868785558	30.28408853	0.666666667	CRA004775;PRJNA827465;Yang2025_Silkie_Wencha	0.787832352
ENSGALG00010005180	WAC	3	0.185678145	0.203099031	0.914224675	0.360598801	0.095775057	9.80309345	79.59827671	0.666666667	CRA004775;PRJNA827465;Yang2025_Silkie_Wencha	0.787832352
ENSGALG00010013696	CETN3	3	0.171160594	0.187168414	0.914473708	0.360467987	0.037164557	3.084252179	35.15445938	0.666666667	CRA004775;PRJNA827465;Yang2025_Silkie_Wencha	0.787832352
ENSGALG00010019872	VTI1A	3	0.158939804	0.173860838	0.914178292	0.360623163	0.072529968	12.56274722	84.07991528	0.666666667	CRA004775;PRJNA827465;Yang2025_Silkie_Wencha	0.787832352
ENSGALG00010013062	ENSGALG0001001306:	3	0.154212054	0.168684813	0.914182854	0.360620772	0.050652743	4.976563229	59.81162284	0.666666667	CRA004775;PRJNA827465;Yang2025_Silkie_Wencha	0.787832352
ENSGALG00010027490	ATP2B4	3	-0.085214401	0.093167507	-0.91463649	0.360382496	0.011825744	3.572689753	44.01976835	0.666666667	CRA004775;PRJNA827465;Yang2025_Silkie_Wencha	0.787832352
ENSGALG00010022518	ECHS1	3	-0.057041575	0.06237882	-0.914438189	0.360486643	0	1.312583817	0	0.666666667	CRA004775;PRJNA827465;Yang2025_Silkie_Wencha	0.787832352
ENSGALG00010005618	PHACTR2	3	0.052676801	0.057618796	0.914229466	0.360596284	0	0.922057293	0	1	CRA004775;PRJNA827465;Yang2025_Silkie_Wencha	0.787832352
ENSGALG00010001849	NDUFV2	3	-0.052363775	0.05723363	-0.914912702	0.360237461	0	1.805894142	0	0.666666667	CRA004775;PRJNA827465;Yang2025_Silkie_Wencha	0.787832352
ENSGALG00010008141	APTX	3	0.30188524	0.330284333	0.914016227	0.360708319	0.287954426	17.91468609	88.83597519	0.666666667	CRA004775;PRJNA827465;Yang2025_Silkie_Wencha	0.787854432
ENSGALG00010014522	FAT2	2	-0.255693396	0.27978367	-0.913896785	0.360771083	0	0.569801936	0	1	PRJNA827465;Yang2025_Silkie_Wenchang	0.787854432
ENSGALG00010021980	GLMN	3	-0.191006292	0.209014817	-0.913840919	0.360800442	0.099844763	10.77948882	81.44624449	0.666666667	CRA004775;PRJNA827465;Yang2025_Silkie_Wencha	0.787854432
ENSGALG00010024206	KRT80	3	-0.422866132	0.463902285	-0.911562173	0.360915411	0.382196851	5.763137411	65.29668031	0.666666667	CRA004775;PRJNA827465;Yang2025_Silkie_Wencha	0.787919028
ENSGALG00010029260	DHX40	3	0.127875261	0.139972744	0.913575585	0.360941477	0.038486931	7.274570197	72.50696679	0.666666667	CRA004775;PRJNA827465;Yang2025_Silkie_Wencha	0.787919028
ENSGALG00010028734	ENSGALG0001002873:	3	0.457228882	0.500670822	0.913233703	0.361120258	0.632771519	29.15765415	93.14073763	0.666666667	CRA004775;PRJNA827465;Yang2025_Silkie_Wencha	0.788065956
ENSGALG00010016463	FREM1	3	0.090620086	0.099222378	0.911302904	0.361083256	0	1.587020588	0	0.666666667	CRA004775;PRJNA827465;Yang2025_Silkie_Wencha	0.788065956
ENSGALG00010000313	ENSGALG0001000031:	3	-2.038624934	2.234379299	-0.912389823	0.361563545	11.65632936	9.644274556	79.26230751	0.666666667	CRA004775;PRJNA827465;Yang2025_Silkie_Wencha	0.788139624
ENSGALG00010019975	ENSGALG0001001997:	3	0.254599416	0.279040807	0.912409257	0.361553318	0.198351213	21.76133296	90.80938652	0.666666667	CRA004775;PRJNA827465;Yang2025_Silkie_Wencha	0.788139624
ENSGALG00010006517	ZBTB20	3	0.214772733	0.235440683	0.912215894	0.361655079	0.068934329	3.371059382	40.67146932	0.666666667	CRA004775;PRJNA827465;Yang2025_Silkie_Wencha	0.788139624
ENSGALG00010027586	HJV	3	-0.165399081	0.181333536	-0.912126262	0.361702255	0.061744997	8.258785717	75.78336491	0.666666667	CRA004775;PRJNA827465;Yang2025_Silkie_Wencha	0.788139624
ENSGALG00010005081	GREB1L	3	0.146058756	0.160151632	0.91200292	0.3617678	0.034573308	3.613652141	44.65432969	0.666666667	CRA004775;PRJNA827465;Yang2025_Silkie_Wencha	0.788139624
ENSGALG00010021519	FNTB	3	0.088397825	0.096921924	0.912051905	0.361741395	0.009684846	2.643598346	24.34554201	0.666666667	CRA004775;PRJNA827465;Yang2025_Silkie_Wencha	0.788139624
ENSGALG00010000047	IQGAP1	3	0.081491637	0.089288585	0.912676996	0.361412446	0.00870261	3.076625831	34.99372008	0.666666667	CRA004775;PRJNA827465;Yang2025_Silkie_Wencha	0.788139624
ENSGALG00010022717	ELOVL1	3	0.06566715	0.07198847	0.912389164	0.361563498	0.000926811	2.043155718	2.11220894	0.666666667	CRA004775;PRJNA827465;Yang2025_Silkie_Wencha	0.788139624
ENSGALG00010018241	RALGAPA2	3	-0.052541798	0.057586862	-0.912392102	0.361562346	0	0.474721043	0	1	CRA004775;PRJNA827465;Yang2025_Silkie_Wencha	0.788139624
ENSGALG00010013269	INPP5B	3	-0.044852938	0.049158046	-0.912423126	0.36154602	0	0.494250105	0	1	CRA004775;PRJNA827465;Yang2025_Silkie_Wencha	0.788139624
ENSGALG00010029253	NUP88	3	-0.040653792	0.044559321	-0.912352141	0.361583375	0	0.17088676	0	0.666666667	CRA004775;PRJNA827465;Yang2025_Silkie_Wencha	0.788139624
ENSGALG00010014641	DOCK11	3	-0.061858496	0.067850617	-0.910718158	0.361939532	0.002135487	2.272445251	11.98907876	0.666666667	CRA004775;PRJNA827465;Yang2025_Silkie_Wencha	0.788263477
ENSGALG00010010915	APP	3	-0.042838783	0.046987088	-0.911713945	0.361919321	0.000641675	2.140949322	6.583505135	0.666666667	CRA004775;PRJNA827465;Yang2025_Silkie_Wencha	0.788263477
ENSGALG00010005272	MBNL2	3	0.257483675	0.282489831	0.911479449	0.362042809	0.188545648	11.95448059	83.2698712	0.666666667	CRA004775;PRJNA827465;Yang2025_Silkie_Wencha	0.788375678
ENSGALG00010022223	DOK4	3	0.20883485	0.22921488	0.911087667	0.362249184	0.114054266	8.622776273	76.80561415	0.666666667	CRA004775;PRJNA827465;Yang2025_Silkie_Wencha	0.788449055
ENSGALG00010006740	BRCA2	3	0.169628336	0.18616413	0.911176263	0.362202509	0.063269893	6.485237679	69.16072935	0.666666667	CRA004775;PRJNA827465;Yang2025_Silkie_Wencha	0.788449055
ENSGALG00010022317	PSME3	3	-0.163988417	0.18001081	-0.91099205	0.362299562	0.084908114	21.24194977	90.58466844	0.666666667	CRA004775;PRJNA827465;Yang2025_Silkie_Wencha	0.788449055
ENSGALG00010016887	TJP2	3	0.126368357	0.138708288	0.91103682	0.362275974	0.037292385	6.986082602	71.37165256	0.666666667	CRA004775;PRJNA827465;Yang2025_Silkie_Wencha	0.788449055
ENSGALG00010009236	DTWD2	3	0.108902134	0.119557635	0.910875609	0.362360919	0	0.634509884	0	1	CRA004775;PRJNA827465;Yang2025_Silkie_Wencha	0.788461223
ENSGALG00010011655	SORCS2	3	-0.122237422	0.134220802	-0.910718907	0.3624435	0.020665892	3.272453365	38.88377383	0.666666667	CRA004775;PRJNA827465;Yang2025_Silkie_Wencha	0.788519563
ENSGALG00010017016	ZIC1	2	1.100723803	1.213277983	0.907231333	0.364284477	2.451807692	5.668317286	82.35808002	1	PRJNA827465;Yang2025_Silkie_Wenchang	0.788777182
ENSGALG00010027121	ELF3	2	1.044699891	1.152722896	0.906288835	0.364782994	2.141769598	5.147854411	80.57443121	0.5	CRA004775;PRJNA827465	0.788777182
ENSGALG00010021053	ENSGALG0001002105:	2	-0.678221333	0.749201823	-0.905258519	0.365328447	0.922547053	5.484167408	81.76569157	0.5	PRJNA827465;Yang2025_Silkie_Wenchang	0.788777182
ENSGALG00010012058	ENSGALG0001001205:	2	0.538731697	0.595063499	0.905334806	0.365288042	0.597217402	6.374926103	84.31354366	0.5	PRJNA827465;Yang2025_Silkie_Wenchang	0.788777182
ENSGALG00010021564	PRKAG3	3	0.470846589	0.519097446	0.907048557	0.36438112	0.661585109	19.17569832	89.57013212	0.666666667	CRA004775;PRJNA827465;Yang2025_Silkie_Wencha	0.788777182
ENSGALG00010002403	ZNFY3	3	-0.405196395	0.447406226	-0.905656585	0.365117648	0.482758833	11.05096563	81.90203403	0.666666667	CRA004775;PRJNA827465;Yang2025_Silkie_Wencha	0.788777182
ENSGALG00010025575	TRPM2	3	0.34008477	0.374869314	0.90720888	0.364296349	0.130109124	2.841443391	29.6132379	0.666666667	CRA004775;PRJNA827465;Yang2025_Silkie_Wencha	0.788777182
ENSGALG00010013295	CA3B	3	-0.332309331	0.365607285	-0.90892426	0.363390107	0.188859035	3.818523007	47.62372791	0.666666667	CRA004775;PRJNA827465;Yang2025_Silkie_Wencha	0.788777182
ENSGALG00010012971	ENSGALG0001001297:	3	0.318811026	0.352422285	0.904627886	0.365662557	0.170072377	3.630147862	44.90582543	0.666666667	CRA004775;PRJNA827465;Yang2025_Silkie_Wencha	0.788777182
ENSGALG00010011662	RIDA	3	-0.31787774	0.351190594	-0.905143091	0.365389586	0.328706572	18.92617568	89.43262477	0.666666667	CRA004775;PRJNA827465;Yang2025_Silkie_Wencha	0.788777182
ENSGALG00010027295	ENSGALG0001002729:	3	0.313676002	0.345363313	0.908249341	0.36374650	0.267784066	9.487272797	78.91912626	0.666666667	CRA004775;PRJNA827465;Yang2025_Silkie_Wencha	0.788777182
ENSGALG00010002919	ENSGALG0001000291:	3	0.285124092	0.315092037	0.904891455	0.365522894	0.249845199	14.6310061	86.33040006	0.666666667	CRA004775;PRJNA827465;Yang2025_Silkie_Wencha	0.788777182
ENSGALG00010016930	C10H15ORF48	3	-0.272196998	0.299662645	-0.908344776	0.363696092	0	1.156309769	0	0.666666667	CRA004775;PRJNA827465;Yang2025_Silkie_Wencha	0.788777182
ENSGALG00010014965	ABHD13	3	-0.250342837	0.276674758	-0.904827166	0.365556958	0.210873455	35.92829248	94.43335638	0.666666667	CRA004775;PRJNA827465;Yang2025_Silkie_Wencha	0.788777182
ENSGALG00010001811	ACADSB	3	-0.249970678	0.276198134	-0.905041153	0.365443586	0.187025075	12.18928677	83.59214909	0.666666667	CRA004775;PRJNA827465;Yang2025_Silkie_Wencha	0.788777182

ENSGALG00010015631	GSKIP	3	-0.24830677	0.273563567	-0.907674852	0.364050034	0.206054066	32.43752536	93.83430155	0.666666667	CRA004775;PRJNA827465;Yang2025_Silkie_Wencha	0.788777182
ENSGALG00010013056	SAG	3	0.248297089	0.273973596	0.906281089	0.364787093	0	1.075158274	0	0.666666667	CRA004775;PRJNA827465;Yang2025_Silkie_Wencha	0.788777182
ENSGALG00010013204	ENSGALG0001001320	2	0.226081795	0.249288029	0.906909953	0.364454418	0	0.095562988	0	1	PRJNA827465;Yang2025_Silkie_Wenchang	0.788777182
ENSGALG00010029603	RHBDL3	3	0.223831887	0.246343631	0.908616494	0.363552597	0	0.884289404	0	0.666666667	CRA004775;PRJNA827465;Yang2025_Silkie_Wencha	0.788777182
ENSGALG00010008214	CCNG2	3	-0.221755182	0.243598562	-0.910330422	0.362648279	0.15145953	15.89948907	87.42097943	0.666666667	CRA004775;PRJNA827465;Yang2025_Silkie_Wencha	0.788777182
ENSGALG00010024997	HES6	2	-0.21567416	0.238193549	-0.905457607	0.365223009	0.000808867	1.00672422	0.667930668	1	PRJNA827465;Yang2025_Silkie_Wenchang	0.788777182
ENSGALG00010028454	C8G	3	0.213804576	0.235767511	0.906844947	0.364488797	0.112768346	6.506191762	69.26005146	0.666666667	CRA004775;PRJNA827465;Yang2025_Silkie_Wencha	0.788777182
ENSGALG00010021510	NTSR1	3	-0.211473235	0.232669316	-0.908900403	0.363402701	0	1.758807597	0	0.666666667	CRA004775;PRJNA827465;Yang2025_Silkie_Wencha	0.788777182
ENSGALG00010017480	DCDC2B	3	0.204511939	0.224815666	0.909687224	0.362987484	0.105529133	6.754252574	70.38902561	0.666666667	CRA004775;PRJNA827465;Yang2025_Silkie_Wencha	0.788777182
ENSGALG00010022478	ATP6V1A	3	-0.186661584	0.205856938	-0.906753912	0.364536947	0.09650875	13.68419585	85.38459971	0.666666667	CRA004775;PRJNA827465;Yang2025_Silkie_Wencha	0.788777182
ENSGALG00010022435	PIGK	3	-0.179145435	0.197659211	-0.906334866	0.364758636	0.084893458	9.695278544	79.37140237	0.666666667	CRA004775;PRJNA827465;Yang2025_Silkie_Wencha	0.788777182
ENSGALG00010022724	LUZP2	3	0.17218001	0.189936238	0.9065148	0.364663435	0	1.555579499	0	0.666666667	CRA004775;PRJNA827465;Yang2025_Silkie_Wencha	0.788777182
ENSGALG00010004039	ALPK1	3	0.17186411	0.189559664	0.906649157	0.364592358	0	1.010410844	0	0.666666667	CRA004775;PRJNA827465;Yang2025_Silkie_Wencha	0.788777182
ENSGALG00010008250	KLHL38	3	0.166359833	0.183077987	0.908682882	0.363517543	0	0.762300034	0	0.666666667	CRA004775;PRJNA827465;Yang2025_Silkie_Wencha	0.788777182
ENSGALG00010027367	SEC22C	3	-0.160783617	0.177728823	-0.904656963	0.365647148	0.075560702	10.6623271	81.24236875	0.666666667	CRA004775;PRJNA827465;Yang2025_Silkie_Wencha	0.788777182
ENSGALG00010016835	CCDC88A	3	0.158859446	0.175088942	0.907307136	0.364244402	0.050703607	5.050943275	60.40343573	0.666666667	CRA004775;PRJNA827465;Yang2025_Silkie_Wencha	0.788777182
ENSGALG00010024512	CCNDBP1	3	-0.148513245	0.164126197	-0.904872272	0.365533058	0.053433305	6.29983678	68.25314576	0.666666667	CRA004775;PRJNA827465;Yang2025_Silkie_Wencha	0.788777182
ENSGALG00010029399	PRKAR1A	3	-0.147889105	0.162891335	-0.907900379	0.363930857	0.069027161	30.4778518	93.43785772	0.666666667	CRA004775;PRJNA827465;Yang2025_Silkie_Wencha	0.788777182
ENSGALG00010028445	SYT11	3	-0.141628058	0.156366903	-0.905741911	0.365072474	0.046688253	6.805708338	70.61290463	0.666666667	CRA004775;PRJNA827465;Yang2025_Silkie_Wencha	0.788777182
ENSGALG00010002088	OZFL1	3	0.138763188	0.153041559	0.906702652	0.364564061	0.035484021	4.105698188	51.28721332	0.666666667	CRA004775;PRJNA827465;Yang2025_Silkie_Wencha	0.788777182
ENSGALG00010029403	FAM20A	3	-0.135354346	0.149456986	-0.90564081	0.365126001	0.033482039	4.080109077	50.98170264	0.666666667	CRA004775;PRJNA827465;Yang2025_Silkie_Wencha	0.788777182
ENSGALG00010004300	VNN2	3	-0.133894275	0.148009444	-0.904633324	0.365659675	0	0.170003068	0	1	CRA004775;PRJNA827465;Yang2025_Silkie_Wencha	0.788777182
ENSGALG00010015974	VPS37A	3	0.133253584	0.147089546	0.905935111	0.364970199	0.043617633	6.706439583	70.17791668	0.666666667	CRA004775;PRJNA827465;Yang2025_Silkie_Wencha	0.788777182
ENSGALG00010012498	KCNT2	3	-0.130005977	0.143719134	-0.904583641	0.365686005	0	0.248149366	0	1	CRA004775;PRJNA827465;Yang2025_Silkie_Wencha	0.788777182
ENSGALG00010029672	KCTD7	3	-0.121675525	0.133764442	-0.909625335	0.363020133	0.004574557	2.153504345	7.128118656	1	CRA004775;PRJNA827465;Yang2025_Silkie_Wencha	0.788777182
ENSGALG00010005800	FRMD4A	3	0.116889256	0.129057287	0.905716053	0.365086163	0.026000848	3.982415231	49.7792198	1	CRA004775;PRJNA827465;Yang2025_Silkie_Wencha	0.788777182
ENSGALG00010020548	CMTR1	3	-0.111789161	0.123016345	-0.90873421	0.363490442	0.022396548	4.039683673	50.49117302	0.666666667	CRA004775;PRJNA827465;Yang2025_Silkie_Wencha	0.788777182
ENSGALG00010021726	ARNTL	3	0.10522315	0.115991366	0.90716364	0.364320268	0.01880015	3.551948975	43.69288484	0.666666667	CRA004775;PRJNA827465;Yang2025_Silkie_Wencha	0.788777182
ENSGALG00010018261	PRNP	3	-0.104684939	0.115138661	-0.90920754	0.363240585	0.021779969	4.853608244	58.79354288	0.666666667	CRA004775;PRJNA827465;Yang2025_Silkie_Wencha	0.788777182
ENSGALG00010010809	CENPH	3	0.103222781	0.114027135	0.905247524	0.36533427	0	1.594568457	0	1	CRA004775;PRJNA827465;Yang2025_Silkie_Wencha	0.788777182
ENSGALG00010019481	PYGL	3	-0.092467391	0.102050421	-0.906095151	0.364885492	0.013186086	3.481231954	42.54907382	1	CRA004775;PRJNA827465;Yang2025_Silkie_Wencha	0.788777182
ENSGALG00010010255	CACNB4	3	0.08898852	0.097851989	0.909419632	0.363128663	0	0.281474417	0	0.666666667	CRA004775;PRJNA827465;Yang2025_Silkie_Wencha	0.788777182
ENSGALG00010024073	RABGGTB	3	-0.08890845	0.097996142	-0.907264801	0.364266783	0.012395948	3.501605893	42.8833495	0.666666667	CRA004775;PRJNA827465;Yang2025_Silkie_Wencha	0.788777182
ENSGALG00010016295	UBE2K	3	-0.086210681	0.095056928	-0.906937382	0.364439912	0.015301143	5.474826967	63.4691651	0.666666667	CRA004775;PRJNA827465;Yang2025_Silkie_Wencha	0.788777182
ENSGALG00010023065	DNA2	3	-0.078903173	0.086691265	-0.910162903	0.362736605	0	1.255810039	0	0.666666667	CRA004775;PRJNA827465;Yang2025_Silkie_Wencha	0.788777182
ENSGALG00010023114	UQCRHL	3	-0.070779967	0.077879237	-0.908842588	0.363433222	0	1.56657823	0	1	CRA004775;PRJNA827465;Yang2025_Silkie_Wencha	0.788777182
ENSGALG00010017838	B3GNT2	3	0.066713252	0.073537625	0.907198893	0.364301629	0	0.646291096	0	1	CRA004775;PRJNA827465;Yang2025_Silkie_Wencha	0.788777182
ENSGALG00010005879	FAM107B	3	0.054141864	0.059534008	0.909427497	0.363124513	0	1.540984123	0	0.666666667	CRA004775;PRJNA827465;Yang2025_Silkie_Wencha	0.788777182
ENSGALG00010026143	RNPEP	3	-0.051016045	0.056077661	-0.90973917	0.362960082	0	1.750741089	0	1	CRA004775;PRJNA827465;Yang2025_Silkie_Wencha	0.788777182
ENSGALG00010027285	PTPRS	3	-0.046158434	0.050796951	-0.908685137	0.363516352	0	0.752974578	0	0.666666667	CRA004775;PRJNA827465;Yang2025_Silkie_Wencha	0.788777182
ENSGALG00010003485	RNGTT	3	-0.043991614	0.04845108	-0.907959398	0.363899674	0	0.965544435	0	1	CRA004775;PRJNA827465;Yang2025_Silkie_Wencha	0.788777182
ENSGALG00010004086	UTP3	3	0.177831502	0.19669948	0.904077129	0.365954505	0.093944676	13.9544487	85.66765307	0.666666667	CRA004775;PRJNA827465;Yang2025_Silkie_Wencha	0.789235928
ENSGALG00010021863	CLDN19	3	0.193882069	0.214556548	0.903640886	0.366185854	0.060906426	3.639474114	45.04700577	0.666666667	CRA004775;PRJNA827465;Yang2025_Silkie_Wencha	0.789614426
ENSGALG00010016141	RRP15	3	0.194876531	0.215696497	0.903475645	0.366273508	0.108693377	9.949412162	79.8983099	0.666666667	CRA004775;PRJNA827465;Yang2025_Silkie_Wencha	0.789683003
ENSGALG00010016750	SYTL4	3	0.075214906	0.083260612	0.903367211	0.366331036	0.002232762	2.202959177	9.213024893	0.666666667	CRA004775;PRJNA827465;Yang2025_Silkie_Wencha	0.789686617
ENSGALG00010020739	PKP3	2	0.903467797	1.002263176	0.901427707	0.367360955	1.360761651	3.086748292	67.60344851	0.5	CRA004775;PRJNA827465	0.790105997
ENSGALG00010004390	PNLDC1	2	-0.496078183	0.55011382	-0.901773714	0.367177086	0.466997081	4.377348656	77.15512109	0.5	PRJNA827465;Yang2025_Silkie_Wenchang	0.790105997
ENSGALG00010020649	MYH11	3	0.417133763	0.463148476	0.90064803	0.367775489	0.563190575	20.29624999	90.14596292	0.666666667	CRA004775;PRJNA827465;Yang2025_Silkie_Wencha	0.790105997
ENSGALG00010022917	CYP4B7	3	0.322442688	0.358063062	0.900519271	0.367843975	0.216022185	4.562739836	56.16668774	0.666666667	CRA004775;PRJNA827465;Yang2025_Silkie_Wencha	0.790105997
ENSGALG00010014796	PRLR	3	0.265327937	0.294153092	0.902006285	0.367053529	0.108201782	3.444925865	41.94359826	0.666666667	CRA004775;PRJNA827465;Yang2025_Silkie_Wencha	0.790105997
ENSGALG00010002810	TRIM27.1	3	-0.250968992	0.278251874	-0.901948972	0.367083975	0.127077417	4.451446066	55.07077992	0.666666667	CRA004775;PRJNA827465;Yang2025_Silkie_Wencha	0.790105997
ENSGALG00010008137	HHLA1	2	0.214947076	0.238090858	0.902794328	0.366635062	0	0.35162714	0	1	PRJNA827465;Yang2025_Silkie_Wenchang	0.790105997
ENSGALG00010012903	RGMB	3	-0.206188997	0.228588003	-0.902011456	0.367050782	0.126322937	13.02281676	84.64233939	0.666666667	CRA004775;PRJNA827465;Yang2025_Silkie_Wencha	0.790105997
ENSGALG00010024651	PSMD3	3	-0.18793125	0.208392852	-0.901812363	0.367156551	0.085517481	6.154119067	67.50144126	0.666666667	CRA004775;PRJNA827465;Yang2025_Silkie_Wencha	0.790105997
ENSGALG00010013861	TCF15	3	0.186853089	0.207284346	0.901433672	0.367357785	0.06517803	4.036580539	50.45311296	0.666666667	CRA004775;PRJNA827465;Yang2025_Silkie_Wencha	0.790105997
ENSGALG00010029273	SLC13A5	3	0.178583898	0.197844701	0.902646857	0.36671335	0	0.858929873	0	0.666666667	CRA004775;PRJNA827465;Yang2025_Silkie_Wencha	0.790105997
ENSGALG00010003714	ORC3	3	-0.1731564	0.192158191	-0.901113813	0.367527809	0.084887471	10.04099895	80.08166308	0.666666667	CRA004775;PRJNA827465;Yang2025_Silkie_Wencha	0.790105997
ENSGALG00010011203	ENSGALG0001001120	3	-0.165546174	0.183864311	-0.900371434	0.367922617	0.061483632	5.104964627	60.82245136	0.666666667	CRA004775;PRJNA827465;Yang2025_Silkie_Wencha	0.790105997
ENSGALG00010016562	GLIS2	3	0.163678294	0.181321415	0.902696981	0.366686739	0.055629835	4.58998705	56.42689231	0.666666667	CRA004775;PRJNA827465;Yang2025_Silkie_Wencha	0.790105997
ENSGALG00010023799	NMT1	3	-0.131733829	0.146032213	-0.902087467	0.367010406	0.044124851	7.797352601	74.35026858	0.666666667	CRA004775;PRJNA827465;Yang2025_Silkie_Wencha	0.790105997
ENSGALG00010016322	EBF4	3	0.127403536	0.141446972	0.900715895	0.367739395	0.03680222	6.134458297	67.39728427	0.666666667	CRA004775;PRJNA827465;Yang2025_Silkie_Wencha	0.790105997
ENSGALG00010024552	CD81	3	-0.097992417	0.108810744	-0.900576666	0.367813445	0.022723179	5.882664959	66.00180337	0.666666667	CRA004775;PRJNA827465;Yang2025_Silkie_Wencha	0.790105997
ENSGALG00010007614	BIRC6	3	-0.097746952	0.108440707	-0.901386153	0.367383041	0.016841957	3.734475244	46.44495225	0.666666667	CRA004775;PRJNA827465;Yang2025_Silkie_Wencha	0.790105997
ENSGALG00010021881	PHF20	3	0.094992923	0.105382615	0.901409817	0.367370464	0.020186942	5.065673539	60.51857696	0.666666667	CRA004775;PRJNA827465;Yang2025_Silkie_Wencha	0.790105997
ENSGALG00010010197	SCCPDH	3	-0.094810174	0.105300885	-0.90037396	0.367921274	0.023102795	6.765759893	70.43938846	0.666666667	CRA004775;PRJNA827465;Yang2025_Silkie_Wencha	0.790105997
ENSGALG00010008394	CD2AP	3	0.091982015	0.102085476	0.901029399	0.367572689	0.016990549	4.763072245	58.01029467	0.666666667	CRA004775;PRJNA827465;Yang2025_Silkie_Wencha	0.790105997
ENSGALG00010001368	WIPF3	3	-0.08617953	0.095586343	-0.901588314	0.367275601	0	0.323088644	0	0.666666667	CRA004775;PRJNA827465;Yang2025_Silkie_Wencha	0.790105997
ENSGALG00010021618	NEO1	3	-0.063438519	0.070337204	-0.901919836	0.367099454	0	1.907499165	0	0.666666667	CRA004775;PRJNA827465;Yang2025_Silkie_Wencha	0.790105997
ENSGALG00010020880	PXK	3	-0.055348697	0.061412002	-0.901268399	0.367445631	0	1.573059178	0	0.666666667	CRA004775;PRJNA827465;Yang2025_Silkie_Wencha	0.790105997
ENSGALG00010027368	EMC3	3	0.047732919	0.052995251	0.900701822	0.36774688	0	1.000395444	0	0.666666667	CRA004775;PRJNA827465;Yang2025_Silkie_Wencha	0.790105997
ENSGALG00010014056	FMO3	3	0.130737815	0.145264185	0.900003137	0.368120071	0	0.231671759	0	1	CRA004775;PRJNA827465;Yang2025_Silkie_Wencha	0.790169985
ENSGALG00010023276	ABCC8	3	0.060549378	0.067264572	0.90016745	0.368031145	0	0.834018891	0	1	CRA004775;PRJNA827465;Yang2025_Silkie_Wencha	0.790169985
ENSGALG00010011621	WASHC4	3	0.043669906	0.048521748	0.900006859	0.368116601	0	1.799434476	0	0.666666667	CRA004775;PRJNA827465;Yang2025_Silkie_Wencha	0.790169985
ENSGALG00010011658	TENM1	3	0.324404173	0.360809904	0.899099968	0.368599415	0.150946649	3.206900182	37.6344792	0.666666667	CRA004775;PRJNA827465;Yang2025_Silkie_Wencha	0.790376063
ENSGALG00010023552	SGPP2	3	-0.216654886	0.240956551	-0.899144857	0.368575508	0.03113596	2.353556054	15.02220664	0.666666667	CRA004775;PRJNA827465;Yang2025_Silkie_Wencha	0.790376063
ENSGALG00010025028	HES6	3	0.173828522	0.193382691	0.898883561	0.368714685	0.072366036	6.663914242	69.98760897	0.666666667	CRA004775;PRJNA827465;Yang2025_Silkie_Wencha	0.790376063
ENSGALG00010014847	PAICS	3	-0.169549179	0.188525276	-0.899344547	0.368469166	0.087025022	15.22455872	86.86333025	0.666666667	CRA004775;PRJNA827465;Yang2025_Silkie_Wencha	0.790376063
ENSGALG00010001453	TAX1BP1	3	0.168838821	0.187833457	0.898875119	0.368719182	0.0839472	15.03436352	86.6971422	0.666666667	CRA004775;PRJNA827465;Yang2025_Silkie_Wencha	0.790376063
ENSGALG00010028536	GPR107	3	-0.152953374	0.170093265	-0.899232406	0.368528883	0.06867764	18.14544492	88.97795007	0.666666667	CRA004775;PRJNA827465;Yang2025_Silkie_Wencha	0.790376063
ENSGALG00010024286	ADA	3	-0.148563201	0.165146624	-0.899583641	0.368341866	0.062295558	8.861663354	77.43087364	0.666666667	CRA004775;PRJNA827465;Yang2025_Silkie_Wencha	0.790376063
ENSGALG00010012599	DMGDH	3	0.107749002	0.119864116	0.89892626	0.368691939	0	0.992908555	0	0.666666667	CRA004775;PRJNA827465;Yang2025_Silkie_Wencha	0.790376063
ENSGALG00010021014	FBXL15	3	-0.057934363	0.064448365	-0.898926804	0.368691649	0	0.803307995	0	1	CRA004775;PRJNA827465;Yang2025_Silkie_Wencha	0.790376063
ENSGALG00010012318	POLR1D	3	-0.191389057	0.212968327	-0.898673806	0.368826433	0.072827944	4.207044696	52.46069047	0.666666667	CRA004775;PRJNA827465;Yang2025_Silkie_Wencha	0.79048612
ENSGALG00010015848	UBE2B	3	-0.17301463	0.192573327	-0.898435069	0.368953647	0.072159098	7.383574492	72.91284862	0.666666667	CRA004775;PRJNA827465;Yang2025_Silkie_Wencha	0.790638924
ENSGALG00010010724	ADCY3	3	0.066312883	0.073856916	0.897856104	0.369262269	0	0.055177288	0	1	CRA004775;PRJNA827465;Yang2025_Silkie_Wencha	0.791060489
ENSGALG00010027614	LONP1	3	-0.036582793	0.040742054	-0.897912335	0.369232288	0	1.734189087	0	0.666666667	CRA004775;PRJNA827465;Yang2025_Silkie_Wencha	0.791060489
ENSGALG00010024938	ENSGALG0001002493	3	0.20492224	0.228569796	0.896541202	0.369963785	0.105921258	6.532130304	69.38211721	0.666666667	CRA004775;PRJNA827465;Yang2025_Silkie_Wencha	0.79136429
ENSGALG00010007966	TMEM74	3	0.150723288	0.168029509	0.897004876	0.369716315	0	1.935037513	0	0.666666667	CRA004775;PRJNA827465;Yang2025_Silkie_Wencha	0.79136429
ENSGALG00010002235	MXRA5	3	-0.119878963	0.133692663	-0.896675708	0.369891987	0	0.595175734	0	0.666666667	CRA004775;PRJNA827465;Yang2025_Silkie_Wencha	0.79136429
ENSGALG00010013389	NAP1L1	3	-0.115724224	0.128961829	-0.897352534	0.369530832	0.040445378	13.85527078	85.56506017	0.666666667	CRA004775;PRJNA827465;Yang2025_Silkie_Wencha	0.79136429
ENSGALG00010005515	PCCA	3	-0.081004145	0.090308518	-0.896971259	0.369734254	0.011941432	4.075875942	50.93079308	0.666666667	CRA004775;PRJNA827465;Yang2025_Silkie_Wencha	0.79136429
ENSGALG00010017733	TRIM62	3	0.068740138	0.076593211	0.897470371	0.369467977	0	1.482630829	0	0.666666667	CRA004775;PRJNA827465;Yang2025_Silkie_Wencha	0.79136429
ENSGALG00010020782	PPIF	3	0.065284486	0.07279747	0.896796083	0.369827738	0	0.058856196	0	1	CRA004775;PRJNA827465;Yang2025_Silkie_Wencha	0.79136429
ENSGALG00010028477	NRARP	3	-0.061714342	0.068799967	-0.897011216	0.369712932	0.001383895	2.113981419	5.391789068	0.666666667	CRA004775;PRJNA827465;Yang2025_Silkie_Wencha	0.79136429
ENSGALG00010021134	CHFR	3	-0.044000319	0.049066015	-0.896757548	0.369848305	0.000691579	2.167263068	7.717709504	0.666666667	CRA004775;PRJNA827465;Yang2025_Silkie_Wencha	0.79136429
ENSGALG00010022737	CYLD	3	-0.043378137	0.048382144	-0.896573267	0.369946668	0	0.2974138	0	0.666666667	CRA004775;PRJNA827465;Yang2025_Silkie_Wencha	0.79136429
ENSGALG00010007344	RAD17	3	0.063137063	0.070437113	0.896360744	0.370060126	0	0.019933312	0	1	CRA004775;PRJNA827465;Yang2025_Silkie_Wencha	0.791450632
ENSGALG00010024747	TULP3	3	-0.150888514	0.168420082	-0.895905714	0.370303122	0.070572772	18.07713274	88.93629853	0.666666667	CRA004775;PRJNA827465;Yang2025_Silkie_Wencha	0.79166905
ENSGALG00010011546	MAOA	3	-0.149066935	0.166401008	-0.89582952	0.370343821	0.057428158	8.849565878	77.40002134	0.666666667	CRA004775;PRJNA827465;Yang2025_Silkie_Wencha	0.79166905
ENSGALG00010016240	EIF4A2	3	-0.0992699	0.110823202	-0.895750149	0.37038622	0.024493819	6.117344069	67.30607307	0.666666667	CRA004775;PRJNA827465;Yang2025_Silkie_Wencha	0.79166905
ENSGALG00010010253	IL10RB	3	-0.070570542	0.078771298	-0.895891568	0.370310678	0.007001532	3.137534544	36.25568191	0.666666667	CRA004775;PRJNA827465;Yang2025_Silkie_Wencha	0.79166905
ENSGALG00010008325	AADAT	3	0.11551713	0.128989009	0.89555793	0.370488913	0.016045619	2.861812538	30.1142205	0.666666667	CRA004775;PRJNA827465;Yang2025_Silkie_Wencha	0.791768855
ENSGALG00010012771	ITGA2	3	0.129329827	0.144483306	0.89511952	0.370723201	0.013314154	2.420149003	17.36046014	0.666666667	CRA004775;PRJNA827465;Yang2025_Silkie_Wencha	0.792130183
ENSGALG00010007403	SDR42E1	3	0.116347342	0.129992397	0.895031897	0.370770037	0	0.251492391	0	0.666666667	CRA004775;PRJNA827465;Yang2025_Silkie_Wencha	0.792130183
ENSGALG00010017737	ENSGALG0001001773	3	0.353782554	0.395348829	0.894861774	0.370860983	0.377901258	10.7594658	81.41171655	0.666666667	CRA004775;PRJNA827465;Yang2025_Silkie_Wencha	0.792162916
ENSGALG00010001245	SERPINI1	3	-0.119819809	0.133907756	-0.894793638	0.370897412	0	1.657736161	0	0.666666667	CRA004775;PRJNA827465;Yang2025_Silkie_Wencha	0.792162916
ENSGALG00010019873	LGR4	3	-0.09059478	0.101273066	-0.894559464	0.371022629	0.014611451	3.846097526	47.99923854	0.666666667	CRA004775;PRJNA827465;Yang2025_Silkie_Wencha	0.792310671
ENSGALG00010013252	PPM1H	3	0.33691169	0.376696491	0.894384996	0.371115938	0.339201141	12.3237023	83.77111073	0.666666667	CRA004775;PRJNA827465;Yang2025_Silkie_Wencha	0.792390251
ENSGALG00010006021	ENSGALG0001000602	3	0.207631336	0.232203564	0.894178077	0.37122662	0.140012668	21.40918372	90.6582146	0.666666667	CRA004775;PRJNA827465;Yang2025_Silkie_Wencha	0.792493131
ENSGALG00010005578	HEATR5A	3	0.053108115	0.059399379	0.89408536	0.371276222	0	1.173994316	0	1	CRA004775;PRJNA827465;Yang2025_Silkie_Wencha	0.792493131
ENSGALG00010021494	SLC2A10	3	-0.22985438	0.257196389	-0.893692098	0.371486655	0.128954613	6.128190185	67.36393716	0.666666667	CRA004775;PRJNA827465;Yang2025_Silkie_Wencha	0.792755738
ENSGALG00010024691	TCTN2	3	-0.189778867	0.21238486	-0.893561181	0.371556724	0.106040983	10.72970731	81.36016257	0.666666667	CRA004775;PRJNA827465;Yang2025_Silkie_Wencha	0.792755738
ENSGALG00010024810	ADIPOR1	3	-0.108558912	0.12149291	-0.893541129	0.371567457	0.024422255	4.300526464	53.49406598	0.666666667	CRA004775;PRJNA827465;Yang2025_Silkie_Wencha	0.792755738
ENSGALG00010020886	GBF1	3	0.066412451	0.074336723	0.893400301	0.371642841	0.007270421	3.428645443	41.66792591	0.666666667	CRA004775;PRJNA827465;Yang2025_Silkie_Wencha	0.792796942
ENSGALG00010005221	ENSGALG0001000522	3	-0.277865332	0.311292086	-0.892619327	0.372061065	0.102611967	3.085866208	35.18837612	0.666666667	CRA004775;PRJNA827465;Yang2025_Silkie_Wencha	0.793304477
ENSGALG00010013183	CLTA	3	0.220100859	0.246601394	0.892536961	0.372105191	0.123383543	6.722040632	70.24713016	0.666666667	CRA004775;PRJNA827465;Yang2025_Silkie_Wencha	0.793304477
ENSGALG00010012228	NHP2	3	0.143043354	0.160263205	0.892552688	0.372096766	0.041544638	4.270692116	53.16918322	0.666666667	CRA004775;PRJNA827465;Yang2025_Silkie_Wencha	0.793304477
ENSGALG00010028005	IER5L	3	0.142820003	0.160012771	0.892553778	0.372096182	0.050147653	6.638275612	69.87169384	0.666666667	CRA004775;PRJNA827465;Yang2025_Silkie_Wencha	0.793304477
ENSGALG00010008716	TATDN1	3	0.180699482	0.202574566	0.892014656	0.372385077	0.098260318	17.01961966	88.24885609	0.666666667	CRA004775;PRJNA827465;Yang2025_Silkie_Wencha	0.793781487
ENSGALG00010013512	ENSGALG0001001351	3	-2.49199841	2.796133544	-0.891230111	0.372805734	21.05118063	20.16076563	90.07974183	0.666666667	CRA004775;PRJNA827465;Yang2025_Silkie_Wencha	0.793840402
ENSGALG00010022174	KCNMA1	3	0.296174639	0.332280514	0.891339176	0.372747238	0.280954739	18.9797441	89.46245013	0.666666667	CRA004775;PRJNA827465;Yang2025_Silkie_Wencha	0.793840402
ENSGALG00010007201	ENSGALG0001000720	3	-0.227358037	0.255084722	-0.891304016	0.372766095	0	1.427497718	0	0.666666667	CRA004775;PRJNA827465;Yang2025_Silkie_Wencha	0.793840402
ENSGALG00010013044	ENSGALG0001001304	3	0.157433169	0.176589103	0.89152256	0.372648894	0.072930522	11.90273911	83.19714494	0.666666667	CRA004775;PRJNA827465;Yang2025_Silkie_Wencha	0.793840402
ENSGALG00010024736	DGKA	3	-0.067167336	0.075340229	-0.89152019	0.372650165	0	1.14649437	0	1	CRA004775;PRJNA827465;Yang2025_Silkie_Wencha	0.793840402
ENSGALG00010005872	STK24	3	0.061013749	0.068433341	0.891579288	0.372618476	0.003120856	2.464642727	18.85233594	0.666666667	CRA004775;PRJNA827465;Yang2025_Silkie_Wencha	0.793840402
ENSGALG00010025304	DDX28	3	-0.06042214	0.067754958	-0.891774448	0.37251384	0	0.775551707	0	1	CRA004775;PRJNA827465;Yang2025_Silkie_Wencha	0.793840402
ENSGALG00010027186	PLXNA2	3	-0.076995855	0.086404387	-0.891110489	0.372869898	0	0.747641961	0	0.666666667	CRA004775;PRJNA827465;Yang2025_Silkie_Wencha	0.793857475
ENSGALG00010004851	MTCL3	2	0.95609518	1.073658014	0.890502532	0.373196109	2.132644047	13.23430499	92.44387974	0.5	PRJNA827465;Yang2025_Silkie_Wenchang	0.793954226
ENSGALG00010025121	DYNC2I1	3	0.258434838	0.290196439	0.890551375	0.373169895	0.189711364	8.721002374	77.06685638	0.666666667	CRA004775;PRJNA827465;Yang2025_Silkie_Wencha	0.793954226
ENSGALG00010020142	SLC35E2B	3	-0.193320884	0.217055374	-0.890652373	0.373115693	0.121181974	21.9759756	90.89915262	0.666666667	CRA004775;PRJNA827465;Yang2025_Silkie_Wencha	0.793954226
ENSGALG00010003087	TWISTNB	3	0.168101035	0.188762414	0.890542937	0.373174424	0.076360034	7.716496173	74.08150079	0.666666667	CRA004775;PRJNA827465;Yang2025_Silkie_Wencha	0.793954226
ENSGALG00010007419	RABL2A	3	-0.08927012	0.100233561	-0.89062105	0.373132503	0	0.860055361	0	1	CRA004775;PRJNA827465;Yang2025_Silkie_Wencha	0.793954226
ENSGALG00010017599	KCNK13	3	-0.142801274	0.160395973	-0.890304613	0.373302345	0.012714466	2.334745239	14.33754885	1	CRA004775;PRJNA827465;Yang2025_Silkie_Wencha	0.793998932
ENSGALG00010019371	TMEM87A	3	-0.095574845	0.10735681	-0.890254147	0.373329436	0.024763514	8.217232598	75.66090559	0.666666667	CRA004775;PRJNA827465;Yang2025_Silkie_Wencha	0.793998932
ENSGALG00010016720	INTS15	3	0.042519574	0.047778274	0.889935321	0.373500617	0	1.119262822	0	0.666666667	CRA004775;PRJNA827465;Yang2025_Silkie_Wencha	0.794243528
ENSGALG00010003262	ENSGALG0001000326	3	-0.878371011	0.987964179	-0.889071719	0.373964537	2.344763103	16.52029649	87.89367975	0.666666667	CRA004775;PRJNA827465;Yang2025_Silkie_Wencha	0.794366713
ENSGALG00010015872	TMC1	2	0.745313163	0.839012091	0.888322315	0.3743674	1.331820556	18.46459329	94.58422948	0.5	PRJNA827465;Yang2025_Silkie_Wenchang	0.794366713
ENSGALG00010015088	ENSGALG0001001508	3	0.534850462	0.601404889	0.889335075	0.373823026	0.829552395	12.05461151	83.40883902	0.666666667	CRA004775;PRJNA827465;Yang2025_Silkie_Wencha	0.794366713
ENSGALG00010005138	ENSGALG0001000513	2	-0.512885259	0.577354921	-0.888336171	0.374359949	0.561164006	6.307845102	84.14672549	0.5	PRJNA827465;Yang2025_Silkie_Wenchang	0.794366713
ENSGALG00010010535	LMX1A	3	-0.383152374	0.431022342	-0.888938546	0.374036108	0.309465295	4.892204495	59.11863451	0.666666667	CRA004775;PRJNA827465;Yang2025_Silkie_Wencha	0.794366713
ENSGALG00010000134	ENSGALG0001000013	3	0.321096232	0.36138299	0.888520602	0.374260779	0.365313216	49.37988433	95.94976775	0.666666667	CRA004775;PRJNA827465;Yang2025_Silkie_Wencha	0.794366713
ENSGALG00010025039	CEBPA	3	-0.251451687	0.28273353	-0.889359273	0.373810026	0.143131284	5.553451823	63.98636265	0.666666667	CRA004775;PRJNA827465;Yang2025_Silkie_Wencha	0.794366713
ENSGALG00010027608	GNAI2	3	0.185652387	0.209056143	0.888050382	0.374513651	0.108232848	14.51226783	86.21855645	0.666666667	CRA004775;PRJNA827465;Yang2025_Silkie_Wencha	0.794366713
ENSGALG00010015042	EFNB2	3	0.166168014	0.186952018	0.888827072	0.374096024	0.074739672	10.87370102	81.60699843	0.666666667	CRA004775;PRJNA827465;Yang2025_Silkie_Wencha	0.794366713
ENSGALG00010004372	SYNJ2	3	-0.162114083	0.182456768	-0.888506821	0.374268189	0.048999971	3.971925715	49.64659101	0.666666667	CRA004775;PRJNA827465;Yang2025_Silkie_Wencha	0.794366713
ENSGALG00010028603	SH3GLB2	3	-0.160837508	0.180892873	-0.88913126	0.373932541	0.054286579	4.542452957	55.97092542	0.666666667	CRA004775;PRJNA827465;Yang2025_Silkie_Wencha	0.794366713
ENSGALG00010000715	PCDH20	2	0.122971757	0.138467431	0.888091561	0.374491502	0.003521778	1.095722873	8.736047709	0.5	PRJNA827465;Yang2025_Silkie_Wenchang	0.794366713
ENSGALG00010011530	ARPC5	3	-0.122797849	0.138205849	-0.88851413	0.374264259	0.035982057	6.764383979	70.43337566	0.666666667	CRA004775;PRJNA827465;Yang2025_Silkie_Wencha	0.794366713
ENSGALG00010000406	NSMCE3	3	-0.11720757	0.131965141	-0.888170686	0.374448945	0.019457592	3.043116598	34.2779044	0.666666667	CRA004775;PRJNA827465;Yang2025_Silkie_Wencha	0.794366713
ENSGALG00010018365	HPSE2	3	-0.113652967	0.127885781	-0.888706831	0.374160659	0	0.422507303	0	1	CRA004775;PRJNA827465;Yang2025_Silkie_Wencha	0.794366713
ENSGALG00010019618	NPHP4	3	-0.099994693	0.112535645	-0.888560177	0.374239501	0.021342691	4.847411622	58.74086717	0.666666667	CRA004775;PRJNA827465;Yang2025_Silkie_Wencha	0.794366713
ENSGALG00010009876	SELENOI	3	-0.099478491	0.111880746	-0.889147544	0.37392379	0.024500151	6.488873374	69.1780085	0.666666667	CRA004775;PRJNA827465;Yang2025_Silkie_Wencha	0.794366713
ENSGALG00010028613	ENSGALG0001002861	3	0.145554482	0.16393927	0.887856107	0.374618158	0	1.371106019	0	0.666666667	CRA004775;PRJNA827465;Yang2025_Silkie_Wencha	0.794433546
ENSGALG00010029563	ADPRM	3	-0.078780363	0.088738318	-0.887782915	0.374657535	0	0.169410074	0	1	CRA004775;PRJNA827465;Yang2025_Silkie_Wencha	0.794433546
ENSGALG00010017422	ENSGALG0001001742	3	0.135882565	0.153088077	0.887610373	0.374750372	0	1.850140794	0	0.666666667	CRA004775;PRJNA827465;Yang2025_Silkie_Wencha	0.794511248
ENSGALG00010007907	IRX2	2	0.40238323	0.453426035	0.887428597	0.374848193	0	0.143109998	0	1	PRJNA827465;Yang2025_Silkie_Wenchang	0.794549869
ENSGALG00010020180	TCF7L1	3	0.197191839	0.222258817	0.887217168	0.374961991	0.106404256	7.660018457	73.89040234	0.666666667	CRA004775;PRJNA827465;Yang2025_Silkie_Wencha	0.794549869
ENSGALG00010009239	STX18	3	-0.124986545	0.140905671	-0.887022819	0.375066615	0.046040932	10.2560437	80.49930307	0.666666667	CRA004775;PRJNA827465;Yang2025_Silkie_Wencha	0.794549869
ENSGALG00010003125	APOO	3	0.060859712	0.068600105	0.887166461	0.374989286	0	1.426650532	0	0.666666667	CRA004775;PRJNA827465;Yang2025_Silkie_Wencha	0.794549869
ENSGALG00010029722	SPECC1	3	-0.059782167	0.067401951	-0.886950108	0.375105762	0	1.090542036	0	0.666666667	CRA004775;PRJNA827465;Yang2025_Silkie_Wencha	0.794549869
ENSGALG00010022649	PATZ1	3	-0.044061848	0.049668368	-0.887120914	0.375013806	0	0.455677876	0	1	CRA004775;PRJNA827465;Yang2025_Silkie_Wencha	0.794549869
ENSGALG00010025369	ENSGALG0001002536	3	0.573325417	0.64685463	0.886328071	0.375440767	0.858205039	6.329366993	68.40126347	0.666666667	CRA004775;PRJNA827465;Yang2025_Silkie_Wencha	0.79457831
ENSGALG00010011090	GRIN2B	2	0.317122398	0.358226603	0.885256415	0.376018352	0.169095908	2.91408672	65.68393133	0.5	PRJNA827465;Yang2025_Silkie_Wenchang	0.79457831
ENSGALG00010023715	CD3E	3	-0.307796968	0.347335596	-0.886165921	0.375528124	0.165359142	3.635461493	44.98635169	0.666666667	CRA004775;PRJNA827465;Yang2025_Silkie_Wencha	0.79457831
ENSGALG00010015606	TMEM131	3	0.229309828	0.258842615	0.885904465	0.37566901	0.168522533	14.47500369	86.18307779	0.666666667	CRA004775;PRJNA827465;Yang2025_Silkie_Wencha	0.79457831
ENSGALG00010000688	HBEGF	3	0.220087652	0.24860808	0.88527956	0.376005871	0.099927718	4.634787556	56.84807608	0.666666667	CRA004775;PRJNA827465;Yang2025_Silkie_Wencha	0.79457831
ENSGALG00010027382	PALM	3	0.212628833	0.240066477	0.885708139	0.375774822	0.133428101	13.52038871	85.20752588	0.666666667	CRA004775;PRJNA827465;Yang2025_Silkie_Wencha	0.79457831
ENSGALG00010021561	TGIF2	3	-0.174563018	0.19714652	-0.885448135	0.375914981	0.09610132	12.90788089	84.5055899	0.666666667	CRA004775;PRJNA827465;Yang2025_Silkie_Wencha	0.79457831
ENSGALG00010014617	GRTP1	3	-0.158599358	0.179100729	-0.885531616	0.375869976	0.067387055	9.291914138	78.47590959	0.666666667	CRA004775;PRJNA827465;Yang2025_Silkie_Wencha	0.79457831
ENSGALG00010029344	ENSGALG0001002934	3	0.149882155	0.169171606	0.885977022	0.375629909	0.037543773	3.404806896	41.25951747	0.666666667	CRA004775;PRJNA827465;Yang2025_Silkie_Wencha	0.79457831
ENSGALG00010013514	LMBRD2	3	0.088359139	0.099751387	0.885793589	0.375728765	0	0.302282872	0	1	CRA004775;PRJNA827465;Yang2025_Silkie_Wencha	0.79457831
ENSGALG00010010688	IQGAP2	3	0.080913756	0.091399523	0.885275472	0.376008076	0	0.641815287	0	0.666666667	CRA004775;PRJNA827465;Yang2025_Silkie_Wencha	0.79457831
ENSGALG00010015695	ASAH1	3	-0.080154654	0.090399606	-0.886670391	0.375256384	0.009661858	3.347854971	40.26025567	0.666666667	CRA004775;PRJNA827465;Yang2025_Silkie_Wencha	0.79457831
ENSGALG00010021966	ADGRA1	3	-0.066736969	0.075318895	-0.88605588	0.375585843	0	0.632585866	0	0.666666667	CRA004775;PRJNA827465;Yang2025_Silkie_Wencha	0.79457831
ENSGALG00010021750	HELLS	3	-0.057734476	0.065116658	-0.886631427	0.375277368	0	1.366208678	0	1	CRA004775;PRJNA827465;Yang2025_Silkie_Wencha	0.79457831
ENSGALG00010005355	FDX1	3	-0.056635224	0.063890444	-0.886442805	0.375378962	0.000562694	2.08625094	4.134255312	0.666666667	CRA004775;PRJNA827465;Yang2025_Silkie_Wencha	0.79457831
ENSGALG00010005082	ENSGALG0001000508	3	0.042180302	0.047613793	0.885884092	0.375679989	0	1.450710493	0	1	CRA004775;PRJNA827465;Yang2025_Silkie_Wencha	0.79457831
ENSGALG00010024753	HOXD8	3	-0.079242536	0.089535923	-0.885036226	0.376137093	0.006782836	2.685540262	25.52708934	0.666666667	CRA004775;PRJNA827465;Yang2025_Silkie_Wencha	0.794710455
ENSGALG00010020693	PTGFR	3	-0.277177655	0.313277105	-0.88476831	0.376281604	0.237510992	13.09397205	84.72579601	0.666666667	CRA004775;PRJNA827465;Yang2025_Silkie_Wencha	0.794787239
ENSGALG00010016374	FRMD5	3	0.134401689	0.151918576	0.884695555	0.376320854	0	0.264051341	0	1	CRA004775;PRJNA827465;Yang2025_Silkie_Wencha	0.794787239
ENSGALG00010009710	ENSGALG0001000971	3	0.131337082	0.148471173	0.88459614	0.376374288	0.040707736	5.532720993	63.85142134	0.666666667	CRA004775;PRJNA827465;Yang2025_Silkie_Wencha	0.794787239

ENSGALG00010021387	SLC2A14	3	-0.057087971	0.064537971	-0.884552037	0.376398285	0.004109943	2.786962685	28.2372882	1	CRA004775;PRJNA827465;Yang2025_Silkie_Wencha	0.794787239
ENSGALG00010006241	MRC1	3	-0.219756269	0.248478332	-0.88440818	0.376475909	0.090879254	3.9532306	49.40846607	0.666666667	CRA004775;PRJNA827465;Yang2025_Silkie_Wencha	0.794832444
ENSGALG00010013831	TCERG1	3	0.157158152	0.177752502	0.884140311	0.376620475	0.075126055	10.7978162	81.47773623	0.666666667	CRA004775;PRJNA827465;Yang2025_Silkie_Wencha	0.794974184
ENSGALG00010012842	DDX17	3	0.055173426	0.062408053	0.884075429	0.376655496	0.004046298	2.890219206	30.80109647	0.666666667	CRA004775;PRJNA827465;Yang2025_Silkie_Wencha	0.794974184
ENSGALG00010001969	PLEKHA8	3	0.120889382	0.13682875	0.883508637	0.376961518	0.021123003	3.22531685	37.99058843	0.666666667	CRA004775;PRJNA827465;Yang2025_Silkie_Wencha	0.795278131
ENSGALG00010029246	DHX33	3	-0.11406441	0.129113385	-0.883443728	0.376996574	0.030145603	5.204506655	61.57176592	0.666666667	CRA004775;PRJNA827465;Yang2025_Silkie_Wencha	0.795278131
ENSGALG00010008332	DHX38	3	-0.056728659	0.064216856	-0.883392034	0.377024494	0.007056891	5.145899148	61.13410033	0.666666667	CRA004775;PRJNA827465;Yang2025_Silkie_Wencha	0.795278131
ENSGALG00010021259	CEP104	3	0.046091328	0.052174614	0.883405248	0.377017357	0	0.985588654	0	0.666666667	CRA004775;PRJNA827465;Yang2025_Silkie_Wencha	0.795278131
ENSGALG00010022536	SMUG1	3	-0.149822226	0.169669106	-0.883025964	0.377222244	0.045785513	3.857552849	48.15365912	0.666666667	CRA004775;PRJNA827465;Yang2025_Silkie_Wencha	0.795379402
ENSGALG00010026919	NICN1	3	0.103865171	0.11762572	0.883014117	0.377228644	0.01184461	2.628445389	23.90939493	0.666666667	CRA004775;PRJNA827465;Yang2025_Silkie_Wencha	0.795379402
ENSGALG00010023046	SLC7A6OS	3	-0.069162724	0.078327801	-0.882990755	0.377241267	0.009114185	3.926697491	49.06661375	0.666666667	CRA004775;PRJNA827465;Yang2025_Silkie_Wencha	0.795379402
ENSGALG00010028890	ENSGALG0001002889	2	0.472740137	0.535503224	0.88279606	0.37734647	0.537736276	15.8925603	93.70774764	0.5	PRJNA827465;Yang2025_Silkie_Wenchang	0.795482591
ENSGALG00010015511	BPIFB4	2	-0.454766331	0.51550189	-0.882181695	0.377678559	0.338139156	2.742684356	63.53936982	0.5	PRJNA827465;Yang2025_Silkie_Wenchang	0.795595521
ENSGALG00010011146	BMS1	3	0.191474837	0.21698998	0.88241327	0.377553362	0.119701769	15.30938277	86.93611604	0.666666667	CRA004775;PRJNA827465;Yang2025_Silkie_Wencha	0.795595521
ENSGALG00010010645	FAM219A	3	-0.169420791	0.192013418	-0.882338291	0.377593895	0.065612335	5.635605201	64.51135364	0.666666667	CRA004775;PRJNA827465;Yang2025_Silkie_Wencha	0.795595521
ENSGALG00010016339	SETD3	3	-0.049872969	0.056534005	-0.882176464	0.377681387	0	0.025800014	0	1	CRA004775;PRJNA827465;Yang2025_Silkie_Wencha	0.795595521
ENSGALG00010005619	CELF2	3	-0.045048041	0.05104156	-0.882575705	0.37746556	0	0.461767634	0	0.666666667	CRA004775;PRJNA827465;Yang2025_Silkie_Wencha	0.795595521
ENSGALG00010014934	MAMDC2	3	-0.155918522	0.176791666	-0.881933662	0.377812682	0.048056419	4.424737035	54.79957376	0.666666667	CRA004775;PRJNA827465;Yang2025_Silkie_Wencha	0.795683066
ENSGALG00010001704	ENSGALG0001000170	3	-0.059505265	0.06751116	-0.881801475	0.377835498	0	0.505498484	0	1	CRA004775;PRJNA827465;Yang2025_Silkie_Wencha	0.795683066
ENSGALG00010021129	TXNRD3	3	-0.055249706	0.062658276	-0.881762301	0.377905362	0	1.530274505	0	1	CRA004775;PRJNA827465;Yang2025_Silkie_Wencha	0.795711678
ENSGALG00010009995	SEPTIN3	3	0.582514782	0.661017028	0.881240207	0.378187822	1.07123765	12.96675863	84.57594487	0.666666667	CRA004775;PRJNA827465;Yang2025_Silkie_Wencha	0.79614087
ENSGALG00010001301	GMPS	3	-0.133506511	0.151509233	-0.881177392	0.378221814	0.053569773	18.47336325	89.17360108	0.666666667	CRA004775;PRJNA827465;Yang2025_Silkie_Wencha	0.79614087
ENSGALG00010013949	PRRC2C	3	0.206146507	0.233993114	0.880993904	0.37832112	0.130827199	10.80658305	81.49762462	0.666666667	CRA004775;PRJNA827465;Yang2025_Silkie_Wencha	0.796231365
ENSGALG00010013632	SLC1A3	2	-0.409060591	0.464583383	-0.880489069	0.378594424	0.211495877	1.790231006	44.14128698	0.5	PRJNA827465;Yang2025_Silkie_Wenchang	0.796289196
ENSGALG00010019253	HBAD	3	-0.296610286	0.337057312	-0.87999956	0.378859548	0.272461933	10.46627782	80.89100984	0.666666667	CRA004775;PRJNA827465;Yang2025_Silkie_Wencha	0.796289196
ENSGALG00010005544	AQP4	3	-0.288276217	0.327533058	-0.880143882	0.378781369	0.200944162	5.767662621	65.32390794	0.666666667	CRA004775;PRJNA827465;Yang2025_Silkie_Wencha	0.796289196
ENSGALG00010015610	ENSGALG0001001561	3	0.266738181	0.302993379	0.880633913	0.378519997	0.189968141	13.79876798	85.50999239	0.666666667	CRA004775;PRJNA827465;Yang2025_Silkie_Wencha	0.796289196
ENSGALG00010016622	KIAA1143	3	-0.249789445	0.284028692	-0.87945145	0.379156546	0.205527179	19.54749753	89.76851131	0.666666667	CRA004775;PRJNA827465;Yang2025_Silkie_Wencha	0.796289196
ENSGALG00010004366	UNC5C	3	0.204994037	0.232873665	0.88028003	0.378707628	0	0.106266343	0	1	CRA004775;PRJNA827465;Yang2025_Silkie_Wencha	0.796289196
ENSGALG00010025005	HIP1R	3	0.194063453	0.22057728	0.879798014	0.378968741	0.128096847	19.60657009	89.79933772	0.666666667	CRA004775;PRJNA827465;Yang2025_Silkie_Wencha	0.796289196
ENSGALG00010017620	UBAP1L	3	-0.154718698	0.175877939	-0.879693611	0.379025311	0	1.402691571	0	0.666666667	CRA004775;PRJNA827465;Yang2025_Silkie_Wencha	0.796289196
ENSGALG00010015660	KLHL3	3	0.129032577	0.146508073	0.880719909	0.378469438	0.041044758	5.8235419	65.65663931	0.666666667	CRA004775;PRJNA827465;Yang2025_Silkie_Wencha	0.796289196
ENSGALG00010016218	N4BP2	3	0.125101018	0.142256488	0.879404657	0.379181908	0.027733002	3.531660918	43.36942174	0.666666667	CRA004775;PRJNA827465;Yang2025_Silkie_Wencha	0.796289196
ENSGALG00010003988	ENOPH1	3	-0.111694354	0.127052404	-0.879120351	0.379336024	0.034855569	7.732306782	74.13449755	0.666666667	CRA004775;PRJNA827465;Yang2025_Silkie_Wencha	0.796289196
ENSGALG00010020338	NECAP2	3	0.091415306	0.104001709	0.878978881	0.379412727	0.016801475	4.444221788	54.9977455	0.666666667	CRA004775;PRJNA827465;Yang2025_Silkie_Wencha	0.796289196
ENSGALG00010024734	ZPR1	3	-0.08942157	0.101630316	-0.87987102	0.378929185	0.01780627	4.721698597	57.64236198	0.666666667	CRA004775;PRJNA827465;Yang2025_Silkie_Wencha	0.796289196
ENSGALG00010011428	UQCRB	3	-0.069168018	0.078692309	-0.878967954	0.379418651	0	0.348163012	0	0.666666667	CRA004775;PRJNA827465;Yang2025_Silkie_Wencha	0.796289196
ENSGALG00010005335	AHSA1	3	0.067557993	0.076817475	0.87946125	0.379151235	0.003021115	2.278419908	12.21986814	1	CRA004775;PRJNA827465;Yang2025_Silkie_Wencha	0.796289196
ENSGALG00010017592	GID4	3	-0.060273786	0.06855151	-0.879248115	0.379266762	0	0.392073749	0	0.666666667	CRA004775;PRJNA827465;Yang2025_Silkie_Wencha	0.796289196
ENSGALG00010024179	ATG4C	3	0.048778329	0.055399132	0.880489038	0.378594441	0.000933028	2.157302261	7.291618968	0.666666667	CRA004775;PRJNA827465;Yang2025_Silkie_Wencha	0.796289196
ENSGALG00010015136	TCEB3	3	-0.038967366	0.044278492	-0.880051772	0.378831264	0	0.444967684	0	1	CRA004775;PRJNA827465;Yang2025_Silkie_Wencha	0.796289196
ENSGALG00010019643	RAB3GAP1	3	-0.02973693	0.033827732	-0.879069571	0.379363556	0	1.866373282	0	0.666666667	CRA004775;PRJNA827465;Yang2025_Silkie_Wencha	0.796289196
ENSGALG00010004001	LARP7	3	0.172432005	0.196206407	0.878829632	0.379493657	0.089707235	9.801778826	79.59554041	0.666666667	CRA004775;PRJNA827465;Yang2025_Silkie_Wencha	0.796328408
ENSGALG00010025799	SC5D	3	0.243329683	0.27706662	0.87823529	0.379816045	0.167062621	7.921688992	74.75285886	0.666666667	CRA004775;PRJNA827465;Yang2025_Silkie_Wencha	0.796464748
ENSGALG00010010693	KCNMB1	3	0.173021488	0.197034873	0.878126215	0.379875228	0	0.994753423	0	0.666666667	CRA004775;PRJNA827465;Yang2025_Silkie_Wencha	0.796464748
ENSGALG00010001066	SLC33A1	3	-0.13859497	0.15778212	-0.878394608	0.379729609	0.052655379	7.230410505	72.33905325	0.666666667	CRA004775;PRJNA827465;Yang2025_Silkie_Wencha	0.796464748
ENSGALG00010016847	EPHB2	3	-0.099205517	0.112990558	-0.877988799	0.379969616	0.018847032	4.072535713	50.89054729	0.666666667	CRA004775;PRJNA827465;Yang2025_Silkie_Wencha	0.796464748
ENSGALG00010020224	MRPL46	3	-0.061632538	0.070168123	-0.878355238	0.379750968	0	0.065394747	0	1	CRA004775;PRJNA827465;Yang2025_Silkie_Wencha	0.796464748
ENSGALG00010011677	EDEM3	3	0.042470976	0.048356015	0.878297666	0.379782202	0	0.897931364	0	0.666666667	CRA004775;PRJNA827465;Yang2025_Silkie_Wencha	0.796464748
ENSGALG00010024611	ENSGALG0001002461	3	-0.254617462	0.290048076	-0.877845719	0.38002745	0.126512976	4.011265985	50.14042929	0.666666667	CRA004775;PRJNA827465;Yang2025_Silkie_Wencha	0.796569122
ENSGALG00010018601	CPNE4	2	0.24386739	0.277820548	0.877787448	0.380059078	0	0.130900388	0	1	PRJNA827465;Yang2025_Silkie_Wenchang	0.796569122
ENSGALG00010006963	ENSGALG0001000696	3	-0.266784462	0.303990836	-0.87760692	0.380157073	0	0.450317142	0	1	CRA004775;PRJNA827465;Yang2025_Silkie_Wencha	0.796656417
ENSGALG00010021315	COX6A1	3	-0.076721504	0.087437431	-0.877444631	0.38024518	0.000548529	2.033705607	1.657349353	0.666666667	CRA004775;PRJNA827465;Yang2025_Silkie_Wencha	0.796722971
ENSGALG00010017148	MOK	2	0.544477072	0.621114633	0.876612856	0.380696954	0.650729977	6.357973994	84.27171925	0.5	PRJNA827465;Yang2025_Silkie_Wenchang	0.796897116
ENSGALG00010021436	ENSGALG0001002143	3	-0.307845891	0.351067632	-0.876884857	0.380549182	0	1.191824577	0	0.666666667	CRA004775;PRJNA827465;Yang2025_Silkie_Wencha	0.796897116
ENSGALG00010005460	TGDS	3	-0.268263424	0.305869483	-0.877051944	0.380458425	0.253578061	29.50204557	93.22080906	0.666666667	CRA004775;PRJNA827465;Yang2025_Silkie_Wencha	0.796897116
ENSGALG00010003793	TRAM1L1	3	-0.145263902	0.165637221	-0.877000356	0.380486445	0.074205396	33.61433289	94.0501571	0.666666667	CRA004775;PRJNA827465;Yang2025_Silkie_Wencha	0.796897116
ENSGALG00010001213	PPIC	3	0.119179532	0.135940641	0.876702733	0.380648122	0	0.764893602	0	0.666666667	CRA004775;PRJNA827465;Yang2025_Silkie_Wencha	0.796897116
ENSGALG00010008852	KHDRBS3	3	0.093215955	0.106393131	0.876484774	0.380766569	0.015557654	3.828873146	47.76531048	0.666666667	CRA004775;PRJNA827465;Yang2025_Silkie_Wencha	0.796897116
ENSGALG00010012803	NUP155	3	-0.081194408	0.092638874	-0.876461521	0.380779186	0	0.076335085	0	1	CRA004775;PRJNA827465;Yang2025_Silkie_Wencha	0.796897116
ENSGALG00010006478	ENSGALG0001000647	3	0.050773128	0.057920749	0.876596543	0.380705818	0	0.486359326	0	1	CRA004775;PRJNA827465;Yang2025_Silkie_Wencha	0.796897116
ENSGALG00010024675	NR1D1	3	0.25179928	0.287334628	0.876327651	0.380851937	0.045626959	2.443303017	18.14359554	0.666666667	CRA004775;PRJNA827465;Yang2025_Silkie_Wencha	0.796931411
ENSGALG00010020887	ARPP19	3	-0.135008496	0.154149775	-0.875826748	0.381124227	0.060720054	19.27325045	89.62292321	0.666666667	CRA004775;PRJNA827465;Yang2025_Silkie_Wencha	0.797045198
ENSGALG00010009113	RPLP1	3	0.072913984	0.083232922	0.876023365	0.381017332	0	0.53237379	0	0.666666667	CRA004775;PRJNA827465;Yang2025_Silkie_Wencha	0.797045198
ENSGALG00010017596	TDP1	3	-0.071830309	0.082015595	-0.875812811	0.381131804	0.007700621	2.902436725	31.09238239	1	CRA004775;PRJNA827465;Yang2025_Silkie_Wencha	0.797045198
ENSGALG00010022034	ENSGALG0001002203	3	-0.063712126	0.07273872	-0.875903866	0.381082298	0	1.038189275	0	0.666666667	CRA004775;PRJNA827465;Yang2025_Silkie_Wencha	0.797045198
ENSGALG00010004852	ENSGALG0001000485	3	-0.284145444	0.324530072	-0.875559674	0.381269456	0.239747003	13.70024564	85.4017216	0.666666667	CRA004775;PRJNA827465;Yang2025_Silkie_Wencha	0.797215149
ENSGALG00010004076	COX7A2	3	-0.281383715	0.321570989	-0.875028295	0.38155851	0.207416119	6.25881117	68.04504968	0.666666667	CRA004775;PRJNA827465;Yang2025_Silkie_Wencha	0.797701579
ENSGALG00010019174	ENSGALG0001001917	3	0.353938878	0.404612592	0.874759919	0.38170455	0.300543206	5.226198079	61.73126296	0.666666667	CRA004775;PRJNA827465;Yang2025_Silkie_Wencha	0.797822596
ENSGALG00010028339	BCAN	3	0.178379925	0.203953476	0.87461086	0.381785677	0	0.246837701	0	0.666666667	CRA004775;PRJNA827465;Yang2025_Silkie_Wencha	0.797822596
ENSGALG00010001886	NEU3	3	-0.129412647	0.147958079	-0.874657524	0.381760278	0.050533851	10.36362496	80.70173316	0.666666667	CRA004775;PRJNA827465;Yang2025_Silkie_Wencha	0.797822596
ENSGALG00010005861	MPC1L	3	-0.26898895	0.307623231	-0.874410391	0.381894801	0.242408079	17.580496	88.62375669	0.666666667	CRA004775;PRJNA827465;Yang2025_Silkie_Wencha	0.797932701
ENSGALG00010003986	FNDC3A	3	-0.06306428	0.072136931	-0.874230144	0.381992933	0.005794617	3.057270211	34.58216443	0.666666667	CRA004775;PRJNA827465;Yang2025_Silkie_Wencha	0.798019811
ENSGALG00010002398	ENSGALG0001000239	2	0.37124798	0.424805701	0.873924194	0.382159538	0	0.266979912	0	1	CRA004775;PRJNA827465	0.79824992
ENSGALG00010012215	RAB17	3	-0.536560273	0.614144017	-0.873671742	0.382297044	0.805731766	6.978189016	71.33926875	0.666666667	CRA004775;PRJNA827465;Yang2025_Silkie_Wencha	0.798349794
ENSGALG00010007068	NCAM2	2	0.522224635	0.59851834	0.872529044	0.382919831	0.59530099	5.831174046	82.85079485	0.5	PRJNA827465;Yang2025_Silkie_Wenchang	0.798349794
ENSGALG00010028085	ENSGALG0001002808	3	0.403353009	0.461757308	0.873517326	0.382381167	0.613096603	57.21690724	96.5045297	0.666666667	CRA004775;PRJNA827465;Yang2025_Silkie_Wencha	0.798349794
ENSGALG00010028357	SPPL2C	3	-0.142555961	0.163338182	-0.87276569	0.382790804	0.065222937	13.76142091	85.46661705	0.666666667	CRA004775;PRJNA827465;Yang2025_Silkie_Wencha	0.798349794
ENSGALG00010015756	PCM1	3	0.141697733	0.162416769	0.872432903	0.382972257	0.059255656	10.04831275	80.09616092	0.666666667	CRA004775;PRJNA827465;Yang2025_Silkie_Wencha	0.798349794
ENSGALG00010013771	PELO	3	0.103655351	0.118818224	0.872385967	0.382997854	0	1.111643039	0	0.666666667	CRA004775;PRJNA827465;Yang2025_Silkie_Wencha	0.798349794
ENSGALG00010023826	DBT	3	-0.102845111	0.117725873	-0.873598204	0.382337105	0.024480316	6.039293399	66.88354302	0.666666667	CRA004775;PRJNA827465;Yang2025_Silkie_Wencha	0.798349794
ENSGALG00010019586	GLYR1	3	-0.100270607	0.114850716	-0.873051656	0.382634923	0.028026791	8.379864659	76.13326609	0.666666667	CRA004775;PRJNA827465;Yang2025_Silkie_Wencha	0.798349794
ENSGALG00010001131	HPS3	3	0.08306202	0.095177549	0.872706023	0.382823334	0.01308951	3.904792213	48.78088536	0.666666667	CRA004775;PRJNA827465;Yang2025_Silkie_Wencha	0.798349794
ENSGALG00010023863	EYA2	3	0.082204788	0.094190468	0.872750601	0.38279903	0	1.970001099	0	0.666666667	CRA004775;PRJNA827465;Yang2025_Silkie_Wencha	0.798349794
ENSGALG00010002868	PPP1R9A	3	-0.051961603	0.059518621	-0.873031025	0.382646167	0	0.902997042	0	0.666666667	CRA004775;PRJNA827465;Yang2025_Silkie_Wencha	0.798349794
ENSGALG00010015085	PSMD2	3	-0.049956071	0.057204713	-0.873285942	0.382507241	0	1.595902876	0	1	CRA004775;PRJNA827465;Yang2025_Silkie_Wencha	0.798349794
ENSGALG00010027386	RHOA	3	-0.03737349	0.042833948	-0.872520322	0.382924587	0.001287271	2.390981663	16.35234887	1	CRA004775;PRJNA827465;Yang2025_Silkie_Wencha	0.798349794
ENSGALG00010005472	PFKP	3	0.035633443	0.04082581	0.87281656	0.382763072	0	1.469034145	0	0.666666667	CRA004775;PRJNA827465;Yang2025_Silkie_Wencha	0.798349794
ENSGALG00010018847	CGRRF1	3	-0.225325341	0.258318671	-0.872276636	0.38305748	0.162313813	11.23094229	82.19205523	0.666666667	CRA004775;PRJNA827465;Yang2025_Silkie_Wencha	0.798356385
ENSGALG00010026851	ENSGALG0001002685	2	-1.748286042	2.004742545	-0.872075093	0.383167413	3.840356352	1.885196648	46.95513589	0.5	PRJNA827465;Yang2025_Silkie_Wenchang	0.79844079
ENSGALG00010016299	SRSF4	3	0.237987976	0.272955492	0.871892972	0.383266768	0.191282521	18.28979821	89.06494223	0.666666667	CRA004775;PRJNA827465;Yang2025_Silkie_Wencha	0.79844079
ENSGALG00010009474	ACVR2A	3	-0.067717421	0.077667228	-0.87189183	0.383267391	0.005548654	2.7151373	26.33890006	0.666666667	CRA004775;PRJNA827465;Yang2025_Silkie_Wencha	0.79844079
ENSGALG00010022831	LRRC8C	3	-0.053156542	0.060986477	-0.87161194	0.383420115	0	1.032676121	0	0.666666667	CRA004775;PRJNA827465;Yang2025_Silkie_Wencha	0.798641279
ENSGALG00010000255	ENSGALG0001000025	3	0.974975899	1.119088951	0.87122288	0.383632469	3.682019949	128.2208832	98.44019168	0.666666667	CRA004775;PRJNA827465;Yang2025_Silkie_Wencha	0.798730597
ENSGALG00010021514	TPR	3	0.19154414	0.21983228	0.871319442	0.383579758	0.117250951	11.80162785	83.05318532	0.666666667	CRA004775;PRJNA827465;Yang2025_Silkie_Wencha	0.798730597
ENSGALG00010005382	RAB32	3	-0.18732702	0.215013471	-0.871233876	0.383626467	0.097888913	8.426359321	76.2649571	0.666666667	CRA004775;PRJNA827465;Yang2025_Silkie_Wencha	0.798730597
ENSGALG00010001122	P2RY14	3	-0.426539735	0.490537153	-0.869536042	0.384554003	0.613212881	13.98772557	85.7017498	0.666666667	CRA004775;PRJNA827465;Yang2025_Silkie_Wencha	0.798852115
ENSGALG00010013154	ENSGALG0001001315	3	-0.364380154	0.418374237	-0.870943099	0.383785222	0	0.726430925	0	0.666666667	CRA004775;PRJNA827465;Yang2025_Silkie_Wencha	0.798852115
ENSGALG00010008273	TMEM144	3	-0.229221745	0.263577373	-0.869656387	0.384488213	0.172705239	12.41073596	83.88492024	0.666666667	CRA004775;PRJNA827465;Yang2025_Silkie_Wencha	0.798852115
ENSGALG00010007590	FBXW7	3	0.228151818	0.262337745	0.869687349	0.384471287	0.178248391	21.08328401	90.5138118	0.666666667	CRA004775;PRJNA827465;Yang2025_Silkie_Wencha	0.798852115
ENSGALG00010016505	KCTD16	2	0.221966832	0.255235928	0.869653556	0.38448976	0	0.007534579	0	1	PRJNA827465;Yang2025_Silkie_Wenchang	0.798852115
ENSGALG00010002196	ENSGALG0001000219	3	-0.187662646	0.215528316	-0.870709933	0.383912554	0.120443197	29.08147209	93.12276905	0.666666667	CRA004775;PRJNA827465;Yang2025_Silkie_Wencha	0.798852115
ENSGALG00010023911	TFCP2	3	-0.179521065	0.206197554	-0.87062655	0.383958095	0.115438922	24.90097091	91.96818467	0.666666667	CRA004775;PRJNA827465;Yang2025_Silkie_Wencha	0.798852115
ENSGALG00010012149	SFXN1	3	-0.161010365	0.185037353	-0.870150608	0.384218104	0.061920426	5.16425699	61.27226039	0.666666667	CRA004775;PRJNA827465;Yang2025_Silkie_Wencha	0.798852115
ENSGALG00010005401	BCLAF1	3	0.150709817	0.173312122	0.869586127	0.384526622	0.066326624	8.567878334	76.65699813	0.666666667	CRA004775;PRJNA827465;Yang2025_Silkie_Wencha	0.798852115
ENSGALG00010018673	NAGPA	3	-0.149078618	0.171454732	-0.869492586	0.384577761	0.07497292	15.13163219	86.7826552	0.666666667	CRA004775;PRJNA827465;Yang2025_Silkie_Wencha	0.798852115
ENSGALG00010017745	SHMT1	3	0.136759371	0.157175374	0.87010686	0.384242009	0.027353664	3.065467536	34.75709735	0.666666667	CRA004775;PRJNA827465;Yang2025_Silkie_Wencha	0.798852115
ENSGALG00010029616	TMEM120A	3	-0.129134341	0.148544688	-0.869329918	0.384666703	0.042228742	5.579659439	64.15551841	0.666666667	CRA004775;PRJNA827465;Yang2025_Silkie_Wencha	0.798852115
ENSGALG00010010805	PTRHD1	3	0.110131344	0.126701932	0.869215978	0.38472901	0	0.212740804	0	0.666666667	CRA004775;PRJNA827465;Yang2025_Silkie_Wencha	0.798852115
ENSGALG00010012102	SON	3	0.105528028	0.121425446	0.869076717	0.384805171	0.0275466	5.663107034	64.6836977	0.666666667	CRA004775;PRJNA827465;Yang2025_Silkie_Wencha	0.798852115
ENSGALG00010029390	RPA1	3	0.080479415	0.092563081	0.869454804	0.384598418	0.013886276	4.85437695	58.80006805	0.666666667	CRA004775;PRJNA827465;Yang2025_Silkie_Wencha	0.798852115
ENSGALG00010011685	NDUFA2	3	-0.078007494	0.089714838	-0.869504922	0.384571017	0	0.003789216	0	1	CRA004775;PRJNA827465;Yang2025_Silkie_Wencha	0.798852115
ENSGALG00010029622	MMP28	3	0.073950063	0.085005567	0.869943766	0.384331136	0	0.287429481	0	0.666666667	CRA004775;PRJNA827465;Yang2025_Silkie_Wencha	0.798852115
ENSGALG00010006285	FZD8	3	0.06154642	0.070745335	0.86997143	0.384316018	0	0.746026422	0	0.666666667	CRA004775;PRJNA827465;Yang2025_Silkie_Wencha	0.798852115
ENSGALG00010028512	NDUFA13	3	-0.055503149	0.063789072	-0.870104346	0.384243383	0	1.163894793	0	0.666666667	CRA004775;PRJNA827465;Yang2025_Silkie_Wencha	0.798852115
ENSGALG00010010994	NAA20	3	-0.051217413	0.058935074	-0.869048081	0.384820833	0	1.212573356	0	1	CRA004775;PRJNA827465;Yang2025_Silkie_Wencha	0.798852115
ENSGALG00010020625	SLC30A1	3	-0.127282136	0.146497407	-0.868835421	0.384937156	0.04751182	15.28222186	86.91289776	0.666666667	CRA004775;PRJNA827465;Yang2025_Silkie_Wencha	0.798976284
ENSGALG00010025215	MYOG	3	0.092719063	0.106732793	0.868702672	0.385009779	0	0.80662191	0	0.666666667	CRA004775;PRJNA827465;Yang2025_Silkie_Wencha	0.799009727
ENSGALG00010017562	SLC30A10	3	0.589571034	0.67929379	0.867917596	0.385439445	1.05459083	8.925033815	77.59112132	0.666666667	CRA004775;PRJNA827465;Yang2025_Silkie_Wencha	0.799014658
ENSGALG00010011497	CPQ	3	-0.336896	0.38790242	-0.868507085	0.385116795	0.381965201	18.74303979	89.32937228	0.666666667	CRA004775;PRJNA827465;Yang2025_Silkie_Wencha	0.799014658
ENSGALG00010027117	RASSF5	3	-0.195298392	0.224998695	-0.867997887	0.385395489	0.047339886	2.82635689	29.23752811	1	CRA004775;PRJNA827465;Yang2025_Silkie_Wencha	0.799014658
ENSGALG00010029532	EVI2A	3	0.164997239	0.190105665	0.867923841	0.385436026	0	1.025981746	0	0.666666667	CRA004775;PRJNA827465;Yang2025_Silkie_Wencha	0.799014658
ENSGALG00010014399	GAS6	3	0.147640393	0.1700373	0.86828239	0.38523976	0.062019523	8.858403233	77.42256762	0.666666667	CRA004775;PRJNA827465;Yang2025_Silkie_Wencha	0.799014658
ENSGALG00010021261	JUP	3	0.114761794	0.132224649	0.867930413	0.385432428	0.032060776	6.476282414	69.11808547	0.666666667	CRA004775;PRJNA827465;Yang2025_Silkie_Wencha	0.799014658
ENSGALG00010024756	CDK2	3	0.063206344	0.072829083	0.867872297	0.385464246	0	1.880350515	0	0.666666667	CRA004775;PRJNA827465;Yang2025_Silkie_Wencha	0.799014658
ENSGALG00010009351	GTF3C2	3	0.059891833	0.068977176	0.868284789	0.385238447	0.00297953	2.254242169	11.2783876	0.666666667	CRA004775;PRJNA827465;Yang2025_Silkie_Wencha	0.799014658
ENSGALG00010023265	RAB5C	3	-0.096846223	0.111607017	-0.867743139	0.385534964	0.027779281	9.311132097	78.52033481	0.666666667	CRA004775;PRJNA827465;Yang2025_Silkie_Wencha	0.799044101
ENSGALG00010014431	ENSGALG0001001443	3	-0.23356553	0.269238737	-0.867503436	0.38566623	0	1.756660895	0	0.666666667	CRA004775;PRJNA827465;Yang2025_Silkie_Wencha	0.799146872
ENSGALG00010025351	CREB1	3	-0.142996277	0.164847435	-0.867446173	0.385697592	0.058919244	11.62860086	82.80102633	0.666666667	CRA004775;PRJNA827465;Yang2025_Silkie_Wencha	0.799146872
ENSGALG00010017780	TAF9	3	0.047081249	0.054303858	0.866996398	0.385943983	0	0.526042391	0	0.666666667	CRA004775;PRJNA827465;Yang2025_Silkie_Wencha	0.799540217
ENSGALG00010029574	SGSM2	3	0.074898066	0.08640503	0.866825291	0.386037742	0.003853003	2.32743155	14.06836435	0.666666667	CRA004775;PRJNA827465;Yang2025_Silkie_Wencha	0.799617293
ENSGALG00010024585	TBCCD1	3	-0.119118585	0.137443263	-0.866674597	0.386120328	0.033429558	5.313277104	62.35844732	0.666666667	CRA004775;PRJNA827465;Yang2025_Silkie_Wencha	0.799671205
ENSGALG00010000123	ENSGALG0001000012	3	-0.244932287	0.282802747	-0.866088781	0.386441477	0.184673405	10.31654877	80.61367184	0.666666667	CRA004775;PRJNA827465;Yang2025_Silkie_Wencha	0.800219105
ENSGALG00010027326	MRPL24	3	-0.055118551	0.063658862	-0.865842543	0.386576516	0	0.862354167	0	0.666666667	CRA004775;PRJNA827465;Yang2025_Silkie_Wencha	0.800264327
ENSGALG00010002920	VCPIP1	3	-0.043720222	0.050494121	-0.865847758	0.386573655	0	1.659472808	0	0.666666667	CRA004775;PRJNA827465;Yang2025_Silkie_Wencha	0.800264327
ENSGALG00010021020	ZDHHC13	3	-0.092228189	0.106552474	-0.865565905	0.38672826	0.013575065	3.331865989	39.97357617	0.666666667	CRA004775;PRJNA827465;Yang2025_Silkie_Wencha	0.800275179
ENSGALG00010016606	VASN	3	0.086264258	0.099659232	0.865592246	0.38671381	0.012188987	3.315649405	39.67999159	0.666666667	CRA004775;PRJNA827465;Yang2025_Silkie_Wencha	0.800275179
ENSGALG00010018043	PSMC6	3	-0.059851173	0.069150263	-0.865523434	0.386751559	0.005242886	2.95375393	32.28955263	0.666666667	CRA004775;PRJNA827465;Yang2025_Silkie_Wencha	0.800275179
ENSGALG00010015092	HSPE1	3	-0.194515056	0.224894745	-0.864915968	0.387084912	0.11057503	7.806631513	74.38075569	0.666666667	CRA004775;PRJNA827465;Yang2025_Silkie_Wencha	0.800624436
ENSGALG00010001472	ENSGALG0001000147	3	0.193291811	0.223512583	0.864791628	0.387153167	0.092371265	6.020249504	66.77878552	0.666666667	CRA004775;PRJNA827465;Yang2025_Silkie_Wencha	0.800624436
ENSGALG00010020790	PRPH2L	3	0.191202182	0.221137813	0.86462907	0.387242411	0	0.012716649	0	1	CRA004775;PRJNA827465;Yang2025_Silkie_Wencha	0.800624436
ENSGALG00010018080	BRICD5	3	-0.147973551	0.171147454	-0.864596859	0.387260097	0.029005667	2.693357064	25.74322852	1	CRA004775;PRJNA827465;Yang2025_Silkie_Wencha	0.800624436
ENSGALG00010010037	NAA15	3	0.141200923	0.163306617	0.864636874	0.387238127	0.061621513	11.24685454	82.21725023	0.666666667	CRA004775;PRJNA827465;Yang2025_Silkie_Wencha	0.800624436
ENSGALG00010027243	HRH1	3	0.091859919	0.106219769	0.864810001	0.387143081	0	0.304808106	0	1	CRA004775;PRJNA827465;Yang2025_Silkie_Wencha	0.800624436
ENSGALG00010015910	C5orf15	3	-0.260368052	0.301195694	-0.864448121	0.387341768	0.261404234	92.94140858	97.84810664	0.666666667	CRA004775;PRJNA827465;Yang2025_Silkie_Wencha	0.800676207
ENSGALG00010000246	ENSGALG0001000024	3	-1.485959416	1.719196071	-0.864333883	0.387404502	5.487100237	5.459431105	63.36614637	0.666666667	CRA004775;PRJNA827465;Yang2025_Silkie_Wencha	0.800688825
ENSGALG00010025422	SSBP3	3	-0.040119324	0.046425641	-0.864163075	0.387498313	0	1.231073581	0	1	CRA004775;PRJNA827465;Yang2025_Silkie_Wencha	0.800765661
ENSGALG00010029229	CBX4	3	0.19145606	0.221818854	0.863118964	0.388072062	0.123096312	16.4482789	87.84067311	0.666666667	CRA004775;PRJNA827465;Yang2025_Silkie_Wencha	0.801160483
ENSGALG00010028058	GLT8D1	3	-0.185686273	0.214993666	-0.863682532	0.387762312	0.110810651	15.3114793	86.93790482	0.666666667	CRA004775;PRJNA827465;Yang2025_Silkie_Wencha	0.801160483
ENSGALG00010006353	ABCE1	3	-0.102413479	0.118610106	-0.863446491	0.387892028	0.029071235	7.09409788	71.80754997	0.666666667	CRA004775;PRJNA827465;Yang2025_Silkie_Wencha	0.801160483
ENSGALG00010016988	ECI1	3	-0.058766069	0.068071177	-0.863303266	0.387970749	0	0.837748024	0	1	CRA004775;PRJNA827465;Yang2025_Silkie_Wencha	0.801160483
ENSGALG00010016894	BTBD7	3	-0.040085636	0.046438724	-0.863194171	0.388030718	0.000826711	2.078040226	3.755472333	0.666666667	CRA004775;PRJNA827465;Yang2025_Silkie_Wencha	0.801160483
ENSGALG00010008170	PICALM	3	0.037807405	0.043804526	0.863093587	0.388086014	0	1.986849053	0	1	CRA004775;PRJNA827465;Yang2025_Silkie_Wencha	0.801160483
ENSGALG00010026964	SRPK1	3	0.030968173	0.035869752	0.863350628	0.387944716	0	1.379760077	0	0.666666667	CRA004775;PRJNA827465;Yang2025_Silkie_Wencha	0.801160483
ENSGALG00010000157	ENSGALG0001000015	3	-1.569305458	1.825553317	-0.859632772	0.389991504	6.060733328	5.152735001	61.1856616	0.666666667	CRA004775;PRJNA827465;Yang2025_Silkie_Wencha	0.801233635
ENSGALG00010029729	RASL10A	2	-0.530064976	0.614850874	-0.862103314	0.388630668	0.43783874	2.37170655	57.83626772	0.5	PRJNA827465;Yang2025_Silkie_Wenchang	0.801233635
ENSGALG00010024706	ENSGALG0001002470	3	0.430656386	0.500604172	0.860273265	0.389638426	0.566820447	8.725617562	77.07898626	0.666666667	CRA004775;PRJNA827465;Yang2025_Silkie_Wencha	0.801233635
ENSGALG00010025849	OBSCN	3	0.376892866	0.438355538	0.85978808	0.389905871	0.467692643	18.67685302	89.29155786	0.666666667	CRA004775;PRJNA827465;Yang2025_Silkie_Wencha	0.801233635
ENSGALG00010022204	ENSGALG0001002220	3	0.311613565	0.361764642	0.861370982	0.389033753	0.199930493	4.084439642	51.03367475	0.666666667	CRA004775;PRJNA827465;Yang2025_Silkie_Wencha	0.801233635
ENSGALG00010012521	HOMER1	3	0.266882373	0.309817824	0.861417106	0.389008358	0.237099249	12.79799798	84.37255574	0.666666667	CRA004775;PRJNA827465;Yang2025_Silkie_Wencha	0.801233635
ENSGALG00010001928	KCTD21	3	-0.242606298	0.281831916	-0.860819106	0.389337681	0.185384133	14.36825679	86.08042695	0.666666667	CRA004775;PRJNA827465;Yang2025_Silkie_Wencha	0.801233635
ENSGALG00010023454	HOXB1	3	-0.228858723	0.266041251	-0.860237732	0.389658009	0.100203348	3.737996861	46.49540719	0.666666667	CRA004775;PRJNA827465;Yang2025_Silkie_Wencha	0.801233635
ENSGALG00010008509	MGARP	3	0.190749295	0.221833932	0.859874289	0.389858343	0	1.286863416	0	0.666666667	CRA004775;PRJNA827465;Yang2025_Silkie_Wencha	0.801233635
ENSGALG00010003829	ENSGALG0001000382	3	0.189475496	0.220146779	0.860678033	0.389415395	0.09602214	6.186038794	67.6691326	0.666666667	CRA004775;PRJNA827465;Yang2025_Silkie_Wencha	0.801233635
ENSGALG00010024020	MAIP1	3	-0.180689696	0.209652751	-0.861852254	0.388768826	0.102726115	13.23761351	84.89153654	0.666666667	CRA004775;PRJNA827465;Yang2025_Silkie_Wencha	0.801233635
ENSGALG00010027088	AGBL4	3	0.149550109	0.173313567	0.862887492	0.388199328	0	0.62928014	0	1	CRA004775;PRJNA827465;Yang2025_Silkie_Wencha	0.801233635
ENSGALG00010027217	SEMA6B	3	0.137952835	0.160094113	0.86169836	0.388853528	0.04287309	5.584217118	64.18477366	0.666666667	CRA004775;PRJNA827465;Yang2025_Silkie_Wencha	0.801233635
ENSGALG00010029274	VMP1	3	-0.132650144	0.15407903	-0.860922762	0.389280584	0.054832763	12.40212075	83.87372579	0.666666667	CRA004775;PRJNA827465;Yang2025_Silkie_Wencha	0.801233635
ENSGALG00010007097	ZCCHC9	3	-0.127310274	0.14767909	-0.862073799	0.388646909	0.017083835	2.684277051	25.49204265	0.666666667	CRA004775;PRJNA827465;Yang2025_Silkie_Wencha	0.801233635
ENSGALG00010005405	CEBPD	3	-0.119319832	0.138689802	-0.860336017	0.389603845	0	0.730568915	0	0.666666667	CRA004775;PRJNA827465;Yang2025_Silkie_Wencha	0.801233635
ENSGALG00010029413	B3GNTL1	3	0.111920624	0.130089496	0.860335594	0.389604078	0	0.549414349	0	0.666666667	CRA004775;PRJNA827465;Yang2025_Silkie_Wencha	0.801233635
ENSGALG00010016852	AHDC1	3	0.11134179	0.129099254	0.86245107	0.388439348	0.027182218	5.061334045	60.48472632	0.666666667	CRA004775;PRJNA827465;Yang2025_Silkie_Wencha	0.801233635
ENSGALG00010021579	C20H20ORF24	3	-0.105487486	0.12270579	-0.859678147	0.389966484	0.030879028	7.95327831	74.85313701	0.666666667	CRA004775;PRJNA827465;Yang2025_Silkie_Wencha	0.801233635
ENSGALG00010025544	CSDE1	3	0.104082742	0.120772492	0.861808349	0.38879299	0.032011112	8.936348272	77.61949357	0.666666667	CRA004775;PRJNA827465;Yang2025_Silkie_Wencha	0.801233635
ENSGALG00010011747	UTP15	3	0.098009287	0.113976735	0.859906078	0.389840818	0	0.611153683	0	0.666666667	CRA004775;PRJNA827465;Yang2025_Silkie_Wencha	0.801233635
ENSGALG00010020019	PEX10	3	0.096132424	0.111469541	0.862409795	0.388462052	0.016767408	3.521020143	43.19828008	0.666666667	CRA004775;PRJNA827465;Yang2025_Silkie_Wencha	0.801233635
ENSGALG00010020321	SZRD1	3	-0.090486374	0.105200346	-0.860133799	0.389715292	0.016718945	3.943502249	49.28366022	0.666666667	CRA004775;PRJNA827465;Yang2025_Silkie_Wencha	0.801233635
ENSGALG00010004345	FAXC	3	-0.085375707	0.099098847	-0.861520687	0.388951333	0	0.485504084	0	1	CRA004775;PRJNA827465;Yang2025_Silkie_Wencha	0.801233635
ENSGALG00010027381	KLHL40	3	-0.077844189	0.090356697	-0.861520967	0.388951178	0.007510461	2.840568934	29.59156963	0.666666667	CRA004775;PRJNA827465;Yang2025_Silkie_Wencha	0.801233635
ENSGALG00010017080	PALB2	3	-0.062436441	0.072630782	-0.8596416	0.389986636	0	1.871556042	0	0.666666667	CRA004775;PRJNA827465;Yang2025_Silkie_Wencha	0.801233635
ENSGALG00010016498	KIF23	3	0.060344699	0.069996236	0.862113485	0.388625071	0	0.936232517	0	1	CRA004775;PRJNA827465;Yang2025_Silkie_Wencha	0.801233635

ENSGALG00010024968	TMEM101	3	-0.058934073	0.068473904	-0.860679322	0.389414685	0	0.198174379	0	1	CRA004775;PRJNA827465;Yang2025_Silkie_Wencha	0.801233635
ENSGALG00010012867	A4GALT	3	-0.045983972	0.053306722	-0.862629904	0.388340983	0	0.157592521	0	1	CRA004775;PRJNA827465;Yang2025_Silkie_Wencha	0.801233635
ENSGALG00010008285	ADSS2	3	0.042929339	0.049787794	0.86224626	0.388552018	0	1.598164191	0	0.666666667	CRA004775;PRJNA827465;Yang2025_Silkie_Wencha	0.801233635
ENSGALG00010024030	STAU1	3	0.041838279	0.048513374	0.862407116	0.388463526	0.000737553	2.156921734	7.275263258	1	CRA004775;PRJNA827465;Yang2025_Silkie_Wencha	0.801233635
ENSGALG00010020237	MIB2	3	0.038259482	0.044483141	0.860089498	0.38973971	0	1.793841248	0	1	CRA004775;PRJNA827465;Yang2025_Silkie_Wencha	0.801233635
ENSGALG00010029929	GDPD1	3	0.027556902	0.032020272	0.860607997	0.38945398	0	0.122796787	0	0.666666667	CRA004775;PRJNA827465;Yang2025_Silkie_Wencha	0.801233635
ENSGALG00010006200	GSTCD	3	-0.136682652	0.159035099	-0.8594496	0.390092515	0.049413315	6.105898302	67.24478691	0.666666667	CRA004775;PRJNA827465;Yang2025_Silkie_Wencha	0.801324723
ENSGALG00010000814	CDH18	2	-0.141703188	0.164940249	-0.859118315	0.390275244	0	0.36245306	0	1	PRJNA827465;Yang2025_Silkie_Wenchang	0.8014672
ENSGALG00010027579	C26H6ORF125	3	-0.063837191	0.07430261	-0.859151399	0.390256993	0	0.266258989	0	1	CRA004775;PRJNA827465;Yang2025_Silkie_Wencha	0.8014672
ENSGALG00010003419	ENSGALG00010003419	2	-0.666101315	0.775993639	-0.85838502	0.390679897	0	0.030219415	0	1	PRJNA827465;Yang2025_Silkie_Wenchang	0.801760195
ENSGALG00010005467	ENSGALG00010005467	3	-0.305169698	0.355553153	-0.858287814	0.390733557	0.300861242	11.99471637	83.32599172	0.666666667	CRA004775;PRJNA827465;Yang2025_Silkie_Wencha	0.801760195
ENSGALG00010003201	MBOAT2	3	-0.221369448	0.258396788	-0.85670356	0.391608735	0.14208794	9.144199364	78.1282109	0.666666667	CRA004775;PRJNA827465;Yang2025_Silkie_Wencha	0.801760195
ENSGALG00010002567	COA4	3	-0.217974468	0.25418471	-0.857543587	0.391144537	0.10915374	4.598203647	56.50475374	0.666666667	CRA004775;PRJNA827465;Yang2025_Silkie_Wencha	0.801760195
ENSGALG00010002379	NHS	3	0.204637235	0.238673721	0.857393239	0.391227595	0.105609895	5.349474554	62.61315051	0.666666667	CRA004775;PRJNA827465;Yang2025_Silkie_Wencha	0.801760195
ENSGALG00010022142	TMEM208	3	-0.200531634	0.233804574	-0.857689096	0.391064163	0.10621729	5.679647642	64.78654793	0.666666667	CRA004775;PRJNA827465;Yang2025_Silkie_Wencha	0.801760195
ENSGALG00010011703	YWHAZ	3	-0.197913151	0.230550465	-0.85843744	0.390650961	0.140015407	30.9480216	93.53755136	0.666666667	CRA004775;PRJNA827465;Yang2025_Silkie_Wencha	0.801760195
ENSGALG00010006506	ZNF827	3	0.196943637	0.229607429	0.857740701	0.39103566	0.114769512	8.535477509	76.56838767	0.666666667	CRA004775;PRJNA827465;Yang2025_Silkie_Wencha	0.801760195
ENSGALG00010000048	MAN2A2	3	0.176074414	0.205467327	0.856946048	0.391474702	0.090660347	15.38020092	86.9962687	0.666666667	CRA004775;PRJNA827465;Yang2025_Silkie_Wencha	0.801760195
ENSGALG00010029726	SEPTIN9	3	-0.148191467	0.172954651	-0.856822675	0.391542892	0.073116529	22.25971274	91.01515809	0.666666667	CRA004775;PRJNA827465;Yang2025_Silkie_Wencha	0.801760195
ENSGALG00010017706	FGFRL1	3	0.144113677	0.167835036	0.858662618	0.39052668	0.048189769	5.361827749	62.69922663	0.666666667	CRA004775;PRJNA827465;Yang2025_Silkie_Wencha	0.801760195
ENSGALG00010022332	KAT6B	3	0.13200409	0.154049533	0.856893803	0.391503578	0.044441128	5.352795814	62.63634801	0.666666667	CRA004775;PRJNA827465;Yang2025_Silkie_Wencha	0.801760195
ENSGALG00010014010	EIF5A2	3	0.117834084	0.137305345	0.858190071	0.390787518	0.031977654	4.655405356	57.03918677	0.666666667	CRA004775;PRJNA827465;Yang2025_Silkie_Wencha	0.801760195
ENSGALG00010016193	TCAIM	3	-0.075380567	0.08792981	-0.857281126	0.391289537	0.006620406	2.684684	25.50333671	0.666666667	CRA004775;PRJNA827465;Yang2025_Silkie_Wencha	0.801760195
ENSGALG00010004342	ZNF438	3	0.074753512	0.087251917	0.856754953	0.391580326	0.006095359	2.541042744	21.29215437	0.666666667	CRA004775;PRJNA827465;Yang2025_Silkie_Wencha	0.801760195
ENSGALG00010008153	SMU1	3	-0.061763972	0.072029684	-0.857479434	0.391179976	0.009784293	6.373705457	68.621079	0.666666667	CRA004775;PRJNA827465;Yang2025_Silkie_Wencha	0.801760195
ENSGALG00010008232	PYURF	3	0.061431437	0.071567088	0.858375527	0.390685137	0.002479753	2.218034927	9.83009438	0.666666667	CRA004775;PRJNA827465;Yang2025_Silkie_Wencha	0.801760195
ENSGALG00010001432	NSMCE4A	3	0.056570591	0.06602577	0.856795517	0.391557904	0	0.791839677	0	0.666666667	CRA004775;PRJNA827465;Yang2025_Silkie_Wencha	0.801760195
ENSGALG00010002649	UFL1	3	0.054652881	0.063783662	0.856847646	0.39152909	0.004962669	3.188482542	37.27423708	0.666666667	CRA004775;PRJNA827465;Yang2025_Silkie_Wencha	0.801760195
ENSGALG00010022888	OSBPL2	3	-0.042812052	0.049959389	-0.856937062	0.391479669	0	1.627542872	0	0.666666667	CRA004775;PRJNA827465;Yang2025_Silkie_Wencha	0.801760195
ENSGALG00010027101	RNF220	3	-0.033550447	0.039150244	-0.856966497	0.391463401	0	1.141540361	0	0.666666667	CRA004775;PRJNA827465;Yang2025_Silkie_Wencha	0.801760195
ENSGALG00010008037	ENSGALG00010008037	3	1.78821818	2.100732561	0.851235523	0.394638534	12.18491688	26.3984582	92.42379996	0.666666667	CRA004775;PRJNA827465;Yang2025_Silkie_Wencha	0.801809775
ENSGALG00010002283	ENSGALG00010002283	2	0.601852338	0.704527958	0.854263242	0.392959149	0.069855919	1.069265617	6.477868182	1	PRJNA827465;Yang2025_Silkie_Wenchang	0.801809775
ENSGALG00010014234	GLRA1	2	0.600511508	0.70471799	0.852130238	0.394141811	0.635935405	2.723695462	63.28517582	0.5	PRJNA827465;Yang2025_Silkie_Wenchang	0.801809775
ENSGALG00010011465	PPP2R2C	3	0.438459129	0.514395514	0.852377436	0.39400464	0.65539626	17.45832665	88.54418436	0.666666667	CRA004775;PRJNA827465;Yang2025_Silkie_Wencha	0.801809775
ENSGALG00010015164	ENSGALG00010015164	3	0.388540046	0.456063086	0.851951203	0.394241176	0.181139829	2.725097809	26.60813886	0.666666667	CRA004775;PRJNA827465;Yang2025_Silkie_Wencha	0.801809775
ENSGALG00010021531	RAB15	3	0.324329494	0.38072976	0.851862732	0.394290284	0.349831406	10.25025971	80.48829926	0.666666667	CRA004775;PRJNA827465;Yang2025_Silkie_Wencha	0.801809775
ENSGALG00010002374	DST	3	0.324239944	0.380104106	0.853029311	0.39364305	0.377343843	20.20285067	90.10040696	0.666666667	CRA004775;PRJNA827465;Yang2025_Silkie_Wencha	0.801809775
ENSGALG00010029439	ENSGALG00010029439	3	0.303295952	0.356055085	0.851823115	0.394312275	0.283677865	8.20989453	75.63915112	0.666666667	CRA004775;PRJNA827465;Yang2025_Silkie_Wencha	0.801809775
ENSGALG00010010275	PDE8B	3	-0.275863591	0.323991059	-0.851454332	0.394517021	0	1.224585193	0	0.666666667	CRA004775;PRJNA827465;Yang2025_Silkie_Wencha	0.801809775
ENSGALG00010014109	AMIGO2	3	0.262439069	0.306860296	0.855239576	0.392418531	0.225672794	11.03972262	81.88360279	0.666666667	CRA004775;PRJNA827465;Yang2025_Silkie_Wencha	0.801809775
ENSGALG00010006457	NCAPH2	3	0.242084432	0.283251202	0.854663387	0.392737525	0.183502809	12.57271568	84.09253776	0.666666667	CRA004775;PRJNA827465;Yang2025_Silkie_Wencha	0.801809775
ENSGALG00010001216	SHOX2	2	0.239623689	0.280468493	0.854369369	0.392900363	0	0.42495934	0	1	PRJNA827465;Yang2025_Silkie_Wenchang	0.801809775
ENSGALG00010001471	NMNAT2	3	-0.203506417	0.238852743	-0.852016242	0.394205077	0	1.533056614	0	0.666666667	CRA004775;PRJNA827465;Yang2025_Silkie_Wencha	0.801809775
ENSGALG00010001758	COL19A1	3	-0.198992976	0.232483264	-0.855945379	0.392027993	0.090694738	5.631212606	64.48367093	0.666666667	CRA004775;PRJNA827465;Yang2025_Silkie_Wencha	0.801809775
ENSGALG00010029547	HIP1	3	0.19874399	0.233375118	0.851607454	0.394432001	0.15253488	35.14881364	94.30990752	0.666666667	CRA004775;PRJNA827465;Yang2025_Silkie_Wencha	0.801809775
ENSGALG00010002418	TRAPPC2	3	-0.195100734	0.228875019	-0.852433503	0.393973532	0.138023266	24.32603409	91.77835568	0.666666667	CRA004775;PRJNA827465;Yang2025_Silkie_Wencha	0.801809775
ENSGALG00010017439	PPP4R4	3	0.19091997	0.22413888	0.851793181	0.39432889	0	1.420246452	0	0.666666667	CRA004775;PRJNA827465;Yang2025_Silkie_Wencha	0.801809775
ENSGALG00010013973	ENSGALG00010013973	3	0.17738553	0.207627967	0.85434314	0.392914891	0	0.626029929	0	0.666666667	CRA004775;PRJNA827465;Yang2025_Silkie_Wencha	0.801809775
ENSGALG00010022807	ADCY6	3	0.176420463	0.207192499	0.851480936	0.394502249	0.105737819	16.96650754	88.21207019	0.666666667	CRA004775;PRJNA827465;Yang2025_Silkie_Wencha	0.801809775
ENSGALG00010021051	GDE1	3	0.169413686	0.198936797	0.851595527	0.394438623	0.088170992	10.64190057	81.20636452	0.666666667	CRA004775;PRJNA827465;Yang2025_Silkie_Wencha	0.801809775
ENSGALG00010026436	PLCD1	3	-0.169212437	0.197798184	-0.855480059	0.392285145	0.092140449	22.02975792	90.92137096	0.666666667	CRA004775;PRJNA827465;Yang2025_Silkie_Wencha	0.801809775
ENSGALG00010002005	COLQ	3	-0.167567442	0.195656896	-0.856435141	0.391757134	0.063851594	5.256213008	61.94979167	0.666666667	CRA004775;PRJNA827465;Yang2025_Silkie_Wencha	0.801809775
ENSGALG00010014295	CACNA1C	3	-0.159546029	0.187149533	-0.852505621	0.393933521	0.048124949	3.729896417	46.37920798	0.666666667	CRA004775;PRJNA827465;Yang2025_Silkie_Wencha	0.801809775
ENSGALG00010001187	GFM1	3	0.15922187	0.186481608	0.853820765	0.393204307	0.080718676	12.75913698	84.32495863	0.666666667	CRA004775;PRJNA827465;Yang2025_Silkie_Wencha	0.801809775
ENSGALG00010020153	AXL	3	-0.158835443	0.186124293	-0.853383725	0.393446544	0.074708127	10.57631948	81.08982994	0.666666667	CRA004775;PRJNA827465;Yang2025_Silkie_Wencha	0.801809775
ENSGALG00010007437	TUSC1	3	0.15453801	0.18149618	0.851467011	0.394509981	0.02537704	2.655736145	24.6913138	0.666666667	CRA004775;PRJNA827465;Yang2025_Silkie_Wencha	0.801809775
ENSGALG00010009330	EIF3K	3	-0.151382527	0.176974789	-0.855390354	0.392335082	0.064228803	6.507550044	69.26646762	0.666666667	CRA004775;PRJNA827465;Yang2025_Silkie_Wencha	0.801809775
ENSGALG00010019726	PEBP4	3	-0.150504025	0.176088834	-0.854705102	0.392714426	0	0.396893489	0	0.666666667	CRA004775;PRJNA827465;Yang2025_Silkie_Wencha	0.801809775
ENSGALG00010024720	BUD13	3	-0.138188153	0.161463022	-0.855850156	0.392080668	0.06215884	10.42880152	80.82234093	0.666666667	CRA004775;PRJNA827465;Yang2025_Silkie_Wencha	0.801809775
ENSGALG00010023883	TBX21	3	-0.133658432	0.156451221	-0.854313773	0.392931158	0.024637231	2.974820296	32.76904817	0.666666667	CRA004775;PRJNA827465;Yang2025_Silkie_Wencha	0.801809775
ENSGALG00010019848	MAD1L1	3	-0.129563501	0.151722105	-0.8539527	0.393131198	0.053088228	9.281173151	78.45100002	0.666666667	CRA004775;PRJNA827465;Yang2025_Silkie_Wencha	0.801809775
ENSGALG00010024514	FAF1	3	-0.129493027	0.151267463	-0.856053405	0.39196824	0.05365546	15.5186655	87.1122939	0.666666667	CRA004775;PRJNA827465;Yang2025_Silkie_Wencha	0.801809775
ENSGALG00010021175	IPO7	3	-0.128241255	0.150172329	-0.853966615	0.393123488	0.056082666	14.78457105	86.47238399	0.666666667	CRA004775;PRJNA827465;Yang2025_Silkie_Wencha	0.801809775
ENSGALG00010029763	GEMIN4	3	-0.125827935	0.147090457	-0.855445945	0.392304318	0.037357575	4.519024261	55.74265849	0.666666667	CRA004775;PRJNA827465;Yang2025_Silkie_Wencha	0.801809775
ENSGALG00010004210	SYNCRIP	3	0.121488618	0.142095583	0.854978139	0.39256325	0.047728049	10.29305876	80.56942988	0.666666667	CRA004775;PRJNA827465;Yang2025_Silkie_Wencha	0.801809775
ENSGALG00010016473	RAB9B	3	-0.118476182	0.139034927	-0.852132518	0.394140545	0.035840517	5.277249662	62.10147088	0.666666667	CRA004775;PRJNA827465;Yang2025_Silkie_Wencha	0.801809775
ENSGALG00010027717	ATXN7L2	3	0.114306999	0.133871748	0.853854538	0.393185592	0.023265831	3.57919545	44.12152039	0.666666667	CRA004775;PRJNA827465;Yang2025_Silkie_Wencha	0.801809775
ENSGALG00010002713	OLFM2	3	0.112389023	0.13128482	0.856070152	0.391958977	0.021313685	3.381270058	40.85062814	0.666666667	CRA004775;PRJNA827465;Yang2025_Silkie_Wencha	0.801809775
ENSGALG00010009499	MXD3	3	0.108817757	0.127510008	0.853358559	0.393460495	0	0.632353786	0	0.666666667	CRA004775;PRJNA827465;Yang2025_Silkie_Wencha	0.801809775
ENSGALG00010023087	SH3GLB1	3	0.106443091	0.12496468	0.85178541	0.394333206	0.028550725	5.73933757	65.15277285	0.666666667	CRA004775;PRJNA827465;Yang2025_Silkie_Wencha	0.801809775
ENSGALG00010009538	CRIM1	3	0.098634911	0.115763113	0.852040952	0.394191363	0.01752545	3.520512276	43.1900859	0.666666667	CRA004775;PRJNA827465;Yang2025_Silkie_Wencha	0.801809775
ENSGALG00010011150	FGD4	3	0.096994915	0.113488761	0.854665381	0.392736421	0	0.495735506	0	0.666666667	CRA004775;PRJNA827465;Yang2025_Silkie_Wencha	0.801809775
ENSGALG00010017334	KDM8	3	-0.091032518	0.106873805	-0.851775772	0.394338556	0	0.516819632	0	0.666666667	CRA004775;PRJNA827465;Yang2025_Silkie_Wencha	0.801809775
ENSGALG00010000058	FURIN	3	0.084989527	0.09984018	0.851255748	0.394627301	0.018175212	6.988785286	71.38272363	0.666666667	CRA004775;PRJNA827465;Yang2025_Silkie_Wencha	0.801809775
ENSGALG00010019236	MPG	3	0.083492874	0.098065752	0.851396994	0.394548861	0.004347154	2.29170413	12.72869942	0.666666667	CRA004775;PRJNA827465;Yang2025_Silkie_Wencha	0.801809775
ENSGALG00010024871	POLQ	3	-0.080545876	0.094539059	-0.851985165	0.394222326	0.003430532	2.235137518	10.52004702	0.666666667	CRA004775;PRJNA827465;Yang2025_Silkie_Wencha	0.801809775
ENSGALG00010027251	ENSGALG00010027251	3	-0.068122339	0.079678819	-0.854961713	0.392572344	0.000930596	2.072937875	3.518575057	1	CRA004775;PRJNA827465;Yang2025_Silkie_Wencha	0.801809775
ENSGALG00010008266	ECSCR	3	0.06324637	0.073976433	0.85495295	0.392577195	0	1.882431923	0	1	CRA004775;PRJNA827465;Yang2025_Silkie_Wencha	0.801809775
ENSGALG00010029446	PRPSAP1	3	-0.061995979	0.072391924	-0.856393586	0.391780111	0.004846199	2.708416457	26.15611256	0.666666667	CRA004775;PRJNA827465;Yang2025_Silkie_Wencha	0.801809775
ENSGALG00010017667	SLC2A1	3	-0.055300652	0.064620624	-0.855774032	0.392122782	0.00259419	2.459406797	18.67957742	0.666666667	CRA004775;PRJNA827465;Yang2025_Silkie_Wencha	0.801809775
ENSGALG00010022733	PCCB	3	0.051328939	0.059994712	0.855557718	0.392242466	0	0.868633754	0	0.666666667	CRA004775;PRJNA827465;Yang2025_Silkie_Wencha	0.801809775
ENSGALG00010022415	DIP2B	3	0.040174113	0.047007335	0.854634987	0.392753252	0	1.5677166	0	0.666666667	CRA004775;PRJNA827465;Yang2025_Silkie_Wencha	0.801809775
ENSGALG00010001861	CBX3	3	-0.030149603	0.03536089	-0.852625671	0.393866922	0	1.415230855	0	0.666666667	CRA004775;PRJNA827465;Yang2025_Silkie_Wencha	0.801809775
ENSGALG00010027305	LSM4	3	-0.138762846	0.163114489	-0.850708277	0.394931424	0.065334032	12.34370345	83.79740725	0.666666667	CRA004775;PRJNA827465;Yang2025_Silkie_Wencha	0.801829965
ENSGALG00010006059	BIVM	3	-0.124253605	0.14605919	-0.850707199	0.394932023	0.055522017	23.9888927	91.66280818	0.666666667	CRA004775;PRJNA827465;Yang2025_Silkie_Wencha	0.801829965
ENSGALG00010029477	FAM104A	3	-0.11881909	0.139624123	-0.85099256	0.394773486	0.045745191	10.368626	80.71104118	0.666666667	CRA004775;PRJNA827465;Yang2025_Silkie_Wencha	0.801829965
ENSGALG00010030014	ENSGALG00010030014	3	0.0966579	0.11359583	0.85089303	0.394828777	0	1.574100209	0	0.666666667	CRA004775;PRJNA827465;Yang2025_Silkie_Wencha	0.801829965
ENSGALG00010007986	MYC	3	-0.041003969	0.048193586	-0.850817957	0.394870485	0	0.303865305	0	0.666666667	CRA004775;PRJNA827465;Yang2025_Silkie_Wencha	0.801829965
ENSGALG00010029583	LIMK1	3	0.168041216	0.197604685	0.850390853	0.39510782	0.081819207	8.678230196	76.95382636	0.666666667	CRA004775;PRJNA827465;Yang2025_Silkie_Wencha	0.802071711
ENSGALG00010011533	ENSGALG00010011533	3	-0.292408291	0.343924801	-0.85020996	0.395208365	0.283818284	10.65135706	81.22304991	0.666666667	CRA004775;PRJNA827465;Yang2025_Silkie_Wencha	0.802078722
ENSGALG00010025229	ENSGALG00010025229	3	-0.136384689	0.160436325	-0.850086094	0.395277222	0.050755331	7.784714131	74.30862629	0.666666667	CRA004775;PRJNA827465;Yang2025_Silkie_Wencha	0.802078722
ENSGALG00010017429	TELO2	3	-0.081512097	0.095887732	-0.850078475	0.395281458	0.011459451	3.321462053	39.78555323	0.666666667	CRA004775;PRJNA827465;Yang2025_Silkie_Wencha	0.802078722
ENSGALG00010016127	ENSGALG00010016127	3	-0.388890264	0.457878684	-0.849330352	0.395697496	0.468757921	10.56564856	81.07073136	0.666666667	CRA004775;PRJNA827465;Yang2025_Silkie_Wencha	0.802279541
ENSGALG00010016648	ADRA2C	3	0.179138574	0.210891547	0.849434589	0.395639513	0.074714431	4.830066651	58.5927039	0.666666667	CRA004775;PRJNA827465;Yang2025_Silkie_Wencha	0.802279541
ENSGALG00010029301	RNF213	3	0.162098017	0.190863354	0.849288318	0.39572088	0.06435853	5.411255618	63.04000141	0.666666667	CRA004775;PRJNA827465;Yang2025_Silkie_Wencha	0.802279541
ENSGALG00010021659	SPOCK2	3	0.130788517	0.153908477	0.849781108	0.395446795	0.039279684	4.550579528	56.04955396	0.666666667	CRA004775;PRJNA827465;Yang2025_Silkie_Wencha	0.802279541
ENSGALG00010015645	MACO1	3	0.110725086	0.130360144	0.849378367	0.395670787	0.033632797	6.571337646	69.56479627	0.666666667	CRA004775;PRJNA827465;Yang2025_Silkie_Wencha	0.802279541
ENSGALG00010004890	ACAT1	3	0.071685304	0.084366834	0.849685835	0.395499776	0	1.890876332	0	0.666666667	CRA004775;PRJNA827465;Yang2025_Silkie_Wencha	0.802279541
ENSGALG00010022548	ENSGALG00010022548	3	0.164148848	0.193311789	0.849140387	0.39580318	0.076815986	8.311522596	75.93702024	0.666666667	CRA004775;PRJNA827465;Yang2025_Silkie_Wencha	0.802331349
ENSGALG00010018147	ISM1	3	0.17751175	0.209102192	0.848923433	0.395923899	0.068982711	4.190962964	52.27827072	0.666666667	CRA004775;PRJNA827465;Yang2025_Silkie_Wencha	0.80246101
ENSGALG00010023627	IFT52	3	0.04437745	0.052283512	0.848784804	0.396001047	0	1.075312332	0	0.666666667	CRA004775;PRJNA827465;Yang2025_Silkie_Wencha	0.802502337
ENSGALG00010014893	SLC9A6	3	-0.081435475	0.095962501	-0.848617682	0.396094065	0.013215105	3.889885642	48.58460675	0.666666667	CRA004775;PRJNA827465;Yang2025_Silkie_Wencha	0.802575807
ENSGALG00010003965	SNTG1	2	0.218683105	0.257819268	0.848203112	0.396324864	0	0.002068804	0	1	PRJNA827465;Yang2025_Silkie_Wenchang	0.802887585
ENSGALG00010019876	MRM2	3	-0.143290634	0.168947449	-0.848137307	0.396361507	0.047100973	4.539783236	55.94503315	0.666666667	CRA004775;PRJNA827465;Yang2025_Silkie_Wencha	0.802887585
ENSGALG00010022789	ENSGALG00010022789	3	-0.319212246	0.376457472	-0.847937071	0.396473018	0.287776119	6.382087283	68.66229008	0.666666667	CRA004775;PRJNA827465;Yang2025_Silkie_Wencha	0.802887585
ENSGALG00010016745	BEGAIN	3	0.228692594	0.269806604	0.847616737	0.39665145	0.019829023	2.174760413	8.035846712	0.666666667	CRA004775;PRJNA827465;Yang2025_Silkie_Wencha	0.80292361
ENSGALG00010021196	ATP6V0A1	3	0.13295324	0.156892997	0.847413471	0.396764698	0.050839647	7.472129752	73.23386951	0.666666667	CRA004775;PRJNA827465;Yang2025_Silkie_Wencha	0.80292361
ENSGALG00010008327	TGFB1	3	0.098203728	0.115889409	0.847391742	0.396776806	0.018910085	3.746697861	46.61966152	0.666666667	CRA004775;PRJNA827465;Yang2025_Silkie_Wencha	0.80292361
ENSGALG00010021245	ZFAND3	3	-0.092403965	0.108988522	-0.847832078	0.396531496	0.025744388	10.08773285	80.17393967	0.666666667	CRA004775;PRJNA827465;Yang2025_Silkie_Wencha	0.80292361
ENSGALG00010019345	ENSGALG00010019345	3	0.077879643	0.091865888	0.847753668	0.396575171	0	1.108052595	0	0.666666667	CRA004775;PRJNA827465;Yang2025_Silkie_Wencha	0.80292361
ENSGALG00010005442	SASH1	3	-0.04678353	0.055199477	-0.847535758	0.396696565	0	0.63879853	0	1	CRA004775;PRJNA827465;Yang2025_Silkie_Wencha	0.80292361
ENSGALG00010008034	DPEP1	3	0.315875665	0.372809909	0.847283448	0.39683715	0.291446644	7.338463421	72.74633823	0.666666667	CRA004775;PRJNA827465;Yang2025_Silkie_Wencha	0.802930805
ENSGALG00010005448	ENSGALG00010005448	3	0.32064833	0.378550619	0.84704215	0.396971628	0.409110336	58.64925603	96.58989707	0.666666667	CRA004775;PRJNA827465;Yang2025_Silkie_Wencha	0.802973083
ENSGALG00010004751	PTER	3	-0.184545763	0.217849898	-0.847123475	0.396926302	0.088672066	6.130208724	67.37468347	0.666666667	CRA004775;PRJNA827465;Yang2025_Silkie_Wencha	0.802973083
ENSGALG00010011431	DRAM1	3	0.242393279	0.286220714	0.84687539	0.397064581	0.125151013	4.05964624	50.73462362	0.666666667	CRA004775;PRJNA827465;Yang2025_Silkie_Wencha	0.802975936
ENSGALG00010006421	LMF2	3	-0.08312628	0.09816103	-0.846835852	0.397086622	0.019088743	6.123307863	67.33791531	0.666666667	CRA004775;PRJNA827465;Yang2025_Silkie_Wencha	0.802975936
ENSGALG00010000149	ENSGALG00010000149	3	0.163192873	0.192823865	0.846331306	0.397367948	0.095660848	14.54340336	86.24806072	0.666666667	CRA004775;PRJNA827465;Yang2025_Silkie_Wencha	0.803315045
ENSGALG00010028562	ACSBG2	3	0.127830709	0.151038621	0.846344517	0.397360581	0.042247327	5.596204074	64.26148915	0.666666667	CRA004775;PRJNA827465;Yang2025_Silkie_Wencha	0.803315045
ENSGALG00010023437	KCNJ5	3	0.100450906	0.11872026	0.846114271	0.397489	0	0.101398371	0	1	CRA004775;PRJNA827465;Yang2025_Silkie_Wencha	0.803444885
ENSGALG00010007175	FOXC2	3	0.50037792	0.591661287	0.845716851	0.39771072	0.778589647	8.490609875	76.44456606	0.666666667	CRA004775;PRJNA827465;Yang2025_Silkie_Wencha	0.803778141
ENSGALG00010010446	C22orf23	3	-0.462166038	0.54662052	-0.84549705	0.397833379	0.553856147	5.453255251	63.32465824	0.666666667	CRA004775;PRJNA827465;Yang2025_Silkie_Wencha	0.803911125
ENSGALG00010022440	SORD	3	-0.161689029	0.191293915	-0.845238748	0.397977552	0.061367758	4.551145158	56.05501625	0.666666667	CRA004775;PRJNA827465;Yang2025_Silkie_Wencha	0.803972654
ENSGALG00010009043	TFAM	3	-0.043926555	0.051969211	-0.845241919	0.397975782	0	1.459088029	0	0.666666667	CRA004775;PRJNA827465;Yang2025_Silkie_Wencha	0.803972654
ENSGALG00010023366	GDAP1L1	3	0.408061804	0.483023585	0.844807203	0.398218493	0.558800667	15.8396062	87.37342346	0.666666667	CRA004775;PRJNA827465;Yang2025_Silkie_Wencha	0.804299038
ENSGALG00010022159	KIAA2013	3	-0.089201629	0.105595847	-0.844745613	0.398252887	0.022650002	9.085269789	77.98634442	0.666666667	CRA004775;PRJNA827465;Yang2025_Silkie_Wencha	0.804299038
ENSGALG00010009324	NKX2-5	2	1.317449841	1.560835556	0.844067036	0.398631947	3.823116176	4.407717597	77.3125211	0.5	CRA004775;PRJNA827465	0.804589825
ENSGALG00010027492	PCP4L1	3	0.428331928	0.509333301	0.840965881	0.400367049	0.402275238	4.138799563	51.67680944	0.666666667	CRA004775;PRJNA827465;Yang2025_Silkie_Wencha	0.804589825
ENSGALG00010012118	ENSGALG00010012118	3	0.396225356	0.470447849	0.842230137	0.399659148	0.241728768	3.126289788	36.02640395	0.666666667	CRA004775;PRJNA827465;Yang2025_Silkie_Wencha	0.804589825
ENSGALG00010022767	CACNB3	3	0.296304588	0.351502704	0.842965315	0.399247843	0.232593235	5.912598301	66.17392391	0.666666667	CRA004775;PRJNA827465;Yang2025_Silkie_Wencha	0.804589825
ENSGALG00010029707	NSRP1	3	0.259555082	0.308336355	0.841792018	0.39990438	0.241418649	14.79198058	86.47916018	0.666666667	CRA004775;PRJNA827465;Yang2025_Silkie_Wencha	0.804589825
ENSGALG00010024686	CMPK1	3	-0.252429439	0.300187315	-0.840906416	0.400400364	0.23958209	25.10545684	92.03360444	0.666666667	CRA004775;PRJNA827465;Yang2025_Silkie_Wencha	0.804589825
ENSGALG00010029498	ENSGALG00010029498	3	-0.240337282	0.28474908	-0.844031813	0.398651629	0.196958897	15.56156634	87.14782332	0.666666667	CRA004775;PRJNA827465;Yang2025_Silkie_Wencha	0.804589825
ENSGALG00010005208	ENSGALG00010005208	3	-0.21838556	0.258862511	-0.84363533	0.398873215	0.16445259	12.58421054	84.10706819	0.666666667	CRA004775;PRJNA827465;Yang2025_Silkie_Wencha	0.804589825
ENSGALG00010018443	PLCB1	3	0.210279287	0.24952638	0.842713652	0.39938861	0.141607476	9.648332841	79.27103021	0.666666667	CRA004775;PRJNA827465;Yang2025_Silkie_Wencha	0.804589825
ENSGALG00010005104	ENPP3	3	-0.206823533	0.245334837	-0.84302554	0.39921416	0.109903947	5.203830889	61.56677565	0.666666667	CRA004775;PRJNA827465;Yang2025_Silkie_Wencha	0.804589825
ENSGALG00010026377	ENSGALG00010026377	3	-0.198686119	0.235654739	-0.8431238	0.399159209	0.095279465	5.016506313	60.13161601	0.666666667	CRA004775;PRJNA827465;Yang2025_Silkie_Wencha	0.804589825
ENSGALG00010027896	TOR4A	3	-0.19096441	0.227142716	-0.840724338	0.400502383	0.106894231	6.794496521	70.56441204	0.666666667	CRA004775;PRJNA827465;Yang2025_Silkie_Wencha	0.804589825
ENSGALG00010020111	ADRA2B	3	0.182210964	0.216543242	0.841453016	0.400094196	0.113485404	14.20076349	85.9162502	0.666666667	CRA004775;PRJNA827465;Yang2025_Silkie_Wencha	0.804589825
ENSGALG00010025472	ZCCHC8	3	0.181693094	0.216019822	0.841094545	0.400294971	0.114268911	12.1773443	83.57605771	0.666666667	CRA004775;PRJNA827465;Yang2025_Silkie_Wencha	0.804589825
ENSGALG00010025404	CERS6	3	-0.176862012	0.209525714	-0.844106473	0.398609911	0.080108928	7.453400613	73.16661074	0.666666667	CRA004775;PRJNA827465;Yang2025_Silkie_Wencha	0.804589825
ENSGALG00010023059	SELENOF	3	-0.17519484	0.207832599	-0.842961312	0.399250081	0.108437703	13.76121583	85.46640046	0.666666667	CRA004775;PRJNA827465;Yang2025_Silkie_Wencha	0.804589825
ENSGALG00010028607	ENSGALG00010028607	3	0.160111238	0.189958105	0.842876578	0.399297474	0.090992245	17.28204113	88.42729291	0.666666667	CRA004775;PRJNA827465;Yang2025_Silkie_Wencha	0.804589825
ENSGALG00010029223	NME2	3	0.158930706	0.188967014	0.841049992	0.400319929	0.082079701	9.31821617	78.53666449	0.666666667	CRA004775;PRJNA827465;Yang2025_Silkie_Wencha	0.804589825
ENSGALG00010008356	ENSGALG00010008356	3	0.154629411	0.183644545	0.842003832	0.399785809	0.026861887	2.645610433	24.40308009	0.666666667	CRA004775;PRJNA827465;Yang2025_Silkie_Wencha	0.804589825
ENSGALG00010020730	RRP12	3	-0.141332439	0.167568121	-0.843432735	0.39898647	0.059358012	7.524565849	73.42039341	0.666666667	CRA004775;PRJNA827465;Yang2025_Silkie_Wencha	0.804589825
ENSGALG00010010330	ALPK2	3	-0.139917879	0.166056047	-0.842594307	0.399455376	0.041693405	4.287303512	53.35063182	0.666666667	CRA004775;PRJNA827465;Yang2025_Silkie_Wencha	0.804589825
ENSGALG00010001854	CHRDL2	3	0.139018011	0.165186621	0.841581544	0.400022224	0	1.611576885	0	1	CRA004775;PRJNA827465;Yang2025_Silkie_Wencha	0.804589825
ENSGALG00010017212	TINAGL1	3	0.130355755	0.155047011	0.84074987	0.400488076	0.040637425	5.221604702	61.69759845	0.666666667	CRA004775;PRJNA827465;Yang2025_Silkie_Wencha	0.804589825
ENSGALG00010027443	MED16	3	-0.121402894	0.14416028	-0.842138304	0.399710543	0.044819398	7.399843335	72.97240077	0.666666667	CRA004775;PRJNA827465;Yang2025_Silkie_Wencha	0.804589825
ENSGALG00010016678	NOX1	3	0.116684456	0.138263376	0.84392888	0.398709149	0.000523903	2.015726577	0.78019398	0.666666667	CRA004775;PRJNA827465;Yang2025_Silkie_Wencha	0.804589825
ENSGALG00010005513	TMEM30CP	3	0.113399448	0.134819146	0.841122733	0.400279181	0	1.335377691	0	1	CRA004775;PRJNA827465;Yang2025_Silkie_Wencha	0.804589825
ENSGALG00010021050	CTR9	3	0.0975861	0.115805346	0.842673533	0.399411053	0.026315	7.117082066	71.89859578	0.666666667	CRA004775;PRJNA827465;Yang2025_Silkie_Wencha	0.804589825
ENSGALG00010017595	CCNF	3	-0.096848755	0.114765388	-0.843884699	0.398733839	0.015599148	3.180036866	37.10764735	0.666666667	CRA004775;PRJNA827465;Yang2025_Silkie_Wencha	0.804589825
ENSGALG00010001199	AFG3L2	3	0.090372512	0.107260857	0.842548851	0.399480808	0.024108631	10.78252422	81.45146759	0.666666667	CRA004775;PRJNA827465;Yang2025_Silkie_Wencha	0.804589825
ENSGALG00010029720	TIMM22	3	-0.072002817	0.085535964	-0.841784127	0.399908798	0	1.086441906	0	0.666666667	CRA004775;PRJNA827465;Yang2025_Silkie_Wencha	0.804589825
ENSGALG00010017475	STUM	3	-0.06824673	0.081019225	-0.842352294	0.399590788	0	1.541925405	0	1	CRA004775;PRJNA827465;Yang2025_Silkie_Wencha	0.804589825
ENSGALG00010018581	SUFU	3	-0.062611806	0.074411432	-0.841427251	0.400108624	0.009459715	5.085277922	60.67078278	0.666666667	CRA004775;PRJNA827465;Yang2025_Silkie_Wencha	0.804589825
ENSGALG00010028585	MINDY1	3	-0.059430341	0.070660999	-0.841062858	0.400312721	0	1.933467467	0	0.666666667	CRA004775;PRJNA827465;Yang2025_Silkie_Wencha	0.804589825
ENSGALG00010027131	UBA52	3	-0.048185804	0.057250482	-0.841666359	0.399974734	0	0.543345505	0	0.666666667	CRA004775;PRJNA827465;Yang2025_Silkie_Wencha	0.804589825
ENSGALG00010029226	MBTD1	3	-0.045568124	0.054071363	-0.842740432	0.399373629	0.000800695	2.128543688	6.039043914	0.666666667	CRA004775;PRJNA827465;Yang2025_Silkie_Wencha	0.804589825
ENSGALG00010027663	COPA	3	-0.03690123	0.043838298	-0.841757828	0.399923522	0.002102108	3.052112288	34.47161141	1	CRA004775;PRJNA827465;Yang2025_Silkie_Wencha	0.804589825
ENSGALG00010021260	KDM4A	3	-0.031998962	0.038058754	-0.840777957	0.400472338	0	1.031597905	0	1	CRA004775;PRJNA827465;Yang2025_Silkie_Wencha	0.804589825
ENSGALG00010006794	PERPA	3	0.520152823	0.619287916	0.839920835	0.400952775	0.491310662	3.487622464	42.65434345	0.666666667	CRA004775;PRJNA827465;Yang2025_Silkie_Wencha	0.80480853
ENSGALG00010001179	GRHPR	3	-0.384705025	0.45799929	-0.839968604	0.40092599	0.518710618	12.22553745	83.64080101	0.666666667	CRA004775;PRJNA827465;Yang2025_Silkie_Wencha	0.80480853
ENSGALG00010024852	CEP89	3	0.243259539	0.289450481	0.8404185	0.40067378	0.198336463	15.99689881	87.49757672	0.666666667	CRA004775;PRJNA827465;Yang2025_Silkie_Wencha	0.80480853
ENSGALG00010002371	BAG3	3	0.198200163	0.235969746	0.839938878	0.400942658	0.109227136	6.717464843	70.22686316	0.666666667	CRA004775;PRJNA827465;Yang2025_Silkie_Wencha	0.80480853
ENSGALG00010024566	MTSS2	3	0.186128461	0.221565364	0.840061182	0.400874084	0.08800166	5.002941279	60.0235164	0.666666667	CRA004775;PRJNA827465;Yang2025_Silkie_Wencha	0.80480853
ENSGALG00010008426	AP1G1	3	-0.080771212	0.096138867	-0.840151489	0.400823454	0.020168159	9.300553454	78.49590339	0.666666667	CRA004775;PRJNA827465;Yang2025_Silkie_Wencha	0.80480853
ENSGALG00010003397	SPATA4	3	-0.185081826	0.2203959	-0.839769825	0.401037455	0	1.444586839	0	0.666666667	CRA004775;PRJNA827465;Yang2025_Silkie_Wencha	0.804864241
ENSGALG00010029426	ZNF750	2	0.746315843	0.891323746	0.837311747	0.402417358	0	0.231714681	0	0.5	PRJNA827465;Yang2025_Silkie_Wenchang	0.805038062
ENSGALG00010024499	CA12	3	-0.531148937	0.636937727	-0.833910309	0.404331527	0.487208494	3.31688026	39.70237563	0.666666667	CRA004775;PRJNA827465;Yang2025_Silkie_Wencha	0.805038062
ENSGALG00010028370	S100A12	3	-0.515267312	0.618423309	-0.833195168	0.404734668	0.925038278	10.91807347	81.68174994	0.666666667	CRA004775;PRJNA827465;Yang2025_Silkie_Wencha	0.805038062
ENSGALG00010007964	RECQL4	3	0.369659221	0.441879163	0.836561783	0.402838935	0.488620468	16.40525404	87.80878373	0.666666667	CRA004775;PRJNA827465;Yang2025_Silkie_Wencha	0.805038062
ENSGALG00010022507	ENSGALG00010022507	3	0.363677356	0.435607491	0.834873972	0.403788671	0.462149265	10.95787763	81.74829044	0.666666667	CRA004775;PRJNA827465;Yang2025_Silkie_Wencha	0.805038062
ENSGALG00010015160	SIM1	2	0.343771268	0.411660205	0.835085012	0.403669845	0.173543455	2.049167105	51.19968509	0.5	PRJNA827465;Yang2025_Silkie_Wenchang	0.805038062
ENSGALG00010008884	FAM135B	2	0.324919632	0.387854378	0.837736147	0.402178907	0	0.007708545	0	1	PRJNA827465;Yang2025_Silkie_Wenchang	0.805038062
ENSGALG00010008846	SLC35D2	3	0.324632921	0.387399536	0.837979633	0.402042142	0.302141708	7.067802571	71.70266175	0.666666667	CRA004775;PRJNA827465;Yang2025_Silkie_Wencha	0.805038062
ENSGALG00010002192	ENSGALG00010002192	3	-0.315546198	0.376798881	-0.837439319	0.402345672	0.367112374	22.6931512	91.18676828	0.666666667	CRA004775;PRJNA827465;Yang2025_Silkie_Wencha	0.805038062
ENSGALG00010013917	TRPC5	2	-0.312677238	0.37335726	-0.837484813	0.40232011	0	0.083985768	0	1	PRJNA827465;Yang2025_Silkie_Wenchang	0.805038062
ENSGALG00010002824	NXPH1	2	-0.298874186	0.357758021	-0.835408763	0.403487598	0	0.000442478	0	1	PRJNA827465;Yang2025_Silkie_Wenchang	0.805038062
ENSGALG00010023250	FZD10	3	-0.290150218	0.34688127	-0.836453976	0.402899558	0	0.662012049	0	1	CRA004775;PRJNA827465;Yang2025_Silkie_Wencha	0.805038062

ENSGALG00010022448	FERRY3	3	0.276954994	0.331013389	0.836688193	0.402767858	0.273974468	12.80485611	84.38092562	0.666666667	CRA004775;PRJNA827465;Yang2025_Silkie_Wencha	0.805038062
ENSGALG00010011772	GRHL2	2	0.272710779	0.325479297	0.837874428	0.402101232	0	0.122131123	0	1	PRJNA827465;Yang2025_Silkie_Wenchang	0.805038062
ENSGALG00010028511	YJEFN3	3	0.22823185	0.273893752	0.833286078	0.404683406	0	1.538994288	0	0.666666667	CRA004775;PRJNA827465;Yang2025_Silkie_Wencha	0.805038062
ENSGALG00010027789	UAP1L1	3	-0.217466888	0.259175763	-0.839071081	0.401429423	0.160677035	29.50810812	93.22220187	0.666666667	CRA004775;PRJNA827465;Yang2025_Silkie_Wencha	0.805038062
ENSGALG00010018854	LYSMD4	3	0.211812888	0.253415467	0.835832519	0.40324913	0.14727906	25.96082863	92.29608566	0.666666667	CRA004775;PRJNA827465;Yang2025_Silkie_Wencha	0.805038062
ENSGALG00010004393	ZBTB45	3	0.206772854	0.247833153	0.834322815	0.404099099	0.145115279	13.9760504	85.68980547	0.666666667	CRA004775;PRJNA827465;Yang2025_Silkie_Wencha	0.805038062
ENSGALG00010015831	LYG2	3	0.204898488	0.244130031	0.839300627	0.401300632	0	0.236416361	0	1	CRA004775;PRJNA827465;Yang2025_Silkie_Wencha	0.805038062
ENSGALG00010029584	TRIM25	3	-0.196823536	0.235846971	-0.834539174	0.403977222	0.136071374	23.98608345	91.66183173	0.666666667	CRA004775;PRJNA827465;Yang2025_Silkie_Wencha	0.805038062
ENSGALG00010003347	GPM6A	2	0.181423683	0.216958235	0.83621478	0.403034085	0	0.306735843	0	1	PRJNA827465;Yang2025_Silkie_Wenchang	0.805038062
ENSGALG00010004346	CEP128	3	0.169656759	0.203484711	0.83375679	0.404418049	0.063718331	4.421183755	54.76324643	0.666666667	CRA004775;PRJNA827465;Yang2025_Silkie_Wencha	0.805038062
ENSGALG00010029743	MYOCD	3	-0.163009109	0.195231619	-0.834952404	0.403744507	0.058395601	4.449692793	55.05307685	0.666666667	CRA004775;PRJNA827465;Yang2025_Silkie_Wencha	0.805038062
ENSGALG00010029362	TADA2A	3	-0.158962969	0.190333088	-0.835183047	0.403614653	0.082586525	10.61253923	81.15436883	0.666666667	CRA004775;PRJNA827465;Yang2025_Silkie_Wencha	0.805038062
ENSGALG00010021254	FNBP1L	3	0.156388494	0.186888635	0.83680045	0.402704745	0.08425535	11.67636475	82.87138127	0.666666667	CRA004775;PRJNA827465;Yang2025_Silkie_Wencha	0.805038062
ENSGALG00010011280	FAM199X	3	-0.154598112	0.185083585	-0.835288081	0.403555527	0.080635183	17.31659903	88.45038799	0.666666667	CRA004775;PRJNA827465;Yang2025_Silkie_Wencha	0.805038062
ENSGALG00010018633	ENSGALG0001001863	3	0.154095514	0.184368085	0.835803622	0.403265388	0.043232516	3.287442156	39.16242766	0.666666667	CRA004775;PRJNA827465;Yang2025_Silkie_Wencha	0.805038062
ENSGALG00010028328	HNRNPM	3	0.152782467	0.183296927	0.83352443	0.404549026	0.083408815	16.4974274	87.8768977	0.666666667	CRA004775;PRJNA827465;Yang2025_Silkie_Wencha	0.805038062
ENSGALG00010023775	ENSGALG0001002377	3	-0.149103466	0.178093262	-0.837221265	0.402468206	0.054037249	4.718012452	57.6092683	0.666666667	CRA004775;PRJNA827465;Yang2025_Silkie_Wencha	0.805038062
ENSGALG00010014902	AASDH	3	0.148488414	0.178182386	0.833350689	0.404646977	0.069442171	8.453945362	76.34240684	0.666666667	CRA004775;PRJNA827465;Yang2025_Silkie_Wencha	0.805038062
ENSGALG00010003831	IRAK1BP1	3	-0.148344403	0.177336029	-0.836515873	0.402864751	0.050233013	4.188014852	52.24467747	0.666666667	CRA004775;PRJNA827465;Yang2025_Silkie_Wencha	0.805038062
ENSGALG00010020949	HPS6	3	-0.147162724	0.176461571	-0.833964714	0.404300868	0.06627091	7.555434704	73.52898836	0.666666667	CRA004775;PRJNA827465;Yang2025_Silkie_Wencha	0.805038062
ENSGALG00010016797	SEC62	3	0.140284063	0.167705404	0.836491011	0.402878732	0.064874878	10.37760557	80.72773158	0.666666667	CRA004775;PRJNA827465;Yang2025_Silkie_Wencha	0.805038062
ENSGALG00010002733	RDH8	3	-0.134752676	0.161800339	-0.832833088	0.404938871	0	0.607241095	0	0.666666667	CRA004775;PRJNA827465;Yang2025_Silkie_Wencha	0.805038062
ENSGALG00010018177	KIF20B	3	0.131332615	0.157390074	0.834440264	0.404032936	0.04197257	5.525497874	63.80416669	0.666666667	CRA004775;PRJNA827465;Yang2025_Silkie_Wencha	0.805038062
ENSGALG00010027674	TREM2	3	0.125806756	0.150778549	0.834381	0.404066321	0.019792448	2.76678868	27.71403128	0.666666667	CRA004775;PRJNA827465;Yang2025_Silkie_Wencha	0.805038062
ENSGALG00010001220	PLXNB2	3	0.124226319	0.148085579	0.838881952	0.401535556	0.042219434	5.943247267	66.34836294	0.666666667	CRA004775;PRJNA827465;Yang2025_Silkie_Wencha	0.805038062
ENSGALG00010024259	ENSGALG0001002425	3	-0.123188372	0.147886073	-0.832995086	0.4048475	0.025213824	2.657397339	24.7383908	0.666666667	CRA004775;PRJNA827465;Yang2025_Silkie_Wencha	0.805038062
ENSGALG00010029632	TMEM132E	3	-0.111021886	0.132950735	-0.83506034	0.403683735	0	0.18107206	0	0.666666667	CRA004775;PRJNA827465;Yang2025_Silkie_Wencha	0.805038062
ENSGALG00010018062	GOLGA7	3	-0.105941023	0.126716507	-0.836047526	0.403128167	0.032275785	7.425020233	73.06404646	0.666666667	CRA004775;PRJNA827465;Yang2025_Silkie_Wencha	0.805038062
ENSGALG00010011064	MYLIP	3	-0.104597685	0.125282489	-0.83489469	0.403777005	0.028797731	5.836074506	65.73038953	0.666666667	CRA004775;PRJNA827465;Yang2025_Silkie_Wencha	0.805038062
ENSGALG00010021408	TTC9	3	0.101109283	0.120758618	0.837284203	0.402432836	0	0.136919015	0	1	CRA004775;PRJNA827465;Yang2025_Silkie_Wencha	0.805038062
ENSGALG00010017024	BUB1	3	-0.095343055	0.113982412	-0.836471632	0.402889629	0.014849447	3.27229666	38.88084707	0.666666667	CRA004775;PRJNA827465;Yang2025_Silkie_Wencha	0.805038062
ENSGALG00010020667	UBTD1	3	0.093626117	0.11167957	0.838345965	0.401836428	0	1.061979844	0	0.666666667	CRA004775;PRJNA827465;Yang2025_Silkie_Wencha	0.805038062
ENSGALG00010014180	SLC19A1	3	-0.089467652	0.106652048	-0.838874211	0.401539901	0.015164089	3.431953405	41.72415054	0.666666667	CRA004775;PRJNA827465;Yang2025_Silkie_Wencha	0.805038062
ENSGALG00010001349	ING3	3	0.088523962	0.105638962	0.837985911	0.402038617	0.014708845	3.441177979	41.88036736	0.666666667	CRA004775;PRJNA827465;Yang2025_Silkie_Wencha	0.805038062
ENSGALG00010009361	LRRK2	3	0.088048116	0.105653339	0.833972727	0.404296353	0.016512786	4.099028874	51.20795531	0.666666667	CRA004775;PRJNA827465;Yang2025_Silkie_Wencha	0.805038062
ENSGALG00010001545	SELENOO	3	-0.087512094	0.105075455	-0.832850009	0.404929327	0	1.489830633	0	0.666666667	CRA004775;PRJNA827465;Yang2025_Silkie_Wencha	0.805038062
ENSGALG00010004297	SULF1	3	0.086677737	0.103659957	0.836173773	0.403057151	0	1.174227145	0	1	CRA004775;PRJNA827465;Yang2025_Silkie_Wencha	0.805038062
ENSGALG00010027139	HSD11B1L	3	-0.083196625	0.099789933	-0.83371761	0.404440132	0	0.072174977	0	1	CRA004775;PRJNA827465;Yang2025_Silkie_Wencha	0.805038062
ENSGALG00010027574	GNG7	3	-0.082718525	0.099115392	-0.834264889	0.404131733	0.01251884	3.096920953	35.41972719	1	CRA004775;PRJNA827465;Yang2025_Silkie_Wencha	0.805038062
ENSGALG00010002664	ZNFB1	3	0.082532962	0.098535342	0.837597558	0.402256765	0	1.735951116	0	0.666666667	CRA004775;PRJNA827465;Yang2025_Silkie_Wencha	0.805038062
ENSGALG00010007905	MRPL32	3	0.074900911	0.089385214	0.837956383	0.402055201	0.005615148	2.433932948	17.82846765	0.666666667	CRA004775;PRJNA827465;Yang2025_Silkie_Wencha	0.805038062
ENSGALG00010023362	GADD45A	3	0.073265905	0.087961891	0.832927806	0.404885446	0	1.628811204	0	0.666666667	CRA004775;PRJNA827465;Yang2025_Silkie_Wencha	0.805038062
ENSGALG00010002370	ENSGALG0001000237	3	-0.072799121	0.086732641	-0.83935091	0.401272423	0	0.136722308	0	1	CRA004775;PRJNA827465;Yang2025_Silkie_Wencha	0.805038062
ENSGALG00010024075	SPATS2L	3	0.071070312	0.085314542	0.833038666	0.404822923	0.005855977	2.512925011	20.41147305	0.666666667	CRA004775;PRJNA827465;Yang2025_Silkie_Wencha	0.805038062
ENSGALG00010000491	ENSGALG0001000049	3	0.066746647	0.079564308	0.838901872	0.401524377	0	1.573440715	0	1	CRA004775;PRJNA827465;Yang2025_Silkie_Wencha	0.805038062
ENSGALG00010004095	ANKRD50	3	0.061170431	0.073420326	0.833153892	0.404757943	0.002739287	2.285413713	12.48849217	0.666666667	CRA004775;PRJNA827465;Yang2025_Silkie_Wencha	0.805038062
ENSGALG00010023261	SNX1	3	0.06069963	0.07318278	0.83338842	0.404625704	0.006930437	3.42514904	41.60838035	0.666666667	CRA004775;PRJNA827465;Yang2025_Silkie_Wencha	0.805038062
ENSGALG00010020063	ENSGALG0001002006	3	-0.060512753	0.072531908	-0.834291489	0.404116747	0.004085837	2.618408609	23.61772746	1	CRA004775;PRJNA827465;Yang2025_Silkie_Wencha	0.805038062
ENSGALG00010020314	THADA	3	-0.059293776	0.070770017	-0.837837532	0.402121957	0	0.414633179	0	1	CRA004775;PRJNA827465;Yang2025_Silkie_Wencha	0.805038062
ENSGALG00010001414	CCDC126	3	-0.051858952	0.061871999	-0.838165131	0.401937967	0.001534182	2.245391877	10.92868821	1	CRA004775;PRJNA827465;Yang2025_Silkie_Wencha	0.805038062
ENSGALG00010005449	TIPRL	3	-0.038758604	0.046245955	-0.838097175	0.401976129	0	1.658146989	0	0.666666667	CRA004775;PRJNA827465;Yang2025_Silkie_Wencha	0.805038062
ENSGALG00010001635	FOXJ2	3	-0.037490239	0.044834727	-0.836187519	0.403049419	0	0.598267082	0	0.666666667	CRA004775;PRJNA827465;Yang2025_Silkie_Wencha	0.805038062
ENSGALG00010010287	STAM2	3	0.03505443	0.041774632	0.839131991	0.401395246	0	0.919310558	0	0.666666667	CRA004775;PRJNA827465;Yang2025_Silkie_Wencha	0.805038062
ENSGALG00010020712	ZC3H7A	3	0.028325657	0.033944975	0.834458016	0.404022936	0	0.003037869	0	1	CRA004775;PRJNA827465;Yang2025_Silkie_Wencha	0.805038062
ENSGALG00010027283	RAB7A	3	-0.027240781	0.032562912	-0.83655851	0.402840775	0	0.847307913	0	1	CRA004775;PRJNA827465;Yang2025_Silkie_Wencha	0.805038062
ENSGALG00010008062	VPS9D1	3	0.24211551	0.290944748	0.832170065	0.405312958	0.202205491	10.73660102	81.37213075	0.666666667	CRA004775;PRJNA827465;Yang2025_Silkie_Wencha	0.805152832
ENSGALG00010003895	THSD7A	3	0.221925782	0.266573233	0.832513375	0.405119232	0.124544494	5.252044607	61.91959228	0.666666667	CRA004775;PRJNA827465;Yang2025_Silkie_Wencha	0.805152832
ENSGALG00010024038	NKD1	3	0.129669752	0.15579366	0.832317257	0.405229892	0.02015646	2.638421328	24.19709549	0.666666667	CRA004775;PRJNA827465;Yang2025_Silkie_Wencha	0.805152832
ENSGALG00010024610	PFKL	3	0.096421668	0.115827277	0.832460835	0.405148876	0.017482706	3.526888431	43.29279082	0.666666667	CRA004775;PRJNA827465;Yang2025_Silkie_Wencha	0.805152832
ENSGALG00010012600	NUP107	3	0.092301535	0.11092729	0.832090424	0.405357907	0.021837316	5.528390963	63.82310851	0.666666667	CRA004775;PRJNA827465;Yang2025_Silkie_Wencha	0.805152832
ENSGALG00010027027	ARHGEF3	3	-0.061016496	0.073314299	-0.832259144	0.405262686	0	0.491392717	0	1	CRA004775;PRJNA827465;Yang2025_Silkie_Wencha	0.805152832
ENSGALG00010015200	TBC1D1	3	-0.042939108	0.051607996	-0.832024316	0.40539522	0	0.442259296	0	0.666666667	CRA004775;PRJNA827465;Yang2025_Silkie_Wencha	0.805152832
ENSGALG00010006492	GAP43	3	0.451937597	0.543381554	0.831713175	0.405570862	0.654405917	8.139164987	75.42745474	0.666666667	CRA004775;PRJNA827465;Yang2025_Silkie_Wencha	0.805181313
ENSGALG00010023914	CCDC117	3	-0.229131719	0.27557518	-0.831467184	0.405709759	0.207973575	34.70970539	94.23792286	0.666666667	CRA004775;PRJNA827465;Yang2025_Silkie_Wencha	0.805181313
ENSGALG00010023161	ODF2L	2	0.214131803	0.257538604	0.831455169	0.405716544	0	0.020775009	0	1	PRJNA827465;Yang2025_Silkie_Wenchang	0.805181313
ENSGALG00010029605	C17orf75	3	-0.158161594	0.190305933	-0.831091241	0.405922088	0.086055441	15.07909712	86.73660642	0.666666667	CRA004775;PRJNA827465;Yang2025_Silkie_Wencha	0.805181313
ENSGALG00010005507	USP53	3	0.140024276	0.168480889	0.831098862	0.405917782	0.047362603	5.066629238	60.52602418	1	CRA004775;PRJNA827465;Yang2025_Silkie_Wencha	0.805181313
ENSGALG00010028493	TOR1A	3	0.078281229	0.094110222	0.831803684	0.405519764	0.014940042	4.594152728	56.46640156	0.666666667	CRA004775;PRJNA827465;Yang2025_Silkie_Wencha	0.805181313
ENSGALG00010005542	KCTD1	3	0.077214826	0.092862286	0.831498226	0.40569223	0	0.919048924	0	0.666666667	CRA004775;PRJNA827465;Yang2025_Silkie_Wencha	0.805181313
ENSGALG00010005236	TMTC4	3	0.06640827	0.079889035	0.831256389	0.405828899	0	0.487038345	0	0.666666667	CRA004775;PRJNA827465;Yang2025_Silkie_Wencha	0.805181313
ENSGALG00010022115	E2F4	3	-0.039142222	0.047083088	-0.831343553	0.405779577	0	1.478567203	0	0.666666667	CRA004775;PRJNA827465;Yang2025_Silkie_Wencha	0.805181313
ENSGALG00010026116	DLX3	3	0.274873066	0.33079364	0.830950275	0.406001721	0	0.985603857	0	0.666666667	CRA004775;PRJNA827465;Yang2025_Silkie_Wencha	0.805226305
ENSGALG00010001864	ENSGALG0001000186	3	-0.154034355	0.185429918	-0.830687715	0.406150068	0.056705745	4.482539915	55.38243857	0.666666667	CRA004775;PRJNA827465;Yang2025_Silkie_Wencha	0.805253854
ENSGALG00010015018	MAP7D3	3	0.106656255	0.128380684	0.830781173	0.406097261	0.03288834	6.047808749	66.93017119	0.666666667	CRA004775;PRJNA827465;Yang2025_Silkie_Wencha	0.805253854
ENSGALG00010016836	HEPH	3	-0.074709948	0.089944435	-0.830623298	0.406186469	0	0.909991548	0	1	CRA004775;PRJNA827465;Yang2025_Silkie_Wencha	0.805253854
ENSGALG00010017493	YPEL5	3	-0.248676865	0.299445352	-0.830458259	0.406279739	0.231834098	41.41310927	95.17061135	0.666666667	CRA004775;PRJNA827465;Yang2025_Silkie_Wencha	0.805325841
ENSGALG00010000382	ENSGALG0001000038	3	0.868648144	1.048176761	0.828722956	0.407261193	3.138071747	50.27685886	96.02202674	0.666666667	CRA004775;PRJNA827465;Yang2025_Silkie_Wencha	0.805387388
ENSGALG00010027899	PMVK	3	0.547808099	0.661052119	0.828691239	0.407279145	1.268208892	70.79246062	97.1748404	0.666666667	CRA004775;PRJNA827465;Yang2025_Silkie_Wencha	0.805387388
ENSGALG00010010537	ENSGALG0001001053	3	0.371338182	0.447957399	0.828958699	0.407127778	0.423627	7.650908599	73.85931391	0.666666667	CRA004775;PRJNA827465;Yang2025_Silkie_Wencha	0.805387388
ENSGALG00010003278	ENSGALG0001000327	2	0.311720104	0.375643548	0.829829516	0.40663518	0	0.078832421	0	1	PRJNA827465;Yang2025_Silkie_Wenchang	0.805387388
ENSGALG00010016762	EML6	3	0.287784273	0.347110123	0.829086373	0.407055534	0.275218844	12.39207185	83.86064878	0.666666667	CRA004775;PRJNA827465;Yang2025_Silkie_Wencha	0.805387388
ENSGALG00010029531	CEP131	3	0.286005843	0.344944713	0.829135312	0.40702784	0.330007608	38.38305894	94.78936787	0.666666667	CRA004775;PRJNA827465;Yang2025_Silkie_Wencha	0.805387388
ENSGALG00010027085	PIM1	3	-0.251608038	0.303399745	-0.829295483	0.406937226	0.22893702	13.63316367	85.32989078	0.666666667	CRA004775;PRJNA827465;Yang2025_Silkie_Wencha	0.805387388
ENSGALG00010002145	ENSGALG0001000214	3	-0.211804428	0.255586775	-0.828698702	0.40727492	0	0.293497308	0	0.666666667	CRA004775;PRJNA827465;Yang2025_Silkie_Wencha	0.805387388
ENSGALG00010005524	STRIP2	3	-0.151652033	0.182692201	-0.830095822	0.406484609	0.050120931	4.161015189	51.93480655	0.666666667	CRA004775;PRJNA827465;Yang2025_Silkie_Wencha	0.805387388
ENSGALG00010022590	ENSGALG0001002259	3	-0.141848942	0.170970078	-0.829671155	0.406724734	0.051733132	5.378932574	62.81790165	0.666666667	CRA004775;PRJNA827465;Yang2025_Silkie_Wencha	0.805387388
ENSGALG00010025390	BCL7A	3	-0.135232097	0.162990359	-0.829693845	0.406711902	0.061064662	13.13999971	84.77929951	0.666666667	CRA004775;PRJNA827465;Yang2025_Silkie_Wencha	0.805387388
ENSGALG00010019854	CCDC14	3	0.12416337	0.149636354	0.82976741	0.4066703	0.04656318	7.062297036	71.68060208	0.666666667	CRA004775;PRJNA827465;Yang2025_Silkie_Wencha	0.805387388
ENSGALG00010023075	PKDREJ	3	0.120658472	0.145462317	0.829482681	0.406831333	0	0.64253337	0	0.666666667	CRA004775;PRJNA827465;Yang2025_Silkie_Wencha	0.805387388
ENSGALG00010019135	KIAA1841	3	0.11349277	0.136902032	0.829007199	0.407100334	0	0.936411728	0	1	CRA004775;PRJNA827465;Yang2025_Silkie_Wencha	0.805387388
ENSGALG00010012086	CIBAR1	3	0.087696222	0.105627689	0.830238947	0.406403699	0.012872852	3.047338691	34.36896247	0.666666667	CRA004775;PRJNA827465;Yang2025_Silkie_Wencha	0.805387388
ENSGALG00010022923	CD63	3	0.057391164	0.069153778	0.829906427	0.406591691	0.001814433	2.126856957	5.964526972	0.666666667	CRA004775;PRJNA827465;Yang2025_Silkie_Wencha	0.805387388
ENSGALG00010016260	INTS6L	3	0.045809627	0.055227037	0.829478275	0.406833825	0.001003445	2.195973182	8.924206517	0.666666667	CRA004775;PRJNA827465;Yang2025_Silkie_Wencha	0.805387388
ENSGALG00010005368	CCDC3	3	-0.094756653	0.114373163	-0.828486774	0.407394882	0	0.769921178	0	0.666666667	CRA004775;PRJNA827465;Yang2025_Silkie_Wencha	0.805503599
ENSGALG00010008087	CPNE8	3	0.128375997	0.154977343	0.828353322	0.407470433	0.027424499	3.181284564	37.1323137	0.666666667	CRA004775;PRJNA827465;Yang2025_Silkie_Wencha	0.805504233
ENSGALG00010020272	ENSGALG0001002027	3	-0.040338684	0.048701457	-0.828284951	0.407509144	0	0.596792452	0	1	CRA004775;PRJNA827465;Yang2025_Silkie_Wencha	0.805504233
ENSGALG00010012061	AZIN1	3	-0.163334241	0.197293938	-0.827872581	0.407742665	0.092320299	17.13968462	88.33117386	0.666666667	CRA004775;PRJNA827465;Yang2025_Silkie_Wencha	0.805710259
ENSGALG00010024339	TOP3B	3	0.098624425	0.119129545	0.827875442	0.407741044	0.031307217	10.42381588	80.8131684	0.666666667	CRA004775;PRJNA827465;Yang2025_Silkie_Wencha	0.805710259
ENSGALG00010019046	SELENOS	3	-0.040466341	0.048884258	-0.827799023	0.407784328	0	0.462431378	0	1	CRA004775;PRJNA827465;Yang2025_Silkie_Wencha	0.805710259
ENSGALG00010009109	ENSGALG0001000910	3	0.107769832	0.13022686	0.827554561	0.40792281	0	0.358386447	0	0.666666667	CRA004775;PRJNA827465;Yang2025_Silkie_Wencha	0.805871261
ENSGALG00010024655	IGF2BP2	3	0.061925427	0.074843565	0.82739814	0.408011435	0.006113077	3.131801543	36.13899308	0.666666667	CRA004775;PRJNA827465;Yang2025_Silkie_Wencha	0.805933735
ENSGALG00010012171	CDH17	3	0.5040787	0.609342348	0.827250398	0.408095151	0.88381951	14.1006142	85.81622069	0.666666667	CRA004775;PRJNA827465;Yang2025_Silkie_Wencha	0.805986499
ENSGALG00010025433	CHPF2	3	-0.073786322	0.089225202	-0.826967268	0.408255614	0.011268158	3.729110575	46.36790838	1	CRA004775;PRJNA827465;Yang2025_Silkie_Wencha	0.8061908
ENSGALG00010024227	MUC6	2	0.598955207	0.724564065	0.826642165	0.408439911	0.871655007	5.835470685	82.86342175	0.5	PRJNA827465;Yang2025_Silkie_Wenchang	0.806228613
ENSGALG00010000007	ND1	3	0.276498376	0.334673464	0.826173587	0.408705629	0.259844655	10.36991195	80.71343315	0.666666667	CRA004775;PRJNA827465;Yang2025_Silkie_Wencha	0.806228613
ENSGALG00010018063	DECR2	3	-0.216501944	0.262082309	-0.826083776	0.40875657	0.168389973	12.20020431	83.60683191	0.666666667	CRA004775;PRJNA827465;Yang2025_Silkie_Wencha	0.806228613
ENSGALG00010005349	ACBD5	3	0.134021946	0.162206644	0.826242025	0.408666813	0.057554522	8.904377386	77.53913706	0.666666667	CRA004775;PRJNA827465;Yang2025_Silkie_Wencha	0.806228613
ENSGALG00010016345	CTSZ	3	0.127097006	0.153757837	0.826605057	0.40846095	0.045766558	6.209160706	67.78952753	0.666666667	CRA004775;PRJNA827465;Yang2025_Silkie_Wencha	0.806228613
ENSGALG00010020037	MPP5	3	0.125081167	0.151401807	0.826153726	0.408716894	0.047662388	7.338888762	72.74791777	0.666666667	CRA004775;PRJNA827465;Yang2025_Silkie_Wencha	0.806228613
ENSGALG00010018471	HHEX	3	-0.121221433	0.14661067	-0.826825445	0.408336006	0.035781785	5.188757616	61.45512764	0.666666667	CRA004775;PRJNA827465;Yang2025_Silkie_Wencha	0.806228613
ENSGALG00010026036	TUB5A	3	0.052094208	0.063065877	0.826028444	0.408787957	0	0.586434282	0	0.666666667	CRA004775;PRJNA827465;Yang2025_Silkie_Wencha	0.806228613
ENSGALG00010005384	MAPRE2	3	0.04719016	0.057105488	0.826368209	0.408595252	0	0.737081973	0	1	CRA004775;PRJNA827465;Yang2025_Silkie_Wencha	0.806228613
ENSGALG00010024465	SENP1	3	0.11403143	0.13810199	0.825704467	0.408971758	0.035505946	5.322707342	62.42513685	0.666666667	CRA004775;PRJNA827465;Yang2025_Silkie_Wencha	0.806478617
ENSGALG00010000628	ENSGALG0001000062	2	0.261159388	0.316331147	0.825588598	0.409037506	0	0.011378612	0	1	PRJNA827465;Yang2025_Silkie_Wenchang	0.806495787
ENSGALG00010003596	CRISPLD1	3	0.083756606	0.101478976	0.825359194	0.409167695	0.010858322	2.91583175	31.40893674	0.666666667	CRA004775;PRJNA827465;Yang2025_Silkie_Wencha	0.806639994
ENSGALG00010019550	ACMSD	3	0.402461964	0.488760491	0.823433914	0.410261284	0.497189759	6.825879377	70.69974593	0.666666667	CRA004775;PRJNA827465;Yang2025_Silkie_Wencha	0.807076251
ENSGALG00010024805	ENSGALG0001002480	3	0.352001421	0.426836899	0.824674301	0.409556525	0.415321697	10.27111431	80.52791605	0.666666667	CRA004775;PRJNA827465;Yang2025_Silkie_Wencha	0.807076251
ENSGALG00010022566	ENSGALG0001002256	2	0.31619921	0.383877373	0.823698482	0.410110902	0	0.658905987	0	0.5	PRJNA827465;Yang2025_Silkie_Wenchang	0.807076251
ENSGALG00010011904	KIF24	3	0.213843549	0.259462796	0.824178081	0.40983838	0.10582196	4.613547531	56.64941162	0.666666667	CRA004775;PRJNA827465;Yang2025_Silkie_Wencha	0.807076251
ENSGALG00010020922	INSIG2	3	-0.197298247	0.239530249	-0.823688228	0.41011673	0.154329096	22.5135397	91.11645691	0.666666667	CRA004775;PRJNA827465;Yang2025_Silkie_Wencha	0.807076251
ENSGALG00010004907	ENSGALG0001000490	3	-0.173910903	0.211218641	-0.823369104	0.410298127	0.094375853	9.612462607	79.19367719	0.666666667	CRA004775;PRJNA827465;Yang2025_Silkie_Wencha	0.807076251
ENSGALG00010013226	DUSP6	3	-0.16812559	0.204033886	-0.824008178	0.409934911	0.097774076	16.83091923	88.11710773	0.666666667	CRA004775;PRJNA827465;Yang2025_Silkie_Wencha	0.807076251
ENSGALG00010013502	PAFAH2	3	-0.118267804	0.14361281	-0.82351849	0.410213207	0	0.071889805	0	1	CRA004775;PRJNA827465;Yang2025_Silkie_Wencha	0.807076251
ENSGALG00010013309	ENSGALG0001001330	3	0.113464953	0.137765062	0.823611961	0.410160078	0	1.433434413	0	0.666666667	CRA004775;PRJNA827465;Yang2025_Silkie_Wencha	0.807076251
ENSGALG00010024263	PHKB	3	-0.102409243	0.124306851	-0.823842303	0.410029167	0.017700181	3.076429478	34.98957104	0.666666667	CRA004775;PRJNA827465;Yang2025_Silkie_Wencha	0.807076251
ENSGALG00010006301	ENSGALG0001000630	3	0.092873253	0.112637322	0.824533568	0.40963645	0.021417195	4.826324612	58.5605992	0.666666667	CRA004775;PRJNA827465;Yang2025_Silkie_Wencha	0.807076251
ENSGALG00010027746	UQCR11	3	-0.090815617	0.110144357	-0.824514478	0.409647293	0	0.106071046	0	0.666666667	CRA004775;PRJNA827465;Yang2025_Silkie_Wencha	0.807076251
ENSGALG00010020934	ABCD3	3	-0.085185664	0.103460794	-0.823361779	0.410302291	0.017672785	5.494560694	63.60036568	0.666666667	CRA004775;PRJNA827465;Yang2025_Silkie_Wencha	0.807076251
ENSGALG00010024439	UFD1	3	-0.060920364	0.073958629	-0.823708675	0.410105109	0.005344481	2.540121108	21.26359669	0.666666667	CRA004775;PRJNA827465;Yang2025_Silkie_Wencha	0.807076251
ENSGALG00010024378	ZDHHC1	3	-0.047956665	0.058193528	-0.824089313	0.409888812	0	0.265928106	0	0.666666667	CRA004775;PRJNA827465;Yang2025_Silkie_Wencha	0.807076251
ENSGALG00010005893	SFT2D1	3	-0.040000931	0.048511567	-0.824564806	0.409618709	0	1.195932514	0	1	CRA004775;PRJNA827465;Yang2025_Silkie_Wencha	0.807076251
ENSGALG00010026984	PGR2/3	2	0.715393464	0.869296091	0.822957184	0.410532342	0.428870681	1.306942897	23.48556298	1	CRA004775;PRJNA827465	0.807416439
ENSGALG00010005516	LARP4B	3	0.194470452	0.236530161	0.822180355	0.410974258	0.139023901	12.46701782	83.95767112	0.666666667	CRA004775;PRJNA827465;Yang2025_Silkie_Wencha	0.807419093
ENSGALG00010009216	NSG1	3	-0.190776042	0.23207313	-0.822051401	0.411047643	0.058194478	3.060672816	34.6548906	0.666666667	CRA004775;PRJNA827465;Yang2025_Silkie_Wencha	0.807419093
ENSGALG00010004340	SLC4A4	3	0.126119681	0.153392177	0.822204128	0.41096073	0.033437461	3.870541336	48.32764137	0.666666667	CRA004775;PRJNA827465;Yang2025_Silkie_Wencha	0.807419093
ENSGALG00010022956	PBLD	3	-0.117367056	0.142691223	-0.822524706	0.410778331	0	0.890571068	0	0.666666667	CRA004775;PRJNA827465;Yang2025_Silkie_Wencha	0.807419093
ENSGALG00010007090	CEBPZ	3	0.110684566	0.134581459	0.82243547	0.410829099	0.039764476	9.251940099	78.38291236	0.666666667	CRA004775;PRJNA827465;Yang2025_Silkie_Wencha	0.807419093
ENSGALG00010002378	ENSGALG0001000237	3	0.09194608	0.11183666	0.82214616	0.410993717	0	0.834202988	0	0.666666667	CRA004775;PRJNA827465;Yang2025_Silkie_Wencha	0.807419093
ENSGALG00010028919	EGFL7	3	-0.062024908	0.075383486	-0.82279173	0.41062644	0.005264895	2.582749299	22.5631384	0.666666667	CRA004775;PRJNA827465;Yang2025_Silkie_Wencha	0.807419093
ENSGALG00010013553	TADA1	3	-0.058089549	0.070658789	-0.822113559	0.411012269	0	0.839112695	0	1	CRA004775;PRJNA827465;Yang2025_Silkie_Wencha	0.807419093
ENSGALG00010029600	RHOT1	3	-0.045345579	0.055128499	-0.822543331	0.410767736	0.002168605	2.566911997	22.08536942	0.666666667	CRA004775;PRJNA827465;Yang2025_Silkie_Wencha	0.807419093
ENSGALG00010008300	CUL1	3	0.156371069	0.190282147	0.821785285	0.41119911	0.090283368	15.04977953	86.71076878	0.666666667	CRA004775;PRJNA827465;Yang2025_Silkie_Wencha	0.807604419
ENSGALG00010001848	HOXA11	3	0.368045813	0.448261183	0.82105216	0.411616558	0.519310607	14.59013555	86.29210816	0.666666667	CRA004775;PRJNA827465;Yang2025_Silkie_Wencha	0.807944877
ENSGALG00010008107	TUBB3	3	-0.132023666	0.160813226	-0.820975176	0.411660408	0.055054383	7.92189946	74.75352963	0.666666667	CRA004775;PRJNA827465;Yang2025_Silkie_Wencha	0.807944877
ENSGALG00010005383	OPTN	3	0.124982738	0.152268698	0.820803879	0.411757989	0.045454703	8.521143121	76.52897068	0.666666667	CRA004775;PRJNA827465;Yang2025_Silkie_Wencha	0.807944877
ENSGALG00010024966	AGAP3	3	0.087265312	0.106284923	0.82105071	0.411617384	0.022652264	8.814119695	77.30913501	0.666666667	CRA004775;PRJNA827465;Yang2025_Silkie_Wencha	0.807944877
ENSGALG00010023134	KNL1	3	-0.055909491	0.068117638	-0.820778481	0.411772458	0	0.154085228	0	0.666666667	CRA004775;PRJNA827465;Yang2025_Silkie_Wencha	0.807944877
ENSGALG00010005342	MAP3K5	3	0.054214573	0.066052377	0.820781561	0.411770704	0	1.66580441	0	0.666666667	CRA004775;PRJNA827465;Yang2025_Silkie_Wencha	0.807944877
ENSGALG00010005590	TBC1D23	3	0.033347655	0.040600118	0.821368437	0.411436436	0	0.238868623	0	1	CRA004775;PRJNA827465;Yang2025_Silkie_Wencha	0.807944877
ENSGALG00010021123	RYR3	3	0.239387633	0.291790324	0.820409769	0.411982548	0.17076256	8.423235858	76.25615579	0.666666667	CRA004775;PRJNA827465;Yang2025_Silkie_Wencha	0.808116573
ENSGALG00010024063	BRD7	3	0.121631632	0.148240058	0.820504481	0.411928576	0.045306461	7.251499854	72.41949886	0.666666667	CRA004775;PRJNA827465;Yang2025_Silkie_Wencha	0.808116573
ENSGALG00010005316	PARP4	3	-0.05868919	0.071543915	-0.820323998	0.412031429	0.004776336	2.807646688	28.76596586	0.666666667	CRA004775;PRJNA827465;Yang2025_Silkie_Wencha	0.808116573
ENSGALG00010007724	RBM11	3	-0.21634805	0.263938082	-0.819692437	0.412391463	0.100185752	3.839982282	47.91642635	0.666666667	CRA004775;PRJNA827465;Yang2025_Silkie_Wencha	0.808459291
ENSGALG00010028311	ENSGALG0001002831	3	0.207914805	0.253673345	0.819616285	0.412434887	0.068792675	3.007827904	33.50683403	0.666666667	CRA004775;PRJNA827465;Yang2025_Silkie_Wencha	0.808459291
ENSGALG00010014984	LPP	3	0.097683309	0.119151053	0.819827494	0.412314455	0	0.614006061	0	1	CRA004775;PRJNA827465;Yang2025_Silkie_Wencha	0.808459291
ENSGALG00010027985	PSMB4	3	-0.037555285	0.045814765	-0.819720123	0.412375676	0	0.779657121	0	0.666666667	CRA004775;PRJNA827465;Yang2025_Silkie_Wencha	0.808459291
ENSGALG00010017039	MAP2K5	3	-0.169274077	0.20666731	-0.819065561	0.412749009	0.11270152	20.80158191	90.38534661	0.666666667	CRA004775;PRJNA827465;Yang2025_Silkie_Wencha	0.808510622
ENSGALG00010015156	CNOT6	3	-0.138217685	0.168791031	-0.818868656	0.412861355	0.066456912	14.48039917	86.18822606	0.666666667	CRA004775;PRJNA827465;Yang2025_Silkie_Wencha	0.808510622
ENSGALG00010017147	SRSF7L	3	-0.124890251	0.15250768	-0.818911223	0.412837066	0.047926596	6.602895836	69.71025971	0.666666667	CRA004775;PRJNA827465;Yang2025_Silkie_Wencha	0.808510622
ENSGALG00010024624	CTSD	3	-0.116406461	0.142118955	-0.819077654	0.41274211	0.036683841	5.339090603	62.54043715	0.666666667	CRA004775;PRJNA827465;Yang2025_Silkie_Wencha	0.808510622
ENSGALG00010016332	ENSGALG0001001633	3	0.081180813	0.099115117	0.819055808	0.412754574	0	0.497967983	0	0.666666667	CRA004775;PRJNA827465;Yang2025_Silkie_Wencha	0.808510622
ENSGALG00010022583	FKBP11	3	-0.079739649	0.097369284	-0.818940493	0.412820365	0	0.827351539	0	1	CRA004775;PRJNA827465;Yang2025_Silkie_Wencha	0.808510622
ENSGALG00010029169	SBDS	3	-0.06963574	0.085020183	-0.819049522	0.41275816	0.012306508	5.1741756	61.34649933	0.666666667	CRA004775;PRJNA827465;Yang2025_Silkie_Wencha	0.808510622
ENSGALG00010006330	STX19	3	0.447791789	0.547035078	0.818579661	0.413026275	0.657160349	8.000596064	75.00186256	0.666666667	CRA004775;PRJNA827465;Yang2025_Silkie_Wencha	0.808513311
ENSGALG00010027227	ENSGALG0001002722	3	0.219188736	0.267771728	0.818565641	0.413034277	0.175032001	19.44695138	89.71561166	0.666666667	CRA004775;PRJNA827465;Yang2025_Silkie_Wencha	0.808513311
ENSGALG00010022864	CSTPP1	3	-0.074566544	0.091089542	-0.818607078	0.413010627	0.002903656	2.225810025	10.1450718	0.666666667	CRA004775;PRJNA827465;Yang2025_Silkie_Wencha	0.808513311
ENSGALG00010015516	SELENON	3	-0.213903293	0.261392782	-0.818321344	0.413173722	0.184747805	32.26405614	93.80115138	0.666666667	CRA004775;PRJNA827465;Yang2025_Silkie_Wencha	0.808669161
ENSGALG00010004730	CLDN12	3	-0.175671041	0.214697517	-0.81822577	0.413228283	0.114023544	18.07890488	88.93738302	0.666666667	CRA004775;PRJNA827465;Yang2025_Silkie_Wencha	0.808669161
ENSGALG00010013129	RASSF3	3	0.072588693	0.088743434	0.81796128	0.413379297	0.008673054	2.971565802	32.69541606	0.666666667	CRA004775;PRJNA827465;Yang2025_Silkie_Wencha	0.808852738
ENSGALG00010012827	ENSGALG0001001282	3	-0.285435376	0.349060823	-0.817723895	0.413514863	0	0.721322952	0	0.666666667	CRA004775;PRJNA827465;Yang2025_Silkie_Wencha	0.809006039
ENSGALG00010020066	ENSGALG0001002006	3	0.210802701	0.257965187	0.817174997	0.413828429	0	1.966655625	0	0.666666667	CRA004775;PRJNA827465;Yang2025_Silkie_Wencha	0.80909382
ENSGALG00010004364	TSHR	2	-0.160918649	0.197048743	-0.816643876	0.414131974	0.010601685	1.149475355	13.00378945	0.5	PRJNA827465;Yang2025_Silkie_Wenchang	0.80909382
ENSGALG00010029709	SLC6A4	3	0.131030845	0.160307212	0.817373365	0.413715092	0.020046454	2.667997697	25.03741654	0.666666667	CRA004775;PRJNA827465;Yang2025_Silkie_Wencha	0.80909382
ENSGALG00010029598	SUPT4H1	3	-0.129252188	0.158219721	-0.816915791	0.413976553	0.039142994	4.255531689	53.00234739	0.666666667	CRA004775;PRJNA827465;Yang2025_Silkie_Wencha	0.80909382
ENSGALG00010022422	RPS6KB2	3	0.083818908	0.102616859	0.816814205	0.414034613	0.015223461	4.026902075	50.33402942	0.666666667	CRA004775;PRJNA827465;Yang2025_Silkie_Wencha	0.80909382
ENSGALG00010023030	HS2ST1	3	-0.083064376	0.101703172	-0.816733391	0.414080805	0.022330007	9.344330471	78.59664739	0.666666667	CRA004775;PRJNA827465;Yang2025_Silkie_Wencha	0.80909382
ENSGALG00010013678	ATP7A	3	-0.07768385	0.095071552	-0.81710931	0.413865963	0.016070074	5.345838926	62.58772425	0.666666667	CRA004775;PRJNA827465;Yang2025_Silkie_Wencha	0.80909382
ENSGALG00010014340	EFNB1	3	0.073986032	0.090535079	0.817208458	0.41380931	0.012138819	4.245575486	52.89213426	0.666666667	CRA004775;PRJNA827465;Yang2025_Silkie_Wencha	0.80909382
ENSGALG00010017425	H6PD	3	-0.05860767	0.071724031	-0.817127382	0.413855636	0	1.781120107	0	0.666666667	CRA004775;PRJNA827465;Yang2025_Silkie_Wencha	0.80909382
ENSGALG00010027569	ATP5PB	3	-0.044785654	0.054820687	-0.81694807	0.413958106	0	0.984130375	0	0.666666667	CRA004775;PRJNA827465;Yang2025_Silkie_Wencha	0.80909382
ENSGALG00010000176	ENSGALG0001000017	3	0.535015784	0.655432858	0.816278552	0.414340838	1.056709471	11.54738012	82.68005401	0.666666667	CRA004775;PRJNA827465;Yang2025_Silkie_Wencha	0.809198177

ENSGALG00010026822	PRELP	3	-0.075583314	0.092598225	-0.816250142	0.414357084	0	1.074674036	0	1	CRA004775;PRJNA827465;Yang2025_Silkie_Wencha	0.809198177
ENSGALG00010005632	ITGB1	3	0.03443206	0.042182003	0.816273838	0.414343534	0	1.236595868	0	1	CRA004775;PRJNA827465;Yang2025_Silkie_Wencha	0.809198177
ENSGALG00010004055	FILIP1	3	0.351277416	0.430463282	0.816045017	0.414474389	0.473178552	17.893488	88.82274937	0.666666667	CRA004775;PRJNA827465;Yang2025_Silkie_Wencha	0.809229624
ENSGALG00010008656	CCDC6	3	0.166249501	0.203756671	0.815921756	0.414544888	0.100539747	11.57609743	82.72302033	0.666666667	CRA004775;PRJNA827465;Yang2025_Silkie_Wencha	0.809229624
ENSGALG00010023005	IRF7	3	-0.126002085	0.154411225	-0.816016355	0.414490782	0.03166341	3.598957664	44.42835435	0.666666667	CRA004775;PRJNA827465;Yang2025_Silkie_Wencha	0.809229624
ENSGALG00010028925	ENSGALG0001002892	3	-0.280497917	0.343972631	-0.8154658	0.414805733	0.198135732	4.537501154	55.92287622	0.666666667	CRA004775;PRJNA827465;Yang2025_Silkie_Wencha	0.809306269
ENSGALG00010012831	ENSGALG0001001283	3	-0.222147077	0.272453576	-0.815357541	0.414867681	0.089127944	3.355254012	40.39199437	0.666666667	CRA004775;PRJNA827465;Yang2025_Silkie_Wencha	0.809306269
ENSGALG00010007252	NFATC1	3	0.144921907	0.177806763	0.815052839	0.415042065	0.044288968	3.709987168	46.09145775	0.666666667	CRA004775;PRJNA827465;Yang2025_Silkie_Wencha	0.809306269
ENSGALG00010011222	RCL1	3	-0.101394432	0.124394503	-0.815103798	0.415012898	0.028557885	6.20178185	67.75120363	0.666666667	CRA004775;PRJNA827465;Yang2025_Silkie_Wencha	0.809306269
ENSGALG00010019204	ARF6	3	0.095812645	0.117468261	0.815647088	0.41470201	0.027097964	5.867775811	65.91553487	0.666666667	CRA004775;PRJNA827465;Yang2025_Silkie_Wencha	0.809306269
ENSGALG00010029658	XYLT2	3	-0.088562002	0.108578111	-0.81565245	0.414698942	0.025208259	7.183521706	72.15850272	0.666666667	CRA004775;PRJNA827465;Yang2025_Silkie_Wencha	0.809306269
ENSGALG00010007650	KLHL36	3	-0.048401285	0.05936421	-0.815327702	0.414884756	0	0.797674824	0	1	CRA004775;PRJNA827465;Yang2025_Silkie_Wencha	0.809306269
ENSGALG00010029607	ZNF207	3	0.037655882	0.046192399	0.815196502	0.41495984	0	1.364104818	0	0.666666667	CRA004775;PRJNA827465;Yang2025_Silkie_Wencha	0.809306269
ENSGALG00010002384	ENSGALG0001000238	2	0.428096772	0.52569113	0.814350381	0.415444255	0.359796775	2.855832708	64.98394331	0.5	PRJNA827465;Yang2025_Silkie_Wenchang	0.809510583
ENSGALG00010027375	FUT7	3	-0.334350465	0.410619175	-0.814259259	0.415496444	0.319499284	5.557290219	64.01123711	0.666666667	CRA004775;PRJNA827465;Yang2025_Silkie_Wencha	0.809510583
ENSGALG00010023764	COX5A	3	-0.31840099	0.390975407	-0.814376005	0.415429581	0.410229018	26.10680903	92.33916333	0.666666667	CRA004775;PRJNA827465;Yang2025_Silkie_Wencha	0.809510583
ENSGALG00010024854	C12orf65	3	0.156592835	0.192318743	0.814235954	0.415509792	0.07901659	7.675457018	73.94291968	0.666666667	CRA004775;PRJNA827465;Yang2025_Silkie_Wencha	0.809510583
ENSGALG00010014603	ENSGALG0001001460	3	-0.133814451	0.164373237	-0.814089039	0.415593945	0.032486735	3.073232495	34.92194283	0.666666667	CRA004775;PRJNA827465;Yang2025_Silkie_Wencha	0.809510583
ENSGALG00010011382	ENSGALG0001001138	3	-0.106596489	0.130942664	-0.814069958	0.415604875	0	1.088034581	0	0.666666667	CRA004775;PRJNA827465;Yang2025_Silkie_Wencha	0.809510583
ENSGALG00010017526	PLEKHA2	3	0.074870584	0.091903739	0.814663082	0.41526519	0	1.386578289	0	0.666666667	CRA004775;PRJNA827465;Yang2025_Silkie_Wencha	0.809510583
ENSGALG00010011496	ACTR6	3	0.042654287	0.052370193	0.814476425	0.415372072	0	1.177060403	0	0.666666667	CRA004775;PRJNA827465;Yang2025_Silkie_Wencha	0.809510583
ENSGALG00010007204	LANCL2	3	-0.066273838	0.081421734	-0.813957587	0.415669249	0.006770828	2.655014148	24.67083455	1	CRA004775;PRJNA827465;Yang2025_Silkie_Wencha	0.80952445
ENSGALG00010008501	ENSGALG0001000850	3	0.187039112	0.229860031	0.813708721	0.415811837	0.100977865	5.557849034	64.01485561	0.666666667	CRA004775;PRJNA827465;Yang2025_Silkie_Wencha	0.809690616
ENSGALG00010000659	ENSGALG0001000065	3	-0.315042875	0.387341547	-0.813346458	0.416019448	0.210206047	3.748308174	46.64259428	0.666666667	CRA004775;PRJNA827465;Yang2025_Silkie_Wencha	0.809983334
ENSGALG00010010912	BAALC	3	0.436906641	0.537872503	0.812286626	0.416627183	0.674368977	11.68155162	82.87898676	0.666666667	CRA004775;PRJNA827465;Yang2025_Silkie_Wencha	0.810124998
ENSGALG00010026016	SLC2A11	3	0.296156071	0.364482587	0.812538327	0.416482804	0.192875392	3.909392651	48.84115825	0.666666667	CRA004775;PRJNA827465;Yang2025_Silkie_Wencha	0.810124998
ENSGALG00010010505	CD38	3	0.233308704	0.287389801	0.811819708	0.416895092	0.197752364	10.15720266	80.30953929	0.666666667	CRA004775;PRJNA827465;Yang2025_Silkie_Wencha	0.810124998
ENSGALG00010022480	PIF1	3	-0.21853072	0.268762227	-0.813100571	0.416160399	0.126160837	5.711473849	64.98276885	0.666666667	CRA004775;PRJNA827465;Yang2025_Silkie_Wencha	0.810124998
ENSGALG00010005336	PEX7	3	-0.201169154	0.247471266	-0.81289904	0.416275945	0.156494453	16.11989064	87.59296794	0.666666667	CRA004775;PRJNA827465;Yang2025_Silkie_Wencha	0.810124998
ENSGALG00010023664	SLC6A9	3	-0.136392888	0.167899903	-0.812346438	0.416592871	0.052961578	7.041960911	71.59881991	0.666666667	CRA004775;PRJNA827465;Yang2025_Silkie_Wencha	0.810124998
ENSGALG00010019731	PANK4	3	0.133103086	0.163907471	0.812062353	0.416755854	0.06035729	11.6470119	82.82821365	0.666666667	CRA004775;PRJNA827465;Yang2025_Silkie_Wencha	0.810124998
ENSGALG00010020818	TMEM62	3	0.130415429	0.160665312	0.811721131	0.416951666	0.052614981	6.461223574	69.04611058	0.666666667	CRA004775;PRJNA827465;Yang2025_Silkie_Wencha	0.810124998
ENSGALG00010024635	ENSGALG0001002463	3	-0.129691321	0.159769121	-0.811742146	0.416939606	0	0.816701891	0	0.666666667	CRA004775;PRJNA827465;Yang2025_Silkie_Wencha	0.810124998
ENSGALG00010013196	MRPS9	3	0.103521043	0.127475855	0.812083535	0.4167437	0.031464376	6.064085834	67.01893649	0.666666667	CRA004775;PRJNA827465;Yang2025_Silkie_Wencha	0.810124998
ENSGALG00010018715	DNAJC13	3	0.079566159	0.097911481	0.812633591	0.416428167	0.01823265	6.37123679	68.60892059	0.666666667	CRA004775;PRJNA827465;Yang2025_Silkie_Wencha	0.810124998
ENSGALG00010025192	CRYAB	3	0.068928128	0.084849073	0.812361596	0.416584176	0	0.302230236	0	1	CRA004775;PRJNA827465;Yang2025_Silkie_Wencha	0.810124998
ENSGALG00010013177	OXR1	3	-0.05990906	0.073694117	-0.812942234	0.416251178	0.008026669	3.981870216	49.77234587	0.666666667	CRA004775;PRJNA827465;Yang2025_Silkie_Wencha	0.810124998
ENSGALG00010010968	RRP7A	3	0.052669238	0.06484099	0.812283074	0.41662922	0	1.835774922	0	0.666666667	CRA004775;PRJNA827465;Yang2025_Silkie_Wencha	0.810124998
ENSGALG00010025790	LRP5	3	0.051505571	0.063438182	0.811901756	0.416848006	0	0.907880191	0	0.666666667	CRA004775;PRJNA827465;Yang2025_Silkie_Wencha	0.810124998
ENSGALG00010002866	ENSGALG0001000286	3	0.369986945	0.456403085	0.81065829	0.417561932	0.519175101	37.09012816	94.60772961	0.666666667	CRA004775;PRJNA827465;Yang2025_Silkie_Wencha	0.810976396
ENSGALG00010026422	P2RX3	2	0.356215239	0.43931367	0.810844878	0.417454758	0.214386838	2.236575799	55.28879457	0.5	PRJNA827465;Yang2025_Silkie_Wenchang	0.810976396
ENSGALG00010022462	ITGA5	3	0.073178187	0.090266354	0.810691731	0.417542723	0	0.194921077	0	0.666666667	CRA004775;PRJNA827465;Yang2025_Silkie_Wencha	0.810976396
ENSGALG00010021502	DNAJB12	3	0.033702949	0.04158031	0.810550694	0.417623742	0	0.079578853	0	0.666666667	CRA004775;PRJNA827465;Yang2025_Silkie_Wencha	0.810985041
ENSGALG00010018721	LRCH2	3	-0.053349368	0.065843459	-0.810245526	0.417799077	0	0.603449802	0	0.666666667	CRA004775;PRJNA827465;Yang2025_Silkie_Wencha	0.81121411
ENSGALG00010011281	ENSGALG0001001128	2	0.481808554	0.595687985	0.808827047	0.418614636	0.159165427	1.183518096	15.50615037	0.5	CRA004775;PRJNA827465	0.811571691
ENSGALG00010023240	PIWIL1	2	0.431012342	0.532493137	0.80942328	0.418271716	0.430892551	4.148261236	75.89351434	0.5	PRJNA827465;Yang2025_Silkie_Wenchang	0.811571691
ENSGALG00010029371	AUTS2	3	0.23673053	0.292583615	0.809103851	0.418455413	0.18887917	8.05326064	75.16533875	0.666666667	CRA004775;PRJNA827465;Yang2025_Silkie_Wencha	0.811571691
ENSGALG00010011595	ENSGALG0001001159	3	0.224003224	0.276862936	0.809076242	0.418471293	0.170138407	8.072702261	75.22514846	0.666666667	CRA004775;PRJNA827465;Yang2025_Silkie_Wencha	0.811571691
ENSGALG00010009085	PAF1	3	0.212820326	0.262996621	0.809213155	0.41839255	0.184950213	21.62458072	90.75126577	0.666666667	CRA004775;PRJNA827465;Yang2025_Silkie_Wencha	0.811571691
ENSGALG00010018399	ABHD12	3	-0.18484972	0.228492582	-0.808996593	0.418517106	0.132661957	19.19721174	89.58182039	0.666666667	CRA004775;PRJNA827465;Yang2025_Silkie_Wencha	0.811571691
ENSGALG00010012767	JOSD1	3	-0.151516826	0.187152788	-0.80958893	0.418176472	0.084241745	11.42376361	82.49263493	0.666666667	CRA004775;PRJNA827465;Yang2025_Silkie_Wencha	0.811571691
ENSGALG00010024590	MRPL23	3	-0.14937364	0.184616297	-0.809103221	0.418455776	0.060082447	4.814348621	58.45751612	0.666666667	CRA004775;PRJNA827465;Yang2025_Silkie_Wencha	0.811571691
ENSGALG00010020370	CEP55	3	-0.08679618	0.107304246	-0.80887927	0.418584594	0	0.68921066	0	1	CRA004775;PRJNA827465;Yang2025_Silkie_Wencha	0.811571691
ENSGALG00010018588	POLR3F	3	0.077245537	0.095477021	0.809048463	0.418487271	0.010748409	3.213781945	37.76802426	0.666666667	CRA004775;PRJNA827465;Yang2025_Silkie_Wencha	0.811571691
ENSGALG00010006804	PSMA6	3	-0.045715045	0.056470814	-0.809534018	0.418208044	0	0.847237799	0	1	CRA004775;PRJNA827465;Yang2025_Silkie_Wencha	0.811571691
ENSGALG00010013174	ADAMTS12	3	0.106376138	0.131574473	0.808486145	0.418810779	0	1.09173504	0	0.666666667	CRA004775;PRJNA827465;Yang2025_Silkie_Wencha	0.81164288
ENSGALG00010005092	ESCO1	3	0.103351764	0.127868753	0.808264422	0.418938379	0.031568815	7.620582736	73.75528789	0.666666667	CRA004775;PRJNA827465;Yang2025_Silkie_Wencha	0.81164288
ENSGALG00010007800	MAF	3	0.087846162	0.10868167	0.808288663	0.418924427	0.019513713	4.757734295	57.96318424	1	CRA004775;PRJNA827465;Yang2025_Silkie_Wencha	0.81164288
ENSGALG00010009289	GPR137B	3	-0.064422825	0.079687849	-0.808439747	0.418837479	0	0.958975579	0	1	CRA004775;PRJNA827465;Yang2025_Silkie_Wencha	0.81164288
ENSGALG00010024880	NAT10	3	-0.061272981	0.075782805	-0.808534083	0.418783193	0.005137315	2.80603008	28.72492656	0.666666667	CRA004775;PRJNA827465;Yang2025_Silkie_Wencha	0.81164288
ENSGALG00010022522	TNS4	3	0.282246966	0.349337609	0.807948982	0.419119952	0.240396848	6.014388915	66.74641384	0.666666667	CRA004775;PRJNA827465;Yang2025_Silkie_Wencha	0.811883409
ENSGALG00010011131	RET	3	-0.140965275	0.174517689	-0.807742047	0.419239093	0	0.066972452	0	1	CRA004775;PRJNA827465;Yang2025_Silkie_Wencha	0.81200295
ENSGALG00010027281	ENSGALG0001002728	3	0.354969436	0.439521185	0.807627592	0.419304998	0.5264642	22.35612661	91.05390645	0.666666667	CRA004775;PRJNA827465;Yang2025_Silkie_Wencha	0.812019363
ENSGALG00010021669	ENSGALG0001002166	3	-0.103586391	0.128294171	-0.807413081	0.419428533	0.033288384	6.345845399	68.48331665	0.666666667	CRA004775;PRJNA827465;Yang2025_Silkie_Wencha	0.812147361
ENSGALG00010017510	HDC	3	-0.561533298	0.695630217	-0.807229594	0.419534218	1.229436296	13.88867284	85.599776	0.666666667	CRA004775;PRJNA827465;Yang2025_Silkie_Wencha	0.812240766
ENSGALG00010017870	RAB11A	3	-0.083471879	0.103440877	-0.806952548	0.419693822	0.023038319	8.668350422	76.92755942	0.666666667	CRA004775;PRJNA827465;Yang2025_Silkie_Wencha	0.812327304
ENSGALG00010018582	RCC2	3	-0.056693574	0.070248652	-0.807041398	0.419642633	0.006742399	3.613986147	44.65944477	0.666666667	CRA004775;PRJNA827465;Yang2025_Silkie_Wencha	0.812327304
ENSGALG00010005262	ENSGALG0001000526	3	-0.057443301	0.071219827	-0.806563334	0.419918106	0	0.962494044	0	1	CRA004775;PRJNA827465;Yang2025_Silkie_Wencha	0.812650164
ENSGALG00010028000	MRPL9	3	0.262821619	0.325898346	0.806452755	0.419981839	0.26506023	15.98615452	87.48917385	0.666666667	CRA004775;PRJNA827465;Yang2025_Silkie_Wencha	0.812662272
ENSGALG00010025809	ENSGALG0001002580	3	0.111790239	0.138636909	0.806352651	0.42003954	0.020675822	3.055325075	34.54051694	0.666666667	CRA004775;PRJNA827465;Yang2025_Silkie_Wencha	0.812662706
ENSGALG00010021892	NDUFV1	3	-0.053537645	0.066432328	-0.806007846	0.420238325	0	0.174258666	0	1	CRA004775;PRJNA827465;Yang2025_Silkie_Wencha	0.812936062
ENSGALG00010018055	PEX5L	2	0.468616294	0.581694874	0.805604992	0.420470646	0.46766301	3.137729794	68.12982425	0.5	PRJNA827465;Yang2025_Silkie_Wenchang	0.812940581
ENSGALG00010017573	ENSGALG0001001757	2	0.401684392	0.498470382	0.80583402	0.420338559	0.2917184	2.421431861	58.702121	0.5	PRJNA827465;Yang2025_Silkie_Wenchang	0.812940581
ENSGALG00010024124	DIDO1	3	0.209055328	0.259447256	0.805771974	0.420374341	0.174893157	18.67500231	89.29049664	0.666666667	CRA004775;PRJNA827465;Yang2025_Silkie_Wencha	0.812940581
ENSGALG00010007862	ATMIN	3	-0.157295685	0.195241421	-0.805647103	0.420446358	0.097809176	22.56409844	91.13636202	0.666666667	CRA004775;PRJNA827465;Yang2025_Silkie_Wencha	0.812940581
ENSGALG00010015040	ZC4H2	3	0.040271585	0.050007642	0.805308616	0.420641611	0	0.70358609	0	0.666666667	CRA004775;PRJNA827465;Yang2025_Silkie_Wencha	0.813159932
ENSGALG00010014408	ENSGALG0001001440	3	0.507379312	0.63039412	0.804860477	0.420900198	1.017629545	18.03359597	88.90958851	0.666666667	CRA004775;PRJNA827465;Yang2025_Silkie_Wencha	0.813326212
ENSGALG00010015165	KLF3	3	-0.048747337	0.060552047	-0.80504855	0.420791664	0	1.625880911	0	0.666666667	CRA004775;PRJNA827465;Yang2025_Silkie_Wencha	0.813326212
ENSGALG00010009016	DNAJC9	3	0.04451609	0.055308487	0.804869075	0.420895236	0	0.43210035	0	1	CRA004775;PRJNA827465;Yang2025_Silkie_Wencha	0.813326212
ENSGALG00010009192	LSM14A	3	-0.029262395	0.036362781	-0.804734774	0.420972748	0	1.400412608	0	0.666666667	CRA004775;PRJNA827465;Yang2025_Silkie_Wencha	0.813355246
ENSGALG00010012315	ATP6V1E1	3	-0.168611939	0.209565796	-0.804577568	0.421063491	0.10758494	15.74520307	87.2977186	0.666666667	CRA004775;PRJNA827465;Yang2025_Silkie_Wencha	0.813419415
ENSGALG00010013871	ENSGALG0001001387	3	0.21056314	0.261943561	0.803849269	0.421484031	0.140746556	6.377597805	68.64023005	0.666666667	CRA004775;PRJNA827465;Yang2025_Silkie_Wencha	0.813519426
ENSGALG00010004246	C8orf34	2	0.179148028	0.222722422	0.804355602	0.421191634	0	0.501333654	0	1	PRJNA827465;Yang2025_Silkie_Wenchang	0.813519426
ENSGALG00010003837	ALKAL2	3	-0.174936993	0.217592808	-0.803964962	0.421417211	0	0.110845711	0	1	CRA004775;PRJNA827465;Yang2025_Silkie_Wencha	0.813519426
ENSGALG00010010534	SYNJ1	3	0.167693802	0.208680481	0.803591218	0.421633096	0.089107354	6.717078672	70.22515147	0.666666667	CRA004775;PRJNA827465;Yang2025_Silkie_Wencha	0.813519426
ENSGALG00010016527	MRPS34	3	0.161964404	0.201541659	0.803627424	0.42161218	0.104814941	17.71162215	88.70797952	0.666666667	CRA004775;PRJNA827465;Yang2025_Silkie_Wencha	0.813519426
ENSGALG00010002835	CYFIP1	3	-0.108564469	0.135022825	-0.80404531	0.421370808	0.040958752	15.33121541	86.93767969	0.666666667	CRA004775;PRJNA827465;Yang2025_Silkie_Wencha	0.813519426
ENSGALG00010018492	ENSGALG0001001849	3	-0.102845954	0.127954909	-0.803767162	0.421531458	0.021971148	3.699373241	45.93678794	0.666666667	CRA004775;PRJNA827465;Yang2025_Silkie_Wencha	0.813519426
ENSGALG00010005389	MCM10	3	0.076332367	0.094921147	0.80416609	0.42130106	0	1.240483259	0	0.666666667	CRA004775;PRJNA827465;Yang2025_Silkie_Wencha	0.813519426
ENSGALG00010013274	MTF1	3	-0.060596147	0.075379215	-0.803884024	0.421463958	0.005748174	2.847312283	29.75831939	0.666666667	CRA004775;PRJNA827465;Yang2025_Silkie_Wencha	0.813519426
ENSGALG00010029533	MLXIPL	3	0.406293753	0.505988156	0.802970877	0.421991568	0.643246469	12.47845172	83.97237058	0.666666667	CRA004775;PRJNA827465;Yang2025_Silkie_Wencha	0.813731865
ENSGALG00010008269	ENSGALG0001000826	2	0.321456967	0.400317193	0.803005647	0.421971471	0.162114652	2.019418292	50.48078924	0.5	PRJNA827465;Yang2025_Silkie_Wenchang	0.813731865
ENSGALG00010019867	PEX11A	3	-0.31238477	0.389214039	-0.802604064	0.422203619	0.268679353	4.940500684	59.51827298	0.666666667	CRA004775;PRJNA827465;Yang2025_Silkie_Wencha	0.813731865
ENSGALG00010023130	DYNLT5	2	-0.243492385	0.303370506	-0.802623788	0.422192215	0	0.800837819	0	0.5	PRJNA827465;Yang2025_Silkie_Wenchang	0.813731865
ENSGALG00010022111	PCGF2	3	0.184037174	0.229138707	0.803171764	0.421875463	0.094324238	4.904011564	59.21706191	0.666666667	CRA004775;PRJNA827465;Yang2025_Silkie_Wencha	0.813731865
ENSGALG00010020976	GDF10	3	-0.164518542	0.204873725	-0.803024117	0.421960795	0	0.002439301	0	1	CRA004775;PRJNA827465;Yang2025_Silkie_Wencha	0.813731865
ENSGALG00010009736	WISP1	3	0.120205152	0.14971348	0.802901332	0.422031766	0.01397951	2.449128243	18.33829015	0.666666667	CRA004775;PRJNA827465;Yang2025_Silkie_Wencha	0.813731865
ENSGALG00010021075	DHRS3	3	-0.110980355	0.138242636	-0.80279397	0.422093828	0.03200493	4.751288226	57.90615293	0.666666667	CRA004775;PRJNA827465;Yang2025_Silkie_Wencha	0.813731865
ENSGALG00010008367	ENSGALG0001000836	2	-1.072969199	1.340447951	-0.800455667	0.42344684	3.192832207	8.866907905	88.71396526	0.5	PRJNA827465;Yang2025_Silkie_Wenchang	0.813751379
ENSGALG00010004942	GABRR1	2	0.266428803	0.332502724	0.801283068	0.422967791	0	0.816625898	0	0.5	PRJNA827465;Yang2025_Silkie_Wenchang	0.813751379
ENSGALG00010000023	ATP6	3	0.216259299	0.270449698	0.799628548	0.423926042	0.158980728	8.496539836	76.46100603	0.666666667	CRA004775;PRJNA827465;Yang2025_Silkie_Wencha	0.813751379
ENSGALG00010017669	ENSGALG0001001766	3	0.206762369	0.258254672	0.800614245	0.423355001	0.165259495	13.78839128	85.49504464	0.666666667	CRA004775;PRJNA827465;Yang2025_Silkie_Wencha	0.813751379
ENSGALG00010023223	CEP164	3	0.191783881	0.239711855	0.799928812	0.423752043	0.117215633	6.862716679	70.83750218	0.666666667	CRA004775;PRJNA827465;Yang2025_Silkie_Wencha	0.813751379
ENSGALG00010026009	ENSGALG0001002600	3	0.188420441	0.235343742	0.800618019	0.423352816	0.107796969	6.316234938	68.33556669	0.666666667	CRA004775;PRJNA827465;Yang2025_Silkie_Wencha	0.813751379
ENSGALG00010003899	LIN54	3	0.175766656	0.219411763	0.801081277	0.423084595	0.100637945	7.502671453	73.34282845	0.666666667	CRA004775;PRJNA827465;Yang2025_Silkie_Wencha	0.813751379
ENSGALG00010013171	SLC9A8	3	0.175740045	0.219078031	0.802180137	0.422448764	0.125048783	19.64041844	89.81691757	0.666666667	CRA004775;PRJNA827465;Yang2025_Silkie_Wencha	0.813751379
ENSGALG00010010022	KMT2E	3	0.13767833	0.171813489	0.801324367	0.422943888	0.050125531	4.327824346	53.78740355	0.666666667	CRA004775;PRJNA827465;Yang2025_Silkie_Wencha	0.813751379
ENSGALG00010004085	RB1CC1	3	0.135204765	0.168834872	0.800810662	0.423241266	0.054691131	7.083117541	71.76384567	0.666666667	CRA004775;PRJNA827465;Yang2025_Silkie_Wencha	0.813751379
ENSGALG00010014019	IRAG2	3	-0.132984719	0.166250052	-0.799907833	0.423764199	0	1.989213925	0	0.666666667	CRA004775;PRJNA827465;Yang2025_Silkie_Wencha	0.813751379
ENSGALG00010010972	EXOSC3	3	0.128540459	0.160636356	0.800195315	0.423597644	0.052909535	7.073547069	71.72564231	0.666666667	CRA004775;PRJNA827465;Yang2025_Silkie_Wencha	0.813751379
ENSGALG00010012871	ENSGALG0001001287	3	-0.121828739	0.152114748	-0.800742327	0.423280833	0.001144505	2.002835351	1.421276034	0.666666667	CRA004775;PRJNA827465;Yang2025_Silkie_Wencha	0.813751379
ENSGALG00010020069	TMEM216	3	0.119363987	0.149286922	0.799560905	0.423965246	0	0.423029944	0	1	CRA004775;PRJNA827465;Yang2025_Silkie_Wencha	0.813751379
ENSGALG00010001116	RNF13	3	-0.116379173	0.145564116	-0.799504553	0.423997907	0.056650789	31.96881693	93.74390362	0.666666667	CRA004775;PRJNA827465;Yang2025_Silkie_Wencha	0.813751379
ENSGALG00010024818	FBXO3	3	-0.113986929	0.14208426	-0.802248813	0.422409045	0.047681245	15.32054771	86.94563642	0.666666667	CRA004775;PRJNA827465;Yang2025_Silkie_Wencha	0.813751379
ENSGALG00010023600	CHST14	3	-0.110739658	0.134107989	-0.800822226	0.42323457	0.043291043	10.88172956	81.62056878	0.666666667	CRA004775;PRJNA827465;Yang2025_Silkie_Wencha	0.813751379
ENSGALG00010018012	DVL1	3	-0.095221351	0.118915336	-0.800749128	0.423276895	0.027629844	7.4287905	73.07771703	0.666666667	CRA004775;PRJNA827465;Yang2025_Silkie_Wencha	0.813751379
ENSGALG00010013427	NEXMIF	3	0.094622491	0.11834792	0.799528125	0.423984245	0	0.499354834	0	0.666666667	CRA004775;PRJNA827465;Yang2025_Silkie_Wencha	0.813751379
ENSGALG00010014824	ANXA6	3	0.090548581	0.113116942	0.800486464	0.423429003	0.024799698	6.944066453	71.19843231	0.666666667	CRA004775;PRJNA827465;Yang2025_Silkie_Wencha	0.813751379
ENSGALG00010020342	CPPED1	3	-0.087853362	0.10955285	-0.80192676	0.422595326	0	1.246000105	0	0.666666667	CRA004775;PRJNA827465;Yang2025_Silkie_Wencha	0.813751379
ENSGALG00010023442	STK25	3	-0.084323677	0.105137274	-0.802034081	0.422533244	0.023087573	9.252187576	78.38349057	0.666666667	CRA004775;PRJNA827465;Yang2025_Silkie_Wencha	0.813751379
ENSGALG00010013261	JADE2	3	-0.080657536	0.100549561	-0.802166964	0.422456384	0.015747467	4.36011483	54.12964846	0.666666667	CRA004775;PRJNA827465;Yang2025_Silkie_Wencha	0.813751379
ENSGALG00010026973	MGLL	3	-0.07859979	0.09823491	-0.800120747	0.423640842	0.009117407	2.876743954	30.47695478	0.666666667	CRA004775;PRJNA827465;Yang2025_Silkie_Wencha	0.813751379
ENSGALG00010021887	NUTF2	3	-0.072628919	0.090644966	-0.801246028	0.422989229	0.010449286	3.031727881	34.03101866	0.666666667	CRA004775;PRJNA827465;Yang2025_Silkie_Wencha	0.813751379
ENSGALG00010017388	PLPBP	3	-0.069299916	0.086586589	-0.800353927	0.423505767	0.008231621	2.795259926	28.45030325	1	CRA004775;PRJNA827465;Yang2025_Silkie_Wencha	0.813751379
ENSGALG00010016861	FGR	3	-0.06785038	0.084705457	-0.801015458	0.423122697	0.010071067	3.761558498	46.83054906	0.666666667	CRA004775;PRJNA827465;Yang2025_Silkie_Wencha	0.813751379
ENSGALG00010006259	PPA2	3	0.05666319	0.070626464	0.802294026	0.422382897	0	0.149649833	0	1	CRA004775;PRJNA827465;Yang2025_Silkie_Wencha	0.813751379
ENSGALG00010000753	TRIO	3	0.046348837	0.057897401	0.800546903	0.42344149	0	1.488741703	0	1	CRA004775;PRJNA827465;Yang2025_Silkie_Wencha	0.813751379
ENSGALG00010003489	SGCE	3	0.037066843	0.046360226	0.799539724	0.423977522	0	1.53243432	0	0.666666667	CRA004775;PRJNA827465;Yang2025_Silkie_Wencha	0.813751379
ENSGALG00010017163	CDV3	3	-0.033186167	0.041428836	-0.801040304	0.423108313	0	1.557287726	0	0.666666667	CRA004775;PRJNA827465;Yang2025_Silkie_Wencha	0.813751379
ENSGALG00010017499	SNRPD1	3	-0.10404836	0.130192834	-0.799186539	0.424182256	0.024261934	3.51153808	43.04490072	0.666666667	CRA004775;PRJNA827465;Yang2025_Silkie_Wencha	0.813994696
ENSGALG00010022845	HYDIN	3	-0.466155328	0.583546163	-0.798831964	0.424387854	0.880911857	16.09469922	87.57354846	0.666666667	CRA004775;PRJNA827465;Yang2025_Silkie_Wencha	0.814111669
ENSGALG00010023871	UQCRFS1	3	-0.156160166	0.195521783	-0.798684237	0.424473529	0.070448597	5.564935289	64.06067823	0.666666667	CRA004775;PRJNA827465;Yang2025_Silkie_Wencha	0.814111669
ENSGALG00010021416	PLEKHH3	3	0.12569882	0.157332894	0.798687922	0.424471392	0.041167599	4.932242741	59.45049534	0.666666667	CRA004775;PRJNA827465;Yang2025_Silkie_Wencha	0.814111669
ENSGALG00010027160	FASTK	3	-0.042194842	0.052825966	-0.798751933	0.424434267	0	0.230916918	0	1	CRA004775;PRJNA827465;Yang2025_Silkie_Wencha	0.814111669
ENSGALG00010021373	CEP250	3	0.280188484	0.350894896	0.798486894	0.424582195	0.326189838	21.30459071	90.61235192	0.666666667	CRA004775;PRJNA827465;Yang2025_Silkie_Wencha	0.81411944
ENSGALG00010007567	TRIP13	3	-0.056981749	0.07136289	-0.798478716	0.42459274	0	1.79925731	0	0.666666667	CRA004775;PRJNA827465;Yang2025_Silkie_Wencha	0.81411944
ENSGALG00010028458	TRAF2	3	-0.146621973	0.183666964	-0.79830346	0.424694411	0.070854076	7.35297291	72.80011739	0.666666667	CRA004775;PRJNA827465;Yang2025_Silkie_Wencha	0.814123086
ENSGALG00010001617	TRIM3	3	0.14178569	0.177614665	0.798276932	0.424709802	0.07632929	13.99181986	85.70593375	0.666666667	CRA004775;PRJNA827465;Yang2025_Silkie_Wencha	0.814123086
ENSGALG00010005888	IMPG2	3	0.308355484	0.3866426	0.79752072	0.425148674	0.365416286	12.28730868	83.72304259	0.666666667	CRA004775;PRJNA827465;Yang2025_Silkie_Wencha	0.814337768
ENSGALG00010019149	COX5B	3	0.240798522	0.302073504	0.797152079	0.425362713	0.225098544	17.59975715	88.63620683	0.666666667	CRA004775;PRJNA827465;Yang2025_Silkie_Wencha	0.814337768
ENSGALG00010009003	ALG12	3	-0.211653107	0.265531674	-0.797091749	0.425397748	0.169228648	35.56321	94.3762107	0.666666667	CRA004775;PRJNA827465;Yang2025_Silkie_Wencha	0.814337768
ENSGALG00010019398	SLC35F5	3	-0.17201389	0.215720907	-0.797390908	0.425224038	0.114485166	15.65669046	87.22590828	0.666666667	CRA004775;PRJNA827465;Yang2025_Silkie_Wencha	0.814337768
ENSGALG00010016757	SRPX2	3	-0.171133543	0.21461716	-0.797389841	0.425224658	0.088928362	7.015033005	71.48979914	0.666666667	CRA004775;PRJNA827465;Yang2025_Silkie_Wencha	0.814337768
ENSGALG00010028211	GATAD2B	3	0.108238397	0.135737034	0.797412421	0.425211548	0.026671263	3.664664001	45.42473745	0.666666667	CRA004775;PRJNA827465;Yang2025_Silkie_Wencha	0.814337768
ENSGALG00010014165	LUZP1	3	0.074568664	0.093549497	0.79710385	0.425390721	0.006441047	2.382398364	16.05098333	0.666666667	CRA004775;PRJNA827465;Yang2025_Silkie_Wencha	0.814337768
ENSGALG00010016257	ENSGALG0001001625	3	0.067531509	0.084652206	0.7977525	0.425014131	0.006205172	2.653409479	24.62527869	0.666666667	CRA004775;PRJNA827465;Yang2025_Silkie_Wencha	0.814337768
ENSGALG00010028528	UHRF1	3	-0.058867889	0.073836128	-0.797277577	0.42528984	0	0.79029293	0	0.666666667	CRA004775;PRJNA827465;Yang2025_Silkie_Wencha	0.814337768
ENSGALG00010016597	FRYL	3	-0.036094871	0.045234312	-0.797953364	0.424897554	0	0.157902491	0	0.666666667	CRA004775;PRJNA827465;Yang2025_Silkie_Wencha	0.814337768
ENSGALG00010003910	THAP9	3	-0.107336614	0.134689439	-0.796919306	0.425497898	0.033867126	5.904366586	66.12676447	0.666666667	CRA004775;PRJNA827465;Yang2025_Silkie_Wencha	0.81441922
ENSGALG00010024739	ASIP	2	-0.37895111	0.475785338	-0.796474964	0.425756021	0.315325716	3.284345692	69.55253516	0.5	PRJNA827465;Yang2025_Silkie_Wenchang	0.814692704
ENSGALG00010013071	ENSGALG0001001307	3	-0.085097623	0.106831407	-0.796559971	0.425706633	0	0.394691251	0	1	CRA004775;PRJNA827465;Yang2025_Silkie_Wencha	0.814692704
ENSGALG00010004377	AGR2	2	1.170151464	1.475024924	0.793309621	0.427597453	3.958752544	10.87098327	90.80120008	0.5	CRA004775;PRJNA827465	0.81503161
ENSGALG00010022994	LIPC	2	-0.965497445	1.215514651	-0.794311647	0.427014025	2.094668674	3.410933388	70.68251161	0.5	PRJNA827465;Yang2025_Silkie_Wenchang	0.81503161
ENSGALG00010000583	CCS	3	0.41906365	0.527196623	0.79489062	0.426677131	0.792986697	51.77387015	96.13704752	0.666666667	CRA004775;PRJNA827465;Yang2025_Silkie_Wencha	0.81503161
ENSGALG00010020369	CNTN3	2	0.41508574	0.522393821	0.794583938	0.426855564	0.463112947	6.499396298	84.6139556	0.5	PRJNA827465;Yang2025_Silkie_Wenchang	0.81503161
ENSGALG00010021758	RASSF10	3	0.322755713	0.405633534	0.795683013	0.426216302	0.221765682	3.597054891	44.39895803	0.666666667	CRA004775;PRJNA827465;Yang2025_Silkie_Wencha	0.81503161
ENSGALG00010008118	ENSGALG0001000811	3	-0.299142436	0.376572904	-0.794381201	0.426973545	0.276351151	5.726922031	65.07722666	0.666666667	CRA004775;PRJNA827465;Yang2025_Silkie_Wencha	0.81503161
ENSGALG00010023428	HOXB3	3	0.213062827	0.268568805	0.793326785	0.427587455	0.151895387	7.092161348	71.79985195	0.666666667	CRA004775;PRJNA827465;Yang2025_Silkie_Wencha	0.81503161
ENSGALG00010017704	ZNF362	3	0.19697251	0.248077045	0.793997324	0.427196989	0.142974941	10.05103394	80.10154963	0.666666667	CRA004775;PRJNA827465;Yang2025_Silkie_Wencha	0.81503161
ENSGALG00010006865	EFCAB2	2	0.19294183	0.243015469	0.793948761	0.427225262	0	0.646908813	0	1	PRJNA827465;Yang2025_Silkie_Wenchang	0.81503161
ENSGALG00010024659	BEND5	3	0.186372145	0.234225162	0.795696516	0.426208453	0.136932112	19.53705343	89.76304176	0.666666667	CRA004775;PRJNA827465;Yang2025_Silkie_Wencha	0.81503161
ENSGALG00010023942	NEGR1	3	-0.136341049	0.171603515	-0.794511985	0.426897435	0.0553329	6.663955249	69.98779366	0.666666667	CRA004775;PRJNA827465;Yang2025_Silkie_Wencha	0.81503161
ENSGALG00010016173	ENSGALG0001001617	2	0.134264265	0.169122214	0.793889001	0.427260054	0	0.142642703	0	1	PRJNA827465;Yang2025_Silkie_Wenchang	0.81503161
ENSGALG00010013139	TMEM185A	3	-0.126411383	0.15925158	-0.793784166	0.427321092	0.060412763	10.72527299	81.352456	0.666666667	CRA004775;PRJNA827465;Yang2025_Silkie_Wencha	0.81503161
ENSGALG00010027648	NFYA	3	-0.124647091	0.157125382	-0.793296981	0.427604815	0.056002951	14.36026655	86.07268192	0.666666667	CRA004775;PRJNA827465;Yang2025_Silkie_Wencha	0.81503161
ENSGALG00010016469	MAVS	3	-0.123690331	0.155597056	-0.794940047	0.426648377	0.047785141	6.352866186	68.51814691	0.666666667	CRA004775;PRJNA827465;Yang2025_Silkie_Wencha	0.81503161
ENSGALG00010007899	APRT	3	-0.108633782	0.136713099	-0.794611363	0.426839607	0.015483946	2.635640693	24.11712244	0.666666667	CRA004775;PRJNA827465;Yang2025_Silkie_Wencha	0.81503161
ENSGALG00010010908	STK17B	3	-0.102179675	0.128741977	-0.793678	0.427382911	0.027416241	5.123763296	60.96619058	0.666666667	CRA004775;PRJNA827465;Yang2025_Silkie_Wencha	0.81503161
ENSGALG00010029168	RFNG	3	-0.098334418	0.123636599	-0.795350394	0.426409707	0.028505707	5.387485775	62.87693214	0.666666667	CRA004775;PRJNA827465;Yang2025_Silkie_Wencha	0.81503161
ENSGALG00010015900	ENSGALG0001001590	3	0.082544362	0.103754844	0.795571159	0.426281335	0	0.388003584	0	1	CRA004775;PRJNA827465;Yang2025_Silkie_Wencha	0.81503161
ENSGALG00010014355	RAB20	3	0.080425791	0.101366533	0.793415621	0.427535712	0.011124213	2.90193341	31.08043096	0.666666667	CRA004775;PRJNA827465;Yang2025_Silkie_Wencha	0.81503161
ENSGALG00010021298	RPGRIP1L	3	0.078965774	0.099363526	0.794715903	0.42677878	0	1.936068688	0	0.666666667	CRA004775;PRJNA827465;Yang2025_Silkie_Wencha	0.81503161
ENSGALG00010028543	ANP32B	3	0.065565009	0.082474062	0.794977323	0.426626694	0.008812775	3.446704844	41.97356343	0.666666667	CRA004775;PRJNA827465;Yang2025_Silkie_Wencha	0.81503161
ENSGALG00010022333	ENSGALG0001002233	3	-0.064912956	0.081567607	-0.795817824	0.42613793	0	1.743038194	0	0.666666667	CRA004775;PRJNA827465;Yang2025_Silkie_Wencha	0.81503161
ENSGALG00010022052	IFFO1	3	-0.063953745	0.080460868	-0.794842835	0.42670493	0.007627479	3.207420532	37.64459695	0.666666667	CRA004775;PRJNA827465;Yang2025_Silkie_Wencha	0.81503161
ENSGALG00010024919	ANKRD52	3	-0.05695772	0.071617659	-0.795302739	0.426437421	0.002142924	2.249388436	11.08694398	0.666666667	CRA004775;PRJNA827465;Yang2025_Silkie_Wencha	0.81503161
ENSGALG00010003246	VWA8	3	0.053685073	0.067511044	0.795204302	0.42649467	0	0.119339726	0	0.666666667	CRA004775;PRJNA827465;Yang2025_Silkie_Wencha	0.81503161
ENSGALG00010018895	SEPTIN7	3	0.05241	0.066058847	0.793383502	0.42755442	0.004911596	2.836299316	29.48558044	0.666666667	CRA004775;PRJNA827465;Yang2025_Silkie_Wencha	0.81503161
ENSGALG00010017322	KHDRBS1	3	0.051606996	0.064844099	0.795862637	0.42611188	0.006557735	4.465710448	55.21429293	0.666666667	CRA004775;PRJNA827465;Yang2025_Silkie_Wencha	0.81503161
ENSGALG00010011597	TBC1D14	3	-0.03226845	0.040672347	-0.793375671	0.427558981	0	1.941703214	0	0.666666667	CRA004775;PRJNA827465;Yang2025_Silkie_Wencha	0.81503161
ENSGALG00010003472	FAM110B	3	-0.230195462	0.290355132	-0.79280659	0.427890516	0.206415219	31.93791158	93.73784978	0.666666667	CRA004775;PRJNA827465;Yang2025_Silkie_Wencha	0.815356336

ENSGALG00010021257	RGS6	3	0.225415114	0.28429647	0.792887489	0.427843377	0.11312232	3.905642952	48.79204207	0.666666667	CRA004775;PRJNA827465;Yang2025_Silkie_Wencha	0.815356336
ENSGALG00010019799	DOK2	3	-0.287127587	0.362273242	-0.792571886	0.428027294	0.262598053	6.850639312	70.80564442	0.666666667	CRA004775;PRJNA827465;Yang2025_Silkie_Wencha	0.815397186
ENSGALG00010020118	GNB1	3	-0.149749211	0.188934259	-0.792599565	0.428011162	0.09177484	38.23481326	94.76916498	0.666666667	CRA004775;PRJNA827465;Yang2025_Silkie_Wencha	0.815397186
ENSGALG00010000791	ACOD1	3	0.640823604	0.810293381	0.790853805	0.429029308	1.776571499	23.45103205	91.47159069	0.666666667	CRA004775;PRJNA827465;Yang2025_Silkie_Wencha	0.815508786
ENSGALG00010018430	LOXL4	3	-0.327869635	0.414643609	-0.790726368	0.429103685	0.371510235	7.887336094	74.64289621	0.666666667	CRA004775;PRJNA827465;Yang2025_Silkie_Wencha	0.815508786
ENSGALG00010027264	ENSGALG0001002726	3	0.262254821	0.331716379	0.790599551	0.429177709	0.295970091	24.26521958	91.75775025	0.666666667	CRA004775;PRJNA827465;Yang2025_Silkie_Wencha	0.815508786
ENSGALG00010018301	ENSGALG0001001830	3	-0.213904389	0.270149521	-0.791799994	0.428477306	0.070642035	2.906945353	31.19925705	0.666666667	CRA004775;PRJNA827465;Yang2025_Silkie_Wencha	0.815508786
ENSGALG00010027952	CIB3	3	-0.187504388	0.236984309	-0.791210136	0.428821377	0.131299823	12.44247467	83.92602715	0.666666667	CRA004775;PRJNA827465;Yang2025_Silkie_Wencha	0.815508786
ENSGALG00010008143	DCTN6	3	-0.158500539	0.200173888	-0.791814261	0.428468986	0.085688696	8.152274899	75.46697057	0.666666667	CRA004775;PRJNA827465;Yang2025_Silkie_Wencha	0.815508786
ENSGALG00010005444	ACTC1	3	-0.139482994	0.176118448	-0.791984005	0.428370003	0.03367765	3.111224776	35.71663431	0.666666667	CRA004775;PRJNA827465;Yang2025_Silkie_Wencha	0.815508786
ENSGALG00010009392	MAPRE3	3	0.128564067	0.162433949	0.791485202	0.428660908	0.055506127	10.81418489	81.5057721	0.666666667	CRA004775;PRJNA827465;Yang2025_Silkie_Wencha	0.815508786
ENSGALG00010005408	APBB1IP	3	-0.127752159	0.161526638	-0.790904586	0.428999672	0.015913525	2.383010881	16.07256116	1	CRA004775;PRJNA827465;Yang2025_Silkie_Wencha	0.815508786
ENSGALG00010007973	CARMIL1	3	0.120426659	0.151984515	0.792361376	0.428149994	0.045924055	6.462432234	69.05189985	0.666666667	CRA004775;PRJNA827465;Yang2025_Silkie_Wencha	0.815508786
ENSGALG00010008187	ENSGALG0001000818	3	0.113958047	0.144009359	0.791323897	0.428755007	0.011711276	2.417094802	17.25603819	0.666666667	CRA004775;PRJNA827465;Yang2025_Silkie_Wencha	0.815508786
ENSGALG00010003858	SUCLA2	3	0.086571002	0.109409354	0.791257769	0.428793586	0.016914363	3.915139913	48.91625728	0.666666667	CRA004775;PRJNA827465;Yang2025_Silkie_Wencha	0.815508786
ENSGALG00010004049	SETDB2	3	0.074905721	0.094746295	0.790592614	0.429181758	0.015052193	5.220875695	61.69225017	0.666666667	CRA004775;PRJNA827465;Yang2025_Silkie_Wencha	0.815508786
ENSGALG00010001178	ZBTB5	3	-0.070761154	0.089469907	-0.790893351	0.429006229	0	1.047191354	0	0.666666667	CRA004775;PRJNA827465;Yang2025_Silkie_Wencha	0.815508786
ENSGALG00010024081	CARS	3	0.056135857	0.070956368	0.791132052	0.428866937	0.006234426	3.045588277	34.33124185	0.666666667	CRA004775;PRJNA827465;Yang2025_Silkie_Wencha	0.815508786
ENSGALG00010020745	ABCC1	3	0.054971605	0.069524842	0.790675719	0.429133249	0.004074431	2.736342168	26.90972557	0.666666667	CRA004775;PRJNA827465;Yang2025_Silkie_Wencha	0.815508786
ENSGALG00010007365	MEMO1	3	-0.047822563	0.060361835	-0.792264897	0.428206235	0	1.640562144	0	0.666666667	CRA004775;PRJNA827465;Yang2025_Silkie_Wencha	0.815508786
ENSGALG00010016262	SPG11	3	-0.038017583	0.048041731	-0.79134499	0.428742702	0.001779543	2.509489112	20.30250338	0.666666667	CRA004775;PRJNA827465;Yang2025_Silkie_Wencha	0.815508786
ENSGALG00010003101	KLHL15	3	0.037778939	0.047776487	0.790743333	0.429093783	0	1.366095214	0	0.666666667	CRA004775;PRJNA827465;Yang2025_Silkie_Wencha	0.815508786
ENSGALG00010009841	GABRA1	3	0.381312741	0.482502097	0.790282039	0.429363073	0.510337893	8.829151186	77.34776585	0.666666667	CRA004775;PRJNA827465;Yang2025_Silkie_Wencha	0.815604193
ENSGALG00010009346	STC2	3	0.321722759	0.407135671	0.790210196	0.429405022	0.382212306	13.76069787	85.46585342	0.666666667	CRA004775;PRJNA827465;Yang2025_Silkie_Wencha	0.815604193
ENSGALG00010024699	FBXL8	3	-0.124152847	0.157105822	-0.790249817	0.429381888	0.058224942	11.9204423	83.22209906	0.666666667	CRA004775;PRJNA827465;Yang2025_Silkie_Wencha	0.815604193
ENSGALG00010021530	MLF2	3	-0.072607709	0.091900798	-0.79006614	0.429489143	0.016943721	6.278531198	68.14541591	0.666666667	CRA004775;PRJNA827465;Yang2025_Silkie_Wencha	0.815654398
ENSGALG00010015929	SLC6A12	2	0.574926782	0.727990794	0.789744578	0.429676952	0.718064677	3.089259116	67.6297791	0.5	PRJNA827465;Yang2025_Silkie_Wenchang	0.815693893
ENSGALG00010000633	VWC2	2	-0.2452703	0.31067889	-0.789465612	0.429839921	0	0.667563496	0	0.5	PRJNA827465;Yang2025_Silkie_Wenchang	0.815693893
ENSGALG00010016049	ZFYVE21	3	-0.183919662	0.233079783	-0.789084578	0.430062575	0.134791784	14.19830881	85.91381532	0.666666667	CRA004775;PRJNA827465;Yang2025_Silkie_Wencha	0.815693893
ENSGALG00010000812	ENSGALG0001000081	3	-0.167864438	0.212604096	-0.789563516	0.429782722	0.117219448	17.54363084	88.59985132	0.666666667	CRA004775;PRJNA827465;Yang2025_Silkie_Wencha	0.815693893
ENSGALG00010020514	CAMKMT	3	-0.161237851	0.204335406	-0.789084249	0.430062767	0.092830196	9.516063306	78.98290569	0.666666667	CRA004775;PRJNA827465;Yang2025_Silkie_Wencha	0.815693893
ENSGALG00010015570	LIMCH1	3	0.123349087	0.156289863	0.789232808	0.42997595	0.04083329	4.456888014	55.12563938	0.666666667	CRA004775;PRJNA827465;Yang2025_Silkie_Wencha	0.815693893
ENSGALG00010029256	RABEP1	3	0.11367715	0.143969386	0.789592519	0.429765778	0.043840736	11.59400335	82.7497031	0.666666667	CRA004775;PRJNA827465;Yang2025_Silkie_Wencha	0.815693893
ENSGALG00010021610	TMEM11	3	0.0725756	0.091979266	0.789043042	0.43008685	0.00837423	2.76978128	27.79213238	0.666666667	CRA004775;PRJNA827465;Yang2025_Silkie_Wencha	0.815693893
ENSGALG00010020982	TLCD4	3	-0.071691418	0.090810725	-0.78945981	0.429843311	0	0.457792096	0	1	CRA004775;PRJNA827465;Yang2025_Silkie_Wencha	0.815693893
ENSGALG00010009484	RTKN2	3	-0.052049781	0.065939994	-0.78935071	0.429907056	0	0.137765639	0	0.666666667	CRA004775;PRJNA827465;Yang2025_Silkie_Wencha	0.815693893
ENSGALG00010005281	TMED8	3	-0.126892802	0.161006075	-0.788124312	0.430623996	0.056987355	13.50325554	85.18875694	0.666666667	CRA004775;PRJNA827465;Yang2025_Silkie_Wencha	0.81572785
ENSGALG00010028559	DYNC2I2	3	-0.114304712	0.144982732	-0.788402254	0.430461453	0.040032376	5.570375903	64.09578034	0.666666667	CRA004775;PRJNA827465;Yang2025_Silkie_Wencha	0.81572785
ENSGALG00010005148	TTC39C	3	-0.113887721	0.144395084	-0.788722986	0.430273931	0.038839116	7.242705157	72.38600831	0.666666667	CRA004775;PRJNA827465;Yang2025_Silkie_Wencha	0.81572785
ENSGALG00010011418	RIPK1	3	0.08113852	0.102939345	0.788216788	0.430569911	0.011955236	2.974504791	32.761917	0.666666667	CRA004775;PRJNA827465;Yang2025_Silkie_Wencha	0.81572785
ENSGALG00010006575	SSBP2	3	-0.078335354	0.099382404	-0.788221564	0.430567118	0	0.136175616	0	0.666666667	CRA004775;PRJNA827465;Yang2025_Silkie_Wencha	0.81572785
ENSGALG00010007951	TGFBR1	3	-0.075641563	0.095884144	-0.788884996	0.430179226	0.012560228	3.805665119	47.4467685	0.666666667	CRA004775;PRJNA827465;Yang2025_Silkie_Wencha	0.81572785
ENSGALG00010013217	TGFBRAP1	3	0.060167469	0.076315717	0.788402064	0.430461564	0	1.253625677	0	1	CRA004775;PRJNA827465;Yang2025_Silkie_Wencha	0.81572785
ENSGALG00010005456	RPL7L1	3	0.038080053	0.048316451	0.78813845	0.430615727	0	0.666942316	0	0.666666667	CRA004775;PRJNA827465;Yang2025_Silkie_Wencha	0.81572785
ENSGALG00010016967	ERLEC1	3	0.032540104	0.041276341	0.788347592	0.430493417	0	1.794243956	0	0.666666667	CRA004775;PRJNA827465;Yang2025_Silkie_Wencha	0.81572785
ENSGALG00010011850	SPDG16	3	0.284459403	0.36124655	0.787438393	0.431025279	0.225240024	5.097358027	60.76398814	0.666666667	CRA004775;PRJNA827465;Yang2025_Silkie_Wencha	0.815798368
ENSGALG00010021634	PLCD4	3	0.27582194	0.350307765	0.787370328	0.431065111	0.299438627	16.56928503	87.92947314	0.666666667	CRA004775;PRJNA827465;Yang2025_Silkie_Wencha	0.815798368
ENSGALG00010023369	ACE	3	-0.148515005	0.188614778	-0.787398564	0.431048587	0.05877929	4.90057591	59.18847016	0.666666667	CRA004775;PRJNA827465;Yang2025_Silkie_Wencha	0.815798368
ENSGALG00010009160	ENSGALG0001000916(	3	0.143025046	0.181594613	0.787606217	0.430927077	0	1.014971471	0	0.666666667	CRA004775;PRJNA827465;Yang2025_Silkie_Wencha	0.815798368
ENSGALG00010006287	ECPAS	3	0.118138726	0.149993207	0.787627174	0.430914815	0.048920814	11.822623	83.08328025	0.666666667	CRA004775;PRJNA827465;Yang2025_Silkie_Wencha	0.815798368
ENSGALG00010013793	METTL13	3	-0.117326726	0.14894831	-0.787700952	0.430871648	0.040452751	5.314596066	62.3677891	0.666666667	CRA004775;PRJNA827465;Yang2025_Silkie_Wencha	0.815798368
ENSGALG00010002501	TCEANC	3	-0.07450623	0.094583104	-0.787732974	0.430852914	0	0.763630203	0	0.666666667	CRA004775;PRJNA827465;Yang2025_Silkie_Wencha	0.815798368
ENSGALG00010005361	ENSGALG0001000536	3	0.393202249	0.499590924	0.787048422	0.431253521	0.450979047	5.078285052	60.6166259	0.666666667	CRA004775;PRJNA827465;Yang2025_Silkie_Wencha	0.815928619
ENSGALG00010010223	ENSGALG0001001022	3	-0.105736813	0.134373825	-0.786885493	0.431348901	0	0.987845949	0	1	CRA004775;PRJNA827465;Yang2025_Silkie_Wencha	0.815928619
ENSGALG00010020828	CAMK2N1	3	-0.090909311	0.115534525	-0.786858393	0.431364766	0	1.849963327	0	0.666666667	CRA004775;PRJNA827465;Yang2025_Silkie_Wencha	0.815928619
ENSGALG00010020323	ARHGAP25	3	-0.06106729	0.0776037	-0.786912088	0.431333331	0	0.055053287	0	1	CRA004775;PRJNA827465;Yang2025_Silkie_Wencha	0.815928619
ENSGALG00010023886	CYP2C18	2	0.414954375	0.527679615	0.786375603	0.431647472	0.270336873	1.922774383	47.99181802	0.5	PRJNA827465;Yang2025_Silkie_Wenchang	0.815975616
ENSGALG00010011452	KITLG	3	0.197383436	0.251020748	0.786323194	0.431678167	0.142110987	11.10779427	81.99462512	0.666666667	CRA004775;PRJNA827465;Yang2025_Silkie_Wencha	0.815975616
ENSGALG00010019985	UMPS	3	-0.166268	0.211420784	-0.786431668	0.431614637	0.107273153	14.11790395	85.83359112	0.666666667	CRA004775;PRJNA827465;Yang2025_Silkie_Wencha	0.815975616
ENSGALG00010016802	TMEM222	3	-0.142553604	0.181263976	-0.786441999	0.431608586	0.083018746	14.398808	86.10996132	0.666666667	CRA004775;PRJNA827465;Yang2025_Silkie_Wencha	0.815975616
ENSGALG00010001620	MOSPD2	3	-0.062867792	0.079923425	-0.786600325	0.431515869	0	1.422930306	0	1	CRA004775;PRJNA827465;Yang2025_Silkie_Wencha	0.815975616
ENSGALG00010023543	ENSGALG0001002354	2	0.532547301	0.6785968	0.784777206	0.432584207	0.228155661	1.324153912	24.48007809	0.5	PRJNA827465;Yang2025_Silkie_Wenchang	0.816036982
ENSGALG00010028152	ENSGALG0001002815	3	-0.348266108	0.444661178	-0.783216806	0.433499807	0.335931606	5.394863869	62.92770219	0.666666667	CRA004775;PRJNA827465;Yang2025_Silkie_Wencha	0.816036982
ENSGALG00010017459	FAM167B	3	0.306163782	0.390787282	0.783453802	0.433360672	0.384723324	12.69465508	84.245338	0.666666667	CRA004775;PRJNA827465;Yang2025_Silkie_Wencha	0.816036982
ENSGALG00010025438	EXOSC6	3	0.278103028	0.35480872	0.783811142	0.433150935	0.287677388	9.103098432	78.02945871	0.666666667	CRA004775;PRJNA827465;Yang2025_Silkie_Wencha	0.816036982
ENSGALG00010027087	RAB7B	3	0.253849948	0.323117022	0.785628519	0.432085151	0.21844798	7.247868026	72.40567857	0.666666667	CRA004775;PRJNA827465;Yang2025_Silkie_Wencha	0.816036982
ENSGALG00010010934	ENSGALG0001001093	3	0.22283214	0.284084469	0.784386913	0.432813115	0.222373463	30.11364644	93.35849279	0.666666667	CRA004775;PRJNA827465;Yang2025_Silkie_Wencha	0.816036982
ENSGALG00010014716	TMEM35A	3	-0.209998549	0.267469535	-0.785130722	0.432376929	0.154217053	7.409972833	73.00934774	0.666666667	CRA004775;PRJNA827465;Yang2025_Silkie_Wencha	0.816036982
ENSGALG00010007143	CKMT2	3	-0.195335281	0.24935953	-0.783347967	0.433422803	0	0.736091425	0	0.666666667	CRA004775;PRJNA827465;Yang2025_Silkie_Wencha	0.816036982
ENSGALG00010002916	SDCBP	3	-0.193882203	0.247119473	-0.784568698	0.432706489	0.152771822	13.96180766	85.67520733	0.666666667	CRA004775;PRJNA827465;Yang2025_Silkie_Wencha	0.816036982
ENSGALG00010002958	ENSGALG0001000295	3	-0.17240807	0.219974216	-0.783764904	0.433178071	0.070762044	4.04426142	50.54721265	0.666666667	CRA004775;PRJNA827465;Yang2025_Silkie_Wencha	0.816036982
ENSGALG00010009266	PCDH7	3	0.161564751	0.206069608	0.784029981	0.433022519	0.039446841	2.744076345	27.11573044	0.666666667	CRA004775;PRJNA827465;Yang2025_Silkie_Wencha	0.816036982
ENSGALG00010015686	CNOT8	3	-0.139427906	0.177592033	-0.785102259	0.432393616	0.080500387	23.38780584	91.44853513	0.666666667	CRA004775;PRJNA827465;Yang2025_Silkie_Wencha	0.816036982
ENSGALG00010005025	THOC1	3	0.130808436	0.166669805	0.784835834	0.432549827	0.065988191	12.65536952	84.19643143	0.666666667	CRA004775;PRJNA827465;Yang2025_Silkie_Wencha	0.816036982
ENSGALG00010014705	ARAP2	3	0.129180132	0.16493502	0.783218334	0.43349891	0	0.438407468	0	0.666666667	CRA004775;PRJNA827465;Yang2025_Silkie_Wencha	0.816036982
ENSGALG00010013477	RFC1	3	0.125965966	0.160534301	0.784666982	0.432648847	0.059302311	9.850007965	79.69544789	0.666666667	CRA004775;PRJNA827465;Yang2025_Silkie_Wencha	0.816036982
ENSGALG00010027743	C1orf74	3	-0.122785487	0.156745521	-0.783342876	0.433425791	0	0.684845266	0	0.666666667	CRA004775;PRJNA827465;Yang2025_Silkie_Wencha	0.816036982
ENSGALG00010016883	TMEM251	3	-0.114960584	0.146366192	-0.785431268	0.432200754	0.047528025	8.75426163	77.15398414	0.666666667	CRA004775;PRJNA827465;Yang2025_Silkie_Wencha	0.816036982
ENSGALG00010017264	PIAS1	3	0.108447578	0.138115664	0.785193907	0.432339887	0.030650903	4.04851495	50.59916971	0.666666667	CRA004775;PRJNA827465;Yang2025_Silkie_Wencha	0.816036982
ENSGALG00010024786	PDCD5	3	-0.089145923	0.113578848	-0.784881384	0.432523118	0.022914716	5.62383661	64.43708915	0.666666667	CRA004775;PRJNA827465;Yang2025_Silkie_Wencha	0.816036982
ENSGALG00010016159	ASPG	3	0.085815929	0.109378561	0.784577241	0.432701478	0	1.557948244	0	0.666666667	CRA004775;PRJNA827465;Yang2025_Silkie_Wencha	0.816036982
ENSGALG00010010383	SLIT2	3	-0.074393746	0.094860373	-0.784244714	0.432896533	0	0.995322156	0	0.666666667	CRA004775;PRJNA827465;Yang2025_Silkie_Wencha	0.816036982
ENSGALG00010029331	TK1	3	-0.07358986	0.093792978	-0.784598822	0.432688821	0.00417844	2.140704496	6.572812645	0.666666667	CRA004775;PRJNA827465;Yang2025_Silkie_Wencha	0.816036982
ENSGALG00010002779	FBXO8	3	-0.072462278	0.092424195	-0.784018485	0.433029264	0.010449278	3.332809394	39.99056761	0.666666667	CRA004775;PRJNA827465;Yang2025_Silkie_Wencha	0.816036982
ENSGALG00010014945	TXN2	3	0.070473898	0.08991313	0.783799843	0.433157566	0	1.398943441	0	0.666666667	CRA004775;PRJNA827465;Yang2025_Silkie_Wencha	0.816036982
ENSGALG00010010867	QSOX1	3	-0.061415648	0.078194068	-0.785425928	0.432203884	0	0.144165971	0	1	CRA004775;PRJNA827465;Yang2025_Silkie_Wencha	0.816036982
ENSGALG00010004477	TULP4	3	-0.05336927	0.068117721	-0.783485845	0.433341863	0	1.602857756	0	0.666666667	CRA004775;PRJNA827465;Yang2025_Silkie_Wencha	0.816036982
ENSGALG00010028111	TRUB2	3	-0.050336397	0.064034165	-0.786086571	0.431816771	0	1.282989842	0	1	CRA004775;PRJNA827465;Yang2025_Silkie_Wencha	0.816036982
ENSGALG00010020890	RPS15A	3	-0.048675635	0.062100528	-0.783819983	0.433145747	0	1.126235539	0	1	CRA004775;PRJNA827465;Yang2025_Silkie_Wencha	0.816036982
ENSGALG00010018969	DIEXF	3	0.046505915	0.059182233	0.785808716	0.431979559	0	0.957291148	0	0.666666667	CRA004775;PRJNA827465;Yang2025_Silkie_Wencha	0.816036982
ENSGALG00010025897	MAPKAPK5	3	0.045673695	0.05824642	0.784145955	0.432954473	0.001003164	2.173212458	7.970341651	1	CRA004775;PRJNA827465;Yang2025_Silkie_Wencha	0.816036982
ENSGALG00010020870	RIOX1	3	-0.038986171	0.049769349	-0.783336965	0.433429261	0	1.046995436	0	1	CRA004775;PRJNA827465;Yang2025_Silkie_Wencha	0.816036982
ENSGALG00010012332	ENSGALG0001001233	2	-0.535390643	0.684084167	-0.782638553	0.433839395	0.495032534	2.117341935	52.77097273	0.5	PRJNA827465;Yang2025_Silkie_Wenchang	0.816237644
ENSGALG00010006087	ENSGALG0001000608	3	0.50173984	0.641200384	0.782500841	0.433920291	0.92904606	9.339144666	78.58476261	0.666666667	CRA004775;PRJNA827465;Yang2025_Silkie_Wencha	0.816237644
ENSGALG00010018654	ENSGALG0001001865	3	0.246494856	0.314871452	0.782842825	0.433719416	0.194685769	5.980797943	66.55964607	0.666666667	CRA004775;PRJNA827465;Yang2025_Silkie_Wencha	0.816237644
ENSGALG00010024961	LSM12	3	0.162787649	0.207999268	0.782635685	0.43384108	0.105732773	11.97868773	83.30368029	0.666666667	CRA004775;PRJNA827465;Yang2025_Silkie_Wencha	0.816237644
ENSGALG00010016919	ARHGDIG	3	0.112148107	0.14333024	0.782445537	0.433952781	0	0.671798868	0	0.666666667	CRA004775;PRJNA827465;Yang2025_Silkie_Wencha	0.816237644
ENSGALG00010023203	COG4	3	-0.081962734	0.104701783	-0.7828208	0.433732351	0.02371473	8.124688269	75.38367093	0.666666667	CRA004775;PRJNA827465;Yang2025_Silkie_Wencha	0.816237644
ENSGALG00010011994	TSPAN8	2	0.970075988	1.240099447	0.782256608	0.434067822	2.554057467	5.894638519	83.03543132	0.5	CRA004775;PRJNA827465	0.81625329
ENSGALG00010005320	ENSGALG0001000532	3	0.532028234	0.680509449	0.781808739	0.434326984	1.085226163	9.43089054	78.79309497	0.666666667	CRA004775;PRJNA827465;Yang2025_Silkie_Wencha	0.81625329
ENSGALG00010021177	OBSL1	3	0.173265807	0.221648935	0.781712787	0.434383385	0.058513831	3.304282169	39.47248154	0.666666667	CRA004775;PRJNA827465;Yang2025_Silkie_Wencha	0.81625329
ENSGALG00010029288	PYCR1	3	-0.155443597	0.198738524	-0.782151312	0.434125654	0.082702076	9.90438238	79.80691856	0.666666667	CRA004775;PRJNA827465;Yang2025_Silkie_Wencha	0.81625329
ENSGALG00010029468	ENSGALG0001002946	3	0.153913614	0.196997799	0.78129611	0.434628357	0	0.530811411	0	0.666666667	CRA004775;PRJNA827465;Yang2025_Silkie_Wencha	0.81625329
ENSGALG00010021483	UNG	3	-0.149203288	0.190919585	-0.781498075	0.434509608	0.056882679	3.878351732	48.43170145	0.666666667	CRA004775;PRJNA827465;Yang2025_Silkie_Wencha	0.81625329
ENSGALG00010022797	EFHD1	3	-0.13619049	0.174223271	-0.781700915	0.434390364	0.0718291	17.87979103	88.81418694	0.666666667	CRA004775;PRJNA827465;Yang2025_Silkie_Wencha	0.81625329
ENSGALG00010016640	OCIAD1	3	-0.124249782	0.158961775	-0.78163308	0.43443024	0.058725903	11.4063255	82.46586949	0.666666667	CRA004775;PRJNA827465;Yang2025_Silkie_Wencha	0.81625329
ENSGALG00010020670	ENSGALG0001002067	3	0.104093557	0.133173859	0.781636559	0.434428195	0.028417335	4.214662738	52.54661822	0.666666667	CRA004775;PRJNA827465;Yang2025_Silkie_Wencha	0.81625329
ENSGALG00010017955	IKBKB	3	0.090405235	0.115606447	0.782008588	0.434209526	0.031738924	10.86433055	81.59113449	0.666666667	CRA004775;PRJNA827465;Yang2025_Silkie_Wencha	0.81625329
ENSGALG00010013755	ANGPT4	3	0.081925767	0.104864615	0.781252729	0.434653867	0.010579092	2.901367462	31.06698733	0.666666667	CRA004775;PRJNA827465;Yang2025_Silkie_Wencha	0.81625329
ENSGALG00010027892	SHE	3	-0.047296848	0.060536987	-0.781288444	0.434632865	0	0.198493707	0	0.666666667	CRA004775;PRJNA827465;Yang2025_Silkie_Wencha	0.81625329
ENSGALG00010010382	SEC11C	3	0.16401919	0.210779819	0.781604051	0.434800112	0.095118201	9.09415692	78.00786364	0.666666667	CRA004775;PRJNA827465;Yang2025_Silkie_Wencha	0.816419494
ENSGALG00010029767	GLOD4	3	-0.125414395	0.160635683	-0.780738082	0.434956558	0.058440399	10.42328658	80.81219407	0.666666667	CRA004775;PRJNA827465;Yang2025_Silkie_Wencha	0.816604803
ENSGALG00010012323	SMAD7B	3	-1.172053947	1.51129488	-0.775529622	0.438026769	3.679202347	4.443180783	54.98720179	0.666666667	CRA004775;PRJNA827465;Yang2025_Silkie_Wencha	0.816680119
ENSGALG00010027191	AOC1	3	0.80915242	1.041717386	0.776748503	0.437307164	2.372282912	7.397361511	72.96333298	0.666666667	CRA004775;PRJNA827465;Yang2025_Silkie_Wencha	0.816680119
ENSGALG00010027197	ENSGALG0001002719	3	-0.573803295	0.736907711	-0.778663713	0.436177837	1.331334056	12.31854201	83.76431238	0.666666667	CRA004775;PRJNA827465;Yang2025_Silkie_Wencha	0.816680119
ENSGALG00010000422	ITIH6	3	0.441852699	0.566916469	0.779396478	0.435746198	0.760513611	9.671656045	79.32101813	0.666666667	CRA004775;PRJNA827465;Yang2025_Silkie_Wencha	0.816680119
ENSGALG00010002181	FIS1	3	0.381197502	0.491594859	0.775430203	0.438085494	0.66925202	37.37614761	94.64899374	0.666666667	CRA004775;PRJNA827465;Yang2025_Silkie_Wencha	0.816680119
ENSGALG00010001262	ENSGALG0001000126	3	0.352818807	0.453030321	0.778797335	0.436099108	0.576182583	58.68218165	96.59181042	0.666666667	CRA004775;PRJNA827465;Yang2025_Silkie_Wencha	0.816680119
ENSGALG00010022264	SCN8A	3	0.302613362	0.389818345	0.776293283	0.437575838	0.351515496	12.71657971	84.27250058	0.666666667	CRA004775;PRJNA827465;Yang2025_Silkie_Wencha	0.816680119
ENSGALG00010005559	FILIP1L	3	0.258878047	0.333285108	0.776746517	0.437308336	0.281313614	19.29596512	89.63513881	0.666666667	CRA004775;PRJNA827465;Yang2025_Silkie_Wencha	0.816680119
ENSGALG00010013262	CA13	3	-0.257714367	0.332848598	-0.774269049	0.438771703	0.259536876	9.866593391	79.72957919	0.666666667	CRA004775;PRJNA827465;Yang2025_Silkie_Wencha	0.816680119
ENSGALG00010016733	PHC3	3	0.25070351	0.32382056	0.774205042	0.438809547	0.232721367	8.254338773	75.77031843	0.666666667	CRA004775;PRJNA827465;Yang2025_Silkie_Wencha	0.816680119
ENSGALG00010027975	IQSEC1	3	0.245739358	0.317131271	0.774882141	0.438409306	0.217956771	7.789404062	74.32409483	0.666666667	CRA004775;PRJNA827465;Yang2025_Silkie_Wencha	0.816680119
ENSGALG00010019335	HIPK3	3	0.230818083	0.296737966	0.777851538	0.436656541	0.202740038	10.59979931	81.13171825	0.666666667	CRA004775;PRJNA827465;Yang2025_Silkie_Wencha	0.816680119
ENSGALG00010020992	KCNH7	3	0.221056369	0.283325005	0.780221882	0.435260284	0.044403733	2.438703863	17.98922245	0.666666667	CRA004775;PRJNA827465;Yang2025_Silkie_Wencha	0.816680119
ENSGALG00010002685	CA8	3	-0.20663642	0.266723683	-0.774720929	0.438504581	0.151186072	9.466813362	78.87356681	0.666666667	CRA004775;PRJNA827465;Yang2025_Silkie_Wencha	0.816680119
ENSGALG00010005979	SUV39H2	3	-0.192177661	0.246382533	-0.779997102	0.435392581	0.166205215	31.16894543	93.58335686	0.666666667	CRA004775;PRJNA827465;Yang2025_Silkie_Wencha	0.816680119
ENSGALG00010009991	LHFPL3	3	-0.187323092	0.241614686	-0.775296795	0.438164303	0	0.385402733	0	0.666666667	CRA004775;PRJNA827465;Yang2025_Silkie_Wencha	0.816680119
ENSGALG00010011013	ENSGALG0001001101	3	0.185226782	0.238214181	0.777564042	0.436826066	0	0.646553341	0	0.666666667	CRA004775;PRJNA827465;Yang2025_Silkie_Wencha	0.816680119
ENSGALG00010009597	CDK1	3	-0.18390058	0.23659612	-0.777276397	0.436995717	0.110048195	7.298064738	72.59547467	0.666666667	CRA004775;PRJNA827465;Yang2025_Silkie_Wencha	0.816680119
ENSGALG00010019132	ENSGALG0001001913	3	-0.174440397	0.225302532	-0.774249609	0.438783197	0.113956114	8.531548801	76.55759761	0.666666667	CRA004775;PRJNA827465;Yang2025_Silkie_Wencha	0.816680119
ENSGALG00010014916	DYNLT2B	3	-0.170158063	0.219807063	-0.774124638	0.438857089	0.097924491	6.268024616	68.09202065	0.666666667	CRA004775;PRJNA827465;Yang2025_Silkie_Wencha	0.816680119
ENSGALG00010013079	ENSGALG0001001307	3	-0.168643838	0.217106539	-0.776779173	0.437289066	0	0.825247902	0	0.666666667	CRA004775;PRJNA827465;Yang2025_Silkie_Wencha	0.816680119
ENSGALG00010022353	SLC39A13	3	-0.166044259	0.214524475	-0.774010792	0.438924409	0.121354399	23.29582003	91.41476884	0.666666667	CRA004775;PRJNA827465;Yang2025_Silkie_Wencha	0.816680119
ENSGALG00010005760	GPR18	3	0.165830799	0.212902456	0.778905056	0.436035646	0.002836484	2.0359828	1.767343019	0.666666667	CRA004775;PRJNA827465;Yang2025_Silkie_Wencha	0.816680119
ENSGALG00010002633	ENSGALG0001000263	3	0.164550101	0.212177037	0.775532091	0.438025311	0.109118355	11.51752734	82.6351617	0.666666667	CRA004775;PRJNA827465;Yang2025_Silkie_Wencha	0.816680119
ENSGALG00010018530	FAM161A	3	0.15624569	0.200874457	0.777827563	0.436670676	0.055093041	3.708509254	46.06997413	0.666666667	CRA004775;PRJNA827465;Yang2025_Silkie_Wencha	0.816680119
ENSGALG00010010625	FAM169A	2	0.156051021	0.201300198	0.775215436	0.438212368	0	0.044569296	0	1	PRJNA827465;Yang2025_Silkie_Wenchang	0.816680119
ENSGALG00010008145	ENSGALG0001000814	3	0.154948249	0.199619621	0.776217525	0.43762056	0.070801019	5.299419615	62.26001817	0.666666667	CRA004775;PRJNA827465;Yang2025_Silkie_Wencha	0.816680119
ENSGALG00010017921	SPATA7	3	0.151398714	0.194864245	0.776944553	0.437191484	0.077405864	7.471198203	73.23053216	0.666666667	CRA004775;PRJNA827465;Yang2025_Silkie_Wencha	0.816680119
ENSGALG00010022248	FIGNL2	3	-0.149783921	0.193496256	-0.77409209	0.438876335	0.057831016	4.181406161	52.16920043	0.666666667	CRA004775;PRJNA827465;Yang2025_Silkie_Wencha	0.816680119
ENSGALG00010024080	TENT4B	3	0.140124902	0.179582386	0.780281993	0.435224909	0.076868596	11.09993568	81.98187758	0.666666667	CRA004775;PRJNA827465;Yang2025_Silkie_Wencha	0.816680119
ENSGALG00010025419	CCND1	3	0.139460888	0.179346002	0.777608009	0.436800138	0.07422534	12.81766108	84.39652923	0.666666667	CRA004775;PRJNA827465;Yang2025_Silkie_Wencha	0.816680119
ENSGALG00010002616	ZNFB3	3	-0.129583432	0.167033475	-0.775793189	0.437871106	0.052094306	5.361227481	62.69511027	0.666666667	CRA004775;PRJNA827465;Yang2025_Silkie_Wencha	0.816680119
ENSGALG00010021382	TPRG1L	3	-0.123352226	0.158772034	-0.776914062	0.437209475	0.054012947	10.21844206	80.42754475	0.666666667	CRA004775;PRJNA827465;Yang2025_Silkie_Wencha	0.816680119
ENSGALG00010010933	ANAPC4	3	-0.118831632	0.153048524	-0.776431084	0.437494497	0.054209339	13.41669387	85.09319793	0.666666667	CRA004775;PRJNA827465;Yang2025_Silkie_Wencha	0.816680119
ENSGALG00010001422	AP1S1	3	0.118540678	0.152900455	0.775280085	0.438174175	0.033563676	3.703974518	46.00394819	0.666666667	CRA004775;PRJNA827465;Yang2025_Silkie_Wencha	0.816680119
ENSGALG00010017228	RAP2C	3	-0.115512882	0.148833827	-0.776119811	0.437678247	0.051914819	12.68153329	84.2290364	0.666666667	CRA004775;PRJNA827465;Yang2025_Silkie_Wencha	0.816680119
ENSGALG00010026023	THY1	3	0.113719664	0.146236628	0.777641455	0.436780415	0	1.783395378	0	0.666666667	CRA004775;PRJNA827465;Yang2025_Silkie_Wencha	0.816680119
ENSGALG00010023655	C1R	3	-0.113651461	0.146001016	-0.778429246	0.436316003	0.039219417	6.135347854	67.4020113	0.666666667	CRA004775;PRJNA827465;Yang2025_Silkie_Wencha	0.816680119
ENSGALG00010014687	CUL4A	3	0.11190742	0.144058312	0.776820289	0.437264805	0.037710907	5.790056333	65.45802174	0.666666667	CRA004775;PRJNA827465;Yang2025_Silkie_Wencha	0.816680119
ENSGALG00010007467	CHODL	3	-0.102587228	0.132342227	-0.775166252	0.438241427	0	0.110325761	0	0.666666667	CRA004775;PRJNA827465;Yang2025_Silkie_Wencha	0.816680119
ENSGALG00010012582	CD24	3	-0.102144268	0.131439414	-0.777120535	0.437087659	0	0.509537104	0	0.666666667	CRA004775;PRJNA827465;Yang2025_Silkie_Wencha	0.816680119
ENSGALG00010005427	ENSGALG0001000542	3	0.098576212	0.126399097	0.779880668	0.435461118	0.019231615	3.266715089	38.77641773	0.666666667	CRA004775;PRJNA827465;Yang2025_Silkie_Wencha	0.816680119
ENSGALG00010009494	UBA2	3	-0.094083461	0.121131848	-0.776702926	0.43733406	0.033738368	18.1243298	88.96510921	0.666666667	CRA004775;PRJNA827465;Yang2025_Silkie_Wencha	0.816680119
ENSGALG00010020936	BET1L	3	-0.093009443	0.119451011	-0.778640905	0.436191276	0.027944196	7.082593546	71.76175666	0.666666667	CRA004775;PRJNA827465;Yang2025_Silkie_Wencha	0.816680119
ENSGALG00010018363	TCTE1	3	-0.092027752	0.118484218	-0.776708944	0.437330509	0	1.065590218	0	1	CRA004775;PRJNA827465;Yang2025_Silkie_Wencha	0.816680119
ENSGALG00010017609	TRIM71	3	-0.090926815	0.116739661	-0.778883385	0.436047234	0	0.325379643	0	0.666666667	CRA004775;PRJNA827465;Yang2025_Silkie_Wencha	0.816680119
ENSGALG00010029284	CYTH1	3	0.083030464	0.106994999	0.776021914	0.437736047	0.017960657	4.114851425	51.3955795	0.666666667	CRA004775;PRJNA827465;Yang2025_Silkie_Wencha	0.816680119
ENSGALG00010012758	NAB1	3	-0.07776335	0.100364114	-0.7748123	0.43845058	0.02026366	8.673667937	76.94170431	0.666666667	CRA004775;PRJNA827465;Yang2025_Silkie_Wencha	0.816680119
ENSGALG00010001152	FEM1C	3	0.073627982	0.094931216	0.775592954	0.437989362	0	0.84354846	0	1	CRA004775;PRJNA827465;Yang2025_Silkie_Wencha	0.816680119
ENSGALG00010017530	TLR1B	3	-0.07046545	0.090882852	-0.77534373	0.438136575	0	1.383483482	0	1	CRA004775;PRJNA827465;Yang2025_Silkie_Wencha	0.816680119
ENSGALG00010011972	IPO11	3	0.070336415	0.090316807	0.778774373	0.436112637	0	0.158547232	0	1	CRA004775;PRJNA827465;Yang2025_Silkie_Wencha	0.816680119
ENSGALG00010008065	CHMP5	3	0.06453383	0.083251222	0.775169762	0.438239353	0.010278705	3.77878052	47.07287207	0.666666667	CRA004775;PRJNA827465;Yang2025_Silkie_Wencha	0.816680119
ENSGALG00010029480	ENSGALG0001002948	3	-0.060417609	0.077797699	-0.776598918	0.43739544	0	0.019895394	0	1	CRA004775;PRJNA827465;Yang2025_Silkie_Wencha	0.816680119
ENSGALG00010010303	ETF1	3	-0.057278848	0.073868571	-0.775415675	0.438094076	0.008253717	4.401612669	54.56210825	0.666666667	CRA004775;PRJNA827465;Yang2025_Silkie_Wencha	0.816680119
ENSGALG00010026106	TESK2	3	-0.054896269	0.070665586	-0.776845878	0.437249706	0	1.979965759	0	0.666666667	CRA004775;PRJNA827465;Yang2025_Silkie_Wencha	0.816680119
ENSGALG00010029941	COX10	3	-0.05293648	0.067829074	-0.780439381	0.435132295	0	0.215634441	0	1	CRA004775;PRJNA827465;Yang2025_Silkie_Wencha	0.816680119
ENSGALG00010005283	ATP5C1	3	-0.052590647	0.067898232	-0.774551048	0.438604993	0	0.516764255	0	0.666666667	CRA004775;PRJNA827465;Yang2025_Silkie_Wencha	0.816680119
ENSGALG00010019059	BRWD3	3	0.051597158	0.066294276	0.778304872	0.436389304	0	1.373925096	0	1	CRA004775;PRJNA827465;Yang2025_Silkie_Wencha	0.816680119
ENSGALG00010008807	BRD1	3	-0.045820556	0.059159795	-0.774521887	0.43862223	0.001577922	2.202010343	9.173905277	0.666666667	CRA004775;PRJNA827465;Yang2025_Silkie_Wencha	0.816680119
ENSGALG00010016504	SNRK	3	-0.045402868	0.058320691	-0.778503604	0.436272183	0	1.88530772	0	0.666666667	CRA004775;PRJNA827465;Yang2025_Silkie_Wencha	0.816680119
ENSGALG00010009323	RNF150	3	-0.044073696	0.05674302	-0.776724548	0.437321301	0	1.332088079	0	0.666666667	CRA004775;PRJNA827465;Yang2025_Silkie_Wencha	0.816680119
ENSGALG00010024398	PHB	3	-0.043558782	0.055976978	-0.778155286	0.436477474	0	0.04959736	0	1	CRA004775;PRJNA827465;Yang2025_Silkie_Wencha	0.816680119
ENSGALG00010018008	ABCF3	3	-0.040680307	0.052506281	-0.774770291	0.438475407	0	1.210322146	0	0.666666667	CRA004775;PRJNA827465;Yang2025_Silkie_Wencha	0.816680119
ENSGALG00010029774	RFLNB	3	-0.039281234	0.050520389	-0.777532289	0.436844792	0	0.031372499	0	1	CRA004775;PRJNA827465;Yang2025_Silkie_Wencha	0.816680119
ENSGALG00010028391	MED26	3	-0.037110104	0.047824311	-0.775967352	0.437768263	0	0.440369224	0	0.666666667	CRA004775;PRJNA827465;Yang2025_Silkie_Wencha	0.816680119
ENSGALG00010015348	CPSF2	3	0.031235897	0.04023556	0.776325649	0.437556732	0	0.464885327	0	0.666666667	CRA004775;PRJNA827465;Yang2025_Silkie_Wencha	0.816680119
ENSGALG00010008375	ENSGALG0001000837	3	0.030029425	0.038579612	0.77837552	0.436347666	0	0.119204359	0	1	CRA004775;PRJNA827465;Yang2025_Silkie_Wencha	0.816680119
ENSGALG00010023063	ENSGALG0001002306	3	-0.028272616	0.036489932	-0.774805946	0.438454335	0	0.214563288	0	1	CRA004775;PRJNA827465;Yang2025_Silkie_Wencha	0.816680119
ENSGALG00010013051	XPOT	3	-0.155323289	0.20070817	-0.773876262	0.439003969	0.108642353	24.34330209	91.78418773	0.666666667	CRA004775;PRJNA827465;Yang2025_Silkie_Wencha	0.816691481

ENSGALG00010022976	MINDY2	3	-0.14787636	0.192132967	-0.769656362	0.441503762	0.090907968	37.26930897	94.63365419	0.666666667	CRA004775;PRJNA827465;Yang2025_Silkie_Wencha	0.817457567
ENSGALG00010016466	TMEM164	3	0.146490262	0.190834124	0.767631381	0.442706214	0.062646521	5.417185759	63.08046116	0.666666667	CRA004775;PRJNA827465;Yang2025_Silkie_Wencha	0.817457567
ENSGALG00010020725	FOPNL	3	-0.142934163	0.186013149	-0.76840892	0.442244282	0.057179412	4.349600469	54.0187653	0.666666667	CRA004775;PRJNA827465;Yang2025_Silkie_Wencha	0.817457567
ENSGALG00010017659	TNRC6A	3	0.138345645	0.180040062	0.768415892	0.442240142	0.068579753	7.28892761	72.56112138	0.666666667	CRA004775;PRJNA827465;Yang2025_Silkie_Wencha	0.817457567
ENSGALG00010020666	TMCO4	3	-0.12669157	0.164692012	-0.769263601	0.44173684	0.035450412	3.499120839	42.84278561	0.666666667	CRA004775;PRJNA827465;Yang2025_Silkie_Wencha	0.817457567
ENSGALG00010009225	HNRNPL	3	0.125912126	0.163398462	0.7705833	0.440953963	0.063564612	15.50223335	87.09863311	0.666666667	CRA004775;PRJNA827465;Yang2025_Silkie_Wencha	0.817457567
ENSGALG00010023833	PIP4K2C	3	0.12048274	0.157037658	0.767221963	0.442949557	0.058664987	10.57675497	81.09060855	0.666666667	CRA004775;PRJNA827465;Yang2025_Silkie_Wencha	0.817457567
ENSGALG00010013404	ENSGALG0001001340	3	-0.118952859	0.154872792	-0.768068152	0.442446697	0.010669008	2.286450351	12.52816842	0.666666667	CRA004775;PRJNA827465;Yang2025_Silkie_Wencha	0.817457567
ENSGALG00010012110	ENSGALG0001001211	3	0.097778251	0.12686748	0.7707117	0.440877835	0.022105821	3.588059066	44.25955752	0.666666667	CRA004775;PRJNA827465;Yang2025_Silkie_Wencha	0.817457567
ENSGALG00010021238	NDUFS8	3	-0.095435248	0.12413847	-0.768780602	0.442023566	0.017655784	2.833127883	29.40664586	0.666666667	CRA004775;PRJNA827465;Yang2025_Silkie_Wencha	0.817457567
ENSGALG00010027118	ABHD6	3	0.075937414	0.098914019	0.767711344	0.442658695	0.004311325	2.333984188	14.30961658	0.666666667	CRA004775;PRJNA827465;Yang2025_Silkie_Wencha	0.817457567
ENSGALG00010027162	SLC4A2	3	0.067233629	0.087241799	0.770658449	0.440909406	0.006525448	2.742692806	27.0789643	0.666666667	CRA004775;PRJNA827465;Yang2025_Silkie_Wencha	0.817457567
ENSGALG00010017089	CRTAP	3	0.066076023	0.085772789	0.77036113	0.441085704	0.007374317	2.978020749	32.84130069	0.666666667	CRA004775;PRJNA827465;Yang2025_Silkie_Wencha	0.817457567
ENSGALG00010001291	ENSGALG0001000129	3	0.060641106	0.078873846	0.768836679	0.441990272	0	0.969421113	0	1	CRA004775;PRJNA827465;Yang2025_Silkie_Wencha	0.817457567
ENSGALG00010019655	RTRAF	3	-0.05953081	0.077213775	-0.770986914	0.440714688	0.005576272	2.52233405	20.70836138	0.666666667	CRA004775;PRJNA827465;Yang2025_Silkie_Wencha	0.817457567
ENSGALG00010021621	ZNF518A	3	-0.056359931	0.073361858	-0.768245686	0.442341236	0	0.537363583	0	0.666666667	CRA004775;PRJNA827465;Yang2025_Silkie_Wencha	0.817457567
ENSGALG00010007803	ME3	3	-0.055647194	0.072201849	-0.770717028	0.440874677	0	0.852853528	0	1	CRA004775;PRJNA827465;Yang2025_Silkie_Wencha	0.817457567
ENSGALG00010002978	DYNLT3	3	0.047623957	0.061964715	0.768565737	0.442151152	0	0.716974711	0	1	CRA004775;PRJNA827465;Yang2025_Silkie_Wencha	0.817457567
ENSGALG00010028502	PLEKHO1	3	-0.046561303	0.060502494	-0.769576579	0.441551103	0	1.134857221	0	0.666666667	CRA004775;PRJNA827465;Yang2025_Silkie_Wencha	0.817457567
ENSGALG00010013897	AMOT	3	0.042314949	0.055143259	0.767363956	0.442865152	0	0.077688306	0	1	CRA004775;PRJNA827465;Yang2025_Silkie_Wencha	0.817457567
ENSGALG00010027202	CHAF1A	3	0.034685306	0.045144476	0.768317827	0.442298386	0	0.213169091	0	0.666666667	CRA004775;PRJNA827465;Yang2025_Silkie_Wencha	0.817457567
ENSGALG00010001988	ENSGALG0001000198	2	0.49162757	0.641847816	0.765956599	0.443702127	0.615307705	3.941283853	74.62755698	0.5	PRJNA827465;Yang2025_Silkie_Wenchang	0.817457997
ENSGALG00010000735	ENSGALG0001000073	3	-0.39677345	0.517817838	-0.766241371	0.443532696	0.262348834	2.916396385	31.42221646	0.666666667	CRA004775;PRJNA827465;Yang2025_Silkie_Wencha	0.817457997
ENSGALG00010004108	PERPB	3	0.316758354	0.413259104	0.766488509	0.443385687	0.409884116	10.95757844	81.74779207	0.666666667	CRA004775;PRJNA827465;Yang2025_Silkie_Wencha	0.817457997
ENSGALG00010027926	CKS1B	3	-0.236438767	0.308646041	-0.766051512	0.443645652	0.175616954	5.380302909	62.82737173	0.666666667	CRA004775;PRJNA827465;Yang2025_Silkie_Wencha	0.817457997
ENSGALG00010029367	DUSP14	3	-0.204560114	0.267081692	-0.765908411	0.443730801	0.189196749	29.43381787	93.20509487	0.666666667	CRA004775;PRJNA827465;Yang2025_Silkie_Wencha	0.817457997
ENSGALG00010003261	PEX2	3	-0.192449079	0.251088904	-0.766457917	0.443403882	0.167289322	37.31553652	94.64030217	0.666666667	CRA004775;PRJNA827465;Yang2025_Silkie_Wencha	0.817457997
ENSGALG00010016307	RAD50	3	0.180157529	0.235185102	0.766024408	0.443661779	0.128879577	11.63929179	82.81682394	0.666666667	CRA004775;PRJNA827465;Yang2025_Silkie_Wencha	0.817457997
ENSGALG00010002838	ENSGALG0001000283	3	0.154804097	0.201824374	0.767023792	0.44306737	0.056003514	3.657478233	45.31751463	0.666666667	CRA004775;PRJNA827465;Yang2025_Silkie_Wencha	0.817457997
ENSGALG00010023019	CABP1	3	-0.145807005	0.19025993	-0.766356872	0.443463987	0	1.689535416	0	0.666666667	CRA004775;PRJNA827465;Yang2025_Silkie_Wencha	0.817457997
ENSGALG00010012676	ENSGALG0001001267	3	0.095920225	0.12519332	0.766176862	0.443571074	0	1.784823669	0	0.666666667	CRA004775;PRJNA827465;Yang2025_Silkie_Wencha	0.817457997
ENSGALG00010020311	SGPL1	3	-0.087358046	0.114059444	-0.765899282	0.443736233	0.020831146	4.703061258	57.47450671	0.666666667	CRA004775;PRJNA827465;Yang2025_Silkie_Wencha	0.817457997
ENSGALG00010002678	CCNI	3	-0.054616275	0.071230303	-0.766756184	0.443226492	0	1.401472135	0	0.666666667	CRA004775;PRJNA827465;Yang2025_Silkie_Wencha	0.817457997
ENSGALG00010028015	DUSP7	3	-0.048553076	0.063393587	-0.765898863	0.443736482	0	0.729385175	0	1	CRA004775;PRJNA827465;Yang2025_Silkie_Wencha	0.817457997
ENSGALG00010023108	TET1	3	0.175485973	0.229420141	0.764910929	0.444324586	0.105642663	6.412065182	68.80880117	0.666666667	CRA004775;PRJNA827465;Yang2025_Silkie_Wencha	0.817970351
ENSGALG00010008093	EFR3A	3	-0.143259971	0.187312208	-0.76481919	0.444379219	0.088091094	24.10243982	91.70208487	0.666666667	CRA004775;PRJNA827465;Yang2025_Silkie_Wencha	0.817970351
ENSGALG00010023658	ENSGALG0001002365	2	-0.137479074	0.179756461	-0.764807414	0.444386232	0	0.031126429	0	1	PRJNA827465;Yang2025_Silkie_Wenchang	0.817970351
ENSGALG00010007203	SLC30A6	3	-0.113339511	0.148186164	-0.764845432	0.444363591	0.049770491	10.43045251	80.82537648	0.666666667	CRA004775;PRJNA827465;Yang2025_Silkie_Wencha	0.817970351
ENSGALG00010019336	DEXI	3	0.105680925	0.138189899	0.764751446	0.444419565	0	1.756265358	0	0.666666667	CRA004775;PRJNA827465;Yang2025_Silkie_Wencha	0.817970351
ENSGALG00010021869	GJC3	3	-0.044188136	0.057767864	-0.764925905	0.444315667	0	0.014183157	0	1	CRA004775;PRJNA827465;Yang2025_Silkie_Wencha	0.817970351
ENSGALG00010029326	NCBP3	3	-0.02948503	0.038525785	-0.76533235	0.444073665	0	0.581782516	0	1	CRA004775;PRJNA827465;Yang2025_Silkie_Wencha	0.817970351
ENSGALG00010021713	RAB27A	3	0.122787043	0.160631898	0.764400125	0.444628834	0.064923088	14.03281735	85.74769449	0.666666667	CRA004775;PRJNA827465;Yang2025_Silkie_Wencha	0.818036056
ENSGALG00010022041	MAPK8IP1	3	-0.066552199	0.087061264	-0.764429508	0.44461133	0	0.54060478	0	0.666666667	CRA004775;PRJNA827465;Yang2025_Silkie_Wencha	0.818036056
ENSGALG00010010404	COP1	3	0.061931406	0.081010311	0.764487937	0.444576523	0.009468157	3.790030826	47.22998065	0.666666667	CRA004775;PRJNA827465;Yang2025_Silkie_Wencha	0.818036056
ENSGALG00010002473	ENSGALG0001000247	3	0.359172305	0.470180774	0.763902577	0.444925302	0.032038648	2.094456565	4.509836417	0.666666667	CRA004775;PRJNA827465;Yang2025_Silkie_Wencha	0.818155656
ENSGALG00010017903	ENSGALG0001001790	2	0.24267344	0.317652414	0.76395906	0.44489164	0	0.249361293	0	1	PRJNA827465;Yang2025_Silkie_Wenchang	0.818155656
ENSGALG00010021678	DNAAF4	3	0.218914075	0.286563224	0.763929413	0.444909308	0.172323389	7.833241431	74.46778556	0.666666667	CRA004775;PRJNA827465;Yang2025_Silkie_Wencha	0.818155656
ENSGALG00010019271	CDKL1	3	0.207078293	0.270993731	0.764144217	0.444781305	0.149676118	6.796654556	70.57375826	0.666666667	CRA004775;PRJNA827465;Yang2025_Silkie_Wencha	0.818155656
ENSGALG00010017219	EARS2	3	-0.125705171	0.164595931	-0.763719797	0.445034241	0.051646585	6.662152345	69.97967179	0.666666667	CRA004775;PRJNA827465;Yang2025_Silkie_Wencha	0.818249562
ENSGALG00010006261	PCLO	2	0.760720888	0.99932528	0.761234508	0.44651701	1.797925719	9.794610277	89.79030332	0.5	PRJNA827465;Yang2025_Silkie_Wenchang	0.818313724
ENSGALG00010011954	ENSGALG0001001195	2	0.547803875	0.717585767	0.763398468	0.445225794	0.906229789	8.311981873	87.96917492	0.5	CRA004775;Yang2025_Silkie_Wenchang	0.818313724
ENSGALG00010006251	SEMA3E	3	0.340987094	0.448061114	0.761028089	0.44664029	0.372697363	5.953509771	66.40637075	0.666666667	CRA004775;PRJNA827465;Yang2025_Silkie_Wencha	0.818313724
ENSGALG00010017937	PTPN21	3	0.283528538	0.371765413	0.762654426	0.445669516	0.335357585	13.61301144	85.30817366	0.666666667	CRA004775;PRJNA827465;Yang2025_Silkie_Wencha	0.818313724
ENSGALG00010026755	ENSGALG0001002675	3	-0.250163193	0.328291627	-0.76201515	0.446050962	0.189649046	4.844575645	58.71671439	0.666666667	CRA004775;PRJNA827465;Yang2025_Silkie_Wencha	0.818313724
ENSGALG00010028915	ADGRD2	2	0.22903596	0.300962275	0.761012187	0.446649788	0	0.013024281	0	1	CRA004775;PRJNA827465	0.818313724
ENSGALG00010019157	ENSGALG0001001915	3	0.205383292	0.269706795	0.761505812	0.446355009	0	1.82389381	0	0.666666667	CRA004775;PRJNA827465;Yang2025_Silkie_Wencha	0.818313724
ENSGALG00010002355	UFM1	3	-0.203103006	0.266810305	-0.761226241	0.446521948	0.18822811	20.87761498	90.4203617	0.666666667	CRA004775;PRJNA827465;Yang2025_Silkie_Wencha	0.818313724
ENSGALG00010020507	ANKRD1	3	0.199004529	0.261335951	0.761489296	0.44636487	0	1.09465552	0	1	CRA004775;PRJNA827465;Yang2025_Silkie_Wencha	0.818313724
ENSGALG00010011099	CCBE1	3	0.177706881	0.233267581	0.761815595	0.446170071	0.10198155	6.126924999	67.35719794	0.666666667	CRA004775;PRJNA827465;Yang2025_Silkie_Wencha	0.818313724
ENSGALG00010025800	ARL2BP	3	-0.175122738	0.230075887	-0.761152073	0.446566241	0.109028768	6.494405418	69.20426319	0.666666667	CRA004775;PRJNA827465;Yang2025_Silkie_Wencha	0.818313724
ENSGALG00010026128	PRDX1	3	-0.157336832	0.206259918	-0.762808565	0.445577572	0.100753855	10.89063097	81.63559113	0.666666667	CRA004775;PRJNA827465;Yang2025_Silkie_Wencha	0.818313724
ENSGALG00010025612	TMEM53	3	-0.143617039	0.188456674	-0.762069264	0.446018666	0.069822602	6.085232842	67.13355016	0.666666667	CRA004775;PRJNA827465;Yang2025_Silkie_Wencha	0.818313724
ENSGALG00010007441	STRN	3	0.141238928	0.185326642	0.762108063	0.445995511	0.071660398	7.005516996	71.45107205	0.666666667	CRA004775;PRJNA827465;Yang2025_Silkie_Wencha	0.818313724
ENSGALG00010002923	SERPINH1	3	-0.127406708	0.166973806	-0.763034102	0.445443058	0.054730324	8.361661367	76.08130834	0.666666667	CRA004775;PRJNA827465;Yang2025_Silkie_Wencha	0.818313724
ENSGALG00010028193	BRINP1	3	-0.106792453	0.140174549	-0.761853373	0.446147521	0	0.846158389	0	0.666666667	CRA004775;PRJNA827465;Yang2025_Silkie_Wencha	0.818313724
ENSGALG00010020460	MAPK8	3	0.101668141	0.133174068	0.763422962	0.44521119	0.038217128	9.184866034	78.22504985	0.666666667	CRA004775;PRJNA827465;Yang2025_Silkie_Wencha	0.818313724
ENSGALG00010027145	CELSR3	3	0.100449531	0.131628915	0.763126632	0.445387879	0.023399161	3.557209866	43.77615954	0.666666667	CRA004775;PRJNA827465;Yang2025_Silkie_Wencha	0.818313724
ENSGALG00010009232	ECH1	3	-0.090502222	0.118731606	-0.762242045	0.445915556	0	0.342514113	0	0.666666667	CRA004775;PRJNA827465;Yang2025_Silkie_Wencha	0.818313724
ENSGALG00010001499	TIPARP	3	-0.09036243	0.118434117	-0.762976349	0.445477501	0.01734757	3.51116952	43.03892226	0.666666667	CRA004775;PRJNA827465;Yang2025_Silkie_Wencha	0.818313724
ENSGALG00010024581	RARA	3	0.081019603	0.106272011	0.762379508	0.445833533	0.01871705	4.907976317	59.25000712	0.666666667	CRA004775;PRJNA827465;Yang2025_Silkie_Wencha	0.818313724
ENSGALG00010016411	CTSH	3	0.074461418	0.097567483	0.763178632	0.445356871	0	0.240455556	0	1	CRA004775;PRJNA827465;Yang2025_Silkie_Wencha	0.818313724
ENSGALG00010023124	NSUN4	3	0.065728555	0.08626184	0.761965606	0.446080532	0	1.851405768	0	0.666666667	CRA004775;PRJNA827465;Yang2025_Silkie_Wencha	0.818313724
ENSGALG00010029650	ACSF2	3	0.058717166	0.077072217	0.761846079	0.446151875	0	0.016312896	0	1	CRA004775;PRJNA827465;Yang2025_Silkie_Wencha	0.818313724
ENSGALG00010007521	SLC12A7	3	-0.052662371	0.069177453	-0.761264948	0.446498832	0	1.675592054	0	0.666666667	CRA004775;PRJNA827465;Yang2025_Silkie_Wencha	0.818313724
ENSGALG00010020847	MIDEAS	3	-0.052056454	0.068341641	-0.76170916	0.446233607	0	1.502031513	0	0.666666667	CRA004775;PRJNA827465;Yang2025_Silkie_Wencha	0.818313724
ENSGALG00010027359	ENSGALG0001002735	3	-0.050815057	0.066778848	-0.760945407	0.446689676	0	0.951892318	0	0.666666667	CRA004775;PRJNA827465;Yang2025_Silkie_Wencha	0.818313724
ENSGALG00010023405	DNAJA2	3	-0.030890301	0.040478211	-0.763134066	0.445383445	0	0.200085697	0	1	CRA004775;PRJNA827465;Yang2025_Silkie_Wencha	0.818313724
ENSGALG00010000177	ENSGALG0001000017	3	-0.102719953	0.135065781	-0.760517967	0.446945034	0.026681548	3.819719281	47.6401313	0.666666667	CRA004775;PRJNA827465;Yang2025_Silkie_Wencha	0.818675455
ENSGALG00010011630	DELE1	3	-0.076519077	0.100633347	-0.760374962	0.447030485	0.012991682	3.478246463	42.49976184	0.666666667	CRA004775;PRJNA827465;Yang2025_Silkie_Wencha	0.818725911
ENSGALG00010018327	SNAP25	2	0.777912209	1.023688157	0.75991131	0.447307601	1.743018775	5.92259142	83.11549913	0.5	PRJNA827465;Yang2025_Silkie_Wenchang	0.81891521
ENSGALG00010007809	ARV1	3	-0.108530798	0.142812753	-0.759951722	0.447283443	0.036222979	5.976132597	66.53354042	0.666666667	CRA004775;PRJNA827465;Yang2025_Silkie_Wencha	0.81891521
ENSGALG00010011419	RNF180	3	0.073440611	0.09663663	0.759966597	0.447274552	0	1.126270666	0	0.666666667	CRA004775;PRJNA827465;Yang2025_Silkie_Wencha	0.81891521
ENSGALG00010028052	ENSGALG0001002805	3	0.256893757	0.338105789	0.759802895	0.447372412	0.26370383	10.26895717	80.52382567	0.666666667	CRA004775;PRJNA827465;Yang2025_Silkie_Wencha	0.818927827
ENSGALG00010027164	ENSGALG0001002716	3	0.622314275	0.820736518	0.758238802	0.448308036	1.74757175	15.89109545	87.41433524	0.666666667	CRA004775;PRJNA827465;Yang2025_Silkie_Wencha	0.81901853
ENSGALG00010020820	VWA5B1	3	0.28422297	0.374373124	0.759197046	0.447734693	0.197726443	3.768060623	46.9222977	0.666666667	CRA004775;PRJNA827465;Yang2025_Silkie_Wencha	0.81901853
ENSGALG00010013103	AIRIM	3	0.232764284	0.306471885	0.759496368	0.447555686	0.244824519	15.87902425	87.40476764	0.666666667	CRA004775;PRJNA827465;Yang2025_Silkie_Wencha	0.81901853
ENSGALG00010017468	AMDHD2	3	-0.223332934	0.294345458	-0.758744284	0.448005541	0.227639883	19.13110042	89.5458183	0.666666667	CRA004775;PRJNA827465;Yang2025_Silkie_Wencha	0.81901853
ENSGALG00010006816	ENSGALG0001000681	3	0.185100531	0.243972854	0.758693142	0.44803614	0	1.165218474	0	0.666666667	CRA004775;PRJNA827465;Yang2025_Silkie_Wencha	0.81901853
ENSGALG00010028170	COPE	3	-0.164970673	0.217556446	-0.758289062	0.448277954	0.112528936	10.32915789	80.63733731	0.666666667	CRA004775;PRJNA827465;Yang2025_Silkie_Wencha	0.81901853

ENSGALG00010008939	RANBP9	3	-0.03874866	0.051429211	-0.753436803	0.451187474	0.001372738	2.261595821	11.56686877	0.666666667	CRA004775;PRJNA827465;Yang2025_Silkie_Wencha	0.821281216
ENSGALG00010001397	KLHL7	3	0.029926961	0.03979565	0.752015897	0.452041499	0	0.002594667	0	1	CRA004775;PRJNA827465;Yang2025_Silkie_Wencha	0.821281216
ENSGALG00010020061	UBA3	3	-0.028009459	0.037242406	-0.752085102	0.451999883	0	0.330646484	0	0.666666667	CRA004775;PRJNA827465;Yang2025_Silkie_Wencha	0.821281216
ENSGALG00010002034	WWC3	3	0.100827512	0.134129234	0.75171914	0.452219978	0.033933917	6.250878065	68.00449506	0.666666667	CRA004775;PRJNA827465;Yang2025_Silkie_Wencha	0.821315127
ENSGALG00010010929	FMC1	3	-0.083384653	0.110943717	-0.75159419	0.452295138	0	0.623862717	0	0.666666667	CRA004775;PRJNA827465;Yang2025_Silkie_Wencha	0.821346129
ENSGALG00010003839	ENSGALG0001000383!	2	0.394439822	0.526394954	0.749322954	0.453662576	0.258440093	1.873093597	46.61238491	0.5	CRA004775;PRJNA827465	0.821388863
ENSGALG00010011213	XKR6	3	0.283538857	0.377864087	0.750372599	0.453030329	0.375638802	16.65400201	87.99087451	0.666666667	CRA004775;PRJNA827465;Yang2025_Silkie_Wencha	0.821388863
ENSGALG00010007821	FAM89A	3	-0.248155871	0.33081439	-0.750136265	0.45317264	0.31066804	54.00309834	96.29650879	0.666666667	CRA004775;PRJNA827465;Yang2025_Silkie_Wencha	0.821388863
ENSGALG00010027170	TLE2	3	0.21489583	0.286420961	0.75027969	0.453086272	0.20654771	16.87030574	88.1448503	0.666666667	CRA004775;PRJNA827465;Yang2025_Silkie_Wencha	0.821388863
ENSGALG00010002113	GDPD4	3	0.211085989	0.280990478	0.751221147	0.452519576	0.119550937	4.161316889	51.93829133	0.666666667	CRA004775;PRJNA827465;Yang2025_Silkie_Wencha	0.821388863
ENSGALG00010011671	SLC41A2	3	-0.205999791	0.274446138	-0.750601894	0.452892281	0.199020968	17.81779704	88.77526781	0.666666667	CRA004775;PRJNA827465;Yang2025_Silkie_Wencha	0.821388863
ENSGALG00010010722	TBC1D19	3	-0.189238891	0.252496045	-0.749472693	0.453572351	0.148906406	14.34700185	86.05980524	0.666666667	CRA004775;PRJNA827465;Yang2025_Silkie_Wencha	0.821388863
ENSGALG00010006821	XRCC4	3	0.142528625	0.189772068	0.751051648	0.452621573	0.047960596	3.595276657	44.37145759	0.666666667	CRA004775;PRJNA827465;Yang2025_Silkie_Wencha	0.821388863
ENSGALG00010022176	ARL2BPL	3	0.140904609	0.187721556	0.750604313	0.452890825	0.027753478	2.697548038	25.8585956	0.666666667	CRA004775;PRJNA827465;Yang2025_Silkie_Wencha	0.821388863
ENSGALG00010007792	ENSGALG0001000779:	3	0.136877116	0.18268809	0.749239405	0.453712923	0.035258013	3.074524924	34.94929949	0.666666667	CRA004775;PRJNA827465;Yang2025_Silkie_Wencha	0.821388863
ENSGALG00010017367	TIMD4	3	-0.132837767	0.177278669	-0.749316135	0.453666685	0.007228756	2.144100901	6.720807811	0.666666667	CRA004775;PRJNA827465;Yang2025_Silkie_Wencha	0.821388863
ENSGALG00010029306	CEP95	3	-0.100521869	0.133927878	-0.750567178	0.45291318	0.034425989	6.620290897	69.78984714	0.666666667	CRA004775;PRJNA827465;Yang2025_Silkie_Wencha	0.821388863
ENSGALG00010015157	CDC42BPB	3	0.09513106	0.126729731	0.750660952	0.452856729	0.031298057	6.304555049	68.2769048	0.666666667	CRA004775;PRJNA827465;Yang2025_Silkie_Wencha	0.821388863
ENSGALG00010029602	MTMR4	3	-0.094945643	0.126647782	-0.749682634	0.453445869	0.036863641	10.56055767	81.06160619	0.666666667	CRA004775;PRJNA827465;Yang2025_Silkie_Wencha	0.821388863
ENSGALG00010024896	RNF41	3	0.077258997	0.103009781	0.750016128	0.453244991	0.015357244	3.7585558	46.78807216	0.666666667	CRA004775;PRJNA827465;Yang2025_Silkie_Wencha	0.821388863
ENSGALG00010026537	FJX1	3	0.071708639	0.095636344	0.749805321	0.453371964	0	1.797705523	0	0.666666667	CRA004775;PRJNA827465;Yang2025_Silkie_Wencha	0.821388863
ENSGALG00010024333	ETV4	3	0.071505721	0.095340459	0.750003953	0.453252324	0.00465786	2.306053983	13.27176143	0.666666667	CRA004775;PRJNA827465;Yang2025_Silkie_Wencha	0.821388863
ENSGALG00010023556	IVD	3	0.064858516	0.086350368	0.751108739	0.452587217	0.006697891	2.754633958	27.39507207	0.666666667	CRA004775;PRJNA827465;Yang2025_Silkie_Wencha	0.821388863
ENSGALG00010028580	PRUNE1	3	0.06275203	0.083552741	0.751046936	0.452624409	0	1.973138365	0	0.666666667	CRA004775;PRJNA827465;Yang2025_Silkie_Wencha	0.821388863
ENSGALG00010013766	DNM3	3	-0.060945662	0.081321624	-0.749439807	0.453592166	0	0.860244052	0	1	CRA004775;PRJNA827465;Yang2025_Silkie_Wencha	0.821388863
ENSGALG00010011267	EVC2	3	0.0570169	0.075987188	0.750348862	0.453044622	0	0.031270696	0	1	CRA004775;PRJNA827465;Yang2025_Silkie_Wencha	0.821388863
ENSGALG00010027236	PEX11G	3	0.054893684	0.073157109	0.750353181	0.4530419	0	1.402466776	0	0.666666667	CRA004775;PRJNA827465;Yang2025_Silkie_Wencha	0.821388863
ENSGALG00010016533	CNIH4	3	-0.053957791	0.071962717	-0.749802027	0.453373948	0	0.939215191	0	1	CRA004775;PRJNA827465;Yang2025_Silkie_Wencha	0.821388863
ENSGALG00010027025	ORAI1	3	-0.053862829	0.071888104	-0.749259285	0.453700942	0	1.517986475	0	0.666666667	CRA004775;PRJNA827465;Yang2025_Silkie_Wencha	0.821388863
ENSGALG00010024870	TPD52L2	3	-0.027685816	0.036959682	-0.749081541	0.45380806	0	1.171739234	0	1	CRA004775;PRJNA827465;Yang2025_Silkie_Wencha	0.821455916
ENSGALG00010009303	TTC1	3	0.03623915	0.048387041	0.748943294	0.453891384	0	0.220934887	0	1	CRA004775;PRJNA827465;Yang2025_Silkie_Wencha	0.821501572
ENSGALG00010018313	MYEF2	3	0.11899425	0.159077208	0.748028277	0.454443102	0.056976719	10.36442206	80.70321732	0.666666667	CRA004775;PRJNA827465;Yang2025_Silkie_Wencha	0.82228961
ENSGALG00010022540	GPR146	3	0.1149962	0.153724122	0.74806867	0.454418738	0.027083446	3.217601964	37.84190767	0.666666667	CRA004775;PRJNA827465;Yang2025_Silkie_Wencha	0.82228961
ENSGALG00010015680	ENSGALG0001001568(	3	0.175580095	0.234801168	0.747782036	0.454591639	0.141110587	21.60118755	90.7413355	0.666666667	CRA004775;PRJNA827465;Yang2025_Silkie_Wencha	0.822453127
ENSGALG00010016813	ENSGALG0001001681:	3	-0.232858426	0.311558818	-0.747407561	0.454817583	0	1.802410991	0	0.666666667	CRA004775;PRJNA827465;Yang2025_Silkie_Wencha	0.822651375
ENSGALG00010008386	SESN3	3	-0.057843757	0.077383552	-0.747494211	0.454765296	0	1.280124124	0	1	CRA004775;PRJNA827465;Yang2025_Silkie_Wencha	0.822651375
ENSGALG00010018283	POF1B	3	0.166040596	0.222242062	0.747115981	0.454993555	0.085501535	5.274562272	62.08216157	0.666666667	CRA004775;PRJNA827465;Yang2025_Silkie_Wencha	0.822759159
ENSGALG00010016576	MRPS26	3	-0.046945381	0.062829074	-0.747192002	0.454947671	0	1.199532357	0	1	CRA004775;PRJNA827465;Yang2025_Silkie_Wencha	0.822759159
ENSGALG00010029297	SH2B2	3	0.167762145	0.22465342	0.746759807	0.455208561	0.075929935	4.15250161	51.8362619	0.666666667	CRA004775;PRJNA827465;Yang2025_Silkie_Wencha	0.822832249
ENSGALG00010026183	SESTD1	3	0.109325066	0.146367026	0.746924143	0.455109352	0.049960734	11.24771651	82.21861301	0.666666667	CRA004775;PRJNA827465;Yang2025_Silkie_Wencha	0.822832249
ENSGALG00010011401	ANKRD46	3	-0.094205216	0.126144419	-0.74680447	0.455181597	0.037881221	16.81456981	88.10555356	0.666666667	CRA004775;PRJNA827465;Yang2025_Silkie_Wencha	0.822832249
ENSGALG00010016319	RBM41	3	-0.065358586	0.08754798	-0.746545901	0.455337715	0.001512183	2.123492845	5.815552681	0.666666667	CRA004775;PRJNA827465;Yang2025_Silkie_Wencha	0.822960494
ENSGALG00010011379	CELF4	2	0.741898283	0.995244603	0.745443161	0.456003865	1.502098084	4.076455536	75.46888489	0.5	PRJNA827465;Yang2025_Silkie_Wenchang	0.823230245
ENSGALG00010015137	AMN	2	0.382595213	0.513433856	0.745169429	0.456169301	0	0.205602313	0	1	CRA004775;PRJNA827465	0.823230245
ENSGALG00010005284	ENSGALG0001000528(	2	0.293704953	0.393867625	0.745694579	0.455851935	0.247810594	4.796596221	79.15188284	0.5	PRJNA827465;Yang2025_Silkie_Wenchang	0.823230245
ENSGALG00010008043	IL17REL	3	0.290981339	0.390215644	0.745693679	0.455852478	0.263693142	4.899385273	59.17855224	0.666666667	CRA004775;PRJNA827465;Yang2025_Silkie_Wencha	0.823230245
ENSGALG00010005223	GRIA4	2	0.214145408	0.287183175	0.745675329	0.455863566	0.050953394	1.377134644	27.38545907	1	PRJNA827465;Yang2025_Silkie_Wenchang	0.823230245
ENSGALG00010001155	CYB5A	3	-0.187065112	0.250929253	-0.745489452	0.455975886	0.164021348	24.26935842	91.75915587	0.666666667	CRA004775;PRJNA827465;Yang2025_Silkie_Wencha	0.823230245
ENSGALG00010012511	OXCT1	3	-0.15562882	0.208822396	-0.745268814	0.45610923	0.107475409	12.47915889	83.97327883	0.666666667	CRA004775;PRJNA827465;Yang2025_Silkie_Wencha	0.823230245
ENSGALG00010012097	GART	3	0.107982276	0.144783363	0.745819639	0.455776374	0.030957879	4.2431731	52.86546288	0.666666667	CRA004775;PRJNA827465;Yang2025_Silkie_Wencha	0.823230245
ENSGALG00010011896	SIK1	3	-0.102996352	0.138143168	-0.745576874	0.455923057	0.029526067	4.661306113	57.09357096	0.666666667	CRA004775;PRJNA827465;Yang2025_Silkie_Wencha	0.823230245
ENSGALG00010009775	KIAA1614	3	0.070348696	0.094409738	0.745142375	0.456185654	0	1.461453149	0	0.666666667	CRA004775;PRJNA827465;Yang2025_Silkie_Wencha	0.823230245
ENSGALG00010007184	VGLL3	3	-0.049161606	0.065900529	-0.745997137	0.455669144	0	1.425609301	0	0.666666667	CRA004775;PRJNA827465;Yang2025_Silkie_Wencha	0.823230245
ENSGALG00010016793	SLC9A1	3	0.047049659	0.063119957	0.745400683	0.456029531	0	0.886619235	0	0.666666667	CRA004775;PRJNA827465;Yang2025_Silkie_Wencha	0.823230245
ENSGALG00010027987	TMEM141	3	0.17713968	0.237771871	0.744997158	0.456273438	0.119861273	7.520435943	73.40579702	0.666666667	CRA004775;PRJNA827465;Yang2025_Silkie_Wencha	0.823283581
ENSGALG00010024859	TARSL2	3	-0.04003303	0.053746331	-0.744851399	0.456361559	0	1.601089435	0	1	CRA004775;PRJNA827465;Yang2025_Silkie_Wencha	0.823337512
ENSGALG00010027173	ENSGALG0001002717:	3	0.119441327	0.160385847	0.744712384	0.456445611	0.056070997	8.875787034	77.46678697	0.666666667	CRA004775;PRJNA827465;Yang2025_Silkie_Wencha	0.823384089
ENSGALG00010008297	ENSGALG0001000829:	3	0.249725937	0.335598585	0.744120948	0.456803307	0.259816623	9.042713327	77.88274462	0.666666667	CRA004775;PRJNA827465;Yang2025_Silkie_Wencha	0.823504011
ENSGALG00010015795	PSMD10	3	-0.151182353	0.203079293	-0.744449868	0.45660436	0.102520831	12.7141407	84.2694835	0.666666667	CRA004775;PRJNA827465;Yang2025_Silkie_Wencha	0.823504011
ENSGALG00010018000	MAP2K1	3	-0.110381082	0.148337362	-0.744121917	0.456802721	0.047529429	7.857866916	74.54780004	0.666666667	CRA004775;PRJNA827465;Yang2025_Silkie_Wencha	0.823504011
ENSGALG00010028480	F11R	3	-0.109040735	0.146479856	-0.744407718	0.456629851	0.032940274	4.093989362	51.14789456	0.666666667	CRA004775;PRJNA827465;Yang2025_Silkie_Wencha	0.823504011
ENSGALG00010025233	WDR12	3	-0.077591777	0.104266224	-0.74416982	0.456773744	0.017890995	4.455883996	55.11552811	0.666666667	CRA004775;PRJNA827465;Yang2025_Silkie_Wencha	0.823504011
ENSGALG00010004765	ENSGALG0001000476!	3	-0.321201482	0.432229011	-0.743126627	0.457402871	0.449909848	14.13638184	85.84209302	0.666666667	CRA004775;PRJNA827465;Yang2025_Silkie_Wencha	0.823548325
ENSGALG00010024023	LHX8	2	-0.25991585	0.349640872	-0.743379482	0.457251963	0.08901367	1.537222267	34.94759857	0.5	PRJNA827465;Yang2025_Silkie_Wenchang	0.823548325
ENSGALG00010029438	ENSGALG0001002943!	3	-0.196777495	0.264640187	-0.743563659	0.457140495	0.102471922	3.693386246	45.84915124	0.666666667	CRA004775;PRJNA827465;Yang2025_Silkie_Wencha	0.823548325
ENSGALG00010008837	ENSGALG0001000883:	3	0.153153209	0.206075792	0.743188741	0.457367419	0.096634422	10.96815905	81.76539937	0.666666667	CRA004775;PRJNA827465;Yang2025_Silkie_Wencha	0.823548325
ENSGALG00010015571	DUSP15	3	0.138948553	0.186763474	0.743981411	0.456887721	0.036239307	3.01553822	33.67684792	0.666666667	CRA004775;PRJNA827465;Yang2025_Silkie_Wencha	0.823548325
ENSGALG00010024649	NCOR2	3	0.130380566	0.175373798	0.743443821	0.457213022	0.058828	5.723258162	65.05487009	0.666666667	CRA004775;PRJNA827465;Yang2025_Silkie_Wencha	0.823548325
ENSGALG00010022891	NTM	3	-0.117144762	0.157628045	-0.74317208	0.457377505	0.027356962	3.15629996	36.63466638	0.666666667	CRA004775;PRJNA827465;Yang2025_Silkie_Wencha	0.823548325
ENSGALG00010011003	FECH	3	-0.102642138	0.138075567	-0.743376545	0.45725374	0.031007983	4.699681529	57.44392492	0.666666667	CRA004775;PRJNA827465;Yang2025_Silkie_Wencha	0.823548325
ENSGALG00010017343	DLG1	3	0.091344588	0.122952571	0.742925399	0.457526847	0.031784903	8.344592572	76.03238285	0.666666667	CRA004775;PRJNA827465;Yang2025_Silkie_Wencha	0.823548325
ENSGALG00010017795	MACF1	3	0.07687215	0.103461817	0.743000195	0.457481563	0.012634088	2.98465339	32.990544	0.666666667	CRA004775;PRJNA827465;Yang2025_Silkie_Wencha	0.823548325
ENSGALG00010011166	SOX7	3	-0.070633416	0.094954732	-0.743864093	0.456958701	0.010270053	3.234298658	38.16279164	0.666666667	CRA004775;PRJNA827465;Yang2025_Silkie_Wencha	0.823548325
ENSGALG00010012786	TEF	3	-0.043509218	0.058563632	-0.742939202	0.45751849	0	0.592806269	0	0.666666667	CRA004775;PRJNA827465;Yang2025_Silkie_Wencha	0.823548325
ENSGALG00010027816	ZMYND19	3	-0.045228982	0.060890099	-0.742796978	0.457604605	0	0.417463965	0	1	CRA004775;PRJNA827465;Yang2025_Silkie_Wencha	0.823583441
ENSGALG00010018532	MFSD13A	3	-0.109873342	0.147978366	-0.742495985	0.457786883	0.043343895	6.952420924	71.2330421	0.666666667	CRA004775;PRJNA827465;Yang2025_Silkie_Wencha	0.823806637
ENSGALG00010025303	TMCC2	3	-0.082607654	0.111274227	-0.742379041	0.457857714	0.016809	3.804289667	47.42776773	0.666666667	CRA004775;PRJNA827465;Yang2025_Silkie_Wencha	0.823829247
ENSGALG00010014453	NR2E1	3	-0.190601799	0.256849951	-0.742074499	0.458042199	0.127794752	6.011849209	66.73236586	0.666666667	CRA004775;PRJNA827465;Yang2025_Silkie_Wencha	0.824056325
ENSGALG00010001181	MFSD1	3	-0.074732681	0.100753261	-0.741737592	0.458246339	0.021923241	8.353376237	76.05758506	0.666666667	CRA004775;PRJNA827465;Yang2025_Silkie_Wencha	0.824213838
ENSGALG00010028270	RAPGEF1	3	-0.062869795	0.084759663	-0.741741918	0.458243717	0.004327427	2.350946931	14.92789678	0.666666667	CRA004775;PRJNA827465;Yang2025_Silkie_Wencha	0.824213838
ENSGALG00010021130	ENSGALG0001002113(	3	-0.996695095	1.348681351	-0.739014516	0.459898183	4.543938491	12.90076832	84.49704738	0.666666667	CRA004775;PRJNA827465;Yang2025_Silkie_Wencha	0.824249006
ENSGALG00010012474	ASB18	2	-0.389490879	0.526026753	-0.740439297	0.459033482	0.325663664	2.307359853	56.660423	0.5	PRJNA827465;Yang2025_Silkie_Wenchang	0.824249006
ENSGALG00010015285	ALG13	3	0.320287282	0.433368234	0.739064973	0.459867545	0.354610526	6.080746454	67.10930122	0.666666667	CRA004775;PRJNA827465;Yang2025_Silkie_Wencha	0.824249006
ENSGALG00010023206	HSPB9	3	0.28108254	0.380101437	0.739493494	0.459607389	0.283660783	6.70197334	70.15804303	0.666666667	CRA004775;PRJNA827465;Yang2025_Silkie_Wencha	0.824249006
ENSGALG00010021516	SRSF5	3	0.268581022	0.36243128	0.741053649	0.458660912	0.350382701	45.08715745	95.56414706	0.666666667	CRA004775;PRJNA827465;Yang2025_Silkie_Wencha	0.824249006
ENSGALG00010027333	PROK1	3	0.246889423	0.334034133	0.739114355	0.459837561	0.123045675	3.112429431	35.74151498	0.666666667	CRA004775;PRJNA827465;Yang2025_Silkie_Wencha	0.824249006
ENSGALG00010021506	MAX	3	0.220404137	0.297448031	0.740983677	0.458703337	0.232127172	17.70250274	88.70216246	0.666666667	CRA004775;PRJNA827465;Yang2025_Silkie_Wencha	0.824249006
ENSGALG00010026113	MFRP	3	-0.170580774	0.230646098	-0.739577976	0.45955611	0	1.743934384	0	0.666666667	CRA004775;PRJNA827465;Yang2025_Silkie_Wencha	0.824249006
ENSGALG00010002072	NEURL4	3	0.152485934	0.206128809	0.739760417	0.459445381	0.111030208	21.07358595	90.50944626	0.666666667	CRA004775;PRJNA827465;Yang2025_Silkie_Wencha	0.824249006
ENSGALG00010019108	KDELR2	3	-0.137047189	0.185263119	-0.739743506	0.459455644	0.084447708	15.70766934	87.2673663	0.666666667	CRA004775;PRJNA827465;Yang2025_Silkie_Wencha	0.824249006
ENSGALG00010007369	ENSGALG0001000736!	3	0.132355505	0.178882228	0.739903041	0.459358829	0	0.960877052	0	0.666666667	CRA004775;PRJNA827465;Yang2025_Silkie_Wencha	0.824249006
ENSGALG00010016692	PRKCI	3	-0.13011601	0.175746292	-0.740362761	0.459079908	0.08186172	22.25385748	91.01279407	0.666666667	CRA004775;PRJNA827465;Yang2025_Silkie_Wencha	0.824249006
ENSGALG00010017133	WDR20	3	0.125560159	0.169695672	0.739913737	0.459352339	0.064658464	8.798733207	77.26945513	0.666666667	CRA004775;PRJNA827465;Yang2025_Silkie_Wencha	0.824249006
ENSGALG00010027327	RBM6	3	0.119251077	0.161131052	0.740087497	0.459246905	0.057619769	8.363361205	76.08616977	0.666666667	CRA004775;PRJNA827465;Yang2025_Silkie_Wencha	0.824249006
ENSGALG00010006041	QKI	3	0.101056269	0.136326815	0.741279467	0.458524009	0.035711783	5.598038953	64.27320323	0.666666667	CRA004775;PRJNA827465;Yang2025_Silkie_Wencha	0.824249006
ENSGALG00010008361	MATR3	3	0.09539912	0.128761941	0.740895325	0.45875691	0.037916224	9.394896644	78.7118467	0.666666667	CRA004775;PRJNA827465;Yang2025_Silkie_Wencha	0.824249006
ENSGALG00010018052	PPP1RL	3	-0.091471225	0.12348092	-0.740772133	0.458831615	0.025533088	4.589556516	56.42280483	0.666666667	CRA004775;PRJNA827465;Yang2025_Silkie_Wencha	0.824249006
ENSGALG00010024698	ENSGALG0001002469!	3	-0.081535133	0.110095586	-0.740584941	0.458945141	0	0.989273582	0	0.666666667	CRA004775;PRJNA827465;Yang2025_Silkie_Wencha	0.824249006
ENSGALG00010027993	CLIC3	3	-0.072853188	0.098552992	-0.739228579	0.459768209	0	1.206339429	0	0.666666667	CRA004775;PRJNA827465;Yang2025_Silkie_Wencha	0.824249006
ENSGALG00010002401	ZRANB1	3	-0.059079131	0.079890006	-0.739505901	0.459599858	0.007464103	3.187992007	37.2645855	0.666666667	CRA004775;PRJNA827465;Yang2025_Silkie_Wencha	0.824249006
ENSGALG00010016529	DCUN1D1	3	-0.057249952	0.077368748	-0.739962242	0.459322906	0.009792706	4.565800871	56.19607476	0.666666667	CRA004775;PRJNA827465;Yang2025_Silkie_Wencha	0.824249006
ENSGALG00010023620	IRS1	3	-0.05570032	0.075174535	-0.740946646	0.458725791	0.004127099	2.57047543	22.19338196	0.666666667	CRA004775;PRJNA827465;Yang2025_Silkie_Wencha	0.824249006
ENSGALG00010010928	HECW2	3	0.053729962	0.072675454	0.739313735	0.459716511	0	1.546233234	0	0.666666667	CRA004775;PRJNA827465;Yang2025_Silkie_Wencha	0.824249006
ENSGALG00010005543	SEC24D	3	0.05179713	0.069903402	0.740981528	0.458704641	0	0.916675544	0	0.666666667	CRA004775;PRJNA827465;Yang2025_Silkie_Wencha	0.824249006
ENSGALG00010019673	NID2	3	0.04699063	0.063568828	0.739208692	0.459780284	0	0.509405055	0	1	CRA004775;PRJNA827465;Yang2025_Silkie_Wencha	0.824249006
ENSGALG00010007787	C1orf198	3	0.043859323	0.059306589	0.739535415	0.459581943	0	0.559364081	0	0.666666667	CRA004775;PRJNA827465;Yang2025_Silkie_Wencha	0.824249006
ENSGALG00010018555	UBN1	3	0.033849137	0.045731223	0.74017563	0.459193433	0	1.656714669	0	0.666666667	CRA004775;PRJNA827465;Yang2025_Silkie_Wencha	0.824249006
ENSGALG00010024723	ITCH	3	0.029576003	0.039973675	0.739887005	0.45936856	0	0.780348317	0	0.666666667	CRA004775;PRJNA827465;Yang2025_Silkie_Wencha	0.824249006
ENSGALG00010012049	IFNAR1	3	-0.131753653	0.178422171	-0.738437673	0.460248529	0.063917215	8.470631171	76.38900857	0.666666667	CRA004775;PRJNA827465;Yang2025_Silkie_Wencha	0.824373494
ENSGALG00010008437	SHKBP1	3	-0.059857961	0.081062198	-0.738420161	0.460259167	0.006667755	2.914805253	31.38478126	0.666666667	CRA004775;PRJNA827465;Yang2025_Silkie_Wencha	0.824373494
ENSGALG00010024787	SARS1	3	-0.044913352	0.060818804	-0.738478057	0.460223997	0	1.743928149	0	1	CRA004775;PRJNA827465;Yang2025_Silkie_Wencha	0.824373494
ENSGALG00010028141	CISH	3	-0.04395702	0.059521588	-0.738505492	0.460207331	0	0.942289816	0	1	CRA004775;PRJNA827465;Yang2025_Silkie_Wencha	0.824373494
ENSGALG00010001112	TSC22D2	3	-0.043113427	0.05836726	-0.738657724	0.460114863	0	1.450920607	0	0.666666667	CRA004775;PRJNA827465;Yang2025_Silkie_Wencha	0.824373494
ENSGALG00010024760	ENSGALG0001002476(	3	-0.07437046	0.100747663	-0.738185461	0.460401757	0	0.150775096	0	1	CRA004775;PRJNA827465;Yang2025_Silkie_Wencha	0.824524438
ENSGALG00010007330	MMP13	3	-0.323371079	0.43819796	-0.737956606	0.460540819	0	0.206509026	0	1	CRA004775;PRJNA827465;Yang2025_Silkie_Wencha	0.8245278
ENSGALG00010016731	C4orf48	3	0.235914483	0.319741357	0.737829116	0.460618298	0.208999298	6.914571024	71.0755737	0.666666667	CRA004775;PRJNA827465;Yang2025_Silkie_Wencha	0.8245278
ENSGALG00010015785	FGL1	3	0.172581497	0.233937844	0.737723725	0.460682352	0.118475249	8.514692041	76.51118807	0.666666667	CRA004775;PRJNA827465;Yang2025_Silkie_Wencha	0.8245278
ENSGALG00010013344	OSMR	3	0.137146494	0.185910285	0.737702565	0.460695213	0.08001932	11.57048663	82.71464231	0.666666667	CRA004775;PRJNA827465;Yang2025_Silkie_Wencha	0.8245278
ENSGALG00010007207	EPM2A	3	-0.048008153	0.065045292	-0.738072684	0.460470282	0	0.301024287	0	0.666666667	CRA004775;PRJNA827465;Yang2025_Silkie_Wencha	0.8245278
ENSGALG00010029689	ENSGALG0001002968!	3	0.300299373	0.407167833	0.737532165	0.460798791	0.450729991	35.39485841	94.34946179	0.666666667	CRA004775;PRJNA827465;Yang2025_Silkie_Wencha	0.824608796
ENSGALG00010011029	HMGCR	3	0.092992376	0.126109524	0.73739376	0.460882929	0.026208058	4.659224334	57.07440001	0.666666667	CRA004775;PRJNA827465;Yang2025_Silkie_Wencha	0.82465499
ENSGALG00010009765	STX10	3	-0.085419345	0.115856504	-0.737285714	0.460948618	0.027728302	6.879307163	70.92730485	0.666666667	CRA004775;PRJNA827465;Yang2025_Silkie_Wencha	0.824668166
ENSGALG00010022098	CDC7	3	0.059749701	0.08106665	0.737044157	0.461095497	0	0.642148647	0	1	CRA004775;PRJNA827465;Yang2025_Silkie_Wencha	0.824722231
ENSGALG00010022167	WDR3	3	-0.044023751	0.059722935	-0.737133078	0.461041426	0	0.755060478	0	1	CRA004775;PRJNA827465;Yang2025_Silkie_Wencha	0.824722231
ENSGALG00010028561	SET	3	0.109518318	0.148665198	0.736677579	0.461318445	0.04222697	5.469178793	63.43143869	0.666666667	CRA004775;PRJNA827465;Yang2025_Silkie_Wencha	0.825016632
ENSGALG00010025695	ENSGALG0001002569!	2	-0.916727391	1.244949002	-0.736357385	0.461513232	0.049766408	1.01168065	1.154578731	1	PRJNA827465;Yang2025_Silkie_Wenchang	0.825054274
ENSGALG00010027040	CLIP1	3	0.241648116	0.32810433	0.736497798	0.461427807	0.289916686	27.82360638	92.81185921	0.666666667	CRA004775;PRJNA827465;Yang2025_Silkie_Wencha	0.825054274
ENSGALG00010023832	ENSGALG0001002383:	3	-0.129236169	0.175530778	-0.736259308	0.461572905	0.054470068	5.099041255	60.77694021	0.666666667	CRA004775;PRJNA827465;Yang2025_Silkie_Wencha	0.825054274
ENSGALG00010026001	ANKS1A	3	0.044787657	0.060825629	0.736328716	0.461530674	0	1.343126374	0	1	CRA004775;PRJNA827465;Yang2025_Silkie_Wencha	0.825054274
ENSGALG00010000627	ENSGALG0001000062'	3	0.304137409	0.413720013	0.735128587	0.462261185	0.390859268	9.040128891	77.87642163	0.666666667	CRA004775;PRJNA827465;Yang2025_Silkie_Wencha	0.825181557
ENSGALG00010012248	H2AC39	3	-0.250358486	0.340581573	-0.735091108	0.462284009	0.252196056	7.444297198	73.13379696	0.666666667	CRA004775;PRJNA827465;Yang2025_Silkie_Wencha	0.825181557
ENSGALG00010026213	AKR1A1	3	-0.137903335	0.187578942	-0.735174927	0.462232967	0.08534426	14.78637148	86.47403116	0.666666667	CRA004775;PRJNA827465;Yang2025_Silkie_Wencha	0.825181557
ENSGALG00010017318	ADSS1	3	-0.13271245	0.180501317	-0.735243666	0.46219111	0.059872312	6.593794887	69.66845293	0.666666667	CRA004775;PRJNA827465;Yang2025_Silkie_Wencha	0.825181557
ENSGALG00010003400	PKIA	3	-0.114034337	0.155004951	-0.735681902	0.461924306	0.043832304	6.532681162	69.38469902	0.666666667	CRA004775;PRJNA827465;Yang2025_Silkie_Wencha	0.825181557
ENSGALG00010020438	HTR7	3	-0.112288906	0.152718493	-0.735267246	0.462176752	0.015893791	2.567601093	22.10628023	0.666666667	CRA004775;PRJNA827465;Yang2025_Silkie_Wencha	0.825181557
ENSGALG00010028565	PKN1	3	0.086552744	0.117744789	0.735087681	0.462286096	0.018995812	3.790619808	47.23818	0.666666667	CRA004775;PRJNA827465;Yang2025_Silkie_Wencha	0.825181557
ENSGALG00010005465	ENSGALG0001000546!	3	0.074550111	0.101415946	0.735092593	0.462283105	0.016135757	4.721204851	57.6379322	0.666666667	CRA004775;PRJNA827465;Yang2025_Silkie_Wencha	0.825181557
ENSGALG00010003729	RARS2	3	0.062455878	0.084862026	0.735969672	0.461749155	0.003959369	2.395999719	16.52753613	0.666666667	CRA004775;PRJNA827465;Yang2025_Silkie_Wencha	0.825181557
ENSGALG00010001164	KPNA4	3	-0.046200043	0.062776633	-0.735943301	0.461765204	0.005529665	3.787708121	47.19762093	0.666666667	CRA004775;PRJNA827465;Yang2025_Silkie_Wencha	0.825181557
ENSGALG00010014396	ING1	3	-0.031750454	0.043180776	-0.735291413	0.462162036	0	0.341077254	0	0.666666667	CRA004775;PRJNA827465;Yang2025_Silkie_Wencha	0.825181557
ENSGALG00010016455	RBM27	3	0.063389673	0.086326105	0.734304793	0.462762998	0.009102139	3.032857079	34.05558033	0.666666667	CRA004775;PRJNA827465;Yang2025_Silkie_Wencha	0.825928556
ENSGALG00010021125	LY75	3	0.187907442	0.256123256	0.733660211	0.463155854	0.154228542	16.8730534	88.14678083	0.666666667	CRA004775;PRJNA827465;Yang2025_Silkie_Wencha	0.826316798
ENSGALG00010027650	CCDC124	3	0.068498033	0.093354658	0.733739849	0.463107307	0.01274762	4.186427255	52.22656747	0.666666667	CRA004775;PRJNA827465;Yang2025_Silkie_Wencha	0.826316798
ENSGALG00010005275	L3MBTL3	3	0.043342197	0.059074109	0.733691931	0.463136517	0	1.281768228	0	0.666666667	CRA004775;PRJNA827465;Yang2025_Silkie_Wencha	0.826316798
ENSGALG00010013346	HMGCS2	3	0.60787113	0.829148698	0.733126798	0.463481097	1.469409883	7.125906738	71.93339636	0.666666667	CRA004775;PRJNA827465;Yang2025_Silkie_Wencha	0.82634984
ENSGALG00010026287	NASP	3	0.157189753	0.214413331	0.733115575	0.463487941	0.109309193	13.10780154	84.74191119	0.666666667	CRA004775;PRJNA827465;Yang2025_Silkie_Wencha	0.82634984
ENSGALG00010002385	PALS2	3	-0.056625438	0.077245852	-0.733054741	0.463525043	0	1.544253055	0	1	CRA004775;PRJNA827465;Yang2025_Silkie_Wencha	0.82634984
ENSGALG00010024008	CCNE1	3	0.050540629	0.068932057	0.733194846	0.463439598	0	0.307007973	0	1	CRA004775;PRJNA827465;Yang2025_Silkie_Wencha	0.82634984
ENSGALG00010028516	GATAD2A	3	-0.047162766	0.064324555	-0.733200043	0.463436429	0.005282607	3.318014552	39.72298889	0.666666667	CRA004775;PRJNA827465;Yang2025_Silkie_Wencha	0.82634984
ENSGALG00010008129	TANGO6	3	0.040194442	0.054808755	0.733358055	0.463340075	0	0.947438405	0	0.666666667	CRA004775;PRJNA827465;Yang2025_Silkie_Wencha	0.82634984
ENSGALG00010018487	ENSGALG0001001848'	3	-0.087259319	0.119098678	-0.732664044	0.463763359	0.017991147	3.406840794	41.29458578	0.666666667	CRA004775;PRJNA827465;Yang2025_Silkie_Wencha	0.826670466
ENSGALG00010024193	GSTK1	3	-0.063501307	0.08670369	-0.732394515	0.463927806	0	0.619223494	0	1	CRA004775;PRJNA827465;Yang2025_Silkie_Wencha	0.826859353
ENSGALG00010010778	UPP2	3	-0.156472421	0.213675347	-0.732290472	0.463991293	0	0.486210549	0	0.666666667	CRA004775;PRJNA827465;Yang2025_Silkie_Wencha	0.826868276
ENSGALG00010012593	TUBA8B	2	-1.598269599	2.185324286	-0.731364955	0.464556265	8.530896835	9.262319172	89.2035679	0.5	CRA004775;PRJNA827465	0.826968056
ENSGALG00010010966	ENSGALG0001001096(	3	-0.346747298	0.473830366	-0.731796277	0.464292922	0.379964162	4.645635701	56.94884126	0.666666667	CRA004775;PRJNA827465;Yang2025_Silkie_Wencha	0.826968056
ENSGALG00010009088	ENSGALG0001000908!	3	0.324320975	0.443479902	0.731309297	0.464590253	0.48138951	12.18892791	83.59166602	0.666666667	CRA004775;PRJNA827465;Yang2025_Silkie_Wencha	0.826968056
ENSGALG00010027939	BSPRY	3	0.268579572	0.367329333	0.731168322	0.464676347	0	0.088791248	0	1	CRA004775;PRJNA827465;Yang2025_Silkie_Wencha	0.826968056
ENSGALG00010003042	TOX	3	0.163346404	0.223534553	0.73074342	0.464935889	0.058202708	3.113924162	35.77236002	0.666666667	CRA004775;PRJNA827465;Yang2025_Silkie_Wencha	0.826968056
ENSGALG00010025506	R3HDM2	3	0.162108081	0.221441149	0.73205943	0.464132295	0.115451708	10.29109595	80.56572391	0.666666667	CRA004775;PRJNA827465;Yang2025_Silkie_Wencha	0.826968056
ENSGALG00010028483	EXD3	3	0.121599028	0.166319678	0.731116303	0.464708117	0.063253453	9.676008226	79.33031935	0.666666667	CRA004775;PRJNA827465;Yang2025_Silkie_Wencha	0.826968056
ENSGALG00010019036	REL	3	0.091393414	0.125014585	0.731062012	0.464741277	0.02324336	3.843720183	47.96707604	0.666666667	CRA004775;PRJNA827465;Yang2025_Silkie_Wencha	0.826968056
ENSGALG00010003449	BORCS6	3	0.079710403	0.109073997	0.730791994	0.464906214	0.011766444	2.773381344	27.88586379	0.666666667	CRA004775;PRJNA827465;Yang2025_Silkie_Wencha	0.826968056
ENSGALG00010007357	NOX4	3	0.070522904	0.096381817	0.731703408	0.464349616	0	0.241697738	0	0.666666667	CRA004775;PRJNA827465;Yang2025_Silkie_Wencha	0.826968056
ENSGALG00010014032	PSD3	3	0.068624757	0.093761464	0.731907903	0.464224782	0	0.211618808	0	0.666666667	CRA004775;PRJNA827465;Yang2025_Silkie_Wencha	0.826968056
ENSGALG00010016910	NSD2	3	0.061100163	0.083589763	0.730952694	0.464808048	0.006847093	2.880731346	30.57318577	0.666666667	CRA004775;PRJNA827465;Yang2025_Silkie_Wencha	0.826968056
ENSGALG00010018904	UGP2	3	0.059603807	0.08143711	0.731899823	0.464229715	0	0.024554337	0	1	CRA004775;PRJNA827465;Yang2025_Silkie_Wencha	0.826968056
ENSGALG00010020030	MRPL33	3	0.057532894	0.078747512	0.730599518	0.465023806	0.00435042	2.410408414	17.02650934	0.666666667	CRA004775;PRJNA827465;Yang2025_Silkie_Wencha	0.826968056
ENSGALG00010029305	SMTNL2	3	0.055200103	0.075557539	0.730570416	0.465041588	0	0.041100838	0	1	CRA004775;PRJNA827465;Yang2025_Silkie_Wencha	0.826968056
ENSGALG00010001674	ENSGALG0001000167-	3	0.053542817	0.073276483	0.730695788	0.464964989	0.006611236	3.193831752	37.37929373	0.666666667	CRA004775;PRJNA827465;Yang2025_Silkie_Wencha	0.826968056
ENSGALG00010004147	SPRYD7	3	0.038915293	0.053251355	0.730785042	0.464910461	0	1.922788241	0	0.666666667	CRA004775;PRJNA827465;Yang2025_Silkie_Wencha	0.826968056
ENSGALG00010029639	ENSGALG0001002963!	2	-0.85663943	1.172870182	-0.730378727	0.465158718	2.190270918	4.501574736	77.78555153	0.5	CRA004775;PRJNA827465	0.827072323
ENSGALG00010008320	GPRIN3	3	-0.082930682	0.113577509	-0.730168172	0.465287395	0.017140493	3.692975331	45.84312592	0.666666667	CRA004775;PRJNA827465;Yang2025_Silkie_Wencha	0.827197092
ENSGALG00010028097	TTC24	3	0.222171116	0.304329144	0.730035624	0.46536841	0.173952294	6.3220732	68.36480792	0.666666667	CRA004775;PRJNA827465;Yang2025_Silkie_Wencha	0.827237106
ENSGALG00010027942	KHDC4	3	0.102332862	0.140209782	0.729855364	0.465478599	0.04645693	12.40527196	83.87782221	0.666666667	CRA004775;PRJNA827465;Yang2025_Silkie_Wencha	0.827313391
ENSGALG00010021062	MYO9A	3	0.04315201	0.05913065	0.729773983	0.46552835	0	0.433016222	0	1	CRA004775;PRJNA827465;Yang2025_Silkie_Wencha	0.827313391
ENSGALG00010020009	MACROD2	3	0.155508652	0.213215119	0.729350963	0.465787005	0.101245408	8.244038987	75.7400468	0.666666667	CRA004775;PRJNA827465;Yang2025_Silkie_Wencha	0.82766903
ENSGALG00010029697	GIT1	3	0.178306464	0.244630193	0.728881673	0.466074046	0.136055054	16.91568101	88.17665101	0.666666667	CRA004775;PRJNA827465;Yang2025_Silkie_Wencha	0.827805087
ENSGALG00010028184	TMEM79	3	-0.131266163	0.180102136	-0.728842902	0.466097765	0.061625674	5.849747015	65.81048728	0.666666667	CRA004775;PRJNA827465;Yang2025_Silkie_Wencha	0.827805087
ENSGALG00010025874	MDH1B	2	-0.129760674	0.178001686	-0.728985648	0.466010442	0	0.009228856	0	1	PRJNA827465;Yang2025_Silkie_Wenchang	0.827805087
ENSGALG00010002570	ADAM12	3	0.071137885	0.097571521	0.729084516	0.465949966	0	1.527716554	0	0.666666667	CRA004775;PRJNA827465;Yang2025_Silkie_Wencha	0.827805087
ENSGALG00010003918	HTR1E	2	0.85848928	1.186456613	0.723574103	0.469327245	2.453763036	7.714783343	87.03787319	0.5	PRJNA827465;Yang2025_Silkie_Wenchang	0.82826404

ENSGALG00010017144	CPB1	3	0.395895828	0.545422468	0.725851704	0.467929686	0.495965361	4.887655047	59.08058198	0.666666667	CRA004775;PRJNA827465;Yang2025_Silkie_Wencha	0.82826404
ENSGALG00010012450	PPFIA2	2	0.283195852	0.390346903	0.725497883	0.468146643	0.251413185	5.674479982	82.3772398	0.5	PRJNA827465;Yang2025_Silkie_Wenchang	0.82826404
ENSGALG00010021455	CFAP58	2	0.265019961	0.366086049	0.723928054	0.469109906	0.150288305	2.212046596	54.79299569	0.5	PRJNA827465;Yang2025_Silkie_Wenchang	0.82826404
ENSGALG00010008180	POU2F1	3	0.213305023	0.2943522	0.724659179	0.468661144	0.181475963	7.399754595	72.97207665	0.666666667	CRA004775;PRJNA827465;Yang2025_Silkie_Wencha	0.82826404
ENSGALG00010018240	SOUL	3	0.195198853	0.268423188	0.727205625	0.467100001	0	0.299739947	0	1	CRA004775;PRJNA827465;Yang2025_Silkie_Wencha	0.82826404
ENSGALG00010008193	CNOT6L	3	-0.194434077	0.267533326	-0.72676582	0.467369425	0.191101709	34.66505585	94.23050115	0.666666667	CRA004775;PRJNA827465;Yang2025_Silkie_Wencha	0.82826404
ENSGALG00010000483	ENSGALG00010000483	3	0.175530238	0.242137081	0.724920931	0.46850054	0.117468866	7.063650802	71.68602956	0.666666667	CRA004775;PRJNA827465;Yang2025_Silkie_Wencha	0.82826404
ENSGALG00010021799	ENSGALG00010021799	3	0.17007494	0.234995865	0.723735881	0.4692279	0.114174055	6.567144484	69.54536321	0.666666667	CRA004775;PRJNA827465;Yang2025_Silkie_Wencha	0.82826404
ENSGALG00010003187	RHOG	3	0.164777235	0.227329861	0.724837616	0.468551656	0.10682813	6.757032146	70.40120637	0.666666667	CRA004775;PRJNA827465;Yang2025_Silkie_Wencha	0.82826404
ENSGALG00010021229	GLO1	3	-0.160746437	0.222043584	-0.723940921	0.469102006	0.101506248	8.134583322	75.41361468	0.666666667	CRA004775;PRJNA827465;Yang2025_Silkie_Wencha	0.82826404
ENSGALG00010025274	PDK2	3	-0.152754602	0.210887922	-0.724340211	0.468856896	0.072925353	4.740697681	57.81211681	0.666666667	CRA004775;PRJNA827465;Yang2025_Silkie_Wencha	0.82826404
ENSGALG00010021145	EIF4G3	3	0.149318765	0.206129717	0.724392228	0.46882497	0.091666925	8.867307652	77.44523954	0.666666667	CRA004775;PRJNA827465;Yang2025_Silkie_Wencha	0.82826404
ENSGALG00010017477	MCF2	3	0.14200926	0.19555985	0.72616777	0.467735927	0.067390666	5.840778971	65.75799205	0.666666667	CRA004775;PRJNA827465;Yang2025_Silkie_Wencha	0.82826404
ENSGALG00010014897	COL8A2	3	0.12000506	0.16504642	0.727098835	0.467165412	0	1.091356066	0	0.666666667	CRA004775;PRJNA827465;Yang2025_Silkie_Wencha	0.82826404
ENSGALG00010029384	WDR81	3	-0.118351492	0.16344526	-0.724104766	0.469001419	0.062119418	12.80716652	84.3837433	0.666666667	CRA004775;PRJNA827465;Yang2025_Silkie_Wencha	0.82826404
ENSGALG00010004969	YTHDC1	3	0.118027991	0.162289667	0.727267439	0.46706214	0.063274986	11.21105409	82.16046427	0.666666667	CRA004775;PRJNA827465;Yang2025_Silkie_Wencha	0.82826404
ENSGALG00010026409	VILL	3	-0.117578626	0.162207677	-0.724864739	0.468535015	0.018628863	2.555866269	21.7486445	0.666666667	CRA004775;PRJNA827465;Yang2025_Silkie_Wencha	0.82826404
ENSGALG00010010577	PBX1	3	0.115957004	0.15983818	0.725464991	0.468166814	0.057284668	8.329335137	75.98847967	0.666666667	CRA004775;PRJNA827465;Yang2025_Silkie_Wencha	0.82826404
ENSGALG00010025720	PHETA1	3	0.113682606	0.156178075	0.727903751	0.466672507	0	1.273302586	0	0.666666667	CRA004775;PRJNA827465;Yang2025_Silkie_Wencha	0.82826404
ENSGALG00010008015	ARHGAP39	3	0.106499725	0.146372059	0.727596005	0.466860927	0.027183149	3.37030076	40.65811503	0.666666667	CRA004775;PRJNA827465;Yang2025_Silkie_Wencha	0.82826404
ENSGALG00010008700	ENSGALG00010008700	3	-0.104616321	0.144306514	-0.724959102	0.468477121	0.034804563	4.595208399	56.47640267	0.666666667	CRA004775;PRJNA827465;Yang2025_Silkie_Wencha	0.82826404
ENSGALG00010022709	RNF5	3	0.100888641	0.139366937	0.723906569	0.469123097	0.043529467	8.396117529	76.17946637	0.666666667	CRA004775;PRJNA827465;Yang2025_Silkie_Wencha	0.82826404
ENSGALG00010016994	ATL2	3	0.099091453	0.136802512	0.724339424	0.468857379	0.040736578	12.56980537	84.08885467	0.666666667	CRA004775;PRJNA827465;Yang2025_Silkie_Wencha	0.82826404
ENSGALG00010017056	AGTR1	3	-0.097798829	0.134791204	-0.725557946	0.468109809	0.017854077	2.8172589	29.00900942	0.666666667	CRA004775;PRJNA827465;Yang2025_Silkie_Wencha	0.82826404
ENSGALG00010021396	MED6	3	-0.097436882	0.134202811	-0.726042036	0.467813001	0.038308559	7.559764488	73.54414938	0.666666667	CRA004775;PRJNA827465;Yang2025_Silkie_Wencha	0.82826404
ENSGALG00010015633	CCM2L	3	0.097184597	0.13354715	0.727715501	0.466787759	0	1.961410095	0	0.666666667	CRA004775;PRJNA827465;Yang2025_Silkie_Wencha	0.82826404
ENSGALG00010017542	PLOD1	3	-0.095944705	0.132060052	-0.726831514	0.467329176	0.037504	9.440742367	78.8152253	0.666666667	CRA004775;PRJNA827465;Yang2025_Silkie_Wencha	0.82826404
ENSGALG00010014162	COL18A1	3	-0.092832699	0.128187039	-0.724197237	0.468944655	0.017522673	2.990158863	33.11392165	0.666666667	CRA004775;PRJNA827465;Yang2025_Silkie_Wencha	0.82826404
ENSGALG00010010347	FBXW11	3	-0.092409957	0.127313828	-0.725843831	0.467934513	0.038290156	18.30770812	89.07563969	0.666666667	CRA004775;PRJNA827465;Yang2025_Silkie_Wencha	0.82826404
ENSGALG00010028606	PRPF3	3	0.083174603	0.114907982	0.723836598	0.468916058	0.025462163	5.960764578	66.44725733	0.666666667	CRA004775;PRJNA827465;Yang2025_Silkie_Wencha	0.82826404
ENSGALG00010008690	YIF1B	3	-0.082476975	0.113766476	-0.724978775	0.468465051	0.02052016	4.074806221	50.9179114	0.666666667	CRA004775;PRJNA827465;Yang2025_Silkie_Wencha	0.82826404
ENSGALG00010022222	ENSGALG00010022222	3	0.079985036	0.109947251	0.727485544	0.466928568	0	1.205876604	0	0.666666667	CRA004775;PRJNA827465;Yang2025_Silkie_Wencha	0.82826404
ENSGALG00010009447	DDX41	3	-0.078242024	0.107926505	-0.724956525	0.468478702	0.025815128	8.94930483	77.65189545	0.666666667	CRA004775;PRJNA827465;Yang2025_Silkie_Wencha	0.82826404
ENSGALG00010028752	MTMR11	3	0.077477624	0.107080262	0.723547204	0.469343764	0	0.160988097	0	1	CRA004775;PRJNA827465;Yang2025_Silkie_Wencha	0.82826404
ENSGALG00010013065	FKTN	3	0.076839654	0.105739847	0.726685879	0.467418406	0.009784999	2.789814426	28.31064384	1	CRA004775;PRJNA827465;Yang2025_Silkie_Wencha	0.82826404
ENSGALG00010013033	MAP3K1	3	0.069459377	0.095581818	0.726700738	0.467409301	0.005006203	2.339999546	14.52989795	0.666666667	CRA004775;PRJNA827465;Yang2025_Silkie_Wencha	0.82826404
ENSGALG00010016266	PDLIM4	3	-0.067459731	0.093206545	-0.723766028	0.469209389	0.012373796	4.022749317	50.28275833	0.666666667	CRA004775;PRJNA827465;Yang2025_Silkie_Wencha	0.82826404
ENSGALG00010006101	ENSGALG00010006101	3	0.06278786	0.086244496	0.72802165	0.466600334	0	0.006234388	0	1	CRA004775;PRJNA827465;Yang2025_Silkie_Wencha	0.82826404
ENSGALG00010021827	CCZ1	3	-0.061647662	0.084915069	-0.725992013	0.467843667	0.011781246	4.747115008	57.86914796	0.666666667	CRA004775;PRJNA827465;Yang2025_Silkie_Wencha	0.82826404
ENSGALG00010002453	TOP2B	3	0.059273675	0.08170249	0.725481869	0.468156464	0.010609422	4.211520518	52.5112132	0.666666667	CRA004775;PRJNA827465;Yang2025_Silkie_Wencha	0.82826404
ENSGALG00010004881	MIPOL1	3	-0.055183611	0.075779791	-0.728210126	0.466484968	0	1.300519113	0	0.666666667	CRA004775;PRJNA827465;Yang2025_Silkie_Wencha	0.82826404
ENSGALG00010019296	ENSGALG00010019296	3	0.05494381	0.075635782	0.726426144	0.467577569	0	1.405366898	0	0.666666667	CRA004775;PRJNA827465;Yang2025_Silkie_Wencha	0.82826404
ENSGALG00010019846	ENSGALG00010019846	3	-0.048169878	0.066270933	-0.726862841	0.467309982	0.001753047	2.268483159	11.83536046	0.666666667	CRA004775;PRJNA827465;Yang2025_Silkie_Wencha	0.82826404
ENSGALG00010020202	MAPKBP1	3	-0.043856586	0.060575615	-0.72399737	0.46906735	0	1.76566972	0	0.666666667	CRA004775;PRJNA827465;Yang2025_Silkie_Wencha	0.82826404
ENSGALG00010018917	DNAJC11	3	-0.043140159	0.059314195	-0.727315936	0.467032438	0	0.794706607	0	0.666666667	CRA004775;PRJNA827465;Yang2025_Silkie_Wencha	0.82826404
ENSGALG00010017500	ACAP2	3	0.039162276	0.054045528	0.724616399	0.468687396	0	0.077816124	0	0.666666667	CRA004775;PRJNA827465;Yang2025_Silkie_Wencha	0.82826404
ENSGALG00010012190	CAST	3	-0.038994255	0.05388979	-0.723592631	0.469315866	0	0.29764661	0	0.666666667	CRA004775;PRJNA827465;Yang2025_Silkie_Wencha	0.82826404
ENSGALG00010029312	SMURF2	3	-0.038484928	0.053006151	-0.726046454	0.467810293	0	0.046430025	0	1	CRA004775;PRJNA827465;Yang2025_Silkie_Wencha	0.82826404
ENSGALG00010024135	FBXO22	3	0.03541548	0.048798501	0.725749336	0.467992451	0	1.62878499	0	0.666666667	CRA004775;PRJNA827465;Yang2025_Silkie_Wencha	0.82826404
ENSGALG00010008825	PPID	3	-0.034006846	0.046837041	-0.726067343	0.467797488	0	0.673225866	0	0.666666667	CRA004775;PRJNA827465;Yang2025_Silkie_Wencha	0.82826404
ENSGALG00010028066	ENSGALG00010028066	3	0.239550034	0.331604533	0.722397645	0.470050034	0.283349304	22.3827351	91.06454153	0.666666667	CRA004775;PRJNA827465;Yang2025_Silkie_Wencha	0.828279178
ENSGALG00010007763	FZD4	3	-0.203172867	0.281151259	-0.722646124	0.469897323	0.211115372	31.37565306	93.62563069	0.666666667	CRA004775;PRJNA827465;Yang2025_Silkie_Wencha	0.828279178
ENSGALG00010018379	SRBD1	3	-0.187651206	0.259600727	-0.722845454	0.469774838	0.15871157	19.15178837	89.557111	0.666666667	CRA004775;PRJNA827465;Yang2025_Silkie_Wencha	0.828279178
ENSGALG00010018542	ZMIZ2	3	-0.177895976	0.246260621	-0.72238905	0.470055317	0.133203185	11.2716467	82.25636365	0.666666667	CRA004775;PRJNA827465;Yang2025_Silkie_Wencha	0.828279178
ENSGALG00010029395	DPH1	3	0.174392296	0.241291575	0.722726797	0.469847749	0.157175472	21.68062947	90.77517559	0.666666667	CRA004775;PRJNA827465;Yang2025_Silkie_Wencha	0.828279178
ENSGALG00010015066	RUFY1	3	0.118139992	0.163474378	0.722682006	0.469875273	0.063081452	14.85964945	86.54073229	0.666666667	CRA004775;PRJNA827465;Yang2025_Silkie_Wencha	0.828279178
ENSGALG00010016854	TAF1A	3	0.110324687	0.152716499	0.722414987	0.470039375	0.045364864	6.083137627	67.12222996	0.666666667	CRA004775;PRJNA827465;Yang2025_Silkie_Wencha	0.828279178
ENSGALG00010023925	NCOA3	3	0.107177378	0.148325505	0.722582259	0.469936571	0.034808109	4.100725213	51.22813903	0.666666667	CRA004775;PRJNA827465;Yang2025_Silkie_Wencha	0.828279178
ENSGALG00010000384	ENSGALG00010000384	3	0.105556154	0.146060824	0.722686275	0.469872649	0.026419122	3.4044736	41.25376681	0.666666667	CRA004775;PRJNA827465;Yang2025_Silkie_Wencha	0.828279178
ENSGALG00010024148	UGGT1	3	-0.083254955	0.115211544	-0.722626063	0.469909172	0.025056313	7.605076905	73.7017781	0.666666667	CRA004775;PRJNA827465;Yang2025_Silkie_Wencha	0.828279178
ENSGALG00010020336	OSBP	3	0.080373869	0.11122025	0.722654998	0.46989187	0.024000154	8.251939447	75.76327344	0.666666667	CRA004775;PRJNA827465;Yang2025_Silkie_Wencha	0.828279178
ENSGALG00010018602	MCTP2	3	-0.071499413	0.098951266	-0.722571994	0.46994288	0	1.05983975	0	0.666666667	CRA004775;PRJNA827465;Yang2025_Silkie_Wencha	0.828279178
ENSGALG00010014042	SGCZ	2	0.559471896	0.775288955	0.721630164	0.470521889	1.084905267	10.24348091	90.24348091	0.5	PRJNA827465;Yang2025_Silkie_Wenchang	0.828894715
ENSGALG00010012054	FAM60A	3	-0.124839927	0.172988673	-0.721665325	0.470500266	0.063204593	11.20054629	82.1437281	0.666666667	CRA004775;PRJNA827465;Yang2025_Silkie_Wencha	0.828894715
ENSGALG00010012708	SPP2	3	0.668154767	0.928264297	0.719789363	0.471654695	2.077565176	10.51307945	80.97607833	0.666666667	CRA004775;PRJNA827465;Yang2025_Silkie_Wencha	0.828963521
ENSGALG00010014889	PVALB	3	-0.507335988	0.704161328	-0.720482606	0.471227906	1.195530421	14.42240543	86.13268772	0.666666667	CRA004775;PRJNA827465;Yang2025_Silkie_Wencha	0.828963521
ENSGALG00010008263	C1orf101	2	-0.35992154	0.499335966	-0.720774374	0.471048345	0.359350142	3.509378681	71.50492749	0.5	PRJNA827465;Yang2025_Silkie_Wenchang	0.828963521
ENSGALG00010023831	SATB2	3	-0.287288227	0.398479566	-0.720961001	0.47093351	0.072649489	2.357115309	15.15052351	0.666666667	CRA004775;PRJNA827465;Yang2025_Silkie_Wencha	0.828963521
ENSGALG00010000020	COX2	3	0.192744755	0.267591543	0.720294645	0.471343601	0.151103569	7.321145946	72.68187228	0.666666667	CRA004775;PRJNA827465;Yang2025_Silkie_Wencha	0.828963521
ENSGALG00010000516	ENSGALG00010000516	3	0.164316152	0.227994362	0.720702698	0.471092452	0.090083796	5.120775407	60.94341499	0.666666667	CRA004775;PRJNA827465;Yang2025_Silkie_Wencha	0.828963521
ENSGALG00010003202	FAM46A	3	0.157504974	0.218830213	0.719756539	0.471674908	0.096190098	8.609240442	76.7691469	0.666666667	CRA004775;PRJNA827465;Yang2025_Silkie_Wencha	0.828963521
ENSGALG00010023519	DGKG	3	0.153451007	0.213185328	0.719800973	0.471647546	0.091689392	6.48709638	69.1695655	0.666666667	CRA004775;PRJNA827465;Yang2025_Silkie_Wencha	0.828963521
ENSGALG00010010982	ZBTB21	3	0.118439083	0.164477149	0.720094453	0.471466843	0.06034161	9.027652286	77.84584589	0.666666667	CRA004775;PRJNA827465;Yang2025_Silkie_Wencha	0.828963521
ENSGALG00010029309	ALKBH4	3	-0.110006149	0.152503173	-0.721336787	0.470702329	0	0.798665289	0	0.666666667	CRA004775;PRJNA827465;Yang2025_Silkie_Wencha	0.828963521
ENSGALG00010028547	ZNF618	3	-0.103383303	0.143469369	-0.720594951	0.471158761	0.039536351	6.29631821	68.23557424	0.666666667	CRA004775;PRJNA827465;Yang2025_Silkie_Wencha	0.828963521
ENSGALG00010011021	PPP1R3G	3	0.098606159	0.136997298	0.719767186	0.471668352	0	1.117483032	0	0.666666667	CRA004775;PRJNA827465;Yang2025_Silkie_Wencha	0.828963521
ENSGALG00010012263	SHLD3	3	0.093418132	0.129649994	0.720540966	0.471191986	0	1.455015935	0	0.666666667	CRA004775;PRJNA827465;Yang2025_Silkie_Wencha	0.828963521
ENSGALG00010022815	LRRC8D	3	0.080733542	0.112121764	0.720052374	0.471492749	0.007786859	2.446726683	18.25813589	0.666666667	CRA004775;PRJNA827465;Yang2025_Silkie_Wencha	0.828963521
ENSGALG00010016468	TTC39B	3	0.075524648	0.104799454	0.720658793	0.471119471	0.005224575	2.317069611	13.68407793	0.666666667	CRA004775;PRJNA827465;Yang2025_Silkie_Wencha	0.828963521
ENSGALG00010028595	SCNM1	3	-0.057026979	0.079226722	-0.719794747	0.47165138	0.004994035	2.569418612	22.16137959	1	CRA004775;PRJNA827465;Yang2025_Silkie_Wencha	0.828963521
ENSGALG00010018909	TTBK1	3	0.055225628	0.076605951	0.720905194	0.470967847	0	0.603541648	0	1	CRA004775;PRJNA827465;Yang2025_Silkie_Wencha	0.828963521
ENSGALG00010027329	MINDY4	3	0.054972328	0.076198817	0.721432828	0.470643255	0	1.151201122	0	0.666666667	CRA004775;PRJNA827465;Yang2025_Silkie_Wencha	0.828963521
ENSGALG00010002834	MRPL53	3	-0.051953445	0.072076743	-0.720807343	0.471028057	0	1.172630655	0	1	CRA004775;PRJNA827465;Yang2025_Silkie_Wencha	0.828963521
ENSGALG00010018491	TIA1	3	0.161978019	0.22515451	0.719408278	0.471889398	0.135360991	26.31205942	92.39892261	0.666666667	CRA004775;PRJNA827465;Yang2025_Silkie_Wencha	0.82913436
ENSGALG00010020415	TSR3	3	0.1093698	0.152026237	0.719413982	0.471885885	0.047854545	8.397876656	76.18445612	0.666666667	CRA004775;PRJNA827465;Yang2025_Silkie_Wencha	0.82913436
ENSGALG00010024290	SOST	3	-0.372065764	0.518660283	-0.717359274	0.473152442	0.494606299	5.415197879	63.06690827	0.666666667	CRA004775;PRJNA827465;Yang2025_Silkie_Wencha	0.829184957
ENSGALG00010013036	ENSGALG00010013036	2	-0.369533715	0.514743827	-0.717898294	0.47282	0.124269321	1.305930596	23.42625227	0.5	CRA004775;Yang2025_Silkie_Wenchang	0.829184957
ENSGALG00010010115	NEB	3	0.363568747	0.506367795	0.717993424	0.472761341	0.6695142	29.57097804	93.23661193	0.666666667	CRA004775;PRJNA827465;Yang2025_Silkie_Wencha	0.829184957
ENSGALG00010019020	KCNK2	3	-0.317815569	0.443405578	-0.716760422	0.473521936	0.457027385	18.21243182	89.01849012	0.666666667	CRA004775;PRJNA827465;Yang2025_Silkie_Wencha	0.829184957
ENSGALG00010004483	STEAP4	2	-0.27675459	0.385536826	-0.717842164	0.472854612	0	0.640475722	0	1	CRA004775;PRJNA827465	0.829184957
ENSGALG00010011433	RNASE6	3	-0.218803107	0.3054143	-0.716414087	0.473735699	0.184265223	5.937160951	66.31386589	0.666666667	CRA004775;PRJNA827465;Yang2025_Silkie_Wencha	0.829184957
ENSGALG00010028746	MYDGF	3	-0.168782996	0.235107354	-0.71789756	0.472820452	0.119269252	7.503844637	73.34699615	0.666666667	CRA004775;PRJNA827465;Yang2025_Silkie_Wencha	0.829184957
ENSGALG00010006877	GSE1	3	0.168569565	0.235250384	0.716553837	0.473649437	0.126504797	9.422724606	78.77471662	0.666666667	CRA004775;PRJNA827465;Yang2025_Silkie_Wencha	0.829184957
ENSGALG00010012865	EP300	3	0.167781617	0.233742543	0.717805217	0.472877396	0.119896814	8.053045575	75.16467551	0.666666667	CRA004775;PRJNA827465;Yang2025_Silkie_Wencha	0.829184957
ENSGALG00010028610	ENSGALG00010028610	3	0.16298916	0.22705346	0.717844861	0.472852949	0.089083425	4.818318653	58.49174486	0.666666667	CRA004775;PRJNA827465;Yang2025_Silkie_Wencha	0.829184957
ENSGALG00010021033	RNF122	3	-0.156127982	0.217757756	-0.716980118	0.473386364	0.086221014	5.06364111	60.50273002	0.666666667	CRA004775;PRJNA827465;Yang2025_Silkie_Wencha	0.829184957
ENSGALG00010024147	ENSGALG00010024147	3	-0.151509214	0.211337907	-0.716905056	0.473432682	0.082720755	5.2522168	61.92084074	0.666666667	CRA004775;PRJNA827465;Yang2025_Silkie_Wencha	0.829184957
ENSGALG00010000205	RNF215	3	0.14360368	0.200127177	0.717562111	0.473027326	0.10037069	13.12281987	84.75937322	0.666666667	CRA004775;PRJNA827465;Yang2025_Silkie_Wencha	0.829184957
ENSGALG00010010930	FAM163A	2	-0.142041565	0.198034372	-0.717257126	0.473215457	0	0.450419151	0	1	PRJNA827465;Yang2025_Silkie_Wenchang	0.829184957
ENSGALG00010001853	ENSGALG00010001853	3	-0.136295702	0.189956605	-0.717509674	0.47305967	0.074958235	7.256701566	72.43926897	0.666666667	CRA004775;PRJNA827465;Yang2025_Silkie_Wencha	0.829184957
ENSGALG00010016460	LBR	3	-0.135138869	0.188044715	-0.718652841	0.472354847	0.079850304	47.38846272	95.77956345	0.666666667	CRA004775;PRJNA827465;Yang2025_Silkie_Wencha	0.829184957
ENSGALG00010010749	NCOA4	3	-0.124002184	0.172721474	-0.717931486	0.472799533	0.068936545	16.10475773	87.58130962	0.666666667	CRA004775;PRJNA827465;Yang2025_Silkie_Wencha	0.829184957
ENSGALG00010028584	LRRC8A	3	-0.115295193	0.160387632	-0.718853393	0.472231256	0.063615372	18.35316161	89.10269499	0.666666667	CRA004775;PRJNA827465;Yang2025_Silkie_Wencha	0.829184957
ENSGALG00010024944	ENSGALG00010024944	3	-0.109252601	0.152391324	-0.716921402	0.473422595	0.036248864	3.924789958	49.04185902	0.666666667	CRA004775;PRJNA827465;Yang2025_Silkie_Wencha	0.829184957
ENSGALG00010008971	ANXA11	3	-0.103445337	0.14423274	-0.717211204	0.473243787	0.049893155	11.78350704	83.02712432	0.666666667	CRA004775;PRJNA827465;Yang2025_Silkie_Wencha	0.829184957
ENSGALG00010005660	UBAC2	3	-0.098383768	0.136951104	-0.718386091	0.47251926	0.034455992	5.109906418	60.86033997	0.666666667	CRA004775;PRJNA827465;Yang2025_Silkie_Wencha	0.829184957
ENSGALG00010017805	DPP8	3	-0.078924505	0.109917274	-0.718035503	0.472735396	0.020607287	5.335857569	62.51774013	0.666666667	CRA004775;PRJNA827465;Yang2025_Silkie_Wencha	0.829184957
ENSGALG00010021615	FLVCR1	3	0.077013821	0.107400124	0.717073856	0.473328526	0	0.70801958	0	0.666666667	CRA004775;PRJNA827465;Yang2025_Silkie_Wencha	0.829184957
ENSGALG00010007038	CENPU	3	-0.067280683	0.093854963	-0.716858018	0.473461708	0	1.315901903	0	0.666666667	CRA004775;PRJNA827465;Yang2025_Silkie_Wencha	0.829184957
ENSGALG00010015521	DKC1	3	0.059891469	0.083437328	0.717801864	0.472879464	0.009550441	3.742403818	46.55841279	0.666666667	CRA004775;PRJNA827465;Yang2025_Silkie_Wencha	0.829184957
ENSGALG00010028071	DPM2	3	-0.058260063	0.081180993	-0.717656453	0.47296914	0	1.032552422	0	0.666666667	CRA004775;PRJNA827465;Yang2025_Silkie_Wencha	0.829184957
ENSGALG00010020635	NDE1	3	0.055274224	0.077151476	0.716437676	0.473721138	0.003582685	2.318808755	13.74881627	0.666666667	CRA004775;PRJNA827465;Yang2025_Silkie_Wencha	0.829184957
ENSGALG00010002338	CD99	3	0.05372203	0.074987485	0.716413282	0.473736196	0.004238291	2.615263513	23.52587072	0.666666667	CRA004775;PRJNA827465;Yang2025_Silkie_Wencha	0.829184957
ENSGALG00010028743	ARRDC1	3	0.044276078	0.061655665	0.718118566	0.472684184	0	1.901792773	0	0.666666667	CRA004775;PRJNA827465;Yang2025_Silkie_Wencha	0.829184957
ENSGALG00010013244	TRIM24	3	-0.040425213	0.056270471	-0.718408998	0.47250514	0	0.758231718	0	0.666666667	CRA004775;PRJNA827465;Yang2025_Silkie_Wencha	0.829184957
ENSGALG00010021649	PXN	3	-0.031264026	0.043521456	-0.718358899	0.472536022	0	0.310598063	0	1	CRA004775;PRJNA827465;Yang2025_Silkie_Wencha	0.829184957
ENSGALG00010009415	HMP19	3	0.314531366	0.43927434	0.7160249	0.473975974	0.366033424	6.154845795	67.5052785	0.666666667	CRA004775;PRJNA827465;Yang2025_Silkie_Wencha	0.829194404
ENSGALG00010012956	FABP3	3	0.310440955	0.43367692	0.715834625	0.474093469	0.494628994	19.23196525	89.60064677	0.666666667	CRA004775;PRJNA827465;Yang2025_Silkie_Wencha	0.829194404
ENSGALG00010000197	ENSGALG00010000197	3	-0.284988345	0.398085626	-0.715897099	0.47405489	0	0.598102771	0	0.666666667	CRA004775;PRJNA827465;Yang2025_Silkie_Wencha	0.829194404
ENSGALG00010015181	VPS13A	3	0.151292284	0.211306811	0.715983942	0.474001265	0.097856258	10.21528896	80.42150341	0.666666667	CRA004775;PRJNA827465;Yang2025_Silkie_Wencha	0.829194404
ENSGALG00010018549	GHSR	2	-0.128525478	0.179433166	-0.716286074	0.473814724	0	0.062127835	0	1	PRJNA827465;Yang2025_Silkie_Wenchang	0.829194404
ENSGALG00010002419	CTBP2	3	0.06992288	0.097649669	0.716058541	0.473955202	0.014328369	4.015955648	50.19865319	0.666666667	CRA004775;PRJNA827465;Yang2025_Silkie_Wencha	0.829194404
ENSGALG00010016932	OGT	3	0.24840785	0.347158873	0.715545156	0.474272249	0.341630233	41.79527998	95.21477066	0.666666667	CRA004775;PRJNA827465;Yang2025_Silkie_Wencha	0.82930192
ENSGALG00010007110	ARL6	3	-0.091672451	0.12811037	-0.715574012	0.474254425	0.032030624	7.085587527	71.7736886	0.666666667	CRA004775;PRJNA827465;Yang2025_Silkie_Wencha	0.82930192
ENSGALG00010023184	SMARCD1	3	-0.070942004	0.099182133	-0.715269996	0.474442225	0.023119995	10.55711097	81.05542315	0.666666667	CRA004775;PRJNA827465;Yang2025_Silkie_Wencha	0.829496552
ENSGALG00010029784	CLDN3	2	1.012062777	1.416278509	0.714593048	0.474860542	3.656658282	11.07214695	90.96832796	0.5	CRA004775;PRJNA827465	0.829547024
ENSGALG00010025881	GAL	3	-0.340925324	0.476975835	-0.714764353	0.474754666	0	0.096142763	0	1	CRA004775;PRJNA827465;Yang2025_Silkie_Wencha	0.829547024
ENSGALG00010015196	PDE4D	3	0.284009357	0.397423295	0.714626849	0.47483965	0.40426645	15.72606414	87.28225968	0.666666667	CRA004775;PRJNA827465;Yang2025_Silkie_Wencha	0.829547024
ENSGALG00010006903	B3GLCT	3	-0.240559818	0.33664316	-0.714584008	0.474866129	0.308494117	53.50678201	96.26215608	0.666666667	CRA004775;PRJNA827465;Yang2025_Silkie_Wencha	0.829547024
ENSGALG00010016912	CCDC88C	3	0.19264871	0.269515194	0.714797212	0.474734358	0.197382957	34.26782232	94.16362096	0.666666667	CRA004775;PRJNA827465;Yang2025_Silkie_Wencha	0.829547024
ENSGALG00010003372	ENOX1	3	0.132666822	0.185687266	0.714463757	0.47494046	0.076600575	11.37895446	82.42369273	0.666666667	CRA004775;PRJNA827465;Yang2025_Silkie_Wencha	0.829547024
ENSGALG00010011846	UBAP1	3	0.057072881	0.07987729	0.714506984	0.474913739	0	0.487448725	0	1	CRA004775;PRJNA827465;Yang2025_Silkie_Wencha	0.829547024
ENSGALG00010025149	IFT81	3	-0.048232691	0.067496498	-0.714595461	0.47485905	0.002713824	2.413301835	17.12599018	0.666666667	CRA004775;PRJNA827465;Yang2025_Silkie_Wencha	0.829547024
ENSGALG00010008814	ENSGALG00010008814	3	-0.387447933	0.543094904	-0.713407417	0.475593685	0.703953467	11.47527106	82.5712178	0.666666667	CRA004775;PRJNA827465;Yang2025_Silkie_Wencha	0.829752617
ENSGALG00010025598	ENSGALG00010025598	3	0.279863697	0.39234436	0.713311382	0.475653096	0.317327364	6.468498295	69.08092251	0.666666667	CRA004775;PRJNA827465;Yang2025_Silkie_Wencha	0.829752617
ENSGALG00010018608	TRIM8	3	0.153278901	0.214665179	0.714037096	0.475204241	0.111095376	10.8697613	81.60033192	0.666666667	CRA004775;PRJNA827465;Yang2025_Silkie_Wencha	0.829752617
ENSGALG00010025170	COMMD9	3	-0.143021508	0.200654269	-0.712775804	0.475984501	0.100688088	13.7079941	85.4099733	0.666666667	CRA004775;PRJNA827465;Yang2025_Silkie_Wencha	0.829752617
ENSGALG00010023740	PPP1R16B	3	-0.131683497	0.184521787	-0.713647417	0.475445229	0.06754377	7.268973411	72.48579838	0.666666667	CRA004775;PRJNA827465;Yang2025_Silkie_Wencha	0.829752617
ENSGALG00010022538	WIPF1	3	0.126332942	0.177189785	0.712980954	0.475857543	0.041890367	3.528694844	43.32182044	0.666666667	CRA004775;PRJNA827465;Yang2025_Silkie_Wencha	0.829752617
ENSGALG00010023601	TPST2	3	-0.124523986	0.174451222	-0.713804031	0.475348367	0.067200147	8.652043631	76.88407404	0.666666667	CRA004775;PRJNA827465;Yang2025_Silkie_Wencha	0.829752617
ENSGALG00010003074	TTPA	3	-0.124439584	0.174524291	-0.71302157	0.47583241	0	0.266571578	0	0.666666667	CRA004775;PRJNA827465;Yang2025_Silkie_Wencha	0.829752617
ENSGALG00010009039	SLC36A4	3	-0.123414104	0.173075892	-0.713063514	0.475806456	0.050139017	4.570537022	56.24146593	0.666666667	CRA004775;PRJNA827465;Yang2025_Silkie_Wencha	0.829752617
ENSGALG00010017553	EDA	3	-0.122801853	0.172022633	-0.713870328	0.475307367	0.057276396	6.461623983	69.04802871	0.666666667	CRA004775;PRJNA827465;Yang2025_Silkie_Wencha	0.829752617
ENSGALG00010000475	RNFT2	3	-0.121342095	0.170244988	-0.712749882	0.476000543	0.063135462	8.565831321	76.65141975	0.666666667	CRA004775;PRJNA827465;Yang2025_Silkie_Wencha	0.829752617
ENSGALG00010022280	PDZD8	3	-0.099435806	0.139527581	-0.712660573	0.476055819	0.040152583	8.128876517	75.39635403	0.666666667	CRA004775;PRJNA827465;Yang2025_Silkie_Wencha	0.829752617
ENSGALG00010024504	CASC3	3	0.091544279	0.128365043	0.713155826	0.475749338	0.03444201	9.454784369	78.84668839	0.666666667	CRA004775;PRJNA827465;Yang2025_Silkie_Wencha	0.829752617
ENSGALG00010017546	NFU1	3	-0.056015809	0.078600253	-0.712667026	0.476051825	0	1.342059587	0	0.666666667	CRA004775;PRJNA827465;Yang2025_Silkie_Wencha	0.829752617
ENSGALG00010020692	TUB	3	0.055650295	0.07808378	0.712699807	0.476031536	0	0.203851014	0	0.666666667	CRA004775;PRJNA827465;Yang2025_Silkie_Wencha	0.829752617
ENSGALG00010028064	SPCS1	3	-0.040841547	0.057286347	-0.712936835	0.475884844	0	1.965745986	0	0.666666667	CRA004775;PRJNA827465;Yang2025_Silkie_Wencha	0.829752617
ENSGALG00010001364	SMCHD1	3	0.036753813	0.051471023	0.714068049	0.475185102	0	1.487430042	0	1	CRA004775;PRJNA827465;Yang2025_Silkie_Wencha	0.829752617
ENSGALG00010010183	NKX3-2	3	0.560444628	0.787226847	0.711922656	0.476512672	1.48104851	10.16334868	80.32144658	0.666666667	CRA004775;PRJNA827465;Yang2025_Silkie_Wencha	0.829753725
ENSGALG00010012348	ENSGALG00010012348	2	0.40055232	0.562395845	0.712224891	0.476325526	0	0.004378212	0	1	PRJNA827465;Yang2025_Silkie_Wenchang	0.829753725
ENSGALG00010018815	ENSGALG00010018815	3	-0.217000508	0.30460671	-0.712395693	0.476219782	0.22830283	11.54192269	82.67186452	0.666666667	CRA004775;PRJNA827465;Yang2025_Silkie_Wencha	0.829753725
ENSGALG00010025647	JHY	2	-0.174010574	0.244430781	-0.711901233	0.476525939	0	0.847232342	0	0.5	PRJNA827465;Yang2025_Silkie_Wenchang	0.829753725
ENSGALG00010018958	PELI3	3	-0.097633781	0.137103265	-0.71211857	0.476391356	0.042975479	15.74787235	87.29987166	0.666666667	CRA004775;PRJNA827465;Yang2025_Silkie_Wencha	0.829753725
ENSGALG00010009464	SPTLC1	3	0.081750562	0.114816619	0.71200983	0.476458689	0	0.005786803	0	1	CRA004775;PRJNA827465;Yang2025_Silkie_Wencha	0.829753725
ENSGALG00010004018	HNRNPD	3	-0.06307235	0.088588523	-0.711969764	0.476483499	0.01104005	3.618117319	44.72263269	0.666666667	CRA004775;PRJNA827465;Yang2025_Silkie_Wencha	0.829753725
ENSGALG00010008038	WRN	3	-0.040855684	0.057339787	-0.712518931	0.476143493	0	0.127799406	0	1	CRA004775;PRJNA827465;Yang2025_Silkie_Wencha	0.829753725
ENSGALG00010029339	PITPNA	3	-0.061787209	0.086812566	-0.711731175	0.476631259	0.010600347	3.852806089	48.08978303	0.666666667	CRA004775;PRJNA827465;Yang2025_Silkie_Wencha	0.829834918
ENSGALG00010002592	RPS6KA3	3	-0.093665416	0.13163488	-0.711554686	0.476740576	0.024615721	3.730405091	46.38651966	0.666666667	CRA004775;PRJNA827465;Yang2025_Silkie_Wencha	0.829923048
ENSGALG00010021388	DPH2	3	-0.199303925	0.280193948	-0.71130703	0.476893996	0.181851736	9.500594672	78.94868617	0.666666667	CRA004775;PRJNA827465;Yang2025_Silkie_Wencha	0.830082934
ENSGALG00010027214	PLIN4	3	0.101199681	0.142290883	0.711216899	0.476949838	0.032482667	4.327001329	53.77861368	0.666666667	CRA004775;PRJNA827465;Yang2025_Silkie_Wencha	0.830082934
ENSGALG00010006069	TTR	3	1.182298013	1.665325998	0.709949892	0.477735209	6.516224651	9.330784455	78.56557496	0.666666667	CRA004775;PRJNA827465;Yang2025_Silkie_Wencha	0.830228067
ENSGALG00010018518	ENSGALG00010018518	2	0.36781394	0.518501704	0.709378459	0.478089651	0	0.906180425	0	0.5	CRA004775;PRJNA827465	0.830228067
ENSGALG00010012087	TMEM171	3	-0.260067055	0.366630397	-0.709343954	0.478111059	0.319031846	11.77479922	83.01457237	0.666666667	CRA004775;PRJNA827465;Yang2025_Silkie_Wencha	0.830228067
ENSGALG00010006342	RFXAP	3	-0.231511758	0.3262103	-0.709700945	0.477889606	0.291878176	34.90614913	94.2703505	0.666666667	CRA004775;PRJNA827465;Yang2025_Silkie_Wencha	0.830228067
ENSGALG00010024964	ITM2C	3	0.20729295	0.292257049	0.709282977	0.47814889	0.196629118	9.072353408	77.9550034	0.666666667	CRA004775;PRJNA827465;Yang2025_Silkie_Wencha	0.830228067
ENSGALG00010021427	RPLP0	3	-0.146197304	0.205985295	-0.709746317	0.477861464	0.087767152	7.868830575	74.58326265	0.666666667	CRA004775;PRJNA827465;Yang2025_Silkie_Wencha	0.830228067
ENSGALG00010025207	BMPR2	3	0.136029235	0.191682157	0.70966039	0.477914761	0.072168014	6.39389513	68.7201626	0.666666667	CRA004775;PRJNA827465;Yang2025_Silkie_Wencha	0.830228067
ENSGALG00010005887	CCNH	3	0.116629608	0.164305608	0.709833396	0.477807457	0.034747416	3.493998844	42.75899651	0.666666667	CRA004775;PRJNA827465;Yang2025_Silkie_Wencha	0.830228067
ENSGALG00010016984	RNF103	3	-0.097673705	0.137685063	-0.709399432	0.47807664	0.038709791	8.360293329	76.0773944	0.666666667	CRA004775;PRJNA827465;Yang2025_Silkie_Wencha	0.830228067
ENSGALG00010009830	RNASEL	3	-0.09087231	0.127842207	-0.710816188	0.477198148	0.010190714	2.466397927	18.91008428	0.666666667	CRA004775;PRJNA827465;Yang2025_Silkie_Wencha	0.830228067
ENSGALG00010023781	COL4A3	3	0.090531837	0.127613371	0.709422822	0.47806213	0	1.221148302	0	0.666666667	CRA004775;PRJNA827465;Yang2025_Silkie_Wencha	0.830228067
ENSGALG00010005454	GPR161	3	-0.087262068	0.122798177	-0.710613713	0.477323643	0.029263894	7.21333825	72.27358637	0.666666667	CRA004775;PRJNA827465;Yang2025_Silkie_Wencha	0.830228067
ENSGALG00010000732	ENSGALG00010000732	3	-0.074598257	0.105022215	-0.710309303	0.477512352	0	0.027562155	0	1	CRA004775;PRJNA827465;Yang2025_Silkie_Wencha	0.830228067
ENSGALG00010016558	NR2F2	3	0.072354146	0.101865836	0.710288642	0.477525161	0.015737551	3.88166898	48.47577137	0.666666667	CRA004775;PRJNA827465;Yang2025_Silkie_Wencha	0.830228067
ENSGALG00010023211	TLK1	3	0.058437784	0.082197676	0.710942039	0.477120154	0.00866053	3.206393192	37.62461807	0.666666667	CRA004775;PRJNA827465;Yang2025_Silkie_Wencha	0.830228067
ENSGALG00010016395	ENSGALG00010016395	3	0.057950287	0.081662225	0.70963395	0.477931161	0.000855846	2.074865593	3.608214126	1	CRA004775;PRJNA827465;Yang2025_Silkie_Wencha	0.830228067
ENSGALG00010006939	FBXO31	3	-0.048209138	0.067856197	-0.710460363	0.477418702	0	1.100689858	0	0.666666667	CRA004775;PRJNA827465;Yang2025_Silkie_Wencha	0.830228067
ENSGALG00010024861	ORC1	3	0.040803977	0.057442471	0.71034509	0.477490165	0.003312057	2.930315507	31.74796382	0.666666667	CRA004775;PRJNA827465;Yang2025_Silkie_Wencha	0.830228067
ENSGALG00010001121	WWTR1	3	0.037117485	0.052295304	0.70976708	0.477848586	0	0.765490901	0	1	CRA004775;PRJNA827465;Yang2025_Silkie_Wencha	0.830228067

ENSGALG00010029780	TEX14	3	0.200081652	0.282181496	0.709053056	0.478291553	0.142702201	5.295675529	62.23333569	0.666666667	CRA004775;PRJNA827465;Yang2025_Silkie_Wencha	0.830373805
ENSGALG00010022769	FXYD6	3	-0.057868582	0.081634398	-0.708874979	0.478402064	0	0.774829991	0	0.666666667	CRA004775;PRJNA827465;Yang2025_Silkie_Wencha	0.830463693
ENSGALG00010012194	LRRFIP1	3	0.227196528	0.320908935	0.707978193	0.4789588	0.265456629	20.94968552	90.45331731	0.666666667	CRA004775;PRJNA827465;Yang2025_Silkie_Wencha	0.831184448
ENSGALG00010025434	SYT6	3	-0.214608133	0.303212482	-0.707781326	0.479081065	0.20235925	14.63943323	86.33826892	0.666666667	CRA004775;PRJNA827465;Yang2025_Silkie_Wencha	0.831184448
ENSGALG00010013569	ENSGALG0001001356(	3	0.197264489	0.278764627	0.707638165	0.479169986	0	0.938061247	0	0.666666667	CRA004775;PRJNA827465;Yang2025_Silkie_Wencha	0.831184448
ENSGALG00010016899	SAXO2	3	-0.168035765	0.237452705	-0.70765993	0.479156466	0.103644634	5.891419483	66.05232396	0.666666667	CRA004775;PRJNA827465;Yang2025_Silkie_Wencha	0.831184448
ENSGALG00010020319	NTRK3	3	0.113700399	0.160629312	0.707843401	0.479042511	0.038385965	4.146527282	51.76686745	0.666666667	CRA004775;PRJNA827465;Yang2025_Silkie_Wencha	0.831184448
ENSGALG00010017813	DDHD2	3	-0.041103121	0.058084268	-0.707646353	0.4791649	0	1.283710426	0	0.666666667	CRA004775;PRJNA827465;Yang2025_Silkie_Wencha	0.831184448
ENSGALG00010011493	ENSGALG0001001149:	3	-0.200037351	0.282730341	-0.707519931	0.479243431	0	0.125359282	0	1	CRA004775;PRJNA827465;Yang2025_Silkie_Wencha	0.831209872
ENSGALG00010007906	NAF1	3	-0.07110444	0.100513027	-0.707415174	0.47930851	0.012363273	3.153743027	36.58329221	0.666666667	CRA004775;PRJNA827465;Yang2025_Silkie_Wencha	0.831220781
ENSGALG00010003253	ITGB1BP1	3	0.196552036	0.277955848	0.707134018	0.479483198	0.210746864	25.0349389	92.01116484	0.666666667	CRA004775;PRJNA827465;Yang2025_Silkie_Wencha	0.831421748
ENSGALG00010026076	C1orf35	3	0.119631231	0.16922171	0.706949662	0.47959776	0.059795556	6.734999402	70.30437747	0.666666667	CRA004775;PRJNA827465;Yang2025_Silkie_Wencha	0.831518422
ENSGALG00010007513	GALNT12	3	-0.07736194	0.109474857	-0.706664004	0.479775304	0.017639794	4.071161364	50.87396884	0.666666667	CRA004775;PRJNA827465;Yang2025_Silkie_Wencha	0.831724255
ENSGALG00010025974	EEF1B2	3	0.134959743	0.191084744	0.706282144	0.480012695	0.083737537	10.396537	80.76282515	0.666666667	CRA004775;PRJNA827465;Yang2025_Silkie_Wencha	0.832033775
ENSGALG00010001501	CAPZA2	3	-0.119987704	0.169915099	-0.706162692	0.480086969	0.062033878	10.98571061	81.79453227	0.666666667	CRA004775;PRJNA827465;Yang2025_Silkie_Wencha	0.832060511
ENSGALG00010020756	MACROH2A2	3	-0.049270862	0.069792689	-0.705960224	0.480212874	0	0.313591229	0	1	CRA004775;PRJNA827465;Yang2025_Silkie_Wencha	0.832176716
ENSGALG00010018865	ENSGALG0001001886:	3	-0.060859021	0.086241973	-0.70567752	0.480388704	0.001364867	2.100114712	4.767106816	0.666666667	CRA004775;PRJNA827465;Yang2025_Silkie_Wencha	0.832379398
ENSGALG00010016479	ENSGALG0001001647(	3	0.257913553	0.365578066	0.70549515	0.480502149	0.248196936	6.307472714	68.29157904	0.666666667	CRA004775;PRJNA827465;Yang2025_Silkie_Wencha	0.832473948
ENSGALG00010022180	PLLP	3	0.174524974	0.247427925	0.70535682	0.480588209	0.086322286	3.835720546	47.85855809	0.666666667	CRA004775;PRJNA827465;Yang2025_Silkie_Wencha	0.83247459
ENSGALG00010012887	KCNJ4	3	-0.166366049	0.235878072	-0.705305279	0.480620276	0.026957436	2.314366257	13.58325441	0.666666667	CRA004775;PRJNA827465;Yang2025_Silkie_Wencha	0.83247459
ENSGALG00010011713	AVPR1A	2	-0.456154392	0.646912388	-0.705125455	0.480732167	0.545782271	2.826313275	64.61821806	0.5	PRJNA827465;Yang2025_Silkie_Wenchang	0.832566402
ENSGALG00010024556	ENSGALG0001002455(	3	0.087592267	0.124267649	0.704867821	0.480892497	0	0.988647747	0	1	CRA004775;PRJNA827465;Yang2025_Silkie_Wencha	0.832586375
ENSGALG00010029165	RAC3	3	-0.047826709	0.067846645	-0.704923715	0.480857711	0	1.104257794	0	1	CRA004775;PRJNA827465;Yang2025_Silkie_Wencha	0.832586375
ENSGALG00010023337	FLI1	3	0.041503111	0.058892345	0.704728451	0.480979242	0	0.831735036	0	0.666666667	CRA004775;PRJNA827465;Yang2025_Silkie_Wencha	0.832586375
ENSGALG00010006716	ENSGALG0001000671(	3	0.041287949	0.058582974	0.704777279	0.48094885	0	1.961734584	0	0.666666667	CRA004775;PRJNA827465;Yang2025_Silkie_Wencha	0.832586375
ENSGALG00010014677	ENSGALG0001001467:	3	0.12529374	0.177849233	0.704494124	0.481125108	0.061958055	5.93052203	66.27615596	0.666666667	CRA004775;PRJNA827465;Yang2025_Silkie_Wencha	0.83273692
ENSGALG00010023077	KANSL2	3	0.157092479	0.223036797	0.704334356	0.481224576	0.131878986	23.86663951	91.6201022	0.666666667	CRA004775;PRJNA827465;Yang2025_Silkie_Wencha	0.832807133
ENSGALG00010024142	DNAJC6	3	-0.17268949	0.245298827	-0.703996397	0.481435017	0.077219876	3.402345543	41.21702294	0.666666667	CRA004775;PRJNA827465;Yang2025_Silkie_Wencha	0.832865497
ENSGALG00010010586	CYTIP	3	-0.171472013	0.243541378	-0.704075534	0.48138449	0	1.59792802	0	0.666666667	CRA004775;PRJNA827465;Yang2025_Silkie_Wencha	0.832865497
ENSGALG00010002380	CMPK2	3	0.142584261	0.202505716	0.704099932	0.481370543	0	0.910588747	0	0.666666667	CRA004775;PRJNA827465;Yang2025_Silkie_Wencha	0.832865497
ENSGALG00010018278	PUSL1	3	0.265428597	0.377132748	0.703806811	0.481553091	0.384662264	23.43284196	91.46497039	0.666666667	CRA004775;PRJNA827465;Yang2025_Silkie_Wencha	0.832967844
ENSGALG00010016324	TUBB1	3	0.170711482	0.242744801	0.703254943	0.481896884	0.126334147	7.468752032	73.22176461	0.666666667	CRA004775;PRJNA827465;Yang2025_Silkie_Wencha	0.833297144
ENSGALG00010018459	SNX12	3	-0.120713423	0.171647005	-0.703265534	0.481890286	0.067824579	9.468414207	78.8771387	0.666666667	CRA004775;PRJNA827465;Yang2025_Silkie_Wencha	0.833297144
ENSGALG00010024712	LRIF1	3	0.033403986	0.047501648	0.703217407	0.481920273	0.000640555	2.093648351	4.47297424	1	CRA004775;PRJNA827465;Yang2025_Silkie_Wencha	0.833297144
ENSGALG00010000056	UNC45A	3	0.127552533	0.181502584	0.702924838	0.482102591	0.083400448	14.00722024	85.72164951	0.666666667	CRA004775;PRJNA827465;Yang2025_Silkie_Wencha	0.833306667
ENSGALG00010004935	SVIL	3	0.117825777	0.167603316	0.703003855	0.482053347	0.066378898	11.91514549	83.21464055	0.666666667	CRA004775;PRJNA827465;Yang2025_Silkie_Wencha	0.833306667
ENSGALG00010017035	PIK3CD	3	0.077263734	0.109905709	0.703000186	0.482055633	0.007940054	2.478798462	19.31574791	0.666666667	CRA004775;PRJNA827465;Yang2025_Silkie_Wencha	0.833306667
ENSGALG00010025499	STAC3	3	0.31395909	0.447058809	0.702276936	0.482506474	0.485694227	27.89881637	92.83123709	0.666666667	CRA004775;PRJNA827465;Yang2025_Silkie_Wencha	0.833653281
ENSGALG00010029577	NOG	3	0.292446444	0.416478441	0.702188674	0.482561508	0	0.675269585	0	0.666666667	CRA004775;PRJNA827465;Yang2025_Silkie_Wencha	0.833653281
ENSGALG00010008686	ENSGALG0001000868(	3	-0.18648455	0.265633992	-0.702035717	0.482656889	0	1.533298363	0	0.666666667	CRA004775;PRJNA827465;Yang2025_Silkie_Wencha	0.833653281
ENSGALG00010001628	MAPK11	3	-0.128097167	0.182452833	-0.702083739	0.482626942	0.064635633	6.558647373	69.50590745	0.666666667	CRA004775;PRJNA827465;Yang2025_Silkie_Wencha	0.833653281
ENSGALG00010005555	PRSS12	3	0.095348985	0.135772784	0.702268764	0.482511569	0.016120012	2.740857681	27.03014046	0.666666667	CRA004775;PRJNA827465;Yang2025_Silkie_Wencha	0.833653281
ENSGALG00010012714	RGS2	3	-0.095324077	0.135692388	-0.702501288	0.482366599	0.028211381	4.381866814	54.35735304	0.666666667	CRA004775;PRJNA827465;Yang2025_Silkie_Wencha	0.833653281
ENSGALG00010008456	NHLRC1	3	-0.047985686	0.068373349	-0.701818564	0.48279232	0	0.557322735	0	1	CRA004775;PRJNA827465;Yang2025_Silkie_Wencha	0.833785344
ENSGALG00010029460	ENSGALG0001002946(	3	-0.220081157	0.313644169	-0.701690574	0.482872153	0.23054708	9.863529304	79.72328222	0.666666667	CRA004775;PRJNA827465;Yang2025_Silkie_Wencha	0.833821368
ENSGALG00010017783	STAR	3	0.159586989	0.227528934	0.701392065	0.483058372	0	1.357832428	0	0.666666667	CRA004775;PRJNA827465;Yang2025_Silkie_Wencha	0.834041071
ENSGALG00010005346	FBXO30	3	-0.17723571	0.252780589	-0.701292424	0.483119407	0.133355017	10.49925244	80.95102474	0.666666667	CRA004775;PRJNA827465;Yang2025_Silkie_Wencha	0.834044603
ENSGALG00010025858	ENSGALG0001002585:	2	-0.80219125	1.146811165	-0.699497244	0.484241334	0	0.290658635	0	1	PRJNA827465;Yang2025_Silkie_Wenchang	0.834565963
ENSGALG00010011949	ABCA12	2	0.204212224	0.291900573	0.699595145	0.484180175	0	0.995742164	0	0.5	PRJNA827465;Yang2025_Silkie_Wenchang	0.834565963
ENSGALG00010016788	ANLN	3	-0.126409239	0.180552408	-0.700124914	0.483849298	0.058023266	6.843357829	70.77458099	0.666666667	CRA004775;PRJNA827465;Yang2025_Silkie_Wencha	0.834565963
ENSGALG00010022788	CYBRD1	3	-0.11995921	0.171511679	-0.69963703	0.484154011	0.056240759	6.065335952	67.01594555	0.666666667	CRA004775;PRJNA827465;Yang2025_Silkie_Wencha	0.834565963
ENSGALG00010027154	PLPP2	3	0.118981986	0.16985219	0.700503098	0.483613171	0.061577781	7.876303088	74.60737636	0.666666667	CRA004775;PRJNA827465;Yang2025_Silkie_Wencha	0.834565963
ENSGALG00010005420	DHRS4	3	0.104996574	0.149892797	0.700477784	0.483628974	0.045268148	6.811693317	70.6387251	0.666666667	CRA004775;PRJNA827465;Yang2025_Silkie_Wencha	0.834565963
ENSGALG00010020990	UBB	3	-0.09245212	0.132077732	-0.699982644	0.483938144	0.030899063	4.722968012	57.65374665	0.666666667	CRA004775;PRJNA827465;Yang2025_Silkie_Wencha	0.834565963
ENSGALG00010019313	MRPL28	3	0.092230744	0.131820313	0.699670209	0.484133285	0.030859344	5.027295764	60.21718049	0.666666667	CRA004775;PRJNA827465;Yang2025_Silkie_Wencha	0.834565963
ENSGALG00010024925	GTF2E1	3	-0.078683619	0.11250157	-0.69940019	0.484301969	0.027391405	7.90195726	74.68981501	0.666666667	CRA004775;PRJNA827465;Yang2025_Silkie_Wencha	0.834565963
ENSGALG00010023311	ETS1	3	-0.076889576	0.109808863	-0.700212844	0.483794391	0.021293164	6.178044755	67.62729829	0.666666667	CRA004775;PRJNA827465;Yang2025_Silkie_Wencha	0.834565963
ENSGALG00010016956	CENPE	3	0.069337196	0.099038613	0.70010265	0.483863201	0.007387754	2.58990665	22.77713949	0.666666667	CRA004775;PRJNA827465;Yang2025_Silkie_Wencha	0.834565963
ENSGALG00010021504	ODR4	3	0.034759911	0.04970015	0.699392471	0.484306792	0	0.708725733	0	0.666666667	CRA004775;PRJNA827465;Yang2025_Silkie_Wencha	0.834565963
ENSGALG00010018961	ASB7	3	-0.03463347	0.049471421	-0.700070246	0.483883436	0.001375913	2.287868157	12.58237526	0.666666667	CRA004775;PRJNA827465;Yang2025_Silkie_Wencha	0.834565963
ENSGALG00010009015	TMEM231	3	0.032572217	0.046527654	0.700061447	0.483888931	0	0.023143675	0	1	CRA004775;PRJNA827465;Yang2025_Silkie_Wencha	0.834565963
ENSGALG00010028904	TRIM32	3	-0.030033568	0.042910611	-0.69991007	0.483983468	0	1.467872062	0	0.666666667	CRA004775;PRJNA827465;Yang2025_Silkie_Wencha	0.834565963
ENSGALG00010010712	ADAMTS5	3	-0.05925837	0.08474705	-0.699238143	0.484403217	0	0.053173835	0	1	CRA004775;PRJNA827465;Yang2025_Silkie_Wencha	0.834630402
ENSGALG00010027889	RBM8A	3	0.142609121	0.204155262	0.698532676	0.48484413	0.098367799	10.18062283	80.3548365	0.666666667	CRA004775;PRJNA827465;Yang2025_Silkie_Wencha	0.834922062
ENSGALG00010001857	HNRNPA2B1	3	0.127077436	0.181873912	0.698711729	0.484732203	0.081313651	15.32590954	86.95020354	0.666666667	CRA004775;PRJNA827465;Yang2025_Silkie_Wencha	0.834922062
ENSGALG00010009489	MROH1	3	-0.095570469	0.136826484	-0.69847932	0.484877487	0.040465444	9.909641925	79.81763605	0.666666667	CRA004775;PRJNA827465;Yang2025_Silkie_Wencha	0.834922062
ENSGALG00010012536	FBXO4	3	0.077352105	0.110756093	0.698400448	0.484926796	0	1.946966811	0	0.666666667	CRA004775;PRJNA827465;Yang2025_Silkie_Wencha	0.834922062
ENSGALG00010023663	PHOSPHO2	3	-0.055079993	0.078853238	-0.698512761	0.48485658	0	1.814602217	0	0.666666667	CRA004775;PRJNA827465;Yang2025_Silkie_Wencha	0.834922062
ENSGALG00010009650	KLHL29	3	-0.045463712	0.065091335	-0.698460279	0.48488939	0	0.857097106	0	0.666666667	CRA004775;PRJNA827465;Yang2025_Silkie_Wencha	0.834922062
ENSGALG00010012464	ENSGALG0001001246	3	-0.253385757	0.362935605	-0.69815624	0.48507949	0	0.560524926	0	0.666666667	CRA004775;PRJNA827465;Yang2025_Silkie_Wencha	0.835083272
ENSGALG00010011636	GRIK1	3	-0.190533992	0.273077164	-0.697729495	0.485346379	0.036486231	2.381262254	16.01093089	0.666666667	CRA004775;PRJNA827465;Yang2025_Silkie_Wencha	0.835135992
ENSGALG00010029717	TRARG1	3	0.171493579	0.245747122	0.697845727	0.485273678	0	0.35979669	0	1	CRA004775;PRJNA827465;Yang2025_Silkie_Wencha	0.835135992
ENSGALG00010009168	MRPS12	3	-0.131508081	0.188447385	-0.69785039	0.485270762	0.049502162	3.325544242	39.85946797	0.666666667	CRA004775;PRJNA827465;Yang2025_Silkie_Wencha	0.835135992
ENSGALG00010009355	GARRE1	3	-0.029467232	0.042230348	-0.697773833	0.485318646	0	0.233673535	0	0.666666667	CRA004775;PRJNA827465;Yang2025_Silkie_Wencha	0.835135992
ENSGALG00010008009	GPLD1	2	0.328629726	0.471197523	0.697435173	0.485530496	0.143858041	1.452490175	31.15271849	0.5	PRJNA827465;Yang2025_Silkie_Wenchang	0.835147894
ENSGALG00010007707	ENSGALG0001000770	2	0.327662886	0.469795057	0.697459203	0.485515463	0.116228532	1.323278294	24.43010633	0.5	CRA004775;PRJNA827465	0.835147894
ENSGALG00010017482	CORO2B	3	-0.141390418	0.202724671	-0.697450478	0.485520921	0.079890171	8.173392691	75.53035715	0.666666667	CRA004775;PRJNA827465;Yang2025_Silkie_Wencha	0.835147894
ENSGALG00010003254	DGKH	3	0.329910904	0.473185241	0.697213006	0.4856695	0.549824416	17.29817835	88.43808892	0.666666667	CRA004775;PRJNA827465;Yang2025_Silkie_Wencha	0.835183783
ENSGALG00010023605	INSYN1	3	-0.141906845	0.203530053	-0.697227965	0.485660141	0.000466982	2.005975413	0.297880672	0.666666667	CRA004775;PRJNA827465;Yang2025_Silkie_Wencha	0.835183783
ENSGALG00010003218	MAP3K7	3	-0.076950627	0.11039502	-0.697047993	0.485772759	0.028340694	12.0519965	83.40523912	0.666666667	CRA004775;PRJNA827465;Yang2025_Silkie_Wencha	0.835259765
ENSGALG00010014104	ALDH1A1	3	0.60462731	0.869288841	0.695542473	0.486715403	1.878378034	11.84303204	83.11243275	0.666666667	CRA004775;PRJNA827465;Yang2025_Silkie_Wencha	0.835456916
ENSGALG00010003871	ENSGALG0001000387	3	0.362115924	0.521607529	0.69423063	0.487537586	0.776154923	49.94108496	95.99528124	0.666666667	CRA004775;PRJNA827465;Yang2025_Silkie_Wencha	0.835456916
ENSGALG00010001150	ENSGALG0001000115(	2	-0.309680565	0.445996452	-0.694356567	0.487458624	0.076874945	1.216387446	17.7893521	0.5	PRJNA827465;Yang2025_Silkie_Wenchang	0.835456916
ENSGALG00010012199	ENSGALG0001001219	3	-0.286609461	0.412223443	-0.695276958	0.486881752	0.330121294	5.7163046	65.01236131	0.666666667	CRA004775;PRJNA827465;Yang2025_Silkie_Wencha	0.835456916
ENSGALG00010003786	ENSGALG0001000378(	3	-0.266276624	0.382410818	-0.696310387	0.48623447	0	0.891556932	0	0.666666667	CRA004775;PRJNA827465;Yang2025_Silkie_Wencha	0.835456916
ENSGALG00010017598	ENSGALG0001001759	3	0.208680152	0.300594953	0.69422374	0.487541907	0.24069472	23.44133229	91.46806173	0.666666667	CRA004775;PRJNA827465;Yang2025_Silkie_Wencha	0.835456916
ENSGALG00010016699	SYNPO	3	0.20568237	0.295540747	0.695952662	0.486458476	0.200591714	12.9079257	84.5056437	0.666666667	CRA004775;PRJNA827465;Yang2025_Silkie_Wencha	0.835456916
ENSGALG00010025594	UPK1B	2	0.162672969	0.233695279	0.696090093	0.48637241	0	0.024938549	0	1	PRJNA827465;Yang2025_Silkie_Wenchang	0.835456916
ENSGALG00010028013	CBARP	3	0.151300848	0.217580727	0.695377986	0.486818453	0.097776663	8.936676508	77.62031558	0.666666667	CRA004775;PRJNA827465;Yang2025_Silkie_Wencha	0.835456916
ENSGALG00010009101	HIKESHI	3	-0.145694159	0.209744354	-0.694627321	0.487288886	0.086034923	5.756570174	65.25708991	0.666666667	CRA004775;PRJNA827465;Yang2025_Silkie_Wencha	0.835456916
ENSGALG00010022375	NMB	3	-0.14193721	0.204353055	-0.694568576	0.487325711	0	0.476685261	0	0.666666667	CRA004775;PRJNA827465;Yang2025_Silkie_Wencha	0.835456916
ENSGALG00010014318	ENSGALG0001001431	3	-0.14131094	0.203252281	-0.695248977	0.486899284	0	1.70748377	0	0.666666667	CRA004775;PRJNA827465;Yang2025_Silkie_Wencha	0.835456916
ENSGALG00010022220	PPP1R14D	3	0.139349169	0.200643392	0.694511628	0.487361411	0	0.198460356	0	1	CRA004775;PRJNA827465;Yang2025_Silkie_Wencha	0.835456916
ENSGALG00010020898	ENSGALG0001002089	3	-0.138263327	0.199049576	-0.694617538	0.487295018	0.070549748	4.954035087	59.6288689	0.666666667	CRA004775;PRJNA827465;Yang2025_Silkie_Wencha	0.835456916
ENSGALG00010029217	CEP112	3	0.136760174	0.196955965	0.694369289	0.487450648	0.076247394	6.077636168	67.0924691	0.666666667	CRA004775;PRJNA827465;Yang2025_Silkie_Wencha	0.835456916
ENSGALG00010027634	OASL	3	0.119961773	0.172406243	0.695808753	0.486548607	0.03239949	3.125443796	36.00908765	0.666666667	CRA004775;PRJNA827465;Yang2025_Silkie_Wencha	0.835456916
ENSGALG00010019584	RAD18	3	0.107581373	0.1548585	0.694707579	0.487238578	0.048196137	6.668304594	70.00736886	0.666666667	CRA004775;PRJNA827465;Yang2025_Silkie_Wencha	0.835456916
ENSGALG00010018157	FBXL2	3	-0.100169635	0.144092002	-0.69517831	0.486943563	0.033191397	4.373983963	54.27509527	0.666666667	CRA004775;PRJNA827465;Yang2025_Silkie_Wencha	0.835456916
ENSGALG00010029555	LYRM9	3	0.096350013	0.138631188	0.69500965	0.487049254	0	0.55917006	0	0.666666667	CRA004775;PRJNA827465;Yang2025_Silkie_Wencha	0.835456916
ENSGALG00010026192	SMIM29	3	-0.092496199	0.132878707	-0.696094969	0.486369357	0	0.941634955	0	0.666666667	CRA004775;PRJNA827465;Yang2025_Silkie_Wencha	0.835456916
ENSGALG00010029409	ENSGALG0001002940	3	-0.086536654	0.124599903	-0.694516222	0.487358531	0	0.704680258	0	0.666666667	CRA004775;PRJNA827465;Yang2025_Silkie_Wencha	0.835456916
ENSGALG00010018951	YKT6	3	-0.076590786	0.110018075	-0.696165476	0.486325206	0.019146444	3.984676707	49.80772226	0.666666667	CRA004775;PRJNA827465;Yang2025_Silkie_Wencha	0.835456916
ENSGALG00010015635	ABTB3	3	0.075180445	0.108166313	0.695044909	0.487027158	0.003940645	2.188932937	8.631280277	0.666666667	CRA004775;PRJNA827465;Yang2025_Silkie_Wencha	0.835456916
ENSGALG00010002787	CEP44	3	-0.072225752	0.103757335	-0.696102614	0.486364569	0.014970046	3.884360947	48.51147906	0.666666667	CRA004775;PRJNA827465;Yang2025_Silkie_Wencha	0.835456916
ENSGALG00010024775	ITFG2	3	0.060302996	0.086812915	0.694631618	0.487286192	0.012266432	5.025836731	60.20563128	0.666666667	CRA004775;PRJNA827465;Yang2025_Silkie_Wencha	0.835456916
ENSGALG00010013331	KIF3B	3	-0.044072449	0.063424973	-0.694875327	0.487133436	0.003781969	2.772063508	27.85158081	0.666666667	CRA004775;PRJNA827465;Yang2025_Silkie_Wencha	0.835456916
ENSGALG00010015507	AMMECR1	3	0.034555065	0.049740674	0.694704396	0.487240572	0	1.314275632	0	0.666666667	CRA004775;PRJNA827465;Yang2025_Silkie_Wencha	0.835456916
ENSGALG00010027936	ENSGALG0001002793(	3	0.032433114	0.046645978	0.695303552	0.486865089	0	0.493914631	0	1	CRA004775;PRJNA827465;Yang2025_Silkie_Wencha	0.835456916
ENSGALG00010005259	SLC27A5	3	0.660895697	0.954267392	0.692568668	0.488580278	2.222407179	11.23528173	82.19893325	0.666666667	CRA004775;PRJNA827465;Yang2025_Silkie_Wencha	0.835615889
ENSGALG00010000312	CABP7	3	0.460427128	0.664044465	0.693367918	0.48807869	1.117344511	13.21650041	84.86740106	0.666666667	CRA004775;PRJNA827465;Yang2025_Silkie_Wencha	0.835615889
ENSGALG00010023246	SP5	2	0.287289239	0.4143434	0.693360241	0.488083507	0	0.019753319	0	1	CRA004775;PRJNA827465	0.835615889
ENSGALG00010029277	WFIKKN2	3	-0.284109739	0.410199276	-0.692613944	0.488551857	0.277074098	4.825455994	58.55313979	0.666666667	CRA004775;PRJNA827465;Yang2025_Silkie_Wencha	0.835615889
ENSGALG00010019878	MCFD2	3	-0.161528607	0.233057203	-0.693085666	0.488255792	0.143936777	21.99097951	90.90536191	0.666666667	CRA004775;PRJNA827465;Yang2025_Silkie_Wencha	0.835615889
ENSGALG00010025333	FZD5	3	-0.1580038	0.22789132	-0.69332961	0.488102725	0.132405034	24.36114421	91.790205	0.666666667	CRA004775;PRJNA827465;Yang2025_Silkie_Wencha	0.835615889
ENSGALG00010022508	USF3	3	0.110053174	0.158778455	0.693124102	0.488231673	0.035347487	3.377784782	40.78959647	0.666666667	CRA004775;PRJNA827465;Yang2025_Silkie_Wencha	0.835615889
ENSGALG00010021118	USMG5	3	-0.109559701	0.158188327	-0.692590301	0.488566699	0.041134783	4.355510404	54.08115664	0.666666667	CRA004775;PRJNA827465;Yang2025_Silkie_Wencha	0.835615889
ENSGALG00010022541	ITPKA	3	-0.102568803	0.147993022	-0.69306513	0.488268679	0.038339045	5.039450382	60.31313242	0.666666667	CRA004775;PRJNA827465;Yang2025_Silkie_Wencha	0.835615889
ENSGALG00010012055	ENSGALG0001001205	3	-0.080818324	0.116553479	-0.693401217	0.488057798	0.017044997	3.46804963	42.33069842	0.666666667	CRA004775;PRJNA827465;Yang2025_Silkie_Wencha	0.835615889
ENSGALG00010000727	ENSGALG0001000072	3	-0.073070338	0.105352348	-0.693580538	0.487945302	0	1.828135871	0	0.666666667	CRA004775;PRJNA827465;Yang2025_Silkie_Wencha	0.835615889
ENSGALG00010029351	ENSGALG0001002935	3	0.071911733	0.103780352	0.692922416	0.488358241	0	0.020547958	0	1	CRA004775;PRJNA827465;Yang2025_Silkie_Wencha	0.835615889
ENSGALG00010017642	MTARC1	3	-0.051131261	0.073699811	-0.693777375	0.487821833	0	0.797861107	0	0.666666667	CRA004775;PRJNA827465;Yang2025_Silkie_Wencha	0.835615889
ENSGALG00010009777	ST3GAL1	3	-0.039937866	0.057607915	-0.693270457	0.488139839	0	1.897652041	0	1	CRA004775;PRJNA827465;Yang2025_Silkie_Wencha	0.835615889
ENSGALG00010001249	PDCD10	3	0.037928473	0.054730981	0.692998236	0.488310658	0	0.03849552	0	1	CRA004775;PRJNA827465;Yang2025_Silkie_Wencha	0.835615889
ENSGALG00010023214	SUPV3L1	3	-0.032789902	0.047327991	-0.692822596	0.48842089	0	1.219975282	0	0.666666667	CRA004775;PRJNA827465;Yang2025_Silkie_Wencha	0.835615889
ENSGALG00010014948	RBM38	3	0.240807484	0.347954251	0.692066511	0.488895562	0.299463889	14.49282904	86.20007182	0.666666667	CRA004775;PRJNA827465;Yang2025_Silkie_Wencha	0.835617792
ENSGALG00010016769	BNC1	2	0.214150804	0.309416676	0.692111386	0.488867382	0	0.001817302	0	1	PRJNA827465;Yang2025_Silkie_Wenchang	0.835617792
ENSGALG00010006175	IL2RA	3	0.190645534	0.275498479	0.692002129	0.488935992	0	0.169259872	0	1	CRA004775;PRJNA827465;Yang2025_Silkie_Wencha	0.835617792
ENSGALG00010000443	TBX3	3	0.154243615	0.222848592	0.692145344	0.488846058	0.106518742	8.25251327	75.76495869	0.666666667	CRA004775;PRJNA827465;Yang2025_Silkie_Wencha	0.835617792
ENSGALG00010029698	NUP85	3	-0.108069068	0.156158709	-0.692046371	0.488908209	0.062242292	14.80816295	86.49393576	0.666666667	CRA004775;PRJNA827465;Yang2025_Silkie_Wencha	0.835617792
ENSGALG00010002368	UVRAG	3	-0.075144782	0.108520037	-0.692450756	0.4886543	0.022273411	5.530274059	63.83542698	0.666666667	CRA004775;PRJNA827465;Yang2025_Silkie_Wencha	0.835617792
ENSGALG00010028753	SV2A	3	0.274743739	0.397281977	0.691558527	0.489214614	0.14326591	2.843116581	29.6546609	0.666666667	CRA004775;PRJNA827465;Yang2025_Silkie_Wencha	0.835689916
ENSGALG00010016915	PLOD2	3	-0.102402196	0.148030911	-0.691762249	0.489086648	0.028485401	3.528882587	43.32483582	0.666666667	CRA004775;PRJNA827465;Yang2025_Silkie_Wencha	0.835689916
ENSGALG00010029628	UNC45B	3	-0.081409387	0.117717236	-0.691567263	0.489209126	0.013287402	2.911583204	31.30884953	0.666666667	CRA004775;PRJNA827465;Yang2025_Silkie_Wencha	0.835689916
ENSGALG00010018299	SHOC2	3	0.030671734	0.044344731	0.691665802	0.489147228	0	0.59566718	0	0.666666667	CRA004775;PRJNA827465;Yang2025_Silkie_Wencha	0.835689916
ENSGALG00010018849	KCNH1	2	0.449626836	0.650387161	0.691321821	0.489363322	0.64090636	4.07679824	75.47094702	0.5	PRJNA827465;Yang2025_Silkie_Wenchang	0.835842958
ENSGALG00010001832	BUB3	3	-0.097050202	0.140422163	-0.691131657	0.489482808	0.043614087	8.424152145	76.25873838	0.666666667	CRA004775;PRJNA827465;Yang2025_Silkie_Wencha	0.835845099
ENSGALG00010019233	ARPC2	3	0.03287006	0.047557345	0.691166839	0.489460701	0.001123582	2.235952767	10.55267226	0.666666667	CRA004775;PRJNA827465;Yang2025_Silkie_Wencha	0.835845099
ENSGALG00010019982	PLCE1	3	0.161965584	0.234416905	0.690929623	0.489609769	0.113992131	7.81070251	74.39410863	0.666666667	CRA004775;PRJNA827465;Yang2025_Silkie_Wencha	0.835926821
ENSGALG00010012209	KIF14	3	0.086362217	0.125005499	0.690867344	0.48964891	0.014413135	2.719509369	26.45732269	0.666666667	CRA004775;PRJNA827465;Yang2025_Silkie_Wencha	0.835926821
ENSGALG00010028024	RBM3	3	-0.100426925	0.145414565	-0.690624944	0.489801268	0.041324739	6.224548007	67.86915294	0.666666667	CRA004775;PRJNA827465;Yang2025_Silkie_Wencha	0.836085975
ENSGALG00010012726	IL7R	3	0.349437438	0.506476564	0.689938021	0.490233164	0.510627509	6.004998114	66.69441085	0.666666667	CRA004775;PRJNA827465;Yang2025_Silkie_Wencha	0.836241665
ENSGALG00010022834	CHRNA7	3	0.206838937	0.299740284	0.690060523	0.490156127	0.145327579	4.831438066	58.60445746	0.666666667	CRA004775;PRJNA827465;Yang2025_Silkie_Wencha	0.836241665
ENSGALG00010027313	BSN	3	-0.201037292	0.291394089	-0.689915479	0.490247341	0.194378819	9.030175631	77.85203653	0.666666667	CRA004775;PRJNA827465;Yang2025_Silkie_Wencha	0.836241665
ENSGALG00010008276	DNAJC18	3	0.130236764	0.188695958	0.690193712	0.490072377	0.085545896	12.55910055	84.07529272	0.666666667	CRA004775;PRJNA827465;Yang2025_Silkie_Wencha	0.836241665
ENSGALG00010023160	ENSGALG0001002316(	3	0.130135408	0.188553305	0.690178345	0.490082039	0.087559906	17.96151251	88.86508027	0.666666667	CRA004775;PRJNA827465;Yang2025_Silkie_Wencha	0.836241665
ENSGALG00010016477	CNIH3	3	0.118056566	0.171039984	0.690192806	0.490072947	0	1.484591785	0	0.666666667	CRA004775;PRJNA827465;Yang2025_Silkie_Wencha	0.836241665
ENSGALG00010000340	NAA10	3	-0.044767033	0.064922809	-0.689542452	0.490481968	0	0.405155761	0	1	CRA004775;PRJNA827465;Yang2025_Silkie_Wencha	0.836540959
ENSGALG00010024713	RND2	3	0.166925751	0.242250523	0.6890625	0.490783937	0	1.522949221	0	0.666666667	CRA004775;PRJNA827465;Yang2025_Silkie_Wencha	0.836897406
ENSGALG00010006183	PRKN	3	-0.066396616	0.096363546	-0.689022129	0.490809342	0	0.727429741	0	0.666666667	CRA004775;PRJNA827465;Yang2025_Silkie_Wencha	0.836897406
ENSGALG00010029323	NOL11	3	-0.112546967	0.163104481	-0.688803684	0.490946817	0.061495351	10.1998001	80.39100359	0.666666667	CRA004775;PRJNA827465;Yang2025_Silkie_Wencha	0.837030875
ENSGALG00010001883	KBTBD11	3	-0.159283828	0.231357296	-0.688475492	0.491153399	0.12597675	12.7328166	84.29255629	0.666666667	CRA004775;PRJNA827465;Yang2025_Silkie_Wencha	0.837174829
ENSGALG00010023110	LIMA1	3	0.057497582	0.083509518	0.688515319	0.491128327	0.012670285	6.972744039	71.31688774	0.666666667	CRA004775;PRJNA827465;Yang2025_Silkie_Wencha	0.837174829
ENSGALG00010014949	AGO3	3	-0.047576184	0.069112518	-0.68838736	0.491208882	0	0.783473635	0	1	CRA004775;PRJNA827465;Yang2025_Silkie_Wencha	0.837174829
ENSGALG00010005784	RASL11A	3	-0.248367606	0.360907838	-0.688174521	0.491342887	0.19190613	3.919951098	48.79389535	0.666666667	CRA004775;PRJNA827465;Yang2025_Silkie_Wencha	0.837206656
ENSGALG00010005493	KLF6	3	0.103021836	0.149704135	0.688169606	0.491345982	0.041688711	7.569751253	73.57905256	0.666666667	CRA004775;PRJNA827465;Yang2025_Silkie_Wencha	0.837206656
ENSGALG00010020900	PDHB	3	-0.125445427	0.182338549	-0.687980835	0.49146485	0.072567796	7.776463513	74.28136843	0.666666667	CRA004775;PRJNA827465;Yang2025_Silkie_Wencha	0.837207412
ENSGALG00010005458	PRKD1	3	0.065138991	0.094674513	0.688030902	0.491433322	0.014060649	4.43039489	54.85729716	0.666666667	CRA004775;PRJNA827465;Yang2025_Silkie_Wencha	0.837207412
ENSGALG00010022900	MMP17	3	-0.19171492	0.278704127	-0.687879734	0.49152852	0.175938628	16.46790061	87.8551611	0.666666667	CRA004775;PRJNA827465;Yang2025_Silkie_Wencha	0.837215003
ENSGALG00010027595	GNAT1	3	-0.212098386	0.308618063	-0.687252016	0.491923931	0.214137213	8.161810153	75.49563194	0.666666667	CRA004775;PRJNA827465;Yang2025_Silkie_Wencha	0.837430972
ENSGALG00010020057	RBKS	3	-0.140293794	0.204106994	-0.687354173	0.491859569	0.094419783	8.95927007	77.67675286	0.666666667	CRA004775;PRJNA827465;Yang2025_Silkie_Wencha	0.837430972
ENSGALG00010015520	UBE3A	3	-0.102479316	0.1491472	-0.687101845	0.492018552	0.054811018	27.98640373	92.85367274	0.666666667	CRA004775;PRJNA827465;Yang2025_Silkie_Wencha	0.837430972
ENSGALG00010015013	SNX18	3	-0.100425561	0.146356171	-0.686971962	0.492100397	0.041662754	7.411905655	73.01638616	0.666666667	CRA004775;PRJNA827465;Yang2025_Silkie_Wencha	0.837430972
ENSGALG00010016985	ENSGALG0001001698:	3	-0.081951955	0.119217961	-0.687418818	0.491822623	0	1.456854322	0	0.666666667	CRA004775;PRJNA827465;Yang2025_Silkie_Wencha	0.837430972
ENSGALG00010022931	RDH5	3	-0.074510672	0.108456148	-0.687001975	0.492075182	0	1.205047147	0	0.666666667	CRA004775;PRJNA827465;Yang2025_Silkie_Wencha	0.837430972
ENSGALG00010000155	MAZ	3	-0.056114548	0.081613399	-0.68756108	0.491726516	0.006670041	2.983473137	32.96403526	0.666666667	CRA004775;PRJNA827465;Yang2025_Silkie_Wencha	0.837430972
ENSGALG00010017933	SIX1	3	-0.049626245	0.07224391	-0.686926348	0.492129143	0	0.231992576	0	1	CRA004775;PRJNA827465;Yang2025_Silkie_Wencha	0.837430972
ENSGALG00010004591	RRS1	3	0.220610247	0.321337684	0.686537115	0.492374468	0.244748583	13.20882243	84.85860484	0.666666667	CRA004775;PRJNA827465;Yang2025_Silkie_Wencha	0.837659126
ENSGALG00010002329	STT3B	3	-0.039614014	0.057702164	-0.686525627	0.49238171	0.005310766	4.283461754	53.30879287	0.666666667	CRA004775;PRJNA827465;Yang2025_Silkie_Wencha	0.837659126
ENSGALG00010022024	MICAL2	3	0.176738844	0.257537431	0.686264683	0.492546216	0.127825029	9.1756073	78.20307763	0.666666667	CRA004775;PRJNA827465;Yang2025_Silkie_Wencha	0.8370752
ENSGALG00010001496	PTP4A1	3	-0.141509295	0.206181698	-0.686332957	0.492503171	0.101611707	11.21499302	82.16672988	0.666666667	CRA004775;PRJNA827465;Yang2025_Silkie_Wencha	0.8370752
ENSGALG00010012293	SRGAP1	3	-0.059201226	0.086274191	-0.686198569	0.4925879	0.008119211	2.795961888	28.46826674	0.666666667	CRA004775;PRJNA827465;Yang2025_Silkie_Wencha	0.83770752
ENSGALG00010005261	ATG3	3	0.133356296	0.194378924	0.686063559	0.492673029	0.092844607	17.56870024	88.61611859	0.666666667	CRA004775;PRJNA827465;Yang2025_Silkie_Wencha	0.837751528
ENSGALG00010011774	ANKRA2	3	-0.103628613	0.151103611	-0.685811624	0.492831906	0.033313179	3.899419931	48.71028066	0.666666667	CRA004775;PRJNA827465;Yang2025_Silkie_Wencha	0.837920913
ENSGALG00010009786	DIPK2B	3	-0.066586497	0.097107933	-0.685695746	0.49290499	0.011554927	3.370625247	40.66382782	0.666666667	CRA004775;PRJNA827465;Yang2025_Silkie_Wencha	0.837944409
ENSGALG00010027311	NFASC	3	0.207146453	0.302187119	0.685490679	0.493034341	0.172999364	5.625843066	64.44977266	0.666666667	CRA004775;PRJNA827465;Yang2025_Silkie_Wencha	0.837952949
ENSGALG00010003778	LCA5	3	0.172365398	0.25146364	0.685448593	0.493060889	0.147907998	10.26318392	80.51286993	0.666666667	CRA004775;PRJNA827465;Yang2025_Silkie_Wencha	0.837952949
ENSGALG00010004891	ANKIB1	3	0.04105392	0.059905446	0.685311978	0.493147075	0.002395153	2.418417256	17.30128476	1	CRA004775;PRJNA827465;Yang2025_Silkie_Wencha	0.837952949
ENSGALG00010013977	SLC38A2	3	-0.036796309	0.053686978	-0.68538612	0.4931003	0	1.799684394	0	0.666666667	CRA004775;PRJNA827465;Yang2025_Silkie_Wencha	0.837952949
ENSGALG00010026630	XIRP1	3	0.232817837	0.33992866	0.684902053	0.493405731	0.23260977	11.46568903	82.55665233	0.666666667	CRA004775;PRJNA827465;Yang2025_Silkie_Wencha	0.838291712
ENSGALG00010013058	ENSGALG0001001305:	3	-0.224807616	0.328516028	-0.684312474	0.493777872	0	1.024423964	0	0.666666667	CRA004775;PRJNA827465;Yang2025_Silkie_Wencha	0.838649948
ENSGALG00010009277	ACTN4	3	0.082601991	0.120702102	0.684345917	0.493756759	0.030681015	9.64877554	79.27198128	0.666666667	CRA004775;PRJNA827465;Yang2025_Silkie_Wencha	0.838649948

ENSGALG00010008200	COG8	3	0.04651381	0.067974215	0.684286093	0.493794527	0	1.230722662	0	0.666666667	CRA004775;PRJNA827465;Yang2025_Silkie_Wencha	0.838649948
ENSGALG00010012157	H2BC32	3	-0.305220933	0.446507538	-0.683573976	0.494244222	0.425080006	6.944569653	71.20051926	0.666666667	CRA004775;PRJNA827465;Yang2025_Silkie_Wencha	0.83880504
ENSGALG00010021385	GDF5	2	0.300156183	0.438906472	0.683872765	0.494055513	0	0.901076081	0	0.5	PRJNA827465;Yang2025_Silkie_Wenchang	0.83880504
ENSGALG00010003733	STAU2	3	0.10830687	0.158504482	0.68330478	0.494414275	0.059381985	11.19763728	82.13908926	0.666666667	CRA004775;PRJNA827465;Yang2025_Silkie_Wencha	0.83880504
ENSGALG00010027043	IL17RD	3	-0.103901953	0.15199429	-0.683591157	0.49423337	0.042329902	7.352397426	72.79798841	0.666666667	CRA004775;PRJNA827465;Yang2025_Silkie_Wencha	0.83880504
ENSGALG00010002566	TMSB4X	3	-0.07849984	0.114825885	-0.683642374	0.49420102	0.0149956	2.789995378	28.31529343	0.666666667	CRA004775;PRJNA827465;Yang2025_Silkie_Wencha	0.83880504
ENSGALG00010018174	BMP4	3	0.07419125	0.108541214	0.683530683	0.494271569	0.001275038	2.061321771	2.974876213	0.666666667	CRA004775;PRJNA827465;Yang2025_Silkie_Wencha	0.83880504
ENSGALG00010016867	FLT4	3	-0.064904833	0.094987862	-0.683296073	0.494419775	0.013978703	4.741503588	57.81928743	0.666666667	CRA004775;PRJNA827465;Yang2025_Silkie_Wencha	0.83880504
ENSGALG00010003913	PSMB5	3	0.050896169	0.07447362	0.68341205	0.494346508	0	1.383128767	0	0.666666667	CRA004775;PRJNA827465;Yang2025_Silkie_Wencha	0.83880504
ENSGALG00010011977	GATD3A	3	0.035933477	0.052531059	0.6840425	0.493948329	0	0.85778139	0	0.666666667	CRA004775;PRJNA827465;Yang2025_Silkie_Wencha	0.83880504
ENSGALG00010005280	TMEM244	2	-0.291390397	0.427167555	-0.682145434	0.495146994	0.191800025	2.106566687	52.52939267	0.5	PRJNA827465;Yang2025_Silkie_Wenchang	0.839154133
ENSGALG00010009347	TESK1	3	0.199620576	0.292499698	0.682464212	0.494945465	0.205281808	14.54652827	86.25101493	0.666666667	CRA004775;PRJNA827465;Yang2025_Silkie_Wencha	0.839154133
ENSGALG00010002429	ENSGALG0001000242	3	-0.193579571	0.283855535	-0.681965111	0.495261012	0	1.04260187	0	0.666666667	CRA004775;PRJNA827465;Yang2025_Silkie_Wencha	0.839154133
ENSGALG00010028592	SEMA6C	3	0.179261505	0.262742046	0.68227186	0.495067063	0.156309534	9.561295837	79.08233325	0.666666667	CRA004775;PRJNA827465;Yang2025_Silkie_Wencha	0.839154133
ENSGALG00010025032	FSTL1	3	-0.17223219	0.252486689	-0.682143643	0.495148126	0.155607572	19.51309953	89.75047508	0.666666667	CRA004775;PRJNA827465;Yang2025_Silkie_Wencha	0.839154133
ENSGALG00010004097	ENSGALG0001000409	3	0.170057451	0.249278662	0.682198187	0.495113641	0.144926854	11.71864118	82.93317485	0.666666667	CRA004775;PRJNA827465;Yang2025_Silkie_Wencha	0.839154133
ENSGALG00010000581	ENSGALG0001000058	3	0.150540497	0.220588021	0.682450917	0.494953869	0.129286425	50.21085566	96.01679761	0.666666667	CRA004775;PRJNA827465;Yang2025_Silkie_Wencha	0.839154133
ENSGALG00010009372	RAB28	3	0.136453146	0.200100005	0.68192475	0.495286535	0.100394419	17.18833616	88.36420244	0.666666667	CRA004775;PRJNA827465;Yang2025_Silkie_Wencha	0.839154133
ENSGALG00010013664	REV3L	3	0.117014121	0.171546246	0.682114143	0.495166778	0.065666086	8.689194005	76.98290545	0.666666667	CRA004775;PRJNA827465;Yang2025_Silkie_Wencha	0.839154133
ENSGALG00010002386	MHCY2B2	3	-0.102661515	0.150590157	-0.681727919	0.49541101	0	1.529161264	0	0.666666667	CRA004775;PRJNA827465;Yang2025_Silkie_Wencha	0.839154133
ENSGALG00010016647	EGR1	3	-0.074628783	0.109388448	-0.682236418	0.49508947	0	1.194871542	0	0.666666667	CRA004775;PRJNA827465;Yang2025_Silkie_Wencha	0.839154133
ENSGALG00010018393	PRKCE	3	0.057009119	0.083527799	0.682516721	0.494912273	0.002620111	2.184362888	8.440121773	0.666666667	CRA004775;PRJNA827465;Yang2025_Silkie_Wencha	0.839154133
ENSGALG00010016661	UBE4B	3	0.029255409	0.042917622	0.681664262	0.49545127	0	1.756508891	0	0.666666667	CRA004775;PRJNA827465;Yang2025_Silkie_Wencha	0.839154133
ENSGALG00010023080	MCRS1	3	-0.026045354	0.038208937	-0.681656077	0.495456447	0	0.812898	0	1	CRA004775;PRJNA827465;Yang2025_Silkie_Wencha	0.839154133
ENSGALG00010029550	CHMP6	3	-0.040286385	0.05911939	-0.681441143	0.495592397	0	1.105890589	0	0.666666667	CRA004775;PRJNA827465;Yang2025_Silkie_Wencha	0.839283855
ENSGALG00010018494	HAO1	3	0.339764196	0.498719768	0.681272768	0.495698911	0.358500554	3.848952919	48.03781594	0.666666667	CRA004775;PRJNA827465;Yang2025_Silkie_Wencha	0.839363701
ENSGALG00010020618	ENSGALG0001002061	2	0.900109368	1.321745727	0.681000399	0.495871238	3.270785122	15.56161708	93.57393262	0.5	PRJNA827465;Yang2025_Silkie_Wenchang	0.839473949
ENSGALG00010028162	CARD9	3	0.152905817	0.224679505	0.680550802	0.496155766	0.09577492	7.491261619	73.30222729	0.666666667	CRA004775;PRJNA827465;Yang2025_Silkie_Wencha	0.839473949
ENSGALG00010002527	LRRC3B	3	-0.140002464	0.205652872	-0.680770767	0.49601655	0.051344061	3.318498492	39.73177915	0.666666667	CRA004775;PRJNA827465;Yang2025_Silkie_Wencha	0.839473949
ENSGALG00010011384	PUM3	3	0.134019922	0.196891636	0.680676595	0.496074884	0.08918378	9.238249352	78.35087662	0.666666667	CRA004775;PRJNA827465;Yang2025_Silkie_Wencha	0.839473949
ENSGALG00010025997	CCDC43	3	0.133132662	0.195662677	0.680419301	0.496239003	0.092967981	14.72279775	86.41562538	0.666666667	CRA004775;PRJNA827465;Yang2025_Silkie_Wencha	0.839473949
ENSGALG00010012899	ENSGALG0001001289	3	0.131964266	0.193851182	0.680750382	0.496029451	0.086144579	10.19955794	80.39130703	0.666666667	CRA004775;PRJNA827465;Yang2025_Silkie_Wencha	0.839473949
ENSGALG00010003507	WDFY3	3	0.109995265	0.161592888	0.680693727	0.496065306	0.048663764	5.318032868	62.39210908	0.666666667	CRA004775;PRJNA827465;Yang2025_Silkie_Wencha	0.839473949
ENSGALG00010019645	ENSGALG0001001964	3	0.054854216	0.080611753	0.680474176	0.496204268	0	1.992506597	0	0.666666667	CRA004775;PRJNA827465;Yang2025_Silkie_Wencha	0.839473949
ENSGALG00010013944	METTL24	3	0.199221951	0.293009945	0.679915321	0.49655808	0.188689834	10.62944182	81.18433654	0.666666667	CRA004775;PRJNA827465;Yang2025_Silkie_Wencha	0.839812763
ENSGALG00010007658	BIRC2	3	0.049763362	0.073187877	0.679939955	0.496542481	0.006937627	3.529595466	43.33628261	0.666666667	CRA004775;PRJNA827465;Yang2025_Silkie_Wencha	0.839812763
ENSGALG00010024598	LRRC3C	3	0.095336461	0.140239257	0.679812933	0.496622917	0.031564827	4.650658603	56.99533828	0.666666667	CRA004775;PRJNA827465;Yang2025_Silkie_Wencha	0.839821962
ENSGALG00010012089	ENSGALG0001001208	2	0.273654312	0.402801907	0.679376902	0.496899081	0.269400309	5.721509603	82.52209523	0.5	PRJNA827465;Yang2025_Silkie_Wenchang	0.839987579
ENSGALG00010010682	SMIM13	3	-0.196880374	0.28976603	-0.679446015	0.496855302	0.221373452	34.07160978	94.13001026	0.666666667	CRA004775;PRJNA827465;Yang2025_Silkie_Wencha	0.839987579
ENSGALG00010024797	IKZF4	3	-0.046375798	0.06824511	-0.679547549	0.49679099	0	1.696682301	0	0.666666667	CRA004775;PRJNA827465;Yang2025_Silkie_Wencha	0.839987579
ENSGALG00010014026	SH3BP1	3	-0.202453045	0.298207311	-0.67890034	0.49720101	0.121961488	3.676695874	45.6033333	0.666666667	CRA004775;PRJNA827465;Yang2025_Silkie_Wencha	0.840196615
ENSGALG00010013936	DLGAP3	3	0.148221216	0.218320523	0.678915631	0.497191321	0.055296729	3.225368155	37.99157479	0.666666667	CRA004775;PRJNA827465;Yang2025_Silkie_Wencha	0.840196615
ENSGALG00010010991	C2CD2	3	-0.114469977	0.168589455	-0.678986579	0.497146366	0.051578181	6.361518075	68.56096333	0.666666667	CRA004775;PRJNA827465;Yang2025_Silkie_Wencha	0.840196615
ENSGALG00010027421	PLPPR3	3	0.149592059	0.220565802	0.678219638	0.497632443	0.099996673	8.347390517	76.04041651	0.666666667	CRA004775;PRJNA827465;Yang2025_Silkie_Wencha	0.840825181
ENSGALG00010028234	EDCH3	2	0.757633318	1.11879505	0.677186869	0.498287398	0	0.564934318	0	0.5	PRJNA827465;Yang2025_Silkie_Wenchang	0.840826533
ENSGALG00010017793	FAM83G	3	0.428931558	0.633260585	0.677338158	0.498191426	0.809191348	6.188731118	67.68319771	0.666666667	CRA004775;PRJNA827465;Yang2025_Silkie_Wencha	0.840826533
ENSGALG00010005450	ENSGALG0001000545	3	-0.220812299	0.325969563	-0.677401586	0.498151192	0.189358189	5.119664206	60.93493792	0.666666667	CRA004775;PRJNA827465;Yang2025_Silkie_Wencha	0.840826533
ENSGALG00010013552	FGF16	3	-0.192635726	0.284440508	-0.677244347	0.498250935	0.161909758	9.445068278	78.82492809	0.666666667	CRA004775;PRJNA827465;Yang2025_Silkie_Wencha	0.840826533
ENSGALG00010023163	CRTAC1	3	0.124862155	0.184333614	0.677370515	0.498170901	0.062592318	5.767896513	65.32531408	0.666666667	CRA004775;PRJNA827465;Yang2025_Silkie_Wencha	0.840826533
ENSGALG00010010100	IRF2BP2	3	0.118538603	0.175007196	0.677335592	0.498193053	0.064288376	9.008260636	77.79815571	0.666666667	CRA004775;PRJNA827465;Yang2025_Silkie_Wencha	0.840826533
ENSGALG00010025465	ENSGALG0001002546	3	-0.106271876	0.156841724	-0.67757401	0.49804183	0.04920884	6.775162875	70.48041447	0.666666667	CRA004775;PRJNA827465;Yang2025_Silkie_Wencha	0.840826533
ENSGALG00010023431	BCAR1	3	0.075967307	0.112084474	0.677768334	0.497918592	0.018572147	4.188699458	52.25248266	0.666666667	CRA004775;PRJNA827465;Yang2025_Silkie_Wencha	0.840826533
ENSGALG00010020065	TTYH3	3	0.05185413	0.076562294	0.677280256	0.498228156	0	0.550596339	0	0.666666667	CRA004775;PRJNA827465;Yang2025_Silkie_Wencha	0.840826533
ENSGALG00010011440	FUNDC1	3	-0.042906811	0.063338283	-0.677423025	0.498137594	0	0.032609319	0	1	CRA004775;PRJNA827465;Yang2025_Silkie_Wencha	0.840826533
ENSGALG00010029183	ENSGALG0001002918	3	0.042218225	0.06229642	0.677699047	0.497962531	0	1.078469036	0	0.666666667	CRA004775;PRJNA827465;Yang2025_Silkie_Wencha	0.840826533
ENSGALG00010014189	DDX4	2	0.392537055	0.58152741	0.675010409	0.499669152	0.368572531	2.17563887	54.03648952	0.5	PRJNA827465;Yang2025_Silkie_Wenchang	0.841415964
ENSGALG00010008164	TG	3	0.347687731	0.515344485	0.674670517	0.49988512	0.456660617	4.712570028	57.56031235	0.666666667	CRA004775;PRJNA827465;Yang2025_Silkie_Wencha	0.841415964
ENSGALG00010023689	WT1	3	0.261206517	0.386377042	0.676040469	0.499014951	0.350940331	12.03342441	83.37962717	0.666666667	CRA004775;PRJNA827465;Yang2025_Silkie_Wencha	0.841415964
ENSGALG00010024243	MPP3	3	0.228134298	0.338089765	0.67477434	0.499819146	0.277279828	12.03699431	83.38455641	0.666666667	CRA004775;PRJNA827465;Yang2025_Silkie_Wencha	0.841415964
ENSGALG00010010006	PPARGC1A	3	0.211058125	0.312660842	0.675038561	0.499651266	0.211167985	10.89586424	81.64441153	0.666666667	CRA004775;PRJNA827465;Yang2025_Silkie_Wencha	0.841415964
ENSGALG00010024469	GTF2H1	3	0.182252135	0.269622139	0.675953893	0.499069919	0.188818046	20.71288635	90.34417528	0.666666667	CRA004775;PRJNA827465;Yang2025_Silkie_Wencha	0.841415964
ENSGALG00010010734	AMN1	3	-0.144304363	0.213795335	-0.674964974	0.499698019	0.089099851	6.236666984	67.93158902	0.666666667	CRA004775;PRJNA827465;Yang2025_Silkie_Wencha	0.841415964
ENSGALG00010022505	ZNF511	3	0.14397271	0.2131274	0.675524168	0.499342802	0.107084995	11.93394724	83.24108562	0.666666667	CRA004775;PRJNA827465;Yang2025_Silkie_Wencha	0.841415964
ENSGALG00010018727	PARK7	3	0.140413151	0.208121702	0.67466847	0.499886421	0.089853791	9.814192105	79.62134857	0.666666667	CRA004775;PRJNA827465;Yang2025_Silkie_Wencha	0.841415964
ENSGALG00010012285	FBLN1	3	-0.121200265	0.17960054	-0.674832411	0.499782246	0.06240561	6.716742181	70.22365983	0.666666667	CRA004775;PRJNA827465;Yang2025_Silkie_Wencha	0.841415964
ENSGALG00010024744	KLF13	3	0.114717755	0.169680506	0.676080933	0.498989261	0.050601751	4.788566713	58.23384908	0.666666667	CRA004775;PRJNA827465;Yang2025_Silkie_Wencha	0.841415964
ENSGALG00010006025	TNFAIP3	3	-0.108806229	0.161126074	-0.675286292	0.499493892	0.050229185	7.198923201	72.21806728	0.666666667	CRA004775;PRJNA827465;Yang2025_Silkie_Wencha	0.841415964
ENSGALG00010002460	LAMTOR1	3	-0.108408544	0.160406027	-0.675838344	0.499143287	0.037308931	3.569305075	43.96668377	0.666666667	CRA004775;PRJNA827465;Yang2025_Silkie_Wencha	0.841415964
ENSGALG00010001234	AASS	3	-0.104143217	0.154298633	-0.674945817	0.49971019	0.047640437	7.870278966	74.58794016	0.666666667	CRA004775;PRJNA827465;Yang2025_Silkie_Wencha	0.841415964
ENSGALG00010026244	CLP1	3	-0.08480087	0.125604623	-0.67514131	0.49958599	0.02698059	4.58177288	56.34877476	0.666666667	CRA004775;PRJNA827465;Yang2025_Silkie_Wencha	0.841415964
ENSGALG00010011875	PSD2	3	-0.081579763	0.120679207	-0.676005135	0.499037385	0	0.032991237	0	1	CRA004775;PRJNA827465;Yang2025_Silkie_Wencha	0.841415964
ENSGALG00010009683	ADAMTS19	3	0.070152021	0.103854679	0.675482522	0.499369252	0	0.943558286	0	0.666666667	CRA004775;PRJNA827465;Yang2025_Silkie_Wencha	0.841415964
ENSGALG00010024941	PDHX	3	0.060132189	0.088974665	0.675834953	0.49914544	0.012726334	5.150473139	61.16861605	0.666666667	CRA004775;PRJNA827465;Yang2025_Silkie_Wencha	0.841415964
ENSGALG00010021699	SYS1	3	-0.055885312	0.082780462	-0.675102678	0.499610532	0	0.57994143	0	1	CRA004775;PRJNA827465;Yang2025_Silkie_Wencha	0.841415964
ENSGALG00010021751	MTCH2	3	-0.04845153	0.071641798	-0.676302547	0.498848576	0.002668651	2.232873171	10.42930578	0.666666667	CRA004775;PRJNA827465;Yang2025_Silkie_Wencha	0.841415964
ENSGALG00010009555	EPC2	3	-0.028379414	0.041976222	-0.676083108	0.498987881	0	1.537213157	0	0.666666667	CRA004775;PRJNA827465;Yang2025_Silkie_Wencha	0.841415964
ENSGALG00010028161	ENSGALG0001002816	3	0.726488325	1.079048717	0.6732674	0.500777188	3.273696891	32.66472373	93.87718685	0.666666667	CRA004775;PRJNA827465;Yang2025_Silkie_Wencha	0.841612597
ENSGALG00010024844	TM4SF19	2	0.55025683	0.8174506	0.673137716	0.500859681	0.93786378	3.306549819	69.75699582	0.5	CRA004775;Yang2025_Silkie_Wenchang	0.841612597
ENSGALG00010003206	ENSGALG0001000320	2	0.515803072	0.76688183	0.672597852	0.501203169	0.842950706	3.389510969	70.49721894	0.5	PRJNA827465;Yang2025_Silkie_Wenchang	0.841612597
ENSGALG00010026137	LPXN	3	0.279888127	0.416443184	0.672091987	0.501525137	0.412432966	9.683050246	79.34535142	0.666666667	CRA004775;PRJNA827465;Yang2025_Silkie_Wencha	0.841612597
ENSGALG00010016736	CENPS	3	-0.224235676	0.333650496	-0.672067564	0.501540685	0.275593985	16.33582038	87.75696627	0.666666667	CRA004775;PRJNA827465;Yang2025_Silkie_Wencha	0.841612597
ENSGALG00010015056	ENSGALG0001001505	3	0.219925851	0.326993217	0.67257007	0.501220849	0.141579464	3.571847137	44.00656235	0.666666667	CRA004775;PRJNA827465;Yang2025_Silkie_Wencha	0.841612597
ENSGALG00010017201	AP5M1	3	-0.210941837	0.313760531	-0.672302012	0.501391449	0.242610751	13.80082785	85.50811573	0.666666667	CRA004775;PRJNA827465;Yang2025_Silkie_Wencha	0.841612597
ENSGALG00010029623	ENSGALG0001002962	3	-0.205813335	0.30578297	-0.673069972	0.500902777	0.049614399	2.409724034	17.00294426	0.666666667	CRA004775;PRJNA827465;Yang2025_Silkie_Wencha	0.841612597
ENSGALG00010025938	ERP29	3	-0.175012687	0.260602127	-0.671570445	0.501857199	0.175110158	21.36663873	90.63961335	0.666666667	CRA004775;PRJNA827465;Yang2025_Silkie_Wencha	0.841612597
ENSGALG00010001454	VEGFD	3	-0.159398784	0.237148385	-0.672147881	0.501489557	0.099984402	5.801861247	65.52830351	0.666666667	CRA004775;PRJNA827465;Yang2025_Silkie_Wencha	0.841612597
ENSGALG00010016923	ENSGALG0001001692	3	-0.157436557	0.234462309	-0.671479171	0.501915324	0.115756349	9.163444511	78.17414622	0.666666667	CRA004775;PRJNA827465;Yang2025_Silkie_Wencha	0.841612597
ENSGALG00010023851	HSCB	3	0.152096673	0.226328185	0.672018259	0.501572073	0.098224733	5.879281264	65.98223643	0.666666667	CRA004775;PRJNA827465;Yang2025_Silkie_Wencha	0.841612597
ENSGALG00010018294	FKBP3	3	0.15198875	0.226284549	0.671670916	0.501793221	0.11797983	11.78641587	83.03131315	0.666666667	CRA004775;PRJNA827465;Yang2025_Silkie_Wencha	0.841612597
ENSGALG00010002716	NHLRC3	3	-0.148097516	0.220642506	-0.67121027	0.502086587	0.102202041	9.894181808	79.78610017	0.666666667	CRA004775;PRJNA827465;Yang2025_Silkie_Wencha	0.841612597
ENSGALG00010029696	SUMO2	3	-0.138952452	0.206566167	-0.672677691	0.501152364	0.110843414	26.88996622	92.56228147	0.666666667	CRA004775;PRJNA827465;Yang2025_Silkie_Wencha	0.841612597
ENSGALG00010009665	DGKQ	3	0.138165477	0.205134415	0.673536309	0.500606157	0.058486581	3.839308836	47.90729046	0.666666667	CRA004775;PRJNA827465;Yang2025_Silkie_Wencha	0.841612597
ENSGALG00010014806	MAP7D1	3	0.133049847	0.197344287	0.674201665	0.500183111	0.076880266	6.691660364	70.11205155	0.666666667	CRA004775;PRJNA827465;Yang2025_Silkie_Wencha	0.841612597
ENSGALG00010014143	NFATC2	3	0.126694685	0.188660877	0.671547206	0.501871997	0.046921099	3.317293639	39.70988951	0.666666667	CRA004775;PRJNA827465;Yang2025_Silkie_Wencha	0.841612597
ENSGALG00010022659	TIE1	3	-0.117475104	0.174885506	-0.67172579	0.50175828	0.067163819	11.40018814	82.45642988	0.666666667	CRA004775;PRJNA827465;Yang2025_Silkie_Wencha	0.841612597
ENSGALG00010028602	ZNF687	3	0.112434801	0.167490143	0.67129205	0.502034498	0.06190948	7.981518077	74.94211025	0.666666667	CRA004775;PRJNA827465;Yang2025_Silkie_Wencha	0.841612597
ENSGALG00010025723	SETD2	3	0.111117628	0.165136056	0.672885321	0.501020252	0.060104617	9.230423119	78.3325209	0.666666667	CRA004775;PRJNA827465;Yang2025_Silkie_Wencha	0.841612597
ENSGALG00010021417	STK16	3	-0.110582978	0.164633699	-0.671691027	0.501780415	0.046271927	4.691040353	57.36553409	0.666666667	CRA004775;PRJNA827465;Yang2025_Silkie_Wencha	0.841612597
ENSGALG00010008013	ENSGALG0001000801	3	0.100735858	0.149973077	0.671692945	0.501779193	0.030118015	3.639308153	45.04449978	0.666666667	CRA004775;PRJNA827465;Yang2025_Silkie_Wencha	0.841612597
ENSGALG00010025538	ANO1	3	0.099329141	0.147702497	0.672494662	0.501268837	0.01880765	2.772872803	27.87263816	0.666666667	CRA004775;PRJNA827465;Yang2025_Silkie_Wencha	0.841612597
ENSGALG00010017221	SPPL2A	3	-0.09772029	0.145353401	-0.672294486	0.501396239	0.055493725	18.20491064	89.01395322	0.666666667	CRA004775;PRJNA827465;Yang2025_Silkie_Wencha	0.841612597
ENSGALG00010006193	RBM17	3	0.094344195	0.140537514	0.671309688	0.502023264	0.046469347	10.14811577	80.29190793	0.666666667	CRA004775;PRJNA827465;Yang2025_Silkie_Wencha	0.841612597
ENSGALG00010011193	FAM20B	3	-0.090955957	0.135476472	-0.671378253	0.501979595	0.038122165	7.918244942	74.74187759	0.666666667	CRA004775;PRJNA827465;Yang2025_Silkie_Wencha	0.841612597
ENSGALG00010018920	ACAD11	3	0.081493995	0.121077497	0.673073003	0.500900848	0.018149831	3.398404745	41.14885807	0.666666667	CRA004775;PRJNA827465;Yang2025_Silkie_Wencha	0.841612597
ENSGALG00010024602	PROCR	3	-0.0782048	0.116040121	-0.673946209	0.500345513	0	0.996411519	0	0.666666667	CRA004775;PRJNA827465;Yang2025_Silkie_Wencha	0.841612597
ENSGALG00010029318	C17orf58	3	-0.067323164	0.100237461	-0.671636767	0.501814965	0	1.195932912	0	0.666666667	CRA004775;PRJNA827465;Yang2025_Silkie_Wencha	0.841612597
ENSGALG00010018163	CCT7	3	-0.060969259	0.090598039	-0.672964446	0.50096991	0.013585869	4.401292728	54.55880525	0.666666667	CRA004775;PRJNA827465;Yang2025_Silkie_Wencha	0.841612597
ENSGALG00010002408	OFD1	3	0.056704812	0.084208615	0.673384929	0.500702434	0	1.951926826	0	0.666666667	CRA004775;PRJNA827465;Yang2025_Silkie_Wencha	0.841612597
ENSGALG00010024322	IDH3A	3	0.050043149	0.074357617	0.673006364	0.500943242	0	0.148142155	0	1	CRA004775;PRJNA827465;Yang2025_Silkie_Wencha	0.841612597
ENSGALG00010009788	PAK1IP1	3	0.035742783	0.053116179	0.672917061	0.501000058	0.001026506	2.157096001	7.282754284	0.666666667	CRA004775;PRJNA827465;Yang2025_Silkie_Wencha	0.841612597
ENSGALG00010028122	SEC16A	3	-0.031730864	0.047195747	-0.672324654	0.501377038	0	0.366247975	0	1	CRA004775;PRJNA827465;Yang2025_Silkie_Wencha	0.841612597
ENSGALG00010027363	VIPR1	3	0.094514621	0.140849439	0.671032998	0.502199508	0.012986111	2.542891471	21.34937637	0.666666667	CRA004775;PRJNA827465;Yang2025_Silkie_Wencha	0.841702092
ENSGALG00010012071	ENSGALG0001001207	3	-0.101608144	0.15149813	-0.670689097	0.50241861	0.043642848	5.900100749	66.10227375	0.666666667	CRA004775;PRJNA827465;Yang2025_Silkie_Wencha	0.841969506
ENSGALG00010019600	MAP3K19	2	0.377615402	0.56311607	0.670581825	0.502486964	0.504504626	4.664040091	78.55936097	0.5	PRJNA827465;Yang2025_Silkie_Wenchang	0.84198426
ENSGALG00010001527	STK32C	3	-0.185264469	0.276359279	-0.670375424	0.502618497	0.178240078	11.4763438	82.57284694	0.666666667	CRA004775;PRJNA827465;Yang2025_Silkie_Wencha	0.842104863
ENSGALG00010026889	ENSGALG0001002688	3	-0.115967889	0.173038567	-0.670185213	0.502739729	0	0.087264479	0	1	CRA004775;PRJNA827465;Yang2025_Silkie_Wencha	0.842208179
ENSGALG00010025451	LRRC43	3	0.436617246	0.652286652	0.669364067	0.503263267	1.100284629	16.6017814	87.95310002	0.666666667	CRA004775;PRJNA827465;Yang2025_Silkie_Wencha	0.8426408
ENSGALG00010017591	ESR2	3	0.264019108	0.394250711	0.669673132	0.503066183	0.255015607	4.64990044	56.9883264	0.666666667	CRA004775;PRJNA827465;Yang2025_Silkie_Wencha	0.8426408
ENSGALG00010019800	EXT2	3	-0.173416726	0.259314303	-0.668751104	0.503654262	0.183440031	56.88376236	96.48405816	0.666666667	CRA004775;PRJNA827465;Yang2025_Silkie_Wencha	0.8426408
ENSGALG00010007251	SPAST	3	-0.13185768	0.197086576	-0.669034306	0.503473594	0.10978815	44.14582781	95.46956055	0.666666667	CRA004775;PRJNA827465;Yang2025_Silkie_Wencha	0.8426408
ENSGALG00010022981	HNRNPH3	3	0.131087287	0.196099372	0.668473773	0.503831218	0.086763647	9.423405693	78.7762507	0.666666667	CRA004775;PRJNA827465;Yang2025_Silkie_Wencha	0.8426408
ENSGALG00010015564	PKD2L2	3	-0.108862469	0.162670857	-0.66921925	0.503355628	0.012969544	2.31081344	13.45039088	0.666666667	CRA004775;PRJNA827465;Yang2025_Silkie_Wencha	0.8426408
ENSGALG00010012509	MICAL3	3	0.102869774	0.153887925	0.668472029	0.503832331	0.037736054	4.263520218	53.09040657	0.666666667	CRA004775;PRJNA827465;Yang2025_Silkie_Wencha	0.8426408
ENSGALG00010004113	ECHDC1	3	-0.092018799	0.137648477	-0.668505754	0.50381081	0.009544413	2.361715474	15.31579388	0.666666667	CRA004775;PRJNA827465;Yang2025_Silkie_Wencha	0.8426408
ENSGALG00010022623	TOP1	3	0.091300092	0.136477214	0.668976817	0.503510267	0.033671893	5.048269932	60.38246712	0.666666667	CRA004775;PRJNA827465;Yang2025_Silkie_Wencha	0.8426408
ENSGALG00010004341	ENSGALG0001000434	3	0.076322411	0.114102245	0.66889491	0.503562517	0	0.316499503	0	0.666666667	CRA004775;PRJNA827465;Yang2025_Silkie_Wencha	0.8426408
ENSGALG00010020440	PAX7	3	0.068700836	0.102691524	0.66900201	0.503494196	0.003927309	2.174169111	8.010835503	0.666666667	CRA004775;PRJNA827465;Yang2025_Silkie_Wencha	0.8426408
ENSGALG00010012031	GRK6	3	-0.042881626	0.064139721	-0.668565826	0.503772478	0.00056102	2.04491215	2.196287514	1	CRA004775;PRJNA827465;Yang2025_Silkie_Wencha	0.8426408
ENSGALG00010021431	CPSF4	3	-0.033086012	0.049491421	-0.668520134	0.503801634	0	1.790059368	0	0.666666667	CRA004775;PRJNA827465;Yang2025_Silkie_Wencha	0.8426408
ENSGALG00010021444	ATG9A	3	-0.030064453	0.044920677	-0.669278716	0.503317702	0.000313595	2.082585795	3.965541077	0.666666667	CRA004775;PRJNA827465;Yang2025_Silkie_Wencha	0.8426408
ENSGALG00010002886	HERC2	3	0.073848286	0.110533424	0.668108195	0.504064531	0.019637429	4.63954248	56.89230116	0.666666667	CRA004775;PRJNA827465;Yang2025_Silkie_Wencha	0.842929439
ENSGALG00010024349	R3HDML	3	-0.214267767	0.321857393	-0.665722682	0.505588373	0	0.45583155	0	1	CRA004775;PRJNA827465;Yang2025_Silkie_Wencha	0.842995974
ENSGALG00010013914	NRG1	3	0.213237727	0.320236788	0.665875174	0.50549089	0.206497318	8.125960824	75.38752594	0.666666667	CRA004775;PRJNA827465;Yang2025_Silkie_Wencha	0.842995974
ENSGALG00010019472	CAPN3	3	0.198195733	0.297064774	0.66718019	0.504657044	0.207187511	14.40029171	86.11139247	0.666666667	CRA004775;PRJNA827465;Yang2025_Silkie_Wencha	0.842995974
ENSGALG00010001166	TRIM59	3	-0.19029925	0.28575909	-0.665942947	0.505447568	0.23246502	44.24989884	95.48021566	0.666666667	CRA004775;PRJNA827465;Yang2025_Silkie_Wencha	0.842995974
ENSGALG00010011628	MATN2	3	0.179382668	0.269168903	0.666431621	0.505135257	0.178878236	15.14028561	86.79020957	0.666666667	CRA004775;PRJNA827465;Yang2025_Silkie_Wencha	0.842995974
ENSGALG00010017905	EIF4E2	3	0.152112701	0.228032753	0.667065142	0.504730524	0.138894313	20.61845006	90.29994983	0.666666667	CRA004775;PRJNA827465;Yang2025_Silkie_Wencha	0.842995974
ENSGALG00010004404	RGS20	3	0.144045329	0.21638556	0.665688267	0.505610375	0	1.763993928	0	1	CRA004775;PRJNA827465;Yang2025_Silkie_Wencha	0.842995974
ENSGALG00010003937	ENSGALG0001000393	3	0.130305523	0.195757718	0.665646926	0.505636805	0.055588186	3.814215102	47.56457235	0.666666667	CRA004775;PRJNA827465;Yang2025_Silkie_Wencha	0.842995974
ENSGALG00010015655	ENSGALG0001001565	3	-0.123763714	0.18556265	-0.666964576	0.504794761	0	1.337474711	0	0.666666667	CRA004775;PRJNA827465;Yang2025_Silkie_Wencha	0.842995974
ENSGALG00010018838	WFIKKN1	3	0.119357152	0.178717209	0.667854834	0.50422626	0	1.969482499	0	0.666666667	CRA004775;PRJNA827465;Yang2025_Silkie_Wencha	0.842995974
ENSGALG00010027063	RAB7L1	3	-0.118327725	0.177700559	-0.665882685	0.505486089	0.073945342	12.85551727	84.44247743	0.666666667	CRA004775;PRJNA827465;Yang2025_Silkie_Wencha	0.842995974
ENSGALG00010001429	MRPS15	3	0.117398838	0.176887667	0.665619262	0.505654491	0.064243244	7.473944461	73.23660851	0.666666667	CRA004775;PRJNA827465;Yang2025_Silkie_Wencha	0.842995974
ENSGALG00010001229	ZNF407	3	0.098510511	0.147963059	0.665777744	0.505553172	0.048249188	8.608357665	76.7667646	0.666666667	CRA004775;PRJNA827465;Yang2025_Silkie_Wencha	0.842995974
ENSGALG00010023096	SPATS2	3	0.088729452	0.133124493	0.666514854	0.505082073	0.039106968	8.104898791	75.32356601	0.666666667	CRA004775;PRJNA827465;Yang2025_Silkie_Wencha	0.842995974
ENSGALG00010003727	FBXO25	3	-0.081983367	0.122882174	-0.667170542	0.504663205	0.025880325	5.021654835	60.17249162	0.666666667	CRA004775;PRJNA827465;Yang2025_Silkie_Wencha	0.842995974
ENSGALG00010020137	NTAN1	3	0.080432928	0.120420495	0.667933876	0.504175802	0.027168132	5.87446289	65.95433425	0.666666667	CRA004775;PRJNA827465;Yang2025_Silkie_Wencha	0.842995974
ENSGALG00010005323	DNAJB14	3	0.071074724	0.106672185	0.666291064	0.505225076	0.018404914	4.225805316	52.67174301	0.666666667	CRA004775;PRJNA827465;Yang2025_Silkie_Wencha	0.842995974
ENSGALG00010020839	PGGHG	3	-0.068380805	0.102469954	-0.66732542	0.504564292	0.006971368	2.520493806	20.65046955	0.666666667	CRA004775;PRJNA827465;Yang2025_Silkie_Wencha	0.842995974
ENSGALG00010026566	ENSGALG0001002656	3	0.066631958	0.099823838	0.667507584	0.504403271	0	1.637403664	0	0.666666667	CRA004775;PRJNA827465;Yang2025_Silkie_Wencha	0.842995974
ENSGALG00010029336	SYNGR2	3	-0.059950941	0.089832532	-0.667363365	0.504540061	0.009935253	3.137330601	36.25153819	0.666666667	CRA004775;PRJNA827465;Yang2025_Silkie_Wencha	0.842995974
ENSGALG00010025464	VPS33A	3	-0.050046489	0.075101253	-0.666386863	0.505163857	0.007128431	3.321194419	39.78070092	0.666666667	CRA004775;PRJNA827465;Yang2025_Silkie_Wencha	0.842995974
ENSGALG00010005170	GPATCH2L	3	-0.043771191	0.065738256	-0.665840458	0.505513082	0	1.287029486	0	0.666666667	CRA004775;PRJNA827465;Yang2025_Silkie_Wencha	0.842995974
ENSGALG00010015388	PSMD1	3	-0.041622063	0.062434764	-0.666658771	0.50499012	0	0.099030082	0	0.666666667	CRA004775;PRJNA827465;Yang2025_Silkie_Wencha	0.842995974
ENSGALG00010023377	OSER1	3	0.035155281	0.05279572	0.6658737	0.505491832	0	0.313110449	0	1	CRA004775;PRJNA827465;Yang2025_Silkie_Wencha	0.842995974
ENSGALG00010002087	POLA1	3	-0.03159512	0.047356473	-0.66717637	0.504659483	0	0.531334149	0	1	CRA004775;PRJNA827465;Yang2025_Silkie_Wencha	0.842995974
ENSGALG00010029716	GOSR1	3	-0.024251896	0.036318378	-0.667758244	0.504287925	0	0.002150117	0	1	CRA004775;PRJNA827465;Yang2025_Silkie_Wencha	0.842995974
ENSGALG00010027540	PPOX	3	0.064075041	0.096299753	0.665370077	0.505813376	0	1.501204313	0	0.666666667	CRA004775;PRJNA827465;Yang2025_Silkie_Wencha	0.843161438
ENSGALG00010005403	ENSGALG0001000540	3	0.274056513	0.412146365	0.664949485	0.506082803	0.438157519	22.7341853	91.20267574	0.666666667	CRA004775;PRJNA827465;Yang2025_Silkie_Wencha	0.843240661
ENSGALG00010024245	TARBP2	3	0.086071337	0.129436063	0.664971841	0.506068504	0.014057711	2.569033898	22.14972321	0.666666667	CRA004775;PRJNA827465;Yang2025_Silkie_Wencha	0.843240661
ENSGALG00010005984	IDH3B	3	0.082248614	0.123713714	0.66483021	0.506159098	0.018554368	3.126849329	36.03785185	0.666666667	CRA004775;PRJNA827465;Yang2025_Silkie_Wencha	0.843240661
ENSGALG00010026167	CABIN1	3	0.07903699	0.118829915	0.665127042	0.505969924	0.023304707	4.352885579	54.05346721	0.666666667	CRA004775;PRJNA827465;Yang2025_Silkie_Wencha	0.843240661
ENSGALG00010017524	ACBD3	3	-0.061539647	0.092560974	-0.664855221	0.506143099	0.01635405	5.555488704	63.6995668	0.666666667	CRA004775;PRJNA827465;Yang2025_Silkie_Wencha	0.843240661
ENSGALG00010000373	ENSGALG0001000037	2	-0.222697633	0.335079715	-0.664610906	0.506299392	0	0.977776348	0	0.5	CRA004775;PRJNA827465	0.843275663
ENSGALG00010023478	TAOK3	3	0.195349752	0.293928257	0.664617122	0.506295415	0.21924884	15.31269247	86.93893968	0.666666667	CRA004775;PRJNA827465;Yang2025_Silkie_Wencha	0.843275663
ENSGALG00010029610	ERN1	3	0.123833487	0.18637997	0.66441437	0.506425287	0.083070225	19.67713407	89.83591821	0.666666667	CRA004775;PRJNA827465;Yang2025_Silkie_Wencha	0.843311198
ENSGALG00010016113	DTWD1	3	-0.039655987	0.059687714	-0.664391118	0.506440016	0	0.052089254	0	1	CRA004775;PRJNA827465;Yang2025_Silkie_Wencha	0.843311198
ENSGALG00010004149	SDAD1	3	-0.071916817	0.108289044	-0.664119054	0.506614116	0.021670831	5.582421438	64.17325309	0.666666667	CRA004775;PRJNA827465;Yang2025_Silkie_Wencha	0.843501765
ENSGALG00010010756	CENPL	3	-0.082057383	0.123632787	-0.663718623	0.506870418	0.012872317	2.69373547	25.75365983	0.666666667	CRA004775;PRJNA827465;Yang2025_Silkie_Wencha	0.843829135
ENSGALG00010018378	ARPP21	3	0.292864379	0.441398992	0.663491272	0.507015967	0.50466499	19.34536538	89.66160649	0.666666667	CRA004775;PRJNA827465;Yang2025_Silkie_Wencha	0.843872721
ENSGALG00010017272	NRXN1	3	0.189521038	0.285622396	0.663537036	0.506986668	0.167923063	6.668037833	70.00616898	0.666666667	CRA004775;PRJNA827465;Yang2025_Silkie_Wencha	0.843872721
ENSGALG00010005459	ZC3H12D	3	0.172243596	0.259799939	0.662985515	0.507339831	0.059816307	2.829210307	29.30889602	0.666666667	CRA004775;PRJNA827465;Yang2025_Silkie_Wencha	0.844184098
ENSGALG00010027148	ELL	3	0.144781321	0.218399579	0.662919414	0.507382167	0.102141297	9.32596504	78.55449821	0.666666667	CRA004775;PRJNA827465;Yang2025_Silkie_Wencha	0.844184098
ENSGALG00010018513	RPUSD1	3	-0.120104788	0.181159226	-0.662979141	0.507343913	0.07314308	9.575944403	79.11433154	0.666666667	CRA004775;PRJNA827465;Yang2025_Silkie_Wencha	0.844184098
ENSGALG00010019295	MAP4K5	3	-0.054656925	0.082480162	-0.662667525	0.507543513	0	0.410057089	0	0.666666667	CRA004775;PRJNA827465;Yang2025_Silkie_Wencha	0.844353186
ENSGALG00010007411	ENSGALG0001000741	2	0.80286106	1.211906949	0.662477478	0.507665264	1.971192151	2.960921457	66.22672994	0.5	PRJNA827465;Yang2025_Silkie_Wenchang	0.844357036
ENSGALG00010007972	EXOC8	3	0.05545662	0.083708801	0.662494498	0.50765436	0.010953589	4.479164185	55.34881247	0.666666667	CRA004775;PRJNA827465;Yang2025_Silkie_Wencha	0.844357036
ENSGALG00010009551	HADHA	3	-0.057356185	0.086597079	-0.66233394	0.50775723	0	0.430828744	0	0.666666667	CRA004775;PRJNA827465;Yang2025_Silkie_Wencha	0.844410665
ENSGALG00010029398	RHBDF2	3	0.062465212	0.094328148	0.662211794	0.507835496	0.013657788	4.234755062	52.7717667	0.666666667	CRA004775;PRJNA827465;Yang2025_Silkie_Wencha	0.844441501
ENSGALG00010029311	ENSGALG0001002931	3	0.078829008	0.119069187	0.662043729	0.507943197	0	1.592345362	0	0.666666667	CRA004775;PRJNA827465;Yang2025_Silkie_Wencha	0.844521268
ENSGALG00010003263	ENSGALG0001000326	3	0.802140698	1.212331392	0.661651347	0.508194692	3.752413274	13.84470014	85.55403888	0.666666667	CRA004775;PRJNA827465;Yang2025_Silkie_Wencha	0.844615845

ENSGALG00010017122	NCBP2	3	0.305049905	0.461121771	0.661538718	0.508266893	0.612318257	57.10376989	96.49760427	0.666666667	CRA004775;PRJNA827465;Yang2025_Silkie_Wencha	0.844615845
ENSGALG00010020950	EFHD2	3	-0.192638724	0.29126087	-0.661395828	0.508358501	0.162070737	5.634347403	64.50343124	0.666666667	CRA004775;PRJNA827465;Yang2025_Silkie_Wencha	0.844615845
ENSGALG00010020681	BCL10	3	-0.166417169	0.251602808	-0.661428109	0.508337805	0.176320667	35.02984988	94.29058358	0.666666667	CRA004775;PRJNA827465;Yang2025_Silkie_Wencha	0.844615845
ENSGALG00010029337	MYO1C	3	0.099305849	0.150057178	0.661786728	0.508107913	0.042253752	5.782877591	65.41514206	0.666666667	CRA004775;PRJNA827465;Yang2025_Silkie_Wencha	0.844615845
ENSGALG00010018485	ATP2C1	3	-0.020892497	0.031577029	-0.661635925	0.508204578	0	1.999537364	0	0.666666667	CRA004775;PRJNA827465;Yang2025_Silkie_Wencha	0.844615845
ENSGALG00010024314	ITFG1	3	0.051567193	0.077990002	0.661202607	0.50848239	0.005801249	2.744771383	27.13418638	0.666666667	CRA004775;PRJNA827465;Yang2025_Silkie_Wencha	0.844722419
ENSGALG00010012226	SLCO4C1	2	-0.5459614	0.826124824	-0.660870349	0.508695463	1.06157824	4.491547095	77.73595648	0.5	CRA004775;PRJNA827465	0.84493276
ENSGALG00010005699	PRPF18	3	-0.035917984	0.054353755	-0.660818803	0.508728523	0.002433943	2.603476213	23.17963228	0.666666667	CRA004775;PRJNA827465;Yang2025_Silkie_Wencha	0.84493276
ENSGALG00010014241	ENSGALG00010014241	3	-0.464487723	0.703722921	-0.660043477	0.50922593	0.41496853	2.768660294	27.76289658	0.666666667	CRA004775;PRJNA827465;Yang2025_Silkie_Wencha	0.845001853
ENSGALG00010002512	DAXX	3	0.169941309	0.257519767	0.659915591	0.509307999	0.16752329	17.74709488	88.73054991	0.666666667	CRA004775;PRJNA827465;Yang2025_Silkie_Wencha	0.845001853
ENSGALG00010004454	SRI	3	-0.159998413	0.24227847	-0.660375803	0.509012696	0.147523012	16.3509641	87.76830536	0.666666667	CRA004775;PRJNA827465;Yang2025_Silkie_Wencha	0.845001853
ENSGALG00010015532	NXT2	3	-0.112841176	0.170849081	-0.660472828	0.50895045	0.06336795	10.72612695	81.35394061	0.666666667	CRA004775;PRJNA827465;Yang2025_Silkie_Wencha	0.845001853
ENSGALG00010025342	TCEANC2	3	-0.087067703	0.131830847	-0.660450158	0.508964994	0.02921252	4.642438883	56.91919592	0.666666667	CRA004775;PRJNA827465;Yang2025_Silkie_Wencha	0.845001853
ENSGALG00010020140	SECISBP2L	3	0.060328936	0.091353654	0.660388871	0.509004312	0.008830449	2.802312149	28.6303633	0.666666667	CRA004775;PRJNA827465;Yang2025_Silkie_Wencha	0.845001853
ENSGALG00010009562	HADHB	3	-0.054732787	0.082910778	-0.660140794	0.509163482	0	0.00556993	0	1	CRA004775;PRJNA827465;Yang2025_Silkie_Wencha	0.845001853
ENSGALG00010010214	FAF2	3	-0.052525101	0.079575568	-0.659966144	0.509275556	0.008141024	3.352940835	40.35087112	0.666666667	CRA004775;PRJNA827465;Yang2025_Silkie_Wencha	0.845001853
ENSGALG00010005502	SCFD1	3	0.0451391	0.068371525	0.660203205	0.509123436	0.00743221	4.551185785	56.05540853	0.666666667	CRA004775;PRJNA827465;Yang2025_Silkie_Wencha	0.845001853
ENSGALG00010003208	ENSGALG00010003208	2	0.807409759	1.225382489	0.658904274	0.509957241	2.628057482	7.995502879	87.4929693	0.5	CRA004775;PRJNA827465	0.845192121
ENSGALG00010015807	ENSGALG00010015807	3	-0.407160995	0.617787923	-0.65906273	0.509855488	0.437834115	3.197455611	37.45026535	0.666666667	CRA004775;PRJNA827465;Yang2025_Silkie_Wencha	0.845192121
ENSGALG00010029936	SERPINF2	3	-0.402999333	0.611703699	-0.658814652	0.510014797	0.86009262	8.90872281	77.55009284	0.666666667	CRA004775;PRJNA827465;Yang2025_Silkie_Wencha	0.845192121
ENSGALG00010023766	ENSGALG00010023766	3	-0.144617998	0.219664594	-0.65835825	0.510307956	0	1.745687471	0	0.666666667	CRA004775;PRJNA827465;Yang2025_Silkie_Wencha	0.845192121
ENSGALG00010023179	KAT2A	3	-0.129063358	0.195987624	-0.658528103	0.510198845	0.102165462	19.40234076	89.69196539	0.666666667	CRA004775;PRJNA827465;Yang2025_Silkie_Wencha	0.845192121
ENSGALG00010011559	MTX3	3	-0.106417764	0.161606398	-0.659390747	0.509644882	0.058986127	9.758840665	79.50576233	0.666666667	CRA004775;PRJNA827465;Yang2025_Silkie_Wencha	0.845192121
ENSGALG00010018516	LRRN4	3	-0.092179371	0.14003612	-0.65825425	0.51037477	0.016584508	2.63359551	24.05819375	0.666666667	CRA004775;PRJNA827465;Yang2025_Silkie_Wencha	0.845192121
ENSGALG00010019231	ENSGALG00010019231	3	-0.091662136	0.139254963	-0.658232452	0.510388775	0	0.328989184	0	1	CRA004775;PRJNA827465;Yang2025_Silkie_Wencha	0.845192121
ENSGALG00010001860	RNF169	3	0.079600305	0.120789753	0.658998826	0.509896523	0.018681265	3.226916164	38.02132134	0.666666667	CRA004775;PRJNA827465;Yang2025_Silkie_Wencha	0.845192121
ENSGALG00010016221	TMEM200B	3	-0.064698145	0.098278527	-0.658314152	0.510336286	0	0.023831005	0	1	CRA004775;PRJNA827465;Yang2025_Silkie_Wencha	0.845192121
ENSGALG00010027440	TWF2	3	0.062226669	0.094367407	0.659408482	0.509633497	0.012182346	3.877079642	48.41478162	0.666666667	CRA004775;PRJNA827465;Yang2025_Silkie_Wencha	0.845192121
ENSGALG00010006739	VCAN	3	0.061876608	0.093929849	0.658753408	0.510054131	0	0.222672541	0	1	CRA004775;PRJNA827465;Yang2025_Silkie_Wencha	0.845192121
ENSGALG00010012808	EYA3	3	-0.056378221	0.085515659	-0.659273659	0.509720054	0.008145741	2.763631457	27.63145045	0.666666667	CRA004775;PRJNA827465;Yang2025_Silkie_Wencha	0.845192121
ENSGALG00010007275	DPY30	3	-0.045066907	0.068357743	-0.659280205	0.509715851	0.001153896	2.122400832	5.76709312	0.666666667	CRA004775;PRJNA827465;Yang2025_Silkie_Wencha	0.845192121
ENSGALG00010011856	NFYB	3	0.037134493	0.056365392	0.658817253	0.510013127	0	0.087961945	0	1	CRA004775;PRJNA827465;Yang2025_Silkie_Wencha	0.845192121
ENSGALG00010021663	CNOT9	3	-0.031213313	0.047414691	-0.658304674	0.510342375	0	0.659614589	0	1	CRA004775;PRJNA827465;Yang2025_Silkie_Wencha	0.845192121
ENSGALG00010012295	CUL4B	3	-0.026526233	0.040303964	-0.658154445	0.510438894	0	0.739180246	0	0.666666667	CRA004775;PRJNA827465;Yang2025_Silkie_Wencha	0.845192121
ENSGALG00010021683	HFM1	2	0.24320510	0.369809297	0.657649297	0.510763509	0.178732847	2.857711242	65.00696133	0.5	PRJNA827465;Yang2025_Silkie_Wenchang	0.845234698
ENSGALG00010018387	NT5DC4	3	0.24259838	0.368705558	0.657973211	0.510555345	0.341232537	12.73379471	84.29376282	0.666666667	CRA004775;PRJNA827465;Yang2025_Silkie_Wencha	0.845234698
ENSGALG00010013994	CHRM2	2	0.236301051	0.359270197	0.65772517	0.510714746	0	0.058177953	0	1	PRJNA827465;Yang2025_Silkie_Wenchang	0.845234698
ENSGALG00010021158	ACTG1L	3	-0.103585142	0.157464351	-0.657832338	0.510645873	0.051821144	7.256444028	72.43829082	0.666666667	CRA004775;PRJNA827465;Yang2025_Silkie_Wencha	0.845234698
ENSGALG00010024199	SLC35B1	3	-0.081543645	0.123944325	-0.657905407	0.510598916	0.029388987	5.619419685	64.40915631	0.666666667	CRA004775;PRJNA827465;Yang2025_Silkie_Wencha	0.845234698
ENSGALG00010028542	ENSGALG00010028542	2	-1.355258627	2.065240285	-0.656223219	0.511680512	4.84959965	2.293616072	56.40072407	0.5	PRJNA827465;Yang2025_Silkie_Wenchang	0.845309013
ENSGALG00010005575	ENSGALG00010005575	3	0.577176189	0.885570868	0.65175607	0.514558545	2.040485448	15.48964513	87.08814835	0.666666667	CRA004775;PRJNA827465;Yang2025_Silkie_Wencha	0.845309013
ENSGALG00010010188	DSP	3	0.48162173	0.737405513	0.653130091	0.513672414	1.187269519	7.386941833	72.92519631	0.666666667	CRA004775;PRJNA827465;Yang2025_Silkie_Wencha	0.845309013
ENSGALG00010011034	FAM180A	3	0.405440612	0.621635512	0.652215976	0.514261855	0.854350907	7.915418768	74.73285926	0.666666667	CRA004775;PRJNA827465;Yang2025_Silkie_Wencha	0.845309013
ENSGALG00010001921	ENSGALG00010001921	3	0.365247301	0.560897141	0.651184102	0.514927652	0	1.512583581	0	0.666666667	CRA004775;PRJNA827465;Yang2025_Silkie_Wencha	0.845309013
ENSGALG00010000269	ENSGALG00010000269	2	-0.361654785	0.550274131	-0.65722658	0.511035237	0.451663278	3.91948113	74.48641882	0.5	PRJNA827465;Yang2025_Silkie_Wenchang	0.845309013
ENSGALG00010022684	ENSGALG00010022684	3	0.316175935	0.481281358	0.65694615	0.511215543	0.517785391	9.314784739	78.52875771	0.666666667	CRA004775;PRJNA827465;Yang2025_Silkie_Wencha	0.845309013
ENSGALG00010013429	PDE4DIP	3	0.214128034	0.328272736	0.652286987	0.514216053	0.276989462	33.28095819	93.99055764	0.666666667	CRA004775;PRJNA827465;Yang2025_Silkie_Wencha	0.845309013
ENSGALG00010019682	TMEM158	3	0.20801472	0.318727342	0.652641593	0.513987365	0.196073479	5.989789286	66.60984378	0.666666667	CRA004775;PRJNA827465;Yang2025_Silkie_Wencha	0.845309013
ENSGALG00010010896	LY86	3	-0.204366924	0.313172863	-0.652569072	0.51403413	0.221858019	10.69949806	81.30753434	0.666666667	CRA004775;PRJNA827465;Yang2025_Silkie_Wencha	0.845309013
ENSGALG00010028442	CHTOP	3	0.19632424	0.300896601	0.652464132	0.514101804	0.254300008	59.39735262	96.63284656	0.666666667	CRA004775;PRJNA827465;Yang2025_Silkie_Wencha	0.845309013
ENSGALG00010005790	MUSK	3	0.194126259	0.298063377	0.65126238	0.514877128	0.184029109	9.704990376	79.39204551	0.666666667	CRA004775;PRJNA827465;Yang2025_Silkie_Wencha	0.845309013
ENSGALG00010013797	IL1RL1	3	-0.184488029	0.281621863	-0.655091287	0.512408982	0.156725018	6.301030996	68.25916265	0.666666667	CRA004775;PRJNA827465;Yang2025_Silkie_Wencha	0.845309013
ENSGALG00010005826	LRP2BP	3	-0.182940927	0.279112186	-0.655438693	0.512185346	0.137831464	5.065292173	60.5156044	0.666666667	CRA004775;PRJNA827465;Yang2025_Silkie_Wencha	0.845309013
ENSGALG00010024330	SDF2L1	3	-0.182330104	0.277580235	-0.656855499	0.511273834	0.190687769	20.70794842	90.3418728	0.666666667	CRA004775;PRJNA827465;Yang2025_Silkie_Wencha	0.845309013
ENSGALG00010021760	CSTA	3	-0.173276913	0.266155176	-0.651037186	0.515022482	0.068600439	2.923745664	31.59459713	0.666666667	CRA004775;PRJNA827465;Yang2025_Silkie_Wencha	0.845309013
ENSGALG00010018517	ECT2	3	-0.167051936	0.254654361	-0.655994796	0.511827473	0.137784405	11.76206335	82.99618068	0.666666667	CRA004775;PRJNA827465;Yang2025_Silkie_Wencha	0.845309013
ENSGALG00010015626	XKR7	3	-0.159969598	0.245495908	-0.651618186	0.514647513	0.005798214	2.060834736	2.951946371	0.666666667	CRA004775;PRJNA827465;Yang2025_Silkie_Wencha	0.845309013
ENSGALG00010013266	KIAA1755	3	0.147300641	0.225858707	0.652180486	0.514284747	0.111308901	11.40243436	82.45988587	0.666666667	CRA004775;PRJNA827465;Yang2025_Silkie_Wencha	0.845309013
ENSGALG00010001918	CNKSR2	3	0.144790477	0.22108043	0.655070329	0.512422474	0.098437778	6.965648509	71.28760981	0.666666667	CRA004775;PRJNA827465;Yang2025_Silkie_Wencha	0.845309013
ENSGALG00010012601	TUBA8A	3	-0.142167877	0.217501524	-0.65364083	0.513343233	0.069409896	4.0151214	50.18830564	0.666666667	CRA004775;PRJNA827465;Yang2025_Silkie_Wencha	0.845309013
ENSGALG00010021230	C1orf174	3	-0.142106314	0.21819498	-0.651281317	0.514864906	0.128246063	31.75884242	93.70254125	0.666666667	CRA004775;PRJNA827465;Yang2025_Silkie_Wencha	0.845309013
ENSGALG00010015050	HIVEP3	3	0.139525633	0.21310281	0.654733895	0.512639098	0.083775022	5.267264681	62.02962788	0.666666667	CRA004775;PRJNA827465;Yang2025_Silkie_Wencha	0.845309013
ENSGALG00010007114	CKLF	3	-0.136820978	0.208826819	-0.655188731	0.512346249	0.070938082	4.467547913	55.23271292	0.666666667	CRA004775;PRJNA827465;Yang2025_Silkie_Wencha	0.845309013
ENSGALG00010003795	LCP1	3	-0.13662453	0.208228657	-0.656127413	0.511742148	0.084392428	8.190917954	75.58271233	0.666666667	CRA004775;PRJNA827465;Yang2025_Silkie_Wencha	0.845309013
ENSGALG00010012673	ENSGALG00010012673	3	0.136578621	0.208039741	0.656502554	0.511500825	0	0.617734715	0	0.666666667	CRA004775;PRJNA827465;Yang2025_Silkie_Wencha	0.845309013
ENSGALG00010010659	ADAMTS1	3	0.136276857	0.208444723	0.653779357	0.513253968	0.090036917	9.654629778	79.28455004	0.666666667	CRA004775;PRJNA827465;Yang2025_Silkie_Wencha	0.845309013
ENSGALG00010029322	BPTF	3	0.136044675	0.209011067	0.650896993	0.515112982	0.089969871	6.55305516	69.47988455	0.666666667	CRA004775;PRJNA827465;Yang2025_Silkie_Wencha	0.845309013
ENSGALG00010024632	IFITM10	3	-0.135938369	0.208799345	-0.651047869	0.515015587	0	1.110158781	0	0.666666667	CRA004775;PRJNA827465;Yang2025_Silkie_Wencha	0.845309013
ENSGALG00010003865	AKAP8L	3	0.129852339	0.199209759	0.65183724	0.514506175	0.087145597	7.881340242	74.6236054	0.666666667	CRA004775;PRJNA827465;Yang2025_Silkie_Wencha	0.845309013
ENSGALG00010003665	RDH14	3	0.127895525	0.19571295	0.653485246	0.513443498	0.08846552	15.45199327	87.05668605	0.666666667	CRA004775;PRJNA827465;Yang2025_Silkie_Wencha	0.845309013
ENSGALG00010021801	VAMP1	3	0.121815697	0.185537795	0.657123421	0.51110156	0.027457117	2.672095031	25.1523626	0.666666667	CRA004775;PRJNA827465;Yang2025_Silkie_Wencha	0.845309013
ENSGALG00010001477	NLGN4	3	0.117006314	0.178676474	0.654850139	0.512564245	0	1.206141673	0	0.666666667	CRA004775;PRJNA827465;Yang2025_Silkie_Wencha	0.845309013
ENSGALG00010008147	CDKAL1	3	-0.112260509	0.171486136	-0.654633148	0.512703977	0.067779105	10.98243597	81.78910393	0.666666667	CRA004775;PRJNA827465;Yang2025_Silkie_Wencha	0.845309013
ENSGALG00010001713	PNPLA4	3	0.111357123	0.169508367	0.656941748	0.511218373	0.031102549	3.095070142	35.38110906	0.666666667	CRA004775;PRJNA827465;Yang2025_Silkie_Wencha	0.845309013
ENSGALG00010016556	ASAP3	3	-0.110686689	0.168685305	-0.656172679	0.511713025	0.048677916	5.232364132	61.77636056	0.666666667	CRA004775;PRJNA827465;Yang2025_Silkie_Wencha	0.845309013
ENSGALG00010001923	RAPGEF5	3	0.109821278	0.168125857	0.653208732	0.513621722	0.05975816	9.7821735	79.55464601	0.666666667	CRA004775;PRJNA827465;Yang2025_Silkie_Wencha	0.845309013
ENSGALG00010020138	ENSGALG00010020138	3	-0.107810209	0.164701658	-0.654578771	0.512738996	0.066363253	13.4494876	85.129545	0.666666667	CRA004775;PRJNA827465;Yang2025_Silkie_Wencha	0.845309013
ENSGALG00010011936	CKAP4	3	-0.107347781	0.164777329	-0.651471788	0.514741983	0.055352689	10.77765999	81.44309616	0.666666667	CRA004775;PRJNA827465;Yang2025_Silkie_Wencha	0.845309013
ENSGALG00010012082	HAUS1	3	0.10534211	0.161031189	0.654172094	0.513000938	0.013266319	2.36288366	15.3576609	0.666666667	CRA004775;PRJNA827465;Yang2025_Silkie_Wencha	0.845309013
ENSGALG00010001677	SDHAF4	3	-0.098149359	0.150790459	-0.650898998	0.515111689	0.033668526	3.800971846	47.38187809	0.666666667	CRA004775;PRJNA827465;Yang2025_Silkie_Wencha	0.845309013
ENSGALG00010013246	LNPEP	3	-0.096908369	0.147798031	-0.655681056	0.51202936	0.039684291	5.696936617	64.89341317	0.666666667	CRA004775;PRJNA827465;Yang2025_Silkie_Wencha	0.845309013
ENSGALG00010021350	CABLES2	3	-0.092437102	0.141907489	-0.65138988	0.514794842	0.036195218	6.089103018	67.15443976	0.666666667	CRA004775;PRJNA827465;Yang2025_Silkie_Wencha	0.845309013
ENSGALG00010024816	NFIA	3	0.091489101	0.139628497	0.655232296	0.512318204	0.02819357	3.536337997	43.44432003	0.666666667	CRA004775;PRJNA827465;Yang2025_Silkie_Wencha	0.845309013
ENSGALG00010022842	PHC1	3	0.090913446	0.139224932	0.652996877	0.513758292	0.038938279	6.141356379	67.4339423	0.666666667	CRA004775;PRJNA827465;Yang2025_Silkie_Wencha	0.845309013
ENSGALG00010011434	NPL	3	-0.090375235	0.138046835	-0.654670819	0.512679717	0.032450292	4.697193345	57.42138223	0.666666667	CRA004775;PRJNA827465;Yang2025_Silkie_Wencha	0.845309013
ENSGALG00010004868	NPAT	3	0.09036805	0.138539242	0.652292083	0.514212766	0.036903869	5.552013343	63.97703182	0.666666667	CRA004775;PRJNA827465;Yang2025_Silkie_Wencha	0.845309013
ENSGALG00010016845	MIA3	3	0.085544529	0.131018272	0.652920599	0.513807468	0.029111071	4.520409975	55.75622541	0.666666667	CRA004775;PRJNA827465;Yang2025_Silkie_Wencha	0.845309013
ENSGALG00010017954	PTGER2	3	0.082273719	0.126368159	0.651063685	0.515005378	0	0.665856174	0	0.666666667	CRA004775;PRJNA827465;Yang2025_Silkie_Wencha	0.845309013
ENSGALG00010021847	P3H1	3	0.076886724	0.118115689	0.65094421	0.515082501	0.021920091	4.209965785	52.49367566	0.666666667	CRA004775;PRJNA827465;Yang2025_Silkie_Wencha	0.845309013
ENSGALG00010012008	ZNF366	3	-0.072975272	0.111646878	-0.653625729	0.513352964	0.004277986	2.186343562	8.523068609	0.666666667	CRA004775;PRJNA827465;Yang2025_Silkie_Wencha	0.845309013
ENSGALG00010029308	GAA	3	0.069876553	0.106473886	0.656278791	0.511644761	0	0.527975443	0	0.666666667	CRA004775;PRJNA827465;Yang2025_Silkie_Wencha	0.845309013
ENSGALG00010013365	CEP85	3	-0.064929669	0.099494477	-0.65259571	0.514016952	0.007075871	2.542342423	21.33239087	0.666666667	CRA004775;PRJNA827465;Yang2025_Silkie_Wencha	0.845309013
ENSGALG00010017533	FAM193A	3	0.060446243	0.092146209	0.655981871	0.511835789	0.010731096	2.983910854	32.97386892	0.666666667	CRA004775;PRJNA827465;Yang2025_Silkie_Wencha	0.845309013
ENSGALG00010013802	PYROXD1	3	-0.057397622	0.087642076	-0.654909433	0.512526067	0	1.067006092	0	0.666666667	CRA004775;PRJNA827465;Yang2025_Silkie_Wencha	0.845309013
ENSGALG00010029283	RPS6KB1	3	-0.055067132	0.084503749	-0.651653123	0.51462497	0.01228621	4.764486726	58.02276058	0.666666667	CRA004775;PRJNA827465;Yang2025_Silkie_Wencha	0.845309013
ENSGALG00010022216	ROMO1	3	-0.051919229	0.07943631	-0.653595689	0.513372322	0	0.213203729	0	1	CRA004775;PRJNA827465;Yang2025_Silkie_Wencha	0.845309013
ENSGALG00010015108	ENSGALG00010015108	3	0.051705792	0.079053301	0.654062402	0.513071603	0	1.197067526	0	0.666666667	CRA004775;PRJNA827465;Yang2025_Silkie_Wencha	0.845309013
ENSGALG00010011802	VPS4B	3	-0.050890754	0.078033985	-0.652161418	0.514297047	0.010210328	4.742875708	57.83149037	0.666666667	CRA004775;PRJNA827465;Yang2025_Silkie_Wencha	0.845309013
ENSGALG00010017890	ANAPC1	3	-0.050500866	0.07742397	-0.652263973	0.514230897	0.009713206	4.930445865	59.43571728	0.666666667	CRA004775;PRJNA827465;Yang2025_Silkie_Wencha	0.845309013
ENSGALG00010004312	PDLIM5	3	-0.048834605	0.074652814	-0.654156262	0.513011137	0	1.078524726	0	0.666666667	CRA004775;PRJNA827465;Yang2025_Silkie_Wencha	0.845309013
ENSGALG00010015693	COA5	3	-0.046770719	0.071477113	-0.654345384	0.512889313	0.001597496	2.143499726	6.694646353	0.666666667	CRA004775;PRJNA827465;Yang2025_Silkie_Wencha	0.845309013
ENSGALG00010021848	LARGE2	3	-0.045992012	0.070093954	-0.656148055	0.511728867	0	0.798832348	0	0.666666667	CRA004775;PRJNA827465;Yang2025_Silkie_Wencha	0.845309013
ENSGALG00010024873	HYOU1	3	0.044569791	0.067898107	0.656421701	0.511552831	0.005194632	3.177451852	37.05648132	0.666666667	CRA004775;PRJNA827465;Yang2025_Silkie_Wencha	0.845309013
ENSGALG00010013402	MARCKS	3	0.042451166	0.064690875	0.656215676	0.511685364	0	0.662457329	0	0.666666667	CRA004775;PRJNA827465;Yang2025_Silkie_Wencha	0.845309013
ENSGALG00010008762	SAE1	3	-0.038984286	0.059853933	-0.651323712	0.514837545	0.002470544	2.269640238	11.88030742	0.666666667	CRA004775;PRJNA827465;Yang2025_Silkie_Wencha	0.845309013
ENSGALG00010011239	RALGPS2	3	0.036940628	0.056718814	0.651294088	0.514856664	0	1.230441237	0	0.666666667	CRA004775;PRJNA827465;Yang2025_Silkie_Wencha	0.845309013
ENSGALG00010021383	MSI1	3	0.034678195	0.053086394	0.653240735	0.513601093	0	0.468823616	0	0.666666667	CRA004775;PRJNA827465;Yang2025_Silkie_Wencha	0.845309013
ENSGALG00010009540	RAB10	3	-0.031870985	0.048594568	-0.655854897	0.511917491	0.000738707	2.129540857	6.083041619	0.666666667	CRA004775;PRJNA827465;Yang2025_Silkie_Wencha	0.845309013
ENSGALG00010027932	POLE3	3	-0.030312128	0.04656959	-0.650899613	0.515111291	0	1.827984157	0	1	CRA004775;PRJNA827465;Yang2025_Silkie_Wencha	0.845309013
ENSGALG00010006720	SRP54	3	-0.029941619	0.045661305	-0.65573289	0.511996003	0	0.697505704	0	0.666666667	CRA004775;PRJNA827465;Yang2025_Silkie_Wencha	0.845309013
ENSGALG00010008262	CYB5B	3	-0.028974313	0.044438478	-0.652009569	0.514394999	0	0.956068694	0	0.666666667	CRA004775;PRJNA827465;Yang2025_Silkie_Wencha	0.845309013
ENSGALG00010022143	MATCAP1	3	-0.078567903	0.120768606	-0.650565615	0.515326933	0.018347806	3.252463404	38.50814747	0.666666667	CRA004775;PRJNA827465;Yang2025_Silkie_Wencha	0.845561971
ENSGALG00010022782	TMPRSS13	3	-0.189283965	0.291406948	-0.649551997	0.515981649	0.166321866	5.800737995	65.52162842	0.666666667	CRA004775;PRJNA827465;Yang2025_Silkie_Wencha	0.845580128
ENSGALG00010022511	IGFBP4	3	0.172584619	0.265457367	0.650140626	0.515601389	0.154960657	12.38311431	83.84897409	0.666666667	CRA004775;PRJNA827465;Yang2025_Silkie_Wencha	0.845580128
ENSGALG00010024031	LIMS2	3	-0.127956408	0.19683809	-0.650059181	0.515653995	0.090096983	12.61326774	84.14368075	0.666666667	CRA004775;PRJNA827465;Yang2025_Silkie_Wencha	0.845580128
ENSGALG00010006465	PLAA	3	0.125423004	0.193088152	0.649563439	0.515974256	0.074446077	6.120950985	67.32533874	0.666666667	CRA004775;PRJNA827465;Yang2025_Silkie_Wencha	0.845580128
ENSGALG00010014312	KIAA1191	3	-0.116716108	0.179579824	-0.649939983	0.51573099	0.072789765	9.564763118	79.08991602	0.666666667	CRA004775;PRJNA827465;Yang2025_Silkie_Wencha	0.845580128
ENSGALG00010005999	PDE10A	3	0.108288484	0.166694891	0.649620891	0.515937135	0.03908483	3.871958578	48.34655486	1	CRA004775;PRJNA827465;Yang2025_Silkie_Wencha	0.845580128
ENSGALG00010017678	YARS	3	-0.103500469	0.159256717	-0.649897043	0.515758728	0.05272584	6.890342739	70.97386769	0.666666667	CRA004775;PRJNA827465;Yang2025_Silkie_Wencha	0.845580128
ENSGALG00010014308	ENSGALG00010014308	3	0.091922579	0.141486037	0.649693646	0.515890129	0.016061189	2.618520958	23.62100469	0.666666667	CRA004775;PRJNA827465;Yang2025_Silkie_Wencha	0.845580128
ENSGALG00010013453	PUM1	3	0.069006599	0.106240817	0.649530016	0.515995852	0.021514864	5.724172637	65.06045281	0.666666667	CRA004775;PRJNA827465;Yang2025_Silkie_Wencha	0.845580128
ENSGALG00010026355	PPP1R12B	3	-0.063999787	0.098402224	-0.650389644	0.515440565	0.014129318	4.286159136	53.33817675	0.666666667	CRA004775;PRJNA827465;Yang2025_Silkie_Wencha	0.845580128
ENSGALG00010002396	SEC23IP	3	-0.052195903	0.080285875	-0.650125606	0.51561109	0.008241695	3.323590235	39.82411011	0.666666667	CRA004775;PRJNA827465;Yang2025_Silkie_Wencha	0.845580128
ENSGALG00010006685	ENSGALG00010006685	3	0.054220296	0.083491986	0.649407192	0.516075216	0	0.944119461	0	0.666666667	CRA004775;PRJNA827465;Yang2025_Silkie_Wencha	0.845612178
ENSGALG00010024291	ENSGALG00010024291	3	0.148544078	0.228773762	0.64930557	0.516140886	0.057272929	3.121473597	35.92769768	0.666666667	CRA004775;PRJNA827465;Yang2025_Silkie_Wencha	0.845621784
ENSGALG00010026205	ENSGALG00010026205	3	0.484066007	0.746552011	0.648402255	0.516724812	1.330912721	10.19080931	80.37447333	0.666666667	CRA004775;PRJNA827465;Yang2025_Silkie_Wencha	0.845666519
ENSGALG00010003249	YJU2B	3	0.362843107	0.559378239	0.648654312	0.516561841	0.874529558	32.19586806	93.78802275	0.666666667	CRA004775;PRJNA827465;Yang2025_Silkie_Wencha	0.845666519
ENSGALG00010014550	SCML4	2	0.277182044	0.427753069	0.647995455	0.51698789	0	0.385570013	0	1	PRJNA827465;Yang2025_Silkie_Wenchang	0.845666519
ENSGALG00010003178	CENPA	3	0.244762044	0.377405367	0.648538853	0.51663649	0.242380773	4.630667002	56.80967776	0.666666667	CRA004775;PRJNA827465;Yang2025_Silkie_Wencha	0.845666519
ENSGALG00010013646	CLDN1	3	0.243941682	0.376612061	0.647726686	0.517161741	0.304227379	7.31755655	72.66847224	0.666666667	CRA004775;PRJNA827465;Yang2025_Silkie_Wencha	0.845666519
ENSGALG00010029207	SPAG5	3	0.198461103	0.306154272	0.64823888	0.516830458	0.218750691	18.59149604	89.24239343	0.666666667	CRA004775;PRJNA827465;Yang2025_Silkie_Wencha	0.845666519
ENSGALG00010024365	ENSGALG00010024365	3	0.153854645	0.237096404	0.64891176	0.516395413	0.135534233	11.35981016	82.39407197	0.666666667	CRA004775;PRJNA827465;Yang2025_Silkie_Wencha	0.845666519
ENSGALG00010005549	RNF20	3	0.142325445	0.219727358	0.647736569	0.517155348	0.107529484	7.860304038	74.55569161	0.666666667	CRA004775;PRJNA827465;Yang2025_Silkie_Wencha	0.845666519
ENSGALG00010015410	HTR2B	3	-0.127496769	0.196682559	-0.648236275	0.516832143	0.050158247	3.50225646	42.89395929	0.666666667	CRA004775;PRJNA827465;Yang2025_Silkie_Wencha	0.845666519
ENSGALG00010009416	COL14A1	3	-0.126886951	0.19582383	-0.647964809	0.517007712	0.059378525	4.500432831	55.55983001	0.666666667	CRA004775;PRJNA827465;Yang2025_Silkie_Wencha	0.845666519
ENSGALG00010011353	ENSGALG00010011353	3	-0.119956106	0.185115838	-0.648005638	0.516981304	0	0.340242219	0	1	CRA004775;PRJNA827465;Yang2025_Silkie_Wencha	0.845666519
ENSGALG00010028700	ANGPTL2	3	0.11152405	0.172231759	0.647523145	0.51729342	0.035111506	3.190854697	37.32086886	0.666666667	CRA004775;PRJNA827465;Yang2025_Silkie_Wencha	0.845666519
ENSGALG00010015584	TTLL9	3	0.111511165	0.172173998	0.647665537	0.517201299	0	0.703497792	0	0.666666667	CRA004775;PRJNA827465;Yang2025_Silkie_Wencha	0.845666519
ENSGALG00010012407	ATF4	3	-0.100084162	0.154596933	-0.647387757	0.517381018	0.053231575	10.02465491	80.04918855	0.666666667	CRA004775;PRJNA827465;Yang2025_Silkie_Wencha	0.845666519
ENSGALG00010001169	ENSGALG00010001169	3	-0.096592083	0.148872431	-0.648824518	0.516451808	0.053115128	10.15066638	80.29686008	0.666666667	CRA004775;PRJNA827465;Yang2025_Silkie_Wencha	0.845666519
ENSGALG00010021661	DUSP16	3	-0.084747075	0.130640764	-0.648703145	0.516530271	0.029858336	5.595843975	64.25918934	0.666666667	CRA004775;PRJNA827465;Yang2025_Silkie_Wencha	0.845666519
ENSGALG00010009359	REEP3	3	-0.077217845	0.119254861	-0.647502703	0.517306646	0.020665779	4.103256452	51.25822567	0.666666667	CRA004775;PRJNA827465;Yang2025_Silkie_Wencha	0.845666519
ENSGALG00010018244	CHM	3	-0.074005783	0.114326252	-0.647320988	0.517424221	0.027314206	7.607202643	73.70912681	0.666666667	CRA004775;PRJNA827465;Yang2025_Silkie_Wencha	0.845666519
ENSGALG00010018053	DIAPH2	3	-0.071077953	0.109679354	-0.64805226	0.51695115	0.018952252	4.277983329	53.24899968	0.666666667	CRA004775;PRJNA827465;Yang2025_Silkie_Wencha	0.845666519
ENSGALG00010029590	UTP6	3	0.050408748	0.077752914	0.648319727	0.516778178	0.007129702	3.282329647	39.06766793	0.666666667	CRA004775;PRJNA827465;Yang2025_Silkie_Wencha	0.845666519
ENSGALG00010013899	WNT5B	3	-0.039101187	0.060280297	-0.648656176	0.516560637	0	0.164969547	0	1	CRA004775;PRJNA827465;Yang2025_Silkie_Wencha	0.845666519
ENSGALG00010025939	TMEM136-1	2	0.255660995	0.395260842	0.646815896	0.517751103	0	0.161307753	0	1	CRA004775;PRJNA827465	0.846102965
ENSGALG00010000231	ENSGALG00010000231	3	0.163533328	0.252885949	0.646668308	0.517846639	0.063102454	2.870004741	30.31370396	0.666666667	CRA004775;PRJNA827465;Yang2025_Silkie_Wencha	0.846161288
ENSGALG00010009297	CTNNA3	3	-0.185868036	0.287989425	-0.645398821	0.518668767	0.153099468	5.667150076	64.70889295	0.666666667	CRA004775;PRJNA827465;Yang2025_Silkie_Wencha	0.846280835
ENSGALG00010023544	SSB	3	0.157789952	0.244279931	0.64593907	0.518318816	0.14425242	13.26513007	84.92287683	0.666666667	CRA004775;PRJNA827465;Yang2025_Silkie_Wencha	0.846280835
ENSGALG00010013249	AURKA	3	-0.135770364	0.210270105	-0.64569504	0.518476873	0.096891533	9.557175889	79.07331597	0.666666667	CRA004775;PRJNA827465;Yang2025_Silkie_Wencha	0.846280835
ENSGALG00010000574	ENSGALG00010000574	3	0.131601156	0.203664669	0.646165861	0.518171946	0.08580575	7.09732007	71.82034937	0.666666667	CRA004775;PRJNA827465;Yang2025_Silkie_Wencha	0.846280835
ENSGALG00010021010	LEO1	3	0.125202638	0.193986139	0.645420536	0.518654699	0.09270845	12.34999399	83.80566013	0.666666667	CRA004775;PRJNA827465;Yang2025_Silkie_Wencha	0.846280835
ENSGALG00010022395	PTGES3L	3	0.124022865	0.1921282	0.645521404	0.518589352	0.071709751	6.973596276	71.32039308	0.666666667	CRA004775;PRJNA827465;Yang2025_Silkie_Wencha	0.846280835
ENSGALG00010001292	LOX	3	-0.077857772	0.120501367	-0.646115258	0.518204715	0	1.00977172	0	0.666666667	CRA004775;PRJNA827465;Yang2025_Silkie_Wencha	0.846280835
ENSGALG00010012653	CBX7	3	-0.077393145	0.119800981	-0.64601428	0.518270107	0	0.166365069	0	1	CRA004775;PRJNA827465;Yang2025_Silkie_Wencha	0.846280835
ENSGALG00010003052	ENSGALG00010003052	3	0.067230383	0.104089237	0.645891786	0.518349439	0.018303853	4.344163515	53.96121731	0.666666667	CRA004775;PRJNA827465;Yang2025_Silkie_Wencha	0.846280835
ENSGALG00010018543	MRPL3	3	-0.061829442	0.095807042	-0.645353843	0.518697908	0.013799769	4.051060913	50.63021656	0.666666667	CRA004775;PRJNA827465;Yang2025_Silkie_Wencha	0.846280835
ENSGALG00010014894	RAB22A	3	-0.056774874	0.087885679	-0.646008249	0.518274013	0	0.997242387	0	0.666666667	CRA004775;PRJNA827465;Yang2025_Silkie_Wencha	0.846280835
ENSGALG00010023730	SP2	3	-0.040277005	0.062350816	-0.645973982	0.518296206	0	1.865281272	0	0.666666667	CRA004775;PRJNA827465;Yang2025_Silkie_Wencha	0.846280835
ENSGALG00010027272	DDX20	3	-0.032437998	0.0502354	-0.645719916	0.51846076	0	1.117033475	0	0.666666667	CRA004775;PRJNA827465;Yang2025_Silkie_Wencha	0.846280835
ENSGALG00010009634	TBC1D31	3	0.123513728	0.191430155	0.645215628	0.51878746	0.075954608	7.553441763	73.52200411	0.666666667	CRA004775;PRJNA827465;Yang2025_Silkie_Wencha	0.846329283
ENSGALG00010029663	GUSB	3	0.036318312	0.056301616	0.645066958	0.518883796	0	0.108566447	0	0.666666667	CRA004775;PRJNA827465;Yang2025_Silkie_Wencha	0.846388785
ENSGALG00010001288	TGIF1	3	-0.134740868	0.208964076	-0.644803982	0.519054221	0.095481661	9.308626663	78.51455352	0.666666667	CRA004775;PRJNA827465;Yang2025_Silkie_Wencha	0.846471469
ENSGALG00010011572	PTCD2	3	-0.066989682	0.10388469	-0.644846531	0.519026645	0	0.442197166	0	0.666666667	CRA004775;PRJNA827465;Yang2025_Silkie_Wencha	0.846471469
ENSGALG00010003775	NCOA2	3	-0.0493321	0.076526492	-0.644640818	0.519159977	0.002733613	2.186690607	8.537586707	0.666666667	CRA004775;PRJNA827465;Yang2025_Silkie_Wencha	0.846546293
ENSGALG00010004328	ENSGALG00010004328	3	-0.246735602	0.383010689	-0.644200304	0.519445554	0.210213223	3.817144151	47.60480819	0.666666667	CRA004775;PRJNA827465;Yang2025_Silkie_Wencha	0.846719009
ENSGALG00010016236	GPATCH2	3	-0.092215282	0.143121546	-0.644314465	0.519371538	0.04297395	10.18240964	80.35828383	0.666666667	CRA004775;PRJNA827465;Yang2025_Silkie_Wencha	0.846719009
ENSGALG00010028642	APH1A	3	-0.037805812	0.058680642	-0.644263768	0.519404406	0.000258487	2.027503418	1.356516487	0.666666667	CRA004775;PRJNA827465;Yang2025_Silkie_Wencha	0.846719009
ENSGALG00010004803	RMC1	3	-0.126797063	0.196908844	-0.643937876	0.519615719	0.090113986	12.26020244	83.68705567	0.666666667	CRA004775;PRJNA827465;Yang2025_Silkie_Wencha	0.846812869
ENSGALG00010029145	RC3H2	3	-0.034346531	0.053339188	-0.643926774	0.519622919	0.000221816	2.016493077	0.817908917	0.666666667	CRA004775;PRJNA827465;Yang2025_Silkie_Wencha	0.846812869
ENSGALG00010029641	ENSGALG00010029641	3	-0.123408186	0.191815222	-0.64337014	0.519983954	0	0.951403926	0	0.666666667	CRA004775;PRJNA827465;Yang2025_Silkie_Wencha	0.846913159
ENSGALG00010014323	KLF4	3	0.072030332	0.111906403	0.643665868	0.519792128	0	1.311039362	0	0.666666667	CRA004775;PRJNA827465;Yang2025_Silkie_Wencha	0.846913159
ENSGALG00010019891	PIGH	3	-0.0568388	0.088336227	-0.64343703	0.519940562	0.001132732	2.076568366	3.687254749	0.666666667	CRA004775;PRJNA827465;Yang2025_Silkie_Wencha	0.846913159
ENSGALG00010001694	ENSGALG00010001694	3	0.052042194	0.080870497	0.643525094	0.519883437	0.001819732	2.18349252	8.403624845	0.666666667	CRA004775;PRJNA827465;Yang2025_Silkie_Wencha	0.846913159
ENSGALG00010017335	THUMPD2	3	-0.045167668	0.070192058	-0.643486874	0.519908229	0	0.822678363	0	0.666666667	CRA004775;PRJNA827465;Yang2025_Silkie_Wencha	0.846913159
ENSGALG00010017759	ENSGALG00010017759	3	0.516091981	0.802656738	0.642979691	0.520237278	1.582667815	13.31431769	84.97857685	0.666666667	CRA004775;PRJNA827465;Yang2025_Silkie_Wencha	0.846935507
ENSGALG00010003898	NDST4	2	0.249827518	0.388438712	0.643158135	0.520121495	0	0.471726466	0	1	CRA004775;PRJNA827465	0.846935507
ENSGALG00010007849	CXADR	3	0.216982137	0.337459308	0.64298756	0.520232172	0.106438485	2.87404158	30.41158438	0.666666667	CRA004775;PRJNA827465;Yang2025_Silkie_Wencha	0.846935507
ENSGALG00010002908	S1PR2	3	-0.06808884	0.105869524	-0.643139193	0.520133785	0	0.946666293	0	0.666666667	CRA004775;PRJNA827465;Yang2025_Silkie_Wencha	0.846935507
ENSGALG00010024931	UBE3C	3	-0.099987406	0.15555331	-0.642785459	0.52036332	0.056964765	12.71979559	84.27647688	0.666666667	CRA004775;PRJNA827465;Yang2025_Silkie_Wencha	0.847038585

ENSGALG00010006901	TUBAL3	3	0.04760113	0.074064596	0.642697489	0.520420411	0	0.033064673	0	0.666666667	CRA004775;PRJNA827465;Yang2025_Silkie_Wencha	0.847038585
ENSGALG00010027707	HIGD1C	3	0.125587645	0.195503003	0.642382181	0.520625067	0.072528156	5.781472707	65.40673802	0.666666667	CRA004775;PRJNA827465;Yang2025_Silkie_Wencha	0.84727415
ENSGALG00010005337	DCTN1	3	0.397993615	0.620023625	0.641900725	0.520937643	0.850591254	8.129332268	75.39773337	0.666666667	CRA004775;PRJNA827465;Yang2025_Silkie_Wencha	0.847489135
ENSGALG00010021194	CYP11A1	3	0.140914787	0.219558612	0.641809428	0.520996928	0.046540974	2.876574711	30.4728644	0.666666667	CRA004775;PRJNA827465;Yang2025_Silkie_Wencha	0.847489135
ENSGALG00010008879	HABP4	3	0.101647254	0.158337067	0.641967522	0.520894271	0.045345478	5.57470813	64.1236823	0.666666667	CRA004775;PRJNA827465;Yang2025_Silkie_Wencha	0.847489135
ENSGALG00010020131	SNRNP200	3	-0.066010765	0.102812114	-0.642052409	0.520839155	0.020373861	5.972597462	66.51373188	0.666666667	CRA004775;PRJNA827465;Yang2025_Silkie_Wencha	0.847489135
ENSGALG00010007774	ENSGALG0001000777	2	0.163399947	0.254674112	0.641604071	0.521130289	0	0.637042312	0	0.5	PRJNA827465;Yang2025_Silkie_Wenchang	0.847608554
ENSGALG00010021919	PARVA	3	-0.144043192	0.224616189	-0.641285889	0.521336956	0.122109544	15.876667	87.40289759	0.666666667	CRA004775;PRJNA827465;Yang2025_Silkie_Wencha	0.847749651
ENSGALG00010001457	NDC80	3	-0.070189046	0.109438821	-0.641354187	0.521292591	0.009938408	2.701394306	25.96415874	0.666666667	CRA004775;PRJNA827465;Yang2025_Silkie_Wencha	0.847749651
ENSGALG00010026242	CCDC141	3	0.170409527	0.265851073	0.6409962	0.521525152	0.134452297	6.271732418	68.11088441	0.666666667	CRA004775;PRJNA827465;Yang2025_Silkie_Wencha	0.847949463
ENSGALG00010006564	SLC10A7	3	-0.154214636	0.240686764	-0.640727533	0.521699722	0.139530542	24.5072606	91.83915317	0.666666667	CRA004775;PRJNA827465;Yang2025_Silkie_Wencha	0.847949463
ENSGALG00010025407	MLXIP	3	-0.086931667	0.135663452	-0.640789142	0.521659688	0.026871246	3.876584258	48.40818962	0.666666667	CRA004775;PRJNA827465;Yang2025_Silkie_Wencha	0.847949463
ENSGALG00010007884	GLI3	3	0.05342204	0.083367664	0.640800485	0.521652318	0	1.820520422	0	0.666666667	CRA004775;PRJNA827465;Yang2025_Silkie_Wencha	0.847949463
ENSGALG00010023248	PRDM10	3	0.070585407	0.110190808	0.640574365	0.521799259	0.017331243	3.528557679	43.31961719	0.666666667	CRA004775;PRJNA827465;Yang2025_Silkie_Wencha	0.848013762
ENSGALG00010027206	ENSGALG0001002720	3	0.43320495	0.677710348	0.639218438	0.522680839	1.026663183	7.850816554	74.5249429	0.666666667	CRA004775;PRJNA827465;Yang2025_Silkie_Wencha	0.848159716
ENSGALG00010023397	ENSGALG0001002339	3	0.290148372	0.453794211	0.63938315	0.522573708	0.528694451	15.93883193	87.45202905	0.666666667	CRA004775;PRJNA827465;Yang2025_Silkie_Wencha	0.848159716
ENSGALG00010002604	BLEC3	3	-0.272922415	0.426934414	-0.639260753	0.522653316	0.252289821	3.715233495	46.16758267	0.666666667	CRA004775;PRJNA827465;Yang2025_Silkie_Wencha	0.848159716
ENSGALG00010011562	ENSGALG0001001156	2	0.245134652	0.3834841	0.639230289	0.522673131	0	0.404025791	0	1	PRJNA827465;Yang2025_Silkie_Wenchang	0.848159716
ENSGALG00010001305	WNT16	3	0.244249867	0.381899378	0.639566025	0.522454777	0.282827807	5.940402476	66.33224755	0.666666667	CRA004775;PRJNA827465;Yang2025_Silkie_Wencha	0.848159716
ENSGALG00010009600	LRRC17	3	-0.188825245	0.2955305	-0.638936573	0.522864194	0.18491041	9.267709486	78.41969472	0.666666667	CRA004775;PRJNA827465;Yang2025_Silkie_Wencha	0.848159716
ENSGALG00010012048	PDE6H	2	-0.152396917	0.238156223	-0.639903148	0.522235567	0	0.448393594	0	1	PRJNA827465;Yang2025_Silkie_Wenchang	0.848159716
ENSGALG00010014314	ENSGALG0001001431	3	-0.128405274	0.200942764	-0.639014173	0.522813712	0.052032045	3.502410597	42.89647246	0.666666667	CRA004775;PRJNA827465;Yang2025_Silkie_Wencha	0.848159716
ENSGALG00010012940	FBN2	3	0.127239156	0.198782715	0.640091649	0.522113018	0.061878954	4.675216287	57.22123048	0.666666667	CRA004775;PRJNA827465;Yang2025_Silkie_Wencha	0.848159716
ENSGALG00010022921	ENSGALG0001002292	3	0.115319191	0.180505525	0.638867932	0.522908851	0.060027461	5.190860468	61.47074242	0.666666667	CRA004775;PRJNA827465;Yang2025_Silkie_Wencha	0.848159716
ENSGALG00010021953	PITPNM1	3	0.096017541	0.150170249	0.639391235	0.52256845	0.04215843	6.392576611	68.71371089	0.666666667	CRA004775;PRJNA827465;Yang2025_Silkie_Wencha	0.848159716
ENSGALG00010001966	ARHGAP6	3	0.087834335	0.137249241	0.639962265	0.522197132	0.024911807	3.278415644	38.99492263	0.666666667	CRA004775;PRJNA827465;Yang2025_Silkie_Wencha	0.848159716
ENSGALG00010004800	ZC3H12C	3	-0.086986474	0.135989724	-0.639654758	0.522397074	0	1.415085049	0	0.666666667	CRA004775;PRJNA827465;Yang2025_Silkie_Wencha	0.848159716
ENSGALG00010014903	MCF2L	3	-0.056708025	0.088695978	-0.639352834	0.522593425	0	1.190338532	0	0.666666667	CRA004775;PRJNA827465;Yang2025_Silkie_Wencha	0.848159716
ENSGALG00010016242	PDS5A	3	-0.053381167	0.083386849	-0.640162895	0.522066703	0.009590989	3.523306195	43.23513515	0.666666667	CRA004775;PRJNA827465;Yang2025_Silkie_Wencha	0.848159716
ENSGALG00010018030	POLB	3	-0.029690243	0.046450003	-0.639187104	0.522701221	0	1.397495445	0	0.666666667	CRA004775;PRJNA827465;Yang2025_Silkie_Wencha	0.848159716
ENSGALG00010029710	BLMH	3	0.026888778	0.04207645	0.639045792	0.522793143	0	1.162338251	0	0.666666667	CRA004775;PRJNA827465;Yang2025_Silkie_Wencha	0.848159716
ENSGALG00010013532	WDR19	3	0.185706872	0.290860207	0.63847466	0.523164744	0.221500332	22.02426079	90.91910499	0.666666667	CRA004775;PRJNA827465;Yang2025_Silkie_Wencha	0.848167051
ENSGALG00010017376	GALNT14	3	0.106838038	0.167258231	0.638761015	0.522978413	0	0.162900628	0	1	CRA004775;PRJNA827465;Yang2025_Silkie_Wencha	0.848167051
ENSGALG00010008497	PAM	3	-0.093133335	0.145885543	-0.638400029	0.523213312	0.041416406	7.216260442	72.28481405	0.666666667	CRA004775;PRJNA827465;Yang2025_Silkie_Wencha	0.848167051
ENSGALG00010024804	WDR53	3	-0.039013321	0.061092198	-0.638597448	0.523084842	0	1.283361271	0	0.666666667	CRA004775;PRJNA827465;Yang2025_Silkie_Wencha	0.848167051
ENSGALG00010009601	ZHX2	3	-0.032963122	0.051621931	-0.638548794	0.523116502	0	1.959749871	0	0.666666667	CRA004775;PRJNA827465;Yang2025_Silkie_Wencha	0.848167051
ENSGALG00010029376	ENSGALG0001002937	3	0.112406854	0.17619283	0.637976325	0.523489091	0.062163841	7.244142583	72.39148764	0.666666667	CRA004775;PRJNA827465;Yang2025_Silkie_Wencha	0.848516825
ENSGALG00010001170	DCAF10	3	-0.05803686	0.091001408	-0.637757825	0.523631337	0	0.244350596	0	1	CRA004775;PRJNA827465;Yang2025_Silkie_Wencha	0.848650101
ENSGALG00010029738	TRPV2	3	-0.082770678	0.129850819	-0.637429007	0.523845439	0.035622695	8.106587117	75.32870527	0.666666667	CRA004775;PRJNA827465;Yang2025_Silkie_Wencha	0.848705244
ENSGALG00010021466	HES4	3	0.054067391	0.084802217	0.637570494	0.523753308	0	1.199389397	0	0.666666667	CRA004775;PRJNA827465;Yang2025_Silkie_Wencha	0.848705244
ENSGALG00010002730	E2F6	3	-0.052799858	0.082823733	-0.637496719	0.523801346	0.007409454	3.038790817	34.18434764	0.666666667	CRA004775;PRJNA827465;Yang2025_Silkie_Wencha	0.848705244
ENSGALG00010001147	PGGT1B	3	0.042734141	0.067053641	0.637312755	0.523921145	0	1.841423451	0	0.666666667	CRA004775;PRJNA827465;Yang2025_Silkie_Wencha	0.848730644
ENSGALG00010024835	ENTREP2	3	0.070452014	0.110581945	0.637102323	0.524058196	0.013015591	3.023284083	33.84677241	0.666666667	CRA004775;PRJNA827465;Yang2025_Silkie_Wencha	0.848790695
ENSGALG00010028459	MCL1	3	-0.035339832	0.055472318	-0.637071491	0.524078278	0	1.425996888	0	0.666666667	CRA004775;PRJNA827465;Yang2025_Silkie_Wencha	0.848790695
ENSGALG00010013019	DPYS	2	0.49709801	0.780841766	0.636618112	0.524373625	0	0.125665766	0	1	CRA004775;PRJNA827465	0.849106701
ENSGALG00010000786	ANKRD33B	3	0.133671	0.209980525	0.636587605	0.524393501	0	0.38316533	0	0.666666667	CRA004775;PRJNA827465;Yang2025_Silkie_Wencha	0.849106701
ENSGALG00010017692	EPCAM	3	0.624884411	0.98468658	0.634602343	0.5256878	2.031610631	6.703827852	70.16629836	0.666666667	CRA004775;PRJNA827465;Yang2025_Silkie_Wencha	0.84911217
ENSGALG00010019247	HBZ	2	-0.480552582	0.757099963	-0.634728048	0.525605797	0.958140315	6.032178178	83.42224035	0.5	PRJNA827465;Yang2025_Silkie_Wenchang	0.84911217
ENSGALG00010010430	ASTN1	2	0.461067177	0.727086487	0.634129757	0.525996145	0.685067573	2.687261336	62.787393	0.5	PRJNA827465;Yang2025_Silkie_Wenchang	0.84911217
ENSGALG00010008108	ENSGALG0001000810	3	0.259491167	0.409406152	0.633823321	0.526196132	0.407910171	11.52637412	82.64848964	0.666666667	CRA004775;PRJNA827465;Yang2025_Silkie_Wencha	0.84911217
ENSGALG00010013202	ENSGALG0001001320	2	0.222099684	0.349311324	0.635821598	0.524892709	0	0.313415048	0	0.5	PRJNA827465;Yang2025_Silkie_Wenchang	0.84911217
ENSGALG00010004983	CLXN	3	-0.197433683	0.310354681	-0.636155003	0.524675398	0.177366127	5.810626249	65.58030212	0.666666667	CRA004775;PRJNA827465;Yang2025_Silkie_Wencha	0.84911217
ENSGALG00010000284	ENSGALG0001000028	3	-0.17452389	0.274652271	-0.635435815	0.525144216	0.179279461	10.37534897	80.72353994	0.666666667	CRA004775;PRJNA827465;Yang2025_Silkie_Wencha	0.84911217
ENSGALG00010029214	ENSGALG0001002921	3	-0.162953707	0.257002277	-0.634055499	0.526044603	0.075504043	3.225838865	38.00062298	0.666666667	CRA004775;PRJNA827465;Yang2025_Silkie_Wencha	0.84911217
ENSGALG00010026013	RABIF	3	0.159139331	0.250726061	0.634713958	0.525614988	0.154833485	16.18827364	87.6453781	0.666666667	CRA004775;PRJNA827465;Yang2025_Silkie_Wencha	0.84911217
ENSGALG00010001372	ENSGALG0001000137	3	-0.147320167	0.232074996	-0.634795516	0.525561788	0.085765433	4.29771547	53.46364798	0.666666667	CRA004775;PRJNA827465;Yang2025_Silkie_Wencha	0.84911217
ENSGALG00010019683	TMEM184A	3	-0.131776024	0.207747157	-0.634309636	0.525878769	0.071719542	4.55020661	56.04595194	0.666666667	CRA004775;PRJNA827465;Yang2025_Silkie_Wencha	0.84911217
ENSGALG00010029731	NCOR1	3	0.131009363	0.206284144	0.635091774	0.525368561	0.081154887	6.212797183	67.80838097	0.666666667	CRA004775;PRJNA827465;Yang2025_Silkie_Wencha	0.84911217
ENSGALG00010002395	CLN8	3	-0.130240132	0.205307968	-0.634364722	0.525842827	0.105355018	26.07492056	92.32979447	0.666666667	CRA004775;PRJNA827465;Yang2025_Silkie_Wencha	0.84911217
ENSGALG00010022546	MTG1	3	0.124234943	0.195955269	0.633996441	0.526083145	0.060477267	4.149563142	51.80215527	0.666666667	CRA004775;PRJNA827465;Yang2025_Silkie_Wencha	0.84911217
ENSGALG00010001297	MYL12A	3	0.120358592	0.189916292	0.633745483	0.526246937	0.084620443	9.979540452	79.95899701	0.666666667	CRA004775;PRJNA827465;Yang2025_Silkie_Wencha	0.84911217
ENSGALG00010029728	TNRC6C	3	0.119783984	0.188230455	0.636368776	0.524536087	0.070118531	6.262133464	68.06200297	0.666666667	CRA004775;PRJNA827465;Yang2025_Silkie_Wencha	0.84911217
ENSGALG00010024647	RPUSD4	3	0.105964826	0.166590712	0.636078833	0.524725041	0.058276617	6.808055083	70.6230344	0.666666667	CRA004775;PRJNA827465;Yang2025_Silkie_Wencha	0.84911217
ENSGALG00010004641	METAP1	3	-0.104178354	0.163876842	-0.63571126	0.524964636	0.068519458	18.75680992	89.33720602	0.666666667	CRA004775;PRJNA827465;Yang2025_Silkie_Wencha	0.84911217
ENSGALG00010011907	BIK	3	-0.10133449	0.15959282	-0.634956448	0.52545682	0	0.204844246	0	1	CRA004775;PRJNA827465;Yang2025_Silkie_Wencha	0.84911217
ENSGALG00010006156	NFKBIZ	3	0.101225108	0.159472577	0.63474931	0.525591928	0	1.70923434	0	0.666666667	CRA004775;PRJNA827465;Yang2025_Silkie_Wencha	0.84911217
ENSGALG00010020181	LEUTX	3	0.077034897	0.121552593	0.633757743	0.526238935	0	0.682578613	0	1	CRA004775;PRJNA827465;Yang2025_Silkie_Wencha	0.84911217
ENSGALG00010005416	PPP3CA	3	-0.075245973	0.118534004	-0.634804954	0.525555632	0.024258641	5.611764445	64.36058535	0.666666667	CRA004775;PRJNA827465;Yang2025_Silkie_Wencha	0.84911217
ENSGALG00010012796	MOCS2	3	0.075141722	0.118209232	0.635667123	0.524993409	0.007966116	2.421828088	17.4177552	0.666666667	CRA004775;PRJNA827465;Yang2025_Silkie_Wencha	0.84911217
ENSGALG00010023880	ENSGALG0001002388	3	-0.069436356	0.109507607	-0.634077924	0.526029969	0	1.40575309	0	0.666666667	CRA004775;PRJNA827465;Yang2025_Silkie_Wencha	0.84911217
ENSGALG00010006515	FBXO33	3	-0.066894866	0.105545776	-0.633799558	0.526211642	0.019355931	5.035285311	60.28030436	0.666666667	CRA004775;PRJNA827465;Yang2025_Silkie_Wencha	0.84911217
ENSGALG00010027576	GADD45B	3	-0.06243615	0.098377073	-0.634661593	0.525649148	0	1.202011804	0	0.666666667	CRA004775;PRJNA827465;Yang2025_Silkie_Wencha	0.84911217
ENSGALG00010003853	ENSGALG0001000385	3	-0.055312912	0.087281833	-0.633727658	0.526258572	0.003630193	2.28856354	12.60893722	0.666666667	CRA004775;PRJNA827465;Yang2025_Silkie_Wencha	0.84911217
ENSGALG00010018950	TTL	3	-0.053563448	0.08433066	-0.635159829	0.52532418	0.013149029	7.588850596	73.64554784	0.666666667	CRA004775;PRJNA827465;Yang2025_Silkie_Wencha	0.84911217
ENSGALG00010008492	ANKRD49	3	-0.04130596	0.064906344	-0.636393267	0.524520128	0	0.852505495	0	1	CRA004775;PRJNA827465;Yang2025_Silkie_Wencha	0.84911217
ENSGALG00010017451	GABPB1	3	0.029762203	0.046894288	0.634665853	0.525646369	0	0.68386375	0	1	CRA004775;PRJNA827465;Yang2025_Silkie_Wencha	0.84911217
ENSGALG00010022237	SUN1	3	0.029545433	0.04661933	0.633759271	0.526237937	0.000708733	2.195056126	8.886156667	0.666666667	CRA004775;PRJNA827465;Yang2025_Silkie_Wencha	0.84911217
ENSGALG00010016510	THG1L	3	-0.054366614	0.085825802	-0.633453027	0.526437846	0	1.429247022	0	0.666666667	CRA004775;PRJNA827465;Yang2025_Silkie_Wencha	0.849304508
ENSGALG00010001810	OPN5SW	3	0.450701946	0.712262176	0.632775348	0.526880358	1.268299858	13.17100915	84.81513468	0.666666667	CRA004775;PRJNA827465;Yang2025_Silkie_Wencha	0.849438172
ENSGALG00010009120	ATG12	3	-0.116384697	0.183910516	-0.632833293	0.526842513	0.078818125	9.591871977	79.14901278	0.666666667	CRA004775;PRJNA827465;Yang2025_Silkie_Wencha	0.849438172
ENSGALG00010022545	CSTF3	3	0.071501546	0.112966241	0.632946137	0.526768818	0.025470017	6.504424419	69.25169898	0.666666667	CRA004775;PRJNA827465;Yang2025_Silkie_Wencha	0.849438172
ENSGALG00010024580	IGF2	3	0.061229299	0.096732669	0.632974362	0.526750386	0	1.048142706	0	0.666666667	CRA004775;PRJNA827465;Yang2025_Silkie_Wencha	0.849438172
ENSGALG00010007360	ROBO1	3	0.058891561	0.093068853	0.632774114	0.526881163	0	1.309008975	0	0.666666667	CRA004775;PRJNA827465;Yang2025_Silkie_Wencha	0.849438172
ENSGALG00010002573	MVB12A	3	0.049483939	0.078161793	0.633096254	0.526670788	0.005150098	2.555122828	21.72587643	0.666666667	CRA004775;PRJNA827465;Yang2025_Silkie_Wencha	0.849438172
ENSGALG00010008191	ENSGALG0001000819	3	0.293691645	0.464879092	0.631759204	0.527544235	0.51278735	10.49801805	80.94878491	0.666666667	CRA004775;PRJNA827465;Yang2025_Silkie_Wencha	0.849493753
ENSGALG00010027350	INSRR	3	0.155367007	0.245876984	0.631889185	0.527459291	0.101410528	4.561300674	56.15285764	0.666666667	CRA004775;PRJNA827465;Yang2025_Silkie_Wencha	0.849493753
ENSGALG00010020309	GXYLT2	3	-0.149854	0.236923127	-0.632500517	0.527059871	0.146966921	30.20157179	93.3778281	0.666666667	CRA004775;PRJNA827465;Yang2025_Silkie_Wencha	0.849493753
ENSGALG00010002349	ZBED1	3	-0.103075826	0.163105214	-0.631959106	0.527413599	0.063730702	17.5854554	88.62696499	0.666666667	CRA004775;PRJNA827465;Yang2025_Silkie_Wencha	0.849493753
ENSGALG00010013162	IDS	3	-0.102092817	0.161432832	-0.632416689	0.527114632	0.051833271	6.340289054	68.45569685	0.666666667	CRA004775;PRJNA827465;Yang2025_Silkie_Wencha	0.849493753
ENSGALG00010009603	HINT1	3	-0.087089833	0.137863676	-0.63170978	0.527576536	0	1.925003031	0	0.666666667	CRA004775;PRJNA827465;Yang2025_Silkie_Wencha	0.849493753
ENSGALG00010024828	TIMM17B	3	-0.067664907	0.107112606	-0.631717493	0.527571496	0.008348838	2.372788417	15.71098436	0.666666667	CRA004775;PRJNA827465;Yang2025_Silkie_Wencha	0.849493753
ENSGALG00010023738	ENSGALG0001002373	3	-0.066827041	0.105780763	-0.631750421	0.527549975	0	0.587548445	0	1	CRA004775;PRJNA827465;Yang2025_Silkie_Wencha	0.849493753
ENSGALG00010008012	CCDC90B	3	-0.043070698	0.068148856	-0.632009104	0.527380928	0.002697016	2.42567046	17.54856923	0.666666667	CRA004775;PRJNA827465;Yang2025_Silkie_Wencha	0.849493753
ENSGALG00010021065	CNNM2	3	0.038171812	0.060403059	0.631951643	0.527418476	0	1.668511692	0	0.666666667	CRA004775;PRJNA827465;Yang2025_Silkie_Wencha	0.849493753
ENSGALG00010015928	CAPN7	3	0.031437482	0.049731802	0.632140423	0.527295123	0	1.962151978	0	0.666666667	CRA004775;PRJNA827465;Yang2025_Silkie_Wencha	0.849493753
ENSGALG00010004358	GC	3	-1.537125637	2.440367219	-0.629874727	0.528776549	15.24216422	15.64137072	87.21339686	0.666666667	CRA004775;PRJNA827465;Yang2025_Silkie_Wencha	0.849590825
ENSGALG00010024721	ENSGALG0001002472	2	0.438062146	0.695044237	0.630265129	0.528521134	0.772383031	4.930512382	79.71813227	0.5	PRJNA827465;Yang2025_Silkie_Wenchang	0.849590825
ENSGALG00010024396	ENSGALG0001002439	3	0.245777815	0.390627159	0.629187728	0.529226162	0.299861769	7.347598612	72.78022242	0.666666667	CRA004775;PRJNA827465;Yang2025_Silkie_Wencha	0.849590825
ENSGALG00010006411	RGS17	2	-0.20225783	0.320415392	-0.631236311	0.527886023	0	0.430297773	0	0.5	PRJNA827465;Yang2025_Silkie_Wenchang	0.849590825
ENSGALG00010000471	ENSGALG0001000047	3	0.201722319	0.320612803	0.629177366	0.529232945	0.266393939	16.13133216	87.60176792	0.666666667	CRA004775;PRJNA827465;Yang2025_Silkie_Wencha	0.849590825
ENSGALG00010002430	NUMA1	3	0.195623792	0.310098494	0.630844056	0.528142494	0.261618312	29.04638527	93.11446164	0.666666667	CRA004775;PRJNA827465;Yang2025_Silkie_Wencha	0.849590825
ENSGALG00010021735	PIGBOS1	3	0.192752952	0.306678225	0.628518545	0.529664302	0.190962744	6.317329442	68.34105268	0.666666667	CRA004775;PRJNA827465;Yang2025_Silkie_Wencha	0.849590825
ENSGALG00010020006	PLB1	3	0.180282647	0.286560926	0.629125015	0.529627215	0.059277005	2.595069653	22.9307777	0.666666667	CRA004775;PRJNA827465;Yang2025_Silkie_Wencha	0.849590825
ENSGALG00010021503	KCNS1	3	0.169365754	0.269406659	0.628662092	0.5295703	0.056054364	2.638803968	24.20808729	0.666666667	CRA004775;PRJNA827465;Yang2025_Silkie_Wencha	0.849590825
ENSGALG00010010859	ENSGALG0001001085	3	-0.147073583	0.233891024	-0.62881243	0.529471862	0.10637121	6.119428126	67.31720745	0.666666667	CRA004775;PRJNA827465;Yang2025_Silkie_Wencha	0.849590825
ENSGALG00010014338	BHLHE41	3	0.139160347	0.220466517	0.631208536	0.527904181	0.106399678	7.984196339	74.95051581	0.666666667	CRA004775;PRJNA827465;Yang2025_Silkie_Wencha	0.849590825
ENSGALG00010022251	WNK4	3	0.132589599	0.210182535	0.630830717	0.528151217	0.091015678	6.883963325	70.94696898	0.666666667	CRA004775;PRJNA827465;Yang2025_Silkie_Wencha	0.849590825
ENSGALG00010013386	ITGAV	3	-0.130575115	0.207372911	-0.629663317	0.528914888	0.094639073	11.97273629	83.29538085	0.666666667	CRA004775;PRJNA827465;Yang2025_Silkie_Wencha	0.849590825
ENSGALG00010023231	ASIC1	3	0.127295473	0.201839748	0.63067594	0.528252434	0.075192515	5.450634883	63.30702674	0.666666667	CRA004775;PRJNA827465;Yang2025_Silkie_Wencha	0.849590825
ENSGALG00010004344	SOSTDC1	2	-0.125130314	0.199021706	-0.628726968	0.529527819	0	0.017221801	0	1	PRJNA827465;Yang2025_Silkie_Wenchang	0.849590825
ENSGALG00010015002	ADAM19	3	-0.121538865	0.193107113	-0.629385751	0.529096544	0.067351049	6.623882851	69.81078693	0.666666667	CRA004775;PRJNA827465;Yang2025_Silkie_Wencha	0.849590825
ENSGALG00010014908	ENSGALG0001001490	3	-0.109514383	0.173986447	-0.629442031	0.529059709	0.066612838	13.31851241	84.98330791	0.666666667	CRA004775;PRJNA827465;Yang2025_Silkie_Wencha	0.849590825
ENSGALG00010006347	PPP6R2	3	0.108038398	0.171864561	0.628625222	0.529594444	0.068067828	10.84687037	81.56150178	0.666666667	CRA004775;PRJNA827465;Yang2025_Silkie_Wencha	0.849590825
ENSGALG00010001334	EBF3	3	0.105195927	0.166930204	0.630179105	0.528577408	0.041071761	3.776771661	47.04472021	0.666666667	CRA004775;PRJNA827465;Yang2025_Silkie_Wencha	0.849590825
ENSGALG00010005174	ST6GAL2	3	0.090170372	0.14320459	0.629661185	0.528916283	0	0.271063501	0	0.666666667	CRA004775;PRJNA827465;Yang2025_Silkie_Wencha	0.849590825
ENSGALG00010022171	FOXP1	3	0.085243141	0.135600669	0.628633634	0.529588936	0.024409962	3.280907165	39.0412499	0.666666667	CRA004775;PRJNA827465;Yang2025_Silkie_Wencha	0.849590825
ENSGALG00010021401	PDAP1	3	0.084313183	0.133785417	0.630212059	0.52855585	0.038182438	7.600600291	73.68628893	0.666666667	CRA004775;PRJNA827465;Yang2025_Silkie_Wencha	0.849590825
ENSGALG00010004433	AHR1A	3	-0.082572465	0.130857946	-0.631008418	0.528035021	0.025428255	4.318771577	53.69053527	0.666666667	CRA004775;PRJNA827465;Yang2025_Silkie_Wencha	0.849590825
ENSGALG00010010205	ARL5A	3	-0.081235801	0.128776368	-0.630828487	0.528152675	0.034192317	6.773024557	70.47109481	0.666666667	CRA004775;PRJNA827465;Yang2025_Silkie_Wencha	0.849590825
ENSGALG00010011342	RASAL2	3	0.078803037	0.125181863	0.629508419	0.529016259	0.021129388	3.538190653	43.47393354	0.666666667	CRA004775;PRJNA827465;Yang2025_Silkie_Wencha	0.849590825
ENSGALG00010008001	ALDH5A1	3	0.074502347	0.118420414	0.629134322	0.529261122	0.010657187	2.633908356	24.06721381	0.666666667	CRA004775;PRJNA827465;Yang2025_Silkie_Wencha	0.849590825
ENSGALG00010009115	COG5	3	-0.074254937	0.117928442	-0.629660969	0.528916424	0.026376708	6.617147148	69.77549455	0.666666667	CRA004775;PRJNA827465;Yang2025_Silkie_Wencha	0.849590825
ENSGALG00010010280	HSP90B1	3	0.070693645	0.112457103	0.628627669	0.529592848	0.025120616	10.75084879	81.3968177	0.666666667	CRA004775;PRJNA827465;Yang2025_Silkie_Wencha	0.849590825
ENSGALG00010026673	DEPDC5	3	-0.063287057	0.100393277	-0.630391389	0.528438543	0.021268264	7.783178116	74.30355608	0.666666667	CRA004775;PRJNA827465;Yang2025_Silkie_Wencha	0.849590825
ENSGALG00010008017	GMNN	3	-0.053341715	0.08482862	-0.628817429	0.529468588	0	1.817077355	0	0.666666667	CRA004775;PRJNA827465;Yang2025_Silkie_Wencha	0.849590825
ENSGALG00010020291	SNX29	3	-0.052405348	0.083382304	-0.628494833	0.52967983	0	0.589202005	0	0.666666667	CRA004775;PRJNA827465;Yang2025_Silkie_Wencha	0.849590825
ENSGALG00010016673	LRPAP1	3	0.048189178	0.076629699	0.628857718	0.529442209	0.003885068	2.40221752	16.7435928	0.666666667	CRA004775;PRJNA827465;Yang2025_Silkie_Wencha	0.849590825
ENSGALG00010010706	SREBF2	3	0.039167116	0.06229907	0.628695033	0.52954873	0	1.484172637	0	0.666666667	CRA004775;PRJNA827465;Yang2025_Silkie_Wencha	0.849590825
ENSGALG00010027700	TCF3	3	-0.031050862	0.049215023	-0.630922435	0.528091242	0	0.297652999	0	0.666666667	CRA004775;PRJNA827465;Yang2025_Silkie_Wencha	0.849590825
ENSGALG00010024759	APOC3	3	0.434859259	0.692466156	0.6279863	0.530012915	0.921800373	5.620369683	64.41515216	0.666666667	CRA004775;PRJNA827465;Yang2025_Silkie_Wencha	0.849646601
ENSGALG00010011874	KCNS2	2	0.258077422	0.411499772	0.6271645	0.530551411	0.220669155	2.734014421	63.42374816	0.5	PRJNA827465;Yang2025_Silkie_Wenchang	0.849646601
ENSGALG00010023743	CD3D	3	0.223995629	0.357113905	0.62723861	0.530502838	0.308820416	12.23781813	83.6572175	0.666666667	CRA004775;PRJNA827465;Yang2025_Silkie_Wencha	0.849646601
ENSGALG00010021218	DPF3	3	0.157699321	0.251625934	0.626721254	0.530841969	0.130820943	6.62993054	69.83377144	0.666666667	CRA004775;PRJNA827465;Yang2025_Silkie_Wencha	0.849646601
ENSGALG00010017381	CA6	3	0.153355112	0.244619305	0.626913367	0.530716024	0.084718293	3.773951053	47.00514205	0.666666667	CRA004775;PRJNA827465;Yang2025_Silkie_Wencha	0.849646601
ENSGALG00010028823	GFI1B	3	0.144162831	0.229940379	0.627056429	0.530622246	0.088017948	4.575078721	56.2849052	0.666666667	CRA004775;PRJNA827465;Yang2025_Silkie_Wencha	0.849646601
ENSGALG00010017563	ALK	3	0.137094124	0.218327749	0.627928076	0.530051058	0.091349345	7.215422318	72.28159473	0.666666667	CRA004775;PRJNA827465;Yang2025_Silkie_Wencha	0.849646601
ENSGALG00010020967	VPS51	3	-0.106972172	0.17027381	-0.628236201	0.529849219	0.062475123	7.573049778	73.59056049	0.666666667	CRA004775;PRJNA827465;Yang2025_Silkie_Wencha	0.849646601
ENSGALG00010017691	LZTS1	3	0.103356475	0.164733759	0.627399268	0.530397547	0.02685383	2.806453656	28.73568405	0.666666667	CRA004775;PRJNA827465;Yang2025_Silkie_Wencha	0.849646601
ENSGALG00010015769	TMEM50A	3	-0.094877499	0.151324286	-0.626981308	0.530671488	0.04679524	6.410308641	68.80025422	0.666666667	CRA004775;PRJNA827465;Yang2025_Silkie_Wencha	0.849646601
ENSGALG00010020287	GALNT16	3	-0.083797794	0.133383602	-0.628246594	0.529842411	0.022040781	3.459466798	42.1875872	0.666666667	CRA004775;PRJNA827465;Yang2025_Silkie_Wencha	0.849646601
ENSGALG00010027108	ENSGALG0001002710	3	0.083187107	0.132445593	0.628085129	0.529948174	0.023935446	3.711836437	46.11831545	0.666666667	CRA004775;PRJNA827465;Yang2025_Silkie_Wencha	0.849646601
ENSGALG00010000649	PAPD7	3	-0.07709111	0.123015819	-0.626676394	0.530871381	0.027752854	6.370936654	68.60744175	0.666666667	CRA004775;PRJNA827465;Yang2025_Silkie_Wencha	0.849646601
ENSGALG00010010473	TRIM35	3	-0.065870609	0.105004852	-0.627310139	0.530455959	0	0.445130324	0	0.666666667	CRA004775;PRJNA827465;Yang2025_Silkie_Wencha	0.849646601
ENSGALG00010008351	IST1	3	-0.04653292	0.074121649	-0.627791221	0.530140718	0.006544612	2.988819305	33.083944	0.666666667	CRA004775;PRJNA827465;Yang2025_Silkie_Wencha	0.849646601
ENSGALG00010022325	MIGA1	3	-0.044378688	0.070823724	-0.62660765	0.530916452	0	0.032838833	0	0.666666667	CRA004775;PRJNA827465;Yang2025_Silkie_Wencha	0.849646601
ENSGALG00010002383	INPP5F	3	0.043155653	0.06873919	0.627874033	0.530086463	0	1.62653711	0	0.666666667	CRA004775;PRJNA827465;Yang2025_Silkie_Wencha	0.849646601
ENSGALG00010005500	KATNA1	3	0.037672639	0.060089393	0.626943251	0.530696435	0	0.418296345	0	0.666666667	CRA004775;PRJNA827465;Yang2025_Silkie_Wencha	0.849646601
ENSGALG00010011210	NUP153	3	-0.033828784	0.053980308	-0.626687485	0.530864109	0.001828445	2.38333384	16.08393391	0.666666667	CRA004775;PRJNA827465;Yang2025_Silkie_Wencha	0.849646601
ENSGALG00010017626	COPS7B	3	-0.029593342	0.047148116	-0.627667542	0.530221753	0	0.136828139	0	1	CRA004775;PRJNA827465;Yang2025_Silkie_Wencha	0.849646601
ENSGALG00010019659	MRPL14	3	-0.14518223	0.231755954	-0.626444449	0.531023463	0.123885043	10.528474	81.00389478	0.666666667	CRA004775;PRJNA827465;Yang2025_Silkie_Wencha	0.849721677
ENSGALG00010020269	ENSGALG0001002026	3	-0.257187719	0.410858676	-0.625988004	0.53132281	0	0.459377216	0	0.666666667	CRA004775;PRJNA827465;Yang2025_Silkie_Wencha	0.849810319
ENSGALG00010024878	RHPN2	3	0.209628613	0.334769621	0.626187682	0.531191846	0.255334417	9.881411337	79.75997627	0.666666667	CRA004775;PRJNA827465;Yang2025_Silkie_Wencha	0.849810319
ENSGALG00010020385	SLC12A8	3	0.18206226	0.290915182	0.625825916	0.531429131	0.106470141	3.459361798	42.1858679	0.666666667	CRA004775;PRJNA827465;Yang2025_Silkie_Wencha	0.849810319
ENSGALG00010021546	OGG1	3	0.148168043	0.236719081	0.625833809	0.531423953	0.094569864	4.528345319	55.83375694	0.666666667	CRA004775;PRJNA827465;Yang2025_Silkie_Wencha	0.849810319
ENSGALG00010024847	PPIL1	3	0.052529029	0.083905063	0.626053151	0.531280079	0	0.299777524	0	0.666666667	CRA004775;PRJNA827465;Yang2025_Silkie_Wencha	0.849810319
ENSGALG00010023322	PRKAB1	3	-0.032308683	0.051626973	-0.625810136	0.531439483	0	1.953046973	0	0.666666667	CRA004775;PRJNA827465;Yang2025_Silkie_Wencha	0.849810319
ENSGALG00010025168	POPDC2	3	-0.070025397	0.111912021	-0.625718281	0.53149774	0.004623236	2.236011368	10.55501645	0.666666667	CRA004775;PRJNA827465;Yang2025_Silkie_Wencha	0.849810565
ENSGALG00010021352	CFAP43	3	0.206400898	0.329911304	0.625625384	0.531560685	0.223878085	7.9262072	74.7672506	0.666666667	CRA004775;PRJNA827465;Yang2025_Silkie_Wencha	0.84981191
ENSGALG00010011882	ENSGALG0001001188	3	-0.161541635	0.258387803	-0.625190638	0.531845946	0	0.718833611	0	0.666666667	CRA004775;PRJNA827465;Yang2025_Silkie_Wencha	0.849856996
ENSGALG00010006146	CCDC142	3	0.144902332	0.231840905	0.625007619	0.531966057	0.104014654	5.694230806	64.87673106	0.666666667	CRA004775;PRJNA827465;Yang2025_Silkie_Wencha	0.849856996
ENSGALG00010016817	GGCT	3	-0.099590002	0.15932489	-0.625074977	0.53192185	0.032339344	3.402615569	41.22168786	0.666666667	CRA004775;PRJNA827465;Yang2025_Silkie_Wencha	0.849856996
ENSGALG00010021513	SDF4	3	-0.083236307	0.133108948	-0.625328152	0.531755707	0.046571203	25.11156027	92.03554069	0.666666667	CRA004775;PRJNA827465;Yang2025_Silkie_Wencha	0.849856996
ENSGALG00010020025	LOXL2	3	-0.06224696	0.099604503	-0.624941219	0.532009638	0.0012603	2.071274356	3.441087172	0.666666667	CRA004775;PRJNA827465;Yang2025_Silkie_Wencha	0.849856996
ENSGALG00010005407	DPH6	3	0.059991262	0.095925219	0.625396142	0.531711094	0	1.595825632	0	0.666666667	CRA004775;PRJNA827465;Yang2025_Silkie_Wencha	0.849856996
ENSGALG00010025705	ACSL3	3	-0.035699604	0.057098237	-0.62523128	0.531819275	0	0.660323831	0	0.666666667	CRA004775;PRJNA827465;Yang2025_Silkie_Wencha	0.849856996
ENSGALG00010027529	ACY1	3	-0.051789982	0.082897722	-0.624745534	0.532138083	0	0.313828815	0	0.666666667	CRA004775;PRJNA827465;Yang2025_Silkie_Wencha	0.849966149
ENSGALG00010028286	KCTD12A	2	-1.464588268	2.345272356	-0.624485367	0.532308877	8.53094063	4.395450108	77.29420144	0.5	PRJNA827465;Yang2025_Silkie_Wenchang	0.849985158
ENSGALG00010003815	RASAL3	2	-0.351905353	0.563757472	-0.624214083	0.532486999	0.369975026	2.389954384	58.15819722	0.5	CRA004775;PRJNA827465	0.849985158
ENSGALG00010013233	LRRCC1	3	0.26071787	0.417963379	0.623781611	0.532771018	0.45827004	21.30902827	90.61430688	0.666666667	CRA004775;PRJNA827465;Yang2025_Silkie_Wencha	0.849985158
ENSGALG00010003030	PPEF1	3	-0.205273012	0.32984388	-0.622333879	0.533722348	0	0.608959292	0	0.666666667	CRA004775;PRJNA827465;Yang2025_Silkie_Wencha	0.849985158
ENSGALG00010014576	HRH2	3	-0.200365435	0.321562506	-0.623099495	0.533219142	0.190105761	5.214238165	61.64348585	0.666666667	CRA004775;PRJNA827465;Yang2025_Silkie_Wencha	0.849985158
ENSGALG00010002998	LECT1	3	0.184120137	0.295051362	0.624027409	0.532609585	0.111354919	3.498769211	42.83704127	0.666666667	CRA004775;PRJNA827465;Yang2025_Silkie_Wencha	0.849985158
ENSGALG00010016977	CLCN7	3	-0.140371577	0.225106944	-0.623577282	0.532905234	0.121165956	11.00100604	81.81984454	0.666666667	CRA004775;PRJNA827465;Yang2025_Silkie_Wencha	0.849985158
ENSGALG00010002411	ENSGALG0001000241	3	0.114660863	0.184001002	0.623153473	0.533183673	0.052496556	4.020308186	50.25257001	0.666666667	CRA004775;PRJNA827465;Yang2025_Silkie_Wencha	0.849985158
ENSGALG00010015980	EXOSC7	3	0.107582218	0.172734404	0.622818707	0.533403664	0.06055009	6.641364242	69.8857053	0.666666667	CRA004775;PRJNA827465;Yang2025_Silkie_Wencha	0.849985158
ENSGALG00010001442	LDLRAD4	3	0.106879838	0.171135518	0.62453335	0.532277375	0.053302627	5.609361635	64.34531895	0.666666667	CRA004775;PRJNA827465;Yang2025_Silkie_Wencha	0.849985158
ENSGALG00010021498	B3GALT6	3	-0.089036614	0.143023556	-0.622531114	0.533592691	0.039869827	7.068844772	71.7068338	0.666666667	CRA004775;PRJNA827465;Yang2025_Silkie_Wencha	0.849985158
ENSGALG00010029394	CYGB	3	-0.080037174	0.128577785	-0.622480576	0.533625912	0.028192526	5.515522929	63.73870573	0.666666667	CRA004775;PRJNA827465;Yang2025_Silkie_Wencha	0.849985158
ENSGALG00010029298	ENDOV	3	-0.079124297	0.127088301	-0.622593082	0.533551959	0.027336612	4.575465321	56.28859887	0.666666667	CRA004775;PRJNA827465;Yang2025_Silkie_Wencha	0.849985158
ENSGALG00010012352	PTPRC	3	-0.069185126	0.111075291	-0.622866936	0.533371967	0.006207199	2.345563692	14.7326501	0.666666667	CRA004775;PRJNA827465;Yang2025_Silkie_Wencha	0.849985158
ENSGALG00010025585	SARNP	3	-0.057587516	0.092313095	-0.623828241	0.532740391	0.013980604	4.693568415	57.38849798	0.666666667	CRA004775;PRJNA827465;Yang2025_Silkie_Wencha	0.849985158

ENSGALG00010019772	ENSGALG00010019772	3	-0.053561998	0.08582326	-0.624096516	0.532564202	0.004577471	2.311242788	13.46646877	0.666666667	CRA004775;PRJNA827465;Yang2025_Silkie_Wencha	0.849985158
ENSGALG00010029703	MIF4GD	3	-0.051074078	0.0820686	-0.622333975	0.533722286	0.010482249	4.154508715	51.85953052	0.666666667	CRA004775;PRJNA827465;Yang2025_Silkie_Wencha	0.849985158
ENSGALG00010017326	STON1	3	0.050484448	0.081034206	0.623001706	0.533283401	0	1.343144656	0	0.666666667	CRA004775;PRJNA827465;Yang2025_Silkie_Wencha	0.849985158
ENSGALG00010026977	ELK4	3	-0.049767618	0.079902493	-0.622854386	0.533380215	0	0.362548067	0	1	CRA004775;PRJNA827465;Yang2025_Silkie_Wencha	0.849985158
ENSGALG00010023999	DGCR14	3	-0.047736218	0.076633752	-0.622913751	0.533341201	0.007734783	3.456339507	42.13531409	0.666666667	CRA004775;PRJNA827465;Yang2025_Silkie_Wencha	0.849985158
ENSGALG00010015862	PDGFRL	3	0.047658949	0.076601759	0.622165207	0.533833291	0	0.73624341	0	0.666666667	CRA004775;PRJNA827465;Yang2025_Silkie_Wencha	0.849985158
ENSGALG00010012081	CHIC1	3	-0.038666142	0.062009779	-0.623549108	0.532923742	0	1.561331853	0	0.666666667	CRA004775;PRJNA827465;Yang2025_Silkie_Wencha	0.849985158
ENSGALG00010010745	ATVR1	3	-0.038130214	0.061286109	-0.622167312	0.533831857	0.007184642	6.781864692	70.50958563	0.666666667	CRA004775;PRJNA827465;Yang2025_Silkie_Wencha	0.849985158
ENSGALG00010010052	ZNF346	3	-0.037979857	0.060833764	-0.624322008	0.532416133	0.003726758	2.921557829	31.54337114	0.666666667	CRA004775;PRJNA827465;Yang2025_Silkie_Wencha	0.849985158
ENSGALG00010007902	C7orf25	3	0.037868648	0.060732398	0.623503256	0.532953863	0.00020001	2.020643531	1.021631504	0.666666667	CRA004775;PRJNA827465;Yang2025_Silkie_Wencha	0.849985158
ENSGALG00010008157	CTSO	3	-0.035400287	0.056892478	-0.62223141	0.533789715	0	0.066494438	0	0.666666667	CRA004775;PRJNA827465;Yang2025_Silkie_Wencha	0.849985158
ENSGALG00010024842	ZFYVE9	3	0.027984502	0.044873404	0.623632249	0.532869127	0	1.141510027	0	0.666666667	CRA004775;PRJNA827465;Yang2025_Silkie_Wencha	0.849985158
ENSGALG00010020732	ARIH2	3	-0.026104039	0.041851581	-0.623728868	0.532805661	0	1.488682842	0	0.666666667	CRA004775;PRJNA827465;Yang2025_Silkie_Wencha	0.849985158
ENSGALG00010005131	IL8L2	2	-0.41494058	0.667252344	-0.621864552	0.534030936	0.487139327	2.157779796	53.65645466	0.5	CRA004775;PRJNA827465	0.850204189
ENSGALG00010015866	ARHGEF6	3	-0.062909024	0.10147117	-0.621762066	0.534098333	0.018924133	6.46641061	69.07094027	0.666666667	CRA004775;PRJNA827465;Yang2025_Silkie_Wencha	0.850215755
ENSGALG00010008368	SNCA	3	0.246154992	0.396157711	0.621356054	0.53436538	0.35974295	10.98429085	81.79217914	0.666666667	CRA004775;PRJNA827465;Yang2025_Silkie_Wencha	0.850263367
ENSGALG00010023759	CALCA	3	0.242970458	0.391093373	0.62125946	0.534428923	0.270032345	5.240012469	61.83215189	0.666666667	CRA004775;PRJNA827465;Yang2025_Silkie_Wencha	0.850263367
ENSGALG00010005969	HECA	3	-0.133366744	0.214647978	-0.621327747	0.534384001	0.121222604	22.99188081	91.3012771	0.666666667	CRA004775;PRJNA827465;Yang2025_Silkie_Wencha	0.850263367
ENSGALG00010017601	ENSGALG00010017601	3	0.131277755	0.211250359	0.621432106	0.534315353	0.101633258	8.785482203	77.235171	0.666666667	CRA004775;PRJNA827465;Yang2025_Silkie_Wencha	0.850263367
ENSGALG00010011688	PDE3A	3	0.093208775	0.149988744	0.621459187	0.53429754	0.026359	3.177033446	37.04819184	0.666666667	CRA004775;PRJNA827465;Yang2025_Silkie_Wencha	0.850263367
ENSGALG00010015031	MYH9	3	0.163380003	0.263101002	0.620978261	0.534613927	0.160949124	15.35877528	86.97812838	0.666666667	CRA004775;PRJNA827465;Yang2025_Silkie_Wencha	0.850368089
ENSGALG00010024817	UCKL1	3	0.079992282	0.128828178	0.62097658	0.534615033	0.026933714	4.974615258	59.79588578	0.666666667	CRA004775;PRJNA827465;Yang2025_Silkie_Wencha	0.850368089
ENSGALG00010023902	PTGER3	3	-0.151672875	0.244313992	-0.620811249	0.534723821	0.121107421	6.525631683	69.35632196	0.666666667	CRA004775;PRJNA827465;Yang2025_Silkie_Wencha	0.850445456
ENSGALG00010015218	PLCL1	3	0.055466083	0.089371541	0.620623551	0.53484734	0	1.92276964	0	0.666666667	CRA004775;PRJNA827465;Yang2025_Silkie_Wencha	0.850546232
ENSGALG00010001210	PANX2	2	0.249762627	0.402575172	0.620412395	0.534986314	0.259475881	4.903111525	79.6047878	0.5	PRJNA827465;Yang2025_Silkie_Wenchang	0.850671558
ENSGALG00010023151	ENSGALG00010023151	2	0.221015703	0.35642728	0.620086384	0.535200916	0	0.187239341	0	1	CRA004775;PRJNA827465	0.850686418
ENSGALG00010005204	ITGBL1	3	0.216322663	0.348940606	0.619941215	0.535296489	0.187997686	4.163792274	51.09666414	0.666666667	CRA004775;PRJNA827465;Yang2025_Silkie_Wencha	0.850686418
ENSGALG00010004494	WTAP	3	-0.10389914	0.167517901	-0.620227089	0.535108289	0.076576955	48.60539725	95.88523063	0.666666667	CRA004775;PRJNA827465;Yang2025_Silkie_Wencha	0.850686418
ENSGALG00010003406	C1GALT1	3	-0.101390906	0.163521267	-0.620047213	0.535226704	0.063960282	15.67583398	87.24150816	0.666666667	CRA004775;PRJNA827465;Yang2025_Silkie_Wencha	0.850686418
ENSGALG00010011635	PCDH12	3	0.047937943	0.077319847	0.619995314	0.535260872	0	1.16643479	0	0.666666667	CRA004775;PRJNA827465;Yang2025_Silkie_Wencha	0.850686418
ENSGALG00010024494	DDAH1	3	0.287740626	0.465014074	0.618778317	0.536062408	0.513087998	10.85643749	81.57775052	0.666666667	CRA004775;PRJNA827465;Yang2025_Silkie_Wencha	0.850714235
ENSGALG00010024683	SHISA4	3	0.239605253	0.386910631	0.619278028	0.535733216	0.360541007	12.12494224	83.50507606	0.666666667	CRA004775;PRJNA827465;Yang2025_Silkie_Wencha	0.850714235
ENSGALG00010024719	PYM1	3	0.179449097	0.290035657	0.618713914	0.536104842	0.22396149	21.03732015	90.49308569	0.666666667	CRA004775;PRJNA827465;Yang2025_Silkie_Wencha	0.850714235
ENSGALG00010014578	ENSGALG00010014578	3	0.178141228	0.287430121	0.619772304	0.535407705	0.182856731	10.16132269	80.31752301	0.666666667	CRA004775;PRJNA827465;Yang2025_Silkie_Wencha	0.850714235
ENSGALG00010004058	TMEM30A	3	-0.143324046	0.231704712	-0.618563365	0.536204043	0.145476835	65.96494744	96.96808672	0.666666667	CRA004775;PRJNA827465;Yang2025_Silkie_Wencha	0.850714235
ENSGALG00010010967	PRXL2A	3	-0.140039112	0.226007738	-0.619620872	0.535507422	0.090083221	4.964094538	59.71067866	0.666666667	CRA004775;PRJNA827465;Yang2025_Silkie_Wencha	0.850714235
ENSGALG00010002211	ACER3	3	-0.134882054	0.218081722	-0.618493165	0.536250302	0.110164527	14.73028899	86.42253386	0.666666667	CRA004775;PRJNA827465;Yang2025_Silkie_Wencha	0.850714235
ENSGALG00010010300	DENND1B	3	0.123486303	0.199600433	0.618667512	0.536135417	0.082153061	6.544958313	69.44212775	0.666666667	CRA004775;PRJNA827465;Yang2025_Silkie_Wencha	0.850714235
ENSGALG00010011251	FRZB	3	-0.118455152	0.19131908	-0.619149706	0.535817741	0.073867429	11.39445995	82.44761042	0.666666667	CRA004775;PRJNA827465;Yang2025_Silkie_Wencha	0.850714235
ENSGALG00010020920	ACOX2	3	-0.078264285	0.126567133	-0.618361837	0.536336848	0	0.981098475	0	0.666666667	CRA004775;PRJNA827465;Yang2025_Silkie_Wencha	0.850714235
ENSGALG00010018342	BNIP3L	3	-0.076803189	0.124194774	-0.618409189	0.536305642	0.031784973	8.077368131	75.23945959	0.666666667	CRA004775;PRJNA827465;Yang2025_Silkie_Wencha	0.850714235
ENSGALG00010010386	PRPF40A	3	0.073423416	0.118639629	0.618877662	0.535996955	0.026690947	6.048134585	66.93195279	0.666666667	CRA004775;PRJNA827465;Yang2025_Silkie_Wencha	0.850714235
ENSGALG00010001482	TRA2A	3	0.055664914	0.089899065	0.619234801	0.535761689	0.012350633	3.842237404	47.94699573	0.666666667	CRA004775;PRJNA827465;Yang2025_Silkie_Wencha	0.850714235
ENSGALG00010016584	EHBP1	3	0.051829773	0.083640748	0.619671319	0.535474202	0.005041872	2.377098932	15.86382994	0.666666667	CRA004775;PRJNA827465;Yang2025_Silkie_Wencha	0.850714235
ENSGALG00010020576	AKR7L	3	-0.045530327	0.073608996	-0.618542963	0.536217486	0	0.034028496	0	1	CRA004775;PRJNA827465;Yang2025_Silkie_Wencha	0.850714235
ENSGALG00010020053	LFNG	3	0.040696765	0.0657168	0.619274914	0.535735268	0	1.636677871	0	0.666666667	CRA004775;PRJNA827465;Yang2025_Silkie_Wencha	0.850714235
ENSGALG00010016394	PDIK1L	3	0.038151044	0.061585831	0.619487531	0.535595233	0.002786193	2.425139949	17.53053259	0.666666667	CRA004775;PRJNA827465;Yang2025_Silkie_Wencha	0.850714235
ENSGALG00010013640	SNPH	2	0.296970623	0.48069182	0.61779837	0.536708258	0	0.519114988	0	1	CRA004775;PRJNA827465	0.850879172
ENSGALG00010003170	C4	3	0.173263622	0.280508508	0.617676886	0.536788351	0.086252175	3.150835618	36.52477493	0.666666667	CRA004775;PRJNA827465;Yang2025_Silkie_Wencha	0.850879172
ENSGALG00010009263	COLEC10	3	-0.109040342	0.17647537	-0.617878528	0.536654414	0.058930791	6.99287413	71.39945661	0.666666667	CRA004775;PRJNA827465;Yang2025_Silkie_Wencha	0.850879172
ENSGALG00010010810	GALT	3	0.108437776	0.175492494	0.617905494	0.536637637	0.033177377	3.103652116	35.55978811	0.666666667	CRA004775;PRJNA827465;Yang2025_Silkie_Wencha	0.850879172
ENSGALG00010004443	ANKRD17	3	0.08427809	0.136448195	0.61765632	0.536801911	0.032781929	4.841107682	58.68714081	0.666666667	CRA004775;PRJNA827465;Yang2025_Silkie_Wencha	0.850879172
ENSGALG00010024789	SUOX	3	-0.045321361	0.073342043	-0.617945168	0.536611484	0	1.035178274	0	1	CRA004775;PRJNA827465;Yang2025_Silkie_Wencha	0.850879172
ENSGALG00010022357	ENSGALG00010022357	3	0.183082783	0.296518822	0.617440681	0.536944096	0	0.887943644	0	0.666666667	CRA004775;PRJNA827465;Yang2025_Silkie_Wencha	0.850913761
ENSGALG00010008410	NAPRT	3	-0.142813357	0.231270217	-0.617517286	0.536893583	0.137343263	16.17438805	87.63477175	0.666666667	CRA004775;PRJNA827465;Yang2025_Silkie_Wencha	0.850913761
ENSGALG00010001903	NARS2	3	-0.092766316	0.150352159	-0.616993572	0.537238966	0.0475244	6.839836087	70.7595332	0.666666667	CRA004775;PRJNA827465;Yang2025_Silkie_Wencha	0.851285637
ENSGALG00010008443	RSFR	3	-0.237263251	0.384810671	-0.616571391	0.537517469	0.357955687	10.47270698	80.90274078	0.666666667	CRA004775;PRJNA827465;Yang2025_Silkie_Wencha	0.851536078
ENSGALG00010020781	SNN	3	-0.062703461	0.101690385	-0.616611501	0.537491007	0.015176482	4.119126391	51.44601524	0.666666667	CRA004775;PRJNA827465;Yang2025_Silkie_Wencha	0.851536078
ENSGALG00010000654	ENSGALG00010000654	3	0.356417757	0.578196945	0.616429678	0.537610977	0.976318313	99.90705603	97.99813939	0.666666667	CRA004775;PRJNA827465;Yang2025_Silkie_Wencha	0.851546023
ENSGALG00010011437	DMRT1	3	-0.174969381	0.283950496	-0.61619678	0.537764653	0	0.836624524	0	0.666666667	CRA004775;PRJNA827465;Yang2025_Silkie_Wencha	0.851546023
ENSGALG00010027221	LRRC61	3	-0.130185466	0.211242285	-0.616285068	0.537706392	0.084074384	5.414760941	63.063928	0.666666667	CRA004775;PRJNA827465;Yang2025_Silkie_Wencha	0.851546023
ENSGALG00010020998	SYT17	3	0.090464092	0.146788744	0.616287662	0.53770468	0.0218554	2.954157047	32.29879223	0.666666667	CRA004775;PRJNA827465;Yang2025_Silkie_Wencha	0.851546023
ENSGALG00010013667	SOWAHA	2	0.316873399	0.514499797	0.615886331	0.537969544	0.284188563	2.09942929	52.3680221	0.5	CRA004775;PRJNA827465	0.851775071
ENSGALG00010001783	SGO1	3	0.055420416	0.09001668	0.615668293	0.538113468	0.000166933	2.009425218	0.469050447	0.666666667	CRA004775;PRJNA827465;Yang2025_Silkie_Wencha	0.851812173
ENSGALG00010025147	COX17	3	-0.046430133	0.075413408	-0.615674774	0.53810919	0	1.250779502	0	1	CRA004775;PRJNA827465;Yang2025_Silkie_Wencha	0.851812173
ENSGALG00010027935	ISY1	3	0.16126083	0.262004757	0.615481177	0.538232375	0.18475914	30.48071524	93.43847418	0.666666667	CRA004775;PRJNA827465;Yang2025_Silkie_Wencha	0.851905021
ENSGALG00010000853	ENSGALG00010000853	3	-0.088818312	0.144370228	-0.615212106	0.538414654	0.019796407	2.806517779	28.73731739	0.666666667	CRA004775;PRJNA827465;Yang2025_Silkie_Wencha	0.852029545
ENSGALG00010024251	ENSGALG00010024251	3	-0.076665826	0.12462209	-0.615186487	0.538431571	0	1.657042694	0	0.666666667	CRA004775;PRJNA827465;Yang2025_Silkie_Wencha	0.852029545
ENSGALG00010026845	ENSGALG00010026845	2	-1.127859834	1.83473267	-0.614727068	0.538734981	0	0.225260199	0	1	PRJNA827465;Yang2025_Silkie_Wenchang	0.852136811
ENSGALG00010028510	CILP2	3	0.425034848	0.691404891	0.614740875	0.538725861	1.108818815	9.462003151	78.86282674	0.666666667	CRA004775;PRJNA827465;Yang2025_Silkie_Wencha	0.852136811
ENSGALG00010003210	EYA1	3	0.20048239	0.326136739	0.614718817	0.538740431	0.235868727	8.686536272	76.97586314	0.666666667	CRA004775;PRJNA827465;Yang2025_Silkie_Wencha	0.852136811
ENSGALG00010008063	KCNV1	3	0.18121356	0.294679695	0.614950955	0.53858711	0.131114064	4.107299998	51.30621087	0.666666667	CRA004775;PRJNA827465;Yang2025_Silkie_Wencha	0.852136811
ENSGALG00010022496	TUBGCP2	3	-0.055681911	0.090610387	-0.614520172	0.538871648	0.015189696	5.695165005	64.88249246	0.666666667	CRA004775;PRJNA827465;Yang2025_Silkie_Wencha	0.852249019
ENSGALG00010003115	UBE3D	3	-0.125803941	0.204782363	-0.614329962	0.538997307	0.076270023	5.279558915	62.11804751	0.666666667	CRA004775;PRJNA827465;Yang2025_Silkie_Wencha	0.852352414
ENSGALG00010028415	FSD1	3	0.048985801	0.0797543	0.614208899	0.539077294	0	1.509955339	0	0.666666667	CRA004775;PRJNA827465;Yang2025_Silkie_Wencha	0.852383567
ENSGALG00010007937	ANGPTL5	3	0.172619598	0.281164377	0.613945481	0.539251355	0.036042823	2.319944513	13.7910416	0.666666667	CRA004775;PRJNA827465;Yang2025_Silkie_Wencha	0.852563447
ENSGALG00010025618	ADIPOQ	3	-0.06681195	0.108853532	-0.613778429	0.539361753	0	0.906639924	0	0.666666667	CRA004775;PRJNA827465;Yang2025_Silkie_Wencha	0.852642646
ENSGALG00010011617	MAMLD1	3	0.102552972	0.167154832	0.613520835	0.539532011	0.056676536	6.344612628	68.4771929	0.666666667	CRA004775;PRJNA827465;Yang2025_Silkie_Wencha	0.852816445
ENSGALG00010005720	COL25A1	3	0.187473407	0.305620224	0.613419506	0.539598992	0.17004499	7.284604179	72.54483633	0.666666667	CRA004775;PRJNA827465;Yang2025_Silkie_Wencha	0.852826978
ENSGALG00010020695	ENSGALG00010020695	2	-0.285858585	0.466120945	-0.613271271	0.539696986	0	0.055352151	0	1	CRA004775;PRJNA827465	0.852886519
ENSGALG00010026886	JUN	3	-0.124012416	0.202310167	-0.612981633	0.539888484	0.089835418	13.3371561	85.00429938	0.666666667	CRA004775;PRJNA827465;Yang2025_Silkie_Wencha	0.852894261
ENSGALG00010018448	KLHDC3	3	-0.115090781	0.187877712	-0.61258347	0.540151791	0.072003868	9.625578265	79.22202755	0.666666667	CRA004775;PRJNA827465;Yang2025_Silkie_Wencha	0.852894261
ENSGALG00010011720	IKZF2	3	-0.095326598	0.155597913	-0.612647021	0.54010976	0	0.061963221	0	1	CRA004775;PRJNA827465;Yang2025_Silkie_Wencha	0.852894261
ENSGALG00010029752	ENSGALG00010029752	3	0.08333088	0.135904629	0.613157041	0.539772507	0.024672888	3.739353607	46.5148202	0.666666667	CRA004775;PRJNA827465;Yang2025_Silkie_Wencha	0.852894261
ENSGALG00010022466	NEDD4	3	0.080738902	0.131811283	0.612534073	0.540184462	0.030119455	4.822407804	58.5269417	0.666666667	CRA004775;PRJNA827465;Yang2025_Silkie_Wencha	0.852894261
ENSGALG00010018322	NT5C1A	3	-0.067019263	0.109344625	-0.612917766	0.539930715	0	1.046996838	0	0.666666667	CRA004775;PRJNA827465;Yang2025_Silkie_Wencha	0.852894261
ENSGALG00010022162	POLE	3	0.044892166	0.073287586	0.612548028	0.540175232	0	1.076884342	0	0.666666667	CRA004775;PRJNA827465;Yang2025_Silkie_Wencha	0.852894261
ENSGALG00010004356	TRAPPC8	3	0.021913155	0.035766588	0.612671094	0.540093839	0	1.194727874	0	0.666666667	CRA004775;PRJNA827465;Yang2025_Silkie_Wencha	0.852894261
ENSGALG00010027100	MIER2	3	-0.091640181	0.149647375	-0.612374131	0.540290253	0.049822997	8.23896464	75.72510519	0.666666667	CRA004775;PRJNA827465;Yang2025_Silkie_Wencha	0.852966044
ENSGALG00010026700	C8B	2	-0.433397581	0.708759092	-0.611487862	0.540876652	0.500898448	1.985906233	49.64515529	0.5	PRJNA827465;Yang2025_Silkie_Wenchang	0.85298597
ENSGALG00010012918	GLRX	3	0.223624557	0.365765497	0.611387784	0.540942888	0.26840562	6.11630931	67.3005419	0.666666667	CRA004775;PRJNA827465;Yang2025_Silkie_Wencha	0.85298597
ENSGALG00010021611	GREM1	3	-0.20326945	0.332644856	-0.611070475	0.541152925	0.136966398	3.393368549	41.06151539	0.666666667	CRA004775;PRJNA827465;Yang2025_Silkie_Wencha	0.85298597
ENSGALG00010023411	ENSGALG00010023411	3	0.201504488	0.329801285	0.610987578	0.541207804	0.237184189	8.818917553	77.32147978	0.666666667	CRA004775;PRJNA827465;Yang2025_Silkie_Wencha	0.85298597
ENSGALG00010005860	ABI3BP	3	0.190523251	0.311590193	0.61145458	0.540898679	0.181965494	5.801169957	65.52419572	0.666666667	CRA004775;PRJNA827465;Yang2025_Silkie_Wencha	0.85298597
ENSGALG00010025487	FGF3	2	-0.175659919	0.287387607	-0.61122997	0.541047345	0	0.001053917	0	1	PRJNA827465;Yang2025_Silkie_Wenchang	0.85298597
ENSGALG00010027473	DNAH1	2	0.149475263	0.244374907	0.611663714	0.540760274	0	0.183985075	0	1	PRJNA827465;Yang2025_Silkie_Wenchang	0.85298597
ENSGALG00010005491	GGACT	3	0.100956019	0.165147693	0.611307471	0.540996046	0.050133672	5.629436231	64.47246371	0.666666667	CRA004775;PRJNA827465;Yang2025_Silkie_Wencha	0.85298597
ENSGALG00010029618	TEX2	3	0.09722884	0.159072058	0.611225133	0.541050547	0.058867266	11.55131996	82.68596137	0.666666667	CRA004775;PRJNA827465;Yang2025_Silkie_Wencha	0.85298597
ENSGALG00010006131	ENSGALG00010006131	3	0.086545198	0.14164517	0.61099999	0.541199587	0.025705501	3.207354766	37.64331837	0.666666667	CRA004775;PRJNA827465;Yang2025_Silkie_Wencha	0.85298597
ENSGALG00010024122	SOCS7	3	0.060442804	0.098912322	0.611074564	0.541150218	0.015395944	4.738293829	57.79071387	0.666666667	CRA004775;PRJNA827465;Yang2025_Silkie_Wencha	0.85298597
ENSGALG00010001856	CHST15	3	0.057803439	0.094516254	0.611571412	0.540821357	0.009535262	3.073795194	34.93385624	0.666666667	CRA004775;PRJNA827465;Yang2025_Silkie_Wencha	0.85298597
ENSGALG00010014298	METTL18	3	-0.048363965	0.079143008	-0.611095865	0.541136117	0	1.016407363	0	0.666666667	CRA004775;PRJNA827465;Yang2025_Silkie_Wencha	0.85298597
ENSGALG00010024669	STIL	3	0.043446093	0.070988104	0.612019354	0.540524953	0	0.246443416	0	0.666666667	CRA004775;PRJNA827465;Yang2025_Silkie_Wencha	0.85298597
ENSGALG00010021086	PSMD14	3	-0.03264839	0.053401854	-0.611371855	0.540953431	0	0.863932243	0	1	CRA004775;PRJNA827465;Yang2025_Silkie_Wencha	0.85298597
ENSGALG00010029449	UBALD2	3	0.208710258	0.341683767	0.610828722	0.541312977	0.292845679	13.52680925	85.21454718	0.666666667	CRA004775;PRJNA827465;Yang2025_Silkie_Wencha	0.853001247
ENSGALG00010005057	FLVCR2	3	0.064174465	0.10506785	0.610790693	0.541338156	0	0.947811222	0	0.666666667	CRA004775;PRJNA827465;Yang2025_Silkie_Wencha	0.853001247
ENSGALG00010023860	TRIP4	3	0.072197818	0.11822564	0.610678178	0.541412656	0.02919094	8.209932107	75.63926262	0.666666667	CRA004775;PRJNA827465;Yang2025_Silkie_Wencha	0.853023572
ENSGALG00010011464	FANCG	3	-0.115298664	0.188970344	-0.610141576	0.541768027	0.049547368	3.765340813	46.8839582	0.666666667	CRA004775;PRJNA827465;Yang2025_Silkie_Wencha	0.853109503
ENSGALG00010021781	RAPGEF4	3	0.100596316	0.164808307	0.610383772	0.541607616	0	1.517225876	0	0.666666667	CRA004775;PRJNA827465;Yang2025_Silkie_Wencha	0.853109503
ENSGALG00010024146	CDKN1C	3	0.095466781	0.156405008	0.610381867	0.541608877	0.032955392	3.672273043	45.53781877	0.666666667	CRA004775;PRJNA827465;Yang2025_Silkie_Wencha	0.853109503
ENSGALG00010021108	PCGF6	3	-0.073360212	0.120228368	-0.610173897	0.541746619	0.027538575	5.767956265	65.32567328	0.666666667	CRA004775;PRJNA827465;Yang2025_Silkie_Wencha	0.853109503
ENSGALG00010019833	MTHFS	3	-0.054444883	0.089233385	-0.610140285	0.541768882	0	1.849473483	0	0.666666667	CRA004775;PRJNA827465;Yang2025_Silkie_Wencha	0.853109503
ENSGALG00010021067	SLC4A10	2	0.17402548	0.285381409	0.609799638	0.541994541	0.10533975	2.616278813	61.77777403	0.5	PRJNA827465;Yang2025_Silkie_Wenchang	0.853369802
ENSGALG00010013580	RANBP3L	3	0.224317184	0.368260253	0.609126784	0.542440405	0.27637257	7.029135896	71.54700052	0.666666667	CRA004775;PRJNA827465;Yang2025_Silkie_Wencha	0.853648767
ENSGALG00010002606	ENSGALG00010002606	3	0.137335078	0.225501925	0.609019539	0.542511488	0.083571834	4.414497835	54.6947337	0.666666667	CRA004775;PRJNA827465;Yang2025_Silkie_Wencha	0.853648767
ENSGALG00010017653	ENSGALG00010017653	3	-0.129149215	0.21213614	-0.608803456	0.542654723	0.0711085	4.113198678	51.37604195	0.666666667	CRA004775;PRJNA827465;Yang2025_Silkie_Wencha	0.853648767
ENSGALG00010021628	REC114	3	-0.102075618	0.167568328	-0.609158183	0.542419595	0	1.270024179	0	0.666666667	CRA004775;PRJNA827465;Yang2025_Silkie_Wencha	0.853648767
ENSGALG00010019318	NOG2	3	-0.091218893	0.149716842	-0.609276093	0.542341451	0	1.648886242	0	0.666666667	CRA004775;PRJNA827465;Yang2025_Silkie_Wencha	0.853648767
ENSGALG00010021736	EDEM1	3	-0.08754805	0.143743301	-0.6090583	0.542485797	0.041674213	8.4511423	76.33456012	0.666666667	CRA004775;PRJNA827465;Yang2025_Silkie_Wencha	0.853648767
ENSGALG00010026234	ZDHHC5	3	-0.037600634	0.061751761	-0.608899794	0.542590861	0	1.946758815	0	0.666666667	CRA004775;PRJNA827465;Yang2025_Silkie_Wencha	0.853648767
ENSGALG00010026112	ZFP91	3	0.019199073	0.031532661	0.608863069	0.542615205	0	0.092351823	0	1	CRA004775;PRJNA827465;Yang2025_Silkie_Wencha	0.853648767
ENSGALG00010023794	MT3	2	0.274044031	0.450996228	0.607641516	0.54342526	0.172541768	1.73606212	42.39837455	0.5	CRA004775;PRJNA827465	0.85409551
ENSGALG00010019110	MBD4	3	0.168323624	0.277038078	0.607582991	0.543464084	0.207578461	23.11410758	91.34727571	0.666666667	CRA004775;PRJNA827465;Yang2025_Silkie_Wencha	0.85409551
ENSGALG00010008294	NOB1	3	0.127556984	0.209869898	0.607790756	0.543326261	0.102798698	10.79727377	81.47680569	0.666666667	CRA004775;PRJNA827465;Yang2025_Silkie_Wencha	0.85409551
ENSGALG00010009769	TMCC3	3	0.123635319	0.203447894	0.607700167	0.543386352	0.049062883	3.27508765	38.93293207	0.666666667	CRA004775;PRJNA827465;Yang2025_Silkie_Wencha	0.85409551
ENSGALG00010024867	TM2D3	3	-0.086983564	0.143042961	-0.608093981	0.543125144	0.038965768	5.545371956	63.93388909	0.666666667	CRA004775;PRJNA827465;Yang2025_Silkie_Wencha	0.85409551
ENSGALG00010003330	HEY1	3	0.081893133	0.134736546	0.607801929	0.54331885	0.034730273	5.803549309	65.53833019	0.666666667	CRA004775;PRJNA827465;Yang2025_Silkie_Wencha	0.85409551
ENSGALG00010013290	AKR1D1	3	0.06324	0.104104872	0.607464368	0.543542782	0	0.915368346	0	0.666666667	CRA004775;PRJNA827465;Yang2025_Silkie_Wencha	0.85409551
ENSGALG00010010959	TMEM263	3	-0.052153538	0.085846718	-0.607519307	0.543506334	0.012953344	5.023648725	60.18829919	0.666666667	CRA004775;PRJNA827465;Yang2025_Silkie_Wencha	0.85409551
ENSGALG00010001127	SIAH2	3	-0.050026365	0.082297234	-0.607874196	0.543270915	0.009109942	3.626736314	44.85400021	0.666666667	CRA004775;PRJNA827465;Yang2025_Silkie_Wencha	0.85409551
ENSGALG00010010769	HEXB	3	0.046512761	0.07650785	0.607947569	0.543222248	0	1.931162015	0	0.666666667	CRA004775;PRJNA827465;Yang2025_Silkie_Wencha	0.85409551
ENSGALG00010010949	FZD3	3	-0.068847659	0.113383851	-0.607208686	0.543712429	0.006669793	2.346026085	14.74945599	0.666666667	CRA004775;PRJNA827465;Yang2025_Silkie_Wencha	0.854203487
ENSGALG00010017585	ENSGALG00010017585	3	-0.047132503	0.077625422	-0.607178698	0.543732328	0.001770911	2.140763908	6.575405515	0.666666667	CRA004775;PRJNA827465;Yang2025_Silkie_Wencha	0.854203487
ENSGALG00010010381	MAST4	3	0.187575718	0.309012367	0.607016864	0.54383972	0.226730755	11.81704781	83.07529908	0.666666667	CRA004775;PRJNA827465;Yang2025_Silkie_Wencha	0.854277281
ENSGALG00010012259	IL1RAPL2	3	0.134323499	0.221376541	0.606764827	0.544006994	0.06748442	3.733092144	46.42511027	0.666666667	CRA004775;PRJNA827465;Yang2025_Silkie_Wencha	0.8542963
ENSGALG00010028392	ASH1L	3	0.103781296	0.17105148	0.606725507	0.544033092	0.063320111	8.723495614	77.07341084	0.666666667	CRA004775;PRJNA827465;Yang2025_Silkie_Wencha	0.8542963
ENSGALG00010029946	METRNL	3	0.031050441	0.051172736	0.606777043	0.543998886	0	1.193004453	0	0.666666667	CRA004775;PRJNA827465;Yang2025_Silkie_Wencha	0.8542963
ENSGALG00010021267	LRRC47	3	-0.05359087	0.088371263	-0.606428695	0.544230119	0.011334091	3.868506212	48.30045784	0.666666667	CRA004775;PRJNA827465;Yang2025_Silkie_Wencha	0.854510788
ENSGALG00010008257	DESI2	3	-0.116773121	0.192611573	-0.606262227	0.544340637	0.101137617	49.90448955	95.99234454	0.666666667	CRA004775;PRJNA827465;Yang2025_Silkie_Wencha	0.854589415
ENSGALG00010005158	IFT43	3	-0.07521681	0.124153968	-0.605834924	0.544624376	0	1.796701345	0	0.666666667	CRA004775;PRJNA827465;Yang2025_Silkie_Wencha	0.854939941
ENSGALG00010012719	COL10A1	3	-0.89926576	1.485321009	-0.605435293	0.544889807	4.756135005	8.873607938	77.46125348	0.666666667	CRA004775;PRJNA827465;Yang2025_Silkie_Wencha	0.855071807
ENSGALG00010025182	PLA1A	3	0.135724626	0.224109725	0.605616853	0.544769209	0.102344624	6.422109888	68.85758676	0.666666667	CRA004775;PRJNA827465;Yang2025_Silkie_Wencha	0.855071807
ENSGALG00010012125	RNF152	3	-0.117529703	0.19409731	-0.605519481	0.544833884	0.081196081	9.435534173	78.80353181	0.666666667	CRA004775;PRJNA827465;Yang2025_Silkie_Wencha	0.855071807
ENSGALG00010006828	GTF2A1	3	0.045834316	0.07573077	0.605227118	0.5450281	0.006600433	2.728138637	26.68994262	1	CRA004775;PRJNA827465;Yang2025_Silkie_Wencha	0.855193907
ENSGALG00010023175	GRK2	3	0.078885437	0.130368628	0.605095248	0.545115711	0.037800958	10.40942969	80.78665153	0.666666667	CRA004775;PRJNA827465;Yang2025_Silkie_Wencha	0.855236467
ENSGALG00010026834	ENSGALG00010026834	2	-1.276718825	2.11068805	-0.604882766	0.545256895	6.042861282	3.071983351	67.44774025	0.5	PRJNA827465;Yang2025_Silkie_Wenchang	0.855318817
ENSGALG00010004275	ADARB2	2	-0.290500102	0.480763391	-0.60424755	0.545679072	0	0.537782595	0	0.5	PRJNA827465;Yang2025_Silkie_Wenchang	0.855318817
ENSGALG00010010084	HK3	3	0.231261106	0.382457044	0.604672106	0.545396886	0.374160712	16.80385329	88.09796798	0.666666667	CRA004775;PRJNA827465;Yang2025_Silkie_Wencha	0.855318817
ENSGALG00010029210	RSKR	3	-0.189105506	0.313023634	-0.604125331	0.545760319	0.198610161	7.729719231	74.12583898	0.666666667	CRA004775;PRJNA827465;Yang2025_Silkie_Wencha	0.855318817
ENSGALG00010019979	ENSGALG00010019979	2	0.172969003	0.286108761	0.604556819	0.545473506	0	0.665000713	0	0.5	PRJNA827465;Yang2025_Silkie_Wenchang	0.855318817
ENSGALG00010016409	GMEB2	3	0.11498142	0.190242147	0.604395092	0.545580998	0.0767876	10.53938154	81.02355445	0.666666667	CRA004775;PRJNA827465;Yang2025_Silkie_Wencha	0.855318817
ENSGALG00010023822	CHEK2	3	0.101645909	0.168087296	0.604720946	0.545364429	0.053870297	6.203198512	67.75856849	0.666666667	CRA004775;PRJNA827465;Yang2025_Silkie_Wencha	0.855318817
ENSGALG00010015858	CHST2	3	-0.091520524	0.151444918	-0.604315584	0.545633848	0.047710851	7.187054728	72.17218908	0.666666667	CRA004775;PRJNA827465;Yang2025_Silkie_Wencha	0.855318817
ENSGALG00010012469	CETN2	3	-0.081623149	0.135172091	-0.603846164	0.545945924	0.025672974	3.735136053	46.45442705	0.666666667	CRA004775;PRJNA827465;Yang2025_Silkie_Wencha	0.855318817
ENSGALG00010008504	RNF139	3	-0.075241834	0.124602637	-0.60385427	0.545940534	0.029621897	6.373408942	68.61961914	0.666666667	CRA004775;PRJNA827465;Yang2025_Silkie_Wencha	0.855318817
ENSGALG00010028128	DNM1	3	0.074760859	0.123662385	0.604556177	0.545473932	0.01849416	3.131970868	36.14244564	0.666666667	CRA004775;PRJNA827465;Yang2025_Silkie_Wencha	0.855318817
ENSGALG00010024563	ESPL1	3	-0.058494603	0.096836272	-0.604056748	0.545805914	0	1.049475669	0	1	CRA004775;PRJNA827465;Yang2025_Silkie_Wencha	0.855318817
ENSGALG00010029423	MYBBP1A	3	-0.030635625	0.050735254	-0.603833091	0.545954617	0	0.076806983	0	1	CRA004775;PRJNA827465;Yang2025_Silkie_Wencha	0.855318817
ENSGALG00010027905	SYN2	3	-0.111556321	0.184851714	-0.603490866	0.54618219	0	0.91804452	0	0.666666667	CRA004775;PRJNA827465;Yang2025_Silkie_Wencha	0.855482603
ENSGALG00010019212	RPL22	3	0.077956937	0.1291765	0.603491632	0.546181681	0.02707684	4.203604671	52.42178662	0.666666667	CRA004775;PRJNA827465;Yang2025_Silkie_Wencha	0.855482603
ENSGALG00010028039	STAB1	3	-0.057711078	0.095642689	-0.603402923	0.546240678	0.007596299	2.678658284	25.33575441	0.666666667	CRA004775;PRJNA827465;Yang2025_Silkie_Wencha	0.855482603
ENSGALG00010022085	MLLT6	3	0.084994238	0.140904614	0.603204079	0.546372934	0.039883846	6.26730406	68.08835217	0.666666667	CRA004775;PRJNA827465;Yang2025_Silkie_Wencha	0.855500212
ENSGALG00010021207	MAD2L2	3	-0.0356849	0.059158614	-0.603207168	0.546370879	0	1.666107245	0	0.666666667	CRA004775;PRJNA827465;Yang2025_Silkie_Wencha	0.855500212
ENSGALG00010005556	IYD	2	0.301222695	0.499632067	0.602889036	0.54658251	0	0.001841488	0	1	PRJNA827465;Yang2025_Silkie_Wenchang	0.855584119
ENSGALG00010013583	FASLG	3	-0.179213602	0.297276957	-0.602850634	0.546608059	0	0.092992015	0	1	CRA004775;PRJNA827465;Yang2025_Silkie_Wencha	0.855584119
ENSGALG00010000785	CLN5	3	-0.087740649	0.145511812	-0.60297956	0.546522287	0.040765145	6.406485377	68.78163482	0.666666667	CRA004775;PRJNA827465;Yang2025_Silkie_Wencha	0.855584119
ENSGALG00010024814	APBA2	3	0.11203804	0.185890497	0.602709887	0.546701703	0.050442487	3.969630061	49.61747142	0.666666667	CRA004775;PRJNA827465;Yang2025_Silkie_Wencha	0.855635973
ENSGALG00010027315	RAB3A	3	0.219469239	0.364550203	0.602027478	0.547155847	0.34641725	20.21080093	90.10430113	0.666666667	CRA004775;PRJNA827465;Yang2025_Silkie_Wencha	0.855657367
ENSGALG00010029773	TRIM65	3	0.180516632	0.299749171	0.602225625	0.547023961	0.219795714	11.01127406	81.83679755	0.666666667	CRA004775;PRJNA827465;Yang2025_Silkie_Wencha	0.855657367
ENSGALG00010009320	GPR20	3	0.174210032	0.289254984	0.602271497	0.546993431	0.1854405	11.26716299	82.24930266	0.666666667	CRA004775;PRJNA827465;Yang2025_Silkie_Wencha	0.855657367
ENSGALG00010028075	RAB25	3	-0.171973733	0.28551406	-0.602330171	0.546954381	0	0.690872648	0	0.666666667	CRA004775;PRJNA827465;Yang2025_Silkie_Wencha	0.855657367
ENSGALG00010017821	ENSGALG00010017821	3	-0.171635757	0.284828923	-0.602592444	0.546779848	0	0.337052706	0	0.666666667	CRA004775;PRJNA827465;Yang2025_Silkie_Wencha	0.855657367
ENSGALG00010017411	RAB11FIP1	3	-0.095512036	0.158667914	-0.601961879	0.547199513	0.049131229	7.025177494	71.53096841	0.666666667	CRA004775;PRJNA827465;Yang2025_Silkie_Wencha	0.855657367
ENSGALG00010020259	ENSGALG00010020259	3	-0.08091425	0.134407114	-0.60200869	0.547168353	0	0.571311399	0	1	CRA004775;PRJNA827465;Yang2025_Silkie_Wencha	0.855657367
ENSGALG00010027545	PPARD	3	-0.059364278	0.098552255	-0.602363468	0.546932222	0.014746067	4.364930916	54.18025992	0.666666667	CRA004775;PRJNA827465;Yang2025_Silkie_Wencha	0.855657367
ENSGALG00010013592	H3C33	3	0.325943615	0.541891409	0.601492493	0.54751201	0.713303682	10.63856868	81.20047855	0.666666667	CRA004775;PRJNA827465;Yang2025_Silkie_Wencha	0.855883699
ENSGALG00010024666	MED24	3	-0.082518795	0.137174268	-0.601561763	0.547465888	0.04803655	14.89985666	86.57705207	0.666666667	CRA004775;PRJNA827465;Yang2025_Silkie_Wencha	0.855883699
ENSGALG00010014810	ENTREP1	3	0.052827578	0.08783053	0.601471701	0.547525855	0	0.760287891	0	0.666666667	CRA004775;PRJNA827465;Yang2025_Silkie_Wencha	0.855883699
ENSGALG00010027159	TMUB1	3	0.207169469	0.344707835	0.601000174	0.54783987	0.308725647	24.2236898	91.74361951	0.666666667	CRA004775;PRJNA827465;Yang2025_Silkie_Wencha	0.856097259
ENSGALG00010017818	SLC38A6	3	0.142581584	0.23733727	0.600755136	0.54800309	0.136273842	12.41094354	83.88518976	0.666666667	CRA004775;PRJNA827465;Yang2025_Silkie_Wencha	0.856097259
ENSGALG00010008111	SYTL2	3	0.136922268	0.227929847	0.600721097	0.548025765	0	0.860526046	0	0.666666667	CRA004775;PRJNA827465;Yang2025_Silkie_Wencha	0.856097259

ENSGALG00010002431	BCKDHB	3	-0.093030581	0.154760674	-0.601125456	0.547756429	0.04554289	6.484881198	69.15903408	0.666666667	CRA004775;PRJNA827465;Yang2025_Silkie_Wencha	0.856097259
ENSGALG00010013179	TBC1D30	3	-0.081920128	0.136346276	-0.600824098	0.547957152	0	0.683170843	0	0.666666667	CRA004775;PRJNA827465;Yang2025_Silkie_Wencha	0.856097259
ENSGALG00010002161	CDKL5	3	-0.078420481	0.130529593	-0.600786985	0.547981874	0.011190252	2.408169227	16.94935815	0.666666667	CRA004775;PRJNA827465;Yang2025_Silkie_Wencha	0.856097259
ENSGALG00010020578	TNKS2	3	-0.102810131	0.171226836	-0.60043235	0.548218133	0.069507589	13.45293898	85.13336005	0.666666667	CRA004775;PRJNA827465;Yang2025_Silkie_Wencha	0.856114003
ENSGALG00010005743	ETNPPL	3	0.072351486	0.120463616	0.600608515	0.548100699	0	1.685980964	0	1	CRA004775;PRJNA827465;Yang2025_Silkie_Wencha	0.856114003
ENSGALG00010003057	DDX1	3	0.013599847	0.022646874	0.600517616	0.548161324	0	0.999243771	0	1	CRA004775;PRJNA827465;Yang2025_Silkie_Wencha	0.856114003
ENSGALG00010005568	PLEKHG1	3	0.111221909	0.185301065	0.600222716	0.548357816	0.06466158	6.684253864	70.07893415	0.666666667	CRA004775;PRJNA827465;Yang2025_Silkie_Wencha	0.856143017
ENSGALG00010029579	GALNT17	3	0.052648892	0.08770364	0.600304525	0.548303304	0	0.442213392	0	0.666666667	CRA004775;PRJNA827465;Yang2025_Silkie_Wencha	0.856143017
ENSGALG00010003220	SPP1	3	-0.353180734	0.588668669	-0.599965232	0.548529407	0	0.271813159	0	0.666666667	CRA004775;PRJNA827465;Yang2025_Silkie_Wencha	0.85620621
ENSGALG00010000298	SLC35E4	3	0.231357717	0.385574263	0.600034129	0.548483491	0.354477064	11.36332845	82.39952309	0.666666667	CRA004775;PRJNA827465;Yang2025_Silkie_Wencha	0.85620621
ENSGALG00010008233	CYRIB	3	-0.032313898	0.053866437	-0.599889381	0.54857996	0.002890394	2.870755502	30.33192835	0.666666667	CRA004775;PRJNA827465;Yang2025_Silkie_Wencha	0.85620621
ENSGALG00010008637	RGS7	2	0.713434874	1.193196523	0.597919002	0.54989398	2.44911523	7.052422505	85.82047517	0.5	PRJNA827465;Yang2025_Silkie_Wenchang	0.85623691
ENSGALG00010029141	C5	2	-0.49227842	0.822367236	-0.598611421	0.549432038	1.087580652	4.956384934	79.82400453	0.5	PRJNA827465;Yang2025_Silkie_Wenchang	0.85623691
ENSGALG00010028891	SLC31A2	3	0.304093288	0.508907071	0.597541879	0.550145655	0.61947388	10.25614825	80.49950185	0.666666667	CRA004775;PRJNA827465;Yang2025_Silkie_Wencha	0.85623691
ENSGALG00010004319	TAAR1	2	0.290762846	0.485095467	0.599391039	0.548910818	0	0.04920751	0	1	CRA004775;PRJNA827465	0.85623691
ENSGALG00010019968	RHCG	2	0.269174197	0.450594676	0.59737545	0.550256741	0	0.052047327	0	1	PRJNA827465;Yang2025_Silkie_Wenchang	0.85623691
ENSGALG00010019473	OXTR	3	0.255203009	0.425926282	0.599171781	0.549058338	0.32890199	5.0721771	60.56920015	0.666666667	CRA004775;PRJNA827465;Yang2025_Silkie_Wencha	0.85623691
ENSGALG00010003743	ARL9	3	0.168483565	0.281281202	0.598986222	0.549182073	0.185690563	10.25881484	80.50457064	0.666666667	CRA004775;PRJNA827465;Yang2025_Silkie_Wencha	0.85623691
ENSGALG00010025486	SLC4A8	3	-0.141209796	0.235967261	-0.598429611	0.549553313	0.105272906	5.998209297	66.65671535	0.666666667	CRA004775;PRJNA827465;Yang2025_Silkie_Wencha	0.85623691
ENSGALG00010012924	GLIPR1	3	0.139857864	0.233776167	0.598255441	0.549669503	0.093455937	4.760997071	57.91199264	0.666666667	CRA004775;PRJNA827465;Yang2025_Silkie_Wencha	0.85623691
ENSGALG00010028875	DUSP23	3	0.133958821	0.223494849	0.59938214	0.548918084	0.095803927	5.820801449	65.64047035	0.666666667	CRA004775;PRJNA827465;Yang2025_Silkie_Wencha	0.85623691
ENSGALG00010011880	U2AF1	3	0.131982403	0.220689358	0.598046069	0.549809194	0.119609952	11.91549499	83.21513288	0.666666667	CRA004775;PRJNA827465;Yang2025_Silkie_Wencha	0.85623691
ENSGALG00010008159	SUPT3H	3	-0.122187618	0.203848935	-0.599402792	0.548904316	0.080603858	5.719742076	65.03338834	0.666666667	CRA004775;PRJNA827465;Yang2025_Silkie_Wencha	0.85623691
ENSGALG00010008039	CHMP1A	3	-0.111215147	0.185699637	-0.598898032	0.549240884	0.083383624	11.44811312	82.52987213	0.666666667	CRA004775;PRJNA827465;Yang2025_Silkie_Wencha	0.85623691
ENSGALG00010001411	FGFR2	3	0.104528921	0.1749641	0.597430676	0.550219878	0	0.887804125	0	0.666666667	CRA004775;PRJNA827465;Yang2025_Silkie_Wencha	0.85623691
ENSGALG00010011651	SERPINB6	3	-0.10377213	0.17336904	-0.59856206	0.549464963	0.063277359	10.30872157	80.5989522	0.666666667	CRA004775;PRJNA827465;Yang2025_Silkie_Wencha	0.85623691
ENSGALG00010021490	ACACB	3	-0.097281123	0.162458483	-0.598806057	0.549302222	0.021106455	2.665655366	24.97154638	0.666666667	CRA004775;PRJNA827465;Yang2025_Silkie_Wencha	0.85623691
ENSGALG00010008086	RHPN1	3	-0.096250101	0.16102775	-0.59772369	0.550024316	0.039216076	4.333266866	53.84544079	0.666666667	CRA004775;PRJNA827465;Yang2025_Silkie_Wencha	0.85623691
ENSGALG00010023472	PTPN6	3	-0.092972295	0.155390856	-0.59831252	0.549631424	0.04071872	5.646912508	64.58241566	0.666666667	CRA004775;PRJNA827465;Yang2025_Silkie_Wencha	0.85623691
ENSGALG00010013775	DBX2	3	0.088488239	0.147590179	0.599553711	0.548803705	0	1.689898042	0	0.666666667	CRA004775;PRJNA827465;Yang2025_Silkie_Wencha	0.85623691
ENSGALG00010023889	SAMD8	3	-0.087024355	0.145647223	-0.597500952	0.55017297	0.037062827	5.881757658	65.99655891	0.666666667	CRA004775;PRJNA827465;Yang2025_Silkie_Wencha	0.85623691
ENSGALG00010021473	SLC8A3	3	0.064386245	0.107792289	0.597317731	0.550295269	0.01595597	3.831253783	47.7977677	0.666666667	CRA004775;PRJNA827465;Yang2025_Silkie_Wencha	0.85623691
ENSGALG00010005463	SKA3	3	-0.056663071	0.094838829	-0.597466997	0.550195635	0	1.575906618	0	0.666666667	CRA004775;PRJNA827465;Yang2025_Silkie_Wencha	0.85623691
ENSGALG00010027317	MPV17L2	3	-0.053055288	0.088757977	-0.597752333	0.550005201	0	0.905352361	0	0.666666667	CRA004775;PRJNA827465;Yang2025_Silkie_Wencha	0.85623691
ENSGALG00010016922	ENSGALG00010016922	3	-0.04645877	0.077626387	-0.598491983	0.549511707	0	0.1265702	0	0.666666667	CRA004775;PRJNA827465;Yang2025_Silkie_Wencha	0.85623691
ENSGALG00010005237	RAP2A	3	-0.039502917	0.066065579	-0.597934922	0.549883357	0	0.996588159	0	1	CRA004775;PRJNA827465;Yang2025_Silkie_Wencha	0.85623691
ENSGALG00010025348	HIPK1	3	-0.03746496	0.062613056	-0.598356989	0.549601758	0	0.048260474	0	1	CRA004775;PRJNA827465;Yang2025_Silkie_Wencha	0.85623691
ENSGALG00010013275	MAPRE1	3	-0.029976628	0.050039378	-0.599060764	0.549132365	0	0.401689406	0	0.666666667	CRA004775;PRJNA827465;Yang2025_Silkie_Wencha	0.85623691
ENSGALG00010024742	PAK2	3	-0.025016984	0.041816015	-0.59826322	0.549664314	0	0.255002397	0	0.666666667	CRA004775;PRJNA827465;Yang2025_Silkie_Wencha	0.85623691
ENSGALG00010006180	CD200L	3	0.328466502	0.550411409	0.596765431	0.550664001	0.331265235	3.142080751	36.34791215	0.666666667	CRA004775;PRJNA827465;Yang2025_Silkie_Wencha	0.856245278
ENSGALG00010028662	FCRL4	3	0.221491353	0.371058032	0.596918362	0.550561887	0.376730899	26.9675941	92.5836914	0.666666667	CRA004775;PRJNA827465;Yang2025_Silkie_Wencha	0.856245278
ENSGALG00010025256	ANAPC5	3	-0.165675564	0.277524193	-0.596977012	0.550522729	0.197757028	17.76007869	88.73878864	0.666666667	CRA004775;PRJNA827465;Yang2025_Silkie_Wencha	0.856245278
ENSGALG00010011692	ARL1	3	0.091216338	0.152796681	0.596978528	0.550521716	0.059527398	16.45903194	87.84861706	0.666666667	CRA004775;PRJNA827465;Yang2025_Silkie_Wencha	0.856245278
ENSGALG00010000485	MED13L	3	-0.072260311	0.121077658	-0.596809622	0.550634493	0.019612694	3.351377346	40.32304353	0.666666667	CRA004775;PRJNA827465;Yang2025_Silkie_Wencha	0.856245278
ENSGALG00010017057	NDUFAB1	3	-0.050181198	0.084056953	-0.596990445	0.55051376	0	0.004942928	0	1	CRA004775;PRJNA827465;Yang2025_Silkie_Wencha	0.856245278
ENSGALG00010013296	AGXT2	3	0.194904834	0.327380847	0.595344644	0.55161312	0	1.432422995	0	0.666666667	CRA004775;PRJNA827465;Yang2025_Silkie_Wencha	0.856309764
ENSGALG00010019825	BCL2A1	3	-0.156524492	0.262729721	-0.595759823	0.550828015	0.100628179	3.891971701	48.61216489	0.666666667	CRA004775;PRJNA827465;Yang2025_Silkie_Wencha	0.856309764
ENSGALG00010027621	TSPO2	3	0.150971323	0.253560622	0.595405241	0.551572623	0.129677914	8.008139399	75.02540977	0.666666667	CRA004775;PRJNA827465;Yang2025_Silkie_Wencha	0.856309764
ENSGALG00010005903	PRR18	3	0.14099943	0.236655874	0.595799409	0.551309238	0.054979908	2.893179081	30.8718906	0.666666667	CRA004775;PRJNA827465;Yang2025_Silkie_Wencha	0.856309764
ENSGALG00010023239	RBPMS2	3	0.130907225	0.219912392	0.59526989	0.55166308	0.094037105	5.843514966	65.77402451	0.666666667	CRA004775;PRJNA827465;Yang2025_Silkie_Wencha	0.856309764
ENSGALG00010019240	ENSGALG00010019240	3	-0.10125979	0.170045416	-0.59548676	0.551518147	0	0.685781469	0	0.666666667	CRA004775;PRJNA827465;Yang2025_Silkie_Wencha	0.856309764
ENSGALG00010027561	ADAMTS4	3	-0.097201246	0.16325644	-0.595389966	0.551582831	0.02849601	2.808584489	28.78975128	0.666666667	CRA004775;PRJNA827465;Yang2025_Silkie_Wencha	0.856309764
ENSGALG00010023453	ARHGAP32	3	0.090643483	0.152175739	0.595650028	0.551409048	0.025597557	2.850813774	29.84459321	0.666666667	CRA004775;PRJNA827465;Yang2025_Silkie_Wencha	0.856309764
ENSGALG00010007337	PRORP	3	-0.089446384	0.149968795	-0.596433304	0.550885798	0.050258201	9.400120519	78.72367704	0.666666667	CRA004775;PRJNA827465;Yang2025_Silkie_Wencha	0.856309764
ENSGALG00010004988	HJURP	3	-0.076133049	0.127823458	-0.595610927	0.551435175	0.025002614	4.904990418	59.22520067	0.666666667	CRA004775;PRJNA827465;Yang2025_Silkie_Wencha	0.856309764
ENSGALG00010008397	ATXN1L	3	-0.074147809	0.124386466	-0.596108336	0.551102856	0.030081099	7.004531722	71.44705629	0.666666667	CRA004775;PRJNA827465;Yang2025_Silkie_Wencha	0.856309764
ENSGALG00010020721	PTDSS2	3	-0.071094967	0.119436591	-0.595252814	0.551674492	0.023388886	4.414423258	54.69396832	0.666666667	CRA004775;PRJNA827465;Yang2025_Silkie_Wencha	0.856309764
ENSGALG00010018915	DOCK5	3	0.053325209	0.089547806	0.59549431	0.551513101	0	0.405829327	0	0.666666667	CRA004775;PRJNA827465;Yang2025_Silkie_Wencha	0.856309764
ENSGALG00010020626	MORN4	3	0.039994988	0.0670801	0.596227313	0.551023382	0	0.828650457	0	0.666666667	CRA004775;PRJNA827465;Yang2025_Silkie_Wencha	0.856309764
ENSGALG00010022239	DEAF1	3	-0.036852966	0.06182807	-0.596055583	0.551138096	0	1.733723531	0	1	CRA004775;PRJNA827465;Yang2025_Silkie_Wencha	0.856309764
ENSGALG00010004965	JCAD	3	0.034209694	0.057392253	0.596068184	0.551129703	0	1.135157272	0	0.666666667	CRA004775;PRJNA827465;Yang2025_Silkie_Wencha	0.856309764
ENSGALG00010016812	LRRC31	3	0.062279942	0.104645425	0.595152082	0.551741817	0	0.074996231	0	1	CRA004775;PRJNA827465;Yang2025_Silkie_Wencha	0.856320259
ENSGALG00010014418	RAC2	3	-0.350380701	0.589925608	-0.593940501	0.552551909	0.949142855	35.06870805	94.29690995	0.666666667	CRA004775;PRJNA827465;Yang2025_Silkie_Wencha	0.8565433
ENSGALG00010000619	ENSGALG00010000619	3	0.338223972	0.568835091	0.594590554	0.552117196	0.884196331	23.9094562	91.63510879	0.666666667	CRA004775;PRJNA827465;Yang2025_Silkie_Wencha	0.8565433
ENSGALG00010000225	LIF	3	0.141606925	0.238226856	0.594420491	0.552230906	0	1.318870612	0	0.666666667	CRA004775;PRJNA827465;Yang2025_Silkie_Wencha	0.8565433
ENSGALG00010001287	PLPP4	3	0.141210483	0.237732579	0.593988775	0.552519621	0	0.253257406	0	0.666666667	CRA004775;PRJNA827465;Yang2025_Silkie_Wencha	0.8565433
ENSGALG00010023076	DNAJC7	3	0.121334906	0.204261258	0.594018208	0.552499935	0.102749604	15.06452108	86.67068419	0.666666667	CRA004775;PRJNA827465;Yang2025_Silkie_Wencha	0.8565433
ENSGALG00010029620	ENSGALG00010029620	3	0.120610007	0.202811699	0.594689595	0.552050978	0	1.095894328	0	0.666666667	CRA004775;PRJNA827465;Yang2025_Silkie_Wencha	0.8565433
ENSGALG00010021135	CLK3	3	0.118477394	0.199180447	0.594824423	0.55196084	0.09722391	16.30444803	87.73340872	0.666666667	CRA004775;PRJNA827465;Yang2025_Silkie_Wencha	0.8565433
ENSGALG00010012088	ATP5F1AZ	3	-0.087708954	0.147647052	-0.594044738	0.552482191	0	0.03565112	0	1	CRA004775;PRJNA827465;Yang2025_Silkie_Wencha	0.8565433
ENSGALG00010017558	ENSGALG00010017558	3	0.057982246	0.097583085	0.594240475	0.552351285	0.018804702	6.66383721	70.09525083	0.666666667	CRA004775;PRJNA827465;Yang2025_Silkie_Wencha	0.8565433
ENSGALG00010025527	KNTC1	3	-0.056800574	0.095611851	-0.594074619	0.552462206	0	0.02353676	0	0.666666667	CRA004775;PRJNA827465;Yang2025_Silkie_Wencha	0.8565433
ENSGALG00010003428	SNX16	3	0.044390338	0.074670006	0.594486862	0.552186527	0.002191028	2.242492602	10.81352963	0.666666667	CRA004775;PRJNA827465;Yang2025_Silkie_Wencha	0.8565433
ENSGALG00010025925	KLHL18	3	-0.027897394	0.046981565	-0.593794475	0.552649585	0	1.827346256	0	0.666666667	CRA004775;PRJNA827465;Yang2025_Silkie_Wencha	0.856600798
ENSGALG00010024707	ENSGALG00010024707	3	0.324867235	0.547961812	0.592864735	0.55327168	0.826938035	26.39894697	92.42394023	0.666666667	CRA004775;PRJNA827465;Yang2025_Silkie_Wencha	0.857002226
ENSGALG00010018899	ACSS1	3	-0.140764549	0.237374452	-0.593006317	0.553176925	0.128955961	14.61917942	86.31934158	0.666666667	CRA004775;PRJNA827465;Yang2025_Silkie_Wencha	0.857002226
ENSGALG00010001221	GRAMD2B	3	-0.107486353	0.181211033	-0.593155677	0.553076972	0.057462697	4.895138351	59.14313638	0.666666667	CRA004775;PRJNA827465;Yang2025_Silkie_Wencha	0.857002226
ENSGALG00010024354	C2CD2L	3	-0.046760896	0.078872903	-0.592863887	0.553272248	0.004955713	2.606791382	23.27732806	0.666666667	CRA004775;PRJNA827465;Yang2025_Silkie_Wencha	0.857002226
ENSGALG00010009306	TBC1D9	3	-0.042346245	0.071426369	-0.592865703	0.55327103	0	1.853769074	0	0.666666667	CRA004775;PRJNA827465;Yang2025_Silkie_Wencha	0.857002226
ENSGALG00010029776	WBP2	3	-0.038255183	0.064518905	-0.592929816	0.553228123	0	1.398233893	0	0.666666667	CRA004775;PRJNA827465;Yang2025_Silkie_Wencha	0.857002226
ENSGALG00010022942	NEU2	3	-0.249950908	0.421820568	-0.592552679	0.553480556	0.381589827	7.589091294	73.6463837	0.666666667	CRA004775;PRJNA827465;Yang2025_Silkie_Wencha	0.857146863
ENSGALG00010023290	SLC16A14	2	-0.118082873	0.199281443	-0.592543247	0.55348687	0.032052899	1.615081353	38.08361428	0.5	PRJNA827465;Yang2025_Silkie_Wenchang	0.857146863
ENSGALG00010029631	LIG3	3	0.026169238	0.044171377	0.59244787	0.553550719	0	1.583145777	0	0.666666667	CRA004775;PRJNA827465;Yang2025_Silkie_Wencha	0.857151858
ENSGALG00010019641	CAPN11	3	0.082993596	0.140234326	0.59182084	0.553970567	0.043709935	12.66062667	84.20299364	0.666666667	CRA004775;PRJNA827465;Yang2025_Silkie_Wencha	0.857708042
ENSGALG00010011351	KANK1	3	0.095279155	0.161048942	0.591616146	0.554107659	0.057017603	8.953154866	77.66150558	0.666666667	CRA004775;PRJNA827465;Yang2025_Silkie_Wencha	0.857826366
ENSGALG00010000076	ENSGALG00010000076	3	-0.103579007	0.175155943	-0.591353086	0.554283867	0.053372686	5.134139844	61.04508134	0.666666667	CRA004775;PRJNA827465;Yang2025_Silkie_Wencha	0.857911286
ENSGALG00010020906	SIRT3	3	0.055919464	0.094558366	0.59137511	0.554269114	0.010795347	3.411775943	41.37950342	0.666666667	CRA004775;PRJNA827465;Yang2025_Silkie_Wencha	0.857911286
ENSGALG00010013351	EDAR	3	0.199732408	0.337853016	0.591181367	0.554398906	0.098050649	2.77500165	27.92797077	0.666666667	CRA004775;PRJNA827465;Yang2025_Silkie_Wencha	0.857975871
ENSGALG00010028346	DENND1A	3	0.073539557	0.124409334	0.591109646	0.554446957	0.025227833	4.342745965	53.94618943	0.666666667	CRA004775;PRJNA827465;Yang2025_Silkie_Wencha	0.857975871
ENSGALG00010005404	BEND7	3	0.087302354	0.147731627	0.590952359	0.554552342	0.041973391	5.875775938	65.96194237	0.666666667	CRA004775;PRJNA827465;Yang2025_Silkie_Wencha	0.858045039
ENSGALG00010010124	ENSGALG00010010124	3	0.29646638	0.503441382	0.588879641	0.555942016	0.662936564	17.2756722	88.42302646	0.666666667	CRA004775;PRJNA827465;Yang2025_Silkie_Wencha	0.858444105
ENSGALG00010002185	ENSGALG00010002185	3	0.263470968	0.446635296	0.589901806	0.555256483	0.387405155	5.821544667	65.64485692	0.666666667	CRA004775;PRJNA827465;Yang2025_Silkie_Wencha	0.858444105
ENSGALG00010010869	ENSGALG00010010869	3	0.259576455	0.441445452	0.58801479	0.556522365	0.500531824	17.31288865	88.44791276	0.666666667	CRA004775;PRJNA827465;Yang2025_Silkie_Wencha	0.858444105
ENSGALG00010006118	NPNT	3	0.2167944	0.368715158	0.587972573	0.556550703	0.334327589	12.50218497	84.00279627	0.666666667	CRA004775;PRJNA827465;Yang2025_Silkie_Wencha	0.858444105
ENSGALG00010027255	ENSGALG00010027255	3	0.18203106	0.308678983	0.589709925	0.55538514	0.224369278	9.79518026	79.58179485	0.666666667	CRA004775;PRJNA827465;Yang2025_Silkie_Wencha	0.858444105
ENSGALG00010021018	F3	3	-0.169056964	0.286908661	-0.589236183	0.555702848	0.183603418	10.97359697	81.77443545	0.666666667	CRA004775;PRJNA827465;Yang2025_Silkie_Wencha	0.858444105
ENSGALG00010017801	SMNDC1	3	-0.143560693	0.243848706	-0.588728541	0.556043389	0.16486663	43.0405147	95.35321542	0.666666667	CRA004775;PRJNA827465;Yang2025_Silkie_Wencha	0.858444105
ENSGALG00010006066	ARHGAP18	3	-0.142712575	0.24281268	-0.58774762	0.556701707	0.146652154	12.90173051	84.49820357	0.666666667	CRA004775;PRJNA827465;Yang2025_Silkie_Wencha	0.858444105
ENSGALG00010007751	HSPA13	3	0.11825594	0.20118245	0.587804453	0.556663555	0.103736949	14.96211441	86.63290531	0.666666667	CRA004775;PRJNA827465;Yang2025_Silkie_Wencha	0.858444105
ENSGALG00010013063	PARP12	3	-0.105340657	0.178817048	-0.589097392	0.555795943	0.062127643	7.57488268	73.59695081	0.666666667	CRA004775;PRJNA827465;Yang2025_Silkie_Wencha	0.858444105
ENSGALG00010019040	HMGN5	3	-0.104360193	0.177164614	-0.589057775	0.555822518	0.078572117	14.13774087	85.85346826	0.666666667	CRA004775;PRJNA827465;Yang2025_Silkie_Wencha	0.858444105
ENSGALG00010010897	CEP170	3	0.095330149	0.161975528	0.588546615	0.556165455	0.055810746	7.325743127	72.69901544	0.666666667	CRA004775;PRJNA827465;Yang2025_Silkie_Wencha	0.858444105
ENSGALG00010027530	PHF19	3	-0.091455534	0.155278479	-0.588977526	0.55587635	0.037871202	4.339698677	53.91385096	0.666666667	CRA004775;PRJNA827465;Yang2025_Silkie_Wencha	0.858444105
ENSGALG00010021263	STOML1	3	0.090673601	0.153705457	0.589917904	0.55524569	0.040309177	4.704480786	57.48733833	0.666666667	CRA004775;PRJNA827465;Yang2025_Silkie_Wencha	0.858444105
ENSGALG00010012760	PLEKHF2	3	-0.090374454	0.153293622	-0.589551301	0.555491509	0.054599268	13.8642616	85.57442107	0.666666667	CRA004775;PRJNA827465;Yang2025_Silkie_Wencha	0.858444105
ENSGALG00010019252	TATDN3	3	-0.088782919	0.150600293	-0.589526871	0.555507892	0.040573691	5.083944333	60.66046618	0.666666667	CRA004775;PRJNA827465;Yang2025_Silkie_Wencha	0.858444105
ENSGALG00010012759	ATP1B4	3	0.088319146	0.14981795	0.589509773	0.555519358	0.030203278	3.84692645	48.01044351	0.666666667	CRA004775;PRJNA827465;Yang2025_Silkie_Wencha	0.858444105
ENSGALG00010025270	NDC1	3	-0.086274112	0.146796143	-0.587713753	0.556724443	0.034642002	4.64466525	56.9398462	0.666666667	CRA004775;PRJNA827465;Yang2025_Silkie_Wencha	0.858444105
ENSGALG00010003838	GAR1	3	0.082619321	0.140004133	0.590120582	0.555109811	0.036144569	6.032911115	66.84850876	0.666666667	CRA004775;PRJNA827465;Yang2025_Silkie_Wencha	0.858444105
ENSGALG00010025357	OLFML3	3	-0.080868939	0.1374907	-0.588177519	0.556413145	0.027998568	3.715285403	46.16833478	0.666666667	CRA004775;PRJNA827465;Yang2025_Silkie_Wencha	0.858444105
ENSGALG00010021162	WIPI2	3	-0.079178084	0.134667872	-0.58795081	0.556565311	0.032444915	5.650454041	64.60461433	0.666666667	CRA004775;PRJNA827465;Yang2025_Silkie_Wencha	0.858444105
ENSGALG00010018670	MRAS	3	-0.078868583	0.134119677	-0.588046325	0.556501199	0.022586807	3.441026239	41.87780443	0.666666667	CRA004775;PRJNA827465;Yang2025_Silkie_Wencha	0.858444105
ENSGALG00010024403	HMBS	3	-0.078847696	0.13378068	-0.589380286	0.555606198	0.029710582	4.49759231	55.53176317	0.666666667	CRA004775;PRJNA827465;Yang2025_Silkie_Wencha	0.858444105
ENSGALG00010029224	FAM222B	3	0.071241678	0.121038206	0.588588352	0.556137449	0.024315002	4.347946226	54.00127103	0.666666667	CRA004775;PRJNA827465;Yang2025_Silkie_Wencha	0.858444105
ENSGALG00010008158	CLPTM1L	3	-0.065358434	0.110864615	-0.58953376	0.555503272	0.026864442	11.55680538	82.69417945	0.666666667	CRA004775;PRJNA827465;Yang2025_Silkie_Wencha	0.858444105
ENSGALG00010010955	GTF3C3	3	-0.061150834	0.103901463	-0.588546419	0.556165586	0.020360309	5.969940386	66.49882795	0.666666667	CRA004775;PRJNA827465;Yang2025_Silkie_Wencha	0.858444105
ENSGALG00010016741	NELFA	3	-0.052820375	0.08954258	-0.589891142	0.555263633	0.012696303	4.624875884	56.75559625	0.666666667	CRA004775;PRJNA827465;Yang2025_Silkie_Wencha	0.858444105
ENSGALG00010021580	KCTD10	3	-0.039486505	0.067209087	-0.587517356	0.556856298	0.005951475	3.662968858	45.3994812	0.666666667	CRA004775;PRJNA827465;Yang2025_Silkie_Wencha	0.858444105
ENSGALG00010023238	NUCB2	3	0.035788066	0.060909519	0.587561134	0.556826905	0	0.332892548	0	0.666666667	CRA004775;PRJNA827465;Yang2025_Silkie_Wencha	0.858444105
ENSGALG00010011973	TRAPPC10	3	-0.035767738	0.060882275	-0.587490164	0.556874555	0.003711705	2.909876096	31.26855118	0.666666667	CRA004775;PRJNA827465;Yang2025_Silkie_Wencha	0.858444105
ENSGALG00010017773	DVL3	3	-0.030875641	0.052489304	-0.588227289	0.556379743	0	1.123723676	0	0.666666667	CRA004775;PRJNA827465;Yang2025_Silkie_Wencha	0.858444105
ENSGALG00010012043	NUP50	3	0.030641479	0.052148785	0.587578005	0.556815578	0	0.931918458	0	0.666666667	CRA004775;PRJNA827465;Yang2025_Silkie_Wencha	0.858444105
ENSGALG00010023809	CDON	3	-0.029648299	0.050409229	-0.588152198	0.556430139	0	0.807058076	0	1	CRA004775;PRJNA827465;Yang2025_Silkie_Wencha	0.858444105
ENSGALG00010024586	DLGAP4	3	0.026276909	0.044516125	0.590278447	0.555003987	0	1.522048487	0	1	CRA004775;PRJNA827465;Yang2025_Silkie_Wencha	0.858444105
ENSGALG00010028479	CASQ1	3	-0.100464039	0.171043957	-0.587358015	0.556963285	0	0.628632398	0	1	CRA004775;PRJNA827465;Yang2025_Silkie_Wencha	0.858487288
ENSGALG00010028537	ENSGALG00010028537	2	-0.715284175	1.21839662	-0.587070059	0.557156655	0.588305702	1.230530271	18.73422187	0.5	PRJNA827465;Yang2025_Silkie_Wenchang	0.858504571
ENSGALG00010003788	NUDT9	3	0.165421572	0.281748462	0.587125022	0.557119744	0.202799422	15.32350507	86.94815585	0.666666667	CRA004775;PRJNA827465;Yang2025_Silkie_Wencha	0.858504571
ENSGALG00010017652	C1orf115	3	-0.118781216	0.202271695	-0.587235976	0.557045234	0.075726506	5.564244561	64.05621683	0.666666667	CRA004775;PRJNA827465;Yang2025_Silkie_Wencha	0.858504571
ENSGALG00010003090	DMA	3	-0.138536221	0.236075282	-0.586830694	0.55731742	0.109277324	5.818445921	65.6265603	0.666666667	CRA004775;PRJNA827465;Yang2025_Silkie_Wencha	0.858658712
ENSGALG00010026706	KRT24	3	0.419670533	0.715264804	0.586734494	0.557382037	1.271551925	12.35979078	83.81849632	0.666666667	CRA004775;PRJNA827465;Yang2025_Silkie_Wencha	0.858664701
ENSGALG00010017660	CLIP4	3	0.113384163	0.19330256	0.586563175	0.557497121	0.070102683	6.706273545	70.17717833	0.666666667	CRA004775;PRJNA827465;Yang2025_Silkie_Wencha	0.858748424
ENSGALG00010000580	ENSGALG00010000580	2	0.159256573	0.271660316	0.586234217	0.557718131	0	0.263056458	0	0.5	PRJNA827465;Yang2025_Silkie_Wenchang	0.858837953
ENSGALG00010028917	NR6A1	3	0.080450367	0.137231259	0.586239372	0.557714667	0.031471893	4.464566417	55.20281673	0.666666667	CRA004775;PRJNA827465;Yang2025_Silkie_Wencha	0.858837953
ENSGALG00010010824	PKP4	3	0.046615961	0.079521543	0.586205435	0.55773747	0.00303758	2.301308737	13.09292979	0.666666667	CRA004775;PRJNA827465;Yang2025_Silkie_Wencha	0.858837953
ENSGALG00010011104	ABCG1	3	-0.096704559	0.165006513	-0.586065107	0.557831764	0.009116471	2.204093926	9.259765364	0.666666667	CRA004775;PRJNA827465;Yang2025_Silkie_Wencha	0.858889612
ENSGALG00010012100	UBE2F	3	-0.037500407	0.064004272	-0.585904747	0.557939527	0	1.322541644	0	0.666666667	CRA004775;PRJNA827465;Yang2025_Silkie_Wencha	0.858961996
ENSGALG00010022391	ENSGALG00010022391	3	0.045970028	0.078490806	0.585674047	0.558094578	0	1.316760617	0	1	CRA004775;PRJNA827465;Yang2025_Silkie_Wencha	0.859054676
ENSGALG00010019263	SNRPB2	3	-0.024681974	0.042145706	-0.585634375	0.558121243	0	1.79798654	0	0.666666667	CRA004775;PRJNA827465;Yang2025_Silkie_Wencha	0.859054676
ENSGALG00010013488	UPRT	3	0.042649052	0.07284724	0.585458721	0.558239315	0.004867491	2.705492742	26.0763125	0.666666667	CRA004775;PRJNA827465;Yang2025_Silkie_Wencha	0.859142883
ENSGALG00010020707	SLC4A5	2	0.16255216	0.277693469	0.58536544	0.558302022	0.049773247	1.435994577	30.361854	0.5	PRJNA827465;Yang2025_Silkie_Wenchang	0.859145873
ENSGALG00010023623	OSBPL6	3	0.086474099	0.147751971	0.585265282	0.558369355	0.015462019	2.552157722	21.63493727	1	CRA004775;PRJNA827465;Yang2025_Silkie_Wencha	0.859155982
ENSGALG00010016377	FRMPD3	3	-0.210219433	0.359291793	-0.585093891	0.558484586	0.288864732	11.83318546	83.09838035	0.666666667	CRA004775;PRJNA827465;Yang2025_Silkie_Wencha	0.859239778
ENSGALG00010027888	PFKFB2	3	-0.087429361	0.149513466	-0.584759104	0.558709706	0.036609446	4.624381409	56.75097222	0.666666667	CRA004775;PRJNA827465;Yang2025_Silkie_Wencha	0.859492606
ENSGALG00010013753	PLEKHA5	3	0.208872059	0.357465002	0.58431471	0.559008597	0.32799859	17.43774375	88.53062628	0.666666667	CRA004775;PRJNA827465;Yang2025_Silkie_Wencha	0.859847529
ENSGALG00010019411	LSM5	3	-0.0599207	0.102578497	-0.584144846	0.559122865	0	0.691346376	0	1	CRA004775;PRJNA827465;Yang2025_Silkie_Wencha	0.859847529
ENSGALG00010027273	HAUS8	3	0.038955097	0.066680456	0.584205627	0.559081976	0	0.772598121	0	0.666666667	CRA004775;PRJNA827465;Yang2025_Silkie_Wencha	0.859847529
ENSGALG00010028756	ENSGALG00010028756	2	0.787256283	1.34953367	0.58335431	0.559654809	2.887143407	4.773563595	79.05128988	0.5	PRJNA827465;Yang2025_Silkie_Wenchang	0.859917338
ENSGALG00010022872	KRT13	3	0.163865699	0.280776937	0.583615238	0.559479206	0	1.824603979	0	0.666666667	CRA004775;PRJNA827465;Yang2025_Silkie_Wencha	0.859917338
ENSGALG00010015574	C14orf132	3	-0.135666199	0.232484878	-0.583548489	0.559524125	0.062673114	3.202023593	37.53949832	0.666666667	CRA004775;PRJNA827465;Yang2025_Silkie_Wencha	0.859917338
ENSGALG00010019172	NEMF	3	0.126647146	0.217006391	0.583610212	0.559482588	0.126107984	23.21469045	91.38476559	0.666666667	CRA004775;PRJNA827465;Yang2025_Silkie_Wencha	0.859917338
ENSGALG00010024587	ENSGALG00010024587	3	-0.103939008	0.178039502	-0.583797458	0.559356588	0.062242862	5.94814019	66.37604468	0.666666667	CRA004775;PRJNA827465;Yang2025_Silkie_Wencha	0.859917338
ENSGALG00010024569	ASCL2	3	-0.094233258	0.161497422	-0.58349698	0.559558789	0	0.990566318	0	0.666666667	CRA004775;PRJNA827465;Yang2025_Silkie_Wencha	0.859917338
ENSGALG00010015039	FAM114A1	3	-0.089311567	0.152963958	-0.583873273	0.559305576	0.056080979	14.14216498	85.85789373	0.666666667	CRA004775;PRJNA827465;Yang2025_Silkie_Wencha	0.859917338
ENSGALG00010012017	N4BP3	3	-0.031677301	0.054298331	-0.583393654	0.559628329	0	0.372683476	0	0.666666667	CRA004775;PRJNA827465;Yang2025_Silkie_Wencha	0.859917338
ENSGALG00010024578	ENSGALG00010024578	2	-0.39845873	0.683367651	-0.583081053	0.559838738	0.424602965	1.799688441	44.43482675	0.5	CRA004775;PRJNA827465	0.859930308
ENSGALG00010002397	ENSGALG00010002397	3	-0.260148371	0.446876046	-0.582148838	0.560466431	0.413332286	6.592584783	69.66288541	0.666666667	CRA004775;PRJNA827465;Yang2025_Silkie_Wencha	0.859930308
ENSGALG00010021764	FAM222A	2	0.219537871	0.37653341	0.583050177	0.559859523	0	0.131208252	0	1	CRA004775;PRJNA827465	0.859930308
ENSGALG00010028866	HORMAD1	2	0.193416411	0.331923765	0.582713356	0.560086281	0.076422452	1.501067788	33.38075681	0.5	PRJNA827465;Yang2025_Silkie_Wenchang	0.859930308
ENSGALG00010011674	ENSGALG00010011674	3	0.188059431	0.323133478	0.581986838	0.560575546	0	1.475246803	0	0.666666667	CRA004775;PRJNA827465;Yang2025_Silkie_Wencha	0.859930308
ENSGALG00010001145	TRIM36	3	-0.155744038	0.267501025	-0.58221847	0.560419534	0.107836168	4.227322206	52.68872581	0.666666667	CRA004775;PRJNA827465;Yang2025_Silkie_Wencha	0.859930308
ENSGALG00010022138	SPAG17	3	0.132824248	0.227994435	0.582576713	0.560178286	0.074801441	3.977553292	49.71783271	0.666666667	CRA004775;PRJNA827465;Yang2025_Silkie_Wencha	0.859930308
ENSGALG00010026095	MIF	3	0.130311858	0.223796059	0.582279502	0.56037843	0.106245731	7.042311111	71.60023225	0.666666667	CRA004775;PRJNA827465;Yang2025_Silkie_Wencha	0.859930308
ENSGALG00010015923	RCAN3	3	-0.10315966	0.176963026	-0.582944709	0.559930522	0.076636583	13.96836694	85.68193398	0.666666667	CRA004775;PRJNA827465;Yang2025_Silkie_Wencha	0.859930308
ENSGALG00010012893	ENSGALG00010012893	3	0.100880723	0.173161541	0.582581575	0.560175012	0.022940096	2.639339096	24.22345415	0.666666667	CRA004775;PRJNA827465;Yang2025_Silkie_Wencha	0.859930308
ENSGALG00010001892	RAB12	3	-0.091698491	0.157483844	-0.58227237	0.560383234	0.056479121	18.06049597	88.926107	0.666666667	CRA004775;PRJNA827465;Yang2025_Silkie_Wencha	0.859930308
ENSGALG00010006496	UBE2H	3	-0.083701321	0.143778609	-0.582154198	0.560462821	0.046097649	12.73377381	84.29373704	0.666666667	CRA004775;PRJNA827465;Yang2025_Silkie_Wencha	0.859930308
ENSGALG00010005324	CENPJ	2	0.057871507	0.099222625	0.583249099	0.559725623	0	0.175609102	0	1	PRJNA827465;Yang2025_Silkie_Wenchang	0.859930308
ENSGALG00010019954	CYB561A3	3	0.054171258	0.093073054	0.582029441	0.560546851	0	1.876019731	0	0.666666667	CRA004775;PRJNA827465;Yang2025_Silkie_Wencha	0.859930308
ENSGALG00010006463	SCO2	3	0.041179693	0.070753336	0.582017691	0.560554765	0	1.408019996	0	0.666666667	CRA004775;PRJNA827465;Yang2025_Silkie_Wencha	0.859930308
ENSGALG00010025305	GPR182	3	0.257189323	0.442478923	0.581246495	0.561074335	0.473269489	10.39903389	80.76744416	0.666666667	CRA004775;PRJNA827465;Yang2025_Silkie_Wencha	0.860025528
ENSGALG00010018839	RPL3L	3	0.141974483	0.244216924	0.581345799	0.561007419	0	1.822714776	0	0.666666667	CRA004775;PRJNA827465;Yang2025_Silkie_Wencha	0.860025528
ENSGALG00010010111	CNOT4	3	0.141807327	0.243887909	0.581444677	0.560940794	0.124516653	6.846611259	70.78846857	0.666666667	CRA004775;PRJNA827465;Yang2025_Silkie_Wencha	0.860025528
ENSGALG00010010945	PI4K2B	3	-0.106079469	0.182537854	-0.581136823	0.561148243	0.069220221	8.793524898	77.25599207	0.666666667	CRA004775;PRJNA827465;Yang2025_Silkie_Wencha	0.860025528
ENSGALG00010027612	BAK1	3	-0.099579356	0.17128665	-0.581360868	0.560997265	0.065924786	9.508309591	78.96576693	0.666666667	CRA004775;PRJNA827465;Yang2025_Silkie_Wencha	0.860025528
ENSGALG00010021492	PARP6	3	0.075550656	0.129971926	0.581284428	0.561048774	0.030345269	6.057244772	66.98168763	0.666666667	CRA004775;PRJNA827465;Yang2025_Silkie_Wencha	0.860025528
ENSGALG00010015152	BOLL	3	0.074916136	0.128805285	0.581623148	0.560820547	0	1.285277162	0	0.666666667	CRA004775;PRJNA827465;Yang2025_Silkie_Wencha	0.860025528
ENSGALG00010002372	SMC6	3	0.053296904	0.091748577	0.580901695	0.56130671	0.007734698	2.836168564	29.4823296	0.666666667	CRA004775;PRJNA827465;Yang2025_Silkie_Wencha	0.860025528
ENSGALG00010019123	PLEKHO2	3	-0.040318017	0.069400496	-0.580947105	0.561276104	0	1.254274013	0	0.666666667	CRA004775;PRJNA827465;Yang2025_Silkie_Wencha	0.860025528
ENSGALG00010028168	PTGES2	3	-0.038023084	0.065442485	-0.58101529	0.561230149	0	0.860566315	0	0.666666667	CRA004775;PRJNA827465;Yang2025_Silkie_Wencha	0.860025528
ENSGALG00010024371	YPEL1	3	-0.036790804	0.063240495	-0.581760205	0.560728213	0	1.517778313	0	0.666666667	CRA004775;PRJNA827465;Yang2025_Silkie_Wencha	0.860025528
ENSGALG00010005445	SFT2D2	3	-0.097930298	0.168624433	-0.580759838	0.561402327	0.060553191	8.358079946	76.07105923	0.666666667	CRA004775;PRJNA827465;Yang2025_Silkie_Wencha	0.860031044

ENSGALG00010021161	ENSGALG00010021161	3	0.077362209	0.133218693	0.58071587	0.561431964	0.032114244	6.084432775	67.12922841	0.666666667	CRA004775;PRJNA827465;Yang2025_Silkie_Wencha	0.860031044
ENSGALG00010016502	YIPF5	3	-0.088284527	0.152114171	-0.580383315	0.561656154	0.042500919	5.733204222	65.11549349	0.666666667	CRA004775;PRJNA827465;Yang2025_Silkie_Wencha	0.860281266
ENSGALG00010024749	APOA5	3	-0.224789671	0.387418722	-0.58022408	0.561763517	0.28075937	5.330384692	62.47925589	0.666666667	CRA004775;PRJNA827465;Yang2025_Silkie_Wencha	0.86035251
ENSGALG00010011893	LIX1	2	-0.359910832	0.620909882	-0.579650675	0.562150213	0.374506285	1.90320495	47.45705133	0.5	CRA004775;PRJNA827465	0.860358932
ENSGALG00010020018	ENSGALG00010020018	3	-0.270951441	0.46751118	-0.579561415	0.56221042	0.465131798	10.33828144	80.6544249	0.666666667	CRA004775;PRJNA827465;Yang2025_Silkie_Wencha	0.860358932
ENSGALG00010003771	SPARCL1	3	0.25241529	0.43573194	0.579290308	0.562393304	0.390572645	6.76308799	70.4277099	0.666666667	CRA004775;PRJNA827465;Yang2025_Silkie_Wencha	0.860358932
ENSGALG00010016996	ZIC4	3	0.232998174	0.402621929	0.578702146	0.562790168	0	0.342627972	0	0.666666667	CRA004775;PRJNA827465;Yang2025_Silkie_Wencha	0.860358932
ENSGALG00010024113	ENSGALG00010024113	3	0.226459357	0.39133484	0.578684374	0.562802162	0.394719774	16.68861212	88.01577995	0.666666667	CRA004775;PRJNA827465;Yang2025_Silkie_Wencha	0.860358932
ENSGALG00010008029	LY6E	3	-0.178020781	0.306963492	-0.579941218	0.561954258	0.250075661	23.35004296	91.43470527	0.666666667	CRA004775;PRJNA827465;Yang2025_Silkie_Wencha	0.860358932
ENSGALG00010008596	ENSGALG00010008596	3	0.155287026	0.268309843	0.578760079	0.562751071	0.158678254	11.37139275	82.41200489	0.666666667	CRA004775;PRJNA827465;Yang2025_Silkie_Wencha	0.860358932
ENSGALG00010027201	ASPN	3	0.1545724	0.266615955	0.579756754	0.562078665	0.097100136	3.670067174	45.50508464	0.666666667	CRA004775;PRJNA827465;Yang2025_Silkie_Wencha	0.860358932
ENSGALG00010004072	SGK3	3	-0.149374606	0.258043605	-0.578873507	0.562674528	0.154474813	12.05623852	83.41107803	0.666666667	CRA004775;PRJNA827465;Yang2025_Silkie_Wencha	0.860358932
ENSGALG00010009227	ENSGALG00010009227	3	-0.088408546	0.152603138	-0.579336358	0.562362238	0.030326242	3.054786681	34.52897996	0.666666667	CRA004775;PRJNA827465;Yang2025_Silkie_Wencha	0.860358932
ENSGALG00010013446	ATP5I	3	-0.083953965	0.144990934	-0.579029065	0.562569562	0	1.300604392	0	0.666666667	CRA004775;PRJNA827465;Yang2025_Silkie_Wencha	0.860358932
ENSGALG00010029665	ASL1	3	-0.083581923	0.144175997	-0.579721483	0.562102453	0.008150674	2.290225282	12.67234643	0.666666667	CRA004775;PRJNA827465;Yang2025_Silkie_Wencha	0.860358932
ENSGALG00010029549	KSR1	3	0.082719463	0.142655668	0.57985402	0.56201306	0.034251717	4.750378601	57.89809259	0.666666667	CRA004775;PRJNA827465;Yang2025_Silkie_Wencha	0.860358932
ENSGALG00010013562	RERGL	3	0.07409869	0.127963454	0.579061345	0.562547782	0.02684725	5.068790388	60.54285447	0.666666667	CRA004775;PRJNA827465;Yang2025_Silkie_Wencha	0.860358932
ENSGALG00010012574	PHAX	3	0.057279229	0.098976957	0.578712771	0.562782997	0	1.247238264	0	0.666666667	CRA004775;PRJNA827465;Yang2025_Silkie_Wencha	0.860358932
ENSGALG00010015161	GFPT2	3	-0.045826609	0.079073963	-0.579541073	0.562224141	0	0.069102393	0	1	CRA004775;PRJNA827465;Yang2025_Silkie_Wencha	0.860358932
ENSGALG00010028581	NR2F6	3	-0.023309565	0.040243856	-0.579208332	0.562448609	0	0.202826587	0	1	CRA004775;PRJNA827465;Yang2025_Silkie_Wencha	0.860358932
ENSGALG00010001767	ENSGALG00010001767	3	0.18940044	0.327415569	0.578471086	0.562946113	0.252580275	10.3959341	80.76170952	0.666666667	CRA004775;PRJNA827465;Yang2025_Silkie_Wencha	0.860461199
ENSGALG00010008308	WWP2	3	0.130316024	0.225302413	0.578404918	0.562990774	0.111813835	7.601198859	73.68836104	0.666666667	CRA004775;PRJNA827465;Yang2025_Silkie_Wencha	0.860461199
ENSGALG00010027241	LRRN2	3	0.034949426	0.060450281	0.578234303	0.563105942	0	0.482097475	0	0.666666667	CRA004775;PRJNA827465;Yang2025_Silkie_Wencha	0.860544198
ENSGALG00010018885	EFEMP1	3	-0.121172146	0.209627312	-0.578036064	0.563239772	0.0972242	8.667465437	76.92520363	0.666666667	CRA004775;PRJNA827465;Yang2025_Silkie_Wencha	0.86056269
ENSGALG00010024542	ZNFX1	3	0.060940487	0.105410721	0.578124189	0.563180277	0	1.658570843	0	0.666666667	CRA004775;PRJNA827465;Yang2025_Silkie_Wencha	0.86056269
ENSGALG00010018476	ENSGALG00010018476	3	0.217597948	0.376522191	0.577915335	0.563321282	0.389113256	25.29723171	92.0939966	0.666666667	CRA004775;PRJNA827465;Yang2025_Silkie_Wencha	0.86059423
ENSGALG00010007772	CLEC3A	3	-0.677942333	1.17972308	-0.577477278	0.563617083	3.188043351	9.188782178	78.22430006	0.666666667	CRA004775;PRJNA827465;Yang2025_Silkie_Wencha	0.860953105
ENSGALG00010002949	ENSGALG00010002949	3	0.707108153	1.225897548	0.576808522	0.564068812	3.818859492	15.64336406	87.21502618	0.666666667	CRA004775;PRJNA827465;Yang2025_Silkie_Wencha	0.86112104
ENSGALG00010016431	B3GNT5	3	-0.173766177	0.30120446	-0.576906388	0.564002695	0.216904804	16.50934032	87.88564557	0.666666667	CRA004775;PRJNA827465;Yang2025_Silkie_Wencha	0.86112104
ENSGALG00010014909	FBXL14	3	0.122781863	0.21284033	0.576873108	0.564025178	0.116159943	15.67961518	87.22448491	0.666666667	CRA004775;PRJNA827465;Yang2025_Silkie_Wencha	0.86112104
ENSGALG00010015083	CEP135	3	0.104949967	0.181844258	0.577142047	0.563843503	0.054940888	5.781490288	65.40684321	0.666666667	CRA004775;PRJNA827465;Yang2025_Silkie_Wencha	0.86112104
ENSGALG00010003841	UBE2W	3	-0.058241376	0.100923186	-0.577086184	0.563881237	0	1.371543073	0	0.666666667	CRA004775;PRJNA827465;Yang2025_Silkie_Wencha	0.86112104
ENSGALG00010008906	DBN1	3	0.044138365	0.076526334	0.576773542	0.564092445	0	0.980406652	0	0.666666667	CRA004775;PRJNA827465;Yang2025_Silkie_Wencha	0.86112104
ENSGALG00010013867	RECQL	3	0.040675497	0.070534398	0.576676028	0.564158329	0.001150668	2.101329508	4.822161746	0.666666667	CRA004775;PRJNA827465;Yang2025_Silkie_Wencha	0.861128642
ENSGALG00010017806	ENSGALG00010017806	3	0.325249555	0.564327542	0.576348895	0.56437938	0.736013506	11.03079746	81.86894459	0.666666667	CRA004775;PRJNA827465;Yang2025_Silkie_Wencha	0.861279534
ENSGALG00010000308	ENSGALG00010000308	3	0.324846245	0.564038116	0.575929598	0.564662769	0.878414761	26.13439982	92.34725108	0.666666667	CRA004775;PRJNA827465;Yang2025_Silkie_Wencha	0.861279534
ENSGALG00010010813	EHD2	3	0.131056529	0.227532861	0.575989456	0.564622309	0.08889441	4.471128253	55.26856116	0.666666667	CRA004775;PRJNA827465;Yang2025_Silkie_Wencha	0.861279534
ENSGALG00010021155	RADIL	3	0.121644975	0.211101252	0.576239951	0.564453005	0.078656229	4.876034526	58.9830632	0.666666667	CRA004775;PRJNA827465;Yang2025_Silkie_Wencha	0.861279534
ENSGALG00010024482	USP3	3	-0.099075334	0.171879103	-0.576424661	0.564328179	0.069298004	26.82767682	92.54501233	0.666666667	CRA004775;PRJNA827465;Yang2025_Silkie_Wencha	0.861279534
ENSGALG00010006883	LRBA	3	-0.047363665	0.082243035	-0.575898795	0.56468359	0	1.364399688	0	0.666666667	CRA004775;PRJNA827465;Yang2025_Silkie_Wencha	0.861279534
ENSGALG00010021160	ZNF143	3	0.027876327	0.048396552	0.575998206	0.564616394	0	0.005024149	0	1	CRA004775;PRJNA827465;Yang2025_Silkie_Wencha	0.861279534
ENSGALG00010024583	ITGB7	2	0.654966438	1.138103016	0.57548959	0.564960229	2.17664849	6.258621237	84.02203997	0.5	CRA004775;PRJNA827465	0.861349401
ENSGALG00010012553	ENSGALG00010012553	3	-0.158209323	0.275007765	-0.575290393	0.565094918	0.088600466	3.280952361	39.04208962	0.666666667	CRA004775;PRJNA827465;Yang2025_Silkie_Wencha	0.861349401
ENSGALG00010015047	NPY2R	3	0.096561807	0.167842622	0.575311594	0.565080582	0	0.316668022	0	0.666666667	CRA004775;PRJNA827465;Yang2025_Silkie_Wencha	0.861349401
ENSGALG00010027258	ENSGALG00010027258	3	0.065082744	0.113074409	0.575574481	0.564902834	0	0.8955193	0	0.666666667	CRA004775;PRJNA827465;Yang2025_Silkie_Wencha	0.861349401
ENSGALG00010017962	TXNDC16	3	-0.060264269	0.104729016	-0.575430488	0.565000189	0.01807389	4.775469331	58.11929966	0.666666667	CRA004775;PRJNA827465;Yang2025_Silkie_Wencha	0.861349401
ENSGALG00010020921	LDB1	3	-0.044293196	0.076954049	-0.575579799	0.564899239	0.006927314	3.303204894	39.45274168	0.666666667	CRA004775;PRJNA827465;Yang2025_Silkie_Wencha	0.861349401
ENSGALG00010009972	PTK2B	3	-0.183508293	0.319605107	-0.574171967	0.565851437	0.153398357	4.012435286	50.15495934	0.666666667	CRA004775;PRJNA827465;Yang2025_Silkie_Wencha	0.861485826
ENSGALG00010009118	ACTN2	3	0.151357288	0.26359457	0.574204878	0.565829169	0.1432565	14.0058649	85.7202678	0.666666667	CRA004775;PRJNA827465;Yang2025_Silkie_Wencha	0.861485826
ENSGALG00010017065	VRK2	3	0.14866703	0.258971378	0.574067418	0.56592218	0.173830123	26.08992976	92.33420704	0.666666667	CRA004775;PRJNA827465;Yang2025_Silkie_Wencha	0.861485826
ENSGALG00010023198	PTK6	2	-0.145628684	0.253647936	-0.574137074	0.565875047	0	0.414981218	0	0.5	PRJNA827465;Yang2025_Silkie_Wenchang	0.861485826
ENSGALG00010018736	EEF2KMT	3	0.129833443	0.225807341	0.574974414	0.565308601	0.131977934	16.51133522	87.8871239	0.666666667	CRA004775;PRJNA827465;Yang2025_Silkie_Wencha	0.861485826
ENSGALG00010017983	SLC4A11	3	-0.110021457	0.19170938	-0.573897098	0.566037437	0.065433745	5.828894016	65.68817353	0.666666667	CRA004775;PRJNA827465;Yang2025_Silkie_Wencha	0.861485826
ENSGALG00010029219	TLCD1	3	-0.102731777	0.178988513	-0.573957377	0.565996645	0.051737432	4.366500991	54.19673546	0.666666667	CRA004775;PRJNA827465;Yang2025_Silkie_Wencha	0.861485826
ENSGALG00010012312	SH3TC2	3	0.097987343	0.170646321	0.574191948	0.565837917	0.040025572	3.81091272	47.30880361	0.666666667	CRA004775;PRJNA827465;Yang2025_Silkie_Wencha	0.861485826
ENSGALG00010026421	POLR2C	3	-0.053599797	0.093325055	-0.574334482	0.56574148	0.012342855	3.75942765	46.80041256	0.666666667	CRA004775;PRJNA827465;Yang2025_Silkie_Wencha	0.861485826
ENSGALG00010017464	SH3BP2	3	0.03432773	0.059776224	0.574270635	0.565784677	0	0.174232321	0	0.666666667	CRA004775;PRJNA827465;Yang2025_Silkie_Wencha	0.861485826
ENSGALG00010024328	YDJC	3	-0.034055802	0.059295567	-0.57433976	0.56573709	0	1.621146651	0	0.666666667	CRA004775;PRJNA827465;Yang2025_Silkie_Wencha	0.861485826
ENSGALG00010005492	TNPO3	3	-0.025882338	0.045073431	-0.574222382	0.565816352	0	1.435502347	0	1	CRA004775;PRJNA827465;Yang2025_Silkie_Wencha	0.861485826
ENSGALG00010016787	PPP2R5C	3	-0.025846692	0.045022972	-0.574077868	0.56591511	0	1.695251818	0	0.666666667	CRA004775;PRJNA827465;Yang2025_Silkie_Wencha	0.861485826
ENSGALG00010024368	DPAGT1	3	0.023871669	0.041541542	0.574645721	0.565530924	0	0.3933053	0	1	CRA004775;PRJNA827465;Yang2025_Silkie_Wencha	0.861485826
ENSGALG00010028669	ZBTB34	3	0.102808839	0.179171772	0.573800425	0.566102861	0.079169822	15.27505204	86.90675492	0.666666667	CRA004775;PRJNA827465;Yang2025_Silkie_Wencha	0.861492665
ENSGALG00010021236	BTBD9	3	-0.094603811	0.16491314	-0.573664783	0.566194664	0.067818952	14.45088499	86.16001718	0.666666667	CRA004775;PRJNA827465;Yang2025_Silkie_Wencha	0.861510439
ENSGALG00010018707	SLC45A1	3	0.064631636	0.112676578	0.573603115	0.566236403	0.018907479	3.926452876	49.06344064	0.666666667	CRA004775;PRJNA827465;Yang2025_Silkie_Wencha	0.861510439
ENSGALG00010019283	NT5C3A	3	-0.088572136	0.154480615	-0.573354373	0.566404777	0.038311751	4.462427687	55.18134656	0.666666667	CRA004775;PRJNA827465;Yang2025_Silkie_Wencha	0.861673891
ENSGALG00010015173	ENS-1	3	0.429039888	0.748428999	0.57325396	0.566472753	1.227902455	7.637448117	73.81324273	0.666666667	CRA004775;PRJNA827465;Yang2025_Silkie_Wencha	0.861677607
ENSGALG00010010447	BTF3	3	0.135629209	0.236666855	0.573080707	0.566590049	0.121317804	7.390016438	72.93646074	0.666666667	CRA004775;PRJNA827465;Yang2025_Silkie_Wencha	0.861677607
ENSGALG00010025097	LMBR1	3	-0.125766136	0.219424047	-0.573164782	0.566533127	0.123640893	23.92719684	91.64131087	0.666666667	CRA004775;PRJNA827465;Yang2025_Silkie_Wencha	0.861677607
ENSGALG00010007572	PDZK1	3	0.353060837	0.617781281	0.571498115	0.567662038	0.94912991	13.50807673	85.19404324	0.666666667	CRA004775;PRJNA827465;Yang2025_Silkie_Wencha	0.861845253
ENSGALG00010000630	ENSGALG00010000630	3	-0.236728001	0.41369374	-0.572230078	0.567166113	0.395094662	8.804233495	77.28365563	0.666666667	CRA004775;PRJNA827465;Yang2025_Silkie_Wencha	0.861845253
ENSGALG00010016735	ENSGALG00010016735	3	0.198732599	0.347234529	0.572310022	0.567111961	0.178626716	4.043491817	50.53780024	0.666666667	CRA004775;PRJNA827465;Yang2025_Silkie_Wencha	0.861845253
ENSGALG00010021769	ENSGALG00010021769	3	0.168310368	0.29406969	0.572348576	0.567085847	0.224538251	17.37622283	88.49001869	0.666666667	CRA004775;PRJNA827465;Yang2025_Silkie_Wencha	0.861845253
ENSGALG00010028870	CACFD1	3	-0.164668588	0.287632919	-0.572495625	0.566986249	0.175335565	7.645475835	73.84073872	0.666666667	CRA004775;PRJNA827465;Yang2025_Silkie_Wencha	0.861845253
ENSGALG00010029350	MRPL12	3	0.157821713	0.275731882	0.572373828	0.567068743	0.174676286	9.954213894	79.90800659	0.666666667	CRA004775;PRJNA827465;Yang2025_Silkie_Wencha	0.861845253
ENSGALG00010017685	PHC2	3	0.138191497	0.241724035	0.571691172	0.567531216	0.142733122	21.83073563	90.81860465	0.666666667	CRA004775;PRJNA827465;Yang2025_Silkie_Wencha	0.861845253
ENSGALG00010009147	HNMT	3	0.131658883	0.230455597	0.571298266	0.567797477	0.112620719	7.551600609	73.51554851	0.666666667	CRA004775;PRJNA827465;Yang2025_Silkie_Wencha	0.861845253
ENSGALG00010003308	MDN1	3	0.115655999	0.202161087	0.572098226	0.567255431	0.088396198	8.381448433	76.13777599	0.666666667	CRA004775;PRJNA827465;Yang2025_Silkie_Wencha	0.861845253
ENSGALG00010021675	DDX42	3	-0.081101678	0.141786667	-0.571997912	0.567323389	0.047435794	12.64806168	84.18730039	0.666666667	CRA004775;PRJNA827465;Yang2025_Silkie_Wencha	0.861845253
ENSGALG00010022293	DNAJC10	3	-0.079323127	0.138584991	-0.572378915	0.567065297	0.045880789	12.97003861	84.57984545	0.666666667	CRA004775;PRJNA827465;Yang2025_Silkie_Wencha	0.861845253
ENSGALG00010016355	MCF2L2	3	-0.072749827	0.12713105	-0.57224279	0.567157502	0	1.06051273	0	0.666666667	CRA004775;PRJNA827465;Yang2025_Silkie_Wencha	0.861845253
ENSGALG00010002530	STAG3	3	0.058886966	0.103045243	0.571467104	0.567683053	0.013044591	3.392478896	41.04605921	0.666666667	CRA004775;PRJNA827465;Yang2025_Silkie_Wencha	0.861845253
ENSGALG00010029519	STX1A	3	0.049395227	0.086381225	0.571828275	0.56743832	0	1.749044868	0	0.666666667	CRA004775;PRJNA827465;Yang2025_Silkie_Wencha	0.861845253
ENSGALG00010009256	CREBRF	3	-0.048275307	0.084450669	-0.57163913	0.56756648	0	0.413861557	0	1	CRA004775;PRJNA827465;Yang2025_Silkie_Wencha	0.861845253
ENSGALG00010020171	RRN3	3	0.043707132	0.076501335	0.571325096	0.567779293	0.005317842	2.781603321	28.0990217	0.666666667	CRA004775;PRJNA827465;Yang2025_Silkie_Wencha	0.861845253
ENSGALG00010027870	HAX1	3	-0.033713912	0.058949232	-0.571914361	0.567379994	0	1.252453639	0	0.666666667	CRA004775;PRJNA827465;Yang2025_Silkie_Wencha	0.861845253
ENSGALG00010013257	STX12	3	0.031996648	0.055936882	0.572013429	0.567312877	0.002517728	2.663642611	24.91485186	0.666666667	CRA004775;PRJNA827465;Yang2025_Silkie_Wencha	0.861845253
ENSGALG00010023668	RCN1	3	-0.045728001	0.080059057	-0.571178366	0.567878741	0.006746746	2.926739496	31.66457066	0.666666667	CRA004775;PRJNA827465;Yang2025_Silkie_Wencha	0.861876076
ENSGALG00010021770	RALGAPB	3	-0.021206823	0.037163558	-0.570634894	0.56824716	0	0.48701783	0	0.666666667	CRA004775;PRJNA827465;Yang2025_Silkie_Wencha	0.862342663
ENSGALG00010019032	GNMT	3	0.054446288	0.095466389	0.570318922	0.56846141	0	0.072902632	0	0.666666667	CRA004775;PRJNA827465;Yang2025_Silkie_Wencha	0.862464706
ENSGALG00010025364	EPHA4	3	-0.046853118	0.082162932	-0.570246417	0.568510578	0	1.961595241	0	0.666666667	CRA004775;PRJNA827465;Yang2025_Silkie_Wencha	0.862464706
ENSGALG00010022436	AFAP1L2	3	0.029691694	0.05205179	0.570425987	0.568388808	0	0.641553939	0	0.666666667	CRA004775;PRJNA827465;Yang2025_Silkie_Wencha	0.862464706
ENSGALG00010013772	KIAA0319L	3	-0.050649603	0.088852694	-0.570040145	0.56865047	0.012874174	5.012824761	60.1023356	0.666666667	CRA004775;PRJNA827465;Yang2025_Silkie_Wencha	0.862584379
ENSGALG00010023253	TACR2	2	-0.270290876	0.474577133	-0.569540455	0.568989425	0	0.036660155	0	1	CRA004775;PRJNA827465	0.863005951
ENSGALG00010028435	ATP8B3	3	0.251336023	0.44139961	0.56940699	0.569079974	0.432664571	8.167681545	75.51324707	0.666666667	CRA004775;PRJNA827465;Yang2025_Silkie_Wencha	0.863050709
ENSGALG00010000635	ENSGALG00010000635	3	0.06566394	0.115392742	0.569047403	0.569323971	0	0.703733304	0	0.666666667	CRA004775;PRJNA827465;Yang2025_Silkie_Wencha	0.863052745
ENSGALG00010003660	IGFBP7	3	-0.062240299	0.109418681	-0.568826996	0.569473553	0.0095775	2.523764267	20.75329594	0.666666667	CRA004775;PRJNA827465;Yang2025_Silkie_Wencha	0.863052745
ENSGALG00010026305	ATP5MC1	3	-0.050290011	0.088384481	-0.568991421	0.569361962	0	1.161921154	0	0.666666667	CRA004775;PRJNA827465;Yang2025_Silkie_Wencha	0.863052745
ENSGALG00010004125	MANEA	3	-0.041974937	0.073769149	-0.569003951	0.569353459	0.002884155	2.316110591	13.64833753	0.666666667	CRA004775;PRJNA827465;Yang2025_Silkie_Wencha	0.863052745
ENSGALG00010007169	QPCT	3	0.037012192	0.065071315	0.568794288	0.569495751	0	0.014731847	0	1	CRA004775;PRJNA827465;Yang2025_Silkie_Wencha	0.863052745
ENSGALG00010001251	ZNF277	3	-0.035987443	0.063271805	-0.568775355	0.569508601	0	1.206023367	0	0.666666667	CRA004775;PRJNA827465;Yang2025_Silkie_Wencha	0.863052745
ENSGALG00010015939	SUN2	3	-0.030399106	0.053442649	-0.568817349	0.5694801	0	0.757040217	0	1	CRA004775;PRJNA827465;Yang2025_Silkie_Wencha	0.863052745
ENSGALG00010024018	KRT18	3	0.671006502	1.183820532	0.566814381	0.570840299	3.269290467	9.146738952	78.13428359	0.666666667	CRA004775;PRJNA827465;Yang2025_Silkie_Wencha	0.863134826
ENSGALG00010005344	ENSGALG00010005344	2	0.500947738	0.882859471	0.567415036	0.570432237	1.093747387	3.091691386	67.65524514	0.5	CRA004775;PRJNA827465	0.863134826
ENSGALG00010021446	HOXB8	3	0.290426713	0.51207831	0.567152927	0.570610286	0.502392758	5.675148725	64.75863282	0.666666667	CRA004775;PRJNA827465;Yang2025_Silkie_Wencha	0.863134826
ENSGALG00010029342	TMC6	3	0.279015205	0.490871463	0.568407875	0.569758044	0.669217534	47.65309855	95.80300115	0.666666667	CRA004775;PRJNA827465;Yang2025_Silkie_Wencha	0.863134826
ENSGALG00010010925	FBXO16	2	-0.175265777	0.308880077	-0.56742338	0.570426569	0	0.000825448	0	1	PRJNA827465;Yang2025_Silkie_Wenchang	0.863134826
ENSGALG00010021034	NPM3	3	0.168408948	0.297358733	0.566349427	0.571156266	0.196464154	8.856215568	77.41699053	0.666666667	CRA004775;PRJNA827465;Yang2025_Silkie_Wencha	0.863134826
ENSGALG00010008388	ADCY1	2	-0.15258961	0.268706796	-0.567866583	0.570125564	0	0.34740251	0	0.5	PRJNA827465;Yang2025_Silkie_Wenchang	0.863134826
ENSGALG00010023703	PITPNB	3	0.147944491	0.260916787	0.567017911	0.570702013	0.18497724	44.58781265	95.51446936	0.666666667	CRA004775;PRJNA827465;Yang2025_Silkie_Wencha	0.863134826
ENSGALG00010020719	RPH3A	3	-0.145423437	0.256942082	-0.565977498	0.571409077	0	0.575588074	0	0.666666667	CRA004775;PRJNA827465;Yang2025_Silkie_Wencha	0.863134826
ENSGALG00010011921	MEGF10	3	0.120107774	0.212381769	0.565527704	0.571714886	0.094578339	8.324682276	75.97505906	0.666666667	CRA004775;PRJNA827465;Yang2025_Silkie_Wencha	0.863134826
ENSGALG00010023274	ZBTB38	3	0.118194467	0.20825671	0.567542181	0.570345877	0.097814978	8.817878231	77.31880677	0.666666667	CRA004775;PRJNA827465;Yang2025_Silkie_Wencha	0.863134826
ENSGALG00010021762	LAMB2	3	-0.107538788	0.189342203	-0.567959946	0.570062165	0.068501384	6.55096082	69.47012729	0.666666667	CRA004775;PRJNA827465;Yang2025_Silkie_Wencha	0.863134826
ENSGALG00010016418	NFIB	3	0.106333852	0.187842569	0.56607963	0.57133965	0.061788656	4.780782901	58.1658477	0.666666667	CRA004775;PRJNA827465;Yang2025_Silkie_Wencha	0.863134826
ENSGALG00010013486	PLPP6	3	-0.104787747	0.18511651	-0.566063756	0.571350441	0.049750155	3.54446551	43.57400307	0.666666667	CRA004775;PRJNA827465;Yang2025_Silkie_Wencha	0.863134826
ENSGALG00010021935	CETP	3	0.094676322	0.16701834	0.566861829	0.57080806	0	0.453256425	0	0.666666667	CRA004775;PRJNA827465;Yang2025_Silkie_Wencha	0.863134826
ENSGALG00010021262	MDGA1	3	0.089419666	0.157813967	0.566614398	0.570976191	0	0.519012929	0	0.666666667	CRA004775;PRJNA827465;Yang2025_Silkie_Wencha	0.863134826
ENSGALG00010025080	BAZ2A	3	0.082468562	0.145349924	0.567379464	0.570456399	0.03006783	3.798468056	47.34719444	0.666666667	CRA004775;PRJNA827465;Yang2025_Silkie_Wencha	0.863134826
ENSGALG00010029768	KCNJ2	3	0.081450933	0.143798704	0.566423274	0.571106077	0.037680143	6.390579864	68.70393544	0.666666667	CRA004775;PRJNA827465;Yang2025_Silkie_Wencha	0.863134826
ENSGALG00010006363	TRAPPC6A	3	0.078596658	0.138931988	0.565720388	0.571583873	0.033449403	4.961147414	59.68674516	0.666666667	CRA004775;PRJNA827465;Yang2025_Silkie_Wencha	0.863134826
ENSGALG00010028032	ENSGALG00010028032	2	-0.075804768	0.134012924	-0.565652672	0.571629914	0	0.008605098	0	1	PRJNA827465;Yang2025_Silkie_Wenchang	0.863134826
ENSGALG00010011979	NEDD1	3	-0.074853698	0.13193463	-0.567354444	0.570473394	0.042711146	15.81439265	87.35329238	0.666666667	CRA004775;PRJNA827465;Yang2025_Silkie_Wencha	0.863134826
ENSGALG00010022042	AIP	3	0.074279549	0.131347644	0.5655187	0.571721009	0.040003121	9.172794859	78.19639455	0.666666667	CRA004775;PRJNA827465;Yang2025_Silkie_Wencha	0.863134826
ENSGALG00010008534	RAB33B	3	-0.070586425	0.124777831	-0.565696838	0.571599885	0.034774846	17.06282152	88.27860915	0.666666667	CRA004775;PRJNA827465;Yang2025_Silkie_Wencha	0.863134826
ENSGALG00010020965	DHX37	3	0.065696715	0.116002823	0.566337207	0.571164572	0.026007681	6.833339638	70.73173432	0.666666667	CRA004775;PRJNA827465;Yang2025_Silkie_Wencha	0.863134826
ENSGALG00010028865	STKLD1	3	0.062974057	0.111197776	0.566324786	0.571173014	0	0.293827017	0	0.666666667	CRA004775;PRJNA827465;Yang2025_Silkie_Wencha	0.863134826
ENSGALG00010029348	HGS	3	-0.061249214	0.107782991	-0.568264187	0.569855593	0.022959691	7.275607747	72.51088748	0.666666667	CRA004775;PRJNA827465;Yang2025_Silkie_Wencha	0.863134826
ENSGALG00010009637	CYBA	3	-0.057988041	0.10230211	-0.566831326	0.570828785	0	1.403462087	0	0.666666667	CRA004775;PRJNA827465;Yang2025_Silkie_Wencha	0.863134826
ENSGALG00010004083	PHF11	3	0.057179739	0.101076871	0.565705474	0.571594013	0.010772239	3.024638349	33.87639217	0.666666667	CRA004775;PRJNA827465;Yang2025_Silkie_Wencha	0.863134826
ENSGALG00010027623	APOBEC2	3	-0.053762464	0.09503758	-0.56569689	0.57159985	0	1.763041879	0	0.666666667	CRA004775;PRJNA827465;Yang2025_Silkie_Wencha	0.863134826
ENSGALG00010002613	ENSGALG00010002613	3	0.048127631	0.085112245	0.56546072	0.571760434	0.008122233	3.067490907	34.80013273	0.666666667	CRA004775;PRJNA827465;Yang2025_Silkie_Wencha	0.863134826
ENSGALG00010029668	CRCP	3	0.043990179	0.07778286	0.565551058	0.571699006	0	1.349322842	0	0.666666667	CRA004775;PRJNA827465;Yang2025_Silkie_Wencha	0.863134826
ENSGALG00010007092	DYNC1LI2	3	0.037408553	0.065922047	0.567466487	0.570397289	0.005358038	2.98690596	33.04107907	0.666666667	CRA004775;PRJNA827465;Yang2025_Silkie_Wencha	0.863134826
ENSGALG00010028745	ENSGALG00010028745	3	0.035577014	0.062811578	0.566408535	0.571116094	0	1.511284083	0	0.666666667	CRA004775;PRJNA827465;Yang2025_Silkie_Wencha	0.863134826
ENSGALG00010016423	TRMT44	3	-0.033849493	0.059855895	-0.565516439	0.571722546	0	0.240126041	0	0.666666667	CRA004775;PRJNA827465;Yang2025_Silkie_Wencha	0.863134826
ENSGALG00010022069	NOP2	3	0.029982674	0.052852924	0.567285058	0.570520527	0	1.963696478	0	0.666666667	CRA004775;PRJNA827465;Yang2025_Silkie_Wencha	0.863134826
ENSGALG00010021583	NFE2L1	3	-0.025746352	0.045512865	-0.565693929	0.571601863	0	0.426662069	0	0.666666667	CRA004775;PRJNA827465;Yang2025_Silkie_Wencha	0.863134826
ENSGALG00010029285	CUX1	3	0.09782387	0.173105945	0.565109826	0.571999066	0.07604847	16.7070819	88.02902857	0.666666667	CRA004775;PRJNA827465;Yang2025_Silkie_Wencha	0.863310716
ENSGALG00010020206	MRPS11	3	-0.034922087	0.061790501	-0.565169188	0.571958692	0	1.491551668	0	0.666666667	CRA004775;PRJNA827465;Yang2025_Silkie_Wencha	0.863310716
ENSGALG00010029644	MYCBPAP	3	0.122589816	0.217024828	0.564865398	0.572165321	0.109562677	10.96772057	81.76467036	0.666666667	CRA004775;PRJNA827465;Yang2025_Silkie_Wencha	0.86346947
ENSGALG00010027262	USE1	3	0.129656888	0.229584814	0.564745052	0.572247186	0.140044974	21.96461205	90.89444423	0.666666667	CRA004775;PRJNA827465;Yang2025_Silkie_Wencha	0.86350085
ENSGALG00010027369	ENSGALG00010027369	3	0.097521358	0.172709662	0.564654908	0.57230851	0.071384614	14.48785003	86.19532922	0.666666667	CRA004775;PRJNA827465;Yang2025_Silkie_Wencha	0.86350123
ENSGALG00010002457	ENSGALG00010002457	3	-0.165188783	0.292636111	-0.5644853	0.572423901	0	0.11063803	0	1	CRA004775;PRJNA827465;Yang2025_Silkie_Wencha	0.863504494
ENSGALG00010027499	MOV10	3	-0.099248007	0.17588448	-0.564279503	0.572563929	0.030727657	2.964816798	32.54220628	0.666666667	CRA004775;PRJNA827465;Yang2025_Silkie_Wencha	0.863504494
ENSGALG00010010697	NEDD9	3	0.087864926	0.155659561	0.564468546	0.572435301	0.042057596	4.913940921	59.29946997	0.666666667	CRA004775;PRJNA827465;Yang2025_Silkie_Wencha	0.863504494
ENSGALG00010027893	DOT1L	3	0.087345801	0.15481274	0.564202927	0.572616036	0.038803655	4.277870256	53.24776395	0.666666667	CRA004775;PRJNA827465;Yang2025_Silkie_Wencha	0.863504494
ENSGALG00010005814	UPF2	3	0.055663951	0.098641914	0.564303234	0.572547781	0.014793092	3.704019844	46.00460894	0.666666667	CRA004775;PRJNA827465;Yang2025_Silkie_Wencha	0.863504494
ENSGALG00010027391	TPM3	3	0.120529039	0.213702839	0.564002984	0.572752101	0.112223947	20.48524724	90.23687644	0.666666667	CRA004775;PRJNA827465;Yang2025_Silkie_Wencha	0.863612324
ENSGALG00010025055	ALS2	3	-0.031036955	0.05503803	-0.563918349	0.572809702	0.00138062	2.169815638	7.826270336	0.666666667	CRA004775;PRJNA827465;Yang2025_Silkie_Wencha	0.863612324
ENSGALG00010022529	CCR7	3	0.159999825	0.283899713	0.563578677	0.573040902	0.091984608	3.17763719	37.06015254	0.666666667	CRA004775;PRJNA827465;Yang2025_Silkie_Wencha	0.863684996
ENSGALG00010025887	ZNF76	3	-0.154559062	0.274245943	-0.563578299	0.573041159	0.209615927	28.86854425	93.07204415	0.666666667	CRA004775;PRJNA827465;Yang2025_Silkie_Wencha	0.863684996
ENSGALG00010023778	FANCF	3	-0.081137511	0.143949304	-0.563653371	0.572990057	0	0.629973092	0	1	CRA004775;PRJNA827465;Yang2025_Silkie_Wencha	0.863684996
ENSGALG00010025337	NEMP1	3	-0.100143433	0.177727842	-0.56346508	0.573118232	0.068616374	7.528438413	73.43406573	0.666666667	CRA004775;PRJNA827465;Yang2025_Silkie_Wencha	0.86370909
ENSGALG00010008729	TMEM254	3	-0.145662277	0.258605474	-0.563260609	0.573257437	0.171077159	14.41510859	86.12566817	0.666666667	CRA004775;PRJNA827465;Yang2025_Silkie_Wencha	0.86376065
ENSGALG00010008391	ENSGALG00010008391	3	-0.075682198	0.134370465	-0.563235363	0.573274626	0.032292444	6.209739304	67.79252877	0.666666667	CRA004775;PRJNA827465;Yang2025_Silkie_Wencha	0.86376065
ENSGALG00010015540	ACSL4	3	0.029795523	0.052911632	0.56311857	0.573354147	0	0.023283213	0	0.666666667	CRA004775;PRJNA827465;Yang2025_Silkie_Wencha	0.863788417
ENSGALG00010000126	ENSGALG00010000126	3	0.300633028	0.534142649	0.562832847	0.573548711	0.740272763	16.15326465	87.61860192	0.666666667	CRA004775;PRJNA827465;Yang2025_Silkie_Wencha	0.863989476
ENSGALG00010001961	ENSGALG00010001961	3	0.295589287	0.525692122	0.562285937	0.573921218	0.6429092	13.25975937	84.91677003	0.666666667	CRA004775;PRJNA827465;Yang2025_Silkie_Wencha	0.864211964
ENSGALG00010023245	TANC2	3	-0.12309047	0.218896831	-0.562321846	0.573896757	0.096179999	6.956363997	71.24934806	0.666666667	CRA004775;PRJNA827465;Yang2025_Silkie_Wencha	0.864211964
ENSGALG00010014131	CDC23	3	0.100197814	0.178233211	0.562172522	0.573998481	0.078936149	21.09653512	90.51977024	0.666666667	CRA004775;PRJNA827465;Yang2025_Silkie_Wencha	0.864211964
ENSGALG00010019004	ECE2	3	0.077816302	0.138421958	0.562167328	0.574002019	0.008487888	2.313220428	13.54044882	0.666666667	CRA004775;PRJNA827465;Yang2025_Silkie_Wencha	0.864211964
ENSGALG00010024623	NRDC	3	-0.061184255	0.108800264	-0.562353921	0.573874907	0.024843751	9.84660731	79.68843545	0.666666667	CRA004775;PRJNA827465;Yang2025_Silkie_Wencha	0.864211964
ENSGALG00010021489	CNPPD1	3	-0.027430481	0.048804445	-0.562048819	0.574082758	0	1.814849237	0	0.666666667	CRA004775;PRJNA827465;Yang2025_Silkie_Wencha	0.864241494
ENSGALG00010017749	TMEM88B	3	-0.22783066	0.406260095	-0.560799996	0.574933889	0.271241892	4.672116666	57.19284977	0.666666667	CRA004775;PRJNA827465;Yang2025_Silkie_Wencha	0.864305482
ENSGALG00010000612	ENSGALG00010000612	3	-0.154534028	0.275668849	-0.560578492	0.575084917	0.136892472	5.015074231	60.12023137	0.666666667	CRA004775;PRJNA827465;Yang2025_Silkie_Wencha	0.864305482
ENSGALG00010029812	SAP30BP	3	0.148628186	0.265188488	0.560462434	0.575164056	0.188861995	25.49484651	92.15527735	0.666666667	CRA004775;PRJNA827465;Yang2025_Silkie_Wencha	0.864305482
ENSGALG00010013836	IL18R1	3	-0.136410516	0.243160993	-0.56098848	0.574805391	0.094800084	4.32798026	53.78906834	0.666666667	CRA004775;PRJNA827465;Yang2025_Silkie_Wencha	0.864305482
ENSGALG00010027275	CPAMD8	3	-0.132759142	0.236869444	-0.560473901	0.575156236	0.101392208	5.558146362	64.0167806	0.666666667	CRA004775;PRJNA827465;Yang2025_Silkie_Wencha	0.864305482
ENSGALG00010025933	SKIC2	3	0.122986561	0.219206185	0.561054245	0.574760558	0.116004696	11.96519057	83.28484625	0.666666667	CRA004775;PRJNA827465;Yang2025_Silkie_Wencha	0.864305482
ENSGALG00010013254	SAR1B	3	-0.120635502	0.214903185	-0.561348134	0.574560234	0.107127033	15.88962201	87.41316817	0.666666667	CRA004775;PRJNA827465;Yang2025_Silkie_Wencha	0.864305482
ENSGALG00010018848	MEF2A	3	0.110712028	0.197170766	0.561503258	0.57445451	0.090615342	9.75449854	79.49663951	0.666666667	CRA004775;PRJNA827465;Yang2025_Silkie_Wencha	0.864305482
ENSGALG00010013146	TNRC6B	3	0.084137797	0.15004998	0.560731814	0.574980376	0.034298063	4.014085542	50.17545144	0.666666667	CRA004775;PRJNA827465;Yang2025_Silkie_Wencha	0.864305482
ENSGALG00010008811	TERF2IP	3	-0.081744309	0.145849496	-0.560470284	0.575158703	0.048080402	9.022910853	77.83420414	0.666666667	CRA004775;PRJNA827465;Yang2025_Silkie_Wencha	0.864305482
ENSGALG00010023091	ENSGALG00010023091	3	-0.077643868	0.138535511	-0.560461844	0.575164459	0.046432919	12.07041595	83.43056272	0.666666667	CRA004775;PRJNA827465;Yang2025_Silkie_Wencha	0.864305482
ENSGALG00010028183	CRTC1	3	0.076303174	0.135821706	0.561789248	0.574259619	0.029614749	4.10820688	51.31695997	0.666666667	CRA004775;PRJNA827465;Yang2025_Silkie_Wencha	0.864305482
ENSGALG00010001475	BMT2	3	0.065897175	0.117443375	0.561097426	0.574731123	0.022973804	4.801630165	58.34747927	0.666666667	CRA004775;PRJNA827465;Yang2025_Silkie_Wencha	0.864305482
ENSGALG00010010547	BST1	3	-0.055962665	0.099739605	-0.561087697	0.574737755	0	1.182301503	0	0.666666667	CRA004775;PRJNA827465;Yang2025_Silkie_Wencha	0.864305482
ENSGALG00010002347	ZNF451	3	-0.052005975	0.092681892	-0.561123365	0.574713441	0.01486494	5.637583866	64.52380936	0.666666667	CRA004775;PRJNA827465;Yang2025_Silkie_Wencha	0.864305482
ENSGALG00010027310	ARHGEF2	3	-0.047618606	0.084830466	-0.561338498	0.574566802	0.008114597	3.015801389	33.68263549	0.666666667	CRA004775;PRJNA827465;Yang2025_Silkie_Wencha	0.864305482
ENSGALG00010012245	ENSGALG00010012245	3	-0.025655364	0.045715148	-0.561200508	0.574660857	0	0.827954266	0	0.666666667	CRA004775;PRJNA827465;Yang2025_Silkie_Wencha	0.864305482
ENSGALG00010008382	ENSGALG00010008382	3	-0.078417298	0.140030738	-0.560000603	0.575479026	0.028645557	3.925558126	49.0518307	0.666666667	CRA004775;PRJNA827465;Yang2025_Silkie_Wencha	0.86448898

ENSGALG00010027918	CSNK1G2	3	0.058716018	0.104863844	0.559926241	0.575529749	0.02086599	6.617591498	69.77752403	0.666666667	CRA004775;PRJNA827465;Yang2025_Silkie_Wencha	0.86448898
ENSGALG00010024149	TCIRG1	3	0.055125364	0.098451476	0.559924204	0.575531139	0.010856438	3.225824474	38.0003464	0.666666667	CRA004775;PRJNA827465;Yang2025_Silkie_Wencha	0.86448898
ENSGALG00010002985	ENSGALG0001000298	3	0.05296175	0.094555849	0.560110778	0.575403879	0	0.003820727	0	1	CRA004775;PRJNA827465;Yang2025_Silkie_Wencha	0.86448898
ENSGALG00010022122	PSMB3	3	-0.07067972	0.126260016	-0.559794958	0.575619303	0.026535451	4.357999218	54.10738048	0.666666667	CRA004775;PRJNA827465;Yang2025_Silkie_Wencha	0.864529565
ENSGALG00010025163	LDLRAD3	3	0.039868043	0.071250746	0.559545618	0.575789407	0	0.805855239	0	1	CRA004775;PRJNA827465;Yang2025_Silkie_Wencha	0.864607482
ENSGALG00010024688	CADM1	3	-0.037947012	0.067818273	-0.559539645	0.575793483	0	1.555566812	0	0.666666667	CRA004775;PRJNA827465;Yang2025_Silkie_Wencha	0.864607482
ENSGALG00010027225	CREB3L3	3	0.483193986	0.86425486	0.559087381	0.576102086	1.884019886	12.67148182	84.21652631	0.666666667	CRA004775;PRJNA827465;Yang2025_Silkie_Wencha	0.86479535
ENSGALG00010016428	ADRA1D	3	0.101262172	0.181120281	0.559087981	0.576101677	0.03778536	3.217859689	37.84688602	0.666666667	CRA004775;PRJNA827465;Yang2025_Silkie_Wencha	0.86479535
ENSGALG00010026715	WDR48	3	-0.071427907	0.127735874	-0.55918439	0.576035885	0.034025189	7.615359509	73.73728715	0.666666667	CRA004775;PRJNA827465;Yang2025_Silkie_Wencha	0.86479535
ENSGALG00010003276	TNFSF11	3	0.240414125	0.43029528	0.558718944	0.576353548	0.340284894	5.212377215	61.6297916	0.666666667	CRA004775;PRJNA827465;Yang2025_Silkie_Wencha	0.865038731
ENSGALG00010027787	DCP1A	3	0.097960785	0.175346248	0.558670549	0.576386582	0.057529769	5.878012171	65.97489182	0.666666667	CRA004775;PRJNA827465;Yang2025_Silkie_Wencha	0.865038731
ENSGALG00010023408	ZBTB44	3	-0.035378603	0.06340023	-0.558020109	0.576830653	0	0.055534676	0	0.666666667	CRA004775;PRJNA827465;Yang2025_Silkie_Wencha	0.865613309
ENSGALG00010019012	ADAM28	3	-0.146662241	0.263014245	-0.557620904	0.577103279	0.095321954	3.898554851	48.69893906	0.666666667	CRA004775;PRJNA827465;Yang2025_Silkie_Wencha	0.865891172
ENSGALG00010028618	DOLPP1	3	-0.078354297	0.140528273	-0.557569631	0.577138299	0.03194806	4.542791276	55.97420443	0.666666667	CRA004775;PRJNA827465;Yang2025_Silkie_Wencha	0.865891172
ENSGALG00010025432	IRX3	3	0.306912273	0.550914461	0.557096056	0.577461802	0.61759698	6.242025057	67.09591613	0.666666667	CRA004775;PRJNA827465;Yang2025_Silkie_Wencha	0.865943918
ENSGALG00010018624	ENSGALG0001001862	3	0.17970034	0.322578383	0.557074961	0.577476214	0.075163555	2.613500367	23.47427898	0.666666667	CRA004775;PRJNA827465;Yang2025_Silkie_Wencha	0.865943918
ENSGALG00010005244	FBXO5	3	-0.078346133	0.140617335	-0.557158425	0.577419193	0.032176172	4.725719725	57.67840422	0.666666667	CRA004775;PRJNA827465;Yang2025_Silkie_Wencha	0.865943918
ENSGALG00010021785	CCDC71	3	-0.027031668	0.048524734	-0.557069887	0.577479681	0.001636022	2.410228745	17.02032414	0.666666667	CRA004775;PRJNA827465;Yang2025_Silkie_Wencha	0.865943918
ENSGALG00010016966	KDM3A	3	-0.02128897	0.038206034	-0.557214872	0.57738063	0	0.030103319	0	0.666666667	CRA004775;PRJNA827465;Yang2025_Silkie_Wencha	0.865943918
ENSGALG00010003822	ENSGALG0001000382	3	0.164378147	0.295145041	0.556940229	0.577568267	0.201982307	9.634559484	79.24139652	0.666666667	CRA004775;PRJNA827465;Yang2025_Silkie_Wencha	0.865984913
ENSGALG00010021167	KIAA1671	3	-0.074359364	0.133563244	-0.55673523	0.577708343	0.029997728	5.247141336	61.88400746	0.666666667	CRA004775;PRJNA827465;Yang2025_Silkie_Wencha	0.866011265
ENSGALG00010013464	ABCB7	3	0.051754822	0.092958934	0.5567493	0.577698728	0.013523656	4.611019176	56.62564124	0.666666667	CRA004775;PRJNA827465;Yang2025_Silkie_Wencha	0.866011265
ENSGALG00010001492	ENSGALG0001000149	3	0.263274294	0.473152768	0.55642556	0.57791997	0.518400416	9.098975415	78.0195032	0.666666667	CRA004775;PRJNA827465;Yang2025_Silkie_Wencha	0.866144844
ENSGALG00010026480	USP49	3	0.104926664	0.188553953	0.556480851	0.577882181	0.050069679	3.823558209	47.66532679	0.666666667	CRA004775;PRJNA827465;Yang2025_Silkie_Wencha	0.866144844
ENSGALG00010019115	DBNL	3	0.16640589	0.299121179	0.556315974	0.577994869	0.252719844	60.86581858	96.71408346	0.666666667	CRA004775;PRJNA827465;Yang2025_Silkie_Wencha	0.866165284
ENSGALG00010015104	CNGA2	3	0.153433338	0.276029805	0.555857863	0.578308026	0.061398195	2.667191708	25.01476389	0.666666667	CRA004775;PRJNA827465;Yang2025_Silkie_Wencha	0.866542728
ENSGALG00010021234	ISLR2	3	-0.300957645	0.542519089	-0.554741116	0.579617749	0.682201271	14.04431458	85.759362	0.666666667	CRA004775;PRJNA827465;Yang2025_Silkie_Wencha	0.866556281
ENSGALG00010021543	PLEKHD1	3	0.27362394	0.492882242	0.555150737	0.578791561	0.605167876	14.50872692	86.21519303	0.666666667	CRA004775;PRJNA827465;Yang2025_Silkie_Wencha	0.866556281
ENSGALG00010018891	GNRH1	2	-0.243607788	0.439820003	-0.553880647	0.579660531	0.255165825	2.800505817	64.29216486	0.5	PRJNA827465;Yang2025_Silkie_Wenchang	0.866556281
ENSGALG00010017490	SCNN1B	2	-0.211355694	0.380608005	-0.555310692	0.578682167	0.086103578	1.245421158	19.7058767	1	CRA004775;PRJNA827465	0.866556281
ENSGALG00010019209	VCPKMT	3	0.160896663	0.290205165	0.554423842	0.579288813	0.238398268	42.73230184	95.31969982	0.666666667	CRA004775;PRJNA827465;Yang2025_Silkie_Wencha	0.866556281
ENSGALG00010008174	SLC26A2	3	-0.151879756	0.273868907	-0.554571008	0.579188124	0.155132264	7.889765778	74.65070503	0.666666667	CRA004775;PRJNA827465;Yang2025_Silkie_Wencha	0.866556281
ENSGALG00010015843	ENSGALG0001001584	3	0.102732274	0.185181028	0.554766732	0.579054225	0	0.663767349	0	0.666666667	CRA004775;PRJNA827465;Yang2025_Silkie_Wencha	0.866556281
ENSGALG00010021057	ZDHHC8	3	0.097917535	0.17628693	0.555444099	0.578590936	0.064457893	8.720657758	77.06595012	0.666666667	CRA004775;PRJNA827465;Yang2025_Silkie_Wencha	0.866556281
ENSGALG00010021805	FUT8	3	-0.095500648	0.17192566	-0.555469946	0.578573261	0.058688786	8.676220799	76.94848891	0.666666667	CRA004775;PRJNA827465;Yang2025_Silkie_Wencha	0.866556281
ENSGALG00010019113	KLHDC1	3	0.091370687	0.164775337	0.554516762	0.579225238	0.057460956	7.206639774	72.24781503	0.666666667	CRA004775;PRJNA827465;Yang2025_Silkie_Wencha	0.866556281
ENSGALG00010024091	ETFA	3	-0.084921394	0.153202912	-0.554306658	0.579368995	0.04026273	4.594081723	56.46572872	0.666666667	CRA004775;PRJNA827465;Yang2025_Silkie_Wencha	0.866556281
ENSGALG00010027727	GSTM2	3	0.083038731	0.14991895	0.553890824	0.579653565	0	1.680160779	0	0.666666667	CRA004775;PRJNA827465;Yang2025_Silkie_Wencha	0.866556281
ENSGALG00010017858	GPR65	3	-0.075716776	0.136699803	-0.553890892	0.579653519	0	0.68412277	0	0.666666667	CRA004775;PRJNA827465;Yang2025_Silkie_Wencha	0.866556281
ENSGALG00010012672	RO60	3	-0.072282806	0.130504176	-0.55387351	0.579665415	0.030001266	5.304611301	62.29695473	0.666666667	CRA004775;PRJNA827465;Yang2025_Silkie_Wencha	0.866556281
ENSGALG00010002295	YAF2	3	0.067066578	0.121047625	0.554051171	0.579543826	0.027329848	5.547537903	63.94797053	0.666666667	CRA004775;PRJNA827465;Yang2025_Silkie_Wencha	0.866556281
ENSGALG00010014427	TMEM267	3	-0.064736985	0.11659635	-0.555223084	0.578742081	0.015021488	3.077789507	35.01829818	0.666666667	CRA004775;PRJNA827465;Yang2025_Silkie_Wencha	0.866556281
ENSGALG00010017545	DICER1	3	0.052775255	0.095271923	0.553943426	0.579617564	0.007719795	2.533667182	21.13151612	0.666666667	CRA004775;PRJNA827465;Yang2025_Silkie_Wencha	0.866556281
ENSGALG00010015038	PCSK5	3	0.04525076	0.08160467	0.554511888	0.579228572	0	0.691255053	0	1	CRA004775;PRJNA827465;Yang2025_Silkie_Wencha	0.866556281
ENSGALG00010021658	STRADA	3	-0.039396446	0.071118093	-0.553958134	0.579607498	0.005404153	3.06501338	34.74743004	0.666666667	CRA004775;PRJNA827465;Yang2025_Silkie_Wencha	0.866556281
ENSGALG00010026643	YWHAH	3	0.037421354	0.067339577	0.555711162	0.578408325	0.001679445	2.199946804	9.088710842	0.666666667	CRA004775;PRJNA827465;Yang2025_Silkie_Wencha	0.866556281
ENSGALG00010029611	YWHAG	3	-0.031419374	0.056691251	-0.554219041	0.579428949	0	1.72460925	0	0.666666667	CRA004775;PRJNA827465;Yang2025_Silkie_Wencha	0.866556281
ENSGALG00010027003	SEPTIN2L	3	0.017759361	0.032011715	0.554776943	0.57904724	0	1.339352263	0	0.666666667	CRA004775;PRJNA827465;Yang2025_Silkie_Wencha	0.866556281
ENSGALG00010005573	CHST9	3	0.146105005	0.263866899	0.553707215	0.579779237	0.132958556	6.186896911	67.67361686	0.666666667	CRA004775;PRJNA827465;Yang2025_Silkie_Wencha	0.866572302
ENSGALG00010013230	ENSGALG0001001323	3	0.097708986	0.17658333	0.553471793	0.57994039	0.058549997	5.875218974	65.9587156	0.666666667	CRA004775;PRJNA827465;Yang2025_Silkie_Wencha	0.866572302
ENSGALG00010001483	ENSGALG0001000148	2	0.096759607	0.17482152	0.553476557	0.579937129	0	0.026918923	0	1	PRJNA827465;Yang2025_Silkie_Wenchang	0.866572302
ENSGALG00010025449	ENSGALG0001002544	3	0.078396624	0.141660699	0.553410166	0.579982579	0.040897291	8.318119387	75.9561037	0.666666667	CRA004775;PRJNA827465;Yang2025_Silkie_Wencha	0.866572302
ENSGALG00010010891	POLR3H	3	-0.069826582	0.126167649	-0.553442835	0.579960214	0.026260077	4.398399883	54.5289184	0.666666667	CRA004775;PRJNA827465;Yang2025_Silkie_Wencha	0.866572302
ENSGALG00010027212	IRF6	3	0.166666204	0.292335105	0.553016731	0.580251953	0	1.35939826	0	0.666666667	CRA004775;PRJNA827465;Yang2025_Silkie_Wencha	0.866883175
ENSGALG00010023617	ENSGALG0001002361	3	-0.15781265	0.28566995	-0.552535015	0.580581852	0.145218391	4.940998745	59.52235361	0.666666667	CRA004775;PRJNA827465;Yang2025_Silkie_Wencha	0.866907391
ENSGALG00010029630	RAD51D	3	-0.144676664	0.261879118	-0.552455899	0.580636042	0.164578574	10.93940767	81.71747447	0.666666667	CRA004775;PRJNA827465;Yang2025_Silkie_Wencha	0.866907391
ENSGALG00010015624	ITGA1	3	0.10301253	0.186457398	0.552472207	0.580624871	0.069917367	7.173029992	72.11778004	0.666666667	CRA004775;PRJNA827465;Yang2025_Silkie_Wencha	0.866907391
ENSGALG00010010593	EIF3L	3	-0.059710561	0.108061376	-0.552561546	0.580563679	0.022062035	5.443123286	63.25638986	0.666666667	CRA004775;PRJNA827465;Yang2025_Silkie_Wencha	0.866907391
ENSGALG00010027919	TSEN2	3	0.052184186	0.094426162	0.552645416	0.580506236	0	0.711440592	0	0.666666667	CRA004775;PRJNA827465;Yang2025_Silkie_Wencha	0.866907391
ENSGALG00010005441	UBE2V2	3	-0.05025843	0.090933546	-0.55269405	0.580472929	0.009869598	2.921234817	31.53580164	0.666666667	CRA004775;PRJNA827465;Yang2025_Silkie_Wencha	0.866907391
ENSGALG00010028541	ENSGALG0001002854	3	0.17210112	0.311849258	0.551872162	0.581035531	0	1.407944678	0	0.666666667	CRA004775;PRJNA827465;Yang2025_Silkie_Wencha	0.867153427
ENSGALG00010022550	TRIM45	3	-0.147130736	0.26665338	-0.551767751	0.581107484	0.163741136	9.017164403	77.82007835	0.666666667	CRA004775;PRJNA827465;Yang2025_Silkie_Wencha	0.867153427
ENSGALG00010027279	F2RL3	3	-0.118723249	0.215117071	-0.551900618	0.581016445	0.067981259	4.022523508	50.27996739	0.666666667	CRA004775;PRJNA827465;Yang2025_Silkie_Wencha	0.867153427
ENSGALG00010011729	FAM129A	3	0.042243866	0.076560258	0.551772776	0.581104041	0	0.269827167	0	0.666666667	CRA004775;PRJNA827465;Yang2025_Silkie_Wencha	0.867153427
ENSGALG00010020117	AKAP13	3	-0.032908564	0.059606427	-0.552097568	0.580881508	0	1.633779006	0	1	CRA004775;PRJNA827465;Yang2025_Silkie_Wencha	0.867153427
ENSGALG00010003879	CASP6	3	-0.126808289	0.229859675	-0.551676968	0.581169693	0.125710802	11.93111773	83.23711118	0.666666667	CRA004775;PRJNA827465;Yang2025_Silkie_Wencha	0.867154737
ENSGALG00010012060	ENSGALG0001001206	3	0.063645254	0.115388173	0.551575194	0.581239436	0	0.365888443	0	0.666666667	CRA004775;PRJNA827465;Yang2025_Silkie_Wencha	0.867167288
ENSGALG00010016658	ZAR1	3	0.21722344	0.393930361	0.551425991	0.581341688	0.085747983	2.447307225	18.27752645	0.666666667	CRA004775;PRJNA827465;Yang2025_Silkie_Wencha	0.867203236
ENSGALG00010022825	SS18L1	3	0.210539025	0.381853284	0.551361045	0.581386199	0.324964742	8.939198663	77.62662991	0.666666667	CRA004775;PRJNA827465;Yang2025_Silkie_Wencha	0.867203236
ENSGALG00010009486	B4GALT1	3	-0.118250403	0.214595045	-0.551039766	0.581606415	0.114754584	21.66931582	90.77035927	0.666666667	CRA004775;PRJNA827465;Yang2025_Silkie_Wencha	0.867440201
ENSGALG00010020525	ENSGALG0001002052	3	-0.309382143	0.561741458	-0.550755402	0.58180136	0.542258174	4.683340448	57.29543854	0.666666667	CRA004775;PRJNA827465;Yang2025_Silkie_Wencha	0.867476027
ENSGALG00010010346	TFB2M	3	-0.08194044	0.148783454	-0.550736242	0.581814497	0.04122109	5.379341842	62.82073052	0.666666667	CRA004775;PRJNA827465;Yang2025_Silkie_Wencha	0.867476027
ENSGALG00010015374	LIMS1	3	-0.044805462	0.081351445	-0.550764183	0.58179534	0.001505422	2.136748746	6.399851478	0.666666667	CRA004775;PRJNA827465;Yang2025_Silkie_Wencha	0.867476027
ENSGALG00010025490	ENSGALG0001002549	3	0.545898558	0.99160415	0.550520647	0.581962319	2.513657637	14.25398169	85.96883283	0.666666667	CRA004775;PRJNA827465;Yang2025_Silkie_Wencha	0.867556515
ENSGALG00010023356	ENSGALG0001002335	3	0.062256213	0.113094716	0.550478531	0.581991198	0	0.412982979	0	0.666666667	CRA004775;PRJNA827465;Yang2025_Silkie_Wencha	0.867556515
ENSGALG00010025565	ENSGALG0001002556	3	-0.129650394	0.235824733	-0.549774372	0.582474139	0.115691109	8.604572957	76.7565455	0.666666667	CRA004775;PRJNA827465;Yang2025_Silkie_Wencha	0.868184888
ENSGALG00010004256	ENSGALG0001000425	2	0.341174527	0.621471114	0.548978897	0.583019932	0	0.007374869	0	1	PRJNA827465;Yang2025_Silkie_Wenchang	0.868305422
ENSGALG00010027718	ENSGALG0001002771	3	-0.179356486	0.326883164	-0.548686828	0.583220387	0.182766167	4.716001815	57.59119528	0.666666667	CRA004775;PRJNA827465;Yang2025_Silkie_Wencha	0.868305422
ENSGALG00010004728	ENSGALG0001000472	3	0.132211843	0.240577939	0.549559344	0.582621651	0.12157353	8.795499474	77.26109807	0.666666667	CRA004775;PRJNA827465;Yang2025_Silkie_Wencha	0.868305422
ENSGALG00010023819	BIN2	3	-0.117651089	0.214568749	-0.548314188	0.583476187	0.047213015	2.910352788	31.27980883	0.666666667	CRA004775;PRJNA827465;Yang2025_Silkie_Wencha	0.868305422
ENSGALG00010013306	ARSB	3	-0.102470604	0.186543738	-0.549311412	0.582791757	0.052500246	4.407804744	54.6259393	0.666666667	CRA004775;PRJNA827465;Yang2025_Silkie_Wencha	0.868305422
ENSGALG00010011128	GCNT4	3	0.094499543	0.172303579	0.548447942	0.583384365	0	1.28762872	0	0.666666667	CRA004775;PRJNA827465;Yang2025_Silkie_Wencha	0.868305422
ENSGALG00010022557	WNT1	3	0.094264409	0.171653567	0.549154969	0.582899105	0	1.714578154	0	0.666666667	CRA004775;PRJNA827465;Yang2025_Silkie_Wencha	0.868305422
ENSGALG00010020304	ZFP36L2	3	0.089500703	0.163014543	0.549035082	0.582981374	0.047153003	4.880097787	59.0172467	0.666666667	CRA004775;PRJNA827465;Yang2025_Silkie_Wencha	0.868305422
ENSGALG00010000529	STIP1	3	0.086195716	0.157045209	0.548859254	0.583102042	0.056661705	16.80489579	88.09870632	0.666666667	CRA004775;PRJNA827465;Yang2025_Silkie_Wencha	0.868305422
ENSGALG00010006657	PDS5B	3	0.081803799	0.149068776	0.548765485	0.5831664	0.04122818	5.317994702	62.39183918	0.666666667	CRA004775;PRJNA827465;Yang2025_Silkie_Wencha	0.868305422
ENSGALG00010002391	REPS2	3	0.07087848	0.129199236	0.548632269	0.583257836	0.026665408	4.823290167	58.5298043	0.666666667	CRA004775;PRJNA827465;Yang2025_Silkie_Wencha	0.868305422
ENSGALG00010016874	ADAMTSL3	3	0.06135866	0.111746556	0.549087705	0.582945262	0.014316983	3.259909998	38.64861295	0.666666667	CRA004775;PRJNA827465;Yang2025_Silkie_Wencha	0.868305422
ENSGALG00010020771	LINGO1	3	0.054759716	0.099775976	0.548826662	0.583124411	0	0.402953137	0	1	CRA004775;PRJNA827465;Yang2025_Silkie_Wencha	0.868305422
ENSGALG00010029609	METTL27	3	0.050721873	0.09248571	0.548429305	0.583397159	0	1.336928255	0	0.666666667	CRA004775;PRJNA827465;Yang2025_Silkie_Wencha	0.868305422
ENSGALG00010019355	ENTPD4	3	-0.031382659	0.057234617	-0.54831605	0.583474909	0	1.785867424	0	0.666666667	CRA004775;PRJNA827465;Yang2025_Silkie_Wencha	0.868305422
ENSGALG00010024519	MIEN1	3	0.067831754	0.123740642	0.548176844	0.58357048	0.019798063	3.42398489	41.58852727	0.666666667	CRA004775;PRJNA827465;Yang2025_Silkie_Wencha	0.868354349
ENSGALG00010022030	ZNF341	3	0.044274722	0.080780743	0.548085098	0.583633472	0.006069002	2.836325543	29.48623246	0.666666667	CRA004775;PRJNA827465;Yang2025_Silkie_Wencha	0.868356694
ENSGALG00010023024	SUGT1	3	-0.086350732	0.157620178	-0.547840597	0.58380136	0.052269746	7.995578047	74.98617376	0.666666667	CRA004775;PRJNA827465;Yang2025_Silkie_Wencha	0.868423717
ENSGALG00010028169	DNLZ	3	0.05746639	0.104895585	0.547843739	0.583799203	0	1.898018233	0	0.666666667	CRA004775;PRJNA827465;Yang2025_Silkie_Wencha	0.868423717
ENSGALG00010014002	ZDHHC18	3	0.064141629	0.117142664	0.547551389	0.583999976	0	0.110730238	0	0.666666667	CRA004775;PRJNA827465;Yang2025_Silkie_Wencha	0.868445063
ENSGALG00010004047	PDE7A	3	0.046981966	0.085789489	0.547642449	0.583937436	0.005519999	2.418570937	17.30653962	0.666666667	CRA004775;PRJNA827465;Yang2025_Silkie_Wencha	0.868445063
ENSGALG00010027358	RNF126	3	0.023007598	0.042012445	0.547637566	0.58394079	0	1.93183253	0	0.666666667	CRA004775;PRJNA827465;Yang2025_Silkie_Wencha	0.868445063
ENSGALG00010018536	ENSGALG0001001853	3	0.257833696	0.471041162	0.547369778	0.584124714	0.546215506	12.23104224	83.64816373	0.666666667	CRA004775;PRJNA827465;Yang2025_Silkie_Wencha	0.868539209
ENSGALG00010009224	ENSGALG0001000922	3	-0.26405721	0.482676094	-0.547069169	0.584331213	0.517639598	8.622938376	76.80605018	0.666666667	CRA004775;PRJNA827465;Yang2025_Silkie_Wencha	0.868578431
ENSGALG00010015970	THPO	3	-0.110898804	0.202716663	-0.547063093	0.584335387	0	0.070837326	0	1	CRA004775;PRJNA827465;Yang2025_Silkie_Wencha	0.868578431
ENSGALG00010009068	AGO2	3	0.041967884	0.076691145	0.547232455	0.584219042	0	0.80872474	0	1	CRA004775;PRJNA827465;Yang2025_Silkie_Wencha	0.868578431
ENSGALG00010001367	ENSGALG0001000136	3	-0.281719476	0.515378246	-0.546626634	0.584635267	0.529483773	6.128131338	67.36362376	0.666666667	CRA004775;PRJNA827465;Yang2025_Silkie_Wencha	0.868699156
ENSGALG00010016614	DNAJC19	3	0.226424129	0.414685965	0.546013485	0.585056667	0.457971352	27.77511929	92.79931085	0.666666667	CRA004775;PRJNA827465;Yang2025_Silkie_Wencha	0.868699156
ENSGALG00010025301	MORN3	3	-0.224220349	0.41082349	-0.545782689	0.585215324	0.40114317	10.833866	81.53936923	0.666666667	CRA004775;PRJNA827465;Yang2025_Silkie_Wencha	0.868699156
ENSGALG00010012804	ENSGALG0001001280	3	0.152773767	0.279821001	0.545969626	0.585086816	0.185139291	11.25282252	82.22668139	0.666666667	CRA004775;PRJNA827465;Yang2025_Silkie_Wencha	0.868699156
ENSGALG00010001330	ENSGALG0001000133	3	0.14992103	0.274658669	0.545845019	0.585172475	0.196266073	16.36348776	87.77766678	0.666666667	CRA004775;PRJNA827465;Yang2025_Silkie_Wencha	0.868699156
ENSGALG00010027093	SLMAP	3	0.130088686	0.238333038	0.54582733	0.585184635	0.148263703	20.39824192	90.19523345	0.666666667	CRA004775;PRJNA827465;Yang2025_Silkie_Wencha	0.868699156
ENSGALG00010023374	ENSGALG0001002337	3	0.093089662	0.170544473	0.545838049	0.585177266	0.002438187	2.04591192	2.244080959	0.666666667	CRA004775;PRJNA827465;Yang2025_Silkie_Wencha	0.868699156
ENSGALG00010010393	FANCC	3	-0.088520588	0.162071823	-0.546181229	0.584941368	0.025107368	2.886977948	30.72340572	0.666666667	CRA004775;PRJNA827465;Yang2025_Silkie_Wencha	0.868699156
ENSGALG00010014783	ENSGALG0001001478	3	-0.073580029	0.134634957	-0.546510344	0.584711949	0.04050755	10.9334934	81.70758488	0.666666667	CRA004775;PRJNA827465;Yang2025_Silkie_Wencha	0.868699156
ENSGALG00010015390	CCNB3	3	-0.047703982	0.087291217	-0.546492345	0.584727549	0	0.90905776	0	0.666666667	CRA004775;PRJNA827465;Yang2025_Silkie_Wencha	0.868699156
ENSGALG00010026237	KLHL22	3	-0.04161644	0.076250519	-0.545785659	0.585213283	0.00686381	3.264617099	38.73707271	0.666666667	CRA004775;PRJNA827465;Yang2025_Silkie_Wencha	0.868699156
ENSGALG00010022497	CORO1C	3	-0.031660786	0.057909604	-0.54672772	0.584565807	0.001797206	2.30605637	13.27185121	0.666666667	CRA004775;PRJNA827465;Yang2025_Silkie_Wencha	0.868699156
ENSGALG00010029581	DGKE	3	-0.02634987	0.04823154	-0.54632031	0.584845778	0	0.961651078	0	0.666666667	CRA004775;PRJNA827465;Yang2025_Silkie_Wencha	0.868699156
ENSGALG00010023218	SSH3	3	0.13113638	0.240410192	0.545469301	0.585430789	0.140871916	11.43693548	82.51279808	0.666666667	CRA004775;PRJNA827465;Yang2025_Silkie_Wencha	0.868745374
ENSGALG00010024889	SLC2A4RG	3	-0.115586677	0.211882543	-0.545522418	0.585394266	0.059478981	3.56623431	43.91843536	0.666666667	CRA004775;PRJNA827465;Yang2025_Silkie_Wencha	0.868745374
ENSGALG00010023802	ENSGALG0001002380	3	-0.084668538	0.155199435	-0.545546691	0.585377577	0	1.33868596	0	0.666666667	CRA004775;PRJNA827465;Yang2025_Silkie_Wencha	0.868745374
ENSGALG00010019376	ENSGALG0001001937	3	0.176362055	0.323376309	0.545377166	0.585494142	0.103854	2.967546819	32.60426466	0.666666667	CRA004775;PRJNA827465;Yang2025_Silkie_Wencha	0.868748208
ENSGALG00010015086	FOXN3	3	0.080347702	0.147351209	0.545280239	0.585560793	0.037509498	4.690756849	57.36295731	0.666666667	CRA004775;PRJNA827465;Yang2025_Silkie_Wencha	0.868755934
ENSGALG00010014300	ELFN2	3	0.238861896	0.438270005	0.545010823	0.585746074	0.218081459	3.104037112	35.56778067	0.666666667	CRA004775;PRJNA827465;Yang2025_Silkie_Wencha	0.868939643
ENSGALG00010026960	SEC61A1	3	-0.122459783	0.224757666	-0.544852531	0.585854946	0.135280622	26.23482957	92.37654663	0.666666667	CRA004775;PRJNA827465;Yang2025_Silkie_Wencha	0.869009975
ENSGALG00010029702	CORO6	3	-0.076250433	0.140025515	-0.544546709	0.586065315	0.004392603	2.125539761	5.906253248	0.666666667	CRA004775;PRJNA827465;Yang2025_Silkie_Wencha	0.869152606
ENSGALG00010021575	ADGRL2	3	-0.057498428	0.105591986	-0.544534017	0.586074047	0.0161408	3.607937749	44.56667107	0.666666667	CRA004775;PRJNA827465;Yang2025_Silkie_Wencha	0.869152606
ENSGALG00010001868	ENSGALG0001000186	3	0.064224777	0.118001408	0.544271278	0.586254809	0.017855415	3.386222402	40.93713399	0.666666667	CRA004775;PRJNA827465;Yang2025_Silkie_Wencha	0.869329495
ENSGALG00010011056	ENSGALG0001001105	3	0.193545322	0.355760734	0.544032278	0.586419261	0	1.38236298	0	0.666666667	CRA004775;PRJNA827465;Yang2025_Silkie_Wencha	0.869482166
ENSGALG00010027970	GRIN3B	3	0.171873807	0.316482219	0.543075715	0.587077673	0.279484387	31.00782735	93.55001569	0.666666667	CRA004775;PRJNA827465;Yang2025_Silkie_Wencha	0.86955283
ENSGALG00010011067	TMTC2	3	-0.141328349	0.260146892	-0.543263644	0.586948292	0.178620867	19.57789075	89.78439493	0.666666667	CRA004775;PRJNA827465;Yang2025_Silkie_Wencha	0.86955283
ENSGALG00010009574	TAF1C	3	-0.132513791	0.243811227	-0.543509799	0.586778846	0.129649358	7.442639117	73.12781167	0.666666667	CRA004775;PRJNA827465;Yang2025_Silkie_Wencha	0.86955283
ENSGALG00010017472	LZTFL1	3	-0.108486885	0.199535622	-0.543696831	0.586650114	0.093227693	11.94469452	83.2561645	0.666666667	CRA004775;PRJNA827465;Yang2025_Silkie_Wencha	0.86955283
ENSGALG00010009831	N6AMT1	3	-0.079829023	0.146833457	-0.543670525	0.586668219	0.040608308	5.628163366	64.46442881	0.666666667	CRA004775;PRJNA827465;Yang2025_Silkie_Wencha	0.86955283
ENSGALG00010008793	C11orf54	3	-0.066246703	0.121985672	-0.543069541	0.587081923	0.021922621	3.885228697	48.52297881	0.666666667	CRA004775;PRJNA827465;Yang2025_Silkie_Wencha	0.86955283
ENSGALG00010005242	CCDC80	3	-0.049364011	0.090836834	-0.543436054	0.586829607	0	0.058750667	0	1	CRA004775;PRJNA827465;Yang2025_Silkie_Wencha	0.86955283
ENSGALG00010027713	ENSGALG0001002771	3	0.048966003	0.090148814	0.543168577	0.58701374	0	0.274030817	0	1	CRA004775;PRJNA827465;Yang2025_Silkie_Wencha	0.86955283
ENSGALG00010002357	RAB31	3	-0.046092314	0.084843903	-0.543260181	0.586950676	0.003700451	2.335260607	14.35645368	0.666666667	CRA004775;PRJNA827465;Yang2025_Silkie_Wencha	0.86955283
ENSGALG00010027320	MON1A	3	0.044703157	0.082299985	0.543173336	0.587010463	0.003543049	2.307053173	13.30932362	0.666666667	CRA004775;PRJNA827465;Yang2025_Silkie_Wencha	0.86955283
ENSGALG00010028115	CACTIN	3	0.094665818	0.174386718	0.542849934	0.58723313	0.07536482	18.29728508	89.06941663	0.666666667	CRA004775;PRJNA827465;Yang2025_Silkie_Wencha	0.869644762
ENSGALG00010018089	TASP1	3	-0.074952257	0.138152492	-0.542532789	0.587451526	0.031943237	4.707228797	57.51215659	0.666666667	CRA004775;PRJNA827465;Yang2025_Silkie_Wencha	0.869644762
ENSGALG00010009127	SCOC	3	-0.072369105	0.133369577	-0.542620785	0.587390925	0.040265295	12.20103152	83.60279434	0.666666667	CRA004775;PRJNA827465;Yang2025_Silkie_Wencha	0.869644762
ENSGALG00010010921	ENSGALG0001001092	3	0.067509145	0.124390715	0.542718517	0.587323623	0.019033942	3.124627828	35.992377	0.666666667	CRA004775;PRJNA827465;Yang2025_Silkie_Wencha	0.869644762
ENSGALG00010024939	COQ10A	3	0.066757994	0.12303048	0.542613452	0.587395975	0.007521525	2.349859542	14.88852996	0.666666667	CRA004775;PRJNA827465;Yang2025_Silkie_Wencha	0.869644762
ENSGALG00010022551	GPER1	2	0.203189448	0.374971202	0.541880142	0.587901077	0.051523701	1.195559397	16.35714609	0.5	PRJNA827465;Yang2025_Silkie_Wenchang	0.869812793
ENSGALG00010022765	KRT12	2	0.183077544	0.337674607	0.542203601	0.587678255	0	0.002071679	0	1	PRJNA827465;Yang2025_Silkie_Wenchang	0.869812793
ENSGALG00010029155	ENSGALG0001002915	3	0.10173102	0.18775486	0.541828957	0.587936342	0.050011166	4.189724902	52.26416897	0.666666667	CRA004775;PRJNA827465;Yang2025_Silkie_Wencha	0.869812793
ENSGALG00010014226	SLC16A7	3	-0.091440309	0.168710186	-0.541996372	0.587821005	0.033804492	3.238733634	38.24746872	0.666666667	CRA004775;PRJNA827465;Yang2025_Silkie_Wencha	0.869812793
ENSGALG00010015198	NME5	3	-0.081025317	0.149443407	-0.542180605	0.587694095	0.024902682	3.147746041	36.46247271	0.666666667	CRA004775;PRJNA827465;Yang2025_Silkie_Wencha	0.869812793
ENSGALG00010004503	ENSGALG0001000450	3	0.061238879	0.113015856	0.541860947	0.587914302	0	1.127879595	0	0.666666667	CRA004775;PRJNA827465;Yang2025_Silkie_Wencha	0.869812793
ENSGALG00010022783	STAG1	3	-0.023258992	0.042940716	-0.541653568	0.588057182	0	1.26578079	0	0.666666667	CRA004775;PRJNA827465;Yang2025_Silkie_Wencha	0.869812793
ENSGALG00010021644	TARDBP	3	0.020479093	0.037805602	0.541694678	0.588028857	0	1.863204301	0	0.666666667	CRA004775;PRJNA827465;Yang2025_Silkie_Wencha	0.869812793
ENSGALG00010001167	SMC4	3	0.109717051	0.202594761	0.541559171	0.588122225	0.076032195	6.078751252	67.00850564	0.666666667	CRA004775;PRJNA827465;Yang2025_Silkie_Wencha	0.869818006
ENSGALG00010003061	ENSGALG0001000306	3	0.31115656	0.57561196	0.540566531	0.588806392	0.705236767	7.630940003	73.79090913	0.666666667	CRA004775;PRJNA827465;Yang2025_Silkie_Wencha	0.87028781
ENSGALG00010027879	ENSGALG0001002787	3	0.308767427	0.571763089	0.540026862	0.589178508	0.893156172	47.27586802	95.76951184	0.666666667	CRA004775;PRJNA827465;Yang2025_Silkie_Wencha	0.87028781
ENSGALG00010017676	SPON2	3	-0.223981629	0.414684351	-0.540125589	0.589110425	0.45793529	23.15812197	91.36372111	0.666666667	CRA004775;PRJNA827465;Yang2025_Silkie_Wencha	0.87028781
ENSGALG00010026916	ENSGALG0001002691	3	0.18799312	0.347927253	0.540323066	0.588974254	0.274204404	8.59664109	76.73509945	0.666666667	CRA004775;PRJNA827465;Yang2025_Silkie_Wencha	0.87028781
ENSGALG00010022120	FOXL3	2	-0.142582615	0.263820958	-0.540452191	0.588885224	0	0.002718547	0	1	PRJNA827465;Yang2025_Silkie_Wenchang	0.87028781
ENSGALG00010008312	ZBTB18	3	0.103948254	0.192310871	0.540521988	0.588837101	0.079059793	9.225787801	78.3216345	0.666666667	CRA004775;PRJNA827465;Yang2025_Silkie_Wencha	0.87028781
ENSGALG00010029314	MED13	3	0.099330292	0.183760362	0.540542537	0.588822934	0.076973461	9.316085182	78.53175491	0.666666667	CRA004775;PRJNA827465;Yang2025_Silkie_Wencha	0.87028781
ENSGALG00010024153	KCNQ1	3	-0.088455811	0.163738396	-0.540226438	0.589040882	0.014311573	2.398095197	16.60047515	0.666666667	CRA004775;PRJNA827465;Yang2025_Silkie_Wencha	0.87028781
ENSGALG00010013116	TARS	3	-0.072136776	0.133404657	-0.540736568	0.58868917	0.013456621	2.648518213	24.48607715	0.666666667	CRA004775;PRJNA827465;Yang2025_Silkie_Wencha	0.87028781
ENSGALG00010022552	GPR155	3	0.0602295	0.111452161	0.540406745	0.588916557	0.025729519	7.836005046	74.4767903	0.666666667	CRA004775;PRJNA827465;Yang2025_Silkie_Wencha	0.87028781
ENSGALG00010024974	CD44	3	0.047858837	0.088615619	0.540072253	0.589147205	0	0.564801221	0	0.666666667	CRA004775;PRJNA827465;Yang2025_Silkie_Wencha	0.87028781
ENSGALG00010018533	PCYOX1	3	-0.04029446	0.074532915	-0.54062638	0.588765131	0	0.874323096	0	0.666666667	CRA004775;PRJNA827465;Yang2025_Silkie_Wencha	0.87028781
ENSGALG00010018060	CASKIN1	3	-0.170738572	0.316239565	-0.539902625	0.589264187	0.203215731	7.10848663	71.86461614	0.666666667	CRA004775;PRJNA827465;Yang2025_Silkie_Wencha	0.870318401
ENSGALG00010025992	MMP11	3	0.142647496	0.264250932	0.539818328	0.589322326	0.132111261	5.892249707	66.05710723	0.666666667	CRA004775;PRJNA827465;Yang2025_Silkie_Wencha	0.870318401
ENSGALG00010000341	EWSR1	3	0.165083943	0.306105973	0.53930324	0.589677634	0.260328969	31.49614105	93.65001574	0.666666667	CRA004775;PRJNA827465;Yang2025_Silkie_Wencha	0.870575699
ENSGALG00010029744	CTNS	3	-0.097131821	0.180107893	-0.53929797	0.589681269	0.07572088	10.09347144	80.18521168	0.666666667	CRA004775;PRJNA827465;Yang2025_Silkie_Wencha	0.870575699
ENSGALG00010017822	VWA3A	2	0.09157888	0.169755511	0.53947515	0.589559039	0.004509666	1.079613882	7.374292154	0.5	PRJNA827465;Yang2025_Silkie_Wenchang	0.870575699
ENSGALG00010019495	EMP2	3	0.126603895	0.234963997	0.538822527	0.590009318	0.147715788	22.67870253	91.18115334	0.666666667	CRA004775;PRJNA827465;Yang2025_Silkie_Wencha	0.870862539
ENSGALG00010005871	EGLN3	3	-0.077941046	0.144637456	-0.538871796	0.58997532	0.034081685	5.087384433	60.68706766	0.666666667	CRA004775;PRJNA827465;Yang2025_Silkie_Wencha	0.870862539
ENSGALG00010005489	G2E3	3	-0.073032991	0.13556043	-0.538748594	0.590060339	0.032672962	6.180258045	67.63889168	0.666666667	CRA004775;PRJNA827465;Yang2025_Silkie_Wencha	0.870862539
ENSGALG00010019871	TAFA1	2	-0.404994013	0.752276738	-0.538357751	0.590330088	0.827201783	3.619109579	72.36889412	0.5	PRJNA827465;Yang2025_Silkie_Wenchang	0.870996255
ENSGALG00010007673	FHDC1	3	0.074170649	0.137774144	0.538349554	0.590335746	0.007278727	2.241389461	10.76963488	0.666666667	CRA004775;PRJNA827465;Yang2025_Silkie_Wencha	0.870996255
ENSGALG00010029864	SLC16A3	3	0.066475686	0.123458903	0.538443844	0.590270664	0	0.253588533	0	0.666666667	CRA004775;PRJNA827465;Yang2025_Silkie_Wencha	0.870996255
ENSGALG00010004420	ENSGALG0001000442	3	-0.112464591	0.209020618	-0.538055012	0.59053907	0	1.003801145	0	0.666666667	CRA004775;PRJNA827465;Yang2025_Silkie_Wencha	0.871023566
ENSGALG00010018488	ENSGALG0001001848	3	-0.09134199	0.169739692	-0.538129818	0.590487428	0.04024657	3.758369996	46.7854415	0.666666667	CRA004775;PRJNA827465;Yang2025_Silkie_Wencha	0.871023566
ENSGALG00010001205	CEP192	3	-0.046716835	0.086797217	-0.538229644	0.590418517	0.009676112	3.529185423	43.32969905	0.666666667	CRA004775;PRJNA827465;Yang2025_Silkie_Wencha	0.871023566
ENSGALG00010011609	ENSGALG0001001160	2	0.232030669	0.431739356	0.537432285	0.590969047	0.122791554	1.394916442	28.31111816	0.5	PRJNA827465;Yang2025_Silkie_Wenchang	0.871053488
ENSGALG00010008071	SMIM18	3	0.184375329	0.343415586	0.536886899	0.591345741	0.260711367	7.743158717	74.17074771	0.666666667	CRA004775;PRJNA827465;Yang2025_Silkie_Wencha	0.871053488
ENSGALG00010008980	ENSGALG0001000898	3	-0.162796977	0.302903161	-0.537455523	0.590953	0.115279628	3.433223968	41.74571718	0.666666667	CRA004775;PRJNA827465;Yang2025_Silkie_Wencha	0.871053488
ENSGALG00010015706	ENSGALG0001001570	3	0.134672464	0.250857485	0.536848496	0.59137227	0.126151775	6.364866406	68.5775023	0.666666667	CRA004775;PRJNA827465;Yang2025_Silkie_Wencha	0.871053488
ENSGALG00010026775	BTG2	3	-0.12955437	0.241356159	-0.536776731	0.591421847	0.116419356	9.819224679	79.63179309	0.666666667	CRA004775;PRJNA827465;Yang2025_Silkie_Wencha	0.871053488

ENSGALG00010015261	IQCG	3	-0.084953477	0.158014344	-0.537631426	0.59083153	0	1.308245265	0	0.666666667	CRA004775;PRJNA827465;Yang2025_Silkie_Wencha	0.871053488
ENSGALG00010024884	ZBTB46	3	-0.07540828	0.140395306	-0.537113968	0.591188893	0.039415294	7.44024218	73.11915457	0.666666667	CRA004775;PRJNA827465;Yang2025_Silkie_Wencha	0.871053488
ENSGALG00010027323	ISG20L2	3	-0.057191303	0.106342987	-0.537800422	0.590714841	0.017693546	4.1077747	51.31183802	0.666666667	CRA004775;PRJNA827465;Yang2025_Silkie_Wencha	0.871053488
ENSGALG00010021031	DDX54	3	-0.055844436	0.103815325	-0.537920928	0.590631639	0.018235024	4.680640368	57.27080393	0.666666667	CRA004775;PRJNA827465;Yang2025_Silkie_Wencha	0.871053488
ENSGALG00010021378	KIF11	3	-0.055484234	0.103206106	-0.537606114	0.590849008	0.004073684	2.241504335	10.7742078	0.666666667	CRA004775;PRJNA827465;Yang2025_Silkie_Wencha	0.871053488
ENSGALG00010017713	ALKBH5	3	-0.048865498	0.0909943	-0.537017127	0.591255784	0.010257226	3.501584511	42.88300072	0.666666667	CRA004775;PRJNA827465;Yang2025_Silkie_Wencha	0.871053488
ENSGALG00010004137	CROT	3	-0.04250633	0.079123576	-0.537214471	0.591119477	0.007589228	3.206021032	37.61737743	0.666666667	CRA004775;PRJNA827465;Yang2025_Silkie_Wencha	0.871053488
ENSGALG00010024626	TSHZ3	3	0.042314394	0.07874366	0.537368895	0.591012825	0	0.646929172	0	0.666666667	CRA004775;PRJNA827465;Yang2025_Silkie_Wencha	0.871053488
ENSGALG00010009514	PRELID1	3	-0.039605573	0.073712181	-0.537300255	0.591060229	0	1.758716548	0	0.666666667	CRA004775;PRJNA827465;Yang2025_Silkie_Wencha	0.871053488
ENSGALG00010025188	PPFIA4	3	0.142610836	0.266077805	0.535974189	0.591976389	0.04139551	2.435816986	17.89202506	0.666666667	CRA004775;PRJNA827465;Yang2025_Silkie_Wencha	0.871144272
ENSGALG00010028495	LINGO3	3	-0.132181143	0.24657539	-0.536067864	0.591911649	0.077256594	3.472398362	42.4029218	0.666666667	CRA004775;PRJNA827465;Yang2025_Silkie_Wencha	0.871144272
ENSGALG00010029293	DNAH17	3	-0.113632341	0.211848029	-0.536386115	0.591691726	0.093100679	7.248394171	72.40768158	0.666666667	CRA004775;PRJNA827465;Yang2025_Silkie_Wencha	0.871144272
ENSGALG00010024807	PPIL3	3	-0.093329763	0.174024355	-0.536302882	0.591749239	0.05876719	5.947057566	66.36992365	0.666666667	CRA004775;PRJNA827465;Yang2025_Silkie_Wencha	0.871144272
ENSGALG00010018226	JAG1	3	0.049634296	0.09251229	0.536515699	0.591602189	0.009633131	2.9527519	32.26657479	1	CRA004775;PRJNA827465;Yang2025_Silkie_Wencha	0.871144272
ENSGALG00010020211	ADD3	3	-0.045261066	0.084425097	-0.536109139	0.591883124	0.005970103	2.557501157	21.79866685	0.666666667	CRA004775;PRJNA827465;Yang2025_Silkie_Wencha	0.871144272
ENSGALG00010008035	TDP2	3	-0.043573037	0.081295297	-0.535984715	0.591969114	0	0.60613246	0	0.666666667	CRA004775;PRJNA827465;Yang2025_Silkie_Wencha	0.871144272
ENSGALG00010023626	LOXL1	3	-0.03576242	0.066668772	-0.536419359	0.591668755	0	1.270315195	0	0.666666667	CRA004775;PRJNA827465;Yang2025_Silkie_Wencha	0.871144272
ENSGALG00010005216	ENSGALG0001000521	3	0.09602959	0.179325179	0.535505334	0.59230047	0.032999489	2.926095572	31.64953259	0.666666667	CRA004775;PRJNA827465;Yang2025_Silkie_Wencha	0.871396861
ENSGALG00010002939	HMGB2	3	-0.074731467	0.139520002	-0.535632641	0.592212465	0.036684626	6.117559885	67.30722645	0.666666667	CRA004775;PRJNA827465;Yang2025_Silkie_Wencha	0.871396861
ENSGALG00010022574	ITPRIPL2	3	-0.046264078	0.086400885	-0.535458387	0.592332925	0	0.345806914	0	1	CRA004775;PRJNA827465;Yang2025_Silkie_Wencha	0.871396861
ENSGALG00010004553	BATF	3	0.301359765	0.563479746	0.534819161	0.592774912	0.814560312	14.56049212	86.26420053	0.666666667	CRA004775;PRJNA827465;Yang2025_Silkie_Wencha	0.871821673
ENSGALG00010001486	WNT2	3	-0.131965849	0.246769735	-0.534773233	0.592806674	0.06453276	3.066933399	34.78828069	0.666666667	CRA004775;PRJNA827465;Yang2025_Silkie_Wencha	0.871821673
ENSGALG00010015384	TAF12	3	-0.039565218	0.073972184	-0.534866158	0.592742412	0.003838295	2.461048766	18.73383302	0.666666667	CRA004775;PRJNA827465;Yang2025_Silkie_Wencha	0.871821673
ENSGALG00010014813	SASH3	3	0.220516909	0.412516692	0.534564816	0.592950819	0.38043432	8.073175863	75.22660185	0.666666667	CRA004775;PRJNA827465;Yang2025_Silkie_Wencha	0.87185229
ENSGALG00010006178	RPS23	3	-0.077723203	0.145380701	-0.534618434	0.592913734	0	1.827997733	0	0.666666667	CRA004775;PRJNA827465;Yang2025_Silkie_Wencha	0.87185229
ENSGALG00010021375	TUBG1	3	-0.068256602	0.127767426	-0.53422538	0.593185612	0.036570832	8.49289955	76.45091658	0.666666667	CRA004775;PRJNA827465;Yang2025_Silkie_Wencha	0.872106828
ENSGALG00010000011	ND2	3	0.193882498	0.363270194	0.533714302	0.593539213	0.294811643	9.841628316	79.67815959	0.666666667	CRA004775;PRJNA827465;Yang2025_Silkie_Wencha	0.872451277
ENSGALG00010022356	TCFL5	3	0.162593377	0.304648348	0.533708383	0.593543308	0.179469283	6.536610642	69.40310339	0.666666667	CRA004775;PRJNA827465;Yang2025_Silkie_Wencha	0.872451277
ENSGALG00010016454	ANKS3	3	-0.044464743	0.083354621	-0.533440652	0.593728584	0.009618442	3.477312946	42.48432536	0.666666667	CRA004775;PRJNA827465;Yang2025_Silkie_Wencha	0.872632894
ENSGALG00010027120	ENSGALG0001002712	3	0.435993909	0.818966869	0.532370631	0.594469322	1.575415811	9.761520192	79.51138797	0.666666667	CRA004775;PRJNA827465;Yang2025_Silkie_Wencha	0.873049915
ENSGALG00010020642	ANKRD2	3	-0.311898157	0.585381633	-0.532811655	0.594163965	0.719505888	6.820510285	70.67668083	0.666666667	CRA004775;PRJNA827465;Yang2025_Silkie_Wencha	0.873049915
ENSGALG00010011487	MAN2B2	3	-0.098645779	0.185253652	-0.532490333	0.594386435	0.077731726	10.27530945	80.535866	0.666666667	CRA004775;PRJNA827465;Yang2025_Silkie_Wencha	0.873049915
ENSGALG00010024510	CNN3	3	-0.091099318	0.171137421	-0.532316762	0.594506624	0.07411588	32.4032933	93.82778787	0.666666667	CRA004775;PRJNA827465;Yang2025_Silkie_Wencha	0.873049915
ENSGALG00010010870	NOL6	3	-0.060313766	0.113314901	-0.532266855	0.594541185	0	0.814655721	0	0.666666667	CRA004775;PRJNA827465;Yang2025_Silkie_Wencha	0.873049915
ENSGALG00010020786	DDX19	3	0.055695638	0.104626626	0.532327577	0.594499136	0.023000147	7.338860568	72.74781308	0.666666667	CRA004775;PRJNA827465;Yang2025_Silkie_Wencha	0.873049915
ENSGALG00010023881	VSTM2B	3	-0.048011003	0.090120854	-0.532740216	0.594213423	0	1.717278295	0	0.666666667	CRA004775;PRJNA827465;Yang2025_Silkie_Wencha	0.873049915
ENSGALG00010014780	EVA1B	3	0.037661269	0.070761525	0.532228064	0.594568048	0	0.046736433	0	1	CRA004775;PRJNA827465;Yang2025_Silkie_Wencha	0.873049915
ENSGALG00010016849	WASF2	3	-0.027497295	0.051664117	-0.532231971	0.594565342	0.000689468	2.118003401	5.571445323	0.666666667	CRA004775;PRJNA827465;Yang2025_Silkie_Wencha	0.873049915
ENSGALG00010027356	ENSGALG0001002735	3	0.206648023	0.388429038	0.532009718	0.594719265	0.414894761	39.28405269	94.90887558	0.666666667	CRA004775;PRJNA827465;Yang2025_Silkie_Wencha	0.873181275
ENSGALG00010028506	SHD	2	0.275770588	0.518486161	0.531876468	0.594811556	0.2680512	1.82354315	45.16170348	0.5	PRJNA827465;Yang2025_Silkie_Wenchang	0.873195927
ENSGALG00010028154	ENSGALG0001002815	3	0.047525569	0.08936452	0.531816982	0.59485276	0	0.768498253	0	0.666666667	CRA004775;PRJNA827465;Yang2025_Silkie_Wencha	0.873195927
ENSGALG00010012497	PLCXD3	2	0.143219978	0.269396806	0.531632056	0.594980858	0	0.071907803	0	1	PRJNA827465;Yang2025_Silkie_Wenchang	0.873293299
ENSGALG00010015825	RUNX3	3	-0.190755732	0.358956277	-0.531417737	0.595129333	0	0.286681914	0	0.666666667	CRA004775;PRJNA827465;Yang2025_Silkie_Wencha	0.873420557
ENSGALG00010005597	ENSGALG0001000559	2	0.251757445	0.473979594	0.531156716	0.595310184	0	0.987031159	0	0.5	CRA004775;Yang2025_Silkie_Wenchang	0.873595298
ENSGALG00010023305	PLCB2	3	-0.136303014	0.256789671	-0.530796326	0.595559926	0.149144276	9.838619807	79.67194546	0.666666667	CRA004775;PRJNA827465;Yang2025_Silkie_Wencha	0.873689749
ENSGALG00010015494	CHRDL1	3	-0.113078634	0.213014125	-0.530850403	0.595522449	0.057441178	3.434506904	41.76747767	0.666666667	CRA004775;PRJNA827465;Yang2025_Silkie_Wencha	0.873689749
ENSGALG00010016767	SLAIN2	3	0.060126703	0.113251234	0.530914329	0.595478148	0.023693627	6.047650382	66.9293052	0.666666667	CRA004775;PRJNA827465;Yang2025_Silkie_Wencha	0.873689749
ENSGALG00010028486	ARHGAP30	3	0.116944638	0.220403453	0.530593494	0.595700505	0.083541532	5.14919283	61.15896091	0.666666667	CRA004775;PRJNA827465;Yang2025_Silkie_Wencha	0.87374446
ENSGALG00010000695	FIGNL1	3	-0.055534666	0.104670964	-0.530564193	0.595720815	0	1.773266548	0	0.666666667	CRA004775;PRJNA827465;Yang2025_Silkie_Wencha	0.87374446
ENSGALG00010016623	DNAJA3	3	0.094703192	0.178689818	0.529986503	0.596121289	0.074910522	13.90506121	85.61674796	0.666666667	CRA004775;PRJNA827465;Yang2025_Silkie_Wencha	0.874150477
ENSGALG00010001458	JAZF1	3	-0.044445584	0.083855585	-0.530025331	0.596094368	0.004770688	2.364651141	15.42092764	0.666666667	CRA004775;PRJNA827465;Yang2025_Silkie_Wencha	0.874150477
ENSGALG00010016287	BCL11B	3	0.2441075	0.460890572	0.529643075	0.596359423	0.36467441	4.680557071	57.27004351	0.666666667	CRA004775;PRJNA827465;Yang2025_Silkie_Wencha	0.874162425
ENSGALG00010015133	RFTN2	3	-0.108305253	0.204515752	-0.529569248	0.596410621	0.090294212	11.39499803	82.44843927	0.666666667	CRA004775;PRJNA827465;Yang2025_Silkie_Wencha	0.874162425
ENSGALG00010003295	IAH1	3	0.088830895	0.167679886	0.52976476	0.596275041	0.042614167	3.824388529	47.70405818	0.666666667	CRA004775;PRJNA827465;Yang2025_Silkie_Wencha	0.874162425
ENSGALG00010016624	MDH1	3	-0.072299208	0.136534949	-0.529528949	0.596438568	0.027549299	3.735080422	46.45362953	0.666666667	CRA004775;PRJNA827465;Yang2025_Silkie_Wencha	0.874162425
ENSGALG00010021000	NFKB2	3	-0.062821661	0.118613027	-0.529635427	0.596364727	0.025854503	5.582614633	64.17449293	0.666666667	CRA004775;PRJNA827465;Yang2025_Silkie_Wencha	0.874162425
ENSGALG00010008010	CERK	3	0.089566907	0.169203593	0.529344003	0.596566836	0.069024746	17.02085698	88.24971032	0.666666667	CRA004775;PRJNA827465;Yang2025_Silkie_Wencha	0.874259793
ENSGALG00010005165	METTL21C	3	0.21124008	0.39916911	0.529199468	0.596667086	0	0.272715946	0	1	CRA004775;PRJNA827465;Yang2025_Silkie_Wencha	0.874316087
ENSGALG00010012376	VGLL2	3	0.137747676	0.260567107	0.528645681	0.597051265	0.125412804	6.510211745	69.27903303	0.666666667	CRA004775;PRJNA827465;Yang2025_Silkie_Wencha	0.874336096
ENSGALG00010023401	LRRC23	3	0.104041058	0.196681531	0.528982347	0.596817696	0	0.602628381	0	0.666666667	CRA004775;PRJNA827465;Yang2025_Silkie_Wencha	0.874336096
ENSGALG00010029624	RASL10B	3	0.080246508	0.151796603	0.52864495	0.597051772	0.010888321	2.318548124	13.73912064	0.666666667	CRA004775;PRJNA827465;Yang2025_Silkie_Wencha	0.874336096
ENSGALG00010007108	MBOAT1	3	0.075080244	0.141962778	0.528872746	0.596893729	0.020589479	2.998073714	33.29049948	0.666666667	CRA004775;PRJNA827465;Yang2025_Silkie_Wencha	0.874336096
ENSGALG00010003442	SPCS3	3	-0.060382985	0.114209415	-0.528704093	0.597010738	0.024266747	5.588384366	64.21148101	0.666666667	CRA004775;PRJNA827465;Yang2025_Silkie_Wencha	0.874336096
ENSGALG00010021003	KAZN	3	0.037940308	0.071752505	0.528766316	0.596967567	0	1.30854356	0	0.666666667	CRA004775;PRJNA827465;Yang2025_Silkie_Wencha	0.874336096
ENSGALG00010017487	C1QTNF8	3	0.208878252	0.395385888	0.5282896	0.597298349	0.155964118	2.971279367	32.6889278	0.666666667	CRA004775;PRJNA827465;Yang2025_Silkie_Wencha	0.874532403
ENSGALG00010008653	ENSGALG0001000865	3	-0.063731896	0.120641859	-0.528273491	0.597309528	0.014274253	2.839144829	29.556253	0.666666667	CRA004775;PRJNA827465;Yang2025_Silkie_Wencha	0.874532403
ENSGALG00010021195	GCLM	3	-0.098771214	0.187061447	-0.52801481	0.597489057	0.078856081	9.784280248	79.5590483	0.666666667	CRA004775;PRJNA827465;Yang2025_Silkie_Wencha	0.874681386
ENSGALG00010021573	NATD1	3	-0.045434956	0.086059431	-0.527948603	0.59753501	0	1.442796881	0	0.666666667	CRA004775;PRJNA827465;Yang2025_Silkie_Wencha	0.874681386
ENSGALG00010004946	SH2D1B	3	0.403050152	0.763656279	0.527790006	0.597645095	1.21942692	6.921226425	71.10338721	0.666666667	CRA004775;PRJNA827465;Yang2025_Silkie_Wencha	0.874751966
ENSGALG00010003401	ENSGALG0001000340	3	0.226322729	0.429158902	0.527363473	0.597941204	0	0.355133869	0	0.666666667	CRA004775;PRJNA827465;Yang2025_Silkie_Wencha	0.874791879
ENSGALG00010000402	PFKFB1	3	0.095618039	0.181242636	0.527569238	0.597798349	0	0.277818709	0	0.666666667	CRA004775;PRJNA827465;Yang2025_Silkie_Wencha	0.874791879
ENSGALG00010028575	SETDB1	3	-0.068158514	0.12921353	-0.527487438	0.597855138	0.040580059	15.84566952	87.378255	0.666666667	CRA004775;PRJNA827465;Yang2025_Silkie_Wencha	0.874791879
ENSGALG00010006229	INTS12	3	-0.068017046	0.128989921	-0.527305122	0.597981718	0.028389452	4.700109636	57.44780112	0.666666667	CRA004775;PRJNA827465;Yang2025_Silkie_Wencha	0.874791879
ENSGALG00010007455	TRPC6	3	0.06540276	0.124029202	0.52731743	0.597973172	0	0.140558423	0	1	CRA004775;PRJNA827465;Yang2025_Silkie_Wencha	0.874791879
ENSGALG00010013074	ENSGALG0001001307	2	0.238788856	0.453144426	0.526959711	0.598221567	0	0.496854264	0	0.5	PRJNA827465;Yang2025_Silkie_Wenchang	0.874843745
ENSGALG00010029570	TNFAIP1	3	-0.086513234	0.164193625	-0.526897644	0.59826467	0.063920331	12.61383829	84.14439797	0.666666667	CRA004775;PRJNA827465;Yang2025_Silkie_Wencha	0.874843745
ENSGALG00010022455	C7orf50	3	-0.063392048	0.120275699	-0.527056156	0.598154592	0	1.524332767	0	0.666666667	CRA004775;PRJNA827465;Yang2025_Silkie_Wencha	0.874843745
ENSGALG00010000526	POLD1	3	-0.059356202	0.112615826	-0.527068028	0.598146348	0.013215834	2.969265398	32.64327259	0.666666667	CRA004775;PRJNA827465;Yang2025_Silkie_Wencha	0.874843745
ENSGALG00010009365	CA5B	3	-0.109268037	0.207456683	-0.526702904	0.598399919	0.092597751	7.366200112	72.84895917	0.666666667	CRA004775;PRJNA827465;Yang2025_Silkie_Wencha	0.874860557
ENSGALG00010023441	ENSGALG0001002344	3	-0.062235486	0.118159646	-0.526706779	0.598397227	0	1.963005923	0	0.666666667	CRA004775;PRJNA827465;Yang2025_Silkie_Wencha	0.874860557
ENSGALG00010011069	ENSGALG0001001106	3	-0.183966558	0.349880545	-0.525798192	0.599028431	0	0.038820475	0	1	CRA004775;PRJNA827465;Yang2025_Silkie_Wencha	0.87497004
ENSGALG00010010080	CDC42EP3	3	-0.159360711	0.303853722	-0.524465227	0.599954999	0.242139528	23.11186925	91.34643772	0.666666667	CRA004775;PRJNA827465;Yang2025_Silkie_Wencha	0.87497004
ENSGALG00010025331	ENSGALG0001002533	3	-0.155150789	0.295681165	-0.524723275	0.599775575	0.051551785	2.474419982	19.17297731	0.666666667	CRA004775;PRJNA827465;Yang2025_Silkie_Wencha	0.87497004
ENSGALG00010022520	TMOD2	3	0.15360297	0.292700399	0.524778819	0.599736958	0.178399852	7.290004137	72.56517332	0.666666667	CRA004775;PRJNA827465;Yang2025_Silkie_Wencha	0.87497004
ENSGALG00010028433	MSTO1	3	0.128037073	0.244141962	0.524436977	0.599974644	0.152173575	19.57203619	89.78133915	0.666666667	CRA004775;PRJNA827465;Yang2025_Silkie_Wencha	0.87497004
ENSGALG00010024412	PIGU	3	-0.118378488	0.22535152	-0.525305922	0.599370542	0.129909641	15.03605439	86.69863816	0.666666667	CRA004775;PRJNA827465;Yang2025_Silkie_Wencha	0.87497004
ENSGALG00010011659	CD14	3	0.099659848	0.189690904	0.525380216	0.599318904	0.056627937	4.437117467	54.92569186	0.666666667	CRA004775;PRJNA827465;Yang2025_Silkie_Wencha	0.87497004
ENSGALG00010008227	DUSP1	3	-0.090043999	0.171107716	-0.526241608	0.598720348	0.050144862	5.500190682	63.6376243	0.666666667	CRA004775;PRJNA827465;Yang2025_Silkie_Wencha	0.87497004
ENSGALG00010020046	BDNF	3	-0.086113597	0.164324167	-0.524047061	0.600245808	0	0.395289861	0	0.666666667	CRA004775;PRJNA827465;Yang2025_Silkie_Wencha	0.87497004
ENSGALG00010024369	MYOD1	3	-0.076113514	0.145178094	-0.524276854	0.600085993	0.027473967	3.655901304	45.29392799	0.666666667	CRA004775;PRJNA827465;Yang2025_Silkie_Wencha	0.87497004
ENSGALG00010012314	ADRB2	3	-0.070772218	0.134511252	-0.526143481	0.59878852	0	1.986456547	0	0.666666667	CRA004775;PRJNA827465;Yang2025_Silkie_Wencha	0.87497004
ENSGALG00010008498	ENSGALG0001000849	3	0.067394047	0.128137771	0.52594989	0.598923024	0.024578306	4.037972642	50.4701944	0.666666667	CRA004775;PRJNA827465;Yang2025_Silkie_Wencha	0.87497004
ENSGALG00010003358	DNAJC15	3	-0.065499256	0.124603898	-0.525659771	0.59912462	0.019101882	3.200376165	37.50734611	0.666666667	CRA004775;PRJNA827465;Yang2025_Silkie_Wencha	0.87497004
ENSGALG00010025102	ARPC3	3	-0.065436681	0.124559706	-0.525343891	0.599344151	0.024486053	4.455356674	55.11021571	0.666666667	CRA004775;PRJNA827465;Yang2025_Silkie_Wencha	0.87497004
ENSGALG00010018121	FERMT2	3	0.061395161	0.116748556	0.525875118	0.598974978	0.024055275	5.287835912	62.17734375	0.666666667	CRA004775;PRJNA827465;Yang2025_Silkie_Wencha	0.87497004
ENSGALG00010027205	OGN	3	0.058033896	0.1106742	0.524366978	0.60002332	0.003847626	2.096955667	4.623639318	0.666666667	CRA004775;PRJNA827465;Yang2025_Silkie_Wencha	0.87497004
ENSGALG00010005526	STRN3	3	-0.056649883	0.107657075	-0.526206778	0.598744545	0.02400402	8.715612044	77.05267295	0.666666667	CRA004775;PRJNA827465;Yang2025_Silkie_Wencha	0.87497004
ENSGALG00010026507	APLNR	3	0.055467575	0.105685555	0.524835918	0.59969726	0.00773375	2.469177632	19.00137219	0.666666667	CRA004775;PRJNA827465;Yang2025_Silkie_Wencha	0.87497004
ENSGALG00010003169	MTRF1	3	-0.047700731	0.091029612	-0.524013339	0.600269262	0.006497903	2.658503806	24.76971462	0.666666667	CRA004775;PRJNA827465;Yang2025_Silkie_Wencha	0.87497004
ENSGALG00010012607	LMNB1	3	0.044375975	0.084670914	0.524099396	0.600209409	0	0.272813056	0	0.666666667	CRA004775;PRJNA827465;Yang2025_Silkie_Wencha	0.87497004
ENSGALG00010015248	ENSGALG0001001524	3	0.043526489	0.082764889	0.525905241	0.598954047	0.006158586	2.77484974	27.92402517	0.666666667	CRA004775;PRJNA827465;Yang2025_Silkie_Wencha	0.87497004
ENSGALG00010023068	CDPF1	3	-0.042386454	0.080681114	-0.525357818	0.599334471	0.008279851	3.446472661	41.96965429	0.666666667	CRA004775;PRJNA827465;Yang2025_Silkie_Wencha	0.87497004
ENSGALG00010010578	GLUD2	3	-0.040592203	0.077464099	-0.524013106	0.600269424	0	1.601389345	0	0.666666667	CRA004775;PRJNA827465;Yang2025_Silkie_Wencha	0.87497004
ENSGALG00010009345	ZFAT	3	-0.035919566	0.068385459	-0.525251524	0.599408351	0	1.270766091	0	0.666666667	CRA004775;PRJNA827465;Yang2025_Silkie_Wencha	0.87497004
ENSGALG00010001201	TUBB6	3	-0.03557569	0.067867444	-0.524193746	0.600143791	0	1.283296934	0	0.666666667	CRA004775;PRJNA827465;Yang2025_Silkie_Wencha	0.87497004
ENSGALG00010029291	RNFT1	3	0.02920441	0.055559957	0.525637737	0.599139932	0.001084834	2.187555051	8.573729416	0.666666667	CRA004775;PRJNA827465;Yang2025_Silkie_Wencha	0.87497004
ENSGALG00010017922	MERTK	3	0.0288091	0.054867344	0.52506824	0.599535754	0	0.689220666	0	0.666666667	CRA004775;PRJNA827465;Yang2025_Silkie_Wencha	0.87497004
ENSGALG00010022044	PXMP4	3	0.025029744	0.047741692	0.524274348	0.600087736	0	0.558748008	0	0.666666667	CRA004775;PRJNA827465;Yang2025_Silkie_Wencha	0.87497004
ENSGALG00010006558	USP10	3	-0.021062111	0.040184414	-0.524136315	0.600183732	0	1.838691433	0	0.666666667	CRA004775;PRJNA827465;Yang2025_Silkie_Wencha	0.87497004
ENSGALG00010026252	ENO2	3	0.225804998	0.431022415	0.523882264	0.600360432	0.33968335	5.31960601	62.40323069	0.666666667	CRA004775;PRJNA827465;Yang2025_Silkie_Wencha	0.875012488
ENSGALG00010013624	SST1	2	1.039790808	1.985636564	0.523656155	0.600517717	7.289465862	13.22804457	92.44030367	0.5	CRA004775;PRJNA827465	0.875105378
ENSGALG00010012207	GRIA3	3	0.138461274	0.264434525	0.523612693	0.600547951	0.125167698	5.816725639	65.61639445	0.666666667	CRA004775;PRJNA827465;Yang2025_Silkie_Wencha	0.875105378
ENSGALG00010013589	DSCAM	2	0.295295713	0.564471232	0.523136869	0.60087901	0.35384043	2.006979255	50.17387461	0.5	PRJNA827465;Yang2025_Silkie_Wenchang	0.875122265
ENSGALG00010009679	ANK3	3	0.144035604	0.275399089	0.523006828	0.600969501	0.177661283	17.53601745	88.59490186	0.666666667	CRA004775;PRJNA827465;Yang2025_Silkie_Wencha	0.875122265
ENSGALG00010005359	MEIS2	3	0.121576265	0.232416607	0.52309629	0.600907247	0.117427919	8.016859656	75.05257562	0.666666667	CRA004775;PRJNA827465;Yang2025_Silkie_Wencha	0.875122265
ENSGALG00010013046	SLC12A5	3	0.109589605	0.209504929	0.523088433	0.600912714	0.011137183	2.159181314	7.372299545	0.666666667	CRA004775;PRJNA827465;Yang2025_Silkie_Wencha	0.875122265
ENSGALG00010023448	FAM219B	3	-0.087444685	0.167205482	-0.52297738	0.600989995	0.064873908	10.32269395	80.62521266	0.666666667	CRA004775;PRJNA827465;Yang2025_Silkie_Wencha	0.875122265
ENSGALG00010005539	IFT88	3	0.042151	0.080562445	0.523209047	0.600828786	0	1.22938894	0	0.666666667	CRA004775;PRJNA827465;Yang2025_Silkie_Wencha	0.875122265
ENSGALG00010022770	HAT1	3	-0.041938763	0.080192926	-0.522973349	0.600992799	0.006709547	2.892414565	30.85361883	0.666666667	CRA004775;PRJNA827465;Yang2025_Silkie_Wencha	0.875122265
ENSGALG00010010796	CAVIN2	3	-0.043530428	0.083300955	-0.522568179	0.601274789	0.005966894	2.653532423	24.62877096	0.666666667	CRA004775;PRJNA827465;Yang2025_Silkie_Wencha	0.875442719
ENSGALG00010011357	UNC80	3	0.219637296	0.420760952	0.522000187	0.6016702	0.362398134	6.433647395	68.91343468	0.666666667	CRA004775;PRJNA827465;Yang2025_Silkie_Wencha	0.875838048
ENSGALG00010000820	KLF5	3	0.165966249	0.317907308	0.522058616	0.601629519	0.234111386	11.54997128	82.68393963	0.666666667	CRA004775;PRJNA827465;Yang2025_Silkie_Wencha	0.875838048
ENSGALG00010005332	ENSGALG0001000533	3	-0.215717233	0.413644036	-0.521504515	0.602015361	0.302762935	4.898872661	59.17428073	0.666666667	CRA004775;PRJNA827465;Yang2025_Silkie_Wencha	0.87607461
ENSGALG00010029634	DDX5	3	0.052882482	0.101401613	0.521515195	0.602007923	0.021554474	8.824448973	77.33569534	0.666666667	CRA004775;PRJNA827465;Yang2025_Silkie_Wencha	0.87607461
ENSGALG00010008199	TPRKB	3	0.037865626	0.072609081	0.521499872	0.602018595	0	0.070664368	0	1	CRA004775;PRJNA827465;Yang2025_Silkie_Wencha	0.87607461
ENSGALG00010002328	ENSGALG0001000232	3	-0.105771026	0.20299931	-0.521041307	0.602337996	0	1.234130507	0	0.666666667	CRA004775;PRJNA827465;Yang2025_Silkie_Wencha	0.876449205
ENSGALG00010018672	PKDCC	3	0.100752256	0.193436644	0.520854035	0.602468458	0.081777548	11.76665378	83.00281424	0.666666667	CRA004775;PRJNA827465;Yang2025_Silkie_Wencha	0.87645864
ENSGALG00010014837	APBA1	3	0.075519085	0.144973164	0.520917686	0.602424114	0.024989068	3.328868133	39.91951859	0.666666667	CRA004775;PRJNA827465;Yang2025_Silkie_Wencha	0.87645864
ENSGALG00010018990	GCK	3	0.19834933	0.381025389	0.520567227	0.602668285	0.324201696	8.001117416	75.00349144	0.666666667	CRA004775;PRJNA827465;Yang2025_Silkie_Wencha	0.876644122
ENSGALG00010015555	PNRC2	3	-0.148669695	0.285632421	-0.520493066	0.60271996	0.215264085	23.07649633	91.33317306	0.666666667	CRA004775;PRJNA827465;Yang2025_Silkie_Wencha	0.876644122
ENSGALG00010020484	TRNT1	3	-0.063586063	0.122220568	-0.520256648	0.602884708	0.025190458	4.910799269	59.27343207	0.666666667	CRA004775;PRJNA827465;Yang2025_Silkie_Wencha	0.876793549
ENSGALG00010015765	ENSGALG0001001576	2	0.478466665	0.923145843	0.518300189	0.604248841	0	0.460435404	0	0.5	CRA004775;Yang2025_Silkie_Wenchang	0.877437813
ENSGALG00010012414	ENSGALG0001001241	3	0.299668149	0.577520545	0.518887427	0.603839246	0.848153417	18.46477261	89.16856415	0.666666667	CRA004775;PRJNA827465;Yang2025_Silkie_Wencha	0.877437813
ENSGALG00010006670	ANKRD16	3	-0.134246346	0.258566923	-0.519193812	0.603625594	0.155190887	9.010576884	77.80386288	0.666666667	CRA004775;PRJNA827465;Yang2025_Silkie_Wencha	0.877437813
ENSGALG00010021630	VTG1	2	0.131088252	0.253043952	0.518045385	0.604426604	0.00258196	1.019597999	1.922130015	0.5	PRJNA827465;Yang2025_Silkie_Wenchang	0.877437813
ENSGALG00010012970	UNC5A	3	0.113108381	0.218347779	0.518019379	0.604444748	0.097344501	8.01321155	75.04121802	0.666666667	CRA004775;PRJNA827465;Yang2025_Silkie_Wencha	0.877437813
ENSGALG00010010463	ACAA2	3	0.095346389	0.183714691	0.518991639	0.603766571	0.05697942	4.621373154	56.72281953	0.666666667	CRA004775;PRJNA827465;Yang2025_Silkie_Wencha	0.877437813
ENSGALG00010022819	PACSIN3	3	-0.091507283	0.176615999	-0.518114349	0.604378489	0.061856599	7.448518673	73.14902348	0.666666667	CRA004775;PRJNA827465;Yang2025_Silkie_Wencha	0.877437813
ENSGALG00010002725	TSPAN7	3	0.085371245	0.164714198	0.518299245	0.6042495	0.043454882	4.737258716	57.78149094	0.666666667	CRA004775;PRJNA827465;Yang2025_Silkie_Wencha	0.877437813
ENSGALG00010024116	MCAM	3	0.084994739	0.16370147	0.519205716	0.603617293	0.03440282	3.581111326	44.15141509	0.666666667	CRA004775;PRJNA827465;Yang2025_Silkie_Wencha	0.877437813
ENSGALG00010022135	CHST1	3	0.072401019	0.139587322	0.518679041	0.60398458	0	0.225484888	0	0.666666667	CRA004775;PRJNA827465;Yang2025_Silkie_Wencha	0.877437813
ENSGALG00010021307	MAP3K9	3	-0.071926701	0.138709542	-0.51854184	0.604080276	0	1.828817655	0	0.666666667	CRA004775;PRJNA827465;Yang2025_Silkie_Wencha	0.877437813
ENSGALG00010002226	OXNAD1	3	0.069959689	0.134833512	0.518859803	0.603858511	0.026277657	3.79172694	47.25358572	0.666666667	CRA004775;PRJNA827465;Yang2025_Silkie_Wencha	0.877437813
ENSGALG00010015063	EEF1AKMT4	3	-0.064929277	0.125285724	-0.518249606	0.604284128	0	1.102254123	0	0.666666667	CRA004775;PRJNA827465;Yang2025_Silkie_Wencha	0.877437813
ENSGALG00010014645	SLC36A1	3	-0.063101916	0.121599547	-0.518932161	0.603808049	0.019195342	3.516585594	43.12665094	0.666666667	CRA004775;PRJNA827465;Yang2025_Silkie_Wencha	0.877437813
ENSGALG00010021448	SZT2	3	-0.055827361	0.107691661	-0.518400034	0.604179191	0.020526572	5.12685359	60.98971884	0.666666667	CRA004775;PRJNA827465;Yang2025_Silkie_Wencha	0.877437813
ENSGALG00010021302	P4HA1	3	0.044363194	0.085506412	0.51882886	0.60388009	0.012177894	4.80776691	58.40064551	0.666666667	CRA004775;PRJNA827465;Yang2025_Silkie_Wencha	0.877437813
ENSGALG00010025057	SCP2	3	-0.030912478	0.059660496	-0.518139813	0.604360724	0	0.716942164	0	0.666666667	CRA004775;PRJNA827465;Yang2025_Silkie_Wencha	0.877437813
ENSGALG00010020708	FAM214A	3	0.027478645	0.053015265	0.518315707	0.604238016	0	0.712591601	0	0.666666667	CRA004775;PRJNA827465;Yang2025_Silkie_Wencha	0.877437813
ENSGALG00010016118	EPRS	3	0.075557004	0.14589697	0.517879185	0.604542566	0.053186215	20.1294847	90.06432589	0.666666667	CRA004775;PRJNA827465;Yang2025_Silkie_Wencha	0.877489718
ENSGALG00010027974	ENSGALG0001002797	3	-0.307706252	0.594689235	-0.517423611	0.604860481	0.820413822	8.847929843	77.39584247	0.666666667	CRA004775;PRJNA827465;Yang2025_Silkie_Wencha	0.877861049
ENSGALG00010005409	PRKCQ	3	0.064245899	0.124200223	0.517276845	0.604962915	0.005235298	2.223409356	10.04805325	0.666666667	CRA004775;PRJNA827465;Yang2025_Silkie_Wencha	0.8779196
ENSGALG00010002579	MATN3	3	-0.503103143	0.973220465	-0.516946737	0.605193341	2.179040642	8.735463525	77.10482112	0.666666667	CRA004775;PRJNA827465;Yang2025_Silkie_Wencha	0.878163859
ENSGALG00010024846	LMO2	3	0.069628993	0.134720286	0.516841192	0.605267023	0.033430277	7.146410576	72.01392253	0.666666667	CRA004775;PRJNA827465;Yang2025_Silkie_Wencha	0.87818065
ENSGALG00010022165	FAM192A	3	0.03896742	0.07544556	0.516497195	0.605507197	0.007481991	3.337011899	40.06614118	0.666666667	CRA004775;PRJNA827465;Yang2025_Silkie_Wencha	0.878348852
ENSGALG00010000141	ENSGALG0001000014	3	-0.032836789	0.063569738	-0.516547492	0.605472078	0.002841982	2.520487095	20.65025829	0.666666667	CRA004775;PRJNA827465;Yang2025_Silkie_Wencha	0.878348852
ENSGALG00010022805	ENSGALG0001002280	3	0.282235123	0.546999532	0.515969588	0.60587565	0.811236973	33.91830234	94.10347847	0.666666667	CRA004775;PRJNA827465;Yang2025_Silkie_Wencha	0.878410994
ENSGALG00010028444	MEF2B	3	0.18739559	0.363083785	0.516122167	0.605769087	0.292595275	10.46750388	80.89324806	0.666666667	CRA004775;PRJNA827465;Yang2025_Silkie_Wencha	0.878410994
ENSGALG00010018521	ENSGALG0001001852	3	0.172553351	0.334717297	0.515519673	0.606189926	0.310461719	44.98073973	95.55365249	0.666666667	CRA004775;PRJNA827465;Yang2025_Silkie_Wencha	0.878410994
ENSGALG00010001239	ENSGALG0001000123	3	-0.128739146	0.249757101	-0.5154574	0.606233431	0.038456781	2.481406913	19.40056306	0.666666667	CRA004775;PRJNA827465;Yang2025_Silkie_Wencha	0.878410994
ENSGALG00010002957	ADGRG2	3	0.110047912	0.213381537	0.515733058	0.606040862	0.053433423	3.240859622	38.2879781	0.666666667	CRA004775;PRJNA827465;Yang2025_Silkie_Wencha	0.878410994
ENSGALG00010029510	PDE6G	3	-0.108601819	0.210471614	-0.515992711	0.6058595	0	0.155421943	0	1	CRA004775;PRJNA827465;Yang2025_Silkie_Wencha	0.878410994
ENSGALG00010022221	CUTC	3	-0.101728058	0.197260752	-0.515703489	0.606061517	0.077040599	5.9767835	66.53718509	0.666666667	CRA004775;PRJNA827465;Yang2025_Silkie_Wencha	0.878410994
ENSGALG00010023496	PLEKHA3	3	-0.086301162	0.167408476	-0.515512498	0.606194939	0.045839113	4.404432474	54.59119848	0.666666667	CRA004775;PRJNA827465;Yang2025_Silkie_Wencha	0.878410994
ENSGALG00010021759	NIFK	3	-0.039288608	0.076199876	-0.515599366	0.606134253	0.007074145	3.098425412	35.45108453	0.666666667	CRA004775;PRJNA827465;Yang2025_Silkie_Wencha	0.878410994
ENSGALG00010002547	PBX4	3	-0.026643871	0.051640617	-0.515947955	0.605890759	0	1.654591227	0	0.666666667	CRA004775;PRJNA827465;Yang2025_Silkie_Wencha	0.878410994
ENSGALG00010015954	CTSA	3	-0.0251788	0.048845523	-0.515478142	0.60621894	0	1.081789518	0	0.666666667	CRA004775;PRJNA827465;Yang2025_Silkie_Wencha	0.878410994
ENSGALG00010029343	SCARF1	3	-0.031699459	0.061519953	-0.515271186	0.606363531	0	1.099238507	0	0.666666667	CRA004775;PRJNA827465;Yang2025_Silkie_Wencha	0.878509474
ENSGALG00010020175	ACAN	3	-1.034553263	2.009436691	-0.514847404	0.606659657	11.29962071	30.51912101	93.44673132	0.666666667	CRA004775;PRJNA827465;Yang2025_Silkie_Wencha	0.878668397
ENSGALG00010009199	SEMA4D	3	0.166559207	0.323472546	0.514909872	0.606616002	0.258582462	18.76728841	89.34315946	0.666666667	CRA004775;PRJNA827465;Yang2025_Silkie_Wencha	0.878668397
ENSGALG00010027966	CLK2	3	0.046914373	0.091104496	0.514951237	0.606587096	0.015311044	6.305935855	68.28385118	0.666666667	CRA004775;PRJNA827465;Yang2025_Silkie_Wencha	0.878668397
ENSGALG00010006291	ENSGALG0001000629	3	0.244703332	0.475611563	0.514502486	0.606900723	0.603928098	26.57552084	92.47427732	0.666666667	CRA004775;PRJNA827465;Yang2025_Silkie_Wencha	0.878771531
ENSGALG00010024820	NUDT19	3	0.053642784	0.104266279	0.51447874	0.606917321	0.015319093	3.880263759	48.45711208	0.666666667	CRA004775;PRJNA827465;Yang2025_Silkie_Wencha	0.878771531
ENSGALG00010006526	LSM6	3	-0.043608316	0.084749847	-0.514553328	0.606865186	0	1.073878971	0	0.666666667	CRA004775;PRJNA827465;Yang2025_Silkie_Wencha	0.878771531
ENSGALG00010000625	ENSGALG0001000062	3	0.143962275	0.279962257	0.514220297	0.607097979	0.150345884	5.687443691	64.83481668	0.666666667	CRA004775;PRJNA827465;Yang2025_Silkie_Wencha	0.878943101
ENSGALG00010010274	MIS18A	3	-0.061194938	0.119100701	-0.513808376	0.607385971	0.024745195	5.479797799	63.50230295	0.666666667	CRA004775;PRJNA827465;Yang2025_Silkie_Wencha	0.879270016
ENSGALG00010027772	ENSGALG0001002777	2	0.609946418	1.189999879	0.512560067	0.608259093	2.133019426	4.043242124	75.26737283	0.5	PRJNA827465;Yang2025_Silkie_Wenchang	0.879273624
ENSGALG00010013778	ENSGALG0001001377	3	-0.459971654	0.896937804	-0.51282447	0.608074111	1.536897818	5.818156099	65.62484805	0.666666667	CRA004775;PRJNA827465;Yang2025_Silkie_Wencha	0.879273624
ENSGALG00010027418	ENSGALG0001002741	3	0.244194616	0.476295119	0.512696029	0.608163968	0	0.976952333	0	0.666666667	CRA004775;PRJNA827465;Yang2025_Silkie_Wencha	0.879273624
ENSGALG00010001751	AVPR2C	2	0.211061624	0.411078393	0.513434001	0.607647766	0.263010289	4.474557678	77.65142229	0.5	PRJNA827465;Yang2025_Silkie_Wenchang	0.879273624
ENSGALG00010013639	ENSGALG0001001363	3	0.118766974	0.231439177	0.513167112	0.607834429	0.071469897	3.660166195	45.35767249	0.666666667	CRA004775;PRJNA827465;Yang2025_Silkie_Wencha	0.879273624

ENSGALG00010021867	CXCL12	3	-0.115792446	0.22548813	-0.51351903	0.607588302	0.116706251	14.17166066	85.8873279	0.666666667	CRA004775;PRJNA827465;Yang2025_Silkie_Wencha	0.879273624
ENSGALG00010018698	MRPS16	3	0.074240918	0.144693148	0.513092145	0.607886866	0.027293218	3.123675873	35.97287039	0.666666667	CRA004775;PRJNA827465;Yang2025_Silkie_Wencha	0.879273624
ENSGALG00010017671	ANTXR1	3	-0.049576366	0.096719841	-0.512577005	0.608247242	0	0.456742197	0	0.666666667	CRA004775;PRJNA827465;Yang2025_Silkie_Wencha	0.879273624
ENSGALG00010020737	POLR3A	3	-0.047082186	0.091851584	-0.512589816	0.608238278	0.014073935	4.840248032	58.67980345	0.666666667	CRA004775;PRJNA827465;Yang2025_Silkie_Wencha	0.879273624
ENSGALG00010006602	EDNRA	3	-0.045814171	0.089258486	-0.513275237	0.607758803	0.011637552	4.149588281	51.80244727	0.666666667	CRA004775;PRJNA827465;Yang2025_Silkie_Wencha	0.879273624
ENSGALG00010027912	RGS3	3	0.037643301	0.073372833	0.513041404	0.607922359	0	1.411721595	0	0.666666667	CRA004775;PRJNA827465;Yang2025_Silkie_Wencha	0.879273624
ENSGALG00010006064	DMTF1	3	0.035483854	0.069175267	0.512955786	0.607982249	0.001774896	2.218666116	9.855746861	0.666666667	CRA004775;PRJNA827465;Yang2025_Silkie_Wencha	0.879273624
ENSGALG00010025459	DIABLO	3	-0.024683241	0.048087249	-0.513301169	0.607740666	0.000321648	2.067448451	3.262400612	0.666666667	CRA004775;PRJNA827465;Yang2025_Silkie_Wencha	0.879273624
ENSGALG00010028428	RABGAP1	3	-0.02205337	0.042951361	-0.513449853	0.607636681	0	0.92089965	0	0.666666667	CRA004775;PRJNA827465;Yang2025_Silkie_Wencha	0.879273624
ENSGALG00010012406	ENSGALG0001001240	3	0.154197248	0.301144784	0.512036923	0.608625169	0.18750369	8.136720163	75.42007148	0.666666667	CRA004775;PRJNA827465;Yang2025_Silkie_Wencha	0.879469821
ENSGALG00010007796	MCUR1	3	-0.081650982	0.159526667	-0.511832808	0.608768027	0.05910797	10.15577515	80.30677156	0.666666667	CRA004775;PRJNA827465;Yang2025_Silkie_Wencha	0.879469821
ENSGALG00010004791	FZD1	3	-0.074193545	0.144888136	-0.512074678	0.608598746	0.041958961	9.067571061	77.9433766	0.666666667	CRA004775;PRJNA827465;Yang2025_Silkie_Wencha	0.879469821
ENSGALG00010024222	ENSGALG0001002422	3	-0.065452174	0.127776996	-0.512237542	0.608484772	0	1.338394652	0	0.666666667	CRA004775;PRJNA827465;Yang2025_Silkie_Wencha	0.879469821
ENSGALG00010018075	MLST8	3	0.057312981	0.111966966	0.511874015	0.608739185	0.016304898	3.284597309	39.10973517	0.666666667	CRA004775;PRJNA827465;Yang2025_Silkie_Wencha	0.879469821
ENSGALG00010014225	ZMPSTE24	3	0.033253078	0.06494661	0.512006363	0.608646556	0.005354153	3.250345171	38.46807355	0.666666667	CRA004775;PRJNA827465;Yang2025_Silkie_Wencha	0.879469821
ENSGALG00010011952	UBE2D2	3	-0.064678845	0.126418024	-0.511626766	0.608912249	0.033527499	8.174576514	75.53390079	0.666666667	CRA004775;PRJNA827465;Yang2025_Silkie_Wencha	0.8795883
ENSGALG00010004362	FBXL4	3	-0.09138317	0.17864776	-0.511527097	0.608982019	0.066087041	7.143759264	72.00353587	0.666666667	CRA004775;PRJNA827465;Yang2025_Silkie_Wencha	0.87959922
ENSGALG00010005227	EXOSC9	3	0.03414567	0.066767262	0.511413369	0.609061635	0.002567668	2.405998071	16.87441383	0.666666667	CRA004775;PRJNA827465;Yang2025_Silkie_Wencha	0.879624358
ENSGALG00010015430	HMGCL	3	0.044079364	0.086223882	0.511219896	0.609197089	0.00407093	2.280456726	12.29827004	0.666666667	CRA004775;PRJNA827465;Yang2025_Silkie_Wencha	0.879730123
ENSGALG00010001744	ENSGALG0001000174	2	1.122504204	2.203019703	0.509529807	0.610380911	8.686440435	9.48497929	89.45701441	0.5	CRA004775;Yang2025_Silkie_Wenchang	0.879760007
ENSGALG00010025313	DIO1	2	0.287318749	0.564430774	0.509041609	0.610723059	0.302559475	1.902330929	47.43291061	0.5	PRJNA827465;Yang2025_Silkie_Wenchang	0.879760007
ENSGALG00010005075	TRDN	3	0.231348125	0.452833998	0.510889479	0.609428449	0.482963487	14.43317079	86.14303102	0.666666667	CRA004775;PRJNA827465;Yang2025_Silkie_Wencha	0.879760007
ENSGALG00010001219	TFEC	2	-0.17329479	0.340540818	-0.508881112	0.61083556	0.022157654	1.049935404	4.756045339	1	CRA004775;PRJNA827465	0.879760007
ENSGALG00010024863	LMOD1	3	0.143297158	0.281381926	0.509262127	0.610568501	0.168529891	7.602598218	73.69320405	0.666666667	CRA004775;PRJNA827465;Yang2025_Silkie_Wencha	0.879760007
ENSGALG00010022841	ENSGALG0001002284	2	0.132681024	0.260044244	0.510224805	0.609893976	0.007501853	1.056883573	5.38219861	0.5	PRJNA827465;Yang2025_Silkie_Wenchang	0.879760007
ENSGALG00010010178	LGI2	3	0.131507633	0.258258132	0.509210037	0.610605008	0.134045848	7.901568617	74.68857012	0.666666667	CRA004775;PRJNA827465;Yang2025_Silkie_Wencha	0.879760007
ENSGALG00010027153	ENSGALG0001002715	3	-0.119103769	0.233635664	-0.509784195	0.610202659	0.124877374	11.35364165	82.38450656	0.666666667	CRA004775;PRJNA827465;Yang2025_Silkie_Wencha	0.879760007
ENSGALG00010021613	ADGRL4	3	-0.116111008	0.227740339	-0.509839443	0.61016395	0.121523636	25.10387676	92.03310302	0.666666667	CRA004775;PRJNA827465;Yang2025_Silkie_Wencha	0.879760007
ENSGALG00010011968	ENSGALG0001001196	3	0.112092187	0.22027054	0.50888415	0.610833431	0.034500641	2.584562727	22.61747108	0.666666667	CRA004775;PRJNA827465;Yang2025_Silkie_Wencha	0.879760007
ENSGALG00010015562	IRF9	3	-0.111677427	0.219200555	-0.509476023	0.610418601	0.05787029	3.245193741	38.3703976	0.666666667	CRA004775;PRJNA827465;Yang2025_Silkie_Wencha	0.879760007
ENSGALG00010001606	PIGA	3	-0.096298689	0.189031997	-0.50943063	0.610450411	0.079078673	10.68512175	81.28238454	0.666666667	CRA004775;PRJNA827465;Yang2025_Silkie_Wencha	0.879760007
ENSGALG00010025591	OTOS	3	0.093632946	0.183734404	0.509610308	0.610324501	0	1.719289194	0	0.666666667	CRA004775;PRJNA827465;Yang2025_Silkie_Wencha	0.879760007
ENSGALG00010013784	ANO6	3	0.088462413	0.173178348	0.510816818	0.609479332	0.057556981	6.172087918	67.59605458	0.666666667	CRA004775;PRJNA827465;Yang2025_Silkie_Wencha	0.879760007
ENSGALG00010005769	TFG	3	0.080850919	0.158820909	0.509069739	0.610703342	0.059327616	11.54833777	82.68149027	0.666666667	CRA004775;PRJNA827465;Yang2025_Silkie_Wencha	0.879760007
ENSGALG00010012763	SMPDL3B	3	-0.080728059	0.158251478	-0.510125146	0.609963789	0.050674802	6.393261843	68.71706417	0.666666667	CRA004775;PRJNA827465;Yang2025_Silkie_Wencha	0.879760007
ENSGALG00010026104	DDX51	3	0.076337634	0.149633793	0.510163063	0.609937227	0.050294624	8.747452503	77.13620052	0.666666667	CRA004775;PRJNA827465;Yang2025_Silkie_Wencha	0.879760007
ENSGALG00010004142	RBBP8	3	0.073975516	0.144907807	0.510500558	0.609700824	0.040059411	7.075898812	71.73503956	0.666666667	CRA004775;PRJNA827465;Yang2025_Silkie_Wencha	0.879760007
ENSGALG00010006748	FAM177A1	3	-0.070487531	0.138393457	-0.509327052	0.610522999	0.042710846	8.62020316	76.79869067	0.666666667	CRA004775;PRJNA827465;Yang2025_Silkie_Wencha	0.879760007
ENSGALG00010004507	CUL2	3	0.054133738	0.105964669	0.51086592	0.609444946	0.020929934	5.789260215	65.45327165	0.666666667	CRA004775;PRJNA827465;Yang2025_Silkie_Wencha	0.879760007
ENSGALG00010018103	BTRC	3	0.045387425	0.089022229	0.509843728	0.610160948	0.008751394	3.0756234	34.97253272	0.666666667	CRA004775;PRJNA827465;Yang2025_Silkie_Wencha	0.879760007
ENSGALG00010007374	CMIP	3	-0.042233316	0.082985656	-0.508923086	0.610806137	0	1.149002638	0	0.666666667	CRA004775;PRJNA827465;Yang2025_Silkie_Wencha	0.879760007
ENSGALG00010021407	SIN3A	3	-0.039387141	0.077152382	-0.510511021	0.609693495	0.007709641	3.472784799	42.40933096	0.666666667	CRA004775;PRJNA827465;Yang2025_Silkie_Wencha	0.879760007
ENSGALG00010022347	SEC11A	3	-0.036899427	0.072432676	-0.509430674	0.61045038	0.00423679	2.595879553	22.95482285	0.666666667	CRA004775;PRJNA827465;Yang2025_Silkie_Wencha	0.879760007
ENSGALG00010020588	PALD1	3	0.03678488	0.0719739	0.511086384	0.609290569	0.005042936	2.749513312	27.2598539	0.666666667	CRA004775;PRJNA827465;Yang2025_Silkie_Wencha	0.879760007
ENSGALG00010022559	SSH1	3	0.02764115	0.054165886	0.510305511	0.609837442	0	0.823785708	0	0.666666667	CRA004775;PRJNA827465;Yang2025_Silkie_Wencha	0.879760007
ENSGALG00010023013	ENSGALG0001002301	2	-0.219527691	0.431782443	-0.508421994	0.611157431	0	0.015038779	0	1	CRA004775;PRJNA827465	0.879775496
ENSGALG00010009525	ENSGALG0001000952	3	-0.174740722	0.343661388	-0.508467719	0.611125372	0.261892742	8.908440852	77.54938229	0.666666667	CRA004775;PRJNA827465;Yang2025_Silkie_Wencha	0.879775496
ENSGALG00010021597	GGT7	3	-0.085855618	0.168850481	-0.508471269	0.611122883	0.055731479	7.083013522	71.76343101	0.666666667	CRA004775;PRJNA827465;Yang2025_Silkie_Wencha	0.879775496
ENSGALG00010020230	ENSGALG0001002023	3	0.057989254	0.114044271	0.508480199	0.611116622	0	1.75495899	0	0.666666667	CRA004775;PRJNA827465;Yang2025_Silkie_Wencha	0.879775496
ENSGALG00010021120	PDCD11	3	0.044569465	0.087604292	0.508758917	0.610921219	0.010663402	3.912548816	48.88242693	0.666666667	CRA004775;PRJNA827465;Yang2025_Silkie_Wencha	0.879775496
ENSGALG00010029548	BAIAP2	3	0.073496364	0.144674177	0.508013012	0.611444218	0.026344079	3.442574579	41.9039456	0.666666667	CRA004775;PRJNA827465;Yang2025_Silkie_Wencha	0.879999049
ENSGALG00010013192	B4GALT5	3	-0.072961402	0.143643394	-0.507934263	0.611499445	0.04389001	12.12561964	83.50599756	0.666666667	CRA004775;PRJNA827465;Yang2025_Silkie_Wencha	0.879999049
ENSGALG00010012998	MAFF	3	0.042322815	0.083303827	0.508053673	0.611415703	0	0.141821981	0	1	CRA004775;PRJNA827465;Yang2025_Silkie_Wencha	0.879999049
ENSGALG00010008048	ENSGALG0001000804	3	0.535337475	1.054530387	0.507654859	0.611695411	2.572849348	9.671144059	79.31992339	0.666666667	CRA004775;PRJNA827465;Yang2025_Silkie_Wencha	0.880049758
ENSGALG00010015110	ENSGALG0001001511	2	0.147930188	0.291420416	0.507617792	0.611721411	0	7.1619E-05	0	1	CRA004775;Yang2025_Silkie_Wenchang	0.880049758
ENSGALG00010006653	CFL2	3	-0.031192302	0.061444811	-0.507647458	0.611700602	0	0.791732196	0	0.666666667	CRA004775;PRJNA827465;Yang2025_Silkie_Wencha	0.880049758
ENSGALG00010014862	ENSGALG0001001486	3	0.258680818	0.509802973	0.507413317	0.611864844	0.532634859	7.04810051	71.62356017	0.666666667	CRA004775;PRJNA827465;Yang2025_Silkie_Wencha	0.880077012
ENSGALG00010027692	PHF2	3	0.084923723	0.16733683	0.507501686	0.611802854	0.061826551	8.012991582	75.04053287	0.666666667	CRA004775;PRJNA827465;Yang2025_Silkie_Wencha	0.880077012
ENSGALG00010024205	ANKRD13D	3	0.147101182	0.290060649	0.507139395	0.612057015	0.215889174	22.79770375	91.22718664	0.666666667	CRA004775;PRJNA827465;Yang2025_Silkie_Wencha	0.880248955
ENSGALG00010018948	POLR3E	3	-0.036278446	0.071545884	-0.507065446	0.612108899	0.006371521	3.171486937	36.9380975	0.666666667	CRA004775;PRJNA827465;Yang2025_Silkie_Wencha	0.880248955
ENSGALG00010013537	LMO3	2	-0.16084458	0.317392609	-0.50676851	0.612317254	0.076317226	1.609459766	37.86735022	0.5	PRJNA827465;Yang2025_Silkie_Wenchang	0.880318157
ENSGALG00010004445	EPHA6	2	0.142299807	0.280819408	0.506730672	0.612343806	0	0.244235063	0	1	PRJNA827465;Yang2025_Silkie_Wenchang	0.880318157
ENSGALG00010005554	CRYL1	3	-0.117851878	0.232523509	-0.506838549	0.612268106	0.121240568	12.51307558	84.01671926	0.666666667	CRA004775;PRJNA827465;Yang2025_Silkie_Wencha	0.880318157
ENSGALG00010025559	SLC6A17	2	0.574639912	1.134264851	0.506618812	0.612422306	1.883153676	3.709526609	73.04238235	0.5	CRA004775;PRJNA827465	0.880341499
ENSGALG00010008903	ENSGALG0001000890	3	0.089307839	0.176367608	0.506373253	0.612594648	0	1.115140155	0	0.666666667	CRA004775;PRJNA827465;Yang2025_Silkie_Wencha	0.880410218
ENSGALG00010003002	PHKA2	3	-0.074278623	0.146685072	-0.506381613	0.61258878	0.019950812	2.825452295	29.21487284	0.666666667	CRA004775;PRJNA827465;Yang2025_Silkie_Wencha	0.880410218
ENSGALG00010023157	STOX1	2	-0.427477566	0.845193236	-0.505774949	0.613014646	1.33340935	14.93558226	93.30457974	0.5	PRJNA827465;Yang2025_Silkie_Wenchang	0.880497952
ENSGALG00010024595	MYBPHL	3	0.163214462	0.322730989	0.505729128	0.613046817	0.201192325	7.186629921	72.17054416	0.666666667	CRA004775;PRJNA827465;Yang2025_Silkie_Wencha	0.880497952
ENSGALG00010019443	NCKAP5	3	0.131949097	0.260987334	0.505576633	0.613153889	0.147614625	9.13751646	78.11221453	0.666666667	CRA004775;PRJNA827465;Yang2025_Silkie_Wencha	0.880497952
ENSGALG00010023226	ENSGALG0001002322	3	0.09991062	0.197446492	0.50601365	0.612847067	0.019500638	2.359223859	15.2263575	0.666666667	CRA004775;PRJNA827465;Yang2025_Silkie_Wencha	0.880497952
ENSGALG00010010371	SOCS1L	3	0.070039597	0.138516963	0.505639129	0.613110008	0.01812974	2.771026577	27.82458254	0.666666667	CRA004775;PRJNA827465;Yang2025_Silkie_Wencha	0.880497952
ENSGALG00010012957	DERA	3	-0.049281402	0.097468648	-0.505612862	0.61312845	0	0.904303004	0	0.666666667	CRA004775;PRJNA827465;Yang2025_Silkie_Wencha	0.880497952
ENSGALG00010020809	ENSGALG0001002080	3	0.04529881	0.089560007	0.505792841	0.613002085	0	1.856713719	0	0.666666667	CRA004775;PRJNA827465;Yang2025_Silkie_Wencha	0.880497952
ENSGALG00010016780	PRORSD1P	3	-0.034065739	0.067363554	-0.505699855	0.61306737	0	0.970165917	0	0.666666667	CRA004775;PRJNA827465;Yang2025_Silkie_Wencha	0.880497952
ENSGALG00010006926	ID4	3	0.123038515	0.243457391	0.505380076	0.61329191	0.102830935	4.740321542	57.80876925	0.666666667	CRA004775;PRJNA827465;Yang2025_Silkie_Wencha	0.880606714
ENSGALG00010019572	NFKBIE	3	-0.066147243	0.131041054	-0.504782591	0.613711545	0.008250637	2.337210306	14.42789745	0.666666667	CRA004775;PRJNA827465;Yang2025_Silkie_Wencha	0.880940866
ENSGALG00010013198	CAMLG	3	-0.051793113	0.10259914	-0.504810399	0.613692011	0.021678073	6.870938783	70.8918961	0.666666667	CRA004775;PRJNA827465;Yang2025_Silkie_Wencha	0.880940866
ENSGALG00010028125	HMG20B	3	0.044767571	0.088665274	0.504905341	0.613625323	0.010463679	3.514961569	43.10037362	0.666666667	CRA004775;PRJNA827465;Yang2025_Silkie_Wencha	0.880940866
ENSGALG00010011446	ENSGALG0001001144	3	0.191924296	0.380356204	0.504590943	0.613846172	0.362519443	13.32015402	84.98515861	0.666666667	CRA004775;PRJNA827465;Yang2025_Silkie_Wencha	0.881044669
ENSGALG00010001818	ADGRB3	2	0.341890132	0.678726622	0.503722884	0.614456125	0.828341804	9.813480124	89.80993504	0.5	PRJNA827465;Yang2025_Silkie_Wenchang	0.881194843
ENSGALG00010008996	CHRM3	3	0.147529172	0.292927848	0.503636554	0.614516801	0.175867455	7.459472961	73.18845433	0.666666667	CRA004775;PRJNA827465;Yang2025_Silkie_Wencha	0.881194843
ENSGALG00010020177	KCNG3	2	-0.135806866	0.26938418	-0.504138241	0.614164236	0.118936398	5.511953438	81.85761162	0.5	PRJNA827465;Yang2025_Silkie_Wenchang	0.881194843
ENSGALG00010024813	RNF168	3	0.093590645	0.185727807	0.503912935	0.61432256	0.075658706	7.584029321	73.62879394	0.666666667	CRA004775;PRJNA827465;Yang2025_Silkie_Wencha	0.881194843
ENSGALG00010025257	LPCAT2	3	-0.088648966	0.176111924	-0.503367197	0.61470613	0.051064982	4.901778265	59.19848079	0.666666667	CRA004775;PRJNA827465;Yang2025_Silkie_Wencha	0.881194843
ENSGALG00010016308	RPS6	3	-0.086478669	0.171717715	-0.503609478	0.614535831	0.036047359	3.350283568	40.30356059	0.666666667	CRA004775;PRJNA827465;Yang2025_Silkie_Wencha	0.881194843
ENSGALG00010000441	SLC7A4	3	-0.076386408	0.151553411	-0.504023018	0.614245202	0.011829407	2.340353524	14.54282527	0.666666667	CRA004775;PRJNA827465;Yang2025_Silkie_Wencha	0.881194843
ENSGALG00010019184	PNKD	3	-0.06520426	0.129381358	-0.503969511	0.614282803	0.017814064	3.055359361	34.54125151	0.666666667	CRA004775;PRJNA827465;Yang2025_Silkie_Wencha	0.881194843
ENSGALG00010011480	BLTP3B	3	-0.052433879	0.104168766	-0.503355092	0.614714639	0.017533998	4.411026312	54.65907799	0.666666667	CRA004775;PRJNA827465;Yang2025_Silkie_Wencha	0.881194843
ENSGALG00010024660	KMT2D	3	-0.048568066	0.09649471	-0.503323612	0.614736768	0.007439232	2.533205027	21.04863292	0.666666667	CRA004775;PRJNA827465;Yang2025_Silkie_Wencha	0.881194843
ENSGALG00010022719	MSL2	3	-0.029395071	0.058368415	-0.503612633	0.614533614	0.001801537	2.288689708	12.6137548	0.666666667	CRA004775;PRJNA827465;Yang2025_Silkie_Wencha	0.881194843
ENSGALG00010005269	KIN	3	-0.02845784	0.056467301	-0.503970247	0.614282285	0	0.229313927	0	1	CRA004775;PRJNA827465;Yang2025_Silkie_Wencha	0.881194843
ENSGALG00010019166	PNPT1	3	-0.021444194	0.0426081	-0.503289126	0.614761011	0	0.945176488	0	0.666666667	CRA004775;PRJNA827465;Yang2025_Silkie_Wencha	0.881194843
ENSGALG00010026315	SYT2	3	0.131600487	0.261759664	0.502753118	0.615137859	0.062930908	2.76882425	27.7671741	0.666666667	CRA004775;PRJNA827465;Yang2025_Silkie_Wencha	0.881230791
ENSGALG00010012685	NPTXR	3	0.102404054	0.203598644	0.502970213	0.614985215	0.064670474	4.290627038	53.3867665	0.666666667	CRA004775;PRJNA827465;Yang2025_Silkie_Wencha	0.881230791
ENSGALG00010002441	USO1	3	0.080350319	0.159743597	0.502995552	0.614967399	0.066937285	28.19166991	92.90570581	0.666666667	CRA004775;PRJNA827465;Yang2025_Silkie_Wencha	0.881230791
ENSGALG00010021595	UBE3B	3	-0.045051752	0.089615714	-0.502721563	0.615160047	0.011176008	3.839092191	47.90435081	0.666666667	CRA004775;PRJNA827465;Yang2025_Silkie_Wencha	0.881230791
ENSGALG00010020373	MS4A4A	3	0.032973071	0.065576439	0.502818867	0.615091627	0	1.829759647	0	0.666666667	CRA004775;PRJNA827465;Yang2025_Silkie_Wencha	0.881230791
ENSGALG00010024729	FOXM1	3	0.023033551	0.045815076	0.502750466	0.615139723	0	1.849733508	0	0.666666667	CRA004775;PRJNA827465;Yang2025_Silkie_Wencha	0.881230791
ENSGALG00010019990	MARCHF8	3	-0.030256862	0.060196737	-0.502632916	0.615222382	0	0.599093247	0	0.666666667	CRA004775;PRJNA827465;Yang2025_Silkie_Wencha	0.881230804
ENSGALG00010012205	SLC6A13	2	0.178629085	0.355594769	0.502338899	0.615429151	0.152978988	2.348645299	57.4222638	0.5	PRJNA827465;Yang2025_Silkie_Wenchang	0.881288843
ENSGALG00010007183	VOPP1	3	0.08793987	0.175083182	0.502335428	0.615417526	0	1.550915183	0	0.666666667	CRA004775;PRJNA827465;Yang2025_Silkie_Wencha	0.881288843
ENSGALG00010017162	BCL11A	3	-0.049716172	0.098975196	-0.502309408	0.615449893	0.008622518	2.730706925	26.75889229	0.666666667	CRA004775;PRJNA827465;Yang2025_Silkie_Wencha	0.881288843
ENSGALG00010021523	CELF6	3	0.129729471	0.258562567	0.501728143	0.615858765	0.153490764	10.40188218	80.7727105	0.666666667	CRA004775;PRJNA827465;Yang2025_Silkie_Wencha	0.881663237
ENSGALG00010028045	ENSGALG0001002804	3	-0.10241093	0.204104359	-0.501757683	0.615837983	0	1.403985012	0	0.666666667	CRA004775;PRJNA827465;Yang2025_Silkie_Wencha	0.881663237
ENSGALG00010021691	PIGB	3	-0.083065527	0.165592227	-0.50162697	0.615929945	0.036176467	3.577051433	44.08802788	0.666666667	CRA004775;PRJNA827465;Yang2025_Silkie_Wencha	0.881663237
ENSGALG00010025232	PRIM1	3	-0.054599057	0.108853452	-0.501583143	0.615960779	0.016933181	3.608589697	44.57668596	0.666666667	CRA004775;PRJNA827465;Yang2025_Silkie_Wencha	0.881663237
ENSGALG00010020986	CFAP20DC	2	-0.106132028	0.211686097	-0.501365177	0.61614143	0.027356829	1.400392234	28.59143491	0.5	PRJNA827465;Yang2025_Silkie_Wenchang	0.881709614
ENSGALG00010001526	PPP1R3A	3	-0.070438744	0.140495386	-0.501359837	0.6161179	0.02624629	3.816776862	47.59976618	0.666666667	CRA004775;PRJNA827465;Yang2025_Silkie_Wencha	0.881709614
ENSGALG00010010884	DNAH7	2	0.092217812	0.183969266	0.501267492	0.616182881	0	0.381321305	0	0.5	PRJNA827465;Yang2025_Silkie_Wenchang	0.881713164
ENSGALG00010000026	ND3	3	0.199870428	0.399054934	0.500859433	0.616470055	0.40128865	14.87783255	86.55718168	0.666666667	CRA004775;PRJNA827465;Yang2025_Silkie_Wencha	0.881768736
ENSGALG00010017301	LHCGR	2	-0.137307443	0.274168317	-0.500804457	0.616508749	0.046205808	1.410999477	29.12802553	0.5	PRJNA827465;Yang2025_Silkie_Wenchang	0.881768736
ENSGALG00010027455	R3HDM4	3	-0.060034609	0.119866591	-0.500845215	0.61648061	0.012779592	2.590934068	22.80776169	0.666666667	CRA004775;PRJNA827465;Yang2025_Silkie_Wencha	0.881768736
ENSGALG00010002206	DPH3P1	3	0.055223211	0.110226724	0.500996569	0.616373537	0.012733998	2.930906515	31.76172662	0.666666667	CRA004775;PRJNA827465;Yang2025_Silkie_Wencha	0.881768736
ENSGALG00010020831	MUL1	3	-0.03485829	0.069609459	-0.500769436	0.616533399	0	0.687181515	0	1	CRA004775;PRJNA827465;Yang2025_Silkie_Wencha	0.881768736
ENSGALG00010018350	CNNM1	3	-0.188383338	0.376402815	-0.500478022	0.616738528	0.237601829	4.539109258	55.93849176	0.666666667	CRA004775;PRJNA827465;Yang2025_Silkie_Wencha	0.881972898
ENSGALG00010014922	MAN1A1	3	-0.112333919	0.224541853	-0.500280537	0.616877557	0.097912445	7.703809478	74.03881799	0.666666667	CRA004775;PRJNA827465;Yang2025_Silkie_Wencha	0.882082501
ENSGALG00010009208	SLC5A6	3	0.189389806	0.379220667	0.499418471	0.617484609	0.372453735	16.42100346	87.82047635	0.666666667	CRA004775;PRJNA827465;Yang2025_Silkie_Wencha	0.882247095
ENSGALG00010010680	ENSGALG0001001068	3	0.173577585	0.347689904	0.499231018	0.617616645	0.109457681	2.790652056	28.3321618	0.666666667	CRA004775;PRJNA827465;Yang2025_Silkie_Wencha	0.882247095
ENSGALG00010001514	MET	3	0.124863471	0.250110952	0.499232321	0.617615728	0.145449374	13.80733319	85.51494359	0.666666667	CRA004775;PRJNA827465;Yang2025_Silkie_Wencha	0.882247095
ENSGALG00010007270	CDH5	3	-0.112032526	0.22409726	-0.499928138	0.617125679	0.116604231	19.31662273	89.64622321	0.666666667	CRA004775;PRJNA827465;Yang2025_Silkie_Wencha	0.882247095
ENSGALG00010022441	FICD	3	-0.09060027	0.181452585	-0.499305482	0.617564194	0.076180787	8.982022737	77.73330063	0.666666667	CRA004775;PRJNA827465;Yang2025_Silkie_Wencha	0.882247095
ENSGALG00010021362	ARPC1B	3	-0.080332459	0.160843157	-0.499445923	0.617465274	0.044171843	4.439283653	54.94768624	0.666666667	CRA004775;PRJNA827465;Yang2025_Silkie_Wencha	0.882247095
ENSGALG00010025962	ENSGALG0001002596	3	0.077673391	0.155528887	0.499414562	0.617487362	0.027247688	3.139710394	36.29985734	0.666666667	CRA004775;PRJNA827465;Yang2025_Silkie_Wencha	0.882247095
ENSGALG00010028124	SUGP2	3	0.054085599	0.108241828	0.499673739	0.617304826	0.020296242	4.829380335	58.5868194	0.666666667	CRA004775;PRJNA827465;Yang2025_Silkie_Wencha	0.882247095
ENSGALG00010018587	SRF	3	0.034839627	0.069783175	0.499255405	0.617599467	0	1.714831306	0	0.666666667	CRA004775;PRJNA827465;Yang2025_Silkie_Wencha	0.882247095
ENSGALG00010011564	ARAP3	3	-0.029611416	0.059301583	-0.499336008	0.617542692	0	1.075686098	0	0.666666667	CRA004775;PRJNA827465;Yang2025_Silkie_Wencha	0.882247095
ENSGALG00010005704	AKAP12	3	0.093102321	0.186648366	0.498811336	0.617912301	0.054066713	4.11217641	51.36395425	0.666666667	CRA004775;PRJNA827465;Yang2025_Silkie_Wencha	0.882455712
ENSGALG00010025805	ATXN2	3	0.08763969	0.175746734	0.498670375	0.618011618	0.063849271	6.704065307	70.16735505	0.666666667	CRA004775;PRJNA827465;Yang2025_Silkie_Wencha	0.882455712
ENSGALG00010013110	ANKRD10	3	0.085456038	0.171368138	0.498669352	0.618012339	0.060922674	7.56963178	73.57863555	0.666666667	CRA004775;PRJNA827465;Yang2025_Silkie_Wencha	0.882455712
ENSGALG00010022005	NLRC5	3	0.064626087	0.129535781	0.498905291	0.617846106	0.01519022	2.827538728	29.26710498	0.666666667	CRA004775;PRJNA827465;Yang2025_Silkie_Wencha	0.882455712
ENSGALG00010015339	ENSGALG0001001533	3	-0.652868013	1.314762036	-0.496567436	0.619494122	4.394854616	13.40022802	85.07488084	0.666666667	CRA004775;PRJNA827465;Yang2025_Silkie_Wencha	0.882522345
ENSGALG00010008442	WDR64	2	-0.463608206	0.932408877	-0.497215564	0.619037047	1.595433194	11.83102152	91.54764448	0.5	PRJNA827465;Yang2025_Silkie_Wenchang	0.882522345
ENSGALG00010027385	CNTN2	3	0.418627732	0.840472078	0.498086424	0.618423129	1.537939421	7.369041434	72.85942795	0.666666667	CRA004775;PRJNA827465;Yang2025_Silkie_Wencha	0.882522345
ENSGALG00010018410	EXOC6B	3	0.157941528	0.318067067	0.496566745	0.619494609	0.262909226	18.56060181	89.22448733	0.666666667	CRA004775;PRJNA827465;Yang2025_Silkie_Wencha	0.882522345
ENSGALG00010019079	CAPG	3	-0.123237488	0.247378613	-0.498173574	0.618361706	0.100336255	4.358162738	54.10910238	0.666666667	CRA004775;PRJNA827465;Yang2025_Silkie_Wencha	0.882522345
ENSGALG00010030105	RAB40B	3	0.095678913	0.19255787	0.49688394	0.619270898	0.058471008	4.671881384	57.19069395	0.666666667	CRA004775;PRJNA827465;Yang2025_Silkie_Wencha	0.882522345
ENSGALG00010000271	RTN4R	3	0.092982549	0.187226912	0.496630252	0.619449816	0	1.242696592	0	0.666666667	CRA004775;PRJNA827465;Yang2025_Silkie_Wencha	0.882522345
ENSGALG00010007975	ANKRD11	3	0.088857535	0.17889688	0.49669695	0.619402773	0.061503884	5.578568758	64.14851037	0.666666667	CRA004775;PRJNA827465;Yang2025_Silkie_Wencha	0.882522345
ENSGALG00010013137	TMEM121	3	-0.086239389	0.17307044	-0.498290689	0.61827917	0.036342441	3.368981425	40.63487602	0.666666667	CRA004775;PRJNA827465;Yang2025_Silkie_Wencha	0.882522345
ENSGALG00010024887	CASP18	3	-0.08161703	0.163972677	-0.497747743	0.618661853	0	0.906464917	0	0.666666667	CRA004775;PRJNA827465;Yang2025_Silkie_Wencha	0.882522345
ENSGALG00010011922	PIK3R1	3	0.075006407	0.150843909	0.497245185	0.619016161	0.033167645	3.854299221	48.10989273	0.666666667	CRA004775;PRJNA827465;Yang2025_Silkie_Wencha	0.882522345
ENSGALG00010010779	DOCK2	3	-0.07198769	0.144918634	-0.496745574	0.61936848	0.013076439	2.453925048	18.49791821	0.666666667	CRA004775;PRJNA827465;Yang2025_Silkie_Wencha	0.882522345
ENSGALG00010024949	PAN2	3	-0.069985648	0.14085571	-0.496860571	0.619287378	0.042313605	8.33131623	75.99418934	0.666666667	CRA004775;PRJNA827465;Yang2025_Silkie_Wencha	0.882522345
ENSGALG00010029755	ACOX1	3	-0.064481067	0.129460225	-0.498076277	0.618430281	0.031428286	5.713507678	64.99523388	0.666666667	CRA004775;PRJNA827465;Yang2025_Silkie_Wencha	0.882522345
ENSGALG00010006355	PTGR1	3	0.064471406	0.129533535	0.497719806	0.618681546	0.032302886	6.374163754	68.62333512	0.666666667	CRA004775;PRJNA827465;Yang2025_Silkie_Wencha	0.882522345
ENSGALG00010024016	CRYZ	3	-0.063693953	0.12804278	-0.497442754	0.618876863	0.029685409	5.334316774	62.50691354	0.666666667	CRA004775;PRJNA827465;Yang2025_Silkie_Wencha	0.882522345
ENSGALG00010012028	TMEM140	3	-0.061330309	0.12321635	-0.497744892	0.618663863	0.017251696	3.068744856	34.82677467	0.666666667	CRA004775;PRJNA827465;Yang2025_Silkie_Wencha	0.882522345
ENSGALG00010016999	ITGB1BP2	3	-0.053074381	0.106466483	-0.498507882	0.618126115	0.009948649	2.721507935	26.51132946	0.666666667	CRA004775;PRJNA827465;Yang2025_Silkie_Wencha	0.882522345
ENSGALG00010001270	DRAP1	3	0.05152272	0.103701612	0.496836252	0.619304529	0.01365306	3.094494132	35.36908086	0.666666667	CRA004775;PRJNA827465;Yang2025_Silkie_Wencha	0.882522345
ENSGALG00010024784	ENSGALG0001002478	3	0.040762248	0.081931702	0.497514967	0.618825951	0	0.965640747	0	0.666666667	CRA004775;PRJNA827465;Yang2025_Silkie_Wencha	0.882522345
ENSGALG00010008241	HERC3	3	0.040431944	0.081376776	0.496848684	0.619295761	0.010137986	4.226443193	52.67888604	0.666666667	CRA004775;PRJNA827465;Yang2025_Silkie_Wencha	0.882522345
ENSGALG00010017888	MPLKIP	3	0.02705683	0.054423367	0.497154648	0.61908	0	1.743175955	0	0.666666667	CRA004775;PRJNA827465;Yang2025_Silkie_Wencha	0.882522345
ENSGALG00010018505	CHTF18	3	-0.022418381	0.045061298	-0.497508555	0.618830472	0	1.062283238	0	1	CRA004775;PRJNA827465;Yang2025_Silkie_Wencha	0.882522345
ENSGALG00010005315	ENSGALG0001000531	3	-0.097057015	0.195570592	-0.496276123	0.61969961	0	1.455189975	0	0.666666667	CRA004775;PRJNA827465;Yang2025_Silkie_Wencha	0.882636525
ENSGALG00010007465	PNAT10	3	-0.084491698	0.170245254	-0.496294001	0.619686998	0.015960394	2.437929414	17.96317037	0.666666667	CRA004775;PRJNA827465;Yang2025_Silkie_Wencha	0.882636525
ENSGALG00010029271	MYL10	3	-0.178245665	0.35927935	-0.496119982	0.619809762	0.254740261	7.807016902	74.38202037	0.666666667	CRA004775;PRJNA827465;Yang2025_Silkie_Wencha	0.882704495
ENSGALG00010023475	ENSGALG0001002347	2	0.436179912	0.880044078	0.495634166	0.620152543	1.297101905	6.092198396	83.58556411	0.5	PRJNA827465;Yang2025_Silkie_Wenchang	0.882898955
ENSGALG00010013335	POPDC3	3	-0.218519787	0.441240091	-0.4952401	0.620430648	0.501002595	37.29353002	94.63713947	0.666666667	CRA004775;PRJNA827465;Yang2025_Silkie_Wencha	0.882898955
ENSGALG00010005232	GATA3	3	-0.19765564	0.39896105	-0.495425906	0.620299512	0.128302274	2.710196325	26.20460809	0.666666667	CRA004775;PRJNA827465;Yang2025_Silkie_Wencha	0.882898955
ENSGALG00010025480	FGF19	2	0.180436387	0.364330516	0.495254664	0.620420368	0.149153829	2.262076248	55.79282525	0.5	PRJNA827465;Yang2025_Silkie_Wenchang	0.882898955
ENSGALG00010019082	POLR3K	3	0.14813768	0.299185661	0.495136295	0.620503915	0.208234958	10.6321136	81.1890648	0.666666667	CRA004775;PRJNA827465;Yang2025_Silkie_Wencha	0.882898955
ENSGALG00010022161	CELF1	3	0.082873614	0.167296474	0.495369758	0.620339138	0.07211816	15.44825236	87.05355173	0.666666667	CRA004775;PRJNA827465;Yang2025_Silkie_Wencha	0.882898955
ENSGALG00010027092	ENSGALG0001002709	3	-0.074823864	0.150946947	-0.49569644	0.620108599	0.04389579	7.03835558	71.58427168	0.666666667	CRA004775;PRJNA827465;Yang2025_Silkie_Wencha	0.882898955
ENSGALG00010018337	TMEM151B	3	-0.05797473	0.116983395	-0.495580849	0.620190167	0	0.080603453	0	1	CRA004775;PRJNA827465;Yang2025_Silkie_Wencha	0.882898955
ENSGALG00010018344	SFXN5	3	0.036829803	0.074384096	0.495130077	0.620508304	0	0.585856225	0	0.666666667	CRA004775;PRJNA827465;Yang2025_Silkie_Wencha	0.882898955
ENSGALG00010021739	EXOSC10	3	0.043693058	0.088276395	0.494957439	0.620630164	0.013071478	5.330703106	62.48149709	0.666666667	CRA004775;PRJNA827465;Yang2025_Silkie_Wencha	0.882925159
ENSGALG00010008165	MRPL1	3	-0.030641544	0.061911233	-0.494927054	0.620651613	0.001351733	2.208721949	9.449896979	0.666666667	CRA004775;PRJNA827465;Yang2025_Silkie_Wencha	0.882925159
ENSGALG00010027284	CTTNBP2NL	3	0.05301095	0.107129139	0.49483223	0.620718552	0.01987802	5.402580563	62.98065384	0.666666667	CRA004775;PRJNA827465;Yang2025_Silkie_Wencha	0.88293155
ENSGALG00010021761	MAFB	3	0.148343533	0.299846269	0.494731961	0.620789337	0.179387584	6.140105274	67.42726858	0.666666667	CRA004775;PRJNA827465;Yang2025_Silkie_Wencha	0.88294341
ENSGALG00010019667	ITPRID1	2	-0.619335978	1.252141747	-0.494621299	0.620867465	2.465769677	4.317496367	76.83842868	0.5	CRA004775;Yang2025_Silkie_Wenchang	0.882965709
ENSGALG00010001700	ENSGALG0001000170	3	0.131284175	0.26562244	0.494251069	0.621128877	0.129803726	5.434883526	63.20068332	0.666666667	CRA004775;PRJNA827465;Yang2025_Silkie_Wencha	0.883248637
ENSGALG00010010742	LCP2	3	-0.063092542	0.127677944	-0.494153807	0.62119756	0	0.868278158	0	1	CRA004775;PRJNA827465;Yang2025_Silkie_Wencha	0.883257472
ENSGALG00010003817	BF2	3	-0.111217343	0.225160909	-0.493946055	0.621344278	0.076432749	4.033542803	50.41579828	0.666666667	CRA004775;PRJNA827465;Yang2025_Silkie_Wencha	0.883377249
ENSGALG00010000798	ENSGALG0001000079	2	-0.103806854	0.210239449	-0.493755358	0.621478964	0	0.420967377	0	0.5	PRJNA827465;Yang2025_Silkie_Wenchang	0.883479899
ENSGALG00010028174	GMPPB	3	0.110254787	0.223437753	0.493447438	0.621696471	0.11458473	9.188363284	78.23333778	0.666666667	CRA004775;PRJNA827465;Yang2025_Silkie_Wencha	0.883700251
ENSGALG00010025648	ENSGALG0001002564	2	0.245536135	0.497867921	0.493175247	0.621888766	0.203608638	1.641568636	39.08265678	0.5	CRA004775;PRJNA827465	0.883707062
ENSGALG00010016900	CISD2	3	0.1383372	0.280455357	0.493259252	0.621829416	0.206307407	22.98991509	91.30053333	0.666666667	CRA004775;PRJNA827465;Yang2025_Silkie_Wencha	0.883707062
ENSGALG00010023666	ENSGALG0001002366	3	-0.061923726	0.125535296	-0.493277412	0.621816586	0	1.788734067	0	0.666666667	CRA004775;PRJNA827465;Yang2025_Silkie_Wencha	0.883707062
ENSGALG00010027874	FRS3	3	0.023781612	0.048238248	0.493003234	0.622010302	0	0.520659317	0	0.666666667	CRA004775;PRJNA827465;Yang2025_Silkie_Wencha	0.883790942
ENSGALG00010000784	FBXL3	3	-0.06372679	0.129287489	-0.492907637	0.622077851	0.034448658	7.36968195	72.8617868	0.666666667	CRA004775;PRJNA827465;Yang2025_Silkie_Wencha	0.883798104
ENSGALG00010000188	ENSGALG0001000018	3	-0.441800335	0.89706105	-0.492497512	0.62236768	0	0.829292857	0	0.666666667	CRA004775;PRJNA827465;Yang2025_Silkie_Wencha	0.883816777

ENSGALG00010011101	ENSGALG0001001110	3	-0.336316213	0.683931863	-0.491739355	0.622903613	1.099392751	9.587890241	79.14035361	0.666666667	CRA004775;PRJNA827465;Yang2025_Silkie_Wencha	0.883816777
ENSGALG00010001231	CNDP1	2	0.191561481	0.38920841	0.492182277	0.622590492	0	0.083505945	0	1	PRJNA827465;Yang2025_Silkie_Wenchang	0.883816777
ENSGALG00010002436	ENSGALG0001000243(	3	0.187102388	0.380165997	0.492159714	0.622606441	0	1.51858132	0	0.666666667	CRA004775;PRJNA827465;Yang2025_Silkie_Wencha	0.883816777
ENSGALG00010023043	CNP	3	0.098593176	0.200461949	0.49182988	0.622839611	0.093859503	9.701581209	79.38480381	0.666666667	CRA004775;PRJNA827465;Yang2025_Silkie_Wencha	0.883816777
ENSGALG00010020571	MRTO4	3	0.096353401	0.195589457	0.492630856	0.622273442	0.093315236	11.57097266	82.71536837	0.666666667	CRA004775;PRJNA827465;Yang2025_Silkie_Wencha	0.883816777
ENSGALG00010001307	CALU	3	0.088585525	0.180130277	0.491785872	0.622870725	0.075204116	9.412353943	78.7513303	0.666666667	CRA004775;PRJNA827465;Yang2025_Silkie_Wencha	0.883816777
ENSGALG00010003810	HMGN2P46	3	0.082849071	0.168213894	0.492522162	0.622350259	0.059928213	8.995127193	77.76573964	0.666666667	CRA004775;PRJNA827465;Yang2025_Silkie_Wencha	0.883816777
ENSGALG00010027968	FKBP15	3	0.063445937	0.128891964	0.49224122	0.622548828	0.034405342	9.347678116	78.60431248	0.666666667	CRA004775;PRJNA827465;Yang2025_Silkie_Wencha	0.883816777
ENSGALG00010001369	PPP2R2D	3	0.057920768	0.117718265	0.492028727	0.622699036	0.029740215	9.888182994	79.7738371	0.666666667	CRA004775;PRJNA827465;Yang2025_Silkie_Wencha	0.883816777
ENSGALG00010009472	ADO	3	-0.029174868	0.059229888	-0.492570033	0.622316427	0	0.910557709	0	0.666666667	CRA004775;PRJNA827465;Yang2025_Silkie_Wencha	0.883816777
ENSGALG00010002487	BCCIP	3	-0.025328574	0.051466808	-0.49213415	0.622624512	0	0.800628806	0	0.666666667	CRA004775;PRJNA827465;Yang2025_Silkie_Wencha	0.883816777
ENSGALG00010022513	NDUFAF1	3	0.021948411	0.044611001	0.491995489	0.622722532	0	0.901723771	0	0.666666667	CRA004775;PRJNA827465;Yang2025_Silkie_Wencha	0.883816777
ENSGALG00010026568	BYSL	3	-0.073353011	0.149202131	-0.491635141	0.622977297	0.033470758	3.739327295	46.51444385	0.666666667	CRA004775;PRJNA827465;Yang2025_Silkie_Wencha	0.883832631
ENSGALG00010000569	SIPA1	3	0.134219885	0.273072125	0.491518075	0.623060071	0.169525852	10.47174777	80.90099146	0.666666667	CRA004775;PRJNA827465;Yang2025_Silkie_Wencha	0.883861377
ENSGALG00010025452	TRIM33	3	0.020984982	0.042715785	0.491269975	0.623235513	0	0.822888458	0	1	CRA004775;PRJNA827465;Yang2025_Silkie_Wencha	0.88402156
ENSGALG00010027271	INKA2	3	-0.093302557	0.190016144	-0.491024369	0.623409212	0.073577199	8.184662689	75.56405101	0.666666667	CRA004775;PRJNA827465;Yang2025_Silkie_Wencha	0.884179241
ENSGALG00010011909	C8orf48	3	0.07687858	0.156731125	0.490512527	0.623771268	0	1.68269506	0	0.666666667	CRA004775;PRJNA827465;Yang2025_Silkie_Wencha	0.884307251
ENSGALG00010025161	ENSGALG0001002516	3	-0.050337825	0.10261439	-0.490553274	0.623742442	0.013955925	3.455825393	42.1267057	0.666666667	CRA004775;PRJNA827465;Yang2025_Silkie_Wencha	0.884307251
ENSGALG00010024101	CNEP1R1	3	-0.033985254	0.069253382	-0.490737822	0.623611892	0.005417529	3.101222743	35.50930824	0.666666667	CRA004775;PRJNA827465;Yang2025_Silkie_Wencha	0.884307251
ENSGALG00010027080	TBC1D22B	3	-0.030466443	0.062093771	-0.490652165	0.623672485	0.003095677	2.552644511	21.64988147	0.666666667	CRA004775;PRJNA827465;Yang2025_Silkie_Wencha	0.884307251
ENSGALG00010025951	TBRG4	3	-0.027685268	0.056448166	-0.490454686	0.623812188	0	1.969899531	0	0.666666667	CRA004775;PRJNA827465;Yang2025_Silkie_Wencha	0.884307251
ENSGALG00010028689	CFAP45	3	-0.521302488	1.067025298	-0.488556821	0.625155489	2.770987366	10.78615293	81.45770774	0.666666667	CRA004775;PRJNA827465;Yang2025_Silkie_Wencha	0.884543315
ENSGALG00010029553	MYH1C	3	0.289327754	0.592569846	0.488259327	0.625366167	0.888856295	37.02874911	94.59879135	0.666666667	CRA004775;PRJNA827465;Yang2025_Silkie_Wencha	0.884543315
ENSGALG00010024571	KCNA3	3	0.198109962	0.405789233	0.488209016	0.625401799	0.184240131	3.181700767	37.14053752	0.666666667	CRA004775;PRJNA827465;Yang2025_Silkie_Wencha	0.884543315
ENSGALG00010009099	CXCR4	3	0.179444005	0.366190549	0.490029047	0.624113345	0.332172344	17.57866271	88.62257026	0.666666667	CRA004775;PRJNA827465;Yang2025_Silkie_Wencha	0.884543315
ENSGALG00010005510	MYOZ2	3	0.136807695	0.280319891	0.488041338	0.625520561	0.147074074	7.324290331	72.6936002	0.666666667	CRA004775;PRJNA827465;Yang2025_Silkie_Wencha	0.884543315
ENSGALG00010021534	KIFC3	3	0.131001553	0.267791839	0.489191731	0.624705963	0.180979894	22.02762686	90.92049265	0.666666667	CRA004775;PRJNA827465;Yang2025_Silkie_Wencha	0.884543315
ENSGALG00010022702	CDC20	3	-0.117477909	0.24035003	-0.488778426	0.624998574	0.120119665	8.318753072	75.95793525	0.666666667	CRA004775;PRJNA827465;Yang2025_Silkie_Wencha	0.884543315
ENSGALG00010000147	ENSGALG0001000014	3	0.113275365	0.232116814	0.488010165	0.625542641	0.132946676	12.50250795	84.00320953	0.666666667	CRA004775;PRJNA827465;Yang2025_Silkie_Wencha	0.884543315
ENSGALG00010000507	TRPT1	3	0.108898479	0.222926221	0.488495605	0.625198838	0.060231219	3.307238769	39.52659183	0.666666667	CRA004775;PRJNA827465;Yang2025_Silkie_Wencha	0.884543315
ENSGALG00010029332	YWHAE	3	0.101728215	0.20796819	0.489152765	0.624733548	0.107839824	13.6343859	85.33120586	0.666666667	CRA004775;PRJNA827465;Yang2025_Silkie_Wencha	0.884543315
ENSGALG00010029328	ENSGALG0001002932	3	-0.090432494	0.185101293	-0.488556793	0.625155509	0.073976688	9.448344198	78.83226989	0.666666667	CRA004775;PRJNA827465;Yang2025_Silkie_Wencha	0.884543315
ENSGALG00010025067	SLC1A2	3	0.090187698	0.184645595	0.488436772	0.625240501	0.03285608	2.850375612	29.83380887	0.666666667	CRA004775;PRJNA827465;Yang2025_Silkie_Wencha	0.884543315
ENSGALG00010023127	CERS5	3	-0.083809912	0.171621576	-0.488341349	0.625308078	0.063568067	9.589740396	79.14437808	0.666666667	CRA004775;PRJNA827465;Yang2025_Silkie_Wencha	0.884543315
ENSGALG00010019049	POLD2	3	0.074643051	0.15262002	0.489077713	0.624786679	0.047702001	6.569015227	69.55403617	0.666666667	CRA004775;PRJNA827465;Yang2025_Silkie_Wencha	0.884543315
ENSGALG00010024068	CASP2	3	-0.062863088	0.128449912	-0.489397674	0.624560182	0.025911762	4.592332313	56.44914471	0.666666667	CRA004775;PRJNA827465;Yang2025_Silkie_Wencha	0.884543315
ENSGALG00010023913	ZMYND8	3	0.061293675	0.12520906	0.489530671	0.624466046	0.025067064	4.035627141	50.44140773	0.666666667	CRA004775;PRJNA827465;Yang2025_Silkie_Wencha	0.884543315
ENSGALG00010003273	NFIX	3	-0.056872204	0.116430396	-0.488465264	0.625220324	0.017124389	3.486475363	42.63547589	0.666666667	CRA004775;PRJNA827465;Yang2025_Silkie_Wencha	0.884543315
ENSGALG00010020843	HMG20A	3	-0.055644385	0.113603767	-0.489811092	0.624267581	0.021067278	5.053165109	60.42084601	0.666666667	CRA004775;PRJNA827465;Yang2025_Silkie_Wencha	0.884543315
ENSGALG00010022731	LRRC8B	3	-0.047777634	0.097888262	-0.488083379	0.625490783	0	1.293913884	0	0.666666667	CRA004775;PRJNA827465;Yang2025_Silkie_Wencha	0.884543315
ENSGALG00010018395	MED12	3	0.039803986	0.081491318	0.488444503	0.625235026	0.009702621	3.90098401	48.73088444	0.666666667	CRA004775;PRJNA827465;Yang2025_Silkie_Wencha	0.884543315
ENSGALG00010019139	DPCD	3	0.03761899	0.076768219	0.490033377	0.62411028	0	0.673369644	0	0.666666667	CRA004775;PRJNA827465;Yang2025_Silkie_Wencha	0.884543315
ENSGALG00010022463	DUSP12	3	-0.037361379	0.07628639	-0.48975157	0.624309705	0	1.365590828	0	0.666666667	CRA004775;PRJNA827465;Yang2025_Silkie_Wencha	0.884543315
ENSGALG00010005309	ASXL3	3	0.033105212	0.067847777	0.487933627	0.625596855	0	0.515957033	0	1	CRA004775;PRJNA827465;Yang2025_Silkie_Wencha	0.884543315
ENSGALG00010021114	SBF2	3	-0.030979703	0.063493182	-0.487921723	0.625605287	0	1.265634588	0	0.666666667	CRA004775;PRJNA827465;Yang2025_Silkie_Wencha	0.884543315
ENSGALG00010019855	TTC7A	3	-0.030726737	0.062868298	-0.488747723	0.625020313	0	0.029332771	0	1	CRA004775;PRJNA827465;Yang2025_Silkie_Wencha	0.884543315
ENSGALG00010024163	HS6ST1	3	0.02733065	0.055904802	0.488878403	0.624927787	0.001418606	2.283150299	12.40173716	0.666666667	CRA004775;PRJNA827465;Yang2025_Silkie_Wencha	0.884543315
ENSGALG00010000647	ENSGALG0001000064	2	0.238532677	0.489302011	0.487495802	0.625907017	0	0.06842837	0	1	PRJNA827465;Yang2025_Silkie_Wenchang	0.884565171
ENSGALG00010028544	ZNF414	3	0.10688895	0.219328531	0.487346311	0.626012934	0.099947305	6.737719953	70.31636794	0.666666667	CRA004775;PRJNA827465;Yang2025_Silkie_Wencha	0.884565171
ENSGALG00010022472	QSER1	3	0.093703403	0.192324874	0.487214165	0.626106568	0.073865659	6.214741467	67.81845213	0.666666667	CRA004775;PRJNA827465;Yang2025_Silkie_Wencha	0.884565171
ENSGALG00010023910	RNF7	3	-0.085150021	0.174837987	-0.487022429	0.626242437	0.071934946	14.34978831	86.06251217	0.666666667	CRA004775;PRJNA827465;Yang2025_Silkie_Wencha	0.884565171
ENSGALG00010028800	ENSGALG0001002880	2	0.083838175	0.172146341	0.487016885	0.626246365	0.014654779	1.304564073	23.34604174	0.5	PRJNA827465;Yang2025_Silkie_Wenchang	0.884565171
ENSGALG00010012896	STK38L	3	-0.069861957	0.143345828	-0.487366513	0.62599862	0.042153112	11.23945477	82.20554253	0.666666667	CRA004775;PRJNA827465;Yang2025_Silkie_Wencha	0.884565171
ENSGALG00010010918	TOR1AIP2	3	-0.066267763	0.135889404	-0.487659528	0.625791023	0.036701197	6.787855636	70.53561379	0.666666667	CRA004775;PRJNA827465;Yang2025_Silkie_Wencha	0.884565171
ENSGALG00010010226	BICC1	3	-0.061315	0.125836561	-0.487259029	0.626074778	0.026051243	4.679790513	57.26304427	0.666666667	CRA004775;PRJNA827465;Yang2025_Silkie_Wencha	0.884565171
ENSGALG00010015874	CLIC4	3	-0.059599962	0.122370214	-0.487046318	0.626225508	0.028801566	7.382100232	72.90743911	0.666666667	CRA004775;PRJNA827465;Yang2025_Silkie_Wencha	0.884565171
ENSGALG00010019125	ENSGALG0001001912	3	0.042093063	0.08640667	0.487150621	0.626151595	0	1.09711687	0	0.666666667	CRA004775;PRJNA827465;Yang2025_Silkie_Wencha	0.884565171
ENSGALG00010008866	WASHC5	3	0.020687159	0.042513773	0.486598978	0.626542548	0.000452614	2.143512154	6.695187337	0.666666667	CRA004775;PRJNA827465;Yang2025_Silkie_Wencha	0.884895124
ENSGALG00010021330	COMMD4	3	0.098616172	0.202723862	0.486455672	0.626644127	0.083428938	6.322373615	68.36631111	0.666666667	CRA004775;PRJNA827465;Yang2025_Silkie_Wencha	0.884912889
ENSGALG00010027705	S100A11	3	0.095314867	0.195993548	0.486316353	0.626742887	0.061544807	4.531129907	55.86089914	0.666666667	CRA004775;PRJNA827465;Yang2025_Silkie_Wencha	0.884912889
ENSGALG00010018726	EBF2	3	-0.036797535	0.075656835	-0.486374236	0.626701854	0	0.389838477	0	0.666666667	CRA004775;PRJNA827465;Yang2025_Silkie_Wencha	0.884912889
ENSGALG00010012387	ERBIN	3	0.051347485	0.105605345	0.48622051	0.626810831	0.014326636	3.399141977	41.16162215	0.666666667	CRA004775;PRJNA827465;Yang2025_Silkie_Wencha	0.884920453
ENSGALG00010019811	UBXN4	3	0.042499668	0.087493106	0.485748768	0.627145302	0.009967871	3.501545132	42.25235838	0.666666667	CRA004775;PRJNA827465;Yang2025_Silkie_Wencha	0.885304256
ENSGALG00010011751	ZNF706	3	-0.139207769	0.286726858	-0.485506556	0.627317063	0.228140821	29.9942054	93.3320454	0.666666667	CRA004775;PRJNA827465;Yang2025_Silkie_Wencha	0.885332848
ENSGALG00010018604	FAM43A	3	0.074540572	0.153547753	0.485455311	0.627353406	0.036002315	4.134097159	51.62184334	0.666666667	CRA004775;PRJNA827465;Yang2025_Silkie_Wencha	0.885332848
ENSGALG00010006010	IPO5	3	-0.065488967	0.134896314	-0.48547633	0.627338499	0.040171484	8.595739751	76.73265992	0.666666667	CRA004775;PRJNA827465;Yang2025_Silkie_Wencha	0.885332848
ENSGALG00010023432	TMOD3	3	-0.084639195	0.174438502	-0.485209229	0.627527936	0.073352306	14.48290348	86.19061431	0.666666667	CRA004775;PRJNA827465;Yang2025_Silkie_Wencha	0.885418131
ENSGALG00010003541	CNR1	3	-0.064252955	0.132427481	-0.485193514	0.627539083	0.024283157	3.988302972	49.85335833	0.666666667	CRA004775;PRJNA827465;Yang2025_Silkie_Wencha	0.885418131
ENSGALG00010008364	ENSGALG0001000836	2	-0.572409034	1.182382732	-0.484114846	0.628304366	2.57956184	12.89388932	92.24438821	0.5	PRJNA827465;Yang2025_Silkie_Wenchang	0.885536634
ENSGALG00010008150	TERT	2	0.406561632	0.838811157	0.484687916	0.627897739	1.106727322	4.631845843	78.41033502	0.5	CRA004775;PRJNA827465	0.885536634
ENSGALG00010014455	C4orf19	2	0.274280676	0.566267062	0.484366293	0.628125936	0.559469863	7.717067602	87.04170999	0.5	PRJNA827465;Yang2025_Silkie_Wenchang	0.885536634
ENSGALG00010019875	PLEKHH1	3	-0.232416536	0.479842629	-0.484359917	0.62813046	0.494192758	9.702557094	79.38687729	0.666666667	CRA004775;PRJNA827465;Yang2025_Silkie_Wencha	0.885536634
ENSGALG00010000768	ENSGALG0001000076	3	0.158909156	0.328214689	0.484162231	0.628270739	0.138881067	3.487483021	42.65205055	0.666666667	CRA004775;PRJNA827465;Yang2025_Silkie_Wencha	0.885536634
ENSGALG00010018284	ENSGALG0001001828	3	0.114724982	0.236972708	0.484127403	0.628295455	0.118387765	12.18052404	83.5803452	0.666666667	CRA004775;PRJNA827465;Yang2025_Silkie_Wencha	0.885536634
ENSGALG00010018753	SYNM	3	0.108281326	0.22391855	0.483574614	0.628687795	0.094196844	6.109098487	67.26194537	0.666666667	CRA004775;PRJNA827465;Yang2025_Silkie_Wencha	0.885536634
ENSGALG00010020147	ABHD2	3	-0.106609142	0.2199334	-0.48473375	0.627865223	0.097224982	8.202373377	75.61681347	0.666666667	CRA004775;PRJNA827465;Yang2025_Silkie_Wencha	0.885536634
ENSGALG00010021246	PECR	3	0.098150983	0.202934731	0.483657887	0.628628686	0.093868124	11.0881061	81.96265456	0.666666667	CRA004775;PRJNA827465;Yang2025_Silkie_Wencha	0.885536634
ENSGALG00010010990	MED18	3	-0.088648514	0.183253768	-0.483747292	0.628565226	0.061119698	5.160739923	61.24586726	0.666666667	CRA004775;PRJNA827465;Yang2025_Silkie_Wencha	0.885536634
ENSGALG00010013181	DYRK1A	3	0.067703754	0.139885218	0.483995059	0.628389373	0.037060839	5.38000073	62.82528386	0.666666667	CRA004775;PRJNA827465;Yang2025_Silkie_Wencha	0.885536634
ENSGALG00010018081	DIO3	3	-0.063058403	0.130076916	-0.484777812	0.627833963	0	1.405841958	0	0.666666667	CRA004775;PRJNA827465;Yang2025_Silkie_Wencha	0.885536634
ENSGALG00010028474	PEX19	3	-0.051016153	0.105390318	-0.484068685	0.628337125	0.003772511	2.15748214	7.299348488	0.666666667	CRA004775;PRJNA827465;Yang2025_Silkie_Wencha	0.885536634
ENSGALG00010020530	SRSF5A	3	0.034146187	0.070605731	0.483617784	0.628657151	0.009579059	7.056445008	71.65711633	0.666666667	CRA004775;PRJNA827465;Yang2025_Silkie_Wencha	0.885536634
ENSGALG00010012769	NEMP2	3	-0.033604246	0.069452486	-0.483845115	0.628495795	0	1.567588258	0	0.666666667	CRA004775;PRJNA827465;Yang2025_Silkie_Wencha	0.885536634
ENSGALG00010001826	HOXA7	3	0.026187741	0.054143088	0.483676524	0.628615456	0	0.814743271	0	0.666666667	CRA004775;PRJNA827465;Yang2025_Silkie_Wencha	0.885536634
ENSGALG00010005909	BRMS1L	3	-0.023138843	0.047749866	-0.484584459	0.62797114	0	1.31171811	0	0.666666667	CRA004775;PRJNA827465;Yang2025_Silkie_Wencha	0.885536634
ENSGALG00010029635	ENSGALG0001002963	3	-0.186378094	0.386065641	-0.482762706	0.629264234	0.301673286	6.312834415	68.31851006	0.666666667	CRA004775;PRJNA827465;Yang2025_Silkie_Wencha	0.885779003
ENSGALG00010014829	ENSGALG0001001482	3	0.149425487	0.309481468	0.482825313	0.629219776	0.091538693	2.900158496	31.03825177	0.666666667	CRA004775;PRJNA827465;Yang2025_Silkie_Wencha	0.885779003
ENSGALG00010021106	ITGB6	3	0.131005243	0.271289768	0.482897838	0.629168277	0.133341786	7.057411406	71.66099743	0.666666667	CRA004775;PRJNA827465;Yang2025_Silkie_Wencha	0.885779003
ENSGALG00010005321	HS6ST3	3	0.104559066	0.21683156	0.48221332	0.629654415	0	1.246913883	0	0.666666667	CRA004775;PRJNA827465;Yang2025_Silkie_Wencha	0.885779003
ENSGALG00010014209	CCDC50	3	-0.093963691	0.19484938	-0.482237569	0.629637192	0.101333978	30.18171537	93.3734714	0.666666667	CRA004775;PRJNA827465;Yang2025_Silkie_Wencha	0.885779003
ENSGALG00010002364	VSNL1	3	-0.087912401	0.182261939	-0.482340974	0.629563745	0	1.188637291	0	0.666666667	CRA004775;PRJNA827465;Yang2025_Silkie_Wencha	0.885779003
ENSGALG00010023304	ENSGALG0001002330	3	-0.075256967	0.156002554	-0.482408561	0.629515741	0.040322724	4.714143412	57.57447695	0.666666667	CRA004775;PRJNA827465;Yang2025_Silkie_Wencha	0.885779003
ENSGALG00010005334	NME7	3	0.070227222	0.145643627	0.482185343	0.629674288	0.030537869	3.749297984	46.65668058	0.666666667	CRA004775;PRJNA827465;Yang2025_Silkie_Wencha	0.885779003
ENSGALG00010014220	C9orf85	3	-0.065414793	0.1355195	-0.482696533	0.629311226	0	1.75241106	0	0.666666667	CRA004775;PRJNA827465;Yang2025_Silkie_Wencha	0.885779003
ENSGALG00010016911	AXIN1	3	0.052526134	0.108907809	0.482299058	0.629593516	0.023676435	6.249001766	67.99488823	0.666666667	CRA004775;PRJNA827465;Yang2025_Silkie_Wencha	0.885779003
ENSGALG00010028744	HOMER3	3	-0.030972524	0.064162274	-0.482721738	0.629293326	0	1.418408882	0	0.666666667	CRA004775;PRJNA827465;Yang2025_Silkie_Wencha	0.885779003
ENSGALG00010018858	EPB41L5	3	0.028787036	0.05968003	0.482356265	0.629552884	0	1.558407134	0	0.666666667	CRA004775;PRJNA827465;Yang2025_Silkie_Wencha	0.885779003
ENSGALG00010021193	USP37	3	-0.022292512	0.04620469	-0.482472918	0.629470033	0	0.009766126	0	1	CRA004775;PRJNA827465;Yang2025_Silkie_Wencha	0.885779003
ENSGALG00010026581	DNER	3	-0.157606309	0.327156154	-0.481746429	0.62998609	0.19530067	6.200175574	67.74284895	0.666666667	CRA004775;PRJNA827465;Yang2025_Silkie_Wencha	0.885800803
ENSGALG00010006408	ENSGALG0001000640	2	0.147712744	0.306575796	0.481814763	0.629937541	0	0.105246938	0	1	PRJNA827465;Yang2025_Silkie_Wenchang	0.885800803
ENSGALG00010023545	PTPRT	3	0.090549082	0.18800308	0.481636164	0.630064431	0.055275521	4.676607906	57.23396016	0.666666667	CRA004775;PRJNA827465;Yang2025_Silkie_Wencha	0.885800803
ENSGALG00010022125	CBFA2T2	3	-0.048076995	0.0997577	-0.481937679	0.629850219	0.010198874	2.938692395	31.94251962	0.666666667	CRA004775;PRJNA827465;Yang2025_Silkie_Wencha	0.885800803
ENSGALG00010018531	ADCY5	3	0.041853369	0.086898629	0.481634404	0.630065682	0.002224874	2.179466345	8.234416878	0.666666667	CRA004775;PRJNA827465;Yang2025_Silkie_Wencha	0.885800803
ENSGALG00010015984	PDCL3	3	0.029238149	0.060682894	0.481818635	0.62993479	0	0.378238413	0	0.666666667	CRA004775;PRJNA827465;Yang2025_Silkie_Wencha	0.885800803
ENSGALG00010022558	NYAP2	2	0.399374391	0.83028332	0.481009773	0.630509553	1.186951776	7.188841384	86.08955259	0.5	PRJNA827465;Yang2025_Silkie_Wenchang	0.885808284
ENSGALG00010011343	PXDC1	3	-0.126753964	0.263347209	-0.481318806	0.630289933	0.184708773	28.86097695	93.07022765	0.666666667	CRA004775;PRJNA827465;Yang2025_Silkie_Wencha	0.885808284
ENSGALG00010002943	SAP30	3	-0.123253005	0.25602678	-0.48140669	0.630227483	0.167658215	18.84710949	89.3882932	0.666666667	CRA004775;PRJNA827465;Yang2025_Silkie_Wencha	0.885808284
ENSGALG00010024531	CEP63	3	0.069696323	0.144891961	0.481022703	0.630500363	0.037151783	5.368992291	62.74906181	0.666666667	CRA004775;PRJNA827465;Yang2025_Silkie_Wencha	0.885808284
ENSGALG00010010766	CENPO	3	0.045232809	0.09396385	0.481385222	0.630242738	0	0.537788044	0	0.666666667	CRA004775;PRJNA827465;Yang2025_Silkie_Wencha	0.885808284
ENSGALG00010020574	DTL	3	-0.036041007	0.074919929	-0.481060351	0.630473607	0	0.218109821	0	0.666666667	CRA004775;PRJNA827465;Yang2025_Silkie_Wencha	0.885808284
ENSGALG00010002507	C2CD3	3	-0.030659612	0.06373178	-0.481072593	0.630464906	0	0.443940311	0	1	CRA004775;PRJNA827465;Yang2025_Silkie_Wencha	0.885808284
ENSGALG00010018192	IQCH	3	0.164839077	0.34283607	0.480810193	0.630651405	0.268707223	10.36549444	80.70521371	0.666666667	CRA004775;PRJNA827465;Yang2025_Silkie_Wencha	0.885919545
ENSGALG00010001191	TOMM5	3	-0.069682033	0.144987074	-0.480608586	0.630794712	0	1.623695601	0	0.666666667	CRA004775;PRJNA827465;Yang2025_Silkie_Wencha	0.886032827
ENSGALG00010028144	FAM78A	3	0.062671406	0.130455215	0.480405523	0.630939068	0.024727881	3.981446419	49.76699949	0.666666667	CRA004775;PRJNA827465;Yang2025_Silkie_Wencha	0.886147559
ENSGALG00010029537	MYH1B	3	0.212498194	0.442720315	0.479982931	0.63123953	0.452462404	17.40160841	88.50680952	0.666666667	CRA004775;PRJNA827465;Yang2025_Silkie_Wencha	0.886481498
ENSGALG00010002282	TSKU	3	-0.074891681	0.156074377	-0.4798461	0.63133683	0.049586961	13.30093327	84.96346114	0.666666667	CRA004775;PRJNA827465;Yang2025_Silkie_Wencha	0.886530087
ENSGALG00010027128	EIF2D	3	0.062395103	0.130087733	0.479638636	0.631484369	0.034124837	6.861574834	70.85217245	0.666666667	CRA004775;PRJNA827465;Yang2025_Silkie_Wencha	0.886649205
ENSGALG00010014240	PIGV	3	0.09195902	0.191970641	0.47902648	0.63191979	0.076203795	6.583066981	69.61902399	0.666666667	CRA004775;PRJNA827465;Yang2025_Silkie_Wencha	0.886776624
ENSGALG00010029907	NTN1	3	0.085933446	0.179388423	0.47903563	0.631913281	0.029228971	2.804903767	28.69630595	0.666666667	CRA004775;PRJNA827465;Yang2025_Silkie_Wencha	0.886776624
ENSGALG00010005755	ZMYM2	3	0.072806755	0.151962815	0.479109018	0.631861074	0.0518864	8.477816709	76.40902052	0.666666667	CRA004775;PRJNA827465;Yang2025_Silkie_Wencha	0.886776624
ENSGALG00010017850	TRMT5	3	-0.064907064	0.135426868	-0.479277594	0.63174116	0.035339386	5.916284309	66.19499849	0.666666667	CRA004775;PRJNA827465;Yang2025_Silkie_Wencha	0.886776624
ENSGALG00010010834	KGD4	3	-0.064685716	0.135048322	-0.478982006	0.631951429	0	0.390435429	0	0.666666667	CRA004775;PRJNA827465;Yang2025_Silkie_Wencha	0.886776624
ENSGALG00010011161	ENSGALG0001001116	3	0.024529587	0.051196368	0.479127479	0.631847942	0	1.183065145	0	0.666666667	CRA004775;PRJNA827465;Yang2025_Silkie_Wencha	0.886776624
ENSGALG00010002827	SBSPON	3	-0.226444519	0.473369425	-0.478367438	0.632388706	0.493598851	7.864977622	74.57081131	0.666666667	CRA004775;PRJNA827465;Yang2025_Silkie_Wencha	0.88697387
ENSGALG00010024215	ENSGALG0001002421	3	0.223052972	0.466302808	0.478343617	0.632405657	0.480981863	9.213595697	78.2929481	0.666666667	CRA004775;PRJNA827465;Yang2025_Silkie_Wencha	0.88697387
ENSGALG00010017344	RERE	3	0.129361314	0.270262767	0.478650148	0.632187536	0.161454534	8.070374646	75.21800303	0.666666667	CRA004775;PRJNA827465;Yang2025_Silkie_Wencha	0.88697387
ENSGALG00010028196	ENSGALG0001002819	3	0.085021671	0.177725851	0.478386627	0.63237505	0.064495071	6.703328902	70.16407774	0.666666667	CRA004775;PRJNA827465;Yang2025_Silkie_Wencha	0.88697387
ENSGALG00010021265	SIPA1L1	3	0.052680701	0.110106362	0.478452839	0.632327934	0.016913593	3.592468501	44.32797395	0.666666667	CRA004775;PRJNA827465;Yang2025_Silkie_Wencha	0.88697387
ENSGALG00010006223	ATG10	3	0.05815293	0.121608208	0.478199059	0.632508532	0	0.105096674	0	1	CRA004775;PRJNA827465;Yang2025_Silkie_Wencha	0.887030166
ENSGALG00010009470	ZNF469	3	-0.052779208	0.110391354	-0.478109979	0.63257193	0	1.804039944	0	0.666666667	CRA004775;PRJNA827465;Yang2025_Silkie_Wencha	0.887031093
ENSGALG00010027193	CAMK1G	3	-0.125518514	0.262841055	-0.477545315	0.632973861	0	0.964159956	0	0.666666667	CRA004775;PRJNA827465;Yang2025_Silkie_Wencha	0.887330698
ENSGALG00010016114	HAUS6	3	-0.054414659	0.113936487	-0.477587647	0.632943724	0	1.332006336	0	0.666666667	CRA004775;PRJNA827465;Yang2025_Silkie_Wencha	0.887330698
ENSGALG00010029859	CSNK1D	3	0.019428317	0.040680854	0.477578897	0.632949954	0	0.531135168	0	0.666666667	CRA004775;PRJNA827465;Yang2025_Silkie_Wencha	0.887330698
ENSGALG00010002365	RAI2	3	0.105234154	0.220540528	0.477164699	0.633244847	0.105257479	7.813638256	74.4037293	0.666666667	CRA004775;PRJNA827465;Yang2025_Silkie_Wencha	0.887622572
ENSGALG00010029509	DNAJC30	3	-0.108764898	0.227991534	-0.477056738	0.63332172	0.097370454	5.647392145	64.5854237	0.666666667	CRA004775;PRJNA827465;Yang2025_Silkie_Wencha	0.887642327
ENSGALG00010019540	UNC5D	3	-0.083330017	0.174905867	-0.476427798	0.633769635	0	0.125377991	0	1	CRA004775;PRJNA827465;Yang2025_Silkie_Wencha	0.888114218
ENSGALG00010017789	IMMT	3	0.073499665	0.15430752	0.476319399	0.633846847	0.050737156	12.07734963	83.44007534	0.666666667	CRA004775;PRJNA827465;Yang2025_Silkie_Wencha	0.888114218
ENSGALG00010024520	MAP3K13	3	-0.052616242	0.110456052	-0.476354539	0.633821817	0	0.26662149	0	0.666666667	CRA004775;PRJNA827465;Yang2025_Silkie_Wencha	0.888114218
ENSGALG00010000650	BCKDK	3	-0.126204704	0.265046596	-0.476160439	0.633960081	0.152826489	8.701888607	77.01648354	0.666666667	CRA004775;PRJNA827465;Yang2025_Silkie_Wencha	0.888184858
ENSGALG00010002598	NMT2	3	-0.059554856	0.125160946	-0.475826193	0.634198208	0.035856412	11.5407286	82.67007163	0.666666667	CRA004775;PRJNA827465;Yang2025_Silkie_Wencha	0.888317978
ENSGALG00010016613	SNIP1	3	0.056109169	0.117917267	0.475835054	0.634191895	0.029735905	7.59483232	73.66630472	0.666666667	CRA004775;PRJNA827465;Yang2025_Silkie_Wencha	0.888317978
ENSGALG00010018033	COQ8A	3	-0.051114342	0.107436674	-0.475762511	0.634243581	0.008317096	2.526022043	20.82412717	0.666666667	CRA004775;PRJNA827465;Yang2025_Silkie_Wencha	0.888317978
ENSGALG00010029742	TAX1BP3	3	-0.039032111	0.082057961	-0.475665129	0.634312967	0.012108565	5.580064712	64.15812176	0.666666667	CRA004775;PRJNA827465;Yang2025_Silkie_Wencha	0.888327164
ENSGALG00010015107	MB	3	-0.449058114	0.945866627	-0.474758387	0.634959196	2.31318571	16.56625418	87.9272648	0.666666667	CRA004775;PRJNA827465;Yang2025_Silkie_Wencha	0.888385993
ENSGALG00010023293	COL11A1	3	0.239803676	0.504706665	0.475134752	0.63469093	0.565029103	8.24919628	75.75521382	0.666666667	CRA004775;PRJNA827465;Yang2025_Silkie_Wencha	0.888385993
ENSGALG00010021718	SST7	2	0.157704403	0.332246012	0.474661537	0.635028237	0	0.622036519	0	1	PRJNA827465;Yang2025_Silkie_Wenchang	0.888385993
ENSGALG00010014818	ENSGALG0001001481	3	-0.123209286	0.259330796	-0.475104724	0.634712332	0	0.514215979	0	0.666666667	CRA004775;PRJNA827465;Yang2025_Silkie_Wencha	0.888385993
ENSGALG00010008031	MAP3K21	3	-0.097557518	0.205312809	-0.475165277	0.634669174	0.059185958	3.939316355	49.22976933	0.666666667	CRA004775;PRJNA827465;Yang2025_Silkie_Wencha	0.888385993
ENSGALG00010007869	GCSH	3	-0.093586964	0.19704518	-0.474951804	0.634821326	0.07654511	6.024029277	66.79963015	0.666666667	CRA004775;PRJNA827465;Yang2025_Silkie_Wencha	0.888385993
ENSGALG00010023249	IL12RB2	3	-0.082897669	0.174655088	-0.474636437	0.63504613	0.017117261	2.389905024	16.31466609	0.666666667	CRA004775;PRJNA827465;Yang2025_Silkie_Wencha	0.888385993
ENSGALG00010011282	CRY1	3	0.064470643	0.135616397	0.475389735	0.63450921	0.031907905	5.532526685	63.85015177	0.666666667	CRA004775;PRJNA827465;Yang2025_Silkie_Wencha	0.888385993
ENSGALG00010005347	DOK1	3	0.056212843	0.11835592	0.474947452	0.634824428	0.012529337	2.671471535	25.13489385	0.666666667	CRA004775;PRJNA827465;Yang2025_Silkie_Wencha	0.888385993
ENSGALG00010021728	TMED5	3	0.055022462	0.115883915	0.474806726	0.634924738	0.029126353	8.04809848	75.14940945	0.666666667	CRA004775;PRJNA827465;Yang2025_Silkie_Wencha	0.888385993
ENSGALG00010024434	B3GNT9	3	0.039464041	0.083046011	0.475206944	0.634639478	0.005912523	2.734177268	26.85185327	0.666666667	CRA004775;PRJNA827465;Yang2025_Silkie_Wencha	0.888385993
ENSGALG00010024096	MYO1D	3	0.103789546	0.218878133	0.474188738	0.635365324	0.107177845	8.604953314	76.75757291	0.666666667	CRA004775;PRJNA827465;Yang2025_Silkie_Wencha	0.88839303
ENSGALG00010027534	KCNN3	3	0.097479043	0.205536079	0.474267309	0.6353093	0.086015269	7.156619628	72.0538452	0.666666667	CRA004775;PRJNA827465;Yang2025_Silkie_Wencha	0.88839303
ENSGALG00010007663	ARFIP1	3	-0.080149298	0.168922868	-0.474472755	0.635162822	0.073130164	22.42319271	91.08066355	0.666666667	CRA004775;PRJNA827465;Yang2025_Silkie_Wencha	0.88839303
ENSGALG00010019403	PACC1	3	0.038658639	0.081510104	0.474280325	0.63530002	0.003750301	2.380434583	15.98172811	0.666666667	CRA004775;PRJNA827465;Yang2025_Silkie_Wencha	0.88839303
ENSGALG00010015942	AP3S2	3	-0.036347789	0.076620711	-0.474385957	0.635224705	0.000405905	2.024566032	1.213397419	0.666666667	CRA004775;PRJNA827465;Yang2025_Silkie_Wencha	0.88839303
ENSGALG00010026149	LASP1	3	0.046375422	0.097842823	0.473978775	0.635515043	0.01700083	6.00093408	66.67185519	0.666666667	CRA004775;PRJNA827465;Yang2025_Silkie_Wencha	0.888514505
ENSGALG00010025782	ENSGALG0001002578	2	-0.925177594	1.953676326	-0.473557253	0.635815665	0	0.083974666	0	1	PRJNA827465;Yang2025_Silkie_Wenchang	0.88855018
ENSGALG00010012042	FNDC7	2	0.224558888	0.473908313	0.473844586	0.635610737	0.343803536	4.242850894	76.43094172	0.5	PRJNA827465;Yang2025_Silkie_Wenchang	0.88855018
ENSGALG00010024129	GID8	3	-0.151844015	0.320587217	-0.473643385	0.635754232	0.292925467	75.49971757	97.35098347	0.666666667	CRA004775;PRJNA827465;Yang2025_Silkie_Wencha	0.88855018
ENSGALG00010016825	ENSGALG0001001682	3	0.093177641	0.196690902	0.473726236	0.635695142	0.0460331	3.259278918	38.63673376	0.666666667	CRA004775;PRJNA827465;Yang2025_Silkie_Wencha	0.88855018
ENSGALG00010020966	PAPLN	3	-0.059969729	0.126651368	-0.473502414	0.635854779	0	1.164369132	0	0.666666667	CRA004775;PRJNA827465;Yang2025_Silkie_Wencha	0.88855018
ENSGALG00010017823	PPIE	3	0.086465258	0.182764947	0.47309541	0.636145111	0.078376429	10.7423027	81.38201785	0.666666667	CRA004775;PRJNA827465;Yang2025_Silkie_Wencha	0.888606998
ENSGALG00010020733	TMEM229B	3	0.080546847	0.170230265	0.47316408	0.636096122	0.054144755	6.131096483	67.37940749	0.666666667	CRA004775;PRJNA827465;Yang2025_Silkie_Wencha	0.888606998
ENSGALG00010002466	UROS	3	-0.076878272	0.162531659	-0.473004907	0.636209678	0.055468785	7.472388893	73.23479775	0.666666667	CRA004775;PRJNA827465;Yang2025_Silkie_Wencha	0.888606998
ENSGALG00010024839	PA2G4	3	0.046647423	0.098572342	0.47323034	0.636048854	0.014113501	3.646475266	45.1525143	0.666666667	CRA004775;PRJNA827465;Yang2025_Silkie_Wencha	0.888606998
ENSGALG00010022377	ZHX3	3	-0.026623893	0.056279412	-0.473066298	0.63616588	0	0.561835216	0	0.666666667	CRA004775;PRJNA827465;Yang2025_Silkie_Wencha	0.888606998
ENSGALG00010022479	ENSGALG0001002247	3	0.507327113	1.075527886	0.471700567	0.637140533	3.195551299	26.27249787	92.38747678	0.666666667	CRA004775;PRJNA827465;Yang2025_Silkie_Wencha	0.888616319
ENSGALG00010003765	ENSGALG0001000376	3	-0.306944312	0.652689539	-0.470276133	0.638157747	0.882893389	6.554640343	69.48726558	0.666666667	CRA004775;PRJNA827465;Yang2025_Silkie_Wencha	0.888616319
ENSGALG00010016777	TNMD	3	0.286107619	0.607288732	0.471122883	0.637552985	0.8893446	16.02291269	87.51787494	0.666666667	CRA004775;PRJNA827465;Yang2025_Silkie_Wencha	0.888616319
ENSGALG00010002362	TRPC4	3	0.226176255	0.480582446	0.470629456	0.637905369	0.502991567	7.595222815	73.66765862	0.666666667	CRA004775;PRJNA827465;Yang2025_Silkie_Wencha	0.888616319
ENSGALG00010013213	SH2D1A	3	0.223061066	0.474012495	0.470580561	0.637940292	0.444721289	5.900530118	66.10474042	0.666666667	CRA004775;PRJNA827465;Yang2025_Silkie_Wencha	0.888616319
ENSGALG00010018119	GRID2IP	2	0.141494411	0.300610089	0.470690826	0.637861536	0	0.786713445	0	0.5	PRJNA827465;Yang2025_Silkie_Wenchang	0.888616319
ENSGALG00010003994	SDR16C5	3	-0.130819771	0.278005761	-0.470564965	0.637951431	0.211381149	24.22610451	91.74444245	0.666666667	CRA004775;PRJNA827465;Yang2025_Silkie_Wencha	0.888616319
ENSGALG00010010781	NAPA	3	0.121101189	0.256940589	0.47131981	0.637412371	0.175467532	26.03003277	92.31656749	0.666666667	CRA004775;PRJNA827465;Yang2025_Silkie_Wencha	0.888616319
ENSGALG00010014702	ENSGALG0001001470	3	-0.117184515	0.248180532	-0.472174485	0.63680225	0.046841506	2.672482525	25.16321506	0.666666667	CRA004775;PRJNA827465;Yang2025_Silkie_Wencha	0.888616319
ENSGALG00010009028	PDLIM7	3	0.115131801	0.245051682	0.46982661	0.638478901	0.154567101	23.45575288	91.47330717	0.666666667	CRA004775;PRJNA827465;Yang2025_Silkie_Wencha	0.888616319
ENSGALG00010003863	HTR1B	3	0.105922807	0.225008029	0.470751232	0.637818394	0.081327773	4.348021068	54.00206281	0.666666667	CRA004775;PRJNA827465;Yang2025_Silkie_Wencha	0.888616319

ENSGALG00010005675	GJA3	3	-0.099123488	0.210101758	-0.471788	0.637078117	0	1.552593172	0	0.666666667	CRA004775;PRJNA827465;Yang2025_Silkie_Wencha	0.888616319
ENSGALG00010011556	LAPTM4B	3	-0.097460751	0.206664298	-0.471589685	0.63721969	0.090674178	9.47072086	78.88228331	0.666666667	CRA004775;PRJNA827465;Yang2025_Silkie_Wencha	0.888616319
ENSGALG00010014898	ENSGALG0001001489	3	0.096805899	0.205966907	0.470007055	0.638349977	0	0.148297185	0	1	CRA004775;PRJNA827465;Yang2025_Silkie_Wencha	0.888616319
ENSGALG00010002310	PRRG1	3	0.096019357	0.203705708	0.471363114	0.637381452	0.105387319	16.31715599	87.74296206	0.666666667	CRA004775;PRJNA827465;Yang2025_Silkie_Wencha	0.888616319
ENSGALG00010009726	PRR16	3	-0.093286572	0.197628718	-0.472029436	0.636905778	0.068309295	5.302615498	62.28276403	0.666666667	CRA004775;PRJNA827465;Yang2025_Silkie_Wencha	0.888616319
ENSGALG00010019418	ZNF703	3	0.092976475	0.197759595	0.470148997	0.638248571	0.073184082	5.556786619	64.00797552	0.666666667	CRA004775;PRJNA827465;Yang2025_Silkie_Wencha	0.888616319
ENSGALG00010013859	MYOC	3	0.086752972	0.183989379	0.471510759	0.637276039	0	1.353076852	0	0.666666667	CRA004775;PRJNA827465;Yang2025_Silkie_Wencha	0.888616319
ENSGALG00010011926	DYRK2	3	0.086088094	0.182259355	0.472338409	0.636685259	0.074298451	12.51929199	84.02465569	0.666666667	CRA004775;PRJNA827465;Yang2025_Silkie_Wencha	0.888616319
ENSGALG00010013482	ENSGALG0001001348	3	0.083841805	0.178448237	0.469838233	0.638470597	0.02986752	2.838806765	29.54786409	0.666666667	CRA004775;PRJNA827465;Yang2025_Silkie_Wencha	0.888616319
ENSGALG00010008279	MAPK15	3	0.076870368	0.163169913	0.471106265	0.637564851	0.039316888	4.003915952	50.04890152	0.666666667	CRA004775;PRJNA827465;Yang2025_Silkie_Wencha	0.888616319
ENSGALG00010024770	CDC27	3	0.076419743	0.162092551	0.471457463	0.637314089	0.061278408	10.75535398	81.40461017	0.666666667	CRA004775;PRJNA827465;Yang2025_Silkie_Wencha	0.888616319
ENSGALG00010005785	RPL21	3	0.069989895	0.148441257	0.471498941	0.637284476	0.043804668	6.45602425	69.02118204	0.666666667	CRA004775;PRJNA827465;Yang2025_Silkie_Wencha	0.888616319
ENSGALG00010003627	MCOLN1	3	-0.065731201	0.139645442	-0.470700653	0.637854518	0.046626449	10.49902864	80.95061868	0.666666667	CRA004775;PRJNA827465;Yang2025_Silkie_Wencha	0.888616319
ENSGALG00010029691	NUFIP2	3	-0.055627263	0.118301492	-0.470216075	0.638200651	0.024399868	5.358759645	62.67793048	0.666666667	CRA004775;PRJNA827465;Yang2025_Silkie_Wencha	0.888616319
ENSGALG00010024781	CEP19	3	-0.050093524	0.106566131	-0.470069841	0.63830512	0	0.19548577	0	1	CRA004775;PRJNA827465;Yang2025_Silkie_Wencha	0.888616319
ENSGALG00010001183	RARRES1	3	0.048886468	0.104042597	0.469869737	0.638448087	0	0.926315494	0	0.666666667	CRA004775;PRJNA827465;Yang2025_Silkie_Wencha	0.888616319
ENSGALG00010012120	ENSGALG0001001212	3	0.046899265	0.099578362	0.470978476	0.637656105	0	1.90171563	0	0.666666667	CRA004775;PRJNA827465;Yang2025_Silkie_Wencha	0.888616319
ENSGALG00010025458	BCAS2	3	0.041215367	0.08731958	0.472006009	0.6369225	0.005021823	2.314292804	13.58051167	0.666666667	CRA004775;PRJNA827465;Yang2025_Silkie_Wencha	0.888616319
ENSGALG00010030102	GPS1	3	-0.037620982	0.07961393	-0.472542715	0.63653946	0.008277511	3.51102678	43.03660652	0.666666667	CRA004775;PRJNA827465;Yang2025_Silkie_Wencha	0.888616319
ENSGALG00010016785	PDGFRB	3	-0.035148395	0.07447096	-0.471974511	0.636944982	0	0.187517127	0	1	CRA004775;PRJNA827465;Yang2025_Silkie_Wencha	0.888616319
ENSGALG00010012431	LAMP2	3	-0.029084974	0.061828196	-0.470416024	0.638057818	0	1.952431283	0	0.666666667	CRA004775;PRJNA827465;Yang2025_Silkie_Wencha	0.888616319
ENSGALG00010027361	FANCD2	3	-0.028867462	0.061150935	-0.472069025	0.636877521	0	0.773499936	0	0.666666667	CRA004775;PRJNA827465;Yang2025_Silkie_Wencha	0.888616319
ENSGALG00010024709	TEAD4	3	0.028331933	0.060094291	0.47145799	0.637313713	0	0.090479885	0	1	CRA004775;PRJNA827465;Yang2025_Silkie_Wencha	0.888616319
ENSGALG00010011570	RGL1	3	-0.027557519	0.058490444	-0.471145662	0.637536719	0	0.314768789	0	0.666666667	CRA004775;PRJNA827465;Yang2025_Silkie_Wencha	0.888616319
ENSGALG00010022431	LARP4	3	-0.025176089	0.05343181	-0.47118167	0.637511007	0	0.482346277	0	0.666666667	CRA004775;PRJNA827465;Yang2025_Silkie_Wencha	0.888616319
ENSGALG00010000469	ENSGALG0001000046	3	-0.064538649	0.137413848	-0.469666267	0.638593472	0	0.316819961	0	0.666666667	CRA004775;PRJNA827465;Yang2025_Silkie_Wencha	0.888688297
ENSGALG00010029146	NEK6	3	-0.086007766	0.183198322	-0.469479007	0.638727287	0.084721774	16.46901726	87.85598456	0.666666667	CRA004775;PRJNA827465;Yang2025_Silkie_Wencha	0.888787039
ENSGALG00010020244	SLC25A37	3	-0.127809433	0.272349272	-0.469285016	0.638865924	0.173872199	10.21265477	80.41645346	0.666666667	CRA004775;PRJNA827465;Yang2025_Silkie_Wencha	0.888825842
ENSGALG00010010685	STIM2	3	0.028697868	0.061155052	0.469264063	0.638880899	0	0.34935191	0	1	CRA004775;PRJNA827465;Yang2025_Silkie_Wencha	0.888825842
ENSGALG00010027355	ADCYAP1R1	3	0.050285755	0.107202572	0.469072282	0.639017971	0	0.992987049	0	0.666666667	CRA004775;PRJNA827465;Yang2025_Silkie_Wencha	0.888929072
ENSGALG00010009530	KIF3C	3	0.123998961	0.264553179	0.468710908	0.639276289	0.166668586	13.66356485	85.36253151	0.666666667	CRA004775;PRJNA827465;Yang2025_Silkie_Wencha	0.88902601
ENSGALG00010026300	HMGA1	3	-0.053803414	0.114761985	-0.468826098	0.639193944	0.024422315	5.665604272	64.69926412	0.666666667	CRA004775;PRJNA827465;Yang2025_Silkie_Wencha	0.88902601
ENSGALG00010008449	SRA1	3	0.035781556	0.076331875	0.46876297	0.639239071	0	1.514770464	0	0.666666667	CRA004775;PRJNA827465;Yang2025_Silkie_Wencha	0.88902601
ENSGALG00010008237	GRIA2	2	0.349129967	0.74614544	0.467911413	0.639847942	0.947613555	6.489949297	84.59155913	0.5	PRJNA827465;Yang2025_Silkie_Wenchang	0.889110624
ENSGALG00010013345	DCN	3	-0.111161129	0.237564207	-0.46792036	0.639841543	0.13132402	15.54473401	87.13390658	0.666666667	CRA004775;PRJNA827465;Yang2025_Silkie_Wencha	0.889110624
ENSGALG00010011468	EFHC2	2	-0.078438007	0.167555673	-0.468131012	0.639690903	0	0.29132648	0	1	PRJNA827465;Yang2025_Silkie_Wenchang	0.889110624
ENSGALG00010017559	DUSP10	3	0.061740029	0.131937539	0.467948919	0.639821119	0.0221684	3.534493053	43.41479895	0.666666667	CRA004775;PRJNA827465;Yang2025_Silkie_Wencha	0.889110624
ENSGALG00010012798	MFSD6	3	-0.05849901	0.124950293	-0.468178254	0.639657122	0.026233083	4.585151262	56.38093739	0.666666667	CRA004775;PRJNA827465;Yang2025_Silkie_Wencha	0.889110624
ENSGALG00010029561	SCO1	3	0.055315478	0.118174619	0.468082557	0.639725553	0.033484244	10.69633889	81.30201352	0.666666667	CRA004775;PRJNA827465;Yang2025_Silkie_Wencha	0.889110624
ENSGALG00010010984	MAT1A	3	-0.052891125	0.113055252	-0.467834308	0.639903084	0.008343843	2.433568667	17.81616737	0.666666667	CRA004775;PRJNA827465;Yang2025_Silkie_Wencha	0.889110624
ENSGALG00010013236	MON2	3	0.044406008	0.094867428	0.46808487	0.639723898	0.01299224	4.005718204	50.07137551	0.666666667	CRA004775;PRJNA827465;Yang2025_Silkie_Wencha	0.889110624
ENSGALG00010023946	ZNRF3	3	-0.033184806	0.070912407	-0.467968965	0.639806783	0	0.667015652	0	0.666666667	CRA004775;PRJNA827465;Yang2025_Silkie_Wencha	0.889110624
ENSGALG00010007957	CDH15	3	0.066235669	0.141614563	0.467717916	0.639986327	0.027354162	3.867962146	48.29318581	0.666666667	CRA004775;PRJNA827465;Yang2025_Silkie_Wencha	0.889138909
ENSGALG00010022987	F2	3	0.404213681	0.865931823	0.466796196	0.640645698	1.902546273	13.75076404	85.45535365	0.666666667	CRA004775;PRJNA827465;Yang2025_Silkie_Wencha	0.889195701
ENSGALG00010023867	ENSGALG0001002386	2	0.242257064	0.518976085	0.466798126	0.640644316	0.326484538	2.503184685	60.05089014	0.5	CRA004775;Yang2025_Silkie_Wenchang	0.889195701
ENSGALG00010017022	CCR4	3	0.158376628	0.339424066	0.466604004	0.640783222	0.236288413	6.617808394	69.77851456	0.666666667	CRA004775;PRJNA827465;Yang2025_Silkie_Wencha	0.889195701
ENSGALG00010026972	TNNT3	3	0.156332188	0.335393034	0.466116383	0.641132197	0.24394683	17.2101117	88.37892493	0.666666667	CRA004775;PRJNA827465;Yang2025_Silkie_Wencha	0.889195701
ENSGALG00010018145	ENSGALG0001001814	3	0.137084986	0.293963464	0.466333416	0.640976863	0.150885054	4.963556643	59.70631255	0.666666667	CRA004775;PRJNA827465;Yang2025_Silkie_Wencha	0.889195701
ENSGALG00010023254	MYO3B	2	-0.082208923	0.176223981	-0.466502472	0.640855879	0	0.045649551	0	1	PRJNA827465;Yang2025_Silkie_Wenchang	0.889195701
ENSGALG00010000227	ENSGALG0001000022	3	-0.081284994	0.174183499	-0.466662997	0.640741007	0	1.679824904	0	0.666666667	CRA004775;PRJNA827465;Yang2025_Silkie_Wencha	0.889195701
ENSGALG00010016115	RD3L	2	-0.0698019	0.149657436	-0.466411171	0.640921217	0	0.086175663	0	1	PRJNA827465;Yang2025_Silkie_Wenchang	0.889195701
ENSGALG00010023489	ETV5	3	-0.068277065	0.146436073	-0.466258507	0.641030475	0.040076426	7.005115796	71.44943698	0.666666667	CRA004775;PRJNA827465;Yang2025_Silkie_Wencha	0.889195701
ENSGALG00010014082	HNRNPR	3	0.061592247	0.132149904	0.466078633	0.641159217	0.035170549	6.218585668	67.8383461	0.666666667	CRA004775;PRJNA827465;Yang2025_Silkie_Wencha	0.889195701
ENSGALG00010024026	TIRAP	3	-0.06129644	0.13143895	-0.466349129	0.640965618	0.026420168	3.910156563	48.85115295	0.666666667	CRA004775;PRJNA827465;Yang2025_Silkie_Wencha	0.889195701
ENSGALG00010028095	CDK9	3	-0.059206353	0.126851205	-0.466738593	0.640686914	0.024892753	3.81634204	47.59379586	0.666666667	CRA004775;PRJNA827465;Yang2025_Silkie_Wencha	0.889195701
ENSGALG00010002463	ASRGL1	3	-0.058951451	0.126342303	-0.466601048	0.640785337	0.022102084	3.664682565	45.42501391	0.666666667	CRA004775;PRJNA827465;Yang2025_Silkie_Wencha	0.889195701
ENSGALG00010017280	SOS1	3	0.057963557	0.124202643	0.466685375	0.640724995	0.024048092	3.828540779	47.76077583	0.666666667	CRA004775;PRJNA827465;Yang2025_Silkie_Wencha	0.889195701
ENSGALG00010029525	SLC38A10	3	0.057624784	0.123503378	0.466584677	0.640797052	0.026935832	5.531720303	63.84488205	0.666666667	CRA004775;PRJNA827465;Yang2025_Silkie_Wencha	0.889195701
ENSGALG00010012709	PPP1R8	3	-0.05208716	0.111723417	-0.466215241	0.641061441	0.018323966	3.881724904	48.47651368	0.666666667	CRA004775;PRJNA827465;Yang2025_Silkie_Wencha	0.889195701
ENSGALG00010015766	TLE1	3	0.050648391	0.108539034	0.466637566	0.640759205	0.015515685	3.575157219	44.05840422	0.666666667	CRA004775;PRJNA827465;Yang2025_Silkie_Wencha	0.889195701
ENSGALG00010024935	KLHL30	3	-0.047220703	0.10107146	-0.467201159	0.640355965	0	0.44242246	0	1	CRA004775;PRJNA827465;Yang2025_Silkie_Wencha	0.889195701
ENSGALG00010005443	BANK1	3	-0.163764371	0.351664166	-0.465683987	0.641441716	0.135707052	3.14670396	36.44143124	0.666666667	CRA004775;PRJNA827465;Yang2025_Silkie_Wencha	0.889240644
ENSGALG00010002369	GEMIN8	3	0.069974506	0.1502576	0.465696946	0.641432439	0.043921931	6.631683929	69.84174726	0.666666667	CRA004775;PRJNA827465;Yang2025_Silkie_Wencha	0.889240644
ENSGALG00010021073	NDUFB8	3	0.056435845	0.121189684	0.465681921	0.641443195	0.018805324	3.247091016	38.40640777	0.666666667	CRA004775;PRJNA827465;Yang2025_Silkie_Wencha	0.889240644
ENSGALG00010008128	RARS	3	0.039154	0.084050029	0.465841598	0.641328887	0.012527946	5.445090236	63.26966288	0.666666667	CRA004775;PRJNA827465;Yang2025_Silkie_Wencha	0.889240644
ENSGALG00010014375	H2BC35	3	0.202376923	0.435020032	0.465212883	0.641779014	0.401606285	6.839276543	70.75714094	0.666666667	CRA004775;PRJNA827465;Yang2025_Silkie_Wencha	0.889357392
ENSGALG00010022218	ENSGALG0001002221	3	0.164765932	0.35411161	0.465293787	0.641721083	0.280870214	9.941278024	79.88186232	0.666666667	CRA004775;PRJNA827465;Yang2025_Silkie_Wencha	0.889357392
ENSGALG00010010699	DNAJC27	3	0.136731467	0.29389464	0.465239745	0.641759779	0.235829278	27.12170962	92.62583359	0.666666667	CRA004775;PRJNA827465;Yang2025_Silkie_Wencha	0.889357392
ENSGALG00010006953	MAP1LC3B	3	-0.067830385	0.145767588	-0.465332423	0.641693419	0.043437883	8.098143279	75.3029808	0.666666667	CRA004775;PRJNA827465;Yang2025_Silkie_Wencha	0.889357392
ENSGALG00010024131	ENSGALG0001002413	2	0.521035255	1.120309272	0.465081624	0.641873005	0	0.378593412	0	0.5	PRJNA827465;Yang2025_Silkie_Wenchang	0.889400472
ENSGALG00010012917	RUNX1	3	-0.288091073	0.6202153	-0.46450172	0.642288328	1.001768996	18.55277517	89.21994159	0.666666667	CRA004775;PRJNA827465;Yang2025_Silkie_Wencha	0.88962722
ENSGALG00010018325	ENSGALG0001001832	3	0.073188258	0.157549947	0.464540038	0.642260881	0	0.611129305	0	0.666666667	CRA004775;PRJNA827465;Yang2025_Silkie_Wencha	0.88962722
ENSGALG00010005270	GSTZ1	3	0.068460509	0.147342565	0.464634972	0.642192883	0.026178574	3.33109583	39.95969788	0.666666667	CRA004775;PRJNA827465;Yang2025_Silkie_Wencha	0.88962722
ENSGALG00010016346	TRAP1	3	0.041933709	0.090241097	0.464685277	0.642156853	0.013511185	5.214619311	61.64628939	0.666666667	CRA004775;PRJNA827465;Yang2025_Silkie_Wencha	0.88962722
ENSGALG00010025628	ENSGALG0001002562	2	0.281101117	0.605859601	0.463970722	0.642668722	0.693414546	17.91989933	94.41961151	0.5	PRJNA827465;Yang2025_Silkie_Wenchang	0.889979731
ENSGALG00010016008	TAF7	3	0.070121779	0.151113219	0.46403471	0.642622878	0.048994768	8.185731635	75.56724201	0.666666667	CRA004775;PRJNA827465;Yang2025_Silkie_Wencha	0.889979731
ENSGALG00010015919	ENSGALG0001001591	3	0.078819883	0.170056893	0.463491256	0.643012281	0	1.121671359	0	0.666666667	CRA004775;PRJNA827465;Yang2025_Silkie_Wencha	0.890368293
ENSGALG00010016368	CCNK	3	0.051448652	0.111083003	0.463155031	0.643253247	0.017965605	3.801603406	47.39061953	0.666666667	CRA004775;PRJNA827465;Yang2025_Silkie_Wencha	0.890614733
ENSGALG00010024783	UBE2T	3	-0.038768558	0.083754708	-0.462882136	0.643448853	0	0.164188549	0	0.666666667	CRA004775;PRJNA827465;Yang2025_Silkie_Wencha	0.890798329
ENSGALG00010009857	ORC5	3	-0.075976297	0.164181648	-0.462757548	0.643538163	0.042433149	4.120997974	51.46806641	0.666666667	CRA004775;PRJNA827465;Yang2025_Silkie_Wencha	0.890834745
ENSGALG00010024576	CSAD	3	0.10523685	0.227545353	0.462487362	0.643731863	0.103293196	6.178537227	67.62987862	0.666666667	CRA004775;PRJNA827465;Yang2025_Silkie_Wencha	0.890953126
ENSGALG00010016347	ANO10	3	-0.082140181	0.17770543	-0.462226621	0.643918814	0.07974731	19.89104934	89.94522629	0.666666667	CRA004775;PRJNA827465;Yang2025_Silkie_Wencha	0.890953126
ENSGALG00010028033	ENSGALG0001002803	3	-0.068933544	0.149199356	-0.462023068	0.644064778	0	0.645221788	0	0.666666667	CRA004775;PRJNA827465;Yang2025_Silkie_Wencha	0.890953126
ENSGALG00010015175	MAPK9	3	-0.049563202	0.107271175	-0.462036537	0.644055119	0.017049833	4.074058465	50.90890282	0.666666667	CRA004775;PRJNA827465;Yang2025_Silkie_Wencha	0.890953126
ENSGALG00010021600	FMN1	3	-0.032446757	0.07020184	-0.462192403	0.643943351	0	1.3013803	0	0.666666667	CRA004775;PRJNA827465;Yang2025_Silkie_Wencha	0.890953126
ENSGALG00010023927	KPNB1	3	0.031777848	0.068732399	0.462341615	0.643836361	0.005793498	3.404588871	41.25575581	0.666666667	CRA004775;PRJNA827465;Yang2025_Silkie_Wencha	0.890953126
ENSGALG00010012412	PURA	3	0.031414176	0.067983874	0.46208275	0.64402198	0	1.194221893	0	0.666666667	CRA004775;PRJNA827465;Yang2025_Silkie_Wencha	0.890953126
ENSGALG00010028546	ENSGALG0001002854	2	-1.083365529	2.346321295	-0.461729402	0.644275383	8.107724316	3.74273998	73.28160638	0.5	PRJNA827465;Yang2025_Silkie_Wenchang	0.890994141
ENSGALG00010025791	FIGN	3	0.126908918	0.274923002	0.461616225	0.644356557	0.153938574	7.238625764	72.3704462	0.666666667	CRA004775;PRJNA827465;Yang2025_Silkie_Wencha	0.890994141
ENSGALG00010016926	ENSGALG0001001692	3	0.100522241	0.217827116	0.461477171	0.644456296	0.072343629	4.296923135	53.45506686	0.666666667	CRA004775;PRJNA827465;Yang2025_Silkie_Wencha	0.890994141
ENSGALG00010029327	CRK	3	-0.088197981	0.191130379	-0.461454541	0.644472529	0.102113683	43.96025501	95.45043585	0.666666667	CRA004775;PRJNA827465;Yang2025_Silkie_Wencha	0.890994141
ENSGALG00010021655	USP22	3	0.056881159	0.123187682	0.461743881	0.644264999	0.027162081	5.076440732	60.60231754	0.666666667	CRA004775;PRJNA827465;Yang2025_Silkie_Wencha	0.890994141
ENSGALG00010029597	ADAP2	3	-0.054815767	0.118785966	-0.461466699	0.644463808	0	1.72324302	0	0.666666667	CRA004775;PRJNA827465;Yang2025_Silkie_Wencha	0.890994141
ENSGALG00010018944	NEFL	3	0.190418046	0.413604338	0.460386964	0.64523849	0.30843152	5.004720733	60.03773024	0.666666667	CRA004775;PRJNA827465;Yang2025_Silkie_Wencha	0.891118567
ENSGALG00010009534	XPA	3	0.127436809	0.276818198	0.460362831	0.645255809	0.148352424	5.760310238	65.27964784	0.666666667	CRA004775;PRJNA827465;Yang2025_Silkie_Wencha	0.891118567
ENSGALG00010005885	SLITRK5	2	-0.088747854	0.192707797	-0.460530687	0.64513535	0.015601687	1.214705663	17.67552994	0.5	PRJNA827465;Yang2025_Silkie_Wenchang	0.891118567
ENSGALG00010014570	KIF27	3	0.076917807	0.167041605	0.460470953	0.645178217	0.035564805	3.437963639	41.82602814	0.666666667	CRA004775;PRJNA827465;Yang2025_Silkie_Wencha	0.891118567
ENSGALG00010021572	MYL9	3	-0.067694417	0.146935843	-0.460707313	0.645008608	0.038047698	5.531008915	63.84023185	0.666666667	CRA004775;PRJNA827465;Yang2025_Silkie_Wencha	0.891118567
ENSGALG00010024934	G6PC3	3	0.06599002	0.143081105	0.461207092	0.644650034	0.027026815	3.515169258	43.10373546	0.666666667	CRA004775;PRJNA827465;Yang2025_Silkie_Wencha	0.891118567
ENSGALG00010013337	RAB19	3	0.06284141	0.136299819	0.461052779	0.644760739	0	0.822679071	0	0.666666667	CRA004775;PRJNA827465;Yang2025_Silkie_Wencha	0.891118567
ENSGALG00010027096	YIPF1	3	-0.061181615	0.132755704	-0.460858655	0.644900016	0.034319902	5.856352152	65.84904821	0.666666667	CRA004775;PRJNA827465;Yang2025_Silkie_Wencha	0.891118567
ENSGALG00010027230	MAP2K2	3	0.042446403	0.092181078	0.46046764	0.645180594	0.010070616	3.220202798	37.89211036	0.666666667	CRA004775;PRJNA827465;Yang2025_Silkie_Wencha	0.891118567
ENSGALG00010011523	PIGO	3	-0.033528429	0.072810525	-0.460488764	0.645165435	0.005031182	2.887872122	30.74485588	0.666666667	CRA004775;PRJNA827465;Yang2025_Silkie_Wencha	0.891118567
ENSGALG00010029286	MAFG	3	-0.030414751	0.066064051	-0.460382774	0.645241497	0	1.113298675	0	0.666666667	CRA004775;PRJNA827465;Yang2025_Silkie_Wencha	0.891118567
ENSGALG00010020773	ZCCHC24	3	-0.097350222	0.211582489	-0.460105288	0.645440649	0.100636141	11.82077845	83.08064051	0.666666667	CRA004775;PRJNA827465;Yang2025_Silkie_Wencha	0.891144422
ENSGALG00010018198	BTBD3	3	-0.050166299	0.109044991	-0.460051378	0.645479343	0.022642545	7.204950572	72.24130853	0.666666667	CRA004775;PRJNA827465;Yang2025_Silkie_Wencha	0.891144422
ENSGALG00010004950	SLIRP	3	0.044276757	0.09620295	0.460243239	0.645341638	0.001869647	2.075830249	3.653008199	0.666666667	CRA004775;PRJNA827465;Yang2025_Silkie_Wencha	0.891144422
ENSGALG00010023228	GORASP2	3	0.014458951	0.031433493	0.459985483	0.64552664	0	1.17779254	0	1	CRA004775;PRJNA827465;Yang2025_Silkie_Wencha	0.891144422
ENSGALG00010015916	COA8	3	-0.098108586	0.213398413	-0.459743748	0.645700164	0.091572715	6.525786952	69.35235528	0.666666667	CRA004775;PRJNA827465;Yang2025_Silkie_Wencha	0.891296947
ENSGALG00010024419	PGAP3	3	-0.072336011	0.157478074	-0.459340209	0.645989877	0.054397017	7.926349905	74.76770488	0.666666667	CRA004775;PRJNA827465;Yang2025_Silkie_Wencha	0.891463277
ENSGALG00010011055	TOR3A	3	0.042229045	0.091911847	0.459451593	0.645909906	0	1.958087782	0	0.666666667	CRA004775;PRJNA827465;Yang2025_Silkie_Wencha	0.891463277
ENSGALG00010021412	ZNF319	3	-0.024671266	0.053713471	-0.459312445	0.646009812	0.000575597	2.100452276	4.782411716	0.666666667	CRA004775;PRJNA827465;Yang2025_Silkie_Wencha	0.891463277
ENSGALG00010025599	COPS9	3	0.088093273	0.191883674	0.459097279	0.646164311	0.072470041	6.29150907	68.21112427	0.666666667	CRA004775;PRJNA827465;Yang2025_Silkie_Wencha	0.891589459
ENSGALG00010015908	CHRD	3	0.041606876	0.090645172	0.459008186	0.646228288	0	0.901908743	0	0.666666667	CRA004775;PRJNA827465;Yang2025_Silkie_Wencha	0.891590726
ENSGALG00010009167	DCTN3	3	-0.06955389	0.151703129	-0.458486848	0.646602709	0.010377354	2.328004139	14.08949981	0.666666667	CRA004775;PRJNA827465;Yang2025_Silkie_Wencha	0.891858958
ENSGALG00010012067	ENSGALG0001001206	3	0.047385308	0.103354407	0.458474001	0.646611937	0.013254862	3.39928757	41.16414222	0.666666667	CRA004775;PRJNA827465;Yang2025_Silkie_Wencha	0.891858958
ENSGALG00010025292	ATP1A1	3	-0.046447749	0.101290951	-0.45855773	0.646551797	0.016690193	4.362293593	54.15255857	0.666666667	CRA004775;PRJNA827465;Yang2025_Silkie_Wencha	0.891858958
ENSGALG00010021725	SH2D6	3	0.085818618	0.187248746	0.458313445	0.646727266	0.044260267	3.157444572	36.65763707	0.666666667	CRA004775;PRJNA827465;Yang2025_Silkie_Wencha	0.891931019
ENSGALG00010011605	APPL2	3	0.095459042	0.208394502	0.45806891	0.646902935	0.106016349	20.95671917	90.45652144	0.666666667	CRA004775;PRJNA827465;Yang2025_Silkie_Wencha	0.891999278
ENSGALG00010021652	BORCS5	3	-0.08628379	0.188361768	-0.458074859	0.646898661	0.090158813	15.46428776	87.0669763	0.666666667	CRA004775;PRJNA827465;Yang2025_Silkie_Wencha	0.891999278
ENSGALG00010007783	NOL7	3	-0.082243802	0.179588718	-0.457956396	0.646983768	0.073048458	8.588011479	76.71172186	0.666666667	CRA004775;PRJNA827465;Yang2025_Silkie_Wencha	0.892023744
ENSGALG00010016615	BCORL1	3	0.058370454	0.127510198	0.457770864	0.647117071	0.028002584	4.725185222	57.67361688	0.666666667	CRA004775;PRJNA827465;Yang2025_Silkie_Wencha	0.892033563
ENSGALG00010012428	CCNE2	3	-0.039941748	0.08723771	-0.457849568	0.647060521	0	1.428689465	0	0.666666667	CRA004775;PRJNA827465;Yang2025_Silkie_Wencha	0.892033563
ENSGALG00010025219	NACA	3	0.08477189	0.1853027	0.457477902	0.647327582	0.084505644	13.5625715	85.25353397	0.666666667	CRA004775;PRJNA827465;Yang2025_Silkie_Wencha	0.892214143
ENSGALG00010028924	TMEM203	3	0.027567742	0.06026883	0.457412925	0.647374276	0	0.087556927	0	0.666666667	CRA004775;PRJNA827465;Yang2025_Silkie_Wencha	0.892214143
ENSGALG00010023267	DSCAML1	2	0.193646955	0.423610459	0.457134499	0.647574375	0.121475595	1.452240318	31.14087336	0.5	PRJNA827465;Yang2025_Silkie_Wenchang	0.892402932
ENSGALG00010025505	ENSGALG0001002550	3	-0.131973118	0.288976699	-0.456691208	0.647893011	0.095037216	3.21599325	37.81081475	0.666666667	CRA004775;PRJNA827465;Yang2025_Silkie_Wencha	0.892755023
ENSGALG00010027194	ENSGALG0001002719	3	0.097677627	0.213995946	0.456446156	0.648069182	0	0.890392424	0	0.666666667	CRA004775;PRJNA827465;Yang2025_Silkie_Wencha	0.892910754
ENSGALG00010021358	MMP15	3	-0.020995694	0.046013327	-0.456295927	0.648177192	0	1.336422192	0	1	CRA004775;PRJNA827465;Yang2025_Silkie_Wencha	0.892972554
ENSGALG00010026681	PLXDC1	3	-0.084346569	0.185008553	-0.455906323	0.648457343	0.058406398	5.453464585	63.32606605	0.666666667	CRA004775;PRJNA827465;Yang2025_Silkie_Wencha	0.89327147
ENSGALG00010009652	CIAO2B	3	0.065699665	0.144164658	0.455726565	0.648586616	0.038477289	5.420922047	63.1059074	0.666666667	CRA004775;PRJNA827465;Yang2025_Silkie_Wencha	0.89336251
ENSGALG00010026885	TULP1	3	0.171272819	0.376107509	0.455382611	0.648834003	0.16979537	3.318045218	39.72354598	0.666666667	CRA004775;PRJNA827465;Yang2025_Silkie_Wencha	0.89337448
ENSGALG00010016729	ELMO1	3	-0.028123366	0.061749513	-0.455442713	0.648790772	0	0.604858485	0	0.666666667	CRA004775;PRJNA827465;Yang2025_Silkie_Wencha	0.89337448
ENSGALG00010022795	TAF4	3	-0.025124856	0.055175435	-0.455363087	0.648848047	0	0.083324991	0	0.666666667	CRA004775;PRJNA827465;Yang2025_Silkie_Wencha	0.89337448
ENSGALG00010018058	ITPKB	3	0.021253416	0.046659521	0.455500087	0.648749505	0	1.207264128	0	0.666666667	CRA004775;PRJNA827465;Yang2025_Silkie_Wencha	0.89337448
ENSGALG00010023212	RIMBP2	3	0.160423278	0.352903162	0.454581584	0.649410286	0.274037768	8.279503745	75.84396286	0.666666667	CRA004775;PRJNA827465;Yang2025_Silkie_Wencha	0.893539513
ENSGALG00010000331	ENSGALG0001000033	3	0.090953816	0.200053319	0.45464787	0.64936259	0.08010362	6.984274655	71.36424183	0.666666667	CRA004775;PRJNA827465;Yang2025_Silkie_Wencha	0.893539513
ENSGALG00010028051	GNL3	3	0.074657564	0.164077401	0.4550143	0.649098951	0.064935285	16.53171792	87.90204376	0.666666667	CRA004775;PRJNA827465;Yang2025_Silkie_Wencha	0.893539513
ENSGALG00010001871	ENSGALG0001000187	3	-0.071442366	0.157126746	-0.454679854	0.649339577	0.031150262	3.415717503	41.44714841	0.666666667	CRA004775;PRJNA827465;Yang2025_Silkie_Wencha	0.893539513
ENSGALG00010029211	SDF2	3	0.059116578	0.129952968	0.454907484	0.649175799	0.023584107	3.523284264	43.23478181	0.666666667	CRA004775;PRJNA827465;Yang2025_Silkie_Wencha	0.893539513
ENSGALG00010028896	ATP6V1G1	3	-0.037329659	0.082088619	-0.454748291	0.649290335	0	1.268237483	0	0.666666667	CRA004775;PRJNA827465;Yang2025_Silkie_Wencha	0.893539513
ENSGALG00010003471	ADI1	3	0.028842313	0.063425753	0.454741362	0.64929532	0.001532039	2.183285834	8.394953647	0.666666667	CRA004775;PRJNA827465;Yang2025_Silkie_Wencha	0.893539513
ENSGALG00010006269	ENSGALG0001000626	2	-0.609076683	1.342840274	-0.45357344	0.650135873	2.384005713	2.904921734	65.57566463	0.5	PRJNA827465;Yang2025_Silkie_Wenchang	0.89355503
ENSGALG00010007995	KIAA0319	2	0.405996652	0.897014938	0.452608574	0.650830623	1.177758577	3.544837264	71.78996028	0.5	PRJNA827465;Yang2025_Silkie_Wenchang	0.89355503
ENSGALG00010002097	PCYT1B	3	-0.318324923	0.703323595	-0.452600944	0.650836119	1.190978227	11.87900539	83.1635736	0.666666667	CRA004775;PRJNA827465;Yang2025_Silkie_Wencha	0.89355503
ENSGALG00010002881	ENSGALG0001000288	3	0.219888143	0.486722015	0.451773571	0.651432111	0.533256932	9.229018741	78.32922377	0.666666667	CRA004775;PRJNA827465;Yang2025_Silkie_Wencha	0.89355503
ENSGALG00010023204	SRGN	3	-0.217266796	0.481404397	-0.451318677	0.651759886	0.408275843	4.875773637	58.98086849	0.666666667	CRA004775;PRJNA827465;Yang2025_Silkie_Wencha	0.89355503
ENSGALG00010004016	ENSGALG0001000401	3	0.210437643	0.466205744	0.451383634	0.651713077	0.471366228	7.220532879	72.30121331	0.666666667	CRA004775;PRJNA827465;Yang2025_Silkie_Wencha	0.89355503
ENSGALG00010006037	METTL21EP	3	0.189736043	0.420259764	0.451473253	0.651648498	0.065640051	2.24783015	11.0253059	0.666666667	CRA004775;PRJNA827465;Yang2025_Silkie_Wencha	0.89355503
ENSGALG00010005521	SYTL3	2	-0.161089398	0.355431174	-0.453222479	0.650388547	0	0.152154415	0	1	CRA004775;PRJNA827465	0.89355503
ENSGALG00010002468	ENSGALG0001000246	3	-0.133101474	0.294249891	-0.452341625	0.651022894	0	0.856723898	0	0.666666667	CRA004775;PRJNA827465;Yang2025_Silkie_Wencha	0.89355503
ENSGALG00010023603	C1S	3	-0.130896921	0.289777339	-0.45171552	0.651473936	0.171090869	6.681400249	70.06615492	0.666666667	CRA004775;PRJNA827465;Yang2025_Silkie_Wencha	0.89355503
ENSGALG00010027868	CACNA2D3	2	0.128138397	0.283718977	0.451638444	0.65152947	0	0.363341674	0	1	PRJNA827465;Yang2025_Silkie_Wenchang	0.89355503
ENSGALG00010021027	PLA2G4A	3	0.10832094	0.238769823	0.453662608	0.650071684	0.125215626	15.73335478	87.28815293	0.666666667	CRA004775;PRJNA827465;Yang2025_Silkie_Wencha	0.89355503
ENSGALG00010015070	EDN2	3	0.107935168	0.239073634	0.451472486	0.651649051	0	0.095568442	0	1	CRA004775;PRJNA827465;Yang2025_Silkie_Wencha	0.89355503
ENSGALG00010012588	PEX26	3	-0.100338246	0.221553031	-0.45288591	0.650630897	0.131444137	22.15354556	90.97209973	0.666666667	CRA004775;PRJNA827465;Yang2025_Silkie_Wencha	0.89355503
ENSGALG00010015131	DOK5	3	0.096284632	0.212520325	0.453060816	0.65050495	0.065246614	4.106009685	51.29090886	0.666666667	CRA004775;PRJNA827465;Yang2025_Silkie_Wencha	0.89355503
ENSGALG00010022531	ENSGALG0001002253	3	0.095518687	0.210995387	0.452705097	0.650761109	0	1.840826035	0	0.666666667	CRA004775;PRJNA827465;Yang2025_Silkie_Wencha	0.89355503
ENSGALG00010013018	ID1	3	0.088618273	0.195004969	0.454441099	0.649511377	0.061874007	4.195461507	52.32943988	0.666666667	CRA004775;PRJNA827465;Yang2025_Silkie_Wencha	0.89355503
ENSGALG00010026697	CHN1	3	-0.081868731	0.181013738	-0.452279103	0.651067929	0.08050035	17.66802504	88.68011566	0.666666667	CRA004775;PRJNA827465;Yang2025_Silkie_Wencha	0.89355503
ENSGALG00010000462	ENSGALG0001000046	3	-0.071386639	0.157630176	-0.452874195	0.650639334	0	1.251965917	0	0.666666667	CRA004775;PRJNA827465;Yang2025_Silkie_Wencha	0.89355503
ENSGALG00010021099	HMCN1	3	0.067730686	0.149326308	0.45357504	0.650134722	0.032306567	3.962855102	49.53133666	0.666666667	CRA004775;PRJNA827465;Yang2025_Silkie_Wencha	0.89355503
ENSGALG00010002102	PAK1	3	-0.064406458	0.142043648	-0.453427233	0.65024113	0	0.526822858	0	0.666666667	CRA004775;PRJNA827465;Yang2025_Silkie_Wencha	0.89355503
ENSGALG00010019026	CHSY1	3	-0.057581804	0.127504594	-0.451605721	0.651553048	0.030960172	10.68963357	81.29028477	0.666666667	CRA004775;PRJNA827465;Yang2025_Silkie_Wencha	0.89355503
ENSGALG00010017583	MTHFD1	3	-0.053050386	0.11711214	-0.45298793	0.650557433	0.017532222	3.470604352	42.37314897	0.666666667	CRA004775;PRJNA827465;Yang2025_Silkie_Wencha	0.89355503
ENSGALG00010024159	NLRX1	3	0.049072314	0.108225776	0.453425381	0.650242464	0.011446745	2.845877352	29.72290255	0.666666667	CRA004775;PRJNA827465;Yang2025_Silkie_Wencha	0.89355503
ENSGALG00010025060	GPR156	3	-0.048871179	0.108230175	-0.451548555	0.651594238	0	0.281062107	0	0.666666667	CRA004775;PRJNA827465;Yang2025_Silkie_Wencha	0.89355503
ENSGALG00010022813	USP24	3	-0.048016963	0.105763375	-0.454003692	0.649826169	0.015713383	4.013888904	50.17301057	0.666666667	CRA004775;PRJNA827465;Yang2025_Silkie_Wencha	0.89355503
ENSGALG00010022296	CIAPIN1	3	-0.047245928	0.104625924	-0.451569997	0.651578789	0.015487251	3.472412014	42.40314825	0.666666667	CRA004775;PRJNA827465;Yang2025_Silkie_Wencha	0.89355503
ENSGALG00010001899	TWF1	3	0.046664138	0.102994687	0.453073258	0.650495991	0.018458432	5.36068473	62.69133327	0.666666667	CRA004775;PRJNA827465;Yang2025_Silkie_Wencha	0.89355503
ENSGALG00010009731	NMI	3	-0.044699906	0.099004518	-0.451493599	0.651633837	0.009846695	2.973774623	32.74540765	0.666666667	CRA004775;PRJNA827465;Yang2025_Silkie_Wencha	0.89355503
ENSGALG00010005132	LNX2	3	0.044133711	0.097732938	0.451574591	0.651575478	0.015134473	3.975852545	49.69632356	0.666666667	CRA004775;PRJNA827465;Yang2025_Silkie_Wencha	0.89355503
ENSGALG00010020866	LCOR	3	0.044017163	0.097406165	0.451892992	0.651346074	0.012730797	3.544152844	43.56902516	0.666666667	CRA004775;PRJNA827465;Yang2025_Silkie_Wencha	0.89355503
ENSGALG00010017824	YAE1	3	0.039044776	0.086266642	0.452605724	0.650832676	0	0.689995192	0	0.666666667	CRA004775;PRJNA827465;Yang2025_Silkie_Wencha	0.89355503
ENSGALG00010016814	HMGXB3	3	0.032252077	0.071210923	0.45290912	0.650614184	0.00577529	3.168529074	36.8792284	0.666666667	CRA004775;PRJNA827465;Yang2025_Silkie_Wencha	0.89355503
ENSGALG00010018150	AAGAB	3	-0.030354342	0.067140987	-0.452098541	0.651197995	0.005584633	3.419997067	41.52041769	0.666666667	CRA004775;PRJNA827465;Yang2025_Silkie_Wencha	0.89355503
ENSGALG00010020316	PDPK1	3	-0.027560546	0.060961767	-0.452095581	0.651200127	0.003264533	2.595780706	22.95188899	0.666666667	CRA004775;PRJNA827465;Yang2025_Silkie_Wencha	0.89355503
ENSGALG00010029653	CCT6A	3	0.025852422	0.05692542	0.454145478	0.649724122	0.002116901	2.43341232	17.81088707	0.666666667	CRA004775;PRJNA827465;Yang2025_Silkie_Wencha	0.89355503
ENSGALG00010007878	PSMB1	3	-0.021219591	0.046740871	-0.453983647	0.649840597	0	1.41873977	0	1	CRA004775;PRJNA827465;Yang2025_Silkie_Wencha	0.89355503
ENSGALG00010009275	CBLL1	3	0.068973576	0.152868052	0.451196802	0.651847714	0.04446084	6.302731311	68.26772551	0.666666667	CRA004775;PRJNA827465;Yang2025_Silkie_Wencha	0.893588795
ENSGALG00010009941	CACNA1E	2	-0.193531574	0.429181973	-0.450931273	0.652039083	0.188011726	1.813017747	44.84334191	0.5	PRJNA827465;Yang2025_Silkie_Wenchang	0.893756754

ENSGALG00010021570	KATNB1	3	-0.068138341	0.151132597	-0.450851391	0.65209666	0.04929585	8.863315105	77.43507958	0.666666667	CRA004775;PRJNA827465;Yang2025_Silkie_Wencha	0.893756754
ENSGALG00010001416	AP4M1	3	0.085067583	0.188894192	0.450345148	0.65246159	0.069902988	5.802631951	65.53288203	0.666666667	CRA004775;PRJNA827465;Yang2025_Silkie_Wencha	0.894030462
ENSGALG00010022072	SMTN	3	-0.045832578	0.101779783	-0.450311213	0.652486055	0.010379322	2.975737234	32.78976459	0.666666667	CRA004775;PRJNA827465;Yang2025_Silkie_Wencha	0.894030462
ENSGALG00010006039	PCNP	3	-0.01452724	0.032253785	-0.450404192	0.65249023	0	0.377151367	0	1	CRA004775;PRJNA827465;Yang2025_Silkie_Wencha	0.894030462
ENSGALG00010025127	PAMR1	3	-0.051829694	0.115134483	-0.450166556	0.652590349	0	0.02681353	0	1	CRA004775;PRJNA827465;Yang2025_Silkie_Wencha	0.894086719
ENSGALG00010022760	ELAVL4	2	-0.16577059	0.368890294	-0.449376394	0.653160157	0.178488699	2.761849244	63.79237563	0.5	PRJNA827465;Yang2025_Silkie_Wenchang	0.894256035
ENSGALG00010002388	ENSGALG00010002388	3	0.157828861	0.351130055	0.449488328	0.653079426	0.098452501	2.70713869	26.1212583	0.666666667	CRA004775;PRJNA827465;Yang2025_Silkie_Wencha	0.894256035
ENSGALG00010001381	MDFIC	3	-0.146504622	0.325866158	-0.44958526	0.653009518	0.281779177	49.3018358	95.94335593	0.666666667	CRA004775;PRJNA827465;Yang2025_Silkie_Wencha	0.894256035
ENSGALG00010022828	PRKG1	3	-0.094321335	0.209920151	-0.449320063	0.653200787	0.094154009	8.574137425	76.67403844	0.666666667	CRA004775;PRJNA827465;Yang2025_Silkie_Wencha	0.894256035
ENSGALG00010027210	ENSGALG00010027210	3	-0.083466366	0.185830534	-0.449153128	0.653321197	0.030089745	2.779863764	28.05402818	0.666666667	CRA004775;PRJNA827465;Yang2025_Silkie_Wencha	0.894256035
ENSGALG00010016848	ENSGALG00010016848	3	-0.076878657	0.171176099	-0.44911818	0.653346406	0.057315044	5.887126492	66.02756875	0.666666667	CRA004775;PRJNA827465;Yang2025_Silkie_Wencha	0.894256035
ENSGALG00010029667	TMEM104	3	-0.074175555	0.165147572	-0.449147113	0.653325536	0.065417307	11.41506639	82.47929594	0.666666667	CRA004775;PRJNA827465;Yang2025_Silkie_Wencha	0.894256035
ENSGALG00010015557	IRS4	3	-0.050151145	0.111587193	-0.449434595	0.65311818	0.016933053	3.686870968	45.75345823	0.666666667	CRA004775;PRJNA827465;Yang2025_Silkie_Wencha	0.894256035
ENSGALG00010026120	C1QTNF5	3	0.046998034	0.104599765	0.449312997	0.653205884	0	0.20962716	0	0.666666667	CRA004775;PRJNA827465;Yang2025_Silkie_Wencha	0.894256035
ENSGALG00010019067	GTF3C1	3	0.043926644	0.097676462	0.449715757	0.652915409	0.019621026	9.145532371	78.13139882	0.666666667	CRA004775;PRJNA827465;Yang2025_Silkie_Wencha	0.894256035
ENSGALG00010022366	AARSD1	3	0.069666501	0.155306146	0.448575301	0.653738055	0.053789873	9.106054506	78.03659094	0.666666667	CRA004775;PRJNA827465;Yang2025_Silkie_Wencha	0.89438074
ENSGALG00010025653	IGSF11	3	-0.039415915	0.087871336	-0.448563967	0.653746232	0	1.529442722	0	0.666666667	CRA004775;PRJNA827465;Yang2025_Silkie_Wencha	0.89438074
ENSGALG00010002647	FAM168A	3	0.036004561	0.080268155	0.448553481	0.653753798	0.008126212	3.181702899	37.14057964	0.666666667	CRA004775;PRJNA827465;Yang2025_Silkie_Wencha	0.89438074
ENSGALG00010024843	BCL9L	3	0.030610565	0.068200717	0.448830552	0.653553897	0	1.545555474	0	0.666666667	CRA004775;PRJNA827465;Yang2025_Silkie_Wencha	0.89438074
ENSGALG00010016402	SEPTIN8	3	0.024693389	0.055041537	0.448631893	0.653697223	0.000378291	2.065658629	3.17858082	0.666666667	CRA004775;PRJNA827465;Yang2025_Silkie_Wencha	0.89438074
ENSGALG00010023048	ARFGAP1	3	-0.050972012	0.113675749	-0.448398295	0.653865772	0.025210639	6.977705096	71.33728106	0.666666667	CRA004775;PRJNA827465;Yang2025_Silkie_Wencha	0.894447383
ENSGALG00010026523	LRRC55	2	0.140431281	0.313330074	0.448189601	0.654016367	0	0.125824179	0	1	PRJNA827465;Yang2025_Silkie_Wenchang	0.894539389
ENSGALG00010024642	RAB3B	3	0.122217163	0.273063513	0.447577788	0.654457936	0.150233802	6.605153782	69.72061415	0.666666667	CRA004775;PRJNA827465;Yang2025_Silkie_Wencha	0.894539389
ENSGALG00010020981	CIT	3	0.113077252	0.252646541	0.447570947	0.654462875	0.125001206	10.23755873	80.46409253	0.666666667	CRA004775;PRJNA827465;Yang2025_Silkie_Wencha	0.894539389
ENSGALG00010005853	COMMD3	3	0.110596279	0.247109498	0.447559806	0.654470917	0.149542916	13.18366579	84.82971253	0.666666667	CRA004775;PRJNA827465;Yang2025_Silkie_Wencha	0.894539389
ENSGALG00010028589	PHYHD1	3	0.090774566	0.202787896	0.447633061	0.654418039	0.056192818	3.645950276	45.14461667	0.666666667	CRA004775;PRJNA827465;Yang2025_Silkie_Wencha	0.894539389
ENSGALG00010022637	DMXL2	3	0.066903159	0.149671162	0.447516138	0.654502439	0.033081468	4.081000888	50.99214449	0.666666667	CRA004775;PRJNA827465;Yang2025_Silkie_Wencha	0.894539389
ENSGALG00010014795	THRAP3	3	0.053912778	0.120361934	0.447922165	0.654209371	0.034749297	13.06546331	84.69246782	0.666666667	CRA004775;PRJNA827465;Yang2025_Silkie_Wencha	0.894539389
ENSGALG00010014328	RASSF8	3	0.051203662	0.114414563	0.447527492	0.654494243	0.02164567	5.157958542	61.22496946	0.666666667	CRA004775;PRJNA827465;Yang2025_Silkie_Wencha	0.894539389
ENSGALG00010018428	PPP2R5D	3	-0.017095626	0.038168805	-0.447895238	0.654228805	0	1.802175545	0	0.666666667	CRA004775;PRJNA827465;Yang2025_Silkie_Wencha	0.894539389
ENSGALG00010013363	HS3ST5	3	-0.051728443	0.115667104	-0.447218281	0.654717463	0	1.817330326	0	0.666666667	CRA004775;PRJNA827465;Yang2025_Silkie_Wencha	0.894539389
ENSGALG00010019423	LYPD1	3	0.044140545	0.098683254	0.447295187	0.654661942	0	0.83331515	0	0.666666667	CRA004775;PRJNA827465;Yang2025_Silkie_Wencha	0.894660309
ENSGALG00010025414	MMP2	3	-0.086551694	0.193585337	-0.4470984	0.654804014	0.079841315	10.07255606	80.14406682	0.666666667	CRA004775;PRJNA827465;Yang2025_Silkie_Wencha	0.894667289
ENSGALG00010020978	BCL2L10	3	-0.061431964	0.137462206	-0.446900755	0.654946719	0.01301715	2.448200776	18.30735372	0.666666667	CRA004775;PRJNA827465;Yang2025_Silkie_Wencha	0.894667289
ENSGALG00010009138	ENSGALG00010009138	3	0.056491503	0.126394326	0.446946508	0.654913683	0.025653284	4.376133163	54.29755161	0.666666667	CRA004775;PRJNA827465;Yang2025_Silkie_Wencha	0.894667289
ENSGALG00010016425	TRIM63	3	-0.050163844	0.112263105	-0.446841766	0.654989313	0.010018094	2.599739614	23.06921858	0.666666667	CRA004775;PRJNA827465;Yang2025_Silkie_Wencha	0.894667289
ENSGALG00010018701	SFXN2	3	-0.033724467	0.075484565	-0.446773019	0.655038954	0.001760588	2.166068462	7.666815004	0.666666667	CRA004775;PRJNA827465;Yang2025_Silkie_Wencha	0.894667289
ENSGALG00010006187	TCF21	3	0.344210738	0.771273817	0.446288634	0.655388766	1.430376758	10.32703968	80.63336579	0.666666667	CRA004775;PRJNA827465;Yang2025_Silkie_Wencha	0.894903895
ENSGALG00010012339	WNK1	3	-0.043705973	0.097936119	-0.446270216	0.655402068	0.007903232	2.441483112	18.08257898	0.666666667	CRA004775;PRJNA827465;Yang2025_Silkie_Wencha	0.894903895
ENSGALG00010015628	UBQLN1	3	0.030447666	0.068218818	0.446323567	0.655363535	0	0.182446719	0	0.666666667	CRA004775;PRJNA827465;Yang2025_Silkie_Wencha	0.894903895
ENSGALG00010011317	BLK	2	-0.115672907	0.259344103	-0.446020964	0.655582103	0	0.131702608	0	0.5	CRA004775;PRJNA827465	0.894980715
ENSGALG00010018349	KCNMB2	3	-0.068856021	0.154379793	-0.446017056	0.655584926	0.028485777	3.254094893	38.53897733	0.666666667	CRA004775;PRJNA827465;Yang2025_Silkie_Wencha	0.894980715
ENSGALG00010020915	EN1	3	0.115696781	0.259626461	0.445627848	0.655866091	0.066323415	2.94673746	32.12832745	0.666666667	CRA004775;PRJNA827465;Yang2025_Silkie_Wencha	0.895125258
ENSGALG00010010317	ENSGALG00010010317	3	0.070147884	0.157440255	0.445552404	0.655920598	0.045025358	5.552583516	63.98073087	0.666666667	CRA004775;PRJNA827465;Yang2025_Silkie_Wencha	0.895125258
ENSGALG00010005981	AREL1	3	-0.047830075	0.107357873	-0.445519957	0.655944041	0.016946831	4.038059823	50.47126373	0.666666667	CRA004775;PRJNA827465;Yang2025_Silkie_Wencha	0.895125258
ENSGALG00010025970	KLHL12	3	-0.036732838	0.082419447	-0.445681684	0.655827197	0.009460845	3.591324313	44.31023695	0.666666667	CRA004775;PRJNA827465;Yang2025_Silkie_Wencha	0.895125258
ENSGALG00010023334	RPS17	3	0.12574568	0.282485683	0.445144153	0.656215583	0.211229779	21.03212041	90.48580906	0.666666667	CRA004775;PRJNA827465;Yang2025_Silkie_Wencha	0.895374969
ENSGALG00010027981	CNN2	3	-0.110807916	0.248955401	-0.44509143	0.656253682	0.13285596	7.653075477	73.86671534	0.666666667	CRA004775;PRJNA827465;Yang2025_Silkie_Wencha	0.895374969
ENSGALG00010029440	FOXK2	3	0.09067779	0.203823017	0.444884937	0.656402909	0.081628431	6.33440926	68.42641645	0.666666667	CRA004775;PRJNA827465;Yang2025_Silkie_Wencha	0.895473228
ENSGALG00010025595	ERI3	3	-0.027031071	0.060769038	-0.444816504	0.656452366	0	1.269449461	0	0.666666667	CRA004775;PRJNA827465;Yang2025_Silkie_Wencha	0.895473228
ENSGALG00010020399	ATP13A2	3	0.167436574	0.377479434	0.443565473	0.657356773	0.407342871	56.65485065	96.46098214	0.666666667	CRA004775;PRJNA827465;Yang2025_Silkie_Wencha	0.895497391
ENSGALG00010002762	PPAN	3	0.15413696	0.347321934	0.443787003	0.657196586	0.342161472	52.14106219	96.16425152	0.666666667	CRA004775;PRJNA827465;Yang2025_Silkie_Wencha	0.895497391
ENSGALG00010025578	ORMDL2	3	0.132765191	0.299126862	0.443842423	0.657156514	0.245797134	27.20331139	92.64795388	0.666666667	CRA004775;PRJNA827465;Yang2025_Silkie_Wencha	0.895497391
ENSGALG00010002148	POLR2A	3	-0.106860866	0.240649059	-0.444052708	0.657004477	0.102404	4.92995556	59.43168299	0.666666667	CRA004775;PRJNA827465;Yang2025_Silkie_Wencha	0.895497391
ENSGALG00010027122	DNASE1L3	3	0.060401926	0.136064618	0.443920884	0.657099785	0	1.887331638	0	0.666666667	CRA004775;PRJNA827465;Yang2025_Silkie_Wencha	0.895497391
ENSGALG00010019697	EIF4EBP1	3	-0.056630775	0.127556014	-0.443967893	0.657065797	0.020610763	3.164721638	36.80328861	0.666666667	CRA004775;PRJNA827465;Yang2025_Silkie_Wencha	0.895497391
ENSGALG00010021719	BHLHE40	3	0.049076389	0.110537589	0.443979191	0.657057629	0.012953206	2.979046073	32.86441529	0.666666667	CRA004775;PRJNA827465;Yang2025_Silkie_Wencha	0.895497391
ENSGALG00010023733	ORC6	3	-0.04134887	0.093102197	-0.444123458	0.656953327	0	1.130636651	0	0.666666667	CRA004775;PRJNA827465;Yang2025_Silkie_Wencha	0.895497391
ENSGALG00010028157	PRRC2B	3	-0.038431053	0.086581413	-0.443624585	0.657314028	0.006014279	2.413781313	17.14245242	0.666666667	CRA004775;PRJNA827465;Yang2025_Silkie_Wencha	0.895497391
ENSGALG00010027537	SGTA	3	-0.036235664	0.081660915	-0.443733255	0.657235449	0.010029549	3.787048376	47.18842218	0.666666667	CRA004775;PRJNA827465;Yang2025_Silkie_Wencha	0.895497391
ENSGALG00010020605	STAMBP	3	-0.035645335	0.080203185	-0.4444379	0.656726017	0.012362587	5.791293709	65.46540202	0.666666667	CRA004775;PRJNA827465;Yang2025_Silkie_Wencha	0.895497391
ENSGALG00010005752	RMND1	3	0.029068288	0.065432001	0.444251861	0.656860501	0	1.947943519	0	0.666666667	CRA004775;PRJNA827465;Yang2025_Silkie_Wencha	0.895497391
ENSGALG00010020767	UBXN10	3	0.024457548	0.055082838	0.444013935	0.657032509	6.85734E-05	2.010288977	0.51815844	0.666666667	CRA004775;PRJNA827465;Yang2025_Silkie_Wencha	0.895497391
ENSGALG00010018919	RALB	3	0.021923912	0.04937882	0.443994253	0.657046739	0.001657682	2.299025942	13.00663627	0.666666667	CRA004775;PRJNA827465;Yang2025_Silkie_Wencha	0.895497391
ENSGALG00010005314	IL20RA	3	-0.233818727	0.527851231	-0.442963306	0.657792276	0.588590099	6.959151873	71.26086574	0.666666667	CRA004775;PRJNA827465;Yang2025_Silkie_Wencha	0.895927203
ENSGALG00010000356	ENSGALG00010000356	3	0.141052006	0.318434894	0.442953987	0.657799016	0.235565841	13.59909344	85.2931373	0.666666667	CRA004775;PRJNA827465;Yang2025_Silkie_Wencha	0.895927203
ENSGALG00010016564	SPEN	3	0.105729385	0.238759025	0.442828854	0.657889531	0.124327676	8.037346853	75.11771453	0.666666667	CRA004775;PRJNA827465;Yang2025_Silkie_Wencha	0.895964177
ENSGALG00010002461	TOMT	3	0.138666806	0.313228695	0.442701477	0.657981674	0.260367736	18.72246441	89.3176456	0.666666667	CRA004775;PRJNA827465;Yang2025_Silkie_Wencha	0.89600336
ENSGALG00010001308	MGMT	3	-0.059835347	0.135192695	-0.442593049	0.658060114	0.032045531	4.929885202	59.43110401	0.666666667	CRA004775;PRJNA827465;Yang2025_Silkie_Wencha	0.896023878
ENSGALG00010024311	KLHL6	3	-0.074366165	0.168073548	-0.442462038	0.658154895	0.04598553	5.007236825	60.05781093	0.666666667	CRA004775;PRJNA827465;Yang2025_Silkie_Wencha	0.8960664
ENSGALG00010023217	CREB3L1	3	-0.041793777	0.094494143	-0.442289604	0.658279653	0	1.773183722	0	0.666666667	CRA004775;PRJNA827465;Yang2025_Silkie_Wencha	0.896150204
ENSGALG00010024537	EPB42	3	-0.070585317	0.159677865	-0.442048225	0.658454309	0.025975298	2.98482764	32.99445592	0.666666667	CRA004775;PRJNA827465;Yang2025_Silkie_Wencha	0.896301673
ENSGALG00010007928	C11orf70	2	0.391790289	0.889219942	0.440599981	0.659502617	1.428977251	10.36439232	90.35158098	0.5	PRJNA827465;Yang2025_Silkie_Wenchang	0.896348668
ENSGALG00010000377	ENSGALG00010000377	3	0.160394322	0.363961817	0.440689969	0.65943746	0.327665844	12.54217219	84.0537989	0.666666667	CRA004775;PRJNA827465;Yang2025_Silkie_Wencha	0.896348668
ENSGALG00010022022	STAMBPL1	3	-0.122484606	0.277690249	-0.441083567	0.6591525	0.138904561	5.231556738	61.77046145	0.666666667	CRA004775;PRJNA827465;Yang2025_Silkie_Wencha	0.896348668
ENSGALG00010018380	SLC20A1	3	-0.112368432	0.254723547	-0.441138769	0.659112538	0.172818049	18.39926085	89.12999812	0.666666667	CRA004775;PRJNA827465;Yang2025_Silkie_Wencha	0.896348668
ENSGALG00010009591	LYPD6	2	-0.111646543	0.252774552	-0.441684268	0.658717695	0	6.70637E-05	0	1	PRJNA827465;Yang2025_Silkie_Wenchang	0.896348668
ENSGALG00010022379	METTL15	3	0.104396702	0.236860153	0.440752489	0.659392193	0.124783057	9.536654821	79.02828573	0.666666667	CRA004775;PRJNA827465;Yang2025_Silkie_Wencha	0.896348668
ENSGALG00010024005	STYK1	3	0.071820545	0.162793066	0.441176932	0.659084912	0.014392817	2.373819467	15.74759464	0.666666667	CRA004775;PRJNA827465;Yang2025_Silkie_Wencha	0.896348668
ENSGALG00010025990	FAM124B	3	0.068981952	0.156202627	0.441618387	0.658765376	0	0.48739067	0	0.666666667	CRA004775;PRJNA827465;Yang2025_Silkie_Wencha	0.896348668
ENSGALG00010029380	RGS9	3	0.066429207	0.150343626	0.441849175	0.658598351	0.030026177	3.566219319	43.91819961	0.666666667	CRA004775;PRJNA827465;Yang2025_Silkie_Wencha	0.896348668
ENSGALG00010008316	ADGRF5	3	0.059548814	0.135181012	0.440511678	0.659566556	0.029254342	4.676702658	57.23482663	0.666666667	CRA004775;PRJNA827465;Yang2025_Silkie_Wencha	0.896348668
ENSGALG00010004117	STBD1	3	0.053673281	0.121558974	0.441541078	0.65882133	0	0.249024501	0	0.666666667	CRA004775;PRJNA827465;Yang2025_Silkie_Wencha	0.896348668
ENSGALG00010014737	RMI1	3	0.050003268	0.113491562	0.440590182	0.659509712	0.001761768	2.089723084	4.293539384	0.666666667	CRA004775;PRJNA827465;Yang2025_Silkie_Wencha	0.896348668
ENSGALG00010011797	SCAF4	3	0.04053233	0.091914631	0.440978002	0.659228923	0.011153177	3.095502893	35.39014277	0.666666667	CRA004775;PRJNA827465;Yang2025_Silkie_Wencha	0.896348668
ENSGALG00010015166	YTHDF2	3	-0.038780597	0.087949488	-0.440941698	0.659255205	0.01326883	4.945084222	59.555795	0.666666667	CRA004775;PRJNA827465;Yang2025_Silkie_Wencha	0.896348668
ENSGALG00010022808	PSMA7	3	-0.037565885	0.085146702	-0.441190133	0.659075356	0.006596024	2.495560955	19.8576979	0.666666667	CRA004775;PRJNA827465;Yang2025_Silkie_Wencha	0.896348668
ENSGALG00010022408	RAD51AP1	3	0.035463494	0.080407689	0.44104605	0.659179659	0.006470105	2.944325708	32.0727325	0.666666667	CRA004775;PRJNA827465;Yang2025_Silkie_Wencha	0.896348668
ENSGALG00010024741	RILPL2	3	0.032912298	0.074524199	0.44163236	0.658755263	0	0.156431368	0	0.666666667	CRA004775;PRJNA827465;Yang2025_Silkie_Wencha	0.896348668
ENSGALG00010020125	NAV2	3	0.081523479	0.185154429	0.440299911	0.659719905	0.062612675	5.564177547	64.05578393	0.666666667	CRA004775;PRJNA827465;Yang2025_Silkie_Wencha	0.896384753
ENSGALG00010012583	NDUFAF6	3	0.030501108	0.069262625	0.440368928	0.659669926	0	1.937189	0	0.666666667	CRA004775;PRJNA827465;Yang2025_Silkie_Wencha	0.896384753
ENSGALG00010023314	CYB561	2	0.696770471	1.588664914	0.438588695	0.66095959	4.3854161	7.607700079	86.85542293	0.5	CRA004775;PRJNA827465	0.896410952
ENSGALG00010025946	KRT5	2	0.320566408	0.728618629	0.439964606	0.659962742	0	0.996524925	0	0.5	PRJNA827465;Yang2025_Silkie_Wenchang	0.896410952
ENSGALG00010009657	MSMP	3	-0.282056501	0.647092038	-0.435883126	0.662921528	0.930416094	9.735292596	79.45619014	0.666666667	CRA004775;PRJNA827465;Yang2025_Silkie_Wencha	0.896410952
ENSGALG00010027887	ERC2	2	0.262926722	0.602041685	0.436725112	0.662310715	0.621050624	6.87270112	85.44968008	0.5	PRJNA827465;Yang2025_Silkie_Wenchang	0.896410952
ENSGALG00010005538	ENSGALG00010005538	3	0.213783447	0.490564199	0.435790968	0.662988398	0.353126871	3.915484714	48.92075577	0.666666667	CRA004775;PRJNA827465;Yang2025_Silkie_Wencha	0.896410952
ENSGALG00010021999	ANKRD22	3	-0.200280595	0.459450935	-0.435912913	0.662899916	0.290176003	3.651145572	45.22267161	0.666666667	CRA004775;PRJNA827465;Yang2025_Silkie_Wencha	0.896410952
ENSGALG00010023278	ENSGALG00010023278	3	0.198387826	0.454651809	0.436351121	0.662581997	0.513197237	14.50407096	86.21076796	0.666666667	CRA004775;PRJNA827465;Yang2025_Silkie_Wencha	0.896410952
ENSGALG00010027216	ENSGALG00010027216	3	0.174074461	0.39699788	0.438477053	0.661040501	0.287271855	5.198396204	61.52659548	0.666666667	CRA004775;PRJNA827465;Yang2025_Silkie_Wencha	0.896410952
ENSGALG00010018799	ENSGALG00010018799	3	0.171310775	0.39187179	0.437160264	0.661995125	0.329385277	7.086729866	71.77823851	0.666666667	CRA004775;PRJNA827465;Yang2025_Silkie_Wencha	0.896410952
ENSGALG00010011007	ENSGALG00010011007	3	0.154194629	0.354124239	0.435425231	0.6632538	0.31262891	15.88268711	87.40767235	0.666666667	CRA004775;PRJNA827465;Yang2025_Silkie_Wencha	0.896410952
ENSGALG00010012843	ENSGALG00010012843	3	0.153282491	0.350031874	0.437910096	0.661451457	0.287624619	10.11650625	80.23032903	0.666666667	CRA004775;PRJNA827465;Yang2025_Silkie_Wencha	0.896410952
ENSGALG00010018160	ENSGALG00010018160	3	0.144822369	0.330196255	0.438594825	0.660955147	0.280911325	16.98115889	88.22224082	0.666666667	CRA004775;PRJNA827465;Yang2025_Silkie_Wencha	0.896410952
ENSGALG00010008100	ENSGALG00010008100	3	0.122828594	0.282155315	0.435322632	0.663328259	0.167657393	7.825635277	74.44296943	0.666666667	CRA004775;PRJNA827465;Yang2025_Silkie_Wencha	0.896410952
ENSGALG00010025003	ENSGALG00010025003	3	0.121458601	0.277193194	0.438173099	0.661260808	0.136281719	5.70354744	64.93410424	0.666666667	CRA004775;PRJNA827465;Yang2025_Silkie_Wencha	0.896410952
ENSGALG00010000532	ENSGALG00010000532	3	-0.120297388	0.27416835	-0.438771974	0.66082677	0	0.964529873	0	0.666666667	CRA004775;PRJNA827465;Yang2025_Silkie_Wencha	0.896410952
ENSGALG00010010774	KPTN	3	-0.117846446	0.270745138	-0.435267083	0.663368575	0.169489733	10.61740687	81.16300878	0.666666667	CRA004775;PRJNA827465;Yang2025_Silkie_Wencha	0.896410952
ENSGALG00010028129	CYB561D2	3	0.115225533	0.265014528	0.434789497	0.66371523	0.175355865	13.6825514	85.38284315	0.666666667	CRA004775;PRJNA827465;Yang2025_Silkie_Wencha	0.896410952
ENSGALG00010009299	MAP4K1	3	0.10628683	0.24299461	0.437404066	0.661818336	0.043047898	2.603674886	23.18549406	0.666666667	CRA004775;PRJNA827465;Yang2025_Silkie_Wencha	0.896410952
ENSGALG00010000338	ENSGALG00010000338	3	0.105838977	0.241804744	0.437704303	0.66160065	0.101281401	4.911172826	59.27652985	0.666666667	CRA004775;PRJNA827465;Yang2025_Silkie_Wencha	0.896410952
ENSGALG00010022689	MPL	3	0.10551476	0.241063747	0.437704803	0.661600287	0.068667339	3.285163403	39.12022768	0.666666667	CRA004775;PRJNA827465;Yang2025_Silkie_Wencha	0.896410952
ENSGALG00010003902	ENSGALG00010003902	3	0.105413488	0.240187083	0.438880753	0.660747943	0.125275701	7.826690718	74.44641583	0.666666667	CRA004775;PRJNA827465;Yang2025_Silkie_Wencha	0.896410952
ENSGALG00010027665	TRAF1	3	0.102311265	0.234162574	0.436924072	0.662166414	0.122351709	8.765016981	77.18201796	0.666666667	CRA004775;PRJNA827465;Yang2025_Silkie_Wencha	0.896410952
ENSGALG00010016057	SOHO1	3	0.101326234	0.231146047	0.438364555	0.661122036	0	0.927813229	0	0.666666667	CRA004775;PRJNA827465;Yang2025_Silkie_Wencha	0.896410952
ENSGALG00010013428	BPGM	3	-0.095881302	0.219981926	-0.435859908	0.662938375	0.101083266	9.958964597	79.91759103	0.666666667	CRA004775;PRJNA827465;Yang2025_Silkie_Wencha	0.896410952
ENSGALG00010005277	ENSGALG00010005277	3	-0.095683901	0.218159513	-0.438596051	0.660954259	0.066687826	3.743052771	46.56767824	0.666666667	CRA004775;PRJNA827465;Yang2025_Silkie_Wencha	0.896410952
ENSGALG00010001967	LAMA1	3	-0.09385118	0.215866709	-0.434764493	0.66373338	0.069561349	4.223923846	52.6506615	0.666666667	CRA004775;PRJNA827465;Yang2025_Silkie_Wencha	0.896410952
ENSGALG00010014210	ENSGALG00010014210	3	0.089489482	0.205147253	0.436220719	0.662676597	0.049038764	3.29312169	39.26735214	0.666666667	CRA004775;PRJNA827465;Yang2025_Silkie_Wencha	0.896410952
ENSGALG00010028556	SPTAN1	3	0.077906681	0.177446767	0.439042548	0.660630707	0.068634611	9.956635536	79.91289334	0.666666667	CRA004775;PRJNA827465;Yang2025_Silkie_Wencha	0.896410952
ENSGALG00010021487	KLHL17	3	-0.07745688	0.176188998	-0.439623816	0.660209588	0.068768732	9.697862245	79.37689823	0.666666667	CRA004775;PRJNA827465;Yang2025_Silkie_Wencha	0.896410952
ENSGALG00010004079	AP1AR	3	0.074687182	0.169796043	0.439864091	0.660035545	0.061716684	7.22273576	72.30966124	0.666666667	CRA004775;PRJNA827465;Yang2025_Silkie_Wencha	0.896410952
ENSGALG00010017470	HSPA2	3	0.074151339	0.170307211	0.435397532	0.663273901	0.053159044	6.479002078	69.13104864	0.666666667	CRA004775;PRJNA827465;Yang2025_Silkie_Wencha	0.896410952
ENSGALG00010028110	COQ4	3	0.071481507	0.164014959	0.435823097	0.662965085	0.049678241	5.23189125	61.77290574	0.666666667	CRA004775;PRJNA827465;Yang2025_Silkie_Wencha	0.896410952
ENSGALG00010013582	MAP4K4	3	0.068011874	0.155752395	0.436666634	0.662353131	0.041654306	5.002231504	60.01784407	0.666666667	CRA004775;PRJNA827465;Yang2025_Silkie_Wencha	0.896410952
ENSGALG00010008756	ITSN2	3	-0.064179924	0.14630289	-0.438678439	0.660894552	0.038472999	4.967112456	59.73515764	0.666666667	CRA004775;PRJNA827465;Yang2025_Silkie_Wencha	0.896410952
ENSGALG00010016577	ZBTB17	3	0.062856397	0.143436627	0.438217199	0.661228842	0.043292464	10.17853247	80.35080199	0.666666667	CRA004775;PRJNA827465;Yang2025_Silkie_Wencha	0.896410952
ENSGALG00010007968	SLC22A31	3	-0.061347287	0.140591473	-0.436351407	0.662581789	0	0.575509881	0	0.666666667	CRA004775;PRJNA827465;Yang2025_Silkie_Wencha	0.896410952
ENSGALG00010019334	ATL1	3	-0.061066196	0.139285122	-0.438425833	0.661077624	0.040848981	9.117428703	78.06399079	0.666666667	CRA004775;PRJNA827465;Yang2025_Silkie_Wencha	0.896410952
ENSGALG00010016475	MCCC1	3	0.059058866	0.134977214	0.437546932	0.661714748	0.039836039	11.42287464	82.49127245	0.666666667	CRA004775;PRJNA827465;Yang2025_Silkie_Wencha	0.896410952
ENSGALG00010024907	PHLDB1	3	0.051175105	0.117674294	0.434887715	0.663643932	0.023191531	5.320054956	62.40640338	0.666666667	CRA004775;PRJNA827465;Yang2025_Silkie_Wencha	0.896410952
ENSGALG00010025167	PTGES3	3	-0.047220288	0.107966664	-0.437359891	0.661850367	0.020862936	5.420668065	63.10417874	0.666666667	CRA004775;PRJNA827465;Yang2025_Silkie_Wencha	0.896410952
ENSGALG00010029770	TLCD3A	3	-0.047208566	0.107866208	-0.437658528	0.661633837	0	1.48355292	0	0.666666667	CRA004775;PRJNA827465;Yang2025_Silkie_Wencha	0.896410952
ENSGALG00010008079	CCDC127	3	0.044288021	0.101501867	0.436327155	0.662599382	0.017630921	4.757274378	57.95912027	0.666666667	CRA004775;PRJNA827465;Yang2025_Silkie_Wencha	0.896410952
ENSGALG00010014159	CDC42EP1	3	0.041265348	0.094837049	0.435118433	0.663476464	0.015955428	5.87573213	65.96168859	0.666666667	CRA004775;PRJNA827465;Yang2025_Silkie_Wencha	0.896410952
ENSGALG00010028578	SPOUT1	3	-0.041066517	0.094187241	-0.436009349	0.662829947	0.01126351	3.292800501	39.2614281	0.666666667	CRA004775;PRJNA827465;Yang2025_Silkie_Wencha	0.896410952
ENSGALG00010013953	DHX29	3	0.040741405	0.092924386	0.4384361	0.661070182	0	0.961033455	0	0.666666667	CRA004775;PRJNA827465;Yang2025_Silkie_Wencha	0.896410952
ENSGALG00010008401	PSMD8	3	0.034682976	0.079263791	0.437563926	0.661702426	0.005512692	2.646546402	24.42981546	0.666666667	CRA004775;PRJNA827465;Yang2025_Silkie_Wencha	0.896410952
ENSGALG00010016746	HDGFL3	3	0.033565926	0.076321668	0.439795503	0.660085225	0.005572046	2.846205016	29.73099307	0.666666667	CRA004775;PRJNA827465;Yang2025_Silkie_Wencha	0.896410952
ENSGALG00010023792	ZNF609	3	0.031256623	0.07125201	0.438677064	0.660895548	0.004385779	2.586257135	22.66816888	0.666666667	CRA004775;PRJNA827465;Yang2025_Silkie_Wencha	0.896410952
ENSGALG00010019477	NCAPH	3	-0.029552833	0.06748774	-0.437899283	0.661459295	0	1.179543656	0	0.666666667	CRA004775;PRJNA827465;Yang2025_Silkie_Wencha	0.896410952
ENSGALG00010009608	AFAP1	3	0.029443482	0.067217325	0.438034128	0.661361544	0	1.502648076	0	0.666666667	CRA004775;PRJNA827465;Yang2025_Silkie_Wencha	0.896410952
ENSGALG00010009172	SPOPL	3	0.029017705	0.06670532	0.435013352	0.663552736	0	1.401383276	0	0.666666667	CRA004775;PRJNA827465;Yang2025_Silkie_Wencha	0.896410952
ENSGALG00010027339	LAMTOR5	3	0.027492525	0.062798642	0.437788521	0.661539593	0	0.38771312	0	0.666666667	CRA004775;PRJNA827465;Yang2025_Silkie_Wencha	0.896410952
ENSGALG00010015782	ATG4A	3	-0.027270433	0.062504771	-0.436293618	0.662623712	0.002304457	2.342231588	14.61134713	0.666666667	CRA004775;PRJNA827465;Yang2025_Silkie_Wencha	0.896410952
ENSGALG00010020005	EOGT	3	-0.026981962	0.061645385	-0.437696387	0.661606389	0.002910982	2.592114688	22.84292015	0.666666667	CRA004775;PRJNA827465;Yang2025_Silkie_Wencha	0.896410952
ENSGALG00010004481	ZNF704	3	-0.026295099	0.060250051	-0.4364328	0.662522745	0	1.171351506	0	0.666666667	CRA004775;PRJNA827465;Yang2025_Silkie_Wencha	0.896410952
ENSGALG00010017428	PSMA3	3	-0.025025658	0.057440558	-0.435679233	0.663069475	0	0.072353734	0	1	CRA004775;PRJNA827465;Yang2025_Silkie_Wencha	0.896410952
ENSGALG00010017552	LARS2	3	-0.023883574	0.054920707	-0.434873754	0.663654067	0	1.97285265	0	0.666666667	CRA004775;PRJNA827465;Yang2025_Silkie_Wencha	0.896410952
ENSGALG00010014205	DPM1	3	0.023268687	0.053365289	0.436026629	0.662817409	0	1.333909892	0	0.666666667	CRA004775;PRJNA827465;Yang2025_Silkie_Wencha	0.896410952
ENSGALG00010002046	FAM126A	3	0.022924745	0.052512828	0.436555138	0.662434004	0	0.819872366	0	0.666666667	CRA004775;PRJNA827465;Yang2025_Silkie_Wencha	0.896410952
ENSGALG00010018087	ENSGALG00010018087	3	0.022476344	0.051652598	0.435144505	0.663457541	0	0.202175775	0	0.666666667	CRA004775;PRJNA827465;Yang2025_Silkie_Wencha	0.896410952
ENSGALG00010029368	DDX52	3	-0.02198163	0.050295179	-0.437052429	0.662073326	0	0.462465793	0	1	CRA004775;PRJNA827465;Yang2025_Silkie_Wencha	0.896410952
ENSGALG00010028498	C9orf78	3	-0.020732732	0.047336221	-0.437988742	0.661394445	0	1.368920664	0	0.666666667	CRA004775;PRJNA827465;Yang2025_Silkie_Wencha	0.896410952
ENSGALG00010028190	ILF2	3	0.018428465	0.042198236	0.43671175	0.662320407	0	1.066438154	0	0.666666667	CRA004775;PRJNA827465;Yang2025_Silkie_Wencha	0.896410952
ENSGALG00010009389	CPEB4	3	0.079383862	0.182673648	0.434566576	0.663877061	0.069409435	7.313501933	72.65331959	0.666666667	CRA004775;PRJNA827465;Yang2025_Silkie_Wencha	0.896510755
ENSGALG00010005547	LRP11	3	-0.065582204	0.150942463	-0.434484787	0.66393644	0.035116291	4.608787847	56.60464169	0.666666667	CRA004775;PRJNA827465;Yang2025_Silkie_Wencha	0.896510755
ENSGALG00010017767	KIF4B	3	0.035002576	0.080576705	0.434400688	0.663997498	0	1.831616408	0	0.666666667	CRA004775;PRJNA827465;Yang2025_Silkie_Wencha	0.896510755
ENSGALG00010022202	CX3CL1	3	0.043676508	0.10057949	0.434248652	0.664107887	0	0.335651117	0	0.666666667	CRA004775;PRJNA827465;Yang2025_Silkie_Wencha	0.896574182
ENSGALG00010003980	CHCHD7	3	0.104247136	0.240113677	0.434157427	0.664174126	0.133247502	9.748296536	79.48359498	0.666666667	CRA004775;PRJNA827465;Yang2025_Silkie_Wencha	0.896574422
ENSGALG00010016823	HSF3	3	0.032519421	0.074916811	0.434073749	0.664234887	0.00966603	5.254639895	61.93840035	0.666666667	CRA004775;PRJNA827465;Yang2025_Silkie_Wencha	0.896574422
ENSGALG00010003018	MOGAT2	3	0.414487217	0.955787141	0.433660591	0.664534928	2.421471878	18.27027031	89.05325446	0.666666667	CRA004775;PRJNA827465;Yang2025_Silkie_Wencha	0.896630081
ENSGALG00010022817	KRT23	2	0.23898757	0.551021295	0.433717484	0.664493609	0	0.002738173	0	1	PRJNA827465;Yang2025_Silkie_Wenchang	0.896630081
ENSGALG00010003731	ENSGALG00010003731	3	-0.14777979	0.341661345	-0.432532951	0.665354109	0	1.049048764	0	0.666666667	CRA004775;PRJNA827465;Yang2025_Silkie_Wencha	0.896630081
ENSGALG00010020307	SHQ1	3	0.146640138	0.338888613	0.432708956	0.665226222	0.264155215	9.206616257	78.27649221	0.666666667	CRA004775;PRJNA827465;Yang2025_Silkie_Wencha	0.896630081
ENSGALG00010003063	GGH	3	-0.129249017	0.298908861	-0.43240276	0.665448712	0.243890089	52.51769219	96.19175954	0.666666667	CRA004775;PRJNA827465;Yang2025_Silkie_Wencha	0.896630081
ENSGALG00010024625	SPRYD3	3	0.129055189	0.298926006	0.431729545	0.665937991	0.240200135	25.28957844	92.09160404	0.666666667	CRA004775;PRJNA827465;Yang2025_Silkie_Wencha	0.896630081
ENSGALG00010025533	INHBC	3	0.124060861	0.286668987	0.432766944	0.66518409	0.054939304	2.566200549	22.06376852	0.666666667	CRA004775;PRJNA827465;Yang2025_Silkie_Wencha	0.896630081
ENSGALG00010000302	ENSGALG00010000302	3	-0.110607001	0.255749815	-0.432481255	0.665391673	0.151738225	12.09322218	83.46181051	0.666666667	CRA004775;PRJNA827465;Yang2025_Silkie_Wencha	0.896630081
ENSGALG00010002405	KCNQ5	3	0.109798684	0.253646158	0.432881321	0.665100991	0.10525872	4.46601917	55.21738883	0.666666667	CRA004775;PRJNA827465;Yang2025_Silkie_Wencha	0.896630081
ENSGALG00010019899	ENSGALG00010019899	3	0.107276741	0.248290304	0.43206174	0.665696541	0.082274645	3.659265646	45.34422496	0.666666667	CRA004775;PRJNA827465;Yang2025_Silkie_Wencha	0.896630081
ENSGALG00010027353	ENSGALG00010027353	3	0.103645397	0.239959017	0.43192958	0.665792595	0	0.369894184	0	0.666666667	CRA004775;PRJNA827465;Yang2025_Silkie_Wencha	0.896630081
ENSGALG00010029355	MCRIP1	3	0.087544898	0.202071236	0.433237802	0.664842019	0.072583609	5.464054748	63.39714567	0.666666667	CRA004775;PRJNA827465;Yang2025_Silkie_Wencha	0.896630081
ENSGALG00010027559	ENSGALG00010027559	3	-0.087032602	0.201275212	-0.432405964	0.665446384	0.06083174	4.013432012	50.16733823	0.666666667	CRA004775;PRJNA827465;Yang2025_Silkie_Wencha	0.896630081
ENSGALG00010028461	ENSGALG00010028461	3	0.083135562	0.192266424	0.432397714	0.665452379	0.058213501	4.571506317	56.25074404	0.666666667	CRA004775;PRJNA827465;Yang2025_Silkie_Wencha	0.896630081
ENSGALG00010011300	EVC	3	0.083040939	0.192418052	0.431565219	0.666057442	0.086657489	10.89995221	81.65129571	0.666666667	CRA004775;PRJNA827465;Yang2025_Silkie_Wencha	0.896630081
ENSGALG00010029203	ALDOC	3	0.076877639	0.178169566	0.43148581	0.666115168	0.059085974	6.046388844	66.92240523	0.666666667	CRA004775;PRJNA827465;Yang2025_Silkie_Wencha	0.896630081
ENSGALG00010008252	ZFP92	3	0.073050283	0.16924366	0.431627886	0.666011888	0.059258951	7.007230778	71.45805436	0.666666667	CRA004775;PRJNA827465;Yang2025_Silkie_Wencha	0.896630081
ENSGALG00010028114	PFKFB4	3	0.071398664	0.165332871	0.431847967	0.665851915	0.06187402	11.65806785	82.84449854	0.666666667	CRA004775;PRJNA827465;Yang2025_Silkie_Wencha	0.896630081
ENSGALG00010010783	F2RL2	3	0.065299572	0.150776376	0.433088879	0.664950201	0	0.362802026	0	0.666666667	CRA004775;PRJNA827465;Yang2025_Silkie_Wencha	0.896630081
ENSGALG00010002402	TXLNG	3	0.065244771	0.151074732	0.431870837	0.665835292	0.048730016	7.792587154	74.33458285	0.666666667	CRA004775;PRJNA827465;Yang2025_Silkie_Wencha	0.896630081
ENSGALG00010028905	COLGALT1	3	0.058653159	0.135878028	0.431660366	0.665988278	0.04065475	8.836326043	77.36615885	0.666666667	CRA004775;PRJNA827465;Yang2025_Silkie_Wencha	0.896630081

ENSGALG00010002504	ENSGALG0001000250(	3	0.048623966	0.112084622	0.433814785	0.664422944	0.022782439	5.324777899	62.43974795	0.666666667	CRA004775;PRJNA827465;Yang2025_Silkie_Wencha	0.896630081
ENSGALG00010029777	ENSGALG0001002977	3	0.045524999	0.1054019	0.431918197	0.665800869	0.009057226	2.70767169	26.1358012	0.666666667	CRA004775;PRJNA827465;Yang2025_Silkie_Wencha	0.896630081
ENSGALG00010000564	NAA40	3	-0.040365829	0.093533335	-0.431566232	0.666056706	0.009282407	2.702823391	26.00330431	0.666666667	CRA004775;PRJNA827465;Yang2025_Silkie_Wencha	0.896630081
ENSGALG00010024479	ERBB2	3	-0.03824867	0.088610494	-0.431649443	0.665996218	0.011841687	4.570381811	56.23997988	0.666666667	CRA004775;PRJNA827465;Yang2025_Silkie_Wencha	0.896630081
ENSGALG00010024798	PTPRK	3	-0.035835615	0.082891289	-0.432320633	0.665508393	0	1.980719803	0	0.666666667	CRA004775;PRJNA827465;Yang2025_Silkie_Wencha	0.896630081
ENSGALG00010016111	EPB41	3	0.031813442	0.073724666	0.431516934	0.666092542	0.000156921	2.013862261	0.688342057	0.666666667	CRA004775;PRJNA827465;Yang2025_Silkie_Wencha	0.896630081
ENSGALG00010024876	CFLAR	3	-0.022136251	0.051059152	-0.433541301	0.664621568	0	1.790498826	0	0.666666667	CRA004775;PRJNA827465;Yang2025_Silkie_Wencha	0.896630081
ENSGALG00010012898	FAM114A2	3	-0.0190291	0.043945015	-0.433020679	0.664999747	0	0.205408215	0	0.666666667	CRA004775;PRJNA827465;Yang2025_Silkie_Wencha	0.896630081
ENSGALG00010012730	CAP2	3	-0.054607029	0.126594017	-0.43135553	0.666209879	0.016358136	2.703887501	26.03242556	0.666666667	CRA004775;PRJNA827465;Yang2025_Silkie_Wencha	0.896672202
ENSGALG00010017054	GALM	3	-0.074575441	0.172924374	-0.431260436	0.666279014	0.072022267	11.19436265	82.13386449	0.666666667	CRA004775;PRJNA827465;Yang2025_Silkie_Wencha	0.896679895
ENSGALG00010015869	SLC7A2	3	0.037590715	0.087207683	0.431048202	0.666433323	0	1.827968778	0	0.666666667	CRA004775;PRJNA827465;Yang2025_Silkie_Wencha	0.896716858
ENSGALG00010022196	PLXNA1	3	-0.030429989	0.070583583	-0.431119918	0.666381179	0	0.069208895	0	1	CRA004775;PRJNA827465;Yang2025_Silkie_Wencha	0.896716858
ENSGALG00010017479	SSTR5	2	-0.158580178	0.36801995	-0.430901038	0.666540329	0.113951702	1.72030982	41.87093579	0.5	PRJNA827465;Yang2025_Silkie_Wenchang	0.896775498
ENSGALG00010009583	LYPD6B	3	-0.096705492	0.224487401	-0.430783605	0.666625721	0	0.121306736	0	1	CRA004775;PRJNA827465;Yang2025_Silkie_Wencha	0.89680505
ENSGALG00010003001	MAP6	3	0.178198195	0.414256848	0.430163548	0.667076676	0.305272652	4.940411349	59.51754097	0.666666667	CRA004775;PRJNA827465;Yang2025_Silkie_Wencha	0.897210552
ENSGALG00010020572	PPP1R3C	3	-0.088434054	0.205532145	-0.430268723	0.667000176	0.072999321	5.346345087	62.59126623	0.666666667	CRA004775;PRJNA827465;Yang2025_Silkie_Wencha	0.897210552
ENSGALG00010017338	MBNL3	3	0.076247623	0.177291532	0.430069175	0.667145322	0.058476701	5.450522579	63.30627071	0.666666667	CRA004775;PRJNA827465;Yang2025_Silkie_Wencha	0.897210552
ENSGALG00010023770	SCRN2	3	-0.065862143	0.153160591	-0.430020168	0.667180971	0.028663	3.245574025	38.37761874	0.666666667	CRA004775;PRJNA827465;Yang2025_Silkie_Wencha	0.897210552
ENSGALG00010023606	LRRC7	2	0.173133421	0.402763905	0.429863298	0.667295085	0.255038003	4.540821514	77.97755325	0.5	PRJNA827465;Yang2025_Silkie_Wenchang	0.897278669
ENSGALG00010018960	RBFOX1	3	0.149819627	0.348579135	0.429578118	0.667502557	0.278978916	9.870309614	79.73721111	0.666666667	CRA004775;PRJNA827465;Yang2025_Silkie_Wencha	0.89738696
ENSGALG00010011038	GSG1	2	0.144089287	0.3354177	0.429581645	0.667499992	0	0.08035443	0	1	PRJNA827465;Yang2025_Silkie_Wenchang	0.89738696
ENSGALG00010028239	EDMPN2	2	-0.694275951	1.618662707	-0.42891947	0.66798183	0	0.103845536	0	1	PRJNA827465;Yang2025_Silkie_Wenchang	0.897394182
ENSGALG00010004109	AGMO	3	0.244653818	0.570658122	0.428722222	0.668125387	0.711190306	7.810726658	74.39418779	0.666666667	CRA004775;PRJNA827465;Yang2025_Silkie_Wencha	0.897394182
ENSGALG00010022279	EP400	3	0.077454368	0.1804605	0.429203996	0.667774775	0.066843786	6.632834806	69.84698009	0.666666667	CRA004775;PRJNA827465;Yang2025_Silkie_Wencha	0.897394182
ENSGALG00010019441	LMCD1	3	0.067475163	0.157179809	0.429286456	0.667714772	0.033156119	3.663335138	45.40494045	0.666666667	CRA004775;PRJNA827465;Yang2025_Silkie_Wencha	0.897394182
ENSGALG00010022776	CDH4	3	-0.06559802	0.152982933	-0.428793059	0.668073831	0.015036523	2.491739624	19.73479167	0.666666667	CRA004775;PRJNA827465;Yang2025_Silkie_Wencha	0.897394182
ENSGALG00010017661	NHSL3	3	0.057432923	0.133817035	0.429189991	0.667784967	0.019514962	2.8972019	30.96787629	0.666666667	CRA004775;PRJNA827465;Yang2025_Silkie_Wencha	0.897394182
ENSGALG00010007944	ACSF3	3	-0.048973336	0.114113161	-0.429164656	0.667803402	0	1.506304229	0	0.666666667	CRA004775;PRJNA827465;Yang2025_Silkie_Wencha	0.897394182
ENSGALG00010005970	SHISA2	3	-0.043151676	0.100625424	-0.428834729	0.668043503	0	1.34427994	0	1	CRA004775;PRJNA827465;Yang2025_Silkie_Wencha	0.897394182
ENSGALG00010024996	COA7	3	-0.035180416	0.082063299	-0.42869854	0.668142623	0	0.268058314	0	0.666666667	CRA004775;PRJNA827465;Yang2025_Silkie_Wencha	0.897394182
ENSGALG00010007551	FEZ2	3	0.024371386	0.056809299	0.429003465	0.667920703	0.001317491	2.129884959	6.098214781	0.666666667	CRA004775;PRJNA827465;Yang2025_Silkie_Wencha	0.897394182
ENSGALG00010025252	PPP1R9B	3	0.099806173	0.232915533	0.428508018	0.668281297	0.143453932	33.8606035	94.09343073	0.666666667	CRA004775;PRJNA827465;Yang2025_Silkie_Wencha	0.89740941
ENSGALG00010009858	ST8SIA5	3	0.077883221	0.181746978	0.428525535	0.668268547	0	0.524529393	0	0.666666667	CRA004775;PRJNA827465;Yang2025_Silkie_Wencha	0.89740941
ENSGALG00010010751	SLC30A5	3	0.060009663	0.140071594	0.428421365	0.668344373	0.036139514	5.22863284	61.74908315	0.666666667	CRA004775;PRJNA827465;Yang2025_Silkie_Wencha	0.89740941
ENSGALG00010021012	KCNIP2	2	0.193896412	0.452875418	0.428145147	0.668545449	0	0.239130341	0	0.5	CRA004775;PRJNA827465	0.89743386
ENSGALG00010017866	RALA	3	-0.088993452	0.207905508	-0.428047593	0.668616471	0.115547154	26.2100165	92.36932949	0.666666667	CRA004775;PRJNA827465;Yang2025_Silkie_Wencha	0.89743386
ENSGALG00010017677	WDR43	3	-0.050000295	0.116806537	-0.428060757	0.668606887	0.027039491	7.003305284	71.44205602	0.666666667	CRA004775;PRJNA827465;Yang2025_Silkie_Wencha	0.89743386
ENSGALG00010029935	SYNRG	3	0.044263929	0.103378485	0.428173513	0.668524799	0.012258206	3.099973047	35.48331004	0.666666667	CRA004775;PRJNA827465;Yang2025_Silkie_Wencha	0.89743386
ENSGALG00010007423	HEATR5B	3	-0.020300566	0.047468643	-0.42766265	0.668896746	0	0.015060089	0	0.666666667	CRA004775;PRJNA827465;Yang2025_Silkie_Wencha	0.897724831
ENSGALG00010029345	RILP	3	0.122787678	0.2873616	0.42729327	0.669165734	0.180298305	12.73852521	84.29959539	0.666666667	CRA004775;PRJNA827465;Yang2025_Silkie_Wencha	0.897915376
ENSGALG00010021169	CAMK2G	3	-0.04063658	0.095100951	-0.427299409	0.669161263	0.014551062	4.713918404	57.57245187	0.666666667	CRA004775;PRJNA827465;Yang2025_Silkie_Wencha	0.897915376
ENSGALG00010025926	ENSGALG0001002592(	2	0.12963232	0.303574671	0.427019553	0.669365086	0	0.970523047	0	0.5	PRJNA827465;Yang2025_Silkie_Wenchang	0.898005902
ENSGALG00010001252	SNCAIP	3	0.067235029	0.157499598	0.42689018	0.669459318	0.018289755	2.624280641	23.78868375	0.666666667	CRA004775;PRJNA827465;Yang2025_Silkie_Wencha	0.898005902
ENSGALG00010022573	AP4E1	3	-0.031749082	0.074379632	-0.426851835	0.669487249	0.004444269	2.639995695	24.2423007	0.666666667	CRA004775;PRJNA827465;Yang2025_Silkie_Wencha	0.898005902
ENSGALG00010005166	CBLB	3	-0.023290795	0.054533723	-0.427089765	0.669313947	0	0.266851646	0	0.666666667	CRA004775;PRJNA827465;Yang2025_Silkie_Wencha	0.898005902
ENSGALG00010021742	MATN4	3	0.283728419	0.664975146	0.426672523	0.6696159	1.052411793	9.815124774	79.62328502	0.666666667	CRA004775;PRJNA827465;Yang2025_Silkie_Wencha	0.898005902
ENSGALG00010017386	ENSGALG0001001738(	2	0.225004353	0.52838936	0.425834157	0.670228696	0	0.021973486	0	1	PRJNA827465;Yang2025_Silkie_Wenchang	0.898082037
ENSGALG00010023333	ENDOU	3	0.17302064	0.406300155	0.425844386	0.670221242	0.189236457	3.218904641	37.86706277	0.666666667	CRA004775;PRJNA827465;Yang2025_Silkie_Wencha	0.898082037
ENSGALG00010001692	ALDH18A1	3	0.066571613	0.156274111	0.425992587	0.670113248	0.038439981	4.715453087	57.58626025	0.666666667	CRA004775;PRJNA827465;Yang2025_Silkie_Wencha	0.898082037
ENSGALG00010025696	MTCH1	3	0.049873807	0.116966297	0.426394684	0.669820275	0.024861467	6.040971835	66.89274417	0.666666667	CRA004775;PRJNA827465;Yang2025_Silkie_Wencha	0.898082037
ENSGALG00010029505	VEZF1	3	0.048168856	0.113013403	0.426151719	0.669997296	0.022595423	5.043986014	60.34881948	0.666666667	CRA004775;PRJNA827465;Yang2025_Silkie_Wencha	0.898082037
ENSGALG00010010939	ZCCHC4	3	-0.041296298	0.097001655	-0.425727767	0.670306227	0	1.793153674	0	0.666666667	CRA004775;PRJNA827465;Yang2025_Silkie_Wencha	0.898082037
ENSGALG00010024806	ANKRD27	3	0.039595127	0.09295865	0.425943436	0.670149063	0.008176741	2.744511952	27.12729859	0.666666667	CRA004775;PRJNA827465;Yang2025_Silkie_Wencha	0.898082037
ENSGALG00010010119	SRPK2	3	-0.031664626	0.07424342	-0.426497405	0.669745439	0.004249479	2.575035385	22.33116439	0.666666667	CRA004775;PRJNA827465;Yang2025_Silkie_Wencha	0.898082037
ENSGALG00010023817	BBS2	3	0.027459175	0.06449027	0.425787872	0.670262425	0	1.505527415	0	0.666666667	CRA004775;PRJNA827465;Yang2025_Silkie_Wencha	0.898082037
ENSGALG00010013316	KDM7A	3	0.026380202	0.061956071	0.425788808	0.670261743	0	1.798975107	0	0.666666667	CRA004775;PRJNA827465;Yang2025_Silkie_Wencha	0.898082037
ENSGALG00010008839	ZNF512	3	-0.01480156	0.034763003	-0.425784858	0.670264622	0	1.858106946	0	0.666666667	CRA004775;PRJNA827465;Yang2025_Silkie_Wencha	0.898082037
ENSGALG00010029234	TOM1L1	3	-0.110511086	0.259670156	-0.425582545	0.670412061	0.106903743	4.496170651	55.51770261	0.666666667	CRA004775;PRJNA827465;Yang2025_Silkie_Wencha	0.898138728
ENSGALG00010005360	CAMK1D	3	-0.051601999	0.121323174	-0.425326818	0.670598446	0.00463262	2.208901537	9.457258904	0.666666667	CRA004775;PRJNA827465;Yang2025_Silkie_Wencha	0.898218211
ENSGALG00010027468	BSG	3	0.022269242	0.052351211	0.425381597	0.670558519	0	0.412000639	0	1	CRA004775;PRJNA827465;Yang2025_Silkie_Wencha	0.898218211
ENSGALG00010016809	SYTL1	2	-0.145899643	0.343729532	-0.424460598	0.671229935	0.045321868	1.200130343	16.67571725	0.5	CRA004775;PRJNA827465	0.898235067
ENSGALG00010008750	PFDN1	3	0.127158911	0.299455165	0.424634221	0.671103342	0.240504158	21.82003112	90.83411023	0.666666667	CRA004775;PRJNA827465;Yang2025_Silkie_Wencha	0.898235067
ENSGALG00010025297	SPSB4	3	0.114117174	0.268479528	0.425049816	0.670800036	0.17153328	21.2858112	90.60396806	0.666666667	CRA004775;PRJNA827465;Yang2025_Silkie_Wencha	0.898235067
ENSGALG00010021329	PSMC3IP	3	0.075321059	0.177403197	0.424575545	0.671146123	0.053321444	4.568236732	56.21943175	0.666666667	CRA004775;PRJNA827465;Yang2025_Silkie_Wencha	0.898235067
ENSGALG00010027894	RSBN1	3	-0.069325306	0.163197329	-0.424794366	0.670986585	0.069461205	19.64969091	89.82172285	0.666666667	CRA004775;PRJNA827465;Yang2025_Silkie_Wencha	0.898235067
ENSGALG00010022755	MINPP1	3	-0.068972401	0.162361971	-0.424806381	0.670977826	0.052120649	7.201616017	72.22845545	0.666666667	CRA004775;PRJNA827465;Yang2025_Silkie_Wencha	0.898235067
ENSGALG00010009142	LGALS8	3	0.059056426	0.139108409	0.424535268	0.67117549	0.028459324	4.215645792	52.55768396	0.666666667	CRA004775;PRJNA827465;Yang2025_Silkie_Wencha	0.898235067
ENSGALG00010000453	ACHE	3	0.054526169	0.128378012	0.424731369	0.671032514	0	0.704819482	0	0.666666667	CRA004775;PRJNA827465;Yang2025_Silkie_Wencha	0.898235067
ENSGALG00010012124	ERAP1	3	-0.053213469	0.125373914	-0.424438127	0.67124632	0	0.675617762	0	0.666666667	CRA004775;PRJNA827465;Yang2025_Silkie_Wencha	0.898235067
ENSGALG00010002800	TUBGCP5	3	-0.021073428	0.049613233	-0.424754171	0.671015889	0	0.081702578	0	0.666666667	CRA004775;PRJNA827465;Yang2025_Silkie_Wencha	0.898235067
ENSGALG00010012195	ITSN1	3	0.079476011	0.187288994	0.424349607	0.671310867	0.073747968	7.259404414	72.44953049	0.666666667	CRA004775;PRJNA827465;Yang2025_Silkie_Wencha	0.898236429
ENSGALG00010005437	PDE7B	3	0.105785309	0.24934115	0.424259329	0.671376697	0.135120195	13.74394836	85.4481409	0.666666667	CRA004775;PRJNA827465;Yang2025_Silkie_Wencha	0.898239507
ENSGALG00010019206	RNF207	3	0.138126296	0.32579418	0.423967967	0.671589175	0.248880367	13.26681703	84.92479398	0.666666667	CRA004775;PRJNA827465;Yang2025_Silkie_Wencha	0.898268787
ENSGALG00010010995	HEBP1	3	0.041880182	0.098770849	0.424013591	0.671555901	0.009924547	2.966748513	32.58612953	0.666666667	CRA004775;PRJNA827465;Yang2025_Silkie_Wencha	0.898268787
ENSGALG00010021097	KATNBL1	3	-0.036280061	0.085540049	-0.424129538	0.671471345	0.01098932	4.153799627	51.85131254	0.666666667	CRA004775;PRJNA827465;Yang2025_Silkie_Wencha	0.898268787
ENSGALG00010029491	ENSGALG00010029491	3	0.064976493	0.153309125	0.42382665	0.671692241	0	0.429189466	0	0.666666667	CRA004775;PRJNA827465;Yang2025_Silkie_Wencha	0.898321661
ENSGALG00010005111	RASSF6	3	-0.089332863	0.210851353	-0.423676974	0.67180141	0	1.338920752	0	0.666666667	CRA004775;PRJNA827465;Yang2025_Silkie_Wencha	0.898382685
ENSGALG00010029436	WDR45B	3	-0.016912646	0.039950199	-0.423343206	0.672044875	0	1.207104063	0	0.666666667	CRA004775;PRJNA827465;Yang2025_Silkie_Wencha	0.898623273
ENSGALG00010026010	ZSWIM5	3	0.07599422	0.179581047	0.423175055	0.672167546	0.075159145	13.68180228	85.38204281	0.666666667	CRA004775;PRJNA827465;Yang2025_Silkie_Wencha	0.89870231
ENSGALG00010012743	ENSGALG0001001274	3	0.25188618	0.595614708	0.422901208	0.672367341	0.978706056	25.35910749	92.11328711	0.666666667	CRA004775;PRJNA827465;Yang2025_Silkie_Wencha	0.898874396
ENSGALG00010028047	ENSGALG0001002804	3	0.093770815	0.221772491	0.422824375	0.672423402	0	1.664312957	0	0.666666667	CRA004775;PRJNA827465;Yang2025_Silkie_Wencha	0.898874396
ENSGALG00010029361	ALYREF	3	0.07938026	0.187788524	0.422710921	0.672506186	0.08858284	17.85610549	88.79934932	0.666666667	CRA004775;PRJNA827465;Yang2025_Silkie_Wencha	0.898875401
ENSGALG00010012768	EPS8	3	-0.063922284	0.151241977	-0.422649093	0.672551302	0.050524877	8.492783849	76.45059576	0.666666667	CRA004775;PRJNA827465;Yang2025_Silkie_Wencha	0.898875401
ENSGALG00010009798	YIPF4	3	-0.091393021	0.216535205	-0.422070033	0.6729739	0.12910856	42.0916247	95.24846091	0.666666667	CRA004775;PRJNA827465;Yang2025_Silkie_Wencha	0.898977206
ENSGALG00010018916	CERS3	3	0.084061239	0.19898152	0.422457517	0.672691103	0	0.472461418	0	0.666666667	CRA004775;PRJNA827465;Yang2025_Silkie_Wencha	0.898977206
ENSGALG00010000686	ENSGALG0001000068(	3	0.071501212	0.169374048	0.422149752	0.672915716	0.053486483	5.50708821	63.68316751	0.666666667	CRA004775;PRJNA827465;Yang2025_Silkie_Wencha	0.898977206
ENSGALG00010021266	COQ5	3	-0.056892914	0.134704964	-0.422352023	0.672768091	0.032218812	4.969212607	59.75217487	0.666666667	CRA004775;PRJNA827465;Yang2025_Silkie_Wencha	0.898977206
ENSGALG00010020015	ATP6V1D	3	0.050229426	0.119045444	0.421934888	0.673072544	0.0263124	5.845666482	65.78662149	0.666666667	CRA004775;PRJNA827465;Yang2025_Silkie_Wencha	0.898977206
ENSGALG00010011425	WASHC3	3	-0.019055862	0.045134118	-0.422205267	0.672875197	0	1.014019067	0	0.666666667	CRA004775;PRJNA827465;Yang2025_Silkie_Wencha	0.898977206
ENSGALG00010019350	SAV1	3	0.014931311	0.03538735	0.421939216	0.673069385	0	1.425001055	0	1	CRA004775;PRJNA827465;Yang2025_Silkie_Wencha	0.898977206
ENSGALG00010029465	ENSGALG0001002946:	3	1.090830755	2.586055061	0.421812657	0.673161767	15.71312744	10.08971584	80.17783621	0.666666667	CRA004775;PRJNA827465;Yang2025_Silkie_Wencha	0.89901145
ENSGALG00010028878	IGSF9	3	0.056614709	0.134264331	0.421666039	0.673268796	0.039175402	7.546604428	73.49801465	0.666666667	CRA004775;PRJNA827465;Yang2025_Silkie_Wencha	0.899069466
ENSGALG00010001218	ENSGALG0001000121ξ	3	0.061463358	0.145824776	0.421487762	0.673398946	0.039466415	5.659656853	64.66216854	0.666666667	CRA004775;PRJNA827465;Yang2025_Silkie_Wencha	0.899073436
ENSGALG00010023026	PHRF1	3	0.036990815	0.087752863	0.421534003	0.673365187	0.012069463	4.066202321	50.81405592	0.666666667	CRA004775;PRJNA827465;Yang2025_Silkie_Wencha	0.899073436
ENSGALG00010003509	GPALPP1	3	-0.026533	0.06297723	-0.421311007	0.673527994	0.0016622	2.255754105	11.3378539	0.666666667	CRA004775;PRJNA827465;Yang2025_Silkie_Wencha	0.899160826
ENSGALG00010027531	UFC1	3	0.035169449	0.083512215	0.421129405	0.67366059	0	0.825750324	0	0.666666667	CRA004775;PRJNA827465;Yang2025_Silkie_Wencha	0.899168044
ENSGALG00010016888	ENSGALG0001001688ξ	3	-0.030504252	0.072423608	-0.421192109	0.673614806	0	0.840709774	0	0.666666667	CRA004775;PRJNA827465;Yang2025_Silkie_Wencha	0.899168044
ENSGALG00010028035	ENSGALG0001002803:	2	-0.64270733	1.533071111	-0.419228648	0.675049037	3.227087912	3.182948087	68.5825853	0.5	CRA004775;Yang2025_Silkie_Wenchang	0.899170887
ENSGALG00010020417	ENSGALG0001002041	2	-0.628111307	1.498711758	-0.419084029	0.675154722	4.1028112	11.48594399	91.29370645	0.5	CRA004775;PRJNA827465	0.899170887
ENSGALG00010028554	ENSGALG0001002855⁄	2	0.3590407	0.864388906	0.415369398	0.677871505	0	0.175170311	0	0.5	PRJNA827465;Yang2025_Silkie_Wenchang	0.899170887
ENSGALG00010028926	ENSGALG0001002892(	3	0.335327303	0.799146612	0.419606738	0.674772766	1.227985051	6.393039324	68.71597532	0.666666667	CRA004775;PRJNA827465;Yang2025_Silkie_Wencha	0.899170887
ENSGALG00010013915	ENSGALG0001001391:	3	0.18173375	0.43378281	0.418951018	0.67525193	0.4484554	10.38071424	80.73350297	0.666666667	CRA004775;PRJNA827465;Yang2025_Silkie_Wencha	0.899170887
ENSGALG00010010320	SYPL1	2	-0.181227818	0.433009364	-0.418530946	0.675558966	0	0.709204849	0	0.5	CRA004775;PRJNA827465	0.899170887
ENSGALG00010009659	APOLD1	3	-0.161092062	0.387647311	-0.415563472	0.677729461	0.388566504	20.84130621	90.40367249	0.666666667	CRA004775;PRJNA827465;Yang2025_Silkie_Wencha	0.899170887
ENSGALG00010000192	ENSGALG0001000019	2	-0.158484193	0.379333349	-0.417796626	0.676095818	0	0.115792392	0	1	PRJNA827465;Yang2025_Silkie_Wenchang	0.899170887
ENSGALG00010001518	SLC17A5	3	-0.157336907	0.37449573	-0.420130043	0.674390457	0.362337052	34.17258176	94.1473547	0.666666667	CRA004775;PRJNA827465;Yang2025_Silkie_Wencha	0.899170887
ENSGALG00010012070	DNAJC28	3	0.141804981	0.338482814	0.418942926	0.675257844	0.294468833	19.75623575	89.87661402	0.666666667	CRA004775;PRJNA827465;Yang2025_Silkie_Wencha	0.899170887
ENSGALG00010012477	ENSGALG0001001247	3	-0.122181418	0.292026959	-0.418390887	0.675661349	0.194891189	9.083701272	77.98254324	0.666666667	CRA004775;PRJNA827465;Yang2025_Silkie_Wencha	0.899170887
ENSGALG00010026105	CNTF	3	-0.114804797	0.276371814	-0.415399803	0.677849251	0.116921892	4.008318645	50.10376726	0.666666667	CRA004775;PRJNA827465;Yang2025_Silkie_Wencha	0.899170887
ENSGALG00010011857	ENSGALG0001001185	3	-0.114617812	0.276023759	-0.415246181	0.677961695	0	0.381312898	0	0.666666667	CRA004775;PRJNA827465;Yang2025_Silkie_Wencha	0.899170887
ENSGALG00010017720	ENSGALG0001001772(	3	-0.112404329	0.269821605	-0.416587578	0.676980097	0.060502404	2.719613381	26.46013532	0.666666667	CRA004775;PRJNA827465;Yang2025_Silkie_Wencha	0.899170887
ENSGALG00010026559	RAB36	2	0.109267757	0.260552291	0.41936978	0.674945906	0	0.292508098	0	1	PRJNA827465;Yang2025_Silkie_Wenchang	0.899170887
ENSGALG00010013260	FAM210B	3	-0.103387522	0.246476443	-0.419462083	0.67487846	0.153438141	17.11127847	88.31180263	0.666666667	CRA004775;PRJNA827465;Yang2025_Silkie_Wencha	0.899170887
ENSGALG00010015035	SLC35F1	3	0.097752725	0.235323933	0.415396443	0.67785171	0.123443168	14.10238651	85.81800322	0.666666667	CRA004775;PRJNA827465;Yang2025_Silkie_Wencha	0.899170887
ENSGALG00010018446	FBN1	3	-0.095528954	0.227957277	-0.419065164	0.675168509	0.088484511	4.944596033	59.55180187	0.666666667	CRA004775;PRJNA827465;Yang2025_Silkie_Wencha	0.899170887
ENSGALG00010021101	TMEM240	2	0.092809968	0.221392979	0.419209175	0.675063267	0	0.60821516	0	0.5	PRJNA827465;Yang2025_Silkie_Wenchang	0.899170887
ENSGALG00010027626	SEMA3B	3	0.09234064	0.220104192	0.41953149	0.674827746	0.111691367	13.82482756	85.53327344	0.666666667	CRA004775;PRJNA827465;Yang2025_Silkie_Wencha	0.899170887
ENSGALG00010028181	BGLAP	3	-0.087019899	0.209535202	-0.415299662	0.677922549	0	0.225948114	0	0.666666667	CRA004775;PRJNA827465;Yang2025_Silkie_Wencha	0.899170887
ENSGALG00010003403	GCDH	3	0.086767564	0.206361338	0.420464243	0.674146346	0.085572565	6.318375415	68.34629365	0.666666667	CRA004775;PRJNA827465;Yang2025_Silkie_Wencha	0.899170887
ENSGALG00010010989	TRIQK	3	-0.084231339	0.202161793	-0.416653105	0.67693216	0.082262041	9.351137381	78.61222738	0.666666667	CRA004775;PRJNA827465;Yang2025_Silkie_Wencha	0.899170887
ENSGALG00010025448	CHD1L	3	0.083357229	0.198082723	0.420820288	0.673886316	0.085596566	9.276089306	78.4391899	0.666666667	CRA004775;PRJNA827465;Yang2025_Silkie_Wencha	0.899170887
ENSGALG00010028464	ENSGALG0001002846⁄	3	0.082789362	0.197360105	0.419483776	0.674862609	0.089676112	11.98972099	83.31904469	0.666666667	CRA004775;PRJNA827465;Yang2025_Silkie_Wencha	0.899170887
ENSGALG00010020736	LRRTM4	3	0.082644527	0.199008342	0.415281722	0.67793568	0.046966671	3.345321123	40.21500698	0.666666667	CRA004775;PRJNA827465;Yang2025_Silkie_Wencha	0.899170887
ENSGALG00010015914	SRRM1	3	0.081128718	0.195433934	0.415120938	0.678053372	0.08917698	10.15638306	80.3079503	0.666666667	CRA004775;PRJNA827465;Yang2025_Silkie_Wencha	0.899170887
ENSGALG00010015964	KLC1	3	0.079828743	0.192309542	0.415105472	0.678064694	0.080061532	7.933254375	74.78966506	0.666666667	CRA004775;PRJNA827465;Yang2025_Silkie_Wencha	0.899170887
ENSGALG00010012353	PIGG	3	-0.077987694	0.187099467	-0.416824777	0.676806578	0.069720416	6.016319098	66.7570824	0.666666667	CRA004775;PRJNA827465;Yang2025_Silkie_Wencha	0.899170887
ENSGALG00010004468	LYPLA1	3	-0.076811872	0.184247368	-0.416895358	0.676754949	0.081531167	12.08719314	83.45356133	0.666666667	CRA004775;PRJNA827465;Yang2025_Silkie_Wencha	0.899170887
ENSGALG00010003978	ANK2	3	0.075510364	0.181704181	0.415567564	0.677726466	0.05817344	6.092462568	67.1725517	0.666666667	CRA004775;PRJNA827465;Yang2025_Silkie_Wencha	0.899170887
ENSGALG00010027578	ENSGALG0001002757ξ	3	0.07493026	0.178900853	0.418836795	0.675335412	0.020532604	2.507522213	20.23998872	0.666666667	CRA004775;PRJNA827465;Yang2025_Silkie_Wencha	0.899170887
ENSGALG00010015182	PHYKPL	3	0.072296576	0.171981122	0.420375069	0.674211478	0.057651179	6.692199928	70.11446129	0.666666667	CRA004775;PRJNA827465;Yang2025_Silkie_Wencha	0.899170887
ENSGALG00010004302	CRPPA	3	-0.07178784	0.172916657	-0.415158613	0.678025794	0.053781149	5.27486926	62.08436832	0.666666667	CRA004775;PRJNA827465;Yang2025_Silkie_Wencha	0.899170887
ENSGALG00010024234	ENSGALG0001002423⁄	3	-0.068574181	0.163087689	-0.420474293	0.674139005	0.034687092	3.51026578	43.02425726	0.666666667	CRA004775;PRJNA827465;Yang2025_Silkie_Wencha	0.899170887
ENSGALG00010023086	PCSK7	3	-0.067141822	0.161685112	-0.415262858	0.677949488	0.064259493	11.63889034	82.81623126	0.666666667	CRA004775;PRJNA827465;Yang2025_Silkie_Wencha	0.899170887
ENSGALG00010012859	FBXL21	3	-0.066668542	0.159888383	-0.416969272	0.676700884	0.057140025	9.141288253	78.12124566	0.666666667	CRA004775;PRJNA827465;Yang2025_Silkie_Wencha	0.899170887
ENSGALG00010003903	COL28A1	3	0.063334677	0.152541637	0.415195996	0.67799843	0	0.717519978	0	1	CRA004775;PRJNA827465;Yang2025_Silkie_Wencha	0.899170887
ENSGALG00010011116	TAPT1	3	-0.061928413	0.147587769	-0.419603965	0.674774791	0.046026833	9.282421359	78.45389772	0.666666667	CRA004775;PRJNA827465;Yang2025_Silkie_Wencha	0.899170887
ENSGALG00010020450	ANKRD39	3	0.058479295	0.139128435	0.420325974	0.674247338	0	1.774337878	0	0.666666667	CRA004775;PRJNA827465;Yang2025_Silkie_Wencha	0.899170887
ENSGALG00010021681	BTBD10	3	-0.057299522	0.137579732	-0.416482287	0.677057126	0.036907836	6.385580183	68.67943174	0.666666667	CRA004775;PRJNA827465;Yang2025_Silkie_Wencha	0.899170887
ENSGALG00010006855	DCLK2	3	0.055347249	0.132556966	0.417535571	0.676286712	0.024520113	3.929393833	49.10156414	0.666666667	CRA004775;PRJNA827465;Yang2025_Silkie_Wencha	0.899170887
ENSGALG00010007418	NME6	3	-0.05345686	0.128639145	-0.415556706	0.677734412	0.028222805	4.738052443	57.78856347	0.666666667	CRA004775;PRJNA827465;Yang2025_Silkie_Wencha	0.899170887
ENSGALG00010014859	TRAPPC3	3	-0.053054008	0.127797056	-0.415142648	0.678037481	0.029585629	5.49558614	63.60715766	0.666666667	CRA004775;PRJNA827465;Yang2025_Silkie_Wencha	0.899170887
ENSGALG00010006283	DNAJC25	3	0.052564289	0.126215548	0.416464451	0.677070175	0.03158323	6.577439816	69.5930323	0.666666667	CRA004775;PRJNA827465;Yang2025_Silkie_Wencha	0.899170887
ENSGALG00010015111	ADAMTS2	3	-0.051672775	0.123330069	-0.418979531	0.675231092	0	0.575717147	0	0.666666667	CRA004775;PRJNA827465;Yang2025_Silkie_Wencha	0.899170887
ENSGALG00010020595	ZFYVE27	3	-0.049796253	0.118902376	-0.418799475	0.675362688	0.025545809	5.131072548	61.02179454	0.666666667	CRA004775;PRJNA827465;Yang2025_Silkie_Wencha	0.899170887
ENSGALG00010009250	ERO1B	3	-0.049774718	0.119928213	-0.415037602	0.678114376	0.023829571	4.776564782	58.12890453	0.666666667	CRA004775;PRJNA827465;Yang2025_Silkie_Wencha	0.899170887
ENSGALG00010021697	PIANP	3	0.049521062	0.119109226	0.41576177	0.677584336	0	1.24403536	0	0.666666667	CRA004775;PRJNA827465;Yang2025_Silkie_Wencha	0.899170887
ENSGALG00010010906	ATP6V1C1	3	-0.049221606	0.117527983	-0.418807543	0.675356792	0.023830552	5.551399866	63.97305097	0.666666667	CRA004775;PRJNA827465;Yang2025_Silkie_Wencha	0.899170887
ENSGALG00010014294	TMPO	3	-0.048164871	0.115202549	-0.41808859	0.675882347	0.02950131	9.465705503	78.87109419	0.666666667	CRA004775;PRJNA827465;Yang2025_Silkie_Wencha	0.899170887
ENSGALG00010024853	TJP1	3	0.047279427	0.11273587	0.419382284	0.67493677	0.019187155	3.84537279	47.98943798	0.666666667	CRA004775;PRJNA827465;Yang2025_Silkie_Wencha	0.899170887
ENSGALG00010000595	MYO10	3	-0.047130309	0.113453512	-0.415415161	0.67783801	0	1.111403229	0	0.666666667	CRA004775;PRJNA827465;Yang2025_Silkie_Wencha	0.899170887
ENSGALG00010025691	ENSGALG0001002569l	3	0.045452178	0.10860914	0.41849312	0.675586616	0	0.046323541	0	1	CRA004775;PRJNA827465;Yang2025_Silkie_Wencha	0.899170887
ENSGALG00010021945	C1QTNF4	3	-0.044061099	0.105246925	-0.418645	0.675475597	0	0.477927277	0	0.666666667	CRA004775;PRJNA827465;Yang2025_Silkie_Wencha	0.899170887
ENSGALG00010001373	IMPA2	3	-0.043310169	0.103220239	-0.419589889	0.674785076	0.0173953	4.808737409	58.40904109	0.666666667	CRA004775;PRJNA827465;Yang2025_Silkie_Wencha	0.899170887
ENSGALG00010002712	PTPRE	3	0.043107875	0.103813524	0.415243343	0.677963772	0.016357147	4.535526156	55.90368281	0.666666667	CRA004775;PRJNA827465;Yang2025_Silkie_Wencha	0.899170887
ENSGALG00010018598	ENSGALG0001001859ξ	3	-0.042772377	0.102727244	-0.416368388	0.677140457	0.018545424	4.926161935	59.40044143	0.666666667	CRA004775;PRJNA827465;Yang2025_Silkie_Wencha	0.899170887
ENSGALG00010004370	ATP6V1H	3	0.041202796	0.099013339	0.416133795	0.677312102	0.012392621	3.262281231	38.69320704	0.666666667	CRA004775;PRJNA827465;Yang2025_Silkie_Wencha	0.899170887
ENSGALG00010021164	SLC41A3	3	-0.039742397	0.095426563	-0.416471004	0.677065381	0	1.850369213	0	0.666666667	CRA004775;PRJNA827465;Yang2025_Silkie_Wencha	0.899170887
ENSGALG00010009498	ORC4	3	-0.039111866	0.093422794	-0.418654427	0.675468707	0.003789667	2.269855688	11.88867157	0.666666667	CRA004775;PRJNA827465;Yang2025_Silkie_Wencha	0.899170887
ENSGALG00010002750	ENSGALG0001000275(	3	-0.037499368	0.089796579	-0.41760353	0.676237015	0	0.748559582	0	0.666666667	CRA004775;PRJNA827465;Yang2025_Silkie_Wencha	0.899170887
ENSGALG00010021642	NPTN	3	-0.035882502	0.085948479	-0.417488498	0.676321135	0.011110284	3.957390841	49.46165086	0.666666667	CRA004775;PRJNA827465;Yang2025_Silkie_Wencha	0.899170887
ENSGALG00010027303	APEH	3	-0.031100856	0.074110227	-0.419656738	0.674736234	0.003851377	2.507582964	20.24192106	0.666666667	CRA004775;PRJNA827465;Yang2025_Silkie_Wencha	0.899170887
ENSGALG00010004824	ATM	3	0.030895657	0.074037152	0.41729938	0.676459442	0.005019081	2.704850096	26.05874894	0.666666667	CRA004775;PRJNA827465;Yang2025_Silkie_Wencha	0.899170887
ENSGALG00010008453	AMOTL1	3	-0.027315877	0.06569184	-0.415818413	0.677542884	0	1.48869455	0	0.666666667	CRA004775;PRJNA827465;Yang2025_Silkie_Wencha	0.899170887
ENSGALG00010009453	GSAP	3	-0.026671508	0.06401295	-0.416658002	0.676928578	0	0.615929195	0	0.666666667	CRA004775;PRJNA827465;Yang2025_Silkie_Wencha	0.899170887
ENSGALG00010008888	FBXO46	3	0.026129247	0.062520668	0.417929746	0.675998483	0	0.560573551	0	0.666666667	CRA004775;PRJNA827465;Yang2025_Silkie_Wencha	0.899170887
ENSGALG00010005376	HTRA2	3	-0.023222065	0.055896465	-0.415447835	0.677814095	0	0.956799672	0	1	CRA004775;PRJNA827465;Yang2025_Silkie_Wencha	0.899170887
ENSGALG00010024662	MOB2	3	-0.016263241	0.038670437	-0.420560049	0.674076372	0	0.6794072	0	0.666666667	CRA004775;PRJNA827465;Yang2025_Silkie_Wencha	0.899170887
ENSGALG00010021210	ENSGALG0001002121(	3	-0.016224309	0.039104969	-0.414891229	0.678221531	0	1.542631988	0	0.666666667	CRA004775;PRJNA827465;Yang2025_Silkie_Wencha	0.899228641
ENSGALG00010018496	GNG13	2	0.138588697	0.334180152	0.414712531	0.678352358	0	1.93213E-05	0	1	PRJNA827465;Yang2025_Silkie_Wenchang	0.899317767
ENSGALG00010025008	ENSGALG0001002500ξ	3	-0.328209804	0.797116114	-0.411746542	0.680525207	1.321478391	7.106277057	71.85586793	0.666666667	CRA004775;PRJNA827465;Yang2025_Silkie_Wencha	0.899418018
ENSGALG00010024003	ERICH3	3	0.219175065	0.532166792	0.411854081	0.680446379	0.778799317	26.24400011	92.37921052	0.666666667	CRA004775;PRJNA827465;Yang2025_Silkie_Wencha	0.899418018
ENSGALG00010028612	DIRAS1	3	0.207879263	0.504975445	0.411662122	0.680587092	0.551457556	7.233461345	72.35071974	0.666666667	CRA004775;PRJNA827465;Yang2025_Silkie_Wencha	0.899418018
ENSGALG00010025319	LDLRAD1	3	0.144001711	0.348053587	0.41373431	0.679068697	0.228911874	5.80688372	65.55811866	0.666666667	CRA004775;PRJNA827465;Yang2025_Silkie_Wencha	0.899418018
ENSGALG00010003868	HBBA	3	-0.135869453	0.328457378	-0.413659313	0.679123628	0.270747814	12.68900582	84.23832388	0.666666667	CRA004775;PRJNA827465;Yang2025_Silkie_Wencha	0.899418018
ENSGALG00010022328	ENSGALG0001002232ξ	3	-0.107346534	0.260528684	-0.412033457	0.680314901	0.136951439	6.394899816	68.7250769	0.666666667	CRA004775;PRJNA827465;Yang2025_Silkie_Wencha	0.899418018
ENSGALG00010005351	DTNA	3	-0.1046683	0.252931671	-0.413820458	0.6790056	0.133653566	8.414936492	76.23273804	0.666666667	CRA004775;PRJNA827465;Yang2025_Silkie_Wencha	0.899418018
ENSGALG00010024181	FOXD3	3	0.080454669	0.195344678	0.411860052	0.680442002	0.056755896	4.360414715	54.13280317	0.666666667	CRA004775;PRJNA827465;Yang2025_Silkie_Wencha	0.899418018
ENSGALG00010018376	PYGB	3	0.060803434	0.147375641	0.412574518	0.679918374	0.042763379	6.432262761	68.90674286	0.666666667	CRA004775;PRJNA827465;Yang2025_Silkie_Wencha	0.899418018
ENSGALG00010002634	RGN	3	0.059965478	0.144726295	0.41433713	0.678627225	0	1.817124897	0	0.666666667	CRA004775;PRJNA827465;Yang2025_Silkie_Wencha	0.899418018
ENSGALG00010009019	ZNF513	3	0.059054194	0.143451852	0.411665607	0.680584537	0.040286991	6.858129828	70.83753078	0.666666667	CRA004775;PRJNA827465;Yang2025_Silkie_Wencha	0.899418018
ENSGALG00010021338	PDIA5	3	0.055775631	0.135199548	0.412543026	0.679941451	0.043944902	10.89424895	81.64168995	0.666666667	CRA004775;PRJNA827465;Yang2025_Silkie_Wencha	0.899418018

ENSGALG00010027547	B4GALT3	3	0.054130361	0.130875211	0.413602853	0.679164983	0.027322176	4.600298396	56.52455932	0.666666667	CRA004775;PRJNA827465;Yang2025_Silkie_Wencha	0.899418018
ENSGALG00010000045	TP53BP1	3	0.052828878	0.128303383	0.41174969	0.6805229	0.031686688	7.271315632	72.4946612	0.666666667	CRA004775;PRJNA827465;Yang2025_Silkie_Wencha	0.899418018
ENSGALG00010026894	IP6K1	3	-0.05098403	0.123060699	-0.41429986	0.678654517	0.032601247	7.46804674	73.21923564	0.666666667	CRA004775;PRJNA827465;Yang2025_Silkie_Wencha	0.899418018
ENSGALG00010010186	TNPO1	3	0.048565351	0.117477687	0.41340064	0.679313106	0.027518641	6.20721981	67.77945584	0.666666667	CRA004775;PRJNA827465;Yang2025_Silkie_Wencha	0.899418018
ENSGALG00010016200	MICU3	3	-0.048442829	0.117311679	-0.412941227	0.679649674	0.025462252	6.265968528	68.0815505	0.666666667	CRA004775;PRJNA827465;Yang2025_Silkie_Wencha	0.899418018
ENSGALG00010017754	PDZD11	3	-0.044448707	0.107938237	-0.411797598	0.680487782	0.01617113	3.686739738	45.75152731	0.666666667	CRA004775;PRJNA827465;Yang2025_Silkie_Wencha	0.899418018
ENSGALG00010001661	TCTN3	3	-0.042559424	0.103050031	-0.412997682	0.679608312	0.018839823	5.862712658	65.88609887	0.666666667	CRA004775;PRJNA827465;Yang2025_Silkie_Wencha	0.899418018
ENSGALG00010022207	ENSGALG0001002220	3	-0.041284669	0.099935638	-0.413112573	0.679524138	0	0.555689449	0	1	CRA004775;PRJNA827465;Yang2025_Silkie_Wencha	0.899418018
ENSGALG00010009699	POLR1C	3	0.041061828	0.099497376	0.412692576	0.679831865	0.016226438	4.409687228	54.64530937	0.666666667	CRA004775;PRJNA827465;Yang2025_Silkie_Wencha	0.899418018
ENSGALG00010022695	PIK3IP1	3	-0.037332387	0.090417493	-0.412888987	0.679687951	0	1.476493055	0	0.666666667	CRA004775;PRJNA827465;Yang2025_Silkie_Wencha	0.899418018
ENSGALG00010021361	WRAP73	3	0.033099507	0.079994861	0.413770423	0.679042247	0	0.325362215	0	0.666666667	CRA004775;PRJNA827465;Yang2025_Silkie_Wencha	0.899418018
ENSGALG00010024930	TMEM237	3	0.032055009	0.077693477	0.412583022	0.679912143	0.006551571	3.065968976	34.76776786	0.666666667	CRA004775;PRJNA827465;Yang2025_Silkie_Wencha	0.899418018
ENSGALG00010013156	ABCC9	3	-0.031518444	0.076261615	-0.413293686	0.679391456	0	1.448367949	0	0.666666667	CRA004775;PRJNA827465;Yang2025_Silkie_Wencha	0.899418018
ENSGALG00010003348	ADAM17	3	0.028411077	0.068655638	0.413820013	0.679005927	0.005056435	2.930799043	31.75922435	0.666666667	CRA004775;PRJNA827465;Yang2025_Silkie_Wencha	0.899418018
ENSGALG00010017974	MXRA8	3	0.027544035	0.066910149	0.411657059	0.680590803	0	1.981564825	0	0.666666667	CRA004775;PRJNA827465;Yang2025_Silkie_Wencha	0.899418018
ENSGALG00010011660	ENSGALG0001001166	3	-0.027489162	0.066568099	-0.412947982	0.679644726	0	1.251027937	0	0.666666667	CRA004775;PRJNA827465;Yang2025_Silkie_Wencha	0.899418018
ENSGALG00010011275	WDR4	3	-0.026897935	0.065086042	-0.413267333	0.679410761	0.00025416	2.026497086	1.307531391	0.666666667	CRA004775;PRJNA827465;Yang2025_Silkie_Wencha	0.899418018
ENSGALG00010013921	PPP2R2B	3	0.024815777	0.06025553	0.411842322	0.680454999	0	1.624462949	0	0.666666667	CRA004775;PRJNA827465;Yang2025_Silkie_Wencha	0.899418018
ENSGALG00010012391	NEURL1B	3	0.023170076	0.05592447	0.414310161	0.678646973	0	1.637721926	0	0.666666667	CRA004775;PRJNA827465;Yang2025_Silkie_Wencha	0.899418018
ENSGALG00010016831	AIDA	3	-0.01893066	0.045899606	-0.412436222	0.680019718	0	0.237438141	0	0.666666667	CRA004775;PRJNA827465;Yang2025_Silkie_Wencha	0.899418018
ENSGALG00010021701	DR1	3	0.018206026	0.044187356	0.4120189	0.680325571	0	1.358360715	0	0.666666667	CRA004775;PRJNA827465;Yang2025_Silkie_Wencha	0.899418018
ENSGALG00010008189	EXOC3	3	-0.016811552	0.040765177	-0.412399822	0.680046394	0	0.847658145	0	0.666666667	CRA004775;PRJNA827465;Yang2025_Silkie_Wencha	0.899418018
ENSGALG00010016631	FAM53A	3	0.072642632	0.176511302	0.411546633	0.680671754	0.077871294	16.63282152	87.9755819	0.666666667	CRA004775;PRJNA827465;Yang2025_Silkie_Wencha	0.899440928
ENSGALG00010014946	PCK1	3	0.47843328	1.166360912	0.41019317	0.68166425	3.561850701	16.85487753	88.13399862	0.666666667	CRA004775;PRJNA827465;Yang2025_Silkie_Wencha	0.899659371
ENSGALG00010011451	CRHBP	2	0.113942728	0.277481801	0.410631355	0.681342869	0	0.030703654	0	0.5	PRJNA827465;Yang2025_Silkie_Wenchang	0.899659371
ENSGALG00010022033	RAPH1	3	0.100352998	0.244392944	0.410621503	0.681350094	0.128200584	7.615511803	73.73781235	0.666666667	CRA004775;PRJNA827465;Yang2025_Silkie_Wencha	0.899659371
ENSGALG00010002472	CDC37	3	0.09847467	0.239813404	0.410630383	0.681343582	0.151079873	26.71052255	92.51231422	0.666666667	CRA004775;PRJNA827465;Yang2025_Silkie_Wencha	0.899659371
ENSGALG00010023234	ENSGALG0001002323	3	0.056857432	0.13829137	0.411142299	0.680968195	0.023996117	2.9513649	32.23474332	0.666666667	CRA004775;PRJNA827465;Yang2025_Silkie_Wencha	0.899659371
ENSGALG00010024102	SGF29	3	0.053318911	0.129801064	0.41077407	0.681238209	0.03801377	11.75351241	82.98381003	0.666666667	CRA004775;PRJNA827465;Yang2025_Silkie_Wencha	0.899659371
ENSGALG00010013814	AMD1	3	-0.048235273	0.117452655	-0.410678437	0.68130834	0.021536287	4.44648379	55.02063891	0.666666667	CRA004775;PRJNA827465;Yang2025_Silkie_Wencha	0.899659371
ENSGALG00010018609	ENSGALG0001001860	3	-0.047657231	0.115934238	-0.41107124	0.681020298	0.020743794	4.059446051	50.73219412	0.666666667	CRA004775;PRJNA827465;Yang2025_Silkie_Wencha	0.899659371
ENSGALG00010019926	KIF7	3	-0.047273826	0.115186339	-0.410411745	0.681503932	0.030705017	10.21256602	80.41628327	0.666666667	CRA004775;PRJNA827465;Yang2025_Silkie_Wencha	0.899659371
ENSGALG00010026344	TIMM10	3	-0.043225617	0.105214015	-0.410835162	0.681193409	0	0.836044721	0	1	CRA004775;PRJNA827465;Yang2025_Silkie_Wencha	0.899659371
ENSGALG00010013313	GAK	3	0.042043818	0.102475469	0.41028178	0.681599256	0	0.315068845	0	0.666666667	CRA004775;PRJNA827465;Yang2025_Silkie_Wencha	0.899659371
ENSGALG00010015544	RTCB	3	-0.028801287	0.070193565	-0.410312362	0.681576825	0.004718122	2.64832163	24.48047179	0.666666667	CRA004775;PRJNA827465;Yang2025_Silkie_Wencha	0.899659371
ENSGALG00010011130	DNM1L	3	0.017543677	0.042763066	0.410253019	0.681620351	0.001602061	2.719399688	26.45435648	0.666666667	CRA004775;PRJNA827465;Yang2025_Silkie_Wencha	0.899659371
ENSGALG00010009655	FAM83A	2	-0.1979099	0.48278396	-0.409934705	0.681853846	0	0.265197938	0	0.5	PRJNA827465;Yang2025_Silkie_Wenchang	0.899694347
ENSGALG00010013898	EMB	3	-0.075721952	0.184773285	-0.409810068	0.68194528	0	0.473805015	0	0.666666667	CRA004775;PRJNA827465;Yang2025_Silkie_Wencha	0.899694347
ENSGALG00010016907	SQSTM1	3	-0.042649791	0.104035133	-0.409955653	0.681838479	0	1.380349806	0	0.666666667	CRA004775;PRJNA827465;Yang2025_Silkie_Wencha	0.899694347
ENSGALG00010029421	TBCD	3	-0.023022362	0.056172189	-0.409853389	0.681913499	0.002304129	2.569483112	22.16333352	0.666666667	CRA004775;PRJNA827465;Yang2025_Silkie_Wencha	0.899694347
ENSGALG00010014801	MPST	3	-0.052077053	0.127129848	-0.409636711	0.682072463	0.024657147	3.98287101	49.7849668	0.666666667	CRA004775;PRJNA827465;Yang2025_Silkie_Wencha	0.899763122
ENSGALG00010022515	CHRNA1	3	-0.050516452	0.123419967	-0.409305344	0.682315595	0	1.955765096	0	0.666666667	CRA004775;PRJNA827465;Yang2025_Silkie_Wencha	0.899763122
ENSGALG00010001423	FAM210A	3	0.049597115	0.121147844	0.409393293	0.682251061	0.022819654	4.322237071	53.7276654	0.666666667	CRA004775;PRJNA827465;Yang2025_Silkie_Wencha	0.899763122
ENSGALG00010021637	ATP6V0D1	3	-0.043903003	0.107205229	-0.409522965	0.682155917	0.022169217	5.658823458	64.65696421	0.666666667	CRA004775;PRJNA827465;Yang2025_Silkie_Wencha	0.899763122
ENSGALG00010005828	LPAR1	3	-0.030729821	0.075069218	-0.409353154	0.682280513	0	1.673487387	0	0.666666667	CRA004775;PRJNA827465;Yang2025_Silkie_Wencha	0.899763122
ENSGALG00010015397	SRD5A3	3	-0.059058932	0.144476404	-0.408779082	0.682701793	0.033025495	4.091714899	51.12073911	0.666666667	CRA004775;PRJNA827465;Yang2025_Silkie_Wencha	0.90018844
ENSGALG00010005239	ENSGALG0001000523	3	0.185470185	0.453852515	0.408657393	0.682791105	0.492116582	12.61140856	84.1413321	0.666666667	CRA004775;PRJNA827465;Yang2025_Silkie_Wencha	0.900222253
ENSGALG00010009728	CCDC149	3	-0.036369638	0.08902921	-0.408513541	0.682896691	0.007034445	2.711535267	26.24104786	0.666666667	CRA004775;PRJNA827465;Yang2025_Silkie_Wencha	0.900277512
ENSGALG00010004330	STX7	3	-0.072237541	0.17687555	-0.408408856	0.682973533	0.078890264	14.65849017	86.35603001	0.666666667	CRA004775;PRJNA827465;Yang2025_Silkie_Wencha	0.90029487
ENSGALG00010005107	TTLL5	3	0.050932418	0.124856729	0.407926897	0.683327345	0.02971396	6.251274422	68.00652371	0.666666667	CRA004775;PRJNA827465;Yang2025_Silkie_Wencha	0.900677294
ENSGALG00010028545	ENSGALG0001002854	2	-0.746445403	1.832835099	-0.407262626	0.683815109	4.538352909	3.029283018	66.98888833	0.5	PRJNA827465;Yang2025_Silkie_Wenchang	0.900710752
ENSGALG00010020468	ENSGALG0001002046	2	0.201417673	0.49429531	0.407484492	0.683652182	0.373698584	4.060644081	75.37336491	0.5	PRJNA827465;Yang2025_Silkie_Wenchang	0.900710752
ENSGALG00010023724	CYP2R1	3	-0.104495954	0.256668994	-0.407123403	0.683917356	0.162961256	15.22707028	86.86549702	0.666666667	CRA004775;PRJNA827465;Yang2025_Silkie_Wencha	0.900710752
ENSGALG00010019735	ENSGALG0001001973	3	0.07759294	0.190357136	0.407617712	0.683554359	0.055744385	4.006087414	50.07597706	0.666666667	CRA004775;PRJNA827465;Yang2025_Silkie_Wencha	0.900710752
ENSGALG00010021394	MAN2C1	3	-0.066858014	0.164228065	-0.407111544	0.683926065	0.059495036	7.853916198	74.53499694	0.666666667	CRA004775;PRJNA827465;Yang2025_Silkie_Wencha	0.900710752
ENSGALG00010018756	EML4	3	0.04609599	0.113079953	0.407640689	0.683537487	0.017868896	3.630065438	44.90457448	0.666666667	CRA004775;PRJNA827465;Yang2025_Silkie_Wencha	0.900710752
ENSGALG00010022002	KIF18B	3	-0.046070106	0.113090499	-0.4073738	0.683733467	0.012062766	2.788855344	28.28599002	0.666666667	CRA004775;PRJNA827465;Yang2025_Silkie_Wencha	0.900710752
ENSGALG00010001528	NR1D2	3	0.038444118	0.094398666	0.407252773	0.683822346	0	0.920821506	0	0.666666667	CRA004775;PRJNA827465;Yang2025_Silkie_Wencha	0.900710752
ENSGALG00010027548	PCBP4	3	-0.02680714	0.065733592	-0.407802519	0.683418664	0	0.559156663	0	0.666666667	CRA004775;PRJNA827465;Yang2025_Silkie_Wencha	0.900710752
ENSGALG00010013669	NPR3	3	0.059738207	0.146771625	0.407014685	0.683997202	0	0.323142153	0	0.666666667	CRA004775;PRJNA827465;Yang2025_Silkie_Wencha	0.900720541
ENSGALG00010009210	PCDH15	3	0.231686845	0.56967548	0.406699696	0.684228564	0.701303878	8.157481539	75.48262916	0.666666667	CRA004775;PRJNA827465;Yang2025_Silkie_Wencha	0.900775029
ENSGALG00010021969	CCDC103	3	-0.08854611	0.217719486	-0.406698141	0.684229706	0	0.14874858	0	0.666666667	CRA004775;PRJNA827465;Yang2025_Silkie_Wencha	0.900775029
ENSGALG00010002981	KLHL35	2	-0.085460565	0.210129068	-0.406703947	0.684225441	0	0.064898594	0	0.5	PRJNA827465;Yang2025_Silkie_Wenchang	0.900775029
ENSGALG00010003716	YLEC8	3	-0.144120144	0.355050979	-0.405913948	0.68480583	0	0.294750496	0	0.666666667	CRA004775;PRJNA827465;Yang2025_Silkie_Wencha	0.900778642
ENSGALG00010001448	GPR37	3	0.119637012	0.294582134	0.406124465	0.68465115	0.151042262	5.586478426	64.19927103	0.666666667	CRA004775;PRJNA827465;Yang2025_Silkie_Wencha	0.900778642
ENSGALG00010029281	KIAA0753	3	0.094163176	0.231896007	0.406057772	0.684700152	0.117429165	9.67641144	79.33118065	0.666666667	CRA004775;PRJNA827465;Yang2025_Silkie_Wencha	0.900778642
ENSGALG00010001878	XK	3	-0.079229315	0.195077461	-0.406142844	0.684637647	0	1.075314979	0	0.666666667	CRA004775;PRJNA827465;Yang2025_Silkie_Wencha	0.900778642
ENSGALG00010012129	CRYZL1	3	-0.070492051	0.173661529	-0.40591633	0.684804079	0.057982737	5.811012879	65.5825922	0.666666667	CRA004775;PRJNA827465;Yang2025_Silkie_Wencha	0.900778642
ENSGALG00010016383	FAM110D	3	-0.06882364	0.1692747	-0.406579598	0.684316784	0.039028734	3.431125462	41.71008836	0.666666667	CRA004775;PRJNA827465;Yang2025_Silkie_Wencha	0.900778642
ENSGALG00010024868	FAAP24	3	0.057046011	0.140442629	0.406187291	0.684604991	0.011233615	2.365358229	15.44621126	0.666666667	CRA004775;PRJNA827465;Yang2025_Silkie_Wencha	0.900778642
ENSGALG00010025325	DPEP2	3	0.047411095	0.116722876	0.406185115	0.68460659	0	0.906007234	0	0.666666667	CRA004775;PRJNA827465;Yang2025_Silkie_Wencha	0.900778642
ENSGALG00010008330	EZH2	3	0.041755558	0.102810728	0.406140084	0.684639675	0.016826655	5.029672209	60.23597728	0.666666667	CRA004775;PRJNA827465;Yang2025_Silkie_Wencha	0.900778642
ENSGALG00010016040	RPSA2	3	-0.050190287	0.12368753	-0.405782918	0.684902111	0.026183874	4.596688759	56.49041941	0.666666667	CRA004775;PRJNA827465;Yang2025_Silkie_Wencha	0.900821483
ENSGALG00010018296	PIK3CA	3	-0.046658407	0.115017452	-0.405663712	0.684989709	0.022153978	4.721034284	57.63640169	0.666666667	CRA004775;PRJNA827465;Yang2025_Silkie_Wencha	0.900852897
ENSGALG00010028449	EFCAB12	3	-0.099811402	0.246365834	-0.405134919	0.68537834	0.079937481	3.486260106	42.63193396	0.666666667	CRA004775;PRJNA827465;Yang2025_Silkie_Wencha	0.901280167
ENSGALG00010017447	CAPN13	3	0.275753366	0.680867293	0.405003103	0.68547523	1.061788555	8.830471319	77.3511523	0.666666667	CRA004775;PRJNA827465;Yang2025_Silkie_Wencha	0.90132375
ENSGALG00010028549	ADAMTS10	3	-0.038084451	0.094100694	-0.404720193	0.685683198	0.009006442	3.010550025	33.56695676	0.666666667	CRA004775;PRJNA827465;Yang2025_Silkie_Wencha	0.901513365
ENSGALG00010027965	ACAD9	3	0.063617592	0.157304654	0.404422821	0.685901821	0.046855451	6.178713743	67.63080338	0.666666667	CRA004775;PRJNA827465;Yang2025_Silkie_Wencha	0.901633121
ENSGALG00010021721	TP53I11	3	-0.044392415	0.109744345	-0.404507543	0.685839532	0.003770247	2.210505666	9.522964336	0.666666667	CRA004775;PRJNA827465;Yang2025_Silkie_Wencha	0.901633121
ENSGALG00010011790	AMY2A	3	0.312592749	0.774561134	0.403574018	0.686525995	1.411872671	12.53885356	84.04957846	0.666666667	CRA004775;PRJNA827465;Yang2025_Silkie_Wencha	0.9018513
ENSGALG00010011095	TNN	3	-0.142090114	0.352444197	-0.403156345	0.686833213	0	0.781065808	0	0.666666667	CRA004775;PRJNA827465;Yang2025_Silkie_Wencha	0.9018513
ENSGALG00010022568	SCRN3	3	0.107247835	0.265568629	0.403842259	0.686328719	0.16345655	14.31017476	86.02393029	0.666666667	CRA004775;PRJNA827465;Yang2025_Silkie_Wencha	0.9018513
ENSGALG00010002554	EGFL6	3	-0.087872011	0.217842695	-0.403373686	0.686673341	0.087382241	6.89498541	70.99341215	0.666666667	CRA004775;PRJNA827465;Yang2025_Silkie_Wencha	0.9018513
ENSGALG00010017431	FYCO1	3	0.071586366	0.177461154	0.403391752	0.686660054	0.059926935	8.266913593	75.80717426	0.666666667	CRA004775;PRJNA827465;Yang2025_Silkie_Wencha	0.9018513
ENSGALG00010014178	LRTM2	3	-0.067475913	0.167225801	-0.403501806	0.686579106	0	0.805347574	0	0.666666667	CRA004775;PRJNA827465;Yang2025_Silkie_Wencha	0.9018513
ENSGALG00010002917	PGLS	3	0.046458073	0.115176139	0.403365436	0.68667941	0.027023727	6.49665234	69.21491415	0.666666667	CRA004775;PRJNA827465;Yang2025_Silkie_Wencha	0.9018513
ENSGALG00010013468	DEPDC1B	3	-0.043266851	0.107302824	-0.403221926	0.686784971	0	0.527609861	0	0.666666667	CRA004775;PRJNA827465;Yang2025_Silkie_Wencha	0.9018513
ENSGALG00010007920	FAT3	3	-0.040171293	0.099614511	-0.403267479	0.686751463	0	0.857707788	0	0.666666667	CRA004775;PRJNA827465;Yang2025_Silkie_Wencha	0.9018513
ENSGALG00010005214	BBS7	3	-0.038571519	0.095567864	-0.403603442	0.686504354	0.014428935	4.395814564	54.5021754	0.666666667	CRA004775;PRJNA827465;Yang2025_Silkie_Wencha	0.9018513
ENSGALG00010027239	ENSGALG0001002723	3	-0.034362206	0.085045797	-0.404043558	0.686180688	0.013939063	6.113604629	67.28607554	0.666666667	CRA004775;PRJNA827465;Yang2025_Silkie_Wencha	0.9018513
ENSGALG00010012569	C6orf203	3	-0.020504476	0.050839715	-0.403316103	0.686715697	0	1.972044556	0	0.666666667	CRA004775;PRJNA827465;Yang2025_Silkie_Wencha	0.9018513
ENSGALG00010029425	SPNS2	3	0.042511317	0.10549188	0.402981885	0.686961551	0	0.039023937	0	1	CRA004775;PRJNA827465;Yang2025_Silkie_Wencha	0.901936055
ENSGALG00010003064	VAV1	3	0.11254553	0.279565657	0.402572802	0.687262521	0.137605524	4.966610795	59.73109063	0.666666667	CRA004775;PRJNA827465;Yang2025_Silkie_Wencha	0.902013776
ENSGALG00010015155	HMOX1	3	-0.090787526	0.225537982	-0.402537634	0.687288397	0.117842973	8.97285962	77.7105618	0.666666667	CRA004775;PRJNA827465;Yang2025_Silkie_Wencha	0.902013776
ENSGALG00010008468	EPPK1	3	-0.089351237	0.222008371	-0.402467872	0.687339728	0	1.799537709	0	0.666666667	CRA004775;PRJNA827465;Yang2025_Silkie_Wencha	0.902013776
ENSGALG00010013268	PTPRR	3	-0.086987743	0.215964361	-0.402787489	0.687104566	0.035054447	2.660106691	24.8150457	0.666666667	CRA004775;PRJNA827465;Yang2025_Silkie_Wencha	0.902013776
ENSGALG00010016451	USP46	3	0.035963592	0.089326827	0.402606843	0.687237474	0	1.649018045	0	0.666666667	CRA004775;PRJNA827465;Yang2025_Silkie_Wencha	0.902013776
ENSGALG00010021636	C1orf159	3	-0.05256481	0.130696662	-0.402189382	0.687544656	0.027390996	4.486446757	55.42129198	0.666666667	CRA004775;PRJNA827465;Yang2025_Silkie_Wencha	0.902198969
ENSGALG00010013423	ZNF804A	2	0.141651543	0.352360016	0.402007995	0.687678142	0.187952009	4.026393016	75.16387506	0.5	PRJNA827465;Yang2025_Silkie_Wenchang	0.902206668
ENSGALG00010023456	MPI	3	-0.026045997	0.064780682	-0.402064256	0.687636738	0	0.290356768	0	1	CRA004775;PRJNA827465;Yang2025_Silkie_Wencha	0.902206668
ENSGALG00010027744	ENSGALG0001002774	2	-0.437112912	1.087952024	-0.401775907	0.687848955	0.789694403	1.496854693	33.19324817	0.5	PRJNA827465;Yang2025_Silkie_Wenchang	0.902263324
ENSGALG00010003439	GMIP	3	-0.060600469	0.150800354	-0.401858928	0.687787851	0	1.880434687	0	0.666666667	CRA004775;PRJNA827465;Yang2025_Silkie_Wencha	0.902263324
ENSGALG00010001731	ENSGALG0001000173	3	-0.038838697	0.096729938	-0.401516817	0.688039659	0	0.430062959	0	1	CRA004775;PRJNA827465;Yang2025_Silkie_Wencha	0.902429754
ENSGALG00010001925	FRMPD4	2	-0.079411879	0.197865234	-0.401343262	0.688167417	0.050959317	2.680237505	62.68987364	0.5	PRJNA827465;Yang2025_Silkie_Wenchang	0.902513598
ENSGALG00010000579	ENSGALG0001000057	3	-0.539116965	1.345921652	-0.400555979	0.688747061	4.874908321	23.69566612	91.559638	0.666666667	CRA004775;PRJNA827465;Yang2025_Silkie_Wencha	0.902758278
ENSGALG00010029442	ENSGALG0001002944	3	0.194885909	0.486073457	0.400939214	0.688464878	0.473503927	6.068110395	67.04081057	0.666666667	CRA004775;PRJNA827465;Yang2025_Silkie_Wencha	0.902758278
ENSGALG00010004622	ENSGALG0001000462	3	0.159769572	0.398779025	0.400646879	0.688680126	0.379258411	11.14262585	82.05090948	0.666666667	CRA004775;PRJNA827465;Yang2025_Silkie_Wencha	0.902758278
ENSGALG00010018480	EME2	3	-0.149842023	0.374396583	-0.400222732	0.688992473	0.308847679	9.446076629	78.82718849	0.666666667	CRA004775;PRJNA827465;Yang2025_Silkie_Wencha	0.902758278
ENSGALG00010022837	ITGB3	3	0.07767871	0.193894836	0.400622889	0.688697791	0.078550314	7.465243764	73.20918026	0.666666667	CRA004775;PRJNA827465;Yang2025_Silkie_Wencha	0.902758278
ENSGALG00010009011	ACP6	3	0.038260698	0.095598063	0.400224611	0.688991089	0.001318172	2.095775669	4.569938963	0.666666667	CRA004775;PRJNA827465;Yang2025_Silkie_Wencha	0.902758278
ENSGALG00010004217	CCDC28A	3	0.032939451	0.082276014	0.40035302	0.688896521	0	1.194251163	0	0.666666667	CRA004775;PRJNA827465;Yang2025_Silkie_Wencha	0.902758278
ENSGALG00010016004	XRCC3	3	0.026348856	0.065823928	0.400292979	0.688940739	0	0.161726184	0	1	CRA004775;PRJNA827465;Yang2025_Silkie_Wencha	0.902758278
ENSGALG00010023808	DHX35	3	0.02595155	0.06476766	0.400686854	0.68865069	0	1.517805823	0	0.666666667	CRA004775;PRJNA827465;Yang2025_Silkie_Wencha	0.902758278
ENSGALG00010001834	ZCRB1	3	-0.025145611	0.062729484	-0.400857933	0.688524723	0	1.711172552	0	0.666666667	CRA004775;PRJNA827465;Yang2025_Silkie_Wencha	0.902758278
ENSGALG00010026298	SNF8	3	0.047223675	0.118140583	0.399724406	0.689359514	0.021633186	4.051259601	50.63263783	0.666666667	CRA004775;PRJNA827465;Yang2025_Silkie_Wencha	0.903155502
ENSGALG00010024255	CFAP97D1	3	0.266981097	0.668309802	0.399487028	0.68953438	1.087074098	12.70446745	84.25750621	0.666666667	CRA004775;PRJNA827465;Yang2025_Silkie_Wencha	0.903263836
ENSGALG00010025957	ENSGALG0001002595	3	-0.053087267	0.13290466	-0.399438714	0.689569973	0.031985725	6.862958304	70.85804822	0.666666667	CRA004775;PRJNA827465;Yang2025_Silkie_Wencha	0.903263836
ENSGALG00010000272	ENSGALG0001000027	3	0.223429453	0.560593525	0.398558746	0.690218364	0.631955525	6.117637965	67.30764371	0.666666667	CRA004775;PRJNA827465;Yang2025_Silkie_Wencha	0.903359941
ENSGALG00010003722	MHCY11	3	-0.211915668	0.531143669	-0.398979937	0.689907987	0.601847854	6.94311168	71.1944717	0.666666667	CRA004775;PRJNA827465;Yang2025_Silkie_Wencha	0.903359941
ENSGALG00010013483	ENSGALG0001001348	3	0.088807785	0.222591735	0.398971618	0.689914117	0	1.145323186	0	0.666666667	CRA004775;PRJNA827465;Yang2025_Silkie_Wencha	0.903359941
ENSGALG00010002801	FGF5	2	-0.075967111	0.190421744	-0.398941369	0.689936406	0	0.221065964	0	0.5	PRJNA827465;Yang2025_Silkie_Wenchang	0.903359941
ENSGALG00010021219	STRA6	2	0.066108673	0.165866067	0.398566593	0.690212581	0	0.000721652	0	1	PRJNA827465;Yang2025_Silkie_Wenchang	0.903359941
ENSGALG00010005146	TGFB3	3	-0.060669458	0.15211365	-0.398842956	0.690008923	0.043656495	7.510117235	73.36925727	0.666666667	CRA004775;PRJNA827465;Yang2025_Silkie_Wencha	0.903359941
ENSGALG00010025056	RBMS2	3	-0.058586612	0.14697841	-0.398606926	0.690182857	0.047937374	8.367364401	76.09761086	0.666666667	CRA004775;PRJNA827465;Yang2025_Silkie_Wencha	0.903359941
ENSGALG00010003214	PRDX4	3	-0.054265697	0.136138212	-0.398607384	0.69018252	0.032278105	4.760329366	57.98610041	0.666666667	CRA004775;PRJNA827465;Yang2025_Silkie_Wencha	0.903359941
ENSGALG00010029948	MYADML2	3	-0.05131721	0.128554209	-0.399187317	0.689755187	0.020632013	3.429935469	41.68986507	0.666666667	CRA004775;PRJNA827465;Yang2025_Silkie_Wencha	0.903359941
ENSGALG00010014173	MND1	3	0.032530301	0.081690257	0.398215192	0.690471569	0	1.584570197	0	0.666666667	CRA004775;PRJNA827465;Yang2025_Silkie_Wencha	0.903607692
ENSGALG00010013508	TUBE1	3	0.091410047	0.22970409	0.397946971	0.690669275	0.098683933	6.124467138	67.34409778	0.666666667	CRA004775;PRJNA827465;Yang2025_Silkie_Wencha	0.90372485
ENSGALG00010013354	WWP1	3	-0.044719743	0.11240816	-0.397833606	0.690752844	0.019539081	4.588499175	56.41276322	0.666666667	CRA004775;PRJNA827465;Yang2025_Silkie_Wencha	0.90372485
ENSGALG00010024387	PPIL2	3	-0.021105483	0.053048824	-0.397850153	0.690740646	0.002746616	2.865289463	30.19902437	0.666666667	CRA004775;PRJNA827465;Yang2025_Silkie_Wencha	0.90372485
ENSGALG00010024415	SIK2	3	-0.015604609	0.039234641	-0.397725282	0.690832699	0	0.081502784	0	1	CRA004775;PRJNA827465;Yang2025_Silkie_Wencha	0.903745701
ENSGALG00010005550	ENSGALG0001000555	3	0.233775061	0.58818851	0.397449215	0.69103623	0.8258152	10.34994217	80.67622053	0.666666667	CRA004775;PRJNA827465;Yang2025_Silkie_Wencha	0.903928324
ENSGALG00010017402	SLC2A5	3	-0.057659597	0.145123668	-0.397313531	0.691136271	0	0.160243261	0	0.666666667	CRA004775;PRJNA827465;Yang2025_Silkie_Wencha	0.903975553
ENSGALG00010021094	ENSGALG0001002109	3	0.130350363	0.328255838	0.397099908	0.691293788	0.245774922	9.02365126	77.83602289	0.666666667	CRA004775;PRJNA827465;Yang2025_Silkie_Wencha	0.904075368
ENSGALG00010012755	CLVS2	2	0.109408142	0.276435225	0.395782203	0.692265707	0	0.095410883	0	0.5	PRJNA827465;Yang2025_Silkie_Wenchang	0.904075368
ENSGALG00010020535	LMLN	3	0.085999255	0.216694066	0.396869451	0.691463733	0.088886269	6.252728565	68.01396416	0.666666667	CRA004775;PRJNA827465;Yang2025_Silkie_Wencha	0.904075368
ENSGALG00010013053	MRTFA	3	0.08189879	0.206785997	0.396055782	0.692063878	0.081152046	5.372877815	62.77600071	0.666666667	CRA004775;PRJNA827465;Yang2025_Silkie_Wencha	0.904075368
ENSGALG00010020682	ZDHHC16	3	0.077459463	0.1955767	0.396056704	0.692063198	0.085990506	8.818702016	77.3209255	0.666666667	CRA004775;PRJNA827465;Yang2025_Silkie_Wencha	0.904075368
ENSGALG00010006581	CEP41	3	-0.056273499	0.141919906	-0.396515896	0.691724483	0.030765912	4.061142049	50.75276915	0.666666667	CRA004775;PRJNA827465;Yang2025_Silkie_Wencha	0.904075368
ENSGALG00010018258	OSCP1	3	0.055969171	0.141079966	0.396719477	0.691574336	0.018387915	2.798739466	28.5392576	0.666666667	CRA004775;PRJNA827465;Yang2025_Silkie_Wencha	0.904075368
ENSGALG00010016982	GLRX5	3	-0.05298345	0.133885757	-0.395736268	0.692299597	0.026938632	3.831657055	47.80326185	0.666666667	CRA004775;PRJNA827465;Yang2025_Silkie_Wencha	0.904075368
ENSGALG00010009467	EGR2	3	0.049317662	0.124285732	0.39680872	0.69150852	0	0.311359794	0	0.666666667	CRA004775;PRJNA827465;Yang2025_Silkie_Wencha	0.904075368
ENSGALG00010018384	LAMP5	3	-0.048257585	0.121881175	-0.39593961	0.692149579	0	0.375791293	0	0.666666667	CRA004775;PRJNA827465;Yang2025_Silkie_Wencha	0.904075368
ENSGALG00010012326	SGTB	3	-0.047728699	0.120597305	-0.395769198	0.692275302	0.031862332	7.923324813	74.7580713	0.666666667	CRA004775;PRJNA827465;Yang2025_Silkie_Wencha	0.904075368
ENSGALG00010010890	NOL12	3	-0.044262779	0.111839672	-0.395769925	0.692274765	0.01742493	3.585374414	44.21782025	0.666666667	CRA004775;PRJNA827465;Yang2025_Silkie_Wencha	0.904075368
ENSGALG00010025145	CPT2	3	-0.040463488	0.102138094	-0.396164509	0.691983671	0.014579173	3.844993291	47.98430456	0.666666667	CRA004775;PRJNA827465;Yang2025_Silkie_Wencha	0.904075368
ENSGALG00010021934	EFTUD2	3	-0.037078743	0.093598967	-0.396144791	0.691998217	0.018079415	7.051308717	71.6364709	0.666666667	CRA004775;PRJNA827465;Yang2025_Silkie_Wencha	0.904075368
ENSGALG00010000818	KLF12	3	-0.034091695	0.086093499	-0.395984543	0.692116431	0	0.965986337	0	0.666666667	CRA004775;PRJNA827465;Yang2025_Silkie_Wencha	0.904075368
ENSGALG00010003160	KIDINS220	3	-0.020042254	0.050620139	-0.395934396	0.692153425	0	0.117323038	0	0.666666667	CRA004775;PRJNA827465;Yang2025_Silkie_Wencha	0.904075368
ENSGALG00010010865	ENSGALG0001001086	3	-0.015944724	0.040161547	-0.397014695	0.691356624	0	0.78047731	0	0.666666667	CRA004775;PRJNA827465;Yang2025_Silkie_Wencha	0.904075368
ENSGALG00010000369	ENSGALG0001000036	2	0.381694724	0.967647305	0.394456453	0.693244072	0	0.349471005	0	0.5	CRA004775;PRJNA827465	0.904091547
ENSGALG00010002294	DAZL	3	-0.305470629	0.773824884	-0.394754207	0.693024293	1.024753638	4.732093197	57.73540552	0.666666667	CRA004775;PRJNA827465;Yang2025_Silkie_Wencha	0.904091547
ENSGALG00010012837	PKIB	3	-0.164992019	0.418156226	-0.394570279	0.693160051	0.380598771	7.904614992	74.69832494	0.666666667	CRA004775;PRJNA827465;Yang2025_Silkie_Wencha	0.904091547
ENSGALG00010001996	ENSGALG0001000199	2	-0.160159314	0.404946191	-0.395507645	0.692468281	0.010660201	1.031450326	3.049136279	0.5	PRJNA827465;Yang2025_Silkie_Wenchang	0.904091547
ENSGALG00010009575	RHOBTB1	3	-0.133976816	0.339258911	-0.394910235	0.692909136	0.228295618	6.391377771	68.70784248	0.666666667	CRA004775;PRJNA827465;Yang2025_Silkie_Wencha	0.904091547
ENSGALG00010007861	INTU	3	-0.109695573	0.277323843	-0.395550457	0.692436691	0.194548429	15.87178236	87.39902076	0.666666667	CRA004775;PRJNA827465;Yang2025_Silkie_Wencha	0.904091547
ENSGALG00010027641	HYAL3	3	-0.100426405	0.254330948	-0.394865059	0.692942478	0.149530311	9.756896329	79.50167827	0.666666667	CRA004775;PRJNA827465;Yang2025_Silkie_Wencha	0.904091547
ENSGALG00010018370	ZBTB8A	3	-0.095853658	0.243077846	-0.394333171	0.693335076	0.161762602	25.60546554	92.18916759	0.666666667	CRA004775;PRJNA827465;Yang2025_Silkie_Wencha	0.904091547
ENSGALG00010026825	ADAM23	3	0.092871926	0.235391315	0.394542706	0.693180404	0.123334857	10.92212393	81.68854325	0.666666667	CRA004775;PRJNA827465;Yang2025_Silkie_Wencha	0.904091547
ENSGALG00010026427	PGC	3	0.091114354	0.230492982	0.395302074	0.692619969	0	0.390260438	0	0.666666667	CRA004775;PRJNA827465;Yang2025_Silkie_Wencha	0.904091547
ENSGALG00010019859	BIN3	3	-0.061904873	0.156764174	-0.394891711	0.692922807	0.058128705	9.958088866	79.91582495	0.666666667	CRA004775;PRJNA827465;Yang2025_Silkie_Wencha	0.904091547
ENSGALG00010020833	RANBP1	3	-0.05751683	0.145781299	-0.394541898	0.693181001	0.052064285	12.57993952	84.10167237	0.666666667	CRA004775;PRJNA827465;Yang2025_Silkie_Wencha	0.904091547
ENSGALG00010004760	THBS2	3	-0.05158094	0.130804277	-0.394336801	0.693332396	0	1.105073267	0	0.666666667	CRA004775;PRJNA827465;Yang2025_Silkie_Wencha	0.904091547
ENSGALG00010015449	HDX	3	-0.031970412	0.080971165	-0.394836995	0.69296319	0.008087274	3.375867024	40.7559603	0.666666667	CRA004775;PRJNA827465;Yang2025_Silkie_Wencha	0.904091547
ENSGALG00010026262	NAV1	3	-0.030903521	0.078343113	-0.39446378	0.693238663	0	1.091419288	0	0.666666667	CRA004775;PRJNA827465;Yang2025_Silkie_Wencha	0.904091547
ENSGALG00010023164	NKIRAS2	3	-0.024101134	0.061073074	-0.394627811	0.693117586	0	1.465641744	0	0.666666667	CRA004775;PRJNA827465;Yang2025_Silkie_Wencha	0.904091547
ENSGALG00010003159	TAP2	3	-0.140712738	0.357132398	-0.394007205	0.69357572	0.303908874	9.926319018	79.8515442	0.666666667	CRA004775;PRJNA827465;Yang2025_Silkie_Wencha	0.904091858
ENSGALG00010009404	GNG4	3	-0.091586549	0.232320639	-0.394224761	0.693415106	0	1.390545067	0	0.666666667	CRA004775;PRJNA827465;Yang2025_Silkie_Wencha	0.904091858
ENSGALG00010000613	ENSGALG0001000061	3	-0.065147395	0.165342572	-0.394014644	0.693570228	0.050588126	5.750679532	65.22150141	0.666666667	CRA004775;PRJNA827465;Yang2025_Silkie_Wencha	0.904091858
ENSGALG00010017364	AKT1	3	0.017931889	0.04551398	0.39398639	0.693591088	0	1.699079832	0	0.666666667	CRA004775;PRJNA827465;Yang2025_Silkie_Wencha	0.904091858
ENSGALG00010000641	ENSGALG0001000064	3	-0.205633974	0.522827777	-0.393311111	0.694089713	0.625542233	10.57853681	81.09379363	0.666666667	CRA004775;PRJNA827465;Yang2025_Silkie_Wencha	0.904226333
ENSGALG00010005490	DCBLD2	3	-0.094709606	0.240764779	-0.393369854	0.694046331	0.157326516	30.4448635	93.43074736	0.666666667	CRA004775;PRJNA827465;Yang2025_Silkie_Wencha	0.904226333
ENSGALG00010008407	GNAI1	3	-0.075696478	0.192368934	-0.393496374	0.693952902	0.094976878	18.94052039	89.44062803	0.666666667	CRA004775;PRJNA827465;Yang2025_Silkie_Wencha	0.904226333
ENSGALG00010000059	MFAP1	3	0.071425575	0.181691028	0.393115586	0.694234113	0.07997727	11.45905417	82.54655253	0.666666667	CRA004775;PRJNA827465;Yang2025_Silkie_Wencha	0.904226333
ENSGALG00010021217	FBXO2	3	-0.058480451	0.148680021	-0.393330932	0.694075075	0	0.719114261	0	0.666666667	CRA004775;PRJNA827465;Yang2025_Silkie_Wencha	0.904226333
ENSGALG00010015153	ERCC6L	3	-0.039351396	0.100060643	-0.393275462	0.69411604	0.00439525	2.235179443	10.5217254	0.666666667	CRA004775;PRJNA827465;Yang2025_Silkie_Wencha	0.904226333
ENSGALG00010023125	COL20A1	3	0.034969338	0.088957349	0.393102291	0.694243932	0	1.657524399	0	1	CRA004775;PRJNA827465;Yang2025_Silkie_Wencha	0.904226333
ENSGALG00010024845	HYCC2	3	0.033973947	0.086432915	0.393067231	0.694269826	0	0.166262094	0	1	CRA004775;PRJNA827465;Yang2025_Silkie_Wencha	0.904226333
ENSGALG00010017529	MFN2	3	0.019893693	0.050599663	0.393158603	0.694202343	0	1.870993688	0	0.666666667	CRA004775;PRJNA827465;Yang2025_Silkie_Wencha	0.904226333
ENSGALG00010023920	ENSGALG0001002392	2	-0.218176555	0.555320455	-0.39288406	0.694405115	0.472529011	4.254190293	76.49376424	0.5	PRJNA827465;Yang2025_Silkie_Wenchang	0.90429405
ENSGALG00010026320	ENSGALG0001002632	3	-0.023381906	0.059522653	-0.392823652	0.694449735	0	0.763575125	0	1	CRA004775;PRJNA827465;Yang2025_Silkie_Wencha	0.90429405

ENSGALG00010007931	GBE1	3	0.081760111	0.208282351	0.392544596	0.694655867	0.108030034	13.40698538	85.08240336	0.666666667	CRA004775;PRJNA827465;Yang2025_Silkie_Wencha	0.90447917
ENSGALG00010015134	PLN	3	-0.296206786	0.756748893	-0.391420177	0.695486681	1.435197551	12.39058883	83.85871707	0.666666667	CRA004775;PRJNA827465;Yang2025_Silkie_Wencha	0.904561326
ENSGALG00010010711	ENSGALG00010010711	3	0.17516908	0.44747825	0.391458312	0.695458498	0.481043197	10.09548631	80.18916634	0.666666667	CRA004775;PRJNA827465;Yang2025_Silkie_Wencha	0.904561326
ENSGALG00010000423	ENSGALG00010000423	3	0.143762163	0.36693638	0.391790432	0.695213065	0.328835917	12.51943128	84.02483343	0.666666667	CRA004775;PRJNA827465;Yang2025_Silkie_Wencha	0.904561326
ENSGALG00010008295	DMAC2	3	0.126366144	0.322541644	0.391782416	0.695218989	0.241012104	9.469050113	78.87855723	0.666666667	CRA004775;PRJNA827465;Yang2025_Silkie_Wencha	0.904561326
ENSGALG00010016445	ZDHHC21	3	-0.079108871	0.201845323	-0.391928185	0.695111277	0.075532051	5.536552307	63.87643629	0.666666667	CRA004775;PRJNA827465;Yang2025_Silkie_Wencha	0.904561326
ENSGALG00010029706	SSH2	3	-0.071184079	0.18164577	-0.391884042	0.695143894	0.063503463	7.167573881	72.0965555	0.666666667	CRA004775;PRJNA827465;Yang2025_Silkie_Wencha	0.904561326
ENSGALG00010020388	MYOF	3	0.060065813	0.153424177	0.391501615	0.695426495	0.0251759	3.051307471	34.45432756	0.666666667	CRA004775;PRJNA827465;Yang2025_Silkie_Wencha	0.904561326
ENSGALG00010026704	WDFY1	3	-0.059930044	0.153065038	-0.391533201	0.695403152	0.056371052	16.15217251	87.61776474	0.666666667	CRA004775;PRJNA827465;Yang2025_Silkie_Wencha	0.904561326
ENSGALG00010017775	PRPSAP2	3	-0.055654661	0.14191706	-0.392163267	0.694937584	0.045919217	11.38480653	82.43272739	0.666666667	CRA004775;PRJNA827465;Yang2025_Silkie_Wencha	0.904561326
ENSGALG00010005415	PRKDC	3	0.051095654	0.130519001	0.391480581	0.69544204	0.028821669	4.958441736	59.66474739	0.666666667	CRA004775;PRJNA827465;Yang2025_Silkie_Wencha	0.904561326
ENSGALG00010005681	EPC1	3	0.032940283	0.084062595	0.391854218	0.695165932	0.010363498	3.834551647	47.84266366	0.666666667	CRA004775;PRJNA827465;Yang2025_Silkie_Wencha	0.904561326
ENSGALG00010006327	DENND6B	3	0.027120197	0.069265405	0.39154029	0.695397914	0.006047577	3.473961765	42.42884247	0.666666667	CRA004775;PRJNA827465;Yang2025_Silkie_Wencha	0.904561326
ENSGALG00010021424	PTCD1	3	-0.018526813	0.047351684	-0.391259856	0.695605169	0	0.230890995	0	0.666666667	CRA004775;PRJNA827465;Yang2025_Silkie_Wencha	0.904632218
ENSGALG00010027166	CDK5	3	-0.057491934	0.147072677	-0.390909327	0.695864999	0.044342624	6.71435496	70.21307316	0.666666667	CRA004775;PRJNA827465;Yang2025_Silkie_Wencha	0.904886896
ENSGALG00010007596	ENSGALG00010007596	3	0.284271696	0.728185344	0.39038371	0.696252832	1.131783276	7.822938425	74.43415899	0.666666667	CRA004775;PRJNA827465;Yang2025_Silkie_Wencha	0.904891882
ENSGALG00010009054	RYR2	2	-0.271798897	0.696227836	-0.390387863	0.696249761	0.764331956	4.452165074	77.53901791	0.5	PRJNA827465;Yang2025_Silkie_Wenchang	0.904891882
ENSGALG00010008396	LRRTM2	2	0.176330056	0.451354403	0.390668741	0.696042108	0.22096605	2.10778898	52.55692058	0.5	PRJNA827465;Yang2025_Silkie_Wenchang	0.904891882
ENSGALG00010001948	EMX2	3	-0.068280402	0.174621082	-0.390608058	0.696086968	0	0.672094717	0	0.666666667	CRA004775;PRJNA827465;Yang2025_Silkie_Wencha	0.904891882
ENSGALG00010012247	TRIM23	3	-0.051447465	0.131735664	-0.390535589	0.696140544	0.018466376	3.065642692	34.76082502	0.666666667	CRA004775;PRJNA827465;Yang2025_Silkie_Wencha	0.904891882
ENSGALG00010011086	OTUD6A	3	0.029299624	0.07504149	0.390445659	0.696207031	0.007688287	3.299168729	39.37866886	0.666666667	CRA004775;PRJNA827465;Yang2025_Silkie_Wencha	0.904891882
ENSGALG00010013463	ENSGALG00010013463	3	-0.050848163	0.130376808	-0.390009266	0.696529695	0.016092102	2.884948377	30.67466939	0.666666667	CRA004775;PRJNA827465;Yang2025_Silkie_Wencha	0.90508532
ENSGALG00010001258	ENSGALG00010001258	3	-0.035150228	0.09011958	-0.390039854	0.696507077	0	1.74799917	0	0.666666667	CRA004775;PRJNA827465;Yang2025_Silkie_Wencha	0.90508532
ENSGALG00010001689	ILK	3	0.047466167	0.121788576	0.389742362	0.696727069	0.036105302	11.51700266	82.6343706	0.666666667	CRA004775;PRJNA827465;Yang2025_Silkie_Wencha	0.905175413
ENSGALG00010001157	TIMM21	3	0.044466328	0.114070341	0.389814979	0.696673366	0.015180712	3.211907565	37.73170742	0.666666667	CRA004775;PRJNA827465;Yang2025_Silkie_Wencha	0.905175413
ENSGALG00010016382	UGDH	3	-0.128120309	0.329013901	-0.389406979	0.69697511	0.305197838	61.55744922	96.75100248	0.666666667	CRA004775;PRJNA827465;Yang2025_Silkie_Wencha	0.905411299
ENSGALG00010021902	LMTK2	3	0.061143694	0.157055159	0.3893237	0.697036707	0.052110643	9.99176636	79.96351915	0.666666667	CRA004775;PRJNA827465;Yang2025_Silkie_Wencha	0.905411299
ENSGALG00010017724	FLII	3	0.061512788	0.15816432	0.388916966	0.697337572	0.056361591	14.56282162	86.26639774	0.666666667	CRA004775;PRJNA827465;Yang2025_Silkie_Wencha	0.905718898
ENSGALG00010009743	CCNG1	3	0.02474476	0.06365976	0.38870331	0.697495634	0	0.919537808	0	0.666666667	CRA004775;PRJNA827465;Yang2025_Silkie_Wencha	0.905840981
ENSGALG00010027178	C4BPM	3	0.051073591	0.131426381	0.388609884	0.697564754	0.031842539	6.984245408	71.36412192	0.666666667	CRA004775;PRJNA827465;Yang2025_Silkie_Wencha	0.905847544
ENSGALG00010017876	COMMD1	3	-0.028525432	0.073468293	-0.388268605	0.697817268	0	1.41131298	0	0.666666667	CRA004775;PRJNA827465;Yang2025_Silkie_Wencha	0.906012669
ENSGALG00010008775	MED17	3	-0.018393978	0.047374826	-0.388264819	0.697820069	0.000805968	2.18700047	8.550545468	0.666666667	CRA004775;PRJNA827465;Yang2025_Silkie_Wencha	0.906012669
ENSGALG00010028894	AKNA	3	0.078246042	0.201676552	0.387977884	0.6980324	0.090339396	12.5163324	84.02087819	0.666666667	CRA004775;PRJNA827465;Yang2025_Silkie_Wencha	0.906121934
ENSGALG00010027497	KCNT1	3	0.045637863	0.117618701	0.388015358	0.698004668	0	1.489757939	0	0.666666667	CRA004775;PRJNA827465;Yang2025_Silkie_Wencha	0.906121934
ENSGALG00010007322	AK6	3	0.051008917	0.131609325	0.387578292	0.698328136	0	1.258648803	0	0.666666667	CRA004775;PRJNA827465;Yang2025_Silkie_Wencha	0.906422612
ENSGALG00010019838	ALX4	3	0.156980034	0.406628477	0.386052732	0.699457616	0.411972178	15.08927737	86.7455548	0.666666667	CRA004775;PRJNA827465;Yang2025_Silkie_Wencha	0.906557084
ENSGALG00010023648	SCN2B	3	0.126907354	0.328134474	0.386754101	0.69893826	0.24789813	19.35312546	89.6657519	0.666666667	CRA004775;PRJNA827465;Yang2025_Silkie_Wencha	0.906557084
ENSGALG00010019804	ELFN1	3	-0.126897031	0.328526173	-0.386261558	0.699302968	0.224751868	7.317229428	72.66725036	0.666666667	CRA004775;PRJNA827465;Yang2025_Silkie_Wencha	0.906557084
ENSGALG00010003420	ENSGALG00010003420	3	-0.114260808	0.295168993	-0.387103019	0.698679943	0	1.843258819	0	0.666666667	CRA004775;PRJNA827465;Yang2025_Silkie_Wencha	0.906557084
ENSGALG00010028099	PCASP2	3	-0.092403576	0.239078714	-0.386498549	0.699127477	0.11535806	6.419520295	68.84502411	0.666666667	CRA004775;PRJNA827465;Yang2025_Silkie_Wencha	0.906557084
ENSGALG00010008938	ZBED4	3	0.082232066	0.212678973	0.386648782	0.699016239	0.116856125	25.22818096	92.07235748	0.666666667	CRA004775;PRJNA827465;Yang2025_Silkie_Wencha	0.906557084
ENSGALG00010005486	IRF5	3	0.066971599	0.173286718	0.386478546	0.699142289	0.03115035	3.012255597	33.60457186	0.666666667	CRA004775;PRJNA827465;Yang2025_Silkie_Wencha	0.906557084
ENSGALG00010015967	ZSWIM1	3	-0.066060077	0.165676218	-0.386054666	0.699456184	0.017162799	2.476442258	19.23898111	0.666666667	CRA004775;PRJNA827465;Yang2025_Silkie_Wencha	0.906557084
ENSGALG00010011235	ENSGALG00010011235	3	0.057255607	0.148138287	0.386501075	0.699125607	0.045640966	7.150646987	72.03050292	0.666666667	CRA004775;PRJNA827465;Yang2025_Silkie_Wencha	0.906557084
ENSGALG00010011699	TIAM1	3	0.054275161	0.140490657	0.386325768	0.699255419	0.028339268	3.958132366	49.47111883	0.666666667	CRA004775;PRJNA827465;Yang2025_Silkie_Wencha	0.906557084
ENSGALG00010007146	ENSGALG00010007146	3	-0.051327887	0.132828799	-0.386421373	0.699184623	0.038349073	8.642062793	76.85737713	0.666666667	CRA004775;PRJNA827465;Yang2025_Silkie_Wencha	0.906557084
ENSGALG00010004416	STXBP6	3	0.051141581	0.132433958	0.386166719	0.6993732	0	0.601610447	0	0.666666667	CRA004775;PRJNA827465;Yang2025_Silkie_Wencha	0.906557084
ENSGALG00010020608	CAPZB	3	0.050008841	0.129125956	0.387287287	0.698543536	0.028831056	5.082082542	60.64605438	0.666666667	CRA004775;PRJNA827465;Yang2025_Silkie_Wencha	0.906557084
ENSGALG00010013890	SFPQ	3	0.0398046	0.103099376	0.386079928	0.699437475	0.021953614	7.685922854	73.97840132	0.666666667	CRA004775;PRJNA827465;Yang2025_Silkie_Wencha	0.906557084
ENSGALG00010018336	JMJD8	3	0.032873277	0.085059497	0.386473922	0.699145712	0.003857536	2.317685929	13.70703102	0.666666667	CRA004775;PRJNA827465;Yang2025_Silkie_Wencha	0.906557084
ENSGALG00010006111	WBP1	3	0.022238174	0.057571174	0.386283153	0.699286976	0	0.487628081	0	1	CRA004775;PRJNA827465;Yang2025_Silkie_Wencha	0.906557084
ENSGALG00010003299	RAD23A	3	0.075206378	0.194955511	0.385761745	0.699673129	0.075770151	7.371960715	72.87017556	0.666666667	CRA004775;PRJNA827465;Yang2025_Silkie_Wencha	0.906670184
ENSGALG00010017060	CLSTN1	3	-0.054954768	0.142432798	-0.385829451	0.699622982	0.040309133	6.182691665	67.65162961	0.666666667	CRA004775;PRJNA827465;Yang2025_Silkie_Wencha	0.906670184
ENSGALG00010019062	PUS10	3	0.06030211	0.156389345	0.385589635	0.699800611	0.050064929	7.189175847	72.1803995	0.666666667	CRA004775;PRJNA827465;Yang2025_Silkie_Wencha	0.906752276
ENSGALG00010003324	ENSGALG00010003324	3	0.16650912	0.432132812	0.385319318	0.70000085	0	1.208050863	0	0.666666667	CRA004775;PRJNA827465;Yang2025_Silkie_Wencha	0.906762561
ENSGALG00010027671	BARX1	2	0.130399303	0.338418915	0.385319192	0.700000944	0	0.010458595	0	1	CRA004775;PRJNA827465	0.906762561
ENSGALG00010020957	RPF1	3	-0.041263056	0.107048215	-0.38546235	0.699894896	0.019244093	4.971587937	59.77140452	0.666666667	CRA004775;PRJNA827465;Yang2025_Silkie_Wencha	0.906893802
ENSGALG00010000278	ENSGALG00010000278	2	0.478871332	1.243603722	0.385067464	0.700187433	0	0.355661538	0	0.5	CRA004775;PRJNA827465	0.906893802
ENSGALG00010015935	EPHX1	3	0.063042569	0.163779815	0.384922705	0.700294684	0.053836269	6.55245182	69.42707431	0.666666667	CRA004775;PRJNA827465;Yang2025_Silkie_Wencha	0.906893802
ENSGALG00010008931	FNIP2	3	0.022229623	0.05774273	0.384976999	0.700254457	0	0.795970266	0	1	CRA004775;PRJNA827465;Yang2025_Silkie_Wencha	0.906893802
ENSGALG00010016372	DNAAF6	3	0.072049558	0.187249782	0.384777793	0.700402053	0.065753097	5.866752179	65.90958781	0.666666667	CRA004775;PRJNA827465;Yang2025_Silkie_Wencha	0.906949778
ENSGALG00010017772	ENSGALG00010017772	3	0.066949622	0.174115213	0.384513342	0.700598009	0	0.959364599	0	0.666666667	CRA004775;PRJNA827465;Yang2025_Silkie_Wencha	0.907120444
ENSGALG00010027229	MDM4	3	0.028081316	0.073069808	0.38430806	0.700750135	0.000747952	2.078626013	3.782595457	0.666666667	CRA004775;PRJNA827465;Yang2025_Silkie_Wencha	0.907234334
ENSGALG00010006847	SEL1L	3	0.03308821	0.086118649	0.384216544	0.700817958	0.010993666	4.121584895	51.47497744	0.666666667	CRA004775;PRJNA827465;Yang2025_Silkie_Wencha	0.907239068
ENSGALG00010013515	RBP4L	3	-0.099448588	0.25917553	-0.383711334	0.701192411	0	1.179085388	0	0.666666667	CRA004775;PRJNA827465;Yang2025_Silkie_Wencha	0.907254657
ENSGALG00010022933	UNC119B	3	-0.053480683	0.139362302	-0.383752869	0.701161623	0.045646531	10.82600929	81.52597189	0.666666667	CRA004775;PRJNA827465;Yang2025_Silkie_Wencha	0.907254657
ENSGALG00010028012	PRRX2	3	0.05052119	0.131665033	0.383709998	0.701193401	0	1.439289627	0	0.666666667	CRA004775;PRJNA827465;Yang2025_Silkie_Wencha	0.907254657
ENSGALG00010001468	ANOS1	3	-0.037066927	0.096545525	-0.38393211	0.701028767	0	1.452751944	0	0.666666667	CRA004775;PRJNA827465;Yang2025_Silkie_Wencha	0.907254657
ENSGALG00010016742	TRMT12	3	-0.03478879	0.090577005	-0.384079714	0.700919367	0	0.888267166	0	0.666666667	CRA004775;PRJNA827465;Yang2025_Silkie_Wencha	0.907254657
ENSGALG00010019931	ZFYVE26	3	0.024978283	0.065101717	0.38368086	0.701215	0	1.731718675	0	0.666666667	CRA004775;PRJNA827465;Yang2025_Silkie_Wencha	0.907254657
ENSGALG00010022811	GIGYF2	3	0.055625335	0.145012613	0.383483857	0.701361038	0.04351793	6.698042745	70.14053095	0.666666667	CRA004775;PRJNA827465;Yang2025_Silkie_Wencha	0.9073096
ENSGALG00010015126	MCM5	3	-0.044395082	0.115777888	-0.383450445	0.701385807	0.014852528	3.038277261	34.17322291	0.666666667	CRA004775;PRJNA827465;Yang2025_Silkie_Wencha	0.9073096
ENSGALG00010029518	FAAP100	3	0.112610271	0.293846635	0.383228042	0.701550688	0.22711632	29.66945871	93.25906138	0.666666667	CRA004775;PRJNA827465;Yang2025_Silkie_Wencha	0.907428231
ENSGALG00010014940	VAMP4	3	0.03427173	0.08944645	0.383153611	0.701605872	0.005316572	2.52075218	20.65860277	0.666666667	CRA004775;PRJNA827465;Yang2025_Silkie_Wencha	0.907428231
ENSGALG00010015892	UBE2C	3	-0.043744946	0.11421024	-0.383021227	0.701704025	0.002882371	2.103239089	4.908575988	0.666666667	CRA004775;PRJNA827465;Yang2025_Silkie_Wencha	0.907472168
ENSGALG00010010841	PMM1	3	-0.122679956	0.320586488	-0.382673507	0.70196186	0.267527995	16.59011542	87.94462878	0.666666667	CRA004775;PRJNA827465;Yang2025_Silkie_Wencha	0.907473598
ENSGALG00010017654	KBTBD13	3	0.083866288	0.219143738	0.382699906	0.701942284	0.076640319	4.510309504	55.65714508	0.666666667	CRA004775;PRJNA827465;Yang2025_Silkie_Wencha	0.907473598
ENSGALG00010021220	SYNPO2L	3	-0.058058608	0.151691803	-0.382740575	0.701912126	0.02164252	2.839663752	29.56912598	0.666666667	CRA004775;PRJNA827465;Yang2025_Silkie_Wencha	0.907473598
ENSGALG00010003183	NAA16	3	-0.036733724	0.095965488	-0.382780565	0.701882473	0.015085965	4.6679024	57.15420271	0.666666667	CRA004775;PRJNA827465;Yang2025_Silkie_Wencha	0.907473598
ENSGALG00010017762	FBXO11	3	-0.011966884	0.031286174	-0.382497515	0.702092372	0	1.793326145	0	0.666666667	CRA004775;PRJNA827465;Yang2025_Silkie_Wencha	0.907559338
ENSGALG00010023194	MRC2	3	-0.028508146	0.074555043	-0.382377163	0.702181627	0	1.055576499	0	0.666666667	CRA004775;PRJNA827465;Yang2025_Silkie_Wencha	0.907591738
ENSGALG00010010937	CDK7	3	-0.072902174	0.190713189	-0.382260787	0.702267937	0.07129969	5.883937271	66.00915496	0.666666667	CRA004775;PRJNA827465;Yang2025_Silkie_Wencha	0.907620326
ENSGALG00010015142	HNRNPAB	3	0.036529657	0.095685905	0.381766329	0.702634696	0.017123089	6.127705007	67.3613531	0.666666667	CRA004775;PRJNA827465;Yang2025_Silkie_Wencha	0.90801133
ENSGALG00010005464	SLC39A8	2	0.181155163	0.474797811	0.381541699	0.702801336	0.126685166	1.370960325	27.05842893	0.5	PRJNA827465;Yang2025_Silkie_Wenchang	0.908024166
ENSGALG00010018932	CDKN3	3	0.139730567	0.366458631	0.381299702	0.702980874	0.326956693	14.78669535	86.47432741	0.666666667	CRA004775;PRJNA827465;Yang2025_Silkie_Wencha	0.908024166
ENSGALG00010024937	ILKAP	3	0.103024943	0.270500245	0.380868205	0.703301046	0.198589249	36.37370412	94.50152233	0.666666667	CRA004775;PRJNA827465;Yang2025_Silkie_Wencha	0.908024166
ENSGALG00010008066	PCNX2	3	0.090076849	0.23646024	0.380938669	0.703248758	0.085900478	4.396117815	54.50531391	0.666666667	CRA004775;PRJNA827465;Yang2025_Silkie_Wencha	0.908024166
ENSGALG00010009596	URB2	3	-0.043551998	0.114369501	-0.380800805	0.703351062	0.022114153	4.99293952	59.94343629	0.666666667	CRA004775;PRJNA827465;Yang2025_Silkie_Wencha	0.908024166
ENSGALG00010013329	LIFR	3	-0.038387399	0.100773309	-0.380928237	0.703256499	0.009267926	2.795639386	28.46001491	0.666666667	CRA004775;PRJNA827465;Yang2025_Silkie_Wencha	0.908024166
ENSGALG00010013206	AFF2	3	-0.03571373	0.093589954	-0.3815979	0.702759642	0	1.476970023	0	0.666666667	CRA004775;PRJNA827465;Yang2025_Silkie_Wencha	0.908024166
ENSGALG00010023166	KDM2A	3	-0.031974016	0.083879288	-0.381190831	0.703061652	0.010505643	3.948314023	49.34546776	0.666666667	CRA004775;PRJNA827465;Yang2025_Silkie_Wencha	0.908024166
ENSGALG00010013737	AFF4	3	-0.023001576	0.060394549	-0.38085516	0.703310727	0.000461755	2.052689198	2.566837611	0.666666667	CRA004775;PRJNA827465;Yang2025_Silkie_Wencha	0.908024166
ENSGALG00010001910	FAM53B	3	0.017720505	0.046487075	0.381192078	0.703060727	0	0.928849233	0	0.666666667	CRA004775;PRJNA827465;Yang2025_Silkie_Wencha	0.908024166
ENSGALG00010007923	YAP1	3	0.016340285	0.042891437	0.380968461	0.703226652	0	0.237370491	0	0.666666667	CRA004775;PRJNA827465;Yang2025_Silkie_Wencha	0.908024166
ENSGALG00010016876	MTURN	3	0.082651637	0.217137301	0.380642278	0.703468705	0.09581136	7.928239507	74.7737187	0.666666667	CRA004775;PRJNA827465;Yang2025_Silkie_Wencha	0.908071372
ENSGALG00010011461	IRF4	3	-0.070916363	0.186338359	-0.380578447	0.703516076	0	0.870511638	0	0.666666667	CRA004775;PRJNA827465;Yang2025_Silkie_Wencha	0.908071372
ENSGALG00010023443	ENSGALG00010023443	3	-0.092825701	0.244053009	-0.380350568	0.703685203	0.137266376	9.186116205	78.22801328	0.666666667	CRA004775;PRJNA827465;Yang2025_Silkie_Wencha	0.908206763
ENSGALG00010017665	CTBP1	3	-0.016625051	0.04372271	-0.38023835	0.703768494	0.000622634	2.185203338	8.4753366	0.666666667	CRA004775;PRJNA827465;Yang2025_Silkie_Wencha	0.908231356
ENSGALG00010024280	ENSGALG00010024280	3	-0.024684444	0.064936257	-0.380133462	0.703846348	0	1.803666133	0	0.666666667	CRA004775;PRJNA827465;Yang2025_Silkie_Wencha	0.908248929
ENSGALG00010009761	PDP2	3	0.064519628	0.169884647	0.379784929	0.704105071	0.067731881	12.6961001	84.24713114	0.666666667	CRA004775;PRJNA827465;Yang2025_Silkie_Wencha	0.908346522
ENSGALG00010020889	PPRC1	3	0.056464071	0.14875697	0.379572609	0.704262696	0.042260859	8.455408063	76.34649936	0.666666667	CRA004775;PRJNA827465;Yang2025_Silkie_Wencha	0.908346522
ENSGALG00010003268	SMS	3	-0.055279045	0.145586443	-0.379699122	0.704168772	0.049873104	13.31538555	84.97978153	0.666666667	CRA004775;PRJNA827465;Yang2025_Silkie_Wencha	0.908346522
ENSGALG00010028394	ZBTB26	3	-0.033114356	0.087245583	-0.379553379	0.704276973	0.01211334	4.416771668	54.71805765	0.666666667	CRA004775;PRJNA827465;Yang2025_Silkie_Wencha	0.908346522
ENSGALG00010011746	SOD1	3	0.021347891	0.056206359	0.379812732	0.70408443	0	1.010840314	0	0.666666667	CRA004775;PRJNA827465;Yang2025_Silkie_Wencha	0.908346522
ENSGALG00010027885	PLEKHJ1	3	0.016624853	0.043805829	0.379512341	0.704307442	0	1.359813636	0	0.666666667	CRA004775;PRJNA827465;Yang2025_Silkie_Wencha	0.908346522
ENSGALG00010020357	RBP4	3	0.280193834	0.739165728	0.379067675	0.70463761	1.279639871	9.630043654	79.23166216	0.666666667	CRA004775;PRJNA827465;Yang2025_Silkie_Wencha	0.908685421
ENSGALG00010007392	NAALAD2	3	-0.038896531	0.102633339	-0.378985338	0.704698751	0	0.436287026	0	0.666666667	CRA004775;PRJNA827465;Yang2025_Silkie_Wencha	0.908685421
ENSGALG00010010438	LDB3	3	0.140364414	0.370819201	0.378525204	0.705040474	0.300737955	12.56994785	84.08903503	0.666666667	CRA004775;PRJNA827465;Yang2025_Silkie_Wencha	0.908913013
ENSGALG00010028116	HYAL1	3	0.03865958	0.10209868	0.378649162	0.704948409	0.011516608	3.075907411	34.97853698	0.666666667	CRA004775;PRJNA827465;Yang2025_Silkie_Wencha	0.908913013
ENSGALG00010005966	PLXDC2	3	-0.033402413	0.088263498	-0.378439721	0.705103966	0.000616907	2.035306973	1.734724696	0.666666667	CRA004775;PRJNA827465;Yang2025_Silkie_Wencha	0.908913013
ENSGALG00010014134	RPAP3	3	0.01733316	0.045806273	0.378401454	0.705132388	0	0.278997894	0	1	CRA004775;PRJNA827465;Yang2025_Silkie_Wencha	0.908913013
ENSGALG00010014717	ELOVL7	3	0.132869643	0.351343306	0.378176106	0.705299774	0.327313836	20.34526411	90.16970245	0.666666667	CRA004775;PRJNA827465;Yang2025_Silkie_Wencha	0.909033172
ENSGALG00010003915	MIOS	3	-0.025163077	0.06655088	-0.378102848	0.705354193	0.002914414	2.382437128	16.05234923	0.666666667	CRA004775;PRJNA827465;Yang2025_Silkie_Wencha	0.909033172
ENSGALG00010011278	ENSGALG00010011278	3	-0.130570018	0.345749324	-0.377643595	0.705695374	0.245249612	7.667681583	73.91649642	0.666666667	CRA004775;PRJNA827465;Yang2025_Silkie_Wencha	0.909089101
ENSGALG00010007536	GABBR2	3	0.103793903	0.274734937	0.377796519	0.70558176	0.124620657	4.836203577	58.64524791	0.666666667	CRA004775;PRJNA827465;Yang2025_Silkie_Wencha	0.909089101
ENSGALG00010022967	ADAMTS15	3	-0.080811525	0.213987225	-0.377646492	0.705693222	0.102886283	16.74184671	88.05388656	0.666666667	CRA004775;PRJNA827465;Yang2025_Silkie_Wencha	0.909089101
ENSGALG00010020097	RETSAT	3	0.051404767	0.136067337	0.377789178	0.705587214	0.010045588	2.388127167	16.25236599	0.666666667	CRA004775;PRJNA827465;Yang2025_Silkie_Wencha	0.909089101
ENSGALG00010010614	IKBIP	3	-0.041934426	0.111051714	-0.377611698	0.705719073	0.015755916	3.480430139	42.53583838	0.666666667	CRA004775;PRJNA827465;Yang2025_Silkie_Wencha	0.909089101
ENSGALG00010001188	PSMG2	3	0.067596631	0.179126015	0.377369144	0.705899294	0.069454802	9.380896233	78.68007544	0.666666667	CRA004775;PRJNA827465;Yang2025_Silkie_Wencha	0.909238418
ENSGALG00010027527	ENSGALG00010027527	3	0.114859387	0.304620293	0.377057567	0.706130824	0	1.116425036	0	0.666666667	CRA004775;PRJNA827465;Yang2025_Silkie_Wencha	0.909288135
ENSGALG00010007894	CD88	3	-0.044832157	0.118861563	-0.377179602	0.706040138	0	0.703069329	0	0.666666667	CRA004775;PRJNA827465;Yang2025_Silkie_Wencha	0.909288135
ENSGALG00010017183	NFYC	3	0.037576639	0.099636537	0.377137146	0.706071688	0.019842533	6.329290734	68.40088275	0.666666667	CRA004775;PRJNA827465;Yang2025_Silkie_Wencha	0.909288135
ENSGALG00010007805	ENSGALG00010007805	3	-0.210193946	0.559121977	-0.375935761	0.706964654	0	0.025868912	0	1	CRA004775;PRJNA827465;Yang2025_Silkie_Wencha	0.909477892
ENSGALG00010024441	SAA	3	-0.187972734	0.499184566	-0.376559586	0.706500926	0.531341682	6.988937843	71.3833483	0.666666667	CRA004775;PRJNA827465;Yang2025_Silkie_Wencha	0.909477892
ENSGALG00010005475	TMEM181	3	-0.067695161	0.179884459	-0.37632579	0.706674709	0.075890122	12.44060965	83.92361744	0.666666667	CRA004775;PRJNA827465;Yang2025_Silkie_Wencha	0.909477892
ENSGALG00010011588	COLGALT2	3	0.063646639	0.169058932	0.37647605	0.706563018	0.056608572	6.631558828	69.84117834	0.666666667	CRA004775;PRJNA827465;Yang2025_Silkie_Wencha	0.909477892
ENSGALG00010024497	ABI3	3	0.059707162	0.158809032	0.375968302	0.706940461	0.002472539	2.063147688	3.060744929	0.666666667	CRA004775;PRJNA827465;Yang2025_Silkie_Wencha	0.909477892
ENSGALG00010008229	PIGY	3	0.052695273	0.140107345	0.376106428	0.706837776	0.03760267	6.392786684	68.71473899	0.666666667	CRA004775;PRJNA827465;Yang2025_Silkie_Wencha	0.909477892
ENSGALG00010002528	MED14	3	0.049869619	0.132598515	0.376094857	0.706846377	0.033282454	5.467300319	63.41887434	0.666666667	CRA004775;PRJNA827465;Yang2025_Silkie_Wencha	0.909477892
ENSGALG00010029319	BRIP1	3	-0.046778775	0.124515819	-0.375685398	0.707150795	0.0222736	4.186647437	52.22907995	0.666666667	CRA004775;PRJNA827465;Yang2025_Silkie_Wencha	0.909477892
ENSGALG00010020724	TACR1	3	0.046729777	0.124396525	0.375651789	0.707175784	0	1.264342687	0	0.666666667	CRA004775;PRJNA827465;Yang2025_Silkie_Wencha	0.909477892
ENSGALG00010013627	MYCBP	3	-0.037085056	0.098721548	-0.375653106	0.707174805	0	0.416520267	0	1	CRA004775;PRJNA827465;Yang2025_Silkie_Wencha	0.909477892
ENSGALG00010002462	RRM2	3	-0.035592561	0.094502897	-0.376629314	0.7064491	0	1.195386626	0	0.666666667	CRA004775;PRJNA827465;Yang2025_Silkie_Wencha	0.909477892
ENSGALG00010001627	ARFIP2	3	0.035277891	0.093912136	0.375647838	0.707178722	0.016161971	5.499750596	63.63471461	0.666666667	CRA004775;PRJNA827465;Yang2025_Silkie_Wencha	0.909477892
ENSGALG00010004757	TRMT10A	3	0.028749387	0.076322864	0.376681181	0.70641055	0	1.188968491	0	0.666666667	CRA004775;PRJNA827465;Yang2025_Silkie_Wencha	0.909477892
ENSGALG00010012993	DOP1B	3	-0.025826711	0.068709253	-0.375884031	0.707003113	0	0.291102939	0	0.666666667	CRA004775;PRJNA827465;Yang2025_Silkie_Wencha	0.909477892
ENSGALG00010009560	RAB24	3	0.071053003	0.189298469	0.375349062	0.707400883	0.071681396	6.446070629	68.97334648	0.666666667	CRA004775;PRJNA827465;Yang2025_Silkie_Wencha	0.909598134
ENSGALG00010016947	CANX	3	0.050366772	0.134181706	0.375362438	0.707390936	0.044500805	21.56200867	90.72442633	0.666666667	CRA004775;PRJNA827465;Yang2025_Silkie_Wencha	0.909598134
ENSGALG00010029363	PIK3R6	3	0.069841811	0.186349751	0.374788861	0.707817499	0.063934054	6.722333494	70.24842636	0.666666667	CRA004775;PRJNA827465;Yang2025_Silkie_Wencha	0.90971108
ENSGALG00010014877	ENSGALG00010014877	3	-0.068256077	0.182156164	-0.374711868	0.707874765	0.076551847	9.005339203	77.79095318	0.666666667	CRA004775;PRJNA827465;Yang2025_Silkie_Wencha	0.90971108
ENSGALG00010002880	RASGEF1B	3	0.065227739	0.173891184	0.375106648	0.707581153	0.041877553	3.769767839	46.94633502	0.666666667	CRA004775;PRJNA827465;Yang2025_Silkie_Wencha	0.90971108
ENSGALG00010018455	RBM20	3	-0.064173922	0.171168851	-0.374915889	0.707723022	0.045557626	4.671616605	57.18826759	0.666666667	CRA004775;PRJNA827465;Yang2025_Silkie_Wencha	0.90971108
ENSGALG00010024178	N4BP1	3	-0.039812291	0.106214733	-0.374828328	0.707788145	0.019669039	5.233661467	61.78583554	0.666666667	CRA004775;PRJNA827465;Yang2025_Silkie_Wencha	0.90971108
ENSGALG00010004248	GRSF1	3	-0.037368944	0.099669812	-0.374927404	0.707714458	0.015932139	4.364387622	54.17455613	0.666666667	CRA004775;PRJNA827465;Yang2025_Silkie_Wencha	0.90971108
ENSGALG00010026893	FKBP5	3	0.175086682	0.467439219	0.374565664	0.707983513	0.53024113	21.49789469	90.69676343	0.666666667	CRA004775;PRJNA827465;Yang2025_Silkie_Wencha	0.909768144
ENSGALG00010001242	ENSGALG00010001242	2	-0.188609074	0.504790087	-0.373638626	0.708673193	0	0.351884122	0	0.5	PRJNA827465;Yang2025_Silkie_Wenchang	0.91004317
ENSGALG00010011902	TP53I3	3	0.1535484	0.410978567	0.373616564	0.708689609	0.416758257	12.88868987	84.48251901	0.666666667	CRA004775;PRJNA827465;Yang2025_Silkie_Wencha	0.91004317
ENSGALG00010001915	EEF1AKMT2	3	0.132402047	0.35410954	0.373901383	0.708477688	0.340152541	23.52356048	91.4978857	0.666666667	CRA004775;PRJNA827465;Yang2025_Silkie_Wencha	0.91004317
ENSGALG00010027982	ENTPD2	3	-0.090724304	0.242896797	-0.373509677	0.708769144	0.11648548	7.051616026	71.63770698	0.666666667	CRA004775;PRJNA827465;Yang2025_Silkie_Wencha	0.91004317
ENSGALG00010022205	GALNT18	3	-0.057254698	0.153292623	-0.373499369	0.708776815	0.047349924	7.508538022	73.36365622	0.666666667	CRA004775;PRJNA827465;Yang2025_Silkie_Wencha	0.91004317
ENSGALG00010015084	FOXO6	3	-0.05644755	0.150895857	-0.374082836	0.708342688	0.033763942	4.249202234	52.93234142	0.666666667	CRA004775;PRJNA827465;Yang2025_Silkie_Wencha	0.91004317
ENSGALG00010000287	IQCD	3	-0.054172808	0.144909233	-0.37383959	0.708523663	0	0.518786597	0	0.666666667	CRA004775;PRJNA827465;Yang2025_Silkie_Wencha	0.91004317
ENSGALG00010015855	NIPAL3	3	0.045488182	0.121641145	0.373953916	0.708438603	0.028774657	7.025059466	71.5304901	0.666666667	CRA004775;PRJNA827465;Yang2025_Silkie_Wencha	0.91004317
ENSGALG00010027903	NDUFA8	3	-0.028758192	0.07686489	-0.374139508	0.708300526	0	0.042431679	0	0.666666667	CRA004775;PRJNA827465;Yang2025_Silkie_Wencha	0.91004317
ENSGALG00010028555	ENSGALG00010028555	2	-0.707402742	1.895482228	-0.373204629	0.708996151	4.601354334	2.746286695	63.58719569	0.5	PRJNA827465;Yang2025_Silkie_Wenchang	0.910046032
ENSGALG00010020813	NPY4R	3	0.088910013	0.238268564	0.373150412	0.709036501	0	1.331161963	0	0.666666667	CRA004775;PRJNA827465;Yang2025_Silkie_Wencha	0.910046032
ENSGALG00010015716	MGAT4A	3	-0.049551149	0.13276775	-0.373216754	0.708987128	0.022184396	3.406328766	41.28576138	0.666666667	CRA004775;PRJNA827465;Yang2025_Silkie_Wencha	0.910046032
ENSGALG00010001462	DHX36	3	0.038880734	0.104183909	0.373193274	0.709004602	0.02390498	7.905585949	74.70143247	0.666666667	CRA004775;PRJNA827465;Yang2025_Silkie_Wencha	0.910046032
ENSGALG00010008144	NTPCR	3	-0.039943429	0.107080894	-0.373021066	0.709132766	0	1.174186087	0	0.666666667	CRA004775;PRJNA827465;Yang2025_Silkie_Wencha	0.910086972
ENSGALG00010013343	SHISAL1	2	0.177822192	0.479369042	0.370950514	0.71067439	0.360287521	4.41839177	77.36733065	0.5	PRJNA827465;Yang2025_Silkie_Wenchang	0.910138998
ENSGALG00010028057	APC2	3	-0.167973164	0.45140514	-0.372111767	0.709809636	0.304796765	3.977588009	49.71827159	0.666666667	CRA004775;PRJNA827465;Yang2025_Silkie_Wencha	0.910138998
ENSGALG00010017699	RNF19B	3	0.107189143	0.288494693	0.371546323	0.71023066	0.207854571	13.2601582	84.91722368	0.666666667	CRA004775;PRJNA827465;Yang2025_Silkie_Wencha	0.910138998
ENSGALG00010011789	TXNRD1	3	0.084039048	0.226461028	0.371097177	0.710565154	0.103390263	6.818106472	70.66634251	0.666666667	CRA004775;PRJNA827465;Yang2025_Silkie_Wencha	0.910138998
ENSGALG00010013531	ARSI	3	0.067381196	0.181648487	0.370942787	0.710680145	0.042546926	3.647878637	45.17361462	0.666666667	CRA004775;PRJNA827465;Yang2025_Silkie_Wencha	0.910138998
ENSGALG00010013967	MLLT3	3	0.06507181	0.174686386	0.372506478	0.70951579	0.050861682	4.65374944	57.02390028	0.666666667	CRA004775;PRJNA827465;Yang2025_Silkie_Wencha	0.910138998
ENSGALG00010029682	CDR2L	3	0.060522605	0.162918229	0.371490688	0.71027209	0.054753617	9.358989901	78.63017247	0.666666667	CRA004775;PRJNA827465;Yang2025_Silkie_Wencha	0.910138998
ENSGALG00010027881	ENSGALG00010027881	3	-0.059074796	0.159297001	-0.370846881	0.710751581	0	1.751260657	0	0.666666667	CRA004775;PRJNA827465;Yang2025_Silkie_Wencha	0.910138998
ENSGALG00010016946	TAF1	3	0.058050302	0.156515024	0.370892843	0.710717346	0.059216459	12.42805442	83.90737655	0.666666667	CRA004775;PRJNA827465;Yang2025_Silkie_Wencha	0.910138998
ENSGALG00010002982	HAND2	3	-0.056732161	0.152463044	-0.372104346	0.70981516	0	1.516363329	0	0.666666667	CRA004775;PRJNA827465;Yang2025_Silkie_Wencha	0.910138998
ENSGALG00010007007	ACSL1	3	0.055373262	0.149080219	0.371432654	0.710315308	0	0.644332438	0	0.666666667	CRA004775;PRJNA827465;Yang2025_Silkie_Wencha	0.910138998
ENSGALG00010028414	KBTBD12	3	0.053941698	0.14531957	0.371193627	0.71049332	0	1.909770505	0	0.666666667	CRA004775;PRJNA827465;Yang2025_Silkie_Wencha	0.910138998
ENSGALG00010013794	GTF3C6	3	-0.050537245	0.136290524	-0.370805273	0.710782574	0.02895929	4.494193249	55.49813083	0.666666667	CRA004775;PRJNA827465;Yang2025_Silkie_Wencha	0.910138998
ENSGALG00010025554	DIP2C	3	0.045196329	0.121748655	0.371226514	0.710468827	0.024849701	4.932166973	59.44987242	0.666666667	CRA004775;PRJNA827465;Yang2025_Silkie_Wencha	0.910138998
ENSGALG00010007960	TSNAX	3	-0.039952951	0.107179558	-0.372766524	0.70932222	0.023247387	7.802506822	74.36721241	0.666666667	CRA004775;PRJNA827465;Yang2025_Silkie_Wencha	0.910138998
ENSGALG00010012944	NCK1	3	-0.039599716	0.106206314	-0.372856515	0.709255238	0.024350183	7.921776136	74.7531366	0.666666667	CRA004775;PRJNA827465;Yang2025_Silkie_Wencha	0.910138998
ENSGALG00010001507	METTL4	3	-0.032835903	0.088257779	-0.372045421	0.709859032	0	1.321771034	0	0.666666667	CRA004775;PRJNA827465;Yang2025_Silkie_Wencha	0.910138998
ENSGALG00010008399	INO80C	3	0.032110711	0.086367545	0.371791407	0.710048162	0.008613604	3.111785295	35.72821355	0.666666667	CRA004775;PRJNA827465;Yang2025_Silkie_Wencha	0.910138998
ENSGALG00010012358	STK10	3	-0.030904483	0.083161285	-0.371621037	0.710175024	0	0.937547542	0	1	CRA004775;PRJNA827465;Yang2025_Silkie_Wencha	0.910138998
ENSGALG00010003844	TMEM18	3	-0.02779922	0.074626046	-0.372513638	0.709510459	0	0.428784143	0	0.666666667	CRA004775;PRJNA827465;Yang2025_Silkie_Wencha	0.910138998
ENSGALG00010001132	SELENOT	3	0.023583221	0.063326967	0.372404088	0.709592011	0.00162878	2.204079873	9.259186803	0.666666667	CRA004775;PRJNA827465;Yang2025_Silkie_Wencha	0.910138998
ENSGALG00010006281	TRMT11	3	0.023274138	0.062665405	0.371403304	0.710337165	0	1.015699238	0	0.666666667	CRA004775;PRJNA827465;Yang2025_Silkie_Wencha	0.910138998

ENSGALG00010018504	SLCO3A1	3	-0.022166805	0.059776563	-0.370827699	0.710765869	0	1.329962542	0	1	CRA004775;PRJNA827465;Yang2025_Silkie_Wencha	0.910138998
ENSGALG00010029357	ARHGDIA	3	-0.019498951	0.052364132	-0.372372276	0.709615693	0.001092604	2.253849089	11.26291419	0.666666667	CRA004775;PRJNA827465;Yang2025_Silkie_Wencha	0.910138998
ENSGALG00010027801	MBD3	3	-0.015906277	0.042856275	-0.371153973	0.710522853	0	1.645591161	0	0.666666667	CRA004775;PRJNA827465;Yang2025_Silkie_Wencha	0.910138998
ENSGALG00010015090	GZMA	2	-0.183734187	0.4957649	-0.370607493	0.7109299	0	0.127973168	0	1	CRA004775;PRJNA827465	0.910238232
ENSGALG00010012101	ENSGALG00010012101	3	0.018953296	0.051152074	0.370528389	0.710988828	0	0.672114749	0	0.666666667	CRA004775;PRJNA827465;Yang2025_Silkie_Wencha	0.910238232
ENSGALG00010022718	C12orf43	3	-0.074346036	0.200718786	-0.370398994	0.711085223	0.083092947	6.660680783	69.97303932	0.666666667	CRA004775;PRJNA827465;Yang2025_Silkie_Wencha	0.910279218
ENSGALG00010012712	EEF1E1	3	-0.072078351	0.194711713	-0.370179841	0.711248496	0.066328694	4.920089081	59.3503295	0.666666667	CRA004775;PRJNA827465;Yang2025_Silkie_Wencha	0.910373335
ENSGALG00010010077	CCT8	3	0.021899886	0.059168496	0.370127465	0.711287519	0	0.556056137	0	0.666666667	CRA004775;PRJNA827465;Yang2025_Silkie_Wencha	0.910373335
ENSGALG00010018623	ARRDC4	3	0.038114222	0.103015966	0.369983635	0.711394684	0.01054781	2.817267427	29.0092243	0.666666667	CRA004775;PRJNA827465;Yang2025_Silkie_Wencha	0.910428081
ENSGALG00010007168	MINA	3	0.069908981	0.189162305	0.369571418	0.71170185	0.084520312	10.98041827	81.7857576	0.666666667	CRA004775;PRJNA827465;Yang2025_Silkie_Wencha	0.910491537
ENSGALG00010020743	PURB	3	0.059840732	0.161846103	0.369738483	0.711577356	0.043459686	4.580425786	56.33593702	0.666666667	CRA004775;PRJNA827465;Yang2025_Silkie_Wencha	0.910491537
ENSGALG00010012995	SERINC2	3	0.058386166	0.157898198	0.369770948	0.711553163	0.040133993	4.814681633	58.46038944	0.666666667	CRA004775;PRJNA827465;Yang2025_Silkie_Wencha	0.910491537
ENSGALG00010027884	SF3A2	3	-0.033018097	0.08933794	-0.369586503	0.711690609	0.010173164	3.240340512	38.27809168	0.666666667	CRA004775;PRJNA827465;Yang2025_Silkie_Wencha	0.910491537
ENSGALG00010006701	LINGO2	2	0.360171132	0.975084815	0.369374157	0.711848858	1.617820165	6.56588346	84.76975709	0.5	PRJNA827465;Yang2025_Silkie_Wenchang	0.910597213
ENSGALG00010016644	NMRAL1	3	0.047014239	0.127397683	0.369035273	0.712101433	0	0.082264312	0	0.666666667	CRA004775;PRJNA827465;Yang2025_Silkie_Wencha	0.910837902
ENSGALG00010011503	NCF2	3	0.151556831	0.411266424	0.368512531	0.712491103	0.251349874	4.033223157	50.41186857	0.666666667	CRA004775;PRJNA827465;Yang2025_Silkie_Wencha	0.911091297
ENSGALG00010018562	ENSGALG00010018562	3	0.096010231	0.260499301	0.368562336	0.712453973	0.059705457	2.715418012	26.34651493	0.666666667	CRA004775;PRJNA827465;Yang2025_Silkie_Wencha	0.911091297
ENSGALG00010005535	AP4S1	3	0.038290493	0.103906201	0.368510182	0.712492854	0.006152823	2.384019371	16.10806422	0.666666667	CRA004775;PRJNA827465;Yang2025_Silkie_Wencha	0.911091297
ENSGALG00010029254	MMD	3	-0.084316402	0.229017565	-0.368165657	0.712749716	0.137483323	27.34761638	92.68674837	0.666666667	CRA004775;PRJNA827465;Yang2025_Silkie_Wencha	0.911172535
ENSGALG00010001378	POLA2	3	0.080923557	0.219761133	0.368234162	0.712698639	0.119210893	18.76217992	89.34025786	0.666666667	CRA004775;PRJNA827465;Yang2025_Silkie_Wencha	0.911172535
ENSGALG00010025846	GRIK4	3	-0.058815855	0.159703342	-0.368281931	0.712663024	0	0.116832041	0	0.666666667	CRA004775;PRJNA827465;Yang2025_Silkie_Wencha	0.911172535
ENSGALG00010011144	ATF7IP	3	0.067692438	0.184018232	0.367857235	0.712979689	0.060963691	4.881123371	59.02582565	0.666666667	CRA004775;PRJNA827465;Yang2025_Silkie_Wencha	0.911219364
ENSGALG00010029396	WIPI1	3	0.019279269	0.052399867	0.367925912	0.712928479	0.001288834	2.167154314	7.713078528	0.666666667	CRA004775;PRJNA827465;Yang2025_Silkie_Wencha	0.911219364
ENSGALG00010024203	SPOP	3	-0.015635321	0.042500895	-0.367882158	0.712961105	0	1.228672285	0	0.666666667	CRA004775;PRJNA827465;Yang2025_Silkie_Wencha	0.911219364
ENSGALG00010008207	CAMK4	3	0.043549257	0.118475939	0.367578912	0.713187242	0	0.058647419	0	0.666666667	CRA004775;PRJNA827465;Yang2025_Silkie_Wencha	0.911319874
ENSGALG00010015813	ENSGALG00010015813	3	-0.022521538	0.061258254	-0.367649038	0.713134944	0	0.721571659	0	0.666666667	CRA004775;PRJNA827465;Yang2025_Silkie_Wencha	0.911319874
ENSGALG00010008366	RPRD1A	3	0.071064299	0.193592054	0.367082728	0.71355731	0.08874268	12.37135628	83.83362377	0.666666667	CRA004775;PRJNA827465;Yang2025_Silkie_Wencha	0.911425526
ENSGALG00010005763	AKAP6	3	-0.055303037	0.150628883	-0.367147629	0.713508902	0.030563379	3.780009732	47.09008331	0.666666667	CRA004775;PRJNA827465;Yang2025_Silkie_Wencha	0.911425526
ENSGALG00010019194	ENSGALG00010019194	3	-0.054701854	0.149051502	-0.366999679	0.713619257	0.00956611	2.251888222	11.18564498	0.666666667	CRA004775;PRJNA827465;Yang2025_Silkie_Wencha	0.911425526
ENSGALG00010022539	LYSMD2	3	-0.047283239	0.128854898	-0.366949491	0.713656694	0.026517074	4.250628221	52.94813153	0.666666667	CRA004775;PRJNA827465;Yang2025_Silkie_Wencha	0.911425526
ENSGALG00010001222	ZNF800	3	0.026887005	0.073233821	0.367139182	0.713515202	0	0.492117847	0	0.666666667	CRA004775;PRJNA827465;Yang2025_Silkie_Wencha	0.911425526
ENSGALG00010004309	MRPS28	3	0.024815561	0.067575527	0.367227037	0.713449673	0	0.828369461	0	0.666666667	CRA004775;PRJNA827465;Yang2025_Silkie_Wencha	0.911425526
ENSGALG00010026666	ENSGALG00010026666	2	0.203032696	0.553525342	0.366799278	0.713768746	0.542467616	8.597755605	88.36905762	0.5	PRJNA827465;Yang2025_Silkie_Wenchang	0.911486299
ENSGALG00010026954	MFSD4A	3	-0.072230981	0.197052875	-0.366556343	0.713949978	0.096475377	19.02238307	89.48607021	0.666666667	CRA004775;PRJNA827465;Yang2025_Silkie_Wencha	0.911635396
ENSGALG00010001987	KCTD14	2	-0.132539115	0.361898464	-0.366232874	0.714191314	0	0.214009167	0	0.5	CRA004775;PRJNA827465	0.91169655
ENSGALG00010019034	CASP7	3	-0.117296178	0.32020642	-0.36631426	0.71413059	0.256379778	13.45358085	85.13406934	0.666666667	CRA004775;PRJNA827465;Yang2025_Silkie_Wencha	0.91169655
ENSGALG00010002972	ME1	3	0.033326063	0.090970271	0.36634015	0.714111274	0	1.151584373	0	0.666666667	CRA004775;PRJNA827465;Yang2025_Silkie_Wencha	0.91169655
ENSGALG00010012147	ARFGAP3	3	0.03658388	0.099993798	0.365861495	0.71446843	0.01510024	4.297501307	53.46132887	0.666666667	CRA004775;PRJNA827465;Yang2025_Silkie_Wencha	0.911967963
ENSGALG00010029514	MRPS23	3	-0.070815858	0.193617617	-0.365751105	0.714550808	0.089100495	12.72024531	84.27703278	0.666666667	CRA004775;PRJNA827465;Yang2025_Silkie_Wencha	0.911990781
ENSGALG00010009398	WTIP	3	0.067833758	0.185623534	0.365437272	0.714785023	0.080384265	9.678607081	79.33586948	0.666666667	CRA004775;PRJNA827465;Yang2025_Silkie_Wencha	0.91212504
ENSGALG00010001935	ABRAXAS2	3	0.034606617	0.094685403	0.365490521	0.714745282	0.008584541	2.75202715	27.32629836	0.666666667	CRA004775;PRJNA827465;Yang2025_Silkie_Wencha	0.91212504
ENSGALG00010019801	FIBIN	3	0.048051694	0.131552783	0.365265507	0.714913224	0	0.202832635	0	0.666666667	CRA004775;PRJNA827465;Yang2025_Silkie_Wencha	0.912184627
ENSGALG00010015436	SLC30A9	3	-0.019053539	0.052172628	-0.365201834	0.714960749	0.001264848	2.266741514	11.7676194	0.666666667	CRA004775;PRJNA827465;Yang2025_Silkie_Wencha	0.912184627
ENSGALG00010024180	HINFP	3	0.029614649	0.081264297	0.36442387	0.715541514	0.002232545	2.206439158	9.356213463	0.666666667	CRA004775;PRJNA827465;Yang2025_Silkie_Wencha	0.912760868
ENSGALG00010013805	HSPA4	3	0.022345408	0.061310312	0.364464112	0.715511468	0.005085041	3.665570318	45.43823125	0.666666667	CRA004775;PRJNA827465;Yang2025_Silkie_Wencha	0.912760868
ENSGALG00010003749	MHCY15	3	-0.295672598	0.812680165	-0.363824061	0.715989394	1.145566814	4.732186171	57.7362359	0.666666667	CRA004775;PRJNA827465;Yang2025_Silkie_Wencha	0.912773397
ENSGALG00010019498	ENSGALG00010019498	3	0.189267037	0.520094512	0.363908928	0.715926017	0.664806994	16.49075145	87.87198991	0.666666667	CRA004775;PRJNA827465;Yang2025_Silkie_Wencha	0.912773397
ENSGALG00010018441	CRIPT	3	-0.113434957	0.311644782	-0.363987988	0.715866979	0.265963404	30.32064911	93.40383515	0.666666667	CRA004775;PRJNA827465;Yang2025_Silkie_Wencha	0.912773397
ENSGALG00010004666	C6orf120	3	0.038973306	0.107101335	0.363891879	0.715938749	0.021792179	5.755131963	65.24840763	0.666666667	CRA004775;PRJNA827465;Yang2025_Silkie_Wencha	0.912773397
ENSGALG00010011321	MAP2	3	0.035065956	0.09634861	0.363948753	0.715896278	0	0.304775935	0	1	CRA004775;PRJNA827465;Yang2025_Silkie_Wencha	0.912773397
ENSGALG00010021251	RNF216	3	-0.027603251	0.075783265	-0.364239398	0.71567925	0.007041597	3.148395349	36.47557634	0.666666667	CRA004775;PRJNA827465;Yang2025_Silkie_Wencha	0.912773397
ENSGALG00010015788	TRPC1	3	0.022805462	0.062685859	0.363805526	0.716003236	0	0.179881301	0	1	CRA004775;PRJNA827465;Yang2025_Silkie_Wencha	0.912773397
ENSGALG00010003223	AREG	2	0.196827318	0.542011089	0.363142603	0.716498361	0	0.554148433	0	0.5	PRJNA827465;Yang2025_Silkie_Wenchang	0.912859184
ENSGALG00010018358	FBXL16	2	0.127371165	0.350374572	0.363528563	0.71621008	0.075231271	1.427282196	29.93677054	0.5	PRJNA827465;Yang2025_Silkie_Wenchang	0.912859184
ENSGALG00010001836	TWSG1	3	-0.079695823	0.219465603	-0.363135828	0.716503422	0.123454192	21.6673629	90.76952738	0.666666667	CRA004775;PRJNA827465;Yang2025_Silkie_Wencha	0.912859184
ENSGALG00010004323	TRDMT1	3	-0.073879942	0.203486005	-0.363071368	0.716551572	0.091690627	9.792663177	79.57654661	0.666666667	CRA004775;PRJNA827465;Yang2025_Silkie_Wencha	0.912859184
ENSGALG00010000601	ENSGALG00010000601	3	0.071388099	0.196542059	0.363220468	0.716440199	0	0.200046476	0	0.666666667	CRA004775;PRJNA827465;Yang2025_Silkie_Wencha	0.912859184
ENSGALG00010002478	PPME1	3	-0.050422587	0.138929132	-0.362937467	0.716651598	0.046150658	11.10208969	81.98537342	0.666666667	CRA004775;PRJNA827465;Yang2025_Silkie_Wencha	0.912859184
ENSGALG00010026334	ENSGALG00010026334	3	-0.031654305	0.08718696	-0.363062379	0.716558287	0.007992519	3.070648341	34.86717534	0.666666667	CRA004775;PRJNA827465;Yang2025_Silkie_Wencha	0.912859184
ENSGALG00010029895	FASN	3	-0.030690945	0.084416794	-0.363564442	0.716183283	0.002429947	2.183070647	8.385924058	0.666666667	CRA004775;PRJNA827465;Yang2025_Silkie_Wencha	0.912859184
ENSGALG00010005119	MIB1	3	-0.023770058	0.065492433	-0.362943583	0.716647029	0.004627983	2.939554354	31.96247598	0.666666667	CRA004775;PRJNA827465;Yang2025_Silkie_Wencha	0.912859184
ENSGALG00010022661	ENSGALG00010022661	3	0.096025627	0.264644859	0.362847128	0.716719084	0	0.416446592	0	0.666666667	CRA004775;PRJNA827465;Yang2025_Silkie_Wencha	0.912862907
ENSGALG00010019898	NUDT1	3	-0.070884603	0.195446008	-0.362681253	0.716843006	0.074710142	6.687421618	70.09310741	0.666666667	CRA004775;PRJNA827465;Yang2025_Silkie_Wencha	0.912938503
ENSGALG00010028508	ENSGALG00010028508	2	-0.902408144	2.490364729	-0.362359832	0.717083153	11.34143281	11.59673873	91.37688601	0.5	CRA004775;PRJNA827465	0.913162091
ENSGALG00010020913	TMEM132B	2	-0.049176896	0.13585058	-0.361992536	0.717357608	0	0.079930958	0	0.5	PRJNA827465;Yang2025_Silkie_Wenchang	0.913264832
ENSGALG00010025531	ENSGALG00010025531	3	0.03287198	0.090802113	0.362017786	0.717338739	0.009623516	3.192966574	37.3623258	0.666666667	CRA004775;PRJNA827465;Yang2025_Silkie_Wencha	0.913264832
ENSGALG00010015709	HNRNPA0	3	0.014715671	0.040641346	0.362086208	0.71728761	0	1.881425276	0	0.666666667	CRA004775;PRJNA827465;Yang2025_Silkie_Wencha	0.913264832
ENSGALG00010007812	POMK	3	-0.05315441	0.14697321	-0.361660539	0.717605719	0.040395423	6.176808575	67.62081946	0.666666667	CRA004775;PRJNA827465;Yang2025_Silkie_Wencha	0.913333987
ENSGALG00010015037	CYFIP2	3	0.046902114	0.129634041	0.361803992	0.717498509	0	1.134887995	0	0.666666667	CRA004775;PRJNA827465;Yang2025_Silkie_Wencha	0.913333987
ENSGALG00010011837	VLDLR	3	0.029137374	0.080549646	0.361731866	0.717552412	0	1.958962236	0	0.666666667	CRA004775;PRJNA827465;Yang2025_Silkie_Wencha	0.913333987
ENSGALG00010004188	BMI1	3	0.027827611	0.076964497	0.361564257	0.717677679	0.007490162	3.278085356	38.98877598	0.666666667	CRA004775;PRJNA827465;Yang2025_Silkie_Wencha	0.913343357
ENSGALG00010024570	TPM1	3	0.065820527	0.182372191	0.36091318	0.71816435	0.055703551	5.68686076	64.83121208	0.666666667	CRA004775;PRJNA827465;Yang2025_Silkie_Wencha	0.913515125
ENSGALG00010014324	COL4A2	3	-0.062596209	0.173461528	-0.360865084	0.718200306	0.058655848	7.687405653	73.98342054	0.666666667	CRA004775;PRJNA827465;Yang2025_Silkie_Wencha	0.913515125
ENSGALG00010023078	TAGLN	3	0.054823586	0.15174309	0.361292143	0.717881067	0.001929529	2.050636064	2.469285737	0.666666667	CRA004775;PRJNA827465;Yang2025_Silkie_Wencha	0.913515125
ENSGALG00010024242	KMT2A	3	-0.045873756	0.127113721	-0.360887525	0.71818353	0.020002054	3.116650639	35.82854701	0.666666667	CRA004775;PRJNA827465;Yang2025_Silkie_Wencha	0.913515125
ENSGALG00010016520	CHMP1B	3	0.033849038	0.093725438	0.361151026	0.71798655	0.014215374	4.184945044	52.20964722	0.666666667	CRA004775;PRJNA827465;Yang2025_Silkie_Wencha	0.913515125
ENSGALG00010002160	CAPN5	3	0.023368771	0.064735664	0.360987583	0.718108729	0	0.742362061	0	1	CRA004775;PRJNA827465;Yang2025_Silkie_Wencha	0.913515125
ENSGALG00010005481	ENSGALG00010005481	3	-0.302145986	0.837547727	-0.360750768	0.718285769	1.479084478	7.215932781	72.28355556	0.666666667	CRA004775;PRJNA827465;Yang2025_Silkie_Wencha	0.913541647
ENSGALG00010003821	RUBCNL	3	-0.05371472	0.149100604	-0.360258234	0.718654403	0.033020551	3.909757268	48.84592923	0.666666667	CRA004775;PRJNA827465;Yang2025_Silkie_Wencha	0.913927804
ENSGALG00010020568	PLA2G4F	3	0.113426068	0.315137619	0.359925508	0.718902841	0	1.959458776	0	0.666666667	CRA004775;PRJNA827465;Yang2025_Silkie_Wencha	0.913976395
ENSGALG00010021509	DDIT4	3	0.088376684	0.245514347	0.359965456	0.718872967	0.115835596	6.371942032	68.61239494	0.666666667	CRA004775;PRJNA827465;Yang2025_Silkie_Wencha	0.913976395
ENSGALG00010009328	ENSGALG00010009328	3	-0.037236609	0.103496507	-0.359786139	0.719007069	0	1.883523557	0	0.666666667	CRA004775;PRJNA827465;Yang2025_Silkie_Wencha	0.913976395
ENSGALG00010024864	MPHOSPH9	3	-0.036240221	0.100730254	-0.359774934	0.71901545	0.010086277	2.864021834	30.16813013	0.666666667	CRA004775;PRJNA827465;Yang2025_Silkie_Wencha	0.913976395
ENSGALG00010012693	UCHL5	3	-0.0275483	0.076528923	-0.359972398	0.718867775	0.00697957	3.113455223	35.76268625	0.666666667	CRA004775;PRJNA827465;Yang2025_Silkie_Wencha	0.913976395
ENSGALG00010027738	ENSGALG00010027738	3	0.169070841	0.470561311	0.359296094	0.719373596	0.598558364	20.31611408	90.15559771	0.666666667	CRA004775;PRJNA827465;Yang2025_Silkie_Wencha	0.914102928
ENSGALG00010013749	NELL2	3	0.086239133	0.239869557	0.359525129	0.719202283	0.085058371	4.016021966	50.19947558	0.666666667	CRA004775;PRJNA827465;Yang2025_Silkie_Wencha	0.914102928
ENSGALG00010011094	CSGALNACT2	3	-0.042359622	0.117862799	-0.359397725	0.719297576	0.02586718	6.455409309	69.0118231	0.666666667	CRA004775;PRJNA827465;Yang2025_Silkie_Wencha	0.914102928
ENSGALG00010000843	DIS3	3	0.020441494	0.056882128	0.359365848	0.71932142	0.001201915	2.227748403	10.22325512	0.666666667	CRA004775;PRJNA827465;Yang2025_Silkie_Wencha	0.914102928
ENSGALG00010012187	ENSGALG00010012187	3	-0.296849617	0.826699174	-0.359078159	0.71953662	1.4594191	7.586721131	73.63815059	0.666666667	CRA004775;PRJNA827465;Yang2025_Silkie_Wencha	0.91414577
ENSGALG00010006961	ZCCHC14	3	0.042543026	0.118469875	0.359104166	0.719517165	0.015062225	2.731181878	26.77162893	0.666666667	CRA004775;PRJNA827465;Yang2025_Silkie_Wencha	0.91414577
ENSGALG00010023007	LMO4	3	0.041969911	0.116916972	0.358971911	0.719616102	0.021169932	4.355798429	54.084193	0.666666667	CRA004775;PRJNA827465;Yang2025_Silkie_Wencha	0.914164607
ENSGALG00010025286	ITGA3	3	0.108953166	0.30388024	0.358539819	0.719939374	0.248905168	24.67288841	91.89393651	0.666666667	CRA004775;PRJNA827465;Yang2025_Silkie_Wencha	0.91449311
ENSGALG00010000294	DUSP21	3	-0.069514399	0.193985916	-0.35834766	0.720083155	0	1.462803016	0	0.666666667	CRA004775;PRJNA827465;Yang2025_Silkie_Wencha	0.91459358
ENSGALG00010016950	ENSGALG00010016950	3	-0.123645248	0.345250683	-0.358131799	0.720244683	0	0.370072013	0	0.666666667	CRA004775;PRJNA827465;Yang2025_Silkie_Wencha	0.914683838
ENSGALG00010009501	HGH1	3	0.056851334	0.158805562	0.357993344	0.720348295	0.037412304	3.717055081	46.19396386	0.666666667	CRA004775;PRJNA827465;Yang2025_Silkie_Wencha	0.914683838
ENSGALG00010013095	ADSL	3	-0.031067255	0.086768093	-0.358049297	0.720306422	0	0.637225396	0	0.666666667	CRA004775;PRJNA827465;Yang2025_Silkie_Wencha	0.914683838
ENSGALG00010027571	RBM10	3	0.068705794	0.192400717	0.357097391	0.721018898	0.095121218	15.68915232	87.25233869	0.666666667	CRA004775;PRJNA827465;Yang2025_Silkie_Wencha	0.914714489
ENSGALG00010014350	PTGER4	3	0.065844894	0.184023473	0.357807041	0.72048772	0.0533196	4.242667705	52.85984811	0.666666667	CRA004775;PRJNA827465;Yang2025_Silkie_Wencha	0.914714489
ENSGALG00010012234	C1GALT1C1	3	-0.06398446	0.178880092	-0.3576787	0.720583775	0.08869776	26.84024683	92.5485037	0.666666667	CRA004775;PRJNA827465;Yang2025_Silkie_Wencha	0.914714489
ENSGALG00010022081	FAS	3	0.063007394	0.176399018	0.357186764	0.720951994	0.049035636	4.253044101	52.97485865	0.666666667	CRA004775;PRJNA827465;Yang2025_Silkie_Wencha	0.914714489
ENSGALG00010016362	NUP62	3	0.061401763	0.171882981	0.35723003	0.720919606	0.071963167	11.53323239	82.65880776	0.666666667	CRA004775;PRJNA827465;Yang2025_Silkie_Wencha	0.914714489
ENSGALG00010011808	SMARCA2	3	0.051247787	0.143391447	0.357397793	0.720794028	0.039050761	5.566935382	64.07359053	0.666666667	CRA004775;PRJNA827465;Yang2025_Silkie_Wencha	0.914714489
ENSGALG00010013042	SLC25A48	3	0.048633022	0.136142261	0.357221821	0.720925751	0	1.343262372	0	0.666666667	CRA004775;PRJNA827465;Yang2025_Silkie_Wencha	0.914714489
ENSGALG00010016514	GASK1A	3	0.043016922	0.120476093	0.357057746	0.721048576	0	0.918324552	0	0.666666667	CRA004775;PRJNA827465;Yang2025_Silkie_Wencha	0.914714489
ENSGALG00010003107	GNG11	3	-0.039543399	0.110566912	-0.357642251	0.720611055	0	1.853211152	0	0.666666667	CRA004775;PRJNA827465;Yang2025_Silkie_Wencha	0.914714489
ENSGALG00010027910	CHRNB2	3	-0.036928915	0.103277457	-0.357569947	0.720665172	0	1.340705837	0	0.666666667	CRA004775;PRJNA827465;Yang2025_Silkie_Wencha	0.914714489
ENSGALG00010014267	SCFD2	3	-0.034765937	0.097380759	-0.35701033	0.721084072	0.014936864	4.355435492	54.08036685	0.666666667	CRA004775;PRJNA827465;Yang2025_Silkie_Wencha	0.914714489
ENSGALG00010011817	KDSR	3	-0.094344233	0.26436761	-0.356867595	0.72119093	0.199938126	48.5455454	95.88015752	0.666666667	CRA004775;PRJNA827465;Yang2025_Silkie_Wencha	0.914767969
ENSGALG00010008166	RUNX2	3	0.255767138	0.717144316	0.356646678	0.721356328	1.194427677	8.998899681	77.77506061	0.666666667	CRA004775;PRJNA827465;Yang2025_Silkie_Wencha	0.914829716
ENSGALG00010016190	THNSL2	3	0.072808098	0.204155994	0.356629733	0.721369015	0.083969632	7.031233284	71.55548793	0.666666667	CRA004775;PRJNA827465;Yang2025_Silkie_Wencha	0.914829716
ENSGALG00010017632	AWAT1	3	0.161238146	0.452495058	0.356331316	0.721592459	0.445026455	7.45131904	73.1586783	0.666666667	CRA004775;PRJNA827465;Yang2025_Silkie_Wencha	0.914859931
ENSGALG00010015132	GCNT1	3	0.158310463	0.444376944	0.356252649	0.721651367	0.441503152	8.341982288	76.02488316	0.666666667	CRA004775;PRJNA827465;Yang2025_Silkie_Wencha	0.914859931
ENSGALG00010001982	DMD	3	0.09922973	0.278537827	0.356252258	0.721651659	0.172855094	11.04488075	81.89206343	0.666666667	CRA004775;PRJNA827465;Yang2025_Silkie_Wencha	0.914859931
ENSGALG00010021212	MYO18B	3	0.089181838	0.250229824	0.356399714	0.721541244	0.15684048	25.7209784	92.22424603	0.666666667	CRA004775;PRJNA827465;Yang2025_Silkie_Wencha	0.914859931
ENSGALG00010017378	ENSGALG00010017378	3	0.102559936	0.288376227	0.355646293	0.722105471	0	0.875020616	0	0.666666667	CRA004775;PRJNA827465;Yang2025_Silkie_Wencha	0.914962651
ENSGALG00010019027	GNLY	3	0.089940536	0.252654183	0.355982772	0.721853467	0.081126091	3.373890432	40.72125221	0.666666667	CRA004775;PRJNA827465;Yang2025_Silkie_Wencha	0.914962651
ENSGALG00010014257	NOP16	3	-0.067802304	0.190519613	-0.355880965	0.721929712	0.066239251	5.082739474	60.65114078	0.666666667	CRA004775;PRJNA827465;Yang2025_Silkie_Wencha	0.914962651
ENSGALG00010014047	HM13	3	-0.051673831	0.145248992	-0.355760339	0.722020053	0.053242216	14.11083752	85.82649685	0.666666667	CRA004775;PRJNA827465;Yang2025_Silkie_Wencha	0.914962651
ENSGALG00010027501	BAP1	3	-0.041074099	0.115498144	-0.355625615	0.722120958	0.028799354	7.724416044	74.10807511	0.666666667	CRA004775;PRJNA827465;Yang2025_Silkie_Wencha	0.914962651
ENSGALG00010017517	LIN9	3	-0.019418599	0.054603341	-0.355630235	0.722117498	0	1.129892325	0	0.666666667	CRA004775;PRJNA827465;Yang2025_Silkie_Wencha	0.914962651
ENSGALG00010027842	SLAMF8	3	0.094318531	0.265528077	0.355211142	0.722431419	0.123191205	4.958236739	59.66307973	0.666666667	CRA004775;PRJNA827465;Yang2025_Silkie_Wencha	0.915002305
ENSGALG00010020878	XYLT1	3	0.091476078	0.257426201	0.355348738	0.722328348	0.133654807	7.4320384	73.08948242	0.666666667	CRA004775;PRJNA827465;Yang2025_Silkie_Wencha	0.915002305
ENSGALG00010004230	TAF1B	3	-0.033702371	0.094895662	-0.355151859	0.722475828	0.008842456	2.829262928	29.31021079	0.666666667	CRA004775;PRJNA827465;Yang2025_Silkie_Wencha	0.915002305
ENSGALG00010011028	HMBOX1	3	0.031307721	0.088121958	0.355277185	0.722381946	0	0.959345142	0	0.666666667	CRA004775;PRJNA827465;Yang2025_Silkie_Wencha	0.915002305
ENSGALG00010014346	AFG1L	3	-0.028705159	0.080765784	-0.355412378	0.722280677	0	1.107553871	0	0.666666667	CRA004775;PRJNA827465;Yang2025_Silkie_Wencha	0.915002305
ENSGALG00010002612	JPH1	3	-0.050356044	0.141843904	-0.355010278	0.722581891	0.028022131	3.861171507	48.20224908	0.666666667	CRA004775;PRJNA827465;Yang2025_Silkie_Wencha	0.915054668
ENSGALG00010001168	DOK6	2	0.186106144	0.524890983	0.354561519	0.722918109	0.492802097	9.399654872	89.35678233	0.5	PRJNA827465;Yang2025_Silkie_Wenchang	0.91510171
ENSGALG00010024891	PKP1	3	0.110217223	0.31109432	0.354288767	0.723122485	0	0.603713725	0	0.666666667	CRA004775;PRJNA827465;Yang2025_Silkie_Wencha	0.91510171
ENSGALG00010027157	ENSGALG00010027157	3	-0.104119842	0.293561566	-0.354678042	0.722830803	0.21091238	11.1038623	81.98824926	0.666666667	CRA004775;PRJNA827465;Yang2025_Silkie_Wencha	0.91510171
ENSGALG00010022157	EPS8L2	3	0.054311512	0.153108701	0.354725184	0.722795482	0	0.211794774	0	0.666666667	CRA004775;PRJNA827465;Yang2025_Silkie_Wencha	0.91510171
ENSGALG00010005150	ALCAM	3	0.052233291	0.147439364	0.354269646	0.723136813	0.026326805	3.201716311	37.53350374	0.666666667	CRA004775;PRJNA827465;Yang2025_Silkie_Wencha	0.91510171
ENSGALG00010009910	ENSGALG00010009910	3	0.040034152	0.112895534	0.354612365	0.722880012	0	1.133258821	0	0.666666667	CRA004775;PRJNA827465;Yang2025_Silkie_Wencha	0.91510171
ENSGALG00010008098	ENSGALG00010008098	3	0.028706104	0.081010772	0.354349223	0.723077183	0	0.605449653	0	0.666666667	CRA004775;PRJNA827465;Yang2025_Silkie_Wencha	0.91510171
ENSGALG00010008317	CEP57	3	0.028020115	0.079045444	0.354481091	0.722978373	0.006586339	3.042398243	34.26238643	0.666666667	CRA004775;PRJNA827465;Yang2025_Silkie_Wencha	0.91510171
ENSGALG00010020108	RPAP1	3	-0.028663143	0.080952042	-0.354075606	0.723282223	0.008548406	3.551204224	43.68107623	0.666666667	CRA004775;PRJNA827465;Yang2025_Silkie_Wencha	0.915203808
ENSGALG00010007585	KCNG4	2	0.171250854	0.484229425	0.353656438	0.723596372	0	0.141669227	0	0.5	CRA004775;PRJNA827465	0.915273672
ENSGALG00010016131	SMYD1	3	0.098592166	0.278730775	0.353718266	0.723550032	0.164319906	18.64048425	89.270665	0.666666667	CRA004775;PRJNA827465;Yang2025_Silkie_Wencha	0.915273672
ENSGALG00010012829	RGS5	3	-0.038681305	0.10934597	-0.353751536	0.723525096	0	1.26322535	0	0.666666667	CRA004775;PRJNA827465;Yang2025_Silkie_Wencha	0.915273672
ENSGALG00010007660	FAM98A	3	0.019012928	0.053747327	0.353746488	0.72352888	0	0.003282139	0	1	CRA004775;PRJNA827465;Yang2025_Silkie_Wencha	0.915273672
ENSGALG00010010850	ENSGALG00010010850	3	-0.041450192	0.117240882	-0.353547254	0.723678209	0	0.926189386	0	0.666666667	CRA004775;PRJNA827465;Yang2025_Silkie_Wencha	0.915295304
ENSGALG00010027270	ENSGALG00010027270	3	0.079359695	0.22469477	0.353188884	0.723946841	0.045686555	2.828273251	29.28547484	0.666666667	CRA004775;PRJNA827465;Yang2025_Silkie_Wencha	0.915307554
ENSGALG00010029608	ENSGALG00010029608	3	0.073243882	0.207227892	0.353446062	0.723754059	0.081833969	5.827036949	65.67723841	0.666666667	CRA004775;PRJNA827465;Yang2025_Silkie_Wencha	0.915307554
ENSGALG00010017537	MANBA	3	0.042121178	0.119248629	0.353221484	0.723922403	0.014919623	2.919402985	31.49284254	0.666666667	CRA004775;PRJNA827465;Yang2025_Silkie_Wencha	0.915307554
ENSGALG00010027099	GNA11	3	-0.033081543	0.093625115	-0.353340476	0.723833205	0.014411631	4.387637741	54.41738544	0.666666667	CRA004775;PRJNA827465;Yang2025_Silkie_Wencha	0.915307554
ENSGALG00010005282	FLT1	3	0.039347918	0.111454378	0.353040577	0.724058021	0.015386736	3.450829695	42.04292368	0.666666667	CRA004775;PRJNA827465;Yang2025_Silkie_Wencha	0.915366269
ENSGALG00010016491	DNAJC8	3	0.049456809	0.14014011	0.352909736	0.724156111	0.037045566	5.306870082	62.31300241	0.666666667	CRA004775;PRJNA827465;Yang2025_Silkie_Wencha	0.915408427
ENSGALG00010015353	RELL1	3	-0.094885013	0.269101966	-0.352598735	0.724389286	0.201581311	45.97308853	95.64962881	0.666666667	CRA004775;PRJNA827465;Yang2025_Silkie_Wencha	0.915490936
ENSGALG00010015983	CNOT7	3	-0.073465695	0.208350411	-0.352606431	0.724383516	0.123542887	51.84222145	96.14214063	0.666666667	CRA004775;PRJNA827465;Yang2025_Silkie_Wencha	0.915490936
ENSGALG00010001858	ADAMTS20	3	0.070023653	0.198626552	0.352539237	0.724433898	0.065077061	5.161022546	61.24798948	0.666666667	CRA004775;PRJNA827465;Yang2025_Silkie_Wencha	0.915490936
ENSGALG00010022685	ATAD1	3	-0.036389505	0.103239303	-0.352477247	0.72448038	0.019449021	5.210239541	61.61404895	0.666666667	CRA004775;PRJNA827465;Yang2025_Silkie_Wencha	0.915490936
ENSGALG00010010417	OTOF	2	-0.638303618	1.813890009	-0.351897643	0.724915027	6.116859325	14.18100834	92.94831527	0.5	PRJNA827465;Yang2025_Silkie_Wenchang	0.915503178
ENSGALG00010026018	FOXL2	2	0.15495951	0.440761735	0.351572058	0.725159223	0.081419236	1.256260717	20.39868906	0.5	CRA004775;PRJNA827465	0.915503178
ENSGALG00010017004	CX3CR1	3	-0.124398165	0.353763952	-0.351641722	0.725106971	0.137877955	3.131545573	36.13377313	0.666666667	CRA004775;PRJNA827465;Yang2025_Silkie_Wencha	0.915503178
ENSGALG00010011331	PMCH	2	-0.092155272	0.262253278	-0.35139798	0.725289797	0	0.698540471	0	0.5	PRJNA827465;Yang2025_Silkie_Wenchang	0.915503178
ENSGALG00010000145	ENSGALG00010000145	3	-0.091209045	0.259601913	-0.351341959	0.72533182	0.150336552	11.5687673	82.71207339	0.666666667	CRA004775;PRJNA827465;Yang2025_Silkie_Wencha	0.915503178
ENSGALG00010021541	CYP27A1	3	-0.060942973	0.173443587	-0.351370574	0.725310356	0.02190953	2.532695134	21.03273809	0.666666667	CRA004775;PRJNA827465;Yang2025_Silkie_Wencha	0.915503178
ENSGALG00010018420	RHOQ	3	0.058946226	0.167752247	0.351388591	0.72529684	0.058537048	7.83623289	74.47753241	0.666666667	CRA004775;PRJNA827465;Yang2025_Silkie_Wencha	0.915503178
ENSGALG00010001347	MYOM1	3	-0.045830669	0.130290361	-0.351757936	0.725019807	0.029142317	6.739366584	70.32362055	0.666666667	CRA004775;PRJNA827465;Yang2025_Silkie_Wencha	0.915503178
ENSGALG00010018438	PPP2R2A	3	0.033817037	0.09622583	0.351434086	0.725262715	0.019086173	7.670297723	73.92539283	0.666666667	CRA004775;PRJNA827465;Yang2025_Silkie_Wencha	0.915503178
ENSGALG00010017496	PARP1	3	-0.03233479	0.091951095	-0.351652033	0.725099238	0.014205904	4.927263897	59.40952135	0.666666667	CRA004775;PRJNA827465;Yang2025_Silkie_Wencha	0.915503178
ENSGALG00010017375	BDH1A	3	0.026297494	0.074751999	0.351796529	0.724990862	0	1.077990514	0	0.666666667	CRA004775;PRJNA827465;Yang2025_Silkie_Wencha	0.915503178
ENSGALG00010016398	SEPTIN6	3	-0.025242583	0.071745229	-0.351836397	0.724960961	0.007337381	3.679020459	45.63770377	0.666666667	CRA004775;PRJNA827465;Yang2025_Silkie_Wencha	0.915503178
ENSGALG00010012877	SLC25A32	3	0.014999523	0.042609463	0.352023285	0.7248208	0	0.681400273	0	0.666666667	CRA004775;PRJNA827465;Yang2025_Silkie_Wencha	0.915503178
ENSGALG00010021646	RPUSD3	3	-0.095980291	0.273404706	-0.351055738	0.725546533	0.17025648	9.569837612	79.10100379	0.666666667	CRA004775;PRJNA827465;Yang2025_Silkie_Wencha	0.915610554
ENSGALG00010022494	FHIP2A	3	-0.080751081	0.230399249	-0.350483267	0.725976046	0.130350586	26.66135579	92.49850602	0.666666667	CRA004775;PRJNA827465;Yang2025_Silkie_Wencha	0.915610554
ENSGALG00010011908	ENSGALG00010011908	3	-0.078307785	0.223467714	-0.350421024	0.72602275	0.098059965	5.882492034	66.00080394	0.666666667	CRA004775;PRJNA827465;Yang2025_Silkie_Wencha	0.915610554
ENSGALG00010002433	GRHL1	3	-0.072048781	0.205287454	-0.350965341	0.72561435	0	0.027803304	0	1	CRA004775;PRJNA827465;Yang2025_Silkie_Wencha	0.915610554
ENSGALG00010017312	PTP4A2	3	0.071825763	0.205154018	0.35010654	0.726258743	0.088695111	8.835057136	77.36290814	0.666666667	CRA004775;PRJNA827465;Yang2025_Silkie_Wencha	0.915610554
ENSGALG00010002619	RAB6A	3	0.069748847	0.198968648	0.350551949	0.725924511	0.099245093	15.23236386	86.87006155	0.666666667	CRA004775;PRJNA827465;Yang2025_Silkie_Wencha	0.915610554
ENSGALG00010007429	C21orf91	3	0.054950916	0.156908823	0.350209214	0.726181692	0.046831692	5.930433463	66.27565232	0.666666667	CRA004775;PRJNA827465;Yang2025_Silkie_Wencha	0.915610554
ENSGALG00010014592	QNG1	3	0.042733671	0.121712713	0.351102771	0.725511249	0.01588286	3.092690673	35.33139225	0.666666667	CRA004775;PRJNA827465;Yang2025_Silkie_Wencha	0.915610554
ENSGALG00010005182	NEPRO	3	0.040376026	0.115306524	0.350162543	0.726216716	0.015840719	3.192711044	37.35731256	0.666666667	CRA004775;PRJNA827465;Yang2025_Silkie_Wencha	0.915610554
ENSGALG00010009137	IPMK	3	-0.036990439	0.105595818	-0.350302117	0.726111977	0.020019659	5.654340725	64.62894443	0.666666667	CRA004775;PRJNA827465;Yang2025_Silkie_Wencha	0.915610554
ENSGALG00010029303	SLC26A11	3	0.032272373	0.092091499	0.350438129	0.726009915	0	1.35545511	0	0.666666667	CRA004775;PRJNA827465;Yang2025_Silkie_Wencha	0.915610554
ENSGALG00010011271	ANGPTL1	3	0.020901746	0.059596007	0.350723943	0.725795461	0	0.635224865	0	1	CRA004775;PRJNA827465;Yang2025_Silkie_Wencha	0.915610554
ENSGALG00010003925	SEC31A	3	0.018024717	0.051372588	0.350862543	0.725691473	0.001402068	2.305086955	13.23537726	0.666666667	CRA004775;PRJNA827465;Yang2025_Silkie_Wencha	0.915610554
ENSGALG00010022593	MANBAL	3	-0.038689616	0.110599464	-0.349817391	0.726475747	0.014247439	3.176864768	37.04484938	0.666666667	CRA004775;PRJNA827465;Yang2025_Silkie_Wencha	0.915802477
ENSGALG00010010567	ERMN	2	0.42858298	1.227375458	0.349186532	0.726949276	2.488019541	5.458509178	81.67997951	0.5	CRA004775;Yang2025_Silkie_Wenchang	0.915834615
ENSGALG00010029730	TTC19	3	0.091239751	0.260936847	0.349662197	0.726592228	0.16728123	18.97508642	89.45986355	0.666666667	CRA004775;PRJNA827465;Yang2025_Silkie_Wencha	0.915834615
ENSGALG00010029397	OVCA2	3	0.087256791	0.249626614	0.349549231	0.726677018	0.135622562	7.442990065	73.12907873	0.666666667	CRA004775;PRJNA827465;Yang2025_Silkie_Wencha	0.915834615
ENSGALG00010026565	RSPH14	2	-0.083961587	0.240592809	-0.348977957	0.727105858	0	0.00924981	0	1	PRJNA827465;Yang2025_Silkie_Wenchang	0.915834615

ENSGALG00010014668	GP5	3	0.061079213	0.175051936	0.348920523	0.727148977	0.050438648	4.605903596	56.57746719	0.666666667	CRA004775;PRJNA827465;Yang2025_Silkie_Wencha	0.915834615
ENSGALG00010004102	PCMTD1	3	0.056843794	0.16271219	0.349351787	0.726825224	0.048380663	5.490039717	63.57039105	0.666666667	CRA004775;PRJNA827465;Yang2025_Silkie_Wencha	0.915834615
ENSGALG00010024141	YTHDF1	3	-0.037646151	0.107827117	-0.349134361	0.726988441	0.02410123	7.363339808	72.83841229	0.666666667	CRA004775;PRJNA827465;Yang2025_Silkie_Wencha	0.915834615
ENSGALG00010012688	NDUFAF2	3	0.035439546	0.101494741	0.34917618	0.726957048	0	0.859319669	0	0.666666667	CRA004775;PRJNA827465;Yang2025_Silkie_Wencha	0.915834615
ENSGALG00010024104	PLPPR5	3	0.026338858	0.075404239	0.349302088	0.726862531	0	0.580093786	0	0.666666667	CRA004775;PRJNA827465;Yang2025_Silkie_Wencha	0.915834615
ENSGALG00010025139	COPB2	3	0.012734062	0.036483331	0.349037823	0.727060914	0	1.888688029	0	0.666666667	CRA004775;PRJNA827465;Yang2025_Silkie_Wencha	0.915834615
ENSGALG00010005777	ENSGALG0001000577	3	-0.288891624	0.833263398	-0.346699045	0.728817428	0.805187147	3.259714596	38.64493528	0.666666667	CRA004775;PRJNA827465;Yang2025_Silkie_Wencha	0.915847734
ENSGALG00010001265	OPN1LW	3	0.260408484	0.749101614	0.347627717	0.728119788	1.467671668	17.09379806	88.29985008	0.666666667	CRA004775;PRJNA827465;Yang2025_Silkie_Wencha	0.915847734
ENSGALG00010011669	ENSGALG0001001166	3	0.205390251	0.590913655	0.347580817	0.728155015	0.604462499	5.323351398	62.42968291	0.666666667	CRA004775;PRJNA827465;Yang2025_Silkie_Wencha	0.915847734
ENSGALG00010016991	PDLIM3	3	0.138610282	0.397922839	0.348334574	0.727588931	0.355660485	15.06688651	86.72585741	0.666666667	CRA004775;PRJNA827465;Yang2025_Silkie_Wencha	0.915847734
ENSGALG00010010234	ENSGALG0001001023	3	-0.129018165	0.371391574	-0.347391202	0.728297442	0.333256456	16.67581916	88.00658618	0.666666667	CRA004775;PRJNA827465;Yang2025_Silkie_Wencha	0.915847734
ENSGALG00010013573	ENSGALG0001001357	3	-0.12526844	0.360462453	-0.347521466	0.728199595	0.233904483	5.213253057	61.63623791	0.666666667	CRA004775;PRJNA827465;Yang2025_Silkie_Wencha	0.915847734
ENSGALG00010028858	ENSGALG0001002885	3	0.122237715	0.351296735	0.347961431	0.727869149	0	1.991878907	0	0.666666667	CRA004775;PRJNA827465;Yang2025_Silkie_Wencha	0.915847734
ENSGALG00010028167	ENSGALG0001002816	3	0.104013965	0.298786059	0.348121883	0.727748651	0.233316625	17.62455215	88.65219393	0.666666667	CRA004775;PRJNA827465;Yang2025_Silkie_Wencha	0.915847734
ENSGALG00010021432	SYT9	3	-0.094766026	0.27262614	-0.347604327	0.728137356	0.115590567	4.404225691	54.5899665	0.666666667	CRA004775;PRJNA827465;Yang2025_Silkie_Wencha	0.915847734
ENSGALG00010010789	ENSGALG0001001078	3	-0.093130474	0.268575512	-0.34675713	0.728773787	0.163704716	8.837588175	77.36939128	0.666666667	CRA004775;PRJNA827465;Yang2025_Silkie_Wencha	0.915847734
ENSGALG00010015103	ITPK1	3	0.086159718	0.248246215	0.347073641	0.728535996	0.123812418	6.129390454	67.37032801	0.666666667	CRA004775;PRJNA827465;Yang2025_Silkie_Wencha	0.915847734
ENSGALG00010027695	TREMB2	3	-0.083918717	0.24213509	-0.34657809	0.728908307	0.060353354	3.010803094	33.5725407	0.666666667	CRA004775;PRJNA827465;Yang2025_Silkie_Wencha	0.915847734
ENSGALG00010001161	B3GALNT1	3	0.076627125	0.220961726	0.346789131	0.728749743	0.110801258	9.471739583	78.8845546	0.666666667	CRA004775;PRJNA827465;Yang2025_Silkie_Wencha	0.915847734
ENSGALG00010016872	EDA2R	3	-0.057899551	0.166623904	-0.347482296	0.728229017	0.019037129	2.526311614	20.84886785	0.666666667	CRA004775;PRJNA827465;Yang2025_Silkie_Wencha	0.915847734
ENSGALG00010024764	ENSGALG0001002476	3	0.052182837	0.150377245	0.347012858	0.728581659	0	0.478820024	0	0.666666667	CRA004775;PRJNA827465;Yang2025_Silkie_Wencha	0.915847734
ENSGALG00010006212	FOS	3	-0.051811625	0.148695426	-0.34844128	0.727508806	0	0.012250374	0	1	CRA004775;PRJNA827465;Yang2025_Silkie_Wencha	0.915847734
ENSGALG00010027276	ENSGALG0001002727	3	0.049907159	0.143223859	0.348459808	0.727494893	0.029340053	3.494736984	42.77108666	0.666666667	CRA004775;PRJNA827465;Yang2025_Silkie_Wencha	0.915847734
ENSGALG00010001556	EPB41L3	3	0.047498229	0.136758167	0.347315488	0.728354316	0.032537855	4.998036615	59.98428675	0.666666667	CRA004775;PRJNA827465;Yang2025_Silkie_Wencha	0.915847734
ENSGALG00010009452	TBCE	3	0.045916086	0.132059019	0.34769367	0.728070246	0.030352779	4.881357088	59.02778748	0.666666667	CRA004775;PRJNA827465;Yang2025_Silkie_Wencha	0.915847734
ENSGALG00010005495	TXN	3	-0.045821072	0.132173413	-0.346673894	0.728836325	0	0.903464792	0	0.666666667	CRA004775;PRJNA827465;Yang2025_Silkie_Wencha	0.915847734
ENSGALG00010024869	NDUFB3	3	-0.043272147	0.124652274	-0.34713484	0.72849002	0.012812507	2.470963575	19.05991571	0.666666667	CRA004775;PRJNA827465;Yang2025_Silkie_Wencha	0.915847734
ENSGALG00010012309	TRAPPC13	3	0.036810733	0.105870791	0.347694885	0.728069338	0	0.680185145	0	0.666666667	CRA004775;PRJNA827465;Yang2025_Silkie_Wencha	0.915847734
ENSGALG00010011544	STOML2	3	0.032117718	0.092495038	0.347237196	0.728413129	0.007291371	2.557959449	21.81267764	0.666666667	CRA004775;PRJNA827465;Yang2025_Silkie_Wencha	0.915847734
ENSGALG00010022181	ENSGALG0001002218	3	0.031044035	0.089121255	0.348334811	0.727588753	0	0.627807366	0	0.666666667	CRA004775;PRJNA827465;Yang2025_Silkie_Wencha	0.915847734
ENSGALG00010005178	EPHA5	3	0.030221159	0.08706281	0.347119045	0.728501886	0	1.111343743	0	0.666666667	CRA004775;PRJNA827465;Yang2025_Silkie_Wencha	0.915847734
ENSGALG00010020994	DHCR7	3	-0.028100134	0.081070366	-0.346614122	0.728881235	0	0.621075862	0	0.666666667	CRA004775;PRJNA827465;Yang2025_Silkie_Wencha	0.915847734
ENSGALG00010011106	USP40	3	-0.023816046	0.068356097	-0.34841144	0.727531213	0	1.343136479	0	0.666666667	CRA004775;PRJNA827465;Yang2025_Silkie_Wencha	0.915847734
ENSGALG00010025954	TUBA1A	3	0.026062727	0.075241502	0.34638765	0.729051405	8.44134E-05	2.005860312	0.292159543	0.666666667	CRA004775;PRJNA827465;Yang2025_Silkie_Wencha	0.915907745
ENSGALG00010011796	METAP2	3	0.014315452	0.041313271	0.346342109	0.729085626	0	1.516476728	0	0.666666667	CRA004775;PRJNA827465;Yang2025_Silkie_Wencha	0.915907745
ENSGALG00010022821	ENSGALG0001002282	2	0.549013031	1.58709253	0.34592377	0.729400005	4.261843753	6.447190914	84.48936888	0.5	PRJNA827465;Yang2025_Silkie_Wenchang	0.916064799
ENSGALG00010017670	FAM179A	3	-0.043150591	0.124689791	-0.346063542	0.729294962	0	1.689727784	0	0.666666667	CRA004775;PRJNA827465;Yang2025_Silkie_Wencha	0.916064799
ENSGALG00010013393	ADCK2	3	0.040831593	0.118038665	0.345917103	0.729405015	0.014520392	2.801749089	28.65160233	0.666666667	CRA004775;PRJNA827465;Yang2025_Silkie_Wencha	0.916064799
ENSGALG00010000468	TESC	3	0.064346508	0.186084758	0.345791394	0.729499493	0	0.467978118	0	0.666666667	CRA004775;PRJNA827465;Yang2025_Silkie_Wencha	0.916102082
ENSGALG00010012882	ENSGALG0001001288	2	-0.397186428	1.14954358	-0.345516633	0.729706008	2.504628851	19.09423037	94.76281588	0.5	PRJNA827465;Yang2025_Silkie_Wenchang	0.916160897
ENSGALG00010001947	TINAG	3	-0.069186607	0.200190562	-0.345603741	0.729640534	0	1.782291329	0	0.666666667	CRA004775;PRJNA827465;Yang2025_Silkie_Wencha	0.916160897
ENSGALG00010019362	ENSGALG0001001936	3	0.06026668	0.174448146	0.345470454	0.729740718	0	1.360251619	0	0.666666667	CRA004775;PRJNA827465;Yang2025_Silkie_Wencha	0.916160897
ENSGALG00010029278	MED31	3	-0.049093589	0.142238621	-0.345149503	0.72998198	0.044028736	9.002425478	77.783765	0.666666667	CRA004775;PRJNA827465;Yang2025_Silkie_Wencha	0.916301066
ENSGALG00010027864	ACTR8	3	0.019039887	0.055160645	0.345171591	0.729965375	0	1.694183941	0	0.666666667	CRA004775;PRJNA827465;Yang2025_Silkie_Wencha	0.916301066
ENSGALG00010029939	ENSGALG0001002993	2	0.811356591	2.351951132	0.344971705	0.730115643	9.308005529	6.302416203	84.13306948	0.5	CRA004775;Yang2025_Silkie_Wenchang	0.916306149
ENSGALG00010011118	GMPR2	3	-0.02449632	0.071000887	-0.345014286	0.73008363	0	0.997702512	0	0.666666667	CRA004775;PRJNA827465;Yang2025_Silkie_Wencha	0.916306149
ENSGALG00010023551	ENSGALG0001002355	3	0.068943912	0.199987249	0.344879498	0.730184964	0.08349933	10.75547658	81.4100074	0.666666667	CRA004775;PRJNA827465;Yang2025_Silkie_Wencha	0.916311814
ENSGALG00010008340	PDIA4	3	-0.075348644	0.218630442	-0.344639306	0.730365552	0.12176221	24.11285776	91.70566998	0.666666667	CRA004775;PRJNA827465;Yang2025_Silkie_Wencha	0.916457095
ENSGALG00010029539	FKBP6	3	0.109018118	0.316482206	0.344468398	0.730494058	0.031477753	2.200054863	9.093176089	0.666666667	CRA004775;PRJNA827465;Yang2025_Silkie_Wencha	0.916537003
ENSGALG00010028862	ENSGALG0001002886	2	-0.921080802	2.675730637	-0.344235249	0.730669375	12.07487905	6.286129967	84.09196111	0.5	PRJNA827465;Yang2025_Silkie_Wenchang	0.916594295
ENSGALG00010006927	UCN3	2	0.144082728	0.418500343	0.344283417	0.730633154	0	0.000119336	0	1	PRJNA827465;Yang2025_Silkie_Wenchang	0.916594295
ENSGALG00010021860	ENSGALG0001002186	3	-0.099502539	0.289248248	-0.344003946	0.730843318	0.187363861	8.255695158	75.7742993	0.666666667	CRA004775;PRJNA827465;Yang2025_Silkie_Wencha	0.916731164
ENSGALG00010002992	ENSGALG0001000299	3	0.129277233	0.375988375	0.343833059	0.730971837	0.320019417	8.420595775	76.24871145	0.666666667	CRA004775;PRJNA827465;Yang2025_Silkie_Wencha	0.916811035
ENSGALG00010027814	ENSGALG0001002781	3	0.079570313	0.231668745	0.343465894	0.731247995	0.143606313	34.36694214	94.18045402	0.666666667	CRA004775;PRJNA827465;Yang2025_Silkie_Wencha	0.916997157
ENSGALG00010021549	ANAPC16	3	0.05300085	0.15431299	0.343463304	0.731249943	0.040613535	4.889185035	59.09338702	0.666666667	CRA004775;PRJNA827465;Yang2025_Silkie_Wencha	0.916997157
ENSGALG00010025355	STAT6	3	0.052088403	0.151846812	0.343032573	0.731573956	0.053665634	16.6341886	87.97657014	0.666666667	CRA004775;PRJNA827465;Yang2025_Silkie_Wencha	0.917322114
ENSGALG00010011916	MCAT	3	-0.043860346	0.127951632	-0.342788481	0.731757592	0.029439147	5.216302197	61.65866308	0.666666667	CRA004775;PRJNA827465;Yang2025_Silkie_Wencha	0.917346163
ENSGALG00010011297	OSR2	3	-0.040038253	0.116788664	-0.342826535	0.731728962	0.017672892	3.69578431	45.88428782	0.666666667	CRA004775;PRJNA827465;Yang2025_Silkie_Wencha	0.917346163
ENSGALG00010008202	KCNG2	3	0.031118004	0.090789651	0.342748362	0.731787777	0.006460046	2.633247924	24.04816948	0.666666667	CRA004775;PRJNA827465;Yang2025_Silkie_Wencha	0.917346163
ENSGALG00010020943	FAM107A	3	0.090664985	0.264657657	0.342574579	0.731918529	0	1.847929489	0	0.666666667	CRA004775;PRJNA827465;Yang2025_Silkie_Wencha	0.917428731
ENSGALG00010029757	ULK2	3	0.046522778	0.135872273	0.342400825	0.732049268	0.032918744	6.44042466	68.9461471	0.666666667	CRA004775;PRJNA827465;Yang2025_Silkie_Wencha	0.917511267
ENSGALG00010027728	CRNN	2	0.630113681	1.849590393	0.340677419	0.73334644	4.228181507	2.484533245	59.75099137	0.5	PRJNA827465;Yang2025_Silkie_Wenchang	0.9175369
ENSGALG00010028567	ENSGALG0001002856	2	0.341825135	1.002802602	0.340869812	0.733201592	0	0.19392119	0	0.5	PRJNA827465;Yang2025_Silkie_Wenchang	0.9175369
ENSGALG00010016570	ENSGALG0001001657	2	0.159906396	0.467646814	0.341938383	0.732397263	0.346101188	4.780000138	79.07949851	0.5	PRJNA827465;Yang2025_Silkie_Wenchang	0.9175369
ENSGALG00010013757	DGKI	3	0.146609109	0.428396413	0.342227677	0.732179558	0.349990621	7.500257897	73.33425027	0.666666667	CRA004775;PRJNA827465;Yang2025_Silkie_Wencha	0.9175369
ENSGALG00010004980	FNDC1	3	0.135483749	0.396607471	0.34160665	0.732646932	0.326520274	7.63272154	73.79702653	0.666666667	CRA004775;PRJNA827465;Yang2025_Silkie_Wencha	0.9175369
ENSGALG00010002942	GABRB3	2	-0.122493936	0.3589015	-0.34130238	0.732875957	0	0.100663044	0	1	PRJNA827465;Yang2025_Silkie_Wenchang	0.9175369
ENSGALG00010028078	LAMTOR2	3	0.113149039	0.332311134	0.340491266	0.733486599	0.280953845	17.70885825	88.70621712	0.666666667	CRA004775;PRJNA827465;Yang2025_Silkie_Wencha	0.9175369
ENSGALG00010013889	ENSGALG0001001388	3	0.097048094	0.284989978	0.340531604	0.733456227	0.21051628	18.51755677	89.19943908	0.666666667	CRA004775;PRJNA827465;Yang2025_Silkie_Wencha	0.9175369
ENSGALG00010027664	TREMB1	3	0.096802785	0.28303971	0.342011319	0.732342374	0.103541501	3.441466848	41.88524579	0.666666667	CRA004775;PRJNA827465;Yang2025_Silkie_Wencha	0.9175369
ENSGALG00010009337	ZNF330	3	0.085847357	0.25161315	0.341187878	0.732962148	0.165088616	22.02547042	90.91960371	0.666666667	CRA004775;PRJNA827465;Yang2025_Silkie_Wencha	0.9175369
ENSGALG00010019919	LIN7C	3	-0.074483845	0.218984979	-0.340132212	0.733756965	0.127916865	22.59354119	91.14791266	0.666666667	CRA004775;PRJNA827465;Yang2025_Silkie_Wencha	0.9175369
ENSGALG00010024370	HERPUD1	3	-0.072822548	0.213154271	-0.341642452	0.732619986	0.109212745	16.51943152	87.89304585	0.666666667	CRA004775;PRJNA827465;Yang2025_Silkie_Wencha	0.9175369
ENSGALG00010011681	MAP1B	3	-0.071506623	0.209641913	-0.341089344	0.733036323	0.064900017	3.76402244	46.86535397	0.666666667	CRA004775;PRJNA827465;Yang2025_Silkie_Wencha	0.9175369
ENSGALG00010010390	KCNK3	3	0.053183628	0.156356925	0.340142451	0.733749254	0	1.614876458	0	0.666666667	CRA004775;PRJNA827465;Yang2025_Silkie_Wencha	0.9175369
ENSGALG00010023926	ZYX	3	0.040033063	0.117384376	0.341042517	0.733071575	0.016722784	3.016821502	33.70506015	0.666666667	CRA004775;PRJNA827465;Yang2025_Silkie_Wencha	0.9175369
ENSGALG00010021152	STK11IP	3	-0.039535268	0.11564312	-0.34187307	0.732446417	0.023371742	5.955966875	66.42022963	0.666666667	CRA004775;PRJNA827465;Yang2025_Silkie_Wencha	0.9175369
ENSGALG00010021897	ENSGALG0001002189	3	0.038744265	0.113731861	0.340663246	0.73335711	0	1.447344482	0	0.666666667	CRA004775;PRJNA827465;Yang2025_Silkie_Wencha	0.9175369
ENSGALG00010018908	ENSGALG0001001890	3	0.038281613	0.112397957	0.340589937	0.733412306	0	1.712266307	0	0.666666667	CRA004775;PRJNA827465;Yang2025_Silkie_Wencha	0.9175369
ENSGALG00010008342	PLD3	3	-0.037567871	0.109897152	-0.341845721	0.732466999	0.016652173	3.686872637	45.75348277	0.666666667	CRA004775;PRJNA827465;Yang2025_Silkie_Wencha	0.9175369
ENSGALG00010010980	POLK	3	0.035917462	0.105543354	0.34031003	0.733623065	0	1.487094191	0	0.666666667	CRA004775;PRJNA827465;Yang2025_Silkie_Wencha	0.9175369
ENSGALG00010013457	SH3BGR	3	0.02651128	0.077910529	0.340278525	0.733646788	0	0.071790622	0	1	CRA004775;PRJNA827465;Yang2025_Silkie_Wencha	0.9175369
ENSGALG00010010913	IFT56	3	0.025957807	0.076310995	0.340158155	0.733737429	0.002395289	2.256796552	11.37880822	0.666666667	CRA004775;PRJNA827465;Yang2025_Silkie_Wencha	0.9175369
ENSGALG00010027913	PYGO2	3	-0.025838716	0.075816154	-0.340807523	0.733248487	0.002200493	2.261528486	11.56423577	0.666666667	CRA004775;PRJNA827465;Yang2025_Silkie_Wencha	0.9175369
ENSGALG00010010800	BICRA	3	-0.023978554	0.070301281	-0.341082746	0.73304129	0.002628294	2.354257757	15.04753488	0.666666667	CRA004775;PRJNA827465;Yang2025_Silkie_Wencha	0.9175369
ENSGALG00010011273	RUNX1T1	3	0.022359783	0.065434613	0.341711857	0.732567748	0	0.930986923	0	0.666666667	CRA004775;PRJNA827465;Yang2025_Silkie_Wencha	0.9175369
ENSGALG00010002588	FAM175A	3	-0.018890555	0.055316452	-0.34149976	0.732727386	0	1.271648753	0	0.666666667	CRA004775;PRJNA827465;Yang2025_Silkie_Wencha	0.9175369
ENSGALG00010028558	ENSGALG0001002855	2	-0.394185921	1.162190062	-0.339175092	0.734477832	0	0.513753613	0	0.5	PRJNA827465;Yang2025_Silkie_Wenchang	0.917626762
ENSGALG00010022409	CARNS1	3	0.114753467	0.338235937	0.339270476	0.734405981	0.207880971	5.497071134	63.61698891	0.666666667	CRA004775;PRJNA827465;Yang2025_Silkie_Wencha	0.917626762
ENSGALG00010017656	FSD2	3	0.062332333	0.183651303	0.339405884	0.734303986	0.061139689	5.672815833	64.74414014	0.666666667	CRA004775;PRJNA827465;Yang2025_Silkie_Wencha	0.917626762
ENSGALG00010006958	KIF26B	3	0.052013772	0.153331391	0.339224552	0.734440575	0	0.943319121	0	0.666666667	CRA004775;PRJNA827465;Yang2025_Silkie_Wencha	0.917626762
ENSGALG00010019905	ENSGALG0001001990	3	-0.050958473	0.150192962	-0.33928669	0.734393768	0.054081587	18.56616196	89.22771435	0.666666667	CRA004775;PRJNA827465;Yang2025_Silkie_Wencha	0.917626762
ENSGALG00010001850	MYOM2	3	-0.041576329	0.122547194	-0.339267896	0.734407925	0.020575146	3.985832015	49.82227066	0.666666667	CRA004775;PRJNA827465;Yang2025_Silkie_Wencha	0.917626762
ENSGALG00010023336	GPR162	3	0.025091545	0.073846249	0.339780906	0.734021528	0	0.227356652	0	0.666666667	CRA004775;PRJNA827465;Yang2025_Silkie_Wencha	0.917626762
ENSGALG00010021082	SENP8	3	-0.023831617	0.070105634	-0.339938684	0.733902704	0	1.212667737	0	0.666666667	CRA004775;PRJNA827465;Yang2025_Silkie_Wencha	0.917626762
ENSGALG00010008323	AKT3	3	0.019127703	0.056326541	0.339585965	0.734168349	0	1.467800604	0	0.666666667	CRA004775;PRJNA827465;Yang2025_Silkie_Wencha	0.917626762
ENSGALG00010006850	IRF2BPL	3	-0.01908354	0.056219024	-0.339449867	0.734270858	0	1.108091149	0	0.666666667	CRA004775;PRJNA827465;Yang2025_Silkie_Wencha	0.917626762
ENSGALG00010024677	LMBR1L	3	-0.095641528	0.282198273	-0.338916063	0.734672963	0.14731661	5.802139321	65.5299556	0.666666667	CRA004775;PRJNA827465;Yang2025_Silkie_Wencha	0.917789453
ENSGALG00010010403	ENSGALG0001001040	2	0.142059423	0.419447209	0.338682486	0.734848936	0.061868562	1.196784635	16.44277749	0.5	PRJNA827465;Yang2025_Silkie_Wenchang	0.91786317
ENSGALG00010027174	SHISA5	3	-0.03606273	0.106484838	-0.338665403	0.734861807	0.020664035	6.736479469	70.31090187	0.666666667	CRA004775;PRJNA827465;Yang2025_Silkie_Wencha	0.91786317
ENSGALG00010004951	AFDN	3	-0.021028171	0.062132613	-0.338440156	0.735031518	0	1.429274394	0	0.666666667	CRA004775;PRJNA827465;Yang2025_Silkie_Wencha	0.917994049
ENSGALG00010010351	NAMPTP1	3	0.070413087	0.208125896	0.338319681	0.735122294	0.090195527	8.125441931	75.38595418	0.666666667	CRA004775;PRJNA827465;Yang2025_Silkie_Wencha	0.918026331
ENSGALG00010017702	ECEL1	2	0.372300207	1.100938674	0.338166163	0.735237973	1.674662524	3.078720986	67.51897932	0.5	PRJNA827465;Yang2025_Silkie_Wenchang	0.918089703
ENSGALG00010016583	VPS41	3	-0.021602501	0.063914042	-0.337993038	0.735368434	0	0.840230237	0	0.666666667	CRA004775;PRJNA827465;Yang2025_Silkie_Wencha	0.91817152
ENSGALG00010009526	ENSGALG0001000952	3	0.099779693	0.295366457	0.337816602	0.735501398	0.18047198	7.508560187	73.36373486	0.666666667	CRA004775;PRJNA827465;Yang2025_Silkie_Wencha	0.918256447
ENSGALG00010015167	RAB3C	2	0.158108567	0.470478626	0.33605898	0.73682639	0.228995847	1.974642507	49.35792193	0.5	PRJNA827465;Yang2025_Silkie_Wenchang	0.918412887
ENSGALG00010006923	ENSGALG0001000692	2	0.137360006	0.407195968	0.33733145	0.735867054	0.271030876	5.424982015	81.56675917	0.5	PRJNA827465;Yang2025_Silkie_Wenchang	0.918412887
ENSGALG00010003726	ENPEP	3	0.094381689	0.280640948	0.336307618	0.736638906	0	1.624832917	0	0.666666667	CRA004775;PRJNA827465;Yang2025_Silkie_Wencha	0.918412887
ENSGALG00010004602	DYNLT2	3	0.080368989	0.238204135	0.33739544	0.735818822	0	0.869743134	0	0.666666667	CRA004775;PRJNA827465;Yang2025_Silkie_Wencha	0.918412887
ENSGALG00010023258	ENSGALG0001002325	3	-0.072687854	0.215595296	-0.337149534	0.736004179	0	0.346677913	0	0.666666667	CRA004775;PRJNA827465;Yang2025_Silkie_Wencha	0.918412887
ENSGALG00010007806	DYNLRB2	3	-0.071404791	0.212298997	-0.336340689	0.73661397	0	1.126618315	0	0.666666667	CRA004775;PRJNA827465;Yang2025_Silkie_Wencha	0.918412887
ENSGALG00010007982	RECK	3	0.068577469	0.203571179	0.33687219	0.736213251	0.103329299	19.5117219	89.74975141	0.666666667	CRA004775;PRJNA827465;Yang2025_Silkie_Wencha	0.918412887
ENSGALG00010025425	ENSGALG0001002542	3	0.059061	0.175476204	0.336575552	0.736436888	0.070121611	13.05260202	84.67738465	0.666666667	CRA004775;PRJNA827465;Yang2025_Silkie_Wencha	0.918412887
ENSGALG00010021480	ENSGALG0001002148	3	-0.051041959	0.151890957	-0.336043433	0.736838114	0.049642971	7.618127631	73.74682997	0.666666667	CRA004775;PRJNA827465;Yang2025_Silkie_Wencha	0.918412887
ENSGALG00010024865	SNAP47	3	-0.038225273	0.113706187	-0.336175846	0.736738266	0.016890433	3.65083625	45.21803053	0.666666667	CRA004775;PRJNA827465;Yang2025_Silkie_Wencha	0.918412887
ENSGALG00010024696	TRADD	3	0.037749807	0.112025647	0.336974683	0.736135985	0.020672717	5.219459173	61.68185374	0.666666667	CRA004775;PRJNA827465;Yang2025_Silkie_Wencha	0.918412887
ENSGALG00010021256	ENSGALG0001002125	3	0.032109456	0.095310131	0.336894475	0.73619645	0	1.188958014	0	0.666666667	CRA004775;PRJNA827465;Yang2025_Silkie_Wencha	0.918412887
ENSGALG00010027869	NSMF	3	-0.030354321	0.089933338	-0.337520231	0.735724764	0.00643687	2.590317728	22.78939458	0.666666667	CRA004775;PRJNA827465;Yang2025_Silkie_Wencha	0.918412887
ENSGALG00010017521	MIIP	3	-0.02752162	0.081746223	-0.336671461	0.73636458	0.005208204	2.597524637	23.0036177	0.666666667	CRA004775;PRJNA827465;Yang2025_Silkie_Wencha	0.918412887
ENSGALG00010022334	PDE1A	3	0.02392271	0.071191143	0.336034928	0.736844528	0	0.519631351	0	1	CRA004775;PRJNA827465;Yang2025_Silkie_Wencha	0.918412887
ENSGALG00010024459	AHCYL1	3	-0.01840576	0.054678111	-0.336620269	0.736403174	0	0.238163912	0	0.666666667	CRA004775;PRJNA827465;Yang2025_Silkie_Wencha	0.918412887
ENSGALG00010008046	RHOB	3	0.016606096	0.049404672	0.336123997	0.736777363	0	1.129447168	0	1	CRA004775;PRJNA827465;Yang2025_Silkie_Wencha	0.918412887
ENSGALG00010003077	EIF2S3	3	-0.01616615	0.048111643	-0.336013261	0.736860866	0	0.106667339	0	1	CRA004775;PRJNA827465;Yang2025_Silkie_Wencha	0.918412887
ENSGALG00010027244	ARHGEF18	3	-0.013965491	0.041464977	-0.336802094	0.736266094	0	0.956685614	0	0.666666667	CRA004775;PRJNA827465;Yang2025_Silkie_Wencha	0.918412887
ENSGALG00010016652	FXR1	3	-0.030630162	0.091262568	-0.335626787	0.737152322	0.013033782	4.770156272	58.07265243	0.666666667	CRA004775;PRJNA827465;Yang2025_Silkie_Wencha	0.918695168
ENSGALG00010000075	ENSGALG0001000007	3	0.113779272	0.340183316	0.334464586	0.73802901	0.092417992	2.706716496	26.10973469	0.666666667	CRA004775;PRJNA827465;Yang2025_Silkie_Wencha	0.919194888
ENSGALG00010004966	LAMA3	3	0.110203011	0.329437244	0.33451898	0.737987971	0.226195172	8.539022221	76.57811459	0.666666667	CRA004775;PRJNA827465;Yang2025_Silkie_Wencha	0.919194888
ENSGALG00010013371	FAT1	3	0.102928776	0.307875376	0.33431961	0.738138394	0.226510512	11.65578143	82.84113328	0.666666667	CRA004775;PRJNA827465;Yang2025_Silkie_Wencha	0.919194888
ENSGALG00010001504	RBMS3	3	0.059969853	0.17926996	0.334522602	0.737985239	0.057362883	4.72646472	57.68507503	0.666666667	CRA004775;PRJNA827465;Yang2025_Silkie_Wencha	0.919194888
ENSGALG00010020038	SKI	3	-0.037694565	0.112713067	-0.334429419	0.738055543	0.012545287	2.628682404	23.91625566	0.666666667	CRA004775;PRJNA827465;Yang2025_Silkie_Wencha	0.919194888
ENSGALG00010022954	ADRM1	3	-0.033785311	0.100968138	-0.334613585	0.737916596	0.013207428	3.420141821	41.52289277	0.666666667	CRA004775;PRJNA827465;Yang2025_Silkie_Wencha	0.919194888
ENSGALG00010009541	PMPCB	3	-0.032910626	0.098332797	-0.334686158	0.737861844	0.014106142	3.890307301	48.59017951	0.666666667	CRA004775;PRJNA827465;Yang2025_Silkie_Wencha	0.919194888
ENSGALG00010028501	USP20	3	0.030725809	0.091892228	0.334367879	0.738101975	0.017528841	6.886466954	70.95753144	0.666666667	CRA004775;PRJNA827465;Yang2025_Silkie_Wencha	0.919194888
ENSGALG00010027289	SMARCC1	3	0.019051577	0.056929114	0.334654376	0.737885821	0	1.674596832	0	0.666666667	CRA004775;PRJNA827465;Yang2025_Silkie_Wencha	0.919194888
ENSGALG00010013151	ENSGALG0001001315	2	-0.621856196	1.860853474	-0.334177948	0.738245283	5.407189298	4.48704087	77.71359725	0.5	PRJNA827465;Yang2025_Silkie_Wenchang	0.919247033
ENSGALG00010006077	TBCK	3	0.03234768	0.096913207	0.333779889	0.73854566	0.012331207	3.649764783	45.2019481	0.666666667	CRA004775;PRJNA827465;Yang2025_Silkie_Wencha	0.919538352
ENSGALG00010017574	DYNC1LI1	3	-0.012544441	0.037592474	-0.333695544	0.738609312	0	0.998721874	0	0.666666667	CRA004775;PRJNA827465;Yang2025_Silkie_Wencha	0.919538352
ENSGALG00010014599	SSTR3	3	0.114825875	0.344618841	0.333196741	0.738985778	0.095937994	2.716146472	26.36626851	0.666666667	CRA004775;PRJNA827465;Yang2025_Silkie_Wencha	0.919620426
ENSGALG00010000098	ENSGALG0001000009	3	0.105261366	0.315931975	0.33317731	0.739000444	0.257618468	17.56961729	88.61671278	0.666666667	CRA004775;PRJNA827465;Yang2025_Silkie_Wencha	0.919620426
ENSGALG00010027319	MST1R	3	-0.098195024	0.294581283	-0.333337619	0.738879445	0.109304903	3.44550832	41.95341255	0.666666667	CRA004775;PRJNA827465;Yang2025_Silkie_Wencha	0.919620426
ENSGALG00010021072	ENSGALG0001002107	3	-0.095490016	0.286387318	-0.333429627	0.738810003	0.236763193	67.49516223	97.03682466	0.666666667	CRA004775;PRJNA827465;Yang2025_Silkie_Wencha	0.919620426
ENSGALG00010017454	ENSGALG0001001745	3	0.045799866	0.137445293	0.333222516	0.738966323	0.03099597	4.347535489	53.99692527	0.666666667	CRA004775;PRJNA827465;Yang2025_Silkie_Wencha	0.919620426
ENSGALG00010027813	LRRC71	3	0.179747251	0.539900731	0.332926481	0.73918978	0.706189181	12.02366292	83.36613382	0.666666667	CRA004775;PRJNA827465;Yang2025_Silkie_Wencha	0.919725577
ENSGALG00010012059	ARHGDIB	3	0.068564337	0.205965081	0.332893017	0.739215041	0.077626919	5.416500037	63.0757872	0.666666667	CRA004775;PRJNA827465;Yang2025_Silkie_Wencha	0.919725577
ENSGALG00010008142	CD247	3	0.096966034	0.291377904	0.332784444	0.739297001	0.160551744	5.528149091	63.82152567	0.666666667	CRA004775;PRJNA827465;Yang2025_Silkie_Wencha	0.919740621
ENSGALG00010019221	VASH2	3	-0.025748869	0.07739257	-0.33270466	0.739357231	0	1.47590661	0	0.666666667	CRA004775;PRJNA827465;Yang2025_Silkie_Wencha	0.919740621
ENSGALG00010012529	BEND3	3	0.014075204	0.042324527	0.332554315	0.739470734	0	1.183973134	0	0.666666667	CRA004775;PRJNA827465;Yang2025_Silkie_Wencha	0.919800889
ENSGALG00010000226	ENSGALG0001000022	3	-0.082818734	0.249236916	-0.332289193	0.7396709	0.090510681	3.889420156	48.57845335	0.666666667	CRA004775;PRJNA827465;Yang2025_Silkie_Wencha	0.919967317
ENSGALG00010001831	CSF2RA	3	-0.066207434	0.199297077	-0.33220474	0.739734665	0.05542224	3.840089409	47.91787932	0.666666667	CRA004775;PRJNA827465;Yang2025_Silkie_Wencha	0.919967317
ENSGALG00010012706	TBC1D8B	3	-0.041973461	0.126386726	-0.332103398	0.739811184	0.030391383	5.712470511	64.98887835	0.666666667	CRA004775;PRJNA827465;Yang2025_Silkie_Wencha	0.91998156
ENSGALG00010014805	SPEF2	2	0.152603918	0.460018571	0.331734256	0.740089932	0.320426428	4.112938007	75.68648012	0.5	PRJNA827465;Yang2025_Silkie_Wenchang	0.920004532
ENSGALG00010017543	ZBTB25	3	-0.06666224	0.200914458	-0.33179414	0.74004471	0.072295958	5.05413198	60.42841762	0.666666667	CRA004775;PRJNA827465;Yang2025_Silkie_Wencha	0.920004532
ENSGALG00010005338	PCGF1	3	0.06364517	0.191819628	0.331796965	0.740042576	0.076538274	6.8986793	71.0089437	0.666666667	CRA004775;PRJNA827465;Yang2025_Silkie_Wencha	0.920004532
ENSGALG00010029804	ITGB4	3	0.037535312	0.113104874	0.331862899	0.739992787	0	0.704647504	0	0.666666667	CRA004775;PRJNA827465;Yang2025_Silkie_Wencha	0.920004532
ENSGALG00010022860	MOB3C	3	-0.033500676	0.101037601	-0.331566422	0.740216678	0.013319282	3.542017578	43.53500636	0.666666667	CRA004775;PRJNA827465;Yang2025_Silkie_Wencha	0.920081197
ENSGALG00010011764	PCYOX1L	3	0.038467562	0.116122016	0.331268464	0.74044171	0.012253532	2.747669312	27.21103695	0.666666667	CRA004775;PRJNA827465;Yang2025_Silkie_Wencha	0.920280005
ENSGALG00010005198	TRPC3	3	0.073070229	0.220938107	0.330727143	0.740850598	0.02947145	2.421831141	17.41785933	0.666666667	CRA004775;PRJNA827465;Yang2025_Silkie_Wencha	0.920626349
ENSGALG00010002792	ENSGALG0001000279	3	-0.068407001	0.20682534	-0.330747675	0.740835087	0.073999237	4.892005461	59.11697123	0.666666667	CRA004775;PRJNA827465;Yang2025_Silkie_Wencha	0.920626349
ENSGALG00010000697	ENSGALG0001000069	3	0.123515675	0.373856583	0.330382507	0.741110958	0.342326362	11.80684018	83.06066679	0.666666667	CRA004775;PRJNA827465;Yang2025_Silkie_Wencha	0.920868955
ENSGALG00010024636	PANX3	3	-0.100572923	0.304668296	-0.330106298	0.741319645	0.227184588	11.0906432	81.96678079	0.666666667	CRA004775;PRJNA827465;Yang2025_Silkie_Wencha	0.92092449
ENSGALG00010024575	TXNRD2	3	0.055958257	0.16958151	0.329978527	0.741416187	0.060295606	7.64233868	73.83000043	0.666666667	CRA004775;PRJNA827465;Yang2025_Silkie_Wencha	0.92092449
ENSGALG00010014301	ARID1A	3	0.043736816	0.13245251	0.330207528	0.741243159	0.0306775	4.822685967	58.52933379	0.666666667	CRA004775;PRJNA827465;Yang2025_Silkie_Wencha	0.92092449
ENSGALG00010009026	RAPGEF2	3	0.014102544	0.042728328	0.330051393	0.74136113	0	0.482454046	0	0.666666667	CRA004775;PRJNA827465;Yang2025_Silkie_Wencha	0.92092449
ENSGALG00010025982	SERPINE2	3	-0.070292985	0.213214201	-0.329682473	0.741639898	0.105891368	17.40682635	88.51025477	0.666666667	CRA004775;PRJNA827465;Yang2025_Silkie_Wencha	0.921121444
ENSGALG00010000721	ENSGALG0001000072	3	0.086328203	0.262658021	0.328671489	0.742404007	0.164571045	10.93164814	81.70449712	0.666666667	CRA004775;PRJNA827465;Yang2025_Silkie_Wencha	0.921665664
ENSGALG00010026073	HPDL	3	-0.0727149	0.221155578	-0.328795232	0.742310467	0.042711869	2.711757463	26.24709151	0.666666667	CRA004775;PRJNA827465;Yang2025_Silkie_Wencha	0.921665664
ENSGALG00010026846	MICAL1	3	0.050437854	0.15343065	0.328733886	0.742356839	0.019301462	2.693625983	25.75064197	0.666666667	CRA004775;PRJNA827465;Yang2025_Silkie_Wencha	0.921665664
ENSGALG00010022569	KIAA1549L	3	0.041704906	0.126775639	0.328966244	0.742181203	0	0.332013732	0	0.666666667	CRA004775;PRJNA827465;Yang2025_Silkie_Wencha	0.921665664
ENSGALG00010002481	CKAP2	3	0.025693223	0.078133092	0.328839198	0.742277234	0	0.029254381	0	1	CRA004775;PRJNA827465;Yang2025_Silkie_Wencha	0.921665664
ENSGALG00010025362	G6PC2	3	-0.033953089	0.103406377	-0.328346183	0.742649929	0	1.694319959	0	0.666666667	CRA004775;PRJNA827465;Yang2025_Silkie_Wencha	0.921890022
ENSGALG00010008222	STYXL2	3	0.033815853	0.103047434	0.328158129	0.742792105	0	1.407039981	0	0.666666667	CRA004775;PRJNA827465;Yang2025_Silkie_Wencha	0.921985565
ENSGALG00010001729	ENSGALG0001000172	3	0.109406924	0.333591786	0.327966482	0.742937006	0.271152059	10.89191357	81.63775367	0.666666667	CRA004775;PRJNA827465;Yang2025_Silkie_Wencha	0.922084474
ENSGALG00010021028	ENSGALG0001002102	3	0.155275258	0.474344492	0.327347025	0.743405429	0.499633353	8.697831725	77.00576347	0.666666667	CRA004775;PRJNA827465;Yang2025_Silkie_Wencha	0.922250782
ENSGALG00010010821	RGS8	3	-0.083805586	0.256090875	-0.327249403	0.743479258	0	1.853861028	0	0.666666667	CRA004775;PRJNA827465;Yang2025_Silkie_Wencha	0.922250782
ENSGALG00010024175	DHRS9	3	-0.083606282	0.255298575	-0.327484328	0.743301594	0	0.583537443	0	0.666666667	CRA004775;PRJNA827465;Yang2025_Silkie_Wencha	0.922250782
ENSGALG00010001402	NUP42	3	-0.048062101	0.146802888	-0.327392064	0.743371368	0.03717676	4.717929383	57.60852192	0.666666667	CRA004775;PRJNA827465;Yang2025_Silkie_Wencha	0.922250782
ENSGALG00010010849	ACBD6	3	-0.04273929	0.130487817	-0.327534715	0.743263491	0.032971541	5.940170794	66.33093442	0.666666667	CRA004775;PRJNA827465;Yang2025_Silkie_Wencha	0.922250782
ENSGALG00010008000	C8orf82	3	-0.039646844	0.121175435	-0.32718549	0.743527595	0.019127851	3.364003468	40.54702919	0.666666667	CRA004775;PRJNA827465;Yang2025_Silkie_Wencha	0.922250782
ENSGALG00010010009	ELF2	3	-0.039458091	0.120504755	-0.327440118	0.743335028	0.032661538	12.71292936	84.26798464	0.666666667	CRA004775;PRJNA827465;Yang2025_Silkie_Wencha	0.922250782
ENSGALG00010025250	GLIS1	3	-0.145945254	0.44624185	-0.327054162	0.743626921	0.310338726	4.241643143	52.8484615	0.666666667	CRA004775;PRJNA827465;Yang2025_Silkie_Wencha	0.922293073
ENSGALG00010002432	RAVER1	3	0.115035064	0.351937562	0.326862139	0.743772158	0.352754372	43.49573283	95.40184779	0.666666667	CRA004775;PRJNA827465;Yang2025_Silkie_Wencha	0.922330698

ENSGALG00010012324	ENSGALG0001001232	3	-0.108607792	0.332294926	-0.326841561	0.743787723	0.202884648	5.189322403	61.45932272	0.666666667	CRA004775;PRJNA827465;Yang2025_Silkie_Wencha	0.922330698
ENSGALG00010024110	CPNE1	3	-0.02764712	0.084672197	-0.326519461	0.744031367	0.011838391	4.845892078	58.72792939	0.666666667	CRA004775;PRJNA827465;Yang2025_Silkie_Wencha	0.922551916
ENSGALG00010009718	ENSGALG0001000971	3	0.164230738	0.504208086	0.325719789	0.74463637	0.675471075	18.3834978	89.12067757	0.666666667	CRA004775;PRJNA827465;Yang2025_Silkie_Wencha	0.922807496
ENSGALG00010025359	CDCP2	3	0.117012827	0.359571621	0.325422865	0.744861052	0.219011231	4.61143811	56.62958166	0.666666667	CRA004775;PRJNA827465;Yang2025_Silkie_Wencha	0.922807496
ENSGALG00010019094	SNRNP25	3	0.103002989	0.316535335	0.325407552	0.74487264	0.260443654	17.24843522	88.40474527	0.666666667	CRA004775;PRJNA827465;Yang2025_Silkie_Wencha	0.922807496
ENSGALG00010010155	BRINP3	2	-0.062418939	0.191703511	-0.32560144	0.744725923	0	0.250586443	0	0.5	PRJNA827465;Yang2025_Silkie_Wenchang	0.922807496
ENSGALG00010011374	ENSGALG0001001137	3	0.060881956	0.187007559	0.325558798	0.74475819	0.074467368	7.174099224	72.12193562	0.666666667	CRA004775;PRJNA827465;Yang2025_Silkie_Wencha	0.922807496
ENSGALG00010002796	PRDM8	3	0.054904798	0.168520181	0.325805474	0.744571537	0	1.752946361	0	0.666666667	CRA004775;PRJNA827465;Yang2025_Silkie_Wencha	0.922807496
ENSGALG00010019029	CENPF	3	0.048602568	0.14935053	0.325426151	0.744858566	0.018817936	2.725079341	26.60764147	0.666666667	CRA004775;PRJNA827465;Yang2025_Silkie_Wencha	0.922807496
ENSGALG00010021165	PTRF	3	-0.027122005	0.083196175	-0.326000631	0.744423878	0.004478694	2.477662951	19.27877038	0.666666667	CRA004775;PRJNA827465;Yang2025_Silkie_Wencha	0.922807496
ENSGALG00010005438	GPR180	3	0.022690786	0.069735321	0.325384402	0.744890159	0	0.511005992	0	0.666666667	CRA004775;PRJNA827465;Yang2025_Silkie_Wencha	0.922807496
ENSGALG00010014361	SSPN	3	-0.018008544	0.055244356	-0.3259798	0.744439638	0	0.871418046	0	0.666666667	CRA004775;PRJNA827465;Yang2025_Silkie_Wencha	0.922807496
ENSGALG00010021529	BTAF1	3	-0.01438002	0.044208239	-0.325279177	0.744969789	0	1.095674754	0	0.666666667	CRA004775;PRJNA827465;Yang2025_Silkie_Wencha	0.922825289
ENSGALG00010008983	ENSGALG0001000898	3	0.122725972	0.377432588	0.325159978	0.745059997	0.197329137	3.696779157	45.898851	0.666666667	CRA004775;PRJNA827465;Yang2025_Silkie_Wencha	0.92285618
ENSGALG00010024553	ASB8	3	0.055775424	0.171648812	0.324939182	0.745227102	0.058215641	6.274796588	68.12645682	0.666666667	CRA004775;PRJNA827465;Yang2025_Silkie_Wencha	0.922941694
ENSGALG00010004007	ENSGALG0001000400	3	-0.02759336	0.084929756	-0.324896259	0.745259588	0	0.363851737	0	0.666666667	CRA004775;PRJNA827465;Yang2025_Silkie_Wencha	0.922941694
ENSGALG00010001250	ENSGALG0001000125	2	-0.134286046	0.413979838	-0.324378226	0.745651702	0	0.041912041	0	1	PRJNA827465;Yang2025_Silkie_Wenchang	0.922962823
ENSGALG00010018583	PPL	3	0.129140063	0.397844214	0.324599575	0.745484149	0.381375528	11.55812126	82.6961497	0.666666667	CRA004775;PRJNA827465;Yang2025_Silkie_Wencha	0.922962823
ENSGALG00010002447	YLEC12	3	0.112472935	0.346630257	0.324475237	0.745578267	0	1.394491082	0	0.666666667	CRA004775;PRJNA827465;Yang2025_Silkie_Wencha	0.922962823
ENSGALG00010016426	HELZ2	3	0.043810399	0.135106673	0.324270047	0.745733594	0.021133904	3.189174355	37.28784388	0.666666667	CRA004775;PRJNA827465;Yang2025_Silkie_Wencha	0.922962823
ENSGALG00010011738	FGD6	3	-0.033599943	0.103576852	-0.324396259	0.745638052	0.014725039	3.672553457	45.54197717	0.666666667	CRA004775;PRJNA827465;Yang2025_Silkie_Wencha	0.922962823
ENSGALG00010012264	MYF5	3	-0.028209691	0.086864703	-0.324754355	0.745366993	0	1.746192292	0	0.666666667	CRA004775;PRJNA827465;Yang2025_Silkie_Wencha	0.922962823
ENSGALG00010020340	DCAF5	3	-0.012858151	0.039646197	-0.324327486	0.745690112	0	0.52156548	0	0.666666667	CRA004775;PRJNA827465;Yang2025_Silkie_Wencha	0.922962823
ENSGALG00010000467	RNASEK	3	-0.038642495	0.119241674	-0.324068707	0.745886018	0.002587583	2.111221325	5.268103504	0.666666667	CRA004775;PRJNA827465;Yang2025_Silkie_Wencha	0.92307067
ENSGALG00010006835	FRY	3	0.072960525	0.225294855	0.323844613	0.746055678	0.111343649	8.238726007	75.72440207	0.666666667	CRA004775;PRJNA827465;Yang2025_Silkie_Wencha	0.923199828
ENSGALG00010024848	ESYT1	3	-0.030893284	0.095489166	-0.323526583	0.746296479	0.015270073	4.646931284	56.9608441	0.666666667	CRA004775;PRJNA827465;Yang2025_Silkie_Wencha	0.923416987
ENSGALG00010017760	ENSGALG0001001776	2	-0.130660834	0.404176529	-0.3232766	0.746485774	0.016262803	1.048555258	4.630681841	0.5	CRA004775;PRJNA827465	0.923489575
ENSGALG00010029760	SHISA6	2	-0.070858587	0.219139405	-0.323349362	0.746430675	0.027639955	1.211212997	17.43813824	0.5	PRJNA827465;Yang2025_Silkie_Wenchang	0.923489575
ENSGALG00010020011	TLE3	3	0.097716372	0.302944853	0.322554982	0.747032292	0.246949183	23.24060929	91.39437364	0.666666667	CRA004775;PRJNA827465;Yang2025_Silkie_Wencha	0.923866711
ENSGALG00010029230	PPM1E	3	0.060443398	0.187495582	0.322372388	0.7471706	0.066544254	6.334910171	68.42891302	0.666666667	CRA004775;PRJNA827465;Yang2025_Silkie_Wencha	0.923866711
ENSGALG00010012741	ENSGALG0001001274	3	0.057899942	0.179689506	0.322188774	0.747309689	0	1.205749799	0	0.666666667	CRA004775;PRJNA827465;Yang2025_Silkie_Wencha	0.923866711
ENSGALG00010001649	UBE2E2	3	-0.042608044	0.132219681	-0.322251902	0.747261868	0	1.345358584	0	0.666666667	CRA004775;PRJNA827465;Yang2025_Silkie_Wencha	0.923866711
ENSGALG00010027158	XRCC2	3	-0.042302763	0.131211774	-0.322400659	0.747149185	0.030209376	5.081844062	60.64420758	0.666666667	CRA004775;PRJNA827465;Yang2025_Silkie_Wencha	0.923866711
ENSGALG00010028626	CRAT	3	-0.038296885	0.118866527	-0.322183929	0.747313359	0.020636374	4.044042788	50.54453909	0.666666667	CRA004775;PRJNA827465;Yang2025_Silkie_Wencha	0.923866711
ENSGALG00010029775	ENSGALG0001002977	3	-0.034113312	0.105833313	-0.322269669	0.747248409	0	1.863285395	0	0.666666667	CRA004775;PRJNA827465;Yang2025_Silkie_Wencha	0.923866711
ENSGALG00010023890	SASS6	3	0.031997894	0.099195033	0.322575569	0.747016699	0.014980024	4.193450876	52.30658331	0.666666667	CRA004775;PRJNA827465;Yang2025_Silkie_Wencha	0.923866711
ENSGALG00010018111	NKX2-2	2	-0.08359669	0.259746671	-0.321839312	0.747574431	0	0.386740337	0	0.5	PRJNA827465;Yang2025_Silkie_Wenchang	0.924032847
ENSGALG00010017449	TRMT6	3	0.058653653	0.182248154	0.321834003	0.747578453	0.075345577	9.638020921	79.24885185	0.666666667	CRA004775;PRJNA827465;Yang2025_Silkie_Wencha	0.924032847
ENSGALG00010024078	KIRREL3	3	0.054405844	0.169132609	0.321675662	0.747698417	0.021361806	2.542398303	21.33411993	0.666666667	CRA004775;PRJNA827465;Yang2025_Silkie_Wencha	0.924100343
ENSGALG00010009677	SLC26A5	3	0.10298097	0.320766436	0.321046587	0.748175087	0.245951751	12.22099382	83.63471883	0.666666667	CRA004775;PRJNA827465;Yang2025_Silkie_Wencha	0.924556673
ENSGALG00010004473	JDP2	3	0.070740777	0.220380514	0.320993794	0.748215094	0.112336583	13.85048776	85.56007533	0.666666667	CRA004775;PRJNA827465;Yang2025_Silkie_Wencha	0.924556673
ENSGALG00010013506	RNF149	3	-0.027294594	0.085071435	-0.320843225	0.748329201	0.008946469	3.418165198	41.4890772	0.666666667	CRA004775;PRJNA827465;Yang2025_Silkie_Wencha	0.924556673
ENSGALG00010028856	ETV3	3	0.022009298	0.068564426	0.320870452	0.748308567	0.004250334	2.770332254	27.80649335	0.666666667	CRA004775;PRJNA827465;Yang2025_Silkie_Wencha	0.924556673
ENSGALG00010025701	ENSGALG0001002570	2	-0.587522084	1.832100279	-0.320682274	0.748451182	0	0.033016627	0	1	PRJNA827465;Yang2025_Silkie_Wenchang	0.924563296
ENSGALG00010005532	GTPBP4	3	-0.017070515	0.053249293	-0.320577311	0.748530734	0	0.068780943	0	0.666666667	CRA004775;PRJNA827465;Yang2025_Silkie_Wencha	0.924563296
ENSGALG00010028507	ANP32E	3	0.014022578	0.04373182	0.320649305	0.748476169	0	0.350559438	0	0.666666667	CRA004775;PRJNA827465;Yang2025_Silkie_Wencha	0.924563296
ENSGALG00010017739	MRPL20	3	0.084915831	0.2651762	0.320226569	0.74879262	0.178101526	17.46679657	88.54970348	0.666666667	CRA004775;PRJNA827465;Yang2025_Silkie_Wencha	0.924566577
ENSGALG00010026111	ENSGALG0001002611	3	-0.086316358	0.269681424	-0.320067867	0.748916884	0.192977081	22.02016214	90.91741474	0.666666667	CRA004775;PRJNA827465;Yang2025_Silkie_Wencha	0.924760066
ENSGALG00010001226	PEF1	3	0.05036728	0.157362755	0.320071165	0.748914384	0.051453597	6.692020203	70.11365867	0.666666667	CRA004775;PRJNA827465;Yang2025_Silkie_Wencha	0.924760066
ENSGALG00010020127	FRMD4B	3	-0.026906379	0.084076658	-0.320021991	0.74895166	0	0.227219023	0	0.666666667	CRA004775;PRJNA827465;Yang2025_Silkie_Wencha	0.924760066
ENSGALG00010021415	SIRT4	3	-0.062408325	0.195486644	-0.31968834	0.749208345	0.076125681	6.699726962	70.14803721	0.666666667	CRA004775;PRJNA827465;Yang2025_Silkie_Wencha	0.924996227
ENSGALG00010016756	NUDT16L1	3	0.01612657	0.050524263	0.319184677	0.749586476	0	1.088592479	0	0.666666667	CRA004775;PRJNA827465;Yang2025_Silkie_Wencha	0.925382274
ENSGALG00010023219	TEX9	3	0.099535542	0.312137561	0.318883577	0.749814798	0.232336632	13.64782227	85.34564738	0.666666667	CRA004775;PRJNA827465;Yang2025_Silkie_Wencha	0.925421738
ENSGALG00010013259	PTPRB	3	-0.024223279	0.075959644	-0.318896683	0.749804859	0	1.044222659	0	0.666666667	CRA004775;PRJNA827465;Yang2025_Silkie_Wencha	0.925421738
ENSGALG00010024389	AAAS	3	-0.020435774	0.064062838	-0.318995771	0.749729719	0	0.645268046	0	0.666666667	CRA004775;PRJNA827465;Yang2025_Silkie_Wencha	0.925421738
ENSGALG00010018470	IQCB1	3	-0.047310815	0.148488215	-0.318616633	0.750017239	0.032458903	4.03567827	50.44203561	0.666666667	CRA004775;PRJNA827465;Yang2025_Silkie_Wencha	0.925590795
ENSGALG00010009091	EXT1	3	-0.027764935	0.087213564	-0.318355697	0.75021514	0.014465789	6.153254928	67.49687729	0.666666667	CRA004775;PRJNA827465;Yang2025_Silkie_Wencha	0.925754221
ENSGALG00010028868	ENSGALG0001002886	2	0.467552271	1.472865772	0.317443911	0.750906792	0	0.04297375	0	0.5	PRJNA827465;Yang2025_Silkie_Wenchang	0.926105027
ENSGALG00010009557	CIDEC	3	-0.113985254	0.35924608	-0.31729018	0.751023428	0	1.688798625	0	0.666666667	CRA004775;PRJNA827465;Yang2025_Silkie_Wencha	0.926105027
ENSGALG00010001415	MC5R	3	-0.085439206	0.268923665	-0.317708022	0.750706425	0.131000301	5.652226663	64.61571485	0.666666667	CRA004775;PRJNA827465;Yang2025_Silkie_Wencha	0.926105027
ENSGALG00010002544	THSD1	3	-0.074689486	0.235347581	-0.317358206	0.750971816	0.13369135	15.40101074	87.01383933	0.666666667	CRA004775;PRJNA827465;Yang2025_Silkie_Wencha	0.926105027
ENSGALG00010012382	SH3PXD2B	3	-0.054219997	0.17087084	-0.317315681	0.75100408	0.057241071	8.571729332	76.66748537	0.666666667	CRA004775;PRJNA827465;Yang2025_Silkie_Wencha	0.926105027
ENSGALG00010009379	RYR1	3	-0.047276065	0.149455324	-0.317810922	0.750628365	0.026758964	3.366868541	40.49762133	0.666666667	CRA004775;PRJNA827465;Yang2025_Silkie_Wencha	0.926105027
ENSGALG00010018149	ENSGALG0001001814	3	0.044823709	0.14126328	0.317306158	0.751011305	0.0198457	2.937201718	31.90797937	0.666666667	CRA004775;PRJNA827465;Yang2025_Silkie_Wencha	0.926105027
ENSGALG00010009111	LRFN1	3	0.038978526	0.122756138	0.31752812	0.750842906	0.014832462	2.939323905	31.95714169	0.666666667	CRA004775;PRJNA827465;Yang2025_Silkie_Wencha	0.926105027
ENSGALG00010021228	ESRP2	3	0.357995258	1.128956251	0.317102862	0.751165554	3.014242264	9.588266637	79.14117248	0.666666667	CRA004775;PRJNA827465;Yang2025_Silkie_Wencha	0.926151622
ENSGALG00010024490	TGM3	3	0.13069551	0.412306514	0.316986285	0.75125409	0.149063567	2.825708552	29.22129217	0.666666667	CRA004775;PRJNA827465;Yang2025_Silkie_Wencha	0.926151622
ENSGALG00010000387	DTX1	3	-0.072629991	0.229130143	-0.31698139	0.751257723	0	0.630351407	0	0.666666667	CRA004775;PRJNA827465;Yang2025_Silkie_Wencha	0.926151622
ENSGALG00010024467	ENSGALG0001002446	3	-0.049218878	0.15540784	-0.316707816	0.751465317	0	0.646952827	0	0.666666667	CRA004775;PRJNA827465;Yang2025_Silkie_Wencha	0.926165283
ENSGALG00010029740	HASPIN	3	0.027455431	0.086683613	0.316731496	0.751447348	0	1.419816546	0	0.666666667	CRA004775;PRJNA827465;Yang2025_Silkie_Wencha	0.926165283
ENSGALG00010020092	SDHAF2	3	0.023554854	0.074339229	0.316856316	0.75135263	0	0.099093586	0	0.666666667	CRA004775;PRJNA827465;Yang2025_Silkie_Wencha	0.926165283
ENSGALG00010012064	RGS14	3	0.076638742	0.24210621	0.316550087	0.751585014	0.125985408	8.414406694	76.23124157	0.666666667	CRA004775;PRJNA827465;Yang2025_Silkie_Wencha	0.926232068
ENSGALG00010011378	TUBB2B	3	-0.03858805	0.121952969	-0.316417471	0.751685658	0	0.333246074	0	0.666666667	CRA004775;PRJNA827465;Yang2025_Silkie_Wencha	0.926275363
ENSGALG00010015838	IFNLR1	3	0.284693034	0.901604297	0.31576273	0.752182609	0	0.017008034	0	1	CRA004775;PRJNA827465;Yang2025_Silkie_Wencha	0.926643515
ENSGALG00010023070	SIDT2	3	0.048755585	0.154425448	0.315722475	0.752213166	0.052433262	11.03524088	81.87624519	0.666666667	CRA004775;PRJNA827465;Yang2025_Silkie_Wencha	0.926643515
ENSGALG00010008025	TBC1D22A	3	0.028151076	0.089176421	0.315678469	0.752246571	0.015354862	5.697672862	64.89794959	0.666666667	CRA004775;PRJNA827465;Yang2025_Silkie_Wencha	0.926643515
ENSGALG00010023103	DGKD	3	0.017919548	0.056748634	0.315770558	0.752176667	0	0.344062248	0	0.666666667	CRA004775;PRJNA827465;Yang2025_Silkie_Wencha	0.926643515
ENSGALG00010013227	CITED4	3	-0.071204134	0.225628699	-0.315581016	0.752320549	0.094872619	6.573210759	69.57346914	0.666666667	CRA004775;PRJNA827465;Yang2025_Silkie_Wencha	0.926653911
ENSGALG00010023380	USP5	3	-0.035305071	0.111936015	-0.315404039	0.7524549	0.020732561	4.50778406	55.63230241	0.666666667	CRA004775;PRJNA827465;Yang2025_Silkie_Wencha	0.926738662
ENSGALG00010012845	HDHD2	3	-0.048565914	0.154120934	-0.315115622	0.752673868	0.025359875	3.086400809	35.19960226	0.666666667	CRA004775;PRJNA827465;Yang2025_Silkie_Wencha	0.926846875
ENSGALG00010000603	ZNF622	3	0.037623424	0.119376859	0.315165139	0.752636273	0.026630771	5.412524816	63.04866827	0.666666667	CRA004775;PRJNA827465;Yang2025_Silkie_Wencha	0.926846875
ENSGALG00010009676	ACO1	3	-0.05149092	0.163478971	-0.314969686	0.75278467	0.048475743	5.393584225	62.91890668	0.666666667	CRA004775;PRJNA827465;Yang2025_Silkie_Wencha	0.926902591
ENSGALG00010003740	PITX2	3	0.055341202	0.175839702	0.314725291	0.75297024	0.055174979	6.324506762	68.37698061	0.666666667	CRA004775;PRJNA827465;Yang2025_Silkie_Wencha	0.926966489
ENSGALG00010001209	CDH7	3	0.034214993	0.108742502	0.314642313	0.753033248	0	1.776834172	0	0.666666667	CRA004775;PRJNA827465;Yang2025_Silkie_Wencha	0.926966489
ENSGALG00010018556	MRPL2	3	-0.028215433	0.08966686	-0.314669577	0.753012546	0	0.49776229	0	0.666666667	CRA004775;PRJNA827465;Yang2025_Silkie_Wencha	0.926966489
ENSGALG00010024173	CBLN1	3	0.215206613	0.685410492	0.313982081	0.753534648	1.127496413	10.99911255	81.81671484	0.666666667	CRA004775;PRJNA827465;Yang2025_Silkie_Wencha	0.926971176
ENSGALG00010024573	ENSGALG0001002457	3	0.192336556	0.6121488	0.314199026	0.753369883	1.02754538	26.66131186	92.49849366	0.666666667	CRA004775;PRJNA827465;Yang2025_Silkie_Wencha	0.926971176
ENSGALG00010009619	ENSGALG0001000961	3	-0.172922337	0.550589994	-0.314067343	0.753469892	0.824503563	23.74550615	91.57735368	0.666666667	CRA004775;PRJNA827465;Yang2025_Silkie_Wencha	0.926971176
ENSGALG00010021776	PMS2	3	0.053592066	0.170664394	0.314020193	0.753505702	0.06630632	10.37886297	80.73006643	0.666666667	CRA004775;PRJNA827465;Yang2025_Silkie_Wencha	0.926971176
ENSGALG00010029586	COIL	3	0.036756833	0.11699246	0.314181217	0.753383407	0.028648338	7.404501704	72.98940455	0.666666667	CRA004775;PRJNA827465;Yang2025_Silkie_Wencha	0.926971176
ENSGALG00010011935	ST8SIA4	3	0.036392634	0.115855751	0.314120222	0.753429732	0.010691294	2.606049281	23.25548045	0.666666667	CRA004775;PRJNA827465;Yang2025_Silkie_Wencha	0.926971176
ENSGALG00010013132	SKIC3	3	-0.02702609	0.085927123	-0.314523385	0.753123558	0	1.004275659	0	0.666666667	CRA004775;PRJNA827465;Yang2025_Silkie_Wencha	0.926971176
ENSGALG00010023359	CDCA3	3	-0.023806058	0.075828352	-0.313946667	0.753561546	0	1.37460922	0	0.666666667	CRA004775;PRJNA827465;Yang2025_Silkie_Wencha	0.926971176
ENSGALG00010028188	ENSGALG0001002818	3	0.088733859	0.282849586	0.313713942	0.753738311	0.205947809	18.23380973	89.03136519	0.666666667	CRA004775;PRJNA827465;Yang2025_Silkie_Wencha	0.926994818
ENSGALG00010021154	CHST13	3	0.060656296	0.193442543	0.313562336	0.753853469	0.071128349	6.392911047	68.71534759	0.666666667	CRA004775;PRJNA827465;Yang2025_Silkie_Wencha	0.926994818
ENSGALG00010025423	NGF	3	0.058405313	0.186315794	0.313474833	0.753919938	0.027344084	2.64936052	24.51008517	0.666666667	CRA004775;PRJNA827465;Yang2025_Silkie_Wencha	0.926994818
ENSGALG00010012052	KLF10	3	0.036682173	0.116972672	0.313596097	0.753827824	0.018962289	3.7893507	47.2205093	0.666666667	CRA004775;PRJNA827465;Yang2025_Silkie_Wencha	0.926994818
ENSGALG00010029407	UBE2O	3	0.025520399	0.081335286	0.313767868	0.753697351	0.008878321	3.656971699	45.30994044	0.666666667	CRA004775;PRJNA827465;Yang2025_Silkie_Wencha	0.926994818
ENSGALG00010022751	SLC25A12	3	-0.023666509	0.075514508	-0.313403477	0.753974143	0	0.186348311	0	0.666666667	CRA004775;PRJNA827465;Yang2025_Silkie_Wencha	0.926994818
ENSGALG00010024496	UEVLD	3	0.078822466	0.251661861	0.313207834	0.754122767	0.152519404	17.38666443	88.49693103	0.666666667	CRA004775;PRJNA827465;Yang2025_Silkie_Wencha	0.927096931
ENSGALG00010000172	ENSGALG0001000017	3	-0.167777254	0.536260354	-0.312865295	0.754383006	0	0.430541698	0	0.666666667	CRA004775;PRJNA827465;Yang2025_Silkie_Wencha	0.927259321
ENSGALG00010026920	GATA2	3	0.084510095	0.270119982	0.312861324	0.754386022	0.13641215	6.086786385	67.14193873	0.666666667	CRA004775;PRJNA827465;Yang2025_Silkie_Wencha	0.927259321
ENSGALG00010013087	GPN2	3	-0.074435298	0.2382647	-0.312405899	0.754732068	0.138033921	11.76313781	82.99773383	0.666666667	CRA004775;PRJNA827465;Yang2025_Silkie_Wencha	0.927441599
ENSGALG00010015310	PGM2	3	-0.060235776	0.192865048	-0.31232085	0.754796696	0.086629844	16.38158537	87.79116945	0.666666667	CRA004775;PRJNA827465;Yang2025_Silkie_Wencha	0.927441599
ENSGALG00010021223	DFFB	3	0.041370375	0.132356647	0.312567415	0.754609338	0.031041727	5.453247741	63.32460774	0.666666667	CRA004775;PRJNA827465;Yang2025_Silkie_Wencha	0.927441599
ENSGALG00010004787	RIOK3	3	-0.040966313	0.13113838	-0.312389959	0.75474418	0.036840596	10.22662781	80.44321122	0.666666667	CRA004775;PRJNA827465;Yang2025_Silkie_Wencha	0.927441599
ENSGALG00010024673	REXO2	3	-0.012380351	0.039657617	-0.312180901	0.754903046	0	1.116565007	0	0.666666667	CRA004775;PRJNA827465;Yang2025_Silkie_Wencha	0.927491672
ENSGALG00010022622	MPPED2	3	-0.031019728	0.099406241	-0.312050109	0.755002442	0.012417716	3.294772594	39.29778329	0.666666667	CRA004775;PRJNA827465;Yang2025_Silkie_Wencha	0.927533193
ENSGALG00010008318	ADPRH	3	0.07929696	0.254559122	0.311507044	0.755415189	0.167142761	16.13008503	87.60080932	0.666666667	CRA004775;PRJNA827465;Yang2025_Silkie_Wencha	0.927782636
ENSGALG00010009666	RRAD	3	-0.04131516	0.132678154	-0.311393841	0.755501235	0	0.605335453	0	0.666666667	CRA004775;PRJNA827465;Yang2025_Silkie_Wencha	0.927782636
ENSGALG00010018396	TBL1XR1	3	0.036980547	0.118688179	0.311577343	0.755361755	0.024032312	4.528829057	55.83847447	0.666666667	CRA004775;PRJNA827465;Yang2025_Silkie_Wencha	0.927782636
ENSGALG00010008658	TSTA3	3	0.027875608	0.089471112	0.311559869	0.755375037	0	1.851228219	0	0.666666667	CRA004775;PRJNA827465;Yang2025_Silkie_Wencha	0.927782636
ENSGALG00010029566	TMEM97	3	-0.018108044	0.058159527	-0.311351291	0.755533579	0	1.088752156	0	0.666666667	CRA004775;PRJNA827465;Yang2025_Silkie_Wencha	0.927782636
ENSGALG00010025268	ZBTB39	3	-0.063664584	0.205014712	-0.310536662	0.756152885	0.104264171	12.41965784	83.89649678	0.666666667	CRA004775;PRJNA827465;Yang2025_Silkie_Wencha	0.928037121
ENSGALG00010000670	ENSGALG0001000067	2	-0.061190753	0.197011531	-0.310594779	0.756108697	0	0.198326264	0	0.5	PRJNA827465;Yang2025_Silkie_Wenchang	0.928037121
ENSGALG00010028028	FAM174C	3	-0.057086399	0.183853941	-0.310498639	0.756181795	0.05554522	4.401260734	54.55847493	0.666666667	CRA004775;PRJNA827465;Yang2025_Silkie_Wencha	0.928037121
ENSGALG00010019257	SLC1A4	3	-0.052416133	0.168712216	-0.310683683	0.756041103	0.054769808	7.287367768	72.55524816	0.666666667	CRA004775;PRJNA827465;Yang2025_Silkie_Wencha	0.928037121
ENSGALG00010007856	PDCD2	3	0.045425508	0.146251038	0.310599556	0.756105064	0.040076958	6.026674477	66.8142023	0.666666667	CRA004775;PRJNA827465;Yang2025_Silkie_Wencha	0.928037121
ENSGALG00010010334	ZBTB41	3	0.03876182	0.124657857	0.310945661	0.755841931	0.022128513	3.568776541	43.95838527	0.666666667	CRA004775;PRJNA827465;Yang2025_Silkie_Wencha	0.928037121
ENSGALG00010005565	PGAP4	3	-0.038105975	0.12280299	-0.310301689	0.756331547	0	1.083898005	0	0.666666667	CRA004775;PRJNA827465;Yang2025_Silkie_Wencha	0.928037121
ENSGALG00010023646	DPH5	3	-0.020937259	0.067461509	-0.31035859	0.756288281	0	0.801227209	0	0.666666667	CRA004775;PRJNA827465;Yang2025_Silkie_Wencha	0.928037121
ENSGALG00010028425	DAP3	3	0.018585848	0.05989113	0.310327232	0.756312125	0	1.649991096	0	0.666666667	CRA004775;PRJNA827465;Yang2025_Silkie_Wencha	0.928037121
ENSGALG00010028465	ENSGALG0001002846	3	-0.030376989	0.097989715	-0.310001806	0.756559583	0	0.22757919	0	0.666666667	CRA004775;PRJNA827465;Yang2025_Silkie_Wencha	0.928236371
ENSGALG00010013380	PHGDH	3	0.061613435	0.199120805	0.309427409	0.756996423	0.058806798	4.103672057	51.26316206	0.666666667	CRA004775;PRJNA827465;Yang2025_Silkie_Wencha	0.928315761
ENSGALG00010011586	ENSGALG0001001158	3	-0.049932028	0.161314437	-0.309532296	0.756916649	0.005637841	2.126832314	5.963437412	0.666666667	CRA004775;PRJNA827465;Yang2025_Silkie_Wencha	0.928315761
ENSGALG00010025413	NECAB3	3	0.042532521	0.137380552	0.309596375	0.756867914	0.037298112	7.233899081	72.35239284	0.666666667	CRA004775;PRJNA827465;Yang2025_Silkie_Wencha	0.928315761
ENSGALG00010013021	SLC25A5	3	-0.040959135	0.132402897	-0.309352257	0.757053584	0.031664175	5.013671916	60.10907707	0.666666667	CRA004775;PRJNA827465;Yang2025_Silkie_Wencha	0.928315761
ENSGALG00010024681	ATP6V0A2	3	-0.035975585	0.116229476	-0.309522043	0.756924447	0.028661443	7.223695961	72.31334194	0.666666667	CRA004775;PRJNA827465;Yang2025_Silkie_Wencha	0.928315761
ENSGALG00010010322	ENSGALG0001001032	3	-0.028834257	0.093164495	-0.309498345	0.756942471	0	1.797753503	0	0.666666667	CRA004775;PRJNA827465;Yang2025_Silkie_Wencha	0.928315761
ENSGALG00010018310	APOOL	3	0.015196258	0.049129157	0.30931242	0.757083884	0.000928886	2.224423312	10.08905591	0.666666667	CRA004775;PRJNA827465;Yang2025_Silkie_Wencha	0.928315761
ENSGALG00010017727	AZIN2	3	-0.031633713	0.102308448	-0.309199421	0.757169834	0.00688661	2.547685762	21.49738284	0.666666667	CRA004775;PRJNA827465;Yang2025_Silkie_Wencha	0.928340642
ENSGALG00010011027	FAM234B	3	0.035544804	0.11503379	0.308994467	0.757325736	0.02076421	4.519353794	55.74588556	0.666666667	CRA004775;PRJNA827465;Yang2025_Silkie_Wencha	0.928451277
ENSGALG00010028219	ENSGALG0001002821	2	-0.326260078	1.056193641	-0.308901763	0.757396255	0	0.890093455	0	0.5	PRJNA827465;Yang2025_Silkie_Wenchang	0.928457227
ENSGALG00010026109	SPDEF	2	0.484006675	1.569754261	0.308332767	0.757829135	4.495982575	11.39997996	91.22805476	0.5	CRA004775;PRJNA827465	0.928544362
ENSGALG00010014145	NTMT2	3	-0.139993385	0.453606322	-0.30862309	0.757608254	0.466802517	11.18812801	82.1239085	0.666666667	CRA004775;PRJNA827465;Yang2025_Silkie_Wencha	0.928544362
ENSGALG00010029687	SLC16A5	3	0.081449977	0.264262298	0.308216412	0.757917665	0.115723386	4.801954971	58.35029666	0.666666667	CRA004775;PRJNA827465;Yang2025_Silkie_Wencha	0.928544362
ENSGALG00010010823	TMEFF2	3	-0.066626839	0.21623822	-0.308117774	0.757992717	0.093485527	6.44877296	68.98634806	0.666666667	CRA004775;PRJNA827465;Yang2025_Silkie_Wencha	0.928544362
ENSGALG00010021931	ITGA6	3	0.051605011	0.167389773	0.308292497	0.757859775	0.058275456	11.84924811	83.1212919	0.666666667	CRA004775;PRJNA827465;Yang2025_Silkie_Wencha	0.928544362
ENSGALG00010007895	GOLGB1	3	0.044284374	0.14370551	0.308160581	0.757960146	0.037600469	5.045992506	60.3645864	0.666666667	CRA004775;PRJNA827465;Yang2025_Silkie_Wencha	0.928544362
ENSGALG00010020186	MGA	3	0.042487322	0.137772001	0.308388653	0.757786615	0.027824595	3.697377802	45.90761055	0.666666667	CRA004775;PRJNA827465;Yang2025_Silkie_Wencha	0.928544362
ENSGALG00010011107	ENSGALG0001001110	3	-0.039793209	0.129001489	-0.308470926	0.75772402	0.008065711	2.343762522	14.66712258	0.666666667	CRA004775;PRJNA827465;Yang2025_Silkie_Wencha	0.928544362
ENSGALG00010004333	DIO2	3	0.079603715	0.258999256	0.307351132	0.75857612	0	1.576031261	0	0.666666667	CRA004775;PRJNA827465;Yang2025_Silkie_Wencha	0.928937101
ENSGALG00010024637	DUSP8	3	0.05934637	0.193079571	0.30736742	0.758563724	0.053806205	4.217247457	52.57570203	0.666666667	CRA004775;PRJNA827465;Yang2025_Silkie_Wencha	0.928937101
ENSGALG00010017811	HACD3	3	-0.040539475	0.131830975	-0.307511001	0.758454451	0.037582978	11.18666039	82.12156327	0.666666667	CRA004775;PRJNA827465;Yang2025_Silkie_Wencha	0.928937101
ENSGALG00010024112	NAP1L4	3	0.036603289	0.11905268	0.307454557	0.758497408	0.028787685	11.01356616	81.8405776	0.666666667	CRA004775;PRJNA827465;Yang2025_Silkie_Wencha	0.928937101
ENSGALG00010006621	DACT2	3	-0.040391975	0.131499275	-0.307164999	0.758717786	0.017249166	2.904601214	31.14373186	0.666666667	CRA004775;PRJNA827465;Yang2025_Silkie_Wencha	0.928949668
ENSGALG00010019013	LRRK1	3	0.032268088	0.105047121	0.307177274	0.758708443	0.016415551	4.119139524	51.44617004	0.666666667	CRA004775;PRJNA827465;Yang2025_Silkie_Wencha	0.928949668
ENSGALG00010006570	ENSGALG0001000657	3	0.193883214	0.632046935	0.306754457	0.759030276	1.025608807	14.18902719	85.90460098	0.666666667	CRA004775;PRJNA827465;Yang2025_Silkie_Wencha	0.929003132
ENSGALG00010017708	MEIS1	3	0.066597104	0.216998985	0.306900531	0.758919085	0.117083456	12.92610023	84.52742928	0.666666667	CRA004775;PRJNA827465;Yang2025_Silkie_Wencha	0.929003132
ENSGALG00010004219	FGF2	3	-0.053942354	0.17589361	-0.30667603	0.759089977	0.061255621	7.836269982	74.47765321	0.666666667	CRA004775;PRJNA827465;Yang2025_Silkie_Wencha	0.929003132
ENSGALG00010009724	ISOC1	3	-0.042497739	0.138554199	-0.306722855	0.759054332	0.033424892	4.919686881	59.34700625	0.666666667	CRA004775;PRJNA827465;Yang2025_Silkie_Wencha	0.929003132
ENSGALG00010004970	RBM48	3	0.024406272	0.079500834	0.306993912	0.758848006	0	0.295596652	0	0.666666667	CRA004775;PRJNA827465;Yang2025_Silkie_Wencha	0.929003132
ENSGALG00010010431	SOCS2	3	0.043198745	0.140910573	0.306568515	0.759171822	0	0.006277407	0	1	CRA004775;PRJNA827465;Yang2025_Silkie_Wencha	0.929022883
ENSGALG00010027557	C6orf132	3	0.188906398	0.617433774	0.305954105	0.75963959	0.363831606	2.913155261	31.34591805	0.666666667	CRA004775;PRJNA827465;Yang2025_Silkie_Wencha	0.92904144
ENSGALG00010011430	ENSGALG0001001143	3	0.120231766	0.392585601	0.30625618	0.759409601	0.305374599	6.617726797	69.77814193	0.666666667	CRA004775;PRJNA827465;Yang2025_Silkie_Wencha	0.92904144
ENSGALG00010005447	MYO3A	2	0.088202896	0.288218963	0.306027388	0.759583793	0.104458634	2.56764157	61.05375409	0.5	PRJNA827465;Yang2025_Silkie_Wenchang	0.92904144
ENSGALG00010025298	ENSGALG0001002529	3	-0.072102703	0.235647343	-0.305977154	0.759622041	0.063176189	2.788989277	28.28943388	0.666666667	CRA004775;PRJNA827465;Yang2025_Silkie_Wencha	0.92904144
ENSGALG00010023263	SRP14	3	-0.060839541	0.198663875	-0.306243606	0.759419174	0.101070769	17.33928829	88.4655012	0.666666667	CRA004775;PRJNA827465;Yang2025_Silkie_Wencha	0.92904144
ENSGALG00010022460	VAT1	3	0.035922496	0.117448219	0.305858154	0.759712648	0.016400245	3.192887657	37.36077762	0.666666667	CRA004775;PRJNA827465;Yang2025_Silkie_Wencha	0.92904144
ENSGALG00010014041	SKIV2L2	3	0.029054469	0.094976267	0.305912939	0.759670934	0	0.456604386	0	0.666666667	CRA004775;PRJNA827465;Yang2025_Silkie_Wencha	0.92904144
ENSGALG00010020315	ANO5	3	-0.018856276	0.06153023	-0.306455482	0.75925787	0	1.775434207	0	0.666666667	CRA004775;PRJNA827465;Yang2025_Silkie_Wencha	0.92904144
ENSGALG00010001162	ENSGALG0001000116	3	0.059883948	0.195868465	0.305735525	0.759806022	0.047759584	3.203685872	37.57190686	0.666666667	CRA004775;PRJNA827465;Yang2025_Silkie_Wencha	0.92907527
ENSGALG00010024340	NETO2	3	0.076134737	0.249242235	0.305464831	0.760012152	0.089483151	3.788429218	47.20767144	0.666666667	CRA004775;PRJNA827465;Yang2025_Silkie_Wencha	0.929117181
ENSGALG00010024017	TYW3	3	0.052141093	0.170654919	0.305535247	0.759958529	0.051658459	4.934519407	59.46920389	0.666666667	CRA004775;PRJNA827465;Yang2025_Silkie_Wencha	0.929117181
ENSGALG00010020109	WDFY4	3	0.039177875	0.128270523	0.305431628	0.760037436	0	0.36030383	0	0.666666667	CRA004775;PRJNA827465;Yang2025_Silkie_Wencha	0.929117181
ENSGALG00010013041	LRP12	3	-0.069082268	0.226317069	-0.305245503	0.760179178	0.140216257	37.6038403	94.68139428	0.666666667	CRA004775;PRJNA827465;Yang2025_Silkie_Wencha	0.929129789
ENSGALG00010023097	LURAP1	3	0.032008376	0.104850377	0.305276692	0.760155426	0	0.914256183	0	0.666666667	CRA004775;PRJNA827465;Yang2025_Silkie_Wencha	0.929129789
ENSGALG00010004181	BBS12	3	-0.025797698	0.084566808	-0.305057018	0.760322727	0.003364349	2.291327933	12.71437097	0.666666667	CRA004775;PRJNA827465;Yang2025_Silkie_Wencha	0.929144601
ENSGALG00010026588	CCND3	3	-0.020356834	0.066719722	-0.305109693	0.760282609	0	0.915627448	0	0.666666667	CRA004775;PRJNA827465;Yang2025_Silkie_Wencha	0.929144601
ENSGALG00010007226	ENSGALG0001000722	3	0.397904634	1.30623119	0.304620374	0.760655301	4.17116673	11.45089287	82.53411308	0.666666667	CRA004775;PRJNA827465;Yang2025_Silkie_Wencha	0.929197997
ENSGALG00010024208	SERPIND1	3	0.229147883	0.752145646	0.304658924	0.760625937	1.344673358	10.14452474	80.28493151	0.666666667	CRA004775;PRJNA827465;Yang2025_Silkie_Wencha	0.929197997
ENSGALG00010027908	PBXIP1	3	0.124122357	0.40775767	0.304402262	0.760821445	0.457777394	34.82875395	94.25761828	0.666666667	CRA004775;PRJNA827465;Yang2025_Silkie_Wencha	0.929197997
ENSGALG00010000593	ATPSCKMT	3	0.08772907	0.288040746	0.304571734	0.760692351	0.190303216	8.815556677	77.31283374	0.666666667	CRA004775;PRJNA827465;Yang2025_Silkie_Wencha	0.929197997
ENSGALG00010002149	ENSGALG0001000214	2	0.060402199	0.198443224	0.304380253	0.760838211	0	0.161184267	0	1	PRJNA827465;Yang2025_Silkie_Wenchang	0.929197997
ENSGALG00010029530	ENSGALG0001002953	3	0.039896612	0.131092444	0.304339526	0.760869235	0	1.183838645	0	0.666666667	CRA004775;PRJNA827465;Yang2025_Silkie_Wencha	0.929197997
ENSGALG00010021947	CDK2AP2	3	0.0326271	0.107204792	0.304343672	0.760866077	0.004639317	2.211667943	9.570511854	0.666666667	CRA004775;PRJNA827465;Yang2025_Silkie_Wencha	0.929197997
ENSGALG00010028158	DDX49	3	-0.016446177	0.05404426	-0.304309418	0.760892171	0	0.193406637	0	0.666666667	CRA004775;PRJNA827465;Yang2025_Silkie_Wencha	0.929197997
ENSGALG00010004257	ENSGALG0001000425	3	0.195811017	0.643919241	0.304092508	0.761057415	0.853156407	6.693710283	70.1212046	0.666666667	CRA004775;PRJNA827465;Yang2025_Silkie_Wencha	0.929319526
ENSGALG00010029463	ENSGALG0001002946	2	0.428455499	1.413996237	0.303010353	0.761881972	3.591442127	9.775787983	89.77064558	0.5	CRA004775;Yang2025_Silkie_Wenchang	0.929547529
ENSGALG00010019580	CD5	2	-0.231535784	0.767046377	-0.301853696	0.762763595	0.854340658	3.65004895	72.60310714	0.5	CRA004775;Yang2025_Silkie_Wenchang	0.929547529
ENSGALG00010005738	ELAVL2	2	0.167798682	0.556184739	0.301695947	0.762883858	0.347358708	2.207303319	54.6958503	0.5	PRJNA827465;Yang2025_Silkie_Wenchang	0.929547529

ENSGALG00010014753	ENSGALG0001001475	2	-0.113536012	0.374817813	-0.302909863	0.761958555	0.135260573	1.918992369	47.88931857	0.5	PRJNA827465;Yang2025_Silkie_Wenchang	0.929547529
ENSGALG00010021664	TYSND1	3	0.112717467	0.371225116	0.303636424	0.761404899	0.342186202	16.89783224	88.1641623	0.666666667	CRA004775;PRJNA827465;Yang2025_Silkie_Wencha	0.929547529
ENSGALG00010012445	SOBP	3	0.069674217	0.230797356	0.301884814	0.762739872	0.097544535	5.293724715	62.21941813	0.666666667	CRA004775;PRJNA827465;Yang2025_Silkie_Wencha	0.929547529
ENSGALG00010008943	ENSGALG0001000894	3	-0.062872012	0.208042957	-0.302206875	0.762494364	0.044160505	2.960684109	32.44804489	0.666666667	CRA004775;PRJNA827465;Yang2025_Silkie_Wencha	0.929547529
ENSGALG00010018638	SEC14L5	3	-0.062632627	0.207147561	-0.302357542	0.762379518	0.084556546	6.39847555	68.74255462	0.666666667	CRA004775;PRJNA827465;Yang2025_Silkie_Wencha	0.929547529
ENSGALG00010021599	ARPC4	3	-0.061801588	0.204219782	-0.302622924	0.762177243	0.097355834	9.859494314	79.71498399	0.666666667	CRA004775;PRJNA827465;Yang2025_Silkie_Wencha	0.929547529
ENSGALG00010016842	NPY7R	3	-0.056238787	0.185337763	-0.303439441	0.761554992	0.032571684	2.872013117	30.36243503	0.666666667	CRA004775;PRJNA827465;Yang2025_Silkie_Wencha	0.929547529
ENSGALG00010028366	MRRF	3	0.054271946	0.179847643	0.301766236	0.762830271	0.075806951	11.93745146	83.24600518	0.666666667	CRA004775;PRJNA827465;Yang2025_Silkie_Wencha	0.929547529
ENSGALG00010015896	LONRF2	3	-0.046882174	0.155064103	-0.3023406	0.762392431	0.05305571	10.32991331	80.6387533	0.666666667	CRA004775;PRJNA827465;Yang2025_Silkie_Wencha	0.929547529
ENSGALG00010016139	LURAP1L	3	0.045195747	0.149581482	0.302148012	0.762539234	0	0.166118726	0	0.666666667	CRA004775;PRJNA827465;Yang2025_Silkie_Wencha	0.929547529
ENSGALG00010022599	RNLS	3	-0.042360418	0.140092202	-0.302375275	0.762366001	0.001156453	2.036508465	1.792698911	0.666666667	CRA004775;PRJNA827465;Yang2025_Silkie_Wencha	0.929547529
ENSGALG00010021680	PIGT	3	0.040796833	0.134840398	0.302556459	0.762227901	0.036433665	6.371554646	68.61048659	0.666666667	CRA004775;PRJNA827465;Yang2025_Silkie_Wencha	0.929547529
ENSGALG00010021024	ENSGALG0001002102	3	0.03955263	0.130337237	0.303463775	0.76153645	0.029235573	5.053583116	60.4241198	0.666666667	CRA004775;PRJNA827465;Yang2025_Silkie_Wencha	0.929547529
ENSGALG00010015618	NCL	3	0.03857065	0.127655767	0.30214577	0.762540942	0.030103799	5.280506297	62.12484395	0.666666667	CRA004775;PRJNA827465;Yang2025_Silkie_Wencha	0.929547529
ENSGALG00010028884	VAV2	3	-0.038086476	0.12602305	-0.302218331	0.762485631	0.030411907	8.382501872	76.14077479	0.666666667	CRA004775;PRJNA827465;Yang2025_Silkie_Wencha	0.929547529
ENSGALG00010017547	RNF4	3	-0.03737384	0.123832581	-0.301809428	0.762797343	0.037599294	18.63457144	89.26726055	0.666666667	CRA004775;PRJNA827465;Yang2025_Silkie_Wencha	0.929547529
ENSGALG00010016754	ENSGALG0001001675	3	-0.036801921	0.12122666	-0.303579432	0.761448323	0	0.521000984	0	0.666666667	CRA004775;PRJNA827465;Yang2025_Silkie_Wencha	0.929547529
ENSGALG00010011212	NCKAP1	3	0.032411064	0.10743137	0.301690877	0.762887724	0.022332888	5.707175338	64.95639469	0.666666667	CRA004775;PRJNA827465;Yang2025_Silkie_Wencha	0.929547529
ENSGALG00010018857	ENSGALG0001001885	3	0.025626082	0.084687651	0.302595264	0.762198324	0.006292979	2.517235665	20.54776485	0.666666667	CRA004775;PRJNA827465;Yang2025_Silkie_Wencha	0.929547529
ENSGALG00010004434	TCP1	3	0.01679556	0.055524283	0.30249036	0.762278281	0	0.531769911	0	0.666666667	CRA004775;PRJNA827465;Yang2025_Silkie_Wencha	0.929547529
ENSGALG00010015668	ZNF217	3	0.016314949	0.053861698	0.302904473	0.761962663	0	0.263249907	0	0.666666667	CRA004775;PRJNA827465;Yang2025_Silkie_Wencha	0.929547529
ENSGALG00010021074	TTC17	3	0.010813106	0.035655032	0.303270118	0.761684017	0	0.30048781	0	1	CRA004775;PRJNA827465;Yang2025_Silkie_Wencha	0.929547529
ENSGALG00010020613	ACTG2	3	0.119603073	0.396686327	0.301505408	0.763029127	0.359083546	9.083129438	77.98115712	0.666666667	CRA004775;PRJNA827465;Yang2025_Silkie_Wencha	0.929559609
ENSGALG00010020493	DEPDC7	3	0.069306463	0.229853189	0.301524913	0.763014256	0.115030373	8.996594726	77.76936651	0.666666667	CRA004775;PRJNA827465;Yang2025_Silkie_Wencha	0.929559609
ENSGALG00010002420	TMEM272	2	0.092512371	0.307217841	0.301129552	0.763315707	0	0.262454	0	0.5	PRJNA827465;Yang2025_Silkie_Wenchang	0.929610339
ENSGALG00010029572	TSPOAP1	3	0.08068923	0.268296088	0.300746948	0.763607466	0.189839776	19.03587947	89.49352457	0.666666667	CRA004775;PRJNA827465;Yang2025_Silkie_Wencha	0.929610339
ENSGALG00010013370	CNKSR1	3	-0.072049029	0.239435741	-0.300911756	0.763481785	0.095680981	5.230742297	61.764509	0.666666667	CRA004775;PRJNA827465;Yang2025_Silkie_Wencha	0.929610339
ENSGALG00010002280	ST7	3	0.059137179	0.196582072	0.300826918	0.763546481	0.073780243	5.964930601	66.47069121	0.666666667	CRA004775;PRJNA827465;Yang2025_Silkie_Wencha	0.929610339
ENSGALG00010017190	STK26	3	0.056074631	0.186525706	0.300626828	0.763699072	0.095165835	34.49174598	94.20151128	0.666666667	CRA004775;PRJNA827465;Yang2025_Silkie_Wencha	0.929610339
ENSGALG00010023171	ENSGALG0001002317	3	-0.043157077	0.14339828	-0.300959518	0.763445364	0.039092601	5.508986454	63.69568129	0.666666667	CRA004775;PRJNA827465;Yang2025_Silkie_Wencha	0.929610339
ENSGALG00010027143	GALNT11	3	-0.041145442	0.136882921	-0.300588571	0.763728248	0.033401262	5.776192661	65.37511615	0.666666667	CRA004775;PRJNA827465;Yang2025_Silkie_Wencha	0.929610339
ENSGALG00010001204	SNX2	3	0.033657309	0.111905134	0.300766441	0.7635926	0.019327827	4.124837548	51.513242	0.666666667	CRA004775;PRJNA827465;Yang2025_Silkie_Wencha	0.929610339
ENSGALG00010003921	COLEC12	3	-0.026343585	0.087494306	-0.30108914	0.763346522	0	0.408796415	0	0.666666667	CRA004775;PRJNA827465;Yang2025_Silkie_Wencha	0.929610339
ENSGALG00010009548	PIK3C3	3	0.025328779	0.08421595	0.300759885	0.7635976	0	1.288599474	0	0.666666667	CRA004775;PRJNA827465;Yang2025_Silkie_Wencha	0.929610339
ENSGALG00010009370	ENSGALG0001000937	3	0.127228908	0.424033186	0.300044695	0.764143063	0.224076938	3.418744965	41.49899977	0.666666667	CRA004775;PRJNA827465;Yang2025_Silkie_Wencha	0.929708224
ENSGALG00010001465	TACC2	3	0.072894256	0.24274379	0.300292981	0.763953687	0.135679208	10.38206806	80.73601533	0.666666667	CRA004775;PRJNA827465;Yang2025_Silkie_Wencha	0.929708224
ENSGALG00010028897	TMEM268	3	0.039729359	0.132484342	0.299879659	0.76426895	0	0.482368575	0	0.666666667	CRA004775;PRJNA827465;Yang2025_Silkie_Wencha	0.929708224
ENSGALG00010021181	DAGLA	3	-0.0363796	0.121304264	-0.299903716	0.7642506	0.022597979	4.371460477	54.24869994	0.666666667	CRA004775;PRJNA827465;Yang2025_Silkie_Wencha	0.929708224
ENSGALG00010027562	SLC39A3	3	-0.029640305	0.09881844	-0.299947105	0.764217503	0.005241162	2.323076701	13.90727654	0.666666667	CRA004775;PRJNA827465;Yang2025_Silkie_Wencha	0.929708224
ENSGALG00010016724	PGD	3	-0.026603795	0.088634235	-0.300152587	0.764060769	0.010018604	3.408303266	41.31977574	0.666666667	CRA004775;PRJNA827465;Yang2025_Silkie_Wencha	0.929708224
ENSGALG00010006202	SACS	3	0.02443462	0.081395896	0.300194736	0.76402862	0	0.553740088	0	0.666666667	CRA004775;PRJNA827465;Yang2025_Silkie_Wencha	0.929708224
ENSGALG00010000184	ENSGALG0001000018	3	0.475278685	1.590546286	0.298814747	0.765081399	0	0.395701113	0	0.666666667	CRA004775;PRJNA827465;Yang2025_Silkie_Wencha	0.929758773
ENSGALG00010020524	ENSGALG0001002052	3	0.139185712	0.466108494	0.298612262	0.765235908	0.546511203	12.74588383	84.30865975	0.666666667	CRA004775;PRJNA827465;Yang2025_Silkie_Wencha	0.929758773
ENSGALG00010021792	KLHDC8B	3	-0.103657511	0.345966735	-0.299616989	0.764469324	0.256705711	7.927384736	74.77099868	0.666666667	CRA004775;PRJNA827465;Yang2025_Silkie_Wencha	0.929758773
ENSGALG00010028138	ENSGALG0001002813	3	-0.077250119	0.257867796	-0.299572572	0.764503208	0.102050654	4.110387564	51.34278778	0.666666667	CRA004775;PRJNA827465;Yang2025_Silkie_Wencha	0.929758773
ENSGALG00010007017	SRD5A2	3	-0.073882685	0.247256368	-0.298810041	0.765084989	0	0.277022821	0	0.666666667	CRA004775;PRJNA827465;Yang2025_Silkie_Wencha	0.929758773
ENSGALG00010018413	SLC15A2	3	0.062550826	0.208974402	0.299322909	0.764693677	0	0.804258509	0	0.666666667	CRA004775;PRJNA827465;Yang2025_Silkie_Wencha	0.929758773
ENSGALG00010019989	ENSGALG0001001998	3	0.056704631	0.189801933	0.298756866	0.765125564	0.020025022	2.400784087	16.69388303	0.666666667	CRA004775;PRJNA827465;Yang2025_Silkie_Wencha	0.929758773
ENSGALG00010024336	TSPAN4	3	0.053916404	0.180537072	0.298644502	0.765211306	0.062229182	5.782422003	65.41241717	0.666666667	CRA004775;PRJNA827465;Yang2025_Silkie_Wencha	0.929758773
ENSGALG00010013250	E2F5	3	0.047934605	0.160149964	0.299310747	0.764702956	0.05990924	9.962848017	79.92541895	0.666666667	CRA004775;PRJNA827465;Yang2025_Silkie_Wencha	0.929758773
ENSGALG00010009219	TNFRSF11B	3	0.038902446	0.130119489	0.298974783	0.764959286	0	0.345249304	0	0.666666667	CRA004775;PRJNA827465;Yang2025_Silkie_Wencha	0.929758773
ENSGALG00010010257	ENSGALG0001001025	3	0.033248469	0.111395749	0.298471614	0.765343238	0	1.078884301	0	0.666666667	CRA004775;PRJNA827465;Yang2025_Silkie_Wencha	0.929758773
ENSGALG00010012925	SETD4	3	-0.031025628	0.103887355	-0.298646815	0.765209541	0	1.286348809	0	0.666666667	CRA004775;PRJNA827465;Yang2025_Silkie_Wencha	0.929758773
ENSGALG00010022093	CHD4	3	0.03004362	0.100561567	0.298758468	0.765124342	0.017559715	4.757979141	57.96534746	0.666666667	CRA004775;PRJNA827465;Yang2025_Silkie_Wencha	0.929758773
ENSGALG00010015149	ENSGALG0001001514	3	0.023072692	0.077127436	0.29915025	0.764825407	0.003510122	2.411283639	17.05662629	0.666666667	CRA004775;PRJNA827465;Yang2025_Silkie_Wencha	0.929758773
ENSGALG00010005123	MTIF3	3	0.021063053	0.070575716	0.29844619	0.76536264	0.001606919	2.15243076	7.081796189	0.666666667	CRA004775;PRJNA827465;Yang2025_Silkie_Wencha	0.929758773
ENSGALG00010016931	SLC30A4	3	-0.010041244	0.033536215	-0.29941494	0.764623464	0	1.916092973	0	0.666666667	CRA004775;PRJNA827465;Yang2025_Silkie_Wencha	0.929758773
ENSGALG00010003920	LPAR6	3	-0.051077906	0.171301077	-0.298176211	0.765568677	0.047136274	4.217114913	52.57421148	0.666666667	CRA004775;PRJNA827465;Yang2025_Silkie_Wencha	0.929779871
ENSGALG00010024143	UBE2Q2	3	-0.040318889	0.135191038	-0.298236401	0.765522741	0.036303259	7.773013381	74.26995295	0.666666667	CRA004775;PRJNA827465;Yang2025_Silkie_Wencha	0.929779871
ENSGALG00010005485	PITRM1	3	0.0182123	0.061081294	0.298164931	0.765577286	0.000953193	2.160885752	7.445361318	0.666666667	CRA004775;PRJNA827465;Yang2025_Silkie_Wencha	0.929779871
ENSGALG00010028475	AMBP	3	0.315832892	1.061165362	0.297628346	0.765986837	2.270179603	6.230092125	67.89774597	0.666666667	CRA004775;PRJNA827465;Yang2025_Silkie_Wencha	0.929877899
ENSGALG00010013111	RFESD	3	-0.091021907	0.305808734	-0.297643256	0.765975456	0.156915133	4.661401916	57.09445278	0.666666667	CRA004775;PRJNA827465;Yang2025_Silkie_Wencha	0.929877899
ENSGALG00010013175	HIPK2	3	0.075050283	0.251967995	0.29785641	0.765812758	0.137376445	8.780090922	77.22119261	0.666666667	CRA004775;PRJNA827465;Yang2025_Silkie_Wencha	0.929877899
ENSGALG00010015241	ENSGALG0001001524	3	-0.070462891	0.236613384	-0.297797572	0.765857668	0.092443829	4.728069902	57.69944096	0.666666667	CRA004775;PRJNA827465;Yang2025_Silkie_Wencha	0.929877899
ENSGALG00010003198	PRKG2	3	0.060913231	0.204656146	0.297636949	0.76598027	0.013153192	2.204221681	9.2650246	0.666666667	CRA004775;PRJNA827465;Yang2025_Silkie_Wencha	0.929877899
ENSGALG00010014707	KPNA5	3	-0.062738542	0.211005794	-0.297330899	0.766213894	0.110904402	29.25230209	93.16293127	0.666666667	CRA004775;PRJNA827465;Yang2025_Silkie_Wencha	0.929914013
ENSGALG00010005594	MRPL50	3	0.043997858	0.147956326	0.297370575	0.766183606	0	0.498474183	0	0.666666667	CRA004775;PRJNA827465;Yang2025_Silkie_Wencha	0.929914013
ENSGALG00010016169	RHOH	3	-0.023589531	0.079319389	-0.297399305	0.766161674	0	0.627040907	0	0.666666667	CRA004775;PRJNA827465;Yang2025_Silkie_Wencha	0.929914013
ENSGALG00010007123	ENSGALG0001000712	3	0.085070615	0.286338769	0.29709779	0.766391852	0.144582114	4.889861121	59.09904289	0.666666667	CRA004775;PRJNA827465;Yang2025_Silkie_Wencha	0.930050158
ENSGALG00010023412	GSS	3	-0.043825201	0.147559278	-0.297000648	0.766466014	0.046832771	7.478368715	73.25619963	0.666666667	CRA004775;PRJNA827465;Yang2025_Silkie_Wencha	0.930060331
ENSGALG00010027590	SEMA3F	3	0.052761846	0.177769306	0.296799526	0.766619567	0.064042352	7.419006264	73.04221173	0.666666667	CRA004775;PRJNA827465;Yang2025_Silkie_Wencha	0.930166829
ENSGALG00010005127	GATA6	3	0.086269177	0.29097791	0.29648016	0.766863416	0.184032446	8.834035218	77.36028949	0.666666667	CRA004775;PRJNA827465;Yang2025_Silkie_Wencha	0.930223219
ENSGALG00010028069	NEK4	3	0.049830736	0.168069494	0.29648888	0.766856758	0.068776572	12.36321471	83.82297771	0.666666667	CRA004775;PRJNA827465;Yang2025_Silkie_Wencha	0.930223219
ENSGALG00010008966	NRBP1	3	0.026065302	0.087910641	0.296497691	0.76685003	0.0097812	3.432770373	41.73801966	0.666666667	CRA004775;PRJNA827465;Yang2025_Silkie_Wencha	0.930223219
ENSGALG00010008500	ENSGALG0001000850	3	-0.049613116	0.167456242	-0.296275105	0.767019997	0.045061812	4.308164415	53.57651642	0.666666667	CRA004775;PRJNA827465;Yang2025_Silkie_Wencha	0.930275172
ENSGALG00010012010	HENMT1	3	0.042958126	0.145047663	0.296165584	0.76710363	0	0.892947791	0	0.666666667	CRA004775;PRJNA827465;Yang2025_Silkie_Wencha	0.930275172
ENSGALG00010000161	CDC42EP2	3	0.027961413	0.094394338	0.296219175	0.767062706	0	1.486888604	0	0.666666667	CRA004775;PRJNA827465;Yang2025_Silkie_Wencha	0.930275172
ENSGALG00010025370	HPD	3	-0.139440131	0.471721607	-0.295598354	0.767536831	0.460483487	6.564400917	69.53263481	0.666666667	CRA004775;PRJNA827465;Yang2025_Silkie_Wencha	0.93036443
ENSGALG00010012894	CLIC6	3	0.137449233	0.46638491	0.294712006	0.768213891	0.357242487	4.670636606	57.17928478	0.666666667	CRA004775;PRJNA827465;Yang2025_Silkie_Wencha	0.93036443
ENSGALG00010029156	ENSGALG0001002915	2	0.130045962	0.441824116	0.294338758	0.768499059	0	0.308531592	0	0.5	PRJNA827465;Yang2025_Silkie_Wenchang	0.93036443
ENSGALG00010024731	AOX1	3	0.096449766	0.328344498	0.293745645	0.768952273	0.216894895	7.199311695	72.21956647	0.666666667	CRA004775;PRJNA827465;Yang2025_Silkie_Wencha	0.93036443
ENSGALG00010006126	ENSGALG0001000612	3	-0.094315952	0.320347906	-0.294417256	0.768439082	0.19660571	6.191464639	67.69746552	0.666666667	CRA004775;PRJNA827465;Yang2025_Silkie_Wencha	0.93036443
ENSGALG00010018128	SEPTIN12	3	-0.090263071	0.306065293	-0.294914427	0.768059251	0.221872946	19.15893093	89.56100418	0.666666667	CRA004775;PRJNA827465;Yang2025_Silkie_Wencha	0.93036443
ENSGALG00010019310	CRELD1	3	0.08836579	0.300826306	0.293743558	0.768953867	0.220641483	21.81873986	90.83356778	0.666666667	CRA004775;PRJNA827465;Yang2025_Silkie_Wencha	0.93036443
ENSGALG00010017409	GPC3	3	0.074899624	0.254720571	0.294046231	0.768722577	0	0.302255037	0	0.666666667	CRA004775;PRJNA827465;Yang2025_Silkie_Wencha	0.93036443
ENSGALG00010012851	ENSGALG0001001285	3	0.055491146	0.188875149	0.293798025	0.768912244	0	1.580628638	0	0.666666667	CRA004775;PRJNA827465;Yang2025_Silkie_Wencha	0.93036443
ENSGALG00010020657	DALRD3	3	-0.055201717	0.186827638	-0.295463333	0.767639959	0.086270769	12.16857893	83.56422708	0.666666667	CRA004775;PRJNA827465;Yang2025_Silkie_Wencha	0.93036443
ENSGALG00010010323	FBP2	3	-0.049318323	0.167500302	-0.294437219	0.76842383	0	0.300658899	0	0.666666667	CRA004775;PRJNA827465;Yang2025_Silkie_Wencha	0.93036443
ENSGALG00010001456	LSM8	3	-0.046728435	0.158776839	-0.294302588	0.768526695	0.029577679	3.153818867	36.58481719	0.666666667	CRA004775;PRJNA827465;Yang2025_Silkie_Wencha	0.93036443
ENSGALG00010017859	EXOC6	3	0.043828955	0.148291863	0.29555873	0.767567095	0.037150724	5.407034616	63.01114859	0.666666667	CRA004775;PRJNA827465;Yang2025_Silkie_Wencha	0.93036443
ENSGALG00010001279	LPIN2	3	0.03870158	0.131231343	0.294911103	0.76806179	0.022338917	3.707022075	46.04833854	0.666666667	CRA004775;PRJNA827465;Yang2025_Silkie_Wencha	0.93036443
ENSGALG00010027135	PRKAG2	3	-0.035347899	0.119879354	-0.294862277	0.76809909	0.030290607	8.83423759	77.36080811	0.666666667	CRA004775;PRJNA827465;Yang2025_Silkie_Wencha	0.93036443
ENSGALG00010005285	MPP7	3	-0.034295069	0.116608069	-0.294105455	0.768677323	0.016887266	3.443243787	41.9152368	0.666666667	CRA004775;PRJNA827465;Yang2025_Silkie_Wencha	0.93036443
ENSGALG00010022438	RPN2	3	-0.032187365	0.108943247	-0.295450761	0.767649562	0.022214995	6.878029748	70.92190535	0.666666667	CRA004775;PRJNA827465;Yang2025_Silkie_Wencha	0.93036443
ENSGALG00010018482	TWNK	3	-0.032160502	0.10878503	-0.295633529	0.767509965	0.010622776	2.626901828	23.86468429	0.666666667	CRA004775;PRJNA827465;Yang2025_Silkie_Wencha	0.93036443
ENSGALG00010017516	AAK1	3	0.03137661	0.106457922	0.29473251	0.768198227	0.016461771	3.597476607	44.40547588	0.666666667	CRA004775;PRJNA827465;Yang2025_Silkie_Wencha	0.93036443
ENSGALG00010029142	ENSGALG0001002914	3	-0.029933615	0.101516008	-0.29486596	0.768096276	0	0.528555911	0	0.666666667	CRA004775;PRJNA827465;Yang2025_Silkie_Wencha	0.93036443
ENSGALG00010012839	TGFBI	3	-0.028501764	0.096357243	-0.295792652	0.767388435	0	0.776033786	0	0.666666667	CRA004775;PRJNA827465;Yang2025_Silkie_Wencha	0.93036443
ENSGALG00010015163	PLK2	3	-0.028469315	0.096800527	-0.294102897	0.768679277	0	0.562837981	0	0.666666667	CRA004775;PRJNA827465;Yang2025_Silkie_Wencha	0.93036443
ENSGALG00010025316	SLC25A36	3	0.027962324	0.094622647	0.295514073	0.767601203	0.012511462	3.585864208	44.22543956	0.666666667	CRA004775;PRJNA827465;Yang2025_Silkie_Wencha	0.93036443
ENSGALG00010012212	RAD54B	3	0.026058057	0.088682286	0.293836099	0.768883149	0	0.677549866	0	0.666666667	CRA004775;PRJNA827465;Yang2025_Silkie_Wencha	0.93036443
ENSGALG00010024629	ENSGALG0001002462	3	-0.023584684	0.080180419	-0.29414518	0.768646968	0.004722248	2.518170953	20.57727465	0.666666667	CRA004775;PRJNA827465;Yang2025_Silkie_Wencha	0.93036443
ENSGALG00010021686	CCPG1	3	-0.022826737	0.077693957	-0.29380325	0.768908251	0	1.584794936	0	0.666666667	CRA004775;PRJNA827465;Yang2025_Silkie_Wencha	0.93036443
ENSGALG00010003072	ELF1	3	-0.017580114	0.059621615	-0.294861423	0.768099742	0	1.991457069	0	0.666666667	CRA004775;PRJNA827465;Yang2025_Silkie_Wencha	0.93036443
ENSGALG00010010666	C12orf75	3	0.033699029	0.115034885	0.292946172	0.769563296	0	1.989282195	0	0.666666667	CRA004775;PRJNA827465;Yang2025_Silkie_Wencha	0.930900774
ENSGALG00010020520	ARHGAP22	3	-0.03196206	0.109120865	-0.292905117	0.769594678	0.014236709	3.279163816	39.00884152	0.666666667	CRA004775;PRJNA827465;Yang2025_Silkie_Wencha	0.930900774
ENSGALG00010019932	KALRN	3	0.019448909	0.066397246	0.292917405	0.769585284	0	1.340914606	0	0.666666667	CRA004775;PRJNA827465;Yang2025_Silkie_Wencha	0.930900774
ENSGALG00010000465	ENSGALG0001000046	3	-0.0362451	0.123789666	-0.292795848	0.769678202	0	0.96750041	0	0.666666667	CRA004775;PRJNA827465;Yang2025_Silkie_Wencha	0.930922165
ENSGALG00010015041	TFAP2C	2	0.110991125	0.379512824	0.292456849	0.769937348	0	0.017236412	0	1	PRJNA827465;Yang2025_Silkie_Wenchang	0.930996679
ENSGALG00010027291	MST1	3	-0.049259508	0.168433073	-0.292457457	0.769936883	0.040237974	3.871928938	48.34615944	0.666666667	CRA004775;PRJNA827465;Yang2025_Silkie_Wencha	0.930996679
ENSGALG00010017010	NONO	3	-0.011780459	0.040272124	-0.29252141	0.769887993	0	1.113662662	0	0.666666667	CRA004775;PRJNA827465;Yang2025_Silkie_Wencha	0.930996679
ENSGALG00010021272	ENSGALG0001002127	3	-0.033787764	0.115573469	-0.29234879	0.770019958	0.008274184	2.456607589	18.58691602	0.666666667	CRA004775;PRJNA827465;Yang2025_Silkie_Wencha	0.931016948
ENSGALG00010008516	MRE11	3	0.034118453	0.116761663	0.292205956	0.770129158	0.026379775	6.964316571	71.28217852	0.666666667	CRA004775;PRJNA827465;Yang2025_Silkie_Wencha	0.93106936
ENSGALG00010017956	TIPIN	3	0.02014051	0.06896034	0.292059323	0.770241267	0	0.09027972	0	1	CRA004775;PRJNA827465;Yang2025_Silkie_Wencha	0.93112528
ENSGALG00010021604	SPTB	3	-0.054035835	0.185235673	-0.29171398	0.770505319	0.082793722	13.30799055	84.97143507	0.666666667	CRA004775;PRJNA827465;Yang2025_Silkie_Wencha	0.931354436
ENSGALG00010027168	SSBP4	3	-0.021405207	0.073396217	-0.291639108	0.770562571	0	0.707655466	0	0.666666667	CRA004775;PRJNA827465;Yang2025_Silkie_Wencha	0.931354436
ENSGALG00010018374	HPS1	3	-0.044861465	0.153938768	-0.291424088	0.770726994	0.034023994	3.644916502	45.12905853	0.666666667	CRA004775;PRJNA827465;Yang2025_Silkie_Wencha	0.931419131
ENSGALG00010021689	MPRIP	3	0.032161142	0.110368887	0.291396816	0.77074784	0.018606831	4.011255455	50.14029841	0.666666667	CRA004775;PRJNA827465;Yang2025_Silkie_Wencha	0.931419131
ENSGALG00010001227	ENSGALG0001000122	2	0.265732563	0.915314175	0.290318418	0.77157265	0	0.188490222	0	0.5	PRJNA827465;Yang2025_Silkie_Wenchang	0.931422391
ENSGALG00010023604	ENSGALG0001002360	3	0.166368783	0.573845435	0.289919154	0.771878088	0.780399231	11.09112941	81.96757132	0.666666667	CRA004775;PRJNA827465;Yang2025_Silkie_Wencha	0.931422391
ENSGALG00010023947	ENSGALG0001002394	2	-0.156728011	0.540593112	-0.289918625	0.771878493	0.063565581	1.120004509	10.71464514	0.5	PRJNA827465;Yang2025_Silkie_Wenchang	0.931422391
ENSGALG00010021971	ICOS	3	-0.111515008	0.38302133	-0.291145686	0.7709399	0.163177282	3.159936285	36.7075846	0.666666667	CRA004775;PRJNA827465;Yang2025_Silkie_Wencha	0.931422391
ENSGALG00010009762	MAN2A1	3	0.063186739	0.217939098	0.289928424	0.771870996	0.117061006	18.203576	89.09314774	0.666666667	CRA004775;PRJNA827465;Yang2025_Silkie_Wencha	0.931422391
ENSGALG00010003585	PNPLA6	3	-0.06155692	0.212001333	-0.290361004	0.771540074	0.119792191	24.01663067	91.67243721	0.666666667	CRA004775;PRJNA827465;Yang2025_Silkie_Wencha	0.931422391
ENSGALG00010016700	CLUAP1	3	0.053900882	0.185581057	0.290443881	0.771476677	0.079615979	9.386110414	78.6919191	0.666666667	CRA004775;PRJNA827465;Yang2025_Silkie_Wencha	0.931422391
ENSGALG00010021115	ALPL	3	0.051987101	0.178660649	0.290657007	0.771313656	0	0.402870058	0	0.666666667	CRA004775;PRJNA827465;Yang2025_Silkie_Wencha	0.931422391
ENSGALG00010013472	ENSGALG0001001347	3	-0.036443962	0.125618693	-0.290115751	0.771727686	0	1.712178089	0	0.666666667	CRA004775;PRJNA827465;Yang2025_Silkie_Wencha	0.931422391
ENSGALG00010018484	NLGN1	2	0.032736811	0.112686949	0.290511109	0.771425254	0	0.709998409	0	0.5	PRJNA827465;Yang2025_Silkie_Wenchang	0.931422391
ENSGALG00010020617	DGUOK	3	0.032114615	0.110701319	0.290101472	0.77173861	0.007958264	2.326978737	14.05164265	0.666666667	CRA004775;PRJNA827465;Yang2025_Silkie_Wencha	0.931422391
ENSGALG00010017290	DACT1	3	0.031090799	0.107258429	0.289868123	0.771917129	0.019135789	5.252852502	61.92544909	0.666666667	CRA004775;PRJNA827465;Yang2025_Silkie_Wencha	0.931422391
ENSGALG00010024449	CACHD1	3	-0.027240471	0.093983552	-0.289843044	0.771936316	0.009855398	2.95161316	32.24044305	0.666666667	CRA004775;PRJNA827465;Yang2025_Silkie_Wencha	0.931422391
ENSGALG00010010338	CNST	3	0.023470591	0.080746052	0.290671687	0.771302427	0.005847091	2.712569417	26.26916799	0.666666667	CRA004775;PRJNA827465;Yang2025_Silkie_Wencha	0.931422391
ENSGALG00010010206	SCARA5	3	-0.022784256	0.078386046	-0.290667243	0.771305827	0.002014116	2.207424368	9.396669322	0.666666667	CRA004775;PRJNA827465;Yang2025_Silkie_Wencha	0.931422391
ENSGALG00010007544	MLYCD	3	0.022591096	0.077557563	0.29128166	0.770835913	0	1.284095407	0	0.666666667	CRA004775;PRJNA827465;Yang2025_Silkie_Wencha	0.931422391
ENSGALG00010005694	ENSGALG0001000569	3	-0.021136237	0.072792409	-0.290363206	0.771538389	0.005382026	2.820312748	29.08587822	0.666666667	CRA004775;PRJNA827465;Yang2025_Silkie_Wencha	0.931422391
ENSGALG00010008731	NDUFB9	3	0.0144343	0.049608572	0.290963822	0.771078989	0	0.325993845	0	0.666666667	CRA004775;PRJNA827465;Yang2025_Silkie_Wencha	0.931422391
ENSGALG00010015159	ENSGALG0001001515	2	-0.338704575	1.169282656	-0.28966869	0.772069712	2.199513867	4.929557341	79.7142029	0.5	CRA004775;Yang2025_Silkie_Wenchang	0.931425539
ENSGALG00010004866	SLC25A21	3	-0.083647361	0.288770338	-0.289667427	0.772070678	0.1630661	8.124233397	75.38229268	0.666666667	CRA004775;PRJNA827465;Yang2025_Silkie_Wencha	0.931425539
ENSGALG00010025890	SCG2	3	-0.166118924	0.57405113	-0.289380014	0.772290588	0.795969318	10.37547748	80.72377869	0.666666667	CRA004775;PRJNA827465;Yang2025_Silkie_Wencha	0.931531873
ENSGALG00010029267	ENSGALG0001002926	3	0.066679972	0.230413472	0.289392681	0.772280895	0.125605183	9.836725937	79.66803169	0.666666667	CRA004775;PRJNA827465;Yang2025_Silkie_Wencha	0.931531873
ENSGALG00010029432	ENSGALG0001002943	3	-0.393406353	1.362616299	-0.288713964	0.772800276	0	0.075171301	0	0.666666667	CRA004775;PRJNA827465;Yang2025_Silkie_Wencha	0.931768237
ENSGALG00010028552	ENSGALG0001002855	2	0.321179688	1.114627316	0.288149845	0.773232039	1.081173566	1.67836768	40.41829975	0.5	PRJNA827465;Yang2025_Silkie_Wenchang	0.931768237
ENSGALG00010019803	FAH	3	-0.180315507	0.625570615	-0.288241651	0.773161768	0.786863566	6.132135682	67.33166163	0.666666667	CRA004775;PRJNA827465;Yang2025_Silkie_Wencha	0.931768237
ENSGALG00010024053	ISL2	3	-0.173583816	0.601437796	-0.288614745	0.772876211	0.834172294	9.071499362	77.95292795	0.666666667	CRA004775;PRJNA827465;Yang2025_Silkie_Wencha	0.931768237
ENSGALG00010017621	SYNE2	3	0.099425936	0.344477891	0.288627917	0.77286613	0.29793974	19.7319964	89.86417816	0.666666667	CRA004775;PRJNA827465;Yang2025_Silkie_Wencha	0.931768237
ENSGALG00010009497	HS3ST1	3	0.089440722	0.284437438	0.288088208	0.773279219	0.17592693	8.293886245	75.88585305	0.666666667	CRA004775;PRJNA827465;Yang2025_Silkie_Wencha	0.931768237
ENSGALG00010002722	TRIM7.1	3	0.073097167	0.253388731	0.288457053	0.772996901	0.13250811	10.17480867	80.34361073	0.666666667	CRA004775;PRJNA827465;Yang2025_Silkie_Wencha	0.931768237
ENSGALG00010006434	MMAA	3	0.053550252	0.186046661	0.287832374	0.773475055	0.083200089	11.31188744	82.31948461	0.666666667	CRA004775;PRJNA827465;Yang2025_Silkie_Wencha	0.931768237
ENSGALG00010011966	SLC25A24	3	0.051003474	0.176779951	0.288513904	0.772953389	0.075346574	16.12593991	87.59762215	0.666666667	CRA004775;PRJNA827465;Yang2025_Silkie_Wencha	0.931768237
ENSGALG00010013048	WDR75	3	0.040969449	0.142221474	0.288064438	0.773297413	0.035134938	4.785591843	58.19041286	0.666666667	CRA004775;PRJNA827465;Yang2025_Silkie_Wencha	0.931768237
ENSGALG00010018922	EEF2K	3	-0.037232575	0.129346445	-0.287851555	0.773460372	0.027966645	4.709107859	57.5291104	0.666666667	CRA004775;PRJNA827465;Yang2025_Silkie_Wencha	0.931768237
ENSGALG00010025907	ARHGEF12	3	0.033004314	0.11448948	0.288273772	0.773137182	0.023368313	5.481165541	63.51141039	0.666666667	CRA004775;PRJNA827465;Yang2025_Silkie_Wencha	0.931768237
ENSGALG00010009252	SIRT1	3	-0.031843501	0.110602949	-0.287908242	0.773416978	0.021922452	5.426763667	63.14562191	0.666666667	CRA004775;PRJNA827465;Yang2025_Silkie_Wencha	0.931768237
ENSGALG00010021248	ANXA7	3	-0.030155723	0.104739047	-0.288844738	0.772700195	0	0.223711176	0	0.666666667	CRA004775;PRJNA827465;Yang2025_Silkie_Wencha	0.931768237
ENSGALG00010001510	AP1S2	3	-0.013673074	0.047315113	-0.288978996	0.772597451	0	1.074107656	0	0.666666667	CRA004775;PRJNA827465;Yang2025_Silkie_Wencha	0.931768237
ENSGALG00010023003	ENSGALG0001002300	3	0.127809036	0.444441903	0.287571975	0.7736744	0	0.600602406	0	0.666666667	CRA004775;PRJNA827465;Yang2025_Silkie_Wencha	0.93184959
ENSGALG00010015676	TGM2	3	-0.040190948	0.139754128	-0.28758326	0.77366576	0.028515089	4.061273668	50.75436517	0.666666667	CRA004775;PRJNA827465;Yang2025_Silkie_Wencha	0.93184959
ENSGALG00010029461	ENSGALG0001002946	3	-0.147736458	0.51393781	-0.287459834	0.773760253	0.705333904	22.98356254	91.20812884	0.666666667	CRA004775;PRJNA827465;Yang2025_Silkie_Wencha	0.931873613
ENSGALG00010027873	RFX2	3	-0.04303035	0.149850304	-0.287155574	0.773993201	0.031835708	3.886399298	48.53848391	0.666666667	CRA004775;PRJNA827465;Yang2025_Silkie_Wencha	0.93207477
ENSGALG00010029921	STX8	3	-0.031907743	0.111258815	-0.286788446	0.77427431	0.020675247	4.362120362	54.15073786	0.666666667	CRA004775;PRJNA827465;Yang2025_Silkie_Wencha	0.932333885
ENSGALG00010029496	TTYH2	3	-0.029055661	0.101395214	-0.286558502	0.774450393	0.014277351	3.919969731	48.97919787	0.666666667	CRA004775;PRJNA827465;Yang2025_Silkie_Wencha	0.9324665
ENSGALG00010000561	ENSGALG0001000056	3	-0.020443963	0.070028287	-0.286404559	0.774568283	0	1.362304142	0	0.666666667	CRA004775;PRJNA827465;Yang2025_Silkie_Wencha	0.932529032
ENSGALG00010000191	NEFH	3	-0.110054596	0.3857467	-0.285302755	0.775412202	0.305571242	6.342194579	68.46517439	0.666666667	CRA004775;PRJNA827465;Yang2025_Silkie_Wencha	0.932613015
ENSGALG00010024601	ENSGALG0001002460	3	0.095923006	0.336165668	0.285344445	0.775380265	0.191341341	5.046361798	60.36748692	0.666666667	CRA004775;PRJNA827465;Yang2025_Silkie_Wencha	0.932613015
ENSGALG00010000634	ENSGALG0001000063	3	0.089022113	0.311476306	0.285807014	0.775025936	0.218939049	8.508753656	76.49479488	0.666666667	CRA004775;PRJNA827465;Yang2025_Silkie_Wencha	0.932613015
ENSGALG00010002309	GADL1	3	-0.036295023	0.127264332	-0.28519399	0.775495525	0	1.792696693	0	0.666666667	CRA004775;PRJNA827465;Yang2025_Silkie_Wencha	0.932613015
ENSGALG00010017423	TMEM204	3	-0.031300892	0.109684425	-0.285372256	0.775358961	0.023383115	9.066009691	77.93957796	0.666666667	CRA004775;PRJNA827465;Yang2025_Silkie_Wencha	0.932613015
ENSGALG00010008862	ZNF367	3	0.029789201	0.104423721	0.28527236	0.775435487	0	0.14244703	0	0.666666667	CRA004775;PRJNA827465;Yang2025_Silkie_Wencha	0.932613015
ENSGALG00010013648	NAV3	3	0.028030606	0.09799689	0.286035667	0.774850804	0.008574151	2.592075902	22.84176562	0.666666667	CRA004775;PRJNA827465;Yang2025_Silkie_Wencha	0.932613015
ENSGALG00010018971	POLR1B	3	-0.024127075	0.084440491	-0.285728745	0.775085887	0.016697334	9.723900166	79.43212121	0.666666667	CRA004775;PRJNA827465;Yang2025_Silkie_Wencha	0.932613015
ENSGALG00010018525	ST8SIA2	3	-0.024051728	0.084217363	-0.285591079	0.775191337	0	0.118441442	0	1	CRA004775;PRJNA827465;Yang2025_Silkie_Wencha	0.932613015
ENSGALG00010001104	MBNL1	3	-0.022024366	0.077102043	-0.285652171	0.775144541	0.005563345	2.770625268	27.81412835	0.666666667	CRA004775;PRJNA827465;Yang2025_Silkie_Wencha	0.932613015
ENSGALG00010009190	ZBTB49	3	0.017283803	0.060534296	0.285520843	0.775245138	0	1.124638701	0	0.666666667	CRA004775;PRJNA827465;Yang2025_Silkie_Wencha	0.932613015
ENSGALG00010019675	MAFK	3	0.016409854	0.057470404	0.285535734	0.775233732	0.003062831	2.833710112	29.42115032	0.666666667	CRA004775;PRJNA827465;Yang2025_Silkie_Wencha	0.932613015
ENSGALG00010023590	S1PR1	3	-0.013641568	0.04776742	-0.285583106	0.775197444	0	1.636392346	0	0.666666667	CRA004775;PRJNA827465;Yang2025_Silkie_Wencha	0.932613015
ENSGALG00010001822	KCNE3	3	-0.082736571	0.290310455	-0.284993425	0.775649179	0.13526105	4.290032232	53.38030364	0.666666667	CRA004775;PRJNA827465;Yang2025_Silkie_Wencha	0.932718468
ENSGALG00010020715	ENSGALG0001002071	2	-0.18810259	0.661816215	-0.28422179	0.776240417	0	0.130452769	0	0.5	PRJNA827465;Yang2025_Silkie_Wenchang	0.932913798
ENSGALG00010012954	RIMS2	3	-0.103076669	0.362563232	-0.28429984	0.776180608	0.154431963	3.245267014	38.3717891	0.666666667	CRA004775;PRJNA827465;Yang2025_Silkie_Wencha	0.932913798
ENSGALG00010023937	XBP1	3	0.059213075	0.208233851	0.284358547	0.776135623	0.099846896	9.250148924	78.37872648	0.666666667	CRA004775;PRJNA827465;Yang2025_Silkie_Wencha	0.932913798
ENSGALG00010005988	NUP58	3	0.050117821	0.17611863	0.284568539	0.775974717	0.080439149	27.25403946	92.66163827	0.666666667	CRA004775;PRJNA827465;Yang2025_Silkie_Wencha	0.932913798
ENSGALG00010002703	PIN1	3	-0.033425808	0.117596618	-0.284241235	0.776225517	0.023506748	4.813623217	58.45125575	0.666666667	CRA004775;PRJNA827465;Yang2025_Silkie_Wencha	0.932913798

ENSGALG00010014728	INTS13	3	0.02717614	0.095630481	0.284178636	0.776273487	0.017471311	6.10387143	67.2339101	0.666666667	CRA004775;PRJNA827465;Yang2025_Silkie_Wencha	0.932913798
ENSGALG00010005552	WDR37	3	-0.022736671	0.07990212	-0.28455654	0.775983912	0	0.546126587	0	0.666666667	CRA004775;PRJNA827465;Yang2025_Silkie_Wencha	0.932913798
ENSGALG00010016404	ESRRG	3	0.036092918	0.127063158	0.284054943	0.776368276	0.028298788	5.761128247	65.2845777	0.666666667	CRA004775;PRJNA827465;Yang2025_Silkie_Wencha	0.93294815
ENSGALG00010027316	TRAIP	3	0.031629422	0.111446172	0.283808954	0.776556792	0	0.450419104	0	0.666666667	CRA004775;PRJNA827465;Yang2025_Silkie_Wencha	0.933017188
ENSGALG00010001586	ZBTB14	3	0.026179742	0.092244536	0.283808051	0.776557484	0.009759902	3.13443516	36.19265042	0.666666667	CRA004775;PRJNA827465;Yang2025_Silkie_Wencha	0.933017188
ENSGALG00010008178	PGR	3	-0.09540954	0.336352809	-0.283659115	0.77667163	0.191658168	4.597206076	56.49531548	0.666666667	CRA004775;PRJNA827465;Yang2025_Silkie_Wencha	0.933075042
ENSGALG00010008247	GASK1B	3	0.028927535	0.102013667	0.28356529	0.77674354	0.008955634	2.747369773	27.20310097	0.666666667	CRA004775;PRJNA827465;Yang2025_Silkie_Wencha	0.933082151
ENSGALG00010002667	ENSGALG0001000266	3	0.428875607	1.513116431	0.283438603	0.77684064	6.060439609	17.76674077	88.7430113	0.666666667	CRA004775;PRJNA827465;Yang2025_Silkie_Wencha	0.933119515
ENSGALG00010010222	PAH	2	-0.584850166	2.067451453	-0.282884595	0.777265304	7.494604668	8.092739524	87.64324495	0.5	PRJNA827465;Yang2025_Silkie_Wenchang	0.933271518
ENSGALG00010027895	LIX1L	3	0.06054027	0.214111808	0.28275073	0.777367925	0.112488641	11.97277503	83.29543489	0.666666667	CRA004775;PRJNA827465;Yang2025_Silkie_Wencha	0.933271518
ENSGALG00010017672	SPRED2	3	-0.052352498	0.185266629	-0.282670758	0.777429234	0.083848772	20.31403372	90.15458954	0.666666667	CRA004775;PRJNA827465;Yang2025_Silkie_Wencha	0.933271518
ENSGALG00010013292	PRDM1	3	0.046030647	0.162802833	0.282738614	0.777377214	0	1.667043115	0	0.666666667	CRA004775;PRJNA827465;Yang2025_Silkie_Wencha	0.933271518
ENSGALG00010006205	CGGBP1	3	-0.04542001	0.160403536	-0.2831609	0.777053499	0.066011138	24.74903608	91.91887719	0.666666667	CRA004775;PRJNA827465;Yang2025_Silkie_Wencha	0.933271518
ENSGALG00010017455	CCR9	3	0.028966468	0.102324215	0.283085176	0.777111545	0.006335334	2.427798853	17.62085243	0.666666667	CRA004775;PRJNA827465;Yang2025_Silkie_Wencha	0.933271518
ENSGALG00010023126	CGNL1	3	0.026412781	0.093335282	0.282988175	0.777185902	0	1.327735935	0	0.666666667	CRA004775;PRJNA827465;Yang2025_Silkie_Wencha	0.933271518
ENSGALG00010018955	ZBTB48	3	-0.016108355	0.057034881	-0.282429882	0.777613905	0	0.859854033	0	0.666666667	CRA004775;PRJNA827465;Yang2025_Silkie_Wencha	0.933413957
ENSGALG00010017246	GGA2	3	0.050515545	0.178931318	0.282318072	0.777699629	0.074455327	11.15191622	82.0658624	0.666666667	CRA004775;PRJNA827465;Yang2025_Silkie_Wencha	0.933437611
ENSGALG00010001388	MPPE1	3	0.062408435	0.22141231	0.281865245	0.77804684	0.116223038	15.20436912	86.84588631	0.666666667	CRA004775;PRJNA827465;Yang2025_Silkie_Wencha	0.933775084
ENSGALG00010008134	SLC2A13	3	0.048350011	0.171745362	0.281521491	0.778310447	0.055026805	6.208915486	67.5582538	0.666666667	CRA004775;PRJNA827465;Yang2025_Silkie_Wencha	0.934012171
ENSGALG00010014648	C1QTNF6	3	-0.086120318	0.30616074	-0.281291187	0.778487069	0.195839169	13.44742924	85.12726883	0.666666667	CRA004775;PRJNA827465;Yang2025_Silkie_Wencha	0.934048242
ENSGALG00010001304	SYVN1	3	0.067301967	0.239318108	0.281223881	0.778538689	0.142703276	13.0858877	84.71635974	0.666666667	CRA004775;PRJNA827465;Yang2025_Silkie_Wencha	0.934048242
ENSGALG00010027976	LRSAM1	3	-0.029792683	0.105909583	-0.281302994	0.77847814	0.02348628	7.012944258	71.48130762	0.666666667	CRA004775;PRJNA827465;Yang2025_Silkie_Wencha	0.934048242
ENSGALG00010021450	OLFML1	3	-0.093783471	0.334105933	-0.280699808	0.778940656	0.237404049	8.925594788	77.59252971	0.666666667	CRA004775;PRJNA827465;Yang2025_Silkie_Wencha	0.934331354
ENSGALG00010006378	CAAP1	3	0.055403415	0.19767142	0.280280351	0.779262425	0.074414723	5.502003305	63.6496038	0.666666667	CRA004775;PRJNA827465;Yang2025_Silkie_Wencha	0.934331354
ENSGALG00010001295	ENSGALG0001000129	3	0.05447273	0.194060682	0.280699667	0.778940917	0.100620582	27.07740107	92.61376675	0.666666667	CRA004775;PRJNA827465;Yang2025_Silkie_Wencha	0.934331354
ENSGALG00010021831	ENSGALG0001002183	3	0.054060367	0.192770396	0.280439158	0.779140598	0	1.518804847	0	0.666666667	CRA004775;PRJNA827465;Yang2025_Silkie_Wencha	0.934331354
ENSGALG00010028030	PFDN2	3	-0.046241008	0.164973839	-0.280292977	0.779252739	0.032122305	3.091160491	35.29938008	0.666666667	CRA004775;PRJNA827465;Yang2025_Silkie_Wencha	0.934331354
ENSGALG00010023720	SCAMP5	3	-0.035164664	0.125467975	-0.280268048	0.779271863	0.029147834	5.37840895	62.81428172	0.666666667	CRA004775;PRJNA827465;Yang2025_Silkie_Wencha	0.934331354
ENSGALG00010000046	TUBGCP4	3	-0.027550584	0.098315224	-0.280227042	0.779303321	0.01577545	4.39160221	54.45853463	0.666666667	CRA004775;PRJNA827465;Yang2025_Silkie_Wencha	0.934331354
ENSGALG00010017779	SNAPC1	3	-0.016551045	0.059012901	-0.280464857	0.779120884	0	0.075895763	0	0.666666667	CRA004775;PRJNA827465;Yang2025_Silkie_Wencha	0.934331354
ENSGALG00010028420	ENSGALG0001002842	3	0.261496625	0.941563645	0.277137242	0.781674726	2.332880132	17.35947197	88.48789124	0.666666667	CRA004775;PRJNA827465;Yang2025_Silkie_Wencha	0.934331354
ENSGALG00010014766	F10	3	-0.164907164	0.58966511	-0.279662408	0.779736522	0.953906627	24.79359908	91.93340187	0.666666667	CRA004775;PRJNA827465;Yang2025_Silkie_Wencha	0.934350616
ENSGALG00010014851	CALHM4	2	-0.107984457	0.389610226	-0.277160223	0.781657081	0.162062877	2.076792897	51.84883378	0.5	PRJNA827465;Yang2025_Silkie_Wenchang	0.934350616
ENSGALG00010021467	LHFPL4	3	0.104166262	0.375695436	0.277262516	0.78157854	0.352499415	13.23711067	84.89096262	0.666666667	CRA004775;PRJNA827465;Yang2025_Silkie_Wencha	0.934350616
ENSGALG00010012674	ENSGALG0001001267	3	0.099571915	0.358939174	0.277406097	0.7814683	0.33641993	21.11220254	90.52680555	0.666666667	CRA004775;PRJNA827465;Yang2025_Silkie_Wencha	0.934350616
ENSGALG00010013799	SCRT2	2	-0.099317195	0.357265749	-0.27799249	0.781018124	0.170960155	2.817391846	64.50617967	0.5	PRJNA827465;Yang2025_Silkie_Wenchang	0.934350616
ENSGALG00010015006	HSPB3	3	-0.088089944	0.317895017	-0.277103885	0.781700339	0.18255399	5.842221926	65.76644939	0.666666667	CRA004775;PRJNA827465;Yang2025_Silkie_Wencha	0.934350616
ENSGALG00010024192	ALG6	3	0.086787175	0.314452722	0.275994351	0.782552405	0.229895608	12.12069393	83.49929458	0.666666667	CRA004775;PRJNA827465;Yang2025_Silkie_Wencha	0.934350616
ENSGALG00010006147	ENSGALG0001000614	3	-0.083788304	0.30043993	-0.278885379	0.780332789	0.148532749	4.611448291	56.62967741	0.666666667	CRA004775;PRJNA827465;Yang2025_Silkie_Wencha	0.934350616
ENSGALG00010005656	CYP2U1	3	0.08312574	0.300672058	0.27646646	0.782189817	0.233717738	15.05419835	86.71466953	0.666666667	CRA004775;PRJNA827465;Yang2025_Silkie_Wencha	0.934350616
ENSGALG00010023972	ENSGALG0001002397	3	0.077544881	0.281276514	0.275689143	0.782786835	0.094852562	3.29013966	39.21230687	0.666666667	CRA004775;PRJNA827465;Yang2025_Silkie_Wencha	0.934350616
ENSGALG00010003473	ENSGALG0001000347	3	0.073332453	0.265271919	0.276442579	0.782208157	0.11375471	4.343164969	53.95063244	0.666666667	CRA004775;PRJNA827465;Yang2025_Silkie_Wencha	0.934350616
ENSGALG00010002027	ENSGALG0001000202	3	-0.072268002	0.26190799	-0.275928968	0.782602624	0	1.746650493	0	0.666666667	CRA004775;PRJNA827465;Yang2025_Silkie_Wencha	0.934350616
ENSGALG00010016674	SLC10A4	3	-0.067805355	0.24618869	-0.275420269	0.782993374	0	0.078828602	0	0.666666667	CRA004775;PRJNA827465;Yang2025_Silkie_Wencha	0.934350616
ENSGALG00010027116	CCDC94	3	-0.062042026	0.223142742	-0.278037394	0.780983654	0.128591566	19.92048458	89.96008359	0.666666667	CRA004775;PRJNA827465;Yang2025_Silkie_Wencha	0.934350616
ENSGALG00010013191	ABRA	3	-0.059232621	0.214675632	-0.275916833	0.782611944	0.068556581	4.365611413	54.18740215	0.666666667	CRA004775;PRJNA827465;Yang2025_Silkie_Wencha	0.934350616
ENSGALG00010001912	TMEM117	3	-0.056736576	0.203110361	-0.279338651	0.779984947	0.081314911	7.425431793	73.0800409	0.666666667	CRA004775;PRJNA827465;Yang2025_Silkie_Wencha	0.934350616
ENSGALG00010021191	MYRF	3	0.052482821	0.189479968	0.27698348	0.781792791	0.048334459	3.378721713	40.80601572	0.666666667	CRA004775;PRJNA827465;Yang2025_Silkie_Wencha	0.934350616
ENSGALG00010027567	PARP3	3	0.051727287	0.185694795	0.278560782	0.780581913	0.067029623	5.832179147	65.70750059	0.666666667	CRA004775;PRJNA827465;Yang2025_Silkie_Wencha	0.934350616
ENSGALG00010012106	TMTC1	3	-0.050787647	0.182484774	-0.278311696	0.780773098	0.068474731	8.106528313	75.32852631	0.666666667	CRA004775;PRJNA827465;Yang2025_Silkie_Wencha	0.934350616
ENSGALG00010001206	RSRC1	3	0.04922506	0.175799654	0.280021138	0.779461288	0.074361286	13.82374493	85.55214046	0.666666667	CRA004775;PRJNA827465;Yang2025_Silkie_Wencha	0.934350616
ENSGALG00010008474	ENSGALG0001000847	3	0.046844342	0.170155571	0.275303017	0.783083447	0.056208358	7.007218467	71.45800421	0.666666667	CRA004775;PRJNA827465;Yang2025_Silkie_Wencha	0.934350616
ENSGALG00010018999	THUMPD3	3	0.046462841	0.168107084	0.276388356	0.782249799	0.071461497	20.28890767	90.14239686	0.666666667	CRA004775;PRJNA827465;Yang2025_Silkie_Wencha	0.934350616
ENSGALG00010018004	CDC42BPA	3	-0.04643513	0.166551547	-0.278803353	0.780395741	0.048762884	4.956084132	59.64555995	0.666666667	CRA004775;PRJNA827465;Yang2025_Silkie_Wencha	0.934350616
ENSGALG00010013523	TMEM178B	3	0.046357507	0.166146127	0.279016479	0.780232178	0	0.426172805	0	0.666666667	CRA004775;PRJNA827465;Yang2025_Silkie_Wencha	0.934350616
ENSGALG00010025936	GOSR2	3	-0.043457324	0.157837688	-0.275329197	0.783063335	0.061164164	19.66573071	89.83002448	0.666666667	CRA004775;PRJNA827465;Yang2025_Silkie_Wencha	0.934350616
ENSGALG00010011745	AFAP1L1	3	0.040488033	0.145537657	0.278196268	0.7808617	0.041341654	8.251600612	75.76227821	0.666666667	CRA004775;PRJNA827465;Yang2025_Silkie_Wencha	0.934350616
ENSGALG00010028186	CCT3	3	0.039861965	0.144381437	0.276087885	0.782480565	0.045370542	9.362435385	78.63803682	0.666666667	CRA004775;PRJNA827465;Yang2025_Silkie_Wencha	0.934350616
ENSGALG00010018544	HACD2	3	-0.039210406	0.140511752	-0.279043129	0.780211726	0.034266392	5.61872593	64.40474184	0.666666667	CRA004775;PRJNA827465;Yang2025_Silkie_Wencha	0.934350616
ENSGALG00010009098	CAD	3	-0.039177556	0.141917939	-0.276056406	0.782504743	0.038258789	6.924154629	71.1156075	0.666666667	CRA004775;PRJNA827465;Yang2025_Silkie_Wencha	0.934350616
ENSGALG00010020984	PSEN1	3	-0.038463066	0.138943568	-0.276825093	0.781914412	0.039672165	7.794596019	74.34119748	0.666666667	CRA004775;PRJNA827465;Yang2025_Silkie_Wencha	0.934350616
ENSGALG00010014758	FXN	3	0.037946749	0.136705618	0.277580024	0.781334769	0.026812126	3.858784369	48.17020572	0.666666667	CRA004775;PRJNA827465;Yang2025_Silkie_Wencha	0.934350616
ENSGALG00010020562	LRRN1	3	-0.035347978	0.128322751	-0.275461503	0.782961609	0	0.294287897	0	0.666666667	CRA004775;PRJNA827465;Yang2025_Silkie_Wencha	0.934350616
ENSGALG00010014515	ETNK1	3	-0.033933825	0.123177239	-0.275487789	0.782941506	0.019638251	3.457945848	42.16219432	0.666666667	CRA004775;PRJNA827465;Yang2025_Silkie_Wencha	0.934350616
ENSGALG00010010472	MMRN2	3	-0.032968347	0.118769116	-0.277583497	0.781332102	0.018457973	3.626469836	44.84994801	0.666666667	CRA004775;PRJNA827465;Yang2025_Silkie_Wencha	0.934350616
ENSGALG00010021335	NFATC3	3	0.03210064	0.115586826	0.277718848	0.781228191	0.018836615	3.363772575	40.54294827	0.666666667	CRA004775;PRJNA827465;Yang2025_Silkie_Wencha	0.934350616
ENSGALG00010020762	TTBK2	3	-0.031562478	0.113835372	-0.277267488	0.781574722	0	0.26634818	0	0.666666667	CRA004775;PRJNA827465;Yang2025_Silkie_Wencha	0.934350616
ENSGALG00010008768	PRXL2C	3	0.03106181	0.111353325	0.278948835	0.78028409	0	0.187391133	0	0.666666667	CRA004775;PRJNA827465;Yang2025_Silkie_Wencha	0.934350616
ENSGALG00010024407	HIRA	3	0.031045152	0.111867937	0.277516082	0.781383859	0.02208059	5.187475131	61.4455983	0.666666667	CRA004775;PRJNA827465;Yang2025_Silkie_Wencha	0.934350616
ENSGALG00010022801	LSM14B	3	0.02904403	0.104782147	0.277184906	0.781639129	0.020831479	6.476861193	69.12084511	0.666666667	CRA004775;PRJNA827465;Yang2025_Silkie_Wencha	0.934350616
ENSGALG00010012401	RIPK2	3	-0.027734138	0.099707149	-0.278356555	0.780736666	0.014024669	3.928798183	49.09384736	0.666666667	CRA004775;PRJNA827465;Yang2025_Silkie_Wencha	0.934350616
ENSGALG00010029166	ZZEF1	3	-0.027256255	0.098867574	-0.275684472	0.782790424	0.014543772	4.110594877	51.34524175	0.666666667	CRA004775;PRJNA827465;Yang2025_Silkie_Wencha	0.934350616
ENSGALG00010022124	ENSGALG0001002212	3	-0.027240193	0.097271863	-0.280041854	0.779445394	0.003475352	2.229207424	10.28201419	0.666666667	CRA004775;PRJNA827465;Yang2025_Silkie_Wencha	0.934350616
ENSGALG00010026484	TNKS1BP1	3	0.024969974	0.089395725	0.279319549	0.779999605	0.00917154	3.123406228	35.9673429	0.666666667	CRA004775;PRJNA827465;Yang2025_Silkie_Wencha	0.934350616
ENSGALG00010011453	GNPTAB	3	0.023242401	0.083871353	0.277119755	0.781688153	0.011080124	4.5657238	56.19533533	0.666666667	CRA004775;PRJNA827465;Yang2025_Silkie_Wencha	0.934350616
ENSGALG00010018719	ERRFI1	3	-0.023177528	0.082884777	-0.279635518	0.779757154	0.007182881	2.826368695	29.23782367	0.666666667	CRA004775;PRJNA827465;Yang2025_Silkie_Wencha	0.934350616
ENSGALG00010028096	TOR2A	3	0.022540203	0.08161417	0.276180023	0.7824098	0	1.132624067	0	0.666666667	CRA004775;PRJNA827465;Yang2025_Silkie_Wencha	0.934350616
ENSGALG00010028590	ENSGALG0001002859	3	-0.019800833	0.071241872	-0.277938132	0.781059852	0.001369992	2.08245042	3.959298093	0.666666667	CRA004775;PRJNA827465;Yang2025_Silkie_Wencha	0.934350616
ENSGALG00010024638	ZNF507	3	0.019536982	0.070101776	0.278694543	0.78047925	0	1.007478099	0	0.666666667	CRA004775;PRJNA827465;Yang2025_Silkie_Wencha	0.934350616
ENSGALG00010012747	TOB2	3	0.017837392	0.063816177	0.279512074	0.779851873	0	1.731705877	0	0.666666667	CRA004775;PRJNA827465;Yang2025_Silkie_Wencha	0.934350616
ENSGALG00010015172	PPP4R3A	3	-0.017258791	0.061902002	-0.278808285	0.780391956	0.002290682	2.246277867	10.96382023	0.666666667	CRA004775;PRJNA827465;Yang2025_Silkie_Wencha	0.934350616
ENSGALG00010029721	EXOC7	3	-0.016614029	0.059735945	-0.278124484	0.780916801	0.004252299	3.15321353	36.57264308	0.666666667	CRA004775;PRJNA827465;Yang2025_Silkie_Wencha	0.934350616
ENSGALG00010010421	POLR2F	3	-0.015769984	0.057032606	-0.276508211	0.782157754	0	0.432315537	0	1	CRA004775;PRJNA827465;Yang2025_Silkie_Wencha	0.934350616
ENSGALG00010028577	CERS2	3	0.013496632	0.048899569	0.27600718	0.782542552	0	1.238591355	0	0.666666667	CRA004775;PRJNA827465;Yang2025_Silkie_Wencha	0.934350616
ENSGALG00010014379	CARS2	3	-0.013116163	0.046965135	-0.279274463	0.780034202	0	1.698282169	0	0.666666667	CRA004775;PRJNA827465;Yang2025_Silkie_Wencha	0.934350616
ENSGALG00010006614	TMEM184C	3	-0.012938836	0.046300981	-0.279450583	0.779899057	0.00105333	2.301222967	13.08969062	0.666666667	CRA004775;PRJNA827465;Yang2025_Silkie_Wencha	0.934350616
ENSGALG00010022407	RUNDC1	3	0.010468533	0.038025977	0.275299523	0.783086131	0	1.073049609	0	0.666666667	CRA004775;PRJNA827465;Yang2025_Silkie_Wencha	0.934350616
ENSGALG00010002965	PRSS35	3	0.046475355	0.16892039	0.275131704	0.783215055	0.052177674	5.601589312	64.29584733	0.666666667	CRA004775;PRJNA827465;Yang2025_Silkie_Wencha	0.934425589
ENSGALG00010001752	ARRB1	3	-0.050981088	0.185394257	-0.274987418	0.783325905	0.066755373	5.713937103	64.99786463	0.666666667	CRA004775;PRJNA827465;Yang2025_Silkie_Wencha	0.934478989
ENSGALG00010022890	MYPN	3	0.102115961	0.371817658	0.274639891	0.783592915	0.311438753	12.88017701	84.47226309	0.666666667	CRA004775;PRJNA827465;Yang2025_Silkie_Wencha	0.934560964
ENSGALG00010007434	ENSGALG0001000743	3	0.059164496	0.215339122	0.274750333	0.783508058	0.045432592	2.921242386	31.53597902	0.666666667	CRA004775;PRJNA827465;Yang2025_Silkie_Wencha	0.934560964
ENSGALG00010001264	FAM3C	3	0.04107318	0.149507994	0.274722299	0.783529598	0.049009269	10.27694442	80.53896256	0.666666667	CRA004775;PRJNA827465;Yang2025_Silkie_Wencha	0.934560964
ENSGALG00010025222	FAM117B	3	0.030987123	0.112925494	0.274403255	0.783774742	0	1.592776775	0	0.666666667	CRA004775;PRJNA827465;Yang2025_Silkie_Wencha	0.934698977
ENSGALG00010023255	SNX22	3	-0.092900216	0.338761184	-0.274235126	0.783903936	0.23934466	6.661469264	69.97659344	0.666666667	CRA004775;PRJNA827465;Yang2025_Silkie_Wencha	0.934732626
ENSGALG00010018767	NDUFB6	3	-0.020447429	0.074572726	-0.27419447	0.783935178	0	0.264235144	0	0.666666667	CRA004775;PRJNA827465;Yang2025_Silkie_Wencha	0.934732626
ENSGALG00010002254	ENSGALG0001000225	3	-0.139053195	0.507674976	-0.273902007	0.784159929	0.517149014	6.04039217	66.88956704	0.666666667	CRA004775;PRJNA827465;Yang2025_Silkie_Wencha	0.934858357
ENSGALG00010000140	SEPHS3	3	0.046275126	0.169011215	0.273799143	0.784238982	0.065362104	11.26206318	82.2412646	0.666666667	CRA004775;PRJNA827465;Yang2025_Silkie_Wencha	0.934858357
ENSGALG00010005712	ENSGALG0001000571	3	0.043724328	0.159661096	0.273857121	0.784194425	0.042555932	4.875293145	58.97682579	0.666666667	CRA004775;PRJNA827465;Yang2025_Silkie_Wencha	0.934858357
ENSGALG00010010517	STMN4	2	0.131869834	0.482233391	0.273456456	0.78450236	0.247116157	1.90696449	47.56063864	0.5	PRJNA827465;Yang2025_Silkie_Wenchang	0.934935845
ENSGALG00010009186	PREB	3	0.034981572	0.127859134	0.273594625	0.784396165	0.021223075	3.211798819	37.72959912	0.666666667	CRA004775;PRJNA827465;Yang2025_Silkie_Wencha	0.934935845
ENSGALG00010023287	RGS19	3	-0.03007291	0.109956043	-0.273499385	0.784469365	0.007764671	2.458299294	18.64294129	0.666666667	CRA004775;PRJNA827465;Yang2025_Silkie_Wencha	0.934935845
ENSGALG00010011394	SERPINC1	3	0.234071503	0.857174657	0.273073289	0.784796879	1.788090785	11.16969623	82.09441009	0.666666667	CRA004775;PRJNA827465;Yang2025_Silkie_Wencha	0.93517542
ENSGALG00010016620	ENSGALG0001001662	3	0.025712435	0.09417687	0.273022826	0.784835669	0	0.463629235	0	0.666666667	CRA004775;PRJNA827465;Yang2025_Silkie_Wencha	0.93517542
ENSGALG00010014148	KCNG1	3	0.073151505	0.268766052	0.272175389	0.785487164	0.107414054	4.389092927	54.43249816	0.666666667	CRA004775;PRJNA827465;Yang2025_Silkie_Wencha	0.93525394
ENSGALG00010004721	LAMA2	3	0.063908852	0.234603355	0.272412355	0.785304974	0.106335894	7.132265475	71.95841901	0.666666667	CRA004775;PRJNA827465;Yang2025_Silkie_Wencha	0.93525394
ENSGALG00010014128	COL4A1	3	-0.060800037	0.223308472	-0.27226928	0.785414975	0.110394342	12.70367706	84.25652675	0.666666667	CRA004775;PRJNA827465;Yang2025_Silkie_Wencha	0.93525394
ENSGALG00010000017	COX1	3	0.055449869	0.203670979	0.272252185	0.785428118	0.057627501	3.615158641	44.67739321	0.666666667	CRA004775;PRJNA827465;Yang2025_Silkie_Wencha	0.93525394
ENSGALG00010007294	TRAPPC11	3	-0.048726024	0.178783791	-0.272541621	0.785205593	0.08244354	18.55714787	89.22248174	0.666666667	CRA004775;PRJNA827465;Yang2025_Silkie_Wencha	0.93525394
ENSGALG00010027984	ARHGAP45	3	0.044369917	0.162907348	0.272362894	0.785343001	0.039652087	4.215692904	52.55821414	0.666666667	CRA004775;PRJNA827465;Yang2025_Silkie_Wencha	0.93525394
ENSGALG00010015213	RUBCN	3	-0.043610968	0.16023857	-0.272162739	0.785496891	0.057007252	9.067840664	77.94403239	0.666666667	CRA004775;PRJNA827465;Yang2025_Silkie_Wencha	0.93525394
ENSGALG00010015931	RBM47	3	0.039787648	0.145995483	0.272526571	0.785217164	0	0.75413256	0	0.666666667	CRA004775;PRJNA827465;Yang2025_Silkie_Wencha	0.93525394
ENSGALG00010011386	CTSB	3	-0.019591837	0.071967685	-0.272231023	0.785444389	0	0.601964282	0	0.666666667	CRA004775;PRJNA827465;Yang2025_Silkie_Wencha	0.93525394
ENSGALG00010006016	MTMR6	3	-0.093017633	0.342034346	-0.27195407	0.785657335	0.332975916	58.93672009	96.60652986	0.666666667	CRA004775;PRJNA827465;Yang2025_Silkie_Wencha	0.935287452
ENSGALG00010003870	MED4	3	-0.021114945	0.077634564	-0.271978659	0.785638428	0.004918242	2.704449151	26.04778688	0.666666667	CRA004775;PRJNA827465;Yang2025_Silkie_Wencha	0.935287452
ENSGALG00010007115	ENSGALG0001000711	3	-0.075592019	0.278099036	-0.271816906	0.785762805	0.147990868	5.568645665	64.08462451	0.666666667	CRA004775;PRJNA827465;Yang2025_Silkie_Wencha	0.935334257
ENSGALG00010002084	HACL1	3	0.019927156	0.073353129	0.271660612	0.78588299	0	0.690679024	0	0.666666667	CRA004775;PRJNA827465;Yang2025_Silkie_Wencha	0.935398568
ENSGALG00010007387	ENSGALG0001000738	3	-0.127440846	0.470279469	-0.270989602	0.786399029	0.346665943	4.296552782	53.4510548	0.666666667	CRA004775;PRJNA827465;Yang2025_Silkie_Wencha	0.935422015
ENSGALG00010019451	CAV3	3	-0.042088572	0.15511638	-0.271335444	0.786133048	0.024772803	3.030434304	34.00285902	0.666666667	CRA004775;PRJNA827465;Yang2025_Silkie_Wencha	0.935422015
ENSGALG00010023638	DCTN2	3	0.039089929	0.144317424	0.270860769	0.786498119	0.04102399	7.425722816	73.066595	0.666666667	CRA004775;PRJNA827465;Yang2025_Silkie_Wencha	0.935422015
ENSGALG00010012458	MRPS30	3	0.033888645	0.124813382	0.271514515	0.785995337	0	0.272007455	0	0.666666667	CRA004775;PRJNA827465;Yang2025_Silkie_Wencha	0.935422015
ENSGALG00010013112	CD40	3	0.031633533	0.116740135	0.270973927	0.786411086	0.013951851	2.971722716	32.69896988	0.666666667	CRA004775;PRJNA827465;Yang2025_Silkie_Wencha	0.935422015
ENSGALG00010016768	GALNT10	3	-0.031526857	0.116157787	-0.271414061	0.786072589	0.014983537	2.979950451	32.8847901	0.666666667	CRA004775;PRJNA827465;Yang2025_Silkie_Wencha	0.935422015
ENSGALG00010021535	SUSD6	3	-0.029251951	0.107872977	-0.271170336	0.786260027	0.023419014	9.495459705	78.93730202	0.666666667	CRA004775;PRJNA827465;Yang2025_Silkie_Wencha	0.935422015
ENSGALG00010010059	ENSGALG0001001005	3	0.02303874	0.085054986	0.270868777	0.78649196	0	0.421785323	0	0.666666667	CRA004775;PRJNA827465;Yang2025_Silkie_Wencha	0.935422015
ENSGALG00010012614	LHFPL2	3	0.019791206	0.073027738	0.27100943	0.786383779	0	0.481422193	0	0.666666667	CRA004775;PRJNA827465;Yang2025_Silkie_Wencha	0.935422015
ENSGALG00010023884	C2orf69	3	-0.0631948	0.233766069	-0.270333501	0.786903695	0.137466328	16.97160181	88.2156085	0.666666667	CRA004775;PRJNA827465;Yang2025_Silkie_Wencha	0.935555919
ENSGALG00010028727	ENSGALG0001002872	3	0.027335059	0.101166665	0.270198279	0.787007716	0	1.098897826	0	0.666666667	CRA004775;PRJNA827465;Yang2025_Silkie_Wencha	0.935555919
ENSGALG00010002470	OXSM	3	-0.0242527	0.089758837	-0.270198464	0.787007574	0	0.085605528	0	1	CRA004775;PRJNA827465;Yang2025_Silkie_Wencha	0.935555919
ENSGALG00010028593	DOLK	3	-0.020412317	0.07553859	-0.270223697	0.786988163	0	1.010808419	0	0.666666667	CRA004775;PRJNA827465;Yang2025_Silkie_Wencha	0.935555919
ENSGALG00010025345	CCM2	3	0.014674558	0.054284643	0.270326145	0.786909353	0	0.022625768	0	1	CRA004775;PRJNA827465;Yang2025_Silkie_Wencha	0.935555919
ENSGALG00010024877	PRPF38A	3	0.014478887	0.053560395	0.270328236	0.786907745	0.001059018	2.164542846	7.601736626	0.666666667	CRA004775;PRJNA827465;Yang2025_Silkie_Wencha	0.935555919
ENSGALG00010002359	CMTM6	3	-0.104258223	0.387630881	-0.268962634	0.787958437	0.406421254	26.46289421	92.44224768	0.666666667	CRA004775;PRJNA827465;Yang2025_Silkie_Wencha	0.936181978
ENSGALG00010005413	GAD2	2	0.082687469	0.307275953	0.269098405	0.787853958	0.047405274	1.261840243	20.75066508	0.5	PRJNA827465;Yang2025_Silkie_Wenchang	0.936181978
ENSGALG00010017886	DDRGK1	3	0.050218506	0.186638448	0.269068388	0.787877056	0.087089501	13.41125669	85.08715442	0.666666667	CRA004775;PRJNA827465;Yang2025_Silkie_Wencha	0.936181978
ENSGALG00010021022	ZFYVE1	3	-0.041642958	0.154806928	-0.268999319	0.787930207	0.052641526	9.440607857	78.81492346	0.666666667	CRA004775;PRJNA827465;Yang2025_Silkie_Wencha	0.936181978
ENSGALG00010015443	PAK3	3	0.029299184	0.108954783	0.268911408	0.787997858	0.01943174	4.494432891	55.50050366	0.666666667	CRA004775;PRJNA827465;Yang2025_Silkie_Wencha	0.936181978
ENSGALG00010028113	PIP5K1C	3	0.028224285	0.104940823	0.268954296	0.787964854	0.023136201	8.996853402	77.77000568	0.666666667	CRA004775;PRJNA827465;Yang2025_Silkie_Wencha	0.936181978
ENSGALG00010013550	KLHL5	3	-0.012416711	0.046112555	-0.269269634	0.787722198	0	0.879926771	0	0.666666667	CRA004775;PRJNA827465;Yang2025_Silkie_Wencha	0.936181978
ENSGALG00010006538	TEK	3	-0.020169345	0.075039411	-0.268783356	0.788096402	0.002123457	2.213868358	9.660391818	0.666666667	CRA004775;PRJNA827465;Yang2025_Silkie_Wencha	0.936220386
ENSGALG00010017513	TNIP2	3	-0.024268749	0.09033224	-0.268660995	0.78819057	0	1.166774471	0	0.666666667	CRA004775;PRJNA827465;Yang2025_Silkie_Wencha	0.936253589
ENSGALG00010028178	ENSGALG0001002817	3	-0.032173772	0.11980423	-0.268552887	0.788273772	0.014805233	3.004553036	33.43435859	0.666666667	CRA004775;PRJNA827465;Yang2025_Silkie_Wencha	0.936273761
ENSGALG00010011906	ENSGALG0001001190	2	-0.543087693	2.027234624	-0.267895825	0.788779507	0	0.358277326	0	0.5	PRJNA827465;Yang2025_Silkie_Wenchang	0.936318738
ENSGALG00010021702	PLD6	3	-0.115090667	0.429591215	-0.267907404	0.788770594	0.434166312	9.379599044	78.67712692	0.666666667	CRA004775;PRJNA827465;Yang2025_Silkie_Wencha	0.936318738
ENSGALG00010015233	ENSGALG0001001523	2	-0.094021501	0.351321483	-0.267622406	0.788989982	0	0.03959037	0	1	PRJNA827465;Yang2025_Silkie_Wenchang	0.936318738
ENSGALG00010021213	FBXO44	3	-0.061821546	0.231131689	-0.267473259	0.789104801	0.113720633	7.168944092	72.10188873	0.666666667	CRA004775;PRJNA827465;Yang2025_Silkie_Wencha	0.936318738
ENSGALG00010009157	ABHD1	3	-0.054763456	0.204480697	-0.267817239	0.788840001	0.074759144	5.4661583	63.41123161	0.666666667	CRA004775;PRJNA827465;Yang2025_Silkie_Wencha	0.936318738
ENSGALG00010009031	SPIN1	3	-0.049576899	0.185091516	-0.267850738	0.788814214	0.063262301	5.295915256	62.23504525	0.666666667	CRA004775;PRJNA827465;Yang2025_Silkie_Wencha	0.936318738
ENSGALG00010025829	ENSGALG0001002582	3	-0.047489776	0.177343675	-0.267783874	0.788865684	0.058043011	7.385873724	72.92128088	0.666666667	CRA004775;PRJNA827465;Yang2025_Silkie_Wencha	0.936318738
ENSGALG00010017589	ATPAF2	3	0.037052545	0.138315116	0.267885001	0.78878784	0.034256536	5.548440334	63.95383424	0.666666667	CRA004775;PRJNA827465;Yang2025_Silkie_Wencha	0.936318738
ENSGALG00010000822	BASP1	3	-0.030322431	0.113248394	-0.26775153	0.788890582	0.013873446	2.865501624	30.20419241	0.666666667	CRA004775;PRJNA827465;Yang2025_Silkie_Wencha	0.936318738
ENSGALG00010010243	ESCO2	3	-0.023623699	0.088302293	-0.267532112	0.789059493	0.004011114	2.293676015	12.80372698	0.666666667	CRA004775;PRJNA827465;Yang2025_Silkie_Wencha	0.936318738
ENSGALG00010016800	RPL14	3	-0.01618664	0.060447691	-0.267779286	0.788869216	0	1.108953142	0	0.666666667	CRA004775;PRJNA827465;Yang2025_Silkie_Wencha	0.936318738
ENSGALG00010005296	PAN3	3	-0.014767027	0.055209763	-0.267471302	0.789106307	0	0.042245764	0	0.666666667	CRA004775;PRJNA827465;Yang2025_Silkie_Wencha	0.936318738
ENSGALG00010011426	NQO2	3	0.040900477	0.153082866	0.26717867	0.789331598	0.039075582	4.499478178	55.55040116	0.666666667	CRA004775;PRJNA827465;Yang2025_Silkie_Wencha	0.936507465
ENSGALG00010021163	INHA	3	0.089084533	0.333607717	0.267033789	0.789443146	0.180359809	4.734074814	57.75309689	0.666666667	CRA004775;PRJNA827465;Yang2025_Silkie_Wencha	0.936517513
ENSGALG00010016810	SH3GL3	3	-0.04088747	0.153139108	-0.266995612	0.789472539	0.036708627	4.557281832	56.11419101	0.666666667	CRA004775;PRJNA827465;Yang2025_Silkie_Wencha	0.936517513
ENSGALG00010010460	SEMA3C	3	-0.058830759	0.220469314	-0.266843301	0.789589813	0.111257318	11.96724594	83.28771707	0.666666667	CRA004775;PRJNA827465;Yang2025_Silkie_Wencha	0.936556388
ENSGALG00010016807	VSIG4	3	0.042884668	0.16080044	0.266694968	0.789704028	0	1.352717957	0	0.666666667	CRA004775;PRJNA827465;Yang2025_Silkie_Wencha	0.936556388
ENSGALG00010022454	ABLIM1	3	-0.023679129	0.088761356	-0.266772955	0.789643979	0	0.591554738	0	0.666666667	CRA004775;PRJNA827465;Yang2025_Silkie_Wencha	0.936556388
ENSGALG00010006372	SBF1	3	-0.026905573	0.100967692	-0.26647705	0.789871832	0.02065714	7.864218314	74.56835606	0.666666667	CRA004775;PRJNA827465;Yang2025_Silkie_Wencha	0.936676829
ENSGALG00010010094	SLC2A9	3	0.065236129	0.244901238	0.266377294	0.789948651	0.030263094	2.348263682	14.83068893	0.666666667	CRA004775;PRJNA827465;Yang2025_Silkie_Wencha	0.936689363
ENSGALG00010022131	ENSGALG0001002213	2	0.43932021	1.662966029	0.264178704	0.79164222	2.683912196	1.810481396	44.76607147	0.5	PRJNA827465;Yang2025_Silkie_Wenchang	0.936723207
ENSGALG00010000454	ENSGALG0001000045	2	0.335087031	1.27886703	0.262018664	0.793307053	2.281825818	3.281302162	69.52429399	0.5	CRA004775;Yang2025_Silkie_Wenchang	0.936723207
ENSGALG00010028245	JAC1	2	-0.250515131	0.948493965	-0.264118845	0.791688343	0.452828355	1.336281335	25.16545926	0.5	PRJNA827465;Yang2025_Silkie_Wenchang	0.936723207
ENSGALG00010010840	RGS16	3	-0.203121399	0.776845527	-0.261469484	0.793730479	1.260557584	6.767528932	70.44711563	0.666666667	CRA004775;PRJNA827465;Yang2025_Silkie_Wencha	0.936723207
ENSGALG00010000408	ENSGALG0001000040	3	0.157601806	0.602825091	0.261438696	0.793754219	0.922242998	30.00257693	93.33390594	0.666666667	CRA004775;PRJNA827465;Yang2025_Silkie_Wencha	0.936723207
ENSGALG00010005864	ENSGALG0001000586	2	0.129965388	0.501326123	0.259243199	0.795447602	0.291302011	2.362772233	57.6768346	0.5	PRJNA827465;Yang2025_Silkie_Wenchang	0.936723207
ENSGALG00010002518	MAST1	3	0.121812743	0.471863701	0.258152393	0.796289298	0.56828004	13.56978753	85.26137572	0.666666667	CRA004775;PRJNA827465;Yang2025_Silkie_Wencha	0.936723207
ENSGALG00010022863	FAM81A	3	-0.106347361	0.411263665	-0.258586814	0.795954058	0.344392239	8.572888877	76.67064127	0.666666667	CRA004775;PRJNA827465;Yang2025_Silkie_Wencha	0.936723207
ENSGALG00010024374	ENSGALG0001002437	3	-0.102021741	0.391842082	-0.260364433	0.794582675	0	1.686297674	0	0.666666667	CRA004775;PRJNA827465;Yang2025_Silkie_Wencha	0.936723207
ENSGALG00010005414	ENSGALG0001000541	3	-0.099654504	0.3851849	-0.258718615	0.795852355	0.352059612	14.72363647	86.41639921	0.666666667	CRA004775;PRJNA827465;Yang2025_Silkie_Wencha	0.936723207
ENSGALG00010024680	BRSK1	3	-0.088298772	0.338994495	-0.260472583	0.794499261	0.205645124	5.410175754	63.03262425	0.666666667	CRA004775;PRJNA827465;Yang2025_Silkie_Wencha	0.936723207
ENSGALG00010021441	ENSGALG0001002144	3	0.078695043	0.303714504	0.259108612	0.795551441	0.178651424	6.186433	67.67119275	0.666666667	CRA004775;PRJNA827465;Yang2025_Silkie_Wencha	0.936723207
ENSGALG00010013959	STK32A	3	-0.072566736	0.276018043	-0.262905769	0.792623209	0	1.276119181	0	0.666666667	CRA004775;PRJNA827465;Yang2025_Silkie_Wencha	0.936723207
ENSGALG00010029692	CRYBA1	3	0.070347719	0.269027571	0.261488883	0.793715521	0.051486404	2.515623985	20.49686235	0.666666667	CRA004775;PRJNA827465;Yang2025_Silkie_Wencha	0.936723207
ENSGALG00010001974	ENSGALG0001000197	3	0.068872511	0.260821665	0.26405978	0.791733855	0.100454314	4.113994462	51.38544744	0.666666667	CRA004775;PRJNA827465;Yang2025_Silkie_Wencha	0.936723207
ENSGALG00010021341	L3MBTL1	3	0.06869401	0.259132189	0.26509254	0.790938174	0.151817581	10.52262098	80.99332852	0.666666667	CRA004775;PRJNA827465;Yang2025_Silkie_Wencha	0.936723207
ENSGALG00010002758	GREB1	2	0.067091381	0.254893057	0.263213844	0.792385761	0	0.028653872	0	1	PRJNA827465;Yang2025_Silkie_Wenchang	0.936723207
ENSGALG00010011723	SPRY3	3	-0.066535292	0.250701207	-0.265396777	0.790703819	0.141090598	10.56121883	81.06279178	0.666666667	CRA004775;PRJNA827465;Yang2025_Silkie_Wencha	0.936723207
ENSGALG00010026083	MUTYH	3	-0.063258658	0.238707736	-0.265004642	0.791005886	0.110675892	5.764437541	65.30450741	0.666666667	CRA004775;PRJNA827465;Yang2025_Silkie_Wencha	0.936723207
ENSGALG00010021384	ARTN	3	0.056121439	0.213867548	0.262412132	0.79300372	0	1.741130383	0	0.666666667	CRA004775;PRJNA827465;Yang2025_Silkie_Wencha	0.936723207

ENSGALG00010023266	RFWD3	3	0.053784926	0.20840946	0.258073342	0.796350305	0.097563721	9.244081143	78.36453435	0.666666667	CRA004775;PRJNA827465;Yang2025_Silkie_Wencha	0.936723207
ENSGALG00010024136	SAP130	3	0.053209748	0.2043448	0.260391985	0.794561425	0.089404227	7.296521112	72.58967706	0.666666667	CRA004775;PRJNA827465;Yang2025_Silkie_Wencha	0.936723207
ENSGALG00010024597	DNAJB11	3	-0.052882875	0.199993247	-0.264423305	0.791453756	0.091374858	9.532220642	79.01853015	0.666666667	CRA004775;PRJNA827465;Yang2025_Silkie_Wencha	0.936723207
ENSGALG00010008344	MFAP3L	3	-0.052642554	0.199147785	-0.264339137	0.791518605	0	1.080490085	0	0.666666667	CRA004775;PRJNA827465;Yang2025_Silkie_Wencha	0.936723207
ENSGALG00010008296	PALLD	3	0.051041188	0.193843774	0.263310947	0.792310923	0.078855689	7.79743598	74.35054286	0.666666667	CRA004775;PRJNA827465;Yang2025_Silkie_Wencha	0.936723207
ENSGALG00010014167	SLC38A9	3	0.050686567	0.19093112	0.265470431	0.790647086	0.068073422	5.337914295	62.53218224	0.666666667	CRA004775;PRJNA827465;Yang2025_Silkie_Wencha	0.936723207
ENSGALG00010010862	STRN4	3	-0.05037886	0.19385284	-0.259881979	0.794954812	0.099886367	19.22631499	89.59759059	0.666666667	CRA004775;PRJNA827465;Yang2025_Silkie_Wencha	0.936723207
ENSGALG00010002551	WDR35	3	-0.049865209	0.192054629	-0.259640758	0.795140894	0.073668083	8.14331067	75.43996439	0.666666667	CRA004775;PRJNA827465;Yang2025_Silkie_Wencha	0.936723207
ENSGALG00010025650	PPP1R1A	3	-0.049711087	0.188339203	-0.26394445	0.791822724	0	1.547664474	0	0.666666667	CRA004775;PRJNA827465;Yang2025_Silkie_Wencha	0.936723207
ENSGALG00010024825	UBXN7	3	-0.048201027	0.183714018	-0.262369891	0.793036284	0.076005393	10.68099882	81.27515943	0.666666667	CRA004775;PRJNA827465;Yang2025_Silkie_Wencha	0.936723207
ENSGALG00010021419	MANSC1	3	-0.047575144	0.179679957	-0.264777135	0.791181152	0.064351205	6.974999582	71.32616316	0.666666667	CRA004775;PRJNA827465;Yang2025_Silkie_Wencha	0.936723207
ENSGALG00010027161	C4BPG	3	-0.047215087	0.178177415	-0.264989178	0.791017798	0.064571225	7.457704375	73.182096	0.666666667	CRA004775;PRJNA827465;Yang2025_Silkie_Wencha	0.936723207
ENSGALG00010028485	USF1	3	0.046951104	0.179886768	0.261003654	0.794089689	0.070419187	7.899955101	74.68340042	0.666666667	CRA004775;PRJNA827465;Yang2025_Silkie_Wencha	0.936723207
ENSGALG00010013398	CHAMP1	3	0.0468772	0.179720615	0.26083374	0.794220724	0.072807653	9.048828888	77.89769235	0.666666667	CRA004775;PRJNA827465;Yang2025_Silkie_Wencha	0.936723207
ENSGALG00010022575	TMEM41B	3	0.046601992	0.179508504	0.259608825	0.795165528	0.078677161	18.51615804	89.19662319	0.666666667	CRA004775;PRJNA827465;Yang2025_Silkie_Wencha	0.936723207
ENSGALG00010024220	SIAH1	3	-0.046002502	0.173918066	-0.26450675	0.791389465	0.062360471	8.802759996	77.27985313	0.666666667	CRA004775;PRJNA827465;Yang2025_Silkie_Wencha	0.936723207
ENSGALG00010016328	USH2A	3	-0.04543262	0.175696764	-0.258585414	0.795955138	0	1.188114349	0	0.666666667	CRA004775;PRJNA827465;Yang2025_Silkie_Wencha	0.936723207
ENSGALG00010030104	P4HB	3	-0.044901703	0.172302435	-0.260598192	0.794402384	0.071565472	16.27699468	87.71271946	0.666666667	CRA004775;PRJNA827465;Yang2025_Silkie_Wencha	0.936723207
ENSGALG00010016866	ZNRF2	3	0.044308029	0.168839316	0.262427202	0.792992104	0.055916645	6.324704225	68.3179679	0.666666667	CRA004775;PRJNA827465;Yang2025_Silkie_Wencha	0.936723207
ENSGALG00010022266	COA3	3	0.044170755	0.167783517	0.263260398	0.792349883	0.037518192	3.216664209	37.82378669	0.666666667	CRA004775;PRJNA827465;Yang2025_Silkie_Wencha	0.936723207
ENSGALG00010029231	CBX8	3	-0.044005381	0.166312257	-0.264594936	0.791321522	0.046790155	4.46368236	55.1939444	0.666666667	CRA004775;PRJNA827465;Yang2025_Silkie_Wencha	0.936723207
ENSGALG00010000674	GRB10	3	-0.043460825	0.16738494	-0.259645969	0.795136874	0.056383489	9.24912293	78.37632806	0.666666667	CRA004775;PRJNA827465;Yang2025_Silkie_Wencha	0.936723207
ENSGALG00010025736	KIF9	3	-0.043263129	0.163324019	-0.264891409	0.791093117	0	1.382080464	0	0.666666667	CRA004775;PRJNA827465;Yang2025_Silkie_Wencha	0.936723207
ENSGALG00010000044	MAP1A	3	0.042133305	0.160773874	0.262065622	0.793270851	0.038116316	3.962840777	49.53115423	0.666666667	CRA004775;PRJNA827465;Yang2025_Silkie_Wencha	0.936723207
ENSGALG00010000046	ENSGALG00010000046	3	0.041844987	0.160830229	0.260181108	0.794724076	0.041740693	4.229332632	52.71121536	0.666666667	CRA004775;PRJNA827465;Yang2025_Silkie_Wencha	0.936723207
ENSGALG00010008310	EXOSC5	3	0.041551705	0.157102127	0.264488495	0.791403529	0.047819546	6.24186293	67.95828389	0.666666667	CRA004775;PRJNA827465;Yang2025_Silkie_Wencha	0.936723207
ENSGALG00010029735	SRP68	3	0.041057952	0.158657696	0.258783235	0.795802494	0.064575266	25.62103279	92.19391343	0.666666667	CRA004775;PRJNA827465;Yang2025_Silkie_Wencha	0.936723207
ENSGALG00010020298	ENSGALG00010020298	3	-0.040205689	0.15582706	-0.258014805	0.796395481	0.032683543	3.257261939	38.59873607	0.666666667	CRA004775;PRJNA827465;Yang2025_Silkie_Wencha	0.936723207
ENSGALG00010023343	TMEM233	3	-0.038720654	0.148538477	-0.260677603	0.794341139	0.00995839	2.217076336	9.791107881	0.666666667	CRA004775;PRJNA827465;Yang2025_Silkie_Wencha	0.936723207
ENSGALG00010012449	SUB1	3	-0.038631621	0.145142998	-0.266162485	0.790114074	0.050998275	12.52199439	84.02810337	0.666666667	CRA004775;PRJNA827465;Yang2025_Silkie_Wencha	0.936723207
ENSGALG00010012041	ICOSLG	3	-0.037731032	0.146130491	-0.258200951	0.796251824	0.035113616	4.889834391	59.09881931	0.666666667	CRA004775;PRJNA827465;Yang2025_Silkie_Wencha	0.936723207
ENSGALG00010000124	ENSGALG00010000124	3	-0.03761424	0.145110907	-0.259210288	0.795472993	0	0.444421187	0	0.666666667	CRA004775;PRJNA827465;Yang2025_Silkie_Wencha	0.936723207
ENSGALG00010003259	VEGFC	3	-0.036894535	0.141248198	-0.261203579	0.793935518	0.033826898	5.339101865	62.54051617	0.666666667	CRA004775;PRJNA827465;Yang2025_Silkie_Wencha	0.936723207
ENSGALG00010027943	RAB43	3	-0.036518988	0.137268685	-0.266040199	0.79020825	0.036714731	6.432116883	68.90603768	0.666666667	CRA004775;PRJNA827465;Yang2025_Silkie_Wencha	0.936723207
ENSGALG00010001895	P4HA3	3	-0.036276357	0.138654212	-0.261631845	0.79360529	0.016772345	2.64408577	24.35948853	0.666666667	CRA004775;PRJNA827465;Yang2025_Silkie_Wencha	0.936723207
ENSGALG00010001283	ENSGALG00010001283	3	-0.035501323	0.133802578	-0.265326149	0.790758223	0.036146834	6.328821383	68.39853933	0.666666667	CRA004775;PRJNA827465;Yang2025_Silkie_Wencha	0.936723207
ENSGALG00010001228	THAP5	3	-0.035161928	0.136228306	-0.258110295	0.796321786	0.035350367	5.812965054	65.59415063	0.666666667	CRA004775;PRJNA827465;Yang2025_Silkie_Wencha	0.936723207
ENSGALG00010013740	GNPDA2	3	0.035068439	0.135077562	0.259617058	0.795159177	0.035054276	6.6789315	70.0550904	0.666666667	CRA004775;PRJNA827465;Yang2025_Silkie_Wencha	0.936723207
ENSGALG00010024795	NRROS	3	-0.034971921	0.1323028	-0.264332437	0.791523767	0.015262126	2.731338922	26.77583935	0.666666667	CRA004775;PRJNA827465;Yang2025_Silkie_Wencha	0.936723207
ENSGALG00010029507	VPS37D	3	0.034093707	0.12922458	0.26383299	0.791908612	0	0.076413485	0	0.666666667	CRA004775;PRJNA827465;Yang2025_Silkie_Wencha	0.936723207
ENSGALG00010029251	ENGASE	3	0.033598572	0.12934717	0.259754985	0.795052776	0	1.8193843	0	0.666666667	CRA004775;PRJNA827465;Yang2025_Silkie_Wencha	0.936723207
ENSGALG00010029734	SEC14L1	3	-0.032167231	0.124092975	-0.25921879	0.795466434	0.027702191	5.921288004	66.22356489	0.666666667	CRA004775;PRJNA827465;Yang2025_Silkie_Wencha	0.936723207
ENSGALG00010008051	ENSGALG00010008051	3	0.031489027	0.121803744	0.25852265	0.796003571	0.030826075	7.440418516	73.11979164	0.666666667	CRA004775;PRJNA827465;Yang2025_Silkie_Wencha	0.936723207
ENSGALG00010000376	ASCC2	3	0.031425046	0.12120496	0.259271944	0.795425425	0.027653867	5.652191716	64.61549607	0.666666667	CRA004775;PRJNA827465;Yang2025_Silkie_Wencha	0.936723207
ENSGALG00010000584	RBM14	3	-0.031069499	0.118131964	-0.263006711	0.792545406	0.026826946	5.972498604	66.51317761	0.666666667	CRA004775;PRJNA827465;Yang2025_Silkie_Wencha	0.936723207
ENSGALG00010002444	KLF11	3	-0.030690677	0.117737822	-0.260669647	0.794347275	0.016953791	3.225254638	37.98939233	0.666666667	CRA004775;PRJNA827465;Yang2025_Silkie_Wencha	0.936723207
ENSGALG00010012290	ENSGALG00010012290	3	-0.030658895	0.116343598	-0.263514338	0.79218238	0.015271471	3.024161585	33.86596767	0.666666667	CRA004775;PRJNA827465;Yang2025_Silkie_Wencha	0.936723207
ENSGALG00010024029	SCAPER	3	-0.030028376	0.115630353	-0.259692855	0.795100704	0.017674272	3.513007336	43.06872122	0.666666667	CRA004775;PRJNA827465;Yang2025_Silkie_Wencha	0.936723207
ENSGALG00010017909	NTHL1	3	-0.029543246	0.111142556	-0.265813985	0.790382472	0	0.927849937	0	0.666666667	CRA004775;PRJNA827465;Yang2025_Silkie_Wencha	0.936723207
ENSGALG00010016247	KIF26A	3	-0.027636695	0.105244498	-0.262595155	0.792862635	0.012050554	3.106717716	35.62337545	0.666666667	CRA004775;PRJNA827465;Yang2025_Silkie_Wencha	0.936723207
ENSGALG00010023948	SULF2	3	-0.02733266	0.104043234	-0.262697058	0.792629924	0	0.81332862	0	0.666666667	CRA004775;PRJNA827465;Yang2025_Silkie_Wencha	0.936723207
ENSGALG00010025685	NCKAP1L	3	-0.027220974	0.102359312	-0.265935492	0.79028889	0.009350259	2.801599492	28.61220865	0.666666667	CRA004775;PRJNA827465;Yang2025_Silkie_Wencha	0.936723207
ENSGALG00010000281	OSBP2	3	-0.027192171	0.104257084	-0.260818455	0.794232512	0.011426648	3.06957792	34.84446226	0.666666667	CRA004775;PRJNA827465;Yang2025_Silkie_Wencha	0.936723207
ENSGALG00010006004	DCLRE1C	3	0.025790717	0.098060645	0.263007827	0.792544546	0	1.365396995	0	0.666666667	CRA004775;PRJNA827465;Yang2025_Silkie_Wencha	0.936723207
ENSGALG00010022932	OPCML	3	-0.02515568	0.096933072	-0.25951971	0.795237661	0	0.761844235	0	0.666666667	CRA004775;PRJNA827465;Yang2025_Silkie_Wencha	0.936723207
ENSGALG00010010876	ENSGALG00010010876	3	-0.024525857	0.094692805	-0.259004446	0.79563181	0	0.519935923	0	0.666666667	CRA004775;PRJNA827465;Yang2025_Silkie_Wencha	0.936723207
ENSGALG00010007328	NOVA1	3	0.024275979	0.091488051	0.265345893	0.790743013	0	1.184324104	0	0.666666667	CRA004775;PRJNA827465;Yang2025_Silkie_Wencha	0.936723207
ENSGALG00010013228	RTFDC1	3	-0.023585578	0.089110397	-0.264678183	0.791257386	0.013179266	4.694988155	57.40138348	0.666666667	CRA004775;PRJNA827465;Yang2025_Silkie_Wencha	0.936723207
ENSGALG00010029245	SKA2	3	-0.023236218	0.089748519	-0.258903634	0.795709594	0.010921799	3.51958026	43.17504213	0.666666667	CRA004775;PRJNA827465;Yang2025_Silkie_Wencha	0.936723207
ENSGALG00010028067	FAM102A	3	-0.02252494	0.086033663	-0.261815425	0.793463747	0	0.781105551	0	0.666666667	CRA004775;PRJNA827465;Yang2025_Silkie_Wencha	0.936723207
ENSGALG00010017597	CNOT10	3	0.022429534	0.085716846	0.261670076	0.793575813	0.01252513	4.85315941	58.78973199	0.666666667	CRA004775;PRJNA827465;Yang2025_Silkie_Wencha	0.936723207
ENSGALG00010013212	ENSGALG00010013212	3	0.021782402	0.083915525	0.259575354	0.795191349	0	0.619848974	0	1	CRA004775;PRJNA827465;Yang2025_Silkie_Wencha	0.936723207
ENSGALG00010005004	CDK6	3	-0.02088165	0.078959171	-0.264461365	0.791424431	0	1.044519847	0	0.666666667	CRA004775;PRJNA827465;Yang2025_Silkie_Wencha	0.936723207
ENSGALG00010023837	STT3A	3	-0.020595492	0.079212606	-0.260002708	0.794861685	0.006893037	3.036575445	34.136331	0.666666667	CRA004775;PRJNA827465;Yang2025_Silkie_Wencha	0.936723207
ENSGALG00010023572	MYBL2	3	0.019578813	0.075307173	0.259986027	0.794874552	0	0.463902278	0	0.666666667	CRA004775;PRJNA827465;Yang2025_Silkie_Wencha	0.936723207
ENSGALG00010020747	DBI	3	-0.019246886	0.074599071	-0.258004361	0.796403541	0	0.920145733	0	0.666666667	CRA004775;PRJNA827465;Yang2025_Silkie_Wencha	0.936723207
ENSGALG00010020329	PPP4R2	3	-0.019180368	0.073283339	-0.261728904	0.793530455	0.007816544	3.779399568	47.08154128	0.666666667	CRA004775;PRJNA827465;Yang2025_Silkie_Wencha	0.936723207
ENSGALG00010018557	TBCC	3	-0.019087138	0.072594916	-0.262926651	0.792607114	0	0.921104038	0	0.666666667	CRA004775;PRJNA827465;Yang2025_Silkie_Wencha	0.936723207
ENSGALG00010001177	SEH1L	3	0.018940589	0.072799921	0.260173211	0.794730168	0.007673218	3.957929977	49.46853503	0.666666667	CRA004775;PRJNA827465;Yang2025_Silkie_Wencha	0.936723207
ENSGALG00010018611	NUDCD3	3	0.018272792	0.07078953	0.258128454	0.796307773	0.005716931	2.962088624	32.48007558	0.666666667	CRA004775;PRJNA827465;Yang2025_Silkie_Wencha	0.936723207
ENSGALG00010001133	EIF2A	3	-0.017535863	0.065976158	-0.26579091	0.790400244	0.005501643	3.441441164	41.88481207	0.666666667	CRA004775;PRJNA827465;Yang2025_Silkie_Wencha	0.936723207
ENSGALG00010001866	PUS7L	3	0.016989123	0.063819725	0.266204899	0.79008141	0	1.450545668	0	0.666666667	CRA004775;PRJNA827465;Yang2025_Silkie_Wencha	0.936723207
ENSGALG00010027592	MAP1S	3	-0.01437708	0.055074419	-0.26104823	0.794055314	0	0.391450722	0	0.666666667	CRA004775;PRJNA827465;Yang2025_Silkie_Wencha	0.936723207
ENSGALG00010025007	ZYG11B	3	0.01350454	0.052055753	0.25942455	0.79530769	0	0.974658633	0	0.666666667	CRA004775;PRJNA827465;Yang2025_Silkie_Wencha	0.936723207
ENSGALG00010025442	ABCF2	3	0.013341787	0.050856325	0.262342734	0.793057219	0	0.763412612	0	0.666666667	CRA004775;PRJNA827465;Yang2025_Silkie_Wencha	0.936723207
ENSGALG00010024751	MTMR10	3	-0.012379421	0.046932947	-0.263768243	0.791958507	0	1.386568019	0	0.666666667	CRA004775;PRJNA827465;Yang2025_Silkie_Wencha	0.936723207
ENSGALG00010021233	COASY	3	-0.009829994	0.037722361	-0.260587982	0.794410259	0	0.586329758	0	1	CRA004775;PRJNA827465;Yang2025_Silkie_Wencha	0.936723207
ENSGALG00010026402	TFEB	3	-0.033617078	0.130346783	-0.257904931	0.796480279	0.032861475	6.111859317	67.2767337	0.666666667	CRA004775;PRJNA827465;Yang2025_Silkie_Wencha	0.936735541
ENSGALG00010014076	PRRX1	3	0.084726086	0.328802586	0.257680717	0.796653329	0.250243207	9.995571159	79.99113839	0.666666667	CRA004775;PRJNA827465;Yang2025_Silkie_Wencha	0.936783219
ENSGALG00010020374	PCNA	3	0.011611956	0.045059504	0.257702711	0.796636353	0	1.885242344	0	0.666666667	CRA004775;PRJNA827465;Yang2025_Silkie_Wencha	0.936783219
ENSGALG00010016781	CDIP1	3	0.038559432	0.149904736	0.257226246	0.797004124	0.038417432	5.447001311	63.28254969	0.666666667	CRA004775;PRJNA827465;Yang2025_Silkie_Wencha	0.937117781
ENSGALG00010020544	BMERB1	3	0.034780924	0.135365281	0.256941249	0.797224127	0.028377646	4.286269405	53.33937718	0.666666667	CRA004775;PRJNA827465;Yang2025_Silkie_Wencha	0.937142661
ENSGALG00010005193	EPB41L2	3	0.024418853	0.095024612	0.256973983	0.797198857	0.010474409	3.144113859	36.38907209	0.666666667	CRA004775;PRJNA827465;Yang2025_Silkie_Wencha	0.937142661
ENSGALG00010026064	ENSGALG00010026064	3	0.020841119	0.081111944	0.25694266	0.797223038	0.001807582	2.081030462	3.893766239	0.666666667	CRA004775;PRJNA827465;Yang2025_Silkie_Wencha	0.937142661
ENSGALG00010016027	COPS2	3	-0.014906025	0.058061154	-0.256729737	0.797387414	0	0.591372065	0	0.666666667	CRA004775;PRJNA827465;Yang2025_Silkie_Wencha	0.937256683
ENSGALG00010004993	SULT1B	3	0.035314608	0.137666917	0.25652211	0.79754771	0	1.342311783	0	0.666666667	CRA004775;PRJNA827465;Yang2025_Silkie_Wencha	0.937305197
ENSGALG00010009466	FAM193B	3	-0.024014311	0.093621386	-0.256504544	0.797561272	0.014751026	4.533251586	55.88155738	0.666666667	CRA004775;PRJNA827465;Yang2025_Silkie_Wencha	0.937305197
ENSGALG00010005439	TBX19	2	-0.072102055	0.281310136	-0.256308057	0.797712976	0	0.75459869	0	0.5	PRJNA827465;Yang2025_Silkie_Wenchang	0.937405566
ENSGALG00010028447	OLFM1	3	-0.05723655	0.223631086	-0.255941833	0.797995752	0.103770078	9.996306572	79.99261041	0.666666667	CRA004775;PRJNA827465;Yang2025_Silkie_Wencha	0.937426217
ENSGALG00010005821	DOCK9	3	0.036379363	0.142106482	0.256000727	0.797950276	0.032418436	4.615036137	56.6633946	0.666666667	CRA004775;PRJNA827465;Yang2025_Silkie_Wencha	0.937426217
ENSGALG00010012819	RANGAP1	3	0.025817719	0.100775577	0.256190233	0.79780395	0.017009177	4.692912164	57.38253924	0.666666667	CRA004775;PRJNA827465;Yang2025_Silkie_Wencha	0.937426217
ENSGALG00010020008	EIF2S1	3	-0.010791595	0.042136179	-0.256112336	0.797864097	0	0.447672226	0	0.666666667	CRA004775;PRJNA827465;Yang2025_Silkie_Wencha	0.937426217
ENSGALG00010017970	AMPH	2	-0.463793169	1.816126552	-0.255374918	0.798433541	5.540140533	6.012237417	83.36725697	0.5	CRA004775;PRJNA827465	0.937826792
ENSGALG00010005037	THNSL1	3	-0.053408952	0.209177418	-0.255328479	0.798469405	0.091808918	7.035090931	71.5710853	0.666666667	CRA004775;PRJNA827465;Yang2025_Silkie_Wencha	0.937826792
ENSGALG00010009531	FBXL13	2	0.112636888	0.441471009	0.255139944	0.798615014	0.257394521	2.913177617	65.67322246	0.5	PRJNA827465;Yang2025_Silkie_Wenchang	0.937842393
ENSGALG00010029739	P2RX5	3	0.040266025	0.157872778	0.255053628	0.798681679	0.0479556	6.592106612	69.66068485	0.666666667	CRA004775;PRJNA827465;Yang2025_Silkie_Wencha	0.937842393
ENSGALG00010021192	ENSGALG00010021192	3	-0.024030233	0.094193961	-0.255114366	0.798634769	0.009770931	3.174562151	36.99918587	0.666666667	CRA004775;PRJNA827465;Yang2025_Silkie_Wencha	0.937842393
ENSGALG00010021657	JAGN1	3	0.037996576	0.149081781	0.254870689	0.798822976	0.030974929	3.635303673	44.98396338	0.666666667	CRA004775;PRJNA827465;Yang2025_Silkie_Wencha	0.937930415
ENSGALG00010000546	FLRT1	2	0.127076736	0.499044924	0.254639873	0.79900126	0	0.221881894	0	0.5	CRA004775;PRJNA827465	0.938061846
ENSGALG00010015049	DCK2	3	-0.068249391	0.268480334	-0.254206294	0.799336189	0.148938414	7.434017849	73.09664786	0.666666667	CRA004775;PRJNA827465;Yang2025_Silkie_Wencha	0.938255775
ENSGALG00010011367	IER3IP1	3	-0.020645191	0.081189742	-0.254283251	0.799276739	0	1.902864952	0	0.666666667	CRA004775;PRJNA827465;Yang2025_Silkie_Wencha	0.938255775
ENSGALG00010000802	SEMA5A	3	0.019914651	0.078352213	0.254168327	0.799365519	0	0.204557946	0	1	CRA004775;PRJNA827465;Yang2025_Silkie_Wencha	0.938255775
ENSGALG00010012662	SLC35E3	3	-0.026578891	0.104607507	-0.254082065	0.799432159	0.016826667	4.203229406	52.41753883	0.666666667	CRA004775;PRJNA827465;Yang2025_Silkie_Wencha	0.938256105
ENSGALG00010008352	ENSGALG00010008352	3	-0.064761802	0.255260807	-0.253708364	0.799720872	0.162078047	15.0841552	86.74105395	0.666666667	CRA004775;PRJNA827465;Yang2025_Silkie_Wencha	0.938283413
ENSGALG00010001225	SOCS6	3	-0.03998515	0.157496723	-0.253879253	0.799588843	0.056715486	11.35501292	82.38663387	0.666666667	CRA004775;PRJNA827465;Yang2025_Silkie_Wencha	0.938283413
ENSGALG00010019000	TRERF1	3	-0.037428284	0.14748944	-0.253769246	0.799673834	0.035312726	4.192067263	52.29084186	0.666666667	CRA004775;PRJNA827465;Yang2025_Silkie_Wencha	0.938283413
ENSGALG00010016041	ENSGALG00010016041	3	0.02699378	0.106324681	0.253880655	0.799587761	0.014511274	3.405543838	41.2722286	0.666666667	CRA004775;PRJNA827465;Yang2025_Silkie_Wencha	0.938283413
ENSGALG00010028519	ENSGALG00010028519	2	-0.301279213	1.193378046	-0.252459155	0.800686182	0	0.43016793	0	0.5	PRJNA827465;Yang2025_Silkie_Wenchang	0.938354512
ENSGALG00010014156	CD164	3	-0.07110652	0.280790731	-0.253236707	0.800085303	0.217427099	51.90586539	96.14687091	0.666666667	CRA004775;PRJNA827465;Yang2025_Silkie_Wencha	0.938354512
ENSGALG00010021488	HYI	3	-0.054159005	0.214168663	-0.252880156	0.800360825	0.093552367	6.718020326	70.22932496	0.666666667	CRA004775;PRJNA827465;Yang2025_Silkie_Wencha	0.938354512
ENSGALG00010013732	FNTA	3	0.045567945	0.18052916	0.252413212	0.800721689	0.071640423	8.750523969	77.1442258	0.666666667	CRA004775;PRJNA827465;Yang2025_Silkie_Wencha	0.938354512
ENSGALG00010005670	SGMS2	3	0.040557833	0.16011906	0.253297968	0.800037967	0.007025253	2.174663815	8.031761702	0.666666667	CRA004775;PRJNA827465;Yang2025_Silkie_Wencha	0.938354512
ENSGALG00010003317	LYRM2	3	0.038194073	0.151358556	0.252341689	0.800776968	0.022063831	2.830586314	29.34326043	0.666666667	CRA004775;PRJNA827465;Yang2025_Silkie_Wencha	0.938354512
ENSGALG00010020023	FOSL2	3	-0.034084035	0.135050548	-0.252379838	0.800747483	0	1.729303057	0	0.666666667	CRA004775;PRJNA827465;Yang2025_Silkie_Wencha	0.938354512
ENSGALG00010014170	CEMIP2	3	-0.026453173	0.104601251	-0.25289538	0.80034906	0.01499723	3.881750802	48.47685742	0.666666667	CRA004775;PRJNA827465;Yang2025_Silkie_Wencha	0.938354512
ENSGALG00010017502	NAT8B	3	-0.026297655	0.104115098	-0.252582531	0.800590831	0	0.324976207	0	0.666666667	CRA004775;PRJNA827465;Yang2025_Silkie_Wencha	0.938354512
ENSGALG00010018383	METRN	3	0.025999849	0.102926259	0.252606572	0.800572251	0.005586558	2.307786822	13.33688271	0.666666667	CRA004775;PRJNA827465;Yang2025_Silkie_Wencha	0.938354512
ENSGALG00010002904	PON1	3	-0.025984526	0.102747487	-0.25289695	0.800347846	0.012455375	3.318242708	39.72713342	0.666666667	CRA004775;PRJNA827465;Yang2025_Silkie_Wencha	0.938354512
ENSGALG00010027536	USP21	3	-0.025875074	0.102338321	-0.252838563	0.800392966	0	1.151345986	0	0.666666667	CRA004775;PRJNA827465;Yang2025_Silkie_Wencha	0.938354512
ENSGALG00010029170	DCXR	3	0.023393851	0.092268374	0.253541382	0.799849887	0.002745889	2.144320432	6.730357558	0.666666667	CRA004775;PRJNA827465;Yang2025_Silkie_Wencha	0.938354512
ENSGALG00010021932	RPAP2	3	-0.021677841	0.085593272	-0.253265708	0.800062893	0.009687481	3.532791583	43.38754628	0.666666667	CRA004775;PRJNA827465;Yang2025_Silkie_Wencha	0.938354512
ENSGALG00010028156	DOHH	3	0.01640791	0.064864337	0.252957339	0.80030118	0	1.650990073	0	0.666666667	CRA004775;PRJNA827465;Yang2025_Silkie_Wencha	0.938354512
ENSGALG00010025349	SETD1A	3	0.03370415	0.133626209	0.252227094	0.800865537	0.024738881	3.324386491	39.83852342	0.666666667	CRA004775;PRJNA827465;Yang2025_Silkie_Wencha	0.938380528
ENSGALG00010027127	S1PR4	2	-0.19961518	0.792470795	-0.251889637	0.80112637	0.784173974	2.626511735	61.92668829	0.5	CRA004775;Yang2025_Silkie_Wenchang	0.938530178
ENSGALG00010010496	MRPL17	3	0.065626123	0.260712508	0.251718351	0.801258772	0.113471955	4.513769445	55.69113522	0.666666667	CRA004775;PRJNA827465;Yang2025_Silkie_Wencha	0.938530178
ENSGALG00010028132	CACNA2D2	3	0.041014589	0.162866382	0.251829683	0.801172712	0.052971589	6.969712388	71.30441131	0.666666667	CRA004775;PRJNA827465;Yang2025_Silkie_Wencha	0.938530178
ENSGALG00010006219	CLEC14A	3	-0.030306939	0.120362266	-0.251797677	0.801197453	0	0.808542957	0	0.666666667	CRA004775;PRJNA827465;Yang2025_Silkie_Wencha	0.938530178
ENSGALG00010013373	ENSGALG00010013373	3	-0.090619556	0.360595433	-0.251305336	0.80157805	0	1.50667802	0	0.666666667	CRA004775;PRJNA827465;Yang2025_Silkie_Wencha	0.938576506
ENSGALG00010023671	ENSGALG00010023671	3	0.059361973	0.23622172	0.251297692	0.801583959	0.037110039	2.549710497	21.55972207	0.666666667	CRA004775;PRJNA827465;Yang2025_Silkie_Wencha	0.938576506
ENSGALG00010015095	RBFOX2	3	0.04296978	0.171020975	0.251254441	0.801617396	0.062961442	10.50523854	80.96187923	0.666666667	CRA004775;PRJNA827465;Yang2025_Silkie_Wencha	0.938576506
ENSGALG00010023118	PIDD1	3	0.023993899	0.095502732	0.251237835	0.801630235	0	1.108357103	0	0.666666667	CRA004775;PRJNA827465;Yang2025_Silkie_Wencha	0.938576506
ENSGALG00010019529	SNAP23	3	0.02176006	0.086565378	0.251371399	0.801526979	0.014167352	6.394164692	68.72148128	0.666666667	CRA004775;PRJNA827465;Yang2025_Silkie_Wencha	0.938576506
ENSGALG00010006678	ENSGALG00010006678	3	0.150546998	0.599707867	0.251033889	0.801787908	0.468402225	3.505986939	42.95472188	0.666666667	CRA004775;PRJNA827465;Yang2025_Silkie_Wencha	0.938611436
ENSGALG00010024004	EIF4B	3	-0.023973107	0.095499918	-0.251027514	0.801792837	0.015187136	5.070073355	60.55283899	0.666666667	CRA004775;PRJNA827465;Yang2025_Silkie_Wencha	0.938611436
ENSGALG00010006072	ENSGALG00010006072	3	-0.028041455	0.111778598	-0.250866049	0.801917674	0.01631938	3.627368971	44.86361835	0.666666667	CRA004775;PRJNA827465;Yang2025_Silkie_Wencha	0.938679857
ENSGALG00010018522	NEK11	3	-0.040725998	0.16250801	-0.250609175	0.802116287	0	0.785187482	0	0.666666667	CRA004775;PRJNA827465;Yang2025_Silkie_Wencha	0.938834618
ENSGALG00010020918	ENSGALG00010020918	3	-0.060593648	0.24228491	-0.250092536	0.802515788	0.03011758	2.378634509	15.9181458	0.666666667	CRA004775;PRJNA827465;Yang2025_Silkie_Wencha	0.939003339
ENSGALG00010017920	AP3M2	3	-0.037512411	0.149912796	-0.25022821	0.802410871	0.0478268	8.095285091	75.29426107	0.666666667	CRA004775;PRJNA827465;Yang2025_Silkie_Wencha	0.939003339
ENSGALG00010025421	NR4A1	3	0.032442597	0.12964292	0.250245803	0.802397266	0	0.257649434	0	0.666666667	CRA004775;PRJNA827465;Yang2025_Silkie_Wencha	0.939003339
ENSGALG00010028437	ENSGALG00010028437	3	-0.009273914	0.037083905	-0.250079217	0.802526088	0	1.425883153	0	1	CRA004775;PRJNA827465;Yang2025_Silkie_Wencha	0.939003339
ENSGALG00010007977	APOB	3	-0.536908924	2.149080555	-0.249831921	0.802717333	12.50161278	20.62831188	90.30458715	0.666666667	CRA004775;PRJNA827465;Yang2025_Silkie_Wencha	0.939071683
ENSGALG00010000093	ENSGALG00010000093	3	0.051216387	0.204959169	0.249885805	0.802675661	0	0.376832837	0	0.666666667	CRA004775;PRJNA827465;Yang2025_Silkie_Wencha	0.939071683
ENSGALG00010014411	ATP13A3	3	0.019167728	0.076770989	0.249674101	0.802839388	0.004055403	2.505338038	20.17045325	0.666666667	CRA004775;PRJNA827465;Yang2025_Silkie_Wencha	0.939111916
ENSGALG00010002422	TRAPPC12	3	0.011930848	0.047796867	0.24961569	0.802884564	0	0.341806436	0	0.666666667	CRA004775;PRJNA827465;Yang2025_Silkie_Wencha	0.939111916
ENSGALG00010020444	ENSGALG00010020444	2	0.215410935	0.86431853	0.24922633	0.803185713	0.553791794	1.552640918	35.59360775	0.5	PRJNA827465;Yang2025_Silkie_Wenchang	0.939345209
ENSGALG00010022416	CCDC186	3	-0.023107354	0.092731338	-0.249186026	0.803216888	0.012708149	3.870385407	48.32555961	0.666666667	CRA004775;PRJNA827465;Yang2025_Silkie_Wencha	0.939345209
ENSGALG00010020285	ADAMTS14	3	0.022198102	0.089203585	0.24884764	0.803478639	0	1.269294503	0	0.666666667	CRA004775;PRJNA827465;Yang2025_Silkie_Wencha	0.939573606
ENSGALG00010006198	ENSGALG00010006198	3	-0.121614337	0.489278837	-0.248558343	0.803702435	0.51204455	7.067383676	71.70098453	0.666666667	CRA004775;PRJNA827465;Yang2025_Silkie_Wencha	0.939746998
ENSGALG00010002450	ENSGALG00010002450	3	-0.092408961	0.372065457	-0.248367484	0.80385009	0.296246963	7.268865409	72.48538957	0.666666667	CRA004775;PRJNA827465;Yang2025_Silkie_Wencha	0.939746998
ENSGALG00010005363	RDX	3	0.027748207	0.111747206	0.248312311	0.803892775	0.023967499	5.65652175	64.6425827	0.666666667	CRA004775;PRJNA827465;Yang2025_Silkie_Wencha	0.939746998
ENSGALG00010020586	C11orf16	3	0.023206903	0.093403304	0.248459119	0.803779196	0.013737172	4.449015527	55.04623466	0.666666667	CRA004775;PRJNA827465;Yang2025_Silkie_Wencha	0.939746998
ENSGALG00010007860	DEFB4A	3	-0.202232096	0.82159807	-0.246144805	0.805570141	1.637850868	10.48424815	80.92376324	0.666666667	CRA004775;PRJNA827465;Yang2025_Silkie_Wencha	0.939864212
ENSGALG00010026786	ENSGALG00010026786	2	0.158666305	0.642997632	0.246760326	0.805093716	0.555355203	3.025504024	66.94765593	0.5	CRA004775;Yang2025_Silkie_Wenchang	0.939864212
ENSGALG00010014982	ENSGALG00010014982	2	0.156590478	0.632896965	0.247418596	0.804584284	0.550973341	2.929278758	65.86190381	0.5	CRA004775;PRJNA827465	0.939864212
ENSGALG00010000242	ENSGALG00010000242	3	-0.096717111	0.392965356	-0.246121216	0.8055884	0.324799218	7.487671224	73.28942551	0.666666667	CRA004775;PRJNA827465;Yang2025_Silkie_Wencha	0.939864212
ENSGALG00010027890	ENSGALG00010027890	3	-0.090891648	0.368709723	-0.246512751	0.805285335	0.216240835	4.348043536	54.00230049	0.666666667	CRA004775;PRJNA827465;Yang2025_Silkie_Wencha	0.939864212
ENSGALG00010021949	ENSGALG00010021949	3	0.078591346	0.318821617	0.246505698	0.805290794	0.231668793	8.893782665	77.51238056	0.666666667	CRA004775;PRJNA827465;Yang2025_Silkie_Wencha	0.939864212
ENSGALG00010013235	GCNT7	3	0.075109382	0.303473189	0.247499236	0.804521883	0.197221656	8.654288571	76.89007035	0.666666667	CRA004775;PRJNA827465;Yang2025_Silkie_Wencha	0.939864212
ENSGALG00010015937	ENSGALG00010015937	3	-0.072099723	0.29222002	-0.246730951	0.805116451	0.174154139	6.348981741	68.49888562	0.666666667	CRA004775;PRJNA827465;Yang2025_Silkie_Wencha	0.939864212
ENSGALG00010004669	ELMOD1	2	0.062609806	0.25354676	0.246935933	0.804957807	0	0.675886217	0	0.5	PRJNA827465;Yang2025_Silkie_Wenchang	0.939864212
ENSGALG00010023981	ENSGALG00010023981	3	-0.056182334	0.226948827	-0.247555075	0.804478674	0.084523535	4.422005058	54.7716483	0.666666667	CRA004775;PRJNA827465;Yang2025_Silkie_Wencha	0.939864212
ENSGALG00010007575	MMP27	3	-0.054747802	0.22219852	-0.246391388	0.805379272	0.052455901	3.059175422	34.62290572	0.666666667	CRA004775;PRJNA827465;Yang2025_Silkie_Wencha	0.939864212
ENSGALG00010019005	ENSGALG00010019005	3	-0.050560504	0.205396964	-0.246159939	0.805558426	0.090193705	11.97208971	83.29447867	0.666666667	CRA004775;PRJNA827465;Yang2025_Silkie_Wencha	0.939864212
ENSGALG00010010408	GAS8	3	0.042466626	0.171715571	0.247307952	0.804669905	0.067238056	8.798588269	77.26908069	0.666666667	CRA004775;PRJNA827465;Yang2025_Silkie_Wencha	0.939864212
ENSGALG00010001117	P2RY13	3	-0.04180397	0.169457128	-0.246693486	0.805145448	0	0.400535822	0	0.666666667	CRA004775;PRJNA827465;Yang2025_Silkie_Wencha	0.939864212
ENSGALG00010006343	SEC23A	3	-0.032393156	0.130687767	-0.247866782	0.804237482	0.040843379	19.63780409	89.81556191	0.666666667	CRA004775;PRJNA827465;Yang2025_Silkie_Wencha	0.939864212
ENSGALG00010015906	BAG5	3	0.028965227	0.117626874	0.246246674	0.805491287	0.018505365	3.466532414	42.30545799	0.666666667	CRA004775;PRJNA827465;Yang2025_Silkie_Wencha	0.939864212
ENSGALG00010028068	POLE4	3	-0.023944464	0.097016824	-0.246807342	0.805057328	0	1.418752982	0	0.666666667	CRA004775;PRJNA827465;Yang2025_Silkie_Wencha	0.939864212
ENSGALG00010006454	SOD2	3	0.021971918	0.088718685	0.247658286	0.80439881	0.006411029	2.499451116	19.98243186	0.666666667	CRA004775;PRJNA827465;Yang2025_Silkie_Wencha	0.939864212
ENSGALG00010015171	NHSL2	3	-0.020501429	0.082672852	-0.247982605	0.804147865	0.000790801	2.054720636	2.663166701	0.666666667	CRA004775;PRJNA827465;Yang2025_Silkie_Wencha	0.939864212
ENSGALG00010019373	DENND5A	3	-0.019559658	0.079254717	-0.246794878	0.805066974	0.005187718	2.37046412	15.62833697	0.666666667	CRA004775;PRJNA827465;Yang2025_Silkie_Wencha	0.939864212
ENSGALG00010015212	GNAQ	3	-0.017131873	0.06944702	-0.246689817	0.805148288	0	0.806232585	0	0.666666667	CRA004775;PRJNA827465;Yang2025_Silkie_Wencha	0.939864212
ENSGALG00010018524	SEC22A	3	-0.015719614	0.063753547	-0.246568461	0.805242216	0.005596632	3.533294067	43.39559736	0.666666667	CRA004775;PRJNA827465;Yang2025_Silkie_Wencha	0.939864212
ENSGALG00010017110	BRF1	3	0.015259137	0.061738342	0.247158191	0.8047858	0	0.381545911	0	0.666666667	CRA004775;PRJNA827465;Yang2025_Silkie_Wencha	0.939864212
ENSGALG00010013381	DENND2A	3	-0.010988453	0.044324041	-0.247911803	0.804202648	0	1.03219017	0	0.666666667	CRA004775;PRJNA827465;Yang2025_Silkie_Wencha	0.939864212
ENSGALG00010011205	CYTL1	3	-0.568084975	2.310214766	-0.24590137	0.805758584	14.91900197	31.5157543	93.65396753	0.666666667	CRA004775;PRJNA827465;Yang2025_Silkie_Wencha	0.939907649
ENSGALG00010002807	P2RY6	3	-0.055103672	0.224085339	-0.245904852	0.805755888	0.102245096	6.743040383	70.33978908	0.666666667	CRA004775;PRJNA827465;Yang2025_Silkie_Wencha	0.939907649
ENSGALG00010024360	FTL	3	0.047168687	0.191898096	0.245800701	0.805836514	0.064371727	4.876011901	58.98287288	0.666666667	CRA004775;PRJNA827465;Yang2025_Silkie_Wencha	0.939921009
ENSGALG00010008897	NSMCE2	3	-0.042402443	0.172575705	-0.245703435	0.805911813	0.061768629	7.905176814	74.70012313	0.666666667	CRA004775;PRJNA827465;Yang2025_Silkie_Wencha	0.939931298
ENSGALG00010018843	MCRIP2	3	0.024415618	0.099426633	0.245564159	0.806019637	0.00197479	2.064714747	3.134319013	0.666666667	CRA004775;PRJNA827465;Yang2025_Silkie_Wencha	0.939979516
ENSGALG00010017462	MCM8	3	0.059824935	0.244087548	0.245096216	0.806381932	0.151461431	13.91029399	85.62215866	0.666666667	CRA004775;PRJNA827465;Yang2025_Silkie_Wencha	0.93999629

ENSGALG00010028503	NDOR1	3	-0.058564743	0.239093923	-0.244944508	0.806499399	0.142528749	12.48577063	83.98176565	0.666666667	CRA004775;PRJNA827465;Yang2025_Silkie_Wencha	0.93999629
ENSGALG00010003652	MYCN	3	-0.04598744	0.187700548	-0.245004294	0.806453106	0.07007863	9.025954081	77.84167765	0.666666667	CRA004775;PRJNA827465;Yang2025_Silkie_Wencha	0.93999629
ENSGALG00010018123	USP13	3	-0.036454394	0.148515568	-0.245458399	0.806101516	0.035898262	5.260831913	61.98319899	0.666666667	CRA004775;PRJNA827465;Yang2025_Silkie_Wencha	0.93999629
ENSGALG00010001940	PTPRM	3	-0.032331323	0.13177042	-0.245361004	0.80617692	0.030947785	5.319409954	62.40184499	0.666666667	CRA004775;PRJNA827465;Yang2025_Silkie_Wencha	0.93999629
ENSGALG00010020744	CHST3	3	0.01692445	0.069079945	0.244998025	0.806457961	0.001278911	2.129281818	6.071616095	0.666666667	CRA004775;PRJNA827465;Yang2025_Silkie_Wencha	0.93999629
ENSGALG00010021565	CBX1	3	-0.012274844	0.050080199	-0.245103734	0.806376112	0	0.580679061	0	0.666666667	CRA004775;PRJNA827465;Yang2025_Silkie_Wencha	0.93999629
ENSGALG00010021715	MMAB	3	0.047292749	0.193359101	0.24458507	0.806777726	0.080866777	8.317793906	75.95516284	0.666666667	CRA004775;PRJNA827465;Yang2025_Silkie_Wencha	0.940088204
ENSGALG00010015810	LIPT1	3	-0.027253442	0.111383417	-0.244681324	0.80670319	0.018776604	3.971627999	49.64281648	0.666666667	CRA004775;PRJNA827465;Yang2025_Silkie_Wencha	0.940088204
ENSGALG00010007945	ENSGALG00010007945	3	0.02239783	0.091569647	0.244598848	0.806767056	0.010324156	3.406659225	41.29145688	0.666666667	CRA004775;PRJNA827465;Yang2025_Silkie_Wencha	0.940088204
ENSGALG00010029545	OMG	3	0.093290172	0.382034595	0.244192995	0.807081352	0.317482696	7.388242801	72.92996381	0.666666667	CRA004775;PRJNA827465;Yang2025_Silkie_Wencha	0.940132084
ENSGALG00010013086	CXCL14	3	0.086064173	0.352371733	0.244242557	0.807042969	0.229925331	5.537184483	63.88056049	0.666666667	CRA004775;PRJNA827465;Yang2025_Silkie_Wencha	0.940132084
ENSGALG00010021791	PLEKHM2	3	-0.038755283	0.15866591	-0.24425715	0.807031668	0.045674071	6.51073229	69.28148922	0.666666667	CRA004775;PRJNA827465;Yang2025_Silkie_Wencha	0.940132084
ENSGALG00010027445	PPM1M	3	-0.020263786	0.082980416	-0.244199621	0.807076221	0	1.497434053	0	0.666666667	CRA004775;PRJNA827465;Yang2025_Silkie_Wencha	0.940132084
ENSGALG00010028247	MVP	3	0.017097975	0.070199457	0.243562789	0.807569451	0	1.926208792	0	0.666666667	CRA004775;PRJNA827465;Yang2025_Silkie_Wencha	0.940623154
ENSGALG00010018936	SYT14	3	0.070171625	0.289118696	0.242708708	0.808231061	0.153393526	6.163149365	67.54905842	0.666666667	CRA004775;PRJNA827465;Yang2025_Silkie_Wencha	0.94095886
ENSGALG00010022231	ZNF503	3	0.04395082	0.181136069	0.242639802	0.808284445	0.053207056	4.496631419	55.5222607	0.666666667	CRA004775;PRJNA827465;Yang2025_Silkie_Wencha	0.94095886
ENSGALG00010023751	PRRG4	3	-0.037453176	0.154220661	-0.242854467	0.808118139	0	1.453769813	0	0.666666667	CRA004775;PRJNA827465;Yang2025_Silkie_Wencha	0.94095886
ENSGALG00010007051	E2F3	3	0.024388506	0.100561025	0.242524435	0.808373826	0	0.799776629	0	0.666666667	CRA004775;PRJNA827465;Yang2025_Silkie_Wencha	0.94095886
ENSGALG00010023142	TLK2	3	-0.01921631	0.079184038	-0.24267909	0.808254007	0.009267603	4.020365148	50.25327486	0.666666667	CRA004775;PRJNA827465;Yang2025_Silkie_Wencha	0.94095886
ENSGALG00010012922	SNRNP40	3	-0.017145364	0.070535373	-0.243074694	0.807947534	0.004190447	2.542377319	21.33347064	0.666666667	CRA004775;PRJNA827465;Yang2025_Silkie_Wencha	0.94095886
ENSGALG00010001838	PPHLN1	3	0.012075971	0.049779441	0.242589518	0.808323402	0	0.501676828	0	0.666666667	CRA004775;PRJNA827465;Yang2025_Silkie_Wencha	0.94095886
ENSGALG00010004243	BLTP1	3	-0.010720141	0.044206136	-0.242503461	0.808390075	0	0.525072597	0	0.666666667	CRA004775;PRJNA827465;Yang2025_Silkie_Wencha	0.94095886
ENSGALG00010008045	DCDC2	2	-0.293989612	1.214862133	-0.241992276	0.808790797	2.446755532	5.831889914	82.85289992	0.5	PRJNA827465;Yang2025_Silkie_Wenchang	0.941025884
ENSGALG00010009089	AGBL5	3	0.047278988	0.19533501	0.242040523	0.808748764	0.098772347	17.26933601	88.41877882	0.666666667	CRA004775;PRJNA827465;Yang2025_Silkie_Wencha	0.941025884
ENSGALG00010021104	INA	3	0.038335537	0.15848342	0.241889889	0.808865485	0	1.386244838	0	0.666666667	CRA004775;PRJNA827465;Yang2025_Silkie_Wencha	0.941025884
ENSGALG00010000061	SERINC4	3	-0.03828678	0.158074241	-0.242207584	0.808619319	0	0.850120508	0	0.666666667	CRA004775;PRJNA827465;Yang2025_Silkie_Wencha	0.941025884
ENSGALG00010019312	KBTBD2	3	-0.032948093	0.136129953	-0.242034119	0.808753726	0.04501213	20.66057048	90.31972519	0.666666667	CRA004775;PRJNA827465;Yang2025_Silkie_Wencha	0.941025884
ENSGALG00010006078	SPATA13	3	0.032337902	0.13372281	0.241827868	0.808913544	0	1.013822974	0	0.666666667	CRA004775;PRJNA827465;Yang2025_Silkie_Wencha	0.941025884
ENSGALG00010002937	GALNT7	3	0.011288677	0.04665013	0.241985972	0.808791033	0	1.550319226	0	0.666666667	CRA004775;PRJNA827465;Yang2025_Silkie_Wencha	0.941025884
ENSGALG00010020164	C10orf71	3	0.049078004	0.203558905	0.241099764	0.809477796	0.070135279	5.162447411	61.25868525	0.666666667	CRA004775;PRJNA827465;Yang2025_Silkie_Wencha	0.941604817
ENSGALG00010022359	ZZZ3	3	-0.013750536	0.057063663	-0.240968329	0.809579663	0.002515185	2.610236968	23.37860414	0.666666667	CRA004775;PRJNA827465;Yang2025_Silkie_Wencha	0.941645842
ENSGALG00010022154	CPN1	3	0.083706845	0.348059215	0.240495989	0.809945773	0.249708805	7.483571469	73.27479255	0.666666667	CRA004775;PRJNA827465;Yang2025_Silkie_Wencha	0.941994183
ENSGALG00010027897	AP3D1	3	-0.008977122	0.037356804	-0.240307559	0.810091836	0	0.137388216	0	0.666666667	CRA004775;PRJNA827465;Yang2025_Silkie_Wencha	0.942086566
ENSGALG00010020556	EMC1	3	-0.014147765	0.058925176	-0.24009712	0.810254967	0.001219561	2.204526009	9.277550298	0.666666667	CRA004775;PRJNA827465;Yang2025_Silkie_Wencha	0.942198781
ENSGALG00010013588	ENSGALG00010013588	2	-0.174607288	0.728373229	-0.239722276	0.810545565	0.873547646	5.641816543	82.27521238	0.5	PRJNA827465;Yang2025_Silkie_Wenchang	0.942226732
ENSGALG00010010911	ENSGALG00010010911	2	0.100822856	0.420532113	0.23975067	0.810523551	0.141851216	1.661378903	39.80903464	0.5	CRA004775;PRJNA827465	0.942226732
ENSGALG00010021732	CASR	3	0.073545036	0.30663477	0.239845715	0.810449866	0.149066602	4.610618732	56.62187406	0.666666667	CRA004775;PRJNA827465;Yang2025_Silkie_Wencha	0.942226732
ENSGALG00010012093	ENSGALG00010012093	3	-0.011868427	0.049456377	-0.239977687	0.810347554	0.001382864	2.230286104	10.32540638	0.666666667	CRA004775;PRJNA827465;Yang2025_Silkie_Wencha	0.942226732
ENSGALG00010012105	DONSON	3	-0.02091507	0.087315512	-0.239534415	0.810691213	0.004910867	2.397524912	16.5806374	0.666666667	CRA004775;PRJNA827465;Yang2025_Silkie_Wencha	0.942299216
ENSGALG00010022429	ADK	3	-0.013597446	0.056781433	-0.239469931	0.81074121	0	0.493127508	0	0.666666667	CRA004775;PRJNA827465;Yang2025_Silkie_Wencha	0.942299216
ENSGALG00010023953	SLC35A3	3	-0.030952568	0.129556855	-0.238911081	0.811174533	0.024550958	4.061129235	50.75261376	0.666666667	CRA004775;PRJNA827465;Yang2025_Silkie_Wencha	0.942725359
ENSGALG00010005596	PARD3	3	-0.014690322	0.061534383	-0.238733561	0.811312191	0.003206268	2.426149828	17.56486031	0.666666667	CRA004775;PRJNA827465;Yang2025_Silkie_Wencha	0.942807847
ENSGALG00010017284	ENSGALG00010017284	3	0.063756296	0.267610838	0.238242577	0.811692956	0.15261255	7.394924097	72.95442152	0.666666667	CRA004775;PRJNA827465;Yang2025_Silkie_Wencha	0.942952014
ENSGALG00010017551	NALF2	3	-0.058563512	0.245779198	-0.238276927	0.811666315	0.120629403	6.583470596	69.62088657	0.666666667	CRA004775;PRJNA827465;Yang2025_Silkie_Wencha	0.942952014
ENSGALG00010015933	ENSGALG00010015933	2	0.057052081	0.239469208	0.238243913	0.81169192	0.015920974	1.152598072	13.23948704	0.5	PRJNA827465;Yang2025_Silkie_Wenchang	0.942952014
ENSGALG00010007846	ENSGALG00010007846	3	-0.030683358	0.128797417	-0.238229604	0.811703017	0.013563977	2.647232896	24.4494127	0.666666667	CRA004775;PRJNA827465;Yang2025_Silkie_Wencha	0.942952014
ENSGALG00010005935	ATP8A2	3	-0.088118303	0.371008297	-0.237510331	0.812260906	0.225086139	4.809013852	58.41143192	0.666666667	CRA004775;PRJNA827465;Yang2025_Silkie_Wencha	0.942975612
ENSGALG00010009334	SS18L2	3	0.040989273	0.172409253	0.237744044	0.812079621	0.04948892	4.576801995	56.30136497	0.666666667	CRA004775;PRJNA827465;Yang2025_Silkie_Wencha	0.942975612
ENSGALG00010027876	ANAPC2	3	-0.038295178	0.160894561	-0.238014126	0.811870138	0.059397862	9.407572874	78.74053141	0.666666667	CRA004775;PRJNA827465;Yang2025_Silkie_Wencha	0.942975612
ENSGALG00010005077	ERG28	3	-0.034231122	0.144026164	-0.237672939	0.812134774	0.036925869	4.919014586	59.3414501	0.666666667	CRA004775;PRJNA827465;Yang2025_Silkie_Wencha	0.942975612
ENSGALG00010009212	NXPH2	3	-0.034217277	0.144117168	-0.237426796	0.812325703	0	1.562864135	0	0.666666667	CRA004775;PRJNA827465;Yang2025_Silkie_Wencha	0.942975612
ENSGALG00010025660	KIF2C	3	0.030582512	0.128853339	0.23734357	0.812390263	0.019574471	3.343732404	40.18660113	0.666666667	CRA004775;PRJNA827465;Yang2025_Silkie_Wencha	0.942975612
ENSGALG00010024943	ABCB9	3	-0.026937692	0.113477999	-0.237382505	0.812360061	0	0.043537219	0	0.666666667	CRA004775;PRJNA827465;Yang2025_Silkie_Wencha	0.942975612
ENSGALG00010008302	ENSGALG00010008302	3	0.021414047	0.090073029	0.237740943	0.812082026	0	1.29926468	0	0.666666667	CRA004775;PRJNA827465;Yang2025_Silkie_Wencha	0.942975612
ENSGALG00010023499	PEBP1	3	-0.013526965	0.056944113	-0.237548081	0.812231623	0	0.583428692	0	0.666666667	CRA004775;PRJNA827465;Yang2025_Silkie_Wencha	0.942975612
ENSGALG00010027252	MAGI3	3	-0.012767261	0.053630619	-0.238059185	0.81183519	0	0.679596714	0	0.666666667	CRA004775;PRJNA827465;Yang2025_Silkie_Wencha	0.942975612
ENSGALG00010004515	ENSGALG00010004515	3	-0.082345191	0.347186942	-0.237178248	0.81251851	0.285919258	12.29869779	83.73811574	0.666666667	CRA004775;PRJNA827465;Yang2025_Silkie_Wencha	0.943047055
ENSGALG00010017878	CHRNG	3	-0.050641737	0.213610987	-0.237074587	0.812598927	0.066416799	4.16928881	52.03018809	0.666666667	CRA004775;PRJNA827465;Yang2025_Silkie_Wencha	0.943062975
ENSGALG00010014654	LAMP1	3	-0.03127367	0.131962889	-0.23698837	0.812665813	0.042061299	13.81314168	85.52103464	0.666666667	CRA004775;PRJNA827465;Yang2025_Silkie_Wencha	0.943063192
ENSGALG00010025271	RNF34	3	-0.022261346	0.093993808	-0.236838429	0.812782138	0.018075436	7.025747464	71.53327799	0.666666667	CRA004775;PRJNA827465;Yang2025_Silkie_Wencha	0.943120775
ENSGALG00010013143	ENSGALG00010013143	3	-0.097687015	0.412735773	-0.236681727	0.812903712	0	1.172021873	0	0.666666667	CRA004775;PRJNA827465;Yang2025_Silkie_Wencha	0.94318444
ENSGALG00010001333	PLCH1	3	0.046208712	0.195340474	0.236554724	0.813002248	0.028450165	2.579458191	22.46433738	0.666666667	CRA004775;PRJNA827465;Yang2025_Silkie_Wencha	0.943203102
ENSGALG00010015091	MSN	3	-0.012811781	0.054174948	-0.236489035	0.813053215	0.002953069	2.947859179	32.15415395	0.666666667	CRA004775;PRJNA827465;Yang2025_Silkie_Wencha	0.943203102
ENSGALG00010009037	ENSGALG00010009037	3	-0.071996139	0.304929907	-0.236107175	0.813349506	0.193291473	6.94127221	71.1868381	0.666666667	CRA004775;PRJNA827465;Yang2025_Silkie_Wencha	0.943310378
ENSGALG00010010974	NCAPG	3	0.049894926	0.211201049	0.236243742	0.813243539	0.086087162	8.422351778	76.25366343	0.666666667	CRA004775;PRJNA827465;Yang2025_Silkie_Wencha	0.943310378
ENSGALG00010008341	DHODH	3	0.04372584	0.185155316	0.236157625	0.81331036	0.070973513	6.628637481	69.82788686	0.666666667	CRA004775;PRJNA827465;Yang2025_Silkie_Wencha	0.943310378
ENSGALG00010024761	NECAB2	3	-0.031969346	0.135448452	-0.236025922	0.813412556	0.011051332	2.354185159	15.04491513	0.666666667	CRA004775;PRJNA827465;Yang2025_Silkie_Wencha	0.943310378
ENSGALG00010017579	P3H2	3	-0.013883762	0.058844633	-0.235939309	0.813479765	0	0.532570637	0	0.666666667	CRA004775;PRJNA827465;Yang2025_Silkie_Wencha	0.943310949
ENSGALG00010012354	DCBLD1	3	-0.020698785	0.087819245	-0.235697601	0.813667331	0	1.066666811	0	0.666666667	CRA004775;PRJNA827465;Yang2025_Silkie_Wencha	0.943451074
ENSGALG00010002951	ENSGALG00010002951	2	-0.480426602	2.043176702	-0.235137079	0.81410234	2.153700797	1.325400508	24.55110784	0.5	PRJNA827465;Yang2025_Silkie_Wenchang	0.943467986
ENSGALG00010013768	FAM110A	3	-0.069320945	0.29477297	-0.235167237	0.814078934	0.195061046	10.62124181	81.16981013	0.666666667	CRA004775;PRJNA827465;Yang2025_Silkie_Wencha	0.943467986
ENSGALG00010013779	SLC52A3	3	0.063918384	0.271408005	0.235506628	0.813815535	0.150007754	6.553768082	69.48320455	0.666666667	CRA004775;PRJNA827465;Yang2025_Silkie_Wencha	0.943467986
ENSGALG00010024196	MRPL21	3	-0.032457961	0.137989961	-0.23521973	0.814038193	0.027039638	3.510167317	43.02265905	0.666666667	CRA004775;PRJNA827465;Yang2025_Silkie_Wencha	0.943467986
ENSGALG00010004241	PREX2	3	0.016989044	0.072270142	0.235076945	0.814149013	0	1.463710347	0	0.666666667	CRA004775;PRJNA827465;Yang2025_Silkie_Wencha	0.943467986
ENSGALG00010007765	NUDT7	3	-0.014108688	0.060002744	-0.235134046	0.814104694	0	0.08590697	0	0.666666667	CRA004775;PRJNA827465;Yang2025_Silkie_Wencha	0.943467986
ENSGALG00010001759	C10orf88	3	0.009689578	0.041137593	0.235540724	0.813789075	0	1.399713695	0	0.666666667	CRA004775;PRJNA827465;Yang2025_Silkie_Wencha	0.943467986
ENSGALG00010016902	MRNIP	3	0.02295863	0.097741784	0.234890635	0.814293618	0	0.176772557	0	0.666666667	CRA004775;PRJNA827465;Yang2025_Silkie_Wencha	0.943501874
ENSGALG00010027831	ATCAY	3	-0.021682074	0.092316268	-0.234867317	0.814311717	0	0.391908365	0	0.666666667	CRA004775;PRJNA827465;Yang2025_Silkie_Wencha	0.943501874
ENSGALG00010022826	SNORC	3	0.370542839	1.584174692	0.233902764	0.815060468	6.680764474	17.87847262	88.81336207	0.666666667	CRA004775;PRJNA827465;Yang2025_Silkie_Wencha	0.943607411
ENSGALG00010016578	GRIK3	2	0.208743499	0.895446537	0.233116653	0.815670824	1.334550777	5.813625527	82.79902971	0.5	PRJNA827465;Yang2025_Silkie_Wenchang	0.943607411
ENSGALG00010002333	ENSGALG00010002333	3	-0.099785632	0.427934182	-0.233179858	0.815621745	0	1.319103088	0	0.666666667	CRA004775;PRJNA827465;Yang2025_Silkie_Wencha	0.943607411
ENSGALG00010028006	ENSGALG00010028006	2	0.090895519	0.388458679	0.233990187	0.814992597	0	0.483334051	0	0.5	CRA004775;Yang2025_Silkie_Wenchang	0.943607411
ENSGALG00010018306	DES	3	0.06662055	0.285161124	0.233624236	0.815276711	0.163739415	8.80999592	77.29851389	0.666666667	CRA004775;PRJNA827465;Yang2025_Silkie_Wencha	0.943607411
ENSGALG00010023660	CRYBB1	2	-0.046572773	0.199686775	-0.23322913	0.815583487	0	0.039084595	0	0.5	PRJNA827465;Yang2025_Silkie_Wenchang	0.943607411
ENSGALG00010018913	ENSGALG00010018913	3	0.0435233	0.186404857	0.233488012	0.815382477	0.048629288	3.74561843	46.6042781	0.666666667	CRA004775;PRJNA827465;Yang2025_Silkie_Wencha	0.943607411
ENSGALG00010015909	TNNC2	3	0.042778186	0.183255912	0.233434141	0.815424305	0.050478582	4.483654771	55.39353269	0.666666667	CRA004775;PRJNA827465;Yang2025_Silkie_Wencha	0.943607411
ENSGALG00010006029	HSDL2	3	0.041722885	0.178966183	0.233132789	0.815658294	0.051044508	4.363361137	54.16377564	0.666666667	CRA004775;PRJNA827465;Yang2025_Silkie_Wencha	0.943607411
ENSGALG00010003862	PCK2	3	0.041469733	0.177764343	0.233284877	0.815540201	0.054363496	4.754027193	57.93040471	0.666666667	CRA004775;PRJNA827465;Yang2025_Silkie_Wencha	0.943607411
ENSGALG00010013751	FUT10	3	0.028826415	0.123631225	0.233164517	0.815633658	0	1.757623569	0	0.666666667	CRA004775;PRJNA827465;Yang2025_Silkie_Wencha	0.943607411
ENSGALG00010009503	IL11RA	3	-0.021544576	0.092381373	-0.233213418	0.815595687	0	1.008044568	0	0.666666667	CRA004775;PRJNA827465;Yang2025_Silkie_Wencha	0.943607411
ENSGALG00010018315	CLASP2	3	0.021510211	0.091797437	0.234322563	0.814734571	0.011686435	3.805948998	47.45068835	0.666666667	CRA004775;PRJNA827465;Yang2025_Silkie_Wencha	0.943607411
ENSGALG00010007859	CENPN	3	-0.019099436	0.081655688	-0.233902094	0.815060987	0	0.621747216	0	0.666666667	CRA004775;PRJNA827465;Yang2025_Silkie_Wencha	0.943607411
ENSGALG00010029257	YPEL2	3	-0.0189673	0.081163406	-0.233692761	0.815223508	0	1.61190801	0	0.666666667	CRA004775;PRJNA827465;Yang2025_Silkie_Wencha	0.943607411
ENSGALG00010001233	CNDP2	3	0.015266759	0.065295026	0.233811982	0.815130947	0	0.584726999	0	0.666666667	CRA004775;PRJNA827465;Yang2025_Silkie_Wencha	0.943607411
ENSGALG00010011663	UTP20	3	-0.014544393	0.062344338	-0.233291317	0.815535201	0	1.749967206	0	0.666666667	CRA004775;PRJNA827465;Yang2025_Silkie_Wencha	0.943607411
ENSGALG00010023979	FAM118B	3	-0.013534415	0.057853685	-0.233942138	0.815029899	0	0.531676219	0	0.666666667	CRA004775;PRJNA827465;Yang2025_Silkie_Wencha	0.943607411
ENSGALG00010029753	AKAP10	3	0.009124787	0.03911087	0.23330566	0.815524064	0	0.053631334	0	0.666666667	CRA004775;PRJNA827465;Yang2025_Silkie_Wencha	0.943607411
ENSGALG00010028386	NCAN	2	0.21075305	0.909030615	0.231843731	0.81665939	1.402960062	6.232740546	83.95569345	0.5	PRJNA827465;Yang2025_Silkie_Wenchang	0.943795853
ENSGALG00010029455	ENSGALG00010029455	3	-0.120251748	0.517542892	-0.232351269	0.816265195	0.680531544	14.38801458	86.09954147	0.666666667	CRA004775;PRJNA827465;Yang2025_Silkie_Wencha	0.943795853
ENSGALG00010023466	CLCF1	3	-0.106753069	0.459210671	-0.232470794	0.816172369	0.466324794	7.830719725	74.45956349	0.666666667	CRA004775;PRJNA827465;Yang2025_Silkie_Wencha	0.943795853
ENSGALG00010025847	DEF6	3	0.073060806	0.315271504	0.231739326	0.816740485	0.251029169	19.19370647	89.57991775	0.666666667	CRA004775;PRJNA827465;Yang2025_Silkie_Wencha	0.943795853
ENSGALG00010003528	GDAP1	3	-0.066734006	0.287998399	-0.231716588	0.816758147	0.159261814	5.876534658	65.96633703	0.666666667	CRA004775;PRJNA827465;Yang2025_Silkie_Wencha	0.943795853
ENSGALG00010029725	TSEN54	3	-0.049092235	0.211533067	-0.232078302	0.816477197	0.118702083	22.39157568	91.0680694	0.666666667	CRA004775;PRJNA827465;Yang2025_Silkie_Wencha	0.943795853
ENSGALG00010021434	ANKZF1	3	0.044460984	0.191911088	0.231674911	0.816790519	0.083166023	9.291418621	78.4747617	0.666666667	CRA004775;PRJNA827465;Yang2025_Silkie_Wencha	0.943795853
ENSGALG00010012307	ENSGALG00010012307	3	0.029749046	0.128440313	0.231617666	0.816834985	0	1.7831348	0	0.666666667	CRA004775;PRJNA827465;Yang2025_Silkie_Wencha	0.943795853
ENSGALG00010022858	HERC4	3	0.02958995	0.127448602	0.232171635	0.816404708	0.032173616	9.784903204	79.56034967	0.666666667	CRA004775;PRJNA827465;Yang2025_Silkie_Wencha	0.943795853
ENSGALG00010007480	TENM3	3	-0.022505788	0.097029339	-0.231948273	0.81657819	0.008952773	2.728428386	26.6977279	0.666666667	CRA004775;PRJNA827465;Yang2025_Silkie_Wencha	0.943795853
ENSGALG00010014367	OPHN1	3	0.021800366	0.093947346	0.232048764	0.816500139	0.013964066	4.37264745	54.26111931	0.666666667	CRA004775;PRJNA827465;Yang2025_Silkie_Wencha	0.943795853
ENSGALG00010023527	TMEM98	3	0.019517437	0.083975561	0.232418061	0.816213322	0.00267513	2.210886387	9.538544742	0.666666667	CRA004775;PRJNA827465;Yang2025_Silkie_Wencha	0.943795853
ENSGALG00010028142	PMPCA	3	0.019349769	0.083157741	0.232687523	0.816004059	0.010541414	4.305760243	53.55059532	0.666666667	CRA004775;PRJNA827465;Yang2025_Silkie_Wencha	0.943795853
ENSGALG00010004394	CCNY	3	-0.015488854	0.066669208	-0.232323956	0.816286407	0.002457072	2.349333398	14.86946884	0.666666667	CRA004775;PRJNA827465;Yang2025_Silkie_Wencha	0.943795853
ENSGALG00010000592	CCT5	3	0.011144958	0.047902832	0.232657604	0.816027293	0	1.595102271	0	0.666666667	CRA004775;PRJNA827465;Yang2025_Silkie_Wencha	0.943795853
ENSGALG00010014739	TRMT2B	3	-0.049363985	0.213542609	-0.231166909	0.817185136	0.107051909	12.33402436	83.78469231	0.666666667	CRA004775;PRJNA827465;Yang2025_Silkie_Wencha	0.944123275
ENSGALG00010020393	SHISA9	3	0.08292699	0.35892393	0.231043359	0.817281118	0.248981058	6.93411563	71.15710054	0.666666667	CRA004775;PRJNA827465;Yang2025_Silkie_Wencha	0.944157017
ENSGALG00010017718	ATOH8	3	0.090111483	0.390573937	0.230715556	0.817535788	0.353024667	16.34126371	87.76104446	0.666666667	CRA004775;PRJNA827465;Yang2025_Silkie_Wencha	0.944350465
ENSGALG00010011295	FAM167A	3	0.066941502	0.290280665	0.230609578	0.817618126	0	1.650195591	0	0.666666667	CRA004775;PRJNA827465;Yang2025_Silkie_Wencha	0.944350465
ENSGALG00010017707	AK2	3	-0.020667063	0.08963469	-0.230569915	0.817648942	0.012358704	3.994410792	49.93003715	0.666666667	CRA004775;PRJNA827465;Yang2025_Silkie_Wencha	0.944350465
ENSGALG00010024989	TIMELESS	3	0.060038027	0.261037896	0.229997362	0.81809382	0.14636234	9.166019476	78.18027765	0.666666667	CRA004775;PRJNA827465;Yang2025_Silkie_Wencha	0.944369139
ENSGALG00010008429	IGFBP3	3	-0.048453288	0.210672096	-0.229993859	0.818096542	0.117015257	20.1993489	90.09869075	0.666666667	CRA004775;PRJNA827465;Yang2025_Silkie_Wencha	0.944369139
ENSGALG00010002994	DUSP4	3	-0.034670938	0.150682726	-0.230092317	0.818020035	0.020581103	2.707838091	26.14034028	0.666666667	CRA004775;PRJNA827465;Yang2025_Silkie_Wencha	0.944369139
ENSGALG00010007993	LRRC14	3	0.030878559	0.13433179	0.229867847	0.818194461	0.029582536	4.798131526	58.31710762	0.666666667	CRA004775;PRJNA827465;Yang2025_Silkie_Wencha	0.944369139
ENSGALG00010022203	DNAAF5	3	-0.030488768	0.132443114	-0.230202739	0.817934233	0.028610331	4.603233142	56.55227667	0.666666667	CRA004775;PRJNA827465;Yang2025_Silkie_Wencha	0.944369139
ENSGALG00010012791	CFAP418	3	-0.029277963	0.127114084	-0.23032824	0.817836718	0.013251514	2.699399507	25.90944782	0.666666667	CRA004775;PRJNA827465;Yang2025_Silkie_Wencha	0.944369139
ENSGALG00010008172	SNTB2	3	-0.026826968	0.116565627	-0.230144755	0.817979289	0.013551093	2.75088404	27.29609933	0.666666667	CRA004775;PRJNA827465;Yang2025_Silkie_Wencha	0.944369139
ENSGALG00010022299	MRGBP	3	-0.013467153	0.058588137	-0.229861434	0.818199445	0.002766188	2.577222718	22.39708326	0.666666667	CRA004775;PRJNA827465;Yang2025_Silkie_Wencha	0.944369139
ENSGALG00010016920	C1QA	3	-0.086429771	0.376280031	-0.229695343	0.818328514	0.298113793	6.798009212	70.5796221	0.666666667	CRA004775;PRJNA827465;Yang2025_Silkie_Wencha	0.944441014
ENSGALG00010028898	TNFSF15	3	-0.069527904	0.302811254	-0.229608057	0.818396345	0.189060577	6.508771446	69.27223491	0.666666667	CRA004775;PRJNA827465;Yang2025_Silkie_Wencha	0.944442207
ENSGALG00010001277	ENSGALG00010001277	3	0.019477058	0.084972649	0.229215619	0.818701334	0.005457208	2.540669681	21.28059719	0.666666667	CRA004775;PRJNA827465;Yang2025_Silkie_Wencha	0.944717062
ENSGALG00010003453	ENSGALG00010003453	2	0.180358058	0.788558315	0.228718732	0.819087534	0.760470248	2.571895551	61.11817217	0.5	PRJNA827465;Yang2025_Silkie_Wenchang	0.944991424
ENSGALG00010024416	VDR	3	0.045778153	0.200122825	0.228750284	0.81906301	0	1.519326807	0	0.666666667	CRA004775;PRJNA827465;Yang2025_Silkie_Wencha	0.944991424
ENSGALG00010002492	RPL8	3	-0.03119294	0.13642118	-0.228651741	0.819139606	0.031666739	4.626521581	56.77097869	0.666666667	CRA004775;PRJNA827465;Yang2025_Silkie_Wencha	0.944991424
ENSGALG00010004995	PIT54	3	-0.305033919	1.335666336	-0.228375838	0.819354071	4.021505161	8.348568957	76.04379852	0.666666667	CRA004775;PRJNA827465;Yang2025_Silkie_Wencha	0.945112619
ENSGALG00010021287	COL17A1	3	-0.18773274	0.822146592	-0.228344606	0.81937835	1.60231887	9.689452575	79.35899903	0.666666667	CRA004775;PRJNA827465;Yang2025_Silkie_Wencha	0.945112619
ENSGALG00010028491	TOR1B	3	-0.020321259	0.089067704	-0.228155188	0.819525598	0.013212439	4.453988161	55.09642308	0.666666667	CRA004775;PRJNA827465;Yang2025_Silkie_Wencha	0.945205353
ENSGALG00010003583	RSAD2	3	-0.061722279	0.271431899	-0.227395083	0.820116544	0	0.650082892	0	0.666666667	CRA004775;PRJNA827465;Yang2025_Silkie_Wencha	0.94523143
ENSGALG00010025830	TBCEL	3	0.053201021	0.233716035	0.227631028	0.819933096	0.097204487	5.603705055	64.30932784	0.666666667	CRA004775;PRJNA827465;Yang2025_Silkie_Wencha	0.94523143
ENSGALG00010021566	ENSGALG00010021566	3	0.034255532	0.150418707	0.227734523	0.819852632	0.044441771	7.578836695	73.61072575	0.666666667	CRA004775;PRJNA827465;Yang2025_Silkie_Wencha	0.94523143
ENSGALG00010013497	FAM172A	3	0.028835043	0.126556524	0.227843196	0.819768144	0	0.517090616	0	0.666666667	CRA004775;PRJNA827465;Yang2025_Silkie_Wencha	0.94523143
ENSGALG00010008505	PTPN3	3	0.026401756	0.115899822	0.227798069	0.819803228	0	0.032845024	0	1	CRA004775;PRJNA827465;Yang2025_Silkie_Wencha	0.94523143
ENSGALG00010012466	PDSS2	3	-0.020604693	0.090628956	-0.227352205	0.820149882	0.013399506	4.99998452	59.99987616	0.666666667	CRA004775;PRJNA827465;Yang2025_Silkie_Wencha	0.94523143
ENSGALG00010027288	DAG1	3	-0.017527112	0.077070411	-0.227416875	0.8200996	0	1.996796476	0	0.666666667	CRA004775;PRJNA827465;Yang2025_Silkie_Wencha	0.94523143
ENSGALG00010001605	APBB1	3	-0.015836397	0.069617388	-0.227477612	0.820052376	0	0.687625501	0	0.666666667	CRA004775;PRJNA827465;Yang2025_Silkie_Wencha	0.94523143
ENSGALG00010011499	SMG7	3	-0.011272791	0.049566542	-0.227427422	0.820091399	0	1.711508385	0	0.666666667	CRA004775;PRJNA827465;Yang2025_Silkie_Wencha	0.94523143
ENSGALG00010005181	IMPACT	3	-0.019951271	0.087861273	-0.227076962	0.820363898	0	0.175195595	0	0.666666667	CRA004775;PRJNA827465;Yang2025_Silkie_Wencha	0.945360885
ENSGALG00010024541	WIPF2	3	-0.016789569	0.073951205	-0.227035767	0.82039593	0	1.415143282	0	0.666666667	CRA004775;PRJNA827465;Yang2025_Silkie_Wencha	0.945360885
ENSGALG00010029656	MRPL27	3	-0.023685442	0.104507023	-0.22663972	0.820703904	0.008047355	2.363234083	15.37021178	0.666666667	CRA004775;PRJNA827465;Yang2025_Silkie_Wencha	0.945561644
ENSGALG00010013466	BRAF	3	-0.011208266	0.049437559	-0.2267156	0.820644896	0	0.524478986	0	0.666666667	CRA004775;PRJNA827465;Yang2025_Silkie_Wencha	0.945561644
ENSGALG00010001727	COL9A1	3	-0.364486209	1.60950883	-0.226458036	0.820845194	6.531554053	13.28000648	84.93976639	0.666666667	CRA004775;PRJNA827465;Yang2025_Silkie_Wencha	0.945578291
ENSGALG00010021974	GFAP	3	-0.110742725	0.489651778	-0.226166287	0.82107209	0.444870248	5.276850314	62.09860274	0.666666667	CRA004775;PRJNA827465;Yang2025_Silkie_Wencha	0.945578291
ENSGALG00010024599	ENSGALG00010024599	3	0.058646074	0.259439507	0.226049128	0.821163211	0.085562506	3.466875662	42.3111702	0.666666667	CRA004775;PRJNA827465;Yang2025_Silkie_Wencha	0.945578291
ENSGALG00010015362	ATP9A	3	-0.056772412	0.251279627	-0.225933205	0.821253371	0.103960498	4.741052655	57.81527552	0.666666667	CRA004775;PRJNA827465;Yang2025_Silkie_Wencha	0.945578291
ENSGALG00010016197	SNRPN	3	0.043745536	0.193226013	0.226395687	0.820893682	0.080180054	7.359355844	72.82370846	0.666666667	CRA004775;PRJNA827465;Yang2025_Silkie_Wencha	0.945578291
ENSGALG00010008373	MACIR	3	-0.028475912	0.12584982	-0.226268989	0.820992216	0.014508067	2.863015943	30.14359543	0.666666667	CRA004775;PRJNA827465;Yang2025_Silkie_Wencha	0.945578291
ENSGALG00010029772	VPS53	3	-0.01568319	0.069375537	-0.226062247	0.821153007	0.007613378	4.649453977	56.98419621	0.666666667	CRA004775;PRJNA827465;Yang2025_Silkie_Wencha	0.945578291
ENSGALG00010017400	GPC4	3	0.010825472	0.047913157	0.225939452	0.821248513	0	0.882916527	0	0.666666667	CRA004775;PRJNA827465;Yang2025_Silkie_Wencha	0.945578291
ENSGALG00010016087	IARS2	3	0.032507914	0.143956419	0.225817744	0.821343176	0.048381802	16.25023937	87.69248899	0.666666667	CRA004775;PRJNA827465;Yang2025_Silkie_Wencha	0.945604687
ENSGALG00010006242	ENSGALG00010006242	3	0.120149674	0.532325269	0.225707252	0.821429117	0.509429382	5.005178253	60.04138317	0.666666667	CRA004775;PRJNA827465;Yang2025_Silkie_Wencha	0.945626632
ENSGALG00010021871	ENSGALG00010021871	3	0.070862257	0.314549881	0.22528146	0.821760321	0.201878077	11.53800131	82.66597528	0.666666667	CRA004775;PRJNA827465;Yang2025_Silkie_Wencha	0.945786129
ENSGALG00010002954	CHST7	3	0.025707372	0.114109528	0.225286811	0.821756159	0	0.049259062	0	1	CRA004775;PRJNA827465;Yang2025_Silkie_Wencha	0.945786129
ENSGALG00010016526	EIF1B	3	-0.014138454	0.062761937	-0.225271149	0.821768343	0	0.287353208	0	0.666666667	CRA004775;PRJNA827465;Yang2025_Silkie_Wencha	0.945786129
ENSGALG00010018502	ARHGEF10L	3	0.034434545	0.153067162	0.224963631	0.822007568	0.050052769	8.840787533	77.37758098	0.666666667	CRA004775;PRJNA827465;Yang2025_Silkie_Wencha	0.945984454
ENSGALG00010015932	ACOT8	3	-0.041102953	0.182893359	-0.224737265	0.822183672	0.060081978	5.08441909	60.66413951	0.666666667	CRA004775;PRJNA827465;Yang2025_Silkie_Wencha	0.946110112
ENSGALG00010026393	SLC43A3	3	-0.031708118	0.141216549	-0.224535424	0.822340707	0.027626748	3.706294388	46.03774577	0.666666667	CRA004775;PRJNA827465;Yang2025_Silkie_Wencha	0.946163019
ENSGALG00010009246	RUSC2	3	0.025365123	0.113025165	0.224420134	0.822430406	0.00193416	2.089052021	4.26279578	0.666666667	CRA004775;PRJNA827465;Yang2025_Silkie_Wencha	0.946163019
ENSGALG00010027977	BOLA1	3	-0.024360385	0.108518276	-0.224481867	0.822382375	0	0.641583453	0	0.666666667	CRA004775;PRJNA827465;Yang2025_Silkie_Wencha	0.946163019
ENSGALG00010024705	SLCO2A1	3	0.035924213	0.160308457	0.224094311	0.822683919	0	0.825886979	0	0.666666667	CRA004775;PRJNA827465;Yang2025_Silkie_Wencha	0.946318659
ENSGALG00010022771	SGMS1	3	-0.015339286	0.068456275	-0.22407422	0.822699552	0.002558371	2.354927458	15.07169389	0.666666667	CRA004775;PRJNA827465;Yang2025_Silkie_Wencha	0.946318659
ENSGALG00010024557	TSPAN32	3	-0.041112142	0.183659192	-0.223850176	0.822873886	0	1.911314551	0	0.666666667	CRA004775;PRJNA827465;Yang2025_Silkie_Wencha	0.946365208
ENSGALG00010020679	OTUD3	3	0.013924392	0.062188019	0.223907949	0.822828931	0	1.199536659	0	0.666666667	CRA004775;PRJNA827465;Yang2025_Silkie_Wencha	0.946365208
ENSGALG00010020343	PATL1	3	0.059617148	0.266838663	0.223420202	0.823208483	0.175645473	13.97113472	85.68477049	0.666666667	CRA004775;PRJNA827465;Yang2025_Silkie_Wencha	0.946473998
ENSGALG00010012019	ENSGALG00010012019	3	-0.049284039	0.220606259	-0.223402721	0.823222087	0	0.123651983	0	0.666666667	CRA004775;PRJNA827465;Yang2025_Silkie_Wencha	0.946473998
ENSGALG00010010826	NCOA1	3	0.04778227	0.213881651	0.223405188	0.823220167	0.098616767	7.729629512	74.12553866	0.666666667	CRA004775;PRJNA827465;Yang2025_Silkie_Wencha	0.946473998
ENSGALG00010007941	MRPL19	3	0.028145413	0.125995355	0.223384532	0.823236242	0.033171917	6.989913326	71.38734192	0.666666667	CRA004775;PRJNA827465;Yang2025_Silkie_Wencha	0.946473998
ENSGALG00010023732	TM2D1	3	-0.017435188	0.078161063	-0.223067434	0.823483023	0	0.640170431	0	0.666666667	CRA004775;PRJNA827465;Yang2025_Silkie_Wencha	0.946680744
ENSGALG00010020121	ENSGALG00010020121	3	0.038515377	0.172772202	0.222925773	0.823593277	0	0.867522815	0	0.666666667	CRA004775;PRJNA827465;Yang2025_Silkie_Wencha	0.946730516
ENSGALG00010008955	KRTCAP3	3	0.101023663	0.453485871	0.222771357	0.823713461	0.503128178	11.13366829	82.0364686	0.666666667	CRA004775;PRJNA827465;Yang2025_Silkie_Wencha	0.946776166
ENSGALG00010001254	NETO1	3	-0.049329533	0.221633247	-0.222572802	0.823868006	0	0.150574618	0	0.666666667	CRA004775;PRJNA827465;Yang2025_Silkie_Wencha	0.946776166
ENSGALG00010010314	CRB1	2	-0.038145366	0.171416256	-0.222530622	0.823900838	0	0.142155723	0	0.5	PRJNA827465;Yang2025_Silkie_Wenchang	0.946776166
ENSGALG00010023083	HARBI1	3	-0.022463067	0.100931865	-0.222556739	0.82388051	0.003462737	2.200914786	9.128694456	0.666666667	CRA004775;PRJNA827465;Yang2025_Silkie_Wencha	0.946776166
ENSGALG00010027706	GSN	3	-0.058952223	0.265663509	-0.22190561	0.824387363	0.161779963	12.1550519	83.54593616	0.666666667	CRA004775;PRJNA827465;Yang2025_Silkie_Wencha	0.94706506
ENSGALG00010021019	VSIG10	3	-0.033046124	0.148847421	-0.222013413	0.824303442	0.039511322	4.975261198	59.8011055	0.666666667	CRA004775;PRJNA827465;Yang2025_Silkie_Wencha	0.94706506

ENSGALG00010027915	GSTM3	3	-0.021570278	0.097223205	-0.221863471	0.824420168	0.007665325	2.504687807	20.14972907	0.666666667	CRA004775;PRJNA827465;Yang2025_Silkie_Wencha	0.94706506
ENSGALG00010024344	YEATS2	3	-0.018695748	0.084207627	-0.222019658	0.824298581	0.009738732	3.505415241	42.94541838	0.666666667	CRA004775;PRJNA827465;Yang2025_Silkie_Wencha	0.94706506
ENSGALG00010025486	B3GALT1	3	-0.042749838	0.192788838	-0.221744363	0.824512893	0.056415573	4.446932015	55.02517256	0.666666667	CRA004775;PRJNA827465;Yang2025_Silkie_Wencha	0.947094629
ENSGALG00010023469	ENSGALG0001002346(	3	-0.051581865	0.232801388	-0.221570266	0.824648432	0.078477929	3.869513907	48.31392138	0.666666667	CRA004775;PRJNA827465;Yang2025_Silkie_Wencha	0.947096432
ENSGALG00010018044	INTS11	3	0.017322547	0.07818063	0.22157083	0.824647993	0.006779555	3.065027079	34.74772168	0.666666667	CRA004775;PRJNA827465;Yang2025_Silkie_Wencha	0.947096432
ENSGALG00010026958	CLTCL1	3	0.010762518	0.048595023	0.221473663	0.824723641	0	1.01436077	0	0.666666667	CRA004775;PRJNA827465;Yang2025_Silkie_Wencha	0.947105878
ENSGALG00010012991	MFSD9	3	-0.021297887	0.096295153	-0.221172985	0.824957743	0.012691804	3.781013363	47.10412771	0.666666667	CRA004775;PRJNA827465;Yang2025_Silkie_Wencha	0.947297777
ENSGALG00010012196	CENPK	3	-0.027494229	0.124402798	-0.221009728	0.825084858	0	0.759364324	0	0.666666667	CRA004775;PRJNA827465;Yang2025_Silkie_Wencha	0.947366803
ENSGALG00010029781	UNC13D	3	-0.070271313	0.318889626	-0.220362493	0.825588854	0.19242857	5.889410486	66.04074373	0.666666667	CRA004775;PRJNA827465;Yang2025_Silkie_Wencha	0.947478006
ENSGALG00010012250	NANS	3	-0.0565797	0.256532407	-0.22055576	0.825438351	0.164531726	14.60120866	86.30250381	0.666666667	CRA004775;PRJNA827465;Yang2025_Silkie_Wencha	0.947478006
ENSGALG00010025386	TPCN2	3	-0.038690821	0.175363269	-0.220632411	0.825378663	0.062912981	7.378568407	72.89447099	0.666666667	CRA004775;PRJNA827465;Yang2025_Silkie_Wencha	0.947478006
ENSGALG00010005462	AHI1	3	0.03801547	0.172481367	0.220403346	0.82555704	0.050915898	5.095744488	60.75156428	0.666666667	CRA004775;PRJNA827465;Yang2025_Silkie_Wencha	0.947478006
ENSGALG00010007887	CDT1	3	0.024620499	0.111683685	0.220448486	0.825521888	0.011272522	2.637854427	24.18080469	0.666666667	CRA004775;PRJNA827465;Yang2025_Silkie_Wencha	0.947478006
ENSGALG00010008334	STX17	3	-0.011970595	0.0543419	-0.220282961	0.825650789	0	1.195599872	0	0.666666667	CRA004775;PRJNA827465;Yang2025_Silkie_Wencha	0.947478006
ENSGALG00010021605	MAP3K3	3	0.010446013	0.047383707	0.220455791	0.825516199	0	0.266908254	0	0.666666667	CRA004775;PRJNA827465;Yang2025_Silkie_Wencha	0.947478006
ENSGALG00010020386	PPM1B	3	-0.023327627	0.106001149	-0.220069571	0.825816973	0.026649095	12.0974587	83.46760217	0.666666667	CRA004775;PRJNA827465;Yang2025_Silkie_Wencha	0.947591801
ENSGALG00010000585	SPTBN2	3	0.155564441	0.707775441	0.2197935	0.826031982	1.252479045	12.98182961	84.59385109	0.666666667	CRA004775;PRJNA827465;Yang2025_Silkie_Wencha	0.947662724
ENSGALG00010024888	PITPNM2	3	0.051164189	0.232848111	0.219732034	0.826079855	0.110608073	7.622010873	73.76020537	0.666666667	CRA004775;PRJNA827465;Yang2025_Silkie_Wencha	0.947662724
ENSGALG00010010623	SMAD2	3	0.02728263	0.124154623	0.219747195	0.826068046	0.015241551	2.982907977	32.95133423	0.666666667	CRA004775;PRJNA827465;Yang2025_Silkie_Wencha	0.947662724
ENSGALG00010020152	ATXN7	3	-0.016378692	0.074599035	-0.219556353	0.826216687	0.002845512	2.240397231	10.7301164	0.666666667	CRA004775;PRJNA827465;Yang2025_Silkie_Wencha	0.947742799
ENSGALG00010022246	RHOV	3	0.127778109	0.582646169	0.219306528	0.826411277	0.702845277	6.653704747	69.94155773	0.666666667	CRA004775;PRJNA827465;Yang2025_Silkie_Wencha	0.947773875
ENSGALG00010001554	ENSGALG0001000155(	3	0.064181812	0.292774236	0.219219468	0.826479091	0.166653453	5.691947609	64.86264215	0.666666667	CRA004775;PRJNA827465;Yang2025_Silkie_Wencha	0.947773875
ENSGALG00010007915	HECW1	3	-0.017315427	0.078962272	-0.219287352	0.826426214	0	0.744085249	0	0.666666667	CRA004775;PRJNA827465;Yang2025_Silkie_Wencha	0.947773875
ENSGALG00010027995	POLR2E	3	-0.016545168	0.075487587	-0.219177337	0.826511909	0.00466918	2.343362988	14.65257368	0.666666667	CRA004775;PRJNA827465;Yang2025_Silkie_Wencha	0.947773875
ENSGALG00010021853	ENSGALG0001002185(	2	0.131911921	0.602918618	0.218788933	0.826814469	0.592035216	5.233389189	80.89192369	0.5	CRA004775;Yang2025_Silkie_Wenchang	0.947887439
ENSGALG00010003604	ODC	3	-0.069471178	0.317544615	-0.218776117	0.826824452	0.249160609	12.90382347	84.50071791	0.666666667	CRA004775;PRJNA827465;Yang2025_Silkie_Wencha	0.947887439
ENSGALG00010009362	IL15	3	-0.055070633	0.251669761	-0.218821018	0.826789473	0.093273137	3.935895152	49.18563826	0.666666667	CRA004775;PRJNA827465;Yang2025_Silkie_Wencha	0.947887439
ENSGALG00010029596	ATAD5	3	-0.016224331	0.074183305	-0.21870596	0.826879106	0	1.35159177	0	0.666666667	CRA004775;PRJNA827465;Yang2025_Silkie_Wencha	0.947887439
ENSGALG00010015154	CYP24A1	2	-0.102358872	0.468615013	-0.218428495	0.827095266	0.083512457	1.233233009	18.91232293	0.5	CRA004775;PRJNA827465	0.948030137
ENSGALG00010023847	PIK3AP1	3	-0.057015812	0.26109244	-0.218374044	0.827137688	0.140435375	7.871807862	74.5928758	0.666666667	CRA004775;PRJNA827465;Yang2025_Silkie_Wencha	0.948030137
ENSGALG00010011634	AVD	3	-0.09910796	0.455492377	-0.217584233	0.82775307	0.386005845	5.300935043	62.27080725	0.666666667	CRA004775;PRJNA827465;Yang2025_Silkie_Wencha	0.948256928
ENSGALG00010024060	FRRS1	3	-0.05586326	0.256821842	-0.217517557	0.827805026	0.164939207	14.84182333	86.52456672	0.666666667	CRA004775;PRJNA827465;Yang2025_Silkie_Wencha	0.948256928
ENSGALG00010021749	CLASP1	3	0.026959939	0.123730719	0.217892045	0.827513225	0.026027369	4.737598056	57.78451493	0.666666667	CRA004775;PRJNA827465;Yang2025_Silkie_Wencha	0.948256928
ENSGALG00010002707	RELT	3	0.026891606	0.123613095	0.217546576	0.827782413	0	0.209150104	0	0.666666667	CRA004775;PRJNA827465;Yang2025_Silkie_Wencha	0.948256928
ENSGALG00010006957	PALM2AKAP2	3	0.026310335	0.120852378	0.217706391	0.827657884	0.022509586	4.270117373	53.16287995	0.666666667	CRA004775;PRJNA827465;Yang2025_Silkie_Wencha	0.948256928
ENSGALG00010012253	MYF6	3	-0.025644905	0.117717483	-0.217851282	0.827544987	0.013111605	2.842438599	29.63788204	0.666666667	CRA004775;PRJNA827465;Yang2025_Silkie_Wencha	0.948256928
ENSGALG00010015299	GALE	3	0.015818984	0.072692173	0.217616056	0.827728274	0.001972735	2.152626245	7.090234341	0.666666667	CRA004775;PRJNA827465;Yang2025_Silkie_Wencha	0.948256928
ENSGALG00010013418	HCN1	2	-0.171236787	0.787923534	-0.217326656	0.827953785	0.995017403	5.034079835	80.13539648	0.5	PRJNA827465;Yang2025_Silkie_Wenchang	0.9483505
ENSGALG00010002693	MSC	3	-0.074977051	0.345775412	-0.216837428	0.828335043	0.276949918	15.58274249	87.16528877	0.666666667	CRA004775;PRJNA827465;Yang2025_Silkie_Wencha	0.948479849
ENSGALG00010017684	TRMT61B	3	0.041327835	0.190422171	0.217032684	0.828182874	0.082373062	9.013925871	77.81210952	0.666666667	CRA004775;PRJNA827465;Yang2025_Silkie_Wencha	0.948479849
ENSGALG00010011793	TLN1	3	-0.032336024	0.149054605	-0.216940788	0.82825449	0.047514552	9.100736524	78.02375671	0.666666667	CRA004775;PRJNA827465;Yang2025_Silkie_Wencha	0.948479849
ENSGALG00010012172	CNOT2	3	0.008435787	0.038900233	0.216856987	0.828319799	0	0.060643813	0	0.666666667	CRA004775;PRJNA827465;Yang2025_Silkie_Wencha	0.948479849
ENSGALG00010025994	INO80D	3	0.017585978	0.081203616	0.216566438	0.828546243	0	1.665864828	0	0.666666667	CRA004775;PRJNA827465;Yang2025_Silkie_Wencha	0.948568043
ENSGALG00010017628	NRDE2	3	0.011870235	0.054809418	0.216572905	0.828541203	0	0.906321731	0	0.666666667	CRA004775;PRJNA827465;Yang2025_Silkie_Wencha	0.948568043
ENSGALG00010009702	ENSGALG0001000970(	3	0.080653128	0.372907132	0.216282075	0.828767881	0.37198928	19.90666899	89.95311571	0.666666667	CRA004775;PRJNA827465;Yang2025_Silkie_Wencha	0.948668156
ENSGALG00010017473	EIF3I	3	-0.009539429	0.044097466	-0.216326022	0.828733626	0	1.674038309	0	0.666666667	CRA004775;PRJNA827465;Yang2025_Silkie_Wencha	0.948668156
ENSGALG00010022214	ENSGALG0001002221(	3	-0.070249622	0.324994486	-0.21615635	0.828865877	0.20948014	6.507449898	69.26599465	0.666666667	CRA004775;PRJNA827465;Yang2025_Silkie_Wencha	0.948703524
ENSGALG00010002584	ENSGALG0001000258(	3	0.101978669	0.4726682	0.215751067	0.829181793	0.511392217	8.631083102	76.82793716	0.666666667	CRA004775;PRJNA827465;Yang2025_Silkie_Wencha	0.948834685
ENSGALG00010023841	OSBPL7	3	-0.03830999	0.177433525	-0.21591179	0.829056507	0.076507186	11.05530803	81.90914269	0.666666667	CRA004775;PRJNA827465;Yang2025_Silkie_Wencha	0.948834685
ENSGALG00010011551	GMDS	3	-0.033513552	0.155278326	-0.215828908	0.829121114	0.056576119	9.895428943	79.78864775	0.666666667	CRA004775;PRJNA827465;Yang2025_Silkie_Wencha	0.948834685
ENSGALG00010016119	FETUB	2	-0.544328489	2.536875577	-0.21456649	0.830105322	11.11883259	7.341717319	86.37920862	0.5	PRJNA827465;Yang2025_Silkie_Wenchang	0.948887498
ENSGALG00010027026	SLC45A3	3	-0.0771903	0.358751576	-0.215163654	0.829639727	0.256676766	6.666665621	69.99999529	0.666666667	CRA004775;PRJNA827465;Yang2025_Silkie_Wencha	0.948887498
ENSGALG00010024488	ADRGS5	3	-0.072165636	0.33612564	-0.214698398	0.830002471	0.17415403	4.173824731	52.08231949	0.666666667	CRA004775;PRJNA827465;Yang2025_Silkie_Wencha	0.948887498
ENSGALG00010014347	KIAA0040	3	0.058405892	0.272178653	0.214586602	0.83008964	0.1250673	4.576743825	56.30080956	0.666666667	CRA004775;PRJNA827465;Yang2025_Silkie_Wencha	0.948887498
ENSGALG00010002848	TRIB2	3	0.057184839	0.266001417	0.214979451	0.82978334	0.190662237	24.62047942	91.87668133	0.666666667	CRA004775;PRJNA827465;Yang2025_Silkie_Wencha	0.948887498
ENSGALG00010022840	MARVELD1	3	-0.045108276	0.210307994	-0.214486742	0.830167505	0.085153213	5.694660363	64.87938046	0.666666667	CRA004775;PRJNA827465;Yang2025_Silkie_Wencha	0.948887498
ENSGALG00010012046	LOXHD1	2	0.038527444	0.179342891	0.214825602	0.829903291	0.002458287	1.039343218	3.785392329	0.5	PRJNA827465;Yang2025_Silkie_Wenchang	0.948887498
ENSGALG00010027397	RFX5	3	0.036262948	0.168713812	0.214937638	0.829815939	0.056880582	6.459503548	69.03786823	0.666666667	CRA004775;PRJNA827465;Yang2025_Silkie_Wencha	0.948887498
ENSGALG00010015972	FSTL4	3	-0.027781329	0.129177433	-0.215063328	0.829717945	0	1.783848432	0	0.666666667	CRA004775;PRJNA827465;Yang2025_Silkie_Wencha	0.948887498
ENSGALG00010017714	ATAD3A	3	0.0189161	0.087806646	0.215429025	0.829432842	0.012897071	4.678038413	57.24703768	0.666666667	CRA004775;PRJNA827465;Yang2025_Silkie_Wencha	0.948887498
ENSGALG00010008192	HNRNPU	3	-0.012006154	0.055881223	-0.214851316	0.829883243	0.00368814	3.243965529	38.34706374	0.666666667	CRA004775;PRJNA827465;Yang2025_Silkie_Wencha	0.948887498
ENSGALG00010004944	ALKBH1	3	0.011964238	0.055749597	0.21460672	0.830073954	0	0.635434323	0	0.666666667	CRA004775;PRJNA827465;Yang2025_Silkie_Wencha	0.948887498
ENSGALG00010016107	UVSSA	3	-0.011797763	0.054945074	-0.214719221	0.829986236	0	1.74733266	0	0.666666667	CRA004775;PRJNA827465;Yang2025_Silkie_Wencha	0.948887498
ENSGALG00010028772	TTF1	3	0.011614113	0.053893652	0.215500578	0.829377062	0.001727498	2.33361839	14.29618448	0.666666667	CRA004775;PRJNA827465;Yang2025_Silkie_Wencha	0.948887498
ENSGALG00010015865	VRK1	3	-0.013938789	0.065028313	-0.214349541	0.830274488	0	0.438144143	0	0.666666667	CRA004775;PRJNA827465;Yang2025_Silkie_Wencha	0.948933068
ENSGALG00010011439	ENSGALG0001001143(	3	0.170856241	0.797985764	0.214108902	0.830462134	1.703747466	22.65533302	91.17205649	0.666666667	CRA004775;PRJNA827465;Yang2025_Silkie_Wencha	0.949050168
ENSGALG00010016312	MECR	3	-0.032581462	0.152217107	-0.214045993	0.830511191	0.034921309	3.956678844	49.37578884	0.666666667	CRA004775;PRJNA827465;Yang2025_Silkie_Wencha	0.949050168
ENSGALG00010024058	SLC44A5	3	0.035269981	0.164923631	0.213856439	0.830659011	0.066732013	12.93085289	84.53311612	0.666666667	CRA004775;PRJNA827465;Yang2025_Silkie_Wencha	0.949065677
ENSGALG00010005679	TMEM45A	3	-0.031140647	0.145569908	-0.21392228	0.830607666	0	1.873576499	0	0.666666667	CRA004775;PRJNA827465;Yang2025_Silkie_Wencha	0.949065677
ENSGALG00010012836	ENSGALG0001001283(	3	0.171012206	0.800093357	0.213740315	0.830749571	1.663416135	15.10002255	86.75498667	0.666666667	CRA004775;PRJNA827465;Yang2025_Silkie_Wencha	0.949092452
ENSGALG00010021539	HEXA	3	-0.033918292	0.158737373	-0.213676566	0.830838281	0.042460092	5.633423103	64.51020689	0.666666667	CRA004775;PRJNA827465;Yang2025_Silkie_Wencha	0.949117109
ENSGALG00010024746	HOXD4	2	0.075051608	0.35166578	0.213417434	0.831001383	0.182349158	3.795551901	73.65337041	0.5	PRJNA827465;Yang2025_Silkie_Wenchang	0.949209517
ENSGALG00010015253	ENSGALG0001001525(	3	-0.023676849	0.110976202	-0.213350686	0.831053441	0.01976762	4.605493967	56.57360504	0.666666667	CRA004775;PRJNA827465;Yang2025_Silkie_Wencha	0.949209517
ENSGALG00010007070	UBL3	3	0.029603707	0.139211273	0.212653085	0.831597563	0.046597646	15.40005611	87.01303433	0.666666667	CRA004775;PRJNA827465;Yang2025_Silkie_Wencha	0.949524181
ENSGALG00010011573	CMAS	3	0.022374402	0.105163863	0.212758373	0.831515434	0.015085735	3.69387346	45.85629363	0.666666667	CRA004775;PRJNA827465;Yang2025_Silkie_Wencha	0.949524181
ENSGALG00010013267	PNPLA3	3	-0.020253185	0.095233138	-0.212669516	0.831584745	0	1.308142788	0	0.666666667	CRA004775;PRJNA827465;Yang2025_Silkie_Wencha	0.949524181
ENSGALG00010010662	MGST3	3	0.015018254	0.070586796	0.212762941	0.831511871	0	1.415342206	0	0.666666667	CRA004775;PRJNA827465;Yang2025_Silkie_Wencha	0.949524181
ENSGALG00010003769	C6orf163	2	-0.080337859	0.378157415	-0.212445546	0.831759456	0	0.814475768	0	0.5	PRJNA827465;Yang2025_Silkie_Wenchang	0.949555668
ENSGALG00010001697	TAF10	3	-0.01941615	0.091374262	-0.212490362	0.831724496	0.004893641	2.218638739	9.854634516	0.666666667	CRA004775;PRJNA827465;Yang2025_Silkie_Wencha	0.949555668
ENSGALG00010012821	ZBTB33	3	0.015866045	0.074759949	0.212226535	0.831930307	0.007997706	3.640711053	45.06567616	0.666666667	CRA004775;PRJNA827465;Yang2025_Silkie_Wencha	0.949674036
ENSGALG00010008261	CRX	2	0.077907629	0.367861653	0.211785133	0.832274668	0	0.001736548	0	1	CRA004775;PRJNA827465	0.949990435
ENSGALG00010014149	LGALS2	2	0.05657198	0.267359295	0.211595344	0.832422742	0	0.647662682	0	0.5	CRA004775;PRJNA827465	0.950082754
ENSGALG00010005724	PROSER2	3	0.04562375	0.215802631	0.21141261	0.832564029	0.098556743	10.8131055	81.50392596	0.666666667	CRA004775;PRJNA827465;Yang2025_Silkie_Wencha	0.950138564
ENSGALG00010019906	ENSGALG0001001990(	3	-0.030885279	0.146163145	-0.211306883	0.832647813	0.040279529	6.934052581	71.15683828	0.666666667	CRA004775;PRJNA827465;Yang2025_Silkie_Wencha	0.950138564
ENSGALG00010007302	CDH11	3	-0.019271963	0.091217735	-0.211274294	0.832673241	0.003802847	2.286275038	12.52146102	0.666666667	CRA004775;PRJNA827465;Yang2025_Silkie_Wencha	0.950138564
ENSGALG00010022401	LCAT	3	0.090038515	0.426795737	0.210963952	0.832915401	0.39058452	7.720614786	74.09532718	0.666666667	CRA004775;PRJNA827465;Yang2025_Silkie_Wencha	0.95033819
ENSGALG00010001793	IKZF5	3	-0.037413413	0.177553149	-0.210716695	0.833108346	0.075950926	12.48017013	83.97457743	0.666666667	CRA004775;PRJNA827465;Yang2025_Silkie_Wencha	0.950404946
ENSGALG00010023887	RASA2	3	-0.013819522	0.065573331	-0.210749118	0.833083045	0	1.924178167	0	0.666666667	CRA004775;PRJNA827465;Yang2025_Silkie_Wencha	0.950404946
ENSGALG00010027325	METTL25B	3	-0.057323606	0.272190646	-0.210600939	0.833198679	0.168112617	8.441546636	76.30765917	0.666666667	CRA004775;PRJNA827465;Yang2025_Silkie_Wencha	0.950431313
ENSGALG00010008358	HPF1	3	0.029524557	0.140271381	0.210481685	0.833291745	0.039263014	7.576513365	73.60263351	0.666666667	CRA004775;PRJNA827465;Yang2025_Silkie_Wencha	0.950460792
ENSGALG00010003742	ZFHX4	3	0.041275133	0.19628397	0.210284884	0.833445332	0.055552059	3.52824773	43.31463796	0.666666667	CRA004775;PRJNA827465;Yang2025_Silkie_Wencha	0.950559293
ENSGALG00010014176	ABHD17B	3	0.02550021	0.121374407	0.210095445	0.83359318	0.020998938	3.822251755	47.6748229	0.666666667	CRA004775;PRJNA827465;Yang2025_Silkie_Wencha	0.950586289
ENSGALG00010019679	RHOBTB2	3	0.015161065	0.072167276	0.210082267	0.833603466	0	0.228030314	0	0.666666667	CRA004775;PRJNA827465;Yang2025_Silkie_Wencha	0.950586289
ENSGALG00010001070	C3orf33	3	-0.057314541	0.272965802	-0.209969676	0.833691341	0.169346769	10.3772394	80.72705155	0.666666667	CRA004775;PRJNA827465;Yang2025_Silkie_Wencha	0.950609828
ENSGALG00010015407	TIMP3	3	-0.024859196	0.118582578	-0.209636155	0.833951659	0.022920861	5.20703856	61.59045152	0.666666667	CRA004775;PRJNA827465;Yang2025_Silkie_Wencha	0.950799282
ENSGALG00010025496	ATG101	3	-0.013640294	0.065082553	-0.20958449	0.833991986	0	0.490766085	0	0.666666667	CRA004775;PRJNA827465;Yang2025_Silkie_Wencha	0.950799282
ENSGALG00010015764	TTI1	3	-0.01100146	0.05269361	-0.208781683	0.834618671	0	1.726510823	0	0.666666667	CRA004775;PRJNA827465;Yang2025_Silkie_Wencha	0.951437022
ENSGALG00010014831	VAPB	3	-0.032905013	0.157697377	-0.208659227	0.834714272	0.064313874	16.84379945	88.12619441	0.666666667	CRA004775;PRJNA827465;Yang2025_Silkie_Wencha	0.951469291
ENSGALG00010011293	NDUFV3	3	-0.02440879	0.117274892	-0.208133129	0.835125021	0.017150344	3.366292962	40.58746453	0.666666667	CRA004775;PRJNA827465;Yang2025_Silkie_Wencha	0.951667054
ENSGALG00010025358	INCENP	3	-0.017282848	0.083053809	-0.208092173	0.835156999	0	0.518848318	0	0.666666667	CRA004775;PRJNA827465;Yang2025_Silkie_Wencha	0.951667054
ENSGALG00010007830	SMIM19	3	-0.011663262	0.056038291	-0.208130225	0.835127287	0	0.554163568	0	0.666666667	CRA004775;PRJNA827465;Yang2025_Silkie_Wencha	0.951667054
ENSGALG00010020582	KANSL3	3	0.010311441	0.049542596	0.208132832	0.835125252	0	1.188115133	0	0.666666667	CRA004775;PRJNA827465;Yang2025_Silkie_Wencha	0.951667054
ENSGALG00010011460	LAMC2	3	0.162425993	0.780967841	0.207984996	0.835240683	1.542629819	13.1970065	84.84504801	0.666666667	CRA004775;PRJNA827465;Yang2025_Silkie_Wencha	0.951685713
ENSGALG00010027605	FCHO1	3	0.057872647	0.278467349	0.207825611	0.835365134	0.142939506	5.209507859	61.60865759	0.666666667	CRA004775;PRJNA827465;Yang2025_Silkie_Wencha	0.951700963
ENSGALG00010029383	GNA13	3	-0.046388527	0.223241307	-0.207795448	0.835388687	0.12133789	21.95203859	90.88922885	0.666666667	CRA004775;PRJNA827465;Yang2025_Silkie_Wencha	0.951700963
ENSGALG00010017471	CRLS1	3	-0.009915165	0.047758266	-0.207611489	0.835532333	0	0.04629837	0	1	CRA004775;PRJNA827465;Yang2025_Silkie_Wencha	0.951787919
ENSGALG00010007633	ENSGALG0001000763(	3	0.061016024	0.294239869	0.207368309	0.83572223	0.236786765	24.61636139	91.8753224	0.666666667	CRA004775;PRJNA827465;Yang2025_Silkie_Wencha	0.951850863
ENSGALG00010003166	PGAP2	3	-0.010862169	0.052378797	-0.207377216	0.835715275	0	1.078292438	0	0.666666667	CRA004775;PRJNA827465;Yang2025_Silkie_Wencha	0.951850863
ENSGALG00010021247	GNAO1	3	-0.082398398	0.398666965	-0.206684789	0.836256037	0.359883924	11.02176084	81.85407914	0.666666667	CRA004775;PRJNA827465;Yang2025_Silkie_Wencha	0.951868777
ENSGALG00010002611	NPS	3	-0.065516705	0.317458691	-0.206378678	0.836495125	0	0.907130152	0	0.666666667	CRA004775;PRJNA827465;Yang2025_Silkie_Wencha	0.951868777
ENSGALG00010028467	ENSGALG0001002846(	3	-0.050911623	0.246144689	-0.206836162	0.836137813	0.077549342	3.411654247	41.37741238	0.666666667	CRA004775;PRJNA827465;Yang2025_Silkie_Wencha	0.951868777
ENSGALG00010015804	CKB	3	-0.030577107	0.147980295	-0.206629584	0.836299154	0.017221819	2.605806364	23.2483262	0.666666667	CRA004775;PRJNA827465;Yang2025_Silkie_Wencha	0.951868777
ENSGALG00010008175	ENSGALG0001000817(	3	0.029084354	0.140757916	0.206626777	0.836301347	0	0.205558868	0	0.666666667	CRA004775;PRJNA827465;Yang2025_Silkie_Wencha	0.951868777
ENSGALG00010007339	ENSGALG0001000733(	3	-0.027555424	0.133213186	-0.206852076	0.836125385	0	1.299147332	0	0.666666667	CRA004775;PRJNA827465;Yang2025_Silkie_Wencha	0.951868777
ENSGALG00010014816	TST	3	0.026886928	0.130320572	0.206313767	0.836545825	0.022339281	3.562639223	43.861843	0.666666667	CRA004775;PRJNA827465;Yang2025_Silkie_Wencha	0.951868777
ENSGALG00010018340	DNPEP	3	0.026267342	0.127013002	0.206808295	0.836159578	0.02787104	5.501865997	63.64869662	0.666666667	CRA004775;PRJNA827465;Yang2025_Silkie_Wencha	0.951868777
ENSGALG00010017849	FBXO48	3	-0.025300723	0.1225733	-0.206413002	0.836468315	0	0.216773064	0	0.666666667	CRA004775;PRJNA827465;Yang2025_Silkie_Wencha	0.951868777
ENSGALG00010018509	ENSGALG0001001850(	3	-0.023691807	0.114458389	-0.206990566	0.836017226	0.027749492	7.26729769	72.47945405	0.666666667	CRA004775;PRJNA827465;Yang2025_Silkie_Wencha	0.951868777
ENSGALG00010028430	NR2C2AP	3	0.015570994	0.075435395	0.206414958	0.836466788	0	1.5980299	0	0.666666667	CRA004775;PRJNA827465;Yang2025_Silkie_Wencha	0.951868777
ENSGALG00010017359	EHD3	3	-0.010205644	0.04945148	-0.206376922	0.836496497	0.000942872	2.206353687	9.35270208	0.666666667	CRA004775;PRJNA827465;Yang2025_Silkie_Wencha	0.951868777
ENSGALG00010021189	ENSGALG0001002118(	3	-0.058802771	0.285317479	-0.206095928	0.836715979	0	0.094451992	0	0.666666667	CRA004775;PRJNA827465;Yang2025_Silkie_Wencha	0.951929305
ENSGALG00010014171	DLC1	3	-0.012405177	0.060197892	-0.206073277	0.836733673	0	1.720723632	0	0.666666667	CRA004775;PRJNA827465;Yang2025_Silkie_Wencha	0.951929305
ENSGALG00010001616	OPNVA	2	-0.066208928	0.322786965	-0.205116486	0.837481117	0	0.274116314	0	0.5	PRJNA827465;Yang2025_Silkie_Wenchang	0.952124475
ENSGALG00010004283	DCK	3	-0.055639558	0.270834467	-0.205437507	0.837230319	0.183592418	14.3690243	86.08117045	0.666666667	CRA004775;PRJNA827465;Yang2025_Silkie_Wencha	0.952124475
ENSGALG00010028195	ENSGALG0001002819(	3	0.036430276	0.177127868	0.205672189	0.837046984	0	0.266453775	0	0.666666667	CRA004775;PRJNA827465;Yang2025_Silkie_Wencha	0.952124475
ENSGALG00010011313	RASSF9	3	-0.034485951	0.168151677	-0.205088356	0.837503094	0	0.586134504	0	0.666666667	CRA004775;PRJNA827465;Yang2025_Silkie_Wencha	0.952124475
ENSGALG00010001621	DLGAP2	2	-0.033725855	0.164308773	-0.205259003	0.837369773	0.027769501	1.924502962	48.03853151	0.5	PRJNA827465;Yang2025_Silkie_Wenchang	0.952124475
ENSGALG00010019006	ENSGALG0001001900(	3	-0.019021688	0.092557699	-0.205511683	0.837172371	0.01779119	6.631589748	69.84131896	0.666666667	CRA004775;PRJNA827465;Yang2025_Silkie_Wencha	0.952124475
ENSGALG00010027129	NCLN	3	0.01364799	0.066338717	0.205731899	0.837000339	0.000734488	2.100588139	4.788570269	0.666666667	CRA004775;PRJNA827465;Yang2025_Silkie_Wencha	0.952124475
ENSGALG00010018251	USP48	3	0.010111045	0.049267621	0.205226983	0.837394789	0.00292136	3.176015515	37.02801544	0.666666667	CRA004775;PRJNA827465;Yang2025_Silkie_Wencha	0.952124475
ENSGALG00010014735	STX16	3	0.008264549	0.040299567	0.205077868	0.837511288	0	1.570981732	0	0.666666667	CRA004775;PRJNA827465;Yang2025_Silkie_Wencha	0.952124475
ENSGALG00010021150	ENSGALG0001002115(	3	0.082785337	0.404347373	0.204738159	0.837776705	0.35954086	10.2297365	80.44915429	0.666666667	CRA004775;PRJNA827465;Yang2025_Silkie_Wencha	0.952327128
ENSGALG00010023695	CLSTN3	3	-0.01228447	0.060018724	-0.204677302	0.837824255	0	1.207490291	0	0.666666667	CRA004775;PRJNA827465;Yang2025_Silkie_Wencha	0.952327128
ENSGALG00010028923	ENSGALG0001002892(	3	0.084523911	0.413234975	0.204542007	0.837929969	0.396504858	9.841470768	79.67783427	0.666666667	CRA004775;PRJNA827465;Yang2025_Silkie_Wencha	0.952370726
ENSGALG00010014317	RAD23B	3	-0.010361418	0.05068738	-0.204418107	0.838026782	0	0.485437796	0	0.666666667	CRA004775;PRJNA827465;Yang2025_Silkie_Wencha	0.952404201
ENSGALG00010007333	QTRT2	3	0.027410982	0.134393118	0.203961202	0.838383818	0.036002812	7.211805467	72.26769345	0.666666667	CRA004775;PRJNA827465;Yang2025_Silkie_Wencha	0.952427189
ENSGALG00010027941	KLF2	3	-0.025657215	0.125717551	-0.204086182	0.838286152	0.023420542	4.060177888	50.74107452	0.666666667	CRA004775;PRJNA827465;Yang2025_Silkie_Wencha	0.952427189
ENSGALG00010003009	TNKS	3	-0.019959408	0.097759567	-0.204168336	0.838221954	0.019513489	7.058360424	71.66480769	0.666666667	CRA004775;PRJNA827465;Yang2025_Silkie_Wencha	0.952427189
ENSGALG00010004249	USP45	3	-0.018592984	0.091134993	-0.204015859	0.838341106	0.011036981	3.613796609	44.65654223	0.666666667	CRA004775;PRJNA827465;Yang2025_Silkie_Wencha	0.952427189
ENSGALG00010005998	YLPM1	3	0.017458229	0.085491741	0.204209538	0.838189758	0.008082623	2.827518675	29.26660334	0.666666667	CRA004775;PRJNA827465;Yang2025_Silkie_Wencha	0.952427189
ENSGALG00010005387	CLDN10	2	0.214403095	1.0522291	0.203760849	0.838540389	1.857298867	6.194631176	83.85698887	0.5	CRA004775;PRJNA827465	0.952518188
ENSGALG00010027195	CENPP	3	0.036460059	0.179076833	0.203600088	0.838666025	0.036396798	3.175530408	37.01839558	0.666666667	CRA004775;PRJNA827465;Yang2025_Silkie_Wencha	0.952518188
ENSGALG00010008167	UTP4	3	0.033596026	0.164982179	0.203634276	0.838639306	0.052631755	6.822674611	70.68598293	0.666666667	CRA004775;PRJNA827465;Yang2025_Silkie_Wencha	0.952518188
ENSGALG00010005286	ENSGALG0001000528(	3	0.145504078	0.715070282	0.203482205	0.838758153	1.190357102	8.953977244	77.66355726	0.666666667	CRA004775;PRJNA827465;Yang2025_Silkie_Wencha	0.952546307
ENSGALG00010018959	UBA5	3	-0.019150177	0.094274523	-0.203132045	0.839031826	0.015569156	6.174392996	67.60815191	0.666666667	CRA004775;PRJNA827465;Yang2025_Silkie_Wencha	0.952780579
ENSGALG00010013431	TMEM175	3	-0.04190093	0.206824325	-0.202591888	0.839454031	0.094352991	7.779420464	74.29114406	0.666666667	CRA004775;PRJNA827465;Yang2025_Silkie_Wencha	0.952830471
ENSGALG00010009352	HSF1	3	-0.038116518	0.187996499	-0.20275121	0.839329495	0.073578912	6.739973625	70.32629338	0.666666667	CRA004775;PRJNA827465;Yang2025_Silkie_Wencha	0.952830471
ENSGALG00010024274	ACSBG1	3	0.033653023	0.166166286	0.202526182	0.839505392	0.048836988	5.171227843	61.32446566	0.666666667	CRA004775;PRJNA827465;Yang2025_Silkie_Wencha	0.952830471
ENSGALG00010007321	CD200	3	0.018255066	0.090160797	0.202472324	0.839547493	0	1.339723951	0	0.666666667	CRA004775;PRJNA827465;Yang2025_Silkie_Wencha	0.952830471
ENSGALG00010001995	LRRC1	3	-0.01740065	0.085729074	-0.202972565	0.839156476	0	1.156860292	0	0.666666667	CRA004775;PRJNA827465;Yang2025_Silkie_Wencha	0.952830471
ENSGALG00010018998	RPS29	3	-0.014836252	0.07323394	-0.202587111	0.839457765	0	0.922524777	0	0.666666667	CRA004775;PRJNA827465;Yang2025_Silkie_Wencha	0.952830471
ENSGALG00010002511	VPS36	3	0.008961447	0.044187867	0.202803344	0.839288744	0	1.316053221	0	0.666666667	CRA004775;PRJNA827465;Yang2025_Silkie_Wencha	0.952830471
ENSGALG00010029520	MKS1	3	0.048875623	0.241644879	0.202262192	0.839711756	0.155777472	19.67220906	89.8333736	0.666666667	CRA004775;PRJNA827465;Yang2025_Silkie_Wencha	0.952863926
ENSGALG00010012879	RCAN1	3	0.039185222	0.193666542	0.202333465	0.83965604	0.079855523	8.59333764	76.72615596	0.666666667	CRA004775;PRJNA827465;Yang2025_Silkie_Wencha	0.952863926
ENSGALG00010024164	SNAP29	3	-0.055861359	0.27672401	-0.201866685	0.840020947	0.207877302	40.17494124	95.02177243	0.666666667	CRA004775;PRJNA827465;Yang2025_Silkie_Wencha	0.952944801
ENSGALG00010001708	AADAC	3	-0.044883761	0.222356856	-0.201854629	0.840030372	0	0.616864157	0	0.666666667	CRA004775;PRJNA827465;Yang2025_Silkie_Wencha	0.952944801
ENSGALG00010023028	TMEM61	3	-0.032759122	0.16238291	-0.201739963	0.840120019	0	1.967204282	0	0.666666667	CRA004775;PRJNA827465;Yang2025_Silkie_Wencha	0.952944801
ENSGALG00010000790	MARCHF6	3	-0.02438846	0.12085393	-0.201801132	0.840072197	0.032779056	11.64401771	82.82379802	0.666666667	CRA004775;PRJNA827465;Yang2025_Silkie_Wencha	0.952944801
ENSGALG00010023312	UBR3	3	-0.01765974	0.087508787	-0.201805333	0.840068912	0.011205932	4.106690987	51.29898971	0.666666667	CRA004775;PRJNA827465;Yang2025_Silkie_Wencha	0.952944801
ENSGALG00010018881	ENSGALG0001001888(	3	0.2234058	1.110636334	0.201151172	0.84058037	2.825558418	8.675399259	76.94630598	0.666666667	CRA004775;PRJNA827465;Yang2025_Silkie_Wencha	0.953008247
ENSGALG00010017891	GOLGA7B	3	-0.077096182	0.38249612	-0.201560691	0.840260178	0.292565874	8.305932545	75.9208254	0.666666667	CRA004775;PRJNA827465;Yang2025_Silkie_Wencha	0.953008247
ENSGALG00010017424	APOD	3	-0.058393551	0.290153633	-0.201250457	0.840502739	0.042406202	2.39817695	16.60331821	0.666666667	CRA004775;PRJNA827465;Yang2025_Silkie_Wencha	0.953008247
ENSGALG00010028001	SBNO2	3	0.033671644	0.167208439	0.201375264	0.840405155	0.054129273	7.793819017	74.33863943	0.666666667	CRA004775;PRJNA827465;Yang2025_Silkie_Wencha	0.953008247
ENSGALG00010012384	NRK	3	0.014247001	0.070754062	0.201359472	0.840417503	0	1.063600044	0	0.666666667	CRA004775;PRJNA827465;Yang2025_Silkie_Wencha	0.953008247
ENSGALG00010002410	RNF121	3	-0.01235528	0.061415103	-0.20117657	0.840560511	0	0.166271756	0	1	CRA004775;PRJNA827465;Yang2025_Silkie_Wencha	0.953008247
ENSGALG00010021983	CTLA4	2	0.175608534	0.874218822	0.200874803	0.84079647	1.003505618	2.909942575	65.63506069	0.5	CRA004775;PRJNA827465	0.9531004
ENSGALG00010008131	OC90	2	0.064541036	0.321289982	0.200880947	0.840791666	0.001659212	1.007696271	0.763749051	0.5	PRJNA827465;Yang2025_Silkie_Wenchang	0.9531004
ENSGALG00010015703	GK5	3	-0.052394778	0.261167768	-0.200617323	0.840997812	0.152346135	11.10017692	81.98226916	0.666666667	CRA004775;PRJNA827465;Yang2025_Silkie_Wencha	0.953252209
ENSGALG00010027767	ENSGALG0001002776(	2	0.091086825	0.455424326	0.200004302	0.841477216	0	0.222376484	0	0.5	PRJNA827465;Yang2025_Silkie_Wenchang	0.953337048
ENSGALG00010008321	ZFHX3	3	0.024758054	0.123636867	0.200248152	0.84128651	0.015108758	2.566352914	22.06839563	0.666666667	CRA004775;PRJNA827465;Yang2025_Silkie_Wencha	0.953337048
ENSGALG00010014769	CCDC69	3	0.023919837	0.119371795	0.200380982	0.841182632	0.002836616	2.111519794	5.281494137	0.666666667	CRA004775;PRJNA827465;Yang2025_Silkie_Wencha	0.953337048
ENSGALG00010024346	PPM1F	3	0.014251241	0.071248464	0.200021726	0.84146359	0	0.810770759	0	0.666666667	CRA004775;PRJNA827465;Yang2025_Silkie_Wencha	0.953337048
ENSGALG00010018579	FNDC3B	3	-0.013396738	0.06690452	-0.200236667	0.841295492	0	1.043925232	0	0.666666667	CRA004775;PRJNA827465;Yang2025_Silkie_Wencha	0.953337048
ENSGALG00010015936	RPL36AL	3	-0.01316029	0.065799443	-0.2000061	0.84147581	0	1.377980281	0	0.666666667	CRA004775;PRJNA827465;Yang2025_Silkie_Wencha	0.953337048
ENSGALG00010015697	FAXDC2	3	0.033927441	0.170052691	0.199511347	0.841862768	0.054086414	5.512829006	63.72098613	0.666666667	CRA004775;PRJNA827465;Yang2025_Silkie_Wencha	0.953391885
ENSGALG00010014614	GHR	3	-0.026703976	0.133756971	-0.199645487	0.84175785	0.024938844	3.856833858	48.14399392	0.666666667	CRA004775;PRJNA827465;Yang2025_Silkie_Wencha	0.953391885
ENSGALG00010014083	ENSGALG0001001408(	3	-0.026403298	0.13223734	-0.199665979	0.841741823	0.00071535	2.026722846	1.318524933	0.666666667	CRA004775;PRJNA827465;Yang2025_Silkie_Wencha	0.953391885
ENSGALG00010029172	LRRC45	3	0.022749846	0.114025465	0.199515485	0.841859532	0.028049194	7.894706881	74.66657052	0.666666667	CRA004775;PRJNA827465;Yang2025_Silkie_Wencha	0.953391885
ENSGALG00010021232	ACTB	3	0.01954455	0.097936291	0.19956392	0.841821648	0.010596677	2.961886554	32.47546915	0.666666667	CRA004775;PRJNA827465;Yang2025_Silkie_Wencha	0.953391885
ENSGALG00010029700	GGA3	3	-0.022185696	0.111358998	-0.199226789	0.842085345	0.02454054	6.841789079	70.76787991	0.666666667	CRA004775;PRJNA827465;Yang2025_Silkie_Wencha	0.953567571
ENSGALG00010016627	SH3RF2	2	0.283154908	1.4270332	0.198422089	0.842714837	3.119649931	3.893092386	74.31347883	0.5	CRA004775;PRJNA827465	0.953616496

ENSGALG00010015020	NIPAL4	2	0.206753221	1.04074721	0.198658444	0.842529933	1.282508687	2.406575508	58.44717954	0.5	PRJNA827465;Yang2025_Silkie_Wenchang	0.953616496
ENSGALG00010010451	RANBP17	2	-0.076729804	0.38541797	-0.199082062	0.842198553	0.107633929	1.546640189	35.34372073	0.5	PRJNA827465;Yang2025_Silkie_Wenchang	0.953616496
ENSGALG00010010791	H-10	3	-0.06176846	0.311359807	-0.198382894	0.8427455	0.229728596	10.10157272	80.20110278	0.666666667	CRA004775;PRJNA827465;Yang2025_Silkie_Wencha	0.953616496
ENSGALG00010027133	CELF5	3	0.056365246	0.283854318	0.198571037	0.842598312	0.153221998	6.396618839	68.7334817	0.666666667	CRA004775;PRJNA827465;Yang2025_Silkie_Wencha	0.953616496
ENSGALG00010023429	BMF	3	-0.041675074	0.210043027	-0.198412082	0.842722665	0.096233526	8.077885446	75.24104528	0.666666667	CRA004775;PRJNA827465;Yang2025_Silkie_Wencha	0.953616496
ENSGALG00010021558	CHURC1	3	0.039711709	0.199702564	0.198854275	0.842376738	0.063460936	4.180135088	52.1546563	0.666666667	CRA004775;PRJNA827465;Yang2025_Silkie_Wencha	0.953616496
ENSGALG00010023710	CDC14A	3	0.01102859	0.055446591	0.198904754	0.842337251	0	0.299826025	0	0.666666667	CRA004775;PRJNA827465;Yang2025_Silkie_Wencha	0.953616496
ENSGALG00010011329	PCDHGC5	3	0.010264217	0.051640059	0.198764632	0.842446864	0	0.694001812	0	0.666666667	CRA004775;PRJNA827465;Yang2025_Silkie_Wencha	0.953616496
ENSGALG00010021102	GOLGA3	3	0.008136186	0.041027742	0.198309386	0.842803008	0	1.722453421	0	0.666666667	CRA004775;PRJNA827465;Yang2025_Silkie_Wencha	0.953616496
ENSGALG00010019785	ACSL5	3	-0.051696045	0.260835013	-0.198194425	0.84289295	0.051579666	2.64371163	24.34878382	0.666666667	CRA004775;PRJNA827465;Yang2025_Silkie_Wencha	0.953641947
ENSGALG00010011352	NTS	2	-0.128770063	0.652986905	-0.196589005	0.844149176	0.473197151	2.197995941	54.50401061	0.5	CRA004775;PRJNA827465	0.953651649
ENSGALG00010027277	KCND3	3	0.117442867	0.596778478	0.196794743	0.843988165	0.839413352	11.03788805	81.88059172	0.666666667	CRA004775;PRJNA827465;Yang2025_Silkie_Wencha	0.953651649
ENSGALG00010011416	ENSGALG0001001141(	3	-0.099110114	0.504651948	-0.196393009	0.844302568	0.415267414	4.390028601	54.44221025	0.666666667	CRA004775;PRJNA827465;Yang2025_Silkie_Wencha	0.953651649
ENSGALG00010000084	ENSGALG00010000084	3	-0.082369351	0.417309681	-0.197381836	0.843528743	0.4162576	11.39436615	82.44746594	0.666666667	CRA004775;PRJNA827465;Yang2025_Silkie_Wencha	0.953651649
ENSGALG00010007936	ENSGALG0001000793(	3	-0.073366929	0.373781141	-0.196283121	0.844388572	0.310422422	10.18628394	80.36575446	0.666666667	CRA004775;PRJNA827465;Yang2025_Silkie_Wencha	0.953651649
ENSGALG00010024526	TGM6	2	0.070866307	0.361232324	0.196179307	0.844469824	0	0.064948743	0	0.5	PRJNA827465;Yang2025_Silkie_Wenchang	0.953651649
ENSGALG00010004096	COL12A1	3	0.061590341	0.312057559	0.197368528	0.843539156	0.182746271	5.851035986	65.81801916	0.666666667	CRA004775;PRJNA827465;Yang2025_Silkie_Wencha	0.953651649
ENSGALG00010011457	ENSGALG0001001145'	3	0.061103492	0.311265241	0.196306828	0.844370017	0.128983266	3.591061402	44.30615977	0.666666667	CRA004775;PRJNA827465;Yang2025_Silkie_Wencha	0.953651649
ENSGALG00010023213	FCSK	3	0.059913062	0.305220205	0.196294546	0.84437963	0.237740931	15.39128637	87.00363454	0.666666667	CRA004775;PRJNA827465;Yang2025_Silkie_Wencha	0.953651649
ENSGALG00010017636	ZBTB8OS	3	0.057292648	0.291427075	0.19659343	0.844145713	0.223420385	21.97198595	90.89750009	0.666666667	CRA004775;PRJNA827465;Yang2025_Silkie_Wencha	0.953651649
ENSGALG00010024025	CLCN1	3	0.048153068	0.243544209	0.197717975	0.843265726	0.093184321	4.391689	54.45943463	0.666666667	CRA004775;PRJNA827465;Yang2025_Silkie_Wencha	0.953651649
ENSGALG00010000092	INPPL1	3	-0.046222934	0.234467268	-0.197140243	0.843717792	0.122797246	10.65441038	81.22843095	0.666666667	CRA004775;PRJNA827465;Yang2025_Silkie_Wencha	0.953651649
ENSGALG00010004927	KIF25	2	0.041045354	0.209050419	0.196341888	0.844342578	0.048861397	2.162295068	53.75284276	0.5	PRJNA827465;Yang2025_Silkie_Wenchang	0.953651649
ENSGALG00010029666	SLC9A3R1	3	-0.031752451	0.161907442	-0.196114836	0.844520284	0.039424291	4.147319384	51.77607956	0.666666667	CRA004775;PRJNA827465;Yang2025_Silkie_Wencha	0.953651649
ENSGALG00010020422	ERCC4	3	0.0317373	0.16130449	0.19675979	0.844020066	0.065018577	16.49514685	87.87522162	0.666666667	CRA004775;PRJNA827465;Yang2025_Silkie_Wencha	0.953651649
ENSGALG00010016569	YY1	3	0.027717741	0.141011775	0.196563311	0.844169284	0.044883022	9.822221437	79.63800742	0.666666667	CRA004775;PRJNA827465;Yang2025_Silkie_Wencha	0.953651649
ENSGALG00010014111	GORAB	3	0.027336514	0.138668143	0.197136226	0.843720935	0.045901111	12.72505703	84.2829781	0.666666667	CRA004775;PRJNA827465;Yang2025_Silkie_Wencha	0.953651649
ENSGALG00010023221	AMY1A	3	-0.02703005	0.137731278	-0.196252081	0.844412866	0.021114799	3.157436922	36.65748361	0.666666667	CRA004775;PRJNA827465;Yang2025_Silkie_Wencha	0.953651649
ENSGALG00010016779	CTNNA2	3	-0.023916997	0.121513767	-0.196825408	0.843964167	0.012886286	2.664728196	24.94544084	0.666666667	CRA004775;PRJNA827465;Yang2025_Silkie_Wencha	0.953651649
ENSGALG00010013149	MCTP1	3	-0.021020009	0.106323397	-0.197698809	0.843280722	0	0.244502679	0	0.666666667	CRA004775;PRJNA827465;Yang2025_Silkie_Wencha	0.953651649
ENSGALG00010022667	PTEN	3	-0.0202924	0.102978526	-0.197054676	0.843784752	0.018407794	4.685925824	57.31900002	0.666666667	CRA004775;PRJNA827465;Yang2025_Silkie_Wencha	0.953651649
ENSGALG00010017414	ENSGALG0001001741	3	-0.018360636	0.092869225	-0.1977042	0.843276504	0	0.255713122	0	0.666666667	CRA004775;PRJNA827465;Yang2025_Silkie_Wencha	0.953651649
ENSGALG00010017657	DIS3L2	3	-0.01432669	0.072701919	-0.197060689	0.843780046	0.004303024	2.679761812	25.3665012	0.666666667	CRA004775;PRJNA827465;Yang2025_Silkie_Wencha	0.953651649
ENSGALG00010000054	VPS33B	3	0.010990379	0.055487041	0.198071102	0.842989434	0	1.047504057	0	0.666666667	CRA004775;PRJNA827465;Yang2025_Silkie_Wencha	0.953651649
ENSGALG00010018217	ENSGALG0001001821'	2	-0.103480219	0.528748514	-0.195707818	0.844838865	0.389876516	3.1895273	68.64739173	0.5	PRJNA827465;Yang2025_Silkie_Wenchang	0.953699909
ENSGALG00010003438	KCNS3	3	0.065447402	0.334147897	0.195863576	0.844716947	0.248857437	8.737177221	77.10931174	0.666666667	CRA004775;PRJNA827465;Yang2025_Silkie_Wencha	0.953699909
ENSGALG00010027213	KCNC4	3	-0.043739371	0.223582856	-0.195629358	0.84490028	0.056464347	3.169218595	36.89296146	0.666666667	CRA004775;PRJNA827465;Yang2025_Silkie_Wencha	0.953699909
ENSGALG00010013173	KCNJ8	3	0.040162331	0.205157407	0.195763495	0.844795284	0.097056116	18.06471403	88.92869272	0.666666667	CRA004775;PRJNA827465;Yang2025_Silkie_Wencha	0.953699909
ENSGALG00010021803	TLL2	3	-0.030398105	0.155343478	-0.195683478	0.844857917	0.035416403	4.25835275	53.03348225	0.666666667	CRA004775;PRJNA827465;Yang2025_Silkie_Wencha	0.953699909
ENSGALG00010018372	NLGN3	3	0.035766026	0.183276424	0.195147993	0.845277092	0.040219219	3.308275379	39.54554047	0.666666667	CRA004775;PRJNA827465;Yang2025_Silkie_Wencha	0.954049078
ENSGALG00010021526	KCNJ12	3	-0.037936798	0.194508072	-0.195039709	0.845361861	0.061804863	5.016634793	60.13263706	0.666666667	CRA004775;PRJNA827465;Yang2025_Silkie_Wencha	0.954068595
ENSGALG00010009313	UNC13B	3	-0.045014482	0.231084058	-0.194797003	0.845551868	0.073740892	3.755535352	46.74527564	0.666666667	CRA004775;PRJNA827465;Yang2025_Silkie_Wencha	0.954074441
ENSGALG00010027001	SLC26A8	3	-0.036379514	0.186721862	-0.194832641	0.845523968	0.049045405	3.827412096	47.74537076	0.666666667	CRA004775;PRJNA827465;Yang2025_Silkie_Wencha	0.954074441
ENSGALG00010002658	FBXL12	3	-0.032202646	0.165332959	-0.194774513	0.845569476	0.053025086	5.769845292	65.33702554	0.666666667	CRA004775;PRJNA827465;Yang2025_Silkie_Wencha	0.954074441
ENSGALG00010024352	MAP6D1	3	0.05120441	0.263011809	0.194684832	0.845639687	0	0.191676814	0	0.666666667	CRA004775;PRJNA827465;Yang2025_Silkie_Wencha	0.954077524
ENSGALG00010004958	TPD52L1	3	0.098639826	0.507383051	0.19440899	0.845855652	0.545631789	7.683601481	73.97053966	0.666666667	CRA004775;PRJNA827465;Yang2025_Silkie_Wencha	0.954082255
ENSGALG00010021823	ENSGALG0001002182:	3	0.079826395	0.412003978	0.193752895	0.846388127	0.463797624	22.63251287	91.16315536	0.666666667	CRA004775;PRJNA827465;Yang2025_Silkie_Wencha	0.954082255
ENSGALG00010012860	ENSGALG0001001286(	2	-0.067780002	0.34881485	-0.194315126	0.845929142	0	0.217359265	0	0.5	PRJNA827465;Yang2025_Silkie_Wenchang	0.954082255
ENSGALG00010002913	NXNL1	3	0.061044214	0.314537921	0.194075848	0.846116493	0.104077721	3.039753355	34.20518817	0.666666667	CRA004775;PRJNA827465;Yang2025_Silkie_Wencha	0.954082255
ENSGALG00010004650	GTPBP10	3	-0.053984925	0.27878252	-0.1936453	0.846643625	0.200227742	20.1302411	90.06469923	0.666666667	CRA004775;PRJNA827465;Yang2025_Silkie_Wencha	0.954082255
ENSGALG00010011019	NECAB1	3	-0.038745074	0.199634517	-0.194079983	0.846113255	0.055515721	3.904677058	48.77937483	0.666666667	CRA004775;PRJNA827465;Yang2025_Silkie_Wencha	0.954082255
ENSGALG00010013184	LDHB	3	-0.036151103	0.185879509	-0.194486758	0.845794763	0.053036063	4.052937782	50.65307914	0.666666667	CRA004775;PRJNA827465;Yang2025_Silkie_Wencha	0.954082255
ENSGALG00010005329	VIPAS39	3	0.033663817	0.173624148	0.193889026	0.846262776	0.077095591	15.15950149	86.80695403	0.666666667	CRA004775;PRJNA827465;Yang2025_Silkie_Wencha	0.954082255
ENSGALG00010027731	OLFML2A	3	0.032928526	0.169608503	0.194144313	0.846062885	0.061822013	8.233927434	75.71025472	0.666666667	CRA004775;PRJNA827465;Yang2025_Silkie_Wencha	0.954082255
ENSGALG00010009704	RGP1	3	-0.024286557	0.125218429	-0.193953354	0.846212261	0.00674083	2.31244993	13.51164087	0.666666667	CRA004775;PRJNA827465;Yang2025_Silkie_Wencha	0.954082255
ENSGALG00010024430	ZNF652	3	0.01129931	0.058309127	0.193782868	0.846345902	0.003258612	2.394245816	16.46638843	0.666666667	CRA004775;PRJNA827465;Yang2025_Silkie_Wencha	0.954082255
ENSGALG00010022079	ABI2	3	-0.010043875	0.051784032	-0.193956986	0.846209562	0	0.510905639	0	0.666666667	CRA004775;PRJNA827465;Yang2025_Silkie_Wencha	0.954082255
ENSGALG00010024150	NHERF4	3	0.036450182	0.188354095	0.193519458	0.846552169	0	0.143637553	0	0.666666667	CRA004775;PRJNA827465;Yang2025_Silkie_Wencha	0.954098089
ENSGALG00010023637	RHBDD1	3	-0.013348638	0.069183348	-0.193455016	0.846602633	0	1.488535292	0	0.666666667	CRA004775;PRJNA827465;Yang2025_Silkie_Wencha	0.954098089
ENSGALG00010021367	GRIP2	3	-0.028125752	0.145766205	-0.192951117	0.846997252	0.03126064	4.27078417	53.17019263	0.666666667	CRA004775;PRJNA827465;Yang2025_Silkie_Wencha	0.954466736
ENSGALG00010001908	FAM83B	2	-0.051499637	0.26745926	-0.192551334	0.847310361	0	0.015269696	0	1	PRJNA827465;Yang2025_Silkie_Wenchang	0.9546674
ENSGALG00010003454	BPNT2	3	-0.025575988	0.132824413	-0.192554874	0.847307589	0.040431547	9.462871176	78.86476564	0.666666667	CRA004775;PRJNA827465;Yang2025_Silkie_Wencha	0.9546674
ENSGALG00010008473	CSMD3	2	-0.168288681	0.87912408	-0.191427692	0.848190526	1.038326958	2.928210238	65.84944664	0.5	PRJNA827465;Yang2025_Silkie_Wenchang	0.954852398
ENSGALG00010028297	ENSGALG0001002829'	2	-0.088032612	0.460279763	-0.191258923	0.848322741	0	0.244482671	0	0.5	PRJNA827465;Yang2025_Silkie_Wenchang	0.954852398
ENSGALG00010007640	TMEM123	3	-0.062691482	0.326580478	-0.191963348	0.847770915	0.226470855	7.402978033	72.98384527	0.666666667	CRA004775;PRJNA827465;Yang2025_Silkie_Wencha	0.954852398
ENSGALG00010003191	CC2D1A	3	0.0547499	0.28631747	0.191220954	0.848352488	0.20718984	13.40365355	85.07869521	0.666666667	CRA004775;PRJNA827465;Yang2025_Silkie_Wencha	0.954852398
ENSGALG00010003407	CHMP4C	3	-0.039470077	0.2063522	-0.191275292	0.848309918	0.076295887	5.236468957	61.80632376	0.666666667	CRA004775;PRJNA827465;Yang2025_Silkie_Wencha	0.954852398
ENSGALG00010012040	B4GALT7	3	0.038142015	0.199322335	0.191358459	0.848244764	0.093512558	10.02565314	80.05117501	0.666666667	CRA004775;PRJNA827465;Yang2025_Silkie_Wencha	0.954852398
ENSGALG00010019598	KCNAB2	3	-0.034135485	0.177748907	-0.192043289	0.847708297	0.059197259	6.585765408	69.63147218	0.666666667	CRA004775;PRJNA827465;Yang2025_Silkie_Wencha	0.954852398
ENSGALG00010002526	ZBTB22	3	-0.033633135	0.17559026	-0.191543278	0.848099977	0.048948919	4.183135028	52.18896864	0.666666667	CRA004775;PRJNA827465;Yang2025_Silkie_Wencha	0.954852398
ENSGALG00010028867	REXO4	3	0.02573369	0.134197552	0.19175976	0.847930393	0.034741628	6.109860698	67.26602947	0.666666667	CRA004775;PRJNA827465;Yang2025_Silkie_Wencha	0.954852398
ENSGALG00010024593	SRSF6	3	0.024597409	0.128253541	0.191787364	0.847908769	0.040158087	16.37761333	87.78820845	0.666666667	CRA004775;PRJNA827465;Yang2025_Silkie_Wencha	0.954852398
ENSGALG00010016168	PLIN2	3	-0.024327802	0.127145623	-0.191338103	0.84826071	0	0.110398923	0	1	CRA004775;PRJNA827465;Yang2025_Silkie_Wencha	0.954852398
ENSGALG00010013480	TBC1D8	3	-0.011340495	0.059075484	-0.191966172	0.847768704	0	1.535032791	0	0.666666667	CRA004775;PRJNA827465;Yang2025_Silkie_Wencha	0.954852398
ENSGALG00010028159	DCAF1	3	-0.010951337	0.057035149	-0.192010308	0.847734131	0.002727527	2.521428719	20.67989133	0.666666667	CRA004775;PRJNA827465;Yang2025_Silkie_Wencha	0.954852398
ENSGALG00010014193	CARD10	3	0.063786768	0.333899321	0.191035933	0.848497441	0.142224359	3.436750559	41.8054943	0.666666667	CRA004775;PRJNA827465;Yang2025_Silkie_Wencha	0.954897265
ENSGALG00010002427	CYB5R4	3	0.010921632	0.057182019	0.190997664	0.848527423	0	1.681944111	0	0.666666667	CRA004775;PRJNA827465;Yang2025_Silkie_Wencha	0.954897265
ENSGALG00010023183	ENSGALG0001002318:	2	0.084220134	0.441897341	0.190587555	0.848848741	0	0.000538622	0	1	PRJNA827465;Yang2025_Silkie_Wenchang	0.955182837
ENSGALG00010025623	ENSGALG0001002562:	3	0.061355629	0.322463229	0.190271706	0.849096224	0.124822036	3.327676916	39.89801141	0.666666667	CRA004775;PRJNA827465;Yang2025_Silkie_Wencha	0.955309263
ENSGALG00010029544	ENSGALG0001002954	3	0.028850553	0.151583069	0.190328335	0.849051851	0.04021426	4.724551535	57.6679398	0.666666667	CRA004775;PRJNA827465;Yang2025_Silkie_Wencha	0.955309263
ENSGALG00010018947	ENSGALG0001001894'	3	-0.061351378	0.322851789	-0.190029542	0.849285981	0.228568354	7.88540549	74.63668796	0.666666667	CRA004775;PRJNA827465;Yang2025_Silkie_Wencha	0.955446729
ENSGALG00010007243	BEAN1	3	0.054891481	0.289007405	0.189931054	0.849363158	0.18665885	10.07701542	80.15285363	0.666666667	CRA004775;PRJNA827465;Yang2025_Silkie_Wencha	0.955457529
ENSGALG00010029638	EPN3	3	0.051518151	0.271477661	0.189769392	0.849489842	0.166701231	13.40145874	85.07625149	0.666666667	CRA004775;PRJNA827465;Yang2025_Silkie_Wencha	0.955524015
ENSGALG00010026154	ZNF385B	3	-0.046637165	0.246271922	-0.189372644	0.849800764	0.11934803	6.828933603	70.71285041	0.666666667	CRA004775;PRJNA827465;Yang2025_Silkie_Wencha	0.955721683
ENSGALG00010009079	KCTD15	3	0.025752178	0.135980935	0.189380795	0.849794375	0.031073559	4.494653147	55.50268431	0.666666667	CRA004775;PRJNA827465;Yang2025_Silkie_Wencha	0.955721683
ENSGALG00010003958	CYSLTR2	3	0.03954354	0.20922725	0.18899804	0.850094353	0.012134663	2.192654509	8.786359567	0.666666667	CRA004775;PRJNA827465;Yang2025_Silkie_Wencha	0.95575853
ENSGALG00010000457	TPCN1	3	0.01699236	0.089909954	0.188993093	0.850098231	0.01186658	3.954323402	49.42244736	0.666666667	CRA004775;PRJNA827465;Yang2025_Silkie_Wencha	0.95575853
ENSGALG00010008208	ILDR2	3	-0.014567396	0.077081943	-0.188985838	0.850103916	0.000557197	2.049470512	2.41381918	0.666666667	CRA004775;PRJNA827465;Yang2025_Silkie_Wencha	0.95575853
ENSGALG00010024086	GABARAPL1	3	0.007348092	0.038855231	0.189114623	0.850002981	0	1.830007057	0	0.666666667	CRA004775;PRJNA827465;Yang2025_Silkie_Wencha	0.95575853
ENSGALG00010016975	MALL	3	0.053146622	0.281798102	0.188598226	0.850407724	0.145113044	5.311929584	62.34889849	0.666666667	CRA004775;PRJNA827465;Yang2025_Silkie_Wencha	0.956016299
ENSGALG00010022930	GTF2B	3	0.016451191	0.087264602	0.188520788	0.850468421	0.009655105	3.399731279	41.17182106	0.666666667	CRA004775;PRJNA827465;Yang2025_Silkie_Wencha	0.956016299
ENSGALG00010023116	PROC	3	0.111709183	0.596261987	0.187349161	0.85138688	0.529760835	3.960470079	49.50094407	0.666666667	CRA004775;PRJNA827465;Yang2025_Silkie_Wencha	0.956569815
ENSGALG00010005301	ENSGALG0001000530	3	-0.070062308	0.374205494	-0.187229502	0.851480694	0.239329383	4.658624866	57.06887638	0.666666667	CRA004775;PRJNA827465;Yang2025_Silkie_Wencha	0.956569815
ENSGALG00010006172	ENSGALG0001000617:	3	0.047764516	0.255149271	0.187202245	0.851502064	0.175260473	22.51162665	91.11570198	0.666666667	CRA004775;PRJNA827465;Yang2025_Silkie_Wencha	0.956569815
ENSGALG00010006068	PACRG	3	0.028444339	0.151922243	0.187229587	0.851480628	0	0.084446663	0	1	CRA004775;PRJNA827465;Yang2025_Silkie_Wencha	0.956569815
ENSGALG00010024532	TMEM86A	3	-0.02355189	0.12556054	-0.187573981	0.851210624	0.024202339	4.959908622	59.67667648	0.666666667	CRA004775;PRJNA827465;Yang2025_Silkie_Wencha	0.956569815
ENSGALG00010024724	HOXD3	3	-0.021488741	0.114459326	-0.187741287	0.851079464	0.006690986	2.355544573	15.09394376	0.666666667	CRA004775;PRJNA827465;Yang2025_Silkie_Wencha	0.956569815
ENSGALG00010018085	PGP	3	0.01869338	0.099852874	0.187209228	0.85149659	0	1.664882174	0	0.666666667	CRA004775;PRJNA827465;Yang2025_Silkie_Wencha	0.956569815
ENSGALG00010007893	MFSD8	3	0.013194728	0.070448515	0.187296046	0.851428523	0	0.573298866	0	0.666666667	CRA004775;PRJNA827465;Yang2025_Silkie_Wencha	0.956569815
ENSGALG00010017855	SDR42E2	2	0.125102124	0.66886044	0.187037709	0.851631067	0.827387733	13.15101174	92.3960223	0.5	PRJNA827465;Yang2025_Silkie_Wenchang	0.956591989
ENSGALG00010020160	SYT7	3	0.059181561	0.31779271	0.18622693	0.852266806	0.130165091	3.395672845	41.10151091	0.666666667	CRA004775;PRJNA827465;Yang2025_Silkie_Wencha	0.956591989
ENSGALG00010021518	CPEB3	3	-0.041046507	0.220512208	-0.186141654	0.852333678	0.105630176	8.185113812	75.5653978	0.666666667	CRA004775;PRJNA827465;Yang2025_Silkie_Wencha	0.956591989
ENSGALG00010024608	DENND2D	3	0.03812799	0.2045577	0.186392348	0.852137093	0	0.543099664	0	0.666666667	CRA004775;PRJNA827465;Yang2025_Silkie_Wencha	0.956591989
ENSGALG00010029615	ENSGALG0001002961:	3	-0.037944173	0.203510571	-0.186448167	0.852093322	0	0.502458946	0	0.666666667	CRA004775;PRJNA827465;Yang2025_Silkie_Wencha	0.956591989
ENSGALG00010017012	SYNGR3	3	0.033919419	0.182128293	0.186239151	0.852257223	0.040162658	3.352888931	40.34994772	0.666666667	CRA004775;PRJNA827465;Yang2025_Silkie_Wencha	0.956591989
ENSGALG00010025738	SOX13	3	-0.027631655	0.148360435	-0.18624679	0.852251232	0.042243997	6.942196813	71.1906756	0.666666667	CRA004775;PRJNA827465;Yang2025_Silkie_Wencha	0.956591989
ENSGALG00010029669	HID1	3	-0.02021804	0.108207419	-0.186845232	0.851781982	0.023761553	6.754738237	70.39115463	0.666666667	CRA004775;PRJNA827465;Yang2025_Silkie_Wencha	0.956591989
ENSGALG00010002194	SHROOM2	3	0.018753924	0.100748697	0.186145576	0.852330602	0	0.660575322	0	0.666666667	CRA004775;PRJNA827465;Yang2025_Silkie_Wencha	0.956591989
ENSGALG00010020106	ENSGALG0001002010(	3	-0.012939992	0.069448271	-0.186325624	0.852189414	0	1.070900419	0	0.666666667	CRA004775;PRJNA827465;Yang2025_Silkie_Wencha	0.956591989
ENSGALG00010000805	FASTKD3	3	0.011929696	0.064045461	0.186269185	0.852233672	0	1.014634532	0	0.666666667	CRA004775;PRJNA827465;Yang2025_Silkie_Wencha	0.956591989
ENSGALG00010006387	OTUD4	3	0.010077038	0.054096594	0.186278608	0.852226282	0.002855276	2.849901089	29.82212583	0.666666667	CRA004775;PRJNA827465;Yang2025_Silkie_Wencha	0.956591989
ENSGALG00010024327	PPP1R1B	3	0.066668003	0.359152772	0.18562575	0.852738259	0.233674581	5.390704707	62.89909931	0.666666667	CRA004775;PRJNA827465;Yang2025_Silkie_Wencha	0.956787923
ENSGALG00010027188	KCNH2	3	-0.061562362	0.331852049	-0.185511472	0.852827883	0.251589854	12.70647742	84.25999643	0.666666667	CRA004775;PRJNA827465;Yang2025_Silkie_Wencha	0.956787923
ENSGALG00010004976	ENSGALG0001000497(	3	0.03474019	0.187281828	0.185496856	0.852839346	0.055358978	4.534043965	55.88926761	0.666666667	CRA004775;PRJNA827465;Yang2025_Silkie_Wencha	0.956787923
ENSGALG00010023744	MT4	3	0.027842018	0.15010178	0.185487596	0.852846608	0	1.816501224	0	0.666666667	CRA004775;PRJNA827465;Yang2025_Silkie_Wencha	0.956787923
ENSGALG00010013539	NSFL1C	3	-0.009955336	0.053636233	-0.185608415	0.852751854	0.000488976	2.063117614	3.059331836	0.666666667	CRA004775;PRJNA827465;Yang2025_Silkie_Wencha	0.956787923
ENSGALG00010022992	SLC7A6	3	0.02179953	0.117661912	0.185272611	0.85301522	0.017577888	3.388292478	40.97321843	0.666666667	CRA004775;PRJNA827465;Yang2025_Silkie_Wencha	0.956898812
ENSGALG00010018752	PLS3	3	-0.016605519	0.089709762	-0.1851027	0.853148484	0.011937485	4.296095141	53.44609618	0.666666667	CRA004775;PRJNA827465;Yang2025_Silkie_Wencha	0.956898812
ENSGALG00010007555	NDUFS6	3	-0.015842549	0.085584322	-0.185110408	0.853142439	0	0.94219182	0	0.666666667	CRA004775;PRJNA827465;Yang2025_Silkie_Wencha	0.956898812
ENSGALG00010023041	CD151	3	-0.025811905	0.139693868	-0.184774788	0.853405683	0.04128285	10.81915942	81.51427553	0.666666667	CRA004775;PRJNA827465;Yang2025_Silkie_Wencha	0.957111363
ENSGALG00010001914	ENSGALG0001000191	3	-0.039492303	0.21396657	-0.184572306	0.853564509	0.073743365	4.401020525	54.55599471	0.666666667	CRA004775;PRJNA827465;Yang2025_Silkie_Wencha	0.957213563
ENSGALG00010023181	PLEKHA7	3	-0.013623142	0.073989925	-0.184121584	0.853918075	0	0.848792677	0	0.666666667	CRA004775;PRJNA827465;Yang2025_Silkie_Wencha	0.957534115
ENSGALG00010002251	GRK5	3	0.022829512	0.124197988	0.183815473	0.854158217	0	0.360482212	0	0.666666667	CRA004775;PRJNA827465;Yang2025_Silkie_Wencha	0.957692799
ENSGALG00010021180	TMEM198	3	0.020766425	0.113006844	0.183762543	0.854199742	0.016223749	3.504952814	42.93789086	0.666666667	CRA004775;PRJNA827465;Yang2025_Silkie_Wencha	0.957692799
ENSGALG00010022532	CBX5	3	-0.02057519	0.112015166	-0.18368218	0.854262789	0.022403826	4.998660932	59.98928458	0.666666667	CRA004775;PRJNA827465;Yang2025_Silkie_Wencha	0.957692799
ENSGALG00010019173	RHO	2	-0.04791799	0.261414057	-0.183303033	0.854560254	0	0.191885441	0	0.5	PRJNA827465;Yang2025_Silkie_Wenchang	0.957750376
ENSGALG00010000479	C12orf49	3	-0.041015882	0.223904112	-0.183185031	0.85465284	0.113705829	9.447180913	78.82966338	0.666666667	CRA004775;PRJNA827465;Yang2025_Silkie_Wencha	0.957750376
ENSGALG00010011040	CDH6	3	-0.040429207	0.220547565	-0.183312869	0.854552537	0.055491373	3.182070075	37.14783293	0.666666667	CRA004775;PRJNA827465;Yang2025_Silkie_Wencha	0.957750376
ENSGALG00010020623	NEK2	3	0.031222499	0.170174993	0.183472895	0.854426984	0.039640929	3.823210019	47.68793789	0.666666667	CRA004775;PRJNA827465;Yang2025_Silkie_Wencha	0.957750376
ENSGALG00010015856	TXNDC9	3	-0.025365088	0.138430547	-0.183233318	0.854614953	0.044633817	12.69038501	84.24003686	0.666666667	CRA004775;PRJNA827465;Yang2025_Silkie_Wencha	0.957750376
ENSGALG00010024899	OGFOD2	3	0.036463787	0.199157519	0.183090184	0.854727257	0.090267604	10.99921179	81.8168789	0.666666667	CRA004775;PRJNA827465;Yang2025_Silkie_Wencha	0.957757861
ENSGALG00010000608	ENSGALG0001000060	2	0.080205401	0.439336072	0.182560473	0.855142901	0	0.000539616	0	1	PRJNA827465;Yang2025_Silkie_Wenchang	0.957759014
ENSGALG00010015979	ENSGALG0001001597	3	-0.071449523	0.392033377	-0.182253674	0.855383652	0.378293281	27.90055296	92.83168329	0.666666667	CRA004775;PRJNA827465;Yang2025_Silkie_Wencha	0.957759014
ENSGALG00010024567	IKZF3	2	0.058040566	0.318648252	0.182146193	0.855467998	0	0.102772583	0	0.5	CRA004775;PRJNA827465	0.957759014
ENSGALG00010024172	ABCG4	3	-0.055194551	0.301948288	-0.182794714	0.854959096	0.148426829	4.648649443	56.97675154	0.666666667	CRA004775;PRJNA827465;Yang2025_Silkie_Wencha	0.957759014
ENSGALG00010027155	ENSGALG0001002715:	3	0.048546914	0.266663665	0.182052975	0.855541152	0.137331793	6.457969828	69.03051495	0.666666667	CRA004775;PRJNA827465;Yang2025_Silkie_Wencha	0.957759014
ENSGALG00010018511	SOCS5	3	-0.038980711	0.214088363	-0.182077676	0.855521768	0.123149937	27.78490475	92.80184684	0.666666667	CRA004775;PRJNA827465;Yang2025_Silkie_Wencha	0.957759014
ENSGALG00010027628	PIERCE1	3	-0.03816437	0.208796821	-0.182782332	0.854968812	0.066779031	4.107599645	51.30976306	0.666666667	CRA004775;PRJNA827465;Yang2025_Silkie_Wencha	0.957759014
ENSGALG00010028440	PSMB7	3	-0.022223668	0.121806373	-0.182450783	0.855228976	0.023168074	3.812978626	47.54756855	0.666666667	CRA004775;PRJNA827465;Yang2025_Silkie_Wencha	0.957759014
ENSGALG00010011515	SERINC5	3	-0.017554896	0.096021826	-0.18282193	0.854937741	0	0.113085572	0	0.666666667	CRA004775;PRJNA827465;Yang2025_Silkie_Wencha	0.957759014
ENSGALG00010019337	XPO1	3	-0.016690868	0.091433976	-0.182545583	0.855154585	0.013727596	4.771256792	58.08232323	0.666666667	CRA004775;PRJNA827465;Yang2025_Silkie_Wencha	0.957759014
ENSGALG00010000412	RFC5	3	0.012364292	0.067810399	0.182336223	0.855318873	0	1.645144829	0	0.666666667	CRA004775;PRJNA827465;Yang2025_Silkie_Wencha	0.957759014
ENSGALG00010027304	PTPN23	3	0.009795383	0.053603703	0.182737065	0.855004332	0	1.719175393	0	0.666666667	CRA004775;PRJNA827465;Yang2025_Silkie_Wencha	0.957759014
ENSGALG00010013029	MYLK2	3	-0.069033593	0.379438346	-0.181936258	0.85563275	0.321349781	16.78044523	88.08136511	0.666666667	CRA004775;PRJNA827465;Yang2025_Silkie_Wencha	0.957768172
ENSGALG00010008383	PLD5	2	0.04653604	0.256118511	0.181697293	0.85582029	0.043552855	1.493532446	33.04464174	0.5	PRJNA827465;Yang2025_Silkie_Wenchang	0.957768172
ENSGALG00010017184	CARD11	3	0.02879989	0.158431087	0.181781814	0.855753957	0.023916454	2.87156661	30.35160693	0.666666667	CRA004775;PRJNA827465;Yang2025_Silkie_Wencha	0.957768172
ENSGALG00010027511	POLRMT	3	-0.008102607	0.044578356	-0.181761016	0.855770279	0	0.926581489	0	0.666666667	CRA004775;PRJNA827465;Yang2025_Silkie_Wencha	0.957768172
ENSGALG00010012939	GJA1	3	-0.031215164	0.172020483	-0.1814619	0.856005036	0.048742811	4.564170599	56.18042848	0.666666667	CRA004775;PRJNA827465;Yang2025_Silkie_Wencha	0.957899106
ENSGALG00010005434	PRDM5	3	-0.02100837	0.115870166	-0.181309567	0.856124597	0.014551282	2.990016527	33.11073762	0.666666667	CRA004775;PRJNA827465;Yang2025_Silkie_Wencha	0.957957081
ENSGALG00010006606	SULT	2	-0.447571503	2.470071455	-0.181197796	0.856212325	10.99391887	10.0964649	90.09554324	0.5	PRJNA827465;Yang2025_Silkie_Wenchang	0.957967139
ENSGALG00010027335	MAST3	3	-0.052204014	0.288357595	-0.18103915	0.856336847	0.207122584	15.39889638	87.01205625	0.666666667	CRA004775;PRJNA827465;Yang2025_Silkie_Wencha	0.957967139
ENSGALG00010016403	GRK4	3	-0.036371463	0.200871935	-0.181067918	0.856314266	0.076159664	7.551426923	73.51493936	0.666666667	CRA004775;PRJNA827465;Yang2025_Silkie_Wencha	0.957967139
ENSGALG00010008991	ENSGALG0001000899	3	0.020395455	0.112780604	0.180841866	0.8564917	0.014315718	2.919671556	31.49914429	0.666666667	CRA004775;PRJNA827465;Yang2025_Silkie_Wencha	0.958064569
ENSGALG00010010887	GABPA	3	-0.025771028	0.142647391	-0.180662458	0.856632528	0.051563775	31.70196594	93.69124299	0.666666667	CRA004775;PRJNA827465;Yang2025_Silkie_Wencha	0.958093528
ENSGALG00010012456	ENSGALG0001001245(	3	0.018249885	0.101031145	0.180636233	0.856653114	0.016308359	4.620376304	56.71348244	0.666666667	CRA004775;PRJNA827465;Yang2025_Silkie_Wencha	0.958093528
ENSGALG00010029197	LHX1	2	-0.080334373	0.445614925	-0.180277564	0.856934668	0.26803077	3.062170081	67.34342072	0.5	PRJNA827465;Yang2025_Silkie_Wenchang	0.958105272
ENSGALG00010015589	FRMD3	3	-0.055080767	0.305500178	-0.180297005	0.856919407	0.208989804	9.477542904	78.89748408	0.666666667	CRA004775;PRJNA827465;Yang2025_Silkie_Wencha	0.958105272
ENSGALG00010010848	RGR	2	-0.053203531	0.294862924	-0.180434793	0.856811241	0	0.353367051	0	0.5	PRJNA827465;Yang2025_Silkie_Wenchang	0.958105272
ENSGALG00010007584	ELP2	3	-0.011638763	0.064538172	-0.180339205	0.856886279	0	1.937263396	0	0.666666667	CRA004775;PRJNA827465;Yang2025_Silkie_Wencha	0.958105272
ENSGALG00010025204	TRAF6	3	-0.0333375	0.185040425	-0.180163333	0.857024344	0.089441998	22.67620132	91.18018061	0.666666667	CRA004775;PRJNA827465;Yang2025_Silkie_Wencha	0.958129769
ENSGALG00010008120	CDC5L	3	-0.006414639	0.035626917	-0.180050343	0.857113046	0	0.072600302	0	0.666666667	CRA004775;PRJNA827465;Yang2025_Silkie_Wencha	0.958153175
ENSGALG00010006635	ENSGALG0001000663:	3	-0.101261292	0.565939435	-0.178926023	0.857995789	0.666783608	6.766631879	70.4431978	0.666666667	CRA004775;PRJNA827465;Yang2025_Silkie_Wencha	0.958328436
ENSGALG00010021716	TFCP2L1	3	-0.049005596	0.273403312	-0.179242877	0.857746998	0.084344283	3.207909589	37.65410326	0.666666667	CRA004775;PRJNA827465;Yang2025_Silkie_Wencha	0.958328436
ENSGALG00010029150	NARF	3	-0.040855384	0.228270713	-0.178977773	0.857955154	0.128737092	18.3076184	89.07558615	0.666666667	CRA004775;PRJNA827465;Yang2025_Silkie_Wencha	0.958328436
ENSGALG00010022084	SELENOM	3	-0.031484706	0.175251905	-0.179653999	0.857424209	0.057349341	5.365197882	62.72271696	0.666666667	CRA004775;PRJNA827465;Yang2025_Silkie_Wencha	0.958328436
ENSGALG00010004425	CREM	3	-0.029824159	0.166184329	-0.179464327	0.857573125	0.049948178	6.257376586	68.0377236	0.666666667	CRA004775;PRJNA827465;Yang2025_Silkie_Wencha	0.958328436
ENSGALG00010025377	MYO1G	3	0.027231776	0.152057515	0.179088659	0.857868087	0.007640717	2.219938006	9.90739406	0.666666667	CRA004775;PRJNA827465;Yang2025_Silkie_Wencha	0.958328436
ENSGALG00010004967	SLC35F2	3	-0.026625347	0.148738871	-0.179007324	0.857931951	0	0.63276983	0	0.666666667	CRA004775;PRJNA827465;Yang2025_Silkie_Wencha	0.958328436
ENSGALG00010018968	GLI2	3	0.022753597	0.126602055	0.179725339	0.8573682	0.017570737	2.828159199	29.28262311	0.666666667	CRA004775;PRJNA827465;Yang2025_Silkie_Wencha	0.958328436
ENSGALG00010022990	SLC25A22	3	-0.021542951	0.120418245	-0.178901054	0.858015395	0.017630871	3.254869532	38.55360467	0.666666667	CRA004775;PRJNA827465;Yang2025_Silkie_Wencha	0.958328436
ENSGALG00010021211	FEN1	3	-0.017611219	0.098360753	-0.179047216	0.857900627	0.010695385	3.082415386	35.1158183	0.666666667	CRA004775;PRJNA827465;Yang2025_Silkie_Wencha	0.958328436
ENSGALG00010009431	SMN	3	0.009083423	0.050734792	0.179037356	0.857908369	0	0.606134869	0	0.666666667	CRA004775;PRJNA827465;Yang2025_Silkie_Wencha	0.958328436
ENSGALG00010022210	EZH1	3	0.021171992	0.118508467	0.178653834	0.858209521	0.033338745	9.84372268	79.68248329	0.666666667	CRA004775;PRJNA827465;Yang2025_Silkie_Wencha	0.958393841
ENSGALG00010012932	ATF6	3	-0.01438395	0.080496019	-0.178691449	0.858179984	0.009271871	3.672599028	45.54265291	0.666666667	CRA004775;PRJNA827465;Yang2025_Silkie_Wencha	0.958393841
ENSGALG00010013675	CHRNA6	3	0.164310315	0.923391915	0.17794212	0.858768432	2.242017613	17.64806178	88.66731075	0.666666667	CRA004775;PRJNA827465;Yang2025_Silkie_Wencha	0.958518835
ENSGALG00010003772	ENSGALG0001000377:	3	-0.0771342	0.432689343	-0.178266927	0.858513351	0.242679459	3.509720516	43.01540561	0.666666667	CRA004775;PRJNA827465;Yang2025_Silkie_Wencha	0.958518835
ENSGALG00010002582	ENSGALG0001000258:	2	-0.043659191	0.244902721	-0.178271563	0.858509711	0.026804488	1.287161663	22.30968118	0.5	PRJNA827465;Yang2025_Silkie_Wenchang	0.958518835
ENSGALG00010027078	PDE12	3	-0.025757347	0.144779822	-0.177907021	0.858795997	0.029420423	3.680128872	45.65407708	0.666666667	CRA004775;PRJNA827465;Yang2025_Silkie_Wencha	0.958518835
ENSGALG00010020728	TMEM37	3	-0.022918987	0.12878305	-0.177965864	0.858749784	0	1.990714536	0	0.666666667	CRA004775;PRJNA827465;Yang2025_Silkie_Wencha	0.958518835
ENSGALG00010022603	ATRNL1	3	0.018922834	0.106346902	0.177934984	0.858774036	0	0.973405168	0	0.666666667	CRA004775;PRJNA827465;Yang2025_Silkie_Wencha	0.958518835
ENSGALG00010024778	RAB5B	3	-0.01491545	0.083764334	-0.178064445	0.858672365	0.007134574	2.645287559	24.39385303	0.666666667	CRA004775;PRJNA827465;Yang2025_Silkie_Wencha	0.958518835

ENSGALG00010016602	HSPB7	3	0.042540971	0.240048072	0.17721855	0.859336726	0	0.752100087	0	0.666666667	CRA004775;PRJNA827465;Yang2025_Silkie_Wencha	0.95859266
ENSGALG00010000611	C7orf57	2	-0.03760754	0.211700931	-0.17764466	0.859002048	0	0.227652925	0	0.5	PRJNA827465;Yang2025_Silkie_Wenchang	0.95859266
ENSGALG00010010528	GPD2	3	0.030290066	0.170898634	0.177239952	0.859319915	0.059743777	10.05683324	80.11302412	0.666666667	CRA004775;PRJNA827465;Yang2025_Silkie_Wencha	0.95859266
ENSGALG00010029508	ENSGALG0001002950	3	-0.0296632	0.167323873	-0.177280141	0.859288349	0.027793846	2.945731046	32.10513897	0.666666667	CRA004775;PRJNA827465;Yang2025_Silkie_Wencha	0.95859266
ENSGALG00010007566	FHIP1A	3	-0.021548595	0.121376818	-0.177534685	0.859088423	0	0.267952905	0	0.666666667	CRA004775;PRJNA827465;Yang2025_Silkie_Wencha	0.95859266
ENSGALG00010013081	NCOA5	3	0.018266052	0.10283619	0.177622801	0.859019217	0.022031016	7.068439871	71.70521308	0.666666667	CRA004775;PRJNA827465;Yang2025_Silkie_Wencha	0.95859266
ENSGALG00010021989	NCAPD2	3	-0.011529939	0.065002884	-0.177375808	0.859213208	0	0.58718001	0	0.666666667	CRA004775;PRJNA827465;Yang2025_Silkie_Wencha	0.95859266
ENSGALG00010018051	GNPNAT1	3	-0.008751709	0.049442542	-0.177007675	0.859502361	0	0.16213828	0	0.666666667	CRA004775;PRJNA827465;Yang2025_Silkie_Wencha	0.95870179
ENSGALG00010018265	PDCD4	3	0.012604445	0.071292527	0.176798963	0.859666303	0.005227037	2.985023593	32.99885452	0.666666667	CRA004775;PRJNA827465;Yang2025_Silkie_Wencha	0.958809013
ENSGALG00010022972	ENSGALG0001002297	3	-0.245918957	1.398144464	-0.175889519	0.86038074	5.220780629	18.45487258	89.16275368	0.666666667	CRA004775;PRJNA827465;Yang2025_Silkie_Wencha	0.959226477
ENSGALG00010029208	CACNG5	2	-0.097773359	0.556536389	-0.175689068	0.860538225	0.532853694	6.866552482	85.43665103	0.5	PRJNA827465;Yang2025_Silkie_Wenchang	0.959226477
ENSGALG00010017809	MOSMO	3	0.035073962	0.199337774	0.175938292	0.860342422	0.094456316	12.71308896	84.26818213	0.666666667	CRA004775;PRJNA827465;Yang2025_Silkie_Wencha	0.959226477
ENSGALG00010020113	CIAO1	3	-0.029997677	0.170432185	-0.176009462	0.860286509	0.059710056	6.944358354	71.19964296	0.666666667	CRA004775;PRJNA827465;Yang2025_Silkie_Wencha	0.959226477
ENSGALG00010011371	ENSGALG0001001137	3	-0.02538485	0.144534601	-0.175631643	0.860583341	0	0.174861624	0	0.666666667	CRA004775;PRJNA827465;Yang2025_Silkie_Wencha	0.959226477
ENSGALG00010011283	LONRF3	3	-0.017910083	0.101689694	-0.17612486	0.860195851	0	1.382380164	0	0.666666667	CRA004775;PRJNA827465;Yang2025_Silkie_Wencha	0.959226477
ENSGALG00010027594	ITPR3	3	-0.013622901	0.077493216	-0.175794761	0.860455185	0	0.252182596	0	0.666666667	CRA004775;PRJNA827465;Yang2025_Silkie_Wencha	0.959226477
ENSGALG00010023178	CFAP20	3	0.011393782	0.064757963	0.175944108	0.860337853	0.004409396	2.862123846	30.12182185	0.666666667	CRA004775;PRJNA827465;Yang2025_Silkie_Wencha	0.959226477
ENSGALG00010004143	TCF24	3	0.053017327	0.302207825	0.175433338	0.860739147	0.121693153	3.644547619	45.12350477	0.666666667	CRA004775;PRJNA827465;Yang2025_Silkie_Wencha	0.959258094
ENSGALG00010013016	QRSL1	3	-0.016746438	0.095473171	-0.175404649	0.860761688	0.007173188	2.560123218	21.87876013	0.666666667	CRA004775;PRJNA827465;Yang2025_Silkie_Wencha	0.959258094
ENSGALG00010018434	HSPBAP1	3	0.015571842	0.088811489	0.175336489	0.860815242	0	0.402061806	0	0.666666667	CRA004775;PRJNA827465;Yang2025_Silkie_Wencha	0.959258094
ENSGALG00010016704	CSTF2	3	0.034959386	0.199774409	0.174994318	0.861084098	0.104426285	19.28532366	89.62941958	0.666666667	CRA004775;PRJNA827465;Yang2025_Silkie_Wencha	0.959482076
ENSGALG00010008081	TOP1MT	3	-0.012203316	0.069886271	-0.174616784	0.861380759	0	0.936036422	0	0.666666667	CRA004775;PRJNA827465;Yang2025_Silkie_Wencha	0.959737002
ENSGALG00010022953	ENSGALG0001002295	2	0.162632091	0.931991483	0.174478056	0.861489775	0	0.197061046	0	0.5	PRJNA827465;Yang2025_Silkie_Wenchang	0.959782833
ENSGALG00010005228	ZNF521	3	-0.012123915	0.069604341	-0.174183321	0.861721393	0	0.065602294	0	0.666666667	CRA004775;PRJNA827465;Yang2025_Silkie_Wencha	0.959962236
ENSGALG00010024528	TBX1	3	0.095392948	0.548067982	0.174053131	0.861823707	0.826911376	27.7367951	92.78936159	0.666666667	CRA004775;PRJNA827465;Yang2025_Silkie_Wencha	0.960003576
ENSGALG00010006735	NR3C2	3	0.02323216	0.13354826	0.173960787	0.86189628	0.030312717	4.827599423	58.57154199	0.666666667	CRA004775;PRJNA827465;Yang2025_Silkie_Wencha	0.960008784
ENSGALG00010021168	IVNS1ABP	3	0.030434934	0.175211066	0.173818552	0.862008064	0.000752854	18.81725308	89.37145613	0.666666667	CRA004775;PRJNA827465;Yang2025_Silkie_Wencha	0.960057662
ENSGALG00010016375	CCNI2	3	0.033002196	0.190113483	0.173592084	0.862186053	0.071303973	8.637105868	76.84409534	0.666666667	CRA004775;PRJNA827465;Yang2025_Silkie_Wencha	0.960104639
ENSGALG00010021187	SEMA7A	3	-0.016274468	0.093733952	-0.173624041	0.862160937	0.010483153	3.391824745	41.03468927	0.666666667	CRA004775;PRJNA827465;Yang2025_Silkie_Wencha	0.960104639
ENSGALG00010000049	FES	3	0.038268099	0.220690195	0.173401902	0.86233553	0.090076496	5.710342454	64.97583085	0.666666667	CRA004775;PRJNA827465;Yang2025_Silkie_Wencha	0.960119857
ENSGALG00010019624	SRGAP3	3	-0.020613091	0.118851166	-0.173436169	0.862308597	0.018573004	3.499581209	42.85030464	0.666666667	CRA004775;PRJNA827465;Yang2025_Silkie_Wencha	0.960119857
ENSGALG00010002922	TMEM106B	3	-0.018172302	0.104898249	-0.173237421	0.862464811	0.015032701	3.815587902	47.58343795	0.666666667	CRA004775;PRJNA827465;Yang2025_Silkie_Wencha	0.960188186
ENSGALG00010021841	CENPT	3	-0.026342335	0.152193087	-0.173084965	0.862584642	0.039213793	5.56814515	64.08139612	0.666666667	CRA004775;PRJNA827465;Yang2025_Silkie_Wencha	0.960245985
ENSGALG00010012869	ENSGALG0001001286	3	0.045356086	0.263198956	0.172326239	0.863181057	0.124983891	5.062097977	60.49068965	0.666666667	CRA004775;PRJNA827465;Yang2025_Silkie_Wencha	0.960253797
ENSGALG00010012978	HSD17B7	3	-0.040697099	0.236348434	-0.172191109	0.863287288	0.116292544	6.540372042	69.42069981	0.666666667	CRA004775;PRJNA827465;Yang2025_Silkie_Wencha	0.960253797
ENSGALG00010003848	ESD	3	0.033645157	0.195009444	0.172530913	0.86302016	0.081108366	7.066037709	71.69559402	0.666666667	CRA004775;PRJNA827465;Yang2025_Silkie_Wencha	0.960253797
ENSGALG00010024474	STRIP1	3	-0.024284146	0.141285419	-0.171880057	0.863531826	0.043327655	8.573852812	76.67326412	0.666666667	CRA004775;PRJNA827465;Yang2025_Silkie_Wencha	0.960253797
ENSGALG00010028185	GLMP	3	0.020394935	0.11866034	0.171876593	0.863534549	0.023659339	4.498076329	55.53654821	0.666666667	CRA004775;PRJNA827465;Yang2025_Silkie_Wencha	0.960253797
ENSGALG00010006155	TNFRSF19	3	-0.017132966	0.099618714	-0.171985411	0.863448998	0.011407984	3.286487149	39.14417491	0.666666667	CRA004775;PRJNA827465;Yang2025_Silkie_Wencha	0.960253797
ENSGALG00010022712	DDX23	3	0.017024324	0.098887669	0.172158209	0.863313152	0.019296653	8.301531249	75.90805913	0.666666667	CRA004775;PRJNA827465;Yang2025_Silkie_Wencha	0.960253797
ENSGALG00010000428	GNL3L	3	-0.015790209	0.091891416	-0.171835522	0.863566838	0.00747423	2.703700309	26.02730438	0.666666667	CRA004775;PRJNA827465;Yang2025_Silkie_Wencha	0.960253797
ENSGALG00010018394	KLHL28	3	-0.014568816	0.084744057	-0.171915489	0.86350397	0.011014106	3.973432224	49.66568228	0.666666667	CRA004775;PRJNA827465;Yang2025_Silkie_Wencha	0.960253797
ENSGALG00010029655	PHKG1	3	-0.013848166	0.080055074	-0.172982994	0.862664795	0	0.642395997	0	0.666666667	CRA004775;PRJNA827465;Yang2025_Silkie_Wencha	0.960253797
ENSGALG00010025869	FASTKD2	3	0.013555513	0.078620045	0.172418022	0.863108905	0.006568592	2.884672193	30.66803206	0.666666667	CRA004775;PRJNA827465;Yang2025_Silkie_Wencha	0.960253797
ENSGALG00010020467	ALDH4A1	3	0.012444612	0.072445001	0.17178012	0.863610389	0.002842449	2.347902883	14.81760107	0.666666667	CRA004775;PRJNA827465;Yang2025_Silkie_Wencha	0.960253797
ENSGALG00010018526	ENSGALG0001001852	3	0.010750913	0.062536121	0.171915245	0.863504161	0	1.119015962	0	0.666666667	CRA004775;PRJNA827465;Yang2025_Silkie_Wencha	0.960253797
ENSGALG00010026011	GJC2	3	-0.010377079	0.060346342	-0.171958708	0.863469992	0	1.715584323	0	0.666666667	CRA004775;PRJNA827465;Yang2025_Silkie_Wencha	0.960253797
ENSGALG00010013251	MTA1	3	-0.007815136	0.045257131	-0.172682977	0.862900625	0.000739271	2.114303405	5.406196873	0.666666667	CRA004775;PRJNA827465;Yang2025_Silkie_Wencha	0.960253797
ENSGALG00010016461	HPCAL4	2	0.03064857	0.178911293	0.171305954	0.863983199	0	0.060371162	0	0.5	PRJNA827465;Yang2025_Silkie_Wenchang	0.960290736
ENSGALG00010017368	ERLIN2	3	-0.021500047	0.12529244	-0.171598916	0.863752859	0.039631392	18.86599371	89.39891516	0.666666667	CRA004775;PRJNA827465;Yang2025_Silkie_Wencha	0.960290736
ENSGALG00010008433	EBAG9	3	-0.01674872	0.097766653	-0.171323736	0.863969217	0.014250414	3.65957202	44.34880337	0.666666667	CRA004775;PRJNA827465;Yang2025_Silkie_Wencha	0.960290736
ENSGALG00010011529	SCYL2	3	-0.013613531	0.079391234	-0.171473984	0.863851086	0.007184464	3.163671997	36.78232125	0.666666667	CRA004775;PRJNA827465;Yang2025_Silkie_Wencha	0.960290736
ENSGALG00010012640	PRRC1	3	0.013165052	0.076816893	0.171382248	0.863923212	0.00949642	4.483683881	55.39382229	0.666666667	CRA004775;PRJNA827465;Yang2025_Silkie_Wencha	0.960290736
ENSGALG00010017207	ENSGALG0001001720	3	0.041994963	0.245526993	0.171040107	0.86419223	0.091664146	4.380580203	54.34394743	0.666666667	CRA004775;PRJNA827465;Yang2025_Silkie_Wencha	0.960400762
ENSGALG00010013651	ENSGALG0001001365	3	0.021654248	0.126623638	0.171007279	0.864218042	0.032368115	6.228418766	67.88912122	0.666666667	CRA004775;PRJNA827465;Yang2025_Silkie_Wencha	0.960400762
ENSGALG00010027553	FGF22	3	0.034083773	0.199681633	0.170690577	0.864467073	0.015175974	2.247738388	11.02167358	0.666666667	CRA004775;PRJNA827465;Yang2025_Silkie_Wencha	0.960602007
ENSGALG00010013579	H2AC32	2	0.271088548	1.596674051	0.169783274	0.865180581	4.171386537	4.700209784	78.72435389	0.5	CRA004775;Yang2025_Silkie_Wenchang	0.96060415
ENSGALG00010005264	ITIH2	3	-0.14440553	0.856389006	-0.16862142	0.866094431	1.88334415	16.54820603	87.91409778	0.666666667	CRA004775;PRJNA827465;Yang2025_Silkie_Wencha	0.96060415
ENSGALG00010003552	PI15	3	0.089101401	0.525592675	0.169525675	0.865383179	0.740713592	30.79106082	93.50460833	0.666666667	CRA004775;PRJNA827465;Yang2025_Silkie_Wencha	0.96060415
ENSGALG00010019205	ENSGALG0001001920	2	-0.057331768	0.337261447	-0.169992059	0.865016382	0	0.706439062	0	0.5	PRJNA827465;Yang2025_Silkie_Wenchang	0.96060415
ENSGALG00010009738	ENSGALG0001000973	3	0.049740337	0.293597689	0.16941665	0.865468928	0.171933411	6.47709299	69.12195019	0.666666667	CRA004775;PRJNA827465;Yang2025_Silkie_Wencha	0.96060415
ENSGALG00010005941	TXLNB	3	-0.045566398	0.268338327	-0.169809502	0.865159954	0.145327115	7.965218771	74.89083404	0.666666667	CRA004775;PRJNA827465;Yang2025_Silkie_Wencha	0.96060415
ENSGALG00010028523	SUGP1	3	-0.043297012	0.256962521	-0.168495437	0.866193533	0.181578797	28.50972995	92.98485112	0.666666667	CRA004775;PRJNA827465;Yang2025_Silkie_Wencha	0.96060415
ENSGALG00010022065	PABPC1L	3	-0.041525031	0.245632854	-0.169053245	0.865754761	0.139648056	10.15027824	80.29610664	0.666666667	CRA004775;PRJNA827465;Yang2025_Silkie_Wencha	0.96060415
ENSGALG00010027619	JAK3	3	-0.039166072	0.23259958	-0.168384104	0.866281113	0.035616668	2.553922602	21.6890912	0.666666667	CRA004775;PRJNA827465;Yang2025_Silkie_Wencha	0.96060415
ENSGALG00010017484	IQCC	3	0.035666809	0.211327374	0.168775149	0.865973506	0.101489428	8.606794266	76.76254436	0.666666667	CRA004775;PRJNA827465;Yang2025_Silkie_Wencha	0.96060415
ENSGALG00010012801	DMC1	3	0.033006388	0.194706395	0.169518765	0.865388614	0	0.802951164	0	0.666666667	CRA004775;PRJNA827465;Yang2025_Silkie_Wencha	0.96060415
ENSGALG00010013494	ASB1	3	-0.032440703	0.19056659	-0.170232901	0.864826978	0.07680481	10.14291873	80.28180987	0.666666667	CRA004775;PRJNA827465;Yang2025_Silkie_Wencha	0.96060415
ENSGALG00010014722	KCTD17	3	-0.030309605	0.180033035	-0.168355796	0.866303382	0	0.321711677	0	0.666666667	CRA004775;PRJNA827465;Yang2025_Silkie_Wencha	0.96060415
ENSGALG00010027776	TKT	3	0.028945401	0.171890061	0.168394852	0.866272658	0.05903689	7.990766212	74.97111107	0.666666667	CRA004775;PRJNA827465;Yang2025_Silkie_Wencha	0.96060415
ENSGALG00010023984	FMNL1	3	0.026147114	0.154387122	0.169360717	0.86551292	0.032097045	3.648496656	45.18290165	0.666666667	CRA004775;PRJNA827465;Yang2025_Silkie_Wencha	0.96060415
ENSGALG00010009570	SHARPIN	3	-0.022123876	0.130084966	-0.170072502	0.864953119	0.026522604	4.735644639	57.76710137	0.666666667	CRA004775;PRJNA827465;Yang2025_Silkie_Wencha	0.96060415
ENSGALG00010000286	TCN2	3	0.02061224	0.122110891	0.168799355	0.865954466	0.018074826	3.254987468	38.55583103	0.666666667	CRA004775;PRJNA827465;Yang2025_Silkie_Wencha	0.96060415
ENSGALG00010022164	PDGFA	3	0.019055286	0.113006535	0.168621099	0.866094683	0.022916971	5.870431234	65.93095259	0.666666667	CRA004775;PRJNA827465;Yang2025_Silkie_Wencha	0.96060415
ENSGALG00010017223	NOC2L	3	0.01903383	0.111930415	0.17005056	0.864970375	0.020591883	4.300857976	53.49765067	0.666666667	CRA004775;PRJNA827465;Yang2025_Silkie_Wencha	0.96060415
ENSGALG00010006065	WDR54	3	-0.018660721	0.11024355	-0.16926814	0.865585735	0	1.780640984	0	0.666666667	CRA004775;PRJNA827465;Yang2025_Silkie_Wencha	0.96060415
ENSGALG00010020187	MMP23B	3	-0.014744374	0.087060418	-0.16935795	0.865515097	0	0.299648853	0	0.666666667	CRA004775;PRJNA827465;Yang2025_Silkie_Wencha	0.96060415
ENSGALG00010007529	CDH13	3	0.014421963	0.085428139	0.168819816	0.865938371	0	1.806304956	0	0.666666667	CRA004775;PRJNA827465;Yang2025_Silkie_Wencha	0.96060415
ENSGALG00010027247	ATG7	3	0.011782769	0.069740313	0.168952059	0.86583435	0.004195417	2.749261877	27.25320143	0.666666667	CRA004775;PRJNA827465;Yang2025_Silkie_Wencha	0.96060415
ENSGALG00010015749	ATR	3	-0.010747378	0.063010537	-0.170564781	0.864565993	0	0.434576739	0	0.666666667	CRA004775;PRJNA827465;Yang2025_Silkie_Wencha	0.96060415
ENSGALG00010020183	XPNPEP1	3	0.010531138	0.062108602	0.169560057	0.865356138	0.002531965	2.464616769	18.8514813	0.666666667	CRA004775;PRJNA827465;Yang2025_Silkie_Wencha	0.96060415
ENSGALG00010024195	PSMA4	3	-0.00865724	0.05094706	-0.169926187	0.865068187	0	1.275608668	0	0.666666667	CRA004775;PRJNA827465;Yang2025_Silkie_Wencha	0.96060415
ENSGALG00010019916	NOC3L	3	-0.007376885	0.04377027	-0.168536428	0.866161288	0	0.453954673	0	0.666666667	CRA004775;PRJNA827465;Yang2025_Silkie_Wencha	0.96060415
ENSGALG00010021913	ENSGALG0001002191	3	-0.025276897	0.150512167	-0.167939226	0.866631091	0.033035391	3.982255278	49.7772026	0.666666667	CRA004775;PRJNA827465;Yang2025_Silkie_Wencha	0.960892173
ENSGALG00010019778	ABHD17C	3	-0.020866349	0.12448683	-0.167618925	0.866883082	0.0275298	5.225812532	61.72843959	0.666666667	CRA004775;PRJNA827465;Yang2025_Silkie_Wencha	0.961096204
ENSGALG00010004778	ENSGALG0001000477	3	-0.166900107	0.997615584	-0.167299018	0.867134777	2.435988961	14.3945377	86.10584069	0.666666667	CRA004775;PRJNA827465;Yang2025_Silkie_Wencha	0.961164993
ENSGALG00010003255	CALCR	3	-0.029522699	0.176851185	-0.166935265	0.867420985	0	1.569698168	0	0.666666667	CRA004775;PRJNA827465;Yang2025_Silkie_Wencha	0.961164993
ENSGALG00010008447	FRRS1L	3	-0.017856412	0.106905225	-0.167030303	0.867346206	0.017295212	4.189926613	52.26646706	0.666666667	CRA004775;PRJNA827465;Yang2025_Silkie_Wencha	0.961164993
ENSGALG00010010218	PBK	3	-0.016226771	0.097103268	-0.167108394	0.867284762	0	1.532058718	0	0.666666667	CRA004775;PRJNA827465;Yang2025_Silkie_Wencha	0.961164993
ENSGALG00010029220	NEK8	3	-0.012813828	0.076749253	-0.166957039	0.867403853	0	1.65468823	0	0.666666667	CRA004775;PRJNA827465;Yang2025_Silkie_Wencha	0.961164993
ENSGALG00010007871	HSPA4L	3	0.011525926	0.068966997	0.167122345	0.867273785	0	0.603823405	0	0.666666667	CRA004775;PRJNA827465;Yang2025_Silkie_Wencha	0.961164993
ENSGALG00010027260	LEMD2	3	0.006491538	0.03882193	0.167213162	0.867202328	0	0.877453754	0	0.666666667	CRA004775;PRJNA827465;Yang2025_Silkie_Wencha	0.961164993
ENSGALG00010006207	ENSGALG0001000620	3	0.023387224	0.140273125	0.166726337	0.867585382	0	1.588127229	0	0.666666667	CRA004775;PRJNA827465;Yang2025_Silkie_Wencha	0.961271821
ENSGALG00010021552	ENSGALG0001002155	3	0.038277051	0.22985669	0.16652572	0.867743244	0.108925526	8.020354354	75.06344593	0.666666667	CRA004775;PRJNA827465;Yang2025_Silkie_Wencha	0.961371394
ENSGALG00010007817	CDYL2	3	0.0230644	0.138704745	0.166284146	0.867933342	0.019752359	2.97945242	32.87357144	0.666666667	CRA004775;PRJNA827465;Yang2025_Silkie_Wencha	0.961506662
ENSGALG00010024132	ENSGALG0001002413	3	-0.022937901	0.13804725	-0.16615978	0.86803121	0.03335534	4.947118808	59.57242837	0.666666667	CRA004775;PRJNA827465;Yang2025_Silkie_Wencha	0.961539743
ENSGALG00010009257	ENSGALG0001000925	3	0.03777944	0.228122107	0.165610603	0.868463403	0.100028668	9.146247444	78.13310855	0.666666667	CRA004775;PRJNA827465;Yang2025_Silkie_Wencha	0.961608869
ENSGALG00010016671	CASS4	3	0.02669538	0.161072243	0.165735444	0.868365151	0.003706679	2.084111574	4.035847931	0.666666667	CRA004775;PRJNA827465;Yang2025_Silkie_Wencha	0.961608869
ENSGALG00010023244	PPIB	3	0.02043051	0.123465432	0.165475552	0.868569692	0.027533439	4.950136663	59.59707507	0.666666667	CRA004775;PRJNA827465;Yang2025_Silkie_Wencha	0.961608869
ENSGALG00010027916	ADAR	3	0.018724755	0.113016448	0.165681677	0.868407467	0.02765068	10.9031749	81.6567191	0.666666667	CRA004775;PRJNA827465;Yang2025_Silkie_Wencha	0.961608869
ENSGALG00010024074	ADCY7	3	-0.018214757	0.110018436	-0.165560949	0.868502482	0	0.390215127	0	1	CRA004775;PRJNA827465;Yang2025_Silkie_Wencha	0.961608869
ENSGALG00010017419	B9D1	3	0.015499751	0.093627852	0.165546374	0.868513953	0.011761046	3.632695652	44.94446572	0.666666667	CRA004775;PRJNA827465;Yang2025_Silkie_Wencha	0.961608869
ENSGALG00010013093	GPATCH3	3	-0.011682952	0.070438708	-0.165859832	0.868267259	0	0.592147797	0	0.666666667	CRA004775;PRJNA827465;Yang2025_Silkie_Wencha	0.961608869
ENSGALG00010022742	IFT46	3	-0.025915959	0.156880133	-0.165195927	0.868789771	0.053224834	7.512549208	73.37787821	0.666666667	CRA004775;PRJNA827465;Yang2025_Silkie_Wencha	0.961701916
ENSGALG00010009700	SH3TC1	3	-0.015278361	0.092454869	-0.165252094	0.868745564	0	0.710605197	0	0.666666667	CRA004775;PRJNA827465;Yang2025_Silkie_Wencha	0.961701916
ENSGALG00010015057	LAS1L	3	-0.038945995	0.235935653	-0.165070409	0.868888564	0.147163405	23.26745212	91.40430164	0.666666667	CRA004775;PRJNA827465;Yang2025_Silkie_Wencha	0.96173598
ENSGALG00010027525	ENSGALG0001002752	3	0.024212647	0.146817738	0.164916359	0.869009816	0	0.419929852	0	0.666666667	CRA004775;PRJNA827465;Yang2025_Silkie_Wencha	0.961777036
ENSGALG00010019300	MESDC2	3	-0.012016139	0.072891155	-0.164850438	0.869061703	0.008800485	4.568300274	56.22004071	0.666666667	CRA004775;PRJNA827465;Yang2025_Silkie_Wencha	0.961777036
ENSGALG00010022833	HRH3	2	0.044139378	0.268045967	0.164670927	0.869203002	0	0.125729109	0	0.5	PRJNA827465;Yang2025_Silkie_Wenchang	0.961782849
ENSGALG00010018864	ABCA3	3	0.008655193	0.052556676	0.164683032	0.869193473	0	0.19369737	0	0.666666667	CRA004775;PRJNA827465;Yang2025_Silkie_Wencha	0.961782849
ENSGALG00010016470	TNIP1	3	0.033256637	0.202356955	0.1643464	0.869458456	0.097816883	24.34181665	91.78368637	0.666666667	CRA004775;PRJNA827465;Yang2025_Silkie_Wencha	0.961839692
ENSGALG00010004281	VIM	3	-0.011386918	0.069264871	-0.164396732	0.869418836	0.004558644	2.884271992	30.65841205	0.666666667	CRA004775;PRJNA827465;Yang2025_Silkie_Wencha	0.961839692
ENSGALG00010012629	NUS1	3	-0.008268557	0.050275914	-0.164463589	0.869366208	0	1.04872638	0	0.666666667	CRA004775;PRJNA827465;Yang2025_Silkie_Wencha	0.961839692
ENSGALG00010012433	ENSGALG0001001243	2	-0.115793635	0.706228295	-0.163960628	0.869762138	0	0.007817609	0	1	CRA004775;Yang2025_Silkie_Wenchang	0.962100366
ENSGALG00010021837	ENSGALG0001002183	3	-0.088668659	0.542404398	-0.163473342	0.870145761	0.710312662	10.97408909	81.77525275	0.666666667	CRA004775;PRJNA827465;Yang2025_Silkie_Wencha	0.962280372
ENSGALG00010021708	CNTNAP5	2	0.08838905	0.541909909	0.163106539	0.870434551	0.43976207	3.671707661	72.76471625	0.5	PRJNA827465;Yang2025_Silkie_Wenchang	0.962280372
ENSGALG00010020521	ENSGALG0001002052	2	-0.079062115	0.485372754	-0.16288948	0.870605454	0	0.08448674	0	0.5	PRJNA827465;Yang2025_Silkie_Wenchang	0.962280372
ENSGALG00010002857	DCAF15	3	0.039053055	0.239381923	0.163141205	0.870407257	0.14217093	12.45038817	83.93624382	0.666666667	CRA004775;PRJNA827465;Yang2025_Silkie_Wencha	0.962280372
ENSGALG00010011932	VAV3	3	0.027185512	0.166741499	0.16303987	0.870487043	0.03202998	3.258313072	38.61854415	0.666666667	CRA004775;PRJNA827465;Yang2025_Silkie_Wencha	0.962280372
ENSGALG00010011976	TNFRSF11A	3	-0.025519231	0.156368643	-0.163199157	0.87036163	0.046867169	5.807983291	65.56463922	0.666666667	CRA004775;PRJNA827465;Yang2025_Silkie_Wencha	0.962280372
ENSGALG00010008099	PANK3	3	-0.024682698	0.150971356	-0.163492587	0.870130609	0.047848485	10.17094246	80.33613888	0.666666667	CRA004775;PRJNA827465;Yang2025_Silkie_Wencha	0.962280372
ENSGALG00010004214	REPS1	3	0.021789603	0.13372563	0.162942609	0.870563622	0.034845143	5.752970586	65.23535155	0.666666667	CRA004775;PRJNA827465;Yang2025_Silkie_Wencha	0.962280372
ENSGALG00010027720	DAB2IP	3	-0.018021881	0.110251432	-0.16346165	0.870154966	0.017388566	3.676365925	45.59845128	0.666666667	CRA004775;PRJNA827465;Yang2025_Silkie_Wencha	0.962280372
ENSGALG00010023588	FARSB	3	0.013089546	0.080270546	0.163067862	0.870465003	0.007479737	3.132731512	36.15795058	0.666666667	CRA004775;PRJNA827465;Yang2025_Silkie_Wencha	0.962280372
ENSGALG00010014944	FGB	3	0.178328869	1.110698953	0.160555539	0.872443475	3.232098653	16.49289319	87.87356483	0.666666667	CRA004775;PRJNA827465;Yang2025_Silkie_Wencha	0.962477257
ENSGALG00010023368	ENSGALG0001002336	3	0.145150366	0.905048767	0.160378502	0.872582923	2.207553866	24.94696547	91.98299287	0.666666667	CRA004775;PRJNA827465;Yang2025_Silkie_Wencha	0.962477257
ENSGALG00010018026	CCDC39	2	0.133361919	0.831777319	0.160333681	0.872618228	1.182343941	6.807967348	85.31132791	0.5	PRJNA827465;Yang2025_Silkie_Wenchang	0.962477257
ENSGALG00010016891	ENSGALG0001001689	3	-0.045433952	0.283174775	-0.160444911	0.872530614	0.150378621	5.377173574	62.80573851	0.666666667	CRA004775;PRJNA827465;Yang2025_Silkie_Wencha	0.962477257
ENSGALG00010015177	TC2N	3	0.036938359	0.22925452	0.1611238	0.871995896	0.1028954	7.743952345	74.17339479	0.666666667	CRA004775;PRJNA827465;Yang2025_Silkie_Wencha	0.962477257
ENSGALG00010008219	NIP7	3	0.034352376	0.214770425	0.159949285	0.872921025	0.094568442	6.505725084	69.25784637	0.666666667	CRA004775;PRJNA827465;Yang2025_Silkie_Wencha	0.962477257
ENSGALG00010020292	NPM2	3	0.030927305	0.194281095	0.159188444	0.873520408	0	0.818777729	0	0.666666667	CRA004775;PRJNA827465;Yang2025_Silkie_Wencha	0.962477257
ENSGALG00010014305	ARL10	3	-0.030747679	0.192963898	-0.159344203	0.873397697	0	1.767561567	0	0.666666667	CRA004775;PRJNA827465;Yang2025_Silkie_Wencha	0.962477257
ENSGALG00010016766	AOAH	3	-0.030400164	0.191498106	-0.158749161	0.873866505	0	1.071707395	0	0.666666667	CRA004775;PRJNA827465;Yang2025_Silkie_Wencha	0.962477257
ENSGALG00010001406	EML3	3	0.030313368	0.189271098	0.160158465	0.872756247	0.088897716	12.77446409	84.34376592	0.666666667	CRA004775;PRJNA827465;Yang2025_Silkie_Wencha	0.962477257
ENSGALG00010027124	IP6K2	3	-0.029421562	0.182913734	-0.160849386	0.872212028	0.084018255	14.50349801	86.21022323	0.666666667	CRA004775;PRJNA827465;Yang2025_Silkie_Wencha	0.962477257
ENSGALG00010029654	EME1	3	-0.027927788	0.173244493	-0.16120448	0.871932353	0.051642741	5.91255904	66.1736993	0.666666667	CRA004775;PRJNA827465;Yang2025_Silkie_Wencha	0.962477257
ENSGALG00010028002	ABCA2	3	-0.027402476	0.170291759	-0.160914866	0.872160454	0.067646443	10.31451869	80.60985626	0.666666667	CRA004775;PRJNA827465;Yang2025_Silkie_Wencha	0.962477257
ENSGALG00010028208	AMIGO3	3	-0.026559028	0.166220086	-0.159782304	0.873052564	0	0.740178184	0	0.666666667	CRA004775;PRJNA827465;Yang2025_Silkie_Wencha	0.962477257
ENSGALG00010006001	NOP56	3	0.026056402	0.162752328	0.160098488	0.872803492	0.063180716	11.48619863	82.58779893	0.666666667	CRA004775;PRJNA827465;Yang2025_Silkie_Wencha	0.962477257
ENSGALG00010016786	DISP1	3	-0.025861813	0.161630665	-0.160005611	0.872876655	0.062812594	11.00011771	81.81837638	0.666666667	CRA004775;PRJNA827465;Yang2025_Silkie_Wencha	0.962477257
ENSGALG00010012913	EGFLAM	3	0.024753788	0.152548608	0.162268202	0.871094654	0.046218139	9.424375063	78.77843372	0.666666667	CRA004775;PRJNA827465;Yang2025_Silkie_Wencha	0.962477257
ENSGALG00010009565	TMEM8B	3	-0.021859509	0.135774396	-0.160998755	0.872094381	0.001392309	2.045030021	2.201924713	0.666666667	CRA004775;PRJNA827465;Yang2025_Silkie_Wencha	0.962477257
ENSGALG00010000051	GDPGP1	3	-0.021593642	0.134609999	-0.160416326	0.87255313	0.016721054	2.871317978	30.34557596	0.666666667	CRA004775;PRJNA827465;Yang2025_Silkie_Wencha	0.962477257
ENSGALG00010009840	ENSGALG0001000984	3	-0.021342916	0.134505035	-0.158677448	0.873923007	0.00354895	2.13356324	6.260102249	0.666666667	CRA004775;PRJNA827465;Yang2025_Silkie_Wencha	0.962477257
ENSGALG00010028446	ENSGALG0001002844	3	0.021141679	0.132123426	0.160014615	0.872869561	0	0.544807552	0	0.666666667	CRA004775;PRJNA827465;Yang2025_Silkie_Wencha	0.962477257
ENSGALG00010002340	RAB23	3	-0.020231244	0.126607746	-0.159794681	0.873042815	0.029771349	6.008324984	66.7128525	0.666666667	CRA004775;PRJNA827465;Yang2025_Silkie_Wencha	0.962477257
ENSGALG00010003582	RDH10	3	0.018816911	0.115777781	0.162526094	0.870891582	0.021548303	4.640532231	56.90149534	0.666666667	CRA004775;PRJNA827465;Yang2025_Silkie_Wencha	0.962477257
ENSGALG00010018371	ENSGALG0001001837	3	0.018346168	0.115237303	0.159203378	0.873508643	0.010214758	2.658117128	24.75877084	0.666666667	CRA004775;PRJNA827465;Yang2025_Silkie_Wencha	0.962477257
ENSGALG00010025405	TAMALIN	3	-0.018008205	0.112403359	-0.160210561	0.872715211	0	0.518811891	0	0.666666667	CRA004775;PRJNA827465;Yang2025_Silkie_Wencha	0.962477257
ENSGALG00010014397	PRKAA1	3	-0.016515097	0.10204781	-0.161836855	0.871434329	0.014771532	3.820822194	47.65524542	0.666666667	CRA004775;PRJNA827465;Yang2025_Silkie_Wencha	0.962477257
ENSGALG00010025931	TMEM136	3	0.015875791	0.099027054	0.160317716	0.872630804	0	1.047624478	0	0.666666667	CRA004775;PRJNA827465;Yang2025_Silkie_Wencha	0.962477257
ENSGALG00010007766	ENSGALG0001000776	3	-0.01536619	0.09671391	-0.158882936	0.873761105	0	1.33781186	0	0.666666667	CRA004775;PRJNA827465;Yang2025_Silkie_Wencha	0.962477257
ENSGALG00010011198	PINX1	3	0.014838704	0.093466888	0.158758944	0.873858796	0.010590916	3.307022441	39.52263597	0.666666667	CRA004775;PRJNA827465;Yang2025_Silkie_Wencha	0.962477257
ENSGALG00010002425	FDX2	3	0.014658442	0.091169268	0.160782709	0.872264544	0	0.107139346	0	0.666666667	CRA004775;PRJNA827465;Yang2025_Silkie_Wencha	0.962477257
ENSGALG00010012792	CBY1	3	-0.014474759	0.090741957	-0.159515613	0.873262658	0	0.538464585	0	0.666666667	CRA004775;PRJNA827465;Yang2025_Silkie_Wencha	0.962477257
ENSGALG00010001285	ENSGALG0001000128	3	0.01427169	0.089963738	0.158638254	0.873953888	0	0.592504746	0	0.666666667	CRA004775;PRJNA827465;Yang2025_Silkie_Wencha	0.962477257
ENSGALG00010016863	MYNN	3	0.014219642	0.089511969	0.158857437	0.873781195	0.009750939	3.344373906	40.19807425	0.666666667	CRA004775;PRJNA827465;Yang2025_Silkie_Wencha	0.962477257
ENSGALG00010011899	PHLPP1	3	0.01414153	0.087959174	0.160773792	0.872271568	0.011516754	4.183806454	52.19664146	0.666666667	CRA004775;PRJNA827465;Yang2025_Silkie_Wencha	0.962477257
ENSGALG00010009477	ZNF365	3	-0.013787031	0.085055628	-0.162094285	0.871231606	0.007767008	2.992524994	33.16680716	0.666666667	CRA004775;PRJNA827465;Yang2025_Silkie_Wencha	0.962477257
ENSGALG00010017807	MRPL35	3	0.013732103	0.085046384	0.161466038	0.871726358	0.007151135	2.805625347	28.71464459	0.666666667	CRA004775;PRJNA827465;Yang2025_Silkie_Wencha	0.962477257
ENSGALG00010017587	MB21D2	3	-0.013297952	0.083845153	-0.158601318	0.873982991	0	1.324122257	0	0.666666667	CRA004775;PRJNA827465;Yang2025_Silkie_Wencha	0.962477257
ENSGALG00010016022	EPHB3	3	0.010752605	0.066844688	0.160859521	0.872204045	0.003115948	2.538350652	21.2086794	0.666666667	CRA004775;PRJNA827465;Yang2025_Silkie_Wencha	0.962477257
ENSGALG00010024977	LRP3	3	-0.009984769	0.061902127	-0.161299291	0.871857682	0	1.07858902	0	0.666666667	CRA004775;PRJNA827465;Yang2025_Silkie_Wencha	0.962477257
ENSGALG00010026232	PSMC5	3	0.009296273	0.058033715	0.160187459	0.872733408	0	1.006839326	0	0.666666667	CRA004775;PRJNA827465;Yang2025_Silkie_Wencha	0.962477257
ENSGALG00010024928	THAP4	3	-0.009089468	0.057031259	-0.159376951	0.873371898	0	0.536899484	0	0.666666667	CRA004775;PRJNA827465;Yang2025_Silkie_Wencha	0.962477257
ENSGALG00010016385	HTT	3	-0.008174761	0.050620273	-0.161491837	0.87170604	0	1.427775328	0	0.666666667	CRA004775;PRJNA827465;Yang2025_Silkie_Wencha	0.962477257
ENSGALG00010007938	BCL9	3	-0.008071366	0.049904693	-0.161735609	0.871514062	0	0.32795645	0	0.666666667	CRA004775;PRJNA827465;Yang2025_Silkie_Wencha	0.962477257
ENSGALG00010029511	CUEDC1	3	-0.008002693	0.050415897	-0.158733525	0.873878824	0	1.189839044	0	0.666666667	CRA004775;PRJNA827465;Yang2025_Silkie_Wencha	0.962477257
ENSGALG00010029521	ACTG1	3	0.007711607	0.048581385	0.158735838	0.873877002	0	1.641057196	0	0.666666667	CRA004775;PRJNA827465;Yang2025_Silkie_Wencha	0.962477257
ENSGALG00010011775	VEZT	3	-0.0073264	0.045525623	-0.160929144	0.872149208	0	0.113821238	0	0.666666667	CRA004775;PRJNA827465;Yang2025_Silkie_Wencha	0.962477257
ENSGALG00010021290	ERCC3	3	0.005122813	0.031953712	0.160319819	0.872629147	0	1.969448832	0	0.666666667	CRA004775;PRJNA827465;Yang2025_Silkie_Wencha	0.962477257
ENSGALG00010001901	PRPS2	3	0.079928359	0.50451618	0.158425759	0.874121317	0.680010251	32.23622885	93.79580034	0.666666667	CRA004775;PRJNA827465;Yang2025_Silkie_Wencha	0.962554619
ENSGALG00010025886	ALDH2	3	0.012886101	0.081421754	0.158263613	0.87424908	0.00631571	2.743136979	27.0907718	0.666666667	CRA004775;PRJNA827465;Yang2025_Silkie_Wencha	0.962620336
ENSGALG00010012168	ENSGALG0001001216	2	-0.161636914	1.025136249	-0.157673592	0.874714011	1.706528735	4.994053456	79.9761855	0.5	PRJNA827465;Yang2025_Silkie_Wenchang	0.962693607
ENSGALG00010024186	HAO2	2	0.122819513	0.779415515	0.157578994	0.874788557	0.833921966	3.185970803	68.61239283	0.5	PRJNA827465;Yang2025_Silkie_Wenchang	0.962693607
ENSGALG00010014303	GABRA2	3	0.109282747	0.693365113	0.157612122	0.874762451	1.173146832	12.4819308	83.97683794	0.666666667	CRA004775;PRJNA827465;Yang2025_Silkie_Wencha	0.962693607
ENSGALG00010016881	C1QB	3	-0.069306863	0.440802799	-0.157228726	0.87506459	0.456395699	9.486051396	78.91641193	0.666666667	CRA004775;PRJNA827465;Yang2025_Silkie_Wencha	0.962693607
ENSGALG00010007577	WFDC1	3	-0.037534366	0.23780781	-0.157834876	0.874586916	0.139922141	18.91699231	89.42749478	0.666666667	CRA004775;PRJNA827465;Yang2025_Silkie_Wencha	0.962693607
ENSGALG00010009791	GABRG2	2	0.037258417	0.23687518	0.157291352	0.875015236	0	0.87640405	0	0.5	PRJNA827465;Yang2025_Silkie_Wenchang	0.962693607
ENSGALG00010000133	USP39	3	0.029393137	0.186686673	0.157446359	0.87489308	0.083423209	11.25224297	82.22576596	0.666666667	CRA004775;PRJNA827465;Yang2025_Silkie_Wencha	0.962693607
ENSGALG00010023762	BBS5	3	-0.024582795	0.155512687	-0.158075819	0.874397055	0.036861149	4.566341725	56.20126306	0.666666667	CRA004775;PRJNA827465;Yang2025_Silkie_Wencha	0.962693607

ENSGALG00010010905	ENC1	3	0.019944686	0.12671615	0.157396561	0.874932324	0.019216916	3.355301441	40.39283698	0.666666667	CRA004775;PRJNA827465;Yang2025_Silkie_Wencha	0.962693607
ENSGALG00010023514	ULK3	3	0.019075429	0.120778256	0.15793761	0.874505961	0.020857719	3.894908897	48.65091706	0.666666667	CRA004775;PRJNA827465;Yang2025_Silkie_Wencha	0.962693607
ENSGALG00010005251	MTRF1L	3	-0.011612516	0.073805735	-0.157338937	0.874977735	0.002701071	2.357734194	15.17279576	0.666666667	CRA004775;PRJNA827465;Yang2025_Silkie_Wencha	0.962693607
ENSGALG00010001865	CYBB	3	-0.03776805	0.240412118	-0.157097113	0.875168313	0.085373537	4.231906197	52.73997326	0.666666667	CRA004775;PRJNA827465;Yang2025_Silkie_Wencha	0.962707058
ENSGALG00010014393	ATP8A1	3	-0.012665069	0.080648469	-0.157040419	0.875212994	0	0.220100365	0	0.666666667	CRA004775;PRJNA827465;Yang2025_Silkie_Wencha	0.962707058
ENSGALG00010028476	ENSGALG0001002847(	3	0.028149748	0.179544591	0.156784162	0.875414956	0.075326639	27.69321299	92.77801387	0.666666667	CRA004775;PRJNA827465;Yang2025_Silkie_Wencha	0.962809047
ENSGALG00010016103	BPNT1	3	0.01292173	0.082435294	0.156749967	0.875441906	0	1.649461726	0	0.666666667	CRA004775;PRJNA827465;Yang2025_Silkie_Wencha	0.962809047
ENSGALG00010023989	ENSGALG0001002398(	3	-0.024898037	0.159315075	-0.156281739	0.875810949	0.057134451	8.711927778	77.04296855	0.666666667	CRA004775;PRJNA827465;Yang2025_Silkie_Wencha	0.963065097
ENSGALG00010028173	GDF3	3	-0.014161267	0.090610773	-0.156286792	0.875806967	0	0.097274493	0	0.666666667	CRA004775;PRJNA827465;Yang2025_Silkie_Wencha	0.963065097
ENSGALG00010028207	ENSGALG0001002820	2	-0.176840898	1.142621884	-0.154767645	0.8770045	2.36982211	10.59223796	90.55912449	0.5	CRA004775;PRJNA827465	0.963326943
ENSGALG00010003131	TAP1	3	0.062370219	0.402218966	0.155065336	0.87676981	0.379433559	9.172632116	78.1960077	0.666666667	CRA004775;PRJNA827465;Yang2025_Silkie_Wencha	0.963326943
ENSGALG00010022101	ENSGALG0001002210	3	0.053307748	0.344221101	0.154864846	0.876927868	0.264110685	8.786292575	77.23727064	0.666666667	CRA004775;PRJNA827465;Yang2025_Silkie_Wencha	0.963326943
ENSGALG00010013720	SLC16A10	3	-0.050963016	0.327973979	-0.155387375	0.876515937	0.264059871	20.20856525	90.10320636	0.666666667	CRA004775;PRJNA827465;Yang2025_Silkie_Wencha	0.963326943
ENSGALG00010002646	FOXO1	3	-0.042919558	0.276424395	-0.155266898	0.876610911	0.190689682	45.99273723	95.65148734	0.666666667	CRA004775;PRJNA827465;Yang2025_Silkie_Wencha	0.963326943
ENSGALG00010003539	ENSGALG0001000353(	3	0.040327133	0.260656811	0.154713522	0.87704717	0.141558082	7.272557842	72.49935933	0.666666667	CRA004775;PRJNA827465;Yang2025_Silkie_Wencha	0.963326943
ENSGALG00010021136	WEE1	3	-0.040093022	0.25939803	-0.154561784	0.8771668	0.165214128	17.60715573	88.64098194	0.666666667	CRA004775;PRJNA827465;Yang2025_Silkie_Wencha	0.963326943
ENSGALG00010000685	ENSGALG0001000068	3	-0.03938266	0.253675758	-0.155248024	0.87662579	0	0.286577388	0	0.666666667	CRA004775;PRJNA827465;Yang2025_Silkie_Wencha	0.963326943
ENSGALG00010003478	COLEC11	3	0.038264236	0.246889077	0.154985537	0.876832719	0.135204455	10.76581364	81.42267675	0.666666667	CRA004775;PRJNA827465;Yang2025_Silkie_Wencha	0.963326943
ENSGALG00010022873	NGEF	3	0.033285392	0.214774613	0.154978242	0.87683847	0	1.102563218	0	0.666666667	CRA004775;PRJNA827465;Yang2025_Silkie_Wencha	0.963326943
ENSGALG00010005266	SLC19A2	3	0.020756565	0.134337672	0.15451038	0.877207327	0	1.778094264	0	0.666666667	CRA004775;PRJNA827465;Yang2025_Silkie_Wencha	0.963326943
ENSGALG00010029140	NPDC1	3	0.019356355	0.124887106	0.154990818	0.876828556	0.026204367	5.374169397	62.7849468	0.666666667	CRA004775;PRJNA827465;Yang2025_Silkie_Wencha	0.963326943
ENSGALG00010009914	RWDD2B	3	0.01829005	0.11830305	0.154603364	0.877134018	0.015571271	3.098045848	35.44317619	0.666666667	CRA004775;PRJNA827465;Yang2025_Silkie_Wencha	0.963326943
ENSGALG00010004781	ENSGALG0001000478	3	0.017814481	0.114836679	0.155128845	0.876719742	0.012855379	2.819543282	29.06652604	0.666666667	CRA004775;PRJNA827465;Yang2025_Silkie_Wencha	0.963326943
ENSGALG00010017104	FANCL	3	-0.013199897	0.085074717	-0.155156515	0.876697929	0	0.079736838	0	0.666666667	CRA004775;PRJNA827465;Yang2025_Silkie_Wencha	0.963326943
ENSGALG00010028582	KYAT1	3	-0.012480585	0.080459125	-0.155117084	0.876729014	0	1.586980292	0	0.666666667	CRA004775;PRJNA827465;Yang2025_Silkie_Wencha	0.963326943
ENSGALG00010012024	UBR5	3	0.006553958	0.042234485	0.155180245	0.876679222	0	1.039049817	0	0.666666667	CRA004775;PRJNA827465;Yang2025_Silkie_Wencha	0.963326943
ENSGALG00010024539	RAPGEFL1	3	0.045046522	0.292765625	0.153865476	0.877715806	0	0.544313005	0	0.666666667	CRA004775;PRJNA827465;Yang2025_Silkie_Wencha	0.963471138
ENSGALG00010021609	ENSGALG0001002160(	3	0.043402799	0.282022917	0.153898127	0.877690061	0.168583537	9.016586007	77.81865555	0.666666667	CRA004775;PRJNA827465;Yang2025_Silkie_Wencha	0.963471138
ENSGALG00010028517	RNF208	3	0.036023003	0.234181268	0.153825296	0.877747488	0.098080945	5.881025777	65.99232726	0.666666667	CRA004775;PRJNA827465;Yang2025_Silkie_Wencha	0.963471138
ENSGALG00010029411	SARM1	3	0.033940452	0.22057204	0.153874678	0.87770855	0.082252956	5.452027134	63.3163968	0.666666667	CRA004775;PRJNA827465;Yang2025_Silkie_Wencha	0.963471138
ENSGALG00010017304	ANKRD9	3	-0.027085223	0.175868648	-0.154008249	0.877603231	0	0.440858129	0	0.666666667	CRA004775;PRJNA827465;Yang2025_Silkie_Wencha	0.963471138
ENSGALG00010028574	ENDOG	3	0.01115029	0.072297682	0.154227492	0.877430366	0	0.893857161	0	0.666666667	CRA004775;PRJNA827465;Yang2025_Silkie_Wencha	0.963471138
ENSGALG00010019047	GHRL	2	-0.084098192	0.547986504	-0.153467633	0.878029513	0.40329785	2.910772609	65.64486017	0.5	PRJNA827465;Yang2025_Silkie_Wenchang	0.963492987
ENSGALG00010012933	ZCCHC17	3	0.036899157	0.240411146	0.153483554	0.878016958	0.151560446	18.28205446	89.06031046	0.666666667	CRA004775;PRJNA827465;Yang2025_Silkie_Wencha	0.963492987
ENSGALG00010000301	UQCR10	3	-0.027176641	0.176968815	-0.153567403	0.877950841	0.047345202	3.709836863	46.08927363	0.666666667	CRA004775;PRJNA827465;Yang2025_Silkie_Wencha	0.963492987
ENSGALG00010010618	APAF1	3	-0.011337821	0.07388399	-0.153454372	0.87803997	0	1.799305386	0	0.666666667	CRA004775;PRJNA827465;Yang2025_Silkie_Wencha	0.963492987
ENSGALG00010003374	SYCE2	3	0.056176942	0.366492402	0.153282692	0.878175349	0.223316531	4.579108451	56.32337558	0.666666667	CRA004775;PRJNA827465;Yang2025_Silkie_Wencha	0.96356676
ENSGALG00010025069	PODN	3	-0.044959297	0.293887457	-0.152981341	0.87841299	0.201426178	9.777115476	79.54406896	0.666666667	CRA004775;PRJNA827465;Yang2025_Silkie_Wencha	0.96360538
ENSGALG00010014476	C5orf34	3	-0.038059124	0.248918796	-0.152897752	0.878478909	0.120886324	6.598736222	69.69116612	0.666666667	CRA004775;PRJNA827465;Yang2025_Silkie_Wencha	0.96360538
ENSGALG00010015820	ENSGALG0001001582(	3	-0.02599543	0.169926202	-0.152980706	0.87841349	0.010798226	2.241642246	10.77969718	0.666666667	CRA004775;PRJNA827465;Yang2025_Silkie_Wencha	0.96360538
ENSGALG00010028742	ENSGALG0001002874	3	0.024480465	0.160115677	0.152892366	0.878483157	0.035044439	3.640732214	45.06599546	0.666666667	CRA004775;PRJNA827465;Yang2025_Silkie_Wencha	0.96360538
ENSGALG00010027222	GUCA1B	2	0.084229517	0.551905843	0.152615737	0.878701314	0.421221817	3.057565108	67.29423692	0.5	PRJNA827465;Yang2025_Silkie_Wenchang	0.963643952
ENSGALG00010018414	EPAS1	3	0.022127448	0.144917193	0.152690291	0.878642518	0.039317485	5.722080248	65.04767648	0.666666667	CRA004775;PRJNA827465;Yang2025_Silkie_Wencha	0.963643952
ENSGALG00010027667	PTPDC1	3	0.012826475	0.084059243	0.152588511	0.878722786	0	0.216627535	0	0.666666667	CRA004775;PRJNA827465;Yang2025_Silkie_Wencha	0.963643952
ENSGALG00010027702	FOXP4	3	-0.050192681	0.329378034	-0.152386243	0.878882307	0.240796204	11.03556002	81.87676931	0.666666667	CRA004775;PRJNA827465;Yang2025_Silkie_Wencha	0.963669402
ENSGALG00010017289	ADGRB2	3	0.022476638	0.14749347	0.152390732	0.878878767	0	1.36163386	0	0.666666667	CRA004775;PRJNA827465;Yang2025_Silkie_Wencha	0.963669402
ENSGALG00010021092	VPS13D	3	-0.021482459	0.141218035	-0.152122629	0.879090217	0.044080576	8.286080191	75.86313487	0.666666667	CRA004775;PRJNA827465;Yang2025_Silkie_Wencha	0.963789978
ENSGALG00010024194	NKAIN4	3	0.010634044	0.069926791	0.152073956	0.879128605	0	1.459617928	0	0.666666667	CRA004775;PRJNA827465;Yang2025_Silkie_Wencha	0.963789978
ENSGALG00010029304	ORAI3	3	0.019816877	0.13069035	0.15163229	0.879476964	0.032695064	6.751972827	70.37902771	0.666666667	CRA004775;PRJNA827465;Yang2025_Silkie_Wencha	0.964022389
ENSGALG00010019256	BBS9	3	-0.013040702	0.086002138	-0.151632296	0.879476959	0	1.818554631	0	0.666666667	CRA004775;PRJNA827465;Yang2025_Silkie_Wencha	0.964022389
ENSGALG00010027865	SELENOK	3	0.029540036	0.195393719	0.151182119	0.879832055	0.083451404	8.197898374	75.60350338	0.666666667	CRA004775;PRJNA827465;Yang2025_Silkie_Wencha	0.964112643
ENSGALG00010018897	CCDC85A	3	-0.024598385	0.162497791	-0.151376732	0.879678543	0.054066205	9.739399411	79.46485286	0.666666667	CRA004775;PRJNA827465;Yang2025_Silkie_Wencha	0.964112643
ENSGALG00010024856	SORT1	3	0.011309933	0.074737857	0.151328028	0.879716961	0.004742697	2.722136347	26.52829452	0.666666667	CRA004775;PRJNA827465;Yang2025_Silkie_Wencha	0.964112643
ENSGALG00010003823	PTPN13	3	-0.008781699	0.0580686	-0.151229733	0.879794497	0	1.819102296	0	0.666666667	CRA004775;PRJNA827465;Yang2025_Silkie_Wencha	0.964112643
ENSGALG00010016206	KNG1	3	0.181023561	1.202224806	0.150573803	0.880311928	3.573991231	12.00593149	83.34156744	0.666666667	CRA004775;PRJNA827465;Yang2025_Silkie_Wencha	0.964488985
ENSGALG00010003968	DSN1	3	0.010375269	0.068895299	0.150594727	0.880295421	0	1.146878136	0	0.666666667	CRA004775;PRJNA827465;Yang2025_Silkie_Wencha	0.964488985
ENSGALG00010026719	ENSGALG0001002671(	2	-0.071185075	0.473763952	-0.150254308	0.880563981	0	0.520364797	0	0.5	CRA004775;PRJNA827465	0.964558251
ENSGALG00010024579	ZNF740	3	-0.015757606	0.104873765	-0.150253083	0.880564947	0.016740716	3.941966217	49.26389802	0.666666667	CRA004775;PRJNA827465;Yang2025_Silkie_Wencha	0.964558251
ENSGALG00010029372	METTL23	3	0.014950988	0.09951784	0.150234246	0.880579808	0	1.764158718	0	0.666666667	CRA004775;PRJNA827465;Yang2025_Silkie_Wencha	0.964558251
ENSGALG00010003280	ENSGALG0001000328(	3	-0.022457727	0.149877492	-0.149840559	0.880890409	0.045365626	7.925779992	74.76589052	0.666666667	CRA004775;PRJNA827465;Yang2025_Silkie_Wencha	0.964823727
ENSGALG00010027635	CD48	3	-0.019878168	0.132989863	-0.1494713	0.881181754	0.017709388	2.945319754	32.09565795	0.666666667	CRA004775;PRJNA827465;Yang2025_Silkie_Wencha	0.964967242
ENSGALG00010021253	TMEM169	3	0.009357936	0.062630506	0.149414989	0.881226185	0	1.807537898	0	0.666666667	CRA004775;PRJNA827465;Yang2025_Silkie_Wencha	0.964967242
ENSGALG00010028600	PSMD4	3	-0.00927852	0.06205877	-0.149511822	0.881149781	0	0.864444433	0	0.666666667	CRA004775;PRJNA827465;Yang2025_Silkie_Wencha	0.964967242
ENSGALG00010014972	FGA	3	0.216594914	1.463168414	0.148031431	0.882317963	4.631518695	7.454712725	73.17133371	0.666666667	CRA004775;PRJNA827465;Yang2025_Silkie_Wencha	0.965090255
ENSGALG00010027082	SLC26A9	3	0.050807614	0.342664266	0.148272286	0.882127886	0.09766856	2.755271438	27.41187047	0.666666667	CRA004775;PRJNA827465;Yang2025_Silkie_Wencha	0.965090255
ENSGALG00010021077	SEC31B	3	0.02468592	0.165652873	0.149021984	0.881536286	0.056968793	6.704678037	70.17008141	0.666666667	CRA004775;PRJNA827465;Yang2025_Silkie_Wencha	0.965090255
ENSGALG00010012982	DDR2	3	0.022775727	0.153836613	0.148051406	0.882302199	0.045712685	7.156764661	72.05441153	0.666666667	CRA004775;PRJNA827465;Yang2025_Silkie_Wencha	0.965090255
ENSGALG00010019912	ARG2	3	-0.021805724	0.14701549	-0.148322628	0.882088159	0	1.302985173	0	0.666666667	CRA004775;PRJNA827465;Yang2025_Silkie_Wencha	0.965090255
ENSGALG00010019223	AAMP	3	0.021352861	0.143567277	0.148730693	0.881766141	0.04223142	6.692581912	70.11616703	0.666666667	CRA004775;PRJNA827465;Yang2025_Silkie_Wencha	0.965090255
ENSGALG00010027960	PPP6C	3	-0.017605021	0.118866803	-0.148107131	0.882258222	0.028069845	7.176776986	72.13233735	0.666666667	CRA004775;PRJNA827465;Yang2025_Silkie_Wencha	0.965090255
ENSGALG00010022666	ENSGALG0001002266(	3	0.017113557	0.115223889	0.148524383	0.881928944	0.029951193	8.980907415	77.73053537	0.666666667	CRA004775;PRJNA827465;Yang2025_Silkie_Wencha	0.965090255
ENSGALG00010023071	POLR2M	3	0.013296518	0.089856427	0.147975146	0.882362383	0.01151844	3.872029966	48.34750718	0.666666667	CRA004775;PRJNA827465;Yang2025_Silkie_Wencha	0.965090255
ENSGALG00010002376	GEN1	3	0.01254883	0.084332119	0.148802497	0.88170948	0	0.149989711	0	0.666666667	CRA004775;PRJNA827465;Yang2025_Silkie_Wencha	0.965090255
ENSGALG00010013220	CSTF1	3	-0.010243822	0.068739511	-0.149023777	0.881534871	0.006995728	3.741738776	46.54891429	0.666666667	CRA004775;PRJNA827465;Yang2025_Silkie_Wencha	0.965090255
ENSGALG00010011111	MRPS14	3	-0.01020194	0.06893688	-0.147989589	0.882350984	0	0.143512698	0	0.666666667	CRA004775;PRJNA827465;Yang2025_Silkie_Wencha	0.965090255
ENSGALG00010003993	ENSGALG0001000399	3	-0.008555616	0.057473468	-0.148862017	0.881662513	0	0.572235968	0	0.666666667	CRA004775;PRJNA827465;Yang2025_Silkie_Wencha	0.965090255
ENSGALG00010026874	TEAD3	3	-0.007789333	0.052471609	-0.148448526	0.881988806	0	1.812933653	0	0.666666667	CRA004775;PRJNA827465;Yang2025_Silkie_Wencha	0.965090255
ENSGALG00010024793	FKBP4	3	0.00587866	0.03953395	0.148699036	0.881791122	0	0.635522172	0	0.666666667	CRA004775;PRJNA827465;Yang2025_Silkie_Wencha	0.965090255
ENSGALG00010012427	C7	3	-0.060555504	0.409918624	-0.147725672	0.882559271	0.361567245	7.862022044	74.56125169	0.666666667	CRA004775;PRJNA827465;Yang2025_Silkie_Wencha	0.965155966
ENSGALG00010017814	ENSGALG0001001781	3	-0.054508181	0.369587364	-0.147483887	0.882750097	0.305882726	8.665460458	76.91986468	0.666666667	CRA004775;PRJNA827465;Yang2025_Silkie_Wencha	0.965155966
ENSGALG00010001470	GPR85	3	0.052790818	0.35716791	0.147803923	0.882497513	0.217826273	4.927495319	59.4114277	0.666666667	CRA004775;PRJNA827465;Yang2025_Silkie_Wencha	0.965155966
ENSGALG00010024679	MSL1	3	0.018380108	0.124639158	0.147466563	0.88276377	0.031894473	7.290145007	72.56570345	0.666666667	CRA004775;PRJNA827465;Yang2025_Silkie_Wencha	0.965155966
ENSGALG00010008201	ENPP4	3	-0.015460884	0.104757882	-0.147586831	0.882668848	0.006398231	2.406521381	16.89248987	0.666666667	CRA004775;PRJNA827465;Yang2025_Silkie_Wencha	0.965155966
ENSGALG00010008932	VASP	3	0.017740225	0.120376102	0.147373314	0.882837368	0.016269778	3.00780047	33.50622756	0.666666667	CRA004775;PRJNA827465;Yang2025_Silkie_Wencha	0.965161799
ENSGALG00010022307	ENSGALG0001002230	3	-0.09005981	0.61691182	-0.1459849	0.883933309	0.970623269	15.82098876	87.35856506	0.666666667	CRA004775;PRJNA827465;Yang2025_Silkie_Wencha	0.965449961
ENSGALG00010017079	ENSGALG0001001707(	2	-0.085588199	0.585849943	-0.146092357	0.88384848	0	0.258737654	0	0.5	PRJNA827465;Yang2025_Silkie_Wenchang	0.965449961
ENSGALG00010007300	CCDC125	3	0.070467688	0.482496162	0.146048184	0.883883351	0.492909675	7.742350145	74.16805024	0.666666667	CRA004775;PRJNA827465;Yang2025_Silkie_Wencha	0.965449961
ENSGALG00010028419	ENSGALG0001002841(	3	-0.062980721	0.429335886	-0.146693353	0.883374065	0.445404599	11.74876867	82.97693949	0.666666667	CRA004775;PRJNA827465;Yang2025_Silkie_Wencha	0.965449961
ENSGALG00010017034	ZMYM3	3	0.031740525	0.216786155	0.146413983	0.883594589	0.12359329	20.35778567	90.17574881	0.666666667	CRA004775;PRJNA827465;Yang2025_Silkie_Wencha	0.965449961
ENSGALG00010008309	ENSGALG0001000830(	3	0.029999706	0.205597382	0.145914826	0.883988627	0	0.838341696	0	0.666666667	CRA004775;PRJNA827465;Yang2025_Silkie_Wencha	0.965449961
ENSGALG00010020894	PSMD13	3	-0.020904709	0.142562001	-0.146635911	0.883419407	0.038688928	6.746716417	70.35594982	0.666666667	CRA004775;PRJNA827465;Yang2025_Silkie_Wencha	0.965449961
ENSGALG00010023137	ETV6	3	-0.02087706	0.142869055	-0.146127233	0.883820948	0.043350408	7.181893897	72.15219232	0.666666667	CRA004775;PRJNA827465;Yang2025_Silkie_Wencha	0.965449961
ENSGALG00010017540	EIF4G1	3	0.018298141	0.125188384	0.146164845	0.883791257	0.028693468	5.623036204	64.43202698	0.666666667	CRA004775;PRJNA827465;Yang2025_Silkie_Wencha	0.965449961
ENSGALG00010001171	POLR1E	3	0.016561137	0.112823173	0.146788432	0.883299016	0.002208945	2.118612474	5.598592247	0.666666667	CRA004775;PRJNA827465;Yang2025_Silkie_Wencha	0.965449961
ENSGALG00010024304	PKIG	3	-0.011059302	0.075776478	-0.145946371	0.883963725	0	0.616065585	0	0.666666667	CRA004775;PRJNA827465;Yang2025_Silkie_Wencha	0.965449961
ENSGALG00010022521	RTF1	3	-0.009027206	0.061446748	-0.146911039	0.883202238	0.005898239	4.385734368	54.39760295	0.666666667	CRA004775;PRJNA827465;Yang2025_Silkie_Wencha	0.965449961
ENSGALG00010002983	NSMAF	3	-0.006987502	0.047887064	-0.145916263	0.883987493	0.001366876	2.431786806	17.75594822	0.666666667	CRA004775;PRJNA827465;Yang2025_Silkie_Wencha	0.965449961
ENSGALG00010007852	SLC20A2	3	-0.03061849	0.210075176	-0.145750157	0.884118625	0.094871018	10.36428033	80.70295346	0.666666667	CRA004775;PRJNA827465;Yang2025_Silkie_Wencha	0.965484607
ENSGALG00010027109	C19orf44	3	-0.013923571	0.095562204	-0.145701649	0.88415692	0.004937502	2.377304001	15.87108762	0.666666667	CRA004775;PRJNA827465;Yang2025_Silkie_Wencha	0.965484607
ENSGALG00010027953	SLC31A1	3	-0.031788847	0.218358705	-0.145580856	0.884252282	0.097361807	7.347992688	72.78168222	0.666666667	CRA004775;PRJNA827465;Yang2025_Silkie_Wencha	0.965514172
ENSGALG00010028678	ENSGALG0001002867	3	-0.082404384	0.567779868	-0.145134389	0.884604767	0.87666749	23.05664778	91.32571214	0.666666667	CRA004775;PRJNA827465;Yang2025_Silkie_Wencha	0.965646209
ENSGALG00010005248	TMEM200A	3	-0.036289526	0.250023026	-0.145144734	0.8845966	0.146615844	15.37788334	86.99430894	0.666666667	CRA004775;PRJNA827465;Yang2025_Silkie_Wencha	0.965646209
ENSGALG00010004846	TTLL2	3	-0.028320523	0.195204007	-0.145081667	0.884646393	0	0.851828515	0	0.666666667	CRA004775;PRJNA827465;Yang2025_Silkie_Wencha	0.965646209
ENSGALG00010019670	ENSGALG0001001967(	3	0.010287704	0.07090313	0.145095196	0.884635711	0	0.578843585	0	0.666666667	CRA004775;PRJNA827465;Yang2025_Silkie_Wencha	0.965646209
ENSGALG00010018054	SMAD6	3	0.014916978	0.1029754	0.144859626	0.884821704	0	0.180001686	0	0.666666667	CRA004775;PRJNA827465;Yang2025_Silkie_Wencha	0.965763013
ENSGALG00010017135	ENSGALG0001001713	3	-0.027995086	0.193523257	-0.144660058	0.884979277	0.073980762	6.208904146	67.78819655	0.666666667	CRA004775;PRJNA827465;Yang2025_Silkie_Wencha	0.96577026
ENSGALG00010024144	HNRNPA3	3	0.025694273	0.17770228	0.144591689	0.885033259	0.076622974	16.22996184	87.67711212	0.666666667	CRA004775;PRJNA827465;Yang2025_Silkie_Wencha	0.96577026
ENSGALG00010016387	CCDC85C	3	0.018828521	0.130071326	0.144755354	0.884904033	0.009818138	2.374790698	15.78205178	0.666666667	CRA004775;PRJNA827465;Yang2025_Silkie_Wencha	0.96577026
ENSGALG00010005243	NGB	2	-0.059531042	0.412331091	-0.144376796	0.885202939	0	0.047999082	0	1	CRA004775;PRJNA827465	0.965791956
ENSGALG00010027232	ARRDC2	3	0.018705082	0.129553733	0.14438088	0.885199714	0.013082172	2.489134646	19.65079095	0.666666667	CRA004775;PRJNA827465;Yang2025_Silkie_Wencha	0.965791956
ENSGALG00010011096	LDB2	3	-0.018056663	0.124965143	-0.144306985	0.885258063	0.004671618	2.603791336	23.13892946	0.666666667	CRA004775;PRJNA827465;Yang2025_Silkie_Wencha	0.965791956
ENSGALG00010000405	ENSGALG0001000040	3	0.09416823	0.656323147	0.143478454	0.885912327	1.138684853	21.44746441	90.67488836	0.666666667	CRA004775;PRJNA827465;Yang2025_Silkie_Wencha	0.965897134
ENSGALG00010001276	ENSGALG0001000127	3	0.062848547	0.436845651	0.143869	0.885603915	0.442000412	9.264861147	78.41306018	0.666666667	CRA004775;PRJNA827465;Yang2025_Silkie_Wencha	0.965897134
ENSGALG00010004707	OTUD1	3	-0.046977385	0.328889884	-0.14283621	0.886419539	0.224769757	10.11182334	80.22117344	0.666666667	CRA004775;PRJNA827465;Yang2025_Silkie_Wencha	0.965897134
ENSGALG00010017645	SYNC	3	0.045991863	0.322656354	0.14254132	0.886652444	0.257480048	18.54317331	89.21435956	0.666666667	CRA004775;PRJNA827465;Yang2025_Silkie_Wencha	0.965897134
ENSGALG00010022322	ENSGALG0001002232	3	-0.044049014	0.307925288	-0.143050979	0.88624992	0.186312506	5.821409418	65.64405874	0.666666667	CRA004775;PRJNA827465;Yang2025_Silkie_Wencha	0.965897134
ENSGALG00010006176	MMR1L3	3	0.042907235	0.298993696	0.143505484	0.88589098	0.147855596	4.529599098	55.84598202	0.666666667	CRA004775;PRJNA827465;Yang2025_Silkie_Wencha	0.965897134
ENSGALG00010010049	ZBTB7C	3	-0.039132503	0.274377894	-0.142622653	0.886588206	0.089632734	3.32388433	39.82943444	0.666666667	CRA004775;PRJNA827465;Yang2025_Silkie_Wencha	0.965897134
ENSGALG00010023850	DAZAP2	3	0.028203559	0.197592167	0.142736222	0.886498509	0.089984205	10.11245024	80.22239959	0.666666667	CRA004775;PRJNA827465;Yang2025_Silkie_Wencha	0.965897134
ENSGALG00010021775	CCDC36	3	-0.026063874	0.182480524	-0.142830989	0.886423662	0	1.968151378	0	0.666666667	CRA004775;PRJNA827465;Yang2025_Silkie_Wencha	0.965897134
ENSGALG00010022451	CHP1	3	0.025561296	0.178119787	0.143506212	0.885890406	0.080512402	13.90349862	85.61513145	0.666666667	CRA004775;PRJNA827465;Yang2025_Silkie_Wencha	0.965897134
ENSGALG00010013004	UAP1	3	-0.022061313	0.153119269	-0.144079272	0.885437872	0.046487826	6.51160711	69.28561619	0.666666667	CRA004775;PRJNA827465;Yang2025_Silkie_Wencha	0.965897134
ENSGALG00010019732	HBA1	3	-0.021387214	0.149409645	-0.143144802	0.886175822	0.01962479	2.787316405	28.24639512	0.666666667	CRA004775;PRJNA827465;Yang2025_Silkie_Wencha	0.965897134
ENSGALG00010001551	FHIP1B	3	0.02045808	0.142806073	0.143257774	0.886086603	0.038221398	7.467771774	73.21824956	0.666666667	CRA004775;PRJNA827465;Yang2025_Silkie_Wencha	0.965897134
ENSGALG00010018834	SAMD4A	3	-0.014181211	0.098716694	-0.143655653	0.885772392	0.010528859	2.961975239	32.47749092	0.666666667	CRA004775;PRJNA827465;Yang2025_Silkie_Wencha	0.965897134
ENSGALG00010011752	RNF2	3	0.014074633	0.098698884	0.142604633	0.886602438	0	0.746703991	0	0.666666667	CRA004775;PRJNA827465;Yang2025_Silkie_Wencha	0.965897134
ENSGALG00010017930	TSC2	3	0.011964023	0.083251552	0.143709313	0.885730017	0.010801473	4.29205259	53.4022485	0.666666667	CRA004775;PRJNA827465;Yang2025_Silkie_Wencha	0.965897134
ENSGALG00010016245	FAM96A	3	0.01108055	0.077212291	0.143507595	0.885889314	0	1.246153354	0	0.666666667	CRA004775;PRJNA827465;Yang2025_Silkie_Wencha	0.965897134
ENSGALG00010021457	USB1	3	-0.009626616	0.067380674	-0.142869097	0.886393565	0	0.266057447	0	0.666666667	CRA004775;PRJNA827465;Yang2025_Silkie_Wencha	0.965897134
ENSGALG00010008148	ABCD2	3	-0.007231083	0.050677803	-0.142687371	0.886537091	0	1.473337812	0	0.666666667	CRA004775;PRJNA827465;Yang2025_Silkie_Wencha	0.965897134
ENSGALG00010000711	SPRY2	3	0.044105908	0.309694845	0.142417314	0.886750387	0.250762634	36.0390022	94.45045679	0.666666667	CRA004775;PRJNA827465;Yang2025_Silkie_Wencha	0.965929408
ENSGALG00010027911	PTPN22	3	0.068267316	0.480015934	0.142218855	0.886907139	0.465405525	6.123981352	67.34150735	0.666666667	CRA004775;PRJNA827465;Yang2025_Silkie_Wencha	0.965969784
ENSGALG00010023002	ATG16L1	3	0.006942361	0.048821999	0.142197392	0.886924092	0	0.669242902	0	0.666666667	CRA004775;PRJNA827465;Yang2025_Silkie_Wencha	0.965969784
ENSGALG00010026121	ENSGALG0001002612	3	-0.06127592	0.43126853	-0.142082984	0.887014459	0.478843871	15.19751562	86.83995431	0.666666667	CRA004775;PRJNA827465;Yang2025_Silkie_Wencha	0.965993794
ENSGALG00010029247	CCDC92B	3	0.050392242	0.355092264	0.14167933	0.887333302	0.21066108	4.67591946	57.22766076	0.666666667	CRA004775;PRJNA827465;Yang2025_Silkie_Wencha	0.966117785
ENSGALG00010018534	NCEH1	3	0.01109212	0.07827599	0.141705263	0.887312818	0	0.799556716	0	0.666666667	CRA004775;PRJNA827465;Yang2025_Silkie_Wencha	0.966117785
ENSGALG00010022544	SPICE1	3	0.007369756	0.05199087	0.14175097	0.887276713	0	0.122221085	0	0.666666667	CRA004775;PRJNA827465;Yang2025_Silkie_Wencha	0.966117785
ENSGALG00010020290	NELL1	2	0.030644572	0.216472575	0.141563299	0.887424958	0	0.00569879	0	0.5	PRJNA827465;Yang2025_Silkie_Wenchang	0.96614318
ENSGALG00010028120	TBXA2R	3	0.044374916	0.313784012	0.141418663	0.887539212	0.247608159	17.12381574	88.32036019	0.666666667	CRA004775;PRJNA827465;Yang2025_Silkie_Wencha	0.966144072
ENSGALG00010009542	WDR1	3	-0.030995415	0.219220437	-0.141389256	0.887562441	0.111250384	10.97846555	81.78251786	0.666666667	CRA004775;PRJNA827465;Yang2025_Silkie_Wencha	0.966144072
ENSGALG00010000452	SLC8B1	3	-0.02966409	0.209983896	-0.141268405	0.887657909	0.093801844	7.354364393	72.80526374	0.666666667	CRA004775;PRJNA827465;Yang2025_Silkie_Wencha	0.966173608
ENSGALG00010012795	TLE6	3	-0.015901372	0.112748366	-0.14103417	0.887842948	0	1.490812572	0	0.666666667	CRA004775;PRJNA827465;Yang2025_Silkie_Wencha	0.966300627
ENSGALG00010025216	DPP6	2	-0.024805263	0.176773593	-0.14088801	0.887958414	0	0.657841708	0	0.5	PRJNA827465;Yang2025_Silkie_Wenchang	0.96635191
ENSGALG00010002416	SH3BGRL2	3	-0.062386931	0.443290348	-0.140736047	0.888078467	0.470153166	11.05263013	81.90475953	0.666666667	CRA004775;PRJNA827465;Yang2025_Silkie_Wencha	0.966408177
ENSGALG00010010946	SH2D4B	2	-0.048816642	0.347633904	-0.14042601	0.888323408	0.057785988	1.309996968	23.66394548	0.5	PRJNA827465;Yang2025_Silkie_Wenchang	0.966441359
ENSGALG00010024833	MAPT	3	0.038333504	0.274989743	0.139399772	0.889134252	0.162063151	7.543649005	73.4876318	0.666666667	CRA004775;PRJNA827465;Yang2025_Silkie_Wencha	0.966441359
ENSGALG00010027990	ENSGALG0001002799	3	0.036812549	0.264004627	0.139438028	0.889103234	0.128711294	5.236269626	61.80486982	0.666666667	CRA004775;PRJNA827465;Yang2025_Silkie_Wencha	0.966441359
ENSGALG00010023093	POP5	3	-0.033856137	0.24217757	-0.139798815	0.888818949	0.125861169	7.564508607	73.5607413	0.666666667	CRA004775;PRJNA827465;Yang2025_Silkie_Wencha	0.966441359
ENSGALG00010022314	DUSP13B	3	0.029821019	0.212963125	0.140029024	0.888637058	0	0.088985329	0	0.666666667	CRA004775;PRJNA827465;Yang2025_Silkie_Wencha	0.966441359
ENSGALG00010016834	ENSGALG0001001683	3	0.028505371	0.203646083	0.139975063	0.888679693	0.107568743	18.39984542	89.31034346	0.666666667	CRA004775;PRJNA827465;Yang2025_Silkie_Wencha	0.966441359
ENSGALG00010029352	AATF	3	0.028483817	0.202650453	0.140556395	0.888220398	0.100098801	11.59311914	82.74838742	0.666666667	CRA004775;PRJNA827465;Yang2025_Silkie_Wencha	0.966441359
ENSGALG00010009441	ZNF518B	3	-0.027138185	0.194501784	-0.139526662	0.889033989	0.098672402	20.7266413	90.35058324	0.666666667	CRA004775;PRJNA827465;Yang2025_Silkie_Wencha	0.966441359
ENSGALG00010018783	ENSGALG0001001878	3	0.025432756	0.182186157	0.139597631	0.888977912	0.062178609	7.503318934	73.34512877	0.666666667	CRA004775;PRJNA827465;Yang2025_Silkie_Wencha	0.966441359
ENSGALG00010020622	ABCG2	3	0.0228313	0.163305094	0.139807644	0.888811973	0.054875179	8.514998858	76.51203443	0.666666667	CRA004775;PRJNA827465;Yang2025_Silkie_Wencha	0.966441359
ENSGALG00010003171	PAQR3	3	-0.020938665	0.149865987	-0.139917977	0.888729538	0.042304431	5.728889406	65.0892196	0.666666667	CRA004775;PRJNA827465;Yang2025_Silkie_Wencha	0.966441359
ENSGALG00010021345	NEIL1	3	0.015199956	0.108964144	0.139495027	0.889058985	0	0.585038714	0	0.666666667	CRA004775;PRJNA827465;Yang2025_Silkie_Wencha	0.966441359
ENSGALG00010017634	PPIH	3	-0.012869825	0.09213925	-0.139677986	0.88891442	0.005130908	2.299602292	13.02843946	0.666666667	CRA004775;PRJNA827465;Yang2025_Silkie_Wencha	0.966441359
ENSGALG00010002577	MRPL48	3	-0.009029917	0.064712714	-0.139539901	0.889033617	0	0.716509145	0	0.666666667	CRA004775;PRJNA827465;Yang2025_Silkie_Wencha	0.966441359
ENSGALG00010004154	COPS5	3	-0.00815096	0.058059261	-0.140390349	0.888351582	0	0.33044787	0	0.666666667	CRA004775;PRJNA827465;Yang2025_Silkie_Wencha	0.966441359
ENSGALG00010002987	PCDH8	3	-0.051749225	0.371666897	-0.139235496	0.88926406	0.308530624	8.037424843	75.11640807	0.666666667	CRA004775;PRJNA827465;Yang2025_Silkie_Wencha	0.966508152
ENSGALG00010023698	ENSGALG0001002369	3	-0.024298724	0.174836941	-0.138979346	0.88946647	0.034540018	3.137038649	36.24560536	0.666666667	CRA004775;PRJNA827465;Yang2025_Silkie_Wencha	0.966619355
ENSGALG00010016970	SYNE3	3	-0.023247193	0.167184359	-0.138932983	0.889503107	0.050194295	5.243279246	61.8857356	0.666666667	CRA004775;PRJNA827465;Yang2025_Silkie_Wencha	0.966619355
ENSGALG00010024917	TRAK2	3	-0.012302936	0.088675186	-0.138741589	0.889654353	0.011320236	3.772794112	46.98889098	0.666666667	CRA004775;PRJNA827465;Yang2025_Silkie_Wencha	0.966709414
ENSGALG00010022258	ENSGALG0001002225	3	0.086588863	0.625160427	0.138506629	0.889840031	0.686689428	4.855712303	58.81139831	0.666666667	CRA004775;PRJNA827465;Yang2025_Silkie_Wencha	0.96678503
ENSGALG00010027130	NCKIPSD	3	-0.01244243	0.089905558	-0.138393988	0.889929048	0.007063403	2.789642186	28.30621754	0.666666667	CRA004775;PRJNA827465;Yang2025_Silkie_Wencha	0.96678503
ENSGALG00010017843	NSD3	3	-0.004750301	0.034321487	-0.138406033	0.889919529	0	0.002859239	0	0.666666667	CRA004775;PRJNA827465;Yang2025_Silkie_Wencha	0.96678503
ENSGALG00010013284	MANEAL	3	-0.050426922	0.365911025	-0.137811977	0.890389018	0.322012451	11.24466219	82.21378317	0.666666667	CRA004775;PRJNA827465;Yang2025_Silkie_Wencha	0.966800272
ENSGALG00010005680	ENSGALG0001000568	3	-0.041501959	0.300888966	-0.137931143	0.890294837	0	1.37945882	0	0.666666667	CRA004775;PRJNA827465;Yang2025_Silkie_Wencha	0.966800272
ENSGALG00010023706	CDK5RAP3	3	-0.035367135	0.256934896	-0.137650183	0.890516893	0.183607785	30.60500698	93.46512157	0.666666667	CRA004775;PRJNA827465;Yang2025_Silkie_Wencha	0.966800272
ENSGALG00010021413	SYNJ2BP	3	0.03040911	0.220344712	0.138006987	0.890234895	0.106660536	7.534877542	73.45676836	0.666666667	CRA004775;PRJNA827465;Yang2025_Silkie_Wencha	0.966800272
ENSGALG00010021184	DRAXIN	3	-0.028945868	0.210338036	-0.137615946	0.890543952	0	0.561499062	0	0.666666667	CRA004775;PRJNA827465;Yang2025_Silkie_Wencha	0.966800272
ENSGALG00010009102	HTRA3	3	0.020448465	0.148529311	0.137672929	0.890498915	0.010334528	2.358058924	15.1844774	0.666666667	CRA004775;PRJNA827465;Yang2025_Silkie_Wencha	0.966800272
ENSGALG00010029592	MNT	3	0.016086126	0.116541358	0.138029337	0.890217232	0.017562805	3.22666842	38.01656261	0.666666667	CRA004775;PRJNA827465;Yang2025_Silkie_Wencha	0.966800272
ENSGALG00010023956	KREMEN1	3	0.013329091	0.096931075	0.137511015	0.890626886	0	0.040642016	0	1	CRA004775;PRJNA827465;Yang2025_Silkie_Wencha	0.966800272
ENSGALG00010021978	NR2C2	3	-0.012446574	0.090463637	-0.137586482	0.89056724	0.01193509	3.808484761	47.4856767	0.666666667	CRA004775;PRJNA827465;Yang2025_Silkie_Wencha	0.966800272
ENSGALG00010023793	RBM45	3	0.006325875	0.045784942	0.138164978	0.890110033	0	1.865588125	0	0.666666667	CRA004775;PRJNA827465;Yang2025_Silkie_Wencha	0.966800272
ENSGALG00010013106	SETD9	3	-0.036582866	0.266565205	-0.137237965	0.890842703	0.152823343	7.427458468	73.07288881	0.666666667	CRA004775;PRJNA827465;Yang2025_Silkie_Wencha	0.966903107
ENSGALG00010024965	STAT2	3	0.031016692	0.226039334	0.137218118	0.89085839	0.134054923	20.49318301	90.2406571	0.666666667	CRA004775;PRJNA827465;Yang2025_Silkie_Wencha	0.966903107
ENSGALG00010010521	SNCG	3	-0.056038082	0.408790338	-0.137082696	0.89096543	0.31365568	6.21436182	67.8164861	0.666666667	CRA004775;PRJNA827465;Yang2025_Silkie_Wencha	0.966913904
ENSGALG00010015109	PPT1	3	0.006368122	0.04647162	0.137032495	0.891000511	0	0.288236159	0	0.666666667	CRA004775;PRJNA827465;Yang2025_Silkie_Wencha	0.966913904
ENSGALG00010002445	EDRF1	3	0.00656067	0.047917268	0.136916621	0.891096701	0.001228719	2.36101957	15.29083345	0.666666667	CRA004775;PRJNA827465;Yang2025_Silkie_Wencha	0.966939083

ENSGALG00010029457	ENSGALG00010029457	3	0.022756882	0.166885147	0.136362539	0.891534687	0.035259779	3.437464255	41.81757681	0.666666667	CRA004775;PRJNA827465;Yang2025_Silkie_Wencha	0.967059839
ENSGALG00010029252	RPAIN	3	-0.020557204	0.150543462	-0.136553286	0.891383903	0.019818461	2.629417551	23.93752756	0.666666667	CRA004775;PRJNA827465;Yang2025_Silkie_Wencha	0.967059839
ENSGALG00010005839	RPS6KA2	3	-0.017386857	0.127522722	-0.136343207	0.891549968	0.022592399	3.873829332	48.3714994	0.666666667	CRA004775;PRJNA827465;Yang2025_Silkie_Wencha	0.967059839
ENSGALG00010027721	SUSD3	3	-0.017152452	0.12551204	-0.136659813	0.891299696	0	0.059972731	0	0.666666667	CRA004775;PRJNA827465;Yang2025_Silkie_Wencha	0.967059839
ENSGALG00010019017	IRAK2	3	0.015479872	0.113446207	0.136451204	0.891464597	0.028971292	8.880259028	77.47813444	0.666666667	CRA004775;PRJNA827465;Yang2025_Silkie_Wencha	0.967059839
ENSGALG00010001825	LIPT2	3	0.044356673	0.326652512	0.135791617	0.891986018	0.243086654	10.50387335	80.95940486	0.666666667	CRA004775;PRJNA827465;Yang2025_Silkie_Wencha	0.9674586
ENSGALG00010028316	TIMM44	3	0.030032633	0.221356174	0.135675607	0.892077732	0.123126737	15.71494226	87.273259	0.666666667	CRA004775;PRJNA827465;Yang2025_Silkie_Wencha	0.967483858
ENSGALG00010018512	FERMT1	3	0.010720612	0.079076025	0.135573475	0.892158476	0.000500539	2.047771838	2.332869188	0.666666667	CRA004775;PRJNA827465;Yang2025_Silkie_Wencha	0.967497215
ENSGALG00010000127	ENSGALG00010000127	3	0.026012315	0.192236557	0.135314093	0.892363543	0.068328755	5.895722904	66.07710314	0.666666667	CRA004775;PRJNA827465;Yang2025_Silkie_Wencha	0.967645382
ENSGALG00010020811	MCOLN2	3	0.09682151	0.716364142	0.135156836	0.892487874	0.843266388	4.42346026	54.78652723	0.666666667	CRA004775;PRJNA827465;Yang2025_Silkie_Wencha	0.967705986
ENSGALG00010014030	WASF1	3	0.013579703	0.100753376	0.134781615	0.892784543	0	0.322111769	0	0.666666667	CRA004775;PRJNA827465;Yang2025_Silkie_Wencha	0.967888228
ENSGALG00010014762	STK40	3	-0.005370756	0.039850948	-0.134771093	0.892792862	0	1.57020622	0	0.666666667	CRA004775;PRJNA827465;Yang2025_Silkie_Wencha	0.967888228
ENSGALG00010016682	GPR141	3	-0.076628425	0.569819857	-0.134478333	0.893024343	0.493614227	4.008676572	50.10822239	0.666666667	CRA004775;PRJNA827465;Yang2025_Silkie_Wencha	0.967916534
ENSGALG00010000458	ENSGALG00010000458	3	0.051488406	0.382671776	0.134549788	0.892967844	0.414441863	52.37593123	96.18145214	0.666666667	CRA004775;PRJNA827465;Yang2025_Silkie_Wencha	0.967916534
ENSGALG00010006634	PRMT9	3	-0.015347869	0.114098115	-0.13451466	0.89299562	0.020932096	4.181481735	52.1700649	0.666666667	CRA004775;PRJNA827465;Yang2025_Silkie_Wencha	0.967916534
ENSGALG00010028418	ENSGALG00010028418	3	-0.014914595	0.111087259	-0.134260177	0.893196842	0.01800942	4.004167217	50.052036	0.666666667	CRA004775;PRJNA827465;Yang2025_Silkie_Wencha	0.968029293
ENSGALG00010019144	IFT122	3	-0.006390462	0.047644438	-0.134128177	0.893301219	0	0.89947487	0	0.666666667	CRA004775;PRJNA827465;Yang2025_Silkie_Wencha	0.96806821
ENSGALG00010006576	CCDC191	3	0.021842399	0.163061162	0.133952184	0.893440385	0.050100751	5.363485557	62.71081596	0.666666667	CRA004775;PRJNA827465;Yang2025_Silkie_Wencha	0.968070626
ENSGALG00010019266	ATP5S	3	0.013465519	0.100472953	0.134021334	0.893385705	0	1.661539165	0	0.666666667	CRA004775;PRJNA827465;Yang2025_Silkie_Wencha	0.968070626
ENSGALG00010017725	ENSGALG00010017725	3	-0.042737648	0.319451358	-0.133784525	0.893572964	0.116650162	3.213968192	37.77163056	0.666666667	CRA004775;PRJNA827465;Yang2025_Silkie_Wencha	0.96808115
ENSGALG00010001386	FKBP14	3	-0.034140194	0.255645215	-0.133545208	0.893762213	0.155391084	14.50875335	86.21521814	0.666666667	CRA004775;PRJNA827465;Yang2025_Silkie_Wencha	0.96808115
ENSGALG00010027187	ENSGALG00010027187	3	-0.020038927	0.150193875	-0.133420403	0.89386091	0.034635807	3.968213067	49.59948052	0.666666667	CRA004775;PRJNA827465;Yang2025_Silkie_Wencha	0.96808115
ENSGALG00010003204	PKD2	3	0.016952575	0.127052731	0.13342944	0.893853764	0.023743332	3.980734801	49.75801956	0.666666667	CRA004775;PRJNA827465;Yang2025_Silkie_Wencha	0.96808115
ENSGALG00010004533	PAG1	3	0.015692105	0.117335601	0.133736946	0.893610589	0.014776866	2.7970912	28.49714732	0.666666667	CRA004775;PRJNA827465;Yang2025_Silkie_Wencha	0.96808115
ENSGALG00010009386	TMEM60	3	-0.008522411	0.063851748	-0.133471851	0.893820225	0	1.853805275	0	0.666666667	CRA004775;PRJNA827465;Yang2025_Silkie_Wencha	0.96808115
ENSGALG00010006912	ENSGALG00010006912	3	0.0232284	0.174653272	0.132997223	0.894195577	0.055780877	5.186755745	61.44025093	0.666666667	CRA004775;PRJNA827465;Yang2025_Silkie_Wencha	0.968170273
ENSGALG00010016567	E2F2	3	-0.014038887	0.105710063	-0.132805587	0.894347136	0	0.248102776	0	0.666666667	CRA004775;PRJNA827465;Yang2025_Silkie_Wencha	0.968170273
ENSGALG00010016774	PEX14	3	-0.013983234	0.105257402	-0.132847991	0.8943136	0.017405844	4.019546592	50.24314425	0.666666667	CRA004775;PRJNA827465;Yang2025_Silkie_Wencha	0.968170273
ENSGALG00010008395	ENDOD1	3	0.010915931	0.082082273	0.132987685	0.89420312	0	1.271131693	0	0.666666667	CRA004775;PRJNA827465;Yang2025_Silkie_Wencha	0.968170273
ENSGALG00010005411	STXBP5	3	0.008509191	0.064076753	0.132796843	0.894354051	0	0.6913693	0	0.666666667	CRA004775;PRJNA827465;Yang2025_Silkie_Wencha	0.968170273
ENSGALG00010027253	VGLL4	3	0.006321232	0.047471233	0.13315922	0.894067461	0.000199945	2.032957659	1.621167985	0.666666667	CRA004775;PRJNA827465;Yang2025_Silkie_Wencha	0.968170273
ENSGALG00010013650	BACE2	3	0.035958608	0.27120701	0.132587312	0.894519767	0.171563186	15.09645173	86.75185377	0.666666667	CRA004775;PRJNA827465;Yang2025_Silkie_Wencha	0.968275532
ENSGALG00010002870	FABP5	3	-0.014922241	0.112934023	-0.132132374	0.89487959	0.007460437	2.370059816	15.61394414	0.666666667	CRA004775;PRJNA827465;Yang2025_Silkie_Wencha	0.968590869
ENSGALG00010001082	P2RY1	3	-0.013258193	0.100471052	-0.131960327	0.895015672	0	0.776376087	0	0.666666667	CRA004775;PRJNA827465;Yang2025_Silkie_Wencha	0.968664007
ENSGALG00010005740	ENSGALG00010005740	3	-0.028197204	0.213942253	-0.131798201	0.895143909	0	1.140281112	0	0.666666667	CRA004775;PRJNA827465;Yang2025_Silkie_Wencha	0.968728644
ENSGALG00010024471	CCDC61	3	-0.024252197	0.184409174	-0.131512962	0.895369533	0.076873678	8.849254902	77.39922714	0.666666667	CRA004775;PRJNA827465;Yang2025_Silkie_Wencha	0.968898656
ENSGALG00010005070	ALB	3	-0.231388985	1.762271303	-0.131301568	0.895536751	8.35126574	19.99453246	89.99726548	0.666666667	CRA004775;PRJNA827465;Yang2025_Silkie_Wencha	0.968931292
ENSGALG00010024184	ENSGALG00010024184	3	-0.018145168	0.138160136	-0.131334325	0.895510839	0.024451792	3.605016194	44.52174713	0.666666667	CRA004775;PRJNA827465;Yang2025_Silkie_Wencha	0.968931292
ENSGALG00010018551	FBXO34	3	-0.00735809	0.056081754	-0.131202918	0.895614787	0	1.290576335	0	0.666666667	CRA004775;PRJNA827465;Yang2025_Silkie_Wencha	0.968941578
ENSGALG00010008960	LRRC2	3	-0.0422559	0.322490176	-0.131030038	0.895751545	0.173277998	4.657996476	57.06308473	0.666666667	CRA004775;PRJNA827465;Yang2025_Silkie_Wencha	0.969015386
ENSGALG00010023285	SLC48A1	3	-0.008938144	0.068272669	-0.13091833	0.895839914	0	1.901823733	0	0.666666667	CRA004775;PRJNA827465;Yang2025_Silkie_Wencha	0.969036841
ENSGALG00010010366	CLU	3	-0.019775014	0.151296356	-0.130703836	0.896009598	0.031093197	3.527354812	43.30028856	0.666666667	CRA004775;PRJNA827465;Yang2025_Silkie_Wencha	0.969146244
ENSGALG00010025882	LDHD	3	0.029144411	0.223209837	0.130569563	0.896115822	0.092505211	5.591509468	64.23148326	0.666666667	CRA004775;PRJNA827465;Yang2025_Silkie_Wencha	0.969152667
ENSGALG00010027307	JUND	3	-0.024481923	0.187567825	-0.130523042	0.896152625	0.050342109	3.739541562	46.51750845	0.666666667	CRA004775;PRJNA827465;Yang2025_Silkie_Wencha	0.969152667
ENSGALG00010009461	ZEB2	3	0.014310496	0.109804164	0.130327441	0.89630737	0.013960742	2.786640601	28.22899374	0.666666667	CRA004775;PRJNA827465;Yang2025_Silkie_Wencha	0.969245882
ENSGALG00010007021	SULT6B1	3	-0.036321465	0.279333969	-0.130028813	0.89654363	0.121504889	4.206099896	52.4500119	0.666666667	CRA004775;PRJNA827465;Yang2025_Silkie_Wencha	0.969353093
ENSGALG00010013528	PRDX6	3	-0.007500019	0.057644162	-0.13010891	0.896480261	0	0.81072873	0	0.666666667	CRA004775;PRJNA827465;Yang2025_Silkie_Wencha	0.969353093
ENSGALG00010023434	ATN1	3	0.029959941	0.230647629	0.12989486	0.896649611	0.108887978	6.457483952	69.02818474	0.666666667	CRA004775;PRJNA827465;Yang2025_Silkie_Wencha	0.96939355
ENSGALG00010014719	ENSGALG00010014719	2	-0.045098987	0.347790848	-0.129672725	0.896825363	0	0.18271233	0	0.5	PRJNA827465;Yang2025_Silkie_Wenchang	0.969509428
ENSGALG00010015934	TMC2	2	0.060965526	0.470665915	0.129530361	0.896938003	0	0.047570709	0	0.5	PRJNA827465;Yang2025_Silkie_Wenchang	0.969557066
ENSGALG00010016534	TRIT1	3	-0.020530555	0.158723513	-0.129347914	0.89708236	0.050163835	6.081121958	67.11133219	0.666666667	CRA004775;PRJNA827465;Yang2025_Silkie_Wencha	0.96963898
ENSGALG00010029578	C17orf67	2	0.055143568	0.426809155	0.129199591	0.89719972	0	0.823104844	0	0.5	PRJNA827465;Yang2025_Silkie_Wenchang	0.969691702
ENSGALG00010005522	SYNPO2	3	0.029309434	0.227455392	0.128857944	0.897470055	0.084897357	4.465289232	55.21006824	0.666666667	CRA004775;PRJNA827465;Yang2025_Silkie_Wencha	0.969761491
ENSGALG00010029377	NCF1C	3	0.0194694	0.150903679	0.129018723	0.897342834	0	0.974892329	0	0.666666667	CRA004775;PRJNA827465;Yang2025_Silkie_Wencha	0.969761491
ENSGALG00010012268	ENSGALG00010012268	3	-0.01841875	0.142867297	-0.128922088	0.897419299	0	0.150478537	0	0.666666667	CRA004775;PRJNA827465;Yang2025_Silkie_Wencha	0.969761491
ENSGALG00010004123	NDUFAF4	3	0.022034991	0.171136023	0.128757177	0.897549791	0.065245632	8.706015264	77.02737775	0.666666667	CRA004775;PRJNA827465;Yang2025_Silkie_Wencha	0.969773537
ENSGALG00010000495	ENSGALG00010000495	3	-0.035535671	0.276820371	-0.128370867	0.897855485	0.012199194	2.102994932	4.897535904	0.666666667	CRA004775;PRJNA827465;Yang2025_Silkie_Wencha	0.970029702
ENSGALG00010009381	INPP4B	3	-0.010494727	0.081815125	-0.128273678	0.897932395	0	0.09878295	0	0.666666667	CRA004775;PRJNA827465;Yang2025_Silkie_Wencha	0.970038671
ENSGALG00010021588	RNF25	3	0.021049116	0.164227593	0.128170397	0.898014126	0.05912115	9.350930214	78.61175354	0.666666667	CRA004775;PRJNA827465;Yang2025_Silkie_Wencha	0.970052848
ENSGALG00010021685	DBNDD2	3	0.013142877	0.102894342	0.127731774	0.898361244	0	1.776835309	0	0.666666667	CRA004775;PRJNA827465;Yang2025_Silkie_Wencha	0.970353677
ENSGALG00010016390	ENSGALG00010016390	3	0.033433561	0.262168631	0.127526933	0.898523358	0	0.142502701	0	0.666666667	CRA004775;PRJNA827465;Yang2025_Silkie_Wencha	0.970454645
ENSGALG00010021624	HSD11B2	3	0.050677617	0.397833403	0.127384018	0.898636465	0.324831466	6.44400308	68.96339162	0.666666667	CRA004775;PRJNA827465;Yang2025_Silkie_Wencha	0.97046621
ENSGALG00010026759	OMA1	3	0.008048019	0.063201052	0.127339952	0.898671341	0.001874547	2.294207154	12.82391406	0.666666667	CRA004775;PRJNA827465;Yang2025_Silkie_Wencha	0.97046621
ENSGALG00010001771	ENSGALG00010001771	3	0.022491704	0.17684837	0.127180727	0.898797359	0.059581137	5.628251252	64.4649837	0.666666667	CRA004775;PRJNA827465;Yang2025_Silkie_Wencha	0.970528171
ENSGALG00010028726	SLC2A8	3	-0.017340969	0.136512124	-0.127028781	0.898917619	0.034456099	5.466468274	63.41330636	0.666666667	CRA004775;PRJNA827465;Yang2025_Silkie_Wencha	0.970583904
ENSGALG00010015583	PRDM4	3	0.017178231	0.135508174	0.126768969	0.899123257	0.026270771	3.652706526	45.24608026	0.666666667	CRA004775;PRJNA827465;Yang2025_Silkie_Wencha	0.970731806
ENSGALG00010017340	SIVA1	3	-0.01200412	0.094858898	-0.126547118	0.899298854	0	1.514089246	0	0.666666667	CRA004775;PRJNA827465;Yang2025_Silkie_Wencha	0.970847255
ENSGALG00010008182	CLIC5	3	0.043453008	0.343827115	0.1263804	0.899430817	0.216556583	5.444701172	63.26703823	0.666666667	CRA004775;PRJNA827465;Yang2025_Silkie_Wencha	0.970915584
ENSGALG00010029497	ENSGALG00010029497	2	0.06578407	0.52097387	0.126271342	0.89951714	0.092172729	1.200939245	16.73184099	0.5	CRA004775;Yang2025_Silkie_Wenchang	0.97093464
ENSGALG00010013263	ATP11A	3	0.009740175	0.077241455	0.126100352	0.899652489	0	1.903760091	0	0.666666667	CRA004775;PRJNA827465;Yang2025_Silkie_Wencha	0.971006606
ENSGALG00010004034	NEDD8	3	-0.030241524	0.2400197	-0.125996008	0.899735085	0.126799929	8.075362823	75.23331095	0.666666667	CRA004775;PRJNA827465;Yang2025_Silkie_Wencha	0.971021629
ENSGALG00010023940	ATP1B3	3	-0.017723049	0.141149612	-0.125562153	0.900078523	0.042817251	10.61522162	81.159131	0.666666667	CRA004775;PRJNA827465;Yang2025_Silkie_Wencha	0.971318137
ENSGALG00010027866	KCNA2	3	-0.010210374	0.081415559	-0.125410604	0.900198493	0	1.597366429	0	0.666666667	CRA004775;PRJNA827465;Yang2025_Silkie_Wencha	0.971373464
ENSGALG00010009424	ENSGALG00010009424	2	0.118645811	0.948729965	0.125057514	0.900478018	0.82370157	1.790712529	44.15630741	0.5	PRJNA827465;Yang2025_Silkie_Wenchang	0.971378553
ENSGALG00010001873	ENSGALG00010001873	3	-0.037735946	0.301581329	-0.125126929	0.900423064	0.208165523	8.646242372	76.86856424	0.666666667	CRA004775;PRJNA827465;Yang2025_Silkie_Wencha	0.971378553
ENSGALG00010012413	NEK7	3	0.014212843	0.113416995	0.125314933	0.900274231	0.020590511	4.239135861	52.82057321	0.666666667	CRA004775;PRJNA827465;Yang2025_Silkie_Wencha	0.971378553
ENSGALG00010012778	TBC1D15	3	0.006558074	0.052397604	0.125159815	0.90039703	0	0.942538758	0	0.666666667	CRA004775;PRJNA827465;Yang2025_Silkie_Wencha	0.971378553
ENSGALG00010009141	MAML3	3	0.01528801	0.122394128	0.124908035	0.900596357	0.023590819	4.768022443	58.05388872	0.666666667	CRA004775;PRJNA827465;Yang2025_Silkie_Wencha	0.971432095
ENSGALG00010017465	TMEM234	3	-0.022036071	0.176793766	-0.124642806	0.900806338	0.058396105	5.673882943	64.75077086	0.666666667	CRA004775;PRJNA827465;Yang2025_Silkie_Wencha	0.97158447
ENSGALG00010004133	CRH	2	0.211799433	1.701629838	0.12446857	0.900944284	5.151943083	9.059752722	88.96217115	0.5	CRA004775;Yang2025_Silkie_Wenchang	0.971585023
ENSGALG00010004872	KIAA0408	3	0.03494206	0.280638769	0.124509026	0.900912254	0.158475619	7.432903861	73.09261579	0.666666667	CRA004775;PRJNA827465;Yang2025_Silkie_Wencha	0.971585023
ENSGALG00010023233	SEZ6L	2	-0.099530774	0.806183078	-0.12345927	0.901743425	0.745611095	2.322471276	56.94241688	0.5	PRJNA827465;Yang2025_Silkie_Wenchang	0.971635051
ENSGALG00010024983	APOF	2	-0.056511527	0.455766133	-0.123992379	0.901321309	0.070694801	1.189815855	15.9533809	0.5	CRA004775;Yang2025_Silkie_Wenchang	0.971635051
ENSGALG00010025920	GTSF1	3	-0.05175984	0.419465721	-0.123394684	0.901794566	0.27579963	4.202485682	52.40911805	0.666666667	CRA004775;PRJNA827465;Yang2025_Silkie_Wencha	0.971635051
ENSGALG00010016816	MAP3K6	3	0.05066555	0.410118542	0.123538794	0.901680456	0.436286695	14.94355753	86.61630608	0.666666667	CRA004775;PRJNA827465;Yang2025_Silkie_Wencha	0.971635051
ENSGALG00010008946	COL22A1	3	-0.036009026	0.290643276	-0.123894233	0.901399019	0.130690563	4.347696033	53.99862399	0.666666667	CRA004775;PRJNA827465;Yang2025_Silkie_Wencha	0.971635051
ENSGALG00010005287	ENSGALG00010005287	2	0.035446297	0.285239105	0.124268714	0.901102518	0	0.050706407	0	0.5	PRJNA827465;Yang2025_Silkie_Wenchang	0.971635051
ENSGALG00010024872	RUNDC3A	3	0.033879226	0.272973319	0.124111858	0.901226709	0.13743206	5.222211043	61.70204568	0.666666667	CRA004775;PRJNA827465;Yang2025_Silkie_Wencha	0.971635051
ENSGALG00010022449	ENSGALG00010022449	3	0.027233086	0.220924639	0.123268666	0.901894352	0.121314043	13.86115154	85.57118437	0.666666667	CRA004775;PRJNA827465;Yang2025_Silkie_Wencha	0.971635051
ENSGALG00010011622	BACH1	3	-0.024862211	0.201177757	-0.123583299	0.901645217	0.094062764	17.19627754	88.36957594	0.666666667	CRA004775;PRJNA827465;Yang2025_Silkie_Wencha	0.971635051
ENSGALG00010012095	RNF38	3	0.022410748	0.181897098	0.12320564	0.901944259	0.061258548	5.230780503	61.76478828	0.666666667	CRA004775;PRJNA827465;Yang2025_Silkie_Wencha	0.971635051
ENSGALG00010017833	ENSGALG00010017833	3	-0.021792956	0.175766694	-0.123987972	0.901324798	0.0349282	3.201168783	37.52281945	0.666666667	CRA004775;PRJNA827465;Yang2025_Silkie_Wencha	0.971635051
ENSGALG00010024710	HSDL1	3	-0.014512014	0.117455344	-0.123553463	0.901668841	0.006662902	2.281309633	12.33105883	0.666666667	CRA004775;PRJNA827465;Yang2025_Silkie_Wencha	0.971635051
ENSGALG00010012699	RNF128	3	-0.011078045	0.08976396	-0.123413064	0.901780012	0	1.977418062	0	0.666666667	CRA004775;PRJNA827465;Yang2025_Silkie_Wencha	0.971635051
ENSGALG00010003900	RB1	3	0.006630897	0.053824437	0.123194908	0.901952758	0	0.011078116	0	0.666666667	CRA004775;PRJNA827465;Yang2025_Silkie_Wencha	0.971635051
ENSGALG00010003754	FAM110C	3	0.044444762	0.36105906	0.123095546	0.902031438	0.313563081	11.99188756	83.32205844	0.666666667	CRA004775;PRJNA827465;Yang2025_Silkie_Wencha	0.97164578
ENSGALG00010009658	NAPEPLD	3	-0.019037391	0.15507408	-0.122763205	0.902294612	0.044084995	5.797477569	65.50223824	0.666666667	CRA004775;PRJNA827465;Yang2025_Silkie_Wencha	0.971661819
ENSGALG00010002474	HPCAL1	3	0.012360866	0.100716246	0.122729609	0.902321217	0.010977763	3.112266274	35.73814629	0.666666667	CRA004775;PRJNA827465;Yang2025_Silkie_Wencha	0.971661819
ENSGALG00010012096	ENSGALG00010012096	3	-0.010076378	0.081998356	-0.122885117	0.902198071	0.002908787	2.237565902	10.61715779	0.666666667	CRA004775;PRJNA827465;Yang2025_Silkie_Wencha	0.971661819
ENSGALG00010029693	HN1	3	0.007280201	0.059281563	0.122807174	0.902259793	0.002008357	2.289613975	12.64903071	0.666666667	CRA004775;PRJNA827465;Yang2025_Silkie_Wencha	0.971661819
ENSGALG00010016434	GPR139	2	0.034709991	0.283427772	0.122465031	0.902530738	0	0.170643699	0	0.5	PRJNA827465;Yang2025_Silkie_Wenchang	0.971813427
ENSGALG00010018011	LIPA	3	0.044777871	0.366275185	0.122251992	0.902699451	0.331462847	36.64880902	94.54279674	0.666666667	CRA004775;PRJNA827465;Yang2025_Silkie_Wencha	0.971874518
ENSGALG00010021782	ADAM11	3	0.04254513	0.348103429	0.122219797	0.902724948	0.230439289	6.055630414	66.97288534	0.666666667	CRA004775;PRJNA827465;Yang2025_Silkie_Wencha	0.971874518
ENSGALG00010017841	GALC	3	0.031429646	0.257663052	0.121979639	0.902915143	0.139017341	8.552264201	76.61438009	0.666666667	CRA004775;PRJNA827465;Yang2025_Silkie_Wencha	0.97200527
ENSGALG00010008306	ENSGALG00010008306	3	-0.035677224	0.292707097	-0.121887115	0.90298842	0.074306703	2.773389795	27.88608353	0.666666667	CRA004775;PRJNA827465;Yang2025_Silkie_Wencha	0.972010146
ENSGALG00010017867	APLF	3	-0.007536781	0.061935036	-0.121688496	0.903145724	0	1.95734873	0	0.666666667	CRA004775;PRJNA827465;Yang2025_Silkie_Wencha	0.972105466
ENSGALG00010000795	ENSGALG00010000795	3	0.061456662	0.507191605	0.121170504	0.903555986	0.66200539	18.23374355	89.03132539	0.666666667	CRA004775;PRJNA827465;Yang2025_Silkie_Wencha	0.972222725
ENSGALG00010016083	CDH22	3	0.049751843	0.410775533	0.121116861	0.903598474	0.260088271	4.151142098	51.82048813	0.666666667	CRA004775;PRJNA827465;Yang2025_Silkie_Wencha	0.972222725
ENSGALG00010013325	CA2	3	0.021733198	0.179224722	0.121262275	0.903483299	0.041617695	3.530012122	43.34297076	0.666666667	CRA004775;PRJNA827465;Yang2025_Silkie_Wencha	0.972222725
ENSGALG00010029147	RABL6	3	-0.010226172	0.08430151	-0.121304726	0.903449677	0.009819591	3.427317797	41.64532972	0.666666667	CRA004775;PRJNA827465;Yang2025_Silkie_Wencha	0.972222725
ENSGALG00010010651	NUAK1	3	0.006501728	0.053673867	0.12113396	0.903584931	0	1.006818242	0	0.666666667	CRA004775;PRJNA827465;Yang2025_Silkie_Wencha	0.972222725
ENSGALG00010014956	FGG	3	-0.19211651	1.591145231	-0.120741027	0.903896162	5.773246538	8.909375282	77.55173694	0.666666667	CRA004775;PRJNA827465;Yang2025_Silkie_Wencha	0.972436606
ENSGALG00010027723	ENSGALG00010027723	3	0.08353791	0.692272313	0.120672036	0.903950809	0.894739672	5.296440145	62.23878784	0.666666667	CRA004775;PRJNA827465;Yang2025_Silkie_Wencha	0.972436606
ENSGALG00010015567	ENSGALG00010015567	3	0.043689447	0.363510602	0.120187545	0.904334584	0.274692967	6.521059272	69.33013615	0.666666667	CRA004775;PRJNA827465;Yang2025_Silkie_Wencha	0.972436606
ENSGALG00010005341	UGGT2	3	0.024247955	0.201778299	0.120171273	0.904347474	0.100370733	13.2421087	84.89666528	0.666666667	CRA004775;PRJNA827465;Yang2025_Silkie_Wencha	0.972436606
ENSGALG00010006885	RHOU	3	-0.016338873	0.135733775	-0.120374413	0.90418656	0.027180825	4.206514159	52.45469468	0.666666667	CRA004775;PRJNA827465;Yang2025_Silkie_Wencha	0.972436606
ENSGALG00010001952	ENSGALG00010001952	3	0.013257385	0.11017261	0.120332855	0.904219479	0.020643486	4.398322379	54.52811714	0.666666667	CRA004775;PRJNA827465;Yang2025_Silkie_Wencha	0.972436606
ENSGALG00010015477	ARMC9	3	0.008253383	0.068669933	0.120189184	0.904333286	0	0.886794161	0	0.666666667	CRA004775;PRJNA827465;Yang2025_Silkie_Wencha	0.972436606
ENSGALG00010008448	TNS3	3	0.007230875	0.060163759	0.120186563	0.904335362	0	1.03011641	0	0.666666667	CRA004775;PRJNA827465;Yang2025_Silkie_Wencha	0.972436606
ENSGALG00010017152	CYP4V2	3	-0.04289304	0.358016184	-0.119807546	0.904635604	0.301443448	9.423513878	78.77649435	0.666666667	CRA004775;PRJNA827465;Yang2025_Silkie_Wencha	0.972672457
ENSGALG00010008956	LVRN	3	0.044332183	0.370897511	0.119526775	0.904858029	0.288408447	7.577219754	73.60509442	0.666666667	CRA004775;PRJNA827465;Yang2025_Silkie_Wencha	0.972763661
ENSGALG00010027372	PIK3C2B	3	0.018939568	0.158405313	0.119563967	0.904828565	0.050733498	11.35630391	82.38863616	0.666666667	CRA004775;PRJNA827465;Yang2025_Silkie_Wencha	0.972763661
ENSGALG00010021360	PTPRN	3	0.042529414	0.356271023	0.119373767	0.904979244	0.276543608	8.387582514	76.15522713	0.666666667	CRA004775;PRJNA827465;Yang2025_Silkie_Wencha	0.972776738
ENSGALG00010028101	MRPL54	3	-0.012515405	0.104873834	-0.119337728	0.905007794	0.003413526	2.111740852	5.291409321	0.666666667	CRA004775;PRJNA827465;Yang2025_Silkie_Wencha	0.972776738
ENSGALG00010023888	CTH	3	-0.018633872	0.156320278	-0.119203168	0.905114397	0.041860349	4.811084949	58.42933514	0.666666667	CRA004775;PRJNA827465;Yang2025_Silkie_Wencha	0.972817367
ENSGALG00010015632	COL4A5	3	-0.02712697	0.227969443	-0.118993885	0.9052802	0.107086166	10.5587394	81.0583449	0.666666667	CRA004775;PRJNA827465;Yang2025_Silkie_Wencha	0.972921614
ENSGALG00010008431	SH3RF1	3	-0.016120999	0.135638541	-0.11885264	0.905392104	0.034327183	5.447303921	63.28458942	0.666666667	CRA004775;PRJNA827465;Yang2025_Silkie_Wencha	0.972967922
ENSGALG00010018973	NEFM	2	0.146864633	1.245445968	0.117921321	0.90613	2.265640877	3.37866566	70.40251683	0.5	PRJNA827465;Yang2025_Silkie_Wenchang	0.973074691
ENSGALG00010019314	GKN2	3	-0.114887451	0.973312646	-0.118037562	0.906037896	2.166231368	8.473728866	76.39763991	0.666666667	CRA004775;PRJNA827465;Yang2025_Silkie_Wencha	0.973074691
ENSGALG00010008168	CAVIN4	3	0.029495259	0.248861101	0.118520971	0.90565488	0.126803227	9.609291764	79.18681159	0.666666667	CRA004775;PRJNA827465;Yang2025_Silkie_Wencha	0.973074691
ENSGALG00010024920	VIPR2	3	-0.026630017	0.225948821	-0.117858625	0.906179677	0.081014536	4.549224546	56.03646336	0.666666667	CRA004775;PRJNA827465;Yang2025_Silkie_Wencha	0.973074691
ENSGALG00010015912	ENSGALG00010015912	2	0.026555538	0.224669213	0.118198383	0.905910471	0.002259825	1.021913345	2.144344781	0.5	PRJNA827465;Yang2025_Silkie_Wenchang	0.973074691
ENSGALG00010024946	KIF1A	3	0.023534335	0.199358411	0.118050372	0.906027746	0	1.751248018	0	0.666666667	CRA004775;PRJNA827465;Yang2025_Silkie_Wencha	0.973074691
ENSGALG00010020619	QARS	3	-0.018787156	0.158929632	-0.118210528	0.905900849	0.05368031	8.043422343	75.13496227	0.666666667	CRA004775;PRJNA827465;Yang2025_Silkie_Wencha	0.973074691
ENSGALG00010003405	GABRR2	2	0.018316332	0.155401776	0.117864366	0.906175128	0	0.063659489	0	0.5	PRJNA827465;Yang2025_Silkie_Wenchang	0.973074691
ENSGALG00010015122	ENSGALG00010015122	3	0.009162676	0.077417603	0.118353908	0.905787245	0	1.613890304	0	0.666666667	CRA004775;PRJNA827465;Yang2025_Silkie_Wencha	0.973074691
ENSGALG00010024438	RBL1	3	0.00568066	0.048124985	0.118039727	0.90603618	0.000430332	2.079214812	3.809842615	0.666666667	CRA004775;PRJNA827465;Yang2025_Silkie_Wencha	0.973074691
ENSGALG00010022320	COL9A3	3	-0.147606752	1.269441079	-0.116276962	0.907433043	4.32609597	19.20521055	89.58615947	0.666666667	CRA004775;PRJNA827465;Yang2025_Silkie_Wencha	0.97313563
ENSGALG00010014142	PPIL6	2	-0.061638935	0.528115009	-0.116714984	0.907085916	0.395653771	3.439939857	70.92972431	0.5	PRJNA827465;Yang2025_Silkie_Wenchang	0.97313563
ENSGALG00010010854	LRIT1	2	-0.050643651	0.43444722	-0.116570318	0.90720056	0.199220254	2.095483588	52.27831868	0.5	PRJNA827465;Yang2025_Silkie_Wenchang	0.97313563
ENSGALG00010013187	KDF1	3	0.044659377	0.383428773	0.116473725	0.907277109	0.333647511	9.02232288	77.83275963	0.666666667	CRA004775;PRJNA827465;Yang2025_Silkie_Wencha	0.97313563
ENSGALG00010007850	ENSGALG00010007850	2	0.03669468	0.314889701	0.116531851	0.907231045	0	0.000127793	0	1	PRJNA827465;Yang2025_Silkie_Wenchang	0.97313563
ENSGALG00010003517	GTF2F2	3	-0.036006399	0.307249227	-0.11718955	0.906709848	0.249508528	18.86241816	89.39690562	0.666666667	CRA004775;PRJNA827465;Yang2025_Silkie_Wencha	0.97313563
ENSGALG00010023536	ENSGALG00010023536	3	0.034024742	0.289629926	0.117476609	0.906482379	0.183104923	7.618809387	73.74917919	0.666666667	CRA004775;PRJNA827465;Yang2025_Silkie_Wencha	0.97313563
ENSGALG00010020240	SV2B	2	0.033391647	0.284124622	0.117524652	0.90644431	0.051668632	1.441653941	30.63522587	0.5	PRJNA827465;Yang2025_Silkie_Wenchang	0.97313563
ENSGALG00010029152	HEXD	3	-0.030097881	0.258578476	-0.116397474	0.907337537	0.171379241	17.23022347	88.39248949	0.666666667	CRA004775;PRJNA827465;Yang2025_Silkie_Wencha	0.97313563
ENSGALG00010017541	SREBF1	3	0.026661554	0.226843062	0.117533036	0.906437666	0.121117713	16.46593918	87.8537144	0.666666667	CRA004775;PRJNA827465;Yang2025_Silkie_Wencha	0.97313563
ENSGALG00010024771	AOX2	3	-0.026487005	0.227548613	-0.116401522	0.907334329	0.065745709	3.477272503	42.48365641	0.666666667	CRA004775;PRJNA827465;Yang2025_Silkie_Wencha	0.97313563
ENSGALG00010024956	VPS37B	3	-0.026259643	0.225035578	-0.116691074	0.907104864	0.131366521	27.29977113	92.67393126	0.666666667	CRA004775;PRJNA827465;Yang2025_Silkie_Wencha	0.97313563
ENSGALG00010024451	PHOSPHO1	3	-0.0199189	0.169948485	-0.117205518	0.906697194	0.021183152	2.644906681	24.3829654	0.666666667	CRA004775;PRJNA827465;Yang2025_Silkie_Wencha	0.97313563
ENSGALG00010008028	KCNK1	3	-0.018721841	0.161370229	-0.116017936	0.907638327	0	1.402996289	0	0.666666667	CRA004775;PRJNA827465;Yang2025_Silkie_Wencha	0.97313563
ENSGALG00010027328	RBM15	3	0.01711892	0.146870069	0.116558265	0.907210112	0.043065975	6.692408305	70.11539181	0.666666667	CRA004775;PRJNA827465;Yang2025_Silkie_Wencha	0.97313563
ENSGALG00010014573	CEBPB	3	0.014953626	0.127971984	0.11685078	0.906978302	0	1.230633209	0	0.666666667	CRA004775;PRJNA827465;Yang2025_Silkie_Wencha	0.97313563
ENSGALG00010019794	RSPH9	3	0.014418846	0.124204969	0.116089121	0.907581911	0	0.450519441	0	0.666666667	CRA004775;PRJNA827465;Yang2025_Silkie_Wencha	0.97313563
ENSGALG00010018022	XPO6	3	0.011217229	0.096803504	0.115876266	0.907750606	0.014368666	4.43782871	54.93291583	0.666666667	CRA004775;PRJNA827465;Yang2025_Silkie_Wencha	0.97313563
ENSGALG00010022104	EXOC3L1	3	-0.010689461	0.092052926	-0.116122993	0.907555066	0	1.602909662	0	0.666666667	CRA004775;PRJNA827465;Yang2025_Silkie_Wencha	0.97313563
ENSGALG00010020382	MFAP2	3	-0.007178827	0.06166939	-0.116408274	0.907328979	0	0.248294358	0	0.666666667	CRA004775;PRJNA827465;Yang2025_Silkie_Wencha	0.97313563
ENSGALG00010023798	ARCN1	3	-0.006857569	0.059171591	-0.115892937	0.907737394	0.005218776	4.059456756	50.73232405	0.666666667	CRA004775;PRJNA827465;Yang2025_Silkie_Wencha	0.97313563
ENSGALG00010011929	ELK3	3	0.006816968	0.058597995	0.116334493	0.90738745	0	1.37898683	0	0.666666667	CRA004775;PRJNA827465;Yang2025_Silkie_Wencha	0.97313563
ENSGALG00010014778	TMPRSS6	2	-0.075692746	0.655187962	-0.115528292	0.908026397	0	0.388866041	0	0.5	CRA004775;PRJNA827465	0.973311841
ENSGALG00010020194	PRICKLE2	3	0.009356428	0.081011429	0.115495162	0.908052655	0	0.334465141	0	0.666666667	CRA004775;PRJNA827465;Yang2025_Silkie_Wencha	0.973311841
ENSGALG00010028649	ENSGALG00010028649	3	0.009581371	0.083272804	0.115060022	0.908397547	0	1.231460832	0	0.666666667	CRA004775;PRJNA827465;Yang2025_Silkie_Wencha	0.973533913
ENSGALG00010022657	MRPS35	3	-0.006444667	0.055979909	-0.115124641	0.908346329	0	1.097803115	0	0.666666667	CRA004775;PRJNA827465;Yang2025_Silkie_Wencha	0.973533913
ENSGALG00010004116	SPRY1	3	0.027392531	0.238548683	0.114829942	0.908579916	0.146496827	20.19255687	90.09536032	0.666666667	CRA004775;PRJNA827465;Yang2025_Silkie_Wencha	0.973650947
ENSGALG00010017726	TTI2	3	0.015457719	0.134709563	0.114748493	0.908644476	0.023924682	3.50378206	42.91882413	0.666666667	CRA004775;PRJNA827465;Yang2025_Silkie_Wencha	0.973650947
ENSGALG00010018806	GCH1	3	-0.035148694	0.306549113	-0.11465926	0.908715207	0.197831968	7.017584396	71.50016463	0.666666667	CRA004775;PRJNA827465;Yang2025_Silkie_Wencha	0.973652949
ENSGALG00010000436	ENSGALG00010000436	3	-0.028424975	0.249106677	-0.114107639	0.909152467	0.095442836	4.269372546	53.15470883	0.666666667	CRA004775;PRJNA827465;Yang2025_Silkie_Wencha	0.974047642
ENSGALG00010008084	CYP2AC1	3	0.154120619	1.357134932	0.113563224	0.909584042	4.888081621	17.55566993	88.60766916	0.666666667	CRA004775;PRJNA827465;Yang2025_Silkie_Wencha	0.974193055
ENSGALG00010016905	C1QC	3	0.057642483	0.506642229	0.113773547	0.909417309	0.620707591	10.49199385	80.93784625	0.666666667	CRA004775;PRJNA827465;Yang2025_Silkie_Wencha	0.974193055
ENSGALG00010029479	ENSGALG00010029479	3	0.055398454	0.48872963	0.113351945	0.909751537	0.356538817	3.987282416	49.84052316	0.666666667	CRA004775;PRJNA827465;Yang2025_Silkie_Wencha	0.974193055
ENSGALG00010020433	GNPTG	3	-0.014197113	0.125121945	-0.11346621	0.90966095	0.028319913	5.061253871	60.48410037	0.666666667	CRA004775;PRJNA827465;Yang2025_Silkie_Wencha	0.974193055
ENSGALG00010019422	IMMP1L	3	-0.0119958	0.105850253	-0.113328025	0.9097705	0	0.496395912	0	0.666666667	CRA004775;PRJNA827465;Yang2025_Silkie_Wencha	0.974193055
ENSGALG00010009502	ARID5B	3	-0.007058597	0.062156562	-0.113561581	0.909585344	0	1.320490576	0	0.666666667	CRA004775;PRJNA827465;Yang2025_Silkie_Wencha	0.974193055
ENSGALG00010019218	RMND5A	3	0.005268851	0.046455944	0.113416083	0.909700689	0	0.144242324	0	0.666666667	CRA004775;PRJNA827465;Yang2025_Silkie_Wencha	0.974193055
ENSGALG00010023243	HK1	3	0.01747993	0.154574458	0.11308421	0.909963793	0.050998066	8.360572019	76.07819183	0.666666667	CRA004775;PRJNA827465;Yang2025_Silkie_Wencha	0.974252466
ENSGALG00010025473	PARS2	3	0.006866515	0.060717514	0.113089536	0.909959571	0	1.019867958	0	0.666666667	CRA004775;PRJNA827465;Yang2025_Silkie_Wencha	0.974252466
ENSGALG00010027923	AMPD2	3	0.029825367	0.264204267	0.112887529	0.910119723	0.17809231	15.67640461	87.24197257	0.666666667	CRA004775;PRJNA827465;Yang2025_Silkie_Wencha	0.974345632
ENSGALG00010013397	MATN1	3	0.156773424	1.392799671	0.112559923	0.91037946	5.198277922	19.31540808	89.64557212	0.666666667	CRA004775;PRJNA827465;Yang2025_Silkie_Wencha	0.974418956
ENSGALG00010011429	UBAP2	3	0.024984754	0.222007045	0.112540366	0.910394965	0.095198903	5.706441661	64.95188913	0.666666667	CRA004775;PRJNA827465;Yang2025_Silkie_Wencha	0.974418956
ENSGALG00010016927	MGAT4B	3	-0.015349826	0.13618629	-0.112711976	0.910258907	0.030478736	4.78007548	58.15965651	0.666666667	CRA004775;PRJNA827465;Yang2025_Silkie_Wencha	0.974418956
ENSGALG00010026457	SSRP1	3	0.011711751	0.104153085	0.112447472	0.910468617	0.023529434	11.45463709	82.53982221	0.666666667	CRA004775;PRJNA827465;Yang2025_Silkie_Wencha	0.974424024

ENSGALG00010026696	CSRNP1	3	-0.019427204	0.172933738	-0.112339006	0.910554615	0.061574281	9.354618288	78.6201859	0.666666667	CRA004775;PRJNA827465;Yang2025_Silkie_Wencha	0.974442303
ENSGALG00010019997	FSHR	3	0.044608425	0.398078569	0.112059347	0.910776351	0.267727023	5.200714782	61.54374766	0.666666667	CRA004775;PRJNA827465;Yang2025_Silkie_Wencha	0.974458329
ENSGALG00010016619	SLC25A47	3	-0.024670602	0.2199358	-0.112171835	0.91068716	0.07931762	4.766697984	58.04223371	0.666666667	CRA004775;PRJNA827465;Yang2025_Silkie_Wencha	0.974458329
ENSGALG00010022991	TAL1	3	0.015063455	0.134401487	0.112078039	0.91076153	0.011272848	2.460250104	18.70745186	0.666666667	CRA004775;PRJNA827465;Yang2025_Silkie_Wencha	0.974458329
ENSGALG00010011937	SLC1A1	3	-0.107114167	0.99644955	-0.107495825	0.914395637	2.717038667	22.8516483	91.24789611	0.666666667	CRA004775;PRJNA827465;Yang2025_Silkie_Wencha	0.974809833
ENSGALG00010004545	TMPRSS7	2	-0.077546211	0.713006392	-0.10875949	0.913393256	0.812831145	4.909519236	79.63140682	0.5	PRJNA827465;Yang2025_Silkie_Wenchang	0.974809833
ENSGALG00010023084	ENSGALG0001002308	3	-0.054858795	0.513786656	-0.106773491	0.914968678	0.71286026	24.0779802	91.69365543	0.666666667	CRA004775;PRJNA827465;Yang2025_Silkie_Wencha	0.974809833
ENSGALG00010021021	GCG	3	0.05297224	0.484407528	0.109354701	0.912921162	0.454616639	5.766311201	65.31578109	0.666666667	CRA004775;PRJNA827465;Yang2025_Silkie_Wencha	0.974809833
ENSGALG00010018902	NOXO1	2	0.051998385	0.474470665	0.109592413	0.912732628	0.019615103	1.041925241	4.023824276	0.5	CRA004775;PRJNA827465	0.974809833
ENSGALG00010010919	CDHR1	2	-0.044744822	0.414486073	-0.107952534	0.914033345	0	0.012119688	0	1	PRJNA827465;Yang2025_Silkie_Wenchang	0.974809833
ENSGALG00010011956	ENSGALG0001001195	2	0.041638143	0.378966959	0.109872753	0.912510292	0	0.053037241	0	0.5	PRJNA827465;Yang2025_Silkie_Wenchang	0.974809833
ENSGALG00010024560	A4GNT	2	0.034463255	0.31623836	0.10897873	0.913219361	0	0.488731161	0	0.5	PRJNA827465;Yang2025_Silkie_Wenchang	0.974809833
ENSGALG00010011348	SLC22A23	3	0.03342375	0.310751929	0.10755766	0.914346584	0.229251915	13.63776663	85.33484217	0.666666667	CRA004775;PRJNA827465;Yang2025_Silkie_Wencha	0.974809833
ENSGALG00010008074	RHAG	3	0.031726789	0.292681388	0.108400433	0.913678058	0.154399971	5.525145263	63.80185669	0.666666667	CRA004775;PRJNA827465;Yang2025_Silkie_Wencha	0.974809833
ENSGALG00010023945	LETMD1	3	0.031543092	0.285684117	0.110412479	0.912082257	0.197870912	12.33003182	83.77944169	0.666666667	CRA004775;PRJNA827465;Yang2025_Silkie_Wencha	0.974809833
ENSGALG00010015440	ENSGALG0001001544	3	0.029020525	0.263966305	0.109940263	0.912456751	0	0.169267686	0	0.666666667	CRA004775;PRJNA827465;Yang2025_Silkie_Wencha	0.974809833
ENSGALG00010013866	KCTD8	3	0.028462431	0.260076609	0.109438644	0.912854585	0	1.206813543	0	0.666666667	CRA004775;PRJNA827465;Yang2025_Silkie_Wencha	0.974809833
ENSGALG00010000639	SCN1B	3	-0.027162685	0.248380417	-0.109359204	0.91291759	0.131223367	7.608341755	73.71306305	0.666666667	CRA004775;PRJNA827465;Yang2025_Silkie_Wencha	0.974809833
ENSGALG00010015886	AFF3	3	0.026228044	0.235722788	0.111266476	0.911405039	0.089853035	4.531416924	55.86369488	0.666666667	CRA004775;PRJNA827465;Yang2025_Silkie_Wencha	0.974809833
ENSGALG00010005065	ENKUR	2	-0.025121737	0.232598416	-0.108004763	0.913991914	0	0.213842098	0	0.5	PRJNA827465;Yang2025_Silkie_Wenchang	0.974809833
ENSGALG00010004999	ADCK1	3	-0.024551632	0.229387177	-0.107031406	0.914764064	0.120797675	9.092336409	78.00345357	0.666666667	CRA004775;PRJNA827465;Yang2025_Silkie_Wencha	0.974809833
ENSGALG00010006139	GAREM1	3	0.024322161	0.227241657	0.107032143	0.91476348	0.107580262	8.028964106	75.09018631	0.666666667	CRA004775;PRJNA827465;Yang2025_Silkie_Wencha	0.974809833
ENSGALG00010001633	B3GAT2	3	-0.023792012	0.218439609	-0.108918031	0.913267505	0	1.475694261	0	0.666666667	CRA004775;PRJNA827465;Yang2025_Silkie_Wencha	0.974809833
ENSGALG00010011285	ENSGALG0001001128	3	-0.023129726	0.216506043	-0.106831779	0.914922436	0	0.403922456	0	0.666666667	CRA004775;PRJNA827465;Yang2025_Silkie_Wencha	0.974809833
ENSGALG00010024480	CDC45	3	0.02271563	0.209766531	0.108290058	0.913765609	0.085505021	6.753761314	70.38687174	0.666666667	CRA004775;PRJNA827465;Yang2025_Silkie_Wencha	0.974809833
ENSGALG00010001203	TMX3	3	-0.021131137	0.193043765	-0.109462933	0.91283532	0.102416922	32.61995981	93.8687846	0.666666667	CRA004775;PRJNA827465;Yang2025_Silkie_Wencha	0.974809833
ENSGALG00010005304	ENSGALG0001000530	3	0.020947222	0.19624409	0.10674065	0.914994733	0.067383827	4.911156408	59.27639371	0.666666667	CRA004775;PRJNA827465;Yang2025_Silkie_Wencha	0.974809833
ENSGALG00010019050	TNS1	3	0.019498025	0.181152341	0.107633304	0.914286577	0.060776697	6.395306277	68.72706461	0.666666667	CRA004775;PRJNA827465;Yang2025_Silkie_Wencha	0.974809833
ENSGALG00010017787	CNRIP1	3	0.018219509	0.168587644	0.108071437	0.913939025	0.042513753	4.045524143	50.56264826	0.666666667	CRA004775;PRJNA827465;Yang2025_Silkie_Wencha	0.974809833
ENSGALG00010021744	LITAF	3	-0.017997127	0.167347357	-0.10754354	0.914357785	0	1.305771092	0	0.666666667	CRA004775;PRJNA827465;Yang2025_Silkie_Wencha	0.974809833
ENSGALG00010005045	UTRN	3	0.017455913	0.157515376	0.110820373	0.91175879	0.049123723	6.169092708	67.58032186	0.666666667	CRA004775;PRJNA827465;Yang2025_Silkie_Wencha	0.974809833
ENSGALG00010023390	HSPB8	3	-0.017102021	0.15487681	-0.110423382	0.91207361	0.03030104	3.589468456	44.28144378	0.666666667	CRA004775;PRJNA827465;Yang2025_Silkie_Wencha	0.974809833
ENSGALG00010018829	PTPN4	3	0.016781161	0.156089509	0.107509858	0.914384505	0.044907479	6.348202141	68.49501709	0.666666667	CRA004775;PRJNA827465;Yang2025_Silkie_Wencha	0.974809833
ENSGALG00010015117	RASD2	3	0.015899312	0.145977491	0.108916188	0.913268967	0.024997039	3.285833731	39.13264749	0.666666667	CRA004775;PRJNA827465;Yang2025_Silkie_Wencha	0.974809833
ENSGALG00010020979	ENSGALG0001002097	3	-0.014866378	0.136146565	-0.109193926	0.913048678	0	0.691541096	0	0.666666667	CRA004775;PRJNA827465;Yang2025_Silkie_Wencha	0.974809833
ENSGALG00010022974	INPP5D	3	0.014817553	0.13638408	0.108645768	0.913483458	0.028154373	4.387235759	54.41320891	0.666666667	CRA004775;PRJNA827465;Yang2025_Silkie_Wencha	0.974809833
ENSGALG00010026005	DERL3	3	-0.01363093	0.12750382	-0.10690605	0.914863514	0.024709642	3.968510769	49.60326136	0.666666667	CRA004775;PRJNA827465;Yang2025_Silkie_Wencha	0.974809833
ENSGALG00010006232	ZNF654	3	0.013528807	0.124268347	0.108867683	0.913307439	0.029414826	6.046044537	66.92052154	0.666666667	CRA004775;PRJNA827465;Yang2025_Silkie_Wencha	0.974809833
ENSGALG00010028031	NISCH	3	-0.013321631	0.120888748	-0.110197443	0.91225279	0.033268365	9.738504932	79.46296671	0.666666667	CRA004775;PRJNA827465;Yang2025_Silkie_Wencha	0.974809833
ENSGALG00010003929	RCBTB2	3	0.013268635	0.121700371	0.109027068	0.913181021	0.021449062	4.174156707	52.08613044	0.666666667	CRA004775;PRJNA827465;Yang2025_Silkie_Wencha	0.974809833
ENSGALG00010024881	CASP10	3	-0.012231791	0.110369806	-0.110825517	0.911754711	0	1.639770757	0	0.666666667	CRA004775;PRJNA827465;Yang2025_Silkie_Wencha	0.974809833
ENSGALG00010021493	UNC93B1	3	-0.011908089	0.110861036	-0.107414559	0.914460105	0.014276345	3.183704946	37.18010828	0.666666667	CRA004775;PRJNA827465;Yang2025_Silkie_Wencha	0.974809833
ENSGALG00010028107	NOTCH1	3	0.011560175	0.104662064	0.110452392	0.912050604	0.01109744	2.864882871	30.18911802	0.666666667	CRA004775;PRJNA827465;Yang2025_Silkie_Wencha	0.974809833
ENSGALG00010008224	ENSGALG0001000822	3	-0.010787699	0.096778421	-0.111468024	0.911245221	0.000950154	2.048127466	2.349827693	0.666666667	CRA004775;PRJNA827465;Yang2025_Silkie_Wencha	0.974809833
ENSGALG00010009280	HSD17B4	3	0.010184814	0.092628785	0.109953018	0.912446635	0	0.892333271	0	0.666666667	CRA004775;PRJNA827465;Yang2025_Silkie_Wencha	0.974809833
ENSGALG00010003666	SLC25A13	3	0.010158177	0.095215149	0.106686561	0.915037645	0.007419668	2.602054791	23.13766771	0.666666667	CRA004775;PRJNA827465;Yang2025_Silkie_Wencha	0.974809833
ENSGALG00010014371	TTC33	3	-0.01011814	0.09196903	-0.110016816	0.912396039	0	1.044117938	0	0.666666667	CRA004775;PRJNA827465;Yang2025_Silkie_Wencha	0.974809833
ENSGALG00010028609	MRPS21	3	-0.009140714	0.08529632	-0.107164223	0.914658698	0	0.182013185	0	0.666666667	CRA004775;PRJNA827465;Yang2025_Silkie_Wencha	0.974809833
ENSGALG00010023131	SOX6	3	-0.008296468	0.076916445	-0.107863386	0.914104061	0	1.355638315	0	0.666666667	CRA004775;PRJNA827465;Yang2025_Silkie_Wencha	0.974809833
ENSGALG00010020032	CHST12	3	-0.008136237	0.075021642	-0.108451861	0.913637265	0.002585944	2.266895407	11.77360922	0.666666667	CRA004775;PRJNA827465;Yang2025_Silkie_Wencha	0.974809833
ENSGALG00010015742	HPRT1	3	-0.007955012	0.073289881	-0.108541752	0.913565963	0	0.103951371	0	0.666666667	CRA004775;PRJNA827465;Yang2025_Silkie_Wencha	0.974809833
ENSGALG00010011868	SNRPF	3	-0.007832041	0.071903813	-0.108923855	0.913262886	0	0.849547211	0	0.666666667	CRA004775;PRJNA827465;Yang2025_Silkie_Wencha	0.974809833
ENSGALG00010024402	GPC1	3	0.007748075	0.071967459	0.107660817	0.914264752	0	0.466367154	0	0.666666667	CRA004775;PRJNA827465;Yang2025_Silkie_Wencha	0.974809833
ENSGALG00010027414	PBX3	3	-0.007641751	0.070030491	-0.109120336	0.913107045	0.00542617	3.052133552	34.47206796	0.666666667	CRA004775;PRJNA827465;Yang2025_Silkie_Wencha	0.974809833
ENSGALG00010010358	ASPM	3	0.007002388	0.065444811	0.106996842	0.914791485	0	0.357710885	0	0.666666667	CRA004775;PRJNA827465;Yang2025_Silkie_Wencha	0.974809833
ENSGALG00010016677	MEAF6	3	-0.006412667	0.059534635	-0.107713215	0.914223186	0.002031798	2.272467281	11.98993196	0.666666667	CRA004775;PRJNA827465;Yang2025_Silkie_Wencha	0.974809833
ENSGALG00010007479	NFX1	3	-0.005929827	0.055625159	-0.106603327	0.915103679	0	0.052533011	0	0.666666667	CRA004775;PRJNA827465;Yang2025_Silkie_Wencha	0.974809833
ENSGALG00010017582	CLCN6	3	0.005848721	0.053516037	0.109289123	0.912973174	0.002578223	2.690403601	25.66171116	0.666666667	CRA004775;PRJNA827465;Yang2025_Silkie_Wencha	0.974809833
ENSGALG00010002348	THAP12	3	0.005407574	0.04909115	0.110153748	0.912287443	0	0.534254592	0	0.666666667	CRA004775;PRJNA827465;Yang2025_Silkie_Wencha	0.974809833
ENSGALG00010017894	KAT6A	3	-0.004802142	0.043304711	-0.110891904	0.911702066	0	1.819133494	0	0.666666667	CRA004775;PRJNA827465;Yang2025_Silkie_Wencha	0.974809833
ENSGALG00010002459	G3BP2	3	-0.004001304	0.03600265	-0.111139165	0.911505992	0	0.370807792	0	0.666666667	CRA004775;PRJNA827465;Yang2025_Silkie_Wencha	0.974809833
ENSGALG00010012945	CLINT1	3	-0.003345856	0.030893558	-0.108302704	0.913755577	0	0.886013543	0	0.666666667	CRA004775;PRJNA827465;Yang2025_Silkie_Wencha	0.974809833
ENSGALG00010023205	DNAJC17	3	0.035902182	0.337815376	0.106277525	0.915362163	0.306238361	27.72936524	92.78742956	0.666666667	CRA004775;PRJNA827465;Yang2025_Silkie_Wencha	0.974817054
ENSGALG00010005609	GJB6	3	-0.033173624	0.31234653	-0.106207755	0.915417519	0	1.099245913	0	0.666666667	CRA004775;PRJNA827465;Yang2025_Silkie_Wencha	0.974817054
ENSGALG00010017881	KCNK10	2	-0.031501079	0.297706051	-0.105812694	0.915730965	0	0.217486055	0	0.5	PRJNA827465;Yang2025_Silkie_Wenchang	0.974817054
ENSGALG00010007914	PAQR8	3	-0.020003626	0.187965655	-0.106421708	0.915247771	0.059020146	4.986948269	59.89531289	0.666666667	CRA004775;PRJNA827465;Yang2025_Silkie_Wencha	0.974817054
ENSGALG00010001635	TIMM10B	3	0.019401058	0.18294904	0.106046243	0.915545663	0.055942651	4.366947309	54.20141673	0.666666667	CRA004775;PRJNA827465;Yang2025_Silkie_Wencha	0.974817054
ENSGALG00010013383	SEPTIN10	3	0.01247585	0.117748937	0.105952976	0.915619662	0.026145639	6.981605559	71.35329426	0.666666667	CRA004775;PRJNA827465;Yang2025_Silkie_Wencha	0.974817054
ENSGALG00010025481	TMEM39A	3	-0.011906865	0.112459711	-0.105876719	0.915680166	0.03025262	10.74053771	81.37895835	0.666666667	CRA004775;PRJNA827465;Yang2025_Silkie_Wencha	0.974817054
ENSGALG00010009749	NDRG1	3	-0.007080589	0.066712904	-0.106135219	0.915475068	0	1.994371413	0	0.666666667	CRA004775;PRJNA827465;Yang2025_Silkie_Wencha	0.974817054
ENSGALG00010000555	OTUB1	3	0.006320747	0.059476012	0.106273892	0.915365045	0	1.785045472	0	0.666666667	CRA004775;PRJNA827465;Yang2025_Silkie_Wencha	0.974817054
ENSGALG00010029194	DHRS11	3	0.014515752	0.137343932	0.105689064	0.915829058	0.031061905	4.488273433	55.43943501	0.666666667	CRA004775;PRJNA827465;Yang2025_Silkie_Wencha	0.97484808
ENSGALG00010012361	WIF1	3	-0.032587979	0.309487392	-0.10529663	0.916140437	0	0.002092106	0	1	CRA004775;PRJNA827465;Yang2025_Silkie_Wencha	0.974980791
ENSGALG00010029569	TMEM220	3	0.030863402	0.292894502	0.105373782	0.916079219	0.20031055	10.85674859	81.57827839	0.666666667	CRA004775;PRJNA827465;Yang2025_Silkie_Wencha	0.974980791
ENSGALG00010020345	AGBL1	3	0.012366476	0.117472534	0.105271213	0.916160604	0	0.424277469	0	0.666666667	CRA004775;PRJNA827465;Yang2025_Silkie_Wencha	0.974980791
ENSGALG00010025629	ST6GAL1	3	0.008703311	0.082795417	0.105118272	0.91628196	0.007574772	3.124599304	35.99179269	0.666666667	CRA004775;PRJNA827465;Yang2025_Silkie_Wencha	0.97503655
ENSGALG00010009309	TRIM54	3	-0.014747565	0.140424799	-0.105021086	0.916359077	0.026366388	3.710780524	46.10298326	0.666666667	CRA004775;PRJNA827465;Yang2025_Silkie_Wencha	0.975045228
ENSGALG00010020509	CRBN	3	-0.015500668	0.148307654	-0.104516976	0.916759097	0.043380873	6.170670778	67.58861278	0.666666667	CRA004775;PRJNA827465;Yang2025_Silkie_Wencha	0.975312608
ENSGALG00010022421	CMKLR1	3	0.014638059	0.139921153	0.104616483	0.916680135	0.021583073	3.116011457	35.81538362	0.666666667	CRA004775;PRJNA827465;Yang2025_Silkie_Wencha	0.975312608
ENSGALG00010010017	CCDC59	3	0.007607316	0.072851742	0.104421881	0.916834559	0.000946436	2.108736487	5.156475806	0.666666667	CRA004775;PRJNA827465;Yang2025_Silkie_Wencha	0.975312608
ENSGALG00010015701	UNC50	3	-0.006806249	0.065221008	-0.104356698	0.916886285	0	0.377832514	0	0.666666667	CRA004775;PRJNA827465;Yang2025_Silkie_Wencha	0.975312608
ENSGALG00010007137	ENSGALG0001000713	3	0.068182385	0.655658199	0.103990746	0.917176692	1.167541851	21.78130262	90.81781271	0.666666667	CRA004775;PRJNA827465;Yang2025_Silkie_Wencha	0.975435564
ENSGALG00010017605	GFPT1	3	-0.008890109	0.085557986	-0.103907411	0.917242826	0.014512024	6.120313236	67.32193398	0.666666667	CRA004775;PRJNA827465;Yang2025_Silkie_Wencha	0.975435564
ENSGALG00010020594	PQLC2	3	-0.008852429	0.085208482	-0.10389141	0.917255524	0	0.114072534	0	0.666666667	CRA004775;PRJNA827465;Yang2025_Silkie_Wencha	0.975435564
ENSGALG00010015097	MFSD2A	3	-0.008197827	0.078929001	-0.103863301	0.917277831	0	0.231147601	0	0.666666667	CRA004775;PRJNA827465;Yang2025_Silkie_Wencha	0.975435564
ENSGALG00010002999	CMTM7	3	0.018641951	0.179652845	0.103766522	0.917354635	0.022439269	2.545975844	21.44465924	0.666666667	CRA004775;PRJNA827465;Yang2025_Silkie_Wencha	0.975443873
ENSGALG00010018061	NDUFAF5	3	-0.008342489	0.080493317	-0.103642006	0.917453451	0	1.827936958	0	0.666666667	CRA004775;PRJNA827465;Yang2025_Silkie_Wencha	0.975475586
ENSGALG00010028384	ENSGALG0001002838	2	0.079316559	0.767539636	0.103338714	0.917694151	0.014994346	1.012550753	1.239518459	0.5	PRJNA827465;Yang2025_Silkie_Wenchang	0.975511435
ENSGALG00010006651	ENSGALG0001000665	3	0.014189343	0.137122955	0.103478978	0.917582833	0.031090099	4.313524984	53.63420851	0.666666667	CRA004775;PRJNA827465;Yang2025_Silkie_Wencha	0.975511435
ENSGALG00010024145	RAP1GAP3	3	0.013719483	0.132661583	0.103417148	0.917631903	0.025933573	3.991003635	49.88729194	0.666666667	CRA004775;PRJNA827465;Yang2025_Silkie_Wencha	0.975511435
ENSGALG00010007678	CDH8	3	-0.026317113	0.254930103	-0.103232661	0.917778318	0.046221468	2.566901933	22.08506393	0.666666667	CRA004775;PRJNA827465;Yang2025_Silkie_Wencha	0.975527563
ENSGALG00010016392	STMN3	3	-0.037634226	0.368097452	-0.102239843	0.918566304	0.192125523	3.813985037	47.56140938	0.666666667	CRA004775;PRJNA827465;Yang2025_Silkie_Wencha	0.975648054
ENSGALG00010008105	MMUT	3	0.02545129	0.247852505	0.102687241	0.918211201	0.164358643	33.64707709	94.05594728	0.666666667	CRA004775;PRJNA827465;Yang2025_Silkie_Wencha	0.975648054
ENSGALG00010023815	PLCD3	3	0.02016976	0.197484296	0.102133488	0.918650721	0.080355003	7.858722183	74.55057001	0.666666667	CRA004775;PRJNA827465;Yang2025_Silkie_Wencha	0.975648054
ENSGALG00010028201	ENSGALG0001002820	3	-0.016843605	0.164026131	-0.102688548	0.918210163	0.034607616	3.27489143	38.92927315	0.666666667	CRA004775;PRJNA827465;Yang2025_Silkie_Wencha	0.975648054
ENSGALG00010008910	TRIB1	3	-0.016265743	0.158334134	-0.102730489	0.918176875	0.040103785	4.594463514	56.46934634	0.666666667	CRA004775;PRJNA827465;Yang2025_Silkie_Wencha	0.975648054
ENSGALG00010012683	MDM2	3	-0.013712046	0.133451031	-0.102749648	0.918161669	0.03740486	8.14766433	75.45308791	0.666666667	CRA004775;PRJNA827465;Yang2025_Silkie_Wencha	0.975648054
ENSGALG00010012682	ENSGALG0001001268	3	0.012008443	0.117327394	0.102349869	0.918478974	0.008965364	2.471056872	19.06297169	0.666666667	CRA004775;PRJNA827465;Yang2025_Silkie_Wencha	0.975648054
ENSGALG00010002929	PDHA2	3	0.007562073	0.073708209	0.102594716	0.918284637	0	0.83338985	0	0.666666667	CRA004775;PRJNA827465;Yang2025_Silkie_Wencha	0.975648054
ENSGALG00010003375	YWHAQ	3	-0.00751738	0.073526071	-0.102241007	0.918565379	0.007647173	3.826683373	47.73541981	0.666666667	CRA004775;PRJNA827465;Yang2025_Silkie_Wencha	0.975648054
ENSGALG00010024230	LONP2	3	-0.007051708	0.068926485	-0.102307664	0.918512473	0.003316183	2.480145206	19.35956026	0.666666667	CRA004775;PRJNA827465;Yang2025_Silkie_Wencha	0.975648054
ENSGALG00010019305	SNRNP27	3	-0.005717348	0.055952157	-0.102182791	0.918611587	0	0.085598426	0	0.666666667	CRA004775;PRJNA827465;Yang2025_Silkie_Wencha	0.975648054
ENSGALG00010026434	ENSGALG0001002643	3	-0.02628308	0.258057937	-0.101849532	0.91887611	0.169634809	13.90515762	85.61684769	0.666666667	CRA004775;PRJNA827465;Yang2025_Silkie_Wencha	0.97571818
ENSGALG00010024535	ENSGALG0001002453	3	-0.013017118	0.127882749	-0.101789479	0.918923777	0.020755707	3.491037255	42.71043665	0.666666667	CRA004775;PRJNA827465;Yang2025_Silkie_Wencha	0.97571818
ENSGALG00010025905	CPT1A	3	-0.010527339	0.103382632	-0.101828897	0.918892488	0.010062328	2.881216572	30.58487794	0.666666667	CRA004775;PRJNA827465;Yang2025_Silkie_Wencha	0.97571818
ENSGALG00010022748	ENSGALG0001002274	3	0.034647971	0.34385812	0.100762403	0.919739073	0.195467668	4.529105509	55.84117005	0.666666667	CRA004775;PRJNA827465;Yang2025_Silkie_Wencha	0.975869927
ENSGALG00010021090	ADM	3	0.031591191	0.312052515	0.101236777	0.919362502	0.205085258	7.549569622	73.50842366	0.666666667	CRA004775;PRJNA827465;Yang2025_Silkie_Wencha	0.975869927
ENSGALG00010025963	OAF	3	0.024271398	0.24053465	0.100906038	0.91962505	0.118997112	9.238139501	78.35061919	0.666666667	CRA004775;PRJNA827465;Yang2025_Silkie_Wencha	0.975869927
ENSGALG00010008830	DHX34	3	-0.020052381	0.198562471	-0.100987768	0.91956017	0.088374182	10.13121147	80.25902425	0.666666667	CRA004775;PRJNA827465;Yang2025_Silkie_Wencha	0.975869927
ENSGALG00010012429	CXCR7	3	0.019185467	0.189644905	0.101165214	0.91941931	0.081099143	8.892360503	77.50878409	0.666666667	CRA004775;PRJNA827465;Yang2025_Silkie_Wencha	0.975869927
ENSGALG00010016472	TFDP1	3	-0.018876594	0.186412258	-0.101262622	0.919341987	0.094661041	32.73116924	93.88961639	0.666666667	CRA004775;PRJNA827465;Yang2025_Silkie_Wencha	0.975869927
ENSGALG00010014767	LRRC15	3	0.018458472	0.182587939	0.101093598	0.91947616	0.042445583	3.561430135	43.84278439	0.666666667	CRA004775;PRJNA827465;Yang2025_Silkie_Wencha	0.975869927
ENSGALG00010029841	SMIM5	3	0.012617381	0.125355216	0.100653018	0.919825908	0.02452829	4.620850406	56.71792366	0.666666667	CRA004775;PRJNA827465;Yang2025_Silkie_Wencha	0.975869927
ENSGALG00010011941	ENSGALG0001001194	3	0.012597213	0.124410988	0.101254828	0.919348174	0	1.117094878	0	0.666666667	CRA004775;PRJNA827465;Yang2025_Silkie_Wencha	0.975869927
ENSGALG00010003985	PRMT5	3	0.010512428	0.104383138	0.100710025	0.919780653	0.014934235	3.678526994	45.63041122	0.666666667	CRA004775;PRJNA827465;Yang2025_Silkie_Wencha	0.975869927
ENSGALG00010016310	HDAC8	3	-0.005797308	0.057542731	-0.100747879	0.919750602	0	1.052218939	0	0.666666667	CRA004775;PRJNA827465;Yang2025_Silkie_Wencha	0.975869927
ENSGALG00010028401	ENSGALG0001002840	2	-0.111299929	1.107809968	-0.10046843	0.919972445	0.824429514	1.47325837	32.12324325	0.5	PRJNA827465;Yang2025_Silkie_Wenchang	0.975952161
ENSGALG00010012277	H3C33	3	-0.035719774	0.357077559	-0.100033658	0.920317604	0.325235135	13.54628334	85.2358027	0.666666667	CRA004775;PRJNA827465;Yang2025_Silkie_Wencha	0.976212571
ENSGALG00010021958	BHLHA15	3	0.035473911	0.355100171	0.09989832	0.92042505	0.12745297	2.915921752	31.41105385	0.666666667	CRA004775;PRJNA827465;Yang2025_Silkie_Wencha	0.976212571
ENSGALG00010018560	ENSGALG0001001856	3	-0.00429399	0.042978998	-0.099909036	0.920416543	0	1.835505054	0	0.666666667	CRA004775;PRJNA827465;Yang2025_Silkie_Wencha	0.976212571
ENSGALG00010028079	ITIH3	2	0.074457221	0.746645154	0.099722366	0.920564744	0.267334678	1.198806443	16.58369823	0.5	CRA004775;PRJNA827465	0.976287497
ENSGALG00010005714	ARHGAP5	3	0.016830777	0.169183057	0.099482638	0.920755072	0.058545991	8.075933713	75.23506171	0.666666667	CRA004775;PRJNA827465;Yang2025_Silkie_Wencha	0.976416108
ENSGALG00010018758	ENSGALG0001001875	3	-0.021362922	0.215369566	-0.099191925	0.920985886	0.102325512	8.20373037	75.62084674	0.666666667	CRA004775;PRJNA827465;Yang2025_Silkie_Wencha	0.976514394
ENSGALG00010016378	WDR76	3	-0.011790593	0.118804984	-0.099243254	0.920945133	0.018629064	3.546883673	43.61247268	0.666666667	CRA004775;PRJNA827465;Yang2025_Silkie_Wencha	0.976514394
ENSGALG00010022067	TMEM130	3	-0.011016439	0.111213998	-0.09905623	0.921093624	0	0.14758807	0	0.666666667	CRA004775;PRJNA827465;Yang2025_Silkie_Wencha	0.976555395
ENSGALG00010009451	ROR2	3	-0.012537407	0.126910602	-0.098789283	0.921305578	0.014590584	2.650003244	24.52839427	0.666666667	CRA004775;PRJNA827465;Yang2025_Silkie_Wencha	0.976706873
ENSGALG00010022439	IFI35	3	0.014112411	0.14308444	0.098629947	0.921432092	0.038648972	5.570059419	64.09374031	0.666666667	CRA004775;PRJNA827465;Yang2025_Silkie_Wencha	0.976767757
ENSGALG00010017365	CD8BP	3	-0.034651633	0.352086333	-0.098418001	0.921600381	0	1.608885353	0	0.666666667	CRA004775;PRJNA827465;Yang2025_Silkie_Wencha	0.976872913
ENSGALG00010009189	ENSGALG0001000918	3	-0.047666737	0.485621513	-0.098156148	0.921808303	0.514949168	11.06824871	81.93029401	0.666666667	CRA004775;PRJNA827465;Yang2025_Silkie_Wencha	0.976946826
ENSGALG00010003896	MCUB	3	-0.020576807	0.209575745	-0.098183153	0.92178686	0.090581156	8.786618269	77.23811438	0.666666667	CRA004775;PRJNA827465;Yang2025_Silkie_Wencha	0.976946826
ENSGALG00010020102	TMEM127	3	-0.02315938	0.237030562	-0.097706302	0.922165511	0.131746474	10.1104717	80.21852926	0.666666667	CRA004775;PRJNA827465;Yang2025_Silkie_Wencha	0.977252148
ENSGALG00010022232	GPR27	3	-0.026632021	0.273452602	-0.097391726	0.922415315	0.197624311	30.20339344	93.3782275	0.666666667	CRA004775;PRJNA827465;Yang2025_Silkie_Wencha	0.977370365
ENSGALG00010024492	NTF3	2	-0.011730642	0.120441041	-0.097397379	0.922410826	0	0.000995872	0	1	PRJNA827465;Yang2025_Silkie_Wenchang	0.977370365
ENSGALG00010003132	TPBG	3	0.026175544	0.269028804	0.097296437	0.922490985	0.145990809	7.846207727	74.50997897	0.666666667	CRA004775;PRJNA827465;Yang2025_Silkie_Wencha	0.977377298
ENSGALG00010009640	NPR2	3	0.028390547	0.29234601	0.097112826	0.922636796	0.190406575	9.758400155	79.50483718	0.666666667	CRA004775;PRJNA827465;Yang2025_Silkie_Wencha	0.977458538
ENSGALG00010011414	PABPC1	3	0.010191815	0.105286421	0.096800846	0.922884552	0.015297074	3.353678174	40.36398557	0.666666667	CRA004775;PRJNA827465;Yang2025_Silkie_Wencha	0.977647762
ENSGALG00010000815	ENSGALG0001000081	3	-0.027877308	0.288792172	-0.09653069	0.923099101	0.220135693	19.50820247	89.74790218	0.666666667	CRA004775;PRJNA827465;Yang2025_Silkie_Wencha	0.977728533
ENSGALG00010008488	CCSER1	3	-0.009773809	0.101216904	-0.096563011	0.923073433	0	1.215968193	0	0.666666667	CRA004775;PRJNA827465;Yang2025_Silkie_Wencha	0.977728533
ENSGALG00010004101	ARID1B	3	-0.010602844	0.109991286	-0.096397129	0.923205172	0.017651493	3.677570715	45.61627349	0.666666667	CRA004775;PRJNA827465;Yang2025_Silkie_Wencha	0.977767635
ENSGALG00010005529	ENSGALG0001000552	3	-0.023893423	0.248496835	-0.096151821	0.923399995	0.057919134	2.851444029	29.86009968	0.666666667	CRA004775;PRJNA827465;Yang2025_Silkie_Wencha	0.977810802
ENSGALG00010014288	ARMC2	3	0.014426432	0.150552365	0.095823349	0.923660873	0	0.589296282	0	0.666666667	CRA004775;PRJNA827465;Yang2025_Silkie_Wencha	0.977810802
ENSGALG00010026433	VPS11	3	-0.010678733	0.111207122	-0.096025623	0.923500223	0.025685579	8.501083638	76.47358754	0.666666667	CRA004775;PRJNA827465;Yang2025_Silkie_Wencha	0.977810802
ENSGALG00010016707	KIF1B	3	-0.010237502	0.106533726	-0.096096346	0.923444053	0.015043257	3.540444565	43.50991908	0.666666667	CRA004775;PRJNA827465;Yang2025_Silkie_Wencha	0.977810802
ENSGALG00010009850	RIF1	3	0.008131638	0.084773085	0.095922405	0.9235822	0.006522472	2.77190606	27.84748267	0.666666667	CRA004775;PRJNA827465;Yang2025_Silkie_Wencha	0.977810802
ENSGALG00010022230	NFS1	3	-0.004202066	0.04382044	-0.095892819	0.923605698	0	1.344118581	0	0.666666667	CRA004775;PRJNA827465;Yang2025_Silkie_Wencha	0.977810802
ENSGALG00010027450	CAMSAP1	3	-0.009583284	0.100473272	-0.095381421	0.924011872	0.016614078	4.561082402	56.15075932	0.666666667	CRA004775;PRJNA827465;Yang2025_Silkie_Wencha	0.978109146
ENSGALG00010001143	CDKN2A	3	0.026447392	0.277629568	0.095261438	0.924107171	0.008639787	2.076741104	3.695265796	0.666666667	CRA004775;PRJNA827465;Yang2025_Silkie_Wencha	0.978136794
ENSGALG00010011477	SDC2	3	0.019426624	0.204580852	0.094958174	0.924348049	0.101745619	14.12961757	85.84533523	0.666666667	CRA004775;PRJNA827465;Yang2025_Silkie_Wencha	0.978318516
ENSGALG00010016150	POLG	3	-0.029791999	0.315836954	-0.094327148	0.924849285	0.283998872	50.00850823	96.00068054	0.666666667	CRA004775;PRJNA827465;Yang2025_Silkie_Wencha	0.978482792
ENSGALG00010008264	CNTNAP2	3	-0.012044267	0.127261855	-0.094641609	0.924599499	0	0.571162767	0	0.666666667	CRA004775;PRJNA827465;Yang2025_Silkie_Wencha	0.978482792
ENSGALG00010018046	CAPN15	3	-0.011791696	0.124666324	-0.09458606	0.924643623	0.025948209	4.50455879	55.60053507	0.666666667	CRA004775;PRJNA827465;Yang2025_Silkie_Wencha	0.978482792
ENSGALG00010018290	RHOT2	3	-0.011748142	0.124491359	-0.09436914	0.924815929	0.028427222	5.36766394	62.73984321	0.666666667	CRA004775;PRJNA827465;Yang2025_Silkie_Wencha	0.978482792
ENSGALG00010017695	FNDC5	3	0.009953705	0.105423475	0.094416401	0.924778388	0.004168175	2.241146131	10.75994677	0.666666667	CRA004775;PRJNA827465;Yang2025_Silkie_Wencha	0.978482792
ENSGALG00010024838	EN2	3	0.11444438	1.216105013	0.094107317	0.925023908	3.523475226	10.32080653	80.62166949	0.666666667	CRA004775;PRJNA827465;Yang2025_Silkie_Wencha	0.978521101
ENSGALG00010024516	EHHADH	3	-0.026501838	0.281569007	-0.094122	0.925012245	0.174765041	7.838069642	74.48351327	0.666666667	CRA004775;PRJNA827465;Yang2025_Silkie_Wencha	0.978521101
ENSGALG00010023216	SLC35D1	3	-0.014652409	0.156300948	-0.093744848	0.925311843	0.046777184	5.804446903	65.54365931	0.666666667	CRA004775;PRJNA827465;Yang2025_Silkie_Wencha	0.978532846
ENSGALG00010004673	RAB18	3	-0.005551364	0.059215305	-0.093748802	0.925308703	0.003555263	2.820915019	29.10101913	0.666666667	CRA004775;PRJNA827465;Yang2025_Silkie_Wencha	0.978532846
ENSGALG00010025338	FADD	3	-0.005315349	0.056637811	-0.093848072	0.925229844	0	1.278202844	0	0.666666667	CRA004775;PRJNA827465;Yang2025_Silkie_Wencha	0.978532846
ENSGALG00010025154	TMEM183A	3	-0.004515547	0.048139419	-0.093801445	0.925266884	0	0.204675355	0	0.666666667	CRA004775;PRJNA827465;Yang2025_Silkie_Wencha	0.978532846
ENSGALG00010000656	ENSGALG0001000065	3	0.03913796	0.419918645	0.093203673	0.925741756	0.470397949	20.30640045	90.15088861	0.666666667	CRA004775;PRJNA827465;Yang2025_Silkie_Wencha	0.978767867
ENSGALG00010018309	DPYSL2	3	-0.005630167	0.060320638	-0.093337326	0.92563558	0	1.412689683	0	0.666666667	CRA004775;PRJNA827465;Yang2025_Silkie_Wencha	0.978767867
ENSGALG00010027902	UBE2Q1	3	0.003308058	0.035473005	0.093255639	0.925700473	0	1.443717918	0	0.666666667	CRA004775;PRJNA827465;Yang2025_Silkie_Wencha	0.978767867
ENSGALG00010025263	SAMD14	3	-0.022059886	0.238012123	-0.092683874	0.926154708	0	0.478760932	0	0.666666667	CRA004775;PRJNA827465;Yang2025_Silkie_Wencha	0.978827179
ENSGALG00010014102	TRIM2	3	0.01445796	0.155735703	0.092836518	0.926033439	0.039241883	4.565870172	56.19673962	0.666666667	CRA004775;PRJNA827465;Yang2025_Silkie_Wencha	0.978827179
ENSGALG00010011545	USP12-like	3	0.014182971	0.153121488	0.092625607	0.926201	0.062833753	23.8811812	91.62520487	0.666666667	CRA004775;PRJNA827465;Yang2025_Silkie_Wencha	0.978827179
ENSGALG00010003352	FARSA	3	0.012521527	0.135334095	0.092523078	0.926282456	0.03693403	6.749889098	70.36988355	0.666666667	CRA004775;PRJNA827465;Yang2025_Silkie_Wencha	0.978827179
ENSGALG00010022365	PLCG1	3	0.009474858	0.102017575	0.092874761	0.926003057	0.01816304	6.283362208	68.16990755	0.666666667	CRA004775;PRJNA827465;Yang2025_Silkie_Wencha	0.978827179
ENSGALG00010012812	DECR1	3	-0.007626417	0.082381404	-0.092574501	0.926241602	0	1.127207756	0	0.666666667	CRA004775;PRJNA827465;Yang2025_Silkie_Wencha	0.978827179
ENSGALG00010025426	ENSGALG0001002542	3	0.007301701	0.07851771	0.092994316	0.925908077	0.00613966	2.941809237	32.0146264	0.666666667	CRA004775;PRJNA827465;Yang2025_Silkie_Wencha	0.978827179
ENSGALG00010007133	BLVRA	3	-0.018539321	0.200915379	-0.092274274	0.926480128	0.054870463	3.684507658	45.7186635	0.666666667	CRA004775;PRJNA827465;Yang2025_Silkie_Wencha	0.978889742
ENSGALG00010021619	FHOD1	3	-0.010797367	0.116931462	-0.09233928	0.926428481	0.013272468	2.873941961	30.40917223	0.666666667	CRA004775;PRJNA827465;Yang2025_Silkie_Wencha	0.978889742
ENSGALG00010011780	SERPINB5	2	0.051432523	0.564201258	0.091159888	0.927365547	0.184730064	1.387048977	27.90449242	0.5	PRJNA827465;Yang2025_Silkie_Wenchang	0.978973428
ENSGALG00010004057	ENSGALG0001000405	3	-0.033284198	0.366997862	-0.090693167	0.9277364	0.301721119	8.080403352	75.24876033	0.666666667	CRA004775;PRJNA827465;Yang2025_Silkie_Wencha	0.978973428
ENSGALG00010025801	PTPN7	3	-0.032141757	0.350310249	-0.091752259	0.926894876	0.148430531	3.349574919	40.29093099	0.666666667	CRA004775;PRJNA827465;Yang2025_Silkie_Wencha	0.978973428
ENSGALG00010009440	DOK3	3	0.029214086	0.32077112	0.091074553	0.927433352	0.210330796	6.336170837	68.43519451	0.666666667	CRA004775;PRJNA827465;Yang2025_Silkie_Wencha	0.978973428
ENSGALG00010029750	TEN1	3	-0.023908987	0.261349248	-0.091482898	0.927108895	0.098852104	4.059593174	50.73397963	0.666666667	CRA004775;PRJNA827465;Yang2025_Silkie_Wencha	0.978973428
ENSGALG00010001271	ENSGALG0001000127	3	0.019073829	0.209465208	0.091059651	0.927445193	0.076660661	4.715365688	57.58547412	0.666666667	CRA004775;PRJNA827465;Yang2025_Silkie_Wencha	0.978973428
ENSGALG00010025753	SORL1	3	0.0119867	0.132164324	0.09069543	0.927734602	0.015683941	2.735367042	26.88366974	0.666666667	CRA004775;PRJNA827465;Yang2025_Silkie_Wencha	0.978973428
ENSGALG00010029648	NIPSNAP2	3	0.010131115	0.111417825	0.090929028	0.927548985	0.01828191	4.056777347	50.69978387	0.666666667	CRA004775;PRJNA827465;Yang2025_Silkie_Wencha	0.978973428
ENSGALG00010018894	SERTAD4	3	0.010114421	0.110553251	0.09148913	0.927103943	0.02208385	5.401168155	62.97097327	0.666666667	CRA004775;PRJNA827465;Yang2025_Silkie_Wencha	0.978973428
ENSGALG00010022071	ACTA2	3	-0.009785205	0.10695072	-0.091492652	0.927101145	0.006714008	2.407972633	16.94257766	0.666666667	CRA004775;PRJNA827465;Yang2025_Silkie_Wencha	0.978973428

ENSGALG00010017825	PLPP5	3	0.007621569	0.083708985	0.091048401	0.927454133	0	0.100463214	0	0.666666667	CRA004775;PRJNA827465;Yang2025_Silkie_Wencha	0.978973428
ENSGALG00010009368	DSCC1	3	-0.007508921	0.082538886	-0.090974346	0.927512976	0	1.00051462	0	0.666666667	CRA004775;PRJNA827465;Yang2025_Silkie_Wencha	0.978973428
ENSGALG00010009809	SMDT1	3	-0.007435476	0.081851229	-0.090841349	0.927618654	0	0.635477961	0	0.666666667	CRA004775;PRJNA827465;Yang2025_Silkie_Wencha	0.978973428
ENSGALG00010005964	HSPA14	3	-0.007224977	0.07912346	-0.091312703	0.927244125	0.00456417	2.472224456	19.10119668	0.666666667	CRA004775;PRJNA827465;Yang2025_Silkie_Wencha	0.978973428
ENSGALG00010012130	TMEM67	3	-0.007217873	0.079432381	-0.09086815	0.927597358	0.002224069	2.16792535	7.745900953	0.666666667	CRA004775;PRJNA827465;Yang2025_Silkie_Wencha	0.978973428
ENSGALG00010014997	RAE1	3	0.004470379	0.048960191	0.091306411	0.927249124	0	0.720139363	0	0.666666667	CRA004775;PRJNA827465;Yang2025_Silkie_Wencha	0.978973428
ENSGALG00010013791	AEBP2	3	-0.004038066	0.043975433	-0.091825503	0.926836682	0	0.599537951	0	0.666666667	CRA004775;PRJNA827465;Yang2025_Silkie_Wencha	0.978973428
ENSGALG00010003269	ENSGALG00010003269	3	0.054988519	0.609407807	0.090232712	0.92810229	0.907880996	15.22031002	86.85966319	0.666666667	CRA004775;PRJNA827465;Yang2025_Silkie_Wencha	0.979286439
ENSGALG00010005479	ENSGALG00010005479	2	-0.081368033	0.924828534	-0.08798175	0.929891181	1.420754022	5.641712518	82.27488556	0.5	PRJNA827465;Yang2025_Silkie_Wenchang	0.979318494
ENSGALG00010002261	NR0B1	3	0.059668711	0.662534554	0.090061281	0.928238517	0.997968153	8.657445805	76.89849818	0.666666667	CRA004775;PRJNA827465;Yang2025_Silkie_Wencha	0.979318494
ENSGALG00010007976	ENSGALG00010007976	3	0.038271629	0.42750772	0.089522662	0.928666544	0.388389165	7.039663103	71.58954952	0.666666667	CRA004775;PRJNA827465;Yang2025_Silkie_Wencha	0.979318494
ENSGALG00010021206	HSD17B1	2	0.0292709	0.327320345	0.089425851	0.92874348	0	0.253091774	0	0.5	CRA004775;Yang2025_Silkie_Wenchang	0.979318494
ENSGALG00010018993	ENSGALG00010018993	2	0.027059042	0.305775751	0.088493092	0.929484774	0	0.090731462	0	0.5	CRA004775;PRJNA827465	0.979318494
ENSGALG00010023767	ENSGALG00010023767	3	-0.022276088	0.250771061	-0.088830479	0.929216714	0.118365371	5.623699489	64.43622203	0.666666667	CRA004775;PRJNA827465;Yang2025_Silkie_Wencha	0.979318494
ENSGALG00010011192	RPL17	3	0.020399964	0.228778234	0.089169166	0.92894747	0.093093225	4.952273668	59.61459974	0.666666667	CRA004775;PRJNA827465;Yang2025_Silkie_Wencha	0.979318494
ENSGALG00010012362	COL6A3	3	-0.01949355	0.219004434	-0.08900984	0.92907409	0.074849017	4.382296505	54.36182838	0.666666667	CRA004775;PRJNA827465;Yang2025_Silkie_Wencha	0.979318494
ENSGALG00010021452	SLC25A1	3	0.017770335	0.199743548	0.088965752	0.929109128	0.086613529	7.338782069	72.74752157	0.666666667	CRA004775;PRJNA827465;Yang2025_Silkie_Wencha	0.979318494
ENSGALG00010003027	SCIN	3	-0.016961284	0.192508165	-0.088106827	0.92979177	0.032148225	2.745465415	27.15260628	0.666666667	CRA004775;PRJNA827465;Yang2025_Silkie_Wencha	0.979318494
ENSGALG00010029612	HSPB1	3	0.014897758	0.167132073	0.089137638	0.928972526	0.041141807	4.325312685	53.76056842	0.666666667	CRA004775;PRJNA827465;Yang2025_Silkie_Wencha	0.979318494
ENSGALG00010003229	STIM1	3	-0.012822622	0.145177559	-0.088323719	0.929619388	0.047389313	8.453739289	76.34183015	0.666666667	CRA004775;PRJNA827465;Yang2025_Silkie_Wencha	0.979318494
ENSGALG00010001136	SERP1	3	-0.012073715	0.134749306	-0.089601312	0.928604042	0.036368517	6.134215535	67.39599402	0.666666667	CRA004775;PRJNA827465;Yang2025_Silkie_Wencha	0.979318494
ENSGALG00010027711	WNK2	3	0.012006795	0.13396596	0.089625718	0.928584647	0.020246276	3.068804688	34.82804533	0.666666667	CRA004775;PRJNA827465;Yang2025_Silkie_Wencha	0.979318494
ENSGALG00010005536	TAF4B	3	-0.011355668	0.126621696	-0.089681851	0.928540039	0	0.889281044	0	0.666666667	CRA004775;PRJNA827465;Yang2025_Silkie_Wencha	0.979318494
ENSGALG00010002358	FREM2	3	-0.01060428	0.120176155	-0.08823947	0.929686348	0	1.706316787	0	0.666666667	CRA004775;PRJNA827465;Yang2025_Silkie_Wencha	0.979318494
ENSGALG00010024463	FBXL22	3	-0.010555966	0.119154699	-0.08859043	0.929407414	0	1.063273757	0	0.666666667	CRA004775;PRJNA827465;Yang2025_Silkie_Wencha	0.979318494
ENSGALG00010016535	BNC2	3	0.010415427	0.118453399	0.087928476	0.929933524	0	1.837806416	0	0.666666667	CRA004775;PRJNA827465;Yang2025_Silkie_Wencha	0.979318494
ENSGALG00010024752	MPP2	3	-0.009673453	0.108831808	-0.088884432	0.929173756	0	0.894386375	0	0.666666667	CRA004775;PRJNA827465;Yang2025_Silkie_Wencha	0.979318494
ENSGALG00010005177	AASDHPPT	3	-0.00950798	0.107432406	-0.08850197	0.929477719	0	0.085491238	0	0.666666667	CRA004775;PRJNA827465;Yang2025_Silkie_Wencha	0.979318494
ENSGALG00010019820	BBOX1	3	-0.009232601	0.103533632	-0.0891749	0.928942913	0	0.458393909	0	0.666666667	CRA004775;PRJNA827465;Yang2025_Silkie_Wencha	0.979318494
ENSGALG00010029167	ENSGALG00010029167	3	-0.008910588	0.100955407	-0.088262609	0.929667957	0.011146222	2.786014208	28.21285713	0.666666667	CRA004775;PRJNA827465;Yang2025_Silkie_Wencha	0.979318494
ENSGALG00010015797	PIP5K1B	3	-0.008950153	0.097788004	-0.089347359	0.928805858	0	0.795218434	0	0.666666667	CRA004775;PRJNA827465;Yang2025_Silkie_Wencha	0.979318494
ENSGALG00010015959	TRNAU1AP	3	0.005931098	0.06691038	0.088642423	0.929366092	0	0.970362766	0	0.666666667	CRA004775;PRJNA827465;Yang2025_Silkie_Wencha	0.979318494
ENSGALG00010010420	NPM1	3	-0.005373099	0.061001829	-0.088080946	0.92981234	0	0.361111721	0	0.666666667	CRA004775;PRJNA827465;Yang2025_Silkie_Wencha	0.979318494
ENSGALG00010003121	ARMC1	3	0.003581953	0.040073272	0.089385092	0.928775871	0	1.571228302	0	0.666666667	CRA004775;PRJNA827465;Yang2025_Silkie_Wencha	0.979318494
ENSGALG00010020350	PDZRN3	3	0.016313673	0.186281633	0.087682684	0.930128883	0.074208274	11.52189774	82.64174839	0.666666667	CRA004775;PRJNA827465;Yang2025_Silkie_Wencha	0.979332751
ENSGALG00010014579	NNT	3	-0.012155772	0.138961673	-0.087475719	0.930293385	0.03601247	5.867488088	65.91386348	0.666666667	CRA004775;PRJNA827465;Yang2025_Silkie_Wencha	0.979332751
ENSGALG00010005476	NFKB1	3	0.008518032	0.09726344	0.087576919	0.930212947	0.015741697	4.678801206	57.25400777	0.666666667	CRA004775;PRJNA827465;Yang2025_Silkie_Wencha	0.979332751
ENSGALG00010011638	GRPEL1	3	-0.008341452	0.095306791	-0.087522118	0.930256505	0.014424116	4.376952196	54.30610364	0.666666667	CRA004775;PRJNA827465;Yang2025_Silkie_Wencha	0.979332751
ENSGALG00010003271	AKAP11	3	0.006441437	0.07344852	0.08770002	0.930115103	0	0.750400233	0	0.666666667	CRA004775;PRJNA827465;Yang2025_Silkie_Wencha	0.979332751
ENSGALG00010004379	ZBTB10	3	-0.006726564	0.07699271	-0.087366243	0.930380401	0.008888897	3.981776897	49.77116871	0.666666667	CRA004775;PRJNA827465;Yang2025_Silkie_Wencha	0.979351436
ENSGALG00010027407	ENSGALG00010027407	2	-0.080243728	0.923380124	-0.086902161	0.930749282	0	0.127799931	0	0.5	PRJNA827465;Yang2025_Silkie_Wenchang	0.979504478
ENSGALG00010010468	ENSGALG00010010468	3	-0.08003091	0.921590404	-0.086839999	0.930798693	1.714793841	6.231454267	67.90476325	0.666666667	CRA004775;PRJNA827465;Yang2025_Silkie_Wencha	0.979504478
ENSGALG00010025604	HVCN1	3	0.020893713	0.242072498	0.0863118	0.931218558	0.13225414	9.056216051	77.91572122	0.666666667	CRA004775;PRJNA827465;Yang2025_Silkie_Wencha	0.979504478
ENSGALG00010002597	ENSGALG00010002597	3	0.020731274	0.239780762	0.086459287	0.931101319	0.151797054	17.91677673	88.83727787	0.666666667	CRA004775;PRJNA827465;Yang2025_Silkie_Wencha	0.979504478
ENSGALG00010024885	SLC4A1	3	0.0194719	0.224275198	0.086821461	0.930813429	0.095421827	5.922114828	66.22828064	0.666666667	CRA004775;PRJNA827465;Yang2025_Silkie_Wencha	0.979504478
ENSGALG00010010993	ENSGALG00010010993	3	-0.015219605	0.176077062	-0.086437182	0.93111889	0	0.634792244	0	0.666666667	CRA004775;PRJNA827465;Yang2025_Silkie_Wencha	0.979504478
ENSGALG00010009439	B3GALNT2	3	-0.010879786	0.12589953	-0.086416411	0.931135402	0.032776347	7.188809369	72.17898128	0.666666667	CRA004775;PRJNA827465;Yang2025_Silkie_Wencha	0.979504478
ENSGALG00010021562	LRP6	3	0.007558547	0.087283054	0.086598101	0.930990975	0	0.97747726	0	0.666666667	CRA004775;PRJNA827465;Yang2025_Silkie_Wencha	0.979504478
ENSGALG00010017633	HLX	3	-0.007371971	0.085089974	-0.086637362	0.930959767	0	0.652924887	0	0.666666667	CRA004775;PRJNA827465;Yang2025_Silkie_Wencha	0.979504478
ENSGALG00010019145	RAC1	3	0.003133629	0.036275405	0.086384392	0.931160854	0	1.235568511	0	0.666666667	CRA004775;PRJNA827465;Yang2025_Silkie_Wencha	0.979504478
ENSGALG00010016972	ASB3	3	0.010752662	0.124756075	0.086189483	0.931315791	0.0236977	4.163041213	51.9581984	0.666666667	CRA004775;PRJNA827465;Yang2025_Silkie_Wencha	0.979533882
ENSGALG00010025762	ENSGALG00010025762	2	-0.143984881	1.678402354	-0.085786868	0.931635846	0	0.039067906	0	0.5	PRJNA827465;Yang2025_Silkie_Wenchang	0.979579031
ENSGALG00010002291	RNASEH1	3	0.011421951	0.132995956	0.085881945	0.931560264	0.03795503	7.81875141	74.42046824	0.666666667	CRA004775;PRJNA827465;Yang2025_Silkie_Wencha	0.979579031
ENSGALG00010028860	SURF2	3	0.005006317	0.058302872	0.085867423	0.931571809	0	0.111173019	0	1	CRA004775;PRJNA827465;Yang2025_Silkie_Wencha	0.979579031
ENSGALG00010009948	WBP2NL	3	-0.004873676	0.056786098	-0.085825154	0.93160541	0.003734721	3.216259594	37.81596473	0.666666667	CRA004775;PRJNA827465;Yang2025_Silkie_Wencha	0.979579031
ENSGALG00010000173	NIPSNAP1	3	0.013483587	0.157826685	0.08543287	0.931917262	0.031922304	3.401994146	41.21095117	0.666666667	CRA004775;PRJNA827465;Yang2025_Silkie_Wencha	0.979802065
ENSGALG00010005154	CWF19L2	3	0.022610991	0.265415457	0.085190936	0.932109597	0.182367187	17.73829948	88.72496204	0.666666667	CRA004775;PRJNA827465;Yang2025_Silkie_Wencha	0.979880297
ENSGALG00010001870	PLP2	3	-0.011986691	0.140746806	-0.085164922	0.932130277	0.01398252	2.548190734	21.51293961	0.666666667	CRA004775;PRJNA827465;Yang2025_Silkie_Wencha	0.979880297
ENSGALG00010013283	PAWR	3	0.022417562	0.263780978	0.084985513	0.932272908	0.147617282	6.990350245	71.3891303	0.666666667	CRA004775;PRJNA827465;Yang2025_Silkie_Wencha	0.979881344
ENSGALG00010021706	ENSGALG00010021706	3	-0.009592231	0.112979842	-0.084902149	0.932339184	0.003802383	2.193792475	8.833673985	0.666666667	CRA004775;PRJNA827465;Yang2025_Silkie_Wencha	0.979881344
ENSGALG00010010671	SIGMAR1	3	-0.009069324	0.106783194	-0.084932127	0.93231535	0.009324085	2.716038426	26.36333931	0.666666667	CRA004775;PRJNA827465;Yang2025_Silkie_Wencha	0.979881344
ENSGALG00010027403	ACKR2	3	-0.036070483	0.42570307	-0.084731555	0.932474809	0.359819514	6.017199468	66.76194614	0.666666667	CRA004775;PRJNA827465;Yang2025_Silkie_Wencha	0.979951043
ENSGALG00010011953	CDK17	3	0.008788927	0.103913015	0.084579658	0.932595572	0.019417918	5.202927238	61.56010052	0.666666667	CRA004775;PRJNA827465;Yang2025_Silkie_Wencha	0.980005113
ENSGALG00010010987	MARCHF7	3	-0.004184506	0.049543369	-0.084461471	0.932689535	0.002640088	3.091983484	35.31660146	0.666666667	CRA004775;PRJNA827465;Yang2025_Silkie_Wencha	0.980031015
ENSGALG00010025932	AP1S3	3	-0.021570544	0.257817968	-0.083665789	0.933322158	0.049342181	2.646030619	24.41508479	0.666666667	CRA004775;PRJNA827465;Yang2025_Silkie_Wencha	0.980554502
ENSGALG00010005172	OSBPL1A	3	0.01871484	0.22370008	0.083660407	0.933326437	0.123891014	12.93928485	84.54319521	0.666666667	CRA004775;PRJNA827465;Yang2025_Silkie_Wencha	0.980554502
ENSGALG00010004172	ST8SIA6	3	-0.008857278	0.106100233	-0.083480285	0.933469653	0	1.190035909	0	0.666666667	CRA004775;PRJNA827465;Yang2025_Silkie_Wencha	0.980632099
ENSGALG00010002565	MRPS18C	3	0.00607581	0.072925688	0.08331509	0.933601002	0	0.610121565	0	0.666666667	CRA004775;PRJNA827465;Yang2025_Silkie_Wencha	0.980697219
ENSGALG00010018523	TEX11	3	0.010406742	0.125311157	0.083047214	0.933813998	0.013233261	2.737216818	26.93308081	0.666666667	CRA004775;PRJNA827465;Yang2025_Silkie_Wencha	0.980848088
ENSGALG00010005601	TMEM215	3	-0.039308194	0.475087619	-0.082738831	0.934059208	0.443048242	6.314447718	68.32660449	0.666666667	CRA004775;PRJNA827465;Yang2025_Silkie_Wencha	0.980887043
ENSGALG00010005836	NEBL	3	0.0116197	0.140239776	0.082855947	0.933966083	0	0.000390011	0	1	CRA004775;PRJNA827465;Yang2025_Silkie_Wencha	0.980887043
ENSGALG00010024620	MSANTD2	3	0.005015623	0.060608495	0.08275446	0.934046781	0.002501149	2.510905308	20.34745422	0.666666667	CRA004775;PRJNA827465;Yang2025_Silkie_Wencha	0.980887043
ENSGALG00010025852	BTBD19	3	0.01289227	0.156585865	0.082333548	0.934381478	0.033313744	3.668513332	45.48200268	0.666666667	CRA004775;PRJNA827465;Yang2025_Silkie_Wencha	0.981079735
ENSGALG00010027644	NAA80	3	0.011059211	0.134237795	0.082385223	0.934340387	0	1.617953282	0	0.666666667	CRA004775;PRJNA827465;Yang2025_Silkie_Wencha	0.981079735
ENSGALG00010015059	ENSGALG00010015059	3	0.018811342	0.229076374	0.082118211	0.934552711	0.129920895	15.73511192	87.28957246	0.666666667	CRA004775;PRJNA827465;Yang2025_Silkie_Wencha	0.981102536
ENSGALG00010029733	ENSGALG00010029733	3	-0.008872019	0.108474066	-0.081789307	0.934814259	0	0.358967367	0	0.666666667	CRA004775;PRJNA827465;Yang2025_Silkie_Wencha	0.981102536
ENSGALG00010005858	CFAP97	3	-0.007915356	0.096690379	-0.081862908	0.93475573	0.009599113	2.992571446	33.16784457	0.666666667	CRA004775;PRJNA827465;Yang2025_Silkie_Wencha	0.981102536
ENSGALG00010010374	CMTM4	3	-0.007360184	0.089881547	-0.081887595	0.934736099	0	1.642037206	0	0.666666667	CRA004775;PRJNA827465;Yang2025_Silkie_Wencha	0.981102536
ENSGALG00010021650	CD276	3	0.004042992	0.049385305	0.08186629	0.934753042	0	0.740868503	0	0.666666667	CRA004775;PRJNA827465;Yang2025_Silkie_Wencha	0.981102536
ENSGALG00010016397	LIAS	3	-0.004039917	0.049461213	-0.081678495	0.93490238	0.000462048	2.094165749	4.496575724	0.666666667	CRA004775;PRJNA827465;Yang2025_Silkie_Wencha	0.981102536
ENSGALG00010029652	LRRC59	3	0.00388524	0.047608472	0.081608161	0.934958312	0	0.340913198	0	0.666666667	CRA004775;PRJNA827465;Yang2025_Silkie_Wencha	0.981102536
ENSGALG00010008301	FAM91A1	3	0.003460597	0.042342816	0.081728077	0.934862951	0	0.32299386	0	0.666666667	CRA004775;PRJNA827465;Yang2025_Silkie_Wencha	0.981102536
ENSGALG00010027944	WDR31	3	0.013409544	0.166007907	0.08077654	0.935619666	0	0.51930431	0	0.666666667	CRA004775;PRJNA827465;Yang2025_Silkie_Wencha	0.98165082
ENSGALG00010021577	ARIH1	3	0.005026956	0.062172971	0.080854365	0.935557772	0.00338544	2.585105446	22.63371681	0.666666667	CRA004775;PRJNA827465;Yang2025_Silkie_Wencha	0.98165082
ENSGALG00010013989	ENSGALG00010013989	3	-0.023445596	0.294672742	-0.079564861	0.936583343	0.208104727	10.0930469	80.18437821	0.666666667	CRA004775;PRJNA827465;Yang2025_Silkie_Wencha	0.981843465
ENSGALG00010007182	ENSGALG00010007182	3	0.022717638	0.286274981	0.079356004	0.936749462	0.149048784	5.095246256	60.74772642	0.666666667	CRA004775;PRJNA827465;Yang2025_Silkie_Wencha	0.981843465
ENSGALG00010003834	MAPK10	2	-0.02151927	0.268738532	-0.08007512	0.93617751	0.029646682	1.25234786	20.14998132	0.5	PRJNA827465;Yang2025_Silkie_Wenchang	0.981843465
ENSGALG00010023702	COL4A4	3	0.015972158	0.201716994	0.079181024	0.936888638	0.053920729	3.675050623	45.57898094	0.666666667	CRA004775;PRJNA827465;Yang2025_Silkie_Wencha	0.981843465
ENSGALG00010001313	EMILIN2	3	-0.014760416	0.186400638	-0.07918651	0.936884274	0.066565943	6.313164047	68.32016426	0.666666667	CRA004775;PRJNA827465;Yang2025_Silkie_Wencha	0.981843465
ENSGALG00010008408	SMARCA4	3	-0.013860058	0.173764262	-0.079763569	0.9364253	0.078377882	16.96614748	88.21182002	0.666666667	CRA004775;PRJNA827465;Yang2025_Silkie_Wencha	0.981843465
ENSGALG00010017927	PCSK2	3	0.013716565	0.171061275	0.080185095	0.936090044	0.058275084	6.158727359	67.52575843	0.666666667	CRA004775;PRJNA827465;Yang2025_Silkie_Wencha	0.981843465
ENSGALG00010024994	NDUFB4	3	0.013602913	0.169381082	0.08030952	0.935991087	0.047665244	4.4962382	55.51837089	0.666666667	CRA004775;PRJNA827465;Yang2025_Silkie_Wencha	0.981843465
ENSGALG00010018535	FAM174B	3	-0.011462788	0.144314122	-0.079429425	0.936691065	0.046017849	13.86891609	85.57926238	0.666666667	CRA004775;PRJNA827465;Yang2025_Silkie_Wencha	0.981843465
ENSGALG00010013509	ENSGALG00010013509	3	0.01081803	0.135561424	0.079801686	0.936394983	0.022126609	3.372394542	40.69495799	0.666666667	CRA004775;PRJNA827465;Yang2025_Silkie_Wencha	0.981843465
ENSGALG00010028869	ADAMTS13	3	-0.010813801	0.13516244	-0.080005958	0.936232517	0	1.108954082	0	0.666666667	CRA004775;PRJNA827465;Yang2025_Silkie_Wencha	0.981843465
ENSGALG00010025312	PLEKHM3	3	0.008793597	0.110938655	0.079265403	0.936821524	0.018100721	4.042123665	50.52105859	0.666666667	CRA004775;PRJNA827465;Yang2025_Silkie_Wencha	0.981843465
ENSGALG00010029187	SRSF1	3	0.006933462	0.087673073	0.079083144	0.93696649	0.011825514	4.069669026	50.85595444	0.666666667	CRA004775;PRJNA827465;Yang2025_Silkie_Wencha	0.981843465
ENSGALG00010009806	WDCP	3	0.006046667	0.075658925	0.079920075	0.936300823	0.002798949	2.312413174	13.51026615	0.666666667	CRA004775;PRJNA827465;Yang2025_Silkie_Wencha	0.981843465
ENSGALG00010014931	RNF145	3	-0.005713758	0.072269891	-0.079061389	0.936983794	0.00619042	3.246193944	38.38938662	0.666666667	CRA004775;PRJNA827465;Yang2025_Silkie_Wencha	0.981843465
ENSGALG00010019693	HAUS2	3	-0.004316904	0.05444577	-0.079288138	0.936803441	0	0.804704962	0	0.666666667	CRA004775;PRJNA827465;Yang2025_Silkie_Wencha	0.981843465
ENSGALG00010013498	KDR	3	-0.004248437	0.053675427	-0.079150503	0.936912914	0	0.474705954	0	0.666666667	CRA004775;PRJNA827465;Yang2025_Silkie_Wencha	0.981843465
ENSGALG00010000596	ENSGALG00010000596	3	-0.075667068	0.958599592	-0.07893501	0.937084316	1.651538653	5.181903353	61.40414315	0.666666667	CRA004775;PRJNA827465;Yang2025_Silkie_Wencha	0.98187603
ENSGALG00010026410	ENSGALG00010026410	3	-0.075236242	0.965762488	-0.077902368	0.937905716	2.387798988	15.86235553	87.39153213	0.666666667	CRA004775;PRJNA827465;Yang2025_Silkie_Wencha	0.981988366
ENSGALG00010012850	CALB1	3	0.006093234	0.76648373	0.078401186	0.93750893	1.105750976	5.765281903	65.30958878	0.666666667	CRA004775;PRJNA827465;Yang2025_Silkie_Wencha	0.981988366
ENSGALG00010017442	LCK	3	0.032882794	0.419190275	0.0784436	0.937475193	0.435270281	12.57480163	84.09517654	0.666666667	CRA004775;PRJNA827465;Yang2025_Silkie_Wencha	0.981988366
ENSGALG00010021142	WNT6	3	0.030183218	0.389193286	0.077553285	0.938183403	0	0.327555213	0	0.666666667	CRA004775;PRJNA827465;Yang2025_Silkie_Wencha	0.981988366
ENSGALG00010000306	ENSGALG00010000306	3	0.029764118	0.380759381	0.078170413	0.937692497	0.406961942	48.47684549	95.87431901	0.666666667	CRA004775;PRJNA827465;Yang2025_Silkie_Wencha	0.981988366
ENSGALG00010019836	MINAR1	2	0.018623023	0.240723985	0.077362556	0.938335127	0	0.443545885	0	0.5	PRJNA827465;Yang2025_Silkie_Wenchang	0.981988366
ENSGALG00010003960	ASPH	3	-0.017511074	0.224062124	-0.078152765	0.937706535	0.110595352	10.14729961	80.29032278	0.666666667	CRA004775;PRJNA827465;Yang2025_Silkie_Wencha	0.981988366
ENSGALG00010009047	SUPT5H	3	0.015585502	0.201139015	0.077486219	0.938236754	0.100710141	17.36388507	88.48184037	0.666666667	CRA004775;PRJNA827465;Yang2025_Silkie_Wencha	0.981988366
ENSGALG00010007569	GPR89A	3	-0.015318803	0.197132482	-0.077708161	0.938060202	0.094994169	13.46140679	85.14271182	0.666666667	CRA004775;PRJNA827465;Yang2025_Silkie_Wencha	0.981988366
ENSGALG00010015180	PITHD1	3	0.010974902	0.140944822	0.077866658	0.937934122	0.030440647	4.06723944	50.826598	0.666666667	CRA004775;PRJNA827465;Yang2025_Silkie_Wencha	0.981988366
ENSGALG00010028027	ENSGALG00010028027	3	-0.010781741	0.13931449	-0.077391384	0.938312195	0.003314091	2.074594951	3.595639286	0.666666667	CRA004775;PRJNA827465;Yang2025_Silkie_Wencha	0.981988366
ENSGALG00010018774	MSRB1	3	-0.00888741	0.113462203	-0.078329256	0.937566146	0.006763359	2.284572472	12.45626811	0.666666667	CRA004775;PRJNA827465;Yang2025_Silkie_Wencha	0.981988366
ENSGALG00010016851	TEC	3	0.008881708	0.114310131	0.0776983	0.938067999	0.014293607	3.104317417	35.57358959	0.666666667	CRA004775;PRJNA827465;Yang2025_Silkie_Wencha	0.981988366
ENSGALG00010002752	NDUFA4	3	-0.008336861	0.107345954	-0.077663489	0.938095738	0.012954449	2.924957143	31.62292976	0.666666667	CRA004775;PRJNA827465;Yang2025_Silkie_Wencha	0.981988366
ENSGALG00010029727	ZSWIM7	3	-0.008236769	0.105068619	-0.078394192	0.937514494	0	1.518094853	0	0.666666667	CRA004775;PRJNA827465;Yang2025_Silkie_Wencha	0.981988366
ENSGALG00010011413	ZBTB37	3	-0.006439027	0.083376179	-0.077228623	0.938441672	0	0.581757036	0	0.666666667	CRA004775;PRJNA827465;Yang2025_Silkie_Wencha	0.981988366
ENSGALG00010016560	DCUN1D4	3	0.006096208	0.078903884	0.077261186	0.938415768	0.003049258	2.339534637	14.51291345	0.666666667	CRA004775;PRJNA827465;Yang2025_Silkie_Wencha	0.981988366
ENSGALG00010005508	SS18	3	0.003726053	0.047997107	0.077630788	0.938121751	0	0.953695502	0	0.666666667	CRA004775;PRJNA827465;Yang2025_Silkie_Wencha	0.981988366
ENSGALG00010006843	STON2	3	-0.010478288	0.135854362	-0.077128828	0.93852106	0.019168937	3.027341525	33.93543531	0.666666667	CRA004775;PRJNA827465;Yang2025_Silkie_Wencha	0.981998761
ENSGALG00010000289	QPRT	3	0.052055677	0.678564825	0.076714376	0.938850768	1.135502051	11.80753536	83.0616641	0.666666667	CRA004775;PRJNA827465;Yang2025_Silkie_Wencha	0.982198372
ENSGALG00010003818	GAB2	3	-0.009817066	0.127947518	-0.07672729	0.938840494	0	0.939829037	0	0.666666667	CRA004775;PRJNA827465;Yang2025_Silkie_Wencha	0.982198372
ENSGALG00010009258	DNAJC12	3	-0.007590718	0.099480517	-0.076303562	0.939177592	0	1.987329949	0	0.666666667	CRA004775;PRJNA827465;Yang2025_Silkie_Wencha	0.982361615
ENSGALG00010010397	PICK1	3	0.006818944	0.089421454	0.076256237	0.939215242	0.014735815	6.320870366	68.35878789	0.666666667	CRA004775;PRJNA827465;Yang2025_Silkie_Wencha	0.982361615
ENSGALG00010015698	TNFAIP2	3	-0.005832684	0.076421141	-0.07632291	0.939162199	0	0.731026828	0	0.666666667	CRA004775;PRJNA827465;Yang2025_Silkie_Wencha	0.982361615
ENSGALG00010029180	CA4	3	-0.033869107	0.445979337	-0.07594322	0.939464272	0.456329593	9.189965853	78.2371335	0.666666667	CRA004775;PRJNA827465;Yang2025_Silkie_Wencha	0.982404063
ENSGALG00010005114	PHLDB2	3	0.010784598	0.141903895	0.075999309	0.939419648	0.037273651	6.288161809	68.19420268	0.666666667	CRA004775;PRJNA827465;Yang2025_Silkie_Wencha	0.982404063
ENSGALG00010020770	B4GALNT4	3	0.00643964	0.084759445	0.075975481	0.939438605	0.005984467	2.526437867	20.83715866	0.666666667	CRA004775;PRJNA827465;Yang2025_Silkie_Wencha	0.982404063
ENSGALG00010001160	TRMT10B	3	-0.0117901	0.155873577	-0.075638863	0.939706417	0.029908118	3.402754884	41.22409436	0.666666667	CRA004775;PRJNA827465;Yang2025_Silkie_Wencha	0.982584605
ENSGALG00010009594	HINT2	3	0.021266129	0.282818772	0.075193486	0.940060767	0.142013165	4.949762231	59.59401873	0.666666667	CRA004775;PRJNA827465;Yang2025_Silkie_Wencha	0.982794256
ENSGALG00010024345	STARD3	3	-0.005430363	0.072284595	-0.07512476	0.940115448	0.003955665	2.314234838	13.57834705	0.666666667	CRA004775;PRJNA827465;Yang2025_Silkie_Wencha	0.982794256
ENSGALG00010016918	RCOR1	3	-0.003462809	0.046091893	-0.075128382	0.940112566	0	1.312840273	0	0.666666667	CRA004775;PRJNA827465;Yang2025_Silkie_Wencha	0.982794256
ENSGALG00010008859	ENSGALG00010008859	3	-0.048464628	0.646776004	-0.074932631	0.940268314	1.013073878	10.66086849	81.23980236	0.666666667	CRA004775;PRJNA827465;Yang2025_Silkie_Wencha	0.98280873
ENSGALG00010000388	ENSGALG00010000388	3	-0.010562835	0.140887768	-0.074973401	0.940235875	0.044894106	11.80723595	83.06123458	0.666666667	CRA004775;PRJNA827465;Yang2025_Silkie_Wencha	0.98280873
ENSGALG00010004158	JCHAIN	3	0.042689322	0.577005527	0.073984251	0.941022917	0.79633025	10.71283213	81.33080052	0.666666667	CRA004775;PRJNA827465;Yang2025_Silkie_Wencha	0.98283974
ENSGALG00010019325	SLITRK4	3	0.0348826	0.469628894	0.074276945	0.940790021	0.481453996	12.10370771	83.47613766	0.666666667	CRA004775;PRJNA827465;Yang2025_Silkie_Wencha	0.98283974
ENSGALG00010008405	LRATD2	3	-0.022092746	0.299523525	-0.073759635	0.941201646	0.232214944	20.08032387	90.04000128	0.666666667	CRA004775;PRJNA827465;Yang2025_Silkie_Wencha	0.98283974
ENSGALG00010015829	SMAD5	3	-0.012018487	0.161167818	-0.07457126	0.940555842	0.06674588	15.87421234	87.40094969	0.666666667	CRA004775;PRJNA827465;Yang2025_Silkie_Wencha	0.98283974
ENSGALG00010010499	NR4A2	3	-0.011587844	0.156319265	-0.07412934	0.940907469	0.017008616	2.529005556	20.91753237	0.666666667	CRA004775;PRJNA827465;Yang2025_Silkie_Wencha	0.98283974
ENSGALG00010019470	TGFB2	3	-0.011481659	0.154839989	-0.074151768	0.940889624	0.044763183	6.479501666	69.13342872	0.666666667	CRA004775;PRJNA827465;Yang2025_Silkie_Wencha	0.98283974
ENSGALG00010012578	NDUFS4	3	-0.010901666	0.146012364	-0.074662622	0.940483148	0	0.833801783	0	0.666666667	CRA004775;PRJNA827465;Yang2025_Silkie_Wencha	0.98283974
ENSGALG00010013117	ARSK	3	-0.009381529	0.126948488	-0.073900282	0.941089731	0	0.062714879	0	0.666666667	CRA004775;PRJNA827465;Yang2025_Silkie_Wencha	0.98283974
ENSGALG00010025590	HRAS	3	0.009072722	0.122700377	0.073942088	0.941056466	0.023852701	4.570873836	56.24469036	0.666666667	CRA004775;PRJNA827465;Yang2025_Silkie_Wencha	0.98283974
ENSGALG00010005424	ENSGALG00010005424	3	0.007993542	0.108312199	0.073800942	0.941168778	0	0.608102181	0	0.666666667	CRA004775;PRJNA827465;Yang2025_Silkie_Wencha	0.98283974
ENSGALG00010024970	CAB39	3	-0.007475473	0.100135153	-0.074653831	0.940490143	0.019646485	6.360388356	68.5553792	0.666666667	CRA004775;PRJNA827465;Yang2025_Silkie_Wencha	0.98283974
ENSGALG00010017610	PSMC1	3	0.006239413	0.084565767	0.07378178	0.941184025	0.009353597	3.299649326	39.38749842	0.666666667	CRA004775;PRJNA827465;Yang2025_Silkie_Wencha	0.98283974
ENSGALG00010012242	CCDC77	3	-0.005501973	0.07399272	-0.074358301	0.940725288	0	0.725377725	0	0.666666667	CRA004775;PRJNA827465;Yang2025_Silkie_Wencha	0.98283974
ENSGALG00010017820	FLRT2	3	0.016733241	0.22737017	0.073594705	0.941332885	0.123353077	11.89019762	83.17942171	0.666666667	CRA004775;PRJNA827465;Yang2025_Silkie_Wencha	0.982904191
ENSGALG00010019038	PLEKHS1	2	0.030023842	0.409244342	0.073364097	0.941516387	0	0.000404774	0	1	CRA004775;PRJNA827465	0.983023202
ENSGALG00010007979	RAB30	3	0.005326407	0.07281535	0.073149504	0.941687149	0.006697675	3.447361265	41.98461239	0.666666667	CRA004775;PRJNA827465;Yang2025_Silkie_Wencha	0.983125481
ENSGALG00010009081	HBP1	3	0.003544873	0.048515884	0.073066231	0.941753414	0	0.486102824	0	0.666666667	CRA004775;PRJNA827465;Yang2025_Silkie_Wencha	0.983125481
ENSGALG00010024061	ENSGALG00010024061	2	0.130521422	1.790902338	0.072880257	0.941901405	4.852361217	4.016040765	75.09985435	0.5	PRJNA827465;Yang2025_Silkie_Wenchang	0.98320738
ENSGALG00010013493	ENSGALG00010013493	3	0.017825207	0.247529398	0.072012487	0.942591971	0.15109222	25.58239856	92.18212477	0.666666667	CRA004775;PRJNA827465;Yang2025_Silkie_Wencha	0.983274891
ENSGALG00010018069	ENSGALG00010018069	3	0.015136145	0.209065707	0.072398985	0.942284393	0	0.071612562	0	0.666666667	CRA004775;PRJNA827465;Yang2025_Silkie_Wencha	0.983274891
ENSGALG00010024735	OTUD7A	3	0.01422684	0.19697131	0.072227979	0.942420479	0.075907253	7.138512276	71.98295776	0.666666667	CRA004775;PRJNA827465;Yang2025_Silkie_Wencha	0.983274891
ENSGALG00010008246	TERF2	3	-0.008907113	0.123122245	-0.07234365	0.942328428	0.036898679	12.24908072	83.6722441	0.666666667	CRA004775;PRJNA827465;Yang2025_Silkie_Wencha	0.983274891
ENSGALG00010011412	KANSL1L	3	-0.007799523	0.107769296	-0.0723724	0.942305549	0.02294553	7.052886804	71.64281725	0.666666667	CRA004775;PRJNA827465;Yang2025_Silkie_Wencha	0.983274891
ENSGALG00010008215	SELENOW	3	-0.007233211	0.099903577	-0.072401917	0.942282059	0.008493239	2.373540903	15.7377066	0.666666667	CRA004775;PRJNA827465;Yang2025_Silkie_Wencha	0.983274891
ENSGALG00010005551	METTL14	3	-0.005027941	0.069817605	-0.072015368	0.942589678	0.004804611	2.713573394	26.29644716	0.666666667	CRA004775;PRJNA827465;Yang2025_Silkie_Wencha	0.983274891
ENSGALG00010023923	CSNK1G1	3	-0.0041586	0.057657536	-0.072125875	0.942501735	0	1.240126668	0	0.666666667	CRA004775;PRJNA827465;Yang2025_Silkie_Wencha	0.983274891
ENSGALG00010021204	NAGLU	3	-0.004141476	0.057043355	-0.072602249	0.942122637	0	1.622389372	0	0.666666667	CRA004775;PRJNA827465;Yang2025_Silkie_Wencha	0.983274891
ENSGALG00010028563	ENSGALG00010028563	2	-0.101007472	1.417548247	-0.07125505	0.943194769	0	0.524482315	0	0.5	PRJNA827465;Yang2025_Silkie_Wenchang	0.983323318
ENSGALG00010013954	LPL	3	-0.020173326	0.282866962	-0.071317362	0.943145178	0.176891569	10.46430872	80.88741403	0.666666667	CRA004775;PRJNA827465;Yang2025_Silkie_Wencha	0.983323318
ENSGALG00010003692	ZNFY2	3	-0.018843849	0.263858638	-0.071416457	0.943066312	0.138130658	6.002642287	66.68133958	0.666666667	CRA004775;PRJNA827465;Yang2025_Silkie_Wencha	0.983323318
ENSGALG00010010544	ENSGALG00010010544	3	0.013545083	0.189811011	0.071360891	0.943110535	0.053883334	4.092215766	51.1267217	0.666666667	CRA004775;PRJNA827465;Yang2025_Silkie_Wencha	0.983323318
ENSGALG00010018140	CHPF	3	0.011191416	0.15705453	0.071258158	0.943192296	0.056968809	9.886151138	79.76968011	0.666666667	CRA004775;PRJNA827465;Yang2025_Silkie_Wencha	0.983323318
ENSGALG00010027393	TEX264	3	-0.009889464	0.138743149	-0.071278935	0.94317576	0.036304065	5.676277477	64.76564072	0.666666667	CRA004775;PRJNA827465;Yang2025_Silkie_Wencha	0.983323318
ENSGALG00010020557	ENSGALG00010020557	3	-0.008165505	0.1139889	-0.071634215	0.94289301	0	1.555559694	0	0.666666667	CRA004775;PRJNA827465;Yang2025_Silkie_Wencha	0.983323318
ENSGALG00010003383	UBE2J1	3	0.003756239	0.052532098	0.071503688	0.94299689	0	1.249014277	0	0.666666667	CRA004775;PRJNA827465;Yang2025_Silkie_Wencha	0.983323318
ENSGALG00010000196	KSR2	2	-0.027224202	0.382699221	-0.071137333	0.943288456	0	0.733686573	0	0.5	PRJNA827465;Yang2025_Silkie_Wenchang	0.983337511
ENSGALG00010025571	SRSF3	3	0.011582524	0.16332163	0.070918496	0.943462623	0.062512429	12.6021095	84.12964116	0.666666667	CRA004775;PRJNA827465;Yang2025_Silkie_Wencha	0.983337511
ENSGALG00010007603	ALG2	3	-0.011453126	0.16171583	-0.070822542	0.943538991	0.056815486	7.478778829	73.25766618	0.666666667	CRA004775;PRJNA827465;Yang2025_Silkie_Wencha	0.983337511
ENSGALG00010017651	PPCS	3	0.009690716	0.137128222	0.070669009	0.943661186	0.025544802	3.326904546	39.88405823	0.666666667	CRA004775;PRJNA827465;Yang2025_Silkie_Wencha	0.983337511
ENSGALG00010020684	P4HTM	3	-0.008110161	0.114811158	-0.070639137	0.943684962	0	0.9935801	0	0.666666667	CRA004775;PRJNA827465;Yang2025_Silkie_Wencha	0.983337511
ENSGALG00010015140	ASCC3	3	-0.007058239	0.100061697	-0.070538867	0.943764766	0.019336674	6.142371129	67.43928431	0.666666667	CRA004775;PRJNA827465;Yang2025_Silkie_Wencha	0.983337511
ENSGALG00010008780	KARS1	3	-0.006024641	0.08510151	-0.070793589	0.943562034	0.013040362	4.974406149	59.79419573	0.666666667	CRA004775;PRJNA827465;Yang2025_Silkie_Wencha	0.983337511
ENSGALG00010013168	VPS26C	3	-0.003458	0.048992845	-0.070581732	0.94373065	0	0.982994644	0	0.666666667	CRA004775;PRJNA827465;Yang2025_Silkie_Wencha	0.983337511
ENSGALG00010020798	C1QL2	3	0.0217376	0.309538632	0.070225805	0.944013936	0	0.993189947	0	0.666666667	CRA004775;PRJNA827465;Yang2025_Silkie_Wencha	0.983524651
ENSGALG00010007759	AGT	3	0.079570185	1.168587851	0.068090889	0.945713283	2.989344157	7.576263617	73.60176334	0.666666667	CRA004775;PRJNA827465;Yang2025_Silkie_Wencha	0.983656792
ENSGALG00010029239	RBFOX3	3	0.028428092	0.40711643	0.069827917	0.944330627	0.369899539	8.378363005	76.12898846	0.666666667	CRA004775;PRJNA827465;Yang2025_Silkie_Wencha	0.983656792

ENSGALG00010027233	ENSGALG00010027233	3	-0.026664983	0.39052083	-0.068280566	0.945562294	0.373207608	14.41498886	86.12555292	0.666666667	CRA004775;PRJNA827465;Yang2025_Silkie_Wencha	0.983656792
ENSGALG00010027555	ENSGALG00010027555	3	0.022453908	0.32515628	0.069055742	0.94494525	0.239733997	8.692143754	76.99071648	0.666666667	CRA004775;PRJNA827465;Yang2025_Silkie_Wencha	0.983656792
ENSGALG00010004212	RSPO3	3	0.021468023	0.310673887	0.069101472	0.944908849	0.180971916	6.471676268	69.0961056	0.666666667	CRA004775;PRJNA827465;Yang2025_Silkie_Wencha	0.983656792
ENSGALG00010028601	ANO8	3	0.019921292	0.285776003	0.069709465	0.944424908	0.181785167	15.84243957	87.37568168	0.666666667	CRA004775;PRJNA827465;Yang2025_Silkie_Wencha	0.983656792
ENSGALG00010002331	ENSGALG00010002331	2	-0.015178069	0.222319137	-0.068271537	0.945569482	0	0.170242135	0	0.5	PRJNA827465;Yang2025_Silkie_Wenchang	0.983656792
ENSGALG00010005087	ENPP1	3	0.014788549	0.217113298	0.068114433	0.945694541	0.10450423	7.896101123	74.67104374	0.666666667	CRA004775;PRJNA827465;Yang2025_Silkie_Wencha	0.983656792
ENSGALG00010002557	ENSGALG00010002557	3	-0.01441884	0.209595122	-0.068793779	0.94515377	0.049044306	3.170112619	36.91075868	0.666666667	CRA004775;PRJNA827465;Yang2025_Silkie_Wencha	0.983656792
ENSGALG00010027261	ENSGALG00010027261	3	0.014051676	0.203955834	0.06889568	0.945072657	0.094708601	10.35004692	80.6764161	0.666666667	CRA004775;PRJNA827465;Yang2025_Silkie_Wencha	0.983656792
ENSGALG00010015759	EIF5	3	0.013661555	0.201256182	0.06788142	0.94588003	0.094548291	13.81115536	85.51895227	0.666666667	CRA004775;PRJNA827465;Yang2025_Silkie_Wencha	0.983656792
ENSGALG00010025320	JAM3	3	-0.013356202	0.193795598	-0.068919018	0.94505408	0.088620534	22.49470838	91.1090201	0.666666667	CRA004775;PRJNA827465;Yang2025_Silkie_Wencha	0.983656792
ENSGALG00010018466	DYSF	3	-0.012654194	0.181844756	-0.069587895	0.944521672	0.058214986	5.782967667	65.41568075	0.666666667	CRA004775;PRJNA827465;Yang2025_Silkie_Wencha	0.983656792
ENSGALG00010010349	RREB1	3	0.012258384	0.176966341	0.069269577	0.944775042	0.061796481	6.430412374	68.89779561	0.666666667	CRA004775;PRJNA827465;Yang2025_Silkie_Wencha	0.983656792
ENSGALG00010012240	TRIM14	3	0.011993788	0.172189964	0.069654398	0.944468739	0	0.342713658	0	0.666666667	CRA004775;PRJNA827465;Yang2025_Silkie_Wencha	0.983656792
ENSGALG00010014344	LSG1	3	0.011648162	0.17157432	0.067889892	0.945873285	0.073743974	15.13267241	86.78356376	0.666666667	CRA004775;PRJNA827465;Yang2025_Silkie_Wencha	0.983656792
ENSGALG00010000660	ENSGALG00010000660	3	0.010900702	0.159690196	0.068261562	0.945577422	0.038117642	4.116645546	51.41675479	0.666666667	CRA004775;PRJNA827465;Yang2025_Silkie_Wencha	0.983656792
ENSGALG00010024185	CHRNA5	3	-0.010751768	0.155299772	-0.06923235	0.944804673	0.037598781	4.448849394	55.04455595	0.666666667	CRA004775;PRJNA827465;Yang2025_Silkie_Wencha	0.983656792
ENSGALG00010011866	KLF8	3	0.010201927	0.149103809	0.068421641	0.945449995	0.041257693	6.092108359	67.17064303	0.666666667	CRA004775;PRJNA827465;Yang2025_Silkie_Wencha	0.983656792
ENSGALG00010014572	IL1RAP	3	0.009524812	0.138650604	0.068696504	0.945231201	0.03048189	4.408849465	54.63669114	0.666666667	CRA004775;PRJNA827465;Yang2025_Silkie_Wencha	0.983656792
ENSGALG00010026584	ENSGALG00010026584	3	0.007668134	0.112529552	0.068143291	0.945671569	0.024475108	6.378311136	68.64373723	0.666666667	CRA004775;PRJNA827465;Yang2025_Silkie_Wencha	0.983656792
ENSGALG00010001898	LHPP	3	-0.007413482	0.105989594	-0.069945379	0.944237134	0.018403609	4.98660021	59.89251362	0.666666667	CRA004775;PRJNA827465;Yang2025_Silkie_Wencha	0.983656792
ENSGALG00010024511	ENSGALG00010024511	3	-0.004858168	0.070602639	-0.068810009	0.945140851	0	1.598822432	0	0.666666667	CRA004775;PRJNA827465;Yang2025_Silkie_Wencha	0.983656792
ENSGALG00010024945	APIP	3	-0.004666241	0.067144077	-0.069495941	0.944594864	0	0.647658054	0	0.666666667	CRA004775;PRJNA827465;Yang2025_Silkie_Wencha	0.983656792
ENSGALG00010024155	SNX7	3	-0.002852289	0.04191629	-0.068047278	0.945747999	0	0.361070319	0	0.666666667	CRA004775;PRJNA827465;Yang2025_Silkie_Wencha	0.983656792
ENSGALG00010009152	RPP25L	3	-0.011451446	0.169774907	-0.067450759	0.946222861	0.030548156	3.04289112	34.27303439	0.666666667	CRA004775;PRJNA827465;Yang2025_Silkie_Wencha	0.983919176
ENSGALG00010021767	CCDC58	3	0.005721124	0.084896194	0.067389642	0.946271515	0	1.344097472	0	0.666666667	CRA004775;PRJNA827465;Yang2025_Silkie_Wencha	0.983919176
ENSGALG00010017834	ENSGALG00010017834	2	0.036754222	0.553703411	0.066378898	0.94707617	0.530142215	7.348410398	86.3916147	0.5	PRJNA827465;Yang2025_Silkie_Wenchang	0.983959877
ENSGALG00010014866	FAM26E	3	0.020450771	0.305069294	0.067036479	0.946552663	0.237383666	25.20888217	92.06628843	0.666666667	CRA004775;PRJNA827465;Yang2025_Silkie_Wencha	0.983959877
ENSGALG00010007946	ENSGALG00010007946	3	-0.017108836	0.255284343	-0.067018745	0.946566781	0.169150085	16.19987976	87.65422936	0.666666667	CRA004775;PRJNA827465;Yang2025_Silkie_Wencha	0.983959877
ENSGALG00010020778	SLIT1	3	0.013102595	0.195553556	0.067002593	0.946579639	0.064685082	4.751269834	57.90598998	0.666666667	CRA004775;PRJNA827465;Yang2025_Silkie_Wencha	0.983959877
ENSGALG00010014089	FIG4	3	0.010538246	0.158304213	0.066569585	0.94692436	0.04910638	7.423507679	73.05855821	0.666666667	CRA004775;PRJNA827465;Yang2025_Silkie_Wencha	0.983959877
ENSGALG00010013099	SIM2	3	-0.010252407	0.153273518	-0.066889618	0.946669578	0.028008864	3.240296823	38.27725948	0.666666667	CRA004775;PRJNA827465;Yang2025_Silkie_Wencha	0.983959877
ENSGALG00010009774	PIAS2	3	-0.006757961	0.101761567	-0.066409757	0.947051602	0.014407948	3.714140554	46.15174167	0.666666667	CRA004775;PRJNA827465;Yang2025_Silkie_Wencha	0.983959877
ENSGALG00010029649	PSPH	3	-0.006728012	0.101356389	-0.066379748	0.947075494	0	1.045584658	0	0.666666667	CRA004775;PRJNA827465;Yang2025_Silkie_Wencha	0.983959877
ENSGALG00010022919	ACLY	3	-0.004654929	0.069893234	-0.066600569	0.946899693	0.003471372	2.54016125	21.26484096	0.666666667	CRA004775;PRJNA827465;Yang2025_Silkie_Wencha	0.983959877
ENSGALG00010003595	SNX13	3	-0.00325198	0.048882676	-0.066526216	0.946958886	0	1.461450539	0	0.666666667	CRA004775;PRJNA827465;Yang2025_Silkie_Wencha	0.983959877
ENSGALG00010020775	UBR1	3	-0.002416675	0.036086767	-0.066968451	0.946606819	0	1.598340868	0	0.666666667	CRA004775;PRJNA827465;Yang2025_Silkie_Wencha	0.983959877
ENSGALG00010024548	CDC6	3	-0.008237205	0.124289746	-0.06627421	0.947159515	0.009219447	2.264361089	11.67486451	0.666666667	CRA004775;PRJNA827465;Yang2025_Silkie_Wencha	0.983974165
ENSGALG00010022419	DUOX2	2	-0.022317396	0.339621827	-0.065712491	0.947606728	0	0.033440064	0	0.5	PRJNA827465;Yang2025_Silkie_Wenchang	0.984004959
ENSGALG00010027980	NUP210	3	-0.019174947	0.290696132	-0.065962169	0.947407944	0.188742413	11.73218998	82.9528843	0.666666667	CRA004775;PRJNA827465;Yang2025_Silkie_Wencha	0.984004959
ENSGALG00010012163	PDP1	3	-0.015905139	0.241831675	-0.065769459	0.947561371	0.129787121	23.60073419	91.52568736	0.666666667	CRA004775;PRJNA827465;Yang2025_Silkie_Wencha	0.984004959
ENSGALG00010029492	SDK2	3	0.010645417	0.161294163	0.066000012	0.947377816	0.033846138	3.612810902	44.64144251	0.666666667	CRA004775;PRJNA827465;Yang2025_Silkie_Wencha	0.984004959
ENSGALG00010022868	VAC14	3	-0.010187691	0.154729081	-0.065842122	0.947503521	0.060283901	18.18836343	89.00395845	0.666666667	CRA004775;PRJNA827465;Yang2025_Silkie_Wencha	0.984004959
ENSGALG00010021141	SGSM1	3	0.007742915	0.117404883	0.065950536	0.947417206	0	0.962152278	0	0.666666667	CRA004775;PRJNA827465;Yang2025_Silkie_Wencha	0.984004959
ENSGALG00010027112	XYLB	3	0.013898403	0.212126736	0.065519334	0.947760513	0.080109196	5.092266432	60.7247573	0.666666667	CRA004775;PRJNA827465;Yang2025_Silkie_Wencha	0.984048438
ENSGALG00010021043	PTPRG	3	-0.006582583	0.100654735	-0.065397653	0.947857393	0.011387454	3.017627054	33.72275751	0.666666667	CRA004775;PRJNA827465;Yang2025_Silkie_Wencha	0.984048438
ENSGALG00010022055	E2F1	3	0.005822572	0.089033016	0.065397902	0.947857194	0	0.706751179	0	0.666666667	CRA004775;PRJNA827465;Yang2025_Silkie_Wencha	0.984048438
ENSGALG00010010874	ENSGALG00010010874	3	-0.018341971	0.282286489	-0.064976438	0.94819276	0.146904356	5.388082764	62.8810453	0.666666667	CRA004775;PRJNA827465;Yang2025_Silkie_Wencha	0.984107571
ENSGALG00010016643	BTBD1	3	-0.009214982	0.141546167	-0.065102306	0.948092544	0.035086974	5.281013873	62.12848426	0.666666667	CRA004775;PRJNA827465;Yang2025_Silkie_Wencha	0.984107571
ENSGALG00010028372	PTGS1	3	-0.006918785	0.106137633	-0.065186916	0.948025179	0.008495677	2.590484278	22.7943587	0.666666667	CRA004775;PRJNA827465;Yang2025_Silkie_Wencha	0.984107571
ENSGALG00010005226	MYCT1	3	-0.004217272	0.064845745	-0.065035446	0.948145778	0	0.841088577	0	0.666666667	CRA004775;PRJNA827465;Yang2025_Silkie_Wencha	0.984107571
ENSGALG00010002126	ENSGALG00010002126	3	-0.010514413	0.162233189	-0.064810494	0.948324886	0.024415481	2.802265225	28.62916822	0.666666667	CRA004775;PRJNA827465;Yang2025_Silkie_Wencha	0.984172458
ENSGALG00010029523	FSCN2	2	0.112717256	1.761750934	0.063980245	0.948985957	5.817395889	15.79397911	93.66847333	0.5	CRA004775;Yang2025_Silkie_Wenchang	0.984191235
ENSGALG00010006106	SHOC1	2	-0.039633324	0.625239342	-0.063389043	0.949456712	0.646914267	5.790310961	82.72977036	0.5	PRJNA827465;Yang2025_Silkie_Wenchang	0.984191235
ENSGALG00010002446	RIMS1	3	0.024868047	0.391844789	0.063464025	0.949397005	0.245695049	4.425987975	54.81234899	0.666666667	CRA004775;PRJNA827465;Yang2025_Silkie_Wencha	0.984191235
ENSGALG00010018168	ENSGALG00010018168	2	0.023796309	0.372233238	0.063928491	0.949027166	0.216806709	4.365925207	77.09534743	0.5	PRJNA827465;Yang2025_Silkie_Wenchang	0.984191235
ENSGALG00010001412	ENSGALG00010001412	3	0.019879965	0.313093159	0.063495368	0.949372047	0.212296514	10.52655938	81.00043967	0.666666667	CRA004775;PRJNA827465;Yang2025_Silkie_Wencha	0.984191235
ENSGALG00010029621	TAF15	3	0.012809751	0.199734584	0.064133865	0.948863637	0.107096878	25.09390677	92.02993771	0.666666667	CRA004775;PRJNA827465;Yang2025_Silkie_Wencha	0.984191235
ENSGALG00010024650	PRKAG1	3	-0.011900168	0.187306988	-0.063532962	0.949342112	0.074536066	8.880529794	77.47882112	0.666666667	CRA004775;PRJNA827465;Yang2025_Silkie_Wencha	0.984191235
ENSGALG00010025984	NDUFS1	3	0.010273671	0.16053854	0.063995042	0.948974175	0.056125376	11.30414137	82.30736918	0.666666667	CRA004775;PRJNA827465;Yang2025_Silkie_Wencha	0.984191235
ENSGALG00010019509	ZNF106	3	0.010247979	0.160416703	0.063883491	0.949062997	0.037674534	4.164875384	51.97935555	0.666666667	CRA004775;PRJNA827465;Yang2025_Silkie_Wencha	0.984191235
ENSGALG00010002790	BCLAF3	3	0.007326302	0.115055292	0.063676358	0.94922793	0.021269691	4.604412786	56.56340791	0.666666667	CRA004775;PRJNA827465;Yang2025_Silkie_Wencha	0.984191235
ENSGALG00010010957	ZMAT2	3	-0.007311448	0.114293672	-0.063970714	0.948993546	0.025813987	7.448833339	73.15015776	0.666666667	CRA004775;PRJNA827465;Yang2025_Silkie_Wencha	0.984191235
ENSGALG00010022862	ACADS	3	-0.006070196	0.095166692	-0.063784883	0.949141515	0.013097535	3.838831342	47.9008109	0.666666667	CRA004775;PRJNA827465;Yang2025_Silkie_Wencha	0.984191235
ENSGALG00010020714	PGAM1	3	-0.004958823	0.077834978	-0.063709446	0.949201584	0	1.159575085	0	0.666666667	CRA004775;PRJNA827465;Yang2025_Silkie_Wencha	0.984191235
ENSGALG00010002393	GPM6B	3	-0.00449147	0.07006047	-0.064108474	0.948883854	0.004286486	2.605876793	23.25040057	0.666666667	CRA004775;PRJNA827465;Yang2025_Silkie_Wencha	0.984191235
ENSGALG00010028130	TMEM115	3	-0.004175712	0.065659587	-0.063596381	0.949291613	0.001214707	2.078454555	3.774658166	0.666666667	CRA004775;PRJNA827465;Yang2025_Silkie_Wencha	0.984191235
ENSGALG00010022235	RAMP2	3	0.003403572	0.052605952	0.064699375	0.94841336	0	0.508902354	0	0.666666667	CRA004775;PRJNA827465;Yang2025_Silkie_Wencha	0.984191235
ENSGALG00010013121	PCBD2	3	-0.00616568	0.097656293	-0.063136539	0.949657778	0	0.139289289	0	0.666666667	CRA004775;PRJNA827465;Yang2025_Silkie_Wencha	0.984327492
ENSGALG00010028514	FNBP1	3	-0.01040144	0.165051693	-0.063019285	0.949751147	0.066763036	12.36477437	83.82501823	0.666666667	CRA004775;PRJNA827465;Yang2025_Silkie_Wencha	0.984352109
ENSGALG00010028853	ENSGALG00010028853	2	0.072335042	1.164099682	0.062138185	0.950452788	0	0.329616466	0	0.5	PRJNA827465;Yang2025_Silkie_Wenchang	0.984455786
ENSGALG00010000797	ENSGALG00010000797	3	0.043482583	0.694628843	0.062598298	0.950086385	1.197390116	12.54612629	84.05882458	0.666666667	CRA004775;PRJNA827465;Yang2025_Silkie_Wencha	0.984455786
ENSGALG00010014414	TMEM255B	3	-0.039420582	0.638837015	-0.06170679	0.950796333	0.951032934	10.24235561	80.47324194	0.666666667	CRA004775;PRJNA827465;Yang2025_Silkie_Wencha	0.984455786
ENSGALG00010002238	ENSGALG00010002238	3	0.026897162	0.436685374	0.061593916	0.950886222	0.281210148	3.911798265	48.87261908	0.666666667	CRA004775;PRJNA827465;Yang2025_Silkie_Wencha	0.984455786
ENSGALG00010028876	ADAMTSL2	3	-0.023882609	0.385548893	-0.061944438	0.950607079	0.393062795	20.39391913	90.19315519	0.666666667	CRA004775;PRJNA827465;Yang2025_Silkie_Wencha	0.984455786
ENSGALG00010010511	GABRP	2	-0.015285431	0.244016478	-0.06264098	0.950052396	0	0.509362557	0	0.5	PRJNA827465;Yang2025_Silkie_Wenchang	0.984455786
ENSGALG00010025212	P2RX4	3	0.014759397	0.239483271	0.061630179	0.950857343	0.12377286	8.009309828	75.0290594	0.666666667	CRA004775;PRJNA827465;Yang2025_Silkie_Wencha	0.984455786
ENSGALG00010004337	BMPR1B	3	0.012854963	0.205610045	0.062521084	0.950147872	0.080074444	7.852938061	74.5318251	0.666666667	CRA004775;PRJNA827465;Yang2025_Silkie_Wencha	0.984455786
ENSGALG00010017143	FAM149A	3	0.012571633	0.20480201	0.061384323	0.951053137	0.062623688	4.029625285	50.36759354	0.666666667	CRA004775;PRJNA827465;Yang2025_Silkie_Wencha	0.984455786
ENSGALG00010015533	PSAT1	3	-0.012207253	0.196732987	-0.062049851	0.950523133	0.080625515	7.583204585	73.62592585	0.666666667	CRA004775;PRJNA827465;Yang2025_Silkie_Wencha	0.984455786
ENSGALG00010013300	GNPDA1	3	0.01132892	0.1851653	0.061182737	0.951213678	0.085333459	13.16600889	84.8093677	0.666666667	CRA004775;PRJNA827465;Yang2025_Silkie_Wencha	0.984455786
ENSGALG00010006544	MOB3B	3	0.009712963	0.157890641	0.061517031	0.950947451	0	1.941109953	0	0.666666667	CRA004775;PRJNA827465;Yang2025_Silkie_Wencha	0.984455786
ENSGALG00010004355	ANKMY2	3	-0.009415921	0.153719897	-0.061253757	0.951157118	0.054886075	10.51895582	80.98670596	0.666666667	CRA004775;PRJNA827465;Yang2025_Silkie_Wencha	0.984455786
ENSGALG00010019269	SOCS1	3	-0.007991907	0.128242739	-0.062318595	0.95030912	0	0.045924163	0	0.666666667	CRA004775;PRJNA827465;Yang2025_Silkie_Wencha	0.984455786
ENSGALG00010020517	UBR4	3	-0.006839391	0.112145618	-0.060986699	0.951369802	0.019687286	4.211882525	52.51529481	0.666666667	CRA004775;PRJNA827465;Yang2025_Silkie_Wencha	0.984455786
ENSGALG00010022312	ZNF592	3	0.005986119	0.096513086	0.062023906	0.950543794	0.014423137	3.954431562	49.42383074	0.666666667	CRA004775;PRJNA827465;Yang2025_Silkie_Wencha	0.984455786
ENSGALG00010011033	DROSHA	3	0.005616934	0.091740876	0.061226075	0.951179164	0.00845791	2.652815892	24.60841304	0.666666667	CRA004775;PRJNA827465;Yang2025_Silkie_Wencha	0.984455786
ENSGALG00010011962	CPSF6	3	0.005394491	0.087743046	0.061480548	0.950976506	0.009644059	3.049748598	34.42082402	0.666666667	CRA004775;PRJNA827465;Yang2025_Silkie_Wencha	0.984455786
ENSGALG00010008117	ENSGALG00010008117	3	0.005304254	0.086839396	0.06108119	0.95129455	0	0.360378413	0	0.666666667	CRA004775;PRJNA827465;Yang2025_Silkie_Wencha	0.984455786
ENSGALG00010011998	MPPED1	3	-0.004865183	0.079102715	-0.061504632	0.950957326	0.002527561	2.270391662	11.90947211	0.666666667	CRA004775;PRJNA827465;Yang2025_Silkie_Wencha	0.984455786
ENSGALG00010018618	FAHD1	3	0.004852275	0.079321449	0.061172295	0.951221994	0	0.397841432	0	0.666666667	CRA004775;PRJNA827465;Yang2025_Silkie_Wencha	0.984455786
ENSGALG00010006615	GRAMD1C	3	-0.00423804	0.069510068	-0.06097016	0.951382974	0	1.078611855	0	0.666666667	CRA004775;PRJNA827465;Yang2025_Silkie_Wencha	0.984455786
ENSGALG00010000793	ENSGALG00010000793	2	-0.04649592	0.78742005	-0.059048432	0.952913532	0	0.66285741	0	0.5	PRJNA827465;Yang2025_Silkie_Wenchang	0.984742353
ENSGALG00010009150	ENSGALG00010009150	3	-0.025497547	0.428577774	-0.059493395	0.952559126	0.375392563	7.114261316	71.88745379	0.666666667	CRA004775;PRJNA827465;Yang2025_Silkie_Wencha	0.984742353
ENSGALG00010009188	ENSGALG00010009188	2	-0.022221512	0.374327335	-0.059363851	0.952662305	0	0.006923895	0	0.5	PRJNA827465;Yang2025_Silkie_Wenchang	0.984742353
ENSGALG00010010171	ENSGALG00010010171	3	0.019219443	0.322502448	0.05959472	0.952478424	0	0.553200273	0	0.666666667	CRA004775;PRJNA827465;Yang2025_Silkie_Wencha	0.984742353
ENSGALG00010003762	EVI5L	3	0.016767655	0.28059523	0.059757447	0.952348818	0.215328753	41.07515302	95.13087633	0.666666667	CRA004775;PRJNA827465;Yang2025_Silkie_Wencha	0.984742353
ENSGALG00010024609	PRR33	3	0.01520966	0.258959958	0.058733636	0.953164268	0.025321991	2.262604958	11.60631056	0.666666667	CRA004775;PRJNA827465;Yang2025_Silkie_Wencha	0.984742353
ENSGALG00010000293	ENSGALG00010000293	3	-0.01332759	0.225541725	-0.059091462	0.952879259	0.061881301	3.370233756	40.65693526	0.666666667	CRA004775;PRJNA827465;Yang2025_Silkie_Wencha	0.984742353
ENSGALG00010018193	ANK1	3	-0.012722844	0.216645881	-0.058726451	0.95316999	0.092986927	7.350414289	72.79064932	0.666666667	CRA004775;PRJNA827465;Yang2025_Silkie_Wencha	0.984742353
ENSGALG00010024223	ENSGALG00010024223	3	-0.011307659	0.187066487	-0.060447271	0.95179941	0	0.118815023	0	0.666666667	CRA004775;PRJNA827465;Yang2025_Silkie_Wencha	0.984742353
ENSGALG00010001705	HTRA1	3	0.011096606	0.186432307	0.059520832	0.952537273	0.074175894	7.485731924	73.2825057	0.666666667	CRA004775;PRJNA827465;Yang2025_Silkie_Wencha	0.984742353
ENSGALG00010021271	ENSGALG00010021271	3	0.010759345	0.183146283	0.058747277	0.953153402	0.077643213	11.1863756	82.1211081	0.666666667	CRA004775;PRJNA827465;Yang2025_Silkie_Wencha	0.984742353
ENSGALG00010025427	SLC35G2	3	-0.01073824	0.183211433	-0.058611188	0.953261799	0.066417709	7.63201314	73.79459438	0.666666667	CRA004775;PRJNA827465;Yang2025_Silkie_Wencha	0.984742353
ENSGALG00010011726	WDR55	3	0.009566754	0.15850646	0.06035561	0.951872412	0.047588976	5.660538955	64.66767536	0.666666667	CRA004775;PRJNA827465;Yang2025_Silkie_Wencha	0.984742353
ENSGALG00010013360	PRR5	3	-0.00893868	0.148209894	-0.060310955	0.951907977	0.037340184	4.830241322	58.59420127	0.666666667	CRA004775;PRJNA827465;Yang2025_Silkie_Wencha	0.984742353
ENSGALG00010002150	EIF3A	3	0.008562721	0.142684197	0.060011701	0.952146315	0.042337508	7.06810865	71.70388715	0.666666667	CRA004775;PRJNA827465;Yang2025_Silkie_Wencha	0.984742353
ENSGALG00010016553	SLC25A14	3	-0.007875484	0.130879481	-0.060173554	0.952017408	0.027433227	4.192362823	52.29420533	0.666666667	CRA004775;PRJNA827465;Yang2025_Silkie_Wencha	0.984742353
ENSGALG00010008238	NTAQ1	3	-0.005768032	0.098181538	-0.058748644	0.953152314	0	1.900375922	0	0.666666667	CRA004775;PRJNA827465;Yang2025_Silkie_Wencha	0.984742353
ENSGALG00010017616	PDCD7	3	-0.005343202	0.090818157	-0.058834076	0.953084267	0.008878159	2.998606292	33.30234764	0.666666667	CRA004775;PRJNA827465;Yang2025_Silkie_Wencha	0.984742353
ENSGALG00010009235	KLHDC4	3	0.004669271	0.077623832	0.06015254	0.952034145	0.005518345	2.676722931	25.28176984	0.666666667	CRA004775;PRJNA827465;Yang2025_Silkie_Wencha	0.984742353
ENSGALG00010020640	PTPN14	3	-0.004186828	0.070754534	-0.059173985	0.95281353	0	1.574899179	0	0.666666667	CRA004775;PRJNA827465;Yang2025_Silkie_Wencha	0.984742353
ENSGALG00010027826	ARHGEF11	3	0.003215901	0.054817037	0.058666081	0.953218076	0.001159394	2.232082031	10.39755833	0.666666667	CRA004775;PRJNA827465;Yang2025_Silkie_Wencha	0.984742353
ENSGALG00010026295	SMPD4	3	-0.003212498	0.054151599	-0.059324162	0.952693916	0	1.421443053	0	0.666666667	CRA004775;PRJNA827465;Yang2025_Silkie_Wencha	0.984742353
ENSGALG00010017810	HIF1A	3	0.002755517	0.04652255	0.059229705	0.95276915	0	1.045001555	0	0.666666667	CRA004775;PRJNA827465;Yang2025_Silkie_Wencha	0.984742353
ENSGALG00010028877	FAM163B	2	-0.029947694	0.519820133	-0.057611647	0.954057972	0.166959172	1.246315958	19.76352434	0.5	PRJNA827465;Yang2025_Silkie_Wenchang	0.984769864
ENSGALG00010024615	SYT8	2	0.026179334	0.454741921	0.057569653	0.954091423	0	0.120522782	0	0.5	PRJNA827465;Yang2025_Silkie_Wenchang	0.984769864
ENSGALG00010027293	ELP6	3	-0.013633336	0.237344856	-0.057441042	0.954193871	0.123129338	7.439334308	73.11587412	0.666666667	CRA004775;PRJNA827465;Yang2025_Silkie_Wencha	0.984769864
ENSGALG00010004987	MAP3K8	3	0.013025305	0.224144632	0.058111164	0.953660082	0.088389682	5.404891162	62.99647967	0.666666667	CRA004775;PRJNA827465;Yang2025_Silkie_Wencha	0.984769864
ENSGALG00010011143	AK3	3	-0.01146037	0.199510721	-0.057442376	0.954192807	0.038903755	2.95198574	32.2489952	0.666666667	CRA004775;PRJNA827465;Yang2025_Silkie_Wencha	0.984769864
ENSGALG00010018665	IL13RA2	3	-0.008262535	0.142013778	-0.058181224	0.953604276	0	1.513419389	0	0.666666667	CRA004775;PRJNA827465;Yang2025_Silkie_Wencha	0.984769864
ENSGALG00010000432	ENSGALG00010000432	3	0.006690079	0.115440249	0.057952745	0.95378627	0.022648016	4.993808267	59.95040472	0.666666667	CRA004775;PRJNA827465;Yang2025_Silkie_Wencha	0.984769864
ENSGALG00010002406	DIAPH3	3	0.005621917	0.096691473	0.058142837	0.953634853	0	1.454505709	0	0.666666667	CRA004775;PRJNA827465;Yang2025_Silkie_Wencha	0.984769864
ENSGALG00010002269	MCPH1	3	-0.004637041	0.080614446	-0.057521217	0.954130005	0	1.668566666	0	0.666666667	CRA004775;PRJNA827465;Yang2025_Silkie_Wencha	0.984769864
ENSGALG00010016875	SESN2	3	0.004486623	0.07795523	0.05755384	0.954104019	0	1.30214972	0	0.666666667	CRA004775;PRJNA827465;Yang2025_Silkie_Wencha	0.984769864
ENSGALG00010016462	RNF24	3	0.004235657	0.073083525	0.05795639	0.953783366	0	0.155201106	0	0.666666667	CRA004775;PRJNA827465;Yang2025_Silkie_Wencha	0.984769864
ENSGALG00010026492	SPECC1L	3	0.003820898	0.065694277	0.058161802	0.953619747	0	1.571937835	0	0.666666667	CRA004775;PRJNA827465;Yang2025_Silkie_Wencha	0.984769864
ENSGALG00010006633	FBXO18	3	0.003127924	0.054360188	0.05754071	0.954114478	0.000586835	2.11246488	5.323869798	0.666666667	CRA004775;PRJNA827465;Yang2025_Silkie_Wencha	0.984769864
ENSGALG00010007160	ENSGALG00010007160	3	0.014461248	0.253051123	0.057147535	0.954427671	0.12796037	6.403927831	68.7691671	0.666666667	CRA004775;PRJNA827465;Yang2025_Silkie_Wencha	0.984939263
ENSGALG00010005330	ENSGALG00010005330	3	-0.016156963	0.28652709	-0.056388954	0.955031957	0	0.232321842	0	0.666666667	CRA004775;PRJNA827465;Yang2025_Silkie_Wencha	0.985131454
ENSGALG00010010154	IQSEC3	2	0.011105174	0.196623806	0.056479295	0.95495999	0.020031002	1.287668191	22.34024209	0.5	PRJNA827465;Yang2025_Silkie_Wenchang	0.985131454
ENSGALG00010010313	GRID1	2	0.010749923	0.189715633	0.056663351	0.954813369	0.013910155	1.234231687	18.97793494	0.5	PRJNA827465;Yang2025_Silkie_Wenchang	0.985131454
ENSGALG00010008255	MSANTD3	3	0.005921917	0.104974613	0.056412851	0.95501292	0.01460982	3.505896627	42.9532524	0.666666667	CRA004775;PRJNA827465;Yang2025_Silkie_Wencha	0.985131454
ENSGALG00010028591	GABPB2	3	-0.004865257	0.086174496	-0.056458203	0.954976792	0.003374499	2.311796769	13.48720499	0.666666667	CRA004775;PRJNA827465;Yang2025_Silkie_Wencha	0.985131454
ENSGALG00010012889	RIC1	3	0.002643083	0.046537448	0.056794754	0.954708693	0	1.340189871	0	0.666666667	CRA004775;PRJNA827465;Yang2025_Silkie_Wencha	0.985131454
ENSGALG00010013572	DENND11	3	-0.01051849	0.18691479	-0.056274249	0.955123332	0.076242753	10.55866636	81.05821388	0.666666667	CRA004775;PRJNA827465;Yang2025_Silkie_Wencha	0.985153837
ENSGALG00010023828	CHEK1	3	0.01257903	0.224343069	0.056070511	0.955285636	0.09632961	7.522432283	73.41285472	0.666666667	CRA004775;PRJNA827465;Yang2025_Silkie_Wencha	0.98524937
ENSGALG00010002947	ENSGALG00010002947	2	-0.030775711	0.550554026	-0.05589953	0.955421845	0.327533607	2.031838011	50.78347809	0.5	PRJNA827465;Yang2025_Silkie_Wenchang	0.985317977
ENSGALG00010020629	TRIM66	3	-0.012695087	0.228793643	-0.055487064	0.955750436	0	1.87671419	0	0.666666667	CRA004775;PRJNA827465;Yang2025_Silkie_Wencha	0.985476441
ENSGALG00010005884	MYB	3	0.01180617	0.212937946	0.055444182	0.955784598	0.043842334	2.868038959	30.2659403	0.666666667	CRA004775;PRJNA827465;Yang2025_Silkie_Wencha	0.985476441
ENSGALG00010008438	SYBU	3	-0.006797686	0.122355382	-0.055556905	0.955694796	0	0.539342855	0	0.666666667	CRA004775;PRJNA827465;Yang2025_Silkie_Wencha	0.985476441
ENSGALG00010017746	MSH6	3	0.006529355	0.118004584	0.055331368	0.955874472	0.025491581	6.058079421	66.98623671	0.666666667	CRA004775;PRJNA827465;Yang2025_Silkie_Wencha	0.985486061
ENSGALG00010005548	EZR	3	0.006266682	0.113408735	0.055257491	0.955933328	0	1.037973331	0	0.666666667	CRA004775;PRJNA827465;Yang2025_Silkie_Wencha	0.985486061
ENSGALG00010027701	NHLH1	3	-0.028879008	0.530689074	-0.054417943	0.956602184	0.607864856	7.312864552	72.65093609	0.666666667	CRA004775;PRJNA827465;Yang2025_Silkie_Wencha	0.985487057
ENSGALG00010000757	SCEL	2	0.026070234	0.478712282	0.054459087	0.956569404	0	0.517474897	0	0.5	PRJNA827465;Yang2025_Silkie_Wenchang	0.985487057
ENSGALG00010024773	APOA1	3	-0.011632777	0.21229812	-0.054794535	0.956302154	0	1.211403789	0	0.666666667	CRA004775;PRJNA827465;Yang2025_Silkie_Wencha	0.985487057
ENSGALG00010018615	HTR2C	2	0.009079357	0.167074674	0.054343106	0.956661807	0	0.239380372	0	0.5	PRJNA827465;Yang2025_Silkie_Wenchang	0.985487057
ENSGALG00010006681	CRISPLD2	3	-0.008854431	0.162442382	-0.054508134	0.956530328	0	1.750117974	0	0.666666667	CRA004775;PRJNA827465;Yang2025_Silkie_Wencha	0.985487057
ENSGALG00010026724	OLA1	3	-0.008371579	0.153060781	-0.054694473	0.956381873	0.046408781	6.850043645	70.80310574	0.666666667	CRA004775;PRJNA827465;Yang2025_Silkie_Wencha	0.985487057
ENSGALG00010015792	MARK3	3	-0.00700023	0.128200203	-0.054603894	0.956454036	0.043644808	18.72820614	89.32092062	0.666666667	CRA004775;PRJNA827465;Yang2025_Silkie_Wencha	0.985487057
ENSGALG00010025929	NELFE	3	-0.006138836	0.113066733	-0.05429392	0.956700994	0.018584801	3.641164264	45.07251377	0.666666667	CRA004775;PRJNA827465;Yang2025_Silkie_Wencha	0.985487057
ENSGALG00010005645	FUCA2	3	0.005600818	0.102436189	0.054676166	0.956396457	0.001227291	2.060365395	2.929839305	0.666666667	CRA004775;PRJNA827465;Yang2025_Silkie_Wencha	0.985487057
ENSGALG00010028087	STIMATE	3	0.004580972	0.084008724	0.054529719	0.956513132	0.009311818	3.642119113	45.08691402	0.666666667	CRA004775;PRJNA827465;Yang2025_Silkie_Wencha	0.985487057
ENSGALG00010017327	CLN6	3	0.004252035	0.078047783	0.054479898	0.956552824	0	0.594297245	0	0.666666667	CRA004775;PRJNA827465;Yang2025_Silkie_Wencha	0.985487057
ENSGALG00010010599	F2RL1	3	0.014981923	0.27811882	0.053868786	0.957039706	0.195864204	16.8531075	88.13275237	0.666666667	CRA004775;PRJNA827465;Yang2025_Silkie_Wencha	0.985764144
ENSGALG00010007595	ATP2C2	3	0.009465365	0.176333931	0.053678636	0.957191204	0.030149408	2.922875354	31.57422888	0.666666667	CRA004775;PRJNA827465;Yang2025_Silkie_Wencha	0.985848371
ENSGALG00010014416	H2BC32	3	-0.037597399	0.705301152	-0.053306873	0.957487404	1.290991666	14.95964986	86.63070313	0.666666667	CRA004775;PRJNA827465;Yang2025_Silkie_Wencha	0.985906358
ENSGALG00010000091	ENSGALG00010000091	2	0.035001109	0.66795411	0.05240047	0.9582096	0	0.916150015	0	0.5	PRJNA827465;Yang2025_Silkie_Wenchang	0.985906358
ENSGALG00010023042	ENSGALG00010023042	3	-0.031820968	0.59513597	-0.0534684	0.957358707	0.518717818	3.906355871	48.80138763	0.666666667	CRA004775;PRJNA827465;Yang2025_Silkie_Wencha	0.985906358
ENSGALG00010021030	CSRP3	3	-0.030290376	0.575521341	-0.052631195	0.958025761	0.731523356	8.028962311	75.09018074	0.666666667	CRA004775;PRJNA827465;Yang2025_Silkie_Wencha	0.985906358
ENSGALG00010019816	ENSGALG00010019816	2	-0.027193551	0.515525825	-0.052749154	0.957931775	0	0.977816538	0	0.5	CRA004775;PRJNA827465	0.985906358
ENSGALG00010014292	NIM1K	3	0.018558539	0.348655334	0.053228899	0.95754953	0.210048504	5.015159483	60.12090928	0.666666667	CRA004775;PRJNA827465;Yang2025_Silkie_Wencha	0.985906358
ENSGALG00010000688	ENSGALG00010000688	3	0.016819858	0.318446994	0.05281839	0.957876609	0.227823906	8.241546852	75.73271091	0.666666667	CRA004775;PRJNA827465;Yang2025_Silkie_Wencha	0.985906358
ENSGALG00010002136	ACAP1	3	-0.014417912	0.274978133	-0.052432941	0.958183727	0.124921258	4.57409299	56.27548447	0.666666667	CRA004775;PRJNA827465;Yang2025_Silkie_Wencha	0.985906358
ENSGALG00010016955	ENSGALG00010016955	3	-0.010378217	0.19503966	-0.053210804	0.957563947	0.03123247	2.64702736	24.44354636	0.666666667	CRA004775;PRJNA827465;Yang2025_Silkie_Wencha	0.985906358
ENSGALG00010013419	ENSGALG00010013419	3	-0.009799605	0.186480758	-0.05255022	0.958090281	0.031433517	2.665429214	24.9651805	0.666666667	CRA004775;PRJNA827465;Yang2025_Silkie_Wencha	0.985906358
ENSGALG00010002288	ANGPT2	3	-0.009113387	0.17286205	-0.052720579	0.957954542	0.051672681	5.111434218	60.87203876	0.666666667	CRA004775;PRJNA827465;Yang2025_Silkie_Wencha	0.985906358
ENSGALG00010005250	SLC35A5	3	0.006701753	0.128152258	0.052295236	0.958293449	0.028508897	5.291613684	62.20434598	0.666666667	CRA004775;PRJNA827465;Yang2025_Silkie_Wencha	0.985906358
ENSGALG00010007324	ENSGALG00010007324	3	0.006614056	0.124662169	0.053055839	0.957687417	0	1.689489781	0	0.666666667	CRA004775;PRJNA827465;Yang2025_Silkie_Wencha	0.985906358
ENSGALG00010001400	SNX24	3	0.005898107	0.112621698	0.052370965	0.958233109	0	0.941853234	0	0.666666667	CRA004775;PRJNA827465;Yang2025_Silkie_Wencha	0.985906358
ENSGALG00010016936	RCC1	3	-0.004645523	0.08795238	-0.052818619	0.957876426	0	0.948946945	0	0.666666667	CRA004775;PRJNA827465;Yang2025_Silkie_Wencha	0.985906358
ENSGALG00010009051	TROJANZ	3	-0.019965128	0.384629397	-0.051907443	0.958602444	0.310904719	6.916404224	71.08324015	0.666666667	CRA004775;PRJNA827465;Yang2025_Silkie_Wencha	0.985956781
ENSGALG00010000292	ABHD16A	3	-0.012806746	0.248935257	-0.051446091	0.958970058	0.149194198	11.13860382	82.04442826	0.666666667	CRA004775;PRJNA827465;Yang2025_Silkie_Wencha	0.985956781
ENSGALG00010002409	CYCS	3	-0.010294648	0.198419473	-0.051883256	0.958621716	0.070490001	5.21305183	61.63475704	0.666666667	CRA004775;PRJNA827465;Yang2025_Silkie_Wencha	0.985956781
ENSGALG00010020088	LMOD3	3	-0.010230406	0.197977919	-0.051674479	0.958788073	0.075039117	9.226021345	78.32218325	0.666666667	CRA004775;PRJNA827465;Yang2025_Silkie_Wencha	0.985956781
ENSGALG00010023122	ENSGALG00010023122	3	0.009141295	0.176272397	0.051858911	0.958641114	0.046958577	4.057500541	50.70857096	0.666666667	CRA004775;PRJNA827465;Yang2025_Silkie_Wencha	0.985956781
ENSGALG00010002312	ENSGALG00010002312	3	0.008053882	0.155299999	0.051860156	0.958640122	0.037695204	4.295953683	53.44456324	0.666666667	CRA004775;PRJNA827465;Yang2025_Silkie_Wencha	0.985956781
ENSGALG00010022524	SOX18	3	0.007425249	0.14409782	0.051529227	0.958903813	0.031853774	4.188975317	52.25562701	0.666666667	CRA004775;PRJNA827465;Yang2025_Silkie_Wencha	0.985956781
ENSGALG00010008764	ETFDH	3	0.007078611	0.137173431	0.051603369	0.958844735	0.038873873	8.384264393	76.14579042	0.666666667	CRA004775;PRJNA827465;Yang2025_Silkie_Wencha	0.985956781
ENSGALG00010016909	MRPS5	3	0.005032047	0.097290654	0.051721788	0.958750376	0.016155957	5.120032547	60.93774831	0.666666667	CRA004775;PRJNA827465;Yang2025_Silkie_Wencha	0.985956781
ENSGALG00010007988	TARBP1	3	0.007691475	0.149896825	0.051311795	0.95907707	0.051328029	11.14618898	82.05664731	0.666666667	CRA004775;PRJNA827465;Yang2025_Silkie_Wencha	0.985995106
ENSGALG00010021041	GLTP	3	0.014860408	0.290668281	0.051124973	0.959225936	0.179573562	7.134142522	71.96579696	0.666666667	CRA004775;PRJNA827465;Yang2025_Silkie_Wencha	0.986076451

ENSGALG00010025044	TMEM9	3	0.024843181	0.487553766	0.050954751	0.959361577	0.642310011	23.34291869	91.43209113	0.666666667	CRA004775;PRJNA827465;Yang2025_Silkie_Wencha	0.986087796
ENSGALG00010012001	EEIG2	3	0.009006018	0.178904356	0.050339849	0.959851569	0.04729643	3.98395768	49.79866352	0.666666667	CRA004775;PRJNA827465;Yang2025_Silkie_Wencha	0.986087796
ENSGALG00010015677	GRHL3	3	0.006503542	0.128559853	0.050587656	0.959654099	0	0.039676799	0	0.666666667	CRA004775;PRJNA827465;Yang2025_Silkie_Wencha	0.986087796
ENSGALG00010023177	LZTS2	3	0.006418871	0.126513712	0.050736567	0.959535438	0.022923895	3.83316289	47.82376702	0.666666667	CRA004775;PRJNA827465;Yang2025_Silkie_Wencha	0.986087796
ENSGALG00010021301	SMURF1	3	0.005594285	0.111166608	0.050323429	0.959864654	0.020821131	4.81621641	58.47362681	0.666666667	CRA004775;PRJNA827465;Yang2025_Silkie_Wencha	0.986087796
ENSGALG00010010843	ZNF395	3	-0.005483747	0.108456016	-0.050561948	0.959674586	0.017961298	4.03626522	50.44924229	0.666666667	CRA004775;PRJNA827465;Yang2025_Silkie_Wencha	0.986087796
ENSGALG00010015333	MED7	3	0.005273854	0.104590583	0.050423797	0.959784673	0.017051689	4.277201878	53.2404582	0.666666667	CRA004775;PRJNA827465;Yang2025_Silkie_Wencha	0.986087796
ENSGALG00010008347	PUF60	3	0.0039281	0.077548646	0.050653368	0.959601736	0.011020963	5.276074216	62.09302754	0.666666667	CRA004775;PRJNA827465;Yang2025_Silkie_Wencha	0.986087796
ENSGALG00010012765	PHF5A	3	0.002639762	0.05220092	0.050569257	0.959668761	0	1.513866802	0	0.666666667	CRA004775;PRJNA827465;Yang2025_Silkie_Wencha	0.986087796
ENSGALG00010020991	ALG14	3	0.004397064	0.087817934	0.050070227	0.960066426	0	0.289793297	0	0.666666667	CRA004775;PRJNA827465;Yang2025_Silkie_Wencha	0.986156305
ENSGALG00010007319	ATP9B	3	0.002793063	0.055789083	0.050064693	0.960070835	0	1.541125515	0	0.666666667	CRA004775;PRJNA827465;Yang2025_Silkie_Wencha	0.986156305
ENSGALG00010014792	ENSGALG0001001479	3	0.006804956	0.136548015	0.049835628	0.960253175	0	0.241524753	0	0.666666667	CRA004775;PRJNA827465;Yang2025_Silkie_Wencha	0.986200514
ENSGALG00010010014	GLUL	3	-0.005920287	0.118704444	-0.049874178	0.960222654	0.007361046	2.33130133	14.21100422	0.666666667	CRA004775;PRJNA827465;Yang2025_Silkie_Wencha	0.986200514
ENSGALG00010027256	PHTF1	3	-0.009665982	0.194996116	-0.049570126	0.960464953	0.082764898	12.51449901	84.01853724	0.666666667	CRA004775;PRJNA827465;Yang2025_Silkie_Wencha	0.986202903
ENSGALG00010008036	SPOCK3	3	-0.007029986	0.141392142	-0.049719778	0.960345696	0.017253981	2.74849716	27.23296102	0.666666667	CRA004775;PRJNA827465;Yang2025_Silkie_Wencha	0.986202903
ENSGALG00010021016	FHIT	3	-0.005493783	0.110651005	-0.049649644	0.960401585	0	0.389696948	0	0.666666667	CRA004775;PRJNA827465;Yang2025_Silkie_Wencha	0.986202903
ENSGALG00010005583	ALDOB	3	0.061899872	1.27375544	0.048596355	0.961240975	4.311304178	17.9259606	88.84299679	0.666666667	CRA004775;PRJNA827465;Yang2025_Silkie_Wencha	0.98647409
ENSGALG00010013341	ENSGALG0001001334	2	-0.041618332	0.887174359	-0.046911108	0.962584075	0	0.550850669	0	0.5	PRJNA827465;Yang2025_Silkie_Wenchang	0.98647409
ENSGALG00010017915	BFSP1	3	0.038623803	0.807285327	0.047844055	0.961840526	1.664823496	13.71674369	85.41927993	0.666666667	CRA004775;PRJNA827465;Yang2025_Silkie_Wencha	0.98647409
ENSGALG00010027697	CATH2	3	0.030496543	0.657283589	0.046397847	0.962993153	0.872945675	6.185918037	67.66850145	0.666666667	CRA004775;PRJNA827465;Yang2025_Silkie_Wencha	0.98647409
ENSGALG00010010099	TNR	2	-0.026221307	0.547478437	-0.047894685	0.961800175	0.222267859	1.511622125	33.84590083	0.5	PRJNA827465;Yang2025_Silkie_Wenchang	0.98647409
ENSGALG00010012454	IQCA1	2	0.022003626	0.448889131	0.049017951	0.96090499	0.321970805	4.973320709	79.89271035	0.5	PRJNA827465;Yang2025_Silkie_Wenchang	0.98647409
ENSGALG00010004586	ENSGALG0001000458	2	-0.020378984	0.438199771	-0.046506149	0.962906833	0.174440149	1.832260257	45.42260051	0.5	PRJNA827465;Yang2025_Silkie_Wenchang	0.98647409
ENSGALG00010014740	TRMT9B	2	0.014307528	0.2940144	0.048662677	0.96118812	0	0.069582156	0	0.5	PRJNA827465;Yang2025_Silkie_Wenchang	0.98647409
ENSGALG00010019595	GRIN2A	3	-0.013478255	0.290937485	-0.046326982	0.963049634	0.147243181	6.458122797	69.03124851	0.666666667	CRA004775;PRJNA827465;Yang2025_Silkie_Wencha	0.98647409
ENSGALG00010024335	AMHR2	3	0.012855079	0.266479625	0.048240384	0.961524666	0	0.587747746	0	0.666666667	CRA004775;PRJNA827465;Yang2025_Silkie_Wencha	0.98647409
ENSGALG00010011558	MYBPC1	3	-0.011715704	0.242618085	-0.048288665	0.961486188	0.108342841	9.184443764	78.22404871	0.666666667	CRA004775;PRJNA827465;Yang2025_Silkie_Wencha	0.98647409
ENSGALG00010001888	SNX10	3	0.010360223	0.219366188	0.047227983	0.962331525	0.10567593	7.707525427	74.05133439	0.666666667	CRA004775;PRJNA827465;Yang2025_Silkie_Wencha	0.98647409
ENSGALG00010024559	ENSGALG0001002455	2	0.009650479	0.202113355	0.047747855	0.961917195	0	0.084745353	0	0.5	PRJNA827465;Yang2025_Silkie_Wenchang	0.98647409
ENSGALG00010017008	RTN1	3	0.009378117	0.191397244	0.048998182	0.960920744	0	1.919220918	0	0.666666667	CRA004775;PRJNA827465;Yang2025_Silkie_Wencha	0.98647409
ENSGALG00010020390	KLHL25	3	-0.008155552	0.168667154	-0.048352936	0.961434967	0.040419868	3.872599242	48.35510015	0.666666667	CRA004775;PRJNA827465;Yang2025_Silkie_Wencha	0.98647409
ENSGALG00010025238	IGSF9B	3	0.007975337	0.166033565	0.048034487	0.961688758	0	1.147258408	0	0.666666667	CRA004775;PRJNA827465;Yang2025_Silkie_Wencha	0.98647409
ENSGALG00010008997	CHST6	3	-0.007589112	0.156060839	-0.048629186	0.96121481	0.056413109	12.26977344	83.69978052	0.666666667	CRA004775;PRJNA827465;Yang2025_Silkie_Wencha	0.98647409
ENSGALG00010016317	CTDSPL2	3	-0.007475813	0.157066761	-0.047596401	0.9620379	0.05848002	13.91928793	85.63144889	0.666666667	CRA004775;PRJNA827465;Yang2025_Silkie_Wencha	0.98647409
ENSGALG00010012963	UIMC1	3	-0.007421556	0.158759439	-0.046747181	0.962714726	0.066004411	10.91271603	81.67275686	0.666666667	CRA004775;PRJNA827465;Yang2025_Silkie_Wencha	0.98647409
ENSGALG00010024452	SLC16A1	3	-0.007117293	0.151472978	-0.046987209	0.962523422	0.034534696	3.976068586	49.69905683	0.666666667	CRA004775;PRJNA827465;Yang2025_Silkie_Wencha	0.98647409
ENSGALG00010027956	RAB8A	3	0.007111113	0.146946354	0.048392577	0.961403375	0.047577704	11.17451496	82.10213144	0.666666667	CRA004775;PRJNA827465;Yang2025_Silkie_Wencha	0.98647409
ENSGALG00010010546	MICALL1	3	-0.006767508	0.146285771	-0.046262242	0.963101234	0.038182209	5.130542955	61.01777107	0.666666667	CRA004775;PRJNA827465;Yang2025_Silkie_Wencha	0.98647409
ENSGALG00010025586	TCTN1	3	0.006566017	0.139136929	0.047191045	0.962460965	0.040847297	9.636209684	79.24495143	0.666666667	CRA004775;PRJNA827465;Yang2025_Silkie_Wencha	0.98647409
ENSGALG00010016459	PAQR5	3	-0.00623811	0.129046401	-0.048340051	0.961445236	0	0.65574263	0	0.666666667	CRA004775;PRJNA827465;Yang2025_Silkie_Wencha	0.98647409
ENSGALG00010005499	PDE5A	3	0.006138488	0.125514381	0.048906653	0.960993687	0.027327592	5.520333129	63.77030238	0.666666667	CRA004775;PRJNA827465;Yang2025_Silkie_Wencha	0.98647409
ENSGALG00010025572	ENSGALG0001002557	3	0.005798831	0.121469258	0.047739086	0.961924184	0.030620821	6.724005208	70.25582316	0.666666667	CRA004775;PRJNA827465;Yang2025_Silkie_Wencha	0.98647409
ENSGALG00010003674	AKIRIN2	3	-0.005333489	0.113459462	-0.047007882	0.962506946	0.02526697	6.247965198	67.98957842	0.666666667	CRA004775;PRJNA827465;Yang2025_Silkie_Wencha	0.98647409
ENSGALG00010021433	ENSGALG0001002143	3	0.0044656	0.096525439	0.046263449	0.963100271	0	0.232127109	0	0.666666667	CRA004775;PRJNA827465;Yang2025_Silkie_Wencha	0.98647409
ENSGALG00010009391	RSBN1L	3	0.004298728	0.0905205	0.047488995	0.962123502	0.008749004	2.953782453	32.29020647	0.666666667	CRA004775;PRJNA827465;Yang2025_Silkie_Wencha	0.98647409
ENSGALG00010027156	ISYNA1	3	-0.002909698	0.062876612	-0.046276313	0.963090019	0	1.901948337	0	0.666666667	CRA004775;PRJNA827465;Yang2025_Silkie_Wencha	0.98647409
ENSGALG00010018099	SMAD3	3	-0.002630793	0.055644163	-0.047440607	0.962162066	0	1.362033739	0	0.666666667	CRA004775;PRJNA827465;Yang2025_Silkie_Wencha	0.98647409
ENSGALG00010026980	LIMK2	3	0.002494555	0.053295195	0.046806368	0.962667553	0	0.347824392	0	0.666666667	CRA004775;PRJNA827465;Yang2025_Silkie_Wencha	0.98647409
ENSGALG00010007866	TBP	3	0.002069338	0.044275312	0.046737969	0.962722068	0	0.024618167	0	0.666666667	CRA004775;PRJNA827465;Yang2025_Silkie_Wencha	0.98647409
ENSGALG00010021970	ERGIC3	3	-0.002002061	0.043108001	-0.046442914	0.962957233	0	1.994638581	0	0.666666667	CRA004775;PRJNA827465;Yang2025_Silkie_Wencha	0.98647409
ENSGALG00010011406	UBE2R2	3	-0.007076198	0.153730269	-0.046029957	0.963286373	0.047333883	6.243476317	67.96656384	0.666666667	CRA004775;PRJNA827465;Yang2025_Silkie_Wencha	0.98652079
ENSGALG00010014062	GGA1	3	-0.003520604	0.07640279	-0.046079525	0.963246865	0.008489361	4.044341463	50.54819139	0.666666667	CRA004775;PRJNA827465;Yang2025_Silkie_Wencha	0.98652079
ENSGALG00010011626	SERPINB1	2	0.016238544	0.354104598	0.045858042	0.963423396	0	0.066042329	0	0.5	CRA004775;PRJNA827465	0.986524523
ENSGALG00010009205	SIRT2	3	-0.004053469	0.088492768	-0.045805656	0.963465151	0.011784914	4.078502321	50.96239152	0.666666667	CRA004775;PRJNA827465;Yang2025_Silkie_Wencha	0.986524523
ENSGALG00010007761	ENSGALG0001000776	3	-0.003356476	0.073345127	-0.045762764	0.963499338	0	1.196297174	0	0.666666667	CRA004775;PRJNA827465;Yang2025_Silkie_Wencha	0.986524523
ENSGALG00010007762	ENSGALG0001000776	2	-0.011936498	0.262173247	-0.045529048	0.963685622	0	0.070239806	0	0.5	PRJNA827465;Yang2025_Silkie_Wenchang	0.98664381
ENSGALG00010026796	DCAKD	3	0.006089394	0.134238874	0.045362375	0.963818471	0	1.236077544	0	0.666666667	CRA004775;PRJNA827465;Yang2025_Silkie_Wencha	0.986708374
ENSGALG00010007601	TMEM154	3	0.014319558	0.320108853	0.044733401	0.96431981	0.14153208	3.710823754	46.10361115	0.666666667	CRA004775;PRJNA827465;Yang2025_Silkie_Wencha	0.986838218
ENSGALG00010022169	TOX2	3	-0.01258401	0.282284377	-0.044579335	0.964442615	0.14534309	5.121264389	60.94714414	0.666666667	CRA004775;PRJNA827465;Yang2025_Silkie_Wencha	0.986838218
ENSGALG00010003814	TPP1	3	-0.007627872	0.170725205	-0.044679239	0.964362982	0.061518791	7.003217505	71.44169807	0.666666667	CRA004775;PRJNA827465;Yang2025_Silkie_Wencha	0.986838218
ENSGALG00010028109	TJP3	3	-0.006759475	0.151504705	-0.04461561	0.9644137	0.023833852	3.041017121	34.23253076	0.666666667	CRA004775;PRJNA827465;Yang2025_Silkie_Wencha	0.986838218
ENSGALG00010013348	MKRN4P	3	0.003937351	0.088474339	0.044502742	0.964503666	0.01551873	7.112752344	71.88148971	0.666666667	CRA004775;PRJNA827465;Yang2025_Silkie_Wencha	0.986838218
ENSGALG00010022481	CORO1B	3	0.003762651	0.083728318	0.044938812	0.964156081	0.00806297	3.232922867	38.13647642	0.666666667	CRA004775;PRJNA827465;Yang2025_Silkie_Wencha	0.986838218
ENSGALG00010016457	SMOX	3	0.002980965	0.066965494	0.044514947	0.964493938	0	1.762262135	0	0.666666667	CRA004775;PRJNA827465;Yang2025_Silkie_Wencha	0.986838218
ENSGALG00010015617	SRSF10	3	0.001649248	0.036701321	0.044937011	0.964157516	0	0.96315476	0	0.666666667	CRA004775;PRJNA827465;Yang2025_Silkie_Wencha	0.986838218
ENSGALG00010028513	ENSGALG0001002851	2	-0.055892649	1.261779532	-0.044296683	0.964667915	0	0.206557236	0	0.5	PRJNA827465;Yang2025_Silkie_Wenchang	0.986918722
ENSGALG00010012775	KCNE2	3	0.005866412	0.133637796	0.043897848	0.964985831	0.026479786	4.25361885	52.9812127	0.666666667	CRA004775;PRJNA827465;Yang2025_Silkie_Wencha	0.986918722
ENSGALG00010009578	KIF5C	3	0.004951293	0.112840655	0.043878623	0.965001155	0	0.999871882	0	0.666666667	CRA004775;PRJNA827465;Yang2025_Silkie_Wencha	0.986918722
ENSGALG00010029364	ANAPC11	3	0.004009651	0.090828362	0.04414536	0.964788536	0	1.411830596	0	0.666666667	CRA004775;PRJNA827465;Yang2025_Silkie_Wencha	0.986918722
ENSGALG00010020155	MFGE8	3	0.003632171	0.082532526	0.04400897	0.964897254	0	0.00345714	0	0.666666667	CRA004775;PRJNA827465;Yang2025_Silkie_Wencha	0.986918722
ENSGALG00010013367	NOL4L	3	-0.003348007	0.075892664	-0.044115034	0.964812709	0.002262043	2.214004387	9.665942329	0.666666667	CRA004775;PRJNA827465;Yang2025_Silkie_Wencha	0.986918722
ENSGALG00010024890	SMARCC2	3	0.006761596	0.154810673	0.043676551	0.965162231	0.060935187	14.44549388	86.15485205	0.666666667	CRA004775;PRJNA827465;Yang2025_Silkie_Wencha	0.987012063
ENSGALG00010018576	PTK7	3	0.004364313	0.100724076	0.043329391	0.965438962	0.019906546	6.610932937	69.74708382	0.666666667	CRA004775;PRJNA827465;Yang2025_Silkie_Wencha	0.987152263
ENSGALG00010013123	PPFIBP1	3	-0.00409803	0.094462488	-0.043382619	0.965396533	0.012452448	3.403761636	41.24147888	0.666666667	CRA004775;PRJNA827465;Yang2025_Silkie_Wencha	0.987152263
ENSGALG00010022308	OGFR	3	0.007718677	0.179073806	0.043103327	0.965619167	0.06329984	6.234532538	67.92061012	0.666666667	CRA004775;PRJNA827465;Yang2025_Silkie_Wencha	0.98717381
ENSGALG00010023677	MN1	3	0.004731695	0.109611147	0.043168009	0.965567607	0.014933865	3.430386708	41.69753528	0.666666667	CRA004775;PRJNA827465;Yang2025_Silkie_Wencha	0.98717381
ENSGALG00010023571	APLP2	3	0.003720851	0.08645061	0.043040194	0.965669494	0.011744791	4.581289119	56.34416541	0.666666667	CRA004775;PRJNA827465;Yang2025_Silkie_Wencha	0.98717381
ENSGALG00010029173	ZNHIT3	3	0.009027455	0.211109915	0.04276187	0.965891359	0.085610387	5.620996351	64.4191194	0.666666667	CRA004775;PRJNA827465;Yang2025_Silkie_Wencha	0.987329231
ENSGALG00010004036	ADCYAP1	2	0.023563102	0.553880199	0.042541874	0.966066731	0.433805058	3.401142852	70.59811824	0.5	PRJNA827465;Yang2025_Silkie_Wenchang	0.98736573
ENSGALG00010007980	MARCHF1	3	0.006203091	0.145671721	0.042582671	0.966034209	0	0.909441502	0	0.666666667	CRA004775;PRJNA827465;Yang2025_Silkie_Wencha	0.98736573
ENSGALG00010022184	PRKAR1B	3	-0.002753483	0.064932271	-0.042405467	0.966175471	0.004164822	2.933924372	31.83191703	0.666666667	CRA004775;PRJNA827465;Yang2025_Silkie_Wencha	0.987365989
ENSGALG00010020215	PRMT3	3	-0.002034693	0.048026147	-0.042366354	0.96620665	0	1.884661763	0	0.666666667	CRA004775;PRJNA827465;Yang2025_Silkie_Wencha	0.987365989
ENSGALG00010027759	ENSGALG0001002775	2	-0.05035298	1.20657584	-0.04173213	0.96671224	2.126069216	3.602030169	72.23787828	0.5	PRJNA827465;Yang2025_Silkie_Wenchang	0.98745444
ENSGALG00010027167	ENSGALG0001002716	3	-0.020816231	0.495206583	-0.042035448	0.96647044	0.528814424	7.470374211	73.22757946	0.666666667	CRA004775;PRJNA827465;Yang2025_Silkie_Wencha	0.98745444
ENSGALG00010003320	OR51E2	3	-0.013976571	0.332641853	-0.042016873	0.966485247	0.171770297	4.496833414	55.52425861	0.666666667	CRA004775;PRJNA827465;Yang2025_Silkie_Wencha	0.98745444
ENSGALG00010023712	ARHGAP40	3	-0.008855757	0.211410161	-0.041888987	0.966587196	0.091928284	10.59657632	81.12597937	0.666666667	CRA004775;PRJNA827465;Yang2025_Silkie_Wencha	0.98745444
ENSGALG00010005141	CCDC170	2	0.008610666	0.206238674	0.041750977	0.966697216	0.036180919	1.740078361	42.53132375	0.5	PRJNA827465;Yang2025_Silkie_Wenchang	0.98745444
ENSGALG00010013697	HDAC4	3	-0.003137064	0.074969808	-0.041844361	0.966622771	0.004848503	2.414052085	17.15174612	0.666666667	CRA004775;PRJNA827465;Yang2025_Silkie_Wencha	0.98745444
ENSGALG00010005279	CCDC181	2	0.017180742	0.417872544	0.041114791	0.967204383	0.196790114	2.266172308	55.87272881	0.5	PRJNA827465;Yang2025_Silkie_Wenchang	0.987512768
ENSGALG00010008058	USP25	3	0.005077524	0.122897448	0.041315128	0.967044673	0.027413653	6.691989446	70.11352131	0.666666667	CRA004775;PRJNA827465;Yang2025_Silkie_Wencha	0.987512768
ENSGALG00010009465	GGPS1	3	0.004953066	0.119736195	0.041366485	0.967003731	0.024334321	4.931143978	59.44146006	0.666666667	CRA004775;PRJNA827465;Yang2025_Silkie_Wencha	0.987512768
ENSGALG00010025645	NBEAL2	3	0.004370196	0.106843566	0.040902756	0.967373421	0.013842692	3.33350589	40.00310587	0.666666667	CRA004775;PRJNA827465;Yang2025_Silkie_Wencha	0.987512768
ENSGALG00010017430	CACNA1H	3	0.004218552	0.102476717	0.041165956	0.967163594	0	1.552340244	0	0.666666667	CRA004775;PRJNA827465;Yang2025_Silkie_Wencha	0.987512768
ENSGALG00010024133	PSMD11	3	-0.003896547	0.095335349	-0.04087201	0.967397931	0.014161888	4.794195662	58.28288745	0.666666667	CRA004775;PRJNA827465;Yang2025_Silkie_Wencha	0.987512768
ENSGALG00010005217	CCNA2	3	-0.003869159	0.094635369	-0.040884919	0.96738764	0.009665938	3.100398567	35.49216474	0.666666667	CRA004775;PRJNA827465;Yang2025_Silkie_Wencha	0.987512768
ENSGALG00010023220	ST3GAL2	3	-0.002105107	0.05081389	-0.041427781	0.966954865	0	1.664422787	0	0.666666667	CRA004775;PRJNA827465;Yang2025_Silkie_Wencha	0.987512768
ENSGALG00010013408	RLIM	3	-0.001290795	0.031317919	-0.041215858	0.967123812	0	1.679986367	0	0.666666667	CRA004775;PRJNA827465;Yang2025_Silkie_Wencha	0.987512768
ENSGALG00010000722	ANKH	3	0.0077884	0.192128055	0.040537545	0.967664575	0.085438204	9.990566392	79.98111497	0.666666667	CRA004775;PRJNA827465;Yang2025_Silkie_Wencha	0.987571057
ENSGALG00010015033	BMP7	3	0.005747086	0.141380734	0.040649709	0.967575155	0	0.021187336	0	0.666666667	CRA004775;PRJNA827465;Yang2025_Silkie_Wencha	0.987571057
ENSGALG00010014736	KIF2A	3	0.001977667	0.048732608	0.040582008	0.967629128	0	0.576617723	0	0.666666667	CRA004775;PRJNA827465;Yang2025_Silkie_Wencha	0.987571057
ENSGALG00010010410	CACNA2D1	3	0.007221499	0.179102467	0.040320489	0.967837619	0.062142316	7.46098116	73.19387414	0.666666667	CRA004775;PRJNA827465;Yang2025_Silkie_Wencha	0.987605088
ENSGALG00010022485	ATF2	3	0.001432374	0.03548526	0.040365321	0.967801877	0	1.346967878	0	0.666666667	CRA004775;PRJNA827465;Yang2025_Silkie_Wencha	0.987605088
ENSGALG00010029406	TMEM199	3	-0.006742941	0.168040307	-0.040126928	0.967991934	0.052021941	5.215906762	61.6557563	0.666666667	CRA004775;PRJNA827465;Yang2025_Silkie_Wencha	0.987691272
ENSGALG00010017055	GPR135	3	0.006994699	0.176319084	0.039670689	0.96835567	0	1.571255416	0	0.666666667	CRA004775;PRJNA827465;Yang2025_Silkie_Wencha	0.987919823
ENSGALG00010022824	PUS1	3	0.001833669	0.046199474	0.039690261	0.968340066	0	0.481203669	0	0.666666667	CRA004775;PRJNA827465;Yang2025_Silkie_Wencha	0.987919823
ENSGALG00010002442	ENSGALG0001000244	3	-0.030135085	0.763099065	-0.039490397	0.968499409	1.470616369	15.26342296	86.89677928	0.666666667	CRA004775;PRJNA827465;Yang2025_Silkie_Wencha	0.987923898
ENSGALG00010026093	TOE1	3	0.002101324	0.053199335	0.039499064	0.9684925	0	1.650973931	0	0.666666667	CRA004775;PRJNA827465;Yang2025_Silkie_Wencha	0.987923898
ENSGALG00010027211	HDGFRP2	3	0.002087355	0.052984241	0.039395768	0.968574854	0.003205523	3.188348383	37.2715977	0.666666667	CRA004775;PRJNA827465;Yang2025_Silkie_Wencha	0.987929582
ENSGALG00010017132	TMEM108	3	-0.019153542	0.495931406	-0.038621354	0.969192277	0.568014567	12.54191045	84.05346611	0.666666667	CRA004775;PRJNA827465;Yang2025_Silkie_Wencha	0.988007166
ENSGALG00010007897	EFHC1	3	-0.009662767	0.247400295	-0.039057218	0.96884477	0.073162057	3.284144777	39.10134492	0.666666667	CRA004775;PRJNA827465;Yang2025_Silkie_Wencha	0.988007166
ENSGALG00010005425	ELAPOR2	3	0.005333227	0.137031714	0.038919656	0.968954445	0	0.194342106	0	0.666666667	CRA004775;PRJNA827465;Yang2025_Silkie_Wencha	0.988007166
ENSGALG00010024654	CCDC92	3	-0.004988486	0.129238077	-0.038599196	0.969209944	0	1.113837061	0	0.666666667	CRA004775;PRJNA827465;Yang2025_Silkie_Wencha	0.988007166
ENSGALG00010024809	KANSL1	3	0.004857449	0.124052216	0.039156491	0.968765622	0.027788004	5.041454357	60.32890792	0.666666667	CRA004775;PRJNA827465;Yang2025_Silkie_Wencha	0.988007166
ENSGALG00010027554	ENSGALG0001002755	3	-0.00432262	0.111725274	-0.03868972	0.96913777	0.009411535	2.628346151	23.90652202	0.666666667	CRA004775;PRJNA827465;Yang2025_Silkie_Wencha	0.988007166
ENSGALG00010017721	VWA1	3	0.00389133	0.100433058	0.038745508	0.969093291	0.011160794	3.171355025	36.93547445	0.666666667	CRA004775;PRJNA827465;Yang2025_Silkie_Wencha	0.988007166
ENSGALG00010024198	PI4KA	3	0.001565821	0.040563624	0.038601614	0.969208015	0	0.552852622	0	0.666666667	CRA004775;PRJNA827465;Yang2025_Silkie_Wencha	0.988007166
ENSGALG00010029416	UNC119	2	-0.005788813	0.15049723	-0.038464585	0.969317268	0	0.431876674	0	0.5	PRJNA827465;Yang2025_Silkie_Wenchang	0.988045336
ENSGALG00010023052	RAD54L	3	0.003111565	0.081718516	0.03807662	0.969626592	0	1.7410049	0	0.666666667	CRA004775;PRJNA827465;Yang2025_Silkie_Wencha	0.988289388
ENSGALG00010000783	DAP	3	-0.006975859	0.184629761	-0.037782963	0.969860728	0.061911194	5.457415094	63.35261354	0.666666667	CRA004775;PRJNA827465;Yang2025_Silkie_Wencha	0.988456775
ENSGALG00010026002	FTH1	3	-0.007016213	0.188259331	-0.037268874	0.970270623	0.065871332	5.21728591	61.6658923	0.666666667	CRA004775;PRJNA827465;Yang2025_Silkie_Wencha	0.98872525
ENSGALG00010016937	DHRS7	3	-0.005352874	0.14426408	-0.037104688	0.970401534	0.020747551	2.89717799	30.96730657	0.666666667	CRA004775;PRJNA827465;Yang2025_Silkie_Wencha	0.98872525
ENSGALG00010021870	ENSGALG0001002187	3	0.004492151	0.121363375	0.037014057	0.970473797	0	0.845787139	0	0.666666667	CRA004775;PRJNA827465;Yang2025_Silkie_Wencha	0.98872525
ENSGALG00010025899	ENSGALG0001002589	3	0.004034631	0.1080432	0.037342757	0.970211714	0	0.887457181	0	0.666666667	CRA004775;PRJNA827465;Yang2025_Silkie_Wencha	0.98872525
ENSGALG00010016549	PTPRA	3	0.001586966	0.042834752	0.037048557	0.97044629	0	0.703198576	0	0.666666667	CRA004775;PRJNA827465;Yang2025_Silkie_Wencha	0.98872525
ENSGALG00010000052	TTLL13	3	0.003509623	0.095095815	0.036906181	0.970559811	0	0.063965713	0	0.666666667	CRA004775;PRJNA827465;Yang2025_Silkie_Wencha	0.988741636
ENSGALG00010013208	ENSGALG0001001320	3	-0.012460844	0.340016784	-0.036647731	0.970765885	0.044665904	2.277727938	12.19320065	0.666666667	CRA004775;PRJNA827465;Yang2025_Silkie_Wencha	0.988832152
ENSGALG00010001971	MLIP	3	-0.008847554	0.245758036	-0.036001078	0.9712815	0.105225982	5.291850036	62.20603406	0.666666667	CRA004775;PRJNA827465;Yang2025_Silkie_Wencha	0.988832152
ENSGALG00010007539	RPS3A	3	-0.006736155	0.185221957	-0.036368013	0.970988919	0.075719896	8.025300987	75.07881632	0.666666667	CRA004775;PRJNA827465;Yang2025_Silkie_Wencha	0.988832152
ENSGALG00010022311	DLL4	3	0.006584062	0.182778773	0.03602203	0.971264793	0.067205304	10.23356948	80.45647706	0.666666667	CRA004775;PRJNA827465;Yang2025_Silkie_Wencha	0.988832152
ENSGALG00010013549	PCP4	3	-0.006174419	0.170181391	-0.0362814	0.971057981	0	1.581974557	0	0.666666667	CRA004775;PRJNA827465;Yang2025_Silkie_Wencha	0.988832152
ENSGALG00010008248	TPK1	3	-0.005541262	0.154231726	-0.035928158	0.971339643	0.048763891	8.060005159	75.1861201	0.666666667	CRA004775;PRJNA827465;Yang2025_Silkie_Wencha	0.988832152
ENSGALG00010002315	ENSGALG0001000231	3	-0.004425604	0.123215312	-0.035917643	0.971348028	0.018406336	3.242653765	38.32212302	0.666666667	CRA004775;PRJNA827465;Yang2025_Silkie_Wencha	0.988832152
ENSGALG00010022250	SLC25A28	3	-0.003500724	0.097031122	-0.036078364	0.971219874	0.010249502	2.921183611	31.53460151	0.666666667	CRA004775;PRJNA827465;Yang2025_Silkie_Wencha	0.988832152
ENSGALG00010003479	SYDE1	3	-0.002068279	0.056882383	-0.03636063	0.970994806	0	0.645614875	0	0.666666667	CRA004775;PRJNA827465;Yang2025_Silkie_Wencha	0.988832152
ENSGALG00010028034	MUM1L1	3	0.001399944	0.038251056	0.036598838	0.97080487	0	1.904590024	0	0.666666667	CRA004775;PRJNA827465;Yang2025_Silkie_Wencha	0.988832152
ENSGALG00010026448	ADORA2A	3	-0.005335306	0.149437256	-0.035702647	0.97151946	0.042648322	6.976382083	71.33184542	0.666666667	CRA004775;PRJNA827465;Yang2025_Silkie_Wencha	0.988870755
ENSGALG00010015143	EXOC1	3	-0.001623646	0.045487079	-0.035694662	0.971525827	0	0.444178132	0	0.666666667	CRA004775;PRJNA827465;Yang2025_Silkie_Wencha	0.988870755
ENSGALG00010024410	MYG1	3	0.003400341	0.096100942	0.035383019	0.971774325	0.007223227	2.326176819	14.02201312	0.666666667	CRA004775;PRJNA827465;Yang2025_Silkie_Wencha	0.989052489
ENSGALG00010010262	HSPA9	3	-0.00376019	0.106932505	-0.035164146	0.971948852	0.021479884	7.431222576	73.0865281	0.666666667	CRA004775;PRJNA827465;Yang2025_Silkie_Wencha	0.989158916
ENSGALG00010024466	ADGRG3	2	0.048909579	1.412559713	0.034624787	0.972378936	3.67663661	12.63358306	92.08458918	0.5	CRA004775;Yang2025_Silkie_Wenchang	0.989455595
ENSGALG00010024517	LACTB	3	-0.003800002	0.109753578	-0.034623039	0.97238033	0.022237329	5.381608291	62.83638846	0.666666667	CRA004775;PRJNA827465;Yang2025_Silkie_Wencha	0.989455595
ENSGALG00010016094	FGF7	3	0.00650569	0.188762797	0.034464894	0.972506437	0.052056999	3.989671724	49.87056233	0.666666667	CRA004775;PRJNA827465;Yang2025_Silkie_Wencha	0.989512702
ENSGALG00010016629	LARS1	3	-0.002330029	0.068200934	-0.03416418	0.97274623	0.006892045	4.047952408	50.5923045	0.666666667	CRA004775;PRJNA827465;Yang2025_Silkie_Wencha	0.989685468
ENSGALG00010001902	WDR45	3	0.003125591	0.09173407	0.034072305	0.972819493	0.011446558	3.641783906	45.08185956	0.666666667	CRA004775;PRJNA827465;Yang2025_Silkie_Wencha	0.98968879
ENSGALG00010020083	CFAP74	2	-0.008112323	0.239068521	-0.033933047	0.97293054	0	0.375872704	0	0.5	PRJNA827465;Yang2025_Silkie_Wenchang	0.98973055
ENSGALG00010019634	GNG2	3	0.005604593	0.165700932	0.033823548	0.973017858	0.043921311	4.647498529	56.96609719	0.666666667	CRA004775;PRJNA827465;Yang2025_Silkie_Wencha	0.989748165
ENSGALG00010018933	PHF13	3	-0.001673977	0.049629203	-0.033729683	0.973092709	0	0.038518853	0	0.666666667	CRA004775;PRJNA827465;Yang2025_Silkie_Wencha	0.989753097
ENSGALG00010007277	FAM151B	3	0.017855502	0.531401042	0.033600804	0.973195481	0.703803438	11.97932051	83.30456224	0.666666667	CRA004775;PRJNA827465;Yang2025_Silkie_Wencha	0.989786427
ENSGALG00010002339	ZNFY4	3	-0.014082846	0.42189872	-0.033379684	0.97337181	0.268525015	4.01229085	50.153165	0.666666667	CRA004775;PRJNA827465;Yang2025_Silkie_Wencha	0.989868902
ENSGALG00010013311	RSPO1	3	0.007280907	0.218793181	0.033277576	0.973453236	0	0.498667986	0	0.666666667	CRA004775;PRJNA827465;Yang2025_Silkie_Wencha	0.989868902
ENSGALG00010007758	DEK	3	0.003035685	0.091338002	0.033235733	0.973486603	0.010777473	3.176014734	37.02799994	0.666666667	CRA004775;PRJNA827465;Yang2025_Silkie_Wencha	0.989868902
ENSGALG00010016680	BPIFB3	2	-0.013571557	0.415970517	-0.032626247	0.973972639	0.156546338	1.744777151	42.68609035	0.5	PRJNA827465;Yang2025_Silkie_Wenchang	0.990081046
ENSGALG00010011411	ENSGALG0001001141	3	-0.011231119	0.342772567	-0.032765512	0.973861581	0.238723959	7.183049827	72.15667372	0.666666667	CRA004775;PRJNA827465;Yang2025_Silkie_Wencha	0.990081046
ENSGALG00010012274	B4GALNT3	3	-0.004690899	0.143791766	-0.032622865	0.973975336	0.037380903	6.059097724	66.99178506	0.666666667	CRA004775;PRJNA827465;Yang2025_Silkie_Wencha	0.990081046
ENSGALG00010026291	SERPING1	3	0.00420945	0.128798122	0.032682542	0.973927746	0.026617747	4.661837117	57.09845819	0.666666667	CRA004775;PRJNA827465;Yang2025_Silkie_Wencha	0.990081046
ENSGALG00010029315	TBC1D16	3	0.002210762	0.067978515	0.032521485	0.974056183	0	0.839722556	0	0.666666667	CRA004775;PRJNA827465;Yang2025_Silkie_Wencha	0.990092046
ENSGALG00010020143	KCNK5	3	0.010392993	0.321768287	0.032299617	0.974233114	0.221805898	8.601975083	76.74952577	0.666666667	CRA004775;PRJNA827465;Yang2025_Silkie_Wencha	0.990165795
ENSGALG00010009462	NRBF2	3	0.00305099	0.094907498	0.032146982	0.974354837	0.012544126	3.786119925	47.17547147	0.666666667	CRA004775;PRJNA827465;Yang2025_Silkie_Wencha	0.990165795
ENSGALG00010008217	ARHGEF37	3	-0.00292483	0.091175158	-0.032079239	0.97440886	0	1.094652733	0	0.666666667	CRA004775;PRJNA827465;Yang2025_Silkie_Wencha	0.990165795
ENSGALG00010013008	STEEP1	3	0.002023518	0.062902242	0.032169249	0.974337079	0	0.82533982	0	0.666666667	CRA004775;PRJNA827465;Yang2025_Silkie_Wencha	0.990165795
ENSGALG00010028909	ENSGALG0001002890	3	0.002914451	0.09251288	0.031503195	0.974868244	0.004690897	2.351611169	14.95192631	0.666666667	CRA004775;PRJNA827465;Yang2025_Silkie_Wencha	0.990561416
ENSGALG00010017950	FBLN7	3	0.015860327	0.507411611	0.031257319	0.975064328	0.562941917	8.230128391	75.69904253	0.666666667	CRA004775;PRJNA827465;Yang2025_Silkie_Wencha	0.990586092
ENSGALG00010010889	CCL19	3	-0.008374305	0.268310551	-0.03121124	0.975101076	0	0.305091938	0	0.666666667	CRA004775;PRJNA827465;Yang2025_Silkie_Wencha	0.990586092
ENSGALG00010002335	TMEM47	3	0.006119501	0.196892899	0.031080352	0.975205459	0.076462989	6.380036894	68.65221889	0.666666667	CRA004775;PRJNA827465;Yang2025_Silkie_Wencha	0.990586092
ENSGALG00010008464	SF3B6	3	0.004764125	0.153289301	0.031079305	0.975206294	0.049253267	7.71147524	74.06462528	0.666666667	CRA004775;PRJNA827465;Yang2025_Silkie_Wencha	0.990586092
ENSGALG00010013638	COMMD8	3	0.003052243	0.098353185	0.031033489	0.975242832	0.013398996	3.603676538	44.50112325	0.666666667	CRA004775;PRJNA827465;Yang2025_Silkie_Wencha	0.990586092
ENSGALG00010028588	ABHD8	3	-0.014906138	0.48369867	-0.030816992	0.975415489	0.500961917	7.147687329	72.01892153	0.666666667	CRA004775;PRJNA827465;Yang2025_Silkie_Wencha	0.990690296
ENSGALG00010025042	BCO2	2	-0.014082885	0.460689061	-0.030569177	0.975613124	0.036661042	1.081957539	7.574931173	0.5	CRA004775;PRJNA827465	0.990819851
ENSGALG00010023351	GNB3	3	0.01501179	0.493267492	0.030433366	0.975721435	0.579699486	10.52703551	81.00129901	0.666666667	CRA004775;PRJNA827465;Yang2025_Silkie_Wencha	0.990858678
ENSGALG00010023389	GNG12	3	-0.003285426	0.109128384	-0.030106067	0.975982462	0.024677581	9.747088632	79.48105249	0.666666667	CRA004775;PRJNA827465;Yang2025_Silkie_Wencha	0.991052574
ENSGALG00010016344	SMIM14	3	-0.007289208	0.243025188	-0.029993631	0.976072133	0.166871627	43.01954298	95.35095015	0.666666667	CRA004775;PRJNA827465;Yang2025_Silkie_Wencha	0.991072452
ENSGALG00010010475	CCDC71L	3	0.002990427	0.10018009	0.029850508	0.976186278	0.010864361	2.966109045	32.57159566	0.666666667	CRA004775;PRJNA827465;Yang2025_Silkie_Wencha	0.991080905
ENSGALG00010008372	CLCN3	3	-0.00137432	0.046106652	-0.02980741	0.976220649	0	0.199121493	0	0.666666667	CRA004775;PRJNA827465;Yang2025_Silkie_Wencha	0.991080905
ENSGALG00010028873	TMEM8C	3	-0.005358587	0.182097053	-0.029427094	0.976523965	0.01756982	2.366272436	15.47887854	0.666666667	CRA004775;PRJNA827465;Yang2025_Silkie_Wencha	0.991317658
ENSGALG00010015796	LARP1	3	-0.001944391	0.066805779	-0.029105131	0.976780744	0	0.635583844	0	0.666666667	CRA004775;PRJNA827465;Yang2025_Silkie_Wencha	0.991507139
ENSGALG00010024582	TLN2	3	-0.002958379	0.102404761	-0.028889076	0.976953058	0.014654327	3.931177201	49.12465407	0.666666667	CRA004775;PRJNA827465;Yang2025_Silkie_Wencha	0.99153968
ENSGALG00010009722	GBA2	3	0.002153747	0.074500492	0.028909162	0.976937038	0	0.44957742	0	0.666666667	CRA004775;PRJNA827465;Yang2025_Silkie_Wencha	0.99153968
ENSGALG00010008078	GUCY1B1	3	-0.002902109	0.101548247	-0.028578622	0.977200662	0.017423981	5.450439439	63.30571099	0.666666667	CRA004775;PRJNA827465;Yang2025_Silkie_Wencha	0.991577448
ENSGALG00010029402	SMG6	3	0.001929597	0.067485736	0.028592661	0.977189465	0.007311305	4.571578578	56.25143556	0.666666667	CRA004775;PRJNA827465;Yang2025_Silkie_Wencha	0.991577448
ENSGALG00010027898	TASOR	3	0.001206	0.041971398	0.028733847	0.977076861	0	1.246716969	0	0.666666667	CRA004775;PRJNA827465;Yang2025_Silkie_Wencha	0.991577448
ENSGALG00010013015	PLA2G6	3	-0.003154115	0.111262034	-0.028348526	0.977384178	0.020639139	5.125168981	60.97689642	0.666666667	CRA004775;PRJNA827465;Yang2025_Silkie_Wencha	0.991621332

ENSGALG00010023746	TTC28	3	0.001856962	0.065445189	0.028374309	0.977363615	0	0.258564325	0	0.666666667	CRA004775;PRJNA827465;Yang2025_Silkie_Wencha	0.991621332
ENSGALG00010028539	ENSGALG00010028539	2	0.031292527	1.10785942	0.028245937	0.977466	0	0.76193312	0	0.5	PRJNA827465;Yang2025_Silkie_Wenchang	0.99163319
ENSGALG00010027862	ENSGALG00010027862	2	0.007225482	0.259348929	0.027860082	0.977773746	0	0.008347281	0	0.5	CRA004775;PRJNA827465	0.991874228
ENSGALG00010019426	GRM7	3	0.009617463	0.347232206	0.027697498	0.977903419	0.27567641	11.10191812	81.98509502	0.666666667	CRA004775;PRJNA827465;Yang2025_Silkie_Wencha	0.991934604
ENSGALG00010019292	ENSGALG00010019292	3	0.00288315	0.104575703	0.027569979	0.978005126	0.009853356	2.808095515	28.77735143	0.666666667	CRA004775;PRJNA827465;Yang2025_Silkie_Wencha	0.991966605
ENSGALG00010013527	ADARB1	3	-0.002730862	0.099610123	-0.027415503	0.978128334	0.01008625	2.879980231	30.55507888	0.666666667	CRA004775;PRJNA827465;Yang2025_Silkie_Wencha	0.991974652
ENSGALG00010009663	FER	3	-0.002498738	0.091247742	-0.027384103	0.978153377	0	1.68822111	0	0.666666667	CRA004775;PRJNA827465;Yang2025_Silkie_Wencha	0.991974652
ENSGALG00010019630	AJAP1	3	-0.006621276	0.245297761	-0.02699281	0.978465469	0	0.627150994	0	0.666666667	CRA004775;PRJNA827465;Yang2025_Silkie_Wencha	0.992155913
ENSGALG00010027649	FBLN2	3	-0.004835656	0.179204247	-0.02698405	0.978472456	0.067121855	8.654790585	76.89141083	0.666666667	CRA004775;PRJNA827465;Yang2025_Silkie_Wencha	0.992155913
ENSGALG00010020176	ENSGALG00010020176	3	0.0038323	0.142625657	0.02686964	0.978563709	0	1.271629497	0	0.666666667	CRA004775;PRJNA827465;Yang2025_Silkie_Wencha	0.992177288
ENSGALG00010005910	ENSGALG00010005910	3	-0.008020357	0.300377428	-0.026700929	0.978698272	0	1.580101658	0	0.666666667	CRA004775;PRJNA827465;Yang2025_Silkie_Wencha	0.992242569
ENSGALG00010014213	ETFRF1	3	0.004648853	0.176398561	0.026354258	0.978974778	0.063821874	7.118113086	71.90266612	0.666666667	CRA004775;PRJNA827465;Yang2025_Silkie_Wencha	0.99230312
ENSGALG00010021200	FTO	3	-0.00366177	0.138508128	-0.026437221	0.978908606	0	0.049890403	0	0.666666667	CRA004775;PRJNA827465;Yang2025_Silkie_Wencha	0.99230312
ENSGALG00010024648	MMP24	3	-0.002123994	0.080839897	-0.026274085	0.979038725	0.008343151	3.489537997	42.68582254	0.666666667	CRA004775;PRJNA827465;Yang2025_Silkie_Wencha	0.99230312
ENSGALG00010000724	OBI1	3	0.001854252	0.070026041	0.026479458	0.978874918	0.00076762	2.081748808	3.926929496	0.666666667	CRA004775;PRJNA827465;Yang2025_Silkie_Wencha	0.99230312
ENSGALG00010024493	JAK1	3	-0.00243836	0.093333795	-0.026125155	0.979157513	0.01424861	4.806367505	58.38853359	0.666666667	CRA004775;PRJNA827465;Yang2025_Silkie_Wencha	0.992352381
ENSGALG00010000022	ATP8	3	0.005575469	0.214645627	0.025975228	0.979277097	0.07386898	4.221290044	52.62111868	0.666666667	CRA004775;PRJNA827465;Yang2025_Silkie_Wencha	0.992402443
ENSGALG00010021540	STK4	3	-0.00262006	0.102544326	-0.025550511	0.97961586	0.017359093	5.566719173	64.07219517	0.666666667	CRA004775;PRJNA827465;Yang2025_Silkie_Wencha	0.992603457
ENSGALG00010008023	ATAD2	3	-0.001865118	0.072838293	-0.025606279	0.979571378	0.003917987	2.572448311	22.25305398	0.666666667	CRA004775;PRJNA827465;Yang2025_Silkie_Wencha	0.992603457
ENSGALG00010004381	ENSGALG00010004381	3	-0.009742629	0.383903142	-0.025377831	0.979753594	0.337363201	8.491022248	76.44571005	0.666666667	CRA004775;PRJNA827465;Yang2025_Silkie_Wencha	0.992671878
ENSGALG00010001517	CAV2	3	-0.005662679	0.226785758	-0.02496929	0.980079459	0.125699569	12.96615244	84.57522377	0.666666667	CRA004775;PRJNA827465;Yang2025_Silkie_Wencha	0.992929234
ENSGALG00010029683	MRPL58	3	0.002640152	0.106101385	0.024883293	0.980148053	0.009606074	2.548090901	21.50986454	0.666666667	CRA004775;PRJNA827465;Yang2025_Silkie_Wencha	0.992929234
ENSGALG00010001695	LMOD2	3	0.011716855	0.480424284	0.024388556	0.980542676	0.506422557	14.08478442	85.80027965	0.666666667	CRA004775;PRJNA827465;Yang2025_Silkie_Wencha	0.993071019
ENSGALG00010000148	ENSGALG00010000148	3	0.007412962	0.307462794	0.024110111	0.980764779	0.23264412	13.23845257	84.89249412	0.666666667	CRA004775;PRJNA827465;Yang2025_Silkie_Wencha	0.993071019
ENSGALG00010011469	CPS1	3	-0.007269668	0.299403987	-0.024280465	0.980628895	0.188563963	8.024795074	75.07724519	0.666666667	CRA004775;PRJNA827465;Yang2025_Silkie_Wencha	0.993071019
ENSGALG00010022406	ITPRID2	3	-0.00290406	0.120619536	-0.024076202	0.980791826	0.024358467	5.621175558	64.42025375	0.666666667	CRA004775;PRJNA827465;Yang2025_Silkie_Wencha	0.993071019
ENSGALG00010013604	FYN	3	0.002529209	0.103629789	0.024406199	0.980528604	0.018744873	6.201003391	67.74715519	0.666666667	CRA004775;PRJNA827465;Yang2025_Silkie_Wencha	0.993071019
ENSGALG00010013390	SLC16A2	3	-0.002218479	0.092423573	-0.024003392	0.980849903	0	1.858872677	0	0.666666667	CRA004775;PRJNA827465;Yang2025_Silkie_Wencha	0.993071019
ENSGALG00010006486	CEP43	3	0.001644079	0.068124834	0.024133326	0.980746261	0.003063068	2.512202573	20.38858565	0.666666667	CRA004775;PRJNA827465;Yang2025_Silkie_Wencha	0.993071019
ENSGALG00010016921	EFL1	3	-0.001355624	0.055829363	-0.024281567	0.980628016	0	1.928917399	0	0.666666667	CRA004775;PRJNA827465;Yang2025_Silkie_Wencha	0.993071019
ENSGALG00010029506	OXLD1	3	-0.003519032	0.149469892	-0.023543419	0.981216805	0.025730388	3.074710869	34.95323347	0.666666667	CRA004775;PRJNA827465;Yang2025_Silkie_Wencha	0.993371359
ENSGALG00010027448	ADAMTSL5	3	0.006092194	0.26149132	0.023297883	0.981412661	0.131500614	7.088612405	71.78573343	0.666666667	CRA004775;PRJNA827465;Yang2025_Silkie_Wencha	0.993498504
ENSGALG00010010761	MEI1	3	0.009527311	0.412593783	0.023091261	0.981577477	0.350242792	7.261609748	72.4578975	0.666666667	CRA004775;PRJNA827465;Yang2025_Silkie_Wencha	0.993504266
ENSGALG00010028009	RRP9	3	-0.005171215	0.227194948	-0.022761136	0.981840809	0.135058348	20.79191693	90.38087731	0.666666667	CRA004775;PRJNA827465;Yang2025_Silkie_Wencha	0.993504266
ENSGALG00010010448	GALNT13	3	0.003665706	0.158809072	0.02308247	0.981584489	0.013779233	2.374286127	15.76415424	0.666666667	CRA004775;PRJNA827465;Yang2025_Silkie_Wencha	0.993504266
ENSGALG00010015840	MTUS1	3	0.003514347	0.153620618	0.022876794	0.981748551	0.047589351	7.659829206	73.88975725	0.666666667	CRA004775;PRJNA827465;Yang2025_Silkie_Wencha	0.993504266
ENSGALG00010001800	ENSGALG00010001800	3	0.00308111	0.135390026	0.022757288	0.981843878	0.029060516	4.57872669	56.31973395	0.666666667	CRA004775;PRJNA827465;Yang2025_Silkie_Wencha	0.993504266
ENSGALG00010027693	RAB14	3	0.002745507	0.120353356	0.022812054	0.981800193	0.035252407	15.58197905	87.16465993	0.666666667	CRA004775;PRJNA827465;Yang2025_Silkie_Wencha	0.993504266
ENSGALG00010000470	FBXW8	3	0.002579532	0.113765463	0.022674118	0.981910221	0.022528698	5.319260069	62.40078556	0.666666667	CRA004775;PRJNA827465;Yang2025_Silkie_Wencha	0.993504266
ENSGALG00010011612	ENSGALG00010011612	2	-0.008672951	0.384549836	-0.022553517	0.982006422	0.187203483	2.711460838	63.1195116	0.5	PRJNA827465;Yang2025_Silkie_Wenchang	0.993530505
ENSGALG00010011911	NUDT2	3	0.002873966	0.128664189	0.022336953	0.982179172	0	0.467097061	0	0.666666667	CRA004775;PRJNA827465;Yang2025_Silkie_Wencha	0.993634181
ENSGALG00010007287	ENSGALG00010007287	3	-0.01544386	0.707904898	-0.021816292	0.982594498	1.064892441	7.016734025	71.49671068	0.666666667	CRA004775;PRJNA827465;Yang2025_Silkie_Wencha	0.993769929
ENSGALG00010014272	PARD6B	3	0.005608039	0.256224677	0.021887192	0.982537941	0.156547146	11.04188005	81.88714249	0.666666667	CRA004775;PRJNA827465;Yang2025_Silkie_Wencha	0.993769929
ENSGALG00010020443	PREPL	3	0.005017217	0.228201012	0.021985953	0.982459161	0.098933432	5.603528404	64.30820269	0.666666667	CRA004775;PRJNA827465;Yang2025_Silkie_Wencha	0.993769929
ENSGALG00010026360	CTDSPL	3	-0.001089103	0.049712324	-0.021908119	0.982521248	0	1.679129399	0	0.666666667	CRA004775;PRJNA827465;Yang2025_Silkie_Wencha	0.993769929
ENSGALG00010023399	SRRM4	2	-0.016050827	0.746292145	-0.021507431	0.982840876	0.808888273	3.630869338	72.45838649	0.5	PRJNA827465;Yang2025_Silkie_Wenchang	0.993948011
ENSGALG00010002065	CLNS1A	3	0.001161482	0.054714695	0.021227963	0.983063808	0	0.047017048	0	0.666666667	CRA004775;PRJNA827465;Yang2025_Silkie_Wencha	0.994102359
ENSGALG00010005016	STX11	3	-0.003619148	0.171644487	-0.021085142	0.983177738	0.04638136	4.488370447	55.44039816	0.666666667	CRA004775;PRJNA827465;Yang2025_Silkie_Wencha	0.994146466
ENSGALG00010023968	TYW5	3	-0.001913099	0.091259656	-0.020963246	0.983274975	0	0.126224826	0	0.666666667	CRA004775;PRJNA827465;Yang2025_Silkie_Wencha	0.994173689
ENSGALG00010002471	OGFRL1	3	-0.003344771	0.160676655	-0.020816785	0.983391808	0.045931817	5.378929938	62.81788343	0.666666667	CRA004775;PRJNA827465;Yang2025_Silkie_Wencha	0.994214724
ENSGALG00010024565	FAM217B	3	-0.002376084	0.114587009	-0.020736071	0.983456194	0.024480768	5.287871852	62.17760082	0.666666667	CRA004775;PRJNA827465;Yang2025_Silkie_Wencha	0.994214724
ENSGALG00010011673	POP1	3	-0.003185177	0.156612783	-0.02033791	0.983773814	0.036614333	4.242941178	52.86288647	0.666666667	CRA004775;PRJNA827465;Yang2025_Silkie_Wencha	0.994283464
ENSGALG00010013076	RTN4IP1	3	-0.003089997	0.150989296	-0.020465005	0.983672429	0.036835642	4.385099965	54.39100554	0.666666667	CRA004775;PRJNA827465;Yang2025_Silkie_Wencha	0.994283464
ENSGALG00010008514	PIEZO1	3	-0.001527807	0.075038114	-0.020360419	0.983755859	0	1.666359612	0	0.666666667	CRA004775;PRJNA827465;Yang2025_Silkie_Wencha	0.994283464
ENSGALG00010017120	ERN2	3	-0.001293096	0.063704914	-0.020298217	0.983805478	0	0.758047274	0	0.666666667	CRA004775;PRJNA827465;Yang2025_Silkie_Wencha	0.994283464
ENSGALG00010029410	VTN	3	0.011531347	0.576830108	0.019990889	0.984050641	0.675535461	6.460238599	69.04139113	0.666666667	CRA004775;PRJNA827465;Yang2025_Silkie_Wencha	0.994303868
ENSGALG00010018520	ENSGALG00010018520	3	0.006782451	0.340479587	0.019920288	0.984106961	0.303040755	18.43956541	89.15375739	0.666666667	CRA004775;PRJNA827465;Yang2025_Silkie_Wencha	0.994303868
ENSGALG00010013304	KCNJ15	3	0.004810863	0.239775748	0.020064012	0.983992309	0.108036033	6.63547719	69.85898764	0.666666667	CRA004775;PRJNA827465;Yang2025_Silkie_Wencha	0.994303868
ENSGALG00010023262	POLD4	3	-0.004478815	0.224420529	-0.019957244	0.98407748	0	0.189734231	0	0.666666667	CRA004775;PRJNA827465;Yang2025_Silkie_Wencha	0.994303868
ENSGALG00010015490	RANBP2	3	-0.001454609	0.074001365	-0.019656516	0.984317379	0.006311972	3.13958796	36.29737323	0.666666667	CRA004775;PRJNA827465;Yang2025_Silkie_Wencha	0.994445404
ENSGALG00010021095	NT5C2	3	0.00250345	0.128926522	0.019417648	0.984507932	0.034739203	6.821880006	70.68256847	0.666666667	CRA004775;PRJNA827465;Yang2025_Silkie_Wencha	0.994566851
ENSGALG00010013310	MYO16	3	0.003179444	0.165676576	0.01919067	0.984689001	0	1.617121487	0	0.666666667	CRA004775;PRJNA827465;Yang2025_Silkie_Wencha	0.994580691
ENSGALG00010022673	KLHL42	3	0.002582564	0.134958848	0.01913594	0.984732661	0.018462365	2.92586081	31.64404836	0.666666667	CRA004775;PRJNA827465;Yang2025_Silkie_Wencha	0.994580691
ENSGALG00010012341	GOPC	3	-0.002134152	0.111041414	-0.019219428	0.98466606	0.024138283	6.909626581	71.05487574	0.666666667	CRA004775;PRJNA827465;Yang2025_Silkie_Wencha	0.994580691
ENSGALG00010023281	B2M	3	-0.00336199	0.179039165	-0.01877796	0.985018236	0.07059499	8.090271184	75.27894981	0.666666667	CRA004775;PRJNA827465;Yang2025_Silkie_Wencha	0.99479806
ENSGALG00010021632	LIMD2	3	-0.002555472	0.137517492	-0.018582889	0.985173853	0.017912776	2.750566836	27.28771488	0.666666667	CRA004775;PRJNA827465;Yang2025_Silkie_Wencha	0.994858497
ENSGALG00010020630	QRICH1	3	-0.000693941	0.037456572	-0.018526541	0.985218804	0	1.168322905	0	0.666666667	CRA004775;PRJNA827465;Yang2025_Silkie_Wencha	0.994858497
ENSGALG00010010931	CYYR1	3	-0.002896432	0.157865678	-0.018347449	0.985361675	0.039509349	4.339921106	53.91621296	0.666666667	CRA004775;PRJNA827465;Yang2025_Silkie_Wencha	0.994931709
ENSGALG00010025927	ENSGALG00010025927	2	-0.020763447	1.146215099	-0.018114791	0.985547279	0	0.161809093	0	0.5	PRJNA827465;Yang2025_Silkie_Wenchang	0.994977006
ENSGALG00010012193	TTLL1	3	-0.003225351	0.177466502	-0.01817442	0.985499709	0	0.32188593	0	0.666666667	CRA004775;PRJNA827465;Yang2025_Silkie_Wencha	0.994977006
ENSGALG00010008270	ENSGALG00010008270	3	0.005222358	0.297012116	0.01758298	0.985971535	0.226874745	14.81989096	86.50462406	0.666666667	CRA004775;PRJNA827465;Yang2025_Silkie_Wencha	0.995175941
ENSGALG00010007230	ENSGALG00010007230	3	0.004845143	0.273292293	0.017728795	0.985855209	0.131944207	4.888013401	59.08358189	0.666666667	CRA004775;PRJNA827465;Yang2025_Silkie_Wencha	0.995175941
ENSGALG00010021275	MAP3K2	3	-0.001630715	0.093104586	-0.017514872	0.986025868	0.012371817	3.721407836	46.25689825	0.666666667	CRA004775;PRJNA827465;Yang2025_Silkie_Wencha	0.995175941
ENSGALG00010017341	TA3	3	0.000922745	0.052505801	0.017574155	0.985978575	0	0.879391426	0	0.666666667	CRA004775;PRJNA827465;Yang2025_Silkie_Wencha	0.995175941
ENSGALG00010015801	VSTM2L	3	-0.002926526	0.168351618	-0.017383408	0.98610746	0.027100269	2.914809479	31.38488075	0.666666667	CRA004775;PRJNA827465;Yang2025_Silkie_Wencha	0.99521075
ENSGALG00010005396	MICU2	3	0.002022201	0.117006606	0.017282793	0.986211013	0.025229566	5.676143983	64.7647562	0.666666667	CRA004775;PRJNA827465;Yang2025_Silkie_Wencha	0.99522072
ENSGALG00010001608	ENSGALG00010001608	2	0.006442119	0.382175533	0.016856439	0.986551144	0	0.132488493	0	0.5	PRJNA827465;Yang2025_Silkie_Wenchang	0.995258001
ENSGALG00010004307	NPC2	3	-0.004583218	0.27350469	-0.016757367	0.986630181	0.175862068	10.81301377	81.50376904	0.666666667	CRA004775;PRJNA827465;Yang2025_Silkie_Wencha	0.995258001
ENSGALG00010018903	CAMTA1	3	0.001735254	0.101399491	0.017113047	0.98634643	0.009283748	2.679758008	25.36639526	0.666666667	CRA004775;PRJNA827465;Yang2025_Silkie_Wencha	0.995258001
ENSGALG00010007141	PRKD3	3	0.001458101	0.086673483	0.016822917	0.986577888	0.009634339	3.583905712	44.19496045	0.666666667	CRA004775;PRJNA827465;Yang2025_Silkie_Wencha	0.995258001
ENSGALG00010010191	RINT1	3	0.001419642	0.0849725	0.016707076	0.986670302	0.009012918	3.394058803	41.07348929	0.666666667	CRA004775;PRJNA827465;Yang2025_Silkie_Wencha	0.995258001
ENSGALG00010020946	CTBS	3	-0.001325318	0.078366157	-0.01691187	0.986506924	0	1.740143371	0	0.666666667	CRA004775;PRJNA827465;Yang2025_Silkie_Wencha	0.995258005
ENSGALG00010006184	LBX2	3	0.006522778	0.401601344	0.016241922	0.987041391	0.352175467	8.364336981	76.08895954	0.666666667	CRA004775;PRJNA827465;Yang2025_Silkie_Wencha	0.995452825
ENSGALG00010019185	ANKDD1A	3	0.003658618	0.228689133	0.015998217	0.987235814	0.093143378	5.307716547	62.31901266	0.666666667	CRA004775;PRJNA827465;Yang2025_Silkie_Wencha	0.995452825
ENSGALG00010024267	MAP3K12	3	0.001831593	0.114104882	0.016051837	0.987193037	0	1.787071959	0	0.666666667	CRA004775;PRJNA827465;Yang2025_Silkie_Wencha	0.995452825
ENSGALG00010018032	ERO1A	3	-0.001529553	0.093931126	-0.016286892	0.987005515	0.010519472	3.201223321	37.52388386	0.666666667	CRA004775;PRJNA827465;Yang2025_Silkie_Wencha	0.995452825
ENSGALG00010002587	DOCK1	3	0.001259082	0.079012273	0.015935469	0.987285874	0.00488656	2.410300683	17.02280077	0.666666667	CRA004775;PRJNA827465;Yang2025_Silkie_Wencha	0.995452825
ENSGALG00010009463	MRPL13	3	0.000811997	0.050306208	0.016141099	0.987121826	0	0.987059579	0	0.666666667	CRA004775;PRJNA827465;Yang2025_Silkie_Wencha	0.995452825
ENSGALG00010001224	DNAJB9	3	0.005174892	0.330862569	0.015640608	0.987521109	0.295970635	27.73081163	92.78780576	0.666666667	CRA004775;PRJNA827465;Yang2025_Silkie_Wencha	0.9955623
ENSGALG00010014319	ENSGALG00010014319	3	-0.002111462	0.135194434	-0.015617965	0.987539174	0.021311359	2.937144868	31.9066614	0.666666667	CRA004775;PRJNA827465;Yang2025_Silkie_Wencha	0.9955623
ENSGALG00010013417	AKR1B1	3	-0.001096459	0.07111658	-0.015417763	0.987698892	0	1.396072546	0	0.666666667	CRA004775;PRJNA827465;Yang2025_Silkie_Wencha	0.995656255
ENSGALG00010008115	ENSGALG00010008115	3	0.003220365	0.210961104	0.015265209	0.987820599	0.090449438	7.298189817	72.59594433	0.666666667	CRA004775;PRJNA827465;Yang2025_Silkie_Wencha	0.995707952
ENSGALG00010028080	MUSTN1	3	-0.005098806	0.347312106	-0.014680761	0.988286868	0.196831318	4.3974492	54.51908802	0.666666667	CRA004775;PRJNA827465;Yang2025_Silkie_Wencha	0.995891318
ENSGALG00010017116	ENSGALG00010017116	3	0.003147598	0.219267842	0.014355036	0.988546731	0.035158387	2.541662706	21.31135278	0.666666667	CRA004775;PRJNA827465;Yang2025_Silkie_Wencha	0.995891318
ENSGALG00010011925	RRP1B	3	0.002244305	0.154660845	0.014511141	0.988422191	0.057962093	15.40272212	87.01528221	0.666666667	CRA004775;PRJNA827465;Yang2025_Silkie_Wencha	0.995891318
ENSGALG00010006091	RTKN	3	-0.00216011	0.149563142	-0.014442798	0.988476715	0.034864064	4.069711957	50.85647287	0.666666667	CRA004775;PRJNA827465;Yang2025_Silkie_Wencha	0.995891318
ENSGALG00010010062	RGS7BP	3	0.001767689	0.123348185	0.014330887	0.988565998	0.010390707	2.409291839	16.98805569	0.666666667	CRA004775;PRJNA827465;Yang2025_Silkie_Wencha	0.995891318
ENSGALG00010015029	TFRC	3	0.001501184	0.1032367	0.014541182	0.988398224	0	1.090094569	0	0.666666667	CRA004775;PRJNA827465;Yang2025_Silkie_Wencha	0.995891318
ENSGALG00010029200	GAS7	3	-0.001333672	0.091345313	-0.01460033	0.988351036	0	0.223997221	0	0.666666667	CRA004775;PRJNA827465;Yang2025_Silkie_Wencha	0.995891318
ENSGALG00010029536	BAZ1B	3	0.00063471	0.043695458	0.014525764	0.988410525	0	0.863870253	0	0.666666667	CRA004775;PRJNA827465;Yang2025_Silkie_Wencha	0.995891318
ENSGALG00010024393	OGFOD1	3	0.001459234	0.1025103	0.014234995	0.988642501	0.015751642	4.075224059	50.92294384	0.666666667	CRA004775;PRJNA827465;Yang2025_Silkie_Wencha	0.995897429
ENSGALG00010008739	VSTM5	3	-0.002273029	0.161119339	-0.014107737	0.988744028	0	0.792570167	0	0.666666667	CRA004775;PRJNA827465;Yang2025_Silkie_Wencha	0.995928746
ENSGALG00010027867	UBAP2L	3	-0.003225742	0.232354989	-0.013882819	0.988923469	0.125534013	10.61959444	81.16688909	0.666666667	CRA004775;PRJNA827465;Yang2025_Silkie_Wencha	0.996038533
ENSGALG00010025998	SMARCB1	3	0.000537015	0.039090023	0.013737915	0.989039075	0	1.825318171	0	0.666666667	CRA004775;PRJNA827465;Yang2025_Silkie_Wencha	0.996084014
ENSGALG00010021249	ENSGALG00010021249	2	-0.004525129	0.341621066	-0.013246048	0.989431492	0	0.278642638	0	0.5	CRA004775;PRJNA827465	0.996173633
ENSGALG00010018943	ACKR4	3	0.002591701	0.193414689	0.01339971	0.989308898	0	0.681141406	0	0.666666667	CRA004775;PRJNA827465;Yang2025_Silkie_Wencha	0.996173633
ENSGALG00010010885	SNRPD2	3	-0.002263683	0.171688543	-0.013184823	0.989480338	0.056422744	5.70502416	64.94318089	0.666666667	CRA004775;PRJNA827465;Yang2025_Silkie_Wencha	0.996173633
ENSGALG00010025103	GSK3B	3	-0.001441381	0.10835206	-0.01330276	0.989386246	0.019703864	4.384984226	54.38980172	0.666666667	CRA004775;PRJNA827465;Yang2025_Silkie_Wencha	0.996173633
ENSGALG00010014754	DIMT1	3	0.001416472	0.104996902	0.013490606	0.98923638	0	0.241209558	0	0.666666667	CRA004775;PRJNA827465;Yang2025_Silkie_Wencha	0.996173633
ENSGALG00010024910	UBTF	3	0.001128631	0.086973159	0.01297677	0.989646326	0.012556327	4.352694832	54.0514537	0.666666667	CRA004775;PRJNA827465;Yang2025_Silkie_Wencha	0.996269805
ENSGALG00010028216	NKX2-3	2	-0.015228352	1.309481924	-0.011629295	0.990721374	2.090128158	2.404264514	58.40723871	0.5	PRJNA827465;Yang2025_Silkie_Wenchang	0.996288016
ENSGALG00010009689	ENSGALG00010009689	3	-0.007009033	0.573929744	-0.012212354	0.990256193	0.636898631	5.657751219	64.65026611	0.666666667	CRA004775;PRJNA827465;Yang2025_Silkie_Wencha	0.996288016
ENSGALG00010009432	BOP1	3	-0.003978004	0.316945864	-0.012551051	0.989985973	0.241603875	11.19185022	82.12985378	0.666666667	CRA004775;PRJNA827465;Yang2025_Silkie_Wencha	0.996288016
ENSGALG00010024664	DNAH10	2	-0.003174624	0.262366005	-0.012099982	0.990345847	0.086795021	2.638700436	62.10255676	0.5	PRJNA827465;Yang2025_Silkie_Wenchang	0.996288016
ENSGALG00010010290	ENSGALG00010010290	3	0.00314066	0.262647625	0.011957694	0.990459368	0.166782844	14.03638997	85.75132207	0.666666667	CRA004775;PRJNA827465;Yang2025_Silkie_Wencha	0.996288016
ENSGALG00010019035	ENSGALG00010019035	3	0.002536515	0.198573893	0.012773657	0.989808373	0	0.410793019	0	0.666666667	CRA004775;PRJNA827465;Yang2025_Silkie_Wencha	0.996288016
ENSGALG00010022959	ADAMTS8	3	-0.002250239	0.192463248	-0.011691785	0.990671518	0.064891233	5.963850877	66.46462091	0.666666667	CRA004775;PRJNA827465;Yang2025_Silkie_Wencha	0.996288016
ENSGALG00010029932	RASA4B	3	-0.002157604	0.174798434	-0.012343385	0.990151654	0.014577408	2.307794334	13.33716481	0.666666667	CRA004775;PRJNA827465;Yang2025_Silkie_Wencha	0.996288016
ENSGALG00010010465	BMPR1A	3	0.002140347	0.175806502	0.012174448	0.990286435	0.075239656	15.31581885	86.94160581	0.666666667	CRA004775;PRJNA827465;Yang2025_Silkie_Wencha	0.996288016
ENSGALG00010006728	ENSGALG00010006728	3	-0.001807081	0.152433248	-0.011854903	0.990541378	0.038009599	5.130186319	61.01506114	0.666666667	CRA004775;PRJNA827465;Yang2025_Silkie_Wencha	0.996288016
ENSGALG00010015370	ENSGALG00010015370	3	0.001626019	0.139271695	0.011675154	0.990684786	0.036188301	6.773888101	70.47485919	0.666666667	CRA004775;PRJNA827465;Yang2025_Silkie_Wencha	0.996288016
ENSGALG00010028468	PHPT1	3	-0.00105217	0.086021817	-0.012231435	0.99024097	0.003000026	2.140297666	6.555053926	0.666666667	CRA004775;PRJNA827465;Yang2025_Silkie_Wencha	0.996288016
ENSGALG00010016945	BDH2	3	-0.000793932	0.062579239	-0.01268682	0.989877653	0	1.844712982	0	0.666666667	CRA004775;PRJNA827465;Yang2025_Silkie_Wencha	0.996288016
ENSGALG00010021574	TTLL4	3	0.000751428	0.063910329	0.011757534	0.990619062	0	1.862129749	0	0.666666667	CRA004775;PRJNA827465;Yang2025_Silkie_Wencha	0.996288016
ENSGALG00010024501	ARL8A	3	0.000729866	0.059385456	0.012290323	0.990193988	0	0.19958255	0	0.666666667	CRA004775;PRJNA827465;Yang2025_Silkie_Wencha	0.996288016
ENSGALG00010000737	ENSGALG00010000737	2	0.006165017	0.545368569	0.011304312	0.990980656	0	0.516284486	0	0.5	PRJNA827465;Yang2025_Silkie_Wenchang	0.996401283
ENSGALG00010020633	HOGA1	3	-0.002453473	0.218608569	-0.011223133	0.991045424	0.082221547	4.632807549	56.82963346	0.666666667	CRA004775;PRJNA827465;Yang2025_Silkie_Wencha	0.996401283
ENSGALG00010020835	PTGR2	3	-0.00189529	0.166872626	-0.011357703	0.990938059	0.05928003	8.106737104	75.32916173	0.666666667	CRA004775;PRJNA827465;Yang2025_Silkie_Wencha	0.996401283
ENSGALG00010029295	SOCS3	3	0.002778903	0.25540212	0.010880501	0.991318788	0.124892174	6.417148073	68.83350708	0.666666667	CRA004775;PRJNA827465;Yang2025_Silkie_Wencha	0.996605257
ENSGALG00010015558	MPP1	3	-0.000906193	0.085432963	-0.010607062	0.991536948	0	0.94701865	0	0.666666667	CRA004775;PRJNA827465;Yang2025_Silkie_Wencha	0.996753708
ENSGALG00010007408	HCLS1	3	-0.002842948	0.271748008	-0.010461708	0.991652917	0.171962092	11.00741522	81.83043012	0.666666667	CRA004775;PRJNA827465;Yang2025_Silkie_Wencha	0.996799416
ENSGALG00010017868	CHRND	3	-0.003450251	0.35541421	-0.009707691	0.992254505	0.278199801	12.41265664	83.8874138	0.666666667	CRA004775;PRJNA827465;Yang2025_Silkie_Wencha	0.997292971
ENSGALG00010005042	SLC46A3	3	-0.002857617	0.295529576	-0.009669478	0.992284993	0.160503618	5.490288467	63.57204158	0.666666667	CRA004775;PRJNA827465;Yang2025_Silkie_Wencha	0.997292971
ENSGALG00010002704	KCNB2	3	0.00176105	0.184019994	0.009569884	0.992364454	0	0.641648974	0	0.666666667	CRA004775;PRJNA827465;Yang2025_Silkie_Wencha	0.997301942
ENSGALG00010024453	HTR3A	3	-0.006525859	0.737723186	-0.008845946	0.992942049	1.364146147	13.36636986	85.03707423	0.666666667	CRA004775;PRJNA827465;Yang2025_Silkie_Wencha	0.997409234
ENSGALG00010020379	COL13A1	3	-0.003365125	0.392575548	-0.008571918	0.993160683	0.351243725	15.50060729	87.09727972	0.666666667	CRA004775;PRJNA827465;Yang2025_Silkie_Wencha	0.997409234
ENSGALG00010005911	TBXT	3	-0.003338357	0.390363923	-0.00855191	0.993176647	0.283768438	6.392210147	68.71191726	0.666666667	CRA004775;PRJNA827465;Yang2025_Silkie_Wencha	0.997409234
ENSGALG00010003371	ENSGALG00010003371	3	0.003272546	0.361979572	0.009040692	0.99278667	0.219897734	4.766826459	58.04336455	0.666666667	CRA004775;PRJNA827465;Yang2025_Silkie_Wencha	0.997409234
ENSGALG00010005211	ESRRB	3	0.003027852	0.329196588	0.0091977	0.9926614	0.251801018	11.47279518	82.56745659	0.666666667	CRA004775;PRJNA827465;Yang2025_Silkie_Wencha	0.997409234
ENSGALG00010014184	SLC44A1	3	0.001954195	0.22158982	0.008818975	0.992963567	0.105777645	9.402193385	78.72836775	0.666666667	CRA004775;PRJNA827465;Yang2025_Silkie_Wencha	0.997409234
ENSGALG00010027073	DNAH12	2	-0.001446377	0.168241728	-0.008597019	0.993140656	0	0.083984613	0	0.5	PRJNA827465;Yang2025_Silkie_Wenchang	0.997409234
ENSGALG00010013133	TXNDC15	3	-0.000931342	0.102008956	-0.009130006	0.99271541	0.010594283	2.732238992	26.79996129	0.666666667	CRA004775;PRJNA827465;Yang2025_Silkie_Wencha	0.997409234
ENSGALG00010011708	IK	3	-0.000861279	0.096700644	-0.008906654	0.992893612	0.018155168	6.349629381	68.50209863	0.666666667	CRA004775;PRJNA827465;Yang2025_Silkie_Wencha	0.997409234
ENSGALG00010010868	SLC39A10	3	0.000653553	0.076409619	0.008553276	0.993175557	0	0.451203808	0	0.666666667	CRA004775;PRJNA827465;Yang2025_Silkie_Wencha	0.997409234
ENSGALG00010009220	GADD45G	3	0.001562965	0.190453899	0.008206525	0.993452214	0.038465989	3.081160063	35.08938324	0.666666667	CRA004775;PRJNA827465;Yang2025_Silkie_Wencha	0.997431123
ENSGALG00010018508	ENSGALG00010018508	3	-0.001536963	0.188101582	-0.00817092	0.993480622	0.085129129	12.65628928	84.19757991	0.666666667	CRA004775;PRJNA827465;Yang2025_Silkie_Wencha	0.997431123
ENSGALG00010023835	INAFM2	3	-0.000719523	0.086061018	-0.008360614	0.993329273	0	0.318840607	0	0.666666667	CRA004775;PRJNA827465;Yang2025_Silkie_Wencha	0.997431123
ENSGALG00010029787	H3-3B	3	-0.000360562	0.043461024	-0.008296225	0.993380646	0	0.710709935	0	0.666666667	CRA004775;PRJNA827465;Yang2025_Silkie_Wencha	0.997431123
ENSGALG00010000729	EDNRB	3	-0.002592556	0.320939866	-0.008078011	0.99355475	0.251355041	15.35285458	86.9731066	0.666666667	CRA004775;PRJNA827465;Yang2025_Silkie_Wencha	0.997434721
ENSGALG00010024663	ICE2	3	0.000639926	0.08136887	0.007864503	0.993725099	0.004193608	2.455129325	18.53789616	0.666666667	CRA004775;PRJNA827465;Yang2025_Silkie_Wencha	0.997534908
ENSGALG00010017139	COL9A2	3	-0.010788581	1.661195643	-0.006494468	0.994818201	7.572363774	23.50773508	91.49216208	0.666666667	CRA004775;PRJNA827465;Yang2025_Silkie_Wencha	0.99757973
ENSGALG00010023642	PAX6	2	-0.003161164	0.489699759	-0.00645531	0.994849444	0.328556379	3.137703057	68.12955267	0.5	PRJNA827465;Yang2025_Silkie_Wenchang	0.99757973
ENSGALG00010028587	ANKLE1	3	-0.002603206	0.380755363	-0.006836951	0.994544945	0.394082773	26.42097942	92.43025791	0.666666667	CRA004775;PRJNA827465;Yang2025_Silkie_Wencha	0.99757973
ENSGALG00010026115	MMACHC	3	-0.001874327	0.26222636	-0.007147743	0.994296975	0.154243104	8.345746026	76.03569539	0.666666667	CRA004775;PRJNA827465;Yang2025_Silkie_Wencha	0.99757973
ENSGALG00010029723	ADORA2B	3	-0.001173481	0.175405048	-0.006690119	0.994662097	0.049160152	4.43261217	54.87987843	0.666666667	CRA004775;PRJNA827465;Yang2025_Silkie_Wencha	0.99757973
ENSGALG00010023896	POP4	3	0.001148761	0.155025053	0.007410163	0.994087599	0.046532862	5.759310377	65.27362012	0.666666667	CRA004775;PRJNA827465;Yang2025_Silkie_Wencha	0.99757973
ENSGALG00010009822	ENSGALG00010009822	3	0.001045499	0.143350832	0.007293291	0.994180847	0	0.529627659	0	0.666666667	CRA004775;PRJNA827465;Yang2025_Silkie_Wencha	0.99757973
ENSGALG00010016353	C1orf232	3	-0.000994121	0.150523611	-0.006604421	0.994730473	0.012876259	2.414883286	17.18026244	0.666666667	CRA004775;PRJNA827465;Yang2025_Silkie_Wencha	0.99757973
ENSGALG00010026148	RPS10	3	-0.000904126	0.128997993	-0.00700884	0.9944078	0.023695672	3.569918857	43.97631766	0.666666667	CRA004775;PRJNA827465;Yang2025_Silkie_Wencha	0.99757973
ENSGALG00010014390	SPARC	3	0.000731493	0.101325994	0.007219205	0.994239957	0	0.00909364	0	0.666666667	CRA004775;PRJNA827465;Yang2025_Silkie_Wencha	0.99757973
ENSGALG00010018404	IL2RG	3	-0.000723992	0.10223272	-0.007081802	0.994349587	0	0.410497249	0	0.666666667	CRA004775;PRJNA827465;Yang2025_Silkie_Wencha	0.99757973
ENSGALG00010026335	IPP	3	-0.000706046	0.107098969	-0.006592466	0.994740011	0.019203217	4.969953093	59.75817151	0.666666667	CRA004775;PRJNA827465;Yang2025_Silkie_Wencha	0.99757973
ENSGALG00010004883	KRIT1	3	0.000697089	0.109028232	0.006393659	0.994898633	0.023336179	8.346751368	76.03858182	0.666666667	CRA004775;PRJNA827465;Yang2025_Silkie_Wencha	0.99757973
ENSGALG00010029671	GRIN2C	3	-0.000639485	0.087083638	-0.007343337	0.994140917	0	0.134311776	0	0.666666667	CRA004775;PRJNA827465;Yang2025_Silkie_Wencha	0.99757973
ENSGALG00010026081	KPNA1	3	0.000540716	0.070201981	0.007702286	0.993854526	0.004212463	2.433713787	17.82106794	0.666666667	CRA004775;PRJNA827465;Yang2025_Silkie_Wencha	0.99757973
ENSGALG00010006085	DLST	3	0.000365275	0.056319391	0.006485777	0.994825135	0	1.04297822	0	0.666666667	CRA004775;PRJNA827465;Yang2025_Silkie_Wencha	0.99757973
ENSGALG00010029554	ENSGALG00010029554	3	-0.003744573	0.606093217	-0.006178213	0.995070531	0.40473304	3.154783983	36.60421724	0.666666667	CRA004775;PRJNA827465;Yang2025_Silkie_Wencha	0.997681338
ENSGALG00010010515	RXRG	3	0.002383701	0.418599451	0.005694467	0.995456497	0.456292492	17.63757516	88.66057277	0.666666667	CRA004775;PRJNA827465;Yang2025_Silkie_Wencha	0.997926788
ENSGALG00010029745	SHPK	3	-0.00098007	0.17174887	-0.005706414	0.995446965	0.041942698	3.767546707	46.91505758	0.666666667	CRA004775;PRJNA827465;Yang2025_Silkie_Wencha	0.997926788
ENSGALG00010005585	ENSGALG00010005585	2	-0.005492254	1.163502469	-0.004720449	0.996233641	2.238359969	5.592065186	82.1175189	0.5	CRA004775;Yang2025_Silkie_Wenchang	0.998635055
ENSGALG00010020969	RBP3	3	-0.001931756	0.43425392	-0.004448449	0.996450663	0.271003104	3.809131746	47.49459632	0.666666667	CRA004775;PRJNA827465;Yang2025_Silkie_Wencha	0.998649476
ENSGALG00010024763	HOXD9	3	-0.001018007	0.226887032	-0.004486845	0.996420028	0.084502992	4.373301606	54.26796091	0.666666667	CRA004775;PRJNA827465;Yang2025_Silkie_Wencha	0.998649476
ENSGALG00010011571	ATOSB	3	-0.000804567	0.181337611	-0.004436847	0.99645992	0.072140314	8.068878905	75.21340916	0.666666667	CRA004775;PRJNA827465;Yang2025_Silkie_Wencha	0.998649476
ENSGALG00010008745	GRAMD4	3	0.000615663	0.142407906	0.004323234	0.996550569	0.036897701	6.434651305	68.91828469	0.666666667	CRA004775;PRJNA827465;Yang2025_Silkie_Wencha	0.998666607
ENSGALG00010021147	ARID3B	3	0.000421032	0.099338194	0.004238374	0.996618277	0.010110331	2.811705467	28.86879427	0.666666667	CRA004775;PRJNA827465;Yang2025_Silkie_Wencha	0.998666607
ENSGALG00010022144	SNTA1	3	0.000868133	0.226150428	0.003838741	0.996937135	0.105468185	8.384901962	76.14760424	0.666666667	CRA004775;PRJNA827465;Yang2025_Silkie_Wencha	0.998876164
ENSGALG00010027642	DIPK1B	3	0.000314503	0.08299427	0.003789455	0.99697646	0	0.83861568	0	0.666666667	CRA004775;PRJNA827465;Yang2025_Silkie_Wencha	0.998876164
ENSGALG00010006853	NET1	3	0.000245161	0.066069679	0.003710639	0.997039346	0	0.311284183	0	0.666666667	CRA004775;PRJNA827465;Yang2025_Silkie_Wencha	0.998876164
ENSGALG00010027110	IKBKE	3	-0.001211568	0.371700993	-0.003259523	0.997399282	0.291229297	7.686582876	73.98063571	0.666666667	CRA004775;PRJNA827465;Yang2025_Silkie_Wencha	0.998882876
ENSGALG00010017256	CARHSP1	3	-0.000591078	0.179417392	-0.003294431	0.997371429	0.071041818	9.069879803	77.94899113	0.666666667	CRA004775;PRJNA827465;Yang2025_Silkie_Wencha	0.998882876
ENSGALG00010021426	SLC38A7	3	-0.000203712	0.057810896	-0.003523766	0.997188447	0	0.931410948	0	0.666666667	CRA004775;PRJNA827465;Yang2025_Silkie_Wencha	0.998882876

ENSGALG00010028818	TSC1	3	-0.000202135	0.061070551	-0.003309857	0.997359121	0	1.86614988	0	0.666666667	CRA004775;PRJNA827465;Yang2025_Silkie_Wencha	0.998882876
ENSGALG00010026943	EEFSEC	3	-0.000161584	0.049302434	-0.0032774	0.997385018	0	1.553901976	0	0.666666667	CRA004775;PRJNA827465;Yang2025_Silkie_Wencha	0.998882876
ENSGALG00010000586	ENSGALG00010000586	3	-0.001123507	0.378900483	-0.002965177	0.997634135	0.264225276	5.142256446	61.10656827	0.666666667	CRA004775;PRJNA827465;Yang2025_Silkie_Wencha	0.999047314
ENSGALG00010006730	HAPLN1	3	-0.003346618	1.505371791	-0.002223117	0.998226211	5.879957899	15.91698293	87.43480464	0.666666667	CRA004775;PRJNA827465;Yang2025_Silkie_Wencha	0.999144867
ENSGALG00010024264	AMER3	3	0.000580734	0.250166365	0.002321392	0.998147799	0.125009588	7.843937381	74.50260115	0.666666667	CRA004775;PRJNA827465;Yang2025_Silkie_Wencha	0.999144867
ENSGALG00010013719	NCDN	3	0.000406106	0.181080207	0.002242685	0.998210598	0.075365954	10.10750719	80.21272741	0.666666667	CRA004775;PRJNA827465;Yang2025_Silkie_Wencha	0.999144867
ENSGALG00010009396	AUH	3	-0.000360119	0.144351155	-0.002494746	0.998009483	0.038793671	5.886498683	66.02394551	0.666666667	CRA004775;PRJNA827465;Yang2025_Silkie_Wencha	0.999144867
ENSGALG00010003247	ENSGALG00010003247	3	-0.000344923	0.144835595	-0.002381481	0.998099855	0	0.870254433	0	0.666666667	CRA004775;PRJNA827465;Yang2025_Silkie_Wencha	0.999144867
ENSGALG00010014488	ENSGALG00010014488	3	0.000328295	0.145724894	0.002252842	0.998202494	0.029370695	3.992991702	49.91224252	0.666666667	CRA004775;PRJNA827465;Yang2025_Silkie_Wencha	0.999144867
ENSGALG00010027821	NMRK2	3	0.000239436	0.107297611	0.002231509	0.998219515	0	1.807710611	0	0.666666667	CRA004775;PRJNA827465;Yang2025_Silkie_Wencha	0.999144867
ENSGALG00010001512	PLCL2	3	-0.000147766	0.078027893	-0.001893764	0.998488996	0	1.44822913	0	0.666666667	CRA004775;PRJNA827465;Yang2025_Silkie_Wencha	0.99933715
ENSGALG00010011443	ENSGALG00010011443	3	-0.000420822	0.233338948	-0.001803481	0.998561031	0	0.306149823	0	0.666666667	CRA004775;PRJNA827465;Yang2025_Silkie_Wencha	0.999338506
ENSGALG00010004296	ENSGALG00010004296	2	-0.001756104	1.103589439	-0.001591265	0.998730354	1.036724621	1.684587966	40.63830323	0.5	PRJNA827465;Yang2025_Silkie_Wenchang	0.999385072
ENSGALG00010018064	TTC8	3	-0.00023761	0.151537925	-0.001567989	0.998748926	0.047369413	6.57460785	69.57993472	0.666666667	CRA004775;PRJNA827465;Yang2025_Silkie_Wencha	0.999385072
ENSGALG00010000591	SBK2	3	-0.000264023	0.190182047	-0.001388263	0.998892327	0	1.869139223	0	0.666666667	CRA004775;PRJNA827465;Yang2025_Silkie_Wencha	0.999457831
ENSGALG00010008457	MARVELD3	3	-0.000112584	0.093556854	-0.001203377	0.999039844	0	1.043824201	0	0.666666667	CRA004775;PRJNA827465;Yang2025_Silkie_Wencha	0.999534699
ENSGALG00010016612	GRIA1	3	0.000559893	0.556275414	0.001006503	0.999196927	0.757123002	15.15881474	86.80635634	0.666666667	CRA004775;PRJNA827465;Yang2025_Silkie_Wencha	0.999621125
ENSGALG00010007873	ENSGALG00010007873	3	-0.000660782	0.857981111	-0.000770159	0.999385502	1.761190056	15.9483814	87.45954244	0.666666667	CRA004775;PRJNA827465;Yang2025_Silkie_Wencha	0.999668314
ENSGALG00010019921	CALM2	3	-5.96637E-05	0.071719206	-0.000831907	0.999336235	0.004753443	2.7552482	27.41125828	0.666666667	CRA004775;PRJNA827465;Yang2025_Silkie_Wencha	0.999668314
ENSGALG00010028106	ARPC5L	3	-0.000103786	0.193370371	-0.000536719	0.99957176	0.077000449	6.678550916	70.05338396	0.666666667	CRA004775;PRJNA827465;Yang2025_Silkie_Wencha	0.999713172
ENSGALG00010015124	UXS1	3	-6.09425E-05	0.105146825	-0.000579595	0.99953755	0.014878548	3.611588706	44.62270865	0.666666667	CRA004775;PRJNA827465;Yang2025_Silkie_Wencha	0.999713172
ENSGALG00010002332	ENSGALG00010002332	2	-0.000193814	0.520695157	-0.000372222	0.99970301	0	0.311499297	0	0.5	PRJNA827465;Yang2025_Silkie_Wenchang	0.99977372
ENSGALG00010007190	ENSGALG00010007190	3	-5.02112E-06	0.184132046	-2.72691E-05	0.999978242	0.03854436	3.135818046	36.22078926	0.666666667	CRA004775;PRJNA827465;Yang2025_Silkie_Wencha	0.999978242
